# Supplementary material for: A novel Drosophila injury model reveals severed axons are cleared through a Draper/MMP-1 signaling cascade
Source: eLife. 2017 Aug 21;6:e23611. doi: 10.7554/eLife.23611 (PMC5565368; doi:10.7554/eLife.23611)
Supplement: Table 1—source code 2. — DOI: http://dx.doi.org/10.7554/eLife.23611.014 [file elife-23611-table1-code2.zip › Table 1 - Source Data 8 - mouse_annokey_report.html]

Annokey Search Report


# Annokey Search Report

- **annokey version:** 1.0.2
- **annokey homepage:** http://bjpop.github.io/annokey/
- **hostname:** barcoo
- **directory:** /vlsci/VLSCI/bjpop/scratch/annokey\_sean\_speese/mouse
- **command:** annokey --email bjpope@unimelb.edu.au --terms ../search\_terms.csv --genes mouse\_genes\_unique.csv --log log --pubmed --organism mouse
- **date:** 29 Apr 2017

## Mthfd2

- NCBI Gene
- GeneRIF
- Pubmed

| Search Term | Rank | Fields |
| --- | --- | --- |
| [Ii]nvasive | 10 | PubMed(1) |

  
hide/show details for Mthfd2

### [Ii]nvasive

#### PubMed

1. In vivo and in vitro investigations of heterozygous nebulin knock-out mice disclose a mild skeletal muscle phenotype. Nemaline myopathy is the most common congenital skeletal muscle disease, and mutations in the nebulin gene account for 50% of all cases. Recent studies suggest that the disease severity might be related to the nebulin expression levels. Considering that mutations in the nebulin gene are typically recessive, one would expect that a single functional nebulin allele would maintain nebulin protein expression which would result in preserved skeletal muscle function. We investigated skeletal muscle function of heterozygous nebulin knock-out (i.e., nebulin(+/-)) mice using a multidisciplinary approach including protein and gene expression analysis and combined in vivo and in vitro force measurements. Skeletal muscle anatomy and energy metabolism were studied strictly non-**invasive**ly using magnetic resonance imaging and 31P-magnetic resonance spectroscopy. Maximal force production was reduced by around 16% in isolated muscle of nebulin(+/-) mice while in vivo force generating capacity was preserved. Muscle weakness was associated with a shift toward a slower proteomic phenotype, but was not related to nebulin protein deficiency or to an impaired energy metabolism. Further studies would be warranted in order to determine the mechanisms leading to a mild skeletal muscle phenotype resulting from the expression of a single nebulin allele.

---

## Tinagl1

- NCBI Gene
- GeneRIF
- Pubmed

| Search Term | Rank | Fields |
| --- | --- | --- |
| [Mm]igration | 6 | PubMed(1) |
| ECM | 7 | PubMed(1) |
| [Ee]xtracellular [Mm]atrix | 8 | PubMed(3); Component(2) |
| [Ii]nvasive | 10 | PubMed(1) |
| [Cc]ell [Ii]nvasion | 13 | PubMed(1) |

  
hide/show details for Tinagl1

### [Mm]igration

#### PubMed

1. Lipocalin-7 is a matricellular regulator of angiogenesis. Matricellular proteins are extracellular regulators of cellular adhesion, signaling and performing a variety of physiological behaviors such as proliferation, **migration** and differentiation. Within vascular microenvironments, matricellular proteins exert both positive and negative regulatory cues to vascular endothelium. The relative balance of these matricellular cues is believed to be critical for vascular homeostasis, angiogenesis activation or angiogenesis resolution. However, our knowledge of matricellular proteins within vascular microenvironments and the mechanisms by which these proteins impact vascular function remain largely undefined. The matricellular protein lipocalin-7 (LCN7) is found throughout vascular microenvironments, and circumstantial evidence suggests that LCN7 may be an important regulator of angiogenesis. Therefore, we hypothesized that LCN7 may be an important regulator of vascular function.To test this hypothesis, we examined the effect of LCN7 overexpression, recombinant protein and gene knockdown in a series of in vitro and in vivo models of angiogenesis. We found that overexpression of LCN7 in MB114 and SVEC murine endothelial cell lines or administration of highly purified recombinant LCN7 protein increased endothelial cell invasion. Similarly, LCN7 increased angiogenic sprouting from quiescent endothelial cell monolayers and ex vivo aortic rings. Moreover, LCN7 increased endothelial cell sensitivity to TGF-β but did not affect sensitivity to other pro-angiogenic growth factors including bFGF and VEGF. Finally, morpholino based knockdown of LCN7 in zebrafish embryos specifically inhibited angiogenic sprouting but did not affect vasculogenesis within injected embryos.No functional analysis has previously been performed to elucidate the function of LCN7 in vascular or other cellular processes. Collectively, our results show for the first time that LCN7 is an important pro-angiogenic matricellular protein of vascular microenvironments.

### ECM

#### PubMed

1. Distribution of tubulointerstitial nephritis antigen-like 1 and structural matrix proteins in mouse embryos during preimplantation development in vivo and in vitro. Summary Tubulointerstitial nephritis antigen-like 1 (TINAGL1) is a novel matricellular protein that interacts with structural matrix proteins and promotes cell adhesion and spreading. We have previously reported unique localization of TINAGL1 to the trophectoderm (TE) of mouse blastocysts. TINAGL1 was found to be upregulated in implantation-competent blastocysts after estrogen treatment using progesterone-treated delayed-implantation models. Moreover, colocalization of TINAGL1 and extracellular matrix (**ECM**) protein laminin 1 was detected in the Reichert membrane on embryonic days 6.5 and 7.5. Although these data suggested a role for TINAGL1 in the embryo development at postimplantation, its relevance to other **ECM** proteins during preimplantation development is not clear. In this study, we examined the expression of TINAGL1 and its relevance to other **ECM** proteins fibronectin (FN) and collagen type IV (ColIV) during in vivo development of preimplantation embryos, particularly at blastocyst stage in detail. Localizations of TINAGL1, FN, and ColIV were similar. In 1-cell to 8-cell embryos, they were expressed in cytoplasm of blastomeres, and in morulae they were localized in the outer cells. FN and ColIV were expressed primarily on outer surface of the cells. In blastocysts, FN and ColIV were distributed in the cytoplasm of TE, but, just prior to implantation, they became localized uniquely to the blastocoelic surface of TE. In in vitro fertilized (IVF) blastocysts, expression levels of TINAGL1 and FN were lower than in in vivo blastocysts. These results suggest that, during preimplantation development, TINAGL1 may be involved in roles of structural matrix proteins, whose expression in blastocysts may be affected by in vitro culture.

### [Ee]xtracellular [Mm]atrix

#### PubMed

1. **Extracellular matrix** secretion by cardiac fibroblasts: role of microRNA-29b and microRNA-30c. MicroRNAs (miRNAs), in particular miR-29b and miR-30c, have been implicated as important regulators of cardiac fibrosis.To perform a proteomics comparison of miRNA effects on **extracellular matrix** secretion by cardiac fibroblasts.Mouse cardiac fibroblasts were transfected with pre-/anti-miR of miR-29b and miR-30c, and their conditioned medium was analyzed by mass spectrometry. miR-29b targeted a cadre of proteins involved in fibrosis, including multiple collagens, matrix metalloproteinases, and leukemia inhibitory factor, insulin-like growth factor 1, and pentraxin 3, 3 predicted targets of miR-29b. miR-29b also attenuated the cardiac fibroblast response to transforming growth factor-β. In contrast, miR-30c had little effect on **extracellular matri**x production but opposite effects regarding leukemia inhibitory factor and insulin-like growth factor 1. Both miRNAs indirectly affected cardiac myocytes. On transfection with pre-miR-29b, the conditioned medium of cardiac fibroblasts lost its ability to support adhesion of rat ventricular myocytes and led to a significant reduction of cardiac myocyte proteins (α-actinin, cardiac myosin-binding protein C, and cardiac troponin I). Similarly, cardiomyocytes derived from mouse embryonic stem cells atrophied under pre-miR-29 conditioned medium, whereas pre-miR-30c conditioned medium had a prohypertrophic effect. Levels of miR-29a, miR-29c, and miR-30c, but not miR-29b, were significantly reduced in a mouse model of pathological but not physiological hypertrophy. Treatment with antagomiRs to miR-29b induced excess fibrosis after aortic constriction without overt deterioration in cardiac function.Our proteomic analysis revealed novel molecular targets of miRNAs that are linked to a fibrogenic cardiac phenotype. Such comprehensive screening methods are essential to define the concerted actions of miRNAs in cardiovascular disease.
2. Tubulointerstitial nephritis antigen-like 1 is expressed in the uterus and binds with integrins in decidualized endometrium during postimplantation in mice. **Extracellular matrix** substrates contribute to both uterine and blastocyst functions during the peri-implantation period. Tubulointerstitial nephritis antigen-like 1 (TINAGL1, also known as adrenocortical zonation factor 1 [AZ-1] or lipocalin 7) is a novel matricellular protein that promotes cell adhesion and spreading. However, the physiological roles of TINAGL1 are still not clearly understood. We examined the expression and localization of TINAGL1 in peri-implantation mouse uteri. During the preimplantation period, TINAGL1 was expressed in the basement membranes of uterine luminal epithelial cells on Days 1 and 2 of pregnancy, while its expression levels declined after Day 3. In the whole uteri, the expression levels of Tinagl1 mRNA and TINAGL1 protein were similar on Days 1-4 of pregnancy. In contrast, the expression of Tinagl1 mRNA and TINAGL1 protein increased in postimplantation uteri. From Days 6 to 8, TINAGL1 was markedly expressed in the decidual endometrium. TINAGL1 is a ligand for integrins and promotes cell adhesion in cultured cells. Therefore, to address whether TINAGL1 interacts with integrins in the uterus, immunohistochemical analysis and immunoprecipitation were performed. Immunohistochemical analysis showed that ITGA2, ITGA5, and ITGB1 were expressed in stromal cells around the implanted embryos on Days 7 and 8. Biacore and immunoprecipitation analysis determined that TINAGL1 linked with ITGA5 and ITGB1 in the decidual endometrium. These results suggest that Tinagl1 functions during the postimplantation period; in particular, it associates with ITGA5B1 in the decidualized uterine endometrium.
3. Distribution of tubulointerstitial nephritis antigen-like 1 and structural matrix proteins in mouse embryos during preimplantation development in vivo and in vitro. Summary Tubulointerstitial nephritis antigen-like 1 (TINAGL1) is a novel matricellular protein that interacts with structural matrix proteins and promotes cell adhesion and spreading. We have previously reported unique localization of TINAGL1 to the trophectoderm (TE) of mouse blastocysts. TINAGL1 was found to be upregulated in implantation-competent blastocysts after estrogen treatment using progesterone-treated delayed-implantation models. Moreover, colocalization of TINAGL1 and **extracellular matrix** (ECM) protein laminin 1 was detected in the Reichert membrane on embryonic days 6.5 and 7.5. Although these data suggested a role for TINAGL1 in the embryo development at postimplantation, its relevance to other ECM proteins during preimplantation development is not clear. In this study, we examined the expression of TINAGL1 and its relevance to other ECM proteins fibronectin (FN) and collagen type IV (ColIV) during in vivo development of preimplantation embryos, particularly at blastocyst stage in detail. Localizations of TINAGL1, FN, and ColIV were similar. In 1-cell to 8-cell embryos, they were expressed in cytoplasm of blastomeres, and in morulae they were localized in the outer cells. FN and ColIV were expressed primarily on outer surface of the cells. In blastocysts, FN and ColIV were distributed in the cytoplasm of TE, but, just prior to implantation, they became localized uniquely to the blastocoelic surface of TE. In in vitro fertilized (IVF) blastocysts, expression levels of TINAGL1 and FN were lower than in in vivo blastocysts. These results suggest that, during preimplantation development, TINAGL1 may be involved in roles of structural matrix proteins, whose expression in blastocysts may be affected by in vitro culture.

#### Component

1. **extracellular matrix**
2. **extracellular matrix**

### [Ii]nvasive

#### PubMed

1. Blimp1/Prdm1 governs terminal differentiation of endovascular trophoblast giant cells and defines multipotent progenitors in the developing placenta. Developmental arrest of Blimp1/Prdm1 mutant embryos at around embryonic day 10.5 (E10.5) has been attributed to placental disturbances. Here we investigate Blimp1/Prdm1 requirements in the trophoblast cell lineage. Loss of function disrupts specification of the **invasive** spiral artery-associated trophoblast giant cells (SpA-TGCs) surrounding maternal blood vessels and severely compromises the ability of the spongiotrophoblast layer to expand appropriately, secondarily causing collapse of the underlying labyrinth layer. Additionally, we identify a population of proliferating Blimp1(+) diploid cells present within the spongiotrophoblast layer. Lineage tracing experiments exploiting a novel Prdm1.Cre-LacZ allele demonstrate that these Blimp1(+) cells give rise to the mature SpA-TGCs, canal TGCs, and glycogen trophoblasts. In sum, the transcriptional repressor Blimp1/Prdm1 is required for terminal differentiation of SpA-TGCs and defines a lineage-restricted progenitor cell population contributing to placental growth and morphogenesis.

### [Cc]ell [Ii]nvasion

#### PubMed

1. Lipocalin-7 is a matricellular regulator of angiogenesis. Matricellular proteins are extracellular regulators of cellular adhesion, signaling and performing a variety of physiological behaviors such as proliferation, migration and differentiation. Within vascular microenvironments, matricellular proteins exert both positive and negative regulatory cues to vascular endothelium. The relative balance of these matricellular cues is believed to be critical for vascular homeostasis, angiogenesis activation or angiogenesis resolution. However, our knowledge of matricellular proteins within vascular microenvironments and the mechanisms by which these proteins impact vascular function remain largely undefined. The matricellular protein lipocalin-7 (LCN7) is found throughout vascular microenvironments, and circumstantial evidence suggests that LCN7 may be an important regulator of angiogenesis. Therefore, we hypothesized that LCN7 may be an important regulator of vascular function.To test this hypothesis, we examined the effect of LCN7 overexpression, recombinant protein and gene knockdown in a series of in vitro and in vivo models of angiogenesis. We found that overexpression of LCN7 in MB114 and SVEC murine endothelial cell lines or administration of highly purified recombinant LCN7 protein increased endothelial **cell invasion**. Similarly, LCN7 increased angiogenic sprouting from quiescent endothelial cell monolayers and ex vivo aortic rings. Moreover, LCN7 increased endothelial cell sensitivity to TGF-β but did not affect sensitivity to other pro-angiogenic growth factors including bFGF and VEGF. Finally, morpholino based knockdown of LCN7 in zebrafish embryos specifically inhibited angiogenic sprouting but did not affect vasculogenesis within injected embryos.No functional analysis has previously been performed to elucidate the function of LCN7 in vascular or other cellular processes. Collectively, our results show for the first time that LCN7 is an important pro-angiogenic matricellular protein of vascular microenvironments.

---

## Gusb

- NCBI Gene
- GeneRIF
- Pubmed

| Search Term | Rank | Fields |
| --- | --- | --- |
| [Mm]igration | 6 | PubMed(2) |
| [Ee]xtracellular [Mm]atrix | 8 | PubMed(1) |
| [Ii]nvasive | 10 | PubMed(2) |

  
hide/show details for Gusb

### [Mm]igration

#### PubMed

1. COX-2 Induces Breast Cancer Stem Cells via EP4/PI3K/AKT/NOTCH/WNT Axis. Cancer stem-like cells (SLC) resist conventional therapies, necessitating searches for SLC-specific targets. We established that cyclo-oxygenase(COX)-2 expression promotes human breast cancer progression by activation of the prostaglandin(PG)E-2 receptor EP4. Present study revealed that COX-2 induces SLCs by EP4-mediated NOTCH/WNT signaling. Ectopic COX-2 over-expression in MCF-7 and SKBR-3 cell lines resulted in: increased **migration**/invasion/proliferation, epithelial-mesenchymal transition (EMT), elevated SLCs (spheroid formation), increased ALDH activity and colocalization of COX-2 and SLC markers (ALDH1A, CD44, β-Catenin, NANOG, OCT3/4, SOX-2) in spheroids. These changes were reversed with COX-2-inhibitor or EP4-antagonist (EP4A), indicating dependence on COX-2/EP4 activities. COX-2 over-expression or EP4-agonist treatments of COX-2-low cells caused up-regulation of NOTCH/WNT genes, blocked with PI3K/AKT inhibitors. NOTCH/WNT inhibitors also blocked COX-2/EP4 induced SLC induction. Microarray analysis showed up-regulation of numerous SLC-regulatory and EMT-associated genes. MCF-7-COX-2 cells showed increased mammary tumorigenicity and spontaneous multiorgan metastases in NOD/SCID/IL-2Rγ-null mice for successive generations with limiting cell inocula. These tumors showed up-regulation of VEGF-A/C/D, Vimentin and phospho-AKT, down-regulation of E-Cadherin and enrichment of SLC marker positive and spheroid forming cells. MCF-7-COX-2 cells also showed increased lung colonization in NOD/SCID/GUSB-null mice, an effect reversed with EP4-knockdown or EP4A treatment of the MCF-7-COX-2 cells. COX-2/EP4/ALDH1A mRNA expression in human breast cancer tissues were highly correlated with one other, more marked in progressive stage of disease. In situ immunostaining of human breast tumor tissues revealed co-localization of SLC markers with COX-2, supporting COX-2 inducing SLCs. High COX-2/EP4 mRNA expression was linked with reduced survival. Thus, EP4 represents a novel SLC-ablative target in human breast cancer. Stem Cells 2016;34:2290-2305.
2. VEGF and PlGF promote adult vasculogenesis by enhancing EPC recruitment and vessel formation at the site of tumor neovascularization. There are growing data to suggest that tissue hypoxia represents a critical force that drives adult vasculogenesis. Vascular endothelial growth factor (VEGF) expression is dramatically up-regulated by hypoxia and results in enhanced neovascularization. Although the role of VEGF in angiogenesis has been well characterized, its role in adult vasculogenesis remains poorly understood. We used two distinct murine bone marrow transplantation (BMT) models to demonstrate that increased VEGF levels at the site of tumor growth promoted vasculogenesis in vivo. This effect of VEGF was downstream of its effect to enhance either mobilization or survival of circulating endothelial progenitor cells (EPCs). Both VEGFR1 (flt1) and VEGFR2 (flk1) are expressed on culture expanded human EPCs. Previous studies suggest that the effect of VEGF on endothelial cell **migration** is primarily mediated via VEGFR2; however, VEGF-induced EPC **migration** in vitro was mediated by both receptors, suggesting that VEGF-VEGFR1 interactions in EPCs are distinct from differentiated endothelial cells. We used specific blocking antibodies to these receptors to demonstrate that VEGFR1 plays an important role in human EPC recruitment to tumors. These findings were further supported by our finding that tumor-associated placental growth factor (PlGF), a VEGFR1-specific agonist, increased tumor vasculogenesis in a murine BMT model. We further showed that both VEGF receptors were necessary for the formation of functional vessels derived from exogenously administered human ex vivo expanded EPCs. Our data suggest local VEGF and/or PlGF expression promote vasculogenesis; VEGF plays a role in EPC recruitment and subsequent formation of functional vessels.

### [Ee]xtracellular [Mm]atrix

#### PubMed

1. Integrated analysis of proteome and transcriptome changes in the mucopolysaccharidosis type VII mouse hippocampus. Mucopolysaccharidosis type VII (MPS VII) is a lysosomal storage disease caused by the deficiency of β-glucuronidase. In this study, we compared the changes relative to normal littermates in the proteome and transcriptome of the hippocampus in the C57Bl/6 mouse model of MPS VII, which has well-documented histopathological and neurodegenerative changes. A completely different set of significant changes between normal and MPS VII littermates were found in each assay. Nevertheless, the functional annotation terms generated by the two methods showed agreement in many of the processes, which also corresponded to known pathology associated with the disease. Additionally, assay-specific changes were found, which in the proteomic analysis included mitochondria, energy generation, and cytoskeletal differences in the mutant, while the transcriptome differences included immune, vesicular, and **extracellular matri**x changes. In addition, the transcriptomic changes in the mutant hippocampus were concordant with those in a MPS VII mouse caused by the same mutation but on a different background inbred strain.

### [Ii]nvasive

#### PubMed

1. Colonic hamartoma development by anomalous duplication in Cdx2 knockout mice. To determine the biological role of caudal-like homeobox gene CDX2, we constructed knockout mice in which its mouse homologue Cdx2 was inactivated by homologous recombination, placing a bacterial lacZ gene under the control of the Cdx2 promoter. Although the homozygous mutants died in utero around implantation, the heterozygotes were viable and fertile and expressed lacZ in the caudal region in early embryos and in the gut tissues in adults. The heterozygotes developed cecal and colonic villi by anteriorization and formed hamartomatous polyps in the proximal colon. The hamartoma started to develop at 11.5 days of gestation as an outpocket of the gut epithelium, which ceased to express the remaining Cdx2 allele. The outpocket then expanded as a partially duplicated gut but was contained as a hamartoma after birth. In adult mice, these hamartomas grew very slowly and took a benign course. None of them progressed into **invasive** adenocarcinomas, even at 1.5 years of age. Whereas the cecal and colonic villi expressed lacZ, the hamartoma epithelium did not, nor did it express Cdx2 mRNA from the wild-type allele. However, genomic DNA analysis of the polyp epithelium did not show a loss of heterozygosity of the Cdx2 gene, suggesting a mechanism of biallelic Cdx2 inactivation other than loss of heterozygosity. These results indicate that the Cdx2 haploin-sufficiency caused cecal and colonic villi, whereas the biallelic inactivation of Cdx2 triggered anomalous duplications of the embryonic gut epithelium, which were contained as hamartomas after birth.
2. Neonatal gene transfer leads to widespread correction of pathology in a murine model of lysosomal storage disease. For many inborn errors of metabolism, early treatment is critical to prevent long-term developmental sequelae. We have used a gene-therapy approach to demonstrate this concept in a murine model of mucopolysaccharidosis type VII (MPS VII). Newborn MPS VII mice received a single intravenous injection with 5.4 x 10(6) infectious units of recombinant adeno-associated virus encoding the human beta-glucuronidase (GUSB) cDNA. Therapeutic levels of GUSB expression were achieved by 1 week of age in liver, heart, lung, spleen, kidney, brain, and retina. GUSB expression persisted in most organs for the 16-week duration of the study at levels sufficient to either reduce or prevent completely lysosomal storage. Of particular significance, neurons, microglia, and meninges of the central nervous system were virtually cleared of disease. In addition, neonatal treatment of MPS VII mice provided access to the central nervous system via an intravenous route, avoiding a more **invasive** procedure later in life. These data suggest that gene transfer mediated by adeno-associated virus can achieve therapeutically relevant levels of enzyme very early in life and that the rapid growth and differentiation of tissues does not limit long-term expression.

---

## Atic

- NCBI Gene
- GeneRIF
- Pubmed

| Search Term | Rank | Fields |
| --- | --- | --- |
| [Mm]etastasis | 12 | PubMed(1) |

  
hide/show details for Atic

### [Mm]etastasis

#### PubMed

1. Lack of periostin leads to suppression of Notch1 signaling and calcific aortic valve disease. The Postn gene encodes protein periostin. During embryonic development, it is highly expressed in the outflow tract (OFT) endocardial cushions of the developing heart, which give rise to several structures of the mature heart including the aortic valve. Periostin was previously implicated in osteoblast differentiation, cancer **metastasis**, and tooth and bone development, but its role in cardiac OFT development is unclear. To elucidate the role that periostin plays in the developing heart we analyzed cardiac OFT phenotype in mice after deletion of the Postn gene. We found that lack of periostin in the embryonic OFT leads to ectopic expression of the proosteogenic growth factor pleiotrophin (Ptn) and overexpression of delta-like 1 homolog (Dlk1), a negative regulator of Notch1, in the distal (prevalvular) cushions of the OFT. This resulted in suppression of Notch1 signaling, strong induction of the central transcriptional regulator of osteoblast cell fate Runx2, upregulation of osteopontin and osteocalcin expression, and subsequent calcification of the aortic valve. Our data suggest that periostin represses a default osteogenic program in the OFT cushion mesenchyme and promotes differentiation along a fibrogenic lineage. Lack of periostin causes derepression of the osteogenic potential of OFT mesenchymal cells, calcium deposition, and calcific aortic valve disease. These results establish periostin as a key regulator of OFT endocardial cushion mesenchymal cell fate during embryonic development.

---

## Pdcd6ip

- NCBI Gene
- GeneRIF
- Pubmed

| Search Term | Rank | Fields |
| --- | --- | --- |
| [Mm]igration | 6 | PubMed(2) |
| [Ee]xtracellular [Mm]atrix | 8 | PubMed(1) |

  
hide/show details for Pdcd6ip

### [Mm]igration

#### PubMed

1. Galectin-3 regulates intracellular trafficking of EGFR through Alix and promotes keratinocyte **migration**. The EGFR-mediated signaling pathways are important in a variety of cellular processes, including cell **migration** and wound re-epithelialization. Intracellular trafficking of EGFR is critical for maintaining EGFR surface expression. Galectin-3, a member of an animal lectin family, has been implicated in a number of physiological and pathological processes. Through studies of galectin-3-deficient mice and cells isolated from these mice, we demonstrated that the absence of galectin-3 impairs keratinocyte **migration** and skin wound re-epithelialization. We have linked this pro-migratory function to a crucial role of cytosolic galectin-3 in controlling intracellular trafficking and cell surface expression of EGFR after EGF stimulation. Without galectin-3, the surface levels of EGFR are markedly reduced, and the receptor accumulates diffusely in the cytoplasm. This is associated with reduced rates of both endocytosis and recycling of the receptor. We have provided evidence that this previously unreported function of galectin-3 may be mediated through interaction with its binding partner Alix, which is a protein component of the ESCRT (endosomal sorting complex required for transport) machinery. Our results suggest that galectin-3 is potentially a critical regulator of a number of important cellular responses through its intracellular control of trafficking of cell surface receptors.
2. Caspase-11 regulates cell **migration** by promoting Aip1-Cofilin-mediated actin depolymerization. Coordinated regulation of cell **migration**, cytokine maturation and apoptosis is critical in inflammatory responses. Caspases, a family of cysteine proteases, are known to regulate cytokine maturation and apoptosis. Here, we show that caspase-11, a mammalian pro-inflammatory caspase, regulates cell **migration** during inflammation. Caspase-11-deficient lymphocytes exhibit a cell-autonomous **migration** defect in vitro and in vivo. We demonstrate that caspase-11 interacts physically and functionally with actin interacting protein 1 (Aip1), an activator of cofilin-mediated actin depolymerization. The caspase-recruitment domain (CARD) of caspase-11 interacts with the carboxy-terminal WD40 propeller domain of Aip1 to promote cofilin-mediated actin depolymerization. Cells with Aip1 or caspase-11 deficiency exhibit defects in actin dynamics. Using in vitro actin depolymerization assays, we found that caspase-11 and Aip1 work cooperatively to promote cofilin-mediated actin depolymerization. These data demonstrate a novel cell autonomous caspase-mediated mechanism that regulates actin dynamics and mammalian cell **migration** distinct from the receptor mediated Rho-Rac-Cdc42 pathway.

### [Ee]xtracellular [Mm]atrix

#### PubMed

1. Extracellular Alix regulates integrin-mediated cell adhesions and **extracellular matrix** assembly. Alix (ALG-2-interacting protein X), a cytoplasmic adaptor protein involved in endosomal sorting and actin cytoskeleton assembly, is required for the maintenance of fibroblast morphology. As Alix has sequence similarity to adhesin in Entamoeba histolytica, and we observed that Alix is secreted, we determined whether extracellular Alix affects fibroblast morphology. Here, we demonstrate that secreted Alix is deposited on the substratum of non-immortalized WI38 fibroblasts. Antibody binding to extracellular Alix retards WI38 cell adhesion and spreading on fibronectin and vitronectin. Alix knockdown in WI38 cells reduces spreading and fibronectin assembly, and the effect is partially complemented by coating recombinant Alix on the cell substratum. Immortalized NIH/3T3 fibroblasts deposit less Alix on the substratum and have defects in alpha5beta1-integrin functions. Coating recombinant Alix on the culture substratum for NIH/3T3 cells promotes alpha5beta1-integrin-mediated cell adhesions and fibronectin assembly, and these effects require the aa 605-709 region of Alix. These findings demonstrate that a sub-population of Alix localizes extracellularly and regulates integrin-mediated cell adhesions and fibronectin matrix assembly.

---

## Hsp90b1

- NCBI Gene
- GeneRIF
- Pubmed

| Search Term | Rank | Fields |
| --- | --- | --- |
| [Mm]igration | 6 | PubMed(1) |
| [Ee]xtracellular [Mm]atrix | 8 | PubMed(3); Component(1) |
| [Ii]nvasive | 10 | PubMed(1) |
| [Mm]etastasis | 12 | PubMed(4); GeneRIFs(1) |
| [Cc]ell [Ii]nvasion | 13 | PubMed(1) |

  
hide/show details for Hsp90b1

### [Mm]igration

#### PubMed

1. Genomic cloning of mouse MIF (macrophage inhibitory factor) and genetic mapping of the human and mouse expressed gene and nine mouse pseudogenes. The single functional mouse gene for MIF (macrophage **migration** inhibitory factor) has been cloned from a P1 library, and its exon/intron structure determined and shown to resemble that of the human gene. The gene was mapped to chromosome 10 using two multilocus crosses between laboratory strains and either Mus musculus musculus or Mus spretus. Nine additional loci containing related sequences, apparently all processed pseudogenes, were also mapped to chromosomes 1, 2, 3, 7, 8, 9, 12, 17, and 19. While most of these pseudogenes were found in inbred mice and M. spretus, some are species specific. This suggests that there have been active phases of pseudogene formation in Mus both before and after the separation of musculus and spretus. The human genome contains no pseudogenes; we assigned the human gene to chromosome 19, consistent with the location of mouse and human functional genes for MIF in a region of conserved linkage.

### [Ee]xtracellular [Mm]atrix

#### PubMed

1. Neural tube opening and abnormal extraembryonic membrane development in SEC23A deficient mice. COPII (coat protein complex-II) vesicles transport proteins from the endoplasmic reticulum (ER) to the Golgi. Higher eukaryotes have two or more paralogs of most COPII components. Here we characterize mice deficient for SEC23A and studied interactions of Sec23a null allele with the previously reported Sec23b null allele. SEC23A deficiency leads to mid-embryonic lethality associated with defective development of extraembryonic membranes and neural tube opening in midbrain. Secretion defects of multiple collagen types are observed in different connective tissues, suggesting that collagens are primarily transported in SEC23A-containing vesicles in these cells. Other **extracellular matrix** proteins, such as fibronectin, are not affected by SEC23A deficiency. Intracellular accumulation of unsecreted proteins leads to strong induction of the unfolded protein response in collagen-producing cells. No collagen secretion defects are observed in SEC23B deficient embryos. We report that E-cadherin is a cargo that accumulates in acini of SEC23B deficient pancreas and salivary glands. Compensatory increase of one paralog is observed in the absence of the second paralog. Haploinsufficiency of the remaining Sec23 paralog on top of homozygous inactivation of the first paralog leads to earlier lethality of embryos. Our results suggest that mammalian SEC23A and SEC23B transport overlapping yet distinct spectra of cargo in vivo.
2. Host airway proteins interact with Staphylococcus aureus during early pneumonia. Staphylococcus aureus is a major cause of hospital-acquired pneumonia and is emerging as an important etiological agent of community-acquired pneumonia. Little is known about the specific host-pathogen interactions that occur when S. aureus first enters the airway. A shotgun proteomics approach was utilized to identify the airway proteins associated with S. aureus during the first 6 h of infection. Host proteins eluted from bacteria recovered from the airways of mice 30 min or 6 h following intranasal inoculation under anesthesia were subjected to liquid chromatography and tandem mass spectrometry. A total of 513 host proteins were associated with S. aureus 30 min and/or 6 h postinoculation. A majority of the identified proteins were host cytosolic proteins, suggesting that S. aureus was rapidly internalized by phagocytes in the airway and that significant host cell lysis occurred during early infection. In addition, **extracellular matrix** and secreted proteins, including fibronectin, antimicrobial peptides, and complement components, were associated with S. aureus at both time points. The interaction of 12 host proteins shown to bind to S. aureus in vitro was demonstrated in vivo for the first time. The association of hemoglobin, which is thought to be the primary staphylococcal iron source during infection, with S. aureus in the airway was validated by immunoblotting. Thus, we used our recently developed S. aureus pneumonia model and shotgun proteomics to validate previous in vitro findings and to identify nearly 500 other proteins that interact with S. aureus in vivo. The data presented here provide novel insights into the host-pathogen interactions that occur when S. aureus enters the airway.
3. The endoplasmic reticulum chaperone protein GRP94 is required for maintaining hematopoietic stem cell interactions with the adult bone marrow niche. Hematopoietic stem cell (HSC) homeostasis in the adult bone marrow (BM) is regulated by both intrinsic gene expression products and interactions with extrinsic factors in the HSC niche. GRP94, an endoplasmic reticulum chaperone, has been reported to be essential for the expression of specific integrins and to selectively regulate early T and B lymphopoiesis. In GRP94 deficient BM chimeras, multipotent hematopoietic progenitors persisted and even increased, however, the mechanism is not well understood. Here we employed a conditional knockout (KO) strategy to acutely eliminate GRP94 in the hematopoietic system. We observed an increase in HSCs and granulocyte-monocyte progenitors in the Grp94 KO BM, correlating with an increased number of colony forming units. Cell cycle analysis revealed that a loss of quiescence and an increase in proliferation led to an increase in Grp94 KO HSCs. This expansion of the HSC pool can be attributed to the impaired interaction of HSCs with the niche, evidenced by enhanced HSC mobilization and severely compromised homing and lodging ability of primitive hematopoietic cells. Transplanting wild-type (WT) hematopoietic cells into a GRP94 null microenvironment yielded a normal hematology profile and comparable numbers of HSCs as compared to WT control, suggesting that GRP94 in HSCs, but not niche cells, is required for maintaining HSC homeostasis. Investigating this, we further determined that there was a near complete loss of integrin α4 expression on the cell surface of Grp94 KO HSCs, which showed impaired binding with fibronectin, an **extracellular matri**x molecule known to play a role in mediating HSC-niche interactions. Furthermore, the Grp94 KO mice displayed altered myeloid and lymphoid differentiation. Collectively, our studies establish GRP94 as a novel cell intrinsic factor required to maintain the interaction of HSCs with their niche, and thus regulate their physiology.

#### Component

1. **extracellular matrix**

### [Ii]nvasive

#### PubMed

1. Glucose-regulated protein 94 deficiency induces squamous cell metaplasia and suppresses PTEN-null driven endometrial epithelial tumor development. Endometrial carcinoma is the most prevalent gynecologic cancer in the United States. The tumor suppressor gene Pten (phosphatase and tensin homolog) is commonly mutated in the more common type 1 (endometrioid) subtype. The glucose-regulated protein 94 (GRP94) is emerging as a novel regulator for cancer development. Here we report that expression profiles from the Cancer Genome Atlas (TCGA) showed significantly increased Grp94 mRNA levels in endometrial tumor versus normal tissues, correlating with highly elevated GRP94 protein expression in patient samples and the requirement of GRP94 for maintaining viability of human endometrioid adenocarcinoma (EAC) cell lines. Through generation of uterus-specific knockout mouse models with deletion of Grp94 alone (c94f/f) or in combination with Pten (cPf/f94f/f), we discovered that c94f/f uteri induced squamous cell metaplasia (SCM) and reduced active nuclear β-catenin. The cPf/f94f/f uteri showed accelerated SCM and suppression of PTEN-null driven EAC, with reduced cellular proliferation, attenuated β-catenin signaling and decreased AKT/S6 activation in the SCM. In contrast to single PTEN knockout uteri (cPf/f), cPf/f94f/f uteri showed no decrease in E-cadherin level and n**o invasi**ve lesion. Collectively, our study implies that GRP94 downregulation induces SCM in EAC and suppresses AKT/S6 signaling, providing a novel mechanism for suppressing EAC progression.

### [Mm]etastasis

#### PubMed

1. α7 helix region of αI domain is crucial for integrin binding to endoplasmic reticulum chaperone gp96: a potential therapeutic target for cance**r metastas**is. Integrins play important roles in regulating a diverse array of cellular functions crucial to the initiation, progression, an**d metastas**is of tumors. Previous studies have shown that a majority of integrins are folded by the endoplasmic reticulum chaperone gp96. Here, we demonstrate that the dimerization of integrin αL and β2 is highly dependent on gp96. The αI domain (AID), a ligand binding domain shared by seven integrin α-subunits, is a critical region for integrin binding to gp96. Deletion of AID significantly reduced the interaction between integrin αL and gp96. Overexpression of AID intracellularly decreased surface expression of gp96 clients (integrins and Toll-like receptors) and cancer cell invasion. The α7 helix region is crucial for AID binding to gp96. A cell-permeable α7 helix peptide competitively inhibited the interaction between gp96 and integrins and blocked cell invasion. Thus, targeting the binding site of α7 helix of AID on gp96 is potentially a new strategy for treatment **of cancer** metastasis.
2. Decreased adhesiveness, resistance to anoikis and suppression of GRP94 are integral to the survival of circulating tumor cells in prostate cancer. The presence of circulating tumor cells (CTC) is common in prostate cancer patients, however until recently their clinical significance was unknown. The CTC stage is essential for the formation of distant metastases, and their continuing presence after radical prostatectomy has been shown to predict recurrent or latent disease. Despite their mechanistic and prognostic importance, due both to their scarcity and difficulties in their isolation, little is known about the characteristics that enable their production and survival. The aim of this study was to investigate the molecular mechanisms underlying the survival of CTC cells. A novel CTC cell line from the bloodstream of an orthotopic mouse model of castration-resistant prostate cancer was established and compared with the primary tumor using attachment assays, detachment culture, Western blot, flow cytometry and 2D gel electrophoresis. Decreased adhesiveness and expression of adhesion molecules E-cadherin, beta4-integrin and gamma-catenin, together with resistance to detachment and drug-induced apoptosis and upregulation of Bcl-2 were integral to the development of CTC and their survival. Using proteomic studies, we observed that the GRP94 glycoprotein was suppressed in CTC. GRP94 was also shown to be suppressed in a tissue microarray study of 79 prostate cancer patients, indicating its possible role in prostate cancer progression. Overall, this study suggests molecular alterations accounting for the release and survival of CTC, which may be used as drug targets for either anti-metastatic therapy or the suppression of latent disease. We also indicate the novel involvement of GRP94 suppression in prostate cancer **metastasis**.
3. Glucose-regulated protein 94/glycoprotein 96 elicits bystander activation of CD4+ T cell Th1 cytokine production in vivo. Glucose-regulated protein 94 (GRP94/gp96), the endoplasmic reticulum heat shock protein 90 paralog, elicits both innate and adaptive immune responses. Regarding the former, GRP94/gp96 stimulates APC cytokine expression and dendritic cell maturation. The adaptive component of GRP94/gp96 function reflects a proposed peptide-binding activity and, consequently, a role for native GRP94/gp96-peptide complexes in cross-presentation. It is by this mechanism that tumor-derived GRP94/gp96 is thought to suppress tumor growth and **metastasis**. Recent data have demonstrated that GRP94/gp96-elicited innate immune responses can be sufficient to suppress tumor growth and **metastasis**. However, the immunological processes activated in response to tumor Ag-negative sources of GRP94/gp96 are currently unknown. We have examined the in vivo immunological response to nontumor sources of GRP94/gp96 and report that administration of syngeneic GRP94/gp96- or GRP94/gp96-N-terminal domain-secreting KBALB fibroblasts to BALB/c mice stimulates CD11b(+) and CD11c(+) APC function and promotes bystander activation of CD4(+) T cell Th1 cytokine production. Only modest activation of CD8(+) T cell or NK cell cytolytic function was observed. The GRP94/gp96-dependent induction of CD4(+) T cell cytokine production was markedly inhibited by carrageenan, indicating an essential role for APC in this response. These results identify the bystander activation of CD4(+) T lymphocytes as a previously unappreciated immunological consequence of GRP94/gp96 administration and demonstrate that GRP94/gp96-elicited alterations in the in vivo cytokine environment influence the development of CD4(+) T cell effector functions, independently of its proposed function as a peptide chaperone.
4. GRP94 (gp96) and GRP94 N-terminal geldanamycin binding domain elicit tissue nonrestricted tumor suppression. In chemical carcinogenesis models, GRP94 (gp96) elicits tumor-specific protective immunity. The tumor specificity of this response is thought to reflect immune responses to GRP94-bound peptide antigens, the cohort of which uniquely identifies the GRP94 tissue of origin. In this study, we examined the apparent tissue restriction of GRP94-elicited protective immunity in a 4T1 mammary carcinoma model. We report that the vaccination of BALB/c mice with irradiated fibroblasts expressing a secretory form of GRP94 markedly suppressed 4T1 tumor growth and **metastasis**. In addition, vaccination with irradiated cells secreting the GRP94 NH(2)-terminal geldanamycin-binding domain (NTD), a region lacking canonical peptide-binding motifs, yielded a similar suppression of tumor growth and metastatic progression. Conditioned media from cultures of GRP94 or GRP94 NTD-secreting fibroblasts elicited the up-regulation of major histocompatibility complex class II and CD86 in dendritic cell cultures, consistent with a natural adjuvant function for GRP94 and the GRP94 NTD. Based on these findings, we propose that GRP94-elicited tumor suppression can occur independent of the GRP94 tissue of origin and suggest a primary role for GRP4 natural adjuvant function in antitumor immune responses.

#### GeneRIFs

1. the novel involvement of GRP94 suppression in prostate cancer **metastasis**.

### [Cc]ell [Ii]nvasion

#### PubMed

1. α7 helix region of αI domain is crucial for integrin binding to endoplasmic reticulum chaperone gp96: a potential therapeutic target for cancer metastasis. Integrins play important roles in regulating a diverse array of cellular functions crucial to the initiation, progression, and metastasis of tumors. Previous studies have shown that a majority of integrins are folded by the endoplasmic reticulum chaperone gp96. Here, we demonstrate that the dimerization of integrin αL and β2 is highly dependent on gp96. The αI domain (AID), a ligand binding domain shared by seven integrin α-subunits, is a critical region for integrin binding to gp96. Deletion of AID significantly reduced the interaction between integrin αL and gp96. Overexpression of AID intracellularly decreased surface expression of gp96 clients (integrins and Toll-like receptors) and **cancer cell i**nvasion. The α7 helix region is crucial for AID binding to gp96. A cell-permeable α7 helix peptide competitively inhibited the interaction between gp96 and integrins and **blocked cell** invasion. Thus, targeting the binding site of α7 helix of AID on gp96 is potentially a new strategy for treatment of cancer metastasis.

---

## Glce

- NCBI Gene
- GeneRIF
- Pubmed

| Search Term | Rank | Fields |
| --- | --- | --- |
| [Mm]igration | 6 | PubMed(2) |

  
hide/show details for Glce

### [Mm]igration

#### PubMed

1. Lack of L-iduronic acid in heparan sulfate affects interaction with growth factors and cell signaling. HSEPI (glucuronyl C5-epimerase) catalyzes the conversion of d-glucuronic acid to l-iduronic acid in heparan sulfate (HS) biosynthesis. Disruption of the Hsepi gene in mice yielded a lethal phenotype with selective organ defects but had remarkably little effect on other organ systems. We have approached the underlying mechanisms by examining the course and effects of FGF2 signaling in a mouse embryonic fibroblast (MEF) cell line derived from the Hsepi(-)(/)(-) mouse. The HS produced by these cells is devoid of l-iduronic acid residues but shows up-regulated N- and 6-O-sulfation compared with wild type (WT) MEF HS. In medium fortified with 10% fetal calf serum, the Hsepi(-)(/)(-) MEFs proliferated and migrated similarly to WT cells. Under starvation conditions, both cell types showed attenuated proliferation and **migration** that could be restored by the addition of FGF2 to WT cells, whereas Hsepi(-)(/)(-) cells were resistant. Moreover, ERK phosphorylation following FGF2 stimulation was delayed in Hsepi(-)(/)(-) compared with WT cells. Assessment of HS-growth factor interaction by nitrocellulose filter trapping revealed a strikingly aberrant binding property of FGF2 and glia-derived neurotropic factor to Hsepi(-)(/)(-) but not to WT HS. glia-derived neurotropic factor has a key role in kidney development, defective in Hsepi(-)(/)(-) mice. By contrast, Hsepi(-)(/)(-) and WT HS interacted similarly and in conventional mode with FGF10. These findings correlate defective function of growth factors with their mode of HS interaction and may help explain the partly modest organ phenotypes observed after genetic ablation of selected enzymes in HS biosynthesis.
2. Defective N-sulfation of heparan sulfate proteoglycans limits PDGF-BB binding and pericyte recruitment in vascular development. During vascular development, endothelial platelet-derived growth factor B (PDGF-B) is critical for pericyte recruitment. Deletion of the conserved C-terminal heparin-binding motif impairs PDGF-BB retention and pericyte recruitment in vivo, suggesting a potential role for heparan sulfate (HS) in PDGF-BB function during vascular development. We studied the participation of HS chains in pericyte recruitment using two mouse models with altered HS biosynthesis. Reduction of N-sulfation due to deficiency in N-deacetylase/N-sulfotransferase-1 attenuated PDGF-BB binding in vitro, and led to pericyte detachment and delayed pericyte **migration** in vivo. Reduced N-sulfation also impaired PDGF-BB signaling and directed cell **migration**, but not proliferation. In contrast, HS from glucuronyl C5-epimerase mutants, which is extensively N- and 6-O-sulfated, but lacks 2-O-sulfated L-iduronic acid residues, retained PDGF-BB in vitro, and pericyte recruitment in vivo was only transiently delayed. These observations were supported by in vitro characterization of the structural features in HS important for PDGF-BB binding. We conclude that pericyte recruitment requires HS with sufficiently extended and appropriately spaced N-sulfated domains to retain PDGF-BB and activate PDGF receptor beta (PDGFRbeta) signaling, whereas the detailed sequence of monosaccharide and sulfate residues does not appear to be important for this interaction.

---

## Pik3cd

- NCBI Gene
- GeneRIF
- Pubmed

| Search Term | Rank | Fields |
| --- | --- | --- |
| [Pp]odosome(s)? | 5 | PubMed(1) |
| [Mm]igration | 6 | Process(1); PubMed(20); GeneRIFs(4) |
| [Ee]xtracellular [Mm]atrix | 8 | PubMed(1); GeneRIFs(1) |
| [Ii]nvasive | 10 | PubMed(2) |
| [Mm]etastasis | 12 | PubMed(1) |

  
hide/show details for Pik3cd

### [Pp]odosome(s)?

#### PubMed

1. Phosphatidylinositol 3-kinase association with the osteoclast cytoskeleton, and its involvement in osteoclast attachment and spreading. Osteoclast activation involves attachment to the mineralized bone matrix and reorganization of the cytoskeleton, leading to polarization of the cell. Signaling molecules, PI3-kinase, rho A, and pp60c-src, were shown to be essential for osteoclastic bone resorption. In this study we have focused on the involvement of these signaling molecules in the early event of osteoclast activation: attachment, spreading, and organization of the cytoskeleton. Highly purified osteoclasts were fractionated into Triton X-100-soluble or cytosolic and Triton X-100-insoluble or cytoskeletal fractions, and the distribution of above-mentioned signaling molecules between the two fractions was examined. PI3-kinase, rho A, and pp60c-src all showed translocation to the cytoskeletal fraction upon osteoclast attachment to plastic. However, PI3-kinase and rho A, but not pp60c-src, showed further translocation of 2.4- and 3.2-fold, respectively, upon attachment of osteoclasts to bone. PI3-kinase translocation to the cytoskeleton was inhibited by either cytochalasin B or colchicine. Furthermore, treatment of osteoclasts with the PI3-kinase inhibitor wortmannin decreased its translocation, suggesting that PI3-kinase activity was needed for its translocation. Moreover, wortmannin inhibited osteoclast attachment to both bone and plastic and caused drastic changes in osteoclast morphology resulting in rounding of the cells, disappearance of F-actin structures or **podosomes**, and appearance of punctate or vesicular structures inside the cells. Osteoblastic MB1.8 cells and IC-21 macrophages did not show additional translocation of PI3-kinase or rho A upon attachment to bone or changes in attachment or morphology in response to wortmannin. Finally, PI3-kinase coimmunoprecipitated with alpha v beta 3 integrin from osteoclasts.

### [Mm]igration

#### Process

1. positive regulation of cell **migration**

#### PubMed

1. The Role of p110δ in the Development and Activation of B Lymphocytes. The phosphatidylinositol-3-kinase (PI3K) pathway has an essential role in signal transduction, where it is required for a number of different cellular processes including proliferation, differentiation, development, **migratio**n and growth. In the immune system, PI3K regulates inflammation by controlling the activation and recruitment of leukocytes. The generation of conditional knockout mice has allowed the study of PI3K isoforms specifically in B and T lymphocytes, and demonstrates the importance of intact signalling in their development and function. PI3K signalling must be tightly regulated in lymphocytes as excessive PI3K can lead to autoimmunity, immunodeficiency or cancer, whilst diminished signalling can result in developmental defects and immunodeficiency. Recent advances in the understanding of PI3K signalling have hastened the application of isoform-specific PI3K inhibitors, which are currently undergoing clinical trials. This review will focus on the p110δ catalytic subunit of the class 1A family of PI3K, and its role in the development and activation of B lymphocytes through various downstream effectors.
2. Mechanisms and implications of phosphoinositide 3-kinase delta in promoting neutrophil trafficking into inflamed tissue. The phosphoinositide 3-kinase (PI3K) catalytic subunit p110 delta is expressed in neutrophils and is thought to play a role in their accumulation at sites of inflammation by contributing to chemoattractant-directed **migration**. We report here that p110 delta is present in endothelial cells and participates in neutrophil trafficking by modulating the proadhesive state of these cells in response to tumor necrosis factor alpha (TNF alpha). Specifically, administration of the selective inhibitor of PI3K delta, IC87114, to animals reduced neutrophil tethering to and increased rolling velocities on cytokine-activated microvessels in a manner similar to that observed in mice deficient in p110 delta. These results were confirmed in vitro as inhibition of this isoform in endothelium, but not neutrophils, diminished cell attachment in flow. A role for PI3K delta in TNF alpha-induced signaling is demonstrated by a reduction in Akt-phosphorylation and phosphatidylinositol-dependent kinase 1 (PDK1) enzyme activity upon treatment of this cell type with IC87114. p110 delta expressed in neutrophils also contributes to trafficking as demonstrated by the impaired movement of these cells across inflamed venules in animals in which this catalytic subunit was blocked or genetically deleted, results corroborated in transwell **migration** assays. Thus, PI3K delta may be a reasonable therapeutic target in specific inflammatory conditions as blockade of its activity reduces neutrophil influx into tissues by diminishing their attachment to and **migration** across vascular endothelium.
3. Distinct roles of class IA PI3K isoforms in primary and immortalised macrophages. The class IA isoforms of phosphoinositide 3-kinase (p110alpha, p110beta and p110delta) often have non-redundant functions in a given cell type. However, for reasons that are unclear, the role of a specific PI3K isoform can vary between cell types. Here, we compare the relative contributions of PI3K isoforms in primary and immortalised macrophages. In primary macrophages stimulated with the tyrosine kinase ligand colony-stimulating factor 1 (CSF1), all class IA PI3K isoforms participate in the regulation of Rac1, whereas p110delta selectively controls the activities of Akt, RhoA and PTEN, in addition to controlling proliferation and chemotaxis. The prominent role of p110delta in these cells correlates with it being the main PI3K isoform that is recruited to the activated CSF1 receptor (CSF1R). In immortalised BAC1.2F5 macrophages, however, the CSF1R also engages p110alpha, which takes up a more prominent role in CSF1R signalling, in processes including Akt phosphorylation and regulation of DNA synthesis. Cell **migration**, however, remains dependent mainly on p110delta. In other immortalised macrophage cell lines, such as IC-21 and J774.2, p110alpha also becomes more prominently involved in CSF1-induced Akt phosphorylation, at the expense of p110delta.These data show that PI3K isoforms can be differentially regulated in distinct cellular contexts, with the dominant role of the p110delta isoform in Akt phosphorylation and proliferation being lost upon cell immortalisation. These findings suggest that p110delta-selective PI3K inhibitors may be more effective in inflammation than in cancer.
4. Phosphoinositide 3-kinase p110 delta regulates natural antibody production, marginal zone and B-1 B cell function, and autoantibody responses. B-1 and marginal zone (MZ) B cells produce natural Abs, make Ab responses to microbial pathogens, and contribute to autoimmunity. Although the delta isoform of the PI3K p110 catalytic subunit is essential for development of these innate-like B cells, its role in the localization, activation, and function of normal B-1 and MZ B cells is not known. Using IC87114, a highly selective inhibitor of p110delta enzymatic activity, we show that p110delta is important for murine B-1 and MZ B cells to respond to BCR clustering, the TLR ligands LPS and CpG DNA, and the chemoattractants CXCL13 and sphingosine 1-phosphate. In these innate-like B cells, p110delta activity mediates BCR-, TLR- and chemoattractant-induced activation of the Akt prosurvival kinase, chemoattractant-induced **migration**, and TLR-induced proliferation. Moreover, we found that TLR-stimulated Ab responses by B-1 and MZ B cells, as well as the localization of MZ B cells in the spleen, depend on p110delta activity. Finally, we show that the in vivo production of natural Abs requires p110delta and that p110delta inhibitors can reduce in vivo autoantibody responses. Thus, targeting p110delta may be a novel approach for regulating innate-like B cells and for treating Ab-mediated autoimmune diseases.
5. The p110delta isoform of PI 3-kinase negatively controls RhoA and PTEN. Inactivation of PI 3-kinase (PI3K) signalling is critical for tumour suppression by PTEN. This is thought to be a unidirectional relationship in which PTEN degrades the lipids produced by PI3K, thus controlling cell proliferation, survival and **migration**. We now show that this relationship is in fact bidirectional, whereby PI3K reciprocally controls PTEN. We report that the p110delta PI3K negatively regulates PTEN, through a pathway involving inhibition of RhoA. Inactivation of p110delta in macrophages led to reduced Akt and Rac1 activation, but paradoxically to increased RhoA and PTEN activity. Partial inactivation of p190RhoGAP and a reduced binding of cytoplasmic RhoA to the cyclin-dependent kinase inhibitor p27 both contributed to the increased RhoA-GTP levels upon p110delta inactivation. Pharmacological inhibition of ROCK, a downstream effector kinase of RhoA, restored all signalling and functional defects of p110delta inactivation, including Akt phosphorylation, chemotaxis and proliferation. This work identifies the RhoA/ROCK pathway as a major target of p110delta-mediated PI3K signalling, and establishes for the first time that PI3K controls itself, via a feedback loop involving PTEN.
6. Structural organization of the mouse phosphatidylinositol 3-kinase p110d gene. Phosphatidylinositol 3-kinases are a family of dual specificity lipid/protein kinases. The products of PI3K's, phosphatidylinositol(3,4,5) triphosphate and phosphatidylinositol(3,4) bisphosphate, act as second messengers connecting activated transmembrane receptors to signaling pathways that control gene transcription, proliferation, transformation, programmed cell death, adhesion, **migration** and vesicular transport. There is evidence that different isoforms of PI3K's activate specific signaling pathways and are thus responsible for integrating cellular responses. The elucidation of the genomic structure of the catalytic subunits is a necessary step for the investigation of the function of PI3K isoforms by inactivation of the gene in vivo. The structural organization of p110alpha, beta, and gamma genes has been previously reported. Here we report the cloning, sequencing, and structural organization of the mouse p110delta gene from a murine 129/Sv genomic library. The p110delta gene consists of 22 exons and spans over 13 kb. Comparison of the genomic structure with that of p110alpha, beta, and gamma demonstrates that the p110delta gene shares its exon structure with p110beta, the most closely related PI3K at the amino acid level.
7. Elastin-derived peptides potentiate atherosclerosis through the immune Neu1-PI3Kγ pathway. Elastin is degraded during vascular ageing and its products, elastin-derived peptides (EP), are present in the human blood circulation. EP binds to the elastin receptor complex (ERC) at the cell surface, composed of elastin-binding protein (EBP), a cathepsin A and a neuraminidase 1. Some in vitro functions have clearly been attributed to this binding, but the in vivo implications for arterial diseases have never been clearly investigated.Here, we demonstrate that chronic doses of EP injected into mouse models of atherosclerosis increase atherosclerotic plaque size formation. Similar effects were observed following an injection of a VGVAPG peptide, suggesting that the ERC mediates these effects. The absence of phosphoinositide 3-kinase γ (PI3Kγ) in bone marrow-derived cells prevented EP-induced atherosclerosis development, demonstrating that PI3Kγ drive EP-induced arterial lesions. Accordingly, in vitro studies showed that PI3Kγ was required for EP-induced mono**cyte migr**ation and ROS production and that this effect was dependent upon neuraminidase activity. Finally, we showed that degradation of elastic lamellae in LDLR(-/-) mice fed an atherogenic diet correlated with atherosclerotic plaque formation. At the same time, the absence of the cathepsin A-neuraminidase 1 complex in cells of the haematopoietic lineage abolished atheroma plaque size progression and decreased leucocytes infiltration, clearly demonstrating the role of this complex in atherogenesis and suggesting the involvement of endogenous EP.Altogether, this work identifies EP as an enhancer of atherogenesis and defines the Neuraminidase 1/PI3Kγ signalling pathway as a key mediator of this function in vitro and in vivo.
8. Genetic or pharmaceutical blockade of phosphoinositide 3-kinase p110δ prevents chronic rejection of heart allografts. Chronic rejection is the major cause of long-term heart allograft failure, characterized by tissue infiltration by recipient T cells with indirect allospecificity. Phosphoinositol-3-kinase p110δ is a key mediator of T cell receptor signaling, regulating both T cell activation an**d migrati**on of primed T cells to non-lymphoid antigen-rich tissue. We investigated the effect of genetic or pharmacologic inactivation of PI3K p110δ on the development of chronic allograft rejection in a murine model in which HY-mismatched male hearts were transplanted into female recipients. We show that suppression of p110δ activity significantly attenuates the development of chronic rejection of heart grafts in the absence of any additional immunosuppressive treatment by impairing the localization of antigen-specific T cells to the grafts, while not inducing specific T cell tolerance. p110δ pharmacologic inactivation is effective when initiated after transplantation. Targeting p110δ activity might be a viable strategy for the treatment of heart chronic rejection in humans.
9. Inactivation of PI3Kδ induces vascular injury and promotes aneurysm development by upregulating the AP-1/MMP-12 pathway in macrophages. An aneurysm is an inflammatory vascular condition. Phosphatidylinositol 3-kinases δ is highly expressed in leukocytes, and play a key role in innate immunity. However, the link between phosphatidylinositol 3-kinases δ and aneurysm development has not yet been elucidated.Carotid ligation unexpectedly induced characteristic aneurysm formation beneath the ligation point in p110δ(D910A/D910A) mice (n=25; P<0.001 versus wild-type). Besides, p110δ inactivation exacerbated CaCl2-induced abdominal aortic aneurysms development. A reverse transcription polymerase chain reaction microarray revealed significant extracellular matrix components degradation and matrix metalloproteinases (MMPs) upregulation in the abdominal aorta of p110δ(D910A/D910A) mice. Similarly, the expression of both collagen I and IV was significantly decreased (n=10; P<0.05 versus wild-type) in carotid artery. Western blot assay confirmed that MMP-12 was significantly upregulated in arteries of p110δ(D910A/D910A) mice (n=10; P<0.01 versus wild-type). In vitro, p110δ inactivation marked increase peritoneal macrophages recruitment and synergistically enhance tumor necrosis factor-α-induced recruitment. A specific phosphatidylinositol 3-kinases δ inhibitor (IC87114) or genetic p110δ inactivation upregulated MMP-12 expression and c-Jun phosphorylation (n=6; P<0.05 versus wild-type macrophages). IC87114 also increased activator protein-1 DNA-binding activity (n=6; P<0.001 versus control) and enhanced the effect of tumor necrosis factor-α on activator protein-1-binding activity (n=5; P<0.01 versus tumor necrosis factor-α treatment groups). Knockdown of c-Jun suppressed the effect of the IC87114 and tumor necrosis factor-α on MMP-12 mRNA expression (n=5 in each group; P<0.01 versus scrRNA treatment groups).Our findings demonstrate that p110δ inactivation leads to extracellular matrix degradation in vessels and promotes aneurysm development by induci**ng macrop**hages migration and upregulating the activator protein-1/MMP-12 pathway in macrophages.
10. p110gamma and p110delta isoforms of phosphoinositide 3-kinase differentially regulate natural killer cell **migration** in health and disease. The mechanisms that regulate NK cell trafficking are unclear. Phosphoinositide-3 kinases (PI3K) control cell motility and the p110gamma and p110delta isoforms are mostly expressed in leukocytes, where they transduce signals downstream of G protein coupled receptors (GPCR) or tyrosine kinase receptors, respectively. Here, we set out to determine the relative contribution of p110gamma and p110delta to NK cell **migration** in mice. Using a combination of single-cell imaging analysis of transgenic cells reporting on PI3K activity in real time and small molecule inhibitors of p110gamma and p110delta, we show here that the tyrosine-kinase coupled p110delta is linked to GPCR signaling and, depending on the GPCR, may even be preferentially activated over p110gamma. Using gene-targeted mice, we showed that both isoforms were essential for NK cell chemotaxis to CXCL12 and to CCL3 and, in vivo, for normal NK cell **migration** during pregnancy and to the inflamed peritoneum. By contrast, only p110delta was indispensable for chemotaxis to S1P and CXCL10 and for NK cell distribution throughout lymphoid and nonlymphoid tissues and for extravasation to tumors. These results implicate p110delta downstream of GPCRs in NK cells and highlight its nonredundant role as a key regulator of NK cell trafficking in health and disease.
11. Essential role for the p110delta phosphoinositide 3-kinase in the allergic response. Inflammatory substances released by mast cells induce and maintain the allergic response. Mast cell differentiation and activation are regulated, respectively, by stem cell factor (SCF; also known as Kit ligand) and by allergen in complex with allergen-specific immunoglobulin E (IgE). Activated SCF receptors and high-affinity receptors for IgE (FcvarepsilonRI) engage phosphoinositide 3-kinases (PI(3)Ks) to generate intracellular lipid second messenger signals. Here, we report that genetic or pharmacological inactivation of the p110delta isoform of PI(3)K in mast cells leads to defective SCF-mediated in vitro proliferation, adhesion and **migration**, and to impaired allergen-IgE-induced degranulation and cytokine release. Inactivation of p110delta protects mice against anaphylactic allergic responses. These results identify p110delta as a new target for therapeutic intervention in allergy and mast-cell-related pathologies.
12. PI3Kδ Regulates the Magnitude of CD8+ T Cell Responses after Challenge with Listeria monocytogenes. PI3Ks regulate diverse immune cell functions by transmitting intracellular signals from Ag, costimulatory receptors, and cytokine receptors to control cell division, differentiation, survival, and **migratio**n. In this study, we report the effect of inhibiting the p110δ subunit of PI3Kδ on CD8(+) T cell responses to infection with the intracellular bacteria Listeria monocytogenes. A strong dependency on PI3Kδ for IFN-γ production by CD8(+) T cells in vitro was not recapitulated after Listeria infection in vivo. Inactivation of PI3Kδ resulted in enhanced bacterial elimination by the innate immune system. However, the magnitudes of the primary and secondary CD8 +: T cell responses were reduced. Moreover, PI3Kδ activity was required for CD8(+) T cells to provide help to other responding CD8(+) cells. These findings identify PI3Kδ as a key regulator of CD8(+) T cell responses that integrates extrinsic cues, including those from other responding cells, to determine the collective behavior of CD8(+) T cell populations responding to infection.
13. Immunomodulation of Selective Naive T Cell Functions by p110δ Inactivation Improves the Outcome of Mismatched Cell Transplantation. Allogeneic hematopoietic stem cell transplantation (HSCT) can treat certain hematologic malignancies due to the graft versus leukemia (GvL) effect but is complicated by graft versus host disease (GvHD). Expression of the p110δ catalytic subunit of the phosphoinositide 3-kinase pathway is restricted to leukocytes, where it regulates proliferation**, migrati**on, and cytokine production. Here, in a mouse model of fully mismatched hematopoietic cell transplantation (HCT), we show that genetic inactivation of p110δ in T cells leads to milder GvHD, whereas GvL is preserved. Inactivation of p110δ in human lymphocytes reduced T cell allorecognition. We demonstrate that both allostimulation and granzyme B expression were dependent on p110δ in naive T cells, which are the main mediators of GvHD, whereas memory T cells were unaffected. Strikingly, p110δ is not mandatory for either naive or memory T cells to mediate GvL. Therefore, immunomodulation of selective naive T cell functions by p110δ inactivation improves the outcome of allogeneic HSCT.
14. Angiogenesis selectively requires the p110alpha isoform of PI3K to control endothelial cell **migration**. Phosphoinositide 3-kinases (PI3Ks) signal downstream of multiple cell-surface receptor types. Class IA PI3K isoforms couple to tyrosine kinases and consist of a p110 catalytic subunit (p110alpha, p110beta or p110delta), constitutively bound to one of five distinct p85 regulatory subunits. PI3Ks have been implicated in angiogenesis, but little is known about potential selectivity among the PI3K isoforms and their mechanism of action in endothelial cells during angiogenesis in vivo. Here we show that only p110alpha activity is essential for vascular development. Ubiquitous or endothelial cell-specific inactivation of p110alpha led to embryonic lethality at mid-gestation because of severe defects in angiogenic sprouting and vascular remodelling. p110alpha exerts this critical endothelial cell-autonomous function by regulating endothelial cell **migration** through the small GTPase RhoA. p110alpha activity is particularly high in endothelial cells and preferentially induced by tyrosine kinase ligands (such as vascular endothelial growth factor (VEGF)-A). In contrast, p110beta in endothelial cells signals downstream of G-protein-coupled receptor (GPCR) ligands such as SDF-1alpha, whereas p110delta is expressed at low level and contributes only minimally to PI3K activity in endothelial cells. These results provide the first in vivo evidence for p110-isoform selectivity in endothelial PI3K signalling during angiogenesis.
15. PDK1 regulation of mTOR and hypoxia-inducible factor 1 integrate metabolism and **migration** of CD8+ T cells. mTORC1 (mammalian target of rapamycin complex 1) controls transcriptional programs that determine CD8+ cytolytic T cell (CTL) fate. In some cell systems, mTORC1 couples phosphatidylinositol-3 kinase (PI3K) and Akt to the control of glucose uptake and glycolysis. However, PI3K-Akt-independent mechanisms control glucose metabolism in CD8+ T cells, and the role of mTORC1 has not been explored. The present study now demonstrates that mTORC1 activity in CD8+ T cells is not dependent on PI3K or Akt but is critical to sustain glucose uptake and glycolysis in CD8+ T cells. We also show that PI3K- and Akt-independent pathways mediated by mTORC1 regulate the expression of HIF1 (hypoxia-inducible factor 1) transcription factor complex. This mTORC1-HIF1 pathway is required to sustain glucose metabolism and glycolysis in effector CTLs and strikingly functions to couple mTORC1 to a diverse transcriptional program that controls expression of glucose transporters, multiple rate-limiting glycolytic enzymes, cytolytic effector molecules, and essential chemokine and adhesion receptors that regulate T cell trafficking. These data reveal a fundamental mechanism linking nutrient and oxygen sensing to transcriptional control of CD8+ T cell differentiation.
16. Reduction in Renal Ischemia-Reperfusion Injury in Mice by a Phosphoinositide 3-Kinase p110gamma-Specific Inhibitor. Although renal ischemia-reperfusion injury (IRI) can cause delayed graft function, a targeted therapy is not yet available. Because phosphoinositide 3-kinases (PI3K) p110γ and p110δ play important roles in immune cel**l migrati**on and function, we investigated the effects of PI3K p110γ- and p110δ-specific inhibitors in a murine renal IRI model.Renal function was assessed by serum creatine and hematoxylin-eosin staining. Immune c**ell migra**tion was assessed by flow cytometry and an in vitro c**ell migra**tion assay using Transwell plates. Gene expression analysis and a multiplex cytokine/chemokine assay were performed to find cytokines/chemokines whose expression was upregulated in renal IRI and affected by p110γ-specific inhibitor.The PI3K p110γ-specific inhibitor, but not p110δ-specific inhibitor, significantly reduced serum creatine levels and acute tubular necrosis. These were accompanied by reduced infiltration of B cells and reduced expression of CXCL9, a CXCR3 ligand, suggesting that p110γ plays an important role in **B-cell m**igration toward injured kidneys. An in vit**ro cell m**igration assay revealed for the first time that **B-cell m**igration to injured kidney cells and to CXCL9 requires p110γ.p110γ-specific inhibitor ameliorates renal IRI by reducing necrosis and im**mune cell** migration. This inhibitor may have the potential to reduce renal graft failure caused by renal IRI.
17. The role of endothelial PI3Kgamma activity in neutrophil trafficking. Phosphoinositide 3-kinase gamma (PI3Kgamma) in neutrophils plays a critical role in the directed **migration** of these cells into inflamed tissues. In this study, we demonstrate the importance of the endothelial component of PI3Kgamma activity relative to its leukocyte counterpart in supporting neutrophil interactions with the inflamed vessel wall. Despite the reconstitution of class-Ib PI3K function in neutrophils of p110gamma-/- mice, we observed a 45% reduction in accumulation of these cells in an acute lung injury model. Mechanistically, this appears to result from a perturbation in selectin-mediated adhesion as manifested by a 70% reduction in wild-type (WT) neutrophil attachment to and 17-fold increase in rolling velocities on p110gamma-/- microvessels in vivo in response to tumor necrosis factor alpha (TNFalpha). This alteration in adhesion was further augmented by a deficiency in p110delta, suggesting that the activity of both catalytic subunits is required for efficient capture of neutrophils by cytokine-stimulated endothelium. Interestingly, E-selectin-mediated adhesion in p110gamma-/-) mice was impaired by more than 95%, but no defect in nuclear factor kappa B (NF-kappaB)-induced gene expression was observed. These findings suggest a previously unrecognized partnership between class-I PI3Ks expressed in leukocytes and endothelium, the combination of which is required for the efficient trafficking of immunocompetent cells to sites of inflammation.
18. Inhibition of PI3Kδ reduces kidney infiltration by macrophages and ameliorates systemic lupus in the mouse. Systemic lupus erythematosus (SLE) is a human chronic inflammatory disease generated and maintained throughout life by autoreactive T and B cells. Class I phosphoinositide 3-kinases (PI3K) are heterodimers composed of a regulatory and a catalytic subunit that catalyze phosphoinositide-3,4,5-P3 formation and regulate cell survival, **migratio**n, and division. Activity of the PI3Kδ isoform is enhanced in human SLE patient PBLs. In this study, we analyzed the effect of inhibiting PI3Kδ in MRL/lpr mice, a model of human SLE. We found that PI3Kδ inhibition ameliorated lupus progression. Treatment of these mice with a PI3Kδ inhibitor reduced the excessive numbers of CD4(+) effector/memory cells and B cells. In addition, this treatment reduced serum TNF-α levels and the number of macrophages infiltrating the kidney. Expression of inactive PI3Kδ, but not deletion of the other hematopoietic isoform PI3Kγ, reduced the ability of macrophages to cross the basement membrane, a process required to infiltrate the kidney, explaining MRL/lpr mice improvement by pharmacologic inhibition of PI3Kδ. The observations that p110δ inhibitor prolonged mouse life span, reduced disease symptoms, and showed no obvious secondary effects indicates that PI3Kδ is a promising target for SLE.
19. The p110δ subunit of PI3K regulates bone marrow-derived eosinophil trafficking and airway eosinophilia in allergen-challenged mice. Trafficking and recruitment of eosinophils during allergic airway inflammation is mediated by the phosphatidylinositol 3-kinase (PI3K) family of signaling molecules. The role played by the p110δ subunit of PI3K (PI3K p110δ) in regulating eosinophil trafficking and recruitment was investigated using a selective pharmacological inhibitor (IC87114). Treatment with the PI3K p110δ inhibitor significantly reduced murine bone marrow-derived eosinophil (BM-Eos) adhesion to VCAM-1 as well as ICAM-1 and inhibited activation-induced changes in cell morphology associated with reduced Mac-1 expression and aberrant cell surface localization/distribution of Mac-1 and α4. Infused BM-Eos demonstrated significantly decreased rolling and adhesion in inflamed cremaster muscle microvessels of mice treated with IC87114 compared with vehicle-treated mice. Furthermore, inhibition of PI3K p110δ significantly attenuated eotaxin-1-induced B**M-Eos mig**ration and prevented eotaxin-1-induced changes in the cytoskeleton and cell morphology. Knockdown of PI3K p110δ with siRNA in BM-Eos resulted in reduced rolling, adhesio**n, and mi**gration, as well as inhibition of activation-induced changes in cell morphology, validating its role in regulating trafficki**ng and mi**gration. Finally, in a mouse model of cockroach antigen-induced allergic airway inflammation, oral administration of the PI3K p110δ inhibitor significantly inhibited airway eosinophil recruitment, resulting in attenuation of airway hyperresponsiveness in response to methacholine, reduced mucus secretion, and expression of proinflammatory molecules (found in inflammatory zone-1 and intelectin-1). Overall, these findings indicate the important role played by PI3K p110δ in mediating BM-Eos traffic**king and** migration by regulating adhesion molecule expression and localization/distribution as well as promoting changes in cell morphology that favor recruitment during inflammation.
20. T cell receptor-induced phosphoinositide-3-kinase p110delta activity is required for T cell localization to antigenic tissue in mice. The establishment of T cell-mediated inflammation requires the **migration** of primed T lymphocytes from the blood stream and their retention in antigenic sites. While naive T lymphocyte recirculation in the lymph and blood is constitutively regulated and occurs in the absence of inflammation, the recruitment of primed T cells to nonlymphoid tissue and their retention at the site are enhanced by various inflammatory signals, including TCR engagement by antigen-displaying endothelium and resident antigen-presenting cells. In this study, we investigated whether signals downstream of TCR ligation mediated by the phosphoinositide-3-kinase (PI3K) subunit p110delta contributed to the regulation of these events. T lymphocytes from mice expressing catalytically inactive p110delta displayed normal constitutive trafficking and migratory responses to nonspecific stimuli. However, these cells lost susceptibility to TCR-induced **migration** and failed to localize efficiently to antigenic tissue. Importantly, we showed that antigen-induced T cell trafficking and subsequent inflammation was abrogated by selective pharmacological inhibition of PI3K p110delta activity. These observations suggest that pharmacological targeting of p110delta activity is a viable strategy for the therapy of T cell-mediated pathology.

#### GeneRIFs

1. p110[gamma] but not p110[delta]-specific inhibitor ameliorates renal ischemia-reperfusion injury by reducing necrosis and immune cell **migration**
2. Pik3cd inactivation leads to extracellular matrix degradation in vessels and promotes aneurysm development by inducing macrophage **migration** and upregulating the activator protein-1/MMP-12 pathway in macrophages.
3. PI3K p110delta mediates bone marrow-derived eosinophil trafficking and **migration** by regulating adhesion molecule expression and localization/distribution as well as promoting changes in cell morphology that favor recruitment during inflammation.
4. genetic or pharmacological inactivation of the p110delta isoform of PI(3)K in mast cells leads to defective SCF-mediated in vitro proliferation, adhesion and **migration**, and to impaired allergen-IgE-induced degranulation and cytokine release

### [Ee]xtracellular [Mm]atrix

#### PubMed

1. Inactivation of PI3Kδ induces vascular injury and promotes aneurysm development by upregulating the AP-1/MMP-12 pathway in macrophages. An aneurysm is an inflammatory vascular condition. Phosphatidylinositol 3-kinases δ is highly expressed in leukocytes, and play a key role in innate immunity. However, the link between phosphatidylinositol 3-kinases δ and aneurysm development has not yet been elucidated.Carotid ligation unexpectedly induced characteristic aneurysm formation beneath the ligation point in p110δ(D910A/D910A) mice (n=25; P<0.001 versus wild-type). Besides, p110δ inactivation exacerbated CaCl2-induced abdominal aortic aneurysms development. A reverse transcription polymerase chain reaction microarray revealed signifi**cant extracellular m**atrix components degradation and matrix metalloproteinases (MMPs) upregulation in the abdominal aorta of p110δ(D910A/D910A) mice. Similarly, the expression of both collagen I and IV was significantly decreased (n=10; P<0.05 versus wild-type) in carotid artery. Western blot assay confirmed that MMP-12 was significantly upregulated in arteries of p110δ(D910A/D910A) mice (n=10; P<0.01 versus wild-type). In vitro, p110δ inactivation marked increase peritoneal macrophages recruitment and synergistically enhance tumor necrosis factor-α-induced recruitment. A specific phosphatidylinositol 3-kinases δ inhibitor (IC87114) or genetic p110δ inactivation upregulated MMP-12 expression and c-Jun phosphorylation (n=6; P<0.05 versus wild-type macrophages). IC87114 also increased activator protein-1 DNA-binding activity (n=6; P<0.001 versus control) and enhanced the effect of tumor necrosis factor-α on activator protein-1-binding activity (n=5; P<0.01 versus tumor necrosis factor-α treatment groups). Knockdown of c-Jun suppressed the effect of the IC87114 and tumor necrosis factor-α on MMP-12 mRNA expression (n=5 in each group; P<0.01 versus scrRNA treatment groups).Our findings demonstrate that p110δ inactiv**ation leads to extra**cellular matrix degradation in vessels and promotes aneurysm development by inducing macrophages migration and upregulating the activator protein-1/MMP-12 pathway in macrophages.

#### GeneRIFs

1. Pik3cd inactivation leads to **extracellular matrix** degradation in vessels and promotes aneurysm development by inducing macrophage migration and upregulating the activator protein-1/MMP-12 pathway in macrophages.

### [Ii]nvasive

#### PubMed

1. p37δ is a new isoform of PI3K p110δ that increases cell proliferation and is overexpressed in tumors. The phosphatidylinositol 3-kinases (PI3Ks) regulate cell growth, proliferation and survival, and are frequently affected in human cancer. PI3K is composed of a catalytic subunit, p110, and a regulatory subunit, p85. The PI3K catalytic subunit p110δ is encoded by PIK3CD and contains p85- and RAS-binding domains, and a kinase domain. Here we present an alternatively spliced PIK3CD transcript encoding a previously unknown protein, p37δ, and demonstrate that this protein is expressed in human ovarian and colorectal tumors. p37δ retains the p85-binding domain and a fraction of the RAS-binding domain, lacks the catalytic domain, and has a unique carboxyl-terminal region. In contrast to p110δ, which stabilizes p85, p37δ promoted p85 sequestering. Despite the truncated RAS-binding domain, p37δ bound to RAS and we found a strong positive correlation between the protein levels of p37δ and RAS. Overexpressing p37δ, but not p110δ, increased the prolife**ration a**nd invasive properties of HEK-293 cells and mouse embryonic fibroblasts. Cells overexpressing p37δ showed a quicker phosphorylation response of AKT and ERK1/2 following serum stimulation. Ubiquitous expression of human p37δ in the fruit fly increased body size, DNA content and phosphorylated ERK1/2 levels. Thus, p37δ appears to be a new tumor-specific isoform of p110δ with growth-promoting properties.
2. Class IA phosphoinositide 3-kinase β and δ regulate neutrophil oxidase activation in response to Aspergillus fumigatus hyphae. An effective immune response to the ubiquitous fungus Aspergillus fumigatus is dependent upon production of reactive oxygen species (ROS) by the NADPH oxidase. This is evidenced by the acute sensitivity of oxidase-deficient humans and mice t**o invasi**ve aspergillosis. Neutrophils are recruited to the lungs shortly postinfection and respond by phagocytosing conidia and mediating extracellular killing of germinated hyphae in a ROS-dependent manner. However, the signaling mechanisms regulating the generation of ROS in response to hyphae are poorly understood. PI3Ks are important regulators of numerous cellular processes, with much recent work describing unique roles for the different class I PI3K isoforms. We showed by live-cell imaging that the lipid products of class I PI3Ks accumulated at the hyphal-bound neutrophil plasma membrane. Further, we used pharmacological and genetic approaches to demonstrate essential, but overlapping, roles for PI3Kβ and PI3Kδ in the ROS and spreading responses of murine neutrophils to Aspergillus hyphae. Hyphal-induced ROS responses were substantially inhibited by deletion of the common β2-integrin subunit CD18, with only a minor, redundant role for Dectin-1. However, addition of soluble algal glucans plus the genetic deletion of CD18 were required to significantly inhibit activation of the PI3K-effector protein kinase B. Hyphal ROS responses were also totally dependent on the presence of Syk, but not its ITAM-containing adaptor proteins FcRγ or DAP12, and the Vav family of Rac-guanine nucleotide exchange factors. These results start to define the signaling network controlling neutrophil ROS responses to A. fumigatus hyphae.

### [Mm]etastasis

#### PubMed

1. The Pten/PI3K pathway governs the homeostasis of Valpha14iNKT cells. The tumor suppressor PTEN is mutated in many human cancers. We previously used the Cre-loxP system to generate mice (LckCrePten mice) with a Pten mutation in T-lineage cells. Here we describe the phenotype of Pten-deficient Valpha14iNKT cells. A failure in the development of Valpha14iNKT cells occurs in the LckCrePten thymus between stage 2 (CD44(high)NK1.1(-)) and stage 3 (CD44(high)NK1.1(+)), resulting in decreased numbers of peripheral Valpha14iNKT cells. In vitro, Pten-deficient Valpha14iNKT cells show reduced proliferation and cytokine secretion in response to alphaGalCer stimulation but enhanced inhibitory Ly49 receptor expression. Following interaction with dendritic cells (DCs) loaded with alphaGalCer, Pten-deficient Valpha14iNKT cells demonstrate activation of PI3K. Indeed, the effects of the Pten mutation require intact function of the PI3K subunits p110gamma and p110delta. In vivo, LckCrePten mice display reduced serum IFNgamma after alphaGalCer administration. Importantly, Valpha14iNKT cell-mediated protection against the **metastasis** of melanoma cells to the lung was impaired in the absence of Pten. Thus, the Pten/PI3K pathway is indispensable for the homeostasis and antitumor surveillance function of Valpha14iNKT cells.

---

## Slc30a7

- NCBI Gene
- GeneRIF
- Pubmed

| Search Term | Rank | Fields |
| --- | --- | --- |
| [Mm]etastasize | 11 | PubMed(1) |
| [Mm]etastasis | 12 | PubMed(1) |

  
hide/show details for Slc30a7

### [Mm]etastasize

#### PubMed

1. A null-mutation in the Znt7 gene accelerates prostate tumor formation in a transgenic adenocarcinoma mouse prostate model. Decrease of cellular zinc in the epithelium of the prostate has been implicated in the development of prostate cancer. To investigate whether ZnT7, a zinc transporter involved in intracellular zinc accumulation, played a role in prostate cancer development, we generated and characterized a transgenic adenocarcinoma mouse prostate (TRAMP) model with a Znt7-null genetic background. TRAMP mice (TRAMP/Znt7(-/-) and TRAMP/Znt7(+/+)) were euthanized at 6, 8, 16, and 28 weeks of age for histopathological analysis of the prostates and for the presence of prostate tumors and metastasis. At 6 and 8 weeks of age, TRAMP/Znt7(-/-) mice displayed higher frequencies of low grade prostatic intraepithelial neoplasia (PIN) and high grade PIN, respectively, in the prostates than the age-matched TRAMP/Znt7(+/+) mice. At 16 weeks of age, 33% TRAMP/Znt7(-/-) mice had prostate tumors and one half of the mice with prostate tumors had tumor **metastasize**d to the draining lymph nodes while no prostate tumor was detected in the control TRAMP mice. By 28 weeks, 67% TRAMP/Znt7(-/-) mice developed prostate tumors while only 22% control TRAMP mice had prostate tumors. Furthermore, apoptosis was reduced in the prostates of TRAMP/Znt7(-/-) mice. In conclusion, a null-mutation of the Znt7 gene accelerates prostate tumor formation in TRAMP mice.

### [Mm]etastasis

#### PubMed

1. A null-mutation in the Znt7 gene accelerates prostate tumor formation in a transgenic adenocarcinoma mouse prostate model. Decrease of cellular zinc in the epithelium of the prostate has been implicated in the development of prostate cancer. To investigate whether ZnT7, a zinc transporter involved in intracellular zinc accumulation, played a role in prostate cancer development, we generated and characterized a transgenic adenocarcinoma mouse prostate (TRAMP) model with a Znt7-null genetic background. TRAMP mice (TRAMP/Znt7(-/-) and TRAMP/Znt7(+/+)) were euthanized at 6, 8, 16, and 28 weeks of age for histopathological analysis of the prostates and for the presence of prostate tumors and **metastasis**. At 6 and 8 weeks of age, TRAMP/Znt7(-/-) mice displayed higher frequencies of low grade prostatic intraepithelial neoplasia (PIN) and high grade PIN, respectively, in the prostates than the age-matched TRAMP/Znt7(+/+) mice. At 16 weeks of age, 33% TRAMP/Znt7(-/-) mice had prostate tumors and one half of the mice with prostate tumors had tumor metastasized to the draining lymph nodes while no prostate tumor was detected in the control TRAMP mice. By 28 weeks, 67% TRAMP/Znt7(-/-) mice developed prostate tumors while only 22% control TRAMP mice had prostate tumors. Furthermore, apoptosis was reduced in the prostates of TRAMP/Znt7(-/-) mice. In conclusion, a null-mutation of the Znt7 gene accelerates prostate tumor formation in TRAMP mice.

---

## Gnmt

- NCBI Gene
- GeneRIF
- Pubmed

| Search Term | Rank | Fields |
| --- | --- | --- |
| [Mm]igration | 6 | PubMed(1) |
| [Ee]xtracellular [Mm]atrix | 8 | PubMed(1) |

  
hide/show details for Gnmt

### [Mm]igration

#### PubMed

1. Niemann-Pick type C2 protein regulates liver cancer progression via modulating ERK1/2 pathway: Clinicopathological correlations and therapeutical implications. Primary hepatocellular carcinoma (HCC) is the fifth most common malignancy worldwide and the third leading cause of cancer-related death. It is important to identify new targets for early diagnosis and treatment of HCC. Niemann-Pick type C2 (NPC2) plays an important role in the regulation of intracellular cholesterol homeostasis via direct binding with free cholesterol. However, little is known about the significance of NPC2 in HCC tumorigenesis. In this study, we showed that NPC2 is abundantly expressed in normal liver, but is downregulated in human HCC tissues. The patients with NPC2 downregulation expressed much higher α-fetoprotein, multiple tumor type, vascular invasion, later pathological stage and shorter survival rate. Knockdown NPC2 in liver cancer cell lines promote cell proliferation, **migratio**n and xenograft tumorigenesis. In contrast, NPC2 overexpression inhibits HuH7 promoted tumor growth. Furthermore, administration of hepatotropic adeno-associated virus 8 (AAV8) delivered NPC2 decreased the inflammatory infiltration, the expression of two early HCC markers-glypican 3 and survivin and suppressed the spontaneous HCC development in mice. To identify the NPC2-dependent mechanism, we emphasized on the status of MAPK/ERK signaling. MEK1/2 inhibitor treatment demonstrated that the expression of NPC2 affected the activation of ERK1/2 but not MEK1/2. In addition, cholesterol trafficking inhibitor treatment did not alter the cell proliferation and the activation of MEK/ERK. In conclusion, our study demonstrates that NPC2 may play an important role in negatively regulate cell proliferation and ERK1/2 activation that were independent of cholesterol accumulation. AAV-NPC2 may thus represent a new treatment strategy for liver cancer.

### [Ee]xtracellular [Mm]atrix

#### PubMed

1. Identification of glucocorticoid-regulated genes that control cell proliferation during murine respiratory development. Glucocorticoids play a vital role in fetal respiratory development and act via the intracellular glucocorticoid receptor (GR) to regulate transcription of key target genes. GR-null mice die at birth due to respiratory dysfunction associated with hypercellularity and atelectasis. To identify events associated with this lung phenotype we examined perinatal cellular proliferation rates and apoptotic indices. We demonstrate that compared to wild-type controls, day 18.5 postcoitum (p.c.) GR-null mouse lungs display significantly increased cell proliferation rates (1.8-fold P < 0.05) and no change in apoptosis. To examine underlying molecular mechanisms, we compared whole genome expression profiles by microarray analysis at 18.5 days p.c. Pathways relating to cell proliferation, division and cell cycle were significantly down-regulated while pathways relating to carbohydrate metabolism, kinase activities and immune responses were significantly up-regulated. Differential levels of gene expression were verified by quantitative-RT-PCR and/or Northern analysis. Key regulators of proliferation differentially expressed in the lung of 18.5 p.c. GR-null lungs included p21 CIP1 (decreased 2.9-fold, P < 0.05), a negative regulator of the cell cycle, and Mdk (increased 6.0-fold, P < 0.05), a lung growth factor. The more under-expressed genes in 18.5 p.c. GR-null lungs included Chi3l3 (11-fold, P < 0.05), a macrophage inflammatory response gene and Ela1 (9.4-fold, P < 0.05), an **extracellular matrix** remodeling enzyme. Our results demonstrate that GR affects the transcriptional status of a number of regulatory processes during late fetal lung development. Amongst these processes is cell proliferation whereby GR induces expression of cell cycle repressors while suppressing induction of a well characterized cell cycle stimulator.

---

## Ldha

- NCBI Gene
- GeneRIF
- Pubmed

| Search Term | Rank | Fields |
| --- | --- | --- |
| [Ff]ilopodia | 1 | PubMed(1) |
| [Mm]igration | 6 | PubMed(3) |
| [Ee]xtracellular [Mm]atrix | 8 | PubMed(2) |
| [Ii]nvasive | 10 | PubMed(2) |
| [Mm]etastasis | 12 | PubMed(1) |

  
hide/show details for Ldha

### [Ff]ilopodia

#### PubMed

1. HIF-1α-PDK1 axis-induced active glycolysis plays an essential role in macrophage migratory capacity. In severely hypoxic condition, HIF-1α-mediated induction of Pdk1 was found to regulate glucose oxidation by preventing the entry of pyruvate into the tricarboxylic cycle. Monocyte-derived macrophages, however, encounter a gradual decrease in oxygen availability during its migration process in inflammatory areas. Here we show that HIF-1α-PDK1-mediated metabolic changes occur in mild hypoxia, where mitochondrial cytochrome c oxidase activity is unimpaired, suggesting a mode of glycolytic reprogramming. In primary macrophages, PKM2, a glycolytic enzyme responsible for glycolytic ATP synthesis localizes **in filopo**dia and lammelipodia, where ATP is rapidly consumed during actin remodelling processes. Remarkably, inhibition of glycolytic reprogramming with dichloroacetate significantly impairs macrophage migration in vitro and in vivo. Furthermore, inhibition of the macrophage HIF-1α-PDK1 axis suppresses systemic inflammation, suggesting a potential therapeutic approach for regulating inflammatory processes. Our findings thus demonstrate that adaptive responses in glucose metabolism contribute to macrophage migratory activity.

### [Mm]igration

#### PubMed

1. Lack of pericytes leads to endothelial hyperplasia and abnormal vascular morphogenesis. The association of pericytes (PCs) to newly formed blood vessels has been suggested to regulate endothelial cell (EC) proliferation, survival, **migration**, differentiation, and vascular branching. Here, we addressed these issues using PDGF-B-- and PDGF receptor-beta (PDGFR-beta)--deficient mice as in vivo models of brain angiogenesis in the absence of PCs. Quantitative morphological analysis showed that these mutants have normal microvessel density, length, and number of branch points. However, absence of PCs correlates with endothelial hyperplasia, increased capillary diameter, abnormal EC shape and ultrastructure, changed cellular distribution of certain junctional proteins, and morphological signs of increased transendothelial permeability. Brain endothelial hyperplasia was observed already at embryonic day (E) 11.5 and persisted throughout development. From E 13.5, vascular endothelial growth factor-A (VEGF-A) and other genes responsive to metabolic stress became upregulated, suggesting that the abnormal microvessel architecture has systemic metabolic consequences. VEGF-A upregulation correlated temporally with the occurrence of vascular abnormalities in the placenta and dilation of the heart. Thus, although PC deficiency appears to have direct effects on EC number before E 13.5, the subsequent increased VEGF-A levels may further abrogate microvessel architecture, promote vascular permeability, and contribute to formation of the edematous phenotype observed in late gestation PDGF-B and PDGFR-beta knock out embryos.
2. HIF-1α-PDK1 axis-induced active glycolysis plays an essential role in macrophage migratory capacity. In severely hypoxic condition, HIF-1α-mediated induction of Pdk1 was found to regulate glucose oxidation by preventing the entry of pyruvate into the tricarboxylic cycle. Monocyte-derived macrophages, however, encounter a gradual decrease in oxygen availability during it**s migrati**on process in inflammatory areas. Here we show that HIF-1α-PDK1-mediated metabolic changes occur in mild hypoxia, where mitochondrial cytochrome c oxidase activity is unimpaired, suggesting a mode of glycolytic reprogramming. In primary macrophages, PKM2, a glycolytic enzyme responsible for glycolytic ATP synthesis localizes in filopodia and lammelipodia, where ATP is rapidly consumed during actin remodelling processes. Remarkably, inhibition of glycolytic reprogramming with dichloroacetate significantly impairs macropha**ge migrat**ion in vitro and in vivo. Furthermore, inhibition of the macrophage HIF-1α-PDK1 axis suppresses systemic inflammation, suggesting a potential therapeutic approach for regulating inflammatory processes. Our findings thus demonstrate that adaptive responses in glucose metabolism contribute to macrophage migratory activity.
3. Relationships between LDH-A, lactate, and metastases in 4T1 breast tumors. To investigate the relationship between lactate dehydrogenase A (LDH-A) expression, lactate concentration, cell metabolism, and metastases in murine 4T1 breast tumors.Inhibition of LDH-A expression and protein levels were achieved in a metastatic breast cancer cell line (4T1) using short hairpin RNA (shRNA) technology. The relationship between tumor LDH-A protein levels and lactate concentration (measured by magnetic resonance spectroscopic imaging, MRSI) and metastases was assessed.LDH-A knockdown cells (KD9) showed a significant reduction in LDH-A protein and LDH activity, less acid production, decreased transwell **migration** and invasion, lower proliferation, reduced glucose consumption and glycolysis, and increase in oxygen consumption, reactive oxygen species (ROS), and cellular ATP levels, compared with control (NC) cells cultured in 25 mmol/L glucose. In vivo studies showed lower lactate levels in KD9, KD5, and KD317 tumors than in NC or 4T1 wild-type tumors (P < 0.01), and a linear relationship between tumor LDH-A protein expression and lactate concentration. Metastases were delayed and primary tumor growth rate decreased.We show for the first time that LDH-A knockdown inhibited the formation of metastases, and was accompanied by in vivo changes in tumor cell metabolism. Lactate MRSI can be used as a surrogate to monitor targeted inhibition of LDH-A in a preclinical setting and provides a noninvasive imaging strategy to monitor LDH-A-targeted therapy. This imaging strategy can be translated to the clinic to identify and monitor patients who are at high risk of developing metastatic disease.

### [Ee]xtracellular [Mm]atrix

#### PubMed

1. Ionizing radiation induces myofibroblast differentiation via lactate dehydrogenase. Pulmonary fibrosis is a common and dose-limiting side-effect of ionizing radiation used to treat cancers of the thoracic region. Few effective therapies are available for this disease. Pulmonary fibrosis is characterized by an accumulation of myofibroblasts and excess deposition of **extracellular matrix** proteins. Although prior studies have reported that ionizing radiation induces fibroblast to myofibroblast differentiation and collagen production, the mechanism remains unclear. Transforming growth factor-β (TGF-β) is a key profibrotic cytokine that drives myofibroblast differentiation an**d extracellular matr**ix production. However, its activation and precise role in radiation-induced fibrosis are poorly understood. Recently, we reported that lactate activates latent TGF-β through a pH-dependent mechanism. Here, we wanted to test the hypothesis that ionizing radiation leads to excessive lactate production via expression of the enzyme lactate dehydrogenase-A (LDHA) to promote myofibroblast differentiation. We found that LDHA expression is increased in human and animal lung tissue exposed to ionizing radiation. We demonstrate that ionizing radiation induces LDHA, lactate production, and extracellular acidification in primary human lung fibroblasts in a dose-dependent manner. We also demonstrate that genetic and pharmacologic inhibition of LDHA protects against radiation-induced myofibroblast differentiation. Furthermore, LDHA inhibition protects from radiation-induced activation of TGF-β. We propose a profibrotic feed forward loop, in which radiation induces LDHA expression and lactate production, which can lead to further activation of TGF-β to drive the fibrotic process. These studies support the concept of LDHA as an important therapeutic target in radiation-induced pulmonary fibrosis.
2. Modulation of gene expression by hypoxia in human umbilical cord vein endothelial cells: A transcriptomic and proteomic study. Hypoxia is a characteristic feature of many human pathologies, including cancer. The sustained proliferation rate of tumor cells leads to alterations of the tumor microenvironment, that progressively becomes more acidic, nutrient-deprived, and hypoxic. The reduced partial pressure of oxygen triggers the onset of an adaptive response, aimed at increasing the local oxygen concentration by several complementary actions. Although directly exposed to the blood stream, endothelial cells lining the vascular lumen in tumors also can be exposed to hypoxia and therefore can contribute to the onset of the adaptive response that leads to tumor angiogenesis. Aiming at getting a detailed insight into the oxygen-dependent regulation of the transcriptional program of vascular endothelial cells and at identifying new relevant markers that may be used as targets for therapeutic intervention in tumor angiogenesis, we have performed a broad-range transcriptomic analysis, using the Affymetrix HG-U133A Gene Chips, of mRNA expression levels in human umbilical cord vein endothelial cells (HUVEC), exposed in vitro to hypoxia for different time periods. The transcriptomic analysis was complemented by a semiquantitative reverse transcriptase-polymerase chain reaction (RT-PCR) analysis of mRNA levels and alternative splicing for some selected **extracellular matrix** protein genes, and by a proteomic analysis, using two-dimensional polyacrylamide gel electrophoresis (2-D PAGE) and tandem mass spectrometry for protein separation and identification, of hypoxic and normoxic HUVEC whole-cell lysates and subcellular fractions. Our analysis confirmed previous findings on genes whose expression is regulated by oxygen concentration but also identified new genes (e.g., CXCR4, claudin 3, CD24, tetranectin, Del-1, procollagen lysyl hydroxylase 1 and 2) which are transcriptionally upregulated in hypoxic conditions.

### [Ii]nvasive

#### PubMed

1. Relationships between LDH-A, lactate, and metastases in 4T1 breast tumors. To investigate the relationship between lactate dehydrogenase A (LDH-A) expression, lactate concentration, cell metabolism, and metastases in murine 4T1 breast tumors.Inhibition of LDH-A expression and protein levels were achieved in a metastatic breast cancer cell line (4T1) using short hairpin RNA (shRNA) technology. The relationship between tumor LDH-A protein levels and lactate concentration (measured by magnetic resonance spectroscopic imaging, MRSI) and metastases was assessed.LDH-A knockdown cells (KD9) showed a significant reduction in LDH-A protein and LDH activity, less acid production, decreased transwell migration and invasion, lower proliferation, reduced glucose consumption and glycolysis, and increase in oxygen consumption, reactive oxygen species (ROS), and cellular ATP levels, compared with control (NC) cells cultured in 25 mmol/L glucose. In vivo studies showed lower lactate levels in KD9, KD5, and KD317 tumors than in NC or 4T1 wild-type tumors (P < 0.01), and a linear relationship between tumor LDH-A protein expression and lactate concentration. Metastases were delayed and primary tumor growth rate decreased.We show for the first time that LDH-A knockdown inhibited the formation of metastases, and was accompanied by in vivo changes in tumor cell metabolism. Lactate MRSI can be used as a surrogate to monitor targeted inhibition of LDH-A in a preclinical setting and provides a non**invasive** imaging strategy to monitor LDH-A-targeted therapy. This imaging strategy can be translated to the clinic to identify and monitor patients who are at high risk of developing metastatic disease.
2. High brain lactate is a hallmark of aging and caused by a shift in the lactate dehydrogenase A/B ratio. At present, there are few means to track symptomatic stages of CNS aging. Thus, although metabolic changes are implicated in mtDNA mutation-driven aging, the manifestations remain unclear. Here, we used normally aging and prematurely aging mtDNA mutator mice to establish a molecular link between mitochondrial dysfunction and abnormal metabolism in the aging process. Using proton magnetic resonance spectroscopy and HPLC, we found that brain lactate levels were increased twofold in both normally and prematurely aging mice during aging. To correlate the striking increase in lactate with tissue pathology, we investigated the respiratory chain enzymes and detected mitochondrial failure in key brain areas from both normally and prematurely aging mice. We used in situ hybridization to show that increased brain lactate levels were caused by a shift in transcriptional activities of the lactate dehydrogenases to promote pyruvate to lactate conversion. Separation of the five tetrameric lactate dehydrogenase (LDH) isoenzymes revealed an increase of those dominated by the Ldh-A product and a decrease of those rich in the Ldh-B product, which, in turn, increases pyruvate to lactate conversion. Spectrophotometric assays measuring LDH activity from the pyruvate and lactate sides of the reaction showed a higher pyruvate → lactate activity in the brain. We argue for the use of lactate proton magnetic resonance spectroscopy as a n**oninvasi**ve strategy for monitoring this hallmark of the aging process. The mtDNA mutator mouse allows us to conclude that the increased LDH-A/LDH-B ratio causes high brain lactate levels, which, in turn, are predictive of aging phenotypes.

### [Mm]etastasis

#### PubMed

1. The miR-34a-LDHA axis regulates glucose metabolism and tumor growth in breast cancer. Lactate dehydrogenase A (LDHA) is involved in a variety of cancers. The purpose of this study was to investigate the expression, prognostic roles and function of LDHA in breast cancer. We found that LDHA was upregulated in both breast cancer cell lines and clinical specimens using quantitative real-time PCR (qRT-PCR). Immunohistochemistry (IHC) analysis of tissue microarrays (TMAs) showed that high LDHA expression was associated with cell proliferation, **metastasis** and poor patient overall survival (OS) and disease free survival (DFS). Furthermore, we found that LDHA promoted glycolysis and cell proliferation in vitro and in vivo. We also performed luciferase reporter assays and found that LDHA was a direct target of miR-34a. Repression of LDHA by miR-34a suppressed glycolysis and cell proliferation in breast cancer cells in vitro. Our findings provide clues regarding the role of miR-34a as a tumor suppressor in breast cancer through the inhibition of LDHA both in vitro and in vivo. Targeting LDHA through miR-34a could be a potential therapeutic strategy in breast cancer.

---

## Ldhb

- NCBI Gene
- GeneRIF
- Pubmed

| Search Term | Rank | Fields |
| --- | --- | --- |
| [Mm]igration | 6 | PubMed(2) |
| [Ii]nvasive | 10 | PubMed(2) |

  
hide/show details for Ldhb

### [Mm]igration

#### PubMed

1. Lack of pericytes leads to endothelial hyperplasia and abnormal vascular morphogenesis. The association of pericytes (PCs) to newly formed blood vessels has been suggested to regulate endothelial cell (EC) proliferation, survival, **migration**, differentiation, and vascular branching. Here, we addressed these issues using PDGF-B-- and PDGF receptor-beta (PDGFR-beta)--deficient mice as in vivo models of brain angiogenesis in the absence of PCs. Quantitative morphological analysis showed that these mutants have normal microvessel density, length, and number of branch points. However, absence of PCs correlates with endothelial hyperplasia, increased capillary diameter, abnormal EC shape and ultrastructure, changed cellular distribution of certain junctional proteins, and morphological signs of increased transendothelial permeability. Brain endothelial hyperplasia was observed already at embryonic day (E) 11.5 and persisted throughout development. From E 13.5, vascular endothelial growth factor-A (VEGF-A) and other genes responsive to metabolic stress became upregulated, suggesting that the abnormal microvessel architecture has systemic metabolic consequences. VEGF-A upregulation correlated temporally with the occurrence of vascular abnormalities in the placenta and dilation of the heart. Thus, although PC deficiency appears to have direct effects on EC number before E 13.5, the subsequent increased VEGF-A levels may further abrogate microvessel architecture, promote vascular permeability, and contribute to formation of the edematous phenotype observed in late gestation PDGF-B and PDGFR-beta knock out embryos.
2. Prox1 ablation in hepatic progenitors causes defective hepatocyte specification and increases biliary cell commitment. The liver has multiple functions that preserve homeostasis. Liver diseases are debilitating, costly and often result in death. Elucidating the developmental mechanisms that establish the liver's architecture or generate the cellular diversity of this organ should help advance the prevention, diagnosis and treatment of hepatic diseases. We previously reported that **migration** of early hepatic precursors away from the gut epithelium requires the activity of the homeobox gene Prox1. Here, we show that Prox1 is a novel regulator of cell differentiation and morphogenesis during hepatogenesis. Prox1 ablation in bipotent hepatoblasts dramatically reduced the expression of multiple hepatocyte genes and led to very defective hepatocyte morphogenesis. As a result, abnormal epithelial structures expressing hepatocyte and cholangiocyte markers or resembling ectopic bile ducts developed in the Prox1-deficient liver parenchyma. By contrast, excessive commitment of hepatoblasts into cholangiocytes, premature intrahepatic bile duct morphogenesis, and biliary hyperplasia occurred in periportal areas of Prox1-deficient livers. Together, these abnormalities indicate that Prox1 activity is necessary to correctly allocate cell fates in liver precursors. These results increase our understanding of differentiation anomalies in pathological conditions and will contribute to improving stem cell protocols in which differentiation is directed towards hepatocytes and cholangiocytes.

### [Ii]nvasive

#### PubMed

1. High brain lactate is a hallmark of aging and caused by a shift in the lactate dehydrogenase A/B ratio. At present, there are few means to track symptomatic stages of CNS aging. Thus, although metabolic changes are implicated in mtDNA mutation-driven aging, the manifestations remain unclear. Here, we used normally aging and prematurely aging mtDNA mutator mice to establish a molecular link between mitochondrial dysfunction and abnormal metabolism in the aging process. Using proton magnetic resonance spectroscopy and HPLC, we found that brain lactate levels were increased twofold in both normally and prematurely aging mice during aging. To correlate the striking increase in lactate with tissue pathology, we investigated the respiratory chain enzymes and detected mitochondrial failure in key brain areas from both normally and prematurely aging mice. We used in situ hybridization to show that increased brain lactate levels were caused by a shift in transcriptional activities of the lactate dehydrogenases to promote pyruvate to lactate conversion. Separation of the five tetrameric lactate dehydrogenase (LDH) isoenzymes revealed an increase of those dominated by the Ldh-A product and a decrease of those rich in the Ldh-B product, which, in turn, increases pyruvate to lactate conversion. Spectrophotometric assays measuring LDH activity from the pyruvate and lactate sides of the reaction showed a higher pyruvate → lactate activity in the brain. We argue for the use of lactate proton magnetic resonance spectroscopy as a n**oninvasi**ve strategy for monitoring this hallmark of the aging process. The mtDNA mutator mouse allows us to conclude that the increased LDH-A/LDH-B ratio causes high brain lactate levels, which, in turn, are predictive of aging phenotypes.
2. High brain lactate is a hallmark of aging and caused by a shift in the lactate dehydrogenase A/B ratio. At present, there are few means to track symptomatic stages of CNS aging. Thus, although metabolic changes are implicated in mtDNA mutation-driven aging, the manifestations remain unclear. Here, we used normally aging and prematurely aging mtDNA mutator mice to establish a molecular link between mitochondrial dysfunction and abnormal metabolism in the aging process. Using proton magnetic resonance spectroscopy and HPLC, we found that brain lactate levels were increased twofold in both normally and prematurely aging mice during aging. To correlate the striking increase in lactate with tissue pathology, we investigated the respiratory chain enzymes and detected mitochondrial failure in key brain areas from both normally and prematurely aging mice. We used in situ hybridization to show that increased brain lactate levels were caused by a shift in transcriptional activities of the lactate dehydrogenases to promote pyruvate to lactate conversion. Separation of the five tetrameric lactate dehydrogenase (LDH) isoenzymes revealed an increase of those dominated by the Ldh-A product and a decrease of those rich in the Ldh-B product, which, in turn, increases pyruvate to lactate conversion. Spectrophotometric assays measuring LDH activity from the pyruvate and lactate sides of the reaction showed a higher pyruvate → lactate activity in the brain. We argue for the use of lactate proton magnetic resonance spectroscopy as a n**oninvasi**ve strategy for monitoring this hallmark of the aging process. The mtDNA mutator mouse allows us to conclude that the increased LDH-A/LDH-B ratio causes high brain lactate levels, which, in turn, are predictive of aging phenotypes.

---

## Flna

- NCBI Gene
- GeneRIF
- Pubmed

| Search Term | Rank | Fields |
| --- | --- | --- |
| [Ff]ilopodium | 2 | PubMed(1) |
| [Pp]odosome(s)? | 5 | PubMed(1); GeneRIFs(1) |
| [Mm]igration | 6 | Process(2); PubMed(22); GeneRIFs(6) |
| ECM | 7 | PubMed(3) |
| [Ee]xtracellular [Mm]atrix | 8 | PubMed(7); Component(1) |
| [Ii]nvasive | 10 | PubMed(2) |
| [Mm]etastasis | 12 | PubMed(2); GeneRIFs(1) |

  
hide/show details for Flna

### [Ff]ilopodium

#### PubMed

1. The Oxygen Sensor PHD2 Controls Dendritic Spines and Synapses via Modification of Filamin A. Neuronal function is highly sensitive to changes in oxygen levels, but how hypoxia affects dendritic spine formation and synaptogenesis is unknown. Here we report that hypoxia, chemical inhibition of the oxygen-sensing prolyl hydroxylase domain proteins (PHDs), and silencing of Phd2 induce immature **filopodium**-like dendritic protrusions, promote spine regression, reduce synaptic density, and decrease the frequency of spontaneous action potentials independently of HIF signaling. We identified the actin cross-linker filamin A (FLNA) as a target of PHD2 mediating these effects. In normoxia, PHD2 hydroxylates the proline residues P2309 and P2316 in FLNA, leading to von Hippel-Lindau (VHL)-mediated ubiquitination and proteasomal degradation. In hypoxia, PHD2 inactivation rapidly upregulates FLNA protein levels because of blockage of its proteasomal degradation. FLNA upregulation induces more immature spines, whereas Flna silencing rescues the immature spine phenotype induced by PHD2 inhibition.

### [Pp]odosome(s)?

#### PubMed

1. Macrophage mesenchymal migration requires **podosome** stabilization by filamin A. Filamin A (FLNa) is a cross-linker of actin filaments and serves as a scaffold protein mostly involved in the regulation of actin polymerization. It is distributed ubiquitously, and null mutations have strong consequences on embryonic development in humans, with organ defects which suggest deficiencies in cell migration. We have reported previously that macrophages, the archetypal migratory cells, use the protease- and **podosome**-dependent mesenchymal migration mode in dense three-dimensional environments, whereas they use the protease- and **podosome**-independent amoeboid mode in more porous matrices. Because FLNa has been shown to localize to **podosomes**, we hypothesized that the defects seen in patients carrying FLNa mutations could be related to the capacity of certain cell types to form **podosomes**. Using strategies based on FLNa knock-out, knockdown, and rescue, we show that FLNa (i) is involved in **podosome** stability and their organization as rosettes and three-dimensional **podosomes**, (ii) regulates the proteolysis of the matrix mediated by **podosomes** in macrophages, (iii) is required for **podosome** rosette formation triggered by Hck, and (iv) is necessary for mesenchymal migration but dispensable for amoeboid migration. These new functions assigned to FLNa, particularly its role in mesenchymal migration, could be directly related to the defects in cell migration described during the embryonic development in FLNa-defective patients.

#### GeneRIFs

1. FLNa is involved in **podosome** stability and their organization as rosettes and three-dimensional **podosomes** in macrophages.

### [Mm]igration

#### Process

1. positive regulation of neuron **migration**
2. regulation of cell **migration**

#### PubMed

1. Filamin A and Filamin B are co-expressed within neurons during periods of neuronal **migration** and can physically interact. Mutations in the X-linked gene Filamin A (FLNA) lead to the human neurological disorder, periventricular heterotopia (PH). Although PH is characterized by a failure in neuronal **migration** into the cerebral cortex with consequent formation of nodules in the ventricular and subventricular zones, many neurons appear to migrate normally, even in males, suggesting compensatory mechanisms. Here we characterize expression patterns for FlnA and a highly homologous protein Filamin B (FlnB) within the nervous system, in order to better understand their potential roles in cortical development. FlnA mRNA was widely expressed in all cortical layers while FlnB mRNA was most highly expressed in the ventricular and subventricular zones during development. In adulthood, widespread but reduced expression of FlnA and FlnB persisted throughout the cerebral cortex. FlnA and FlnB proteins were highly expressed in both the leading processes and somata of migratory neurons during corticogenesis. Postnatally, FlnA immunoreactivity was largely localized to the cell body with FlnB in the soma and neuropil during neuronal differentiation. In adulthood, diminished expression of both proteins localized to the cell soma and nucleus. Moreover, the putative FLNB homodimerization domain strongly interacted with itself or the corresponding homologous region of FLNA by yeast two-hybrid interaction, the two proteins co-localized within neuronal precursors by immunocytochemistry and the existence of FLNA-FLNB heterodimers could be detected by co-immunoprecipitation. These results suggest that FLNA and FLNB may form both homodimers and heterodimers and that their interaction could potentially compensate for the loss of FLNA function during cortical development within PH individuals.
2. Macrophage mesenchymal **migration** requires podosome stabilization by filamin A. Filamin A (FLNa) is a cross-linker of actin filaments and serves as a scaffold protein mostly involved in the regulation of actin polymerization. It is distributed ubiquitously, and null mutations have strong consequences on embryonic development in humans, with organ defects which suggest deficiencies in cell **migration**. We have reported previously that macrophages, the archetypal migratory cells, use the protease- and podosome-dependent mesenchymal **migration** mode in dense three-dimensional environments, whereas they use the protease- and podosome-independent amoeboid mode in more porous matrices. Because FLNa has been shown to localize to podosomes, we hypothesized that the defects seen in patients carrying FLNa mutations could be related to the capacity of certain cell types to form podosomes. Using strategies based on FLNa knock-out, knockdown, and rescue, we show that FLNa (i) is involved in podosome stability and their organization as rosettes and three-dimensional podosomes, (ii) regulates the proteolysis of the matrix mediated by podosomes in macrophages, (iii) is required for podosome rosette formation triggered by Hck, and (iv) is necessary for mesenchymal **migration** but dispensable for amoeboid **migration**. These new functions assigned to FLNa, particularly its role in mesenchymal **migration**, could be directly related to the defects in cell **migration** described during the embryonic development in FLNa-defective patients.
3. Androgen-induced cell **migration**: role of androgen receptor/filamin A association. Androgen receptor (AR) controls male morphogenesis, gametogenesis and prostate growth as well as development of prostate cancer. These findings support a role for AR in cell **migration** and invasiveness. However, the molecular mechanism involved in AR-mediated cell **migration** still remains elusive.Mouse embryo NIH3T3 fibroblasts and highly metastatic human fibrosarcoma HT1080 cells harbor low levels of transcriptionally incompetent AR. We now report that, through extra nuclear action, AR triggers **migration** of both cell types upon stimulation with physiological concentrations of the androgen R1881. We analyzed the initial events leading to androgen-induced cell **migration** and observed that challenging NIH3T3 cells with 10 nM R1881 rapidly induces interaction of AR with filamin A (FlnA) at cytoskeleton. AR/FlnA complex recruits integrin beta 1, thus activating its dependent cascade. Silencing of AR, FlnA and integrin beta 1 shows that this ternary complex controls focal adhesion kinase (FAK), paxillin and Rac, thereby driving cell **migration**. FAK-null fibroblasts migrate poorly and Rac inhibition by EHT impairs motility of androgen-treated NIH3T3 cells. Interestingly, FAK and Rac activation by androgens are independent of each other. Findings in human fibrosarcoma HT1080 cells strengthen the role of Rac in androgen signaling. The Rac inhibitor significantly impairs androgen-induced **migration** in these cells. A mutant AR, deleted of the sequence interacting with FlnA, fails to mediate FAK activation and paxillin tyrosine phosphorylation in androgen-stimulated cells, further reinforcing the role of AR/FlnA interaction in androgen-mediated motility.The present report, for the first time, indicates that the extra nuclear AR/FlnA/integrin beta 1 complex is the key by which androgen activates signaling leading to cell **migration**. Assembly of this ternary complex may control organ development and prostate cancer metastasis.
4. Filamin A (FLNA) is required for cell-cell contact in vascular development and cardiac morphogenesis. Mutations in the human Filamin A (FLNA) gene disrupt neuronal **migration** to the cerebral cortex and cause cardiovascular defects. Complete loss of Flna in mice results in embryonic lethality with severe cardiac structural defects involving ventricles, atria, and outflow tracts, as well as widespread aberrant vascular patterning. Despite these widespread developmental defects, **migration** and motility of many cell types does not appear to be affected. Instead, Flna-null embryos display abnormal epithelial and endothelial organization and aberrant adherens junctions in developing blood vessels, heart, brain, and other tissues. Essential roles for FLNA in intercellular junctions provide a mechanism for the diverse developmental defects seen in patients with FLNA mutations.
5. The Amot/Patj/Syx signaling complex spatially controls RhoA GTPase activity in migrating endothelial cells. Controlled regulation of Rho GTPase activity is an essential component mediating growth factor-stimulated **migration**. We have previously shown that angiomotin (Amot), a membrane-associated scaffold protein, plays a critical role during vascular patterning and endothelial **migration** during embryogenesis. However, the signaling pathways by which Amot controls directional **migration** are not known. Here we have used peptide pull-down and yeast 2-hybrid (Y2H) screening to identify proteins that interact with the C-terminal PDZ-binding motifs of Amot and its related proteins AmotL1 and 2. We report that Amot and its related proteins bind to the RhoA GTPase exchange factor (RhoGEF) protein Syx. We show that Amot forms a ternary complex together with Patj (or its paralogue Mupp1) and Syx. Using FRET analysis, we provide evidence that Amot controls targeting of RhoA activity to lamellipodia in vitro. We also report that, similar to Amot, morpholino knockdown of Syx in zebrafish results in inhibition of **migration** of intersegmental arteries. Taken together, our results indicate that the directional **migration** of capillaries in the embryo is governed by the Amot:Patj/Mupp1:Syx signaling that controls local GTPase activity.
6. Filamin A promotes dynamin-dependent internalization of hyperpolarization-activated cyclic nucleotide-gated type 1 (HCN1) channels and restricts Ih in hippocampal neurons. The actin-binding protein filamin A (FLNa) regulates neuronal **migration** during development, yet its roles in the mature brain remain largely obscure. Here, we probed the effects of FLNa on the regulation of ion channels that influence neuronal properties. We focused on the HCN1 channels that conduct Ih, a hyperpolarization-activated current crucial for shaping intrinsic neuronal properties. Whereas regulation of HCN1 channels by FLNa has been observed in melanoma cell lines, its physiological relevance to neuronal function and the underlying cellular pathways that govern this regulation remain unknown. Using a combination of mutational, pharmacological, and imaging approaches, we find here that FLNa facilitates a selective and reversible dynamin-dependent internalization of HCN1 channels in HEK293 cells. This internalization is accompanied by a redistribution of HCN1 channels on the cell surface, by accumulation of the channels in endosomal compartments, and by reduced Ih density. In hippocampal neurons, expression of a truncated dominant-negative FLNa enhances the expression of native HCN1. Furthermore, acute abrogation of HCN1-FLNa interaction in neurons, with the use of decoy peptides that mimic the FLNa-binding domain of HCN1, abolishes the punctate distribution of HCN1 channels in neuronal cell bodies, augments endogenous Ih, and enhances the rebound-response ("voltage-sag") of the neuronal membrane to transient hyperpolarizing events. Together, these results support a major function of FLNa in modulating ion channel abundance and membrane trafficking in neurons, thereby shaping their biophysical properties and function.
7. Filamin A and FILIP (Filamin A-Interacting Protein) regulate cell polarity and motility in neocortical subventricular and intermediate zones during radial **migration**. In the developing neocortex, most excitatory neurons are supplied and arranged through radial **migration**. Because neurons show global morphological changes and complicated behavior during that **migration**, precise regulation of cell shape and polarity is essential for proper **migration** and correct neocortical formation; however, how cell shape and polarity are regulated in migrating neuron remains elusive. We show here that Filamin A, a well known actin-binding protein, determines the shape of neocortical neurons during radial **migration** in vivo. Dysfunction of Filamin A, caused by a mutant Filamin A expression, prevents cells from acquiring consistent polarity toward specific direction and decreases motility in the subventricular and intermediate zones. In contrast, Filamin A overexpression, achieved by a short interfering RNA for Filamin A-interacting protein that induces Filamin A degradation (FILIP), promotes the development and maintenance of a bipolar shape also in the subventricular and intermediate zones. These results suggest that the amount of Filamin A helps migrating neurons determine their mode of **migration**, multipolar or bipolar, before entering the cortical plate and that FILIP is responsible, at least in part, for Filamin A content. In addition, our results also give a possible clue to understanding the pathogenesis of human malformation periventricular heterotopia, which is caused by various "loss-of-function" mutations in the filamin A gene.
8. Targeted deletion of the mitogen-activated protein kinase kinase 4 gene in the nervous system causes severe brain developmental defects and premature death. The c-Jun NH2-terminal protein kinase (JNK) is a mitogen-activated protein kinase (MAPK) involved in the regulation of various physiological processes. Its activity is increased upon phosphorylation by the MAPK kinases MKK4 and MKK7. The early embryonic death of mice lacking an mkk4 or mkk7 gene has provided genetic evidence that MKK4 and MKK7 have nonredundant functions in vivo. To elucidate the physiological role of MKK4, we generated a novel mouse model in which the mkk4 gene could be specifically deleted in the brain. At birth, the mutant mice were indistinguishable from their control littermates, but they stopped growing a few days later and died prematurely, displaying severe neurological defects. Decreased JNK activity in the absence of MKK4 correlated with impaired phosphorylation of a subset of physiologically relevant JNK substrates and with altered gene expression. These defects resulted in the misalignment of the Purkinje cells in the cerebellum and delayed radial **migration** in the cerebral cortex. Together, our data demonstrate for the first time that MKK4 is an essential activator of JNK required for the normal development of the brain.
9. Brefeldin A-inhibited guanine exchange factor 2 regulates filamin A phosphorylation and neuronal **migration**. Periventricular heterotopia (PH) is a human malformation of cortical development associated with gene mutations in ADP-ribosylation factor guanine exchange factor 2 (ARFGEF2 encodes for Big2 protein) and Filamin A (FLNA). PH is thought to derive from neuroependymal disruption, but the extent to which neuronal **migration** contributes to this phenotype is unknown. Here, we show that Arfgef2 null mice develop PH and exhibit impaired neural **migration** with increased protein expression for both FlnA and phosphoFlnA at Ser2152. Big2 physically interacts with FlnA and overexpression of phosphomimetic Ser2512 FLNA impairs neuronal **migration**. FlnA phosphorylation directs FlnA localization toward the cell cytoplasm, diminishes its binding affinity to actin skeleton, and alters the number and size of paxillin focal adhesions. Collectively, our results demonstrate a molecular mechanism whereby Big2 inhibition promotes phosphoFlnA (Ser2152) expression, and increased phosphoFlnA impairs its actin binding affinity and the distribution of focal adhesions, thereby disrupting cell intrinsic neuronal **migration**.
10. Upregulation of neurovascular communication through filamin abrogation promotes ectopic periventricular neurogenesis. Neuronal fate-restricted intermediate progenitors (IPs) are derived from the multipotent radial glia (RGs) and serve as the direct precursors for cerebral cortical neurons, but factors that control their neurogenic plasticity remain elusive. Here we report that IPs' neuron production is enhanced by abrogating filamin function, leading to the generation of periventricular neurons independent of normal neocortical neurogenesis and neuronal **migration**. Loss of Flna in neural progenitor cells (NPCs) led RGs to undergo changes resembling epithelial-mesenchymal transition (EMT) along with exuberant angiogenesis that together changed the microenvironment and increased neurogenesis of IPs. We show that by collaborating with β-arrestin, Flna maintains the homeostatic signaling between the vasculature and NPCs, and loss of this function results in escalated Vegfa and Igf2 signaling, which exacerbates both EMT and angiogenesis to further potentiate IPs' neurogenesis. These results suggest that the neurogenic potential of IPs may be boosted in vivo by manipulating Flna-mediated neurovascular communication.
11. Filamin A regulates neuronal **migration** through brefeldin A-inhibited guanine exchange factor 2-dependent Arf1 activation. Periventricular heterotopias is a malformation of cortical development, characterized by ectopic neuronal nodules around ventricle lining and caused by an initial **migration** defect during early brain development. Human mutations in the Filamin A (FLNA) and ADP-ribosylation factor guanine exchange factor 2 [ARFGEF2; encoding brefeldin-A-inhibited guanine exchange factor-2 (BIG2)] genes give rise to this disorder. Previously, we have reported that Big2 inhibition impairs neuronal **migration** and binds to FlnA, and its loss promotes FlnA phosphorylation. FlnA phosphorylation dictates FlnA-actin binding affinity and consequently alters focal adhesion size and number to effect neuronal **migration**. Here we show that FlnA loss similarly impairs **migration**, reciprocally enhances Big2 expression, but also alters Big2 subcellular localization in both null and conditional FlnA mice. FlnA phosphorylation promotes relocalization of Big2 from the Golgi toward the lipid ruffles, thereby activating Big2-dependent Arf1 at the cell membrane. Loss of FlnA phosphorylation or Big2 function impairs Arf1-dependent vesicle trafficking at the periphery, and Arf1 is required for maintenance of cell-cell junction connectivity and focal adhesion assembly. Loss of Arf1 activity disrupts neuronal **migration** and cell adhesion. Collectively, these studies demonstrate a potential mechanism whereby coordinated interactions between actin (through FlnA) and vesicle trafficking (through Big2-Arf) direct the assembly and disassembly of membrane protein complexes required for neuronal **migration** and neuroependymal integrity.
12. Receptor tyrosine kinase Ror2 mediates Wnt5a-induced polarized cell **migration** by activating c-Jun N-terminal kinase via actin-binding protein filamin A. The receptor tyrosine kinase Ror2 has recently been shown to act as an alternative receptor or coreceptor for Wnt5a and to mediate Wnt5a-induced **migration** of cultured cells. However, little is known about the molecular mechanism underlying this migratory process. Here we show by wound-healing assays that Ror2 plays critical roles in Wnt5a-induced cell **migration** by regulating formation of lamellipodia and reorientation of microtubule-organizing center (MTOC). Wnt5a stimulation induces activation of the c-Jun N-terminal kinase JNK at the wound edge in a Ror2-dependent manner, and inhibiting JNK activity abrogates Wnt5a-induced lamellipodia formation and MTOC reorientation. Additionally, the association of Ror2 with the actin-binding protein filamin A is required for Wnt5a-induced JNK activation and polarized cell **migration**. We further show that Wnt5a-induced JNK activation and MTOC reorientation can be suppressed by inhibiting PKCzeta. Taken together, our findings indicate that Wnt5a/Ror2 activates JNK, through a process involving filamin A and PKCzeta, to regulate polarized cell **migration**.
13. Trouble making the first move: interpreting arrested neuronal **migration** in the cerebral cortex. Postmitotic cortical neurons that fail to initiate **migration** can remain near their site of origin and form persistent periventricular nodular heterotopia (PH). In human telencephalon, this malformation is most commonly associated with Filamin-A (FLNa) mutations. The lack of genetic animal models that reliably produce PH has delayed our understanding of the underlying molecular mechanisms. This review examines PH pathogenesis using a new mouse model. Although PH have not been observed in Flna-deficient mice generated thus far, the loss of MEKK4, a regulator of Flna, produces striking PH in mice and offers insight into the mechanisms involved in neuronal **migration** initiation. Elucidating the basic functions of FLNa and associated molecules is crucial for understanding the causes of PH and for developing prevention for at-risk patients.
14. IKAP localizes to membrane ruffles with filamin A and regulates actin cytoskeleton organization and cell **migration**. Loss-of-function mutations in the IKBKAP gene, which encodes IKAP (ELP1), cause familial dysautonomia (FD), with defective neuronal development and maintenance. Molecular mechanisms leading to FD are poorly understood. We demonstrate that various RNA-interference-based depletions of IKAP lead to defective adhesion and **migration** in several cell types, including rat primary neurons. The defects could be rescued by reintroduction of wild-type IKAP but not by FD-IKAP, a truncated form of IKAP constructed according to the mutation found in the majority of FD patients. Cytosolic IKAP co-purified with proteins involved in cell **migration**, including filamin A, which is also involved in neuronal **migration**. Immunostaining of IKAP and filamin A revealed a distinct co-localization of these two proteins in membrane ruffles. Depletion of IKAP resulted in a significant decrease in filamin A localization in membrane ruffles and defective actin cytoskeleton organization, which both could be rescued by the expression of wild-type IKAP but not by FD-IKAP. No downregulation in the protein levels of paxillin or beclin 1, which were recently described as specific transcriptional targets of IKAP, was detected. These results provide evidence for the role of the cytosolic interactions of IKAP in cell adhesion and **migration**, and support the notion that cell-motility deficiencies could contribute to FD.
15. MEKK4 signaling regulates filamin expression and neuronal **migration**. Periventricular heterotopia (PVH) is a congenital malformation of human cerebral cortex frequently associated with Filamin-A (FLN-A) mutations but the pathogenetic mechanisms remain unclear. Here, we show that the MEKK4 (MAP3K4) pathway is involved in Fln-A regulation and PVH formation. MEKK4(-/-) mice developed PVH associated with breaches in the neuroependymal lining which were largely comprised of neurons that failed to reach the cortical plate. RNA interference (RNAi) targeting MEKK4 also impaired neuronal **migration**. Expression of Fln was elevated in MEKK4(-/-) forebrain, most notably near sites of failed neuronal **migration**. Importantly, recombinant MKK4 protein precipitated a complex containing MEKK4 and Fln-A, and MKK4 mediated signaling between MEKK4 and Fln-A, suggesting that MKK4 may bridge these molecules during development. Finally, we showed that wild-type FLN-A overexpression inhibited neuronal **migration**. Collectively, our results demonstrate a link between MEKK4 and Fln-A that impacts neuronal **migration** initiation and provides insight into the pathogenesis of human PVH.
16. Filamin A regulates monocyte **migration** through Rho small GTPases during osteoclastogenesis. Osteoclastogenesis (OCG) results from the fusion of monocytes after stimulation with macrophage colony-stimulating factor (M-CSF) and receptor activator of NF-kappaB ligand (RANKL). **Migration** of monocytes into close proximity precedes critical fusion events that are required for osteoclast formation. Cellular **migration** requires leading-edge actin cytoskeleton assembly that drives cellular locomotion. Filamin A (FLNa) cross-links F-actin filaments in the leading edge of migrating cells and also has been shown to regulate signal transduction during cell **migration**. However, little is known about the possible role of FLNa in osteoclastogenesis. Our objective in this study was to investigate the role of FLNa in osteoclastogenesis. Bone marrow monocytes isolated from the tibiae and femora of wild type (WT) and Flna-null mice were cultured for 6 days with M-CSF and RANKL, and osteoclasts were identified by tartrate-resistant acid phosphatase (TRACP) staining. The Flna-null mouse skeletal phenotype was characterized using dual-energy X-ray absorptiometry (DXA) to analyze the skeleton, as well as tests on blood chemistry. Osteoclast levels in vivo were quantified by counting of TRACP-stained histologic sections of distal femora. To elucidate the mechanisms by which Flna regulates osteoclastogenesis, **migration**, actin polymerization, and activation of Rho GTPases, Rac1, Cdc42, and RhoA were assessed in monocytes during in vitro OCG. Deficiencies in **migration** were rescued using constitutively active Rac1 and Cdc42 TAT fusion proteins. The RANKL signaling pathway was evaluated for activation by monitoring nuclear translocation of NF kappaB and c-jun and expression of key osteoclast genes using quantitative real-time polymerase chain reaction (qRT-PCR). Our results show that Flna-null monocytes formed fewer osteoclasts in vitro, and those that were formed were smaller with fewer nuclei. Decreased OCG was reflected in vivo in TRACP-stained histologic bone sections. Flna-null monocytes experienced impaired migratory ability. When OCG was performed at increasing starting cellular plating densities in order to decrease intercellular distances, there was progressive rescue of Flna-null osteoclast formation comparable with WT levels, confirming that Flna regulates monocyte **migration** prefusion. Activation of the actin cytoskeleton regulators Rac1, Cdc42, and RhoA and actin free-barbed end generation were partially or completely abrogated in Flna-null monocytes; however, monocyte **migration** was restored on rescuing with constitutively active Rac1 and Cdc42 TAT fusion proteins. We conclude that filamin A is required for osteoclastogenesis by regulating actin dynamics via Rho GTPases that control monocyte **migration**.
17. Deficiency of filamin A in endothelial cells impairs left ventricular remodelling after myocardial infarction. Actin-binding protein filamin A (FLNA) regulates signal transduction important for cell locomotion, but the role of FLNA after myocardial infarction (MI) has not been explored. The main purpose of this study was to determine the impact of endothelial deletion of FLNA on post-MI remodelling of the left ventricle (LV).We found that FLNA is expressed in human and mouse endothelial cells (ECs) during MI. To determine the biological significance of endothelial expression of FLNA, we used mice that are deficient for endothelial FLNA by cross-breeding adult mice expressing floxed Flna (Flna(o/fl)) with mice expressing Cre under the vascular endothelial-specific cadherin promoter (VECadCre+). Male Flna(o/fl) and Flna(o/fl)/VECadCre+ mice were subjected to permanent coronary artery ligation to induce MI. Flna(o/fl)/VECadCre+ mice that were deficient for endothelial FLNA exhibited larger and thinner LV with impaired cardiac function as well as elevated plasma levels of NT-proBNP and decreased secretion of VEGF-A. The number of capillary structures within the infarcted areas was reduced in Flna(o/fl)/VECadCre+ hearts. ECs silenced for Flna mRNA expression exhibited impaired tubular formation and **migration**, secreted less VEGF-A, and produced lower levels of phosphorylated AKT and ERK1/2 as well as active RAC1.Deletion of FLNA in ECs aggravated MI-induced LV dysfunction and cardiac failure as a result of defective endothelial response and increased scar formation by impaired endothelial function and signalling.
18. Filamin a regulates neural progenitor proliferation and cortical size through Wee1-dependent Cdk1 phosphorylation. Cytoskeleton-associated proteins play key roles not only in regulating cell morphology and **migration** but also in proliferation. Mutations in the cytoskeleton-associated gene filamin A (FlnA) cause the human disorder periventricular heterotopia (PH). PH is a disorder of neural stem cell development that is characterized by disruption of progenitors along the ventricular epithelium and subsequent formation of ectopic neuronal nodules. FlnA-dependent regulation of cytoskeletal dynamics is thought to direct neural progenitor **migration** and proliferation. Here we show that embryonic FlnA-null mice exhibited a reduction in brain size and decline in neural progenitor numbers over time. The drop in the progenitor population was not attributable to cell death or changes in premature differentiation, but to prolonged cell cycle duration. Suppression of FlnA led to prolongation of the entire cell cycle length, principally in M phase. FlnA loss impaired degradation of cyclin B1-related proteins, thereby delaying the onset and progression through mitosis. We found that the cdk1 kinase Wee1 bound FlnA, demonstrated increased expression levels after loss of FlnA function, and was associated with increased phosphorylation of cdk1. Phosphorylation of cdk1 inhibited activation of the anaphase promoting complex degradation system, which was responsible for cyclin B1 degradation and progression through mitosis. Collectively, our results demonstrate a molecular mechanism whereby FlnA loss impaired G2 to M phase entry, leading to cell cycle prolongation, compromised neural progenitor proliferation, and reduced brain size.
19. The filamins: organizers of cell structure and function. Filamin A (FLNa), the first non-muscle actin filament cross-linking protein, was identified in 1975. Thirty five years of FLNa research has revealed its structure in great detail, discovered its isoforms (FLNb and c), and identified over 90 binding partners including channels, receptors, intracellular signaling molecules, and even transcription factors. Due to this diversity, mutations in human FLN genes result in a wide range of anomalies with moderate to lethal consequences. This review focuses on the structure and functions of FLNa in cell **migration** and adhesion.
20. Binding of the P2Y2 nucleotide receptor to filamin A regulates **migration** of vascular smooth muscle cells. The functional expression of the G protein-coupled P2Y(2) nucleotide receptor (P2Y(2)R) has been associated with proliferation and **migration** of vascular smooth muscle cells (SMCs), two processes involved in atherosclerosis and restenosis. Activation of the P2Y(2)R causes dynamic reorganization of the actin cytoskeleton, which transmits biochemical signals and forces necessary for cell locomotion, suggesting that P2Y(2)Rs may be linked to the actin cytoskeleton. Here, we identified filamin A (FLNa) as a P2Y(2)R-interacting protein using a yeast 2-hybrid system screen with the C-terminal region of the P2Y(2)R as bait. The FLNa binding site in the P2Y(2)R is localized between amino acids 322 and 333. Deletion of this region led to selective loss of FLNa binding to the P2Y(2)R and abolished Tyr phosphorylation of FLNa induced by the P2Y(2)R agonist UTP. Using both time-lapse microscopy and the Transwell cell **migration** assay, we showed that UTP significantly increased SMC spreading on collagen I (6.8 fold; P < or = 0.01) and **migration** (3.6 fold; P < or = 0.01) of aortic SMCs isolated from wild-type mice, as compared with unstimulated SMCs. UTP-induced spreading and **migration** of aortic SMCs did not occur with cells isolated from P2Y(2)R knockout mice. Expression of the full-length P2Y(2)R in SMCs isolated from P2Y(2)R knockout mice restored both UTP-induced spreading and **migration**. In contrast, UTP-induced spreading and **migration** did not occur in SMCs isolated from P2Y(2)R knockout mice transfected with a mutant P2Y(2)R that does not bind FLNa. Furthermore, ex vivo studies showed that both ATP and UTP (10 micromol/L) promoted **migration** of SMCs out of aortic explants isolated from wild-type but not P2Y(2)R knockout mice. Thus, this study demonstrates that P2Y(2)R/FLNa interaction selectively regulates spreading and **migration** of vascular SMCs.
21. Epicardially derived fibroblasts preferentially contribute to the parietal leaflets of the atrioventricular valves in the murine heart. The importance of the epicardium for myocardial and valvuloseptal development has been well established; perturbation of epicardial development results in cardiac abnormalities, including thinning of the ventricular myocardial wall and malformations of the atrioventricular valvuloseptal complex. To determine the spatiotemporal contribution of epicardially derived cells to the developing fibroblast population in the heart, we have used a mWt1/IRES/GFP-Cre mouse to trace the fate of EPDCs from embryonic day (ED)10 until birth. EPDCs begin to populate the compact ventricular myocardium around ED12. The **migration** of epicardially derived fibroblasts toward the interface between compact and trabecular myocardium is completed around ED14. Remarkably, epicardially derived fibroblasts do not migrate into the trabecular myocardium until after ED17. **Migration** of EPDCs into the atrioventricular cushion mesenchyme commences around ED12. As development progresses, the number of EPDCs increases significantly, specifically in the leaflets which derive from the lateral atrioventricular cushions. In these developing leaflets the epicardially derived fibroblasts eventually largely replace the endocardially derived cells. Importantly, the contribution of EPDCs to the leaflets derived from the major AV cushions is very limited. The differential contribution of EPDCs to the various leaflets of the atrioventricular valves provides a new paradigm in valve development and could lead to new insights into the pathogenesis of abnormalities that preferentially affect individual components of this region of the heart. The notion that there is a significant difference in the contribution of epicardially and endocardially derived cells to the individual leaflets of the atrioventricular valves has also important pragmatic consequences for the use of endocardial and epicardial cre-mouse models in studies of heart development.
22. A meckelin-filamin A interaction mediates ciliogenesis. MKS3, encoding the transmembrane receptor meckelin, is mutated in Meckel-Gruber syndrome (MKS), an autosomal-recessive ciliopathy. Meckelin localizes to the primary cilium, basal body and elsewhere within the cell. Here, we found that the cytoplasmic domain of meckelin directly interacts with the actin-binding protein filamin A, potentially at the apical cell surface associated with the basal body. Mutations in FLNA, the gene for filamin A, cause periventricular heterotopias. We identified a single consanguineous patient with an MKS-like ciliopathy that presented with both MKS and cerebellar heterotopia, caused by an unusual in-frame deletion mutation in the meckelin C-terminus at the region of interaction with filamin A. We modelled this mutation and found it to abrogate the meckelin-filamin A interaction. Furthermore, we found that loss of filamin A by siRNA knockdown, in patient cells, and in tissues from Flna(Dilp2) null mouse embryos results in cellular phenotypes identical to those caused by meckelin loss, namely basal body positioning and ciliogenesis defects. In addition, morpholino knockdown of flna in zebrafish embryos significantly increases the frequency of dysmorphology and severity of ciliopathy developmental defects caused by mks3 knockdown. Our results suggest that meckelin forms a functional complex with filamin A that is disrupted in MKS and causes defects in neuronal **migration** and Wnt signalling. Furthermore, filamin A has a crucial role in the normal processes of ciliogenesis and basal body positioning. Concurrent with these processes, the meckelin-filamin A signalling axis may be a key regulator in maintaining correct, normal levels of Wnt signalling.

#### GeneRIFs

1. coordinated interactions between actin (through FlnA) and vesicle trafficking (through Big2-Arf) direct the assembly and disassembly of membrane protein complexes required for neuronal **migration** and neuroependymal integrity.
2. Filamin A interacts with Big2 and overexpression of Filamin A interfers with neuronal **migration**.
3. filamin A is required for osteoclastogenesis by regulating actin dynamics via Rho GTPases that control monocyte **migration**.
4. Wnt5a/Ror2 activates JNK, through a process involving filamin A and PKCzeta, to regulate polarized cell **migration**.
5. This study demonstrates that P2Y(2)R/FLNa interaction selectively regulates spreading and **migration** of vascular SMCs.
6. Results suggest that the amount of Filamin A helps migrating neurons determine their mode of **migration**, multipolar or bipolar, before entering the cortical plate and that FILIP is responsible, at least in part, for Filamin A content.

### ECM

#### PubMed

1. Developmental basis for filamin-A-associated myxomatous mitral valve disease. We hypothesized that the structure and function of the mature valves is largely dependent upon how these tissues are built during development, and defects in how the valves are built can lead to the pathological progression of a disease phenotype. Thus, we sought to uncover potential developmental origins and mechanistic underpinnings causal to myxomatous mitral valve disease. We focus on how filamin-A, a cytoskeletal binding protein with strong links to human myxomatous valve disease, can function as a regulatory interface to control proper mitral valve development.Filamin-A-deficient mice exhibit abnormally enlarged mitral valves during foetal life, which progresses to a myxomatous phenotype by 2 months of age. Through expression studies, in silico modelling, 3D morphometry, biochemical studies, and 3D matrix assays, we demonstrate that the inception of the valve disease occurs during foetal life and can be attributed, in part, to a deficiency of interstitial cells to efficiently organize the extracellular matrix (**ECM**). This **ECM** organization during foetal valve gestation is due, in part, to molecular interactions between filamin-A, serotonin, and the cross-linking enzyme, transglutaminase-2 (TG2). Pharmacological and genetic perturbations that inhibit serotonin-TG2-filamin-A interactions lead to impaired **ECM** remodelling and engender progression to a myxomatous valve phenotype.These findings illustrate a molecular mechanism by which valve interstitial cells, through a serotonin, TG, and filamin-A pathway, regulate matrix organization during foetal valve development. Additionally, these data indicate that disrupting key regulatory interactions during valve development can set the stage for the generation of postnatal myxomatous valve disease.
2. Filamin A-beta1 integrin complex tunes epithelial cell response to matrix tension. The physical properties of the extracellular matrix (**ECM**) regulate the behavior of several cell types; yet, mechanisms by which cells recognize and respond to changes in these properties are not clear. For example, breast epithelial cells undergo ductal morphogenesis only when cultured in a compliant collagen matrix, but not when the tension of the matrix is increased by loading collagen gels or by increasing collagen density. We report that the actin-binding protein filamin A (FLNa) is necessary for cells to contract collagen gels, and pull on collagen fibrils, which leads to collagen remodeling and morphogenesis in compliant, low-density gels. In stiffer, high-density gels, cells are not able to contract and remodel the matrix, and morphogenesis does not occur. However, increased FLNa-beta1 integrin interactions rescue gel contraction and remodeling in high-density gels, resulting in branching morphogenesis. These results suggest morphogenesis can be "tuned" by the balance between cell-generated contractility and opposing matrix stiffness. Our findings support a role for FLNa-beta1 integrin as a mechanosensitive complex that bidirectionally senses the tension of the matrix and, in turn, regulates cellular contractility and response to this matrix tension.
3. Filamin B mutations cause chondrocyte defects in skeletal development. Filamin B (FLNB) is a cytoplasmic protein that regulates the cytoskeletal network by cross-linking actin, linking cell membrane to the cytoskeleton and regulating intracellular signaling pathways responsible for skeletal development (Stossel, T.P., Condeelis, J., Cooley, L., Hartwig, J.H., Noegel, A., Schleicher, M. and Shapiro, S.S. (2001) Filamins as integrators of cell mechanics and signalling. Nat. Rev. Mol. Cell Biol., 2, 138-145). Mutations in FLNB cause human skeletal disorders [boomerang dysplasia, spondylocarpotarsal (SCT), Larsen, and atelosteogenesis I/III syndromes], which are characterized by disrupted vertebral segmentation, joint formation and endochondral ossification [Krakow, D., Robertson, S.P., King, L.M., Morgan, T., Sebald, E.T., Bertolotto, C., Wachsmann-Hogiu, S., Acuna, D., Shapiro, S.S., Takafuta, T. et al. (2004) Mutations in the gene encoding filamin B disrupt vertebral segmentation, joint formation and skeletogenesis. Nat. Genet., 36, 405-410; Bicknell, L.S., Morgan, T., Bonafe, L., Wessels, M.W., Bialer, M.G., Willems, P.J., Cohn, D.H., Krakow, D. and Robertson, S.P. (2005) Mutations in FLNB cause boomerang dysplasia. J. Med. Genet., 42, e43]. Here we show that Flnb deficient mice have shortened distal limbs with small body size, and develop fusion of the ribs and vertebrae, abnormal spinal curvatures, and dysmorphic facial/calvarial bones, similar to the human phenotype. Characterization of the mutant mice demonstrated increased apoptosis along the bone periphery of the distal appendages, consistent with reduced bone width. No changes in the initial proliferative rate of chondrocytes were observed, but the progressive differentiation of chondrocyte precursors was impaired, consistent with reduced bone length. The extracellular matrix appeared disrupted and phosphorylated beta1-integrin (a collagen receptor and Flnb binding partner) expression was diminished in the mutant growth plate. Like integrin-deficient chondrocytes, adhesion to the **ECM** was decreased in Flnb(-/-) chondrocytes, and inhibition of beta1-integrin in these cells led to further impairments in cell spreading. These data suggest that disruption of the **ECM**-beta1-integrin-Flnb pathway contributes to defects in vertebral and distal limb development, similar to those seen in the human autosomal recessive SCT due to Flnb mutations.

### [Ee]xtracellular [Mm]atrix

#### PubMed

1. Different splice variants of filamin-B affect myogenesis, subcellular distribution, and determine binding to integrin [beta] subunits. Integrins connect the **extracellular matrix** with the cell interior, and transduce signals through interactions of their cytoplasmic tails with cytoskeletal and signaling proteins. Using the yeast two-hybrid system, we isolated a novel splice variant (filamin-Bvar-1) of the filamentous actin cross-linking protein, filamin-B, that interacts with the cytoplasmic domain of the integrin beta1A and beta1D subunits. RT-PCR analysis showed weak, but wide, expression of filamin-Bvar-1 and a similar splice variant of filamin-A (filamin-Avar-1) in human tissues. Furthermore, alternative splice variants of filamin-B and filamin-C, from which the flexible hinge-1 region is deleted (DeltaH1), were induced during in vitro differentiation of C2C12 mouse myoblasts. We show that both filamin-Avar-1 and filamin-Bvar-1 bind more strongly than their wild-type isoforms to different integrin beta subunits. The mere presence of the high-affinity binding site for beta1A is not sufficient for targeting the filamin-Bvar-1 construct to focal contacts. Interestingly, the simultaneous deletion of the H1 region is required for the localization of filamin-B at the tips of actin stress fibers. When expressed in C2C12 cells, filamin-Bvar-1(DeltaH1) accelerates their differentiation into myotubes. Furthermore, filamin-B variants lacking the H1 region induce the formation of thinner myotubes than those in cells containing variants with this region. These findings suggest that specific combinations of filamin mRNA splicing events modulate the organization of the actin cytoskeleton and the binding affinity for integrins.
2. Filamin A Mediates Wound Closure by Promoting Elastic Deformation and Maintenance of Tension in the Collagen Matrix. Cell-mediated remodeling and wound closure are critical for efficient wound healing, but the contribution of actin-binding proteins to contraction of the **extracellular matrix** is not defined. We examined the role of filamin A (FLNa), an actin filament cross-linking protein, in wound contraction and maintenance of matrix tension. Conditional deletion of FLNa in fibroblasts in mice was associated with ~4 day delay of full-thickness skin wound contraction compared with wild-type (WT) mice. We modeled the healing wound matrix using cultured fibroblasts plated on grid-supported collagen gels that create lateral boundaries, which are analogues to wound margins. In contrast to WT cells, FLNa knockdown (KD) cells could not completely maintain tension when matrix compaction was resisted by boundaries, which manifested as relaxed matrix tension. Similarly, WT cells on cross-linked collagen, which requires higher levels of sustained tension, exhibited approximately fivefold larger deformation fields and approximately twofold greater fiber alignment compared with FLNa KD cells. Maintenance of boundary-resisted tension markedly influenced the elongation of cell extensions: in WT cells, the number (~50%) and length (~300%) of cell extensions were greater than FLNa KD cells. We conclude that FLNa is required for wound contraction, in part by enabling elastic deformation and maintenance of tension in the matrix.
3. Developmental basis for filamin-A-associated myxomatous mitral valve disease. We hypothesized that the structure and function of the mature valves is largely dependent upon how these tissues are built during development, and defects in how the valves are built can lead to the pathological progression of a disease phenotype. Thus, we sought to uncover potential developmental origins and mechanistic underpinnings causal to myxomatous mitral valve disease. We focus on how filamin-A, a cytoskeletal binding protein with strong links to human myxomatous valve disease, can function as a regulatory interface to control proper mitral valve development.Filamin-A-deficient mice exhibit abnormally enlarged mitral valves during foetal life, which progresses to a myxomatous phenotype by 2 months of age. Through expression studies, in silico modelling, 3D morphometry, biochemical studies, and 3D matrix assays, we demonstrate that the inception of the valve disease occurs during foetal life and can be attributed, in part, to a deficiency of interstitial cells to efficiently organize the **extracellular matrix** (ECM). This ECM organization during foetal valve gestation is due, in part, to molecular interactions between filamin-A, serotonin, and the cross-linking enzyme, transglutaminase-2 (TG2). Pharmacological and genetic perturbations that inhibit serotonin-TG2-filamin-A interactions lead to impaired ECM remodelling and engender progression to a myxomatous valve phenotype.These findings illustrate a molecular mechanism by which valve interstitial cells, through a serotonin, TG, and filamin-A pathway, regulate matrix organization during foetal valve development. Additionally, these data indicate that disrupting key regulatory interactions during valve development can set the stage for the generation of postnatal myxomatous valve disease.
4. Filamin A-beta1 integrin complex tunes epithelial cell response to matrix tension. The physical properties of the **extracellular matrix** (ECM) regulate the behavior of several cell types; yet, mechanisms by which cells recognize and respond to changes in these properties are not clear. For example, breast epithelial cells undergo ductal morphogenesis only when cultured in a compliant collagen matrix, but not when the tension of the matrix is increased by loading collagen gels or by increasing collagen density. We report that the actin-binding protein filamin A (FLNa) is necessary for cells to contract collagen gels, and pull on collagen fibrils, which leads to collagen remodeling and morphogenesis in compliant, low-density gels. In stiffer, high-density gels, cells are not able to contract and remodel the matrix, and morphogenesis does not occur. However, increased FLNa-beta1 integrin interactions rescue gel contraction and remodeling in high-density gels, resulting in branching morphogenesis. These results suggest morphogenesis can be "tuned" by the balance between cell-generated contractility and opposing matrix stiffness. Our findings support a role for FLNa-beta1 integrin as a mechanosensitive complex that bidirectionally senses the tension of the matrix and, in turn, regulates cellular contractility and response to this matrix tension.
5. Structural basis of the migfilin-filamin interaction and competition with integrin beta tails. A link between sites of cell adhesion and the cytoskeleton is essential for regulation of cell shape, motility, and signaling. Migfilin is a recently identified adaptor protein that localizes at cell-cell and cell-**extracellular matrix** adhesion sites, where it is thought to provide a link to the cytoskeleton by interacting with the actin cross-linking protein filamin. Here we have used x-ray crystallography, NMR spectroscopy, and protein-protein interaction studies to investigate the molecular basis of migfilin binding to filamin. We report that the N-terminal portion of migfilin can bind all three human filamins (FLNa, -b, or -c) and that there are multiple migfilin-binding sites in FLNa. Human filamins are composed of an N-terminal actin-binding domain followed by 24 immunoglobulin-like (IgFLN) domains and we find that migfilin binds preferentially to IgFLNa21 and more weakly to IgFLNa19 and -22. The filamin-binding site in migfilin is localized between Pro(5) and Pro(19) and binds to the CD face of the IgFLNa21 beta-sandwich. This interaction is similar to the previously characterized beta 7 integrin-IgFLNa21 interaction and migfilin and integrin beta tails can compete with one another for binding to IgFLNa21. This suggests that competition between filamin ligands for common binding sites on IgFLN domains may provide a general means of modulating filamin interactions and signaling. In this specific case, displacement of integrin tails from filamin by migfilin may provide a mechanism for switching between different integrin-cytoskeleton linkages.
6. Filamin A regulates the organization and remodeling of the pericellular collagen matrix. **Extracellular matrix** remodeling by cell adhesion-related processes is critical for proliferation and tissue homeostasis, but how adhesions and the cytoskeleton interact to organize the pericellular matrix (PCM) is not understood. We examined the role of the actin-binding protein, filamin A (FLNa), in pericellular collagen remodeling. Compared with wild-type (WT), mice with fibroblast-specific deletion of FLNa exhibited higher density but reduced organization of collagen fibers after increased loading of the periodontal ligament for 2 wk. In cultured fibroblasts, FLNa knockdown (KD) did not affect collagen mRNA, but after 24 h of culture, FLNa WT cells exhibited ∼2-fold higher cell-surface collagen KD cells and 13-fold higher levels of activated β1 integrins. In FLNa WT cells, there was 3-fold more colocalization of talin with pericellular cleaved collagen than in FLNa KD cells. MMP-9 mRNA and protein expression were >2-fold higher in FLNa KD cells than in WT cells. Cathepsin B, which is necessary for intracellular collagen digestion, was >3-fold higher in FLNa WT cells than in KD cells. FLNa WT cells exhibited 2-fold more collagen phagocytosis than KD cells, which involved the FLNa actin-binding domain. Evidently, FLNa regulates PCM remodeling through its effects on degradation pathways that affect the abundance and organization of collagen.-Mezawa, M., Pinto, V. I., Kazembe, M. P., Lee, W. S., McCulloch, C. A. Filamin A regulates the organization and remodeling of the pericellular collagen matrix.
7. Filamin B mutations cause chondrocyte defects in skeletal development. Filamin B (FLNB) is a cytoplasmic protein that regulates the cytoskeletal network by cross-linking actin, linking cell membrane to the cytoskeleton and regulating intracellular signaling pathways responsible for skeletal development (Stossel, T.P., Condeelis, J., Cooley, L., Hartwig, J.H., Noegel, A., Schleicher, M. and Shapiro, S.S. (2001) Filamins as integrators of cell mechanics and signalling. Nat. Rev. Mol. Cell Biol., 2, 138-145). Mutations in FLNB cause human skeletal disorders [boomerang dysplasia, spondylocarpotarsal (SCT), Larsen, and atelosteogenesis I/III syndromes], which are characterized by disrupted vertebral segmentation, joint formation and endochondral ossification [Krakow, D., Robertson, S.P., King, L.M., Morgan, T., Sebald, E.T., Bertolotto, C., Wachsmann-Hogiu, S., Acuna, D., Shapiro, S.S., Takafuta, T. et al. (2004) Mutations in the gene encoding filamin B disrupt vertebral segmentation, joint formation and skeletogenesis. Nat. Genet., 36, 405-410; Bicknell, L.S., Morgan, T., Bonafe, L., Wessels, M.W., Bialer, M.G., Willems, P.J., Cohn, D.H., Krakow, D. and Robertson, S.P. (2005) Mutations in FLNB cause boomerang dysplasia. J. Med. Genet., 42, e43]. Here we show that Flnb deficient mice have shortened distal limbs with small body size, and develop fusion of the ribs and vertebrae, abnormal spinal curvatures, and dysmorphic facial/calvarial bones, similar to the human phenotype. Characterization of the mutant mice demonstrated increased apoptosis along the bone periphery of the distal appendages, consistent with reduced bone width. No changes in the initial proliferative rate of chondrocytes were observed, but the progressive differentiation of chondrocyte precursors was impaired, consistent with reduced bone length. The **extracellular matrix** appeared disrupted and phosphorylated beta1-integrin (a collagen receptor and Flnb binding partner) expression was diminished in the mutant growth plate. Like integrin-deficient chondrocytes, adhesion to the ECM was decreased in Flnb(-/-) chondrocytes, and inhibition of beta1-integrin in these cells led to further impairments in cell spreading. These data suggest that disruption of the ECM-beta1-integrin-Flnb pathway contributes to defects in vertebral and distal limb development, similar to those seen in the human autosomal recessive SCT due to Flnb mutations.

#### Component

1. **extracellular matrix**

### [Ii]nvasive

#### PubMed

1. Androgen-induced cell migration: role of androgen receptor/filamin A association. Androgen receptor (AR) controls male morphogenesis, gametogenesis and prostate growth as well as development of prostate cancer. These findings support a role for AR in cell migration and **invasive**ness. However, the molecular mechanism involved in AR-mediated cell migration still remains elusive.Mouse embryo NIH3T3 fibroblasts and highly metastatic human fibrosarcoma HT1080 cells harbor low levels of transcriptionally incompetent AR. We now report that, through extra nuclear action, AR triggers migration of both cell types upon stimulation with physiological concentrations of the androgen R1881. We analyzed the initial events leading to androgen-induced cell migration and observed that challenging NIH3T3 cells with 10 nM R1881 rapidly induces interaction of AR with filamin A (FlnA) at cytoskeleton. AR/FlnA complex recruits integrin beta 1, thus activating its dependent cascade. Silencing of AR, FlnA and integrin beta 1 shows that this ternary complex controls focal adhesion kinase (FAK), paxillin and Rac, thereby driving cell migration. FAK-null fibroblasts migrate poorly and Rac inhibition by EHT impairs motility of androgen-treated NIH3T3 cells. Interestingly, FAK and Rac activation by androgens are independent of each other. Findings in human fibrosarcoma HT1080 cells strengthen the role of Rac in androgen signaling. The Rac inhibitor significantly impairs androgen-induced migration in these cells. A mutant AR, deleted of the sequence interacting with FlnA, fails to mediate FAK activation and paxillin tyrosine phosphorylation in androgen-stimulated cells, further reinforcing the role of AR/FlnA interaction in androgen-mediated motility.The present report, for the first time, indicates that the extra nuclear AR/FlnA/integrin beta 1 complex is the key by which androgen activates signaling leading to cell migration. Assembly of this ternary complex may control organ development and prostate cancer metastasis.
2. S6K is a morphogenic protein with a mechanism involving Filamin-A phosphorylation and phosphatidic acid binding. Change of cell shape in vivo plays many roles that are central to life itself, such as embryonic development, inflammation, wound healing, and pathologic processes such as cancer metastasis. Nonetheless, the spatiotemporal mechanisms that control the concerted regulation of cell shape remain understudied. Here, we show that ribosomal S6K, which is normally considered a protein involved in protein translation, is a morphogenic protein. Its presence in cells alters the overall organization of the cell surface and cell circularity [(4π × area)/(perimeter)(2)] from 0.47 ± 0.06 units in mock-treated cells to 0.09 ± 0.03 units in S6K-overexpressing macrophages causing stellation and arborization of cell shape. This effect was partially reversed in cells expressing a kinase-inactive S6K mutant and was fully reversed in cells silenced with small interference RNA. Equally important is that S6K is itself regulated by phospholipids, specifically phosphatidic acid, whereby 300 nM 1,2-dioleoyl-sn-glycero-3-phosphate (DOPA), but not the control 1,2-dioleoyl-sn-glycero-3-phosphocholine (DOPC), binds directly to S6K and causes an ∼ 2.9-fold increase in S6K catalytic activity. This was followed by an increase in Filamin A (FLNA) functionality as measured by phospho-FLNA (S(2152)) expression and by a subsequent elevation of actin nucleation. This reliance of S6K on phosphatidic acid (PA), a curvature-inducing phospholipid, explained the extra-large perimeter of cells that overexpressed S6K. Furthermore, the diversity of the response to S6K in several unrelated cell types (fibroblasts, leukocytes**, and in**vasive cancer cells) that we report here indicates the existence of an underlying common mechanism in mammalian cells. This new signaling set, PA-S6K-FLNA-actin, sheds light for the first time into the morphogenic pathway of cytoskeletal structures that are crucial for adhesion and cell locomotion during inflammation and metastasis.

### [Mm]etastasis

#### PubMed

1. Androgen-induced cell migration: role of androgen receptor/filamin A association. Androgen receptor (AR) controls male morphogenesis, gametogenesis and prostate growth as well as development of prostate cancer. These findings support a role for AR in cell migration and invasiveness. However, the molecular mechanism involved in AR-mediated cell migration still remains elusive.Mouse embryo NIH3T3 fibroblasts and highly metastatic human fibrosarcoma HT1080 cells harbor low levels of transcriptionally incompetent AR. We now report that, through extra nuclear action, AR triggers migration of both cell types upon stimulation with physiological concentrations of the androgen R1881. We analyzed the initial events leading to androgen-induced cell migration and observed that challenging NIH3T3 cells with 10 nM R1881 rapidly induces interaction of AR with filamin A (FlnA) at cytoskeleton. AR/FlnA complex recruits integrin beta 1, thus activating its dependent cascade. Silencing of AR, FlnA and integrin beta 1 shows that this ternary complex controls focal adhesion kinase (FAK), paxillin and Rac, thereby driving cell migration. FAK-null fibroblasts migrate poorly and Rac inhibition by EHT impairs motility of androgen-treated NIH3T3 cells. Interestingly, FAK and Rac activation by androgens are independent of each other. Findings in human fibrosarcoma HT1080 cells strengthen the role of Rac in androgen signaling. The Rac inhibitor significantly impairs androgen-induced migration in these cells. A mutant AR, deleted of the sequence interacting with FlnA, fails to mediate FAK activation and paxillin tyrosine phosphorylation in androgen-stimulated cells, further reinforcing the role of AR/FlnA interaction in androgen-mediated motility.The present report, for the first time, indicates that the extra nuclear AR/FlnA/integrin beta 1 complex is the key by which androgen activates signaling leading to cell migration. Assembly of this ternary complex may control organ development and prostate cancer **metastasis**.
2. S6K is a morphogenic protein with a mechanism involving Filamin-A phosphorylation and phosphatidic acid binding. Change of cell shape in vivo plays many roles that are central to life itself, such as embryonic development, inflammation, wound healing, and pathologic processes such as cancer **metastasis**. Nonetheless, the spatiotemporal mechanisms that control the concerted regulation of cell shape remain understudied. Here, we show that ribosomal S6K, which is normally considered a protein involved in protein translation, is a morphogenic protein. Its presence in cells alters the overall organization of the cell surface and cell circularity [(4π × area)/(perimeter)(2)] from 0.47 ± 0.06 units in mock-treated cells to 0.09 ± 0.03 units in S6K-overexpressing macrophages causing stellation and arborization of cell shape. This effect was partially reversed in cells expressing a kinase-inactive S6K mutant and was fully reversed in cells silenced with small interference RNA. Equally important is that S6K is itself regulated by phospholipids, specifically phosphatidic acid, whereby 300 nM 1,2-dioleoyl-sn-glycero-3-phosphate (DOPA), but not the control 1,2-dioleoyl-sn-glycero-3-phosphocholine (DOPC), binds directly to S6K and causes an ∼ 2.9-fold increase in S6K catalytic activity. This was followed by an increase in Filamin A (FLNA) functionality as measured by phospho-FLNA (S(2152)) expression and by a subsequent elevation of actin nucleation. This reliance of S6K on phosphatidic acid (PA), a curvature-inducing phospholipid, explained the extra-large perimeter of cells that overexpressed S6K. Furthermore, the diversity of the response to S6K in several unrelated cell types (fibroblasts, leukocytes, and invasive cancer cells) that we report here indicates the existence of an underlying common mechanism in mammalian cells. This new signaling set, PA-S6K-FLNA-actin, sheds light for the first time into the morphogenic pathway of cytoskeletal structures that are crucial for adhesion and cell locomotion during inflammatio**n and meta**stasis.

#### GeneRIFs

1. A new signaling set, phosphatidic acid-S6K1-FLNA-actin, sheds light into the morphogenic pathway of cytoskeletal structures that are crucial for adhesion and cell locomotion during inflammation and **metastasis**.

---

## Flna

- NCBI Gene
- GeneRIF
- Pubmed

| Search Term | Rank | Fields |
| --- | --- | --- |
| [Mm]igration | 6 | PubMed(1) |
| [Ee]xtracellular [Mm]atrix | 8 | PubMed(1) |

  
hide/show details for Flna

### [Mm]igration

#### PubMed

1. Filamin A and Filamin B are co-expressed within neurons during periods of neuronal **migration** and can physically interact. Mutations in the X-linked gene Filamin A (FLNA) lead to the human neurological disorder, periventricular heterotopia (PH). Although PH is characterized by a failure in neuronal **migration** into the cerebral cortex with consequent formation of nodules in the ventricular and subventricular zones, many neurons appear to migrate normally, even in males, suggesting compensatory mechanisms. Here we characterize expression patterns for FlnA and a highly homologous protein Filamin B (FlnB) within the nervous system, in order to better understand their potential roles in cortical development. FlnA mRNA was widely expressed in all cortical layers while FlnB mRNA was most highly expressed in the ventricular and subventricular zones during development. In adulthood, widespread but reduced expression of FlnA and FlnB persisted throughout the cerebral cortex. FlnA and FlnB proteins were highly expressed in both the leading processes and somata of migratory neurons during corticogenesis. Postnatally, FlnA immunoreactivity was largely localized to the cell body with FlnB in the soma and neuropil during neuronal differentiation. In adulthood, diminished expression of both proteins localized to the cell soma and nucleus. Moreover, the putative FLNB homodimerization domain strongly interacted with itself or the corresponding homologous region of FLNA by yeast two-hybrid interaction, the two proteins co-localized within neuronal precursors by immunocytochemistry and the existence of FLNA-FLNB heterodimers could be detected by co-immunoprecipitation. These results suggest that FLNA and FLNB may form both homodimers and heterodimers and that their interaction could potentially compensate for the loss of FLNA function during cortical development within PH individuals.

### [Ee]xtracellular [Mm]atrix

#### PubMed

1. Different splice variants of filamin-B affect myogenesis, subcellular distribution, and determine binding to integrin [beta] subunits. Integrins connect the **extracellular matrix** with the cell interior, and transduce signals through interactions of their cytoplasmic tails with cytoskeletal and signaling proteins. Using the yeast two-hybrid system, we isolated a novel splice variant (filamin-Bvar-1) of the filamentous actin cross-linking protein, filamin-B, that interacts with the cytoplasmic domain of the integrin beta1A and beta1D subunits. RT-PCR analysis showed weak, but wide, expression of filamin-Bvar-1 and a similar splice variant of filamin-A (filamin-Avar-1) in human tissues. Furthermore, alternative splice variants of filamin-B and filamin-C, from which the flexible hinge-1 region is deleted (DeltaH1), were induced during in vitro differentiation of C2C12 mouse myoblasts. We show that both filamin-Avar-1 and filamin-Bvar-1 bind more strongly than their wild-type isoforms to different integrin beta subunits. The mere presence of the high-affinity binding site for beta1A is not sufficient for targeting the filamin-Bvar-1 construct to focal contacts. Interestingly, the simultaneous deletion of the H1 region is required for the localization of filamin-B at the tips of actin stress fibers. When expressed in C2C12 cells, filamin-Bvar-1(DeltaH1) accelerates their differentiation into myotubes. Furthermore, filamin-B variants lacking the H1 region induce the formation of thinner myotubes than those in cells containing variants with this region. These findings suggest that specific combinations of filamin mRNA splicing events modulate the organization of the actin cytoskeleton and the binding affinity for integrins.

---

## Chrd

- NCBI Gene
- GeneRIF
- Pubmed

| Search Term | Rank | Fields |
| --- | --- | --- |
| [Mm]igration | 6 | Process(1); PubMed(1) |
| ECM | 7 | PubMed(2) |
| [Ee]xtracellular [Mm]atrix | 8 | PubMed(4) |
| [Ii]nvasive | 10 | PubMed(1) |

  
hide/show details for Chrd

### [Mm]igration

#### Process

1. negative regulation of cell **migration**

#### PubMed

1. Endogenous bone morphogenetic protein antagonists regulate mammalian neural crest generation and survival. We demonstrate here that Chordin and Noggin function as bone morphogenetic protein (BMP) antagonists in vivo to promote mammalian neural crest development. Using Chrd and Nog single and compound mutants, we find that Noggin has a major role in promoting neural crest formation, in which Chordin is partially redundant. BMP signaling is increased in dorsal tissues lacking Noggin and is further increased when Chordin is also absent. The early neural crest domain is expanded with decreased BMP antagonism in vivo. Noggin and Chordin also regulate subsequent neural crest cell e**migration** from the neural tube. However, reduced levels of these BMP antagonists ultimately result in perturbation of neural crest cell derived peripheral nervous system and craniofacial skeletal elements. Such defects reflect, at least in part, a function to limit apoptosis in neural crest cells. Noggin and Chordin, therefore, function together to regulate both the generation and survival of neural crest cells in mammalian development.

### ECM

#### PubMed

1. Nell1-deficient mice have reduced expression of extracellular matrix proteins causing cranial and vertebral defects. The mammalian Nell1 gene encodes a protein kinase C-beta1 (PKC-beta1) binding protein that belongs to a new class of cell-signaling molecules controlling cell growth and differentiation. Over-expression of Nell1 in the developing cranial sutures in both human and mouse induces craniosynostosis, the premature fusion of the growing cranial bone fronts. Here, we report the generation, positional cloning and characterization of Nell1(6R), a recessive, neonatal-lethal point mutation in the mouse Nell1 gene, induced by N-ethyl-N-nitrosourea. Nell1(6R) has a T-->A base change that converts a codon for cysteine into a premature stop codon [Cys(502)Ter], resulting in severe truncation of the predicted protein product and marked reduction in steady-state levels of the transcript. In addition to the expected alteration of cranial morphology, Nell1(6R) mutants manifest skeletal defects in the vertebral column and ribcage, revealing a hitherto undefined role for Nell1 in signal transduction in endochondral ossification. Real-time quantitative reverse transcription-PCR assays of 219 genes showed an association between the loss of Nell1 function and reduced expression of genes for extracellular matrix (**ECM**) proteins critical for chondrogenesis and osteogenesis. Several affected genes are involved in the human cartilage disorder Ehlers-Danlos Syndrome and other disorders associated with spinal curvature anomalies. Nell1(6R) mutant mice are a new tool for elucidating basic mechanisms in osteoblast and chrondrocyte differentiation in the developing skull and vertebral column and understanding how perturbations in the production of **ECM** proteins can lead to anomalies in these structures.
2. Use of Bmp1/Tll1 doubly homozygous null mice and proteomics to identify and validate in vivo substrates of bone morphogenetic protein 1/tolloid-like metalloproteinases. Bone morphogenetic protein 1 (BMP-1) and mammalian Tolloid (mTLD), two proteinases encoded by Bmp1, provide procollagen C-proteinase (pCP) activity that converts procollagens I to III into the major fibrous components of mammalian extracellular matrix (**ECM**). Yet, although Bmp1(-/-) mice have aberrant collagen fibrils, they have residual pCP activity, indicative of genetic redundancy. Mammals possess two additional proteinases structurally similar to BMP-1 and mTLD: the genetically distinct mammalian Tolloid-like 1 (mTLL-1) and mTLL-2. Mice lacking the mTLL-1 gene Tll1 are embryonic lethal but have pCP activity levels similar to those of the wild type, suggesting that mTLL-1 might not be an in vivo pCP. In vitro studies have shown BMP-1 and mTLL-1 capable of cleaving Chordin, an extracellular antagonist of BMP signaling, suggesting that these proteases might also serve to modulate BMP signaling and to coordinate the latter with **ECM** formation. However, in vivo evidence of roles for BMP-1 and mTLL-1 in BMP signaling in mammals is lacking. To remove functional redundancy obscuring the in vivo functions of BMP-1-related proteases in mammals, we here characterize Bmp1 Tll1 doubly null mouse embryos. Although these appear morphologically indistinguishable from Tll1(-/-) embryos, biochemical analysis of cells derived from doubly null embryos shows functional redundancy removed to an extent enabling us to demonstrate that (i) products of Bmp1 and Tll1 are responsible for in vivo cleavage of Chordin in mammals and (ii) mTLL-1 is an in vivo pCP that provides residual activity observed in Bmp1(-/-) embryos. Removal of functional redundancy also enabled use of Bmp1(-/-) Tll1(-/-) cells in a proteomics approach for identifying novel substrates of Bmp1 and Tll1 products.

### [Ee]xtracellular [Mm]atrix

#### PubMed

1. Loss of procollagen IIA from the anterior mesendoderm disrupts the development of mouse embryonic forebrain. Morphogenesis of the mammalian forebrain is influenced by the patterning activity of signals emanating from the anterior mesendoderm. In this study, we show that procollagen IIA (IIA), an isoform of the cartilage **extracellular matrix** protein encoded by an alternatively spliced transcript of Col2a1, is expressed in the prechordal plate and the anterior definitive endoderm. In the absence of IIA activity, the null mutants displayed a partially penetrant phenotype of loss of head tissues, holoprosencephaly, and loss of mid-facial structures, which is associated with reduced sonic hedgehog (Shh) expression in the prechordal mesoderm. Genetic interaction studies reveal that IIA function in forebrain and face development does not involve bone morphogenetic protein receptor 1A (BMPR1A)- or NODAL-mediated signaling activity.
2. Nell1-deficient mice have reduced expression of **extracellular matrix** proteins causing cranial and vertebral defects. The mammalian Nell1 gene encodes a protein kinase C-beta1 (PKC-beta1) binding protein that belongs to a new class of cell-signaling molecules controlling cell growth and differentiation. Over-expression of Nell1 in the developing cranial sutures in both human and mouse induces craniosynostosis, the premature fusion of the growing cranial bone fronts. Here, we report the generation, positional cloning and characterization of Nell1(6R), a recessive, neonatal-lethal point mutation in the mouse Nell1 gene, induced by N-ethyl-N-nitrosourea. Nell1(6R) has a T-->A base change that converts a codon for cysteine into a premature stop codon [Cys(502)Ter], resulting in severe truncation of the predicted protein product and marked reduction in steady-state levels of the transcript. In addition to the expected alteration of cranial morphology, Nell1(6R) mutants manifest skeletal defects in the vertebral column and ribcage, revealing a hitherto undefined role for Nell1 in signal transduction in endochondral ossification. Real-time quantitative reverse transcription-PCR assays of 219 genes showed an association between the loss of Nell1 function and reduced expression of genes for **extracellular matrix** (ECM) proteins critical for chondrogenesis and osteogenesis. Several affected genes are involved in the human cartilage disorder Ehlers-Danlos Syndrome and other disorders associated with spinal curvature anomalies. Nell1(6R) mutant mice are a new tool for elucidating basic mechanisms in osteoblast and chrondrocyte differentiation in the developing skull and vertebral column and understanding how perturbations in the production of ECM proteins can lead to anomalies in these structures.
3. Man1, an inner nuclear membrane protein, regulates vascular remodeling by modulating transforming growth factor beta signaling. A growing number of integral inner nuclear membrane (INM) proteins have been implicated in diverse cellular functions. Man1, an INM protein, has recently been shown to regulate transforming growth factor (Tgf) beta superfamily signaling by interacting with receptor-associated Smads. However, the in vivo roles of Man1 have not been fully characterized. Here, we show that Man1 regulates vascular remodeling by analyzing Man1-deficient embryos lacking the Smad interacting domain. Man1-deficient embryos die at midgestation because of defects in embryonic vasculature; the primary capillary plexus forms, but subsequent remodeling is perturbed. It has been proposed that the angiogenesis process is divided into two balanced phases, the activation and resolution/maturation phases, both of which are regulated by Tgfbeta1. We have demonstrated, in Man1-deficient embryos, the expression of Tgfb1 is upregulated and Smad2/3 signaling is abnormally activated, resulting in increased **extracellular matrix** deposition, a hallmark of the resolution phase of angiogenesis. We have also showed that the recruitment of mural cells to the vascular wall is severely disturbed in mutants, which may lead to disruption of intercellular communication between endothelial and mural cells required for proper vascular remodeling. These results have revealed a novel role for Man1 in angiogenesis and provide the first evidence that vascular remodeling can be regulated at the INM through the interaction between Man1 and Smads.
4. Use of Bmp1/Tll1 doubly homozygous null mice and proteomics to identify and validate in vivo substrates of bone morphogenetic protein 1/tolloid-like metalloproteinases. Bone morphogenetic protein 1 (BMP-1) and mammalian Tolloid (mTLD), two proteinases encoded by Bmp1, provide procollagen C-proteinase (pCP) activity that converts procollagens I to III into the major fibrous components of mammalian **extracellular matrix** (ECM). Yet, although Bmp1(-/-) mice have aberrant collagen fibrils, they have residual pCP activity, indicative of genetic redundancy. Mammals possess two additional proteinases structurally similar to BMP-1 and mTLD: the genetically distinct mammalian Tolloid-like 1 (mTLL-1) and mTLL-2. Mice lacking the mTLL-1 gene Tll1 are embryonic lethal but have pCP activity levels similar to those of the wild type, suggesting that mTLL-1 might not be an in vivo pCP. In vitro studies have shown BMP-1 and mTLL-1 capable of cleaving Chordin, an extracellular antagonist of BMP signaling, suggesting that these proteases might also serve to modulate BMP signaling and to coordinate the latter with ECM formation. However, in vivo evidence of roles for BMP-1 and mTLL-1 in BMP signaling in mammals is lacking. To remove functional redundancy obscuring the in vivo functions of BMP-1-related proteases in mammals, we here characterize Bmp1 Tll1 doubly null mouse embryos. Although these appear morphologically indistinguishable from Tll1(-/-) embryos, biochemical analysis of cells derived from doubly null embryos shows functional redundancy removed to an extent enabling us to demonstrate that (i) products of Bmp1 and Tll1 are responsible for in vivo cleavage of Chordin in mammals and (ii) mTLL-1 is an in vivo pCP that provides residual activity observed in Bmp1(-/-) embryos. Removal of functional redundancy also enabled use of Bmp1(-/-) Tll1(-/-) cells in a proteomics approach for identifying novel substrates of Bmp1 and Tll1 products.

### [Ii]nvasive

#### PubMed

1. Mammary epithelial-specific expression of the integrin-linked kinase (ILK) results in the induction of mammary gland hyperplasias and tumors in transgenic mice. The integrin linked kinase (ILK) is a cytoplasmic effector of integrin receptors, involved in the regulation of integrin binding properties as well as the activation of cell survival and proliferative pathways, including those involving MAP kinase, PKB/Akt and GSK-3beta. Overexpression of ILK in cultured intestinal and mammary epithelial cells has been previously shown to induce changes characteristic of oncogenic transformation, including anchorage-independent growth, **invasive**ness, suppression of anoikis and tumorigenicity in nude mice. In order to determine if ILK overexpression can result in the formation of mammary tumors in vivo, we generated transgenic mice expressing ILK in the mammary epithelium, under the transcriptional control of the mouse mammary tumor virus (MMTV) long terminal repeat (LTR). By the age of 6 months, female MMTV/ILK mice developed a hyperplastic mammary phenotype, which was accompanied by the constitutive phosphorylation of PKB/Akt, GSK-3beta and MAP kinase. Focal mammary tumors subsequently appeared in 34% of the animals at an average age of 18 months. Given the focal nature and long latency of the tumors, however, additional genetic events are likely required for tumor induction in the MMTV/ILK mice. These results provide the first direct demonstration of a potential oncogenic role for ILK, which is upregulated in human tumors and tumor cell lines.

---

## Gch1

- NCBI Gene
- GeneRIF
- Pubmed

| Search Term | Rank | Fields |
| --- | --- | --- |
| [Mm]igration | 6 | PubMed(2) |
| [Ee]xtracellular [Mm]atrix | 8 | PubMed(1) |

  
hide/show details for Gch1

### [Mm]igration

#### PubMed

1. GTP cyclohydrolase I prevents diabetic-impaired endothelial progenitor cells and wound healing by suppressing oxidative stress/thrombospondin-1. Endothelial progenitor cell (EPC) dysfunction is a key contributor to diabetic refractory wounds. Endothelial nitric oxide synthase (eNOS), which critically regulates the mobilization and function of EPCs, is uncoupled in diabetes due to decreased cofactor tetrahydrobiopterin (BH4). We tested whether GTP cyclohydrolase I (GTPCH I), the rate-limiting enzyme of BH4 synthesis, preserves EPC function in type 1 diabetic mice. Type 1 diabetes was induced in wild-type (WT) and GTPCH I transgenic (Tg-GCH) mice by intraperitoneal injection of streptozotocin (STZ). EPCs were isolated from the peripheral blood and bone marrow of WT, Tg-GCH, and GTPCH I-deficient hph-1 mice. The number of EPCs was significantly lower in STZ-WT mice and hph-1 mice and was rescued in STZ Tg-GCH mice. Furthermore, GTPCH I overexpression improved impaired diabetic EPC **migration** and tube formation. EPCs from WT, Tg-GCH, and STZ-Tg-GCH mice were administered to diabetic excisional wounds and accelerated wound healing significantly, with a concomitant augmentation of angiogenesis. Flow cytometry measurements showed that intracellular nitric oxide (NO) levels were reduced significantly in STZ-WT and hph-1 mice, paralleled by increased superoxide anion levels; both were rescued in STZ-Tg-GCH mice. Western blot analysis revealed that thrombospondin-1 (TSP-1) was significantly upregulated in the EPCs of STZ-WT mice and hph-1 mice and suppressed in STZ-treated Tg-GCH mice. Our results demonstrate that the GTPCH I/BH4 pathway is critical to preserve EPC quantity, function, and regenerative capacity during wound healing in type 1 diabetic mice at least partly through the attenuation of superoxide and TSP-1 levels and augmentation of NO level.
2. Regulation of iNOS function and cellular redox state by macrophage Gch1 reveals specific requirements for tetrahydrobiopterin in NRF2 activation. Inducible nitric oxide synthase (iNOS) is a key enzyme in the macrophage inflammatory response, which is the source of nitric oxide (NO) that is potently induced in response to proinflammatory stimuli. However, the specific role of NO production, as distinct from iNOS induction, in macrophage inflammatory responses remains unproven. We have generated a novel mouse model with conditional deletion of Gch1, encoding GTP cyclohydrolase 1 (GTPCH), an essential enzyme in the biosynthesis of tetrahydrobiopterin (BH4) that is a required cofactor for iNOS NO production. Mice with a floxed Gch1 allele (Gch1(fl/fl)) were crossed with Tie2cre transgenic mice, causing Gch1 deletion in leukocytes (Gch1(fl/fl)Tie2cre). Macrophages from Gch1(fl/fl)Tie2cre mice lacked GTPCH protein and de novo biopterin biosynthesis. When activated with LPS and IFNγ, macrophages from Gch1(fl/fl)Tie2cre mice induced iNOS protein in a manner indistinguishable from wild-type controls, but produced no detectable NO, as judged by L-citrulline production, EPR spin trapping of NO, and by nitrite accumulation. Incubation of Gch1(fl/fl)Tie2cre macrophages with dihydroethidium revealed significantly increased production of superoxide in the presence of iNOS expression, and an iNOS-independent, BH4-dependent increase in other ROS species. Normal BH4 levels, nitric oxide production, and cellular redox state were restored by sepiapterin, a precursor of BH4 production by the salvage pathway, demonstrating that the effects of BH4 deficiency were reversible. Gch1(fl/fl)Tie2cre macrophages showed only minor alterations in cytokine production and normal cell **migratio**n, and minimal changes in basal gene expression. However, gene expression analysis after iNOS induction identified 78 genes that were altered between wild-type and Gch1(fl/fl)Tie2cre macrophages. Pathway analysis identified decreased NRF2 activation, with reduced induction of archetypal NRF2 genes (gclm, prdx1, gsta3, nqo1, and catalase) in BH4-deficient Gch1(fl/fl)Tie2cre macrophages. These findings identify BH4-dependent iNOS regulation and NO generation as specific requirements for NRF2-dependent responses in macrophage inflammatory activation.

### [Ee]xtracellular [Mm]atrix

#### PubMed

1. Endothelium-specific GTP cyclohydrolase I overexpression attenuates blood pressure progression in salt-sensitive low-renin hypertension. Tetrahydrobiopterin (BH4) is an essential cofactor of endothelial nitric oxide synthase (eNOS). When BH4 levels are decreased, eNOS becomes uncoupled to produce superoxide anion (O2(-)) instead of NO, which contributes to endothelial dysfunction. Deoxycorticosterone acetate (DOCA)-salt hypertension is characterized by a suppressed plasma renin level due to sodium retention but manifests in eNOS uncoupling; however, how endogenous BH4 regulates blood pressure is unknown. GTP cyclohydrolase I (GTPCH I) is the rate-limiting enzyme for de novo BH4 synthesis. This study tested the hypothesis that endothelium-specific GTPCH I overexpression retards the progression of hypertension through preservation of the structure and function of resistance mesenteric arteries.During 3 weeks of DOCA-salt treatment, arterial blood pressure was increased significantly in wild-type mice, as determined by radiotelemetry, but this increase was attenuated in transgenic mice with endothelium-specific GTPCH I overexpression (Tg-GCH). Arterial GTPCH I activity and BH4 levels were decreased significantly in wild-type DOCA-salt mice, but both were preserved in Tg-GCH mice despite DOCA-salt treatment. Significant remodeling of resistance mesenteric arteries (approximately 100-microm outside diameter) in wild-type DOCA-salt mice exists, evidenced by increased medial cross-sectional area, media thickness, and media-lumen ratio and overexpression of tenascin C, an **extracellular matrix** glycoprotein that contributes to hypertrophic remodeling; all of these effects were prevented in DOCA-salt-treated Tg-GCH mice. Furthermore, NO-mediated relaxation in mesenteric arteries was significantly improved in DOCA-salt-treated Tg-GCH mice, in parallel with reduced O2(-) levels. Finally, phosphorylation of eNOS at serine residue 1177 (eNOS-S1177), but not its dimer-monomer ratio, was decreased significantly in wild-type DOCA-salt mice compared with sham controls but was preserved in DOCA-salt-treated Tg-GCH mice.These results demonstrate that endothelium-specific GTPCH I overexpression abrogates O2(-) production and preserves eNOS phosphorylation, which results in preserved structural and functional integrity of resistance mesenteric arteries and lowered blood pressure in low-renin hypertension.

---

## Vcl

- NCBI Gene
- GeneRIF
- Pubmed

| Search Term | Rank | Fields |
| --- | --- | --- |
| [Ff]ilopodia | 1 | PubMed(2) |
| [Ii]nvadopodia | 3 | PubMed(2) |
| [Ii]nvadopodium | 4 | PubMed(1) |
| [Pp]odosome(s)? | 5 | PubMed(2); Component(1) |
| [Mm]igration | 6 | Process(1); PubMed(27); GeneRIFs(3) |
| ECM | 7 | PubMed(10); GeneRIFs(1) |
| [Ee]xtracellular [Mm]atrix | 8 | PubMed(22); GeneRIFs(1) |
| [Ii]nvasive | 10 | PubMed(3) |
| [Mm]etastasis | 12 | PubMed(4) |
| [Cc]ell [Ii]nvasion | 13 | PubMed(2); GeneRIFs(1) |

  
hide/show details for Vcl

### [Ff]ilopodia

#### PubMed

1. Muscle costameric protein, Chisel/Smpx, associates with focal adhesion complexes and modulates cell spreading in vitro via a Rac1/p38 pathway. The murine X-linked gene Chisel (Csl/Smpx) encodes a 9-kDa protein that associates in heart and skeletal muscle cells with the costameric cytoskeleton, implicated in maintaining muscle integrity and responses to biomechanical stress. After expression in C2C12 myoblasts, MYC epitope-tagged Csl co-localized with actin networks at peripheral membranes, and with focal adhesion proteins vinculin, paxillin, integrin beta1, and the small GTPase Rac1. Csl could be co-immunoprecipitated with vinculin from extracts of C2C12 cells and native muscle. MYC-Csl induced cell spreading and lamellipodia formation in C2C12 cells at the expense of **filopodia**, suggestive of modulation of Rac1 activity. Lamellipodia formation was indeed Rac1-dependent, and in MYC-Csl cells replated on fibronectin, Rac1 activity was increased relative to controls. Expression of MYC-Csl led to an increased association between vinculin and p34, a subunit of the Arp2/3 actin nucleation complex, a Rac1-dependent event. Induced cell spreading was also dependent upon p38 kinases that act downstream of Rac1 to control the actin capping activity of heat shock protein 27. Our data suggest that Csl localizes to the costameric cytoskeleton of muscle cells through an association with focal adhesion proteins, where it may participate in regulation of cytoskeletal dynamics through the Rac1-p38 pathway.
2. Mammalian Fat1 cadherin regulates actin dynamics and cell-cell contact. Fat cadherins form a distinct subfamily of the cadherin gene superfamily, and are featured by their unusually large extracellular domain. In this work, we investigated the function of a mammalian Fat cadherin. Fat1 was localized at **filopodia**l tips, lamellipodial edges, and cell-cell boundaries, overlapping with dynamic actin structures. RNA interference-mediated knockdown of Fat1 resulted in disorganization of cell junction-associated F-actin and other actin fibers/cables, disturbance of cell-cell contacts, and also inhibition of cell polarity formation at wound margins. Furthermore, we identified Ena/vasodilator-stimulated phosphoproteins as a potential downstream effector of Fat1. These results suggest that Fat1 regulates actin cytoskeletal organization at cell peripheries, thereby modulating cell contacts and polarity.

### [Ii]nvadopodia

#### PubMed

1. Src-dependent phosphorylation of ASAP1 regulates podosomes. **Invadopodia** are Src-induced cellular structures that are thought to mediate tumor invasion. ASAP1, an Arf GTPase-activating protein (GAP) containing Src homology 3 (SH3) and Bin, amphiphysin, and RVS161/167 (BAR) domains, is a substrate of Src that controls **invadopodia**. We have examined the structural requirements for ASAP1-dependent formation of **invadopodia** and related structures in NIH 3T3 fibroblasts called podosomes. We found that both predominant splice variants of ASAP1 (ASAP1a and ASAP1b) associated with **invadopodia** and podosomes. Podosomes were highly dynamic, with rapid turnover of both ASAP1 and actin. Reduction of ASAP1 levels by small interfering RNA blocked formation of **invadopodia** and podosomes. Podosomes were formed in NIH 3T3 fibroblasts in which endogenous ASAP1 was replaced with either recombinant ASAP1a or ASAP1b. ASAP1 mutants that lacked the Src binding site or GAP activity functioned as well as wild-type ASAP1 in the formation of podosomes. Recombinant ASAP1 lacking the BAR domain, the SH3 domain, or the Src phosphorylation site did not support podosome formation. Based on these results, we conclude that ASAP1 is a critical target of tyrosine kinase signaling involved in the regulation of podosomes and **invadopodia** and speculate that ASAP1 may function as a coincidence detector of simultaneous protein association through the ASAP1 SH3 domain and phosphorylation by Src.
2. Phosphoinositide 3-kinase p85beta regulates invadopodium formation. The acquisition of invasiveness is characteristic of tumor progression. Numerous genetic changes are associated with metastasis, but the mechanism by which a cell becomes invasive remains unclear. Expression of p85β, a regulatory subunit of phosphoinositide-3-kinase, markedly increases in advanced carcinoma, but its mode of action is unknown. We postulated that p85β might facilitate cell invasion. We show that p85β localized at cell adhesions in complex with focal adhesion kinase and enhanced stability and maturation of cell adhesions. In addition, p85β induced development at cell adhesions of an F-actin core that extended several microns into the cell z-axis resembling the skeleton **of invadop**odia. p85β lead to F-actin polymerization at cell adhesions by recruiting active Cdc42/Rac at these structures. In accordance with p85β function in invadopodium-like formation, p85β levels increased in metastatic melanoma and p85β depletion reduced invadopodium formation and invasion. These results show that p85β enhances invasion by inducing cell adhesion developm**ent into in**vadopodia-like structures explaining the metastatic potential of tumors with increased p85β levels.

### [Ii]nvadopodium

#### PubMed

1. Phosphoinositide 3-kinase p85beta regulates **invadopodium** formation. The acquisition of invasiveness is characteristic of tumor progression. Numerous genetic changes are associated with metastasis, but the mechanism by which a cell becomes invasive remains unclear. Expression of p85β, a regulatory subunit of phosphoinositide-3-kinase, markedly increases in advanced carcinoma, but its mode of action is unknown. We postulated that p85β might facilitate cell invasion. We show that p85β localized at cell adhesions in complex with focal adhesion kinase and enhanced stability and maturation of cell adhesions. In addition, p85β induced development at cell adhesions of an F-actin core that extended several microns into the cell z-axis resembling the skeleton of invadopodia. p85β lead to F-actin polymerization at cell adhesions by recruiting active Cdc42/Rac at these structures. In accordance with p85β functi**on in invado**podium-like formation, p85β levels increased in metastatic melanoma and p85β depletion **reduced inva**dopodium formation and invasion. These results show that p85β enhances invasion by inducing cell adhesion development into invadopodia-like structures explaining the metastatic potential of tumors with increased p85β levels.

### [Pp]odosome(s)?

#### PubMed

1. Src-dependent phosphorylation of ASAP1 regulates **podosomes**. Invadopodia are Src-induced cellular structures that are thought to mediate tumor invasion. ASAP1, an Arf GTPase-activating protein (GAP) containing Src homology 3 (SH3) and Bin, amphiphysin, and RVS161/167 (BAR) domains, is a substrate of Src that controls invadopodia. We have examined the structural requirements for ASAP1-dependent formation of invadopodia and related structures in NIH 3T3 fibroblasts called **podosomes**. We found that both predominant splice variants of ASAP1 (ASAP1a and ASAP1b) associated with invadopodia and **podosomes**. **Podosomes** were highly dynamic, with rapid turnover of both ASAP1 and actin. Reduction of ASAP1 levels by small interfering RNA blocked formation of invadopodia and **podosomes**. **Podosomes** were formed in NIH 3T3 fibroblasts in which endogenous ASAP1 was replaced with either recombinant ASAP1a or ASAP1b. ASAP1 mutants that lacked the Src binding site or GAP activity functioned as well as wild-type ASAP1 in the formation of **podosomes**. Recombinant ASAP1 lacking the BAR domain, the SH3 domain, or the Src phosphorylation site did not support **podosome** formation. Based on these results, we conclude that ASAP1 is a critical target of tyrosine kinase signaling involved in the regulation of **podosomes** and invadopodia and speculate that ASAP1 may function as a coincidence detector of simultaneous protein association through the ASAP1 SH3 domain and phosphorylation by Src.
2. The transient appearance of zipper-like actin superstructures during the fusion of osteoclasts. Multinucleated osteoclasts are responsible for bone resorption. Hypermultinucleated osteoclasts are often observed in some bone-related diseases such as Paget's disease and cherubism. The cellular mechanics controlling the size of osteoclasts is poorly understood. We introduced EGFP-actin into RAW 264.7 cells to monitor actin dynamics during osteoclast differentiation. Before their terminal differentiation into osteoclasts, syncytia displayed two main types of actin assembly, **podosome** clusters and clusters of zipper-like structures. The zipper-like structures morphologically resembled the adhesion zippers found at the initial stage of cell-cell adhesion in keratinocytes. In the zipper-like structure, Arp3 and cortactin overlapped with the distribution of dense F-actin, whereas integrin β3, paxillin and vinculin were localized to the periphery of the structure. The structure was negative for WGA-lectin staining and biotin labeling. The zipper-like structure broke down and transformed into a large actin ring, called a **podosom**e belt. Syncytia containing clusters of zipper-like structures had more nuclei than those with **podosom**e clusters. Differentiated osteoclasts with a **podosom**e belt also formed the zipper-like structure at the cell contact site during cell fusion. The breakdown of the cell contact site resulted in the fusion of the **podosom**e belts following plasma membrane fusion. Additionally, osteoclasts in mouse calvariae formed the zipper-like structure in the sealing zone. Therefore, we propose that the zipper-like actin superstructures might be involved in cell-cell interaction to achieve efficient multinucleation of osteoclasts. Understanding of the zipper-like structure might lead to selective therapeutics for bone diseases caused by hypermultinucleated osteoclasts.

#### Component

1. **podosome**

### [Mm]igration

#### Process

1. regulation of cell **migration**

#### PubMed

1. Brefeldin A-inhibited guanine exchange factor 2 regulates filamin A phosphorylation and neuronal **migration**. Periventricular heterotopia (PH) is a human malformation of cortical development associated with gene mutations in ADP-ribosylation factor guanine exchange factor 2 (ARFGEF2 encodes for Big2 protein) and Filamin A (FLNA). PH is thought to derive from neuroependymal disruption, but the extent to which neuronal **migration** contributes to this phenotype is unknown. Here, we show that Arfgef2 null mice develop PH and exhibit impaired neural **migration** with increased protein expression for both FlnA and phosphoFlnA at Ser2152. Big2 physically interacts with FlnA and overexpression of phosphomimetic Ser2512 FLNA impairs neuronal **migration**. FlnA phosphorylation directs FlnA localization toward the cell cytoplasm, diminishes its binding affinity to actin skeleton, and alters the number and size of paxillin focal adhesions. Collectively, our results demonstrate a molecular mechanism whereby Big2 inhibition promotes phosphoFlnA (Ser2152) expression, and increased phosphoFlnA impairs its actin binding affinity and the distribution of focal adhesions, thereby disrupting cell intrinsic neuronal **migration**.
2. Loss of migfilin expression has no overt consequences on murine development and homeostasis. Migfilin is a LIM-domain-containing protein of the zyxin family of adaptor proteins and is found at cell-matrix and cell-cell adhesion sites and in the nucleus. In vitro studies have suggested that migfilin promotes β1 integrin activity, regulates cell spreading and **migratio**n and induces cardiomyocyte differentiation. To test directly the function of migfilin in vivo, we generated a migfilin-null mouse strain. Here, we report that loss of migfilin expression permits normal development and normal postnatal aging. Fibroblasts and keratinocytes from migfilin-null mice display normal spreading and adhesion, and normal integrin expression and activation. The **migratio**n velocity and directionality of migfilin-null embryonic fibroblasts were normal, whereas the velocity of migfilin-null keratinocytes in wound scratch assays was slightly but significantly reduced. Our findings indicate that the roles of migfilin are functionally redundant during mouse development and tissue homeostasis.
3. β3 integrin in cardiac fibroblast is critical for extracellular matrix accumulation during pressure overload hypertrophy in mouse. The adhesion receptor β3 integrin regulates diverse cellular functions in various tissues. As β3 integrin has been implicated in extracellular matrix (ECM) remodeling, we sought to explore the role of β3 integrin in cardiac fibrosis by using wild type (WT) and β3 integrin null (β3-/-) mice for in vivo pressure overload (PO) and in vitro primary cardiac fibroblast phenotypic studies. Compared to WT mice, β3-/- mice upon pressure overload hypertrophy for 4 wk by transverse aortic constriction (TAC) showed a substantially reduced accumulation of interstitial fibronectin and collagen. Moreover, pressure overloaded LV from β3-/- mice exhibited reduced levels of both fibroblast proliferation and fibroblast-specific protein-1 (FSP1) expression in early time points of PO. To test if the observed impairment of ECM accumulation in β3-/- mice was due to compromised cardiac fibroblast function, we analyzed primary cardiac fibroblasts from WT and β3-/- mice for adhesion to ECM proteins, cell spreading, prolifera**tion, and** migration in response to platelet derived growth factor-BB (PDGF, a growth factor known to promote fibrosis) stimulation. Our results showed that β3-/- cardiac fibroblasts exhibited a significant reduction in cell-matrix adhesion, cell spreading, prolife**ration an**d migration. In addition, the activation of PDGF receptor associated tyrosine kinase and non-receptor tyrosine kinase Pyk2, upon PDGF stimulation were impaired in β3-/- cells. Adenoviral expression of a dominant negative form of Pyk2 (Y402F) resulted in reduced accumulation of fibronectin. These results indicate that β3 integrin-mediated Pyk2 signaling in cardiac fibroblasts plays a critical role in PO-induced cardiac fibrosis.
4. Contractility modulates cell adhesion strengthening through focal adhesion kinase and assembly of vinculin-containing focal adhesions. Actin-myosin contractility modulates focal adhesion assembly, stress fiber formation, and cell **migration**. We analyzed the contributions of contractility to fibroblast adhesion strengthening using a hydrodynamic adhesion assay and micropatterned substrates to control cell shape and adhesive area. Serum addition resulted in adhesion strengthening to levels 30-40% higher than serum-free cultures. Inhibition of myosin light chain kinase or Rho-kinase blocked phosphorylation of myosin light chain to similar extents and eliminated the serum-induced enhancements in strengthening. Blebbistatin-induced inhibition of myosin II reduced serum-induced adhesion strength to similar levels as those obtained by blocking myosin light chain phosphorylation. Reductions in adhesion strengthening by inhibitors of contractility correlated with loss of vinculin and talin from focal adhesions without changes in integrin binding. In vinculin-null cells, inhibition of contractility did not alter adhesive force, whereas controls displayed a 20% reduction in adhesion strength, indicating that the effects of contractility on adhesive force are vinculin-dependent. Furthermore, in cells expressing FAK, inhibitors of contractility reduced serum-induced adhesion strengthening as well as eliminated focal adhesion assembly. In contrast, in the absence of FAK, these inhibitors did not alter adhesion strength or focal adhesion assembly. These results indicate that contractility modulates adhesion strengthening via FAK-dependent, vinculin-containing focal adhesion assembly.
5. Phosphorylation of mouse LASP-1 on threonine 156 by cAMP- and cGMP-dependent protein kinase. LIM and SH3 domain protein (LASP-1) is a specific focal adhesion protein involved in cell **migration**. Overlay studies demonstrate that LASP-1 directly binds to the proline-rich domains of zyxin, lipoma preferred partner (LPP), and vasodilator-stimulated phosphoprotein (VASP), with zyxin being the most prominent interacting partner. Despite the LIM/zinc-finger domain, hypothesized to be involved in homodimerization, LASP-1 exists as a monomer. In vitro phosphorylation of recombinant mouse LASP-1 by cAMP- and cGMP-dependent protein kinase (PKA and PKG, respectively) occurs at serine 61, serine 99, and threonine 156 whereas in intact cells mouse LASP-1 is phosphorylated only at threonine 156. This site is different from the known in vivo phosphorylation sites in human (serine 146) and rabbit (serine 99 and serine 146). Nevertheless, immunofluorescence of LASP-1 in human and mouse mesangial cells revealed no difference in subcellular distribution. Exposure of the cells to forskolin induced a translocation of both, human and mouse LASP-1, from the focal contacts to the cell interior without affecting F-actin structure. Immunoblotting of LASP-1 in various mouse and human tissues detected a similar prominent expression in non-muscle tissue. Altogether, our data suggest so far no functional differences between human and mouse LASP-1.
6. Vinculin controls focal adhesion formation by direct interactions with talin and actin. Focal adhesions (FAs) regulate cell **migration**. Vinculin, with its many potential binding partners, can interconnect signals in FAs. Despite the well-characterized structure of vinculin, the molecular mechanisms underlying its action have remained unclear. Here, using vinculin mutants, we separate the vinculin head and tail regions into distinct functional domains. We show that the vinculin head regulates integrin dynamics and clustering and the tail regulates the link to the mechanotransduction force machinery. The expression of vinculin constructs with unmasked binding sites in the head and tail regions induces dramatic FA growth, which is mediated by their direct interaction with talin. This interaction leads to clustering of activated integrin and an increase in integrin residency time in FAs. Surprisingly, paxillin recruitment, induced by active vinculin constructs, occurs independently of its potential binding site in the vinculin tail. The vinculin tail, however, is responsible for the functional link of FAs to the actin cytoskeleton. We propose a new model that explains how vinculin orchestrates FAs.
7. Alpha5beta1, alphaVbeta3 and the platelet-associated integrin alphaIIbbeta3 coordinately regulate adhesion and **migration** of differentiating mouse trophoblast cells. During blastocyst implantation, interaction between integrins on the apical surface of the trophoblast and extracellular matrix (ECM) in the endometrium anchors the embryo to the uterine wall. Strong adhesion of the blastocyst to fibronectin (FN) requires integrin signaling initiated by exogenous fibronectin. However, it is not known how integrin signaling enhances blastocyst adhesion. We present new evidence that the integrin, alphaIIbbeta3, plays a key role in trophoblast adhesion to fibronectin during mouse peri-implantation development. Trafficking of alphaIIb to the apical surface of the trophoblast increased dramatically after blastocysts were exposed to fibronectin, whereas other fibronectin-binding integrins, alpha5beta1 and alphaVbeta3, were resident at the apical surface before ligand exposure. Functional comparisons among the three integrins revealed that ligation of alpha5beta1 most efficiently strengthened blastocyst fibronectin-binding activity, while subsequent trophoblast cell **migration** was dependent primarily on the beta3-class integrins. In vivo, alphaIIb was highly expressed by invasive trophoblast cells in the ectoplacental cone and trophoblast giant cells of the parietal yolk sac. These data demonstrate that trafficking of alphaIIb regulates adhesion between trophoblast cells and fibronectin as invasion of the endometrium commences.
8. Clathrin mediates integrin endocytosis for focal adhesion disassembly in migrating cells. Focal adhesion disassembly is regulated by microtubules (MTs) through an unknown mechanism that involves dynamin. To test whether endocytosis may be involved, we interfered with the function of clathrin or its adaptors autosomal recessive hypercholesteremia (ARH) and Dab2 (Disabled-2) and found that both treatments prevented MT-induced focal adhesion disassembly. Surface labeling experiments showed that integrin was endocytosed in an extracellular matrix-, clathrin-, and ARH- and Dab2-dependent manner before entering Rab5 endosomes. Clathrin colocalized with a subset of focal adhesions in an ARH- and Dab2-dependent fashion. Direct imaging showed that clathrin rapidly accumulated on focal adhesions during MT-stimulated disassembly and departed from focal adhesions with integrin upon their disassembly. In migrating cells, depletion of clathrin or Dab2 and ARH inhibited focal adhesion disassembly and decreased the rate of **migration**. These results show that focal adhesion disassembly occurs through a targeted mechanism involving MTs, clathrin, and specific clathrin adaptors and that direct endocytosis of integrins from focal adhesions mediates their disassembly in migrating cells.
9. PTB deficiency causes the loss of adherens junctions in the dorsal telencephalon and leads to lethal hydrocephalus. Polypyrimidine tract-binding protein (PTB) is a well-characterized RNA-binding protein and known to be preferentially expressed in neural stem cells (NSCs) in the central nervous system; however, its role in NSCs in the developing brain remains unclear. To explore the role of PTB in embryonic NSCs in vivo, Nestin-Cre-mediated conditional Ptb knockout mice were generated for this study. In the mutant forebrain, despite the depletion of PTB protein, neither abnormal neurogenesis nor flagrant morphological abnormalities were observed at embryonic day 14.5 (E14.5). Nevertheless, by 10 weeks, nearly all mutant mice succumbed to hydrocephalus (HC), which was caused by a lack of the ependymal cell layer in the dorsal cortex. Upon further analysis, a gradual loss of adherens junctions (AJs) was observed in the ventricular zone (VZ) of the dorsal telencephalon in the mutant brains, beginning at E14.5. In the AJs-deficient VZ, impaired interkinetic nuclear **migration** and precocious differentiation of NSCs were observed after E14.5. These findings demonstrated that PTB depletion in the dorsal telencephalon is causally involved in the development of HC and that PTB is important for the maintenance of AJs in the NSCs of the dorsal telencephalon.
10. Cell adhesion strengthening: contributions of adhesive area, integrin binding, and focal adhesion assembly. Mechanical interactions between a cell and its environment regulate **migration**, contractility, gene expression, and cell fate. We integrated micropatterned substrates to engineer adhesive area and a hydrodynamic assay to analyze fibroblast adhesion strengthening on fibronectin. Independently of cell spreading, integrin binding and focal adhesion assembly resulted in rapid sevenfold increases in adhesion strength to steady-state levels. Adhesive area strongly modulated adhesion strength, integrin binding, and vinculin and talin recruitment, exhibiting linear increases for small areas. However, above a threshold area, adhesion strength and focal adhesion assembly reached a saturation limit, whereas integrin binding transitioned from a uniform distribution to discrete complexes. Adhesion strength exhibited exponential increases with bound integrin numbers as well as vinculin and talin recruitment, and the relationship between adhesion strength and these biochemical events was accurately described by a simple mechanical model. Furthermore, adhesion strength was regulated by the position of an adhesive patch, comprised of bound integrins and cytoskeletal elements, which generated a constant 200-nN adhesive force. Unexpectedly, focal adhesion assembly, in particular vinculin recruitment, contributed only 30% of the adhesion strength. This work elucidates the roles of adhesive complex size and position in the generation of cell-extracellular matrix forces.
11. Vinculin controls talin engagement with the actomyosin machinery. The link between extracellular-matrix-bound integrins and intracellular F-actin is essential for cell spreading and **migration**. Here, we demonstrate how the actin-binding proteins talin and vinculin cooperate to provide this link. By expressing structure-based talin mutants in talin null cells, we show that while the C-terminal actin-binding site (ABS3) in talin is required for adhesion complex assembly, the central ABS2 is essential for focal adhesion (FA) maturation. Thus, although ABS2 mutants support cell spreading, the cells lack FAs, fail to polarize and exert reduced force on the surrounding matrix. ABS2 is inhibited by the preceding mechanosensitive vinculin-binding R3 domain, and deletion of R2R3 or expression of constitutively active vinculin generates stable force-independent FAs, although cell polarity is compromised. Our data suggest a model whereby force acting on integrin-talin complexes via ABS3 promotes R3 unfolding and vinculin binding, activating ABS2 and locking talin into an actin-binding configuration that stabilizes FAs.
12. Mice carrying a complete deletion of the talin2 coding sequence are viable and fertile. Mice homozygous for several Tln2 gene targeted alleles are viable and fertile. Here we show that although the expression of talin2 protein is drastically reduced in muscle from these mice, other tissues continue to express talin2 albeit at reduced levels. We therefore generated a Tln2 allele lacking the entire coding sequence (Tln2(cd)). Tln2(cd/cd) mice were viable and fertile, and the genotypes of Tln2(cd/+) intercrosses were at the expected Mendelian ratio. Tln2(cd/cd) mice showed no major difference in body mass or the weight of the major organs compared to wild-type, although they displayed a mildly dystrophic phenotype. Moreover, Tln2(cd/cd) mouse embryo fibroblasts showed no obvious defects in cell adhesion, **migration** or proliferation. However, the number of Tln2(cd/cd) pups surviving to adulthood was variable suggesting that such mice have an underlying defect.
13. Rac1 regulates pancreatic islet morphogenesis. Pancreatic islets of Langerhans originate from endocrine progenitors within the pancreatic ductal epithelium. Concomitant with differentiation of these progenitors into hormone-producing cells such cells delaminate, aggregate and migrate away from the ductal epithelium. The cellular and molecular mechanisms regulating islet cell delamination and cell **migration** are poorly understood. Extensive biochemical and cell biological studies using cultured cells demonstrated that Rac1, a member of the Rho family of small GTPases, acts as a key regulator of cell **migration**.To address the functional role of Rac1 in islet morphogenesis, we generated transgenic mice expressing dominant negative Rac1 under regulation of the Rat Insulin Promoter. Blocking Rac1 function in beta cells inhibited their **migration** away from the ductal epithelium in vivo. Consistently, transgenic islet cell spreading was compromised in vitro. We also show that the EGF-receptor ligand betacellulin induced actin remodelling and cell spreading in wild-type islets, but not in transgenic islets. Finally, we demonstrate that cell-cell contact E-cadherin increased as a consequence of blocking Rac1 activity.Our data support a model where Rac1 signalling controls islet cell **migration** by modulating E-cadherin-mediated cell-cell adhesion. Furthermore, in vitro experiments show that betacellulin stimulated islet cell spreading and actin remodelling is compromised in transgenic islets, suggesting that betacellulin may act as a regulator of Rac1 activity and islet **migration** in vivo. Our results further emphasize Rac1 as a key regulator of cell **migration** and cell adhesion during tissue and organ morphogenesis.
14. Deletion of integrin-linked kinase from neural crest cells in mice results in aortic aneurysms and embryonic lethality. Neural crest cells (NCCs) participate in the remodeling of the cardiac outflow tract and pharyngeal arch arteries during cardiovascular development. Integrin-linked kinase (ILK) is a serine/threonine kinase and a major regulator of integrin signaling. It links integrins to the actin cytoskeleton and recruits other adaptor molecules into a large complex to regulate actin dynamics and integrin function. Using the Cre-lox system, we deleted Ilk from NCCs of mice to investigate its role in NCC morphogenesis. The resulting mutants developed a severe aneurysmal arterial trunk that resulted in embryonic lethality during late gestation. Ilk mutants showed normal cardiac NCC **migration** but reduced differentiation into smooth muscle within the aortic arch arteries and the outflow tract. Within the conotruncal cushions, Ilk-deficient NCCs exhibited disorganization of F-actin stress fibers and a significantly rounder morphology, with shorter cellular projections. Additionally, absence of ILK resulted in reduced in vivo phosphorylation of Smad3 in NCCs, which correlated with reduced αSMA levels. Our findings resemble those seen in Pinch1 and β1 integrin conditional mutant mice, and therefore support that, in neural crest-derived cells, ILK and Pinch1 act as cytoplasmic effectors of β1 integrin in a pathway that protects against aneurysms. In addition, our conditional Ilk mutant mice might prove useful as a model to study aortic aneurysms caused by reduced Smad3 signaling, as occurs in the newly described aneurysms-osteoarthritis syndrome, for example.
15. Vinculin knockout results in heart and brain defects during embryonic development. The vinculin gene codes for a cytoskeletal protein, found in focal adhesion plaques and in cell-cell adherens junctions. Vinculin was inactivated by homologous recombination using a targeting vector in embryonic stem (ES) cells. The heterozygous ES cells were introduced into mice by established procedures to produce heterozygous animals that were normal and fertile. No homozygous vinculin-/- embryos were born and analyses during the gestational period showed that the vinculin null embryos were small and abnormal from day E8 but some survived until E10. The most prominent defect was lack of midline fusion of the rostral neural tube, producing a cranial bilobular appearance and attenuation of cranial and spinal nerve development. Heart development was curtailed at E9.5, with severely reduced and akinetic myocardial and endocardial structures. Mutant embryos were 30-40% smaller, somites and limbs were retarded and ectodermal tissues were sparse and fragile. Fibroblasts (MEF) isolated from mutant embryos were shown to have reduced adhesion to fibronectin, vitronectin, laminin and collagen compared to wild-type levels. In addition, **migration** rates over these substrata were two-fold higher and the level of focal adhesion kinase (FAK) activity was three-fold higher. We conclude that vinculin is necessary for normal embryonic development, probably because of its role in the regulation of cell adhesion and locomotion, cell behaviors essential for normal embryonic morphogenesis, although specific roles in neural and cardiac development cannot be ruled out.
16. Vinculin arrests motile B cells by stabilizing integrin clustering at the immune synapse. Lymphocytes use integrin-based platforms to move and adhere firmly to the surface of other cells. The molecular mechanisms governing lymphocyte adhesion dynamics are however poorly understood. In this study, we show that in mouse B lymphocytes, the actin binding protein vinculin localizes to the ring-shaped integrin-rich domain of the immune synapse (IS); the assembly of this platform, triggered by cognate immune interactions, is needed for chemokine-mediated B cell motility arrest and leads to firm, long-lasting B cell adhesion to the APC. Vinculin is recruited early in IS formation, in parallel to a local phosphatidylinositol (4,5)-bisphosphate wave, and requires spleen tyrosine kinase activity. Lack of vinculin at the IS impairs firm adhesion, promoting, in turn, cell **migration** with Ag clustered at the uropod. Vinculin localization to the B cell contact area depends on actomyosin. These results identify vinculin as a major controller of integrin-mediated adhesion dynamics in B cells.
17. Role of vinculin in regulating focal adhesion turnover. Although vinculin (-/-) mouse embryo fibroblasts assemble focal adhesions (FAs), they spread more slowly, less extensively, and close a wound more rapidly than vinculin (+/+) cells. To investigate the structure and dynamics of FAs in these cells, we used real-time interference reflection microscopy (IRM) thus avoiding the need to express exogenous GFP-tagged FA proteins which may be misregulated. This showed that the FAs were smaller, less abundant and turned over more rapidly in vinculin null compared to wild-type cells. Expression of vinculin rescued the spreading defect and resulted in larger and more stable FAs. Phosphatidylinositol 4,5-bisphosphate (PIP2) is thought to play a role in vinculin activation by relieving an intramolecular association between the vinculin head (Vh) and tail (Vt) that masks the ligand binding sites in Vh and Vt. To investigate the role of the vinculin/PIP2 interaction in FA dynamics, we used a vinculin mutant lacking the C-terminal arm (residues 1053-1066) and referred to as the deltaC mutation. This mutation reduced PIP2 binding to a Vt deltaC polypeptide by >90% compared to wild type without affecting binding to Vh or F-actin. Interestingly, cells expressing the vinculin deltaC mutant assembled remarkably stable FAs. The results suggest that vinculin inhibits cell **migration** by stabilising FAs, and that binding of inositol phospholipids to Vt plays an important role in FA turnover.
18. Vinculin-dependent actin bundling regulates cell **migration** and traction forces. Vinculin binding to actin filaments is thought to be critical for force transduction within a cell, but direct experimental evidence to support this conclusion has been limited. In the present study, we found mutation (R1049E) of the vinculin tail impairs its ability to bind F-actin, stimulate actin polymerization, and bundle F-actin in vitro. Further, mutant (R1049E) vinculin expressing cells are altered in cell **migration**, which is accompanied by changes in cell adhesion, cell spreading and cell generation of traction forces, providing direct evidence for the critical role of vinculin in mechanotransduction at adhesion sites. Lastly, we discuss the viability of models detailing the F-actin-binding surface on vinculin in the context of our mutational analysis.
19. Mutations in the paxillin-binding site of integrin-linked kinase (ILK) destabilize the pseudokinase domain and cause embryonic lethality in mice. Integrin-linked kinase (ILK) localizes to focal adhesions (FAs) where it regulates cell spreading, **migration**, and growth factor receptor signaling. Previous reports showed that overexpressed ILK in which Val(386) and Thr(387) were substituted with glycine residues (ILK-VT/GG) could neither interact with paxillin nor localize to FA in cells expressing endogenous wild-type ILK, implying that paxillin binding to ILK is required for its localization to FAs. Here, we show that introducing this mutation into the germ line of mice (ILK-VT/GG) caused vasculogenesis defects, resulting in a general developmental delay and death at around embryonic day 12.5. Fibroblasts isolated from ILK-VT/GG mice contained mutant ILK in FAs, showed normal adhesion to and spreading on extracellular matrix substrates but displayed impaired **migration**. Biochemical analysis revealed that VT/GG substitutions decreased ILK protein stability leading to decreased ILK levels and reduced binding to paxillin and α-parvin. Because paxillin depletion did not affect ILK localization to FAs, the embryonic lethality and the in vitro **migratio**n defects are likely due to the reduced levels of ILK-VT/GG and diminished binding to parvins.
20. Conditional knockout of focal adhesion kinase in endothelial cells reveals its role in angiogenesis and vascular development in late embryogenesis. Focal adhesion kinase (FAK) is a critical mediator of signal transduction by integrins and growth factor receptors in a variety of cells including endothelial cells (ECs). Here, we describe EC-specific knockout of FAK using a Cre-loxP approach. In contrast to the total FAK knockout, deletion of FAK specifically in ECs did not affect early embryonic development including normal vasculogenesis. However, in late embryogenesis, FAK deletion in the ECs led to defective angiogenesis in the embryos, yolk sac, and placenta, impaired vasculature and associated hemorrhage, edema, and developmental delay, and late embryonic lethal phenotype. Histologically, ECs and blood vessels in the mutant embryos present a disorganized, detached, and apoptotic appearance. Consistent with these phenotypes, deletion of FAK in ECs isolated from the floxed FAK mice led to reduced tubulogenesis, cell survival, proliferation, and **migration** in vitro. Together, these results strongly suggest a role of FAK in angiogenesis and vascular development due to its essential function in the regulation of multiple EC activities.
21. Vinculin is required for cell polarization, **migration**, and extracellular matrix remodeling in 3D collagen. Vinculin is filamentous (F)-actin-binding protein enriched in integrin-based adhesions to the extracellular matrix (ECM). Whereas studies in 2-dimensional (2D) tissue culture models have suggested that vinculin negatively regulates cell **migration** by promoting cytoskeleton-ECM coupling to strengthen and stabilize adhesions, its role in regulating cell **migration** in more physiologic, 3-dimensional (3D) environments is unclear. To address the role of vinculin in 3D cell **migration**, we analyzed the morphodynamics, **migration**, and ECM remodeling of primary murine embryonic fibroblasts (MEFs) with cre/loxP-mediated vinculin gene disruption in 3D collagen I cultures. We found that vinculin promoted 3D cell **migration** by increasing directional persistence. Vinculin was necessary for persistent cell protrusion, cell elongation, and stable cell orientation in 3D collagen, but was dispensable for lamellipodia formation, suggesting that vinculin-mediated cell adhesion to the ECM is needed to convert actin-based cell protrusion into persistent cell shape change and **migration**. Consistent with this finding, vinculin was necessary for efficient traction force generation in 3D collagen without affecting myosin II activity and promoted 3D collagen fiber alignment and macroscopical gel contraction. Our results suggest that vinculin promotes directionally persistent cell **migration** and tension-dependent ECM remodeling in complex 3D environments by increasing cell-ECM adhesion and traction force generation.
22. Force fluctuations within focal adhesions mediate ECM-rigidity sensing to guide directed cell **migration**. Cell **migration** toward areas of higher extracellular matrix (ECM) rigidity via a process called "durotaxis" is thought to contribute to development, immune response, and cancer metastasis. To understand how cells sample ECM rigidity to guide durotaxis, we characterized cell-generated forces on the nanoscale within single mature integrin-based focal adhesions (FAs). We found that individual FAs act autonomously, exhibiting either stable or dynamically fluctuating ("tugging") traction. We show that a FAK/phosphopaxillin/vinculin pathway is essential for high FA traction and to enable tugging FA traction over a broad range of ECM rigidities. We show that tugging FA traction is dispensable for FA maturation, chemotaxis, and haptotaxis but is critical to direct cell **migratio**n toward rigid ECM. We conclude that individual FAs dynamically sample rigidity by applying fluctuating pulling forces to the ECM to act as sensors to guide durotaxis, and that FAK/phosphopaxillin/vinculin signaling defines the rigidity range over which this dynamic sensing process operates.
23. Endothelial cell **migration** during murine yolk sac vascular remodeling occurs by means of a Rac1 and FAK activation pathway in vivo. The molecular mechanism(s) controlling cell **migration** during vascular morphogenesis in vivo remain largely undefined. To address this within a physiological context, we used retinaldehyde dehydrogenase-2 (Raldh2) null mouse embryos and demonstrate that retinoic acid (RA) deficiency results in abnormal yolk sac vascular remodeling due to decreased Rac1 activation, increased RhoA activation, and increased focal adhesions. Vinculin was increased in Raldh2-/- yolk sacs, and molecular events important for focal adhesion turnover, FAK phosphorylation (Tyr397) and FAK-paxillin association, were decreased. RA-rescue of vascular remodeling down-regulated vinculin and restored FAK phosphorylation (Tyr397) and FAK-paxillin association. Furthermore, vascular rescue with vascular endothelial growth factor-A, Indian hedgehog, and basic fibroblast growth factor restored FAK phosphorylation (Tyr397) in the endothelium of Raldh2-/- yolk sacs. Our results provide new insights into the regulation of endothelial cell **migration** during vascular remodeling in vivo by adding the Rac1 and FAK activation pathway as a critical mediator of focal adhesion formation and turnover during vascular remodeling.
24. Role of FIP200 in cardiac and liver development and its regulation of TNFalpha and TSC-mTOR signaling pathways. Focal adhesion kinase family interacting protein of 200 kD (FIP200) has been shown to regulate diverse cellular functions such as cell size, proliferation, and **migration** in vitro. However, the function of FIP200 in vivo has not been investigated. We show that targeted deletion of FIP200 in the mouse led to embryonic death at mid/late gestation associated with heart failure and liver degeneration. We found that FIP200 knockout (KO) embryos show reduced S6 kinase activation and cell size as a result of increased tuberous sclerosis complex function. Furthermore, FIP200 KO embryos exhibited significant apoptosis in heart and liver. Consistent with this, FIP200 KO mouse embryo fibroblasts and liver cells showed increased apoptosis and reduced c-Jun N-terminal kinase phosphorylation in response to tumor necrosis factor (TNF) alpha stimulation, which might be mediated by FIP200 interaction with apoptosis signal-regulating kinase 1 (ASK1) and TNF receptor-associated factor 2 (TRAF2), regulation of TRAF2-ASK1 interaction, and ASK1 phosphorylation. Together, our results reveal that FIP200 functions as a regulatory node to couple two important signaling pathways to regulate cell growth and survival during mouse embryogenesis.
25. Anchorage of vinculin to lipid membranes influences cell mechanical properties. The focal adhesion protein vinculin (1066 residues) can be separated into a 95-kDa head and a 30-kDa tail domain. Vinculin's lipid binding sites localized on the tail, helix 3 (residues 944-978) and the unstructured C-terminal arm (residues 1052-1066, the so-called lipid anchor), influence focal adhesion turnover and are important for cell **migration** and adhesion. Using magnetic tweezers, we characterized the cell mechanical behavior in mouse embryonic fibroblast (MEF)-vin(-/-) cells transfected with EGFP-linked-vinculin deficient of the lipid anchor (vinDeltaC, residues 1-1051). MEF-vinDeltaC cells incubated with fibronectin-coated paramagnetic beads were less stiff, and more beads detached during these experiments compared to MEF-rescue cells. Cells expressing vinDeltaC formed fewer focal contacts as determined by confocal microscopy. Two-dimensional traction measurements showed that MEF-vinDeltaC cells generate less force compared to rescue cells. Attenuated traction forces were also found in cells that expressed vinculin with point mutations (R1060 and K1061 to Q) of the lipid anchor that impaired lipid binding. However, traction generation was not diminished in cells that expressed vinculin with impaired lipid binding caused by point mutations on helix 3. Mutating the src-phosphorylation site (Y1065 to F) resulted in reduced traction generation. These observations show that both the lipid binding and the src-phosphorylation of vinculin's C-terminus are important for cell mechanical behavior.
26. Recruitment of the Arp2/3 complex to vinculin: coupling membrane protrusion to matrix adhesion. Cell **migration** involves many steps, including membrane protrusion and the development of new adhesions. Here we have investigated whether there is a link between actin polymerization and integrin engagement. In response to signals that trigger membrane protrusion, the actin-related protein (Arp)2/3 complex transiently binds to vinculin, an integrin-associated protein. The interaction is regulated, requiring phosphatidylinositol-4,5-bisphosphate and Rac1 activation, and is sufficient to recruit the Arp2/3 complex to new sites of integrin aggregation. Binding of the Arp2/3 complex to vinculin is direct and does not depend on the ability of vinculin to associate with actin. We have mapped the binding site for the Arp2/3 complex to the hinge region of vinculin, and a point mutation in this region selectively blocks binding to the Arp2/3 complex. Compared with WT vinculin, expression of this mutant in vinculin-null cells results in diminished lamellipodial protrusion and spreading on fibronectin. The recruitment of the Arp2/3 complex to vinculin may be one mechanism through which actin polymerization and membrane protrusion are coupled to integrin-mediated adhesion.
27. Inactivation of Cdc42 in neural crest cells causes craniofacial and cardiovascular morphogenesis defects. Neural crest cells (NCCs) are physically responsible for craniofacial skeleton formation, pharyngeal arch artery remodeling and cardiac outflow tract septation during vertebrate development. Cdc42 (cell division cycle 42) is a Rho family small GTP-binding protein that works as a molecular switch to regulate cytoskeleton remodeling and the establishment of cell polarity. To investigate the role of Cdc42 in NCCs during embryonic development, we deleted Cdc42 in NCCs by crossing Cdc42 flox mice with Wnt1-cre mice. We found that the inactivation of Cdc42 in NCCs caused embryonic lethality with craniofacial deformities and cardiovascular developmental defects. Specifically, Cdc42 NCC knockout embryos showed fully penetrant cleft lips and short snouts. Alcian Blue and Alizarin Red staining of the cranium exhibited an unfused nasal capsule and palatine in the mutant embryos. India ink intracardiac injection analysis displayed a spectrum of cardiovascular developmental defects, including persistent truncus arteriosus, hypomorphic pulmonary arteries, interrupted aortic arches, and right-sided aortic arches. To explore the underlying mechanisms of Cdc42 in the formation of the great blood vessels, we generated Wnt1Cre-Cdc42-Rosa26 reporter mice. By beta-galactosidase staining, a subpopulation of Cdc42-null NCCs was observed halting in their **migration** midway from the pharyngeal arches to the conotruncal cushions. Phalloidin staining revealed dispersed, shorter and disoriented stress fibers in Cdc42-null NCCs. Finally, we demonstrated that the inactivation of Cdc42 in NCCs impaired bone morphogenetic protein 2 (BMP2)-induced NCC cytoskeleton remodeling and **migration**. In summary, our results demonstrate that Cdc42 plays an essential role in NCC **migration**, and inactivation of Cdc42 in NCCs impairs craniofacial and cardiovascular development in mice.

#### GeneRIFs

1. results suggest that vinculin promotes directionally persistent cell **migration** and tension-dependent ECM remodeling in complex 3D environments by increasing cell-ECM adhesion and traction force generation.
2. Mutant vinculin expressing cells are altered in cell **migration**, which is accompanied by changes in cell adhesion.
3. The results suggest that vinculin inhibits cell **migration** by stabilising FAs, and that binding of inositol phospholipids to Vt plays an important role in FA turnover.

### ECM

#### PubMed

1. β3 integrin in cardiac fibroblast is critical for extracellular matrix accumulation during pressure overload hypertrophy in mouse. The adhesion receptor β3 integrin regulates diverse cellular functions in various tissues. As β3 integrin has been implicated in extracellular matri**x (**ECM) remodeling, we sought to explore the role of β3 integrin in cardiac fibrosis by using wild type (WT) and β3 integrin null (β3-/-) mice for in vivo pressure overload (PO) and in vitro primary cardiac fibroblast phenotypic studies. Compared to WT mice, β3-/- mice upon pressure overload hypertrophy for 4 wk by transverse aortic constriction (TAC) showed a substantially reduced accumulation of interstitial fibronectin and collagen. Moreover, pressure overloaded LV from β3-/- mice exhibited reduced levels of both fibroblast proliferation and fibroblast-specific protein-1 (FSP1) expression in early time points of PO. To test if the observed impair**men**t of ECM accumulation in β3-/- mice was due to compromised cardiac fibroblast function, we analyzed primary cardiac fibroblasts from WT and β3-/- mice for ad**hes**ion to ECM proteins, cell spreading, proliferation, and migration in response to platelet derived growth factor-BB (PDGF, a growth factor known to promote fibrosis) stimulation. Our results showed that β3-/- cardiac fibroblasts exhibited a significant reduction in cell-matrix adhesion, cell spreading, proliferation and migration. In addition, the activation of PDGF receptor associated tyrosine kinase and non-receptor tyrosine kinase Pyk2, upon PDGF stimulation were impaired in β3-/- cells. Adenoviral expression of a dominant negative form of Pyk2 (Y402F) resulted in reduced accumulation of fibronectin. These results indicate that β3 integrin-mediated Pyk2 signaling in cardiac fibroblasts plays a critical role in PO-induced cardiac fibrosis.
2. Myosin II activity regulates vinculin recruitment to focal adhesions through FAK-mediated paxillin phosphorylation. Focal adhesions (FAs) are mechanosensitive adhesion and signaling complexes that grow and change composition in response to myosin II-mediated cytoskeletal tension in a process known as FA maturation. To understand tension-mediated FA maturation, we sought to identify proteins that are recruited to FAs in a myosin II-dependent manner and to examine the mechanism for their myosin II-sensitive FA association. We find that FA recruitment of both the cytoskeletal adapter protein vinculin and the tyrosine kinase FA kinase (FAK) are myosin II and extracellular matrix (**ECM**) stiffness dependent. Myosin II activity promotes FAK/Src-mediated phosphorylation of paxillin on tyrosines 31 and 118 and vinculin association with paxillin. We show that phosphomimic mutations of paxillin can specifically induce the recruitment of vinculin to adhesions independent of myosin II activity. These results reveal an important role for paxillin in adhesion mechanosensing via myosin II-mediated FAK phosphorylation of paxillin that promotes vinculin FA recruitment to reinforce the cytoskeletal **ECM** linkage and drive FA maturation.
3. Alpha5beta1, alphaVbeta3 and the platelet-associated integrin alphaIIbbeta3 coordinately regulate adhesion and migration of differentiating mouse trophoblast cells. During blastocyst implantation, interaction between integrins on the apical surface of the trophoblast and extracellular matrix (**ECM**) in the endometrium anchors the embryo to the uterine wall. Strong adhesion of the blastocyst to fibronectin (FN) requires integrin signaling initiated by exogenous fibronectin. However, it is not known how integrin signaling enhances blastocyst adhesion. We present new evidence that the integrin, alphaIIbbeta3, plays a key role in trophoblast adhesion to fibronectin during mouse peri-implantation development. Trafficking of alphaIIb to the apical surface of the trophoblast increased dramatically after blastocysts were exposed to fibronectin, whereas other fibronectin-binding integrins, alpha5beta1 and alphaVbeta3, were resident at the apical surface before ligand exposure. Functional comparisons among the three integrins revealed that ligation of alpha5beta1 most efficiently strengthened blastocyst fibronectin-binding activity, while subsequent trophoblast cell migration was dependent primarily on the beta3-class integrins. In vivo, alphaIIb was highly expressed by invasive trophoblast cells in the ectoplacental cone and trophoblast giant cells of the parietal yolk sac. These data demonstrate that trafficking of alphaIIb regulates adhesion between trophoblast cells and fibronectin as invasion of the endometrium commences.
4. How vinculin regulates force transmission. Focal adhesions mediate force transfer between **ECM**-integrin complexes and the cytoskeleton. Although vinculin has been implicated in force transmission, few direct measurements have been made, and there is little mechanistic insight. Using vinculin-null cells expressing vinculin mutants, we demonstrate that vinculin is not required for transmission of adhesive and traction forces but is necessary for myosin contractility-dependent adhesion strength and traction force and for the coupling of cell area and traction force. Adhesion strength and traction forces depend differentially on vinculin head (V(H)) and tail domains. V(H) enhances adhesion strength by increasing **ECM**-bound integrin-talin complexes, independently from interactions with vinculin tail ligands and contractility. A full-length, autoinhibition-deficient mutant (T12) increases adhesion strength compared with VH, implying roles for both vinculin activation and the actin-binding tail. In contrast to adhesion strength, vinculin-dependent traction forces absolutely require a full-length and activated molecule; V(H) has no effect. Physical linkage of the head and tail domains is required for maximal force responses. Residence times of vinculin in focal adhesions, but not T12 or V(H), correlate with applied force, supporting a mechanosensitive model for vinculin activation in which forces stabilize vinculin's active conformation to promote force transfer.
5. Transient induction of vinculin gene expression in 3T3 fibroblasts stimulated by serum-growth factors. When stimulated with serum, quiescent Balb/C-3T3 fibroblasts were found to induce vinculin transcription transiently within 30 min, followed by accumulation of vinculin mRNA and protein synthesis between 2 and 4 h after stimulation and a decrease to the basal level by 6-8 h. Platelet-derived growth factor (PDGF), fibroblast growth factor (FGF), and 12-O-tetradecanoylphorbol-13-acetate (TPA) each could elicit a similar response, albeit to a lesser extent, whereas epidermal growth factor (EGF) was inefficient in inducing vinculin expression. In cells stimulated with serum and cycloheximide, vinculin expression was superinduced and vinculin mRNA levels persisted longer than in cells stimulated with serum alone. Cells arrested in the presence of serum by anchorage denial in methyl cellulose suspension culture also induced vinculin expression and formed large vinculin positive plaques when reattaching and spreading on the substrate in the presence of serum. Cells replated from suspension culture in the absence of serum on either plastic or extracellular matrix (**ECM**) components were capable of extensive spreading, but failed to elevate vinculin expression and displayed diffuse vinculin staining. The results indicate that the changes in vinculin organization and expression in response to growth factor stimulation may reflect either a necessary step in the progression through the cell cycle or a response related to complex cellular processes such as wound repair and embryogenesis.
6. Nanoscale architecture of integrin-based cell adhesions. Cell adhesions to the extracellular matrix (**ECM**) are necessary for morphogenesis, immunity and wound healing. Focal adhesions are multifunctional organelles that mediate cell-**ECM** adhesion, force transmission, cytoskeletal regulation and signalling. Focal adhesions consist of a complex network of trans-plasma-membrane integrins and cytoplasmic proteins that form a <200-nm plaque linking th**e E**CM to the actin cytoskeleton. The complexity of focal adhesion composition and dynamics implicate an intricate molecular machine. However, focal adhesion molecular architecture remains unknown. Here we used three-dimensional super-resolution fluorescence microscopy (interferometric photoactivated localization microscopy) to map nanoscale protein organization in focal adhesions. Our results reveal that integrins and actin are vertically separated by a ∼40-nm focal adhesion core region consisting of multiple protein-specific strata: a membrane-apposed integrin signalling layer containing integrin cytoplasmic tails, focal adhesion kinase and paxillin; an intermediate force-transduction layer containing talin and vinculin; and an uppermost actin-regulatory layer containing zyxin, vasodilator-stimulated phosphoprotein and α-actinin. By localizing amino- and carboxy-terminally tagged talins, we reveal talin's polarized orientation, indicative of a role in organizing the focal adhesion strata. The composite multilaminar protein architecture provides a molecular blueprint for understanding focal adhesion functions.
7. Vinculin-actin interaction couples actin retrograde flow to focal adhesions, but is dispensable for focal adhesion growth. In migrating cells, integrin-based focal adhesions (FAs) assemble in protruding lamellipodia in association with rapid filamentous actin (F-actin) assembly and retrograde flow. How dynamic F-actin is coupled to FA is not known. We analyzed the role of vinculin in integrating F-actin and FA dynamics by vinculin gene disruption in primary fibroblasts. Vinculin slowed F-actin flow in maturing FA to establish a lamellipodium-lamellum border and generate high extracellular matrix (**ECM**) traction forces. In addition, vinculin promoted nascent FA formation and turnover in lamellipodia and inhibited the frequency and rate of FA maturation. Characterization of a vinculin point mutant that specifically disrupts F-actin binding showed that vinculin-F-actin interaction is critical for these functions. However, FA growth rate correlated with F-actin flow speed independently of vinculin. Thus, vinculin functions as a molecular clutch, organizing leading edge F-actin, generating **ECM** traction, and promoting FA formation and turnover, but vinculin is dispensible for FA growth.
8. Vinculin facilitates cell invasion into three-dimensional collagen matrices. The cytoskeletal protein vinculin contributes to the mechanical link of the contractile actomyosin cytoskeleton to the extracellular matrix (**ECM**) through integrin receptors. In addition, vinculin modulates the dynamics of cell adhesions and is associated with decreased cell motility on two-dimensional **ECM** substrates. The effect of vinculin on cell invasion through dense three-dimensional **ECM** gels is unknown. Here, we report how vinculin expression affects cell invasion into three-dimensional collagen matrices. Cell motility was investigated in vinculin knockout and vinculin expressing wild-type mouse embryonic fibroblasts. Vinculin knockout cells were 2-fold more motile on two-dimensional collagen-coated substrates compared with wild-type cells, but 3-fold less invasive in 2.4 mg/ml three-dimensional collagen matrices. Vinculin knockout cells were softer and remodeled their cytoskeleton more dynamically, which is consistent with their enhanced two-dimensional motility but does not explain their reduced three-dimensional invasiveness. Importantly, vinculin-expressing cells adhered more strongly to collagen and generated 3-fold higher traction forces compared with vinculin knockout cells. Moreover, vinculin-expressing cells were able to migrate into dense (5.8 mg/ml) three-dimensional collagen matrices that were impenetrable for vinculin knockout cells. These findings suggest that vinculin facilitates three-dimensional matrix invasion through up-regulation or enhanced transmission of traction forces that are needed to overcome the steric hindrance of **ECM**s.
9. Vinculin is required for cell polarization, migration, and extracellular matrix remodeling in 3D collagen. Vinculin is filamentous (F)-actin-binding protein enriched in integrin-based adhesions to the extracellular matrix (**ECM**). Whereas studies in 2-dimensional (2D) tissue culture models have suggested that vinculin negatively regulates cell migration by promoting cytoskeleton-**ECM** coupling to strengthen and stabilize adhesions, its role in regulating cell migration in more physiologic, 3-dimensional (3D) environments is unclear. To address the role of vinculin in 3D cell migration, we analyzed the morphodynamics, migration, and **ECM** remodeling of primary murine embryonic fibroblasts (MEFs) with cre/loxP-mediated vinculin gene disruption in 3D collagen I cultures. We found that vinculin promoted 3D cell migration by increasing directional persistence. Vinculin was necessary for persistent cell protrusion, cell elongation, and stable cell orientation in 3D collagen, but was dispensable for lamellipodia formation, suggesting that vinculin-mediated cell adhesion to the **ECM** is needed to convert actin-based cell protrusion into persistent cell shape change and migration. Consistent with this finding, vinculin was necessary for efficient traction force generation in 3D collagen without affecting myosin II activity and promoted 3D collagen fiber alignment and macroscopical gel contraction. Our results suggest that vinculin promotes directionally persistent cell migration and tension-dependent **ECM** remodeling in complex 3D environments by increasing cell-**ECM** adhesion and traction force generation.
10. Force fluctuations within focal adhesions mediate **ECM**-rigidity sensing to guide directed cell migration. Cell migration toward areas of higher extracellular matrix (**ECM**) rigidity via a process called "durotaxis" is thought to contribute to development, immune response, and cancer metastasis. To understand how cells sample **ECM** rigidity to guide durotaxis, we characterized cell-generated forces on the nanoscale within single mature integrin-based focal adhesions (FAs). We found that individual FAs act autonomously, exhibiting either stable or dynamically fluctuating ("tugging") traction. We show that a FAK/phosphopaxillin/vinculin pathway is essential for high FA traction and to enable tugging FA traction over a broad range of **ECM** rigidities. We show that tugging FA traction is dispensable for FA maturation, chemotaxis, and haptotaxis but is critical to direct cell migration toward rigid **EC**M. We conclude that individual FAs dynamically sample rigidity by applying fluctuating pulling forces to the **EC**M to act as sensors to guide durotaxis, and that FAK/phosphopaxillin/vinculin signaling defines the rigidity range over which this dynamic sensing process operates.

#### GeneRIFs

1. results suggest that vinculin promotes directionally persistent cell migration and tension-dependent **ECM** remodeling in complex 3D environments by increasing cell-**ECM** adhesion and traction force generation.

### [Ee]xtracellular [Mm]atrix

#### PubMed

1. Different splice variants of filamin-B affect myogenesis, subcellular distribution, and determine binding to integrin [beta] subunits. Integrins connect the **extracellular matrix** with the cell interior, and transduce signals through interactions of their cytoplasmic tails with cytoskeletal and signaling proteins. Using the yeast two-hybrid system, we isolated a novel splice variant (filamin-Bvar-1) of the filamentous actin cross-linking protein, filamin-B, that interacts with the cytoplasmic domain of the integrin beta1A and beta1D subunits. RT-PCR analysis showed weak, but wide, expression of filamin-Bvar-1 and a similar splice variant of filamin-A (filamin-Avar-1) in human tissues. Furthermore, alternative splice variants of filamin-B and filamin-C, from which the flexible hinge-1 region is deleted (DeltaH1), were induced during in vitro differentiation of C2C12 mouse myoblasts. We show that both filamin-Avar-1 and filamin-Bvar-1 bind more strongly than their wild-type isoforms to different integrin beta subunits. The mere presence of the high-affinity binding site for beta1A is not sufficient for targeting the filamin-Bvar-1 construct to focal contacts. Interestingly, the simultaneous deletion of the H1 region is required for the localization of filamin-B at the tips of actin stress fibers. When expressed in C2C12 cells, filamin-Bvar-1(DeltaH1) accelerates their differentiation into myotubes. Furthermore, filamin-B variants lacking the H1 region induce the formation of thinner myotubes than those in cells containing variants with this region. These findings suggest that specific combinations of filamin mRNA splicing events modulate the organization of the actin cytoskeleton and the binding affinity for integrins.
2. β3 integrin in cardiac fibroblast is critical for **extracellular matri**x accumulation during pressure overload hypertrophy in mouse. The adhesion receptor β3 integrin regulates diverse cellular functions in various tissues. As β3 integrin has been implicated **in extracellular mat**rix (ECM) remodeling, we sought to explore the role of β3 integrin in cardiac fibrosis by using wild type (WT) and β3 integrin null (β3-/-) mice for in vivo pressure overload (PO) and in vitro primary cardiac fibroblast phenotypic studies. Compared to WT mice, β3-/- mice upon pressure overload hypertrophy for 4 wk by transverse aortic constriction (TAC) showed a substantially reduced accumulation of interstitial fibronectin and collagen. Moreover, pressure overloaded LV from β3-/- mice exhibited reduced levels of both fibroblast proliferation and fibroblast-specific protein-1 (FSP1) expression in early time points of PO. To test if the observed impairment of ECM accumulation in β3-/- mice was due to compromised cardiac fibroblast function, we analyzed primary cardiac fibroblasts from WT and β3-/- mice for adhesion to ECM proteins, cell spreading, proliferation, and migration in response to platelet derived growth factor-BB (PDGF, a growth factor known to promote fibrosis) stimulation. Our results showed that β3-/- cardiac fibroblasts exhibited a significant reduction in cell-matrix adhesion, cell spreading, proliferation and migration. In addition, the activation of PDGF receptor associated tyrosine kinase and non-receptor tyrosine kinase Pyk2, upon PDGF stimulation were impaired in β3-/- cells. Adenoviral expression of a dominant negative form of Pyk2 (Y402F) resulted in reduced accumulation of fibronectin. These results indicate that β3 integrin-mediated Pyk2 signaling in cardiac fibroblasts plays a critical role in PO-induced cardiac fibrosis.
3. Myosin II activity regulates vinculin recruitment to focal adhesions through FAK-mediated paxillin phosphorylation. Focal adhesions (FAs) are mechanosensitive adhesion and signaling complexes that grow and change composition in response to myosin II-mediated cytoskeletal tension in a process known as FA maturation. To understand tension-mediated FA maturation, we sought to identify proteins that are recruited to FAs in a myosin II-dependent manner and to examine the mechanism for their myosin II-sensitive FA association. We find that FA recruitment of both the cytoskeletal adapter protein vinculin and the tyrosine kinase FA kinase (FAK) are myosin II and **extracellular matrix** (ECM) stiffness dependent. Myosin II activity promotes FAK/Src-mediated phosphorylation of paxillin on tyrosines 31 and 118 and vinculin association with paxillin. We show that phosphomimic mutations of paxillin can specifically induce the recruitment of vinculin to adhesions independent of myosin II activity. These results reveal an important role for paxillin in adhesion mechanosensing via myosin II-mediated FAK phosphorylation of paxillin that promotes vinculin FA recruitment to reinforce the cytoskeletal ECM linkage and drive FA maturation.
4. Tyrosine phosphorylation of vinculin at position 1065 modifies focal adhesion dynamics and cell tractions. Focal adhesions (FAs) connect the cellular actin cytoskeleton via integrin with the **extracellular matrix**. They comprise of many structural and signaling proteins which are highly dynamic, well regulated, and responsible for the sensing of physical properties from the environment. Vinculin is a protein that incorporates all these functions. Here, we investigated the phosphorylation of Y1065 in the activation/regulation of vinculin. We used different vinculin mutants mimicking either a permanently activated or inhibited phosphorylation site at position 1065. Using these mutants, we determined their influence on the exchange dynamics and cell forces using fluorescence recovery after photobleaching and traction microscopy. The results indicate that phosphorylation at Y1065 significantly increases the amount of freely exchanging vinculin within FAs whereas inhibition of this phosphorylation site leads to an uncontrolled exchange of vinculin and reduced adhesive cell forces. In conclusion, we show that phosphorylation on position Y1065 is essential for accurate incorporation of vinculin into FAs and mechanical behavior of cells.
5. Alpha5beta1, alphaVbeta3 and the platelet-associated integrin alphaIIbbeta3 coordinately regulate adhesion and migration of differentiating mouse trophoblast cells. During blastocyst implantation, interaction between integrins on the apical surface of the trophoblast and **extracellular matrix** (ECM) in the endometrium anchors the embryo to the uterine wall. Strong adhesion of the blastocyst to fibronectin (FN) requires integrin signaling initiated by exogenous fibronectin. However, it is not known how integrin signaling enhances blastocyst adhesion. We present new evidence that the integrin, alphaIIbbeta3, plays a key role in trophoblast adhesion to fibronectin during mouse peri-implantation development. Trafficking of alphaIIb to the apical surface of the trophoblast increased dramatically after blastocysts were exposed to fibronectin, whereas other fibronectin-binding integrins, alpha5beta1 and alphaVbeta3, were resident at the apical surface before ligand exposure. Functional comparisons among the three integrins revealed that ligation of alpha5beta1 most efficiently strengthened blastocyst fibronectin-binding activity, while subsequent trophoblast cell migration was dependent primarily on the beta3-class integrins. In vivo, alphaIIb was highly expressed by invasive trophoblast cells in the ectoplacental cone and trophoblast giant cells of the parietal yolk sac. These data demonstrate that trafficking of alphaIIb regulates adhesion between trophoblast cells and fibronectin as invasion of the endometrium commences.
6. Clathrin mediates integrin endocytosis for focal adhesion disassembly in migrating cells. Focal adhesion disassembly is regulated by microtubules (MTs) through an unknown mechanism that involves dynamin. To test whether endocytosis may be involved, we interfered with the function of clathrin or its adaptors autosomal recessive hypercholesteremia (ARH) and Dab2 (Disabled-2) and found that both treatments prevented MT-induced focal adhesion disassembly. Surface labeling experiments showed that integrin was endocytosed in an **extracellular matrix**-, clathrin-, and ARH- and Dab2-dependent manner before entering Rab5 endosomes. Clathrin colocalized with a subset of focal adhesions in an ARH- and Dab2-dependent fashion. Direct imaging showed that clathrin rapidly accumulated on focal adhesions during MT-stimulated disassembly and departed from focal adhesions with integrin upon their disassembly. In migrating cells, depletion of clathrin or Dab2 and ARH inhibited focal adhesion disassembly and decreased the rate of migration. These results show that focal adhesion disassembly occurs through a targeted mechanism involving MTs, clathrin, and specific clathrin adaptors and that direct endocytosis of integrins from focal adhesions mediates their disassembly in migrating cells.
7. Vinculin regulates the recruitment and release of core focal adhesion proteins in a force-dependent manner. Cells sense the extracellular environment using adhesion receptors (integrins) linked to the intracellular actin cytoskeleton through a complex network of regulatory proteins that, all together, form focal adhesions (FAs). The molecular basis of how these sensing units are regulated, how they are implicated in transducing mechanical stimuli, and how this leads to a spatiotemporal coordination of FAs is unclear.Here we show that vinculin, through its links to the talin-integrin complex and F-actin, regulates the transmission of mechanical signals from the **extracellular matrix** to the actomyosin machinery. We demonstrate that the vinculin interaction with the talin-integrin complex drives the recruitment and release of core FA components. The activation state of vinculin is itself regulated by force, as underscored by our observation that vinculin localization to FAs is dependent on actomyosin contraction. Using a variety of vinculin mutants, we establish which components of the cell-matrix adhesion network are coordinated through direct and indirect associations with vinculin. Moreover, using cyclic stretching, we demonstrate that vinculin plays a key role in the transmission of extracellular mechanical stimuli leading to the reorganization of cell polarity. Of particular importance is the actin-binding tail region of vinculin, without which the cell's ability to repolarize in response to cyclic stretching is perturbed.Overall our data promote a model whereby vinculin controls the transmission of intracellular and extracellular mechanical cues that are important for the spatiotemporal assembly, disassembly, and reorganization of FAs to coordinate polarized cell motility.
8. Cell adhesion strengthening: contributions of adhesive area, integrin binding, and focal adhesion assembly. Mechanical interactions between a cell and its environment regulate migration, contractility, gene expression, and cell fate. We integrated micropatterned substrates to engineer adhesive area and a hydrodynamic assay to analyze fibroblast adhesion strengthening on fibronectin. Independently of cell spreading, integrin binding and focal adhesion assembly resulted in rapid sevenfold increases in adhesion strength to steady-state levels. Adhesive area strongly modulated adhesion strength, integrin binding, and vinculin and talin recruitment, exhibiting linear increases for small areas. However, above a threshold area, adhesion strength and focal adhesion assembly reached a saturation limit, whereas integrin binding transitioned from a uniform distribution to discrete complexes. Adhesion strength exhibited exponential increases with bound integrin numbers as well as vinculin and talin recruitment, and the relationship between adhesion strength and these biochemical events was accurately described by a simple mechanical model. Furthermore, adhesion strength was regulated by the position of an adhesive patch, comprised of bound integrins and cytoskeletal elements, which generated a constant 200-nN adhesive force. Unexpectedly, focal adhesion assembly, in particular vinculin recruitment, contributed only 30% of the adhesion strength. This work elucidates the roles of adhesive complex size and position in the generation of cell-**extracellular matrix** forces.
9. Transient induction of vinculin gene expression in 3T3 fibroblasts stimulated by serum-growth factors. When stimulated with serum, quiescent Balb/C-3T3 fibroblasts were found to induce vinculin transcription transiently within 30 min, followed by accumulation of vinculin mRNA and protein synthesis between 2 and 4 h after stimulation and a decrease to the basal level by 6-8 h. Platelet-derived growth factor (PDGF), fibroblast growth factor (FGF), and 12-O-tetradecanoylphorbol-13-acetate (TPA) each could elicit a similar response, albeit to a lesser extent, whereas epidermal growth factor (EGF) was inefficient in inducing vinculin expression. In cells stimulated with serum and cycloheximide, vinculin expression was superinduced and vinculin mRNA levels persisted longer than in cells stimulated with serum alone. Cells arrested in the presence of serum by anchorage denial in methyl cellulose suspension culture also induced vinculin expression and formed large vinculin positive plaques when reattaching and spreading on the substrate in the presence of serum. Cells replated from suspension culture in the absence of serum on either plastic or **extracellular matrix** (ECM) components were capable of extensive spreading, but failed to elevate vinculin expression and displayed diffuse vinculin staining. The results indicate that the changes in vinculin organization and expression in response to growth factor stimulation may reflect either a necessary step in the progression through the cell cycle or a response related to complex cellular processes such as wound repair and embryogenesis.
10. Vinculin phosphorylation at residues Y100 and Y1065 is required for cellular force transmission. The focal adhesion protein vinculin connects the actin cytoskeleton, through talin and integrins, with the **extracellular matrix**. Vinculin consists of a globular head and tail domain, which undergo conformational changes from a closed auto-inhibited conformation in the cytoplasm to an open conformation in focal adhesions. Src-mediated phosphorylation has been suggested to regulate this conformational switch. To explore the role of phosphorylation in vinculin activation, we used knock-out mouse embryonic fibroblasts re-expressing different vinculin mutants in traction microscopy, magnetic tweezer microrheology, FRAP and actin-binding assays. Compared to cells expressing wild-type or constitutively active vinculin, we found reduced tractions, cytoskeletal stiffness, adhesion strength, and increased vinculin dynamics in cells expressing constitutively inactive vinculin or vinculin where Src-mediated phosphorylation was blocked by replacing tyrosine at position 100 and/or 1065 with a non-phosphorylatable phenylalanine residue. Replacing tyrosine residues with phospho-mimicking glutamic acid residues restored cellular tractions, stiffness and adhesion strength, as well as vinculin dynamics, and facilitated vinculin-actin binding. These data demonstrate that Src-mediated phosphorylation is necessary for vinculin activation, and that phosphorylation controls cytoskeletal mechanics by regulating force transmission between the actin cytoskeleton and focal adhesion proteins.
11. Nanoscale architecture of integrin-based cell adhesions. Cell adhesions to the **extracellular matrix** (ECM) are necessary for morphogenesis, immunity and wound healing. Focal adhesions are multifunctional organelles that mediate cell-ECM adhesion, force transmission, cytoskeletal regulation and signalling. Focal adhesions consist of a complex network of trans-plasma-membrane integrins and cytoplasmic proteins that form a <200-nm plaque linking the ECM to the actin cytoskeleton. The complexity of focal adhesion composition and dynamics implicate an intricate molecular machine. However, focal adhesion molecular architecture remains unknown. Here we used three-dimensional super-resolution fluorescence microscopy (interferometric photoactivated localization microscopy) to map nanoscale protein organization in focal adhesions. Our results reveal that integrins and actin are vertically separated by a ∼40-nm focal adhesion core region consisting of multiple protein-specific strata: a membrane-apposed integrin signalling layer containing integrin cytoplasmic tails, focal adhesion kinase and paxillin; an intermediate force-transduction layer containing talin and vinculin; and an uppermost actin-regulatory layer containing zyxin, vasodilator-stimulated phosphoprotein and α-actinin. By localizing amino- and carboxy-terminally tagged talins, we reveal talin's polarized orientation, indicative of a role in organizing the focal adhesion strata. The composite multilaminar protein architecture provides a molecular blueprint for understanding focal adhesion functions.
12. Vinculin is indispensable for repopulation by hematopoietic stem cells, independent of integrin function. Vinculin is a highly conserved actin-binding protein that is localized in integrin-mediated focal adhesion complexes. Although critical roles have been proposed for integrins in hematopoietic stem cell (HSC) function, little is known about the involvement of intracellular focal adhesion proteins in HSC functions. This study showed that the ability of c-Kit(+)Sca1(+)Lin(-) HSCs to support reconstitution of hematopoiesis after competitive transplantation was severely impaired by lentiviral transduction with short hairpin RNA sequences for vinculin. The potential of these HSCs to differentiate into granulocytic and monocytic lineages, to migrate toward stromal cell-derived factor 1α, and to home to the bone marrow in vivo were not inhibited by the loss of vinculin. However, the capacities to form long term culture-initiating cells and cobblestone-like areas were abolished in vinculin-silenced c-Kit(+)Sca1(+)Lin(-) HSCs. In contrast, adhesion to the **extracellular matri**x was inhibited by silencing of talin-1, but not of vinculin. Whole body in vivo luminescence analyses to detect transduced HSCs confirmed the role of vinculin in long term HSC reconstitution. Our results suggest that vinculin is an indispensable factor determining HSC repopulation capacity, independent of integrin functions.
13. Vinculin-actin interaction couples actin retrograde flow to focal adhesions, but is dispensable for focal adhesion growth. In migrating cells, integrin-based focal adhesions (FAs) assemble in protruding lamellipodia in association with rapid filamentous actin (F-actin) assembly and retrograde flow. How dynamic F-actin is coupled to FA is not known. We analyzed the role of vinculin in integrating F-actin and FA dynamics by vinculin gene disruption in primary fibroblasts. Vinculin slowed F-actin flow in maturing FA to establish a lamellipodium-lamellum border and generate high **extracellular matrix** (ECM) traction forces. In addition, vinculin promoted nascent FA formation and turnover in lamellipodia and inhibited the frequency and rate of FA maturation. Characterization of a vinculin point mutant that specifically disrupts F-actin binding showed that vinculin-F-actin interaction is critical for these functions. However, FA growth rate correlated with F-actin flow speed independently of vinculin. Thus, vinculin functions as a molecular clutch, organizing leading edge F-actin, generating ECM traction, and promoting FA formation and turnover, but vinculin is dispensible for FA growth.
14. Serum response factor is required for cell contact maintenance but dispensable for proliferation in visceral yolk sac endothelium. Endothelial-specific knockout of the transcription factor serum response factor (SRF) results in embryonic lethality by mid-gestation. The associated phenotype exhibits vascular failure in embryos as well as visceral yolk sac (VYS) tissues. Previous data suggest that this vascular failure is caused by alterations in cell-cell and cell-matrix contacts. In the current study, we sought to more carefully address the role of SRF in endothelial function and cell contact interactions in VYS tissues.Tie2-Cre recombinase-mediated knockout of SRF expression resulted in loss of detectable SRF from VYS mesoderm by E12.5. This loss was accompanied by decreased expression of smooth muscle alpha-actin as well as vascular endothelial cadherin and claudin 5, endothelial-specific components of adherens and tight junctions, respectively. Focal adhesion (FA) integrins alpha5 and beta1 were largely unchanged in contrast to loss of the FA-associated molecule vinculin. The integrin binding partner fibronectin-1 was also profoundly decreased in the **extracellular matrix**, indicating another aspect of impaired adhesive function and integrin signaling. Additionally, cells in SRF-null VYS mesoderm failed to reduce proliferation, suggesting not only that integrin-mediated contact inhibition is impaired but also that SRF protein is not required for proliferation in these cells.Our data support a model in which SRF is critical in maintaining functional cell-cell and cell-matrix adhesion in endothelial cells. Furthermore, we provide evidence that supports a model in which loss of SRF protein results in a sustained proliferation defect due in part to failed integrin signaling.
15. Vinculin facilitates cell invasion into three-dimensional collagen matrices. The cytoskeletal protein vinculin contributes to the mechanical link of the contractile actomyosin cytoskeleton to the **extracellular matrix** (ECM) through integrin receptors. In addition, vinculin modulates the dynamics of cell adhesions and is associated with decreased cell motility on two-dimensional ECM substrates. The effect of vinculin on cell invasion through dense three-dimensional ECM gels is unknown. Here, we report how vinculin expression affects cell invasion into three-dimensional collagen matrices. Cell motility was investigated in vinculin knockout and vinculin expressing wild-type mouse embryonic fibroblasts. Vinculin knockout cells were 2-fold more motile on two-dimensional collagen-coated substrates compared with wild-type cells, but 3-fold less invasive in 2.4 mg/ml three-dimensional collagen matrices. Vinculin knockout cells were softer and remodeled their cytoskeleton more dynamically, which is consistent with their enhanced two-dimensional motility but does not explain their reduced three-dimensional invasiveness. Importantly, vinculin-expressing cells adhered more strongly to collagen and generated 3-fold higher traction forces compared with vinculin knockout cells. Moreover, vinculin-expressing cells were able to migrate into dense (5.8 mg/ml) three-dimensional collagen matrices that were impenetrable for vinculin knockout cells. These findings suggest that vinculin facilitates three-dimensional matrix invasion through up-regulation or enhanced transmission of traction forces that are needed to overcome the steric hindrance of ECMs.
16. Mutations in the paxillin-binding site of integrin-linked kinase (ILK) destabilize the pseudokinase domain and cause embryonic lethality in mice. Integrin-linked kinase (ILK) localizes to focal adhesions (FAs) where it regulates cell spreading, migration, and growth factor receptor signaling. Previous reports showed that overexpressed ILK in which Val(386) and Thr(387) were substituted with glycine residues (ILK-VT/GG) could neither interact with paxillin nor localize to FA in cells expressing endogenous wild-type ILK, implying that paxillin binding to ILK is required for its localization to FAs. Here, we show that introducing this mutation into the germ line of mice (ILK-VT/GG) caused vasculogenesis defects, resulting in a general developmental delay and death at around embryonic day 12.5. Fibroblasts isolated from ILK-VT/GG mice contained mutant ILK in FAs, showed normal adhesion to and spreading on **extracellular matrix** substrates but displayed impaired migration. Biochemical analysis revealed that VT/GG substitutions decreased ILK protein stability leading to decreased ILK levels and reduced binding to paxillin and α-parvin. Because paxillin depletion did not affect ILK localization to FAs, the embryonic lethality and the in vitro migration defects are likely due to the reduced levels of ILK-VT/GG and diminished binding to parvins.
17. Vinculin is required for cell polarization, migration, and **extracellular matrix** remodeling in 3D collagen. Vinculin is filamentous (F)-actin-binding protein enriched in integrin-based adhesions to the **extracellular matrix** (ECM). Whereas studies in 2-dimensional (2D) tissue culture models have suggested that vinculin negatively regulates cell migration by promoting cytoskeleton-ECM coupling to strengthen and stabilize adhesions, its role in regulating cell migration in more physiologic, 3-dimensional (3D) environments is unclear. To address the role of vinculin in 3D cell migration, we analyzed the morphodynamics, migration, and ECM remodeling of primary murine embryonic fibroblasts (MEFs) with cre/loxP-mediated vinculin gene disruption in 3D collagen I cultures. We found that vinculin promoted 3D cell migration by increasing directional persistence. Vinculin was necessary for persistent cell protrusion, cell elongation, and stable cell orientation in 3D collagen, but was dispensable for lamellipodia formation, suggesting that vinculin-mediated cell adhesion to the ECM is needed to convert actin-based cell protrusion into persistent cell shape change and migration. Consistent with this finding, vinculin was necessary for efficient traction force generation in 3D collagen without affecting myosin II activity and promoted 3D collagen fiber alignment and macroscopical gel contraction. Our results suggest that vinculin promotes directionally persistent cell migration and tension-dependent ECM remodeling in complex 3D environments by increasing cell-ECM adhesion and traction force generation.
18. Integrin-β5 and zyxin mediate formation of ventral stress fibers in response to transforming growth factor β. Cell adhesion to th**e extracellular matr**ix is an essential element of various biological processes. TGF-β cytokines regulate the matrix components and cell-matrix adhesions. The present study investigates the molecular organization of TGF-β-induced matrix adhesions. The study demonstrates that in various mouse and human epithelial cells TGF-β induces cellular structures containing 2 matrix adhesions bridged by a stretch of actin fibers. These structures are similar to ventral stress fibers (VSFs). Suppression of integrin-β5 by RNA interference reduces VSFs in majority of cells (> 75%), while overexpression of integrin-β5 fragments revealed a critical role of a distinct sequence in the cytoplasmic domain of integrin-β5 in the VSF structures. In addition, the integrity of actin fibers and Src kinase activity contribute to integrin-β5-mediated signaling and VSF formation. TGF-β-Smad signaling upregulates actin-regulatory proteins, such as caldesmon, zyxin, and zyxin-binding protein Csrp1 in mouse and human epithelial cells. Suppression of zyxin markedly inhibits formation of VSFs in response to TGF-β and integrin-β5. Zyxin is localized at actin fibers and matrix adhesions of VSFs and might bridge integrin-β5-mediated adhesions to actin fibers. These findings provide a platform for defining the molecular mechanism regulating the organization and activities of VSFs in response to TGF-β.
19. Force fluctuations within focal adhesions mediate ECM-rigidity sensing to guide directed cell migration. Cell migration toward areas of higher **extracellular matrix** (ECM) rigidity via a process called "durotaxis" is thought to contribute to development, immune response, and cancer metastasis. To understand how cells sample ECM rigidity to guide durotaxis, we characterized cell-generated forces on the nanoscale within single mature integrin-based focal adhesions (FAs). We found that individual FAs act autonomously, exhibiting either stable or dynamically fluctuating ("tugging") traction. We show that a FAK/phosphopaxillin/vinculin pathway is essential for high FA traction and to enable tugging FA traction over a broad range of ECM rigidities. We show that tugging FA traction is dispensable for FA maturation, chemotaxis, and haptotaxis but is critical to direct cell migration toward rigid ECM. We conclude that individual FAs dynamically sample rigidity by applying fluctuating pulling forces to the ECM to act as sensors to guide durotaxis, and that FAK/phosphopaxillin/vinculin signaling defines the rigidity range over which this dynamic sensing process operates.
20. Deletion of the cell adhesion adaptor protein vinculin disturbs the localization of GFAP in Bergmann glial cells. Astrocytes operate in close spatial relationship to other cells including neurons. Structural interaction is controlled by a dynamic interplay between actin-based cell motility and contact formation via cell-cell and cell-**extracellular matrix** adhesions. A central player in the control of cell adhesion is the cytoskeletal adaptor protein Vinculin. Incorporation of Vinculin affects mechanical properties and turnover of cell adhesion sites. To study the in vivo function of Vinculin in astrocytes, a mouse line with astrocyte specific and inducible deletion of vinculin was generated. Deletion of vinculin decreased the expression of the glial acidic fibrillary protein (GFAP) in Bergmann glial cells in the cerebellum. In addition, localization of GFAP to Bergmann glial endfeet was disturbed, indicating a role for vinculin in controlling its expression and localization. In contrast, vimentin expression, morphology, activation state and polarity of the targeted cells as well as the localization of the **extracellular matrix** protein laminin was not compromised. Furthermore, stab wound lesions were performed in the cerebellar cortex. In both wildtype and vinculin knockout mice GFAP expression was upregulated in Bergmann glial cells of the lesioned area with no differences observed between genotypes in expression and localization of GFAP. These results propose a selective requirement for vinculin in cellular events related to cell adhesion in vivo. As in vitro data suggested a major role for vinculin in the control of the cytoskeletal connection affecting mechanical stability and cell motility, our data add a note of caution to the extrapolation of in vitro data to in vivo function.
21. Abl knockout differentially affects p130 Crk-associated substrate, vinculin, and paxillin in blood vessels of mice. Actin polymerization has recently emerged as an important cellular process that regulates smooth muscle contraction. Abelson tyrosine kinase (Abl) has been implicated in the regulation of actin dynamics and force development in vascular smooth muscle. In the present study, the systolic blood pressure was lower in Abl(-/-) knockout mice compared with wild-type mice. The knockout of Abl diminished the tyrosine phosphorylation of p130 Crk-associated substrate (CAS, an adapter protein associated with smooth muscle contraction) in resistance arteries upon stimulation with phenylephrine or angiotensin II. The agonist-elicited enhancement of F-actin-to-G-actin ratios in arteries assessed by fluorescent microscopy was also reduced in Abl(-/-) mice. It has been known that vinculin is a structural protein that links actin filaments to **extracellular matrix** via transmembrane integrins, whereas paxillin is a signaling protein associated with focal contacts mediating actin cytoskeleton remodeling. The expression of vinculin and paxillin at protein and messenger levels was lower in arterial vessels from Abl knockout mice. However, the agonist-induced increase in myosin phosphorylation was not attenuated in arteries from Abl knockout mice. These results indicate that Abl differentially regulates Crk-associated substrate, vinculin, and paxillin in arterial vessels. The Abl-regulated cellular process and blood pressure are independent of myosin activation in vascular smooth muscle.
22. What is vinculin needed for in platelets? Summary. Vinculin links integrins to the cell cytoskeleton by virtue of its binding to proteins such as talin and F-actin. It has been implicated in the transmission of mechanical forces from th**e extracellular matr**ix to the cytoskeleton of migrating cells. Vinculin's function in platelets is unknown.To determine whether vinculin is required for the functions of platelets and their major integrin, α(IIb) β(3) .The murine vinculin gene (Vcl) was deleted in the megakaryocyte/platelet lineage by breeding Vcl fl/fl mice with Pf4-Cre mice. Platelet and integrin functions were studied in vivo and ex vivo.Vinculin was undetectable in platelets from Vcl fl/fl Cre(+) mice, as determined by immunoblotting and fluorescence microscopy. Vinculin-deficient megakaryocytes exhibited increased membrane tethers in response to mechanical pulling on α(IIb) β(3) with laser tweezers, suggesting that vinculin helps to maintain membrane cytoskeleton integrity. Surprisingly, vinculin-deficient platelets displayed normal agonist-induced fibrinogen binding to α(IIb) β(3) , aggregation, spreading, actin polymerization/organization, clot retraction and the ability to form a procoagulant surface. Furthermore, vinculin-deficient platelets adhered to immobilized fibrinogen or collagen normally, under both static and flow conditions. Tail bleeding times were prolonged in 59% of vinculin-deficient mice. However, these mice exhibited no spontaneous bleeding and they formed occlusive platelet thrombi comparable to those in wild-type littermates in response to carotid artery injury with FeCl(3) .Despite promoting membrane cytoskeleton integrity when mechanical force is applied to α(IIb) β(3) , vinculin is not required for the traditional functions of α(IIb) β(3) or the platelet actin cytoskeleton.

#### GeneRIFs

1. Vinculin organizes lamellipodia and lamella F-actin and mediates F-actin retrograde flow engagement at focal adhesion to generate **extracellular matrix** traction.

### [Ii]nvasive

#### PubMed

1. Alpha5beta1, alphaVbeta3 and the platelet-associated integrin alphaIIbbeta3 coordinately regulate adhesion and migration of differentiating mouse trophoblast cells. During blastocyst implantation, interaction between integrins on the apical surface of the trophoblast and extracellular matrix (ECM) in the endometrium anchors the embryo to the uterine wall. Strong adhesion of the blastocyst to fibronectin (FN) requires integrin signaling initiated by exogenous fibronectin. However, it is not known how integrin signaling enhances blastocyst adhesion. We present new evidence that the integrin, alphaIIbbeta3, plays a key role in trophoblast adhesion to fibronectin during mouse peri-implantation development. Trafficking of alphaIIb to the apical surface of the trophoblast increased dramatically after blastocysts were exposed to fibronectin, whereas other fibronectin-binding integrins, alpha5beta1 and alphaVbeta3, were resident at the apical surface before ligand exposure. Functional comparisons among the three integrins revealed that ligation of alpha5beta1 most efficiently strengthened blastocyst fibronectin-binding activity, while subsequent trophoblast cell migration was dependent primarily on the beta3-class integrins. In vivo, alphaIIb was highly expressed by **invasive** trophoblast cells in the ectoplacental cone and trophoblast giant cells of the parietal yolk sac. These data demonstrate that trafficking of alphaIIb regulates adhesion between trophoblast cells and fibronectin as invasion of the endometrium commences.
2. Phosphoinositide 3-kinase p85beta regulates invadopodium formation. The acquisition of **invasive**ness is characteristic of tumor progression. Numerous genetic changes are associated with metastasis, but the mechanism by which a cell becomes **invasive** remains unclear. Expression of p85β, a regulatory subunit of phosphoinositide-3-kinase, markedly increases in advanced carcinoma, but its mode of action is unknown. We postulated that p85β might facilitate cell invasion. We show that p85β localized at cell adhesions in complex with focal adhesion kinase and enhanced stability and maturation of cell adhesions. In addition, p85β induced development at cell adhesions of an F-actin core that extended several microns into the cell z-axis resembling the skeleton of invadopodia. p85β lead to F-actin polymerization at cell adhesions by recruiting active Cdc42/Rac at these structures. In accordance with p85β function in invadopodium-like formation, p85β levels increased in metastatic melanoma and p85β depletion reduced invadopodium formation and invasion. These results show that p85β enhances invasion by inducing cell adhesion development into invadopodia-like structures explaining the metastatic potential of tumors with increased p85β levels.
3. Vinculin facilitates cell invasion into three-dimensional collagen matrices. The cytoskeletal protein vinculin contributes to the mechanical link of the contractile actomyosin cytoskeleton to the extracellular matrix (ECM) through integrin receptors. In addition, vinculin modulates the dynamics of cell adhesions and is associated with decreased cell motility on two-dimensional ECM substrates. The effect of vinculin on cell invasion through dense three-dimensional ECM gels is unknown. Here, we report how vinculin expression affects cell invasion into three-dimensional collagen matrices. Cell motility was investigated in vinculin knockout and vinculin expressing wild-type mouse embryonic fibroblasts. Vinculin knockout cells were 2-fold more motile on two-dimensional collagen-coated substrates compared with wild-type cells, but 3-fold less **invasive** in 2.4 mg/ml three-dimensional collagen matrices. Vinculin knockout cells were softer and remodeled their cytoskeleton more dynamically, which is consistent with their enhanced two-dimensional motility but does not explain their reduced three-dimensional **invasive**ness. Importantly, vinculin-expressing cells adhered more strongly to collagen and generated 3-fold higher traction forces compared with vinculin knockout cells. Moreover, vinculin-expressing cells were able to migrate into dense (5.8 mg/ml) three-dimensional collagen matrices that were impenetrable for vinculin knockout cells. These findings suggest that vinculin facilitates three-dimensional matrix invasion through up-regulation or enhanced transmission of traction forces that are needed to overcome the steric hindrance of ECMs.

### [Mm]etastasis

#### PubMed

1. Vinculin modulation of paxillin-FAK interactions regulates ERK to control survival and motility. Cells lacking vinculin are highly metastatic and motile. The reasons for this finding have remained unclear. Both enhanced survival and motility are critical to **metastasis**. Here, we show that vinculin null (vin-/-) cells and cells expressing a vinculin Y822F mutant have increased survival due to up-regulated activity of extracellular signal-regulated kinase (ERK). This increase is shown to result from vinculin's modulation of paxillin-FAK interactions. A vinculin fragment (amino acids 811-1066) containing the paxillin binding site restored apoptosis and suppressed ERK activity in vin-/- cells. Both vinY822F and vin-/- cells exhibit increased interaction between paxillin and focal adhesion kinase (FAK) and increased paxillin and FAK phosphorylation. Transfection with paxillin Y31FY118F dominant-negative mutant in these cells inhibits ERK activation and restores apoptosis. The enhanced motility of vin-/- and vinY822F cells is also shown to be due to a similar mechanism. Thus, vinculin regulates survival and motility via ERK by controlling the accessibility of paxillin for FAK interaction.
2. Phosphatase of regenerating liver 2 (PRL2) is essential for placental development by down-regulating PTEN (Phosphatase and Tensin Homologue Deleted on Chromosome 10) and activating Akt protein. The PRL (phosphatase of regenerating liver) phosphatases are implicated in the control of cell proliferation and invasion. Aberrant PRL expression is associated with progression and **metastasis** of multiple cancers. However, the specific in vivo function of the PRLs remains elusive. Here we show that deletion of PRL2, the most ubiquitously expressed PRL family member, leads to impaired placental development and retarded growth at both embryonic and adult stages. Ablation of PRL2 inactivates Akt and blocks glycogen cell proliferation, resulting in reduced spongiotrophoblast and decidual layers in the placenta. These structural defects cause placental hypotrophy and insufficiency, leading to fetal growth retardation. We demonstrate that the tumor suppressor PTEN is elevated in PRL2-deficient placenta. Biochemical analyses indicate that PRL2 promotes Akt activation by down-regulating PTEN through the proteasome pathway. This study provides the first evidence that PRL2 is required for extra-embryonic development and associates the oncogenic properties of PRL2 with its ability to negatively regulate PTEN, thereby activating the PI3K-Akt pathway.
3. Phosphoinositide 3-kinase p85beta regulates invadopodium formation. The acquisition of invasiveness is characteristic of tumor progression. Numerous genetic changes are associated with **metastasis**, but the mechanism by which a cell becomes invasive remains unclear. Expression of p85β, a regulatory subunit of phosphoinositide-3-kinase, markedly increases in advanced carcinoma, but its mode of action is unknown. We postulated that p85β might facilitate cell invasion. We show that p85β localized at cell adhesions in complex with focal adhesion kinase and enhanced stability and maturation of cell adhesions. In addition, p85β induced development at cell adhesions of an F-actin core that extended several microns into the cell z-axis resembling the skeleton of invadopodia. p85β lead to F-actin polymerization at cell adhesions by recruiting active Cdc42/Rac at these structures. In accordance with p85β function in invadopodium-like formation, p85β levels increased in metastatic melanoma and p85β depletion reduced invadopodium formation and invasion. These results show that p85β enhances invasion by inducing cell adhesion development into invadopodia-like structures explaining the metastatic potential of tumors with increased p85β levels.
4. Force fluctuations within focal adhesions mediate ECM-rigidity sensing to guide directed cell migration. Cell migration toward areas of higher extracellular matrix (ECM) rigidity via a process called "durotaxis" is thought to contribute to development, immune response, and cancer **metastasis**. To understand how cells sample ECM rigidity to guide durotaxis, we characterized cell-generated forces on the nanoscale within single mature integrin-based focal adhesions (FAs). We found that individual FAs act autonomously, exhibiting either stable or dynamically fluctuating ("tugging") traction. We show that a FAK/phosphopaxillin/vinculin pathway is essential for high FA traction and to enable tugging FA traction over a broad range of ECM rigidities. We show that tugging FA traction is dispensable for FA maturation, chemotaxis, and haptotaxis but is critical to direct cell migration toward rigid ECM. We conclude that individual FAs dynamically sample rigidity by applying fluctuating pulling forces to the ECM to act as sensors to guide durotaxis, and that FAK/phosphopaxillin/vinculin signaling defines the rigidity range over which this dynamic sensing process operates.

### [Cc]ell [Ii]nvasion

#### PubMed

1. Phosphoinositide 3-kinase p85beta regulates invadopodium formation. The acquisition of invasiveness is characteristic of tumor progression. Numerous genetic changes are associated with metastasis, but the mechanism by which a cell becomes invasive remains unclear. Expression of p85β, a regulatory subunit of phosphoinositide-3-kinase, markedly increases in advanced carcinoma, but its mode of action is unknown. We postulated that p85β might facilitat**e cell invasi**on. We show that p85β localized at cell adhesions in complex with focal adhesion kinase and enhanced stability and maturation of cell adhesions. In addition, p85β induced development at cell adhesions of an F-actin core that extended several microns into the cell z-axis resembling the skeleton of invadopodia. p85β lead to F-actin polymerization at cell adhesions by recruiting active Cdc42/Rac at these structures. In accordance with p85β function in invadopodium-like formation, p85β levels increased in metastatic melanoma and p85β depletion reduced invadopodium formation and invasion. These results show that p85β enhances invasion by inducing cell adhesion development into invadopodia-like structures explaining the metastatic potential of tumors with increased p85β levels.
2. Vinculin facilitates **cell invasion** into three-dimensional collagen matrices. The cytoskeletal protein vinculin contributes to the mechanical link of the contractile actomyosin cytoskeleton to the extracellular matrix (ECM) through integrin receptors. In addition, vinculin modulates the dynamics of cell adhesions and is associated with decreased cell motility on two-dimensional ECM substrates. The effect of vinculin on **cell invasion** through dense three-dimensional ECM gels is unknown. Here, we report how vinculin expression affects **cell invasion** into three-dimensional collagen matrices. Cell motility was investigated in vinculin knockout and vinculin expressing wild-type mouse embryonic fibroblasts. Vinculin knockout cells were 2-fold more motile on two-dimensional collagen-coated substrates compared with wild-type cells, but 3-fold less invasive in 2.4 mg/ml three-dimensional collagen matrices. Vinculin knockout cells were softer and remodeled their cytoskeleton more dynamically, which is consistent with their enhanced two-dimensional motility but does not explain their reduced three-dimensional invasiveness. Importantly, vinculin-expressing cells adhered more strongly to collagen and generated 3-fold higher traction forces compared with vinculin knockout cells. Moreover, vinculin-expressing cells were able to migrate into dense (5.8 mg/ml) three-dimensional collagen matrices that were impenetrable for vinculin knockout cells. These findings suggest that vinculin facilitates three-dimensional matrix invasion through up-regulation or enhanced transmission of traction forces that are needed to overcome the steric hindrance of ECMs.

#### GeneRIFs

1. Vinculin facilitates **cell invasion** into three-dimensional collagen matrices.

---

## Smpd1

- NCBI Gene
- GeneRIF
- Pubmed

| Search Term | Rank | Fields |
| --- | --- | --- |
| [Mm]igration | 6 | PubMed(3) |
| [Ii]nvasive | 10 | PubMed(2) |
| [Mm]etastasis | 12 | PubMed(3); GeneRIFs(2) |

  
hide/show details for Smpd1

### [Mm]igration

#### PubMed

1. [siRNAs targetting sphingomyelin phosphodiesterase 1 protect mouse oocytes from apoptosis]. To investigate the potential of siRNAs targeting sphingomyelin phosphodiesterase 1 (SMPD1) in protecting the oocytes from apoptosis, and explore new approaches to female fertility preservation.Chemically synthesized siRNA targeting SMPD1 were introduced into mouse oocytes retrieved by hyperstimulation, and the cell apoptosis was analyzed by comic assay 48 and 72 h later.In the oocytes without any siRNA injection, oocyte DNA damage occurred after 24 h, and large amount of DNA fragments migrated from the cells 48 h later. In oocytes injected with siRNA003, DNA **migration** decreased significantly as compared with the control and the other two groups injected with siRNA001 and siRNA002 (P<0.01).siRNA targeting SMPD1 may protect the oocytes from apoptosis, and has the potential for use in future female fertility preservation.
2. Role of Acid Sphingomyelinase in Shifting the Balance Between Proinflammatory and Reparative Bone Marrow Cells in Diabetic Retinopathy. The metabolic insults associated with diabetes lead to low-grade chronic inflammation, retinal endothelial cell damage, and inadequate vascular repair. This is partly due to the increased activation of bone marrow (BM)-derived proinflammatory monocytes infiltrating the retina, and the compromised function of BM-derived reparative circulating angiogenic cells (CACs), which home to sites of endothelial injury and foster vascular repair. We now propose that a metabolic link leading to activated monocytes and dysfunctional CACs in diabetes involves upregulation of a central enzyme of sphingolipid signaling, acid sphingomyelinase (ASM). Selective inhibition of ASM in the BM prevented diabetes-induced activation of BM-derived microglia-like cells and normalized proinflammatory cytokine levels in the retina. ASM upregulation in diabetic CACs caused accumulation of ceramide on their cell membrane, thereby reducing membrane fluidity and impairing CAC **migration**. Replacing sphingomyelin with ceramide in synthetic membrane vesicles caused a similar decrease in membrane fluidity. Inhibition of ASM in diabetic CACs improved membrane fluidity and homing of these cells to damaged retinal vessels. Collectively, these findings indicate that selective modulation of sphingolipid metabolism in BM-derived cell populations in diabetes normalizes the reparative/proinflammatory cell balance and can be explored as a novel therapeutic strategy for treating diabetic retinopathy.
3. Liver acid sphingomyelinase inhibits growth of metastatic colon cancer. Acid sphingomyelinase (ASM) regulates the homeostasis of sphingolipids, including ceramides and sphingosine-1-phosphate (S1P). These sphingolipids regulate carcinogenesis and proliferation, survival, and apoptosis of cancer cells. However, the role of ASM in host defense against liver metastasis remains unclear. In this study, the involvement of ASM in liver metastasis of colon cancer was examined using Asm-/- and Asm+/+ mice that were inoculated with SL4 colon cancer cells to produce metastatic liver tumors. Asm-/- mice demonstrated enhanced tumor growth and reduced macrophage accumulation in the tumor, accompanied by decreased numbers of hepatic myofibroblasts (hMFs), which express tissue inhibitor of metalloproteinase 1 (TIMP1), around the tumor margin. Tumor growth was increased by macrophage depletion or by Timp1 deficiency, but was decreased by hepatocyte-specific ASM overexpression, which was associated with increased S1P production. S1P stimulated macrophage **migration** and TIMP1 expression in hMFs in vitro. These findings indicate that ASM in the liver inhibits tumor growth through cytotoxic macrophage accumulation and TIMP1 production by hMFs in response to S1P. Targeting ASM may represent a new therapeutic strategy for treating liver metastasis of colon cancer.

### [Ii]nvasive

#### PubMed

1. Sperm abnormalities in heterozygous acid sphingomyelinase knockout mice reveal a novel approach for the prevention of genetic diseases. Acid sphingomyelinase knockout mice are a model of the inherited human disorder types A and B Niemann-Pick disease. Herein, we show that heterozygous (ASMKO(+/-)) mice have two distinct sperm populations resembling those found in normal and mutant animals, respectively, and that these two populations could be distinguished by their morphology, ability to undergo capacitation or the acrosome reaction, and/or mitochondrial membrane potential (MMP). The abnormal morphology of the mutant sperm could be normalized by demembranation with detergents or by the addition of recombinant acid sphingomyelinase to the culture media, and the corrected sperm also had an enhanced fertilization capacity. Methods were then explored to enrich for normal sperm from the mixed ASMKO(+/-) population, and flow cytometric sorting based on MMP provided the best results. In vitro fertilization was performed using ASMKO(+/-) oocytes and sperm before and after MMP sorting, and it was found that the sorted sperm produced significantly more wild-type pups than nonsorted sperm. Sperm sorting is much less **invasive** and more cost-effective than egg isolation, and offers several advantages over the existing assisted reproduction options for Niemann-Pick disease carrier couples. It therefore could have a major impact on the prevention of this and perhaps other genetic diseases.
2. Acid sphingomyelinase determines melanoma progression and metastatic behaviour via the microphtalmia-associated transcription factor signalling pathway. Melanoma is a rapidly growing and highly metastatic cancer with high mortality rates, for which a resolutive treatment is lacking. Identification of novel therapeutic strategies and biomarkers of tumour stage is thus of particular relevance. We report here on a novel biomarker and possible candidate therapeutic target, the sphingolipid metabolising enzyme acid sphingomyelinase (A-SMase). A-SMase expression correlates inversely with tumour stage in human melanoma biopsies. Studies in a mouse model of melanoma and on cell lines derived from mouse and human melanomas demonstrated that A-SMase levels of expression actually determine the malignant phenotype of melanoma cells in terms of pigmentation, tumour progression, **invasive**ness and metastatic ability. The action of A-SMase is mediated by the activation of the extracellular signal-regulated kinase, the subsequent proteasomal degradation of the Microphtalmia-associated transcription factor (Mitf) and inhibition of cyclin-dependent kinase 2, Bcl-2 and c-Met, downstream targets of Mitf involved in tumour cell proliferation, survival and metastatisation.

### [Mm]etastasis

#### PubMed

1. Regulation of hematogenous tumor **metastasis** by acid sphingomyelinase. Metastatic dissemination of cancer cells is the ultimate hallmark of malignancy and accounts for approximately 90% of human cancer deaths. We investigated the role of acid sphingomyelinase (Asm) in the hematogenous **metastasis** of melanoma cells. Intravenous injection of B16F10 melanoma cells into wild-type mice resulted in multiple lung metastases, while Asm-deficient mice (Smpd1(-/-) mice) were protected from pulmonary tumor spread. Transplanting wild-type platelets into Asm-deficient mice reinstated tumor **metastasis**. Likewise, Asm-deficient mice were protected from hematogenous MT/ret melanoma **metastasis** to the spleen in a mouse model of spontaneous tumor **metastasis**. Human and mouse melanoma cells triggered activation and release of platelet secretory Asm, in turn leading to ceramide formation, clustering, and activation of α5β1 integrins on melanoma cells finally leading to adhesion of the tumor cells. Clustering of integrins by applying purified Asm or C16 ceramide to B16F10 melanoma cells before intravenous injection restored trapping of tumor cells in the lung in Asm-deficient mice. This effect was revertable by arginine-glycine-aspartic acid peptides, which are known inhibitors of integrins, and by antibodies neutralizing β1 integrins. These findings indicate that melanoma cells employ platelet-derived Asm for adhesion a**nd metasta**sis.
2. Melanoma cell **metastasis** via P-selectin-mediated activation of acid sphingomyelinase in platelets. Metastatic dissemination of cancer cells is one of the hallmarks of malignancy and accounts for approximately 90 % of human cancer deaths. Within the blood vasculature, tumor cells may aggregate with platelets to form clots, adhere to and spread onto endothelial cells, and finally extravasate to form metastatic colonies. We have previously shown that sphingolipids play a central role in the interaction of tumor cells with platelets; this interaction is a prerequisite for hematogenous tumor **metastasi**s in at least some tumor models. Here we show that the interaction between melanoma cells and platelets results in rapid and transient activation and secretion of acid sphingomyelinase (Asm) in WT but not in P-selectin-deficient platelets. Stimulation of P-selectin resulted in activation of p38 MAPK, and inhibition of p38 MAPK in platelets prevented the secretion of Asm after interaction with tumor cells. Intravenous injection of melanoma cells into WT mice resulted in multiple lung metastases, while in P-selectin-deficient mice pulmonary tumor **metastasi**s and trapping of tumor cells in the lung was significantly reduced. Pre-incubation of tumor cells with recombinant ASM restored trapping of B16F10 melanoma cells in the lung in P-selectin-deficient mice. These findings indicate a novel pathway in tumor **metastasi**s, i.e., tumor cell mediated activation of P-selectin in platelets, followed by activation and secretion of Asm and in turn release of ceramide and tumor **metastasi**s. The data suggest that p38 MAPK acts downstream from P-selectin and is necessary for the secretion of Asm.
3. Liver acid sphingomyelinase inhibits growth of metastatic colon cancer. Acid sphingomyelinase (ASM) regulates the homeostasis of sphingolipids, including ceramides and sphingosine-1-phosphate (S1P). These sphingolipids regulate carcinogenesis and proliferation, survival, and apoptosis of cancer cells. However, the role of ASM in host defense against liver **metastasis** remains unclear. In this study, the involvement of ASM in liver **metastasis** of colon cancer was examined using Asm-/- and Asm+/+ mice that were inoculated with SL4 colon cancer cells to produce metastatic liver tumors. Asm-/- mice demonstrated enhanced tumor growth and reduced macrophage accumulation in the tumor, accompanied by decreased numbers of hepatic myofibroblasts (hMFs), which express tissue inhibitor of metalloproteinase 1 (TIMP1), around the tumor margin. Tumor growth was increased by macrophage depletion or by Timp1 deficiency, but was decreased by hepatocyte-specific ASM overexpression, which was associated with increased S1P production. S1P stimulated macrophage migration and TIMP1 expression in hMFs in vitro. These findings indicate that ASM in the liver inhibits tumor growth through cytotoxic macrophage accumulation and TIMP1 production by hMFs in response to S1P. Targeting ASM may represent a new therapeutic strategy for treating liver **metastasis** of colon cancer.

#### GeneRIFs

1. These findings indicate a novel pathway in tumor **metastasis**, i.e., tumor cell mediated activation of P-selectin in platelets, followed by activation and secretion of Asm and in turn release of ceramide and tumor **metastasis**. The data suggest that p38 MAPK acts downstream from P-selectin and is necessary for the secretion of Asm.
2. the involvement of ASM in liver **metastasis** of colon cancer was examined using Asm-/- and Asm+/+ mice that were inoculated with SL4 colon cancer cells to produce metastatic liver tumors

---

## Slc39a13

- NCBI Gene
- GeneRIF
- Pubmed

| Search Term | Rank | Fields |
| --- | --- | --- |
| [Ee]xtracellular [Mm]atrix | 8 | PubMed(1); GeneRIFs(1) |

  
hide/show details for Slc39a13

### [Ee]xtracellular [Mm]atrix

#### PubMed

1. Comparative study of dermal components and plasma TGF-β1 levels in Slc39a13/Zip13-KO mice. Ehlers-Danlos syndrome (EDS) is a group of disorders caused by abnormalities that are identified in the **extracellular matri**x. Transforming growth factor-β1 (TGF-β1) plays a crucial role in formation of t**he extracellular mat**rix. It has been reported that the loss of function of zinc transporter ZRT/IRT-like protein 13 (ZIP13) causes the spondylocheiro dysplastic form of EDS (SCD-EDS: OMIM 612350), in which dysregulation of the TGF-β1 signaling pathway is observed, although the relationship between the dermis abnormalities and peripheral TGF-β1 level has been unclear. We investigated the characteristics of the dermis of the Zip13-knockout (KO) mouse, an animal model for SCD-EDS. Both the ratio of dermatan sulfate (DS) in glycosaminoglycan (GAG) components and the amount of collagen were decreased, and there were very few collagen fibrils with diameters of more than 150 nm in Zip13-KO mice dermis. We also found that the TGF-β1 level was significantly higher in Zip13-KO mice serum. These results suggest that collagen synthesis and collagen fibril fusion might be impaired in Zip13-KO mice and that the possible decrease of decorin level by reduction of the DS ratio probably caused an increase of free TGF-β1 in Zip13-KO mice. In conclusion, skin fragility due to defective ZIP13 protein may be attributable to im**paired extracellular** matrix synthesis accompanied by abnormal peripheral TGF-β homeostasis.

#### GeneRIFs

1. This study concluded that skin fragility due to defective ZIP13 protein may be attributable to impaired **extracellular matrix** synthesis accompanied by abnormal peripheral TGF-beta homeostasis.

---

## Itgb1

- NCBI Gene
- GeneRIF
- Pubmed

| Search Term | Rank | Fields |
| --- | --- | --- |
| [Ff]ilopodia | 1 | PubMed(6) |
| [Ff]ilopodium | 2 | Component(1) |
| [Ii]nvadopodium | 4 | Component(1) |
| [Pp]odosome(s)? | 5 | PubMed(2) |
| [Mm]igration | 6 | Process(4); PubMed(179); GeneRIFs(35) |
| ECM | 7 | PubMed(65); GeneRIFs(6) |
| [Ee]xtracellular [Mm]atrix | 8 | PubMed(205); GeneRIFs(14) |
| [Ii]nvadosome | 9 | PubMed(1); GeneRIFs(1) |
| [Ii]nvasive | 10 | PubMed(12); GeneRIFs(3) |
| [Mm]etastasize | 11 | PubMed(1) |
| [Mm]etastasis | 12 | PubMed(21); GeneRIFs(7) |
| [Cc]ell [Ii]nvasion | 13 | PubMed(7) |

  
hide/show details for Itgb1

### [Ff]ilopodia

#### PubMed

1. Serum response factor is required for sprouting angiogenesis and vascular integrity. Serum response factor (SRF) is a transcription factor that controls the expression of cytoskeletal proteins and immediate early genes in different cell types. Here, we found that SRF expression is restricted to endothelial cells (ECs) of small vessels such as capillaries in the mouse embryo. EC-specific Srf deletion led to aneurysms and hemorrhages from 11.5 days of mouse development (E11.5) and lethality at E14.5. Mutant embryos presented a reduced capillary density and defects in EC migration, with fewer numbers of **filopodia** in tip cells and ECs showing defects in actin polymerization and intercellular junctions. We show that SRF is essential for the expression of VE-cadherin and beta-actin in ECs both in vivo and in vitro. Moreover, knockdown of SRF in ECs impaired VEGF- and FGF-induced in vitro angiogenesis. Taken together, our results demonstrate that SRF plays an important role in sprouting angiogenesis and small vessel integrity in the mouse embryo.
2. Cadherin-11 coordinates cellular migration and extracellular matrix remodeling during aortic valve maturation. Proper remodeling of the endocardial cushions into thin fibrous valves is essential for gestational progression and long-term function. This process involves dynamic interactions between resident cells and their local environment, much of which is not understood. In this study, we show that deficiency of the cell-cell adhesion protein cadherin-11 (Cad-11) results in significant embryonic and perinatal lethality primarily due to valve related cardiac dysfunction. While endocardial to mesenchymal transformation is not abrogated, mesenchymal cells do not homogeneously cellularize the cushions. These cushions remain thickened with disorganized ECM, resulting in pronounced aortic valve insufficiency. Mice that survive to adulthood maintain thickened and stenotic semilunar valves, but interestingly do not develop calcification. Cad-11 (-/-) aortic valve leaflets contained reduced Sox9 activity, β1 integrin expression, and RhoA-GTP activity, suggesting that remodeling defects are due to improper migration and/or cellular contraction. Cad-11 deletion or siRNA knockdown reduced migration, eliminated collective migration, and impaired 3D matrix compaction by aortic valve interstitial cells (VIC). Cad-11 depleted cells in culture contained few **filopodi**a, stress fibers, or contact inhibited locomotion. Transfection of Cad-11 depleted cells with constitutively active RhoA restored cell phenotypes. Together, these results identify cadherin-11 mediated adhesive signaling for proper remodeling of the embryonic semilunar valves.
3. Endothelial basement membrane limits tip cell formation by inducing Dll4/Notch signalling in vivo. How individual components of the vascular basement membrane influence endothelial cell behaviour remains unclear. Here we show that laminin α4 (Lama4) regulates tip cell numbers and vascular density by inducing endothelial Dll4/Notch signalling in vivo. Lama4 deficiency leads to reduced Dll4 expression, excessive **filopodi**a and tip cell formation in the mouse retina, phenocopying the effects of Dll4/Notch inhibition. Lama4-mediated Dll4 expression requires a combination of integrins in vitro and integrin β1 in vivo. We conclude that appropriate laminin/integrin-induced signalling is necessary to induce physiologically functional levels of Dll4 expression and regulate branching frequency during sprouting angiogenesis in vivo.
4. Integrins control dendritic spine plasticity in hippocampal neurons through NMDA receptor and Ca2+/calmodulin-dependent protein kinase II-mediated actin reorganization. The formation of dendritic spines during development and their structural plasticity in the adult brain are critical aspects of synaptogenesis and synaptic plasticity. Many different factors and proteins have been shown to control dendritic spine development and remodeling (Ethell and Pasquale, 2005). The extracellular matrix (ECM) components and their cell surface receptors, integrins, have been found in the vicinity of synapses and shown to regulate synaptic efficacy and play an important role in long-term potentiation (Bahr et al., 1997; Chavis and Westbrook, 2001; Chan et al., 2003; Lin et al., 2003; Bernard-Trifilo et al., 2005). Although molecular mechanisms by which integrins affect synaptic efficacy have begun to emerge, their role in structural plasticity is poorly understood. Here, we show that integrins are involved in spine remodeling in cultured hippocampal neurons. The treatment of 14 d in vitro hippocampal neurons with arginine-glycine-aspartate (RGD)-containing peptide, an established integrin ligand, induced elongation of existing dendritic spines and promoted formation of new **filopodia**. These effects were also accompanied by integrin-dependent actin reorganization and synapse remodeling, which were partially inhibited by function-blocking antibodies against beta1 and beta3 integrins. This actin reorganization was blocked with the NMDA receptor (NMDAR) antagonist MK801 [(+)-5-methyl-10,11-dihydro-5H-dibenzo[a,d]cyclohepten-5,10-imine hydrogen maleate]. The Ca2+/calmodulin-dependent protein kinase II (CaMKII) inhibitor KN93 (N-[2-[N-(4-chlorocinnamyl)-N-methylaminomethyl]phenyl]-N-(2-hydroxyethyl)-4-methoxybenzenesulfonamide) also suppressed RGD-induced actin reorganization and synapse remodeling. Our findings show that integrins control ECM-mediated spine remodeling in hippocampal neurons through NMDAR/CaMKII-dependent actin reorganization.
5. Laminin alpha5 is required for dental epithelium growth and polarity and the development of tooth bud and shape. In tooth development, the oral ectoderm and mesenchyme coordinately and reciprocally interact through the basement membrane for their growth and differentiation to form the proper shape and size of the tooth. Laminin alpha5 subunit-containing laminin-10/11 (LM-511/521) is the major laminin in the tooth germ basement membrane. Here, we have examined the role of laminin alpha5 (Lama5) in tooth development using laminin alpha5-null mouse primary dental epithelium and tooth germ organ cultures. Lama5-null mice develop a small tooth germ with defective cusp formation and have reduced proliferation of dental epithelium. Also, cell polarity and formation of the monolayer of the inner dental epithelium are disturbed. The enamel knot, a signaling center for tooth germ development, is defective, and there is a significant reduction of Shh and Fgf4 expression in the dental epithelium. In the absence of laminin alpha5, the basement membrane in the inner dental epithelium becomes discontinuous. In normal mice, integrin alpha6beta4, a receptor for laminin alpha5, is strongly localized at the basal layer of the epithelium, whereas in mutant mice, integrin alpha6beta4 is expressed around the cell surface. In primary dental epithelium culture, laminin-10/11 promotes cell growth, spreading, and **filopodia**-like microspike formation. This promotion is inhibited by anti-integrin alpha6 and beta4 antibodies and by phosphatidylinositol 3-kinase inhibitors and dominant negative Rho-GTPase family proteins Cdc42 and Rac. In organ culture, anti-integrin alpha6 antibody and wortmannin reduce tooth germ size and shape. Our studies demonstrate that laminin alpha5 is required for the proliferation and polarity of basal epithelial cells and suggest that the interaction between laminin-10/11-integrin alpha6beta4 and the phosphatidylinositol 3-kinase-Cdc42/Rac pathways play an important role in determining the size and shape of tooth germ.
6. Interactions between ICAM-5 and β1 integrins regulate neuronal synapse formation. Intercellular adhesion molecule-5 (ICAM-5) is a dendrite-specific adhesion molecule, which functions in both the immune and nervous systems. ICAM-5 is the only negative regulator that has been identified for maturation of dendritic spines so far. Shedding of the ICAM-5 ectodomain promotes spine maturation and enhances synaptic activity. However, the mechanism by which ICAM-5 regulates spine development remains poorly understood. In this study, we found that ablation of ICAM5 expression resulted in a significant increase in the formation of synaptic contacts and the frequency of miniature excitatory post-synaptic currents, an indicator of pre-synaptic release probability. Antibodies against ICAM-5 and β1 integrins altered spine maturation. Furthermore, we found that β1 integrins serve as binding partners for ICAM-5. β1 integrins were immunoprecipitated with ICAM-5 from mouse brain and the binding region in ICAM-5 was localized to the two first Ig domains. β1 integrins were juxtapose**d to filo**podia tips at the early stage of synaptic formation, but as synapses matured, β1 integrins covered the mushroom spines. Loss of β1 integrins from the pre-synaptic sites affected the morphology of the post-synaptic structures. ICAM-5 ectodomain cleavage decreased or increased when the interaction between ICAM-5 and β1 integrins was potentiated or weakened, respectively, using antibodies. These results suggest that the interaction between ICAM-5 and β1 integrins is important in formation of functional synapses.

### [Ff]ilopodium

#### Component

1. **filopodium**

### [Ii]nvadopodium

#### Component

1. **invadopodium** membrane

### [Pp]odosome(s)?

#### PubMed

1. Ameloblastin modulates osteoclastogenesis through the integrin/ERK pathway. Proteins of the extracellular matrix often have multiple functions to facilitate complex tasks ranging from signaling to structural support. Here we have focused on the function of one of the matrix proteins expressed in bones and teeth, the matrix adhesion protein ameloblastin (AMBN). Transgenic mice with 5-fold elevated AMBN levels in mandibles suffered from root cementum resorption, delamination, and reduced alveolar bone thickness. AMBN gain of function also resulted in a significant reduction in trabecular bone volume and bone mass dentistry in 42 days postnatal mouse jaws. In an in vitro model of osteoclastogenesis, AMBN modulated osteoclast differentiation from bone marrow derived monocytes (BMMCs), and dramatically increased osteoclast numbers and resorption pits. Furthermore, AMBN more than doubled BMMC adhesion, accelerated cell spreading, and promoted **podosome** belt and actin ring formation. These effects were associated with elevated ERK1/2 and AKT phosphorylation as well as higher expression of osteoclast activation related genes. Blocking integrin α2β1 and ERK 1/2 pathways alleviated the effects of AMBN on osteoclast differentiation. Together, our data indicate that AMBN increases osteoclast number and differentiation as well as mineralized tissue resorption by regulating cell adhesion and actin cytoskeleton polymerization, initiating integrin-dependent extracellular matrix signaling cascades and enhancing osteoclastogenesis.
2. Kindlin-3-mediated signaling from multiple integrin classes is required for osteoclast-mediated bone resorption. The blood cell-specific kindlin-3 protein is required to activate leukocyte and platelet integrins. In line with this function, mutations in the KINDLIN-3 gene in man cause immunodeficiency and severe bleeding. Some patients also suffer from osteopetrosis, but the underlying mechanism leading to abnormal bone turnover is unknown. Here we show that kindlin-3-deficient mice develop severe osteopetrosis because of profound adhesion and spreading defects in bone-resorbing osteoclasts. Mechanistically, loss of kindlin-3 impairs the activation of β1, β2, and β3 integrin classes expressed on osteoclasts, which in turn abrogates the formation **of podoso**mes and sealing zones required for bone resorption. In agreement with these findings, genetic ablation of all integrin classes abolishes the development **of podoso**mes, mimicking kindlin-3 deficiency. Although loss of single integrin classes gives rise **to podoso**mes, their resorptive activity is impaired. These findings show that osteoclasts require their entire integrin repertoire to be regulated by kindlin-3 to orchestrate bone homeostasis.

### [Mm]igration

#### Process

1. cell **migration**
2. cell **migration** involved in sprouting angiogenesis
3. germ cell **migration**
4. positive regulation of cell **migration**

#### PubMed

1. Cell adhesion strengthening: contributions of adhesive area, integrin binding, and focal adhesion assembly. Mechanical interactions between a cell and its environment regulate **migration**, contractility, gene expression, and cell fate. We integrated micropatterned substrates to engineer adhesive area and a hydrodynamic assay to analyze fibroblast adhesion strengthening on fibronectin. Independently of cell spreading, integrin binding and focal adhesion assembly resulted in rapid sevenfold increases in adhesion strength to steady-state levels. Adhesive area strongly modulated adhesion strength, integrin binding, and vinculin and talin recruitment, exhibiting linear increases for small areas. However, above a threshold area, adhesion strength and focal adhesion assembly reached a saturation limit, whereas integrin binding transitioned from a uniform distribution to discrete complexes. Adhesion strength exhibited exponential increases with bound integrin numbers as well as vinculin and talin recruitment, and the relationship between adhesion strength and these biochemical events was accurately described by a simple mechanical model. Furthermore, adhesion strength was regulated by the position of an adhesive patch, comprised of bound integrins and cytoskeletal elements, which generated a constant 200-nN adhesive force. Unexpectedly, focal adhesion assembly, in particular vinculin recruitment, contributed only 30% of the adhesion strength. This work elucidates the roles of adhesive complex size and position in the generation of cell-extracellular matrix forces.
2. Androgen-induced cell **migration**: role of androgen receptor/filamin A association. Androgen receptor (AR) controls male morphogenesis, gametogenesis and prostate growth as well as development of prostate cancer. These findings support a role for AR in cell **migration** and invasiveness. However, the molecular mechanism involved in AR-mediated cell **migration** still remains elusive.Mouse embryo NIH3T3 fibroblasts and highly metastatic human fibrosarcoma HT1080 cells harbor low levels of transcriptionally incompetent AR. We now report that, through extra nuclear action, AR triggers **migration** of both cell types upon stimulation with physiological concentrations of the androgen R1881. We analyzed the initial events leading to androgen-induced cell **migration** and observed that challenging NIH3T3 cells with 10 nM R1881 rapidly induces interaction of AR with filamin A (FlnA) at cytoskeleton. AR/FlnA complex recruits integrin beta 1, thus activating its dependent cascade. Silencing of AR, FlnA and integrin beta 1 shows that this ternary complex controls focal adhesion kinase (FAK), paxillin and Rac, thereby driving cell **migration**. FAK-null fibroblasts migrate poorly and Rac inhibition by EHT impairs motility of androgen-treated NIH3T3 cells. Interestingly, FAK and Rac activation by androgens are independent of each other. Findings in human fibrosarcoma HT1080 cells strengthen the role of Rac in androgen signaling. The Rac inhibitor significantly impairs androgen-induced **migration** in these cells. A mutant AR, deleted of the sequence interacting with FlnA, fails to mediate FAK activation and paxillin tyrosine phosphorylation in androgen-stimulated cells, further reinforcing the role of AR/FlnA interaction in androgen-mediated motility.The present report, for the first time, indicates that the extra nuclear AR/FlnA/integrin beta 1 complex is the key by which androgen activates signaling leading to cell **migration**. Assembly of this ternary complex may control organ development and prostate cancer metastasis.
3. Rac1 regulates pancreatic islet morphogenesis. Pancreatic islets of Langerhans originate from endocrine progenitors within the pancreatic ductal epithelium. Concomitant with differentiation of these progenitors into hormone-producing cells such cells delaminate, aggregate and migrate away from the ductal epithelium. The cellular and molecular mechanisms regulating islet cell delamination and cell **migration** are poorly understood. Extensive biochemical and cell biological studies using cultured cells demonstrated that Rac1, a member of the Rho family of small GTPases, acts as a key regulator of cell **migration**.To address the functional role of Rac1 in islet morphogenesis, we generated transgenic mice expressing dominant negative Rac1 under regulation of the Rat Insulin Promoter. Blocking Rac1 function in beta cells inhibited their **migration** away from the ductal epithelium in vivo. Consistently, transgenic islet cell spreading was compromised in vitro. We also show that the EGF-receptor ligand betacellulin induced actin remodelling and cell spreading in wild-type islets, but not in transgenic islets. Finally, we demonstrate that cell-cell contact E-cadherin increased as a consequence of blocking Rac1 activity.Our data support a model where Rac1 signalling controls islet cell **migration** by modulating E-cadherin-mediated cell-cell adhesion. Furthermore, in vitro experiments show that betacellulin stimulated islet cell spreading and actin remodelling is compromised in transgenic islets, suggesting that betacellulin may act as a regulator of Rac1 activity and islet **migration** in vivo. Our results further emphasize Rac1 as a key regulator of cell **migration** and cell adhesion during tissue and organ morphogenesis.
4. Role of β1 integrin in tissue homing of neutrophils during sepsis. Aberrant activation of neutrophils during sepsis results in the widespread release of proinflammatory mediators, leading to multiorgan system failure and death. However, aberrant activation of neutrophils during sepsis results in the widespread release of harmful inflammatory mediators causing host tissue injuries that can lead to multiorgan system failure and death. One of the pivotal components of neutrophil **migratio**n during inflammation is the expression of surface integrins. In this study, we show that administration of a cyclic analog of RGD peptide (Arg-Gly-Asp) significantly reduced the number of tissue-invading neutrophils and the degree of sepsis-induced lethality in mice as compared with control peptide. Second, β1 integrin (CD29) was highly upregulated on the neutrophils isolated from both septic patients and animals. Finally, conditional genetic ablation of β1 integrin from granulocytes also improved survival and bacterial clearance in septic animals Thus, our results indicate that expression of β1 integrin is important for modulating neutrophil trafficking during sepsis and that therapeutics designed against β1 integrins may be beneficial.
5. The effects of increased extracellular deformation, pressure, and integrin phosphorylation on fibroblast **migration**. Wound healing requires fibroblast **migration**. Increased pressure slows **migration** and ulcer healing. Pressure also induces beta1 integrin phosphorylation. We hypothesized that beta1 phosphorylation influences cell adhesion and **migration**. We compared the effects of increased pressure on the adhesion and motility of GD25 beta1-integrin null fibroblasts transfected with wild-type beta1A-integrin, S785A or TT788/9AA (phosphorylation-deficient), or T788D (constitutively phosphomimetic) mutants. GD25 beta1 null cells adhered less than wild type beta1A cells, suggesting adherence by non-integrin mechanisms. Preventing Ser-785 or Thr 788/789 phosphorylation reduced adhesion, suggesting that phosphorylation regulates adhesiveness. Substituting Asp for Thr788 stimulated adhesion on both substrates. Pressure decreased **migration** in all lines and on all matrixes, the most in wild type beta1A integrin cells and only slightly in beta1A TT788/9AA cells. In comparison, another physical force, repetitive deformation, increased **migration** in the beta1A integrin T788D, S785A, and wild type cells on fibronectin, and decreased **migration** on collagen. Deformation did not affect the **migration** of GD25 beta1-integrin null or TT788/9AA cells. Extracellular signal-regulated kinase (ERK) blockade neither altered basal **migration** nor prevented pressure inhibition, while the cellular deformation response on fibronectin was altered. beta1-Integrin phosphorylation regulates cellular adhesion and the deformation effects on motility. The pressure-induced motility response is independently regulated.
6. Caveolin-1-dependent beta1 integrin endocytosis is a critical regulator of fibronectin turnover. beta1 integrins are major cell surface receptors for fibronectin. Some integrins, including beta1 integrins, are known to undergo constitutive endocytosis and recycling. Integrin endocytosis/recycling has been implicated in the regulation of cell **migration**. However, the mechanisms by which integrin endocytosis/recycling regulates cell **migration**, and other biological consequences of integrin trafficking are not completely understood. We previously showed that turnover of extracellular matrix (ECM) fibronectin occurs via receptor-mediated endocytosis. Here, we investigate the biological relevance of beta1 integrin endocytosis to fibronectin matrix turnover. First, we demonstrate that beta1 integrins, including alpha5beta1 play an important role in endocytosis and turnover of matrix fibronectin. Second, we show that caveolin-1 constitutively regulates endocytosis of alpha5beta1 integrins, and that alpha5beta1 integrin endocytosis can occur in the absence of fibronectin and fibronectin matrix. We also show that downregulation of caveolin-1 expression by siRNA results in marked reduction of beta1 integrin and fibronectin endocytosis. Hence, caveolin-1-dependent beta1 integrin and fibronectin endocytosis plays a critical role in fibronectin matrix turnover, and may contribute to abnormal ECM remodeling that occurs in fibrotic disorders.
7. Consequences of lack of beta 1 integrin gene expression in mice. beta 1 integrins are cell-surface receptors that mediate cell-cell and cell-matrix interactions. We have generated a null mutation in the gene for the beta 1 integrin subunit in mice and embryonic stem (ES) cells. Heterozygous mice are indistinguishable from normal littermates. Homozygous null embryos develop normally to the blastocyst stage, implant, and invade the uterine basement membrane but die shortly thereafter. Using beta 1 integrin-deficient ES cells we have established chimeric embryos and adult mice. Analysis of the chimeric embryos demonstrated the presence of beta 1 integrin-deficient cells in all germ layers indicating that beta 1-null cells can differentiate and migrate in a context of normal tissue. When evaluated at embryonic day 9.5 (E9.5), embryos with a beta 1-null cell contribution below 25% were developing normally, whereas embryos with a contribution above this threshold were distorted and showed abnormal morphogenesis. In adult chimeric mice beta 1 integrin-deficient cells failed to colonize liver and spleen but were found in all other tissues analyzed at levels from 2%-25%. Immunostaining of chimeric mice showed that in cardiac muscle, there were small, scattered patches of myocytes that were beta 1-null. In contrast, many myotubes showed some beta 1-null contribution as a result of fusion between wild-type and mutant myoblasts to form mixed myotubes. The adult chimeric brain contained beta 1-null cells in all regions analyzed. Also, tissues derived from the neural crest contained beta 1 integrin-deficient cells indicating that **migration** of neuronal cells as well as neural crest cells can occur in the absence of beta 1 integrins.
8. Deletion of beta 1 integrins in mice results in inner cell mass failure and peri-implantation lethality. Integrin receptors for extracellular matrix receptors are important effectors of cell adhesion, differentiation, and **migration** in cultured cells and are believed to be critical effectors of these processes during development. To determine when beta 1 integrins become critical during embryonic development, we generated mutant mice with a targeted disruption of the beta 1 integrin subunit gene. Heterozygous mutant mice were normal. Homozygous loss of beta 1 integrin expression was lethal during early postimplantation development. Homozygous embryos lacking beta 1 integrins formed normal-looking blastocysts and initiated implantation at E4.5. However, the E4.5 beta 1-null embryos in situ had collapsed blastocoeles, and whereas the trophoblast penetrated the uterine epithelium, extensive invasion of the decidua was not observed. Laminin-positive endoderm cells were detected in the inner cell mass area, but endoderm morphogenesis and **migration** were defective. By E5.5 beta 1-null embryos had degenerated extensively. In vitro analysis showed that trophoblast function in beta 1-null peri-implantation embryos was largely normal, including expression of tissue-specific markers, and outgrowth on fibronectin- and vitronectin-coated, although not on laminin-coated substrates. In contrast, the inner cell mass region of beta 1-null blastocyst outgrowths, and inner cell masses isolated from beta 1-null blastocysts, showed highly retarded growth and defective extraembryonic endoderm morphogenesis and **migration**. These data suggest that beta 1 integrins are required for normal morphogenesis of the inner cell mass and are essential mediators of growth and survival of cells of the inner cell mass. Failure of continued trophoblast development in beta 1-null embryos after inner cell mass failure could be attributable to either an intrinsic requirement for beta 1 integrins for later stages of trophoblast development, or to the lack of trophic signals from the beta 1-null inner cell mass.
9. Serum response factor is required for sprouting angiogenesis and vascular integrity. Serum response factor (SRF) is a transcription factor that controls the expression of cytoskeletal proteins and immediate early genes in different cell types. Here, we found that SRF expression is restricted to endothelial cells (ECs) of small vessels such as capillaries in the mouse embryo. EC-specific Srf deletion led to aneurysms and hemorrhages from 11.5 days of mouse development (E11.5) and lethality at E14.5. Mutant embryos presented a reduced capillary density and defects in EC **migration**, with fewer numbers of filopodia in tip cells and ECs showing defects in actin polymerization and intercellular junctions. We show that SRF is essential for the expression of VE-cadherin and beta-actin in ECs both in vivo and in vitro. Moreover, knockdown of SRF in ECs impaired VEGF- and FGF-induced in vitro angiogenesis. Taken together, our results demonstrate that SRF plays an important role in sprouting angiogenesis and small vessel integrity in the mouse embryo.
10. Inhibition of pathologic retinal neovascularization by alpha-defensins. Proliferative retinopathies, such as those complicating prematurity and diabetes, are major causes of blindness. A prominent feature of these retinopathies is excessive neovascularization, which is orchestrated by the hypoxia-induced vascular endothelial growth factor (VEGF) stimulating endothelial cells and the integrin-mediated adhesive interactions of endothelial cells with extracellular matrix components such as fibronectin (FN). Recently, we demonstrated that alpha-defensins interfere with alpha5beta1-FN interactions and dependent endothelial cell functions. Here, alpha-defensins were studied in hypoxia-induced proliferative retinopathy. In vitro, alpha-defensins specifically inhibited alpha5beta1-integrin-dependent **migration** of bovine retinal endothelial cells (BRECs) to FN, attenuated the VEGF-stimulated increase in endothelial permeability, and blocked BREC proliferation and capillary sprout formation in 3-dimensional fibrin-matrices. An up-regulation of beta1-integrin and FN was observed in the retinal vessels in the mouse model of hypoxia-induced retinal angiogenesis. Systemic and local administration of alpha-defensins reduced retinal neovascularization by 45% and 60%, respectively, and this effect was comparable to the inhibitory effect of alpha5beta1-blocking antibody. alpha-Defensins were detected in human diabetic retinas associated with normal retinal vessels but were absent from proliferative lesions. Together, these data show that alpha-defensins inhibit pathologic retinal neovascularization in vivo and may provide a clinically efficient strategy against proliferative retinopathies.
11. Junctional adhesion molecule A interacts with Afadin and PDZ-GEF2 to activate Rap1A, regulate beta1 integrin levels, and enhance cell **migration**. Junctional adhesion molecule-A (JAM-A) is a transmembrane tight junction protein that has been shown to regulate barrier function and cell **migration** through incompletely understood mechanisms. We have previously demonstrated that JAM-A regulates cell **migration** by dimerization of the membrane-distal immunoglobulin-like loop and a C-terminal postsynaptic density 95/disc-large/zona occludens (PDZ) binding motif. Disruption of dimerization resulted in decreased epithelial cell **migration** secondary to diminished levels of beta1 integrin and active Rap1. Here, we report that JAM-A is physically and functionally associated with the PDZ domain-containing molecules Afadin and PDZ-guanine nucleotide exchange factor (GEF) 2, but not zonula occludens (ZO)-1, in epithelial cells, and these interactions mediate outside-in signaling events. Both Afadin and PDZ-GEF2 colocalized and coimmunoprecipitated with JAM-A. Furthermore, association of PDZ-GEF2 with Afadin was dependent on the expression of JAM-A. Loss of JAM-A, Afadin, or PDZ-GEF2, but not ZO-1 or PDZ-GEF1, similarly decreased cellular levels of activated Rap1, beta1 integrin protein, and epithelial cell **migration**. The functional effects observed were secondary to decreased levels of Rap1A because knockdown of Rap1A, but not Rap1B, resulted in decreased beta1 integrin levels and reduced cell **migration**. These findings suggest that JAM-A dimerization facilitates formation of a complex with Afadin and PDZ-GEF2 that activates Rap1A, which regulates beta1 integrin levels and cell **migration**.
12. Balancing neural crest cell intrinsic processes with those of the microenvironment in Tcof1 haploinsufficient mice enables complete enteric nervous system formation. The enteric nervous system (ENS) comprises a complex neuronal network that regulates peristalsis of the gut wall and secretions into the lumen. The ENS is formed from a multipotent progenitor cell population called the neural crest, which is derived from the neuroepithelium. Neural crest cells (NCCs) migrate over incredible distances to colonize the entire length of the gut and during their **migration** they must survive, proliferate and ultimately differentiate. The absence of an ENS from variable lengths of the colon results in Hirschsprung's disease (HSCR) or colonic aganglionosis. Mutations in about 12 different genes have been identified in HSCR patients but the complex pattern of inheritance and variable penetrance suggests that additional genes or modifiers must be involved in the etiology and pathogenesis of this disease. We discovered that Tcof1 haploinsufficiency in mice models many of the early features of HSCR. Neuroepithelial apoptosis diminished the size of the neural stem cell pool resulting in reduced NCC numbers and their delayed **migration** along the gut from E10.5 to E14.5. Surprisingly however, we observe continued and complete colonization of the entire colon throughout E14.5-E18.5, a period in which the gut is considered to be non- or less-permissive to NCC. Thus, we reveal for the first time that reduced NCC progenitor numbers and delayed **migration** do not unequivocally equate with a predisposition for the pathogenesis of HSCR. In fact, these deficiencies can be overcome by balancing NCC intrinsic processes of proliferation and differentiation with extrinsic influences of the gut microenvironment.
13. Wound healing in the alpha2beta1 integrin-deficient mouse: altered keratinocyte biology and dysregulated matrix metalloproteinase expression. The alpha2beta1 integrin, a collagen/laminin receptor, is expressed at high level in the basal cell layer of the epidermis. To define the role of the alpha2beta1 integrin in wound healing, wound repair was extensively evaluated in wild-type and alpha2-null mice in vivo. In addition, the impact of alpha2beta1 integrin-deficiency on the function of primary murine keratinocytes in vitro was analyzed. Our in vivo findings demonstrate that genetic deletion of the alpha2beta1 integrin does not significantly alter the rate of re-epithelialization, collagen deposition, or tensile strength during wound closure in mice. In marked contrast to the observed similarities in wound healing, deletion of the alpha2beta1 integrin resulted in a dramatic increase in neoangiogenesis in the wound microenvironment. In contrast to in vivo studies, primary keratinocytes from alpha2-null mice adhered poorly and displayed impaired **migration** on type I collagen in vitro. We demonstrate that alpha2beta1 integrin-ligation negatively regulates expression of genes including matrix metalloproteinases both in vivo and in vitro. Furthermore, the changes in gene expression could potentially account for relatively normal wound healing in the alpha2-deficient mouse and our recent observation that suggests an antiangiogenic role for the alpha2beta1 integrin in vivo.
14. Epithelial β1 integrin is required for lung branching morphogenesis and alveolarization. Integrin-dependent interactions between cells and extracellular matrix regulate lung development; however, specific roles for β1-containing integrins in individual cell types, including epithelial cells, remain incompletely understood. In this study, the functional importance of β1 integrin in lung epithelium during mouse lung development was investigated by deleting the integrin from E10.5 onwards using surfactant protein C promoter-driven Cre. These mutant mice appeared normal at birth but failed to gain weight appropriately and died by 4 months of age with severe hypoxemia. Defects in airway branching morphogenesis in association with impaired epithelial cell adhesion **and migr**ation, as well as alveolarization defects and persistent macrophage-mediated inflammation were identified. Using an inducible system to delete β1 integrin after completion of airway branching, we showed that alveolarization defects, characterized by disrupted secondary septation, abnormal alveolar epithelial cell differentiation, excessive collagen I and elastin deposition, and hypercellularity of the mesenchyme occurred independently of airway branching defects. By depleting macrophages using liposomal clodronate, we found that alveolarization defects were secondary to persistent alveolar inflammation. β1 integrin-deficient alveolar epithelial cells produced excessive monocyte chemoattractant protein 1 and reactive oxygen species, suggesting a direct role for β1 integrin in regulating alveolar homeostasis. Taken together, these studies define distinct functions of epithelial β1 integrin during both early and late lung development that affect airway branching morphogenesis, epithelial cell differentiation, alveolar septation and regulation of alveolar homeostasis.
15. CBAP functions as a novel component in chemokine-induced ZAP70-mediated T-cell adhesion and **migration**. Activated chemokine receptor initiates inside-out signaling to transiently trigger activation of integrins, a process involving multiple components that have not been fully characterized. Here we report that GM-CSF/IL-3/IL-5 receptor common beta-chain-associated protein (CBAP) is required to optimize this inside-out signaling and activation of integrins. First, knockdown of CBAP expression in human Jurkat T cells caused attenuated CXC chemokine ligand-12 (CXCL12)-induced cell **migration** and integrin α4β1- and αLβ2-mediated cell adhesion in vitro, which could be rescued sufficiently upon expression of murine CBAP proteins. Freshly isolated CBAP-deficient primary T cells also exhibited diminution of chemotaxis toward CC chemokine ligand-21 (CCL21) and CXCL12, and these chemokines-induced T-cell adhesions in vitro. Adoptive transfer of isolated naive T cells demonstrated that CBAP deficiency significantly reduced lymph node homing ability in vivo. Final**ly, migra**tion of T cell-receptor-activated T cells induced by inflammatory chemokines was also attenuated in CBAP-deficient cells. Further analyses revealed that CBAP constitutively associated with both integrin β1 and ZAP70 and that CBAP is required for chemokine-induced initial binding of the talin-Vav1 complex to integrin β1 and to facilitate subsequent ZAP70-mediated dissociation of the talin-Vav1 complex and Vav1 phosphorylation. Within such an integrin signaling complex, CBAP likely functions as an adaptor and ultimately leads to activation of both integrin α4β1 and Rac1. Taken together, our data suggest that CBAP indeed can function as a novel signaling component within the ZAP70/Vav1/talin complex and plays an important role in regulating chemokine-promoted T-cell trafficking.
16. Rap1 GTPase is required for mouse lens epithelial maintenance and morphogenesis. Rap1, a Ras-like small GTPase, plays a crucial role in cell-matrix adhesive interactions, cell-cell junction formation, cell polarity and **migration**. The role of Rap1 in vertebrate organ development and tissue architecture, however, remains elusive. We addressed this question in a mouse lens model system using a conditional gene targeting approach. While individual germline deficiency of either Rap1a or Rap1b did not cause overt defects in mouse lens, conditional double deficiency (Rap1 cKO) prior to lens placode formation led to an ocular phenotype including microphthalmia and lens opacification in embryonic mice. The embryonic Rap1 cKO mouse lens exhibited striking defects including loss of E-cadherin- and ZO-1-based cell-cell junctions, disruption of paxillin and β1-integrin-based cell adhesive interactions along with abnormalities in cell shape and apical-basal polarity of epithelium. These epithelial changes were accompanied by increased levels of α-smooth muscle actin, vimentin and N-cadherin, and expression of transcriptional suppressors of E-cadherin (Snai1, Slug and Zeb2), and a mesenchymal metabolic protein (Dihydropyrimidine dehydrogenase). Additionally, while lens differentiation was not overtly affected, increased apoptosis and dysregulated cell cycle progression were noted in epithelium and fibers in Rap1 cKO mice. Collectively these observations uncover a requirement for Rap1 in maintenance of lens epithelial phenotype and morphogenesis.
17. Integrin alpha9beta1 mediates enhanced cell **migration** through nitric oxide synthase activity regulated by Src tyrosine kinase. Integrins are important mediators of cell adhesion and **migration**, which in turn are essential for diverse biological functions, including wound healing and cancer metastasis. The integrin alpha9beta1 is expressed on numerous mammalian tissues and can mediate accelerated cell **migration**. As the molecular signaling mechanisms that transduce this effect are poorly defined, we investigated the pathways by which activated integrin alpha9beta1 signals **migration**. We found for the first time that specific ligation of integrin alpha9beta1 rapidly activates Src tyrosine kinase, with concomitant tyrosine phosphorylation of p130Cas and activation of Rac-1. Furthermore, activation of integrin alpha9beta1 also enhanced NO production through activation of inducible nitric oxide synthase (iNOS). Inhibition of Src tyrosine kinase or NOS decreased integrin-alpha9beta1-dependent cell **migration**. Src appeared to function most proximal in the signaling cascade, in a FAK-independent manner to facilitate iNOS activation and NO-dependent cell **migration**. The cytoplasmic domain of integrin alpha9 was crucial for integrin-alpha9beta1-induced Src activation, subsequent signaling events and cell **migration**. When taken together, our results describe a novel and unique mechanism of coordinated interactions of the integrin alpha9 cytoplasmic domain, Src tyrosine kinase and iNOS to transduce integrin-alpha9beta1-mediated cell **migration**.
18. Adhesion molecules involved in macrophage responses to Wallerian degeneration in the murine peripheral nervous system. When a peripheral nerve is damaged the severed axon undergoes Wallerian degeneration. The distal nerve is infiltrated by large numbers of monocyte-derived macrophages which participate in the phagocytosis of degenerating myelin. In other tissues, adhesion molecules play a crucial role in leukocyte recruitment during inflammation. Blood-borne cells enter damaged tissue by interacting with adhesion molecules expressed on activated endothelium. Having crossed the endothelium, leukocytes must adhere and migrate within the tissue. We investigated the adhesion molecules involved in both stages of the macrophage response to transection of one sciatic nerve of BALB/c mice. By injecting monoclonal antibodies in vivo, before and after peripheral nerve injury, we showed that intercellular adhesion molecule-1 (ICAM-1) and integrins alpha4beta1 (VLA-4) and alphaMbeta2 (type 3 complement receptor) are unlikely to be involved in the transendothelial **migration** of monocytes responding to peripheral nerve degeneration. We also studied the adhesion of macrophages within the endoneurium, using an in vitro adhesion assay. Macrophages showed much greater levels of adhesion to cryostat sections of transected nerves than to control nerves. This increased adhesion was partially inhibited by antibodies to the beta1-integrin chain, and more strongly inhibited by the extracellular matrix molecules fibronectin and collagen. Adhesion was unaffected by laminin-1 and by antibodies to other adhesion molecules, including alpha4beta1- and alpha5beta1-integrins. Thus we conclude that monocyte entry into a degenerating peripheral nerve is independent of alphaLbeta2/alphaMbeta2-ICAM-1 or alpha4beta1/VCAM-1 interactions, and that adhesion within the endoneurium is mediated in part by a beta1-integrin other than alpha4beta1 or alpha5beta1.
19. Vimocin and vidapin, cyclic KTS peptides, are dual antagonists of α1β1/α2β1 integrins with antiangiogenic activity. Obtustatin and viperistatin, members of the disintegrin protein family, served as lead compounds for the synthesis of linear and cyclic peptides containing the KTS binding motif. The most active linear peptide, a viperistatin analog, indicated the importance of Cys(19) and Cys(29), as well as the presence of Arg at position 24 for their biologic activity, and was used as the basic sequence for the synthesis of cyclic peptides. Vimocin (compound 6) and vidapin (compound 10) showed a high potency (IC50 = 0.17 nM) and intermediate efficacy (20 and 40%) in inhibition of adhesion of α1/α2 integrin overexpressor cells to respective collagens. Vimocin was more active in inhibition of the wound healing (53%) and corneal micropocket (17%) vascularization, whereas vidapin was more potent in inhibiti**on of mig**ration in the Matrigel tube formation assay (90%). Both compounds similarly inhibited proliferation (50-90%) of endothelial cells, and angiogenesis induced by vascular endothelial growth factor (80%) and glioma (55%) in the chorioallantoic membrane assay. These peptides were not toxic to endothelial cell cultures and caused no acute toxicity upon intravenous injection in mice, and were stable for 10-30 hours in human serum. The in vitro and in vivo potency of the peptides are consistent with conformational ensembles and "bioactive" space shared by obtustatin and viperistatin. These findings suggest that vimocin and vidapin can serve as dual α1β1/α2β1 integrin antagonists in antiangiogenesis and cancer therapy.
20. Dual functions of [alpha]4[beta]1 integrin in epicardial development: initial **migration** and long-term attachment. The epicardium of the mammalian heart arises from progenitor cells outside the developing heart. The epicardial progenitor (EPP) cells migrate onto the heart through a cyst-mediated mechanism in which the progenitors are released from the tissue of origin as cysts; the cysts float in the fluid of the pericardial cavity and attach to the naked myocardial surface of the heart, and cells in the cysts then migrate out to form an epithelial sheet. In this paper, we show that the gene encoding the alpha4 subunit of alpha4beta1 integrin (alpha4beta1) is essential for this migratory process. We have generated a knockin mutation in mice replacing the alpha4 integrin gene with the lacZ reporter gene, placing lacZ under the control of the alpha4 integrin promoter. We show that in homozygous mutant embryos, the **migration** of EPP progenitor cells is impaired due to inefficient budding of the cysts and a failure of the cells in the cysts to migrate on the heart. This study provides direct genetic evidence for essential roles for alpha4beta1 integrin-mediated cell adhesion in the **migration** of progenitor cells to form the epicardium, in addition to a previous finding that alpha4beta1 is essential for maintaining the epicardium (Yang, J.T., H. Rayburn, and R.O. Hynes. 1995. Development. 121:549-560).
21. Deletion of core fucosylation on alpha3beta1 integrin down-regulates its functions. The core fucosylation (alpha1,6-fucosylation) of glycoprotein is widely distributed in mammalian tissues. Recently alpha1,6-fucosylation has been further reported to be very crucial by the study of alpha1,6-fucosyltransferase (Fut8)-knock-out mice, which shows the phenotype of emphysema-like changes in the lung and severe growth retardation. In this study, we extensively investigated the effect of core fucosylation on alpha3beta1 integrin and found for the first time that Fut8 makes an important contribution to the functions of this integrin. The role of core fucosylation in alpha3beta1 integrin-mediated events has been studied by using Fut8(+/+) and Fut8(-/-) embryonic fibroblasts, respectively. We found that the core fucosylation of alpha3beta1 integrin, the major receptor for laminin 5, was abundant in Fut8(+/+) cells but was totally abolished in Fut8(-/-) cells, which was associated with the deficient **migration** mediated by alpha3beta1 integrin in Fut8(-/-) cells. Moreover integrin-mediated cell signaling was reduced in Fut8(-/-) cells. The reintroduction of Fut8 potentially restored laminin 5-induced **migration** and intracellular signaling. Collectively, these results suggested that core fucosylation is essential for the functions of alpha3beta1 integrin.
22. Control of the collective **migration** of enteric neural crest cells by the Complement anaphylatoxin C3a and N-cadherin. We analyzed the cellular and molecular mechanisms governing the adhesive and migratory behavior of enteric neural crest cells (ENCCs) during their collective **migration** within the developing mouse gut. We aimed to decipher the role of the complement anaphylatoxin C3a during this process, because this well-known immune system attractant has been implicated in cephalic NCC co-attraction, a process controlling directional **migration**. We used the conditional Ht-PA-cre transgenic mouse model allowing a specific ablation of the N-cadherin gene and the expression of a fluorescent reporter in migratory ENCCs without affecting the central nervous system. We performed time-lapse videomicroscopy of ENCCs from control and N-cadherin mutant gut explants cultured on fibronectin (FN) and micropatterned FN-stripes with C3a or C3aR antagonist, and studied cell **migration** behavior with the use of triangulation analysis to quantify cell dispersion. We performed ex vivo gut cultures with or without C3aR antagonist to determine the effect on ENCC behavior. Confocal microscopy was used to analyze the cell-matrix adhesion properties. We provide the first demonstration of the localization of the complement anaphylatoxin C3a and its receptor on ENCCs during their **migration** in the embryonic gut. C3aR receptor inhibition alters ENCC adhesion and **migration**, perturbing directionality and increasing cell dispersion both in vitro and ex vivo. N-cadherin-null ENCCs do not respond to C3a co-attraction. These findings indicate that C3a regulates cell **migration** in a N-cadherin-dependent process. Our results shed light on the role of C3a in regulating collective and directional cell **migration**, and in ganglia network organization during enteric nervous system ontogenesis. The detection of an immune system chemokine in ENCCs during ENS development may also shed light on new mechanisms for gastrointestinal disorders.
23. Role of hypoxia-induced fibronectin-integrin β1 expression in embryonic stem cell proliferation and **migratio**n: Involvement of PI3K/Akt and FAK. Cell **migratio**n is largely dependent on integrin (IN) binding to the extracellular matrix, and several signaling pathways involved in these processes have been shown to be modified by hypoxia. Therefore, the aim of this study was to determine the influence of hypoxia on fibronectin (FN) and IN β1 expression in mouse embryonic stem cells (mESCs) and their signaling pathways to modulate proliferation. FN and IN β1 expression were significantly increased in hypoxic mESCs by 24 h. Hypoxia also increased cell attachment, which was accompanied by concomitant increases in the binding level of FN and IN β1. Hypoxia-induced FN expression was mediated by increased phosphatidylinositol 3 kinase (PI3K)/Akt and mammalian target of rapamycin (mTOR) phosphorylation, and hypoxia-inducible factor-1α (HIF-1α) expression. Moreover, under hypoxic conditions, focal adhesion kinase (FAK) and Src phosphorylation were increased in a time-dependent fashion; these increases were blocked by IN β1 antibody. In addition, the hypoxia induced increase of F-actin distribution **and cell** migration (activation of matrix metalloproteinase-2 and -9) was inhibited by IN β1 antibody. Indeed, hypoxia increased the level of cell-cycle regulatory protein and DNA synthesis. In conclusion, hypoxia increases the prolifer**ation and** migration of mESCs via FN-IN β1 production through the PI3K/Akt, mTOR, and HIF-1α pathways, followed by FAK activation.
24. Regulation of neural progenitor proliferation and survival by beta1 integrins. Neural stem cells give rise to undifferentiated nestin-positive progenitors that undergo extensive cell division before differentiating into neuronal and glial cells. The precise control of this process is likely to be, at least in part, controlled by instructive cues originating from the extracellular environment. Some of these cues are interpreted by the integrin family of extracellular matrix receptors. Using neurosphere cell cultures as a model system, we show that beta1-integrin signalling plays a crucial role in the regulation of progenitor cell proliferation, survival and **migration**. Following conditional genetic ablation of the beta1-integrin allele, and consequent loss of beta1-integrin cell surface protein, mutant nestin-positive progenitor cells proliferate less and die in higher numbers than their wild-type counterparts. Mutant progenitor cell **migration** on different ECM substrates is also impaired. These effects can be partially compensated by the addition of exogenous growth factors. Thus, beta1-integrin signalling and growth factor signalling tightly interact to control the number and migratory capacity of nestin-positive progenitor cells.
25. Close homolog of L1 modulates area-specific neuronal positioning and dendrite orientation in the cerebral cortex. We show that the neural cell recognition molecule Close Homolog of L1 (CHL1) is required for neuronal positioning and dendritic growth of pyramidal neurons in the posterior region of the developing mouse neocortex. CHL1 was expressed in pyramidal neurons in a high-caudal to low-rostral gradient within the developing cortex. Deep layer pyramidal neurons of CHL1-minus mice were shifted to lower laminar positions in the visual and somatosensory cortex and developed misoriented, often inverted apical dendrites. Impaired **migration** of CHL1-minus cortical neurons was suggested by strikingly slower rates of radial **migration** in cortical slices, failure to potentiate integrin-dependent haptotactic cell **migration** in vitro, and accumulation of migratory cells in the intermediate and ventricular/subventricular zones in vivo. The restriction of CHL1 expression and effects of its deletion in posterior neocortical areas suggests that CHL1 may regulate area-specific neuronal connectivity and, by extension, function in the visual and somatosensory cortex.
26. Alpha3beta1 integrin regulates MMP-9 mRNA stability in immortalized keratinocytes: a novel mechanism of integrin-mediated MMP gene expression. Matrix metalloproteinases facilitate cell **migration** and tumor invasion through their ability to proteolyse the extracellular matrix. The laminin-binding integrin alpha3beta1 is expressed at high levels in squamous cell carcinomas and in normal keratinocytes during cutaneous wound healing. We showed previously that alpha3beta1 is required for MMP-9/gelatinase B secretion in immortalized mouse keratinocytes (MK cells) and that this regulation was acquired as part of the immortalized phenotype, suggesting a possible role for alpha3beta1 during malignant conversion. In the current study, we identify a novel mechanism whereby alpha3beta1 regulates the induction of MMP-9 expression that occurs in response to activation of a MAPK kinase (MEK)/extracellular signal-regulated kinase (ERK) pathway. Inhibition of MEK/ERK signaling in wild-type MK cells with a pharmacological inhibitor, U0126, showed that ERK activation was necessary for high levels of endogenous MMP-9 gene expression and activity of a transfected MMP-9 promoter. Furthermore, activation of MEK/ERK signaling in these cells with an oncogenic mutant of Ras, RasV12, increased both endogenous MMP-9 gene expression and MMP-9 promoter activity. Experiments with alpha3beta1-deficient MK cells revealed that alpha3beta1 was required for both baseline levels and RasV12-induced levels of MMP-9 mRNA expression. However, alpha3beta1 was not required for RasV12-mediated activation of ERK or for ERK-dependent MMP-9 promoter activity. Direct comparison of mRNA turnover in the wild type and alpha3-null MK cells identified a requirement for alpha3beta1 in stabilization of MMP-9 mRNA transcripts. These results identify a novel function for integrins in promoting mRNA stability as a mechanism to potentiate MAPK-mediated gene expression. They also suggest a role for alpha3beta1 in maintaining high levels of MMP-9 mRNA expression in response to oncogenic activation of MEK/ERK signaling pathways.
27. A novel multistep mechanism for initial lymphangiogenesis in mouse embryos based on ultramicroscopy. During mammalian development, a subpopulation of endothelial cells in the cardinal vein (CV) expresses lymphatic-specific genes and subsequently develops into the first lymphatic structures, collectively termed as lymph sacs. Budding, sprouting and ballooning of lymphatic endothelial cells (LECs) have been proposed to underlie the emergence of LECs from the CV, but the exact mechanisms of lymph vessel formation remain poorly understood. Applying selective plane illumination-based ultramicroscopy to entire wholemount-immunostained mouse embryos, we visualized the complete developing vascular system with cellular resolution. Here, we report emergence of the earliest detectable LECs as strings of loosely connected cells between the CV and superficial venous plexus. Subsequent aggregation of LECs resulted in formation of two distinct, previously unidentified lymphatic structures, the dorsal peripheral longitudinal lymphatic vessel (PLLV) and the ventral primordial thoracic duct (pTD), which at later stages formed a direct contact with the CV. Providing new insights into their function, we found vascular endothelial growth factor C (VEGF-C) and the matrix component CCBE1 indispensable for LEC budding and **migration**. Altogether, we present a significantly more detailed view and novel model of early lymphatic development.
28. Genetic deletion or antibody blockade of alpha1beta1 integrin induces a stable plaque phenotype in ApoE-/- mice. Adhesive interactions between cells and the extracellular matrix play an important role in inflammatory diseases like atherosclerosis. We investigated the role of the collagen-binding integrin alpha1beta1 in atherosclerosis.ApoE-/- mice were alpha1-deficient or received early or delayed anti-alpha1 antibody treatment. Deficiency in alpha1 integrin reduced the area of atherosclerotic plaques and altered plaque composition by reducing inflammation and increasing extracellular matrix. In advanced plaques, alpha1-deficient mice had a reduced macrophage and CD3+ cell content, collagen and smooth muscle cell content increased, lipid core sizes decreased, and cartilaginous metaplasia occurred. Anti-alpha1 antibody treatment reduced the macrophage content in initial plaques after early and delayed treatment, decreased the CD3+ cell content in advanced plaques after delayed treatment, and increased the collagen content in initial and advanced plaques after delayed treatment. **Migration** assays performed on alpha1-deficient macrophages on collagen I and IV substrata revealed that alpha1-deficient cells can migrate on collagen I, but not IV. Anti-alpha1 antibody treatment of ApoE-/- macrophages also inhibited **migration** of cells on collagen IV.Our results suggest that alpha1beta1 integrin is involved in atherosclerosis by mediating the **migration** of leukocytes to lesions by adhesion to collagen IV. Blocking this integrin reduces atherosclerosis and induces a stable plaque phenotype.
29. Involvement of beta1-integrin up-regulation in basic fibroblast growth factor- and epidermal growth factor-induced proliferation of mouse neuroepithelial cells. In neural stem cells, basic fibroblast growth factor (bFGF) and epidermal growth factor (EGF) promote cell proliferation and self-renewal. In the bFGF- and EGF-responsive neural stem cells, beta1-integrin also plays important roles in crucial cellular processes, including proliferation, **migration**, and apoptosis. The cross-talk of the signaling pathways mediated by these growth factors and beta1-integrin, however, has not been fully elucidated. Here we report a novel molecular mechanism through which bFGF or EGF promotes the proliferation of mouse neuroepithelial cells (NECs). In the NECs, total beta1-integrin expression levels and proliferation were dose-dependently increased by bFGF but not by EGF. EGF rather than bFGF strongly induced the increase of beta1-integrin localization on the NEC surface. bFGF- and EGF-induced beta1-integrin up-regulation and proliferation were inhibited after treatment with a mitogen-activated protein kinase kinase inhibitor, U0126, which indicates the dependence on the mitogen-activated protein kinase pathway. Involvement of beta1-integrin in bFGF- and EGF-induced proliferation was confirmed by the finding that NEC proliferation and adhesion to fibronectin-coated dishes were inhibited by knockdown of beta1-integrin using small interfering RNA. On the other hand, apoptosis was induced in NECs treated with RGD peptide, a small beta1-integrin inhibitor peptide with the Arg-Gly-Asp motif, but it was independent of beta1-integrin expression levels. Those results suggest that regulation of beta1-integrin expression/localization is involved in cellular processes, such as proliferation, induced by bFGF and EGF in NECs. The mechanism underlying the proliferation through beta1-integrin would not be expected to be completely identical, however, for bFGF and EGF.
30. alpha5beta1-integrin expression is essential for tumor progression in experimental lung cancer. The matrix glycoprotein, fibronectin, stimulates the proliferation of non-small cell lung carcinoma in vitro through α5β1 integrin receptor-mediated signals. However, the true role of fibronectin and its receptor in lung carcinogenesis in vivo remains unclear. To test this, we generated mouse Lewis lung carcinoma cells stably transfected with short hairpin RNA shRNA targeting the α5 integrin subunit. These cells were characterized and tested in proliferation, cell adhesio**n, migrat**ion, and soft agar colony formation assays in vitro. In addition, their growth and metastatic potential was tested in vivo in a murine model of lung cancer. We found that transfected Lewis lung carcinoma cells showed decreased expression of the α5 gene, which was associated with decreased adhesion to fibronectin and reduced c**ell migra**tion, proliferation, and colony formation when compared with control cells and cells stably transfected with α2 integrin subunit in vitro. C57BL/6 mice injected with α5-silenced cells showed lower burden of implanted tumors, and a dramatic decrease in lung metastases resulting in higher survival as compared with mice injected with wild-type or α2 integrin-silenced cells. These observations reveal that recognition of host- and/or tumor-derived fibronectin via α5β1 is important for tumor growth both in vitro and in vivo, and unveil α5β1 as a potential target for the development of anti-lung cancer therapies.
31. CD98 modulates integrin beta1 function in polarized epithelial cells. The type II transmembrane protein CD98, best known as the heavy chain of the heterodimeric amino acid transporters (HAT), is required for the surface expression and basolateral localization of this transporter complex in polarized epithelial cells. CD98 also interacts with beta1 integrins resulting in an increase in their affinity for ligand. In this study we explored the role of the transmembrane and cytoplasmic domains of CD98 on integrin-dependent cell adhesion and **migration** in polarized renal epithelial cells. We demonstrate that the transmembrane domain of CD98 was sufficient, whereas the five N-terminal amino acids of this domain were required for CD98 interactions with beta1 integrins. Overexpression of either full-length CD98 or CD98 lacking its cytoplasmic tail increased cell adhesion and **migration**, whereas deletion of the five N-terminal amino acids of the transmembrane domain of CD98 abrogated this effect. CD98 and mutants that interacted with beta1 integrins increased both focal adhesion formation and FAK and AKT phosphorylation. CD98-induced cell adhesion and **migration** was inhibited by addition of phosphoinositol 3-OH kinase (PI3-K) inhibitors suggesting these cell functions are PI3-K-dependent. Finally, CD98 and mutants that interacted with beta1, induced marked changes in polarized renal epithelial cell branching morphogenesis in collagen gels. Thus, in polarized renal epithelial cells, CD98 might be viewed as a scaffolding protein that interacts with basolaterally expressed amino acid transporters and beta1 integrins and can alter diverse cellular functions such as amino acid transport as well as cell adhesion, **migration** and branching morphogenesis.
32. Semaphorin 3C regulates endothelial cell function by increasing integrin activity. Class 3 semaphorins (sema 3) are secreted guidance proteins. Sema 3A expressed by endothelial cells controls vascular morphogenesis through integrin inhibition. Sema 3C is required for normal cardiovascular patterning. Here we examined the potential role of sema 3C as regulator of endothelial cell function in vitro using mouse glomerular endothelial cells (MGEC). We determined that MGEC express sema 3C mRNA and protein and its receptors mRNA. Recombinant sema 3C induced MGEC proliferation 18 +/- 2% above control, as assessed by bromodeoxyuridine (BrdU) incorporation, and reduced starvation-induced apoptosis by 46 +/- 3%, as indicated by an in situ marker of activated caspase 3. Sema 3C increased MGEC adhesion to fibronectin 79 +/- 13% and to collagen 55 +/- 12% as compared with control. Sema 3C-induced MGEC adhesion was prevented by integrin blocking antibodies and involved beta1 integrin serine phosphorylation. Sema 3C-induced MGEC adhesion and proliferation were similar to those induced by vascular endothelial growth factor (VEGF)-A. Sema 3C induced a 44 +/- 11% increase in MGEC directional **migration** and stimulated MGEC capillary-like network formation on collagen I gels. Collectively, our data indicate that sema 3C promotes glomerular endothelial cell proliferation, adhesion, directional **migration**, and tube formation in vitro by stimulating integrin phosphorylation and VEGF120 secretion, functions that are similar to VEGF-A and opposite to sema 3A.
33. Collagen XIII induced in vascular endothelium mediates alpha1beta1 integrin-dependent trans**migration** of monocytes in renal fibrosis. Alport syndrome is a common hereditary basement membrane disorder caused by mutations in the collagen IV α3, α4, or α5 genes that results in progressive glomerular and interstitial renal disease. Interstitial monocytes that accumulate in the renal cortex from Alport mice are immunopositive for integrin α1β1, while only a small fraction of circulating monocytes are immunopositive for this integrin. We surmised that such a disparity might be due to the selective recruitment of α1β1-positive monocytes. In this study, we report the identification of collagen XIII as a ligand that facilitates this selective recruitment of α1β1 integrin-positive monocytes. Collagen XIII is absent in the vascular endothelium from normal renal cortex and abundant in Alport renal cortex. Neutralizing antibodies against the binding site in collagen XIII for α1β1 integrin selectively block VLA1-positiv**e monocyt**e migration in transwell assays. Injection of these antibodies into Alport mice slows monocyte recruitment and protects against renal fibrosis. Thus, the induction of collagen XIII in endothelial cells of Alport kidneys mediates the selective recruitment of α1β1 integrin-positive monocytes and may potentially serve as a therapeutic target for inflammatory diseases in which lymphocyte/monocyte recruitment involves the interaction with α1β1 integrin.
34. Deletion of JAM-A causes morphological defects in the corneal epithelium. Junctional adhesion molecule-A (JAM-A, JAM-1, F11R) is an Ig domain containing transmembrane protein that has been proposed to function in diverse processes including platelet activation and adhesion, leukocyte trans**migration**, angiogenesis, epithelial cell shape and endothelial cell **migration** although its function in vivo is less well established. In the mouse eye, JAM-A protein expression is first detected at 12.5 dpc in the blood vessels of the tunica vasculosa, while it is first detected in both the corneal epithelium and lens between 13.5 and 14.5 dpc. In the corneal epithelium, JAM-A levels remain appreciable throughout life, while JAM-A immunostaining becomes stronger in the lens as the animals age. Both the cornea and lens of mice lacking an intact JAM-A gene are transparent until at least a year of age, although the cells of the JAM-A null corneal epithelium are irregularly shaped. In wild-type mice, JAM-A protein is found at the leading edge of repairing corneal epithelial wounds, however, corneal epithelial wound repair was qualitatively normal in JAM-A null animals. In summary, JAM-A is expressed in the corneal epithelium where it appears to regulate cell shape.
35. Expression and functional importance of collagen-binding integrins, alpha 1 beta 1 and alpha 2 beta 1, on virus-activated T cells. Adhesive interactions are crucial to cell **migration** into inflammatory sites. Using murine lymphocytic choriomeningitis virus as an Ag model system, we have investigated expression and function of collagen-binding integrins, alpha(1)beta(1) and alpha(2)beta(1), on activated and memory T cells. Using this system and MHC tetramers to define Ag-specific T cells, we demonstrate that contrary to being VLAs, expression of alpha(1)beta(1) and alpha(2)beta(1) can be rapidly induced on acutely activated T cells, that expression of alpha(1)beta(1) remains elevated on memory T cells, and that expression of alpha(1)beta(1) parallels that of viral-specific effector CD8(+) T cells (defined by tetramer and IFN-gamma staining). In an adoptive transfer model, mAb-mediated blockade of these integrins on activated effector and memory T cells inhibited Ag-specific delayed-type hypersensitivity responses; similar decreased responses were seen upon transfer of alpha(1)-deficient activated/memory T cells. Thus, expression of alpha(1)beta(1) and alpha(2)beta(1) integrins on activated T cells is directly functionally important for generation of inflammatory responses within tissues. Finally, the inhibitory effect of alpha(1)beta(1) blockade on the delayed-type hypersensitivity response could be bypassed by direct injection of Ag-specific T cells to inflammatory sites, demonstrating for the first time in vivo that collagen-binding integrins are involved in leukocyte **migration** into tissues.
36. Deletion of integrin alpha 1 by homologous recombination permits normal murine development but gives rise to a specific deficit in cell adhesion. Integrin alpha 1 is a receptor for laminin and collagen which is expressed widely and dynamically in embryogenesis and has been implicated in various developmental processes including establishment of the placenta and formation of the central and peripheral nervous system. In the adult it is the sole collagen receptor in smooth muscle and liver and is thought to be important for the stability of these tissues. We have generated a null allele of the alpha 1 gene in the germline of mice by homologous recombination in embryonic stem cells. Mice homozygous for the mutation are viable and fertile and have no overt phenotype, demonstrating that the molecule is not required for development. Embryonic fibroblasts derived from mutant animals are unable to spread on or migrate into substrata of collagen IV and are deficient in spreading on and migrating into laminin. Further in vitro analysis of cell spreading and **migration** suggests that alpha 1 beta 1 is not required for binding to collagen I and implicates a third receptor, possibly integrin alpha 3 beta 1, in collagen I binding.
37. Hyperhomocysteinemia suppresses bone marrow CD34+/VEGF receptor 2+ cells and inhibits progenitor cell mobilization and homing to injured vasculature-a role of β1-integrin in progenitor cell **migratio**n and adhesion. Hyperhomocysteinemia (HHcy) impairs re-endothelialization and accelerates vascular remodeling. The role of CD34(+)/VEGF receptor (VEGFR) 2(+) progenitor cells (PCs) in vascular repair in HHcy is unknown. We studied the effect of HHcy on PCs and its role in vascular repair in severe HHcy (∼150 μM), which was induced in cystathionine-β synthase heterozygous mice fed a high-methionine diet for 8 weeks. Vascular injury was introduced by carotid air-dry endothelium denudation. CD34(+)/VEGFR2(+) cells were examined by flow cytometry. HHcy reduced bone marrow (BM) CD34(+)/VEGFR2(+) cells and suppressed replenishment of postinjury CD34(+)/VEGFR2(+) cells in peripheral blood (PB). Donor green fluorescent protein-positive PC homing to the injured vessel was reduced in HHcy after CD34(+) PCs from enhanced green fluorescent protein mice were adoptively transferred following carotid injury. CD34(+) PC transfusion partially reversed HHcy-suppressed re-endothelialization and HHcy-induced neointimal formation. Furthermore, homocysteine (Hcy) inhibited proliferation, adhesion, **and migr**ation and suppressed β1-integrin expression and activity in human CD34(+) endothelial colony-forming cells (ECFCs) isolated from PBs in a dose-dependent manner. A functional-activating β1-integrin antibody rescued Hcy-suppressed adhesi**on and mi**gration in CD34(+) ECFCs. In conclusion, HHcy reduces BM CD34(+)/VEGFR2(+) generation and suppresses CD34(+)/VEGFR2(+) cell mobilization and homing to the injured vessel via β1-integrin inhibition, which partially contributes to impaired re-endothelialization and vascular remodeling.
38. Persistent cell **migration** and adhesion rely on retrograde transport of β(1) integrin. Integrins have key functions in cell adhesion and **migratio**n. How integrins are dynamically relocalized to the leading edge in highly polarized migratory cells has remained unexplored. Here, we demonstrate that β1 integrin (known as PAT-3 in Caenorhabditis elegans), but not β3, is transported from the plasma membrane to the trans-Golgi network, to be resecreted in a polarized manner. This retrograde trafficking is restricted to the non-ligand-bound conformation of β1 integrin. Retrograde trafficking inhibition abrogates several β1-integrin-specific functions such as cell adhesion in early embryonic development of mice, and persisten**t cell mi**gration in the developing posterior gonad arm of C. elegans. Our results establish a paradigm according to which retrograde trafficking, and not endosomal recycling, is the key driver for β1 integrin function in highly polarized cells. These data more generally suggest that the retrograde route is used to relocalize plasma membrane machinery from previous sites of function to the leading edge of migratory cells.
39. p190RhoGAP is the convergence point of adhesion signals from alpha 5 beta 1 integrin and syndecan-4. The fibronectin receptors alpha(5)beta(1) integrin and syndecan-4 cocluster in focal adhesions and coordinate cell **migration** by making individual contributions to the suppression of RhoA activity during matrix engagement. p190Rho-guanosine triphosphatase-activating protein (GAP) is known to inhibit RhoA during the early stages of cell spreading in an Src-dependent manner. This paper dissects the mechanisms of p190RhoGAP regulation and distinguishes the contributions of alpha(5)beta(1) integrin and syndecan-4. Matrix-induced tyrosine phosphorylation of p190RhoGAP is stimulated solely by engagement of alpha(5)beta(1) integrin and is independent of syndecan-4. Parallel engagement of syndecan-4 causes redistribution of the tyrosine-phosphorylated pool of p190RhoGAP between membrane and cytosolic fractions by a mechanism that requires direct activation of protein kinase C alpha by syndecan-4. Activation of both pathways is necessary for the efficient regulation of RhoA and, as a consequence, focal adhesion formation. Accordingly, we identify p190RhoGAP as the convergence point for adhesive signals mediated by alpha(5)beta(1) integrin and syndecan-4. This molecular mechanism explains the cooperation between extracellular matrix receptors during cell adhesion.
40. Band 4.1 proteins regulate integrin-dependent cell spreading. Integrins link the extracellular matrix (ECM) to the cytoskeleton to control cell behaviors including adhesion, spreading and **migration**. Band 4.1 proteins contain 4.1, ezrin, radixin, moesin (FERM) domains that likely mediate signaling events and cytoskeletal reorganization via integrins. However, the mechanisms by which Band 4.1 proteins and integrins are functionally interconnected remain enigmatic. Here we have investigated roles for Band 4.1 proteins in integrin-mediated cell spreading using primary astrocytes as a model system. We demonstrate that Proteins 4.1B and 4.1G show dynamic patterns of sub-cellular localization in astrocytes spreading on fibronectin. During early stages of cell spreading Proteins 4.1B and 4.1G are enriched in ECM adhesion sites but become more diffusely localized at later stages of spreading. Combinatorial inactivation of Protein 4.1B and 4.1G expression leads to impaired astrocyte spreading. Furthermore, in exogenous expression systems we show that the isolated Protein 4.1 FERM domain significantly enhances integrin-mediated cell spreading. Protein 4.1B is dispensable for reactive astrogliosis in experimental models of cortical injury, likely due to functional compensation by related Protein 4.1 family members. Collectively, these findings reveal that Band 4.1 proteins are important intracellular components for integrin-mediated cell spreading.
41. Integrin alpha4beta1 (VLA-4) expression and activity in retinal and peripheral neurons. The integrin alpha4beta1 fulfills important roles in inflammation and hematopoesis, but its functions in neurons are not well understood. Here we show that the alpha4 subunit is expressed on mouse retinal ganglion cells (RGCs) and undifferentiated retinal neuroblasts during the period of axon extension and **migration**. To determine if alpha4 integrins expressed by retinal neurons were active, neurons were cultured on known alpha4 ligands in vitro. Recombinant soluble vascular cell adhesion molecule 1 (rsVCAM-1), fibronectin, and osteopontin (OPN) induced neurite outgrowth that was diminished by function blocking antibodies specific for alpha4. Neurite outgrowth on OPN was also blocked by antibodies to the integrin beta1 subunit, implicating the alpha4beta1 heterodimer as one integrin receptor mediating outgrowth on OPN. OPN immunoreactivity was detected in the RGC fiber layer and optic nerve, suggesting that it may act as an alpha4 ligand in vivo. Neurons from chick lumbar sympathetic ganglia, chick dorsal root ganglia, and mouse superior cervical ganglia also extended neurites on rsVCAM-1, suggesting that integrin alpha4beta1 may play a role in the development of multiple neuronal cell types.
42. Mouse models of Hirschsprung disease and other developmental disorders of the enteric nervous system: Old and new players. Hirschsprung disease (HSCR, intestinal aganglionosis) is a multigenic disorder with variable penetrance and severity that has a general population incidence of 1/5000 live births. Studies using animal models have contributed to our understanding of the developmental origins of HSCR and the genetic complexity of this disease. This review summarizes recent progress in understanding control of enteric nervous system (ENS) development through analyses in mouse models. An overview of signaling pathways that have long been known to control the **migration**, proliferation and differentiation of enteric neural progenitors into and along the developing gut is provided as a framework for the latest information on factors that influence enteric ganglia formation and maintenance. Newly identified genes and additional factors beyond discrete genes that contribute to ENS pathology including regulatory sequences, miRNAs and environmental factors are also introduced. Finally, because HSCR has become a paradigm for complex oligogenic diseases with non-Mendelian inheritance, the importance of gene interactions, modifier genes, and initial studies on genetic background effects are outlined.
43. CCR7/CCL21 **migration** on fibronectin is mediated by phospholipase Cgamma1 and ERK1/2 in primary T lymphocytes. CCR7 binds to its cognate ligand, CCL21, to mediate the **migration** of circulating naive T lymphocytes to the lymph nodes. T lymphocytes can bind to fibronectin, a constituent of lymph nodes, via their β1 integrins, which is a primary mechanism of T lymphocyte **migratio**n; however, the signaling pathways involved are unclear. We report that rapid (within 2 min) and transient phosphorylation of ERK1/2 is required for T cell **migratio**n on fibronectin in response to CCL21. Conversely, prevention of ERK1/2 phosphorylation by inhibition of its kinase, MAPK/MEK, prevented T lymphocyte **migratio**n. Previous studies have suggested that phospholipase Cγ1 (PLCγ1) can mediate phosphorylation of ERK1/2, which is required for β1 integrin activation. Paradoxically, we found that inhibition of PLCγ1 phosphorylation by the general PLC inhibitor U73122 was associated with a delayed and reduced phosphorylation of ERK1/2 and red**uced migr**ation of T lymphocytes on fibronectin. To further characterize the relationship between ERK1/2 and PLCγ1, we reduced PLCγ1 levels by 85% using shRNA and observed a reduced phosphorylation of ERK1/2 and a significant loss of CCR7-me**diated mi**gration of T lymphocytes on fibronectin. In addition, we found that inhibition of ERK1/2 phosphorylation by U0126 resulted in a decreased phosphorylation of PLCγ1, suggesting a feedback loop between ERK1/2 and PLCγ1. Overall, these results suggest that the CCR7 signaling pathway leading to T ly**mphocyte** migration on fibronectin is a β1 integrin-dependent pathway involving transient ERK1/2 phosphorylation, which is modulated by PLCγ1.
44. Integrin alpha5beta1 is necessary for regulation of radial **migration** of cortical neurons during mouse brain development. During cerebral cortex development, post-mitotic neurons interact with radial glial fibers and the extracellular environment to migrate away from the ventricular region and form a correct laminar structure. Integrin receptors are major mediators of cell-cell and cell-extracellular matrix interactions. Several integrin heterodimers are present during formation of the cortical layers. The alpha5beta1 receptor is expressed in the neural progenitors of the ventricular zone during cerebral cortex formation. Using in utero electroporation to introduce short hairpin RNAs in the brain at embryonic day 15.5, we were able to inhibit acutely the expression of alpha5 integrin in the developing cortex. The knockdown of alpha5 integrin expression level in neural precursors resulted in an inhibition of radial **migration**, without perturbing the glial scaffold. Moreover, the same inhibitory effect on neuronal **migration** was observed after electroporation of a Cre recombinase expression plasmid into the neural progenitors of conditional knockout mice for alpha5 integrin. In both types of experiments, the electroporated cells expressing reduced levels of alpha5 integrin accumulated in the premigratory region with an abnormal morphology. At postnatal day 2, ectopic neurons were observed in cortical layer V, while a deficit of neurons was observed in cortical layer II-IV. We show that these neurons do not express a layer V-specific marker, suggesting that they have not undergone premature differentiation. Overall, these results indicate that alpha5beta1 integrin functions in the regulation of neural morphology and **migration** during cortical development, playing a role in cortical lamination.
45. Cardiac myocyte-specific excision of the beta1 integrin gene results in myocardial fibrosis and cardiac failure. Integrins link the extracellular matrix to the cellular cytoskeleton and serve important roles in cell growth, differentiation, **migration**, and survival. Ablation of beta1 integrin in all murine tissues results in peri-implantation embryonic lethality. To investigate the role of beta1 integrin in the myocardium, we used Cre-LoxP technology to inactivate the beta1 integrin gene exclusively in ventricular cardiac myocytes. Animals with homozygous ventricular myocyte beta1 integrin gene excision were born in appropriate numbers and grew into adulthood. These animals had 18% of control levels of beta1D integrin protein in the heart and displayed myocardial fibrosis. High-fidelity micromanometer-tipped catheterization of the intact 5-week-old beta1 integrin knockout mice showed depressed left ventricular basal and dobutamine-stimulated contractility and relaxation (LV dP/dt(max) and LV dP/dt(min)) as compared with control groups (n=8 to 10 of each, P<0.01). Hemodynamic loading imposed by 7 days of transverse aortic constriction showed that the beta1 integrin knockout mice were intolerant of this stress as they had 53% survival versus 88% in controls (n=15 each). By 6 months of age, mice with depressed ventricular expression of beta1 integrin developed a dilated cardiomyopathy that was not evident in any control animals and had patchy decrease in glucose metabolism as determined by positron emission tomography. Myocyte membrane integrity as determined via Evan's blue dye staining was disrupted in the beta1 integrin knockout mice. This model provides strong evidence for the importance of beta1 integrin in cardiac form and function and indicates that integrins can be linked to development of cardiomyopathies.
46. TSC2 modulates cell adhesion and **migration** via integrin-α1β1. Recent evidence suggests that the rare and progressive lung disease lymphangioleiomyomatosis (LAM) is metastatic in nature. Dysfunction of the tumor suppressor genes tuberous sclerosis complex (TSC), in particular mutational inactivation of TSC2, enhances both cell proliferation an**d migrati**on. Although substantial progress has been made in understanding the role of TSC2 in abnormal LAM cell proliferation and its pharmacological targeting, the mechanisms underlying the enhanced migratory capacity in LAM are not well understood. In this study, we examined the role of TSC2 in cell attachment, spreading, an**d migrati**on, processes that contribute to the metastatic phenotype. Here we show that loss of TSC2 increased both the attachment and spreading of mouse embryonic fibroblasts to the extracellular matrix proteins collagen type I and fibronectin and that reexpression of TSC2 reduced these effects. Integrin-α1β1 modulated c**ell migra**tion with the β1-subunit involved in cell attachment and spreading as shown by using functional blocking antibodies. Loss of TSC2 increased integrin-α1 expression, and inhibition of this integrin subunit reduced **cell mig**ration. The enhanced attachment and spreading were independent of the intracellular signaling pathways mammalian target of rapamycin complex 1 and Rho-associated kinase, as pharmacological inhibition with rapamycin or Y27632, respectively, was without effect. Together, these data demonstrate that TSC2 controls **cell mig**ration, attachment, and spreading through the α1β1-integrin receptor and thus suggest a potential therapeutic target for the treatment of increased cell invasiveness in LAM.
47. Fibronectin induces endothelial cell **migration** through β1 integrin and Src-dependent phosphorylation of fibroblast growth factor receptor-1 at tyrosines 653/654 and 766. The extracellular matrix microenvironment regulates cell phenotype and function. One mechanism by which this is achieved is the transactivation of receptor tyrosine kinases by specific matrix molecules. Here, we demonstrate that the provisional matrix protein, fibronectin (FN), activates fibroblast growth factor (FGF) receptor-1 (FGFR1) independent of FGF ligand in liver endothelial cells. FN activation of FGFR1 requires β1 integrin, as evidenced by neutralizing antibody and siRNA-based studies. Complementary genetic and pharmacologic approaches identify that the non-receptor tyrosine kinase Src is required for FN transactivation of FGFR1. Whereas FGF ligand-induced phosphorylation of FGFR1 preferentially activates ERK, FN-induced phosphorylation of FGFR1 preferentially activates AKT, indicating differential downstream signaling of FGFR1 in response to alternate stimuli. Mutation analysis of known tyrosine residues of FGFR1 reveals that tyrosine 653/654 and 766 residues are required for FN-FGFR1 activation of AKT and chemotaxis. Thus, our study mechanistically dissects a new signaling pathway by which FN achieves endothelial cell chemotaxis, demonstrates how differential phosphorylation profiles of FGFR1 can achieve alternate downstream signals, and, more broadly, highlights the diversity of mechanisms by which the extracellular matrix microenvironment regulates cell behavior through transactivation of receptor tyrosine kinases.
48. Cadherin-11 coordinates cellular **migration** and extracellular matrix remodeling during aortic valve maturation. Proper remodeling of the endocardial cushions into thin fibrous valves is essential for gestational progression and long-term function. This process involves dynamic interactions between resident cells and their local environment, much of which is not understood. In this study, we show that deficiency of the cell-cell adhesion protein cadherin-11 (Cad-11) results in significant embryonic and perinatal lethality primarily due to valve related cardiac dysfunction. While endocardial to mesenchymal transformation is not abrogated, mesenchymal cells do not homogeneously cellularize the cushions. These cushions remain thickened with disorganized ECM, resulting in pronounced aortic valve insufficiency. Mice that survive to adulthood maintain thickened and stenotic semilunar valves, but interestingly do not develop calcification. Cad-11 (-/-) aortic valve leaflets contained reduced Sox9 activity, β1 integrin expression, and RhoA-GTP activity, suggesting that remodeling defects are due to improper **migratio**n and/or cellular contraction. Cad-11 deletion or siRNA knockdown reduced **migratio**n, eliminated collective **migratio**n, and impaired 3D matrix compaction by aortic valve interstitial cells (VIC). Cad-11 depleted cells in culture contained few filopodia, stress fibers, or contact inhibited locomotion. Transfection of Cad-11 depleted cells with constitutively active RhoA restored cell phenotypes. Together, these results identify cadherin-11 mediated adhesive signaling for proper remodeling of the embryonic semilunar valves.
49. Cell adaptive response to extracellular matrix density is controlled by ICAP-1-dependent beta1-integrin affinity. Cell **migration** is an integrated process requiring the continuous coordinated assembly and disassembly of adhesion structures. How cells orchestrate adhesion turnover is only partially understood. We provide evidence for a novel mechanistic insight into focal adhesion (FA) dynamics by demonstrating that integrin cytoplasmic domain-associated protein 1 (ICAP-1) slows down FA assembly. Live cell imaging, which was performed in both Icap-1-deficient mouse embryonic fibroblasts and cells expressing active beta(1) integrin, shows that the integrin high affinity state favored by talin is antagonistically controlled by ICAP-1. This affinity switch results in modulation in the speed of FA assembly and, consequently, of cell spreading and **migration**. Unexpectedly, the ICAP-1-dependent decrease in integrin affinity allows cell sensing of matrix surface density, suggesting that integrin conformational changes are important in mechanotransduction. Our results clarify the function of ICAP-1 in cell adhesion and highlight the central role it plays in the cell's integrated response to the extracellular microenvironment.
50. α1β1 integrin-mediated adhesion inhibits macrophage exit from a peripheral inflammatory lesion. Integrins are adhesion molecules critical for the recruitment of leukocytes from blood into peripheral tissues. However, whether integrins are also involved in leukocyte exit from peripheral tissues via afferent lymphatics to the draining lymph node remains poorly understood. In this article, we show that adhesion by the collagen IV-binding integrin α1β1 unexpectedly inhibited macrophage exit from inflamed skin. We monitored macrophages exiting mouse footpads using a newly developed in situ pulse labeling technique. Blockade of α1β1 integrin or genetic deletion (Itga1(-/-)) increased macrophage exit efficiency. Chemotaxis assays through collagen IV showed more effi**cient mig**ration of Itga1(-/-) macrophages relative to wild type. Given that macrophages are key orchestrators of inflammation, α1β1 integrin adhesion may represent a mechanism for regulating inflammatory responses by controlling macrophage exit or persistence in inflamed tissues.
51. Epidermal growth factor induction of front-rear polarity and **migration** in keratinocytes is mediated by integrin-linked kinase and ELMO2. Epidermal growth factor (EGF) is a potent chemotactic and mitogenic factor for epidermal keratinocytes, and these properties are central for normal epidermal regeneration after injury. The involvement of mitogen-activated protein kinases as mediators of the proliferative effects of EGF is well established. However, the molecular mechanisms that mediate motogenic responses to this growth factor are not clearly understood. An obligatory step for forward cell **migration** is the development of front-rear polarity and formation of lamellipodia at the leading edge. We show that stimulation of epidermal keratinocytes with EGF, but not with other growth factors, induces development of front-rear polarity and directional **migration** through a pathway that requires integrin-linked kinase (ILK), Engulfment and Cell Motility-2 (ELMO2), integrin β1, and Rac1. Furthermore, EGF induction of front-rear polarity and chemotaxis require the tyrosine kinase activity of the EGF receptor and are mediated by complexes containing active RhoG, ELMO2, and ILK. Our findings reveal a novel link between EGF receptor stimulation, ILK-containing complexes, and activation of small Rho GTPases necessary for acquisition of front-rear polarity and forward movement.
52. Impaired integrin-mediated adhesion contributes to reduced barrier properties in VASP-deficient microvascular endothelium. Recent studies point to a significant role of vasodilator-stimulated phosphoprotein (VASP) in the maintenance of endothelial barrier functions in vivo and in vitro. Moreover, it has been reported that VASP is required for activation of the small GTPase Rac 1. However, little is known whether VASP is involved in the regulation of cell adhesion molecules that are critical for maintenance of the endothelial barrier. Here we demonstrate that impaired barrier properties in VASP-deficient (VASP-/-) microvascular myocardial endothelial cells (MyEnd) correlated with both impaired integrin-mediated adhesion as revealed by laser tweezer trapping and reduced integrin-dependent cell **migration**. This was paralleled by reduction of focal adhesions at the cell periphery as well as of beta(1)-integrin and VE-cadherin cytoskeletal anchorage. Incubation of MyEnd VASP wt with RGD peptide to block interaction of integrins with extracellular matrix (ECM) reduced barrier properties and Rac 1 activity in wt endothelial monolayers mimicking the situation in VASP (-/-) cells under resting conditions. Moreover, cAMP-mediated Rac 1 activation was reduced under conditions of impaired integrin-mediated adhesion in wt cells and cAMP-induced increase in VE-cadherin cytoskeletal anchorage was abolished in VASP (-/-) endothelium. In summary, these data indicate that VASP is required for integrin-mediated adhesion which stabilizes endothelial barrier properties at least in part by facilitating Rac 1 activation.
53. Netrin-4 activates endothelial integrin {alpha}6{beta}1. Netrin-4 regulates vascular development. Identity of netrin-4 endothelial receptor and its subsequent cell functions is controversial. We previously demonstrated that the inhibition of netrin-1 canonical receptors, Unc5B and neogenin, expressed by lymphatic endothelial cells, do not suppress netrin-4-induced cell signaling and functions. Netrin family members were shown to signal through a range of receptors, including integrins (such as α3β1, α6β1, and α6β4) in nonendothelial cells.We tested whether integrins are netrin-4 receptors in the endothelium.The α6β1 integrin is expressed by endothelial cells, and binds netrin-4 in a dose-dependent manner. Inhibition of α6 or β1 integrin subunits suppresses netrin-4-induced endothe**lial cell** migration, adhesion, and focal adhesion contact. Netrin-4-stimulated phosphorylation of Src kinase family, effectors of endothe**lial cell** migration, is also abolished by α6 or β1 inhibition. Finally, netrin-4 and α6β1 integrin expression colocalize in mouse embryonic, intestine, and tumor vasculature.The α6β1 integrin is a netrin-4 receptor in lymphatic endothelium and consequently represents a potential target to inhibit netrin-4-induced metastatic dissemination.
54. Dependence of fibroblast infiltration in tumor stroma on type IV collagen-initiated integrin signal through induction of platelet-derived growth factor. Cancer-associated fibroblasts play a crucial role in accelerating tumor progression, but there is a knowledge gap regarding the chemotactic signal activated in a tumor microenvironment. In this study, the expression of type IV collagen was knocked down using a lentiviral-mediated short hairpin RNA strategy. Although there was no obvious effect on cell growth in vitro, silencing the Col4-α1 gene decreased the tumorigenicity of B16F10 in C57BL/6 mice, which was accompanied by a reduction in the infiltration of alpha-smooth muscle actin-positive (α-SMA+) fibroblasts. Silencing the Col4-α1 gene or disrupting integrin engagement by blocking the antibody reduced the expression of platelet-derived growth factor A (PDGF-A), a potent chemotactic factor for fibroblasts. Furthermore, ectopic expression of the autoclustering integrin mutant significantly stimulated PDGF-A expression in murine B16F10 and human U118MG and Huh7 cells. PDGF-A-specific sh-RNA and neutralizing anti-PDGF-A antibody effectively inhibited the transwe**ll migrat**ion of fibroblasts. Adding recombinant PDGF-A back to shCol cell-conditioned media restored the fibroblast-attraction ability indicating that PDGF-A is a major chemotactic factor for fibroblasts in the current study model. The integrin-associated PDGF-A production correlated with the activation of Src and ERK. High type IV collagen staining intensity colocalized with elevated PDGF-A expression was observed in tumor tissues obtained from hepatoma and glioma patients. The integrin signal pathway was activated by collagen engagement through Src and ERK, leading to enhanced PDGF-A production, which serves as a key regulator of fibroblast recruitment.
55. Mast cell-mediated inflammatory responses require the alpha 2 beta 1 integrin. Although the alpha 2 beta 1 integrin is widely expressed and has been extensively studied, it has not been previously implicated in mast cell biology. We observed that alpha 2 integrin subunit-deficient mice exhibited markedly diminished neutrophil and interleukin-6 responses during Listeria monocytogenes- and zymosan-induced peritonitis. Since exudative neutrophils of wild-type mice expressed little alpha 2 beta 1 integrin, it seemed unlikely that this integrin mediated neutrophil **migration** directly. Here, we demonstrate constitutive alpha 2 beta 1 integrin expression on peritoneal mast cells. Although alpha 2-null mice contain normal numbers of peritoneal mast cells, these alpha 2-null cells do not support in vivo mast cell-dependent inflammatory responses. We conclude that alpha 2 beta 1 integrin provides a costimulatory function required for mast cell activation and cytokine production in response to infection.
56. Arhgef1 regulates alpha5beta1 integrin-mediated matrix metalloproteinase expression and is required for homeostatic lung immunity. Pulmonary immunity depends on the ability of leukocytes to neutralize potentially harmful and frequent insults to the lung, and appropriate regulation of leukocyte **migration** and adhesion is integral to this process. Arhgef1 is a hematopoietic-restricted signaling molecule that regulates leukocyte **migration** and integrin-mediated adhesion. To explore a possible regulatory role for Arhgef1 in pulmonary immunity we examined the lung and its leukocytes in wild-type and Arhgef1-deficient animals. Here we report that the lungs of Arhgef1-/- mice harbored significantly more leukocytes, increased expression and activity of matrix metalloproteinases (MMPs), airspace enlargement, and decreased lung elastance compared with wild-type lungs. Transfer of Arhgef1-/- lung leukocytes to wild-type mice led to airspace enlargement and impaired lung function, indicating that loss of Arhgef1 in leukocytes was sufficient to induce pulmonary pathology. Furthermore, we showed that Arhgef1-deficient peritoneal macrophages when either injected into the lungs of wild-type mice or cultured on fibronectin significantly increased expression and activity of MMPs relative to control macrophages, and the in vitro fibronectin induction was dependent on the alpha5beta1 integrin pair. Together these data demonstrate that Arhgef1 regulates alpha5beta1-mediated MMP expression by macrophages and that loss of Arhgef1 by leukocytes leads to pulmonary pathology.
57. Discovery of a functional protein complex of netrin-4, laminin gamma1 chain, and integrin alpha6beta1 in mouse neural stem cells. Molecular and cellular interactions coordinating the origin and fate of neural stem cells (NSCs) in the adult brain are far from being understood. We present a protein complex that controls proliferation and **migration** of adult NSCs destined for the mouse olfactory bulb (OB). Combinatorial selection based on phage display technology revealed a previously unrecognized complex between the soluble protein netrin-4 and laminin gamma1 subunit that in turn activates an alpha6beta1 integrin-mediated signaling pathway in NSCs. Differentiation of NSCs is accompanied by a decrease in netrin-4 receptors, indicating that netrin-4 participates in the continual propagation of this stem cell population. Notably, the stem cells themselves do not synthesize netrin-4. Further, we show that netrin-4 is produced by selected GFAP-positive astrocytes positioned close to newborn neurons migrating in the anterior part of the rostral migratory stream (RMS) and within the OB. Our findings present a unique molecular mechanism mediating astrocytic/neuronal crosstalk that regulates ongoing neurogenesis in the adult olfactory system.
58. An essential requirement for β1 integrin in the assembly of extracellular matrix proteins within the vascular wall. β1 integrin has been shown to contribute to vascular smooth muscle cell differentiation, adhesion and mechanosensation in vitro. Here we showed that deletion of β1 integrin at the onset of smooth muscle differentiation resulted in interrupted aortic arch, aneurysms and failure to assemble extracellular matrix proteins. These defects result in lethality prior to birth. Our data indicates that β1 integrin is not required for the acquisition, but it is essential for the maintenance of the smooth muscle cell phenotype, as levels of critical smooth muscle proteins are gradually reduced in mutant mice. Furthermore, while deposition of extracellular matrix was not affected, its structure was disrupted. Interestingly, defects in extracellular matrix and vascular wall assembly, were restricted to the aortic arch and its branches, compromising the brachiocephalic and carotid arteries and to the exclusion of the descending aorta. Additional analysis of β1 integrin in the pharyngeal arch smooth muscle progenitors was performed using wnt1Cre. Neural crest cells deleted for β1 integrin were able to migrate to the pharyngeal arches and associate with endothelial lined arteries; but exhibited vascular remodeling defects and early lethality. This work demonstrates that β1 integrin is dispensab**le for mi**gration and initiation of the smooth muscle differentiation program, however, it is essential for remodeling of the pharyngeal arch arteries and for the assembly of the vessel wall of their derivatives. It further establishes a critical role of β1 integrin in the protection against aneurysms that is particularly confined to the ascending aorta and its branches.
59. Under-expression of α8 integrin aggravates experimental atherosclerosis. Integrins play an important role in vascular biology. The α8 integrin chain attenuates smooth muscle cel**l migrati**on but its functional role in the development of atherosclerosis is unclear. Therefore, we studied the contribution of α8 integrin to atherosclerosis and vascular remodelling. We hypothesized that α8 integrin expression is reduced in atherosclerotic lesions, and that its under-expression leads to a more severe course of atherosclerosis. α8 Integrin was detected by immunohistochemistry and qPCR and α8 integrin-deficient mice were used to induce two models of atherosclerotic lesions. First, ligation of the carotid artery led to medial thickening and neointima formation, which was quantified in carotid cross-sections. Second, after crossing into ApoE-deficient mice, the formation of advanced vascular lesions with atherosclerotic plaques was quantified in aortic en face preparations stained with Sudan IV. Parameters of renal physiology and histopathology were assessed: α8 integrin was detected in the media of human and murine vascular tissue and was down-regulated in arteries with advanced atherosclerotic lesions. In α8 integrin-deficient mice (α8(-/-) ) as well as α8(+/-) and α8(+/+) littermates, carotid artery ligation increased media:lumen ratios in all genotypes, with higher values in ligated α8(-/-) and α8(+/-) compared to ligated α8(+/+) animals. Carotid artery ligation increased smooth muscle cell number in the media of α8(+/+) mice and, more prominently, of α8(-/-) or α8(+/-) mice. On an ApoE(-/-) background, α8(+/-) and α8(-/-) mice developed more atherosclerotic plaques than α8(+/+) mice. α8 Integrin expression was reduced in α8(+/-) animals. Renal damage with increased serum creatinine and glomerulosclerosis was detected in α8(-/-) mice only. Thus, under-expression of α8 integrin aggravates vascular lesions, while a complete loss of α8 integrin results in reduced renal mass and additional renal disease in the presence of generalized atherosclerosis. Our data support the hypothesis that integrin α8β1 has a protective role in arterial remodelling and atherosclerosis.
60. The alpha9beta1 integrin enhances cell **migration** by polyamine-mediated modulation of an inward-rectifier potassium channel. The alpha9beta1 integrin accelerates cell **migration** through binding of spermidine/spermine acetyltransferase (SSAT) to the alpha9 cytoplasmic domain. We now show that SSAT enhances alpha9-mediated **migration** specifically through catabolism of spermidine and/or spermine. Because spermine and spermidine are effective blockers of K(+) ion efflux through inward-rectifier K(+) (Kir) channels, we examined the involvement of Kir channels in this pathway. The Kir channel inhibitor, barium, or knockdown of a single subunit, Kir4.2, specifically inhibited alpha9-dependent cell **migration**. alpha9beta1 and Kir4.2 colocalized in focal adhesions at the leading edge of migrating cells and inhibition or knockdown of Kir4.2 caused reduced persistence and an increased number of lamellipodial extensions in cells migrating on an alpha9beta1 ligand. These results identify a pathway through which the alpha9 integrin subunit stimulates cell **migration** by localized polyamine catabolism and modulation of Kir channel function.
61. Knockout and knockin of the beta1 exon D define distinct roles for integrin splice variants in heart function and embryonic development. The beta1D integrin is a recently characterized isoform of the beta1 subunit that is specifically expressed in heart and skeletal muscle. In this study we have assessed the function of the beta1D integrin splice variant in mice by generating, for the first time, Cre-mediated exon-specific knockout and knockin strains for this splice variant. We show that removal of the exon for beta1D leads to a mildly disturbed heart phenotype, whereas replacement of beta1A by beta1D results in embryonic lethality with a plethora of developmental defects, in part caused by the abnormal **migration** of neuroepithelial cells. Our data demonstrate that the splice variants A and D are not functionally equivalent. We propose that beta1D is less efficient than beta1A in mediating the signaling that regulates cell motility and responses of the cells to mechanical stress.
62. Inhibition of β1 integrin and IL-3Rβ common subunit interaction hinders tumour angiogenesis. Integrin/cytokine receptor interaction provides permissive signals leading to neoangiogenesis, and integrins are crucial for differentiation of endothelial progenitor cells (EPCs). It is known that the inflammatory interleukin-3 (IL-3), released in the tumoral microenvironment, contributes to both angiogenesis and vasculogenic processes. Herein, we generated IL-3 receptor beta common (IL-3Rβc) extracellular domain-derived fusion proteins (Fc) to elucidate the molecular mechanisms regulating these processes. Three different Fc were generated, containing the entire extracellular domain of IL-3Rβc (Fc1.4), a fragment corresponding to domains 1-3 (Fc1.3) and a fragment corresponding to domain 4 (Fc4), respectively. The ability of the fusion proteins to interfere with IL-3Rβc/β1 integrin interaction was assessed on endothelial cells (ECs), EPCs and murine-derived ECs. Pull-down experiments showed that Fc1.4 and Fc4 fusion proteins specifically interacted with β1 integrin. Fc4 and Fc1.4 fragments prevented IL-3-mediated EPC expansion, arterial morphogenesis and tumour-deri**ved EC mi**gration, without affecting cell adhesion. Fc4 in vivo inhibited the IL-3-mediated vasculogenic process, as well as inflammatory and tumour vascular growth. In conclusion, these data identify the β1 integrin-interacting domain in the juxta-membrane IL-3Rβc extracellular domain, and provide the rational for targeting this interaction to impair vascular growth.
63. Inactivation of integrin-β1 prevents the development of polycystic kidney disease after the loss of polycystin-1. Dysregulation of polycystin-1 (PC1) leads to autosomal dominant polycystic kidney disease (ADPKD), a disorder characterized by the formation of multiple bilateral renal cysts, the progressive accumulation of extracellular matrix (ECM), and the development of tubulointerstitial fibrosis. Correspondingly, cystic epithelia express higher levels of integrins (ECM receptors that control various cellular responses, such as cell proliferation, **migratio**n, and survival) that are characteristically altered in cystic cells. To determine whether the altered expression of ECM and integrins could establish a pathologic autostimulatory loop, we tested the role of integrin-β1 in vitro and on the cystic development of ADPKD in vivo. Compared with wild-type cells, PC1-depleted immortalized renal collecting duct cells had higher levels of integrin-β1 and fibronectin and displayed increased integrin-mediated signaling in the presence of Mn(2+). In mice, conditional inactivation of integrin-β1 in collecting ducts resulted in a dramatic inhibition of Pkd1-dependent cystogenesis with a concomitant suppression of fibrosis and preservation of normal renal function. Our data provide genetic evidence that a functional integrin-β1 is required for the early events leading to renal cystogenesis in ADPKD and suggest that the integrin signaling pathway may be an effective therapeutic target for slowing disease progression.
64. Beta1 integrin expression on endothelial cells is required for angiogenesis but not for vasculogenesis. Integrins are a family of cell adhesion receptors that are involved in cell-matrix and cell-cell communications. They facilitate cell proliferation, **migration**, and survival. Using the Cre-Lox system, we deleted beta1 integrin on Tie2-positive (Tie2-cre beta1 Int (fl/fl)) vascular endothelial cells. Deletion of beta1 integrin on vascular endothelial cells results in embryonic lethality. Blood vessel defects are encountered in the Tie2-Cre beta1 Int (fl/fl) embryos at embryonic age (E9.5), and embryos die before reaching E10.5. The embryos exhibit growth retardation and both histological evaluation and PECAM-1 staining of E9.5 embryos revealed defects in angiogenic sprouting and vascular branching morphogenesis. Large and medium-size vessel formation is not affected in these embryos. Angiogenic defects were observed in several regions of the embryo and yolk sacs. These results indicate that beta1 integrin expression on vascular endothelial cells is crucial for embryonic angiogenesis but dispensable for vasculogenesis.
65. Type VIII collagen signals via β1 integrin and RhoA to regulate MMP-2 expression and smooth muscle cell **migratio**n. The extracellular matrix signals and regulates the behavior of vascular cells during the pathogenesis of atherosclerosis. Type VIII collagen, a short chain collagen, is scarcely present in normal arteries, but is dramatically upregulated in atherosclerosis and after other types of vascular injury. Cell culture studies have revealed that this protein supports smooth muscle cell (SMC) adhesion and stimulates **migratio**n, however little is known about the signaling or the mechanisms by which this occurs. SMCs isolated from wild-type C57BL/6 and type VIII collagen deficient mice were studied using assays to measure chemotactic and haptotactic **migratio**n, and remodeling and contraction of 3-dimensional type I collagen gels. Col8(-/-) SMCs exhibited impairments in **migratio**n, and a strongly adhesive phenotype with prominent stress fibers, stable microtubules and pronounced central basal focal adhesions. The addition of exogenous type VIII collagen to the Col8(-/-) SMCs rescued the impairments in **migratio**n, and restored cytoskeletal architecture so that it was similar to Col8(+/+) cells. We measured elevated levels of active GTP-RhoA in the Col8(-/-) cells, and this too was reversed by treatment with exogenous type VIII collagen. We showed that type VIII collagen normally suppresses RhoA activation through a beta-1 integrin dependent mechanism. MMP-2 levels were reduced in the Col8(-/-) SMCs, and knockdown of MMP-2 in Col8(+/+) SMCs partially recapitulated the decreases in **migratio**n and 3D gel contraction seen in Col8(-/-) cells, showing that type VIII collagen-stimulated **migratio**n was dependent on MMP-2. Inhibition of Rho restored MMP-2 activity in the Col8(-/-) cells, and partially rescued **migratio**n, demonstrating that the elevations in RhoA activity were responsible for the suppression of **migratio**n of these cells. In conclusion, we have shown that type VIII collagen signals through beta-1 integrin receptors to suppress RhoA, allowing optimal configuration of the cytoskeleton, and the stimulation of MMP-2-dependent cell **migratio**n.
66. Dysregulation of Semaphorin7A/β1-integrin signaling leads to defective GnRH-1 cell **migratio**n, abnormal gonadal development and altered fertility. Reproduction in mammals is dependent on the function of specific neurons that secrete gonadotropin-releasing hormone-1 (GnRH-1). These neurons originate prenatally in the nasal placode and migrate into the forebrain along the olfactory-vomeronasal nerves. Alterations in this migratory process lead to defective GnRH-1 secretion, resulting in heterogeneous genetic disorders such as idiopathic hypogonadotropic hypogonadism (IHH), and other reproductive diseases characterized by the reduction or failure of sexual competence. Combining mouse genetics with in vitro models, we demonstrate that Semaphorin 7A (Sema7A) is essential for the development of the GnRH-1 neuronal system. Loss of Sema7A signaling alters the **migratio**n of GnRH-1 neurons, resulting in significantly reduced numbers of these neurons in the adult brain as well as in reduced gonadal size and subfertility. We also show that GnRH-1 cells differentially express the Sema7 receptors β1-integrin and Plexin C1 as a function of their migratory stage, whereas the ligand is robustly expressed along developing olfactory/vomeronasal fibers. Disruption of Sema7A function in vitro inhibits β1-integrin-mediat**ed migrat**ion. Analysis of Plexin C1(-/-) mice did not reveal any difference in the migratory process of GnRH-1 neurons, indicating that Sema7A mainly signals through β1-integrin to regulate GnRH-1 cell motility. In conclusion, we have identified Sema7A as a gene implicated in the normal development of the GnRH-1 system in mice and as a genetic marker for the elucidation of some forms of GnRH-1 deficiency in humans.
67. NG2 proteoglycan promotes endothelial cell motility and angiogenesis via engagement of galectin-3 and alpha3beta1 integrin. The NG2 proteoglycan is expressed by microvascular pericytes in newly formed blood vessels. We have used in vitro and in vivo models to investigate the role of NG2 in cross-talk between pericytes and endothelial cells (EC). Binding of soluble NG2 to the EC surface induces cell motility and multicellular network formation in vitro and stimulates corneal angiogenesis in vivo. Biochemical data demonstrate the involvement of both galectin-3 and alpha3beta1 integrin in the EC response to NG2 and show that NG2, galectin-3, and alpha3beta1 form a complex on the cell surface. Transmembrane signaling via alpha3beta1 is responsible for EC motility and morphogenesis in this system. Galectin-3-dependent oligomerization may potentiate NG2-mediated activation of alpha3beta1. In conjunction with recent studies demonstrating the early involvement of pericytes in angiogenesis, these data suggest that pericyte-derived NG2 is an important factor in promoting EC **migration** and morphogenesis during the early stages of neovascularization.
68. Mesenchymal stem cells use integrin beta1 not CXC chemokine receptor 4 for myocardial **migration** and engraftment. Recent evidence has demonstrated the importance of bone marrow-derived mesenchymal stem cells (BM-MSCs) in the repair of damaged myocardium. The molecular mechanisms of engraftment and **migration** of BM-MSCs in the ischemic myocardium are unknown. In this study, we developed a functional genomics approach toward the identification of mediators of engraftment and **migration** of BM-MSCs within the ischemic myocardium. Our strategy involves microarray profiling (>22,000 probes) of ischemic hearts, complemented by reverse transcription-polymerase chain reaction and fluorescence-activated cell sorting of corresponding adhesion molecule and cytokine receptors in BM-MSCs to focus on the coexpressed pairs only. Our data revealed nine complementary adhesion molecules and cytokine receptors, including integrin beta1, integrin alpha4, and CXC chemokine receptor 4 (CXCR4). To examine their functional contributions, we first blocked selectively these receptors by preincubation of BM-MSCs with specific neutralizing antibodies, and then we administered these cells intramyocardially. A significant reduction in the total number of BM-MSC in the infarcted myocardium was observed after integrin beta1 blockade but not integrin alpha4 or CXCR4 blockade. The latter observation is distinctively different from that reported for hematopoietic stem cells (HSCs). Thus, our data show that BM-MSCs use a different pathway from HSCs for intramyocardial trafficking and engraftment.
69. Beta1 integrins are required for the invasion of the caecum and proximal hindgut by enteric neural crest cells. Integrins are the major adhesive receptors for extracellular matrix and have various roles in development. To determine their role in cell **migration**, the gene encoding the beta1 integrin subunit (Itgb1) was conditionally deleted in mouse neural crest cells just after their e**migration** from the neural tube. We previously identified a major defect in gut colonisation by conditional Itgb1-null enteric neural crest cells (ENCCs) resulting from their impaired migratory abilities and enhanced aggregation properties. Here, we show that the **migration** defect occurs primarily during the invasion of the caecum, when Itgb1-null ENCCs stop their normal progression before invading the caecum and proximal hindgut by becoming abnormally aggregated. We found that the caecum and proximal hindgut express high levels of fibronectin and tenascin-C, two well-known ligands of integrins. In vitro, tenascin-C and fibronectin have opposite effects on ENCCs, with tenascin-C decreasing **migration** and adhesion and fibronectin strongly promoting them. Itgb1-null ENCCs exhibited an enhanced response to the inhibitory effect of tenascin-C, whereas they were insensitive to the stimulatory effect of fibronectin. These findings suggest that beta1 integrins are required to overcome the tenascin-C-mediated inhibition of **migration** within the caecum and proximal hindgut and to enhance fibronectin-dependent **migration** in these regions.
70. MicroRNA-223 antagonizes angiogenesis by targeting β1 integrin and preventing growth factor signaling in endothelial cells. Endothelial cells in situ are largely quiescent, and their isolation and culture are associated with the switch to a proliferative phenotype.To identify antiangiogenic microRNAs expressed by native endothelial cells that are altered after isolation and culture, as well as the protein targets that regulate responses to growth factors.Profiling studies revealed that miR-223 was highly expressed in freshly isolated human, murine, and porcine endothelial cells, but those levels decreased in culture. In primary cultures of endothelial cells, vascular endothelial cell growth factor and basic fibroblast growth factor further decreased miR-223 expression. The overexpression of precursor-miR-223 did not affect basal endothelial cell proliferation but abrogated vascular endothelial cell growth factor-induced and basic fibroblast growth factor-induced proliferation, as well as **migratio**n and sprouting. Inhibition of miR-223 in vivo using specific antagomirs potentiated postnatal retinal angiogenesis in wild-type mice, whereas recovery of perfusion after femoral artery ligation and endothelial sprouting from aortic rings from adult miR-223(-/y) animals were enhanced. MiR-223 overexpression had no effect on the growth factor-induced activation of ERK1/2 but inhibited the vascular endothelial cell growth factor-induced and basic fibroblast growth factor-induced phosphorylation of their receptors and activation of Akt. β1 integrin was identified as a target of miR-223 and its downregulation reproduced the defects in growth factor receptor phosphorylation and Akt signaling seen after miR-223 overexpression. Reintroduction of β1 integrin into miR-223-ovexpressing cells was sufficient to rescue growth factor signaling and angiogenesis.These results indicate that miR-223 is an antiangiogenic microRNA that prevents endothelial cell proliferation at least partly by targeting β1 integrin.
71. Beta1-class integrins regulate the development of laminae and folia in the cerebral and cerebellar cortex. Mice that lack all beta1-class integrins in neurons and glia die prematurely after birth with severe brain malformations. Cortical hemispheres and cerebellar folia fuse, and cortical laminae are perturbed. These defects result from disorganization of the cortical marginal zone, where beta1-class integrins regulate glial endfeet anchorage, meningeal basement membrane remodeling, and formation of the Cajal-Retzius cell layer. Surprisingly, beta1-class integrins are not essential for neuron-glia interactions and neuronal **migration** during corticogenesis. The phenotype of the beta1-deficient mice resembles pathological changes observed in human cortical dysplasias, suggesting that defective integrin-mediated signal transduction contributes to the development of some of these diseases.
72. Cell-extracellular matrix versus cell-cell interactions during the development of the cochlear-vestibular ganglion. Cells destined to become the neurones of the cochlear-vestibular ganglion (CVG) originate within the otic epithelium. Early in development they detach from their neighbors and migrate out of the epithelium, where they coalesce to form the CVG. To accomplish this process, the neuroblasts must modify their interactions with other cells within the epithelium and with proteins in the extracellular matrix to allow for repositioning. The aim of this study was to investigate the roles of the major families of adhesion molecules that mediate cellular interactions with the extracellular matrix, the integrins, and with other cells, the cadherins, in neuroblast segregation from the otic epithelium. The expression of classical cadherins increased in migrating neuroblasts compared with the otic epithelium. Quantitative RT-PCR revealed that this was concomitant with down-regulation of E-cadherin and up-regulation of N-cadherin in the migrating cells. In contrast, the level of β1 integrin expression by the epithelium was maintained in migrating neuroblasts. However, although multiple integrin ligands were expressed within the otic basement membrane at this stage of development, only fibronectin (FN) supported neuroblast **migratio**n along the substrate in vitro. Inhibition of β1 integrins resulted in significantly reduced linea**r migrati**on on FN. Importantly, neuroblasts retained the ability to segregate from the epithelium but remained compacted immediately adjacent to the originating tissue, suggesting dominance of cell-cell over cell-matrix interactions. These data suggest that the balance between cell-cell and cell-substratum interactions directs otic neuroblas**t migrati**on and gangliogenesis.
73. Expression of sialyl-Tn epitopes on beta1 integrin alters epithelial cell phenotype, proliferation and haptotaxis. Sialyl-Tn (STn) is a tumor-associated carbohydrate antigen overexpressed in various carcinomas. To obtain its expression, murine carcinoma cells were transfected with the cDNA encoding ST6GalNAc I, a glycosyltransferase that acts exclusively on O-glycans. Overexpression of this enzyme led to the expected expression of cell surface STn epitopes. Surprisingly, the transfectants (STn+ cells) presented dramatic morphological changes and altered behavior. These STn+ cells lost the epithelial appearance of parental cells, became larger, more elongated and presented disorganized actin stress fibers. Additionally, their proliferation was impaired and their ability to migrate on fibronectin and hyaluronic acid was severely reduced. By contrast their adhesion on fibronectin remained unchanged. The major glycoprotein carrying the STn epitope was shown to be the integrin beta1 subunit. Anti-STn antibodies could restore **migration** of STn+ cells on fibronectin. A constitutively active permeant form of RhoA (TAT-RhoA(Val-14)) also restored motility on fibronectin of STn+ cells as well as a parental STn-cellular phenotype. These observations indicate that overexpression of ST6GalNAc I leads to a major change of the O-glycosylation of the integrin beta1 chain which in turn impairs the integrin-mediated signalling and leads to major alterations in morphology and cell behavior.
74. β1 integrins with individually disrupted cytoplasmic NPxY motifs are embryonic lethal but partially active in the epidermis. β1 Integrin adhesion is believed to require binding of talins and kindlins to the membrane proximal and distal NPxY motifs of the β1 cytoplasmic tail, respectively. To test this hypothesis, we substituted the membrane proximal and distal tyrosines (Y) of the β1 tail with alanine (A) residues (β1 Y783A; β1 Y795A) in the germline of mice. We report that β1 Y783A or β1 Y795A substitutions blocked talin or kindlin binding, respectively, and led to β1 null-like peri-implantation lethality. Expression of β1 Y783A or β1 Y795A in the epidermis, however, resulted in skin blister and hair follicle phenotypes that were considerably milder than those observed with β1 integrin gene deletion or a β1 double Y-to-A substitution (β1 YY783/795AA). In culture, defects in adhesion, s**preading,** and migration were more severe with the β1 Y783A than with the β1 Y795A substitution despite markedly reduced β1 Y795A integrin surface levels owing to diminished protein stability. We conclude that regulation of β1 integrin adhesion through talins and kindlins may differ substantially between stably adherent keratinocytes and cells of the developing embryo, and that β1 cytoplasmic NPxY motifs contribute individually and independent of each other to β1 function in keratinocytes.
75. Integrin alpha3beta1 inhibits directional **migration** and wound re-epithelialization in the skin. Re-epithelialization after skin wounding requires both **migration** and hyperproliferation of keratinocytes. Laminin-332 is deposited during **migration** over the provisional matrix. To investigate the function of the laminin-332 binding integrin alpha3beta1 in wound re-epithelialization, we generated Itga3flox/flox; K14-Cre mice lacking the alpha3 subunit specifically in the basal layer of the epidermis. These mice are viable but display several skin defects, including local inflammation, hair loss, basement membrane duplication and microblistering at the dermal-epidermal junction, whereas hemidesmosome assembly and keratinocyte differentiation are not impaired. Wound healing is slightly faster in the absence of integrin alpha3beta1, whereas proliferation, the distribution of other integrins and the deposition of basement membrane proteins in the wound bed are unaltered. In vitro, cell spreading is rescued by increased surface expression of alpha6beta1 integrin in the absence of integrin alpha3. The alpha3-deficient keratinocytes migrate with an increased velocity and persistence, whereas proliferation, growth factor signaling, hemidesmosome assembly, and laminin-332 deposition appeared to be normal. We suggest that integrin alpha3beta1 delays keratinocyte **migration** during wound re-epithelialization, by binding to the laminin-332 that is newly deposited on the wound bed.
76. CD9 of mouse brain is implicated in neurite outgrowth and cell **migration** in vitro and is associated with the alpha 6/beta 1 integrin and the neural adhesion molecule L1. We describe here a novel monoclonal antibody (mab H6) which recognizes CD9, an integral cell surface constituent previously described in cells of the hematopoietic lineage and involved in the aggregation of platelets. Mab H6 was raised against membranes of immature mouse astrocytes and reacted with a protein of 25-27 kD in detergent extracts of adult mouse brain membranes. Sequence analysis of the N-terminal amino acids revealed an identity of 96% with CD9 from mouse kidney. CD9 was localized in the central and peripheral mouse nervous systems: in the spinal cord of 11-day-old mouse embryos, CD9 was strongly expressed in the floor and roof plates. In the adult mouse sciatic nerve, myelin sheaths were highly CD9-immunoreactive. Mab H6 reacted with the cell surfaces of both glial cells and neurons in culture and inhibited **migration** of neuronal cell bodies, neurite fasciculation and outgrowth of astrocytic processes from cerebellar microexplants. Neurite outgrowth from isolated small cerebellar neurons was increased in the presence of mab H6 on substrate-coated laminin, but not on substrate-coated poly-L-lysine. Addition of mab H6 elicited an increase in intracellular Ca2+ concentration in these cells on substrate-coated laminin. Immunoprecipitates of CD9 from cultured mouse neuroblastoma N2A cells contained the alpha 6/beta 1 integrin. Moreover, preparations of CD9 immunoaffinity-purified from adult mouse brain using a mab H6 column contained the neural adhesion molecule L1, but not other neural adhesion molecules. CD9 bound to L1, but not to NCAM or MAG. Both the alpha 6/beta 1 integrin and L1 could be induced to coredistribute with CD9 on the surface of cultured neuroblastoma N2A cells. The combined observations suggest that CD9 can associate with L1 and alpha 6/beta 1 integrin to influence neural cell interactions in vitro.
77. A role for alphabeta1 integrins in focal adhesion function and polarized cytoskeletal dynamics. alphabeta1 integrins have been implicated in the survival, spreading, and **migration** of cells and tissues. To explore the underlying biology, we identified conditions where primary beta1 null keratinocytes adhere, proliferate, and display robust alphavbeta6 integrin-induced, peripheral focal contacts associated with elaborate stress fibers. Mechanistically, this appears to be due to reduced FAK and Src and elevated RhoA and Rock activities. Visualization on a genetic background of GFPactin shows that beta1 null keratinocytes spread, but do so aberrantly, and when induced to migrate from skin explants in vitro, the cells are not able to rapidly reorient their actin cytoskeleton toward the polarized movement. As judged by RFPzyxin/GFPactin videomicroscopy, the alphavbeta6-actin network does not undergo efficient turnover. Without the ability to remodel their integrin-actin network efficiently, alphabeta1-deficient keratinocytes cannot respond dynamically to their environment and polarize movements.
78. β1 integrins regulate fibroblast chemotaxis through control of N-WASP stability. Chemotactic **migratio**n of fibroblasts towards growth factors, such as during development and wound healing, requires precise spatial coordination of receptor signalling. However, the mechanisms regulating this remain poorly understood. Here, we demonstrate that β1 integrins are required both for fibroblast chemotaxis towards platelet-derived growth factor (PDGF) and growth factor-induced dorsal ruffling. Mechanistically, we show that β1 integrin stabilises and spatially regulates the actin nucleating endocytic protein neuronal Wiskott–Aldrich syndrome protein (N-WASP) to facilitate PDGF receptor traffic and directed motility. Furthermore, we show that in intact cells, PDGF binding leads to rapid activation of β1 integrin within newly assembled actin-rich membrane ruffles. Active β1 in turn controls assembly of N-WASP complexes with both Cdc42 and WASP-interacting protein (WIP), the latter of which acts to stabilise the N-WASP. Both of these protein complexes are required for PDGF internalisation and fibroblast chemotaxis downstream of β1 integrins. This represents a novel mechanism by which integrins cooperate with growth factor receptors to promote localised signalling and directed cell motility.
79. Tetraspanin CD63 promotes vascular endothelial growth factor receptor 2-β1 integrin complex formation, thereby regulating activation and downstream signaling in endothelial cells in vitro and in vivo. CD63 is a member of the transmembrane-4 glycoprotein superfamily (tetraspanins) implicated in the regulation of membrane protein trafficking, leukocyte recruitment, and adhesion processes. We have investigated the involvement of CD63 in endothelial cell (EC) signaling downstream of β1 integrin and VEGF. We report that silencing of CD63 in primary ECs arrested capillary sprouting and tube formation in vitro because of impaired adhesion an**d migrati**on of ECs. Mechanistically, CD63 associated with both β1 integrin and the main VEGF receptor on ECs, VEGFR2. Our data suggest that CD63 serves to bridge between β1 integrin and VEGFR2 because CD63 silencing disrupted VEGFR2-β1 integrin complex formation identified using proximity ligation assays. Signaling downstream of β1 integrin and VEGFR2 was attenuated in CD63-silenced cells, although their cell surface expression levels remained unaffected. CD63 was furthermore required for efficient internalization of VEGFR2 in response to VEGF. Importantly, systemic delivery of VEGF failed to potently induce VEGFR2 phosphorylation and downstream signaling in CD63-deficient mouse lungs. Taken together, our findings demonstrate a previously unrecognized role for CD63 in coordinated integrin and receptor tyrosine kinase signaling in vitro and in vivo.
80. Spatiotemporal localization of periostin and its potential role in epithelial-mesenchymal transition during palatal fusion. The medial epithelial seam (MES) between the palatal shelves degrades during palatal fusion to achieve the confluence of palatal mesenchyme. Cellular mechanisms underlying the degradation of MES have been proposed, such as apoptosis, epithelial-mesenchymal transition (EMT) and **migration** of medial edge epithelia (MEE). Extracellular matrix components have been shown to play an important role in EMT in many model systems. Periostin (also known as osteoblast-specific factor-2) is a secreted mesenchymal extracellular matrix component that affects the ability of cells to migrate and/or facilitates EMT during both embryonic development and pathologic conditions. In this study, we evaluated the spatiotemporal expression patterns of periostin during mouse palatal fusion by in situ hybridization and immunofluorescence. Periostin mRNA and protein were present in the palatal mesenchyme, the protein being distributed in a fine fibrillar network and in the basement membrane, but absent from the epithelium. During MES degradation, the protein was strongly expressed in the basement membrane underlying the MES and in some select MEE. Confocal microscopic analysis using an EMT marker, twist1, and an epithelial marker, cytokeratin 14, provided evidence that select MEE were undergoing EMT in association with periostin. Moreover, the major extracellular matrix molecules in basement membrane, laminin and collagen type IV were degraded earlier than periostin. The result is that select MEE establish interactions with periostin in the mesenchymal extracellular matrix, and these new cell-matrix interactions may regulate MEE transdifferentiation during palatal fusion.
81. Ric-8a, a guanine nucleotide exchange factor for heterotrimeric G proteins, regulates bergmann glia-basement membrane adhesion during cerebellar foliation. The cerebellum consists of an intricate array of lobules that arises during the process of foliation. Foliation not only increases surface area, but may also facilitate organization of cerebellar neural circuitry. Defects in cerebellar foliation are associated with a number of diseases. Yet, little is known about how foliation, a process involving large-scale and simultaneous movement of several different cell types, is coordinated by cell-cell signaling at the molecular level. Here we show that Ric-8a, a guanine nucleotide exchange factor in the G-protein-coupled receptor pathway, is specifically required in Bergmann glia during cerebellar foliation. We find that ric-8a mutation in mice results in disorganized Bergmann glial scaffolding, defective granule cell **migration**, and disrupted Purkinje cell positioning. These abnormalities result from primary defects in Bergmann glia since mutations in granule cells do not show similar effects. They first arise during late embryogenesis, at the onset of foliation, when ric-8a mutant Bergmann glia fail to maintain adhesion to the basement membrane specifically at emerging fissures. This suggests that Ric-8a is essential for the enhanced Bergmann glia-basement membrane adhesion required for fissure formation. Indeed, we find that ric-8a-deficient cerebellar glia show decreased affinity for basement membrane components. We also find that weakening Bergmann glia-basement membrane interaction by β1 integrin deletion results in a similar phenotype. These results thus reveal a novel role of Ric-8a in modulating Bergmann glia-basement membrane adhesion during foliation, and provide new insights into the signaling pathways that coordinate cellular movement during cerebellar morphogenesis.
82. Tlx acts as a proangiogenic switch by regulating extracellular assembly of fibronectin matrices in retinal astrocytes. In response to hypoxia, hypoxia-inducible factors act as the primary proangiogenic triggers by regulating transcription levels of target genes, including VEGF. However, little is known about the specific factors that control other components of the angiogenic process, particularly formation of matrix scaffolds that promote adhesion and **migration** of endothelial cells. We show that in the postnatal mouse retina, the orphan nuclear receptor tailless (Tlx) is strongly expressed in the proangiogenic astrocytes, which secrete VEGF and fibronectin. Tlx expression by retinal astrocytes is controlled by oxygen concentration and rapidly downregulated upon contact with blood vessels. In mice null for Tlx, retinal astrocytes maintain VEGF expression; however, the extracellular assembly of fibronectin matrices by astrocytes is severely impaired, leading to defective scaffold formation and a complete failure of normal retinal vascular development. This work identifies Tlx as an essential component of the molecular network involved in the hypoxia-inducible proangiogenic switch in retinal astrocytes.
83. miR379-410 cluster miRNAs regulate neurogenesis and neuronal **migration** by fine-tuning N-cadherin. N-cadherin-mediated adhesion is essential for maintaining the tissue architecture and stem cell niche in the developing neocortex. N-cadherin expression level is precisely and dynamically controlled throughout development; however, the underlying regulatory mechanisms remain largely unknown. MicroRNAs (miRNAs) play an important role in the regulation of protein expression and subcellular localisation. In this study, we show that three miRNAs belonging to the miR379-410 cluster regulate N-cadherin expression levels in neural stem cells and migrating neurons. The overexpression of these three miRNAs in radial glial cells repressed N-cadherin expression and increased neural stem cell differentiation and neuronal **migration**. This phenotype was rescued when N-cadherin was expressed from a miRNA-insensitive construct. Transient abrogation of the miRNAs reduced stem cell differentiation and increased cell proliferation. The overexpression of these miRNAs specifically in newborn neurons delayed **migration** into the cortical plate, whereas the knockdown increased **migration**. Collectively, our results indicate a novel role for miRNAs of the miR379-410 cluster in the fine-tuning of N-cadherin expression level and in the regulation of neurogenesis and neuronal **migration** in the developing neocortex.
84. Mouse primordial germ cells lacking beta1 integrins enter the germline but fail to migrate normally to the gonads. Primordial germ cells are the founder cells of the gametes. They are set aside at the initial stages of gastrulation in mammals, become embedded in the hind-gut endoderm, then actively migrate to the sites of gonad formation. The molecular basis of this **migration** is poorly understood. Here we sought to determine if members of the integrin family of cell surface receptors are required for primordial germ cell **migration**, as integrins have been implicated in the **migration** of several other motile cell types. We have established a line of mice which express green fluorescent protein in germline cells that has enabled us to efficiently purify primordial germ cells at different stages by flow cytometry. We have catalogued the spectrum of integrin subunit expression by primordial germ cells during and after **migration**, using flow cytometry, immunocytochemistry and RT-PCR. Through analysis of integrin beta1(-/-)-->wild-type chimeras, we show that embryonic cells lacking beta1 integrins can enter the germline. However, integrin beta1(-/-) primordial germ cells do not colonize the gonad efficiently. Embryos with targeted deletion of integrin subunit alpha3, alpha6, or alphaV show no major defects in primordial germ cell **migration**. These results demonstrate a role for beta1-containing integrins in the development of the germline, although an equivalent role for \* integrin subunit(s) has yet to be established.
85. Impaired **migration** but not differentiation of haematopoietic stem cells in the absence of beta1 integrins. Adhesive interactions mediated by integrins of the beta1 subfamily are thought to be critical in controlling differentiation and **migration** of blood cell precursors. Here we report that chimaeric mice generated with beta1-integrin-deficient embryonic stem (ES) cells lack beta1(-/-) cells in blood and in haematopoietic organs such as spleen, thymus and bone marrow. Chimaeric embryos contain beta1-null haematopoietic cells in the yolk sac and in fetal blood but not in fetal liver. We show that such beta1(-/-) haematopoietic stem cells derived from yolk sac of 10.5-day-old chimaeric embryos readily generate erythroid and myeloid colonies and that beta1(-/-) ES cells can differentiate into mature B lymphocytes in vitro. Our results indicate that haematopoietic stem cells lacking beta1 integrins can form and differentiate into different lineages but cannot colonize the fetal liver.
86. C1GALT1 promotes invasive phenotypes of hepatocellular carcinoma cells by modulating integrin β1 glycosylation and activity. Cancer cell invasion and metastasis are the primary causes of treatment failure and death in hepatocellular carcinoma (HCC). We previously reported that core 1 β1,3-galactosyltransferase (C1GALT1) is frequently overexpressed in HCC tumors and its expression is associated with advanced tumor stage, metastasis, and poor survival. However, the underlying mechanisms of C1GALT1 in HCC malignancy remain unclear. In this study, we found that overexpression of C1GALT1 enhanced HCC cell adhesion to extracellular matrix (ECM) proteins**, migrati**on, and invasion, whereas RNAi-mediated knockdown of C1GALT1 suppressed these phenotypes. The promoting effect of C1GALT1 on the metastasis of HCC cells was demonstrated in a mouse xenograft model. Mechanistic investigations showed that the C1GALT1-enhanced phenotypic changes in HCC cells were significantly suppressed by anti-integrin β1 blocking antibody. Moreover, C1GALT1 was able to modify O-glycans on integrin β1 and regulate integrin β1 activity as well as its downstream signaling. These results suggest that C1GALT1 could enhance HCC invasiveness through integrin β1 and provide novel insights into the roles of O-glycosylation in HCC metastasis.
87. Regulation of Integrin β 1 recycling to lipid rafts by Rab1a to promote cell **migratio**n. Rab1a is a member of the Rab family of small GTPases with a well characterized function in the regulation of vesicle trafficking from the endoplasmic reticulum to the Golgi apparatus and within Golgi compartments. The integrin family heterodimeric transmembrane proteins serve as major receptors for extracellular matrix proteins, which play essential roles in cell adhesion and **migratio**n. Although effects on intracellular trafficking of integrins or other key cargos by Rab1a could influence cell **migratio**n, the regulatory mechanisms linking Rab1a to cell **migratio**n are not well understood. Here, we report identification of Rab1a as a novel regulator of cell **migratio**n using an unbiased RNAi screen targeting GTPases. Inhibition of Rab1a reduced integrin-mediated cell adhesion and spreading on fibronectins, reduced integrin β1 localization to lipid rafts, and decreased recycling of integrin β1 to the plasma membrane. Analysis of Rab1a effector molecules showed that p115 mediated Rab1a regulation of integrin recycling and lipid raft localization in ce**ll migrat**ion. Taken together, these results suggest a novel function for Rab1a in the regulation of ce**ll migrat**ion through controlling integrin β1 recycling and localization to lipid rafts via a specific downstream effector pathway.
88. Polymerase delta-interacting protein 2 regulates collagen accumulation via activation of the Akt/mTOR pathway in vascular smooth muscle cells. Polymerase delta interacting protein 2 (Poldip2) has previously been implicated in **migration**, proliferation and extracellular matrix (ECM) production in vascular smooth muscle cells. To better understand the role of Poldip2 in ECM regulation, we investigated the mechanism responsible for collagen I accumulation in Poldip2(+/-) mouse aortic smooth muscle cells (MASMs).Protein degradation and protein synthesis pathways were investigated. Depletion of Poldip2 had no effect on proteasome activity, but caused a partial reduction in autophagic flux. However, the rate of collagen I degradation was increased in Poldip2(+/-) vs. Poldip2(+/+) MASMs. Conversely, activation of the PI3K/Akt/mTOR signaling pathway, involved in regulation of protein synthesis, was significantly elevated in Poldip2(+/-) MASMs as was β1-integrin expression. Suppressing mTOR signaling using Akt inhibitor or rapamycin and reducing β1-integrin expression using siRNA prevented the increase in collagen I production. While collagen I and fibronectin were increased in Poldip2(+/-) MASMs, overall protein synthesis was not different from that in Poldip2(+/)(+)MASMs, suggesting selectivity of Poldip2 for ECM proteins.Poldip2(+/-) MASMs exhibit higher β1-integrin expression and activity of the PI3K/Akt/mTOR signaling pathway, leading to increased ECM protein synthesis. These findings have important implications for vascular diseases in which ECM accumulation plays a role.
89. miRNA-29c suppresses lung cancer cell adhesion to extracellular matrix and metastasis by targeting integrin β1 and matrix metalloproteinase2 (MMP2). Our pilot study using miRNA arrays found that miRNA-29c (miR-29c) is differentially expressed in the paired low-metastatic lung cancer cell line 95C compared to the high-metastatic lung cancer cell line 95D. Bioinformatics analysis shows that integrin β1 and matrix metalloproteinase 2 (MMP2) could be important target genes of miR-29c. Therefore, we hypothesized that miR-29c suppresses lung cancer cell adhesion to extracellular matrix (ECM) and metastasis by targeting integrin β1 and MMP2. The gain-of-function studies that raised miR-29c expression in 95D cells by using its mimics showed reductions in cell proliferation, adhesion to ECM, invasion a**nd migrat**ion. In contrasts, loss-of-function studies that reduced miR-29c by using its inhibitor in 95C cells promoted proliferation, adhesion to ECM, invasion a**nd migrat**ion. Furthermore, the dual-luciferase reporter assay demonstrated that miR-29c inhibited the expression of the luciferase gene containing the 3'-UTRs of integrin β1 and MMP2 mRNA. Western blotting indicated that miR-29c downregulated the expression of integrin β1 and MMP2 at the protein level. Gelatin zymography analysis further confirmed that miR-29c decreased MMP2 enzyme activity. Nude mice with xenograft models of lung cancer cells confirmed that miR-29c inhibited lung cancer metastasis in vivo, including bone and liver metastasis. Taken together, our results demonstrate that miR-29c serves as a tumor metastasis suppressor, which suppresses lung cancer cell adhesion to ECM and metastasis by directly inhibiting integrin β1 and MMP2 expression and by further reducing MMP2 enzyme activity. The results show that miR-29c may be a novel therapeutic candidate target to slow lung cancer metastasis.
90. Disruption of genital ridge development causes aberrant primordial germ cell proliferation but does not affect their directional **migration**. The directional **migration** and the following development of primordial germ cells (PGCs) during gonad formation are key steps for germline development. It has been proposed that the interaction between germ cells and genital ridge (GR) somatic cells plays essential roles in this process. However, the in vivo functional requirements of GR somatic cells in germ cell development are largely unknown.Wt1 mutation (Wt1(R394W/R394W)) results in GR agenesis through mitotic arrest of coelomic epitheliums. In this study, we employed the GR-deficient mouse model, Wt1(R394W/R394W), to investigate the roles of GR somatic cells in PGC **migration** and proliferation. We found that the number of PGCs was dramatically reduced in GR-deficient embryos at embryonic day (E) 11.5 and E12.5 due to decreased proliferation of PGCs, involving low levels of BMP signaling. In contrast, the germ cells in Wt1(R394W/R394W) embryos were still mitotically active at E13.5, while all the germ cells in control embryos underwent mitotic arrest at this stage. Strikingly, the directional **migration** of PGCs was not affected by the absence of GR somatic cells. Most of the PGCs reached the mesenchyme under the coelomic epithelium at E10.5 and no ectopic PGCs were noted in GR-deficient embryos. However, the precise positioning of PGCs was disrupted.Our work provides in vivo evidence that the proliferation of germ cells is precisely regulated by GR somatic cells during different stages of gonad development. GR somatic cells are probably dispensable for the directional **migration** of PGCs, but they are required for precise positioning of PGCs at the final step of **migration**.
91. Netrin-1-alpha3beta1 integrin interactions regulate the **migration** of interneurons through the cortical marginal zone. Cortical GABAergic interneurons, most of which originate in the ganglionic eminences, take distinct tangential migratory trajectories into the developing cerebral cortex. However, the ligand-receptor systems that modulate the tangential **migration** of distinct groups of interneurons into the emerging cerebral wall remain unclear. Here, we show that netrin-1, a diffusible guidance cue expressed along the migratory routes traversed by GABAergic interneurons, interacts with alpha3beta1 integrin to promote interneuronal **migration**. In vivo analysis of interneuron-specific alpha3beta1 integrin, netrin-1-deficient mice (alpha3(lox/-)Dlx5/6-CIE, netrin-1(-/-)) reveals specific deficits in the patterns of interneuronal **migration** along the top of the developing cortical plate, resulting in aberrant interneuronal positioning throughout the cerebral cortex and hippocampus of conditional alpha3(lox/-)Dlx5/6-CIE, netrin-1(-/-) mice. These results indicate that specific guidance mechanisms, such as netrin-1-alpha3beta1 integrin interactions, modulate distinct routes of interneuronal **migration** and the consequent positioning of groups of cortical interneurons in the developing cerebral cortex.
92. Radial glia regulate Cajal-Retzius cell positioning in the early embryonic cerebral cortex. The organization of neocortex, along its radial axis, into a six-layered structure is one of the most exquisite features of the brain. Because of their strategic localization in the marginal zone, and their expression of reelin, a signal that controls spatial ordering of cortical layers, Cajal-Retzius (C-R) cells play a crucial role in cortical patterning along this axis. Yet, it remains less well understood how C-R cell targeting itself is regulated. At the onset of corticogenesis when C-R cells first arrive in the cortex via tangential **migration**, radial glia (RG) are the main cell type present. This suggests that RG may play a role in C-R cell localization. To test this, we used genetic approaches to perturb RG scaffold during early corticogenesis. We found that disrupting RG endfoot adhesion to basal lamina consistently results in C-R cell displacement. These displacements do not appear to result from primary defects in neural progenitor cell proliferation, deficits in the meninges or basement membrane, or cell autonomous defects in C-R cells. Instead, they show close temporal and spatial correlation with RG endfoot retraction. Moreover, ablation of RG via cell cycle blockade similarly results in local displacement of C-R cells. These lines of evidence thus indicate that, during early corticogenesis, RG play a primary role in regulating spatial targeting of C-R cells. Since RG are also neural progenitors as well as neuronal **migration** scaffolds, these findings suggest that, during nervous system development, neuroepithelial stem cells may not only be responsible for generating a diverse array of neuronal cell types and facilitating their radial **migration**. They may also, through regulating the placement of guidepost cells, coordinate spatial patterning of the nervous system along its radial axis.
93. Fibronectin-integrin (alpha5beta1) modulates **migration** and invasion of murine melanoma cell line B16F10 by involving MMP-9. Cell adhesion to extracellular matrix (ECM) initiates signaling cascade regulated by cell surface integrin receptors, which affects the proliferation and invasion of cells. Cells cultured in the presence of ECM ligand fibronectin (FN) stimulate secretion of matrix metalloproteinases (MMPs), facilitating cancer cell invasion and metastasis. Among all the members of the MMP family, MMP-9 is of crucial importance in tumor invasion and metastasis. The present study aims at studying the effects of integrin receptor alpha5beta1 and its ligand FN on expression of MMP-9 in murine melanoma cell line B16F10 and understanding the molecular mechanism(s) involved. The main experimental methods performed in the study were gelatin zymography, immunoblot, real-time RT-PCR, immunocytochemistry, enzyme linked immunosorbent assay (ELISA), transwell chamber assay, and in vivo metastasis assay in syngenic (C57BL6J) mice. The study reports that FN induces the activity, mRNA, and protein expression of MMP-9 and initiates its proteolytic activation in B16F10 cells. Blockage of the alpha5 receptor abrogated the FN-mediated stimulatory response on MMP-9 in B16F10 cells. Inhibitor studies and immunoblot analysis strongly suggest the involvement of focal adhesion kinase (FAK), extracellular regulated kinase (ERK), and phosphatidylinositol-3-kinase (PI-3K) in the FN-mediated responses. Immunocytochemical analysis showed the nuclear localization of nuclear factor-kappaB (NF-kappaB) might lead to activation of MMP-9 gene upon FN treatment. This study demonstrates that integrin receptor alpha5beta1 and FN interaction induces the invasive potential of B16F10 cells and MMP-9 induction is the downstream effectors in the process. This system serves as a novel model system to understand the molecular mechanism of melanoma growth and invasion.
94. Knock-in of integrin beta 1D affects primary but not secondary myogenesis in mice. Integrins are extracellular matrix receptors composed of alpha and beta subunits involved in cell adhesion, **migration** and signal transduction. The beta1 subunit has two isoforms, beta 1A ubiquitously expressed and beta 1D restricted to striated muscle. They are not functionally equivalent. Replacement of beta 1A by beta 1D (beta 1D knock-in) in the mouse leads to midgestation lethality on a 50% Ola/50% FVB background [Baudoin, C., Goumans, M. J., Mummery, C. and Sonnenberg, A. (1998). Genes Dev. 12, 1202-1216]. We crossed the beta 1D knock-in line into a less penetrant genetic background. This led to an attenuation of the midgestation lethality and revealed a second period of lethality around birth. Midgestation death was apparently not caused by failure in cell **migration**, but rather by abnormal placentation. The beta 1D knock-in embryos that survived midgestation developed until birth, but exhibited severely reduced skeletal muscle mass. Quantification of myotube numbers showed that substitution of beta 1A with beta 1D impairs primary myogenesis with no direct effect on secondary myogenesis. Furthermore, long-term primary myotube survival was affected in beta 1D knock-in embryos. Finally, overexpression of beta 1D in C2C12 cells impaired myotube formation while overexpression of beta 1A primarily affected myotube maturation. Together these results demonstrate for the first time distinct roles for beta1 integrins in primary versus secondary myogenesis and that the beta 1A and beta 1D variants are not functionally equivalent in this process.
95. Integrin alpha2beta1 is required for regulation of murine wound angiogenesis but is dispensable for reepithelialization. The alpha2beta1 integrin functions as the major receptor for collagen type I on a large number of different cell types, including keratinocytes, fibroblasts, endothelial cells, and a variety of inflammatory cells. Recently, we demonstrated that adhesion of keratinocytes to collagen critically depends on alpha2beta1, whereas fibroblasts can partly compensate for loss of alpha2beta1 in simple adhesion to collagen. However, in three-dimensional collagen matrices, alpha2beta1-null fibroblasts are hampered in generating mechanical forces. These data suggested a pivotal role for alpha2beta1 during wound healing in vivo. Unexpectedly, reepithelialization of excisional wounds of alpha2beta1-null mice was not impaired, indicating that keratinocytes do not require adhesion to or **migration** on collagen for wound closure. Whereas wound contraction and myofibroblast differentiation were similar, wound tensile strain was reduced in alpha2beta1-null mice, suggesting subtle changes in organization of the extracellular matrix. In addition, we observed reduced influx of mast cells into the granulation tissue, whereas infiltration of other inflammatory cells was not impaired. Interestingly, ablation of alpha2beta1 resulted in strong enhancement of neovascularization of granulation tissue and sponge implants. Both ultrastructurally and functionally, these new blood vessels appeared intact. In conclusion, our data show unique and overlapping functions of alpha2beta1 integrin during murine cutaneous wound healing.
96. The integrin beta1 subunit transmembrane domain regulates phosphatidylinositol 3-kinase-dependent tyrosine phosphorylation of Crk-associated substrate. Our previous studies on the transmembrane domain of human integrin subunits have shown that a conserved basic amino acid in both subunits of integrin heterodimers is positioned in the plasma membrane in the absence of interacting proteins. To investigate the possible functional role of the lipid-embedded lysine in the mouse integrin beta1 subunit, this amino acid was replaced with leucine, and the mutated beta1 subunit (beta1A(K756L)) was stably expressed in beta1-deficient GD25 cells. The extracellular domain of beta1A(K756L) integrins possesses a competent conformation for ligand binding as determined by the ability to mediate cell adhesion, and by the presence of the monoclonal antibody 9EG7 epitope. However, the spreading of GD25-beta1A(K756L) cells on fibronectin and laminin-1 was impaired, and the rate of **migration** of GD25-beta1A(K756L) cells on fibronectin was reduced compared with GD25-beta1A cells. Phosphorylation of tyrosines in focal adhesion kinase (FAK) and the Y416 in c-Src in response to beta1A(K756L)-mediated adhesion was similar to that induced by wild-type beta1. The tyrosine phosphorylation level of paxillin, a downstream target of FAK/Src, was unaffected by the beta1 mutation, whereas tyrosine phosphorylation of CAS was strongly reduced. The results demonstrate that CAS is a target for phosphorylation both by FAK-dependent and -independent pathways after integrin ligation. The latter pathway was inhibited by wortmannin and LY294002, implicating that it required an active phosphatidylinositol 3-kinase. Furthermore, the K756L mutation in the beta1 subunit was found to interfere with beta1-induced activation of Akt. The results from this study identify phosphatidylinositol 3-kinase as an early component of a FAK-independent integrin signaling pathway triggered by the membrane proximal part of the beta1 subunit.
97. Alteration of podocytes in a murine model of crescentic glomerulonephritis. Recent observations suggest a central role of podocytes in crescent formation. In experimental glomerulonephritis podocytes disrupt the parietal epithelial layer and attach on its basement membrane, thus forming bridges between the tuft and Bowman's capsule, and they are a major constituent of crescents. In order to explain these findings we hypothesize that inflammation triggers motility in podocytes. In the present study we asked whether podocytes display alterations which suggest a migratory behavior in glomerulonephritis. Glomerulonephritis was induced in mice by injection of a rabbit serum against the glomerular basement membrane. The kidneys were perfusion-fixed 6 days later and examined by light and electron microscopy as well as by immunohistochemistry. In glomerulonephritis the apical cytoplasm of podocytes displayed numerous actin-containing microprotrusions. Cortactin, a protein involved in the regulation of actin polymerization, was predominantly expressed in foot processes of podocytes in control mice. It was redistributed to the cell body in glomerulonephritis. In untreated mice betal-integrin was restricted to the foot processes. In glomerulonephritis it was additionally found in the cytoplasm and in the apical cell membrane. Recycling of integrins is a crucial event in initiation of cell **migration**. ICAM-1 and CD44, the ligation of which induces migratory behaviors, were absent from healthy podocytes but expressed by some podocytes in glomerulonephritis. Thus, in glomerulonephritis podocytes display some characteristic features of migrating cells. This might explain their ability to break through the parietal epithelium and to become a constituent of early crescents.
98. CASK deletion in intestinal epithelia causes mislocalization of LIN7C and the DLG1/Scrib polarity complex without affecting cell polarity. CASK is the mammalian ortholog of LIN2, a component of the LIN2/7/10 protein complex that targets epidermal growth factor receptor (EGFR) to basolateral membranes in Caenorhabditis elegans. A member of the MAGUK family of scaffolding proteins, CASK resides at basolateral membranes in polarized epithelia. Its interaction with LIN7 is evolutionarily conserved. In addition, CASK forms a complex with another MAGUK, the DLG1 tumor suppressor. Although complete knockout of CASK is lethal, the gene is X-linked, enabling us to generate heterozygous female adults that are mosaic for its expression. We also generated intestine-specific CASK knockout mice. Immunofluorescence analysis revealed that in intestine, CASK is not required for epithelial polarity or differentiation but is necessary for the basolateral localization of DLG1 and LIN7C. However, the subcellular distributions of DLG1 and LIN7C are independent of CASK in the stomach. Moreover, CASK and LIN7C show normal localization in dlg1(-/-) intestine. Despite the disappearance of basolateral LIN7C in CASK-deficient intestinal crypts, this epithelium retains normal localization of LIN7A/B, EGFR and ErbB-2. Finally, crypt-to-villus **migration** rates are unchanged in CASK-deficient intestinal epithelium. Thus, CASK expression and the appropriate localization of DLG1 are not essential for either epithelial polarity or intestinal homeostasis in vivo.
99. The opposing roles of laminin-binding integrins in cancer. Integrins play an important role in cell adhesion by linking the cytoskeleton of cells to components in the extracellular matrix. In this capacity, integrins cooperate with different cell surface receptors, including growth factor receptors and G-protein coupled receptors, to regulate intracellular signaling pathways that control cell polarization, spreading, **migration**, survival, and gene expression. A distinct subfamily of molecules in the integrin family of adhesion receptors is formed by receptors that mediate cell adhesion to laminins, major components of the basement membrane that lie under clusters of cells or surround them, separating them from other cells and/or adjacent connective tissue. During the past decades, many studies have provided evidence for a role of laminin-binding integrins in tumorigenesis, and both tumor-promoting and suppressive activities have been identified. In this review we discuss the dual role of the laminin-binding integrins α3β1 and α6β4 in tumor development and progression, and examine the factors and mechanisms involved in these opposing effects.
100. Genetic analysis of beta1 integrin function: confirmed, new and revised roles for a crucial family of cell adhesion molecules. Integrins are heterodimeric cell adhesion proteins connecting the extracellular matrix to the cytoskeleton and transmitting signals in both directions. These integrins are suggested to be involved in many different biological processes such as growth, differentiation, **migration**, and cell death. Of more than 20 known integrins, 10 contain the nearly ubiquitously expressed beta1 integrin subunit. Disruption of the beta1 integrin gene by homologous recombination allows us to assess the supposed functions of beta1 containing integrins in vivo in a new way. This review will present and discuss recent findings derived from such studies concerning the biological roles of beta1 integrins in early development, differentiation and **migration**, hematopoiesis, tumorigenesis, and supramolecular assembly of extracellular matrix proteins. While several former results were confirmed, others were contradicted and new functions found, significantly changing the previous view of beta1 integrin function in vivo.
101. Cytohesin-1 controls the activation of RhoA and modulates integrin-dependent adhesion and **migration** of dendritic cells. Adhesion and motility of mammalian leukocytes are essential requirements for innate and adaptive immune defense mechanisms. We show here that the guanine nucleotide exchange factor cytohesin-1, which had previously been demonstrated to be an important component of beta-2 integrin activation in lymphocytes, regulates the activation of the small GTPase RhoA in primary dendritic cells (DCs). Cytohesin-1 and RhoA are both required for the induction of chemokine-dependent conformational changes of the integrin beta-2 subunit of DCs during adhesion under physiological flow conditions. Furthermore, use of RNAi in murine bone marrow DCs (BM-DCs) revealed that interference with cytohesin-1 signaling impairs **migration** of wild-type dendritic cells in complex 3D environments and in vivo. This phenotype was not observed in the complete absence of integrins. We thus demonstrate an essential role of cytohesin-1/RhoA during ameboid **migration** in the presence of integrins and further suggest that DCs without integrins switch to a different **migration** mode.
102. **Migration** of encephalitogenic CD8 T cells into the central nervous system is dependent on the α4β1-integrin. Although CD8 T cells are key players in neuroinflammation, little is known about their trafficking cues into the central nervous system (CNS). We used a murine model of CNS autoimmunity to define the molecules involved in cytotoxic CD8 T-cel**l migrati**on into the CNS. Using a panel of mAbs, we here show that the α4β1-integrin is essential for CD8 T-cell interaction with CNS endothelium. We also investigated which α4β1-integrin ligands expressed by endothelial cells are implicated. The blockade of VCAM-1 did not protect against autoimmune encephalomyelitis, and only partly decreased the CD8(+) T-cell infiltration into the CNS. In addition, inhibition of junctional adhesion molecule-B expressed by CNS endothelial cells also decreases CD8 T-cell infiltration. CD8 T cells may use additional and possibly unidentified adhesion molecules to gain access to the CNS.
103. Integrin alpha3beta1 directs the stabilization of a polarized lamellipodium in epithelial cells through activation of Rac1. Epithelial cell **migration** is a crucial event in wound healing, yet little is known about mechanisms whereby integrins regulate epithelial cell polarization and **migration**. In the present work, we demonstrate the importance of adhesion through the alpha3beta1 integrin in promoting the stabilization of leading lamellipodia in migrating keratinocytes. We demonstrate that this integrin is found at the leading edge of migrating keratinocytes and that inhibition of alpha3beta1 binding to laminin-5 prevents the formation of stable leading lamellipodia. Consistent with this observation, keratinocytes derived from alpha3beta1-deficient mice fail to form stable leading lamellipodia but retain the ability to form actin-containing protrusions that rapidly extend and retract from the cell membrane. Formation of a leading lamellipodium also requires alpha3beta1-dependent activation of Rac1, because alpha3beta1-deficient keratinocytes show decreased activation of Rac1 compared with alpha3beta1-expressing cells, and formation of stable leading lamellipodia can be inhibited in the latter cells by expression of the dominant negative Rac1 mutant Rac1N17. Furthermore, alpha3beta1-deficient keratinocytes expressing constitutively active Rac1L61 failed to form stable lamellipodia when plated onto laminin-5, demonstrating that alpha3beta1 is required for Rac1-mediated formation of a stable lamellipodium. These observations identify a crucial role for integrin-mediated adhesion and signaling in the formation of large, polarized, stable lamellipodia by migrating epithelial cells. To our knowledge, this study is the first to demonstrate that signal transduction through a specific integrin is required to direct the development of a lamellipodium from an initial protrusion and promote persistent epithelial cell **migration**.
104. The role of integrin alpha8beta1 in fetal lung morphogenesis and injury. Prenatal inflammation prevents normal lung morphogenesis and leads to bronchopulmonary dysplasia (BPD), a common complication of preterm birth. We previously demonstrated in a bacterial endotoxin mouse model of BPD that disrupting fibronectin localization in the fetal lung mesenchyme causes arrested saccular airway branching. In this study we show that expression of the fibronectin receptor, integrin alpha8beta1 is decreased in the lung mesenchyme in the same inflammation model suggesting it is required for normal lung development. We verified a role for integrin alpha8beta1 in lung development using integrin alpha8-null mice, which develop fusion of the medial and caudal lobes as well as abnormalities in airway division. We further show in vivo and in vitro that alpha8-null fetal lung mesenchymal cells fail to form stable adhesions and have increased **migration**. Thus we propose that integrin alpha8beta1 plays a critical role in lung morphogenesis by regulating mesenchymal cell adhesion and **migration**. Furthermore, our data suggest that disruption of the interactions between extracellular matrix and integrin alpha8beta1 may contribute to the pathogenesis of BPD.
105. The major laminin receptor of mouse embryonic stem cells is a novel isoform of the alpha 6 beta 1 integrin. Laminin is the first extracellular matrix protein expressed in the developing mouse embryo. It is known to influence morphogenesis and affect cell **migration** and polarization. Several laminin receptors are included in the integrin family of extracellular matrix receptors. Ligand binding by integrin heterodimers results in signal transduction events controlling cell motility. We report that the major laminin receptor on murine embryonic stem (ES) cells is the integrin heterodimer alpha 6 beta 1, an important receptor for laminin in neurons, lymphocytes, macrophages, fibroblasts, platelets and other cell types. However, the cytoplasmic domain of the ES cell alpha 6 (alpha 6 B) differs totally from the reported cytoplasmic domain amino acid sequence of alpha 6 (alpha 6 A). Comparisons of alpha 6 cDNAs from ES cells and other cells suggest that the alpha 6 A and alpha 6 B cytoplasmic domains derive from alternative mRNA splicing. Anti-peptide antibodies to alpha 6 A are unreactive with ES cells, but react with mouse melanoma cells and embryonic fibroblasts. When ES cells are cultured under conditions that permit their differentiation, they become positive for alpha 6 A, concurrent with the morphologic appearance of differentiated cell types. Thus, expression of the alpha 6 B beta 1 laminin receptor may be favored in undifferentiated, totipotent cells, while the expression of alpha 6 A beta 1 receptor occurs in committed lineages. While the functions of integrin alpha chain cytoplasmic domains are not understood, it is possible that they contribute to transferring signals to the cell interior, e.g., by delivering cytoskeleton organizing signals in response to integrin engagement with extracellular matrix ligands. It is therefore reasonable to propose that the cellular responses to laminin may vary, according to what alpha subunit isoform (alpha 6 A or alpha 6 B) is expressed as part of the alpha 6 beta 1 laminin receptor. The switch from alpha 6 B to alpha 6 A, if confirmed in early embryos, could then be of striking potential relevance to the developmental role of laminin.
106. Enhanced stem cell **migration** mediated by VCAM-1/VLA-4 interaction improves cardiac function in virus-induced dilated cardiomyopathy. Endogenous circulation of bone marrow-derived cells (BMCs) was observed in patients with dilated cardiomyopathy (DCM) who showed cardiac upregulation of Vascular Cell Adhesion Protein-1 (VCAM-1). However, the underlying pathophysiology is currently unknown. Thus, we aimed to analyze circulation, **migration** and G-CSF-based mobilization of BMCs in a murine model of virus-induced DCM. Mice with coxsackievirus B3 (CVB3) induced DCM and healthy controls were analyzed regarding their myocardial homing factors by PCR. To determine cardiac VCAM-1 expression ELISA and immunohistochemistry were applied. Flow cytometry was performed to analyze BMCs. Cardiac diameters and function were evaluated by echocardiography before and 4 weeks after G-CSF treatment. In murine CVB3-induced DCM an increase of BMCs in peripheral blood and a decrease of BMCs in bone marrow was observed. We found an enhanced **migration** of Very Late Antigen-4 (VLA-4⁺) BMCs to the diseased heart overexpressing VCAM-1 and higher numbers of CD45⁻CD34⁻Sca-1⁺ and CD45⁻CD34⁻c-kit⁺ cells. Mobilization of BMCs by **G-CSF boo**sted migration along the VCAM-1/VLA-4 axis and reduced apoptosis of cardiomyocytes. Significant improvement of cardiac function was detected by echocardiography in G-CSF-treated mice. Blocking VCAM-1 by a neutralizing antibody reduced the G-CSF-dependent effects **on stem** cell migration and cardiac function. This is the first study showing that in virus-induced DCM VCAM-1/VLA-4 interaction is crucial for recruitment of circulating BMCs leading to beneficial anti-apoptotic effects resulting in improved cardiac function after G-CSF-induced mobilization.
107. Neutrophil swarms require LTB4 and integrins at sites of cell death in vivo. Neutrophil recruitment from blood to extravascular sites of sterile or infectious tissue damage is a hallmark of early innate immune responses, and the molecular events leading to cell exit from the bloodstream have been well defined. Once outside the vessel, individual neutrophils often show extremely coordinated chemotaxis and cluster formation reminiscent of the swarming behaviour of insects. The molecular players that direct this response at the single-cell and population levels within the complexity of an inflamed tissue are unknown. Using two-photon intravital microscopy in mouse models of sterile injury and infection, we show a critical role for intercellular signal relay among neutrophils mediated by the lipid leukotriene B4, which acutely amplifies local cell death signals to enhance the radius of highly directed interstitial neutrophil recruitment. Integrin receptors are dispensable for long-distance **migration**, but have a previously unappreciated role in maintaining dense cellular clusters when congregating neutrophils rearrange the collagenous fibre network of the dermis to form a collagen-free zone at the wound centre. In this newly formed environment, integrins, in concert with neutrophil-derived leukotriene B4 and other chemoattractants, promote local neutrophil interaction while forming a tight wound seal. This wound seal has borders that cease to grow in kinetic concert with late recruitment of monocytes and macrophages at the edge of the displaced collagen fibres. Together, these data provide an initial molecular map of the factors that contribute to neutrophil swarming in the extravascular space of a damaged tissue. They reveal how local events are propagated over large-range distances, and how auto-signalling produces coordinated, self-organized neutrophil-swarming behaviour that isolates the wound or infectious site from surrounding viable tissue.
108. Alpha11 beta1 integrin-dependent regulation of periodontal ligament function in the erupting mouse incisor. The fibroblast integrin alpha11beta1 is a key receptor for fibrillar collagens. To study the potential function of alpha11 in vivo, we generated a null allele of the alpha11 gene. Integrin alpha11(-/-) mice are viable and fertile but display dwarfism with increased mortality, most probably due to severely defective incisors. Mutant incisors are characterized by disorganized periodontal ligaments, whereas molar ligaments appear normal. The primary defect in the incisor ligament leads to halted tooth eruption. alpha11beta1-defective embryonic fibroblasts displayed severe defects in vitro, characterized by (i) greatly reduced cell adhesion and spreading on collagen I, (ii) reduced ability to retract collagen lattices, and (iii) reduced cell proliferation. Analysis of matrix metalloproteinase in vitro and in vivo revealed disturbed MMP13 and MMP14 synthesis in alpha11(-/-) cells. We show that alpha11beta1 is the major receptor for collagen I on mouse embryonic fibroblasts and suggest that alpha11beta1 integrin is specifically required on periodontal ligament fibroblasts for cell **migration** and collagen reorganization to help generate the forces needed for axial tooth movement. Our data show a unique role for alpha11beta1 integrin during tooth eruption.
109. VCAM-1 and VLA-4 modulate dendritic cell IL-12p40 production in experimental visceral leishmaniasis. Vascular cell adhesion molecule-1 (VCAM-1) interacts with its major ligand very late antigen-4 (VLA-4) to mediate cell adhesion and transendothelial **migration** of leukocytes. We report an important role for VCAM-1/VLA-4 interactions in the generation of immune responses during experimental visceral leishmaniasis caused by Leishmania donovani. Our studies demonstrate that these molecules play no direct role in the recruitment of leukocytes to the infected liver, but instead contribute to IL-12p40-production by splenic CD8(+) dendritic cells (DC). Blockade of VCAM-1/VLA-4 interactions using whole antibody or anti-VCAM-1 Fab' fragments reduced IL-12p40 mRNA accumulation by splenic DC 5 hours after L. donovani infection. This was associated with reduced anti-parasitic CD4(+) T cell activation in the spleen and lowered hepatic IFNgamma, TNF and nitric oxide production by 14 days post infection. Importantly, these effects were associated with enhanced parasite growth in the liver in studies with either anti-VCAM-1 or anti-VLA-4 antibodies. These data indicate a role for VCAM-1 and VLA-4 in DC activation during infectious disease.
110. Introduction of bisecting GlcNAc into integrin alpha5beta1 reduces ligand binding and down-regulates cell adhesion and cell **migration**. The enzyme beta1,4-N-acetylglucosaminyltransferase III (GnT-III) catalyzes the addition of a bisecting GlcNAc residue to glycoproteins, resulting in a modulation in biological function. Our previous studies showed that the transfection of the GnT-III gene into B16 melanoma cells results in a suppression of invasive ability and lung colonization. The suppression has been postulated to be due to an increased level of E-cadherin expression on the cell surface, which in turn leads to the up-regulation of cell-cell adhesion. In this study, we report on the effects of overexpression of GnT-III on cell-matrix adhesion. The overexpression of GnT-III, but not that of an enzymatic inactive GnT-III (D323A), inhibits cell spreading and **migration** on fibronectin, a specific ligand for integrin alpha(5)beta(1), and the focal adhesion kinase phosphorylation. E(4)-PHA lectin blot analyses showed that the levels of bisecting GlcNAc structures on the integrin alpha(5) subunit as well as alpha(2) and alpha(3) subunits immunoprecipitated from GnT-III transfectants were substantially increased. In addition, the affinity of the binding of integrin alpha(5)beta(1) to fibronectin was significantly reduced by the introduction of the bisecting GlcNAc, to the alpha(5) subunit. These findings suggest that the modification of N-glycan of integrin by GnT-III inhibits its ligand binding ability, subsequently leading to the down-regulation of integrin-mediated signaling.
111. The lymphangiogenic vascular endothelial growth factors VEGF-C and -D are ligands for the integrin alpha9beta1. Mice homozygous for a null mutation of the integrin alpha9 subunit die 6-12 days after birth from bilateral chylothoraces suggesting an underlying defect in lymphatic development. However, until now the mechanisms by which the integrin alpha9beta1 modulates lymphangiogenesis have not been described. In this study we show that adhesion to and **migration** on the lymphangiogenic vascular endothelial growth factors (VEGF-C and -D) are alpha9beta1-dependent. Mouse embryonic fibroblasts and human colon carcinoma cells (SW-480) transfected to express alpha9beta1 adhered and/or migrated on both growth factors in a concentration-dependent fashion, and both adhesion and **migration** were abrogated by anti-alpha9beta1 function-blocking antibody. In SW-480 cells, which lack cognate receptors for VEGF-C and -D, both growth factors induced alpha9beta1-dependent Erk and paxillin phosphorylation. Human microvascular endothelial cells, which express both alpha9beta1 and VEGF-R3, also adhered to and migrated on both growth factors, and both responses were blocked by anti-alpha9beta1 antibody. Furthermore, in a solid phase binding assay recombinant VEGF-C and -D bound to purified alpha9beta1 integrin in a dose- and cation-dependent fashion showing that VEGF-C and VEGF-D are ligands for the integrin alpha9beta1. The interaction between alpha9beta1 and VEGF-C and/or -D may begin to explain the abnormal lymphatic phenotype of the alpha9 knock-out mice.
112. JAM-C regulates tight junctions and integrin-mediated cell adhesion and **migration**. Junctional Adhesion Molecules (JAMs) have been described as major components of tight junctions in endothelial and epithelial cells. Tight junctions are crucial for the establishment and maintenance of cell polarity. During tumor development, they are remodeled, enabling neoplastic cells to escape from constraints imposed by intercellular junctions and to adopt a migratory behavior. Using a carcinoma cell line we tested whether JAM-C could affect tight junctions and migratory properties of tumor cells. We show that transfection of JAM-C improves the tight junctional barrier in tumor cells devoid of JAM-C expression. This is dependent on serine 281 in the cytoplasmic tail of JAM-C because serine mutation into alanine abolishes the specific localization of JAM-C in tight junctions and establishment of cell polarity. More importantly, the same mutation stimulates integrin-mediated cell **migration** and adhesion via the modulation of beta1 and beta3 integrin activation. These results highlight an unexpected function for JAM-C in controlling epithelial cell conversion from a static, polarized state to a pro-migratory phenotype.
113. Redundant role of protein kinase C delta and epsilon during mouse embryonic development. Protein Kinase C delta and epsilon are mediators of important cellular events, such as cell proliferation, **migration** or apoptosis. The formation of blood vessels, i.e., vasculo- and angiogenesis, is a process where these isoforms have also been shown to participate. However, mice deficient in either Protein Kinase C delta or epsilon are viable and therefore their individual contribution to the formation of the vasculature appeared so far dispensable. In this study, we show that double null mutation of Protein Kinase C delta and epsilon causes embryonic lethality at approximately E9.5. At this stage, whole mount staining of the endothelial marker CD31 in double null embryos revealed defective blood vessel formation. Moreover, culture of double deficient mouse allantois showed impaired endothelial cell organization, and analyses of double deficient embryo sections showed dilated vessels, decreased endothelial-specific adherent junctions, and decreased contact of endothelial cells with mural cells. Protein kinase C delta and epsilon also appeared essential for vascular smooth muscle cell differentiation, since α-smooth muscle actin, a classical marker for vascular smooth muscle cells, was almost undetectable in double deficient embryonic aorta at E9.5. Subsequent qPCR analyses showed decreased VE-cadherin, Vegfr2, Cd31, Cdh2, Ets1, and Fli-1, among other angiogenesis related transcripts in double deficient embryos. Taken together, these data suggest for the first time an in vivo redundant role between members of the novel Protein Kinase C subfamily that allows for mutual compensation during mouse embryonic development, with vasculogenesis/angiogenesis as an obvious common function of these two Protein Kinase Cs. Protein Kinase C delta and epsilon might therefore be useful targets for inhibiting vasculo- and/or angiogenesis.
114. Angiopoietin-like 4 interacts with integrins beta1 and beta5 to modulate keratinocyte **migration**. Adipose tissue secretes adipocytokines for energy homeostasis, but recent evidence indicates that some adipocytokines also have a profound local impact on wound healing. Upon skin injury, keratinocytes use various signaling molecules to promote reepithelialization for efficient wound closure. In this study, we identify a novel function of adipocytokine angiopoietin-like 4 (ANGPTL4) in keratinocytes during wound healing through the control of both integrin-mediated signaling and internalization. Using two different in vivo models based on topical immuno-neutralization of ANGPTL4 as well as ablation of the ANGPTL4 gene, we show that ANGPTL4-deficient mice exhibit delayed wound reepithelialization with impaired keratinocyte **migration**. Human keratinocytes in which endogenous ANGPTL4 expression was suppressed by either siRNA or a neutralizing antibody show impaired **migration** associated with diminished integrin-mediated signaling. Importantly, we identify integrins β1 and β5, but not β3, as novel binding partners of ANGPTL4. ANGPTL4-bound integrin β1 activated the FAK-Src-PAK1 signaling pathway, which is important for c**ell migra**tion. The findings presented herein reveal an unpredicted role of ANGPTL4 during wound healing and demonstrate how ANGPTL4 stimulates intracellular signaling mechanisms to coordinate cellular behavior. Our findings provide insight into a novel c**ell migra**tion control mechanism and underscore the physiological importance of the modulation of integrin activity in cancer metastasis.
115. Lack of beta 1 integrin gene in embryonic stem cells affects morphology, adhesion, and **migration** but not integration into the inner cell mass of blastocysts. A gene trap-type targeting vector was designed to inactivate the beta 1 integrin gene in embryonic stem (ES) cells. Using this vector more than 50% of the ES cell clones acquired a disruption in the beta 1 integrin gene and a single clone was mutated in both alleles. The homozygous mutant did not produce beta 1 integrin mRNA or protein, while alpha 3, alpha 5, and alpha 6 integrin subunits were transcribed but not detectable on the cell surface. Heterozygous mutants showed reduced beta 1 expression and surface localization of alpha/beta 1 heterodimers. The alpha V subunit expression was not impaired on any of the mutants. Homozygous ES cell mutants lacked adhesiveness for laminin and fibronectin but not for vitronectin and showed a reduced association with a fibroblast feeder layer. Furthermore, they did not migrate towards chemoattractants in fibroblast medium. None of these functions were impaired in heterozygous mutants. Scanning electron microscopy revealed that homozygous cells showed fewer cell-cell junctions and had many microvilli not usually found on wild type and heterozygous cells. This profound change in cell shape is not associated with gross alterations in the expression and distribution of cytoskeletal components. Unexpectedly, microinjection into blastocysts demonstrated full integration of homozygous and heterozygous mutants into the inner cell mass. This will allow studies of the consequences of beta 1 integrin deficiency in several in vivo situations.
116. Transmembrane collagen XVII modulates integrin dependent keratinocyte **migration** via PI3K/Rac1 signaling. The hemidesmosomal transmembrane component collagen XVII (ColXVII) plays an important role in the anchorage of the epidermis to the underlying basement membrane. However, this adhesion protein seems to be also involved in the regulation of keratinocyte **migration**, since its expression in these cells is strongly elevated during reepithelialization of acute wounds and in the invasive front of squamous cell carcinoma, while its absence in ColXVII-deficient keratinocytes leads to altered cell motility. Using a genetic model of murine Col17a1⁻/⁻ keratinocytes we elucidated ColXVII mediated signaling pathways in cell adhesion **and migra**tion. Col17a1⁻/⁻ keratinocytes exhibited increased spreading on laminin 332 and accelerated, but less directed cell motility. These effects were accompanied by increased expression of the integrin subunits β4 and β1. The migratory phenotype, as evidenced by formation of multiple unstable lamellipodia, was associated with enhanced phosphoinositide 3-kinase (PI3K) activity. Dissection of the signaling pathway uncovered enhanced phosphorylation of the β4 integrin subunit and the focal adhesion kinase (FAK) as activators of PI3K. This resulted in elevated Rac1 activity as a downstream consequence. These results provide mechanistic evidence that ColXVII coordinates keratinocyte adhesion and directed motility by interfering integrin dependent PI3K activation and by stabilizing lamellipodia at the leading edge of reepithelializing wounds and in invasive squamous cell carcinoma.
117. Role of integrins in mouse eyelid development: studies in normal embryos and embryos in which there is a failure of eyelid fusion. Eyelid fusion normally occurs between E15.5 and E16.5 of mouse embryonic development and results from the **migration** of a population of periderm-derived epithelial cells over the corneal surface. Cell **migration** is known to depend on extracellular matrix receptors of the integrin family and to be regulated by growth factors. We were therefore interested that a failure of eyelid fusion has been reported in mice that are homozygous null for the transforming growth factor alpha (TGF-alpha) gene and in mice (invalpha5beta1) in which a transgenic alpha5beta1 integrin under the control of the involucrin promoter is misexpressed in differentiating keratinocytes. We examined expression of the alpha2beta1, alpha3beta1, alpha5beta1 and alpha6beta4 integrins during eyelid fusion in wild-type embryos and found selective upregulation of the alpha5beta1 integrin and its ligand, fibronectin, in the migrating eyelid tip cells. In TGF-alpha null embryos, the failure of eyelid fusion was correlated with a failure to upregulate the alpha5beta1 integrin and fibronectin in the tip cells. Using beta-galactosidase as a reporter gene in transgenic mice, we observed specific activity of the involucrin promoter in the eyelid tip cells. In invalpha5beta1 mice the transgenic human integrin was overexpressed not only in the tip cells but throughout the eyelid epidermis. In contrast, the endogenous, murine, alpha5beta1 integrin was only weakly expressed in the tip cells. We speculate that selective and coordinated expression of the alpha5beta1 integrin and fibronectin in eyelid tip cells is required for eyelid fusion and may be under the control of growth factors that include TGF-alpha.
118. Lck tyrosine kinase mediates β1-integrin signalling to regulate Schwann cell **migratio**n and myelination. The interaction between laminin and β1-integrin on the surface of Schwann cells regulates Schwann cell proliferation, maturation and differentiation. However, the signalling mediators that fine-tune these outcomes are not fully elucidated. Here we show that lymphoid cell kinase is the crucial effector of β1-integrin signalling in Schwann cells. Lymphoid cell kinase is activated after laminin treatment of Schwann cells, while downregulation of β1-integrin with short interfering RNAs inhibits lymphoid cell kinase phosphorylation. Treatment of Schwann cells with a selective lymphoid cell kinase inhibitor reveals a pathway that involves paxillin and CrkII, which ultimately elevates Rac-GTP levels to induce radial lamellipodia formation. Inhibition of lymphoid cell kinase in Schwann cell-dorsal root ganglion cocultures and dorsal root ganglions from Lck(-/-) mice show a reduction of Schwann cell longitudi**nal migra**tion, reduced myelin formation and internode length. Finally, Lck(-/-) mice exhibit delays in myelination, thinner myelin with abnormal g-ratios and aberrant myelin outfoldings. Our data implicate lymphoid cell kinase as a major regulator of cytoskeletal dynami**cs, migra**tion and myelination in the peripheral nervous system.
119. Reelin, Disabled 1, and beta 1 integrins are required for the formation of the radial glial scaffold in the hippocampus. The extracellular matrix molecule Reelin is required for the correct positioning of neurons during the development of the forebrain. However, the mechanism of Reelin action on neuronal **migration** is poorly understood. Reelin is assumed to act on neurons directly, but it may also affect the differentiation of glial cells necessary for neuronal **migration**. Here we show that a regular glial scaffold fails to form in vivo in the dentate gyrus of mice deficient of Reelin or Disabled 1, a neuronal adaptor protein in the Reelin signaling pathway. A subset of these defects is observed in mice that lack beta(1)-class integrins, known to bind Reelin. Moreover, recombinant Reelin induced branching of glial processes in vitro. Our data suggest that Reelin affects glial differentiation via Disabled 1 and beta(1)-class integrin-dependent signaling pathways.
120. Targeted deletion of beta 1 integrins in F9 embryonal carcinoma cells affects morphological differentiation but not tissue-specific gene expression. The integrin superfamily of heterodimeric transmembrane adhesion receptors mediates many cell-cell and cell-matrix interactions whose functions are believed to be critical for normal morphogenesis and differentiation. By eliminating the beta 1 integrin gene through homologous recombination, we have assessed the role of the beta 1 integrin family in the F9 embryonal carcinoma model for endodermal differentiation. F9 cells were unexpectedly found to maintain three copies of the beta 1 gene and complete elimination required three sequential rounds of targeting to generate triple knockout lines (beta 1 TKO). Elimination of the beta 1 integrin family of adhesion receptors from F9 cells resulted in reduced adhesion to fibronectin, laminin and collagen, but strongly enhanced adhesion to vitronectin. The absence of beta 1 integrins did not promote significant compensatory upregulation of either beta 3 or beta 5 subunits, both of which are known to act as vitronectin receptors when associated with alpha v. The loss of beta 1 integrins severely affected morphological differentiation when the beta 1-deficient cells were induced to differentiate to either parietal or visceral endoderm. Parietal endoderm derived from beta 1-deficient cells retained a rounded morphology and migrated poorly on both fibronectin and vitronectin. Visceral endoderm derived from beta 1-deficient cells were also unable to form a normal, confluent epithelial monolayer; instead, a non-contiguous layer containing clumps of disorganized cells was observed. However, loss of beta 1 integrins did not interfere with induction by differentiating agents of tissue-specific gene products for either visceral or parietal endoderm. These results suggest that beta 1 integrins mediate morphological differentiation (**migration** and epithelial formation) but not tissue-specific gene expression in induced F9 cells, and that these two processes are not necessarily linked in this system.
121. beta1 integrin maintains integrity of the embryonic neocortical stem cell niche. During embryogenesis, the neural stem cells (NSC) of the developing cerebral cortex are located in the ventricular zone (VZ) lining the cerebral ventricles. They exhibit apical and basal processes that contact the ventricular surface and the pial basement membrane, respectively. This unique architecture is important for VZ physical integrity and fate determination of NSC daughter cells. In addition, the shorter apical process is critical for interkinetic nuclear **migration** (INM), which enables VZ cell mitoses at the ventricular surface. Despite their importance, the mechanisms required for NSC adhesion to the ventricle are poorly understood. We have shown previously that one class of candidate adhesion molecules, laminins, are present in the ventricular region and that their integrin receptors are expressed by NSC. However, prior studies only demonstrate a role for their interaction in the attachment of the basal process to the overlying pial basement membrane. Here we use antibody-blocking and genetic experiments to reveal an additional and novel requirement for laminin/integrin interactions in apical process adhesion and NSC regulation. Transient abrogation of integrin binding and signalling using blocking antibodies to specifically target the ventricular region in utero results in abnormal INM and alterations in the orientation of NSC divisions. We found that these defects were also observed in laminin alpha2 deficient mice. More detailed analyses using a multidisciplinary approach to analyse stem cell behaviour by expression of fluorescent transgenes and multiphoton time-lapse imaging revealed that the transient embryonic disruption of laminin/integrin signalling at the VZ surface resulted in apical process detachment from the ventricular surface, dystrophic radial glia fibers, and substantial layering defects in the postnatal neocortex. Collectively, these data reveal novel roles for the laminin/integrin interaction in anchoring embryonic NSCs to the ventricular surface and maintaining the physical integrity of the neocortical niche, with even transient perturbations resulting in long-lasting cortical defects.
122. The perlecan fragment LG3 regulates homing of mesenchymal stem cells and neointima formation during vascular rejection. Transplant vasculopathy is associated with neointimal accumulation of recipient-derived mesenchymal stem cells. Increased circulating levels of LG3, a C-terminal fragment of perlecan, were found in renal transplant patients with vascular rejection. Here, we evaluated whether LG3 regulates the **migration** and homing of mesenchymal stem cells and the accumulation of recipient-derived neointimal cells. Mice were transplanted with a fully-MHC mismatched aortic graft followed by intravenous injection of recombinant LG3. LG3 injections increased neointimal accumulation of α-smooth muscle actin positive cells. When green fluorescent protein (GFP)-transgenic mice were used as recipients, LG3 injection favored accumulation of GFP+ cells to sites of neointima formation. LG3 increased horizontal **migratio**n and tran**smigratio**n of mouse and human MSC in vitro and led to increased ERK1/2 phosphorylation. Neutralizing β1 integrin antibodies or use of mesenchymal stem cells from α2 integrin-/- mice decreas**ed migrat**ion in response to recombinant LG3. Reduced intima-media ratios and decreased numbers of neointimal cells showing ERK1/2 phosphorylation were found in α2-/- recipients injected with recombinant LG3. Collectively, our results suggest that LG3, through interactions with α2β1 integrins on recipient-derived cells leading to activation of ERK1/2 and incr**eased mig**ration, favors myointimal thickening.
123. The LIM-only proteins FHL2 and FHL3 interact with alpha- and beta-subunits of the muscle alpha7beta1 integrin receptor. FHL1, FHL2, and FHL3 are members of the four and one-half LIM domain protein subclass that are expressed in striated muscles. Here we show that FHL2 and FHL3 are novel alpha(7)beta(1) integrin-interacting proteins. They bind both the alpha- and the beta-subunit as well as different splice isoforms. The minimal binding sites for FHL2 and FHL3 on beta(1A)-chain overlap, whereas on alpha(7A) and alpha(7B) subunits they are situated adjacent. Determining the binding sites for integrins on FHL2 or FHL3 revealed that the suprastructure of the whole molecule is important for these associations, rather than any single LIM domain. Immunofluorescence studies with cells expressing full-length FHL proteins or their deletion mutants showed that FHL2 and FHL3 but not FHL1 colocalize with integrins at cell adhesion sites. Further, their recruitment to the membrane results from binding to either the alpha- or the beta-chain of the integrin receptor. The association of FHL2 or FHL3 with integrin receptors neither influences attachment of cells to different substrates nor changes their **migration** capacity. However, in cardiac and skeletal muscles, FHL2 and FHL3, respectively, are colocalized with alpha(7)beta(1) integrin receptor at the periphery of Z-discs, suggesting a role in mechanical stabilization of muscle cells.
124. Phagocytic cells of the thymic reticulum interact with thymocytes via extracellular matrix ligands and receptors. We previously showed that, in the context of thymic epithelial cells, thymocyte **migration** is partially controlled by extracellular matrix (ECM)-mediated interactions. Herein we evaluated whether these interactions could be involved in cell **migration** related events in the context of non-epithelial cells of the thymic microenvironment, the phagocytic cells of the thymic reticulum (PTR). We first showed, by immunocytochemistry, cytofluorometry, and RT-PCR, that PTR produce ECM components, including fibronectin and laminin, and express the corresponding integrin-type receptors, VLA-4, VLA-5, and VLA-6. Thymocytes adhere onto PTR monolayers, with immature CD4(+)CD8(+) cells being predominant. Importantly, such an adhesion is partially mediated by ECM ligands and receptors, since it was impaired by anti-ECM or anti-ECM receptor antibodies. Conjointly, our data reveal that the ECM-dependence for thymocyte adhesion onto the thymic microenvironment is not restricted to the epithelial cells, being also seen when they encounter non-epithelial phagocytic cells.
125. Endothelial beta1 integrins regulate sprouting and network formation during vascular development. beta1 integrins are important regulators of vascular differentiation and development, as their endothelial-specific deletion results in embryonic lethality. In the present study, we investigated the molecular mechanisms underlying the prominent vascular abnormalities that occur in the absence of beta1 integrins. Because of the early embryonic lethality of knockout mice, we studied endothelial cell and vessel development in beta1-integrin-deficient murine embryonic stem cells to gain novel insights into the role of beta1 integrins in vasculo-angiogenesis. We found that vessel development was strongly defective in the mutant embryoid bodies (EBs), as only primitive and short sprouts developed from clusters of vascular precursors in beta1 integrin(-/-) EBs, whereas complex network formation of endothelial tubes was observed in wild-type EBs. The vascular defect was due to deficient beta1 integrin expression in endothelial cells, as its endothelial-specific re-expression rescued the phenotype entirely. The mechanism responsible for defective vessel formation was found to be reduced endothelial cell maturation, **migration** and elongation. Moreover, the lower number of endothelial cells in beta1 integrin(-/-) EBs was due to an increased apoptosis versus proliferation rate. The enhanced apoptosis and proliferation of beta1 integrin(-/-) endothelial cells was related to the elevation of peNOS and pAKT signaling molecules, respectively. Our data demonstrate that endothelial beta1 integrins are determinants of vessel formation and that this effect is mediated via different signaling pathways.
126. Laminin α1 is essential for mouse cerebellar development. Laminin α1 (Lama1), which is a subunit of laminin-1 (laminin-111), a heterotrimeric ECM protein, is essential for embryonic development and promotes neurite outgrowth in culture. Because the deletion of Lama1 causes lethality at early embryonic stages in mice, the in vivo role of Lama1 in neural development and functions has not yet been possible to determine. In this study, we generated conditional Lama1 knockout (Lama1(CKO)) mice in the epiblast lineage using Sox2-Cre mice. These Lama1(CKO) mice survived, but displayed behavioral disorders and impaired formation of the cerebellum. Deficiency of Lama1 in the pial basement membrane of the meninges resulted in defects in the conformation of the meninges. During cerebellar development, Lama1 deficiency also caused a decrease in the proliferation an**d migrati**on of granule cell precursors, disorganization of Bergmann glial fibers and endfeet, and a transient reduction in the activity of Akt. A marked reduction in numbers of dendritic processes in Purkinje cells was observed in Lama1(CKO) mice. Together, these results indicate that Lama1 is required for cerebellar development and functions.
127. Beta1 integrins in radial glia but not in migrating neurons are essential for the formation of cell layers in the cerebral cortex. Radial glial cells in the cerebral cortex serve as progenitors for neurons and glia and guide the **migration** of cortical neurons. The integrin alpha3beta1 is thought to mediate interactions of migrating neurons with radial glial cells and to function as a receptor for the reelin signaling molecule. Here, we challenge this view and demonstrate that beta1 integrins in migrating neurons are not essential for the formation of cell layers in the cerebral cortex. Cortical cell layers also form normally in mice deficient in the integrin alpha3beta1. However, we provide evidence that beta1 integrins in radial glia control the morphological differentiation of both glia and neurons. We conclude that beta1 integrins in radial glia are required for the proper development of the cerebral cortex, whereas beta1 integrins in migrating neurons are not essential for glial-guided **migration** and reelin signaling.
128. Conditional beta1-integrin gene deletion in neural crest cells causes severe developmental alterations of the peripheral nervous system. Integrins are transmembrane receptors that are known to interact with the extracellular matrix and to be required for **migration**, proliferation, differentiation and apoptosis. We have generated mice with a neural crest cell-specific deletion of the beta1-integrin gene to analyse the role of beta1-integrins in neural crest cell **migration** and differentiation. This targeted mutation caused death within a month of birth. The loss of beta1-integrins from the embryo delayed the **migration** of Schwann cells along axons and induced multiple defects in spinal nerve arborisation and morphology. There was an almost complete absence of Schwann cells and sensory axon segregation and defective maturation in neuromuscular synaptogenesis. Thus, beta1-integrins are important for the control of embryonic and postnatal peripheral nervous system development.
129. Mena binds α5 integrin directly and modulates α5β1 function. Mena is an Ena/VASP family actin regulator with roles in ce**ll migrat**ion, chemotaxis, cell-cell adhesion, tumor cell invasion, and metastasis. Although enriched in focal adhesions, Mena has no established function within these structures. We find that Mena forms an adhesion-regulated complex with α5β1 integrin, a fibronectin receptor involved in cell adhesion, motility, fibronectin fibrillogenesis, signaling, and growth factor receptor trafficking. Mena bound directly to the carboxy-terminal portion of the α5 cytoplasmic tail via a 91-residue region containing 13 five-residue "LERER" repeats. In fibroblasts, the Mena-α5 complex was required for "outside-in" α5β1 functions, including normal phosphorylation of FAK and paxillin and formation of fibrillar adhesions. It also supported fibrillogenesis and cell spreading and control**led cell** migration speed. Thus, fibroblasts require Mena for multiple α5β1-dependent processes involving bidirectional interactions between the extracellular matrix and cytoplasmic focal adhesion proteins.
130. The integrins Mac-1 and alpha4beta1 perform crucial roles in neutrophil and T cell recruitment to lungs during Streptococcus pneumoniae infection. Neutrophils and T cells play an important role in host protection against pulmonary infection caused by Streptococcus pneumoniae. However, the role of the integrins in recruitment of these cells to infected lungs is not well understood. In this study we used the twin approaches of mAb blockade and gene-deficient mice to investigate the relative impact of specific integrins on cellular recruitment and bacterial loads following pneumococcal infection. We find that both Mac-1 (CD11b/CD18) and α(4)β(1) (CD49d/CD29) integrins, but surprisingly not LFA-1 (CD11a/CD18), contribute to two aspects of the response. In terms of recruitment from the circulation into lungs, neutrophils depend on Mac-1 and α(4)β(1), whereas the T cells are entirely dependent on α(4)β(1). Second, immunohistochemistry results indicate that adhesion also plays a role within infected lung tissue itself. There is widespread expression of ICAM-1 within lung tissue. Use of ICAM-1(-/-) mice revealed that neutrophils make use of this Mac-1 ligand, not for lung entry o**r for mig**ration within lung tissue, but for combating the pneumococcal infection. In contrast to ICAM-1, there is restricted and constitutive expression of the α(4)β(1) ligand, VCAM-1, on the bronchioles, allowing direct access of the leukocytes to the airways via this integrin at an early stage of pneumococcal infection. Therefore, integrins Mac-1 and α(4)β(1) have a pivotal role in prevention of pneumococcal outgrowth during disease both in regulating neutrophil and T cell recruitment into infected lungs and by influencing their behavior within the lung tissue itself.
131. Lack of beta1 integrins in enteric neural crest cells leads to a Hirschsprung-like phenotype. The enteric nervous system arises mainly from vagal and sacral neural crest cells that colonise the gut between 9.5 and 14 days of development in mice. Using the Cre-LoxP system, we removed beta1 integrins in the neural crest cells when they emerge from the neural tube. beta1-null enteric neural crest cells fail to colonise the gut completely, leading to an aganglionosis of the descending colon, which resembles the human Hirschsprung's disease. Moreover, beta1-null enteric neural crest cells form abnormal aggregates in the gut wall, leading to a severe alteration of the ganglia network organisation. Organotypic cultures of gut explants reveal that beta1-null enteric neural crest cells show impaired adhesion on extracellular matrix and enhanced intercellular adhesion properties. They display **migration** defects in collagen gels and gut tissue environments. We also provide evidence that beta1 integrins are required for the villi innervation in the small intestine. Our findings highlight the crucial roles played by beta1 integrins at various steps of enteric nervous system development.
132. Cas adaptor proteins organize the retinal ganglion cell layer downstream of integrin signaling. Stratification of retinal neuronal cell bodies and lamination of their processes provide a scaffold upon which neural circuits can be built. However, the molecular mechanisms that direct retinal ganglion cells (RGCs) to resolve into a single-cell retinal ganglion cell layer (GCL) are not well understood. The extracellular matrix protein laminin conveys spatial information that instructs the **migration**, process outgrowth, and reorganization of GCL cells. Here, we show that the β1-Integrin laminin receptor is required for RGC positioning and reorganization into a single-cell GCL layer. β1-Integrin signaling within migrating GCL cells requires Cas signaling-adaptor proteins, and in the absence of β1-Integrin or Cas function retinal neurons form ectopic cell clusters beyond the inner-limiting membrane (ILM), phenocopying laminin mutants. These data reveal an essential role for Cas adaptor proteins in β1-Integrin-mediated signaling events critical for the formation of the single-cell GCL in the mammalian retina.
133. VEGF-induced endothelial cell **migration** requires urokinase receptor (uPAR)-dependent integrin redistribution. Vascular endothelial growth factor (VEGF)-initiated angiogenesis requires coordinated proteolytic degradation of extracellular matrix provided by the urokinase plasminogen activator/urokinase receptor (uPA/uPAR) system and regulation of cell **migration** provided by integrin-matrix interaction. In this study, we investigated the mechanisms underlying the uPAR-dependent modulation of VEGF-induced endothelial **migration**.We used flow cytometry to quantify integrins at the cell surface. Stimulation of human and murine endothelial cells with VEGF resulted in internalization of α5β1-integrins. Micropatterning and immunocytochemistry revealed co-clustering of uPAR and α5β1-integrins and retrieval via clathrin-coated vesicles. It was also contingent on receptors of the low-density lipoprotein receptor (LDL-R) family. VEGF-induced integrin redistribution was inhibited by elimination of uPAR from the endothelial cell surface or by inhibitory peptides that block the uPAR-integrin interaction. Under these conditions, the migratory response of endothelial cells upon VEGF stimulation was impaired both in vitro and in vivo.The observations indicate that uPAR is an essential component of the network through which VEGF controls endothelial c**ell migra**tion. uPAR is a bottleneck through which the VEGF-induced signal must be funnelled for both focused proteolytic activity at the leading edge and for redistribution of integrins.
134. Deletion of mouse embryo fibroblast N-acetylglucosaminyltransferase V stimulates alpha5beta1 integrin expression mediated by the protein kinase C signaling pathway. An N-linked glycan often increased during oncogenic transformation contains beta(1,6)-linked GlcNAc, synthesized by the N-acetylglucosaminyltransferase V (GnT-V). The progression of polyoma middle T-antigen oncoprotein-induced mammary carcinomas in GnT-V null mice was significantly retarded compared with that observed in wild-type mice. The matrix adhesion of mouse embryonic fibroblasts (MEF) from GnT-V null and wild-type mice was investigated to understand the mechanism by which deletion of GnT-V could retard tumor progression. GnT-V null MEF displayed enhanced adhesion to and spreading on fibronectin-coated plates with concomitant inhibition of cell **migration**. GnT-V null MEF also showed increased focal adhesion kinase tyrosine phosphorylation, consistent with decreased cell motility on fibronectin-coated plates. Expression of GnT-V cDNA in the null MEF reversed these abnormal characteristics, indicating the direct involvement of N-glycosylation events in these phenotypic changes. The alpha5beta1 fibronectin receptors exhibited increased clustering on the null MEF cell surfaces, consistent with previous studies that observed less integrin clustering in cells overexpressing GnT-V. Most surprisingly, GnT-V null MEF displayed increased expression levels of both alpha5 and beta1 subunits in lysates and on the cell surface. Increased alpha5beta1 expression in the null MEF was because of increased alpha5beta1 transcript levels that declined after re-expression of GnT-V cDNA, confirming that increased alpha5beta1 expression in null MEF was because of changes in GnT-V expression. The increased null MEF transcripts were shown to be caused at least in part by increased integrin promoter activity. Moreover, increased alpha5beta1 integrin transcripts in GnT-V null MEF were not due to a differential response to fibronectin; rather, they appeared to be mediated by activation of a protein kinase C signaling pathway. These results demonstrate that deletion of MEF GnT-V resulted in enhanced integrin clustering and activation of alpha5beta1 transcription by protein kinase C signaling, which in turn up-regulated levels of cell surface alpha5beta1 fibronectin receptors that resulted in increased matrix adhesion and inhibition of **migration**.
135. Interactions of primary fibroblasts and keratinocytes with extracellular matrix proteins: contribution of alpha2beta1 integrin. The alpha2beta1 integrin is a collagen-binding protein with very high affinity for collagen I. It also binds several other collagens and laminins and it is expressed by many cells, including keratinocytes and fibroblasts in the skin. In the past, alpha2beta1 integrin was suggested to be responsible for cell attachment, spreading and **migration** on monomeric collagen I and contraction of three-dimensional collagen lattices. In view of these functions, normal development and fertility in integrin alpha2-deficient mice, which we generated by targeting the integrin alpha2 gene, came as a surprise. This suggested the existence of compensatory mechanisms that we investigate here using primary fibroblasts and keratinocytes isolated from wild-type and alpha2-deficient mice, antibodies blocking integrin function and downregulation of integrin alpha2 expression. The results show that the alpha2beta1 integrin is absolutely required for keratinocyte adhesion to collagens whereas for fibroblasts other collagen-binding integrins partially back-up the lack of alpha2beta1 in simple adhesion to collagen monomers. A prominent requirement for alpha2beta1 integrins became apparent when fibroblasts executed mechanical tasks of high complexity in three-dimensional surroundings, such as contracting free-floating collagen gels and developing isometric forces in tethered lattices. The deficits observed for alpha2-deficient fibroblasts appeared to be linked to alterations in the distribution of force-bearing focal adhesions and deregulation of Rho-GTPase activation.
136. Calcium and calmodulin-dependent serine/threonine protein kinase type II (CaMKII)-mediated intramolecular opening of integrin cytoplasmic domain-associated protein-1 (ICAP-1α) negatively regulates β1 integrins. Focal adhesion turnover during cel**l migrati**on is an integrated cyclic process requiring tight regulation of integrin function. Interaction of integrin with its ligand depends on its activation state, which is regulated by the direct recruitment of proteins onto the β integrin chain cytoplasmic domain. We previously reported that ICAP-1α, a specific cytoplasmic partner of β1A integrins, limits both talin and kindlin interaction with β1 integrin, thereby restraining focal adhesion assembly. Here we provide evidence that the calcium and calmodulin-dependent serine/threonine protein kinase type II (CaMKII) is an important regulator of ICAP-1α for controlling focal adhesion dynamics. CaMKII directly phosphorylates ICAP-1α and disrupts an intramolecular interaction between the N- and the C-terminal domains of ICAP-1α, unmasking the PTB domain, thereby permitting ICAP-1α binding onto the β1 integrin tail. ICAP-1α direct interaction with the β1 integrin tail and the modulation of β1 integrin affinity state are required for down-regulating focal adhesion assembly. Our results point to a molecular mechanism for the phosphorylation-dependent control of ICAP-1α function by CaMKII, allowing the dynamic control of β1 integrin activation and cell adhesion.
137. Integrin alpha3beta1 potentiates TGFbeta-mediated induction of MMP-9 in immortalized keratinocytes. Transforming growth factor-beta (TGFbeta) signaling pathways regulate a number of keratinocyte functions during epidermal carcinogenesis and wound healing, including proliferation, survival, and **migration**. TGFbeta can induce expression of the matrix metalloproteinase-9 (MMP-9), which has critical roles in promoting extracellular matrix remodeling and angiogenesis during tumorigenesis and tissue repair. Integrin alpha3beta1 is a cell adhesion receptor for laminin-332/laminin-5 with important roles in the survival and motility of epidermal keratinocytes. We previously reported that alpha3beta1 induces the expression of MMP-9 in immortalized keratinocytes. In this study, we show that endogenous TGFbeta is required for maximal MMP-9 expression, and that alpha3beta1 is required for full induction of MMP-9 protein and mRNA in response to TGFbeta. This regulation was not observed in non-immortalized, primary keratinocytes, indicating that coordinate regulation of MMP-9 by alpha3beta1 and TGFbeta is a property of immortalized cells. Alpha3beta1 did not regulate endogenous TGFbeta gene expression, TGFbeta bioavailability, or TGFbeta-Smad signaling. However, the combined inductive effects of TGFbeta and alpha3beta1 on MMP-9 were suppressed by a Src family kinase (SFK) inhibitor, indicating involvement of a SFK pathway. These findings provide early evidence of a role for alpha3beta1 in augmenting TGFbeta-mediated induction of MMP-9 in immortalized or transformed keratinocytes during skin carcinogenesis.
138. Abnormal Wnt and PI3Kinase signaling in the malformed intestine of lama5 deficient mice. Laminins are major constituents of basement membranes and are essential for tissue homeostasis. Laminin-511 is highly expressed in the intestine and its absence causes severe malformation of the intestine and embryonic lethality. To understand the mechanistic role of laminin-511 in tissue homeostasis, we used RNA profiling of embryonic intestinal tissue of lama5 knockout mice and identified a lama5 specific gene expression signature. By combining cell culture experiments with mediated knockdown approaches, we provide a mechanistic link between laminin α5 gene deficiency and the physiological phenotype. We show that laminin α5 plays a crucial role in both epithelial and mesenchymal cell behavior by inhibiting Wnt and activating PI3K signaling. We conclude that conflicting signals are elicited in the absence of lama5, which alter cell adhesion**, migrati**on as well as epithelial and muscle differentiation. Conversely, adhesion to laminin-511 may serve as a potent regulator of known interconnected PI3K/Akt and Wnt signaling pathways. Thus deregulated adhesion to laminin-511 may be instrumental in diseases such as human pathologies of the gut where laminin-511 is abnormally expressed as it is shown here.
139. Neuregulin-1/glial growth factor stimulates Schwann cell **migration** by inducing α5 β1 integrin-ErbB2-focal adhesion kinase complex formation. After peripheral nerve injury, Schwann cells gain a migratory phenotype and remodel their extracellular matrix to provide a supportive environment for axonal regeneration. The soluble neuregulin-1 isoform, that is, glial growth factor (GGF), is expressed in regenerating axons of injured peripheral nerves and regulates Schwann cell motility by activating the ErbB family of tyrosine kinase receptors, but how GGF/ErbB signaling contributes to Schwann cell motility remains unclear. Here, we show that GGF stimulates Schwann cel**l migrati**on by inducing the formation of a protein complex containing the fibronectin receptor α5β1 integrin, ErbB2, and focal adhesion kinase (FAK). ErbB2 co-localizes and co-immunoprecipitates with the focal complex members including α5β1 integrin and FAK after GGF treatment. These effects of GGF appear to involve FAK activation, which occurs downstream of ErbB2 stimulation. RNAi-mediated down-regulation of α5 integrin expression in primary cultured Schwann cells resulted in significantly decreased interaction between FAK and ErbB2, as well as decreased GGF-i**nduced mi**gration. An increase in the α5β1 integrin-ErbB2-FAK complex formation was observed in injured nerve Schwann cells, but not uninjured control. Taken together, these data suggest that GGF plays an important modulatory role in Schw**ann cell** migration after nerve crush by inducing α5β1 integrin-ErbB2-FAK complex formation.
140. alpha2beta1 integrin controls association of Rac with the membrane and triggers quiescence of endothelial cells. Integrin receptors and their extracellular matrix ligands provide cues to cell proliferation, survival, differentiation and **migration**. Here, we show that alpha2beta1 integrin, when ligated to the basement membrane component laminin-1, triggers a proliferation arrest in primary endothelial cells. Indeed, in the presence of strong growth signals supplied by growth factors and fibronectin, alpha2beta1 engagement alters assembly of mature focal adhesions by alpha5beta1 and leads to impairment of downstream signaling and cell-cycle arrest in the G1 phase. Although the capacity of alpha5beta1 to signal for GTP loading of Rac is preserved, the joint engagement of alpha2beta1 interferes with membrane anchorage of Rac. Adapting the 'split-ubiquitin' sensor to screen for membrane-proximal alpha2 integrin partners, we identified the CD9 tetraspanin and further establish its requirement for destabilization of focal adhesions, control of Rac subcellular localization and growth arrest induced by alpha2beta1 integrin. Altogether, our data establish that alpha2beta1 integrin controls endothelial cell commitment towards quiescence by triggering a CD9-dependent dominant signaling.
141. Close homolog of L1 is an enhancer of integrin-mediated cell **migration**. Close homolog of L1 (CHL1) is a member of the L1 family of cell adhesion molecules expressed by subpopulations of neurons and glia in the central and peripheral nervous system. It promotes neurite outgrowth and neuronal survival in vitro. This study describes a novel function for CHL1 in potentiating integrin-dependent cell **migration** toward extracellular matrix proteins. Expression of CHL1 in HEK293 cells stimulated their haptotactic **migration** toward collagen I, fibronectin, laminin, and vitronectin substrates in Transwell assays. CHL1-potentiated cell **migration** to collagen I was dependent on alpha1beta1 and alpha2beta1 integrins, as shown with function blocking antibodies. Potentiated **migration** relied on the early integrin signaling intermediates c-Src, phosphatidylinositol 3-kinase, and mitogen-activated protein kinase. Enhancement of **migration** was disrupted by mutation of a potential integrin interaction motif Asp-Gly-Glu-Ala (DGEA) in the sixth immunoglobulin domain of CHL1, suggesting that CHL1 functionally interacts with beta1 integrins through this domain. CHL1 was shown to associate with beta1 integrins on the cell surface by antibody-induced co-capping. Through a cytoplasmic domain sequence containing a conserved tyrosine residue (Phe-Ile-Gly-Ala-Tyr), CHL1 recruited the actin cytoskeletal adapter protein ankyrin to the plasma membrane, and this sequence was necessary for promoting integrin-dependent **migration** to extracellular matrix proteins. These results support a role for CHL1 in integrin-dependent cell **migration** that may be physiologically important in regulating cell **migration** in nerve regeneration and cortical development.
142. Sox10 and Itgb1 interaction in enteric neural crest cell **migration**. SOX10 involvement in syndromic form of Hirschsprung disease (intestinal aganglionosis, HSCR) in humans as well as developmental defects in animal models highlight the importance of this transcription factor in control of the pool of enteric progenitors and their differentiation. Here, we characterized the role of SOX10 in cell **migration** and its interactions with β1-integrins. To this end, we crossed the Sox10(lacZ/+) mice with the conditional Ht-PA::Cre; beta1(neo/+) and beta1(fl/fl) mice and compared the phenotype of embryos of different genotypes during enteric nervous system (ENS) development. The Sox10(lacZ/+); Ht-PA::Cre; beta1(neo/fl) double mutant embryos presented with increased intestinal aganglionosis length and more severe neuronal network disorganization compared to single mutants. These defects, detected by E11.5, are not compensated after birth, showing that a coordinated and balanced interaction between these two genes is required for normal ENS development. Use of video-microscopy revealed that defects observed result from reduced **migratio**n speed and altered directionality of enteric neural crest cells. Expression of β1-integrins upon SOX10 overexpression or in Sox10(lacZ/+) mice was also analyzed. The modulation of SOX10 expression altered β1-integrins, suggesting that SOX10 levels are critical for proper expression and function of this adhesion molecule. Together with previous studies, our results strongly indicate that SOX10 mediates ENCC adhesion a**nd migrat**ion, and contribute to the understanding of the molecular and cellular basis of ENS defects observed both in mutant mouse models and in patients carrying SOX10 mutations.
143. Specific β-containing integrins exert differential control on proliferation and two-dimensional collective cell **migratio**n in mammary epithelial cells. Understanding how cell cycle is regulated in normal mammary epithelia is essential for deciphering defects of breast cancer and therefore for developing new therapies. Signals provided by both the extracellular matrix and growth factors are essential for epithelial cell proliferation. However, the mechanisms by which adhesion controls cell cycle in normal epithelia are poorly established. In this study, we describe the consequences of removing the β1-integrin gene from primary cultures of mammary epithelial cells in situ, using CreER. Upon β1-integrin gene deletion, the cells were unable to progress efficiently through S-phase, but were still able to undergo collective two-dimension**al migrat**ion. These responses are explained by the presence of β3-integrin in β1-integrin-null cells, indicating that integrins containing different β-subunits exert differential control on mammary epithelial proliferatio**n and mig**ration. β1-Integrin deletion did not inhibit growth factor signaling to Erk or prevent the recruitment of core adhesome components to focal adhesions. Instead the S-phase arrest resulted from defective Rac activation and Erk translocation to the nucleus. Rac inhibition prevented Erk translocation and blocked proliferation. Activated Rac1 rescued the proliferation defect in β1-integrin-depleted cells, indicating that this GTPase is essential in propagating proliferative β1-integrin signals. These results show that β1-integrins promote cell cycle in mammary epithelial cells, whereas β3-integrins are i**nvolved i**n migration.
144. MMP7 shedding of syndecan-1 facilitates re-epithelialization by affecting alpha(2)beta(1) integrin activation. Lung injury promotes the expression of matrix metalloproteinase-7 (MMP7, matrilysin), which is required for neutrophil recruitment and re-epithelialization. MMP7 governs the lung inflammatory response through the shedding of syndecan-1. Because inflammation and repair are related events, we evaluated the role of syndecan-1 shedding in lung re-epithelialization.Epithelial injury induced syndecan-1 shedding from wild-type epithelium but not from Mmp7(-/-) mice in vitro and in vivo. Moreover, cell **migration** and wound closure was enhanced by MMP7 shedding of syndecan-1. Additionally, we found that syndecan-1 augmented cell adhesion to collagen by controlling the affinity state of the alpha(2)beta(1) integrin.MMP7 shedding of syndecan-1 facilitates wound closure by causing the alpha(2)beta(1) integrin to assume a less active conformation thereby removing restrictions to **migration**. MMP7 acts in the lungs to regulate inflammation and repair, and our data now show that both these functions are controlled through the shedding of syndecan-1.
145. Spontaneous phosphoinositide 3-kinase signaling dynamics drive spreading and random **migration** of fibroblasts. During directed cell **migration** (chemotaxis), cytoskeletal dynamics are stimulated and spatially biased by phosphoinositide 3-kinase (PI3K) and other signal transduction pathways. Live-cell imaging using total internal reflection fluorescence (TIRF) microscopy revealed that, in the absence of soluble cues, 3'-phosphoinositides are enriched in a localized and dynamic fashion during active spreading and random **migration** of mouse fibroblasts on adhesive surfaces. Surprisingly, we found that PI3K activation is uncoupled from classical integrin-mediated pathways and feedback from the actin cytoskeleton. Inhibiting PI3K significantly impairs cell motility, both in the context of normal spreading and when microtubules are dissociated, which induces a dynamic protrusion phenotype as seen by TIRF in our cells. Accordingly, during random **migration**, 3'-phosphoinositides are frequently localized to regions of membrane protrusion and correlate quantitatively with the direction and persistence of cell movement. These results underscore the importance of localized PI3K signaling not only in chemotaxis but also in basal motility/**migration** of fibroblasts.
146. β1 integrin signaling promotes neuronal **migratio**n along vascular scaffolds in the post-stroke brain. Cerebral ischemic stroke is a main cause of chronic disability. However, there is currently no effective treatment to promote recovery from stroke-induced neurological symptoms. Recent studies suggest that after stroke, immature neurons, referred to as neuroblasts, generated in a neurogenic niche, the ventricular-subventricular zone, migrate toward the injured area, where they differentiate into mature neurons. Interventions that increase the number of neuroblasts distributed at and around the lesion facilitate neuronal repair in rodent models for ischemic stroke, suggesting that promoting neuroblast **migratio**n in the post-stroke brain could improve efficient neuronal regeneration. To move toward the lesion, neuroblasts form chain-like aggregates and migrate along blood vessels, which are thought to increase their **migratio**n efficiency. However, the molecular mechanisms regulating these **migratio**n processes are largely unknown. Here we studied the role of β1-class integrins, transmembrane receptors for extracellular matrix proteins, in these migrating neuroblasts. We found that the neuroblast chain formation and blood vessel-guide**d migrati**on critically depend on β1 integrin signaling. β1 integrin facilitated the adhesion of neuroblasts to laminin and the efficient translocation of their soma dur**ing migra**tion. Moreover, artificial laminin-containing scaffolds promoted neuroblast chain formation **and migra**tion toward the injured area. These data suggest that laminin signaling via β1 integrin supports vasculature-guided neur**onal migr**ation to efficiently supply neuroblasts to injured areas. This study also highlights the importance of vascular scaffolds for **cell migr**ation in development and regeneration.
147. Tissue inhibitor of metalloproteinase-2 (TIMP-2) regulates myogenesis and beta1 integrin expression in vitro. Myogenesis in vitro involves myoblast cell cycle arrest, **migration**, and fusion to form multinucleated myotubes. Extracellular matrix (ECM) integrity during these processes is maintained by the opposing actions of matrix metalloproteinase (MMP) proteases and their inhibitors, the tissue inhibitor of metalloproteinases (TIMPs). Here, we report that TIMP-2, MMP-2, and MT1-MMP are differentially expressed during mouse myoblast differentiation in vitro. A specific role for TIMP-2 in myogenesis is demonstrated by altered TIMP-2(-/-) myotube formation. When differentiated in horse serum-containing medium, TIMP-2(-/-) myotubes are larger than wild-type myotubes. In contrast, when serum-free medium is used, TIMP-2(-/-) myotubes are smaller than wild-type myotubes. Regardless of culture condition, myotube size is directly correlated with MMP activity and inversely correlated with beta1 integrin expression. Treatment with recombinant TIMP-2 rescues reduced TIMP-2(-/-) myotube size and induces increased MMP-9 activation and decreased beta1 integrin expression. Treatment with either MMP-2 or MMP-9 similarly rescues reduced myotube size, but has no effect on beta1 integrin expression. These data suggest a specific regulatory relationship between TIMP-2 and beta1 integrin during myogenesis. Elucidating the role of TIMP-2 in myogenesis in vitro may lead to new therapeutic options for the use of TIMP-2 in myopathies and muscular dystrophies in vivo.
148. Protogenin prevents premature apoptosis of rostral cephalic neural crest cells by activating the α5β1-integrin. The bones and connective tissues of the murine jaws and skull are partly derived from cephalic neural crest cells (CNCCs). Here, we report that mice deficient of protogenin (Prtg) protein, an immunoglobulin domain-containing receptor expressed in the developing nervous system, have impairments of the palatine and skull. Data from lineage tracing experiments, expression patterns of neural crest cell (NCC) marker genes and detection of apoptotic cells indicate that the malformation of bones in Prtg-deficient mice is due to increased apoptosis of rostral CNCCs (R-CNCCs). Using a yeast two-hybrid screening, we found that Prtg interacts with Radil, a protein previously shown to affect th**e migrati**on and survival of NCCs in zebrafish with unknown mechanism. Overexpression of Prtg induces translocation of Radil from cytoplasm to cell membrane in cultured AD293 cells. In addition, overexpression of Prtg and Radil activates α5β1-integrins to high-affinity conformational forms, which is further enhanced by the addition of Prtg ligand ERdj3 into cultured cells. Blockage of Radil by RNA interference abolishes the effect of ERdj3 and Prtg on the α5β1-integrin, suggesting that Radil acts downstream of Prtg. Prtg-deficient R-CNCCs display fewer activated α5β1-integrins in embryos, and these cells show reduced migratory ability in in vitro transwell assay. These results suggest that the inside-out activation of the α5β1-integrin mediated by ERdj3/Prtg/Radil signaling is crucial for proper functions of R-CNCCs, and the deficiency of this pathway causes premature apoptosis of a subset of R-CNCCs and malformation of craniofacial structures.
149. Suppression of β1-integrin in gonadotropin-releasing hormone cells disrupts **migratio**n and axonal extension resulting in severe reproductive alterations. Reproduction in mammals is dependent on the function of hypothalamic neurons whose axons project to the hypothalamic median eminence (ME) where they release gonadotropin-releasing hormone (GnRH) into a specialized capillary network for delivery to the anterior pituitary. These neurons originate prenatally in the nasal placode and migrate into the forebrain along the olfactory-vomeronasal nerves. The complex developmental events leading to the correct establishment of the GnRH system are tightly regulated by the specific spatiotemporal expression patterns of guidance cues and extracellular matrix molecules, the functions of which, in part, are mediated by their binding to β1-subunit-containing integrins. To determine the biological role of these cell-surface proteins in reproduction, Cre/LoxP technology was used to generate GnRH neuron-specific β1-integrin conditional KO (GnRH-Itgb1(-/-)) mice. Loss of β1-integrin signaling impai**red migra**tion of GnRH neurons, their axonal extension to the ME, timing of pubertal onset, and fertility in these mice. These results identify β1-integrin as a gene involved in normal development of the GnRH system and demonstrate a fundamental role for this protein in acquisition of normal reproductive competence in female mice.
150. NG2 proteoglycan promotes tumor vascularization via integrin-dependent effects on pericyte function. The NG2 proteoglycan stimulates the proliferation and **migration** of various immature cell types, including pericytes. However, the role of NG2 in mediating pericyte/endothelial cell interaction has been less clear. In this study, we show that pericyte-specific NG2 ablation causes several structural deficits in blood vessels in intracranial B16F10 melanomas, including decreased pericyte ensheathment of endothelial cells, diminished formation of endothelial junctions, and reduced assembly of the vascular basal lamina. These deficits result in decreased tumor vessel patency, increased vessel leakiness, and increased intratumoral hypoxia. NG2-dependent mechanisms of pericyte interaction with endothelial cells are further explored in pericyte/endothelial cell co-cultures. siRNA-mediated NG2 knockdown in pericytes leads to reduced formation of pericyte/endothelial networks, reduced formation of ZO-1 positive endothelial cell junctions, and increased permeability of endothelial cell monolayers. We also show that NG2 knockdown results in loss of β1 integrin activation in endothelial cells, revealing a mechanism for NG2-dependent cross talk between pericytes and endothelial cells.
151. N-cadherin and β1-integrins cooperate during the development of the enteric nervous system. Cell adhesion controls various embryonic morphogenetic processes, including the development of the enteric nervous system (ENS). Ablation of β1-integrin (β1-/-) expression in enteric neural crest cells (ENCC) in mice leads to major alterations in the ENS structure caused by reduc**ed migrat**ion and increased aggregation properties of ENCC during gut colonization, which gives rise to a Hirschsprung's disease-like phenotype. In the present study, we examined the role of N-cadherin in ENS development and the interplay with β1 integrins during this process. The Ht-PA-Cre mouse model was used to target gene disruption of N-cadherin and β1 integrin in migratory NCC and to produce single- and double-conditional mutants for these two types of adhesion receptors. Double mutation of N-cadherin and β1 integrin led to embryonic lethality with severe defects in ENS development. N-cadherin-null (Ncad-/-) ENCC exhibited a delayed colonization in the developing gut at E12.5, although this was to a lesser extent than in β1-/- mutants. This delay of Ncad-/**- ENCC mi**gration was recovered at later stages of development. The double Ncad-/-; β1-/- mutant ENCC failed to colonize the distal part of the gut and there was more severe aganglionosis in the proximal hindgut than in the single mutants for N-cadherin or β1-integrin. This was due to an altered speed of locomotion and directionality in the gut wall. The abnormal aggregation defect of ENCC and the disorganized ganglia network in the β1-/- mutant was not observed in the double mutant. This indicates that N-cadherin enhances the effect of the β1-integrin mutation and demonstrates cooperation between these two adhesion receptors during ENS ontogenesis. In conclusion, our data reveal that N-cadherin is not essential for ENS development but it does modulate the mod**es of ENC**C migration and acts in concert with β1-integrin to control the proper development of the ENS.
152. Integrin Cytoplasmic domain-Associated Protein-1 (ICAP-1) promotes **migration** of myoblasts and affects focal adhesions. Integrin Cytoplasmic domain-Associated Protein-1 (ICAP-1) binds specifically to the beta1 integrin subunit cytoplasmic domain. We observed that RNAi-induced knockdown of ICAP-1 reduced **migration** of C2C12 myoblasts on the beta1 integrin ligand laminin and that overexpression of ICAP-1 increased this **migration**. In contrast, **migration** on the beta3 integrin ligand vitronectin was not affected. ICAP-1 knockdown also greatly diminished **migration** of microvascular endothelial cells on collagen. The number of central focal adhesions in C2C12 cells on laminin was reduced by ICAP-1 knockdown and increased by ICAP-1 overexpression. Previously, we demonstrated that ICAP-1 binds to the ROCK-I kinase and translocates ROCK-I to the plasma membrane. We show here that the ROCK kinase inhibitor Y27362 reduces **migration** on laminin and causes a loss of central focal adhesions, similarly as ICAP-1 knockdown. ICAP-1 and ROCK were co-immune-precipitated from C2C12 cells, and in cells that overexpressed ICAP-1, YFP-ROCK was translocated to membrane ruffles. These results indicate that ICAP-1 regulates beta1 integrin-dependent cell **migration** by affecting the pattern of focal adhesion formation. This is likely due to ICAP-1-induced translocation of ROCK to beta1 integrin attachment sites.
153. A new in vitro model of murine mesoderm **migration**: the role of fibronectin and laminin. Examination of the factors involved in primary mesodermal **migration** in the mouse has been complicated by the lack of a suitable in vitro model. We have developed a new culture system using primitive streak stage embryos denuded of primitive endoderm, which allows easy observation and manipulation of the outgrowing cells. The cells migrating away from these explants were shown by immunocytochemistry to express vimentin and an epitope of the I antigen recognised by the antibody C6, both of which are present on the newly emerged mesoderm and not on the embryonic ectoderm in sections of embryos in utero. Conversely, cytokeratin, stage-specific embryonic antigen 1 (SSEA-1), E-cadherin and desmoplakin are expressed by the embryonic ectoderm but lost during mesoderm formation in vivo. They are absent or expressed very weakly by the migrated cells in vitro. In addition, only explants of the ectoplacental cone (EPC) and visceral endoderm alone, expressed a carbohydrate epitope (recognised by monoclonal antibody BOO6), characteristic of the EPC and primitive endoderm in utero, but absent from mesoderm. Thus we conclude that the cells which outgrow in this system are indeed mesodermal in phenotype. We have confirmed the work of others in demonstrating the presence of fibronectin (FN) and laminin (LN) in the migratory path of the mesoderm, at the ectoderm-visceral endoderm interface. We also report that the beta 1 integrin subunit of the FN and LN receptor is expressed by mesodermal cells at this interface. Using our in vitro model we have examined the role of the extracellular matrix (ECM) in mesodermal **migration**. Mesodermal cells migrate further and faster on substrates coated with FN or LN, and this increased **migration** is abolished by appropriate blocking antibodies. We conclude that the ECM, in particular FN and LN, plays an important role in the **migration** of primary mesodermal cells during gastrulation in the mouse embryo.
154. A crucial role of beta 1 integrins for keratinocyte **migration** in vitro and during cutaneous wound repair. Integrins are ubiquitous transmembrane receptors that play crucial roles in cell-cell and cell-matrix interactions. In this study, we have determined the effects of the loss of beta 1 integrins in keratinocytes in vitro and during cutaneous wound repair. Flow cytometry of cultured beta 1-deficient keratinocytes confirmed the absence of beta 1 integrins and showed downregulation of alpha 6 beta 4 but not of alpha v integrins. beta 1-null keratinocytes were characterised by poor adhesion to various substrates, by a reduced proliferation rate and by a strongly impaired migratory capacity. In vivo, the loss of beta 1 integrins in keratinocytes caused a severe defect in wound healing. beta 1-null keratinocytes showed impaired **migration** and were more densely packed in the hyperproliferative epithelium. Surprisingly, their proliferation rate was not reduced in early wounds and even increased in late wounds. The failure in re-epithelialisation resulted in a prolonged inflammatory response, leading to dramatic alterations in the expression of important wound-regulated genes. Ultimately, beta 1-deficient epidermis did cover the wound bed, but the epithelial architecture was abnormal. These findings demonstrate a crucial role of beta 1 integrins in keratinocyte **migration** and wound re-epithelialisation. Movies available on-line
155. Beta1 integrins control the formation of cell chains in the adult rostral migratory stream. The subventricular zone (SVZ) of the lateral ventricle is the major site of neurogenesis in the adult brain. Neuroblasts that are born in the SVZ migrate as chains along the rostral migratory stream (RMS) to the olfactory bulb. Little is known about the mechanisms that control interactions between neuroblasts during their **migration**. Here we show that migrating neuroblasts express beta1 integrins and that the integrin ligand laminin is localized to cell chains. Using genetically modified mice and time-lapse video recordings of SVZ explants, we demonstrate that beta1 integrins and laminin promote the formation of cell chains. Laminin also induces the aggregation of purified neuroblasts. We conclude that the formation of cell chains in the RMS is controlled in part by beta1 integrins via binding to laminin. In addition, we provide evidence that beta1 class integrins are required for the maintenance of the glial tubes and that defects in the glial tubes lead to the ectopic **migration** of neuroblasts into the surrounding tissue.
156. Spironolactone inhibits podocyte motility via decreasing integrin β1 and increasing integrin β3 in podocytes under high-glucose conditions. Integrin β1 and β3 expression by podocytes is required to maintain glomerular structural integrity. Previous studies have shown that aldosterone (ALD) is involved in glomerular podocyte injury, and mineralocorticoid receptor (MR) blocker spironolactone effectively reduces proteinuria in patients with diabetic nephropathy. The present study was designed to observe the effects of spironolactone on β1 and β3 integrin expression and podocyte motility under in vitro diabetic conditions. Immortalized mouse podocytes were cultured in media containing normal glucose (NG) levels, high glucose (HG) or HG plus spironolacton. The expression of β1 and β3 integrin in podocytes was detected by reverse transcription quantitative polymerase chain reaction, immunofluorescence and western blot analyses. The effects of spironolacton on podocyte motility was further evaluated using a wound healing assay. HG stimulation markedly decreased mRNA and protein expression of integrin β1, and significantly increased mRNA and protein expression of integrin β3 in cultured podocytes. However, simultaneous treatment with spironolacton (10‑7 mol/l) significantly attenuated HG-mediated increases in integrin β3 and decreases in integrin β1 expression. Fur**thermore,** the migration of podocytes induced by HG was abrogated by concomitant treatment with spironolacton. In conclusion, the present study suggested that HG decreased the expression of integrin β1 in cultured podocytes, accompanied with an increase of integrin β3. Spironolactone inhibited cell motility and stabilized podoctyes treated with HG, probably through partly normalizing the expression of integrin β1 and decreasing the expression of integrin β3.
157. Epigenetic regulation of galectin-3 expression by β1 integrins promotes cell adhesion and **migratio**n. Introduction of the integrin β1- but not the β3-subunit in GE11 cells induces an epithelial-to-mesenchymal-transition (EMT)-like phenomenon that is characterized by the loss of cell-cell contacts, cell scattering, increased ce**ll migrat**ion and RhoA activity, and fibronectin fibrillogenesis. Because galactose-binding lectins (galectins) have been implicated in these phenomena, we investigated whether galectins are involved in the β1-induced phenotype. We examined 9 galectins and, intriguingly, found that the expression of galectin-3 (Gal-3) is specifically induced by β1 but not by β3. Using β1-β3 chimeric integrins, we show that the induction of Gal-3 expression requires the hypervariable region in the extracellular domain of β1, but not its cytoplasmic tail. Furthermore, Gal-3 expression does not depend on RhoA signaling, serum factors, or any of the major signal transduction pathways involving protein kinase C (PKC), p38 mitogen-activated protein kinase (p38MAPK), extracellular signal-regulated kinase-1/-2 (ERK-1/2), phosphatidylinositol-3-OH kinase (PI3-K), or Src kinases. Instead, Gal-3 expression is controlled in an epigenetic manner. Whereas DNA methylation of the Lgals3 promoter maintains Gal-3 silencing in GE11 cells, expression of β1 causes its demethylation, leading to transcriptional activation of the Lgals3 gene. In turn, Gal-3 expression enhances β1 integrin-mediated cell adhesion to fibronectin (FN) and laminin (LN), as we**ll as cel**l migration. Gal-3 also promotes β1-mediated cell adhesion to LN and Collagen-1 (Col)-1 in cells that endogenously express Gal-3 and β1 integrins. In conclusion, we identify a functional feedback-loop between β1 integrins and Gal-3 that involves the epigenetic induction of Gal-3 expression during integrin-induced EMT and cell scattering.
158. OXPHOS dysfunction regulates integrin-β1 modifications and enhances cell motility and **migratio**n. Mitochondria are central organelles for cellular metabolism. In cancer cells, mitochondrial oxidative phosphorylation (OXPHOS) dysfunction has been shown to promote **migratio**n, invasion, metastization and apoptosis resistance. With the purpose of analysing the effects of OXPHOS dysfunction in cancer cells and the molecular players involved, we generated cybrid cell lines harbouring either wild-type (WT) or mutant mitochondrial DNA (mtDNA) [tRNAmut cybrids, which harbour the pathogenic A3243T mutation in the leucine transfer RNA gene (tRNAleu)]. tRNAmut cybrids exhibited lower oxygen consumption and higher glucose consumption and lactate production than WT cybrids. tRNAmut cybrids displayed increased motility and **migratio**n capacities, which were associated with altered integrin-β1 N-glycosylation, in particular with higher levels of β-1,6-N-acetylglucosamine (GlcNAc) branched N-glycans. This integrin-β1 N-glycosylation pattern was correlated with higher levels of membrane-bound integrin-β1 and also with increased binding to fibronectin. When cultured in vitro, tRNAmut cybrids presented lower growth rate than WT cybrids, however, when injected in nude mice, tRNAmut cybrids produced larger tumours and showed higher metastatic potential than WT cybrids. We conclude that mtDNA-driven OXPHOS dysfunction correlates with increased motility **and migr**ation capacities, through a mechanism that may involve the cross talk between cancer cell mitochondria and the extracellular matrix.
159. Integrin alpha4beta1 signaling is required for lymphangiogenesis and tumor metastasis. Recent studies have shown that lymphangiogenesis or the growth of lymphatic vessels at the periphery of tumors promotes tumor metastasis to lymph nodes. We show here that the fibronectin-binding integrin alpha4beta1 and its ligand fibronectin are novel functional markers of proliferative lymphatic endothelium. Tumors and lymphangiogenic growth factors, such as vascular endothelial growth factor-C (VEGF-C) and VEGF-A, induce lymphatic vessel expression of integrin alpha4beta1. Integrin alpha4beta1 then promotes growth factor and tumor-induced lymphangiogenesis, as genetic loss of integrin alpha4beta1 expression in Tie2Cre+ alpha4(loxp/loxp) mice or genetic loss of alpha4 signaling in alpha4Y991A knock-in mice blocks growth factor and tumor-induced lymphangiogenesis, as well as tumor metastasis to lymph nodes. In addition, antagonists of integrin alpha4beta1 suppress lymphangiogenesis and tumor metastasis. Our studies show that integrin alpha4beta1 and the signals it transduces regulate the adhesion, **migration**, invasion, and survival of proliferating lymphatic endothelial cells. As suppression of alpha4beta1 expression, signal transduction, or function in tumor lymphatic endothelium not only inhibits tumor lymphangiogenesis but also prevents metastatic disease, these results show that integrin alpha4beta1-mediated tumor lymphangiogenesis promotes metastasis and is a useful target for the suppression of metastatic disease.
160. Defective blood vessel development and pericyte/pvSMC distribution in alpha 4 integrin-deficient mouse embryos. Blood vessel development is in part regulated by pericytes/presumptive vascular smooth muscle cells (PC/pvSMCs). Here, we demonstrate that interactions between PC/pvSMCs and extracellular matrix play a critical role in this event. We show that the cranial vessels in alpha4 integrin-deficient mouse embryos at the stage of vessel remodeling are increased in diameter. This defect is accompanied by a failure of PC/pvSMCs, which normally express alpha4beta1 integrin, to spread uniformly along the vessels. We also find that fibronectin but not VCAM-1 is localized in the cranial vessels at this stage. Furthermore, cultured alpha4 integrin-null PC/pvSMCs plated on fibronectin display a delay in initiating **migration**, a reduction in **migration** speed, and a decrease in directional persistence in response to a polarized force of shear flow. These results suggest that specific motile activities of PC/pvSMCs regulated by mechanical signals imposed by the interstitial extracellular matrix may also be required in vivo for the distribution and function of the PC/pvSMCs during blood vessel development.
161. Mouse myoblasts can fuse and form a normal sarcomere in the absence of beta1 integrin expression. Antibody perturbation experiments suggested that **migration**, terminal differentiation and fusion of myoblasts are dependent on beta1 integrin expression. In addition, several studies have postulated that beta1 integrins have a role in the formation of sarcomeres. In the present report we have analysed skeletal myogenesis in wild-type/beta1-null chimeric mice and beta1-null embryoid bodies. Trunk and limbs of beta1-null chimeric mice contained muscle tissue composed of normal and beta1-null myoblasts indicating that all myotomic sublineages can form, migrate to their peripheral targets and fuse in the absence of beta1 integrin expression. Pure populations of beta1-null myoblasts and satellite cells isolated from beta1-null chimeric embryos and chimeric newborn mice, respectively, were able to differentiate in vitro and to fuse into multinucleated myotubes. Quantitative and qualitative comparisons between normal and beta1-null myoblasts revealed no apparent difference in their capacity to terminally differentiate and fuse. Furthermore, beta1-null myotubes developed sarcomeres which were indistinguishable from wild-type controls. When normal and beta1-null ES cells were differentiated into embryoid bodies, they contained fully differentiated myotubes with normal sarcomeres and normal deposition of costameric components. However, formation of beta1-null myotubes was delayed and was less efficient in beta1-null embryoid bodies than in wild-type controls. High expression of alphav integrin subunit at the tips of normal as well as beta1-null myotubes indicated that the lack of beta1 integrins did not result in a significant redistribution of alphav-containing receptors.
162. Reelin, integrin and DAB1 interactions during embryonic cerebral cortical development. Extracellular matrix-like molecule reelin and cell surface adhesion receptors such as alpha3beta1 integrin can regulate neuronal **migration** and position in the developing cerebral cortex. Here we show that alpha3beta1 integrin binds to the N-terminal region of reelin, a site distinct from the region of reelin shown to associate with other reelin receptors such as VLDLR/ApoER2. Furthermore, Dab1, a member of the reelin signaling pathway, can complex with the cytoplasmic region of beta1 integrin in a reelin-dependent manner. Thus, alpha3beta1 integrin-reelin interactions may contribute to appropriate neuronal placement in the developing cerebral cortex.
163. Low density lipoprotein receptor-related protein 1 mediated endocytosis of β1-integrin influences cell adhesion and cell **migratio**n. The low density lipoprotein receptor-related protein 1 (LRP1) has been shown to interact with β1-integrin and regulate its surface expression. LRP1 knock-out cells exhibit altered cytoskeleton organization and decreased cel**l migrati**on. Here we demonstrate coupled endocytosis of LRP1 and β1-integrin and the involvement of the intracellular NPxY2 motif of LRP1 in this process. Mouse embryonic fibroblasts harboring a knock in replacement of the NPxY2 motif of LRP1 by a multiple alanine cassette (AAxA) showed elevated surface expression of β1-integrin and decreased β1-integrin internalization rates. As a consequence, cell spreading was altered and adhesion rates were increased in our cell model. Cells formed more focal adhesion complexes, whereby in vitro **cell migr**ation rates were decreased. Similar results could be observed in a corresponding mouse model, the C57Bl6 LRP1 NPxYxxL knock in mice, therefore, the biochemistry of cellular adhesion was altered in primary cortical neurons. In vivo **cell migr**ation experiments demonstrated a disturbance of neuroblast **cell migr**ation along the rostral migratory stream. In summary, our results indicate that LRP1 interacts with β1-integrin mediating integrin internalization and thus correlates with downstream signaling of β1-integrin such as focal adhesion dynamics. Consequently, the disturbance of this interaction resulted in a dysfunction in in vivo and in vitro cell adhesion an**d cell mi**gration.
164. Analysis of Netrin 1 receptors during inner ear development. Netrin 1 plays key roles in axon guidance and neuronal **migration** during central nervous system (CNS) development. Outside the CNS, Netrin 1 has been shown to be involved in epithelial morphogenesis of various organs. We have shown that Netrin 1 is essential for inner ear semicircular duct formation, but the involvement of Netrin 1 receptors in this process has remained unknown. Netrin 1 receptors include members of the Deleted in colorectal cancer (Dcc), Unc5-homologue and integrin families. Here we have analysed the expression of these receptor genes during inner ear development and verified the inner ear phenotypes of several receptor mutant mice. Special interest was directed to receptors that could cooperate with Netrin 1 during semicircular duct formation. We show that Neogenin (Neo1), Unc5c as well as integrin b1 (Itgb1) are expressed in periotic mesenchyme, while Dcc, Unc5b, Unc5c, Itga3, Itga6 and Itgb1 are expressed in different parts of the otic epithelium. In spite of the broad and strong expression of several receptors in ear region, none of the analysed receptor mutant embryos showed any defects in inner ear development.
165. SNX17 protects integrins from degradation by sorting between lysosomal and recycling pathways. The FERM-like domain-containing sorting nexins of the SNX17/SNX27/SNX31 family have been proposed to mediate retrieval of transmembrane proteins from the lysosomal pathway. In this paper, we describe a stable isotope labeling with amino acids in culture-based quantitative proteomic approach that allows an unbiased, global identification of transmembrane cargoes that are rescued from lysosomal degradation by SNX17. This screen revealed that several integrins required SNX17 for their stability, as depletion of SNX17 led to a loss of β1 and β5 integrins and associated a subunits from HeLa cells as a result of increased lysosomal degradation. SNX17 bound to the membrane distal NPXY motif in β integrin cytoplasmic tails, thereby preventing lysosomal degradation of β integrins and their associated a subunits. Furthermore, SNX17-dependent retrieval of integrins did not depend on the retromer complex. Consistent with an effect on integrin recycling, depletion of SNX17 also caused alterations in c**ell migra**tion. Our data provide mechanistic insight into the retrieval of internalized integrins from the lysosomal degradation pathway, a prerequisite for subsequent recycling of these matrix receptors.
166. Alpha4beta1 integrin mediates the recruitment of immature dendritic cells across the blood-brain barrier during experimental autoimmune encephalomyelitis. Dendritic cells (DCs) within the CNS are recognized to play an important role in the effector phase and propagation of the immune response in experimental autoimmune encephalomyelitis (EAE), a mouse model for multiple sclerosis. However, the mechanisms regulating DC trafficking into the CNS still need to be characterized. In this study, we show by performing intravital fluorescence videomicroscopy of the inflamed spinal cord white-matter microvasculature in SJL mice with EAE that immature, and to a lesser extent, LPS-matured, bone marrow-derived DCs efficiently interact with the CNS endothelium by rolling, capturing, and firm adhesion. Immature but not LPS-matured DCs efficiently migrated across the wall of inflamed parenchymal microvessels into the CNS. Blocking alpha4 integrins interfered with the adhesion but not the rolling or capturing of immature and LPS-matured DCs to the CNS microvascular endothelium, inhibiting their **migration** across the vascular wall. Functional absence of beta1 integrins but not of beta7 integrins or alpha4beta7 integrin similarly reduced the adhesion of immature DCs to the CNS microvascular endothelium, demonstrating that alpha4beta1 but not alpha4beta7 integrin mediates this step of immature DCs interaction with the inflamed blood-brain barrier during EAE. Our study shows that during EAE, especially immature DCs migrate into the CNS, where they may be crucial for the perpetuation of the CNS-targeted autoimmune response. Thus therapeutic targeting of alpha4 integrins affects DC trafficking into the CNS and may therefore lead to the resolution of the CNS autoimmune inflammation by reducing the number of CNS professional APCs.
167. Protein 4.1G Regulates Cell Adhesion, Spreading, and **Migration** of Mouse Embryonic Fibroblasts through the β1 Integrin Pathway. Protein 4.1G is a membrane skeletal protein that can serve as an adapter between transmembrane proteins and the underlying membrane skeleton. The function of 4.1G remains largely unexplored. Here, using 4.1G knockout mouse embryonic fibroblasts (MEFs) as a model system, we explored the function of 4.1G in motile cells. We show that the adhesion, spreading, and **migratio**n of 4.1G(-/-) MEF cells are impaired significantly. We further show that, although the total cellular expression of β1 integrin is unchanged, the surface expression of β1 integrin and its active form are decreased significantly in 4.1G(-/-) MEF cells. Moreover, the phosphorylation of focal adhesion kinase, a downstream component of the integrin-mediated signal transduction pathway, is suppressed in 4.1G(-/-) MEF cells. Co-immunoprecipitation experiments and in vitro binding assays showed that 4.1G binds directly to β1 integrin via its membrane-binding domain. These findings identified a novel role of 4.1G in cell adhesion, spreading, **and migra**tion in MEF cells by modulating the surface expression of β1 integrin and subsequent downstream signal transduction.
168. Upregulation of alpha 9 integrin and tenascin during epithelial regeneration after debridement in the cornea. Stratified epithelia are exposed to abrasive forces and are required to respond rapidly to injury to minimize fluid loss and the risk for microbial infection. Healing involves a cell migratory phase to reestablish barrier function and cell proliferation to restratify the epithelium. Cell **migration** during re-epithelialization involves cell sliding, termed sheet movement, during which cells retain their cell-cell junctions while dynamically altering their shape and cell-substrate interactions to permit movement across the exposed wound bed. Proteins of the integrin family of receptor molecules modulate cell shape, cell **migration**, and signal transduction in many cell types. In epithelial cells, integrins of the beta 1 family have been implicated in regulating cell proliferation and differentiation, alpha 9 beta b1 is one of the newer members of the integrin beta family and has been recently shown to function as a tenascin receptor. Although little is known about its function in vivo, studies in developing mouse cornea and eyelid suggest that it may play a role in epithelial differentiation. Using a debridement wound model in the mouse cornea, we show in this study that (a) in response to small debridement wounds that close without cell proliferation, alpha 9 integrin protein and mRNA are not induced during **migration** but are induced during restratification, (b) larger debridement wounds that require cell proliferation to generate the cells necessary for sheet movement result in a dramatic induction of alpha 9 protein and its mRNA during both **migration** and restratification, and (c) tenascin, an alpha 9 beta 1 ligand, accumulates beneath epithelial cells during restratification but not during cell **migration**. Therefore, alpha 9 integrin protein production and tenascin accumulation are dynamically regulated in response to corneal epithelial injury.
169. Beta1 integrin activates Rac1 in Schwann cells to generate radial lamellae during axonal sorting and myelination. Myelin is a multispiraled extension of glial membrane that surrounds axons. How glia extend a surface many-fold larger than their body is poorly understood. Schwann cells are peripheral glia and insert radial cytoplasmic extensions into bundles of axons to sort, ensheath, and myelinate them. Laminins and beta1 integrins are required for axonal sorting, but the downstream signals are largely unknown. We show that Schwann cells devoid of beta1 integrin migrate to and elongate on axons but cannot extend radial lamellae of cytoplasm, similar to cells with low Rac1 activation. Accordingly, active Rac1 is decreased in beta1 integrin-null nerves, inhibiting Rac1 activity decreases radial lamellae in Schwann cells, and ablating Rac1 in Schwann cells of transgenic mice delays axonal sorting and impairs myelination. Finally, expressing active Rac1 in beta1 integrin-null nerves improves sorting. Thus, increased activation of Rac1 by beta1 integrins allows Schwann cells to switch from **migration**/elongation to the extension of radial membranes required for axonal sorting and myelination.
170. Rac1 GTPase-deficient mouse lens exhibits defects in shape, suture formation, fiber cell **migration** and survival. Morphogenesis and shape of the ocular lens depend on epithelial cell elongation and differentiation into fiber cells, followed by the symmetric and compact organization of fiber cells within an enclosed extracellular matrix-enriched elastic capsule. The cellular mechanisms orchestrating these different events however, remain obscure. We investigated the role of the Rac1 GTPase in these processes by targeted deletion of expression using the conditional gene knockout (cKO) approach. Rac1 cKO mice were derived from two different Cre (Le-Cre and MLR-10) transgenic mice in which lens-specific Cre expression starts at embryonic day 8.75 and 10.5, respectively, in both the lens epithelium and fiber cells. The Le-Cre/Rac1 cKO mice exhibited an early-onset (E12.5) and severe lens phenotype compared to the MLR-10/Rac1 cKO (E15.5) mice. While the Le-Cre/Rac1 cKO lenses displayed delayed primary fiber cell elongation, lenses from both Rac1 cKO strains were characterized by abnormal shape, impaired secondary fiber cell **migration**, sutural defects and thinning of the posterior capsule which often led to rupture. Lens fiber cell N-cadherin/β-catenin/Rap1/Nectin-based cell-cell junction formation and WAVE-2/Abi-2/Nap1-regulated actin polymerization were impaired in the Rac1 deficient mice. Additionally, the Rac1 cKO lenses were characterized by a shortened epithelial sheet, reduced levels of extracellular matrix (ECM) proteins and increased apoptosis. Taken together, these data uncover the essential role of Rac1 GTPase activity in establishment and maintenance of lens shape, suture formation and capsule integrity, and in fiber cell **migratio**n, adhesion and survival, via regulation of actin cytoskeletal dynamics, cell adhesive interactions and ECM turnover.
171. A critical role of CD29 and CD49f in mediating metastasis for cancer-initiating cells isolated from a Brca1-associated mouse model of breast cancer. Cancer metastasis is a lethal problem that claims the lives of over 90% of cancer patients. In this study, we have investigated metastatic potential of cancer stem cells (CSCs) isolated from mammary tumors of a Brca1-mutant mouse model. Our data indicated that CSCs, which are enriched in CD24(+)CD29(+)/CD49f(+) cell population, displayed much higher **migration** ability than CD24(-)CD29(-)/CD49f(-) cells in tissue culture and enhanced metastatic potential in allograft-nude mice. CD24(+)CD29(+) cells maintained the ability to differentiate and reconstitute heterogeneity in the metastatic tumors whereas CD24(-)CD29(-) cells could not. Corresponding to their enhanced metastatic ability, CD24(+)CD29(+) cells exhibited features of the epithelial to mesenchymal transition. Finally, using short hairpin RNA to knock down CD29 and/or CD49f in metastatic cancer cells, we demonstrated that while acute knockdown of CD29 or CD49f alone slightly decreased cell **migration** ability, knockdown of both genes generated a profound effect to block their **migration**, revealing an overlapping, yet critical function of both genes in the **migration** of CSCs. Our findings indicate that in addition to serving as markers of CSCs, CD29 and CD49f may also serve as potential therapeutic targets for cancer metastasis.
172. alpha6beta1 integrin directs **migration** of neuronal precursors in adult mouse forebrain. New neuroblasts are constantly generated in the adult mammalian subventricular zone (SVZ) and migrate via the very-restricted rostral migratory stream (RMS) to the olfactory bulb, where they differentiate into functional neurons. Several facilitating and repulsive molecules for this **migration** have been identified, but little is known about chemoattractive molecules involved in the directed nature of this **migration** in vivo. Here, we investigated the role of the alpha6beta1 integrin, and its ligand, laminin, in controlling guidance of the migrating neuroblasts in adult mice. Immunostaining for the alpha6beta1 integrin was present in neuroblasts and their processes in the anterior/rostral SVZ and the RMS. Inhibition of the endogenous alpha6 or beta1 subunit with locally injected antibodies disrupted the cohesive nature of the RMS, but did not kill the neuroblasts. Infusion of a 15 a.a. peptide, representing the E8 domain of the laminin alpha chains that bind alpha6beta1 integrin, into the neostriatum redirected the neuroblasts away from the RMS towards the site of infusion. Injection of a narrow tract of intact laminin also drew the neuroblasts away from the RMS, but in a more restricted localization. These results suggest a critical role for integrins and laminins in adult SVZ-derived neuroblast **migration**. They also suggest that integrin-based strategies could be used to direct or restrict neuroblasts to CNS regions where they are needed for cell replacement therapies in the nervous system.
173. Essential role of VLA-4/VCAM-1 pathway in the establishment of CD8+ T-cell-mediated Trypanosoma cruzi-elicited meningoencephalitis. Central nervous system (CNS) damage can occur during Trypanosoma cruzi infection, especially in immunosuppressed patients. The enhanced susceptibility of C3H/He mice to CD8-mediated acute meningoencephalitis is associated with higher up-regulation of vascular cell adhesion molecule-1 (VCAM-1) on CNS vascular endothelia than in the less susceptible C57BL/6. Further, in vitro adhesion of activated peripheral blood cells to CNS blood vessels was abrogated by anti-VLA-4 antibodies that also inhibited cell **migration** into the CNS of T. cruzi-infected mice. Lastly, the reactivation of meningoencephalitis in immunosuppressed chronically infected mice was associated with VCAM-1 up-regulation. Therefore, we hypothesize that VLA-4/VCAM-1 pathway plays a pivotal role in the establishment of T. cruzi-elicited encephalitis.
174. Dense fibrillar collagen matrices to analyse extracellular matrix receptor function. The goal of this study was to understand whether dense fibrillar collagen matrices, with a hierarchical structure resembling native collagen matrices, could be useful to study collagen receptor function, in a more physiological context. The receptor analysed here was integrin α11β1, already shown to be involved in cell attachment an**d migrati**on on collagen-coated plastic, and also in contraction of loose fibrillar collagen hydrogels.Collagen matrices prepared here corresponded to dense fibrillar hydrogels concentrated at 5mg/ml. The behaviour of α11β1 deficient fibroblasts seeded on these concentrated matrices was assessed in terms of adhesion, morphology **and migra**tion, then compared to that observed on classical hydrogels at 1mg/ml, corresponding to loose collagen matrices.Short-term attachment assays showed disturbed interactions between α11β1 deficient cells and collagen matrices in a concentration-dependent manner. Long-term assays revealed reduced cell spreading of alpha 11(-/-) cells on the dense collagen matrices, associated with a disturbed cytoskeleton network. Moreover, anoikis was observed when alpha 11(-/-) cells were seeded on 5mg/ml matrices, and not on looser 1mg/ml matrices. In scratch wound in vitro assays, carried out with cells on 5mg/ml fibrillar collagen matrices, alpha 11(-/-) cells migrated much better than their wild-type counterparts. In contrast, no significant difference was observed between wild and knock-out cells seeded on plastic.The present study demonstrates the validity of in vivo-like dense fibrillar collagen matrices to evaluate cell receptor functions more significantly than with 2D cell cultures or loose hydrogels.
175. Conditional ablation of beta1 integrin in skin. Severe defects in epidermal proliferation, basement membrane formation, and hair follicle invagination. The major epidermal integrins are alpha3beta1 and hemidesmosome-specific alpha6beta4; both share laminin 5 as ligand. Keratinocyte culture studies implicate both integrins in adhesion, proliferation, and stem cell maintenance and suggest unique roles for alphabeta1 integrins in **migration** and terminal differentiation. In mice, however, whereas ablation of alpha6 or beta4 results in loss of hemidesmosomes, epidermal polarity, and basement membrane (BM) attachment, ablation of alpha3 only generates microblistering due to localized internal shearing of BM. Using conditional knockout technology to ablate beta1 in skin epithelium, we have uncovered biological roles for alphabeta1 integrins not predicted from either the alpha3 knockout or from in vitro studies. In contrast to alpha3 null mice, beta1 mutant mice exhibit severe skin blistering and hair defects, accompanied by massive failure of BM assembly/organization, hemidesmosome instability, and a failure of hair follicle keratinocytes to remodel BM and invaginate into the dermis. Although epidermal proliferation is impaired, a spatial and temporal program of terminal differentiation is executed. These results indicate that beta1's minor partners in skin are important, and together, alphabeta1 integrins are required not only for extracellular matrix assembly but also for BM formation. This, in turn, is required for hemidesmosome stability, epidermal proliferation, and hair follicle morphogenesis. However, beta1 downregulation does not provide the trigger to terminally differentiate.
176. Cell-autonomous requirement for beta1 integrin in endothelial cell adhesion, **migration** and survival during angiogenesis in mice. beta1 integrin (encoded by Itgb1) is established as a regulator of angiogenesis based upon the phenotypes of complete knockouts of beta1 heterodimer partners or ligands and upon antibody inhibition studies in mice. Its direct function in endothelial cells (ECs) in vivo has not been determined because Itgb1(-/-) embryos die before vascular development. Excision of Itgb1 from ECs and a subset of hematopoietic cells, using Tie2-Cre, resulted in abnormal vascular development by embryonic day (e) 8.5 and lethality by e10.5. Tie1-Cre mediated a more restricted excision of Itgb1 from ECs and hematopoietic cells and resulted in embryonic lethal vascular defects by e11.5. Capillaries of the yolk sacs were disorganized, and the endothelium of major blood vessels and of the heart was frequently discontinuous in mutant embryos. We also found similar vascular morphogenesis defects characterized by EC disorganization in embryonic explants and isolated ECs. Itgb1-null ECs were deficient in adhesion and **migration** in a ligand-specific fashion, with impaired responses to laminin and collagens, but not to fibronectin. Deletion of Itgb1 reduced EC survival, but did not affect proliferation. Our findings demonstrate that beta1 integrin is essential for EC adhesion, **migration** and survival during angiogenesis, and further validate that therapies targeting beta1 integrins may effectively impair neovascularization.
177. Enhanced trafficking to the pancreatic lymph nodes and auto-antigen presentation capacity distinguishes peritoneal B lymphocytes in non-obese diabetic mice. NOD.Igmicro ( null ) mice lacking mature B cells are highly resistant to diabetes and display poor CD4 T cell responses to autoantigens. Nevertheless, the degree to which different B cell subsets contribute to diabetes in NOD mice remains unresolved. Due to their role in the recognition of microbial and autoantigens, peritoneal B cell characteristics were examined in NOD mice to see if they differ developmentally, phenotypically or functionally in aspects relevant to diabetogenesis.The population dynamics, activation state, migratory behaviour and antigen presentation function were investigated in NOD peritoneal B cells.NOD peritoneal B cells were found to express abnormally high levels of co-stimulatory molecules (CD40, CD86 and CD69). In contrast, the expression of L-selectin and integrin alpha4beta1 was markedly reduced in NOD mice compared with BALB/c and C57BL/6 mice. The number of B cells in the peritoneum was lower in NOD than in control mice throughout development; **migration** of B cells from the peritoneum to the pancreatic lymph nodes in NOD mice was enhanced tenfold. NOD B cells showed no chemotactic response to sphingosine-1-phosphate, which normally acts to retain B cells in the peritoneum. Peritoneal B cells of NOD mice also presented insulin autoantigen to CD4 T cells, inducing T cell proliferation.NOD peritoneal B cells are hyperactivated, migrate to the pancreatic lymph nodes and are capable of driving insulin-specific CD4 T cell activation. These characteristics could make them important for inducing or amplifying T cell responses against islet-antigens.
178. Angiopoietin-2 regulates cortical neurogenesis in the developing telencephalon. Vascular-specific growth factor angiopoietin-2 (Ang2) is mainly involved during vascular network setup. Recently, Ang2 was suggested to play a role in adult neurogenesis, affecting **migration** and differentiation of adult neuroblasts in vitro. However, to date, no data have reported an effect of Ang2 on neurogenesis during embryonic development. As we detected Ang2 expression in the developing cerebral cortex at embryonic day E14.5 and E16.5, we used in utero electroporation to knock down Ang2 expression in neuronal progenitors located in the cortical ventricular zone (VZ) to examine the role of Ang2 in cortical embryonic neurogenesis. Using this strategy, we showed that radial **migration** from the VZ toward the cortical plate of Ang2-knocked down neurons is altered as well as their morphology. In parallel, we observed a perturbation of intermediate progenitor population and the surrounding vasculature. Taken together, our results show for the first time that, in addition to its role during brain vasculature setup, Ang2 is also involved in embryonic cortical neurogenesis and especially in the radial **migration** of projection neurons.
179. Rapid leukocyte **migration** by integrin-independent flowing and squeezing. All metazoan cells carry transmembrane receptors of the integrin family, which couple the contractile force of the actomyosin cytoskeleton to the extracellular environment. In agreement with this principle, rapidly migrating leukocytes use integrin-mediated adhesion when moving over two-dimensional surfaces. As **migration** on two-dimensional substrates naturally overemphasizes the role of adhesion, the contribution of integrins during three-dimensional movement of leukocytes within tissues has remained controversial. We studied the interplay between adhesive, contractile and protrusive forces during interstitial leukocyte chemotaxis in vivo and in vitro. We ablated all integrin heterodimers from murine leukocytes, and show here that functional integrins do not contribute to **migration** in three-dimensional environments. Instead, these cells migrate by the sole force of actin-network expansion, which promotes protrusive flowing of the leading edge. Myosin II-dependent contraction is only required on passage through narrow gaps, where a squeezing contraction of the trailing edge propels the rigid nucleus.

#### GeneRIFs

1. Data show that beta1 integrin facilitate blood vessel-guided neuronal **migration** in injured brain.
2. 4.1G has a role in cell adhesion, spreading, and **migration** in MEF cells by modulating the surface expression of beta1 integrin and subsequent downstream signal transduction
3. LG3, through interactions with alpha2beta1 integrins on recipient-derived cells leading to activation of ERK1/2 and increased **migration**, favors myointimal thickening
4. Mobilization of bone marrow-derived cells by G-CSF boosted **migration** along the VCAM-1/VLA-4 axis and reduced apoptosis of cardiomyocytes
5. Type VIII collagen signals via beta1 integrin and RhoA to regulate MMP-2 expression and smooth muscle cell **migration**.
6. Interaction between Sox10 and Itgb1 controls neural crest cells **migration**.
7. beta1 integrin is dispensable for **migration** and initiation of the smooth muscle differentiation program, however, it is essential for remodeling of the pharyngeal arch arteries and for the assembly of the vessel wall of their derivatives
8. Gal-3 expression enhances beta1 integrin-mediated cell adhesion and cell **migration**.
9. TSC2 controls cell **migration**, attachment, and spreading through the alpha1beta1-integrin receptor.
10. The results show that beta1-integrins promote cell cycle in mammary epithelial cells, whereas beta3-integrins are involved in **migration**.
11. uPAR is an essential component of the network through which VEGF controls integrin redistribution and endothelial cell **migration**.
12. The data showed that N-cadherin is not essential for enteric nervous system development but it does modulate the modes of enteric neural crest cell **migration** and acts in concert with beta1-integrin to control the development of the enteric nervous system.
13. Fibronectin induces endothelial cell **migration** through beta1 integrin and Src-dependent phosphorylation of fibroblast growth factor receptor-1 at tyrosines 653/654 and 766.
14. Angiopoietin-like 4 interacts with integrins beta1 and beta5 to modulate keratinocyte **migration**.
15. the CCR7/CCL21 signaling pathway leading to T lymphocyte **migration** on fibronectin is a beta1 integrin-dependent pathway involving transient ERK1/2 phosphorylation, which is modulated by PLCgamma1
16. Data show that hypoxia increases the proliferation and **migration** of mESCs via FN-IN beta1 production through the PI3K/Akt, mTOR, and HIF-1alpha pathways, followed by FAK activation.
17. Results suggest a novel function for Rab1a in the regulation of cell **migration** through controlling integrin beta1 recycling and localization to lipid rafts via a specific downstream effector pathway.
18. Results indicate that alpha5beta1 integrin functions in the regulation of neural morphology and **migration** during cortical development, playing a role in cortical lamination.
19. MMP7 shedding of syndecan-1 facilitates wound closure by causing the alpha(2)beta(1) integrin to assume a less active conformation thereby removing restrictions to **migration**
20. we propose that integrin alpha8beta1 plays a critical role in lung morphogenesis by regulating mesenchymal cell adhesion and **migration**
21. Results suggest that beta1 integrins are required to overcome the tenascin-C-mediated inhibition of **migration** within the caecum and proximal hindgut and to enhance fibronectin-dependent **migration** in these regions.
22. A novel and unique mechanism of coordinated interactions of the integrin alpha9 cytoplasmic domain, Src tyrosine kinase and iNOS to transduce integrin-alpha9beta1-mediated cell **migration**.
23. JAM-A dimerization facilitates formation of a complex with Afadin and PDZ-GEF2 that activates Rap1A, which regulates beta1 integrin levels and cell **migration**.
24. netrin-1-alpha3beta1 integrin interactions modulate distinct routes of interneuronal **migration** and the consequent positioning of groups of cortical interneurons in the developing cerebral cortex.
25. integrin alpha3beta1 delays keratinocyte **migration** during wound re-epithelialization, by binding to the laminin-332 that is newly deposited on the wound bed.
26. Cell-autonomous requirement for Itgb1 in endothelial cell adhesion, **migration** and survival during angiogenesis is reported.
27. alpha9beta1 integrin enhances cell **migration** by polyamine-mediated modulation of an inward-rectifier potassium channel
28. Integrin Cytoplasmic domain-Associated Protein-1(ICAP-1) regulates beta1 integrin-dependent cell **migration** by affecting the pattern of focal adhesion formation; this is likely due to ICAP-1-induced translocation of ROCK to beta1 integrin attachment sites
29. increased activation of Rac1 by beta1 integrins allows Schwann cells to switch from **migration**/elongation to the extension of radial membranes required for axonal sorting and myelination
30. A crucial role of beta 1 integrins for keratinocyte **migration** in vitro and during cutaneous wound repair.
31. alpha1beta1 integrin is involved in atherosclerosis by mediating the **migration** of leukocytes to lesions by adhesion to collagen IV
32. Dual functions of [alpha]4[beta]1 integrin in epicardial development: initial **migration** and long-term attachment.
33. beta 1 integrin has roles in cell adhesion and **migration** in development and metastasis [review]
34. Inhibition of the endogenous integrin alpha6 or beta1 subunit with locally injected antibodies disrupted the neuronal rostral migratory stream suggesting a role for integrins and laminins in adult neuroblast **migration**
35. sema 3C promotes glomerular endothelial cell proliferation, adhesion, directional **migration**, and tube formation in vitro by stimulating integrin beta1 phosphorylation and VEGF120 secretion

### ECM

#### PubMed

1. Caveolin-1-dependent beta1 integrin endocytosis is a critical regulator of fibronectin turnover. beta1 integrins are major cell surface receptors for fibronectin. Some integrins, including beta1 integrins, are known to undergo constitutive endocytosis and recycling. Integrin endocytosis/recycling has been implicated in the regulation of cell migration. However, the mechanisms by which integrin endocytosis/recycling regulates cell migration, and other biological consequences of integrin trafficking are not completely understood. We previously showed that turnover of extracellular matrix (**ECM**) fibronectin occurs via receptor-mediated endocytosis. Here, we investigate the biological relevance of beta1 integrin endocytosis to fibronectin matrix turnover. First, we demonstrate that beta1 integrins, including alpha5beta1 play an important role in endocytosis and turnover of matrix fibronectin. Second, we show that caveolin-1 constitutively regulates endocytosis of alpha5beta1 integrins, and that alpha5beta1 integrin endocytosis can occur in the absence of fibronectin and fibronectin matrix. We also show that downregulation of caveolin-1 expression by siRNA results in marked reduction of beta1 integrin and fibronectin endocytosis. Hence, caveolin-1-dependent beta1 integrin and fibronectin endocytosis plays a critical role in fibronectin matrix turnover, and may contribute to abnormal **ECM** remodeling that occurs in fibrotic disorders.
2. Laminin/β1 integrin signal triggers axon formation by promoting microtubule assembly and stabilization. Axon specification during neuronal polarization is closely associated with increased microtubule stabilization in one of the neurites of unpolarized neuron, but how this increased microtubule stability is achieved is unclear. Here, we show that extracellular matrix **(EC**M) component laminin promotes neuronal polarization via regulating directional microtubule assembly through β1 integrin (Itgb1). Contact with laminin coated on culture substrate or polystyrene beads was sufficient for axon specification of undifferentiated neurites in cultured hippocampal neurons and cortical slices. Active Itgb1 was found to be concentrated in laminin-contacting neurites. Axon formation was promoted and abolished by enhancing and attenuating Itgb1 signaling, respectively. Interestingly, laminin contact promoted plus-end microtubule assembly in a manner that required Itgb1. Moreover, stabilizing microtubules partially prevented polarization defects caused by Itgb1 downregulation. Finally, genetic ablation of Itgb1 in dorsal telencephalic progenitors caused deficits in axon development of cortical pyramidal neurons. Thus, laminin/Itgb1 signaling plays an instructive role in axon initiation and growth, both in vitro and in vivo, through the regulation of microtubule assembly. This study has established a linkage between an extrinsic factor and intrinsic cytoskeletal dynamics during neuronal polarization.
3. Increased expression of fibronectin and the alpha 5 beta 1 integrin in angiogenic cerebral blood vessels of mice subject to hypobaric hypoxia. The extracellular matrix (**ECM**) is an important regulator of angiogenesis and vascular remodeling. We showed previously that angiogenic capillaries in the developing CNS express high levels of fibronectin and its receptor alpha5beta1 integrin, and that this expression is developmentally downregulated. As cerebral hypoxia leads to an angiogenic response, we sought to determine whether angiogenic vessels in the adult CNS re-express fibronectin and the alpha5beta1 integrin. Ten-week old mice were subject to hypobaric hypoxia for 0, 4, 7 and 14 days, and fibronectin/integrin expression examined. Fibronectin and the alpha5 integrin subunit were strongly upregulated on capillaries in the hypoxic CNS, with the effect maximal at the earliest time point examined (4 days). Immunofluorescent studies demonstrated that the alpha5 integrin was expressed by angiogenic endothelial cells. In light of the defined angiogenic role for fibronectin in other systems, this work suggests that induction of fibronectin-alpha5beta1 integrin expression may be an important molecular switch driving angiogenesis in the hypoxic CNS.
4. Patterned collagen fibers orient branching mammary epithelium through distinct signaling modules. For decades, the work of cell and developmental biologists has demonstrated the striking ability of the mesenchyme and the stroma to instruct epithelial form and function in the mammary gland, but the role of extracellular matrix (**ECM**) molecules in mammary pattern specification has not been elucidated. Here, we show that stromal collagen I (Col-I) fibers in the mammary fat pad are axially oriented prior to branching morphogenesis. Upon puberty, the branching epithelium orients along these fibers, thereby adopting a similar axial bias. To establish a causal relationship from Col-I fiber to epithelial orientation, we embedded mammary organoids within axially oriented Col-I fiber gels and observed dramatic epithelial co-orientation. Whereas a constitutively active form of Rac1, a molecule implicated in cell motility, prevented a directional epithelial response to Col-I fiber orientation, inhibition of the RhoA/Rho-associated kinase (ROCK) pathway did not. However, time-lapse studies revealed that, within randomly oriented Col-I matrices, the epithelium axially aligns fibers at branch sites via RhoA/ROCK-mediated contractions. Our data provide an explanation for how the stromal **ECM** encodes architectural cues for branch orientation as well as how the branching epithelium interprets and reinforces these cues through distinct signaling processes.
5. Regulation of neural progenitor proliferation and survival by beta1 integrins. Neural stem cells give rise to undifferentiated nestin-positive progenitors that undergo extensive cell division before differentiating into neuronal and glial cells. The precise control of this process is likely to be, at least in part, controlled by instructive cues originating from the extracellular environment. Some of these cues are interpreted by the integrin family of extracellular matrix receptors. Using neurosphere cell cultures as a model system, we show that beta1-integrin signalling plays a crucial role in the regulation of progenitor cell proliferation, survival and migration. Following conditional genetic ablation of the beta1-integrin allele, and consequent loss of beta1-integrin cell surface protein, mutant nestin-positive progenitor cells proliferate less and die in higher numbers than their wild-type counterparts. Mutant progenitor cell migration on different **ECM** substrates is also impaired. These effects can be partially compensated by the addition of exogenous growth factors. Thus, beta1-integrin signalling and growth factor signalling tightly interact to control the number and migratory capacity of nestin-positive progenitor cells.
6. Alteration of cartilage mechanical properties in absence of β1 integrins revealed by rheometry and FRAP analyses. Mechanical properties are essential for biological functions of the hyaline cartilage such as energy dissipation and diffusion of solutes. Mechanical properties are primarily dependent on the hierarchical organization of the two major extracellular matrix **(EC**M) macromolecular components of the cartilage: the fibrillar collagen network and the glycosaminoglycan (GAG)-substituted proteoglycan, mainly aggrecan, aggregates. Interaction of chondrocytes, the only cell type in the tissue, with the **EC**M through adhesion receptors is involved in establishing mechanical stability via bidirectional transduction of both mechanical forces and chemical signals. In this study, we aimed to determine the role of the transmembrane β1 integrin adhesion receptors in cartilage biomechanical properties by the use of genetic modification in mice.Costal cartilages of wild type and mutant mice lacking β1 integrins in chondrocytes were investigated. Cartilage compressive properties and solute diffusion were characterized by rheometric analysis and Fluorescence Recovery After Photobleaching (FRAP), respectively. Cartilage tissue sections were analyzed by histology, immunohistochemistry and transmission electron microscopy (TEM).At the histological level, the mutant costal cartilage was characterized by chondrocyte rounding and loss of tissue polarity. Immunohistochemistry and safranin orange staining demonstrated apparently normal aggrecan and GAG levels, respectively. Antibody staining for collagen II and TEM showed comparable expression and organization of the collagen fibrils between mutant and control cartilages. Despite the lack of gross histological and ultrastructural abnormalities, rheological measurements revealed that the peak elastic modulus in compression of mutant cartilage was 1.6-fold higher than the peak elastic modulus of wild-type sample. Interestingly, the diffusion coefficient within the mutant cartilage tissue was found to be 1.2-fold lower in the extracellular space and 14-fold lower in the pericellular (PCM) space compared to control.The results demonstrate that the absence of β1 integrins on the surface of chondrocytes increases the stiffness and modifies the diffusion properties of costal cartilage. Our data imply that β1 integrins-mediated chondrocyte-matrix interactions directly affect cartilage biomechanics probably by modifying physical properties of individual cells. This study thus highlights the crucial role of β1 integrins in the cartilage function.
7. Tissue inhibitor of metalloproteinase-2 (TIMP-2) regulates neuromuscular junction development via a beta1 integrin-mediated mechanism. Extracellular matrix (**ECM**) molecules play critical roles in muscle function by participating in neuromuscular junction (NMJ) development and the establishment of stable, cytoskeleton-associated adhesions required for muscle contraction. Matrix metalloproteinases (MMPs) are neutral endopeptidases that degrade all **ECM** components. While the role of MMPs and their inhibitors, the tissue inhibitor of metalloproteinases (TIMPs), has been investigated in many tissues, little is known about their role in muscle development and mature function. TIMP-2 -/- mice display signs of muscle weakness. Here, we report that TIMP-2 is expressed at the NMJ and its expression is greater in fast-twitch (extensor digitorum longus, EDL) than slow-twitch (soleus) muscle. EDL muscle mass is reduced in TIMP-2-/- mice without a concomitant change in fiber diameter or number. The TIMP-2-/- phenotype is not likely due to increased **ECM** proteolysis because net MMP activity is actually reduced in TIMP-2-/- muscle. Most strikingly, TIMP-2 colocalizes with beta1 integrin at costameres in the wild-type EDL and beta1 integrin expression is significantly reduced in TIMP-2-/- EDL. We propose that reduced beta1 integrin in fast-twitch muscle may be associated with destabilized **ECM**-cytoskeletal interactions required for muscle contraction in TIMP-2-/- muscle; thus, explaining the muscle weakness. Given that fast-twitch fibers are lost in muscular dystrophies and age-related sarcopenia, if TIMP-2 regulates mechanotransduction in an MMP-independent manner it opens new potential therapeutic avenues.
8. In vivo analysis of Arg-Gly-Asp sequence/integrin α5β1-mediated signal involvement in embryonic enchondral ossification by exo utero development system. Enchondral ossification is a fundamental mechanism for longitudinal bone growth during vertebrate development. In vitro studies suggested that functional blockade with RGD peptides or with an antibody that interferes with integrin α5β1-ligand interactions inhibited pre-hypertrophic chondrocyte differentiation. The purpose of this study is to elucidate in vivo the roles of the integrin α5β1-mediated signal through the Arg-Gly-Asp (RGD) sequence in the cell-extracellular ma**tri**x (ECM) interaction in embryonic enchondral ossification by an exo utero development system. We injected Arg-Gly-Asp-Ser (RGDS) peptides and anti-integrin α5β1 antibody (α5β1 ab) in the upper limbs of mouse embryos at embryonic day (E) 15.5 (RGDS-injected limbs, α5β1 ab-injected limbs), and compared the effects on enchondral ossification with those found in the control limbs (Arg-Gly-Glu-Ser peptide-, mouse IgG-, or vehicle-injected, and no surgery) at E16.5. In the RGDS-injected limbs, the humeri were shorter and there were fewer BrdU-positive cells than in the control limbs. The ratios of cartilage length and area to those of the humerus were higher in the RGDS-injected limbs. The ratios of type X collagen to type 2 collagen mRNA and protein (Coll X/Coll 2) were significantly lower in the RGDS-injected limbs. In those limbs, TUNEL-positive cells were hardly observed, and the ratios of fractin to the Coll X/Coll 2 ratio were lower than in the control limbs. Furthermore, the α5β1 ab-injected limbs showed results similar to those of RGDS-injected limbs. The present in vivo study by exo utero development system showed that RGDS and α5β1 ab injection decreased chondrocyte proliferation, differentiation, and apoptosis in enchondral ossification, and suggested that the integri**n �**�5β1-mediated ECM signal through the RGD sequence is involved in embryonic enchondral ossification.
9. The spatial and temporal expression of the alpha 2 beta 1 integrin and its ligands, collagen I, collagen IV, and laminin, suggest important roles in mouse mammary morphogenesis. To begin to determine the role of the alpha 2 beta 1 integrin and its ligands, collagen I, collagen IV, and laminin, in mammary epithelial differentiation in vivo, we determined the expression of these molecules by in situ hybridization and immunofluorescence in the developing mouse mammary gland. Expression of collagen I, collagen IV, and laminin mRNAs in the mammary gland during puberty corresponded to the period of greatest growth of the gland, 4-7 weeks postnatally. Collagen I expression preceded collagen IV expression, both of which preceded laminin expression, suggesting an important temporal sequence of extracellular matrix (**ECM**) production. When growth of the epithelium ceased in the adult virgin gland, expression of all three mRNAs became undetectable. Following the onset of pregnancy these molecules were re-expressed with the same chronology observed during puberty. Collagen I, collagen IV, and laminin were expressed by stromal cells immediately surrounding the developing ductal epithelium. Surprisingly, we found no expression of **ECM** components in the epithelial cells, suggesting the mammary epithelium does not synthesize its own basement membrane. The distribution of collagen I was consistent with a role in duct formation, since collagen I was strikingly abundant around larger mammary ducts, but was sparse around growing endbuds or alveoli. Conversely, there was abundant laminin near growing endbuds and around alveoli, and less around large ducts, suggesting its role is different than collagen I. The alpha 2 beta 1 integrin was present on the basal, lateral, and apical surfaces of the mammary epithelium throughout postnatal development and pregnancy. The alpha 2 beta 1 integrin expression was strongest at midpregnancy, suggesting a role for alpha 2 beta 1 integrin in the alveolar formation that occurs at this time. The alpha 2 beta 1 integrin expression decreased dramatically in the lactating gland. Our results suggest that alpha 2 beta 1 integrin interactions with its temporally and spatially regulated ligands, collagen I, collagen IV, and laminin, could play an important role in mammary morphogenesis in vivo.
10. Beta1 integrin expression by podocytes is required to maintain glomerular structural integrity. Integrins are transmembrane heteromeric receptors that mediate interactions between cells and extracellular matrix (**ECM**). beta1, the most abundantly expressed integrin subunit, binds at least 12 alpha subunits. beta1 containing integrins are highly expressed in the glomerulus of the kidney; however their role in glomerular morphogenesis and maintenance of glomerular filtration barrier integrity is poorly understood. To study these questions we selectively deleted beta1 integrin in the podocyte by crossing beta1(flox/flox) mice with podocyte specific podocin-cre mice (pod-Cre), which express cre at the time of glomerular capillary formation. We demonstrate that podocyte abnormalities are visualized during glomerulogenesis of the pod-Cre;beta1(flox/flox) mice and proteinuria is present at birth, despite a grossly normal glomerular basement membrane. Following the advent of glomerular filtration there is progressive podocyte loss and the mice develop capillary loop and mesangium degeneration with little evidence of glomerulosclerosis. By 3 weeks of age the mice develop severe end stage renal failure characterized by both tubulointerstitial and glomerular pathology. Thus, expression of beta1 containing integrins by the podocyte is critical for maintaining the structural integrity of the glomerulus.
11. Patterns of laminins and integrins in the embryonic ventricular zone of the CNS. The extracellular matrix (**ECM**) provides both a physical framework and a microenvironment that supplies instructive signals from the earliest stages of multicellular development. As a first step toward understanding the role of the **ECM** in regulating the behavior of neural stem cells (NSCs), here we show the localization of laminins, a heterotrimeric family of **ECM** molecules expressed in many different stem cell microenvironments, and their corresponding receptors in the embryonic murine ventricular zone (VZ) within which the NSCs undergo symmetrical and asymmetrical divisions required for cortical development. In addition to the presence of laminins containing both the alpha2 and alpha4 chains, we find distinct patterns of **ECM** receptor expression in the VZ and in the overlying cortex. Neural stem cells derived from the VZ express high levels of the integrin laminin receptor alpha6beta1. At developmental stages at which NSCs undergo asymmetrical divisions, integrin beta1 was unevenly distributed in some mitotic pairs at the ventricular wall. These results suggest a significant role in the regulation of NSC fate for laminin/integrin signaling within the microenvironment of the VZ and provide a framework for future molecular and cellular analyses of the role of the **ECM** in neural development.
12. Beta1 integrin deletion from the basal compartment of the mammary epithelium affects stem cells. The mammary gland epithelium comprises two major cell types: basal and luminal. Basal cells interact directly with the extracellular matrix (**ECM**) and express higher levels of the **ECM** receptors, integrins, than luminal cells. We show that deletion of beta1 integrin from basal cells abolishes the regenerative potential of the mammary epithelium and affects mammary gland development. The mutant epithelium was characterized by an abnormal ductal branching pattern and aberrant morphogenesis in pregnancy, although at the end of gestation, the secretory alveoli developed from beta1 integrin-positive progenitors. Lack of beta1 integrin altered the orientation of the basal-cell division axis and in mutant epithelium, in contrast to control tissue, the progeny of beta1 integrin-null basal cells, identified by a genetic marker, was found in the luminal compartment. These results reveal, for the first time, the essential role of the basal mammary epithelial cell-**ECM** interactions mediated by beta1 integrins in the maintenance of a functional stem cell population, mammary morphogenesis and segregation of the two major mammary cell lineages.
13. TGF-beta-1 up-regulates extra-cellular matrix production in mouse hepatoblasts. Fetal liver is the major embryonic hematopoietic organ and is extrinsically colonized by circulating hematopoietic stem cells (HSCs). Integrin beta-1 expression on HSCs is crucial for colonization, suggesting that interaction of Integrin beta-1 with extra-cellular matrix (**ECM**) factors promotes HSC adherence to fetal liver. However, little is known about how **ECM** production is regulated in fetal liver. Here we used flow cytometry to sort fetal liver compartments and detected **ECM** gene and protein expression predominantly in sorted hepatoblasts. mRNA and protein analysis suggested that TGF-beta-1 expressed by hepatoblasts, sinusoid endothelial cells and hematopoietic cells, binds to the TGF-beta receptor type-2 expressed on hepatoblasts to stimulate **ECM** production. Intra-cardiac injection of TGF-inhibitors into mouse embryos dramatically decreased fetal liver **ECM** gene expression. Taken together, our observations suggest that hepatoblasts predominantly produce **ECM** factors under control of TGF-beta-1 in fetal liver.
14. β1A integrin is a master regulator of invadosome organization and function. Invadosomes are adhesion structures involved in tissue invasion that are characterized by an intense actin polymerization-depolymerization associated with β1 and β3 integrins and coupled to extracellular matri**x (**ECM) degradation activity. We induced the formation of invadosomes by expressing the constitutive active form of Src, SrcYF, in different cell types. Use **of** ECM surfaces micropatterned at the subcellular scale clearly showed that in mesenchymal cells, integrin signaling controls invadosome activity. Using β1⁻/⁻ or β3⁻/⁻ cells, it seemed that β1A but not β3 integrins are essential for initiation of invadosome formation. Protein kinase C activity was shown to regulate autoassembly of invadosomes into a ring-like metastructure (rosette), probably by phosphorylation of Ser785 on the β1A tail. Moreover, our study clearly showed that β1A links ac**tin** dynamics and ECM degradation in invadosomes. Finally, a new strategy based on fusion of the photosensitizer KillerRed to the β1A cytoplasmic domain allowed specific and immediate loss of function of β1A, resulting in disorganization and disassembly of invadosomes and formation of focal adhesions.
15. Loss of integrin alpha9beta1 results in defects in proliferation, causing poor re-epithelialization during cutaneous wound healing. The wound microenvironment comprises constituents, such as the extracellular matrix (**ECM**), that regulate with temporal and spatial precision, the migratory, proliferative, and contractility of wound cells. Prompt closure of the wound is an early and critical phase of healing and beta1 integrins are important in this process. We previously reported a marked increase in integrin alpha9beta1 expression in epidermal keratinocytes in cutaneous and corneal wounds. However, the functional role of keratinocyte alpha9beta1 during re-epithelialization is unknown and analysis has been precluded by the lethal phenotype of integrin alpha9beta1 knockout mice. We now report that in conditional integrin alpha9 knockout (K14-alpha9 null) mice, normal proliferation occurs in epidermal keratinocytes and corneal basal cells. Normal epidermal keratinocyte morphology is also retained. However, corneal basal cell morphology and epithelial thickness are altered, suggesting that loss of integrin alpha9beta1 results in abnormal corneal differentiation. In cutaneous wounds, the number of proliferating epidermal keratinocytes is significantly reduced in K14-alpha9 null mice compared with alpha9(fl/-) mice, but not in Cre (control) mice. The decreased keratinocyte proliferation observed in K14-alpha9 null mice negatively impacts healing, resulting in a thinner migrating epithelium, demonstrating that alpha9beta1 is crucial for efficient and proper re-epithelialization during cutaneous wound healing.
16. Band 4.1 proteins regulate integrin-dependent cell spreading. Integrins link the extracellular matrix (**ECM**) to the cytoskeleton to control cell behaviors including adhesion, spreading and migration. Band 4.1 proteins contain 4.1, ezrin, radixin, moesin (FERM) domains that likely mediate signaling events and cytoskeletal reorganization via integrins. However, the mechanisms by which Band 4.1 proteins and integrins are functionally interconnected remain enigmatic. Here we have investigated roles for Band 4.1 proteins in integrin-mediated cell spreading using primary astrocytes as a model system. We demonstrate that Proteins 4.1B and 4.1G show dynamic patterns of sub-cellular localization in astrocytes spreading on fibronectin. During early stages of cell spreading Proteins 4.1B and 4.1G are enriched in **ECM** adhesion sites but become more diffusely localized at later stages of spreading. Combinatorial inactivation of Protein 4.1B and 4.1G expression leads to impaired astrocyte spreading. Furthermore, in exogenous expression systems we show that the isolated Protein 4.1 FERM domain significantly enhances integrin-mediated cell spreading. Protein 4.1B is dispensable for reactive astrogliosis in experimental models of cortical injury, likely due to functional compensation by related Protein 4.1 family members. Collectively, these findings reveal that Band 4.1 proteins are important intracellular components for integrin-mediated cell spreading.
17. Integrins protect cardiomyocytes from ischemia/reperfusion injury. Ischemic damage is recognized to cause cardiomyocyte (CM) death and myocardial dysfunction, but the role of cell-matrix interactions and integrins in this process has not been extensively studied. Expression of α7β1D integrin, the dominant integrin in normal adult CMs, increases during ischemia/reperfusion (I/R), while deficiency of β1 integrins increases ischemic damage. We hypothesized that the forced overexpression of integrins on the CM would offer protection from I/R injury. Tg mice with CM-specific overexpression of integrin α7β1D exposed to I/R had a substantial reduction in infarct size compared with that of α5β1D-overexpressing mice and WT littermate controls. Using isolated CMs, we found that α7β1D preserved mitochondrial membrane potential during hypoxia/reoxygenation (H/R) injury via inhibition of mitochondrial Ca2+ overload but did not alter H/R effects on oxidative stress. Therefore, we assessed Ca2+ handling proteins in the CM and found that β1D integrin colocalized with ryanodine receptor 2 (RyR2) in CM T-tubules, complexed with RyR2 in human and rat heart, and specifically bound to RyR2 amino acids 165-175. Integrins stabilized the RyR2 interdomain interaction, and this stabilization required integrin receptor bindi**ng** to its ECM ligand. These data suggest that α7β1D integrin modifies Ca2+ regulatory pathways and offers a means to protect the myocardium from ischemic injury.
18. Cardiomyocyte contractile status is associated with differences in fibronectin and integrin interactions. Integrins link the extracellular matrix (**ECM**) with the intracellular cytoskeleton and other cell adhesion-associated signaling proteins to function as mechanotransducers. However, direct quantitative measurements of the cardiomyocyte mechanical state and its relationship to the interactions between specific **ECM** proteins and integrins are lacking. The purpose of this study was to characterize the interactions between the **ECM** protein fibronectin (FN) and integrins in cardiomyocytes and to test the hypothesis that these interactions would vary during contraction and relaxation states in cardiomyocytes. Using atomic force microscopy, we quantified the unbinding force (adhesion force) and adhesion probability between integrins and FN and correlated these measurements with the contractile state as indexed by cell stiffness on freshly isolated mouse cardiomyocytes. Experiments were performed in normal physiological (control), high-K(+) (tonically contracted), or low-Ca(2+) (fully relaxed) solutions. Under control conditions, the initial peak of adhesion force between FN and myocyte alpha(3)beta(1)- and/or alpha(5)beta(1)-integrins was 39.6 +/- 1.3 pN. The binding specificity between FN and alpha(3)beta(1)- and alpha(5)beta(1)-integrins was verified by using monoclonal antibodies against alpha(3)-, alpha(5)-, alpha(3) + alpha(5)-, or beta(1)-integrin subunits, which inhibited binding by 48%, 65%, 70%, or 75%, respectively. Cytochalasin D or 2,3-butanedione monoxime (BDM), to disrupt the actin cytoskeleton or block myofilament function, respectively, significantly decreased the cell stiffness; however, the adhesion force and binding probability were not altered. Tonic contraction with high-K(+) solution increased total cell adhesion (1.2-fold) and cell stiffness (27.5-fold) compared with fully relaxed cells with low-Ca(2+) solution. However, it could be partially prevented by high-K(+) bath solution containing BDM, which suppresses contraction by inhibiting the actin-myosin interactions. Thus, our results demonstrate that integrin binding to FN is modulated by the contractile state of cardiac myocytes.
19. Cadherin-11 coordinates cellular migration and extracellular matrix remodeling during aortic valve maturation. Proper remodeling of the endocardial cushions into thin fibrous valves is essential for gestational progression and long-term function. This process involves dynamic interactions between resident cells and their local environment, much of which is not understood. In this study, we show that deficiency of the cell-cell adhesion protein cadherin-11 (Cad-11) results in significant embryonic and perinatal lethality primarily due to valve related cardiac dysfunction. While endocardial to mesenchymal transformation is not abrogated, mesenchymal cells do not homogeneously cellularize the cushions. These cushions remain thickened with disorganized **ECM**, resulting in pronounced aortic valve insufficiency. Mice that survive to adulthood maintain thickened and stenotic semilunar valves, but interestingly do not develop calcification. Cad-11 (-/-) aortic valve leaflets contained reduced Sox9 activity, β1 integrin expression, and RhoA-GTP activity, suggesting that remodeling defects are due to improper migration and/or cellular contraction. Cad-11 deletion or siRNA knockdown reduced migration, eliminated collective migration, and impaired 3D matrix compaction by aortic valve interstitial cells (VIC). Cad-11 depleted cells in culture contained few filopodia, stress fibers, or contact inhibited locomotion. Transfection of Cad-11 depleted cells with constitutively active RhoA restored cell phenotypes. Together, these results identify cadherin-11 mediated adhesive signaling for proper remodeling of the embryonic semilunar valves.
20. Impaired integrin-mediated adhesion contributes to reduced barrier properties in VASP-deficient microvascular endothelium. Recent studies point to a significant role of vasodilator-stimulated phosphoprotein (VASP) in the maintenance of endothelial barrier functions in vivo and in vitro. Moreover, it has been reported that VASP is required for activation of the small GTPase Rac 1. However, little is known whether VASP is involved in the regulation of cell adhesion molecules that are critical for maintenance of the endothelial barrier. Here we demonstrate that impaired barrier properties in VASP-deficient (VASP-/-) microvascular myocardial endothelial cells (MyEnd) correlated with both impaired integrin-mediated adhesion as revealed by laser tweezer trapping and reduced integrin-dependent cell migration. This was paralleled by reduction of focal adhesions at the cell periphery as well as of beta(1)-integrin and VE-cadherin cytoskeletal anchorage. Incubation of MyEnd VASP wt with RGD peptide to block interaction of integrins with extracellular matrix (**ECM**) reduced barrier properties and Rac 1 activity in wt endothelial monolayers mimicking the situation in VASP (-/-) cells under resting conditions. Moreover, cAMP-mediated Rac 1 activation was reduced under conditions of impaired integrin-mediated adhesion in wt cells and cAMP-induced increase in VE-cadherin cytoskeletal anchorage was abolished in VASP (-/-) endothelium. In summary, these data indicate that VASP is required for integrin-mediated adhesion which stabilizes endothelial barrier properties at least in part by facilitating Rac 1 activation.
21. Tissue-type plasminogen activator promotes murine myofibroblast activation through LDL receptor-related protein 1-mediated integrin signaling. The activation of interstitial fibroblasts to become alpha-SMA-positive myofibroblasts is an essential step in the evolution of chronic kidney fibrosis, as myofibroblasts are responsible for the production and deposition of the **ECM** components that are a hallmark of the disease. Here we describe a signaling pathway that leads to this activation. Tissue-type plasminogen activator (tPA) promoted TGF-beta1-mediated alpha-SMA and type I collagen expression in rat kidney interstitial fibroblasts. This fibrogenic effect was independent of its protease activity but required its membrane receptor, the LDL receptor-related protein 1 (LRP-1). In rat kidney fibroblasts, tPA induced rapid LRP-1 tyrosine phosphorylation and enhanced beta1 integrin recruitment by facilitating the LRP-1/beta1 integrin complex formation. Blockade or knockdown of beta1 integrin abolished type I collagen and alpha-SMA expression. Furthermore, inhibition of the integrin-linked kinase (ILK), a downstream effector of beta1 integrin, or disruption of beta1 integrin/ILK engagement, abrogated the tPA action, whereas ectopic expression of ILK mimicked tPA in promoting myofibroblast activation. In murine renal interstitium after obstructive injury, tPA and alpha-SMA colocalized with LRP-1, and tPA deficiency reduced LRP-1/beta1 integrin interaction and myofibroblast activation. These findings show that tPA induces LRP-1 tyrosine phosphorylation, which in turn facilitates the LRP-1-mediated recruitment of beta1 integrin and downstream ILK signaling, thereby leading to myofibroblast activation. This study implicates tPA as a fibrogenic cytokine that promotes the progression of kidney fibrosis.
22. Talin depletion reveals independence of initial cell spreading from integrin activation and traction. Cell spreading, adhesion and remodelling of the extracellular matrix (**ECM**) involve bi-directional signalling and physical linkages between the **ECM**, integrins and the cell cytoskeleton. The actin-binding proteins talin1 and 2 link ligand-bound integrins to the actin cytoskeleton and increase the affinity of integrin for the **ECM**. Here we report that depletion of talin2 in talin1-null (talin1(-/-)) cells did not affect the initiation of matrix-activated spreading or Src family kinase (SFK) activation, but abolished the **ECM**-integrin-cytoskeleton linkage and sustained cell spreading and adhesion. Specifically, focal adhesion assembly, focal adhesion kinase (FAK) signalling and traction force generation on substrates were severely affected. The talin1 head domain restored beta1 integrin activation but only full-length talin1 restored the **ECM**-cytoskeleton linkage and normal cytoskeleton organization. Our results demonstrate three biochemically distinct steps in fibronectin-activated cell spreading and adhesion: (1) fibronectin-integrin binding and initiation of spreading, (2) fast cell spreading and (3) focal adhesion formation and substrate traction. We suggest that talin is not required for initial cell spreading. However, talin provides the important mechanical linkage between ligand-bound integrins and the actin cytoskeleton required to catalyse focal adhesion-dependent pathways.
23. ADAMTS9-Mediated Extracellular Matrix Dynamics Regulates Umbilical Cord Vascular Smooth Muscle Differentiation and Rotation. Despite the significance for fetal nourishment in mammals, mechanisms of umbilical cord vascular growth remain poorly understood. Here, the secreted metalloprotease ADAMTS9 is shown to be necessary for murine umbilical cord vascular development. Restricting it to the cell surface using a gene trap allele, Adamts9(Gt), impaired umbilical vessel elongation and radial growth via reduced versican proteolysis and accumulation of extracellular matrix (**ECM**). Both Adamts9(Gt) and conditional Adamts9 deletion revealed that ADAMTS9 produced by mesenchymal cells acted non-autonomously to regulate smooth muscle cell (SMC) proliferation, differentiation, and orthogonal reorientation during growth of the umbilical vasculature. In Adamts9(Gt/Gt), we observed interference with PDGFRβ signaling via the mitogen-activated protein kinase/extracellular signal-regulated kinase (MAPK/ERK) pathway, which regulates cytoskeletal dynamics during SMC rotation. In addition, we observed disrupted Shh signaling and perturbed orientation of the mesenchymal primary cilium. Thus, **EC**M dynamics is a major influence on umbilical vascular SMC fate, with ADAMTS9 acting as its principal mediator.
24. Inactivation of integrin-β1 prevents the development of polycystic kidney disease after the loss of polycystin-1. Dysregulation of polycystin-1 (PC1) leads to autosomal dominant polycystic kidney disease (ADPKD), a disorder characterized by the formation of multiple bilateral renal cysts, the progressive accumulation of extracellular matrix **(EC**M), and the development of tubulointerstitial fibrosis. Correspondingly, cystic epithelia express higher levels of integrins **(EC**M receptors that control various cellular responses, such as cell proliferation, migration, and survival) that are characteristically altered in cystic cells. To determine whether the altered expression of **EC**M and integrins could establish a pathologic autostimulatory loop, we tested the role of integrin-β1 in vitro and on the cystic development of ADPKD in vivo. Compared with wild-type cells, PC1-depleted immortalized renal collecting duct cells had higher levels of integrin-β1 and fibronectin and displayed increased integrin-mediated signaling in the presence of Mn(2+). In mice, conditional inactivation of integrin-β1 in collecting ducts resulted in a dramatic inhibition of Pkd1-dependent cystogenesis with a concomitant suppression of fibrosis and preservation of normal renal function. Our data provide genetic evidence that a functional integrin-β1 is required for the early events leading to renal cystogenesis in ADPKD and suggest that the integrin signaling pathway may be an effective therapeutic target for slowing disease progression.
25. Fibrillar Type I Collagen Enhances the Differentiation and Proliferation of Myofibroblasts by Lowering α2β1 Integrin Expression in Cardiac Fibrosis. Many studies have shown that α2β1 integrin plays an important role in the development of cardiac fibrosis. However, the mechanism of how α2β1 integrin regulates the differentiation and proliferation of myofibroblasts in cardiac fibrosis through fibrillar collagen (FC) remains uncertain. We established that FC mimicked the 3-dimensional extracellular ma**tri**x (ECM) of fibroblasts from post-myocardial infarction (MI) patients in vivo. This allowed us to explore the differentiation and proliferation of cardiac fibroblasts on FC. Here, we report that low expression of α2β1 integrin increased protein kinase B (AKT) activation and α-smooth muscle actin (α-SMA) expression. This occurred due to the instability of phosphatase and tensin homolog (PTEN) in myofibroblasts on FC. We also demonstrated that FC reduced protein phosphatase type 2A (PP2A) activity of myofibroblasts, which was coincident with low α2β1 integrin expression and activation of AKT, but not mitogen-activated protein kinase (ERK). In addition, knock-down of both β1 integrin and PP2A in fibroblasts promoted differentiation and proliferation via AKT activation and increased α-SMA expression. In summary, our study demonstrated that low α2β1 integrin expression regulated its downstream targets PTEN and AKT via crosstalk with PP2A, a critical cell signaling pathway that permits aberrant differentiation and proliferation of myofibroblasts on FC.
26. The effect of conditional inactivation of beta 1 integrins using twist 2 Cre, Osterix Cre and osteocalcin Cre lines on skeletal phenotype. Skeletal development and growth are complex processes regulated by multiple microenvironmental cues, including integrin-**ECM** interactions. The β1 sub-family of integrins is the largest integrin sub-family and constitutes the main integrin binding partners of collagen I, the major **EC**M component of bone. As complete β1 integrin knockout results in embryonic lethality, studies of β1 integrin function in vivo rely on tissue-specific gene deletions. While multiple in vitro studies indicate that β1 integrins are crucial regulators of osteogenesis and mineralization, in vivo osteoblast-specific perturbations of β1 integrins have resulted in mild and sometimes contradictory skeletal phenotypes. To further investigate the role of β1 integrins on skeletal phenotype, we used the Twist2-Cre, Osterix-Cre and osteocalcin-Cre lines to generate conditional β1 integrin deletions, where Cre is expressed primarily in mesenchymal condensation, pre-osteoblast, and mature osteoblast lineage cells respectively within these lines. Mice with Twist2-specific β1 integrin disruption were smaller, had impaired skeletal development, especially in the craniofacial and vertebral tissues at E19.5, and did not survive beyond birth. Osterix-specific β1 integrin deficiency resulted in viable mice which were normal at birth but displayed early defects in calvarial ossification, incisor eruption and growth as well as femoral bone mineral density, structure, and mechanical properties. Although these defects persisted into adulthood, they became milder with age. Finally, a lack of β1 integrins in mature osteoblasts and osteocytes resulted in minor alterations to femur structure but had no effect on mineral density, biomechanics or fracture healing. Taken together, our data indicate that β1 integrin expression in early mesenchymal condensations play an important role in skeletal ossification, while �**�1** integrin-ECM interactions in pre-osteoblast, odontoblast- and hypertrophic chondryocyte-lineage cells regulate incisor eruption and perinatal bone formation in both intramembranously and endochondrally formed bones in young, rapidly growing mice. In contrast, the osteocalcin-specific β1 integrin deletion had only minor effects on skeletal phenotype.
27. Islet Endothelial Cells Induce Glycosylation and Increase Cell-surface Expression of Integrin β1 in β Cells. The co-culturing of insulinoma and islet-derived endothelial cell (iEC) lines results in the spontaneous formation of free-floating pseudoislets (PIs). We previously showed that iEC-induced PIs display improved insulin expression and secretion in response to glucose stimulation. This improvement was associated with a de novo deposition of extracellular matrix **(E**CM) proteins by iECs in and around the PIs. Here, iEC-induced PIs were used to study the expression and posttranslational modification of th**e E**CM receptor integrin β1. A wide array of integrin β subunits was detected in βTC3 and NIT-1 insulinomas as well as in primary islets, with integrin β1 mRNA and protein detected in all three cell types. Interestingly, the formation of iEC-induced PIs altered the glycosylation patterns of integrin β1, resulting in a higher molecular weight form of the receptor. This form was found in native pancreas but was completely absent in monolayer β-cells. Fluorescence-activated cell sorting analysis of monolayers and PIs revealed a higher expression of integrin β1 in PIs. Antibody-mediated blocking of integrin β1 led to alterations in β-cell morphology, reduced insulin gene expression, and enhanced glucose secretion under baseline conditions. These results suggest that iEC-induced PI formation may alter integrin β1 expression and posttranslational modification by enhancing glycosylation, thereby providing a more physiological culture system for studyi**ng** integrin-ECM interactions in β cells.
28. Sterile Inflammation Enhances **ECM** Degradation in Integrin β1 KO Embryonic Skin. Epidermal knockout of integrin β1 results in complete disorganization of the basement membrane (BM), resulting in neonatal lethality. Here, we report that this disorganization is exacerbated by an early embryonic inflammatory response involving the recruitment of tissue-resident and monocyte-derived macrophages to the dermal-epidermal junction, associated with increased matrix metalloproteinase activity. Remarkably, the skin barrier in the integrin β1 knockout animals is intact, suggesting that this inflammatory response is initiated in a sterile environment. We demonstrate that the molecular mechanism involves de novo expression of integrin αvβ6 in the basal epidermal cells, which activates a TGF-β1 driven inflammatory cascade resulting in upregulation of dermal NF-κB in a Tenascin C-dependent manner. Importantly, treatment of β1 KO embryos in utero with small molecule inhibitors of TGF-βR1 and NF-κB results in marked rescue of the BM defects and amelioration of immune response, revealing an unconventional immuno-protective role for integrin β1 during BM remodeling.
29. Integrin endosomal signalling suppresses anoikis. Integrin-containing focal adhesions transmit extracellular signals across the plasma membrane to modulate cell adhesion, signalling and survival. Although integrins are known to undergo continuous endo/exocytic traffic, the potential impact of endocytic traffic on integrin-induced signals is unknown. Here, we demonstrate that integrin signalling is not restricted to cell-**ECM** adhesions and identify an endosomal signalling platform that supports integrin signalling away from the plasma membrane. We show that active focal adhesion kinase (FAK), an established marker of integrin-**ECM** downstream signalling, localizes with active integrins on endosomes. Integrin endocytosis positively regulates adhesion-induced FAK activation, which is early endosome antigen-1 and small GTPase Rab21 dependent. FAK binds directly to purified endosomes and becomes activated on them, suggesting a role for endocytosis in enhancing distinct integrin downstream signalling events. Finally, endosomal integrin signalling contributes to cancer-related processes such as anoikis resistance, anchorage independence and metastasis.
30. The **ECM** protein nephronectin promotes kidney development via integrin alpha8beta1-mediated stimulation of Gdnf expression. Development of the metanephric kidney crucially depends on proper interactions between cells and the surrounding extracellular matrix. For example, we showed previously that in the absence of alpha8beta1 integrin, invasion by the ureteric bud into the metanephric mesenchyme is inhibited, resulting in renal agenesis. Here we present genetic evidence that the extracellular matrix protein nephronectin is an essential ligand that engages alpha8beta1 integrin during early kidney development. We show that embryos lacking a functional nephronectin gene frequently display kidney agenesis or hypoplasia, which can be traced to a delay in the invasion of the metanephric mesenchyme by the ureteric bud at an early stage of kidney development. Significantly, we detected no defects in extracellular matrix organization in the nascent kidneys of the nephronectin mutants. Instead, we found that Gdnf expression was dramatically reduced in both nephronectin- and alpha8 integrin-null mutants specifically in the metanephric mesenchyme at the time of ureteric bud invasion. We show that this reduction is sufficient to explain the agenesis and hypoplasia observed in both mutants. Interestingly, the reduction in Gdnf expression is transient, and its resumption presumably enables the nephronectin-deficient ureteric buds to invade the metanephric mesenchyme and begin branching. Our results thus place nephronectin and alpha8beta1 integrin in a pathway that regulates Gdnf expression and is essential for kidney development.
31. Impact of extracellular matrix derived from osteoarthritis subchondral bone osteoblasts on osteocytes: role of integrinβ1 and focal adhesion kinase signaling cues. Our recent study indicated that subchondral bone pathogenesis in osteoarthritis (OA) is associated with osteocyte morphology and phenotypic abnormalities. However, the mechanism underlying this abnormality needs to be identified. In this study we investigated the effect of extracellular matrix **(EC**M) produced from normal and OA bone on osteocytic cells function.De-cellularized matrices, resembling the bone provisional **EC**M secreted from primary human subchondral bone osteoblasts (SBOs) of normal and OA patients were used as a model to study the effect on osteocytic cells. Osteocytic cells (MLOY4 osteocyte cell line) cultured on normal and OA derived **EC**Ms were analyzed by confocal microscopy, scanning electron microscopy (SEM), cell attachment assays, zymography, apoptosis assays, qRT-PCR and western blotting. The role of integrinβ1 and focal adhesion kinase (FAK) signaling pathways during these interactions were monitored using appropriate blocking antibodies.Th**e E**CM produced by OA SBOs contained less mineral content, showed altered organization of matrix proteins and matrix structure compared with the matrices produced by normal SBOs. Culture of osteocytic cells on these defective O**A E**CM resulted in a decrease of integrinβ1 expression and the de-activation of FAK cell signaling pathway, which subsequently affected the initial osteocytic cell's attachment and functions including morphological abnormalities of cytoskeletal structures, focal adhesions, increased apoptosis, altered osteocyte specific gene expression and increased Matrix metalloproteinases (MMP-2) and -9 expression.This study provides new insights in understanding how altered OA bone matrix can lead to the abnormal osteocyte phenotypic changes, which is typical in OA pathogenesis.
32. Deletion of hensin/DMBT1 blocks conversion of beta- to alpha-intercalated cells and induces distal renal tubular acidosis. Acid-base transport in the renal collecting tubule is mediated by two canonical cell types: the β-intercalated cell secretes HCO(3) by an apical Cl:HCO(3) named pendrin and a basolateral vacuolar (V)-ATPase. Acid secretion is mediated by the α-intercalated cell, which has an apical V-ATPase and a basolateral Cl:HCO(3) exchanger (kAE1). We previously suggested that the β-cell converts to the α-cell in response to acid feeding, a process that depended on the secretion and deposition of an extracellular matrix protein termed hensin (DMBT1). Here, we show that deletion of hensin from intercalated cells results in the absence of typical α-intercalated cells and the consequent development of complete distal renal tubular acidosis (dRTA). Essentially all of the intercalated cells in the cortex of the mutant mice are canonical β-type cells, with apical pendrin and basolateral or diffuse/bipolar V-ATPase. In the medulla, however, a previously undescribed cell type has been uncovered, which resembles the cortical β-intercalated cell in ultrastructure, but does not express pendrin. Polymerization and deposition of hensin (in response to acidosis) requires the activation of β1 integrin, and deletion of this gene from the intercalated cell caused a phenotype that was identical to the deletion of hensin itself, supporting its critical role in hensin function. Because previous studies suggested that the conversion of β- to α-intercalated cells is a manifestation of terminal differentiation, the present results demonstrate that this differentiation proceeds from HCO(3) secreting to acid secreting phenotypes, a process that requires deposition of hens**in** in the ECM.
33. Loss of fibulin-5 binding to beta1 integrins inhibits tumor growth by increasing the level of ROS. Tumor survival depends in part on the ability of tumor cells to transform the surrounding extracellular matrix (**ECM**) into an environment conducive to tumor progression. Matricellular proteins are secreted into the **ECM** and impact signaling pathways that are required for pro-tumorigenic activities such as angiogenesis. Fibulin-5 (Fbln5) is a matricellular protein that was recently shown to regulate angiogenesis; however, its effect on tumor angiogenesis and thus tumor growth is currently unknown. We report that the growth of pancreatic tumors and tumor angiogenesis is suppressed in Fbln5-null (Fbln5(-/-)) mice compared with wild-type (WT) littermates. Furthermore, we observed an increase in the level of reactive oxygen species (ROS) in tumors grown in Fbln5(-/-) animals. Increased ROS resulted in elevated DNA damage, increased apoptosis of endothelial cells within the tumor, and represented the underlying cause for the reduction in angiogenesis and tumor growth. In vitro, we identified a novel pathway by which Fbln5 controls ROS production through a mechanism that is dependent on beta1 integrins. These results were validated in Fbln5(RGE/RGE) mice, which harbor a point mutation in the integrin-binding RGD motif of Fbln5, preventing its interaction with integrins. Tumor growth and angiogenesis was reduced in Fbln5(RGE/RGE) mice, however treatment with an antioxidant rescued angiogenesis and elevated tumor growth to WT levels. These findings introduce a novel function for Fbln5 in the regulation of integrin-induced ROS production and establish a rationale for future studies to examine whether blocking Fbln5 function could be an effective anti-tumor strategy, alone or in combination with other therapies.
34. Adhesion to the extracellular matrix is required for interleukin-1 beta actions leading to reactive phenotype in rat astrocytes. The extracellular matrix (**ECM**) of the brain is essential for homeostasis and normal functions, but is rapidly remodelled during acute brain injury alongside the development of an inflammatory response driven by the cytokine interleukin (IL)-1. Whether the **ECM** regulates IL-1 actions in astrocytes is completely unknown. The aim of this study was to test the hypothesis that cellular attachment to the **ECM** is a critical mediator of IL-1beta-induced signalling pathways and development of reactive phenotype in astrocytes. Primary rat astrocytes adhered to fibronectin, laminin and fibrillin-1 in an integrin-dependent manner. Attachment to these **ECM** molecules significantly increased IL-1beta-induced activation of extracellular signal-regulated kinase 1/2 (ERK1/2) and inhibition of RhoA and Rho kinase (ROCK), coincident with loss of focal adhesions and cellular morphological changes. Our data demonstrate that the **ECM** regulates IL-1 actions in astrocytes via cross-talk mechanisms between ERK1/2 and RhoA/ROCK, which could have important implications in brain inflammatory disorders.
35. Ephrin B1 maintains apical adhesion of neural progenitors. Apical neural progenitors are polarized cells for which the apical membrane is the site of cell-cell and cell-extracellular matrix adhesion events that are essential for maintaining the integrity of the developing neuroepithelium. Apical adhesion is important for several aspects of the nervous system development, including morphogenesis and neurogenesis, yet the mechanisms underlying its regulation remain poorly understood. Here, we show that ephrin B1, a cell surface protein that engages in cell signaling upon binding cognate Eph receptors, controls normal morphogenesis of the developing cortex. Efnb1-deficient embryos exhibit morphological alterations of the neuroepithelium that correlate with neural tube closure defects. Using loss-of-function experiments by ex vivo electroporation, we demonstrate that ephrin B1 is required in apical progenitors (APs) to maintain their apical adhesion. Mechanistically, we show that ephrin B1 controls cell-**ECM** adhesion by promoting apical localization of integrin β1 and we identify ADP-ribosylation factor 6 (Arf6) as an important effector of ephrin B1 reverse signaling in apical adhesion of APs. Our results provide evidence for an important role for ephrin B1 in maintaining the structural integrity of the developing cortex and highlight the importance of tightly controlling apical cell**-EC**M adhesion for neuroepithelial development.
36. C1GALT1 promotes invasive phenotypes of hepatocellular carcinoma cells by modulating integrin β1 glycosylation and activity. Cancer cell invasion and metastasis are the primary causes of treatment failure and death in hepatocellular carcinoma (HCC). We previously reported that core 1 β1,3-galactosyltransferase (C1GALT1) is frequently overexpressed in HCC tumors and its expression is associated with advanced tumor stage, metastasis, and poor survival. However, the underlying mechanisms of C1GALT1 in HCC malignancy remain unclear. In this study, we found that overexpression of C1GALT1 enhanced HCC cell adhesion to extracellular matrix **(E**CM) proteins, migration, and invasion, whereas RNAi-mediated knockdown of C1GALT1 suppressed these phenotypes. The promoting effect of C1GALT1 on the metastasis of HCC cells was demonstrated in a mouse xenograft model. Mechanistic investigations showed that the C1GALT1-enhanced phenotypic changes in HCC cells were significantly suppressed by anti-integrin β1 blocking antibody. Moreover, C1GALT1 was able to modify O-glycans on integrin β1 and regulate integrin β1 activity as well as its downstream signaling. These results suggest that C1GALT1 could enhance HCC invasiveness through integrin β1 and provide novel insights into the roles of O-glycosylation in HCC metastasis.
37. Polymerase delta-interacting protein 2 regulates collagen accumulation via activation of the Akt/mTOR pathway in vascular smooth muscle cells. Polymerase delta interacting protein 2 (Poldip2) has previously been implicated in migration, proliferation and extracellular matrix (**ECM**) production in vascular smooth muscle cells. To better understand the role of Poldip2 in **ECM** regulation, we investigated the mechanism responsible for collagen I accumulation in Poldip2(+/-) mouse aortic smooth muscle cells (MASMs).Protein degradation and protein synthesis pathways were investigated. Depletion of Poldip2 had no effect on proteasome activity, but caused a partial reduction in autophagic flux. However, the rate of collagen I degradation was increased in Poldip2(+/-) vs. Poldip2(+/+) MASMs. Conversely, activation of the PI3K/Akt/mTOR signaling pathway, involved in regulation of protein synthesis, was significantly elevated in Poldip2(+/-) MASMs as was β1-integrin expression. Suppressing mTOR signaling using Akt inhibitor or rapamycin and reducing β1-integrin expression using siRNA prevented the increase in collagen I production. While collagen I and fibronectin were increased in Poldip2(+/-) MASMs, overall protein synthesis was not different from that in Poldip2(+/)(+)MASMs, suggesting selectivity of Poldip2 fo**r E**CM proteins.Poldip2(+/-) MASMs exhibit higher β1-integrin expression and activity of the PI3K/Akt/mTOR signaling pathway, leading to increas**ed** ECM protein synthesis. These findings have important implications for vascular diseases in whi**ch** ECM accumulation plays a role.
38. miRNA-29c suppresses lung cancer cell adhesion to extracellular matrix and metastasis by targeting integrin β1 and matrix metalloproteinase2 (MMP2). Our pilot study using miRNA arrays found that miRNA-29c (miR-29c) is differentially expressed in the paired low-metastatic lung cancer cell line 95C compared to the high-metastatic lung cancer cell line 95D. Bioinformatics analysis shows that integrin β1 and matrix metalloproteinase 2 (MMP2) could be important target genes of miR-29c. Therefore, we hypothesized that miR-29c suppresses lung cancer cell adhesion to extracellular matrix **(E**CM) and metastasis by targeting integrin β1 and MMP2. The gain-of-function studies that raised miR-29c expression in 95D cells by using its mimics showed reductions in cell proliferation, adhesion **to** ECM, invasion and migration. In contrasts, loss-of-function studies that reduced miR-29c by using its inhibitor in 95C cells promoted proliferation, adhesion **to** ECM, invasion and migration. Furthermore, the dual-luciferase reporter assay demonstrated that miR-29c inhibited the expression of the luciferase gene containing the 3'-UTRs of integrin β1 and MMP2 mRNA. Western blotting indicated that miR-29c downregulated the expression of integrin β1 and MMP2 at the protein level. Gelatin zymography analysis further confirmed that miR-29c decreased MMP2 enzyme activity. Nude mice with xenograft models of lung cancer cells confirmed that miR-29c inhibited lung cancer metastasis in vivo, including bone and liver metastasis. Taken together, our results demonstrate that miR-29c serves as a tumor metastasis suppressor, which suppresses lung cancer cell adhesio**n t**o ECM and metastasis by directly inhibiting integrin β1 and MMP2 expression and by further reducing MMP2 enzyme activity. The results show that miR-29c may be a novel therapeutic candidate target to slow lung cancer metastasis.
39. β1-integrin controls cell fate specification in early lens development. Integrins are heterodimeric cell surface molecules that mediate cell-extracellular matrix **(EC**M) adhesion, **EC**M assembly, and regulation of both **EC**M and growth factor induced signaling. However, the developmental context of these diverse functions is not clear. Loss of β1-integrin from the lens vesicle (mouse E10.5) results in abnormal exit of anterior lens epithelial cells (LECs) from the cell cycle and their aberrant elongation toward the presumptive cornea by E12.5. These cells lose expression of LEC markers and initiate expression of the Maf (also known as c-Maf) and Prox1 transcription factors as well as other lens fiber cell markers. β1-integrin null LECs also upregulate the ERK, AKT and Smad1/5/8 phosphorylation indicative of BMP and FGF signaling. By E14.5, β1-integrin null lenses have undergone a complete conversion of all lens epithelial cells into fiber cells. These data suggest that shortly after lens vesicle closure, β1-integrin blocks inappropriate differentiation of the lens epithelium into fibers, potentially by inhibiting BMP and/or FGF receptor activation. Thus, β1-integrin has an important role in fine-tuning the response of the early lens to the gradient of growth factors that regulate lens fiber cell differentiation.
40. Expression of integrin beta1 by fibroblasts is required for tissue repair in vivo. In tissue repair, fibroblasts migrate into the wound to produce and remodel extracellular matrix (**ECM**). Integrins are believed to be crucial for tissue repair, but their tissue-specific role in this process is poorly understood. Here, we show that mice containing a fibroblast-specific deletion of integrin β1 exhibit delayed cutaneous wound closure and less granulation tissue formation, including reduced production of new **EC**M and reduced expression of α-smooth muscle actin (α-SMA). Integrin-β1-deficient fibroblasts showed reduced expression of type I collagen and connective tissue growth factor, and failed to differentiate into myofibroblasts as a result of reduced α-SMA stress fiber formation. Loss of integrin β1 in adult fibroblasts reduced their ability to adhere to, to spread on and to con**tra**ct ECM. Within stressed collagen matrices, integrin-β1-deficient fibroblasts showed reduced activation of latent TGFβ. Addition of active TGFβ alleviated the phenotype of integrin-β1-deficient mice. Thus integrin β1 is essential for normal wound healing, where it acts, at least in part, through a TGFβ-dependent mechanism in vivo.
41. Transmembrane/cytoplasmic, rather than catalytic, domains of Mmp14 signal to MAPK activation and mammary branching morphogenesis via binding to integrin β1. Epithelial cell invasion through the extracellular matrix **(EC**M) is a crucial step in branching morphogenesis. The mechanisms by which the mammary epithelium integrates cues from the **EC**M with intracellular signaling in order to coordinate invasion through the stroma to make the mammary tree are poorly understood. Because the cell membrane-bound matrix metalloproteinase Mmp14 is known to play a key role in cancer cell invasion, we hypothesized that it could also be centrally involved in integrating signals for mammary epithelial cells (MECs) to navigate the collagen 1 (CL-1)-rich stroma of the mammary gland. Expression studies in nulliparous mice that carry a NLS-lacZ transgene downstream of the Mmp14 promoter revealed that Mmp14 is expressed in MECs at the tips of the branches. Using both mammary organoids and 3D organotypic cultures, we show that MMP activity is necessary for invasion through dense CL-1 (3 mg/ml) gels, but dispensable for MEC branching in sparse CL-1 (1 mg/ml) gels. Surprisingly, however, Mmp14 without its catalytic activity was still necessary for branching. Silencing Mmp14 prevented cell invasion through CL-1 and disrupted branching altogether; it also reduced integrin β1 (Itgb1) levels and attenuated MAPK signaling, disrupting Itgb1-dependent invasion/branching within CL-1 gels. FRET imaging revealed that Mmp14 associates directly with Itgb1. We identified a domain of Mmp14 that is required for modulating the levels of Itgb1, MEC signaling and the rate of invasion within CL-1. These results shed light on hitherto undescribed non-proteolytic activities of Mmp14 that are necessary for the Itgb1-dependent biochemical and mechanical signals that regulate branching in the mammary epithelium.
42. Fibronectin-integrin (alpha5beta1) modulates migration and invasion of murine melanoma cell line B16F10 by involving MMP-9. Cell adhesion to extracellular matrix (**ECM**) initiates signaling cascade regulated by cell surface integrin receptors, which affects the proliferation and invasion of cells. Cells cultured in the presence of **ECM** ligand fibronectin (FN) stimulate secretion of matrix metalloproteinases (MMPs), facilitating cancer cell invasion and metastasis. Among all the members of the MMP family, MMP-9 is of crucial importance in tumor invasion and metastasis. The present study aims at studying the effects of integrin receptor alpha5beta1 and its ligand FN on expression of MMP-9 in murine melanoma cell line B16F10 and understanding the molecular mechanism(s) involved. The main experimental methods performed in the study were gelatin zymography, immunoblot, real-time RT-PCR, immunocytochemistry, enzyme linked immunosorbent assay (ELISA), transwell chamber assay, and in vivo metastasis assay in syngenic (C57BL6J) mice. The study reports that FN induces the activity, mRNA, and protein expression of MMP-9 and initiates its proteolytic activation in B16F10 cells. Blockage of the alpha5 receptor abrogated the FN-mediated stimulatory response on MMP-9 in B16F10 cells. Inhibitor studies and immunoblot analysis strongly suggest the involvement of focal adhesion kinase (FAK), extracellular regulated kinase (ERK), and phosphatidylinositol-3-kinase (PI-3K) in the FN-mediated responses. Immunocytochemical analysis showed the nuclear localization of nuclear factor-kappaB (NF-kappaB) might lead to activation of MMP-9 gene upon FN treatment. This study demonstrates that integrin receptor alpha5beta1 and FN interaction induces the invasive potential of B16F10 cells and MMP-9 induction is the downstream effectors in the process. This system serves as a novel model system to understand the molecular mechanism of melanoma growth and invasion.
43. RECK-Mediated β1-Integrin Regulation by TGF-β1 Is Critical for Wound Contraction in Mice. Fibroblasts are critical for wound contraction; a pivotal step in wound healing. They produce and modify the extracellular matrix **(E**CM) required for the proper tissue remodeling. Reversion-inducing cysteine-rich protein with Kazal motifs (RECK) is a key regulator o**f E**CM homeostasis and turnover. However, its role in wound contraction is presently unknown. Here we describe that Transforming growth factor type β1 (TGF-β1), one of the main pro-fibrotic wound-healing promoting factors, decreases RECK expression in fibroblasts through the Smad and JNK dependent pathways. This TGF-β1 dependent downregulation of RECK occurs with the concomitant increase of β1-integrin, which is required for fibroblasts adhesion and wound contraction through the activation of focal adhesion kinase (FAK). Loss and gain RECK expression experiments performed in different types of fibroblasts indicate that RECK downregulation mediates TGF-β1 dependent β1-integrin expression. Also, reduced levels of RECK potentiate TGF-β1 effects over fibroblasts FAK-dependent contraction, without affecting its cognate signaling. The above results were confirmed on fibroblasts derived from the Reck+/- mice compared to wild type-derived fibroblasts. We observed that Reck+/- mice heal dermal wounds more efficiently than wild type mice. Our results reveal a critical role for RECK in skin wound contraction as a key mediator in the axis: TGF-β1-RECK-β1-integrin.
44. Integrin shedding as a mechanism of cellular adaptation during cardiac growth. Integrin-mediated cell-extracellular matrix (**ECM**) interactions are essential for multiple cellular processes; however, little is known regarding integrin turnover during these events. Recent studies have demonstrated shedding of cell surface molecules and suggested this as a potential mechanism for integrin turnover. Confocal microscopy of mouse hearts under different physiological conditions demonstrated the presence of beta(1)-integrin-immunoreactive material in the interstitium. Culture media from neonatal rat cardiac myocytes and fibroblasts contained a 55-kDa fragment of beta(1)-integrin. Attachment to **ECM** components, response to phorbol 12-myristate 13-acetate stimulation, and matrix metalloproteinase inhibition assays demonstrated that fibroblasts responded differently to the fragment compared with myocytes. The beta(1)-integrin fragment stimulated myocyte attachment to collagen and the fragment itself bound a variety of **ECM** proteins. These studies indicate that as myocytes and fibroblasts change size and shape, cellular contacts with the **ECM** are altered, resulting in the liberation of a beta(1)-integrin fragment from the cell surface. Integrin shedding may represent a novel mechanism of rapidly modifying cell-**ECM** contacts during various cellular processes.
45. Integrin beta1-focal adhesion kinase signaling directs the proliferation of metastatic cancer cells disseminated in the lungs. The development of metastases is an extended and inefficient process involving multiple steps. The last of these involves the growth of micrometastases into macroscopic tumors. We show here that intravenously injected, nonmetastatic cancer cells cease proliferating after extravasating into the parenchyma of the lungs; this response is attributable to the cells inability to trigger adhesion-related signaling events when they are scattered sparsely within the extracellular matrix (**ECM**) of the parenchyma. We recapitulate this situation by culturing these nonmetastatic cells at low seeding density in **ECM**-derived gels in vitro, in which they undergo cell-cycle arrest resulting, in part, from insufficient activation of focal adhesion kinase (FAK). Metastatic cancer cells, in contrast, show sufficient FAK activation to enable their proliferation within **ECM** gels in vitro and continue cell-cycle progression within the lung parenchyma in vivo. Activation of FAK in these metastatic cells depends on expression of the beta(1) subunit of integrins, and proliferation of these cells after extravasation in the lungs is diminished by knocking down the expression of either FAK or integrin beta(1). These results demonstrate the critical role of integrin beta(1)-FAK signaling axis in controlling the initial proliferation of micrometastatic cancer cells disseminated in the lungs.
46. Beta1 integrins regulate myoblast fusion and sarcomere assembly. The mechanisms that regulate the formation of multinucleated muscle fibers from mononucleated myoblasts are not well understood. We show here that extracellular matrix (**ECM**) receptors of the beta1 integrin family regulate myoblast fusion. beta1-deficient myoblasts adhere to each other, but plasma membrane breakdown is defective. The integrin-associated tetraspanin CD9 that regulates cell fusion is no longer expressed at the cell surface of beta1-deficient myoblasts, suggesting that beta1 integrins regulate the formation of a protein complex important for fusion. Subsequent to fusion, beta1 integrins are required for the assembly of sarcomeres. Other **ECM** receptors such as the dystrophin glycoprotein complex are still expressed but cannot compensate for the loss of beta1 integrins, providing evidence that different **ECM** receptors have nonredundant functions in skeletal muscle fibers.
47. β1 integrin is a crucial regulator of pancreatic β-cell expansion. Development of the endocrine compartment of the pancreas, as represented by the islets of Langerhans, occurs through a series of highly regulated events encompassing branching of the pancreatic epithelium, delamination and differentiation of islet progenitors from ductal domains, followed by expansion and three-dimensional organization into islet clusters. Cellular interactions with the extracellular matrix **(E**CM) mediated by receptors of the integrin family are postulated to regulate key functions in these processes. Yet, specific events regulated by these receptors in the developing pancreas remain unknown. Here, we show that ablation of the β1 integrin gene in developing pancreatic β-cells reduces their ability to expand during embryonic life, during the first week of postnatal life, and thereafter. Mice lacking β1 integrin in insulin-producing cells exhibit a dramatic reduction of the number of β-cells to only ∼18% of wild-type levels. Despite the significant reduction in β-cell mass, these mutant mice are not diabetic. A thorough phenotypic analysis of β-cells lacking β1 integrin revealed a normal expression repertoire of β-cell markers, normal architectural organization within islet clusters, and a normal ultrastructure. Global gene expression analysis revealed that ablat**ion** of this ECM receptor in β-cells inhibits the expression of genes regulating cell cycle progression. Collectively, our results demonstrate that β1 integrin receptors function as crucial positive regulators of β-cell expansion.
48. Phagocytic cells of the thymic reticulum interact with thymocytes via extracellular matrix ligands and receptors. We previously showed that, in the context of thymic epithelial cells, thymocyte migration is partially controlled by extracellular matrix (**ECM**)-mediated interactions. Herein we evaluated whether these interactions could be involved in cell migration related events in the context of non-epithelial cells of the thymic microenvironment, the phagocytic cells of the thymic reticulum (PTR). We first showed, by immunocytochemistry, cytofluorometry, and RT-PCR, that PTR produce **ECM** components, including fibronectin and laminin, and express the corresponding integrin-type receptors, VLA-4, VLA-5, and VLA-6. Thymocytes adhere onto PTR monolayers, with immature CD4(+)CD8(+) cells being predominant. Importantly, such an adhesion is partially mediated by **ECM** ligands and receptors, since it was impaired by anti-**ECM** or anti-**ECM** receptor antibodies. Conjointly, our data reveal that the **ECM**-dependence for thymocyte adhesion onto the thymic microenvironment is not restricted to the epithelial cells, being also seen when they encounter non-epithelial phagocytic cells.
49. Laminin α1 is essential for mouse cerebellar development. Laminin α1 (Lama1), which is a subunit of laminin-1 (laminin-111), a heterotrimeri**c E**CM protein, is essential for embryonic development and promotes neurite outgrowth in culture. Because the deletion of Lama1 causes lethality at early embryonic stages in mice, the in vivo role of Lama1 in neural development and functions has not yet been possible to determine. In this study, we generated conditional Lama1 knockout (Lama1(CKO)) mice in the epiblast lineage using Sox2-Cre mice. These Lama1(CKO) mice survived, but displayed behavioral disorders and impaired formation of the cerebellum. Deficiency of Lama1 in the pial basement membrane of the meninges resulted in defects in the conformation of the meninges. During cerebellar development, Lama1 deficiency also caused a decrease in the proliferation and migration of granule cell precursors, disorganization of Bergmann glial fibers and endfeet, and a transient reduction in the activity of Akt. A marked reduction in numbers of dendritic processes in Purkinje cells was observed in Lama1(CKO) mice. Together, these results indicate that Lama1 is required for cerebellar development and functions.
50. β1 integrins restrict the growth of foci and spheroids. Extracellular matrices **(EC**M) have important roles for tissue architecture, both as structural and signaling components. Members of the integrin family are the main regulators of **EC**M assembly and transmitters of signals from the **EC**M to cells. In this study, we have analyzed the role of integrin subunit β1 in two-dimensional (2D) and three-dimensional (3D) cell cultures using integrin β1 null cells (MEFβ1(-/-) and GD25) and their β1 integrin-expressing counterparts. GD25 and GD25β1 cells proliferated with similar kinetics in sub-confluent 2D cultures, whereas GD25 cells attained higher cell numbers in confluent culture and formed foci with fivefold higher frequency than GD25β1 cells. Fibronectin fibrils were abundantly deposited throughout the GD25β1 colonies but strictly limited to the central multilayered area (focus) of GD25 colonies. During 3D growth as spheroids, GD25 continuously increased in size for >21 days while the growth of GD25β1 spheroids ceased after 14 days. Similarly, MEFβ1(-/-) cells formed foci and grew as spheroids, while the β1 integrin-expressing MEF did not. Expression levels of the cell cycle markers Ki67, PCNA, and histone H3-pSer10 were similar between GD25β1 and GD25 spheroids. Apoptotic cells accumulated earlier in GD25 spheroids; however, cell death increased with spheroid volumes in both spheroid types. In both cell systems, the presence of β1 integrins resulted in higher levels of active myosin light chain and inactive myosin light chain phosphatase, and a more compact spheroid structure. In conclusion, our results reveal that regulation of 3D growth in spheroids and foci is dependent on the β1 subfamily of integrins, and suggest that myosin-based spheroid contraction in combination with cell death limits the growth of β1-expressing spheroids.
51. Essential role of alpha 6 integrins in cortical and retinal lamination. Extracellular matrix (**ECM**) is believed to play important roles in many aspects of nervous system development [1]. The laminins are **ECM** glycoproteins expressed in neural tissues and are potent stimulators of neurite outgrowth in vitro [1-3]. Genetic approaches using Drosophila and Caenorhabditis elegans have demonstrated a role for laminin and a laminin receptor in vivo in axon pathfinding and fasciculation, respectively [4,5]. In higher organisms, however, the role of laminins in the development of the nervous system is poorly understood. Integrins alpha 6 beta 1 and alpha 6 beta 4 are major laminin receptors. A role for the alpha 6 integrin in neurulation has been reported in amphibians [6]. We previously described mice lacking integrin alpha 6; these mice died at birth with severe skin blistering [7]. Detailed analyses of integrin alpha 6-/- mice reported here revealed abnormalities in the laminar organization of the developing cerebral cortex and retina. Ectopic neuroblastic outgrowths were found on the brain surface and in the vitreous body in the eye. Alterations of laminin deposition were found in mutant brains. Thus, this study provides evidence for an essential role of integrin-laminin interactions in the proper development of the nervous system. These observations are particularly significant given the recent report that human patients suffering from epidermolysis bullosa can carry mutations in ITGA6, the gene encoding the alpha 6 integrin chain [8,9].
52. Collagen-binding integrin alpha1beta1 regulates intestinal inflammation in experimental colitis. Central to inflammatory responses are the integrin-mediated adhesive interactions of cells with their **ECM**-rich environment. We investigated the role of the collagen-binding integrin alpha(1)beta(1) in intestinal inflammation using the mouse model of colitis induced by dextran sodium sulfate (DSS). mAb's directed against murine alpha(1) were found to significantly attenuate inflammation and injury in DSS-treated wild-type mice; similar protection was seen in mice deficient for alpha(1)beta(1) integrin. Blockade or loss of alpha(1)beta(1) was also associated with decreased mucosal inflammatory cell infiltrate and cytokine production. Importantly, we demonstrated that development and alpha(1)-mediated inhibition of DSS-induced colitis occurred independently of lymphocytes (Rag-2(-/-) mice), and identified the monocyte as a key alpha(1)beta(1)-expressing cell type involved in the development of colitis in this model. In response to DSS, both alpha(1) deficiency and anti-alpha(1) mAb treatment significantly reduced monocyte accumulation and activation within the lamina propria. In summary, the data demonstrate that engagement of leukocyte-associated alpha(1)beta(1) receptors with **ECM** plays a pivotal role in mediating intestinal inflammation via promotion of monocyte movement and/or activation within the inflamed interstitium. Therapeutic strategies designed to disrupt such interactions may prove beneficial in treating intestinal inflammation.
53. Epithelial cell alpha3beta1 integrin links beta-catenin and Smad signaling to promote myofibroblast formation and pulmonary fibrosis. Pulmonary fibrosis, in particular idiopathic pulmonary fibrosis (IPF), results from aberrant wound healing and scarification. One population of fibroblasts involved in the fibrotic process is thought to originate from lung epithelial cells via epithelial-mesenchymal transition (EMT). Indeed, alveolar epithelial cells (AECs) undergo EMT in vivo during experimental fibrosis and ex vivo in response to TGF-beta1. As the **ECM** critically regulates AEC responses to TGF-beta1, we explored the role of the prominent epithelial integrin alpha3beta1 in experimental fibrosis by generating mice with lung epithelial cell-specific loss of alpha3 integrin expression. These mice had a normal acute response to bleomycin injury, but they exhibited markedly decreased accumulation of lung myofibroblasts and type I collagen and did not progress to fibrosis. Signaling through beta-catenin has been implicated in EMT; we found that in primary AECs, alpha3 integrin was required for beta-catenin phosphorylation at tyrosine residue 654 (Y654), formation of the pY654-beta-catenin/pSmad2 complex, and initiation of EMT, both in vitro and in vivo during the fibrotic phase following bleomycin injury. Finally, analysis of lung tissue from IPF patients revealed the presence of pY654-beta-catenin/pSmad2 complexes and showed accumulation of pY654-beta-catenin in myofibroblasts. These findings demonstrate epithelial integrin-dependent profibrotic crosstalk between beta-catenin and Smad signaling and support the hypothesis that EMT is an important contributor to pathologic fibrosis.
54. Integrins control dendritic spine plasticity in hippocampal neurons through NMDA receptor and Ca2+/calmodulin-dependent protein kinase II-mediated actin reorganization. The formation of dendritic spines during development and their structural plasticity in the adult brain are critical aspects of synaptogenesis and synaptic plasticity. Many different factors and proteins have been shown to control dendritic spine development and remodeling (Ethell and Pasquale, 2005). The extracellular matrix (**ECM**) components and their cell surface receptors, integrins, have been found in the vicinity of synapses and shown to regulate synaptic efficacy and play an important role in long-term potentiation (Bahr et al., 1997; Chavis and Westbrook, 2001; Chan et al., 2003; Lin et al., 2003; Bernard-Trifilo et al., 2005). Although molecular mechanisms by which integrins affect synaptic efficacy have begun to emerge, their role in structural plasticity is poorly understood. Here, we show that integrins are involved in spine remodeling in cultured hippocampal neurons. The treatment of 14 d in vitro hippocampal neurons with arginine-glycine-aspartate (RGD)-containing peptide, an established integrin ligand, induced elongation of existing dendritic spines and promoted formation of new filopodia. These effects were also accompanied by integrin-dependent actin reorganization and synapse remodeling, which were partially inhibited by function-blocking antibodies against beta1 and beta3 integrins. This actin reorganization was blocked with the NMDA receptor (NMDAR) antagonist MK801 [(+)-5-methyl-10,11-dihydro-5H-dibenzo[a,d]cyclohepten-5,10-imine hydrogen maleate]. The Ca2+/calmodulin-dependent protein kinase II (CaMKII) inhibitor KN93 (N-[2-[N-(4-chlorocinnamyl)-N-methylaminomethyl]phenyl]-N-(2-hydroxyethyl)-4-methoxybenzenesulfonamide) also suppressed RGD-induced actin reorganization and synapse remodeling. Our findings show that integrins control **ECM**-mediated spine remodeling in hippocampal neurons through NMDAR/CaMKII-dependent actin reorganization.
55. MT1-MMP-dependent control of skeletal stem cell commitment via a β1-integrin/YAP/TAZ signaling axis. In vitro, topographical and biophysical cues arising from the extracellular matrix **(EC**M) direct skeletal stem cell (SSC) commitment and differentiation. However, the mechanisms by which the SSC**-EC**M interface is regulated and the outcome of such interactions on stem cell fate in vivo remain unknown. Here we demonstrate that conditional deletion of the membrane-anchored metalloproteinase MT1-MMP (Mmp14) in mesenchymal progenitors, but not in committed osteoblasts, redirects SSC fate decisions from osteogenesis to adipo- and chondrogenesis. By effecting **EC**M remodeling, MT1-MMP regulates stem cell shape, thereby activating a β1-integrin/RhoGTPase signaling cascade and triggering the nuclear localization of the transcriptional coactivators YAP and TAZ, which serve to control SSC lineage commitment. These data identify a critical MT1-MMP/integrin/YAP/TAZ axis operative in the stem cell niche that oversees SSC fate determination.
56. β1 integrin inhibition elicits a prometastatic switch through the TGFβ-miR-200-ZEB network in E-cadherin-positive triple-negative breast cancer. Interactions with the extracellular matrix **(E**CM) through integrin adhesion receptors provide cancer cells with physical and chemical cues that act together with growth factors to support survival and proliferation. Antagonists that target integrins containing the β1 subunit inhibit tumor growth and sensitize cells to irradiation or cytotoxic chemotherapy in preclinical breast cancer models and are under clinical investigation. We found that the loss of β1 integrins attenuated breast tumor growth but markedly enhanced tumor cell dissemination to the lungs. When cultured in three-dimensio**nal** ECM scaffolds, antibodies that blocked β1 integrin function or knockdown of β1 switched the migratory behavior of human and mouse E-cadherin-positive triple-negative breast cancer (TNBC) cells from collective to single cell movement. This switch involved activation of the transforming growth factor-β (TGFβ) signaling network that led to a shift in the balance between miR-200 microRNAs and the transcription factor zinc finger E-box-binding homeobox 2 (ZEB2), resulting in suppressed transcription of the gene encoding E-cadherin. Reducing the abundance of a TGFβ receptor, restoring the ZEB/miR-200 balance, or increasing the abundance of E-cadherin reestablished cohesion in β1 integrin-deficient cells and reduced dissemination to the lungs without affecting growth of the primary tumor. These findings reveal that β1 integrins control a signaling network that promotes an epithelial phenotype and suppresses dissemination and indicate that targeting β1 integrins may have undesirable effects in TNBC.
57. Localization of extracellular matrix molecules, integrins and their regulators, TGF betas, is correlated with axon pathfinding in the spinal cord of normal and Danforth's short tail mice. To clarify the roles of extracellular matrix molecules (**ECM**s) and their receptors, integrins, in axonal pathfinding, their spatiotemporal localization was studied immunohistochemically in the developing mouse spinal cord. Immunoreactivities for collagen type IV (Col IV), laminin, integrins (alpha2, alpha3 and beta1 subunits), and transforming growth factor (TGF) betas (TGF beta2 and TGF beta3), putative regulators of gene expression of these molecules, were localized on the midline of the ventral spinal cord, or the 'floor plate', in normal ICR mice. These immunoreactions appeared at embryonic day 9 (E9), when commissural axons began to cross the floor plate, and peaked at E13-E14 when more axons crossed, turned rostrally, and projected longitudinally. At this stage, TAG-1-immunopositive commissural axons passed through the floor plate region. Longitudinal axons, on the other hand, projected ipsilaterally parallel to the midline. Immunoreactions for **ECM**s, integrins and TGF betas were weakly positive at the midline until postnatal day 0 (P0), after which they decreased markedly. In Danforth's short tail (Sd) heterozygous mutants with discontinuous floor plates, immunoreactions for **ECM**s, integrins, and TGF betas were not detected at P0. Few crossing axons were seen at this stage, and longitudinally ascending axons crossed via aberrant paths in these regions. These results suggest that the restricted localization of **ECM**s, integrins, and TGF betas at the floor plate is involved in the pathfinding of commissural and longitudinal axons.
58. Tissue inhibitor of metalloproteinase-2 (TIMP-2) regulates myogenesis and beta1 integrin expression in vitro. Myogenesis in vitro involves myoblast cell cycle arrest, migration, and fusion to form multinucleated myotubes. Extracellular matrix (**ECM**) integrity during these processes is maintained by the opposing actions of matrix metalloproteinase (MMP) proteases and their inhibitors, the tissue inhibitor of metalloproteinases (TIMPs). Here, we report that TIMP-2, MMP-2, and MT1-MMP are differentially expressed during mouse myoblast differentiation in vitro. A specific role for TIMP-2 in myogenesis is demonstrated by altered TIMP-2(-/-) myotube formation. When differentiated in horse serum-containing medium, TIMP-2(-/-) myotubes are larger than wild-type myotubes. In contrast, when serum-free medium is used, TIMP-2(-/-) myotubes are smaller than wild-type myotubes. Regardless of culture condition, myotube size is directly correlated with MMP activity and inversely correlated with beta1 integrin expression. Treatment with recombinant TIMP-2 rescues reduced TIMP-2(-/-) myotube size and induces increased MMP-9 activation and decreased beta1 integrin expression. Treatment with either MMP-2 or MMP-9 similarly rescues reduced myotube size, but has no effect on beta1 integrin expression. These data suggest a specific regulatory relationship between TIMP-2 and beta1 integrin during myogenesis. Elucidating the role of TIMP-2 in myogenesis in vitro may lead to new therapeutic options for the use of TIMP-2 in myopathies and muscular dystrophies in vivo.
59. Collagen I regulates the self-renewal of mouse embryonic stem cells through α2β1 integrin- and DDR1-dependent Bmi-1. Adhesion of cells to extracellular matrix **(E**CM) influences vital aspects of anchorage-dependent cell behavior including survival, proliferation, and differentiation. However, the role of collagen I in mouse embryonic stem cells (mESCs) is not well-known. Therefore, in the present study we examined the effect of collagen I on mESC self-renewal and related signal pathways. Collagen I (10 µg/ml) maintained mESCs in an undifferentiated state (Nanog, OCT4, and SSEA-1) and did not affect differentiation (GATA4, Tbx5, Fgf5, and Cdx2) in the presence of leukemia inhibitory factor (LIF). Treatment with collagen I bound both α2β1 integrin and discoidin domain receptor 1 (DDR1), and stimulated intracellular signaling pathways. Collagen I-bound α2β1 integrin increased integrin-linked kinase (ILK) phosphorylation, cleaved Notch protein expression in the nuclear fraction, and Gli-1 mRNA expression. In addition, collagen I-bound DDR1 increased GTP-bound Ras, phosphoinositide 3-kinase (PI3K) p85α catalytic subunit protein expression, and Akt and ERK phosphorylation. Importantly, collagen I increased Bmi-1 protein expression in the nucleus which was blocked by small interfering RNA (siRNA) specific for Gli-1 and ERK, showing that parallel pathways of integrins and DDR1 merge at Bmi-1. Furthermore, collagen I-induced p16 decrease and p-Rb increase were reversed by Bmi-1-specific siRNA. Moreover, Bmi-1 silencing abolished the collagen I-induced increase of proliferation indices and undifferentiation markers. These results indicate that collagen I stimulates the self-renewal of mESCs mediated by Bmi-1 through α2β1 integrin-dependent ILK, Notch, Gli-1, and DDR1-dependent Ras, PI3K/Akt, and ERK.
60. A new in vitro model of murine mesoderm migration: the role of fibronectin and laminin. Examination of the factors involved in primary mesodermal migration in the mouse has been complicated by the lack of a suitable in vitro model. We have developed a new culture system using primitive streak stage embryos denuded of primitive endoderm, which allows easy observation and manipulation of the outgrowing cells. The cells migrating away from these explants were shown by immunocytochemistry to express vimentin and an epitope of the I antigen recognised by the antibody C6, both of which are present on the newly emerged mesoderm and not on the embryonic ectoderm in sections of embryos in utero. Conversely, cytokeratin, stage-specific embryonic antigen 1 (SSEA-1), E-cadherin and desmoplakin are expressed by the embryonic ectoderm but lost during mesoderm formation in vivo. They are absent or expressed very weakly by the migrated cells in vitro. In addition, only explants of the ectoplacental cone (EPC) and visceral endoderm alone, expressed a carbohydrate epitope (recognised by monoclonal antibody BOO6), characteristic of the EPC and primitive endoderm in utero, but absent from mesoderm. Thus we conclude that the cells which outgrow in this system are indeed mesodermal in phenotype. We have confirmed the work of others in demonstrating the presence of fibronectin (FN) and laminin (LN) in the migratory path of the mesoderm, at the ectoderm-visceral endoderm interface. We also report that the beta 1 integrin subunit of the FN and LN receptor is expressed by mesodermal cells at this interface. Using our in vitro model we have examined the role of the extracellular matrix (**ECM**) in mesodermal migration. Mesodermal cells migrate further and faster on substrates coated with FN or LN, and this increased migration is abolished by appropriate blocking antibodies. We conclude that the **ECM**, in particular FN and LN, plays an important role in the migration of primary mesodermal cells during gastrulation in the mouse embryo.
61. Functionally distinct PI 3-kinase pathways regulate myelination in the peripheral nervous system. The PI 3-kinase (PI 3-K) signaling pathway is essential for Schwann cell myelination. Here we have characterized PI 3-K effectors activated during myelination by probing myelinating cultures and developing nerves with an antibody that recognizes phosphorylated substrates for this pathway. We identified a discrete number of phospho-proteins including the S6 ribosomal protein (S6rp), which is down-regulated at the onset of myelination, and N-myc downstream-regulated gene-1 (NDRG1), which is up-regulated strikingly with myelination. We show that type III Neuregulin1 on the axon is the primary activator of S6rp, an effector of mTORC1. In contrast, laminin-2 in the extracellular matrix (**ECM**), signaling through the α6β4 integrin and Sgk1 (serum and glucocorticoid-induced kinase 1), drives phosphorylation of NDRG1 in the Cajal bands of the abaxonal compartment. Unexpectedly, mice deficient in α6β4 integrin signaling or Sgk1 exhibit hypermyelination during development. These results identify functionally and spatially distinct PI 3-K pathways: an early, pro-myelinating pathway driven by axonal Neuregulin1 and a later-acting, laminin-integrin-dependent pathway that negatively regulates myelination.
62. Integrin-laminin interactions controlling neurite outgrowth from adult DRG neurons in vitro. A prerequisite for axon regeneration is the interaction between the growth cone and the extracellular matrix (**ECM**). Laminins are prominent constituents of **ECM** throughout the body, known to support axon growth in vitro and in vivo. The regenerative capacity of adult neurons is greatly diminished compared to embryonic or early postnatal neurons. Since most lesions in the nervous system occur in the adult, we have examined neurite outgrowth from adult mouse DRG neurons on four laminin isoforms (laminin-1/LM-111, laminin-2/LM-211, laminin-8/LM-411 and laminin-10/LM-511) in vitro. The growth on laminin-1 and -10 was trophic factor-independent and superior to the one on laminin-2 and -8, where growth was very poor in the absence of neurotrophins. Among other **ECM** proteins, laminins were by far the most active molecules. Using function-blocking antibodies to laminin-binding integrins, we identified non-overlapping functions of integrins alpha3beta1, alpha7beta1 and alpha6beta1 on different laminin isoforms, in that alpha3beta1 and alpha7beta1 integrins appeared to be specific receptors for both laminin-1 and-2, whereas integrin alpha6beta1 was a receptor for laminin-8 and-10. Lastly, by use of immunohistochemistry, expression of subunits of laminin-1, -2, -8 and -10 in sensory organs in the human epidermis could be demonstrated, supporting an important role for these laminins in relation to primary sensory axons.
63. Integrin β1 is necessary for the maintenance of corneal structural integrity. The precise role of a normal keratocyte in maintaining corneal structural integrity is unclear; it is generally considered to remain quiescent at the end of cell division. Given that integrins are essential for cell/extracellular matrix interactions, the authors tested the hypothesis that integrin expression by keratocytes is essential for corneal structure and function.Using a tamoxifen-dependent cre recombinase expressed under the control of a fibroblast-specific promoter/enhancer, the authors conditionally deleted the integrin β1 (Itgb1) gene in mouse keratocytes during the postnatal matrix maturation phase of the cornea. The effects of this deletion were monitored histologically and by macroscopic observation of the cornea.The resultant cornea shows an initial thinning of the stroma, reduced space between collagen fibrils, loss of epithelial layers and subsequent edema, thickening of Descemet's membrane, and degenerative changes in the endothelial cell layer, with eventual scarring. These pathologic changes have some similarities to human corneal disease keratoconus. The phenotype did not develop when Itgb1 was deleted after complete corneal maturation.Loss of integrin β1 expression in keratocytes during the phase of stromal maturation results in corneal thinning and edema. Keratocy**te-**ECM interaction is essential for matrix maturation and thus in the maintenance of corneal structural integrity. This model has relevance in understanding corneal diseases such as keratoconus.
64. Rac1 GTPase-deficient mouse lens exhibits defects in shape, suture formation, fiber cell migration and survival. Morphogenesis and shape of the ocular lens depend on epithelial cell elongation and differentiation into fiber cells, followed by the symmetric and compact organization of fiber cells within an enclosed extracellular matrix-enriched elastic capsule. The cellular mechanisms orchestrating these different events however, remain obscure. We investigated the role of the Rac1 GTPase in these processes by targeted deletion of expression using the conditional gene knockout (cKO) approach. Rac1 cKO mice were derived from two different Cre (Le-Cre and MLR-10) transgenic mice in which lens-specific Cre expression starts at embryonic day 8.75 and 10.5, respectively, in both the lens epithelium and fiber cells. The Le-Cre/Rac1 cKO mice exhibited an early-onset (E12.5) and severe lens phenotype compared to the MLR-10/Rac1 cKO (E15.5) mice. While the Le-Cre/Rac1 cKO lenses displayed delayed primary fiber cell elongation, lenses from both Rac1 cKO strains were characterized by abnormal shape, impaired secondary fiber cell migration, sutural defects and thinning of the posterior capsule which often led to rupture. Lens fiber cell N-cadherin/β-catenin/Rap1/Nectin-based cell-cell junction formation and WAVE-2/Abi-2/Nap1-regulated actin polymerization were impaired in the Rac1 deficient mice. Additionally, the Rac1 cKO lenses were characterized by a shortened epithelial sheet, reduced levels of extracellular matrix **(EC**M) proteins and increased apoptosis. Taken together, these data uncover the essential role of Rac1 GTPase activity in establishment and maintenance of lens shape, suture formation and capsule integrity, and in fiber cell migration, adhesion and survival, via regulation of actin cytoskeletal dynamics, cell adhesive interactions and **EC**M turnover.
65. Gradient of integrin alpha 6A distribution in the myocardium during early heart development. The interactions of cells with extracellular matrices (**ECM**)1 are likely to be key determinants of embryonic development. Integrin adhesion receptors are ideally positioned to mediate some of these interactions since, in addition to mechanical adhesion, they transduce signals affecting cell proliferation and differentiation. We investigated expression of the integrin alpha 6 beta 1, a receptor for the **ECM** component, laminin in the early mouse embryo. An intriguing feature of this integrin is the existence of alpha 6 subunit isoforms. The A and B isoforms, which differ in the cytoplasmic tails, are expressed in cell-type specific fashion, and are likely to implement distinct cellular interactions with laminin. By RT-PCR, alpha 6B but not alpha 6A mRNA was detectable in embryo extracts from fertilized oocytes to 6.5 d.p.c. In subsequent stages, up to 11.5 d.p.c., alpha 6A mRNA was observed in mRNA extracts from whole embryos, but still in significantly lower amounts than alpha 6B. However, in extracts from isolated heart (9.5 to 11.5 d.p.c.), alpha 6A was the predominant alpha 6 isoform, while in extracts from other embryo parts no alpha 6A mRNA was detectable. At the protein level, immunostaining with specific antibodies showed alpha 6A protein in myocardial cells, at the early stage of heart tube development (8.5 d.p.c.). Localization to the myocardium was tightly restricted, since other structures of the embryonic heart, e.g., endocardium, or of the remaining embryo did not stain with anti-alpha 6A antibody. In the ventricular myocardium, expression of alpha 6A appeared more intense than in the subendocardial layer. Quantitation by confocal microscopy unveiled a gradient of expression of alpha 6A, increasing from the outer to the inner layers of the myocardium. This is the first demonstration of a gradient distribution of integrin molecules in a tissue, which appears to be directly connected with the process of organogenesis. The mechanism underlying our observations is not the turning on of a gene, rather it is the activation of a splicing mechanism that substitutes the cytoplasmic domain of a laminin receptor. Because integrin cytoplasmic domains are thought to be an important functional end of the molecule, this may be a mechanism to modulate cellular responses to laminin.

#### GeneRIFs

1. results suggest that iEC-induced PI formation may alter integrin beta1 expression and posttranslational modification by enhancing glycosylation, thereby providing a more physiological culture system for studying integrin-**ECM** interactions in beta cells
2. Data indicate that osteocytic cells on defective osteoarthritis (OA) extracellular matrix (**ECM**) resulted in a decrease of integrinbeta1 expression and the de-activation of focal adhesion kinase cell signaling pathway.
3. miR-29c serves as a tumor metastasis suppressor, which suppresses lung cancer cell adhesion to **ECM** and metastasis by directly inhibiting integrin beta1 and MMP2 expression
4. analysis of the essential role of the basal mammary epithelial cell-**ECM** interactions mediated by beta1 integrins in the maintenance of a functional stem cell populati
5. Data demonstrate that engagement of leukocyte-associated alpha(1)beta(1) integrins with **ECM** plays a pivotal role in mediating intestinal inflammation via promotion of monocyte movement and/or activation within the inflamed interstitium.
6. We propose that reduced beta1 integrin in fast-twitch muscle may be associated with destabilized **ECM**-cytoskeletal interactions required for muscle contraction in TIMP-2-/- muscle; thus, explaining the muscle weakness.

### [Ee]xtracellular [Mm]atrix

#### PubMed

1. Cell adhesion strengthening: contributions of adhesive area, integrin binding, and focal adhesion assembly. Mechanical interactions between a cell and its environment regulate migration, contractility, gene expression, and cell fate. We integrated micropatterned substrates to engineer adhesive area and a hydrodynamic assay to analyze fibroblast adhesion strengthening on fibronectin. Independently of cell spreading, integrin binding and focal adhesion assembly resulted in rapid sevenfold increases in adhesion strength to steady-state levels. Adhesive area strongly modulated adhesion strength, integrin binding, and vinculin and talin recruitment, exhibiting linear increases for small areas. However, above a threshold area, adhesion strength and focal adhesion assembly reached a saturation limit, whereas integrin binding transitioned from a uniform distribution to discrete complexes. Adhesion strength exhibited exponential increases with bound integrin numbers as well as vinculin and talin recruitment, and the relationship between adhesion strength and these biochemical events was accurately described by a simple mechanical model. Furthermore, adhesion strength was regulated by the position of an adhesive patch, comprised of bound integrins and cytoskeletal elements, which generated a constant 200-nN adhesive force. Unexpectedly, focal adhesion assembly, in particular vinculin recruitment, contributed only 30% of the adhesion strength. This work elucidates the roles of adhesive complex size and position in the generation of cell-**extracellular matrix** forces.
2. Integrin-β5 and zyxin mediate formation of ventral stress fibers in response to transforming growth factor β. Cell adhesion to th**e extracellular matr**ix is an essential element of various biological processes. TGF-β cytokines regulate the matrix components and cell-matrix adhesions. The present study investigates the molecular organization of TGF-β-induced matrix adhesions. The study demonstrates that in various mouse and human epithelial cells TGF-β induces cellular structures containing 2 matrix adhesions bridged by a stretch of actin fibers. These structures are similar to ventral stress fibers (VSFs). Suppression of integrin-β5 by RNA interference reduces VSFs in majority of cells (> 75%), while overexpression of integrin-β5 fragments revealed a critical role of a distinct sequence in the cytoplasmic domain of integrin-β5 in the VSF structures. In addition, the integrity of actin fibers and Src kinase activity contribute to integrin-β5-mediated signaling and VSF formation. TGF-β-Smad signaling upregulates actin-regulatory proteins, such as caldesmon, zyxin, and zyxin-binding protein Csrp1 in mouse and human epithelial cells. Suppression of zyxin markedly inhibits formation of VSFs in response to TGF-β and integrin-β5. Zyxin is localized at actin fibers and matrix adhesions of VSFs and might bridge integrin-β5-mediated adhesions to actin fibers. These findings provide a platform for defining the molecular mechanism regulating the organization and activities of VSFs in response to TGF-β.
3. Serum response factor is required for cell contact maintenance but dispensable for proliferation in visceral yolk sac endothelium. Endothelial-specific knockout of the transcription factor serum response factor (SRF) results in embryonic lethality by mid-gestation. The associated phenotype exhibits vascular failure in embryos as well as visceral yolk sac (VYS) tissues. Previous data suggest that this vascular failure is caused by alterations in cell-cell and cell-matrix contacts. In the current study, we sought to more carefully address the role of SRF in endothelial function and cell contact interactions in VYS tissues.Tie2-Cre recombinase-mediated knockout of SRF expression resulted in loss of detectable SRF from VYS mesoderm by E12.5. This loss was accompanied by decreased expression of smooth muscle alpha-actin as well as vascular endothelial cadherin and claudin 5, endothelial-specific components of adherens and tight junctions, respectively. Focal adhesion (FA) integrins alpha5 and beta1 were largely unchanged in contrast to loss of the FA-associated molecule vinculin. The integrin binding partner fibronectin-1 was also profoundly decreased in the **extracellular matrix**, indicating another aspect of impaired adhesive function and integrin signaling. Additionally, cells in SRF-null VYS mesoderm failed to reduce proliferation, suggesting not only that integrin-mediated contact inhibition is impaired but also that SRF protein is not required for proliferation in these cells.Our data support a model in which SRF is critical in maintaining functional cell-cell and cell-matrix adhesion in endothelial cells. Furthermore, we provide evidence that supports a model in which loss of SRF protein results in a sustained proliferation defect due in part to failed integrin signaling.
4. The spatial and temporal expression patterns of integrin alpha9beta1 and one of its ligands, the EIIIA segment of fibronectin, in cutaneous wound healing. The fibronectins (FN) comprise a family of adhesive **extracellular matrix** proteins thought to mediate important functions in cutaneous wounds. Plasma fibronectin (pFN) extravasates for days from intact hyperpermeable vessels following injury whereas mRNAs encoding the cellular fibronectins (cFN) that include two segments, termed EIIIA (EDA) and EIIIB (EDB), are expressed by wound cells. Wounds in mice null for pFN appear to heal normally whereas those in EIIIA null mice exhibit defects, suggesting that cFN may play a role when pFN is missing. Integrin alpha9beta1, a receptor for several **extracellular matrix** proteins as well as the EIIIA segment, is expressed normally in the basal layer of squamous epithelia. We report results from immunohistochemistry on healing wounds demonstrating that EIIIA-containing cFN are deposited abundantly but transiently from day 4 to 7 whereas EIIIB-containing cFN persist at least through day 14. Elevated expression of alpha9beta1 is seen in basal and suprabasal epidermal keratinocytes in wounds. The spatial expression patterns of cFN and alpha9beta1 are distinct, but overlap in the dermal-epidermal junction, and both are expressed contemporaneously. These observations suggest a role for alpha9beta1-EIIIA interactions in wound keratinocyte function.
5. Integrin expression patterns during early limb muscle development in the mouse. Cell-**extracellular matrix** interactions play crucial roles in limb muscle development but practically nothing is known on what integrins are involved before the differentiation of muscle precursor cells (MPCs) in the limb muscle masses. In this study we determine the expression patterns of integrins during early forelimb muscle development in the mouse. alpha6beta1 integrin is downregulated in the lateral dermomyotome when delamination of MPCs occurs. In late E9.5 embryos, alpha1beta1 and alpha5beta1 are expressed in a pattern very similar to pax3, which marks MPCs migrating to the limb bud. After myf5 upregulation in the limb bud, alpha1beta1 and alpha5beta1 expression is maintained and the alpha4beta1 integrin starts being expressed.
6. Caveolin-1-dependent beta1 integrin endocytosis is a critical regulator of fibronectin turnover. beta1 integrins are major cell surface receptors for fibronectin. Some integrins, including beta1 integrins, are known to undergo constitutive endocytosis and recycling. Integrin endocytosis/recycling has been implicated in the regulation of cell migration. However, the mechanisms by which integrin endocytosis/recycling regulates cell migration, and other biological consequences of integrin trafficking are not completely understood. We previously showed that turnover of **extracellular matrix** (ECM) fibronectin occurs via receptor-mediated endocytosis. Here, we investigate the biological relevance of beta1 integrin endocytosis to fibronectin matrix turnover. First, we demonstrate that beta1 integrins, including alpha5beta1 play an important role in endocytosis and turnover of matrix fibronectin. Second, we show that caveolin-1 constitutively regulates endocytosis of alpha5beta1 integrins, and that alpha5beta1 integrin endocytosis can occur in the absence of fibronectin and fibronectin matrix. We also show that downregulation of caveolin-1 expression by siRNA results in marked reduction of beta1 integrin and fibronectin endocytosis. Hence, caveolin-1-dependent beta1 integrin and fibronectin endocytosis plays a critical role in fibronectin matrix turnover, and may contribute to abnormal ECM remodeling that occurs in fibrotic disorders.
7. Laminin/β1 integrin signal triggers axon formation by promoting microtubule assembly and stabilization. Axon specification during neuronal polarization is closely associated with increased microtubule stabilization in one of the neurites of unpolarized neuron, but how this increased microtubule stability is achieved is unclear. Here, we show that **extracellular matri**x (ECM) component laminin promotes neuronal polarization via regulating directional microtubule assembly through β1 integrin (Itgb1). Contact with laminin coated on culture substrate or polystyrene beads was sufficient for axon specification of undifferentiated neurites in cultured hippocampal neurons and cortical slices. Active Itgb1 was found to be concentrated in laminin-contacting neurites. Axon formation was promoted and abolished by enhancing and attenuating Itgb1 signaling, respectively. Interestingly, laminin contact promoted plus-end microtubule assembly in a manner that required Itgb1. Moreover, stabilizing microtubules partially prevented polarization defects caused by Itgb1 downregulation. Finally, genetic ablation of Itgb1 in dorsal telencephalic progenitors caused deficits in axon development of cortical pyramidal neurons. Thus, laminin/Itgb1 signaling plays an instructive role in axon initiation and growth, both in vitro and in vivo, through the regulation of microtubule assembly. This study has established a linkage between an extrinsic factor and intrinsic cytoskeletal dynamics during neuronal polarization.
8. Deletion of beta 1 integrins in mice results in inner cell mass failure and peri-implantation lethality. Integrin receptors for **extracellular matrix** receptors are important effectors of cell adhesion, differentiation, and migration in cultured cells and are believed to be critical effectors of these processes during development. To determine when beta 1 integrins become critical during embryonic development, we generated mutant mice with a targeted disruption of the beta 1 integrin subunit gene. Heterozygous mutant mice were normal. Homozygous loss of beta 1 integrin expression was lethal during early postimplantation development. Homozygous embryos lacking beta 1 integrins formed normal-looking blastocysts and initiated implantation at E4.5. However, the E4.5 beta 1-null embryos in situ had collapsed blastocoeles, and whereas the trophoblast penetrated the uterine epithelium, extensive invasion of the decidua was not observed. Laminin-positive endoderm cells were detected in the inner cell mass area, but endoderm morphogenesis and migration were defective. By E5.5 beta 1-null embryos had degenerated extensively. In vitro analysis showed that trophoblast function in beta 1-null peri-implantation embryos was largely normal, including expression of tissue-specific markers, and outgrowth on fibronectin- and vitronectin-coated, although not on laminin-coated substrates. In contrast, the inner cell mass region of beta 1-null blastocyst outgrowths, and inner cell masses isolated from beta 1-null blastocysts, showed highly retarded growth and defective extraembryonic endoderm morphogenesis and migration. These data suggest that beta 1 integrins are required for normal morphogenesis of the inner cell mass and are essential mediators of growth and survival of cells of the inner cell mass. Failure of continued trophoblast development in beta 1-null embryos after inner cell mass failure could be attributable to either an intrinsic requirement for beta 1 integrins for later stages of trophoblast development, or to the lack of trophic signals from the beta 1-null inner cell mass.
9. Inhibition of pathologic retinal neovascularization by alpha-defensins. Proliferative retinopathies, such as those complicating prematurity and diabetes, are major causes of blindness. A prominent feature of these retinopathies is excessive neovascularization, which is orchestrated by the hypoxia-induced vascular endothelial growth factor (VEGF) stimulating endothelial cells and the integrin-mediated adhesive interactions of endothelial cells with **extracellular matrix** components such as fibronectin (FN). Recently, we demonstrated that alpha-defensins interfere with alpha5beta1-FN interactions and dependent endothelial cell functions. Here, alpha-defensins were studied in hypoxia-induced proliferative retinopathy. In vitro, alpha-defensins specifically inhibited alpha5beta1-integrin-dependent migration of bovine retinal endothelial cells (BRECs) to FN, attenuated the VEGF-stimulated increase in endothelial permeability, and blocked BREC proliferation and capillary sprout formation in 3-dimensional fibrin-matrices. An up-regulation of beta1-integrin and FN was observed in the retinal vessels in the mouse model of hypoxia-induced retinal angiogenesis. Systemic and local administration of alpha-defensins reduced retinal neovascularization by 45% and 60%, respectively, and this effect was comparable to the inhibitory effect of alpha5beta1-blocking antibody. alpha-Defensins were detected in human diabetic retinas associated with normal retinal vessels but were absent from proliferative lesions. Together, these data show that alpha-defensins inhibit pathologic retinal neovascularization in vivo and may provide a clinically efficient strategy against proliferative retinopathies.
10. Increased expression of fibronectin and the alpha 5 beta 1 integrin in angiogenic cerebral blood vessels of mice subject to hypobaric hypoxia. The **extracellular matrix** (ECM) is an important regulator of angiogenesis and vascular remodeling. We showed previously that angiogenic capillaries in the developing CNS express high levels of fibronectin and its receptor alpha5beta1 integrin, and that this expression is developmentally downregulated. As cerebral hypoxia leads to an angiogenic response, we sought to determine whether angiogenic vessels in the adult CNS re-express fibronectin and the alpha5beta1 integrin. Ten-week old mice were subject to hypobaric hypoxia for 0, 4, 7 and 14 days, and fibronectin/integrin expression examined. Fibronectin and the alpha5 integrin subunit were strongly upregulated on capillaries in the hypoxic CNS, with the effect maximal at the earliest time point examined (4 days). Immunofluorescent studies demonstrated that the alpha5 integrin was expressed by angiogenic endothelial cells. In light of the defined angiogenic role for fibronectin in other systems, this work suggests that induction of fibronectin-alpha5beta1 integrin expression may be an important molecular switch driving angiogenesis in the hypoxic CNS.
11. Ablation of beta1 integrin in mammary epithelium reveals a key role for integrin in glandular morphogenesis and differentiation. Integrin-mediated adhesion regulates the development and function of a range of tissues; however, little is known about its role in glandular epithelium. To assess the contribution of beta1 integrin, we conditionally deleted its gene in luminal epithelia during different stages of mouse mammary gland development and in cultured primary mammary epithelia. Loss of beta1 integrin in vivo resulted in impaired alveologenesis and lactation. Cultured beta1 integrin-null cells displayed abnormal focal adhesion function and signal transduction and could not form or maintain polarized acini. In vivo, epithelial cells became detached from the **extracellular matrix** but remained associated with each other and did not undergo overt apoptosis. beta1 integrin-null mammary epithelial cells did not differentiate in response to prolactin stimulation because of defective Stat5 activation. In mice where beta1 integrin was deleted after the initiation of differentiation, fewer defects in alveolar morphology occurred, yet major deficiencies were also observed in milk protein and milk fat production and Stat5 activation, indicating a permissive role for beta1 integrins in prolactin signaling. This study demonstrates that beta1 integrin is critical for the alveolar morphogenesis of a glandular epithelium and for maintenance of its differentiated function. Moreover, it provides genetic evidence for the cooperation between integrin and cytokine signaling pathways.
12. Integrin α3β1 signaling through MEK/ERK determines alternative polyadenylation of the MMP-9 mRNA transcript in immortalized mouse keratinocytes. Integrin α3β1 is highly expressed in both normal and tumorigenic epidermal keratinocytes where it regulates genes that control cellular function **and extracellular ma**trix remodeling during normal and pathological tissue remodeling processes, including wound healing and development of squamous cell carcinoma (SCC). Previous studies identified a role for α3β1 in immortalized and transformed keratinocytes in the regulation of genes that promote tumorigenesis, invasion, and pro-angiogenic crosstalk to endothelial cells. One such gene, matrix metalloproteinase-9 (MMP-9), is induced by α3β1 through a post-transcriptional mechanism of enhanced mRNA stability. In the current study, we sought to investigate the mechanism through which α3β1 controls MMP-9 mRNA stability. First, we utilized a luciferase reporter assay to show that AU-rich elements (AREs) residing within the 3'-untranslated region (3'-UTR) of the MMP-9 mRNA renders the transcript unstable in a manner that is independent of α3β1. Next, we cloned a truncated variant of the MMP-9 mRNA which is generated through usage of an alternative, upstream polyadenylation signal and lacks the 3'-UTR region containing the destabilizing AREs. Using an RNase protection assay to distinguish "long" (full-length 3'-UTR) and "short" (truncated 3'-UTR) MMP-9 mRNA variants, we demonstrated that the shorter, more stable mRNA that lacks 3'-UTR AREs was preferentially generated in α3β1-expressing keratinocytes compared with α3β1-deficient (i.e., α3-null) keratinocytes. Moreover, we determined that α3β1-dependent alternative polyadenylation was acquired by immortalized keratinocytes, as primary neonatal keratinocytes did not display α3β1-dependent differences in the long and short transcripts. Finally, pharmacological inhibition of the extracellular signal-regulated kinase (ERK)/mitogen-activated protein kinase (MAPK) pathway in α3β1-expressing keratinocytes caused a shift towards long variant expression, while Raf-1-mediated activation of ERK in α3-null keratinocytes dramatically enhanced short variant expression, indicating a role for ERK/MAPK signaling in α3β1-mediated selection of the proximal polyadenylation site. These findings identify a novel mode of integrin α3β1-mediated gene regulation through alternative polyadenylation.
13. Epidermal growth factor system is a physiological regulator of development of the mouse fetal submandibular gland and regulates expression of the alpha6-integrin subunit. Epidermal growth factor (EGF) and transforming growth factor-alpha (TGF-alpha) regulate branching morphogenesis of fetal mouse submandibular gland (SMG) rudiments in vitro. The EGF system (EGF, TGF-alpha, and their shared receptor, EGFR) also regulates expression of integrins and their ligands in the **extracellular matrix**. We show here that inhibition of EGFR tyrosine-kinase activity by a tyrphostin retards in vitro development of SMGs. Using total RNA isolated from pooled SMGs taken from intact mouse fetuses, mRNA transcripts for EGF, TGF-alpha, and EGFR were detected by reverse transcription-polymerase chain reaction (RT-PCR), and age-dependent variations in the levels of these mRNA were quantitatively determined by nuclease protection assays. These findings suggest that the EGF system is operative in the in vivo development of this gland. alpha6-Integrin subunit was localized by immunofluorescence at the basal surface of epithelial cells. Branching morphogenesis of cultured SMG rudiments was inhibited by anti-alpha6 antibodies. Synthesis of alpha6-subunit in cultured SMGs, detected by metabolic labeling and immunoprecipitation, was increased by EGF and drastically reduced by tyrphostin. RT-PCR revealed that mRNAs for alpha6- and beta1- and beta4-integrin subunits are expressed at all ages between embryonic day 13 and postnatal day 7. These findings suggest that 1) the EGF system is a physiologic regulator of development of fetal mouse SMG, and 2) one mechanism by which it acts may be by regulating expression of integrins, which in turn control interaction of epithelial cells with the **extracellular matrix**.
14. Expression patterns of beta1-related alpha integrin subunits in murine lens during embryonic development and wound healing. To study the expression patterns of b1-related alpha integrin subunits in murine lens epithelial cells, comparing embryonic fiber differentiation with injury-induced epithelial mesenchymal transition (EMT).Adult mice type C57BL/6, pregnant as well as with an eye injured, were sacrificed at different time-course intervals. The embryonic and the injured eyes were obtained and deparaffinized sections of these eyes were processed for immunohistochemistry staining for detection of integrin a subunits.Embryonic lens epithelial cells expressed primarily a3 and a5 subunits, whereas embryonic fiber cells expressed a2, a5, and a6 subunits. Adult lens epithelial cells expressed a3, and a6 subunits,whereas injured lens cells expressed a2, a3, and a6 integrin subunits.The phenotypic changes of lens epithelial cells during embryonic fiber differentiation and EMT are characterized by different expression of integrin subunits as a result both of the altered **extracellular matrix** conditions and of the altered cell signaling pathways recruited in each process.
15. Interaction of reelin with amyloid precursor protein promotes neurite outgrowth. The processing of amyloid precursor protein (APP) to Abeta is an important event in the pathogenesis of Alzheimer's disease, but the physiological function of APP is not well understood. Our previous work has shown that APP processing and Abeta production are regulated by the **extracellular matrix** protein Reelin. In the present study, we examined whether Reelin interacts with APP, and the functional consequences of that interaction in vitro. Using coimmunoprecipitation, we found that Reelin interacted with APP through the central domain of Reelin (repeats 3-6) and the E1 extracellular domain of APP. Reelin increased cell surface levels of APP and decreased endocytosis of APP in hippocampal neurons in vitro. In vivo, Reelin levels were increased in brains of APP knock-out mice and decreased in APP-overexpressing mice. RNA interference knockdown of APP decreased neurite outgrowth in vitro and prevented Reelin from increasing neurite outgrowth. Knock-out of APP or Reelin decreased dendritic arborization in cortical neurons in vivo, and APP overexpression increased dendritic arborization. APP and Reelin have previously been shown to promote neurite outgrowth through interactions with integrins. We confirmed that APP interacted with alpha3beta1 integrin, and alpha3beta1 integrin altered APP trafficking and processing. Addition of an alpha3beta1 integrin antibody prevented APP and Reelin-induced neurite outgrowth. These findings demonstrate that Reelin interacts with APP, potentially having important effects on neurite development.
16. beta1 integrin is necessary for ureteric bud branching morphogenesis and maintenance of collecting duct structural integrity. The kidney collecting system develops from branching morphogenesis of the ureteric bud (UB). This process requires signaling by growth factors such as glial cell line derived neurotrophic factor (GDNF) and fibroblast growth factors (FGFs) as well as cell **extracellular matrix** interactions mediated by integrins. The importance of integrin signaling in UB development was investigated by deleting integrin beta1 at initiation (E10.5) and late (E18.5) stages of development. Deletion at E10.5 resulted in a severe branching morphogenesis phenotype. Deletion at E18.5 did not alter renal development but predisposed the collecting system to severe injury following ureteric obstruction. beta1 integrin was required for renal tubular epithelial cells to mediate GDNF- and FGF-dependent signaling despite normal receptor localization and activation in vitro. Aberrations in the same signaling molecules were present in the beta1-null UBs in vivo. Thus beta1 integrins can regulate organ branching morphogenesis during development by mediating growth-factor-dependent signaling in addition to their well-defined role as adhesion receptors.
17. Epithelial β1 integrin is required for lung branching morphogenesis and alveolarization. Integrin-dependent interactions between cells and **extracellular matri**x regulate lung development; however, specific roles for β1-containing integrins in individual cell types, including epithelial cells, remain incompletely understood. In this study, the functional importance of β1 integrin in lung epithelium during mouse lung development was investigated by deleting the integrin from E10.5 onwards using surfactant protein C promoter-driven Cre. These mutant mice appeared normal at birth but failed to gain weight appropriately and died by 4 months of age with severe hypoxemia. Defects in airway branching morphogenesis in association with impaired epithelial cell adhesion and migration, as well as alveolarization defects and persistent macrophage-mediated inflammation were identified. Using an inducible system to delete β1 integrin after completion of airway branching, we showed that alveolarization defects, characterized by disrupted secondary septation, abnormal alveolar epithelial cell differentiation, excessive collagen I and elastin deposition, and hypercellularity of the mesenchyme occurred independently of airway branching defects. By depleting macrophages using liposomal clodronate, we found that alveolarization defects were secondary to persistent alveolar inflammation. β1 integrin-deficient alveolar epithelial cells produced excessive monocyte chemoattractant protein 1 and reactive oxygen species, suggesting a direct role for β1 integrin in regulating alveolar homeostasis. Taken together, these studies define distinct functions of epithelial β1 integrin during both early and late lung development that affect airway branching morphogenesis, epithelial cell differentiation, alveolar septation and regulation of alveolar homeostasis.
18. Patterned collagen fibers orient branching mammary epithelium through distinct signaling modules. For decades, the work of cell and developmental biologists has demonstrated the striking ability of the mesenchyme and the stroma to instruct epithelial form and function in the mammary gland, but the role of **extracellular matrix** (ECM) molecules in mammary pattern specification has not been elucidated. Here, we show that stromal collagen I (Col-I) fibers in the mammary fat pad are axially oriented prior to branching morphogenesis. Upon puberty, the branching epithelium orients along these fibers, thereby adopting a similar axial bias. To establish a causal relationship from Col-I fiber to epithelial orientation, we embedded mammary organoids within axially oriented Col-I fiber gels and observed dramatic epithelial co-orientation. Whereas a constitutively active form of Rac1, a molecule implicated in cell motility, prevented a directional epithelial response to Col-I fiber orientation, inhibition of the RhoA/Rho-associated kinase (ROCK) pathway did not. However, time-lapse studies revealed that, within randomly oriented Col-I matrices, the epithelium axially aligns fibers at branch sites via RhoA/ROCK-mediated contractions. Our data provide an explanation for how the stromal ECM encodes architectural cues for branch orientation as well as how the branching epithelium interprets and reinforces these cues through distinct signaling processes.
19. Ameloblastin modulates osteoclastogenesis through the integrin/ERK pathway. Proteins of the **extracellular matrix** often have multiple functions to facilitate complex tasks ranging from signaling to structural support. Here we have focused on the function of one of the matrix proteins expressed in bones and teeth, the matrix adhesion protein ameloblastin (AMBN). Transgenic mice with 5-fold elevated AMBN levels in mandibles suffered from root cementum resorption, delamination, and reduced alveolar bone thickness. AMBN gain of function also resulted in a significant reduction in trabecular bone volume and bone mass dentistry in 42 days postnatal mouse jaws. In an in vitro model of osteoclastogenesis, AMBN modulated osteoclast differentiation from bone marrow derived monocytes (BMMCs), and dramatically increased osteoclast numbers and resorption pits. Furthermore, AMBN more than doubled BMMC adhesion, accelerated cell spreading, and promoted podosome belt and actin ring formation. These effects were associated with elevated ERK1/2 and AKT phosphorylation as well as higher expression of osteoclast activation related genes. Blocking integrin α2β1 and ERK 1/2 pathways alleviated the effects of AMBN on osteoclast differentiation. Together, our data indicate that AMBN increases osteoclast number and differentiation as well as mineralized tissue resorption by regulating cell adhesion and actin cytoskeleton polymerization, initiating integrin-dependen**t extracellular matr**ix signaling cascades and enhancing osteoclastogenesis.
20. Differential expression of alpha-6 and other subunits of laminin binding integrins during development of the murine heart. The development of the heart from a single heart tube to a four chambered organ with two separated unidirectional flows is a highly complex process. Events like looping, septation, tissue remodelling, and development of valves take place in a time period in which the heart already exerts its pump function. Adhesion of cells to each other and to their **extracellular matrix** as well as the capability to migrate in such a dynamic environment are extremely important. Integrins and **extracellular matrix** components have already been implicated in this process. In this report, we describe in detail the differential expression of the alpha-6 integrin subunit during late murine heart development, e.g., in the process from looping to the end of septation. We compare mRNA and protein expression patterns with those of beta-1 and other subunits of laminin-binding integrins, alpha-3 and alpha-7. We show a constant and high expression of alpha-6 in the atrial myocardium and a decrease in expression in the ventricular trabecular myocardium. The compact myocardial wall and the ventricular septum do not express alpha-6, except for the myocardium of the distal outflow tract at early stages. Moreover, we describe expression of this integrin subunit in the endocardial cushions that contribute to the development of the atrioventricular and semilunar valves. We propose a role for the alpha-6-beta-1 laminin receptor in the adhesion of cells to their **extracellular matrix** at sites of high stress due to cardiac contraction or blood flow induced shear stress. Moreover, site specific endothelial expression within the heart and surrounding extracardiac tissue is discussed. This study suggests a distinct role for alpha-6-beta-1 in the heart and provides insight concerning probably important roles of integrins and their **extracellular matrix** ligands during embryonic development.
21. The tetraspan protein epithelial membrane protein-2 interacts with beta1 integrins and regulates adhesion. The growth arrest-specific-3 (GAS3)/PMP22 proteins are members of the four-transmembrane (tetraspan) superfamily. Although the function of these proteins is poorly understood, GAS3/PMP22 proteins have been implicated in the control of growth and progression of certain cancers. Epithelial membrane protein-2 (EMP2), a GAS3/PMP22 family member, was recently identified as a putative tumor suppressor gene. Here, we addressed the normal function of EMP2 by testing the prediction that it influences integrin-related cell functions. We observed that EMP2 associates with the beta(1) integrin subunit. Co-immunoprecipitation and immunodepletion experiments indicated that approximately 60% of beta(1) integrins and EMP2 can be isolated in common protein complexes. Whereas this association between EMP2 and beta(1) integrin may be direct or indirect, it has features of integrin heterodimer selectivity. Thus, by laser confocal microscopy, EMP2 colocalized with alpha(6)beta(1) but not alpha(5)beta(1) integrin. Increased expression of EMP2 also influenced the integrin heterodimer repertoire present on the plasma membrane. EMP2 specifically increased the surface expression of the alpha(6)beta(1) integrin while decreasing that of the alpha(5)beta(1) protein. Reciprocally, reduction in EMP2 expression using a specific ribozyme decreased surface expression of alpha(6)beta(1) integrin. Accordingly, these EMP2-mediated changes resulted in a dramatic alteration in cellular adhesion to **extracellular matrix** proteins. This study demonstrates for the first time the interaction of a GAS3/PMP22 family member with an integrin protein and suggests that such interactions and their functional consequences are a physiologic role of GAS3/PMP22 proteins.
22. Adhesion molecules involved in macrophage responses to Wallerian degeneration in the murine peripheral nervous system. When a peripheral nerve is damaged the severed axon undergoes Wallerian degeneration. The distal nerve is infiltrated by large numbers of monocyte-derived macrophages which participate in the phagocytosis of degenerating myelin. In other tissues, adhesion molecules play a crucial role in leukocyte recruitment during inflammation. Blood-borne cells enter damaged tissue by interacting with adhesion molecules expressed on activated endothelium. Having crossed the endothelium, leukocytes must adhere and migrate within the tissue. We investigated the adhesion molecules involved in both stages of the macrophage response to transection of one sciatic nerve of BALB/c mice. By injecting monoclonal antibodies in vivo, before and after peripheral nerve injury, we showed that intercellular adhesion molecule-1 (ICAM-1) and integrins alpha4beta1 (VLA-4) and alphaMbeta2 (type 3 complement receptor) are unlikely to be involved in the transendothelial migration of monocytes responding to peripheral nerve degeneration. We also studied the adhesion of macrophages within the endoneurium, using an in vitro adhesion assay. Macrophages showed much greater levels of adhesion to cryostat sections of transected nerves than to control nerves. This increased adhesion was partially inhibited by antibodies to the beta1-integrin chain, and more strongly inhibited by the **extracellular matrix** molecules fibronectin and collagen. Adhesion was unaffected by laminin-1 and by antibodies to other adhesion molecules, including alpha4beta1- and alpha5beta1-integrins. Thus we conclude that monocyte entry into a degenerating peripheral nerve is independent of alphaLbeta2/alphaMbeta2-ICAM-1 or alpha4beta1/VCAM-1 interactions, and that adhesion within the endoneurium is mediated in part by a beta1-integrin other than alpha4beta1 or alpha5beta1.
23. The **extracellular matrix** glycoprotein tenascin-R regulates neurogenesis during development and in the adult dentate gyrus of mice. Abnormal generation of inhibitory neurons that synthesize γ-aminobutyric acid (GABAergic) is characteristic of neuropsychological disorders. We provide evidence that the **extracellular matri**x molecule tenascin-R (TNR) - which is predominantly expressed by a subpopulation of interneurons - plays a role in the generation of GABAergic and granule neurons in the murine dentate gyrus by regulating fate determination of neural stem or progenitor cells (NSCs). During development, absence of TNR in constitutively TNR-deficient (TNR(-/-)) mice results in increased numbers of dentate gyrus GABAergic neurons, decreased expression of its receptor β1 integrin, increased activation of p38 MAPK and increased expression of the GABAergic specification gene Ascl1. Postnatally, increased GABAergic input to adult hippocampal NSCs in TNR(-/-) mice is associated not only with increased numbers of GABAergic and, particularly, parvalbumin-immunoreactive neurons, as seen during development, but also with increased numbers of granule neurons, thus contributing to the increased differentiation of NSCs into granule cells. These findings indicate the importance of TNR in the regulation of hippocampal neurogenesis and suggest that TNR acts through distinct direct and indirect mechanisms during development and in the adult.
24. Role of hypoxia-induced fibronectin-integrin β1 expression in embryonic stem cell proliferation and migration: Involvement of PI3K/Akt and FAK. Cell migration is largely dependent on integrin (IN) binding to the **extracellular matri**x, and several signaling pathways involved in these processes have been shown to be modified by hypoxia. Therefore, the aim of this study was to determine the influence of hypoxia on fibronectin (FN) and IN β1 expression in mouse embryonic stem cells (mESCs) and their signaling pathways to modulate proliferation. FN and IN β1 expression were significantly increased in hypoxic mESCs by 24 h. Hypoxia also increased cell attachment, which was accompanied by concomitant increases in the binding level of FN and IN β1. Hypoxia-induced FN expression was mediated by increased phosphatidylinositol 3 kinase (PI3K)/Akt and mammalian target of rapamycin (mTOR) phosphorylation, and hypoxia-inducible factor-1α (HIF-1α) expression. Moreover, under hypoxic conditions, focal adhesion kinase (FAK) and Src phosphorylation were increased in a time-dependent fashion; these increases were blocked by IN β1 antibody. In addition, the hypoxia induced increase of F-actin distribution and cell migration (activation of matrix metalloproteinase-2 and -9) was inhibited by IN β1 antibody. Indeed, hypoxia increased the level of cell-cycle regulatory protein and DNA synthesis. In conclusion, hypoxia increases the proliferation and migration of mESCs via FN-IN β1 production through the PI3K/Akt, mTOR, and HIF-1α pathways, followed by FAK activation.
25. Regulation of neural progenitor proliferation and survival by beta1 integrins. Neural stem cells give rise to undifferentiated nestin-positive progenitors that undergo extensive cell division before differentiating into neuronal and glial cells. The precise control of this process is likely to be, at least in part, controlled by instructive cues originating from the extracellular environment. Some of these cues are interpreted by the integrin family of **extracellular matrix** receptors. Using neurosphere cell cultures as a model system, we show that beta1-integrin signalling plays a crucial role in the regulation of progenitor cell proliferation, survival and migration. Following conditional genetic ablation of the beta1-integrin allele, and consequent loss of beta1-integrin cell surface protein, mutant nestin-positive progenitor cells proliferate less and die in higher numbers than their wild-type counterparts. Mutant progenitor cell migration on different ECM substrates is also impaired. These effects can be partially compensated by the addition of exogenous growth factors. Thus, beta1-integrin signalling and growth factor signalling tightly interact to control the number and migratory capacity of nestin-positive progenitor cells.
26. Dwarfism in mice lacking collagen-binding integrins α2β1 and α11β1 is caused by severely diminished IGF-1 levels. Mice with a combined deficiency in the α2β1 and α11β1 integrins lack the major receptors for collagen I. These mutants are born with inconspicuous differences in size but develop dwarfism within the first 4 weeks of life. Dwarfism correlates with shorter, less mineralized and functionally weaker bones that do not result from growth plate abnormalities or osteoblast dysfunction. Besides skeletal dwarfism, internal organs are correspondingly smaller, indicating proportional dwarfism and suggesting a systemic cause for the overall size reduction. In accordance with a critical role of insulin-like growth factor (IGF)-1 in growth control and bone mineralization, circulating IGF-1 levels in the sera of mice lacking either α2β1 or α11β1 or both integrins were sharply reduced by 39%, 64%, or 81% of normal levels, respectively. Low hepatic IGF-1 production resulted from diminished growth hormone-releasing hormone expression in the hypothalamus and, subsequently, reduced growth hormone expression in the pituitary glands of these mice. These findings point out a novel role of collagen-binding integrin receptors in the control of growth hormone/IGF-1-dependent biological activities. Thus, coupling hormone s**ecretion to extracel**lular matrix signaling via integrins represents a novel concept in the control of endocrine homeostasis.
27. Alteration of cartilage mechanical properties in absence of β1 integrins revealed by rheometry and FRAP analyses. Mechanical properties are essential for biological functions of the hyaline cartilage such as energy dissipation and diffusion of solutes. Mechanical properties are primarily dependent on the hierarchical organization of the two major **extracellular matri**x (ECM) macromolecular components of the cartilage: the fibrillar collagen network and the glycosaminoglycan (GAG)-substituted proteoglycan, mainly aggrecan, aggregates. Interaction of chondrocytes, the only cell type in the tissue, with the ECM through adhesion receptors is involved in establishing mechanical stability via bidirectional transduction of both mechanical forces and chemical signals. In this study, we aimed to determine the role of the transmembrane β1 integrin adhesion receptors in cartilage biomechanical properties by the use of genetic modification in mice.Costal cartilages of wild type and mutant mice lacking β1 integrins in chondrocytes were investigated. Cartilage compressive properties and solute diffusion were characterized by rheometric analysis and Fluorescence Recovery After Photobleaching (FRAP), respectively. Cartilage tissue sections were analyzed by histology, immunohistochemistry and transmission electron microscopy (TEM).At the histological level, the mutant costal cartilage was characterized by chondrocyte rounding and loss of tissue polarity. Immunohistochemistry and safranin orange staining demonstrated apparently normal aggrecan and GAG levels, respectively. Antibody staining for collagen II and TEM showed comparable expression and organization of the collagen fibrils between mutant and control cartilages. Despite the lack of gross histological and ultrastructural abnormalities, rheological measurements revealed that the peak elastic modulus in compression of mutant cartilage was 1.6-fold higher than the peak elastic modulus of wild-type sample. Interestingly, the diffusion coefficient within the mutant cartilage tissue was found to be 1.2-fold lower in the extracellular space and 14-fold lower in the pericellular (PCM) space compared to control.The results demonstrate that the absence of β1 integrins on the surface of chondrocytes increases the stiffness and modifies the diffusion properties of costal cartilage. Our data imply that β1 integrins-mediated chondrocyte-matrix interactions directly affect cartilage biomechanics probably by modifying physical properties of individual cells. This study thus highlights the crucial role of β1 integrins in the cartilage function.
28. Skin and hair follicle integrity is crucially dependent on beta 1 integrin expression on keratinocytes. beta 1 integrins are ubiquitously expressed receptors that mediate cell-cell and cell-**extracellular matrix** interactions. To analyze the function of beta1 integrin in skin we generated mice with a keratinocyte-restricted deletion of the beta 1 integrin gene using the cre-loxP system. Mutant mice developed severe hair loss due to a reduced proliferation of hair matrix cells and severe hair follicle abnormalities. Eventually, the malformed hair follicles were removed by infiltrating macrophages. The epidermis of the back skin became hyperthickened, the basal keratinocytes showed reduced expression of alpha 6 beta 4 integrin, and the number of hemidesmosomes decreased. Basement membrane components were atypically deposited and, at least in the case of laminin-5, improperly processed, leading to disruption of the basement membrane and blister formation at the dermal-epidermal junction. In contrast, the integrity of the basement membrane surrounding the beta 1-deficient hair follicle was not affected. Finally, the dermis became fibrotic. These results demonstrate an important role of beta 1 integrins in hair follicle morphogenesis, in the processing of basement membrane components, in the maintenance of some, but not all basement membranes, in keratinocyte differentiation and proliferation, and in the formation and/or maintenance of hemidesmosomes.
29. Tissue inhibitor of metalloproteinase-2 (TIMP-2) regulates neuromuscular junction development via a beta1 integrin-mediated mechanism. **Extracellular matrix** (ECM) molecules play critical roles in muscle function by participating in neuromuscular junction (NMJ) development and the establishment of stable, cytoskeleton-associated adhesions required for muscle contraction. Matrix metalloproteinases (MMPs) are neutral endopeptidases that degrade all ECM components. While the role of MMPs and their inhibitors, the tissue inhibitor of metalloproteinases (TIMPs), has been investigated in many tissues, little is known about their role in muscle development and mature function. TIMP-2 -/- mice display signs of muscle weakness. Here, we report that TIMP-2 is expressed at the NMJ and its expression is greater in fast-twitch (extensor digitorum longus, EDL) than slow-twitch (soleus) muscle. EDL muscle mass is reduced in TIMP-2-/- mice without a concomitant change in fiber diameter or number. The TIMP-2-/- phenotype is not likely due to increased ECM proteolysis because net MMP activity is actually reduced in TIMP-2-/- muscle. Most strikingly, TIMP-2 colocalizes with beta1 integrin at costameres in the wild-type EDL and beta1 integrin expression is significantly reduced in TIMP-2-/- EDL. We propose that reduced beta1 integrin in fast-twitch muscle may be associated with destabilized ECM-cytoskeletal interactions required for muscle contraction in TIMP-2-/- muscle; thus, explaining the muscle weakness. Given that fast-twitch fibers are lost in muscular dystrophies and age-related sarcopenia, if TIMP-2 regulates mechanotransduction in an MMP-independent manner it opens new potential therapeutic avenues.
30. Alpha3beta1 integrin regulates MMP-9 mRNA stability in immortalized keratinocytes: a novel mechanism of integrin-mediated MMP gene expression. Matrix metalloproteinases facilitate cell migration and tumor invasion through their ability to proteolyse the **extracellular matrix**. The laminin-binding integrin alpha3beta1 is expressed at high levels in squamous cell carcinomas and in normal keratinocytes during cutaneous wound healing. We showed previously that alpha3beta1 is required for MMP-9/gelatinase B secretion in immortalized mouse keratinocytes (MK cells) and that this regulation was acquired as part of the immortalized phenotype, suggesting a possible role for alpha3beta1 during malignant conversion. In the current study, we identify a novel mechanism whereby alpha3beta1 regulates the induction of MMP-9 expression that occurs in response to activation of a MAPK kinase (MEK)/extracellular signal-regulated kinase (ERK) pathway. Inhibition of MEK/ERK signaling in wild-type MK cells with a pharmacological inhibitor, U0126, showed that ERK activation was necessary for high levels of endogenous MMP-9 gene expression and activity of a transfected MMP-9 promoter. Furthermore, activation of MEK/ERK signaling in these cells with an oncogenic mutant of Ras, RasV12, increased both endogenous MMP-9 gene expression and MMP-9 promoter activity. Experiments with alpha3beta1-deficient MK cells revealed that alpha3beta1 was required for both baseline levels and RasV12-induced levels of MMP-9 mRNA expression. However, alpha3beta1 was not required for RasV12-mediated activation of ERK or for ERK-dependent MMP-9 promoter activity. Direct comparison of mRNA turnover in the wild type and alpha3-null MK cells identified a requirement for alpha3beta1 in stabilization of MMP-9 mRNA transcripts. These results identify a novel function for integrins in promoting mRNA stability as a mechanism to potentiate MAPK-mediated gene expression. They also suggest a role for alpha3beta1 in maintaining high levels of MMP-9 mRNA expression in response to oncogenic activation of MEK/ERK signaling pathways.
31. In vivo analysis of Arg-Gly-Asp sequence/integrin α5β1-mediated signal involvement in embryonic enchondral ossification by exo utero development system. Enchondral ossification is a fundamental mechanism for longitudinal bone growth during vertebrate development. In vitro studies suggested that functional blockade with RGD peptides or with an antibody that interferes with integrin α5β1-ligand interactions inhibited pre-hypertrophic chondrocyte differentiation. The purpose of this study is to elucidate in vivo the roles of the integrin α5β1-mediated signal through the Arg-Gly-Asp (RGD) sequence in the **cell-extracellular** matrix (ECM) interaction in embryonic enchondral ossification by an exo utero development system. We injected Arg-Gly-Asp-Ser (RGDS) peptides and anti-integrin α5β1 antibody (α5β1 ab) in the upper limbs of mouse embryos at embryonic day (E) 15.5 (RGDS-injected limbs, α5β1 ab-injected limbs), and compared the effects on enchondral ossification with those found in the control limbs (Arg-Gly-Glu-Ser peptide-, mouse IgG-, or vehicle-injected, and no surgery) at E16.5. In the RGDS-injected limbs, the humeri were shorter and there were fewer BrdU-positive cells than in the control limbs. The ratios of cartilage length and area to those of the humerus were higher in the RGDS-injected limbs. The ratios of type X collagen to type 2 collagen mRNA and protein (Coll X/Coll 2) were significantly lower in the RGDS-injected limbs. In those limbs, TUNEL-positive cells were hardly observed, and the ratios of fractin to the Coll X/Coll 2 ratio were lower than in the control limbs. Furthermore, the α5β1 ab-injected limbs showed results similar to those of RGDS-injected limbs. The present in vivo study by exo utero development system showed that RGDS and α5β1 ab injection decreased chondrocyte proliferation, differentiation, and apoptosis in enchondral ossification, and suggested that the integrin α5β1-mediated ECM signal through the RGD sequence is involved in embryonic enchondral ossification.
32. Glycoprotein VI but not alpha2beta1 integrin is essential for platelet interaction with collagen. Platelet adhesion on and activation by components of the **extracellular matrix** are crucial to arrest post-traumatic bleeding, but can also harm tissue by occluding diseased vessels. Integrin alpha2beta1 is thought to be essential for platelet adhesion to subendothelial collagens, facilitating subsequent interactions with the activating platelet collagen receptor, glycoprotein VI (GPVI). Here we show that Cre/loxP-mediated loss of beta1 integrin on platelets has no significant effect on the bleeding time in mice. Aggregation of beta1-null platelets to native fibrillar collagen is delayed, but not reduced, whereas aggregation to enzymatically digested soluble collagen is abolished. Furthermore, beta1-null platelets adhere to fibrillar, but not soluble collagen under static as well as low (150 s(-1)) and high (1000 s(-1)) shear flow conditions, probably through binding of alphaIIbbeta3 to von Willebrand factor. On the other hand, we show that platelets lacking GPVI can not activate integrins and consequently fail to adhere to and aggregate on fibrillar as well as soluble collagen. These data show that GPVI plays the central role in platelet-collagen interactions by activating different adhesive receptors, including alpha2beta1 integrin, which strengthens adhesion without being essential.
33. Genetic deletion or antibody blockade of alpha1beta1 integrin induces a stable plaque phenotype in ApoE-/- mice. Adhesive interactions between cells and the **extracellular matrix** play an important role in inflammatory diseases like atherosclerosis. We investigated the role of the collagen-binding integrin alpha1beta1 in atherosclerosis.ApoE-/- mice were alpha1-deficient or received early or delayed anti-alpha1 antibody treatment. Deficiency in alpha1 integrin reduced the area of atherosclerotic plaques and altered plaque composition by reducing inflammation and increasing **extracellular matrix**. In advanced plaques, alpha1-deficient mice had a reduced macrophage and CD3+ cell content, collagen and smooth muscle cell content increased, lipid core sizes decreased, and cartilaginous metaplasia occurred. Anti-alpha1 antibody treatment reduced the macrophage content in initial plaques after early and delayed treatment, decreased the CD3+ cell content in advanced plaques after delayed treatment, and increased the collagen content in initial and advanced plaques after delayed treatment. Migration assays performed on alpha1-deficient macrophages on collagen I and IV substrata revealed that alpha1-deficient cells can migrate on collagen I, but not IV. Anti-alpha1 antibody treatment of ApoE-/- macrophages also inhibited migration of cells on collagen IV.Our results suggest that alpha1beta1 integrin is involved in atherosclerosis by mediating the migration of leukocytes to lesions by adhesion to collagen IV. Blocking this integrin reduces atherosclerosis and induces a stable plaque phenotype.
34. The spatial and temporal expression of the alpha 2 beta 1 integrin and its ligands, collagen I, collagen IV, and laminin, suggest important roles in mouse mammary morphogenesis. To begin to determine the role of the alpha 2 beta 1 integrin and its ligands, collagen I, collagen IV, and laminin, in mammary epithelial differentiation in vivo, we determined the expression of these molecules by in situ hybridization and immunofluorescence in the developing mouse mammary gland. Expression of collagen I, collagen IV, and laminin mRNAs in the mammary gland during puberty corresponded to the period of greatest growth of the gland, 4-7 weeks postnatally. Collagen I expression preceded collagen IV expression, both of which preceded laminin expression, suggesting an important temporal sequence of **extracellular matrix** (ECM) production. When growth of the epithelium ceased in the adult virgin gland, expression of all three mRNAs became undetectable. Following the onset of pregnancy these molecules were re-expressed with the same chronology observed during puberty. Collagen I, collagen IV, and laminin were expressed by stromal cells immediately surrounding the developing ductal epithelium. Surprisingly, we found no expression of ECM components in the epithelial cells, suggesting the mammary epithelium does not synthesize its own basement membrane. The distribution of collagen I was consistent with a role in duct formation, since collagen I was strikingly abundant around larger mammary ducts, but was sparse around growing endbuds or alveoli. Conversely, there was abundant laminin near growing endbuds and around alveoli, and less around large ducts, suggesting its role is different than collagen I. The alpha 2 beta 1 integrin was present on the basal, lateral, and apical surfaces of the mammary epithelium throughout postnatal development and pregnancy. The alpha 2 beta 1 integrin expression was strongest at midpregnancy, suggesting a role for alpha 2 beta 1 integrin in the alveolar formation that occurs at this time. The alpha 2 beta 1 integrin expression decreased dramatically in the lactating gland. Our results suggest that alpha 2 beta 1 integrin interactions with its temporally and spatially regulated ligands, collagen I, collagen IV, and laminin, could play an important role in mammary morphogenesis in vivo.
35. Requirement of alpha(4)beta(1) and alpha(5)beta(1) integrin expression in bone-marrow-derived progenitor cells in preventing endotoxin-induced lung vascular injury and edema in mice. The goal of this study was to determine the role of integrin-mediated adhesion of bone-marrow-derived progenitor cells (BMPCs) as a requirement for the endothelial barrier protection in a lung injury model. C57BL mice were used as the source for BMPCs, which were characterized as CD34(+) and fetal liver kinase-1 (Flk1)(+) and also an expression of a repertoire of integrins. We used a mouse model of bacterial lipopolysaccharide (LPS)-induced lung vascular injury and edema formation to test the effects of BMPC integrin expression in preventing endothelial barrier injury. Adhesion of BMPCs to purified **extracellular matrix** proteins induced focal adhesion kinase (Fak) phosphorylation and formation of branching point structures in a alpha(4) and alpha(5) integrin-dependent manner. BMPCs expressing red fluorescent protein (RFP) were administered via the retro-orbital venous route in mice treated intraperitonially with LPS (7.5 mg/kg body weight). We observed increased retention of RFP-labeled Flk1(+) and CD34(+) BMPCs for up to 8 weeks in mice injured with LPS. BMPC transplantation increased survival by 50% (at 72-96 hours after LPS) and reduced lung vascular injury and extravascular water content induced by LPS. However, blocking with anti-alpha(4) or anti-alpha(5) integrin antibody or shRNA-mediated silencing of alpha(4) or alpha(5) integrins in donor BMPCs failed to prevent the vascular injury or edema formation and mortality. Thus, alpha(4) and alpha(5) integrin-dependent adhesion of BMPCs in lung tissue plays a critical role in preventing lung vascular injury and increasing survival in a mouse model of LPS-induced acute lung injury.
36. Complex patterns of expression suggest extensive roles for the alpha 2 beta 1 integrin in murine development. The **extracellular matrix** plays important roles in embryogenesis. The integrin family of adhesion receptors may mediate critical cellular interactions with the **extracellular matrix** during development. In this study, we elucidated the developmental spatial and temporal expression pattern of the alpha 2 beta 1 integrin heterodimer, a cell surface receptor for collagens and laminin. We generated reagents for studying the alpha 2 beta 1 integrin and examined the developmental expression of the integrin in postimplantation mice. A partial length murine alpha 2 cDNA was isolated and the protein encoding region was found to be 82% homologous to that of the human alpha 2 cDNA. A synthetic peptide corresponding to the carboxy-terminus of murine alpha 2 was used to generate alpha 2-specific antiserum. The antiserum and riboprobes derived from both the alpha 2 cDNA and the previously characterized murine beta 1 subunit cDNA were used to determine the spatiotemporal expression of the alpha 2 subunit by immunocytochemistry and of the alpha 2 and beta 1 mRNAs by in situ hybridization. Both approaches gave concordant results. Expression of the alpha 2 integrin subunit was observed in both the maternal and embryonic components of the placenta, namely the perivascular and basal zone decidual cells and decidual cells and spongiotrophoblasts at the maternal/embryonic junction. Expression was also observed in cells actively producing and remodeling the **extracellular matrix** in the maternal uterus and in the developing gut, lens, cartilage, bone, and tooth of the embryo. Generally, expression of the alpha 2 integrin subunit was found in cells entering their later stages of differentiation such as in chondrocytes as they became hypertrophic, ameloblasts and odontoblasts as they became columnar and began to secrete the matrix of the tooth, endothelial cells after they formed tubules, in the lens just prior to and during lens fiber production, and in the collecting ducts of the kidney only after full gestation. Throughout embryogenesis, beta 1 mRNA was widely distributed and present in cell types expressing alpha 2 mRNA and protein. The developmental expression pattern of the alpha 2 beta 1 integrin suggests roles for the integrin in placental development and matrix assembly and remodeling.
37. Loss of nidogen-1 and -2 results in syndactyly and changes in limb development. Nidogens are two ubiquitous basement membrane proteins produced mainly by mesenchymal cells. Nidogen-mediated interactions, in particular with laminin, collagen IV, and perlecan have been considered important in the formation and maintenance of the basement membrane. However, whereas mice lacking both nidogen isoforms or carrying mutations in the high affinity nidogen-binding site upon the laminin gamma1 chain have specific basement membrane defects in certain organs, particularly in the lung, characterization of these mice has also shown that basement membrane formation per se does not need nidogens or the laminin-nidogen interaction. Limb development requires the complex interplay of numerous growth factors whose expression is dependent upon the apical ectodermal ridge. Here, we show that lack of nidogen-1 and -2 results in a specific and time-limited failure in the ectodermal basement membrane of the limb bud. The absence of this basement membrane leads to aberrant apical ectodermal ridge formation. It also causes altered distribution of growth factors, such as fibroblast growth factors and leads to a fully penetrant soft tissue syndactyly caused by the dysregulation of interdigital apoptosis. Further, in certain animals more severe changes in bone formation occur, providing evidence for the interplay between growth factors and the **extracellular matrix**.
38. Beta1 integrin expression by podocytes is required to maintain glomerular structural integrity. Integrins are transmembrane heteromeric receptors that mediate interactions between cells and **extracellular matrix** (ECM). beta1, the most abundantly expressed integrin subunit, binds at least 12 alpha subunits. beta1 containing integrins are highly expressed in the glomerulus of the kidney; however their role in glomerular morphogenesis and maintenance of glomerular filtration barrier integrity is poorly understood. To study these questions we selectively deleted beta1 integrin in the podocyte by crossing beta1(flox/flox) mice with podocyte specific podocin-cre mice (pod-Cre), which express cre at the time of glomerular capillary formation. We demonstrate that podocyte abnormalities are visualized during glomerulogenesis of the pod-Cre;beta1(flox/flox) mice and proteinuria is present at birth, despite a grossly normal glomerular basement membrane. Following the advent of glomerular filtration there is progressive podocyte loss and the mice develop capillary loop and mesangium degeneration with little evidence of glomerulosclerosis. By 3 weeks of age the mice develop severe end stage renal failure characterized by both tubulointerstitial and glomerular pathology. Thus, expression of beta1 containing integrins by the podocyte is critical for maintaining the structural integrity of the glomerulus.
39. Patterns of laminins and integrins in the embryonic ventricular zone of the CNS. The **extracellular matrix** (ECM) provides both a physical framework and a microenvironment that supplies instructive signals from the earliest stages of multicellular development. As a first step toward understanding the role of the ECM in regulating the behavior of neural stem cells (NSCs), here we show the localization of laminins, a heterotrimeric family of ECM molecules expressed in many different stem cell microenvironments, and their corresponding receptors in the embryonic murine ventricular zone (VZ) within which the NSCs undergo symmetrical and asymmetrical divisions required for cortical development. In addition to the presence of laminins containing both the alpha2 and alpha4 chains, we find distinct patterns of ECM receptor expression in the VZ and in the overlying cortex. Neural stem cells derived from the VZ express high levels of the integrin laminin receptor alpha6beta1. At developmental stages at which NSCs undergo asymmetrical divisions, integrin beta1 was unevenly distributed in some mitotic pairs at the ventricular wall. These results suggest a significant role in the regulation of NSC fate for laminin/integrin signaling within the microenvironment of the VZ and provide a framework for future molecular and cellular analyses of the role of the ECM in neural development.
40. The beta1 and beta3 integrins promote T cell receptor-mediated cytotoxic T lymphocyte activation. Recognition by CD8+ cytotoxic T lymphocytes (CTLs) of antigenic peptides bound to major histocompatibility class (MHC) I molecules on target cells leads to sustained calcium mobilization and CTL degranulation resulting in perforin-dependent killing. We report that beta1 and beta3 integrin-mediated adhesion to **extracellular matrix** proteins on target cells and/or surfaces dramatically promotes CTL degranulation. CTLs, when adhered to fibronectin but not CTL in suspension, efficiently degranulate upon exposure to soluble MHC.peptide complexes, even monomeric ones. This adhesion induces recruitment and activation of the focal adhesion kinase Pyk2, the cytoskeleton linker paxillin, and the Src kinases Lck and Fyn in the contact site. The T cell receptor, by association with Pyk2, becomes part of this adhesion-induced activation cluster, which greatly increases its signaling.
41. Beta1 integrin deletion from the basal compartment of the mammary epithelium affects stem cells. The mammary gland epithelium comprises two major cell types: basal and luminal. Basal cells interact directly with the **extracellular matrix** (ECM) and express higher levels of the ECM receptors, integrins, than luminal cells. We show that deletion of beta1 integrin from basal cells abolishes the regenerative potential of the mammary epithelium and affects mammary gland development. The mutant epithelium was characterized by an abnormal ductal branching pattern and aberrant morphogenesis in pregnancy, although at the end of gestation, the secretory alveoli developed from beta1 integrin-positive progenitors. Lack of beta1 integrin altered the orientation of the basal-cell division axis and in mutant epithelium, in contrast to control tissue, the progeny of beta1 integrin-null basal cells, identified by a genetic marker, was found in the luminal compartment. These results reveal, for the first time, the essential role of the basal mammary epithelial cell-ECM interactions mediated by beta1 integrins in the maintenance of a functional stem cell population, mammary morphogenesis and segregation of the two major mammary cell lineages.
42. Immunolocalization of **extracellular matrix** components and integrins during mouse liver development. Intrahepatic biliary cell differentiation takes place in periportal hepatoblasts under the influence of the subjacent connective tissue, the mechanism of which is still unclear. This study was undertaken to analyze the immunolocalization of **extracellular matrix** components and their cellular receptors during mouse liver development, with special attention given to biliary differentiation and vascular development. In young fetal mouse liver, primitive structures of sinusoids were developed between hepatic cords associated with hematopoietic cells demonstrated by immunohistochemistry of basal laminar components, the alpha6 integrin subunit, and PECAM-1. Portal veins and hepatic veins showed different staining intensities of alpha2, alpha3, and alpha6 integrin subunits from early stages of development. Anti-beta4 integrin subunit antibodies reacted with portal veins, but not with hepatic veins after perinatal stages. Their different phenotypes may be related to the preferential differentiation of periportal bile ducts. In intrahepatic bile duct development, periportal hepatoblasts adjacent to the connective tissue were immunostained for each basal laminar component on the basal side at almost the same time; alpha3, alpha5, alpha6, and beta4 integrin subunits were immunohistochemically detectable later than the basal laminar components. These staining patterns of intrahepatic bile duct cells clearly differed from those of extrahepatic bile duct cells from the beginning of their development, suggesting that these ducts are of different origins. In conclusion, the vascular structures, including sinusoids, portal veins, and hepatic veins, develop from early stages of liver development, and the **extracellular matrix** components may play important roles in biliary differentiation and vascular development. Supplementary material for this article can be found on the HEPATOLOGY website (http://interscience.wiley.com/jpages/0270-9139/suppmat/index.html).
43. Integrin beta1-mediated matrix assembly and signaling are critical for the normal development and function of the kidney glomerulus. The human kidneys filter 180 l of blood every day via about 2.5 million glomeruli. The three layers of the glomerular filtration apparatus consist of fenestrated endothelium, specialized **extracellular matrix** known as the glomerular basement membrane (GBM) and the podocyte foot processes with their modified adherens junctions known as the slit diaphragm (SD). In this study we explored the contribution of podocyte beta1 integrin signaling for normal glomerular function. Mice with podocyte specific deletion of integrin beta1 (podocin-Cre beta1-fl/fl mice) are born normal but cannot complete postnatal renal development. They exhibit detectable proteinuria on day 1 and die within a week. The kidneys of podocin-Cre beta1-fl/fl mice exhibit normal glomerular endothelium but show severe GBM defects with multilaminations and splitting including podocyte foot process effacement. The integrin linked kinase (ILK) is a downstream mediator of integrin beta1 activity in epithelial cells. To further explore whether integrin beta1-mediated signaling facilitates proper glomerular filtration, we generated mice deficient of ILK in the podocytes (podocin-Cre ILK-fl/fl mice). These mice develop normally but exhibit postnatal proteinuria at birth and die within 15 weeks of age due to renal failure. Collectively, our studies demonstrate that podocyte beta1 integrin and ILK signaling is critical for postnatal development and function of the glomerular filtration apparatus.
44. Increased tumor cell dissemination and cellular senescence in the absence of beta1-integrin function. Integrins are transmembrane receptors that bind **extracellular matrix** proteins and enable cell adhesion and cytoskeletal organization, as well as transduction of signals into cells, to promote various aspects of cellular behavior, such as proliferation or survival. Integrins participate in many aspects of tumor biology. Here, we have employed the Rip1Tag2 transgenic mouse model of pancreatic beta cell carcinogenesis to investigate the role of beta(1)-integrin in tumor progression. Specific ablation of beta(1)-integrin function in pancreatic beta cells resulted in a defect in sorting between insulin-expressing beta cells and glucagon-expressing alpha cells in islets of Langerhans. Ablation of beta(1)-integrin in beta tumor cells of Rip1Tag2 mice led to the dissemination of tumor cell emboli into lymphatic blood vessels in the absence of ongoing lymphangiogenesis. Yet, disseminating beta(1)-integrin-deficient beta tumor cells did not elicit metastasis. Rather, primary tumor growth was significantly impaired by reduced tumor cell proliferation and the acquisition of cellular senescence by beta(1)-integrin-deficient beta tumor cells. The results indicate a critical role of beta(1)-integrin function in mediating metastatic dissemination and preventing tumor cell senescence.
45. β1A integrin is a master regulator of invadosome organization and function. Invadosomes are adhesion structures involved in tissue invasion that are characterized by an intense actin polymerization-depolymerization associated with β1 and β3 integrins and coupled **to extracellular mat**rix (ECM) degradation activity. We induced the formation of invadosomes by expressing the constitutive active form of Src, SrcYF, in different cell types. Use of ECM surfaces micropatterned at the subcellular scale clearly showed that in mesenchymal cells, integrin signaling controls invadosome activity. Using β1⁻/⁻ or β3⁻/⁻ cells, it seemed that β1A but not β3 integrins are essential for initiation of invadosome formation. Protein kinase C activity was shown to regulate autoassembly of invadosomes into a ring-like metastructure (rosette), probably by phosphorylation of Ser785 on the β1A tail. Moreover, our study clearly showed that β1A links actin dynamics and ECM degradation in invadosomes. Finally, a new strategy based on fusion of the photosensitizer KillerRed to the β1A cytoplasmic domain allowed specific and immediate loss of function of β1A, resulting in disorganization and disassembly of invadosomes and formation of focal adhesions.
46. β1-integrin restricts astrocytic differentiation of adult hippocampal neural stem cells. Integrins are transmembrane receptors that mediate cell**-extracellular matri**x and cell-cell interactions. The β1-integrin subunit is highly expressed by embryonic neural stem cells (NSCs) and is critical for NSC maintenance in the developing nervous system, but its role in the adult hippocampal niche remains unexplored. We show that β1-integrin expression in the adult mouse dentate gyrus (DG) is localized to radial NSCs and early progenitors, but is lost in more mature progeny. Although NSCs in the hippocampal subgranular zone (SGZ) normally only infrequently differentiate into astrocytes, deletion of β1-integrin significantly enhanced astrocyte differentiation. Ablation of β1-integrin also led to reduced neurogenesis as well as depletion of the radial NSC population. Activation of integrin-linked kinase (ILK) in cultured adult NSCs from β1-integrin knockout mice reduced astrocyte differentiation, suggesting that at least some of the inhibitory effects of β1-integrin on astrocytic differentiation are mediated through ILK. In addition, β1-integrin conditional knockout also resulted in extensive cellular disorganization of the SGZ as well as non-neurogenic regions of the DG. The effects of β1-integrin ablation on DG structure and astrogliogenesis show sex-specific differences, with the effects following a substantially slower time-course in males. β1-integrin thus plays a dual role in maintaining the adult hippocampal NSC population by supporting the structural integrity of the NSC niche and by inhibiting astrocytic lineage commitment. GLIA 2016;64:1235-1251.
47. p190RhoGAP is the convergence point of adhesion signals from alpha 5 beta 1 integrin and syndecan-4. The fibronectin receptors alpha(5)beta(1) integrin and syndecan-4 cocluster in focal adhesions and coordinate cell migration by making individual contributions to the suppression of RhoA activity during matrix engagement. p190Rho-guanosine triphosphatase-activating protein (GAP) is known to inhibit RhoA during the early stages of cell spreading in an Src-dependent manner. This paper dissects the mechanisms of p190RhoGAP regulation and distinguishes the contributions of alpha(5)beta(1) integrin and syndecan-4. Matrix-induced tyrosine phosphorylation of p190RhoGAP is stimulated solely by engagement of alpha(5)beta(1) integrin and is independent of syndecan-4. Parallel engagement of syndecan-4 causes redistribution of the tyrosine-phosphorylated pool of p190RhoGAP between membrane and cytosolic fractions by a mechanism that requires direct activation of protein kinase C alpha by syndecan-4. Activation of both pathways is necessary for the efficient regulation of RhoA and, as a consequence, focal adhesion formation. Accordingly, we identify p190RhoGAP as the convergence point for adhesive signals mediated by alpha(5)beta(1) integrin and syndecan-4. This molecular mechanism explains the cooperation between **extracellular matrix** receptors during cell adhesion.
48. Insulin resistance in striated muscle-specific integrin receptor beta1-deficient mice. Integrin receptor plays key roles in mediating both inside-out and outside-in signaling between cells and the **extracellular matrix**. We have observed that the tissue-specific loss of the integrin beta1 subunit in striated muscle results in a near complete loss of integrin beta1 subunit protein expression concomitant with a loss of talin and to a lesser extent, a reduction in F-actin content. Muscle-specific integrin beta1-deficient mice had no significant difference in food intake, weight gain, fasting glucose, and insulin levels with their littermate controls. However, dynamic analysis of glucose homeostasis using euglycemichyperinsulinemic clamps demonstrated a 44 and 48% reduction of insulin-stimulated glucose infusion rate and glucose clearance, respectively. The whole body insulin resistance resulted from a specific inhibition of skeletal muscle glucose uptake and glycogen synthesis without any significant effect on the insulin suppression of hepatic glucose output or insulin-stimulated glucose uptake in adipose tissue. The reduction in skeletal muscle insulin responsiveness occurred without any change in GLUT4 protein expression levels but was associated with an impairment of the insulin-stimulated protein kinase B/Akt serine 473 phosphorylation but not threonine 308. The inhibition of insulin-stimulated serine 473 phosphorylation occurred concomitantly with a decrease in integrin-linked kinase expression but with no change in the mTOR.Rictor.LST8 complex (mTORC2). These data demonstrate an in vivo crucial role of integrin beta1 signaling events in mediating cross-talk to that of insulin action.
49. Loss of integrin alpha9beta1 results in defects in proliferation, causing poor re-epithelialization during cutaneous wound healing. The wound microenvironment comprises constituents, such as the **extracellular matrix** (ECM), that regulate with temporal and spatial precision, the migratory, proliferative, and contractility of wound cells. Prompt closure of the wound is an early and critical phase of healing and beta1 integrins are important in this process. We previously reported a marked increase in integrin alpha9beta1 expression in epidermal keratinocytes in cutaneous and corneal wounds. However, the functional role of keratinocyte alpha9beta1 during re-epithelialization is unknown and analysis has been precluded by the lethal phenotype of integrin alpha9beta1 knockout mice. We now report that in conditional integrin alpha9 knockout (K14-alpha9 null) mice, normal proliferation occurs in epidermal keratinocytes and corneal basal cells. Normal epidermal keratinocyte morphology is also retained. However, corneal basal cell morphology and epithelial thickness are altered, suggesting that loss of integrin alpha9beta1 results in abnormal corneal differentiation. In cutaneous wounds, the number of proliferating epidermal keratinocytes is significantly reduced in K14-alpha9 null mice compared with alpha9(fl/-) mice, but not in Cre (control) mice. The decreased keratinocyte proliferation observed in K14-alpha9 null mice negatively impacts healing, resulting in a thinner migrating epithelium, demonstrating that alpha9beta1 is crucial for efficient and proper re-epithelialization during cutaneous wound healing.
50. β1 integrin**-extracellular matri**x interactions are essential for maintaining exocrine pancreas architecture and function. Integrin receptors are responsible for integrating **extracellular matri**x signals inside the cell. The most prominent integrin receptor, β1 integrin, has a role in cell function, survival and differentiation. Recently, we demonstrated a profound in vivo role of β1 integrin expression in the pancreas on glucose homeostasis and islet function. Here, we extend these results by examining the role of β1 integrin in exocrine pancreatic structure and function. Adult C57Bl/6 mice hemizygous for a collagen type Iα2 (Col1a2) promoter-controlled tamoxifen-inducible Cre recombinase gene and homozygous for loxP-β1 integrin were injected with tamoxifen or corn oil to generate mice deleted or not for β1 integrin. Pancreata derived from these male mice were analyzed by quantitative reverse transcriptase-polymerase chain reaction, western blot and immunofluorescence. Our results showed that β1 integrin-deficient mice displayed a significant decrease in pancreas weight with a significant reduction of amylase, regenerating islet-derived protein II and carboxypeptidase-A expression (P<0.05-0.01). Compared with control pancreata, β1 integrin-deficient pancreata showed reduced mRNA expre**ssion of extracellul**ar matrix (collagen type Iα2, fibronectin and laminin) genes (P<0.05), detached acini clusters and lost focal adhesion structure. Moreover, β1 integrin-deficient pancreatic acinar cells displayed decreased proliferation (P<0.05) and increased apoptosis (P<0.001). Apoptosis was reduced to that of controls when isolated exocrine clusters were cultured in media supplem**ented with extracell**ular matrix proteins. Taken together, these results implicate β1 integrin as an essential component for maintaining exocrine pancreatic structure and function.
51. Perlecan domain V is neuroprotective and proangiogenic following ischemic stroke in rodents. Stroke is the leading cause of long-term disability and the third leading cause of death in the United States. While most research thus far has focused on acute stroke treatment and neuroprotection, the exploitation of endogenous brain self-repair mechanisms may also yield therapeutic strategies. Here, we describe a distinct type of stroke treatment, the naturally occurring **extracellular matrix** fragment of perlecan, domain V, which we found had neuroprotective properties and enhanced post-stroke angiogenesis, a key component of brain repair, in rodent models of stroke. In both rat and mouse models, Western blot analysis revealed elevated levels of perlecan domain V. When systemically administered 24 hours after stroke, domain V was well tolerated, reached infarct and peri-infarct brain vasculature, and restored stroke-affected motor function to baseline pre-stroke levels in these multiple stroke models in both mice and rats. Post-stroke domain V administration increased VEGF levels via a mechanism involving brain endothelial cell α5β1 integrin, and the subsequent neuroprotective and angiogenic actions of domain V were in turn mediated via VEGFR. These results suggest that perlecan domain V represents a promising approach for stroke treatment.
52. Band 4.1 proteins regulate integrin-dependent cell spreading. Integrins link the **extracellular matrix** (ECM) to the cytoskeleton to control cell behaviors including adhesion, spreading and migration. Band 4.1 proteins contain 4.1, ezrin, radixin, moesin (FERM) domains that likely mediate signaling events and cytoskeletal reorganization via integrins. However, the mechanisms by which Band 4.1 proteins and integrins are functionally interconnected remain enigmatic. Here we have investigated roles for Band 4.1 proteins in integrin-mediated cell spreading using primary astrocytes as a model system. We demonstrate that Proteins 4.1B and 4.1G show dynamic patterns of sub-cellular localization in astrocytes spreading on fibronectin. During early stages of cell spreading Proteins 4.1B and 4.1G are enriched in ECM adhesion sites but become more diffusely localized at later stages of spreading. Combinatorial inactivation of Protein 4.1B and 4.1G expression leads to impaired astrocyte spreading. Furthermore, in exogenous expression systems we show that the isolated Protein 4.1 FERM domain significantly enhances integrin-mediated cell spreading. Protein 4.1B is dispensable for reactive astrogliosis in experimental models of cortical injury, likely due to functional compensation by related Protein 4.1 family members. Collectively, these findings reveal that Band 4.1 proteins are important intracellular components for integrin-mediated cell spreading.
53. Intravenous Administration of Endothelial Colony-Forming Cells Overexpressing Integrin β1 Augments Angiogenesis in Ischemic Legs. When injected directly into ischemic tissue in patients with peripheral artery disease, the reparative capacity of endothelial progenitor cells (EPCs) appears to be limited by their poor survival. We, therefore, attempted to improve the survival of transplanted EPCs through intravenous injection and gene modification. We anticipated that overexpression of integrin β1 will enable injected EPCs to home to ischemic tissue, which abundantly expres**s extracellular matr**ix proteins, the ligands for integrins. In addition, integrin β1 has an independent angiogenesis-stimulating function. Human endothelial colony-forming cells (ECFCs; late-outgrowth EPCs) were transduced using a lentiviral vector encoding integrin β1 (ITGB1) or enhanced green fluorescent protein (GFP). We then locally or systemically injected phosphate-buffered saline or the genetically modified ECFCs (GFP-ECFCs or ITGB1-ECFCs; 1 × 10(5) cells each) into NOD/Shi-scid, IL-2Rγnull mice whose right femoral arteries had been occluded 24 hours earlier. Upregulati**on of extracellular** matrix proteins, including fibronectin, was apparent in the ischemic legs. Four weeks later, blood perfusion of the ischemic limb was significantly augmented only in the ITGB1-ECFC group. Scanning electron microscopy of vascular casts revealed increases in the perfused blood vessels in the ischemic legs of mice in the ITGB1-ECFC group and significant increases in the density of both capillaries and arterioles. Transplanted ECFC-derived vessels accounted for 28% ± 4.2% of the vessels in the ITGB1-ECFC group, with no cell fusion. Intravenous administration of ECFCs engineered to home to ischemic tissue appears to efficiently mediate therapeutic angiogenesis in a mouse model of peripheral artery disease. Significance: The intravenous administration of endothelial colony-forming cells (ECFCs) genetically modified to overexpress integrin β1 effectively stimulated angiogenesis in ischemic mouse hindlimbs. Transplanted ECFCs were observed in the ischemic leg tissue, even at the chronic stage. Moreover, the cells appeared functional, as evidenced by the improved blood flow. The cell type used (ECFCs), the route of administration (intravenous, not directly injected into the affected area), and the use of ligand-receptor intera**ctions (extracellula**r matrix and integrins) for homing represent substantial advantages over previously reported cell therapies for the treatment of peripheral artery disease.
54. Integrators of epidermal growth and differentiation: distinct functions for beta 1 and beta 4 integrins. Mammalian epithelia are critically dependent on interactions with components in the underlying basal lamina for proper morphogenesis and function. Substratum attachment is essential for survival, proliferation, movement, and differentiation; detachment compromises the cell's ability to perform these functions, often resulting in human disease. Interactions with the **extracellular matrix** are mediated through transmembrane integrin receptors that transmit signals to the cytoskeleton and to signaling molecules within the proliferating cells of the epithelium. In the past year, novel insights have emerged regarding the specific role of integrins in their attachment to **extracellular matrix** and in their signal transduction pathways within the epidermis.
55. Integrin alpha5beta1 is necessary for regulation of radial migration of cortical neurons during mouse brain development. During cerebral cortex development, post-mitotic neurons interact with radial glial fibers and the extracellular environment to migrate away from the ventricular region and form a correct laminar structure. Integrin receptors are major mediators of cell-cell and cell-**extracellular matrix** interactions. Several integrin heterodimers are present during formation of the cortical layers. The alpha5beta1 receptor is expressed in the neural progenitors of the ventricular zone during cerebral cortex formation. Using in utero electroporation to introduce short hairpin RNAs in the brain at embryonic day 15.5, we were able to inhibit acutely the expression of alpha5 integrin in the developing cortex. The knockdown of alpha5 integrin expression level in neural precursors resulted in an inhibition of radial migration, without perturbing the glial scaffold. Moreover, the same inhibitory effect on neuronal migration was observed after electroporation of a Cre recombinase expression plasmid into the neural progenitors of conditional knockout mice for alpha5 integrin. In both types of experiments, the electroporated cells expressing reduced levels of alpha5 integrin accumulated in the premigratory region with an abnormal morphology. At postnatal day 2, ectopic neurons were observed in cortical layer V, while a deficit of neurons was observed in cortical layer II-IV. We show that these neurons do not express a layer V-specific marker, suggesting that they have not undergone premature differentiation. Overall, these results indicate that alpha5beta1 integrin functions in the regulation of neural morphology and migration during cortical development, playing a role in cortical lamination.
56. Notch, epidermal growth factor receptor, and beta1-integrin pathways are coordinated in neural stem cells. Notch1 and beta1-integrins are cell surface receptors involved in the recognition of the niche that surrounds stem cells through cell-cell and cell-**extracellular matrix** interactions, respectively. Notch1 is also involved in the control of cell fate choices in the developing central nervous system (Lewis, J. (1998) Semin. Cell Dev. Biol. 9, 583-589). Here we report that Notch and beta1-integrins are co-expressed and that these proteins cooperate with the epidermal growth factor receptor in neural progenitors. We describe data that suggests that beta1-integrins may affect Notch signaling through 1) physical interaction (sequestration) of the Notch intracellular domain fragment by the cytoplasmic tail of the beta1-integrin and 2) affecting trafficking of the Notch intracellular domain via caveolin-mediated mechanisms. Our findings suggest that caveolin 1-containing lipid rafts play a role in the coordination and coupling of beta1-integrin, Notch1, and tyrosine kinase receptor signaling pathways. We speculate that this will require the presence of the adequate beta1-activating **extracellular matrix** or growth factors in restricted regions of the central nervous system and namely in neurogenic niches.
57. Cardiac myocyte-specific excision of the beta1 integrin gene results in myocardial fibrosis and cardiac failure. Integrins link the **extracellular matrix** to the cellular cytoskeleton and serve important roles in cell growth, differentiation, migration, and survival. Ablation of beta1 integrin in all murine tissues results in peri-implantation embryonic lethality. To investigate the role of beta1 integrin in the myocardium, we used Cre-LoxP technology to inactivate the beta1 integrin gene exclusively in ventricular cardiac myocytes. Animals with homozygous ventricular myocyte beta1 integrin gene excision were born in appropriate numbers and grew into adulthood. These animals had 18% of control levels of beta1D integrin protein in the heart and displayed myocardial fibrosis. High-fidelity micromanometer-tipped catheterization of the intact 5-week-old beta1 integrin knockout mice showed depressed left ventricular basal and dobutamine-stimulated contractility and relaxation (LV dP/dt(max) and LV dP/dt(min)) as compared with control groups (n=8 to 10 of each, P<0.01). Hemodynamic loading imposed by 7 days of transverse aortic constriction showed that the beta1 integrin knockout mice were intolerant of this stress as they had 53% survival versus 88% in controls (n=15 each). By 6 months of age, mice with depressed ventricular expression of beta1 integrin developed a dilated cardiomyopathy that was not evident in any control animals and had patchy decrease in glucose metabolism as determined by positron emission tomography. Myocyte membrane integrity as determined via Evan's blue dye staining was disrupted in the beta1 integrin knockout mice. This model provides strong evidence for the importance of beta1 integrin in cardiac form and function and indicates that integrins can be linked to development of cardiomyopathies.
58. TSC2 modulates cell adhesion and migration via integrin-α1β1. Recent evidence suggests that the rare and progressive lung disease lymphangioleiomyomatosis (LAM) is metastatic in nature. Dysfunction of the tumor suppressor genes tuberous sclerosis complex (TSC), in particular mutational inactivation of TSC2, enhances both cell proliferation and migration. Although substantial progress has been made in understanding the role of TSC2 in abnormal LAM cell proliferation and its pharmacological targeting, the mechanisms underlying the enhanced migratory capacity in LAM are not well understood. In this study, we examined the role of TSC2 in cell attachment, spreading, and migration, processes that contribute to the metastatic phenotype. Here we show that loss of TSC2 increased both the attachment and spreading of mouse embryonic fibroblasts to th**e extracellular matr**ix proteins collagen type I and fibronectin and that reexpression of TSC2 reduced these effects. Integrin-α1β1 modulated cell migration with the β1-subunit involved in cell attachment and spreading as shown by using functional blocking antibodies. Loss of TSC2 increased integrin-α1 expression, and inhibition of this integrin subunit reduced cell migration. The enhanced attachment and spreading were independent of the intracellular signaling pathways mammalian target of rapamycin complex 1 and Rho-associated kinase, as pharmacological inhibition with rapamycin or Y27632, respectively, was without effect. Together, these data demonstrate that TSC2 controls cell migration, attachment, and spreading through the α1β1-integrin receptor and thus suggest a potential therapeutic target for the treatment of increased cell invasiveness in LAM.
59. Cardiomyocyte contractile status is associated with differences in fibronectin and integrin interactions. Integrins link the **extracellular matrix** (ECM) with the intracellular cytoskeleton and other cell adhesion-associated signaling proteins to function as mechanotransducers. However, direct quantitative measurements of the cardiomyocyte mechanical state and its relationship to the interactions between specific ECM proteins and integrins are lacking. The purpose of this study was to characterize the interactions between the ECM protein fibronectin (FN) and integrins in cardiomyocytes and to test the hypothesis that these interactions would vary during contraction and relaxation states in cardiomyocytes. Using atomic force microscopy, we quantified the unbinding force (adhesion force) and adhesion probability between integrins and FN and correlated these measurements with the contractile state as indexed by cell stiffness on freshly isolated mouse cardiomyocytes. Experiments were performed in normal physiological (control), high-K(+) (tonically contracted), or low-Ca(2+) (fully relaxed) solutions. Under control conditions, the initial peak of adhesion force between FN and myocyte alpha(3)beta(1)- and/or alpha(5)beta(1)-integrins was 39.6 +/- 1.3 pN. The binding specificity between FN and alpha(3)beta(1)- and alpha(5)beta(1)-integrins was verified by using monoclonal antibodies against alpha(3)-, alpha(5)-, alpha(3) + alpha(5)-, or beta(1)-integrin subunits, which inhibited binding by 48%, 65%, 70%, or 75%, respectively. Cytochalasin D or 2,3-butanedione monoxime (BDM), to disrupt the actin cytoskeleton or block myofilament function, respectively, significantly decreased the cell stiffness; however, the adhesion force and binding probability were not altered. Tonic contraction with high-K(+) solution increased total cell adhesion (1.2-fold) and cell stiffness (27.5-fold) compared with fully relaxed cells with low-Ca(2+) solution. However, it could be partially prevented by high-K(+) bath solution containing BDM, which suppresses contraction by inhibiting the actin-myosin interactions. Thus, our results demonstrate that integrin binding to FN is modulated by the contractile state of cardiac myocytes.
60. Fibronectin induces endothelial cell migration through β1 integrin and Src-dependent phosphorylation of fibroblast growth factor receptor-1 at tyrosines 653/654 and 766. The **extracellular matri**x microenvironment regulates cell phenotype and function. One mechanism by which this is achieved is the transactivation of receptor tyrosine kinases by specific matrix molecules. Here, we demonstrate that the provisional matrix protein, fibronectin (FN), activates fibroblast growth factor (FGF) receptor-1 (FGFR1) independent of FGF ligand in liver endothelial cells. FN activation of FGFR1 requires β1 integrin, as evidenced by neutralizing antibody and siRNA-based studies. Complementary genetic and pharmacologic approaches identify that the non-receptor tyrosine kinase Src is required for FN transactivation of FGFR1. Whereas FGF ligand-induced phosphorylation of FGFR1 preferentially activates ERK, FN-induced phosphorylation of FGFR1 preferentially activates AKT, indicating differential downstream signaling of FGFR1 in response to alternate stimuli. Mutation analysis of known tyrosine residues of FGFR1 reveals that tyrosine 653/654 and 766 residues are required for FN-FGFR1 activation of AKT and chemotaxis. Thus, our study mechanistically dissects a new signaling pathway by which FN achieves endothelial cell chemotaxis, demonstrates how differential phosphorylation profiles of FGFR1 can achieve alternate downstream signals, and, more broadly, highlights the diversity of mechanisms by which th**e extracellular matr**ix microenvironment regulates cell behavior through transactivation of receptor tyrosine kinases.
61. Cadherin-11 coordinates cellular migration and **extracellular matrix** remodeling during aortic valve maturation. Proper remodeling of the endocardial cushions into thin fibrous valves is essential for gestational progression and long-term function. This process involves dynamic interactions between resident cells and their local environment, much of which is not understood. In this study, we show that deficiency of the cell-cell adhesion protein cadherin-11 (Cad-11) results in significant embryonic and perinatal lethality primarily due to valve related cardiac dysfunction. While endocardial to mesenchymal transformation is not abrogated, mesenchymal cells do not homogeneously cellularize the cushions. These cushions remain thickened with disorganized ECM, resulting in pronounced aortic valve insufficiency. Mice that survive to adulthood maintain thickened and stenotic semilunar valves, but interestingly do not develop calcification. Cad-11 (-/-) aortic valve leaflets contained reduced Sox9 activity, β1 integrin expression, and RhoA-GTP activity, suggesting that remodeling defects are due to improper migration and/or cellular contraction. Cad-11 deletion or siRNA knockdown reduced migration, eliminated collective migration, and impaired 3D matrix compaction by aortic valve interstitial cells (VIC). Cad-11 depleted cells in culture contained few filopodia, stress fibers, or contact inhibited locomotion. Transfection of Cad-11 depleted cells with constitutively active RhoA restored cell phenotypes. Together, these results identify cadherin-11 mediated adhesive signaling for proper remodeling of the embryonic semilunar valves.
62. Cell adaptive response to **extracellular matrix** density is controlled by ICAP-1-dependent beta1-integrin affinity. Cell migration is an integrated process requiring the continuous coordinated assembly and disassembly of adhesion structures. How cells orchestrate adhesion turnover is only partially understood. We provide evidence for a novel mechanistic insight into focal adhesion (FA) dynamics by demonstrating that integrin cytoplasmic domain-associated protein 1 (ICAP-1) slows down FA assembly. Live cell imaging, which was performed in both Icap-1-deficient mouse embryonic fibroblasts and cells expressing active beta(1) integrin, shows that the integrin high affinity state favored by talin is antagonistically controlled by ICAP-1. This affinity switch results in modulation in the speed of FA assembly and, consequently, of cell spreading and migration. Unexpectedly, the ICAP-1-dependent decrease in integrin affinity allows cell sensing of matrix surface density, suggesting that integrin conformational changes are important in mechanotransduction. Our results clarify the function of ICAP-1 in cell adhesion and highlight the central role it plays in the cell's integrated response to the extracellular microenvironment.
63. Focal adhesion kinase is essential for costamerogenesis in cultured skeletal muscle cells. A central question in muscle biology is how costameres are formed and become aligned with underlying myofibrils in mature tissues. Costameres are composed of focal adhesion proteins, including vinculin and paxillin, and anchor myofibril Z-bands to the sarcolemma. In the present study, we investigated the process of costamere formation ("costamerogenesis") in differentiating primary mouse myoblasts. Using vinculin and paxillin as costameric markers, we found that two additional focal adhesion components, alpha5beta1 integrin and focal adhesion kinase (FAK), are associated with costameres. We have characterized costamerogenesis as occurring in three distinct stages based on the organizational pattern of these costameric proteins. We show that both costamerogenesis and myofibrillogenesis are initiated at sites of membrane contacts with the **extracellular matrix** and that their maturation is tightly coupled. To test the importance of FAK signaling in these processes, we analyzed cells expressing a dominant negative form of FAK (dnFAK). When cells expressing dnFAK were induced to differentiate, both costamerogenesis and myofibrillogenesis were disrupted although the expression of constituent proteins was not inhibited. Likewise, inhibiting FAK activity by reducing FAK levels using an siRNA approach also resulted in an inhibition of costamerogenesis and myofibrillogenesis. The relationship between costamere and myofibril formation was tested further by treating myotube cultures with potassium or tetrodotoxin to block contraction and disrupt myofibril organization. This also resulted in inhibition of costamere maturation. We present a model of costamerogenesis whereby signaling through FAK is essential for both normal costamerogenesis and normal myofibrillogenesis which are tightly coupled during skeletal myogenesis.
64. Impaired integrin-mediated adhesion contributes to reduced barrier properties in VASP-deficient microvascular endothelium. Recent studies point to a significant role of vasodilator-stimulated phosphoprotein (VASP) in the maintenance of endothelial barrier functions in vivo and in vitro. Moreover, it has been reported that VASP is required for activation of the small GTPase Rac 1. However, little is known whether VASP is involved in the regulation of cell adhesion molecules that are critical for maintenance of the endothelial barrier. Here we demonstrate that impaired barrier properties in VASP-deficient (VASP-/-) microvascular myocardial endothelial cells (MyEnd) correlated with both impaired integrin-mediated adhesion as revealed by laser tweezer trapping and reduced integrin-dependent cell migration. This was paralleled by reduction of focal adhesions at the cell periphery as well as of beta(1)-integrin and VE-cadherin cytoskeletal anchorage. Incubation of MyEnd VASP wt with RGD peptide to block interaction of integrins with **extracellular matrix** (ECM) reduced barrier properties and Rac 1 activity in wt endothelial monolayers mimicking the situation in VASP (-/-) cells under resting conditions. Moreover, cAMP-mediated Rac 1 activation was reduced under conditions of impaired integrin-mediated adhesion in wt cells and cAMP-induced increase in VE-cadherin cytoskeletal anchorage was abolished in VASP (-/-) endothelium. In summary, these data indicate that VASP is required for integrin-mediated adhesion which stabilizes endothelial barrier properties at least in part by facilitating Rac 1 activation.
65. Tbx1 genetically interacts with the transforming growth factor-β/bone morphogenetic protein inhibitor Smad7 during great vessel remodeling. Growth and remodeling of the pharyngeal arch arteries are vital for the development of a mature great vessel system. Dysmorphogenesis of the fourth arch arteries can result in interruption of the aortic arch type B, typically found in DiGeorge syndrome. Tbx1 haploinsufficient embryos, which model DiGeorge syndrome, display fourth arch artery defects during formation of the vessels. Recovery from such defects is a documented yet unexplained phenotype in Tbx1 haploinsufficiency.To understand the nature of fourth arch artery growth recovery in Tbx1 haploinsufficiency and its underlying genetic control.We categorized vessel phenotypes of Tbx1 heterozygotes as hypoplastic or aplastic at the conclusion of pharyngeal artery formation and compared these against the frequency of vessel defects scored at the end of great vessel development. The frequency of hypoplastic vessels decreased during embryogenesis, whereas no reduction of vessel aplasia was seen, implying recovery is attributable to remodeling of hypoplastic vessels. We showed that Smad7, an inhibitory Smad within the transforming growth factor-β pathway, is regulated by Tbx1, is required for arch artery remodeling, and genetically interacts with Tbx1 in this process. Tbx1 and Tbx1;Smad7 haploinsufficiency affected several remodeling processes; however, concurrent haploinsufficiency particularly impacted on the earliest stage of vascular smooth muscle cell vessel coverage and subsequent fibronectin deposition. Conditional reconstitution of Smad7 with a Tbx1Cre driver indicated that the interaction between the 2 genes is cell autonomous.Tbx1 acts upstream of Smad7 controlling vascular smooth muscle an**d extracellular matr**ix investment of the fourth arch artery.
66. Peripheral myelin protein 22 is in complex with alpha6beta4 integrin, and its absence alters the Schwann cell basal lamina. Peripheral myelin protein 22 (PMP22) is a tetraspan membrane glycoprotein, the misexpression of which is associated with hereditary demyelinating neuropathies. Myelinating Schwann cells (SCs) produce the highest levels of PMP22, yet the function of the protein in peripheral nerve biology is unresolved. To investigate the potential roles of PMP22, we engineered a novel knock-out (-/-) mouse line by replacing the first two coding exons of pmp22 with the lacZ reporter. PMP22-deficient mice show strong beta-galactosidase reactivity in peripheral nerves, cartilage, intestines, and lungs, whereas phenotypically they display the characteristics of tomaculous neuropathy. In the absence of PMP22, myelination of peripheral nerves is delayed, and numerous axon-SC profiles show loose basal lamina, suggesting altered interactions of the glial cells with the **extracellular matrix**. The levels of beta4 integrin, a molecule involved in the linkage between SCs and the basal lamina, are severely reduced in nerves of PMP22-deficient mice. During early stages of myelination, PMP22 and beta4 integrin are coexpressed at the cell surface and can be coimmunoprecipitated together with laminin and alpha6 integrin. In agreement, in clone A colonic carcinoma cells, epitope-tagged PMP22 forms a complex with beta4 integrin. Together, these data indicate that PMP22 is a binding partner in the integrin/laminin complex and is involved in mediating the interaction of SCs with the extracellular environment.
67. An essential requirement for β1 integrin in the assembly of **extracellular matri**x proteins within the vascular wall. β1 integrin has been shown to contribute to vascular smooth muscle cell differentiation, adhesion and mechanosensation in vitro. Here we showed that deletion of β1 integrin at the onset of smooth muscle differentiation resulted in interrupted aortic arch, aneurysms and failure to assemb**le extracellular mat**rix proteins. These defects result in lethality prior to birth. Our data indicates that β1 integrin is not required for the acquisition, but it is essential for the maintenance of the smooth muscle cell phenotype, as levels of critical smooth muscle proteins are gradually reduced in mutant mice. Furthermore, while deposition **of extracellular ma**trix was not affected, its structure was disrupted. Interestingly, defects **in extracellular ma**trix and vascular wall assembly, were restricted to the aortic arch and its branches, compromising the brachiocephalic and carotid arteries and to the exclusion of the descending aorta. Additional analysis of β1 integrin in the pharyngeal arch smooth muscle progenitors was performed using wnt1Cre. Neural crest cells deleted for β1 integrin were able to migrate to the pharyngeal arches and associate with endothelial lined arteries; but exhibited vascular remodeling defects and early lethality. This work demonstrates that β1 integrin is dispensable for migration and initiation of the smooth muscle differentiation program, however, it is essential for remodeling of the pharyngeal arch arteries and for the assembly of the vessel wall of their derivatives. It further establishes a critical role of β1 integrin in the protection against aneurysms that is particularly confined to the ascending aorta and its branches.
68. Integrin α6β1 Expressed in ESCs Instructs the Differentiation to Endothelial Cells. Adhesion of embryonic stem cells (ESCs) to th**e extracellular matr**ix may influence differentiation potential and cell fate decisions. Here, we investigated the inductive role of binding of integrin α6β1 expressed in mouse (m)ESCs to laminin-1 (LN1) in mediating the differentiation of ESCs to endothelial cells (ECs). We observed that α6β1 binding to LN1 was required for differentiation to ECs. α6β1 functioned by recruiting the adaptor tetraspanin protein CD151, which activated FAK and Akt signaling and mediated the EC lineage-specifying transcription factor Er71. In contrast, association of the ESC-expressed α3β1, another highly expressed LN1 binding integrin, with CD151, prevented α6β1-mediated differentiation. CD151 thus functioned as a bifurcation router to direct ESCs toward ECs when α6β1 associated with CD151, or prevented transition to ECs when α3β1 associated with CD151. These observations were recapitulated in mice in which α6 integrin or CD151 knockdown reduced the expression of Er71-regulated angiogenesis genes and development of blood vessels. Thus, interaction of α6β1 in ESCs with LN1 activates α6β1/CD151 signaling which programs ESCs toward the EC lineage fate.
69. Influence of beta1 integrins on epidermal squamous cell carcinoma formation in a transgenic mouse model: alpha3beta1, but not alpha2beta1, suppresses malignant conversion. Although aberrant integrin expression has been documented in many epithelial tumors, little is known about how integrins influence neoplastic progression. To examine this issue, transgenic mice in which the alpha2beta1 or alpha3beta1 integrin was expressed in the suprabasal epidermal layers via the involucrin promoter were subjected to skin carcinogenesis. Equal numbers of benign squamous papillomas were observed in transgenic and wild-type animals. However, the frequency of conversion of papillomas to malignant squamous cell carcinomas was much lower in alpha3beta1 transgenic than in alpha2beta1 transgenic and wild-type mice. No differences were observed in apoptosis or in the expression of endogenous integrins in transgenic and wild-type papillomas. However, alpha3beta1 transgenic papillomas displayed a diminished proliferative capacity and were more highly differentiated as judged by BrdUrd incorporation and keratin 10 expression, respectively, than alpha2beta1 transgenic and wild-type papillomas. Two proteins that associate with alpha3beta1 and not alpha2beta1 are **extracellular matrix** metalloproteinase inducer and CD81. **Extracellular matrix** metalloproteinase inducer expression correlated inversely with the degree of differentiation in normal epidermis and in transgenic and wild-type papillomas. Up-regulation of CD81 was observed in 100% of wild-type and 88% of alpha2beta1 transgenic papillomas but in only 25% of alpha3beta1 transgenic papillomas. CD81 was undetectable in untreated epidermis and strongly expressed in all transgenic and wild-type squamous cell carcinomas. Our results demonstrate that the alpha3beta1 integrin can suppress malignant conversion, and that the mechanism may involve CD81.
70. Fibronectin signals through integrin α5β1 to regulate cardiovascular development in a cell type-specific manner. Fibronectin (Fn1) is an evolutionarily conserve**d extracellular matr**ix glycoprotein essential for embryonic development. Global deletion of Fn1 leads to mid-gestation lethality from cardiovascular defects. However, severe morphogenetic defects that occur early in embryogenesis in these embryos precluded assigning a direct role for Fn1 in cardiovascular development. We noticed that Fn1 is expressed in strikingly non-uniform patterns during mouse embryogenesis, and that its expression is particularly enriched in the pharyngeal region corresponding with the pharyngeal arches 3, 4, and 6. This region bears a special importance for the developing cardiovascular system, and we hypothesized that the localized enrichment of Fn1 in the pharyngeal region may be essential for cardiovascular morphogenesis. To test this hypothesis, we ablated Fn1 using the Isl1(Cre) knock-in strain of mice. Deletion of Fn1 using the Isl1(Cre) strain resulted in defective formation of the 4th pharyngeal arch arteries (PAAs), aberrant development of the cardiac outflow tract (OFT), and ventricular septum defects. To determine the cell types responding to Fn1 signaling during cardiovascular development, we deleted a major Fn1 receptor, integrin α5 using the Isl1(Cre) strain, and observed the same spectrum of abnormalities seen in the Fn1 conditional mutants. Additional conditional mutagenesis studies designed to ablate integrin α5 in distinct cell types within the Isl1(+) tissues and their derivatives, suggested that the expression of integrin α5 in the pharyngeal arch mesoderm, endothelium, surface ectoderm and the neural crest were not required for PAA formation. Our studies suggest that an (as yet unknown) integrin α5-dependent signal extrinsic to the pharyngeal endothelium mediates the formation of the 4th PAAs.
71. Talin depletion reveals independence of initial cell spreading from integrin activation and traction. Cell spreading, adhesion and remodelling of the **extracellular matrix** (ECM) involve bi-directional signalling and physical linkages between the ECM, integrins and the cell cytoskeleton. The actin-binding proteins talin1 and 2 link ligand-bound integrins to the actin cytoskeleton and increase the affinity of integrin for the ECM. Here we report that depletion of talin2 in talin1-null (talin1(-/-)) cells did not affect the initiation of matrix-activated spreading or Src family kinase (SFK) activation, but abolished the ECM-integrin-cytoskeleton linkage and sustained cell spreading and adhesion. Specifically, focal adhesion assembly, focal adhesion kinase (FAK) signalling and traction force generation on substrates were severely affected. The talin1 head domain restored beta1 integrin activation but only full-length talin1 restored the ECM-cytoskeleton linkage and normal cytoskeleton organization. Our results demonstrate three biochemically distinct steps in fibronectin-activated cell spreading and adhesion: (1) fibronectin-integrin binding and initiation of spreading, (2) fast cell spreading and (3) focal adhesion formation and substrate traction. We suggest that talin is not required for initial cell spreading. However, talin provides the important mechanical linkage between ligand-bound integrins and the actin cytoskeleton required to catalyse focal adhesion-dependent pathways.
72. ADAMTS9-Mediated **Extracellular Matrix** Dynamics Regulates Umbilical Cord Vascular Smooth Muscle Differentiation and Rotation. Despite the significance for fetal nourishment in mammals, mechanisms of umbilical cord vascular growth remain poorly understood. Here, the secreted metalloprotease ADAMTS9 is shown to be necessary for murine umbilical cord vascular development. Restricting it to the cell surface using a gene trap allele, Adamts9(Gt), impaired umbilical vessel elongation and radial growth via reduced versican proteolysis and accumulation of **extracellular matrix** (ECM). Both Adamts9(Gt) and conditional Adamts9 deletion revealed that ADAMTS9 produced by mesenchymal cells acted non-autonomously to regulate smooth muscle cell (SMC) proliferation, differentiation, and orthogonal reorientation during growth of the umbilical vasculature. In Adamts9(Gt/Gt), we observed interference with PDGFRβ signaling via the mitogen-activated protein kinase/extracellular signal-regulated kinase (MAPK/ERK) pathway, which regulates cytoskeletal dynamics during SMC rotation. In addition, we observed disrupted Shh signaling and perturbed orientation of the mesenchymal primary cilium. Thus, ECM dynamics is a major influence on umbilical vascular SMC fate, with ADAMTS9 acting as its principal mediator.
73. Inactivation of integrin-β1 prevents the development of polycystic kidney disease after the loss of polycystin-1. Dysregulation of polycystin-1 (PC1) leads to autosomal dominant polycystic kidney disease (ADPKD), a disorder characterized by the formation of multiple bilateral renal cysts, the progressive accumulation of **extracellular matri**x (ECM), and the development of tubulointerstitial fibrosis. Correspondingly, cystic epithelia express higher levels of integrins (ECM receptors that control various cellular responses, such as cell proliferation, migration, and survival) that are characteristically altered in cystic cells. To determine whether the altered expression of ECM and integrins could establish a pathologic autostimulatory loop, we tested the role of integrin-β1 in vitro and on the cystic development of ADPKD in vivo. Compared with wild-type cells, PC1-depleted immortalized renal collecting duct cells had higher levels of integrin-β1 and fibronectin and displayed increased integrin-mediated signaling in the presence of Mn(2+). In mice, conditional inactivation of integrin-β1 in collecting ducts resulted in a dramatic inhibition of Pkd1-dependent cystogenesis with a concomitant suppression of fibrosis and preservation of normal renal function. Our data provide genetic evidence that a functional integrin-β1 is required for the early events leading to renal cystogenesis in ADPKD and suggest that the integrin signaling pathway may be an effective therapeutic target for slowing disease progression.
74. Type VIII collagen signals via β1 integrin and RhoA to regulate MMP-2 expression and smooth muscle cell migration. The **extracellular matri**x signals and regulates the behavior of vascular cells during the pathogenesis of atherosclerosis. Type VIII collagen, a short chain collagen, is scarcely present in normal arteries, but is dramatically upregulated in atherosclerosis and after other types of vascular injury. Cell culture studies have revealed that this protein supports smooth muscle cell (SMC) adhesion and stimulates migration, however little is known about the signaling or the mechanisms by which this occurs. SMCs isolated from wild-type C57BL/6 and type VIII collagen deficient mice were studied using assays to measure chemotactic and haptotactic migration, and remodeling and contraction of 3-dimensional type I collagen gels. Col8(-/-) SMCs exhibited impairments in migration, and a strongly adhesive phenotype with prominent stress fibers, stable microtubules and pronounced central basal focal adhesions. The addition of exogenous type VIII collagen to the Col8(-/-) SMCs rescued the impairments in migration, and restored cytoskeletal architecture so that it was similar to Col8(+/+) cells. We measured elevated levels of active GTP-RhoA in the Col8(-/-) cells, and this too was reversed by treatment with exogenous type VIII collagen. We showed that type VIII collagen normally suppresses RhoA activation through a beta-1 integrin dependent mechanism. MMP-2 levels were reduced in the Col8(-/-) SMCs, and knockdown of MMP-2 in Col8(+/+) SMCs partially recapitulated the decreases in migration and 3D gel contraction seen in Col8(-/-) cells, showing that type VIII collagen-stimulated migration was dependent on MMP-2. Inhibition of Rho restored MMP-2 activity in the Col8(-/-) cells, and partially rescued migration, demonstrating that the elevations in RhoA activity were responsible for the suppression of migration of these cells. In conclusion, we have shown that type VIII collagen signals through beta-1 integrin receptors to suppress RhoA, allowing optimal configuration of the cytoskeleton, and the stimulation of MMP-2-dependent cell migration.
75. beta1 integrins are not required for the maintenance of lymphocytes within intestinal epithelia. beta(1) integrins are thought to play a central role in maintaining lymphocytes within mucosal epithelia via their interactions with **extracellular matrix** proteins and subepithelial cellular components within and underlying the basement membrane. In the current study type a (CD8alphabetaTCRalphabeta) and type b (CD8alphaalphaTCRgammadelta and CD8alphaalphaTCRalphabeta) intraepithelial lymphocyte (IEL) subsets within the mouse small intestine were found to express functional beta(1) integrin and the beta(1) integrin alpha chain partners alpha(1), alpha(2), and alpha(4). Using inducible beta(1) integrin-knockout bone marrow-chimeric mice we demonstrate that IEL expression of alpha(1) and alpha(2) but not alpha(4) is dependent on expression of the beta(1) chain. Importantly, deletion of the beta(1) chain in IEL did not alter the number or composition of lymphocytes within the intestinal epithelium. Thus, while IEL express functional beta(1) integrins, these are not required to maintain lymphocytes within intestinal epithelia. This result is discussed in the light of conventional views of intestinal lymphocyte homing and localization.
76. Fibrillar Type I Collagen Enhances the Differentiation and Proliferation of Myofibroblasts by Lowering α2β1 Integrin Expression in Cardiac Fibrosis. Many studies have shown that α2β1 integrin plays an important role in the development of cardiac fibrosis. However, the mechanism of how α2β1 integrin regulates the differentiation and proliferation of myofibroblasts in cardiac fibrosis through fibrillar collagen (FC) remains uncertain. We established that FC mimicked the 3-dimens**ional extracellular** matrix (ECM) of fibroblasts from post-myocardial infarction (MI) patients in vivo. This allowed us to explore the differentiation and proliferation of cardiac fibroblasts on FC. Here, we report that low expression of α2β1 integrin increased protein kinase B (AKT) activation and α-smooth muscle actin (α-SMA) expression. This occurred due to the instability of phosphatase and tensin homolog (PTEN) in myofibroblasts on FC. We also demonstrated that FC reduced protein phosphatase type 2A (PP2A) activity of myofibroblasts, which was coincident with low α2β1 integrin expression and activation of AKT, but not mitogen-activated protein kinase (ERK). In addition, knock-down of both β1 integrin and PP2A in fibroblasts promoted differentiation and proliferation via AKT activation and increased α-SMA expression. In summary, our study demonstrated that low α2β1 integrin expression regulated its downstream targets PTEN and AKT via crosstalk with PP2A, a critical cell signaling pathway that permits aberrant differentiation and proliferation of myofibroblasts on FC.
77. Molecular cloning and characterization of the pig homologue to human CD29, the integrin beta1 subunit. CD29 is the beta1 subunit, a member of the integrin gene superfamily that function as receptor for cell adhesion molecules of the **extracellular matrix**. Porcine integrin beta1 subunit is involved in rejection of pig-to-human tissue xenografts as target of the natural antibodies present in the human serum. Moreover since CD29, as part of the beta1 integrins very late antigen 4 (VLA-4) and VLA-6, is involved in homing and differentiation of haematopoietic progenitor cells, its characterization in pig is critical to study the interaction of porcine adhesion molecules with human ligands in the induction of donor-specific tolerance toward porcine antigens, a process extremely desirable to prevent rejection of xenogeneic organs.The porcine CD29 cDNA has been isolated from a cDNA library and its structure determined. In addition, reverse transcription-polymerase chain reaction (RT-PCR) was performed to determine the expression of CD29 in different tissues.The nucleotide sequence of the porcine cDNA includes an open reading frame encoding a polypeptide of 798 amino acids. Expression analysis showed that porcine CD29 is expressed in all lymphoid tissues tested and, in lower amounts, in nonlymphoid tissues. Pig CD29 deduced amino acid sequence displays extensive conservation compared with CD29 sequences from other species and a common structural feature with all the other CD29 molecules analyzed in mammals, including the 12 potential N-glycosilation sites. Punctual changes between human and swine CD29 molecule into the ligand binding domain, and/or into the regulatory domain, suggest potential differences between human and porcine CD29 relative to the human CD29 ligands.Cloning of the swine CD29 gene offers a new tool for an alternative protocol of removing xenoreactive antibodies in the recipient. In addition, the determination of the differences between human and swine CD29 will help to understand the adhesion molecule-ligand interactions and their function across the swine-human barrier in xenotransplantation.
78. Islet Endothelial Cells Induce Glycosylation and Increase Cell-surface Expression of Integrin β1 in β Cells. The co-culturing of insulinoma and islet-derived endothelial cell (iEC) lines results in the spontaneous formation of free-floating pseudoislets (PIs). We previously showed that iEC-induced PIs display improved insulin expression and secretion in response to glucose stimulation. This improvement was associated with a de novo deposition o**f extracellular matr**ix (ECM) proteins by iECs in and around the PIs. Here, iEC-induced PIs were used to study the expression and posttranslational modification of the ECM receptor integrin β1. A wide array of integrin β subunits was detected in βTC3 and NIT-1 insulinomas as well as in primary islets, with integrin β1 mRNA and protein detected in all three cell types. Interestingly, the formation of iEC-induced PIs altered the glycosylation patterns of integrin β1, resulting in a higher molecular weight form of the receptor. This form was found in native pancreas but was completely absent in monolayer β-cells. Fluorescence-activated cell sorting analysis of monolayers and PIs revealed a higher expression of integrin β1 in PIs. Antibody-mediated blocking of integrin β1 led to alterations in β-cell morphology, reduced insulin gene expression, and enhanced glucose secretion under baseline conditions. These results suggest that iEC-induced PI formation may alter integrin β1 expression and posttranslational modification by enhancing glycosylation, thereby providing a more physiological culture system for studying integrin-ECM interactions in β cells.
79. Integrin-dependent force transmission to the **extracellular matrix** by α-actinin triggers adhesion maturation. Focal adhesions are mechanosensitive elements that enable mechanical communication between cells and the **extracellular matri**x. Here, we demonstrate a major mechanosensitive pathway in which α-actinin triggers adhesion maturation by linking integrins to actin in nascent adhesions. We show that depletion of the focal adhesion protein α-actinin enhances force generation in initial adhesions on fibronectin, but impairs mechanotransduction in a subsequent step, preventing adhesion maturation. Expression of an α-actinin fragment containing the integrin binding domain, however, dramatically reduces force generation in depleted cells. This behavior can be explained by a competition between talin (which mediates initial adhesion and force generation) and α-actinin for integrin binding. Indeed, we show in an in vitro assay that talin and α-actinin compete for binding to β3 integrins, but cooperate in binding to β1 integrins. Consistently, we find opposite effects of α-actinin depletion and expression of mutants on substrates that bind β3 integrins (fibronectin and vitronectin) versus substrates that only bind β1 integrins (collagen). We thus suggest that nascent adhesions composed of β3 integrins are initially linked to the actin cytoskeleton by talin, and then α-actinin competes with talin to bind β3 integrins. Force transmitted through α-actinin then triggers adhesion maturation. Once adhesions have matured, α-actinin recruitment correlates with force generation, suggesting that α-actinin is the main link transmitting force between integrins and the cytoskeleton in mature adhesions. Such a multistep process enables cells to adjust forces on matrices, unveiling a role of α-actinin that is different from its well-studied function as an actin cross-linker.
80. Clustering of alpha(5)beta(1) integrins determines adhesion strength whereas alpha(v)beta(3) and talin enable mechanotransduction. A key molecular link between cells and the **extracellular matrix** is the binding between fibronectin and integrins alpha(5)beta(1) and alpha(v)beta(3). However, the roles of these different integrins in establishing adhesion remain unclear. We tested the adhesion strength of fibronectin-integrin-cytoskeleton linkages by applying physiological nanonewton forces to fibronectin-coated magnetic beads bound to cells. We report that the clustering of fibronectin domains within 40 nm led to integrin alpha(5)beta(1) recruitment, and increased the ability to sustain force by over six-fold. This force was supported by alpha(5)beta(1) integrin clusters. Importantly, we did not detect a role of either integrin alpha(v)beta(3) or talin 1 or 2 in maintaining adhesion strength. Instead, these molecules enabled the connection to the cytoskeleton and reinforcement in response to an applied force. Thus, high matrix forces are primarily supported by clustered alpha(5)beta(1) integrins, while less stable links to alpha(v)beta(3) integrins initiate mechanotransduction, resulting in reinforcement of integrin-cytoskeleton linkages through talin-dependent bonds.
81. Beta1 integrins are required for the invasion of the caecum and proximal hindgut by enteric neural crest cells. Integrins are the major adhesive receptors for **extracellular matrix** and have various roles in development. To determine their role in cell migration, the gene encoding the beta1 integrin subunit (Itgb1) was conditionally deleted in mouse neural crest cells just after their emigration from the neural tube. We previously identified a major defect in gut colonisation by conditional Itgb1-null enteric neural crest cells (ENCCs) resulting from their impaired migratory abilities and enhanced aggregation properties. Here, we show that the migration defect occurs primarily during the invasion of the caecum, when Itgb1-null ENCCs stop their normal progression before invading the caecum and proximal hindgut by becoming abnormally aggregated. We found that the caecum and proximal hindgut express high levels of fibronectin and tenascin-C, two well-known ligands of integrins. In vitro, tenascin-C and fibronectin have opposite effects on ENCCs, with tenascin-C decreasing migration and adhesion and fibronectin strongly promoting them. Itgb1-null ENCCs exhibited an enhanced response to the inhibitory effect of tenascin-C, whereas they were insensitive to the stimulatory effect of fibronectin. These findings suggest that beta1 integrins are required to overcome the tenascin-C-mediated inhibition of migration within the caecum and proximal hindgut and to enhance fibronectin-dependent migration in these regions.
82. Coordinate integrin and c-Met signaling regulate Wnt gene expression during epithelial morphogenesis. Integrin receptors for the **extracellular matrix** and receptor tyrosine kinase growth factor receptors represent two of the major families of receptors that transduce into cells information about the surrounding environment. Wnt proteins are a major family of signaling molecules that regulate morphogenetic events. There is presently little understanding of how the expression of Wnt genes themselves is regulated. In this study, we demonstrate that alpha3beta1 integrin, a major laminin receptor involved in the development of the kidney, and c-Met, the receptor for hepatocyte growth factor, signal coordinately to regulate the expression of Wnt7b in the mouse. Wnt signals in turn appear to regulate epithelial cell survival in the papilla of the developing kidney, allowing for the elongation of epithelial tubules to form a mature papilla. Together, these results demonstrate how signals from integrins and growth factor receptors can be integrated to regulate the expression of an important family of signaling molecules so as to regulate morphogenetic events.
83. Mouse polymorphonuclear granulocyte binding to **extracellular matrix** molecules involves beta 1 integrins. The mechanism of adhesion of purified mouse polymorphonuclear granulocytes (PMN) to **extracellular matrix** proteins characteristic of basement membranes and the interstitium has been investigated and compared with the adhesion of a mouse progranulocytic cell line, 32DC13, and a mouse monocytic cell line, WEHI 78/24. All three cell types bound specifically to fibronectin and vitronectin to different degrees under different cellular activation states. 32DC13 bound to fibronectin and vitronectin strongly, and this binding increased upon cellular activation with phorbol 12-myristate-13-acetate (PMA) but not with formyl-Met-Leu-Phe. Only 32DC13 showed significant binding to laminin-1. By contrast, WEHI 78/24 and PMN bound only fibronectin and vitronectin; this binding was weak and was altered only marginally upon activation with PMA. In the case of WEHI 78/24, a slight increase in adhesion both to fibronectin and to vitronectin was observed after cellular activation with PMA, while PMN adhesion to both substrates was slightly reduced. The mechanism of binding to fibronectin and vitronectin was similar in the three cell types. The integrin alpha5 beta1 mediated fibronectin adhesion, demonstrating for the first time the existence of a functionally active beta1 integrin on mouse PMN. Vitronectin binding was mediated by alpha(v) beta3, as demonstrated by the ability of alpha(v)-specific cyclic L-Arg-L-Gly-L-Asp-D-Phe-L-Val (RGDfV) peptide (EMD66203), and anti-beta3 antibody to inhibit cell adhesion. 32DC13 adhesion to laminin-1 was via the alpha6 beta1 integrin. None of the three cell types tested bound to the basement membrane proteins collagen type IV and perlecan, or to the interstitial stromal constituents tenascin, collagen types I, V and VI. Interestingly, perlecan and collagen type IV were found to repel all three cell types. The relative inability of PMN, WEHI 78/24, and 32DC13 to bind to **extracellular matrix** proteins characteristic of basement membranes and their ability to bind inflammatory markers of the interstitium is discussed with respect to leukocyte extravasation processes.
84. Cell-**extracellular matrix** versus cell-cell interactions during the development of the cochlear-vestibular ganglion. Cells destined to become the neurones of the cochlear-vestibular ganglion (CVG) originate within the otic epithelium. Early in development they detach from their neighbors and migrate out of the epithelium, where they coalesce to form the CVG. To accomplish this process, the neuroblasts must modify their interactions with other cells within the epithelium and with proteins in the **extracellular matrix** to allow for repositioning. The aim of this study was to investigate the roles of the major families of adhesion molecules that mediate cellular interactions with the **extracellular matrix**, the integrins, and with other cells, the cadherins, in neuroblast segregation from the otic epithelium. The expression of classical cadherins increased in migrating neuroblasts compared with the otic epithelium. Quantitative RT-PCR revealed that this was concomitant with down-regulation of E-cadherin and up-regulation of N-cadherin in the migrating cells. In contrast, the level of β1 integrin expression by the epithelium was maintained in migrating neuroblasts. However, although multiple integrin ligands were expressed within the otic basement membrane at this stage of development, only fibronectin (FN) supported neuroblast migration along the substrate in vitro. Inhibition of β1 integrins resulted in significantly reduced linear migration on FN. Importantly, neuroblasts retained the ability to segregate from the epithelium but remained compacted immediately adjacent to the originating tissue, suggesting dominance of cell-cell over cell-matrix interactions. These data suggest that the balance between cell-cell and cell-substratum interactions directs otic neuroblast migration and gangliogenesis.
85. Alpha5beta1 integrin engagement increases large conductance, Ca2+-activated K+ channel current and Ca2+ sensitivity through c-src-mediated channel phosphorylation. Large conductance, calcium-activated K(+) (BK) channels are important regulators of cell excitability and recognized targets of intracellular kinases. BK channel modulation by tyrosine kinases, including focal adhesion kinase and c-src, suggests their potential involvement in integrin signaling. Recently, we found that fibronectin, an endogenous alpha5beta1 integrin ligand, enhances BK channel current through both Ca(2+)- and phosphorylation-dependent mechanisms in vascular smooth muscle. Here, we show that macroscopic currents from HEK 293 cells expressing murine BK channel alpha-subunits (mSlo) are acutely potentiated following alpha5beta1 integrin activation. The effect occurs in a Ca(2+)-dependent manner, 1-3 min after integrin engagement. After integrin activation, normalized conductance-voltage relations for mSlo are left-shifted at free Ca(2+) concentrations >or=1 microm. Overexpression of human c-src with mSlo, in the absence of integrin activation, leads to similar shifts in mSlo Ca(2+) sensitivity, whereas overexpression of catalytically inactive c-src blocks integrin-induced potentiation. However, neither integrin activation nor c-src overexpression potentiates current in BK channels containing a point mutation at Tyr-766. Biochemical tests confirmed the critical importance of residue Tyr-766 in integrin-induced channel phosphorylation. Thus, BK channel activity is enhanced by alpha5beta1 integrin activation, likely through an intracellular signaling pathway involving c-src phosphorylation of the channel alpha-subunit at Tyr-766. The net result is increased current amplitude, enhanced Ca(2+) sensitivity, and rate of activation of the BK channel, which would collectively promote smooth muscle hyperpolarization in response to integrin-**extracellular matrix** interactions.
86. IpaA targets beta1 integrins and rho to promote actin cytoskeleton rearrangements necessary for Shigella entry. Shigella invasion into the colonic epithelium involves many steps including the formation of large membrane protrusions by the epithelial cells that facilitate bacterial engulfment. IpaA, a Shigella protein secreted into target cells upon cell contact induces a loss of actin stress fibers in cells and promotes the reorganization of actin at the site of entry. The mechanism for this is not known but is thought to involve recruitment of the focal adhesion protein vinculin to IpaA. Here we have examined the mechanism for the effects of IpaA on the actin cytoskeleton. We show that IpaA-induced loss of actin stress fibers and cell rounding do not require vinculin expression or an intact vinculin binding site on IpaA. Rather, we find that cells expressing IpaA exhibited elevated Rho activity and increased myosin light chain phosphorylation. In addition, IpaA decreases integrin affinity for **extracellular matrix** ligands by interfering with talin recruitment to the integrin cytoplasmic tail. The combination of these two effects, namely weakened adhesion and increased contractility, account for the loss of actin stress fibers and cell rounding observed in cells exposed to IpaA.
87. Biologically-active laminin-111 fragment that modulates the epithelial-to-mesenchymal transition in embryonic stem cells. The dynamic interplay between the **extracellular matrix** and embryonic stem cells (ESCs) constitutes one of the key steps in understanding stem cell differentiation in vitro. Here we report a biologically-active laminin-111 fragment generated by matrix metalloproteinase 2 (MMP2) processing, which is highly up-regulated during differentiation. We show that the β1-chain-derived fragment interacts via α3β1-integrins, thereby triggering the down-regulation of MMP2 in mouse and human ESCs. Additionally, the expression of MMP9 and E-cadherin is up-regulated in mouse ESCs--key players in the epithelial-to-mesenchymal transition. We also demonstrate that the fragment acts through the α3β1-inte**grin/extracellular m**atrix metalloproteinase inducer complex. This study reveals a previously unidentified role of laminin-111 in early stem cell differentiation that goes far beyond basement membrane assembly and a mechanism by which an MMP2-cleaved laminin fragment regulates the expression of E-cadherin, MMP2, and MMP9.
88. The ECM protein nephronectin promotes kidney development via integrin alpha8beta1-mediated stimulation of Gdnf expression. Development of the metanephric kidney crucially depends on proper interactions between cells and the surrounding **extracellular matrix**. For example, we showed previously that in the absence of alpha8beta1 integrin, invasion by the ureteric bud into the metanephric mesenchyme is inhibited, resulting in renal agenesis. Here we present genetic evidence that the **extracellular matrix** protein nephronectin is an essential ligand that engages alpha8beta1 integrin during early kidney development. We show that embryos lacking a functional nephronectin gene frequently display kidney agenesis or hypoplasia, which can be traced to a delay in the invasion of the metanephric mesenchyme by the ureteric bud at an early stage of kidney development. Significantly, we detected no defects in **extracellular matrix** organization in the nascent kidneys of the nephronectin mutants. Instead, we found that Gdnf expression was dramatically reduced in both nephronectin- and alpha8 integrin-null mutants specifically in the metanephric mesenchyme at the time of ureteric bud invasion. We show that this reduction is sufficient to explain the agenesis and hypoplasia observed in both mutants. Interestingly, the reduction in Gdnf expression is transient, and its resumption presumably enables the nephronectin-deficient ureteric buds to invade the metanephric mesenchyme and begin branching. Our results thus place nephronectin and alpha8beta1 integrin in a pathway that regulates Gdnf expression and is essential for kidney development.
89. Binding of laminin-1 to monosialoganglioside GM1 in lipid rafts is crucial for neurite outgrowth. Laminin-1, an **extracellular matrix** molecule, promotes neurite outgrowth through the interaction of integrin and actin. Monosialoganglioside GM1 in the lipid rafts associates with and activates the NGF receptor TrkA, and enhances neurite outgrowth. However, the role of GM1 in laminin-1-induced neurite outgrowth was still unclear. Here, we describe that laminin-1 binds to GM1 through a carbohydrate moiety and a specific conformation of GM1, induces focal formation of large clusters of GM1, and enhances the relocation of TrkA in the membrane of dorsal root ganglion (DRG) and PC12 cells. We found that laminin-1-mediated clustering of GM1 causes the translocation and enrichment of beta1 integrin in lipid rafts--where TrkA colocalizes with beta1 integrin--and the activation of Lyn, Akt and MAPK to promote the outgrowth of neurites. Our results suggest that the binding of laminin-1 to GM1 facilitates the formation of a focal microdomain in the membrane, and enhances signal transduction that promotes neurite outgrowth by linking NGF-TrkA signaling with the laminin-integrin signaling pathways.
90. Down-regulation of integrin β1 and focal adhesion kinase in renal glomeruli under various hemodynamic conditions. Given that integrin β1 is an important component of the connection to maintain glomerular structural integrity, by binding with multipl**e extracellular matr**ix proteins and mediating intracellular signaling. Focal adhesion kinase (FAK) is the most essential intracellular integrator in the integrin β1-FAK signalling pathway. Here, we investigated the changes of the two molecules and visualized the possible interaction between them under various hemodynamic conditions in podocytes. Mice kidney tissues were prepared using in vivo cryotechnique (IVCT) and then were stained and observed using light microscopy, confocal laser scanning microscopy and immunoelectron microscopy. The expression of these molecules were examined by western blot. Under the normal condition, integrin β1 stained continually and evenly at the membrane, and FAK was located in the cytoplasm and nuclei of the podocytes. There were significant colocalized plaques of two molecules. But under acute hypertensive and cardiac arrest conditions, integrin β1 decreased and stained intermittently. Similarly, FAK decreased and appeared uneven. Additionally, FAK translocated to the nuclei of the podocytes. As a result, the colocalization of integrin β1 and FAK reduced obviously under these conditions. Western blot assay showed a consistent result with the immunostaining. Collectively, the abnormal redistribution and decreased expressions of integrin β1 and FAK are important molecular events in regulating the functions of podocytes under abnormal hemodynamic conditions. IVCT could offer considerable advantages for morphological analysis when researching renal diseases.
91. Impact of **extracellular matrix** derived from osteoarthritis subchondral bone osteoblasts on osteocytes: role of integrinβ1 and focal adhesion kinase signaling cues. Our recent study indicated that subchondral bone pathogenesis in osteoarthritis (OA) is associated with osteocyte morphology and phenotypic abnormalities. However, the mechanism underlying this abnormality needs to be identified. In this study we investigated the effect of **extracellular matri**x (ECM) produced from normal and OA bone on osteocytic cells function.De-cellularized matrices, resembling the bone provisional ECM secreted from primary human subchondral bone osteoblasts (SBOs) of normal and OA patients were used as a model to study the effect on osteocytic cells. Osteocytic cells (MLOY4 osteocyte cell line) cultured on normal and OA derived ECMs were analyzed by confocal microscopy, scanning electron microscopy (SEM), cell attachment assays, zymography, apoptosis assays, qRT-PCR and western blotting. The role of integrinβ1 and focal adhesion kinase (FAK) signaling pathways during these interactions were monitored using appropriate blocking antibodies.The ECM produced by OA SBOs contained less mineral content, showed altered organization of matrix proteins and matrix structure compared with the matrices produced by normal SBOs. Culture of osteocytic cells on these defective OA ECM resulted in a decrease of integrinβ1 expression and the de-activation of FAK cell signaling pathway, which subsequently affected the initial osteocytic cell's attachment and functions including morphological abnormalities of cytoskeletal structures, focal adhesions, increased apoptosis, altered osteocyte specific gene expression and increased Matrix metalloproteinases (MMP-2) and -9 expression.This study provides new insights in understanding how altered OA bone matrix can lead to the abnormal osteocyte phenotypic changes, which is typical in OA pathogenesis.
92. Deletion of hensin/DMBT1 blocks conversion of beta- to alpha-intercalated cells and induces distal renal tubular acidosis. Acid-base transport in the renal collecting tubule is mediated by two canonical cell types: the β-intercalated cell secretes HCO(3) by an apical Cl:HCO(3) named pendrin and a basolateral vacuolar (V)-ATPase. Acid secretion is mediated by the α-intercalated cell, which has an apical V-ATPase and a basolateral Cl:HCO(3) exchanger (kAE1). We previously suggested that the β-cell converts to the α-cell in response to acid feeding, a process that depended on the secretion and deposition of **an extracellular ma**trix protein termed hensin (DMBT1). Here, we show that deletion of hensin from intercalated cells results in the absence of typical α-intercalated cells and the consequent development of complete distal renal tubular acidosis (dRTA). Essentially all of the intercalated cells in the cortex of the mutant mice are canonical β-type cells, with apical pendrin and basolateral or diffuse/bipolar V-ATPase. In the medulla, however, a previously undescribed cell type has been uncovered, which resembles the cortical β-intercalated cell in ultrastructure, but does not express pendrin. Polymerization and deposition of hensin (in response to acidosis) requires the activation of β1 integrin, and deletion of this gene from the intercalated cell caused a phenotype that was identical to the deletion of hensin itself, supporting its critical role in hensin function. Because previous studies suggested that the conversion of β- to α-intercalated cells is a manifestation of terminal differentiation, the present results demonstrate that this differentiation proceeds from HCO(3) secreting to acid secreting phenotypes, a process that requires deposition of hensin in the ECM.
93. Differential expression of laminin chain-specific mRNA transcripts during mouse preimplantation embryo development. Laminin is the first **extracellular matrix** protein that has been shown to be synthesized in preimplantation mouse embryos. In the present study, chain-specific expression patterns of laminin mRNAs were examined by semiquantitative reverse transcription-polymerase chain reaction (RT-PCR). During preimplantation mouse embryo development, temporal expression patterns of laminin chain mRNAs were somewhat differential: B1 chain mRNA was first detectable at the late two-cell stage and its level was gradually increased by the blastocyst stage. In contrast, B2 and A chain mRNAs first appeared at the morula and blastocyst stages, respectively. At the blastocyst stage, all of the laminin chain mRNAs were highly detected compared to the earlier stages. When embryos were flushed at the morula stage and cultured in vitro, all laminin chain mRNA levels were decreased or not changed in the process of blastocoele expansion. In contrast, in the in vivo condition where embryos at different stages of blastocyst were flushed at different time points, laminin chain mRNA levels were increased as a function of blastocoele expansion. These changes in laminin mRNAs were parallel with its receptors such as integrin alpha 3 and alpha 6. 3-Isobutyl-1-methylxanthine (IBMX), which is known to be a potent activator of blastocoele expansion and regulates cAMP metabolism, upregulated laminin expression (except B1 chain) in blastocysts cultured in vitro. In vitro cultured embryos normally developed up to the late blastocyst, although their development was delayed compared with the in vivo condition where laminin gene expression was gradually increased as the blastocoele expanded. These results indicate that laminin expression may not be involved directly in the regulation of blastocoele expansion. The uterine environment enclosing the preimplantation embryos appears, therefore, to play an important role in the regulation of laminin gene expression during blastocyst development.
94. Loss of fibulin-5 binding to beta1 integrins inhibits tumor growth by increasing the level of ROS. Tumor survival depends in part on the ability of tumor cells to transform the surrounding **extracellular matrix** (ECM) into an environment conducive to tumor progression. Matricellular proteins are secreted into the ECM and impact signaling pathways that are required for pro-tumorigenic activities such as angiogenesis. Fibulin-5 (Fbln5) is a matricellular protein that was recently shown to regulate angiogenesis; however, its effect on tumor angiogenesis and thus tumor growth is currently unknown. We report that the growth of pancreatic tumors and tumor angiogenesis is suppressed in Fbln5-null (Fbln5(-/-)) mice compared with wild-type (WT) littermates. Furthermore, we observed an increase in the level of reactive oxygen species (ROS) in tumors grown in Fbln5(-/-) animals. Increased ROS resulted in elevated DNA damage, increased apoptosis of endothelial cells within the tumor, and represented the underlying cause for the reduction in angiogenesis and tumor growth. In vitro, we identified a novel pathway by which Fbln5 controls ROS production through a mechanism that is dependent on beta1 integrins. These results were validated in Fbln5(RGE/RGE) mice, which harbor a point mutation in the integrin-binding RGD motif of Fbln5, preventing its interaction with integrins. Tumor growth and angiogenesis was reduced in Fbln5(RGE/RGE) mice, however treatment with an antioxidant rescued angiogenesis and elevated tumor growth to WT levels. These findings introduce a novel function for Fbln5 in the regulation of integrin-induced ROS production and establish a rationale for future studies to examine whether blocking Fbln5 function could be an effective anti-tumor strategy, alone or in combination with other therapies.
95. The muscle integrin binding protein (MIBP) interacts with alpha7beta1 integrin and regulates cell adhesion and laminin matrix deposition. Integrins are alphabeta transmembrane receptors that function in key cellular processes, including cell adhesion, differentiation, and **extracellular matrix** deposition through interactions with extracellular, membrane, and cytoplasmic proteins. We previously identified and cloned a muscle beta1 integrin cytoplasmic binding protein termed MIBP and found that the expression level of MIBP is critical in the decision-making process of terminal myogenic differentiation. We report here that MIBP interacts with the alpha7beta1 integrin but not the alpha5beta1 integrin in C2C12 myoblasts, suggesting an important role of integrin alpha chains in the regulation of the beta1-MIBP interaction. Furthermore, consistent with its selective binding activity toward the alpha7beta1 laminin receptor, we have found that overexpression of MIBP in C2C12 myoblasts resulted in a significant reduction of cell adhesion to laminin and inhibition of laminin matrix deposition. By contrast, neither cell adhesion to fibronectin nor fibronectin matrix deposition was significantly altered in cells overexpressing MIBP. Finally, we show that both the protein level and tyrosine phosphorylation of paxillin, a key signaling molecule involved in the cellular control of myogenic differentiation, are increased by MIBP. These results suggest that MIBP functions in the control of myogenic differentiation by regulating alpha7beta1 integrin-mediated cell interactions with laminin matrix and intracellular signaling through paxillin.
96. Beta1 integrins activate a MAPK signalling pathway in neural stem cells that contributes to their maintenance. The emerging evidence that stem cells develop in specialised niches highlights the potential role of environmental factors in their regulation. Here we examine the role of beta1 integrin/**extracellular matrix** interactions in neural stem cells. We find high levels of beta1 integrin expression in the stem-cell containing regions of the embryonic CNS, with associated expression of the laminin alpha2 chain. Expression levels of laminin alpha2 are reduced in the postnatal CNS, but a population of cells expressing high levels of beta1 remains. Using neurospheres - aggregate cultures, derived from single stem cells, that have a three-dimensional architecture that results in the localisation of the stem cell population around the edge of the sphere - we show directly that beta1 integrins are expressed at high levels on neural stem cells and can be used for their selection. MAPK, but not PI3K, signalling is required for neural stem cell maintenance, as assessed by neurosphere formation, and inhibition or genetic ablation of beta1 integrin using cre/lox technology reduces the level of MAPK activity. We conclude that integrins are therefore an important part of the signalling mechanisms that control neural stem cell behaviour in specific areas of the CNS.
97. Fibronectin receptor functions in embryonic cells deficient in alpha 5 beta 1 integrin can be replaced by alpha V integrins. alpha 5 beta 1 integrin mediates cell adhesion to **extracellular matrix** by interacting with fibronectin (FN). Mouse lines carrying null mutations in genes encoding either the alpha 5 integrin subunit or FN have been generated previously. Both mutations are embryonic lethal with overlapping defects, but the defects of alpha 5-null embryos are less severe. Primary embryonic cells lacking alpha 5 beta 1 are able to adhere to FN, form focal contacts, migrate on FN, and assemble FN matrix. These results suggest the involvement of (an)other FN receptors(s). In this study, we examined functions of alpha 4 beta 1 and alpha V integrins in embryonic cells lacking alpha 5 beta 1. Our analysis of cells lacking both alpha 4 beta 1 and alpha 5 beta 1 showed that alpha 4 beta 1 is also not required for these FN-dependent functions. Using alpha V-specific blocking reagents, we showed that alpha V integrins are required for alpha 5-null cells, but not wild-type cells, to adhere and spread on FN. Our data also showed that, although the expression levels of alpha V integrins on the wild-type and alpha 5-null cells are similar, there is an increase in recruitment of alpha V integrins into focal contacts in alpha 5-null cells plated on FN, indicating that alpha V integrins can compensate functionally for the loss of alpha 5 beta 1 in focal contacts of alpha 5-null cells. Finally, our data suggested possible roles for alpha V integrins in replacing the role of alpha 5 beta 1 in FN matrix assembly in vitro and in FN-dependent embryonic functions in vivo.
98. Spatiotemporal localization of periostin and its potential role in epithelial-mesenchymal transition during palatal fusion. The medial epithelial seam (MES) between the palatal shelves degrades during palatal fusion to achieve the confluence of palatal mesenchyme. Cellular mechanisms underlying the degradation of MES have been proposed, such as apoptosis, epithelial-mesenchymal transition (EMT) and migration of medial edge epithelia (MEE). **Extracellular matrix** components have been shown to play an important role in EMT in many model systems. Periostin (also known as osteoblast-specific factor-2) is a secreted mesenchymal **extracellular matrix** component that affects the ability of cells to migrate and/or facilitates EMT during both embryonic development and pathologic conditions. In this study, we evaluated the spatiotemporal expression patterns of periostin during mouse palatal fusion by in situ hybridization and immunofluorescence. Periostin mRNA and protein were present in the palatal mesenchyme, the protein being distributed in a fine fibrillar network and in the basement membrane, but absent from the epithelium. During MES degradation, the protein was strongly expressed in the basement membrane underlying the MES and in some select MEE. Confocal microscopic analysis using an EMT marker, twist1, and an epithelial marker, cytokeratin 14, provided evidence that select MEE were undergoing EMT in association with periostin. Moreover, the major **extracellular matrix** molecules in basement membrane, laminin and collagen type IV were degraded earlier than periostin. The result is that select MEE establish interactions with periostin in the mesenchymal **extracellular matrix**, and these new cell-matrix interactions may regulate MEE transdifferentiation during palatal fusion.
99. Regulation of fibulin-2 gene expression by integrin α3β1 contributes to the invasive phenotype of transformed keratinocytes. The laminin-binding integrin α3β1 is highly expressed in epidermal keratinocytes, where it regulates both cell-autonomous and paracrine functions that promote wound healing and skin tumorigenesis. However, the roles for α3β1 in regulating gene expression programs that control the behaviors of immortalized or transformed keratinocytes remain underexplored. In the current study, we used a microarray approach to identify genes that are regulated by α3β1 in immortalized keratinocytes. α3β1-Responsive genes included several genes that are in**volved in extracellu**lar matrix proteolysis or remodeling, including fibulin-2 and secreted protein acidic and rich in cysteine. However, α3β1-dependent induction of specific target genes was influenced by the genetic lesion that triggered immortalization, as α3β1-dependent fibulin-2 expression occurred in cells immortalized by either SV40 large T antigen or p53-null mutation, whereas α3β1-dependent expression of secreted protein acidic and rich in cysteine occurred only in the former cells. Interestingly, quantitative PCR arrays did not reveal strong patterns of α3β1-dependent gene expression in freshly isolated primary keratinocytes, suggesting that this regulation is acquired during immortalization. p53-null keratinocytes transformed with oncogenic RasV12 retained α3β1-dependent fibulin-2 expression, and RNAi-mediated knockdown of fibulin-2 in these cells reduced invasion, although not their tumorigenic potential. These findings demonstrate a prominent role for α3β1 in immortalized/transformed keratinocytes in regulating fibulin-2 and other genes that promote matrix remodeling and invasion.
100. The forkhead box m1 transcription factor is essential for embryonic development of pulmonary vasculature. Transgenic and gene knock-out studies demonstrated that the mouse Forkhead Box m1 (Foxm1 or Foxm1b) transcription factor (previously called HFH-11B, Trident, Win, or MPP2) is essential for hepatocyte entry into mitosis during liver development, regeneration, and liver cancer. Targeted deletion of Foxm1 gene in mice produces an embryonic lethal phenotype due to severe abnormalities in the development of liver and heart. In this study, we show for the first time that Foxm1(-/-) lungs exhibit severe hypertrophy of arteriolar smooth muscle cells and defects in the formation of peripheral pulmonary capillaries as evidenced by significant reduction in platelet endothelial cell adhesion molecule 1 staining of the distal lung. Consistent with these findings, significant reduction in proliferation of the embryonic Foxm1(-/-) lung mesenchyme was found, yet proliferation levels were normal in the Foxm1-deficient epithelial cells. Severe abnormalities of the lung vasculature in Foxm1(-/-) embryos were associated with diminished expression of the transforming growth factor beta receptor II, a disintegrin and metalloprotease domain 17 (ADAM-17), vascular endothelial growth factor receptors, Polo-like kinase 1, Aurora B kinase, laminin alpha4 (Lama4), and the Forkhead Box f1 transcription factor. Cotransfection studies demonstrated that Foxm1 stimulates transcription of the Lama4 promoter, and this stimulation requires the Foxm1 binding sites located between -1174 and -1145 bp of the mouse Lama4 promoter. In summary, development of mouse lungs depends on the Foxm1 transcription factor, which regulates expression of genes essential for mesenchyme proliferation, **extracellular matrix** remodeling, and vasculogenesis.
101. Adhesion to the **extracellular matrix** is required for interleukin-1 beta actions leading to reactive phenotype in rat astrocytes. The **extracellular matrix** (ECM) of the brain is essential for homeostasis and normal functions, but is rapidly remodelled during acute brain injury alongside the development of an inflammatory response driven by the cytokine interleukin (IL)-1. Whether the ECM regulates IL-1 actions in astrocytes is completely unknown. The aim of this study was to test the hypothesis that cellular attachment to the ECM is a critical mediator of IL-1beta-induced signalling pathways and development of reactive phenotype in astrocytes. Primary rat astrocytes adhered to fibronectin, laminin and fibrillin-1 in an integrin-dependent manner. Attachment to these ECM molecules significantly increased IL-1beta-induced activation of extracellular signal-regulated kinase 1/2 (ERK1/2) and inhibition of RhoA and Rho kinase (ROCK), coincident with loss of focal adhesions and cellular morphological changes. Our data demonstrate that the ECM regulates IL-1 actions in astrocytes via cross-talk mechanisms between ERK1/2 and RhoA/ROCK, which could have important implications in brain inflammatory disorders.
102. Ephrin B1 maintains apical adhesion of neural progenitors. Apical neural progenitors are polarized cells for which the apical membrane is the site of cell-cell and cell-**extracellular matrix** adhesion events that are essential for maintaining the integrity of the developing neuroepithelium. Apical adhesion is important for several aspects of the nervous system development, including morphogenesis and neurogenesis, yet the mechanisms underlying its regulation remain poorly understood. Here, we show that ephrin B1, a cell surface protein that engages in cell signaling upon binding cognate Eph receptors, controls normal morphogenesis of the developing cortex. Efnb1-deficient embryos exhibit morphological alterations of the neuroepithelium that correlate with neural tube closure defects. Using loss-of-function experiments by ex vivo electroporation, we demonstrate that ephrin B1 is required in apical progenitors (APs) to maintain their apical adhesion. Mechanistically, we show that ephrin B1 controls cell-ECM adhesion by promoting apical localization of integrin β1 and we identify ADP-ribosylation factor 6 (Arf6) as an important effector of ephrin B1 reverse signaling in apical adhesion of APs. Our results provide evidence for an important role for ephrin B1 in maintaining the structural integrity of the developing cortex and highlight the importance of tightly controlling apical cell-ECM adhesion for neuroepithelial development.
103. C1GALT1 promotes invasive phenotypes of hepatocellular carcinoma cells by modulating integrin β1 glycosylation and activity. Cancer cell invasion and metastasis are the primary causes of treatment failure and death in hepatocellular carcinoma (HCC). We previously reported that core 1 β1,3-galactosyltransferase (C1GALT1) is frequently overexpressed in HCC tumors and its expression is associated with advanced tumor stage, metastasis, and poor survival. However, the underlying mechanisms of C1GALT1 in HCC malignancy remain unclear. In this study, we found that overexpression of C1GALT1 enhanced HCC cell adhesion t**o extracellular matr**ix (ECM) proteins, migration, and invasion, whereas RNAi-mediated knockdown of C1GALT1 suppressed these phenotypes. The promoting effect of C1GALT1 on the metastasis of HCC cells was demonstrated in a mouse xenograft model. Mechanistic investigations showed that the C1GALT1-enhanced phenotypic changes in HCC cells were significantly suppressed by anti-integrin β1 blocking antibody. Moreover, C1GALT1 was able to modify O-glycans on integrin β1 and regulate integrin β1 activity as well as its downstream signaling. These results suggest that C1GALT1 could enhance HCC invasiveness through integrin β1 and provide novel insights into the roles of O-glycosylation in HCC metastasis.
104. Regulation of Integrin β 1 recycling to lipid rafts by Rab1a to promote cell migration. Rab1a is a member of the Rab family of small GTPases with a well characterized function in the regulation of vesicle trafficking from the endoplasmic reticulum to the Golgi apparatus and within Golgi compartments. The integrin family heterodimeric transmembrane proteins serve as major receptors for **extracellular matri**x proteins, which play essential roles in cell adhesion and migration. Although effects on intracellular trafficking of integrins or other key cargos by Rab1a could influence cell migration, the regulatory mechanisms linking Rab1a to cell migration are not well understood. Here, we report identification of Rab1a as a novel regulator of cell migration using an unbiased RNAi screen targeting GTPases. Inhibition of Rab1a reduced integrin-mediated cell adhesion and spreading on fibronectins, reduced integrin β1 localization to lipid rafts, and decreased recycling of integrin β1 to the plasma membrane. Analysis of Rab1a effector molecules showed that p115 mediated Rab1a regulation of integrin recycling and lipid raft localization in cell migration. Taken together, these results suggest a novel function for Rab1a in the regulation of cell migration through controlling integrin β1 recycling and localization to lipid rafts via a specific downstream effector pathway.
105. Interaction between fibronectin and β1 integrin is essential for tooth development. The dental epithelium and **extracellular matri**x interact to ensure that cell growth and differentiation lead to the formation of teeth of appropriate size and quality. To determine the role of fibronectin in differentiation of the dental epithelium and tooth formation, we analyzed its expression in developing incisors. Fibronectin mRNA was expressed during the presecretory stage in developing dental epithelium, decreased in the secretory and early maturation stages, and then reappeared during the late maturation stage. The binding of dental epithelial cells derived from postnatal day-1 molars to a fibronectin-coated dish was inhibited by the RGD but not RAD peptide, and by a β1 integrin-neutralizing antibody, suggesting that fibronectin-β1 integrin interactions contribute to dental epithelial-cell binding. Because fibronectin and β1 integrin are highly expressed in the dental mesenchyme, it is difficult to determine precisely how their interactions influence dental epithelial differentiation in vivo. Therefore, we analyzed β1 integrin conditional knockout mice (Intβ1lox-/lox-/K14-Cre) and found that they exhibited partial enamel hypoplasia, and delayed eruption of molars and differentiation of ameloblasts, but not of odontoblasts. Furthermore, a cyst-like structure was observed during late ameloblast maturation. Dental epithelial cells from knockout mice did not bind to fibronectin, and induction of ameloblastin expression in these cells by neurotrophic factor-4 was inhibited by treatment with RGD peptide or a fibronectin siRNA, suggesting that the epithelial interaction between fibronectin and β1 integrin is important for ameloblast differentiation and enamel formation.
106. Polymerase delta-interacting protein 2 regulates collagen accumulation via activation of the Akt/mTOR pathway in vascular smooth muscle cells. Polymerase delta interacting protein 2 (Poldip2) has previously been implicated in migration, proliferation and **extracellular matrix** (ECM) production in vascular smooth muscle cells. To better understand the role of Poldip2 in ECM regulation, we investigated the mechanism responsible for collagen I accumulation in Poldip2(+/-) mouse aortic smooth muscle cells (MASMs).Protein degradation and protein synthesis pathways were investigated. Depletion of Poldip2 had no effect on proteasome activity, but caused a partial reduction in autophagic flux. However, the rate of collagen I degradation was increased in Poldip2(+/-) vs. Poldip2(+/+) MASMs. Conversely, activation of the PI3K/Akt/mTOR signaling pathway, involved in regulation of protein synthesis, was significantly elevated in Poldip2(+/-) MASMs as was β1-integrin expression. Suppressing mTOR signaling using Akt inhibitor or rapamycin and reducing β1-integrin expression using siRNA prevented the increase in collagen I production. While collagen I and fibronectin were increased in Poldip2(+/-) MASMs, overall protein synthesis was not different from that in Poldip2(+/)(+)MASMs, suggesting selectivity of Poldip2 for ECM proteins.Poldip2(+/-) MASMs exhibit higher β1-integrin expression and activity of the PI3K/Akt/mTOR signaling pathway, leading to increased ECM protein synthesis. These findings have important implications for vascular diseases in which ECM accumulation plays a role.
107. miRNA-29c suppresses lung cancer cell adhesion to **extracellular matrix** and metastasis by targeting integrin β1 and matrix metalloproteinase2 (MMP2). Our pilot study using miRNA arrays found that miRNA-29c (miR-29c) is differentially expressed in the paired low-metastatic lung cancer cell line 95C compared to the high-metastatic lung cancer cell line 95D. Bioinformatics analysis shows that integrin β1 and matrix metalloproteinase 2 (MMP2) could be important target genes of miR-29c. Therefore, we hypothesized that miR-29c suppresses lung cancer cell adhesion t**o extracellular matr**ix (ECM) and metastasis by targeting integrin β1 and MMP2. The gain-of-function studies that raised miR-29c expression in 95D cells by using its mimics showed reductions in cell proliferation, adhesion to ECM, invasion and migration. In contrasts, loss-of-function studies that reduced miR-29c by using its inhibitor in 95C cells promoted proliferation, adhesion to ECM, invasion and migration. Furthermore, the dual-luciferase reporter assay demonstrated that miR-29c inhibited the expression of the luciferase gene containing the 3'-UTRs of integrin β1 and MMP2 mRNA. Western blotting indicated that miR-29c downregulated the expression of integrin β1 and MMP2 at the protein level. Gelatin zymography analysis further confirmed that miR-29c decreased MMP2 enzyme activity. Nude mice with xenograft models of lung cancer cells confirmed that miR-29c inhibited lung cancer metastasis in vivo, including bone and liver metastasis. Taken together, our results demonstrate that miR-29c serves as a tumor metastasis suppressor, which suppresses lung cancer cell adhesion to ECM and metastasis by directly inhibiting integrin β1 and MMP2 expression and by further reducing MMP2 enzyme activity. The results show that miR-29c may be a novel therapeutic candidate target to slow lung cancer metastasis.
108. β1-integrin controls cell fate specification in early lens development. Integrins are heterodimeric cell surface molecules that mediate cell**-extracellular matri**x (ECM) adhesion, ECM assembly, and regulation of both ECM and growth factor induced signaling. However, the developmental context of these diverse functions is not clear. Loss of β1-integrin from the lens vesicle (mouse E10.5) results in abnormal exit of anterior lens epithelial cells (LECs) from the cell cycle and their aberrant elongation toward the presumptive cornea by E12.5. These cells lose expression of LEC markers and initiate expression of the Maf (also known as c-Maf) and Prox1 transcription factors as well as other lens fiber cell markers. β1-integrin null LECs also upregulate the ERK, AKT and Smad1/5/8 phosphorylation indicative of BMP and FGF signaling. By E14.5, β1-integrin null lenses have undergone a complete conversion of all lens epithelial cells into fiber cells. These data suggest that shortly after lens vesicle closure, β1-integrin blocks inappropriate differentiation of the lens epithelium into fibers, potentially by inhibiting BMP and/or FGF receptor activation. Thus, β1-integrin has an important role in fine-tuning the response of the early lens to the gradient of growth factors that regulate lens fiber cell differentiation.
109. Expression of integrin beta1 by fibroblasts is required for tissue repair in vivo. In tissue repair, fibroblasts migrate into the wound to produce and remodel **extracellular matrix** (ECM). Integrins are believed to be crucial for tissue repair, but their tissue-specific role in this process is poorly understood. Here, we show that mice containing a fibroblast-specific deletion of integrin β1 exhibit delayed cutaneous wound closure and less granulation tissue formation, including reduced production of new ECM and reduced expression of α-smooth muscle actin (α-SMA). Integrin-β1-deficient fibroblasts showed reduced expression of type I collagen and connective tissue growth factor, and failed to differentiate into myofibroblasts as a result of reduced α-SMA stress fiber formation. Loss of integrin β1 in adult fibroblasts reduced their ability to adhere to, to spread on and to contract ECM. Within stressed collagen matrices, integrin-β1-deficient fibroblasts showed reduced activation of latent TGFβ. Addition of active TGFβ alleviated the phenotype of integrin-β1-deficient mice. Thus integrin β1 is essential for normal wound healing, where it acts, at least in part, through a TGFβ-dependent mechanism in vivo.
110. Transmembrane/cytoplasmic, rather than catalytic, domains of Mmp14 signal to MAPK activation and mammary branching morphogenesis via binding to integrin β1. Epithelial cell invasion through the **extracellular matri**x (ECM) is a crucial step in branching morphogenesis. The mechanisms by which the mammary epithelium integrates cues from the ECM with intracellular signaling in order to coordinate invasion through the stroma to make the mammary tree are poorly understood. Because the cell membrane-bound matrix metalloproteinase Mmp14 is known to play a key role in cancer cell invasion, we hypothesized that it could also be centrally involved in integrating signals for mammary epithelial cells (MECs) to navigate the collagen 1 (CL-1)-rich stroma of the mammary gland. Expression studies in nulliparous mice that carry a NLS-lacZ transgene downstream of the Mmp14 promoter revealed that Mmp14 is expressed in MECs at the tips of the branches. Using both mammary organoids and 3D organotypic cultures, we show that MMP activity is necessary for invasion through dense CL-1 (3 mg/ml) gels, but dispensable for MEC branching in sparse CL-1 (1 mg/ml) gels. Surprisingly, however, Mmp14 without its catalytic activity was still necessary for branching. Silencing Mmp14 prevented cell invasion through CL-1 and disrupted branching altogether; it also reduced integrin β1 (Itgb1) levels and attenuated MAPK signaling, disrupting Itgb1-dependent invasion/branching within CL-1 gels. FRET imaging revealed that Mmp14 associates directly with Itgb1. We identified a domain of Mmp14 that is required for modulating the levels of Itgb1, MEC signaling and the rate of invasion within CL-1. These results shed light on hitherto undescribed non-proteolytic activities of Mmp14 that are necessary for the Itgb1-dependent biochemical and mechanical signals that regulate branching in the mammary epithelium.
111. Fibronectin-integrin (alpha5beta1) modulates migration and invasion of murine melanoma cell line B16F10 by involving MMP-9. Cell adhesion to **extracellular matrix** (ECM) initiates signaling cascade regulated by cell surface integrin receptors, which affects the proliferation and invasion of cells. Cells cultured in the presence of ECM ligand fibronectin (FN) stimulate secretion of matrix metalloproteinases (MMPs), facilitating cancer cell invasion and metastasis. Among all the members of the MMP family, MMP-9 is of crucial importance in tumor invasion and metastasis. The present study aims at studying the effects of integrin receptor alpha5beta1 and its ligand FN on expression of MMP-9 in murine melanoma cell line B16F10 and understanding the molecular mechanism(s) involved. The main experimental methods performed in the study were gelatin zymography, immunoblot, real-time RT-PCR, immunocytochemistry, enzyme linked immunosorbent assay (ELISA), transwell chamber assay, and in vivo metastasis assay in syngenic (C57BL6J) mice. The study reports that FN induces the activity, mRNA, and protein expression of MMP-9 and initiates its proteolytic activation in B16F10 cells. Blockage of the alpha5 receptor abrogated the FN-mediated stimulatory response on MMP-9 in B16F10 cells. Inhibitor studies and immunoblot analysis strongly suggest the involvement of focal adhesion kinase (FAK), extracellular regulated kinase (ERK), and phosphatidylinositol-3-kinase (PI-3K) in the FN-mediated responses. Immunocytochemical analysis showed the nuclear localization of nuclear factor-kappaB (NF-kappaB) might lead to activation of MMP-9 gene upon FN treatment. This study demonstrates that integrin receptor alpha5beta1 and FN interaction induces the invasive potential of B16F10 cells and MMP-9 induction is the downstream effectors in the process. This system serves as a novel model system to understand the molecular mechanism of melanoma growth and invasion.
112. RECK-Mediated β1-Integrin Regulation by TGF-β1 Is Critical for Wound Contraction in Mice. Fibroblasts are critical for wound contraction; a pivotal step in wound healing. They produce and modify th**e extracellular matr**ix (ECM) required for the proper tissue remodeling. Reversion-inducing cysteine-rich protein with Kazal motifs (RECK) is a key regulator of ECM homeostasis and turnover. However, its role in wound contraction is presently unknown. Here we describe that Transforming growth factor type β1 (TGF-β1), one of the main pro-fibrotic wound-healing promoting factors, decreases RECK expression in fibroblasts through the Smad and JNK dependent pathways. This TGF-β1 dependent downregulation of RECK occurs with the concomitant increase of β1-integrin, which is required for fibroblasts adhesion and wound contraction through the activation of focal adhesion kinase (FAK). Loss and gain RECK expression experiments performed in different types of fibroblasts indicate that RECK downregulation mediates TGF-β1 dependent β1-integrin expression. Also, reduced levels of RECK potentiate TGF-β1 effects over fibroblasts FAK-dependent contraction, without affecting its cognate signaling. The above results were confirmed on fibroblasts derived from the Reck+/- mice compared to wild type-derived fibroblasts. We observed that Reck+/- mice heal dermal wounds more efficiently than wild type mice. Our results reveal a critical role for RECK in skin wound contraction as a key mediator in the axis: TGF-β1-RECK-β1-integrin.
113. Coordinate induction of fibronectin, fibronectin receptor, tropomyosin, and actin genes in serum-stimulated fibroblasts. From a collection of more than 80 nonoverlapping clones, isolated by differential screening of a lambda cDNA library prepared from serum-stimulated cells in the presence of cycloheximide, we have identified four clones that encoded for components of the cytoskeleton and **extracellular matrix**. DNA sequencing of clones B2, V58, TT1, and P38 demonstrated that they corresponded to beta-actin, alpha-tropomyosin, fibronectin, and the beta-subunit of fibronectin receptor. All four mRNA levels showed a detectable increase 30 min after stimulation and remained at high levels for at least 8 h. The half-lives of these mRNAs were found to be very long in contrast to those of other growth factor-inducible genes. An increase in transcription was observed for the four genes. Actin and fibronectin showed nearly maximal increase at 15 min, while fibronectin receptor and tropomyosin reached their maximum transcription at 1 h. These results demonstrated that four interacting components of the cytoskeleton and **extracellular matrix** are rapidly induced in stimulated quiescent cells, possibly reflecting part of the coordinate changes in gene expression that occur during embryogenesis and wound healing.
114. β1-Integrin cytoskeletal signaling regulates sensory neuron response to matrix dimensionality. Neuronal differentiation, pathfinding and morphology are directed by biochemical cues that in vivo are presented in a complex scaffold of **extracellular matri**x. This microenvironment is three-dimensional (3D) and heterogeneous. Therefore, it is not surprising that more physiologically-relevant cellular responses are found in 3D culture environments rather than on two-dimensional (2D) flat substrates. One key difference between 2D and 3D environments is the spatial arrangement of cell-matrix interactions. Integrins and other receptor proteins link the various molecules presented in the extracellular environment to intracellular signaling cascades and thus influence a number of neuronal responses including the availability and activation of integrins themselves. We have previously reported that a 3D substrate induces an important morphological transformation of embryonic mouse dorsal root ganglion (DRG) neurons. Here, we investigate the hypothesis that β1-integrin signaling via focal adhesion kinase (FAK) and the RhoGTPases Rac and Rho influences neuronal morphology in 2D vs 3D environments. We report that β1-integrin activity and FAK phosphorylation at tyrosine 397 (FAKpY397) are linked to neuronal polarization as well as neurite outgrowth and branching. Rac and Rho expression are decreased in 3D vs 2D culture but not correlated with β1-integrin function. These results suggest that proper β1-integrin activity is required for the elaboration of physiologic DRG morphology and that 3D culture provides a more appropriate milieu to the mimic in vivo scenario. We propose that neuronal morphology may be directed during development and regeneration by factors that influence how β1-integrin, FAK and RhoGTPase molecules integrate substrate signals in the 3D microenvironment.
115. Knock-in of integrin beta 1D affects primary but not secondary myogenesis in mice. Integrins are **extracellular matrix** receptors composed of alpha and beta subunits involved in cell adhesion, migration and signal transduction. The beta1 subunit has two isoforms, beta 1A ubiquitously expressed and beta 1D restricted to striated muscle. They are not functionally equivalent. Replacement of beta 1A by beta 1D (beta 1D knock-in) in the mouse leads to midgestation lethality on a 50% Ola/50% FVB background [Baudoin, C., Goumans, M. J., Mummery, C. and Sonnenberg, A. (1998). Genes Dev. 12, 1202-1216]. We crossed the beta 1D knock-in line into a less penetrant genetic background. This led to an attenuation of the midgestation lethality and revealed a second period of lethality around birth. Midgestation death was apparently not caused by failure in cell migration, but rather by abnormal placentation. The beta 1D knock-in embryos that survived midgestation developed until birth, but exhibited severely reduced skeletal muscle mass. Quantification of myotube numbers showed that substitution of beta 1A with beta 1D impairs primary myogenesis with no direct effect on secondary myogenesis. Furthermore, long-term primary myotube survival was affected in beta 1D knock-in embryos. Finally, overexpression of beta 1D in C2C12 cells impaired myotube formation while overexpression of beta 1A primarily affected myotube maturation. Together these results demonstrate for the first time distinct roles for beta1 integrins in primary versus secondary myogenesis and that the beta 1A and beta 1D variants are not functionally equivalent in this process.
116. Integrin alpha2beta1 is required for regulation of murine wound angiogenesis but is dispensable for reepithelialization. The alpha2beta1 integrin functions as the major receptor for collagen type I on a large number of different cell types, including keratinocytes, fibroblasts, endothelial cells, and a variety of inflammatory cells. Recently, we demonstrated that adhesion of keratinocytes to collagen critically depends on alpha2beta1, whereas fibroblasts can partly compensate for loss of alpha2beta1 in simple adhesion to collagen. However, in three-dimensional collagen matrices, alpha2beta1-null fibroblasts are hampered in generating mechanical forces. These data suggested a pivotal role for alpha2beta1 during wound healing in vivo. Unexpectedly, reepithelialization of excisional wounds of alpha2beta1-null mice was not impaired, indicating that keratinocytes do not require adhesion to or migration on collagen for wound closure. Whereas wound contraction and myofibroblast differentiation were similar, wound tensile strain was reduced in alpha2beta1-null mice, suggesting subtle changes in organization of the **extracellular matrix**. In addition, we observed reduced influx of mast cells into the granulation tissue, whereas infiltration of other inflammatory cells was not impaired. Interestingly, ablation of alpha2beta1 resulted in strong enhancement of neovascularization of granulation tissue and sponge implants. Both ultrastructurally and functionally, these new blood vessels appeared intact. In conclusion, our data show unique and overlapping functions of alpha2beta1 integrin during murine cutaneous wound healing.
117. β1-integrin is essential for vasoregulation and smooth muscle survival in vivo. Integrins contribute to vascular morphogenesis through regulation of adhesion and assembly of the **extracellular matri**x. However, the role of β1-integrin in the mature vascular wall is less clear.We sought to determine the function of β1-integrin in mature smooth muscle cells in vivo using a loss of function approach by crossing a tamoxifen-inducible sm22αCre line to a floxed β1-integrin transgenic line. Adult mice lacking smooth muscle β1-integrin survived only 10 weeks post induction. The deletion of β1-integrin resulted in profound loss of vasomotor control. Histological analysis revealed progressive fibrosis in arteries with associated apoptosis of smooth muscle cells, which was not rescued by adventitial stem cells. Smooth muscle cell apoptosis was detected in arteries with dead cells replaced primarily by collagen. Despite the catastrophic effects on vascular smooth muscle, the deleted visceral smooth muscle remained viable with the exception of a short portion of the colon, indicating that vascular but not visceral smooth muscle is particularly sensitive to changes in β1-integrin.This study reveals an essential function of β1-integrin in the maintenance of vasomotor control and highlights a critical role for β1-integrin in vascular, but not visceral, smooth muscle survival.
118. Integrin shedding as a mechanism of cellular adaptation during cardiac growth. Integrin-mediated cell-**extracellular matrix** (ECM) interactions are essential for multiple cellular processes; however, little is known regarding integrin turnover during these events. Recent studies have demonstrated shedding of cell surface molecules and suggested this as a potential mechanism for integrin turnover. Confocal microscopy of mouse hearts under different physiological conditions demonstrated the presence of beta(1)-integrin-immunoreactive material in the interstitium. Culture media from neonatal rat cardiac myocytes and fibroblasts contained a 55-kDa fragment of beta(1)-integrin. Attachment to ECM components, response to phorbol 12-myristate 13-acetate stimulation, and matrix metalloproteinase inhibition assays demonstrated that fibroblasts responded differently to the fragment compared with myocytes. The beta(1)-integrin fragment stimulated myocyte attachment to collagen and the fragment itself bound a variety of ECM proteins. These studies indicate that as myocytes and fibroblasts change size and shape, cellular contacts with the ECM are altered, resulting in the liberation of a beta(1)-integrin fragment from the cell surface. Integrin shedding may represent a novel mechanism of rapidly modifying cell-ECM contacts during various cellular processes.
119. Α2 integrin, **extracellular matri**x metalloproteinase inducer, and matrix metalloproteinase-3 act sequentially to induce differentiation of mouse embryonic stem cells into odontoblast-like cells. We previously reported that interleukin 1β acts via matrix metalloproteinase (MMP)-3 to regulate cell proliferation and suppress apoptosis in α2 integrin-positive odontoblast-like cells differentiated from mouse embryonic stem (ES) cells. Here we characterize the signal cascade underpinning odontoblastic differentiation in mouse ES cells. The expression of α2 integr**in, extracellular ma**trix metalloproteinase inducer (Emmprin), and MMP-3 mRNA and protein were all potently increased during odontoblastic differentiation. Small interfering RNA (siRNA) disruption of the expression of these effectors potently suppressed the expression of the odontoblastic biomarkers dentin sialophosphoprotein, dentin matrix protein-1 and alkaline phosphatase, and blocked odontoblast calcification. Our siRNA, western blot and blocking antibody analyses revealed a unique sequential cascade involving α2 integrin, Emmprin and MMP-3 that drives ES cell differentiation into odontoblasts. This cascade requires the interaction between α2 integrin and Emmprin and is potentiated by exogenous MMP-3. Finally, although odontoblast-like cells potently express α2, α6, αV, β1, and β3, integrins, we confirmed that β1 integrin acts as the trigger for ES cell differentiation, apparently in complex with α2 integrin. These results demonstrate a unique and unanticipated role for an α2 integrin-, Emmprin-, and MMP-3-mediated signaling cascade in driving mouse ES cell differentiation into odontoblast-like cells.
120. Reciprocal expressions between α-dystroglycan and integrin β1, perlecan receptors, in the murine enamel organ development. Signals of perlecan, a**n extracellular matr**ix molecule, which accumulates within the intercellular spaces of the stellate reticulum of the enamel organ, are mediated by at least two receptors, dystroglycan (DG) and integrin β1, in a case-dependent manner in various events in embryogenesis and pathogenesis. This study aims to understand the expression profiles of these two perlecan receptors at both protein and gene levels in murine enamel organ development. Before birth, α-DG was immunolocalized in stellate reticulum cells, in which perlecan was colocalized, while integrin β1 was mainly distributed in the peripheral enamel organ cells as well as the dental mesenchymal cells. On and after postnatal Day 1, the expression of α-DG was dramatically decreased in the stellate reticulum, while integrin β1 was enhanced around blood vessels within the enamel organ. Furthermore, biosyntheses of α-DG and integrin β1 by dental epithelial and pulp mesenchymal cells were confirmed in vitro by using immunofluorescence and reverse-transcriptase polymerase chain reaction. The results suggest that DG is a perlecan receptor that specifically functions in the stellate reticulum of the embryonic stage, but that dental epithelial and mesenchymal cells are maturated by capturing perlecan signals differentially through integrin β1.
121. Beta1 integrins regulate mammary gland proliferation and maintain the integrity of mammary alveoli. Integrin-**extracellular matrix** interactions play important roles in the coordinated integration of external and internal cues that are essential for proper development. To study the role of beta1 integrin in the mammary gland, Itgbeta1(flox/flox) mice were crossed with WAPiCre transgenic mice, which led to specific ablation of beta1 integrin in luminal alveolar epithelial cells. In the beta1 integrin mutant mammary gland, individual alveoli were disorganized resulting from alterations in cell-basement membrane associations. Activity of focal adhesion kinase (FAK) was also decreased in mutant mammary glands. Luminal cell proliferation was strongly inhibited in beta1 integrin mutant glands, which correlated with a specific increase of p21 Cip1 expression. In a p21 Cip1 null background, there was a partial rescue of BrdU incorporation, providing in vivo evidence linking p21 Cip1 to the proliferative defect observed in beta1 integrin mutant glands. A connection between p21 Cip1 and beta1 integrin as well as FAK was also established in primary mammary cells. These results point to the essential role of beta1 integrin signaling in mammary epithelial cell proliferation.
122. Integrin beta1-focal adhesion kinase signaling directs the proliferation of metastatic cancer cells disseminated in the lungs. The development of metastases is an extended and inefficient process involving multiple steps. The last of these involves the growth of micrometastases into macroscopic tumors. We show here that intravenously injected, nonmetastatic cancer cells cease proliferating after extravasating into the parenchyma of the lungs; this response is attributable to the cells inability to trigger adhesion-related signaling events when they are scattered sparsely within the **extracellular matrix** (ECM) of the parenchyma. We recapitulate this situation by culturing these nonmetastatic cells at low seeding density in ECM-derived gels in vitro, in which they undergo cell-cycle arrest resulting, in part, from insufficient activation of focal adhesion kinase (FAK). Metastatic cancer cells, in contrast, show sufficient FAK activation to enable their proliferation within ECM gels in vitro and continue cell-cycle progression within the lung parenchyma in vivo. Activation of FAK in these metastatic cells depends on expression of the beta(1) subunit of integrins, and proliferation of these cells after extravasation in the lungs is diminished by knocking down the expression of either FAK or integrin beta(1). These results demonstrate the critical role of integrin beta(1)-FAK signaling axis in controlling the initial proliferation of micrometastatic cancer cells disseminated in the lungs.
123. Efficient dendritic cell priming of T lymphocytes depends on the **extracellular matrix** protein mindin. Rho guanosine triphosphatases (GTPases) regulate multiple aspects of dendritic cell (DC) function, but what regulates the expression of Rho GTPases in DCs is unknown. Here, we show that the **extracellular matrix** protein mindin regulates the expression of Rho GTPases in DCs. Mindin(-/-) mice displayed defective CD4+ T-cell priming and impaired humoral immune responses to T-dependent antigens. Mindin(-/-) DCs had reduced expression of Rac1/2 and impaired priming capacity owing to inefficient engagement with T lymphocytes. Ectopic Rac1 expression restored the priming capability of Mindin(-/-) DCs. Furthermore, we show that DC adhesion to mindin matrix was blocked by antibodies to alpha4, alpha5 and beta1 integrins. DCs lacking beta1 integrin had reduced adhesion to mindin matrix, decreased expression of Rac1/2 and impaired priming capacity. These results suggest that mindin-integrin interactions play a key role in regulating Rho GTPase expression in DCs and DC priming of T lymphocytes.
124. The opposing roles of laminin-binding integrins in cancer. Integrins play an important role in cell adhesion by linking the cytoskeleton of cells to components in the **extracellular matrix**. In this capacity, integrins cooperate with different cell surface receptors, including growth factor receptors and G-protein coupled receptors, to regulate intracellular signaling pathways that control cell polarization, spreading, migration, survival, and gene expression. A distinct subfamily of molecules in the integrin family of adhesion receptors is formed by receptors that mediate cell adhesion to laminins, major components of the basement membrane that lie under clusters of cells or surround them, separating them from other cells and/or adjacent connective tissue. During the past decades, many studies have provided evidence for a role of laminin-binding integrins in tumorigenesis, and both tumor-promoting and suppressive activities have been identified. In this review we discuss the dual role of the laminin-binding integrins α3β1 and α6β4 in tumor development and progression, and examine the factors and mechanisms involved in these opposing effects.
125. Genetic analysis of beta1 integrin function: confirmed, new and revised roles for a crucial family of cell adhesion molecules. Integrins are heterodimeric cell adhesion proteins connecting the **extracellular matrix** to the cytoskeleton and transmitting signals in both directions. These integrins are suggested to be involved in many different biological processes such as growth, differentiation, migration, and cell death. Of more than 20 known integrins, 10 contain the nearly ubiquitously expressed beta1 integrin subunit. Disruption of the beta1 integrin gene by homologous recombination allows us to assess the supposed functions of beta1 containing integrins in vivo in a new way. This review will present and discuss recent findings derived from such studies concerning the biological roles of beta1 integrins in early development, differentiation and migration, hematopoiesis, tumorigenesis, and supramolecular assembly of **extracellular matrix** proteins. While several former results were confirmed, others were contradicted and new functions found, significantly changing the previous view of beta1 integrin function in vivo.
126. Beta1 integrins regulate myoblast fusion and sarcomere assembly. The mechanisms that regulate the formation of multinucleated muscle fibers from mononucleated myoblasts are not well understood. We show here that **extracellular matrix** (ECM) receptors of the beta1 integrin family regulate myoblast fusion. beta1-deficient myoblasts adhere to each other, but plasma membrane breakdown is defective. The integrin-associated tetraspanin CD9 that regulates cell fusion is no longer expressed at the cell surface of beta1-deficient myoblasts, suggesting that beta1 integrins regulate the formation of a protein complex important for fusion. Subsequent to fusion, beta1 integrins are required for the assembly of sarcomeres. Other ECM receptors such as the dystrophin glycoprotein complex are still expressed but cannot compensate for the loss of beta1 integrins, providing evidence that different ECM receptors have nonredundant functions in skeletal muscle fibers.
127. The mouse KRAB zinc-finger protein CHATO is required in embryonic-derived tissues to control yolk sac and placenta morphogenesis. Yolk sac and placenta are required to sustain embryonic development in mammals, yet our understanding of the genes and processes that control morphogenesis of these extraembryonic tissues is still limited. The chato mutation disrupts ZFP568, a Krüppel-Associated-Box (KRAB) domain Zinc finger protein, and causes a unique set of extraembryonic malformations, including ruffling of the yolk sac membrane, defective extraembryonic mesoderm morphogenesis and vasculogenesis, failure to close the ectoplacental cavity, and incomplete placental development. Phenotypic analysis of chato embryos indicated that ZFP568 does not control proliferation or differentiation of extraembryonic lineages but rather regulates the morphogenetic events that shape extraembryonic tissues. Analysis of chimeric embryos showed that Zfp568 function is required in embryonic-derived lineages, including the extraembryonic mesoderm. Depleting Zfp568 affects the ability of extraembryonic mesoderm cells to migrate. However, explanted Zfp568 mutant cells could migrate properly when plated on appropriate **extracellular matri**x conditions. We show that expression of Fibronectin and Indian Hedgehog are reduced in chato mutant yolk sacs. These data suggest that ZFP568 controls the production of secreted factors required to promote morphogenesis of extraembryonic tissues. Our results support previously undescribed roles of the extraembryonic mesoderm in yolk sac morphogenesis and in the closure of the ectoplacental cavity and identify a novel role of ZFP568 in the development of extraembryonic tissues.
128. The dermatan sulfate proteoglycan decorin modulates α2β1 integrin and the vimentin intermediate filament system during collagen synthesis. Decorin, a small leucine-rich proteoglycan harboring a dermatan sulfate chain at its N-terminus, is involved in regulating matrix organization and cell signaling. Loss of the dermatan sulfate of decorin leads to an Ehlers-Danlos syndrome characterized by delayed wound healing. Decorin-null (Dcn(-/-)) mice display a phenotype similar to that of EDS patients. The fibrillar collagen phenotype of Dcn(-/-) mice could be rescued in vitro by decorin but not with decorin lacking the glycosaminoglycan chain. We utilized a 3D cell culture model to investigate the impact of the altere**d extracellular matr**ix on Dcn(-/-) fibroblasts. Using 2D gel electrophoresis followed by mass spectrometry, we identified vimentin as one of the proteins that was differentially upregulated by the presence of decorin. We discovered that a decorin-deficient matrix leads to abnormal nuclear morphology in the Dcn(-/-) fibroblasts. This phenotype could be rescued by the decorin proteoglycan but less efficiently by the decorin protein core. Decorin treatment led to a significant reduction of the α2β1 integrin at day 6 in Dcn(-/-) fibroblasts, whereas the protein core had no effect on β1. Interestingly, only the decorin core induced mRNA synthesis, phosphorylation and de novo synthesis of vimentin indicating that the proteoglycan decorin in **the extracellular m**atrix stabilizes the vimentin intermediate filament system. We could support these results in vivo, because the dermis of wild-type mice have more vimentin and less β1 integrin compared to Dcn(-/-). Furthermore, the α2β1 null fibroblasts also showed a reduced amount of vimentin compared to wild-type. These data show for the first time that decorin has an impact on the biology of α2β1 integrin and the vimentin intermediate filament system. Moreover, our findings provide a mechanistic explanation for the reported defects in wound healing associated with the Dcn(-/-) phenotype.
129. Foxf2 is required for secondary palate development and Tgfβ signaling in palatal shelf mesenchyme. The secondary palate separates the oral from the nasal cavity and its closure during embryonic development is sensitive to genetic perturbations. Mice with deleted Foxf2, encoding a forkhead transcription factor, are born with cleft palate, and an abnormal tongue morphology has been proposed as the underlying cause. Here, we show that Foxf2(-/-) maxillary explants cultured in vitro, in the absence of tongue and mandible, failed to close the secondary palate. Proliferation and collagen content were decreased in Foxf2(-/-) palatal shelf mesenchyme. Phosphorylation of Smad2/3 was reduced in mutant palatal shelf, diagnostic of attenuated canonical Tgfβ signaling, whereas phosphorylation of p38 was increased. The amount of Tgfβ2 protein was diminished, whereas the Tgfb2 mRNA level was unaltered. Expression of several genes encoding extracellular proteins important for Tgfβ signaling were reduced in Foxf2(-)(/)(-) palatal shelves: a fibronectin splice-isoform essential for formation of extracellular Tgfβ latency complexes; Tgfbr3 - or betaglycan - which acts as a co-receptor and an extracellular reservoir of Tgfβ; and integrins αV and β1, which are both Tgfβ targets and required for activation of latent Tgfβ. Decreased proliferation an**d reduced extracellu**lar matrix content are consistent with diminished Tgfβ signaling. We therefore propose that gene expression changes in palatal shelf mesenchyme that lead to reduced Tgfβ signaling contribute to cleft palate in Foxf2(-)(/)(-) mice.
130. Endothelial cell laminin isoforms, laminins 8 and 10, play decisive roles in T cell recruitment across the blood-brain barrier in experimental autoimmune encephalomyelitis. An active involvement of blood-brain barrier endothelial cell basement membranes in development of inflammatory lesions in the central nervous system (CNS) has not been considered to date. Here we investigated the molecular composition and possible function of the **extracellular matrix** encountered by extravasating T lymphocytes during experimental autoimmune encephalomyelitis (EAE). Endothelial basement membranes contained laminin 8 (alpha4beta1gamma1) and/or 10 (alpha5beta1gamma1) and their expression was influenced by proinflammatory cytokines or angiostatic agents. T cells emigrating into the CNS during EAE encountered two biochemically distinct basement membranes, the endothelial (containing laminins 8 and 10) and the parenchymal (containing laminins 1 and 2) basement membranes. However, inflammatory cuffs occurred exclusively around endothelial basement membranes containing laminin 8, whereas in the presence of laminin 10 no infiltration was detectable. In vitro assays using encephalitogenic T cell lines revealed adhesion to laminins 8 and 10, whereas binding to laminins 1 and 2 could not be induced. Downregulation of integrin alpha6 on cerebral endothelium at sites of T cell infiltration, plus a high turnover of laminin 8 at these sites, suggested two possible roles for laminin 8 in the endothelial basement membrane: one at the level of the endothelial cells resulting in reduced adhesion and, thereby, increased penetrability of the monolayer; and secondly at the level of the T cells providing direct signals to the transmigrating cells.
131. Deletion of integrin-linked kinase from skeletal muscles of mice resembles muscular dystrophy due to alpha 7 beta 1-integrin deficiency. Integrin-linked kinase (Ilk) is a serine/threonine kinase and an adaptor protein that links integrins to the actin cytoskeleton and to a number of signaling pathways involved in integrin action. We hypothesized that Ilk may act as an important effector of integrins in skeletal muscle, where these receptors provide a critical link between the sarcolemma and the **extracellular matrix**. Using the cre/lox system, we deleted Ilk from skeletal muscles of mice. The resulting mutants developed a progressive muscular dystrophy with multiple degenerating and regenerating muscle fibers, increased central nuclei, and endomysial fibrosis. These defects were widespread but were most severe near myofascial junctions where Ilk mutants showed displacement of focal adhesion-related proteins, including vinculin, paxillin, focal adhesion kinase, dystrophin, and the alpha 7 beta 1D-integrin subunits. Distal ends of mutant muscle fibers appeared irregular, and there was restructuring of the actin cytoskeleton. These findings resemble those seen in humans and mice lacking the alpha 7-integrin subunit and suggest that Ilk may act as a cytoplasmic effector of alpha 7 beta1-integrin in the pathogenesis of these deficiencies.
132. Differential mRNA regulation of integrin subunits alpha V, beta 1, beta 3, and beta 5 during mouse embryonic organogenesis. Cell interactions with extracellular matrices play important roles in morphogenetic processes during embryonic development. **Extracellular matrix** receptors of the integrin family have been implicated in these steps. Recent studies indicate that a variety of integrins can be differentially expressed during development, consistent with diverse roles for integrins in embryogenesis. The present study compares the expression patterns of several major members of the alpha V integrin subfamily, focusing on mRNA expression of alpha V, beta 1, beta 3, and beta 5 subunits during mouse embryonic organogenesis using Northern blot analysis and in situ hybridization. The alpha V and beta 1 subunits showed widespread tissue expression, although most tissues expressed alpha V at relatively low or basal levels. The mRNA for beta 5 was also expressed in a variety of embryonic organs and showed unusual localization patterns in certain organs. Striking, high-level expression of beta 5 transcripts was detected in the ependymal layer of the central nervous system, glomeruli of the kidney, epicardial region of the heart, and in the tooth germs, suggesting specific functions for this molecule during morphogenetic events in these organs. In contrast, few beta 3 transcripts were expressed during mid-gestation mouse embryogenesis except in megakaryocytes within the embryonic liver. These observations of differing expression spectra suggest that members of the alpha V integrin subfamily have distinct roles and also suggests that they have different transcriptional regulation. The beta 5 integrin is unique in its degree of tissue-specific mRNA regulation associated with morphogenesis of embryonic organs.
133. The role of integrin alpha8beta1 in fetal lung morphogenesis and injury. Prenatal inflammation prevents normal lung morphogenesis and leads to bronchopulmonary dysplasia (BPD), a common complication of preterm birth. We previously demonstrated in a bacterial endotoxin mouse model of BPD that disrupting fibronectin localization in the fetal lung mesenchyme causes arrested saccular airway branching. In this study we show that expression of the fibronectin receptor, integrin alpha8beta1 is decreased in the lung mesenchyme in the same inflammation model suggesting it is required for normal lung development. We verified a role for integrin alpha8beta1 in lung development using integrin alpha8-null mice, which develop fusion of the medial and caudal lobes as well as abnormalities in airway division. We further show in vivo and in vitro that alpha8-null fetal lung mesenchymal cells fail to form stable adhesions and have increased migration. Thus we propose that integrin alpha8beta1 plays a critical role in lung morphogenesis by regulating mesenchymal cell adhesion and migration. Furthermore, our data suggest that disruption of the interactions between **extracellular matrix** and integrin alpha8beta1 may contribute to the pathogenesis of BPD.
134. CCN6 as a profibrotic mediator that stimulates the proliferation of lung fibroblasts via the integrin β1/focal adhesion kinase pathway. Idiopathic pulmonary fibrosis is a progressive and lethal disease of the lung that is characterized by the proliferation of fibroblasts and increased deposition of the **extracellular matri**x. The CCN6/WISP-3 is a member of the CCN family of matricellular proteins, which consists of six members that are involved in many vital biological functions. However, the regulation of lung fibroblasts mediated by CCN6 protein has not been fully elucidated. Here, we demonstrated that CCN6 induced the proliferation of lung fibroblasts by binding to integrin β1, leading to the phosphorylation of FAK(Y397). Furthermore, CCN6 showed a weak, but significant, ability to stimulate the expression of fibronectin. CCN6 was highly expressed in the lung tissues of mice treated with bleomycin. Our results suggest that CCN6 plays a role in the fibrogenesis of the lungs mainly by stimulating the growth of lung fibroblasts and is a potential target for the treatment of pulmonary fibrosis.
135. The major laminin receptor of mouse embryonic stem cells is a novel isoform of the alpha 6 beta 1 integrin. Laminin is the first **extracellular matrix** protein expressed in the developing mouse embryo. It is known to influence morphogenesis and affect cell migration and polarization. Several laminin receptors are included in the integrin family of **extracellular matrix** receptors. Ligand binding by integrin heterodimers results in signal transduction events controlling cell motility. We report that the major laminin receptor on murine embryonic stem (ES) cells is the integrin heterodimer alpha 6 beta 1, an important receptor for laminin in neurons, lymphocytes, macrophages, fibroblasts, platelets and other cell types. However, the cytoplasmic domain of the ES cell alpha 6 (alpha 6 B) differs totally from the reported cytoplasmic domain amino acid sequence of alpha 6 (alpha 6 A). Comparisons of alpha 6 cDNAs from ES cells and other cells suggest that the alpha 6 A and alpha 6 B cytoplasmic domains derive from alternative mRNA splicing. Anti-peptide antibodies to alpha 6 A are unreactive with ES cells, but react with mouse melanoma cells and embryonic fibroblasts. When ES cells are cultured under conditions that permit their differentiation, they become positive for alpha 6 A, concurrent with the morphologic appearance of differentiated cell types. Thus, expression of the alpha 6 B beta 1 laminin receptor may be favored in undifferentiated, totipotent cells, while the expression of alpha 6 A beta 1 receptor occurs in committed lineages. While the functions of integrin alpha chain cytoplasmic domains are not understood, it is possible that they contribute to transferring signals to the cell interior, e.g., by delivering cytoskeleton organizing signals in response to integrin engagement with **extracellular matrix** ligands. It is therefore reasonable to propose that the cellular responses to laminin may vary, according to what alpha subunit isoform (alpha 6 A or alpha 6 B) is expressed as part of the alpha 6 beta 1 laminin receptor. The switch from alpha 6 B to alpha 6 A, if confirmed in early embryos, could then be of striking potential relevance to the developmental role of laminin.
136. β1 integrin is a crucial regulator of pancreatic β-cell expansion. Development of the endocrine compartment of the pancreas, as represented by the islets of Langerhans, occurs through a series of highly regulated events encompassing branching of the pancreatic epithelium, delamination and differentiation of islet progenitors from ductal domains, followed by expansion and three-dimensional organization into islet clusters. Cellular interactions with th**e extracellular matr**ix (ECM) mediated by receptors of the integrin family are postulated to regulate key functions in these processes. Yet, specific events regulated by these receptors in the developing pancreas remain unknown. Here, we show that ablation of the β1 integrin gene in developing pancreatic β-cells reduces their ability to expand during embryonic life, during the first week of postnatal life, and thereafter. Mice lacking β1 integrin in insulin-producing cells exhibit a dramatic reduction of the number of β-cells to only ∼18% of wild-type levels. Despite the significant reduction in β-cell mass, these mutant mice are not diabetic. A thorough phenotypic analysis of β-cells lacking β1 integrin revealed a normal expression repertoire of β-cell markers, normal architectural organization within islet clusters, and a normal ultrastructure. Global gene expression analysis revealed that ablation of this ECM receptor in β-cells inhibits the expression of genes regulating cell cycle progression. Collectively, our results demonstrate that β1 integrin receptors function as crucial positive regulators of β-cell expansion.
137. Integrin-linked kinase stabilizes myotendinous junctions and protects muscle from stress-induced damage. Skeletal muscle expresses high levels of integrin-linked kinase (ILK), predominantly at myotendinous junctions (MTJs) and costameres. ILK binds the cytoplasmic domain of beta1 integrin and mediates phosphorylation of protein kinase B (PKB)/Akt, which in turn plays a central role during skeletal muscle regeneration. We show that mice with a skeletal muscle-restricted deletion of ILK develop a mild progressive muscular dystrophy mainly restricted to the MTJs with detachment of basement membranes and accumulation of **extracellular matrix**. Endurance exercise training enhances the defects at MTJs, leads to disturbed subsarcolemmal myofiber architecture, and abrogates phosphorylation of Ser473 as well as phosphorylation of Thr308 of PKB/Akt. The reduction in PKB/Akt activation is accompanied by an impaired insulin-like growth factor 1 receptor (IGF-1R) activation. Coimmunoprecipitation experiments reveal that the beta1 integrin subunit is associated with the IGF-1R in muscle cells. Our data identify the beta1 integrin-ILK complex as an important component of IGF-1R/insulin receptor substrate signaling to PKB/Akt during mechanical stress in skeletal muscle.
138. Combined deficiency of dystrophin and beta1 integrin in the cardiac myocyte causes myocardial dysfunction, fibrosis and calcification. The dystrophin-glycoprotein complex is a large complex of membrane-associated proteins linking the cytoskeleton to the **extracellular matrix** in muscle. Transmembrane heterodimeric (alphabeta) integrins serve also as cellular adhesion molecules and mechanotransducers. In the animal model for Duchenne muscular dystrophy, the mdx mouse, loss of dystrophin causes more severe abnormalities in skeletal than in cardiac muscle. We hypothesized that ablation of cardiac myocyte integrins in the mdx background would lead to a severe cardiomyopathic phenotype. Mdx mice were crossed to ones with cardiac myocyte-specific deletion of beta1 integrin (beta1KO) to generate beta1KOmdx. Unstressed beta1KOmdx mice were viable and had normal cardiac function; however, high mortality was seen in peri- and postpartum females by 6 months of age, when severe myocardial necrosis and fibrosis and extensive dystrophic calcification was seen. Decreased ventricular function and blunted adrenergic responsiveness was found in the beta1KOmdx mice compared with control (Lox/Lox, no Cre), beta1KO, and mdx. Similarly, adult beta1KOmdx males were more prone to isoproterenol-induced heart failure and death compared with control groups. Given the extensive calcification, we analyzed transcript levels of genes linked to fibrosis and calcification and found matrix gamma-carboxyglutamic acid protein, decorin, periostin, and the osteoblast transcription factor Runx2/Cbfa1 significantly increased in beta1KOmdx cardiac muscle. Our data show that combined deficiency of dystrophin and integrins in murine cardiac myocytes results in more severe cardiomyopathic changes in the stressed myocardium than reduction of either dystrophin or integrins alone and predisposes to myocardial calcification.
139. Coordination between extrinsic **extracellular matrix** cues and intrinsic responses to orient the centrosome in polarizing cerebellar granule neurons. Successful axon targeting during development is critically dependent on directionality of axon extension and requires coordination between the extrinsic cues that provide spatial information to the axon and the intrinsic responses that regulate structural specification of the axon during neuronal polarization. How these responses are coordinated is unclear but are known to involve aligning the centrosome with the base of the emerging axon. We have used a novel in vitro micropatterning assay that spatially segregates the extrinsic cues used by polarizing cerebellar granule cells to orient axon extension and used it to investigate the signaling mechanisms responsible for coordinating centrosome positioning with intrinsic responses. The results show that, when laminin and/or vitronectin are used as spatially restricted cues in association with substrate-associated sonic hedgehog, they are sufficient to induce cell cycle arrest, that laminin and vitronectin then induce integrin-mediated signaling that upregulates phosphoinositide-3 kinase and PKC function to produce phosphatidylinositol 3,4,5-trisphosphate (PIP3) that is associated with the centrosome, that this PIP3 can interact with PKC-phosphorylated growth-associated protein GAP-43, and that PKC-phosphorylated GAP-43 in turn is required for positioning Par6, Cdc42, and IQGAP1, all intrinsic response components, in proximity to the centrosome, such that, in the absence of GAP-43, they are mislocalized and microtubules are not oriented appropriately. We conclude from these results that GAP-43 plays an important role in coordinating extrinsic signaling and intrinsic responses in polarizing cerebellar granule neurons.
140. Integrin signalling regulates YAP and TAZ to control skin homeostasis. The skin is a squamous epithelium that is continuously renewed by a population of basal layer stem/progenitor cells and can heal wounds. Here, we show that the transcription regulators YAP and TAZ localise to the nucleus in the basal layer of skin and are elevated upon wound healing. Skin-specific deletion of both YAP and TAZ in adult mice slows proliferation of basal layer cells, leads to hair loss and impairs regeneration after wounding. Contact with the basal **extracellular matrix** and consequent integrin-Src signalling is a key determinant of the nuclear localisation of YAP/TAZ in basal layer cells and in skin tumours. Contact with the basement membrane is lost in differentiating daughter cells, where YAP and TAZ become mostly cytoplasmic. In other types of squamous epithelia and squamous cell carcinomas, a similar control mechanism is present. By contrast, columnar epithelia differentiate an apical domain that recruits CRB3, Merlin (also known as NF2), KIBRA (also known as WWC1) and SAV1 to induce Hippo signalling and retain YAP/TAZ in the cytoplasm despite contact with the basal layer **extracellular matrix**. When columnar epithelial tumours lose their apical domain and become invasive, YAP/TAZ becomes nuclear and tumour growth becomes sensitive to the Src inhibitor Dasatinib.
141. Akt1 signaling regulates integrin activation, matrix recognition, and fibronectin assembly. Akt, a serine-threonine kinase, regulates multiple cellular processes in vascular cells. We have previously documented that Akt activates integrins and Akt1 deficiency results in matrix abnormalities in skin and blood vessels in vivo. Based on these observations, we hypothesized that Akt1 is necessary for integrin activation and matrix assembly by fibroblasts. In this study, using various cell systems, we show that Akt1 is essential for the inside-out activation of integrins in endothelial cells and fibroblasts, which in turn, mediates matrix assembly. Fibronectin is a major **extracellular matrix** component of the skin and the vascular basement membrane, which possesses binding sites for many integrins and **extracellular matrix** proteins. Akt1(-/-) fibroblasts and NIH fibroblasts expressing dominant negative Akt1 (K179M-Akt1) showed impaired fibronectin assembly compared with control fibroblasts. In contrast, expression of constitutively active Akt1 (myrAkt1) resulted in enhanced fibronectin assembly. Although increased fibronectin assembly by myrAkt1-expressing human foreskin fibroblasts was abolished by treatment with anti-integrin beta(1) blocking antibodies, treatment with beta(1)-stimulating antibodies rescued the impaired fibronectin assembly that was due to lack of Akt activity. Finally, expression of myrAkt1 corrected the phenotype of Akt1(-/-) fibroblasts thus showing that Akt1 regulates fibronectin assembly through activation of integrin alpha(5)beta(1).
142. Role of integrins in mouse eyelid development: studies in normal embryos and embryos in which there is a failure of eyelid fusion. Eyelid fusion normally occurs between E15.5 and E16.5 of mouse embryonic development and results from the migration of a population of periderm-derived epithelial cells over the corneal surface. Cell migration is known to depend on **extracellular matrix** receptors of the integrin family and to be regulated by growth factors. We were therefore interested that a failure of eyelid fusion has been reported in mice that are homozygous null for the transforming growth factor alpha (TGF-alpha) gene and in mice (invalpha5beta1) in which a transgenic alpha5beta1 integrin under the control of the involucrin promoter is misexpressed in differentiating keratinocytes. We examined expression of the alpha2beta1, alpha3beta1, alpha5beta1 and alpha6beta4 integrins during eyelid fusion in wild-type embryos and found selective upregulation of the alpha5beta1 integrin and its ligand, fibronectin, in the migrating eyelid tip cells. In TGF-alpha null embryos, the failure of eyelid fusion was correlated with a failure to upregulate the alpha5beta1 integrin and fibronectin in the tip cells. Using beta-galactosidase as a reporter gene in transgenic mice, we observed specific activity of the involucrin promoter in the eyelid tip cells. In invalpha5beta1 mice the transgenic human integrin was overexpressed not only in the tip cells but throughout the eyelid epidermis. In contrast, the endogenous, murine, alpha5beta1 integrin was only weakly expressed in the tip cells. We speculate that selective and coordinated expression of the alpha5beta1 integrin and fibronectin in eyelid tip cells is required for eyelid fusion and may be under the control of growth factors that include TGF-alpha.
143. PAR3 is essential for cyst-mediated epicardial development by establishing apical cortical domains. Epithelial cysts are one of the fundamental architectures for mammalian organogenesis. Although in vitro studies using cultured epithelial cells have revealed proteins required for cyst formation, the mechanisms that orchestrate the functions of these proteins in vivo remain to be clarified. We show that the targeted disruption of the mouse Par3 gene results in midgestational embryonic lethality with defective epicardial development. The epicardium is mainly derived from epicardial cysts and essential for cardiomyocyte proliferation during cardiac morphogenesis. PAR3-deficient epicardial progenitor (EPP) cells do not form cell cysts and show defects in the establishment of apical cortical domains, but not in basolateral domains. In PAR3-deficient EPP cells, the localizations of aPKC, PAR6beta and ezrin to the apical cortical domains are disturbed. By contrast, ZO1 and alpha4/beta1 integrins normally localize to cell-cell junctions and basal domains, respectively. Our observations indicate that EPP cell cyst formation requires PAR3 to interpret the polarity cues from cell-cell and cell-**extracellular matrix** interactions so that each EPP cell establishes apical cortical domains. These results also provide a clear example of the proper organization of epithelial tissues through the regulation of individual cell polarity.
144. Reelin, Disabled 1, and beta 1 integrins are required for the formation of the radial glial scaffold in the hippocampus. The **extracellular matrix** molecule Reelin is required for the correct positioning of neurons during the development of the forebrain. However, the mechanism of Reelin action on neuronal migration is poorly understood. Reelin is assumed to act on neurons directly, but it may also affect the differentiation of glial cells necessary for neuronal migration. Here we show that a regular glial scaffold fails to form in vivo in the dentate gyrus of mice deficient of Reelin or Disabled 1, a neuronal adaptor protein in the Reelin signaling pathway. A subset of these defects is observed in mice that lack beta(1)-class integrins, known to bind Reelin. Moreover, recombinant Reelin induced branching of glial processes in vitro. Our data suggest that Reelin affects glial differentiation via Disabled 1 and beta(1)-class integrin-dependent signaling pathways.
145. Beta1 integrins and neural stem cells: making sense of the extracellular environment. Neural Stem Cells (NSC) are present in the developing and adult CNS. In both the embryonic and adult neurogenic regions, beta1 integrins may act as sensors for the changing **extracellular matrix**. Here we highlight the integrative functions that beta1 integrins may play in the "niche" by regulating NSC growth factor responsiveness in a timely and spatially controlled manner. beta1 integrins may provide NSC with the capacity to react to a dynamic "niche", and to respond adequately by either remaining as stem cells or by differentiating and migrating away to shape the developing cortex.
146. Phagocytic cells of the thymic reticulum interact with thymocytes via **extracellular matrix** ligands and receptors. We previously showed that, in the context of thymic epithelial cells, thymocyte migration is partially controlled by **extracellular matrix** (ECM)-mediated interactions. Herein we evaluated whether these interactions could be involved in cell migration related events in the context of non-epithelial cells of the thymic microenvironment, the phagocytic cells of the thymic reticulum (PTR). We first showed, by immunocytochemistry, cytofluorometry, and RT-PCR, that PTR produce ECM components, including fibronectin and laminin, and express the corresponding integrin-type receptors, VLA-4, VLA-5, and VLA-6. Thymocytes adhere onto PTR monolayers, with immature CD4(+)CD8(+) cells being predominant. Importantly, such an adhesion is partially mediated by ECM ligands and receptors, since it was impaired by anti-ECM or anti-ECM receptor antibodies. Conjointly, our data reveal that the ECM-dependence for thymocyte adhesion onto the thymic microenvironment is not restricted to the epithelial cells, being also seen when they encounter non-epithelial phagocytic cells.
147. Age-related bone deterioration is diminished by disrupted collagen sensing in integrin α2β1 deficient mice. Collagen binding integrins are of essential importance in the crosstalk between cells and th**e extracellular matr**ix. Integrin α2β1 is a major receptor for collagen I, the most abundant protein in bone. In this study we show for the first time that integrin α2 deficiency is linked to collagen type I expression in bone. Investigating the femurs of wild type and integrin α2β1 deficient mice, we found that loss of integrin α2 results in altered bone properties. Histomorphometric analysis of integrin α2 long bones displayed more trabecular network compared to wild type bones. During age related bone loss the integrin α2β1 deficient bones retain trabecular structure even at old age. These findings were supported by functional, biomechanical testing, wherein the bones of integrin α2β1 deficient mice do not undergo age-related alteration of biomechanical properties. These results might be explained by the increased presence of collagen in integrin α2β1 deficient bone. Collagen type I could be detected in higher quantities in the integrin α2β1 deficient bones, forming abnormal, amorphous fibrils. This was linked to higher expression levels of collagen type I and other bone formation related proteins as alkaline phosphatase of integrin α2β1 deficient osteoblasts. Osteoclasts of integrin α2β1 deficient mice did not show any differences. Consequently these results indicate that the absence of integrin α2β1 alleviates the effects of age related bone degradation through over-expression of collagen type I and demonstrate a molecular mechanism how collagen binding integrins might directly impact bone aging.
148. Genetic compensation for sarcoglycan loss by integrin alpha7beta1 in muscle. Disruption of the sarcoglycan complex leads to muscle membrane instability and muscular dystrophy in humans and mice. Through the dystrophin glycoprotein complex, sarcoglycan participates in connecting the internal cytoskeleton to the membrane and the **extracellular matrix**. Integrin alpha7beta1 is also a transmembrane protein of skeletal and cardiac muscle that similarly links the cytoskeleton to the **extracellular matrix**. Mice lacking integrin alpha7 develop mild muscle degeneration, while sarcoglycan mutant mice display overt muscle degeneration and muscular dystrophy. In sarcoglycan-deficient muscle, integrin alpha7 protein was upregulated at the plasma membrane. To ascertain whether integrin alpha7 upregulation compensates for the loss of the transmembrane sarcoglycan linkage in sarcoglycan-deficient muscle, we generated mice lacking both integrin alpha7 and gamma-sarcoglycan (gxi). These double-mutant gxi mice exhibit profound, rapid muscle degeneration leading to death before one month of age consistent with a weakened cellular attachment to the **extracellular matrix**. The regenerative capacity of gxi muscle was intact with increased embryonic myosin heavy chain expression, myofiber central nucleation and normal in vivo myoblast differentiation. Therefore, upregulation of integrin alpha7beta1 compensates as a transmembrane muscle cell attachment for sarcoglycan consistent with overlapping roles for sarcoglycan and integrins in mediating cytoskeletal-membrane-**extracellular matrix** interaction.
149. Beta 1-integrins are required for hippocampal AMPA receptor-dependent synaptic transmission, synaptic plasticity, and working memory. Integrins comprise a large family of cell adhesion receptors that mediate diverse biological events through cell-cell and cell-**extracellular matrix** interactions. Recent studies have shown that several integrins are localized to synapses with suggested roles in synaptic plasticity and memory formation. We generated a postnatal forebrain and excitatory neuron-specific knock-out of beta1-integrin in the mouse. Electrophysiological studies demonstrated that these mutants have impaired synaptic transmission through AMPA receptors and diminished NMDA receptor-dependent long-term potentiation. Despite the impairment in hippocampal synaptic transmission, the mutants displayed normal hippocampal-dependent spatial and contextual memory but were impaired in a hippocampal-dependent, nonmatching-to-place working memory task. These phenotypes parallel those observed in animals carrying knock-outs of the GluR1 (glutamate receptor subunit 1) subunit of the AMPA receptor. These observations suggest a new function of beta1-integrins as regulators of synaptic glutamate receptor function and working memory.
150. Perturbation of beta1-integrin function in involuting mammary gland results in premature dedifferentiation of secretory epithelial cells. To study the mechanism of beta1-integrin function in vivo, we have generated transgenic mouse expressing a dominant negative mutant of beta1-integrin under the control of mouse mammary tumor virus (MMTV) promoter (MMTV-beta1-cyto). Mammary glands from MMTV-beta1-cyto transgenic females present significant growth defects during pregnancy and lactation and impaired differentiation of secretory epithelial cells at the onset of lactation. We report herein that perturbation of beta1-integrin function in involuting mammary gland induced precocious dedifferentiation of the secretory epithelium, as shown by the premature decrease in beta-casein and whey acidic protein mRNA levels, accompanied by inactivation of STAT5, a transcription factor essential for mammary gland development and up-regulation of nuclear factor-kappaB, a negative regulator of STAT5 signaling. This is the first study demonstrating in vivo that cell-**extracellular matrix** interactions involving beta1-integrins play an important role in the control of milk gene transcription and in the maintenance of the mammary epithelial cell differentiated state.
151. Conditional beta1-integrin gene deletion in neural crest cells causes severe developmental alterations of the peripheral nervous system. Integrins are transmembrane receptors that are known to interact with the **extracellular matrix** and to be required for migration, proliferation, differentiation and apoptosis. We have generated mice with a neural crest cell-specific deletion of the beta1-integrin gene to analyse the role of beta1-integrins in neural crest cell migration and differentiation. This targeted mutation caused death within a month of birth. The loss of beta1-integrins from the embryo delayed the migration of Schwann cells along axons and induced multiple defects in spinal nerve arborisation and morphology. There was an almost complete absence of Schwann cells and sensory axon segregation and defective maturation in neuromuscular synaptogenesis. Thus, beta1-integrins are important for the control of embryonic and postnatal peripheral nervous system development.
152. Immunohistochemical localization of **extracellular matrix** proteins in developing lung tissues. To examine the laminin specific receptor, known as beta1 integrin, and fibronectin distributions in 13, 15, 17, and 20 days prenatally old and 2 and 6 days postnatally old murine lung tissue by immunohistochemical methods at light microscope level.Male and female swiss albino mice were used for experimental procedures from July 2001 to March 2002 at Gazi University Faculty of Medicine. The study was conducted on samples of 13, 15, 17, and 20 prenatally old and 2 and 6 days postnatally old lung tissues. Indirect immunohistochemical methods and fibronectin and laminin beta1 integrin antibodies were used; tissues were examined with a light microscope.In the prenatal group, fibronectin immunoreactivity was determined to be irregular in bronchiole epithelium cells, while it was strongly evident in mesenchymal cells and vascular endothelial cells. Laminin beta1 integrin immunoreactivity was also observed irregularly. Development period of lungs, immunoreactivity of fibronectin was clearly identified in the bronchiolus, ductus alveolaris and alveolar epithelial cells, mesenchymal cells, and vascular endothelial cells, and laminin immunoreactivity was strongly involved both in the apical and basal membranes of all the epithelial cells and within the basal lamina. The decreased immunoreactivity of fibronectin and laminin beta1 integrin was established after birth on the fetal period.In this study we concluded: 1. During the development of the lung, fibronectin is necessary for the shaping of lung parenchyma and stroma. 2. beta1 integrin as the receptor of laminin is important in the process of lung maturation and the modelling of basal lamina.
153. Mena binds α5 integrin directly and modulates α5β1 function. Mena is an Ena/VASP family actin regulator with roles in cell migration, chemotaxis, cell-cell adhesion, tumor cell invasion, and metastasis. Although enriched in focal adhesions, Mena has no established function within these structures. We find that Mena forms an adhesion-regulated complex with α5β1 integrin, a fibronectin receptor involved in cell adhesion, motility, fibronectin fibrillogenesis, signaling, and growth factor receptor trafficking. Mena bound directly to the carboxy-terminal portion of the α5 cytoplasmic tail via a 91-residue region containing 13 five-residue "LERER" repeats. In fibroblasts, the Mena-α5 complex was required for "outside-in" α5β1 functions, including normal phosphorylation of FAK and paxillin and formation of fibrillar adhesions. It also supported fibrillogenesis and cell spreading and controlled cell migration speed. Thus, fibroblasts require Mena for multiple α5β1-dependent processes involving bidirectional interactions b**etween the extracell**ular matrix and cytoplasmic focal adhesion proteins.
154. Essential role of alpha 6 integrins in cortical and retinal lamination. **Extracellular matrix** (ECM) is believed to play important roles in many aspects of nervous system development [1]. The laminins are ECM glycoproteins expressed in neural tissues and are potent stimulators of neurite outgrowth in vitro [1-3]. Genetic approaches using Drosophila and Caenorhabditis elegans have demonstrated a role for laminin and a laminin receptor in vivo in axon pathfinding and fasciculation, respectively [4,5]. In higher organisms, however, the role of laminins in the development of the nervous system is poorly understood. Integrins alpha 6 beta 1 and alpha 6 beta 4 are major laminin receptors. A role for the alpha 6 integrin in neurulation has been reported in amphibians [6]. We previously described mice lacking integrin alpha 6; these mice died at birth with severe skin blistering [7]. Detailed analyses of integrin alpha 6-/- mice reported here revealed abnormalities in the laminar organization of the developing cerebral cortex and retina. Ectopic neuroblastic outgrowths were found on the brain surface and in the vitreous body in the eye. Alterations of laminin deposition were found in mutant brains. Thus, this study provides evidence for an essential role of integrin-laminin interactions in the proper development of the nervous system. These observations are particularly significant given the recent report that human patients suffering from epidermolysis bullosa can carry mutations in ITGA6, the gene encoding the alpha 6 integrin chain [8,9].
155. Lack of beta1 integrins in enteric neural crest cells leads to a Hirschsprung-like phenotype. The enteric nervous system arises mainly from vagal and sacral neural crest cells that colonise the gut between 9.5 and 14 days of development in mice. Using the Cre-LoxP system, we removed beta1 integrins in the neural crest cells when they emerge from the neural tube. beta1-null enteric neural crest cells fail to colonise the gut completely, leading to an aganglionosis of the descending colon, which resembles the human Hirschsprung's disease. Moreover, beta1-null enteric neural crest cells form abnormal aggregates in the gut wall, leading to a severe alteration of the ganglia network organisation. Organotypic cultures of gut explants reveal that beta1-null enteric neural crest cells show impaired adhesion on **extracellular matrix** and enhanced intercellular adhesion properties. They display migration defects in collagen gels and gut tissue environments. We also provide evidence that beta1 integrins are required for the villi innervation in the small intestine. Our findings highlight the crucial roles played by beta1 integrins at various steps of enteric nervous system development.
156. Cas adaptor proteins organize the retinal ganglion cell layer downstream of integrin signaling. Stratification of retinal neuronal cell bodies and lamination of their processes provide a scaffold upon which neural circuits can be built. However, the molecular mechanisms that direct retinal ganglion cells (RGCs) to resolve into a single-cell retinal ganglion cell layer (GCL) are not well understood. The **extracellular matrix** protein laminin conveys spatial information that instructs the migration, process outgrowth, and reorganization of GCL cells. Here, we show that the β1-Integrin laminin receptor is required for RGC positioning and reorganization into a single-cell GCL layer. β1-Integrin signaling within migrating GCL cells requires Cas signaling-adaptor proteins, and in the absence of β1-Integrin or Cas function retinal neurons form ectopic cell clusters beyond the inner-limiting membrane (ILM), phenocopying laminin mutants. These data reveal an essential role for Cas adaptor proteins in β1-Integrin-mediated signaling events critical for the formation of the single-cell GCL in the mammalian retina.
157. VEGF-induced endothelial cell migration requires urokinase receptor (uPAR)-dependent integrin redistribution. Vascular endothelial growth factor (VEGF)-initiated angiogenesis requires coordinated proteolytic degradation of **extracellular matrix** provided by the urokinase plasminogen activator/urokinase receptor (uPA/uPAR) system and regulation of cell migration provided by integrin-matrix interaction. In this study, we investigated the mechanisms underlying the uPAR-dependent modulation of VEGF-induced endothelial migration.We used flow cytometry to quantify integrins at the cell surface. Stimulation of human and murine endothelial cells with VEGF resulted in internalization of α5β1-integrins. Micropatterning and immunocytochemistry revealed co-clustering of uPAR and α5β1-integrins and retrieval via clathrin-coated vesicles. It was also contingent on receptors of the low-density lipoprotein receptor (LDL-R) family. VEGF-induced integrin redistribution was inhibited by elimination of uPAR from the endothelial cell surface or by inhibitory peptides that block the uPAR-integrin interaction. Under these conditions, the migratory response of endothelial cells upon VEGF stimulation was impaired both in vitro and in vivo.The observations indicate that uPAR is an essential component of the network through which VEGF controls endothelial cell migration. uPAR is a bottleneck through which the VEGF-induced signal must be funnelled for both focused proteolytic activity at the leading edge and for redistribution of integrins.
158. Interactions of primary fibroblasts and keratinocytes with **extracellular matrix** proteins: contribution of alpha2beta1 integrin. The alpha2beta1 integrin is a collagen-binding protein with very high affinity for collagen I. It also binds several other collagens and laminins and it is expressed by many cells, including keratinocytes and fibroblasts in the skin. In the past, alpha2beta1 integrin was suggested to be responsible for cell attachment, spreading and migration on monomeric collagen I and contraction of three-dimensional collagen lattices. In view of these functions, normal development and fertility in integrin alpha2-deficient mice, which we generated by targeting the integrin alpha2 gene, came as a surprise. This suggested the existence of compensatory mechanisms that we investigate here using primary fibroblasts and keratinocytes isolated from wild-type and alpha2-deficient mice, antibodies blocking integrin function and downregulation of integrin alpha2 expression. The results show that the alpha2beta1 integrin is absolutely required for keratinocyte adhesion to collagens whereas for fibroblasts other collagen-binding integrins partially back-up the lack of alpha2beta1 in simple adhesion to collagen monomers. A prominent requirement for alpha2beta1 integrins became apparent when fibroblasts executed mechanical tasks of high complexity in three-dimensional surroundings, such as contracting free-floating collagen gels and developing isometric forces in tethered lattices. The deficits observed for alpha2-deficient fibroblasts appeared to be linked to alterations in the distribution of force-bearing focal adhesions and deregulation of Rho-GTPase activation.
159. Fibronectin-tissue transglutaminase matrix rescues RGD-impaired cell adhesion through syndecan-4 and beta1 integrin co-signaling. Heterotropic association of tissue transglutaminase (TG2) with **extracellular matrix**-associated fibronectin (FN) can restore the adhesion of fibroblasts when the integrin-mediated direct binding to FN is impaired using RGD-containing peptide. We demonstrate that the compensatory effect of the TG-FN complex in the presence of RGD-containing peptides is mediated by TG2 binding to the heparan sulfate chains of the syndecan-4 cell surface receptor. This binding mediates activation of protein kinase Calpha (PKCalpha) and its subsequent interaction with beta(1) integrin since disruption of PKCalpha binding to beta(1) integrins with a cell-permeant competitive peptide inhibits cell adhesion and the associated actin stress fiber formation. Cell signaling by this process leads to the activation of focal adhesion kinase and ERK1/2 mitogen-activated protein kinases. Fibroblasts deficient in Raf-1 do not respond fully to the TG-FN complex unless either the full-length kinase competent Raf-1 or the kinase-inactive domain of Raf-1 is reintroduced, indicating the involvement of the Raf-1 protein in the signaling mechanism. We propose a model for a novel RGD-independent cell adhesion process that could be important during tissue injury and/or remodeling whereby TG-FN binding to syndecan-4 activates PKCalpha leading to its association with beta(1) integrin, reinforcement of actin-stress fiber organization, and MAPK pathway activation.
160. Integrin alpha3beta1 potentiates TGFbeta-mediated induction of MMP-9 in immortalized keratinocytes. Transforming growth factor-beta (TGFbeta) signaling pathways regulate a number of keratinocyte functions during epidermal carcinogenesis and wound healing, including proliferation, survival, and migration. TGFbeta can induce expression of the matrix metalloproteinase-9 (MMP-9), which has critical roles in promoting **extracellular matrix** remodeling and angiogenesis during tumorigenesis and tissue repair. Integrin alpha3beta1 is a cell adhesion receptor for laminin-332/laminin-5 with important roles in the survival and motility of epidermal keratinocytes. We previously reported that alpha3beta1 induces the expression of MMP-9 in immortalized keratinocytes. In this study, we show that endogenous TGFbeta is required for maximal MMP-9 expression, and that alpha3beta1 is required for full induction of MMP-9 protein and mRNA in response to TGFbeta. This regulation was not observed in non-immortalized, primary keratinocytes, indicating that coordinate regulation of MMP-9 by alpha3beta1 and TGFbeta is a property of immortalized cells. Alpha3beta1 did not regulate endogenous TGFbeta gene expression, TGFbeta bioavailability, or TGFbeta-Smad signaling. However, the combined inductive effects of TGFbeta and alpha3beta1 on MMP-9 were suppressed by a Src family kinase (SFK) inhibitor, indicating involvement of a SFK pathway. These findings provide early evidence of a role for alpha3beta1 in augmenting TGFbeta-mediated induction of MMP-9 in immortalized or transformed keratinocytes during skin carcinogenesis.
161. The C-terminal region of NELL1 mediates osteoblastic cell adhesion through integrin α3β1. NELL1 is a secretory osteogenic protein containing several structural motifs that suggest that it functions as a**n extracellular matr**ix component. To determine the mechanisms underlying NELL1-induced osteoblast differentiation, we examined the cell-adhesive activity of NELL1 using a series of recombinant NELL1 proteins. We demonstrated that NELL1 promoted osteoblastic cell adhesion through at least three cell-binding domains located in the C-terminal region of NELL1. Adhesion of cells to NELL1 was strongly inhibited by function-blocking antibodies against integrin α3 and β1 subunits, suggesting that osteoblastic cells adhered to NELL1 through integrin α3β1. Further, focal adhesion kinase activation is involved in NELL1 signaling.
162. Epithelial cell integrin β1 is required for developmental angiogenesis in the pituitary gland. As a key component of the vertebrate neuroendocrine system, the pituitary gland relies on the progressive and coordinated development of distinct hormone-producing cell types and an invading vascular network. The molecular mechanisms that drive formation of the pituitary vasculature, which is necessary for regulated synthesis and secretion of hormones that maintain homeostasis, metabolism, and endocrine function, remain poorly understood. Here, we report that expression of integrin β1 in embryonic pituitary epithelial cells is required for angiogenesis in the developing mouse pituitary gland. Deletion of pituitary epithelial integrin β1 before the onset of angiogenesis resulted in failure of invading endothelial cells to recruit pericytes efficiently, whereas deletion later in embryogenesis led to decreased vascular density and lumen formation. In both cases, lack of epithelial integrin β1 was associated with a complete absence of vasculature in the pituitary gland at birth. Within pituitary epithelial cells, integrin β1 directs a large transcriptional program that includes components of **the extracellular m**atrix and associated signaling factors that are linked to the observed non-cell-autonomous effects on angiogenesis. We conclude that epithelial integrin β1 functions as a critical and canonical regulator of developmental angiogenesis in the pituitary gland, thus providing insight into the long-standing systems biology conundrum of how vascular invasion is coordinated with tissue development.
163. Neuregulin-1/glial growth factor stimulates Schwann cell migration by inducing α5 β1 integrin-ErbB2-focal adhesion kinase complex formation. After peripheral nerve injury, Schwann cells gain a migratory phenotype and remodel thei**r extracellular matr**ix to provide a supportive environment for axonal regeneration. The soluble neuregulin-1 isoform, that is, glial growth factor (GGF), is expressed in regenerating axons of injured peripheral nerves and regulates Schwann cell motility by activating the ErbB family of tyrosine kinase receptors, but how GGF/ErbB signaling contributes to Schwann cell motility remains unclear. Here, we show that GGF stimulates Schwann cell migration by inducing the formation of a protein complex containing the fibronectin receptor α5β1 integrin, ErbB2, and focal adhesion kinase (FAK). ErbB2 co-localizes and co-immunoprecipitates with the focal complex members including α5β1 integrin and FAK after GGF treatment. These effects of GGF appear to involve FAK activation, which occurs downstream of ErbB2 stimulation. RNAi-mediated down-regulation of α5 integrin expression in primary cultured Schwann cells resulted in significantly decreased interaction between FAK and ErbB2, as well as decreased GGF-induced migration. An increase in the α5β1 integrin-ErbB2-FAK complex formation was observed in injured nerve Schwann cells, but not uninjured control. Taken together, these data suggest that GGF plays an important modulatory role in Schwann cell migration after nerve crush by inducing α5β1 integrin-ErbB2-FAK complex formation.
164. alpha2beta1 integrin controls association of Rac with the membrane and triggers quiescence of endothelial cells. Integrin receptors and their **extracellular matrix** ligands provide cues to cell proliferation, survival, differentiation and migration. Here, we show that alpha2beta1 integrin, when ligated to the basement membrane component laminin-1, triggers a proliferation arrest in primary endothelial cells. Indeed, in the presence of strong growth signals supplied by growth factors and fibronectin, alpha2beta1 engagement alters assembly of mature focal adhesions by alpha5beta1 and leads to impairment of downstream signaling and cell-cycle arrest in the G1 phase. Although the capacity of alpha5beta1 to signal for GTP loading of Rac is preserved, the joint engagement of alpha2beta1 interferes with membrane anchorage of Rac. Adapting the 'split-ubiquitin' sensor to screen for membrane-proximal alpha2 integrin partners, we identified the CD9 tetraspanin and further establish its requirement for destabilization of focal adhesions, control of Rac subcellular localization and growth arrest induced by alpha2beta1 integrin. Altogether, our data establish that alpha2beta1 integrin controls endothelial cell commitment towards quiescence by triggering a CD9-dependent dominant signaling.
165. Close homolog of L1 is an enhancer of integrin-mediated cell migration. Close homolog of L1 (CHL1) is a member of the L1 family of cell adhesion molecules expressed by subpopulations of neurons and glia in the central and peripheral nervous system. It promotes neurite outgrowth and neuronal survival in vitro. This study describes a novel function for CHL1 in potentiating integrin-dependent cell migration toward **extracellular matrix** proteins. Expression of CHL1 in HEK293 cells stimulated their haptotactic migration toward collagen I, fibronectin, laminin, and vitronectin substrates in Transwell assays. CHL1-potentiated cell migration to collagen I was dependent on alpha1beta1 and alpha2beta1 integrins, as shown with function blocking antibodies. Potentiated migration relied on the early integrin signaling intermediates c-Src, phosphatidylinositol 3-kinase, and mitogen-activated protein kinase. Enhancement of migration was disrupted by mutation of a potential integrin interaction motif Asp-Gly-Glu-Ala (DGEA) in the sixth immunoglobulin domain of CHL1, suggesting that CHL1 functionally interacts with beta1 integrins through this domain. CHL1 was shown to associate with beta1 integrins on the cell surface by antibody-induced co-capping. Through a cytoplasmic domain sequence containing a conserved tyrosine residue (Phe-Ile-Gly-Ala-Tyr), CHL1 recruited the actin cytoskeletal adapter protein ankyrin to the plasma membrane, and this sequence was necessary for promoting integrin-dependent migration to **extracellular matrix** proteins. These results support a role for CHL1 in integrin-dependent cell migration that may be physiologically important in regulating cell migration in nerve regeneration and cortical development.
166. Perlecan proteolysis induces an alpha2beta1 integrin- and Src family kinase-dependent anti-apoptotic pathway in fibroblasts in the absence of focal adhesion kinase activation. Dysregulation of apoptosis in endothelial cells (EC) and fibroblasts contributes to fibrosis. We have shown previously that apoptosis of EC triggers the proteolysis of **extracellular matrix** components and the release of a C-terminal fragment of perlecan, which in turn inhibits apoptosis of fibroblasts. Here we have defined the receptors and pathways implicated in this anti-apoptotic response in fibroblasts. Neutralizing alpha2beta1 integrin activity in fibroblasts exposed to either medium conditioned by apoptotic EC (SSC) or a recombinant perlecan C-terminal fragment (LG3) prevented resistance to apoptosis and is associated with decreased levels of Akt phosphorylation. Co-incubation of fibroblasts for 24 h with SSC or LG3 in the presence of PP2 (AG1879), a biochemical inhibitor of Src family kinases (SFKs) and focal adhesion kinase, showed a significantly decreased anti-apoptotic response. However, focal adhesion kinase gene silencing with RNA interference did not inhibit the anti-apoptotic response in fibroblasts. Src phosphorylation was increased in fibroblasts exposed to SSC, and transfection of fibroblasts with constitutively active Src mutants induced an anti-apoptotic response that was not further increased by SSC. Also, Src(-/-)Fyn(-/-) fibroblasts failed to mount an anti-apoptotic response in presence of SSC for 24 h but developed a complete anti-apoptotic response when exposed to SSC for 7 days. These results suggest that **extracellular matrix** fragments produced by apoptotic EC initiate a state of resistance to apoptosis in fibroblasts via an alpha2beta1 integrin/SFK (Src and Fyn)/phosphatidylinositol 3-kinase (PI3K)-dependent pathway. In the long term, additional SFK members are recruited for sustaining the anti-apoptotic response, which could play crucial roles in abnormal fibrogenic healing.
167. Specific β-containing integrins exert differential control on proliferation and two-dimensional collective cell migration in mammary epithelial cells. Understanding how cell cycle is regulated in normal mammary epithelia is essential for deciphering defects of breast cancer and therefore for developing new therapies. Signals provided by both the **extracellular matri**x and growth factors are essential for epithelial cell proliferation. However, the mechanisms by which adhesion controls cell cycle in normal epithelia are poorly established. In this study, we describe the consequences of removing the β1-integrin gene from primary cultures of mammary epithelial cells in situ, using CreER. Upon β1-integrin gene deletion, the cells were unable to progress efficiently through S-phase, but were still able to undergo collective two-dimensional migration. These responses are explained by the presence of β3-integrin in β1-integrin-null cells, indicating that integrins containing different β-subunits exert differential control on mammary epithelial proliferation and migration. β1-Integrin deletion did not inhibit growth factor signaling to Erk or prevent the recruitment of core adhesome components to focal adhesions. Instead the S-phase arrest resulted from defective Rac activation and Erk translocation to the nucleus. Rac inhibition prevented Erk translocation and blocked proliferation. Activated Rac1 rescued the proliferation defect in β1-integrin-depleted cells, indicating that this GTPase is essential in propagating proliferative β1-integrin signals. These results show that β1-integrins promote cell cycle in mammary epithelial cells, whereas β3-integrins are involved in migration.
168. Integrin β1 signals through Arg to regulate postnatal dendritic arborization, synapse density, and behavior. Integrins are heterodimeric **extracellular matri**x receptors that are essential for the proper development of the vertebrate nervous system. We report here that selective loss of integrin β1 in excitatory neurons leads to reductions in the size and complexity of hippocampal dendritic arbors, hippocampal synapse loss, impaired hippocampus-dependent learning, and exaggerated psychomotor sensitivity to cocaine in mice. Our biochemical and genetic experiments demonstrate that the intracellular tail of integrin β1 binds directly to Arg kinase and that this interaction stimulates activity of the Arg substrate p190RhoGAP, an inactivator of the RhoA GTPase. Moreover, genetic manipulations that reduce integrin β1 signaling through Arg recapitulate the integrin β1 knock-out phenotype in a gene dose-sensitive manner. Together, these results describe a novel integrin β1-Arg-p190RhoGAP pathway that regulates dendritic arbor size, promotes synapse maintenance, supports proper hippocampal function, and mitigates the behavioral consequences of cocaine exposure.
169. Integrins control dendritic spine plasticity in hippocampal neurons through NMDA receptor and Ca2+/calmodulin-dependent protein kinase II-mediated actin reorganization. The formation of dendritic spines during development and their structural plasticity in the adult brain are critical aspects of synaptogenesis and synaptic plasticity. Many different factors and proteins have been shown to control dendritic spine development and remodeling (Ethell and Pasquale, 2005). The **extracellular matrix** (ECM) components and their cell surface receptors, integrins, have been found in the vicinity of synapses and shown to regulate synaptic efficacy and play an important role in long-term potentiation (Bahr et al., 1997; Chavis and Westbrook, 2001; Chan et al., 2003; Lin et al., 2003; Bernard-Trifilo et al., 2005). Although molecular mechanisms by which integrins affect synaptic efficacy have begun to emerge, their role in structural plasticity is poorly understood. Here, we show that integrins are involved in spine remodeling in cultured hippocampal neurons. The treatment of 14 d in vitro hippocampal neurons with arginine-glycine-aspartate (RGD)-containing peptide, an established integrin ligand, induced elongation of existing dendritic spines and promoted formation of new filopodia. These effects were also accompanied by integrin-dependent actin reorganization and synapse remodeling, which were partially inhibited by function-blocking antibodies against beta1 and beta3 integrins. This actin reorganization was blocked with the NMDA receptor (NMDAR) antagonist MK801 [(+)-5-methyl-10,11-dihydro-5H-dibenzo[a,d]cyclohepten-5,10-imine hydrogen maleate]. The Ca2+/calmodulin-dependent protein kinase II (CaMKII) inhibitor KN93 (N-[2-[N-(4-chlorocinnamyl)-N-methylaminomethyl]phenyl]-N-(2-hydroxyethyl)-4-methoxybenzenesulfonamide) also suppressed RGD-induced actin reorganization and synapse remodeling. Our findings show that integrins control ECM-mediated spine remodeling in hippocampal neurons through NMDAR/CaMKII-dependent actin reorganization.
170. Developmental regulation of integrin expression at the time of implantation in the mouse embryo. The trophectoderm layer of the mouse blastocyst differentiates at the late blastocyst stage to form the invasive trophoblast that mediates implantation of the embryo into the uterine wall. The first sign that trophoblast cells have developed an invasion-specific cell behavior appears about 10-15 hours after the embryo hatches from the zona pellucida, when the quiescent, non-adherent trophectoderm cells initiate protrusive activity and become adhesive to **extracellular matrix**. Our previous findings that trophoblast outgrowth on extracellular-matrix-coated substrata involves the integrin family of adhesion receptors (Sutherland, A. E., Calarco, P. G. and Damsky, C. H., 1988, J. Cell Biol. 106, 1331-1348), suggested that the onset of trophoblast adhesive and migratory behavior at the time of implantation may be due to changes in expression or distribution of integrin receptors. We have thus examined the mRNA and protein expression of individual integrin subunits during pre- and periimplantation development (E0-E7.5). A basic repertoire of integrins, including receptors for fibronectin (alpha 5 beta 1), laminin (alpha 6B beta 1) and vitronectin (alpha v beta 3), was expressed continuously throughout this period, whereas the expression of five other integrin subunits was developmentally regulated. The mRNA for three of these (alpha 2, alpha 6A and alpha 7) was first detected in the late blastocyst, coincident with endoderm differentiation and development of attachment competence. The mRNA for another (alpha 1) was not detected until after trophoblast outgrowth had begun, suggesting that its expression may be induced by contact with matrix. At E7.5, three of the temporally regulated integrins (alpha 1, apha 6A, alpha 7), all of which can form receptors for laminin, were detected only in the ectoplacental cone (differentiating trophoblast), and may thus play specific roles in trophoblast adhesion and/or differentiation. Because laminin expression is upregulated in decidualized uterine stroma in response to the implanting embryo, we examined trophoblast-laminin interactions, using laminin fragments and integrin antibodies to determine which integrin receptors were involved. Trophoblast cells attached and spread on both the E8 and P1' fragments of laminin; however, the P1' binding site was cryptic in intact laminin. Interaction with P1' was RGD- and alpha v beta 3-dependent, whereas outgrowth on E8 was RGD-independent and not inhibited by antibodies to the laminin receptor alpha 6 beta 1, suggesting that alpha 7 beta 1 is the major trophoblast integrin E8 receptor.(ABSTRACT TRUNCATED AT 400 WORDS)
171. Bradykinin B2 receptor interacts with integrin alpha5beta1 to transactivate epidermal growth factor receptor in kidney cells. We have shown previously that the vasoactive peptide bradykinin (BK) stimulates proliferation of a cultured murine cell model of the inner medullary collecting duct (mIMCD-3 cells) via transactivation of epidermal growth factor receptor (EGFR) by a mechanism that involves matrix metalloproteinases (collagenase-2 and -3). Because collagenases lack an integral membrane domain, we hypothesized that receptors for **extracellular matrix** proteins, integrins, may play a role in BK-induced signaling by targeting collagenases to the membrane, thus forming a functional signaling complex. BK-induced phosphorylation of extracellular signal-regulated protein kinase (ERK) in mIMCD-3 cells was reduced by approximately 65% by synthetic peptides containing an Arg-Gly-Asp sequence, supporting roles for integrins in BK-induced signaling. Neutralizing antibody against alpha5beta1 integrin partially (approximately 60%) blocked BK-induced ERK activation but did not affect EGF-induced ERK activation. Silencing of alpha5 and beta1 expression by transfecting cells with small interfering RNAs (siRNA) significantly decreased BK-induced ERK activation (approximately 80%) and EGFR phosphorylation (approximately 50%). This effect was even more pronounced in cells that were cotransfected with siRNAs directed against both collagenases and alpha5beta1 integrin. On the basis of our results, we suggested that integrin alpha5beta1 is involved in BK-induced signaling in mIMCD-3 cells. Using immunoprecipitation/Western blotting, we demonstrated association of BK B(2) receptor with alpha5beta1 integrin upon BK treatment. Furthermore, BK induced association of alpha5beta1 integrin with EGFR. These data provide the first evidence that specific integrins are involved in BK B(2) receptor-induced signaling in kidney cells, and ultimately might lead to development of new strategies for treatment of renal tubulointerstitial fibrosis.
172. MT1-MMP-dependent control of skeletal stem cell commitment via a β1-integrin/YAP/TAZ signaling axis. In vitro, topographical and biophysical cues arising from the **extracellular matri**x (ECM) direct skeletal stem cell (SSC) commitment and differentiation. However, the mechanisms by which the SSC-ECM interface is regulated and the outcome of such interactions on stem cell fate in vivo remain unknown. Here we demonstrate that conditional deletion of the membrane-anchored metalloproteinase MT1-MMP (Mmp14) in mesenchymal progenitors, but not in committed osteoblasts, redirects SSC fate decisions from osteogenesis to adipo- and chondrogenesis. By effecting ECM remodeling, MT1-MMP regulates stem cell shape, thereby activating a β1-integrin/RhoGTPase signaling cascade and triggering the nuclear localization of the transcriptional coactivators YAP and TAZ, which serve to control SSC lineage commitment. These data identify a critical MT1-MMP/integrin/YAP/TAZ axis operative in the stem cell niche that oversees SSC fate determination.
173. β1 integrin signaling promotes neuronal migration along vascular scaffolds in the post-stroke brain. Cerebral ischemic stroke is a main cause of chronic disability. However, there is currently no effective treatment to promote recovery from stroke-induced neurological symptoms. Recent studies suggest that after stroke, immature neurons, referred to as neuroblasts, generated in a neurogenic niche, the ventricular-subventricular zone, migrate toward the injured area, where they differentiate into mature neurons. Interventions that increase the number of neuroblasts distributed at and around the lesion facilitate neuronal repair in rodent models for ischemic stroke, suggesting that promoting neuroblast migration in the post-stroke brain could improve efficient neuronal regeneration. To move toward the lesion, neuroblasts form chain-like aggregates and migrate along blood vessels, which are thought to increase their migration efficiency. However, the molecular mechanisms regulating these migration processes are largely unknown. Here we studied the role of β1-class integrins, transmembrane receptors fo**r extracellular matr**ix proteins, in these migrating neuroblasts. We found that the neuroblast chain formation and blood vessel-guided migration critically depend on β1 integrin signaling. β1 integrin facilitated the adhesion of neuroblasts to laminin and the efficient translocation of their soma during migration. Moreover, artificial laminin-containing scaffolds promoted neuroblast chain formation and migration toward the injured area. These data suggest that laminin signaling via β1 integrin supports vasculature-guided neuronal migration to efficiently supply neuroblasts to injured areas. This study also highlights the importance of vascular scaffolds for cell migration in development and regeneration.
174. BCR/ABL expression of myeloid progenitors increases beta1-integrin mediated adhesion to stromal cells. The expression of the fusion protein BCR/ABL is a hallmark of chronic myeloid leukemia. BCR/ABL is a constitutively active tyrosine kinase influencing cell proliferation, apoptosis, and differentiation. To what extent and by which mechanism BCR/ABL affects the adhesion of leukemic cells to bone marrow stromal cells (BMSC) is controversial. To characterize adhesion of BCR/ABL-transformed 32D cells (32D-BCR/ABL) to the BMSC line M2-10B4, we used washing assays and single-cell force spectroscopy (SCFS). Compared to control 32D cells (32D-V), 32D-BCR/ABL developed threefold higher adhesion forces. This enhanced cell adhesion could be reduced to control levels after specifically inhibiting the activity of the tyrosine kinase BCR/ABL using imatinib mesylate (IM). SCFS showed that the adhesion forces of 32D-BCR/ABL were strongest to fibronectin and collagen type I, suggesting that beta1-integrin has a major role in mediating the adhesion of leukemic cells to BMSC. Indeed, the beta1-integrin blocking antibody Ha2/5 abrogated the attachment of 32D-V and 32D-BCR/ABL cells to BMSC. Although 32D-BCR/ABL cells show significantly increased beta1-integrin expression, no significant difference of beta1-integrin mRNA levels could be detected, indicating a post-transcriptional regulation of beta1-comprising integrin heterodimers by BCR/ABL. The data presented here argue that the interaction of beta1-integrin and **extracellular matrix** components is functionally important in leukemic cells expressing high-levels of BCR/ABL, and could provide a rationale for the development of optimized targeted therapies.
175. Integrin β1 is required for dermal homeostasis. Integrins are heterodimers that mediate cell attachment to the **extracellular matri**x. Previously, we used fibroblast-specific knockout mice to show that the integrin β1 subunit was required in vivo for dermal fibrogenesis and cutaneous tissue repair. Here, we show that integrin β1 expression by fibroblasts is required for dermal homeostasis; at 8 weeks after deletion of integrin β1, the dermis is significantly thinner, expressing less collagen and displaying reduced generation of reactive oxygen species (ROS). Cultured integrin β1-deficient fibroblasts show reduced rac1 activation and ROS generation. Overexpressing rac1 in integrin β1-deficient fibroblasts restored ROS generation; adding hydrogen peroxide to integrin β1-deficient fibroblasts restored Col1a2 (collagen, type I, α2) and α-smooth muscle actin (α-SMA) mRNA expression and α-SMA protein expression and stress fiber formation. Thus integrin β1 expression by fibroblasts is required for the maintenance of dermal connective tissue via a rac/ROS pathway.
176. Beta 1D integrin displaces the beta 1A isoform in striated muscles: localization at junctional structures and signaling potential in nonmuscle cells. The cytoplasmic domains of integrins provide attachment of these **extracellular matrix** receptors to the cytoskeleton and play a critical role in integrin-mediated signal transduction. In this report we describe the identification, expression, localization, and initial functional characterization of a novel form of beta 1 integrin, termed beta 1D. This isoform contains a unique alternatively spliced cytoplasmic domain of 50 amino acids, with the last 24 amino acids encoded by an additional exon. Of these 24 amino acids, 11 are conserved when compared to the beta 1A isoform, but 13 are unique (Zhidkova, N. I., A. M. Belkin, and R. Mayne. 1995. Biochem. Biophys. Res. Commun. 214:279-285; van der Flier, A., I. Kuikman, C. Baudoin, R, van der Neuf, and A. Sonnenberg. 1995. FEBS Lett. 369:340-344). Using an anti-peptide antibody against the beta 1D integrin subunit, we demonstrated that the beta 1D isoform is synthesized only in skeletal and cardiac muscles, while very low amounts of beta 1A were detected by immunoblot in striated muscles. Whereas beta 1A could not be detected in adult skeletal muscle fibers and cardiomyocytes by immunofluorescence, beta 1D was localized to the sarcolemma of both cell types. In skeletal muscle, beta 1D was concentrated in costameres, myotendinous, and neuromuscular junctions. In cardiac muscle this beta 1 isoform was found in costamers and intercalated discs. beta 1D was associated with alpha 7A and alpha 7B in adult skeletal muscle. In cardiomyocytes of adult heart, alpha 7B was the major partner for the beta 1D isoform. beta 1D could not be detected in proliferating C2C12 myoblasts, but it appeared immediately after myoblast fusion and its amount continued to rise during myotube growth and maturation. In contrast, expression of the beta 1A isoform was downregulated during myodifferentiation in culture and it was completely displaced by beta 1D in mature differentiated myotubes. We also analyzed some functional properties of the beta 1D integrin subunit. Expression of human beta 1D in CHO cells led to its localization at focal adhesions. Clustering of this integrin isoform on the cell surface stimulated tyrosine phosphorylation of pp125FAK (focal adhesion kinase) and caused transient activation of mitogen-activated protein (MAP) kinases. These data indicate that beta 1D and beta 1A integrin isoforms are functionally similar with regard to integrin-mediated signaling.
177. β1 integrin inhibition elicits a prometastatic switch through the TGFβ-miR-200-ZEB network in E-cadherin-positive triple-negative breast cancer. Interactions with th**e extracellular matr**ix (ECM) through integrin adhesion receptors provide cancer cells with physical and chemical cues that act together with growth factors to support survival and proliferation. Antagonists that target integrins containing the β1 subunit inhibit tumor growth and sensitize cells to irradiation or cytotoxic chemotherapy in preclinical breast cancer models and are under clinical investigation. We found that the loss of β1 integrins attenuated breast tumor growth but markedly enhanced tumor cell dissemination to the lungs. When cultured in three-dimensional ECM scaffolds, antibodies that blocked β1 integrin function or knockdown of β1 switched the migratory behavior of human and mouse E-cadherin-positive triple-negative breast cancer (TNBC) cells from collective to single cell movement. This switch involved activation of the transforming growth factor-β (TGFβ) signaling network that led to a shift in the balance between miR-200 microRNAs and the transcription factor zinc finger E-box-binding homeobox 2 (ZEB2), resulting in suppressed transcription of the gene encoding E-cadherin. Reducing the abundance of a TGFβ receptor, restoring the ZEB/miR-200 balance, or increasing the abundance of E-cadherin reestablished cohesion in β1 integrin-deficient cells and reduced dissemination to the lungs without affecting growth of the primary tumor. These findings reveal that β1 integrins control a signaling network that promotes an epithelial phenotype and suppresses dissemination and indicate that targeting β1 integrins may have undesirable effects in TNBC.
178. Localization of **extracellular matrix** molecules, integrins and their regulators, TGF betas, is correlated with axon pathfinding in the spinal cord of normal and Danforth's short tail mice. To clarify the roles of **extracellular matrix** molecules (ECMs) and their receptors, integrins, in axonal pathfinding, their spatiotemporal localization was studied immunohistochemically in the developing mouse spinal cord. Immunoreactivities for collagen type IV (Col IV), laminin, integrins (alpha2, alpha3 and beta1 subunits), and transforming growth factor (TGF) betas (TGF beta2 and TGF beta3), putative regulators of gene expression of these molecules, were localized on the midline of the ventral spinal cord, or the 'floor plate', in normal ICR mice. These immunoreactions appeared at embryonic day 9 (E9), when commissural axons began to cross the floor plate, and peaked at E13-E14 when more axons crossed, turned rostrally, and projected longitudinally. At this stage, TAG-1-immunopositive commissural axons passed through the floor plate region. Longitudinal axons, on the other hand, projected ipsilaterally parallel to the midline. Immunoreactions for ECMs, integrins and TGF betas were weakly positive at the midline until postnatal day 0 (P0), after which they decreased markedly. In Danforth's short tail (Sd) heterozygous mutants with discontinuous floor plates, immunoreactions for ECMs, integrins, and TGF betas were not detected at P0. Few crossing axons were seen at this stage, and longitudinally ascending axons crossed via aberrant paths in these regions. These results suggest that the restricted localization of ECMs, integrins, and TGF betas at the floor plate is involved in the pathfinding of commissural and longitudinal axons.
179. Tissue inhibitor of metalloproteinase-2 (TIMP-2) regulates myogenesis and beta1 integrin expression in vitro. Myogenesis in vitro involves myoblast cell cycle arrest, migration, and fusion to form multinucleated myotubes. **Extracellular matrix** (ECM) integrity during these processes is maintained by the opposing actions of matrix metalloproteinase (MMP) proteases and their inhibitors, the tissue inhibitor of metalloproteinases (TIMPs). Here, we report that TIMP-2, MMP-2, and MT1-MMP are differentially expressed during mouse myoblast differentiation in vitro. A specific role for TIMP-2 in myogenesis is demonstrated by altered TIMP-2(-/-) myotube formation. When differentiated in horse serum-containing medium, TIMP-2(-/-) myotubes are larger than wild-type myotubes. In contrast, when serum-free medium is used, TIMP-2(-/-) myotubes are smaller than wild-type myotubes. Regardless of culture condition, myotube size is directly correlated with MMP activity and inversely correlated with beta1 integrin expression. Treatment with recombinant TIMP-2 rescues reduced TIMP-2(-/-) myotube size and induces increased MMP-9 activation and decreased beta1 integrin expression. Treatment with either MMP-2 or MMP-9 similarly rescues reduced myotube size, but has no effect on beta1 integrin expression. These data suggest a specific regulatory relationship between TIMP-2 and beta1 integrin during myogenesis. Elucidating the role of TIMP-2 in myogenesis in vitro may lead to new therapeutic options for the use of TIMP-2 in myopathies and muscular dystrophies in vivo.
180. Alteration of medial-edge epithelium cell adhesion in two Tgf-beta3 null mouse strains. Although palatal shelf adhesion is a crucial event during palate development, little work has been carried out to determine which molecules are responsible for this process. Furthermore, whether altered palatal shelf adhesion causes the cleft palate presented by Tgf-beta3 null mutant mice has not yet been clarified. Here, we study the presence/distribution of some **extracellular matrix** and cell adhesion molecules at the time of the contact of palatal shelves in both wild-type and Tgf-beta3 null mutant palates of two strains of mice (C57/BL/6J (C57), and MF1) that develop cleft palates of different severity. We have performed immunohistochemistry with antibodies against collagens IV and IX, laminin, fibronectin, the alpha5- and beta1-integrins, and ICAM-1; in situ hybridization with a Nectin-1 riboprobe; and palatal shelf cultures treated or untreated with TGF-beta3 or neutralizing antibodies against fibronectin or the alpha5-integrin. Our results show the location of these molecules in the wild-type mouse medial edge epithelium (MEE) of both strains at the time of the contact of palatal shelves; the heavier (C57) and milder (MF1) alteration of their presence in the Tgf-beta3 null mutants; the importance of TGF-beta3 to restore their normal pattern of expression; and the crucial role of fibronectin and the alpha5-integrin in palatal shelf adhesion. We thus provide insight into the molecular bases of this important process and the cleft palate presented by Tgf-beta3 null mutant mice.
181. Collagen I regulates the self-renewal of mouse embryonic stem cells through α2β1 integrin- and DDR1-dependent Bmi-1. Adhesion of cells t**o extracellular matr**ix (ECM) influences vital aspects of anchorage-dependent cell behavior including survival, proliferation, and differentiation. However, the role of collagen I in mouse embryonic stem cells (mESCs) is not well-known. Therefore, in the present study we examined the effect of collagen I on mESC self-renewal and related signal pathways. Collagen I (10 µg/ml) maintained mESCs in an undifferentiated state (Nanog, OCT4, and SSEA-1) and did not affect differentiation (GATA4, Tbx5, Fgf5, and Cdx2) in the presence of leukemia inhibitory factor (LIF). Treatment with collagen I bound both α2β1 integrin and discoidin domain receptor 1 (DDR1), and stimulated intracellular signaling pathways. Collagen I-bound α2β1 integrin increased integrin-linked kinase (ILK) phosphorylation, cleaved Notch protein expression in the nuclear fraction, and Gli-1 mRNA expression. In addition, collagen I-bound DDR1 increased GTP-bound Ras, phosphoinositide 3-kinase (PI3K) p85α catalytic subunit protein expression, and Akt and ERK phosphorylation. Importantly, collagen I increased Bmi-1 protein expression in the nucleus which was blocked by small interfering RNA (siRNA) specific for Gli-1 and ERK, showing that parallel pathways of integrins and DDR1 merge at Bmi-1. Furthermore, collagen I-induced p16 decrease and p-Rb increase were reversed by Bmi-1-specific siRNA. Moreover, Bmi-1 silencing abolished the collagen I-induced increase of proliferation indices and undifferentiation markers. These results indicate that collagen I stimulates the self-renewal of mESCs mediated by Bmi-1 through α2β1 integrin-dependent ILK, Notch, Gli-1, and DDR1-dependent Ras, PI3K/Akt, and ERK.
182. Integrin subunit expression associated with epithelial-mesenchymal interactions during murine tooth development. The initial information for patterning of early tooth development resides in the epithelium. Later, this is shifted to the mesenchyme. The process is governed by multiple epithelial-mesenchymal interactions. Integrins are cell surface receptors for **extracellular matrix** components. Expression of the beta 5 integrin subunit alternates between epithelium and mesenchyme during early tooth development (Yamada et al. [1994] Int. J. Dev. Biol. 38: 553-556). By immunofluorescence and in situ hybridization we show here a remarkably similar oscillating expression pattern of the alpha v integrin subunit. This subunit is known to associate with beta 5, and we therefore suggest that integrin alpha v beta 5 is involved in epithelial-mesenchymal interactions during tooth development. We also demonstrate that the developing tooth epithelium expresses the alpha 6, beta 1 and beta 4 subunits. The laminin receptors alpha 6 beta 1 and alpha 6 beta 4 may thus in part mediate the effect of basement membranes on tooth epithelial development. Interestingly, the enamel knot region expressed very little alpha 6 integrin subunit, whereas some expression was seen transiently in the condensing mesenchyme. During early tooth development, integrins possessing the alpha 6 subunit might also be involved in cell-cell interactions independently of laminins.
183. Suppression of β1-integrin in gonadotropin-releasing hormone cells disrupts migration and axonal extension resulting in severe reproductive alterations. Reproduction in mammals is dependent on the function of hypothalamic neurons whose axons project to the hypothalamic median eminence (ME) where they release gonadotropin-releasing hormone (GnRH) into a specialized capillary network for delivery to the anterior pituitary. These neurons originate prenatally in the nasal placode and migrate into the forebrain along the olfactory-vomeronasal nerves. The complex developmental events leading to the correct establishment of the GnRH system are tightly regulated by the specific spatiotemporal expression patterns of guidance cues and **extracellular matri**x molecules, the functions of which, in part, are mediated by their binding to β1-subunit-containing integrins. To determine the biological role of these cell-surface proteins in reproduction, Cre/LoxP technology was used to generate GnRH neuron-specific β1-integrin conditional KO (GnRH-Itgb1(-/-)) mice. Loss of β1-integrin signaling impaired migration of GnRH neurons, their axonal extension to the ME, timing of pubertal onset, and fertility in these mice. These results identify β1-integrin as a gene involved in normal development of the GnRH system and demonstrate a fundamental role for this protein in acquisition of normal reproductive competence in female mice.
184. Beta 1 integrin-mediated effects of tenascin-R domains EGFL and FN6-8 on neural stem/progenitor cell proliferation and differentiation in vitro. Neural stem/progenitor cells (NSCs) have the capacity for self-renewal and differentiation into major classes of central nervous system cell types, such as neurons, astrocytes, and oligodendrocytes. The determination of fate of NSCs appears to be regulated by both intrinsic and extrinsic factors. Mounting evidence has shown that **extracellular matrix** molecules contribute to NSC proliferation and differentiation as extrinsic factors. Here we explore the effects of the epidermal growth factor-like (EGFL) and fibronectin type III homologous domains 6-8 (FN6-8) of the **extracellular matrix** molecule tenascin-R on NSC proliferation and differentiation. Our results show that domain FN6-8 inhibited NSC proliferation and promoted NSCs differentiation into astrocytes and less into oligodendrocytes or neurons. The EGFL domain did not affect NSC proliferation, but promoted NSC differentiation into neurons and reduced NSC differentiation into astrocytes and oligodendrocytes. Treatment of NSCs with beta 1 integrin function-blocking antibody resulted in attenuation of inhibition of the effect of FN6-8 on NSC proliferation. The influence of EGFL or FN6-8 on NSCs differentiation was inhibited by beta 1 integrin antibody application, implicating beta 1 integrin in proliferation and differentiation induced by EGFL and FN6-8 mediated triggering of NSCs.
185. β1- and αv-class integrins cooperate to regulate myosin II during rigidity sensing of fibronectin-based microenvironments. How different integrins that bind to the same type o**f extracellular matr**ix protein mediate specific functions is unclear. We report the functional analysis of β1- and αv-class integrins expressed in pan-integrin-null fibroblasts seeded on fibronectin. Reconstitution with β1-class integrins promotes myosin-II-independent formation of small peripheral adhesions and cell protrusions, whereas expression of αv-class integrins induces the formation of large focal adhesions. Co-expression of both integrin classes leads to full myosin activation and traction-force development on stiff fibronectin-coated substrates, with αv-class integrins accumulating in adhesion areas exposed to high traction forces. Quantitative proteomics linked αv-class integrins to a GEF-H1-RhoA pathway coupled to the formin mDia1 but not myosin II, and α5β1 integrins to a RhoA-Rock-myosin II pathway. Our study assigns specific functions to distinct fibronectin-binding integrins, demonstrating that α5β1integrins accomplish force generation, whereas αv-class integrins mediate the structural adaptations to forces, which cooperatively enable cells to sense the rigidity of fibronectin-based microenvironments.
186. Chemerin contributes to inflammation by promoting macrophage adhesion to VCAM-1 and fibronectin through clustering of VLA-4 and VLA-5. Chemerin is a potent macrophage chemoattractant protein. We used murine peritoneal exudate cells (PECs) in adhesion, flow cytometry, and confocal microscopy assays to test the hypothesis that chemerin can also contribute to inflammation by promoting macrophage adhesion. Chemerin stimulated the adhesion of PECs to the **extracellular matrix** protein fibronectin and to the adhesion molecule VCAM-1 within a minute, with an EC(50) of 322 and 196 pM, respectively. Experiments using pertussis toxin and PECs from ChemR23(-/-) mice demonstrated that chemerin stimulated the adhesion of macrophages via the Gi protein-coupled receptor ChemR23. Blocking Abs against integrin subunits revealed that 89% of chemerin-stimulated adhesion to fibronectin was dependent on increased avidity of the integrin VLA-5 (alpha(5)beta(1)) and that 88% of adhesion to VCAM-1 was dependent on increased avidity of VLA-4 (alpha(4)beta(1)). Although chemerin was unable to induce an increase in integrin affinity as judged by the binding of soluble ligand, experiments using confocal microscopy revealed an increase in valency resulting from integrin clustering as the mechanism responsible for chemerin-stimulated macrophage adhesion. PI3K, Akt, and p38 were identified as key signaling mediators in chemerin-stimulated adhesion. The finding that chemerin can rapidly stimulate macrophage adhesion to **extracellular matrix** proteins and adhesion molecules, taken together with its ability to promote chemotaxis, suggests a novel role for chemerin in the recruitment and retention of macrophages at sites of inflammation.
187. A new in vitro model of murine mesoderm migration: the role of fibronectin and laminin. Examination of the factors involved in primary mesodermal migration in the mouse has been complicated by the lack of a suitable in vitro model. We have developed a new culture system using primitive streak stage embryos denuded of primitive endoderm, which allows easy observation and manipulation of the outgrowing cells. The cells migrating away from these explants were shown by immunocytochemistry to express vimentin and an epitope of the I antigen recognised by the antibody C6, both of which are present on the newly emerged mesoderm and not on the embryonic ectoderm in sections of embryos in utero. Conversely, cytokeratin, stage-specific embryonic antigen 1 (SSEA-1), E-cadherin and desmoplakin are expressed by the embryonic ectoderm but lost during mesoderm formation in vivo. They are absent or expressed very weakly by the migrated cells in vitro. In addition, only explants of the ectoplacental cone (EPC) and visceral endoderm alone, expressed a carbohydrate epitope (recognised by monoclonal antibody BOO6), characteristic of the EPC and primitive endoderm in utero, but absent from mesoderm. Thus we conclude that the cells which outgrow in this system are indeed mesodermal in phenotype. We have confirmed the work of others in demonstrating the presence of fibronectin (FN) and laminin (LN) in the migratory path of the mesoderm, at the ectoderm-visceral endoderm interface. We also report that the beta 1 integrin subunit of the FN and LN receptor is expressed by mesodermal cells at this interface. Using our in vitro model we have examined the role of the **extracellular matrix** (ECM) in mesodermal migration. Mesodermal cells migrate further and faster on substrates coated with FN or LN, and this increased migration is abolished by appropriate blocking antibodies. We conclude that the ECM, in particular FN and LN, plays an important role in the migration of primary mesodermal cells during gastrulation in the mouse embryo.
188. An integrin-ILK-microtubule network orients cell polarity and lumen formation in glandular epithelium. The **extracellular matrix** has a crucial role in determining the spatial orientation of epithelial polarity and the formation of lumens in glandular tissues; however, the underlying mechanisms remain elusive. By using Cre–Lox deletion we show that β1 integrins are required for normal mammary gland morphogenesis and lumen formation, both in vivo and in a three-dimensional primary culture model in which epithelial cells directly contact a basement membrane. Downstream of basement membrane β1 integrins, Rac1 is not involved; however, ILK is needed to polarize microtubule plus ends at the basolateral membrane and disrupting each of these components prevents lumen formation. The integrin–microtubule axis is necessary for the endocytic removal of apical proteins from the basement-membrane–cell interface and for internal Golgi positioning. We propose that this integrin signalling network controls the delivery of apical components to the correct surface and thereby governs the orientation of polarity and development of lumens.
189. Functionally distinct PI 3-kinase pathways regulate myelination in the peripheral nervous system. The PI 3-kinase (PI 3-K) signaling pathway is essential for Schwann cell myelination. Here we have characterized PI 3-K effectors activated during myelination by probing myelinating cultures and developing nerves with an antibody that recognizes phosphorylated substrates for this pathway. We identified a discrete number of phospho-proteins including the S6 ribosomal protein (S6rp), which is down-regulated at the onset of myelination, and N-myc downstream-regulated gene-1 (NDRG1), which is up-regulated strikingly with myelination. We show that type III Neuregulin1 on the axon is the primary activator of S6rp, an effector of mTORC1. In contrast, laminin-2 in the **extracellular matrix** (ECM), signaling through the α6β4 integrin and Sgk1 (serum and glucocorticoid-induced kinase 1), drives phosphorylation of NDRG1 in the Cajal bands of the abaxonal compartment. Unexpectedly, mice deficient in α6β4 integrin signaling or Sgk1 exhibit hypermyelination during development. These results identify functionally and spatially distinct PI 3-K pathways: an early, pro-myelinating pathway driven by axonal Neuregulin1 and a later-acting, laminin-integrin-dependent pathway that negatively regulates myelination.
190. Integrin-laminin interactions controlling neurite outgrowth from adult DRG neurons in vitro. A prerequisite for axon regeneration is the interaction between the growth cone and the **extracellular matrix** (ECM). Laminins are prominent constituents of ECM throughout the body, known to support axon growth in vitro and in vivo. The regenerative capacity of adult neurons is greatly diminished compared to embryonic or early postnatal neurons. Since most lesions in the nervous system occur in the adult, we have examined neurite outgrowth from adult mouse DRG neurons on four laminin isoforms (laminin-1/LM-111, laminin-2/LM-211, laminin-8/LM-411 and laminin-10/LM-511) in vitro. The growth on laminin-1 and -10 was trophic factor-independent and superior to the one on laminin-2 and -8, where growth was very poor in the absence of neurotrophins. Among other ECM proteins, laminins were by far the most active molecules. Using function-blocking antibodies to laminin-binding integrins, we identified non-overlapping functions of integrins alpha3beta1, alpha7beta1 and alpha6beta1 on different laminin isoforms, in that alpha3beta1 and alpha7beta1 integrins appeared to be specific receptors for both laminin-1 and-2, whereas integrin alpha6beta1 was a receptor for laminin-8 and-10. Lastly, by use of immunohistochemistry, expression of subunits of laminin-1, -2, -8 and -10 in sensory organs in the human epidermis could be demonstrated, supporting an important role for these laminins in relation to primary sensory axons.
191. OXPHOS dysfunction regulates integrin-β1 modifications and enhances cell motility and migration. Mitochondria are central organelles for cellular metabolism. In cancer cells, mitochondrial oxidative phosphorylation (OXPHOS) dysfunction has been shown to promote migration, invasion, metastization and apoptosis resistance. With the purpose of analysing the effects of OXPHOS dysfunction in cancer cells and the molecular players involved, we generated cybrid cell lines harbouring either wild-type (WT) or mutant mitochondrial DNA (mtDNA) [tRNAmut cybrids, which harbour the pathogenic A3243T mutation in the leucine transfer RNA gene (tRNAleu)]. tRNAmut cybrids exhibited lower oxygen consumption and higher glucose consumption and lactate production than WT cybrids. tRNAmut cybrids displayed increased motility and migration capacities, which were associated with altered integrin-β1 N-glycosylation, in particular with higher levels of β-1,6-N-acetylglucosamine (GlcNAc) branched N-glycans. This integrin-β1 N-glycosylation pattern was correlated with higher levels of membrane-bound integrin-β1 and also with increased binding to fibronectin. When cultured in vitro, tRNAmut cybrids presented lower growth rate than WT cybrids, however, when injected in nude mice, tRNAmut cybrids produced larger tumours and showed higher metastatic potential than WT cybrids. We conclude that mtDNA-driven OXPHOS dysfunction correlates with increased motility and migration capacities, through a mechanism that may involve the cross talk between cancer cell mitochondria and **the extracellular m**atrix.
192. Defective blood vessel development and pericyte/pvSMC distribution in alpha 4 integrin-deficient mouse embryos. Blood vessel development is in part regulated by pericytes/presumptive vascular smooth muscle cells (PC/pvSMCs). Here, we demonstrate that interactions between PC/pvSMCs and **extracellular matrix** play a critical role in this event. We show that the cranial vessels in alpha4 integrin-deficient mouse embryos at the stage of vessel remodeling are increased in diameter. This defect is accompanied by a failure of PC/pvSMCs, which normally express alpha4beta1 integrin, to spread uniformly along the vessels. We also find that fibronectin but not VCAM-1 is localized in the cranial vessels at this stage. Furthermore, cultured alpha4 integrin-null PC/pvSMCs plated on fibronectin display a delay in initiating migration, a reduction in migration speed, and a decrease in directional persistence in response to a polarized force of shear flow. These results suggest that specific motile activities of PC/pvSMCs regulated by mechanical signals imposed by the interstitial **extracellular matrix** may also be required in vivo for the distribution and function of the PC/pvSMCs during blood vessel development.
193. Differentiation and integrity of cardiac muscle cells are impaired in the absence of beta 1 integrin. Cellular interactions with substrata of the microenvironment are one of the major mechanisms for differentiation and morphogenesis. Many of these interactions are mediated via the beta 1 integrin subfamily of cell surface receptors, which are believed to transduce signals upon cell adhesion. We have used beta 1 integrin-deficient embryonic stem cells to test their ability to differentiate into cardiac muscle cells. We show here by several approaches that beta 1 integrin is important for normal cardiogenesis. First, the in vitro differentiation of beta 1 integrin-deficient embryonic stem cells into cardiac muscle cells is retarded. This is demonstrated by the delayed expression of cardiac muscle-specific genes and action potentials. Second, the specification of cardiac precursor cells into pacemaker-, atrial- and ventricular-like cells is significantly impaired in beta 1 integrin-deficient cells. The occurrence of atrial- and ventricular-like cells is reduced and transient. Only cells exhibiting peacemaker-like action potentials of high frequency and arrhythmias survive. Third, the sarcomeric architecture is incomplete and disarranged in the absence of beta 1 integrin. Fourth, beta 1-deficient embryonic stem cells can contribute to the developing heart in chimaeric mice but many areas with beta 1-null cells contain cell debris. The number of beta 1-null cells decrease from prenatal to postnatal stages and is lost completely in 6-month-old hearts. Thus, we conclude that interactions with the **extracellular matrix** via beta 1 integrin is necessary for differentiation and the maintenance of a specialized phenotype of cardiac muscle cells.
194. Reelin, integrin and DAB1 interactions during embryonic cerebral cortical development. **Extracellular matrix**-like molecule reelin and cell surface adhesion receptors such as alpha3beta1 integrin can regulate neuronal migration and position in the developing cerebral cortex. Here we show that alpha3beta1 integrin binds to the N-terminal region of reelin, a site distinct from the region of reelin shown to associate with other reelin receptors such as VLDLR/ApoER2. Furthermore, Dab1, a member of the reelin signaling pathway, can complex with the cytoplasmic region of beta1 integrin in a reelin-dependent manner. Thus, alpha3beta1 integrin-reelin interactions may contribute to appropriate neuronal placement in the developing cerebral cortex.
195. Modulation of morphogenesis by noncanonical Wnt signaling requires ATF/CREB family-mediated transcriptional activation of TGFbeta2. Transcriptional readout downstream of canonical Wnt signaling is known to be mediated by beta-catenin activation of well-described targets, but potential transcriptional readout in response to noncanonical Wnt signaling remains poorly understood. Here, we define a transcriptional pathway important in noncanonical Wnt signaling. We have found that Wnt11 is a direct target of a canonical beta-catenin pathway in developing heart and that Wnt11 mutants show cardiac outflow tract defects. We provide genetic and biochemical evidence thatWnt11 signaling affects **extracellular matrix** composition, cytoskeletal rearrangements and polarized cell movement required for morphogenesis of the cardiac outflow tract. Notably, transforming growth factor beta2 (TGFbeta2), a key effector of organ morphogenesis, is regulated by Wnt11-mediated noncanonical signaling in developing heart and somites via one or more activating transcription factor (ATF)/cyclic AMP response element binding protein (CREB) family members. Thus, we propose that transcriptional readout mediated at least in part by a Wnt11 --> ATF/CREB --> TGFbeta2 pathway is critical in regulating morphogenesis in response to noncanonical Wnt signaling.
196. Involvement of alpha7beta1 integrin in the conditioning-lesion effect on sensory axon regeneration. Conditioning lesions of peripheral nerves improve axonal regeneration after injury and involve changes in expression of proteins required for axonal growth. Integrin alpha7beta1 expression in motor and sensory neurons increases following nerve lesions and motor axon regeneration is impaired in alpha7 integrin KO mice (J. Neurosci. 20, 1822-1830). To investigate the role of alpha7beta1 integrin in sensory axon regeneration, dorsal root ganglia of adult mice were cultured in gels of laminin-rich **extracellular matrix** (Matrigel) or collagen. Normal dorsal root ganglia in Matrigel or collagen supplemented with laminin showed spontaneous axonal outgrowth, which was greatly increased in conditioned preparations, but only in the presence of laminin. Conditioned dorsal root ganglia from normal mice cultured with a blocking antibody to beta1 integrin and from alpha7 integrin KO mice showed reduced axonal growth in both Matrigel- and laminin-supplemented collagen gels. Enhanced axonal regeneration after conditioning lesions therefore involves increased responsiveness to laminin and integrin alpha7beta1 expression.
197. Integrin beta1 subunit controls mural cell adhesion, spreading, and blood vessel wall stability. Growth, maturation, and integrity of the blood vessel network require extensive communication between the endothelial cells, which line the vascular lumen, and associated mural cells, namely vascular smooth muscle cells and pericytes. Pericytes extend long processes, make direct contact with the capillary endothelium, and promote vascular quiescence by suppressing angiogenic sprouting. Vascular smooth muscle cells are highly contractile, **extracellular matrix**-secreting cells that cover arteries and veins and provide them with mechanical stability and elasticity. In the damaged blood vessel wall, for example in atherosclerotic lesions, vascular smooth muscle cells lose their differentiated state and acquire a highly mitotic, so-called "synthetic" phenotype, which is thought to promote pathogenesis. Among other factors, **extracellular matrix** molecules and integrin family cell-matrix receptors may regulate this phenotypic transition. Here we show that the inactivation of the gene encoding the integrin beta1 subunit (Itgb1) with a Cre-loxP approach in mice leads to mural cell defects and postnatal lethality. Integrin beta1-deficient vascular smooth muscle cells display several hallmarks of the synthetic phenotype: Cell proliferation is enhanced, whereas differentiation and their ability to support blood vessels are compromised. Similarly, mutant pericytes are poorly spread but present in larger numbers. Our analysis of this mutant model shows that integrin beta1-mediated cell-matrix adhesion is a major determinant of the mural cell phenotype.
198. Integrin α3β1 regulates kidney collecting duct development via TRAF6-dependent K63-linked polyubiquitination of Akt. The collecting system of the kidney develops from the ureteric bud (UB), which undergoes branching morphogenesis, a process regulated by multiple factors, including integri**n-extracellular matr**ix interactions. The laminin (LM)-binding integrin α3β1 is crucial for this developmental program; however, the LM types and LM/integrin α3β1-dependent signaling pathways are poorly defined. We show that α3 chain-containing LMs promote normal UB branching morphogenesis and that LM-332 is a better substrate than LM-511 for stimulating integrin α3β1-dependent collecting duct cell functions. We demonstrate that integrin α3β1-mediated cell adhesion to LM-332 modulates Akt activation in the developing collecting system and that Akt activation is PI3K independent but requires decreased PTEN activity and K63-linked polyubiquitination. We identified the ubiquitin-modifying enzyme TRAF6 as an interactor with the integrin β1 subunit and regulator of integrin α3β1-dependent Akt activation. Finally, we established that the developmental defects of TRAF6- and integrin α3-null mouse kidneys are similar. Thus K63-linked polyubiquitination plays a previously unrecognized role in integrin α3β1-dependent cell signaling required for UB development and may represent a novel mechanism whereby integrins regulate signaling pathways.
199. Integrin β1 mediates vaccinia virus entry through activation of PI3K/Akt signaling. Vaccinia virus has a broad range of infectivity in many cell lines and animals. Although it is known that the vaccinia mature virus binds to cell surface glycosaminoglycans and **extracellular matri**x proteins, whether additional cellular receptors are required for virus entry remains unclear. Our previous studies showed that the vaccinia mature virus enters through lipid rafts, suggesting the involvement of raft-associated cellular proteins. Here we demonstrate that one lipid raft-associated protein, integrin β1, is important for vaccinia mature virus entry into HeLa cells. Vaccinia virus associates with integrin β1 in lipid rafts on the cell surface, and the knockdown of integrin β1 in HeLa cells reduces vaccinia mature virus entry. Additionally, vaccinia mature virus infection is reduced in a mouse cell line, GD25, that is deficient in integrin β1 expression. Vaccinia mature virus infection triggers the activation of phosphatidylinositol 3-kinase (PI3K)/Akt signaling, and the treatment of cells with inhibitors to block P13K activation reduces virus entry in an integrin β1-dependent manner, suggesting that integrin β1-mediates PI3K/Akt activation induced by vaccinia virus and that this signaling pathway is essential for virus endocytosis. The inhibition of integrin β1-mediated cell adhesion results in a reduction of vaccinia virus entry and the disruption of focal adhesion and PI3K/Akt activation. In summary, our results show that the binding of vaccinia mature virus to cells mimics the outside-in activation process of integrin functions to facilitate vaccinia virus entry into HeLa cells.
200. Integrin β1 is necessary for the maintenance of corneal structural integrity. The precise role of a normal keratocyte in maintaining corneal structural integrity is unclear; it is generally considered to remain quiescent at the end of cell division. Given that integrins are essential for cell**/extracellular matri**x interactions, the authors tested the hypothesis that integrin expression by keratocytes is essential for corneal structure and function.Using a tamoxifen-dependent cre recombinase expressed under the control of a fibroblast-specific promoter/enhancer, the authors conditionally deleted the integrin β1 (Itgb1) gene in mouse keratocytes during the postnatal matrix maturation phase of the cornea. The effects of this deletion were monitored histologically and by macroscopic observation of the cornea.The resultant cornea shows an initial thinning of the stroma, reduced space between collagen fibrils, loss of epithelial layers and subsequent edema, thickening of Descemet's membrane, and degenerative changes in the endothelial cell layer, with eventual scarring. These pathologic changes have some similarities to human corneal disease keratoconus. The phenotype did not develop when Itgb1 was deleted after complete corneal maturation.Loss of integrin β1 expression in keratocytes during the phase of stromal maturation results in corneal thinning and edema. Keratocyte-ECM interaction is essential for matrix maturation and thus in the maintenance of corneal structural integrity. This model has relevance in understanding corneal diseases such as keratoconus.
201. Rac1 GTPase-deficient mouse lens exhibits defects in shape, suture formation, fiber cell migration and survival. Morphogenesis and shape of the ocular lens depend on epithelial cell elongation and differentiation into fiber cells, followed by the symmetric and compact organization of fiber cells within an enclosed **extracellular matrix**-enriched elastic capsule. The cellular mechanisms orchestrating these different events however, remain obscure. We investigated the role of the Rac1 GTPase in these processes by targeted deletion of expression using the conditional gene knockout (cKO) approach. Rac1 cKO mice were derived from two different Cre (Le-Cre and MLR-10) transgenic mice in which lens-specific Cre expression starts at embryonic day 8.75 and 10.5, respectively, in both the lens epithelium and fiber cells. The Le-Cre/Rac1 cKO mice exhibited an early-onset (E12.5) and severe lens phenotype compared to the MLR-10/Rac1 cKO (E15.5) mice. While the Le-Cre/Rac1 cKO lenses displayed delayed primary fiber cell elongation, lenses from both Rac1 cKO strains were characterized by abnormal shape, impaired secondary fiber cell migration, sutural defects and thinning of the posterior capsule which often led to rupture. Lens fiber cell N-cadherin/β-catenin/Rap1/Nectin-based cell-cell junction formation and WAVE-2/Abi-2/Nap1-regulated actin polymerization were impaired in the Rac1 deficient mice. Additionally, the Rac1 cKO lenses were characterized by a shortened epithelial sheet, reduced levels of **extracellular matri**x (ECM) proteins and increased apoptosis. Taken together, these data uncover the essential role of Rac1 GTPase activity in establishment and maintenance of lens shape, suture formation and capsule integrity, and in fiber cell migration, adhesion and survival, via regulation of actin cytoskeletal dynamics, cell adhesive interactions and ECM turnover.
202. Dense fibrillar collagen matrices to analyse **extracellular matrix** receptor function. The goal of this study was to understand whether dense fibrillar collagen matrices, with a hierarchical structure resembling native collagen matrices, could be useful to study collagen receptor function, in a more physiological context. The receptor analysed here was integrin α11β1, already shown to be involved in cell attachment and migration on collagen-coated plastic, and also in contraction of loose fibrillar collagen hydrogels.Collagen matrices prepared here corresponded to dense fibrillar hydrogels concentrated at 5mg/ml. The behaviour of α11β1 deficient fibroblasts seeded on these concentrated matrices was assessed in terms of adhesion, morphology and migration, then compared to that observed on classical hydrogels at 1mg/ml, corresponding to loose collagen matrices.Short-term attachment assays showed disturbed interactions between α11β1 deficient cells and collagen matrices in a concentration-dependent manner. Long-term assays revealed reduced cell spreading of alpha 11(-/-) cells on the dense collagen matrices, associated with a disturbed cytoskeleton network. Moreover, anoikis was observed when alpha 11(-/-) cells were seeded on 5mg/ml matrices, and not on looser 1mg/ml matrices. In scratch wound in vitro assays, carried out with cells on 5mg/ml fibrillar collagen matrices, alpha 11(-/-) cells migrated much better than their wild-type counterparts. In contrast, no significant difference was observed between wild and knock-out cells seeded on plastic.The present study demonstrates the validity of in vivo-like dense fibrillar collagen matrices to evaluate cell receptor functions more significantly than with 2D cell cultures or loose hydrogels.
203. Conditional ablation of beta1 integrin in skin. Severe defects in epidermal proliferation, basement membrane formation, and hair follicle invagination. The major epidermal integrins are alpha3beta1 and hemidesmosome-specific alpha6beta4; both share laminin 5 as ligand. Keratinocyte culture studies implicate both integrins in adhesion, proliferation, and stem cell maintenance and suggest unique roles for alphabeta1 integrins in migration and terminal differentiation. In mice, however, whereas ablation of alpha6 or beta4 results in loss of hemidesmosomes, epidermal polarity, and basement membrane (BM) attachment, ablation of alpha3 only generates microblistering due to localized internal shearing of BM. Using conditional knockout technology to ablate beta1 in skin epithelium, we have uncovered biological roles for alphabeta1 integrins not predicted from either the alpha3 knockout or from in vitro studies. In contrast to alpha3 null mice, beta1 mutant mice exhibit severe skin blistering and hair defects, accompanied by massive failure of BM assembly/organization, hemidesmosome instability, and a failure of hair follicle keratinocytes to remodel BM and invaginate into the dermis. Although epidermal proliferation is impaired, a spatial and temporal program of terminal differentiation is executed. These results indicate that beta1's minor partners in skin are important, and together, alphabeta1 integrins are required not only for **extracellular matrix** assembly but also for BM formation. This, in turn, is required for hemidesmosome stability, epidermal proliferation, and hair follicle morphogenesis. However, beta1 downregulation does not provide the trigger to terminally differentiate.
204. beta1 integrin cytoplasmic domain residues selectively modulate fibronectin matrix assembly and cell spreading through talin and Akt-1. The integrin beta(1) cytoplasmic domain (tail) serves as a scaffold for numerous intracellular proteins. The mechanisms by which the tail coordinates these proteins to facilitate **extracellular matrix** assembly and cell spreading are not clear. This study demonstrates that the beta(1) cytoplasmic domain can regulate cell spreading on fibronectin and fibronectin matrix assembly through Akt- and talin-dependent mechanisms, respectively. To identify these mechanisms, we characterized GD25 cells expressing the beta(1) integrin cytoplasmic domain mutants W775A and R760A. Although cell spreading appears normal in R760A mutant-integrin cells compared with wild type, it is inhibited in W775A mutant cells. In contrast, both mutant cell lines show defective fibronectin matrix assembly. Inhibition of cell spreading, but not matrix assembly, in the W775A mutant cells is due to a specific defect in Akt-1 activation. In addition, we find that both W775A and R760A mutant integrins have reduced surface expression of the 9EG7 epitope that correlates with reduced recruitment of talin to beta(1) integrin cytoplasmic complexes. Down-regulation of talin with small interfering RNA or expression of green fluorescent protein-talin head domain inhibits matrix assembly in beta(1) wild-type cells, mimicking the defect seen with the W775A and R760A mutant cells. These results demonstrate distinct mechanisms by which integrins regulate cell spreading and matrix assembly through the beta(1) integrin cytoplasmic tail.
205. Osteoblast mineralization requires beta1 integrin/ICAP-1-dependent fibronectin deposition. The morphogenetic and differentiation events required for bone formation are orchestrated by diffusible and insoluble factors that are localized within the **extracellular matrix**. In mice, the deletion of ICAP-1, a modulator of β1 integrin activation, leads to severe defects in osteoblast proliferation, differentiation, and mineralization and to a delay in bone formation. Deposition of fibronectin and maturation of fibrillar adhesions, adhesive structures that accompany fibronectin deposition, are impaired upon ICAP-1 loss, as are type I collagen deposition and mineralization. Expression of β1 integrin with a mutated binding site for ICAP-1 recapitulates the ICAP-1-null phenotype. Follow-up experiments demonstrated that ICAP-1 negatively regulates kindlin-2 recruitment onto the β1 integrin cytoplasmic domain, whereas an excess of kindlin-2 binding has a deleterious effect on fibrillar adhesion formation. These results suggest that ICAP-1 works in concert with kindlin-2 to control the dynamics of β1 integrin-containing fibrillar adhesions and, thereby, regulates fibronectin deposition and osteoblast mineralization.

#### GeneRIFs

1. The integrin alpha5beta1-mediated **extracellular matrix** signal through the RGD sequence is involved in embryonic enchondral ossification.
2. Data indicate that osteocytic cells on defective osteoarthritis (OA) **extracellular matrix** (ECM) resulted in a decrease of integrinbeta1 expression and the de-activation of focal adhesion kinase cell signaling pathway.
3. MT1-MMP-dependent **extracellular matrix** remodeling controls skeletal stem cell differentiation via beta1-integrin activation.
4. alpha-actinin has a role in adhesion maturation through integrin-dependent force transmission to the **extracellular matrix**
5. Fibroblasts require Mena for multiple integrin alpha5beta1-dependent processes involving bidirectional interactions between the **extracellular matrix** and cytoplasmic focal adhesion proteins.
6. Depletion of talin2 in talin1-null cells did not affect the initiation of matrix-activated spreading or Src family kinase activation, but abolished the **extracellular matrix**-integrin-cytoskeleton linkage, sustained cell spreading and adhesion.
7. The interaction of beta1-integrin and **extracellular matrix** components is functionally important in leukemic cells expressing high-levels of BCR/ABL.
8. beta1 and beta3 integrin-mediated adhesion to **extracellular matrix** proteins on target cells and/or surfaces dramatically promotes CTL degranulation.
9. **extracellular matrix** fragments produced by apoptotic EC initiate a state of resistance to apoptosis in fibroblasts via an alpha2beta1 integrin/SFK (Src and Fyn)/phosphatidylinositol 3-kinase (PI3K)-dependent pathway
10. myocytes and fibroblasts change size and shape, cellular contacts with the **extracellular matrix** are altered, resulting in the liberation of a beta(1)-integrin fragment from the cell surface
11. data demonstrate in vivo that cell-**extracellular matrix** interactions involving beta1-integrins play an important role in the control of milk gene transcription and in the maintenance of the mammary epithelial cell differentiated state
12. sarcoglycan and integrin alpha7beta1 have overlapping roles in mediating cytoskeletal-membrane-**extracellular matrix** interaction
13. Beta1 integrins act as sensors for the changing **extracellular matrix** in neural stem cells [review]
14. Results describe the role of alpha2beta1 integrin in the interactions of primary fibroblasts and keratinocytes with the **extracellular matrix**.

### [Ii]nvadosome

#### PubMed

1. β1A integrin is a master regulator of **invadosom**e organization and function. **Invadosom**es are adhesion structures involved in tissue invasion that are characterized by an intense actin polymerization-depolymerization associated with β1 and β3 integrins and coupled to extracellular matrix (ECM) degradation activity. We induced the formation **of invados**omes by expressing the constitutive active form of Src, SrcYF, in different cell types. Use of ECM surfaces micropatterned at the subcellular scale clearly showed that in mesenchymal cells, integrin signaling contro**ls invados**ome activity. Using β1⁻/⁻ or β3⁻/⁻ cells, it seemed that β1A but not β3 integrins are essential for **initiatio**n of invadosome formation. Protein kinase C activity was shown to regulate a**utoassembl**y of invadosomes into a ring-like metastructure (rosette), probably by phosphorylation of Ser785 on the β1A tail. Moreover, our study clearly showed that β1A links actin dynamics and EC**M degradat**ion in invadosomes. Finally, a new strategy based on fusion of the photosensitizer KillerRed to the β1A cytoplasmic domain allowed specific and immediate loss of function of β1A, resulting in disorganization **and disass**embly of invadosomes and formation of focal adhesions.

#### GeneRIFs

1. results support a central role of beta1 integrin-dependent outside-in signaling pathways in the regulation of the multiple cell compartments involved in **invadosome** functions

### [Ii]nvasive

#### PubMed

1. Androgen-induced cell migration: role of androgen receptor/filamin A association. Androgen receptor (AR) controls male morphogenesis, gametogenesis and prostate growth as well as development of prostate cancer. These findings support a role for AR in cell migration and **invasive**ness. However, the molecular mechanism involved in AR-mediated cell migration still remains elusive.Mouse embryo NIH3T3 fibroblasts and highly metastatic human fibrosarcoma HT1080 cells harbor low levels of transcriptionally incompetent AR. We now report that, through extra nuclear action, AR triggers migration of both cell types upon stimulation with physiological concentrations of the androgen R1881. We analyzed the initial events leading to androgen-induced cell migration and observed that challenging NIH3T3 cells with 10 nM R1881 rapidly induces interaction of AR with filamin A (FlnA) at cytoskeleton. AR/FlnA complex recruits integrin beta 1, thus activating its dependent cascade. Silencing of AR, FlnA and integrin beta 1 shows that this ternary complex controls focal adhesion kinase (FAK), paxillin and Rac, thereby driving cell migration. FAK-null fibroblasts migrate poorly and Rac inhibition by EHT impairs motility of androgen-treated NIH3T3 cells. Interestingly, FAK and Rac activation by androgens are independent of each other. Findings in human fibrosarcoma HT1080 cells strengthen the role of Rac in androgen signaling. The Rac inhibitor significantly impairs androgen-induced migration in these cells. A mutant AR, deleted of the sequence interacting with FlnA, fails to mediate FAK activation and paxillin tyrosine phosphorylation in androgen-stimulated cells, further reinforcing the role of AR/FlnA interaction in androgen-mediated motility.The present report, for the first time, indicates that the extra nuclear AR/FlnA/integrin beta 1 complex is the key by which androgen activates signaling leading to cell migration. Assembly of this ternary complex may control organ development and prostate cancer metastasis.
2. TSC2 modulates cell adhesion and migration via integrin-α1β1. Recent evidence suggests that the rare and progressive lung disease lymphangioleiomyomatosis (LAM) is metastatic in nature. Dysfunction of the tumor suppressor genes tuberous sclerosis complex (TSC), in particular mutational inactivation of TSC2, enhances both cell proliferation and migration. Although substantial progress has been made in understanding the role of TSC2 in abnormal LAM cell proliferation and its pharmacological targeting, the mechanisms underlying the enhanced migratory capacity in LAM are not well understood. In this study, we examined the role of TSC2 in cell attachment, spreading, and migration, processes that contribute to the metastatic phenotype. Here we show that loss of TSC2 increased both the attachment and spreading of mouse embryonic fibroblasts to the extracellular matrix proteins collagen type I and fibronectin and that reexpression of TSC2 reduced these effects. Integrin-α1β1 modulated cell migration with the β1-subunit involved in cell attachment and spreading as shown by using functional blocking antibodies. Loss of TSC2 increased integrin-α1 expression, and inhibition of this integrin subunit reduced cell migration. The enhanced attachment and spreading were independent of the intracellular signaling pathways mammalian target of rapamycin complex 1 and Rho-associated kinase, as pharmacological inhibition with rapamycin or Y27632, respectively, was without effect. Together, these data demonstrate that TSC2 controls cell migration, attachment, and spreading through the α1β1-integrin receptor and thus suggest a potential therapeutic target for the treatment of increas**ed cell** invasiveness in LAM.
3. Regulation of fibulin-2 gene expression by integrin α3β1 contributes to th**e invasi**ve phenotype of transformed keratinocytes. The laminin-binding integrin α3β1 is highly expressed in epidermal keratinocytes, where it regulates both cell-autonomous and paracrine functions that promote wound healing and skin tumorigenesis. However, the roles for α3β1 in regulating gene expression programs that control the behaviors of immortalized or transformed keratinocytes remain underexplored. In the current study, we used a microarray approach to identify genes that are regulated by α3β1 in immortalized keratinocytes. α3β1-Responsive genes included several genes that are involved in extracellular matrix proteolysis or remodeling, including fibulin-2 and secreted protein acidic and rich in cysteine. However, α3β1-dependent induction of specific target genes was influenced by the genetic lesion that triggered immortalization, as α3β1-dependent fibulin-2 expression occurred in cells immortalized by either SV40 large T antigen or p53-null mutation, whereas α3β1-dependent expression of secreted protein acidic and rich in cysteine occurred only in the former cells. Interestingly, quantitative PCR arrays did not reveal strong patterns of α3β1-dependent gene expression in freshly isolated primary keratinocytes, suggesting that this regulation is acquired during immortalization. p53-null keratinocytes transformed with oncogenic RasV12 retained α3β1-dependent fibulin-2 expression, and RNAi-mediated knockdown of fibulin-2 in these cells reduced invasion, although not their tumorigenic potential. These findings demonstrate a prominent role for α3β1 in immortalized/transformed keratinocytes in regulating fibulin-2 and other genes that promote matrix remodeling and invasion.
4. C1GALT1 promotes **invasive** phenotypes of hepatocellular carcinoma cells by modulating integrin β1 glycosylation and activity. Cancer cell invasion and metastasis are the primary causes of treatment failure and death in hepatocellular carcinoma (HCC). We previously reported that core 1 β1,3-galactosyltransferase (C1GALT1) is frequently overexpressed in HCC tumors and its expression is associated with advanced tumor stage, metastasis, and poor survival. However, the underlying mechanisms of C1GALT1 in HCC malignancy remain unclear. In this study, we found that overexpression of C1GALT1 enhanced HCC cell adhesion to extracellular matrix (ECM) proteins, migration, and invasion, whereas RNAi-mediated knockdown of C1GALT1 suppressed these phenotypes. The promoting effect of C1GALT1 on the metastasis of HCC cells was demonstrated in a mouse xenograft model. Mechanistic investigations showed that the C1GALT1-enhanced phenotypic changes in HCC cells were significantly suppressed by anti-integrin β1 blocking antibody. Moreover, C1GALT1 was able to modify O-glycans on integrin β1 and regulate integrin β1 activity as well as its downstream signaling. These results suggest that C1GALT1 could enhance **HCC inv**asiveness through integrin β1 and provide novel insights into the roles of O-glycosylation in HCC metastasis.
5. Fibronectin-integrin (alpha5beta1) modulates migration and invasion of murine melanoma cell line B16F10 by involving MMP-9. Cell adhesion to extracellular matrix (ECM) initiates signaling cascade regulated by cell surface integrin receptors, which affects the proliferation and invasion of cells. Cells cultured in the presence of ECM ligand fibronectin (FN) stimulate secretion of matrix metalloproteinases (MMPs), facilitating cancer cell invasion and metastasis. Among all the members of the MMP family, MMP-9 is of crucial importance in tumor invasion and metastasis. The present study aims at studying the effects of integrin receptor alpha5beta1 and its ligand FN on expression of MMP-9 in murine melanoma cell line B16F10 and understanding the molecular mechanism(s) involved. The main experimental methods performed in the study were gelatin zymography, immunoblot, real-time RT-PCR, immunocytochemistry, enzyme linked immunosorbent assay (ELISA), transwell chamber assay, and in vivo metastasis assay in syngenic (C57BL6J) mice. The study reports that FN induces the activity, mRNA, and protein expression of MMP-9 and initiates its proteolytic activation in B16F10 cells. Blockage of the alpha5 receptor abrogated the FN-mediated stimulatory response on MMP-9 in B16F10 cells. Inhibitor studies and immunoblot analysis strongly suggest the involvement of focal adhesion kinase (FAK), extracellular regulated kinase (ERK), and phosphatidylinositol-3-kinase (PI-3K) in the FN-mediated responses. Immunocytochemical analysis showed the nuclear localization of nuclear factor-kappaB (NF-kappaB) might lead to activation of MMP-9 gene upon FN treatment. This study demonstrates that integrin receptor alpha5beta1 and FN interaction induces the **invasive** potential of B16F10 cells and MMP-9 induction is the downstream effectors in the process. This system serves as a novel model system to understand the molecular mechanism of melanoma growth and invasion.
6. hTERT mediates gastric cancer metastasis partially through the indirect targeting of ITGB1 by microRNA-29a. Human telomerase reverse transcriptase (hTERT) plays a key role in tumor invasion and metastasis, but the mechanism of its involvement in these processes is not clear. The purpose of this study is to investigate the possible molecular mechanism of hTERT in the promotion of gastric cancer (GC) metastasis. We found that the up-regulation of hTERT in gastric cancer cells could inhibit the expression of miR-29a and enhance the expression of Integrin β1 (ITGB1). In addition, the **invasiv**e capacity of gastric cancer cells was also highly increased after hTERT overexpression. Our study also found that the restoration of miR-29a suppressed the expression of ITGB1 and inhibited GC cell metastasis both in vitro and in vivo. Taken together, our results suggested that hTERT may promote GC metastasis through the hTERT-miR-29a-ITGB1 regulatory pathway.
7. Introduction of bisecting GlcNAc into integrin alpha5beta1 reduces ligand binding and down-regulates cell adhesion and cell migration. The enzyme beta1,4-N-acetylglucosaminyltransferase III (GnT-III) catalyzes the addition of a bisecting GlcNAc residue to glycoproteins, resulting in a modulation in biological function. Our previous studies showed that the transfection of the GnT-III gene into B16 melanoma cells results in a suppression of **invasive** ability and lung colonization. The suppression has been postulated to be due to an increased level of E-cadherin expression on the cell surface, which in turn leads to the up-regulation of cell-cell adhesion. In this study, we report on the effects of overexpression of GnT-III on cell-matrix adhesion. The overexpression of GnT-III, but not that of an enzymatic inactive GnT-III (D323A), inhibits cell spreading and migration on fibronectin, a specific ligand for integrin alpha(5)beta(1), and the focal adhesion kinase phosphorylation. E(4)-PHA lectin blot analyses showed that the levels of bisecting GlcNAc structures on the integrin alpha(5) subunit as well as alpha(2) and alpha(3) subunits immunoprecipitated from GnT-III transfectants were substantially increased. In addition, the affinity of the binding of integrin alpha(5)beta(1) to fibronectin was significantly reduced by the introduction of the bisecting GlcNAc, to the alpha(5) subunit. These findings suggest that the modification of N-glycan of integrin by GnT-III inhibits its ligand binding ability, subsequently leading to the down-regulation of integrin-mediated signaling.
8. Integrin signalling regulates YAP and TAZ to control skin homeostasis. The skin is a squamous epithelium that is continuously renewed by a population of basal layer stem/progenitor cells and can heal wounds. Here, we show that the transcription regulators YAP and TAZ localise to the nucleus in the basal layer of skin and are elevated upon wound healing. Skin-specific deletion of both YAP and TAZ in adult mice slows proliferation of basal layer cells, leads to hair loss and impairs regeneration after wounding. Contact with the basal extracellular matrix and consequent integrin-Src signalling is a key determinant of the nuclear localisation of YAP/TAZ in basal layer cells and in skin tumours. Contact with the basement membrane is lost in differentiating daughter cells, where YAP and TAZ become mostly cytoplasmic. In other types of squamous epithelia and squamous cell carcinomas, a similar control mechanism is present. By contrast, columnar epithelia differentiate an apical domain that recruits CRB3, Merlin (also known as NF2), KIBRA (also known as WWC1) and SAV1 to induce Hippo signalling and retain YAP/TAZ in the cytoplasm despite contact with the basal layer extracellular matrix. When columnar epithelial tumours lose their apical domain and become **invasive**, YAP/TAZ becomes nuclear and tumour growth becomes sensitive to the Src inhibitor Dasatinib.
9. Transmembrane collagen XVII modulates integrin dependent keratinocyte migration via PI3K/Rac1 signaling. The hemidesmosomal transmembrane component collagen XVII (ColXVII) plays an important role in the anchorage of the epidermis to the underlying basement membrane. However, this adhesion protein seems to be also involved in the regulation of keratinocyte migration, since its expression in these cells is strongly elevated during reepithelialization of acute wounds and in the **invasive** front of squamous cell carcinoma, while its absence in ColXVII-deficient keratinocytes leads to altered cell motility. Using a genetic model of murine Col17a1⁻/⁻ keratinocytes we elucidated ColXVII mediated signaling pathways in cell adhesion and migration. Col17a1⁻/⁻ keratinocytes exhibited increased spreading on laminin 332 and accelerated, but less directed cell motility. These effects were accompanied by increased expression of the integrin subunits β4 and β1. The migratory phenotype, as evidenced by formation of multiple unstable lamellipodia, was associated with enhanced phosphoinositide 3-kinase (PI3K) activity. Dissection of the signaling pathway uncovered enhanced phosphorylation of the β4 integrin subunit and the focal adhesion kinase (FAK) as activators of PI3K. This resulted in elevated Rac1 activity as a downstream consequence. These results provide mechanistic evidence that ColXVII coordinates keratinocyte adhesion and directed motility by interfering integrin dependent PI3K activation and by stabilizing lamellipodia at the leading edge of reepithelializing wou**nds and** in invasive squamous cell carcinoma.
10. **Invasive** potential of melanoma cells correlates with the expression of MT1-MMP and regulated by modulating its association with motility receptors via N-glycosylation on the receptors. Matrix remodeling and invasion of basement membrane are the major determinants of malignant progression. Matrix degrading enzymes play a pivotal role in this process and have been shown to be regulated at multiple levels. Using high metastatic B16F10 and its **invasive** variant B16BL6 cells, we previously demonstrated that the expression of β1,6 branched N-oligosaccharides promotes cellular adhesion on different matrix components which in turn induces secretion of MMP9. The present investigations report that although the two cell lines do not differ in the expression of uPAR, expression of MT1-MMP is significantly higher on B16BL6 cells. Analysis of the transcripts of tissue inhibitors of matrix metalloproteinases (TIMPs) showed that expression of both TIMP1 and TIMP2 correlates negatively with the **invasiv**e potential of cells. CD44 and β1 integrin, the two important receptors involved in motility, were identified to carry β1,6 branched N-oligosaccharides in **an invas**ive potential dependent manner. However, their glycosylation status did not appear to influence their surface expression. Although glycosylation on CD44 had no effect, that on β1 integrin significantly affected association of β1 integrin with MT1-MMP. The results thus demonstrate that the cancer cells use multiple mechanisms for degradation of matrix in a controlled manner to couple it with movement for effective invasion.
11. Developmental regulation of integrin expression at the time of implantation in the mouse embryo. The trophectoderm layer of the mouse blastocyst differentiates at the late blastocyst stage to form the **invasive** trophoblast that mediates implantation of the embryo into the uterine wall. The first sign that trophoblast cells have developed an invasion-specific cell behavior appears about 10-15 hours after the embryo hatches from the zona pellucida, when the quiescent, non-adherent trophectoderm cells initiate protrusive activity and become adhesive to extracellular matrix. Our previous findings that trophoblast outgrowth on extracellular-matrix-coated substrata involves the integrin family of adhesion receptors (Sutherland, A. E., Calarco, P. G. and Damsky, C. H., 1988, J. Cell Biol. 106, 1331-1348), suggested that the onset of trophoblast adhesive and migratory behavior at the time of implantation may be due to changes in expression or distribution of integrin receptors. We have thus examined the mRNA and protein expression of individual integrin subunits during pre- and periimplantation development (E0-E7.5). A basic repertoire of integrins, including receptors for fibronectin (alpha 5 beta 1), laminin (alpha 6B beta 1) and vitronectin (alpha v beta 3), was expressed continuously throughout this period, whereas the expression of five other integrin subunits was developmentally regulated. The mRNA for three of these (alpha 2, alpha 6A and alpha 7) was first detected in the late blastocyst, coincident with endoderm differentiation and development of attachment competence. The mRNA for another (alpha 1) was not detected until after trophoblast outgrowth had begun, suggesting that its expression may be induced by contact with matrix. At E7.5, three of the temporally regulated integrins (alpha 1, apha 6A, alpha 7), all of which can form receptors for laminin, were detected only in the ectoplacental cone (differentiating trophoblast), and may thus play specific roles in trophoblast adhesion and/or differentiation. Because laminin expression is upregulated in decidualized uterine stroma in response to the implanting embryo, we examined trophoblast-laminin interactions, using laminin fragments and integrin antibodies to determine which integrin receptors were involved. Trophoblast cells attached and spread on both the E8 and P1' fragments of laminin; however, the P1' binding site was cryptic in intact laminin. Interaction with P1' was RGD- and alpha v beta 3-dependent, whereas outgrowth on E8 was RGD-independent and not inhibited by antibodies to the laminin receptor alpha 6 beta 1, suggesting that alpha 7 beta 1 is the major trophoblast integrin E8 receptor.(ABSTRACT TRUNCATED AT 400 WORDS)
12. Integrin α5β1-fimbriae binding and actin rearrangement are essential for Porphyromonas gingivalis invasion of osteoblasts and subsequent activation of the JNK pathway. Chronic periodontitis is an infectious disease of the periodontium, which includes the gingival epithelium, periodontal ligament and alveolar bone. The signature clinical feature of periodontitis is resorption of alveolar bone and subsequent tooth loss. The Gram-negative oral anaerobe, Porphyromonas gingivalis, is strongly associated with periodontitis, and it has been shown previously that P. gingivalis is capable of invading osteoblasts in a dose- and time-dependent manner resulting in inhibition of osteoblast differentiation and mineralization in vitro. It is not yet clear which receptors and cytoskeletal components mediate th**e invasi**ve process, nor how the signaling pathways and viability of osteoblasts are affected by bacterial internalization. This study aimed to investigate these issues using an in vitro model system involving the inoculation of P. gingivalis ATCC 33277 into primary osteoblast cultures.It was found that binding between P. gingivalis fimbriae and integrin α5β1 on osteoblasts, and subsequent peripheral condensation of actin, are essential for entry of P. gingivalis into osteoblasts. The JNK pathway was activated in invaded osteoblasts, and apoptosis was induced by repeated infections.These observations indicate that P. gingivalis manipulates osteoblast function to promote its initial intracellular persistence by prolonging the host cell life span prior to its intercellular dissemination via host cell lysis. The identification of molecules critical to the interaction between P. gingivalis and osteoblasts will facilitate the development of new therapeutic strategies for the prevention of periodontal bone loss.

#### GeneRIFs

1. C1GALT1 promotes **invasive** phenotypes of hepatocellular carcinoma cells by modulating integrin beta1 glycosylation and activity.
2. of the transcripts of tissue inhibitors of matrix metalloproteinases (TIMPs) showed that expression of both TIMP1 and TIMP2 correlates negatively with the **invasive** potential of cells.
3. demonstrates that integrin receptor alpha5beta1 and FN interaction induces the **invasive** potential of B16F10 cells and MMP-9 induction is the downstream effectors in the process

### [Mm]etastasize

#### PubMed

1. beta1-integrin is dispensable for the induction of ErbB2 mammary tumors but plays a critical role in the metastatic phase of tumor progression. Cross-talk between integrin receptors and activated growth factor receptors has been hypothesized to play a critical role in the initiation and progression of cancer. Despite in vitro evidence documenting the important role of integrin receptors in the regulation of cancer cell proliferation, the relative contribution of the integrin receptors to the initiation and progression of tumors remains unclear. Previous studies with a polyomavirus middle T mammary tumor model have indicated that targeted disruption of beta1-integrin in the mammary glands of these mice completely blocks tumor induction. To further explore the general significance of these observations, we have crossed these conditional beta1-integrin strains to a strain of mice carrying mouse mammary tumor virus/activated erbB2 (herein referred to as the NIC strain). In contrast to the tumor induction block in the polyomavirus middle T model, tumor onset in the beta1-integrin-deficient NIC mice was delayed by only 30 d and was 100% penetrant. This modest effect on tumor induction was not a result of inefficient excision, as all tumors were confirmed as beta1-integrin-null. Animals bearing beta1-integrin-deficient ErbB2 tumors exhibited significantly reduced tumor volume, which was associated with increased tumor cell apoptosis and a reduction in tumor angiogenesis. In addition, beta1-integrin-deficient tumors were compromised in their capacity to **metastasize** to the lung, a deficiency associated with abrogation of adhesion signaling. Taken together, these observations suggest that, although beta1-integrin is dispensable for the initiation of ErbB2 tumor induction, it plays a critical role in metastatic phase of tumor progression.

### [Mm]etastasis

#### PubMed

1. Androgen-induced cell migration: role of androgen receptor/filamin A association. Androgen receptor (AR) controls male morphogenesis, gametogenesis and prostate growth as well as development of prostate cancer. These findings support a role for AR in cell migration and invasiveness. However, the molecular mechanism involved in AR-mediated cell migration still remains elusive.Mouse embryo NIH3T3 fibroblasts and highly metastatic human fibrosarcoma HT1080 cells harbor low levels of transcriptionally incompetent AR. We now report that, through extra nuclear action, AR triggers migration of both cell types upon stimulation with physiological concentrations of the androgen R1881. We analyzed the initial events leading to androgen-induced cell migration and observed that challenging NIH3T3 cells with 10 nM R1881 rapidly induces interaction of AR with filamin A (FlnA) at cytoskeleton. AR/FlnA complex recruits integrin beta 1, thus activating its dependent cascade. Silencing of AR, FlnA and integrin beta 1 shows that this ternary complex controls focal adhesion kinase (FAK), paxillin and Rac, thereby driving cell migration. FAK-null fibroblasts migrate poorly and Rac inhibition by EHT impairs motility of androgen-treated NIH3T3 cells. Interestingly, FAK and Rac activation by androgens are independent of each other. Findings in human fibrosarcoma HT1080 cells strengthen the role of Rac in androgen signaling. The Rac inhibitor significantly impairs androgen-induced migration in these cells. A mutant AR, deleted of the sequence interacting with FlnA, fails to mediate FAK activation and paxillin tyrosine phosphorylation in androgen-stimulated cells, further reinforcing the role of AR/FlnA interaction in androgen-mediated motility.The present report, for the first time, indicates that the extra nuclear AR/FlnA/integrin beta 1 complex is the key by which androgen activates signaling leading to cell migration. Assembly of this ternary complex may control organ development and prostate cancer **metastasis**.
2. Integrin alpha9beta1 mediates enhanced cell migration through nitric oxide synthase activity regulated by Src tyrosine kinase. Integrins are important mediators of cell adhesion and migration, which in turn are essential for diverse biological functions, including wound healing and cancer **metastasis**. The integrin alpha9beta1 is expressed on numerous mammalian tissues and can mediate accelerated cell migration. As the molecular signaling mechanisms that transduce this effect are poorly defined, we investigated the pathways by which activated integrin alpha9beta1 signals migration. We found for the first time that specific ligation of integrin alpha9beta1 rapidly activates Src tyrosine kinase, with concomitant tyrosine phosphorylation of p130Cas and activation of Rac-1. Furthermore, activation of integrin alpha9beta1 also enhanced NO production through activation of inducible nitric oxide synthase (iNOS). Inhibition of Src tyrosine kinase or NOS decreased integrin-alpha9beta1-dependent cell migration. Src appeared to function most proximal in the signaling cascade, in a FAK-independent manner to facilitate iNOS activation and NO-dependent cell migration. The cytoplasmic domain of integrin alpha9 was crucial for integrin-alpha9beta1-induced Src activation, subsequent signaling events and cell migration. When taken together, our results describe a novel and unique mechanism of coordinated interactions of the integrin alpha9 cytoplasmic domain, Src tyrosine kinase and iNOS to transduce integrin-alpha9beta1-mediated cell migration.
3. Increased tumor cell dissemination and cellular senescence in the absence of beta1-integrin function. Integrins are transmembrane receptors that bind extracellular matrix proteins and enable cell adhesion and cytoskeletal organization, as well as transduction of signals into cells, to promote various aspects of cellular behavior, such as proliferation or survival. Integrins participate in many aspects of tumor biology. Here, we have employed the Rip1Tag2 transgenic mouse model of pancreatic beta cell carcinogenesis to investigate the role of beta(1)-integrin in tumor progression. Specific ablation of beta(1)-integrin function in pancreatic beta cells resulted in a defect in sorting between insulin-expressing beta cells and glucagon-expressing alpha cells in islets of Langerhans. Ablation of beta(1)-integrin in beta tumor cells of Rip1Tag2 mice led to the dissemination of tumor cell emboli into lymphatic blood vessels in the absence of ongoing lymphangiogenesis. Yet, disseminating beta(1)-integrin-deficient beta tumor cells did not elicit **metastasis**. Rather, primary tumor growth was significantly impaired by reduced tumor cell proliferation and the acquisition of cellular senescence by beta(1)-integrin-deficient beta tumor cells. The results indicate a critical role of beta(1)-integrin function in mediating metastatic dissemination and preventing tumor cell senescence.
4. Melanoma cell adhesion can be blocked by heparin in vitro: suggestion of VLA-4 as a novel target for antimetastatic approaches. The clinical benefit of heparin in cancer patients to prolong survival can be attributed to non-anticoagulant mechanisms. Since adhesion molecules are crucially involved in tumour cell **metastasis**, their inhibition offers an attractive approach for interfering with the metastatic cascade. Heparin is known to attenuate **metastasis** in a selectin-dependent manner and possesses a variety of additional effects that are thought to influence tumour cell dissemination, proliferation, and angiogenesis. We investigated the adhesion behaviour of B16F10 melanoma cells in vitro regarding selectin- and VLA-4/VCAM-1-mediated binding to get an insight into underlying mechanisms of melanoma cell **metastasis**. We show that B16F10 cells display binding ability to P- and L-selectin as well as to isolated platelets. In contrast, B16F10 cells did not adhere to immobilized P-selectin under flow. This contributes to recent findings that elucidate a major role of platelet P-selectin for microemboli formation and thus, facilitating **metastasis**. In contrast, B16F10 cells adhered to endothelial cells under flow, which could partly be inhibited by a function-blocking anti-VCAM-1 mAb. To emphasize VCAM-1 function, we analyzed cell adhesion at immobilized VCAM-1 and observed an integrin dependency. Inhibition experiments reveal that heparin influences VLA-4-mediated binding pathways. By a combination of different techniques we prove that the site of heparin action is rather VLA-4 than VCAM-1. To our knowledge, this is the first time that heparin is shown to interfere with the VLA-4/VCAM-1 interaction leading to the suggestion of a novel heparin target. Our results may contribute to the understanding of how heparin exerts its anti-metastatic activity.
5. Integrin endosomal signalling suppresses anoikis. Integrin-containing focal adhesions transmit extracellular signals across the plasma membrane to modulate cell adhesion, signalling and survival. Although integrins are known to undergo continuous endo/exocytic traffic, the potential impact of endocytic traffic on integrin-induced signals is unknown. Here, we demonstrate that integrin signalling is not restricted to cell-ECM adhesions and identify an endosomal signalling platform that supports integrin signalling away from the plasma membrane. We show that active focal adhesion kinase (FAK), an established marker of integrin-ECM downstream signalling, localizes with active integrins on endosomes. Integrin endocytosis positively regulates adhesion-induced FAK activation, which is early endosome antigen-1 and small GTPase Rab21 dependent. FAK binds directly to purified endosomes and becomes activated on them, suggesting a role for endocytosis in enhancing distinct integrin downstream signalling events. Finally, endosomal integrin signalling contributes to cancer-related processes such as anoikis resistance, anchorage independence and **metastasis**.
6. Integrin α11β1 regulates cancer stromal stiffness and promotes tumorigenicity an**d metastas**is in non-small cell lung cancer. Integrin α11β1 is a stromal cell-specific receptor for fibrillar collagens and is overexpressed in carcinoma-associated fibroblasts (CAFs). We have investigated its direct role in cancer progression by generating severe combined immune deficient (SCID) mice deficient in integrin α11 (α11) expression. The growth of A549 lung adenocarcinoma cells and two patient-derived non-small cell lung carcinoma (NSCLC) xenografts in these α11 knockout (α11(-/-)) mice was significantly impeded, as compared with wild-type (α11(+/+)) SCID mice. Orthotopic implantation of a spontaneously metastatic NCI-H460SM cell line into the lungs of α11(-/-) and α11(+/+) mice showed significant reduction in the metastatic potential of these cells in the α11(-/-) mice. We identified that collagen cross-linking is associated with stromal α11 expression, and the loss of tumor stromal α11 expression was correlated with decreased collagen reorganization and stiffness. This study shows the role of integrin α11β1, a receptor for fibrillar collagen in differentiation of fibroblasts into CAFs. Furthermore, our data support an important role for α11 signaling pathway in CAFs, promoting tumor growth and metastatic potential of NSCLC cells and being closely associated with collagen cross-linking and the organization and stiffness of fibrillar collagen matrices.
7. The α₂β₁ integrin **is a meta**stasis suppressor in mouse models and human cancer. Integrins regulate cell-cell and cell-matrix adhesion and thereby play critical roles in tumor progressio**n and meta**stasis. Although work in preclinical models suggests that β1 integrins may sti**mulate met**astasis of a number of cancers, expression of the β1 subunit alone has not been shown to be a useful prognostic indicator in human cancer patients. Here we have demonstrated that the α2β1 integrin s**uppresses** metastasis in a clinically relevant spontaneous mouse model of breast cancer. These data are consistent with previous studies indicating high expression of α2β1 integrin in normal breast epithelium and loss of α2β1 in poorly differentiated breast cancer. They are also consistent with our systematic analysis of microarray databases of human breast and prostate cancer, which revealed that decreased expression of the gene encoding α2 integrin, but not genes encoding α1, α3, or β1 integrin, was predictive of metastatic dissemination and decreased survival. The predictive value of α2 expression persisted within both good-risk and poor-risk cohorts defined by estrogen receptor and lymph node status. Thus, the α2β1 integrin functionally i**nhibits br**east tumor metastasis, and α2 expression may serve as an important biomarker of metastatic potential and patient survival.
8. C1GALT1 promotes invasive phenotypes of hepatocellular carcinoma cells by modulating integrin β1 glycosylation and activity. Cancer cell invasion and **metastasi**s are the primary causes of treatment failure and death in hepatocellular carcinoma (HCC). We previously reported that core 1 β1,3-galactosyltransferase (C1GALT1) is frequently overexpressed in HCC tumors and its expression is associated with advanced tumor stage**, metastas**is, and poor survival. However, the underlying mechanisms of C1GALT1 in HCC malignancy remain unclear. In this study, we found that overexpression of C1GALT1 enhanced HCC cell adhesion to extracellular matrix (ECM) proteins, migration, and invasion, whereas RNAi-mediated knockdown of C1GALT1 suppressed these phenotypes. The promoting effect of C1GALT1 on th**e metastas**is of HCC cells was demonstrated in a mouse xenograft model. Mechanistic investigations showed that the C1GALT1-enhanced phenotypic changes in HCC cells were significantly suppressed by anti-integrin β1 blocking antibody. Moreover, C1GALT1 was able to modify O-glycans on integrin β1 and regulate integrin β1 activity as well as its downstream signaling. These results suggest that C1GALT1 could enhance HCC invasiveness through integrin β1 and provide novel insights into the roles of O-glycosylation i**n HCC meta**stasis.
9. miRNA-29c suppresses lung cancer cell adhesion to extracellular matrix and **metastasis** by targeting integrin β1 and matrix metalloproteinase2 (MMP2). Our pilot study using miRNA arrays found that miRNA-29c (miR-29c) is differentially expressed in the paired low-metastatic lung cancer cell line 95C compared to the high-metastatic lung cancer cell line 95D. Bioinformatics analysis shows that integrin β1 and matrix metalloproteinase 2 (MMP2) could be important target genes of miR-29c. Therefore, we hypothesized that miR-29c suppresses lung cancer cell adhesion to extracellular matrix (ECM) an**d metastas**is by targeting integrin β1 and MMP2. The gain-of-function studies that raised miR-29c expression in 95D cells by using its mimics showed reductions in cell proliferation, adhesion to ECM, invasion and migration. In contrasts, loss-of-function studies that reduced miR-29c by using its inhibitor in 95C cells promoted proliferation, adhesion to ECM, invasion and migration. Furthermore, the dual-luciferase reporter assay demonstrated that miR-29c inhibited the expression of the luciferase gene containing the 3'-UTRs of integrin β1 and MMP2 mRNA. Western blotting indicated that miR-29c downregulated the expression of integrin β1 and MMP2 at the protein level. Gelatin zymography analysis further confirmed that miR-29c decreased MMP2 enzyme activity. Nude mice with xenograft models of lung cancer cells confirmed that miR-29c inhibited lung ca**ncer metas**tasis in vivo, including bone and l**iver metas**tasis. Taken together, our results demonstrate that miR-29c serves as a t**umor metas**tasis suppressor, which suppresses lung cancer cell adhesion to ECM **and metas**tasis by directly inhibiting integrin β1 and MMP2 expression and by further reducing MMP2 enzyme activity. The results show that miR-29c may be a novel therapeutic candidate target to slow lung c**ancer meta**stasis.
10. Fibronectin-integrin (alpha5beta1) modulates migration and invasion of murine melanoma cell line B16F10 by involving MMP-9. Cell adhesion to extracellular matrix (ECM) initiates signaling cascade regulated by cell surface integrin receptors, which affects the proliferation and invasion of cells. Cells cultured in the presence of ECM ligand fibronectin (FN) stimulate secretion of matrix metalloproteinases (MMPs), facilitating cancer cell invasion and **metastasis**. Among all the members of the MMP family, MMP-9 is of crucial importance in tumor invasion and **metastasis**. The present study aims at studying the effects of integrin receptor alpha5beta1 and its ligand FN on expression of MMP-9 in murine melanoma cell line B16F10 and understanding the molecular mechanism(s) involved. The main experimental methods performed in the study were gelatin zymography, immunoblot, real-time RT-PCR, immunocytochemistry, enzyme linked immunosorbent assay (ELISA), transwell chamber assay, and in vivo **metastasis** assay in syngenic (C57BL6J) mice. The study reports that FN induces the activity, mRNA, and protein expression of MMP-9 and initiates its proteolytic activation in B16F10 cells. Blockage of the alpha5 receptor abrogated the FN-mediated stimulatory response on MMP-9 in B16F10 cells. Inhibitor studies and immunoblot analysis strongly suggest the involvement of focal adhesion kinase (FAK), extracellular regulated kinase (ERK), and phosphatidylinositol-3-kinase (PI-3K) in the FN-mediated responses. Immunocytochemical analysis showed the nuclear localization of nuclear factor-kappaB (NF-kappaB) might lead to activation of MMP-9 gene upon FN treatment. This study demonstrates that integrin receptor alpha5beta1 and FN interaction induces the invasive potential of B16F10 cells and MMP-9 induction is the downstream effectors in the process. This system serves as a novel model system to understand the molecular mechanism of melanoma growth and invasion.
11. hTERT mediates gastric cancer **metastasis** partially through the indirect targeting of ITGB1 by microRNA-29a. Human telomerase reverse transcriptase (hTERT) plays a key role in tumor invasion and **metastasis**, but the mechanism of its involvement in these processes is not clear. The purpose of this study is to investigate the possible molecular mechanism of hTERT in the promotion of gastric cancer (GC) **metastasis**. We found that the up-regulation of hTERT in gastric cancer cells could inhibit the expression of miR-29a and enhance the expression of Integrin β1 (ITGB1). In addition, the invasive capacity of gastric cancer cells was also highly increased after hTERT overexpression. Our study also found that the restoration of miR-29a suppressed the expression of ITGB1 and inhibited GC cell **metastasi**s both in vitro and in vivo. Taken together, our results suggested that hTERT may promote GC **metastasi**s through the hTERT-miR-29a-ITGB1 regulatory pathway.
12. Integrin β1, Osmosensing, and Chemoresistance in Mouse Ehrlich Carcinoma Cells. Altered expression of the integrin family of cell adhesion receptors has been associated with initiation, progression, and **metastasi**s of solid tumors as well as in the development of chemoresistance. Here, we investigated the role of integrins, in particular integrin β1, in cell volume regulation and drug-induced apoptosis in adherent and non-adherent Ehrlich ascites cell lines.Adhesion phenotypes were verified by colorimetric cell-adhesion-assay. Quantitative real-time PCR and western blot were used to compare expression levels of integrin subunits. Small interfering RNA was used to silence integrin β1 expression. Regulatory volume decrease (RVD) after cell swelling was studied with calcein-fluorescence-self-quenching and Coulter counter analysis. Taurine efflux was estimated with tracer technique. Caspase assay was used to determine apoptosis.We show that adherent cells have stronger fibronectin binding and a significantly increased expression of integrin α5, αv, and β1 at mRNA and protein level, compared to non-adherent cells. Knockdown of integrin β1 reduced RVD of the adherent but not of the non-adherent cells. Efflux of taurine was unaffected. In contrast to non-adherent, adherent cells exhibited chemoresistance to chemotherapeutic drugs (cisplatin and gemcitabine). However, knockdown of integrin β1 promoted cisplatin-induced caspase activity in adherent cells.Our data identifies integrin β1 as a part of the osmosensing machinery and regulator of cisplatin resistance in adherent Ehrlich cells.
13. Bone homing of mesenchymal stem cells by ectopic alpha 4 integrin expression. The pluripotent nature of mesenchymal stem cells (MSC) widens their potential for tissue regeneration and as vehicles for cell therapy in molecular medicine. Although the MSC are relatively easier to obtain and propagate in culture, a major impediment remains in their engraftment to target tissues on autologous transfer. We report here that transient, ectopic expression of alpha4 integrin (CD49d) on MSC greatly increases bone homing in an immunocompetent mouse model. Heterodimerization of the alpha4 integrin with endogenous beta1 integrin (CD29) was confirmed to influence this targeting. In addition to retaining their stem cell property, the engrafted MSC were also found to form osteoblasts and osteocytes in the growth plate of recipient mouse limb bones (femur/tibia) in vivo. These findings provide evidence for a novel strategy to achieve bone homing of genetically engineered MSC, which may broadly benefit in targeted therapies for osteopenic bone defects and cancer bone **metastasis**.
14. Angiopoietin-like 4 interacts with integrins beta1 and beta5 to modulate keratinocyte migration. Adipose tissue secretes adipocytokines for energy homeostasis, but recent evidence indicates that some adipocytokines also have a profound local impact on wound healing. Upon skin injury, keratinocytes use various signaling molecules to promote reepithelialization for efficient wound closure. In this study, we identify a novel function of adipocytokine angiopoietin-like 4 (ANGPTL4) in keratinocytes during wound healing through the control of both integrin-mediated signaling and internalization. Using two different in vivo models based on topical immuno-neutralization of ANGPTL4 as well as ablation of the ANGPTL4 gene, we show that ANGPTL4-deficient mice exhibit delayed wound reepithelialization with impaired keratinocyte migration. Human keratinocytes in which endogenous ANGPTL4 expression was suppressed by either siRNA or a neutralizing antibody show impaired migration associated with diminished integrin-mediated signaling. Importantly, we identify integrins β1 and β5, but not β3, as novel binding partners of ANGPTL4. ANGPTL4-bound integrin β1 activated the FAK-Src-PAK1 signaling pathway, which is important for cell migration. The findings presented herein reveal an unpredicted role of ANGPTL4 during wound healing and demonstrate how ANGPTL4 stimulates intracellular signaling mechanisms to coordinate cellular behavior. Our findings provide insight into a novel cell migration control mechanism and underscore the physiological importance of the modulation of integrin activity in can**cer metast**asis.
15. Mena binds α5 integrin directly and modulates α5β1 function. Mena is an Ena/VASP family actin regulator with roles in cell migration, chemotaxis, cell-cell adhesion, tumor cell invasion, a**nd metasta**sis. Although enriched in focal adhesions, Mena has no established function within these structures. We find that Mena forms an adhesion-regulated complex with α5β1 integrin, a fibronectin receptor involved in cell adhesion, motility, fibronectin fibrillogenesis, signaling, and growth factor receptor trafficking. Mena bound directly to the carboxy-terminal portion of the α5 cytoplasmic tail via a 91-residue region containing 13 five-residue "LERER" repeats. In fibroblasts, the Mena-α5 complex was required for "outside-in" α5β1 functions, including normal phosphorylation of FAK and paxillin and formation of fibrillar adhesions. It also supported fibrillogenesis and cell spreading and controlled cell migration speed. Thus, fibroblasts require Mena for multiple α5β1-dependent processes involving bidirectional interactions between the extracellular matrix and cytoplasmic focal adhesion proteins.
16. Targeted disruption of the beta1 integrin gene in a lymphoma cell line greatly reduces metastatic capacity. Integrins have been implicated in tumor **metastasis**. To investigate this, we generated beta1 integrin-negative double knockout (DKO) mutants of the highly metastatic ESb murine T-lymphoma cell line. The in vivo growth capacity of the mutants, which had lost alpha4beta1 and alpha6beta1 expression, was not altered, but their metastatic capacity was greatly reduced. Tail vein injection of 10(4) ESb and single-knockout cells led to death of all animals within 9-11 days. In contrast, only one-half of the animals injected with 10(4) DKO cells died, but much later, after 20-60 days. The other one-half remained disease-free for up to 100 days. Whereas ESb and single-knockout cells disseminated predominantly to liver and spleen, **metastasis** of DKO cells to these organs was rare, even after this prolonged period. Instead, skeletal muscles were invaded extensively. Metastatic capacity was largely restored in a DKO clone, which had been transfected with beta1 cDNA and expressed beta1 at similar levels as ESb cells. We conclude that beta1 integrins are essential for efficient liver and spleen colonization by the ESb lymphoma.
17. Integrin alpha4beta1 signaling is required for lymphangiogenesis and tumor **metastasis**. Recent studies have shown that lymphangiogenesis or the growth of lymphatic vessels at the periphery of tumors promotes tumor **metastasis** to lymph nodes. We show here that the fibronectin-binding integrin alpha4beta1 and its ligand fibronectin are novel functional markers of proliferative lymphatic endothelium. Tumors and lymphangiogenic growth factors, such as vascular endothelial growth factor-C (VEGF-C) and VEGF-A, induce lymphatic vessel expression of integrin alpha4beta1. Integrin alpha4beta1 then promotes growth factor and tumor-induced lymphangiogenesis, as genetic loss of integrin alpha4beta1 expression in Tie2Cre+ alpha4(loxp/loxp) mice or genetic loss of alpha4 signaling in alpha4Y991A knock-in mice blocks growth factor and tumor-induced lymphangiogenesis, as well as tumor **metastasis** to lymph nodes. In addition, antagonists of integrin alpha4beta1 suppress lymphangiogenesis and tumor **metastasis**. Our studies show that integrin alpha4beta1 and the signals it transduces regulate the adhesion, migration, invasion, and survival of proliferating lymphatic endothelial cells. As suppression of alpha4beta1 expression, signal transduction, or function in tumor lymphatic endothelium not only inhibits tumor lymphangiogenesis but also prevents metastatic disease, these results show that integrin alpha4beta1-mediated tumor lymphangiogenesis promotes **metastasis** and is a useful target for the suppression of metastatic disease.
18. Targeted inactivation of β1 integrin induces β3 integrin switching, which drives breast cance**r metastas**is by TGF-β. Mammary tumorigenesis and epithelial-mesenchymal transition (EMT) programs cooperate in converting transforming growth factor-β (TGF-β) from a suppressor to a promoter of breast ca**ncer metas**tasis. Although previous reports associated β1 and β3 integrins with TGF-β stimulation of **EMT and me**tastasis, the functional interplay and plasticity exhibited by these adhesion molecules in shaping the oncogenic activities of TGF-β remain unknown. We demonstrate that inactivation of β1 integrin impairs TGF-β from stimulating the motility of normal and malignant mammary epithelial cells (MECs) and elicits robust compensatory expression of β3 integrin solely in malignant MECs, but not in their normal counterparts. Compensatory β3 integrin expression also 1) enhances the growth of malignant MECs in rigid and compliant three-dimensional organotypic cultures and 2) restores the induction of the EMT phenotypes by TGF-β. Of importance, compensatory expression of β3 integrin rescues the growth **and pulmo**nary metastasis of β1 integrin-deficient 4T1 tumors in mice, a process that is prevented by genetic depletion or functional inactivation of β3 integrin. Collectively our findings demonstrate that inactivation of β1 integrin elicits metastatic progression via a β3 integrin-specific mechanism, indicating that dual β1 and β3 integrin targeting is necessary to alleviate metastatic disease in breast cancer patients.
19. Trimeric Tn antigen on syndecan 1 produced by ppGalNAc-T13 enhances cancer **metastasis** via a complex formation with integrin α5β1 and matrix metalloproteinase 9. We demonstrated previously that ppGalNAc-T13 (T13), identified as an up-regulated gene with increase**d metastas**is in a DNA microarray, generated trimeric Tn (tTn) antigen (GalNAcα1-Ser/Thr)3 on Syndecan 1 in highly metastatic sublines of Lewis lung cancer. However, it is not known how tTn antigen regulates canc**er metasta**sis. Here, we analyzed the roles of tTn antigen in cancer properties. tTn antigen on Syndecan 1 increased cell adhesion to fibronectin in an integrin-dependent manner. Furthermore, cell adhesion to fibronectin induced phosphorylation of focal adhesion kinase and paxillin in T13-transfectant cells. In the search of Syndecan 1-interacting molecules, it was demonstrated that tTn antigen-carrying Syndecan 1 interacted with integrin α5β1 and matrix metalloproteinase 9 and that these molecules shifted to a glycolipid-enriched microdomain/rafts along with increased metastatic potential in T13-transfectant cells. We also identified a tTn substitution site on Syndecan 1, demonstrating that tTn on Syndecan 1 is essential for the interaction with integrin α5β1 as well as for the reaction with mAb MLS128. These data suggest that high expression of the ppGalNAc-T13 gene generates tTn antigen on Syndecan 1 under reduced expression of GM1, leading to enhanced invasi**on and met**astasis via the formation of a molecular complex consisting of integrin α5β1, Syndecan 1, and MMP-9 in the glycolipid-enriched microdomain/rafts.
20. A critical role of CD29 and CD49f in mediating **metastasis** for cancer-initiating cells isolated from a Brca1-associated mouse model of breast cancer. Cancer **metastasis** is a lethal problem that claims the lives of over 90% of cancer patients. In this study, we have investigated metastatic potential of cancer stem cells (CSCs) isolated from mammary tumors of a Brca1-mutant mouse model. Our data indicated that CSCs, which are enriched in CD24(+)CD29(+)/CD49f(+) cell population, displayed much higher migration ability than CD24(-)CD29(-)/CD49f(-) cells in tissue culture and enhanced metastatic potential in allograft-nude mice. CD24(+)CD29(+) cells maintained the ability to differentiate and reconstitute heterogeneity in the metastatic tumors whereas CD24(-)CD29(-) cells could not. Corresponding to their enhanced metastatic ability, CD24(+)CD29(+) cells exhibited features of the epithelial to mesenchymal transition. Finally, using short hairpin RNA to knock down CD29 and/or CD49f in metastatic cancer cells, we demonstrated that while acute knockdown of CD29 or CD49f alone slightly decreased cell migration ability, knockdown of both genes generated a profound effect to block their migration, revealing an overlapping, yet critical function of both genes in the migration of CSCs. Our findings indicate that in addition to serving as markers of CSCs, CD29 and CD49f may also serve as potential therapeutic targets for cancer **metastasis**.
21. Matrix compliance regulates Rac1b localization, NADPH oxidase assembly, and epithelial-mesenchymal transition. Epithelial-mesenchymal transition (EMT) is a form of epithelial plasticity implicated in fibrosis and tumor **metastasis**. Here we show that the mechanical rigidity of the microenvironment plays a pivotal role in the promotion of EMT by controlling the subcellular localization and downstream signaling of Rac GTPases. Soft substrata, with compliances comparable to that of normal mammary tissue, are protective against EMT, whereas stiffer substrata, with compliances characteristic of breast tumors, promote EMT. Rac1b, a highly activated splice variant of Rac1 found in tumors, localizes to the plasma membrane in cells cultured on stiff substrata or in collagen-rich regions of human breast tumors. At the membrane, Rac1b forms a complex with NADPH oxidase and promotes the production of reactive oxygen species, expression of Snail, and activation of the EMT program. In contrast, soft microenvironments inhibit the membrane localization of Rac1b and subsequent redox changes. These results reveal a novel mechanotransduction pathway in the regulation of epithelial plasticity via EMT.

#### GeneRIFs

1. TERT may promote gastric cancer **metastasis** through the TERT-miR-29a-ITGB1 regulatory pathway.
2. CD29 and CD49f have a critical role of in mediating cancer **metastasis**.
3. miR-29c serves as a tumor **metastasis** suppressor, which suppresses lung cancer cell adhesion to ECM and **metastasis** by directly inhibiting integrin beta1 and MMP2 expression
4. high expression of the ppGalNAc-T13 gene generates tTn antigen on Syndecan 1, leading to enhanced invasion and **metastasis** via the formation of a complex consisting of integrin alpha5beta1, Syndecan 1, and MMP-9 in the glycolipid-enriched microdomain/rafts.
5. alpha2beta1 integrin suppresses **metastasis** in a clinically relevant spontaneous mouse model of breast cancer
6. alpha4beta1-mediated tumor lymphangiogenesis promotes **metastasis**
7. beta 1 integrin has roles in cell adhesion and migration in development and **metastasis** [review]

### [Cc]ell [Ii]nvasion

#### PubMed

1. Caveolin limits membrane microdomain mobility and integrin-mediated uptake of fibronectin-binding pathogens. Staphylococcus aureus, which is a leading cause of hospital-acquired infections, binds via fibronectin to integrin α5β1, a process that can promote host colonization in vivo. Integrin engagement induces actin cytoskeleton rearrangements that result in the uptake of S. aureus by non-professional phagocytic cells. Interestingly, we found that fibronectin-binding S. aureus trigger the redistribution of membrane microdomain components. In particular, ganglioside GM1 and GPI-linked proteins were recruited upon integrin β1 engagement, and disruption of membrane microdomains blocked bacterial internalization. Several membrane-microdomain-associated proteins, such as flotillin-1 and flotillin-2, as well as caveolin, were recruited to sites of bacterial attachment. Whereas dominant-negative versions of flotillin-2 did not affect bacterial attachment or internalization, cells deficient for caveolin-1 (Cav1(-/-)) showed increased uptake of S. aureus and other Fn-binding pathogens. Recruitment of membrane microdomains to cell-associated bacteria was unaltered in Cav1(-/-) cells. However, fluorescence recovery after photobleaching (FRAP) revealed an enhanced mobility of membrane-microdomain-associated proteins in the absence of caveolin-1. Enhanced membrane microdomain mobility and increased uptake of S. aureus was repressed by expression of wild-type caveolin-1, but not caveolin-1 G83S, which harbors a point mutation in the caveolin scaffolding domain. Similarly, chemical or physical stimulation of membrane fluidity led to increased uptake of S. aureus. These results highlight a crucial role for caveolin-1 in negative regulation of membrane microdomain mobility, thereby affecting endocytosis of bacteria-engaged integrins. This process might not only limit ho**st cell invas**ion by integrin-binding bacterial pathogens, but might also be physiologically relevant for integrin-mediated cell adhesion.
2. Molecular mechanisms underlying postoperative peritoneal tumor dissemination may differ between a laparotomy and carbon dioxide pneumoperitoneum: a syngeneic mouse model with controlled respiratory support. The mechanisms promoting postoperative peritoneal tumor dissemination are unclear. This study aimed to investigate postoperative tumor dissemination over time on both tissue and molecular levels.For this study, C57BL6 mice were randomized into four groups: anesthesia alone (control), carbon dioxide (CO(2)) pneumoperitoneum at low (2 mmHg) or high (8 mmHg) intraperitoneal pressure (IPP), and laparotomy. A mouse ovarian cancer cell line (ID8) was injected intraperitoneally just before surgery. The groups were further subdivided into three groups, and a laparotomy was performed to evaluate tumor dissemination on postoperative day (POD) 7, 14, or 42.The incidence of cancer **cell invasion** into the muscle layers of the abdominal wall was significantly higher in the laparotomy and high-IPP groups than in the low-IPP and control groups on PODs 7 and 42. Expression levels of beta 1 integrin, cMet, urokinase-type plasminogen activator (uPA), urokinase-type plasminogen activator receptor (uPAR), and type-1 plasminogen activator inhibitor (PAI-1) mRNA in the disseminated nodules were not significantly different among the four groups on POD 7. However, the expression levels of all these genes in the disseminated nodules in the laparotomy group were significantly higher on POD 14 than on POD 7. They then returned to control levels on POD 42. There were no significant differences in the expression levels of any of these genes among the groups on POD 42.The current study suggests that the molecular mechanisms underlying postoperative peritoneal tumor dissemination may differ between a laparotomy and CO(2) pneumoperitoneum. Therefore, strategies targeting postoperative tumor dissemination likely will need to account for the surgical environment.
3. Integrin-mediated host **cell invasion** by type 1-piliated uropathogenic Escherichia coli. Uropathogenic Escherichia coli (UPEC), the primary causative agent of urinary tract infections, typically express filamentous adhesive organelles called type 1 pili that mediate both bacterial attachment to and invasion of bladder urothelial cells. Several host proteins have previously been identified as receptors for type 1 pili, but none have been conclusively shown to promote UPEC entry into host bladder cells. Using overlay assays with FimH, the purified type 1 pilus adhesin, and mass spectroscopy, we have identified beta1 and alpha3 integrins as key host receptors for UPEC. FimH recognizes N-linked oligosaccharides on these receptors, which are expressed throughout the urothelium. In a bladder cell culture system, beta1 and alpha3 integrin receptors co-localize with invading type 1-piliated bacteria and F-actin. FimH-mediated bacterial invasion of host bladder cells is inhibited by beta1 and alpha3 integrin-specific antibodies and by disruption of the beta1 integrin gene in the GD25 fibroblast cell line. Phosphorylation site mutations within the cytoplasmic tail of beta1 integrin that alter integrin signaling also variably affect UPEC entry into host cells, by either attenuating or boosting invasion frequencies. Furthermore, focal adhesion and Src family kinases, which propagate integrin-linked signaling and downstream cytoskeletal rearrangements, are shown to be required for FimH-dependent bacterial invasion of target host cells. Cumulatively, these results indicate that beta1 and alpha3 integrins are functionally important receptors for type 1 pili-expressing bacteria within the urinary tract and possibly at other sites within the host.
4. C1GALT1 promotes invasive phenotypes of hepatocellular carcinoma cells by modulating integrin β1 glycosylation and activity. Cancer **cell invasio**n and metastasis are the primary causes of treatment failure and death in hepatocellular carcinoma (HCC). We previously reported that core 1 β1,3-galactosyltransferase (C1GALT1) is frequently overexpressed in HCC tumors and its expression is associated with advanced tumor stage, metastasis, and poor survival. However, the underlying mechanisms of C1GALT1 in HCC malignancy remain unclear. In this study, we found that overexpression of C1GALT1 enhanced HCC cell adhesion to extracellular matrix (ECM) proteins, migration, and invasion, whereas RNAi-mediated knockdown of C1GALT1 suppressed these phenotypes. The promoting effect of C1GALT1 on the metastasis of HCC cells was demonstrated in a mouse xenograft model. Mechanistic investigations showed that the C1GALT1-enhanced phenotypic changes in HCC cells were significantly suppressed by anti-integrin β1 blocking antibody. Moreover, C1GALT1 was able to modify O-glycans on integrin β1 and regulate integrin β1 activity as well as its downstream signaling. These results suggest that C1GALT1 could enhance HCC invasiveness through integrin β1 and provide novel insights into the roles of O-glycosylation in HCC metastasis.
5. Transmembrane/cytoplasmic, rather than catalytic, domains of Mmp14 signal to MAPK activation and mammary branching morphogenesis via binding to integrin β1. Epithelial **cell invasio**n through the extracellular matrix (ECM) is a crucial step in branching morphogenesis. The mechanisms by which the mammary epithelium integrates cues from the ECM with intracellular signaling in order to coordinate invasion through the stroma to make the mammary tree are poorly understood. Because the cell membrane-bound matrix metalloproteinase Mmp14 is known to play a key role in cancer **cell invasio**n, we hypothesized that it could also be centrally involved in integrating signals for mammary epithelial cells (MECs) to navigate the collagen 1 (CL-1)-rich stroma of the mammary gland. Expression studies in nulliparous mice that carry a NLS-lacZ transgene downstream of the Mmp14 promoter revealed that Mmp14 is expressed in MECs at the tips of the branches. Using both mammary organoids and 3D organotypic cultures, we show that MMP activity is necessary for invasion through dense CL-1 (3 mg/ml) gels, but dispensable for MEC branching in sparse CL-1 (1 mg/ml) gels. Surprisingly, however, Mmp14 without its catalytic activity was still necessary for branching. Silencing Mmp14 prevented **cell invasio**n through CL-1 and disrupted branching altogether; it also reduced integrin β1 (Itgb1) levels and attenuated MAPK signaling, disrupting Itgb1-dependent invasion/branching within CL-1 gels. FRET imaging revealed that Mmp14 associates directly with Itgb1. We identified a domain of Mmp14 that is required for modulating the levels of Itgb1, MEC signaling and the rate of invasion within CL-1. These results shed light on hitherto undescribed non-proteolytic activities of Mmp14 that are necessary for the Itgb1-dependent biochemical and mechanical signals that regulate branching in the mammary epithelium.
6. Fibronectin-integrin (alpha5beta1) modulates migration and invasion of murine melanoma cell line B16F10 by involving MMP-9. Cell adhesion to extracellular matrix (ECM) initiates signaling cascade regulated by cell surface integrin receptors, which affects the proliferation and invasion of cells. Cells cultured in the presence of ECM ligand fibronectin (FN) stimulate secretion of matrix metalloproteinases (MMPs), facilitating cancer **cell invasion** and metastasis. Among all the members of the MMP family, MMP-9 is of crucial importance in tumor invasion and metastasis. The present study aims at studying the effects of integrin receptor alpha5beta1 and its ligand FN on expression of MMP-9 in murine melanoma cell line B16F10 and understanding the molecular mechanism(s) involved. The main experimental methods performed in the study were gelatin zymography, immunoblot, real-time RT-PCR, immunocytochemistry, enzyme linked immunosorbent assay (ELISA), transwell chamber assay, and in vivo metastasis assay in syngenic (C57BL6J) mice. The study reports that FN induces the activity, mRNA, and protein expression of MMP-9 and initiates its proteolytic activation in B16F10 cells. Blockage of the alpha5 receptor abrogated the FN-mediated stimulatory response on MMP-9 in B16F10 cells. Inhibitor studies and immunoblot analysis strongly suggest the involvement of focal adhesion kinase (FAK), extracellular regulated kinase (ERK), and phosphatidylinositol-3-kinase (PI-3K) in the FN-mediated responses. Immunocytochemical analysis showed the nuclear localization of nuclear factor-kappaB (NF-kappaB) might lead to activation of MMP-9 gene upon FN treatment. This study demonstrates that integrin receptor alpha5beta1 and FN interaction induces the invasive potential of B16F10 cells and MMP-9 induction is the downstream effectors in the process. This system serves as a novel model system to understand the molecular mechanism of melanoma growth and invasion.
7. Mena binds α5 integrin directly and modulates α5β1 function. Mena is an Ena/VASP family actin regulator with roles in cell migration, chemotaxis, cell-cell adhesion, tum**or cell invas**ion, and metastasis. Although enriched in focal adhesions, Mena has no established function within these structures. We find that Mena forms an adhesion-regulated complex with α5β1 integrin, a fibronectin receptor involved in cell adhesion, motility, fibronectin fibrillogenesis, signaling, and growth factor receptor trafficking. Mena bound directly to the carboxy-terminal portion of the α5 cytoplasmic tail via a 91-residue region containing 13 five-residue "LERER" repeats. In fibroblasts, the Mena-α5 complex was required for "outside-in" α5β1 functions, including normal phosphorylation of FAK and paxillin and formation of fibrillar adhesions. It also supported fibrillogenesis and cell spreading and controlled cell migration speed. Thus, fibroblasts require Mena for multiple α5β1-dependent processes involving bidirectional interactions between the extracellular matrix and cytoplasmic focal adhesion proteins.

---

## Gmfb

- NCBI Gene
- GeneRIF
- Pubmed

| Search Term | Rank | Fields |
| --- | --- | --- |
| [Mm]igration | 6 | PubMed(1); GeneRIFs(1) |

  
hide/show details for Gmfb

### [Mm]igration

#### PubMed

1. GMFβ controls branched actin content and lamellipodial retraction in fibroblasts. The lamellipodium is an important structure for cell **migratio**n containing branched actin nucleated via the Arp2/3 complex. The formation of branched actin is relatively well studied, but less is known about its disassembly and how this influences **migratio**n. GMF is implicated in both Arp2/3 debranching and inhibition of Arp2/3 activation. Modulation of GMFβ, a ubiquitous GMF isoform, by depletion or overexpression resulted in changes in lamellipodial dynamics, branched actin content, an**d migrati**on. Acute pharmacological inhibition of Arp2/3 by CK-666, coupled to quantitative live-cell imaging of the complex, showed that depletion of GMFβ decreased the rate of branched actin disassembly. These data, along with mutagenesis studies, suggest that debranching (not inhibition of Arp2/3 activation) is a primary activity of GMFβ in vivo. Furthermore, depletion or overexpression of GMFβ disrupted the ability of cells to directionally migrate to a gradient of fibronectin (haptotaxis). These data suggest that debranching by GMFβ plays an important role in branched actin regulation, lamellipodial dynamics, and direct**ional mig**ration.

#### GeneRIFs

1. The data suggest that debranching by GMFbeta plays an important role in branched actin regulation, lamellipodial dynamics, and directional **migration**.

---

## Strap

- NCBI Gene
- GeneRIF
- Pubmed

| Search Term | Rank | Fields |
| --- | --- | --- |
| [Mm]igration | 6 | Process(1) |
| [Ee]xtracellular [Mm]atrix | 8 | PubMed(1) |

  
hide/show details for Strap

### [Mm]igration

#### Process

1. negative regulation of epithelial cell **migration**

### [Ee]xtracellular [Mm]atrix

#### PubMed

1. Serine threonine receptor-associated protein (STRAP) plays a role in the maintenance of mesenchymal morphology. The stromal tissue, made of **extracellular matrix** and mesenchymal cells, is vital for the functional design of all complex tissues. Fibroblasts are key components of stromal tissue and play a crucial role during organ development, wound repair, angiogenesis and fibrosis. We have previously reported the identification of a novel WD-domain protein, STRAP(1) that inhibits transforming growth factor-beta (TGF-beta) signaling and enhances tumorigenicity via TGF-beta-dependent and TGF-beta-independent mechanisms. Here, we report, for the first time, that deletion of STRAP from Mouse Embryonic Fibroblasts (MEFs) results in a loss of mesenchymal morphology. These cells lose their spindle shape and exhibit features of an epithelial morphology. Gene expression profiling has confirmed that deletion of STRAP affects expression of sets of genes important for diverse functions including cell-cell adhesion and cell polarization, and upregulates E-cadherin expression leading to the formation of adherens junctions, subsequent localization of beta-catenin to the cell membrane and downregulation of the mesenchymal markers like LEF1 (lymphoid enhancer-binding factor 1). Upregulation of WT1 (Wilms tumor homolog 1) in STRAP null MEFs plays a role in E-cadherin induction. Finally, stable expression of STRAP in these cells results in a loss of WT1 and E-cadherin expressions, and a reversal from epithelial to the mesenchymal morphology. Thus, these results provide a novel TGF-beta-independent function of STRAP and describe a mechanism for the role of STRAP in the maintenance of mesenchymal morphology.

---

## Myl12a

- NCBI Gene
- GeneRIF
- Pubmed

| Search Term | Rank | Fields |
| --- | --- | --- |
| [Mm]igration | 6 | PubMed(1) |

  
hide/show details for Myl12a

### [Mm]igration

#### PubMed

1. Myosin regulatory light chains are required to maintain the stability of myosin II and cellular integrity. Myosin II is an actin-binding protein composed of MHC (myosin heavy chain) IIs, RLCs (regulatory light chains) and ELCs (essential light chains). Myosin II expressed in non-muscle tissues plays a central role in cell adhesion, **migration** and division. The regulation of myosin II activity is known to involve the phosphorylation of RLCs, which increases the Mg2+-ATPase activity of MHC IIs. However, less is known about the details of RLC-MHC II interaction or the loss-of-function phenotypes of non-muscle RLCs in mammalian cells. In the present paper, we investigate three highly conserved non-muscle RLCs of the mouse: MYL (myosin light chain) 12A (referred to as MYL12A), MYL12B and MYL9 (MYL12A/12B/9). Proteomic analysis showed that all three are associated with the MHCs MYH9 (NMHC IIA) and MYH10 (NMHC IIB), as well as the ELC MYL6, in NIH 3T3 fibroblasts. We found that knockdown of MYL12A/12B in NIH 3T3 cells results in striking changes in cell morphology and dynamics. Remarkably, the levels of MYH9, MYH10 and MYL6 were reduced significantly in knockdown fibroblasts. Comprehensive interaction analysis disclosed that MYL12A, MYL12B and MYL9 can all interact with a variety of MHC IIs in diverse cell and tissue types, but do so optimally with non-muscle types of MHC II. Taken together, our study provides direct evidence that normal levels of non-muscle RLCs are essential for maintaining the integrity of myosin II, and indicates that the RLCs are critical for cell structure and dynamics.

---

## Lcp1

- NCBI Gene
- GeneRIF
- Pubmed

| Search Term | Rank | Fields |
| --- | --- | --- |
| [Ff]ilopodium | 2 | Component(1) |
| [Pp]odosome(s)? | 5 | Process(1); PubMed(1); Component(1) |
| [Mm]igration | 6 | Process(1); PubMed(5); GeneRIFs(1) |
| [Ee]xtracellular [Mm]atrix | 8 | Process(1) |

  
hide/show details for Lcp1

### [Ff]ilopodium

#### Component

1. **filopodium**

### [Pp]odosome(s)?

#### Process

1. positive regulation of **podosome** assembly

#### PubMed

1. Regulation of sealing ring formation by L-plastin and cortactin in osteoclasts. The aim of this study is to identify the exact mechanism(s) by which cytoskeletal structures are modulated during bone resorption. In this study, we have shown the possible role of different actin-binding and signaling proteins in the regulation of sealing ring formation. Our analyses have demonstrated a significant increase in cortactin and a corresponding decrease in L-plastin protein levels in osteoclasts subjected to bone resorption for 18 h in the presence of RANKL, M-CSF, and native bone particles. Time-dependent changes in the localization of L-plastin (in actin aggregates) and cortactin (in the sealing ring) suggest that these proteins may be involved in the initial and maturation phases of sealing ring formation, respectively. siRNA to cortactin inhibits this maturation process but not the formation of actin aggregates. Osteoclasts treated as above but with TNF-α demonstrated very similar effects as observed with RANKL. Osteoclasts treated with a neutralizing antibody to TNF-α displaye**d podoso**me-like structures in the entire subsurface and at the periphery of osteoclast. It is possible that TNF-α and RANKL-mediated signaling may play a role in the early phase of sealing ring configuration (i.e. either in the disassembly **of podoso**mes or formation of actin aggregates). Furthermore, osteoclasts treated with alendronate or αv reduced the formation of the sealing ring but not actin aggregates. The present study demonstrates a novel mechanistic link between L-plastin and cortactin in sealing ring formation. These results suggest that actin aggregates formed by L-plastin independent of integrin signaling function as a core in assembling signaling molecules (integrin αvβ3, Src, cortactin, etc.) involved in the maturation process.

#### Component

1. **podosome**

### [Mm]igration

#### Process

1. cell **migration**

#### PubMed

1. Intrinsic T- and B-cell defects impair T-cell-dependent antibody responses in mice lacking the actin-bundling protein L-plastin. Germinal center development, critical for long-term humoral immunity, requires the trafficking of T and B lymphocytes to defined tissues and locations after antigenic challenge. The molecular mechanisms that support lymphocyte trafficking through the linkage of extracellular chemotactic and adhesive cues to the actin cytoskeleton are not yet fully defined. We have previously identified the actin-bundling protein L-plastin (LPL) as a requisite intermediary in both naive B and T lymphocyte **migration** and in T-cell activation. We tested the hypothesis that humoral immunity would require LPL. We show that mice lacking LPL demonstrated defective germinal center formation and reduced production of T-cell-dependent antibodies. T cells from LPL(-/-) mice exhibited defective expansion of the follicular helper T population. Reduced expansion of LPL(-/-) follicular helper T cells correlated with impaired trafficking to or retention of cells in the spleen following challenge, highlighting the importance of initial lymphocyte recruitment to the eventual success of the immune response. Furthermore, LPL(-/-) B cells demonstrated cell-intrinsic defects in population expansion and in differentiation into germinal center B cells. LPL thus modulates both T- and B-cell function during the germinal center reaction and the production of T-cell-dependent antibody responses.
2. The actin-bundling protein L-plastin is essential for marginal zone B cell development. B cell development is exquisitely sensitive to location within specialized niches in the bone marrow and spleen. Location within these niches is carefully orchestrated through chemotactic and adhesive cues. In this article, we demonstrate the requirement for the actin-bundling protein L-plastin (LPL) in B cell motility toward the chemokines CXCL12 and CXCL13 and the lipid chemoattractant sphingosine-1-phosphate, which guide normal B cell development. Impaired motility of B cells in LPL(-/-) mice correlated with diminished splenic maturation of B cells, with a moderate (40%) loss of follicular B cells and a profound (>80%) loss of marginal zone B cells. Entry of LPL(-/-) B cells into the lymph nodes and bone marrow of mice was also impaired. Furthermore, LPL was required for the integrin-mediated enhancement of Transwell **migration** but was dispensable for integrin-mediated lymphocyte adhesion. These results suggest that LPL may participate in signaling that enables lymphocyte trans**migration**. In support of this hypothesis, the phosphorylation of Pyk-2, a tyrosine kinase that integrates chemotactic and adhesive cues, is diminished in LPL(-/-) B cells stimulated with chemokine. Finally, a well-characterized role of marginal zone B cells is the generation of a rapid humoral response to polysaccharide Ags. LPL(-/-) mice exhibited a defective Ab response to Streptococcus pneumoniae, indicating a functional consequence of defective marginal zone B cell development in LPL(-/-) mice.
3. Microglia/macrophage-specific protein Iba1 binds to fimbrin and enhances its actin-bundling activity. Ionized calcium binding adaptor molecule 1 (Iba1) is a microglia/macrophage-specific calcium-binding protein. Iba1 has the actin-bundling activity and participates in membrane ruffling and phagocytosis in activated microglia. In order to understand the Iba1-related intracellular signalling pathway in greater detail, we employed a yeast two-hybrid screen to isolate an Iba1-interacting molecule and identified another actin-bundling protein, L-fimbrin. In response to stimulation, L-fimbrin accumulated and co-localized with Iba1 in membrane ruffles induced by M-CSF-stimulation and phagocytic cups formed by IgG-opsonized beads in microglial cell line MG5. L-fimbrin was shown to associate with Iba1 in cell lysate of COS-7 expressing L-fimbrin and Iba1. By using purified proteins, direct binding of Iba1 to L-fimbrin was demonstrated by immunoprecipitation, glutathione S-transferase pull-down assays and ligand overlay assays. The binding of Iba1 was also found to increase the actin-bundling activity of L-fimbrin. These results indicate that Iba1 forms complexes with L-fimbrin in membrane ruffles and phagocytic cups, and suggest that Iba1 co-operates with L-fimbrin in modulating actin reorganization to facilitate cell **migration** and phagocytosis by microglia.
4. The actin-bundling protein L-plastin dissociates CCR7 proximal signaling from CCR7-induced motility. Chemokines promote lymphocyte motility by triggering F-actin rearrangements and inducing cellular polarization. Chemokines can also enhance cell-cell adhesion and costimulate T cells. In this study, we establish a requirement for the actin-bundling protein L-plastin (LPL) in CCR7- and sphingosine-1-phosphate-mediated T cell chemotaxis using LPL(-/-) mice. Disrupted motility of mature LPL(-/-) thymocytes manifested in vivo as diminished thymic egress. Two-photon microscopy of LPL(-/-) lymphocytes revealed reduced velocity and motility in lymph nodes. Defective **migration** resulted from defective cellular polarization following CCR7 ligation, as CCR7 did not polarize to the leading edge in chemokine-stimulated LPL(-/-) T cells. However, CCR7 signaling to F-actin polymerization and CCR7-mediated costimulation was intact in LPL(-/-) lymphocytes. The differential requirement for LPL in CCR7-induced cellular adhesion and CCR7-induced motility allowed assessment of the contribution of CCR7-mediated motility to positive selection of thymocytes and lineage commitment. Results suggest that normal motility is not required for CCR7 to function in positive selection and lineage commitment. We thus identify LPL as a molecule critical for CCR7-mediated motility but dispensable for early CCR7 signaling. The requirement for actin bundling by LPL for polarization reveals a novel mechanism of regulating actin dynamics during T cell motility.
5. Alveolar macrophage development in mice requires L-plastin for cellular localization in alveoli. Alveolar macrophages are lung-resident sentinel cells that develop perinatally and protect against pulmonary infection. Molecular mechanisms controlling alveolar macrophage generation have not been fully defined. Here, we show that the actin-bundling protein L-plastin (LPL) is required for the perinatal development of alveolar macrophages. Mice expressing a conditional allele of LPL (CD11c.Cre(pos)-LPL(fl/fl)) exhibited significant reductions in alveolar macrophages and failed to effectively clear pulmonary pneumococcal infection, showing that immunodeficiency results from reduced alveolar macrophage numbers. We next identified the phase of alveolar macrophage development requiring LPL. In mice, fetal monocytes arrive in the lungs during a late fetal stage, maturing to alveolar macrophages through a prealveolar macrophage intermediate. LPL was required for the transition from prealveolar macrophages to mature alveolar macrophages. The transition from prealveolar macrophage to alveolar macrophage requires the upregulation of the transcription factor peroxisome proliferator-activated receptor-γ (PPAR-γ), which is induced by exposure to granulocyte-macrophage colony-stimulating factor (GM-CSF). Despite abundant lung GM-CSF and intact GM-CSF receptor signaling, PPAR-γ was not sufficiently upregulated in developing alveolar macrophages in LPL(-/-) pups, suggesting that precursor cells were not correctly localized to the alveoli, where GM-CSF is produced. We found that LPL supports 2 actin-based processes essential for correct localization of alveolar macrophage precursors: (1) tr**ansmigrat**ion into the alveoli, and (2) engraftment in the alveoli. We thus identify a molecular pathway governing neonatal alveolar macrophage development and show that genetic disruption of alveolar macrophage development results in immunodeficiency.

#### GeneRIFs

1. L-plastin may participate in signaling that enables lymphocyte trans**migration**

### [Ee]xtracellular [Mm]atrix

#### Process

1. **extracellular matrix** disassembly

---

## Wdr1

- NCBI Gene
- GeneRIF
- Pubmed

| Search Term | Rank | Fields |
| --- | --- | --- |
| [Mm]igration | 6 | Process(1); PubMed(1) |

  
hide/show details for Wdr1

### [Mm]igration

#### Process

1. neutrophil **migration**

#### PubMed

1. Caspase-11 regulates cell **migration** by promoting Aip1-Cofilin-mediated actin depolymerization. Coordinated regulation of cell **migration**, cytokine maturation and apoptosis is critical in inflammatory responses. Caspases, a family of cysteine proteases, are known to regulate cytokine maturation and apoptosis. Here, we show that caspase-11, a mammalian pro-inflammatory caspase, regulates cell **migration** during inflammation. Caspase-11-deficient lymphocytes exhibit a cell-autonomous **migration** defect in vitro and in vivo. We demonstrate that caspase-11 interacts physically and functionally with actin interacting protein 1 (Aip1), an activator of cofilin-mediated actin depolymerization. The caspase-recruitment domain (CARD) of caspase-11 interacts with the carboxy-terminal WD40 propeller domain of Aip1 to promote cofilin-mediated actin depolymerization. Cells with Aip1 or caspase-11 deficiency exhibit defects in actin dynamics. Using in vitro actin depolymerization assays, we found that caspase-11 and Aip1 work cooperatively to promote cofilin-mediated actin depolymerization. These data demonstrate a novel cell autonomous caspase-mediated mechanism that regulates actin dynamics and mammalian cell **migration** distinct from the receptor mediated Rho-Rac-Cdc42 pathway.

---

## Mapk14

- NCBI Gene
- GeneRIF
- Pubmed

| Search Term | Rank | Fields |
| --- | --- | --- |
| [Ff]ilopodia | 1 | PubMed(1) |
| [Pp]odosome(s)? | 5 | PubMed(1) |
| [Mm]igration | 6 | PubMed(37); GeneRIFs(6) |
| ECM | 7 | PubMed(6) |
| [Ee]xtracellular [Mm]atrix | 8 | PubMed(20); GeneRIFs(1) |
| [Ii]nvasive | 10 | PubMed(6) |
| [Mm]etastasize | 11 | PubMed(1) |
| [Mm]etastasis | 12 | PubMed(14); GeneRIFs(4) |
| [Cc]ell [Ii]nvasion | 13 | PubMed(1) |

  
hide/show details for Mapk14

### [Ff]ilopodia

#### PubMed

1. Regulation of myotube formation by the actin-binding factor drebrin. Myogenic differentiation involves cell-cycle arrest, activation of the muscle-specific transcriptome, and elongation, alignment and fusion of myoblasts into multinucleated myotubes. This process is controlled by promyogenic transcription factors and regulated by signaling pathways in response to extracellular cues. The p38 mitogen-activated protein kinase (p38 MAPK) pathway promotes the activity of several such transcription factors, including MyoD and MEF2, thereby controlling the muscle-specific transcription program. However, few p38-regulated genes that play a role in the regulation of myogenesis have been identified.RNA interference (RNAi), chemical inhibition and immunofluorescence approaches were used to assess the role of drebrin in differentiation of primary mouse myoblasts and C2C12 cells.In a search for p38-regulated genes that promote myogenic differentiation, we identified Dbn1, which encodes the actin-binding protein drebrin. Drebrin is an F-actin side-binding protein that remodels actin to facilitate the change of **filopodia** into dendritic spines during synaptogenesis in developing neurons. Dbn1 mRNA and protein are induced during differentiation of primary mouse and C2C12 myoblasts, and induction is substantially reduced by the p38 MAPK inhibitor SB203580. Primary myoblasts and C2C12 cells depleted of drebrin by RNAi display reduced levels of myogenin and myosin heavy chain and form multinucleated myotubes very inefficiently. Treatment of myoblasts with BTP2, a small-molecule inhibitor of drebrin, produces a phenotype similar to that produced by knockdown of drebrin, and the inhibitory effects of BTP2 are rescued by expression of a mutant form of drebrin that is unable to bind BTP2. Drebrin in myoblasts is enriched in cellular projections and cell cortices and at regions of cell-cell contact, all sites where F-actin, too, was concentrated.Our findings reveal that Dbn1 expression is a target of p38 MAPK signaling during myogenesis and that drebrin promotes myoblast differentiation.

### [Pp]odosome(s)?

#### PubMed

1. Osteoprotegerin induces **podosome** disassembly in osteoclasts through calcium, ERK, and p38 MAPK signaling pathways. Osteoclasts are critical for bone resorption and use **podosomes** to attach to bone matrix. Osteoprotegerin (OPG) is a negative regulator of osteoclast function that can affect the formation and function of **podosomes**. However, the signaling pathways that link OPG to **podosome** function have not been well characterized. Therefore, this study examined the roles of intracellular calcium and MAPKs in OPG-induced **podosome** disassembly in osteoclasts. We assessed the effects of the intracellular calcium chelator Bapta-AM, ERK inhibitor U0126, and p38 inhibitor SB202190 on OPG-treated osteoclast differentiation, adhesion structures, intracellular free Ca(2+) concentration and the phosphorylation state of **podosome** associated proteins (Pyk2 and Src). Mouse monocytic RAW 264.7 cells were differentiated to osteoclasts using RANKL (30ng/mL) and M-CSF (25ng/mL). The cells were pretreated with Bapta-AM (5μM), U0126 (5μM), or SB202190 (10μM) for 30min, followed by 40ng/mL OPG for 3h. Osteoclastogenesis, adhesion structure, viability and morphology, intracellular free Ca(2+) concentration and the phosphorylation state of Pyk2 and Src were measured by TRAP staining, scanning electron microscopy, real-time cell analyzer, flow cytometry and western blotting, respectively. OPG significantly inhibited osteoclastogenesis, the formation of adhesion structures, and reduced the amount of phosphorylated Pyk2 and Src-pY527, but increased phosphorylation of Src-pY416. Bapta-AM, U0126, and SB202190 partially restored osteoclast differentiation and adhesion structures. Both Bapta-AM and U0126, but not SB202190, restored the levels of intracellular free Ca(2+) concentration, phosphorylated Pyk2 and Src-pY527. All three inhibitors blocked OPG-induced phosphorylation at Src-pY416. These results suggest OPG disrupts the attachment structures of osteoclasts and activates Src as an adaptor protein that competes for the reduced amount of phosphorylated Pyk2 through calcium- and ERK-dependent signaling pathways. p38 MAPK signaling may have a different role in OPG-induced osteoclast retraction. Our findings potentially offer novel insights into the signaling mechanisms downstream of OPG that affect osteoclast attachment to the extracellular matrix.

### [Mm]igration

#### PubMed

1. Targeted deletion of the mitogen-activated protein kinase kinase 4 gene in the nervous system causes severe brain developmental defects and premature death. The c-Jun NH2-terminal protein kinase (JNK) is a mitogen-activated protein kinase (MAPK) involved in the regulation of various physiological processes. Its activity is increased upon phosphorylation by the MAPK kinases MKK4 and MKK7. The early embryonic death of mice lacking an mkk4 or mkk7 gene has provided genetic evidence that MKK4 and MKK7 have nonredundant functions in vivo. To elucidate the physiological role of MKK4, we generated a novel mouse model in which the mkk4 gene could be specifically deleted in the brain. At birth, the mutant mice were indistinguishable from their control littermates, but they stopped growing a few days later and died prematurely, displaying severe neurological defects. Decreased JNK activity in the absence of MKK4 correlated with impaired phosphorylation of a subset of physiologically relevant JNK substrates and with altered gene expression. These defects resulted in the misalignment of the Purkinje cells in the cerebellum and delayed radial **migration** in the cerebral cortex. Together, our data demonstrate for the first time that MKK4 is an essential activator of JNK required for the normal development of the brain.
2. Accumulating microglia phagocytose injured neurons in hippocampal slice cultures: involvement of p38 MAP kinase. In this study, microglial **migration** and phagocytosis were examined in mouse organotypic hippocampal slice cultures, which were treated with N-methyl-D-aspartate (NMDA) to selectively injure neuronal cells. Microglial cells were visualized by the expression of enhanced green fluorescent protein. Daily observation revealed microglial accumulation in the pyramidal cell layer, which peaked 5 to 6 days after NMDA treatment. Time-lapse imaging showed that microglia migrated to the pyramidal cell layer from adjacent and/or remote areas. There was no difference in the number of proliferating microglia between control and NMDA-treated slices in both the pyramidal cell layer and stratum radiatum, suggesting that microglial accumulation in the injured areas is mainly due to microglial **migration**, not to proliferation. Time-lapse imaging also showed that the injured neurons, which were visualized by propidium iodide (PI), disappeared just after being surrounded by microglia. Daily observation revealed that the intensity of PI fluorescence gradually attenuated, and this attenuation was suppressed by pretreatment with clodronate, a microglia toxin. These findings suggest that accumulating microglia phagocytosed injured neurons, and that PI fluorescence could be a useful indicator for microglial phagocytosis. Using this advantage to examine microglial phagocytosis in living slice cultures, we investigated the involvements of mitogen-activated protein (MAP) kinases in microglial accumulation and phagocytosis. p38 MAP kinase inhibitor SB203580, but not MAP kinase/extracellular signal-regulated kinase inhibitor PD98059 or c-Jun N-terminal kinase inhibitor SP600125, suppressed the attenuation of PI fluorescence. On the other hand, microglial accumulation in the injured areas was not inhibited by any of these inhibitors. These data suggest that p38 MAP kinase plays an important role in microglial phagocytosis of injured neurons.
3. Mammary gland specific expression of Brk/PTK6 promotes delayed involution and tumor formation associated with activation of p38 MAPK. Protein tyrosine kinases (PTKs) are frequently overexpressed and/or activated in human malignancies, and regulate cancer cell proliferation, cellular survival, and **migration**. As such, they have become promising molecular targets for new therapies. The non-receptor PTK termed breast tumor kinase (Brk/PTK6) is overexpressed in approximately 86% of human breast tumors. The role of Brk in breast pathology is unclear.We expressed a WAP-driven Brk/PTK6 transgene in FVB/n mice, and analyzed mammary glands from wild-type (wt) and transgenic mice after forced weaning. Western blotting and immunohistochemistry (IHC) studies were conducted to visualize markers of mammary gland involution, cell proliferation and apoptosis, as well as Brk, STAT3, and activated p38 mitogen-activated protein kinase (MAPK) in mammary tissues and tumors from WAP-Brk mice. Human (HMEC) or mouse (HC11) mammary epithelial cells were stably or transiently transfected with Brk cDNA to assay p38 MAPK signaling and cell survival in suspension or in response to chemotherapeutic agents.Brk-transgenic dams exhibited delayed mammary gland involution and aged mice developed infrequent tumors with reduced latency relative to wt mice. Consistent with delayed involution, mammary glands of transgenic animals displayed decreased STAT3 phosphorylation, a marker of early-stage involution. Notably, p38 MAPK, a pro-survival signaling mediator downstream of Brk, was activated in mammary glands of Brk transgenic relative to wt mice. Brk-dependent signaling to p38 MAPK was recapitulated by Brk overexpression in the HC11 murine mammary epithelial cell (MEC) line and human MEC, while Brk knock-down in breast cancer cells blocked EGF-stimulated p38 signaling. Additionally, human or mouse MECs expressing Brk exhibited increased anchorage-independent survival and resistance to doxorubicin. Finally, breast tumor biopsies were subjected to IHC analysis for co-expression of Brk and phospho-p38 MAPK; ductal and lobular carcinomas expressing Brk were significantly more likely to express elevated phospho-p38 MAPK.These studies illustrate that forced expression of Brk/PTK6 in non-transformed mammary epithelial cells mediates p38 MAPK phosphorylation and promotes increased cellular survival, delayed involution, and latent tumor formation. Brk expression in human breast tumors may contribute to progression by inducing p38-driven pro-survival signaling pathways.
4. The goya mouse mutant reveals distinct newly identified roles for MAP3K1 in the development and survival of cochlear sensory hair cells. Mitogen-activated protein kinase, MAP3K1, plays an important role in a number of cellular processes, including epithelial **migration** during eye organogenesis. In addition, studies in keratinocytes indicate that MAP3K1 signalling through JNK is important for actin stress fibre formation and cell **migration**. However, MAP3K1 can also act independently of JNK in the regulation of cell proliferation and apoptosis. We have identified a mouse mutant, goya, which exhibits the eyes-open-at-birth and microphthalmia phenotypes. In addition, these mice also have hearing loss. The goya mice carry a splice site mutation in the Map3k1 gene. We show that goya and kinase-deficient Map3k1 homozygotes initially develop supernumerary cochlear outer hair cells (OHCs) that subsequently degenerate, and a progressive profound hearing loss is observed by 9 weeks of age. Heterozygote mice also develop supernumerary OHCs, but no cellular degeneration or hearing loss is observed. MAP3K1 is expressed in a number of inner-ear cell types, including outer and inner hair cells, stria vascularis and spiral ganglion. Investigation of targets downstream of MAP3K1 identified an increase in p38 phosphorylation (Thr180/Tyr182) in multiple cochlear tissues. We also show that the extra OHCs do not arise from aberrant control of proliferation via p27KIP1. The identification of the goya mutant reveals a signalling molecule involved with hair-cell development and survival. Mammalian hair cells do not have the ability to regenerate after damage, which can lead to irreversible sensorineural hearing loss. Given the observed goya phenotype, and the many diverse cellular processes that MAP3K1 is known to act upon, further investigation of this model might help to elaborate upon the mechanisms underlying sensory hair cell specification, and pathways important for their survival. In addition, MAP3K1 is revealed as a new candidate gene for human sensorineural hearing loss.
5. NADPH oxidase contributes to conversion of cardiac myocytes to a proinflammatory phenotype in sepsis. It has been reported that polymorphonuclear leukocyte (PMN) infiltration into the myocardial interstitium is involved in sepsis-induced myocardial dysfunction. The aim of this study was to evaluate the role of NADPH oxidase in the sepsis-induced conversion of cardiomyocytes to a proinflammatory phenotype. Using an in vitro approach we evaluated the role of NADPH oxidase in cardiomyocyte CXC chemokine production and its ability to promote PMN transendothelial **migration** under septic conditions. Treatment of cardiac myocytes with septic plasma (1) activated NADPH oxidase (p47phox phosphorylation) and increased its activity (O(2)(-) production) and (2) converted them to a proinflammatory phenotype; both effects were prevented by blockade of NADPH oxidase. NF-kappaB nuclear translocation was increased in cardiomyocytes conditioned with septic plasma, a response prevented by blockade of NADPH oxidase. The increase in NF-kappaB activation/translocation was associated with phosphorylation of both IKK and the p65 subunit of NF-kappaB. Blockade of NADPH oxidase prevented phosphorylation of IKK, but not p65. Blockade approaches indicated that p38 MAP kinase (previously implicated in NF-kappaB activation) did not play a role in the NADPH oxidase pathway, either upstream or downstream. Collectively, the results of this study and those of previous reports indicate that the conversion of cardiomyocytes to a proinflammatory phenotype in sepsis involves two distinct pathways: NADPH oxidase-mediated phosphorylation of IKK and p38 MAP kinase-mediated phosphorylation of p65.
6. Structural and functional basis for p38-MK2-activated Rsk signaling in toll-like receptor-stimulated dendritic cells. Rsk kinases play important roles in several cellular processes such as proliferation, metabolism, and **migration**. Until recently, Rsk activation was thought to be exclusively initiated by Erk1/2, but in dendritic cells (DC) Rsk is also activated by p38 mitogen-activated protein (MAP) kinase via its downstream substrates, MK2/3. How and why this noncanonical configuration of the MAP kinase pathway is adopted by these key immune cells are not known. We demonstrate that the Erk1/2-activated C-terminal kinase domain of Rsk is dispensable for p38-MK2/3 activation and show that compared with fibroblasts, a greater fraction of p38 and MK2/3 is located in the cytosol of DC prior to stimulation, suggesting a partial explanation for the operation of the noncanonical pathway of Rsk activation in these cells. p38/MK2/3-activated Rsk phosphorylated downstream targets and is physiologically important because in plasmacytoid DC (pDC) stimulated with Toll-like receptor 7 (TLR7) agonists, Erk1/2 activation is very weak relative to p38. As a result, Rsk activation is entirely p38 dependent. We show that this unusual configuration of MAP kinase signaling contributes substantially to production of type I interferons, a hallmark of pDC activation.
7. APC and Smad7 link TGFβ type I receptors to the microtubule system to promote cell **migratio**n. Cell **migratio**n occurs by activation of complex regulatory pathways that are spatially and temporally integrated in response to extracellular cues. Binding of adenomatous polyposis coli (APC) to the microtubule plus ends in polarized cells is regulated by glycogen synthase kinase 3β (GSK-3β). This event is crucial for establishment of cell polarity during direction**al migrat**ion. However, the role of APC for cellular extension in response to extracellular signals is less clear. Smad7 is a direct target gene for transforming growth factor-β (TGFβ) and is known to inhibit various TGFβ-induced responses. Here we report a new function for Smad7. We show that Smad7 and p38 mitogen-activated protein kinase together regulate the expression of APC and **cell mig**ration in prostate cancer cells in response to TGFβ stimulation. In addition, Smad7 forms a complex with APC and acts as an adaptor protein for p38 and GSK-3β kinases to facilitate local TGFβ/p38-dependent inactivation of GSK-3β, accumulation of β-catenin, and recruitment of APC to the microtubule plus end in the leading edge of migrating prostate cancer cells. Moreover, the Smad7-APC complex links the TGFβ type I receptor to the microtubule system to regulate directed cellular extension and migratory responses evoked by TGFβ.
8. Human lysyl-tRNA synthetase is secreted to trigger proinflammatory response. Although aminoacyl-tRNA synthetases (ARSs) are essential for protein synthesis, they also function as regulators and signaling molecules in diverse biological processes. Here, we screened 11 different human ARSs to identify the enzyme that is secreted as a signaling molecule. Among them, we found that lysyl-tRNA synthetase (KRS) was secreted from intact human cells, and its secretion was induced by TNF-alpha. The secreted KRS bound to macrophages and peripheral blood mononuclear cells to enhance the TNF-alpha production and their **migration**. The mitogen-activated protein kinases, extracellular signal-regulated kinase and p38 mitogen-activated protein kinase, and Galphai were determined to be involved in the signal transduction triggered by KRS. All of these activities demonstrate that human KRS may work as a previously uncharacterized signaling molecule, inducing immune response through the activation of monocyte/macrophages.
9. Cripto is required for mesoderm and endoderm cell allocation during mouse gastrulation. During mouse gastrulation, cells in the primitive streak undergo epithelial-mesenchymal transformation and the resulting mesenchymal cells migrate out laterally to form mesoderm and definitive endoderm across the entire embryonic cylinder. The mechanisms underlying mesoderm and endoderm specification, **migration**, and allocation are poorly understood. In this study, we focused on the function of mouse Cripto, a member of the EGF-CFC gene family that is highly expressed in the primitive streak and migrating mesoderm cells on embryonic day 6.5. Conditional inactivation of Cripto during gastrulation leads to varied defects in mesoderm and endoderm development. Mutant embryos display accumulation of mesenchymal cells around the shortened primitive streak indicating a functional requirement of Cripto during the formation of mesoderm layer in gastrulation. In addition, some mutant embryos showed poor formation and abnormal allocation of definitive endoderm cells on embryonic day 7.5. Consistently, many mutant embryos that survived to embryonic day 8.5 displayed defects in ventral closure of the gut endoderm causing cardia bifida. Detailed analyses revealed that both the Fgf8-Fgfr1 pathway and p38 MAP kinase activation are partially affected by the loss of Cripto function. These results demonstrate a critical role for Cripto during mouse gastrulation, especially in mesoderm and endoderm formation and allocation.
10. Important role of p38 MAP kinase/NF-kappaB signaling pathway in the sepsis-induced conversion of cardiac myocytes to a proinflammatory phenotype. Septic plasma can convert murine cardiac myocytes to a proinflammatory phenotype. These myocytes 1) have increased nuclear levels of nuclear factor-kappaB (NF-kappaB), 2) release CXC chemokines, and 3) promote polymorphonuclear neutrophil (PMN) transendothelial **migration**. The purpose of the present study was to evaluate the role of the mitogen-activated protein (MAP) kinases [p38 MAP kinase, extracellular signal-regulated kinase (ERK) 1/2, and c-Jun NH(2)-terminal kinase (JNK)] as upstream intracellular signaling components involved in this phenomenon. Feces-induced peritonitis (FIP) was employed as a model of sepsis. In vitro, cardiac myocytes were treated with plasma (20%) obtained 6 h after either sham (saline) or FIP procedures. Myocyte supernatants were used for 1) detection of the CXC chemokines (enzyme-linked immunosorbent assay) and 2) assessment of their ability to promote PMN transendothelial **migration**. In vivo, myocardial PMN accumulation was assessed by measuring myeloperoxidase (MPO) activity and function (dF/dt and heart work). Treatment of cardiac myocytes with septic plasma activated p38 MAP kinase and ERK1/2, but not JNK. Blockade approaches (inhibitors or small-interference RNA) indicated that only p38 MAP kinase played a role in the conversion of the myocytes to a proinflammatory phenotype. Time course studies indicated that phosphorylation of p38 MAP kinase preceded the phosphorylation of NF-kappaB p65. Inhibition of p38 MAP kinase (SB-202190) blocked both NF-kappaB p65 phosphorylation and NF-kappaB nuclear translocation. Confirmatory studies in vivo indicated that FIP resulted in an increase in myocardial MPO activity and dysfunction, events reversed by the inhibitor of p38 MAP kinase. Collectively, these data indicate that the cardiomyocyte p38 MAP kinase/NF-kappaB signaling pathway plays an important role in the sepsis-induced conversion of myocytes to a proinflammatory phenotype.
11. Grhl3 and Lmo4 play coordinate roles in epidermal **migration**. In addition to its role in formation of the epidermal barrier, the mammalian transcription factor Grainy head-like 3 (Grhl3) is also essential for neural tube closure and wound repair, processes that are dependent in part on epidermal **migration**. Here, we demonstrate that the LIM-only domain protein, LMO4 serves as a functional partner of GRHL3 in its established roles, and define a new cooperative role for these factors in another developmental epidermal **migration** event, eyelid fusion. GRHL3 and LMO4 interact biochemically and genetically, with mutant mice exhibiting fully penetrant exencephaly, thoraco-lumbo-sacral spina bifida, defective skin barrier formation, and a co-incident eyes-open-at-birth (EOB) phenotype, which is not observed in the original individual null lines. The two genes are co-expressed in the surface ectoderm of the migrating eyelid root, and electron microscopy of Grhl3/Lmo4-null eyes reveals a failure in epithelial extension and a lack of peridermal clump formation at the eyelid margins. Accumulation of actin fibers is also absent in the circumference of these eyelids, and ERK1/2 phosphorylation is lost in the epidermis and eyelids of Grhl3(-/-)/Lmo4(-/-) embryos. Keratinocytes from mutant mice fail to "heal" in in vitro scratch assays, consistent with a general epidermal migratory defect that is dependent on ERK activation and actin cable formation.
12. Sensitized mast cells migrate toward the antigen: a response regulated by p38 mitogen-activated protein kinase and Rho-associated coiled-coil-forming protein kinase. Although mast cells accumulate within the mucosal epithelial layer of patients with allergic rhinitis and bronchial asthma, the responsible chemotactic factors are undefined. We investigated whether mast cells sensitized with Ag-specific IgE migrate toward the Ag. MC/9 mast cells sensitized with anti-DNP IgE migrated toward DNP-conjugated human serum albumin. This **migration** was directional, and the degree was stronger than that induced by stem cell factor. IL-3 and stem cell factor-dependent cultured mast cells derived from mouse bone marrow also migrated toward the Ag. Subsequent **migration** mediated by the Fc(epsilon)RI was significantly inhibited by incubating the cells with Y-27632, a Rho-associated coiled-coil-forming protein kinase inhibitor, or with SB203580, a p38 mitogen-activated protein kinase (MAPK) inhibitor. Both p38 MAPK and MAPK-activated protein kinase (MAPKAPK)2 were activated following Fc(epsilon)RI aggregation, and activation of MAPKAPK2 was almost completely inhibited by 10 microM SB203580. Wortmannin or a low concentration of SB203580 partially inhibited MAPKAPK2, but did not block mast cell **migration**. In contrast, Y-27632 did not affect the activation of MAPKAPK2. These results indicate that Ag works not only as a stimulant for allergic mediators from IgE-sensitized mast cells, but also as a chemotactic factor for mast cells. Both p38 MAPK activation and Rho-dependent activation of Rho-associated coiled-coil-forming protein kinase may be required for Fc(epsilon)RI-mediated cell **migration**.
13. Wnt5a and Wnt11 regulate mammalian anterior-posterior axis elongation. Mesoderm formation and subsequent anterior-posterior (A-P) axis elongation are fundamental aspects of gastrulation, which is initiated by formation of the primitive streak (PS). Convergent extension (CE) movements and epithelial-mesenchymal transition (EMT) are important for A-P axis elongation in vertebrate embryos. The evolutionarily conserved planar cell polarity (PCP) pathway regulates CE, and Wnts regulate many aspects of gastrulation including CE and EMT. However, the Wnt ligands that regulate A-P axis elongation in mammalian development remain unknown. Wnt11 and Wnt5a regulate axis elongation in lower vertebrates, but only Wnt5a, not Wnt11, regulates mammalian PCP signaling and A-P axis elongation in development. Here, by generating Wnt5a; Wnt11 compound mutants, we show that Wnt11 and Wnt5a play redundant roles during mouse A-P axis elongation. Both genes regulate trunk notochord extension through PCP-controlled CE of notochord cells, establishing a role for Wnt11 in mammalian PCP. We show that Wnt5a and Wnt11 are required for proper patterning of the neural tube and somites by regulating notochord formation, and provide evidence that both genes are required for the generation and **migration** of axial and paraxial mesodermal precursor cells by regulating EMT. Axial and paraxial mesodermal precursors ectopically accumulate in the PS at late gastrula stages in Wnt5a(-/-); Wnt11(-/-) embryos and these cells ectopically express epithelial cell adhesion molecules. Our data suggest that Wnt5a and Wnt11 regulate EMT by inducing p38 (Mapk14) phosphorylation. Our findings provide new insights into the role of Wnt5a and Wnt11 in mouse early development and also in cancer metastasis, during which EMT plays a crucial role.
14. SASH1 is a scaffold molecule in endothelial TLR4 signaling. Recognition of microbial products by TLRs is critical for mediating innate immune responses to invading pathogens. In this study, we identify a novel scaffold protein in TLR4 signaling called SAM and SH3 domain containing protein 1 (SASH1). Sash1 is expressed across all microvascular beds and functions as a scaffold molecule to independently bind TRAF6, TAK1, IκB kinase α, and IκB kinase β. This interaction fosters ubiquitination of TRAF6 and TAK1 and promotes LPS-induced NF-κB, JNK, and p38 activation, culminating in increased production of proinflammatory cytokines and increased LPS-induced endothe**lial migr**ation. Our findings suggest that SASH1 acts to assemble a signaling complex downstream of TLR4 to activate early endothelial responses to receptor activation.
15. CXCL12 and C5a trigger cell **migration** via a PAK1/2-p38alpha MAPK-MAPKAP-K2-HSP27 pathway. Cell **migration** is critical for many processes, such as angiogenesis, inflammation, development and wound healing, and is also involved in tumour progression and metastasis. Here we show that CXCL12, complement factor 5a (C5a), hepatocyte growth factor (HGF) and platelet-derived growth factor (PDGF)-BB, which stimulate cell **migration**, also activate p38alpha MAPK. Pharmacological inhibition of this protein kinase with SB 203580 or BIRB 0796, or the genetic ablation of p38alpha MAPK, blocked cell **migration** induced by the aforementioned chemo-attractants. Macrophages from mice lacking one or more of the other p38 MAPK isoforms showed normal cell **migration** in response to C5a. We also show that the activation of p38alpha MAPK in response to CXCL12 requires the p21-activated protein kinases (PAK)-1 and PAK-2. MAPKAP-K2 is a protein kinase that is activated by p38alpha MAPK. Reducing its expression using RNA interference blocked CXCL12-induced HeLa cell **migration**, while macrophages from mice that do not express MAPKAP-K2 failed to migrate in response to C5a. Moreover, RNA interference against the small heat shock protein 27 (HSP27), a physiological substrate of MAPKAP-K2, blocked the CXCL12-induced cell **migration**. These results demonstrate a general and essential role of the PAK-p38alpha MAPK-MAPKAP-K2-HSP27 signalling pathway in mediating the effects of chemotactic stimuli on cell **migration**.
16. Epidermal growth factor-stimulated intestinal epithelial cell **migration** requires Src family kinase-dependent p38 MAPK signaling. Members of the epidermal growth factor (EGF) family of ligands and their receptors regulate **migration** and growth of intestinal epithelial cells. However, our understanding of the signal transduction pathways determining these responses is incomplete. In this study we tested the hypothesis that p38 is required for EGF-stimulated intestinal epithelial monolayer restitution. EGF-stimulated **migration** in a wound closure model required continuous presence of ligand for several hours for maximal response, suggesting a requirement for sustained signal transduction pathway activation. In this regard, prolonged exposure of cells to EGF activated p38 for up to 5 h. Furthermore genetic or pharmacological blockade of p38 signaling inhibited the ability of EGF to accelerate wound closure. Interestingly p38 inhibition was associated with increased EGF-stimulated ERK1/ERK2 phosphorylation and cell proliferation, suggesting that p38 regulates the balance of proliferation/**migration** signaling in response to EGF receptor activity. Activation of p38 in intestinal epithelial cells through EGF receptor was abolished by blockade of Src family tyrosine kinase signaling but not inhibition of phosphatidylinositol 3-kinase or protein kinase C. Taken together, these data suggest that Src family kinase-dependent p38 activation is a key component of a signaling switch routing EGF-stimulated responses to epithelial cell **migration**/restitution rather than proliferation during wound closure.
17. Mechanisms of kringle fragment of urokinase-induced vascular smooth muscle cell **migration**. Urokinase plasminogen activator (uPA) is involved in vessel remodeling and mediates smooth muscle cell **migration**. **Migration** in response to uPA is dependent on both the growth factor binding domain at the aminoterminal end and the kringle (K) domain of the molecule. uPA is readily degraded in vivo into these constitutive domains. The aim of this study was to examine cell signaling during the **migration** of smooth muscle cell in response to the kringle domain of urokinase.Murine arterial smooth muscle cells were cultured in vitro. **Migration** assays were performed in the presence of K with and without the plasmin inhibitors (aprotinin and -aminocaproic acid), the Galphai inhibitor Pertussis toxin, the MMP inhibitor (GM6001), the PI3-K inhibitors, Wortmannin and LY294002, and the MAPK inhibitors PD98089 (MEK1 inhibitor) and SB203580 (p38(MAPK) inhibitor). Western blotting was performed for ERK 1/2 and p38(MAPK) phosphorylation after stimulation with K in the presence and absence of the inhibitors. Statistics were analyzed by one-way ANOVA (n = 6).The kringle domain (K) induced a plasmin-independent, MMP-dependent increase in cell **migration** (2-fold, P < 0.05) compared to control. This migratory response to K was Galphai mediated and dependent on both ERK 1/2 and p38(MAPK) activation. K induced time-dependent increases in the phosphorylation of ERK 1/2 (3-fold, P < 0.05) and p38(MAPK) (3-fold, P < 0.05). Activation of p38(MAPK) and ERK 1/2 was completely inhibited by the PI3-K inhibitors. We explored a potential role for the epidermal growth factor receptor (EGFR). K induced EGFR phosphorylation and the presence of AG1478, the EGFR inhibitor, inhibited both cell **migration** and akt activation in response to K.Kringle domain of uPA induces smooth muscle cell **migration** through a G-protein-coupled PI3-K-dependent process involving both ERK 1/2 and p38(MAPK) and is mediated in part through EGFR. Defining the differences in response to key molecular domains of uPA is vital to understand its role in vessel remodeling.
18. PTEN functions to 'prioritize' chemotactic cues and prevent 'distraction' in migrating neutrophils. Neutrophils encounter and 'prioritize' many chemoattractants in their pursuit of bacteria. Here we tested the possibility that the phosphatase PTEN is responsible for the prioritization of chemoattractants. Neutrophils induced chemotaxis by two separate pathways, the phosphatidylinositol-3-OH kinase (PI(3)K) phosphatase and tensin homolog (PTEN) pathway, and the p38 mitogen-activated protein kinase pathway, with the p38 pathway dominating over the PI(3)K pathway. Pten(-/-) neutrophils could not prioritize chemoattractants and were 'distracted' by chemokines when moving toward bacterial chemoattractants. In opposing gradients, PTEN became distributed throughout the cell circumference, which inhibited all PI(3)K activity, thus permitting 'preferential' **migration** toward bacterial products via phospholipase A(2) and p38. Such prioritization was defective in Pten(-/-) neutrophils, which resulted in defective bacterial clearance in vivo. Our data identify a PTEN-dependent mechanism in neutrophils to prioritize, 'triage' and integrate responses to multiple chemotactic cues.
19. MAPK-upstream protein kinase (MUK) regulates the radial **migration** of immature neurons in telencephalon of mouse embryo. The radial **migration** of differentiating neurons provides an essential step in the generation of laminated neocortex, although its molecular mechanism is not fully understood. We show that the protein levels of a JNK activator kinase, MUK/DLK/ZPK, and JNK activity increase potently and temporally in newly generated neurons in developing mouse telencephalon during radial **migration**. The ectopic expression of MUK/DLK/ZPK in neural precursor cells in utero impairs radial **migration**, whereas it allows these cells to leave the ventricular zone and differentiate into neural cells. The MUK/DLK/ZPK protein is associated with dotted structures that are frequently located along microtubules and with Golgi apparatus in cultured embryonic cortical cells. In COS-1 cells, MUK/DLK/ZPK overexpression impairs the radial organization of microtubules without massive depolymerization. These results suggest that MUK/DLK/ZPK and JNK regulate radial cell **migration** via microtubule-based events.
20. p38 MAPK-mediated regulation of Xbp1s is crucial for glucose homeostasis. Here we show that p38 mitogen-activated protein kinase (p38 MAPK) phosphorylates the spliced form of X-box binding protein 1 (Xbp1s) on its Thr48 and Ser61 residues and greatly enhances its nuclear **migration** in mice, whereas mutation of either residue to alanine substantially reduces its nuclear translocation and activity. We also show that p38 MAPK activity is markedly reduced in the livers of obese mice compared with lean mice. Further, we show that activation of p38 MAPK by expression of constitutively active MAP kinase kinase 6 (MKK6Glu) greatly enhances nuclear translocation of Xbp1s, reduces endoplasmic reticulum stress and establishes euglycemia in severely obese and diabetic mice. Hence, our results define a crucial role for phosphorylation on Thr48 and Ser61 of Xbp1s in the maintenance of glucose homeostasis in obesity, and they suggest that p38 MAPK activation in the livers of obese mice could lead to a new therapeutic approach to the treatment of type 2 diabetes.
21. Negative regulation of Odd-skipped related 2 by TGF-beta achieves the induction of cellular **migration** and the arrest of cell cycle. The transcription factor Odd-skipped related 2 (Osr2) functions in craniofacial and limb developments in mammals. We previously found that Osr2 gene expression is regulated by intracellular transcription factors such as Runx2, and C/EBP, whereas it remains unclear if extracellular factors would functionally regulate the Osr2 expression. In this study, we showed that TGF-β down-regulated the Osr2 expression, which is involved in regulation of cellular **migratio**n and cell cycle. Furthermore, the down-regulation was found to be mediated by Smad3/Smad4 and p38/ATF2 signaling molecules. The Osr2 promoter was shown to possess Smad3/4 binding element and ATF2 binding element between -647 and -64 of promoter. TGF-β induced cellula**r migrati**on in C3H10T1/2 cells and arrested cell cycle at G1 phase in NMuMG-Fucci cells. In contrast, the Osr2 reduced th**e migrati**on and also stimulated the cell-cycle progression. These results suggest that Osr2 is involved in TGF-β regulating ce**ll migrat**ion and cell cycle via a Smad3-ATF2 transcriptional complex mediating pathway.
22. p38 and a p38-interacting protein are critical for downregulation of E-cadherin during mouse gastrulation. During vertebrate gastrulation, an epithelial to mesenchymal transition (EMT) is necessary for **migration** of mesoderm from the primitive streak. We demonstrate that p38 MAP kinase and a p38-interacting protein (p38IP) are critically required for downregulation of E-cadherin during gastrulation. In an ENU-mutagenesis screen we identified the droopy eye (drey) mutation, which affects splicing of p38IP. p38IP(drey) mutant embryos display incompletely penetrant defects in neural tube closure, eye development, and gastrulation. A stronger allele (p38IP(RRK)) exhibits gastrulation defects in which mesoderm **migration** is defective due to deficiency in E-cadherin protein downregulation in the primitive streak. We show that p38IP binds directly to p38 and is required for p38 activation in vivo. Moreover, both p38 and p38IP are required for E-cadherin downregulation during gastrulation. Finally, p38 regulates E-cadherin protein expression downstream from NCK-interacting kinase (NIK) and independently of the regulation of transcription by Fibroblast Growth Factor (Fgf) signaling and Snail.
23. Inhibitor of NF-kappa B kinases alpha and beta are both essential for high mobility group box 1-mediated chemotaxis [corrected]. Inhibitor of NF-kappaB kinases beta (IKKbeta) and alpha (IKKalpha) activate distinct NF-kappaB signaling modules. The IKKbeta/canonical NF-kappaB pathway rapidly responds to stress-like conditions, whereas the IKKalpha/noncanonical pathway controls adaptive immunity. Moreover, IKKalpha can attenuate IKKbeta-initiated inflammatory responses. High mobility group box 1 (HMGB1), a chromatin protein, is an extracellular signal of tissue damage-attracting cells in inflammation, tissue regeneration, and scar formation. We show that IKKalpha and IKKbeta are each critically important for HMGB1-elicited chemotaxis of fibroblasts, macrophages, and neutrophils in vitro and neutrophils in vivo. By time-lapse microscopy we dissected different parameters of the HMGB1 **migration** response and found that IKKalpha and IKKbeta are each essential to polarize cells toward HMGB1 and that each kinase also differentially affects cellular velocity in a time-dependent manner. In addition, HMGB1 modestly induces noncanonical IKKalpha-dependent p52 nuclear translocation and p52/RelB target gene expression. Akin to IKKalpha and IKKbeta, p52 and RelB are also required for HMGB1 chemotaxis, and p52 is essential for cellular orientation toward an HMGB1 gradient. RAGE, a ubiquitously expressed HMGB1 receptor, is required for HMGB1 chemotaxis. Moreover, IKKbeta, but not IKKalpha, is required for HMGB1 to induce RAGE mRNA, suggesting that RAGE is at least one IKKbeta target involved in HMGB1 **migration** responses, and in accord with these results enforced RAGE expression rescues the HMGB1 **migration** defect of IKKbeta, but not IKKalpha, null cells. Thus, proinflammatory HMGB1 chemotactic responses mechanistically require the differential collaboration of both IKK-dependent NF-kappaB signaling pathways.
24. The phosphatidylinositol 3-kinase, p38, and extracellular signal-regulated kinase pathways are involved in osteoclast differentiation. Phosphatidylinositol 3-kinase (PI 3-kinase) and mitogen-activated protein kinases (MAPKs) have been implicated in diverse cellular functions, including proliferation, **migration**, and survival. In this study, we examined the involvement of these kinases in osteoclast differentiation by employing specific inhibitors of the kinases. The osteoclast differentiation was assessed in three different culture systems: a coculture of mouse bone marrow cells with mouse calvarial osteoblasts, a mouse bone marrow cell culture in the presence of receptor activator of NF-kappaB ligand (RANKL) and macrophage-colony stimulating factor (M-CSF), and a culture of bone-resident osteoclast precursor cells driven by RANKL and M-CSF. LY294002, a specific inhibitor of PI 3-kinase, potently inhibited osteoclast differentiation in all culture systems when assessed by both tartrate-resistant acid phosphatase (TRAP) staining and dentine resorption assays. Inhibition of p38 MAPK by SB202190 resulted in a strong suppression in the exogenous RANKL dependent mouse bone marrow and bone resident precursor cell cultures. Another MAPK pathway inhibitor (PD98059), which blocks the activation of extracellular signal-regulated kinase (ERK) by inhibiting the upstream kinase MAPK-ERK kinase (MEK) 1, exerted an inhibitory effect on osteoclast differentiation only at the highest concentration tested (30 micromol/L) in many cases. Whether the signaling pathways involving these kinases are activated by RANKL was also examined. The RANKL-stimulated phosphorylation of Akt, a downstream target of PI 3-kinase, and that of ERK were observed. RANKL also stimulated the activity of p38. These results suggest that PI 3 kinase, p38, and ERK play roles in osteoclast differentiation, at least in part, by participating in RANKL signaling.
25. Loss of mitogen-activated protein kinase kinase kinase 4 (MAP3K4) reveals a requirement for MAPK signalling in mouse sex determination. Sex determination in mammals is controlled by the presence or absence of the Y-linked gene SRY. In the developing male (XY) gonad, sex-determining region of the Y (SRY) protein acts to up-regulate expression of the related gene, SOX9, a transcriptional regulator that in turn initiates a downstream pathway of testis development, whilst also suppressing ovary development. Despite the requirement for a number of transcription factors and secreted signalling molecules in sex determination, intracellular signalling components functioning in this process have not been defined. Here we report a role for the phylogenetically ancient mitogen-activated protein kinase (MAPK) signalling pathway in mouse sex determination. Using a forward genetic screen, we identified the recessive boygirl (byg) mutation. On the C57BL/6J background, embryos homozygous for byg exhibit consistent XY gonadal sex reversal. The byg mutation is an A to T transversion causing a premature stop codon in the gene encoding MAP3K4 (also known as MEKK4), a mitogen-activated protein kinase kinase kinase. Analysis of XY byg/byg gonads at 11.5 d post coitum reveals a growth deficit and a failure to support mesonephric cell **migration**, both early cellular processes normally associated with testis development. Expression analysis of mutant XY gonads at the same stage also reveals a dramatic reduction in Sox9 and, crucially, Sry at the transcript and protein levels. Moreover, we describe experiments showing the presence of activated MKK4, a direct target of MAP3K4, and activated p38 in the coelomic region of the XY gonad at 11.5 d post coitum, establishing a link between MAPK signalling in proliferating gonadal somatic cells and regulation of Sry expression. Finally, we provide evidence that haploinsufficiency for Map3k4 accounts for T-associated sex reversal (Tas). These data demonstrate that MAP3K4-dependent signalling events are required for normal expression of Sry during testis development, and create a novel entry point into the molecular and cellular mechanisms underlying sex determination in mice and disorders of sexual development in humans.
26. TAK1 kinase signaling regulates embryonic angiogenesis by modulating endothelial cell survival and **migration**. TGF-β activated kinase 1 (TAK1) is a mediator of various cytokine signaling pathways. Germline deficiency of Tak1 causes multiple abnormalities, including dilated blood vessels at midgestation. However, the mechanisms by which TAK1 regulates vessel formation have not been elucidated. TAK1 binding proteins 1 and 2 (TAB1 and TAB2) are activators of TAK1, but their roles in embryonic TAK1 signaling have not been determined. In the present study, we characterized mouse embryos harboring endothelial-specific deletions of Tak1, Tab1, or Tab2 and found that endothelial TAK1 and TAB2, but not TAB1, were critically involved in vascular formation. TAK1 deficiency in endothelial cells caused increased cell death and vessel regression at embryonic day 10.5 (E10.5). Deletion of TNF signaling largely rescued endothelial cell death in TAK1-deficient embryos at E10.5. However, embryos deficient in both TAK1 and TNF signaling still exhibited dilated capillary networks at E12.5. TAB2 deficiency caused reduced TAK1 activity, resulting in abnormal capillary blood vessels, similar to the compound deficiency of TAK1 and TNF signaling. Ablation of either TAK1 or TAB2 impaired cell **migratio**n and tube formation. Our results show that endothelial TAK1 signaling is important for 2 biologic processes in angiogenesis: inhibiting TNF-dependent endothelial cell death and promoting TNF-independent angiogenic cell **migratio**n.
27. Fbxo45 forms a novel ubiquitin ligase complex and is required for neuronal development. Fbxo45 is an F-box protein that is restricted to the nervous system. Unlike other F-box proteins, Fbxo45 was found not to form an SCF complex as a result of an amino acid substitution in the consensus sequence for Cul1 binding. Proteomics analysis revealed that Fbxo45 specifically associates with PAM (protein associated with Myc), a RING finger-type ubiquitin ligase. Mice deficient in Fbxo45 were generated and found to die soon after birth as a result of respiratory distress. Fbxo45(-)(/)(-) embryos show abnormal innervation of the diaphragm, impaired synapse formation at neuromuscular junctions, and aberrant development of axon fiber tracts in the brain. Similar defects are also observed in mice lacking Phr1 (mouse ortholog of PAM), suggesting that Fbxo45 and Phr1 function in the same pathway. In addition, neuronal **migration** was impaired in Fbxo45(-)(/)(-) mice. These results suggest that Fbxo45 forms a novel Fbxo45-PAM ubiquitin ligase complex that plays an important role in neural development.
28. p38 kinase regulates epidermal growth factor receptor downregulation and cellular **migration**. Internalization and proteolytic degradation of epidermal growth factor (EGF) receptor (R) following ligand binding is an important mechanism for regulating EGF-stimulated signals. Using pharmacological and RNA interference inhibition of p38 mitogen-activated protein kinase, we show that p38 is required for efficient EGF-induced EGFR destruction but not internalization. In the absence of p38 activity, EGF fails to stimulate the ubiquitin ligase Cbl or ubiquitinylation of EGFR, and internalized EGFR accumulates in intracellular vesicles containing caveolin-1. These effects are accompanied by loss of EGFR phosphorylation on Y1045, a phosphorylation site required for Cbl activation. Furthermore, similar to cells treated with p38 inhibitors, intestinal epithelial cells expressing Y1045F EGFR mutants show increased proliferation but not **migration** in response to EGF, thus uncoupling these biological responses. Together these data position p38 as a modulator of ligand-stimulated EGFR processing and demonstrate that this processing has a profound impact on the cellular outcome of EGFR signaling.
29. MEKK1 transduces activin signals in keratinocytes to induce actin stress fiber formation and **migration**. Activins and other members of the transforming growth factor beta family play a critical role in morphological changes of the epidermis that require epithelial cell movement. We investigated the molecular pathways in the transmission of activin signals that lead to actin reorganization and epithelial cell **migration**. We found that activins cause the activation of RhoA but not of Rac and CDC42, leading to MEKK1-dependent phosphorylation of JNK and transcription factor c-Jun. Through a RhoA-independent mechanism, the activins also induce p38 activity in keratinocytes from wild-type but not from MEKK1-deficient mice. Although neither pathway is dependent on Smad activation, the MEKK1-mediated JNK and p38 activities are both essential for activin-stimulated and transcription-dependent keratinocyte **migration**. Only JNK is involved in transcription-independent actin stress fiber formation, which needs also the activity of ROCK. Because ROCK is required for JNK activation by RhoA and its overexpression leads to MEKK1 activation, we propose a RhoA-ROCK-MEKK1-JNK pathway and a MEKK1-p38 pathway as Smad-independent mechanisms in the transmission of activin signals. Together, these pathways lead to the control of actin cytoskeleton reorganization and epithelial cell **migration**, contributing to the physiologic and pathological effects of activins on epithelial morphogenesis.
30. The p38α mitogen-activated protein kinase is a key regulator of myelination and remyelination in the CNS. The p38α mitogen-activated protein kinase (MAPK) is one of the serine/threonine kinases regulating a variety of biological processes, including cell-type specification, differentiation an**d migrati**on. Previous in vitro studies using pharmacological inhibitors suggested that p38 MAPK is essential for oligodendrocyte (OL) differentiation and myelination. To investigate the specific roles of p38α MAPK in OL development and myelination in vivo, we generated p38α conditional knockout (CKO) mice under the PLP and nerve/glial antigen 2 (NG2) gene promoters, as these genes are specifically expressed in OL progenitor cells (OPCs). Our data revealed that myelin synthesis was completely inhibited in OLs differentiated from primary OPC cultures derived from the NG2 Cre-p38α CKO mouse brains. Although an in vivo myelination defect was not obvious after gross examination of these mice, electron microscopic analysis showed that the ultrastructure of myelin bundles was severely impaired. Moreover, the onset of myelination in the corpus callosum was delayed in the knockout mice compared with p38α fl/fl control mice. A delay in OL differentiation in the central nervous system was observed with concomitant downregulation in the expression of OPC- and OL-specific genes such as Olig1 and Zfp488 during early postnatal development. OPC proliferation was not affected during this time. These data indicate that p38α is a positive regulator of OL differentiation and myelination. Unexpectedly, we observed an opposite effect of p38α on remyelination in the cuprizone-induced demyelination model. The p38α CKO mice exhibited better remyelination capability compared with p38α fl/fl mice following demyelination. The opposing roles of p38α in myelination and remyelination could be due to a strong anti-inflammatory effect of p38α or a dual reciprocal regulatory action of p38α on myelin formation during development and on remyelination after demyelination.
31. Bidirectional regulation of neutrophil **migration** by mitogen-activated protein kinases. To kill invading bacteria, neutrophils must interpret spatial cues, migrate and reach target sites. Although the initiation of chemotactic **migration** has been extensively studied, little is known about its termination. Here we found that two mitogen-activated protein kinases (MAPKs) had opposing roles in neutrophil trafficking. The extracellular signal-regulated kinase Erk potentiated activity of the G protein-coupled receptor kinase GRK2 and inhibited neutrophil **migration**, whereas the MAPK p38 acted as a noncanonical GRK that phosphorylated the formyl peptide receptor FPR1 and facilitated neutrophil **migration** by blocking GRK2 function. Therefore, the dynamic balance between Erk and p38 controlled neutrophil 'stop' and 'go' activity, which ensured that neutrophils reached their final destination as the first line of host defense.
32. Real-time imaging reveals that P2Y2 and P2Y12 receptor agonists are not chemoattractants and macrophage chemotaxis to complement C5a is phosphatidylinositol 3-kinase (PI3K)- and p38 mitogen-activated protein kinase (MAPK)-independent. Adenosine 5'-triphosphate (ATP) has been implicated in the recruitment of professional phagocytes (neutrophils and macrophages) to sites of infection and tissue injury in two distinct ways. First, ATP itself is thought to be a chemotactic "find me" signal released by dying cells, and second, autocrine ATP signaling is implicated as an amplifier mechanism for chemotactic navigation to end-target chemoattractants, such as complement C5a. Here we show using real-time chemotaxis assays that mouse peritoneal macrophages do not directionally migrate to stable analogs of ATP (adenosine-5'-(γ-thio)-triphosphate (ATPγS)) or its hydrolysis product ADP (adenosine-5'-(β-thio)-diphosphate (ADPβS)). HPLC revealed that these synthetic P2Y(2) (ATPγS) and P2Y(12) (ADPβS) receptor ligands were in fact slowly degraded. We also found that ATPγS, but not ADPβS, promoted chemokinesis (increased **random m**igration). Furthermore, we found that photorelease of ATP or ADP induced lamellipodial membrane extensions. At the cell signaling level, C5a, but not ATPγS, activated Akt, whereas both ligands induced p38 MAPK activation. p38 MAPK and Akt activation are strongly implicated in neutrophil chemotaxis. However, we found that inhibitors of phosphatidylinositol 3-kinase (PI3K; upstream of Akt) and p38 MAPK (or conditional deletion of p38α MAPK) did not impair macrophage chemotactic effi**ciency or** migration velocity. Our results suggest that PI3K and p38 MAPK are redundant for macrophage chemotaxis and that purinergic P2Y(2) and P2Y(12) receptor ligands are not chemotactic. We propose that ATP signaling is strictly autocrine or paracrine and that ATP and ADP may act as short-range "touch me" (rather than long-range find me) signals to promote phagocytic clearance via cell spreading.
33. Eosinophil **migration** induced by mast cell chymase is mediated by extracellular signal-regulated kinase pathway. Mast cell chymase is known to induce eosinophil **migration** in vivo and in vitro. In the present study, we investigated possible involvement of mitogen-activated protein (MAP) kinases; extracellular signal-regulated kinase (ERK), c-Jun amino-terminal kinase (JNK), and p38, in the chymase-induced eosinophil **migration**. Human chymase induced a rapid phosphorylation of ERK1/2 and p38 in human eosinophilic leukemia EoL-1 cells, while no phosphorylation was detected in JNK. The chymase-induced phosphorylation of ERK and p38 was inhibited by pertussis toxin. Similar results were obtained in the experiments using mouse chymase and eosinophils. U0126 (the inhibitor for MAP/ERK kinase) suppressed chymase-induced **migration** of EoL-1 cells and mouse eosinophils. However, SB203580 (p38 inhibitor) and SP600125 (JNK inhibitor) showed little effect on the **migration**. It is suggested therefore that chymase activates ERK and p38 probably through G-protein-coupled receptor, and that ERK but not p38 cascade may have a crucial role in chymase-induced **migration** of eosinophils.
34. Lipopolysaccharide: a p38 MAPK-dependent disrupter of neutrophil chemotaxis. In sepsis, and in models of sepsis including endotoxemia, impaired neutrophil recruitment and chemotaxis have been reported. The inability of the endotoxemic neutrophil to chemotax could be attributed to the fact that intracellular signaling via LPS overrides signals from endogenous chemokines or, alternatively, that sequestration of neutrophils into lungs prevents access to peripheral tissues. Using both in vitro and in vivo chemotaxis assays the authors established that neutrophils from healthy mice chemotaxed in vivo toward MIP-2, whereas endotoxemic neutrophils did not. Since LPS activates leukocytes via the p38 MAPK pathway, SKF86002, a p38 MAPK inhibitor, was given to endotoxemic animals. SKF86002 significantly reversed the LPS-induced impairment in e**migration** of endotoxic neutrophils in response to MIP-2. Neutrophil chemotaxis in vitro was also impaired by LPS, via a p38 MAPK-dependent pathway, and this impairment could be reversed via p38 MAPK inhibition. Although neutrophil numbers dropped in the circulation and trapped in lungs during endotoxemia, SKF86002 did not reverse these parameters, demonstrating that p38 MAPK inhibition did not release trapped neutrophils from the lungs. In conclusion, the data suggest that the impaired e**migration** and chemotaxis of neutrophils at peripheral sites during endotoxemia may be partially due to a p38 MAPK-mediated inhibition of neutrophil responses to endogenous chemokines.
35. Genetic evidence for critical roles of P38α protein in regulating mast cell differentiation and chemotaxis through distinct mechanisms. Mast cells mediate a range of immune responses. However, the mechanisms that contribute to their development remain poorly understood. Here, using a P38α conditional knockout system, we provide evidence to suggest that P38α plays critical roles in regulating mast cell differentiation a**nd migrat**ion via distinct mechanisms. Induced deletion of P38α in bone marrow cells retards the maturation of mast cells in part by inhibiting the activation of cAMP response element-binding protein and expression of microphthalmia-associated transcription factor, which encourages the generation of basophils over mast cells. In fully differentiated mast cells, absence of P38α inhibits stem cell factor-induced activation of Akt and ERK, which is associated with reduced chemotaxis. In vivo, conditional deletion of P38α results in reduced numbers of mast cells in certain tissues and a failure to reconstitute these cells in W(sh) mice transplanted with P38α(-/-) Lin(-)c-kit(+)Sca-1(+) (LKS(+)) cells. Our findings suggest that P38α plays a dual role in mast cell development by regulating IL-3-induced differentiation of mast cell progenitor cells as well as by regulating stem cell factor-**induced m**igration of fully differentiated mast cells.
36. Apolipoprotein B is a new target of the GDNF/RET and ET-3/EDNRB signalling pathways. GDNF/RET and Endothelin-3 (ET-3)/EDNRB regulate survival, differentiation, **migration**, and proliferation of neural crest-derived cells. Although several RET and EDNRB signalling mediators have been characterized, most of the genes targeted by these two pathways are still largely unknown. We focused our study on apolipoprotein B (APOB) as a novel target gene of the RET and EDNRB pathways, based on previous data obtained using a Caenorhabditis elegans strain mutant for the homologue of mammalian ECE1.Molecular and cellular studies of Apob were performed in the murine Neuro2a cells, an in vitro model for studying neural crest-derived cell development, along with a mouse knock-in for the Hirschsprung-associated mutation Ret(C620R). Silencing for Apob and Ret has been performed via shRNA.GDNF/RET and ET-3/EDNRB cooperated in inducing neuronal differentiation resulting in Apob activation in Neuro2a cell line. Apob expression was downregulated in mouse embryos homozygous for the Ret(C620R) mutation and presenting a severe Hirschsprung phenotype. Ret silencing prevented Apob expression increase. MAPK P38 kinase activation evoked Apob expression via GDNF/RET signalling in Neuro2a cells. A p53-dependent repressor element in Apob promoter resulted in a reduced Apob expression. Silencing of Apob reduced HuD protein expression.Apob is a novel downstream target of the RET/EDNRB pathways with a role in neuronal survival and maintenance, as indicated by its effect on HuD expression. Our data provide a conceptual framework to investigate and establish the role of APOB gene in severe gut dysmotility.
37. Casein kinase 2- and protein kinase A-regulated adenomatous polyposis coli and beta-catenin cellular localization is dependent on p38 MAPK. Skin cancer is the most common form of malignancy in the world with epidemic proportions. Identifying the biochemical and molecular mechanisms underlying the events leading to tumors is paramount to designing new and effective treatments that may aid in treating and/or preventing skin cancers. Herein we identify p38 MAPK, along with its positive modulator, Gadd45a, as important regulators of nucleocytoplasmic shuttling of the adenomatous polyposis coli (APC) tumor suppressor. APC normally functions to block beta-catenin from promoting cell proliferation and **migration**/invasion. Keratinocytes lacking proper p38 MAPK activation, either due to lack of Gadd45a or through the use of p38 MAPK-specific inhibitors, are unable to effectively transport APC into the nucleus. We also show that p38 MAPK is able to directly associate with and modulate both casein kinase 2 (CK2) and protein kinase A (PKA), which promote and block APC nuclear import, respectively. We demonstrate that p38 MAPK is able to not only enhance CK2 kinase activity but also suppress PKA kinase activity. Moreover, lack of normal p38 MAPK activity in either Gadd45a-null keratinocytes or in p38 MAPK inhibitor treated keratinocytes leads to decreased CK2 activity and increased PKA activity. In either case, disruption of APC nuclear import results in elevated levels of free cellular, and potentially oncogenic, beta-catenin. Numerous tumors, including skin cancers, are associated with high levels of beta-catenin, and our data indicate that p38 MAPK signaling, along with Gadd45a, may provide tumor suppressor-like functions in part by promoting APC nuclear localization and effective beta-catenin regulation.

#### GeneRIFs

1. Src family kinase-dependent p38 activation is a key component of a signaling switch routing EGF-stimulated responses to epithelial cell **migration**
2. mast cell chymase activates ERK and p38 probably through G-protein-coupled receptor, and the ERK but not p38 cascade may have a crucial role in chymase-induced **migration** of eosinophils
3. Here we show that CXCL12, complement factor 5a (C5a), hepatocyte growth factor (HGF) and platelet-derived growth factor (PDGF)-BB, which stimulate cell **migration**, also activate p38alpha MAPK
4. the impaired e**migration** and chemotaxis of neutrophils at peripheral sites during endotoxemia may be partially due to a p38 MAPK-mediated inhibition of neutrophil responses to endogenous chemokines
5. Kringle domain of uPA induces smooth muscle cell **migration** through a G-protein-coupled PI3-K-dependent process involving both ERK 1/2 and p38(MAPK) and is mediated in part through EGFR.
6. p38 MAP kinase regulates the **migration** of IgE-sensitized mast cells toward a specific antigen, and its activation may be required for Fc epsilon RI-mediated cell **migration**.

### ECM

#### PubMed

1. FGF signalling regulates bone growth through autophagy. Skeletal growth relies on both biosynthetic and catabolic processes. While the role of the former is clearly established, how the latter contributes to growth-promoting pathways is less understood. Macroautophagy, hereafter referred to as autophagy, is a catabolic process that plays a fundamental part in tissue homeostasis. We investigated the role of autophagy during bone growth, which is mediated by chondrocyte rate of proliferation, hypertrophic differentiation and extracellular matrix (**ECM**) deposition in growth plates. Here we show that autophagy is induced in growth-plate chondrocytes during post-natal development and regulates the secretion of type II collagen (Col2), the major component of cartilage **ECM**. Mice lacking the autophagy related gene 7 (Atg7) in chondrocytes experience endoplasmic reticulum storage of type II procollagen (PC2) and defective formation of the Col2 fibrillary network in the **ECM**. Surprisingly, post-natal induction of chondrocyte autophagy is mediated by the growth factor FGF18 through FGFR4 and JNK-dependent activation of the autophagy initiation complex VPS34-beclin-1. Autophagy is completely suppressed in growth plates from Fgf18(-/-) embryos, while Fgf18(+/-) heterozygous and Fgfr4(-/-) mice fail to induce autophagy during post-natal development and show decreased Col2 levels in the growth plate. Strikingly, the Fgf18(+/-) and Fgfr4(-/-) phenotypes can be rescued in vivo by pharmacological activation of autophagy, pointing to autophagy as a novel effector of FGF signalling in bone. These data demonstrate that autophagy is a developmentally regulated process necessary for bone growth, and identify FGF signalling as a crucial regulator of autophagy in chondrocytes.
2. Hydroureternephrosis due to loss of Sox9-regulated smooth muscle cell differentiation of the ureteric mesenchyme. Congenital ureter anomalies, including hydroureter, affect up to 1% of the newborn children. Despite the prevalence of these developmental abnormalities in young children, the underlying molecular causes are only poorly understood. Here, we show that the high mobility group domain transcription factor Sox9 plays an important role in ureter development in the mouse. Transient Sox9 expression was detected in the undifferentiated ureteric mesenchyme and inactivation of Sox9 in this domain resulted in strong proximal hydroureter formation due to functional obstruction. Loss of Sox9 did not affect condensation, proliferation and apoptosis of the undifferentiated mesenchyme, but perturbed cyto-differentiation into smooth muscle cells (SMCs). Expression of genes encoding extracellular matrix (**ECM**) components was strongly reduced, suggesting that deficiency in **ECM** composition and/or signaling may underlie the observed defects. Prolonged expression of Sox9 in the ureteric mesenchyme led to increased deposition of **ECM** components and SMC dispersal. Furthermore, Sox9 genetically interacts with the T-box transcription factor 18 gene (Tbx18) during ureter development at two levels--as a downstream mediator of Tbx18 function and in a converging pathway. Together, our results argue that obstructive uropathies in campomelic dysplasia patients that are heterozygous for mutations in and around SOX9 arise from a primary requirement of Sox9 in the development of the ureteric mesenchyme.
3. Requirement of mitogen-activated protein kinase kinase 3 (MKK3) for activation of p38alpha and p38delta MAPK isoforms by TGF-beta 1 in murine mesangial cells. Transforming growth factor-beta1 (TGF-beta1) is a potent inducer of extracellular matrix (**ECM**) synthesis that leads to renal fibrosis. Intracellular signaling mechanisms involved in this process remain incompletely understood. Mitogen-activated protein kinase (MAPK) is a major stress signal-transducing pathway, and we have previously reported activation of p38 MAPK by TGF-beta1 in rat mesangial cells and its role in the stimulation of pro-alpha1(I) collagen. In this study, we further investigated the mechanism of p38 MAPK activation by TGF-beta1 and the role of MKK3, an upstream MAPK kinase of p38 MAPK, by examining the effect of targeted disruption of the Mkk3 gene. We first isolated glomerular mesangial cells from MKK3-null (Mkk3-/-) and wild-type (Mkk3+/+) control mice. Treatment with TGF-beta1 induced rapid phosphorylation of MKK3 as well as p38 MAPK within 15 min in cultured wild-type (Mkk3+/+) mouse mesangial cells. In contrast, TGF-beta1 failed to induce phosphorylation of either MKK3 or p38 MAPK in MKK3-deficient (Mkk3-/-) mouse mesangial cells, indicating that MKK3 is required for TGF-beta1-induced p38 MAPK activation. TGF-beta1 selectively activated the p38 MAPK isoforms p38alpha and p38delta in wild-type (Mkk3+/+) mesangial cells, but not in MKK3-deficient (Mkk3-/-) mesangial cells. Thus, activation of p38alpha and p38delta is dependent on the activation of upstream MKK3 by TGF-beta1. Furthermore, MKK3 deficiency resulted in a selective disruption of TGF-beta1-stimulated up-regulation of pro-alpha1(I) collagen expression but not TGF-beta1 induction of fibronectin and PAI-1. These data demonstrate that the MKK3 is a critical component of the TGF-beta1 signaling pathway, and its activation is required for subsequent p38alpha and p38delta MAPK activation and collagen stimulation by TGF-beta1.
4. Apoptosis commitment and activation of mitochondrial Bax during anoikis is regulated by p38MAPK. Most cells undergo apoptosis through the intrinsic pathway. This is dependent on mitochondrial outer membrane permeabilisation (MOMP), which is mediated by the pro-apoptotic Bcl-2 family proteins, Bax and Bak. During apoptosis, Bax translocates from the cytosol to the outer mitochondrial membrane (OMM), wherein it contributes to the formation of pores to release cytochrome-c. However, it remains unclear whether Bax translocation is sufficient to bring about MOMP or whether Bax requires further signals on the OMM to be fully activated. We have previously shown that during mammary epithelial cell anoikis, Bax translocation does not commit cells to MOMP and detached cells are rescued if survival signals from the extracellular matrix (**ECM**) are restored. These findings implied that a second signal is required for mitochondrial Bax to fully activate and cause MOMP. We now identify p38MAPK (mitogen-activated protein kinase) as this necessary signal to activate Bax after its translocation to mitochondria. The inhibition of p38MAPK did not prevent Bax translocation, but its activity was required for mitochondrial Bax to bring about MOMP. p38MAPK was activated and recruited to a high molecular weight mitochondrial complex after loss of **ECM** attachment. Artificially targeting p38MAPK to the OMM increased the kinetics of anoikis, supporting a requirement for its mitochondrial localisation to regulate Bax activation and drive commitment to apoptosis.
5. Angiotensin II-induced pro-fibrotic effects require p38MAPK activity and transforming growth factor beta 1 expression in skeletal muscle cells. Fibrotic disorders are typically characterised by excessive connective tissue and extracellular matrix (**ECM**) deposition that preclude the normal healing of different tissues. Several skeletal muscle dystrophies are characterised by extensive fibrosis. Among the factors involved in skeletal muscle fibrosis is angiotensin II (Ang-II), a key protein of the renin-angiotensin system (RAS). We previously demonstrated that myoblasts responded to Ang-II by increasing the **ECM** protein levels mediated by AT-1 receptors, implicating an Ang-II-induced reactive oxygen species (ROS) by a NAD(P)H oxidase-dependent mechanism. In this paper, we show that in myoblasts, Ang-II induced the increase of transforming growth factor beta 1 (TGF-β1) and connective tissue growth factor (CTGF) expression through its AT-1 receptor. This effect is dependent of the NAD(P)H oxidase (NOX)-induced ROS, as indicated by a decrease of the expression of both pro-fibrotic factors when the ROS production was inhibited via the NOX inhibitor apocynin. The increase in pro-fibrotic factors levels was paralleled by enhanced p38MAPK and ERK1/2 phosphorylation in response to Ang-II. However, only the p38MAPK activity was critical for the Ang-II-induced fibrotic effects, as indicated by the decrease in the Ang-II-induced TGF-β1 and CTGF expression and fibronectin levels by SB-203580, an inhibitor of the p38MAPK, but not by U0126, an inhibitor of ERK1/2 phosphorylation. Furthermore, we showed that the Ang-II-dependent p38MAPK activation, but not the ERK1/2 phosphorylation, was necessary for the NOX-derived ROS. In addition, we demonstrated that TGF-β1 expression was required for the Ang-II-induced pro-fibrotic effects evaluated by using SB-431542, an inhibitor of TGF-βRI kinase activity, and by knocking down TGF-β1 levels by shRNA technique. These results strongly suggest that the fibrotic response to Ang-II is mediated by the AT-1 receptor and requires the p38MAPK phosphorylation, NOX-induced ROS, and TGF-β1 expression increase mediated by Ang-II in skeletal muscle cells.
6. Leptin regulates MMP-2, TIMP-1 and collagen synthesis via p38 MAPK in HL-1 murine cardiomyocytes. A clear association between obesity and heart failure exists and a significant role for leptin, the product of the obese gene, has been suggested. One aspect of myocardial remodeling which characterizes heart failure is a disruption in the balance of extracellular matrix synthesis and degradation. Here we investigated the effects of leptin on matrix metalloproteinase (MMP) activity, tissue inhibitor of metalloproteinase (TIMP) expression, as well as collagen synthesis in HL-1 cardiac muscle cells. Gelatin zymographic analysis of MMP activity in conditioned media showed that leptin enhanced MMP-2 activity in a dose- and time-dependent manner. Leptin is known to stimulate phosphorylation of p38 MAPK in cardiac cells and utilization of the p38 MAPK inhibitor, SB203580, demonstrated that this kinase also plays a role in regulating several extracellular matrix components, such that inhibition of p38 MAPK signaling prevented the leptin-induced increase in MMP-2 activation. We also observed that leptin enhanced collagen synthesis determined by both proline incorporation and picrosirius red staining of conditioned media. Pro-collagen type-I and pro-collagen type-III expression, measured by real-time PCR and Western blotting were also increased by leptin, effects which were again attenuated by SB203580. In summary, these results demonstrate the potential for leptin to play a role in mediating myocardial **ECM** remodeling and that the p38 MAPK pathway plays an important role in mediating these effects.

### [Ee]xtracellular [Mm]atrix

#### PubMed

1. beta1 integrin is necessary for ureteric bud branching morphogenesis and maintenance of collecting duct structural integrity. The kidney collecting system develops from branching morphogenesis of the ureteric bud (UB). This process requires signaling by growth factors such as glial cell line derived neurotrophic factor (GDNF) and fibroblast growth factors (FGFs) as well as cell **extracellular matrix** interactions mediated by integrins. The importance of integrin signaling in UB development was investigated by deleting integrin beta1 at initiation (E10.5) and late (E18.5) stages of development. Deletion at E10.5 resulted in a severe branching morphogenesis phenotype. Deletion at E18.5 did not alter renal development but predisposed the collecting system to severe injury following ureteric obstruction. beta1 integrin was required for renal tubular epithelial cells to mediate GDNF- and FGF-dependent signaling despite normal receptor localization and activation in vitro. Aberrations in the same signaling molecules were present in the beta1-null UBs in vivo. Thus beta1 integrins can regulate organ branching morphogenesis during development by mediating growth-factor-dependent signaling in addition to their well-defined role as adhesion receptors.
2. Foxf2 is required for secondary palate development and Tgfβ signaling in palatal shelf mesenchyme. The secondary palate separates the oral from the nasal cavity and its closure during embryonic development is sensitive to genetic perturbations. Mice with deleted Foxf2, encoding a forkhead transcription factor, are born with cleft palate, and an abnormal tongue morphology has been proposed as the underlying cause. Here, we show that Foxf2(-/-) maxillary explants cultured in vitro, in the absence of tongue and mandible, failed to close the secondary palate. Proliferation and collagen content were decreased in Foxf2(-/-) palatal shelf mesenchyme. Phosphorylation of Smad2/3 was reduced in mutant palatal shelf, diagnostic of attenuated canonical Tgfβ signaling, whereas phosphorylation of p38 was increased. The amount of Tgfβ2 protein was diminished, whereas the Tgfb2 mRNA level was unaltered. Expression of several genes encoding extracellular proteins important for Tgfβ signaling were reduced in Foxf2(-)(/)(-) palatal shelves: a fibronectin splice-isoform essential for formation of extracellular Tgfβ latency complexes; Tgfbr3 - or betaglycan - which acts as a co-receptor and an extracellular reservoir of Tgfβ; and integrins αV and β1, which are both Tgfβ targets and required for activation of latent Tgfβ. Decreased proliferation an**d reduced extracellu**lar matrix content are consistent with diminished Tgfβ signaling. We therefore propose that gene expression changes in palatal shelf mesenchyme that lead to reduced Tgfβ signaling contribute to cleft palate in Foxf2(-)(/)(-) mice.
3. Genetic ablation of Rac1 in cartilage results in chondrodysplasia. Small GTPases of the Rho family have been implicated in the regulation of many intracellular processes. However, their tissue-specific roles in mammalian growth and development in vivo remain largely unknown. Here we describe the effects of cartilage-specific inactivation of the Rac1 gene in mice. Mice carrying this mutation show increased lethality, skeletal deformities, severe kyphosis and dwarfism. Rac1-deficient growth plates are disorganized and hypocellular, with chondrocytes of abnormal shape and size. Rac1-deficient chondrocytes also display reduced adhesion and spreading on collagen II and fibronectin as well as altered organization of the actin cytoskeleton, suggesting that Rac1 is required for normal cell-**extracellular matrix** interactions in cartilage. This phenotype is accompanied by reduced proliferation, increased apoptosis and deregulated expression of the cell cycle genes cyclin D1 and p57 in vivo. Moreover, phosphorylation of p38 MAP kinases is greatly reduced and expression of a key regulator of cartilage development, Indian hedgehog, is increased in mutant mice. In summary, these data identify a novel, essential and tissue-specific role of Rac1 in skeletal development and demonstrate that Rac1 deficiency affects numerous regulatory pathways in cartilage.
4. Palatal adhesion is dependent on Src family kinases and p38MAPK. During secondary palate development, palatal shelves adhere to each other in the midline to form a midline epithelial seam leading to palatal closure. Cell-cell and cell-**extracellular matrix** adhesions, which are mediated by cell adhesion receptors, E-cadherin and integrins, are implicated in the process of adhesion of the palatal shelves. Src family kinases (SFK) function downstream of both receptors. In this study, we focused on the role of SFK in the process of palatal adhesion. During palatal adhesion, the expression of SFK mRNA, as well as localization and quantitation of the protein in the activated form, were examined by real-time qPCR and immunofluorescence. Palatal organ cultures were performed to identify the effect of pharmacological inhibition of SFK on palatal adhesion. Activated SFKs were found to be co-localized with adhesion receptors, E-cadherin and integrins in the palatal medial edge epithelium. Src, Fyn and Yes subfamily members were expressed in the palatal tissue. The expression of SFK mRNA and the quantity of the activated form of the protein were upregulated during palatal adhesion. An SFK inhibitor, PP2, blocked palatal adhesion, but another SFK inhibitor, SU6656 was not inhibitory. However, the combination of SU6656 and either of the p38MAPK inhibitors, SB203580 or BIRB0796, showed similar inhibitory effects on palatal adhesion compared to PP2 alone. The p38MAPK inhibitors alone did not alter palatal adhesion. Real-time qPCR revealed that p38MAPK alpha and delta were elevated during palatal adhesion. This study indicates that palatal cell adhesion is dependent on signaling from integrin receptors and E-cadherin through SFK and p38MAPK.
5. FGF signalling regulates bone growth through autophagy. Skeletal growth relies on both biosynthetic and catabolic processes. While the role of the former is clearly established, how the latter contributes to growth-promoting pathways is less understood. Macroautophagy, hereafter referred to as autophagy, is a catabolic process that plays a fundamental part in tissue homeostasis. We investigated the role of autophagy during bone growth, which is mediated by chondrocyte rate of proliferation, hypertrophic differentiation and **extracellular matrix** (ECM) deposition in growth plates. Here we show that autophagy is induced in growth-plate chondrocytes during post-natal development and regulates the secretion of type II collagen (Col2), the major component of cartilage ECM. Mice lacking the autophagy related gene 7 (Atg7) in chondrocytes experience endoplasmic reticulum storage of type II procollagen (PC2) and defective formation of the Col2 fibrillary network in the ECM. Surprisingly, post-natal induction of chondrocyte autophagy is mediated by the growth factor FGF18 through FGFR4 and JNK-dependent activation of the autophagy initiation complex VPS34-beclin-1. Autophagy is completely suppressed in growth plates from Fgf18(-/-) embryos, while Fgf18(+/-) heterozygous and Fgfr4(-/-) mice fail to induce autophagy during post-natal development and show decreased Col2 levels in the growth plate. Strikingly, the Fgf18(+/-) and Fgfr4(-/-) phenotypes can be rescued in vivo by pharmacological activation of autophagy, pointing to autophagy as a novel effector of FGF signalling in bone. These data demonstrate that autophagy is a developmentally regulated process necessary for bone growth, and identify FGF signalling as a crucial regulator of autophagy in chondrocytes.
6. TEF-1 and C/EBPbeta are major p38alpha MAPK-regulated transcription factors in proliferating cardiomyocytes. p38 MAPKs (mitogen-activated protein kinases) play important roles in the regulation of cellular responses to environmental stress. Recently, this signalling pathway has also been implicated in the regulation of processes unrelated to stress, for example, in T lymphocytes and cardiomyocytes. In order to identify molecular targets responsible for the housekeeping functions of p38 MAPKs, we have analysed the differences in the transcriptomes of normally proliferating wild-type and p38alpha knockout immortalized embryonic cardiomyocytes. Interestingly, many potential components of the myocardium **extracellular matrix** were found to be upregulated in the absence of p38alpha. Further analysis of the microarray data identified TEF-1 (transcriptional enhancer factor-1), a known regulator of heart-specific gene expression, and C/EBPbeta (CCAAT/enhancer-binding protein beta), as the two transcription factors the binding sites of which were most enriched in the promoters of p38alpha-regulated genes. We have focused on the study of the **extracellular matrix** component COL1A1 (alpha1 chain of type I collagen) and found evidence for the involvement of both TEF-1 and C/EBPbeta in the p38alpha-dependent inhibition of COL1A1 transcription. Our data therefore show that p38 MAPKs regulate TEF-1 and C/EBPbeta transcriptional activity in the absence of environmental stress and suggests a role for p38alpha in the expression of **extracellular matrix** components that maintain organ architecture.
7. Transcriptome analysis in prenatal IGF1-deficient mice identifies molecular pathways and target genes involved in distal lung differentiation. Insulin-like Growth Factor 1 (IGF1) is a multifunctional regulator of somatic growth and development throughout evolution. IGF1 signaling through IGF type 1 receptor (IGF1R) controls cell proliferation, survival and differentiation in multiple cell types. IGF1 deficiency in mice disrupts lung morphogenesis, causing altered prenatal pulmonary alveologenesis. Nevertheless, little is known about the cellular and molecular basis of IGF1 activity during lung development.Prenatal Igf1(-/-) mutant mice with a C57Bl/6J genetic background displayed severe disproportional lung hypoplasia, leading to lethal neonatal respiratory distress. Immuno-histological analysis of their lungs showed a thickened mesenchyme, alterations in **extracellular matrix** deposition, thinner smooth muscles and dilated blood vessels, which indicated immature and delayed distal pulmonary organogenesis. Transcriptomic analysis of Igf1(-/-) E18.5 lungs using RNA microarrays identified deregulated genes related to vascularization, morphogenesis and cellular growth, and to MAP-kinase, Wnt and cell-adhesion pathways. Up-regulation of immunity-related genes was verified by an increase in inflammatory markers. Increased expression of Nfib and reduced expression of Klf2, Egr1 and Ctgf regulatory proteins as well as activation of ERK2 MAP-kinase were corroborated by Western blot. Among IGF-system genes only IGFBP2 revealed a reduction in mRNA expression in mutant lungs. Immuno-staining patterns for IGF1R and IGF2, similar in both genotypes, correlated to alterations found in specific cell compartments of Igf1(-/-) lungs. IGF1 addition to Igf1(-/-) embryonic lungs cultured ex vivo increased airway septa remodeling and distal epithelium maturation, processes accompanied by up-regulation of Nfib and Klf2 transcription factors and Cyr61 matricellular protein.We demonstrated the functional tissue specific implication of IGF1 on fetal lung development in mice. Results revealed novel target genes and gene networks mediators of IGF1 action on pulmonary cellular proliferation, differentiation, adhesion and immunity, and on vascular and distal epithelium maturation during prenatal lung development.
8. Tumor necrosis factor-related weak inducer of apoptosis augments matrix metalloproteinase 9 (MMP-9) production in skeletal muscle through the activation of nuclear factor-kappaB-inducing kinase and p38 mitogen-activated protein kinase: a potential role of MMP-9 in myopathy. Destruction of skeletal muscle **extracellular matrix** is an important pathological consequence of many diseases involving muscle wasting. However, the underlying mechanisms leading to **extracellular matrix** breakdown in skeletal muscle tissues remain unknown. Using a microarray approach, we investigated the effect of tumor necrosis factor-related weak inducer of apoptosis (TWEAK), a recently identified muscle-wasting cytokine, on the expression of extracellular proteases in skeletal muscle. Among several other matrix metalloproteinases (MMPs), we found that the expression of MMP-9, a type IV collagenase, was drastically increased in myotubes in response to TWEAK. The level of MMP-9 was also higher in myofibers of TWEAK transgenic mice. TWEAK increased the activation of both classical and alternative nuclear factor-kappaB (NF-kappaB) signaling pathways. Inhibition of NF-kappaB activity blocked the TWEAK-induced production of MMP-9 in myotubes. TWEAK also increased the activation of AP-1, and its inhibition attenuated the TWEAK-induced MMP-9 production. Overexpression of a kinase-dead mutant of NF-kappaB-inducing kinase or IkappaB kinase-beta but not IkappaB kinase-alpha significantly inhibited the TWEAK-induced activation of MMP-9 promoter. The activation of MMP-9 also involved upstream recruitment of TRAF2 and cIAP2 proteins. TWEAK increased the activity of ERK1/2, JNK1, and p38 MAPK. However, the inhibition of only p38 MAPK blocked the TWEAK-induced expression of MMP-9 in myotubes. Furthermore the loss of body and skeletal muscle weights, inflammation, fiber necrosis, and degradation of basement membrane around muscle fibers were significantly attenuated in Mmp9 knock-out mice on chronic administration of TWEAK protein. The study unveils a novel mechanism of skeletal muscle tissue destruction in pathological conditions.
9. Hydroureternephrosis due to loss of Sox9-regulated smooth muscle cell differentiation of the ureteric mesenchyme. Congenital ureter anomalies, including hydroureter, affect up to 1% of the newborn children. Despite the prevalence of these developmental abnormalities in young children, the underlying molecular causes are only poorly understood. Here, we show that the high mobility group domain transcription factor Sox9 plays an important role in ureter development in the mouse. Transient Sox9 expression was detected in the undifferentiated ureteric mesenchyme and inactivation of Sox9 in this domain resulted in strong proximal hydroureter formation due to functional obstruction. Loss of Sox9 did not affect condensation, proliferation and apoptosis of the undifferentiated mesenchyme, but perturbed cyto-differentiation into smooth muscle cells (SMCs). Expression of genes encoding **extracellular matrix** (ECM) components was strongly reduced, suggesting that deficiency in ECM composition and/or signaling may underlie the observed defects. Prolonged expression of Sox9 in the ureteric mesenchyme led to increased deposition of ECM components and SMC dispersal. Furthermore, Sox9 genetically interacts with the T-box transcription factor 18 gene (Tbx18) during ureter development at two levels--as a downstream mediator of Tbx18 function and in a converging pathway. Together, our results argue that obstructive uropathies in campomelic dysplasia patients that are heterozygous for mutations in and around SOX9 arise from a primary requirement of Sox9 in the development of the ureteric mesenchyme.
10. Requirement of mitogen-activated protein kinase kinase 3 (MKK3) for activation of p38alpha and p38delta MAPK isoforms by TGF-beta 1 in murine mesangial cells. Transforming growth factor-beta1 (TGF-beta1) is a potent inducer of **extracellular matrix** (ECM) synthesis that leads to renal fibrosis. Intracellular signaling mechanisms involved in this process remain incompletely understood. Mitogen-activated protein kinase (MAPK) is a major stress signal-transducing pathway, and we have previously reported activation of p38 MAPK by TGF-beta1 in rat mesangial cells and its role in the stimulation of pro-alpha1(I) collagen. In this study, we further investigated the mechanism of p38 MAPK activation by TGF-beta1 and the role of MKK3, an upstream MAPK kinase of p38 MAPK, by examining the effect of targeted disruption of the Mkk3 gene. We first isolated glomerular mesangial cells from MKK3-null (Mkk3-/-) and wild-type (Mkk3+/+) control mice. Treatment with TGF-beta1 induced rapid phosphorylation of MKK3 as well as p38 MAPK within 15 min in cultured wild-type (Mkk3+/+) mouse mesangial cells. In contrast, TGF-beta1 failed to induce phosphorylation of either MKK3 or p38 MAPK in MKK3-deficient (Mkk3-/-) mouse mesangial cells, indicating that MKK3 is required for TGF-beta1-induced p38 MAPK activation. TGF-beta1 selectively activated the p38 MAPK isoforms p38alpha and p38delta in wild-type (Mkk3+/+) mesangial cells, but not in MKK3-deficient (Mkk3-/-) mesangial cells. Thus, activation of p38alpha and p38delta is dependent on the activation of upstream MKK3 by TGF-beta1. Furthermore, MKK3 deficiency resulted in a selective disruption of TGF-beta1-stimulated up-regulation of pro-alpha1(I) collagen expression but not TGF-beta1 induction of fibronectin and PAI-1. These data demonstrate that the MKK3 is a critical component of the TGF-beta1 signaling pathway, and its activation is required for subsequent p38alpha and p38delta MAPK activation and collagen stimulation by TGF-beta1.
11. Ginsenoside Rb1 attenuates angiotensin II-induced abdominal aortic aneurysm through inactivation of the JNK and p38 signaling pathways. Abdominal aortic aneurysm (AAA), a life-threatening vascular disease, accounts for approximately 10% of the morbidity in people over 65 years old. No satisfactory approach is available to treat AAA. Ginsenosides Rb1 and Rg1 are primary ingredients of Panax notoginseng for the treatment of cardiovascular diseases, but their impact on AAA is unknown.An AAA model was established using an Ang II infusion in ApoE(-/-) mice. After continuous stimulation of Ang II for 28 days, suprarenal aortic aneurysms developed in 77% mice and 12% mice died suddenly due to AAA rupture. Administration of ginsenoside Rb1 (20 mg/kg/day), but not ginsenoside Rg1, significantly reduced the incidence and mortality of AAA. Ginsenoside Rb1 treatment dramatically suppressed Ang II-induced diameter enlargement, **extracellular matrix** degradation, matrix metalloproteinase (MMP) production, inflammatory cell infiltration, and vascular smooth muscle cell (VSMC) dysfunction. Mechanistic studies indicated that the protective effects of ginsenoside Rb1 were associated with the inactivation of JNK and p38 MAPK signaling pathways. A specific activator of JNK and p38, anisomycin, nearly abolished ginsenoside Rb1-driven suppression of MMP secretion by VSMCs.Ginsenoside Rb1, as a potential anti-AAA agent, suppressed AAA through inhibiting the JNK and p38 signaling pathways.
12. Apoptosis commitment and activation of mitochondrial Bax during anoikis is regulated by p38MAPK. Most cells undergo apoptosis through the intrinsic pathway. This is dependent on mitochondrial outer membrane permeabilisation (MOMP), which is mediated by the pro-apoptotic Bcl-2 family proteins, Bax and Bak. During apoptosis, Bax translocates from the cytosol to the outer mitochondrial membrane (OMM), wherein it contributes to the formation of pores to release cytochrome-c. However, it remains unclear whether Bax translocation is sufficient to bring about MOMP or whether Bax requires further signals on the OMM to be fully activated. We have previously shown that during mammary epithelial cell anoikis, Bax translocation does not commit cells to MOMP and detached cells are rescued if survival signals from the **extracellular matrix** (ECM) are restored. These findings implied that a second signal is required for mitochondrial Bax to fully activate and cause MOMP. We now identify p38MAPK (mitogen-activated protein kinase) as this necessary signal to activate Bax after its translocation to mitochondria. The inhibition of p38MAPK did not prevent Bax translocation, but its activity was required for mitochondrial Bax to bring about MOMP. p38MAPK was activated and recruited to a high molecular weight mitochondrial complex after loss of ECM attachment. Artificially targeting p38MAPK to the OMM increased the kinetics of anoikis, supporting a requirement for its mitochondrial localisation to regulate Bax activation and drive commitment to apoptosis.
13. Transforming growth factor-beta1 stimulates vascular endothelial growth factor 164 via mitogen-activated protein kinase kinase 3-p38alpha and p38delta mitogen-activated protein kinase-dependent pathway in murine mesangial cells. Transforming growth factor-beta1 (TGF-beta1) is a potent inducer of **extracellular matrix** synthesis leading to progressive glomerular fibrosis. The intracellular signaling mechanisms involved in this process remain incompletely understood. The p38 mitogen-activated protein kinase (MAPK) is a major stress signal transducing pathway that is rapidly activated by TGF-beta1 in mesangial cells. We have previously demonstrated MKK3 as the immediate upstream MAPK kinase required for selective activation of p38 MAPK isoforms, p38alpha and p38delta, and stimulation of pro-alpha1(I) collagen by TGF-beta1 in murine mesangial cells. In this study, we further sought to determine MAPK kinase 3 (MKK3)-dependent TGF-beta1 responses by gene expression profiling analysis utilizing mesangial cells isolated from Mkk3-/- mice compared with Mkk3+/+ controls. Interestingly, vascular endothelial growth factor (VEGF) was identified as a TGF-beta1-induced gene affected by deletion of Mkk3. VEGF is a well known endothelial mitogen, whose actions in nonendothelial cell types are still not well understood. We confirmed that TGF-beta1 increased VEGF mRNA and protein synthesis of VEGF164 and VEGF188 isoforms in wild-type mesangial cells. However, in the Mkk3-/- mesangial cells, both TGF-beta1-induced VEGF mRNA and VEGF164 protein expression were inhibited, whereas TGF-beta1-induced VEGF188 protein expression was unaffected. Furthermore, transfection of dominant negative mutants of p38alpha and p38delta resulted in marked inhibition of TGF-beta1-induced VEGF164 expression but not VEGF188, and treatment with recombinant mouse VEGF164 increased collagen and fibronectin mRNA expression in mesangial cells. Taken together, our findings suggest a critical role for the MKK3-p38alpha and p38delta MAPK pathway in mediating VEGF164 isoform-specific stimulation by TGF-beta1 in mesangial cells. Further, VEGF164 stimulates collagen and fibronectin expression in mesangial cells and thus in turn enhances TGF-beta1-induced **extracellular matrix** and may play an important role in progressive glomerular fibrosis.
14. Osteoprotegerin induces podosome disassembly in osteoclasts through calcium, ERK, and p38 MAPK signaling pathways. Osteoclasts are critical for bone resorption and use podosomes to attach to bone matrix. Osteoprotegerin (OPG) is a negative regulator of osteoclast function that can affect the formation and function of podosomes. However, the signaling pathways that link OPG to podosome function have not been well characterized. Therefore, this study examined the roles of intracellular calcium and MAPKs in OPG-induced podosome disassembly in osteoclasts. We assessed the effects of the intracellular calcium chelator Bapta-AM, ERK inhibitor U0126, and p38 inhibitor SB202190 on OPG-treated osteoclast differentiation, adhesion structures, intracellular free Ca(2+) concentration and the phosphorylation state of podosome associated proteins (Pyk2 and Src). Mouse monocytic RAW 264.7 cells were differentiated to osteoclasts using RANKL (30ng/mL) and M-CSF (25ng/mL). The cells were pretreated with Bapta-AM (5μM), U0126 (5μM), or SB202190 (10μM) for 30min, followed by 40ng/mL OPG for 3h. Osteoclastogenesis, adhesion structure, viability and morphology, intracellular free Ca(2+) concentration and the phosphorylation state of Pyk2 and Src were measured by TRAP staining, scanning electron microscopy, real-time cell analyzer, flow cytometry and western blotting, respectively. OPG significantly inhibited osteoclastogenesis, the formation of adhesion structures, and reduced the amount of phosphorylated Pyk2 and Src-pY527, but increased phosphorylation of Src-pY416. Bapta-AM, U0126, and SB202190 partially restored osteoclast differentiation and adhesion structures. Both Bapta-AM and U0126, but not SB202190, restored the levels of intracellular free Ca(2+) concentration, phosphorylated Pyk2 and Src-pY527. All three inhibitors blocked OPG-induced phosphorylation at Src-pY416. These results suggest OPG disrupts the attachment structures of osteoclasts and activates Src as an adaptor protein that competes for the reduced amount of phosphorylated Pyk2 through calcium- and ERK-dependent signaling pathways. p38 MAPK signaling may have a different role in OPG-induced osteoclast retraction. Our findings potentially offer novel insights into the signaling mechanisms downstream of OPG that affect osteoclast attachment to t**he extracellular mat**rix.
15. Angiotensin II-induced pro-fibrotic effects require p38MAPK activity and transforming growth factor beta 1 expression in skeletal muscle cells. Fibrotic disorders are typically characterised by excessive connective tissue and **extracellular matrix** (ECM) deposition that preclude the normal healing of different tissues. Several skeletal muscle dystrophies are characterised by extensive fibrosis. Among the factors involved in skeletal muscle fibrosis is angiotensin II (Ang-II), a key protein of the renin-angiotensin system (RAS). We previously demonstrated that myoblasts responded to Ang-II by increasing the ECM protein levels mediated by AT-1 receptors, implicating an Ang-II-induced reactive oxygen species (ROS) by a NAD(P)H oxidase-dependent mechanism. In this paper, we show that in myoblasts, Ang-II induced the increase of transforming growth factor beta 1 (TGF-β1) and connective tissue growth factor (CTGF) expression through its AT-1 receptor. This effect is dependent of the NAD(P)H oxidase (NOX)-induced ROS, as indicated by a decrease of the expression of both pro-fibrotic factors when the ROS production was inhibited via the NOX inhibitor apocynin. The increase in pro-fibrotic factors levels was paralleled by enhanced p38MAPK and ERK1/2 phosphorylation in response to Ang-II. However, only the p38MAPK activity was critical for the Ang-II-induced fibrotic effects, as indicated by the decrease in the Ang-II-induced TGF-β1 and CTGF expression and fibronectin levels by SB-203580, an inhibitor of the p38MAPK, but not by U0126, an inhibitor of ERK1/2 phosphorylation. Furthermore, we showed that the Ang-II-dependent p38MAPK activation, but not the ERK1/2 phosphorylation, was necessary for the NOX-derived ROS. In addition, we demonstrated that TGF-β1 expression was required for the Ang-II-induced pro-fibrotic effects evaluated by using SB-431542, an inhibitor of TGF-βRI kinase activity, and by knocking down TGF-β1 levels by shRNA technique. These results strongly suggest that the fibrotic response to Ang-II is mediated by the AT-1 receptor and requires the p38MAPK phosphorylation, NOX-induced ROS, and TGF-β1 expression increase mediated by Ang-II in skeletal muscle cells.
16. Inhibition of p38 MAPK attenuates renal atrophy and fibrosis in a murine renal artery stenosis model. Renal artery stenosis (RAS) is an important cause of chronic renal dysfunction. Recent studies have underscored a critical role for CCL2 (MCP-1)-mediated inflammation in the progression of chronic renal damage in RAS and other chronic renal diseases. In vitro studies have implicated p38 MAPK as a critical intermediate for the production of CCL2. However, a potential role of p38 signaling in the development and progression of chronic renal disease in RAS has not been previously defined. We sought to test the hypothesis that inhibition of p38 MAPK ameliorates chronic renal injury in mice with RAS. We established a murine RAS model by placing a cuff on the right renal artery and treated mice with the p38 inhibitor SB203580 or vehicle for 2 wk. In mice treated with vehicle, the cuffed kidney developed interstitial fibrosis, tubular atrophy, and interstitial inflammation. In mice treated with SB203580, the RAS-induced renal atrophy was reduced (70% vs. 39%, P < 0.05). SB203580 also reduced interstitial inflammation and **extracellular matrix** deposition but had no effect on the development of hypertension. SB203580 partially blocked the induction of CCL2, CCL7 (MCP-3), CC chemokine receptor 2 (CCR2), and collagen 4 mRNA expression in the cuffed kidneys. In vitro, blockade of p38 hindered both TNF-α and TGF-β-induced CCL2 upregulation. Based on these observations, we conclude that p38 MAPK plays a critical role in the induction of CCL2/CCL7/CCR2 system and the development of interstitial inflammation in RAS.
17. Trophoblast stem cell maintenance by fibroblast growth factor 4 requires MEKK4 activation of Jun N-terminal kinase. Trophoblast differentiation during placentation involves an epithelial-mesenchymal transition (EMT) with loss of E-cadherin and gain of trophoblast invasiveness. Mice harboring a point mutation that renders inactive the mitogen-activated protein kinase kinase kinase MEKK4 exhibit dysregulated placental development with increased trophoblast invasion. Isolated MEKK4 kinase-inactive trophoblast stem (TS) cells cultured under undifferentiating, self-renewing conditions in the presence of fibroblast growth factor 4 (FGF4) display increased expression of Slug, Twist, and matrix metalloproteinase 2 (MMP2), loss of E-cadherin, and hyperinvasion of **extracellular matrix**, each a hallmark of EMT. MEKK4 kinase-inactive TS cells show a preferential differentiation to Tpbp alpha- and Gcm1-positive trophoblasts, which are indicative of spongiotrophoblast and syncytiotrophoblast differentiation, respectively. FGF4-stimulated Jun N-terminal kinase (JNK) and p38 activity is markedly reduced in MEKK4 kinase-inactive TS cells. Chemical inhibition of JNK in wild-type TS cells induced a similar EMT response as loss of MEKK4 kinase activity, including inhibition of E-cadherin expression and increased expression of Slug, MMP2, Tpbp alpha, and Gcm1. Chromatin immunoprecipitation analyses revealed changes in AP-1 composition with increased Fra-2 and decreased Fra-1 and JunB binding to the regulatory regions of Gcm1 and MMP2 genes in MEKK4 kinase-inactive TS cells. Our results define MEKK4 as a signaling hub for FGF4 activation of JNK that is required for maintenance of TS cells in an undifferentiated state.
18. Leptin regulates MMP-2, TIMP-1 and collagen synthesis via p38 MAPK in HL-1 murine cardiomyocytes. A clear association between obesity and heart failure exists and a significant role for leptin, the product of the obese gene, has been suggested. One aspect of myocardial remodeling which characterizes heart failure is a disruption in the balance of **extracellular matrix** synthesis and degradation. Here we investigated the effects of leptin on matrix metalloproteinase (MMP) activity, tissue inhibitor of metalloproteinase (TIMP) expression, as well as collagen synthesis in HL-1 cardiac muscle cells. Gelatin zymographic analysis of MMP activity in conditioned media showed that leptin enhanced MMP-2 activity in a dose- and time-dependent manner. Leptin is known to stimulate phosphorylation of p38 MAPK in cardiac cells and utilization of the p38 MAPK inhibitor, SB203580, demonstrated that this kinase also plays a role in regulating several **extracellular matrix** components, such that inhibition of p38 MAPK signaling prevented the leptin-induced increase in MMP-2 activation. We also observed that leptin enhanced collagen synthesis determined by both proline incorporation and picrosirius red staining of conditioned media. Pro-collagen type-I and pro-collagen type-III expression, measured by real-time PCR and Western blotting were also increased by leptin, effects which were again attenuated by SB203580. In summary, these results demonstrate the potential for leptin to play a role in mediating myocardial ECM remodeling and that the p38 MAPK pathway plays an important role in mediating these effects.
19. Phosphorylation of c-Fos by members of the p38 MAPK family. Role in the AP-1 response to UV light. Exposure to sources of UV radiation, such as sunlight, induces a number of cellular alterations that are highly dependent on its ability to affect gene expression. Among them, the rapid activation of genes coding for two subfamilies of proto-oncoproteins, Fos and Jun, which constitute the AP-1 transcription factor, plays a key role in the subsequent regulation of expression of genes involved in DNA repair, cell proliferation, cell cycle arrest, death by apoptosis, and tissue and **extracellular matrix** remodeling proteases. Besides being regulated at the transcriptional level, Jun and Fos transcriptional activities are also regulated by phosphorylation as a result of the activation of intracellular signaling cascades. In this regard, the phosphorylation of c-Jun by UV-induced JNK has been readily documented, whereas a role for Fos proteins in UV-mediated responses and the identification of Fos-activating kinases has remained elusive. Here we identify p38 MAPKs as proteins that can associate with c-Fos and phosphorylate its transactivation domain both in vitro and in vivo. This phosphorylation is transduced into changes in its transcriptional ability as p38-activated c-Fos enhances AP1-driven gene expression. Our findings indicate that as a consequence of the activation of stress pathways induced by UV light, endogenous c-Fos becomes a substrate of p38 MAPKs and, for the first time, provide evidence that support a critical role for p38 MAPKs in mediating stress-induced c-Fos phosphorylation and gene transcription activation. Using a specific pharmacological inhibitor for p38alpha and -beta, we found that most likely these two isoforms mediate UV-induced c-Fos phosphorylation in vivo. Thus, these newly described pathways act concomitantly with the activation of c-Jun by JNK/MAPKs, thereby contributing to the complexity of AP1-driven gene transcription regulation.
20. Myostatin directly regulates skeletal muscle fibrosis. Skeletal muscle fibrosis is a major pathological hallmark of chronic myopathies in which myofibers are replaced by progressive deposition of collagen and other **extracellular matrix** proteins produced by muscle fibroblasts. Recent studies have shown that in the absence of the endogenous muscle growth regulator myostatin, regeneration of muscle is enhanced, and muscle fibrosis is correspondingly reduced. We now demonstrate that myostatin not only regulates the growth of myocytes but also directly regulates muscle fibroblasts. Our results show that myostatin stimulates the proliferation of muscle fibroblasts and the production of **extracellular matrix** proteins both in vitro and in vivo. Further, muscle fibroblasts express myostatin and its putative receptor activin receptor IIB. Proliferation of muscle fibroblasts, induced by myostatin, involves the activation of Smad, p38 MAPK and Akt pathways. These results expand our understanding of the function of myostatin in muscle tissue and provide a potential target for anti-fibrotic therapies.

#### GeneRIFs

1. p38 MAPKs regulate TEF-1 and C/EBPbeta transcriptional activity in the absence of environmental stress, suggesting a role for p38alpha in the expression of **extracellular matrix** components that maintain organ architecture.

### [Ii]nvasive

#### PubMed

1. Role of P38 MAPK on MMP Activity in Photothrombotic Stroke Mice as Measured using an Ultrafast MMP Activatable Probe. Matrix metalloproteinases (MMPs) exert a dual effect in ischemic stroke and thus represent an ideal target for detection and therapy. However, to date, all clinical trials of MMP inhibitors have failed, and alternative drug candidates and therapeutic targets are urgently required. Nonetheless, further investigations are limited by the lack of non-**invasive** imaging techniques. Here, we report a novel, fast and ultrasensitive MMP activatable optical imaging probe for the dynamic visualization of MMP activity in photothrombotic stroke mice. This probe provides a significant signal enhancement in as little as 15 min, with the highest signal intensity occurring at 1 h post-injection, and shows high sensitivity in measuring MMP activity alterations, which makes it specifically suitable for the real-time visualization of MMP activity and drug discovery in preclinical research. Moreover, using this probe, we successfully demonstrate that the regulation of the p38 mitogen-activated protein kinase (MAPK) signal pathway is capable of modulating MMP activity after stroke, revealing a novel regulatory mechanism of postischemic brain damage and overcoming the limitations of traditional therapeutic strategies associated with MMP inhibitors by using a **non-inva**sive molecular imaging method.
2. Trophoblast stem cell maintenance by fibroblast growth factor 4 requires MEKK4 activation of Jun N-terminal kinase. Trophoblast differentiation during placentation involves an epithelial-mesenchymal transition (EMT) with loss of E-cadherin and gain of trophoblast **invasive**ness. Mice harboring a point mutation that renders inactive the mitogen-activated protein kinase kinase kinase MEKK4 exhibit dysregulated placental development with increased trophoblast invasion. Isolated MEKK4 kinase-inactive trophoblast stem (TS) cells cultured under undifferentiating, self-renewing conditions in the presence of fibroblast growth factor 4 (FGF4) display increased expression of Slug, Twist, and matrix metalloproteinase 2 (MMP2), loss of E-cadherin, and hyperinvasion of extracellular matrix, each a hallmark of EMT. MEKK4 kinase-inactive TS cells show a preferential differentiation to Tpbp alpha- and Gcm1-positive trophoblasts, which are indicative of spongiotrophoblast and syncytiotrophoblast differentiation, respectively. FGF4-stimulated Jun N-terminal kinase (JNK) and p38 activity is markedly reduced in MEKK4 kinase-inactive TS cells. Chemical inhibition of JNK in wild-type TS cells induced a similar EMT response as loss of MEKK4 kinase activity, including inhibition of E-cadherin expression and increased expression of Slug, MMP2, Tpbp alpha, and Gcm1. Chromatin immunoprecipitation analyses revealed changes in AP-1 composition with increased Fra-2 and decreased Fra-1 and JunB binding to the regulatory regions of Gcm1 and MMP2 genes in MEKK4 kinase-inactive TS cells. Our results define MEKK4 as a signaling hub for FGF4 activation of JNK that is required for maintenance of TS cells in an undifferentiated state.
3. Synthetic cationic peptide IDR-1002 provides protection against bacterial infections through chemokine induction and enhanced leukocyte recruitment. With the rapid rise in the incidence of multidrug resistant infections, there is substantial interest in host defense peptides as templates for production of new antimicrobial therapeutics. Natural peptides are multifunctional mediators of the innate immune response, with some direct antimicrobial activity and diverse immunomodulatory properties. We have previously developed an innate defense regulator (IDR) 1, with protective activity against bacterial infection mediated entirely through its effects on the immunity of the host, as a novel approach to anti-infective therapy. In this study, an immunomodulatory peptide IDR-1002 was selected from a library of bactenecin derivatives based on its substantially more potent ability to induce chemokines in human PBMCs. The enhanced chemokine induction activity of the peptide in vitro correlated with stronger protective activity in vivo in the Staphylococcus aureus-**invasive** infection model, with a >5-fold reduction in the protective dose in direct comparison with IDR-1. IDR-1002 also afforded protection against the Gram-negative bacterial pathogen Escherichia coli. Chemokine induction by IDR-1002 was found to be mediated through a Gi-coupled receptor and the PI3K, NF-kappaB, and MAPK signaling pathways. The protective activity of the peptide was associated with in vivo augmentation of chemokine production and recruitment of neutrophils and monocytes to the site of infection. These results highlight the importance of the chemokine induction activity of host defense peptides and demonstrate that the optimization of the ex vivo chemokine-induction properties of peptides is a promising method for the rational development of immunomodulatory IDR peptides with enhanced anti-infective activity.
4. Phenotype-assisted transcriptome analysis identifies FOXM1 downstream from Ras-MKK3-p38 to regulate in vitro cellular invasion. The Ras oncogene is known to activate three major MAPK pathways, ERK, JNK, p38 and exert distinct cellular phenotypes, that is, apoptosis and invasion through the Ras-MKK3-p38-signaling cascade. We attempted to identify the molecular targets of this pathway that selectively govern the **invasive** phenotype. Stable transfection of NIH3T3 fibroblasts with MKK3(act) cDNA construct revealed similar p38-dependent in vitro characteristics observed in Ha-Ras(EJ)-transformed NIH3T3 cells, including enhanced **invasive**ness and anchorage-independent growth correlating with p38 phosphorylation status. To identify the consensus downstream targets of the Ras-MKK3-p38 cascade involved in invasion, in vitro invasion assays were used to isolate highly **invasive** cells from both, MKK3 and Ha-Ras(EJ) transgenic cell lines. Subsequently a genome-wide transcriptome analysis was employed to investigate differentially regulated genes in **invasive** Ha-Ras(EJ)- and MKK3(act)-transfected NIH3T3 fibroblasts. Using this phenotype-assisted approach combined with system level protein-interaction network analysis, we identified FOXM1, PLK1 and CDK1 to be differentially regulated in **invasive** Ha-Ras(EJ)-NIH3T3 and MKK3(act)-NIH3T3 cells. Finally, a FOXM1 RNA-knockdown approach revealed its requirement for both invasion and anchorage-independent growth of Ha-Ras(EJ)- and MKK3(act)-NIH3T3 cells. Together, we identified FOXM1 as a key downstream target of Ras and MKK3-induced cellular in vitro invasion and anchorage-independent growth signaling.
5. Glypican-3 reexpression regulates apoptosis in murine adenocarcinoma mammary cells modulating PI3K/Akt and p38MAPK signaling pathways. Glypican-3 (GPC3) is a proteoglycan involved in proliferation and cell survival. Several reports demonstrated that GPC3 is downregulated in some tumors, such as breast cancer. Previously, we determined that GPC3 reexpression in the murine mammary adenocarcinoma LM3 cells induced an impairment of their **invasive** and metastatic capacities, associated with a decrease of their motility and an increase of their cell death. We demonstrated that GPC3 inhibits canonical Wnt signaling, as well as it activates non canonical pathway. Now, we identified signaling pathways responsible for the pro-apoptotic role of GPC3 in LM3 cells. We found for the first time that GPC3 inhibits the PI3K/Akt anti-apoptotic pathway while it stimulates the p38MAPK stress-activated one. We report a concomitant modulation of CDK inhibitors as well as of pro- and anti-apoptotic molecules. Our results provide new clues regarding the mechanism involved in the modulation induced by GPC3 of mammary tumor cell growth and survival.
6. The pore-forming toxin β hemolysin/cytolysin triggers p38 MAPK-dependent IL-10 production in macrophages and inhibits innate immunity. Group B Streptococcus (GBS) is a leading cause of **invasiv**e bacterial infections in human newborns and immune-compromised adults. The pore-forming toxin (PFT) β hemolysin/cytolysin (βh/c) is a major virulence factor for GBS, which is generally attributed to its cytolytic functions. Here we show βh/c has immunomodulatory properties on macrophages at sub-lytic concentrations. βh/c-mediated activation of p38 MAPK drives expression of the anti-inflammatory and immunosuppressive cytokine IL-10, and inhibits both IL-12 and NOS2 expression in GBS-infected macrophages, which are critical factors in host defense. Isogenic mutant bacteria lacking βh/c fail to activate p38-mediated IL-10 production in macrophages and promote increased IL-12 and NOS2 expression. Furthermore, targeted deletion of p38 in macrophages increases resistan**ce to in**vasive GBS infection in mice, associated with impaired IL-10 induction and increased IL-12 production in vivo. These data suggest p38 MAPK activation by βh/c contributes to evasion of host defense through induction of IL-10 expression and inhibition of macrophage activation, a new mechanism of action for a PFT and a novel anti-inflammatory role for p38 in the pathogene**sis of i**nvasive bacterial infection. Our studies suggest p38 MAPK may represent a new therapeutic target to blunt virulence and improve clinical outc**ome of i**nvasive GBS infection.

### [Mm]etastasize

#### PubMed

1. Breast cancer-derived factors stimulate osteoclastogenesis through the Ca2+/protein kinase C and transforming growth factor-beta/MAPK signaling pathways. Breast cancer commonly **metastasize**s to bone where its growth depends on the action of bone-resorbing osteoclasts. We have previously shown that breast cancer cells secrete factors able to directly stimulate osteoclastogenesis from receptor activator of nuclear factor kappaB ligand (RANKL)-primed precursors and that transforming growth factor-beta (TGFbeta) plays a permissive role in this process. Now, we evaluate the signaling events triggered in osteoclast precursors by soluble factors produced by MDA-MB-231 human breast carcinoma cells. In mouse bone marrow cultures and RAW 264.7 murine monocytic cells, MDA-MB-231-derived factors increased osteoclast number, size, and nucleation. These factors failed to induce Smad2 phosphorylation, and short interfering RNAs against Smad4 did not affect their ability to induce osteoclastogenesis. In contrast, MDA-MB-231 factors induced phosphorylation of p38 and ERK1/2, and pharmacological inhibitors against p38 (SB203580) and MEK1/2 (PD98059) impeded the osteoclastogenic effects of cancer-derived factors. Neutralizing antibodies against TGFbeta attenuated p38 activation, whereas activation of ERK1/2 was shortened in duration, but not decreased in amplitude. ERK1/2 phosphorylation induced by cancer-derived factors was blocked by MEK1/2 inhibitor, but not by Ras (manumycin A) or Raf (GW5074) inhibitors. Inhibition of protein kinase Calpha using Gö6976 prevented both ERK1/2 phosphorylation and osteoclast formation in response to MDA-MB-231-derived factors. Using microspectrofluorimetry of fura-2-AM-loaded osteoclast precursors, we have found that cancer-derived factors, similar to RANKL, induced sustained oscillations in cytosolic free calcium. The calcium chelator BAPTA prevented calcium elevations and osteoclast formation in response to MDA-MB-231-derived factors. Thus, we have shown that breast cancer-derived factors induce osteoclastogenesis through the activation of calcium/protein kinase Calpha and TGFbeta-dependent ERK1/2 and p38 signaling pathways.

### [Mm]etastasis

#### PubMed

1. Melanoma cell **metastasis** via P-selectin-mediated activation of acid sphingomyelinase in platelets. Metastatic dissemination of cancer cells is one of the hallmarks of malignancy and accounts for approximately 90 % of human cancer deaths. Within the blood vasculature, tumor cells may aggregate with platelets to form clots, adhere to and spread onto endothelial cells, and finally extravasate to form metastatic colonies. We have previously shown that sphingolipids play a central role in the interaction of tumor cells with platelets; this interaction is a prerequisite for hematogenous tumor **metastasi**s in at least some tumor models. Here we show that the interaction between melanoma cells and platelets results in rapid and transient activation and secretion of acid sphingomyelinase (Asm) in WT but not in P-selectin-deficient platelets. Stimulation of P-selectin resulted in activation of p38 MAPK, and inhibition of p38 MAPK in platelets prevented the secretion of Asm after interaction with tumor cells. Intravenous injection of melanoma cells into WT mice resulted in multiple lung metastases, while in P-selectin-deficient mice pulmonary tumor **metastasi**s and trapping of tumor cells in the lung was significantly reduced. Pre-incubation of tumor cells with recombinant ASM restored trapping of B16F10 melanoma cells in the lung in P-selectin-deficient mice. These findings indicate a novel pathway in tumor **metastasi**s, i.e., tumor cell mediated activation of P-selectin in platelets, followed by activation and secretion of Asm and in turn release of ceramide and tumor **metastasi**s. The data suggest that p38 MAPK acts downstream from P-selectin and is necessary for the secretion of Asm.
2. Phosphatase of regenerating liver 2 (PRL2) is essential for placental development by down-regulating PTEN (Phosphatase and Tensin Homologue Deleted on Chromosome 10) and activating Akt protein. The PRL (phosphatase of regenerating liver) phosphatases are implicated in the control of cell proliferation and invasion. Aberrant PRL expression is associated with progression and **metastasis** of multiple cancers. However, the specific in vivo function of the PRLs remains elusive. Here we show that deletion of PRL2, the most ubiquitously expressed PRL family member, leads to impaired placental development and retarded growth at both embryonic and adult stages. Ablation of PRL2 inactivates Akt and blocks glycogen cell proliferation, resulting in reduced spongiotrophoblast and decidual layers in the placenta. These structural defects cause placental hypotrophy and insufficiency, leading to fetal growth retardation. We demonstrate that the tumor suppressor PTEN is elevated in PRL2-deficient placenta. Biochemical analyses indicate that PRL2 promotes Akt activation by down-regulating PTEN through the proteasome pathway. This study provides the first evidence that PRL2 is required for extra-embryonic development and associates the oncogenic properties of PRL2 with its ability to negatively regulate PTEN, thereby activating the PI3K-Akt pathway.
3. Melanoma cell **metastasis** via P-selectin-mediated activation of acid sphingomyelinase in platelets. Metastatic dissemination of cancer cells is one of the hallmarks of malignancy and accounts for approximately 90 % of human cancer deaths. Within the blood vasculature, tumor cells may aggregate with platelets to form clots, adhere to and spread onto endothelial cells, and finally extravasate to form metastatic colonies. We have previously shown that sphingolipids play a central role in the interaction of tumor cells with platelets; this interaction is a prerequisite for hematogenous tumor **metastasi**s in at least some tumor models. Here we show that the interaction between melanoma cells and platelets results in rapid and transient activation and secretion of acid sphingomyelinase (Asm) in WT but not in P-selectin-deficient platelets. Stimulation of P-selectin resulted in activation of p38 MAPK, and inhibition of p38 MAPK in platelets prevented the secretion of Asm after interaction with tumor cells. Intravenous injection of melanoma cells into WT mice resulted in multiple lung metastases, while in P-selectin-deficient mice pulmonary tumor **metastasi**s and trapping of tumor cells in the lung was significantly reduced. Pre-incubation of tumor cells with recombinant ASM restored trapping of B16F10 melanoma cells in the lung in P-selectin-deficient mice. These findings indicate a novel pathway in tumor **metastasi**s, i.e., tumor cell mediated activation of P-selectin in platelets, followed by activation and secretion of Asm and in turn release of ceramide and tumor **metastasi**s. The data suggest that p38 MAPK acts downstream from P-selectin and is necessary for the secretion of Asm.
4. Piperine alleviates osteoclast formation through the p38/c-Fos/NFATc1 signaling axis. Increased bone fracture is one of the health risk factors in patients with bone loss related disorders such as osteoporosis and breast cancer **metastasis** to bone. Over activity of osteoclasts leads to uncoupling of bone remodeling favoring bone loss over bone formation. Receptor activator of nuclear factor-κβ ligand (RANKL) triggers the differentiation pathway leading to multinucleated osteoclast formation. Modulation of RANKL or its downstream signaling pathways involved in osteoclast formation is of significant interest in the development of anti-resorptive agents. In this study, the effects of piperine, an alkaloid present in Piper nigrum L. on osteoclast formation was investigated. Piperine inhibited tartrate-resistant acid phosphatase-positive multinucleated osteoclast formation in murine RAW264.7 macrophages and human CD14+ monocytes induced by RANKL and breast cancer cells. Piperine attenuated the p38-mitogen activated protein kinase pathway activation, while the extracellular-signal-regulated kinase, c-Jun N-terminal kinase, or NF-κβ pathways downstream of RANKL remained unaffected. Concomitantly, expression of c-Fos and nuclear factor of activated T-cells, cytoplasmic 1 (NFATc1), the key transcription factors involved in osteoclastogenesis were remarkably inhibited by piperine. Furthermore, piperine disrupted the actin ring structure and bone resorption, a characteristic hallmark of osteoclasts. Collectively, these results suggested that piperine inhibited osteoclast differentiation by suppressing the p38/NFATc1/c-Fos signaling axis..
5. LY2228820 dimesylate, a selective inhibitor of p38 mitogen-activated protein kinase, reduces angiogenic endothelial cord formation in vitro and in vivo. LY2228820 dimesylate is a highly selective small molecule inhibitor of p38α and p38β mitogen-activated protein kinases (MAPKs) that is currently under clinical investigation for human malignancies. p38 MAPK is implicated in a wide range of biological processes, in particular those that support tumorigenesis. One such process, angiogenesis, is required for tumor growth an**d metastas**is, and many new cancer therapies are therefore directed against the tumor vasculature. Using an in vitro co-culture endothelial cord formation assay, a surrogate of angiogenesis, we investigated the role of p38 MAPK in growth factor- and tumor-driven angiogenesis using LY2228820 dimesylate treatment and by shRNA gene knockdown. p38 MAPK was activated in endothelial cells upon growth factor stimulation, with inhibition by LY2228820 dimesylate treatment causing a significant decrease in VEGF-, bFGF-, EGF-, and IL-6-induced endothelial cord formation and an even more dramatic decrease in tumor-driven cord formation. In addition to involvement in downstream cytokine signaling, p38 MAPK was important for VEGF, bFGF, EGF, IL-6, and other proangiogenic cytokine secretion in stromal and tumor cells. LY2228820 dimesylate results were substantiated using p38α MAPK-specific shRNA and shRNA against the downstream p38 MAPK effectors MAPKAPK-2 and HSP27. Using in vivo models of functional neoangiogenesis, LY2228820 dimesylate treatment reduced hemoglobin content in a plug assay and decreased VEGF-A-stimulated vascularization in a mouse ear model. Thus, p38α MAPK is implicated in tumor angiogenesis through direct tumoral effects and through reduction of proangiogenic cytokine secretion via the microenvironment.
6. Sirt7 Contributes to Myocardial Tissue Repair by Maintaining Transforming Growth Factor-β Signaling Pathway. Sirt7, 1 of the 7 members of the mammalian sirtuin family, promotes oncogenic transformation. Tumor growth and **metastasi**s require fibrotic and angiogenic responses. Here, we investigated the role of Sirt7 in cardiovascular tissue repair process.In wild-type mice, Sirt7 expression increased in response to acute cardiovascular injury, including myocardial infarction and hind-limb ischemia, particularly at the active wound healing site. Compared with wild-type mice, homozygous Sirt7-deficient (Sirt7(-/-)) mice showed susceptibility to cardiac rupture after myocardial infarction, delayed blood flow recovery after hind-limb ischemia, and impaired wound healing after skin injury. Histological analysis showed reduced fibrosis, fibroblast differentiation, and inflammatory cell infiltration in the border zone of infarction in Sirt7(-/-) mice. In vitro, Sirt7(-/-) mouse-derived or Sirt7 siRNA-treated cardiac fibroblasts showed reduced transforming growth factor-β signal activation and low expression levels of fibrosis-related genes compared with wild-type mice-derived or control siRNA-treated cells. These changes were accompanied by reduction in transforming growth factor receptor I protein. Loss of Sirt7 activated autophagy in cardiac fibroblasts. Transforming growth factor-β receptor I downregulation induced by loss of Sirt7 was blocked by autophagy inhibitor, and interaction of Sirt7 with protein interacting with protein kinase-Cα was involved in this process.Sirt7 maintains transforming growth factor receptor I by modulating autophagy and is involved in the tissue repair process.
7. Wnt5a and Wnt11 regulate mammalian anterior-posterior axis elongation. Mesoderm formation and subsequent anterior-posterior (A-P) axis elongation are fundamental aspects of gastrulation, which is initiated by formation of the primitive streak (PS). Convergent extension (CE) movements and epithelial-mesenchymal transition (EMT) are important for A-P axis elongation in vertebrate embryos. The evolutionarily conserved planar cell polarity (PCP) pathway regulates CE, and Wnts regulate many aspects of gastrulation including CE and EMT. However, the Wnt ligands that regulate A-P axis elongation in mammalian development remain unknown. Wnt11 and Wnt5a regulate axis elongation in lower vertebrates, but only Wnt5a, not Wnt11, regulates mammalian PCP signaling and A-P axis elongation in development. Here, by generating Wnt5a; Wnt11 compound mutants, we show that Wnt11 and Wnt5a play redundant roles during mouse A-P axis elongation. Both genes regulate trunk notochord extension through PCP-controlled CE of notochord cells, establishing a role for Wnt11 in mammalian PCP. We show that Wnt5a and Wnt11 are required for proper patterning of the neural tube and somites by regulating notochord formation, and provide evidence that both genes are required for the generation and migration of axial and paraxial mesodermal precursor cells by regulating EMT. Axial and paraxial mesodermal precursors ectopically accumulate in the PS at late gastrula stages in Wnt5a(-/-); Wnt11(-/-) embryos and these cells ectopically express epithelial cell adhesion molecules. Our data suggest that Wnt5a and Wnt11 regulate EMT by inducing p38 (Mapk14) phosphorylation. Our findings provide new insights into the role of Wnt5a and Wnt11 in mouse early development and also in cancer **metastasis**, during which EMT plays a crucial role.
8. CXCL12 and C5a trigger cell migration via a PAK1/2-p38alpha MAPK-MAPKAP-K2-HSP27 pathway. Cell migration is critical for many processes, such as angiogenesis, inflammation, development and wound healing, and is also involved in tumour progression and **metastasis**. Here we show that CXCL12, complement factor 5a (C5a), hepatocyte growth factor (HGF) and platelet-derived growth factor (PDGF)-BB, which stimulate cell migration, also activate p38alpha MAPK. Pharmacological inhibition of this protein kinase with SB 203580 or BIRB 0796, or the genetic ablation of p38alpha MAPK, blocked cell migration induced by the aforementioned chemo-attractants. Macrophages from mice lacking one or more of the other p38 MAPK isoforms showed normal cell migration in response to C5a. We also show that the activation of p38alpha MAPK in response to CXCL12 requires the p21-activated protein kinases (PAK)-1 and PAK-2. MAPKAP-K2 is a protein kinase that is activated by p38alpha MAPK. Reducing its expression using RNA interference blocked CXCL12-induced HeLa cell migration, while macrophages from mice that do not express MAPKAP-K2 failed to migrate in response to C5a. Moreover, RNA interference against the small heat shock protein 27 (HSP27), a physiological substrate of MAPKAP-K2, blocked the CXCL12-induced cell migration. These results demonstrate a general and essential role of the PAK-p38alpha MAPK-MAPKAP-K2-HSP27 signalling pathway in mediating the effects of chemotactic stimuli on cell migration.
9. Endothelial CCR2 signaling induced by colon carcinoma cells enables extravasation via the JAK2-Stat5 and p38MAPK pathway. Increased expression of the chemokine CCL2 in tumor cells correlates with enhanced **metastasis**, poor prognosis, and recruitment of CCR2(+)Ly6C(hi) monocytes. However, the mechanisms driving tumor cell extravasation through the endothelium remain elusive. Here, we describe CCL2 upregulation in metastatic UICC stage IV colon carcinomas and demonstrate that tumor cell-derived CCL2 activates the CCR2(+) endothelium to increase vascular permeability in vivo. CCR2 deficiency prevents colon carcinoma extravasation and **metastasi**s. Of note, CCR2 expression on radio-resistant cells or endothelial CCR2 expression restores extravasation and **metastasi**s in Ccr2(-/-) mice. Reduction of CCR2 expression on myeloid cells decreases but does not prevent **metastasi**s. CCL2-induced vascular permeability and **metastasi**s is dependent on JAK2-Stat5 and p38MAPK signaling. Our study identifies potential targets for treating CCL2-dependent **metastasi**s.
10. The p38 SAPK pathway is required for Ha-ras induced in vitro invasion of NIH3T3 cells. Constitutive activation of the ras oncoprotein plays a critical role in cancer invasion and **metastasis**. Particularly, ras-related protease expression such as the serine protease urokinase plasminogen activator (u-PA) has been implicated in mediating cancer cell invasion. Previous studies have shown that ras-mediated u-PA expression is regulated through the mitogen- (MAPK) and stress-activated protein kinase (SAPK) signal transduction pathways extracellular signal-regulated kinase (ERK) and c-Jun-activating kinase (JNK). We therefore asked the question, if ras-related cell invasion might additionally require the third MAPK/SAPK signal transduction cascade, p38. Indeed, we found that ras induces invasion based on the activation of certain p38 protein kinase isoforms, in particular, p38alpha. Moreover, ras activation through transient or stable expression of a Ha-rasEJ mutant induced the expression of u-PA. This was found to be a consequence of an increase of u-PA m-RNA, which was paralleled by only a modest activation of the u-PA promoter. In conclusion, we provide evidence for the requirement of a novel ras-p38alpha-u-PA pathway for ras-dependent cellular invasion.
11. Involvement of p38alpha mitogen-activated protein kinase in lung **metastasis** of tumor cells. To study the role of p38 mitogen-activated protein kinase (p38) activity during the process of **metastasis**, p38alpha(+/-) mice were subjected to an in vivo **metastasis** assay. The number of lung colonies of tumor cells intravenously injected in p38alpha(+/-) mice was markedly decreased compared with that in wild-type (WT) mice. On the other hand, the time-dependent increase in tumor volume after subcutaneous tumor cells transplantation was comparable between WT and p38alpha(+/-) mice. Platelets of p38alpha(+/-) mice were poorly bound to tumor cells in vitro and in vivo compared with those of WT mice. E- and P-selectin mRNAs were markedly induced in the lung after intravenous injection of tumor cells. However, the induction of these selectin mRNAs in p38alpha(+/-) mice was weaker than that in WT mice. Furthermore, the resting expression levels of E-selectin in lung endothelial cells and P-selectin in platelets of p38alpha(+/-) mice were suppressed compared with those of WT mice. The number of tumor cells attached on lung endothelial cells of p38alpha(+/-) mice was significantly reduced compared with that of WT mice. The transmigrating activity of tumor cells through lung endothelial cells of p38alpha(+/-) mice was similar to that of WT mice. These results suggest that p38alpha plays an important role in extravasation of tumor cells, possibly through regulating the formation of tumor-platelet aggregates and their interaction with the endothelium involved in a step of hematogenous **metastasis**.
12. Extracellular galectin-3 induces MMP9 expression by activating p38 MAPK pathway via lysosome-associated membrane protein-1 (LAMP1). Matrix metalloproteinases (MMPs) play a key role in matrix remodelling and thus invasion and **metastasis**. Extracellular galectin-3 has been shown to induce MMP9 secretion. Here, we demonstrate that galectin-3 induces MMP9 at transcript level and it is dependent on the surface levels of poly-N-acetyllactosamine (polyLacNAc). By employing signalling pathway inhibitors, MMP9 expression was shown to be induced via p38 MAP-kinase pathway. Using clones of melanoma cells expressing shRNAs to lysosome-associated membrane protein-1 (LAMP1), a major carrier of polyLacNAc, surface LAMP1 was demonstrated to serve as one of the key mediators of galectin-3-induced MMP9 expression via p38 MAPK pathway.
13. Transcriptional activation of MMP-13 by periodontal pathogenic LPS requires p38 MAP kinase. Matrix metalloprotease-13 (MMP-13) is induced by pro-inflammatory cytokines and increased expression is associated with a number of pathological conditions such as tumor **metastasis**, osteoarthritis, rheumatoid arthritis and periodontal diseases. MMP-13 gene regulation and the signal transduction pathways activated in response to bacterial LPS are largely unknown. In these studies, the role of the mitogen-activated protein kinase (MAPK) pathways in the regulation of MMP-13 induced by lipopolysaccharide was investigated. Lipopolysaccharide from Escherichia coli and Actinobacillus actinomycetemcomitans significantly (P < 0.05) increased MMP-13 steady-state mRNA (average of 27% and 46% increase, respectively) in murine periodontal ligament fibroblasts. MMP-13 mRNA induction was significantly reduced by inhibition of p38 MAP kinase. Immunoblot analysis indicated that p38 signaling was required for LPS-induced MMP-13 expression. Lipopolysaccharide induced proximal promoter reporter (-660/+32 mMMP-13) gene activity required p38 signaling. Collectively, these results indicate that lipopolysaccharide-induced murine MMP-13 is regulated by p38 signaling through a transcriptional mechanism.
14. TGF-β2 dictates disseminated tumour cell fate in target organs through TGF-β-RIII and p38α/β signalling. In patients, non-proliferative disseminated tumour cells (DTCs) can persist in the bone marrow (BM) while other organs (such as lung) present grow**ing metast**asis. This suggested that the BM might b**e a metast**asis 'restrictive soil' by encoding dormancy-inducing cues in DTCs. Here we show in a head and neck squamous cell carcinoma (HNSCC) model that strong and specific transforming growth factor-β2 (TGF-β2) signalling in the BM activates the MAPK p38α/β, inducing an (ERK/p38)(low) signalling ratio. This results in induction of DEC2/SHARP1 and p27, downregulation of cyclin-dependent kinase 4 (CDK4) and dormancy of malignant DTCs. TGF-β2-induced dormancy required TGF-β receptor-I (TGF-β-RI), TGF-β-RIII and SMAD1/5 activation to induce p27. **In lungs,** a metastasis 'permissive soil' with low TGF-β2 levels, DTC dormancy was short-lived and followed by metastatic growth. Importantly, systemic inhibition of TGF-β-RI or p38α/β activities awakened dormant DTCs, fuel**ling multi**-organ metastasis. Our work reveals a 'seed and soil' mechanism where TGF-β2 and TGF-β-RIII signalling through p38α/β regulates DTC dormancy and defines restrictive (BM) and permissive (lung) microen**vironments** for HNSCC metastasis.

#### GeneRIFs

1. These findings indicate a novel pathway in tumor **metastasis**, i.e., tumor cell mediated activation of P-selectin in platelets, followed by activation and secretion of Asm and in turn release of ceramide and tumor **metastasis**. The data suggest that p38 MAPK acts downstream from P-selectin and is necessary for the secretion of Asm.
2. work reveals a 'seed and soil' mechanism where TGF-beta2 and TGF-beta-RIII signalling through p38alpha/beta regulates DTC dormancy and defines restrictive (BM) and permissive (lung) microenvironments for HNSCC **metastasis**
3. CCL2-induced vascular permeability and **metastasis** is dependent on JAK2-Stat5 and p38MAPK signaling
4. p38alpha plays an important role in extravasation of tumor cells, possibly through regulating the formation of tumor-platelet aggregates and their interaction with the endothelium involved in a step of hematogenous **metastasis**

### [Cc]ell [Ii]nvasion

#### PubMed

1. The p38 SAPK pathway is required for Ha-ras induced in vitro invasion of NIH3T3 cells. Constitutive activation of the ras oncoprotein plays a critical role in cancer invasion and metastasis. Particularly, ras-related protease expression such as the serine protease urokinase plasminogen activator (u-PA) has been implicated in mediating cancer **cell invasion**. Previous studies have shown that ras-mediated u-PA expression is regulated through the mitogen- (MAPK) and stress-activated protein kinase (SAPK) signal transduction pathways extracellular signal-regulated kinase (ERK) and c-Jun-activating kinase (JNK). We therefore asked the question, if ras-related **cell invasion** might additionally require the third MAPK/SAPK signal transduction cascade, p38. Indeed, we found that ras induces invasion based on the activation of certain p38 protein kinase isoforms, in particular, p38alpha. Moreover, ras activation through transient or stable expression of a Ha-rasEJ mutant induced the expression of u-PA. This was found to be a consequence of an increase of u-PA m-RNA, which was paralleled by only a modest activation of the u-PA promoter. In conclusion, we provide evidence for the requirement of a novel ras-p38alpha-u-PA pathway for ras-dependent cellular invasion.

---

## Fermt2

- NCBI Gene
- GeneRIF
- Pubmed

| Search Term | Rank | Fields |
| --- | --- | --- |
| [Pp]odosome(s)? | 5 | PubMed(1) |
| [Mm]igration | 6 | PubMed(8); GeneRIFs(2) |
| ECM | 7 | PubMed(1) |
| [Ee]xtracellular [Mm]atrix | 8 | PubMed(2) |
| [Mm]etastasis | 12 | PubMed(1) |

  
hide/show details for Fermt2

### [Pp]odosome(s)?

#### PubMed

1. The Kindlins: subcellular localization and expression during murine development. The three Kindlins are a novel family of focal adhesion proteins. The Kindlin-1 (URP1) gene is mutated in Kindler syndrome, the first skin blistering disease affecting actin attachment in basal keratinocytes. Kindlin-2 (Mig-2), the best studied member of this family, binds ILK and Migfilin, which links Kindlin-2 to the actin cytoskeleton. Kindlin-3 is expressed in hematopoietic cells. Here we describe the genomic organization, gene expression and subcellular localization of murine Kindlins-1 to -3. In situ hybridizations showed that Kindlin-1 is preferentially expressed in epithelia, and Kindlin-2 in striated and smooth muscle cells. Kindlins-1 and -2 are both expressed in the epidermis. While both localize to integrin-mediated adhesion sites in cultured keratinocytes Kindlin-2, but not Kindlin-1, colocalizes with E-cadherin to cell-cell contacts in differentiated keratinocytes. Using a Kindlin-3-specific antiserum and an EGFP-tagged Kindlin-3 construct, we could show that Kindlin-3 is present in the F-actin surrounding ring structure of **podosomes**, which are specialized adhesion structures of hematopoietic cells.

### [Mm]igration

#### PubMed

1. Distinct roles of talin and kindlin in regulating integrin α5β1 function and trafficking. Integrins are heterodimeric αβ transmembrane receptors that play key roles in cellular physiology and pathology. Accumulating data indicate that the two NPxY motifs in the cytoplasmic domain of the β1 subunit synergistically promote integrin activation through the binding of talin and kindlin. However, it is unclear how the individual motifs regulate integrin function and trafficking.To investigate how the two NPxY motifs individually control integrin α5β1 function and trafficking, we introduced Y > A mutations in either motif. Disruption of the membrane-proximal NPxY completely prevented α5β1-induced morphological changes, cell scatte**ring and** migration, and fibronectin fibrillogenesis. In addition, it reduced α5β1 internalization but not its recycling. In contrast, disruption of the membrane-distal NPxY promoted degradation of α5β1 in late endosomes/lysosomes but did not prevent α5β1-dependent ce**ll scatte**ring, migration, or fibronectin fibrillogenesis. Whereas depletion of either talin-1 or kindlin-2 reduced α5β1 binding to fibronectin and cell adhesion, talin-1 depletion recapitulated the loss-of-function phenotype of the membrane-proximal NPxY mutation, whereas kindlin-2 depletion induced α5β1 accumulation in lysosomes and degradation.The two NPxY motifs of β1 play distinct and separable roles in controlling the function and trafficking of α5β1. Whereas talin binding to the membrane-proximal NPxY is crucial for connecting α5β1 to the actin cytoskeleton and thus permit the tension required for fibronectin f**ibrilloge**nesis and cell migration, kindlin binding to the membrane-distal NPxY is dispensable for these events but regulates α5β1 surface expression and degradation.
2. Kindlin-2 tyrosine phosphorylation and interaction with Src serve as a regulatable switch in the integrin outside-in signaling circuit. Integrin-mediated cell-extracellular matrix (ECM) adhesion is critical for control of intracellular signaling; however, the mechanisms underlying this "outside-in" signaling are incompletely understood. Here we show that depletion of kindlin-2 impairs integrin outside-in signaling. Kindlin-2 is tyrosine-phosphorylated upon cell-ECM adhesion. Furthermore, kindlin-2 binds Src in a cell-ECM adhesion-regulatable fashion. At the molecular level, the kindlin-2·Src interaction is mediated by the kindlin-2 F0 and the Src SH2 and SH3 domains. Src activation increases kindlin-2 tyrosine phosphorylation and the kindlin-2·Src interaction. Conversely, inhibition of Src reduces kindlin-2 tyrosine phosphorylation and diminishes the kindlin-2·Src interaction. Finally, disruption of the kindlin-2·Src interaction, unlike depletion of kindlin-2, impairs neither cell-ECM adhesion nor cell-ECM adhesion-induced focal adhesion kinase Tyr-397 phosphorylation. However, it markedly inhibits cell-ECM adhesion-induced paxillin tyrosine phosphorylation, c**ell migra**tion, and proliferation. These results suggest that kindlin-2 tyrosine phosphorylation and interaction with Src serve as a regulatable switch downstream of focal adhesion kinase in the integrin outside-in signaling circuit, relaying signals from cell-ECM adhesion to paxillin that control c**ell migra**tion and proliferation.
3. Kindlin-2 induced by TGF-β signaling promotes pancreatic ductal adenocarcinoma progression through downregulation of transcriptional factor HOXB9. Pancreatic ductal adenocarcinoma (PDAC) is the fourth leading cause of cancer-related deaths with no effective therapeutics. Invasion and metastasis are the major characteristics of PDAC. However, mechanisms underlying PDAC invasion and metastasis are elusive. In this report, we found that Kindlin-2 is a target protein of transforming growth factor β (TGF-β) signaling and is upregulated by TGF-β1 in PDAC cells. TGF-β1-upregulated Kindlin-2 promotes PDAC cell gro**wth, migr**ation and invasion, whereas Kindlin-2 upregulates transforming growth factor receptor I (TβRI), a key component of TGF-β signaling. Thereby Kindlin-2 and TGF-β signaling constitute a positive feedback loop. Mechanistically, Kindlin-2 promotes PDAC progression by downregulation of HOXB9 and E-cadherin. For clinical relevance, enhanced expression of Kindlin-2 predicts a poor overall survival for PDAC patients. Gene expression levels of Kindlin-2, TGF-β, TβRI and HOXB9 are all correlated with the overall survival of PDAC patients in an Oncomine dataset. Taken together, our findings demonstrated that TGF-β1-induced Kindlin-2 expression promotes PDAC progression by downregulation of HOXB9 and E-cadherin.
4. Interaction of kindlin-2 with integrin β3 promotes outside-in signaling responses by the αVβ3 vitronectin receptor. The bidirectional signaling and hemostatic functions of platelet αIIbβ3 are regulated by kindlin-3 through interactions with the β3 cytoplasmic tail. Little is known about kindlin regulation of the related "vitronectin receptor," αVβ3. These relationships were investigated in endothelial cells, which express αVβ3 and kindlin-2 endogenously. "β3ΔRGT" knock-in mice lack the 3 C-terminal β3 tail residues, whereas in "β3/β1(EGK)" mice, RGT is replaced by the corresponding residues of β1. The wild-type β3 tail pulled down kindlin-2 and c-Src in vitro, whereas β3ΔRGT bound neither protein and β3/β1(EGK) bound kindlin-2, but not c-Src. β3ΔRGT endothelial cells, but not β3/β1(EGK) endo**thelial c**ells, exhibited migration and spreading defects on vitronectin and reduced sprouting in 3-dimensional fibrin. Short hairpin RNA silencing of kindlin-2, but not c-Src, blocked sprouting by β3 wild-type endothelial cells. Moreover, defective sprouting by β3ΔRGT endothelial cells could be rescued by conditional, forced interaction of αVβ3ΔRGT with kindlin-2. Stimulation of β3ΔRGT endothelial cells led to normal extracellular ligand binding to αVβ3, pin-pointing their defect to one of outside-in αVβ3 signaling. β3ΔRGT mice, but not β3/β1(EGK) mice, exhibited defects in both developmental and tumor angiogenesis, responses that require endothelial cell function. Thus, the β3/kindlin-2 interaction promotes outside-in αVβ3 signaling selectively, with biological consequences in vivo.
5. Role of kindlin-2 in fibroblast functions: implications for wound healing. Kindlins are a protein family that regulates cell-matrix interactions. Although kindlin-2 is known to be essential for the early developmental processes, its role in the homeostasis of adult tissues has remained elusive. In this study, we used new domain-specific antibodies and small interfering RNA technology to uncover physiological functions of kindlin-2 in human dermal fibroblasts in vitro and in adult skin in situ. In primary dermal fibroblasts, kindlin-2 is essential for the integrin clustering and activation, and it regulates cell adhesion and directed **migration** by guiding formation and maturation of focal adhesions (FAs) and organization of the cytoskeleton. These functions are linked to the transmission of mechanical cues between cells and their microenvironment, typically in processes wherein fibroblasts differentiate to myofibroblasts under mechanical tension. In concert, kindlin-2 was shown to be required for the stabilization and maturation of FAs and stress fibers in activated fibroblasts and myofibroblasts. These findings implicated that in physiological wound closure and tissue repair, the prediction was validated by strong upregulation of kindlin-2 in contractile myofibroblasts during middle stages of wound healing in human skin. Taken together, the data reveal a physiological role for kindlin-2 in skin fibroblasts under normal steady-state conditions and during tissue regeneration.
6. The integrin coactivator kindlin-2 plays a critical role in angiogenesis in mice and zebrafish. Kindlin-2, a widely distributed cytoskeletal protein, has been implicated in integrin activation, and its absence is embryonically lethal in mice and causes severe developmental defects in zebrafish. Knockdown of kindlin-2 levels in endothelial cells resulted in defective adhesive and migratory responses, suggesting that angiogenesis might be aberrant even with partial reduction of kindlin-2. This hypothesis has now been tested in the kindlin-2(+/-) mice. RM1 prostate tumors grown in kindlin-2(+/-) mice had fewer blood vessels, which were thinner and shorter and supported less tumor growth compared with wild-type littermates. The vessels that did form in the kindlin-2(+/-) mice lacked smooth muscle cells and pericytes and had thinner basement membranes, indicative of immature vessels. VEGF-induced angiogenesis in matrigel implants was also abnormal in the kindlin-2(+/-) mice. Vessels in the kindlin-2(+/-) mice were leaky, and BM transplantation from kindlin-2(+/-) to WT mice did not correct this defect. Endothelial cells derived from kindlin-2(+/-) mice had integrin expression levels similar to WT mice but reduced αVβ3-dependent signaling**, migrati**on, adhesion, spreading, and tube formation. Developmental angiogenesis was markedly impaired by kindlin-2 morpholinos in zebrafish. Taken together, kindlin-2 plays an important role in pathologic and developmental angiogenesis, which arises from defective activation of integrin αVβ3.
7. Kindlin-2 inhibited the growth and **migration** of colorectal cancer cells. Activation of Wingless (Wnt)/beta-catenin signaling is a hallmark of colorectal carcinoma (CRC). It is very important to find out the molecular mechanism for the hyperactivation of Wnt/beta-catenin signaling and identify novel therapeutic targets. Kindlin-2, a regulator of integrins, recently has been found to be involved in the tumorigenesis. However, its expression profile and functions in the progression of CRC remain poorly understood. Here, we found that the expression of Kindlin-2 was downregulated in the CRC tissues. Moreover, overexpression of Kindlin-2 in CRC cells and normal colon epithelial cells inhibited cell proliferation and **migration**, while downregulation of Kindlin-2 promoted the tumorigenecity of CRC cells in vitro and in vivo. Mechanistically, Kindlin-2 decreased the phosphorylation of Ser9 in glycogen synthase kinase (GSK) 3beta and promoted the ubiquitination of beta-catenin. Taken together, our study suggests the suppressive roles of Kindlin-2 in the pathogenesis of CRC.
8. Kindlin binds migfilin tandem LIM domains and regulates migfilin focal adhesion localization and recruitment dynamics. Focal adhesions (FAs), sites of tight adhesion to the extracellular matrix, are composed of clusters of transmembrane integrin adhesion receptors and intracellular proteins that link integrins to the actin cytoskeleton and signaling pathways. Two integrin-binding proteins present in FAs, kindlin-1 and kindlin-2, are important for integrin activation, FA formation, and signaling. Migfilin, originally identified in a yeast two-hybrid screen for kindlin-2-interacting proteins, is a LIM domain-containing adaptor protein found in FAs and implicated in control of cell adhesion, spreading, and **migration**. By binding filamin, migfilin provides a link between kindlin and the actin cytoskeleton. Here, using a combination of kindlin knockdown, biochemical pulldown assays, fluorescence microscopy, fluorescence resonance energy transfer (FRET), and fluorescence recovery after photobleaching (FRAP), we have established that the C-terminal LIM domains of migfilin dictate its FA localization, shown that these domains mediate an interaction with kindlin in vitro and in cells, and demonstrated that kindlin is important for normal migfilin dynamics in cells. We also show that when the C-terminal LIM domain region is deleted, then the N-terminal filamin-binding region of the protein, which is capable of targeting migfilin to actin-rich stress fibers, is the predominant driver of migfilin localization. Our work details a correlation between migfilin domains that drive kindlin binding and those that drive FA localization as well as a kindlin dependence on migfilin FA recruitment and mobility. We therefore suggest that the kindlin interaction with migfilin LIM domains drives migfilin FA recruitment, localization, and mobility.

#### GeneRIFs

1. kindlin-2 tyrosine phosphorylation and interaction with Src serve as a regulatable switch downstream of focal adhesion kinase in the integrin outside-in signaling circuit controlling cell **migration** and proliferation
2. Endothelial cells derived from kindlin-2(+/-) mice had integrin expression levels similar to WT mice but reduced alphaVbeta3-dependent signaling, **migration**, adhesion, spreading, and tube formation.

### ECM

#### PubMed

1. Kindlin-2 tyrosine phosphorylation and interaction with Src serve as a regulatable switch in the integrin outside-in signaling circuit. Integrin-mediated cell-extracellular matrix (**ECM**) adhesion is critical for control of intracellular signaling; however, the mechanisms underlying this "outside-in" signaling are incompletely understood. Here we show that depletion of kindlin-2 impairs integrin outside-in signaling. Kindlin-2 is tyrosine-phosphorylated upon cell-**ECM** adhesion. Furthermore, kindlin-2 binds Src in a cell-**ECM** adhesion-regulatable fashion. At the molecular level, the kindlin-2·Src interaction is mediated by the kindlin-2 F0 and the Src SH2 and SH3 domains. Src activation increases kindlin-2 tyrosine phosphorylation and the kindlin-2·Src interaction. Conversely, inhibition of Src reduces kindlin-2 tyrosine phosphorylation and diminishes the kindlin-2·Src interaction. Finally, disruption of the kindlin-2·Src interaction, unlike depletion of kindlin-2, impairs neither c**ell**-ECM adhesion nor c**ell**-ECM adhesion-induced focal adhesion kinase Tyr-397 phosphorylation. However, it markedly inhibits c**ell**-ECM adhesion-induced paxillin tyrosine phosphorylation, cell migration, and proliferation. These results suggest that kindlin-2 tyrosine phosphorylation and interaction with Src serve as a regulatable switch downstream of focal adhesion kinase in the integrin outside-in signaling circuit, relaying signals from c**ell**-ECM adhesion to paxillin that control cell migration and proliferation.

### [Ee]xtracellular [Mm]atrix

#### PubMed

1. Kindlin-2 tyrosine phosphorylation and interaction with Src serve as a regulatable switch in the integrin outside-in signaling circuit. Integrin-mediated cell-**extracellular matrix** (ECM) adhesion is critical for control of intracellular signaling; however, the mechanisms underlying this "outside-in" signaling are incompletely understood. Here we show that depletion of kindlin-2 impairs integrin outside-in signaling. Kindlin-2 is tyrosine-phosphorylated upon cell-ECM adhesion. Furthermore, kindlin-2 binds Src in a cell-ECM adhesion-regulatable fashion. At the molecular level, the kindlin-2·Src interaction is mediated by the kindlin-2 F0 and the Src SH2 and SH3 domains. Src activation increases kindlin-2 tyrosine phosphorylation and the kindlin-2·Src interaction. Conversely, inhibition of Src reduces kindlin-2 tyrosine phosphorylation and diminishes the kindlin-2·Src interaction. Finally, disruption of the kindlin-2·Src interaction, unlike depletion of kindlin-2, impairs neither cell-ECM adhesion nor cell-ECM adhesion-induced focal adhesion kinase Tyr-397 phosphorylation. However, it markedly inhibits cell-ECM adhesion-induced paxillin tyrosine phosphorylation, cell migration, and proliferation. These results suggest that kindlin-2 tyrosine phosphorylation and interaction with Src serve as a regulatable switch downstream of focal adhesion kinase in the integrin outside-in signaling circuit, relaying signals from cell-ECM adhesion to paxillin that control cell migration and proliferation.
2. Kindlin binds migfilin tandem LIM domains and regulates migfilin focal adhesion localization and recruitment dynamics. Focal adhesions (FAs), sites of tight adhesion to the **extracellular matrix**, are composed of clusters of transmembrane integrin adhesion receptors and intracellular proteins that link integrins to the actin cytoskeleton and signaling pathways. Two integrin-binding proteins present in FAs, kindlin-1 and kindlin-2, are important for integrin activation, FA formation, and signaling. Migfilin, originally identified in a yeast two-hybrid screen for kindlin-2-interacting proteins, is a LIM domain-containing adaptor protein found in FAs and implicated in control of cell adhesion, spreading, and migration. By binding filamin, migfilin provides a link between kindlin and the actin cytoskeleton. Here, using a combination of kindlin knockdown, biochemical pulldown assays, fluorescence microscopy, fluorescence resonance energy transfer (FRET), and fluorescence recovery after photobleaching (FRAP), we have established that the C-terminal LIM domains of migfilin dictate its FA localization, shown that these domains mediate an interaction with kindlin in vitro and in cells, and demonstrated that kindlin is important for normal migfilin dynamics in cells. We also show that when the C-terminal LIM domain region is deleted, then the N-terminal filamin-binding region of the protein, which is capable of targeting migfilin to actin-rich stress fibers, is the predominant driver of migfilin localization. Our work details a correlation between migfilin domains that drive kindlin binding and those that drive FA localization as well as a kindlin dependence on migfilin FA recruitment and mobility. We therefore suggest that the kindlin interaction with migfilin LIM domains drives migfilin FA recruitment, localization, and mobility.

### [Mm]etastasis

#### PubMed

1. Kindlin-2 induced by TGF-β signaling promotes pancreatic ductal adenocarcinoma progression through downregulation of transcriptional factor HOXB9. Pancreatic ductal adenocarcinoma (PDAC) is the fourth leading cause of cancer-related deaths with no effective therapeutics. Invasion and **metastasi**s are the major characteristics of PDAC. However, mechanisms underlying PDAC invasion and **metastasi**s are elusive. In this report, we found that Kindlin-2 is a target protein of transforming growth factor β (TGF-β) signaling and is upregulated by TGF-β1 in PDAC cells. TGF-β1-upregulated Kindlin-2 promotes PDAC cell growth, migration and invasion, whereas Kindlin-2 upregulates transforming growth factor receptor I (TβRI), a key component of TGF-β signaling. Thereby Kindlin-2 and TGF-β signaling constitute a positive feedback loop. Mechanistically, Kindlin-2 promotes PDAC progression by downregulation of HOXB9 and E-cadherin. For clinical relevance, enhanced expression of Kindlin-2 predicts a poor overall survival for PDAC patients. Gene expression levels of Kindlin-2, TGF-β, TβRI and HOXB9 are all correlated with the overall survival of PDAC patients in an Oncomine dataset. Taken together, our findings demonstrated that TGF-β1-induced Kindlin-2 expression promotes PDAC progression by downregulation of HOXB9 and E-cadherin.

---

## Hspa5

- NCBI Gene
- GeneRIF
- Pubmed

| Search Term | Rank | Fields |
| --- | --- | --- |
| [Mm]igration | 6 | Process(1); PubMed(5) |
| ECM | 7 | PubMed(1) |
| [Ee]xtracellular [Mm]atrix | 8 | PubMed(7); Component(1) |
| [Ii]nvasive | 10 | PubMed(2) |
| [Mm]etastasize | 11 | PubMed(1) |
| [Mm]etastasis | 12 | PubMed(4) |
| [Cc]ell [Ii]nvasion | 13 | PubMed(1) |

  
hide/show details for Hspa5

### [Mm]igration

#### Process

1. positive regulation of cell **migration**

#### PubMed

1. Lack of pericytes leads to endothelial hyperplasia and abnormal vascular morphogenesis. The association of pericytes (PCs) to newly formed blood vessels has been suggested to regulate endothelial cell (EC) proliferation, survival, **migration**, differentiation, and vascular branching. Here, we addressed these issues using PDGF-B-- and PDGF receptor-beta (PDGFR-beta)--deficient mice as in vivo models of brain angiogenesis in the absence of PCs. Quantitative morphological analysis showed that these mutants have normal microvessel density, length, and number of branch points. However, absence of PCs correlates with endothelial hyperplasia, increased capillary diameter, abnormal EC shape and ultrastructure, changed cellular distribution of certain junctional proteins, and morphological signs of increased transendothelial permeability. Brain endothelial hyperplasia was observed already at embryonic day (E) 11.5 and persisted throughout development. From E 13.5, vascular endothelial growth factor-A (VEGF-A) and other genes responsive to metabolic stress became upregulated, suggesting that the abnormal microvessel architecture has systemic metabolic consequences. VEGF-A upregulation correlated temporally with the occurrence of vascular abnormalities in the placenta and dilation of the heart. Thus, although PC deficiency appears to have direct effects on EC number before E 13.5, the subsequent increased VEGF-A levels may further abrogate microvessel architecture, promote vascular permeability, and contribute to formation of the edematous phenotype observed in late gestation PDGF-B and PDGFR-beta knock out embryos.
2. A critical role for GRP78/BiP in the tumor microenvironment for neovascularization during tumor growth and metastasis. Glucose-regulated protein 78 (GRP78)/BiP is a multifunctional protein which plays a major role in endoplasmic reticulum (ER) protein processing, protein quality control, maintaining ER homeostasis, and controlling cell signaling and viability. Previously, using a transgene-induced mammary tumor model, we showed that Grp78 heterozygosity impeded cancer growth through suppression of tumor cell proliferation and promotion of apoptosis and the Grp78(+/-) mice exhibited dramatic reduction (70%) in the microvessel density (MVD) of the endogenous mammary tumors, while having no effect on the MVD of normal organs. This observation suggests that GRP78 may critically regulate the function of the host vasculature within the tumor microenvironment. In this article, we interrogated the role of GRP78 in the tumor microenvironment. In mouse tumor models in which wild-type (WT), syngeneic mammary tumor cells were injected into the host, we showed that Grp78(+/-) mice suppressed tumor growth and angiogenesis during the early phase but not during the late phase of tumor growth. Growth of metastatic lesions of WT, syngeneic melanoma cells in the Grp78(+/-) mice was potently suppressed. We created conditional heterozygous knockout of GRP78 in the host endothelial cells and showed severe reduction of tumor angiogenesis and metastatic growth, with minimal effect on normal tissue MVD. Furthermore, knockdown of GRP78 expression in immortalized human endothelial cells showed that GRP78 is a critical mediator of angiogenesis by regulating cell proliferation, survival, and **migration**. Our findings suggest that concomitant use of current chemotherapeutic agents and novel therapies against GRP78 may offer a powerful dual approach to arrest cancer initiation, progression, and metastasis.
3. Altered quality control in the endoplasmic reticulum causes cortical dysplasia in knock-in mice expressing a mutant BiP. Binding immunoglobulin protein (BiP) is an endoplasmic reticulum (ER) molecular chaperone that is central to ER function. We examined knock-in mice expressing a mutant BiP in order to elucidate physiological processes that are sensitive to BiP functions during development and adulthood. The mutant BiP lacked the retrieval sequence that normally functions to return BiP to the ER from the secretory pathway. This allowed us to examine the effects of a defect in ER function without completely eliminating BiP function. The homozygous mutant BiP neonates died after birth due to respiratory failure. Besides that, the mutant BiP mice displayed disordered layer formation in the cerebral cortex and cerebellum, a neurological phenotype of reeler mutant-like malformation. Consistent with the phenotype, Cajal-Retzius (CR) cells did not secrete reelin, and the expression of reelin was markedly reduced posttranscriptionally. Furthermore, the reduction in the size of the whole brain and the apparent scattering of CR cells throughout the cortex, which were distinct from the reeler phenotype, were also seen. These findings suggest that the maturation and secretion of reelin in CR cells and other factors related to neural **migration** may be sensitive to aberrant ER quality control, which may cause various neurological disorders.
4. Protein 600 is a microtubule/endoplasmic reticulum-associated protein in CNS neurons. There is an increasing body of literature pointing to cytoskeletal proteins as spatial organizers and interactors of organelles. In this study, we identified protein 600 (p600) as a novel microtubule-associated protein (MAP) developmentally regulated in neurons. p600 exhibits the unique feature to interact with the endoplasmic reticulum (ER). Silencing of p600 by RNA interference (RNAi) destabilizes neuronal processes in young primary neurons undergoing neurite extension and containing scarce staining of the ER marker Bip. Furthermore, in utero electroporation of p600 RNAi alters neuronal **migration**, a process that depends on synergistic actions of microtubule dynamics and ER functions. p600-depleted migrating neurons display thin, crooked, and "zigzag" leading process with very few ER membranes. Thus, p600 constitutes the only known MAP to associate with the ER in neurons, and this interaction may impact on multiple cellular processes ranging from neuronal development to neuronal maturation and plasticity.
5. Mild endoplasmic reticulum stress promotes retinal neovascularization via induction of BiP/GRP78. Endoplasmic reticulum (ER) stress occurs as a result of accumulation of unfolded or misfolded proteins in the ER and is involved in the mechanisms of various diseases, such as cancer and neurodegeneration. The goal of the present study was to clarify the relationship between ER stress and pathological neovascularization in the retina. Proliferation and **migration** of human retinal microvascular endothelial cells (HRMEC) were assessed in the presence of ER stress inducers, such as tunicamycin and thapsigargin. The expression of ER chaperone immunoglobulin heavy-chain binding protein (BiP), known as Grp78, was evaluated by real time RT-PCR, immunostaining, and Western blotting. Tunicamycin or thapsigargin was injected into the intravitreal body of oxygen-induced retinopathy (OIR) model mice at postnatal day 14 (P14) and retinal neovascularization was quantified at P17. The expression and localization of BiP in the retina was also evaluated in the OIR model. Exposure to tunicamycin and thapsigargin increased the proliferation and **migration** of HRMEC. Tunicamycin enhanced the expression of BiP in HRMEC at both the mRNA level and at the protein level on the cell surface, and increased the formation of a BiP/T-cadherin immunocomplex. In OIR model mice, retinal neovascularization was accelerated by treatments with ER stress inducers. BiP was particularly observed in the pathological vasculature and retinal microvascular endothelial cells, and the increase of BiP expression was correlated with retinal neovascularization. In conclusion, ER stress may contribute to the formation of abnormal vasculature in the retina via BiP complexation with T-cadherin, which then promotes endothelial cell proliferation and **migration**.

### ECM

#### PubMed

1. Adamtsl2 deletion results in bronchial fibrillin microfibril accumulation and bronchial epithelial dysplasia--a novel mouse model providing insights into geleophysic dysplasia. Mutations in the secreted glycoprotein ADAMTSL2 cause recessive geleophysic dysplasia (GD) in humans and Musladin-Lueke syndrome (MLS) in dogs. GD is a severe, often lethal, condition presenting with short stature, brachydactyly, stiff skin, joint contractures, tracheal-bronchial stenosis and cardiac valve anomalies, whereas MLS is non-lethal and characterized by short stature and severe skin fibrosis. Although most mutations in fibrillin-1 (FBN1) cause Marfan syndrome (MFS), a microfibril disorder leading to transforming growth factor-β (TGFβ) dysregulation, domain-specific FBN1 mutations result in dominant GD. ADAMTSL2 has been previously shown to bind FBN1 and latent TGFβ-binding protein-1 (LTBP1). Here, we investigated mice with targeted Adamtsl2 inactivation as a new model for GD (Adamtsl2(-/-) mice). An intragenic lacZ reporter in these mice showed that ADAMTSL2 was produced exclusively by bronchial smooth muscle cells during embryonic lung development. Adamtsl2(-/-) mice, which died at birth, had severe bronchial epithelial dysplasia with abnormal glycogen-rich inclusions in bronchial epithelium resembling the cellular anomalies described previously in GD. An increase in microfibrils in the bronchial wall was associated with increased FBN2 and microfibril-associated glycoprotein-1 (MAGP1) staining, whereas LTBP1 staining was increased in bronchial epithelium. ADAMTSL2 was shown to bind directly to FBN2 with an affinity comparable to FBN1. The observed extracellular matri**x (**ECM) alterations were associated with increased bronchial epithelial TGFβ signaling at 17.5 days of gestation; however, treatment with TGFβ-neutralizing antibody did not correct the epithelial dysplasia. These investigations reveal a new function of ADAMTSL2 in modulating microfibril formation, and a previously unsuspected association with FBN2. Our studies suggest that the bronchial epithelial dysplasia accompanying microfibril dysregulation in Adamtsl2(-/-) mice cannot be reversed by TGFβ neutralization, and thus might be mediated by other mechanisms.

### [Ee]xtracellular [Mm]atrix

#### PubMed

1. Neural tube opening and abnormal extraembryonic membrane development in SEC23A deficient mice. COPII (coat protein complex-II) vesicles transport proteins from the endoplasmic reticulum (ER) to the Golgi. Higher eukaryotes have two or more paralogs of most COPII components. Here we characterize mice deficient for SEC23A and studied interactions of Sec23a null allele with the previously reported Sec23b null allele. SEC23A deficiency leads to mid-embryonic lethality associated with defective development of extraembryonic membranes and neural tube opening in midbrain. Secretion defects of multiple collagen types are observed in different connective tissues, suggesting that collagens are primarily transported in SEC23A-containing vesicles in these cells. Other **extracellular matrix** proteins, such as fibronectin, are not affected by SEC23A deficiency. Intracellular accumulation of unsecreted proteins leads to strong induction of the unfolded protein response in collagen-producing cells. No collagen secretion defects are observed in SEC23B deficient embryos. We report that E-cadherin is a cargo that accumulates in acini of SEC23B deficient pancreas and salivary glands. Compensatory increase of one paralog is observed in the absence of the second paralog. Haploinsufficiency of the remaining Sec23 paralog on top of homozygous inactivation of the first paralog leads to earlier lethality of embryos. Our results suggest that mammalian SEC23A and SEC23B transport overlapping yet distinct spectra of cargo in vivo.
2. Lethal skeletal dysplasia in mice and humans lacking the golgin GMAP-210. Establishing the genetic basis of phenotypes such as skeletal dysplasia in model organisms can provide insights into biologic processes and their role in human disease.We screened mutagenized mice and observed a neonatal lethal skeletal dysplasia with an autosomal recessive pattern of inheritance. Through genetic mapping and positional cloning, we identified the causative mutation.Affected mice had a nonsense mutation in the thyroid hormone receptor interactor 11 gene (Trip11), which encodes the Golgi microtubule-associated protein 210 (GMAP-210); the affected mice lacked this protein. Golgi architecture was disturbed in multiple tissues, including cartilage. Skeletal development was severely impaired, with chondrocytes showing swelling and stress in the endoplasmic reticulum, abnormal cellular differentiation, and increased cell death. Golgi-mediated glycosylation events were altered in fibroblasts and chondrocytes lacking GMAP-210, and these chondrocytes had intracellular accumulation of perlecan, an **extracellular matrix** protein, but not of type II collagen or aggrecan, two other **extracellular matrix** proteins. The similarities between the skeletal and cellular phenotypes in these mice and those in patients with achondrogenesis type 1A, a neonatal lethal form of skeletal dysplasia in humans, suggested that achondrogenesis type 1A may be caused by GMAP-210 deficiency. Sequence analysis revealed loss-of-function mutations in the 10 unrelated patients with achondrogenesis type 1A whom we studied.GMAP-210 is required for the efficient glycosylation and cellular transport of multiple proteins. The identification of a mutation affecting GMAP-210 in mice, and then in humans, as the cause of a lethal skeletal dysplasia underscores the value of screening for abnormal phenotypes in model organisms and identifying the causative mutations.
3. Severe **Extracellular Matrix** Abnormalities and Chondrodysplasia in Mice Lacking Collagen Prolyl 4-Hydroxylase Isoenzyme II in Combination with a Reduced Amount of Isoenzyme I. Collagen prolyl 4-hydroxylases (C-P4H-I, C-P4H-II, and C-P4H-III) catalyze formation of 4-hydroxyproline residues required to form triple-helical collagen molecules. Vertebrate C-P4Hs are α2β2 tetramers differing in their catalytic α subunits. C-P4H-I is the major isoenzyme in most cells, and inactivation of its catalytic subunit (P4ha1(-/-)) leads to embryonic lethality in mouse, whereas P4ha1(+/-) mice have no abnormalities. To study the role of C-P4H-II, which predominates in chondrocytes, we generated P4ha2(-/-) mice. Surprisingly, they had no apparent phenotypic abnormalities. To assess possible functional complementarity, we established P4ha1(+/-);P4ha2(-/-) mice. They were smaller than their littermates, had moderate chondrodysplasia, and developed kyphosis. A transient inner cell death phenotype was detected in their developing growth plates. The columnar arrangement of proliferative chondrocytes was impaired, the amount of 4-hydroxyproline and the Tm of collagen II were reduced, and t**he extracellular mat**rix was softer in the growth plates of newborn P4ha1(+/-);P4ha2(-/-) mice. No signs of uncompensated ER stress were detected in the mutant growth plate chondrocytes. Some of these defects were also found in P4ha2(-/-) mice, although in a much milder form. Our data show that C-P4H-I can to a large extent compensate for the lack of C-P4H-II in proper endochondral bone development, but their combined partial and complete inactivation, respectively, leads to biomechanically impair**ed extracellular mat**rix, moderate chondrodysplasia, and kyphosis. Our mouse data suggest that inactivating mutations in human P4HA2 are not likely to lead to skeletal disorders, and a simultaneous decrease in P4HA1 function would most probably be required to generate such a disease phenotype.
4. Adamtsl2 deletion results in bronchial fibrillin microfibril accumulation and bronchial epithelial dysplasia--a novel mouse model providing insights into geleophysic dysplasia. Mutations in the secreted glycoprotein ADAMTSL2 cause recessive geleophysic dysplasia (GD) in humans and Musladin-Lueke syndrome (MLS) in dogs. GD is a severe, often lethal, condition presenting with short stature, brachydactyly, stiff skin, joint contractures, tracheal-bronchial stenosis and cardiac valve anomalies, whereas MLS is non-lethal and characterized by short stature and severe skin fibrosis. Although most mutations in fibrillin-1 (FBN1) cause Marfan syndrome (MFS), a microfibril disorder leading to transforming growth factor-β (TGFβ) dysregulation, domain-specific FBN1 mutations result in dominant GD. ADAMTSL2 has been previously shown to bind FBN1 and latent TGFβ-binding protein-1 (LTBP1). Here, we investigated mice with targeted Adamtsl2 inactivation as a new model for GD (Adamtsl2(-/-) mice). An intragenic lacZ reporter in these mice showed that ADAMTSL2 was produced exclusively by bronchial smooth muscle cells during embryonic lung development. Adamtsl2(-/-) mice, which died at birth, had severe bronchial epithelial dysplasia with abnormal glycogen-rich inclusions in bronchial epithelium resembling the cellular anomalies described previously in GD. An increase in microfibrils in the bronchial wall was associated with increased FBN2 and microfibril-associated glycoprotein-1 (MAGP1) staining, whereas LTBP1 staining was increased in bronchial epithelium. ADAMTSL2 was shown to bind directly to FBN2 with an affinity comparable to FBN1. The observ**ed extracellular mat**rix (ECM) alterations were associated with increased bronchial epithelial TGFβ signaling at 17.5 days of gestation; however, treatment with TGFβ-neutralizing antibody did not correct the epithelial dysplasia. These investigations reveal a new function of ADAMTSL2 in modulating microfibril formation, and a previously unsuspected association with FBN2. Our studies suggest that the bronchial epithelial dysplasia accompanying microfibril dysregulation in Adamtsl2(-/-) mice cannot be reversed by TGFβ neutralization, and thus might be mediated by other mechanisms.
5. Expression and distribution of grp-78/bip in mineralizing tissues and mesenchymal cells. Glucose-regulated protein 78 (GRP-78) is one of the many endoplasmic reticulum chaperone proteins that have been shown to possess multifunctional roles. We have previously demonstrated that GRP-78 functions as a receptor for dentin matrix protein 1 (DMP1) and is required for DMP1-mediated calcium release; that it is a secreted protein and can bind to type I collagen and DMP1 extracellularly and aid in the nucleation of calcium phosphate. We provide evidence in this study that tyrosine phosphorylation is required for DMP1/GRP-78-mediated calcium release in mesenchymal cells. We further demonstrate that GRP-78 is localized in the nucleus of mesenchymal cells and that the cell surface GRP-78 is not associated with the G-protein Gαq in mesenchymal cells. Results from this study show that during development of mineralized tissues, increased expression of GRP-78 can be observed in condensing cartilage and mesenchymal cells of the alveolar bone, endochondral bone and dental pulp. Additionally, we show that GRP-78 is present in the mineralizing matrices of teeth, bone and in the **extracellular matri**x of differentiating human marrow stromal cells and dental pulp stem cells. Collectively, our observations provide a new perspective on GRP-78 with respect to mineralized matrix formation.
6. Abnormal expression of collagen IV in lens activates unfolded protein response resulting in cataract. Human diseases caused by mutations in **extracellular matrix** genes are often associated with an increased risk of cataract and lens capsular rupture. However, the underlying mechanisms of cataract pathogenesis in these conditions are still unknown. Using two different mouse models, we show that the accumulation of collagen chains in the secretory pathway activates the stress signaling pathway termed unfolded protein response (UPR). Transgenic mice expressing ectopic Col4a3 and Col4a4 genes in the lens exhibited activation of IRE1, ATF6, and PERK associated with expansion of the endoplasmic reticulum and attenuation of general protein translation. The expression of the transgenes had adverse effects on lens fiber cell differentiation and eventually induced cell death in a group of transgenic fiber cells. In Col4a1(+/Deltaex40) mutant mice, the accumulation of mutant chains also caused low levels of UPR activation. However, cell death was not induced in mutant lenses, suggesting that low levels of UPR activation are not proapoptotic. Collectively, the results provide in vivo evidence for a role of UPR in cataract formation in response to accumulation of terminally unfolded proteins in the endoplasmic reticulum.
7. Regulation of endoplasmic reticulum stress response by a BBF2H7-mediated Sec23a pathway is essential for chondrogenesis. Many tissues have a specific signal transduction system for endoplasmic reticulum (ER) dysfunction; however, the mechanisms underlying the ER stress response in cartilage remain unclear. BBF2H7 (BBF2 human homologue on chromosome 7), an ER-resident basic leucine zipper transcription factor, is activated in response to ER stress and is highly expressed in chondrocytes. In this study, we generated Bbf2h7(-/-) mice to assess the in vivo function of BBF2H7. The mice showed severe chondrodysplasia and died by suffocation shortly after birth because of an immature chest cavity. The cartilage showed a lack of typical columnar structure in the proliferating zone and a decrease in the size of the hypertrophic zone, resulting in a significant reduction of **extracellular matrix** proteins. Interestingly, proliferating chondrocytes showed abnormally expanded ER, containing aggregated type II collagen (Col2) and cartilage oligomeric matrix protein (COMP). We identified Sec23a, which encodes a coat protein complex II component responsible for protein transport from the ER to the Golgi, as a target of BBF2H7, which directly bound to a CRE-like sequence in the promoter region of Sec23a to activate its transcription. When Sec23a was introduced to Bbf2h7(-/-) chondrocytes, the impaired transport and secretion of cartilage matrix proteins was totally restored, indicating that by activating protein secretion the BBF2H7-Sec23a pathway has a crucial role in chondrogenesis. Our findings provide a new link by which ER stress is converted to signalling for the activation of ER-to-Golgi trafficking.

#### Component

1. **extracellular matrix**

### [Ii]nvasive

#### PubMed

1. Murine Models of Acute Alcoholic Hepatitis and Their Relevance to Human Disease. Alcohol-induced liver damage is a major burden for most societies, and murine studies can provide a means to better understand its pathogenesis and test new therapies. However, there are many models reported with widely differing phenotypes, not all of which fully regenerate the spectrum of human disease. Thus, it is important to understand the implications of these variations to efficiently model human disease. This review critically appraises key articles in the field, detailing the spectrum of liver damage seen in different models, and how they relate to the phenotype of disease seen in patients. A range of different methods of alcohol administration have been studied, ranging from ad libitum consumption of alcohol and water to modified diets (eg, Lieber deCarli liquid diet). Other feeding regimens have taken more **invasive** routes using intragastric feeding tubes to infuse alcohol directly into the stomach. Notably, models using wild-type mice generally produce a milder phenotype of liver damage than those using genetically modified mice, with the exception of the chronic binge-feeding model. We recommend panels of tests for consideration to standardize end points for the evaluation of the severity of liver damage-key for comparison of models of injury, testing of new therapies, and subsequent translation of findings into clinical practice.
2. Pten null prostate tumorigenesis and AKT activation are blocked by targeted knockout of ER chaperone GRP78/BiP in prostate epithelium. GRP78/BiP has recently emerged as a novel biomarker for aggressive prostate cancer. Here, we report that homozygous deletion of Grp78 specifically in mouse prostate epithelium suppresses prostate tumorigenesis without affecting postnatal prostate development and growth. Mouse prostates with double conditional knockout of Grp78 and Pten exhibit normal histology and cytology, in contrast to the **invasive** adenocarcinoma in mouse prostates with Pten inactivation. AKT activation in Pten null prostate epithelium is inhibited by Grp78 homozygous deletion, corresponding with suppression of AKT phosphorylation by GRP78 knockdown in prostate cancer cell line. Thus, inactivation of GRP78 may represent a previously undescribed approach to stop prostate cancer and potentially other cancers resulting from the loss of PTEN tumor suppression and/or activation of the oncogenic AKT.

### [Mm]etastasize

#### PubMed

1. Identification of genes differentially expressed in B16 murine melanoma sublines with different metastatic potentials. B16-F10 and B16-BL6 are B16 mouse melanoma sublines that preferentially **metastasize** to the lung following i.v. and s.c. injections, respectively. To study molecular mechanisms underlying the different metastatic behaviors exhibited by the B16 melanoma sublines, we performed differential hybridization of the genes transcribed in these cells and compared their expression levels. We isolated four genes that were highly expressed in B16-F10 cells but not in B16-BL6 cells: TI-225 (polyubiquitin), TI-229 (pyruvate kinase), TI-241 (LRF-1 homologue), and TI-227 (novel gene). Triosephosphate isomerase, 10-formyltetrahydrofolate dehydrogenase, tyrosinase-related protein 2, cytochrome c oxidase, ATP synthetase alpha subunit, RNA helicase, and ribosomal protein (L37, J1, acidic phosphoprotein), however, showed higher expression in B16-BL6 cells than in B16-F10 cells. Among these clones, transfection of TI-241 into the low metastatic clone F1 converted the parental cells from low- into high-metastatic cells. TI-241 may regulate the expression of various genes as a transcription factor in the complex process of metastasis.

### [Mm]etastasis

#### PubMed

1. A critical role for GRP78/BiP in the tumor microenvironment for neovascularization during tumor growth and **metastasis**. Glucose-regulated protein 78 (GRP78)/BiP is a multifunctional protein which plays a major role in endoplasmic reticulum (ER) protein processing, protein quality control, maintaining ER homeostasis, and controlling cell signaling and viability. Previously, using a transgene-induced mammary tumor model, we showed that Grp78 heterozygosity impeded cancer growth through suppression of tumor cell proliferation and promotion of apoptosis and the Grp78(+/-) mice exhibited dramatic reduction (70%) in the microvessel density (MVD) of the endogenous mammary tumors, while having no effect on the MVD of normal organs. This observation suggests that GRP78 may critically regulate the function of the host vasculature within the tumor microenvironment. In this article, we interrogated the role of GRP78 in the tumor microenvironment. In mouse tumor models in which wild-type (WT), syngeneic mammary tumor cells were injected into the host, we showed that Grp78(+/-) mice suppressed tumor growth and angiogenesis during the early phase but not during the late phase of tumor growth. Growth of metastatic lesions of WT, syngeneic melanoma cells in the Grp78(+/-) mice was potently suppressed. We created conditional heterozygous knockout of GRP78 in the host endothelial cells and showed severe reduction of tumor angiogenesis and metastatic growth, with minimal effect on normal tissue MVD. Furthermore, knockdown of GRP78 expression in immortalized human endothelial cells showed that GRP78 is a critical mediator of angiogenesis by regulating cell proliferation, survival, and migration. Our findings suggest that concomitant use of current chemotherapeutic agents and novel therapies against GRP78 may offer a powerful dual approach to arrest cancer initiation, progression, and **metastasis**.
2. Inhibition of established micrometastases by targeted drug delivery via cell surface-associated GRP78. The major cause of morbidity in breast cancer is development of metastatic disease, for which few effective therapies exist. Because tumor cell dissemination is often an early event in breast cancer progression and can occur before diagnosis, new therapies need to focus on targeting established metastatic disease in secondary organs. We report an effective therapy based on targeting cell surface-localized glucose-regulated protein 78 (GRP78). GRP78 is expressed normally in the endoplasmic reticulum, but many tumors and disseminated tumor cells are subjected to environmental stresses and exhibit elevated levels of GRP78, some of which are localized at the plasma membrane.Here, we show that matched primary tumors and metastases from patients who died from advanced breast cancer also express high levels of GRP78. We used a peptidomimetic targeting strategy that uses a known GRP78-binding peptide fused to a proapoptotic moiety [designated bone **metastasis** targeting peptide 78 (BMTP78)] and show that it can selectively kill breast cancer cells that express surface-localized GRP78. Furthermore, in preclinical **metastasis** models, we show that administration of BMTP78 can inhibit primary tumor growth as well as prolong overall survival by reducing the extent of outgrowth of established lung and bone micrometastases.The data presented here provide strong evidence that it is possible to induce cell death in established micrometastases by peptide-mediated targeting of cell surface-localized GRP in advanced breast cancers. The significance to patients with advanced breast cancer of a therapy that can reduce established metastatic disease should not be underestimated.
3. STAT3 and STAT6 Signaling Pathways Synergize to Promote Cathepsin Secretion from Macrophages via IRE1α Activation. Tumor-associated macrophages play critical roles during tumor progression by promoting angiogenesis, cancer cell proliferation, invasion, and **metastasi**s. Cysteine cathepsin proteases, produced by macrophages and cancer cells, modulate these processes, but it remains unclear how these typically lysosomal enzymes are regulated and secreted within the tumor microenvironment. Here, we identify a STAT3 and STAT6 synergy that potently upregulates cathepsin secretion by macrophages via engagement of an unfolded protein response (UPR) pathway. Whole-genome expression analyses revealed that the TH2 cytokine interleukin (IL)-4 synergizes with IL-6 or IL-10 to activate UPR via STAT6 and STAT3. Pharmacological inhibition of the UPR sensor IRE1α blocks cathepsin secretion and blunts macrophage-mediated cancer cell invasion. Similarly, genetic deletion of STAT3 and STAT6 signaling components impairs tumor development and invasion in vivo. Together, these findings demonstrate that cytokine-activated STAT3 and STAT6 cooperate in macrophages to promote a secretory phenotype that enhances tumor progression in a cathepsin-dependent manner.
4. Identification of genes differentially expressed in B16 murine melanoma sublines with different metastatic potentials. B16-F10 and B16-BL6 are B16 mouse melanoma sublines that preferentially metastasize to the lung following i.v. and s.c. injections, respectively. To study molecular mechanisms underlying the different metastatic behaviors exhibited by the B16 melanoma sublines, we performed differential hybridization of the genes transcribed in these cells and compared their expression levels. We isolated four genes that were highly expressed in B16-F10 cells but not in B16-BL6 cells: TI-225 (polyubiquitin), TI-229 (pyruvate kinase), TI-241 (LRF-1 homologue), and TI-227 (novel gene). Triosephosphate isomerase, 10-formyltetrahydrofolate dehydrogenase, tyrosinase-related protein 2, cytochrome c oxidase, ATP synthetase alpha subunit, RNA helicase, and ribosomal protein (L37, J1, acidic phosphoprotein), however, showed higher expression in B16-BL6 cells than in B16-F10 cells. Among these clones, transfection of TI-241 into the low metastatic clone F1 converted the parental cells from low- into high-metastatic cells. TI-241 may regulate the expression of various genes as a transcription factor in the complex process of **metastasis**.

### [Cc]ell [Ii]nvasion

#### PubMed

1. STAT3 and STAT6 Signaling Pathways Synergize to Promote Cathepsin Secretion from Macrophages via IRE1α Activation. Tumor-associated macrophages play critical roles during tumor progression by promoting angiogenesis, cancer cell proliferation, invasion, and metastasis. Cysteine cathepsin proteases, produced by macrophages and cancer cells, modulate these processes, but it remains unclear how these typically lysosomal enzymes are regulated and secreted within the tumor microenvironment. Here, we identify a STAT3 and STAT6 synergy that potently upregulates cathepsin secretion by macrophages via engagement of an unfolded protein response (UPR) pathway. Whole-genome expression analyses revealed that the TH2 cytokine interleukin (IL)-4 synergizes with IL-6 or IL-10 to activate UPR via STAT6 and STAT3. Pharmacological inhibition of the UPR sensor IRE1α blocks cathepsin secretion and blunts macrophage-mediated cance**r cell invasi**on. Similarly, genetic deletion of STAT3 and STAT6 signaling components impairs tumor development and invasion in vivo. Together, these findings demonstrate that cytokine-activated STAT3 and STAT6 cooperate in macrophages to promote a secretory phenotype that enhances tumor progression in a cathepsin-dependent manner.

---

## Ugcg

- NCBI Gene
- GeneRIF
- Pubmed

| Search Term | Rank | Fields |
| --- | --- | --- |
| [Ee]xtracellular [Mm]atrix | 8 | PubMed(1) |
| [Ii]nvasive | 10 | PubMed(1); Alternative Name(1) |

  
hide/show details for Ugcg

### [Ee]xtracellular [Mm]atrix

#### PubMed

1. Differentiation of epidermal keratinocytes is dependent on glucosylceramide:ceramide processing. Skin barrier function is primarily assigned to the outer epidermal layer, the stratum corneum (SC), mainly composed of corneocytes and lipid-enriched **extracellular matrix**. Epidermal ceramides (Cers) are essential barrier lipids, containing ultra-long-chain (ULC) fatty acids (FAs) with a unique ω-hydroxy group, which is necessary for binding to corneocyte proteins. In the SC, Cers are believed to derive from glucosylated intermediates, namely glucosylceramides (GlcCers), as surmised from human Gaucher's disease and related mouse models. Tamoxifen (TAM)-induced deletion of the endogenous GlcCer-synthesizing enzyme UDP-glucose:ceramide glucosyltransferase (UGCG) in keratin K14-positive cells resulted in epidermal GlcCer depletion. Although free extractable Cers were elevated in total epidermis and as well in SC, protein-bound Cers decreased significantly in Ugcg(f/fK14CreERT2) mice, indicating glucosylation to be required for regular Cer processing as well as arrangement and extrusion of lipid lamellae. The almost complete loss of protein-bound Cers led to a disruption of the water permeability barrier (WPB). UGCG-deficient mice developed an ichthyosis-like skin phenotype marked by impaired keratinocyte differentiation associated with delayed wound healing. Gene expression profiling of Ugcg-mutant skin revealed a subset of differentially expressed genes involved in lipid signaling and epidermal differentiation/proliferation, correlating to human skin diseases such as psoriasis and atopic dermatitis. Peroxisome proliferator-activated receptor beta/delta (PPARβ/δ), a Cer-sensitive transcription factor was identified as potential mediator of the altered gene sets.

### [Ii]nvasive

#### PubMed

1. cDNA subtraction cloning reveals novel genes whose temporal and spatial expression indicates association with trophoblast invasion. Trophoblast invasion is a critical process in development of most mammals that shares similarities with the **invasive** behavior of tumor cells. In the present investigation, a cDNA subtraction library was constructed between **invasive** trophoblast at day 8 of murine development and mature non**invasive** placenta at day 18 of gestation. One of the differentially expressed clones, Epcs26, was mapped to the X chromosome and revealed no homology to any known gene. It was predominantly expressed in parietal endoderm, undifferentiated cells of the ectoplacental cone, and a few trophoblast giant cells. Another gene, designated Epcs50, was mapped to chromosome 19. It exhibited homologies to the mouse Mps1 gene and, like Mps1, may have a distant relationship to the lytic protein perforin. High expression was detected in parietal endoderm cells and in a subset of secondary trophoblast giant cells. Two sequences, Epcs24 and Epcs68, exhibited an extensive open reading frame that shared the common features of the cysteine proteinase cathepsin L. Expression was confined to an undefined subpopulation of trophoblast giant cells. Both genes were mapped to chromosome 13 in close proximity to cathepsins L and J. The known functions of MPS1 and cathepsin L proteins indicate that the related proteins EPCS50, EPCS24, and EPCS68 participate in conferring **invasive** properties to the mouse trophoblast.

#### Alternative Name

1. ectoplacental cone, **invasive** trophoblast giant cells, extraembryonic ectoderm and chorion sequence 21

---

## Gpsm2

- NCBI Gene
- GeneRIF
- Pubmed

| Search Term | Rank | Fields |
| --- | --- | --- |
| [Mm]igration | 6 | PubMed(3) |
| [Ee]xtracellular [Mm]atrix | 8 | PubMed(1) |

  
hide/show details for Gpsm2

### [Mm]igration

#### PubMed

1. Primary cilium **migration** depends on G-protein signalling control of subapical cytoskeleton. In ciliated mammalian cells, the precise **migration** of the primary cilium at the apical surface of the cells, also referred to as translational polarity, defines planar cell polarity (PCP) in very early stages. Recent research has revealed a co-dependence between planar polarization of some cell types and cilium positioning at the surface of cells. This important role of the primary cilium in mammalian cells is in contrast with its absence from Drosophila melanogaster PCP establishment. Here, we show that deletion of GTP-binding protein alpha-i subunit 3 (Gαi3) and mammalian Partner of inscuteable (mPins) disrupts the **migratio**n of the kinocilium at the surface of cochlear hair cells and affects hair bundle orientation and shape. Inhibition of G-protein function in vitro leads to kinocilium **migratio**n defects, PCP phenotype and abnormal hair bundle morphology. We show that Gαi3/mPins are expressed in an apical and distal asymmetrical domain, which is opposite and complementary to an aPKC/Par-3/Par-6b expression domain, and non-overlapping with the core PCP protein Vangl2. Thus G-protein-dependent signalling controls th**e migrati**on of the cilium cell autonomously, whereas core PCP signalling controls long-range tissue PCP.
2. The GPSM2/LGN GoLoco motifs are essential for hearing. The planar cell polarity (PCP) pathway is responsible for polarizing and orienting cochlear hair cells during development through movement of a primary cilium, the kinocilium. GPSM2/LGN, a mitotic spindle-orienting protein associated with deafness in humans, is a PCP effector involved in kinocilium **migration**. Here, we link human and mouse truncating mutations in the GPSM2/LGN gene, both leading to hearing loss. The human variant, p.(Trp326\*), was identified by targeted genomic enrichment of genes associated with deafness, followed by massively parallel sequencing. Lgn (ΔC) mice, with a targeted deletion truncating the C-terminal GoLoco motifs, are profoundly deaf and show misorientation of the hair bundle and severe malformations in stereocilia shape that deteriorates over time. Full-length protein levels are greatly reduced in mutant mice, with upregulated mRNA levels. The truncated Lgn (ΔC) allele is translated in vitro, suggesting that mutant mice may have partially functioning Lgn. Gαi and aPKC, known to function in the same pathway as Lgn, are dependent on Lgn for proper localization. The polarization of core PCP proteins is not affected in Lgn mutants; however, Lgn and Gαi are misoriented in a PCP mutant, supporting the role of Lgn as a PCP effector. The kinocilium, previously shown to be dependent on Lgn for robust localization, is essential for proper localization of Lgn, as well as Gαi and aPKC, suggesting that cilium function plays a role in positioning of apical proteins. Taken together, our data provide a mechanism for the loss of hearing found in human patients with GPSM2/LGN variants.
3. Pins homolog LGN regulates meiotic spindle organization in mouse oocytes. Mouse oocytes undergo polarization during meiotic maturation, and this polarization is essential for asymmetric cell divisions that maximize retention of maternal components required for early development. Without conventional centrosomes, the meiotic spindle has less focused poles and is barrel-shaped. The **migration** of meiotic spindles to the cortex is accompanied by a local reorganization and polarization of the cortex. LGN is a conserved protein involved in cell polarity and regulation of spindle organization. In the present study, we characterized the localization dynamics of LGN during mouse oocyte maturation and analyzed the effects of LGN upregulation and downregulation on meiotic spindle organization. At the germinal vesicle stage, LGN is distributed both cytoplasmically and at the cortex. During maturation, LGN localizes to the meiotic spindle apparatus and cortical LGN becomes less concentrated at the actin cap region. Excessive LGN induces meiotic spindle organization defects by elongating the spindle and enhancing pole focusing, whereas depletion of LGN by RNA interference results in meiotic spindle deformation and chromosome misalignment. Furthermore, the N-terminus of LGN has the ability of full-length LGN to regulate spindle organization, whereas the C-terminus of LGN controls cortical localization and polarization. Our results reveal that LGN is cortically polarized in mouse oocytes and is critical for meiotic spindle organization.

### [Ee]xtracellular [Mm]atrix

#### PubMed

1. Asymmetric cell divisions promote stratification and differentiation of mammalian skin. The epidermis is a stratified squamous epithelium forming the barrier that excludes harmful microbes and retains body fluids. To perform these functions, proliferative basal cells in the innermost layer periodically detach from an underlying basement membrane of **extracellular matrix**, move outward and eventually die. Once suprabasal, cells stop dividing and enter a differentiation programme to form the barrier. The mechanism of stratification is poorly understood. Although studies in vitro have led to the view that stratification occurs through the delamination and subsequent movement of epidermal cells, most culture conditions favour keratinocytes that lack the polarity and cuboidal morphology of basal keratinocytes in tissue. These features could be important in considering an alternative mechanism, that stratification occurs through asymmetric cell divisions in which the mitotic spindle orients perpendicularly to the basement membrane. Here we show that basal epidermal cells use their polarity to divide asymmetrically, generating a committed suprabasal cell and a proliferative basal cell. We further demonstrate that integrins and cadherins are essential for the apical localization of atypical protein kinase C, the Par3-LGN-Inscuteable complex and NuMA-dynactin to align the spindle.

---

## Coro1c

- NCBI Gene
- GeneRIF
- Pubmed

| Search Term | Rank | Fields |
| --- | --- | --- |
| [Mm]igration | 6 | Process(4); PubMed(3) |
| [Ee]xtracellular [Mm]atrix | 8 | PubMed(2) |

  
hide/show details for Coro1c

### [Mm]igration

#### Process

1. negative regulation of epithelial cell **migration**
2. neural crest cell **migration**
3. regulation of epithelial cell **migration**
4. regulation of fibroblast **migration**

#### PubMed

1. Coronin-1C Protein and Caveolin Protein Provide Constitutive and Inducible Mechanisms of Rac1 Protein Trafficking. Sustained directional fibroblast **migration** requires both polarized activation of the protrusive signal, Rac1, and redistribution of inactive Rac1 from the rear of the cell so that it can be redistributed or degraded. In this work, we determine how alternative endocytic mechanisms dictate the fate of Rac1 in response to the extracellular matrix environment. We discover that both coronin-1C and caveolin retrieve Rac1 from similar locations at the rear and sides of the cell. We find that coronin-1C-mediated extraction, which is responsible for Rac1 recycling, is a constitutive process that maintains Rac1 protein levels within the cell. In the absence of coronin-1C, the effect of caveolin-mediated endocytosis, which targets Rac1 for proteasomal degradation, becomes apparent. Unlike constitutive coronin-1C-mediated trafficking, caveolin-mediated Rac1 endocytosis is induced by engagement of the fibronectin receptor syndecan-4. Such an inducible endocytic/degradation mechanism would predict that, in the presence of fibronectin, caveolin defines regions of the cell that are resistant to Rac1 activation but, in the absence of fibronectin leaves more of the membrane susceptible to Rac1 activation and protrusion. Indeed, we demonstrate that fibronectin-stimulated activation of Rac1 is accelerated in the absence of caveolin and that, when caveolin is knocked down, polarization of active Rac1 is lost in FRET experiments and culminates in shunting **migration** in a fibrous fibronectin matrix. Although the concept of polarized Rac1 activity in response to chemoattractants has always been apparent, our understanding of the balance between recycling and degradation explains how polarity can be maintained when the chemotactic gradient has faded.
2. Coronin 3 and its role in murine brain morphogenesis. Coronins belong to the fundamental WD40-repeat proteins. They are mainly found at the submembraneous area, they bind F-actin in vitro, and most of the seven mammalian coronins have unclear roles. Coronin 3 is abundantly expressed in the adult CNS. All murine brain areas express coronin 3 during embryogenesis and the first postnatal stages. Expression in grey matter decreases postnatally, except for hippocampal pyramidal and dentate gyrus neurons, and cerebellar Purkinje cells, while levels in white matter increase in the course of myelination. Consistently, coronin 3 is abundant in differentiating neuro-2a and PC-12 cells and in primary oligodendrocytes. Treatment with PKC activator PMA reduced coronin 3 protein levels. To address its functions, neuro-2a and PC-12 cells were transfected with GFP-tagged coronin 3 versions. Full-length coronin 3 among other areas localized to outgrowing neurites, whereas truncated proteins efficiently suppressed neurite formation. Our results favour a role for coronin 3 in neuron morphogenesis and possibly **migration**.
3. Coronin-1C and RCC2 guide mesenchymal **migration** by trafficking Rac1 and controlling GEF exposure. Sustained forward **migration** through a fibrillar extracellular matrix requires localization of protrusive signals. Contact with fibronectin at the tip of a cell protrusion activates Rac1, and for linear **migration** it is necessary to dampen Rac1 activity in off-axial positions and redistribute Rac1 from non-protrusive membrane to the leading edge. Here, we identify interactions between coronin-1C (Coro1C), RCC2 and Rac1 that focus active Rac1 to a single protrusion. Coro1C mediates release of inactive Rac1 from non-protrusive membrane and is necessary for Rac1 redistribution to a protrusive tip and fibronectin-dependent Rac1 activation. The second component, RCC2, attenuates Rac1 activation outside the protrusive tip by binding to the Rac1 switch regions and competitively inhibiting GEF action, thus preventing off-axial protrusion. Depletion of Coro1C or RCC2 by RNA interference causes loss of cell polarity that results in shunting **migration** in 1D or 3D culture systems. Furthermore, morpholinos against Coro1C or RCC2, or mutation of any of the binding sites in the Rac1-RCC2-Coro1C complex delays the arrival of neural crest derivatives at the correct location in developing zebrafish, demonstrating the crucial role in **migration** guidance in vivo.

### [Ee]xtracellular [Mm]atrix

#### PubMed

1. Coronin-1C Protein and Caveolin Protein Provide Constitutive and Inducible Mechanisms of Rac1 Protein Trafficking. Sustained directional fibroblast migration requires both polarized activation of the protrusive signal, Rac1, and redistribution of inactive Rac1 from the rear of the cell so that it can be redistributed or degraded. In this work, we determine how alternative endocytic mechanisms dictate the fate of Rac1 in response to the **extracellular matrix** environment. We discover that both coronin-1C and caveolin retrieve Rac1 from similar locations at the rear and sides of the cell. We find that coronin-1C-mediated extraction, which is responsible for Rac1 recycling, is a constitutive process that maintains Rac1 protein levels within the cell. In the absence of coronin-1C, the effect of caveolin-mediated endocytosis, which targets Rac1 for proteasomal degradation, becomes apparent. Unlike constitutive coronin-1C-mediated trafficking, caveolin-mediated Rac1 endocytosis is induced by engagement of the fibronectin receptor syndecan-4. Such an inducible endocytic/degradation mechanism would predict that, in the presence of fibronectin, caveolin defines regions of the cell that are resistant to Rac1 activation but, in the absence of fibronectin leaves more of the membrane susceptible to Rac1 activation and protrusion. Indeed, we demonstrate that fibronectin-stimulated activation of Rac1 is accelerated in the absence of caveolin and that, when caveolin is knocked down, polarization of active Rac1 is lost in FRET experiments and culminates in shunting migration in a fibrous fibronectin matrix. Although the concept of polarized Rac1 activity in response to chemoattractants has always been apparent, our understanding of the balance between recycling and degradation explains how polarity can be maintained when the chemotactic gradient has faded.
2. Coronin-1C and RCC2 guide mesenchymal migration by trafficking Rac1 and controlling GEF exposure. Sustained forward migration through a fibrillar **extracellular matrix** requires localization of protrusive signals. Contact with fibronectin at the tip of a cell protrusion activates Rac1, and for linear migration it is necessary to dampen Rac1 activity in off-axial positions and redistribute Rac1 from non-protrusive membrane to the leading edge. Here, we identify interactions between coronin-1C (Coro1C), RCC2 and Rac1 that focus active Rac1 to a single protrusion. Coro1C mediates release of inactive Rac1 from non-protrusive membrane and is necessary for Rac1 redistribution to a protrusive tip and fibronectin-dependent Rac1 activation. The second component, RCC2, attenuates Rac1 activation outside the protrusive tip by binding to the Rac1 switch regions and competitively inhibiting GEF action, thus preventing off-axial protrusion. Depletion of Coro1C or RCC2 by RNA interference causes loss of cell polarity that results in shunting migration in 1D or 3D culture systems. Furthermore, morpholinos against Coro1C or RCC2, or mutation of any of the binding sites in the Rac1-RCC2-Coro1C complex delays the arrival of neural crest derivatives at the correct location in developing zebrafish, demonstrating the crucial role in migration guidance in vivo.

---

## Mpp6

- NCBI Gene
- GeneRIF
- Pubmed

| Search Term | Rank | Fields |
| --- | --- | --- |
| [Mm]igration | 6 | PubMed(1) |

  
hide/show details for Mpp6

### [Mm]igration

#### PubMed

1. The Amot/Patj/Syx signaling complex spatially controls RhoA GTPase activity in migrating endothelial cells. Controlled regulation of Rho GTPase activity is an essential component mediating growth factor-stimulated **migration**. We have previously shown that angiomotin (Amot), a membrane-associated scaffold protein, plays a critical role during vascular patterning and endothelial **migration** during embryogenesis. However, the signaling pathways by which Amot controls directional **migration** are not known. Here we have used peptide pull-down and yeast 2-hybrid (Y2H) screening to identify proteins that interact with the C-terminal PDZ-binding motifs of Amot and its related proteins AmotL1 and 2. We report that Amot and its related proteins bind to the RhoA GTPase exchange factor (RhoGEF) protein Syx. We show that Amot forms a ternary complex together with Patj (or its paralogue Mupp1) and Syx. Using FRET analysis, we provide evidence that Amot controls targeting of RhoA activity to lamellipodia in vitro. We also report that, similar to Amot, morpholino knockdown of Syx in zebrafish results in inhibition of **migration** of intersegmental arteries. Taken together, our results indicate that the directional **migration** of capillaries in the embryo is governed by the Amot:Patj/Mupp1:Syx signaling that controls local GTPase activity.

---

## Utp20

- NCBI Gene
- GeneRIF
- Pubmed

| Search Term | Rank | Fields |
| --- | --- | --- |
| [Mm]etastasis | 12 | PubMed(1); Conserved Domains(1); Alternative Name(1) |

  
hide/show details for Utp20

### [Mm]etastasis

#### PubMed

1. Isolation of novel mouse genes that were differentially expressed in W/W(V) mouse fundus. Using cDNA microarray analysis that displays a total of 8734 mouse genes, we have identified 21 known and novel transcripts, including mouse expressed sequence tags (ESTs) that were consistently up- or downregulated in the fundus of wild-type mice and W/W(V) mice, where intramuscular interstitial cells of Cajal (ICC; IC-IM) are lost.By using these tags as part of the full-length mRNA sequence of the murine genes, we screened a mouse-brain cDNA library and the FASTA database (http://www.ebi.ac.uk/fasta33/).Three of the queries were identified as novel mouse genes, whereas six transcripts had their human counterparts that were known to encode functional proteins. Four transcripts, DRIM (downregulated in **metastasis**), SLP8 (tumor antigen), PTK7/CCK4 (receptor protein tyrosine kinase-like molecule-7/colon carcinoma kinase-4), and a novel gene AWMS2 were increased in W/W(V) mice. Expression of another five genes was decreased in W/W(V) mice: BB1 (overexpressed in bladder and breast carcinoma), HTATIP/CPLA2 (HIV-1 TAT interactive protein/cytosolic phospholipase A2), Tenascin-receptor like (Hexabrachion, Cytotactin, Neuronectin, Myotendinous antigen), and two novel transcripts: DRWMS1 and DRWMS2. Molecular profiling generated by cDNA microarray analysis from the fundus of W/W(V) mice and its complete list of full-length cDNAs will be shown on our web site (http://www.yamanashi.ac.jp/).Differential gene comparisons between control and mutant animals with losses in specific populations of ICCs will contribute significantly to our understanding of motility disorders associated with the loss of these cells and electrical slow waves in the gastrointestinal tract.

#### Conserved Domains

1. pfam07539: DRIM; Down-regulated in **metastasis**

#### Alternative Name

1. down-regulated in **metastasis** protein

---

## Bmpr1b

- NCBI Gene
- GeneRIF
- Pubmed

| Search Term | Rank | Fields |
| --- | --- | --- |
| [Mm]igration | 6 | PubMed(3) |
| ECM | 7 | PubMed(1) |
| [Ee]xtracellular [Mm]atrix | 8 | PubMed(3) |
| [Ii]nvasive | 10 | PubMed(1) |
| [Mm]etastasis | 12 | PubMed(3) |
| [Cc]ell [Ii]nvasion | 13 | PubMed(1) |

  
hide/show details for Bmpr1b

### [Mm]igration

#### PubMed

1. DCC is specifically required for the survival of retinal ganglion and displaced amacrine cells in the developing mouse retina. Netrin-1 and DCC are well known for their roles in neurite growth, axonal guidance, and neuronal **migration**. Recently, a number of studies showed that DCC is involved in the induction of apoptosis, and this proapoptotic activity can be blocked in the presence of Netrin-1. However, here, we found that DCC is required for the survival of two types of neurons selectively in the developing mouse retina where DCC is abundantly expressed. Our results showed that the DCC(-/-) retina displayed a reduced ganglion cell layer with relatively normal neuroblastic layer. Immunostaining assays revealed that in DCC(-/-) mice, initial neurogenesis within retina was unchanged while the numbers of differentiated retinal ganglion cells and displaced amacrine cells in ganglion cell layer were greatly reduced due to increased apoptosis. By contrast, other neuronal types including horizontal cells, bipolar cells, amacrine cells, photoreceptors, and Müller cells appeared normal in DCC mutant retinas. Moreover, DCC(kanga) mice that lack the intracellular P3 domain of DCC receptor displayed the same defects as DCC(-/-) mice. Thus, our findings suggest that DCC is a key regulator for the survival of specific types of neurons during retinal development and that DCC-P3 domain is essential for this developing event.
2. Neural crest cell-specific inactivation of Nipbl or Mau2 during mouse development results in a late onset of craniofacial defects. Nipbl (Scc2) and Mau2 (Scc4) encode evolutionary conserved proteins that play a vital role for loading the cohesin complex onto chromosomes, thereby ensuring accurate chromosome segregation during cell division. While mutations in human NIPBL are known to cause the developmental disorder Cornelia de Lange syndrome, the functions of Nipbl and Mau2 in mammalian development are poorly defined. Here we generated conditional alleles for both genes in mice and show that neural crest cell-specific inactivation of Nipbl or Mau2 strongly affects craniofacial development. Surprisingly, the early phase of neural crest cell proliferation and **migration** is only moderately affected in these mutants. Moreover, we found that Mau2 single homozygous mutants exhibited a more severe craniofacial phenotype when compared to that of Nipbl;Mau2 double homozygous mutants. This raises the possibility that the Mau2/Nipbl protein interaction is not only required for cohesin loading, but may also be required to restrict the level of Nipbl involved in regulating gene expression. Together, the data suggest that proliferating neural crest cells tolerate a substantial reduction of cohesin loading proteins and we propose that the successive decrease of cohesin loading proteins in neural crest cells may alter developmental gene regulation in a highly dynamic manner.
3. Timing of cortical interneuron **migration** is influenced by the cortical hem. Cerebral cortical γ-aminobutyric acid (GABA)ergic interneurons originate from the basal forebrain and migrate into the cortex in 2 phases. First, interneurons cross the boundary between the developing striatum and the cortex to migrate tangentially through the cortical primordium. Second, interneurons migrate radially to their correct neocortical layer position. A previous study demonstrated that mice in which the cortical hem was genetically ablated displayed a massive reduction of Cajal-Retzius (C-R) cells in the neocortical marginal zone (MZ), thereby losing C-R cell-generated reelin in the MZ. Surprisingly, pyramidal cell **migratio**n and subsequent layering were almost normal. In contrast, we find that the timing of **migratio**n of cortical GABAergic interneurons is abnormal in hem-ablated mice. Migrating interneurons both advance precociously along their tangential path and switch prematurely from tangential to radial **migratio**n to invade the cortical plate (CP). We propose that the cortical hem is responsible for establishing cues that control the timing of interneuron **migratio**n. In particular, we suggest that loss of a repellant signal from the medial neocortex, which is greatly decreased in size in hem-ablated mice, allows the early advance of interneurons and that reduction of another secreted molecule from C-R cells, the chemokine SDF-1/CXCL12, permits early radial **migratio**n into the CP.

### ECM

#### PubMed

1. Nell1-deficient mice have reduced expression of extracellular matrix proteins causing cranial and vertebral defects. The mammalian Nell1 gene encodes a protein kinase C-beta1 (PKC-beta1) binding protein that belongs to a new class of cell-signaling molecules controlling cell growth and differentiation. Over-expression of Nell1 in the developing cranial sutures in both human and mouse induces craniosynostosis, the premature fusion of the growing cranial bone fronts. Here, we report the generation, positional cloning and characterization of Nell1(6R), a recessive, neonatal-lethal point mutation in the mouse Nell1 gene, induced by N-ethyl-N-nitrosourea. Nell1(6R) has a T-->A base change that converts a codon for cysteine into a premature stop codon [Cys(502)Ter], resulting in severe truncation of the predicted protein product and marked reduction in steady-state levels of the transcript. In addition to the expected alteration of cranial morphology, Nell1(6R) mutants manifest skeletal defects in the vertebral column and ribcage, revealing a hitherto undefined role for Nell1 in signal transduction in endochondral ossification. Real-time quantitative reverse transcription-PCR assays of 219 genes showed an association between the loss of Nell1 function and reduced expression of genes for extracellular matrix (**ECM**) proteins critical for chondrogenesis and osteogenesis. Several affected genes are involved in the human cartilage disorder Ehlers-Danlos Syndrome and other disorders associated with spinal curvature anomalies. Nell1(6R) mutant mice are a new tool for elucidating basic mechanisms in osteoblast and chrondrocyte differentiation in the developing skull and vertebral column and understanding how perturbations in the production of **ECM** proteins can lead to anomalies in these structures.

### [Ee]xtracellular [Mm]atrix

#### PubMed

1. Nell1-deficient mice have reduced expression of **extracellular matrix** proteins causing cranial and vertebral defects. The mammalian Nell1 gene encodes a protein kinase C-beta1 (PKC-beta1) binding protein that belongs to a new class of cell-signaling molecules controlling cell growth and differentiation. Over-expression of Nell1 in the developing cranial sutures in both human and mouse induces craniosynostosis, the premature fusion of the growing cranial bone fronts. Here, we report the generation, positional cloning and characterization of Nell1(6R), a recessive, neonatal-lethal point mutation in the mouse Nell1 gene, induced by N-ethyl-N-nitrosourea. Nell1(6R) has a T-->A base change that converts a codon for cysteine into a premature stop codon [Cys(502)Ter], resulting in severe truncation of the predicted protein product and marked reduction in steady-state levels of the transcript. In addition to the expected alteration of cranial morphology, Nell1(6R) mutants manifest skeletal defects in the vertebral column and ribcage, revealing a hitherto undefined role for Nell1 in signal transduction in endochondral ossification. Real-time quantitative reverse transcription-PCR assays of 219 genes showed an association between the loss of Nell1 function and reduced expression of genes for **extracellular matrix** (ECM) proteins critical for chondrogenesis and osteogenesis. Several affected genes are involved in the human cartilage disorder Ehlers-Danlos Syndrome and other disorders associated with spinal curvature anomalies. Nell1(6R) mutant mice are a new tool for elucidating basic mechanisms in osteoblast and chrondrocyte differentiation in the developing skull and vertebral column and understanding how perturbations in the production of ECM proteins can lead to anomalies in these structures.
2. An FGF autocrine loop initiated in second heart field mesoderm regulates morphogenesis at the arterial pole of the heart. In order to understand how secreted signals regulate complex morphogenetic events, it is crucial to identify their cellular targets. By conditional inactivation of Fgfr1 and Fgfr2 and overexpression of the FGF antagonist sprouty 2 in different cell types, we have dissected the role of FGF signaling during heart outflow tract development in mouse. Contrary to expectation, cardiac neural crest and endothelial cells are not primary paracrine targets. FGF signaling within second heart field mesoderm is required for remodeling of the outflow tract: when disrupted, outflow myocardium fails to produce **extracellular matrix** and TGFbeta and BMP signals essential for endothelial cell transformation and invasion of cardiac neural crest. We conclude that an autocrine regulatory loop, initiated by the reception of FGF signals by the mesoderm, regulates correct morphogenesis at the arterial pole of the heart. These findings provide new insight into how FGF signaling regulates context-dependent cellular responses during development.
3. Bmpr1a and Bmpr1b have overlapping functions and are essential for chondrogenesis in vivo. Previous studies have demonstrated the ability of bone morphogenetic proteins (BMPs) to promote chondrogenic differentiation in vitro. However, the in vivo role of BMP signaling during chondrogenesis has been unclear. We report here that BMP signaling is essential for multiple aspects of early chondrogenesis. Whereas mice deficient in type 1 receptors Bmpr1a or Bmpr1b in cartilage are able to form intact cartilaginous elements, double mutants develop a severe generalized chondrodysplasia. The majority of skeletal elements that form through endochondral ossification are absent, and the ones that form are rudimentary. The few cartilage condensations that form in double mutants are delayed in the prechondrocytic state and never form an organized growth plate. The reduced size of mutant condensations results from increased apoptosis and decreased proliferation. Moreover, the expression of cartilage-specific **extracellular matrix** proteins is severely reduced in mutant elements. We demonstrate that this defect in chondrocytic differentiation can be attributed to lack of Sox9, L-Sox5, and Sox6 expression in precartilaginous condensations in double mutants. In summary, our study demonstrates that BMPR1A and BMPR1B are functionally redundant during early chondrogenesis and that BMP signaling is required for chondrocyte proliferation, survival, and differentiation in vivo.

### [Ii]nvasive

#### PubMed

1. BID-dependent release of mitochondrial SMAC dampens XIAP-mediated immunity against Shigella. The X-linked inhibitor of apoptosis protein (XIAP) is a potent caspase inhibitor, best known for its anti-apoptotic function in cancer. During apoptosis, XIAP is antagonized by SMAC, which is released from the mitochondria upon caspase-mediated activation of BID. Recent studies suggest that XIAP is involved in immune signaling. Here, we explore XIAP as an important mediator of an immune response against the entero**invasive** bacterium Shigella flexneri, both in vitro and in vivo. Our data demonstrate for the first time that Shigella evades the XIAP-mediated immune response by inducing the BID-dependent release of SMAC from the mitochondria. Unlike apoptotic stimuli, Shigella activates the calpain-dependent cleavage of BID to trigger the release of SMAC, which antagonizes the inflammatory action of XIAP without inducing apoptosis. Our results demonstrate how the cellular death machinery can be subverted by an **invasive** pathogen to ensure bacterial colonization.

### [Mm]etastasis

#### PubMed

1. Notch promotes epithelial-mesenchymal transition during cardiac development and oncogenic transformation. Epithelial-to-mesenchymal transition (EMT) is fundamental to both embryogenesis and tumor **metastasis**. The Notch intercellular signaling pathway regulates cell fate determination throughout metazoan evolution, and overexpression of activating alleles is oncogenic in mammals. Here we demonstrate that Notch activity promotes EMT during both cardiac development and oncogenic transformation via transcriptional induction of the Snail repressor, a potent and evolutionarily conserved mediator of EMT in many tissues and tumor types. In the embryonic heart, Notch functions via lateral induction to promote a selective transforming growth factor-beta (TGFbeta)-mediated EMT that leads to cellularization of developing cardiac valvular primordia. Embryos that lack Notch signaling elements exhibit severely attenuated cardiac snail expression, abnormal maintenance of intercellular endocardial adhesion complexes, and abortive endocardial EMT in vivo and in vitro. Accordingly, transient ectopic expression of activated Notch1 (N1IC) in zebrafish embryos leads to hypercellular cardiac valves, whereas Notch inhibition prevents valve development. Overexpression of N1IC in immortalized endothelial cells in vitro induces EMT accompanied by oncogenic transformation, with corresponding induction of snail and repression of VE-cadherin expression. Notch is expressed in embryonic regions where EMT occurs, suggesting an intimate and fundamental role for Notch, which may be reactivated during tumor **metastasis**.
2. Hepatocyte growth factor up-regulates the expression of the bone morphogenetic protein (BMP) receptors, BMPR-IB and BMPR-II, in human prostate cancer cells. Hepatocyte growth factor (HGF) plays multiple roles in cancer, by acting as a motility, invasion and angiogenesis stimulating factor, which promotes **metastasis** and tumour growth. Bone morphogenetic proteins (BMPs) are members of the TGF-beta superfamily. The effects of BMPs are mediated by two subgroups of receptors, type I and type II. Recent studies have shown that some BMPs, via their signaling pathways, affect the growth of prostate cancer cells. BMPR-IB and BMPR-II have been reported to be expressed at low levels in prostate cancer. However, little is known about the crosstalk between HGF and BMP pathways. In this study, prostate cancer cells (PC-3 and DU-145) were exposed to HGF at different concentrations (1-75 ng/ml) for 18 h, or were treated with HGF at 40 ng/ml over various time periods (up to 24 h). The effect of HGF on BMP receptor expression was further investigated in a nude mouse PC-3 xenograft model. Mice were treated with either HGF, the HGF antagonist NK4, or a combination of both. The expression of BMPR-IB and BMPR-II mRNA was up-regulated by HGF, as shown by both conventional PCR and quantitative PCR. An elevation of BMPR-IB and BMPR-II at the protein level was confirmed by both Western blot analysis and immunocytochemical staining. In a murine prostate tumour model, infusion of recombinant HGF resulted in an increase in the levels of both BMPR-IB and BMPR-II transcript in prostate tumours. Concomitant delivery of NK4, an HGF antagonist, prevented this effect. In conclusion, HGF up-regulates the expression of the bone morphogenetic protein receptors, BMPR-IB and BMPR-II, in prostate cancer cells, both in vitro and in vivo. This may have important implications in the development of bone **metastasis** in prostate cancer.
3. Cloning of human bone morphogenetic protein type IB receptor (BMPR-IB) and its expression in prostate cancer in comparison with other BMPRs. Bone **metastasis** is a common event in prostate cancer, and it is known that some of the bone morphogenetic proteins (BMPs) are expressed in prostate cancer cells, while no study on the expression of their receptors, BMPRs, has been reported. Here we report cloning and sequence analysis of the human BMPR-IB cDNA. We also analysed the expression of transcripts of three types of the BMPR genes in human tissues and prostate cancer cell lines. The BMPR-IB mRNA was present in various organs, but the highest level was found in the prostate. Moreover, the amount of BMPR-IB mRNA was significantly low in prostate cancer tissues after androgen withdrawal and was also low in prostate cancer cell lines. RT-PCR analysis showed that the BMPR-IB message was upregulated by androgen stimulation in the LNCaP cell line which expresses the androgen receptor. By contrast, the mRNA levels of BMPR-IA and BMPR-II were not significantly different among non-cancerous and cancerous prostate tissues. It was also suggested that human BMPR-IA and BMPR-IB might have different biological functions in the prostate, although their sequences were 85.3% identical in the serine-threonine kinase domain.

### [Cc]ell [Ii]nvasion

#### PubMed

1. Thyroid follicle development requires Smad1/5- and endothelial cell-dependent basement membrane assembly. Thyroid follicles, the functional units of the thyroid gland, are delineated by a monolayer of thyrocytes resting on a continuous basement membrane. The developmental mechanisms of folliculogenesis, whereby follicles are formed by the reorganization of a non-structured mass of non-polarized epithelial cells, are largely unknown. Here we show that assembly of the epithelial basement membrane is crucial for folliculogenesis and is controlled by endothelial **cell invasion** and by BMP-Smad signaling in thyrocytes. Thyroid-specific Smad1 and Smad5 double-knockout (Smad1/5(dKO)) mice displayed growth retardation, hypothyroidism and defective follicular architecture. In Smad1/5(dKO) embryonic thyroids, epithelial cells remained associated in large clusters and formed small follicles. Although similar follicular defects are found in Vegfa knockout (Vegfa(KO)) thyroids, Smad1/5(dKO) thyroids had normal endothelial cell density yet impaired endothelial differentiation. Interestingly, both Vegfa(KO) and Smad1/5(dKO) thyroids displayed impaired basement membrane assembly. Furthermore, conditioned medium (CM) from embryonic endothelial progenitor cells (eEPCs) rescued the folliculogenesis defects of both Smad1/5(dKO) and Vegfa(KO) thyroids. Laminin α1, β1 and γ1, abundantly released by eEPCs into CM, were crucial for folliculogenesis. Thus, epithelial Smad signaling and endotheli**al cell invas**ion promote folliculogenesis via assembly of the basement membrane.

---

## Dock3

- NCBI Gene
- GeneRIF
- Pubmed

| Search Term | Rank | Fields |
| --- | --- | --- |
| [Ii]nvasive | 10 | PubMed(1) |

  
hide/show details for Dock3

### [Ii]nvasive

#### PubMed

1. Dock3 protects myelin in the cuprizone model for demyelination. Dedicator of cytokinesis 3 (Dock3) belongs to an atypical family of the guanine nucleotide exchange factors. It is predominantly expressed in the neural tissues and causes cellular morphological changes by activating the small GTPase Rac1. We previously reported that Dock3 overexpression protects retinal ganglion cells from excitotoxic cell death. Oligodendrocytes are the myelinating cells of axons in the central nervous system and these cells are damaged in demyelinating disorders including multiple sclerosis (MS) and optic neuritis. In this study, we examined if Dock3 is expressed in oligodendrocytes and if increasing Dock3 signals can suppress demyelination in a cuprizone-induced demyelination model, an animal model of MS. We demonstrate that Dock3 is expressed in oligodendrocytes and Dock3 overexpression protects myelin in the corpus callosum following cuprizone treatment. Furthermore, we show that cuprizone demyelinates optic nerves and the extent of demyelination is ameliorated in mice overexpressing Dock3. Cuprizone treatment impairs visual function, which was demonstrated by multifocal electroretinograms, an established non-**invasive** method, and Dock3 overexpression prevented this effect. In mice overexpressing Dock3, Erk activation is increased, suggesting this may at least partly explain the observed protective effects. Our findings suggest that Dock3 may be a therapeutic target for demyelinating disorders including optic neuritis.Cell Death and Disease (2014) 5, e1395; doi:10.1038/cddis.2014.357; published online 28 August 2014.

---

## Akt2

- NCBI Gene
- GeneRIF
- Pubmed

| Search Term | Rank | Fields |
| --- | --- | --- |
| [Mm]igration | 6 | Process(1); PubMed(19); GeneRIFs(5) |
| ECM | 7 | PubMed(2) |
| [Ee]xtracellular [Mm]atrix | 8 | PubMed(4) |
| [Ii]nvasive | 10 | PubMed(5) |
| [Mm]etastasis | 12 | PubMed(8); GeneRIFs(1) |

  
hide/show details for Akt2

### [Mm]igration

#### Process

1. positive regulation of cell **migration**

#### PubMed

1. Abnormal Wnt and PI3Kinase signaling in the malformed intestine of lama5 deficient mice. Laminins are major constituents of basement membranes and are essential for tissue homeostasis. Laminin-511 is highly expressed in the intestine and its absence causes severe malformation of the intestine and embryonic lethality. To understand the mechanistic role of laminin-511 in tissue homeostasis, we used RNA profiling of embryonic intestinal tissue of lama5 knockout mice and identified a lama5 specific gene expression signature. By combining cell culture experiments with mediated knockdown approaches, we provide a mechanistic link between laminin α5 gene deficiency and the physiological phenotype. We show that laminin α5 plays a crucial role in both epithelial and mesenchymal cell behavior by inhibiting Wnt and activating PI3K signaling. We conclude that conflicting signals are elicited in the absence of lama5, which alter cell adhesion**, migrati**on as well as epithelial and muscle differentiation. Conversely, adhesion to laminin-511 may serve as a potent regulator of known interconnected PI3K/Akt and Wnt signaling pathways. Thus deregulated adhesion to laminin-511 may be instrumental in diseases such as human pathologies of the gut where laminin-511 is abnormally expressed as it is shown here.
2. Angiogenesis selectively requires the p110alpha isoform of PI3K to control endothelial cell **migration**. Phosphoinositide 3-kinases (PI3Ks) signal downstream of multiple cell-surface receptor types. Class IA PI3K isoforms couple to tyrosine kinases and consist of a p110 catalytic subunit (p110alpha, p110beta or p110delta), constitutively bound to one of five distinct p85 regulatory subunits. PI3Ks have been implicated in angiogenesis, but little is known about potential selectivity among the PI3K isoforms and their mechanism of action in endothelial cells during angiogenesis in vivo. Here we show that only p110alpha activity is essential for vascular development. Ubiquitous or endothelial cell-specific inactivation of p110alpha led to embryonic lethality at mid-gestation because of severe defects in angiogenic sprouting and vascular remodelling. p110alpha exerts this critical endothelial cell-autonomous function by regulating endothelial cell **migration** through the small GTPase RhoA. p110alpha activity is particularly high in endothelial cells and preferentially induced by tyrosine kinase ligands (such as vascular endothelial growth factor (VEGF)-A). In contrast, p110beta in endothelial cells signals downstream of G-protein-coupled receptor (GPCR) ligands such as SDF-1alpha, whereas p110delta is expressed at low level and contributes only minimally to PI3K activity in endothelial cells. These results provide the first in vivo evidence for p110-isoform selectivity in endothelial PI3K signalling during angiogenesis.
3. Opposing roles for Akt1 and Akt2 in Rac/Pak signaling and cell **migration**. The Akt/PKB isoforms have different roles in animals, with Akt2 primarily regulating metabolic signaling and Akt1 regulating growth and survival. Here we show distinct roles for Akt1 and Akt2 in mouse embryo fibroblast cell **migration** and regulation of the cytoskeleton. Akt1-deficient cells responded poorly to platelet-derived growth factor while Akt2-deficient cells had a dramatically enhanced response, resulting in a substantial increase in dorsal ruffling. Swapping domains between Akt1 and Akt2 demonstrated that the N-terminal region containing the pleckstrin homology domain and a linker region distinguishes the two isoforms, while the catalytic domains are interchangeable. Akt2 knock-out cells also migrated faster than wild-type cells, especially through extracellular matrix (ECM), while Akt1 knock-out cells migrated more slowly than wild-type cells. Consistently, Akt2 knock-out cells had elevated Pak1 and Rac activities, suggesting that Akt2 inhibits Rac and Pak1. Both Akt2 and Akt1 associated in complexes with Pak1, but only Akt2 inhibited Pak1 in kinase assays, suggesting an underlying molecular basis for the different cellular phenotypes. Together these data provide evidence for an unexpected functional link between Akt2 and Pak1 that opposes the actions of Akt1 on cell **migration**.
4. Dosage-dependent effects of Akt1/protein kinase Balpha (PKBalpha) and Akt3/PKBgamma on thymus, skin, and cardiovascular and nervous system development in mice. Akt/protein kinase B (PKB) plays a critical role in the regulation of metabolism, transcription, cell **migration**, cell cycle progression, and cell survival. The existence of viable knockout mice for each of the three isoforms suggests functional redundancy. We generated mice with combined mutant alleles of Akt1 and Akt3 to study their effects on mouse development. Here we show that Akt1-/- Akt3+/- mice display multiple defects in the thymus, heart, and skin and die within several days after birth, while Akt1+/- Akt3-/- mice survive normally. Double knockout (Akt1-/-) Akt3-/-) causes embryonic lethality at around embryonic days 11 and 12, with more severe developmental defects in the cardiovascular and nervous systems. Increased apoptosis was found in the developing brain of double mutant embryos. These data indicate that the Akt1 gene is more essential than Akt3 for embryonic development and survival but that both are required for embryo development. Our results indicate isoform-specific and dosage-dependent effects of Akt on animal survival and development.
5. Endothelial Akt1 mediates angiogenesis by phosphorylating multiple angiogenic substrates. The PI3K/Akt pathway is necessary for several key endothelial cell (EC) functions, including cell growth, **migration**, survival, and vascular tone. However, existing literature supports the idea that Akt can be either pro- or antiangiogenic, possibly due to compensation by multiple isoforms in the EC when a single isoform is deleted. Thus, biochemical, genetic, and proteomic studies were conducted to examine isoform-substrate specificity for Akt1 vs. Akt2. In vitro, Akt1 preferentially phosphorylates endothelial nitric oxide synthase (eNOS) and promotes NO release, whereas nonphysiological overexpression of Akt2 can bypass the loss of Akt1. Conditional deletion of Akt1 in the EC, in the absence or presence of Akt2, retards retinal angiogenesis, implying that Akt1 exerts a nonredundant function during physiological angiogenesis. Finally, proteomic analysis of Akt substrates isolated from Akt1- or Akt2-deficient ECs documents that phosphorylation of multiple Akt substrates regulating angiogenic signaling is reduced in Akt1-deficient, but not Akt2-deficient, ECs, including eNOS and Forkhead box proteins. Therefore, Akt1 promotes angiogenesis largely due to phosphorylation and regulation of important downstream effectors that promote aspects of angiogenic signaling.
6. Extracellular heat shock protein 90 signals through subdomain II and the NPVY motif of LRP-1 receptor to Akt1 and Akt2: a circuit essential for promoting skin cell **migration** in vitro and wound healing in vivo. Normal cells secrete heat shock protein 90 alpha (Hsp90α) in response to tissue injury. Tumor cells have managed to constitutively secrete Hsp90α during invasion and metastasis. The sole function of extracellular Hsp90α (eHsp90α) is to promote cell motility, a critical event for both wound healing and tumor progression. The mechanism of promotility action by eHsp90α, however, has remained elusive. A key issue is whether eHsp90α still acts as a chaperone outside the cells or is a new and bona fide signaling molecule. Here, we have provided evidence that eHsp90α utilizes a unique transmembrane signaling mechanism to promote cell motility and wound healing. First, subdomain II in the extracellular part of low-density lipoprotein receptor-related protein 1 (LRP-1) receives the eHsp90α signal. Then, the NPVY but not the NPTY motif in the cytoplasmic tail of LRP-1 connects eHsp90α signaling to serine 473 but not threonine 308 phosphorylation in Akt kinases. Individual knockdown of Akt1, Akt2, or Akt3 revealed the importance of Akt1 and Akt2 in eHsp90α-induced cell motility. Akt gene rescue experiments suggest that Akt1 and Akt2 work in concert, rather than independently, to mediate eHsp90α promotility signaling. Finally, Akt1 and Akt2 knockout mice showed impaired wound healing that cannot be corrected by topical application with the eHsp90α protein.
7. Protein kinase B alpha/Akt1 regulates placental development and fetal growth. Protein kinase B alpha (PKB alpha/Akt1) is implicated in the regulation of metabolism, transcription, cell survival, angiogenesis, cell **migration**, growth, and tumorigenesis. Previously, it was reported that PKB alpha-deficient mice are small with increased neonatal mortality (Cho, H., Thorvaldsen, J. L., Chu, Q., Feng, F., and Birnbaum, M. J. (2001) J. Biol. Chem. 276, 38349-38352 and Chen, W. S., Xu, P. Z., Gottlob, K., Chen, M. L., Sokol, K., Shiyanova, T., Roninson, I., Wenig, W., Suzuki, R., Tobe, K., Kadowaki, T., and Hay, N. (2001) Genes Dev. 15, 2203-2208). Here we show that PKB alpha is widely expressed in placenta including all types of trophoblast and vascular endothelial cells. Pkb alpha-/- placentae display significant hypotrophy, with marked reduction of the decidual basalis and nearly complete loss of glycogen-containing cells in the spongiotrophoblast, and exhibit decreased vascularization. Pkb alpha-/- placentae also show significant reduction of phosphorylation of PKB and endothelial nitric-oxide synthase. These defects may cause placental insufficiency, fetal growth impairment, and neonatal mortality. These data represent the first evidence for the role of PKB alpha and endothelial nitricoxide synthase in regulating placental development and provide an animal model for intrauterine growth retardation.
8. Akt2/LDLr double knockout mice display impaired glucose tolerance and develop more complex atherosclerotic plaques than LDLr knockout mice. To characterize the phenotype of Akt2/low-density-lipoprotein receptor double knockout (dKO) (Akt2/LDLr dKO) mice with respect to insulin resistance and features of atherosclerotic plaque progression.Metabolic profile and atherosclerotic plaque progression were compared between LDLr KO mice and Akt2/LDLr dKO mice. Total cholesterol, glucose, and insulin levels were significantly higher and oral glucose tolerance test (GTT) was more impaired in Akt2/LDLr dKO mice than in LDLr KO mice. Although atherosclerotic plaques at both the carotid artery and the aortic root of Akt2/LDLr dKO mice were significantly smaller (P < 0.05) compared with LDLr KO controls, plaque composition in these mice was more complex, showing 34-50% reduced collagen content (P < 0.01), 1.4-fold larger necrotic cores (P < 0.05) and six-fold more TUNEL-positive cells (P < 0.01). In situ zymography revealed a more than two-fold higher gelatinolytic activity in Akt2/LDLr dKO mice (P < 0.05). In vitro analyses showed that deletion of Akt2 caused decreased **migration**, proliferation, and collagen content of vascular smooth muscle cells (VSMCs) and disturbed the balance of metalloproteinases (MMPs) and tissue inhibitor of metalloproteinase (TIMP) mRNA expression in macrophages and VSMCs.Akt2/LDLr dKO mice develop insulin resistance and complex atherosclerotic lesions. These phenotypic characteristics make Akt2/LDLr dKO mice an interesting mouse model to study the effects of insulin resistance on the development and progression of atherosclerosis.
9. Macrophage deficiency of Akt2 reduces atherosclerosis in Ldlr null mice. Macrophages play crucial roles in the formation of atherosclerotic lesions. Akt, a serine/threonine protein kinase B, is vital for cell proliferation, **migration**, and survival. Macrophages express three Akt isoforms, Akt1, Akt2, and Akt3, but the roles of Akt1 and Akt2 in atherosclerosis in vivo remain unclear. To dissect the impact of macrophage Akt1 and Akt2 on early atherosclerosis, we generated mice with hematopoietic deficiency of Akt1 or Akt2. After 8 weeks on Western diet, Ldlr(-/-) mice reconstituted with Akt1(-/-) fetal liver cells (Akt1(-/-)→Ldlr(-/-)) had similar atherosclerotic lesion areas compared with control mice transplanted with WT cells (WT→Ldlr(-/-)). In contrast, Akt2(-/-)→Ldlr(-/-) mice had dramatically reduced atherosclerotic lesions compared with WT→Ldlr(-/-) mice of both genders. Similarly, in the setting of advanced atherosclerotic lesions, Akt2(-/-)→Ldlr(-/-) mice had smaller aortic lesions compared with WT→Ldlr(-/-) and Akt1(-/-)→Ldlr(-/-) mice. Importantly, Akt2(-/-)→Ldlr(-/-) mice had reduced numbers of proinflammatory blood monocytes expressing Ly-6C(hi) and chemokine C-C motif receptor 2. Peritoneal macrophages isolated from Akt2(-/-) mice were skewed toward an M2 phenotype and showed decreased expression of proinflammatory genes a**nd reduce**d cell migration. Our data demonstrate that loss of Akt2 suppresses the ability of macrophages to undergo M1 polarization reducing both early and advanced atherosclerosis.
10. Akt1/protein kinase Balpha is critical for ischemic and VEGF-mediated angiogenesis. Akt, or protein kinase B, is a multifunctional serine-threonine protein kinase implicated in a diverse range of cellular functions including cell metabolism, survival, **migration**, and gene expression. However, the in vivo roles and effectors of individual Akt isoforms in signaling are not explicitly clear. Here we show that the genetic loss of Akt1, but not Akt2, in mice results in defective ischemia and VEGF-induced angiogenesis as well as severe peripheral vascular disease. Akt1 knockout (Akt1-/-) mice also have reduced endothelial progenitor cell (EPC) mobilization in response to ischemia, and reintroduction of WT EPCs, but not EPCs isolated from Akt1-/- mice, into WT mice improves limb blood flow after ischemia. Mechanistically, the loss of Akt1 reduces the basal phosphorylation of several Akt substrates, the **migration** of fibroblasts and ECs, and NO release. Reconstitution of Akt1-/- ECs with Akt1 rescues the defects in substrate phosphorylation, cell **migration**, and NO release. Thus, the Akt1 isoform exerts an essential role in blood flow control, cellular **migration**, and NO synthesis during postnatal angiogenesis.
11. Deficiency of Akt1, but not Akt2, attenuates the development of pulmonary hypertension. Pulmonary vascular remodeling, mainly attributable to enhanced pulmonary arterial smooth muscle cell proliferation and **migration**, is a major cause for elevated pulmonary vascular resistance and pulmonary arterial pressure in patients with pulmonary hypertension. The signaling cascade through Akt, comprised of three isoforms (Akt1-3) with distinct but overlapping functions, is involved in regulating cell proliferation and **migration**. This study aims to investigate whether the Akt/mammalian target of rapamycin (mTOR) pathway, and particularly which Akt isoform, contributes to the development and progression of pulmonary vascular remodeling in hypoxia-induced pulmonary hypertension (HPH). Compared with the wild-type littermates, Akt1(-/-) mice were protected against the development and progression of chronic HPH, whereas Akt2(-/-) mice did not demonstrate any significant protection against the development of HPH. Furthermore, pulmonary vascular remodeling was significantly attenuated in the Akt1(-/-) mice, with no significant effect noted in the Akt2(-/-) mice after chronic exposure to normobaric hypoxia (10% O2). Overexpression of the upstream repressor of Akt signaling, phosphatase and tensin homolog deleted on chromosome 10 (PTEN), and conditional and inducible knockout of mTOR in smooth muscle cells were also shown to attenuate the rise in right ventricular systolic pressure and the development of right ventricular hypertrophy. In conclusion, Akt isoforms appear to have a unique function within the pulmonary vasculature, with the Akt1 isoform having a dominant role in pulmonary vascular remodeling associated with HPH. The PTEN/Akt1/mTOR signaling pathway will continue to be a critical area of study in the pathogenesis of pulmonary hypertension, and specific Akt isoforms may help specify therapeutic targets for the treatment of pulmonary hypertension.
12. Akt2 is required for macrophage chemotaxis. Tumor-associated macrophages play an important role in tumorigenesis and metastasis. Trafficking of macrophages to the proximity of tumors is mediated by CSF-1, a growth factor. In this study, we investigated the role of PKB/Akt in CSF-1-induced macrophage **migration**. Disruption of Akt2 expression by small interference RNA impaired chemotaxis of both THP-1 cells and mouse peritoneal macrophages. Phosphorylation of PKCzeta, an essential component in chemotaxis signaling pathway, was reduced. LIMK/Cofilin, downstream of PKCzeta, regulated cytoskeleton rearrangement during cell **migration**. Disruption of Akt2 expression inhibited CSF-1-induced LIMK/Cofilin phosphorylation, which contributed to defects in actin polymerization and chemotaxis. Furthermore, MCP-1, a chemokine, -induced macrophage chemotaxis was also impaired. Taken together, our results demonstrated that Akt2 plays an essential role in both CSF-1- and chemokine-induced chemotaxis of macrophages.
13. Hematopoietic Akt2 deficiency attenuates the progression of atherosclerosis. Atherosclerosis is the major cause of death and disability in diabetic and obese subjects with insulin resistance. Akt2, a phosphoinositide-dependent serine-threonine protein kinase, is highly express in insulin-responsive tissues; however, its role during the progression of atherosclerosis remains unknown. Thus, we aimed to investigate the contribution of Akt2 during the progression of atherosclerosis. We found that germ-line Akt2-deficient mice develop similar atherosclerotic plaques as wild-type mice despite higher plasma lipids and glucose levels. It is noteworthy that transplantation of bone marrow cells isolated from Akt2(-/-) mice to Ldlr(-/-) mice results in marked reduction of the progression of atherosclerosis compared with Ldlr(-/-) mice transplanted with wild-type bone marrow cells. In vitro studies indicate that Akt2 is required for macrophage **migration** in response to proatherogenic cytokines (monocyte chemotactic protein-1 and macrophage colony-stimulating factor). Moreover, Akt2(-/-) macrophages accumulate less cholesterol and have an alternative activated or M2-type phenotype when stimulated with proinflammatory cytokines. Together, these results provide evidence that macrophage Akt2 regulates **migration**, the inflammatory response and cholesterol metabolism and suggest that targeting Akt2 in macrophages might be beneficial for treating atherosclerosis.
14. Appl1 is dispensable for mouse development, and loss of Appl1 has growth factor-selective effects on Akt signaling in murine embryonic fibroblasts. The adaptor protein APPL1 (adaptor protein containing pleckstrin homology (PH), phosphotyrosine binding (PTB), and leucine zipper motifs) was first identified as a binding protein of AKT2 by yeast two-hybrid screening. APPL1 was subsequently found to bind to several membrane-bound receptors and was implicated in their signal transduction through AKT and/or MAPK pathways. To determine the unambiguous role of Appl1 in vivo, we generated Appl1 knock-out mice. Here we report that Appl1 knock-out mice are viable and fertile. Appl1-null mice were born at expected Mendelian ratios, without obvious phenotypic abnormalities. Moreover, Akt activity in various fetal tissues was unchanged compared with that observed in wild-type littermates. Studies of isolated Appl1(-/-) murine embryonic fibroblasts (MEFs) showed that Akt activation by epidermal growth factor, insulin, or fetal bovine serum was similar to that observed in wild-type MEFs, although Akt activation by HGF was diminished in Appl1(-/-) MEFs. To rule out a possible redundant role played by the related Appl2, we used small interfering RNA to knock down Appl2 expression in Appl1(-/-) MEFs. Unexpectedly, cell survival was unaffected under normal culture conditions, and activation of Akt was unaltered following epidermal growth factor stimulation, although Akt activity did decrease further after HGF stimulation. Furthermore, we found that Appl proteins are required for HGF-induced cell survival and **migration** via activation of Akt. Our studies suggest that Appl1 is dispensable for development and only participate in Akt signaling under certain conditions.
15. Myocardial injury after ischemia-reperfusion in mice deficient in Akt2 is associated with increased cardiac macrophage density. Akt2 protein kinase has been shown to promote cell **migration** and actin polymerization in several cell types, including macrophages. Because migrating macrophages constitute an important inflammatory response after myocardial ischemia, we determined cardiac macrophage expression after ischemia-reperfusion (I/R) injury and cryo-injury in mice lacking Akt2 (Akt2-KO). At 7 days post-I/R, Akt2-KO cardiac tissues showed an increase in immunohistochemical staining for macrophage markers (Galectin 3 and F4/80) compared with wild-type (WT) mice, indicating macrophage density was increased in the injured Akt2-KO myocardium. This change was time dependent because macrophage density was similar between WT and Akt2-KO myocardium at 3 days post-I/R, but by 7 and 14 days post-I/R, macrophage density was significantly increased in Akt2-KO myocardium. Concomitantly, infarct size was larger and cardiac function was reduced in Akt2-KO mice subjected to I/R. However, when cryo-infarction produced similar infarct sizes in the anterior wall in both WT and Akt2-KO mice, macrophage density remained higher in Akt2-KO mouse myocardium, suggesting Akt2 regulates myocardial macrophage density independent of infarct size. Consistently, bone marrow from Akt2-KO mice enhanced myocardial macrophage density in both C57/B6 WT and Akt2-KO recipient mice. Finally, reciprocal ex-vivo coculturing of macrophages and cardiac myocytes showed that activated Akt2-KO peritoneal macrophages had reduced mobility and adhesion when compared with WT littermate controls. Thus, although Akt-2 KO mice did not affect the initial inflammation response after injury and Akt2 deficiency has been shown to impair cell **migration** or motility in macrophages, our data suggested a novel mechanism in which increasing retention of Akt2-KO macrophages resulted in increasing cardiac Akt2-KO macrophage density in the myocardial space.
16. Akt isoforms differentially regulate neutrophil functions. In neutrophils, the phosphoinositide 3-kinase/Akt signaling cascade is involved in **migration**, degranulation, and O(2)(-) production. However, it is unclear whether the Akt kinase isoforms have distinct functions in neutrophil activation. Here we report functional differences between the 2 major Akt isoforms in neutrophil activation on the basis of studies in which we used individual Akt1 and Akt2 knockout mice. Akt2(-/-) neutrophils exhibited decreased cell **migration**, granule enzyme release, and O(2)(-) production compared with wild-type and Akt1(-/-) neutrophils. Surprisingly, Akt2 deficiency and pharmacologic inhibition of Akt also abrogated phorbol ester-induced O(2)(-) production, which was unaffected by treatment with the phosphoinositide 3-kinase inhibitor LY294002. The decreased O(2)(-) production in Akt2(-/-) neutrophils was accompanied by reduced p47(phox) phosphorylation and its membrane translocation, suggesting that Akt2 is important for the assembly of phagocyte nicotinamide adenine dinucleotide phosphate oxidase. In wild-type neutrophils, Akt2 but not Akt1 translocated to plasma membrane upon chemoattractant stimulation and to the leading edge in polarized neutrophils. In the absence of Akt2, chemoattractant-induced Akt protein phosphorylation was significantly reduced. These results demonstrate a predominant role of Akt2 in regulating neutrophil functions and provide evidence for differential activation of the 2 Akt isoforms in neutrophils.
17. Akt2- and ETS1-dependent IP3 receptor 2 expression in dendritic cell **migration**. The protein kinase Akt2/PKBβ is a known regulator of macrophage and dendritic cell (DC) **migratio**n. The mechanisms linking Akt2 activity to **migratio**n remained, however, elusive. DC **migratio**n is governed by Ca(2+) signaling. We thus explored whether Akt2 regulates DC Ca(2+) signaling.DCs were derived from bone marrow of Akt2-deficient mice (akt2(-/-)) and their wild type littermates (akt2(+/+)). DC maturation was induced by lipopolysaccharides (LPS) and evaluated by flow cytometry. Cytosolic Ca(2+) concentration was determined by Fura-2 fluorescence, channel activity by whole cell recording, transcript levels by RT-PCR, **migratio**n utilizing transwells.Upon maturation, chemokine CCL21 stimulated **migratio**n of akt2(+/+) but not akt2(-/-) DCs. CCL21-induced increase in cytosolic Ca(2+) concentration, thapsigargin-induced release of Ca(2+) from intracellular stores with subsequent store-operated Ca(2+) entry (SOCE), ATP-induced inositol 1,4,5-trisphosphate (IP3)-dependent Ca(2+) release as well as Ca(2+) release-activated Ca(2+) (CRAC) channel activity were all significantly lower in mature akt2(-/-) than in mature akt2(+/+) DCs. Transcript levels of IP3 receptor IP3R2 and of IP3R2 regulating transcription factor ETS1 were significantly higher in akt2(+/+) than in akt2(-/-) DCs prior to maturation and were upregulated by LPS stimulation (1h) in akt2(+/+) and to a lower extent in akt2(-/-) DCs. Following maturation, protein abundance of IP3R2 and ETS1 were similarly higher in akt2(+/+) than in akt2(-/-) DCs. The IP3R inhibitor Xestospongin C significantly decreased CCL21-induced **migratio**n of akt2(+/+)DCs and abrogated the differences between genotypes. Finally, knock-down of ETS1 with siRNA decreased IP3R2 mRNA abundance, thapsigargin- and ATP-induced Ca(2+) release, SOCE and CRAC channel activation, as well as DC **migratio**n.Akt2 upregulates DC **migratio**n at least in part by ETS1-dependent stimulation of IP3R2 transcription.
18. Akt1 is critical for acute inflammation and histamine-mediated vascular leakage. Akt1 is implicated in cell metabolism, survival **migration**, and gene expression; however, little is known about the role of specific Akt isoforms during inflammation in vivo. Thus, we directly explored the roles of the isoforms Akt1 and Akt2 in acute inflammation models by using mice deficient in either Akt1 or Akt2. Akt1(-/-) mice showed a markedly reduced edema versus Akt2(-/-) and WT controls, and the reduced inflammation was associated with a dramatic decrease in neutrophil and monocyte infiltration. The loss of Akt1 did not affect leukocyte functions in vitro, and bone marrow transplant experiments suggest that host Akt1 regulates leukocyte e**migration** into inflamed tissues. Moreover, carrageenan-induced edema and the direct propermeability actions of bradykinin and histamine were reduced dramatically in Akt1(-/-) versus WT mice. These findings are supported by in vitro experiments showing that Akt1 deficiency or blockade of nitric oxide synthase markedly reduces histamine-stimulated changes in transendothelial electrical resistance of microvascular endothelial cells. Collectively, these results suggest that Akt1 is necessary for acute inflammation and exerts its actions primarily via regulation of vascular permeability, leading to edema and leukocyte extravasation.
19. Regulation of Na+/H+ exchanger in dendritic cells by Akt2. Dendritic cells (DCs) are antigen-presenting cells decisive in primary immune responses and establishment of immunological memory. They are activated by bacterial lipopolysaccharides (LPS), which lead to activation of Na(+)/H(+) exchanger activity, cell swelling, reactive oxygen species (ROS) formation, and **migration**. The effects require functional phosphoinositide 3 kinase and are paralleled by Akt phosphorylation. The present study explored the putative involvement of the Akt isoform Akt2. To this end, experiments were performed in DCs isolated from bone marrow of mice lacking functional Akt2/PKBß (akt2 (-/-)) and respective wild-type animals (akt2 (+/+)). Based on BCECF fluorescence, cytosolic pH (pH(i)) was significantly lower in akt2 (-/-) than in akt2 (+/+) DCs. Transient exposure to NH(4)Cl was followed by profound cytosolic acidification in both genotypes. Subsequent re-alkalinization was largely dependent on Na(+) thus reflecting Na(+)/H(+) exchanger activity and was significantly lower in akt2 (-/-) than in akt2 (+/+) DCs. According to forward scatter in FACS analysis, cell volume was significantly lower in akt2 (-/-) than in akt2 (+/+) DCs. Exposure of DCs to LPS led within 4 h to significant increases of Na(+)/H(+) exchanger activity, cell volume, ROS production, an**d migrati**on in akt2 (+/+) mice, and its effects were significantly blunted in akt2 (-/-) DCs. The present observations disclose a role of Akt2 in the regulation of pH(i), cell volume, ROS production, an**d migrati**on in dendritic cells.

#### GeneRIFs

1. Akt2 upregulates dendritic cell **migration** at least in part by ETS1-dependent stimulation of IP3R2 transcription.
2. macrophage Akt2 regulates **migration**, the inflammatory response and cholesterol metabolism in atherosclerosis
3. There is a role of Akt2 in the regulation of pH(i), cell volume, ROS production, and **migration** in dendritic cells.
4. Akt2(-/-) neutrophils exhibited decreased cell **migration**, granule enzyme release, and O(2)(-) production compared with wild-type and Akt1(-/-) neutrophils.
5. Akt1 and Akt2 have opposing roles in Rac/Pak signaling and cell **migration**

### ECM

#### PubMed

1. Functionally distinct PI 3-kinase pathways regulate myelination in the peripheral nervous system. The PI 3-kinase (PI 3-K) signaling pathway is essential for Schwann cell myelination. Here we have characterized PI 3-K effectors activated during myelination by probing myelinating cultures and developing nerves with an antibody that recognizes phosphorylated substrates for this pathway. We identified a discrete number of phospho-proteins including the S6 ribosomal protein (S6rp), which is down-regulated at the onset of myelination, and N-myc downstream-regulated gene-1 (NDRG1), which is up-regulated strikingly with myelination. We show that type III Neuregulin1 on the axon is the primary activator of S6rp, an effector of mTORC1. In contrast, laminin-2 in the extracellular matrix (**ECM**), signaling through the α6β4 integrin and Sgk1 (serum and glucocorticoid-induced kinase 1), drives phosphorylation of NDRG1 in the Cajal bands of the abaxonal compartment. Unexpectedly, mice deficient in α6β4 integrin signaling or Sgk1 exhibit hypermyelination during development. These results identify functionally and spatially distinct PI 3-K pathways: an early, pro-myelinating pathway driven by axonal Neuregulin1 and a later-acting, laminin-integrin-dependent pathway that negatively regulates myelination.
2. Opposing roles for Akt1 and Akt2 in Rac/Pak signaling and cell migration. The Akt/PKB isoforms have different roles in animals, with Akt2 primarily regulating metabolic signaling and Akt1 regulating growth and survival. Here we show distinct roles for Akt1 and Akt2 in mouse embryo fibroblast cell migration and regulation of the cytoskeleton. Akt1-deficient cells responded poorly to platelet-derived growth factor while Akt2-deficient cells had a dramatically enhanced response, resulting in a substantial increase in dorsal ruffling. Swapping domains between Akt1 and Akt2 demonstrated that the N-terminal region containing the pleckstrin homology domain and a linker region distinguishes the two isoforms, while the catalytic domains are interchangeable. Akt2 knock-out cells also migrated faster than wild-type cells, especially through extracellular matrix (**ECM**), while Akt1 knock-out cells migrated more slowly than wild-type cells. Consistently, Akt2 knock-out cells had elevated Pak1 and Rac activities, suggesting that Akt2 inhibits Rac and Pak1. Both Akt2 and Akt1 associated in complexes with Pak1, but only Akt2 inhibited Pak1 in kinase assays, suggesting an underlying molecular basis for the different cellular phenotypes. Together these data provide evidence for an unexpected functional link between Akt2 and Pak1 that opposes the actions of Akt1 on cell migration.

### [Ee]xtracellular [Mm]atrix

#### PubMed

1. Functionally distinct PI 3-kinase pathways regulate myelination in the peripheral nervous system. The PI 3-kinase (PI 3-K) signaling pathway is essential for Schwann cell myelination. Here we have characterized PI 3-K effectors activated during myelination by probing myelinating cultures and developing nerves with an antibody that recognizes phosphorylated substrates for this pathway. We identified a discrete number of phospho-proteins including the S6 ribosomal protein (S6rp), which is down-regulated at the onset of myelination, and N-myc downstream-regulated gene-1 (NDRG1), which is up-regulated strikingly with myelination. We show that type III Neuregulin1 on the axon is the primary activator of S6rp, an effector of mTORC1. In contrast, laminin-2 in the **extracellular matrix** (ECM), signaling through the α6β4 integrin and Sgk1 (serum and glucocorticoid-induced kinase 1), drives phosphorylation of NDRG1 in the Cajal bands of the abaxonal compartment. Unexpectedly, mice deficient in α6β4 integrin signaling or Sgk1 exhibit hypermyelination during development. These results identify functionally and spatially distinct PI 3-K pathways: an early, pro-myelinating pathway driven by axonal Neuregulin1 and a later-acting, laminin-integrin-dependent pathway that negatively regulates myelination.
2. Opposing roles for Akt1 and Akt2 in Rac/Pak signaling and cell migration. The Akt/PKB isoforms have different roles in animals, with Akt2 primarily regulating metabolic signaling and Akt1 regulating growth and survival. Here we show distinct roles for Akt1 and Akt2 in mouse embryo fibroblast cell migration and regulation of the cytoskeleton. Akt1-deficient cells responded poorly to platelet-derived growth factor while Akt2-deficient cells had a dramatically enhanced response, resulting in a substantial increase in dorsal ruffling. Swapping domains between Akt1 and Akt2 demonstrated that the N-terminal region containing the pleckstrin homology domain and a linker region distinguishes the two isoforms, while the catalytic domains are interchangeable. Akt2 knock-out cells also migrated faster than wild-type cells, especially through **extracellular matrix** (ECM), while Akt1 knock-out cells migrated more slowly than wild-type cells. Consistently, Akt2 knock-out cells had elevated Pak1 and Rac activities, suggesting that Akt2 inhibits Rac and Pak1. Both Akt2 and Akt1 associated in complexes with Pak1, but only Akt2 inhibited Pak1 in kinase assays, suggesting an underlying molecular basis for the different cellular phenotypes. Together these data provide evidence for an unexpected functional link between Akt2 and Pak1 that opposes the actions of Akt1 on cell migration.
3. Akt Regulates Axon Wrapping and Myelin Sheath Thickness in the PNS. The signaling pathways that regulate myelination in the PNS remain poorly understood. Phosphatidylinositol-4,5-bisphosphate 3-kinase 1A, activated in Schwann cells by neuregulin and the **extracellular matrix**, has an essential role in the early events of myelination. Akt/PKB, a key effector of phosphatidylinositol-4,5-bisphosphate 3-kinase 1A, was previously implicated in CNS, but not PNS myelination. Here we demonstrate that Akt plays a crucial role in axon ensheathment and in the regulation of myelin sheath thickness in the PNS. Pharmacological inhibition of Akt in DRG neuron-Schwann cell cocultures dramatically decreased MBP and P0 levels and myelin sheath formation without affecting expression of Krox20/Egr2, a key transcriptional regulator of myelination. Conversely, expression of an activated form of Akt in purified Schwann cells increased expression of myelin proteins, but not Krox20/Egr2, and the levels of activated Rac1. Transgenic mice expressing a membrane-targeted, activated form of Akt under control of the 2',3'-cyclic nucleotide 3'-phosphodiesterase promoter, exhibited thicker PNS and CNS myelin sheaths, and PNS myelin abnormalities, such as tomacula and myelin infoldings/outfoldings, centered around the paranodes and Schmidt Lanterman incisures. These effects were corrected by rapamycin treatmentin vivo Importantly, Akt activity in the transgenic mice did not induce myelination of nonmyelinating Schwann cells in the sympathetic trunk or Remak fibers of the dorsal roots, although, in those structures, they wrapped membranes redundantly around axons. Together, our data indicate that Akt is crucial for PNS myelination driving axonal wrapping by unmyelinated and myelinated Schwann cells and enhancing myelin protein synthesis in myelinating Schwann cells.Although the role of the key serine/threonine kinase Akt in promoting CNS myelination has been demonstrated, its role in the PNS has not been established and remains uncertain. This work reveals that Akt controls several key steps of the PNS myelination. First, its activity promotes membrane production and axonal wrapping independent of a transcriptional effect. In myelinated axons, it also enhances myelin thickness through the mTOR pathway. Finally, sustained Akt activation in Schwann cells leads to hypermyelination/dysmyelination, mimicking some features present in neuropathies, such as hereditary neuropathy with liability to pressure palsies or demyelinating forms of Charcot-Marie-Tooth disease. Together, these data demonstrate the role of Akt in regulatory mechanisms underlying axonal wrapping and myelination in the PNS.
4. Integrin linked kinase (ILK) is required for lens epithelial cell survival, proliferation and differentiation. While the role of growth factors in lens development has been investigated extensively, the role of **extracellular matrix** signalling is less well understood. The developing lens expresses predominantly laminin-binding integrins (such as α3β1, α6β1), which are cooperatively required in the lens epithelium during development. We investigated the role of ILK, a downstream mediator of integrin signalling in mice conditionally null for Ilk. Mutant lenses showed epithelial thinning at E17.5 with reduced proliferation and epithelial cell number and aberrant fibre differentiation. There was complete loss of the central epithelium from postnatal day (P) 2 due to cell death followed by fibre cell degeneration and death by P10 as well as rupture of the lens capsule between P10 and P21. At E17.5 there was significant inhibition (∼50%) of epithelial cell cycle progression, as shown by BrdU incorporation, cyclin D1/D2 and phospho-histone H3 immunostaining. The epithelial marker, E-cadherin, was decreased progressively from E17.5 to P2, in the central epithelium, but there was no significant change in Pax6 expression. Analyses of ERK and Akt phosphorylation indicated marked depression of MAPK and PI3K-Akt signalling, which correlated with decreased phosphorylation of FRS2α and Shp2, indicating altered activation of FGF receptors. At later postnatal stages there was reduced or delayed expression of fibre cell markers (β-crystallin and p57(kip2)). Loss of Ilk also affected deposi**tion of extracellula**r matrix, with marked retention of collagen IV within differentiating fibre cells. By quantitative RT-PCR array there was significantly decreased expression of 19 genes associated with focal adhesions, actin filament stability and MAPK and PI3K/Akt signalling. Overall, these data indicate that ILK is required for complete activation of signalling cascades downstream of the FGF receptor in lens epithelium and fibre cells during development and thus is involved in epithelial proliferation, survival and subsequent fibre differentiation.

### [Ii]nvasive

#### PubMed

1. Optimising experimental design for high-throughput phenotyping in mice: a case study. To further the functional annotation of the mammalian genome, the Sanger Mouse Genetics Programme aims to generate and characterise knockout mice in a high-throughput manner. Annually, approximately 200 lines of knockout mice will be characterised using a standardised battery of phenotyping tests covering key disease indications ranging from obesity to sensory acuity. From these findings secondary centres will select putative mutants of interest for more in-depth, confirmatory experiments. Optimising experimental design and data analysis is essential to maximise output using the resources with greatest efficiency, thereby attaining our biological objective of understanding the role of genes in normal development and disease. This study uses the example of the non**invasive** blood pressure test to demonstrate how statistical investigation is important for generating meaningful, reliable results and assessing the design for the defined research objectives. The analysis adjusts for the multiple-testing problem by applying the false discovery rate, which controls the number of false calls within those highlighted as significant. A variance analysis finds that the variation between mice dominates this assay. These variance measures were used to examine the interplay between days, readings, and number of mice on power, the ability to detect change. If an experiment is underpowered, we cannot conclude whether failure to detect a biological difference arises from low power or lack of a distinct phenotype, hence the mice are subjected to testing without gain. Consequently, in confirmatory studies, a power analysis along with the 3Rs can provide justification to increase the number of mice used.
2. MicroRNAs differentially regulated by Akt isoforms control EMT and stem cell renewal in cancer cells. Although Akt is known to play a role in human cancer, the relative contribution of its three isoforms to oncogenesis remains to be determined. We expressed each isoform individually in an Akt1(-/-)/Akt2(-/-)/Akt3(-/-) cell line. MicroRNA profiling of growth factor-stimulated cells revealed unique microRNA signatures for cells with each isoform. Among the differentially regulated microRNAs, the abundance of the miR-200 family was decreased in cells bearing Akt2. Knockdown of Akt1 in transforming growth factor-beta (TGFbeta)-treated MCF10A cells also decreased the abundance of miR-200; however, knockdown of Akt2, or of both Akt1 and Akt2, did not. Furthermore, Akt1 knockdown in MCF10A cells promoted TGFbeta-induced epithelial-mesenchymal transition (EMT) and a stem cell-like phenotype. Carcinomas developing in MMTV-cErbB2/Akt1(-/-) mice showed increased **invasive**ness because of miR-200 down-regulation. Finally, the ratio of Akt1 to Akt2 and the abundance of miR-200 and of the messenger RNA encoding E-cadherin in a set of primary and metastatic human breast cancers were consistent with the hypothesis that in many cases breast cancer metastasis may be under the control of the Akt-miR-200-E-cadherin axis. We conclude that induction of EMT is controlled by microRNAs whose abundance depends on the balance between Akt1 and Akt2 rather than on the overall activity of Akt.
3. Loss of PKBβ/Akt2 predisposes mice to ovarian cyst formation and increases the severity of polycystic ovary formation in vivo. Ovarian cysts affect women of all ages and decrease fertility. In particular, polycystic ovarian syndrome (PCOS), in which multiple follicular cysts develop, affects 5-10% of women of reproductive age and can result in infertility. Current non**-invasiv**e treatments for PCOS can resolve cysts and restore fertility, but unresponsive patients must undergo severe ovarian wedge resection and resort to in vitro fertilization. PCOS is related to the deregulation of leutinizing hormone (LH) signaling at various levels of the hypothalamic-pituitary-ovarian axis and resultant hyperproduction of androgens. Because insulin resistance and compensatory hyperinsulinemia are observed in 50-70% of individuals with PCOS, deregulated insulin signaling in the ovary is considered an important factor in the disease. Here we report that aged mice specifically lacking the PKBβ (also known as Akt2) isoform that is crucial for insulin signaling develop increased testosterone levels and ovarian cysts, both of which are also observed in insulin-resistant PCOS patients. Young PKBβ knockout mice were used to model PCOS by treatment with LH and exhibited a cyst area that was threefold greater than in controls, but without hyperinsulinemia. Thus, loss of PKBβ might predispose mice to ovarian cysts independently of hyperactive insulin signaling. Targeted therapeutic augmentation of specific PKBβ signaling could therefore provide a new avenue for the treatment and management of ovarian cysts.
4. Akt1 ablation inhibits, whereas Akt2 ablation accelerates, the development of mammary adenocarcinomas in mouse mammary tumor virus (MMTV)-ErbB2/neu and MMTV-polyoma middle T transgenic mice. Ample evidence to date links the phosphatidylinositol 3-kinase-regulated protein kinase Akt with the induction and progression of human cancer, including breast cancer. However, there are three Akt isoforms with limited information about their specificity during oncogenesis. This study addresses the role of the three isoforms in polyoma middle T (PyMT) and ErbB2/Neu-driven mammary adenocarcinomas in mice. The effects of ablation of Akt1, Akt2, and Akt3 on the induction and the biology of these tumors were dramatically different, with ablation of Akt1 inhibiting, ablation of Akt2 accelerating, and ablation of Akt3 having a small, not statistically significant, inhibitory effect on tumor induction by both transgenes. Whereas PyMT-induced tumors are all **invasive**, Akt1(-/-)Neu-induced tumors are more **invasive** than Akt2(-/-)Neu-induced tumors. **Invasive**ness, however, does not always correlate with metastasis. Ablation of individual Akt isoforms does not affect the development of the mammary gland during puberty or the expression of the transgenes. Akt ablation, therefore, influences tumor induction by modulating transgene-induced oncogenic signaling. Immunostaining for Ki-67 and cyclin D1 and terminal deoxynucleotidyl transferase-mediated dUTP nick end labeling assays on tissue sections revealed that the delay of tumor induction in Akt1 knockout mice is due to the inhibitory effects of Akt1 ablation on cell proliferation and survival. Given that these animal models exhibit significant similarities to human breast cancer, the results of the present study may have significant translational implications because they may influence how Akt inhibitors will be used in the treatment of human cancer.
5. APC haploinsufficiency coupled with p53 loss sufficiently induces mucinous cystic neoplasms and **invasive** pancreatic carcinoma in mice. Adenomatous polyposis coli (APC), a tumor-suppressor gene critically involved in familial adenomatous polyposis, is integral in Wnt/β-catenin signaling and is implicated in the development of sporadic tumors of the distal gastrointestinal tract including pancreatic cancer (PC). Here we report for the first time that functional APC is required for the growth and maintenance of pancreatic islets and maturation. Subsequently, a non-Kras mutation-induced premalignancy mouse model was developed; in this model, APC haploinsufficiency coupled with p53 deletion resulted in the development of a distinct type of pancreatic premalignant precursors, mucinous cystic neoplasms (MCNs), exhibiting pathomechanisms identical to those observed in human MCNs, including accumulation of cystic fluid secreted by neoplastic and ovarian-like stromal cells, with 100% penetrance and the presence of hepatic and gastric metastases in >30% of the mice. The major clinical implications of this study suggest targeting the Wnt signaling pathway as a novel strategy for managing MCN.

### [Mm]etastasis

#### PubMed

1. MiR-194 deregulation contributes to colorectal carcinogenesis via targeting AKT2 pathway. Recent studies have increasingly linked microRNAs to colorectal cancer (CRC). MiR-194 has been reported deregulated in different tumor types, whereas the function of miR-194 in CRC largely remains unexplored. Here we investigated the biological effects, mechanisms and clinical significance of miR-194. Functional assay revealed that overexpression of miR-194 inhibited CRC cell viability and invasion in vitro and suppressed CRC xenograft tumor growth in vivo. Conversely, block of miR-194 in APC(Min/+) mice promoted tumor growth. Furthermore, miR-194 reduced the expression of AKT2 both in vitro and in vivo. Clinically, the expression of miR-194 gradually decreased from 20 normal colorectal mucosa (N-N) cases through 40 colorectal adenomas (CRA) cases and then to 40 CRC cases, and was negatively correlated with AKT2 and pAKT2 expression. Furthermore, expression of miR-194 in stool samples was gradually decreased from 20 healthy cases, 20 CRA cases, then to 28 CRC cases. Low expression of miR-194 in CRC tissues was associated with large tumor size (P=0.006), lymph node **metastasis** (P=0.012) and shorter survival (HR =2.349, 95% CI = 1.242 to 4.442; P=0.009). In conclusion, our data indicated that miR-194 acted as a tumor suppressor in the colorectal carcinogenesis via targeting PDK1/AKT2/XIAP pathway, and could be a significant diagnostic and prognostic biomarker for CRC.
2. MicroRNAs differentially regulated by Akt isoforms control EMT and stem cell renewal in cancer cells. Although Akt is known to play a role in human cancer, the relative contribution of its three isoforms to oncogenesis remains to be determined. We expressed each isoform individually in an Akt1(-/-)/Akt2(-/-)/Akt3(-/-) cell line. MicroRNA profiling of growth factor-stimulated cells revealed unique microRNA signatures for cells with each isoform. Among the differentially regulated microRNAs, the abundance of the miR-200 family was decreased in cells bearing Akt2. Knockdown of Akt1 in transforming growth factor-beta (TGFbeta)-treated MCF10A cells also decreased the abundance of miR-200; however, knockdown of Akt2, or of both Akt1 and Akt2, did not. Furthermore, Akt1 knockdown in MCF10A cells promoted TGFbeta-induced epithelial-mesenchymal transition (EMT) and a stem cell-like phenotype. Carcinomas developing in MMTV-cErbB2/Akt1(-/-) mice showed increased invasiveness because of miR-200 down-regulation. Finally, the ratio of Akt1 to Akt2 and the abundance of miR-200 and of the messenger RNA encoding E-cadherin in a set of primary and metastatic human breast cancers were consistent with the hypothesis that in many cases breast cancer **metastasis** may be under the control of the Akt-miR-200-E-cadherin axis. We conclude that induction of EMT is controlled by microRNAs whose abundance depends on the balance between Akt1 and Akt2 rather than on the overall activity of Akt.
3. Akt1 ablation inhibits, whereas Akt2 ablation accelerates, the development of mammary adenocarcinomas in mouse mammary tumor virus (MMTV)-ErbB2/neu and MMTV-polyoma middle T transgenic mice. Ample evidence to date links the phosphatidylinositol 3-kinase-regulated protein kinase Akt with the induction and progression of human cancer, including breast cancer. However, there are three Akt isoforms with limited information about their specificity during oncogenesis. This study addresses the role of the three isoforms in polyoma middle T (PyMT) and ErbB2/Neu-driven mammary adenocarcinomas in mice. The effects of ablation of Akt1, Akt2, and Akt3 on the induction and the biology of these tumors were dramatically different, with ablation of Akt1 inhibiting, ablation of Akt2 accelerating, and ablation of Akt3 having a small, not statistically significant, inhibitory effect on tumor induction by both transgenes. Whereas PyMT-induced tumors are all invasive, Akt1(-/-)Neu-induced tumors are more invasive than Akt2(-/-)Neu-induced tumors. Invasiveness, however, does not always correlate with **metastasis**. Ablation of individual Akt isoforms does not affect the development of the mammary gland during puberty or the expression of the transgenes. Akt ablation, therefore, influences tumor induction by modulating transgene-induced oncogenic signaling. Immunostaining for Ki-67 and cyclin D1 and terminal deoxynucleotidyl transferase-mediated dUTP nick end labeling assays on tissue sections revealed that the delay of tumor induction in Akt1 knockout mice is due to the inhibitory effects of Akt1 ablation on cell proliferation and survival. Given that these animal models exhibit significant similarities to human breast cancer, the results of the present study may have significant translational implications because they may influence how Akt inhibitors will be used in the treatment of human cancer.
4. Akt1 and akt2 play distinct roles in the initiation and metastatic phases of mammary tumor progression. The phosphatidylinositol 3-kinase (PI3K)/Akt survival pathway is often dysregulated in cancer. Our previous studies have shown that coexpression of activated Akt1 with activated ErbB2 or polyoma virus middle T antigen uncoupled from the PI3K pathway (PyVmT Y315/322F) accelerates mammary tumor development but cannot rescue the metastatic phenotype associated with these models. Here, we report the generation of transgenic mice expressing activated Akt2 in the mammary epithelium. Like the mouse mammary tumor virus-Akt1 strain, mammary-specific expression of Akt2 delayed mammary gland involution. However, in contrast to Akt1, coexpression of Akt2 with activated ErbB2 or PyVmT Y315/322F in the mammary glands of transgenic mice did not affect the latency of tumor development. Strikingly, Akt2 coexpresssion markedly increased the incidence of pulmonary metastases in both tumor models, demonstrating a unique role in tumor progression. Together, these observations argue that these highly conserved kinases have distinct biological and biochemical outputs that play opposing roles in mammary tumor induction and **metastasis**.
5. Extracellular heat shock protein 90 signals through subdomain II and the NPVY motif of LRP-1 receptor to Akt1 and Akt2: a circuit essential for promoting skin cell migration in vitro and wound healing in vivo. Normal cells secrete heat shock protein 90 alpha (Hsp90α) in response to tissue injury. Tumor cells have managed to constitutively secrete Hsp90α during invasion an**d metastas**is. The sole function of extracellular Hsp90α (eHsp90α) is to promote cell motility, a critical event for both wound healing and tumor progression. The mechanism of promotility action by eHsp90α, however, has remained elusive. A key issue is whether eHsp90α still acts as a chaperone outside the cells or is a new and bona fide signaling molecule. Here, we have provided evidence that eHsp90α utilizes a unique transmembrane signaling mechanism to promote cell motility and wound healing. First, subdomain II in the extracellular part of low-density lipoprotein receptor-related protein 1 (LRP-1) receives the eHsp90α signal. Then, the NPVY but not the NPTY motif in the cytoplasmic tail of LRP-1 connects eHsp90α signaling to serine 473 but not threonine 308 phosphorylation in Akt kinases. Individual knockdown of Akt1, Akt2, or Akt3 revealed the importance of Akt1 and Akt2 in eHsp90α-induced cell motility. Akt gene rescue experiments suggest that Akt1 and Akt2 work in concert, rather than independently, to mediate eHsp90α promotility signaling. Finally, Akt1 and Akt2 knockout mice showed impaired wound healing that cannot be corrected by topical application with the eHsp90α protein.
6. Spontaneous Hepatocellular Carcinoma after the Combined Deletion of Akt Isoforms. Akt is frequently hyperactivated in human cancers and is targeted for cancer therapy. However, the physiological consequences of systemic Akt isoform inhibition were not fully explored. We showed that while combined Akt1 and Akt3 deletion in adult mice is tolerated, combined Akt1 and Akt2 deletion induced rapid mortality. Akt2(-/-) mice survived hepatic Akt1 deletion but all developed spontaneous hepatocellular carcinoma (HCC), which is associated with FoxO-dependent liver injury and inflammation. The gene expression signature of HCC-bearing livers is similar to aggressive human HCC. Consistently, neither Akt1(-/-) nor Akt2(-/-) mice are resistant to diethylnitrosamine-induced hepatocarcinogenesis, and Akt2(-/-) mice display a high incidence of lung **metastasis**. Thus, in contrast to other cancers, hepatic Akt inhibition induces liver injury that could promote HCC.
7. Akt2 is required for macrophage chemotaxis. Tumor-associated macrophages play an important role in tumorigenesis and **metastasis**. Trafficking of macrophages to the proximity of tumors is mediated by CSF-1, a growth factor. In this study, we investigated the role of PKB/Akt in CSF-1-induced macrophage migration. Disruption of Akt2 expression by small interference RNA impaired chemotaxis of both THP-1 cells and mouse peritoneal macrophages. Phosphorylation of PKCzeta, an essential component in chemotaxis signaling pathway, was reduced. LIMK/Cofilin, downstream of PKCzeta, regulated cytoskeleton rearrangement during cell migration. Disruption of Akt2 expression inhibited CSF-1-induced LIMK/Cofilin phosphorylation, which contributed to defects in actin polymerization and chemotaxis. Furthermore, MCP-1, a chemokine, -induced macrophage chemotaxis was also impaired. Taken together, our results demonstrated that Akt2 plays an essential role in both CSF-1- and chemokine-induced chemotaxis of macrophages.
8. Loss of Akt1 or Akt2 delays mammary tumor onset and suppresses tumor growth rate in MTB-IGFIR transgenic mice. Akt is a serine/threonine kinase that mediates signaling downstream of tyrosine kinase receptors like the type I insulin-like growth factor receptor (IGF-IR). In fact, we have previously shown that mammary tumors induced by elevated expression of the IGF-IR are associated with hyperactivation of Akt. However, there are three mammalian isoforms of Akt (Akt1, Akt2 and Akt3) and these isoforms regulate distinct physiologic properties within cells. In this manuscript, the impact of disrupting Akt1 or Akt2 in mammary tumors induced by IGF-IR overexpression were examined to determine whether specific Akt isoforms regulate different aspects of mammary tumorigenesis.Akt1 and Akt2 levels were stably ablated in mammary tumors of MTB-IGFIR transgenic mice by crossing MTB-IGFIR transgenic mice with either Akt1(-/-) or Akt2(-/-) mice. Tumor onset, growth rate, and **metastasis** were determined.Ablation of Akt1 or Akt2 significantly delayed tumor onset and tumor growth rate but did not significantly alter lung **metastasis**. Despite the absence of Akt1 or Akt2, mammary tumors that developed in the MTB-IGFIR mice maintained detectable levels of phosphorylated Akt. Disruption of Akt1 or Akt2 did not affect cell morphology or the expression of luminal or basal cytokeratins in mammary tumors.Although loss of Akt1 or Akt2 significantly inhibited mammary tumor onset and growth rates the effects were less dramatic than anticipated. Despite the complete loss of Akt1 or Akt2, the level of total phosphorylated Akt remained largely unaffected in the mammary tumors suggesting that loss of one Akt isoform is compensated by enhanced activation of the remaining Akt isoforms. These findings indicate that therapeutic strategies targeting the activation of individual Akt isoforms will prove less effective than simultaneously inhibiting the activity of all three Akt isoforms for the treatment of breast cancer.

#### GeneRIFs

1. Akt2(-/-) mice survive hepatic Akt1 deletion but all develop spontaneous hepatocellular carcinoma. They are insulin resistant. Akt2(-/-) mice are not resistant to diethylnitrosamine hepatocarcinogenesis. They have a high incidence of lung **metastasis**.

---

## Llgl1

- NCBI Gene
- GeneRIF
- Pubmed

| Search Term | Rank | Fields |
| --- | --- | --- |
| [Mm]igration | 6 | PubMed(3) |
| [Ii]nvasive | 10 | PubMed(1) |
| [Cc]ell [Ii]nvasion | 13 | PubMed(1) |

  
hide/show details for Llgl1

### [Mm]igration

#### PubMed

1. The tumor suppressor Lgl1 forms discrete complexes with NMII-A and Par6α-aPKCζ that are affected by Lgl1 phosphorylation. Non-muscle myosin IIA (NMII-A) and the tumor suppressor lethal giant larvae 1 (Lgl1) play a central role in the polarization of migrating cells. Mammalian Lgl1 interacts directly with NMII-A, inhibiting its ability to assemble into filaments in vitro. Lgl1 also regulates the cellular localization of NMII-A, the maturation of focal adhesions and cel**l migrati**on. In Drosophila, phosphorylation of Lgl affects its association with the cytoskeleton. Here we show that phosphorylation of mammalian Lgl1 by aPKCζ prevents its interaction with NMII-A both in vitro and in vivo, and affects its inhibition of NMII-A filament assembly. Phosphorylation of Lgl1 affects its cellular localization and is important for the cellular organization of the acto-NMII cytoskeleton. We further show that Lgl1 forms two distinct complexes in vivo, Lgl1-NMIIA and Lgl1-Par6α-aPKCζ, and that the formation of these complexes is affected by the phosphorylation state of Lgl1. The complex Lgl1-Par6α-aPKCζ resides in the leading edge of the cell. Finally, we show that aPKCζ and NMII-A compete to bind directly to Lgl1 at the same domain. These results provide new insights into the mechanism regulating the interaction between Lgl1, NMII-A, Par6α and aPKCζ in polarized migrating cells.
2. The tumor suppressor Lgl1 regulates NMII-A cellular distribution and focal adhesion morphology to optimize cell **migration**. The Drosophila tumor suppressor Lethal (2) giant larvae (Lgl) regulates the apical-basal polarity in epithelia and asymmetric cell division. However, little is known about the role of Lgl in cell polarity in migrating cells. In this study we show direct physiological interactions between the mammalian homologue of Lgl (Lgl1) and the nonmuscle myosin II isoform A (NMII-A). We demonstrate that Lgl1 and NMII-A form a complex in vivo and provide data that Lgl1 inhibits NMII-A filament assembly in vitro. Furthermore, depletion of Lgl1 results in the unexpected presence of NMII-A in the cell leading edge, a region that is not usually occupied by this protein, suggesting that Lgl1 regulates the cellular localization of NMII-A. Finally, we show that depletion of Lgl1 affects the size and number of focal adhesions, as well as cell polarity, membrane dynamics, and the rate of migrating cells. Collectively these findings indicate that Lgl1 regulates the polarity of migrating cells by controlling the assembly state of NMII-A, its cellular localization, and focal adhesion assembly.
3. Stability and function of mammalian lethal giant larvae-1 oncoprotein are regulated by the scaffolding protein RanBPM. The evolutionarily conserved lethal giant larvae (Lgl) tumor suppressor gene has an essential role in establishing apical-basal cell polarity, cell proliferation, differentiation, and tissue organization. However, the precise molecular mechanism by which the Lgl carries out its function remains obscure. In the current study, we have identified Ran-binding protein M (RanBPM) as a novel binding partner of Mgl-1, a mammalian homolog of Drosophila tumor suppressor protein lethal (2) giant larvae (L(2)gl) by yeast two-hybrid screening. RanBPM seems to act as a scaffolding protein with a modulatory function with respect to Mgl-1. The Mgl-1 and RanBPM association was confirmed by co-immunoprecipitation and GST pull-down experiments. Additionally, expression of RanBPM resulted in inhibition of Mgl-1 degradation, and thereby extended the half-life of Mgl-1. Furthermore, the ability of Mgl-1 activity in cell **migration** and colony formation assay was enhanced by RanBPM. Taken together, our findings reveal that RanBPM plays a novel role in regulating Mgl-1 stability and contributes to its biological function as a tumor suppressor.

### [Ii]nvasive

#### PubMed

1. Differential regulation of Dlg1, Scrib, and Lgl1 expression in a transgenic mouse model of ocular cancer. Discs large (dlg), scribble (scrib), and lethal giant larvae (lgl) are major suppressor genes in Drosophila melanogaster. They encode proteins that regulate cell polarity and cell proliferation in Drosophila and mammals. However, their basic oncogenic roles have not yet been established in mouse epithelial ocular cancer. We evaluated the potential implication of these proteins in tumorigenesis of adenocarcinomas originating from the retinal pigmented epithelium of the Trp1/Tag transgenic mouse model. We examined the changes in the distribution and levels of these proteins in mouse ocular tissues from the Trp1/Tag mouse model.The expression patterns of theses genes and their corresponding proteins in normal mouse ocular tissues were studied by in situ hibridization and immunohistofluorescence experiments. In addition, variations in mRNA and proteins levels and protein distributions for Dlg1, Scrib, and Lgl1 were analyzed in the ocular tissues from Trp1/Tag transgenic mouse model by reverse transcription polymerase chain reaction (RT-PCR), western blot analysis, and immunohistofluorescence.We found that mouse Dlg1, Scrib, and Lgl1 are widely distributed in normal ocular tissues, particularly in retinal neurons. We found that the three proteins are mislocalized in retinal layers during ocular carcinogenesis. These mislocalizations were correlated to the early dysplastic stages of ocular tumorigenesis. Additionally, the mislocalization of each protein was associated with its downregulation. Decreased levels of these proteins may be considered as late-stage markers of the disease but also as markers of the **invasive** stage of this cancerous process. This downregulation may be involved in epithelial-mesenchymal transition in this mouse ocular tumoral model. This would be consistent with the downregulation of E-cadherin and upregulation of N-cadherin expression observed in this model.This is the first study to demonstrate the involvement of Dlg1, Scrib, and Lgl1 in a mouse with ocular adenocarcinoma and the simultaneous involvement of these proteins in the same cancer. Our results indicate that both the mislocalization and downregulation of these proteins may be involved together in ocular carcinogenesis.

### [Cc]ell [Ii]nvasion

#### PubMed

1. Mammalian Llgl2 is necessary for proper branching morphogenesis during placental development. Cell polarity plays a critical role in the development of all metazoans; however, the mechanisms of cell polarity and the specific role of cell polarity pathways in mammalian organisms are still poorly understood. Lethal giant larvae (Lgl) is an apical-basal polarity gene identified in Drosophila, where it functions as a tumor suppressor controlling self-renewal and differentiation of progenitor cells. There are two orthologs of Lgl in mammalian genomes: Llgl1 and Llgl2. While mammalian Lgls are assumed to be tumor suppressor genes, little is known about their function in vivo. Here we report the functional analysis of murine Llgl2. We generated Llgl2(-/-) mice and found that Llgl2 functions as a polarity protein required for proper branching morphogenesis during placental development. Llgl2(-/-) pups are born as runts but quickly catch up in size and grow into normal-size adults. Surprisingly, no prominent phenotypes or spontaneous tumors were observed in adult Llgl2(-/-) mice. Analyses of placental trophoblasts reveal a critical role for Llgl2 in cell polarization and polarized **cell invasion**. We conclude that mammalian Llgl2 is required for proper polarized invasion of trophoblasts and efficient branching morphogenesis during placental development, but, unlike its Drosophila ortholog, it does not function as a canonical tumor suppressor gene.

---

## Eif4ebp1

- NCBI Gene
- GeneRIF
- Pubmed

| Search Term | Rank | Fields |
| --- | --- | --- |
| [Mm]igration | 6 | PubMed(1) |
| ECM | 7 | PubMed(1) |
| [Ee]xtracellular [Mm]atrix | 8 | PubMed(2) |
| [Mm]etastasis | 12 | PubMed(1) |

  
hide/show details for Eif4ebp1

### [Mm]igration

#### PubMed

1. Role of FIP200 in cardiac and liver development and its regulation of TNFalpha and TSC-mTOR signaling pathways. Focal adhesion kinase family interacting protein of 200 kD (FIP200) has been shown to regulate diverse cellular functions such as cell size, proliferation, and **migration** in vitro. However, the function of FIP200 in vivo has not been investigated. We show that targeted deletion of FIP200 in the mouse led to embryonic death at mid/late gestation associated with heart failure and liver degeneration. We found that FIP200 knockout (KO) embryos show reduced S6 kinase activation and cell size as a result of increased tuberous sclerosis complex function. Furthermore, FIP200 KO embryos exhibited significant apoptosis in heart and liver. Consistent with this, FIP200 KO mouse embryo fibroblasts and liver cells showed increased apoptosis and reduced c-Jun N-terminal kinase phosphorylation in response to tumor necrosis factor (TNF) alpha stimulation, which might be mediated by FIP200 interaction with apoptosis signal-regulating kinase 1 (ASK1) and TNF receptor-associated factor 2 (TRAF2), regulation of TRAF2-ASK1 interaction, and ASK1 phosphorylation. Together, our results reveal that FIP200 functions as a regulatory node to couple two important signaling pathways to regulate cell growth and survival during mouse embryogenesis.

### ECM

#### PubMed

1. FGF signalling regulates bone growth through autophagy. Skeletal growth relies on both biosynthetic and catabolic processes. While the role of the former is clearly established, how the latter contributes to growth-promoting pathways is less understood. Macroautophagy, hereafter referred to as autophagy, is a catabolic process that plays a fundamental part in tissue homeostasis. We investigated the role of autophagy during bone growth, which is mediated by chondrocyte rate of proliferation, hypertrophic differentiation and extracellular matrix (**ECM**) deposition in growth plates. Here we show that autophagy is induced in growth-plate chondrocytes during post-natal development and regulates the secretion of type II collagen (Col2), the major component of cartilage **ECM**. Mice lacking the autophagy related gene 7 (Atg7) in chondrocytes experience endoplasmic reticulum storage of type II procollagen (PC2) and defective formation of the Col2 fibrillary network in the **ECM**. Surprisingly, post-natal induction of chondrocyte autophagy is mediated by the growth factor FGF18 through FGFR4 and JNK-dependent activation of the autophagy initiation complex VPS34-beclin-1. Autophagy is completely suppressed in growth plates from Fgf18(-/-) embryos, while Fgf18(+/-) heterozygous and Fgfr4(-/-) mice fail to induce autophagy during post-natal development and show decreased Col2 levels in the growth plate. Strikingly, the Fgf18(+/-) and Fgfr4(-/-) phenotypes can be rescued in vivo by pharmacological activation of autophagy, pointing to autophagy as a novel effector of FGF signalling in bone. These data demonstrate that autophagy is a developmentally regulated process necessary for bone growth, and identify FGF signalling as a crucial regulator of autophagy in chondrocytes.

### [Ee]xtracellular [Mm]atrix

#### PubMed

1. FGF signalling regulates bone growth through autophagy. Skeletal growth relies on both biosynthetic and catabolic processes. While the role of the former is clearly established, how the latter contributes to growth-promoting pathways is less understood. Macroautophagy, hereafter referred to as autophagy, is a catabolic process that plays a fundamental part in tissue homeostasis. We investigated the role of autophagy during bone growth, which is mediated by chondrocyte rate of proliferation, hypertrophic differentiation and **extracellular matrix** (ECM) deposition in growth plates. Here we show that autophagy is induced in growth-plate chondrocytes during post-natal development and regulates the secretion of type II collagen (Col2), the major component of cartilage ECM. Mice lacking the autophagy related gene 7 (Atg7) in chondrocytes experience endoplasmic reticulum storage of type II procollagen (PC2) and defective formation of the Col2 fibrillary network in the ECM. Surprisingly, post-natal induction of chondrocyte autophagy is mediated by the growth factor FGF18 through FGFR4 and JNK-dependent activation of the autophagy initiation complex VPS34-beclin-1. Autophagy is completely suppressed in growth plates from Fgf18(-/-) embryos, while Fgf18(+/-) heterozygous and Fgfr4(-/-) mice fail to induce autophagy during post-natal development and show decreased Col2 levels in the growth plate. Strikingly, the Fgf18(+/-) and Fgfr4(-/-) phenotypes can be rescued in vivo by pharmacological activation of autophagy, pointing to autophagy as a novel effector of FGF signalling in bone. These data demonstrate that autophagy is a developmentally regulated process necessary for bone growth, and identify FGF signalling as a crucial regulator of autophagy in chondrocytes.
2. The AMPK stress response pathway mediates anoikis resistance through inhibition of mTOR and suppression of protein synthesis. Suppression of anoikis after detachment of cancer cells from the **extracellular matrix** is a key step during metastasis. Here we show that, after detachment, mouse embryonic fibroblasts (MEFs) transformed by K-Ras(V12) or ETV6-NTRK3 (EN) activate a transcriptional response overrepresented by genes related to bioenergetic stress and the AMP-activated protein kinase (AMPK) energy-sensing pathway. Accordingly, AMPK is activated in both transformed and non-transformed cells after detachment, and AMPK deficiency restores anoikis to transformed MEFs. However, AMPK activation represses the mTOR complex-1 (mTORC1) pathway only in transformed cells, suggesting a key role for AMPK-mediated mTORC1 inhibition in the suppression of anoikis. Consistent with this, AMPK-/- MEFs transformed by EN or K-Ras show sustained mTORC1 activation after detachment and fail to suppress anoikis. Transformed TSC1-/- MEFs, which are incapable of suppressing mTORC1, also undergo anoikis after detachment, which is reversed by mTORC1 inhibitors. Furthermore, transformed AMPK-/- and TSC1-/- MEFs both have higher total protein synthesis rates than wild-type controls, and translation inhibition using cycloheximide partially restores their anoikis resistance, indicating a mechanism whereby mTORC1 inhibition suppresses anoikis. Finally, breast carcinoma cell lines show similar detachment-induced AMPK/mTORC1 activation and restoration of anoikis by AMPK inhibition. Our data implicate AMPK-mediated mTORC1 inhibition and suppression of protein synthesis as a means for bioenergetic conservation during detachment, thus promoting anoikis resistance.

### [Mm]etastasis

#### PubMed

1. The AMPK stress response pathway mediates anoikis resistance through inhibition of mTOR and suppression of protein synthesis. Suppression of anoikis after detachment of cancer cells from the extracellular matrix is a key step during **metastasis**. Here we show that, after detachment, mouse embryonic fibroblasts (MEFs) transformed by K-Ras(V12) or ETV6-NTRK3 (EN) activate a transcriptional response overrepresented by genes related to bioenergetic stress and the AMP-activated protein kinase (AMPK) energy-sensing pathway. Accordingly, AMPK is activated in both transformed and non-transformed cells after detachment, and AMPK deficiency restores anoikis to transformed MEFs. However, AMPK activation represses the mTOR complex-1 (mTORC1) pathway only in transformed cells, suggesting a key role for AMPK-mediated mTORC1 inhibition in the suppression of anoikis. Consistent with this, AMPK-/- MEFs transformed by EN or K-Ras show sustained mTORC1 activation after detachment and fail to suppress anoikis. Transformed TSC1-/- MEFs, which are incapable of suppressing mTORC1, also undergo anoikis after detachment, which is reversed by mTORC1 inhibitors. Furthermore, transformed AMPK-/- and TSC1-/- MEFs both have higher total protein synthesis rates than wild-type controls, and translation inhibition using cycloheximide partially restores their anoikis resistance, indicating a mechanism whereby mTORC1 inhibition suppresses anoikis. Finally, breast carcinoma cell lines show similar detachment-induced AMPK/mTORC1 activation and restoration of anoikis by AMPK inhibition. Our data implicate AMPK-mediated mTORC1 inhibition and suppression of protein synthesis as a means for bioenergetic conservation during detachment, thus promoting anoikis resistance.

---

## Ptprb

- NCBI Gene
- GeneRIF
- Pubmed

| Search Term | Rank | Fields |
| --- | --- | --- |
| [Ff]ilopodia | 1 | PubMed(1) |
| [Mm]igration | 6 | PubMed(5); GeneRIFs(2) |

  
hide/show details for Ptprb

### [Ff]ilopodia

#### PubMed

1. Promotion of cell spreading and migration by vascular endothelial-protein tyrosine phosphatase (VE-PTP) in cooperation with integrins. Vascular endothelial-protein tyrosine phosphatase (VE-PTP) is a receptor-type protein tyrosine phosphatase with a single catalytic domain in its cytoplasmic region and multiple fibronectin type III-like domains in its extracellular region. VE-PTP is expressed specifically in endothelial cells and is implicated in regulation of angiogenesis. The molecular basis for such regulation by VE-PTP has remained largely unknown, however. We now show that forced expression of VE-PTP promoted cell spreading as well as formation of lamellipodia and **filopodia** in cultured fibroblasts plated on fibronectin. These effects of VE-PTP on cell morphology required its catalytic activity as well as activation of integrins and Ras. In addition, VE-PTP-induced cell spreading and lamellipodium formation were prevented by inhibition of Src family kinases or of Rac or Cdc42. Indeed, forced expression of VE-PTP increased the level of c-Src phosphorylation at tyrosine-416. Moreover, the VE-PTP-induced changes in cell morphology were suppressed by expression of dominant negative forms of FRG or Vav2, both of which are guanine nucleotide exchange factors for Rho family proteins and are activated by tyrosine phosphorylation. Forced expression of VE-PTP also enhanced fibronectin-dependent migration of cultured fibroblasts. Conversely, depletion of VE-PTP by RNA interference in human umbilical vein endothelial cells or mouse endothelioma cells inhibited cell spreading on fibronectin. These results suggest that VE-PTP, in cooperation with integrins, regulates the spreading and migration of endothelial cells during angiogenesis.

### [Mm]igration

#### PubMed

1. Promotion of cell spreading and **migration** by vascular endothelial-protein tyrosine phosphatase (VE-PTP) in cooperation with integrins. Vascular endothelial-protein tyrosine phosphatase (VE-PTP) is a receptor-type protein tyrosine phosphatase with a single catalytic domain in its cytoplasmic region and multiple fibronectin type III-like domains in its extracellular region. VE-PTP is expressed specifically in endothelial cells and is implicated in regulation of angiogenesis. The molecular basis for such regulation by VE-PTP has remained largely unknown, however. We now show that forced expression of VE-PTP promoted cell spreading as well as formation of lamellipodia and filopodia in cultured fibroblasts plated on fibronectin. These effects of VE-PTP on cell morphology required its catalytic activity as well as activation of integrins and Ras. In addition, VE-PTP-induced cell spreading and lamellipodium formation were prevented by inhibition of Src family kinases or of Rac or Cdc42. Indeed, forced expression of VE-PTP increased the level of c-Src phosphorylation at tyrosine-416. Moreover, the VE-PTP-induced changes in cell morphology were suppressed by expression of dominant negative forms of FRG or Vav2, both of which are guanine nucleotide exchange factors for Rho family proteins and are activated by tyrosine phosphorylation. Forced expression of VE-PTP also enhanced fibronectin-dependent **migration** of cultured fibroblasts. Conversely, depletion of VE-PTP by RNA interference in human umbilical vein endothelial cells or mouse endothelioma cells inhibited cell spreading on fibronectin. These results suggest that VE-PTP, in cooperation with integrins, regulates the spreading and **migration** of endothelial cells during angiogenesis.
2. How T cells trigger the dissociation of the endothelial receptor phosphatase VE-PTP from VE-cadherin. The vascular endothelial (VE) receptor protein tyrosine phosphatase (VE-PTP) associates with VE-cadherin and supports endothelial cell contact integrity. This complex is rapidly dissociated by adhesion of leukocytes to endothelial cells or by vascular endothelial growth factor. We have shown recently that this dissociation is indeed required for the opening of endothelial cell contacts during leukocyte extravasation in vivo. The leukocyte receptor and signaling mechanism that stimulates VE-cadherin/VE-PTP dissociation are unknown. Here, we identify vascular cell adhesion molecule 1 as the relevant receptor for lymphocytes in this process. As signaling steps downstream of this receptor, we determined the activation of Rac1, the generation of reactive oxygen species by nicotinamide adenine dinucleotide phosphate oxidase and the activation of the redox-sensitive tyrosine kinase Pyk2 as essential for VE-cadherin/VE-PTP dissociation. These signaling steps are also required for the dissociation induced by VE growth factor. Searching for the molecular mechanism of complex dissociation, we found that a model substrate of VE-PTP represented by a tyrosine-phosphorylated peptide of Tie-2 dissociates VE-PTP from VE-cadherin when introduced with the help of a Tat peptide. We suggest that lymphocyte binding to vascular cell adhesion molecule 1 triggers a signaling process that enables a VE-PTP substrate to dissociate VE-PTP from VE-cadherin, thereby facilitating efficient trans**migration**.
3. Insulin-like growth factor (IGF) binding protein 2 functions coordinately with receptor protein tyrosine phosphatase β and the IGF-I receptor to regulate IGF-I-stimulated signaling. Insulin-like growth factor I (IGF-I) is a mitogen for vascular smooth muscle cells (VSMC) and has been implicated in the development and progression of atherosclerosis. IGF binding proteins (IGFBPs) modify IGF-I actions independently of IGF binding, but a receptor-based mechanism by which they function has not been elucidated. We investigated the role of IGFBP-2 and receptor protein tyrosine phosphatase β (RPTPβ) in regulating IGF-I signaling and cellular proliferation. IGFBP-2 bound RPTPβ, which led to its dimerization and inactivation. This enhanced PTEN tyrosine phosphorylation and inhibited PTEN activity. Utilization of substrate trapping and phosphatase-dead mutants showed that RPTPβ bound specifically to PTEN and dephosphorylated it. IGFBP-2 knockdown led to decreased PTEN tyrosine phosphorylation and decreased AKT Ser473 activation. IGFBP-2 enhanced IGF-I-stimulated **VSMC migr**ation and proliferation. Analysis of aortas obtained from IGFBP-2(-/-) mice showed that RPTPβ was activated, and this was associated with inhibition of IGF-I stimulated AKT Ser473 phosphorylation and VSMC proliferation. These changes were rescued following administration of IGFBP-2. These findings present a novel mechanism for coordinate regulation of IGFBP-2 and IGF-I signaling functions that lead to stimulation of VSMC proliferation. The results have important implications for understanding how IGFBPs modulate the cellular response to IGF-I.
4. VE-PTP maintains the endothelial barrier via plakoglobin and becomes dissociated from VE-cadherin by leukocytes and by VEGF. We have shown recently that vascular endothelial protein tyrosine phosphatase (VE-PTP), an endothelial-specific membrane protein, associates with vascular endothelial (VE)-cadherin and enhances VE-cadherin function in transfected cells (Nawroth, R., G. Poell, A. Ranft, U. Samulowitz, G. Fachinger, M. Golding, D.T. Shima, U. Deutsch, and D. Vestweber. 2002. EMBO J. 21:4885-4895). We show that VE-PTP is indeed required for endothelial cell contact integrity, because down-regulation of its expression enhanced endothelial cell permeability, augmented leukocyte trans**migration**, and inhibited VE-cadherin-mediated adhesion. Binding of neutrophils as well as lymphocytes to endothelial cells triggered rapid (5 min) dissociation of VE-PTP from VE-cadherin. This dissociation was only seen with tumor necrosis factor alpha-activated, but not resting, endothelial cells. Besides leukocytes, vascular endothelial growth factor also rapidly dissociated VE-PTP from VE-cadherin, indicative of a more general role of VE-PTP in the regulation of endothelial cell contacts. Dissociation of VE-PTP and VE-cadherin in endothelial cells was accompanied by tyrosine phoshorylation of VE-cadherin, beta-catenin, and plakoglobin. Surprisingly, only plakoglobin but not beta-catenin was necessary for VE-PTP to support VE-cadherin adhesion in endothelial cells. In addition, inhibiting the expression of VE-PTP preferentially increased tyrosine phosphorylation of plakoglobin but not beta-catenin. In conclusion, leukocytes interacting with endothelial cells rapidly dissociate VE-PTP from VE-cadherin, weakening endothelial cell contacts via a mechanism that requires plakoglobin but not beta-catenin.
5. Interfering with VE-PTP stabilizes endothelial junctions in vivo via Tie-2 in the absence of VE-cadherin. Vascular endothelial (VE)-protein tyrosine phosphatase (PTP) associates with VE-cadherin, thereby supporting its adhesive activity and endothelial junction integrity. VE-PTP also associates with Tie-2, dampening the tyrosine kinase activity of this receptor that can support stabilization of endothelial junctions. Here, we have analyzed how interference with VE-PTP affects the stability of endothelial junctions in vivo. Blocking VE-PTP by antibodies, a specific pharmacological inhibitor (AKB-9778), and gene ablation counteracted vascular leak induction by inflammatory mediators. In addition, leukocyte trans**migration** through the endothelial barrier was attenuated. Interference with Tie-2 expression in vivo reversed junction-stabilizing effects of AKB-9778 into junction-destabilizing effects. Furthermore, lack of Tie-2 was sufficient to weaken the vessel barrier. Mechanistically, inhibition of VE-PTP stabilized endothelial junctions via Tie-2, which triggered activation of Rap1, which then caused the dissolution of radial stress fibers via Rac1 and suppression of nonmuscle myosin II. Remarkably, VE-cadherin gene ablation did not abolish the junction-stabilizing effect of the VE-PTP inhibitor. Collectively, we conclude that inhibition of VE-PTP stabilizes challenged endothelial junctions in vivo via Tie-2 by a VE-cadherin-independent mechanism. In the absence of Tie-2, however, VE-PTP inhibition destabilizes endothelial barrier integrity in agreement with the VE-cadherin-supportive effect of VE-PTP.

#### GeneRIFs

1. We suggest that lymphocyte binding to vascular cell adhesion molecule 1 triggers a signaling process that enables a VE-PTP substrate to dissociate VE-PTP from VE-cadherin, thereby facilitating efficient trans**migration**.
2. Suggest that VE-PTP, in cooperation with integrins, regulates the spreading and **migration** of endothelial cells during angiogenesis.

---

## Cers6

- NCBI Gene
- GeneRIF
- Pubmed

| Search Term | Rank | Fields |
| --- | --- | --- |
| [Mm]igration | 6 | PubMed(1) |

  
hide/show details for Cers6

### [Mm]igration

#### PubMed

1. Exacerbation of experimental autoimmune encephalomyelitis in ceramide synthase 6 knockout mice is associated with enhanced activation/**migration** of neutrophils. Ceramides are mediators of inflammatory processes. In experimental autoimmune encephalomyelitis (EAE), an animal model of multiple sclerosis (MS), we observed that CerS6 mRNA expression was upregulated 15-fold in peripheral blood leukocytes before the onset of EAE symptoms. In peripheral blood leukocytes from MS patients, a 3.9-fold upregulation was found. Total genetic deletion of CerS6 and the selective deletion of CerS6 in peripheral blood leucocytes exacerbated the progression of clinical symptoms in EAE mice. This was associated with enhanced leukocyte, predominantly neutrophil infiltration and enhanced demyelination in the lumbar spinal cord of EAE mice. Interferon-gamma/tumor necrosis factor alpha (IFN-γ/TNF-α) and granulocyte colony-stimulating factor (G-CSF) both drive EAE development and induce expression of the integrin CD11b and the chemokine receptor C-X-C motif chemokine receptor 2 (CXCR2), and we found they also induce CerS6 expression. In vivo, the genetic deletion of CerS6 enhanced the activatio**n/migrati**on of neutrophils, as reflected by an enhanced upregulation of CD11b and CXCR2. In vitro, the genetic deletion of CerS6 enhanced the activation status of IFN-γ/TNF-α-stimulated neutrophils, as shown by increased expression of nitric oxide and CD11b and an increased adhesion capacity. In G-CSF-stimulated neutrophils, **the migra**tion status was enhanced, as reflected by an elevated level of CXCR2 and an increa**sed migra**tion capacity. These data suggest that CerS6/C16-Cer mediates feedback regulation by inhibiting the formation of CD11b and CXCR2, which are induced either by IFN-γ/TNF-α or by G-CSF, respectively. We conclude that CerS6/C16-Cer mediates anti-inflammatory effects during the development of EAE and MS possibly by suppressin**g the mig**ration and deactivation of neutrophils.

---

## Fmnl2

- NCBI Gene
- GeneRIF
- Pubmed

| Search Term | Rank | Fields |
| --- | --- | --- |
| [Ff]ilopodia | 1 | PubMed(1) |
| [Mm]igration | 6 | PubMed(1); GeneRIFs(1) |

  
hide/show details for Fmnl2

### [Ff]ilopodia

#### PubMed

1. FMNL2 drives actin-based protrusion and migration downstream of Cdc42. Cell migration entails protrusion of lamellipodia, densely packed networks of actin filaments at the cell front. Filaments are generated by nucleation, likely mediated by Arp2/3 complex and its activator Scar/WAVE. It is unclear whether formins contribute to lamellipodial actin filament nucleation or serve as elongators of filaments nucleated by Arp2/3 complex. Here we show that the Diaphanous-related formin FMNL2, also known as FRL3 or FHOD2, accumulates at lamellipodia and **filopodia** tips. FMNL2 is cotranslationally modified by myristoylation and regulated by interaction with the Rho-guanosine triphosphatase Cdc42. Abolition of myristoylation or Cdc42 binding interferes with proper FMNL2 activation, constituting an essential prerequisite for subcellular targeting. In vitro, C-terminal FMNL2 drives elongation rather than nucleation of actin filaments in the presence of profilin. In addition, filament ends generated by Arp2/3-mediated branching are captured and efficiently elongated by the formin. Consistent with these biochemical properties, RNAi-mediated silencing of FMNL2 expression decreases the rate of lamellipodia protrusion and, accordingly, the efficiency of cell migration. Our data establish that the FMNL subfamily member FMNL2 is a novel elongation factor of actin filaments that constitutes the first Cdc42 effector promoting cell migration and actin polymerization at the tips of lamellipodia.

### [Mm]igration

#### PubMed

1. FMNL2 drives actin-based protrusion and **migration** downstream of Cdc42. Cell **migration** entails protrusion of lamellipodia, densely packed networks of actin filaments at the cell front. Filaments are generated by nucleation, likely mediated by Arp2/3 complex and its activator Scar/WAVE. It is unclear whether formins contribute to lamellipodial actin filament nucleation or serve as elongators of filaments nucleated by Arp2/3 complex. Here we show that the Diaphanous-related formin FMNL2, also known as FRL3 or FHOD2, accumulates at lamellipodia and filopodia tips. FMNL2 is cotranslationally modified by myristoylation and regulated by interaction with the Rho-guanosine triphosphatase Cdc42. Abolition of myristoylation or Cdc42 binding interferes with proper FMNL2 activation, constituting an essential prerequisite for subcellular targeting. In vitro, C-terminal FMNL2 drives elongation rather than nucleation of actin filaments in the presence of profilin. In addition, filament ends generated by Arp2/3-mediated branching are captured and efficiently elongated by the formin. Consistent with these biochemical properties, RNAi-mediated silencing of FMNL2 expression decreases the rate of lamellipodia protrusion and, accordingly, the efficiency of cell **migration**. Our data establish that the FMNL subfamily member FMNL2 is a novel elongation factor of actin filaments that constitutes the first Cdc42 effector promoting cell **migration** and actin polymerization at the tips of lamellipodia.

#### GeneRIFs

1. FMNL2 is a novel elongation factor of actin filaments that constitutes the first Cdc42 effector promoting cell **migration** and actin polymerization at the tips of lamellipodia.

---

## Ist1

- NCBI Gene
- GeneRIF
- Pubmed

| Search Term | Rank | Fields |
| --- | --- | --- |
| [Ee]xtracellular [Mm]atrix | 8 | PubMed(1) |

  
hide/show details for Ist1

### [Ee]xtracellular [Mm]atrix

#### PubMed

1. Identification of tooth-specific downstream targets of Runx2. Odontogenesis or tooth development is a highly regulated process that involves complex epithelial-mesenchymal signaling interactions that lead to cuspal morphogenesis, cell differentiation and the subsequent formation of the specialized matrices of enamel, dentin, cementum and bone. Although studies on tooth epithelial-mesenchymal signaling interactions have greatly increased our understanding of molecules that regulate tooth initiation and early morphogenesis (review: Jernvall and Thesleff, Mech. Dev. 92 (2000) 19), the precise nature of the molecular events controlling late morphogenesis and terminal cytodifferentiation is not known. We have recently reported a unique phenotype involving dentition in mice lacking a functional Runx2 gene (D'Souza et al., Development 126 (1999) 2911). The markedly hypoplastic tooth organs as well as defects in the maturation of ameloblasts and odontoblasts point to an important and non-redundant role for Runx2 in tooth morphogenesis and cytodifferentiation. In order to identify genes that are affected by the absence of Runx2, a cDNA library was generated from Runx2(-/-) and Runx2(+/+) first molar organs. Thus far, our analysis has revealed several tooth-specific downstream target genes of Runx2 that include **extracellular matrix** proteins, kinases, receptors, growth factors, mitochondrial proteins and transcription molecules. Sequence analysis of 61 differentially expressed genes revealed that 96.03% of the clones matched previously described genes in the GenBank/EBML database and 3.96% did not match any entries in the database. Our preliminary expression analysis of one of the differentially expressed clones which encodes for a zinc finger transcription factor termed Zfp reveals that the gene is temporally regulated during tooth development. In conclusion, we have successfully generated a library enriched in genes expressed in Runx2(+/+) molar tooth organs and performed preliminary studies to assess the role of Zfp in tooth development.

---

## Mylip

- NCBI Gene
- GeneRIF
- Pubmed

| Search Term | Rank | Fields |
| --- | --- | --- |
| [Mm]igration | 6 | PubMed(1) |

  
hide/show details for Mylip

### [Mm]igration

#### PubMed

1. The zebrafish band 4.1 member Mir is involved in cell movements associated with gastrulation. Cellular processes rely on dynamic events occurring between the cortical cytoskeleton and plasma membrane. Members of the Band 4.1 superfamily, which are best known for their ability to tether the cytoskeleton to the plasma membrane, play prominent structural and regulatory roles that influence cell-cell and cell-substrate interactions, endo- and exocytosis, cell polarity, **migration**, proliferation, and differentiation. We have identified a new member of the zebrafish Band 4.1 superfamily, which is the homolog of human myosin regulatory light chain interacting protein (MIR), and have examined its role in embryonic development. Zebrafish Mir contains the conserved amino-terminal plasma membrane-binding FERM (Band 4.1/ezrin/radixin/moesin) domain as well as other putative protein-protein interacting domains, including a RING finger. Overall, zebrafish Mir is 71% identical to human MIR located at chromosome 6p23-p22.3, and maps on linkage group 19 to a region of synteny with human chromosome 6. In situ hybridization and RT-PCR revealed that mir is expressed maternally and ubiquitously throughout development. Blocking Mir translation using a mir-specific, morpholino-based, knock-down strategy or expressing Mir constructs lacking the RING finger domain disrupts gastrulation and leads to subsequent trunk and tail defects. In severe cases, morphants exogastrulate. The synergistic effect seen when two mir-specific morpholinos are used in conjunction reflects the specific knock-down of mir. In addition, morphant phenotypes induced by mir-specific morpholinos are rescued by overexpression of the full-length Mir. In situ hybridization analysis with mesodermal- and neural-specific markers shows that morphants exhibit a delay in cell movements associated with gastrulation, epiboly, convergence, and extension. A yeast two-hybrid analysis was performed to identify binding partners that may participate with Mir during gastrulation, and Annexin V, a calcium channel protein, was isolated. At early developmental stages, annexin V transcripts colocalize with mir, but after gastrulation, annexin V mRNA becomes localized to the distal tail region and an area in the olfactory placode. At the protein level, Mir colocalizes with Annexin V when expressed in COS cells. Together, these results indicate that Mir is essential for embryonic development and that its role in early embryonic development likely involves calcium-dependent mechanisms essential during the extensive cell movements associated with gastrulation.

---

## Ect2

- NCBI Gene
- GeneRIF
- Pubmed

| Search Term | Rank | Fields |
| --- | --- | --- |
| [Mm]igration | 6 | PubMed(1) |
| [Ii]nvasive | 10 | PubMed(1) |

  
hide/show details for Ect2

### [Mm]igration

#### PubMed

1. The ect2 rho Guanine nucleotide exchange factor is essential for early mouse development and normal cell cytokinesis and **migration**. Ect2 is a member of the human Dbl family of guanine nucleotide exchange factors (RhoGEFs) that serve as activators of Rho family small GTPases. Although Ect2 is one of at least 25 RhoGEFs that can activate the RhoA small GTPase, cell culture studies using established cell lines determined that Ect2 is essential for mammalian cell cytokinesis and proliferation. To address the function of Ect2 in normal mammalian development, we performed gene targeting to generate Ect2 knockout mice. The heterozygous Ect2(+/-) mice showed normal development and life span, indicating that Ect2 haplodeficiency was not deleterious for development or growth. In contrast, Ect2(-/-) embryos were not found at birth or postimplantation stages. Ect2(-/-) blastocysts were recovered at embryonic day 3.5 but did not give rise to viable outgrowths in culture, indicating that Ect2 is required for peri-implantation development. To further assess the importance of Ect2 in normal cell physiology, we isolated primary fibroblasts from Ect2(fl/fl) embryos (MEFs) and ablated Ect2 using adenoviral delivery of Cre recombinase. We observed a significant increase in multinucleated cells and accumulation of cells in G2/M phase, consistent with a role for Ect2 in cytokinesis. Ect2 deficiency also caused enlargement of the cytoplasm and impaired cell **migration**. Finally, although Ect2-dependent activation of RhoA has been implicated in cytokinesis, Ect2 can also activate Rac1 and Cdc42 to cause growth transformation. Surprisingly, ectopic expression of constitutively activated RhoA, Rac1, or Cdc42, known substrates of Ect2, failed to phenocopy Ect2 and did not rescue the defect in cytokinesis caused by loss of Ect2. In summary, our results establish the unique role of Ect2 in development and normal cell proliferation.

### [Ii]nvasive

#### PubMed

1. A chemically induced model for squamous cell carcinoma of the lung in mice: histopathology and strain susceptibility. Lung cancer, primarily associated with tobacco use, is the leading cause of cancer morbidity and mortality in the United States. Squamous cell carcinoma (SCC) is one of the four major histological types of lung cancer. Although there are several established models for lung adenoma and adenocarcinomas, there is no well-established mouse model for lung SCC. We treated eight different inbred strains of mice with N-nitroso-tris-chloroethylurea by skin painting and found that this regimen induced lung SCCs in five strains of mouse (SWR/J, NIH Swiss, A/J, BALB/cJ, and FVB/J) but not in the others (AKR/J, 129/svJ, and C57BL/6J). Mouse lung SCCs have similar histopathological features and keratin staining to human SCC. Moreover, a wide spectrum of abnormal lung squamous phenotypes including hyperplasia, metaplasia, carcinoma in situ, and **invasive** carcinoma, were observed. There are strain-specific differences in susceptibility to Lscc induction by N-nitroso-tris-chloroethylurea with NIH Swiss, A/J, and SWR/J mice developing scores of SCCs whereas the resistant strains AKR/J, 129/svJ, and C57BL/6J failed to develop any SCCs. FVB/J and BALB/cJ mice had an intermediate response. We conducted whole-genome linkage disequilibrium analysis in seven strains of mice, divided into three phenotype categories of susceptibility, using Fisher's exact test applied to 6,128 markers in publically available databases. Three markers were found significantly associated with susceptibility to SCC with the P < 0.05. They were D1Mit169, D3Mit178, and D18Mit91. Interestingly, none of these sites overlap with the major susceptibility loci associated with lung adenoma/adenocarcinoma development in mice. The mouse SCC described here is highly significant for preclinical studies of lung cancer chemopreventive agents because most human trials have been conducted against precancerous lesions for SCC. Furthermore, this model can be used in determining genetic modifiers that contribute to susceptibility or resistance to lung SCC development.

---

## Rock2

- NCBI Gene
- GeneRIF
- Pubmed

| Search Term | Rank | Fields |
| --- | --- | --- |
| [Ff]ilopodia | 1 | PubMed(2) |
| [Ii]nvadopodia | 3 | PubMed(1); GeneRIFs(1) |
| [Mm]igration | 6 | Process(1); PubMed(32); GeneRIFs(8) |
| ECM | 7 | PubMed(2) |
| [Ee]xtracellular [Mm]atrix | 8 | PubMed(4) |
| [Ii]nvasive | 10 | PubMed(2); GeneRIFs(2) |
| [Mm]etastasis | 12 | PubMed(4) |
| [Cc]ell [Ii]nvasion | 13 | PubMed(3); GeneRIFs(1) |

  
hide/show details for Rock2

### [Ff]ilopodia

#### PubMed

1. Vangl2 acts via RhoA signaling to regulate polarized cell movements during development of the proximal outflow tract. Loop-tail (Lp) mice develop double outlet right ventricle and aortic arch defects, and the defects are caused by mutations in the Vangl2 gene. Vangl2 mRNA is found in the outflow tract myocardium, including the myocardializing cells that migrate into the outflow tract cushions. Analysis of muscularization of the outflow tract septum showed that this process is compromised in Lp/Lp fetuses. Vangl2 is a component of the noncanonical Wnt, planar cell polarity (PCP) pathway that signals via RhoA. We therefore looked for evidence of polarization in myocardializing cells. In wild-type fetuses, myocardializing cells extend lamellipodia and **filopodia** into the cushion tissue and reorganize their actin cytoskeleton from a cortical form to stress fibers; behaviors that are characteristic of polarized cells. In contrast, Lp/Lp littermates do not extend lamellipodia or **filopodia** into the cushion tissue, and their actin remains in a cortical form, suggesting that polarized cell migration of myocardializing cells is inhibited in Lp/Lp. Several other components of the PCP pathway are also localized in the outflow tract myocardium. In wild-type fetuses, the myocardializing cells coexpress RhoA and one of its downstream mediators, ROCK1. RhoA expression is disrupted in Lp/Lp, and is lost from the myocardial-cushion tissue interface, including the presumptive myocardializing cells. These data suggest that Vangl2 is required for the polarization and movement of myocardializing cells into the outflow tract cushions, and that RhoA and ROCK1 are downstream mediators of the PCP signaling pathway in the developing outflow tract.
2. Rho-associated kinases play a role in endocardial cell differentiation and migration. Development of the endocardial cushions in the heart involves cell migration and cell differentiation, which is known as epithelial-mesenchymal transformation (EMT). These processes are regulated by cell signaling systems. Yet, the roles of intracellular GTPases and their effectors on these cellular activities remain to be addressed. This study investigated the role Rho GTPase-associated kinases (ROCKs) in endocardial cushion development. Using reverse transcription (RT) and polymerase chain reaction (PCR), expression of the rock1 and rock2 genes was found in the endocardial cushions during development. To investigate the role of ROCKs in development, the ROCK inhibitor Y27632 and adenoviruses containing a dominant negative form of the rock gene were used to treat cultured endocardial cushions and cells. In monolayer cell culture and three-dimensional tissue culture, blockade of ROCK inhibited EMT development. Using three-dimensional collagen gel assays and confocal microscopy, we also observed inhibition of cell migration with ROCK inhibition. Examination of cell morphology and actin cytoskeleton revealed that inhibition of ROCK activity disturbed cytoskeletal organization and blocked the formation of lamellipodia and **filopodia**. Collectively, these data show that ROCKs play an essential role in endothelial cell differentiation and migration during endocardial cushion development.

### [Ii]nvadopodia

#### PubMed

1. ROCK-II mediates colon cancer invasion via regulation of MMP-2 and MMP-13 at the site of **invadopodia** as revealed by multiphoton imaging. The ROCK-II isoform of Rho's downstream effector, Rho kinase, has been linked with greater invasion and metastasis in solid tumors. We have previously shown that ROCK-II is overexpressed at the advancing edge of colon cancers. The mechanism whereby ROCK-II contributes invasion, particularly in the setting of colon cancer, remains to be elucidated fully. To better understand its contribution, we evaluated ROCK-II expression in both non-malignant (NCM460 and IEC-6) and malignant (Caco-2 E, SW620, and HCT-116) intestinal epithelial cell lines grown in type I collagen scaffolds. Using multiphoton microscopy, we observed that ROCK-II localized to the actin cytoskeleton in non-malignant cells but localized to the cell periphery as focal collections with an absence of adjacent collagen in all colon cancer cell lines. By transmission electron microscopy, these collections corresponded with finger-like projections previously described as **invadopodia**. Immunogold staining with cortactin, matrix metalloprotease (MMP)-2, -9, and -13 confirmed that these were indeed **invadopodia**. To further link ROCK-II to colon cancer invasion, we treated non-malignant and malignant intestinal epithelial cell lines with ROCK-II siRNA and evaluated depth of invasion, proliferation, and MMP-2, -9, and -13 activities. The most striking effect was seen in the highly tumorigenic cell lines, SW620 and HCT-116, wherein ROCK-II knockdown resulted in a two-fold or more reduction in invasion. This reduction in invasion was not due to a decrease in cell proliferation, as a significant reduction in proliferation was only observed in the two non-malignant intestinal cell lines. Finally, both MMP-2 and -13 activities were significantly decreased in all colon cancer cell lines. Taken together, these data suggest for the first time that ROCK-II is a critical mediator of colon cancer cell invasion through its modulation of MMP-2 and -13 at the site of **invadopodia** but regulates proliferation in non-malignant intestinal cells.

#### GeneRIFs

1. ROCK-II is a critical mediator of colon cancer cell invasion through its modulation of MMP-2 and -13 at the site of **invadopodia** but regulates proliferation in non-malignant intestinal cells.

### [Mm]igration

#### Process

1. positive regulation of endothelial cell **migration**

#### PubMed

1. Targeted deletion of the mitogen-activated protein kinase kinase 4 gene in the nervous system causes severe brain developmental defects and premature death. The c-Jun NH2-terminal protein kinase (JNK) is a mitogen-activated protein kinase (MAPK) involved in the regulation of various physiological processes. Its activity is increased upon phosphorylation by the MAPK kinases MKK4 and MKK7. The early embryonic death of mice lacking an mkk4 or mkk7 gene has provided genetic evidence that MKK4 and MKK7 have nonredundant functions in vivo. To elucidate the physiological role of MKK4, we generated a novel mouse model in which the mkk4 gene could be specifically deleted in the brain. At birth, the mutant mice were indistinguishable from their control littermates, but they stopped growing a few days later and died prematurely, displaying severe neurological defects. Decreased JNK activity in the absence of MKK4 correlated with impaired phosphorylation of a subset of physiologically relevant JNK substrates and with altered gene expression. These defects resulted in the misalignment of the Purkinje cells in the cerebellum and delayed radial **migration** in the cerebral cortex. Together, our data demonstrate for the first time that MKK4 is an essential activator of JNK required for the normal development of the brain.
2. Vangl2 acts via RhoA signaling to regulate polarized cell movements during development of the proximal outflow tract. Loop-tail (Lp) mice develop double outlet right ventricle and aortic arch defects, and the defects are caused by mutations in the Vangl2 gene. Vangl2 mRNA is found in the outflow tract myocardium, including the myocardializing cells that migrate into the outflow tract cushions. Analysis of muscularization of the outflow tract septum showed that this process is compromised in Lp/Lp fetuses. Vangl2 is a component of the noncanonical Wnt, planar cell polarity (PCP) pathway that signals via RhoA. We therefore looked for evidence of polarization in myocardializing cells. In wild-type fetuses, myocardializing cells extend lamellipodia and filopodia into the cushion tissue and reorganize their actin cytoskeleton from a cortical form to stress fibers; behaviors that are characteristic of polarized cells. In contrast, Lp/Lp littermates do not extend lamellipodia or filopodia into the cushion tissue, and their actin remains in a cortical form, suggesting that polarized cell **migration** of myocardializing cells is inhibited in Lp/Lp. Several other components of the PCP pathway are also localized in the outflow tract myocardium. In wild-type fetuses, the myocardializing cells coexpress RhoA and one of its downstream mediators, ROCK1. RhoA expression is disrupted in Lp/Lp, and is lost from the myocardial-cushion tissue interface, including the presumptive myocardializing cells. These data suggest that Vangl2 is required for the polarization and movement of myocardializing cells into the outflow tract cushions, and that RhoA and ROCK1 are downstream mediators of the PCP signaling pathway in the developing outflow tract.
3. Raf-1 sets the threshold of Fas sensitivity by modulating Rok-alpha signaling. Ablation of the Raf-1 protein causes fetal liver apoptosis, embryonic lethality, and selective hypersensitivity to Fas-induced cell death. Furthermore, Raf-1-deficient cells show defective **migration** as a result of the deregulation of the Rho effector kinase Rok-alpha. In this study, we show that the kinase-independent modulation of Rok-alpha signaling is also the basis of the antiapoptotic function of Raf-1. Fas activation stimulates the formation of Raf-1-Rok-alpha complexes, and Rok-alpha signaling is up-regulated in Raf-1-deficient cells. This leads to increased clustering and membrane expression of Fas, which is rescued both by kinase-dead Raf-1 and by interfering with Rok-alpha or its substrate ezrin. Increased Fas clustering and membrane expression are also evident in the livers of Raf-1-deficient embryos, and genetically reducing Fas expression counteracts fetal liver apoptosis, embryonic lethality, and the apoptotic defects of embryonic fibroblasts. Thus, Raf-1 has an essential function in regulating Fas expression and setting the threshold of Fas sensitivity during embryonic life.
4. Role of Rho-kinase and p27 in angiotensin II-induced vascular injury. Angiotensin II enhances the development of atherosclerotic lesion in which cellular proliferation and/or **migration** are critical steps. Although cyclin-dependent kinase inhibitor, p27, and Rho/Rho-kinase pathway have recently been implicated as factors regulating these events cooperatively, their role in vivo has not been fully elucidated. We evaluated the contribution of p27 and Rho-kinase to angiotensin II-induced vascular injury using p27-deficient mice. Two-week angiotensin II (1500 ng/kg per minute SC) infusion elicited similar degrees of elevation in systolic blood pressure in wild-type mice (159+/-5 mm Hg) and p27-deficient mice (157+/-5 mm Hg; P>0.05). Angiotensin II infusion to wild-type mice resulted in increases in the medial thickness of aorta, proliferating cell number, and monocyte/macrophage infiltration within the vasculature. In p27-deficient mice, however, these changes were more prominent than those in wild-type mice. Treatment of wild-type mice with fasudil, a selective Rho-kinase inhibitor, did not alter blood pressure but significantly upregulated p27 expression, decreased medial thickness of aorta, reduced proliferating cell number, and prevented monocyte/macrophage infiltration. These protective effects of fasudil were attenuated in p27-deficient mice. In conclusion, p27 constitutes an important modulator of angiotensin II-induced monocyte/macrophage infiltration and vascular remodeling, which is mediated in part by Rho-kinase stimulation. Inhibition of Rho-kinase activity improves angiotensin II-induced vascular injury through p27-dependent and p27-independent mechanisms.
5. Wnt activity guides facial branchiomotor neuron **migration**, and involves the PCP pathway and JNK and ROCK kinases. Wnt proteins play roles in many biological processes, including axon guidance and cell **migration**. In the mammalian hindbrain, facial branchiomotor (FBM) neurons undergo a striking rostral to caudal **migration**, yet little is known of the underlying molecular mechanisms. In this study, we investigated a possible role of Wnts and the planar cell polarity (PCP) pathway in this process.Here we demonstrate a novel role for Wnt proteins in guiding FBM neurons during their rostral to caudal **migration** in the hindbrain. We found that Wnt5a is expressed in a caudal high to rostral low gradient in the hindbrain. Wnt-coated beads chemoattracted FBM neurons to ectopic positions in an explant **migration** assay. The rostrocaudal FBM **migration** was moderately perturbed in Wnt5a mutant embryos and severely disrupted in Frizzled3 mutant mouse embryos, and was aberrant following inhibition of Wnt function by secreted Frizzled-related proteins. We also show the involvement of the Wnt/PCP pathway in mammalian FBM neuron **migration**. Thus, mutations in two PCP genes, Vangl2 and Scribble, caused severe defects in FBM **migration**. Inhibition of JNK and ROCK kinases strongly and specifically reduced the FBM **migration**, as well as blocked the chemoattractant effects of ectopic Wnt proteins.These results provide in vivo evidence that Wnts chemoattract mammalian FBM neurons and that Wnt5a is a candidate to mediate this process. Molecules of the PCP pathway and the JNK and ROCK kinases also play a role in the FBM **migration** and are likely mediators of Wnt signalling.
6. Cooperation of matrix metalloproteinases with the RhoA/Rho kinase and mitogen-activated protein kinase kinase-1/extracellular signal-regulated kinase signaling pathways is required for the sphingosine-1-phosphate-induced mobilization of marrow-derived stromal cells. The ease of isolation and ex vivo culture of marrow-derived stromal cells (MSCs) from adult bone marrow renders them a very promising source of adult stem cells for gene transfer and cell therapy. However, little is known about the signaling pathways that control their in vivo mobilization and subsequent biodistribution. Platelet-derived sphingosine-1-phosphate (S1P), a bioactive lipid that acts via G-protein-coupled-receptors, exerts strong chemoattraction upon MSCs through yet-uncharacterized signaling pathways. We show that the S1P-induced **migration** and morphological changes of MSCs in vitro require the activities of extracellular signal-regulated kinase (ERK), Rho kinase (ROCK), and matrix metalloproteinase (MMP) signaling molecules. Specifically, S1P-induced remodeling of the MSC cytoskeleton led to the rapid (<1 minute) formation of actin stress fibers via activation of the RhoA/ROCK pathway and required the catalytic activity of MMPs. S1P-induced activation of the mitogen-activated protein kinase kinase-1 (MEK1)/ERK pathway also contributed to the induction of the actin stress fibers and to the redistribution of paxillin at the focal adhesions through tyrosine phosphorylation of focal adhesion kinase in an MMP-dependent manner. Moreover, MMP- and ROCK-dependent molecular events are implicated in the regulation of the S1P-induced activation of ERK. Our results demonstrate that MSC mobilization in response to S1P requires cooperation between MMP-mediated signaling events and the RhoA/ROCK and MEK1/ERK intracellular pathways. Therefore, the characterization of the cellular factors and the intracellular signaling pathways underlying MSC mobilization is crucial to achieve high efficacy in therapeutic use.
7. Inhibition of Rho kinases increases directional motility of microvascular endothelial cells. Rho kinases are major regulators of actin cytoskeletal organization and cell motility. Depending on the model system, inhibitors of Rho kinases (ROCK) have been reported to increase or decrease endothelial cell **migration**. In the present study we investigated the effect of Rho kinase inhibitors on microvascular endothelial cell **migration** with a special focus on the isoform ROCK2. **Migration** of microvascular endothelial cells was analyzed in a wound-healing, a spheroid-on-collagen **migration** assay and in cells embedded in collagen-1 gels. The non-selective Rho kinase inhibitor H1152 was compared to the selective ROCK2 inhibitor SLX2119 and to siRNA knock down. Non-selective inhibition of Rho kinases decreased cell-spanning F-actin fibers, loosened cell-cell contacts visualized by VE cadherin staining, and reduced cell-matrix interactions as shown by reduced Hic-5 expression in focal contacts. Rho kinase inhibitors facilitated directed **migration** of endothelial cells away from spheroids on fibronectin-coated plates and in collagen-1 gels. By contrast, **migration** of firmly attached endothelial cells, resembling intact vessels, was not promoted by Rho kinase inhibition. Selective inhibition of ROCK2 mimicked the cytoskeletal effects of H1152 and also increased cell motility, although to a lesser extent. In summary, Rho kinase inhibition enhanced the **migration** and cytoskeletal restructuring preferentially in freshly attached endothelial cells. ROCK2 may be a potential target to manipulate endothelial cell **migration** after vessel injury.
8. Rho kinase in the regulation of cell death and survival. Rho kinase (ROCK) belongs to a family of serine/threonine kinases that are activated via interaction with Rho GTPases. ROCK is involved in a wide range of fundamental cellular functions, such as contraction, adhesion, **migration**, and proliferation. Recent studies have shown that ROCK plays an important role in the regulation of apoptosis in various cell types and animal disease models. Two ROCK isoforms, ROCK1 and ROCK2, are assumed to be function redundant, this based largely on kinase construct overexpression and chemical inhibitors (Y27632 and fasudil) which inhibit both ROCK1 and ROCK2. Gene targeting and RNA interference approaches allow further dissection of distinct cellular, physiological, and patho-physiological functions of the two ROCK isoforms. This review, based on recent molecular, cellular, and animal studies, focuses on the current understanding of ROCK signaling in the regulation of apoptosis and highlights new findings from recently generated ROCK-deficient mice.
9. Inhibition of Rho-dependent kinases ROCK I/II activates VEGF-driven retinal neovascularization and sprouting angiogenesis. Vascular endothelial growth factor (VEGF) is an endothelial-specific growth factor that activates the small GTPase RhoA. While the role of RhoA for VEGF-driven endothelial **migration** and angiogenesis has been studied in detail, the function of its target proteins, the Rho-dependent kinases ROCK I and II, are controversially discussed. Using the mouse model of oxygen-induced proliferative retinopathy, ROCK I/II inhibition by H-1152 resulted in increased angiogenesis. This enhanced angiogenesis, however, was completely blocked by the VEGF-receptor antagonist PTK787/ZK222584. Loss-of-function experiments in endothelial cells revealed that inhibition of ROCK I/II using the pharmacological inhibitor H-1152 and ROCK I/II-specific small-interfering RNAs resulted in a rise of VEGF-driven sprouting angiogenesis. These functional data were biochemically substantiated by showing an enhanced VEGF-receptor kinase insert domain receptor phosphorylation and extracellular signal-regulated kinase 1/2 activation after inhibition of ROCK I/II. Thus our data identify that the inhibition of Rho-dependent kinases ROCK I/II activates angiogenesis both, in vitro and in vivo.
10. Autotaxin/lysophospholipase D-mediated lysophosphatidic acid signaling is required to form distinctive large lysosomes in the visceral endoderm cells of the mouse yolk sac. Autotaxin, a lysophospholipase D encoded by the Enpp2 gene, is an exoenzyme that produces lysophosphatidic acid in the extracellular space. Lysophosphatidic acid acts on specific G protein-coupled receptors, thereby regulating cell growth, **migration**, and survival. Previous studies have revealed that Enpp2(-/-) mouse embryos die at about embryonic day (E) 9.5 because of angiogenic defects in the yolk sac. However, what cellular defects occur in Enpp2(-/-) embryos and what intracellular signaling pathways are involved in the phenotype manifestation remain unknown. Here, we show that Enpp2 is required to form distinctive large lysosomes in the yolk sac visceral endoderm cells. From E7.5 to E9.5, Enpp2 mRNA is abundantly expressed in the visceral endoderm cells. In Enpp2(-/-) mouse embryos, lysosomes in the visceral endoderm cells are fragmented. By using a whole embryo culture system combined with specific pharmacological inhibitors for intracellular signaling molecules, we show that lysophosphatidic acid receptors and the Rho-Rho-associated coiled-coil containing protein kinase (ROCK)-LIM kinase pathway are required to form large lysosomes. In addition, electroporation of dominant negative forms of Rho, ROCK, or LIM kinase also leads to the size reduction of lysosomes in wild-type visceral endoderm cells. In Enpp2(-/-) visceral endoderm cells, the steady-state levels of cofilin phosphorylation and actin polymerization are reduced. In addition, perturbations of actin turnover dynamics by actin inhibitors cytochalasin B and jasplakinolide result in the defect in lysosome formation. These results suggest that constitutive activation of the Rho-ROCK-LIM kinase pathway by extracellular production of lysophosphatidic acid by the action of autotaxin is required to maintain the large size of lysosomes in visceral endoderm cells.
11. From autoinhibition to inhibition in trans: the Raf-1 regulatory domain inhibits Rok-alpha kinase activity. The activity of Raf-1 and Rok-alpha kinases is regulated by intramolecular binding of the regulatory region to the kinase domain. Autoinhibition is relieved upon binding to the small guanosine triphosphatases Ras and Rho. Downstream of Ras, Raf-1 promotes **migration** and tumorigenesis by antagonizing Rok-alpha, but the underlying mechanism is unknown. In this study, we show that Rok-alpha inhibition by Raf-1 relies on an intermolecular interaction between the Rok-alpha kinase domain and the cysteine-rich Raf-1 regulatory domain (Raf-1reg), which is similar to Rok-alpha's own autoinhibitory region. Thus, Raf-1 mediates Rok-alpha inhibition in trans, which is a new concept in kinase regulation. This mechanism is physiologically relevant because Raf-1reg is sufficient to rescue all Rok-alpha-dependent defects of Raf-1-deficient cells. Downstream of Ras and Rho, the Raf-1-Rok-alpha interaction represents a novel paradigm of pathway cross talk that contributes to tumorigenesis and cell motility.
12. Rho kinases play an obligatory role in vertebrate embryonic organogenesis. Rho-associated kinases (Rho kinases), which are downstream effectors of RhoA GTPase, regulate diverse cellular functions including actin cytoskeletal organization. We have demonstrated that Rho kinases also direct the early stages of chick and mouse embryonic morphogenesis. We observed that Rho kinase transcripts were enriched in cardiac mesoderm, lateral plate mesoderm and the neural plate. Treatment of neurulating embryos with Y27632, a specific inhibitor of Rho kinases, blocked **migration** and fusion of the bilateral heart primordia, formation of the brain and neural tube, caudalward movement of Hensen's node, and establishment of normal left-right asymmetry. Moreover, Y27632 induced precocious expression of cardiac alpha-actin, an early marker of cardiomyocyte differentiation, coincident with the upregulated expression of serum response factor and GATA4. In addition, specific antisense oligonucleotides significantly diminished Rho kinase mRNA levels and replicated many of the teratologies induced by Y27632. Thus, our study reveals new biological functions for Rho kinases in regulating major morphogenetic events during early chick and mouse development.
13. FHL2 prevents cardiac hypertrophy in mice with cardiac-specific deletion of ROCK2. The Rho-associated coiled-coil containing kinases, ROCK1 and ROCK2, are important regulators of cell shape, **migration**, and proliferation through effects on the actin cytoskeleton. However, it is not known whether ROCK2 plays an important role in the development of cardiac hypertrophy. To determine whether the loss of ROCK2 could prevent cardiac hypertrophy, cardiomyocyte-specific ROCK2-null (c-ROCK2(-/-)) were generated using conditional ROCK2(flox/flox) mice and α-myosin heavy-chain promoter-driven Cre recombinase transgenic mice. Cardiac hypertrophy was induced by Ang II infusion (400 ng/kg/min, 28 d) or transverse aortic constriction (TAC). Under basal conditions, hemodynamic parameters, cardiac anatomy, and function of c-ROCK2(-/-) mice were comparable to wild-type (WT) mice. However, following Ang II infusion or TAC, c-ROCK2(-/-) mice exhibited a substantially smaller increase in heart-to-body weight ratio, left ventricular mass, myocyte cross-sectional area, hypertrophy-related fetal gene expression, intraventricular fibrosis, cardiac apoptosis, and oxidative stress compared to control mice. Deletion of ROCK2 in cardiomyocytes leads to increased expression of four-and-a-half LIM-only protein-2 (FHL2) and FHL2-mediated inhibition of serum response factor (SRF) and extracellular signal-regulated mitogen-activated protein kinase (ERK). Knockdown of FHL2 expression in ROCK2-deficient cardiomyocytes or placing ROCK2-haploinsufficient (ROCK2(+/-)) mice on FHL2(+/-)-haploinsufficient background restored the hypertrophic response to Ang II. These results indicate that cardiomyocyte ROCK2 is essential for the development of cardiac hypertrophy and that up-regulation of FHL2 may contribute to the antihypertrophic phenotype that is observed in cardiac-specific ROCK2-deficient mice.
14. Rho-GTPase effector ROCK phosphorylates cofilin in actin-meditated cytokinesis during mouse oocyte meiosis. During oocyte meiosis, a spindle forms in the central cytoplasm and migrates to the cortex. Subsequently, the oocyte extrudes a small body and forms a highly polarized egg; this process is regulated primarily by actin. ROCK is a Rho-GTPase effector that is involved in various cellular functions, such as stress fiber formation, cell **migration**, tumor cell invasion, and cell motility. In this study, we investigated possible roles for ROCK in mouse oocyte meiosis. ROCK was localized around spindles after germinal vesicle breakdown and was colocalized with cytoplasmic actin and mitochondria. Disrupting ROCK activity by RNAi or an inhibitor resulted in cell cycle progression and polar body extrusion failure. Time-lapse microscopy showed that this may have been due to spindle **migration** and cytokinesis defects, as chromosomes segregated but failed to extrude a polar body and then realigned. Actin expression at oocyte membranes and in cytoplasm was significantly decreased after these treatments. Actin caps were also disrupted, which was confirmed by a failure to form cortical granule-free domains. The mitochondrial distribution was also disrupted, which indicated that mitochondria were involved in the ROCK-mediated actin assembly. In addition, the phosphorylation levels of Cofilin, a downstream molecule of ROCK, decreased after disrupting ROCK activity. Thus, our results indicated that a ROCK-Cofilin-actin pathway regulated meiotic spindle **migration** and cytokinesis during mouse oocyte maturation.
15. Long-term inhibition of Rho-kinase suppresses left ventricular remodeling after myocardial infarction in mice. Rho-kinase has been implicated as an important regulator of inflammatory responses mediated by cytokines and chemokines. Because proinflammatory cytokines play a critical role in left ventricular (LV) remodeling after myocardial infarction (MI), we examined whether long-term blockade of Rho-kinase suppresses LV remodeling in a mouse model of MI in vivo.Mice underwent ligation of the left coronary artery and were treated with a Rho-kinase inhibitor, fasudil (100 mg x kg(-1) x d(-1) in tap water), for 4 weeks, starting 1 day after the surgery. At 4 weeks, LV infarct size was histologically comparable between the 2 groups. LV cavity dilatation and dysfunction evaluated by echocardiography were significantly suppressed in the fasudil group (P<0.05, n=15 to 28). The beneficial effects of fasudil were accompanied by suppression of cardiomyocyte hypertrophy and interstitial fibrosis (both P<0.01, n=6). The expression of inflammatory cytokines, including transforming growth factor (TGF)-beta2, TGF-beta3, and macrophage **migration** inhibitory factor, was upregulated in the noninfarcted LV in the control group and was significantly suppressed in the fasudil group (both P<0.05, n=10 to 11). Rho-kinase activity as evaluated by the extent of phosphorylation of the ERM family, a substrate of Rho-kinase, was significantly increased in the noninfarcted LV in the control group and was significantly suppressed in the fasudil group (P<0.05, n=5).These results indicate that Rho-kinase is substantially involved in the pathogenesis of LV remodeling after MI associated with upregulation of proinflammatory cytokines, suggesting a therapeutic importance of the molecule for the prevention of post-MI heart failure.
16. Effects of pharmacological inhibition of small GTPases on axon extension and **migration** of enteric neural crest-derived cells. In the developing enteric nervous system, there is a close association between migrating neural crest-derived cells and the axons of early differentiating neurons. We used pharmacological inhibitors of small GTPases to determine if crest cell **migration** and axon growth could be uncoupled in cultured intact explants of embryonic mouse gut and slices of embryonic gut grown on collagen gels containing GDNF. Inhibition of the Rho effectors, ROCKI/II, or Rac/Cdc42 inhibited both cell **migration** and neurite growth in intact explants of embryonic gut. The effects of both ROCKI/II and Rac/Cdc42 inhibitors were more severe on cell **migration** and axon extension in gut explants from Ret(+/-) mice than in explants from wildtype mice, indicating that Rho GTPases probably act downstream of the receptor tyrosine kinase, Ret. Inhibition of ROCKI/II had different effects on **migration** and axon extension in gut slices grown on collagen gels containing GDNF from that seen in intact explants of gut. We conclude that ROCKI/II and Rac/Cdc42 are required for both neural crest-derived cell **migration** and axon growth in the developing gut.
17. RhoA and RhoC have distinct roles in **migration** and invasion by acting through different targets. Several studies suggest that RhoA and RhoC, despite their sequence similarity, have different roles in cell **migration** and invasion, but the molecular basis for this is not known. Using RNAi, we show that RhoA-depleted cells became elongated and extended multiple Rac1-driven narrow protrusions in 2D and 3D environments, leading to increased invasion. These phenotypes were caused by combined but distinct effects of the Rho-regulated kinases ROCK1 and ROCK2. Depletion of ROCK2 induced multiple delocalized protrusions and reduced migratory polarity, whereas ROCK1 depletion selectively led to cell elongation and defective tail retraction. In contrast, RhoC depletion increased cell spreading and induced Rac1 activation around the periphery in broad lamellipodia, thereby inhibiting directed **migration** and invasion. These effects of RhoC depletion are mediated by the formin FMNL3, which we identify as a new target of RhoC but not RhoA. We propose that RhoA contributes to migratory cell polarity through ROCK2-mediated suppression of Rac1 activity in lamellipodia, whereas RhoC promotes polarized **migration** through FMNL3 by restricting lamellipodial broadening.
18. Rho-associated kinases play a role in endocardial cell differentiation and **migration**. Development of the endocardial cushions in the heart involves cell **migration** and cell differentiation, which is known as epithelial-mesenchymal transformation (EMT). These processes are regulated by cell signaling systems. Yet, the roles of intracellular GTPases and their effectors on these cellular activities remain to be addressed. This study investigated the role Rho GTPase-associated kinases (ROCKs) in endocardial cushion development. Using reverse transcription (RT) and polymerase chain reaction (PCR), expression of the rock1 and rock2 genes was found in the endocardial cushions during development. To investigate the role of ROCKs in development, the ROCK inhibitor Y27632 and adenoviruses containing a dominant negative form of the rock gene were used to treat cultured endocardial cushions and cells. In monolayer cell culture and three-dimensional tissue culture, blockade of ROCK inhibited EMT development. Using three-dimensional collagen gel assays and confocal microscopy, we also observed inhibition of cell **migration** with ROCK inhibition. Examination of cell morphology and actin cytoskeleton revealed that inhibition of ROCK activity disturbed cytoskeletal organization and blocked the formation of lamellipodia and filopodia. Collectively, these data show that ROCKs play an essential role in endothelial cell differentiation and **migration** during endocardial cushion development.
19. ROCK inhibition prevents early mouse embryo development. ROCK is a Rho-GTPase effector that is important for actin assembly and is involved in various cellular functions, including cell contraction, **migration**, motility, and tumor cell invasion. In this study, we investigated ROCK expression and function during early mouse embryo development. Inhibiting ROCK by Y-27632 treatment at the zygote stage resulted in first cleavage failure, and most embryos failed to develop to the 8-cell stage. When adding Y-27632 at the 8-cell stage, embryos failed to undergo compaction and could not develop into blastocysts. In addition, fluorescence staining intensity analysis indicated that actin expression at blastomere membranes was significantly reduced. After ROCK inhibition, two or more nuclei were observed in a cell, which indicated possible cytokinesis failure. Moreover, after ROCK inhibition with Y-27632, the phosphorylation levels of LIMK1/2, a downstream molecule of ROCK, were decreased at blastomere membranes. Thus, our results showed conserved roles for ROCK in this mammalian embryo model and indicated that a ROCK-LIMK1/2-actin pathway might regulate cleavage and blastocyst formation during early mouse embryo development.
20. Tubers from patients with tuberous sclerosis complex are characterized by changes in microtubule biology through ROCK2 signalling. Most patients with tuberous sclerosis complex (TSC) develop cortical tubers that cause severe neurological disabilities. It has been suggested that defects in neuronal differentiation and/or **migration** underlie the appearance of tubers. However, the precise molecular alterations remain largely unknown. Here, by combining cytological and immunohistochemical analyses of tubers from nine TSC patients (four of them diagnosed with TSC2 germline mutations), we show that alteration of microtubule biology through ROCK2 signalling contributes to TSC neuropathology. All tubers showed a larger number of binucleated neurons than expected relative to control cortex. An excess of normal and altered cytokinetic figures was also commonly observed. Analysis of centrosomal markers suggested increased microtubule nucleation capacity, which was supported by the analysis of an expression dataset from cortical tubers and control cortex, and subsequently linked to under-expression of Rho-associated coiled-coil containing kinase 2 (ROCK2). Thus, augmented microtubule nucleation capacity was observed in mouse embryonic fibroblasts and human fibroblasts deficient in the Tsc2/TSC2 gene product, tuberin. Consistent with ROCK2 under-expression, microtubule acetylation was found to be increased with tuberin deficiency; this alteration was abrogated by rapamycin treatment and mimicked by HDAC6 inhibition. Together, the results of this study support the hypothesis that loss of TSC2 expression can alter microtubule organization and dynamics, which, in turn, deregulate cell division and potentially impair neuronal differentiation.
21. Src-dependent phosphorylation of ROCK participates in regulation of focal adhesion dynamics. When a cell migrates, the RhoA-ROCK-mediated contractile signal is suppressed in the leading edge to allow dynamic adhesions for protrusion. However, several studies have reported that RhoA is indeed active in the leading edge of a migrating cell during serum stimulation. Here, we present evidence that regulation of ROCKII phosphorylation at the Y722 site in peripheral focal contacts is crucial for controlling the turnover of the focal adhesion (FA) complex uncoupled from RhoA activation during serum-stimulated **migration**. However, this phosphorylation control is dispensable for **migration** when RhoA is downregulated in cells treated with platelet-derived growth factor (PDGF). We further present evidence that ROCKII is phosphorylated by Src in FAs and this phosphorylation event decreases RhoA binding activity of ROCKII. Lack of this regulatory control leads to sustained myosin-mediated contractility and FA elongation during lysophosphatidic acid (LPA) stimulation. Altogether, our data suggest that Src-dependent ROCKII phosphorylation provides a means of tuning contractility required for FAs dynamics when RhoA is active.
22. Crucial role of ROCK2 in vascular smooth muscle cells for hypoxia-induced pulmonary hypertension in mice. Rho/Rho-kinase (ROCK) pathway in vascular smooth muscle cells (VSMCs) plays an important role in the pathogenesis of cardiovascular diseases, including pulmonary arterial hypertension (PAH). Rho-kinase has 2 isoforms, ROCK1 and ROCK2, with different functions in different cells; ROCK1 for circulating inflammatory cells and ROCK2 for the vasculature. In the present study, we aimed to examine whether ROCK2 in VSMC is involved in the pathogenesis of PAH.In patients with PAH, the expression of ROCK2 was increased in pulmonary arterial media and primary pulmonary arterial smooth muscle cells when compared with controls. To investigate the role of ROCK2 in VSMC, we generated VSMC-specific heterozygous ROCK2-deficient (ROCK2(+/-)) mice and VSMC-specific ROCK2-overexpressing transgenic (ROCK2-Tg) mice. The extent of hypoxia-induced pulmonary hypertension was reduced in ROCK2(+/-) mice and was enhanced in ROCK2-Tg mice compared with respective littermates. The protein expression of ROCK activity and phosphorylated extracellular signal-regulated kinase and the number of Ki67-positive proliferating cells in the lung were reduced in ROCK2(+/-) mice and were increased in ROCK2-Tg mice compared with respective littermates. In cultured mouse aortic VSMC, **migration** and proliferation activities were reduced in ROCK2(+/-) mice, and **migration** activity was increased in ROCK2-Tg mice compared with respective littermates. In addition, in primary pulmonary arterial smooth muscle cells from a patient with PAH, ROCK2 was required for **migration** and proliferation through ROCK and extracellular signal-regulated kinase activation.ROCK2 in VSMC contributes to the pathogenesis of PAH.
23. Disruption of c-Jun reduces cellular **migration** and invasion through inhibition of c-Src and hyperactivation of ROCK II kinase. The spread of metastatic tumors to different organs is associated with poor prognosis. The metastatic process requires **migration** and cellular invasion. The protooncogene c-jun encodes the founding member of the activator protein-1 family and is required for cellular proliferation and DNA synthesis in response to oncogenic signals and plays an essential role in chemical carcinogenesis. The role of c-Jun in cellular invasion remains to be defined. Genetic deletion of c-Jun in transgenic mice is embryonic lethal; therefore, transgenic mice encoding a c-Jun gene flanked by LoxP sites (c-jun(f/f)) were used. c-jun gene deletion reduced c-Src expression, hyperactivated ROCK II signaling, and reduced cellular polarity, **migration**, and invasiveness. c-Jun increased c-Src mRNA abundance and c-Src promoter activity involving an AP-1 site in the c-Src promoter. Transduction of c-jun(-/-) cells with either c-Jun or c-Src retroviral expression systems restored the defective cellular **migration** of c-jun(-/-) cells. As c-Src is a critical component of pathways regulating proliferation, survival, and metastasis, the induction of c-Src abundance, by c-Jun, provides a novel mechanism of cooperative signaling in cellular invasion.
24. Signaling Rho-kinase mediates inflammation and apoptosis in T cells and renal tubules in cisplatin nephrotoxicity. Nephrotoxicity is a frequent complication of cisplatin-induced chemotherapy, in which T cells are known to promote acute kidney injury (AKI). Apoptosis and necrosis of tubules and inflammatory events also contribute to the nephrotoxicity. A delineation of the mechanisms that underlie the inappropriate renal and tubular inflammation can thus provide important insights into potential therapies for cisplatin-induced AKI. Rho-kinases are known to act as molecular switches controlling several critical cellular functions, including cell **migration**, cytokine production, and apoptosis. Here, we show that the Rho-kinase inhibitor fasudil attenuated cisplatin nephrotoxicity, resulting in less histological damage, improved renal function, and the infiltration of fewer leukocytes into the kidney. Renal nuclear factor-κB activation and apoptosis were reduced, and the expressions of proinflammatory renal cytokine and chemokine mRNA were decreased. Urinary and renal kidney injury molecule-1 (Kim-1) expression was also reduced, a finding that is consistent with diminished kidney injury. In the current study, we also showed that fasudil could be protective of the impaired tubules. In vitro, fasudil reduced the apoptosis (annexin-V+PI cells) and cytokine production (tumor necrosis factor+ cells) in T cells and the apoptosis (annexin-V+PI cells) and tubular damage (Kim-1+ cells) in proximal tubular cells by flow cytometric analysis. As Rho-kinase plays an important role in promoting cisplatin nephrotoxicity, inhibiting Rho-kinase may be a therapeutic strategy for preventing cisplatin-induced AKI.
25. B-Raf acts via the ROCKII/LIMK/cofilin pathway to maintain actin stress fibers in fibroblasts. Recent data have shown that the BRAF gene is mutated at a high frequency in human malignancies. We have analyzed the migratory characteristics of B-raf(-/-) mouse embryonic fibroblasts (MEFs) and compared these with the organization of the actin cytoskeleton and the activity of signaling pathways that are known to influence this organization. Disruption of B-raf significantly reduced the levels of phospho-ERK1/2 and, surprisingly, induced an approximately 1.5-fold increase in cell **migration**. Consistent with these findings, the high level of actin stress fibers normally present in MEFs was considerably reduced following disruption of B-raf, and the F-actin content of B-raf(-/-) cells was less than half that of B-raf(+/+) cells. Phosphorylation of the myosin light chain on Thr18/Ser19 residues was not reduced in B-raf(-/-) cells. Rather, reduced ROCKII expression and attenuated phosphorylation of ADF/cofilin on serine 3 occurred. Normal stress fiber and phosphocofilin levels were restored by the expression of human B-Raf and catalytically active MEK and by the overexpression of LIM kinase (LIMK). These results have important implications for the role of the B-Raf/ERK signaling pathway in regulating cell motility in normal and malignant cells. They suggest that B-Raf is involved in invasiveness by regulating the proper assembly of actin stress fibers and contractility through a ROCKII/LIMK/cofilin signaling pathway.
26. ROCK-2 is associated with focal adhesion maturation during myoblast **migration**. Satellite cell **migration** is critical for skeletal muscle growth and regeneration. Controlled cell **migration** is dependent on the formation of mature focal adhesions between the cell and the underlying extracellular matrix (ECM). These cell-ECM interactions trigger the activation of signalling events such as the Rho/ROCK pathway. We have previously identified a specific role for ROCK-2 during myoblast **migration**. In this study we report that ROCK inhibition with Y-27632 increases C2C12 myoblast velocity, but at the expense of directional **migration**. In response to Y-27632 an increased number of smaller focal adhesions were distributed across adhesion sites that in turn were clearly larger than sites in untreated cells, suggesting a reduction in focal adhesion maturation. We also confirm ROCK-2 localisation to the focal adhesion sites in migrating myoblasts and demonstrate a change in the distribution of these ROCK-2 containing adhesions in response to Y-27632. Taken together, our observations provide further proof that ROCK-2 regulates directional myoblast **migration** through focal adhesion formation and maturation.
27. The Rho kinase pathway regulates mouse adult neural precursor cell **migration**. Adult neural precursor cells (NPCs) in the subventricular zone (SVZ) normally migrate via the rostral migratory stream (RMS) to the olfactory bulb (OB). Following neural injury, they also migrate to the site of damage. This study investigated the role of Rho-dependent kinase (ROCK) on the **migration** of NPCs in vitro and in vivo. In vitro, using neurospheres or SVZ explants, inhibition of ROCK using Y27632 promoted cell body elongation, process protrusion, and **migration**, while inhibiting NPC chain formation. It had no effect on proliferation, apoptosis, or differentiation. Both isoforms of ROCK were involved. Using siRNA, knockdown of both ROCK1 and ROCK2 was required to promote NPC **migration** and morphological changes; knockdown of ROCK2 alone was partially effective, with little/no effect of knockdown of ROCK1 alone. In vivo, infusion of Y27632 plus Bromodeoxyuridine (BrdU) into the lateral ventricle for 1 week reduced the number of BrdU-labeled NPCs in the OB compared with BrdU infusion alone. However, ROCK inhibition did not affect the tangential-to-radial switch of NPC **migration**, as labeled cells were present in all OB layers. The decrease in NPC number at the OB was not attributed to a decrease in NPCs at the SVZ. However, ROCK inhibition decreased the density of BrdU-labeled cells in the RMS and increased the distribution of these cells to ectopic brain regions, such as the accessory olfactory nucleus, where the majority differentiated into neurons. These findings suggest that ROCK signaling regulates NPC **migration** via regulation of cell-cell contact and chain **migration**.
28. Rho Kinase Inhibitor Fasudil Suppresses the Vasculogenic Mimicry of B16 Mouse Melanoma Cells Both In Vitro and In Vivo. The aim of this study was to investigate the biologic role of the Rho kinase inhibitor fasudil in the vasculogenic mimicry (VM) of B16 mouse melanoma cells. It was previously reported that RhoA plays a critical role in angiogenesis by coordinating endothelial cell cytoskeleton remodeling and promoting endothelial cell motility. Although RhoA has been implicated in the regulation of angiogenesis, little has been described regarding its control of these tumor cell-lined channels. In this study, we established an in vitro model of VM using 3-dimensional cell culturing of mouse B16 melanoma cells and studied VM in vivo by transplanting B16 cells into C57/BL mice. Next, we explored the effect of RhoA and Rho-associated, coiled-coil containing protein kinase (ROCK) on VM formation using the Rho kinase inhibitor fasudil. We provide direct evidence that fasudil leads to reduced vascular-like channels in Matrigel. Additional experiments suggested that fasudil prevents both initial cellular architecture changes and cell **migration** in vitro. Finally, we provide in-depth evidence for the underlying mechanisms of fasudil-induced VM destruction using the Rho-GTPase agonist lysophosphatidic acid. In vivo studies revealed that fasudil reduced B16 melanoma cell xenograft tumor growth without causing significant toxicity in mice. Fasudil-treated tumors also displayed fewer VM channels. These results suggest that fasudil may be an emerging therapeutic option for targeting cancer VM.
29. Distinct roles of Rac1/Cdc42 and Rho/Rock for axon outgrowth and nucleokinesis of precerebellar neurons toward netrin 1. During embryonic development, tangentially migrating precerebellar neurons emit a leading process and then translocate their nuclei inside it (nucleokinesis). Netrin 1 (also known as netrin-1) acts as a chemoattractant factor for neurophilic **migration** of precerebellar neurons (PCN) both in vivo and in vitro. In the present work, we analyzed Rho GTPases that could direct axon outgrowth and/or nuclear **migration**. We show that the expression pattern of Rho GTPases in developing PCN is consistent with their involvement in the **migration** of PCN from the rhombic lips. We report that pharmacological inhibition of Rho enhances axon outgrowth of PCN and prevents nuclei **migration** toward a netrin 1 source, whereas inhibition of Rac and Cdc42 sub-families impair neurite outgrowth of PCN without affecting **migration**. We show, through pharmacological inhibition, that Rho signaling directs neurophilic **migration** through Rock activation. Altogether, our results indicate that Rho/Rock acts on signaling pathways favoring nuclear translocation during tangential **migration** of PCN. Thus, axon extension and nuclear **migration** of PCN in response to netrin 1 are not strictly dependent processes because: (1) distinct small GTPases are involved; (2) axon extension can occur when **migration** is blocked; and (3) **migration** can occur when axon outgrowth is impaired.
30. ROCK insufficiency attenuates ozone-induced airway hyperresponsiveness in mice. Ozone causes airway hyperresponsiveness (AHR) and pulmonary inflammation. Rho kinase (ROCK) is a key regulator of smooth muscle cell contraction and inflammatory cell **migration**. To determine the contribution of the two ROCK isoforms ROCK1 and ROCK2 to ozone-induced AHR, we exposed wild-type, ROCK1(+/-), and ROCK2(+/-) mice to air or ozone (2 ppm for 3 h) and evaluated mice 24 h later. ROCK1 or ROCK2 haploinsufficiency did not affect airway responsiveness in air-exposed mice but significantly reduced ozone-induced AHR, with a greater reduction in ROCK2(+/-) mice despite increased bronchoalveolar lavage (BAL) inflammatory cells in ROCK2(+/-) mice. Compared with wild-type mice, ozone-induced increases in BAL hyaluronan, a matrix protein implicated in ozone-induced AHR, were lower in ROCK1(+/-) but not ROCK2(+/-) mice. Ozone-induced increases in other inflammatory moieties reported to contribute to ozone-induced AHR (IL-17A, osteopontin, TNFα) were not different in wild-type vs. ROCK1(+/-) or ROCK2(+/-) mice. We also observed a dose-dependent reduction in ozone-induced AHR after treatment with the ROCK1/ROCK2 inhibitor fasudil, even though fasudil was administered after induction of inflammation. Ozone increased pulmonary expression of ROCK2 but not ROCK1 or RhoA. A ROCK2 inhibitor, SR3677, reduced contractile forces in primary human airway smooth muscle cells, confirming a role for ROCK2 in airway smooth muscle contraction. Our results demonstrate that ozone-induced AHR requires ROCK. Whereas ROCK1-dependent changes in hyaluronan may contribute to ROCK1's role in O3-induced AHR, the role of ROCK2 is downstream of inflammation, likely at the level of airway smooth muscle contraction.
31. ROCK1 & 2 perform overlapping and unique roles in angiogenesis and angiosarcoma tumor progression. The serine/threonine protein kinase paralogs ROCK1 & 2 have been implicated as essential modulators of angiogenesis; however their paralog-specific roles in endothelial function are unknown. shRNA knockdown of ROCK1 or 2 in endothelial cells resulted in a significant disruption of in vitro capillary network formation, cell polarization, and cell **migration** compared to cells harboring non-targeting control shRNA plasmids. Knockdowns led to alterations in cytoskeletal dynamics due to ROCK1 & 2-mediated reductions in actin isoform expression, and ROCK2-specific reduction in myosin phosphatase and cofilin phosphorylation. Knockdowns enhanced cell survival and led to ROCK1 & 2-mediated reduction in caspase 6 and 9 cleavage, and a ROCK2-specific reduction in caspase 3 cleavage. Microarray analysis of ROCK knockdown lines revealed overlapping and unique control of global transcription by the paralogs, and a reduction in the transcriptional regulation of just under 50% of VEGF responsive genes. Finally, paralog knockdown in xenograft angiosarcoma tumors resulted in a significant reduction in tumor formation. Our data reveals that ROCK1 & 2 exhibit overlapping and unique roles in normal and dysfunctional endothelial cells, that alterations in cytoskeletal dynamics are capable of overriding mitogen activated transcription, and that therapeutic targeting of ROCK signaling may have profound impacts for targeting angiogenesis.
32. Raf-1 regulates Rho signaling and cell **migration**. Raf kinases relay signals inducing proliferation, differentiation, and survival. The Raf-1 isoform has been extensively studied as the upstream kinase linking Ras activation to the MEK/ERK module. Recently, however, genetic experiments have shown that Raf-1 plays an essential role in counteracting apoptosis, and that it does so independently of its ability to activate MEK. By conditional gene ablation, we now show that Raf-1 is required for normal wound healing in vivo and for the **migration** of keratinocytes and fibroblasts in vitro. Raf-1-deficient cells show a symmetric, contracted appearance, characterized by cortical actin bundles and by a disordered vimentin cytoskeleton. These defects are due to the hyperactivity and incorrect localization of the Rho-effector Rok-alpha to the plasma membrane. Raf-1 physically associates with Rok-alpha in wild-type (WT) cells, and reintroduction of either WT or kinase-dead Raf-1 in knockout fibroblasts rescues their defects in shape and **migration**. Thus, Raf-1 plays an essential, kinase-independent function as a spatial regulator of Rho downstream signaling during **migration**.

#### GeneRIFs

1. ROCK-2 regulates directional myoblast **migration** through focal adhesion formation and maturation.
2. Using siRNA, knockdown of both ROCK1 and ROCK2 was required to promote NPC **migration** and morphological changes
3. Data show that inhibition of JNK and ROCK kinases strongly and specifically reduce the facial branchiomotor neurons **migration**, as well as block the chemoattractant effects of ectopic Wnt proteins.
4. Raf-1-mediated Rok-alpha inhibition is essential for cell **migration** in vivo and in vitro
5. c-jun gene deletion reduced c-Src expression, hyperactivated ROCK II signaling, and reduced cellular polarity, **migration**, and invasiveness.
6. ROCKI/II and Rac/Cdc42 are required for both neural crest-derived cell **migration** and axon growth in the developing gut.
7. Comparison of cell **migration**, actin cytoskeleton activity, and signal pathways implies that B-Raf is involved in cell invasiveness by regulating the proper assembly of actin stress fibers and contractility through a ROCKII/LIMK/cofilin signaling pathway
8. Plays an essential role in endothelial cell differentiation and **migration** during endocardial cushion development.

### ECM

#### PubMed

1. Rho-ROCK-myosin signaling mediates membrane type 1 matrix metalloproteinase-induced cellular aggregation of keratinocytes. Membrane type 1-matrix metalloproteinase (MT1-MMP, MMP14), which is associated with extracellular matrix (**ECM**) breakdown in squamous cell carcinoma (SCC), promotes tumor formation and epithelial-mesenchymal transition. However, in this report we demonstrate that MT1-MMP, by cleaving the underlying **ECM**, causes cellular aggregation of keratinocytes and SCC cells. Treatment with an MMP inhibitor abrogated MT1-MMP-induced phenotypic changes, but decreasing E-cadherin expression did not affect MT1-MMP-induced cellular aggregation. As ROCK1/2 can regulate cell-cell and cell-**ECM** interaction, we examined its role in mediating MT1-MMP-induced phenotypic changes. Blocking ROCK1/2 expression or activity abrogated the cellular aggregation resulting from MT1-MMP expression. Additionally, blocking Rho and non-muscle myosin attenuated MT1-MMP-induced phenotypic changes. Moreover, SCC cells expressing only the catalytically active MT1-MMP protein demonstrated increased cellular aggregation and increased myosin II activity in vivo when injected subcutaneously into nude mice. Together, these results demonstrate that expression of MT1-MMP may be anti-tumorigenic in keratinocytes by promoting cellular aggregation.
2. ROCK-2 is associated with focal adhesion maturation during myoblast migration. Satellite cell migration is critical for skeletal muscle growth and regeneration. Controlled cell migration is dependent on the formation of mature focal adhesions between the cell and the underlying extracellular matrix (**ECM**). These cell-**ECM** interactions trigger the activation of signalling events such as the Rho/ROCK pathway. We have previously identified a specific role for ROCK-2 during myoblast migration. In this study we report that ROCK inhibition with Y-27632 increases C2C12 myoblast velocity, but at the expense of directional migration. In response to Y-27632 an increased number of smaller focal adhesions were distributed across adhesion sites that in turn were clearly larger than sites in untreated cells, suggesting a reduction in focal adhesion maturation. We also confirm ROCK-2 localisation to the focal adhesion sites in migrating myoblasts and demonstrate a change in the distribution of these ROCK-2 containing adhesions in response to Y-27632. Taken together, our observations provide further proof that ROCK-2 regulates directional myoblast migration through focal adhesion formation and maturation.

### [Ee]xtracellular [Mm]atrix

#### PubMed

1. Identification of tooth-specific downstream targets of Runx2. Odontogenesis or tooth development is a highly regulated process that involves complex epithelial-mesenchymal signaling interactions that lead to cuspal morphogenesis, cell differentiation and the subsequent formation of the specialized matrices of enamel, dentin, cementum and bone. Although studies on tooth epithelial-mesenchymal signaling interactions have greatly increased our understanding of molecules that regulate tooth initiation and early morphogenesis (review: Jernvall and Thesleff, Mech. Dev. 92 (2000) 19), the precise nature of the molecular events controlling late morphogenesis and terminal cytodifferentiation is not known. We have recently reported a unique phenotype involving dentition in mice lacking a functional Runx2 gene (D'Souza et al., Development 126 (1999) 2911). The markedly hypoplastic tooth organs as well as defects in the maturation of ameloblasts and odontoblasts point to an important and non-redundant role for Runx2 in tooth morphogenesis and cytodifferentiation. In order to identify genes that are affected by the absence of Runx2, a cDNA library was generated from Runx2(-/-) and Runx2(+/+) first molar organs. Thus far, our analysis has revealed several tooth-specific downstream target genes of Runx2 that include **extracellular matrix** proteins, kinases, receptors, growth factors, mitochondrial proteins and transcription molecules. Sequence analysis of 61 differentially expressed genes revealed that 96.03% of the clones matched previously described genes in the GenBank/EBML database and 3.96% did not match any entries in the database. Our preliminary expression analysis of one of the differentially expressed clones which encodes for a zinc finger transcription factor termed Zfp reveals that the gene is temporally regulated during tooth development. In conclusion, we have successfully generated a library enriched in genes expressed in Runx2(+/+) molar tooth organs and performed preliminary studies to assess the role of Zfp in tooth development.
2. Rho-ROCK-myosin signaling mediates membrane type 1 matrix metalloproteinase-induced cellular aggregation of keratinocytes. Membrane type 1-matrix metalloproteinase (MT1-MMP, MMP14), which is associated with **extracellular matrix** (ECM) breakdown in squamous cell carcinoma (SCC), promotes tumor formation and epithelial-mesenchymal transition. However, in this report we demonstrate that MT1-MMP, by cleaving the underlying ECM, causes cellular aggregation of keratinocytes and SCC cells. Treatment with an MMP inhibitor abrogated MT1-MMP-induced phenotypic changes, but decreasing E-cadherin expression did not affect MT1-MMP-induced cellular aggregation. As ROCK1/2 can regulate cell-cell and cell-ECM interaction, we examined its role in mediating MT1-MMP-induced phenotypic changes. Blocking ROCK1/2 expression or activity abrogated the cellular aggregation resulting from MT1-MMP expression. Additionally, blocking Rho and non-muscle myosin attenuated MT1-MMP-induced phenotypic changes. Moreover, SCC cells expressing only the catalytically active MT1-MMP protein demonstrated increased cellular aggregation and increased myosin II activity in vivo when injected subcutaneously into nude mice. Together, these results demonstrate that expression of MT1-MMP may be anti-tumorigenic in keratinocytes by promoting cellular aggregation.
3. Role of the Rho-ROCK (Rho-associated kinase) signaling pathway in the regulation of pancreatic beta-cell function. **Extracellular matrix** has a beneficial impact on beta-cell spreading and function, but the underlying signaling pathways have yet to be fully elucidated. In other cell types, Rho, a well-characterized member of the family of Rho GTPases, and its effector Rho-associated kinase (ROCK), play an important role as downstream mediators of outside in signaling from **extracellular matrix**. Therefore, a possible role of the Rho-ROCK pathway in beta-cell spreading, actin cytoskeleton dynamics, and function was investigated. Rho was inhibited using a new cell-permeable version of C3 transferase, whereas the activity of ROCK was repressed using the specific ROCK inhibitors H-1152 and Y-27632. Inhibition of Rho and of ROCK increased spreading and improved both short-term and prolonged glucose-stimulated insulin secretion but had no impact on basal secretion. Inhibition of this pathway led to a depolymerization of the actin cytoskeleton. Furthermore, the impact of the inhibition of ROCK on stimulated insulin secretion was acute and reversible, suggesting that rapid signaling such as phosphorylation is involved. Finally, quantification of the activity of RhoA indicated that the **extracellular matrix** represses RhoA activity. Overall these results show for the first time that the Rho-ROCK signaling pathway contributes to the stabilization of the actin cytoskeleton and inhibits glucose-stimulated insulin secretion in primary pancreatic beta-cells. Furthermore, they indicate that inhibition of this pathway might be one of the mechanisms by which the **extracellular matrix** exerts its beneficial effects on pancreatic beta-cell function.
4. ROCK-2 is associated with focal adhesion maturation during myoblast migration. Satellite cell migration is critical for skeletal muscle growth and regeneration. Controlled cell migration is dependent on the formation of mature focal adhesions between the cell and the underlying **extracellular matrix** (ECM). These cell-ECM interactions trigger the activation of signalling events such as the Rho/ROCK pathway. We have previously identified a specific role for ROCK-2 during myoblast migration. In this study we report that ROCK inhibition with Y-27632 increases C2C12 myoblast velocity, but at the expense of directional migration. In response to Y-27632 an increased number of smaller focal adhesions were distributed across adhesion sites that in turn were clearly larger than sites in untreated cells, suggesting a reduction in focal adhesion maturation. We also confirm ROCK-2 localisation to the focal adhesion sites in migrating myoblasts and demonstrate a change in the distribution of these ROCK-2 containing adhesions in response to Y-27632. Taken together, our observations provide further proof that ROCK-2 regulates directional myoblast migration through focal adhesion formation and maturation.

### [Ii]nvasive

#### PubMed

1. Disruption of c-Jun reduces cellular migration and invasion through inhibition of c-Src and hyperactivation of ROCK II kinase. The spread of metastatic tumors to different organs is associated with poor prognosis. The metastatic process requires migration and cellular invasion. The protooncogene c-jun encodes the founding member of the activator protein-1 family and is required for cellular proliferation and DNA synthesis in response to oncogenic signals and plays an essential role in chemical carcinogenesis. The role of c-Jun in cellular invasion remains to be defined. Genetic deletion of c-Jun in transgenic mice is embryonic lethal; therefore, transgenic mice encoding a c-Jun gene flanked by LoxP sites (c-jun(f/f)) were used. c-jun gene deletion reduced c-Src expression, hyperactivated ROCK II signaling, and reduced cellular polarity, migration, and **invasive**ness. c-Jun increased c-Src mRNA abundance and c-Src promoter activity involving an AP-1 site in the c-Src promoter. Transduction of c-jun(-/-) cells with either c-Jun or c-Src retroviral expression systems restored the defective cellular migration of c-jun(-/-) cells. As c-Src is a critical component of pathways regulating proliferation, survival, and metastasis, the induction of c-Src abundance, by c-Jun, provides a novel mechanism of cooperative signaling in cellular invasion.
2. B-Raf acts via the ROCKII/LIMK/cofilin pathway to maintain actin stress fibers in fibroblasts. Recent data have shown that the BRAF gene is mutated at a high frequency in human malignancies. We have analyzed the migratory characteristics of B-raf(-/-) mouse embryonic fibroblasts (MEFs) and compared these with the organization of the actin cytoskeleton and the activity of signaling pathways that are known to influence this organization. Disruption of B-raf significantly reduced the levels of phospho-ERK1/2 and, surprisingly, induced an approximately 1.5-fold increase in cell migration. Consistent with these findings, the high level of actin stress fibers normally present in MEFs was considerably reduced following disruption of B-raf, and the F-actin content of B-raf(-/-) cells was less than half that of B-raf(+/+) cells. Phosphorylation of the myosin light chain on Thr18/Ser19 residues was not reduced in B-raf(-/-) cells. Rather, reduced ROCKII expression and attenuated phosphorylation of ADF/cofilin on serine 3 occurred. Normal stress fiber and phosphocofilin levels were restored by the expression of human B-Raf and catalytically active MEK and by the overexpression of LIM kinase (LIMK). These results have important implications for the role of the B-Raf/ERK signaling pathway in regulating cell motility in normal and malignant cells. They suggest that B-Raf is involved in **invasive**ness by regulating the proper assembly of actin stress fibers and contractility through a ROCKII/LIMK/cofilin signaling pathway.

#### GeneRIFs

1. c-jun gene deletion reduced c-Src expression, hyperactivated ROCK II signaling, and reduced cellular polarity, migration, and **invasive**ness.
2. Comparison of cell migration, actin cytoskeleton activity, and signal pathways implies that B-Raf is involved in cell **invasive**ness by regulating the proper assembly of actin stress fibers and contractility through a ROCKII/LIMK/cofilin signaling pathway

### [Mm]etastasis

#### PubMed

1. ROCK-II mediates colon cancer invasion via regulation of MMP-2 and MMP-13 at the site of invadopodia as revealed by multiphoton imaging. The ROCK-II isoform of Rho's downstream effector, Rho kinase, has been linked with greater invasion and **metastasis** in solid tumors. We have previously shown that ROCK-II is overexpressed at the advancing edge of colon cancers. The mechanism whereby ROCK-II contributes invasion, particularly in the setting of colon cancer, remains to be elucidated fully. To better understand its contribution, we evaluated ROCK-II expression in both non-malignant (NCM460 and IEC-6) and malignant (Caco-2 E, SW620, and HCT-116) intestinal epithelial cell lines grown in type I collagen scaffolds. Using multiphoton microscopy, we observed that ROCK-II localized to the actin cytoskeleton in non-malignant cells but localized to the cell periphery as focal collections with an absence of adjacent collagen in all colon cancer cell lines. By transmission electron microscopy, these collections corresponded with finger-like projections previously described as invadopodia. Immunogold staining with cortactin, matrix metalloprotease (MMP)-2, -9, and -13 confirmed that these were indeed invadopodia. To further link ROCK-II to colon cancer invasion, we treated non-malignant and malignant intestinal epithelial cell lines with ROCK-II siRNA and evaluated depth of invasion, proliferation, and MMP-2, -9, and -13 activities. The most striking effect was seen in the highly tumorigenic cell lines, SW620 and HCT-116, wherein ROCK-II knockdown resulted in a two-fold or more reduction in invasion. This reduction in invasion was not due to a decrease in cell proliferation, as a significant reduction in proliferation was only observed in the two non-malignant intestinal cell lines. Finally, both MMP-2 and -13 activities were significantly decreased in all colon cancer cell lines. Taken together, these data suggest for the first time that ROCK-II is a critical mediator of colon cancer cell invasion through its modulation of MMP-2 and -13 at the site of invadopodia but regulates proliferation in non-malignant intestinal cells.
2. Small molecule inhibition of cytoskeletal dynamics in melanoma tumors results in altered transcriptional expression patterns of key genes involved in tumor initiation and progression. Rho kinase signaling plays an important role in the oncogenic process largely through its regulation of F-actin dynamics, and inhibition of this pathway results in reduction in tumor volume and **metastasis** across a number of tumor types. While the cytoskeletal-regulatory role of Rho kinase has been a topic of in-depth study, the mechanisms linking Rho kinase to altered gene expression are largely unknown.Global gene expression analysis was performed on melanoma tumors treated with sham or the small molecule inhibitor Y27632.Inhibition of Rho kinase activity in melanoma tumors results in a statistically significant change in gene transcription of 94 genes, many of which are critically involved in tumor initiation and progression.In addition to regulating tumorigenesis through modulation of the phosphoproteome, Rho kinase signaling also contributes to the regulation of the tumor transcriptome.
3. Disruption of c-Jun reduces cellular migration and invasion through inhibition of c-Src and hyperactivation of ROCK II kinase. The spread of metastatic tumors to different organs is associated with poor prognosis. The metastatic process requires migration and cellular invasion. The protooncogene c-jun encodes the founding member of the activator protein-1 family and is required for cellular proliferation and DNA synthesis in response to oncogenic signals and plays an essential role in chemical carcinogenesis. The role of c-Jun in cellular invasion remains to be defined. Genetic deletion of c-Jun in transgenic mice is embryonic lethal; therefore, transgenic mice encoding a c-Jun gene flanked by LoxP sites (c-jun(f/f)) were used. c-jun gene deletion reduced c-Src expression, hyperactivated ROCK II signaling, and reduced cellular polarity, migration, and invasiveness. c-Jun increased c-Src mRNA abundance and c-Src promoter activity involving an AP-1 site in the c-Src promoter. Transduction of c-jun(-/-) cells with either c-Jun or c-Src retroviral expression systems restored the defective cellular migration of c-jun(-/-) cells. As c-Src is a critical component of pathways regulating proliferation, survival, and **metastasis**, the induction of c-Src abundance, by c-Jun, provides a novel mechanism of cooperative signaling in cellular invasion.
4. Cytoplasmic p21Cip1 is involved in Ras-induced inhibition of the ROCK/LIMK/cofilin pathway. Accumulating evidence suggests that p21(Cip1) located in the cytoplasm might play a role in promoting transformation and tumor progression. Here we show that oncogenic H-RasV12 contributes to the loss of actin stress fibers by inducing cytoplasmic localization of p21(Cip1), which uncouples Rho-GTP from stress fiber formation by inhibiting Rho kinase (ROCK). Concomitant with the loss of stress fibers in Ras-transformed cells, there is a decrease in the phosphorylation level of cofilin, which is indicative of a compromised ROCK/LIMK/cofilin pathway. Inhibition of MEK in Ras-transformed NIH3T3 results in restoration of actin stress fibers accompanied by a loss of cytoplasmic p21(Cip1), and increased phosphorylation of cofilin. Ectopic expression of cytoplasmic but not nuclear p21(Cip1) in Ras-transformed cells was effective in preventing stress fibers from being restored upon MEK inhibition and inhibited phosphorylation of cofilin. p21(Cip1) was also found to form a complex with ROCK in Ras-transformed cells in vivo. Furthermore, inhibition of the PI 3-kinase pathway resulted in loss of p21(Cip1) expression accompanied by restoration of phosphocofilin, which was not accompanied by stress fiber formation. These results suggest that restoration of cofilin phosphorylation in Ras-transformed cells is necessary but not sufficient for stress fiber formation. Our findings define a novel mechanism for coupling cytoplasmic p21(Cip1) to the control of actin polymerization by compromising the Rho/ROCK/LIMK/cofilin pathway by oncogenic Ras. These studies suggest that localization of p21(Cip1) to the cytoplasm in transformed cells contributes to pathways that favor not only cell proliferation, but also cell motility thereby contributing to invasion and **metastasis**.

### [Cc]ell [Ii]nvasion

#### PubMed

1. ROCK-II mediates colon cancer invasion via regulation of MMP-2 and MMP-13 at the site of invadopodia as revealed by multiphoton imaging. The ROCK-II isoform of Rho's downstream effector, Rho kinase, has been linked with greater invasion and metastasis in solid tumors. We have previously shown that ROCK-II is overexpressed at the advancing edge of colon cancers. The mechanism whereby ROCK-II contributes invasion, particularly in the setting of colon cancer, remains to be elucidated fully. To better understand its contribution, we evaluated ROCK-II expression in both non-malignant (NCM460 and IEC-6) and malignant (Caco-2 E, SW620, and HCT-116) intestinal epithelial cell lines grown in type I collagen scaffolds. Using multiphoton microscopy, we observed that ROCK-II localized to the actin cytoskeleton in non-malignant cells but localized to the cell periphery as focal collections with an absence of adjacent collagen in all colon cancer cell lines. By transmission electron microscopy, these collections corresponded with finger-like projections previously described as invadopodia. Immunogold staining with cortactin, matrix metalloprotease (MMP)-2, -9, and -13 confirmed that these were indeed invadopodia. To further link ROCK-II to colon cancer invasion, we treated non-malignant and malignant intestinal epithelial cell lines with ROCK-II siRNA and evaluated depth of invasion, proliferation, and MMP-2, -9, and -13 activities. The most striking effect was seen in the highly tumorigenic cell lines, SW620 and HCT-116, wherein ROCK-II knockdown resulted in a two-fold or more reduction in invasion. This reduction in invasion was not due to a decrease in cell proliferation, as a significant reduction in proliferation was only observed in the two non-malignant intestinal cell lines. Finally, both MMP-2 and -13 activities were significantly decreased in all colon cancer cell lines. Taken together, these data suggest for the first time that ROCK-II is a critical mediator of colon cancer **cell invasion** through its modulation of MMP-2 and -13 at the site of invadopodia but regulates proliferation in non-malignant intestinal cells.
2. Rho-GTPase effector ROCK phosphorylates cofilin in actin-meditated cytokinesis during mouse oocyte meiosis. During oocyte meiosis, a spindle forms in the central cytoplasm and migrates to the cortex. Subsequently, the oocyte extrudes a small body and forms a highly polarized egg; this process is regulated primarily by actin. ROCK is a Rho-GTPase effector that is involved in various cellular functions, such as stress fiber formation, cell migration, tumor **cell invasion**, and cell motility. In this study, we investigated possible roles for ROCK in mouse oocyte meiosis. ROCK was localized around spindles after germinal vesicle breakdown and was colocalized with cytoplasmic actin and mitochondria. Disrupting ROCK activity by RNAi or an inhibitor resulted in cell cycle progression and polar body extrusion failure. Time-lapse microscopy showed that this may have been due to spindle migration and cytokinesis defects, as chromosomes segregated but failed to extrude a polar body and then realigned. Actin expression at oocyte membranes and in cytoplasm was significantly decreased after these treatments. Actin caps were also disrupted, which was confirmed by a failure to form cortical granule-free domains. The mitochondrial distribution was also disrupted, which indicated that mitochondria were involved in the ROCK-mediated actin assembly. In addition, the phosphorylation levels of Cofilin, a downstream molecule of ROCK, decreased after disrupting ROCK activity. Thus, our results indicated that a ROCK-Cofilin-actin pathway regulated meiotic spindle migration and cytokinesis during mouse oocyte maturation.
3. ROCK inhibition prevents early mouse embryo development. ROCK is a Rho-GTPase effector that is important for actin assembly and is involved in various cellular functions, including cell contraction, migration, motility, and tumor **cell invasion**. In this study, we investigated ROCK expression and function during early mouse embryo development. Inhibiting ROCK by Y-27632 treatment at the zygote stage resulted in first cleavage failure, and most embryos failed to develop to the 8-cell stage. When adding Y-27632 at the 8-cell stage, embryos failed to undergo compaction and could not develop into blastocysts. In addition, fluorescence staining intensity analysis indicated that actin expression at blastomere membranes was significantly reduced. After ROCK inhibition, two or more nuclei were observed in a cell, which indicated possible cytokinesis failure. Moreover, after ROCK inhibition with Y-27632, the phosphorylation levels of LIMK1/2, a downstream molecule of ROCK, were decreased at blastomere membranes. Thus, our results showed conserved roles for ROCK in this mammalian embryo model and indicated that a ROCK-LIMK1/2-actin pathway might regulate cleavage and blastocyst formation during early mouse embryo development.

#### GeneRIFs

1. ROCK-II is a critical mediator of colon cancer **cell invasion** through its modulation of MMP-2 and -13 at the site of invadopodia but regulates proliferation in non-malignant intestinal cells.

---

## Capza1

- NCBI Gene
- GeneRIF
- Pubmed

| Search Term | Rank | Fields |
| --- | --- | --- |
| [Mm]igration | 6 | PubMed(2) |

  
hide/show details for Capza1

### [Mm]igration

#### PubMed

1. Actin-capping protein promotes microtubule stability by antagonizing the actin activity of mDia1. In migrating fibroblasts, RhoA and its effector mDia1 regulate the selective stabilization of microtubules (MTs) polarized in the direction of **migration**. The conserved formin homology 2 domain of mDia1 is involved both in actin polymerization and MT stabilization, and the relationship between these two activities is unknown. We found that latrunculin A (LatA) and jasplakinolide, actin drugs that release mDia1 from actin filament barbed ends, stimulated stable MT formation in serum-starved fibroblasts and caused a redistribution of mDia1 onto MTs. Knockdown of mDia1 by small interfering RNA (siRNA) prevented stable MT induction by LatA, whereas blocking upstream Rho or integrin signaling had no effect. In search of physiological regulators of mDia1, we found that actin-capping protein induced stable MTs in an mDia1-dependent manner and inhibited the translocation of mDia on the ends of growing actin filaments. Knockdown of capping protein by siRNA reduced stable MT levels in proliferating cells and in starved cells stimulated with lysophosphatidic acid. These results show that actin-capping protein is a novel regulator of MT stability that functions by antagonizing mDia1 activity toward actin filaments and suggest a novel form of actin-MT cross-talk in which a single factor acts sequentially on actin and MTs.
2. Mammalian CARMIL inhibits actin filament capping by capping protein. Actin polymerization in cells occurs via filament elongation at the barbed end. Proteins that cap the barbed end terminate this elongation. Heterodimeric capping protein (CP) is an abundant and ubiquitous protein that caps the barbed end. We find that the mouse homolog of the adaptor protein CARMIL (mCARMIL) binds CP with high affinity and decreases its affinity for the barbed end. Addition of mCARMIL to cell extracts increases the rate and extent of Arp2/3 or spectrin-actin seed-induced polymerization. In cells, GFP-mCARMIL concentrates in lamellipodia and increases the fraction of cells with large lamellipodia. Decreasing mCARMIL levels by siRNA transfection lowers the F-actin level and slows cell **migration** through a mechanism that includes decreased lamellipodia protrusion. This phenotype is reversed by full-length mCARMIL but not mCARMIL lacking the domain that binds CP. Thus, mCARMIL is a key regulator of CP and has profound effects on cell behavior.

---

## Capza2

- NCBI Gene
- GeneRIF
- Pubmed

| Search Term | Rank | Fields |
| --- | --- | --- |
| [Ff]ilopodia | 1 | PubMed(1) |

  
hide/show details for Capza2

### [Ff]ilopodia

#### PubMed

1. Capzb2 interacts with beta-tubulin to regulate growth cone morphology and neurite outgrowth. Capping protein (CP) is a heterodimer that regulates actin assembly by binding to the barbed end of F-actin. In cultured nonneuronal cells, each CP subunit plays a critical role in the organization and dynamics of lamellipodia and **filopodia**. Mutations in either alpha or beta CP subunit result in retinal degeneration in Drosophila. However, the function of CP subunits in mammalian neurons remains unclear. Here, we investigate the role of the beta CP subunit expressed in the brain, Capzb2, in growth cone morphology and neurite outgrowth. We found that silencing Capzb2 in hippocampal neurons resulted in short neurites and misshapen growth cones in which microtubules overgrew into the periphery and completely overlapped with F-actin. In searching for the mechanisms underlying these cytoskeletal abnormalities, we identified beta-tubulin as a novel binding partner of Capzb2 and demonstrated that Capzb2 decreases the rate and the extent of tubulin polymerization in vitro. We mapped the region of Capzb2 that was required for the subunit to interact with beta-tubulin and inhibit microtubule polymerization. A mutant Capzb2 lacking this region was able to bind F-actin and form a CP heterodimer with alpha2-subunit. However, this mutant was unable to rescue the growth cone and neurite outgrowth phenotypes caused by Capzb2 knockdown. Together, these data suggest that Capzb2 plays an important role in growth cone formation and neurite outgrowth and that the underlying mechanism may involve direct interaction between Capzb2 and microtubules.

---

## Hmgcr

- NCBI Gene
- GeneRIF
- Pubmed

| Search Term | Rank | Fields |
| --- | --- | --- |
| [Mm]igration | 6 | PubMed(3); GeneRIFs(1) |

  
hide/show details for Hmgcr

### [Mm]igration

#### PubMed

1. Inhibition of HMG CoA reductase reveals an unexpected role for cholesterol during PGC **migration** in the mouse. Primordial germ cells (PGCs) are the embryonic precursors of the sperm and eggs. Environmental or genetic defects that alter PGC development can impair fertility or cause formation of germ cell tumors.We demonstrate a novel role for cholesterol during germ cell **migration** in mice. Cholesterol was measured in living tissue dissected from mouse embryos and was found to accumulate within the developing gonads as germ cells migrate to colonize these structures. Cholesterol synthesis was blocked in culture by inhibiting the activity of HMG CoA reductase (HMGCR) resulting in germ cell survival and **migration** defects. These defects were rescued by co-addition of isoprenoids and cholesterol, but neither compound alone was sufficient. In contrast, loss of the last or penultimate enzyme in cholesterol biosynthesis did not alter PGC numbers or position in vivo. However embryos that lack these enzymes do not exhibit cholesterol defects at the stage at which PGCs are migrating. This demonstrates that during gestation, the cholesterol required for PGC **migration** can be supplied maternally.In the mouse, cholesterol is required for PGC survival and motility. It may act cell-autonomously by regulating clustering of growth factor receptors within PGCs or non cell-autonomously by controlling release of growth factors required for PGC guidance and survival.
2. Modified methylenedioxyphenol analogs lower LDL cholesterol through induction of LDL receptor expression. Although statin therapy is a cornerstone of current low density lipoprotein (LDL)-lowering strategies, there is a need for additional therapies to incrementally lower plasma LDL cholesterol. In this study, we investigated the effect of several methylenedioxyphenol derivatives in regulating LDL cholesterol through induction of LDL receptor (LDLR). INV-403, a modified methylenedioxyphenol derivative, increased LDLR mRNA and protein expression in HepG2 cells in a dose- and time-dependent fashion. These effects were apparent even under conditions of HMG-CoA reductase inhibition. Electrophoresis **migration** shift assays demonstrated that INV-403 activates SREBP2 but not SREBP1c, with immunoblot analysis showing an increased expression of the mature form of SREBP2. Knockdown of SREBP2 reduced the effect of INV-403 on LDLR expression. The activation of SREBP2 by INV-403 is partly mediated by Akt/GSK3β pathways through inhibition of phosphorylation-dependent degradation by ubiquitin-proteosome pathway. Treatment of C57Bl/6j mice with INV-403 for two weeks increased hepatic SREBP2 levels (mature form) and upregulated LDLR with concomitant lowering of plasma LDL levels. Transient expression of a LDLR promoter-reporter construct, a SRE-mutant LDLR promoter construct, and a SRE-only construct in HepG2 cells revealed an effect predominantly through a SRE-dependent mechanism. INV-403 lowered plasma LDL cholesterol levels through LDLR upregulation. These results indicate a role for small molecule approaches other than statins for lowering LDL cholesterol.
3. Analysis of hedgehog signaling in cerebellar granule cell precursors in a conditional Nsdhl allele demonstrates an essential role for cholesterol in postnatal CNS development. NSDHL is a 3β-hydroxysterol dehydrogenase that is involved in the removal of two C-4 methyl groups in one of the later steps of cholesterol biosynthesis. Mutations in the gene encoding the enzyme are responsible for the X-linked, male lethal mouse mutations bare patches and striated, as well as most cases of human CHILD syndrome. Rare, hypomorphic NSDHL mutations are also associated with X-linked intellectual disability in males with CK syndrome. Since hemizygous male mice with Nsdhl mutations die by midgestation, we generated a conditional targeted Nsdhl mutation (Nsdhl(tm1.1Hrm)) to investigate the essential role of cholesterol in the early postnatal CNS. Ablation of Nsdhl in radial glia using GFAP-cre resulted in live-born, normal appearing affected male pups. However, the pups develop overt ataxia by postnatal day 8-10 and die shortly thereafter. Histological abnormalities include progressive loss of cortical and hippocampal neurons, as well as deficits in the proliferation and **migratio**n of cerebellar granule precursors and subsequent massive apoptosis of the cerebellar cortex. We replicated the granule cell precursor proliferation defect in vitro and demonstrate that it results from defective signaling by SHH. Furthermore, this defect is almost completely rescued by supplementation of the culture media with exogenous cholesterol, while methylsterol accumulation above the enzymatic block appears to be associated with increased cell death. These data support the absolute requirement for cholesterol synthesis in situ once the blood-brain-barrier forms and cholesterol transport to the fetus is abolished. They further emphasize the complex ramifications of cholesterogenic enzyme deficiency on cellular metabolism.

#### GeneRIFs

1. Cholesterol synthesis was blocked in culture by inhibiting the activity of HMG CoA reductase (HMGCR) resulting in germ cell survival and **migration** defects

---

## Cap1

- NCBI Gene
- GeneRIF
- Pubmed

| Search Term | Rank | Fields |
| --- | --- | --- |
| [Mm]igration | 6 | Process(1); PubMed(2) |
| [Ee]xtracellular [Mm]atrix | 8 | PubMed(1) |

  
hide/show details for Cap1

### [Mm]igration

#### Process

1. ameboidal-type cell **migration**

#### PubMed

1. COUP-TFI is required for the formation of commissural projections in the forebrain by regulating axonal growth. The transcription factor COUP-TFI (NR2F1), an orphan member of the nuclear receptor superfamily, is an important regulator of neurogenesis, cellular differentiation and cell **migration**. In the forebrain, COUP-TFI controls the connectivity between thalamus and cortex and neuronal tangential **migration** in the basal telencephalon. Here, we show that COUP-TFI is required for proper axonal growth and guidance of all major forebrain commissures. Fibres of the corpus callosum, the hippocampal commissure and the anterior commissure project aberrantly and fail to cross the midline in COUP-TFI null mutants. Moreover, hippocampal neurons lacking COUP-TFI have a defect in neurite outgrowth and show an abnormal axonal morphology. To search for downstream effectors, we used microarray analysis and showed that, in the absence of COUP-TFI, expression of various cytoskeleton molecules involved in neuronal morphogenesis is affected. Diminished protein levels of the microtubule-associated protein MAP1B and increased levels of the GTP-binding protein RND2 were confirmed in the developing cortex in vivo and in primary hippocampal neurons in vitro. Therefore, based on morphological studies, gene expression profiling and primary cultured neurons, the present data uncover a previously unappreciated intrinsic role for COUP-TFI in axonal growth in vivo and supply one of the premises for COUP-TFI coordination of neuronal morphogenesis in the developing forebrain.
2. Cyclase-associated protein 1 (CAP1) promotes cofilin-induced actin dynamics in mammalian nonmuscle cells. Cyclase-associated proteins (CAPs) are highly conserved actin monomer binding proteins present in all eukaryotes. However, the mechanism by which CAPs contribute to actin dynamics has been elusive. In mammals, the situation is further complicated by the presence of two CAP isoforms whose differences have not been characterized. Here, we show that CAP1 is widely expressed in mouse nonmuscle cells, whereas CAP2 is the predominant isoform in developing striated muscles. In cultured NIH3T3 and B16F1 cells, CAP1 is a highly abundant protein that colocalizes with cofilin-1 to dynamic regions of the cortical actin cytoskeleton. Analysis of CAP1 knockdown cells demonstrated that this protein promotes rapid actin filament depolymerization and is important for cell morphology, **migration**, and endocytosis. Interestingly, depletion of CAP1 leads to an accumulation of cofilin-1 into abnormal cytoplasmic aggregates and to similar cytoskeletal defects to those seen in cofilin-1 knockdown cells, demonstrating that CAP1 is required for proper subcellular localization and function of ADF/cofilin. Together, these data provide the first direct in vivo evidence that CAP promotes rapid actin dynamics in conjunction with ADF/cofilin and is required for several central cellular processes in mammals.

### [Ee]xtracellular [Mm]atrix

#### PubMed

1. **Extracellular matrix** secretion by cardiac fibroblasts: role of microRNA-29b and microRNA-30c. MicroRNAs (miRNAs), in particular miR-29b and miR-30c, have been implicated as important regulators of cardiac fibrosis.To perform a proteomics comparison of miRNA effects on **extracellular matrix** secretion by cardiac fibroblasts.Mouse cardiac fibroblasts were transfected with pre-/anti-miR of miR-29b and miR-30c, and their conditioned medium was analyzed by mass spectrometry. miR-29b targeted a cadre of proteins involved in fibrosis, including multiple collagens, matrix metalloproteinases, and leukemia inhibitory factor, insulin-like growth factor 1, and pentraxin 3, 3 predicted targets of miR-29b. miR-29b also attenuated the cardiac fibroblast response to transforming growth factor-β. In contrast, miR-30c had little effect on **extracellular matri**x production but opposite effects regarding leukemia inhibitory factor and insulin-like growth factor 1. Both miRNAs indirectly affected cardiac myocytes. On transfection with pre-miR-29b, the conditioned medium of cardiac fibroblasts lost its ability to support adhesion of rat ventricular myocytes and led to a significant reduction of cardiac myocyte proteins (α-actinin, cardiac myosin-binding protein C, and cardiac troponin I). Similarly, cardiomyocytes derived from mouse embryonic stem cells atrophied under pre-miR-29 conditioned medium, whereas pre-miR-30c conditioned medium had a prohypertrophic effect. Levels of miR-29a, miR-29c, and miR-30c, but not miR-29b, were significantly reduced in a mouse model of pathological but not physiological hypertrophy. Treatment with antagomiRs to miR-29b induced excess fibrosis after aortic constriction without overt deterioration in cardiac function.Our proteomic analysis revealed novel molecular targets of miRNAs that are linked to a fibrogenic cardiac phenotype. Such comprehensive screening methods are essential to define the concerted actions of miRNAs in cardiovascular disease.

---

## Glb1l

- NCBI Gene
- GeneRIF
- Pubmed

| Search Term | Rank | Fields |
| --- | --- | --- |
| [Ee]xtracellular [Mm]atrix | 8 | PubMed(1) |

  
hide/show details for Glb1l

### [Ee]xtracellular [Mm]atrix

#### PubMed

1. **Extracellular matrix** secretion by cardiac fibroblasts: role of microRNA-29b and microRNA-30c. MicroRNAs (miRNAs), in particular miR-29b and miR-30c, have been implicated as important regulators of cardiac fibrosis.To perform a proteomics comparison of miRNA effects on **extracellular matrix** secretion by cardiac fibroblasts.Mouse cardiac fibroblasts were transfected with pre-/anti-miR of miR-29b and miR-30c, and their conditioned medium was analyzed by mass spectrometry. miR-29b targeted a cadre of proteins involved in fibrosis, including multiple collagens, matrix metalloproteinases, and leukemia inhibitory factor, insulin-like growth factor 1, and pentraxin 3, 3 predicted targets of miR-29b. miR-29b also attenuated the cardiac fibroblast response to transforming growth factor-β. In contrast, miR-30c had little effect on **extracellular matri**x production but opposite effects regarding leukemia inhibitory factor and insulin-like growth factor 1. Both miRNAs indirectly affected cardiac myocytes. On transfection with pre-miR-29b, the conditioned medium of cardiac fibroblasts lost its ability to support adhesion of rat ventricular myocytes and led to a significant reduction of cardiac myocyte proteins (α-actinin, cardiac myosin-binding protein C, and cardiac troponin I). Similarly, cardiomyocytes derived from mouse embryonic stem cells atrophied under pre-miR-29 conditioned medium, whereas pre-miR-30c conditioned medium had a prohypertrophic effect. Levels of miR-29a, miR-29c, and miR-30c, but not miR-29b, were significantly reduced in a mouse model of pathological but not physiological hypertrophy. Treatment with antagomiRs to miR-29b induced excess fibrosis after aortic constriction without overt deterioration in cardiac function.Our proteomic analysis revealed novel molecular targets of miRNAs that are linked to a fibrogenic cardiac phenotype. Such comprehensive screening methods are essential to define the concerted actions of miRNAs in cardiovascular disease.

---

## Glb1

- NCBI Gene
- GeneRIF
- Pubmed

| Search Term | Rank | Fields |
| --- | --- | --- |
| [Mm]igration | 6 | PubMed(1) |

  
hide/show details for Glb1

### [Mm]igration

#### PubMed

1. Molecular heterogeneity of progenitors and radial **migration** in the developing cerebral cortex revealed by transgene expression. We have analyzed the developmental pattern of beta-galactosidase (beta-gal) expression in the cerebral cortex of the beta 2nZ3'1 transgenic mouse line, which was generated using regulatory elements of the beta 2-microglobulin gene and shows ectopic expression in nervous tissue. From embryonic day 10 onward, beta-gal was expressed in the medial and dorsal cortices, including the hippocampal region, whereas lateral cortical areas were devoid of labeling. During the period of cortical neurogenesis (embryonic days 11-17), beta-gal was expressed by selective precursors in the proliferative ventricular zone of the neocortex and hippocampus, as well as by a number of migrating and postmigratory neurons arranged into narrow radial stripes above the labeled progenitors. Thus, the transgene labels a subset of cortical progenitors and their progeny. Postnatally, radial clusters of beta-gal-positive neurons were discernible until postpartum day 10. At this age, the clusters were 250 to 500 microns wide, composed of neurons spanning all the cortical layers and exhibiting several neuronal phenotypes. These data suggest molecular heterogeneity of cortical progenitors and of the cohorts of postmitotic neurons originating from them, which implies intrinsic molecular mosaicism in both cortical progenitors and developing neurons. Furthermore, the data show that neurons committed to the expression of the transgene migrate along very narrow, radial stripes.

---

## Hmgcs2

- NCBI Gene
- GeneRIF
- Pubmed

| Search Term | Rank | Fields |
| --- | --- | --- |
| [Mm]igration | 6 | PubMed(2) |

  
hide/show details for Hmgcs2

### [Mm]igration

#### PubMed

1. Abnormal Wnt and PI3Kinase signaling in the malformed intestine of lama5 deficient mice. Laminins are major constituents of basement membranes and are essential for tissue homeostasis. Laminin-511 is highly expressed in the intestine and its absence causes severe malformation of the intestine and embryonic lethality. To understand the mechanistic role of laminin-511 in tissue homeostasis, we used RNA profiling of embryonic intestinal tissue of lama5 knockout mice and identified a lama5 specific gene expression signature. By combining cell culture experiments with mediated knockdown approaches, we provide a mechanistic link between laminin α5 gene deficiency and the physiological phenotype. We show that laminin α5 plays a crucial role in both epithelial and mesenchymal cell behavior by inhibiting Wnt and activating PI3K signaling. We conclude that conflicting signals are elicited in the absence of lama5, which alter cell adhesion**, migrati**on as well as epithelial and muscle differentiation. Conversely, adhesion to laminin-511 may serve as a potent regulator of known interconnected PI3K/Akt and Wnt signaling pathways. Thus deregulated adhesion to laminin-511 may be instrumental in diseases such as human pathologies of the gut where laminin-511 is abnormally expressed as it is shown here.
2. Relationships between hematopoiesis and hepatogenesis in the midtrimester fetal liver characterized by dynamic transcriptomic and proteomic profiles. In fetal hematopoietic organs, the switch from hematopoiesis is hypothesized to be a critical time point for organogenesis, but it is not yet evidenced. The transient coexistence of hematopoiesis will be useful to understand the development of fetal liver (FL) around this time and its relationship to hematopoiesis. Here, the temporal and the comparative transcriptomic and proteomic profiles were observed during the critical time points corresponding to the initiation (E11.5), peak (E14.5), recession (E15.5), and disappearance (3 ddp) of mouse FL hematopoiesis. We found that E11.5-E14.5 corresponds to a FL hematopoietic expansion phase with distinct molecular features, including the expression of new transcription factors, many of which are novel KRAB (Kruppel-associated box)-containing zinc finger proteins. This time period is also characterized by extensive depression of some liver functions, especially catabolism/utilization, immune and defense, classical complement cascades, and intrinsic blood coagulation. Instead, the other liver functions increased, such as xenobiotic and sterol metabolism, synthesis of carbohydrate and glycan, the alternate and lectin complement cascades and extrinsic blood coagulation, and etc. Strikingly, all of the liver functions were significantly increased at E14.5-E15.5 and thereafter, and the depression of the key pathways attributes to build the hematopoietic microenvironment. These findings signal hematopoiesis e**migration** is the key to open the door of liver maturation.

---

## Tln1

- NCBI Gene
- GeneRIF
- Pubmed

| Search Term | Rank | Fields |
| --- | --- | --- |
| [Ff]ilopodia | 1 | PubMed(1) |
| [Pp]odosome(s)? | 5 | PubMed(1) |
| [Mm]igration | 6 | PubMed(25); GeneRIFs(2) |
| ECM | 7 | PubMed(5) |
| [Ee]xtracellular [Mm]atrix | 8 | PubMed(13); GeneRIFs(1) |
| [Mm]etastasis | 12 | PubMed(3) |

  
hide/show details for Tln1

### [Ff]ilopodia

#### PubMed

1. Mechanisms and consequences of agonist-induced talin recruitment to platelet integrin alphaIIbbeta3. Platelet aggregation requires agonist-induced alphaIIbbeta3 activation, a process mediated by Rap1 and talin. To study mechanisms, we engineered alphaIIbbeta3 Chinese hamster ovary (CHO) cells to conditionally express talin and protease-activated receptor (PAR) thrombin receptors. Human PAR1 or murine PAR4 stimulation activates alphaIIbbeta3, which was measured with antibody PAC-1, indicating complete pathway reconstitution. Knockdown of Rap1-guanosine triphosphate-interacting adaptor molecule (RIAM), a Rap1 effector, blocks this response. In living cells, RIAM overexpression stimulates and RIAM knockdown blocks talin recruitment to alphaIIbbeta3, which is monitored by bimolecular fluorescence complementation. Mutations in talin or beta3 that disrupt their mutual interaction block both talin recruitment and alphaIIbbeta3 activation. However, one talin mutant (L325R) is recruited to alphaIIbbeta3 but cannot activate it. In platelets, RIAM localizes to **filopodia** and lamellipodia, and, in megakaryocytes, RIAM knockdown blocks PAR4-mediated alphaIIbbeta3 activation. The RIAM-related protein lamellipodin promotes talin recruitment and alphaIIbbeta3 activity in CHO cells but is not expressed in megakaryocytes or platelets. Thus, talin recruitment to alphaIIbbeta3 by RIAM mediates agonist-induced alphaIIbbeta3 activation, with implications for hemostasis and thrombosis.

### [Pp]odosome(s)?

#### PubMed

1. New isoform-specific monoclonal antibodies reveal different sub-cellular localisations for talin1 and talin2. Talins are adaptor proteins that connect the integrin family of cell adhesion receptors to cytoskeletal actin. Vertebrates express two closely related talins encoded by separate genes, and while it is well established that talin1 plays a key role in cell adhesion and spreading, little is known about the role of talin2. To facilitate such studies, we report the characterisation of 4 new isoform-specific talin mouse monoclonal antibodies that work in Western blotting, immuno-precipitation, immuno-fluorescence and immuno-histochemistry. Using these antibodies, we show that talin1 and talin2 do not form heterodimers, and that they are differentially localised within the cell. Talin1 was concentrated in peripheral focal adhesions while talin2 was observed in both focal and fibrillar adhesions, and knock-down of talin2 compromised fibronectin fibrillogenesis. Although differentiated human macrophages express both isoforms, only talin1 showed discrete staining and was localised to the ring structure of **podosomes**. However, siRNA-mediated knock-down of macrophage talin2 led to a significant reduction in podosomal matrix degradation. We have also used the antibodies to localise each isoform in tissue sections using both cryostat and paraffin-embedded material. In skeletal muscle talin2 was localised to both myotendinous junctions and costameres while talin1 was restricted to the former structure. In contrast, both isoforms co-localised in kidney with staining of the glomerulus, and the tubular epithelial and interstitial cells of the cortex and medulla. We anticipate that these antibodies will form a valuable resource for future studies on the function of the two major talin isoforms.

### [Mm]igration

#### PubMed

1. Distinct roles of talin and kindlin in regulating integrin α5β1 function and trafficking. Integrins are heterodimeric αβ transmembrane receptors that play key roles in cellular physiology and pathology. Accumulating data indicate that the two NPxY motifs in the cytoplasmic domain of the β1 subunit synergistically promote integrin activation through the binding of talin and kindlin. However, it is unclear how the individual motifs regulate integrin function and trafficking.To investigate how the two NPxY motifs individually control integrin α5β1 function and trafficking, we introduced Y > A mutations in either motif. Disruption of the membrane-proximal NPxY completely prevented α5β1-induced morphological changes, cell scatte**ring and** migration, and fibronectin fibrillogenesis. In addition, it reduced α5β1 internalization but not its recycling. In contrast, disruption of the membrane-distal NPxY promoted degradation of α5β1 in late endosomes/lysosomes but did not prevent α5β1-dependent ce**ll scatte**ring, migration, or fibronectin fibrillogenesis. Whereas depletion of either talin-1 or kindlin-2 reduced α5β1 binding to fibronectin and cell adhesion, talin-1 depletion recapitulated the loss-of-function phenotype of the membrane-proximal NPxY mutation, whereas kindlin-2 depletion induced α5β1 accumulation in lysosomes and degradation.The two NPxY motifs of β1 play distinct and separable roles in controlling the function and trafficking of α5β1. Whereas talin binding to the membrane-proximal NPxY is crucial for connecting α5β1 to the actin cytoskeleton and thus permit the tension required for fibronectin f**ibrilloge**nesis and cell migration, kindlin binding to the membrane-distal NPxY is dispensable for these events but regulates α5β1 surface expression and degradation.
2. Cell adhesion strengthening: contributions of adhesive area, integrin binding, and focal adhesion assembly. Mechanical interactions between a cell and its environment regulate **migration**, contractility, gene expression, and cell fate. We integrated micropatterned substrates to engineer adhesive area and a hydrodynamic assay to analyze fibroblast adhesion strengthening on fibronectin. Independently of cell spreading, integrin binding and focal adhesion assembly resulted in rapid sevenfold increases in adhesion strength to steady-state levels. Adhesive area strongly modulated adhesion strength, integrin binding, and vinculin and talin recruitment, exhibiting linear increases for small areas. However, above a threshold area, adhesion strength and focal adhesion assembly reached a saturation limit, whereas integrin binding transitioned from a uniform distribution to discrete complexes. Adhesion strength exhibited exponential increases with bound integrin numbers as well as vinculin and talin recruitment, and the relationship between adhesion strength and these biochemical events was accurately described by a simple mechanical model. Furthermore, adhesion strength was regulated by the position of an adhesive patch, comprised of bound integrins and cytoskeletal elements, which generated a constant 200-nN adhesive force. Unexpectedly, focal adhesion assembly, in particular vinculin recruitment, contributed only 30% of the adhesion strength. This work elucidates the roles of adhesive complex size and position in the generation of cell-extracellular matrix forces.
3. Vinculin controls talin engagement with the actomyosin machinery. The link between extracellular-matrix-bound integrins and intracellular F-actin is essential for cell spreading and **migration**. Here, we demonstrate how the actin-binding proteins talin and vinculin cooperate to provide this link. By expressing structure-based talin mutants in talin null cells, we show that while the C-terminal actin-binding site (ABS3) in talin is required for adhesion complex assembly, the central ABS2 is essential for focal adhesion (FA) maturation. Thus, although ABS2 mutants support cell spreading, the cells lack FAs, fail to polarize and exert reduced force on the surrounding matrix. ABS2 is inhibited by the preceding mechanosensitive vinculin-binding R3 domain, and deletion of R2R3 or expression of constitutively active vinculin generates stable force-independent FAs, although cell polarity is compromised. Our data suggest a model whereby force acting on integrin-talin complexes via ABS3 promotes R3 unfolding and vinculin binding, activating ABS2 and locking talin into an actin-binding configuration that stabilizes FAs.
4. Mice carrying a complete deletion of the talin2 coding sequence are viable and fertile. Mice homozygous for several Tln2 gene targeted alleles are viable and fertile. Here we show that although the expression of talin2 protein is drastically reduced in muscle from these mice, other tissues continue to express talin2 albeit at reduced levels. We therefore generated a Tln2 allele lacking the entire coding sequence (Tln2(cd)). Tln2(cd/cd) mice were viable and fertile, and the genotypes of Tln2(cd/+) intercrosses were at the expected Mendelian ratio. Tln2(cd/cd) mice showed no major difference in body mass or the weight of the major organs compared to wild-type, although they displayed a mildly dystrophic phenotype. Moreover, Tln2(cd/cd) mouse embryo fibroblasts showed no obvious defects in cell adhesion, **migration** or proliferation. However, the number of Tln2(cd/cd) pups surviving to adulthood was variable suggesting that such mice have an underlying defect.
5. Neutrophil swarms require LTB4 and integrins at sites of cell death in vivo. Neutrophil recruitment from blood to extravascular sites of sterile or infectious tissue damage is a hallmark of early innate immune responses, and the molecular events leading to cell exit from the bloodstream have been well defined. Once outside the vessel, individual neutrophils often show extremely coordinated chemotaxis and cluster formation reminiscent of the swarming behaviour of insects. The molecular players that direct this response at the single-cell and population levels within the complexity of an inflamed tissue are unknown. Using two-photon intravital microscopy in mouse models of sterile injury and infection, we show a critical role for intercellular signal relay among neutrophils mediated by the lipid leukotriene B4, which acutely amplifies local cell death signals to enhance the radius of highly directed interstitial neutrophil recruitment. Integrin receptors are dispensable for long-distance **migration**, but have a previously unappreciated role in maintaining dense cellular clusters when congregating neutrophils rearrange the collagenous fibre network of the dermis to form a collagen-free zone at the wound centre. In this newly formed environment, integrins, in concert with neutrophil-derived leukotriene B4 and other chemoattractants, promote local neutrophil interaction while forming a tight wound seal. This wound seal has borders that cease to grow in kinetic concert with late recruitment of monocytes and macrophages at the edge of the displaced collagen fibres. Together, these data provide an initial molecular map of the factors that contribute to neutrophil swarming in the extravascular space of a damaged tissue. They reveal how local events are propagated over large-range distances, and how auto-signalling produces coordinated, self-organized neutrophil-swarming behaviour that isolates the wound or infectious site from surrounding viable tissue.
6. Rapid leukocyte **migration** by integrin-independent flowing and squeezing. All metazoan cells carry transmembrane receptors of the integrin family, which couple the contractile force of the actomyosin cytoskeleton to the extracellular environment. In agreement with this principle, rapidly migrating leukocytes use integrin-mediated adhesion when moving over two-dimensional surfaces. As **migration** on two-dimensional substrates naturally overemphasizes the role of adhesion, the contribution of integrins during three-dimensional movement of leukocytes within tissues has remained controversial. We studied the interplay between adhesive, contractile and protrusive forces during interstitial leukocyte chemotaxis in vivo and in vitro. We ablated all integrin heterodimers from murine leukocytes, and show here that functional integrins do not contribute to **migration** in three-dimensional environments. Instead, these cells migrate by the sole force of actin-network expansion, which promotes protrusive flowing of the leading edge. Myosin II-dependent contraction is only required on passage through narrow gaps, where a squeezing contraction of the trailing edge propels the rigid nucleus.
7. Vinculin controls talin engagement with the actomyosin machinery. The link between extracellular-matrix-bound integrins and intracellular F-actin is essential for cell spreading and **migration**. Here, we demonstrate how the actin-binding proteins talin and vinculin cooperate to provide this link. By expressing structure-based talin mutants in talin null cells, we show that while the C-terminal actin-binding site (ABS3) in talin is required for adhesion complex assembly, the central ABS2 is essential for focal adhesion (FA) maturation. Thus, although ABS2 mutants support cell spreading, the cells lack FAs, fail to polarize and exert reduced force on the surrounding matrix. ABS2 is inhibited by the preceding mechanosensitive vinculin-binding R3 domain, and deletion of R2R3 or expression of constitutively active vinculin generates stable force-independent FAs, although cell polarity is compromised. Our data suggest a model whereby force acting on integrin-talin complexes via ABS3 promotes R3 unfolding and vinculin binding, activating ABS2 and locking talin into an actin-binding configuration that stabilizes FAs.
8. Distinct roles of talin and kindlin in regulating integrin α5β1 function and trafficking. Integrins are heterodimeric αβ transmembrane receptors that play key roles in cellular physiology and pathology. Accumulating data indicate that the two NPxY motifs in the cytoplasmic domain of the β1 subunit synergistically promote integrin activation through the binding of talin and kindlin. However, it is unclear how the individual motifs regulate integrin function and trafficking.To investigate how the two NPxY motifs individually control integrin α5β1 function and trafficking, we introduced Y > A mutations in either motif. Disruption of the membrane-proximal NPxY completely prevented α5β1-induced morphological changes, cell scatte**ring and** migration, and fibronectin fibrillogenesis. In addition, it reduced α5β1 internalization but not its recycling. In contrast, disruption of the membrane-distal NPxY promoted degradation of α5β1 in late endosomes/lysosomes but did not prevent α5β1-dependent ce**ll scatte**ring, migration, or fibronectin fibrillogenesis. Whereas depletion of either talin-1 or kindlin-2 reduced α5β1 binding to fibronectin and cell adhesion, talin-1 depletion recapitulated the loss-of-function phenotype of the membrane-proximal NPxY mutation, whereas kindlin-2 depletion induced α5β1 accumulation in lysosomes and degradation.The two NPxY motifs of β1 play distinct and separable roles in controlling the function and trafficking of α5β1. Whereas talin binding to the membrane-proximal NPxY is crucial for connecting α5β1 to the actin cytoskeleton and thus permit the tension required for fibronectin f**ibrilloge**nesis and cell migration, kindlin binding to the membrane-distal NPxY is dispensable for these events but regulates α5β1 surface expression and degradation.
9. Arginylation-dependent neural crest cell **migration** is essential for mouse development. Coordinated cell **migration** during development is crucial for morphogenesis and largely relies on cells of the neural crest lineage that migrate over long distances to give rise to organs and tissues throughout the body. Recent studies of protein arginylation implicated this poorly understood posttranslational modification in the functioning of actin cytoskeleton and in cell **migration** in culture. Knockout of arginyltransferase (Ate1) in mice leads to embryonic lethality and severe heart defects that are reminiscent of cell **migration**-dependent phenotypes seen in other mouse models. To test the hypothesis that arginylation regulates cell **migration** during morphogenesis, we produced Wnt1-Cre Ate1 conditional knockout mice (Wnt1-Ate1), with Ate1 deletion in the neural crest cells driven by Wnt1 promoter. Wnt1-Ate1 mice die at birth and in the first 2-3 weeks after birth with severe breathing problems and with growth and behavioral retardation. Wnt1-Ate1 pups have prominent defects, including short palate and altered opening to the nasopharynx, and cranial defects that likely contribute to the abnormal breathing and early death. Analysis of neural crest cell movement patterns in situ and cell motility in culture shows an overall delay in the **migration** of Ate1 knockout cells that is likely regulated by intracellular mechanisms rather than extracellular signaling events. Taken together, our data suggest that arginylation plays a general role in the **migration** of the neural crest cells in development by regulating the molecular machinery that underlies cell **migration** through tissues and organs during morphogenesis.
10. Blocking neutrophil integrin activation prevents ischemia-reperfusion injury. Neutrophil recruitment, mediated by β2 integrins, combats pyogenic infections but also plays a key role in ischemia-reperfusion injury and other inflammatory disorders. Talin induces allosteric rearrangements in integrins that increase affinity for ligands (activation). Talin also links integrins to actin and other proteins that enable formation of adhesions. Structural studies have identified a talin1 mutant (L325R) that perturbs activation without impairing talin's capacity to link integrins to actin and other proteins. Here, we found that mice engineered to express only talin1(L325R) in myeloid cells were protected from renal ischemia-reperfusion injury. Dissection of neutrophil function in vitro and in vivo revealed that talin1(L325R) neutrophils had markedly impaired chemokine-induced, β2 integrin-mediated arrest, spreading, an**d migrati**on. Surprisingly, talin1(L325R) neutrophils exhibited normal selectin-induced, β2 integrin-mediated slow rolling, in sharp contrast to the defective slow rolling of neutrophils lacking talin1 or expressing a talin1 mutant (W359A) that blocks talin interaction with integrins. These studies reveal the importance of talin-mediated activation of integrins for renal ischemia-reperfusion injury. They further show that neutrophil arrest requires talin recruitment to and activation of integrins. However, although neutrophil slow rolling requires talin recruitment to integrins, talin-mediated integrin activation is dispensable.
11. The mechanisms and dynamics of (alpha)v(beta)3 integrin clustering in living cells. During cell **migration**, the physical link between the extracellular substrate and the actin cytoskeleton mediated by receptors of the integrin family is constantly modified. We analyzed the mechanisms that regulate the clustering and incorporation of activated alphavbeta3 integrins into focal adhesions. Manganese (Mn2+) or mutational activation of integrins induced the formation of de novo F-actin-independent integrin clusters. These clusters recruited talin, but not other focal adhesion adapters, and overexpression of the integrin-binding head domain of talin increased clustering. Integrin clustering required immobilized ligand and was prevented by the sequestration of phosphoinositole-4,5-bisphosphate (PI(4,5)P2). Fluorescence recovery after photobleaching analysis of Mn(2+)-induced integrin clusters revealed increased integrin turnover compared with mature focal contacts, whereas stabilization of the open conformation of the integrin ectodomain by mutagenesis reduced integrin turnover in focal contacts. Thus, integrin clustering requires the formation of the ternary complex consisting of activated integrins, immobilized ligands, talin, and PI(4,5)P2. The dynamic remodeling of this ternary complex controls cell motility.
12. Talin-1 and kindlin-3 regulate alpha4beta1 integrin-mediated adhesion stabilization, but not G protein-coupled receptor-induced affinity upregulation. Chemokine/chemoattractant G protein-coupled receptors trigger an inside-out signaling network that rapidly activates integrins, a key step in inflammatory leukocyte recruitment. Integrins mediate leukocyte arrest and adhesion to endothelium through multivalent binding, and they transmit outside-in signals to stabilize adhesion and coordinate cell spreading and **migration**. In the present study, we used RNA interference in the U937 monocytic cell line to investigate the role of talin-1, kindlin-3, and α-actinin-1 in the fMLF- and SDF-1α-induced upregulation of α(4)β(1) integrin affinity and consequent adhesive events. Affinity upregulation of α(4)β(1) integrin was not impaired by small interfering RNA knockdown of talin-1, kindlin-3, or α-actinin-1. Only kindlin-3 knockdown increased flow-induced detachment from VCAM-1-coated surfaces in response to fluid flow, whereas knockdown of either talin-1 or kindlin-3 increased detachment from ICAM-1-coated surfaces. Biochemical analyses revealed that α(4)β(1) expression was highly enriched in U937 cell microridges and murine lymphocyte microvilli. Kindlin-3 was present throughout the cell, whereas talin-1 was largely excluded from microridges/microvilli. The subcellular colocalization of α(4)β(1) and kindlin-3 in microridges may explain why kindlin-3 rapidly associates with α(4)β(1) after G protein-coupled receptor signaling and contributes to adhesion strengthening. Talin-1 contributed to α(4)β(1)-dependent chemotaxis, suggesting that it participates in a later stage of the leukocyte adhesion cascade when the leukocyte cytoskeleton undergoes dramatic rearrangement.
13. Disruption of the talin gene arrests mouse development at the gastrulation stage. Studies on cultured cells show that the cytoskeletal protein talin plays a key role in cell spreading and the assembly of cell-extracellular matrix junctions. To examine the role of talin in vivo, we have generated mice with a targeted disruption of the talin gene. Heterozygotes are normal, but no surviving homozygous mutant animals were obtained, proving that talin is required for embryogenesis. Mutant embryos develop normally to the blastocyst stage and implant, but there is a gross disorganization of the embryos at gastrulation (6.5-7.5 days post coitum), and they die around 8.5-9.5 days post coitum. The embryonic ectoderm is reduced in size, with fewer cells, and is incompletely organised compared with wild-type embryos. The mutant embryos show disorganised extraembryonic tissues, and the ectoplacental and excocoelomic cavities are not formed. This seems to be because embryonic mesoderm accumulates as a mass on the posterior side of the embryos and fails to migrate to extraembryonic regions, although mesodermal cells are evident in the embryo proper. Spreading of trophoblast cells derived from cultured mutant blastocysts on fibronectin and laminin is also considerably reduced. Therefore, the fundamental deficit in these embryos seems to be a failure of cell **migration** at gastrulation.
14. The methyltransferase Ezh2 controls cell adhesion and **migration** through direct methylation of the extranuclear regulatory protein talin. A cytosolic role for the histone methyltransferase Ezh2 in regulating lymphocyte activation has been suggested, but the molecular mechanisms underpinning this extranuclear function have remained unclear. Here we found that Ezh2 regulated the integrin signaling and adhesion dynamics of neutrophils and dendritic cells (DCs). Ezh2 deficiency impaired the integrin-dependent transendothelial **migration** of innate leukocytes and restricted disease progression in an animal model of multiple sclerosis. Direct methylation of talin, a key regulatory molecule in cell **migration**, by Ezh2 disrupted the binding of talin to F-actin and thereby promoted the turnover of adhesion structures. This regulatory effect was abolished by targeted disruption of the interactions of Ezh2 with the cytoskeletal-reorganization effector Vav1. Our studies reveal an unforeseen extranuclear function for Ezh2 in regulating adhesion dynamics, with implications for leukocyte **migration**, immune responses and potentially pathogenic processes.
15. An integrin phosphorylation switch: the effect of beta3 integrin tail phosphorylation on Dok1 and talin binding. Integrins play a fundamental role in cell **migration** and adhesion; knowledge of how they are regulated and controlled is vital for understanding these processes. Recent work showed that Dok1 negatively regulates integrin activation, presumably by competition with talin. To understand how this occurs, we used NMR spectroscopy and x-ray crystallography to investigate the molecular details of interactions with integrins. The binding affinities of beta3 integrin tails for the Dok1 and talin phosphotyrosine binding domains were quantified using 15N-1H hetero-nuclear single quantum correlation titrations, revealing that the unphosphorylated integrin tail binds more strongly to talin than Dok1. Chemical shift mapping showed that unlike talin, Dok1 exclusively interacts with the canonical NPXY motif of the beta3 integrin tail. Upon phosphorylation of Tyr 747 in the beta3 integrin tail, however, Dok1 then binds much more strongly than talin. Thus, we show that phosphorylation of Tyr 747 provides a switch for integrin ligand binding. This switch may represent an in vivo mechanism for control of integrin receptor activation. These results have implications for the control of integrin signaling by proteins containing phosphotyrosine binding domains.
16. The small muscle-specific protein Csl modifies cell shape and promotes myocyte fusion in an insulin-like growth factor 1-dependent manner. We have isolated a murine cDNA encoding a 9-kD protein, Chisel (Csl), in a screen for transcriptional targets of the cardiac homeodomain factor Nkx2-5. Csl transcripts were detected in atria and ventricles of the heart and in all skeletal muscles and smooth muscles of the stomach and pulmonary veins. Csl protein was distributed throughout the cytoplasm in fetal muscles, although costameric and M-line localization to the muscle cytoskeleton became obvious after further maturation. Targeted disruption of Csl showed no overt muscle phenotype. However, ectopic expression in C2C12 myoblasts induced formation of lamellipodia in which Csl protein became tethered to membrane ruffles. **Migration** of these cells was retarded in a monolayer wound repair assay. Csl-expressing myoblasts differentiated and fused normally, although in the presence of insulin-like growth factor (IGF)-1 they showed dramatically enhanced fusion, leading to formation of large dysmorphogenic "myosacs." The activities of transcription factors nuclear factor of activated T cells (NFAT) and myocyte enhancer-binding factor (MEF)2, were also enhanced in an IGF-1 signaling-dependent manner. The dynamic cytoskeletal localization of Csl and its dominant effects on cell shape and behavior and transcription factor activity suggest that Csl plays a role in the regulatory network through which muscle cells coordinate their structural and functional states during growth, adaptation, and repair.
17. Tiam1 interaction with the PAR complex promotes talin-mediated Rac1 activation during polarized cell **migration**. Migrating cells acquire front-rear polarity with a leading edge and a trailing tail for directional movement. The Rac exchange factor Tiam1 participates in polarized cell **migration** with the PAR complex of PAR3, PAR6, and atypical protein kinase C. However, it remains largely unknown how Tiam1 is regulated and contributes to the establishment of polarity in migrating cells. We show here that Tiam1 interacts directly with talin, which binds and activates integrins to mediate their signaling. Tiam1 accumulated at adhesions in a manner dependent on talin and the PAR complex. The interactions of talin with Tiam1 and the PAR complex were required for adhesion-induced Rac1 activation, cell spreading, and **migration** toward integrin substrates. Furthermore, Tiam1 acted with talin to regulate adhesion turnover. Thus, we propose that Tiam1, with the PAR complex, binds to integrins through talin and, together with the PAR complex, thereby regulates Rac1 activity and adhesion turnover for polarized **migration**.
18. Conformational activation of talin by RIAM triggers integrin-mediated cell adhesion. The membrane localization and activation of cytoskeletal protein talin are key steps to initiate the integrin transmembrane receptors' activation, which mediates many cellular adhesive responses such as cell **migration**, spreading and proliferation. RIAM, a membrane anchor and small GTPase RAP1 effector, is known to bind to the C-terminal rod domain of talin (talin-R) and promote localizations of talin to the membrane. Through systematic mapping analysis, we find that RIAM also binds to the N-terminal head of talin (talin-H), a crucial domain involved in binding and activating integrins. We show that the RIAM binding to talin-H sterically occludes the binding of a talin-R domain that otherwise masks the integrin-binding site on talin-H. We further provide functional evidence that such RIAM-mediated steric unmasking of talin triggers integrin activation. Our findings thus uncover a novel role for RIAM in conformational regulation of talin during integrin activation and cell adhesion.
19. Talin1 is required for integrin-dependent B lymphocyte homing to lymph nodes and the bone marrow but not for follicular B-cell maturation in the spleen. Talin1 is a key integrin coactivator. We investigated the roles of this cytoskeletal adaptor and its target integrins in B-cell lymphogenesis, differentiation, **migration**, and function. Using CD19 Cre-mediated depletion of talin1 selectively in B cells, we found that talin1 was not required for B-cell generation in the bone marrow or for the entry of immature B cells to the white pulp of the spleen. Loss of talin1 also did not affect B-cell maturation into follicular B cells but compromised differentiation of marginal zone B cells. Nevertheless, serum IgM and IgG levels remained normal. Ex vivo analysis of talin1-deficient spleen B cells indicated a necessary role for talin1 in LFA-1 and VLA-4 activation stimulated by canonical agonists, but not in B-cell chemotaxis. Consequently, talin1 null B splenocytes could not enter lymph nodes nor return to the bone marrow. Talin1 deficiency in B cells was also impaired in the humoral response to a T cell-dependent antigen. Collectively, these results indicate that talin1 is not required for follicular B-cell maturation in the spleen or homeostatic humoral immunity but is critical for integrin-dependent B lymphocyte e**migration** to lymph nodes and optimal immunity against T-dependent antigens.
20. Structural basis for the interaction between the cytoplasmic domain of the hyaluronate receptor layilin and the talin F3 subdomain. Talin is a large cytoskeletal protein that is involved in coupling the integrin family of cell adhesion molecules to the actin cytoskeleton, colocalising with the integrins in focal adhesions (FAs). However, at the leading edge of motile cells, talin colocalises with the hyaluronan receptor layilin in what are thought to be transient adhesions, some of which subsequently mature into more stable FAs. During this maturation process, layilin is replaced with integrins, which are highly clustered in FAs, where localised production of PI(4,5)P(2) by type 1 phosphatidyl inositol phosphate kinase type 1gamma (PIPK1gamma) is thought to play a role in FA assembly. The talin FERM F3 subdomain binds both the integrin beta-subunit cytoplasmic domain and PIPK1gamma, and these interactions are understood in detail at the atomic level. The talin F3 domain also binds to short sequences in the layilin cytoplasmic domain, and here we report the structure of the talin/layilin complex, which shows that talin binds integrins, PIPK1gamma and layilin in similar although subtly different ways. Based on structure comparisons, we designed a set of talin F3 mutations that selectively affected the affinity of talin for its targets, as determined by stopped-flow fluorescence measurements. Such mutations will help to assess the importance of the interactions between talin and its various ligands in cell adhesion and **migration**.
21. Structural model and functional significance of pH-dependent talin-actin binding for focal adhesion remodeling. Actin filament binding by the focal adhesion (FA)-associated protein talin stabilizes cell-substrate adhesions and is thought to be rate-limiting in cell **migration**. Although F-actin binding by talin is known to be pH-sensitive in vitro, with lower affinity at higher pH, the functional significance of this pH dependence is unknown. Because increased intracellular pH (pH(i)) promotes cell **migration** and is a hallmark of metastatic carcinomas, we asked whether it increases FA remodeling through lower-affinity talin-actin binding. Talin contains several actin binding sites, but we found that only the COOH-terminal USH-I/LWEQ module showed pH-dependent actin binding, with lower affinity and decreased maximal binding at higher pH. Molecular dynamics simulations and NMR of this module revealed a structural mechanism for pH-dependent actin binding. A cluster of titratable amino acids with upshifted pK(a) values, including His-2418, was identified at one end of the five-helix bundle distal from the actin binding site. Protonation of His-2418 induces changes in the conformation and dynamics of the remote actin binding site. Structural analyses of a mutant talin-H2418F at pH 6.0 and 8.0 suggested changes different from the WT protein, and we confirmed that actin binding by talin-H2418F was relatively pH-insensitive. In motile fibroblasts, increasing pH(i) decreased FA lifetime and increased the migratory rate. However, expression of talin-H2418F increased lifetime 2-fold and decreased the migratory rate. These data identify a molecular mechanism for pH-sensitive actin binding by talin and suggest that FA turnover is pH-dependent and in part mediated by pH-dependent affinity of talin for binding actin.
22. Activation of a vinculin-binding site in the talin rod involves rearrangement of a five-helix bundle. The interaction between the cytoskeletal proteins talin and vinculin plays a key role in integrin-mediated cell adhesion and **migration**. We have determined the crystal structures of two domains from the talin rod spanning residues 482-789. Talin 482-655, which contains a vinculin-binding site (VBS), folds into a five-helix bundle whereas talin 656-789 is a four-helix bundle. We show that the VBS is composed of a hydrophobic surface spanning five turns of helix 4. All the key side chains from the VBS are buried and contribute to the hydrophobic core of the talin 482-655 fold. We demonstrate that the talin 482-655 five-helix bundle represents an inactive conformation, and mutations that disrupt the hydrophobic core or deletion of helix 5 are required to induce an active conformation in which the VBS is exposed. We also report the crystal structure of the N-terminal vinculin head domain in complex with an activated form of talin. Activation of the VBS in talin and the recruitment of vinculin may support the maturation of small integrin/talin complexes into more stable adhesions.
23. Talin phosphorylation by Cdk5 regulates Smurf1-mediated talin head ubiquitylation and cell **migration**. Cell **migration** is a dynamic process that requires temporal and spatial regulation of integrin activation and focal adhesion assembly/disassembly. Talin, an actin and beta-integrin tail-binding protein, is essential for integrin activation and focal adhesion formation. Calpain-mediated cleavage of talin has a key role in focal adhesion turnover; however, the talin head domain, one of the two cleavage products, stimulates integrin activation, localizes to focal adhesions and maintains cell edge protrusions, suggesting that other steps, downstream of talin proteolysis, are required for focal adhesion disassembly. Here we show that talin head binds Smurf1, an E3 ubiquitin ligase involved in cell polarity and **migration**, more tightly than full-length talin does and that this interaction leads to talin head ubiquitylation and degradation. We found that talin head is a substrate for Cdk5, a cyclin-dependent protein kinase that is essential for cell **migration**, synaptic transmission and cancer metastasis. Cdk5 phosphorylated talin head at Ser 425, inhibiting its binding to Smurf1, thus preventing talin head ubiquitylation and degradation. Expression of the mutant tal(S425A), which resists Cdk5 phosphorylation thereby increasing its susceptibility to Smurf1-mediated ubiqitylation, resulted in extensive focal adhesion turnover and inhibited cell **migration**. Thus, talin head produced by calpain-induced cleavage of talin is degraded through Smurf1-mediated ubiquitylation; moreover, phosphorylation by Cdk5 regulates the binding of Smurf1 to talin head, controlling talin head turnover, adhesion stability and ultimately, cell **migration**.
24. Competition for talin results in trans-dominant inhibition of integrin activation. The ability of integrin adhesion receptors to undergo rapid changes in affinity for their extracellular ligands (integrin activation) is essential for the development and function of multicellular animals and is dependent on interactions between the integrin beta subunit-cytoplasmic tail and the cytoskeletal protein talin. Cross-talk among different integrins and between integrins and other receptors impacts many cellular processes including adhesion, spreading, **migration**, clot retraction, proliferation, and differentiation. One form of integrin cross-talk, transdominant inhibition of integrin activation, occurs when ligand binding to one integrin inhibits the activation of a second integrin. This may be relevant clinically in a number of settings such as during platelet adhesion, leukocyte trans-**migration**, and angiogenesis. Here we report that competition for talin underlies the trans-dominant inhibition of integrin activation. This conclusion is based on our observations that (i). beta tails selectively defective in talin binding are unable to mediate trans-dominant inhibition, (ii). trans-dominant inhibition can be reversed by overexpression of integrin binding and activating fragments of talin, and (iii). expression of another non-integrin talin-binding protein, phosphatidylinositol phosphate kinase type Igamma-90, also inhibits integrin activation. Thus, the sequestration of talin by the suppressive species is both necessary and sufficient for trans-dominant inhibition of integrin activation.
25. PIP2 and Talin Join Forces to Activate Integrin. Integrins are major players in cell adhesion and **migration**, and malfunctions in controlling their activity are associated with various diseases. Nevertheless, the details of integrin activation are not completely understood, and the role of lipids in the process is largely unknown. Herein, we show using atomistic molecular dynamics simulations that the interplay of phosphatidylinositol 4,5-bisphosphate (PIP2) and talin may directly alter the conformation of integrin αIIbβ3. Our results provide a new perspective on the role of PIP2 in integrin activation and indicate that the charged PIP2 lipid headgroup can perturb a clasp at the cytoplasmic face of the integrin heterodimer.

#### GeneRIFs

1. Direct methylation of talin, a key regulatory molecule in cell **migration**, by Ezh2 disrupted the binding of talin to F-actin and thereby promoted the turnover of adhesion structures.
2. Talin head produced by calpain-induced cleavage of talin is degraded through Smurf1-mediated ubiquitylation. Phosphorylation by Cdk5 regulates the binding of Smurf1 to talin head, controlling talin head turnover, adhesion stability and cell **migration**.

### ECM

#### PubMed

1. Myosin II activity regulates vinculin recruitment to focal adhesions through FAK-mediated paxillin phosphorylation. Focal adhesions (FAs) are mechanosensitive adhesion and signaling complexes that grow and change composition in response to myosin II-mediated cytoskeletal tension in a process known as FA maturation. To understand tension-mediated FA maturation, we sought to identify proteins that are recruited to FAs in a myosin II-dependent manner and to examine the mechanism for their myosin II-sensitive FA association. We find that FA recruitment of both the cytoskeletal adapter protein vinculin and the tyrosine kinase FA kinase (FAK) are myosin II and extracellular matrix (**ECM**) stiffness dependent. Myosin II activity promotes FAK/Src-mediated phosphorylation of paxillin on tyrosines 31 and 118 and vinculin association with paxillin. We show that phosphomimic mutations of paxillin can specifically induce the recruitment of vinculin to adhesions independent of myosin II activity. These results reveal an important role for paxillin in adhesion mechanosensing via myosin II-mediated FAK phosphorylation of paxillin that promotes vinculin FA recruitment to reinforce the cytoskeletal **ECM** linkage and drive FA maturation.
2. Talin depletion reveals independence of initial cell spreading from integrin activation and traction. Cell spreading, adhesion and remodelling of the extracellular matrix (**ECM**) involve bi-directional signalling and physical linkages between the **ECM**, integrins and the cell cytoskeleton. The actin-binding proteins talin1 and 2 link ligand-bound integrins to the actin cytoskeleton and increase the affinity of integrin for the **ECM**. Here we report that depletion of talin2 in talin1-null (talin1(-/-)) cells did not affect the initiation of matrix-activated spreading or Src family kinase (SFK) activation, but abolished the **ECM**-integrin-cytoskeleton linkage and sustained cell spreading and adhesion. Specifically, focal adhesion assembly, focal adhesion kinase (FAK) signalling and traction force generation on substrates were severely affected. The talin1 head domain restored beta1 integrin activation but only full-length talin1 restored the **ECM**-cytoskeleton linkage and normal cytoskeleton organization. Our results demonstrate three biochemically distinct steps in fibronectin-activated cell spreading and adhesion: (1) fibronectin-integrin binding and initiation of spreading, (2) fast cell spreading and (3) focal adhesion formation and substrate traction. We suggest that talin is not required for initial cell spreading. However, talin provides the important mechanical linkage between ligand-bound integrins and the actin cytoskeleton required to catalyse focal adhesion-dependent pathways.
3. Talin depletion reveals independence of initial cell spreading from integrin activation and traction. Cell spreading, adhesion and remodelling of the extracellular matrix (**ECM**) involve bi-directional signalling and physical linkages between the **ECM**, integrins and the cell cytoskeleton. The actin-binding proteins talin1 and 2 link ligand-bound integrins to the actin cytoskeleton and increase the affinity of integrin for the **ECM**. Here we report that depletion of talin2 in talin1-null (talin1(-/-)) cells did not affect the initiation of matrix-activated spreading or Src family kinase (SFK) activation, but abolished the **ECM**-integrin-cytoskeleton linkage and sustained cell spreading and adhesion. Specifically, focal adhesion assembly, focal adhesion kinase (FAK) signalling and traction force generation on substrates were severely affected. The talin1 head domain restored beta1 integrin activation but only full-length talin1 restored the **ECM**-cytoskeleton linkage and normal cytoskeleton organization. Our results demonstrate three biochemically distinct steps in fibronectin-activated cell spreading and adhesion: (1) fibronectin-integrin binding and initiation of spreading, (2) fast cell spreading and (3) focal adhesion formation and substrate traction. We suggest that talin is not required for initial cell spreading. However, talin provides the important mechanical linkage between ligand-bound integrins and the actin cytoskeleton required to catalyse focal adhesion-dependent pathways.
4. Loss of alpha3beta1 integrin function results in an altered differentiation program in the mouse submandibular gland. Mammalian submandibular gland (SMG) development leads to the establishment of highly organized secretory acinar and nonsecretory ductal epithelial cells. The ability of maturing salivary epithelial cells to attain their differentiated state has been shown to depend, in part, on interactions between extracellular matrix (**ECM**) proteins and their integrin receptors. In a search for key regulators of salivary cell lineage, we have studied alpha3beta1 integrin, a receptor for the basement membrane protein laminin, by characterizing embryonic day 18 (E18) SMGs isolated from mice carrying a targeted mutation in the alpha3 integrin gene. Transmission electron microscopy studies showed that the mutant SMGs exhibited an aberrant differentiation phenotype with defects in the apical-basal polarity axis and in the basement membrane. Based on immunohistochemistry and Western blot analyses, the alpha3beta1-deficient SMGs had altered expression and/or localization of several **ECM** and adhesive molecules, including laminin beta1, fibronectin, alpha5 integrin, and E-cadherin. These changes correlated with alterations in the activation state of Ras-extracellular signal-regulated kinase (ERK), as well as the expression and/or localization of Cdc42 and RhoA, two Rho GTPases that regulate the organization of the actin cytoskeleton. We conclude that alpha3beta1 is required for normal salivary cell differentiation and that its absence affects multiple components of adhesive complexes and their associated signalling pathways.
5. Progressive myopathy and defects in the maintenance of myotendinous junctions in mice that lack talin 1 in skeletal muscle. The development and function of skeletal muscle depend on molecules that connect the muscle fiber cytoskeleton to the extracellular matrix (**ECM**). beta1 integrins are **ECM** receptors in skeletal muscle, and mutations that affect the alpha7beta1 integrin cause myopathy in humans. In mice, beta1 integrins control myoblast fusion, the assembly of the muscle fiber cytoskeleton, and the maintenance of myotendinous junctions (MTJs). The effector molecules that mediate beta1 integrin functions in muscle are not known. Previous studies have shown that talin 1 controls the force-dependent assembly of integrin adhesion complexes and regulates the affinity of integrins for ligands. Here we show that talin 1 is essential in skeletal muscle for the maintenance of integrin attachment sites at MTJs. Mice with a skeletal muscle-specific ablation of the talin 1 gene suffer from a progressive myopathy. Surprisingly, myoblast fusion and the assembly of integrin-containing adhesion complexes at costameres and MTJs advance normally in the mutants. However, with progressive ageing, the muscle fiber cytoskeleton detaches from MTJs. Mechanical measurements on isolated muscles show defects in the ability of talin 1-deficient muscle to generate force. Collectively, our findings show that talin 1 is essential for providing mechanical stability to integrin-dependent adhesion complexes at MTJs, which is crucial for optimal force generation by skeletal muscle.

### [Ee]xtracellular [Mm]atrix

#### PubMed

1. Myosin II activity regulates vinculin recruitment to focal adhesions through FAK-mediated paxillin phosphorylation. Focal adhesions (FAs) are mechanosensitive adhesion and signaling complexes that grow and change composition in response to myosin II-mediated cytoskeletal tension in a process known as FA maturation. To understand tension-mediated FA maturation, we sought to identify proteins that are recruited to FAs in a myosin II-dependent manner and to examine the mechanism for their myosin II-sensitive FA association. We find that FA recruitment of both the cytoskeletal adapter protein vinculin and the tyrosine kinase FA kinase (FAK) are myosin II and **extracellular matrix** (ECM) stiffness dependent. Myosin II activity promotes FAK/Src-mediated phosphorylation of paxillin on tyrosines 31 and 118 and vinculin association with paxillin. We show that phosphomimic mutations of paxillin can specifically induce the recruitment of vinculin to adhesions independent of myosin II activity. These results reveal an important role for paxillin in adhesion mechanosensing via myosin II-mediated FAK phosphorylation of paxillin that promotes vinculin FA recruitment to reinforce the cytoskeletal ECM linkage and drive FA maturation.
2. Different splice variants of filamin-B affect myogenesis, subcellular distribution, and determine binding to integrin [beta] subunits. Integrins connect the **extracellular matrix** with the cell interior, and transduce signals through interactions of their cytoplasmic tails with cytoskeletal and signaling proteins. Using the yeast two-hybrid system, we isolated a novel splice variant (filamin-Bvar-1) of the filamentous actin cross-linking protein, filamin-B, that interacts with the cytoplasmic domain of the integrin beta1A and beta1D subunits. RT-PCR analysis showed weak, but wide, expression of filamin-Bvar-1 and a similar splice variant of filamin-A (filamin-Avar-1) in human tissues. Furthermore, alternative splice variants of filamin-B and filamin-C, from which the flexible hinge-1 region is deleted (DeltaH1), were induced during in vitro differentiation of C2C12 mouse myoblasts. We show that both filamin-Avar-1 and filamin-Bvar-1 bind more strongly than their wild-type isoforms to different integrin beta subunits. The mere presence of the high-affinity binding site for beta1A is not sufficient for targeting the filamin-Bvar-1 construct to focal contacts. Interestingly, the simultaneous deletion of the H1 region is required for the localization of filamin-B at the tips of actin stress fibers. When expressed in C2C12 cells, filamin-Bvar-1(DeltaH1) accelerates their differentiation into myotubes. Furthermore, filamin-B variants lacking the H1 region induce the formation of thinner myotubes than those in cells containing variants with this region. These findings suggest that specific combinations of filamin mRNA splicing events modulate the organization of the actin cytoskeleton and the binding affinity for integrins.
3. Cell adhesion strengthening: contributions of adhesive area, integrin binding, and focal adhesion assembly. Mechanical interactions between a cell and its environment regulate migration, contractility, gene expression, and cell fate. We integrated micropatterned substrates to engineer adhesive area and a hydrodynamic assay to analyze fibroblast adhesion strengthening on fibronectin. Independently of cell spreading, integrin binding and focal adhesion assembly resulted in rapid sevenfold increases in adhesion strength to steady-state levels. Adhesive area strongly modulated adhesion strength, integrin binding, and vinculin and talin recruitment, exhibiting linear increases for small areas. However, above a threshold area, adhesion strength and focal adhesion assembly reached a saturation limit, whereas integrin binding transitioned from a uniform distribution to discrete complexes. Adhesion strength exhibited exponential increases with bound integrin numbers as well as vinculin and talin recruitment, and the relationship between adhesion strength and these biochemical events was accurately described by a simple mechanical model. Furthermore, adhesion strength was regulated by the position of an adhesive patch, comprised of bound integrins and cytoskeletal elements, which generated a constant 200-nN adhesive force. Unexpectedly, focal adhesion assembly, in particular vinculin recruitment, contributed only 30% of the adhesion strength. This work elucidates the roles of adhesive complex size and position in the generation of cell-**extracellular matrix** forces.
4. Talin depletion reveals independence of initial cell spreading from integrin activation and traction. Cell spreading, adhesion and remodelling of the **extracellular matrix** (ECM) involve bi-directional signalling and physical linkages between the ECM, integrins and the cell cytoskeleton. The actin-binding proteins talin1 and 2 link ligand-bound integrins to the actin cytoskeleton and increase the affinity of integrin for the ECM. Here we report that depletion of talin2 in talin1-null (talin1(-/-)) cells did not affect the initiation of matrix-activated spreading or Src family kinase (SFK) activation, but abolished the ECM-integrin-cytoskeleton linkage and sustained cell spreading and adhesion. Specifically, focal adhesion assembly, focal adhesion kinase (FAK) signalling and traction force generation on substrates were severely affected. The talin1 head domain restored beta1 integrin activation but only full-length talin1 restored the ECM-cytoskeleton linkage and normal cytoskeleton organization. Our results demonstrate three biochemically distinct steps in fibronectin-activated cell spreading and adhesion: (1) fibronectin-integrin binding and initiation of spreading, (2) fast cell spreading and (3) focal adhesion formation and substrate traction. We suggest that talin is not required for initial cell spreading. However, talin provides the important mechanical linkage between ligand-bound integrins and the actin cytoskeleton required to catalyse focal adhesion-dependent pathways.
5. Clustering of alpha(5)beta(1) integrins determines adhesion strength whereas alpha(v)beta(3) and talin enable mechanotransduction. A key molecular link between cells and the **extracellular matrix** is the binding between fibronectin and integrins alpha(5)beta(1) and alpha(v)beta(3). However, the roles of these different integrins in establishing adhesion remain unclear. We tested the adhesion strength of fibronectin-integrin-cytoskeleton linkages by applying physiological nanonewton forces to fibronectin-coated magnetic beads bound to cells. We report that the clustering of fibronectin domains within 40 nm led to integrin alpha(5)beta(1) recruitment, and increased the ability to sustain force by over six-fold. This force was supported by alpha(5)beta(1) integrin clusters. Importantly, we did not detect a role of either integrin alpha(v)beta(3) or talin 1 or 2 in maintaining adhesion strength. Instead, these molecules enabled the connection to the cytoskeleton and reinforcement in response to an applied force. Thus, high matrix forces are primarily supported by clustered alpha(5)beta(1) integrins, while less stable links to alpha(v)beta(3) integrins initiate mechanotransduction, resulting in reinforcement of integrin-cytoskeleton linkages through talin-dependent bonds.
6. Clustering of alpha(5)beta(1) integrins determines adhesion strength whereas alpha(v)beta(3) and talin enable mechanotransduction. A key molecular link between cells and the **extracellular matrix** is the binding between fibronectin and integrins alpha(5)beta(1) and alpha(v)beta(3). However, the roles of these different integrins in establishing adhesion remain unclear. We tested the adhesion strength of fibronectin-integrin-cytoskeleton linkages by applying physiological nanonewton forces to fibronectin-coated magnetic beads bound to cells. We report that the clustering of fibronectin domains within 40 nm led to integrin alpha(5)beta(1) recruitment, and increased the ability to sustain force by over six-fold. This force was supported by alpha(5)beta(1) integrin clusters. Importantly, we did not detect a role of either integrin alpha(v)beta(3) or talin 1 or 2 in maintaining adhesion strength. Instead, these molecules enabled the connection to the cytoskeleton and reinforcement in response to an applied force. Thus, high matrix forces are primarily supported by clustered alpha(5)beta(1) integrins, while less stable links to alpha(v)beta(3) integrins initiate mechanotransduction, resulting in reinforcement of integrin-cytoskeleton linkages through talin-dependent bonds.
7. Talin depletion reveals independence of initial cell spreading from integrin activation and traction. Cell spreading, adhesion and remodelling of the **extracellular matrix** (ECM) involve bi-directional signalling and physical linkages between the ECM, integrins and the cell cytoskeleton. The actin-binding proteins talin1 and 2 link ligand-bound integrins to the actin cytoskeleton and increase the affinity of integrin for the ECM. Here we report that depletion of talin2 in talin1-null (talin1(-/-)) cells did not affect the initiation of matrix-activated spreading or Src family kinase (SFK) activation, but abolished the ECM-integrin-cytoskeleton linkage and sustained cell spreading and adhesion. Specifically, focal adhesion assembly, focal adhesion kinase (FAK) signalling and traction force generation on substrates were severely affected. The talin1 head domain restored beta1 integrin activation but only full-length talin1 restored the ECM-cytoskeleton linkage and normal cytoskeleton organization. Our results demonstrate three biochemically distinct steps in fibronectin-activated cell spreading and adhesion: (1) fibronectin-integrin binding and initiation of spreading, (2) fast cell spreading and (3) focal adhesion formation and substrate traction. We suggest that talin is not required for initial cell spreading. However, talin provides the important mechanical linkage between ligand-bound integrins and the actin cytoskeleton required to catalyse focal adhesion-dependent pathways.
8. Disruption of the talin gene arrests mouse development at the gastrulation stage. Studies on cultured cells show that the cytoskeletal protein talin plays a key role in cell spreading and the assembly of cell-**extracellular matrix** junctions. To examine the role of talin in vivo, we have generated mice with a targeted disruption of the talin gene. Heterozygotes are normal, but no surviving homozygous mutant animals were obtained, proving that talin is required for embryogenesis. Mutant embryos develop normally to the blastocyst stage and implant, but there is a gross disorganization of the embryos at gastrulation (6.5-7.5 days post coitum), and they die around 8.5-9.5 days post coitum. The embryonic ectoderm is reduced in size, with fewer cells, and is incompletely organised compared with wild-type embryos. The mutant embryos show disorganised extraembryonic tissues, and the ectoplacental and excocoelomic cavities are not formed. This seems to be because embryonic mesoderm accumulates as a mass on the posterior side of the embryos and fails to migrate to extraembryonic regions, although mesodermal cells are evident in the embryo proper. Spreading of trophoblast cells derived from cultured mutant blastocysts on fibronectin and laminin is also considerably reduced. Therefore, the fundamental deficit in these embryos seems to be a failure of cell migration at gastrulation.
9. Extracellular rigidity sensing by talin isoform-specific mechanical linkages. The ability of cells to adhere and sense differences in tissue stiffness is crucial for organ development and function. The central mechanisms by which adherent cells detect **extracellular matrix** compliance, however, are still unknown. Using two single-molecule-calibrated biosensors that allow the analysis of a previously inaccessible but physiologically highly relevant force regime in cells, we demonstrate that the integrin activator talin establishes mechanical linkages following cell adhesion, which are indispensable for cells to probe tissue stiffness. Talin linkages are exposed to a range of piconewton forces and bear, on average, 7-10 pN during cell adhesion depending on their association with F-actin and vinculin. Disruption of talin's mechanical engagement does not impair integrin activation and initial cell adhesion but prevents focal adhesion reinforcement and thus extracellular rigidity sensing. Intriguingly, talin mechanics are isoform specific so that expression of either talin-1 or talin-2 modulates extracellular rigidity sensing.
10. Localisation of the human gene encoding the cytoskeletal protein talin to chromosome 9p. The cytoskeletal protein talin is localised on the cytoplasmic face of the integrin family of adhesion receptors in cellular junctions with the **extracellular matrix**. Using polymerase chain reaction amplification and DNA from a panel of human-rodent somatic cell hybrids, we have assigned the talin gene to chromosome 9p. Deletions in 9p have been implicated in a variety of cancers, including malignant melanoma, and the concept that talin might be a candidate tumour suppressor gene is discussed.
11. Loss of alpha3beta1 integrin function results in an altered differentiation program in the mouse submandibular gland. Mammalian submandibular gland (SMG) development leads to the establishment of highly organized secretory acinar and nonsecretory ductal epithelial cells. The ability of maturing salivary epithelial cells to attain their differentiated state has been shown to depend, in part, on interactions between **extracellular matrix** (ECM) proteins and their integrin receptors. In a search for key regulators of salivary cell lineage, we have studied alpha3beta1 integrin, a receptor for the basement membrane protein laminin, by characterizing embryonic day 18 (E18) SMGs isolated from mice carrying a targeted mutation in the alpha3 integrin gene. Transmission electron microscopy studies showed that the mutant SMGs exhibited an aberrant differentiation phenotype with defects in the apical-basal polarity axis and in the basement membrane. Based on immunohistochemistry and Western blot analyses, the alpha3beta1-deficient SMGs had altered expression and/or localization of several ECM and adhesive molecules, including laminin beta1, fibronectin, alpha5 integrin, and E-cadherin. These changes correlated with alterations in the activation state of Ras-extracellular signal-regulated kinase (ERK), as well as the expression and/or localization of Cdc42 and RhoA, two Rho GTPases that regulate the organization of the actin cytoskeleton. We conclude that alpha3beta1 is required for normal salivary cell differentiation and that its absence affects multiple components of adhesive complexes and their associated signalling pathways.
12. Progressive myopathy and defects in the maintenance of myotendinous junctions in mice that lack talin 1 in skeletal muscle. The development and function of skeletal muscle depend on molecules that connect the muscle fiber cytoskeleton to the **extracellular matrix** (ECM). beta1 integrins are ECM receptors in skeletal muscle, and mutations that affect the alpha7beta1 integrin cause myopathy in humans. In mice, beta1 integrins control myoblast fusion, the assembly of the muscle fiber cytoskeleton, and the maintenance of myotendinous junctions (MTJs). The effector molecules that mediate beta1 integrin functions in muscle are not known. Previous studies have shown that talin 1 controls the force-dependent assembly of integrin adhesion complexes and regulates the affinity of integrins for ligands. Here we show that talin 1 is essential in skeletal muscle for the maintenance of integrin attachment sites at MTJs. Mice with a skeletal muscle-specific ablation of the talin 1 gene suffer from a progressive myopathy. Surprisingly, myoblast fusion and the assembly of integrin-containing adhesion complexes at costameres and MTJs advance normally in the mutants. However, with progressive ageing, the muscle fiber cytoskeleton detaches from MTJs. Mechanical measurements on isolated muscles show defects in the ability of talin 1-deficient muscle to generate force. Collectively, our findings show that talin 1 is essential for providing mechanical stability to integrin-dependent adhesion complexes at MTJs, which is crucial for optimal force generation by skeletal muscle.
13. Type I gamma phosphatidylinositol phosphate kinase targets and regulates focal adhesions. The ability of cells to form cell contacts, adhere to the **extracellular matrix**, change morphology, and migrate is essential for development, wound healing, metastasis, cell survival and the immune response. These events depend on the binding of integrin to the **extracellular matrix**, and assembly of focal adhesions, which are complexes comprising scaffolding and signalling proteins organized by adhesion to the **extracellular matrix**. Phosphatidylinositol-4,5-bisphosphate (PtdIns(4,5)P(2)) regulates interactions between these proteins, including the interaction of vinculin with actin and talin. The binding of talin to beta-integrin is strengthened by PtdIns(4,5)P(2), suggesting that the basis of focal adhesion assembly is regulated by this lipid mediator. Here we show that the type I phosphatidylinositol phosphate kinase isoform-gamma 661 (PIPKI gamma 661), an enzyme that makes PtdIns(4,5)P(2), is targeted to focal adhesions by an association with talin. PIPKI gamma 661 is tyrosine phosphorylated by focal adhesion associated kinase signalling, increasing both the activity of phosphatidylinositol phosphate kinase and its association with talin. This defines a mechanism for spatial generation of PtdIns(4,5)P(2) at focal adhesions.

#### GeneRIFs

1. Depletion of talin2 in talin1-null cells did not affect the initiation of matrix-activated spreading or Src family kinase activation, but abolished the **extracellular matrix**-integrin-cytoskeleton linkage, sustained cell spreading and adhesion.

### [Mm]etastasis

#### PubMed

1. Targeted downregulation of platelet CLEC-2 occurs through Syk-independent internalization. Platelet aggregation at sites of vascular injury is not only essential for hemostasis, but may also cause acute ischemic disease states such as myocardial infarction or stroke. The hemi-immunoreceptor tyrosine-based activation motif-containing C-type lectinlike receptor 2 (CLEC-2) mediates powerful platelet activation through a Src- and spleen tyrosine kinase (Syk)-dependent tyrosine phosphorylation cascade. Thereby, CLEC-2 not only contributes to thrombus formation and stabilization but also plays a central role in blood-lymphatic vessel development, tumor **metastasis**, and prevention of inflammatory bleeding, making it a potential pharmacologic target to modulate these processes. We have previously shown that injection of the anti-CLEC-2 antibody, INU1, results in virtually complete immunodepletion of platelet CLEC-2 in mice, which is, however, preceded by a severe transient thrombocytopenia thereby limiting its potential therapeutic use. The mechanisms underlying this targeted CLEC-2 downregulation have remained elusive. Here, we show that INU1-induced CLEC-2 immunodepletion occurs through Src-family kinase-dependent receptor internalization in vitro and in vivo, presumably followed by intracellular degradation. In mice with platelet-specific Syk deficiency, INU1-induced CLEC-2 internalization/degradation was fully preserved whereas the associated thrombocytopenia was largely prevented. These results show for the first time that CLEC-2 can be downregulated from the platelet surface through internalization in vitro and in vivo and that this can be mechanistically uncoupled from the associated antibody-induced thrombocytopenia.
2. Talin phosphorylation by Cdk5 regulates Smurf1-mediated talin head ubiquitylation and cell migration. Cell migration is a dynamic process that requires temporal and spatial regulation of integrin activation and focal adhesion assembly/disassembly. Talin, an actin and beta-integrin tail-binding protein, is essential for integrin activation and focal adhesion formation. Calpain-mediated cleavage of talin has a key role in focal adhesion turnover; however, the talin head domain, one of the two cleavage products, stimulates integrin activation, localizes to focal adhesions and maintains cell edge protrusions, suggesting that other steps, downstream of talin proteolysis, are required for focal adhesion disassembly. Here we show that talin head binds Smurf1, an E3 ubiquitin ligase involved in cell polarity and migration, more tightly than full-length talin does and that this interaction leads to talin head ubiquitylation and degradation. We found that talin head is a substrate for Cdk5, a cyclin-dependent protein kinase that is essential for cell migration, synaptic transmission and cancer **metastasis**. Cdk5 phosphorylated talin head at Ser 425, inhibiting its binding to Smurf1, thus preventing talin head ubiquitylation and degradation. Expression of the mutant tal(S425A), which resists Cdk5 phosphorylation thereby increasing its susceptibility to Smurf1-mediated ubiqitylation, resulted in extensive focal adhesion turnover and inhibited cell migration. Thus, talin head produced by calpain-induced cleavage of talin is degraded through Smurf1-mediated ubiquitylation; moreover, phosphorylation by Cdk5 regulates the binding of Smurf1 to talin head, controlling talin head turnover, adhesion stability and ultimately, cell migration.
3. Type I gamma phosphatidylinositol phosphate kinase targets and regulates focal adhesions. The ability of cells to form cell contacts, adhere to the extracellular matrix, change morphology, and migrate is essential for development, wound healing, **metastasis**, cell survival and the immune response. These events depend on the binding of integrin to the extracellular matrix, and assembly of focal adhesions, which are complexes comprising scaffolding and signalling proteins organized by adhesion to the extracellular matrix. Phosphatidylinositol-4,5-bisphosphate (PtdIns(4,5)P(2)) regulates interactions between these proteins, including the interaction of vinculin with actin and talin. The binding of talin to beta-integrin is strengthened by PtdIns(4,5)P(2), suggesting that the basis of focal adhesion assembly is regulated by this lipid mediator. Here we show that the type I phosphatidylinositol phosphate kinase isoform-gamma 661 (PIPKI gamma 661), an enzyme that makes PtdIns(4,5)P(2), is targeted to focal adhesions by an association with talin. PIPKI gamma 661 is tyrosine phosphorylated by focal adhesion associated kinase signalling, increasing both the activity of phosphatidylinositol phosphate kinase and its association with talin. This defines a mechanism for spatial generation of PtdIns(4,5)P(2) at focal adhesions.

---

## Arpc1a

- NCBI Gene
- GeneRIF
- Pubmed

| Search Term | Rank | Fields |
| --- | --- | --- |
| [Mm]igration | 6 | PubMed(1) |

  
hide/show details for Arpc1a

### [Mm]igration

#### PubMed

1. Recruitment of the Arp2/3 complex to vinculin: coupling membrane protrusion to matrix adhesion. Cell **migration** involves many steps, including membrane protrusion and the development of new adhesions. Here we have investigated whether there is a link between actin polymerization and integrin engagement. In response to signals that trigger membrane protrusion, the actin-related protein (Arp)2/3 complex transiently binds to vinculin, an integrin-associated protein. The interaction is regulated, requiring phosphatidylinositol-4,5-bisphosphate and Rac1 activation, and is sufficient to recruit the Arp2/3 complex to new sites of integrin aggregation. Binding of the Arp2/3 complex to vinculin is direct and does not depend on the ability of vinculin to associate with actin. We have mapped the binding site for the Arp2/3 complex to the hinge region of vinculin, and a point mutation in this region selectively blocks binding to the Arp2/3 complex. Compared with WT vinculin, expression of this mutant in vinculin-null cells results in diminished lamellipodial protrusion and spreading on fibronectin. The recruitment of the Arp2/3 complex to vinculin may be one mechanism through which actin polymerization and membrane protrusion are coupled to integrin-mediated adhesion.

---

## Hgs

- NCBI Gene
- GeneRIF
- Pubmed

| Search Term | Rank | Fields |
| --- | --- | --- |
| [Mm]etastasis | 12 | PubMed(1) |

  
hide/show details for Hgs

### [Mm]etastasis

#### PubMed

1. Inhibition of tumor growth and **metastasis** by depletion of vesicular sorting protein Hrs: its regulatory role on E-cadherin and beta-catenin. Abnormally high signals from receptor tyrosine kinases (RTK) are associated with carcinogenesis, and impaired deactivation of RTKs may also be a mechanism in cancer. Hepatocyte growth factor-regulated tyrosine kinase substrate (Hrs) is one of the master regulators that sort activated receptors toward lysosomes and shut down their signals. Hrs contains a ubiquitin-interacting motif and is involved in the endosomal sorting of monoubiquitinated membrane proteins, such as growth factor receptor and E-cadherin. Here, we investigated the role of Hrs in determining the malignancy of cancer cells and discovered that the targeted disruption of Hrs by small interfering RNA effectively attenuated the proliferation, anchorage-independent growth, tumorigenesis, and metastatic potential of HeLa cells in vitro and in vivo. The restoration of Hrs expression increased cell proliferation and anchorage-independent growth in a mouse embryonic fibroblast line established from a Hrs knockout mouse. Further analysis revealed that Hrs depletion was associated with the up-regulation of E-cadherin and reduced beta-catenin signaling. The aberrant accumulation of E-cadherin most likely resulted from impaired E-cadherin degradation in lysosomes. These results suggest that Hrs may play a critical role in determining the malignancy of cancer cells by regulating the degradation of E-cadherin.

---

## Ano10

- NCBI Gene
- GeneRIF
- Pubmed

| Search Term | Rank | Fields |
| --- | --- | --- |
| [Mm]igration | 6 | PubMed(1) |

  
hide/show details for Ano10

### [Mm]igration

#### PubMed

1. Expression and functional significance of the Ca(2+)-activated Cl(-) channel ANO6 in dendritic cells. **Migration** of dendritic cells (DCs), antigen presenting cells that link innate and adaptive immunity, is critical for initiation of immune responses. DC **migration** is controlled by the activity of different ion channels, which mediate Ca(2+) flux or set the membrane potential. Moreover, cell **migration** requires local volume changes at the leading and rear end of travelling cells, which might be mediated by the fluxes of osmotically active solutes, including Cl(-). The present study explored the functional expression, regulation and role of Cl(-) channels in mouse bone marrow-derived DCs.In whole-cell patch clamp experiments we detected outwardly rectifying Cl(-) currents which were activated by elevation of cytosolic Ca(2+), triggered either by ionomycin in the presence of extracellular Ca(2+) or mobilization of Ca(2+) by IP(3) Most importantly, Ca(2+)-activated Cl(-) channels (CaCCs) were activated by CCL21 (75 ng/ml), an agonist of the chemokine receptor CCR7. The currents showed sensitivity to Cl(-) channel blockers such as tannic acid (10 µM), digallic acid (100 µM) and more specific CaCC blockers niflumic acid (300 µM) and AO1 (20 µM). According to RT-PCR and Western blot data, Anoctamin 6 (ANO6) is expressed in DCs. Knock-down of ANO6 with siRNA led to inhibition of CaCC currents in DCs. Moreover, chemokine-indu**ced migra**tion of both immature and LPS-matured DCs was reduced upon ANO6 knock-down.Our data identify ANO6 as a Ca(2+)-activated Cl(-) channel in mouse DCs, show its activation upon chemokine receptor ligation and establish an important role of ANO6 in chemokine-induced **DC migra**tion.

---

## Rab6a

- NCBI Gene
- GeneRIF
- Pubmed

| Search Term | Rank | Fields |
| --- | --- | --- |
| [Mm]igration | 6 | PubMed(1) |

  
hide/show details for Rab6a

### [Mm]igration

#### PubMed

1. Persistent cell **migration** and adhesion rely on retrograde transport of β(1) integrin. Integrins have key functions in cell adhesion and **migratio**n. How integrins are dynamically relocalized to the leading edge in highly polarized migratory cells has remained unexplored. Here, we demonstrate that β1 integrin (known as PAT-3 in Caenorhabditis elegans), but not β3, is transported from the plasma membrane to the trans-Golgi network, to be resecreted in a polarized manner. This retrograde trafficking is restricted to the non-ligand-bound conformation of β1 integrin. Retrograde trafficking inhibition abrogates several β1-integrin-specific functions such as cell adhesion in early embryonic development of mice, and persisten**t cell mi**gration in the developing posterior gonad arm of C. elegans. Our results establish a paradigm according to which retrograde trafficking, and not endosomal recycling, is the key driver for β1 integrin function in highly polarized cells. These data more generally suggest that the retrograde route is used to relocalize plasma membrane machinery from previous sites of function to the leading edge of migratory cells.

---

## Shc1

- NCBI Gene
- GeneRIF
- Pubmed

| Search Term | Rank | Fields |
| --- | --- | --- |
| [Mm]igration | 6 | PubMed(8); GeneRIFs(2) |
| ECM | 7 | PubMed(1) |
| [Ee]xtracellular [Mm]atrix | 8 | PubMed(5) |
| [Ii]nvasive | 10 | PubMed(1) |
| [Mm]etastasis | 12 | PubMed(2); GeneRIFs(1) |
| [Cc]ell [Ii]nvasion | 13 | PubMed(1) |

  
hide/show details for Shc1

### [Mm]igration

#### PubMed

1. The redox enzyme p66Shc contributes to diabetes and ischemia-induced delay in cutaneous wound healing. The redox enzyme p66Shc produces hydrogen peroxide and triggers proapoptotic signals. Genetic deletion of p66Shc prolongs life span and protects against oxidative stress. In the present study, we evaluated the role of p66Shc in an animal model of diabetic wound healing.Skin wounds were created in wild-type (WT) and p66Shc(-/-) control and streptozotocin-induced diabetic mice with or without hind limb ischemia. Wounds were assessed for collagen content, thickness and vascularity of granulation tissue, apoptosis, reepithelialization, and expression of c-myc and beta-catenin. Response to hind limb ischemia was also evaluated.Diabetes delayed wound healing in WT mice with reduced granulation tissue thickness and vascularity, increased apoptosis, epithelial expression of c-myc, and nuclear localization of beta-catenin. These nonhealing features were worsened by hind limb ischemia. Diabetes induced p66Shc expression and activation; wound healing was significantly faster in p66Shc(-/-) than in WT diabetic mice, with or without hind limb ischemia, at 1 and 3 months of diabetes duration and in both SV129 and C57BL/6 genetic backgrounds. Deletion of p66Shc reversed nonhealing features, with increased collagen content and granulation tissue thickness, and reduced apoptosis and expression of c-myc and beta-catenin. p66Shc deletion improved response to hind limb ischemia in diabetic mice in terms of tissue damage, capillary density, and perfusion. **Migration** of p66Shc(-/-) dermal fibroblasts in vitro was significantly faster than WT fibroblasts under both high glucose and hypoxia.p66Shc is involved in the delayed wound-healing process in the setting of diabetes and ischemia. Thus, p66Shc may represent a potential therapeutic target against this disabling diabetes complication.
2. The adaptor protein Shc integrates growth factor and ECM signaling during postnatal angiogenesis. Angiogenesis requires integration of cues from growth factors, extracellular matrix (ECM) proteins, and their receptors in endothelial cells. In the present study, we show that the adaptor protein Shc is required for angiogenesis in zebrafish, mice, and cell-culture models. Shc knockdown zebrafish embryos show defects in intersegmental vessel sprouting in the trunk. Shc flox/flox; Tie2-Cre mice display reduced angiogenesis in the retinal neovascularization model and in response to VEGF in the Matrigel plug assay in vivo. Functional studies reveal a model in which Shc is required for integrin-mediated spreading and **migration** specifically on fibronectin, as well as endothelial cell survival in response to VEGF. Mechanistically, Shc is required for activation of the Akt pathway downstream of both integrin and VEGF signaling, as well as for integration of signals from these 2 receptors when cells are grown on fibronectin. Therefore, we have identified a unique mechanism in which signals from 2 critical angiogenic signaling axes, integrins and VEGFR-2, converge at Shc to regulate postnatal angiogenesis.
3. The mitochondrial reactive oxygen species regulator p66Shc controls PDGF-induced signaling and **migration** through protein tyrosine phosphatase oxidation. Growth factor receptors induce a transient increase in reactive oxygen species (ROS) levels upon receptor binding to promote signaling through oxidation of protein tyrosine phosphatases (PTPs). Most studies have focused on NADPH oxidases as the dominant source of ROS to induce PTP oxidation. A potential additional regulator of growth factor-induced PTP oxidation is p66Shc, which stimulates mitochondrial ROS production. This study explores the contribution of p66Shc-induced ROS to PTP oxidation and growth factor receptor-induced signaling and **migration** through analyses of p66Shc-KO fibroblasts and cells with siRNA-mediated p66Shc downregulation. Analyses of PDGFβR phosphorylation in two independent cell systems demonstrated a decrease in PDGFβR phosphorylation after p66Shc deletion or downregulation, which occurred in a partially site-selective and antioxidant-sensitive manner. Deletion of p66Shc also reduced PDGF-induced activation of downstream signaling of Erk, Akt, PLCγ-1, and FAK. Importantly, reduced levels of p66Shc led to decreased oxidation of DEP1, PTP1B, and SHP2 after PDGF stimulation. The cell biological relevance of these findings was indicated by demonstration of a significantly reduced migratory response in PDGF-stimulated p66Shc-KO fibroblasts, consistent with reduced PDGFβR-Y1021 and PLCγ-1 phosphorylation. Downregulation of p66Shc also reduced EGFR phosphorylation and signaling, indicating that the positive role of p66Shc in receptor tyrosine kinase signaling is potentially general. Moreover, downregulation of the mitochondrial hydrogen peroxide scavenger peroxiredoxin 3 increased PDGFβR phosphorylation, showing that mitochondrial ROS in general promote PDGFβR signaling. This study thus identifies a previously unrecognized role for p66Shc in the regulation of PTP oxidation controlling growth factor-induced signali**ng and mi**gration. In more general terms, the study indicates a regulatory role for mitochondrial-derived ROS in the control of PTP oxidation influencing growth factor signaling.
4. EphB1 recruits c-Src and p52Shc to activate MAPK/ERK and promote chemotaxis. Eph receptors and their ligands (ephrins) play an important role in axonal guidance, topographic mapping, and angiogenesis. The signaling pathways mediating these activities are starting to emerge and are highly cell- and receptor-type specific. Here we demonstrate that activated EphB1 recruits the adaptor proteins Grb2 and p52Shc and promotes p52Shc and c-Src tyrosine phosphorylation as well as MAPK/extracellular signal-regulated kinase (ERK) activation. EphB1-mediated increase of cell **migration** was abrogated by the MEK inhibitor PD98059 and Src inhibitor PP2. In contrast, cell adhesion, which we previously showed to be c-jun NH2-terminal kinase (JNK) dependent, was unaffected by ERK1/2 and Src inhibition. Expression of dominant-negative c-Src significantly reduced EphB1-dependent ERK1/2 activation and chemotaxis. Site-directed mutagenesis experiments demonstrate that tyrosines 600 and 778 of EphB1 are required for its interaction with c-Src and p52Shc. Furthermore, phosphorylation of p52Shc by c-Src is essential for its recruitment to EphB1 signaling complexes through its phosphotyrosine binding domain. Together these findings highlight a new aspect of EphB1 signaling, whereby the concerted action of c-Src and p52Shc activates MAPK/ERK and regulates events involved in cell motility.
5. Sequence-specific peptide aptamers, interacting with the intracellular domain of the epidermal growth factor receptor, interfere with Stat3 activation and inhibit the growth of tumor cells. Receptor tyrosine kinases of the epidermal growth factor (EGF) receptor family regulate essential cellular functions such as proliferation, survival, **migration**, and differentiation but also play central roles in the etiology and progression of tumors. We have identified short peptide sequences from a random peptide library integrated into the thioredoxin scaffold protein, which specifically bind to the intracellular domain of the EGF receptor (EGFR). These molecules have the potential to selectively inhibit specific aspects of EGF receptor signaling and might become valuable as anticancer agents. Intracellular expression of the aptamer encoding gene construct KDI1 or introduction of bacterially expressed KDI1 via a protein transduction domain into EGFR-expressing cells results in KDI1.EGF receptor complex formation, a slower proliferation, and reduced soft agar colony formation. Aptamer KDI1 did not summarily block the EGF receptor tyrosine kinase activity but selectively interfered with the EGF-induced phosphorylation of the tyrosine residues 845, 1068, and 1148 as well as the phosphorylation of tyrosine 317 of p46 Shc. EGF-induced phosphorylation of Stat3 at tyrosine 705 and Stat3-dependent transactivation were also impaired. Transduction of a short synthetic peptide aptamer sequence not embedded into the scaffold protein resulted in the same impairment of EGF-induced Stat3 activation.
6. Distinct phosphotyrosine-dependent functions of the ShcA adaptor protein are required for transforming growth factor β (TGFβ)-induced breast cancer cel**l migrati**on, invasion, and metastasis. The ErbB2 and TGFβ signaling pathways cooperate to promote the migratory, invasive, and metastatic behavior of breast cancer cells. We previously demonstrated that ShcA is necessary for these synergistic interactions. Through a structure/function approach, we now show that the phosphotyrosine-binding, but not the Src homology 2, domain of ShcA is required for TGFβ-indu**ced migra**tion and invasion of ErbB2-expressing breast cancer cells. We further demonstrate that the tyrosine phosphorylation sites within ShcA (Tyr(239)/Tyr(240) and Tyr(313)) transduce distinct and non-redundant signals that promote these TGFβ-mediated effects. We demonstrate that Grb2 is required specifically downstream of Tyr(313), whereas the Tyr(239)/Tyr(240) phosphorylation sites require the Crk adaptor proteins to augment TGFβ-in**duced mig**ration and invasion. Furthermore, ShcA Tyr(313) phosphorylation enhances tumor cell survival, and ShcA Tyr(239)/Tyr(240) signaling promotes endothelial cell recruitment into ErbB2-expressing breast tumors in vivo, whereas all three ShcA tyrosine residues are required for efficient breast cancer metastasis to the lungs. Our data uncover a novel ShcA-dependent signaling axis downstream of TGFβ and ErbB2 that requires both the Grb2 and Crk adaptor proteins to increase the migratory and invasive properties of breast cancer cells. In addition, signaling downstream of specific ShcA tyrosine residues facilitates the survival, vascularization, and metastatic spread of breast tumors.
7. Negative regulation of chemokine receptor signaling and B-cell chemotaxis by p66Shc. Shc (Src homology 2 domain containing) adaptors are ubiquitous components of the signaling pathways triggered by tyrosine kinase-coupled receptors. In lymphocytes, similar to other cell types, the p52 and p66 isoforms of ShcA/Shc participate in a self-limiting loop where p52Shc acts as a positive regulator of antigen receptor signaling by promoting Ras activation, whereas p66Shc limits this activity by competitively inhibiting p52Shc. Based on the fact that many signaling mediators are shared by antigen and chemokine receptors, including p52Shc, we have assessed the potential implication of p66Shc in the regulation of B-cell responses to chemokines, focusing on the homing receptors CXCR4 (C-X-C chemokine receptor type 4) and CXCR5 (C-X-C chemokine receptor type 5). The results identify p66Shc as a negative regulator of the chemotactic responses triggered by these receptors, including adhesion, polarization and **migration**. We also provide evidence that this function is dependent on the ability of p66Shc to interact with the chemokine receptors and promote the assembly of an inhibitory complex, which includes the phosphatases SHP-1 (Src homology phosphatase-1) and SHIP-1 (SH2 domain-containing inositol 5'-phosphatase-1), that results in impaired Vav-dependent reorganization of the actin cytoskeleton. This function maps to the phosphorylatable tyrosine residues in the collagen homology 1 (CH1) domain. The results identify p66Shc as a negative regulator of B-cell chemotaxis and suggest a role for this adaptor in the control of B-cell homing.
8. Mutant p53 disrupts role of ShcA protein in balancing Smad protein-dependent and -independent signaling activity of transforming growth factor-β (TGF-β). Biomarkers are lacking for identifying the switch of transforming growth factor-β (TGF-β) from tumor-suppressing to tumor-promoting. Mutated p53 (mp53) has been suggested to switch TGF-β to a tumor promoter. However, we found that mp53 does not always promote the oncogenic role of TGF-β. Here, we show that endogenous mp53 knockdown enhanced **cell mig**ration and phosphorylation of ERK in DU145 prostate cancer cells. Furthermore, ectopic expression of mp53 in p53-null PC-3 prostate cancer cells enhanced Smad-dependent signaling but inhibited TGF-β-induce**d cell mi**gration by down-regulating activated ERK. Reactivation of ERK by the expression of its activator, MEK-1, restored TGF-β-induc**ed cell m**igration. Because TGF-β is known to activate the MAPK/ERK pathway through direct phosphorylation of the adaptor protein ShcA and MAPK/ERK signaling is pivotal to tumor progression, we investigated whether ShcA contributed to mp53-induced ERK inhibition and the conversion of the role of TGF-β during carcinogenesis. We found that mp53 expression led to a decrease of phosphorylated p52ShcA/ERK levels and an increase of phosphorylated Smad levels in a panel of mp53-expressing cancer cell lines and in mammary glands and tumors from mp53 knock-in mice. By manipulating ShcA levels to regulate ERK and Smad signaling in human untransformed and cancer cell lines, we showed that the role of TGF-β in regulating anchorage-dependent and -independent **growth an**d migration can be shifted between growth suppr**ession an**d migration promotion. Thus, our results for the first time suggest that mp53 disrupts the role of ShcA in balancing the Smad-dependent and -independent signaling activity of TGF-β and that ShcA/ERK signaling is a major pathway regulating the tumor-promoting activity of TGF-β.

#### GeneRIFs

1. This study thus identifies a previously unrecognized role for p66Shc in the regulation of PTP oxidation controlling growth factor-induced signaling and **migration**.
2. Distinct phosphotyrosine-dependent functions of the ShcA adaptor protein are required for transforming growth factor beta (TGFbeta)-induced breast cancer cell **migration**, invasion, and metastasis

### ECM

#### PubMed

1. The adaptor protein Shc integrates growth factor and **ECM** signaling during postnatal angiogenesis. Angiogenesis requires integration of cues from growth factors, extracellular matrix (**ECM**) proteins, and their receptors in endothelial cells. In the present study, we show that the adaptor protein Shc is required for angiogenesis in zebrafish, mice, and cell-culture models. Shc knockdown zebrafish embryos show defects in intersegmental vessel sprouting in the trunk. Shc flox/flox; Tie2-Cre mice display reduced angiogenesis in the retinal neovascularization model and in response to VEGF in the Matrigel plug assay in vivo. Functional studies reveal a model in which Shc is required for integrin-mediated spreading and migration specifically on fibronectin, as well as endothelial cell survival in response to VEGF. Mechanistically, Shc is required for activation of the Akt pathway downstream of both integrin and VEGF signaling, as well as for integration of signals from these 2 receptors when cells are grown on fibronectin. Therefore, we have identified a unique mechanism in which signals from 2 critical angiogenic signaling axes, integrins and VEGFR-2, converge at Shc to regulate postnatal angiogenesis.

### [Ee]xtracellular [Mm]atrix

#### PubMed

1. Ablation of the gene encoding p66Shc protects mice against AGE-induced glomerulopathy by preventing oxidant-dependent tissue injury and further AGE accumulation. AGEs have been implicated in renal disease associated with ageing, diabetes and other age-related disorders. Reactive oxygen species (ROS) promote formation of AGEs, which cause AGE-receptor-mediated ROS generation with activation of signalling pathways leading to tissue injury and further AGE accumulation. ROS generation is regulated by the Src homology 2 domain-containing transforming protein C1 (Shc1) isoform p66(Shc), whose deletion has been shown to protect from tissue injury induced by ageing, diabetes, hyperlipidaemia and ischaemia-reperfusion by preventing oxidative stress. This study was aimed at assessing the role of p66(Shc) in the modulation of oxidative stress and oxidant-dependent renal injury induced by AGEs.For 10 weeks, male p66 (shc) knockout (KO) and wild-type (WT) mice were injected with 60 microg/day albumin modified or unmodified by N epsilon-(carboxymethyl) lysine (CML). Mice were then killed for the assessment of renal function and structure, as well as systemic and renal tissue oxidative stress.Upon CML injection, KO mice, in contrast to WT mice, showed no or only mild forms of proteinuria, glomerular hypertrophy, mesangial expansion, glomerular sclerosis, renal/glomerular cell apoptosis and **extracellular matrix** upregulation. Moreover, KO mice had lower circulating and tissue AGEs than WT mice and unchanged plasma isoprostane 8-epi-prostaglandin-F(2alpha) levels, renal/glomerular CML, 4-hydroxy-2-nonenal, AGE receptor and NAD(P)H oxidase 4 (NOX4) content (and expression of the corresponding genes), and nuclear factor kappaB activation (NFkappaB). Mesangial cells from KO mice exposed to CML showed no or slight increase in ROS levels and NFkappaB activation, again at variance with WT cells.These data indicate that p66(Shc) participates in the pathogenesis of AGE-dependent glomerulopathy by mediating AGE-induced tissue injury and further AGE formation through ROS-dependent mechanisms involving NFkappaB activation and upregulation of Nox4 expression and NOX4 production.
2. The adaptor protein Shc integrates growth factor and ECM signaling during postnatal angiogenesis. Angiogenesis requires integration of cues from growth factors, **extracellular matrix** (ECM) proteins, and their receptors in endothelial cells. In the present study, we show that the adaptor protein Shc is required for angiogenesis in zebrafish, mice, and cell-culture models. Shc knockdown zebrafish embryos show defects in intersegmental vessel sprouting in the trunk. Shc flox/flox; Tie2-Cre mice display reduced angiogenesis in the retinal neovascularization model and in response to VEGF in the Matrigel plug assay in vivo. Functional studies reveal a model in which Shc is required for integrin-mediated spreading and migration specifically on fibronectin, as well as endothelial cell survival in response to VEGF. Mechanistically, Shc is required for activation of the Akt pathway downstream of both integrin and VEGF signaling, as well as for integration of signals from these 2 receptors when cells are grown on fibronectin. Therefore, we have identified a unique mechanism in which signals from 2 critical angiogenic signaling axes, integrins and VEGFR-2, converge at Shc to regulate postnatal angiogenesis.
3. The ShcA phosphotyrosine docking protein sensitizes cardiovascular signaling in the mouse embryo. The ShcA gene products have served as a model for the analysis of phosphotyrosine-recognition domains, and for the functions of docking proteins during tyrosine kinase signaling. Here we show that ShcA is primarily expressed in the cardiovascular system during early mouse embryogenesis and regulates both heart development and establishment of mature blood vessels. Targeted mutation suggests that the ShcA adaptor is a pivotal target of tyrosine kinases that selectively potentiates activation of the MAP kinase pathway in the remodeling vasculature. Biochemical analysis of mutant cells shows that ShcA sensitizes cells to growth factor-induced MAP kinase activation, and also organizes cytoskeletal rearrangement in response to the **extracellular matrix**. ShcA may therefore orchestrate complex interactions within the vascular compartment by rendering cells permissive to respond to soluble and adhesive external cues.
4. beta1 integrins modulate p66ShcA expression and EGF-induced MAP kinase activation in fetal lung cells. ShcA proteins mediate Erk1/Erk2 activation by integrins and epidermal growth factor (EGF), and are expressed as p46ShcA, p52ShcA, and p66ShcA. Although p52ShcA and p46ShcA mediate Erk1/Erk2 activation, p66ShcA antagonizes Erk activation. p66ShcA is spatially regulated during lung development, leading us to hypothesize that integrin signaling regulates p66ShcA expression and, consequently, EGF signaling. Fetal lung mesenchymal cells were isolated from E16 Swiss-Webster mice, stimulated with oligopeptide **extracellular matrix** analogs or anti-integrin antibodies, and subjected to ShcA Western analyses and EGF-stimulated Erk1/Erk2 kinase assays. p66ShcA expression was decreased by anti-alpha1 integrin antibody and DGEA collagen analog, and increased by anti-beta1, anti-alpha4, and anti-alpha5 integrin antibodies and RGDS fibronectin analog. Paradoxically, beta1 integrin stimulation increased EGF-induced Erk activation while increasing expression of the inhibitory p66ShcA isoform. This paradox was resolved by demonstrating that Erk inhibition attenuates integrin-mediated p66ShcA induction. These results suggest that p66ShcA is up-regulated as inhibitory feedback on integrin-mediated Erk activation.
5. The ShcA phosphotyrosine docking protein uses distinct mechanisms to regulate myocyte and global heart function. Although tyrosine kinases (TKs) are important for cardiac function, their relevant downstream targets in the adult heart are unknown. The ShcA docking protein binds specific phosphotyrosine (pTyr) sites on activated TKs through its N-terminal pTyr-binding (PTB) and C-terminal SH2 domains and stimulates downstream pathways through motifs such as pTyr sites in its central CH1 region. Therefore, ShcA could be a potential hub for downstream TK signaling in the myocardium.To define the role of ShcA, a TK scaffold, in the adult heart using a myocardial-specific knockout of murine ShcA (ShcA CKO) and domain knock-in models.ShcA CKO mice developed a dilated cardiomyopathy phenotype involving impaired systolic function with enhanced cardiomyocyte contractility. This uncoupling of global heart and intrinsic myocyte functions was associated with altered collagen and **extracellular matrix** compliance properties, suggesting disruption of mechanical coupling. In vivo dissection of ShcA signaling properties revealed that selective inactivation of the PTB domain in the myocardium had effects resembling those seen in ShcA CKO mice, whereas disruption of the SH2 domain caused a less severe cardiac phenotype. Downstream signaling through the CH1 pTyr sites was dispensable for baseline cardiac function but necessary to prevent adverse remodeling after hemodynamic overload.These data demonstrate a requirement for TK-ShcA PTB domain signaling to maintain cardiac function. In addition, analysis of the SH2 domain and CH1 pTyr sites reveals that ShcA mediates pTyr signaling in the adult heart through multiple distinct signaling elements that control myocardial functions and response to stresses.

### [Ii]nvasive

#### PubMed

1. Distinct phosphotyrosine-dependent functions of the ShcA adaptor protein are required for transforming growth factor β (TGFβ)-induced breast cancer cell migration, invasion, and metastasis. The ErbB2 and TGFβ signaling pathways cooperate to promote the migrator**y, invas**ive, and metastatic behavior of breast cancer cells. We previously demonstrated that ShcA is necessary for these synergistic interactions. Through a structure/function approach, we now show that the phosphotyrosine-binding, but not the Src homology 2, domain of ShcA is required for TGFβ-induced migration and invasion of ErbB2-expressing breast cancer cells. We further demonstrate that the tyrosine phosphorylation sites within ShcA (Tyr(239)/Tyr(240) and Tyr(313)) transduce distinct and non-redundant signals that promote these TGFβ-mediated effects. We demonstrate that Grb2 is required specifically downstream of Tyr(313), whereas the Tyr(239)/Tyr(240) phosphorylation sites require the Crk adaptor proteins to augment TGFβ-induced migration and invasion. Furthermore, ShcA Tyr(313) phosphorylation enhances tumor cell survival, and ShcA Tyr(239)/Tyr(240) signaling promotes endothelial cell recruitment into ErbB2-expressing breast tumors in vivo, whereas all three ShcA tyrosine residues are required for efficient breast cancer metastasis to the lungs. Our data uncover a novel ShcA-dependent signaling axis downstream of TGFβ and ErbB2 that requires both the Grb2 and Crk adaptor proteins to increase the migrato**ry and i**nvasive properties of breast cancer cells. In addition, signaling downstream of specific ShcA tyrosine residues facilitates the survival, vascularization, and metastatic spread of breast tumors.

### [Mm]etastasis

#### PubMed

1. Distinct phosphotyrosine-dependent functions of the ShcA adaptor protein are required for transforming growth factor β (TGFβ)-induced breast cancer cell migration, invasion, an**d metastas**is. The ErbB2 and TGFβ signaling pathways cooperate to promote the migratory, invasive, and metastatic behavior of breast cancer cells. We previously demonstrated that ShcA is necessary for these synergistic interactions. Through a structure/function approach, we now show that the phosphotyrosine-binding, but not the Src homology 2, domain of ShcA is required for TGFβ-induced migration and invasion of ErbB2-expressing breast cancer cells. We further demonstrate that the tyrosine phosphorylation sites within ShcA (Tyr(239)/Tyr(240) and Tyr(313)) transduce distinct and non-redundant signals that promote these TGFβ-mediated effects. We demonstrate that Grb2 is required specifically downstream of Tyr(313), whereas the Tyr(239)/Tyr(240) phosphorylation sites require the Crk adaptor proteins to augment TGFβ-induced migration and invasion. Furthermore, ShcA Tyr(313) phosphorylation enhances tumor cell survival, and ShcA Tyr(239)/Tyr(240) signaling promotes endothelial cell recruitment into ErbB2-expressing breast tumors in vivo, whereas all three ShcA tyrosine residues are required for efficient breast c**ancer meta**stasis to the lungs. Our data uncover a novel ShcA-dependent signaling axis downstream of TGFβ and ErbB2 that requires both the Grb2 and Crk adaptor proteins to increase the migratory and invasive properties of breast cancer cells. In addition, signaling downstream of specific ShcA tyrosine residues facilitates the survival, vascularization, and metastatic spread of breast tumors.
2. p66(Shc) restrains Ras hyperactivation and suppresses metastatic behavior. Normal tissue cells survive and proliferate only while anchored to solid substrate. Conversely, transformed cells both survive and proliferate following detachment, having lost attachment context through unclear mechanisms. p66(Shc) is a focal adhesion-associated protein that reports cell attachment through a RhoA-dependent mechanosensory test. We find that human small cell lung cancer (SCLC) cells and mouse Lewis lung carcinoma (LLC), which display aggressive metastatic behavior, lack both p66(Shc) and retinoblastoma (pRB) and bypass anoikis. Re-expression of p66(Shc) in these cells restores anoikis and provides striking protection from **metastasis** by LLC cells in vivo. Notably, knockdown of p66(Shc) in normal epithelial cells leads to unrestrained Ras activation, preventing anoikis through downstream suppression of RhoA but blocking proliferation in a pRB-dependent manner, thus mimicking oncogenic Ras. Conversely, LLC and SCLC cells display constitutive Ras activation necessary to bypass anoikis, which is reversed by re-expression of p66(Shc). p66(Shc) therefore coordinates Ras-dependent control of proliferation and anchorage sensation, which can be defeated in the evolution of highly metastatic tumors by combined loss of both p66(Shc) and pRB.

#### GeneRIFs

1. Distinct phosphotyrosine-dependent functions of the ShcA adaptor protein are required for transforming growth factor beta (TGFbeta)-induced breast cancer cell migration, invasion, and **metastasis**

### [Cc]ell [Ii]nvasion

#### PubMed

1. Temporal regulation of EGF signalling networks by the scaffold protein Shc1. Cell-surface receptors frequently use scaffold proteins to recruit cytoplasmic targets, but the rationale for this is uncertain. Activated receptor tyrosine kinases, for example, engage scaffolds such as Shc1 that contain phosphotyrosine (pTyr)-binding (PTB) domains. Using quantitative mass spectrometry, here we show that mammalian Shc1 responds to epidermal growth factor (EGF) stimulation through multiple waves of distinct phosphorylation events and protein interactions. After stimulation, Shc1 rapidly binds a group of proteins that activate pro-mitogenic or survival pathways dependent on recruitment of the Grb2 adaptor to Shc1 pTyr sites. Akt-mediated feedback phosphorylation of Shc1 Ser 29 then recruits the Ptpn12 tyrosine phosphatase. This is followed by a sub-network of proteins involved in cytoskeletal reorganization, trafficking and signal termination that binds Shc1 with delayed kinetics, largely through the SgK269 pseudokinase/adaptor protein. Ptpn12 acts as a switch to convert Shc1 from pTyr/Grb2-based signalling to SgK269-mediated pathways that regulat**e cell invasi**on and morphogenesis. The Shc1 scaffold therefore directs the temporal flow of signalling information after EGF stimulation.

---

## Msn

- NCBI Gene
- GeneRIF
- Pubmed

| Search Term | Rank | Fields |
| --- | --- | --- |
| [Ff]ilopodia | 1 | PubMed(1) |
| [Ff]ilopodium | 2 | Component(2) |
| [Pp]odosome(s)? | 5 | Process(1); PubMed(1) |
| [Mm]igration | 6 | Process(2); PubMed(5); GeneRIFs(2) |
| [Ee]xtracellular [Mm]atrix | 8 | PubMed(3); GeneRIFs(1) |
| [Ii]nvasive | 10 | PubMed(2) |

  
hide/show details for Msn

### [Ff]ilopodia

#### PubMed

1. Structure and localization on the X chromosome of the gene coding for the human **filopodia**l protein moesin (MSN). Moesin is a member of a recently discovered family of closely related proteins that includes ezrin, radixin, and merlin. It is widely expressed in different tissues and cells and has been localized to **filopodia** and other membranous protrusions that are important for cell-cell recognition and signaling and cell movement. Here, we have localized the coding gene (MSN) to Xq11.2-q12 by Southern and Western blot analyses of Chinese hamster x human somatic cell hybrids and by fluorescence chromosomal in situ hybridization. Moesin-like sequences were identified on chromosomes 5 and 6. The murine Msn locus was mapped to the X chromosome as well by studying a rodent x mouse hybrid panel. The structure of the human moesin gene has been determined. The 12 exons are distributed over > 30 kb, and the exon/intron junctions demarcate individual highly conserved domains. Primer extension analysis revealed two major start transcription sites, 184 and 133 bp upstream of the initiation codon. The 5'-flanking region is GC-rich, lacks a TATA box, and contains four SP1 and one AP1 binding sites.

### [Ff]ilopodium

#### Component

1. **filopodium**
2. **filopodium** membrane

### [Pp]odosome(s)?

#### Process

1. positive regulation of **podosome** assembly

#### PubMed

1. Phosphorylation of moesin by Jun N-terminal kinase is important for **podosome** rosette formation in Src-transformed fibroblasts. **Podosomes** are actin-based membrane protrusions that facilitate extracellular matrix degradation and motility of invasive cells. **Podosomes** can self-organize into large rosette-like structures in Src-transformed fibroblasts, osteoclasts and some highly invasive cancer cells. However, the mechanism of this assembly remains obscure. In this study, we show that the suppression of Jun N-terminal kinase (JNK) by the JNK inhibitor SP600125 or short-hairpin RNA inhibited **podosome** rosette formation in SrcY527F-transformed NIH3T3 fibroblasts. In addition, SrcY527F was less able to induce **podosome** rosettes in JNK1-null or JNK2-null mouse embryo fibroblasts than in wild-type counterparts. The kinase activity of JNK was essential for promoting **podosome** rosette formation but not for its localization to **podosome** rosettes. Moesin, a member of the ERM (ezrin, radixin and moesin) protein family, was identified as a substrate of JNK. We show that the phosphorylation of moesin at Thr558 by JNK was important for **podosome** rosette formation in SrcY527F-transformed NIH3T3 fibroblasts. Taken together, our results unveil a novel role of JNK in **podosome** rosette formation through the phosphorylation of moesin.

### [Mm]igration

#### Process

1. T cell **migration**
2. regulation of lymphocyte **migration**

#### PubMed

1. CD43 interaction with ezrin-radixin-moesin (ERM) proteins regulates T-cell trafficking and CD43 phosphorylation. Cell polarization is a key feature of cell motility, driving cell **migration** to tissues. CD43 is an abundantly expressed molecule on the T-cell surface that shows distinct localization to the migrating T-cell uropod and the distal pole complex (DPC) opposite the immunological synapse via association with the ezrin-radixin-moesin (ERM) family of actin regulatory proteins. CD43 regulates multiple T-cell functions, including T-cell activation, proliferation, apoptosis, and **migration**. We recently demonstrated that CD43 regulates T-cell trafficking through a phosphorylation site at Ser-76 (S76) within its cytoplasmic tail. Using a phosphorylation-specific antibody, we now find that CD43 phosphorylation at S76 is enhanced by **migration** signals. We further show that CD43 phosphorylation and normal T-cell trafficking depend on CD43 association with ERM proteins. Interestingly, mutation of S76 to mimic phosphorylation enhances T-cell **migration** and CD43 movement to the DPC while blocking ERM association, showing that CD43 movement can occur in the absence of ERM binding. We also find that protein kinase CΘ can phosphorylate CD43. These results show that while CD43 binding to ERM proteins is crucial for S76 phosphorylation, CD43 movement and regulation of T-cell **migratio**n can occur through an ERM-independent, phosphorylation-dependent mechanism.
2. Moesin-deficient mice reveal a non-redundant role for moesin in lymphocyte homeostasis. Moesin is a member of the ezrin-radixin-moesin (ERM) family of cytoskeletal proteins. These proteins organize membrane domains by interacting with plasma membrane proteins and the actin cytoskeleton. Because of their high sequence similarity, ERM proteins are usually thought to be functionally redundant. Lymphocytes express two ERM proteins, ezrin and moesin. Whether each ERM plays a specialized role in lymphocytes, particularly in vivo, remains unknown. Here, we show that moesin has a crucial, non-redundant role in lymphocyte homeostasis. Moesin-deficient mice exhibited decreases in both T and B cells in the peripheral blood and lymph nodes, but not in the spleen. This phenotype was recapitulated in bone marrow (BM) chimeras with a hematopoietic moesin deficiency. Although the T and B cells apparently developed without major defects in the moesin-deficient mice, T cell egress from the thymus and immature B cell egress from the BM were impaired. In the periphery, both T and B cells showed delayed egress from lymphoid organs. We showed that moesin is the primary phosphorylated ERM subject to dynamic regulation during cell shape changes and **migration**. Our findings identify a previously unknown, non-redundant function of moesin in lymphocyte homeostasis in regulating lymphocyte egress from lymphoid organs.
3. Attenuated response to liver injury in moesin-deficient mice: impaired stellate cell **migration** and decreased fibrosis. Hepatic stellate cells (HSCs) respond to injury with a coordinated set of events (termed activation), which includes **migration** and upregulation of matrix protein production. Cell **migration** requires an intact actin cytoskeleton that is linked to the plasma membrane by ezrin-radixin-moesin (ERM) proteins. We have previously found that the linker protein in HSCs is exclusively moesin. Here, we describe HSC **migration** and fibrogenesis in moesin-deficient mice. We developed an acute liver injury model that involved focal thermal denaturation and common bile duct ligation. HSC **migration** and collagen deposition were assessed by immunohistology and quantitative real-time PCR. Activated HSCs were isolated from wild-type or moesin-deficient mice for direct examination of **migration**. Activated HSCs from wild-type mice were positive for moesin. **Migration** of moesin-deficient HSCs was significantly reduced. In a culture assay, 22.1% of normal HSCs migrated across a filter in 36 h. In contrast, only 1.3% of activated moesin-deficient HSCs migrated. Collagen deposition around the injury area similarly was reduced in moesin-deficient liver. The linker protein moesin is essential for HSC activation and **migration** in response to injury. Fibrogenesis is coupled to **migration** and reduced in moesin-deficient mice. Agents that target moesin may be beneficial for chronic progressive fibrosis.
4. LOK is a major ERM kinase in resting lymphocytes and regulates cytoskeletal rearrangement through ERM phosphorylation. ERM (ezrin-radixin-moesin) proteins mediate linkage of actin cytoskeleton to plasma membrane in many cells. ERM activity is regulated in part by phosphorylation at a C-terminal threonine, but the identity of ERM kinases is unknown in lymphocytes and incompletely defined in other mammalian cells. Our studies show that lymphocyte-oriented kinase (LOK) is an ERM kinase in vitro and in vivo. Mass spectrometric analysis indicates LOK is abundant at the lymphocyte plasma membrane and immunofluorescence studies show LOK enrichment at the plasma membrane near ERM. In vitro peptide specificity analyses characterize LOK as a basophilic kinase whose optimal substrate sequence resembles the ERM site, including unusual preference for tyrosine at P-2. LOK's activity on moesin peptide and protein was comparable to reported ERM kinases ROCK and PKC but unlike them LOK displayed preferential specificity for moesin compared to traditional basophilic kinase substrates. Two genetic approaches demonstrate a role for LOK in ERM phosphorylation: cell transfection with LOK kinase domain augments ERM phosphorylation and lymphocytes from LOK knockout mice have >50% reduction in ERM phosphorylation. The findings on localization and specificity argue that LOK is a direct ERM kinase. The knockout mice have normal hematopoietic cell development but notably lymphocyte **migration** and polarization in response to chemokine are enhanced. These functional alterations fit the current understanding of the role of ERM phosphorylation in regulating cortical reorganization. Thus, these studies identify a new ERM kinase of importance in lymphocytes and confirm the role of ERM phosphorylation in regulating cell shape and motility.
5. Induction of ezrin-radixin-moesin molecules after cryogenic traumatic brain injury of the mouse cortex. Traumatic brain injury promotes rapid induction of microglial cells and infiltration of peripheral macrophages to the injury sites. Such inflammatory responses are mediated by the activation and **migration** of immune cells, which are influenced by the actin cytoskeleton remodeling. In this study, we observed that the phosphorylation and expressions of ezrin-radixin-moesin (ERM) proteins, which are linkers for cell surface with actin cytoskeleton, are induced in the activated microglia/macrophages, whereas ERM molecules are only marginally expressed in quiescent microglia in the normal brain. These results suggest that ERM activation in the injury penumbra is implicated in the inflammatory immune responses after traumatic brain injury.

#### GeneRIFs

1. Results show that while CD43 binding to ezrin-radixin-moesin proteins is crucial for S76 phosphorylation, CD43 movement and regulation of T-cell **migration** can occur through an ERM-independent, phosphorylation-dependent mechanism.
2. Hepatic stellate cell **migration** and fibrogenesis in moesin-deficient mice, is described.

### [Ee]xtracellular [Mm]atrix

#### PubMed

1. Phosphorylation of moesin by Jun N-terminal kinase is important for podosome rosette formation in Src-transformed fibroblasts. Podosomes are actin-based membrane protrusions that facilitate **extracellular matrix** degradation and motility of invasive cells. Podosomes can self-organize into large rosette-like structures in Src-transformed fibroblasts, osteoclasts and some highly invasive cancer cells. However, the mechanism of this assembly remains obscure. In this study, we show that the suppression of Jun N-terminal kinase (JNK) by the JNK inhibitor SP600125 or short-hairpin RNA inhibited podosome rosette formation in SrcY527F-transformed NIH3T3 fibroblasts. In addition, SrcY527F was less able to induce podosome rosettes in JNK1-null or JNK2-null mouse embryo fibroblasts than in wild-type counterparts. The kinase activity of JNK was essential for promoting podosome rosette formation but not for its localization to podosome rosettes. Moesin, a member of the ERM (ezrin, radixin and moesin) protein family, was identified as a substrate of JNK. We show that the phosphorylation of moesin at Thr558 by JNK was important for podosome rosette formation in SrcY527F-transformed NIH3T3 fibroblasts. Taken together, our results unveil a novel role of JNK in podosome rosette formation through the phosphorylation of moesin.
2. Vitronectin induces phosphorylation of ezrin/radixin/moesin actin-binding proteins through binding to its novel neuronal receptor telencephalin. Vitronectin (VN) is an **extracellular matrix** protein abundantly present in blood and a wide variety of tissues and plays important roles in a number of biological phenomena mainly through its binding to αV integrins. However, its definite function in the brain remains largely unknown. Here we report the identification of telencephalin (TLCN/ICAM-5) as a novel VN receptor on neuronal dendrites. VN strongly binds to TLCN, a unique neuronal member of the ICAM family, which is specifically expressed on dendrites of spiny neurons in the mammalian telencephalon. VN-coated microbeads induce the formation of phagocytic cup-like plasma membrane protrusions on dendrites of cultured hippocampal neurons and trigger the activation of TLCN-dependent intracellular signaling cascade including the phosphorylation of ezrin/radixin/moesin actin-binding proteins and recruitment of F-actin and phosphatidylinositol 4,5-bisphosphate for morphological transformation of the dendritic protrusions. These results suggest that the **extracellular matri**x molecule VN and its neuronal receptor TLCN play a pivotal role in the phosphorylation of ezrin/radixin/moesin proteins and the formation of phagocytic cup-like structures on neuronal dendrites.
3. Tumor necrosis factor-alpha regulates transforming growth factor-beta-dependent epithelial-mesenchymal transition by promoting hyaluronan-CD44-moesin interaction. Aberrant epithelial-mesenchymal transition (EMT) is involved in development of fibrotic disorders and cancer invasion. Alterations of cell-**extracellular matrix** interaction also contribute to those pathological conditions. However, the functional interplay between EMT and cell-**extracellular matrix** interactions remains poorly understood. We now show that the inflammatory mediator tumor necrosis factor-alpha (TNF-alpha) induces the formation of fibrotic foci by cultured retinal pigment epithelial cells through activation of transforming growth factor-beta (TGF-beta) signaling in a manner dependent on hyaluronan-CD44-moesin interaction. TNF-alpha promoted CD44 expression and moesin phosphorylation by protein kinase C, leading to the pericellular interaction of hyaluronan and CD44. Formation of the hyaluronan-CD44-moesin complex resulted in both cell-cell dissociation and increased cellular motility through actin remodeling. Furthermore, this complex was found to be associated with TGF-beta receptor II and clathrin at actin microdomains, leading to activation of TGF-beta signaling. We established an in vivo model of TNF-alpha-induced fibrosis in the mouse eye, and such ocular fibrosis was attenuated in CD44-null mice. The production of hyaluronan and its interaction with CD44, thus, play an essential role in TNF-alpha-induced EMT and are potential therapeutic targets in fibrotic disorders.

#### GeneRIFs

1. the **extracellular matrix** molecule VN and its neuronal receptor TLCN play a pivotal role in the phosphorylation of ezrin/radixin/moesin proteins and the formation of phagocytic cup-like structures on neuronal dendrites

### [Ii]nvasive

#### PubMed

1. Dynamic actin remodeling during epithelial-mesenchymal transition depends on increased moesin expression. Remodeling of actin filaments is necessary for epithelial-mesenchymal transition (EMT); however, understanding of how this is regulated in real time is limited. We used an actin filament reporter and high-resolution live-cell imaging to analyze the regulated dynamics of actin filaments during transforming growth factor-β-induced EMT of mammary epithelial cells. Progressive changes in cell morphology were accompanied by reorganization of actin filaments from thin cortical bundles in epithelial cells to thick, parallel, contractile bundles that disassembled more slowly but remained dynamic in transdifferentiated cells. We show that efficient actin filament remodeling during EMT depends on increased expression of the ezrin/radixin/moesin (ERM) protein moesin. Cells suppressed for moesin expression by short hairpin RNA had fewer, thinner, and less stable actin bundles, incomplete morphological transition, and decreased **invasiv**e capacity. These cells also had less α-smooth muscle actin and phosphorylated myosin light chain in cortical patches, decreased abundance of the adhesion receptor CD44 at membrane protrusions, and attenuated autophosphorylation of focal adhesion kinase. Our findings suggest that increased moesin expression promotes EMT by regulating adhesion and contractile elements for changes in actin filament organization. We propose that the transciptional program driving EMT controls progressive remodeling of actin filament architectures.
2. Phosphorylation of moesin by Jun N-terminal kinase is important for podosome rosette formation in Src-transformed fibroblasts. Podosomes are actin-based membrane protrusions that facilitate extracellular matrix degradation and motility of **invasive** cells. Podosomes can self-organize into large rosette-like structures in Src-transformed fibroblasts, osteoclasts and some highly **invasive** cancer cells. However, the mechanism of this assembly remains obscure. In this study, we show that the suppression of Jun N-terminal kinase (JNK) by the JNK inhibitor SP600125 or short-hairpin RNA inhibited podosome rosette formation in SrcY527F-transformed NIH3T3 fibroblasts. In addition, SrcY527F was less able to induce podosome rosettes in JNK1-null or JNK2-null mouse embryo fibroblasts than in wild-type counterparts. The kinase activity of JNK was essential for promoting podosome rosette formation but not for its localization to podosome rosettes. Moesin, a member of the ERM (ezrin, radixin and moesin) protein family, was identified as a substrate of JNK. We show that the phosphorylation of moesin at Thr558 by JNK was important for podosome rosette formation in SrcY527F-transformed NIH3T3 fibroblasts. Taken together, our results unveil a novel role of JNK in podosome rosette formation through the phosphorylation of moesin.

---

## Carmil1

- NCBI Gene
- GeneRIF
- Pubmed

| Search Term | Rank | Fields |
| --- | --- | --- |
| [Mm]igration | 6 | Process(3); PubMed(3); GeneRIFs(1) |

  
hide/show details for Carmil1

### [Mm]igration

#### Process

1. cell **migration**
2. positive regulation of cell **migration**
3. positive regulation of cell **migration**

#### PubMed

1. Mammalian CARMIL inhibits actin filament capping by capping protein. Actin polymerization in cells occurs via filament elongation at the barbed end. Proteins that cap the barbed end terminate this elongation. Heterodimeric capping protein (CP) is an abundant and ubiquitous protein that caps the barbed end. We find that the mouse homolog of the adaptor protein CARMIL (mCARMIL) binds CP with high affinity and decreases its affinity for the barbed end. Addition of mCARMIL to cell extracts increases the rate and extent of Arp2/3 or spectrin-actin seed-induced polymerization. In cells, GFP-mCARMIL concentrates in lamellipodia and increases the fraction of cells with large lamellipodia. Decreasing mCARMIL levels by siRNA transfection lowers the F-actin level and slows cell **migration** through a mechanism that includes decreased lamellipodia protrusion. This phenotype is reversed by full-length mCARMIL but not mCARMIL lacking the domain that binds CP. Thus, mCARMIL is a key regulator of CP and has profound effects on cell behavior.
2. Mammalian CARMIL inhibits actin filament capping by capping protein. Actin polymerization in cells occurs via filament elongation at the barbed end. Proteins that cap the barbed end terminate this elongation. Heterodimeric capping protein (CP) is an abundant and ubiquitous protein that caps the barbed end. We find that the mouse homolog of the adaptor protein CARMIL (mCARMIL) binds CP with high affinity and decreases its affinity for the barbed end. Addition of mCARMIL to cell extracts increases the rate and extent of Arp2/3 or spectrin-actin seed-induced polymerization. In cells, GFP-mCARMIL concentrates in lamellipodia and increases the fraction of cells with large lamellipodia. Decreasing mCARMIL levels by siRNA transfection lowers the F-actin level and slows cell **migration** through a mechanism that includes decreased lamellipodia protrusion. This phenotype is reversed by full-length mCARMIL but not mCARMIL lacking the domain that binds CP. Thus, mCARMIL is a key regulator of CP and has profound effects on cell behavior.
3. Actin-capping proteins play essential roles in the asymmetric division of maturing mouse oocytes. Actin polymerization is essential for various stages of mammalian oocyte maturation, including spindle **migration**, actin cap formation, polar body extrusion and cytokinesis. The heterodimeric actin-capping protein is an essential element of the actin cytoskeleton. It binds to the fast-growing (barbed) ends of actin filaments and plays essential roles in various actin-mediated cellular processes. However, the roles of capping protein in mammalian oocyte maturation are poorly understood. We investigated the roles of capping protein in mouse oocytes and found that it is essential for correct asymmetric spindle **migration** and polar body extrusion. Capping protein mainly localized in the cytoplasm during maturation. By knocking down or ectopically overexpressing this protein, we revealed that it is crucial for efficient spindle **migration** and maintenance of the cytoplasmic actin mesh density. Expression of the capping-protein-binding region of CARMIL (also known as LRRC16A) impaired spindle **migration** and polar body extrusion during oocyte maturation and decreased the density of the cytoplasmic actin mesh. Taken together, these findings show that capping protein is an essential component of the actin cytoskeleton machinery that plays crucial roles in oocyte maturation, presumably by controlling the cytoplasmic actin mesh density.

#### GeneRIFs

1. Expression of the capping-protein-binding region of CARMIL (also known as LRRC16A) impaired spindle **migration** and polar body extrusion during oocyte maturation and decreased the density of the cytoplasmic actin mesh

---

## Sphk2

- NCBI Gene
- GeneRIF
- Pubmed

| Search Term | Rank | Fields |
| --- | --- | --- |
| [Mm]igration | 6 | PubMed(10) |
| [Mm]etastasis | 12 | PubMed(1) |

  
hide/show details for Sphk2

### [Mm]igration

#### PubMed

1. Sphingosine-kinase 1 and 2 contribute to oral sensitization and effector phase in a mouse model of food allergy. Sphingosine-1-phosphate (S1P) influences activation, **migration** and death of immune cells. Further, S1P was proposed to play a major role in the induction and promotion of allergic diseases. However, to date only limited information is available on the role of S1P in food allergy.We aimed to investigate the role of sphingosine-kinase (SphK) 1 and 2, the enzymes responsible for endogenous S1P production, on the induction of food allergy.Human epithelial colorectal CaCo2 cells stimulated in vitro with S1P revealed a decrease of transepithelial resistance and enhanced transport of FITC labeled OVA. We studied the effect of genetic deletion of the enzymes involved in S1P production on food allergy induction using a mouse model of food allergy based on intragastrically (i.g.) administered ovalbumin (OVA) with concomitant acid-suppression. Wild-type (WT), SphK1(-/-) and SphK2(-/-) mice immunized with OVA alone i.g. or intraperitoneally (i.p.) were used as negative or positive controls, respectively. SphK1- and SphK2-deficient mice fed with OVA under acid-suppression showed reduced induction of OVA specific IgE and IgG compared to WT mice, but had normal responses when immunized by the intraperitoneal route. Flow cytometric analysis of spleen cells revealed a significantly reduced proportion of CD4(+) effector T-cells in both SphK deficient animals after oral sensitization. This was accompanied by a reduced accumulation of mast cells in the gastric mucosa in SphK-deficient animals compared to WT mice. Furthermore, mouse mast cell protease-1 (mMCP-1) levels, an IgE-mediated anaphylaxis marker, were reliably elevated in allergic WT animals.Modulation of the S1P homeostasis by deletion of either SphK1 or SphK2 alters the sensitization and effector phase of food allergy.
2. Sphingosine 1-phosphate modulates spinal nociceptive processing. Sphingosine 1-Phosphate (S1P) modulates various cellular functions such as apoptosis, cell differentiation, and **migration**. Although S1P is an abundant signaling molecule in the central nervous system, very little is known about its influence on neuronal functions. We found that S1P concentrations were selectively decreased in the cerebrospinal fluid of adult rats in an acute and an inflammatory pain model. Pharmacological inhibition of sphingosine kinases (SPHK) decreased basal pain thresholds and SphK2 knock-out mice, but not SphK1 knock-out mice, had a significant decrease in withdrawal latency. Intrathecal application of S1P or sphinganine 1-phosphate (dihydro-S1P) reduced the pain-related (nociceptive) behavior in the formalin assay. S1P and dihydro-S1P inhibited cyclic AMP (cAMP) synthesis, a key second messenger of spinal nociceptive processing, in spinal cord neurons. By combining fluorescence resonance energy transfer (FRET)-based cAMP measurements with Multi Epitope Ligand Cartography (MELC), we showed that S1P decreased cAMP synthesis in excitatory dorsal horn neurons. Accordingly, intrathecal application of dihydro-S1P abolished the cAMP-dependent phosphorylation of NMDA receptors in the outer laminae of the spinal cord. Taken together, the data show that S1P modulates spinal nociceptive processing through inhibition of neuronal cAMP synthesis.
3. Podoplanin maintains high endothelial venule integrity by interacting with platelet CLEC-2. Circulating lymphocytes continuously enter lymph nodes for immune surveillance through specialized blood vessels named high endothelial venules, a process that increases markedly during immune responses. How high endothelial venules (HEVs) permit lymphocyte trans**migration** while maintaining vascular integrity is unknown. Here we report a role for the transmembrane O-glycoprotein podoplanin (PDPN, also known as gp38 and T1α) in maintaining HEV barrier function. Mice with postnatal deletion of Pdpn lost HEV integrity and exhibited spontaneous bleeding in mucosal lymph nodes, and bleeding in the draining peripheral lymph nodes after immunization. Blocking lymphocyte homing rescued bleeding, indicating that PDPN is required to protect the barrier function of HEVs during lymphocyte trafficking. Further analyses demonstrated that PDPN expressed on fibroblastic reticular cells, which surround HEVs, functions as an activating ligand for platelet C-type lectin-like receptor 2 (CLEC-2, also known as CLEC1B). Mice lacking fibroblastic reticular cell PDPN or platelet CLEC-2 exhibited significantly reduced levels of VE-cadherin (also known as CDH5), which is essential for overall vascular integrity, on HEVs. Infusion of wild-type platelets restored HEV integrity in Clec-2-deficient mice. Activation of CLEC-2 induced release of sphingosine-1-phosphate from platelets, which promoted expression of VE-cadherin on HEVs ex vivo. Furthermore, draining peripheral lymph nodes of immunized mice lacking sphingosine-1-phosphate had impaired HEV integrity similar to Pdpn- and Clec-2-deficient mice. These data demonstrate that local sphingosine-1-phosphate release after PDPN-CLEC-2-mediated platelet activation is critical for HEV integrity during immune responses.
4. Dynamic regulation of sphingosine-1-phosphate homeostasis during development of mouse metanephric kidney. Branching morphogenesis of the metanephric kidney is critically dependent on the delicate orchestration of diverse cellular processes including proliferation, apoptosis, **migration**, and differentiation. Sphingosine-1-phosphate (S1P) is a potent lipid mediator influencing many of these cellular events. We report increased expression and activity of both sphingosine kinases and S1P phosphatases during development of the mouse metanephric kidney from induction at embryonic day 11.5 to maturity. Sphingosine kinase activity exceeded S1P phosphatase activity in embryonic kidneys, resulting in a net accumulation of S1P, while kinase and phosphatase activities were similar in adult tissue, resulting in reduced S1P content. Sphingosine kinase expression was greater in the metanephric mesenchyme than in the ureteric bud, while the S1P phosphatase SPP2 was expressed at greater levels in the ureteric bud. Treatment of cultured embryonic kidneys with sphingosine kinase inhibitors resulted in a dose-dependent reduction of ureteric bud tip numbers and increased apoptosis. Exogenous S1P rescued kidneys from apoptosis induced by kinase inhibitors. Ureteric bud tip number was unaffected by exogenous S1P in kidneys treated with N,N-dimethylsphingosine, although tip number increased in those treated with d,l-threo-dihydrosphingosine. S1P1 and S1P2 were the predominant S1P receptors expressed in the embryonic kidney. S1P1 expression increased during renal development while expression of S1P2 decreased, and both receptors were expressed predominantly in the metanephric mesenchyme. These results demonstrate dynamic regulation of S1P homeostasis during renal morphogenesis and suggest that differential expression of S1P metabolic enzymes and receptors provides a novel mechanism contributing to the regulation of kidney development.
5. Sphingosine-1-phosphate inhibits cell **migration** and endothelial to mesenchymal cell transformation during cardiac development. Sphingosine-1-phosphate (S1P) is a biologically active sphingolipid metabolite that exerts important effects on numerous cellular events via cell surface receptors, S1P(1-5). S1P influences differentiation, proliferation, and **migration** during vascular development. However, the effects of S1P signaling on early cardiac development are not well understood. To address this issue, we examined the expression of S1P regulatory enzymes and S1P receptors during cardiac development. We observed that enzymes that regulate S1P levels, sphingosine kinase and sphingosine-1-phosphate phosphatase, are expressed in the developing heart. In addition, RT-PCR revealed that four of the five known S1P receptors (S1P(1-4)) are also expressed in the developing heart. Next, effects of altered S1P levels on whole embryo and atrioventricular (AV) canal cultures were investigated. We demonstrate that inactivation of the S1P producing enzyme, sphingosine kinase, leads to cell death in cardiac tissue which is rescued by exogenous S1P treatment. Other experiments reveal that increased S1P concentration prevents alterations in cell morphology that are required for cell **migration**. This effect results in reduced cell **migration** and inhibited mesenchymal cell formation in AV canal cushion tissue. These data indicate that S1P, locally maintained within a specific concentration range, is an important and necessary component of early heart development.
6. Sphingosine kinase type 2 inhibition elevates circulating sphingosine 1-phosphate. S1P (sphingosine 1-phosphate) is a pleiotropic lipid mediator involved in numerous cellular and physiological functions. Of note among these are cell survival and **migration**, as well as lymphocyte trafficking. S1P, which exerts its effects via five GPCRs (G-protein-coupled receptors) (S1P1-S1P5), is formed by the action of two SphKs (sphingosine kinases). Although SphK1 is the more intensively studied isotype, SphK2 is unique in it nuclear localization and has been reported to oppose some of the actions ascribed to SphK1. Although several scaffolds of SphK1 inhibitors have been described, there is a scarcity of selective SphK2 inhibitors that are necessary to evaluate the downstream effects of inhibition of this isotype. In the present paper we report a cationic amphiphilic small molecule that is a selective SphK2 inhibitor. In the course of characterizing this compound in wild-type and SphK-null mice, we discovered that administration of the inhibitor to wild-type mice resulted in a rapid increase in blood S1P, which is in contrast with our SphK1 inhibitor that drives circulating S1P levels down. Using a cohort of F2 hybrid mice, we confirmed, compared with wild-type mice, that circulating S1P levels were higher in SphK2-null mice and lower in SphK1-null mice. Thus both SphK1 and SphK2 inhibitors recapitulate the blood S1P levels observed in the corresponding null mice. Moreover, circulating S1P levels mirror SphK2 inhibitor levels, providing a convenient biomarker of target engagement.
7. The sphingosine 1-phosphate receptor S1P₂ maintains the homeostasis of germinal center B cells and promotes niche confinement. Mice deficient in sphingosine 1-phosphate receptor type 2 (S1P(2)) develop diffuse large B cell lymphoma. However, the role of S1P(2) in normal germinal center (GC) physiology is unknown. Here we show that S1P(2)-deficient GC B cells outgrew their wild-type counterparts in chronically established GCs. We found that antagonism of the kinase Akt mediated by S1P(2) and its downstream mediators Gα(12), Gα(13) and p115RhoGEF regulated cell viability and was required for growth control in chronically proliferating GCs. Moreover, S1P(2) inhibited GC B cell responses to follicular chemoattractants and helped confine cells to the GC. In addition, S1P(2) overexpression promoted the centering of activated B cells in the follicle. We suggest that by inhibiting Akt activation **and migra**tion, S1P(2) helps restrict GC B cell survival and localization to an S1P-low niche at the follicle center.
8. Normal neutrophil functions in sphingosine kinase type 1 and 2 knockout mice. Sphingosine kinase (SPHK) has been implicated as an important element in neutrophil responses to diverse stimulatory agents. To get more insight into the role of the type 1 and 2 isoforms of SPHK in neutrophil functions, we made use of the respective SPHK knockout mice. Neutrophils isolated from the bone marrow of these mice showed normal increase of intracellular Ca(2+) when stimulated in vitro by fMLP, platelet-activating factor, the anaphylatoxin C5a, or ATP, and normal **migration** towards fMLP and C5a. Also, recruitment of neutrophils into the peritoneum towards the chemokines KC and MIP-2 or to LPS, and into the peripheral blood after fMLP injection was similar in SPHK knockout strains and wild-type animals. An in vivo model of bacterial lung infection revealed an accelerated progression of disease in SPHK2 (but not SPHK1) knockout mice as compared to wild-type controls. However, effector functions of SPHK-deficient neutrophils, such as superoxide production, beta-glucuronidase release and their capacity to kill bacteria were unchanged as compared to wild-type cells. To conclude, the data derived from SPHK knockout mice do not support the hypothesis that any of the two lipid kinases plays a crucial role in signalling downstream of various neutrophil stimuli; SPHKs appear not to be essential for neutrophil recruitment and effector functions.
9. Sphingosine kinase type 2 is essential for lymphopenia induced by the immunomodulatory drug FTY720. FTY720, a potent immunomodulatory drug in phase 2/3 clinical trials, induces rapid and reversible sequestration of lymphocytes into secondary lymphoid organs, thereby preventing their **migration** to sites of inflammation. As prerequisite for its function, phosphorylation of FTY720 to yield a potent agonist of the sphingosine-1-phosphate receptor S1P(1) is required in vivo, catalyzed by an as-yet-unknown kinase. Here, we report on the generation of sphingosine kinase 2 (SPHK2) knockout mice and demonstrate that this enzyme is essential for FTY720 phosphate formation in vivo. Consequently, administration of FTY720 does not induce lymphopenia in SPHK2-deficient mice. After direct dosage of FTY720 phosphate, lymphopenia is only transient in this strain, indicating that SPHK2 is constantly required to maintain FTY720 phosphate levels in vivo.
10. Lysophosphatidic acid stimulates cell **migration** of satellite cells. A role for the sphingosine kinase/sphingosine 1-phosphate axis. Regulation of the motility of skeletal muscle precursor cells, such as satellite cells, is critically important for their proper recruitment at the site of tissue damage, and ultimately for its correct repair. Here we show that lysophosphatidic acid (LPA), which is well-recognized as a powerful bioactive agent, strongly stimulates cell **migration** of activated murine satellite cells. The biological effect exerted by LPA was found to be induced via activation of LPA1 and LPA3 , being abolished by cell treatment with the antagonist Ki16425, and severely impaired by siRNA-mediated down-regulation of the two receptor isoforms. In contrast, silencing of LPA2 potentiated the stimulation of cell motility by LPA, suggesting that it is negatively coupled to cell **migration**. Pharmacological inhibition of both sphingosine kinase (SK) isoforms using VPC96047, or the selective blocking of SK1 using VPC96091, abolished cell responsiveness to LPA; in agreement, gene silencing of SK1 or SK2 significantly reduced the biological effect of LPA. Moreover, the LPA-dependent stimulation of cell chemotaxis was found to be impaired by down-regulation of the sphingosine 1-phosphate (S1P) receptors S1P1 or S1P4 by specific siRNAs. In summary, the results obtained support the notion that the sphingosine kinase/sphingosine 1-phosphate (SK/S1P) axis is critically involved in the mechanism by which LPA elicits its pro-migratory action. This study provides compelling new information on the regulatory mechanisms of satellite cell motility, and reinforces the view that the SK/S1P signaling pathway plays a crucial role in the control of skeletal muscle precursor cell biology.

### [Mm]etastasis

#### PubMed

1. Role for sphingosine kinase 1 in colon carcinogenesis. Sphingosine kinase 1 (SphK1) phosphorylates sphingosine to form sphingosine-1-phosphate (S1P) and is a critical regulator of sphingolipid-mediated functions. Cell-based studies suggest a tumor-promoting function for the SphK1/S1P pathway. Also, our previous studies implicated the SphK1/S1P pathway in the induction of the arachidonic acid cascade, a major inflammatory pathway involved in colon carcinogenesis. Therefore, we investigated whether the SphK1/S1P pathway is necessary for mediating carcinogenesis in vivo. Here, we report that 89% (42/47) of human colon cancer samples stained positively for SphK1, whereas normal colon mucosa had negative or weak staining. Adenomas had higher expression of SphK1 vs. normal mucosa, and colon cancers with **metastasis** had higher expression of SphK1 than those without **metastasis**. In the azoxymethane (AOM) murine model of colon cancer, SphK1 and S1P were significantly elevated in colon cancer tissues compared to normal mucosa. Moreover, blood levels of S1P were higher in mice with colon cancers than in those without cancers. Notably, SphK1(-/-) mice subjected to AOM had significantly less aberrant crypt foci (ACF) formation and significantly reduced colon cancer development. These results are the first in vivo evidence that the SphK1/S1P pathway contributes to colon carcinogenesis and that inhibition of this pathway is a potential target for chemoprevention.

---

## Lims1

- NCBI Gene
- GeneRIF
- Pubmed

| Search Term | Rank | Fields |
| --- | --- | --- |
| [Mm]igration | 6 | PubMed(7) |
| ECM | 7 | PubMed(2) |
| [Ee]xtracellular [Mm]atrix | 8 | PubMed(7) |

  
hide/show details for Lims1

### [Mm]igration

#### PubMed

1. Mutations in the paxillin-binding site of integrin-linked kinase (ILK) destabilize the pseudokinase domain and cause embryonic lethality in mice. Integrin-linked kinase (ILK) localizes to focal adhesions (FAs) where it regulates cell spreading, **migration**, and growth factor receptor signaling. Previous reports showed that overexpressed ILK in which Val(386) and Thr(387) were substituted with glycine residues (ILK-VT/GG) could neither interact with paxillin nor localize to FA in cells expressing endogenous wild-type ILK, implying that paxillin binding to ILK is required for its localization to FAs. Here, we show that introducing this mutation into the germ line of mice (ILK-VT/GG) caused vasculogenesis defects, resulting in a general developmental delay and death at around embryonic day 12.5. Fibroblasts isolated from ILK-VT/GG mice contained mutant ILK in FAs, showed normal adhesion to and spreading on extracellular matrix substrates but displayed impaired **migration**. Biochemical analysis revealed that VT/GG substitutions decreased ILK protein stability leading to decreased ILK levels and reduced binding to paxillin and α-parvin. Because paxillin depletion did not affect ILK localization to FAs, the embryonic lethality and the in vitro **migratio**n defects are likely due to the reduced levels of ILK-VT/GG and diminished binding to parvins.
2. Role of PINCH and its partner tumor suppressor Rsu-1 in regulating liver size and tumorigenesis. Particularly interesting new cysteine-histidine-rich protein (PINCH) protein is part of the ternary complex known as the IPP (integrin linked kinase (ILK)-PINCH-Parvin-α) complex. PINCH itself binds to ILK and to another protein known as Rsu-1 (Ras suppressor 1). We generated PINCH 1 and PINCH 2 Double knockout mice (referred as PINCH DKO mice). PINCH2 elimination was systemic whereas PINCH1 elimination was targeted to hepatocytes. The genetically modified mice were born normal. The mice were sacrificed at different ages after birth. Soon after birth, they developed abnormal hepatic histology characterized by disorderly hepatic plates, increased proliferation of hepatocytes and biliary cells and increased deposition of extracellular matrix. After a sustained and prolonged proliferation of all epithelial components, proliferation subsided and final liver weight by the end of 30 weeks in livers with PINCH DKO deficient hepatocytes was 40% larger than the control mice. The livers of the PINCH DKO mice were also very stiff due to increased ECM deposition throughout the liver, with no observed nodularity. Mice developed liver cancer by one year. These mice regenerated normally when subjected to 70% partial hepatectomy and did not show any termination defect. Ras suppressor 1 (Rsu-1) protein, the binding partner of PINCH is frequently deleted in human liver cancers. Rsu-1 expression is dramatically decreased in PINCH DKO mouse livers. Increased expression of Rsu-1 suppressed cell proliferation and **migratio**n in HCC cell lines. These changes were brought about not by affecting activation of Ras (as its name suggests) but by suppression of Ras downstream signaling via RhoGTPase proteins. In conclusion, our studies suggest that removal of PINCH results in enlargement of liver and tumorigenesis. Decreased levels of Rsu-1, a partner for PINCH and a protein often deleted in human liver cancer, may play an important role in the development of the observed phenotype.
3. Thymosin beta4 activates integrin-linked kinase and promotes cardiac cell **migration**, survival and cardiac repair. Heart disease is a leading cause of death in newborn children and in adults. Efforts to promote cardiac repair through the use of stem cells hold promise but typically involve isolation and introduction of progenitor cells. Here, we show that the G-actin sequestering peptide thymosin beta4 promotes myocardial and endothelial cell **migration** in the embryonic heart and retains this property in postnatal cardiomyocytes. Survival of embryonic and postnatal cardiomyocytes in culture was also enhanced by thymosin beta4. We found that thymosin beta4 formed a functional complex with PINCH and integrin-linked kinase (ILK), resulting in activation of the survival kinase Akt (also known as protein kinase B). After coronary artery ligation in mice, thymosin beta4 treatment resulted in upregulation of ILK and Akt activity in the heart, enhanced early myocyte survival and improved cardiac function. These findings suggest that thymosin beta4 promotes cardiomyocyte **migration**, survival and repair and the pathway it regulates may be a new therapeutic target in the setting of acute myocardial damage.
4. The focal adhesion protein PINCH-1 associates with EPLIN at integrin adhesion sites. PINCH-1 is a LIM-only domain protein that forms a ternary complex with integrin-linked kinase (ILK) and parvin (to form the IPP complex) downstream of integrins. Here, we demonstrate that PINCH-1 (also known as Lims1) gene ablation in the epidermis of mice caused epidermal detachment from the basement membrane, epidermal hyperthickening and progressive hair loss. PINCH-1-deficient keratinocytes also displayed profound adhesion, spreading and **migration** defects in vitro that were substantially more severe than those of ILK-deficient keratinocytes indicating that PINCH-1 also exerts functions in an ILK-independent manner. By isolating the PINCH-1 interactome, the LIM-domain-containing and actin-binding protein epithelial protein lost in neoplasm (EPLIN, also known as LIMA1) was identified as a new PINCH-1-associated protein. EPLIN localized, in a PINCH-1-dependent manner, to integrin adhesion sites of keratinocytes in vivo and in vitro and its depletion severely attenuated keratinocyte spreading and **migration** on collagen and fibronectin without affecting PINCH-1 levels in focal adhesions. Given that the low PINCH-1 levels in ILK-deficient keratinocytes were sufficient to recruit EPLIN to integrin adhesions, our findings suggest that PINCH-1 regulates integrin-mediated adhesion of keratinocytes through the interactions with ILK as well as EPLIN.
5. Expression analysis of primary mouse megakaryocyte differentiation and its application in identifying stage-specific molecular markers and a novel transcriptional target of NF-E2. Megakaryocyte (MK) differentiation is well described in morphologic terms but its molecular counterparts and the basis for platelet release are incompletely understood. We profiled mRNA expression in populations of primary mouse MKs representing successive differentiation stages. Genes associated with DNA replication are highly expressed in young MKs, in parallel with endomitosis. Intermediate stages are characterized by disproportionate expression of genes associated with the cytoskeleton, cell **migration**, and G-protein signaling, whereas terminally mature MKs accumulate hemostatic factors, including many membrane proteins. We used these expression profiles to extract a reliable panel of molecular markers for MKs of early, intermediate, or advanced differentiation and establish the value of this marker panel using mouse models of defective thrombopoiesis resulting from absence of GATA1, NF-E2, or tubulin beta1. Computational analysis of the promoters of late-expressed MK genes identified new candidate targets for NF-E2, a critical transcriptional regulator of platelet release. One such gene encodes the kinase adaptor protein LIMS1/PINCH1, which is highly expressed in MKs and platelets and significantly reduced in NF-E2-deficient cells. Transactivation studies and chromatin immunoprecipitation implicate Lims1 as a direct target of NF-E2 regulation. Attribution of stage-specific genes, in combination with various applications, thus constitutes a powerful way to study MK differentiation and platelet biogenesis.
6. Integrin-linked kinase is required for radial sorting of axons and Schwann cell remyelination in the peripheral nervous system. During development, Schwann cells (SCs) interpret different extracellular cues to regulate their **migration**, proliferation, and the remarkable morphological changes associated with the sorting, ensheathment, and myelination of axons. Although interactions between extracellular matrix proteins and integrins are critical to some of these processes, the downstream signaling pathways they control are still poorly understood. Integrin-linked kinase (ILK) is a focal adhesion protein that associates with multiple binding partners to link integrins to the actin cytoskeleton and is thought to participate in integrin and growth factor-mediated signaling. Using SC-specific gene ablation, we report essential functions for ILK in radial sorting of axon bundles and in remyelination in the peripheral nervous system. Our in vivo and in vitro experiments show that ILK negatively regulates Rho/Rho kinase signaling to promote SC process extension and to initiate radial sorting. ILK also facilitates axon remyelination, likely by promoting the activation of downstream molecules such as AKT/protein kinase B.
7. Pinch1 is required for normal development of cranial and cardiac neural crest-derived structures. Pinch1, an adaptor protein composed of 5 LIM domains, has been suggested to play an important role in multiple cellular processes. We found that Pinch1 is highly expressed in neural crest cells and their derivatives. To examine the requirement for Pinch1 in neural crest development, we generated neural crest conditional Pinch1 knockout mice using the Wnt1-Cre/loxP system. Neural crest conditional Pinch1 mutant embryos die perinatally from severe cardiovascular defects with an unusual aneurysmal common arterial trunk. Pinch1 mutants also exhibit multiple deficiencies in cranial neural crest-derived structures. Fate mapping demonstrated that initial **migration** of neural crest cells to the pharyngeal arch region occurs normally in the mutant embryos. However, in the cardiac outflow tract of mutants, neural crest cells exhibited hyperplasia and failed to differentiate into smooth muscle. Markedly increased apoptosis is observed in outflow tract cushions of mutants between embryonic days 11.5 and 13.5, likely contributing to the observed defects in cushion/valve remodeling and ventricular septation. Expression of transforming growth factor-beta(2), which plays a crucial role in outflow tract development, was decreased or absent in the outflow tract of the mutants. The decrease in transforming growth factor-beta(2) expression preceded neural crest cell death. Together, our results demonstrate that Pinch1 plays an essential role in neural crest development, perhaps in part through transforming growth factor-beta signaling.

### ECM

#### PubMed

1. Role of PINCH and its partner tumor suppressor Rsu-1 in regulating liver size and tumorigenesis. Particularly interesting new cysteine-histidine-rich protein (PINCH) protein is part of the ternary complex known as the IPP (integrin linked kinase (ILK)-PINCH-Parvin-α) complex. PINCH itself binds to ILK and to another protein known as Rsu-1 (Ras suppressor 1). We generated PINCH 1 and PINCH 2 Double knockout mice (referred as PINCH DKO mice). PINCH2 elimination was systemic whereas PINCH1 elimination was targeted to hepatocytes. The genetically modified mice were born normal. The mice were sacrificed at different ages after birth. Soon after birth, they developed abnormal hepatic histology characterized by disorderly hepatic plates, increased proliferation of hepatocytes and biliary cells and increased deposition of extracellular matrix. After a sustained and prolonged proliferation of all epithelial components, proliferation subsided and final liver weight by the end of 30 weeks in livers with PINCH DKO deficient hepatocytes was 40% larger than the control mice. The livers of the PINCH DKO mice were also very stiff due to increased **EC**M deposition throughout the liver, with no observed nodularity. Mice developed liver cancer by one year. These mice regenerated normally when subjected to 70% partial hepatectomy and did not show any termination defect. Ras suppressor 1 (Rsu-1) protein, the binding partner of PINCH is frequently deleted in human liver cancers. Rsu-1 expression is dramatically decreased in PINCH DKO mouse livers. Increased expression of Rsu-1 suppressed cell proliferation and migration in HCC cell lines. These changes were brought about not by affecting activation of Ras (as its name suggests) but by suppression of Ras downstream signaling via RhoGTPase proteins. In conclusion, our studies suggest that removal of PINCH results in enlargement of liver and tumorigenesis. Decreased levels of Rsu-1, a partner for PINCH and a protein often deleted in human liver cancer, may play an important role in the development of the observed phenotype.
2. PINCH1 regulates Akt1 activation and enhances radioresistance by inhibiting PP1alpha. Tumor cell resistance to ionizing radiation and chemotherapy is a major obstacle in cancer therapy. One factor contributing to this is integrin-mediated adhesion to **ECM**. The adapter protein particularly interesting new cysteine-histidine-rich 1 (PINCH1) is recruited to integrin adhesion sites and promotes cell survival, but the mechanisms underlying this effect are not well understood. Here we have shown that PINCH1 is expressed at elevated levels in human tumors of diverse origins relative to normal tissue. Furthermore, PINCH1 promoted cell survival upon treatment with ionizing radiation in vitro and in vivo by perpetuating Akt1 phosphorylation and activity. Mechanistically, PINCH1 was found to directly bind to protein phosphatase 1alpha (PP1alpha) - an Akt1-regulating protein - and inhibit PP1alpha activity, resulting in increased Akt1 phosphorylation and enhanced radioresistance. Thus, our data suggest that targeting signaling molecules such as PINCH1 that function downstream of focal adhesions (the complexes that mediate tumor cell adhesion to **ECM**) may overcome radio- and chemoresistance, providing new therapeutic approaches for cancer.

### [Ee]xtracellular [Mm]atrix

#### PubMed

1. Mutations in the paxillin-binding site of integrin-linked kinase (ILK) destabilize the pseudokinase domain and cause embryonic lethality in mice. Integrin-linked kinase (ILK) localizes to focal adhesions (FAs) where it regulates cell spreading, migration, and growth factor receptor signaling. Previous reports showed that overexpressed ILK in which Val(386) and Thr(387) were substituted with glycine residues (ILK-VT/GG) could neither interact with paxillin nor localize to FA in cells expressing endogenous wild-type ILK, implying that paxillin binding to ILK is required for its localization to FAs. Here, we show that introducing this mutation into the germ line of mice (ILK-VT/GG) caused vasculogenesis defects, resulting in a general developmental delay and death at around embryonic day 12.5. Fibroblasts isolated from ILK-VT/GG mice contained mutant ILK in FAs, showed normal adhesion to and spreading on **extracellular matrix** substrates but displayed impaired migration. Biochemical analysis revealed that VT/GG substitutions decreased ILK protein stability leading to decreased ILK levels and reduced binding to paxillin and α-parvin. Because paxillin depletion did not affect ILK localization to FAs, the embryonic lethality and the in vitro migration defects are likely due to the reduced levels of ILK-VT/GG and diminished binding to parvins.
2. Role of PINCH and its partner tumor suppressor Rsu-1 in regulating liver size and tumorigenesis. Particularly interesting new cysteine-histidine-rich protein (PINCH) protein is part of the ternary complex known as the IPP (integrin linked kinase (ILK)-PINCH-Parvin-α) complex. PINCH itself binds to ILK and to another protein known as Rsu-1 (Ras suppressor 1). We generated PINCH 1 and PINCH 2 Double knockout mice (referred as PINCH DKO mice). PINCH2 elimination was systemic whereas PINCH1 elimination was targeted to hepatocytes. The genetically modified mice were born normal. The mice were sacrificed at different ages after birth. Soon after birth, they developed abnormal hepatic histology characterized by disorderly hepatic plates, increased proliferation of hepatocytes and biliary cells and increased deposition of **extracellular matri**x. After a sustained and prolonged proliferation of all epithelial components, proliferation subsided and final liver weight by the end of 30 weeks in livers with PINCH DKO deficient hepatocytes was 40% larger than the control mice. The livers of the PINCH DKO mice were also very stiff due to increased ECM deposition throughout the liver, with no observed nodularity. Mice developed liver cancer by one year. These mice regenerated normally when subjected to 70% partial hepatectomy and did not show any termination defect. Ras suppressor 1 (Rsu-1) protein, the binding partner of PINCH is frequently deleted in human liver cancers. Rsu-1 expression is dramatically decreased in PINCH DKO mouse livers. Increased expression of Rsu-1 suppressed cell proliferation and migration in HCC cell lines. These changes were brought about not by affecting activation of Ras (as its name suggests) but by suppression of Ras downstream signaling via RhoGTPase proteins. In conclusion, our studies suggest that removal of PINCH results in enlargement of liver and tumorigenesis. Decreased levels of Rsu-1, a partner for PINCH and a protein often deleted in human liver cancer, may play an important role in the development of the observed phenotype.
3. Formation and phosphorylation of the PINCH-1-integrin linked kinase-alpha-parvin complex are important for regulation of renal glomerular podocyte adhesion, architecture, and survival. Alterations in the cellular architecture, adhesion, and/or loss of glomerular podocytes are causal factors in the development of proteinuria and the progression to end-stage renal failure. With the use of an inducible podocyte differentiation system, it was found that the cellular levels of PINCH-1, integrin linked kinase (ILK), and alpha-parvin, cytoplasmic components of cell-**extracellular matrix** adhesions, were significantly increased during podocyte differentiation. Concomitantly, an increased amount of the PINCH-1-ILK-alpha-parvin complex was detected in the differentiated, foot process-containing podocytes. Overexpression of the PINCH-1-binding ankyrin repeat domain of ILK but not that of a PINCH-1-binding defective mutant form of the ankyrin domain effectively inhibited the formation of the PINCH-1-ILK-alpha-parvin complex. Disruption of the PINCH-1-ILK-alpha-parvin complex significantly reduced the podocyte-matrix adhesion and foot process formation. Furthermore, a marked increase of apoptosis in the podocytes in which the assembly of the PINCH-1-ILK-alpha-parvin complex was compromised was detected. Inhibition of ILK with a small compound inhibitor also altered podocyte cytoskeleton and increased apoptosis. Finally, it is shown that alpha-parvin is phosphorylated in podocytes. Mutations at the alpha-parvin N-terminal proline-directed serine phosphorylation sites reduced its complex formation with ILK and resulted in defects in podocyte adhesion, architecture, and survival. These results provide important evidence for a crucial role of the PINCH-1-ILK-alpha-parvin complex in the control of podocyte adhesion, morphology, and survival.
4. Integrin-linked kinase is required for radial sorting of axons and Schwann cell remyelination in the peripheral nervous system. During development, Schwann cells (SCs) interpret different extracellular cues to regulate their migration, proliferation, and the remarkable morphological changes associated with the sorting, ensheathment, and myelination of axons. Although interactions between **extracellular matrix** proteins and integrins are critical to some of these processes, the downstream signaling pathways they control are still poorly understood. Integrin-linked kinase (ILK) is a focal adhesion protein that associates with multiple binding partners to link integrins to the actin cytoskeleton and is thought to participate in integrin and growth factor-mediated signaling. Using SC-specific gene ablation, we report essential functions for ILK in radial sorting of axon bundles and in remyelination in the peripheral nervous system. Our in vivo and in vitro experiments show that ILK negatively regulates Rho/Rho kinase signaling to promote SC process extension and to initiate radial sorting. ILK also facilitates axon remyelination, likely by promoting the activation of downstream molecules such as AKT/protein kinase B.
5. Integrin-linked kinase is an adaptor with essential functions during mouse development. The development of multicellular organisms requires integrin-mediated interactions between cells and their extracellular environment. Integrin binding to **extracellular matrix** catalyses assembly of multiprotein complexes, which transduce mechanical and chemical signals that regulate many aspects of cell physiology. Integrin-linked kinase (Ilk) is a multifunctional protein that binds beta-integrin cytoplasmic domains and regulates actin dynamics by recruiting actin binding regulatory proteins such as alpha- and beta-parvin. Ilk has also been shown to possess serine/threonine kinase activity and to phosphorylate signalling proteins such as Akt1 and glycogen synthase kinase 3beta (Gsk3beta) in mammalian cells; however, these functions have been shown by genetic studies not to occur in flies and worms. Here we show that mice carrying point mutations in the proposed autophosphorylation site of the putative kinase domain and in the pleckstrin homology domain are normal. In contrast, mice with point mutations in the conserved lysine residue of the potential ATP-binding site of the kinase domain, which mediates Ilk binding to alpha-parvin, die owing to renal agenesis. Similar renal defects occur in alpha-parvin-null mice. Thus, we provide genetic evidence that the kinase activity of Ilk is dispensable for mammalian development; however, an interaction between Ilk and alpha-parvin is critical for kidney development.
6. Assembly of the PINCH-ILK-CH-ILKBP complex precedes and is essential for localization of each component to cell-matrix adhesion sites. PINCH, integrin-linked kinase (ILK) and calponin homology-containing ILK-binding protein (CH-ILKBP) form a ternary complex that plays crucial roles at cell-**extracellular matrix** adhesion sites. To understand the mechanism underlying the complex formation and recruitment to cell-adhesion sites we have undertaken a combined structural, mutational and cell biological analysis. Three-dimensional structure-based point mutations identified specific PINCH and ILK sites that mediate the complex formation. Analyses of the binding defective point mutants revealed that the assembly of the PINCH-ILK-CH-ILKBP complex is essential for their localization to cell-**extracellular matrix** adhesion sites. The formation of the PINCH-ILK-CH-ILKBP complex precedes integrin-mediated cell adhesion and spreading. Furthermore, inhibition of protein kinase C, but not that of actin polymerization, inhibited the PINCH-ILK-CH-ILKBP complex formation, suggesting that the PINCH-ILK-CH-ILKBP complex likely serves as a downstream effector of protein kinase C in the cellular control of focal adhesion assembly. Finally, we provide evidence that the formation of the PINCH-ILK-CH-ILKBP complex, while necessary, is not sufficient for ILK localization to cell-**extracellular matrix** adhesion sites. These results provide new insights into the molecular mechanism underlying the assembly and regulation of cell-matrix adhesion structures.
7. The role of the focal adhesion protein PINCH1 for the radiosensitivity of adhesion and suspension cell cultures. Focal adhesion (FA) signaling mediated by adhesion to **extracellular matrix** and growth factor receptors contributes to the regulation of the cellular stress response to external stimuli. Critical to focal adhesion assembly and signaling is the adapter protein PINCH1. To evaluate whether the prosurvival function of PINCH1 in radiation cell survival depends on cell adhesion, we examined PINCH1(fl/fl) and PINCH1(-/-) mouse embryonic fibroblasts and human cancer cell lines. Here, we found that the enhanced cellular radiosensitivity mediated by PINCH1 depletion observed under adhesion conditions is conserved when cells are irradiated under suspension conditions. This unsuspected finding could not be explained by the observed modification of adhesion and growth factor associated signaling involving FAK, Paxillin, p130(CAS), Src, AKT, GSK3β and ERK1/2 under suspension and serum withdrawal relative to adhesion conditions with serum. Our data suggest that the adapter protein PINCH1 critically participates in the regulation of the cellular radiosensitivity of normal and malignant cells similarly under adhesion and suspension conditions.

---

## Lims2

- NCBI Gene
- GeneRIF
- Pubmed

| Search Term | Rank | Fields |
| --- | --- | --- |
| [Mm]igration | 6 | PubMed(5) |
| ECM | 7 | PubMed(2) |
| [Ee]xtracellular [Mm]atrix | 8 | PubMed(4) |

  
hide/show details for Lims2

### [Mm]igration

#### PubMed

1. Role of PINCH and its partner tumor suppressor Rsu-1 in regulating liver size and tumorigenesis. Particularly interesting new cysteine-histidine-rich protein (PINCH) protein is part of the ternary complex known as the IPP (integrin linked kinase (ILK)-PINCH-Parvin-α) complex. PINCH itself binds to ILK and to another protein known as Rsu-1 (Ras suppressor 1). We generated PINCH 1 and PINCH 2 Double knockout mice (referred as PINCH DKO mice). PINCH2 elimination was systemic whereas PINCH1 elimination was targeted to hepatocytes. The genetically modified mice were born normal. The mice were sacrificed at different ages after birth. Soon after birth, they developed abnormal hepatic histology characterized by disorderly hepatic plates, increased proliferation of hepatocytes and biliary cells and increased deposition of extracellular matrix. After a sustained and prolonged proliferation of all epithelial components, proliferation subsided and final liver weight by the end of 30 weeks in livers with PINCH DKO deficient hepatocytes was 40% larger than the control mice. The livers of the PINCH DKO mice were also very stiff due to increased ECM deposition throughout the liver, with no observed nodularity. Mice developed liver cancer by one year. These mice regenerated normally when subjected to 70% partial hepatectomy and did not show any termination defect. Ras suppressor 1 (Rsu-1) protein, the binding partner of PINCH is frequently deleted in human liver cancers. Rsu-1 expression is dramatically decreased in PINCH DKO mouse livers. Increased expression of Rsu-1 suppressed cell proliferation and **migratio**n in HCC cell lines. These changes were brought about not by affecting activation of Ras (as its name suggests) but by suppression of Ras downstream signaling via RhoGTPase proteins. In conclusion, our studies suggest that removal of PINCH results in enlargement of liver and tumorigenesis. Decreased levels of Rsu-1, a partner for PINCH and a protein often deleted in human liver cancer, may play an important role in the development of the observed phenotype.
2. Integrin-linked kinase is required for radial sorting of axons and Schwann cell remyelination in the peripheral nervous system. During development, Schwann cells (SCs) interpret different extracellular cues to regulate their **migration**, proliferation, and the remarkable morphological changes associated with the sorting, ensheathment, and myelination of axons. Although interactions between extracellular matrix proteins and integrins are critical to some of these processes, the downstream signaling pathways they control are still poorly understood. Integrin-linked kinase (ILK) is a focal adhesion protein that associates with multiple binding partners to link integrins to the actin cytoskeleton and is thought to participate in integrin and growth factor-mediated signaling. Using SC-specific gene ablation, we report essential functions for ILK in radial sorting of axon bundles and in remyelination in the peripheral nervous system. Our in vivo and in vitro experiments show that ILK negatively regulates Rho/Rho kinase signaling to promote SC process extension and to initiate radial sorting. ILK also facilitates axon remyelination, likely by promoting the activation of downstream molecules such as AKT/protein kinase B.
3. Mutations in the paxillin-binding site of integrin-linked kinase (ILK) destabilize the pseudokinase domain and cause embryonic lethality in mice. Integrin-linked kinase (ILK) localizes to focal adhesions (FAs) where it regulates cell spreading, **migration**, and growth factor receptor signaling. Previous reports showed that overexpressed ILK in which Val(386) and Thr(387) were substituted with glycine residues (ILK-VT/GG) could neither interact with paxillin nor localize to FA in cells expressing endogenous wild-type ILK, implying that paxillin binding to ILK is required for its localization to FAs. Here, we show that introducing this mutation into the germ line of mice (ILK-VT/GG) caused vasculogenesis defects, resulting in a general developmental delay and death at around embryonic day 12.5. Fibroblasts isolated from ILK-VT/GG mice contained mutant ILK in FAs, showed normal adhesion to and spreading on extracellular matrix substrates but displayed impaired **migration**. Biochemical analysis revealed that VT/GG substitutions decreased ILK protein stability leading to decreased ILK levels and reduced binding to paxillin and α-parvin. Because paxillin depletion did not affect ILK localization to FAs, the embryonic lethality and the in vitro **migratio**n defects are likely due to the reduced levels of ILK-VT/GG and diminished binding to parvins.
4. Role of PINCH and its partner tumor suppressor Rsu-1 in regulating liver size and tumorigenesis. Particularly interesting new cysteine-histidine-rich protein (PINCH) protein is part of the ternary complex known as the IPP (integrin linked kinase (ILK)-PINCH-Parvin-α) complex. PINCH itself binds to ILK and to another protein known as Rsu-1 (Ras suppressor 1). We generated PINCH 1 and PINCH 2 Double knockout mice (referred as PINCH DKO mice). PINCH2 elimination was systemic whereas PINCH1 elimination was targeted to hepatocytes. The genetically modified mice were born normal. The mice were sacrificed at different ages after birth. Soon after birth, they developed abnormal hepatic histology characterized by disorderly hepatic plates, increased proliferation of hepatocytes and biliary cells and increased deposition of extracellular matrix. After a sustained and prolonged proliferation of all epithelial components, proliferation subsided and final liver weight by the end of 30 weeks in livers with PINCH DKO deficient hepatocytes was 40% larger than the control mice. The livers of the PINCH DKO mice were also very stiff due to increased ECM deposition throughout the liver, with no observed nodularity. Mice developed liver cancer by one year. These mice regenerated normally when subjected to 70% partial hepatectomy and did not show any termination defect. Ras suppressor 1 (Rsu-1) protein, the binding partner of PINCH is frequently deleted in human liver cancers. Rsu-1 expression is dramatically decreased in PINCH DKO mouse livers. Increased expression of Rsu-1 suppressed cell proliferation and **migratio**n in HCC cell lines. These changes were brought about not by affecting activation of Ras (as its name suggests) but by suppression of Ras downstream signaling via RhoGTPase proteins. In conclusion, our studies suggest that removal of PINCH results in enlargement of liver and tumorigenesis. Decreased levels of Rsu-1, a partner for PINCH and a protein often deleted in human liver cancer, may play an important role in the development of the observed phenotype.
5. Arid3b is essential for second heart field cell deployment and heart patterning. Arid3b, a member of the conserved ARID family of transcription factors, is essential for mouse embryonic development but its precise roles are poorly understood. Here, we show that Arid3b is expressed in the myocardium of the tubular heart and in second heart field progenitors. Arid3b-deficient embryos show cardiac abnormalities, including a notable shortening of the poles, absence of myocardial differentiation and altered patterning of the atrioventricular canal, which also lacks epithelial-to-mesenchymal transition. Proliferation and death of progenitors as well as early patterning of the heart appear normal. However, DiI labelling of second heart field progenitors revealed a defect in the addition of cells to the heart. RNA microarray analysis uncovered a set of differentially expressed genes in Arid3b-deficient tissues, including Bhlhb2, a regulator of cardiomyocyte differentiation, and Lims2, a gene involved in cell **migration**. Arid3b is thus required for heart development by regulating the motility and differentiation of heart progenitors. These findings identify Arid3b as a candidate gene involved in the aetiology of human congenital malformations.

### ECM

#### PubMed

1. Role of PINCH and its partner tumor suppressor Rsu-1 in regulating liver size and tumorigenesis. Particularly interesting new cysteine-histidine-rich protein (PINCH) protein is part of the ternary complex known as the IPP (integrin linked kinase (ILK)-PINCH-Parvin-α) complex. PINCH itself binds to ILK and to another protein known as Rsu-1 (Ras suppressor 1). We generated PINCH 1 and PINCH 2 Double knockout mice (referred as PINCH DKO mice). PINCH2 elimination was systemic whereas PINCH1 elimination was targeted to hepatocytes. The genetically modified mice were born normal. The mice were sacrificed at different ages after birth. Soon after birth, they developed abnormal hepatic histology characterized by disorderly hepatic plates, increased proliferation of hepatocytes and biliary cells and increased deposition of extracellular matrix. After a sustained and prolonged proliferation of all epithelial components, proliferation subsided and final liver weight by the end of 30 weeks in livers with PINCH DKO deficient hepatocytes was 40% larger than the control mice. The livers of the PINCH DKO mice were also very stiff due to increased **EC**M deposition throughout the liver, with no observed nodularity. Mice developed liver cancer by one year. These mice regenerated normally when subjected to 70% partial hepatectomy and did not show any termination defect. Ras suppressor 1 (Rsu-1) protein, the binding partner of PINCH is frequently deleted in human liver cancers. Rsu-1 expression is dramatically decreased in PINCH DKO mouse livers. Increased expression of Rsu-1 suppressed cell proliferation and migration in HCC cell lines. These changes were brought about not by affecting activation of Ras (as its name suggests) but by suppression of Ras downstream signaling via RhoGTPase proteins. In conclusion, our studies suggest that removal of PINCH results in enlargement of liver and tumorigenesis. Decreased levels of Rsu-1, a partner for PINCH and a protein often deleted in human liver cancer, may play an important role in the development of the observed phenotype.
2. Role of PINCH and its partner tumor suppressor Rsu-1 in regulating liver size and tumorigenesis. Particularly interesting new cysteine-histidine-rich protein (PINCH) protein is part of the ternary complex known as the IPP (integrin linked kinase (ILK)-PINCH-Parvin-α) complex. PINCH itself binds to ILK and to another protein known as Rsu-1 (Ras suppressor 1). We generated PINCH 1 and PINCH 2 Double knockout mice (referred as PINCH DKO mice). PINCH2 elimination was systemic whereas PINCH1 elimination was targeted to hepatocytes. The genetically modified mice were born normal. The mice were sacrificed at different ages after birth. Soon after birth, they developed abnormal hepatic histology characterized by disorderly hepatic plates, increased proliferation of hepatocytes and biliary cells and increased deposition of extracellular matrix. After a sustained and prolonged proliferation of all epithelial components, proliferation subsided and final liver weight by the end of 30 weeks in livers with PINCH DKO deficient hepatocytes was 40% larger than the control mice. The livers of the PINCH DKO mice were also very stiff due to increased **EC**M deposition throughout the liver, with no observed nodularity. Mice developed liver cancer by one year. These mice regenerated normally when subjected to 70% partial hepatectomy and did not show any termination defect. Ras suppressor 1 (Rsu-1) protein, the binding partner of PINCH is frequently deleted in human liver cancers. Rsu-1 expression is dramatically decreased in PINCH DKO mouse livers. Increased expression of Rsu-1 suppressed cell proliferation and migration in HCC cell lines. These changes were brought about not by affecting activation of Ras (as its name suggests) but by suppression of Ras downstream signaling via RhoGTPase proteins. In conclusion, our studies suggest that removal of PINCH results in enlargement of liver and tumorigenesis. Decreased levels of Rsu-1, a partner for PINCH and a protein often deleted in human liver cancer, may play an important role in the development of the observed phenotype.

### [Ee]xtracellular [Mm]atrix

#### PubMed

1. Role of PINCH and its partner tumor suppressor Rsu-1 in regulating liver size and tumorigenesis. Particularly interesting new cysteine-histidine-rich protein (PINCH) protein is part of the ternary complex known as the IPP (integrin linked kinase (ILK)-PINCH-Parvin-α) complex. PINCH itself binds to ILK and to another protein known as Rsu-1 (Ras suppressor 1). We generated PINCH 1 and PINCH 2 Double knockout mice (referred as PINCH DKO mice). PINCH2 elimination was systemic whereas PINCH1 elimination was targeted to hepatocytes. The genetically modified mice were born normal. The mice were sacrificed at different ages after birth. Soon after birth, they developed abnormal hepatic histology characterized by disorderly hepatic plates, increased proliferation of hepatocytes and biliary cells and increased deposition of **extracellular matri**x. After a sustained and prolonged proliferation of all epithelial components, proliferation subsided and final liver weight by the end of 30 weeks in livers with PINCH DKO deficient hepatocytes was 40% larger than the control mice. The livers of the PINCH DKO mice were also very stiff due to increased ECM deposition throughout the liver, with no observed nodularity. Mice developed liver cancer by one year. These mice regenerated normally when subjected to 70% partial hepatectomy and did not show any termination defect. Ras suppressor 1 (Rsu-1) protein, the binding partner of PINCH is frequently deleted in human liver cancers. Rsu-1 expression is dramatically decreased in PINCH DKO mouse livers. Increased expression of Rsu-1 suppressed cell proliferation and migration in HCC cell lines. These changes were brought about not by affecting activation of Ras (as its name suggests) but by suppression of Ras downstream signaling via RhoGTPase proteins. In conclusion, our studies suggest that removal of PINCH results in enlargement of liver and tumorigenesis. Decreased levels of Rsu-1, a partner for PINCH and a protein often deleted in human liver cancer, may play an important role in the development of the observed phenotype.
2. Integrin-linked kinase is required for radial sorting of axons and Schwann cell remyelination in the peripheral nervous system. During development, Schwann cells (SCs) interpret different extracellular cues to regulate their migration, proliferation, and the remarkable morphological changes associated with the sorting, ensheathment, and myelination of axons. Although interactions between **extracellular matrix** proteins and integrins are critical to some of these processes, the downstream signaling pathways they control are still poorly understood. Integrin-linked kinase (ILK) is a focal adhesion protein that associates with multiple binding partners to link integrins to the actin cytoskeleton and is thought to participate in integrin and growth factor-mediated signaling. Using SC-specific gene ablation, we report essential functions for ILK in radial sorting of axon bundles and in remyelination in the peripheral nervous system. Our in vivo and in vitro experiments show that ILK negatively regulates Rho/Rho kinase signaling to promote SC process extension and to initiate radial sorting. ILK also facilitates axon remyelination, likely by promoting the activation of downstream molecules such as AKT/protein kinase B.
3. Mutations in the paxillin-binding site of integrin-linked kinase (ILK) destabilize the pseudokinase domain and cause embryonic lethality in mice. Integrin-linked kinase (ILK) localizes to focal adhesions (FAs) where it regulates cell spreading, migration, and growth factor receptor signaling. Previous reports showed that overexpressed ILK in which Val(386) and Thr(387) were substituted with glycine residues (ILK-VT/GG) could neither interact with paxillin nor localize to FA in cells expressing endogenous wild-type ILK, implying that paxillin binding to ILK is required for its localization to FAs. Here, we show that introducing this mutation into the germ line of mice (ILK-VT/GG) caused vasculogenesis defects, resulting in a general developmental delay and death at around embryonic day 12.5. Fibroblasts isolated from ILK-VT/GG mice contained mutant ILK in FAs, showed normal adhesion to and spreading on **extracellular matrix** substrates but displayed impaired migration. Biochemical analysis revealed that VT/GG substitutions decreased ILK protein stability leading to decreased ILK levels and reduced binding to paxillin and α-parvin. Because paxillin depletion did not affect ILK localization to FAs, the embryonic lethality and the in vitro migration defects are likely due to the reduced levels of ILK-VT/GG and diminished binding to parvins.
4. Role of PINCH and its partner tumor suppressor Rsu-1 in regulating liver size and tumorigenesis. Particularly interesting new cysteine-histidine-rich protein (PINCH) protein is part of the ternary complex known as the IPP (integrin linked kinase (ILK)-PINCH-Parvin-α) complex. PINCH itself binds to ILK and to another protein known as Rsu-1 (Ras suppressor 1). We generated PINCH 1 and PINCH 2 Double knockout mice (referred as PINCH DKO mice). PINCH2 elimination was systemic whereas PINCH1 elimination was targeted to hepatocytes. The genetically modified mice were born normal. The mice were sacrificed at different ages after birth. Soon after birth, they developed abnormal hepatic histology characterized by disorderly hepatic plates, increased proliferation of hepatocytes and biliary cells and increased deposition of **extracellular matri**x. After a sustained and prolonged proliferation of all epithelial components, proliferation subsided and final liver weight by the end of 30 weeks in livers with PINCH DKO deficient hepatocytes was 40% larger than the control mice. The livers of the PINCH DKO mice were also very stiff due to increased ECM deposition throughout the liver, with no observed nodularity. Mice developed liver cancer by one year. These mice regenerated normally when subjected to 70% partial hepatectomy and did not show any termination defect. Ras suppressor 1 (Rsu-1) protein, the binding partner of PINCH is frequently deleted in human liver cancers. Rsu-1 expression is dramatically decreased in PINCH DKO mouse livers. Increased expression of Rsu-1 suppressed cell proliferation and migration in HCC cell lines. These changes were brought about not by affecting activation of Ras (as its name suggests) but by suppression of Ras downstream signaling via RhoGTPase proteins. In conclusion, our studies suggest that removal of PINCH results in enlargement of liver and tumorigenesis. Decreased levels of Rsu-1, a partner for PINCH and a protein often deleted in human liver cancer, may play an important role in the development of the observed phenotype.

---

## Gnpat

- NCBI Gene
- GeneRIF
- Pubmed

| Search Term | Rank | Fields |
| --- | --- | --- |
| [Mm]igration | 6 | PubMed(2) |

  
hide/show details for Gnpat

### [Mm]igration

#### PubMed

1. Combined deficiency of peroxisomal beta-oxidation and ether lipid synthesis in mice causes only minor cortical neuronal **migration** defects but severe hypotonia. The metabolic factors causing cortical neuronal **migration** defects, hypotonia and malformation of cerebellum in patients and mice with severe peroxisome biogenesis disorders are still not identified. In the present investigation, we tested the hypothesis that the combined inactivity of peroxisomal beta-oxidation and ether lipid biosynthesis could be at the origin of these pathologies. Double MFP2/DAPAT knockout mice were generated and their postnatal phenotypes were compared with single knockouts and control mice. Cortical neuronal **migration** was not affected in DAPAT knockouts and only mildly in double MFP2/DAPAT knockout mice. The latter mice were severely hypotonic and usually died in the postnatal period. Both DAPAT and MFP2 single knockout mice exhibited delays in the formation of cerebellar folia. We conclude that the combined defect of peroxisomal beta-oxidation and ether lipid synthesis does not solely account for the typical cortical neuronal **migration** defect of mice with peroxisome biogenesis disorders but contributes to their hypotonia.
2. Defects in myelination, paranode organization and Purkinje cell innervation in the ether lipid-deficient mouse cerebellum. Ether lipids (ELs), particularly plasmalogens, are essential constituents of the mammalian central nervous system. The physiological role of ELs, in vivo, however is still enigmatic. In the present study, we characterized a mouse model carrying a targeted deletion of the peroxisomal dihydroxyacetonephosphate acyltransferase gene that results in the complete lack of ELs. Investigating the cerebellum of these mice, we observed: (i) defects in foliation patterning and delay in precursor granule cell **migration**, (ii) defects in myelination and concomitant reduction in the level of myelin basic protein, (iii) disturbances in paranode organization by extending the Caspr distribution and disrupting axo-glial septate-like junctions, (iv) impaired innervation of Purkinje cells by both parallel fibers and climbing fibers and (v) formation of axon swellings by the accumulation of inositol-tris-phosphate receptor 1 containing smooth ER-like tubuli. Functionally, conduction velocity of myelinated axons in the corpus callosum was significantly reduced. Most of these phenotypes were already apparent at P20 but still persisted in 1-year-old animals. In summary, these data show that EL deficiency results in severe developmental and lasting structural alterations at the cellular and network level of the cerebellum, and reveal an important role of ELs for proper brain function. Common molecular mechanisms that may underlie these phenotypes are discussed.

---

## Ifrd2

- NCBI Gene
- GeneRIF
- Pubmed

| Search Term | Rank | Fields |
| --- | --- | --- |
| [Ee]xtracellular [Mm]atrix | 8 | PubMed(1) |

  
hide/show details for Ifrd2

### [Ee]xtracellular [Mm]atrix

#### PubMed

1. Characterization of the murine hyaluronidase gene region reveals complex organization and cotranscription of Hyal1 with downstream genes, Fus2 and Hyal3. Hyaluronidases are required for the breakdown of hyaluronan (HA), an abundant component of the **extracellular matrix** of vertebrate tissues. Multiple hyaluronidase genes have been identified, but the only clue to the function of their products has come from the identification of hyaluronidase 1 deficiency in a single patient with a mild clinical phenotype. As a first step in the generation of mice with hyaluronidase deficiency, we have used experimental and bioinformatic approaches to examine the organization of the mouse chromosome 9 region containing, in order, Hyal2, Hyal1, and Hyal3. This region was found to be complex, with Fus2 partially embedded in Hyal3, and Ifrd2 immediately downstream from Hyal3. The Hyal genes were all found to have four exons, and exons 2-4 exhibited the highest sequence conservation. Northern blot analysis demonstrated that the tissue expression profile for Hyal1 was similar in mice and humans, but a greater number of transcripts was detected in mouse tissues. Hyal3 was expressed more broadly in mice compared with humans and again exhibited additional transcripts. Reverse transcription-PCR demonstrated that some of the larger Hyal1 transcripts, seen on the Northern blot, were the result of cotranscription of Hyal1 with downstream genes, Fus2 or Hyal3. In vitro transcription/translation of one of the high abundance bicistronic transcripts produced Hyal 1, suggesting that Hyal 1 could be produced from all of the bicistronic transcripts. Characterization of the region including mouse Hyal1 and Hyal3 revealed complex organization and transcription that must be considered in the development and interpretation of mouse models involving genes in this region.

---

## Uso1

- NCBI Gene
- GeneRIF
- Pubmed

| Search Term | Rank | Fields |
| --- | --- | --- |
| ECM | 7 | PubMed(1) |
| [Ee]xtracellular [Mm]atrix | 8 | PubMed(1) |

  
hide/show details for Uso1

### ECM

#### PubMed

1. FGF signalling regulates bone growth through autophagy. Skeletal growth relies on both biosynthetic and catabolic processes. While the role of the former is clearly established, how the latter contributes to growth-promoting pathways is less understood. Macroautophagy, hereafter referred to as autophagy, is a catabolic process that plays a fundamental part in tissue homeostasis. We investigated the role of autophagy during bone growth, which is mediated by chondrocyte rate of proliferation, hypertrophic differentiation and extracellular matrix (**ECM**) deposition in growth plates. Here we show that autophagy is induced in growth-plate chondrocytes during post-natal development and regulates the secretion of type II collagen (Col2), the major component of cartilage **ECM**. Mice lacking the autophagy related gene 7 (Atg7) in chondrocytes experience endoplasmic reticulum storage of type II procollagen (PC2) and defective formation of the Col2 fibrillary network in the **ECM**. Surprisingly, post-natal induction of chondrocyte autophagy is mediated by the growth factor FGF18 through FGFR4 and JNK-dependent activation of the autophagy initiation complex VPS34-beclin-1. Autophagy is completely suppressed in growth plates from Fgf18(-/-) embryos, while Fgf18(+/-) heterozygous and Fgfr4(-/-) mice fail to induce autophagy during post-natal development and show decreased Col2 levels in the growth plate. Strikingly, the Fgf18(+/-) and Fgfr4(-/-) phenotypes can be rescued in vivo by pharmacological activation of autophagy, pointing to autophagy as a novel effector of FGF signalling in bone. These data demonstrate that autophagy is a developmentally regulated process necessary for bone growth, and identify FGF signalling as a crucial regulator of autophagy in chondrocytes.

### [Ee]xtracellular [Mm]atrix

#### PubMed

1. FGF signalling regulates bone growth through autophagy. Skeletal growth relies on both biosynthetic and catabolic processes. While the role of the former is clearly established, how the latter contributes to growth-promoting pathways is less understood. Macroautophagy, hereafter referred to as autophagy, is a catabolic process that plays a fundamental part in tissue homeostasis. We investigated the role of autophagy during bone growth, which is mediated by chondrocyte rate of proliferation, hypertrophic differentiation and **extracellular matrix** (ECM) deposition in growth plates. Here we show that autophagy is induced in growth-plate chondrocytes during post-natal development and regulates the secretion of type II collagen (Col2), the major component of cartilage ECM. Mice lacking the autophagy related gene 7 (Atg7) in chondrocytes experience endoplasmic reticulum storage of type II procollagen (PC2) and defective formation of the Col2 fibrillary network in the ECM. Surprisingly, post-natal induction of chondrocyte autophagy is mediated by the growth factor FGF18 through FGFR4 and JNK-dependent activation of the autophagy initiation complex VPS34-beclin-1. Autophagy is completely suppressed in growth plates from Fgf18(-/-) embryos, while Fgf18(+/-) heterozygous and Fgfr4(-/-) mice fail to induce autophagy during post-natal development and show decreased Col2 levels in the growth plate. Strikingly, the Fgf18(+/-) and Fgfr4(-/-) phenotypes can be rescued in vivo by pharmacological activation of autophagy, pointing to autophagy as a novel effector of FGF signalling in bone. These data demonstrate that autophagy is a developmentally regulated process necessary for bone growth, and identify FGF signalling as a crucial regulator of autophagy in chondrocytes.

---

## Rab7

- NCBI Gene
- GeneRIF
- Pubmed

| Search Term | Rank | Fields |
| --- | --- | --- |
| [Mm]igration | 6 | PubMed(1) |

  
hide/show details for Rab7

### [Mm]igration

#### PubMed

1. Ablation of vacuole protein sorting 18 (Vps18) gene leads to neurodegeneration and impaired neuronal **migration** by disrupting multiple vesicle transport pathways to lysosomes. Intracellular vesicle transport pathways are critical for neuronal survival and central nervous system development. The Vps-C complex regulates multiple vesicle transport pathways to the lysosome in lower organisms. However, little is known regarding its physiological function in mammals. We deleted Vps18, a central member of Vps-C core complex, in neural cells by generating Vps18(F/F); Nestin-Cre mice (Vps18 conditional knock-out mice). These mice displayed severe neurodegeneration and neuronal **migration** defects. Mechanistic studies revealed that Vps18 deficiency caused neurodegeneration by blocking multiple vesicle transport pathways to the lysosome, including autophagy, endocytosis, and biosynthetic pathways. Our study also showed that ablation of Vps18 resulted in up-regulation of β1 integrin in mouse brain probably due to lysosome dysfunction but had no effects on the reelin pathway, expression of N-cadherin, or activation of JNK, which are implicated in the regulation of neuronal **migratio**n. Finally, we demonstrated that knocking down β1 integrin partially rescued th**e migrati**on defects, suggesting that Vps18 deficiency-mediated up-regulation of β1 integrin may contribute to the defect of neuron**al migrat**ion in the Vps18-deficient brain. Our results demonstrate important roles of Vps18 in neuron survival a**nd migrat**ion, which are disrupted in multiple neural disorders.

---

## Ptbp1

- NCBI Gene
- GeneRIF
- Pubmed

| Search Term | Rank | Fields |
| --- | --- | --- |
| [Mm]igration | 6 | PubMed(1) |

  
hide/show details for Ptbp1

### [Mm]igration

#### PubMed

1. PTB deficiency causes the loss of adherens junctions in the dorsal telencephalon and leads to lethal hydrocephalus. Polypyrimidine tract-binding protein (PTB) is a well-characterized RNA-binding protein and known to be preferentially expressed in neural stem cells (NSCs) in the central nervous system; however, its role in NSCs in the developing brain remains unclear. To explore the role of PTB in embryonic NSCs in vivo, Nestin-Cre-mediated conditional Ptb knockout mice were generated for this study. In the mutant forebrain, despite the depletion of PTB protein, neither abnormal neurogenesis nor flagrant morphological abnormalities were observed at embryonic day 14.5 (E14.5). Nevertheless, by 10 weeks, nearly all mutant mice succumbed to hydrocephalus (HC), which was caused by a lack of the ependymal cell layer in the dorsal cortex. Upon further analysis, a gradual loss of adherens junctions (AJs) was observed in the ventricular zone (VZ) of the dorsal telencephalon in the mutant brains, beginning at E14.5. In the AJs-deficient VZ, impaired interkinetic nuclear **migration** and precocious differentiation of NSCs were observed after E14.5. These findings demonstrated that PTB depletion in the dorsal telencephalon is causally involved in the development of HC and that PTB is important for the maintenance of AJs in the NSCs of the dorsal telencephalon.

---

## Adam10

- NCBI Gene
- GeneRIF
- Pubmed

| Search Term | Rank | Fields |
| --- | --- | --- |
| [Mm]igration | 6 | Process(1); PubMed(16); GeneRIFs(4); Summary(1) |
| [Ee]xtracellular [Mm]atrix | 8 | PubMed(6); GeneRIFs(2) |
| [Mm]etastasis | 12 | PubMed(2) |

  
hide/show details for Adam10

### [Mm]igration

#### Process

1. positive regulation of cell **migration**

#### PubMed

1. The expression pattern of Adam10 in the central nervous system of adult mice: Detection by in situ hybridization combined with immunohistochemistry staining. ADAM10 (a disintegrin and metalloprotease 10) is a member of the ADAMs family, which is key in the development of the nervous system, by regulating proliferation, **migratio**n, differentiation and survival of various cells, including axonal growth and myelination. Previous studies have investigated the embryonic or postnatal expression of ADAM10, however, detailed information regarding its cellular distribution in the adult stage, to the best of our knowledge, is not available. The present study investigated the expression pattern of the ADAM10 gene in the adult mouse central nervous system (CNS) using an ADAM10 complementary RNA probe for in situ hybridization (ISH). Immunohistochemical staining was used to identify the type of the ISH staining‑positive cells with neuron‑ or astrocyte‑specific antibodies. The results of the current study demonstrated that the ADAM10 gene was predominantly expressed in the neurons of the cerebral cortex, hippocampus, thalamus and cerebellar granular cells in adult mouse CNS.
2. ADAM10 is required for SCF-induced mast cell **migration**. A Disintegrin and Metalloproteinase (ADAM)-10 plays critical roles in neuronal **migration** and distribution. Recently, ADAM10 deletion was shown to disrupt myelopoiesis. We found that inducible deletion of ADAM10 using Mx1-driven Cre recombinase for a period of three weeks resulted in mast cell hyperplasia in the skin, intestine and spleen. Mast cells express surface ADAM10 in vitro and in vivo, at high levels compared to other immune cells tested. ADAM10 is important for mast cell **migration**, since ADAM10-deficiency reduced c-Kit-mediated **migration**. As with some mast cell proteases, ADAM10 expression could be altered by the cytokine microenvironment, being inhibited by IL-10 or TGFβ1, but not by several other T cell-derived cytokines. Collectively these data show that the ADAM10 protease is an important factor in mast cell **migratio**n and tissue distribution, and can be manipulated by environmental cues.
3. Comprehensive gene expression profiling and functional analysis of matrix metalloproteinases and TIMPs, and identification of ADAM-10 gene expression, in a corneal model of epithelial resurfacing. This study provides a comprehensive expression analysis for the entire matrix metalloproteinase (MMP) gene family during the process of epithelial resurfacing following corneal abrasion injury in the mouse. The mRNA levels for all known MMP genes expressed in mouse, the related enzyme ADAM-10, and the known tissue inhibitors of metalloproteinases (TIMPs) were determined semi-quantitatively by reverse transcriptase-polymerase chain reaction (RT-PCR) in the uninjured epithelium, and in the epithelial tissue resurfacing the abraded area or residing in its periphery at two time points: during the epithelial **migration** phase and immediately following wound closure. The mRNA levels for MMP-1a, -1b, -9, -10, -12, and -13 as well as TIMP-1 were significantly up-regulated in the migrating corneal epithelium. After wound resurfacing, the mRNA levels for all of these MMPs were down-regulated, although MMP-1a, -1b, and -13 remained significantly elevated in comparison to the uninjured epithelium. The only gene found to be down-regulated was TIMP-3, which occurred throughout the wound-healing process. During resurfacing, MMP-9 was localized to the front of the migrating epithelium, MMP-10 and -13 were localized throughout the migrating epithelium, and MMP-13 could also be found in the periphery. Following epithelial closure, immunoreactive MMPs-9 and -10 became undetectable, but MMP-13 continued to be found throughout the epithelium. Functional analysis of MMP-10 revealed no effects on epithelial **migration** or cell proliferation. In conclusion, distinct MMP temporal-spatial profiles define the uninjured corneal epithelium and the corneal epithelium at different stages of regeneration. An extensive review of the literature is also provided in the discussion.
4. Regulated release and functional modulation of junctional adhesion molecule A by disintegrin metalloproteinases. Junctional adhesion molecule A (JAM-A) is a transmembrane adhesive glycoprotein that participates in the organization of endothelial tight junctions and contributes to leukocyte transendothelial **migration**. We demonstrate here that cultured endothelial cells not only express a cellular 43-kDa variant of JAM-A but also release considerable amounts of a 33-kDa soluble JAM-A variant. This release is enhanced by treatment with proinflammatory cytokines and is associated with the down-regulation of surface JAM-A. Inhibition experiments, loss/gain-of-function experiments, and cleavage experiments with recombinant proteases indicated that cleavage of JAM-A is mediated predominantly by the disintegrin and metalloproteinase (ADAM) 17 and, to a lesser extent, by ADAM10. Cytokine treatment of mice increased JAM-A serum level and in excised murine aortas increased ADAM10/17 activity correlated with enhanced JAM-A release. Functionally, soluble JAM-A blocked **migration** of cultured endothelial cells, reduced transendothelial **migration** of isolated neutrophils in vitro, and decreased neutrophil infiltration in a murine air pouch model by LFA-1- and JAM-A-dependent mechanisms. Therefore, shedding of JAM-A by inflamed vascular endothelium via ADAM17 and ADAM10 may not only generate a biomarker for vascular inflammation but could also be instrumental in controlling JAM-A functions in the molecular zipper guiding transendothelial diapedesis of leukocytes.
5. ADAM10-Initiated Release of Notch Intracellular Domain Regulates Microtubule Stability and Radial **Migration** of Cortical Neurons. Proper neuronal **migration** is orchestrated by combined membrane signal paradigms, whereas the role and mechanism of regulated intramembrane proteolysis (RIP) remain to be illustrated. We show here that the disintegrin and metalloprotease-domain containing protein 10 (ADAM10) regulates cortical neurons **migration** by initiating the RIP of Notch. We found that Notch intracellular domain (NICD) significantly rescued the **migration** defect of ADAM10-deficient neurons. Moreover, ADAM10 deficiency led to reduced neuronal motility and disrupted microtubule (MT) structure, which were associated with downregulated expression of acetylated tubulin and MT-associated proteins. Specifically, the NICD/RBPJ complex bound directly to the promoter, and regulated the neuronal expression level of doublecortin (DCX), a modulator of the MT cytoskeleton. Functionally, DCX overexpression largely restored neuron motility and reversed **migration** defect caused by ADAM10 knockout. Taken together, these findings demonstrate the direct requirement of ADAM10 in cortical radial **migration** and reveal the underlying mechanism by linking ADAM10-initiated RIP of Notch to the regulation of MT cytoskeleton through transcriptional control of Dcx expression.
6. GPR124, an orphan G protein-coupled receptor, is required for CNS-specific vascularization and establishment of the blood-brain barrier. Every organ in the body requires blood vessels for efficient delivery of oxygen and nutrients, but independent vascular beds are highly specialized to meet the individual needs of specific organs. The vasculature of the brain is tightly sealed, with blood-brain barrier (BBB) properties developing coincident with neural vascularization. G protein-coupled receptor 124 (GPR124) (tumor endothelial marker 5, TEM5), an orphan member of the adhesion family of G protein-coupled receptors, was previously identified on the basis of its overexpression in tumor vasculature. Here, we show that global deletion or endothelial-specific deletion of GPR124 in mice results in embryonic lethality associated with abnormal angiogenesis of the forebrain and spinal cord. Expression of GPR124 was found to be required for invasion and **migration** of blood vessels into neuroepithelium, establishment of BBB properties, and expansion of the cerebral cortex. Thus, GPR124 is an important regulator of neurovasculature development and a potential drug target for cerebrovascular diseases.
7. L1 is sequentially processed by two differently activated metalloproteases and presenilin/gamma-secretase and regulates neural cell adhesion, cell **migration**, and neurite outgrowth. The immunoglobulin superfamily recognition molecule L1 plays important functional roles in the developing and adult nervous system. Metalloprotease-mediated cleavage of this adhesion molecule has been shown to stimulate cellular **migration** and neurite outgrowth. We demonstrate here that L1 cleavage is mediated by two distinct members of the disintegrin and metalloprotease family, ADAM10 and ADAM17. This cleavage is differently regulated and leads to the generation of a membrane bound C-terminal fragment, which is further processed through gamma-secretase activity. Pharmacological approaches with two hydroxamate-based inhibitors with different preferences in blocking ADAM10 and ADAM17, as well as loss of function and gain of function studies in murine embryonic fibroblasts, showed that constitutive shedding of L1 is mediated by ADAM10 while phorbol ester stimulation or cholesterol depletion led to ADAM17-mediated L1 cleavage. In contrast, N-methyl-d-aspartate treatment of primary neurons stimulated ADAM10-mediated L1 shedding. Both proteases were able to affect L1-mediated adhesion and haptotactic **migration** of neuronal cells. In particular, both proteases were involved in L1-dependent neurite outgrowth of cerebellar neurons. Thus, our data identify ADAM10 and ADAM17 as differentially regulated L1 membrane sheddases, both critically affecting the physiological functions of this adhesion protein.
8. Human metalloprotease-disintegrin Kuzbanian regulates sympathoadrenal cell fate in development and neoplasia. The development of the sympathetic nervous system involves cell-cell interactions that regulate the fate and **migration** of progenitor neural cells. Recent evidence shows that focal membrane-bound protease activity is critical for such interactions. The Drosophila kuzbanian (kuz) gene is required in neurogenesis and encodes a highly conserved, membrane-bound metalloprotease- disintegrin closley related to theTNF-alphaconvertingenzyme (TACE). We have characterized the human and mouse kuz homologs and mapped human kuz to chromosome 15q22. During mouse embryonic development Kuz is expressed mainly in the sympathoadrenal and olfactory neural precursors. Once sympathoadrenal cells differentiate into chromaffin cells in the adult adrenal medulla, they no longer express Kuz. However, we found that tumors of sympathoadrenal origin, such as pheochromocytomas and neuroblastomas, overexpress Kuz. Further, transfection of a kuz construct lacking the protease domain, but not the full-length construct, induces neurite formation in PC12 chromaffin tumor cells. Taken together our results suggest a critical role for Kuz in regulation of sympathoadrenal cell fate.
9. Leukocytes require ADAM10 but not ADAM17 for their **migration** and inflammatory recruitment into the alveolar space. Inflammation is a key process in various diseases, characterized by leukocyte recruitment to the inflammatory site. This study investigates the role of a disintegrin and a metalloproteinase (ADAM) 10 and ADAM17 for leukocyte **migration** in vitro and in a murine model of acute pulmonary inflammation. Inhibition experiments or RNA knockdown indicated that monocytic THP-1 cells and primary human neutrophils require ADAM10 but not ADAM17 for efficient chemokine-induced cell **migration**. Signaling and adhesion events that are linked to cell **migration** such as p38 and ρ GTPase-family activation, F-actin polymerization, adhesion to fibronectin, and up-regulation of α5 integrin were also dependent on ADAM10 but not ADAM17. This was confirmed with leukocytes isolated from mice lacking either ADAM10 or ADAM17 in all hematopoietic cells (vav 1 guanine nucleotide exchange factor [Vav]-Adam10(-/-) or Vav-Adam17(-/-) mice). In lipopolysaccharide-induced acute pulmonary inflammation, alveolar recruitment of neutrophils and monocytes was transiently increased in Vav-Adam17(-/-) but steadily reduced in Vav-Adam10(-/-) mice. This deficit in alveolar leukocyte recruitment was also observed in LysM-Adam10(-/-) mice lacking ADAM10 in myeloid cells and correlated with protection against edema formation. Thus, with regard to leukocyt**e migrati**on, leukocyte-expressed ADAM10 but not ADAM17 displays proinflammatory activities and may therefore serve as a target to limit inflammatory cell recruitment.
10. Cleavage of E-cadherin by ADAM10 mediates epithelial cell sorting downstream of EphB signalling. The formation and maintenance of complex organs requires segregation of distinct cell populations into defined territories (that is, cell sorting) and the establishment of boundaries between them. Here we have investigated the mechanism by which Eph/ephrin signalling controls the compartmentalization of cells in epithelial tissues. We show that EphB/ephrin-B signalling in epithelial cells regulates the formation of E-cadherin-based adhesions. EphB receptors interact with E-cadherin and with the metalloproteinase ADAM10 at sites of adhesion and their activation induces shedding of E-cadherin by ADAM10 at interfaces with ephrin-B1-expressing cells. This process results in asymmetric localization of E-cadherin and, as a consequence, in differences in cell affinity between EphB-positive and ephrin-B-positive cells. Furthermore, genetic inhibition of ADAM10 activity in the intestine of mice results in a lack of compartmentalization of Paneth cells within the crypt stem cell niche, a defect that phenocopies that of EphB3-null mice. These results provide important insights into the regulation of cell **migration** in the intestinal epithelium and may help in the understanding of the nature of the cell sorting process in other epithelial tissues where Eph-ephrin interactions play a central role.
11. Formation of the collateral circulation is regulated by vascular endothelial growth factor-A and a disintegrin and metalloprotease family members 10 and 17. The density of native (preexisting) collaterals varies widely and is a significant determinant of variation in severity of stroke, myocardial infarction, and peripheral artery disease. However, little is known about mechanisms responsible for formation of the collateral circulation in healthy tissues.We previously found that variation in vascular endothelial growth factor (VEGF) expression causes differences in collateral density of newborn and adult mice. Herein, we sought to determine mechanisms of collaterogenesis in the embryo and the role of VEGF in this process.Pial collaterals begin forming between embryonic day 13.5 and 14.5 as sprout-like extensions from arterioles of existing cerebral artery trees. Global VEGF-A overexpressing mice (Vegf(hi/+)) formed more, and Vegf(lo/+) formed fewer, collaterals during embryogenesis, in association with differences in vascular patterning. Conditional global reduction of Vegf or Flk1 only during collaterogenesis significantly reduced collateral formation, but now without affecting vascular patterning, and the effects remained in adulthood. Endothelial-specific Vegf reduction had no effect on collaterogenesis. Endothelial-specific reduction of a disintegrin-and-metalloprotease-domain-10 (Adam10) and inhibition of γ-secretase increased collateral formation, consistent with their roles in VEGF-induced Notch1 activation and suppression of prosprouting signals. Endothelial-specific knockdown of Adam17 reduced collateral formation, consistent with its roles in endothelial cell **migratio**n and embryonic vascular stabilization, but not in activation of ligand-bound Notch1. These effects also remained in adulthood.Formation of pial collaterals occurs during a narrow developmental window via a sprouting angiogenesis-like mechanism, requires paracrine VEGF stimulation of fetal liver kinase 1-Notch signaling, and adult collateral number is dependent on embryonic collaterogenesis.
12. ADAM10 releases a soluble form of the GPNMB/Osteoactivin extracellular domain with angiogenic properties. Glycoprotein non-metastatic melanoma protein B (GPNMB)/Osteoactivin (OA) is a transmembrane protein expressed in approximately 40-75% of breast cancers. GPNMB/OA promotes the **migration**, invasion and metastasis of breast cancer cells; it is commonly expressed in basal/triple-negative breast tumors and is associated with shorter recurrence-free and overall survival times in patients with breast cancer. Thus, GPNMB/OA represents an attractive target for therapeutic intervention in breast cancer; however, little is known about the functions of GPNMB/OA within the primary tumor microenvironment.We have employed mouse and human breast cancer cells to investigate the effects of GPNMB/OA on tumor growth and angiogenesis. GPNMB/OA-expressing tumors display elevated endothelial recruitment and reduced apoptosis when compared to vector control-derived tumors. Primary human breast cancers characterized by high vascular density also display elevated levels of GPNMB/OA when compared to those with low vascular density. Using immunoblot and ELISA assays, we demonstrate the GPNMB/OA ectodomain is shed from the surface of breast cancer cells. Transient siRNA-mediated knockdown studies of known sheddases identified ADAM10 as the protease responsible for GPNMB/OA processing. Finally, we demonstrate that the shed extracellular domain (ECD) of GPNMB/OA can promote endothelial **migration** in vitro.GPNMB/OA expression promotes tumor growth, which is associated with enhanced endothelial recruitment. We identify ADAM10 as a sheddase capable of releasing the GPNMB/OA ectodomain from the surface of breast cancer cells, which induces endothelial cell **migration**. Thus, ectodomain shedding may serve as a novel mechanism by which GPNMB/OA promotes angiogenesis in breast cancer.
13. Myeloid A disintegrin and metalloproteinase domain 10 deficiency modulates atherosclerotic plaque composition by shifting the balance from inflammation toward fibrosis. A disintegrin and metalloproteinase domain 10 (ADAM10) is a metalloprotease involved in cleavage of various cell surface molecules, such as adhesion molecules, chemokines, and growth factor receptors. Although we have previously shown an association of ADAM10 expression with atherosclerotic plaque progression, a causal role of ADAM10 in atherosclerosis has not been investigated. Bone marrow from conditional knockout mice lacking Adam10 in the myeloid lineage or from littermate controls was transplanted into lethally irradiated low density lipoprotein receptor Ldlr(-/-) mice on an atherogenic diet. Myeloid Adam10 deficiency did not affect plaque size, but it increased plaque collagen content. Matrix metalloproteinase 9 and 13 expression and matrix metalloproteinase 2 gelatinase activity were significantly impaired in Adam10-deficient macrophages, whereas their capacity to stimulate collagen production was unchanged. Furthermore, relative macrophage content in advanced atherosclerotic lesions was decreased. In vitro, Adam10-deficient macrophages showed reduced **migration** toward monocyte chemoattractant protein-1 and trans**migration** through collagen. In addition, Adam10-deficient macrophages displayed increased anti-inflammatory phenotype with elevated IL-10, and reduced production of proinflammatory tumor necrosis factor, IL-12, and nitric oxide in response to lipopolysaccharide. These data suggest a critical role of Adam10 for leukocyte recruitment, inflammatory mediator production, and extracellular matrix degradation. Thereby, myeloid ADAM10 may play a causal role in modulating atherosclerotic plaque stability.
14. The disintegrin/metalloproteinase ADAM10 is essential for the establishment of the brain cortex. The metalloproteinase and major amyloid precursor protein (APP) alpha-secretase candidate ADAM10 is responsible for the shedding of proteins important for brain development, such as cadherins, ephrins, and Notch receptors. Adam10(-/-) mice die at embryonic day 9.5, due to major defects in development of somites and vasculogenesis. To investigate the function of ADAM10 in brain, we generated Adam10 conditional knock-out (cKO) mice using a Nestin-Cre promotor, limiting ADAM10 inactivation to neural progenitor cells (NPCs) and NPC-derived neurons and glial cells. The cKO mice die perinatally with a disrupted neocortex and a severely reduced ganglionic eminence, due to precocious neuronal differentiation resulting in an early depletion of progenitor cells. Premature neuronal differentiation is associated with aberrant neuronal **migration** and a disorganized laminar architecture in the neocortex. Neurospheres derived from Adam10 cKO mice have a disrupted sphere organization and segregated more neurons at the expense of astrocytes. We found that Notch-1 processing was affected, leading to downregulation of several Notch-regulated genes in Adam10 cKO brains, in accordance with the central role of ADAM10 in this signaling pathway and explaining the neurogenic phenotype. Finally, we found that alpha-secretase-mediated processing of APP was largely reduced in these neurons, demonstrating that ADAM10 represents the most important APP alpha-secretase in brain. Our study reveals that ADAM10 plays a central role in the developing brain by controlling mainly Notch-dependent pathways but likely also by reducing surface shedding of other neuronal membrane proteins including APP.
15. N-cadherin promotes recruitment and **migration** of neural progenitor cells from the SVZ neural stem cell niche into demyelinated lesions. Discrete cellular microenvironments regulate stem cell pools and their development, as well as function in maintaining tissue homeostasis. Although the signaling elements modulating neural progenitor cells (NPCs) of the adult subventricular zone (SVZ) niche are fairly well understood, the pathways activated following injury and the resulting outcomes, are less clear. In the present study, we used mouse models of demyelination and proteomics analysis to identify molecular cues present in the adult SVZ niche during injury, and analyzed their role on NPCs in the context of promoting myelin repair. Proteomic analysis of SVZ tissue from mice with experimental demyelination identified several proteins that are known to play roles in NPC proliferation, adhesion, and **migration**. Among the proteins found to be upregulated were members of the N-cadherin signaling pathway. During the onset of demyelination in the subcortical white matter (SCWM), activation of epidermal growth factor receptor (EGFR) signaling in SVZ NPCs stimulates the interaction between N-cadherin and ADAM10. Upon cleavage and activation of N-cadherin signaling by ADAM10, NPCs undergo cytoskeletal rearrangement and polarization, leading to enhanced **migration** out of the SVZ into demyelinated lesions of the SCWM. Genetically disrupting either EGFR signaling or ADAM10 inhibits this pathway, preventing N-cadherin regulated NPC polarization and **migration**. Additionally, in vivo experiments using N-cadherin gain- and loss-of-function approaches demonstrated that N-cadherin enhances the recruitment of SVZ NPCs into demyelinated lesions. Our data revealed that EGFR-dependent N-cadherin signaling physically initiated by ADAM10 cleavage is the response of the SVZ niche to promote repair of the injured brain.
16. ADAM10 mediates E-cadherin shedding and regulates epithelial cell-cell adhesion, **migration**, and beta-catenin translocation. E-cadherin controls a wide array of cellular behaviors, including cell-cell adhesion, differentiation, and tissue development. We show here that E-cadherin is cleaved specifically by ADAM (a disintegrin and metalloprotease) 10 in its ectodomain. Analysis of ADAM10-deficient fibroblasts, inhibitor studies, and RNA interference-mediated down-regulation of ADAM10 demonstrated that ADAM10 is responsible not only for the constitutive shedding but also for the regulated shedding of this adhesion molecule in fibroblasts and keratinocytes. ADAM10-mediated E-cadherin shedding affects epithelial cell-cell adhesion as well as cell **migration**. Furthermore, the shedding of E-cadherin by ADAM10 modulates the beta-catenin subcellular localization and downstream signaling. ADAM10 overexpression in epithelial cells increased the expression of the beta-catenin downstream gene cyclin D1 dose-dependently and enhanced cell proliferation. In ADAM10-deficient mouse embryos, the C-terminal E-cadherin fragment is not generated, and the full-length protein accumulates, highlighting the in vivo relevance for ADAM10 in E-cadherin shedding. Our data strongly suggest that this protease constitutes a major regulatory element for the multiple functions of E-cadherin under physiological as well as pathological conditions.

#### GeneRIFs

1. Findings demonstrate the direct requirement of ADAM10 in cortical radial **migration** and reveal the underlying mechanism by linking ADAM10-initiated regulated intramembrane proteolysis of Notch to the regulation of microtubule cytoskeleton through transcriptional control of Dcx expression
2. Collectively these data show that the ADAM10 protease is an important factor in mast cell **migration** and tissue distribution, and can be manipulated by environmental cues.
3. Thus, with regard to leukocyte **migration**, leukocyte-expressed ADAM10 but not ADAM17 displays proinflammatory activities and may therefore serve as a target to limit inflammatory cell recruitment.
4. ADAM10 has a role in E-cadherin shedding and epithelial cell-cell adhesion, **migration**, and beta-catenin translocation

#### Summary

1. This gene encodes a member of a disintegrin and metalloprotease (ADAM) family of endoproteases that play important roles in various biological processes including cell signaling, adhesion and **migration**. The encoded preproprotein undergoes proteolytic processing to generate a mature enzyme that is involved in the proteolytic release of membrane-bound proteins in a process called ectodomain shedding. Mice lacking the encoded protein die in utero with multiple defects of the developing central nervous system, somites, and cardiovascular system. [provided by RefSeq, May 2016]

### [Ee]xtracellular [Mm]atrix

#### PubMed

1. **Extracellular matrix** secretion by cardiac fibroblasts: role of microRNA-29b and microRNA-30c. MicroRNAs (miRNAs), in particular miR-29b and miR-30c, have been implicated as important regulators of cardiac fibrosis.To perform a proteomics comparison of miRNA effects on **extracellular matrix** secretion by cardiac fibroblasts.Mouse cardiac fibroblasts were transfected with pre-/anti-miR of miR-29b and miR-30c, and their conditioned medium was analyzed by mass spectrometry. miR-29b targeted a cadre of proteins involved in fibrosis, including multiple collagens, matrix metalloproteinases, and leukemia inhibitory factor, insulin-like growth factor 1, and pentraxin 3, 3 predicted targets of miR-29b. miR-29b also attenuated the cardiac fibroblast response to transforming growth factor-β. In contrast, miR-30c had little effect on **extracellular matri**x production but opposite effects regarding leukemia inhibitory factor and insulin-like growth factor 1. Both miRNAs indirectly affected cardiac myocytes. On transfection with pre-miR-29b, the conditioned medium of cardiac fibroblasts lost its ability to support adhesion of rat ventricular myocytes and led to a significant reduction of cardiac myocyte proteins (α-actinin, cardiac myosin-binding protein C, and cardiac troponin I). Similarly, cardiomyocytes derived from mouse embryonic stem cells atrophied under pre-miR-29 conditioned medium, whereas pre-miR-30c conditioned medium had a prohypertrophic effect. Levels of miR-29a, miR-29c, and miR-30c, but not miR-29b, were significantly reduced in a mouse model of pathological but not physiological hypertrophy. Treatment with antagomiRs to miR-29b induced excess fibrosis after aortic constriction without overt deterioration in cardiac function.Our proteomic analysis revealed novel molecular targets of miRNAs that are linked to a fibrogenic cardiac phenotype. Such comprehensive screening methods are essential to define the concerted actions of miRNAs in cardiovascular disease.
2. MicroRNA-140 plays dual roles in both cartilage development and homeostasis. Osteoarthritis (OA), the most prevalent aging-related joint disease, is characterized by insufficient **extracellular matrix** synthesis and articular cartilage degradation, mediated by several proteinases, including Adamts-5. miR-140 is one of a very limited number of noncoding microRNAs (miRNAs) specifically expressed in cartilage; however, its role in development and/or tissue maintenance is largely uncharacterized. To examine miR-140 function in tissue development and homeostasis, we generated a mouse line through a targeted deletion of miR-140. miR-140(-/-) mice manifested a mild skeletal phenotype with a short stature, although the structure of the articular joint cartilage appeared grossly normal in 1-mo-old miR-140(-/-) mice. Interestingly, miR-140(-/-) mice showed age-related OA-like changes characterized by proteoglycan loss and fibrillation of articular cartilage. Conversely, transgenic (TG) mice overexpressing miR-140 in cartilage were resistant to antigen-induced arthritis. OA-like changes in miR-140-deficient mice can be attributed, in part, to elevated Adamts-5 expression, regulated directly by miR-140. We show that miR-140 regulates cartilage development and homeostasis, and its loss contributes to the development of age-related OA-like changes.
3. Oligodendrocyte precursor cells modulate the neuronal network by activity-dependent ectodomain cleavage of glial NG2. The role of glia in modulating neuronal network activity is an important question. Oligodendrocyte precursor cells (OPC) characteristically express the transmembrane proteoglycan nerve-glia antigen 2 (NG2) and are unique glial cells receiving synaptic input from neurons. The development of NG2+ OPC into myelinating oligodendrocytes has been well studied, yet the retention of a large population of synapse-bearing OPC in the adult brain poses the question as to additional functional roles of OPC in the neuronal network. Here we report that activity-dependent processing of NG2 by OPC-expressed secretases functionally regulates the neuronal network. NG2 cleavage by the α-secretase ADAM10 yields an ectodomain present in the **extracellular matri**x and a C-terminal fragment that is subsequently further processed by the γ-secretase to release an intracellular domain. ADAM10-dependent NG2 ectodomain cleavage and release (shedding) in acute brain slices or isolated OPC is increased by distinct activity-increasing stimuli. Lack of NG2 expression in OPC (NG2-knockout mice), or pharmacological inhibition of NG2 ectodomain shedding in wild-type OPC, results in a striking reduction of N-methyl-D-aspartate (NMDA) receptor-dependent long-term potentiation (LTP) in pyramidal neurons of the somatosensory cortex and alterations in the subunit composition of their α-amino-3-hydroxy-5-methyl-4-isoxazolepr opionicacid (AMPA) receptors. In NG2-knockout mice these neurons exhibit diminished AMPA and NMDA receptor-dependent current amplitudes; strikingly AMPA receptor currents can be rescued by application of conserved LNS protein domains of the NG2 ectodomain. Furthermore, NG2-knockout mice exhibit altered behavior in tests measuring sensorimotor function. These results demonstrate for the first time a bidirectional cross-talk between OPC and the surrounding neuronal network and demonstrate a novel physiological role for OPC in regulating information processing at neuronal synapses.
4. Upregulation of the alpha-secretase ADAM10--risk or reason for hope? A decade ago, a disintegrin and metalloproteinase 10 (ADAM10) was identified as an alpha-secretase and as a key proteinase in the processing of the amyloid precursor protein. Accordingly, the important role that it plays in Alzheimer's disease was manifested. Animal models with an overexpression of ADAM10 revealed a beneficial profile of the metalloproteinase with respect to learning and memory, plaque load and synaptogenesis. Therefore, ADAM10 presents a worthwhile target with respect to the treatment of a neurodegenerative disease such as Morbus Alzheimer. Initially, ADAM10 was suggested to be an enzyme, shaping the **extracellular matrix** by cleavage of collagen type IV, or to be a tumour necrosis factor alpha convertase. In a relatively short time, a wide variety of additional substrates (with amyloid precursor protein probably being the most prominent) has been identified and the search is still ongoing. Hence, any side effects concerning the therapeutic enhancement of ADAM10 alpha-secretase activity have to be considered. The present review summarizes our knowledge about the structure and function of ADAM10 and highlights the opportunities for enhancing the expression and/or activity of the alpha-secretase as a therapeutic target.
5. Myeloid A disintegrin and metalloproteinase domain 10 deficiency modulates atherosclerotic plaque composition by shifting the balance from inflammation toward fibrosis. A disintegrin and metalloproteinase domain 10 (ADAM10) is a metalloprotease involved in cleavage of various cell surface molecules, such as adhesion molecules, chemokines, and growth factor receptors. Although we have previously shown an association of ADAM10 expression with atherosclerotic plaque progression, a causal role of ADAM10 in atherosclerosis has not been investigated. Bone marrow from conditional knockout mice lacking Adam10 in the myeloid lineage or from littermate controls was transplanted into lethally irradiated low density lipoprotein receptor Ldlr(-/-) mice on an atherogenic diet. Myeloid Adam10 deficiency did not affect plaque size, but it increased plaque collagen content. Matrix metalloproteinase 9 and 13 expression and matrix metalloproteinase 2 gelatinase activity were significantly impaired in Adam10-deficient macrophages, whereas their capacity to stimulate collagen production was unchanged. Furthermore, relative macrophage content in advanced atherosclerotic lesions was decreased. In vitro, Adam10-deficient macrophages showed reduced migration toward monocyte chemoattractant protein-1 and transmigration through collagen. In addition, Adam10-deficient macrophages displayed increased anti-inflammatory phenotype with elevated IL-10, and reduced production of proinflammatory tumor necrosis factor, IL-12, and nitric oxide in response to lipopolysaccharide. These data suggest a critical role of Adam10 for leukocyte recruitment, inflammatory mediator production, and **extracellular matrix** degradation. Thereby, myeloid ADAM10 may play a causal role in modulating atherosclerotic plaque stability.
6. The substrate degradome of meprin metalloproteases reveals an unexpected proteolytic link between meprin β and ADAM10. The in vivo roles of meprin metalloproteases in pathophysiological conditions remain elusive. Substrates define protease roles. Therefore, to identify natural substrates for human meprin α and β we employed TAILS (terminal amine isotopic labeling of substrates), a proteomics approach that enriches for N-terminal peptides of proteins and cleavage fragments. Of the 151 new extracellular substrates we identified, it was notable that ADAM10 (a disintegrin and metalloprotease domain-containing protein 10)-the constitutive α-secretase-is activated by meprin β through cleavage of the propeptide. To validate this cleavage event, we expressed recombinant proADAM10 and after preincubation with meprin β, this resulted in significantly elevated ADAM10 activity. Cellular expression in murine primary fibroblasts confirmed activation. Other novel substrates inc**luding extracellular** matrix proteins, growth factors and inhibitors were validated by western analyses and enzyme activity assays with Edman sequencing confirming the exact cleavage sites identified by TAILS. Cleavages in vivo were confirmed by comparing wild-type and meprin(-/-) mice. Our finding of cystatin C, elafin and fetuin-A as substrates and natural inhibitors for meprins reveal new mechanisms in the regulation of protease activity important for understanding pathophysiological processes.

#### GeneRIFs

1. NG2 cleavage by ADAM10 yields an ectodomain present in the **extracellular matrix** and a C-terminal fragment that is subsequently further processed by the gamma-secretase to release an intracellular domain.
2. These data suggest a critical role of Adam10 for leukocyte recruitment, inflammatory mediator production, and **extracellular matrix** degradation.

### [Mm]etastasis

#### PubMed

1. Cutting edge: mast cells critically augment myeloid-derived suppressor cell activity. Myeloid-derived suppressor cells (MDSCs) are primarily recognized for their immunosuppressive properties in malignant disease. However, their interaction with other innate immune cells and their regulation of immune responses, such as in parasitic infection, necessitate further characterization. We used our previously published mouse model of MDSC accumulation to examine the immunoregulatory role of MDSCs in B16 melanoma **metastasis** and Nippostrongylus brasiliensis infection. In this study, we demonstrate that the activity of MDSCs is dependent on the immune stimuli and subset induced. Monocytic MDSCs predictably suppressed antitumor immune responses but granulocytic MDSCs surprisingly enhanced the clearance of N. brasiliensis infection. Intriguingly, both results were dependent on MDSC interaction with mast cells (MCs), as demonstrated by adoptive-transfer studies in MC-deficient (Kit(Wsh)(/)(Wsh)) mice. These findings were further supported by ex vivo cocultures of MCs and MDSCs, indicating a synergistic increase in cytokine production. Thus, MCs can enhance both immunosuppressive and immunosupportive functions of MDSCs.
2. ADAM10 releases a soluble form of the GPNMB/Osteoactivin extracellular domain with angiogenic properties. Glycoprotein non-metastatic melanoma protein B (GPNMB)/Osteoactivin (OA) is a transmembrane protein expressed in approximately 40-75% of breast cancers. GPNMB/OA promotes the migration, invasion and **metastasis** of breast cancer cells; it is commonly expressed in basal/triple-negative breast tumors and is associated with shorter recurrence-free and overall survival times in patients with breast cancer. Thus, GPNMB/OA represents an attractive target for therapeutic intervention in breast cancer; however, little is known about the functions of GPNMB/OA within the primary tumor microenvironment.We have employed mouse and human breast cancer cells to investigate the effects of GPNMB/OA on tumor growth and angiogenesis. GPNMB/OA-expressing tumors display elevated endothelial recruitment and reduced apoptosis when compared to vector control-derived tumors. Primary human breast cancers characterized by high vascular density also display elevated levels of GPNMB/OA when compared to those with low vascular density. Using immunoblot and ELISA assays, we demonstrate the GPNMB/OA ectodomain is shed from the surface of breast cancer cells. Transient siRNA-mediated knockdown studies of known sheddases identified ADAM10 as the protease responsible for GPNMB/OA processing. Finally, we demonstrate that the shed extracellular domain (ECD) of GPNMB/OA can promote endothelial migration in vitro.GPNMB/OA expression promotes tumor growth, which is associated with enhanced endothelial recruitment. We identify ADAM10 as a sheddase capable of releasing the GPNMB/OA ectodomain from the surface of breast cancer cells, which induces endothelial cell migration. Thus, ectodomain shedding may serve as a novel mechanism by which GPNMB/OA promotes angiogenesis in breast cancer.

---

## Esyt2

- NCBI Gene
- GeneRIF
- Pubmed

| Search Term | Rank | Fields |
| --- | --- | --- |
| [Mm]igration | 6 | PubMed(1) |

  
hide/show details for Esyt2

### [Mm]igration

#### PubMed

1. Loss of Extended Synaptotagmins ESyt2 and ESyt3 does not affect mouse development or viability, but in vitro cell **migration** and survival under stress are affected. The Extended Synaptotagmins (Esyts) are a family of multi-C2 domain membrane proteins with orthologs in organisms from yeast to human. Three Esyt genes exist in mouse and human and these have most recently been implicated in the formation of junctions between endoplasmic reticulum and plasma membrane, as well as the Ca(2+) dependent replenishment of membrane phospholipids. The data are consistent with a function in extracellular signal transduction and cell adhesion, and indeed Esyt2 was previously implicated in both these functions in Xenopus. Despite this, little is known of the function of the Esyts in vivo. We have generated mouse lines carrying homozygous deletions in one or both of the genes encoding the highly homologous Esyt2 and Esyt3 proteins. Surprisingly, esyt2(-/-)/esyt3(-/-) mice develop normally and are both viable and fertile. In contrast, esyt2(-/-)/esyt3(-/-) mouse embryonic fibroblasts display a reduced ability to migrate in standard in vitro assays, and are less resistant to stringent culture conditions and to oxidative stress than equivalent wild type fibroblasts.

---

## Stat5b

- NCBI Gene
- GeneRIF
- Pubmed

| Search Term | Rank | Fields |
| --- | --- | --- |
| [Mm]igration | 6 | Process(1); PubMed(4) |

  
hide/show details for Stat5b

### [Mm]igration

#### Process

1. mast cell **migration**

#### PubMed

1. Transcription factor achaete-scute homologue 2 initiates follicular T-helper-cell development. In immune responses, activated T cells migrate to B-cell follicles and develop into follicular T-helper (TFH) cells, a recently identified subset of CD4(+) T cells specialized in providing help to B lymphocytes in the induction of germinal centres. Although Bcl6 has been shown to be essential in TFH-cell function, it may not regulate the initial **migration** of T cells or the induction of the TFH program, as exemplified by C-X-C chemokine receptor type 5 (CXCR5) upregulation. Here we show that expression of achaete-scute homologue 2 (Ascl2)--a basic helix-loop-helix (bHLH) transcription factor--is selectively upregulated in TFH cells. Ectopic expression of Ascl2 upregulates CXCR5 but not Bcl6, and downregulates C-C chemokine receptor 7 (CCR7) expression in T cells in vitro, as well as accelerating T-cell **migration** to the follicles and TFH-cell development in vivo in mice. Genome-wide analysis indicates that Ascl2 directly regulates TFH-related genes whereas it inhibits expression of T-helper cell 1 (TH1) and TH17 signature genes. Acute deletion of Ascl2, as well as blockade of its function with the Id3 protein in CD4(+) T cells, results in impaired TFH-cell development and germinal centre response. Conversely, mutation of Id3, known to cause antibody-mediated autoimmunity, greatly enhances TFH-cell generation. Thus, Ascl2 directly initiates TFH-cell development.
2. Oncostatin M-induced and constitutive activation of the JAK2/STAT5/CIS pathway suppresses CCL1, but not CCL7 and CCL8, chemokine expression. The recruitment of leukocytes to injured tissue is crucial for the initiation of inflammatory responses as well as for immune surveillance to fight tumor progression. In this study, we show that oncostatin M, a member of the IL-6-type cytokine family and potent proinflammatory cytokine stimulates the expression of the chemokines CCL1, CCL7, and CCL8 in primary human dermal fibroblasts at a faster kinetic than IL-1beta or TNF-alpha. The production of CCL1 and CCL8 is important for **migration** of monocytes, while specific Abs against CCL1 additionally inhibit the **migration** of T lymphocytes. We identify the mitogen-activated protein kinases ERK1/2 and p38 as crucial factors for the enhanced expression of CCL1 and CCL8. Depletion of the ERK1/2 target genes c-Jun or c-Fos strongly decrease CCL1 and CCL8 expression, while p38 MAPK prolongs the half-life of CCL1, CCL7, and CCL8 mRNA through inhibition of tristetraprolin. None of the STAT transcription factors STAT1, STAT3, or STAT5 stimulate transcription of CCL1 or CCL8. However, we identify a negative regulatory function of activated STAT5 for the gene expression of CCL1. Importantly, not STAT5 itself, but its target gene cytokine inducible SH2-domain containing protein is required for the STAT5 inhibitory effect on CCL1 expression. Finally, we show that constitutive activation of STAT5 through a mutated form of JAK2 (JAK2 V617F) occurring in patients with myeloproliferative disorders similarly suppresses CCL1 expression. Taken together, we identify novel important inflammatory target genes of OSM which are independent of STAT signaling per se, but depend on MAPK activation and are partly repressed through STAT5-dependent expression of cytokine inducible SH2-domain containing protein.
3. Genotype-dependent effects of TGF-β1 on mast cell function: targeting the Stat5 pathway. We previously demonstrated that TGF-β1 suppresses IgE-mediated signaling in human and mouse mast cells in vitro, an effect that correlated with decreased expression of the high-affinity IgE receptor, FcεRI. The in vivo effects of TGF-β1 and the means by which it suppresses mast cells have been less clear. This study shows that TGF-β1 suppresses FcεRI and c-Kit expression in vivo. By examining changes in cytokine production concurrent with FcεRI expression, we found that TGF-β1 suppresses TNF production independent of FcεRI levels. Rather, IgE-mediated signaling was altered. TGF-β1 significantly reduced expression of Fyn and Stat5, proteins critical for cytokine induction. These changes may partly explain the effects of TGF-β1, because Stat5B overexpression blocked TGF-mediated suppression of IgE-induced cytokine production. We also found that Stat5B is required for **mast cel**l migration toward stem cell factor, and that TGF-β1 r**educed th**is migration. We found evidence that genetic background may alter TGF responses. TGF-β1 greatly reduced mast cell numbers in Th1-prone C57BL/6, but not Th2-prone 129/Sv mice. Furthermore, TGF-β1 did not suppress IgE-induced cytokine release and did increase c**-Kit-medi**ated migration in 129/Sv mast cells. These data correlated with high basal Fyn and Stat5 expression in 129/Sv cells, which was not reduced by TGF-β1 treatment. Finally, primary human mast cell populations also showed variable sensitivity to TGF-β1-mediated changes in Stat5 and IgE-mediated IL-6 secretion. We propose that TGF-β1 regulates mast cell homeostasis, and that this feedback suppression may be dependent on genetic context, predisposing some individuals to atopic disease.
4. STAT5A/B activity is required in the developing forebrain and spinal cord. Formation of the CNS requires the coordination and integration of processes such as cell proliferation, neuronal differentiation, neuronal **migration**, axon tract formation and synaptogenesis, all of which must occur at precise times and places during development. Although growth factors are known to play a role in regulating many of these processes, very little is known of the signaling events immediately downstream of ligand-receptor interactions in the developing CNS. Here we present evidence that STAT5, an important mediator of cytokine signaling, is required for some aspects of CNS development. We show that phosphorylated and hence activated forms of STAT5 (pSTAT5) are expressed in a temporally restricted manner in a subset of early-born telencephalic neurons and axons. Accordingly, Stat5 mutants have reduced numbers of interneurons in the cortical marginal zone, suggestive of **migration** defects. Moreover, corticofugal axons develop aberrantly in Stat5 mutants, indicative of a role for pSTAT5 in axon guidance. Notably, pSTAT5 is also expressed in commissural axons in the embryonic spinal cord, where it is also required for their guidance. Taken together, we provide the first evidence that STAT5 is a key effector molecule in the developing mammalian CNS.

---

## Serac1

- NCBI Gene
- GeneRIF
- Pubmed

| Search Term | Rank | Fields |
| --- | --- | --- |
| ECM | 7 | PubMed(1) |
| [Ee]xtracellular [Mm]atrix | 8 | Process(1); PubMed(1); Component(1) |

  
hide/show details for Serac1

### ECM

#### PubMed

1. Transcriptome-based systematic identification of extracellular matrix proteins. Extracellular matrix (**ECM**), which provides critical scaffolds for all adhesive cells, regulates proliferation, differentiation, and apoptosis. Different cell types employ customized **ECM**s, which are thought to play important roles in the generation of so-called niches that contribute to cell-specific functions. The molecular entities of these customized **ECM**s, however, have not been elucidated. Here, we describe a strategy for transcriptome-wide identification of **ECM** proteins based on computational screening of >60,000 full-length mouse cDNAs for secreted proteins, followed by in vitro functional assays. These assays screened the candidate proteins for **ECM**-assembling activities, interactions with other **ECM** molecules, modifications with glycosaminoglycans, and cell-adhesive activities, and were then complemented with immunohistochemical analysis. We identified 16 **ECM** proteins, of which seven were localized in basement membrane (BM) zones. The identification of these previously unknown BM proteins allowed us to construct a body map of BM proteins, which represents the comprehensive immunohistochemistry-based expression profiles of the tissue-specific customization of BMs.

### [Ee]xtracellular [Mm]atrix

#### Process

1. **extracellular matrix** organization

#### PubMed

1. Transcriptome-based systematic identification of **extracellular matrix** proteins. **Extracellular matrix** (ECM), which provides critical scaffolds for all adhesive cells, regulates proliferation, differentiation, and apoptosis. Different cell types employ customized ECMs, which are thought to play important roles in the generation of so-called niches that contribute to cell-specific functions. The molecular entities of these customized ECMs, however, have not been elucidated. Here, we describe a strategy for transcriptome-wide identification of ECM proteins based on computational screening of >60,000 full-length mouse cDNAs for secreted proteins, followed by in vitro functional assays. These assays screened the candidate proteins for ECM-assembling activities, interactions with other ECM molecules, modifications with glycosaminoglycans, and cell-adhesive activities, and were then complemented with immunohistochemical analysis. We identified 16 ECM proteins, of which seven were localized in basement membrane (BM) zones. The identification of these previously unknown BM proteins allowed us to construct a body map of BM proteins, which represents the comprehensive immunohistochemistry-based expression profiles of the tissue-specific customization of BMs.

#### Component

1. proteinaceous **extracellular matrix**

---

## Anxa11

- NCBI Gene
- GeneRIF
- Pubmed

| Search Term | Rank | Fields |
| --- | --- | --- |
| [Mm]etastasis | 12 | PubMed(1) |
| [Cc]ell [Ii]nvasion | 13 | PubMed(1) |

  
hide/show details for Anxa11

### [Mm]etastasis

#### PubMed

1. Annexin A11 knockdown inhibits in vitro proliferation and enhances survival of Hca-F cell via Akt2/FoxO1 pathway and MMP-9 expression. Annexin A11 (Anxa11), a Ca(2+)-regulated phospholipid-binding protein, is involved in cell apoptosis, differentiation, vesicle trafficking, cancer progression and autoimmune diseases. Previous study from our group indicated that Anxa11 was associated with lymphatic metastatic potential of murine hepatocarcinoma cells. Herein, we investigated the effects and action mechanism of Anxa11 knockdown on in vitro cell proliferation and apoptosis of Hca-F, a murine hepatocarcinoma cell with∼75% lymph node metastatic potential. Real-time PCR and western blotting assays indicated that Anxa11 was significantly downregulated in monoclonal Anxa11-shRNA-transfected Hca-F cells. Anxa11 knockdown in Hca-F suppressed its in vitro proliferation and cell apoptosis capacities. Following Anxa11 knockdown in Hca-F cells, Bax/Bcl-2 expression level ratio, Akt2 and FoxO1 (pSer319) expression levels as well as MMP-9 mRNA and active MMP-9 protein levels were significantly elevated in Hca-F cells. In conclusion, Annexin A11 knockdown inhibits the in vitro proliferation and cell apoptosis of Hca-F cell via Akt2/FoxO1 and/or MMP-9 expression pathway. Anxa11 might play an important role in hepatocarcinoma cell invasion an**d metastas**is and hepatocarcinoma malignancy.

### [Cc]ell [Ii]nvasion

#### PubMed

1. Annexin A11 knockdown inhibits in vitro proliferation and enhances survival of Hca-F cell via Akt2/FoxO1 pathway and MMP-9 expression. Annexin A11 (Anxa11), a Ca(2+)-regulated phospholipid-binding protein, is involved in cell apoptosis, differentiation, vesicle trafficking, cancer progression and autoimmune diseases. Previous study from our group indicated that Anxa11 was associated with lymphatic metastatic potential of murine hepatocarcinoma cells. Herein, we investigated the effects and action mechanism of Anxa11 knockdown on in vitro cell proliferation and apoptosis of Hca-F, a murine hepatocarcinoma cell with∼75% lymph node metastatic potential. Real-time PCR and western blotting assays indicated that Anxa11 was significantly downregulated in monoclonal Anxa11-shRNA-transfected Hca-F cells. Anxa11 knockdown in Hca-F suppressed its in vitro proliferation and cell apoptosis capacities. Following Anxa11 knockdown in Hca-F cells, Bax/Bcl-2 expression level ratio, Akt2 and FoxO1 (pSer319) expression levels as well as MMP-9 mRNA and active MMP-9 protein levels were significantly elevated in Hca-F cells. In conclusion, Annexin A11 knockdown inhibits the in vitro proliferation and cell apoptosis of Hca-F cell via Akt2/FoxO1 and/or MMP-9 expression pathway. Anxa11 might play an important role in hepatocarcinom**a cell invasi**on and metastasis and hepatocarcinoma malignancy.

---

## Rac1

- NCBI Gene
- GeneRIF
- Pubmed

| Search Term | Rank | Fields |
| --- | --- | --- |
| [Ff]ilopodia | 1 | PubMed(17); GeneRIFs(4) |
| [Ff]ilopodium | 2 | PubMed(1) |
| [Pp]odosome(s)? | 5 | PubMed(1) |
| [Mm]igration | 6 | Process(6); PubMed(146); GeneRIFs(40) |
| ECM | 7 | PubMed(15); GeneRIFs(1) |
| [Ee]xtracellular [Mm]atrix | 8 | PubMed(36); Component(1) |
| [Ii]nvasive | 10 | PubMed(9) |
| [Mm]etastasize | 11 | PubMed(1) |
| [Mm]etastasis | 12 | PubMed(14); GeneRIFs(4) |
| [Cc]ell [Ii]nvasion | 13 | PubMed(1) |

  
hide/show details for Rac1

### [Ff]ilopodia

#### PubMed

1. Muscle costameric protein, Chisel/Smpx, associates with focal adhesion complexes and modulates cell spreading in vitro via a Rac1/p38 pathway. The murine X-linked gene Chisel (Csl/Smpx) encodes a 9-kDa protein that associates in heart and skeletal muscle cells with the costameric cytoskeleton, implicated in maintaining muscle integrity and responses to biomechanical stress. After expression in C2C12 myoblasts, MYC epitope-tagged Csl co-localized with actin networks at peripheral membranes, and with focal adhesion proteins vinculin, paxillin, integrin beta1, and the small GTPase Rac1. Csl could be co-immunoprecipitated with vinculin from extracts of C2C12 cells and native muscle. MYC-Csl induced cell spreading and lamellipodia formation in C2C12 cells at the expense of **filopodia**, suggestive of modulation of Rac1 activity. Lamellipodia formation was indeed Rac1-dependent, and in MYC-Csl cells replated on fibronectin, Rac1 activity was increased relative to controls. Expression of MYC-Csl led to an increased association between vinculin and p34, a subunit of the Arp2/3 actin nucleation complex, a Rac1-dependent event. Induced cell spreading was also dependent upon p38 kinases that act downstream of Rac1 to control the actin capping activity of heat shock protein 27. Our data suggest that Csl localizes to the costameric cytoskeleton of muscle cells through an association with focal adhesion proteins, where it may participate in regulation of cytoskeletal dynamics through the Rac1-p38 pathway.
2. Rac1-dependent collective cell migration is required for specification of the anterior-posterior body axis of the mouse. Cell migration and cell rearrangements are critical for establishment of the body plan of vertebrate embryos. The first step in organization of the body plan of the mouse embryo, specification of the anterior-posterior body axis, depends on migration of the anterior visceral endoderm from the distal tip of the embryo to a more proximal region overlying the future head. The anterior visceral endoderm (AVE) is a cluster of extra-embryonic cells that secretes inhibitors of the Wnt and Nodal pathways to inhibit posterior development. Because Rac proteins are crucial regulators of cell migration and mouse Rac1 mutants die early in development, we tested whether Rac1 plays a role in AVE migration. Here we show that Rac1 mutant embryos fail to specify an anterior-posterior axis and, instead, express posterior markers in a ring around the embryonic circumference. Cells that express the molecular markers of the AVE are properly specified in Rac1 mutants but remain at the distal tip of the embryo at the time when migration should take place. Using tissue specific deletions, we show that Rac1 acts autonomously within the visceral endoderm to promote cell migration. High-resolution imaging shows that the leading wild-type AVE cells extend long lamellar protrusions that span several cell diameters and are polarized in the direction of cell movement. These projections are tipped by **filopodia**-like structures that appear to sample the environment. Wild-type AVE cells display hallmarks of collective cell migration: they retain tight and adherens junctions as they migrate and exchange neighbors within the plane of the visceral endoderm epithelium. Analysis of mutant embryos shows that Rac1 is not required for intercellular signaling, survival, proliferation, or adhesion in the visceral endoderm but is necessary for the ability of visceral endoderm cells to extend projections, change shape, and exchange neighbors. The data show that Rac1-mediated epithelial migration of the AVE is a crucial step in the establishment of the mammalian body plan and suggest that Rac1 is essential for collective migration in mammalian tissues.
3. Lamellipodia nucleation by **filopodia** depends on integrin occupancy and downstream Rac1 signaling. Time-lapse video-microscopy unambiguously shows that fibroblast **filopodia** are the scaffold of lamellipodia nucleation that allows anisotropic cell spreading. This process was dissected into elementary stages by monitoring cell adhesion on micropatterned extracellular matrix arrays of various pitches. Adhesion structures are stabilized by contact with the adhesive plots and subsequently converted into lamellipodia-like extensions starting at the **filopodia** tips. This mechanism progressively leads to full cell spreading. Stable expression of the dominant-negative Rac1 N17 impairs this change in membrane extension mode and stops cell spreading on matrix arrays. Similar expression of the dominant-negative Cdc42 N17 impairs cell spreading on homogenous and structured substrate, suggesting that **filopodia** extension is a prerequisite for cell spreading in this model. The differential polarity of the nucleation of lamellipodial structures by **filopodia** on homogenous and structured surfaces starting from the cell body and of **filopodia** tip, respectively, suggested that this process is triggered by areas that are in contact with extracellular matrix proteins for longer times. Consistent with this view, wild-type cells cannot spread on microarrays made of function blocking or neutral anti-beta 1 integrin antibodies. However, stable expression of a constitutively active Rac1 mutant rescues the cell ability to spread on these integrin microarrays. Thereby, lamellipodia nucleation by **filopodia** requires integrin occupancy by matrix substrate and downstream Rac1 signaling.
4. The Rho GTPase Rac1 is required for recycling endosome-mediated secretion of TNF in macrophages. Rho GTPases are required for many cellular events such as adhesion, motility, and membrane trafficking. Here we show that in macrophages, the Rho GTPases Rac1 and Cdc42 are involved in lamellipodia and **filopodia** formation, respectively, and that both of these Rho GTPases are essential for the efficient surface delivery of tumor necrosis factor (TNF) to the plasma membrane following TLR4 stimulation. We have previously demonstrated intracellular trafficking of TNF via recycling endosomes in lipopolysaccharide (LPS)-activated macrophages. Here, we further define a specific role for Rac1 in intracellular TNF trafficking, demonstrating impairment in TNF release following TLR4 stimulation in the presence of a Rac inhibitor, in cells expressing a dominant negative (DN) form of Rac1, and following small interfering RNA (siRNA) knockdown of Rac1. Rac1 activity was required for TNF trafficking but not for TLR4 signaling following LPS stimulation. Reduced TNF secretion was due to a defect in Rac1 activity, but not of the closely related Rho GTPase Rac2, demonstrated by the additional use of macrophages derived from Rac2-deficient mice. Labeling recycling endosomes by the uptake of fluorescent transferrin enabled us to show that Rac1 was required for the final stages of TNF trafficking and delivery from recycling endosomes to the plasma membrane. Thus, actin remodeling by the Rho GTPase Rac1 is required for TNF cell surface delivery and release from macrophages.
5. The cytotoxic necrotizing factor of Yersinia pseudotuberculosis (CNFY) enhances inflammation and Yop delivery during infection by activation of Rho GTPases. Some isolates of Yersinia pseudotuberculosis produce the cytotoxic necrotizing factor (CNFY), but the functional consequences of this toxin for host-pathogen interactions during the infection are unknown. In the present study we show that CNFY has a strong influence on virulence. We demonstrate that the CNFY toxin is thermo-regulated and highly expressed in all colonized lymphatic tissues and organs of orally infected mice. Most strikingly, we found that a cnfY knock-out variant of a naturally toxin-expressing Y. pseudotuberculosis isolate is strongly impaired in its ability to disseminate into the mesenteric lymph nodes, liver and spleen, and has fully lost its lethality. The CNFY toxin contributes significantly to the induction of acute inflammatory responses and to the formation of necrotic areas in infected tissues. The analysis of the host immune response demonstrated that presence of CNFY leads to a strong reduction of professional phagocytes and natural killer cells in particular in the spleen, whereas loss of the toxin allows efficient tissue infiltration of these immune cells and rapid killing of the pathogen. Addition of purified CNFY triggers formation of actin-rich membrane ruffles and **filopodia**, which correlates with the activation of the Rho GTPases, RhoA, Rac1 and Cdc42. The analysis of type III effector delivery into epithelial and immune cells in vitro and during the course of the infection further demonstrated that CNFY enhances the Yop translocation process and supports a role for the toxin in the suppression of the antibacterial host response. In summary, we highlight the importance of CNFY for pathogenicity by showing that this toxin modulates inflammatory responses, protects the bacteria from attacks of innate immune effectors and enhances the severity of a Yersinia infection.
6. Serine-71 phosphorylation of Rac1 modulates downstream signaling. The Rho GTPases Rac1 and Cdc42 regulate a variety of cellular functions by signaling to different signal pathways. It is believed that the presence of a specific effector at the location of GTPase activation determines the route of downstream signaling. We previously reported about EGF-induced Ser-71 phosphorylation of Rac1/Cdc42. By using the phosphomimetic S71E-mutants of Rac1 and Cdc42 we investigated the impact of Ser-71 phosphorylation on binding to selected effector proteins. Binding of the constitutively active (Q61L) variants of Rac1 and Cdc42 to their specific interaction partners Sra-1 and N-WASP, respectively, as well as to their common effector protein PAK was abrogated when Ser-71 was exchanged to glutamate as phosphomimetic substitution. Interaction with their common effector proteins IQGAP1/2/3 or MRCK alpha was, however, hardly affected. This ambivalent behaviour was obvious in functional assays. In contrast to Rac1 Q61L, phosphomimetic Rac1 Q61L/S71E was not able to induce increased membrane ruffling. Instead, Rac1 Q61L/S71E allowed **filopodia** formation, which is in accordance with abrogation of the dominant Sra-1/Wave signalling pathway. In addition, in contrast to Rac1 transfected cells Rac1 S71E failed to activate PAK1/2. On the other hand, Rac1 Q61L/S71E was as effective in activation of NF-kappaB as Rac1 Q61L, illustrating positive signal transduction of phosphorylated Rac1. Together, these data suggest that phosphorylation of Rac1 and Cdc42 at serine-71 represents a reversible mechanism to shift specificity of GTPase/effector coupling, and to preferentially address selected downstream pathways.
7. Regulation of cell protrusions by small GTPases during fusion of the neural folds. Epithelial fusion is a crucial process in embryonic development, and its failure underlies several clinically important birth defects. For example, failure of neural fold fusion during neurulation leads to open neural tube defects including spina bifida. Using mouse embryos, we show that cell protrusions emanating from the apposed neural fold tips, at the interface between the neuroepithelium and the surface ectoderm, are required for completion of neural tube closure. By genetically ablating the cytoskeletal regulators Rac1 or Cdc42 in the dorsal neuroepithelium, or in the surface ectoderm, we show that these protrusions originate from surface ectodermal cells and that Rac1 is necessary for the formation of membrane ruffles which typify late closure stages, whereas Cdc42 is required for the predominance of **filopodia** in early neurulation. This study provides evidence for the essential role and molecular regulation of membrane protrusions prior to fusion of a key organ primordium in mammalian development.
8. Sequential activation of Rap1 and Rac1 small G proteins by PDGF locally at leading edges of NIH3T3 cells. Moving cells form protrusions, such as **filopodia** and lamellipodia, and focal complexes at leading edges, which eventually enhance cell movement. The Rho family small G proteins, Rac1, Cdc42 and RhoA, are involved in the formation of these leading edge structures. We investigated the role of another small G protein Rap1 in the platelet-derived growth factor (PDGF)-induced formation of leading edge structures and cell movement. Upon stimulation of NIH3T3 cells by PDGF, leading edge structures were formed and Necl-5, integrin alpha(V)beta(3), and PDGF receptor were accumulated at leading edges. Rap1, upstream regulators of Rap1 such as Crk and C3G, and a downstream effector RalGDS, were accumulated at peripheral ruffles over lamellipodia. Over-expression of Rap1GAP, which inactivates Rap1, and knockdown of Rap1 inhibited the PDGF-induced formation of leading edge structures, accumulation of these molecules, and cell movement. In addition, Rap1 activation subsequently induced accumulation of Rac1, Vav2 and PAK at peripheral ruffles, which was inhibited by Rap1GAP and knockdown of Rap1. These results indicate that Rap1, activated by PDGF, is recruited to leading edges and that Rac1 is thereby activated locally at peripheral ruffles. This process is pivotal for the PDGF-induced formation of leading edge structures and cell movement.
9. Tiam1 and Rac1 are required for platelet-activating factor-induced endothelial junctional disassembly and increase in vascular permeability. It is known that platelet-activating factor (PAF) induces severe endothelial barrier leakiness, but the signaling mechanisms remain unclear. Here, using a wide range of biochemical and morphological approaches applied in both mouse models and cultured endothelial cells, we addressed the mechanisms of PAF-induced disruption of interendothelial junctions (IEJs) and of increased endothelial permeability. The formation of interendothelial gaps filled with **filopodia** and lamellipodia is the cellular event responsible for the disruption of endothelial barrier. We observed that PAF ligation of its receptor induced the activation of the Rho GTPase Rac1. Following PAF exposure, both Rac1 and its guanine nucleotide exchange factor Tiam1 were found associated with a membrane fraction from which they co-immunoprecipitated with PAF receptor. In the same time frame with Tiam1-Rac1 translocation, the junctional proteins ZO-1 and VE-cadherin were relocated from the IEJs, and formation of numerous interendothelial gaps was recorded. Notably, the response was independent of myosin light chain phosphorylation and thus distinct from other mediators, such as histamine and thrombin. The changes in actin status are driven by the PAF-induced localized actin polymerization as a consequence of Rac1 translocation and activation. Tiam1 was required for the activation of Rac1, actin polymerization, relocation of junctional associated proteins, and disruption of IEJs. Thus, PAF-induced IEJ disruption and increased endothelial permeability requires the activation of a Tiam1-Rac1 signaling module, suggesting a novel therapeutic target against increased vascular permeability associated with inflammatory diseases.
10. Tubulin tyrosination is required for the proper organization and pathfinding of the growth cone. During development, neuronal growth cones integrate diffusible and contact guidance cues that are conveyed to both actin and microtubule (MT) cytoskeletons and ensure axon outgrowth and pathfinding. Although several post-translational modifications of tubulin have been identified and despite their strong conservation among species, their physiological roles during development, especially in the nervous sytem, are still poorly understood.Here, we have dissected the role of a post-translational modification of the last amino acid of the alpha-tubulin on axonal growth by analyzing the phenotype of precerebellar neurons in Tubulin tyrosin ligase knock-out mice (TTL(-/-)) through in vivo, ex vivo and in vitro analyses. TTL(-/-) neurons are devoid of tyrosinated tubulin. Their pathway shows defects in vivo, ex vivo, in hindbrains open-book preparations or in vitro, in a collagen matrix. Their axons still orient toward tropic cues, but they emit supernumerary branches and their growth cones are enlarged and exhibit an emission of mis-oriented **filopodia**. Further analysis of the TTL(-/-) growth cone intracellular organization also reveals that the respective localization of actin and MT filaments is disturbed, with a decrease in the distal accumulation of Myosin IIB, as well as a concomitant Rac1 over-activation in the hindbrain. Pharmacological inhibition of Rac1 over-activation in TTL(-/-) neurons can rescue Myosin IIB localization.In the growth cone, we propose that tubulin tyrosination takes part in the relative arrangement of actin and MT cytoskeletons, in the regulation of small GTPases activity, and consequently, in the proper morphogenesis, organization and pathfinding of the growth cone during development.
11. Rac1 is essential for platelet lamellipodia formation and aggregate stability under flow. The role of Rac family proteins in platelet spreading on matrix proteins under static and flow conditions has been investigated by using Rac-deficient platelets. Murine platelets form **filopodia** and undergo limited spreading on fibrinogen independent of Rac1 and Rac2. In the presence of thrombin, marked lamellipodia formation is observed on fibrinogen, which is abrogated in the absence of Rac1. However, Rac1 is not required for thrombin-induced aggregation or elevation of F-actin levels. Formation of lamellipodia on collagen and laminin is also Rac1-dependent. Analysis of platelet adhesion dynamics on collagen under flow conditions in vitro revealed that Rac1 is required for platelet aggregate stability at arterial rates of shear, as evidenced by a dramatic increase in platelet embolization. Furthermore, studies employing intravital microscopy demonstrated that Rac1 plays a critical role in the development of stable thrombi at sites of vascular injury in vivo. Thus, our data demonstrated that Rac1 is essential for lamellipodia formation in platelets and indicated that Rac1 is required for aggregate integrity leading to thrombus formation under physiologically relevant levels of shear both in vitro and in vivo.
12. Membrane targeting of WAVE2 is not sufficient for WAVE2-dependent actin polymerization: a role for IRSp53 in mediating the interaction between Rac and WAVE2. Wiskott-Aldrich syndrome protein (WASP)-family verprolin homologous (WAVE) proteins play a major role in Rac-induced actin dynamics, but Rac does not bind directly to WAVE proteins. It has been proposed that either the insulin receptor substrate protein 53 (IRSp53) or a complex of proteins containing Abelson interactor protein 1 (Abi1) mediates the interaction of WAVE2 and Rac. Depletion of endogenous IRSp53 by RNA-mediated interference (RNAi) in a RAW/LR5 macrophage cell line resulted in a significant reduction of Rac1Q61L-induced surface ruffles and colony-stimulating factor 1 (CSF-1)-induced actin polymerization, protrusion and cell migration. However, IRSp53 was not essential for Fcgamma-R-mediated phagocytosis, formation of podosomes or for formation of Cdc42V12-induced **filopodia**. IRSp53 was found to be present in an immunoprecipitable complex with WAVE2 and Abi1 in a Rac1-activation-dependent manner in RAW/LR5 cells in vivo. Importantly, reduction of endogenous IRSp53 or expression of IRSp53 lacking the WAVE2-binding site (IRSp53DeltaSH3) resulted in a significant reduction in the association of Rac1 with WAVE2 and Abi1, indicating that the association of Rac1 with WAVE2 and Abi1 is IRSp53 dependent. While it has been proposed that WAVE2 activity is regulated by membrane recruitment, membrane targeting of WAVE2 in RAW/LR5 and Cos-7 cells did not induce actin polymerization or protrusion, suggesting that membrane recruitment was insufficient for regulation of WAVE2. Combined, these data suggest that IRSp53 links Rac1 to WAVE2 in vivo and its function is crucial for production of CSF-1-induced F-actin-rich protrusions and cell migration in macrophages. This study indicates that Rac1, along with IRSp53 and Abi1, is involved in a more complex and tight regulation of WAVE2 than one operating solely through membrane localization.
13. Cdc42- and IRSp53-dependent contractile **filopodia** tether presumptive lens and retina to coordinate epithelial invagination. The vertebrate lens provides an excellent model with which to study the mechanisms required for epithelial invagination. In the mouse, the lens forms from the head surface ectoderm. A domain of ectoderm first thickens to form the lens placode and then invaginates to form the lens pit. The epithelium of the lens placode remains in close apposition to the epithelium of the presumptive retina as these structures undergo a coordinated invagination. Here, we show that F-actin-rich basal **filopodia** that link adjacent presumptive lens and retinal epithelia function as physical tethers that coordinate invagination. The **filopodia**, most of which originate in the presumptive lens, form at E9.5 when presumptive lens and retinal epithelia first come into close contact, and have retracted by E11.5 when invagination is complete. At E10.5--the lens pit stage--there is approximately one filopodium per epithelial cell. Formation of **filopodia** is dependent on the Rho family GTPase Cdc42 and the Cdc42 effector IRSp53 (Baiap2). Loss of **filopodia** results in reduced lens pit invagination. Pharmacological manipulation of the actin-myosin contraction pathway showed that the **filopodia** can respond rapidly in length to change inter-epithelial distance. These data suggest that the lens-retina inter-epithelial **filopodia** are a fine-tuning mechanism to assist in lens pit invagination by transmitting the forces between presumptive lens and retina. Although invagination of the archenteron in sea urchins and dorsal closure in Drosophila are known to be partly dependent on **filopodia**, this mechanism of morphogenesis has not previously been identified in vertebrates.
14. Imaging **filopodia** dynamics in the mouse blastocyst. During mammalian development, the first cell lineage diversification event occurs in the blastocyst, when the trophectoderm (TE) and the inner cell mass (ICM) become established. Part of the TE (polar) remains in contact with the ICM and differs from the mural TE (mTE) which is separated from the ICM by a cavity known as the blastocoele. The presence of **filopodia** connecting ICM cells with the distant mural TE cells through the blastocoelic fluid was investigated in this work. We describe two types of actin-based cell projections found in freshly dissected and in vitro cultured expanding blastocysts: abundant short **filopodia** projecting into the blastocoelic cavity that present a continuous undulating behavior; and long, thin traversing **filopodia** connecting the mural TE with the ICM. Videomicroscopy analyses revealed the presence of vesicle-like structures moving along traversing **filopodia** and dynamic cytoskeletal rearrangements. These observations, together with immunolocalization of the FGFR2 and the ErbB3 receptors to these cell extensions, suggest that they display signal transduction activity. We propose that traversing **filopodia** are employed by mitotic mTE cells to receive the required signals for cell division after they become distant to the ICM.
15. Functional analysis of ARHGAP6, a novel GTPase-activating protein for RhoA. Microphthalmia with linear skin defects (MLS) is an X-linked dominant, male-lethal syndrome characterized by microphthalmia, aplastic skin and agenesis of the corpus callosum, and is caused by the deletion of a 500 kb critical region in Xp22.3. Our laboratory isolated a novel rho GTPase-activating protein (rhoGAP) gene named ARHGAP6 from the MLS region. ARHGAP6 contains 14 exons encoding a 974 amino acid protein with three putative SH3-binding domains. Because exons 2-14 are deleted in all MLS patients, we hypothesized that ARHGAP6 may be responsible for some of the phenotypic features of MLS. We pursued two approaches to study the function of ARHGAP6 and its role in the pathogenesis of MLS: gene targeting of the rhoGAP domain in mouse embryonic stem cells and in vitro expression studies. Surprisingly, loss of the rhoGAP function of Arhgap6 does not cause any detectable phenotypic or behavioral abnormalities in the mutant mice. Transfected mammalian cells expressing ARHGAP6 lose their actin stress fibers, retract from the growth surface and extend thin, branching processes resembling **filopodia**. The ARHGAP6 protein co-localizes with actin filaments through an N-terminal domain and recruits F-actin into the growing processes. Mutation of a conserved arginine residue in the rhoGAP domain prevents the loss of stress fibers but has little effect on process outgrowth. These results suggest that ARHGAP6 has two independent functions: one as a GAP with specificity for RhoA and the other as a cytoskeletal protein that promotes actin remodeling.
16. Abba promotes PDGF-mediated membrane ruffling through activation of the small GTPase Rac1. Abba is a member of the I-BAR-domain protein family that is characterized by a convex-shaped membrane-binding motif. Overexpression of GFP-tagged Abba in murine fibroblasts potentiated PDGF-mediated formation of membrane ruffles and lamellipodia. Immunofluorescent microscopy and pull-down analysis revealed that GFP-Abba colocalized with an active form of Rac1 in the membrane ruffles and enhanced the Rac GTPase activity in response to PDGF stimulation. Further immunoprecipitation assays demonstrated that GFP-Abba bound to both wild-type and constitutively active Rac1 and that the binding to either of the Rac1 forms was significantly enhanced upon PDGF stimulation. On the other hand, an Abba mutant deficient in Rac1 binding failed to promote membrane ruffling and Rac1 activation in response to PDGF. However, the cells overexpressing a truncated mutant carrying the I-BAR domain alone displayed numerous **filopodia**-like microspikes in a manner independent of growth factors. Also, the Rac-binding activity of the mutant was not affected by PDGF treatment. Our data indicates that the interaction between full-length Abba and Rac1 is implicated in membrane deformation and subjected to a growth factor-mediated regulation through the C-terminal sequence.
17. The Arp2/3 Complex Is Essential for Distinct Stages of Spine Synapse Maturation, Including Synapse Unsilencing. Dendritic **filopodia** are actin-rich structures that are thought to contribute to early spine synapse formation; however, the actin regulatory proteins important for early synaptogenesis are poorly defined. Using organotypic hippocampal slice cultures and primary neuron hippocampal cultures from Arp2/3 conditional knock-out mice, we analyze the roles of the Arp2/3 complex, an actin regulator that creates branched actin networks, and demonstrate it is essential for distinct stages of both structural and functional maturation of excitatory spine synapses. Our data show that initially the Arp2/3 complex inhibits the formation of dendritic **filopodia** but that later during development, the Arp2/3 complex drives the morphological maturation from **filopodia** to typical spine morphology. Furthermore, we demonstrate that although the Arp2/3 complex is not required for key spine maturation steps, such as presynaptic contact and recruitment of MAGUK (membrane-associated guanylate kinase) scaffolding proteins or NMDA receptors, it is necessary for the recruitment of AMPA receptors. This latter process, also known as synapse unsilencing, is a final and essential step in the neurodevelopment of excitatory postsynaptic synaptogenesis, setting the stage for neuronal interconnectivity. These findings provide the first evidence that the Arp2/3 complex is directly involved in functional maturation of dendritic spines during the developmental period of spinogenesis.Excitatory spine synapse formation (spinogenesis) is a poorly understood yet pivotal period of neurodevelopment that occurs within 2-3 weeks after birth. Neurodevelopmental disorders such as intellectual disability and autism are characterized by abnormal spine structure, which may arise from abnormal excitatory synaptogenesis. The initial stage of spinogenesis is thought to begin with the emergence of actin-rich dendritic **filopodia** that initiate contact with presynaptic axonal boutons. However, it remains enigmatic how actin cytoskeletal regulation directs dendritic **filopodia**l emergence or their subsequent maturation into dendritic spines during development and on into adulthood. In this study, we provide the first evidence that the Arp2/3 complex, a key actin nucleator, is involved in distinct stages of spine formation and is required for synapse unsilencing.

#### GeneRIFs

1. These data show that membrane protrusions originate from surface ectodermal cells and that Rac1 is necessary for the formation of membrane ruffles which typify late neural fold closure stages, whereas Cdc42 is required for the predominance of **filopodia** in early neurulation.
2. Addition of purified CNFY triggers formation of actin-rich membrane ruffles and **filopodia**, which correlates with the activation of the Rho GTPases, RhoA, Rac1 and Cdc42.
3. Thereby, lamellipodia nucleation by **filopodia** requires integrin occupancy by matrix substrate and downstream Rac1 signaling.
4. platelets form **filopodia** and undergo limited spreading on fibrinogen independent of Rac1 and Rac2

### [Ff]ilopodium

#### PubMed

1. Cdc42- and IRSp53-dependent contractile filopodia tether presumptive lens and retina to coordinate epithelial invagination. The vertebrate lens provides an excellent model with which to study the mechanisms required for epithelial invagination. In the mouse, the lens forms from the head surface ectoderm. A domain of ectoderm first thickens to form the lens placode and then invaginates to form the lens pit. The epithelium of the lens placode remains in close apposition to the epithelium of the presumptive retina as these structures undergo a coordinated invagination. Here, we show that F-actin-rich basal filopodia that link adjacent presumptive lens and retinal epithelia function as physical tethers that coordinate invagination. The filopodia, most of which originate in the presumptive lens, form at E9.5 when presumptive lens and retinal epithelia first come into close contact, and have retracted by E11.5 when invagination is complete. At E10.5--the lens pit stage--there is approximately one **filopodium** per epithelial cell. Formation of filopodia is dependent on the Rho family GTPase Cdc42 and the Cdc42 effector IRSp53 (Baiap2). Loss of filopodia results in reduced lens pit invagination. Pharmacological manipulation of the actin-myosin contraction pathway showed that the filopodia can respond rapidly in length to change inter-epithelial distance. These data suggest that the lens-retina inter-epithelial filopodia are a fine-tuning mechanism to assist in lens pit invagination by transmitting the forces between presumptive lens and retina. Although invagination of the archenteron in sea urchins and dorsal closure in Drosophila are known to be partly dependent on filopodia, this mechanism of morphogenesis has not previously been identified in vertebrates.

### [Pp]odosome(s)?

#### PubMed

1. Membrane targeting of WAVE2 is not sufficient for WAVE2-dependent actin polymerization: a role for IRSp53 in mediating the interaction between Rac and WAVE2. Wiskott-Aldrich syndrome protein (WASP)-family verprolin homologous (WAVE) proteins play a major role in Rac-induced actin dynamics, but Rac does not bind directly to WAVE proteins. It has been proposed that either the insulin receptor substrate protein 53 (IRSp53) or a complex of proteins containing Abelson interactor protein 1 (Abi1) mediates the interaction of WAVE2 and Rac. Depletion of endogenous IRSp53 by RNA-mediated interference (RNAi) in a RAW/LR5 macrophage cell line resulted in a significant reduction of Rac1Q61L-induced surface ruffles and colony-stimulating factor 1 (CSF-1)-induced actin polymerization, protrusion and cell migration. However, IRSp53 was not essential for Fcgamma-R-mediated phagocytosis, formation of **podosomes** or for formation of Cdc42V12-induced filopodia. IRSp53 was found to be present in an immunoprecipitable complex with WAVE2 and Abi1 in a Rac1-activation-dependent manner in RAW/LR5 cells in vivo. Importantly, reduction of endogenous IRSp53 or expression of IRSp53 lacking the WAVE2-binding site (IRSp53DeltaSH3) resulted in a significant reduction in the association of Rac1 with WAVE2 and Abi1, indicating that the association of Rac1 with WAVE2 and Abi1 is IRSp53 dependent. While it has been proposed that WAVE2 activity is regulated by membrane recruitment, membrane targeting of WAVE2 in RAW/LR5 and Cos-7 cells did not induce actin polymerization or protrusion, suggesting that membrane recruitment was insufficient for regulation of WAVE2. Combined, these data suggest that IRSp53 links Rac1 to WAVE2 in vivo and its function is crucial for production of CSF-1-induced F-actin-rich protrusions and cell migration in macrophages. This study indicates that Rac1, along with IRSp53 and Abi1, is involved in a more complex and tight regulation of WAVE2 than one operating solely through membrane localization.

### [Mm]igration

#### Process

1. cell **migration**
2. cerebral cortex radially oriented cell **migration**
3. embryonic olfactory bulb interneuron precursor **migration**
4. regulation of cell **migration**
5. regulation of cell **migration**
6. regulation of fibroblast **migration**

#### PubMed

1. CBAP functions as a novel component in chemokine-induced ZAP70-mediated T-cell adhesion and **migration**. Activated chemokine receptor initiates inside-out signaling to transiently trigger activation of integrins, a process involving multiple components that have not been fully characterized. Here we report that GM-CSF/IL-3/IL-5 receptor common beta-chain-associated protein (CBAP) is required to optimize this inside-out signaling and activation of integrins. First, knockdown of CBAP expression in human Jurkat T cells caused attenuated CXC chemokine ligand-12 (CXCL12)-induced cell **migration** and integrin α4β1- and αLβ2-mediated cell adhesion in vitro, which could be rescued sufficiently upon expression of murine CBAP proteins. Freshly isolated CBAP-deficient primary T cells also exhibited diminution of chemotaxis toward CC chemokine ligand-21 (CCL21) and CXCL12, and these chemokines-induced T-cell adhesions in vitro. Adoptive transfer of isolated naive T cells demonstrated that CBAP deficiency significantly reduced lymph node homing ability in vivo. Final**ly, migra**tion of T cell-receptor-activated T cells induced by inflammatory chemokines was also attenuated in CBAP-deficient cells. Further analyses revealed that CBAP constitutively associated with both integrin β1 and ZAP70 and that CBAP is required for chemokine-induced initial binding of the talin-Vav1 complex to integrin β1 and to facilitate subsequent ZAP70-mediated dissociation of the talin-Vav1 complex and Vav1 phosphorylation. Within such an integrin signaling complex, CBAP likely functions as an adaptor and ultimately leads to activation of both integrin α4β1 and Rac1. Taken together, our data suggest that CBAP indeed can function as a novel signaling component within the ZAP70/Vav1/talin complex and plays an important role in regulating chemokine-promoted T-cell trafficking.
2. Coronin-1C Protein and Caveolin Protein Provide Constitutive and Inducible Mechanisms of Rac1 Protein Trafficking. Sustained directional fibroblast **migration** requires both polarized activation of the protrusive signal, Rac1, and redistribution of inactive Rac1 from the rear of the cell so that it can be redistributed or degraded. In this work, we determine how alternative endocytic mechanisms dictate the fate of Rac1 in response to the extracellular matrix environment. We discover that both coronin-1C and caveolin retrieve Rac1 from similar locations at the rear and sides of the cell. We find that coronin-1C-mediated extraction, which is responsible for Rac1 recycling, is a constitutive process that maintains Rac1 protein levels within the cell. In the absence of coronin-1C, the effect of caveolin-mediated endocytosis, which targets Rac1 for proteasomal degradation, becomes apparent. Unlike constitutive coronin-1C-mediated trafficking, caveolin-mediated Rac1 endocytosis is induced by engagement of the fibronectin receptor syndecan-4. Such an inducible endocytic/degradation mechanism would predict that, in the presence of fibronectin, caveolin defines regions of the cell that are resistant to Rac1 activation but, in the absence of fibronectin leaves more of the membrane susceptible to Rac1 activation and protrusion. Indeed, we demonstrate that fibronectin-stimulated activation of Rac1 is accelerated in the absence of caveolin and that, when caveolin is knocked down, polarization of active Rac1 is lost in FRET experiments and culminates in shunting **migration** in a fibrous fibronectin matrix. Although the concept of polarized Rac1 activity in response to chemoattractants has always been apparent, our understanding of the balance between recycling and degradation explains how polarity can be maintained when the chemotactic gradient has faded.
3. Distinct roles of Rac1/Cdc42 and Rho/Rock for axon outgrowth and nucleokinesis of precerebellar neurons toward netrin 1. During embryonic development, tangentially migrating precerebellar neurons emit a leading process and then translocate their nuclei inside it (nucleokinesis). Netrin 1 (also known as netrin-1) acts as a chemoattractant factor for neurophilic **migration** of precerebellar neurons (PCN) both in vivo and in vitro. In the present work, we analyzed Rho GTPases that could direct axon outgrowth and/or nuclear **migration**. We show that the expression pattern of Rho GTPases in developing PCN is consistent with their involvement in the **migration** of PCN from the rhombic lips. We report that pharmacological inhibition of Rho enhances axon outgrowth of PCN and prevents nuclei **migration** toward a netrin 1 source, whereas inhibition of Rac and Cdc42 sub-families impair neurite outgrowth of PCN without affecting **migration**. We show, through pharmacological inhibition, that Rho signaling directs neurophilic **migration** through Rock activation. Altogether, our results indicate that Rho/Rock acts on signaling pathways favoring nuclear translocation during tangential **migration** of PCN. Thus, axon extension and nuclear **migration** of PCN in response to netrin 1 are not strictly dependent processes because: (1) distinct small GTPases are involved; (2) axon extension can occur when **migration** is blocked; and (3) **migration** can occur when axon outgrowth is impaired.
4. Integrin alpha3beta1 directs the stabilization of a polarized lamellipodium in epithelial cells through activation of Rac1. Epithelial cell **migration** is a crucial event in wound healing, yet little is known about mechanisms whereby integrins regulate epithelial cell polarization and **migration**. In the present work, we demonstrate the importance of adhesion through the alpha3beta1 integrin in promoting the stabilization of leading lamellipodia in migrating keratinocytes. We demonstrate that this integrin is found at the leading edge of migrating keratinocytes and that inhibition of alpha3beta1 binding to laminin-5 prevents the formation of stable leading lamellipodia. Consistent with this observation, keratinocytes derived from alpha3beta1-deficient mice fail to form stable leading lamellipodia but retain the ability to form actin-containing protrusions that rapidly extend and retract from the cell membrane. Formation of a leading lamellipodium also requires alpha3beta1-dependent activation of Rac1, because alpha3beta1-deficient keratinocytes show decreased activation of Rac1 compared with alpha3beta1-expressing cells, and formation of stable leading lamellipodia can be inhibited in the latter cells by expression of the dominant negative Rac1 mutant Rac1N17. Furthermore, alpha3beta1-deficient keratinocytes expressing constitutively active Rac1L61 failed to form stable lamellipodia when plated onto laminin-5, demonstrating that alpha3beta1 is required for Rac1-mediated formation of a stable lamellipodium. These observations identify a crucial role for integrin-mediated adhesion and signaling in the formation of large, polarized, stable lamellipodia by migrating epithelial cells. To our knowledge, this study is the first to demonstrate that signal transduction through a specific integrin is required to direct the development of a lamellipodium from an initial protrusion and promote persistent epithelial cell **migration**.
5. Epidermal growth factor induction of front-rear polarity and **migration** in keratinocytes is mediated by integrin-linked kinase and ELMO2. Epidermal growth factor (EGF) is a potent chemotactic and mitogenic factor for epidermal keratinocytes, and these properties are central for normal epidermal regeneration after injury. The involvement of mitogen-activated protein kinases as mediators of the proliferative effects of EGF is well established. However, the molecular mechanisms that mediate motogenic responses to this growth factor are not clearly understood. An obligatory step for forward cell **migration** is the development of front-rear polarity and formation of lamellipodia at the leading edge. We show that stimulation of epidermal keratinocytes with EGF, but not with other growth factors, induces development of front-rear polarity and directional **migration** through a pathway that requires integrin-linked kinase (ILK), Engulfment and Cell Motility-2 (ELMO2), integrin β1, and Rac1. Furthermore, EGF induction of front-rear polarity and chemotaxis require the tyrosine kinase activity of the EGF receptor and are mediated by complexes containing active RhoG, ELMO2, and ILK. Our findings reveal a novel link between EGF receptor stimulation, ILK-containing complexes, and activation of small Rho GTPases necessary for acquisition of front-rear polarity and forward movement.
6. Rac1 regulates pancreatic islet morphogenesis. Pancreatic islets of Langerhans originate from endocrine progenitors within the pancreatic ductal epithelium. Concomitant with differentiation of these progenitors into hormone-producing cells such cells delaminate, aggregate and migrate away from the ductal epithelium. The cellular and molecular mechanisms regulating islet cell delamination and cell **migration** are poorly understood. Extensive biochemical and cell biological studies using cultured cells demonstrated that Rac1, a member of the Rho family of small GTPases, acts as a key regulator of cell **migration**.To address the functional role of Rac1 in islet morphogenesis, we generated transgenic mice expressing dominant negative Rac1 under regulation of the Rat Insulin Promoter. Blocking Rac1 function in beta cells inhibited their **migration** away from the ductal epithelium in vivo. Consistently, transgenic islet cell spreading was compromised in vitro. We also show that the EGF-receptor ligand betacellulin induced actin remodelling and cell spreading in wild-type islets, but not in transgenic islets. Finally, we demonstrate that cell-cell contact E-cadherin increased as a consequence of blocking Rac1 activity.Our data support a model where Rac1 signalling controls islet cell **migration** by modulating E-cadherin-mediated cell-cell adhesion. Furthermore, in vitro experiments show that betacellulin stimulated islet cell spreading and actin remodelling is compromised in transgenic islets, suggesting that betacellulin may act as a regulator of Rac1 activity and islet **migration** in vivo. Our results further emphasize Rac1 as a key regulator of cell **migration** and cell adhesion during tissue and organ morphogenesis.
7. Rac1 GTPase-deficient mouse lens exhibits defects in shape, suture formation, fiber cell **migration** and survival. Morphogenesis and shape of the ocular lens depend on epithelial cell elongation and differentiation into fiber cells, followed by the symmetric and compact organization of fiber cells within an enclosed extracellular matrix-enriched elastic capsule. The cellular mechanisms orchestrating these different events however, remain obscure. We investigated the role of the Rac1 GTPase in these processes by targeted deletion of expression using the conditional gene knockout (cKO) approach. Rac1 cKO mice were derived from two different Cre (Le-Cre and MLR-10) transgenic mice in which lens-specific Cre expression starts at embryonic day 8.75 and 10.5, respectively, in both the lens epithelium and fiber cells. The Le-Cre/Rac1 cKO mice exhibited an early-onset (E12.5) and severe lens phenotype compared to the MLR-10/Rac1 cKO (E15.5) mice. While the Le-Cre/Rac1 cKO lenses displayed delayed primary fiber cell elongation, lenses from both Rac1 cKO strains were characterized by abnormal shape, impaired secondary fiber cell **migration**, sutural defects and thinning of the posterior capsule which often led to rupture. Lens fiber cell N-cadherin/β-catenin/Rap1/Nectin-based cell-cell junction formation and WAVE-2/Abi-2/Nap1-regulated actin polymerization were impaired in the Rac1 deficient mice. Additionally, the Rac1 cKO lenses were characterized by a shortened epithelial sheet, reduced levels of extracellular matrix (ECM) proteins and increased apoptosis. Taken together, these data uncover the essential role of Rac1 GTPase activity in establishment and maintenance of lens shape, suture formation and capsule integrity, and in fiber cell **migratio**n, adhesion and survival, via regulation of actin cytoskeletal dynamics, cell adhesive interactions and ECM turnover.
8. Coronin-1C and RCC2 guide mesenchymal **migration** by trafficking Rac1 and controlling GEF exposure. Sustained forward **migration** through a fibrillar extracellular matrix requires localization of protrusive signals. Contact with fibronectin at the tip of a cell protrusion activates Rac1, and for linear **migration** it is necessary to dampen Rac1 activity in off-axial positions and redistribute Rac1 from non-protrusive membrane to the leading edge. Here, we identify interactions between coronin-1C (Coro1C), RCC2 and Rac1 that focus active Rac1 to a single protrusion. Coro1C mediates release of inactive Rac1 from non-protrusive membrane and is necessary for Rac1 redistribution to a protrusive tip and fibronectin-dependent Rac1 activation. The second component, RCC2, attenuates Rac1 activation outside the protrusive tip by binding to the Rac1 switch regions and competitively inhibiting GEF action, thus preventing off-axial protrusion. Depletion of Coro1C or RCC2 by RNA interference causes loss of cell polarity that results in shunting **migration** in 1D or 3D culture systems. Furthermore, morpholinos against Coro1C or RCC2, or mutation of any of the binding sites in the Rac1-RCC2-Coro1C complex delays the arrival of neural crest derivatives at the correct location in developing zebrafish, demonstrating the crucial role in **migration** guidance in vivo.
9. Integrin-linked kinase is required for radial sorting of axons and Schwann cell remyelination in the peripheral nervous system. During development, Schwann cells (SCs) interpret different extracellular cues to regulate their **migration**, proliferation, and the remarkable morphological changes associated with the sorting, ensheathment, and myelination of axons. Although interactions between extracellular matrix proteins and integrins are critical to some of these processes, the downstream signaling pathways they control are still poorly understood. Integrin-linked kinase (ILK) is a focal adhesion protein that associates with multiple binding partners to link integrins to the actin cytoskeleton and is thought to participate in integrin and growth factor-mediated signaling. Using SC-specific gene ablation, we report essential functions for ILK in radial sorting of axon bundles and in remyelination in the peripheral nervous system. Our in vivo and in vitro experiments show that ILK negatively regulates Rho/Rho kinase signaling to promote SC process extension and to initiate radial sorting. ILK also facilitates axon remyelination, likely by promoting the activation of downstream molecules such as AKT/protein kinase B.
10. GPR124, an orphan G protein-coupled receptor, is required for CNS-specific vascularization and establishment of the blood-brain barrier. Every organ in the body requires blood vessels for efficient delivery of oxygen and nutrients, but independent vascular beds are highly specialized to meet the individual needs of specific organs. The vasculature of the brain is tightly sealed, with blood-brain barrier (BBB) properties developing coincident with neural vascularization. G protein-coupled receptor 124 (GPR124) (tumor endothelial marker 5, TEM5), an orphan member of the adhesion family of G protein-coupled receptors, was previously identified on the basis of its overexpression in tumor vasculature. Here, we show that global deletion or endothelial-specific deletion of GPR124 in mice results in embryonic lethality associated with abnormal angiogenesis of the forebrain and spinal cord. Expression of GPR124 was found to be required for invasion and **migration** of blood vessels into neuroepithelium, establishment of BBB properties, and expansion of the cerebral cortex. Thus, GPR124 is an important regulator of neurovasculature development and a potential drug target for cerebrovascular diseases.
11. Transmembrane collagen XVII modulates integrin dependent keratinocyte **migration** via PI3K/Rac1 signaling. The hemidesmosomal transmembrane component collagen XVII (ColXVII) plays an important role in the anchorage of the epidermis to the underlying basement membrane. However, this adhesion protein seems to be also involved in the regulation of keratinocyte **migration**, since its expression in these cells is strongly elevated during reepithelialization of acute wounds and in the invasive front of squamous cell carcinoma, while its absence in ColXVII-deficient keratinocytes leads to altered cell motility. Using a genetic model of murine Col17a1⁻/⁻ keratinocytes we elucidated ColXVII mediated signaling pathways in cell adhesion **and migra**tion. Col17a1⁻/⁻ keratinocytes exhibited increased spreading on laminin 332 and accelerated, but less directed cell motility. These effects were accompanied by increased expression of the integrin subunits β4 and β1. The migratory phenotype, as evidenced by formation of multiple unstable lamellipodia, was associated with enhanced phosphoinositide 3-kinase (PI3K) activity. Dissection of the signaling pathway uncovered enhanced phosphorylation of the β4 integrin subunit and the focal adhesion kinase (FAK) as activators of PI3K. This resulted in elevated Rac1 activity as a downstream consequence. These results provide mechanistic evidence that ColXVII coordinates keratinocyte adhesion and directed motility by interfering integrin dependent PI3K activation and by stabilizing lamellipodia at the leading edge of reepithelializing wounds and in invasive squamous cell carcinoma.
12. Opposing roles for Akt1 and Akt2 in Rac/Pak signaling and cell **migration**. The Akt/PKB isoforms have different roles in animals, with Akt2 primarily regulating metabolic signaling and Akt1 regulating growth and survival. Here we show distinct roles for Akt1 and Akt2 in mouse embryo fibroblast cell **migration** and regulation of the cytoskeleton. Akt1-deficient cells responded poorly to platelet-derived growth factor while Akt2-deficient cells had a dramatically enhanced response, resulting in a substantial increase in dorsal ruffling. Swapping domains between Akt1 and Akt2 demonstrated that the N-terminal region containing the pleckstrin homology domain and a linker region distinguishes the two isoforms, while the catalytic domains are interchangeable. Akt2 knock-out cells also migrated faster than wild-type cells, especially through extracellular matrix (ECM), while Akt1 knock-out cells migrated more slowly than wild-type cells. Consistently, Akt2 knock-out cells had elevated Pak1 and Rac activities, suggesting that Akt2 inhibits Rac and Pak1. Both Akt2 and Akt1 associated in complexes with Pak1, but only Akt2 inhibited Pak1 in kinase assays, suggesting an underlying molecular basis for the different cellular phenotypes. Together these data provide evidence for an unexpected functional link between Akt2 and Pak1 that opposes the actions of Akt1 on cell **migration**.
13. Beta1 integrin activates Rac1 in Schwann cells to generate radial lamellae during axonal sorting and myelination. Myelin is a multispiraled extension of glial membrane that surrounds axons. How glia extend a surface many-fold larger than their body is poorly understood. Schwann cells are peripheral glia and insert radial cytoplasmic extensions into bundles of axons to sort, ensheath, and myelinate them. Laminins and beta1 integrins are required for axonal sorting, but the downstream signals are largely unknown. We show that Schwann cells devoid of beta1 integrin migrate to and elongate on axons but cannot extend radial lamellae of cytoplasm, similar to cells with low Rac1 activation. Accordingly, active Rac1 is decreased in beta1 integrin-null nerves, inhibiting Rac1 activity decreases radial lamellae in Schwann cells, and ablating Rac1 in Schwann cells of transgenic mice delays axonal sorting and impairs myelination. Finally, expressing active Rac1 in beta1 integrin-null nerves improves sorting. Thus, increased activation of Rac1 by beta1 integrins allows Schwann cells to switch from **migration**/elongation to the extension of radial membranes required for axonal sorting and myelination.
14. Endothelial cell **migration** during murine yolk sac vascular remodeling occurs by means of a Rac1 and FAK activation pathway in vivo. The molecular mechanism(s) controlling cell **migration** during vascular morphogenesis in vivo remain largely undefined. To address this within a physiological context, we used retinaldehyde dehydrogenase-2 (Raldh2) null mouse embryos and demonstrate that retinoic acid (RA) deficiency results in abnormal yolk sac vascular remodeling due to decreased Rac1 activation, increased RhoA activation, and increased focal adhesions. Vinculin was increased in Raldh2-/- yolk sacs, and molecular events important for focal adhesion turnover, FAK phosphorylation (Tyr397) and FAK-paxillin association, were decreased. RA-rescue of vascular remodeling down-regulated vinculin and restored FAK phosphorylation (Tyr397) and FAK-paxillin association. Furthermore, vascular rescue with vascular endothelial growth factor-A, Indian hedgehog, and basic fibroblast growth factor restored FAK phosphorylation (Tyr397) in the endothelium of Raldh2-/- yolk sacs. Our results provide new insights into the regulation of endothelial cell **migration** during vascular remodeling in vivo by adding the Rac1 and FAK activation pathway as a critical mediator of focal adhesion formation and turnover during vascular remodeling.
15. Coronin-1C Protein and Caveolin Protein Provide Constitutive and Inducible Mechanisms of Rac1 Protein Trafficking. Sustained directional fibroblast **migration** requires both polarized activation of the protrusive signal, Rac1, and redistribution of inactive Rac1 from the rear of the cell so that it can be redistributed or degraded. In this work, we determine how alternative endocytic mechanisms dictate the fate of Rac1 in response to the extracellular matrix environment. We discover that both coronin-1C and caveolin retrieve Rac1 from similar locations at the rear and sides of the cell. We find that coronin-1C-mediated extraction, which is responsible for Rac1 recycling, is a constitutive process that maintains Rac1 protein levels within the cell. In the absence of coronin-1C, the effect of caveolin-mediated endocytosis, which targets Rac1 for proteasomal degradation, becomes apparent. Unlike constitutive coronin-1C-mediated trafficking, caveolin-mediated Rac1 endocytosis is induced by engagement of the fibronectin receptor syndecan-4. Such an inducible endocytic/degradation mechanism would predict that, in the presence of fibronectin, caveolin defines regions of the cell that are resistant to Rac1 activation but, in the absence of fibronectin leaves more of the membrane susceptible to Rac1 activation and protrusion. Indeed, we demonstrate that fibronectin-stimulated activation of Rac1 is accelerated in the absence of caveolin and that, when caveolin is knocked down, polarization of active Rac1 is lost in FRET experiments and culminates in shunting **migration** in a fibrous fibronectin matrix. Although the concept of polarized Rac1 activity in response to chemoattractants has always been apparent, our understanding of the balance between recycling and degradation explains how polarity can be maintained when the chemotactic gradient has faded.
16. Transmembrane collagen XVII modulates integrin dependent keratinocyte **migration** via PI3K/Rac1 signaling. The hemidesmosomal transmembrane component collagen XVII (ColXVII) plays an important role in the anchorage of the epidermis to the underlying basement membrane. However, this adhesion protein seems to be also involved in the regulation of keratinocyte **migration**, since its expression in these cells is strongly elevated during reepithelialization of acute wounds and in the invasive front of squamous cell carcinoma, while its absence in ColXVII-deficient keratinocytes leads to altered cell motility. Using a genetic model of murine Col17a1⁻/⁻ keratinocytes we elucidated ColXVII mediated signaling pathways in cell adhesion **and migra**tion. Col17a1⁻/⁻ keratinocytes exhibited increased spreading on laminin 332 and accelerated, but less directed cell motility. These effects were accompanied by increased expression of the integrin subunits β4 and β1. The migratory phenotype, as evidenced by formation of multiple unstable lamellipodia, was associated with enhanced phosphoinositide 3-kinase (PI3K) activity. Dissection of the signaling pathway uncovered enhanced phosphorylation of the β4 integrin subunit and the focal adhesion kinase (FAK) as activators of PI3K. This resulted in elevated Rac1 activity as a downstream consequence. These results provide mechanistic evidence that ColXVII coordinates keratinocyte adhesion and directed motility by interfering integrin dependent PI3K activation and by stabilizing lamellipodia at the leading edge of reepithelializing wounds and in invasive squamous cell carcinoma.
17. Rac1 GTPase-deficient mouse lens exhibits defects in shape, suture formation, fiber cell **migration** and survival. Morphogenesis and shape of the ocular lens depend on epithelial cell elongation and differentiation into fiber cells, followed by the symmetric and compact organization of fiber cells within an enclosed extracellular matrix-enriched elastic capsule. The cellular mechanisms orchestrating these different events however, remain obscure. We investigated the role of the Rac1 GTPase in these processes by targeted deletion of expression using the conditional gene knockout (cKO) approach. Rac1 cKO mice were derived from two different Cre (Le-Cre and MLR-10) transgenic mice in which lens-specific Cre expression starts at embryonic day 8.75 and 10.5, respectively, in both the lens epithelium and fiber cells. The Le-Cre/Rac1 cKO mice exhibited an early-onset (E12.5) and severe lens phenotype compared to the MLR-10/Rac1 cKO (E15.5) mice. While the Le-Cre/Rac1 cKO lenses displayed delayed primary fiber cell elongation, lenses from both Rac1 cKO strains were characterized by abnormal shape, impaired secondary fiber cell **migration**, sutural defects and thinning of the posterior capsule which often led to rupture. Lens fiber cell N-cadherin/β-catenin/Rap1/Nectin-based cell-cell junction formation and WAVE-2/Abi-2/Nap1-regulated actin polymerization were impaired in the Rac1 deficient mice. Additionally, the Rac1 cKO lenses were characterized by a shortened epithelial sheet, reduced levels of extracellular matrix (ECM) proteins and increased apoptosis. Taken together, these data uncover the essential role of Rac1 GTPase activity in establishment and maintenance of lens shape, suture formation and capsule integrity, and in fiber cell **migratio**n, adhesion and survival, via regulation of actin cytoskeletal dynamics, cell adhesive interactions and ECM turnover.
18. Endothelial cell **migration** during murine yolk sac vascular remodeling occurs by means of a Rac1 and FAK activation pathway in vivo. The molecular mechanism(s) controlling cell **migration** during vascular morphogenesis in vivo remain largely undefined. To address this within a physiological context, we used retinaldehyde dehydrogenase-2 (Raldh2) null mouse embryos and demonstrate that retinoic acid (RA) deficiency results in abnormal yolk sac vascular remodeling due to decreased Rac1 activation, increased RhoA activation, and increased focal adhesions. Vinculin was increased in Raldh2-/- yolk sacs, and molecular events important for focal adhesion turnover, FAK phosphorylation (Tyr397) and FAK-paxillin association, were decreased. RA-rescue of vascular remodeling down-regulated vinculin and restored FAK phosphorylation (Tyr397) and FAK-paxillin association. Furthermore, vascular rescue with vascular endothelial growth factor-A, Indian hedgehog, and basic fibroblast growth factor restored FAK phosphorylation (Tyr397) in the endothelium of Raldh2-/- yolk sacs. Our results provide new insights into the regulation of endothelial cell **migration** during vascular remodeling in vivo by adding the Rac1 and FAK activation pathway as a critical mediator of focal adhesion formation and turnover during vascular remodeling.
19. Rac1 regulates pancreatic islet morphogenesis. Pancreatic islets of Langerhans originate from endocrine progenitors within the pancreatic ductal epithelium. Concomitant with differentiation of these progenitors into hormone-producing cells such cells delaminate, aggregate and migrate away from the ductal epithelium. The cellular and molecular mechanisms regulating islet cell delamination and cell **migration** are poorly understood. Extensive biochemical and cell biological studies using cultured cells demonstrated that Rac1, a member of the Rho family of small GTPases, acts as a key regulator of cell **migration**.To address the functional role of Rac1 in islet morphogenesis, we generated transgenic mice expressing dominant negative Rac1 under regulation of the Rat Insulin Promoter. Blocking Rac1 function in beta cells inhibited their **migration** away from the ductal epithelium in vivo. Consistently, transgenic islet cell spreading was compromised in vitro. We also show that the EGF-receptor ligand betacellulin induced actin remodelling and cell spreading in wild-type islets, but not in transgenic islets. Finally, we demonstrate that cell-cell contact E-cadherin increased as a consequence of blocking Rac1 activity.Our data support a model where Rac1 signalling controls islet cell **migration** by modulating E-cadherin-mediated cell-cell adhesion. Furthermore, in vitro experiments show that betacellulin stimulated islet cell spreading and actin remodelling is compromised in transgenic islets, suggesting that betacellulin may act as a regulator of Rac1 activity and islet **migration** in vivo. Our results further emphasize Rac1 as a key regulator of cell **migration** and cell adhesion during tissue and organ morphogenesis.
20. Beta1 integrin activates Rac1 in Schwann cells to generate radial lamellae during axonal sorting and myelination. Myelin is a multispiraled extension of glial membrane that surrounds axons. How glia extend a surface many-fold larger than their body is poorly understood. Schwann cells are peripheral glia and insert radial cytoplasmic extensions into bundles of axons to sort, ensheath, and myelinate them. Laminins and beta1 integrins are required for axonal sorting, but the downstream signals are largely unknown. We show that Schwann cells devoid of beta1 integrin migrate to and elongate on axons but cannot extend radial lamellae of cytoplasm, similar to cells with low Rac1 activation. Accordingly, active Rac1 is decreased in beta1 integrin-null nerves, inhibiting Rac1 activity decreases radial lamellae in Schwann cells, and ablating Rac1 in Schwann cells of transgenic mice delays axonal sorting and impairs myelination. Finally, expressing active Rac1 in beta1 integrin-null nerves improves sorting. Thus, increased activation of Rac1 by beta1 integrins allows Schwann cells to switch from **migration**/elongation to the extension of radial membranes required for axonal sorting and myelination.
21. Integrin alpha3beta1 directs the stabilization of a polarized lamellipodium in epithelial cells through activation of Rac1. Epithelial cell **migration** is a crucial event in wound healing, yet little is known about mechanisms whereby integrins regulate epithelial cell polarization and **migration**. In the present work, we demonstrate the importance of adhesion through the alpha3beta1 integrin in promoting the stabilization of leading lamellipodia in migrating keratinocytes. We demonstrate that this integrin is found at the leading edge of migrating keratinocytes and that inhibition of alpha3beta1 binding to laminin-5 prevents the formation of stable leading lamellipodia. Consistent with this observation, keratinocytes derived from alpha3beta1-deficient mice fail to form stable leading lamellipodia but retain the ability to form actin-containing protrusions that rapidly extend and retract from the cell membrane. Formation of a leading lamellipodium also requires alpha3beta1-dependent activation of Rac1, because alpha3beta1-deficient keratinocytes show decreased activation of Rac1 compared with alpha3beta1-expressing cells, and formation of stable leading lamellipodia can be inhibited in the latter cells by expression of the dominant negative Rac1 mutant Rac1N17. Furthermore, alpha3beta1-deficient keratinocytes expressing constitutively active Rac1L61 failed to form stable lamellipodia when plated onto laminin-5, demonstrating that alpha3beta1 is required for Rac1-mediated formation of a stable lamellipodium. These observations identify a crucial role for integrin-mediated adhesion and signaling in the formation of large, polarized, stable lamellipodia by migrating epithelial cells. To our knowledge, this study is the first to demonstrate that signal transduction through a specific integrin is required to direct the development of a lamellipodium from an initial protrusion and promote persistent epithelial cell **migration**.
22. Opposing roles for Akt1 and Akt2 in Rac/Pak signaling and cell **migration**. The Akt/PKB isoforms have different roles in animals, with Akt2 primarily regulating metabolic signaling and Akt1 regulating growth and survival. Here we show distinct roles for Akt1 and Akt2 in mouse embryo fibroblast cell **migration** and regulation of the cytoskeleton. Akt1-deficient cells responded poorly to platelet-derived growth factor while Akt2-deficient cells had a dramatically enhanced response, resulting in a substantial increase in dorsal ruffling. Swapping domains between Akt1 and Akt2 demonstrated that the N-terminal region containing the pleckstrin homology domain and a linker region distinguishes the two isoforms, while the catalytic domains are interchangeable. Akt2 knock-out cells also migrated faster than wild-type cells, especially through extracellular matrix (ECM), while Akt1 knock-out cells migrated more slowly than wild-type cells. Consistently, Akt2 knock-out cells had elevated Pak1 and Rac activities, suggesting that Akt2 inhibits Rac and Pak1. Both Akt2 and Akt1 associated in complexes with Pak1, but only Akt2 inhibited Pak1 in kinase assays, suggesting an underlying molecular basis for the different cellular phenotypes. Together these data provide evidence for an unexpected functional link between Akt2 and Pak1 that opposes the actions of Akt1 on cell **migration**.
23. Kalirin promotes neointimal hyperplasia by activating Rac in smooth muscle cells. Kalirin is a multifunctional protein that contains 2 guanine nucleotide exchange factor domains for the GTPases Rac1 and RhoA. Variants of KALRN have been associated with atherosclerosis in humans, but Kalirin's activity has been characterized almost exclusively in the central nervous system. We therefore tested the hypothesis that Kalirin functions as a Rho-guanine nucleotide exchange factor in arterial smooth muscle cells (SMCs).Kalirin-9 protein is expressed abundantly in aorta and bone marrow, as well as in cultured SMCs, endothelial cells, and macrophages. Moreover, arterial Kalirin was upregulated during early atherogenesis in apolipoprotein E-deficient mice. In cultured SMCs, signaling was affected similarly in 3 models of Kalirin loss-of-function: heterozygous Kalrn deletion, Kalirin RNAi, and treatment with the Kalirin Rho-guanine nucleotide exchange factor -1 inhibitor 1-(3-nitrophenyl)-1H-pyrrole-2,5-dione. With reduced Kalirin function, SMCs showed normal RhoA activation but diminished Rac1 activation, assessed as reduced Rac-GTP levels, p21-activated kinase autophosphorylation, and SMC **migration**. Kalrn(-/+) SMCs proliferated 30% less rapidly than wild-type SMCs. Neointimal hyperplasia engendered by carotid endothelial denudation was ≈60% less in Kalrn(-/+) and SMC-specific Kalrn(-/+) mice than in control mice.Kalirin functions as a guanine nucleotide exchange factor for Rac1 in SMCs, and promotes SM**C migrati**on and proliferation both in vitro and in vivo.
24. Epithelial-specific knockout of the Rac1 gene leads to enamel defects. The Ras-related C3 botulinum toxin substrate 1 (Rac1) gene encodes a 21-kDa GTP-binding protein belonging to the RAS superfamily. RAS members play important roles in controlling focal adhesion complex formation and cytoskeleton contraction, activities with consequences for cell growth, adhesion, **migration**, and differentiation. To examine the role(s) played by RAC1 protein in cell-matrix interactions and enamel matrix biomineralization, we used the Cre/loxP binary recombination system to characterize the expression of enamel matrix proteins and enamel formation in Rac1 knockout mice (Rac1(-/-)). Mating between mice bearing the floxed Rac1 allele and mice bearing a cytokeratin 14-Cre transgene generated mice in which Rac1 was absent from epithelial organs. Enamel of the Rac1 conditional knockout mouse was characterized by light microscopy, backscattered electron imaging in the scanning electron microscope, microcomputed tomography, and histochemistry. Enamel matrix protein expression was analyzed by western blotting. Major findings showed that the Tomes' processes of Rac1(-/-) ameloblasts lose contact with the forming enamel matrix in unerupted teeth, the amounts of amelogenin and ameloblastin are reduced in Rac1(-/-) ameloblasts, and after eruption, the enamel from Rac1(-/-) mice displays severe structural defects with a complete loss of enamel. These results support an essential role for RAC1 in the dental epithelium involving cell-matrix interactions and matrix biomineralization.
25. Rac1-dependent collective cell **migration** is required for specification of the anterior-posterior body axis of the mouse. Cell **migration** and cell rearrangements are critical for establishment of the body plan of vertebrate embryos. The first step in organization of the body plan of the mouse embryo, specification of the anterior-posterior body axis, depends on **migration** of the anterior visceral endoderm from the distal tip of the embryo to a more proximal region overlying the future head. The anterior visceral endoderm (AVE) is a cluster of extra-embryonic cells that secretes inhibitors of the Wnt and Nodal pathways to inhibit posterior development. Because Rac proteins are crucial regulators of cell **migration** and mouse Rac1 mutants die early in development, we tested whether Rac1 plays a role in AVE **migration**. Here we show that Rac1 mutant embryos fail to specify an anterior-posterior axis and, instead, express posterior markers in a ring around the embryonic circumference. Cells that express the molecular markers of the AVE are properly specified in Rac1 mutants but remain at the distal tip of the embryo at the time when **migration** should take place. Using tissue specific deletions, we show that Rac1 acts autonomously within the visceral endoderm to promote cell **migration**. High-resolution imaging shows that the leading wild-type AVE cells extend long lamellar protrusions that span several cell diameters and are polarized in the direction of cell movement. These projections are tipped by filopodia-like structures that appear to sample the environment. Wild-type AVE cells display hallmarks of collective cell **migration**: they retain tight and adherens junctions as they migrate and exchange neighbors within the plane of the visceral endoderm epithelium. Analysis of mutant embryos shows that Rac1 is not required for intercellular signaling, survival, proliferation, or adhesion in the visceral endoderm but is necessary for the ability of visceral endoderm cells to extend projections, change shape, and exchange neighbors. The data show that Rac1-mediated epithelial **migration** of the AVE is a crucial step in the establishment of the mammalian body plan and suggest that Rac1 is essential for collective **migration** in mammalian tissues.
26. Rac-GTPases Regulate Microtubule Stability and Axon Growth of Cortical GABAergic Interneurons. Cortical interneurons are characterized by extraordinary functional and morphological diversity. Although tremendous progress has been made in uncovering molecular and cellular mechanisms implicated in interneuron generation and function, several questions still remain open. Rho-GTPases have been implicated as intracellular mediators of numerous developmental processes such as cytoskeleton organization, vesicle trafficking, transcription, cell cycle progression, and apoptosis. Specifically in cortical interneurons, we have recently shown a cell-autonomous and stage-specific requirement for Rac1 activity within proliferating interneuron precursors. Conditional ablation of Rac1 in the medial ganglionic eminence leads to a 50% reduction of GABAergic interneurons in the postnatal cortex. Here we examine the additional role of Rac3 by analyzing Rac1/Rac3 double-mutant mice. We show that in the absence of both Rac proteins, the embryonic **migration** of medial ganglionic eminence-derived interneurons is further impaired. Postnatally, double-mutant mice display a dramatic loss of cortical interneurons. In addition, Rac1/Rac3-deficient interneurons show gross cytoskeletal defects in vitro, with the length of their leading processes significantly reduced and a clear multipolar morphology. We propose that in the absence of Rac1/Rac3, cortical interneurons fail to migrate tangentially towards the pallium due to defects in actin and microtubule cytoskeletal dynamics.
27. Rac1 in cortical projection neurons is selectively required for midline crossing of commissural axonal formation. Rac1 is a member of Rho family GTPases and regulates multiple cellular functions through actin cytoskeleton reorganization. During cerebral corticogenesis, Rac1 has been assumed to be involved in neuronal **migration**, neurite formation, polarization and axonal guidance. Here we show the specific role of Rac1, regulating midline crossing of commissural axons during cortical development by using cortex-restricted Rac1-knockout mice. In the knockout mice, Rac1 was eliminated from the beginning of corticogenesis exclusively in the dorsal telencephalon where progenitors of cortical projection neurons are located. Cortical lamination was distorted only mildly in the knockout mice, being preserved with six layers of neurons. However, cortex-restricted Rac1 deletion exhibited striking agenesis of commissural axons including the corpus callosum and anterior commissure without affecting other corticofugal axons including corticospinal and corticothalamic projections. Of note, the commissural axons of the knockout mice were potent in extending their process, but failed to cross the midline. Therefore, these findings indicate that Rac1 specifically controls the midline crossing of the commissural fibers, but not axonal formation of corticospinal or corticothalamic fibers during cortical development.
28. A Novel Rac1-GSPT1 Signaling Pathway Controls Astrogliosis Following Central Nervous System Injury. Astrogliosis (i.e. glial scar), which is comprised primarily of proliferated astrocytes at the lesion site and migrated astrocytes from neighboring regions, is one of the key reactions in determining outcomes after CNS injury. In an effort to identify potential molecules/pathways that regulate astrogliosis, we sought to determine whether Rac/Rac-mediated signaling in astrocytes represents a novel candidate for therapeutic intervention following CNS injury. For these studies, we generated mice with Rac1 deletion under the control of the GFAP (glial fibrillary acidic protein) promoter (GFAP-Cre;Rac1(flox/flox)). GFAP-Cre;Rac1(flox/flox) (Rac1-KO) mice exhibited better recovery after spinal cord injury and exhibited reduced astrogliosis at the lesion site relative to control. Reduced astrogliosis was also observed in Rac1-KO mice following microbeam irradiation-induced injury. Moreover, knockdown (KD) or KO of Rac1 in astrocytes (LN229 cells, primary astrocytes, or primary astrocytes from Rac1-KO mice) led to delayed cell cycle progression and reduced cell **migration**. Rac1-KD or Rac1-KO astrocytes additionally had decreased levels of GSPT1 (G1 to S phase transition 1) expression and reduced responses of IL-1β and GSPT1 to LPS treatment, indicating that IL-1β and GSPT1 are downstream molecules of Rac1 associated with inflammatory condition. Furthermore, GSPT1-KD astrocytes had cell cycle delay, with no effect on cel**l migrati**on. The cell cycle delay induced by Rac1-KD was rescued by overexpression of GSPT1. Based on these results, we propose that Rac1-GSPT1 represents a novel signaling axis in astrocytes that accelerates proliferation in response to inflammation, which is one important factor in the development of astrogliosis/glial scar following CNS injury.
29. The small GTPases RhoA and Rac1 regulate cerebellar development by controlling cell morphogenesis, **migration** and foliation. The small GTPases RhoA and Rac1 are key cytoskeletal regulators that function in a mutually antagonistic manner to control the **migration** and morphogenesis of a broad range of cell types. However, their role in shaping the cerebellum, a unique brain structure composed of an elaborate set of folia separated by fissures of different lengths, remains largely unexplored. Here we show that dysregulation of both RhoA and Rac1 signaling results in abnormal cerebellar ontogenesis. Ablation of RhoA from neuroprogenitor cells drastically alters the timing and placement of fissure formation, the **migration** and positioning of granule and Purkinje cells, the alignment of Bergmann glia, and the integrity of the basement membrane, primarily in the anterior lobules. Furthermore, in the absence of RhoA, granule cell precursors located at the base of fissures fail to undergo cell shape changes required for fissure initiation. Many of these abnormalities can be recapitulated by deleting RhoA specifically from granule cell precursors but not postnatal glia, indicating that RhoA functions in granule cell precursors to control cerebellar morphogenesis. Notably, mice with elevated Rac1 activity due to loss of the Rac1 inhibitors Bcr and Abr show similar anterior cerebellar deficits, including ectopic neurons and defects in fissure formation, Bergmann glia organization and basement membrane integrity. Together, our results suggest that RhoA and Rac1 play indispensable roles in patterning cerebellar morphology.
30. Podocyte-specific deletion of Rac1 leads to aggravation of renal injury in STZ-induced diabetic mice. Rac1, a GTPase of the Rho subfamily, has a crucial role in cytoskeletal architecture, as well as the regulation of cell **migration** and growth. However, renal injury in mice with podocyte-specific deletion of Rac1 has yet to be elucidated fully due to conflicting findings. Herein, we identified a possible role for Rac1 in podocytes of streptozotocin- (STZ) induced diabetic mice. The urinary albumin/creatinine ratio (ACR) in the knockout (KO) group was significantly higher than that in the wild type (WT) group at any week of age. A more marked ACR increase was observed in STZ/KO group than STZ/WT group, although ACR did increase with weeks of age in both diabetic groups. The kidney sections from diabetic mice revealed a glomerular hypertrophy with mesangial expansion, but there was no appreciable difference in glomerular findings under a light microscope between STZ/WT and STZ/KO mice. However, an electron microscopy analysis revealed that regardless of the presence or absence of diabetes, both KO (KO and STZ/KO) groups had a higher rate of foot process effacement compared with both WT (WT and STZ/WT) groups. The expression levels of the slit diaphragm protein, podocin, was reduced with the induction of diabetes, and the levels in the STZ/KO group experienced a further reduction compared with the STZ/WT group. The number of WT1-positive cells in the STZ/KO group was more significantly decreased than that in the other three groups. In contrast, the numbers of cleaved caspase 3- and TUNEL-positive cells in the glomeruli of the STZ/KO group were more increased than those in the STZ/WT group. Thus, this study provides evidence that podocyte-specific deletion of Rac1 results in morphological alteration in podocytes, and that the induction of apoptosis or decreased expression of the slit diaphragm proteins by hyperglycemic stimuli are associated with the progression of diabetic nephropathy.
31. The LIM protein Ajuba influences p130Cas localization and Rac1 activity during cell **migration**. Cell **migration** requires extension of lamellipodia that are stabilized by formation of adhesive complexes at the leading edge. Both processes are regulated by signaling proteins recruited to nascent adhesive sites that lead to activation of Rho GTPases. The Ajuba/Zyxin family of LIM proteins are components of cellular adhesive complexes. We show that cells from Ajuba null mice are inhibited in their **migration**, without associated abnormality in adhesion to extracellular matrix proteins, cell spreading, or integrin activation. Lamellipodia production, or function, is defective and there is a selective reduction in the level and tyrosine phosphorylation of FAK, p130Cas, Crk, and Dock180 at nascent focal complexes. In response to migratory cues Rac activation is blunted in Ajuba null cells, as detected biochemically and by FRET analysis. Ajuba associates with the focal adhesion-targeting domain of p130Cas, and rescue experiments suggest that Ajuba acts upstream of p130Cas to localize p130Cas to nascent adhesive sites in migrating cells thereby leading to the activation of Rac.
32. Calcium mobilization and Rac1 activation are required for VCAM-1 (vascular cell adhesion molecule-1) stimulation of NADPH oxidase activity. VCAM-1 (vascular cell adhesion molecule-1) plays an important role in the regulation of inflammation in atherosclerosis, asthma, inflammatory bowel disease and transplantation. VCAM-1 activates endothelial cell NADPH oxidase, and this oxidase activity is required for VCAM-1-dependent lymphocyte **migration**. We reported previously that a mouse microvascular endothelial cell line promotes lymphocyte **migration** that is dependent on VCAM-1, but not on other known adhesion molecules. Here we have investigated the signalling mechanisms underlying VCAM-1 function. Lymphocyte binding to VCAM-1 on the endothelial cell surface activated an endothelial cell calcium flux that could be inhibited with anti-alpha4-integrin and mimicked by anti-VCAM-1-coated beads. VCAM-1 stimulation of calcium responses could be blocked by an inhibitor of intracellular calcium mobilization, a calcium channel inhibitor or a calcium chelator, resulting in the inhibition of NADPH oxidase activity. Addition of ionomycin overcame the calcium channel blocker suppression of VCAM-1-stimulated NADPH oxidase activity, but could not reverse the inhibitory effect imposed by intracellular calcium blockage, indicating that both intracellular and extracellular calcium mobilization are required for VCAM-1-mediated activation of NADPH oxidase. Furthermore, VCAM-1 specifically activated the Rho-family GTPase Rac1, and VCAM-1 activation of NADPH oxidase was blocked by a dominant negative Rac1. Thus VCAM-1 stimulates the mobilization of intracellular and extracellular calcium and Rac1 activity that are required for the activation of NADPH oxidase.
33. Rac1 deletion in mouse neutrophils has selective effects on neutrophil functions. Defects in myeloid cell function in Rac2 knockout mice underline the importance of this isoform in activation of NADPH oxidase and cell motility. However, the specific role of Rac1 in neutrophil function has been difficult to assess since deletion of Rac1 results in embryonic lethality in mice. To elucidate the specific role of Rac1 in neutrophils, we generated mice with a conditional Rac1 deficiency restricted to cells of the granulocyte/monocyte lineage. As observed in Rac2-deficient neutrophils, Rac1-deficient neutrophils demonstrated profound defects in inflammatory recruitment in vivo, **migration** to chemotactic stimuli, and chemoattractant-mediated actin assembly. In contrast, superoxide production is normal in Rac1-deficient neutrophils but markedly diminished in Rac2 null cells. These data demonstrate that although Rac1 and Rac2 are both required for actin-mediated functions, Rac2 is specifically required for activation of the neutrophil NADPH oxidase.
34. N-acetylglucosaminyltransferase V mediates cell **migration** and invasion of mouse mammary tumor cells 4TO7 via RhoA and Rac1 signaling pathway. In tumor cells, alterations in cellular glycosylation may play a key role in their metastatic behavior. Using small interfering RNA against GnT-V, we found that the expression of GnT-V and beta1,6GlcNAc branching were significantly reduced which was particularly accompanied by the arrest in both cell **migration** and invasion as compared to the negative control. Moreover, the suppressed GnT-V expression by siRNA technique inactivated the signaling molecules including Rac1, cofilin, Erk and Akt, and activated RhoA levels in cells lacking GnT-V, but revealed no impact on Cdc42 activity. All these notions disclose for the first time that GnT-V and beta1, 6GlcNAc branching mediate the cell **migration** and invasion in Rac1-positive and RhoA-negative regulatory manners.
35. The N-BAR domain protein, Bin3, regulates Rac1- and Cdc42-dependent processes in myogenesis. Actin dynamics are necessary at multiple steps in the formation of multinucleated muscle cells. BAR domain proteins can regulate actin dynamics in several cell types, but have been little studied in skeletal muscle. Here, we identify novel functions for the N-BAR domain protein, Bridging integrator 3 (Bin3), during myogenesis in mice. Bin3 plays an important role in regulating myofiber size in vitro and in vivo. During early myogenesis, Bin3 promotes **migration** of differentiated muscle cells, where it colocalizes with F-actin in lamellipodia. In addition, Bin3 forms a complex with Rac1 and Cdc42, Rho GTPases involved in actin polymerization, which are known to be essential for myotube formation. Importantly, a Bin3-dependent pathway is a major regulator of Rac1 and Cdc42 activity in differentiated muscle cells. Overall, these data classify N-BAR domain proteins as novel regulators of actin-dependent processes in myogenesis, and further implicate BAR domain proteins in muscle growth and repair.
36. Differential Rac1 signalling by guanine nucleotide exchange factors implicates FLII in regulating Rac1-driven cell **migration**. The small GTPase Rac1 has been implicated in the formation and dissemination of tumours. Upon activation by guanine nucleotide exchange factors (GEFs), Rac1 associates with a variety of proteins in the cell thereby regulating various functions, including cell **migration**. However, activation of Rac1 can lead to opposing migratory phenotypes raising the possibility of exacerbating tumour progression when targeting Rac1 in a clinical setting. This calls for the identification of factors that influence Rac1-driven cell motility. Here we show that Tiam1 and P-Rex1, two Rac GEFs, promote Rac1 anti- and pro-migratory signalling cascades, respectively, through regulating the Rac1 interactome. In particular, we demonstrate that P-Rex1 stimulates **migration** through enhancing the interaction between Rac1 and the actin-remodelling protein flightless-1 homologue, to modulate cell contraction in a RhoA-ROCK-independent manner.
37. Rac1 is required for epithelial stem cell function during dermal and oral mucosal wound healing but not for tissue homeostasis in mice. The regenerative capacity of the skin, including the continuous replacement of exfoliated cells and healing of injuries relies on the epidermal stem cells and their immediate cell descendants. The relative contribution of the hair follicle stem cells and the interfollicular stem cells to dermal wound healing is an area of active investigation. Recent studies have revealed that the small GTPase Rac1, which regulates cell **migration** and nuclear gene expression, is required for hair follicle stem function but not for the normal homeostasis of the interfollicular skin.Here we explored whether Rac1 contributes to wound healing in the skin and in the oral mucosa, the latter an anatomical site that presents similar architecture to that of the skin but is devoid of any hair follicle structures, and hence lacks hair follicle stem cells. Epidermal Rac1 gene excision led to the clearly delayed closure of cutaneous wounds. Remarkably, genetic ablation of Rac1 from the oral mucosa resulted in the complete inability of oral wounds to heal. We present evidence that the lack of oral mucosal re-epithelization may result from the reduced migratory capacity of cells lacking Rac1 together with altered expression of injury-induced proliferative and cellular stress-related expression programs.Together, these observations support that while the normal development and homeostasis of the interfollicular skin and oral mucosa do not require Rac1 function, the interfollicular and oral epithelial stem cells may require a Rac1-dependent program to orchestrate the tissue response to injury and ultimate for wound closure. Ultimately, these findings may enable the molecular characterization of the acute tissue regenerative response of these stem cell populations, thus facilitating the identification of novel molecular-targeted strategies aimed at accelerating wound closure.
38. Therapeutic ultrasound bypasses canonical syndecan-4 signaling to activate rac1. The application of pulsed, low intensity ultrasound is emerging as a potent therapy for the treatment of complex bone fractures and tissue damage. Ultrasonic stimuli accelerate fracture healing by up to 40% and enhance tendon and ligament healing by promoting cell proliferation, **migration**, and matrix synthesis through an unresolved mechanism. Ultrasound treatment also induces closure of nonunion fractures, at a success rate (85% of cases) similar to that of surgical intervention (68-96%) while avoiding the complications associated with surgery. The regulation of cell adhesion necessary for wound healing depends on cooperative engagement of the extracellular matrix receptors, integrin and syndecan, as exemplified by the wound healing defects observed in syndecan- and integrin-knock-out mice. This report distinguishes the influence of ultrasound on signals downstream of the prototypic fibronectin receptors, alpha(5)beta(1) integrin and syndecan-4, which cooperate to regulate Rac1 and RhoA. Ultrasonic stimulation fails to activate integrins or induce cell spreading on poor, electrostatic ligands. By contrast, ultrasound treatment overcomes the necessity of engagement or expression of syndecan-4 during the process of focal adhesion formation, which normally requires simultaneous engagement of both receptors. Ultrasound exerts an influence downstream of syndecan-4 and PKCalpha to specifically activate Rac1, itself a critical regulator of tissue repair, and to a lesser extent RhoA. The ability of ultrasound to bypass syndecan-4 signaling, which is known to facilitate efficient tissue repair, explains the reduction in healing times observed in ultrasound-treated patients. By substituting for one of the key axes of adhesion-dependent signaling, ultrasound therapy has considerable potential as a clinical technique.
39. Rac1 links leading edge and uropod events through Rho and myosin activation during chemotaxis. Chemotactic responsiveness is crucial to neutrophil recruitment to sites of infection. During chemotaxis, highly divergent cytoskeletal programs are executed at the leading and trailing edge of motile neutrophils. The Rho family of small GTPases plays a critical role in cell **migration**, and recent work has focused on elucidating the specific roles played by Rac1, Rac2, Cdc42, and Rho during cellular chemotaxis. Rac GTPases regulate actin polymerization and extension of the leading edge, whereas Rho GTPases control myosin-based contraction of the trailing edge. Rac and Rho signaling are thought to crosstalk with one another, and previous research has focused on mutual inhibition of Rac and Rho signaling during chemotaxis. Indeed, polarization of neutrophils has been proposed to involve the activity of a negative feedback system where Rac activation at the front of the cell inhibits local Rho activation, and vice versa. Using primary human neutrophils and neutrophils derived from a Rac1/Rac2-null transgenic mouse model, we demonstrate here that Rac1 (and not Rac2) is essential for Rho and myosin activation at the trailing edge to regulate uropod function. We conclude that Rac plays both positive and negative roles in the organization of the Rhomyosin "backness" program, thereby promoting stable polarity in chemotaxing neutrophils.
40. Integrin alpha3beta1-dependent activation of FAK/Src regulates Rac1-mediated keratinocyte polarization on laminin-5. Upon epidermal wounding, keratinocytes at the wound edge become activated, deposit newly synthesized laminin-5 into the extracellular matrix, and migrate into the wound bed. The interaction between integrin alpha3beta1 and laminin-5 is essential for establishment of a stable, leading lamellipodium and persistent keratinocyte **migration**. We previously showed that integrin alpha3beta1 activates the Rho family GTPase Rac1 and regulates Rac1-dependent formation of polarized, leading lamellipodia in migrating keratinocytes. In the present study, we explored the role of focal adhesion kinase (FAK) and src signaling in this process. We show that overexpression of the FAK inhibitor FAK-related non-kinase or of the FAK(Y397F) auto-phosphorylation mutant, induced abnormal, non-polarized spreading of keratinocytes on laminin-5. Integrin alpha3beta1 was required for full FAK auto-phosphorylation at Y397, and subsequent src kinase-dependent phosphorylation of FAK at residues Y861 and Y925, sites responsible for promoting signal transduction downstream of FAK, indicating that alpha3beta1 regulates the coordination of FAK/src signal transduction. Inhibiting either src kinase activity or FAK signaling interfered with alpha3beta1-mediated Rac1 activation and polarized cell spreading. These findings reveal a novel pathway in migratory keratinocytes wherein alpha3beta1-laminin-5 interactions regulate src kinase signaling through FAK, promoting Rac1 activation and polarized lamellipodium extension.
41. Involvement of the phosphatidylinositol 3-kinase/rac1 and cdc42 pathways in radial **migration** of cortical neurons. During cortical development, newly generated neurons migrate radially toward their final positions. Although several candidate genes essential for this radial **migration** have been reported, the signaling pathways regulating it are largely unclear. Here we studied the role of phosphatidylinositol (PI) 3-kinase and its downstream signaling molecules in the radial **migration** of cortical neurons in vivo and in vitro. The expression of constitutively active and dominant-negative PI 3-kinases markedly inhibited radial **migration**. In the neocortical slice culture, a PI 3-kinase inhibitor suppressed the formation of GTP-bound Rac1 and Cdc42 and radial **migration**. Constitutively active and dominant-negative forms of Rac1 and Cdc42 but not Akt also significantly inhibited radial **migration**. In migrating neurons, wild-type Rac1 and Cdc42 showed different localizations; Rac1 localized to the plasma membrane and Cdc42 to the perinuclear region on the side of the leading processes. These results suggest that both the PI 3-kinase/Rac1 and Cdc42 pathways are involved in the radial **migration** of cortical neurons and that they have different roles.
42. A Novel c-Jun N-terminal Kinase (JNK) Signaling Complex Involved in Neuronal **Migration** during Brain Development. Disturbance of neuronal **migration** may cause various neurological disorders. Both the transforming growth factor-β (TGF-β) signaling and microcephaly-associated protein WDR62 are important for neurona**l migrati**on during brain development; however, the underlying molecular mechanisms involved remain unclear. We show here that knock-out or knockdown of Tak1 (TGFβ-activated kinase 1) and Jnk2 (c-Jun N-terminal kinase 2) perturbs neuron**al migrat**ion during cortical development and that t**he migrat**ion defects incurred by knock-out and/or knockdown of Tβr2 (type II TGF-β receptor) or Tak1 can be partially rescued by expression of TAK1 and JNK2, respectively. Furthermore, TAK1 forms a protein complex with RAC1 and two scaffold proteins of the JNK pathway, the microcephaly-associated protein WDR62 and the RAC1-interacting protein POSH (plenty of Src homology). Components of the complex coordinate with each other in the regulation of TAK1 as well as JNK activities. We suggest that unique JNK protein complexes are involved in the diversified biological and pathological functions during brain development and pathogenesis of diseases.
43. Melanoblasts on the move: Rac1 sets the pace. Cell **migration** is fundamental to development and many cell types have a migratory phase during embryonic development when tissues and body structures are forming. Cancer metastasis is in many ways thought to be analogous to embryonic development. Some of the mechanisms that tumor cells use to hijack the adult body are thought to derive from their abilities to reactivate embryonic signaling and motility pathways and thus enhance their growth and motility. Melanomas are notorious for their ability to become highly invasive and metastatic if not removed early. While adult melanin producing cells, melanocytes, have limited mobility, melanoblasts are highly motile cells that move through the dermis and epidermis during embryogenesis and could serve as a useful paradigm for some aspects of melanoma invasion and metastasis. Recent findings from our laboratory using ex-vivo imaging of mouse melanoblast **migration** in the epidermis provide the first insights into the role of Rac1 in developing mouse melanoblasts in vivo. Melanoblasts do not move as a collective group, or use an invasive or blebbing mode of **migration** as revealed by other in vivo systems, but rather they extend short and long dynamic pseudopodia and squeeze between epidermal keratinocytes using myosin motors. Melanoblasts can initiate short actin-based protrusions independently of Rac1. Rac1 is required to control the rate of formation of long actin-based protrusions for effective translocation in skin. Our results reveal a novel mode of in vivo **migration** controlled by Rac1 that is important for normal development and likely in melanoma.
44. Temporal-specific roles of Rac1 during vascular development and retinal angiogenesis. Angiogenesis, the formation of new blood vessels by remodeling and growth of pre-existing vessels, is a highly orchestrated process that requires a tight balance between pro-angiogenic and anti-angiogenic factors and the integration of their corresponding signaling networks. The family of Rho GTPases, including RhoA, Rac1, and Cdc42, play a central role in many cell biological processes that involve cytoskeletal changes and cell movement. Specifically for Rac1, we have shown that excision of Rac1 using a Tie2-Cre animal line results in embryonic lethality in midgestation (embryonic day (E) 9.5), with multiple vascular defects. However, Tie2-Cre can be also expressed during vasculogenesis, prior to angiogenesis, and is active in some hematopoietic precursors that can affect vessel formation. To circumvent these limitations, we have now conditionally deleted Rac1 in a temporally controlled and endothelial-restricted fashion using Cdh5(PAC)-iCreERT2 transgenic mice. In this highly controlled experimental in vivo system, we now show that Rac1 is required for embryonic vascular integrity and angiogenesis, and for the formation of superficial and deep vascular networks in the post-natal developing retina, the latter involving a novel specific function for Rac1 in vertical blood vessel sprouting. Aligned with these findings, we show that RAC1 is spatially involved in endothelial cell **migration**, invasion, and radial sprouting activities in 3D collagen matrix in vitro models. Hence, Rac1 and its downstream molecules may represent potential anti-angiogeneic therapeutic targets for the treatment of many human diseases that involve aberrant neovascularization and blood vessel overgrowth.
45. Ras-related C3 botulinum toxin substrate 1 (RAC1) regulates glucose-stimulated insulin secretion via modulation of F-actin. The small G-protein ras-related C3 botulinum toxin substrate 1 (RAC1) plays various roles in mammalian cells, such as in the regulation of cytoskeletal organisation, cell adhesion, **migration** and morphological changes. The present study examines the effects of RAC1 ablation on pancreatic beta cell function.Isolated islets from pancreatic beta cell-specific Rac1-knockout (betaRac1(-/-)) mice and RAC1 knockdown INS-1 insulinoma cells treated with small interfering RNA were used to investigate insulin secretion and cytoskeletal organisation in pancreatic beta cells.BetaRac1(-/-) mice showed decreased glucose-stimulated insulin secretion, while there were no apparent differences in islet morphology. Isolated islets from the mice had blunted insulin secretion in response to high glucose levels. In RAC1 knockdown INS-1 cells, insulin secretion was also decreased in response to high glucose levels, consistent with the phenotype of betaRac1(-/-) mice. Even under high glucose levels, RAC1 knockdown INS-1 cells remained intact with F-actin, which inhibits the recruitment of the insulin granules, resulting in an inhibition of insulin secretion.In RAC1-deficient pancreatic beta cells, F-actin acts as a barrier for insulin granules and reduces glucose-stimulated insulin secretion.
46. SrGAP3 interacts with lamellipodin at the cell membrane and regulates Rac-dependent cellular protrusions. SrGAP3/MEGAP is a member of the Slit-Robo GAP (srGAP) family and is implicated in repulsive axon guidance and neuronal **migration** through Slit-Robo-mediated signal transduction. Here we describe an inhibitory role of srGAP3 on actin dynamics, specifically on lamellipodia formation. We show that the F-BAR domain localizes srGAP3 to the leading edge of cellular protrusions whereas the SH3 domain is important for focal adhesion targeting. We report on a novel srGAP3 interaction partner, lamellipodin, which localizes with srGAP3 at the leading edge. Live-cell analyses revealed that srGAP3 influences lamellipodin-evoked lamellipodial dynamics. Furthermore, we show that mouse embryonic fibroblasts derived from homozygous srGAP3-knockout embryos display an increased cell area and lamellipodia formation that can be blocked by shRNA-mediated knockdown of lamellipodin.
47. 8-Hydroxy-2-deoxyguanosine prevents plaque formation and inhibits vascular smooth muscle cell activation through Rac1 inactivation. 8-Hydroxy-2-deoxyguanosine (8-OHdG), a marker of oxidative stress, has been recently rediscovered to inhibit Rac1 in neutrophils and macrophages, thereby inhibiting Rac1-linked functions of these cells, including reactive oxygen species production through NADPH oxidase activation, phagocytosis, chemotaxis, and cytokine release. In vascular smooth muscle cells (VSMCs), reactive oxygen species also induce abnormal proliferation and **migration** leading to progression of atherosclerosis. Based upon the involvement of reactive oxygen species in phagocytic cells and VSMCs during the atherosclerotic process, we hypothesized that 8-OHdG could have antiatherosclerotic action and tested this hypothesis in an experimentally induced atherosclerosis in mice. Partially ligated ApoE knockout mice, a more physiologically relevant model of low and oscillatory flow, developed an advanced lesion in 2 weeks, and orally administered 8-OHdG significantly reduced plaque formation along with reduced superoxide formation, monocyte/macrophage infiltration, and extracellular matrix (ECM) accumulation. The effects of 8-OHdG observed in primary VSMCs were consistent with the in vivo effects of 8-OHdG and were inhibitory to angiotensin II or platelet-derived growth factor-induced production of reactive oxygen species, proliferation, **migration**, and ECM production. Also, angiotensin II-induced Rac1 activity in VSMCs was significantly inhibited by 8-OHdG, and transfection of constitutively active Rac1 reversed the inhibitory effect of 8-OHdG on VSMC activation. Molecular docking study showed that 8-OHdG stabilizes Rac1-GEF complex, indicating the physical contact of 8-OHdG with Rac1. These findings highly suggest that the antiatherosclerotic effect of 8-OHdG is mediated by inhibition of Rac1 activity. In conclusion, our results show a novel action of orally active 8-OHdG in suppressing atherosclerotic plaque formation in vivo and VSMC activation in vitro through inhibition of Rac1, which emphasizes a new therapeutic avenue to benefit atherosclerosis.
48. Rac1 and Rac3 have opposing functions in cell adhesion and differentiation of neuronal cells. Rac1 and Rac3 are highly homologous members of the Rho small GTPase family. Rac1 is ubiquitously expressed and regulates cell adhesion, **migration** and differentiation in various cell types. Rac3 is primarily expressed in brain and may therefore have a specific function in neuronal cells. We found that depletion of Rac1 by short interference RNA leads to decreased cell-matrix adhesions and cell rounding in neuronal N1E-115 cells. By contrast, depletion of Rac3 induces stronger cell adhesions and dramatically increases the outgrowth of neurite-like protrusions, suggesting opposite functions for Rac1 and Rac3 in neuronal cells. Consistent with this, overexpression of Rac1 induces cell spreading, whereas overexpression of Rac3 results in a contractile round morphology. Rac1 is mainly found at the plasma membrane, whereas Rac3 is predominantly localized in the perinuclear region. Residues 185-187, present in the variable polybasic rich region at the carboxyl terminus are responsible for the difference in phenotype induced by Rac1 and Rac3 as well as for their different intracellular localization. The Rac1-opposing function of Rac3 is not mediated by or dependent on components of the RhoA signaling pathway. It rather seems that Rac3 exerts its function through negatively affecting integrin-mediated cell-matrix adhesions. Together, our data reveal that Rac3 opposes Rac1 in the regulation of cell adhesion and differentiation of neuronal cells.
49. Epidermal growth factor stimulates Rac activation through Src and phosphatidylinositol 3-kinase to promote colonic epithelial cell **migration**. Regulated intestinal epithelial cell **migration** plays a key role in wound healing and maintenance of a healthy gastrointestinal tract. Epidermal growth factor (EGF) stimulates cell **migration** and wound closure in intestinal epithelial cells through incompletely understood mechanisms. In this study we investigated the role of the small GTPase Rac in EGF-induced cell **migration** using an in vitro wound-healing assay. In mouse colonic epithelial (MCE) cell lines, EGF-stimulated wound closure was accompanied by a doubling of the number of cells containing lamellipodial extensions at the wound margin, increased Rac membrane translocation in cells at the wound margin, and rapid Rac activation. Either Rac1 small interfering (si)RNA or a Rac1 inhibitor completely blocked EGF-stimulated wound closure. Whereas EGF failed to activate Rac in colon cells from EGF receptor (EGFR) knockout mice, stable expression of wild-type EGFR restored EGF-stimulated Rac activation and **migration**. Pharmacological inhibition of either phosphatidylinositol 3-kinase (PI3K) or Src family kinases reduced EGF-stimulated Rac activation. Cotreatment of cells with both inhibitors completely blocked EGF-stimulated Rac activation and localization to the leading edge of cells and lamellipodial extension. Our results present a novel mechanism by which the PI3K and Src signaling cascades cooperate to activate Rac and promote intestinal epithelial cell **migration** downstream of EGFR.
50. ApoER2 and Reelin are expressed in regenerating peripheral nerve and regulate Schwann cell **migration** by activating the Rac1 GEF protein, Tiam1. ApoER2 and its ligand Reelin participate in neuronal **migration** during development. Upon receptor binding, Reelin induces the proteolytic processing of ApoER2 as well as the activation of signaling pathway, including small Rho GTPases. Besides its presence in the central nervous system (CNS), Reelin is also secreted by Schwann cells (SCs), the glial cells of the peripheral nervous system (PNS). Reelin deficient mice (reeler) show decreased axonal regeneration in the PNS; however neither the presence of ApoER2 nor the role of the Reelin signaling pathway in the PNS have been evaluated. Interestingly SC **migration** occurs during PNS development and during injury-induced regeneration and involves activation of small Rho GTPases. Thus, Reelin-ApoER2 might regulate SC **migration** during axon regeneration in the PNS. Here we demonstrate the presence of ApoER2 in PNS. After sciatic nerve injury Reelin was induced and its receptor ApoER2 was proteolytically processed. In vitro, SCs express both Reelin and ApoER2 and Reelin induces SC **migration**. To elucidate the molecular mechanism underlying Reelin-dependent SC **migration**, we examined the involvement of Rac1, a conspicuous small GTPase family member. FRET experiments revealed that Reelin activates Rac1 at the leading edge of SCs. In addition, Tiam1, a major Rac1-specific GEF was required for Reelin-induced SC **migration**. Moreover, Reelin-induced SC **migration** was decreased after suppression of the polarity protein PAR3, consistent with its association to Tiam1. Even more interesting, we demonstrated that PAR3 binds preferentially to the full-length cytoplasmic tail of ApoER2 corresponding to the splice-variant containing the exon 19 that encodes a proline-rich insert and that ApoER2 was required for SC **migration**. Our study reveals a novel function for Reelin/ApoER2 in PNS, inducing cell **migration** of SCs, a process relevant for PNS development and regeneration.
51. Brain-derived neurotrophic factor (BDNF) induces polarized signaling of small GTPase (Rac1) protein at the onset of Schwann cell myelination through partitioning-defective 3 (Par3) protein. Brain-derived neurotrophic factor (BDNF) was shown to play a role in Schwann cell myelination by recruiting Par3 to the axon-glial interface, but the underlying mechanism has remained unclear. Here we report that Par3 regulates Rac1 activation by BDNF but not by NRG1-Type III in Schwann cells, although both ligands activate Rac1 in vivo. During development, active Rac1 signaling is localized to the axon-glial interface in Schwann cells by a Par3-dependent polarization mechanism. Knockdown of p75 and Par3 individually inhibits Rac1 activation, whereas constitutive activation of Rac1 disturbs the polarized activation of Rac1 in vivo. Polarized Rac1 activation is necessary for myelination as Par3 knockdown attenuates myelination in mouse sciatic nerves as well as in zebrafish. Specifically, Par3 knockdown in zebrafish disrupts proper alignment between the axon and Schwann cells without perturbing Schwann cell **migration**, suggesting that localized Rac1 activation at the axon-glial interface helps identify the initial wrapping sites. We therefore conclude that polarization of Rac1 activation is critical for myelination.
52. Regulation of Rac1 activation by the low density lipoprotein receptor-related protein. The low density lipoprotein receptor-related protein (LRP-1) binds and mediates the endocytosis of multiple ligands, transports the urokinase-type plasminogen activator receptor (uPAR) and other membrane proteins into endosomes, and binds intracellular adaptor proteins involved in cell signaling. In this paper, we show that in murine embryonic fibroblasts (MEFs) and L929 cells, LRP-1 functions as a major regulator of Rac1 activation, and that this activity depends on uPAR. LRP-1-deficient MEFs demonstrated increased Rac1 activation compared with LRP-1-expressing MEFs, and this property was reversed by expressing the VLDL receptor, a member of the same gene family as LRP-1, with overlapping ligand-binding specificity. Neutralizing the activity of LRP-1 with receptor-associated protein (RAP) increased Rac1 activation and cell **migration** in MEFs and L929 cells. The same parameters were unaffected by RAP in uPAR-/- MEFs, prepared from uPAR gene knockout embryos, and in uPAR-deficient LM-TK- cells. Untreated uPAR+/+ MEFs demonstrated substantially increased Rac1 activation compared with uPAR-/- MEFs. In addition to Rac1, LRP-1 suppressed activation of extracellular signal-regulated kinase (ERK) in MEFs; however, it was Rac1 (and not ERK) that was responsible for the effects of LRP-1 on MEF **migration**. Thus, LRP-1 regulates two signaling proteins in the same cell (Rac1 and ERK), both of which may impact on cell **migration**. In uPAR-negative cells, LRP-1 neutralization does not affect Rac1 activation, and other mechanisms by which LRP-1 may regulate cell **migration** are not unmasked.
53. Diacylglycerol kinase zeta regulates actin cytoskeleton reorganization through dissociation of Rac1 from RhoGDI. Activation of Rac1 GTPase signaling is stimulated by phosphorylation and release of RhoGDI by the effector p21-activated kinase 1 (PAK1), but it is unclear what initiates this potential feed-forward mechanism for regulation of Rac activity. Phosphatidic acid (PA), which is produced from the lipid second messenger diacylglycerol (DAG) by the action of DAG kinases (DGKs), is known to activate PAK1. Here, we investigated whether PA produced by DGKzeta initiates RhoGDI release and Rac1 activation. In DGKzeta-deficient fibroblasts PAK1 phosphorylation and Rac1-RhoGDI dissociation were attenuated, leading to reduced Rac1 activation after platelet-derived growth factor stimulation. The cells were defective in Rac1-regulated behaviors, including lamellipodia formation, membrane ruffling, **migration**, and spreading. Wild-type DGKzeta, but not a kinase-dead mutant, or addition of exogenous PA rescued Rac activation. DGKzeta stably associated with PAK1 and RhoGDI, suggesting these proteins form a complex that functions as a Rac1-selective RhoGDI dissociation factor. These results define a pathway that links diacylglycerol, DGKzeta, and PA to the activation of Rac1: the PA generated by DGKzeta activates PAK1, which dissociates RhoGDI from Rac1 leading to changes in actin dynamics that facilitate the changes necessary for cell motility.
54. Novel Activities of Select NSAID R-Enantiomers against Rac1 and Cdc42 GTPases. Rho family GTPases (including Rac, Rho and Cdc42) collectively control cell proliferation, adhesion and **migration** and are of interest as functional therapeutic targets in numerous epithelial cancers. Based on high throughput screening of the Prestwick Chemical Library® and cheminformatics we identified the R-enantiomers of two approved drugs (naproxen and ketorolac) as inhibitors of Rac1 and Cdc42. The corresponding S-enantiomers are considered the active component in racemic drug formulations, acting as non-steroidal anti-inflammatory drugs (NSAIDs) with selective activity against cyclooxygenases. Here, we show that the S-enantiomers of naproxen and ketorolac are inactive against the GTPases. Additionally, more than twenty other NSAIDs lacked inhibitory action against the GTPases, establishing the selectivity of the two identified NSAIDs. R-naproxen was first identified as a lead compound and tested in parallel with its S-enantiomer and the non-chiral 6-methoxy-naphthalene acetic acid (active metabolite of nabumetone, another NSAID) as a structural series. Cheminformatics-based substructure analyses-using the rotationally constrained carboxylate in R-naproxen-led to identification of racemic [R/S] ketorolac as a suitable FDA-approved candidate. Cell based measurement of GTPase activity (in animal and human cell lines) demonstrated that the R-enantiomers specifically inhibit epidermal growth factor stimulated Rac1 and Cdc42 activation. The GTPase inhibitory effects of the R-enantiomers in cells largely mimic those of established Rac1 (NSC23766) and Cdc42 (CID2950007/ML141) specific inhibitors. Docking predicts that rotational constraints position the carboxylate moieties of the R-enantiomers to preferentially coordinate the magnesium ion, thereby destabilizing nucleotide binding to Rac1 and Cdc42. The S-enantiomers can be docked but are less favorably positioned in proximity to the magnesium. R-naproxen and R-ketorolac have potential for rapid translation and efficacy in the treatment of several epithelial cancer types on account of established human toxicity profiles and novel activities against Rho-family GTPases.
55. Paxillin and Hic-5 interaction with vinculin is differentially regulated by Rac1 and RhoA. Cell **migration** is of paramount importance to organism development and maintenance as well as multiple pathological processes, including cancer metastasis. The RhoGTPases Rac1 and RhoA are indispensable for cell **migration** as they regulate cell protrusion, cell-extracellular matrix (ECM) interactions and force transduction. However, the consequences of their activity at a molecular level within the cell remain undetermined. Using a combination of FRET, FRAP and biochemical analyses we show that the interactions between the focal adhesion proteins vinculin and paxillin, as well as the closely related family member Hic-5 are spatially and reciprocally regulated by the activity of Rac1 and RhoA. Vinculin in its active conformation interacts with either paxillin or Hic-5 in adhesions in response to Rac1 and RhoA activation respectively, while inactive vinculin interacts with paxillin in the membrane following Rac1 inhibition. Additionally, Rac1 specifically regulates the dynamics of paxillin as well as its binding partner and F-actin interacting protein actopaxin (α-parvin) in adhesions. Furthermore, FRET analysis of protein:protein interactions within cell adhesions formed in 3D matrices revealed that, in contrast to 2D systems vinculin interacts preferentially with Hic-5. This study provides new insight into the complexity of cell-ECM adhesions in both 2D and 3D matrices by providing the first description of RhoGTPase-coordinated protein:protein interactions in a cellular microenvironment. These data identify discrete roles for paxillin and Hic-5 in Rac1 and RhoA-dependent cell adhesion formation and maturation; processes essential for productive cell **migratio**n.
56. Cdc42 and Rac family GTPases regulate mode and speed but not direction of primary fibroblast **migration** during platelet-derived growth factor-dependent chemotaxis. Cdc42 and Rac family GTPases are important regulators of morphology, motility, and polarity in a variety of mammalian cell types. However, comprehensive analysis of their roles in the morphological and behavioral aspects of chemotaxis within a single experimental system is still lacking. Here we demonstrate using a direct viewing chemotaxis assay that of all of the Cdc42/Rac1-related GTPases expressed in primary fibroblasts, Cdc42, Rac1, and RhoG are required for efficient **migration** towards platelet-derived growth factor (PDGF). During **migration**, Cdc42-, Rac1-, and RhoG-deficient cells show aberrant morphology characterized as cell elongation and cell body rounding, loss of lamellipodia, and formation of thick membrane extensions, respectively. Analysis of individual cell trajectories reveals that cell speed is significantly reduced, as well as persistence, but to a smaller degree, while the directional response to the gradient of PDGF is not affected. Combined knockdown of Cdc42, Rac1, and RhoG results in greater inhibition of cell speed than when each protein is knocked down alone, but the cells are still capable of migrating toward PDGF. We conclude that, Cdc42, Rac1, and RhoG function cooperatively during cell **migration** and that, while each GTPase is implicated in the control of morphology and cell speed, these and other Cdc42/Rac-related GTPases are not essential for the directional response toward PDGF.
57. Regulation of lymphatic-blood vessel separation by endothelial Rac1. Sprouting angiogenesis and lymphatic-blood vessel segregation both involve the **migration** of endothelial cells, but the precise migratory molecules that govern the decision of blood vascular endothelial cells to segregate into lymphatic vasculature are unknown. Here, we deleted endothelial Rac1 in mice (Tie1-Cre(+);Rac1(fl/fl)) and revealed, unexpectedly, that whereas blood vessel morphology appeared normal, lymphatic-blood vessel separation was impaired, with corresponding edema, haemorrhage and embryonic lethality. Importantly, normal levels of Rac1 were essential for directed endothelial cell migratory responses to lymphatic-inductive signals. Our studies identify Rac1 as a crucial part of the migratory machinery required for endothelial cells to separate and form lymphatic vasculature.
58. Rac1-dependent cell cycle exit of MGE precursors and GABAergic interneuron **migration** to the cortex. Cortical γ-aminobutyric acid (GABA)ergic interneurons are characterized by extraordinary neurochemical and functional diversity. Although recent studies have uncovered some of the molecular components underlying interneuron development, including the cellular and molecular mechanisms guiding their **migratio**n to the cortex, the intracellular components involved are still unknown. Rac1, a member of the Rac subfamily of Rho-GTPases, has been implicated in various cellular processes such as cell cycle dynamics, axonogenesis, and **migratio**n. In this study, we have addressed the specific role of Rac1 in interneuron progenitors originating in the medial ganglionic eminence, via Cre/loxP technology. We show that ablation of Rac1 from Nkx2.1-positive progenitors, results in a migratory impairment. As a consequence, only half of GABAergic interneurons are found in the postnatal cortex. The rest remain aggregated in the ventral telencephalon and show morphological defects in their growing processes in vitro. Ablation of Rac1 from postmitotic progenitors does not result in similar defects, thus underlying a novel cell autonomous and stage-specific requirement for Rac1 activity, within proliferating progenitors of cortical interneurons. Rac1 is necessary for their transition from G1 to S phase, at least in part by regulating cyclin D levels and retinoblastoma protein phosphorylation.
59. Genetic deletion of Cdc42GAP reveals a role of Cdc42 in erythropoiesis and hematopoietic stem/progenitor cell survival, adhesion, and engraftment. Rho family GTPases are key signal transducers in cell regulation. Although a body of literature has implicated the Rho family members Rac1 and Rac2 in multiple hematopoietic-cell functions, the role of Cdc42 in hematopoiesis remains unclear. Here we have examined the hematopoietic properties and the hematopoietic stem/progenitor cell (HSP) functions of gene-targeted mice carrying null alleles of cdc42gap, a negative regulator of Cdc42. The Cdc42GAP-/- fetal liver and bone marrow cells showed a 3-fold increase in Cdc42 activity but normal Rac and RhoA activities, indicating that Cdc42GAP knockout resulted in a gain of Cdc42 activity in the hematopoietic tissues. Cdc42GAP-/- mice were anemic. The cellularity of fetal liver and bone marrow, the number and composition percentage of HSPs, and the erythroid blast-forming unit and colony-forming unit (BFU-E/CFU-E) activities were significantly reduced in the homozygous mice. The decrease in HSP number was associated with increased apoptosis of the Cdc42GAP-/- HSPs and the activation of JNK-mediated apoptotic machinery. Moreover, homozygous HSPs showed impaired cortical F-actin assembly, deficiency in adhesion and **migration**, and defective engraftment. These results provide evidence that Cdc42 activity is important for erythropoiesis and for multiple HSP functions, including survival, adhesion, and engraftment.
60. Vav GEFs regulate macrophage morphology and adhesion-induced Rac and Rho activation. The Vav family of proteins have the potential to act as both signalling adapters and GEFs for Rho GTPases. They have therefore been proposed as regulators of the cytoskeleton in various cell types. We have used macrophages from mice deficient in all three Vav isoforms to determine how their function affects cell morphology and **migration**. Macrophages lacking Vav proteins adopt an elongated morphology and have enhanced migratory persistence in culture. To investigate the pathways through which Vav proteins exert their effects we analysed the responses of macrophages to the chemoattractant CSF-1 and to adhesion. We found that morphological and signalling responses of macrophages to CSF-1 did not require Vav proteins. In contrast, adhesion-induced cell spreading, RhoA and Rac1 activation and cell signalling were all dependent on Vav proteins. We propose that Vav proteins affect macrophage morphology and motile behaviour by coupling adhesion receptors to Rac1 and RhoA activity and regulating adhesion signalling events such as paxillin and ERK1/2 phosphorylation by acting as adapters.
61. 12/15-Lipoxygenase gene knockout severely impairs ischemia-induced angiogenesis due to lack of Rac1 farnesylation. To understand the mechanisms by which 15(S)-hydroxyeicosatetraenoic acid (15(S)-HETE) activates Rac1 in the induction of angiogenesis, we studied the role of 3-hydroxy-3-methylglutaryl-coenzyme A (HMG-CoA) reductase and αPix. 15(S)-HETE stimulated Rac1 in a sustained manner in human dermal microvascular endothelial cells (HDMVECs). Simvastatin, a potent inhibitor of HMG-CoA reductase, suppressed 15(S)-HETE-induced Rac1 activation in HDMVECs affecting their **migratio**n and tube formation. 15(S)-HETE by inducing HMG-CoA reductase expression caused increased farnesylation and membrane translocation of Rac1 where it became activated by Src-dependent αPix stimulation. Mevalonate rescued 15(S)-HETE-induced Rac1 farnesylation and membrane translocation in HDMVECs and th**e migrati**on and tube formation of these cells from inhibition by simvastatin. Down-regulation of αPix inhibited 15(S)-HETE-induced HDMV**EC migrat**ion and tube formation. Hind-limb ischemia induced Rac1 farnesylation and activation leading to increased angiogenesis and these effects were blocked by simvastatin and rescued by mevalonate in WT mice. In contrast, hind-limb ischemia failed to induce Rac1 farnesylation and activation as well as angiogenic response in 12/15-Lox(-/-) mice. Activation of Src and αPix were also compromised at least to some extent in 12/15-Lox(-/-) mice compared with WT mice in response to hind-limb ischemia. Together, these findings demonstrate for the first time that HMG-CoA reductase plays a determinant role in 12/15-Lox-induced angiogenesis.
62. Rho GTPases in hematopoietic stem/progenitor cell **migration**. Rho GTPases including RhoA, Rac1, and Cdc42 are a class of intracellular signaling proteins critical for the regulation of cytoskeleton organization, adhesion, and **migration**. Molecular mechanisms of mammalian cell **migration** were first revealed in fibroblasts where RhoA, Rac1, and Cdc42 facilitate in the multistep process including establishment and maintenance of polarity, formation of actin-rich protrusions, remodeling of adhesive contacts, and generation of force. In hematopoietic stem/progenitor cells, Rho GTPases relay signals from chemokines and cytokines such as SDF-1α and SCF to the actin and microtubule cytoskeleton through effector kinases and/or adaptor molecules that affect adhesion or transcription. Comprehensive use of murine conditional gene knockout technology combined with biochemical approaches in recent studies allows for physiologically relevant investigations of the involvement of Rho GTPases in hematopoietic stem/progenitor cell **migratio**n, providing important mechanisms for the stem/progenitor maintenance.
63. Rac1 expression by fibroblasts is required for tissue repair in vivo. Tissue repair requires that fibroblasts migrate into the wound to produce and remodel extracellular matrix, a process that requires adhesion. Failure to suppress the tissue repair program results in fibrotic disorders that are characterized by excessive adhesive signaling. The role of specific components of adhesive signaling in fibrogenic responses is unclear, but may involve small GTPases such as Rac1. To address the functions of Rac1 in fibroblasts, we generated mice containing a fibroblast-specific deletion of Rac1. These mice show delayed cutaneous wound closure, including reduced collagen production and myofibroblast formation. In cultured Rac1-deficient fibroblasts, adhesion, spreading, and **migration** were significantly inhibited. Rac1-deficient fibroblasts possessed impaired myofibroblast formation and function as visualized by reduced alpha-smooth muscle actin expression as well as matrix contraction. Both in vivo and in vitro, Rac1- deficient fibroblasts showed a reduced generation of reactive oxygen species; in vitro, hydrogen peroxide alleviated the phenotype of Rac1-deficient fibroblasts. Thus, Rac1 is an essential signaling integrator that is required for normal wound healing and dermal homeostasis.
64. RACK1 promotes the proliferation, **migration** and invasion capacity of mouse hepatocellular carcinoma cell line in vitro probably by PI3K/Rac1 signaling pathway. Hca-P and Hca-F is a pair of synogenetic mouse hepatocarcinoma ascites cell lines, possessing different capacity of lymphatic metastasis. Receptor of activated C-kinase 1 (Rack1), together with Jnk1 and gelsolin (Gsn) were previously identified as differentially expressed proteins for lymphatic metastatic potential between the two cell lines. As an intracellular scaffold protein, Rack1 could recruit such signaling molecules as integrins, Src, PKC which are involved in many important biological processes and play key roles in cancer progression. In our present studies, pCDNA3.1(+)-Rack1, a eukaryotic expression plasmid, was constructed and stably transfected into Hca-P cells with a low metastatic potential. CCK8 assay and transwell system were used to evaluate the effects of Rack1 on proliferation, **migration** and invasion of Hca-P cells in vitro. Then, LY294002, an inhibitor of PI3K, was added into the culture medium of pCDNA3.1(+)-Rack1-Hca-P cells and their biological behaviors observed further. Moreover, the expression of Jnk1, Rac1 and Gsn of pCDNA3.1(+)-Rack1-Hca-P cells were detected by western blot after pretreated with various doses of LY294002. As a result, the proliferation, **migration** and invasion of pCDNA3.1(+)-Rack1-Hca-P cells were significantly enhanced and could be inhibited by LY294002. In addition, the expression of Gsn, Rac1 and Jnk1 of pCDNA3.1(+)-Rack1-Hca-P cells also decreased after pretreated with LY294002. The expression of Gsn can be inhibited by NSC33766 (an inhibitor of Rac1). Taken together, Rack1/PI3K/Rac1 signaling pathway may play a crucial role in malignant biological behaviors of mouse hepatocarcinoma cells with lymphatic metastasis potential. It may be a potential target for therapy of cancer lymphatic metastasis.
65. Inactivation of Cdc42 in embryonic brain results in hydrocephalus with ependymal cell defects in mice. The establishment of a polarized cellular morphology is essential for a variety of processes including neural tube morphogenesis and the development of the brain. Cdc42 is a Ras-related GTPase that plays an essential role in controlling cell polarity through the regulation of the actin and microtubule cytoskeleton architecture. Previous studies have shown that Cdc42 plays an indispensable role in telencephalon development in earlier embryo developmental stage (before E12.5). However, the functions of Cdc42 in other parts of brain in later embryo developmental stage or in adult brain remain unclear. Thus, in order to address the role of Cdc42 in the whole brain in later embryo developmental stage or in adulthood, we used Cre/loxP technology to generate two lines of tissue-specific Cdc42-knock-out mice. Inactivation of Cdc42 was achieved in neuroepithelial cells by crossing Cdc42/ flox mice with Nestin-Cre mice and resulted in hydrocephalus, causing death to occur within the postnatal stage. Histological analyses of the brains from these mice showed that ependymal cell differentiation was disrupted, resulting in aqueductal stenosis. Deletion of Cdc42 in the cerebral cortex also induced obvious defects in interkinetic nuclear **migration** and hypoplasia. To further explore the role of Cdc42 in adult mice brain, we examined the effects of knocking-out Cdc42 in radial glial cells by crossing Cdc42/flox mice with human glial fibrillary acidic protein (GFAP)-Cre mice. Inactivation of Cdc42 in radial glial cells resulted in hydrocephalus and ependymal cell denudation. Taken together, these results highlight the importance of Cdc42 for ependymal cell differentiation and maintaining, and suggest that these functions likely contribute to the essential roles played by Cdc42 in the development of the brain.
66. IRGM1 enhances B16 melanoma cell metastasis through PI3K-Rac1 mediated epithelial mesenchymal transition. Melanoma is one of the most aggressive skin cancers and is well known for its high metastatic rate. Studies have shown that epithelial mesenchymal transition (EMT) is essential for melanoma cell metastasis. However, the molecular mechanisms underlying EMT are still not fully understood. We have shown that IRGM1, a member of immunity-related GTPase family that regulates immune cell motility, is highly expressed by melanoma cells. The current study aimed to explore whether and how IRGM1 may regulate melanoma cell metastasis. To test this, we modified IRGM1 expression in B16 melanoma cells. We found that over-expression of IRGM1 substantially enhanced pulmonary metastasis in vivo. In keeping with that, knocking-in IRGM1 strongly enhanced while knocking-down IRGM1 impaired B16 cell **migration** and invasion ability in vitro. Interestingly, we observed that IRGM1 enhanced F-actin polymerization and triggers epithelial mesenchymal transition (EMT) through a mechanism involved in PIK3CA mediated Rac1 activation. Together, these data reveals a novel molecular mechanism that involved in melanoma metastasis.
67. Tuberous sclerosis complex 2 loss-of-function mutation regulates reactive oxygen species production through Rac1 activation. The products of the TSC1 (hamartin) and TCS2 (tuberin) tumor suppressor genes negatively regulate cell growth by inhibiting mTOR signaling. Recent research has led to the postulation that tuberin and/or hamartin are involved in tumor **migration**, presumably through Rho activation. Here we show that LEF-8 cells, which contain a Y1571 missense mutation in tuberin, express higher Rac1 activity than tuberin negative and positive cells. We also provide evidence of obvious lamellipodia formation in LEF-8 cells. Since the production of TSC2(Y1571H) cannot form a hetero-complex with hamartin, we further analyzed another mutant, TSC2(R611Q), which also lacks the ability to form a complex with hamartin. Introducing both forms of mutated TSC2 into COS-1 cells increased Rac1 activity as well as cell motility. We also found these two mutants interacted with Rac1. We further demonstrated that the introduction of mutated TSC2 into COS-1 cells can generate higher reactive oxygen species (ROS). These results indicate that loss-of-function mutated tuberin can activate Rac1 and thereby increase ROS production.
68. ACF7 regulates cytoskeletal-focal adhesion dynamics and **migration** and has ATPase activity. Coordinated interactions between microtubule (MT) and actin cytoskeletons are involved in many polarized cellular processes. Spectraplakins are enormous (>500 kDa) proteins able to bind both MTs and actin filaments (F-actin) directly. To elucidate the physiological significance and functions of mammalian spectraplakin ACF7, we've conditionally targeted it in skin epidermis. Intriguingly, ACF7 deficiency compromises the targeting of microtubules along F-actin to focal adhesions (FAs), stabilizes FA-actin networks, and impairs epidermal **migration**. Exploring underlying mechanisms, we show that ACF7's binding domains for F-actin, MTs, and MT plus-end proteins are not sufficient to rescue the defects in FA-cytoskeletal dynamics and **migration** functions of ACF7 null keratinocytes. We've uncovered an intrinsic actin-regulated ATPase domain in ACF7 and demonstrate that it is both functional and essential for these roles. Our findings provide insight into the functions of this important cytoskeletal crosslinking protein in regulating dynamic interactions between MTs and F-actin to sustain directional cell movement.
69. Angiotensin-II and MARCKS: a hydrogen peroxide- and RAC1-dependent signaling pathway in vascular endothelium. MARCKS is an actin-binding protein that modulates vascular endothelial cell **migration** and cytoskeleton signaling (Kalwa, H., and Michel, T. (2011) J. Biol. Chem. 286, 2320-2330). Angiotensin-II is a vasoactive peptide implicated in vascular physiology as well as pathophysiology; the pathways connecting angiotensin-II and cytoskeletal remodeling are incompletely understood. Here we show that MARCKS is expressed in intact arterial preparations, with prominent staining of the endothelium. In endothelial cells, angiotensin-II-promoted MARCKS phosphorylation is abrogated by PEG-catalase, implicating endogenous H(2)O(2) in the angiotensin-II response. Studies using the H(2)O(2) biosensor HyPer2 reveal that angiotensin-II promotes increases in intracellular H(2)O(2). We used a Rac1 FRET biosensor to show that angiotensin-II promotes Rac1 activation that is attenuated by PEG-catalase. siRNA-mediated Rac1 knockdown blocks angiotensin-II-stimulated MARCKS phosphorylation. Cell imaging studies using a phosphoinositide 4,5-bisphosphate (PIP(2)) biosensor revealed that angiotensin-II PIP(2) regulation depends on MARCKS and H(2)O(2). siRNA-mediated knockdown of MARCKS or Rac1 attenuates receptor-mediated activation of the tyrosine kinase c-Abl and disrupts actin fiber formation. These studies establish a critical role for H(2)O(2) in angiotensin-II signaling to the endothelial cytoskeleton in a novel pathway that is critically dependent on MARCKS, Rac1, and c-Abl.
70. Essential role of Cdc42 in cardiomyocyte proliferation and cell-cell adhesion during heart development. Cdc42 is a member of the Rho GTPase family and functions as a molecular switch in regulating cell **migration**, proliferation, differentiation and survival. However, the role of Cdc42 in heart development remains largely unknown. To determine the function of Cdc42 in heart formation, we have generated a Cdc42 cardiomyocyte knockout (CCKO) mouse line by crossing Cdc42 flox mice with myosin light chain (MLC) 2a-Cre mice. The inactivation of Cdc42 in embryonic cardiomyocytes induced lethality after embryonic day 12.5. Histological analysis of CCKO embryos showed cardiac developmental defects that included thin ventricular walls and ventricular septum defects. Microarray and real-time PCR data also revealed that the expression level of p21 was significantly increased and cyclin B1 was dramatically decreased, suggesting that Cdc42 is required for cardiomyocyte proliferation. Phosphorylated Histone H3 staining confirmed that the inactivation of Cdc42 inhibited cardiomyocytes proliferation. In addition, transmission electron microscope studies showed disorganized sarcomere structure and disruption of cell-cell contact among cardiomyocytes in CCKO hearts. Accordingly, we found that the distribution of N-cadherin/β-Catenin in CCKO cardiomyocytes was impaired. Taken together, our data indicate that Cdc42 is essential for cardiomyocyte proliferation, sarcomere organization and cell-cell adhesion during heart development.
71. Requirement of Rac1 and Rac2 expression by mature dendritic cells for T cell priming. Upon maturation, dendritic cells (DCs) acquire the unique ability to activate naïve T cells. We used time-lapse video microscopy and two-photon imaging of intact lymph nodes to show that after establishing initial contact between their dendrites and naïve T lymphocytes, mature DCs migrate toward the contacted lymphocytes. Subsequently, the DCs tightly entrap the T cells within a complex net of membrane extensions. The Rho family guanosine triphosphatases Rac1 and Rac2 but not Rho itself control the formation of dendrites in mature DCs, their polarized short-rang**e migrati**on toward T cells, and T cell priming.
72. Rac1 and Rac3 GTPases regulate the development of hilar mossy cells by affecting the **migration** of their precursors to the hilus. We have previously shown that double deletion of the genes for Rac1 and Rac3 GTPases during neuronal development affects late developmental events that perturb the circuitry of the hippocampus, with ensuing epileptic phenotype. These effects include a defect in mossy cells, the major class of excitatory neurons of the hilus. Here, we have addressed the mechanisms that affect the loss of hilar mossy cells in the dorsal hippocampus of mice depleted of the two Rac GTPases. Quantification showed that the loss of mossy cells was evident already at postnatal day 8, soon after these cells become identifiable by a specific marker in the dorsal hilus. Comparative analysis of the hilar region from control and double mutant mice revealed that synaptogenesis was affected in the double mutants, with strongly reduced presynaptic input from dentate granule cells. We found that apoptosis was equally low in the hippocampus of both control and double knockout mice. Labelling with bromodeoxyuridine at embryonic day 12.5 showed no evident difference in the proliferation of neuronal precursors in the hippocampal primordium, while differences in the number of bromodeoxyuridine-labelled cells in the developing hilus revealed a defect in the **migration** of immature, developing mossy cells in the brain of double knockout mice. Overall, our data show that Rac1 and Rac3 GTPases participate in the normal development of hilar mossy cells, and indicate that they are involved in the regulation of the **migration** of the mossy cell precursor by preventing their arrival to the dorsal hilus.
73. Endothelial Rac1 is essential for hematogenous metastasis to the lung. A variety of vasoactive stimuli induce endothelial permeability through Rac1, a membrane of Rho small GTPases. Here, we determine whether tumor-secreted vasoactive stimulant through Rac1 inducing permeability contributes to hematogenous metastasis. Activation of Rac1 was assayed in human umbilical vein endothelial cells (HUVEC), transendothelial passages were measured by Transwell chambers, and hematogenously metastatic mouse model was generated by intravenous injection with Lewis lung carcinoma cells (LLC). LLC secreted abundant vascular endothelial growth factor (VEGF) in the culture media and sera of mice bearing LLC xenografts or metastatic LLC, and VEGF activated Rac1 through VEGF receptors/PI3Kβ signaling cascade, resulting in hyperoxidative stress and consequent hyperpermeability in HUVEC. Moreover, in co-culture of LLC and HUVEC, significant increases in endothelial permeability and transendothelial **migratio**n of LLC were robustly attenuated by either anti-VEGF neutralizing antibody or Rac1 knockdown in HUVEC. Finally, in metastatic mouse model, deletion of one copy of Rac1 in endothelium not only significantly attenuated LLC-induced vascular permeability, but robustly reduced the metastasis of LLC to lungs. This study supports that tumor-secreted vasoactive stimuli activate Rac1 to induce permeability and consequent transendothelial **migratio**n of tumor cells, and that loss of Rac1 function in endothelium is an effective therapeutic intervention for hematogenous metastasis.
74. Suppression of RhoG activity is mediated by a syndecan 4-synectin-RhoGDI1 complex and is reversed by PKCalpha in a Rac1 activation pathway. Fibroblast growth factor 2 (FGF2) is a major regulator of developmental, pathological, and therapeutic angiogenesis. Its activity is partially mediated by binding to syndecan 4 (S4), a proteoglycan receptor. Angiogenesis requires polarized activation of the small guanosine triphosphatase Rac1, which involves localized dissociation from RhoGDI1 and association with the plasma membrane. Previous work has shown that genetic deletion of S4 or its adapter, synectin, leads to depolarized Rac activation, decreased endothelial **migration**, and other physiological defects. In this study, we show that Rac1 activation downstream of S4 is mediated by the RhoG activation pathway. RhoG is maintained in an inactive state by RhoGDI1, which is found in a ternary complex with synectin and S4. Binding of S4 to synectin increases the latter's binding to RhoGDI1, which in turn enhances RhoGDI1's affinity for RhoG. S4 clustering activates PKCalpha, which phosphorylates RhoGDI1 at Ser(96). This phosphorylation triggers release of RhoG, leading to polarized activation of Rac1. Thus, FGF2-induced Rac1 activation depends on the suppression of RhoG by a previously uncharacterized ternary S4-synectin-RhoGDI1 protein complex and activation via PKCalpha.
75. FAT1 mutations cause a glomerulotubular nephropathy. Steroid-resistant nephrotic syndrome (SRNS) causes 15% of chronic kidney disease (CKD). Here we show that recessive mutations in FAT1 cause a distinct renal disease entity in four families with a combination of SRNS, tubular ectasia, haematuria and facultative neurological involvement. Loss of FAT1 results in decreased cell adhesion and **migration** in fibroblasts and podocytes and the decreased **migration** is partially reversed by a RAC1/CDC42 activator. Podocyte-specific deletion of Fat1 in mice induces abnormal glomerular filtration barrier development, leading to podocyte foot process effacement. Knockdown of Fat1 in renal tubular cells reduces **migration**, decreases active RAC1 and CDC42, and induces defects in lumen formation. Knockdown of fat1 in zebrafish causes pronephric cysts, which is partially rescued by RAC1/CDC42 activators, confirming a role of the two small GTPases in the pathogenesis. These findings provide new insights into the pathogenesis of SRNS and tubulopathy, linking FAT1 and RAC1/CDC42 to podocyte and tubular cell function.
76. c-Cbl facilitates cytoskeletal effects in v-Abl transformed fibroblast through Rac1- and Rap1-mediated signaling. c-Cbl functions as a multifunctional adaptor and an E3 ubiquitin ligase. Several studies have shown that c-Cbl is involved in cytoskeleton-mediated events, but the molecular mechanisms linking c-Cbl to cytoskeletal rearrangements remain to be elucidated. Our previous results indicated that c-Cbl facilitates spreading and **migration** of v-Abl-transformed NIH 3T3 fibroblasts and suggested that small GTPases play important roles in the cytoskeletal effects of c-Cbl in this system. To elucidate the individual contributions of small GTPases to these effects, we assessed the roles of endogenous Rac1, RhoA and Rap1 in the c-Cbl-dependent spreading and **migration** of v-Abl-transformed fibroblasts overexpressing c-Cbl, using RNAi. Furthermore, since it has been shown that Rap1 can act as an upstream regulator of Rac1 in inducing cell spreading, we analyzed the interplay between Rap1 and Rac1 in the signaling pathways connecting c-Cbl to the cytoskeletal events. Our results indicate that Rac1 is essential for cell **migration** and spreading, whereas activation of RhoA exerts a negative effect. We have also shown that Rap1 is essential for cell spreading, although not for **migration** in our experimental system. Furthermore, we provide evidence that Rap1 is located upstream of Rac1 in one of the signaling pathways that regulate c-Cbl-facilitated cell spreading. Overall, our findings are consistent with the model describing the connection of c-Cbl to the cytoskeletal rearrangements via two pathways, one of which is mediated by PI3K and Rac1, and the other, by CrkL/C3G, Rap1 and Rac1.
77. Regulation of ephexin1, a guanine nucleotide exchange factor of Rho family GTPases, by fibroblast growth factor receptor-mediated tyrosine phosphorylation. Fibroblast growth factor (FGF) signal is implicated in not only cell proliferation, but cell **migration** and morphological changes. Several different Rho family GTPases downstream of the Ras/ERK pathway are postulated to mediate the latter functions. However, none have been recognized to be directly coupled to FGF receptors (FGFRs). We have previously reported that EphA4 and FGFRs hetero-oligomerize through their cytoplasmic domains, trans-activate each other, and transduce a signal for cell proliferation through a docking protein, FRS2alpha (Yokote, H., Fujita, K., Jing, X., Sawada, T., Liang, S., Yao, L., Yan, X., Zhang, Y., Schlessinger, J., and Sakaguchi, K. (2005) Proc. Natl. Acad. Sci. U. S. A. 102, 18866-18871). Here, we have found that ephexin1, a guanine nucleotide exchange factor for Rho family GTPases, constitutes another downstream component of the receptor complex. Ephexin1 directly binds to the kinase domain of FGFR mainly through its DH and PH domains. The binding appears to become weaker and limited to the DH domain when FGFRs become activated. FGFR-mediated phosphorylation of ephexin1 enhances the guanine nucleotide exchange activity toward RhoA without affecting the activity to Rac1 or Cdc42. The FGFR-mediated tyrosine phosphorylation includes, but is not limited to, the residue (Tyr-87) phosphorylated by Src family kinase, which is known to be activated following EphA4 activation. The Tyr-to-Asp mutations that mimic the tyrosine phosphorylation in some of the putative FGFR-mediated phosphorylation sites increase the nucleotide exchange activity for RhoA without changing the activity for Rac1 or Cdc42. From these results, we conclude that ephexin1 is located immediately downstream of the EphA4-FGFR complex and the function is altered by the FGFR-mediated tyrosine phosphorylation at multiple sites.
78. Human neutrophils coordinate chemotaxis by differential activation of Rac1 and Rac2. Rac1 and Rac2, members of the small Rho GTPase family, play essential roles in coordinating directional **migration** and superoxide production during neutrophil responses to chemoattractants. Although earlier studies in Rac1 and Rac2 knockout mice have demonstrated unique roles for each Rac isoform in chemotaxis and NADPH oxidase activation, it is still unclear how human neutrophils use Rac1 and Rac2 to achieve their immunological responses to foreign agent stimulation. In the current study, we used TAT dominant-negative Rac1-T17N and Rac2-T17N fusion proteins to acutely alter the activity of Rac1 and Rac2 individually in human neutrophils. We demonstrate distinct activation kinetics and different roles for Rac1 and Rac2 in response to low vs high concentrations of fMLP. These observations were verified using neutrophils from mice in which Rac1 or Rac2 was genetically absent. Based on these results, we propose a model to explain how human neutrophils kill invading microbes while limiting oxidative damage to the adjacent surrounding healthy tissue through the differential activation of Rac1 and Rac2 in response to different concentrations of chemoattractant.
79. Loss of protein kinase Cepsilon results in impaired cutaneous wound closure and myofibroblast function. Cutaneous wound repair requires the de novo induction of a specialized form of fibroblast, the alpha-smooth muscle actin (alpha-SMA)-expressing myofibroblast, which migrates into the wound where it adheres to and contracts extracellular matrix (ECM), resulting in wound closure. Persistence of the myofibroblast results in scarring and fibrotic disease. In this report, we show that, compared with wild-type littermates, PKCepsilon-/- mice display delayed impaired cutaneous wound closure and a reduction in myofibroblasts. Moreover, both in the presence and absence of TGFbeta, dermal fibroblasts from PKCepsilon-/- mice cultured on fibronectin show impaired abilities to form ;supermature' focal adhesions and alpha-SMA stress fibers, and reduced pro-fibrotic gene expression. Smad3 phosphorylation in response to TGFbeta1 was impaired in PKCepsilon-/- fibroblasts. PKCepsilon-/- fibroblasts show reduced FAK and Rac activation, and adhesive, contractile and migratory abilities. Overexpressing constitutively active Rac1 rescues the defective FAK phosphorylation, cell **migration**, adhesion and stress fiber formation of these PKCepsilon-/- fibroblasts, indicating that Rac1 operates downstream of PKCepsilon, yet upstream of FAK. These results suggest that loss of PKCepsilon severely impairs myofibroblast formation and function, and that targeting PKCepsilon may be beneficial in selectively modulating wound healing and fibrotic responses in vivo.
80. Vav GEFs are required for beta2 integrin-dependent functions of neutrophils. Integrin regulation of neutrophils is essential for appropriate adhesion and trans**migration** into tissues. Vav proteins are Rho family guanine nucleotide exchange factors that become tyrosine phosphorylated in response to adhesion. Using Vav1/Vav3-deficient neutrophils (Vav1/3ko), we show that Vav proteins are required for multiple beta2 integrin-dependent functions, including sustained adhesion, spreading, and complement-mediated phagocytosis. These defects are not attributable to a lack of initial beta2 activation as Vav1/3ko neutrophils undergo chemoattractant-induced arrest on intercellular adhesion molecule-1 under flow. Accordingly, in vivo, Vav1/3ko leukocytes arrest on venular endothelium yet are unable to sustain adherence. Thus, Vav proteins are specifically required for stable adhesion. beta2-induced activation of Cdc42, Rac1, and RhoA is defective in Vav1/3ko neutrophils, and phosphorylation of Pyk2, paxillin, and Akt is also significantly reduced. In contrast, Vav proteins are largely dispensable for G protein-coupled receptor-induced signaling events and chemotaxis. Thus, Vav proteins play an essential role coupling beta2 to Rho GTPases and regulating multiple integrin-induced events important in leukocyte adhesion and phagocytosis.
81. Inhibition of Rac activity alleviates lipopolysaccharide-induced acute pulmonary injury in mice. Rac small GTPases play important roles in cytoskeleton and many cell functions including cell cycle, cell growth, cell adhesion and gene transcription. Here, we investigated the roles of Rac including Rac1 and Rac2 in lipopolysaccharide (LPS)-induced pulmonary injury.After LPS was intratracheally instilled to lungs in mice, Rac, CDC42 and RhoA activation assay by pull-down and West blot, inflammatory cell infiltration assay by counting cell numbers and lung histological examination, pro-inflammatory mediator mRNA expression assay by quantitative RT-PCR, measurement of myeloperoxidase (MPO) activity, Evans Blue and albumin accumulation by spectrophotometry were performed to evaluate the roles of Rac in pulmonary injury by using its specific inhibitor, NSC23766.LPS challenge led to increases of both Rac1 and Rac2, but not CDC42 or RhoA activities in lungs, and intraperitoneal administration with NSC23766 inhibited both Rac1 and Rac2, but not CDC42 or RhoA activities. Treatment with NSC23766 at 1 or 3mg/kg not only reduced the inflammatory cells infiltration and MPO activities, but also inhibited pro-inflammatory mediators, tumor necrosis factor-α and interleukin-1β, mRNA expression. Moreover, in vitro neutrophi**l migrati**on assay and in vivo microvascular permeability assay indicated that NSC23766 not only inhibited neutrophil transwel**l migrati**on toward a chemoattractant, fMLP, but also reduced Evans Blue and albumin accumulation in LPS-challenged lungs. LPS activated both Rac1 and Rac2, but not CDC42 or RhoA activities in lungs, and specific inhibition of Rac activities by NSC23766 effectively alleviated LPS-induced injury.Rac could be a potential target for therapeutic intervention of pulmonary inflammation.
82. Cdc42 and Rac1 signaling are both required for and act synergistically in the correct formation of myelin sheaths in the CNS. The formation of myelin sheaths in the CNS is the result of a complex series of events involving oligodendrocyte progenitor cell (OPC) proliferation, directed **migration**, and the morphological changes associated with axon ensheathment and myelination. To examine the role of Rho GTPases in oligodendrocyte biology, we have used a conditional tissue-specific gene-targeting approach. Ablation of Cdc42 in cells of the oligodendrocyte lineage did not affect OPC proliferation, directed **migration**, or in vitro differentiation, but it led to the formation of a unique and stage-specific myelination phenotype. This was characterized by the extraordinary enlargement of the inner tongue of the oligodendrocyte process and concomitant formation of a myelin outfolding as a result of abnormal accumulation of cytoplasm in this region. Ablation of Rac1 also resulted in the abnormal accumulation of cytoplasm in the inner tongue of the oligodendrocyte process, and we provide genetic evidence that rac1 synergizes with cdc42 in a gene dosage-dependent way to regulate myelination.
83. Calpain-6, a microtubule-stabilizing protein, regulates Rac1 activity and cell motility through interaction with GEF-H1. Crosstalk between microtubules and actin filaments is crucial for various cellular functions, including cell **migration**, spreading and cytokinesis. The Rac1 GTPase plays a key role in such crosstalk at the leading edge of migrating cells in order to promote lamellipodial formation. However, the mechanism underlying the link between microtubules and Rac1 activation remains unclear. Here, we show that calpain-6 (CAPN6), a non-proteolytic calpain with microtubule-binding and -stabilizing activity, might participate in this crosstalk. Small interfering RNA (siRNA)-induced knockdown of Capn6 in NIH 3T3 cells resulted in Rac1 activation, which promoted cell **migration**, spreading and lamellipodial protrusion. This increase in Rac1 activity was abolished by knockdown of the Rho guanine nucleotide exchange factor GEF-H1 (officially known as Arhgef2). CAPN6 and GEF-H1 colocalized with microtubules and also interacted with each other through specific domains. Upon knockdown of Capn6, GEF-H1 was shown to translocate from microtubules to the lamellipodial region and to interact with Rac1. By contrast, RhoA activity was decreased upon knockdown of Capn6, although low levels of active RhoA or the presence of RhoA molecules appeared to be required for the Capn6-knockdown-induced Rac1 activation. We suggest that CAPN6 acts as a potential regulator of Rac1 activity, through a mechanism involving interaction with GEF-H1, to control lamellipodial formation and cell motility.
84. Impaired Rho GTPase activation abrogates cell polarization and **migration** in macrophages with defective lipolysis. Infiltration of monocytes and macrophages into the site of inflammation is critical in the progression of inflammatory diseases such as atherosclerosis. Cell **migration** is dependent on the continuous organization of the actin cytoskeleton, which is regulated by members of the small Rho GTPase family (RhoA, Cdc42, Rac) that are also important for the regulation of signal transduction pathways. We have recently reported on reduced plaque formation in an atherosclerotic mouse model transplanted with bone marrow from adipose triglyceride lipase-deficient (Atgl-/-) mice. Here we provide evidence that defective lipolysis in macrophages lacking ATGL, the major enzyme responsible for triacylglycerol hydrolysis, favors an anti-inflammatory M2-like macrophage phenotype. Our data implicate an as yet unrecognized principle that insufficient lipolysis influences macrophage polarization and actin polymerization, resulting in impaired macrophage **migration**. Sustained phosphorylation of focal adhesion kinase [due to inactivation of its phosphatase by elevated levels of reactive oxygen species (ROS)] results in defective Cdc42, Rac1 and RhoA activation and in increased and sustained activation of Rac2. Inhibition of ROS production restores the migratory capacity of Atgl-/- macrophages. Since monocyte and macrophage **migration** are a prerequisite for infiltrating the arterial wall, our results provide a molecular link between lipolysis and the development of atherosclerosis.
85. Rac regulates PtdInsP₃ signaling and the chemotactic compass through a redox-mediated feedback loop. Directional cel**l migrati**on is an essential requirement for efficient neutrophil translocation to sites of infection and requires the establishment of a polarized cell characterized by an actin-rich leading edge facing the chemoattractant gradient. The asymmetrical accumulation of phosphatidylinositol(3,4,5)-trisphosphate [PtdIns(3,4,5)P(3)] in the up-gradient leading edge is a hallmark of polarization and regulates the recruitment and localization of various effector proteins at the leading-edge plasma membrane. How shallow gradients of chemoattractants trigger and maintain a much steeper intracellular gradient of PtdIns(3,4,5)P(3) is a critical question in the study of leukocyte chemotaxis. Our data demonstrate that th**e migrati**on of neutrophils toward the chemoattractant N-formyl-L-methionyl-L-leucyl-L-phenylalanine depends on the generation of reactive oxygen species by the phagocytic NADPH oxidase (NOX2) and subsequent oxidation and inhibition of phosphatase and tensin homolog. Moreover, we show that events downstream of PtdIns(3,4,5)P(3), including phosphorylation of AKT, Rac activation, uncapping of actin filaments, and directiona**l migrati**on, can be attenuated by ROS scavengers or genetic ablation of NOX2. Using Rac mutants that are defective in their ability to activate NOX2, we show that Rac regulates a redox-mediated feedback loop that mediates directiona**l migrati**on of neutrophils.
86. Rac1 controls the formation of midline commissures and the competency of tangential **migration** in ventral telencephalic neurons. Previous studies using dominant-mutant constructs have implicated Rac1 GTPase in neuritogenesis and neuronal **migration**. However, overexpression of dominant mutants generally blocks Rho-GTPase activity; thus, it may not reveal the specific or physiological functions of Rac1. To address this issue, we have applied a conditional gene-targeting strategy, using Foxg1-Cre mice to delete Rac1 in the ventricular zone (VZ) of telencephalon and Dlx5/6-Cre-IRES (internal ribosomal entry site)-EGFP (enhanced green fluorescent protein) (Dlx5/6-CIE) in the subventricular zone (SVZ) of ventral telencephalon, respectively. Surprisingly, the deletion of Rac1 in VZ progenitors did not prevent axonal outgrowth of telencephalic neurons. However, the anterior commissure was absent, and the corpus callosal as well as hippocampal commissural axons failed to cross the midline in Rac1/Foxg1-Cre knock-out embryos. The thalamocortical and corticothalamic axons also showed defasciculation or projection defects. These results suggest that Rac1 controls axon guidance rather than neuritogenesis. In addition, although Rac1/Foxg1-Cre knock-out embryos showed delayed radial **migration** of cortical projection neurons and severe impairment of tangential **migration** by the ventral telencephalon-derived interneurons, deletion of Rac1 in the SVZ by Dlx5/6-CIE mice produced no discernible defects in tangential **migration**. These contrasting effects of Rac1 deletion on tangential **migration** suggest that Rac1 is dispensable for cellular motility per se during neuronal **migration**. Together, these results underscore the challenge of deciphering the biological functions of Rac1, and Rho-GTPases in general, during mammalian brain development. Moreover, they indicate that Rac1 has a critical role in axon guidance and in acquisition of migratory competency during differentiation of the progenitors for the ventral telencephalon-derived interneurons.
87. Rac1/PAK1 signaling promotes epithelial-mesenchymal transition of podocytes in vitro via triggering β-catenin transcriptional activity under high glucose conditions. Ras-related C3 botulinum toxin substrate 1 (Rac1), together with its major downstream effector p21-activated kinase 1 (PAK1), has been identified a central role in cellular events such as cell cytoskeletal remodeling that contributed to cell **migratio**n and epithelial-mesenchymal transition (EMT). And there are data implicating that podocytes underwent EMT under pathological conditions. However, little is known about mechanisms of podocytes undergoing EMT. To address this, we assessed the cellular changes of podocytes after high glucose stimulation in vitro, detected the effects of Rac1/PAK1 signaling on podocytes in response to the stimuli, and investigated interactions of Rac1/PAK1 axis with β-catenin and Snail under high glucose conditions. We found that in vitro high glucose treatment led to remarkable down-regulation of nephrin and P-cadherin, as well as significant up-regulation of α-SMA and FSP-1, suggesting that in the presence of high glucose, podocytes underwent EMT, during which Rac1/PAK1 signaling was activated. And these were notably ameliorated by Rac1 gene knockdown. Furthermore, β-catenin and Snail nuclear translocation were triggered by Rac1/PAK1 axis, which were both markedly reversed via Rac1 gene knockdown or pretreatment of IPA-3, a PAK1 inhibitor. These findings elaborated that Rac1/PAK1 signaling contributed to high glucose-induced podocyte EMT via promoting β-catenin and Snail transcriptional activities, which could be a potential mechanism involved in podocytes injury in response to stimuli under diabetic conditions.
88. Neural crest cell-specific deletion of Rac1 results in defective cell-matrix interactions and severe craniofacial and cardiovascular malformations. The small GTP-binding protein Rac1, a member of the Rho family of small GTPases, has been implicated in regulation of many cellular processes including adhesion, **migration** and cytokinesis. These functions have largely been attributed to its ability to reorganize cytoskeleton. While the function of Rac1 is relatively well known in vitro, its role in vivo has been poorly understood. It has previously been shown that in neural crest cells (NCCs) Rac1 is required in a stage-specific manner to acquire responsiveness to mitogenic EGF signals. Here we demonstrate that mouse embryos lacking Rac1 in neural crest cells (Rac1/Wnt1-Cre) showed abnormal craniofacial development including regional ectodermal detachment associated with mesenchymal acellularity culminating in cleft face at E12. Rac1/Wnt1-Cre mutants also displayed inappropriate remodelling of pharyngeal arch arteries and defective outflow tract septation resulting in the formation of a common arterial trunk ('persistent truncus arteriosus' or PTA). The mesenchyme around the aortic sac also developed acellular regions, and the distal aortic sac became grossly dysmorphic, forming a pair of bilateral, highly dilated arterial structures connecting to the dorsal aortas. Smooth muscle cells lacking Rac1 failed to differentiate appropriately, and subpopulations of post-migratory NCCs demonstrated aberrant cell death and attenuated proliferation. These novel data demonstrate that while Rac1 is not required for normal NCC **migration** in vivo, it plays a critical cell-autonomous role in post-migratory NCCs during craniofacial and cardiac development by regulating the integrity of the craniofacial and pharyngeal mesenchyme.
89. Interaction between Tiam1 and the Arp2/3 complex links activation of Rac to actin polymerization. The Rac-specific GEF (guanine-nucleotide exchange factor) Tiam1 (T-lymphoma invasion and metastasis 1) regulates **migration**, cell-matrix and cell-cell adhesion by modulating the actin cytoskeleton through the GTPase, Rac1. Using yeast two-hybrid screening and biochemical assays, we found that Tiam1 interacts with the p21-Arc [Arp (actin-related protein) complex] subunit of the Arp2/3 complex. Association occurred through the N-terminal pleckstrin homology domain and the adjacent coiled-coil region of Tiam1. As a result, Tiam1 co-localizes with the Arp2/3 complex at sites of actin polymerization, such as epithelial cell-cell contacts and membrane ruffles. Deletion of the p21-Arc-binding domain in Tiam1 impairs its subcellular localization and capacity to activate Rac1, suggesting that binding to the Arp2/3 complex is important for the function of Tiam1. Indeed, blocking Arp2/3 activation with a WASP (Wiskott-Aldrich syndrome protein) inhibitor leads to subcellular relocalization of Tiam1 and decreased Rac activation. Conversely, functionally active Tiam1, but not a GEF-deficient mutant, promotes activation of the Arp2/3 complex and its association with cytoskeletal components, indicating that Tiam1 and Arp2/3 are mutually dependent for their correct localization and signalling. Our data suggests a model in which the Arp2/3 complex acts as a scaffold to localize Tiam1, and thereby Rac activity, which are both required for activation of the Arp2/3 complex and further Arp2/3 recruitment. This 'self-amplifying' signalling module involving Tiam1, Rac and the Arp2/3 complex could thus drive actin polymerization at specific sites in cells that are required for dynamic morphological changes.
90. Rac mediates mouse spermatogonial stem cell homing to germline niches by regulating trans**migration** through the blood-testis barrier. The homing ability of spermatogonial stem cells (SSCs) allows them to migrate into niches after being transplantated into infertile testes. Transplanted SSCs attach to Sertoli cells and transmigrate through the blood-testis barrier (BTB), formed by inter-Sertoli tight junctions, toward niches on the basement membrane. The most critical step is the passage through the BTB, which limits the homing efficiency to <10%. Here we demonstrated the involvement of Rac1 in SSC trans**migration**. Rac1-deficient SSCs did not colonize the adult testes, but they reinitiated spermatogenesis when transplanted into pup testes without a BTB. Moreover, a dominant-negative Rac1 construct not only reduced the expression of several claudin proteins, which comprise the BTB, but also increased SSC proliferation both in vitro and in vivo. Short hairpin RNA (shRNA) -mediated suppression of claudin3, which was downregulated by Rac inhibition, reduced the SSC homing efficiency. Thus, Rac1 is a critical regulator of SSC homing and proliferation.
91. Novel insights into a retinoic-acid-induced cleft palate based on Rac1 regulation of the fibronectin arrangement. Retinoic acid (RA)-induced cleft palate results from both extrinsic obstructions by the tongue and internal factors within the palatal shelves. Our previous study showed that the spatiotemporal expression of Rac1 regulates the fibronectin (FN) arrangement through cell density alterations that play an important role in palate development. In this study, we investigate the involvement of the Rac1 regulation of the FN arrangement in RA-induced cleft palate. Our results demonstrate that RA-induced intrinsic alterations in palatal shelves, including a delayed progress of cell condensation, delay palate development, even after the removal of the tongue. Further analysis shows that RA treatment diminishes the region-distinctive expression of Rac1 within the palatal shelves, which reversely alters the fibrillar arrangement of FN. Furthermore, RA treatment disrupts the formation of lamellipodia, which are indicative structures of cell **migration** that are regulated by Rac1. These results suggest that the Rac1 regulation of the FN arrangement is involved in RA-induced cleft palate through the regulation of cell **migration**, which delays the progress of cell condensation and subsequently influences the FN arrangement, inducing a delay in palate development. Our study provides new insights into the RA-induced impairment of palatal shelf elevation based on cell **migration** dynamics.
92. Rac1 modulates mammalian lung branching morphogenesis in part through canonical Wnt signaling. Lung branching morphogenesis relies on a number of factors, including proper epithelial cell proliferation and differentiation, cell polarity, and **migration**. Rac1, a small Rho GTPase, orchestrates a number of these cellular processes, including cell proliferation and differentiation, cellular alignment, and polarization. Furthermore, Rac1 modulates both noncanonical and canonical Wnt signaling, important pathways in lung branching morphogenesis. Culture of embryonic mouse lung explants in the presence of the Rac1 inhibitor (NSC23766) resulted in a dose-dependent decrease in branching. Increased cell death and BrdU uptake were notably seen in the mesenchyme, while no direct effect on the epithelium was observed. Moreover, vasculogenesis was impaired following Rac1 inhibition as shown by decreased Vegfa expression and impaired LacZ staining in Flk1-Lacz reporter mice. Rac1 inhibition decreased Fgf10 expression in conjunction with many of its associated factors. Moreover, using the reporter lines TOPGAL and Axin2-LacZ, there was an evident decrease in canonical Wnt signaling in the explants treated with the Rac1 inhibitor. Activation of canonical Wnt pathway using WNT3a or WNT7b only partially rescued the branching inhibition. Moreover, these results were validated on human explants, where Rac1 inhibition resulted in impaired branching and decreased AXIN2 and FGFR2b expression. We therefore conclude that Rac1 regulates lung branching morphogenesis, in part through canonical Wnt signaling. However, the exact mechanisms by which Rac1 interacts with canonical Wnt in human and mouse lung requires further investigation.
93. Critical roles for Rac1 and Rac2 GTPases in B cell development and signaling. The Rac1 guanosine triphosphatase (GTPase) has been implicated in multiple cellular functions, including actin dynamics, proliferation, apoptosis, adhesion, and **migration** resulting from signaling by multiple receptors, including the B cell antigen receptor (BCR). We used conditional gene targeting to generate mice with specific Rac1 deficiency in the B cell lineage. In the absence of both Rac1 and the highly related Rac2, B cell development was almost completely blocked. Both GTPases were required to transduce BCR signals leading to proliferation, survival and up-regulation of BAFF-R, a receptor for BAFF, a key survival molecule required for B cell development and maintenance.
94. The in vivo roles of STEF/Tiam1, Rac1 and JNK in cortical neuronal **migration**. The coordinated **migration** of neurons is a pivotal step for functional architectural formation of the mammalian brain. To elucidate its molecular mechanism, gene transfer by means of in utero electroporation was applied in the developing murine brain, revealing the crucial roles of Rac1, its activators, STEF/Tiam1, and its downstream molecule, c-Jun N-terminal kinase (JNK), in the cerebral cortex. Functional repression of these molecules resulted in inhibition of radial **migration** of neurons without affecting their proper differentiation. Interestingly, distinct morphological phenotypes were observed; suppression of Rac1 activity caused loss of the leading process, whereas repression of JNK activity did not, suggesting the complexity of the signaling cascade. In cultured neurons from the intermediate zone, activated JNK was detected along microtubules in the processes. Application of a JNK inhibitor caused irregular morphology and increased stable microtubules in processes, and decreased phosphorylation of microtubule associated protein 1B, raising a possibility of the involvement of JNK in controlling tubulin dynamics in migrating neurons. Our data thus provide important clues for understanding the intracellullar signaling machinery for cortical neuronal **migration**.
95. CD81 is essential for the formation of membrane protrusions and regulates Rac1-activation in adhesion-dependent immune cell **migration**. CD81 (TAPA-1) is a member of the widely expressed and evolutionary conserved tetraspanin family that forms complexes with a variety of other cell surface receptors and facilitates hepatitis C virus entry. Here, we show that CD81 is specifically required for the formation of lamellipodia in migrating dendritic cells (DCs). Mouse CD81(-/-) DCs, or murine and human CD81 RNA interference knockdown DCs lacked the ability to form actin protrusions, thereby impairing their motility dramatically. Moreover, we observed a selective loss of Rac1 activity in the absence of CD81, the latter of which is exclusively required for integrin-dependent **migration** on 2-dimensional substrates. Neither integrin affinity for substrate nor the size of basal integrin clusters was affected by CD81 deficiency in adherent DCs. However, the use of total internal reflection fluorescence microscopy revealed an accumulation of integrin clusters above the basal layer in CD81 knockdown cells. Furthermore, β1- or β2-integrins, actin, and Rac are strongly colocalized at the leading edge of DCs, but the very fronts of these cells protrude CD81-containing membranes that project outward from the actin-integrin area. Taken together, these data suggest a thus far unappreciated role for CD81 in the mobilization of preformed integrin clusters into the leading edge of migratory DCs on 2-dimensional surfaces.
96. Endogenous RhoG is dispensable for integrin-mediated cell spreading but contributes to Rac-independent **migration**. Rac activation by integrins is essential for cell spreading, **migration**, growth and survival. Based mainly on overexpression of dominant-negative mutants, RhoG has been proposed to mediate integrin-dependent Rac activation upstream of ELMO and Dock180. RhoG-knockout mice, however, display no significant developmental or functional abnormalities. To clarify the role of RhoG in integrin-mediated signaling, we developed a RhoG-specific antibody, which, together with shRNA-mediated knockdown, allowed analysis of the endogenous protein. Despite dramatic effects of dominant-negative constructs, nearly complete RhoG depletion did not substantially inhibit cell adhesion, spreading, **migration** or Rac activation. Additionally, RhoG was not detectably activated by adhesion to fibronectin. Using Rac1(-/-) cells, we found that constitutively active RhoG induced membrane ruffling via both Rac-dependent and -independent pathways. Additionally, endogenous RhoG was important for Rac-independent cell **migration**. However, RhoG did not significantly contribute to cell spreading even in these cells. These data therefore clarify the role of RhoG in integrin signaling and cell motility.
97. Rac1 mutations produce aberrant epithelial differentiation in the developing and adult mouse small intestine. The mouse small intestinal epithelium undergoes continuous renewal throughout life. Previous studies suggest that differentiation of this epithelium is regulated by instructions that are received as cells migrate along crypt-villus units. The nature of the instructions and their intracellular processing remain largely undefined. In this report, we have used genetic mosaic analysis to examine the role of Rac1 GTPase-mediated signaling in controlling differentiation. A constitutively active mutation (Rac1Leu61) or a dominant negative mutation (Rac1Asn17) was expressed in the 129/Sv embryonic stem cell-derived component of the small intestine of C57Bl/6-ROSA26<->129/Sv mice. Rac1Leu61 induces precocious differentiation of members of the Paneth cell and enterocytic lineages in the proliferative compartment of the fetal gut, without suppressing cell division. Forced expression of the dominant negative mutation inhibits epithelial differentiation, without affecting cell division, and slows enterocytic **migration** along crypt-villus units. The effects produced by Rac1Leu61 or Rac1Asn17 in the 129/Sv epithelium do not spread to adjacent normal C57Bl/6 epithelial cells. These results provide in vivo evidence that Rac1 is involved in the import and intracellular processing of signals that control differentiation of a mammalian epithelium.
98. Loss of Either Rac1 or Rac3 GTPase Differentially Affects the Behavior of Mutant Mice and the Development of Functional GABAergic Networks. Rac GTPases regulate the development of cortical/hippocampal GABAergic interneurons by affecting the early development and **migration** of GABAergic precursors. We have addressed the function of Rac1 and Rac3 proteins during the late maturation of hippocampal interneurons. We observed specific phenotypic differences between conditional Rac1 and full Rac3 knockout mice. Rac1 deletion caused greater generalized hyperactivity and cognitive impairment compared with Rac3 deletion. This phenotype matched with a more evident functional impairment of the inhibitory circuits in Rac1 mutants, showing higher excitability and reduced spontaneous inhibitory currents in the CA hippocampal pyramidal neurons. Morphological analysis confirmed a differential modification of the inhibitory circuits: deletion of either Rac caused a similar reduction of parvalbumin-positive inhibitory terminals in the pyramidal layer. Intriguingly, cannabinoid receptor-1-positive terminals were strongly increased only in the CA1 of Rac1-depleted mice. This increase may underlie the stronger electrophysiological defects in this mutant. Accordingly, incubation with an antagonist for cannabinoid receptors partially rescued the reduction of spontaneous inhibitory currents in the pyramidal cells of Rac1 mutants. Our results show that Rac1 and Rac3 have independent roles in the formation of GABAergic circuits, as highlighted by the differential effects of their deletion on the late maturation of specific populations of interneurons.
99. Rac1 regulates neuronal polarization through the WAVE complex. Neuronal **migration** and axon growth, key events during neuronal development, require distinct changes in the cytoskeleton. Although many molecular regulators of polarity have been identified and characterized, relatively little is known about their physiological role in this process. To study the physiological function of Rac1 in neuronal development, we have generated a conditional knock-out mouse, in which Rac1 is ablated in the whole brain. Rac1-deficient cerebellar granule neurons, which do not express other Rac isoforms, showed impaired neuronal **migration** and axon formation both in vivo and in vitro. In addition, Rac1 ablation disrupts lamellipodia formation in growth cones. The analysis of Rac1 effectors revealed the absence of the Wiskott-Aldrich syndrome protein (WASP) family verprolin-homologous protein (WAVE) complex from the plasma membrane of knock-out growth cones. Loss of WAVE function inhibited axon growth, whereas overexpression of a membrane-tethered WAVE mutant partially rescued axon growth in Rac1-knock-out neurons. In addition, pharmacological inhibition of the WAVE complex effector Arp2/3 also reduced axon growth. We propose that Rac1 recruits the WAVE complex to the plasma membrane to enable actin remodeling necessary for axon growth.
100. Cross-talk between RhoH and Rac1 in regulation of actin cytoskeleton and chemotaxis of hematopoietic progenitor cells. RhoH, a hematopoietic-specific and constitutively active member of the Rho guanosine triphosphatase (GTPase) family, has been implicated in the negative regulation of Rac GTPase-mediated signaling in hematopoietic cells. However, the molecular mechanisms underlying the functional interaction between RhoH and Rac in primary cells are poorly understood. Here we show that deletion of Rhoh in hematopoietic progenitor cells (HPCs) leads to increased stromal-derived factor-1alpha (SDF-1alpha)-induced chemotaxis and chemokinesis (random **migration**). The abnormally enhanced **migration** of Rhoh(-/-) HPCs is associated with increased Rac1 activity and translocation of Rac1 protein to the cell membrane, where it colocalizes with cortical filamentous-actin (F-actin) and lipid rafts. Expression of the dominant-negative mutant Rac1N17 inhibits the cortical F-actin assembly and chemotaxis of wild-type and Rhoh(-/-) HPCs to the same extent. Conversely, overexpression of RhoH in HPCs blocks the membrane translocation of Rac1-enhanced green fluorescence protein (EGFP) and active Rac1V12-EGFP proteins and impairs cortical F-actin assembly and chemotaxis in response to SDF-1alpha stimulation. Furthermore, we demonstrate that the subcellular localization and inhibitory function of RhoH in HPCs are regulated by C-terminal motifs, including a CKIF prenylation site. Together, we have identified an antagonistic role of RhoH in regulation of cortical F-actin assembly and chemotaxis via suppressing Rac1 membrane targeting and activation in primary HPCs.
101. Rac1 drives melanoblast organization during mouse development by orchestrating pseudopod- driven motility and cell-cycle progression. During embryogenesis, melanoblasts proliferate and migrate ventrally through the developing dermis and epidermis as single cells. Targeted deletion of Rac1 in melanoblasts during embryogenesis causes defects in **migratio**n, cell-cycle progression, and cytokinesis. Rac1 null cells migrate markedly less efficiently, but surprisingly, global steering, crossing the dermal/epidermal junction, and homing to hair follicles occur normally. Melanoblasts navigate in the epidermis using two classes of protrusion: short stubs and long pseudopods. Short stubs are distinct from blebs and are driven by actin assembly but are independent of Rac1, Arp2/3 complex, myosin, or microtubules. Rac1 positively regulates the frequency of initiation of long pseudopods, which promot**e migrati**on speed and directional plasticity. Scar/WAVE and Arp2/3 complex drive actin assembly for long pseudopod extension, which also depends on microtubule dynamics. Myosin contractility balances the extension of long pseudopods by effecting retraction and allowing force generation for movement through the complex 3D epidermal environment.
102. A palmitoylation switch mechanism regulates Rac1 function and membrane organization. The small GTPase Rac1 plays important roles in many processes, including cytoskeletal reorganization, cell **migration**, cell-cycle progression and gene expression. The initiation of Rac1 signalling requires at least two mechanisms: GTP loading via the guanosine triphosphate (GTP)/guanosine diphosphate (GDP) cycle, and targeting to cholesterol-rich liquid-ordered plasma membrane microdomains. Little is known about the molecular mechanisms governing this specific compartmentalization. We show that Rac1 can incorporate palmitate at cysteine 178 and that this post-translational modification targets Rac1 for stabilization at actin cytoskeleton-linked ordered membrane regions. Palmitoylation of Rac1 requires its prior prenylation and the intact C-terminal polybasic region and is regulated by the triproline-rich motif. Non-palmitoylated Rac1 shows decreased GTP loading and lower association with detergent-resistant (liquid-ordered) membranes (DRMs). Cells expressing no Rac1 or a palmitoylation-deficient mutant have an increased content of disordered membrane domains, and markers of ordered membranes isolated from Rac1-deficient cells do not correctly partition in DRMs. Importantly, cells lacking Rac1 palmitoylation show spreading and **migration** defects. These data identify palmitoylation as a mechanism for Rac1 function in actin cytoskeleton remodelling by controlling its membrane partitioning, which in turn regulates membrane organization.
103. The rho GTPase Rac1 is required for proliferation and survival of progenitors in the developing forebrain. Progenitor cells in the ventricular zone (VZ) and subventricular zone (SVZ) of the developing forebrain give rise to neurons and glial cells, and are characterized by distinct morphologies and proliferative behaviors. The mechanisms that distinguish VZ and SVZ progenitors are not well understood, although the homeodomain transcription factor Cux2 and Cyclin D2, a core component of the cell cycle machinery, are specifically involved in controlling SVZ cell proliferation. Rho GTPases have been implicated in regulating the proliferation, differentiation, and **migration** of many cell types, and one family member, Cdc42, affects the polarity and proliferation of radial glial cells in the VZ. Here, we show that another family member, Rac1, is required for the normal proliferation and differentiation of SVZ progenitors and for survival of both VZ and SVZ progenitors. A forebrain-specific loss of Rac1 leads to an SVZ-specific reduction in proliferation, a concomitant increase in cell cycle exit, and premature differentiation. In Rac1 mutants, the SVZ and VZ can no longer be delineated, but rather fuse to become a single compact zone of intermingled cells. Cyclin D2 expression, which is normally expressed by both VZ and SVZ progenitors, is reduced in Rac1 mutants, suggesting that the mutant cells differentiate precociously. Rac1-deficient mice can still generate SVZ-derived upper layer neurons, indicating that Rac1 is not required for the acquisition of upper layer neuronal fates, but instead is needed for the normal regulation of proliferation by progenitor cells in the SVZ.
104. A novel Rac-dependent checkpoint in B cell development controls entry into the splenic white pulp and cell survival. Rac1 and Rac2 GTPases transduce signals from multiple receptors leading to cell **migration**, adhesion, proliferation, and survival. In the absence of Rac1 and Rac2, B cell development is arrested at an IgD- transitional B cell stage that we term transitional type 0 (T0). We show that T0 cells cannot enter the white pulp of the spleen until they mature into the T1 and T2 stages, and that this entry into the white pulp requires integrin and chemokine receptor signaling and is required for cell survival. In the absence of Rac1 and Rac2, transitional B cells are unable to migrate in response to chemokines and cannot enter the splenic white pulp. We propose that loss of Rac1 and Rac2 causes arrest at the T0 stage at least in part because transitional B cells need to migrate into the white pulp to receive survival signals. Finally, we show that in the absence of Syk, a kinase that transduces B cell antigen receptor signals required for positive selection, development is arrested at the same T0 stage, with transitional B cells excluded from the white pulp. Thus, these studies identify a novel developmental checkpoint that coincides with B cell positive selection.
105. HER2/ErbB2-induced breast cancer cell **migration** and invasion require p120 catenin activation of Rac1 and Cdc42. Breast cancers that overexpress the receptor tyrosine kinase ErbB2/HER2/Neu result in poor patient outcome because of extensive metastatic progression. Herein, we delineate a molecular mechanism that may govern this malignant phenotype. ErbB2 induction of **migration** requires activation of the small GTPases Rac1 and Cdc42. The ability of ErbB2 to activate these small GTPases necessitated expression of p120 catenin, which is itself up-regulated by signaling through ErbB2 and the tyrosine kinase Src. Silencing p120 in ErbB2-dependent breast cancer cell lines dramatically inhibited **migration** and invasion as well as activation of Rac1 and Cdc42. In contrast, overexpression of constitutively active mutants of these GTPases reversed the effects of p120 silencing. Lastly, ectopic expression of p120 promoted **migration** and invasion and potentiated metastatic progression of a weakly metastatic, ErbB2-dependent breast cancer cell line. These results suggest that p120 acts as an obligate intermediate between ErbB2 and Rac1/Cdc42 to modulate the metastatic potential of breast cancer cells.
106. Membrane targeting of WAVE2 is not sufficient for WAVE2-dependent actin polymerization: a role for IRSp53 in mediating the interaction between Rac and WAVE2. Wiskott-Aldrich syndrome protein (WASP)-family verprolin homologous (WAVE) proteins play a major role in Rac-induced actin dynamics, but Rac does not bind directly to WAVE proteins. It has been proposed that either the insulin receptor substrate protein 53 (IRSp53) or a complex of proteins containing Abelson interactor protein 1 (Abi1) mediates the interaction of WAVE2 and Rac. Depletion of endogenous IRSp53 by RNA-mediated interference (RNAi) in a RAW/LR5 macrophage cell line resulted in a significant reduction of Rac1Q61L-induced surface ruffles and colony-stimulating factor 1 (CSF-1)-induced actin polymerization, protrusion and cell **migration**. However, IRSp53 was not essential for Fcgamma-R-mediated phagocytosis, formation of podosomes or for formation of Cdc42V12-induced filopodia. IRSp53 was found to be present in an immunoprecipitable complex with WAVE2 and Abi1 in a Rac1-activation-dependent manner in RAW/LR5 cells in vivo. Importantly, reduction of endogenous IRSp53 or expression of IRSp53 lacking the WAVE2-binding site (IRSp53DeltaSH3) resulted in a significant reduction in the association of Rac1 with WAVE2 and Abi1, indicating that the association of Rac1 with WAVE2 and Abi1 is IRSp53 dependent. While it has been proposed that WAVE2 activity is regulated by membrane recruitment, membrane targeting of WAVE2 in RAW/LR5 and Cos-7 cells did not induce actin polymerization or protrusion, suggesting that membrane recruitment was insufficient for regulation of WAVE2. Combined, these data suggest that IRSp53 links Rac1 to WAVE2 in vivo and its function is crucial for production of CSF-1-induced F-actin-rich protrusions and cell **migration** in macrophages. This study indicates that Rac1, along with IRSp53 and Abi1, is involved in a more complex and tight regulation of WAVE2 than one operating solely through membrane localization.
107. Rac1 is required for the formation of three germ layers during gastrulation. The Rac1, a member of the Rho family proteins, regulates actin organization of cytoskeleton and cell adhesion. We used genetic analysis to elucidate the role of Rac1 in mouse embryonic development. The rac1 deficient embryos showed numerous cell deaths in the space between the embryonic ectoderm and endoderm at the primitive streak stage. Investigation of the primary epiblast culture isolated from rac1 deficient embryos indicated that Rac1 is involved in lamellipodia formation, cell adhesion and cell **migration** in vivo. These results suggest that Rac1-mediated cell adhesion is essential for the formation of three germ layers during gastrulation.
108. The atypical Guanine-nucleotide exchange factor, dock7, negatively regulates schwann cell differentiation and myelination. In development of the peripheral nervous system, Schwann cells proliferate, migrate, and ultimately differentiate to form myelin sheath. In all of the myelination stages, Schwann cells continuously undergo morphological changes; however, little is known about their underlying molecular mechanisms. We previously cloned the dock7 gene encoding the atypical Rho family guanine-nucleotide exchange factor (GEF) and reported the positive role of Dock7, the target Rho GTPases Rac/Cdc42, and the downstream c-Jun N-terminal kinase in Schwann cell **migration** (Yamauchi et al., 2008). We investigated the role of Dock7 in Schwann cell differentiation and myelination. Knockdown of Dock7 by the specific small interfering (si)RNA in primary Schwann cells promotes dibutyryl cAMP-induced morphological differentiation, indicating the negative role of Dock7 in Schwann cell differentiation. It also results in a shorter duration of activation of Rac/Cdc42 and JNK, which is the negative regulator of myelination, and the earlier activation of Rho and Rho-kinase, which is the positive regulator of myelination. To obtain the in vivo evidence, we generated Dock7 short hairpin (sh)RNA transgenic mice. They exhibited a decreased expression of Dock7 in the sciatic nerves and enhanced myelin thickness, consistent with in vitro observation. The effects of the in vivo knockdown on the signals to Rho GTPases are similar to those of the in vitro knockdown. Collectively, the signaling through Dock7 negatively regulates Schwann cell differentiation and the onset of myelination, demonstrating the unexpected role of Dock7 in the interplay between Schwann cell **migration** and myelination.
109. Fetal endocannabinoids orchestrate the organization of pancreatic islet microarchitecture. Endocannabinoids are implicated in the control of glucose utilization and energy homeostasis by orchestrating pancreatic hormone release. Moreover, in some cell niches, endocannabinoids regulate cell proliferation, fate determination, and **migration**. Nevertheless, endocannabinoid contributions to the development of the endocrine pancreas remain unknown. Here, we show that α cells produce the endocannabinoid 2-arachidonoylglycerol (2-AG) in mouse fetuses and human pancreatic islets, which primes the recruitment of β cells by CB1 cannabinoid receptor (CB1R) engagement. Using subtractive pharmacology, we extend these findings to anandamide, a promiscuous endocannabinoid/endovanilloid ligand, which impacts both the determination of islet size by cell proliferation and α/β cell sorting by differential activation of transient receptor potential cation channel subfamily V member 1 (TRPV1) and CB1Rs. Accordingly, genetic disruption of TRPV1 channels increases islet size whereas CB1R knockout augments cellular heterogeneity and favors insulin over glucagon release. Dietary enrichment in ω-3 fatty acids during pregnancy and lactation in mice, which permanently reduces endocannabinoid levels in the offspring, phenocopies CB1R(-/-) islet microstructure and improves coordinated hormone secretion. Overall, our data mechanistically link endocannabinoids to cell proliferation and sorting during pancreatic islet formation, as well as to life-long programming of hormonal determinants of glucose homeostasis.
110. Aquaporin 9 phosphorylation mediates membrane localization and neutrophil polarization. Neutrophils are of prime importance in the host innate defense against invading microorganisms by using two primary mechanisms-locomotion toward and phagocytosis of the prey. Recent research points to pivotal roles for water channels known as AQPs in cell motility. Here, we focused on the role of AQP9 in chemoattractant-induced polarization and **migration** of primary mouse neutrophils and neutrophil-like HL60 cells. We found that AQP9 is phosphorylated downstream of fMLFR or PMA stimulation in primary human neutrophils. The dynamics of AQP9 were assessed using GFP-tagged AQP9 constructs and other fluorescent markers through various live-cell imaging techniques. Expression of WT or the phosphomimic S11D AQP9 changed cell volume regulation as a response to hyperosmotic changes and enhanced neutrophil polarization and chemotaxis. WT AQP9 and S11D AQP9 displayed a very dynamic distribution at the cell membrane, whereas the phosphorylation-deficient S11A AQP9 failed to localize to the plasma membrane. Furthermore, we found that Rac1 regulated the translocation of AQP9 to the plasma membrane. Our results show that AQP9 plays an active role in neutrophil volume regulation and **migration**. The display of AQP9 at the plasma membrane depends on AQP9 phosphorylation, which appeared to be regulated through a Rac1-dependent pathway.
111. Rac GTPase isoforms Rac1 and Rac2 play a redundant and crucial role in T-cell development. Rac GTPases have been implicated in the regulation of diverse functions in various blood cell lineages, but their role in T-cell development is not well understood. We have carried out conditional gene targeting to achieve hematopoietic stem cell (HSC)- or T-cell lineage-specific deletion of Rac1 or Rac1/Rac2 by crossbreeding the Mx-Cre or Lck-Cre transgenic mice with Rac1(loxp/loxp) or Rac1(loxp/loxp);Rac2(-/-) mice. We found that (1) HSC deletion of both Rac1 and Rac2 inhibited production of common lymphoid progenitors (CLPs) in bone marrow and suppressed T-cell development in thymus and peripheral organs, whereas deletion of Rac1 moderately affected CLP production and T-cell development. (2) T cell-specific deletion of Rac1 did not affect T-cell development, whereas deletion of both Rac1 and Rac2 reduced immature CD4(+)CD8(+) and mature CD4(+) populations in thymus as well as CD4(+) and CD8(+) populations in spleen. (3) The developmental defects of Rac1/Rac2 knockout T cells were associated with proliferation, survival, adhesion, and **migration** defects. (4) Rac1/Rac2 deletion suppressed T-cell receptor-mediated proliferation, IL-2 production, and Akt activation in thymocytes. Thus, Rac1 and Rac2 have unique roles in CLP production and share a redundant but essential role in later stages of T-cell development by regulating survival and proliferation signals.
112. Activated Rac1 requires gp130 for Stat3 activation, cell proliferation and **migration**. Rac1 (Rac) is a member of the Rho family of small GTPases which controls cell **migration** by regulating the organization of actin filaments. Previous results suggested that mutationally activated forms of the Rho GTPases can activate the Signal Transducer and Activator of Transcription-3 (Stat3), but the exact mechanism is a matter of controversy. We recently demonstrated that Stat3 activity of cultured cells increases dramatically following E-cadherin engagement. To better understand this pathway, we now compared Stat3 activity levels in mouse HC11 cells before and after expression of the mutationally activated Rac1 (Rac(V12)), at different cell densities. The results revealed for the first time a dramatic increase in protein levels and activity of both the endogenous Rac and Rac(V12) with cell density, which was due to inhibition of proteasomal degradation. In addition, Rac(V12)-expressing cells had higher Stat3, tyrosine-705 phosphorylation and activity levels at all densities, indicating that Rac(V12) is able to activate Stat3. Further examination of the mechanism of Stat3 activation showed that Rac(V12) expression caused a surge in mRNA of Interleukin-6 (IL6) family cytokines, known potent Stat3 activators. Knockdown of gp130, the common subunit of this family reduced Stat3 activity, indicating that these cytokines may be responsible for the Stat3 activation by Rac(V12). The upregulation of IL6 family cytokines was required for cell **migration** and proliferation induced by Rac(V12), as shown by gp130 knockdown experiments, thus demonstrating that the gp130/Stat3 axis represents an essential effector of activated Rac for the regulation of key cellular functions.
113. Tyr724 phosphorylation of ELMO1 by Src is involved in cell spreading and **migration** via Rac1 activation. The complex of Dock180/ELMO1 that functions as a bipartite guanine nucleotide exchange factor for Rac is essential for diverse physiological and pathological processes of cells such as cell **migration**, phagocytosis, and invasion of cancer cells. Among the Src-family tyrosine kinases (SFKs), it has been reported that Hck directly phosphorylates ELMO1, regulating phagocytosis by promoting activation of Rac1; however, the involvement of other SFKs in ELMO1 phosphorylation has remained unknown. Here, we identified novel tyrosine (Y) residues of ELMO1 phosphorylated by SFKs, and examined the effects on Rac1 activity, cell adhesion, spreading, and cell motility on extracellular matrix (ECM).In this study, we unveiled that Src and Fyn can induce tyrosine phosphorylation of ELMO1 in in vivo and in vitro phosphorylation assays. Mutational analyses identified both Y720 and Y724 residues of ELMO1 as Src-mediated phosphorylation sites, preferentially on Y724. Single substitution of Y724 to Phe abrogated Rac1 activation triggered by Src. To elucidate the biological function of pY724, we established NIH3T3 cells stably expressing wild-type ELMO1 or its Y724F mutant together with Dock180. Among them, Y724-deficient cells exhibited a depletion of Rac1 activity with diminished phosphorylation of ELMO1 even upon the ECM-stimulation. It is noteworthy that NIH3T3 cells with ELMO1 Y724F were strikingly defective to promote cell spreading on fibronectin-coated dish, concomitantly exhibiting immature assemblies of actin stress fibers and focal adhesions. Eventually, ELMO1 Y724F significantly impaired cell **migration**.These results define that Src-mediated Y724 phosphorylation in ELMO1 plays a critical role for cell spreading via activation of Rac1, leading to promotion of cell **migration**. As the overexpression and/or hyperactivation of Src have been shown in a wide variety of human cancers, Src-mediated phosphorylation of Y724 in ELMO1 may regulate cancer cell adhesion to the ECM, invasion into surrounding tissues, and subsequent distant metastasis.
114. Rac is involved in the interkinetic nuclear **migration** of cortical progenitor cells. The small GTPase Rac regulates neuronal behavior, but whether it also functions in neural progenitor cells has not yet been explored. Here we report that Rac contributes to the regulation of nuclear **migration** in neocortical progenitor cells. Rac1 is expressed by progenitor cells in a unique spatiotemporal pattern. Cross-sectional immunohistochemical examination revealed intense Rac1 immunoreactivity at the ventricular surface. Similar staining patterns were obtained by immunofluorescence for a Rac-activator, Tiam1, and by reactions to detect the GTP-bound (active) form of Rac. En face inspection of the ventricular surface revealed that apical Rac1 localization was most frequent in M-phase cells, and the endfeet of cells in other cell cycle phases also showed apical Rac1 distribution at lower frequencies. To ask whether progenitor cell behavior prior to and during M phase is Rac-dependent, we monitored individual DiI-labeled progenitor cells live in the presence of a Rac inhibitor, NSC23766. We observed significantly retarded adventricular nuclear **migration**, as well as cytokinesis failures. Similar inhibitory effects were obtained by forced expression of a dominant-negative Rac1. These results suggest that Rac may play a role in interkinetic nuclear **migration** in the developing mouse brain.
115. Rac activity is polarized and regulates meiotic spindle stability and anchoring in mammalian oocytes. Mammalian meiotic divisions are asymmetrical and generate a large oocyte and two small polar bodies. This asymmetry results from the anchoring of the meiotic spindle to the oocyte cortex and subsequent cortical reorganization, but the mechanisms involved are poorly understood. We investigated the role of Rac in oocyte meiosis by using a fluorescent reporter for Rac-GTP. We find that Rac-GTP is polarized in the cortex overlying the meiotic spindle. Polarization of Rac activation occurs during spindle **migration** and is promoted by the proximity of chromatin to the cortex. Inhibition of Rac during oocyte maturation caused a permanent block at prometaphase I and spindle elongation. In metaphase II-arrested oocytes, Rac inhibition caused the spindle to detach from the cortex and prevented polar body emission after activation. These results demonstrate that Rac-GTP plays a major role in oocyte meiosis, via the regulation of spindle stability and anchoring to the cortex.
116. The interaction between caveolin-1 and Rho-GTPases promotes metastasis by controlling the expression of alpha5-integrin and the activation of Src, Ras and Erk. Proteins containing a caveolin-binding domain (CBD), such as the Rho-GTPases, can interact with caveolin-1 (Cav1) through its caveolin scaffold domain. Rho-GTPases are important regulators of p130(Cas), which is crucial for both normal cell **migration** and Src kinase-mediated metastasis of cancer cells. However, although Rho-GTPases (particularly RhoC) and Cav1 have been linked to cancer progression and metastasis, the underlying molecular mechanisms are largely unknown. To investigate the function of Cav1-Rho-GTPase interaction in metastasis, we disrupted Cav1-Rho-GTPase binding in melanoma and mammary epithelial tumor cells by overexpressing CBD, and examined the loss-of-function of RhoC in metastatic cancer cells. Cancer cells overexpressing CBD or lacking RhoC had reduced p130(Cas) phosphorylation and Rac1 activation, resulting in an inhibition of **migration** and invasion in vitro. The activity of Src and the activation of its downstream targets FAK, Pyk2, Ras and extracellular signal-regulated kinase (Erk)1/2 were also impaired. A reduction in α5-integrin expression, which is required for binding to fibronectin and thus cell **migratio**n and survival, was observed in CBD-expressing cells and cells lacking RhoC. As a result of these defects, CBD-expressing melanoma cells had a reduced ability to metastasize in recipient mice, and impaired extravasation and survival in secondary sites in chicken embryos. Our data indicate that interaction between Cav1 and Rho-GTPases (most likely RhoC but not RhoA) promotes metastasis by stimulating α5-integrin expression and regulating the Src-dependent activation of p130(Cas)/Rac1, FAK/Pyk2 and Ras/Erk1/2 signaling cascades.
117. Rac1 mediates morphogenetic responses to intercellular signals in the gastrulating mouse embryo. The establishment of the mammalian body plan depends on signal-regulated cell **migration** and adhesion, processes that are controlled by the Rho family of GTPases. Here we use a conditional allele of Rac1, the only Rac gene expressed early in development, to define its roles in the gastrulating mouse embryo. Embryos that lack Rac1 in the epiblast (Rac1Δepi) initiate development normally: the signaling pathways required for gastrulation are active, definitive endoderm and all classes of mesoderm are specified, and the neural plate is formed. After the initiation of gastrulation, Rac1Δepi embryos have an enlarged primitive streak, make only a small amount of paraxial mesoderm, and the lateral anlage of the heart do not fuse at the midline. Because these phenotypes are also seen in Nap1 mutants, we conclude that Rac1 acts upstream of the Nap1/WAVE complex to promot**e migrati**on of the nascent mesoderm. In addition t**o migrati**on phenotypes, Rac1Δepi cells fail to adhere to matrix, which leads to extensive cell death. Cell death is largely rescued in Rac1Δepi mutants that are heterozygous for a null mutation in Pten, providing evidence that Rac1 is required to link signals from the basement membrane to activation of the PI3K-Akt pathway in vivo. Surprisingly, the frequency of apoptosis is greater in the anterior half of the embryo, suggesting that cell survival can be promoted either by matrix adhesion or by signals from the posterior primitive streak. Rac1 also has essential roles in morphogenesis of the posterior notochordal plate (the node) and the midline.
118. Rac1-deficient macrophages exhibit defects in cell spreading and membrane ruffling but not **migration**. Rac GTPases are activated by extracellular stimuli and contribute to cellular responses including cytoskeletal changes and cell **migration**. Dominant-negative Rac1 has been used to implicate Rac GTPases in these responses, but which of the three mammalian Rac isoforms it inhibits is not known. We show that mouse bone marrow-derived macrophages express Rac1, low levels of Rac2 but not Rac3. As Rac1-null mice die early in development, we have used mice with a loxP-flanked allele of Rac1 and the type I interferon-inducible Mx1-Cre transgene to address for the first time the specific role of Rac1 in cell motility. Bone marrow-derived macrophages isolated from mice treated with polyIC to induce interferon lack detectable Rac1, and there is no compensatory increase in Rac2 or Cdc42 expression. Rac1-deficient macrophages have an altered morphology: they are significantly more elongated than control cells and have a reduced adhesive area. Re-expression of Rac1 reverts the morphology to that of control cells. Loss of Rac1 reduces but does not completely prevent membrane ruffling in response to CSF-1. However, Rac1-deficient macrophages show normal **migration** and chemotaxis. Thus in macrophages Rac1 is primarily responsible for regulating cell morphology, contributes to membrane ruffling, but is not required for **migration**.
119. Endothelial-Rac1 is not required for tumor angiogenesis unless alphavbeta3-integrin is absent. Endothelial cell **migration** is an essential aspect of tumor angiogenesis. Rac1 activity is needed for cell **migration** in vitro implying a requirement for this molecule in angiogenesis in vivo. However, a precise role for Rac1 in tumor angiogenesis has never been addressed. Here we show that depletion of endothelial Rac1 expression in adult mice, unexpectedly, has no effect on tumor growth or tumor angiogenesis. In addition, repression of Rac1 expression does not inhibit VEGF-mediated angiogenesis in vivo or ex vivo, nor does it affect chemotactic migratory responses to VEGF in 3-dimensions. In contrast, the requirement for Rac1 in tumor growth and angiogenesis becomes important when endothelial beta3-integrin levels are reduced or absent: the enhanced tumor growth, tumor angiogenesis and VEGF-mediated responses in beta3-null mice are all Rac1-dependent. These data indicate that in the presence of alphavbeta3-integrin Rac1 is not required for tumor angiogenesis.
120. Plexin-B2 negatively regulates macrophage motility, Rac, and Cdc42 activation. Plexins are cell surface receptors widely studied in the nervous system, where they mediate **migration** and morphogenesis though the Rho family of small GTPases. More recently, plexins have been implicated in immune processes including cell-cell interaction, immune activation, **migration**, and cytokine production. Plexin-B2 facilitates ligand induced cell guidance and **migration** in the nervous system, and induces cytoskeletal changes in overexpression assays through RhoGTPase. The function of Plexin-B2 in the immune system is unknown. This report shows that Plexin-B2 is highly expressed on cells of the innate immune system in the mouse, including macrophages, conventional dendritic cells, and plasmacytoid dendritic cells. However, Plexin-B2 does not appear to regulate the production of proinflammatory cytokines, phagocytosis of a variety of targets, or directional **migration** towards chemoattractants or extracellular matrix in mouse macrophages. Instead, Plxnb2(-/-) macrophages have greater cellular motility than wild type in the unstimulated state that is accompanied by more active, GTP-bound Rac and Cdc42. Additionally, Plxnb2(-/-) macrophages demonstrate faster in vitro wound closure activity. Studies have shown that a closely related family member, Plexin-B1, binds to active Rac and sequesters it from downstream signaling. The interaction of Plexin-B2 with Rac has only been previously confirmed in yeast and bacterial overexpression assays. The data presented here show that Plexin-B2 functions in mouse macrophages as a negative regulator of the GTPases Rac and Cdc42 and as a negative regulator of basal cell motility and wound healing.
121. p19Arf-p53 tumor suppressor pathway regulates cell motility by suppression of phosphoinositide 3-kinase and Rac1 GTPase activities. The p19(Arf)-p53 tumor suppressor pathway plays a critical role in cell-cycle checkpoint control and apoptosis, whereas Rho family small GTPases are key regulators of actin structure and cell motility. By using primary mouse embryonic fibroblasts that lack Arf, p53, or both, we studied the involvement of the p19(Arf)-p53 pathway in the regulation of cell motility and its relationship with Rho GTPases. Deletion of Arf and/or p53 led to actin cytoskeleton reorganization and a significant increase in cell motility. The endogenous phosphoinositide (PI) 3- kinase and Rac1 activities were elevated in Arf(-/-) and p53(-/-) cells, and these activities are required for p19(Arf)- and p53-regulated **migration**. Reintroduction of the wild type Arf or p53 genes into Arf(-/-) or p53(-/-) cells reversed the PI 3-kinase and Rho GTPase activities as well as the **migration** phenotype. These results suggest a functional relationship between an established tumor suppressor pathway and a signaling module that controls actin structure and cell motility and show that p19(Arf) and p53 negatively regulate cell **migration** by suppression of PI 3-kinase and Rac1 activities.
122. Lack of a significant role of P-Rex1, a major regulator of macrophage Rac1 activation and chemotaxis, in atherogenesis. Rho GTPases are known to play important roles in regulating multiple cellular processes that include cell polarization and **migration**. Among these Rho GTPases, Rac has been shown to be essential for F actin formation and cell **migration**. P-Rex1 is a guanine nucleotide exchange factor (GEF) that was previously found to mediate the activation of Rac2, but not Rac1, in mouse neutrophils.Here we examined the role of P-Rex1 in mouse macrophages and atherogenesis.PBD (p21 binding domain) pull down assay was performed to compare the Rac1 activation in WT and P-Rex1-deficient macrophage. In addition, transwell assay was conducted to compare chemotaxis of WT and P-Rex1-deficient macrophage. We found that P-Rex1 is a major Rac1 regulator in mouse macrophages as its deficiency significantly compromises macrophage chemotaxis, superoxide production (SOD), and Rac1 activation in response to chemoattractants. The potential role of P-Rex1 in atherogenesis is also investigated by transferring P-Rex1-deficient bone marrow cells to LDLR deficient mice. Contrary to our prediction, P-Rex1 deficiency did not alter atherogenesis, suggesting chemoattractant-induced macrophage **migration** may not have a significant role in atherogenesis.P-Rex1 is one of the major GEFs in macrophage regulating Rac1 activation and chemotaxis.
123. 14-3-3beta-Rac1-p21 activated kinase signaling regulates Akt1-mediated cytoskeletal organization, lamellipodia formation and fibronectin matrix assembly. Akt1 belongs to the three-gene Akt family and functions as a serine-threonine kinase regulating phosphorylation of an array of substrates and mediating cellular processes such as cell **migration**, proliferation, survival, and cell cycle. Our previous studies have established the importance of Akt1 in angiogenesis and absence of Akt1 resulted in impaired integrin activation, adhesion, **migration**, and extracellular matrix assembly by endothelial cells and fibroblasts. In this study, we identify the downstream signaling pathways activated by Akt1 in the regulation of these cellular events. We demonstrate here that Akt1 is necessary for the growth factor stimulated activation of 14-3-3beta-Rac1-p21 activated kinase (Pak) pathway in endothelial cells and fibroblasts. While activation of Akt1 resulted in translocation of Rac1 to membrane ruffles, enhanced Rac1 activity, Pak1 phosphorylation, and lamellipodia formation, resulting in enhanced adhesion and assembly of fibronectin, inhibition of Akt1 resulted in inhibition of these processes due to impaired Rac1-Pak signaling. Formation of lamellipodia, adhesion, and fibronectin assembly by myristoylated Akt1 expression in NIH 3T3 fibroblasts was inhibited by co-expression with either dominant negative Rac1 or dominant negative Pak1. In contrast, impaired lamellipodia formation, adhesion, and fibronectin assembly by dominant negative-Akt1 expression was rescued by co-expression with either constitutively active-Rac1 or -Pak1. Moreover, previously reported defects in adhesion and extracellular matrix assembly by Akt1(-/-) fibroblasts could be rescued by expression with either active-Rac1 or -Pak1, implying the importance of Rac1-Pak signaling in growth factor stimulated cytoskeletal assembly, lamellipodia formation and cell **migration** in endothelial cells and fibroblasts downstream of Akt1 activation.
124. MT1-MMP is required for myeloid cell fusion via regulation of Rac1 signaling. Cell fusion is essential for fertilization, myotube formation, and inflammation. Macrophages fuse under various circumstances, but the molecular signals involved in the distinct steps of their fusion are not fully characterized. Using null mice and derived cells, we show that the protease MT1-MMP is necessary for macrophage fusion during osteoclast and giant-cell formation in vitro and in vivo. Specifically, MT1-MMP is required for lamellipodia formation and for proper cell morphology and motility of bone marrow myeloid progenitors prior to membrane fusion. These functions of MT1-MMP do not depend on MT1-MMP catalytic activity or downstream pro-MMP-2 activation. Instead, MT1-MMP null cells show a decreased Rac1 activity and reduced membrane targeting of Rac1 and the adaptor protein p130Cas. Retroviral rescue experiments and protein binding assays delineate a signaling pathway in which MT1-MMP, via its cytosolic tail, contributes to macrophage **migration** and fusion by regulating Rac1 activity through an association with p130Cas.
125. Involvement of phosphoinositide 3-kinase gamma, Rac, and PAK signaling in chemokine-induced macrophage **migration**. In macrophages, chemotactic stimuli cause the activation of Rac and PAK, but little is known about the signaling pathways involved and their role in chemotactic gradient sensing. Herein, we report that in macrophages, the chemokine RANTES (regulated on activation normal T cell expressed and secreted)/CCL5 activates the small GTPase Rac and its downstream target PAK2 within seconds. This response depends on Gi activation and largely on the subsequent triggering of phosphoinositide 3-kinase gamma (PI3Kgamma) and Rac. Retroviral transduction of tagged Rac1 and -2 indicates that RANTES/CCL5-mediated activation of PI3Kgamma triggers Rac1 but not Rac2. In agreement, silencing of Rac1 by shRNA blocks PAK2 activity and inhibits RANTES/CCL5-induced macrophage polarization and directional **migration**. On the other hand, the tyrosine kinase receptor agonist CSF-1 activates PAK2 independently of PI3Kgamma and Rac. Our results thus demonstrate a chemokine-specific signaling pathway in which Gi and PI3Kgamma coordinate to drive Rac1 and PAK2 activation that eventually controls the chemotactic response.
126. Rac1-mediated indentation of resting neurons promotes the chain **migration** of new neurons in the rostral migratory stream of post-natal mouse brain. New neurons generated in the ventricular-subventricular zone in the post-natal brain travel toward the olfactory bulb by using a collective cell **migration** process called 'chain **migration**.' These new neurons show a saltatory movement of their soma, suggesting that each neuron cycles through periods of 'rest' during **migration**. Here, we investigated the role of the resting neurons in chain **migration** using post-natal mouse brain, and found that they undergo a dynamic morphological change, in which a deep indentation forms in the cell body. Inhibition of Rac1 activity resulted in less indentation of the new neurons in vivo. Live cell imaging using a Förster resonance energy transfer biosensor revealed that Rac1 was activated at the sites of contact between actively migrating and resting new neurons. On the cell surface of resting neurons, Rac1 activation coincided with the formation of the indentation. Furthermore, Rac1 knockdown prevented the indentation from forming and impaired **migratio**n along the resting neurons. These results suggest that Rac1 regulates a morphological change in the resting neurons, which allows them to serve as a migratory scaffold, and thereby non-cell-autonomously promotes chain **migratio**n.
127. The role of chemokine activation of Rac GTPases in hematopoietic stem cell marrow homing, retention, and peripheral mobilization. Signaling downstream from the chemokine receptor CXCR4, the tyrosine kinase receptor c-kit and beta1-integrins has been shown to be crucial in the regulation of **migration**, homing, and engraftment of hematopoietic stem cells and progenitors. Each of these receptors signal through Rac-type Rho guanosine triphosphatases (GTPases). Rac GTPases play a major role in the organization of the actin cytoskeleton and also in the control of gene expression and the activation of proliferation and survival pathways. Here we review the specific roles of the members of the Rac subfamily of the Rho GTPase family in regulating the intracellular signaling of hematopoietic cells responsible for regulation of homing, marrow retention, and peripheral mobilization.
128. Rac1 is essential for intraembryonic hematopoiesis and for the initial seeding of fetal liver with definitive hematopoietic progenitor cells. Definitive hematopoietic stem and progenitor cells (HSCs/Ps) originating from the yolk sac and/or para-aorta-splanchno-pleura/aorta-gonad-mesonephros are hypothesized to colonize the fetal liver, but mechanisms involved are poorly defined. The Rac subfamily of Rho GTPases has been shown to play essential roles in HSC/P localization to the bone marrow following transplantation. Here, we study the role of Rac1 in HSC/P **migration** during ontogeny and seeding of fetal liver. Using a triple-transgenic approach, we have deleted Rac1 in HSCs/Ps during very early embryonic development. Without Rac1, there was a decrease in circulating HSCs/Ps in the blood of embryonic day (E) 10.5 embryos, while yolk sac definitive hematopoiesis was quantitatively normal. Intraembryonic hematopoiesis was significantly impaired in Rac1-deficient embryos, culminating with absence of intra-aortic clusters and fetal liver hematopoiesis. At E10.5, Rac1-deficient HSCs/Ps displayed decreased transwell **migration** and impaired inter-action with the microenvironment in **migration**-dependent assays. These data suggest that Rac1 plays an important role in HSC/P **migration** during embryonic development and is essential for the emergence of intraembryonic hematopoiesis.
129. Impaired epidermal wound healing in vivo upon inhibition or deletion of Rac1. To address the functions of Rac1 in keratinocytes of the basal epidermal layer and in the outer root sheath of hair follicles, we generated transgenic mice expressing a dominant inhibitory mutant of Rac, N17Rac1, under the control of the keratin 14 promoter. These mice do not exhibit an overt skin phenotype but show protracted skin wound re-epithelialization. Investigation into the underlying mechanisms revealed that in vivo both proliferation of wound-edge keratinocytes and centripetal **migration** of the neo-epidermis were impaired. Similar results were obtained in mice with an epidermis-specific deletion of Rac1. Primary epidermal keratinocytes that expressed the N17Rac1 transgene were less proliferative than control cells and showed reduced ERK1/2 phosphorylation upon growth factor stimulation. Adhesion, spreading, random **migration** and closure of scratch wounds in vitro were significantly inhibited on collagen I and, to a lesser extent, on fibronectin. Stroboscopic analysis of cell dynamics (SACED) of N17Rac1 transgenic and control keratinocytes identified decreased lamella-protrusion persistence in connection with increased ruffle frequency as a probable mechanism for the observed impairment of keratinocyte adhesion and **migration**. We conclude that Rac1 is functionally required for normal epidermal wound healing and, in this context, exerts a dual function - namely the regulation of keratinocyte proliferation and **migration**.
130. Rho GTPase Rac1 is critical for neutrophil **migration** into the lung. Neutrophils are critical in the inflammatory process by moving rapidly to tissue sites of inflammation. Members of the small Rho GTPase family, Rac1, Rac2, CDC42, and RhoA, are central regulators of cell **migration** by cytoskeleton rearrangement. The role of Rac1 in neutrophil **migration** related to inflammatory processes has remained elusive and has yet to be determined in physiologic in vivo models. We previously demonstrated a role for Rac1 in tail retraction. Here, we present evidence that Rac1-mediated uropod formation may be due to crosstalk with a related Rho GTPase RhoA. To assess the physiologic relevance of these findings, we used adoptive transfer of Rac1flox/flox bone marrow cells which allows postengraftment in vivo deletion of Rac1 only in blood cells. We examined the specific role of Rac1 in neutrophil **migration** into the lung during the inflammatory process induced by formyl-methionyl-leucyl-phenylalanine exposure. The loss of Rac1 activity in neutrophils is associated with a significant decreased neutrophil recruitment into lung alveolar and attenuation of emphysematous lesions. Overall, this study suggests that Rac1 is a physiologic integrator of signals for neutrophil recruitment into lung tissue during an inflammatory response.
131. EphA2 receptor tyrosine kinase regulates endothelial cell **migration** and vascular assembly through phosphoinositide 3-kinase-mediated Rac1 GTPase activation. Angiogenesis is critical for vascular remodeling during development and contributes to the pathogenesis of diseases such as cancer. Targeted disruption of several EphB class receptor tyrosine kinases results in vascular remodeling defects during embryogenesis. The role of EphA class receptors in vascular remodeling, however, is not well-characterized. We recently demonstrated that global inhibition of EphA receptors disrupts endothelial **migration** induced by ephrin, VEGF or tumor-derived signals, though the specific target remained undefined. Here, we report that EphA2 regulates endothelial cell assembly and **migration** through phosphoinositide (PI) 3-kinase-mediated activation of Rac1 GTPase in two model systems: primary bovine and murine pulmonary microvascular endothelial cells. EphA2-deficient endothelial cells fail to undergo vascular assembly and **migration** in response to ephrin-A1 in vitro. Ephrin-A1 stimulation induces PI3-kinase-dependent activation of Rac1 in wild-type endothelial cells, whereas EphA2-deficient cells fail to activate Rac1 upon stimulation. Expression of dominant negative PI3-kinase or Rac1 inhibits ephrin-A1-induced endothelial cell **migration**. Consistent with in vitro data, EphA2-deficient mice show a diminished angiogenic response to ephrin-A1 in vivo. Moreover, EphA2-deficient endothelial cells fail to assemble in vivo when transplanted into recipient mice. These data suggest that EphA2 is an essential regulator of post-natal angiogenesis.
132. Stat3 promotes directional cell **migration** by regulating Rac1 activity via its activator betaPIX. Stat3 is a member of the signal transducer and activator of transcription family, which is important for cytokine signaling as well as for a number of cellular processes including cell proliferation, anti-apoptosis and immune responses. In recent years, evidence has emerged suggesting that Stat3 also participates in cell invasion and motility. However, how Stat3 regulates these processes remains poorly understood. Here, we find that loss of Stat3 expression in mouse embryonic fibroblasts leads to an elevation of Rac1 activity, which promotes a random mode of **migration** by reducing directional persistence and formation of actin stress fibers. Through rescue experiments, we demonstrate that Stat3 can regulate the activation of Rac1 to mediate persistent directional **migration** and that this function is not dependent on Stat3 transcriptional activity. We find that Stat3 binds to betaPIX, a Rac1 activator, and that this interaction could represent a mechanism by which cytoplasmic Stat3 regulates Rac1 activity to modulate the organization of actin cytoskeleton and directional **migration**.
133. Critical roles for Rac GTPases in T-cell **migration** to and within lymph nodes. Naive T cells continuously recirculate between secondary lymphoid tissue via the blood and lymphatic systems, a process that maximizes the chances of an encounter between a T cell and its cognate antigen. This recirculation depends on signals from chemokine receptors, integrins, and the sphingosine-1-phosphate receptor. The authors of previous studies in other cell types have shown that Rac GTPases transduce signals leading to cell **migration** and adhesion; however, their roles in T cells are unknown. By using both 3-dimensional intravital and in vitro approaches, we show that Rac1- and Rac2-deficient T cells have multiple defects in this recirculation process. Rac-deficient T cells home very inefficiently to lymph nodes and the white pulp of the spleen, show reduced interstitial **migration** within lymph node parenchyma, and are defective in egress from lymph nodes. These mutant T cells show defective chemokine-induced chemotaxis, chemokinesis, and adhesion to integrin ligands. They have reduced lateral motility on endothelial cells and transmigrate in-efficiently. These multiple defects stem from critical roles for Rac1 and Rac2 in transducing chemokine and sphingosine-1-phosphate receptor 1 signals leading to motility and adhesion.
134. Rac1 and rac3 GTPases control synergistically the development of cortical and hippocampal GABAergic interneurons. The intracellular mechanisms driving postmitotic development of cortical γ-aminobutyric acid (GABA)ergic interneurons are poorly understood. We have addressed the function of Rac GTPases in cortical and hippocampal interneuron development. Developing neurons express both Rac1 and Rac3. Previous work has shown that Rac1 ablation does not affect the development of migrating cortical interneurons. Analysis of mice with double deletion of Rac1 and Rac3 shows that these GTPases are required during postmitotic interneuron development. The number of parvalbumin-positive cells was affected in the hippocampus and cortex of double knockout mice. Rac depletion also influences the maturation of interneurons that reach their destination, with reduction of inhibitory synapses in both hippocampal CA1 and cortical pyramidal cells. The decreased number of cortical migrating interneurons and their altered morphology indicate a role of Rac1 and Rac3 in regulating the motility of cortical interneurons, thus interfering with their final localization. While electrophysiological passive and active properties of pyramidal neurons including membrane capacity, resting potential, and spike amplitude and duration were normal, these cells showed reduced spontaneous inhibitory currents and increased excitability. Our results show that Rac1 and Rac3 contribute synergistically to postmitotic development of specific populations of GABAergic cells, suggesting that these proteins regulate their **migratio**n and differentiation.
135. Inhibition of Rac GTPase signaling and downstream prosurvival Bcl-2 proteins as combination targeted therapy in MLL-AF9 leukemia. The Rac family of small Rho GTPases coordinates diverse cellular functions in hematopoietic cells including adhesion, **migration**, cytoskeleton rearrangements, gene transcription, proliferation, and survival. The integrity of Rac signaling has also been found to critically regulate cellular functions in the initiation and maintenance of hematopoietic malignancies. Using an in vivo gene targeting approach, we demonstrate that Rac2, but not Rac1, is critical to the initiation of acute myeloid leukemia in a retroviral expression model of MLL-AF9 leukemogenesis. However, loss of either Rac1 or Rac2 is sufficient to impair survival and growth of the transformed MLL-AF9 leukemia. Rac2 is known to positively regulate expression of Bcl-2 family proteins toward a prosurvival balance. We demonstrate that disruption of downstream survival signaling through antiapoptotic Bcl-2 proteins is implicated in mediating the effects of Rac2 deficiency in MLL-AF9 leukemia. Indeed, overexpression of Bcl-xL is able to rescue the effects of Rac2 deficiency and MLL-AF9 cells are exquisitely sensitive to direct inhibition of Bcl-2 family proteins by the BH3-mimetic, ABT-737. Furthermore, concurrent exposure to NSC23766, a small-molecule inhibitor of Rac activation, increases the apoptotic effect of ABT-737, indicating the Rac/Bcl-2 survival pathway may be targeted synergistically.
136. SCF E3 ligase F-box protein complex SCF(FBXL19) regulates cell **migration** by mediating Rac1 ubiquitination and degradation. Rac1, a member of the Rho family of GTPases, regulates diverse cellular functions, including cytoskeleton reorganization and cell **migration**. F-box proteins are major subunits within the Skp1-Cul1-F-box (SCF) E3 ubiquitin ligases that recognize specific substrates for ubiquitination. The role of F-box proteins in regulating Rac1 stability has not been studied. Mouse lung epithelial (MLE12) cells were used to investigate Rac1 stability and cell **migration**. Screening of an F-box protein library and in vitro ubiquitination assays identified FBXL19, a relatively new member of the F-box protein family that targets Rac1 for its polyubiquitination and proteasomal degradation. Overexpression of FBXL19 decreased both Rac1 active and inactive forms and significantly reduced cellular **migration**. Protein kinase AKT-mediated phosphorylation of Rac1 at serine(71) was essential for FBXL19-mediated Rac1 ubiquitination and depletion. Lysine(166) within Rac1 was identified as a polyubiquitination acceptor site. Rac1(S71A) and Rac1(K166R) mutant proteins were resistant to FBXL19-mediated ubiquitination and degradation. Further, ectopically expressed FBXL19 reduced cell **migration** in Rac1-overexpressing cells (P<0.01, Rac1 cells vs. FBXL19+Rac1 cells), but not in Rac1 lysine(166) mutant-overexpressing cells. FBXL19 diminished formation of the migratory leading edge. Thus, SCF(FBXL19) targets Rac1 for its disposal, a process regulated by AKT. These findings provide the first evidence of an F-box protein targeting a small G protein for ubiquitination and degradation to modulate cell **migration**.
137. The C-terminal domain of Rac1 contains two motifs that control targeting and signaling specificity. Rho-like GTPases control a wide range of cellular functions such as integrin- and cadherin-mediated adhesion, cell motility, and gene expression. The hypervariable C-terminal domain of these GTPases has been implicated in membrane association and effector binding. We found that cell-permeable peptides, encoding the C termini of Rac1, Rac2, RhoA, and Cdc42, interfere with GTPase signaling in a specific fashion in a variety of cellular models. Pull-down assays showed that the C terminus of Rac1 does not associate to either RhoGDI or to Pak. In contrast, the C terminus of Rac1 (but not Rac2 or Cdc42) binds to phosphatidylinositol 4,5-phosphate kinase (PIP5K) via amino acids 185-187 (RKR). Moreover, Rac1 associates to the adapter protein Crk via the N-terminal Src homology 3 (SH3) domain of Crk and the proline-rich stretch in the Rac1 C terminus. These differential interactions mediate Rac1 localization, as well as Rac1 signaling, toward membrane ruffling, cell-cell adhesion, and **migration**. These data show that the C-terminal, hypervariable domain of Rac1 encodes two distinct binding motifs for signaling proteins and regulates intracellular targeting and differential signaling in a unique and non-redundant fashion.
138. Rac1-mediated membrane raft localization of PI3K/p110β is required for its activation by GPCRs or PTEN loss. We aimed to understand how spatial compartmentalization in the plasma membrane might contribute to the functions of the ubiquitous class IA phosphoinositide 3-kinase (PI3K) isoforms, p110α and p110β. We found that p110β localizes to membrane rafts in a Rac1-dependent manner. This localization potentiates Akt activation by G-protein-coupled receptors (GPCRs). Thus genetic targeting of a Rac1 binding-deficient allele of p110β to rafts alleviated the requirement for p110β-Rac1 association for GPCR signaling, cell growt**h and mig**ration. In contrast, p110α, which does not play a physiological role in GPCR signaling, is found to reside in nonraft regions of the plasma membrane. Raft targeting of p110α allowed its EGFR-mediated activation by GPCRs. Notably, p110β dependent, PTEN null tumor cells critically rely upon raft-associated PI3K activity. Collectively, our findings provide a mechanistic account of how membrane raft localization regulates differential activation of distinct PI3K isoforms and offer insight into why PTEN-deficient cancers depend on p110β.
139. Transgenic expression of constitutively active RAC1 disrupts mouse rod morphogenesis. Dominant-active RAC1 rescues photoreceptor structure in Drosophila rhodopsin-null mutants, indicating an important role in morphogenesis. This report assesses the morphogenetic effect of activated RAC1 during mammalian rod photoreceptor development using transgenic mice that express constitutively active (CA) RAC1.Transgenic mice were generated by expressing CA RAC1 under control of the Rhodopsin promoter, and morphological features of the photoreceptors were evaluated by histology, immunohistochemistry, and transmission electron microscopy. Function was evaluated by electroretinography. Potential protein partners of CA RAC1 were identified by co-immunoprecipitation of retinal extracts.Constitutively active RAC1 expression in differentiating rods disrupted outer retinal lamination as early as postnatal day (P)6, and many photoreceptor cell nuclei were displaced apically into the presumptive subretinal space. These photoreceptors did not develop normal inner and outer segments and had abnormal placement of synaptic elements. Some photoreceptor nuclei were also mislocalized into the inner nuclear layer. Extensive photoreceptor degeneration was subsequently observed in the adult animal. Constitutively active RAC1 formed a complex with the polarity protein PAR6 and with microtubule motor dynein in mouse retina. The normal localization of the PAR6 complex was disrupted in CA RAC1-expressing rod photoreceptors.Constitutively active RAC1 had a profound negative effect on mouse rod cell viability and development. Rod photoreceptors in the CA RAC1 retina exhibited a defect in polarity and **migration**. Constitutively active RAC1 disrupted rod morphogenesis and gave a phenotype resembling that found in the Crumbs mutant. PAR6 and dynein are two potential downstream effectors that may be involved in CA RAC1-mediated defective mouse photoreceptor morphogenesis.
140. Redundant and nonredundant roles for Cdc42 and Rac1 in lymphomas developed in NPM-ALK transgenic mice. Increasing evidence suggests that Rho family GTPases could have a critical role in the biology of T-cell lymphoma. In ALK-rearranged anaplastic large cell lymphoma (ALCL), a specific subtype of T-cell lymphoma, the Rho family GTPases Cdc42 and Rac1 are activated by the ALK oncogenic activity. In vitro studies have shown that Cdc42 and Rac1 control rather similar phenotypes of ALCL biology such as the proliferation, survival, and **migration** of lymphoma cells. However, their role and possible redundancy in ALK-driven lymphoma development in vivo are still undetermined. We genetically deleted Cdc42 or Rac1 in a mouse model of ALK-rearranged ALCL to show that either Cdc42 or Rac1 deletion impaired lymphoma development, modified lymphoma morphology, actin filament distribution, and **migration** properties of lymphoma cells. Cdc42 or Rac1 deletion primarily affected survival rather than proliferation of lymphoma cells. Apoptosis of lymphoma cells was equally induced following Cdc42 or Rac1 deletion, was associated with upregulation of the proapoptotic molecule Bid, and was blocked by Bcl2 overexpression. Remarkably, Cdc42/Rac1 double deletion, but not Cdc42 or Rac1 single deletions, completely prevented NPM-ALK lymphoma dissemination in vivo. Thus, Cdc42 and Rac1 have nonredundant roles in controlling ALK-rearranged lymphoma survival and morphology but are redundant for lymphoma dissemination, suggesting that targeting both GTPases could represent a preferable therapeutic option for ALCL treatment.
141. Hematopoietic cell regulation by Rac1 and Rac2 guanosine triphosphatases. The Rho guanosine triphosphatases (GTPases) Rac1 and Rac2 are critical signaling regulators in mammalian cells. The deletion of both Rac1 and Rac2 murine alleles leads to a massive egress of hematopoietic stem/progenitor cells (HSC/Ps) into the blood from the marrow, whereas Rac1-/- but not Rac2-/- HSC/Ps fail to engraft in the bone marrow of irradiated recipient mice. In contrast, Rac2, but not Rac1, regulates superoxide production and directed **migration** in neutrophils, and in each cell type, the two GTPases play distinct roles in actin organization, cell survival, and proliferation. Thus, Rac1 and Rac2 regulate unique aspects of hematopoietic development and function.
142. Deletion of ABCA1 and ABCG1 impairs macrophage **migration** because of increased Rac1 signaling. Reduced plasma cholesterol and increased high-density lipoprotein (HDL) levels promote regression of atherosclerosis, in a process characterized by lipid unloading and e**migration** of macrophages from lesions. In contrast free cholesterol loading of macrophages leads to imbalanced Rac1/Rho activities and impaired chemotaxis.To study the role of HDL and the ATP-binding cassette transporters ABCA1 and ABCG1 in modulating the chemotaxis of macrophages.Abca1(-/-)Abcg1(-/-) mouse macrophages displayed profoundly impaired chemotaxis both in a Transwell chamber assay and in the peritoneal cavity of wild-type (WT) mice. HDL reversed impaired chemotaxis in free cholesterol-loaded WT macrophages but was without effect in Abca1(-/-)Abcg1(-/-) cells, whereas cyclodextrin was effective in both. Abca1(-/-)Abcg1(-/-) macrophages had markedly increased Rac1 activity and increased association of Rac1 with the plasma membrane (PM). Their defective chemotaxis was reversed by a Rac1 inhibitor. To gain a better understanding of the role of transporters in PM cholesterol movement, we measured transbilayer PM sterol distribution. In WT macrophages, the majority of cholesterol was located on the inner leaflet, whereas on upregulation of transporters by liver X receptor activation, PM sterol was shifted to the outer leaflet, where it could be removed by HDL. Abca1(-/-)Abcg1(-/-) macrophages showed increased PM sterol content and defective redistribution of sterol to the outer leaflet.Deletion of ABCA1 and ABCG1 causes an increased cholesterol content on the inner leaflet of the PM, associated with increased Rac1 PM localization, activation, and impairment of **migration**. ABCA1 and ABCG1 facilitate macrophage chemotaxis by promoting PM transbilayer cholesterol movement and may contribute to the ability of HDL to promote regression of atherosclerosis.
143. Regulation of focal adhesions by flightless i involves inhibition of paxillin phosphorylation via a Rac1-dependent pathway. Flightless I (Flii) is an actin-remodeling protein that influences diverse processes including cell **migration** and gene transcription and links signal transduction with cytoskeletal regulation. Here, we show that Flii modulation of focal adhesions and filamentous actin stress fibers is Rac1-dependent. Using primary skin fibroblasts from Flii overexpressing (Flii(Tg/Tg)), wild-type, and Flii deficient (Flii(+/-)) mice, we show that elevated expression of Flii increases stress fiber formation by impaired focal adhesion turnover and enhanced formation of fibrillar adhesions. Conversely, Flii knockdown increases the percentage of focal complex positive cells. We further show that a functional effect of Flii at both the cellular level and in in vivo mouse wounds is through inhibiting paxillin tyrosine phosphorylation and suppression of signaling proteins Src and p130Cas, both of which regulate adhesion signaling pathways. Flii is upregulated in response to wounding, and overexpression of Flii inhibits paxillin activity and reduces adhesion signaling by modulating the activity of the Rho family GTPases. Overexpression of constitutively active Rac1 GTPase restores the spreading ability of Flii(Tg/Tg) fibroblasts and may explain the reduced adhesion, **migration**, and proliferation observed in Flii(Tg/Tg) mice and their impaired wound healing, a process dependent on effective cellular motility and adhesion.
144. POSH localizes activated Rac1 to control the formation of cytoplasmic dilation of the leading process and neuronal **migration**. The formation of proximal cytoplasmic dilation in the leading process (PCDLP) of migratory neocortical neurons is crucial for somal translocation and neuronal **migration**, processes that require the elaborate coordination of F-actin dynamics, centrosomal movement, and nucleokinesis. However, the underlying molecular mechanisms remain poorly understood. Here, we show that the Rac1-interacting scaffold protein POSH is essential for neuronal **migration** in vivo. We demonstrate that POSH is concentrated in the PCDLP and that knockdown of POSH impairs PCDLP formation, centrosome translocation, and nucleokinesis. Furthermore, POSH colocalizes with F-actin and the activated form of Rac1. Knockdown of POSH impairs F-actin assembly and delocalizes activated Rac1. Interference of Rac1 activity also disrupts F-actin assembly and PCDLP formation and perturbs neuronal **migration**. Thus, we have uncovered a mechanism by which POSH regulates the localization of activated Rac1 and F-actin assembly to control PCDLP formation and subsequent somal translocation of migratory neurons.
145. Rac1 plays an essential role in axon growth and guidance and in neuronal survival in the central and peripheral nervous systems. Rac1 is a critical regulator of cytoskeletal dynamics in multiple cell types. In the nervous system, it has been implicated in the control of cell proliferation, neuronal **migration**, and axon development.To systematically investigate the role of Rac1 in axon growth and guidance in the developing nervous system, we have examined the phenotypes associated with deleting Rac1 in the embryonic mouse forebrain, in cranial and spinal motor neurons, in cranial sensory and dorsal root ganglion neurons, and in the retina. We observe a widespread requirement for Rac1 in axon growth and guidance and a cell-autonomous defect in axon growth in Rac1 (-/-) motor neurons in culture. Neuronal death, presumably a secondary consequence of the axon growth and/or guidance defects, was observed in multiple locations. Following deletion of Rac1 in the forebrain, thalamocortical axons were misrouted inferiorly, with the majority projecting to the contralateral thalamus and a minority projecting ipsilaterally to the ventral cortex, a pattern of misrouting that is indistinguishable from the pattern previously observed in Frizzled3 (-/-) and Celsr3 (-/-) forebrains. In the limbs, motor-neuron-specific deletion of Rac1 produced a distinctive stalling of axons within the dorsal nerve of the hindlimb but a much milder loss of axons in the ventral hindlimb and forelimb nerves, a pattern that is virtually identical to the one previously observed in Frizzled3 (-/-) limbs.The similarities in axon growth and guidance phenotypes caused by Rac1, Frizzled3, and Celsr3 loss-of-function mutations suggest a mechanistic connection between tissue polarity/planar cell polarity signaling and Rac1-dependent cytoskeletal regulation.
146. An essential role for Rac1 in endothelial cell function and vascular development. Numerous cell surface receptors, including tyrosine kinase and G protein-coupled receptors, play critical roles in endothelial cell function and blood vessel development. These receptors share the ability of stimulating an intricate network of intracellular signaling pathways, including the activation of members of the Ras and Rho family of small GTPases. However, the contribution of these signaling molecules to the numerous biological activities performed by endothelial cells is still not fully understood. Here, we have used a conditional Cre/Flox approach, enabling the deletion of the Rac1 gene in endothelial cells, to examine the role of the Rho-related GTPase Rac1 in endothelial cell function and vascular development. Rac1 excision in primary endothelial cells in vitro revealed that Rac1 plays a central role in endothelial cell **migration**, tubulogenesis, adhesion, and permeability in response to vascular endothelial growth factor (VEGF) and sphingosine-1-phosphate (S1P), which is likely due to the inability of Rac1-deficient endothelial cells to form lamellipodial structures and focal adhesions, and to remodel their cell-cell contacts. Importantly, endothelial-specific excision of Rac1 results in embryonic lethality in midgestation (around E9.5), and defective development of major vessels and complete lack of small branched vessels was readily observed in these endothelial Rac1-deficient embryos and their yolk sacs. These findings provide direct evidence that the activity of Rac1 in endothelial cells is essential for vascular development and suggest that Rac1 and its downstream targets may represent promising therapeutic targets for the treatment of numerous human diseases that involve aberrant neovascularization.

#### GeneRIFs

1. reveals a novel function for Reelin/ApoER2 in peripheral nervous system, inducing cell **migration** of Schwann cells, a process relevant for peripheral nervous system development and regeneration
2. This study supports that tumor-secreted vasoactive stimuli activate Rac1 to induce permeability and consequent transendothelial **migration** of tumor cells
3. This study showed that in the absence of both Rac1 and rac3, the embryonic **migration** of medial ganglionic eminence-derived interneurons is further impaired.
4. Src contributed to tyrosine phosphorylation of ELMO1 at Y720 and Y724 residues, which was crucial for activation of Rac1, followed by the promoting cell adhesion, spreading, and **migration**.
5. Transmembrane collagen XVII modulates integrin dependent keratinocyte **migration** via PI3K/Rac1 signaling.
6. Constitutively active RAC1 had a profound negative effect on mouse rod cell viability and development. Rod photoreceptors in the CA RAC1 retina exhibited a defect in polarity and **migration**.
7. These results suggest that Rac1 regulates a morphological change in the resting neurons, which allows them to serve as a migratory scaffold, and thereby non-cell-autonomously promotes chain **migration**.
8. Data indicate that overexpression of FBXL19 decreased both Rac1 active and inactive forms and significantly reduced cellular **migration**.
9. Kalirin functions as a guanine nucleotide exchange factor for Rac1 in smooth muscle cells, promoting **migration** and proliferation.
10. Rac1 is required for mouse melanoblast **migration**.
11. These data identify discrete roles for paxillin and Hic-5 in Rac1 and RhoA-dependent cell adhesion formation and maturation; processes essential for productive cell **migration**.
12. Using Rac mutants that are defective in their ability to activate NOX2, we show that Rac regulates a redox-mediated feedback loop that mediates directional **migration** of neutrophils.
13. role of Rac1 activity in establishment and maintenance of lens shape, suture formation and capsule integrity, and in fiber cell **migration**, adhesion and survival, via regulation of actin cytoskeletal dynamics, cell adhesive interactions and ECM turnover
14. The cell **migration** is dependent on the continuous organization of the actin cytoskeleton, which is regulated by Rac1.
15. Loss of Rac1 in melanoblasts during embryogenesis causes defects in **migration**, cell-cycle progression, and cytokinesis.
16. CD81 is required for the formation of actin membrane protrusions via Rac1 activation in adhesion-dependent immune cell **migration**.
17. Hematopoietic stem cell **migration** is essentially regulated by Rho GTPase, including RhoA, Rac1, and Cdc42, and necessitates the rearrangement of cytoskeleton structures, i.e., F-actin and microtubules.
18. Deletion of ABCA1 and ABCG1 impairs macrophage **migration** because of increased Rac1 signaling.
19. Results provide new insights into the regulation of endothelial cell **migration** during vascular remodeling in vivo by adding the Rac1 and FAK activation pathway as a critical mediator of focal adhesion formation and turnover during vascular remodeling.
20. Rac1-deficient T cells home very inefficiently to lymph nodes and show reduced interstitial **migration** within lymph node parenchyma, and are defective in egress from lymph nodes.
21. The data show that Rac1-mediated epithelial **migration** of the AVE is a crucial step in the establishment of the mammalian body plan and suggest that Rac1 is essential for collective **migration** in mammalian tissues.
22. cytoplasmic Stat3 regulates Rac1 activity to modulate the organization of actin cytoskeleton and directional **migration**
23. Retroviral rescue experiments and protein binding assays delineate a signaling pathway in which MT1-MMP, via its cytosolic tail, contributes to macrophage **migration** and fusion by regulating Rac1 activity through an association with p130Cas.
24. Data suggest that Cdc42, Rac1, and RhoG function cooperatively during cell **migration** and but are not essential for the directional response toward PDGF.
25. This study suggested that Rac1 may play a role in interkinetic nuclear **migration** in the developing mouse brain.
26. Results suggest that Rac1 is a key regulator of cell **migration** and cell adhesion during tissue and organ morphogenesis.
27. Rac1 excision in primary endothelial cells in vitro revealed that Rac1 plays a central role in endothelial cell **migration**, tubulogenesis, adhesion, and permeability in response to vascular endothelial growth factor and sphingosine-1-phosphate
28. GnT-V and beta1, 6GlcNAc branching mediate the cell **migration** and invasion in Rac1-positive and RhoA-negative regulatory manners.
29. Rac1 plays an important role in HSC/P **migration** during embryonic development and is essential for the emergence of intraembryonic hematopoiesis.
30. Present a novel mechanism by which the PI3K and Src signaling cascades cooperate to activate Rac1 and promote intestinal epithelial cell **migration** downstream of EGFR.
31. increased activation of Rac1 by beta1 integrins allows Schwann cells to switch from **migration**/elongation to the extension of radial membranes required for axonal sorting and myelination
32. Rac1 has a critical role in axon guidance and cell **migration** during differentiation of the ventral telecephalon.
33. Rac1 GTPase activation is mediated by phosphoinositide 3-kinase and has a role in EphA2 receptor tyrosine kinase regulated endothelial cell **migration**
34. Rac1 and Rac2 control the formation of dendrites in mature dendritic cells, their polarized short-range **migration** toward T cells, and T cell priming
35. Akt1 and Akt2 have opposing roles in Rac/Pak signaling and cell **migration**
36. the PI 3-kinase/Rac1 and Cdc42 pathways are involved in the radial **migration** of cortical neurons but have different roles
37. in macrophages Rac1 is primarily responsible for regulating cell morphology, contributes to membrane ruffling, but is not required for **migration**
38. Rac1 has a crucial role in cortical neuronal **migration**.
39. Rac1-deficient neutrophils demonstrate profound defects in inflammatory recruitment in vivo, **migration** to chemotactic stimuli, and chemoattractant-mediated actin assembly.
40. Ras is suppressed by p19(Arf) and p53, which negatively regulate cell **migration**

### ECM

#### PubMed

1. Rac1 GTPase-deficient mouse lens exhibits defects in shape, suture formation, fiber cell migration and survival. Morphogenesis and shape of the ocular lens depend on epithelial cell elongation and differentiation into fiber cells, followed by the symmetric and compact organization of fiber cells within an enclosed extracellular matrix-enriched elastic capsule. The cellular mechanisms orchestrating these different events however, remain obscure. We investigated the role of the Rac1 GTPase in these processes by targeted deletion of expression using the conditional gene knockout (cKO) approach. Rac1 cKO mice were derived from two different Cre (Le-Cre and MLR-10) transgenic mice in which lens-specific Cre expression starts at embryonic day 8.75 and 10.5, respectively, in both the lens epithelium and fiber cells. The Le-Cre/Rac1 cKO mice exhibited an early-onset (E12.5) and severe lens phenotype compared to the MLR-10/Rac1 cKO (E15.5) mice. While the Le-Cre/Rac1 cKO lenses displayed delayed primary fiber cell elongation, lenses from both Rac1 cKO strains were characterized by abnormal shape, impaired secondary fiber cell migration, sutural defects and thinning of the posterior capsule which often led to rupture. Lens fiber cell N-cadherin/β-catenin/Rap1/Nectin-based cell-cell junction formation and WAVE-2/Abi-2/Nap1-regulated actin polymerization were impaired in the Rac1 deficient mice. Additionally, the Rac1 cKO lenses were characterized by a shortened epithelial sheet, reduced levels of extracellular matrix **(EC**M) proteins and increased apoptosis. Taken together, these data uncover the essential role of Rac1 GTPase activity in establishment and maintenance of lens shape, suture formation and capsule integrity, and in fiber cell migration, adhesion and survival, via regulation of actin cytoskeletal dynamics, cell adhesive interactions and **EC**M turnover.
2. Opposing roles for Akt1 and Akt2 in Rac/Pak signaling and cell migration. The Akt/PKB isoforms have different roles in animals, with Akt2 primarily regulating metabolic signaling and Akt1 regulating growth and survival. Here we show distinct roles for Akt1 and Akt2 in mouse embryo fibroblast cell migration and regulation of the cytoskeleton. Akt1-deficient cells responded poorly to platelet-derived growth factor while Akt2-deficient cells had a dramatically enhanced response, resulting in a substantial increase in dorsal ruffling. Swapping domains between Akt1 and Akt2 demonstrated that the N-terminal region containing the pleckstrin homology domain and a linker region distinguishes the two isoforms, while the catalytic domains are interchangeable. Akt2 knock-out cells also migrated faster than wild-type cells, especially through extracellular matrix (**ECM**), while Akt1 knock-out cells migrated more slowly than wild-type cells. Consistently, Akt2 knock-out cells had elevated Pak1 and Rac activities, suggesting that Akt2 inhibits Rac and Pak1. Both Akt2 and Akt1 associated in complexes with Pak1, but only Akt2 inhibited Pak1 in kinase assays, suggesting an underlying molecular basis for the different cellular phenotypes. Together these data provide evidence for an unexpected functional link between Akt2 and Pak1 that opposes the actions of Akt1 on cell migration.
3. Patterned collagen fibers orient branching mammary epithelium through distinct signaling modules. For decades, the work of cell and developmental biologists has demonstrated the striking ability of the mesenchyme and the stroma to instruct epithelial form and function in the mammary gland, but the role of extracellular matrix (**ECM**) molecules in mammary pattern specification has not been elucidated. Here, we show that stromal collagen I (Col-I) fibers in the mammary fat pad are axially oriented prior to branching morphogenesis. Upon puberty, the branching epithelium orients along these fibers, thereby adopting a similar axial bias. To establish a causal relationship from Col-I fiber to epithelial orientation, we embedded mammary organoids within axially oriented Col-I fiber gels and observed dramatic epithelial co-orientation. Whereas a constitutively active form of Rac1, a molecule implicated in cell motility, prevented a directional epithelial response to Col-I fiber orientation, inhibition of the RhoA/Rho-associated kinase (ROCK) pathway did not. However, time-lapse studies revealed that, within randomly oriented Col-I matrices, the epithelium axially aligns fibers at branch sites via RhoA/ROCK-mediated contractions. Our data provide an explanation for how the stromal **ECM** encodes architectural cues for branch orientation as well as how the branching epithelium interprets and reinforces these cues through distinct signaling processes.
4. Patterned collagen fibers orient branching mammary epithelium through distinct signaling modules. For decades, the work of cell and developmental biologists has demonstrated the striking ability of the mesenchyme and the stroma to instruct epithelial form and function in the mammary gland, but the role of extracellular matrix (**ECM**) molecules in mammary pattern specification has not been elucidated. Here, we show that stromal collagen I (Col-I) fibers in the mammary fat pad are axially oriented prior to branching morphogenesis. Upon puberty, the branching epithelium orients along these fibers, thereby adopting a similar axial bias. To establish a causal relationship from Col-I fiber to epithelial orientation, we embedded mammary organoids within axially oriented Col-I fiber gels and observed dramatic epithelial co-orientation. Whereas a constitutively active form of Rac1, a molecule implicated in cell motility, prevented a directional epithelial response to Col-I fiber orientation, inhibition of the RhoA/Rho-associated kinase (ROCK) pathway did not. However, time-lapse studies revealed that, within randomly oriented Col-I matrices, the epithelium axially aligns fibers at branch sites via RhoA/ROCK-mediated contractions. Our data provide an explanation for how the stromal **ECM** encodes architectural cues for branch orientation as well as how the branching epithelium interprets and reinforces these cues through distinct signaling processes.
5. Rac1 GTPase-deficient mouse lens exhibits defects in shape, suture formation, fiber cell migration and survival. Morphogenesis and shape of the ocular lens depend on epithelial cell elongation and differentiation into fiber cells, followed by the symmetric and compact organization of fiber cells within an enclosed extracellular matrix-enriched elastic capsule. The cellular mechanisms orchestrating these different events however, remain obscure. We investigated the role of the Rac1 GTPase in these processes by targeted deletion of expression using the conditional gene knockout (cKO) approach. Rac1 cKO mice were derived from two different Cre (Le-Cre and MLR-10) transgenic mice in which lens-specific Cre expression starts at embryonic day 8.75 and 10.5, respectively, in both the lens epithelium and fiber cells. The Le-Cre/Rac1 cKO mice exhibited an early-onset (E12.5) and severe lens phenotype compared to the MLR-10/Rac1 cKO (E15.5) mice. While the Le-Cre/Rac1 cKO lenses displayed delayed primary fiber cell elongation, lenses from both Rac1 cKO strains were characterized by abnormal shape, impaired secondary fiber cell migration, sutural defects and thinning of the posterior capsule which often led to rupture. Lens fiber cell N-cadherin/β-catenin/Rap1/Nectin-based cell-cell junction formation and WAVE-2/Abi-2/Nap1-regulated actin polymerization were impaired in the Rac1 deficient mice. Additionally, the Rac1 cKO lenses were characterized by a shortened epithelial sheet, reduced levels of extracellular matrix **(EC**M) proteins and increased apoptosis. Taken together, these data uncover the essential role of Rac1 GTPase activity in establishment and maintenance of lens shape, suture formation and capsule integrity, and in fiber cell migration, adhesion and survival, via regulation of actin cytoskeletal dynamics, cell adhesive interactions and **EC**M turnover.
6. Opposing roles for Akt1 and Akt2 in Rac/Pak signaling and cell migration. The Akt/PKB isoforms have different roles in animals, with Akt2 primarily regulating metabolic signaling and Akt1 regulating growth and survival. Here we show distinct roles for Akt1 and Akt2 in mouse embryo fibroblast cell migration and regulation of the cytoskeleton. Akt1-deficient cells responded poorly to platelet-derived growth factor while Akt2-deficient cells had a dramatically enhanced response, resulting in a substantial increase in dorsal ruffling. Swapping domains between Akt1 and Akt2 demonstrated that the N-terminal region containing the pleckstrin homology domain and a linker region distinguishes the two isoforms, while the catalytic domains are interchangeable. Akt2 knock-out cells also migrated faster than wild-type cells, especially through extracellular matrix (**ECM**), while Akt1 knock-out cells migrated more slowly than wild-type cells. Consistently, Akt2 knock-out cells had elevated Pak1 and Rac activities, suggesting that Akt2 inhibits Rac and Pak1. Both Akt2 and Akt1 associated in complexes with Pak1, but only Akt2 inhibited Pak1 in kinase assays, suggesting an underlying molecular basis for the different cellular phenotypes. Together these data provide evidence for an unexpected functional link between Akt2 and Pak1 that opposes the actions of Akt1 on cell migration.
7. Arhgap28 is a RhoGAP that inactivates RhoA and downregulates stress fibers. The small GTPase RhoA is a major regulator of actin reorganization during the formation of stress fibers; thus identifying molecules that regulate Rho activity is necessary for a complete understanding of the mechanisms that determine cell contractility. Here, we have identified Arhgap28 as a Rho GTPase activating protein (RhoGAP) that switches RhoA to its inactive form. We generated an Arhgap28-LacZ reporter mouse that revealed gene expression in soft tissues at E12.5, pre-bone structures of the limb at E15.5, and prominent expression restricted mostly to ribs and limb long bones at E18.5 days of development. Expression of recombinant Arhgap28-V5 in human osteosarcoma SaOS-2 cells caused a reduction in the basal level of RhoA activation and disruption of actin stress fibers. Extracellular matrix assembly studies using a 3-dimensional cell culture system showed that Arhgap28 was upregulated during Rho-dependent assembly of the **ECM**. Taken together, these observations led to the hypothesis that an Arhgap28 knockout mouse model would show a connective tissue phenotype, perhaps affecting bone. Arhgap28-null mice were viable and appeared normal, suggesting that there could be compensation from other RhoGAPs. Indeed, we showed that expression of Arhgap6 (a closely related RhoGAP) was upregulated in Arhgap28-null bone tissue. An upregulation in RhoA expression was also detected suggesting that Arhgap28 may be able to additionally regulate Rho signaling at a transcriptional level. Microarray analyses revealed that Col2a1, Col9a1, Matn3, and Comp that encode extracellular matrix proteins were downregulated in Arhgap28-null bone. Although mutations in these genes cause bone dysplasias no bone phenotype was detected in the Arhgap-28 null mice. Together, these data suggest that the regulation of Rho by RhoGAPs, including Arhgap28, during the assembly and development of mechanically strong tissues is complex and may involve multiple RhoGAPs.
8. 8-Hydroxy-2-deoxyguanosine prevents plaque formation and inhibits vascular smooth muscle cell activation through Rac1 inactivation. 8-Hydroxy-2-deoxyguanosine (8-OHdG), a marker of oxidative stress, has been recently rediscovered to inhibit Rac1 in neutrophils and macrophages, thereby inhibiting Rac1-linked functions of these cells, including reactive oxygen species production through NADPH oxidase activation, phagocytosis, chemotaxis, and cytokine release. In vascular smooth muscle cells (VSMCs), reactive oxygen species also induce abnormal proliferation and migration leading to progression of atherosclerosis. Based upon the involvement of reactive oxygen species in phagocytic cells and VSMCs during the atherosclerotic process, we hypothesized that 8-OHdG could have antiatherosclerotic action and tested this hypothesis in an experimentally induced atherosclerosis in mice. Partially ligated ApoE knockout mice, a more physiologically relevant model of low and oscillatory flow, developed an advanced lesion in 2 weeks, and orally administered 8-OHdG significantly reduced plaque formation along with reduced superoxide formation, monocyte/macrophage infiltration, and extracellular matrix (**ECM**) accumulation. The effects of 8-OHdG observed in primary VSMCs were consistent with the in vivo effects of 8-OHdG and were inhibitory to angiotensin II or platelet-derived growth factor-induced production of reactive oxygen species, proliferation, migration, and **ECM** production. Also, angiotensin II-induced Rac1 activity in VSMCs was significantly inhibited by 8-OHdG, and transfection of constitutively active Rac1 reversed the inhibitory effect of 8-OHdG on VSMC activation. Molecular docking study showed that 8-OHdG stabilizes Rac1-GEF complex, indicating the physical contact of 8-OHdG with Rac1. These findings highly suggest that the antiatherosclerotic effect of 8-OHdG is mediated by inhibition of Rac1 activity. In conclusion, our results show a novel action of orally active 8-OHdG in suppressing atherosclerotic plaque formation in vivo and VSMC activation in vitro through inhibition of Rac1, which emphasizes a new therapeutic avenue to benefit atherosclerosis.
9. Paxillin and Hic-5 interaction with vinculin is differentially regulated by Rac1 and RhoA. Cell migration is of paramount importance to organism development and maintenance as well as multiple pathological processes, including cancer metastasis. The RhoGTPases Rac1 and RhoA are indispensable for cell migration as they regulate cell protrusion, cell-extracellular matrix (**ECM**) interactions and force transduction. However, the consequences of their activity at a molecular level within the cell remain undetermined. Using a combination of FRET, FRAP and biochemical analyses we show that the interactions between the focal adhesion proteins vinculin and paxillin, as well as the closely related family member Hic-5 are spatially and reciprocally regulated by the activity of Rac1 and RhoA. Vinculin in its active conformation interacts with either paxillin or Hic-5 in adhesions in response to Rac1 and RhoA activation respectively, while inactive vinculin interacts with paxillin in the membrane following Rac1 inhibition. Additionally, Rac1 specifically regulates the dynamics of paxillin as well as its binding partner and F-actin interacting protein actopaxin (α-parvin) in adhesions. Furthermore, FRET analysis of protein:protein interactions within cell adhesions formed in 3D matrices revealed that, in contrast to 2D systems vinculin interacts preferentially with Hic-5. This study provides new insight into the complexity of cell**-EC**M adhesions in both 2D and 3D matrices by providing the first description of RhoGTPase-coordinated protein:protein interactions in a cellular microenvironment. These data identify discrete roles for paxillin and Hic-5 in Rac1 and RhoA-dependent cell adhesion formation and maturation; processes essential for productive cell migration.
10. Changes in dermal matrix in the absence of Rac1 in keratinocytes. Keratinocytes, in response to irritants, secrete pro-inflammatory mediators which recruit and activate immune and mesenchymal cells, including fibroblasts, to repair the skin. Fibroblasts respond by synthesising collagen and promoting the crosslinking extracellular matrix (**ECM**). We recently showed that the deletion of Rac1 in keratinocytes causes heightened inflammation due to aberrant crosstalk with immune cells. Indeed, the skin of these mice shows a higher inflammatory response to the induction of irritant contact dermatitis (ICD), and also even to treatment with a vehicle alone, compared with controls. As inflammation is intimately linked with fibrotic disease in the skin, this raised the question as to whether this deletion may also affect the deposition and arrangement of the dermal **ECM**. This study assessed the effects of Rac1 deletion in keratinocytes and of the heightened inflammatory status by induction of ICD on the tissue localisation and arrangements of dermal collagen. Qualitative analysis did not reveal evidence for the formation of pathologies in the dermis. However, quantitative analysis did reveal some perturbations in the dermal matrix, namely that only the combination of the lack of Rac1 and ICD affects the architectural organisation of the dermal collagen, and that a higher inflammatory state in the tissue (i.e. when Rac1 is deleted in the keratinocytes or ICD is induced in the skin, or a combination of both) influences the diameter of the collagen fibrils. It is proposed that this increase in the diameter of collagen fibrils due to inflammation may serve as pre-fibrotic marker enabling earlier determination of fibrosis and earlier treatment. This study has revealed previously unknown effects on the **ECM** due to the deletion of Rac1 in keratinocytes.
11. Loss of protein kinase Cepsilon results in impaired cutaneous wound closure and myofibroblast function. Cutaneous wound repair requires the de novo induction of a specialized form of fibroblast, the alpha-smooth muscle actin (alpha-SMA)-expressing myofibroblast, which migrates into the wound where it adheres to and contracts extracellular matrix (**ECM**), resulting in wound closure. Persistence of the myofibroblast results in scarring and fibrotic disease. In this report, we show that, compared with wild-type littermates, PKCepsilon-/- mice display delayed impaired cutaneous wound closure and a reduction in myofibroblasts. Moreover, both in the presence and absence of TGFbeta, dermal fibroblasts from PKCepsilon-/- mice cultured on fibronectin show impaired abilities to form ;supermature' focal adhesions and alpha-SMA stress fibers, and reduced pro-fibrotic gene expression. Smad3 phosphorylation in response to TGFbeta1 was impaired in PKCepsilon-/- fibroblasts. PKCepsilon-/- fibroblasts show reduced FAK and Rac activation, and adhesive, contractile and migratory abilities. Overexpressing constitutively active Rac1 rescues the defective FAK phosphorylation, cell migration, adhesion and stress fiber formation of these PKCepsilon-/- fibroblasts, indicating that Rac1 operates downstream of PKCepsilon, yet upstream of FAK. These results suggest that loss of PKCepsilon severely impairs myofibroblast formation and function, and that targeting PKCepsilon may be beneficial in selectively modulating wound healing and fibrotic responses in vivo.
12. EMMPRIN activates multiple transcription factors in cardiomyocytes, and induces interleukin-18 expression via Rac1-dependent PI3K/Akt/IKK/NF-kappaB andMKK7/JNK/AP-1 signaling. The transmembrane glycoprotein extracellular matrix metalloproteinase inducer (EMMPRIN), and the pleiotropic proinflammatory cytokine interleukin (IL)-18, play critical roles in myocardial remodeling, by inducing matrix degrading metalloproteinases (MMPs). Previously we showed that IL-18 induces EMMPRIN expression in cardiomyocytes via MyD88/IRAK4/TRAF6/JNK-dependent Sp1 activation. Here in reciprocal studies we demonstrate that EMMPRIN is a potent inducer of IL-18 transcription, protein expression and protein secretion in primary mouse cardiomyocytes. We show for the first time that EMMPRIN stimulates the activation of NF-kappaB, AP-1, CREB, and ATF-2 in cardiomyocytes, and induces IL-18 expression via Rac1-dependent PI3K/Akt/IKK/NF-kappaB and MKK7/JNK/AP-1 signaling. Moreover, EMMPRIN induces robust time-dependent induction of various MMP mRNAs. EMMPRIN also induces the mRNA of TIMPs 1 and 3, but in a delayed fashion. These results suggest that IL-18-induced EMMPRIN expression may favor net MMP expression and **ECM** destruction, and thus identify both as potential therapeutic targets in countering adverse myocardial remodeling.
13. Plakoglobin regulates cell motility through Rho- and fibronectin-dependent Src signaling. We previously showed that the cell-cell junction protein plakoglobin (PG) not only suppresses motility of keratinocytes in contact with each other, but also, unexpectedly, of single cells. Here we show that PG deficiency results in extracellular matrix (**ECM**)-dependent disruption of mature focal adhesions and cortical actin organization. Plating PG⁻/⁻ cells o**nto** ECM deposited by PG+/⁻ cells partially restored normal cell morphology and inhibited PG⁻/⁻ cell motility. In over 70 adhesion molecules whose expression we previously showed to be altered in PG⁻/⁻ cells, a substantial decrease in fibronectin (FN) in PG⁻/⁻ cells stood out. Re-introduction of PG into PG⁻/⁻ cells restored FN expression, and keratinocyte motility was reversed by plating PG⁻/⁻ cells onto FN. Somewhat surprisingly, based on previously reported roles for PG in regulating gene transcription, PG-null cells exhibited an increase, not a decrease, in FN promoter activity. Instead, PG was required for maintenance of FN mRNA stability. PG⁻/⁻ cells exhibited an increase in activated Src, one of the kinases controlled by FN, a phenotype rever**sed** by plating PG⁻/⁻ cells on ECM deposited by PG+/⁻ keratinocytes. PG⁻/⁻ cells also exhibited Src-independent activation of the small GTPases Rac1 and RhoA. Both Src and RhoA inhibition attenuated PG⁻/⁻ keratinocyte motility. We propose a novel role for PG i**n r**egulating cell motility through distinct ECM-Src and RhoGTPase-dependent pathways, influenced in part by PG-dependent regulation of FN mRNA stability.
14. Rac1 links integrin-mediated adhesion to the control of lactational differentiation in mammary epithelia. The expression of tissue-specific genes during mammary gland differentiation relies on the coincidence of two distinct signaling events: the continued engagement of beta1 integrins with the extracellular matrix (**ECM**) and a hormonal stimulus from prolactin (Prl). How the integrin and Prl receptor (PrlR) systems integrate to regulate milk protein gene synthesis is unknown. In this study, we identify Rac1 as a key link. Dominant-negative Rac1 prevents Prl-induced synthesis of the milk protein beta-casein in primary mammary epithelial cells cultured as three-dimensional acini on basement membrane. Conversely, activated Rac1 rescues the defective beta-casein synthesis that occurs under conditions not normally permissive for mammary differentiation, either in beta1 integrin-null cells or in wild-type cells cultured on collagen. Rac1 is required downstream of integrins for activation of the PrlR/Stat5 signaling cascade. Cdc42 is also necessary for milk protein synthesis but functions via a distinct mechanism to Rac1. This study identifies the integration of signals provided by **ECM** and hormones as a novel role for Rho family guanosine triphosphatases.
15. Tyr724 phosphorylation of ELMO1 by Src is involved in cell spreading and migration via Rac1 activation. The complex of Dock180/ELMO1 that functions as a bipartite guanine nucleotide exchange factor for Rac is essential for diverse physiological and pathological processes of cells such as cell migration, phagocytosis, and invasion of cancer cells. Among the Src-family tyrosine kinases (SFKs), it has been reported that Hck directly phosphorylates ELMO1, regulating phagocytosis by promoting activation of Rac1; however, the involvement of other SFKs in ELMO1 phosphorylation has remained unknown. Here, we identified novel tyrosine (Y) residues of ELMO1 phosphorylated by SFKs, and examined the effects on Rac1 activity, cell adhesion, spreading, and cell motility on extracellular matrix (**ECM**).In this study, we unveiled that Src and Fyn can induce tyrosine phosphorylation of ELMO1 in in vivo and in vitro phosphorylation assays. Mutational analyses identified both Y720 and Y724 residues of ELMO1 as Src-mediated phosphorylation sites, preferentially on Y724. Single substitution of Y724 to Phe abrogated Rac1 activation triggered by Src. To elucidate the biological function of pY724, we established NIH3T3 cells stably expressing wild-type ELMO1 or its Y724F mutant together with Dock180. Among them, Y724-deficient cells exhibited a depletion of Rac1 activity with diminished phosphorylation of ELMO1 even upon the **ECM**-stimulation. It is noteworthy that NIH3T3 cells with ELMO1 Y724F were strikingly defective to promote cell spreading on fibronectin-coated dish, concomitantly exhibiting immature assemblies of actin stress fibers and focal adhesions. Eventually, ELMO1 Y724F significantly impaired cell migration.These results define that Src-mediated Y724 phosphorylation in ELMO1 plays a critical role for cell spreading via activation of Rac1, leading to promotion of cell migration. As the overexpression and/or hyperactivation of Src have been shown in a wide variety of human cancers, Src-mediated phosphorylation of Y724 in ELMO1 may regulate cancer cell adhesion to the **ECM**, invasion into surrounding tissues, and subsequent distant metastasis.

#### GeneRIFs

1. role of Rac1 activity in establishment and maintenance of lens shape, suture formation and capsule integrity, and in fiber cell migration, adhesion and survival, via regulation of actin cytoskeletal dynamics, cell adhesive interactions and **ECM** turnover

### [Ee]xtracellular [Mm]atrix

#### PubMed

1. Coronin-1C Protein and Caveolin Protein Provide Constitutive and Inducible Mechanisms of Rac1 Protein Trafficking. Sustained directional fibroblast migration requires both polarized activation of the protrusive signal, Rac1, and redistribution of inactive Rac1 from the rear of the cell so that it can be redistributed or degraded. In this work, we determine how alternative endocytic mechanisms dictate the fate of Rac1 in response to the **extracellular matrix** environment. We discover that both coronin-1C and caveolin retrieve Rac1 from similar locations at the rear and sides of the cell. We find that coronin-1C-mediated extraction, which is responsible for Rac1 recycling, is a constitutive process that maintains Rac1 protein levels within the cell. In the absence of coronin-1C, the effect of caveolin-mediated endocytosis, which targets Rac1 for proteasomal degradation, becomes apparent. Unlike constitutive coronin-1C-mediated trafficking, caveolin-mediated Rac1 endocytosis is induced by engagement of the fibronectin receptor syndecan-4. Such an inducible endocytic/degradation mechanism would predict that, in the presence of fibronectin, caveolin defines regions of the cell that are resistant to Rac1 activation but, in the absence of fibronectin leaves more of the membrane susceptible to Rac1 activation and protrusion. Indeed, we demonstrate that fibronectin-stimulated activation of Rac1 is accelerated in the absence of caveolin and that, when caveolin is knocked down, polarization of active Rac1 is lost in FRET experiments and culminates in shunting migration in a fibrous fibronectin matrix. Although the concept of polarized Rac1 activity in response to chemoattractants has always been apparent, our understanding of the balance between recycling and degradation explains how polarity can be maintained when the chemotactic gradient has faded.
2. Genetic ablation of Rac1 in cartilage results in chondrodysplasia. Small GTPases of the Rho family have been implicated in the regulation of many intracellular processes. However, their tissue-specific roles in mammalian growth and development in vivo remain largely unknown. Here we describe the effects of cartilage-specific inactivation of the Rac1 gene in mice. Mice carrying this mutation show increased lethality, skeletal deformities, severe kyphosis and dwarfism. Rac1-deficient growth plates are disorganized and hypocellular, with chondrocytes of abnormal shape and size. Rac1-deficient chondrocytes also display reduced adhesion and spreading on collagen II and fibronectin as well as altered organization of the actin cytoskeleton, suggesting that Rac1 is required for normal cell-**extracellular matrix** interactions in cartilage. This phenotype is accompanied by reduced proliferation, increased apoptosis and deregulated expression of the cell cycle genes cyclin D1 and p57 in vivo. Moreover, phosphorylation of p38 MAP kinases is greatly reduced and expression of a key regulator of cartilage development, Indian hedgehog, is increased in mutant mice. In summary, these data identify a novel, essential and tissue-specific role of Rac1 in skeletal development and demonstrate that Rac1 deficiency affects numerous regulatory pathways in cartilage.
3. Rac1 GTPase-deficient mouse lens exhibits defects in shape, suture formation, fiber cell migration and survival. Morphogenesis and shape of the ocular lens depend on epithelial cell elongation and differentiation into fiber cells, followed by the symmetric and compact organization of fiber cells within an enclosed **extracellular matrix**-enriched elastic capsule. The cellular mechanisms orchestrating these different events however, remain obscure. We investigated the role of the Rac1 GTPase in these processes by targeted deletion of expression using the conditional gene knockout (cKO) approach. Rac1 cKO mice were derived from two different Cre (Le-Cre and MLR-10) transgenic mice in which lens-specific Cre expression starts at embryonic day 8.75 and 10.5, respectively, in both the lens epithelium and fiber cells. The Le-Cre/Rac1 cKO mice exhibited an early-onset (E12.5) and severe lens phenotype compared to the MLR-10/Rac1 cKO (E15.5) mice. While the Le-Cre/Rac1 cKO lenses displayed delayed primary fiber cell elongation, lenses from both Rac1 cKO strains were characterized by abnormal shape, impaired secondary fiber cell migration, sutural defects and thinning of the posterior capsule which often led to rupture. Lens fiber cell N-cadherin/β-catenin/Rap1/Nectin-based cell-cell junction formation and WAVE-2/Abi-2/Nap1-regulated actin polymerization were impaired in the Rac1 deficient mice. Additionally, the Rac1 cKO lenses were characterized by a shortened epithelial sheet, reduced levels of **extracellular matri**x (ECM) proteins and increased apoptosis. Taken together, these data uncover the essential role of Rac1 GTPase activity in establishment and maintenance of lens shape, suture formation and capsule integrity, and in fiber cell migration, adhesion and survival, via regulation of actin cytoskeletal dynamics, cell adhesive interactions and ECM turnover.
4. Coronin-1C and RCC2 guide mesenchymal migration by trafficking Rac1 and controlling GEF exposure. Sustained forward migration through a fibrillar **extracellular matrix** requires localization of protrusive signals. Contact with fibronectin at the tip of a cell protrusion activates Rac1, and for linear migration it is necessary to dampen Rac1 activity in off-axial positions and redistribute Rac1 from non-protrusive membrane to the leading edge. Here, we identify interactions between coronin-1C (Coro1C), RCC2 and Rac1 that focus active Rac1 to a single protrusion. Coro1C mediates release of inactive Rac1 from non-protrusive membrane and is necessary for Rac1 redistribution to a protrusive tip and fibronectin-dependent Rac1 activation. The second component, RCC2, attenuates Rac1 activation outside the protrusive tip by binding to the Rac1 switch regions and competitively inhibiting GEF action, thus preventing off-axial protrusion. Depletion of Coro1C or RCC2 by RNA interference causes loss of cell polarity that results in shunting migration in 1D or 3D culture systems. Furthermore, morpholinos against Coro1C or RCC2, or mutation of any of the binding sites in the Rac1-RCC2-Coro1C complex delays the arrival of neural crest derivatives at the correct location in developing zebrafish, demonstrating the crucial role in migration guidance in vivo.
5. Integrin-linked kinase is required for radial sorting of axons and Schwann cell remyelination in the peripheral nervous system. During development, Schwann cells (SCs) interpret different extracellular cues to regulate their migration, proliferation, and the remarkable morphological changes associated with the sorting, ensheathment, and myelination of axons. Although interactions between **extracellular matrix** proteins and integrins are critical to some of these processes, the downstream signaling pathways they control are still poorly understood. Integrin-linked kinase (ILK) is a focal adhesion protein that associates with multiple binding partners to link integrins to the actin cytoskeleton and is thought to participate in integrin and growth factor-mediated signaling. Using SC-specific gene ablation, we report essential functions for ILK in radial sorting of axon bundles and in remyelination in the peripheral nervous system. Our in vivo and in vitro experiments show that ILK negatively regulates Rho/Rho kinase signaling to promote SC process extension and to initiate radial sorting. ILK also facilitates axon remyelination, likely by promoting the activation of downstream molecules such as AKT/protein kinase B.
6. Opposing roles for Akt1 and Akt2 in Rac/Pak signaling and cell migration. The Akt/PKB isoforms have different roles in animals, with Akt2 primarily regulating metabolic signaling and Akt1 regulating growth and survival. Here we show distinct roles for Akt1 and Akt2 in mouse embryo fibroblast cell migration and regulation of the cytoskeleton. Akt1-deficient cells responded poorly to platelet-derived growth factor while Akt2-deficient cells had a dramatically enhanced response, resulting in a substantial increase in dorsal ruffling. Swapping domains between Akt1 and Akt2 demonstrated that the N-terminal region containing the pleckstrin homology domain and a linker region distinguishes the two isoforms, while the catalytic domains are interchangeable. Akt2 knock-out cells also migrated faster than wild-type cells, especially through **extracellular matrix** (ECM), while Akt1 knock-out cells migrated more slowly than wild-type cells. Consistently, Akt2 knock-out cells had elevated Pak1 and Rac activities, suggesting that Akt2 inhibits Rac and Pak1. Both Akt2 and Akt1 associated in complexes with Pak1, but only Akt2 inhibited Pak1 in kinase assays, suggesting an underlying molecular basis for the different cellular phenotypes. Together these data provide evidence for an unexpected functional link between Akt2 and Pak1 that opposes the actions of Akt1 on cell migration.
7. Patterned collagen fibers orient branching mammary epithelium through distinct signaling modules. For decades, the work of cell and developmental biologists has demonstrated the striking ability of the mesenchyme and the stroma to instruct epithelial form and function in the mammary gland, but the role of **extracellular matrix** (ECM) molecules in mammary pattern specification has not been elucidated. Here, we show that stromal collagen I (Col-I) fibers in the mammary fat pad are axially oriented prior to branching morphogenesis. Upon puberty, the branching epithelium orients along these fibers, thereby adopting a similar axial bias. To establish a causal relationship from Col-I fiber to epithelial orientation, we embedded mammary organoids within axially oriented Col-I fiber gels and observed dramatic epithelial co-orientation. Whereas a constitutively active form of Rac1, a molecule implicated in cell motility, prevented a directional epithelial response to Col-I fiber orientation, inhibition of the RhoA/Rho-associated kinase (ROCK) pathway did not. However, time-lapse studies revealed that, within randomly oriented Col-I matrices, the epithelium axially aligns fibers at branch sites via RhoA/ROCK-mediated contractions. Our data provide an explanation for how the stromal ECM encodes architectural cues for branch orientation as well as how the branching epithelium interprets and reinforces these cues through distinct signaling processes.
8. An integrin-ILK-microtubule network orients cell polarity and lumen formation in glandular epithelium. The **extracellular matrix** has a crucial role in determining the spatial orientation of epithelial polarity and the formation of lumens in glandular tissues; however, the underlying mechanisms remain elusive. By using Cre–Lox deletion we show that β1 integrins are required for normal mammary gland morphogenesis and lumen formation, both in vivo and in a three-dimensional primary culture model in which epithelial cells directly contact a basement membrane. Downstream of basement membrane β1 integrins, Rac1 is not involved; however, ILK is needed to polarize microtubule plus ends at the basolateral membrane and disrupting each of these components prevents lumen formation. The integrin–microtubule axis is necessary for the endocytic removal of apical proteins from the basement-membrane–cell interface and for internal Golgi positioning. We propose that this integrin signalling network controls the delivery of apical components to the correct surface and thereby governs the orientation of polarity and development of lumens.
9. Coronin-1C Protein and Caveolin Protein Provide Constitutive and Inducible Mechanisms of Rac1 Protein Trafficking. Sustained directional fibroblast migration requires both polarized activation of the protrusive signal, Rac1, and redistribution of inactive Rac1 from the rear of the cell so that it can be redistributed or degraded. In this work, we determine how alternative endocytic mechanisms dictate the fate of Rac1 in response to the **extracellular matrix** environment. We discover that both coronin-1C and caveolin retrieve Rac1 from similar locations at the rear and sides of the cell. We find that coronin-1C-mediated extraction, which is responsible for Rac1 recycling, is a constitutive process that maintains Rac1 protein levels within the cell. In the absence of coronin-1C, the effect of caveolin-mediated endocytosis, which targets Rac1 for proteasomal degradation, becomes apparent. Unlike constitutive coronin-1C-mediated trafficking, caveolin-mediated Rac1 endocytosis is induced by engagement of the fibronectin receptor syndecan-4. Such an inducible endocytic/degradation mechanism would predict that, in the presence of fibronectin, caveolin defines regions of the cell that are resistant to Rac1 activation but, in the absence of fibronectin leaves more of the membrane susceptible to Rac1 activation and protrusion. Indeed, we demonstrate that fibronectin-stimulated activation of Rac1 is accelerated in the absence of caveolin and that, when caveolin is knocked down, polarization of active Rac1 is lost in FRET experiments and culminates in shunting migration in a fibrous fibronectin matrix. Although the concept of polarized Rac1 activity in response to chemoattractants has always been apparent, our understanding of the balance between recycling and degradation explains how polarity can be maintained when the chemotactic gradient has faded.
10. Patterned collagen fibers orient branching mammary epithelium through distinct signaling modules. For decades, the work of cell and developmental biologists has demonstrated the striking ability of the mesenchyme and the stroma to instruct epithelial form and function in the mammary gland, but the role of **extracellular matrix** (ECM) molecules in mammary pattern specification has not been elucidated. Here, we show that stromal collagen I (Col-I) fibers in the mammary fat pad are axially oriented prior to branching morphogenesis. Upon puberty, the branching epithelium orients along these fibers, thereby adopting a similar axial bias. To establish a causal relationship from Col-I fiber to epithelial orientation, we embedded mammary organoids within axially oriented Col-I fiber gels and observed dramatic epithelial co-orientation. Whereas a constitutively active form of Rac1, a molecule implicated in cell motility, prevented a directional epithelial response to Col-I fiber orientation, inhibition of the RhoA/Rho-associated kinase (ROCK) pathway did not. However, time-lapse studies revealed that, within randomly oriented Col-I matrices, the epithelium axially aligns fibers at branch sites via RhoA/ROCK-mediated contractions. Our data provide an explanation for how the stromal ECM encodes architectural cues for branch orientation as well as how the branching epithelium interprets and reinforces these cues through distinct signaling processes.
11. Rac1 GTPase-deficient mouse lens exhibits defects in shape, suture formation, fiber cell migration and survival. Morphogenesis and shape of the ocular lens depend on epithelial cell elongation and differentiation into fiber cells, followed by the symmetric and compact organization of fiber cells within an enclosed **extracellular matrix**-enriched elastic capsule. The cellular mechanisms orchestrating these different events however, remain obscure. We investigated the role of the Rac1 GTPase in these processes by targeted deletion of expression using the conditional gene knockout (cKO) approach. Rac1 cKO mice were derived from two different Cre (Le-Cre and MLR-10) transgenic mice in which lens-specific Cre expression starts at embryonic day 8.75 and 10.5, respectively, in both the lens epithelium and fiber cells. The Le-Cre/Rac1 cKO mice exhibited an early-onset (E12.5) and severe lens phenotype compared to the MLR-10/Rac1 cKO (E15.5) mice. While the Le-Cre/Rac1 cKO lenses displayed delayed primary fiber cell elongation, lenses from both Rac1 cKO strains were characterized by abnormal shape, impaired secondary fiber cell migration, sutural defects and thinning of the posterior capsule which often led to rupture. Lens fiber cell N-cadherin/β-catenin/Rap1/Nectin-based cell-cell junction formation and WAVE-2/Abi-2/Nap1-regulated actin polymerization were impaired in the Rac1 deficient mice. Additionally, the Rac1 cKO lenses were characterized by a shortened epithelial sheet, reduced levels of **extracellular matri**x (ECM) proteins and increased apoptosis. Taken together, these data uncover the essential role of Rac1 GTPase activity in establishment and maintenance of lens shape, suture formation and capsule integrity, and in fiber cell migration, adhesion and survival, via regulation of actin cytoskeletal dynamics, cell adhesive interactions and ECM turnover.
12. Genetic ablation of Rac1 in cartilage results in chondrodysplasia. Small GTPases of the Rho family have been implicated in the regulation of many intracellular processes. However, their tissue-specific roles in mammalian growth and development in vivo remain largely unknown. Here we describe the effects of cartilage-specific inactivation of the Rac1 gene in mice. Mice carrying this mutation show increased lethality, skeletal deformities, severe kyphosis and dwarfism. Rac1-deficient growth plates are disorganized and hypocellular, with chondrocytes of abnormal shape and size. Rac1-deficient chondrocytes also display reduced adhesion and spreading on collagen II and fibronectin as well as altered organization of the actin cytoskeleton, suggesting that Rac1 is required for normal cell-**extracellular matrix** interactions in cartilage. This phenotype is accompanied by reduced proliferation, increased apoptosis and deregulated expression of the cell cycle genes cyclin D1 and p57 in vivo. Moreover, phosphorylation of p38 MAP kinases is greatly reduced and expression of a key regulator of cartilage development, Indian hedgehog, is increased in mutant mice. In summary, these data identify a novel, essential and tissue-specific role of Rac1 in skeletal development and demonstrate that Rac1 deficiency affects numerous regulatory pathways in cartilage.
13. Opposing roles for Akt1 and Akt2 in Rac/Pak signaling and cell migration. The Akt/PKB isoforms have different roles in animals, with Akt2 primarily regulating metabolic signaling and Akt1 regulating growth and survival. Here we show distinct roles for Akt1 and Akt2 in mouse embryo fibroblast cell migration and regulation of the cytoskeleton. Akt1-deficient cells responded poorly to platelet-derived growth factor while Akt2-deficient cells had a dramatically enhanced response, resulting in a substantial increase in dorsal ruffling. Swapping domains between Akt1 and Akt2 demonstrated that the N-terminal region containing the pleckstrin homology domain and a linker region distinguishes the two isoforms, while the catalytic domains are interchangeable. Akt2 knock-out cells also migrated faster than wild-type cells, especially through **extracellular matrix** (ECM), while Akt1 knock-out cells migrated more slowly than wild-type cells. Consistently, Akt2 knock-out cells had elevated Pak1 and Rac activities, suggesting that Akt2 inhibits Rac and Pak1. Both Akt2 and Akt1 associated in complexes with Pak1, but only Akt2 inhibited Pak1 in kinase assays, suggesting an underlying molecular basis for the different cellular phenotypes. Together these data provide evidence for an unexpected functional link between Akt2 and Pak1 that opposes the actions of Akt1 on cell migration.
14. The LIM protein Ajuba influences p130Cas localization and Rac1 activity during cell migration. Cell migration requires extension of lamellipodia that are stabilized by formation of adhesive complexes at the leading edge. Both processes are regulated by signaling proteins recruited to nascent adhesive sites that lead to activation of Rho GTPases. The Ajuba/Zyxin family of LIM proteins are components of cellular adhesive complexes. We show that cells from Ajuba null mice are inhibited in their migration, without associated abnormality in adhesion to **extracellular matrix** proteins, cell spreading, or integrin activation. Lamellipodia production, or function, is defective and there is a selective reduction in the level and tyrosine phosphorylation of FAK, p130Cas, Crk, and Dock180 at nascent focal complexes. In response to migratory cues Rac activation is blunted in Ajuba null cells, as detected biochemically and by FRET analysis. Ajuba associates with the focal adhesion-targeting domain of p130Cas, and rescue experiments suggest that Ajuba acts upstream of p130Cas to localize p130Cas to nascent adhesive sites in migrating cells thereby leading to the activation of Rac.
15. Arhgap28 is a RhoGAP that inactivates RhoA and downregulates stress fibers. The small GTPase RhoA is a major regulator of actin reorganization during the formation of stress fibers; thus identifying molecules that regulate Rho activity is necessary for a complete understanding of the mechanisms that determine cell contractility. Here, we have identified Arhgap28 as a Rho GTPase activating protein (RhoGAP) that switches RhoA to its inactive form. We generated an Arhgap28-LacZ reporter mouse that revealed gene expression in soft tissues at E12.5, pre-bone structures of the limb at E15.5, and prominent expression restricted mostly to ribs and limb long bones at E18.5 days of development. Expression of recombinant Arhgap28-V5 in human osteosarcoma SaOS-2 cells caused a reduction in the basal level of RhoA activation and disruption of actin stress fibers. **Extracellular matrix** assembly studies using a 3-dimensional cell culture system showed that Arhgap28 was upregulated during Rho-dependent assembly of the ECM. Taken together, these observations led to the hypothesis that an Arhgap28 knockout mouse model would show a connective tissue phenotype, perhaps affecting bone. Arhgap28-null mice were viable and appeared normal, suggesting that there could be compensation from other RhoGAPs. Indeed, we showed that expression of Arhgap6 (a closely related RhoGAP) was upregulated in Arhgap28-null bone tissue. An upregulation in RhoA expression was also detected suggesting that Arhgap28 may be able to additionally regulate Rho signaling at a transcriptional level. Microarray analyses revealed that Col2a1, Col9a1, Matn3, and Comp that encode **extracellular matrix** proteins were downregulated in Arhgap28-null bone. Although mutations in these genes cause bone dysplasias no bone phenotype was detected in the Arhgap-28 null mice. Together, these data suggest that the regulation of Rho by RhoGAPs, including Arhgap28, during the assembly and development of mechanically strong tissues is complex and may involve multiple RhoGAPs.
16. Therapeutic ultrasound bypasses canonical syndecan-4 signaling to activate rac1. The application of pulsed, low intensity ultrasound is emerging as a potent therapy for the treatment of complex bone fractures and tissue damage. Ultrasonic stimuli accelerate fracture healing by up to 40% and enhance tendon and ligament healing by promoting cell proliferation, migration, and matrix synthesis through an unresolved mechanism. Ultrasound treatment also induces closure of nonunion fractures, at a success rate (85% of cases) similar to that of surgical intervention (68-96%) while avoiding the complications associated with surgery. The regulation of cell adhesion necessary for wound healing depends on cooperative engagement of the **extracellular matrix** receptors, integrin and syndecan, as exemplified by the wound healing defects observed in syndecan- and integrin-knock-out mice. This report distinguishes the influence of ultrasound on signals downstream of the prototypic fibronectin receptors, alpha(5)beta(1) integrin and syndecan-4, which cooperate to regulate Rac1 and RhoA. Ultrasonic stimulation fails to activate integrins or induce cell spreading on poor, electrostatic ligands. By contrast, ultrasound treatment overcomes the necessity of engagement or expression of syndecan-4 during the process of focal adhesion formation, which normally requires simultaneous engagement of both receptors. Ultrasound exerts an influence downstream of syndecan-4 and PKCalpha to specifically activate Rac1, itself a critical regulator of tissue repair, and to a lesser extent RhoA. The ability of ultrasound to bypass syndecan-4 signaling, which is known to facilitate efficient tissue repair, explains the reduction in healing times observed in ultrasound-treated patients. By substituting for one of the key axes of adhesion-dependent signaling, ultrasound therapy has considerable potential as a clinical technique.
17. Integrin alpha3beta1-dependent activation of FAK/Src regulates Rac1-mediated keratinocyte polarization on laminin-5. Upon epidermal wounding, keratinocytes at the wound edge become activated, deposit newly synthesized laminin-5 into the **extracellular matrix**, and migrate into the wound bed. The interaction between integrin alpha3beta1 and laminin-5 is essential for establishment of a stable, leading lamellipodium and persistent keratinocyte migration. We previously showed that integrin alpha3beta1 activates the Rho family GTPase Rac1 and regulates Rac1-dependent formation of polarized, leading lamellipodia in migrating keratinocytes. In the present study, we explored the role of focal adhesion kinase (FAK) and src signaling in this process. We show that overexpression of the FAK inhibitor FAK-related non-kinase or of the FAK(Y397F) auto-phosphorylation mutant, induced abnormal, non-polarized spreading of keratinocytes on laminin-5. Integrin alpha3beta1 was required for full FAK auto-phosphorylation at Y397, and subsequent src kinase-dependent phosphorylation of FAK at residues Y861 and Y925, sites responsible for promoting signal transduction downstream of FAK, indicating that alpha3beta1 regulates the coordination of FAK/src signal transduction. Inhibiting either src kinase activity or FAK signaling interfered with alpha3beta1-mediated Rac1 activation and polarized cell spreading. These findings reveal a novel pathway in migratory keratinocytes wherein alpha3beta1-laminin-5 interactions regulate src kinase signaling through FAK, promoting Rac1 activation and polarized lamellipodium extension.
18. Phosphoinositide-3 kinase-Rac1-c-Jun NH2-terminal kinase signaling mediates collagen I-induced cell scattering and up-regulation of N-cadherin expression in mouse mammary epithelial cells. During epithelial-to-mesenchymal transitions (EMTs), cells must change their interactions with one another and with their **extracellular matrix** in a synchronized manner. To characterize signaling pathways cells use to coordinate these changes, we used NMuMG mammary epithelial cells. We showed that these cells become fibroblastic and scattered, with increased N-cadherin expression when cultured on collagen I. Rac1 and c-Jun NH2-terminal kinase (JNK) were activated when cells were plated on collagen I, and dominant inhibitory Rac1 (RacN17) or inhibition of JNK signaling prevented collagen I-induced morphological changes and N-cadherin up-regulation. Furthermore, inhibiting phosphoinositide-3 kinase (PI3K) activity prevented Rac1 and JNK activation as well as collagen I-induced N-cadherin up-regulation. These data implicate PI3K-Rac1-JNK signaling in collagen I-induced changes in NMuMG cells. To establish a role for N-cadherin in collagen I-induced cell scattering, we generated N-cadherin overexpressing and knockdown NMuMG cells and showed that knocking down N-cadherin expression prevented collagen I-induced morphological changes. Motility assays showed that cells overexpressing N-cadherin were significantly more motile than mock-transfected cells and that N-cadherin-mediated motility was collagen I dependent. In addition, we showed that cord formation and branching in three-dimensional culture (EMT-dependent events) required N-cadherin expression and PI3K-Rac1-JNK signaling.
19. 8-Hydroxy-2-deoxyguanosine prevents plaque formation and inhibits vascular smooth muscle cell activation through Rac1 inactivation. 8-Hydroxy-2-deoxyguanosine (8-OHdG), a marker of oxidative stress, has been recently rediscovered to inhibit Rac1 in neutrophils and macrophages, thereby inhibiting Rac1-linked functions of these cells, including reactive oxygen species production through NADPH oxidase activation, phagocytosis, chemotaxis, and cytokine release. In vascular smooth muscle cells (VSMCs), reactive oxygen species also induce abnormal proliferation and migration leading to progression of atherosclerosis. Based upon the involvement of reactive oxygen species in phagocytic cells and VSMCs during the atherosclerotic process, we hypothesized that 8-OHdG could have antiatherosclerotic action and tested this hypothesis in an experimentally induced atherosclerosis in mice. Partially ligated ApoE knockout mice, a more physiologically relevant model of low and oscillatory flow, developed an advanced lesion in 2 weeks, and orally administered 8-OHdG significantly reduced plaque formation along with reduced superoxide formation, monocyte/macrophage infiltration, and **extracellular matrix** (ECM) accumulation. The effects of 8-OHdG observed in primary VSMCs were consistent with the in vivo effects of 8-OHdG and were inhibitory to angiotensin II or platelet-derived growth factor-induced production of reactive oxygen species, proliferation, migration, and ECM production. Also, angiotensin II-induced Rac1 activity in VSMCs was significantly inhibited by 8-OHdG, and transfection of constitutively active Rac1 reversed the inhibitory effect of 8-OHdG on VSMC activation. Molecular docking study showed that 8-OHdG stabilizes Rac1-GEF complex, indicating the physical contact of 8-OHdG with Rac1. These findings highly suggest that the antiatherosclerotic effect of 8-OHdG is mediated by inhibition of Rac1 activity. In conclusion, our results show a novel action of orally active 8-OHdG in suppressing atherosclerotic plaque formation in vivo and VSMC activation in vitro through inhibition of Rac1, which emphasizes a new therapeutic avenue to benefit atherosclerosis.
20. Enhanced cell-substratum adhesion of E-cadherin-expressing cells is mediated by activation of the small GTPase protein, Rac1. L cell transfectants stably expressing E-cadherin demonstrate a remarkable increase in adherence to **extracellular matrix** proteins, such as type IV collagen and fibronectin. This enhanced adhesion is mediated by integrin-type cell surface receptors, as assessed by inhibition with anti-receptor antibodies. Both the rate and efficiency of adhesion were enhanced 4- to 5-fold. In contrast, non-specific adhesion processes, such as cell attachment to polylysine-coated substrata, are unaffected by E-cadherin expression. Thus, integrin-mediated but not non-specific adhesion is modulated by E-cadherin expression. L cells expressing mutant E-cadherin molecules either lacking the cytoplasmic domain or bearing an amino acid substitution in the Ca2+-binding motif did not exhibit enhanced adhesion. The amount of collagen receptor, the alpha1 and beta1 integrin, did not change following expression of E-cadherin. Pull-down assays with the Cdc42/Rac interactive binding (CRIB) domain of the Rac effector, p21-activated kinase, revealed increased Rac-GTP levels in cells expressing wild-type E-cadherin. These results suggest that the activation of Rac is involved in the enhancement of integrin-mediated adhesion induced by E-cadherin expression.
21. Non-small cell lung carcinoma cell motility, rac activation and metastatic dissemination are mediated by protein kinase C epsilon. Protein kinase C (PKC) ε, a key signaling transducer implicated in mitogenesis, survival, and cancer progression, is overexpressed in human primary non-small cell lung cancer (NSCLC). The role of PKCε in lung cancer metastasis has not yet been established.Here we show that RNAi-mediated knockdown of PKCε in H358, H1299, H322, and A549 NSCLC impairs activation of the small GTPase Rac1 in response to phorbol 12-myristate 13-acetate (PMA), serum, or epidermal growth factor (EGF). PKCε depletion markedly impaired the ability of NSCLC cells to form membrane ruffles and migrate. Similar results were observed by pharmacological inhibition of PKCε with εV1-2, a specific PKCε inhibitor. PKCε was also required for invasiveness of NSCLC cells and modulated the secre**tion of extracellula**r matrix proteases and protease inhibitors. Finally, we found that PKCε-depleted NSCLC cells fail to disseminate to lungs in a mouse model of metastasis.Our results implicate PKCε as a key mediator of Rac signaling and motility of lung cancer cells, highlighting its potential as a therapeutic target.
22. Paxillin and Hic-5 interaction with vinculin is differentially regulated by Rac1 and RhoA. Cell migration is of paramount importance to organism development and maintenance as well as multiple pathological processes, including cancer metastasis. The RhoGTPases Rac1 and RhoA are indispensable for cell migration as they regulate cell protrusion, cell-**extracellular matrix** (ECM) interactions and force transduction. However, the consequences of their activity at a molecular level within the cell remain undetermined. Using a combination of FRET, FRAP and biochemical analyses we show that the interactions between the focal adhesion proteins vinculin and paxillin, as well as the closely related family member Hic-5 are spatially and reciprocally regulated by the activity of Rac1 and RhoA. Vinculin in its active conformation interacts with either paxillin or Hic-5 in adhesions in response to Rac1 and RhoA activation respectively, while inactive vinculin interacts with paxillin in the membrane following Rac1 inhibition. Additionally, Rac1 specifically regulates the dynamics of paxillin as well as its binding partner and F-actin interacting protein actopaxin (α-parvin) in adhesions. Furthermore, FRET analysis of protein:protein interactions within cell adhesions formed in 3D matrices revealed that, in contrast to 2D systems vinculin interacts preferentially with Hic-5. This study provides new insight into the complexity of cell-ECM adhesions in both 2D and 3D matrices by providing the first description of RhoGTPase-coordinated protein:protein interactions in a cellular microenvironment. These data identify discrete roles for paxillin and Hic-5 in Rac1 and RhoA-dependent cell adhesion formation and maturation; processes essential for productive cell migration.
23. Lamellipodia nucleation by filopodia depends on integrin occupancy and downstream Rac1 signaling. Time-lapse video-microscopy unambiguously shows that fibroblast filopodia are the scaffold of lamellipodia nucleation that allows anisotropic cell spreading. This process was dissected into elementary stages by monitoring cell adhesion on micropatterned **extracellular matrix** arrays of various pitches. Adhesion structures are stabilized by contact with the adhesive plots and subsequently converted into lamellipodia-like extensions starting at the filopodia tips. This mechanism progressively leads to full cell spreading. Stable expression of the dominant-negative Rac1 N17 impairs this change in membrane extension mode and stops cell spreading on matrix arrays. Similar expression of the dominant-negative Cdc42 N17 impairs cell spreading on homogenous and structured substrate, suggesting that filopodia extension is a prerequisite for cell spreading in this model. The differential polarity of the nucleation of lamellipodial structures by filopodia on homogenous and structured surfaces starting from the cell body and of filopodia tip, respectively, suggested that this process is triggered by areas that are in contact with **extracellular matrix** proteins for longer times. Consistent with this view, wild-type cells cannot spread on microarrays made of function blocking or neutral anti-beta 1 integrin antibodies. However, stable expression of a constitutively active Rac1 mutant rescues the cell ability to spread on these integrin microarrays. Thereby, lamellipodia nucleation by filopodia requires integrin occupancy by matrix substrate and downstream Rac1 signaling.
24. Changes in dermal matrix in the absence of Rac1 in keratinocytes. Keratinocytes, in response to irritants, secrete pro-inflammatory mediators which recruit and activate immune and mesenchymal cells, including fibroblasts, to repair the skin. Fibroblasts respond by synthesising collagen and promoting the crosslinking **extracellular matrix** (ECM). We recently showed that the deletion of Rac1 in keratinocytes causes heightened inflammation due to aberrant crosstalk with immune cells. Indeed, the skin of these mice shows a higher inflammatory response to the induction of irritant contact dermatitis (ICD), and also even to treatment with a vehicle alone, compared with controls. As inflammation is intimately linked with fibrotic disease in the skin, this raised the question as to whether this deletion may also affect the deposition and arrangement of the dermal ECM. This study assessed the effects of Rac1 deletion in keratinocytes and of the heightened inflammatory status by induction of ICD on the tissue localisation and arrangements of dermal collagen. Qualitative analysis did not reveal evidence for the formation of pathologies in the dermis. However, quantitative analysis did reveal some perturbations in the dermal matrix, namely that only the combination of the lack of Rac1 and ICD affects the architectural organisation of the dermal collagen, and that a higher inflammatory state in the tissue (i.e. when Rac1 is deleted in the keratinocytes or ICD is induced in the skin, or a combination of both) influences the diameter of the collagen fibrils. It is proposed that this increase in the diameter of collagen fibrils due to inflammation may serve as pre-fibrotic marker enabling earlier determination of fibrosis and earlier treatment. This study has revealed previously unknown effects on the ECM due to the deletion of Rac1 in keratinocytes.
25. Rac1 expression by fibroblasts is required for tissue repair in vivo. Tissue repair requires that fibroblasts migrate into the wound to produce and remodel **extracellular matrix**, a process that requires adhesion. Failure to suppress the tissue repair program results in fibrotic disorders that are characterized by excessive adhesive signaling. The role of specific components of adhesive signaling in fibrogenic responses is unclear, but may involve small GTPases such as Rac1. To address the functions of Rac1 in fibroblasts, we generated mice containing a fibroblast-specific deletion of Rac1. These mice show delayed cutaneous wound closure, including reduced collagen production and myofibroblast formation. In cultured Rac1-deficient fibroblasts, adhesion, spreading, and migration were significantly inhibited. Rac1-deficient fibroblasts possessed impaired myofibroblast formation and function as visualized by reduced alpha-smooth muscle actin expression as well as matrix contraction. Both in vivo and in vitro, Rac1- deficient fibroblasts showed a reduced generation of reactive oxygen species; in vitro, hydrogen peroxide alleviated the phenotype of Rac1-deficient fibroblasts. Thus, Rac1 is an essential signaling integrator that is required for normal wound healing and dermal homeostasis.
26. Integrins regulate Rac targeting by internalization of membrane domains. Translocation of the small GTP-binding protein Rac1 to the cell plasma membrane is essential for activating downstream effectors and requires integrin-mediated adhesion of cells to **extracellular matrix**. We report that active Rac1 binds preferentially to low-density, cholesterol-rich membranes, and specificity is determined at least in part by membrane lipids. Cell detachment triggered internalization of plasma membrane cholesterol and lipid raft markers. Preventing internalization maintained Rac1 membrane targeting and effector activation in nonadherent cells. Regulation of lipid rafts by integrin signals may regulate the location of membrane domains such as lipid rafts and thereby control domain-specific signaling events in anchorage-dependent cells.
27. First step of the cell-penetrating peptide mechanism involves Rac1 GTPase-dependent actin-network remodelling. Application of CPPs (cell-penetrating peptides) constitutes a promising strategy for the intracellular delivery of therapeutic molecules. The non-covalent approach based on the amphipathic peptide MPG has been successfully used to improve the delivery of biologically active macromolecules, both in cellulo and in vivo, through a mechanism independent of the endosomal pathway and mediated by the membrane potential.In the present study, we have investigated the first step of the cellular uptake mechanism of MPG and shown that both MPG and MPG-cargo complexes interact with the **extracellular matrix** through the negatively charged heparan sulfate proteoglycans. We demonstrated that initiation of cellular uptake constitutes a highly dynamic mechanism where the binding of MPG or the MPG-cargo to the **extracellular matrix** is rapidly followed by a remodelling of the actin network associated with the activation of the GTPase Rac1. We suggest that MPG-induced clustering of the glycosaminoglycan platform constitutes the 'onset' of the cellular uptake mechanism, thereby increasing membrane dynamics and membrane fusion processes. This process favours cell entry of MPG or MPG-DNA complexes, which is further controlled by the ability of MPG to induce a local membrane destabilization.Although CPPs are taken up through different pathways and mechanisms, the initial step involves electrostatic interactions with the glycosaminoglycan platform, and the dynamics of associated membrane microdomains can be generalized to most non-viral delivery systems.
28. Loss of protein kinase Cepsilon results in impaired cutaneous wound closure and myofibroblast function. Cutaneous wound repair requires the de novo induction of a specialized form of fibroblast, the alpha-smooth muscle actin (alpha-SMA)-expressing myofibroblast, which migrates into the wound where it adheres to and contracts **extracellular matrix** (ECM), resulting in wound closure. Persistence of the myofibroblast results in scarring and fibrotic disease. In this report, we show that, compared with wild-type littermates, PKCepsilon-/- mice display delayed impaired cutaneous wound closure and a reduction in myofibroblasts. Moreover, both in the presence and absence of TGFbeta, dermal fibroblasts from PKCepsilon-/- mice cultured on fibronectin show impaired abilities to form ;supermature' focal adhesions and alpha-SMA stress fibers, and reduced pro-fibrotic gene expression. Smad3 phosphorylation in response to TGFbeta1 was impaired in PKCepsilon-/- fibroblasts. PKCepsilon-/- fibroblasts show reduced FAK and Rac activation, and adhesive, contractile and migratory abilities. Overexpressing constitutively active Rac1 rescues the defective FAK phosphorylation, cell migration, adhesion and stress fiber formation of these PKCepsilon-/- fibroblasts, indicating that Rac1 operates downstream of PKCepsilon, yet upstream of FAK. These results suggest that loss of PKCepsilon severely impairs myofibroblast formation and function, and that targeting PKCepsilon may be beneficial in selectively modulating wound healing and fibrotic responses in vivo.
29. EMMPRIN activates multiple transcription factors in cardiomyocytes, and induces interleukin-18 expression via Rac1-dependent PI3K/Akt/IKK/NF-kappaB andMKK7/JNK/AP-1 signaling. The transmembrane glycoprotein **extracellular matrix** metalloproteinase inducer (EMMPRIN), and the pleiotropic proinflammatory cytokine interleukin (IL)-18, play critical roles in myocardial remodeling, by inducing matrix degrading metalloproteinases (MMPs). Previously we showed that IL-18 induces EMMPRIN expression in cardiomyocytes via MyD88/IRAK4/TRAF6/JNK-dependent Sp1 activation. Here in reciprocal studies we demonstrate that EMMPRIN is a potent inducer of IL-18 transcription, protein expression and protein secretion in primary mouse cardiomyocytes. We show for the first time that EMMPRIN stimulates the activation of NF-kappaB, AP-1, CREB, and ATF-2 in cardiomyocytes, and induces IL-18 expression via Rac1-dependent PI3K/Akt/IKK/NF-kappaB and MKK7/JNK/AP-1 signaling. Moreover, EMMPRIN induces robust time-dependent induction of various MMP mRNAs. EMMPRIN also induces the mRNA of TIMPs 1 and 3, but in a delayed fashion. These results suggest that IL-18-induced EMMPRIN expression may favor net MMP expression and ECM destruction, and thus identify both as potential therapeutic targets in countering adverse myocardial remodeling.
30. Plakoglobin regulates cell motility through Rho- and fibronectin-dependent Src signaling. We previously showed that the cell-cell junction protein plakoglobin (PG) not only suppresses motility of keratinocytes in contact with each other, but also, unexpectedly, of single cells. Here we show that PG deficiency results in **extracellular matrix** (ECM)-dependent disruption of mature focal adhesions and cortical actin organization. Plating PG⁻/⁻ cells onto ECM deposited by PG+/⁻ cells partially restored normal cell morphology and inhibited PG⁻/⁻ cell motility. In over 70 adhesion molecules whose expression we previously showed to be altered in PG⁻/⁻ cells, a substantial decrease in fibronectin (FN) in PG⁻/⁻ cells stood out. Re-introduction of PG into PG⁻/⁻ cells restored FN expression, and keratinocyte motility was reversed by plating PG⁻/⁻ cells onto FN. Somewhat surprisingly, based on previously reported roles for PG in regulating gene transcription, PG-null cells exhibited an increase, not a decrease, in FN promoter activity. Instead, PG was required for maintenance of FN mRNA stability. PG⁻/⁻ cells exhibited an increase in activated Src, one of the kinases controlled by FN, a phenotype reversed by plating PG⁻/⁻ cells on ECM deposited by PG+/⁻ keratinocytes. PG⁻/⁻ cells also exhibited Src-independent activation of the small GTPases Rac1 and RhoA. Both Src and RhoA inhibition attenuated PG⁻/⁻ keratinocyte motility. We propose a novel role for PG in regulating cell motility through distinct ECM-Src and RhoGTPase-dependent pathways, influenced in part by PG-dependent regulation of FN mRNA stability.
31. Atypical protein kinase C and Par3 are required for proteoglycan-induced axon growth inhibition. When the CNS is injured, damaged axons do not regenerate. This failure is due in part to the growth-inhibitory environment that forms at the injury site. Myelin-associated molecules, repulsive axon guidance molecules, and **extracellular matrix** molecules including chondroitin sulfate proteoglycans (CSPGs) found within the glial scar inhibit axon regeneration but the intracellular signaling mechanisms triggered by these diverse molecules remain largely unknown. Here we provide biochemical and functional evidence that atypical protein kinase C (PKCζ) and polarity (Par) complex proteins mediate axon growth inhibition. Treatment of postnatal rat neurons in vitro with the NG2 CSPG, a major component of the glial scar, activates PKCζ, and this activation is both necessary and sufficient to inhibit axonal growth. NG2 treatment also activates Cdc42, increases the association of Par6 with PKCζ, and leads to a Par3-dependent activation of Rac1. Transfection of neurons with kinase-dead forms of PKCζ, dominant-negative forms of Cdc42, or mutant forms of Par6 that do not bind to Cdc42 prevent NG2-induced growth inhibition. Similarly, transfection with either a phosphomutant Par3 (S824A) or dominant-negative Rac1 prevent inhibition, whereas expression of constitutively active Rac1 inhibits axon growth on control surfaces. These results suggest a model in which NG2 binding to neurons activates PKCζ and modifies Par complex function. They also identify the Par complex as a novel therapeutic target for promoting axon regeneration after CNS injury.
32. Rac1 links integrin-mediated adhesion to the control of lactational differentiation in mammary epithelia. The expression of tissue-specific genes during mammary gland differentiation relies on the coincidence of two distinct signaling events: the continued engagement of beta1 integrins with the **extracellular matrix** (ECM) and a hormonal stimulus from prolactin (Prl). How the integrin and Prl receptor (PrlR) systems integrate to regulate milk protein gene synthesis is unknown. In this study, we identify Rac1 as a key link. Dominant-negative Rac1 prevents Prl-induced synthesis of the milk protein beta-casein in primary mammary epithelial cells cultured as three-dimensional acini on basement membrane. Conversely, activated Rac1 rescues the defective beta-casein synthesis that occurs under conditions not normally permissive for mammary differentiation, either in beta1 integrin-null cells or in wild-type cells cultured on collagen. Rac1 is required downstream of integrins for activation of the PrlR/Stat5 signaling cascade. Cdc42 is also necessary for milk protein synthesis but functions via a distinct mechanism to Rac1. This study identifies the integration of signals provided by ECM and hormones as a novel role for Rho family guanosine triphosphatases.
33. Regulation of endothelial cell cytoskeletal reorganization by a secreted frizzled-related protein-1 and frizzled 4- and frizzled 7-dependent pathway: role in neovessel formation. Consistent with findings of Wnt pathway members involved in vascular cells, a role for Wnt/Frizzled signaling has recently emerged in vascular cell development. Among the few Wnt family members implicated in vessel formation in adult, Wnt7b and Frizzled 4 have been shown as involved in vessel formation in the lung and in the retina, respectively. Our previous work has shown a role for secreted Frizzled-related protein-1 (sFRP-1), a proposed Wnt signaling inhibitor, in neovascularization after an ischemic event and demonstrated its role as a potent angiogenic factor. However the mechanisms involved have not been investigated. Here, we show that sFRP-1 treatment increases endothelial cell spreading on **extracellular matrix** as revealed by actin stress fiber reorganization in an integrin-dependent manner. We demonstrate that sFRP-1 can interact with Wnt receptors Frizzled 4 and 7 on endothelial cells to transduce downstream to cellular machineries requiring Rac-1 activity in cooperation with GSK-3beta. sFRP-1 overexpression in endothelium specifically reversed the inactivation of GSK-3 beta and increased neovascularization in ischemia-induced angiogenesis in mouse hindlimb. This study illustrates a regulated pathway by sFRP-1 involving GSK-3beta and Rac-1 in endothelial cell cytoskeletal reorganization and in neovessel formation.
34. Tyr724 phosphorylation of ELMO1 by Src is involved in cell spreading and migration via Rac1 activation. The complex of Dock180/ELMO1 that functions as a bipartite guanine nucleotide exchange factor for Rac is essential for diverse physiological and pathological processes of cells such as cell migration, phagocytosis, and invasion of cancer cells. Among the Src-family tyrosine kinases (SFKs), it has been reported that Hck directly phosphorylates ELMO1, regulating phagocytosis by promoting activation of Rac1; however, the involvement of other SFKs in ELMO1 phosphorylation has remained unknown. Here, we identified novel tyrosine (Y) residues of ELMO1 phosphorylated by SFKs, and examined the effects on Rac1 activity, cell adhesion, spreading, and cell motility on **extracellular matrix** (ECM).In this study, we unveiled that Src and Fyn can induce tyrosine phosphorylation of ELMO1 in in vivo and in vitro phosphorylation assays. Mutational analyses identified both Y720 and Y724 residues of ELMO1 as Src-mediated phosphorylation sites, preferentially on Y724. Single substitution of Y724 to Phe abrogated Rac1 activation triggered by Src. To elucidate the biological function of pY724, we established NIH3T3 cells stably expressing wild-type ELMO1 or its Y724F mutant together with Dock180. Among them, Y724-deficient cells exhibited a depletion of Rac1 activity with diminished phosphorylation of ELMO1 even upon the ECM-stimulation. It is noteworthy that NIH3T3 cells with ELMO1 Y724F were strikingly defective to promote cell spreading on fibronectin-coated dish, concomitantly exhibiting immature assemblies of actin stress fibers and focal adhesions. Eventually, ELMO1 Y724F significantly impaired cell migration.These results define that Src-mediated Y724 phosphorylation in ELMO1 plays a critical role for cell spreading via activation of Rac1, leading to promotion of cell migration. As the overexpression and/or hyperactivation of Src have been shown in a wide variety of human cancers, Src-mediated phosphorylation of Y724 in ELMO1 may regulate cancer cell adhesion to the ECM, invasion into surrounding tissues, and subsequent distant metastasis.
35. Plexin-B2 negatively regulates macrophage motility, Rac, and Cdc42 activation. Plexins are cell surface receptors widely studied in the nervous system, where they mediate migration and morphogenesis though the Rho family of small GTPases. More recently, plexins have been implicated in immune processes including cell-cell interaction, immune activation, migration, and cytokine production. Plexin-B2 facilitates ligand induced cell guidance and migration in the nervous system, and induces cytoskeletal changes in overexpression assays through RhoGTPase. The function of Plexin-B2 in the immune system is unknown. This report shows that Plexin-B2 is highly expressed on cells of the innate immune system in the mouse, including macrophages, conventional dendritic cells, and plasmacytoid dendritic cells. However, Plexin-B2 does not appear to regulate the production of proinflammatory cytokines, phagocytosis of a variety of targets, or directional migration towards chemoattractants or **extracellular matrix** in mouse macrophages. Instead, Plxnb2(-/-) macrophages have greater cellular motility than wild type in the unstimulated state that is accompanied by more active, GTP-bound Rac and Cdc42. Additionally, Plxnb2(-/-) macrophages demonstrate faster in vitro wound closure activity. Studies have shown that a closely related family member, Plexin-B1, binds to active Rac and sequesters it from downstream signaling. The interaction of Plexin-B2 with Rac has only been previously confirmed in yeast and bacterial overexpression assays. The data presented here show that Plexin-B2 functions in mouse macrophages as a negative regulator of the GTPases Rac and Cdc42 and as a negative regulator of basal cell motility and wound healing.
36. 14-3-3beta-Rac1-p21 activated kinase signaling regulates Akt1-mediated cytoskeletal organization, lamellipodia formation and fibronectin matrix assembly. Akt1 belongs to the three-gene Akt family and functions as a serine-threonine kinase regulating phosphorylation of an array of substrates and mediating cellular processes such as cell migration, proliferation, survival, and cell cycle. Our previous studies have established the importance of Akt1 in angiogenesis and absence of Akt1 resulted in impaired integrin activation, adhesion, migration, and **extracellular matrix** assembly by endothelial cells and fibroblasts. In this study, we identify the downstream signaling pathways activated by Akt1 in the regulation of these cellular events. We demonstrate here that Akt1 is necessary for the growth factor stimulated activation of 14-3-3beta-Rac1-p21 activated kinase (Pak) pathway in endothelial cells and fibroblasts. While activation of Akt1 resulted in translocation of Rac1 to membrane ruffles, enhanced Rac1 activity, Pak1 phosphorylation, and lamellipodia formation, resulting in enhanced adhesion and assembly of fibronectin, inhibition of Akt1 resulted in inhibition of these processes due to impaired Rac1-Pak signaling. Formation of lamellipodia, adhesion, and fibronectin assembly by myristoylated Akt1 expression in NIH 3T3 fibroblasts was inhibited by co-expression with either dominant negative Rac1 or dominant negative Pak1. In contrast, impaired lamellipodia formation, adhesion, and fibronectin assembly by dominant negative-Akt1 expression was rescued by co-expression with either constitutively active-Rac1 or -Pak1. Moreover, previously reported defects in adhesion and **extracellular matrix** assembly by Akt1(-/-) fibroblasts could be rescued by expression with either active-Rac1 or -Pak1, implying the importance of Rac1-Pak signaling in growth factor stimulated cytoskeletal assembly, lamellipodia formation and cell migration in endothelial cells and fibroblasts downstream of Akt1 activation.

#### Component

1. **extracellular matrix**

### [Ii]nvasive

#### PubMed

1. Transmembrane collagen XVII modulates integrin dependent keratinocyte migration via PI3K/Rac1 signaling. The hemidesmosomal transmembrane component collagen XVII (ColXVII) plays an important role in the anchorage of the epidermis to the underlying basement membrane. However, this adhesion protein seems to be also involved in the regulation of keratinocyte migration, since its expression in these cells is strongly elevated during reepithelialization of acute wounds and in the **invasive** front of squamous cell carcinoma, while its absence in ColXVII-deficient keratinocytes leads to altered cell motility. Using a genetic model of murine Col17a1⁻/⁻ keratinocytes we elucidated ColXVII mediated signaling pathways in cell adhesion and migration. Col17a1⁻/⁻ keratinocytes exhibited increased spreading on laminin 332 and accelerated, but less directed cell motility. These effects were accompanied by increased expression of the integrin subunits β4 and β1. The migratory phenotype, as evidenced by formation of multiple unstable lamellipodia, was associated with enhanced phosphoinositide 3-kinase (PI3K) activity. Dissection of the signaling pathway uncovered enhanced phosphorylation of the β4 integrin subunit and the focal adhesion kinase (FAK) as activators of PI3K. This resulted in elevated Rac1 activity as a downstream consequence. These results provide mechanistic evidence that ColXVII coordinates keratinocyte adhesion and directed motility by interfering integrin dependent PI3K activation and by stabilizing lamellipodia at the leading edge of reepithelializing wou**nds and** in invasive squamous cell carcinoma.
2. Transmembrane collagen XVII modulates integrin dependent keratinocyte migration via PI3K/Rac1 signaling. The hemidesmosomal transmembrane component collagen XVII (ColXVII) plays an important role in the anchorage of the epidermis to the underlying basement membrane. However, this adhesion protein seems to be also involved in the regulation of keratinocyte migration, since its expression in these cells is strongly elevated during reepithelialization of acute wounds and in the **invasive** front of squamous cell carcinoma, while its absence in ColXVII-deficient keratinocytes leads to altered cell motility. Using a genetic model of murine Col17a1⁻/⁻ keratinocytes we elucidated ColXVII mediated signaling pathways in cell adhesion and migration. Col17a1⁻/⁻ keratinocytes exhibited increased spreading on laminin 332 and accelerated, but less directed cell motility. These effects were accompanied by increased expression of the integrin subunits β4 and β1. The migratory phenotype, as evidenced by formation of multiple unstable lamellipodia, was associated with enhanced phosphoinositide 3-kinase (PI3K) activity. Dissection of the signaling pathway uncovered enhanced phosphorylation of the β4 integrin subunit and the focal adhesion kinase (FAK) as activators of PI3K. This resulted in elevated Rac1 activity as a downstream consequence. These results provide mechanistic evidence that ColXVII coordinates keratinocyte adhesion and directed motility by interfering integrin dependent PI3K activation and by stabilizing lamellipodia at the leading edge of reepithelializing wou**nds and** in invasive squamous cell carcinoma.
3. Activated mutant NRas(Q61K) drives aberrant melanocyte signaling, survival, and **invasive**ness via a Rac1-dependent mechanism. Around a fifth of melanomas exhibit an activating mutation in the oncogene NRas that confers constitutive signaling to proliferation and promotes tumor initiation. NRas signals downstream of the major melanocyte tyrosine kinase receptor c-kit and activated NRas results in increased signaling via the extracellular signal-regulated kinase (ERK)/MAPK/ERK kinase/mitogen-activated protein kinase (MAPK) pathways to enhance proliferation. The Ras oncogene also activates signaling via the related Rho GTPase Rac1, which can mediate growth, survival, and motility signaling. We tested the effects of activated NRas(Q61K) on the proliferation, motility, and **invasive**ness of melanoblasts and melanocytes in the developing mouse and ex vivo explant culture as well as in a melanoma transplant model. We find an important role for Rac1 downstream of NRas(Q61K) in mediating dermal melanocyte survival in vivo in mouse, but surprisingly NRas(Q61K) does not appear to affect melanoblast motility or proliferation during mouse embryogenesis. We also show that genetic deletion or pharmacological inhibition of Rac1 in NRas(Q61K) induced melanoma suppresses tumor growth, lymph node spread, and tumor cell **invasive**ness, suggesting a potential value for Rac1 as a therapeutic target for activated NRas-driven tumor growth and **invasive**ness.
4. Melanoblasts on the move: Rac1 sets the pace. Cell migration is fundamental to development and many cell types have a migratory phase during embryonic development when tissues and body structures are forming. Cancer metastasis is in many ways thought to be analogous to embryonic development. Some of the mechanisms that tumor cells use to hijack the adult body are thought to derive from their abilities to reactivate embryonic signaling and motility pathways and thus enhance their growth and motility. Melanomas are notorious for their ability to become highly **invasive** and metastatic if not removed early. While adult melanin producing cells, melanocytes, have limited mobility, melanoblasts are highly motile cells that move through the dermis and epidermis during embryogenesis and could serve as a useful paradigm for some aspects of melanoma invasion and metastasis. Recent findings from our laboratory using ex-vivo imaging of mouse melanoblast migration in the epidermis provide the first insights into the role of Rac1 in developing mouse melanoblasts in vivo. Melanoblasts do not move as a collective group, or use an **invasive** or blebbing mode of migration as revealed by other in vivo systems, but rather they extend short and long dynamic pseudopodia and squeeze between epidermal keratinocytes using myosin motors. Melanoblasts can initiate short actin-based protrusions independently of Rac1. Rac1 is required to control the rate of formation of long actin-based protrusions for effective translocation in skin. Our results reveal a novel mode of in vivo migration controlled by Rac1 that is important for normal development and likely in melanoma.
5. Non-small cell lung carcinoma cell motility, rac activation and metastatic dissemination are mediated by protein kinase C epsilon. Protein kinase C (PKC) ε, a key signaling transducer implicated in mitogenesis, survival, and cancer progression, is overexpressed in human primary non-small cell lung cancer (NSCLC). The role of PKCε in lung cancer metastasis has not yet been established.Here we show that RNAi-mediated knockdown of PKCε in H358, H1299, H322, and A549 NSCLC impairs activation of the small GTPase Rac1 in response to phorbol 12-myristate 13-acetate (PMA), serum, or epidermal growth factor (EGF). PKCε depletion markedly impaired the ability of NSCLC cells to form membrane ruffles and migrate. Similar results were observed by pharmacological inhibition of PKCε with εV1-2, a specific PKCε inhibitor. PKCε was also requi**red for** invasiveness of NSCLC cells and modulated the secretion of extracellular matrix proteases and protease inhibitors. Finally, we found that PKCε-depleted NSCLC cells fail to disseminate to lungs in a mouse model of metastasis.Our results implicate PKCε as a key mediator of Rac signaling and motility of lung cancer cells, highlighting its potential as a therapeutic target.
6. The adenomatous polyposis coli-associated exchange factors Asef and Asef2 are required for adenoma formation in Apc(Min/+)mice. Sporadic and familial colorectal tumours usually harbour biallelic adenomatous polyposis coli (APC)-associated mutations that result in constitutive activation of Wnt signalling. Furthermore, APC activates Asef and Asef2, which are guanine-nucleotide exchange factors specific for Rac1 and Cdc42. Here, we show that Asef and Asef2 expression is aberrantly enhanced in intestinal adenomas and tumours. We also show that deficiency of either Asef or Asef2 significantly reduces the number and size of adenomas in Apc(Min/+) mice, which are heterozygous for an APC mutation and spontaneously develop adenomas in the intestine. We observed that the APC-Asef/Asef2 complex induces c-Jun amino-terminal kinase-mediated transactivation of matrix metalloproteinase 9, and is required for the **invasive** activity of colorectal tumour cells. Furthermore, we show that Asef and Asef2 are required for tumour angiogenesis. These results suggest that Asef and Asef2 have a crucial role in intestinal adenoma formation and tumour progression, and might be promising molecular targets for the treatment of colorectal tumours.
7. Staphylococcus aureus keratinocyte invasion is mediated by integrin-linked kinase and Rac1. Staphylococcus aureus is a major component of the skin microbiota and causes a large number of serious infections. S. aureus first interacts with epidermal keratinocytes to breach the epidermal barrier through mechanisms not fully understood. By use of primary keratinocytes from mice with epidermis-restricted Ilk gene inactivation and control integrin-linked kinase (ILK)-expressing littermates, we investigated the role of ILK in epidermal S. aureus invasion. Heat-killed, but not live, bacteria were internalized to Rab5- and Rab7-positive phagosomes, and incubation with keratinocyte growth factor increased their uptake 2.5-fold. ILK-deficient mouse keratinocytes internalized bacteria 2- to 4-fold less efficiently than normal cells. The reduced invasion by live S. aureus of ILK-deficient cells was restored in the presence of exogenous, constitutively active Rac1. Thus, Rac1 functions downstream from ILK during invasion. Further, invasion by S. aureus of Rac1-deficient cells was 2.5-fold lower than in normal cells. Paradoxically, staphylococcal cutaneous penetration of mouse skin explants with ILK-deficient epidermis was 35-fold higher than that of normal skin, indicating defects in epidermal barrier function in the absence of ILK. Thus, we identified an ILK-Rac1 pathway essential for bacterial invasion of keratinocytes, and established ILK as a key contributor to prevent **invasive** staphylococcal cutaneous infection.
8. Cdc42 and Rac1 are necessary for autotaxin-induced tumor cell motility in A2058 melanoma cells. Autotaxin (ATX) is a strong motogen that can increase **invasive**ness and angiogenesis. In the present study, we investigated the signal transduction mechanism of ATX-induced tumor cell motility. Unlike N19RhoA expressing cells, the cells expressing N17Cdc42 or N17Rac1 showed reduced motility against ATX. ATX activated Cdc42 and Rac1 and increased complex formation between these small G proteins and p21-activated kinase (PAK). Furthermore, ATX phosphorylated focal adhesion kinase (FAK) that was not shown in cells expressing dominant negative mutants of Cdc42 or Rac1. Collectively, these data strongly indicate that Cdc42 and Rac1 are essential for ATX-induced tumor cell motility in A2058 melanoma cells, and that PAK and FAK might be also involved in the process.
9. A role for Rac1 activity in malignant progression of sebaceous skin tumors. The small GTPase Rac1 is crucial for maintaining stem cells (SCs) in mammalian epidermis, and Rac1 activation leads to SC expansion. Loss or inhibition of Rac1 correlates with decreased frequency of skin cancer formation in a chemical carcinogenesis model. Here, we have addressed whether Rac1 activation would enhance carcinogenesis and result in tumor progression. We used K14ΔNLef1 mice, a model for differentiated sebaceous adenomas (SAs), and activated Rac1 in an epidermis-specific manner (K14L61Rac1). Surprisingly, Rac1 activation did not change the incidence and frequency of sebaceous tumors. However, tumors, which occurred exclusively in K14ΔNLef1/K14L61Rac1 double-transgenic mice, were poorly differentiated resembling malignant sebaceous tumors and were termed sebaceous carcinoma-like tumors (SCLTs). Compared with SAs, SCLTs showed an aberrant pattern of cell proliferation**, invasi**ve growth and less abundant expression of sebocyte differentiation markers, including stearoyl-CoA desaturase-1 and adipophilin. Interestingly, the adnexal SC marker Lrig1 was upregulated in SCLTs, showing that active Rac1 leads to the accumulation of sebocyte precursors in the context of K14ΔNLef1-induced skin tumors. In a search for targets of Rac1, we found cancer progression-related proteins, Dhcr24/Seladin1 and Nuclear protein 1/P8, to be strongly regulated in SCLTs. At last, Rac1 and Dhcr24/Seladin1 were detected in human sebaceous tumors demonstrating a potential high impact of our findings for human skin disease. This is the first study showing that Rac1 activity can lead to malignant progression of skin tumors.

### [Mm]etastasize

#### PubMed

1. The interaction between caveolin-1 and Rho-GTPases promotes metastasis by controlling the expression of alpha5-integrin and the activation of Src, Ras and Erk. Proteins containing a caveolin-binding domain (CBD), such as the Rho-GTPases, can interact with caveolin-1 (Cav1) through its caveolin scaffold domain. Rho-GTPases are important regulators of p130(Cas), which is crucial for both normal cell migration and Src kinase-mediated metastasis of cancer cells. However, although Rho-GTPases (particularly RhoC) and Cav1 have been linked to cancer progression and metastasis, the underlying molecular mechanisms are largely unknown. To investigate the function of Cav1-Rho-GTPase interaction in metastasis, we disrupted Cav1-Rho-GTPase binding in melanoma and mammary epithelial tumor cells by overexpressing CBD, and examined the loss-of-function of RhoC in metastatic cancer cells. Cancer cells overexpressing CBD or lacking RhoC had reduced p130(Cas) phosphorylation and Rac1 activation, resulting in an inhibition of migration and invasion in vitro. The activity of Src and the activation of its downstream targets FAK, Pyk2, Ras and extracellular signal-regulated kinase (Erk)1/2 were also impaired. A reduction in α5-integrin expression, which is required for binding to fibronectin and thus cell migration and survival, was observed in CBD-expressing cells and cells lacking RhoC. As a result of these defects, CBD-expressing melanoma cells had a reduced ability to **metastasiz**e in recipient mice, and impaired extravasation and survival in secondary sites in chicken embryos. Our data indicate that interaction between Cav1 and Rho-GTPases (most likely RhoC but not RhoA) promotes metastasis by stimulating α5-integrin expression and regulating the Src-dependent activation of p130(Cas)/Rac1, FAK/Pyk2 and Ras/Erk1/2 signaling cascades.

### [Mm]etastasis

#### PubMed

1. Pro-metastatic signaling by c-Met through RAC-1 and reactive oxygen species (ROS). Overexpression of the c-Met/hepatocyte growth factor receptor(HGF-R) proto-oncogene and abnormal generation of intracellular oxygen species (reactive oxygen species (ROS)) have been linked, by independent lines of evidence, to cell transformation and to malignant growth. By comparing two subpopulations of the B16 mouse melanoma (B16-F0 and B16-F10) endowed with different lung **metastasis** capacities (low and high, respectively) we found that both the expression/phosphorylation of c-Met and the steady-state levels of ROS positively correlated with metastatic growth. shRNA-mediated downregulation of c-Met in F10 cells led to a parallel decrease in the generation of oxygen species and in metastatic capacity, suggesting that oxidants may mediate the pro-metastatic activity of the HGF receptor. c-Met activation by a ligand elicits the formation of oxidant species through the oxidase-coupled small GTPase Rac-1, a relevant downstream target of the HGF-R. Moreover, cell treatment with the catalytic ROS scavengers EUK-134 and EUK-189 attenuates Met signaling to ERKs and inhibits the anchorage-independent growth of F10 cells, consistent with a critical role for oxygen species in HGF signaling and in aggressive cell behavior. Finally, genetic manipulation of the Rac-ROS cascade at different levels demonstrated its crucial role in the pro-metastatic activity of c-Met in vivo. Thus, we have outlined a novel cascade triggered by c-Met and mediated by ROS, linked to **metastasis** and potentially targetable by new antimetastatic, redox-based therapies.
2. Melanoblasts on the move: Rac1 sets the pace. Cell migration is fundamental to development and many cell types have a migratory phase during embryonic development when tissues and body structures are forming. Cancer **metastasis** is in many ways thought to be analogous to embryonic development. Some of the mechanisms that tumor cells use to hijack the adult body are thought to derive from their abilities to reactivate embryonic signaling and motility pathways and thus enhance their growth and motility. Melanomas are notorious for their ability to become highly invasive and metastatic if not removed early. While adult melanin producing cells, melanocytes, have limited mobility, melanoblasts are highly motile cells that move through the dermis and epidermis during embryogenesis and could serve as a useful paradigm for some aspects of melanoma invasion and **metastasis**. Recent findings from our laboratory using ex-vivo imaging of mouse melanoblast migration in the epidermis provide the first insights into the role of Rac1 in developing mouse melanoblasts in vivo. Melanoblasts do not move as a collective group, or use an invasive or blebbing mode of migration as revealed by other in vivo systems, but rather they extend short and long dynamic pseudopodia and squeeze between epidermal keratinocytes using myosin motors. Melanoblasts can initiate short actin-based protrusions independently of Rac1. Rac1 is required to control the rate of formation of long actin-based protrusions for effective translocation in skin. Our results reveal a novel mode of in vivo migration controlled by Rac1 that is important for normal development and likely in melanoma.
3. The interferon-gamma-induced GTPase, mGBP-2, inhibits tumor necrosis factor alpha (TNF-alpha) induction of matrix metalloproteinase-9 (MMP-9) by inhibiting NF-kappaB and Rac protein. Matrix metalloproteinase-9 (MMP-9) is important in numerous normal and pathological processes, including the angiogenic switch during tumor development and tumor **metastasis**. Whereas TNF-α and other cytokines up-regulate MMP-9 expression, interferons (IFNs) inhibit MMP-9 expression. We found that IFN-γ treatment or forced expression of the IFN-induced GTPase, mGBP-2, inhibit TNF-α-induced MMP-9 expression in NIH 3T3 fibroblasts, by inhibiting MMP-9 transcription. The NF-κB transcription factor is required for full induction of MMP-9 by TNF-α. Both IFN-γ and mGBP-2 inhibit the transcription of a NF-κB-dependent reporter construct, suggesting that mGBP-2 inhibits MMP-9 induction via inhibition of NF-κB-mediated transcription. Interestingly, mGBP-2 does not inhibit TNF-α-induced degradation of IκBα or p65/RelA translocation into the nucleus. However, mGBP-2 inhibits p65 binding to a κB oligonucleotide probe in gel shift assays and to the MMP-9 promoter in chromatin immunoprecipitation assays. In addition, TNF-α activation of NF-κB in NIH 3T3 cells is dependent on Rac activation, as evidenced by the inhibition of TNF-α induction of NF-κB-mediated transcription by a dominant inhibitory form of Rac1. A role for Rac in the inhibitory action of mGBP-2 on NF-κB is further shown by the findings that mGBP-2 inhibits TNF-α activation of endogenous Rac and constitutively activate Rac can restore NF-κB transcription in the presence of mGBP-2. This is a novel mechanism by which IFNs can inhibit the cytokine induction of MMP-9 expression.
4. Non-small cell lung carcinoma cell motility, rac activation and metastatic dissemination are mediated by protein kinase C epsilon. Protein kinase C (PKC) ε, a key signaling transducer implicated in mitogenesis, survival, and cancer progression, is overexpressed in human primary non-small cell lung cancer (NSCLC). The role of PKCε in lung cance**r metastas**is has not yet been established.Here we show that RNAi-mediated knockdown of PKCε in H358, H1299, H322, and A549 NSCLC impairs activation of the small GTPase Rac1 in response to phorbol 12-myristate 13-acetate (PMA), serum, or epidermal growth factor (EGF). PKCε depletion markedly impaired the ability of NSCLC cells to form membrane ruffles and migrate. Similar results were observed by pharmacological inhibition of PKCε with εV1-2, a specific PKCε inhibitor. PKCε was also required for invasiveness of NSCLC cells and modulated the secretion of extracellular matrix proteases and protease inhibitors. Finally, we found that PKCε-depleted NSCLC cells fail to disseminate to lungs in a mouse **model of m**etastasis.Our results implicate PKCε as a key mediator of Rac signaling and motility of lung cancer cells, highlighting its potential as a therapeutic target.
5. Paxillin and Hic-5 interaction with vinculin is differentially regulated by Rac1 and RhoA. Cell migration is of paramount importance to organism development and maintenance as well as multiple pathological processes, including cancer **metastasis**. The RhoGTPases Rac1 and RhoA are indispensable for cell migration as they regulate cell protrusion, cell-extracellular matrix (ECM) interactions and force transduction. However, the consequences of their activity at a molecular level within the cell remain undetermined. Using a combination of FRET, FRAP and biochemical analyses we show that the interactions between the focal adhesion proteins vinculin and paxillin, as well as the closely related family member Hic-5 are spatially and reciprocally regulated by the activity of Rac1 and RhoA. Vinculin in its active conformation interacts with either paxillin or Hic-5 in adhesions in response to Rac1 and RhoA activation respectively, while inactive vinculin interacts with paxillin in the membrane following Rac1 inhibition. Additionally, Rac1 specifically regulates the dynamics of paxillin as well as its binding partner and F-actin interacting protein actopaxin (α-parvin) in adhesions. Furthermore, FRET analysis of protein:protein interactions within cell adhesions formed in 3D matrices revealed that, in contrast to 2D systems vinculin interacts preferentially with Hic-5. This study provides new insight into the complexity of cell-ECM adhesions in both 2D and 3D matrices by providing the first description of RhoGTPase-coordinated protein:protein interactions in a cellular microenvironment. These data identify discrete roles for paxillin and Hic-5 in Rac1 and RhoA-dependent cell adhesion formation and maturation; processes essential for productive cell migration.
6. RACK1 promotes the proliferation, migration and invasion capacity of mouse hepatocellular carcinoma cell line in vitro probably by PI3K/Rac1 signaling pathway. Hca-P and Hca-F is a pair of synogenetic mouse hepatocarcinoma ascites cell lines, possessing different capacity of lymphatic **metastasis**. Receptor of activated C-kinase 1 (Rack1), together with Jnk1 and gelsolin (Gsn) were previously identified as differentially expressed proteins for lymphatic metastatic potential between the two cell lines. As an intracellular scaffold protein, Rack1 could recruit such signaling molecules as integrins, Src, PKC which are involved in many important biological processes and play key roles in cancer progression. In our present studies, pCDNA3.1(+)-Rack1, a eukaryotic expression plasmid, was constructed and stably transfected into Hca-P cells with a low metastatic potential. CCK8 assay and transwell system were used to evaluate the effects of Rack1 on proliferation, migration and invasion of Hca-P cells in vitro. Then, LY294002, an inhibitor of PI3K, was added into the culture medium of pCDNA3.1(+)-Rack1-Hca-P cells and their biological behaviors observed further. Moreover, the expression of Jnk1, Rac1 and Gsn of pCDNA3.1(+)-Rack1-Hca-P cells were detected by western blot after pretreated with various doses of LY294002. As a result, the proliferation, migration and invasion of pCDNA3.1(+)-Rack1-Hca-P cells were significantly enhanced and could be inhibited by LY294002. In addition, the expression of Gsn, Rac1 and Jnk1 of pCDNA3.1(+)-Rack1-Hca-P cells also decreased after pretreated with LY294002. The expression of Gsn can be inhibited by NSC33766 (an inhibitor of Rac1). Taken together, Rack1/PI3K/Rac1 signaling pathway may play a crucial role in malignant biological behaviors of mouse hepatocarcinoma cells with lymphatic **metastasis** potential. It may be a potential target for therapy of cancer lymphatic **metastasis**.
7. IRGM1 enhances B16 melanoma cell **metastasis** through PI3K-Rac1 mediated epithelial mesenchymal transition. Melanoma is one of the most aggressive skin cancers and is well known for its high metastatic rate. Studies have shown that epithelial mesenchymal transition (EMT) is essential for melanoma cell **metastasis**. However, the molecular mechanisms underlying EMT are still not fully understood. We have shown that IRGM1, a member of immunity-related GTPase family that regulates immune cell motility, is highly expressed by melanoma cells. The current study aimed to explore whether and how IRGM1 may regulate melanoma cell **metastasis**. To test this, we modified IRGM1 expression in B16 melanoma cells. We found that over-expression of IRGM1 substantially enhanced pulmonary **metastasis** in vivo. In keeping with that, knocking-in IRGM1 strongly enhanced while knocking-down IRGM1 impaired B16 cell migration and invasion ability in vitro. Interestingly, we observed that IRGM1 enhanced F-actin polymerization and triggers epithelial mesenchymal transition (EMT) through a mechanism involved in PIK3CA mediated Rac1 activation. Together, these data reveals a novel molecular mechanism that involved in melanoma **metastasis**.
8. Endothelial Rac1 is essential for hematogenous **metastasis** to the lung. A variety of vasoactive stimuli induce endothelial permeability through Rac1, a membrane of Rho small GTPases. Here, we determine whether tumor-secreted vasoactive stimulant through Rac1 inducing permeability contributes to hematogenous **metastasis**. Activation of Rac1 was assayed in human umbilical vein endothelial cells (HUVEC), transendothelial passages were measured by Transwell chambers, and hematogenously metastatic mouse model was generated by intravenous injection with Lewis lung carcinoma cells (LLC). LLC secreted abundant vascular endothelial growth factor (VEGF) in the culture media and sera of mice bearing LLC xenografts or metastatic LLC, and VEGF activated Rac1 through VEGF receptors/PI3Kβ signaling cascade, resulting in hyperoxidative stress and consequent hyperpermeability in HUVEC. Moreover, in co-culture of LLC and HUVEC, significant increases in endothelial permeability and transendothelial migration of LLC were robustly attenuated by either anti-VEGF neutralizing antibody or Rac1 knockdown in HUVEC. Finally, in metastatic mouse model, deletion of one copy of Rac1 in endothelium not only significantly attenuated LLC-induced vascular permeability, but robustly reduced the **metastasi**s of LLC to lungs. This study supports that tumor-secreted vasoactive stimuli activate Rac1 to induce permeability and consequent transendothelial migration of tumor cells, and that loss of Rac1 function in endothelium is an effective therapeutic intervention for hematogenous **metastasi**s.
9. The simultaneous induction of tumorigenesis and Cre-loxP recombination in mice. To investigate the role of Rac1 for tumorigenesis, we generated inducible transgenic (Tg) mice that simultaneously express polyomavirus middle T antigen (mT) and Cre recombinase under the control of mouse mammary tumor virus long terminal repeat (MMTV-LTR) promoter (MMTV-LTR-tTA/mT-TRE-cre Tg). MMTV-LTR-tTA/mT- TRE-cre Tg mice formed tumors in the subcutaneous tissue and developed lung **metastasis**. We examined tumor latency and types in rac1 deficient (rac1(flox/-)) and control (rac1(+/+), rac1(+/-) or rac1(flox/+)) MMTV-LTR-tTA/mT-TRE-cre Tg mice and found that formation of cutaneous appendage tumor was suppressed although tumor latency in these mice was not affected by loss of Rac1. These results suggested that Rac1 may play a pivotal role in induction and growth of the mT-mediated epithelial tumors. MMTV-LTR-tTA/mT-TRE-cre Tg mice would provide a versatile animal model to investigate genetic interaction in the tumorigenesis.
10. Interaction between Tiam1 and the Arp2/3 complex links activation of Rac to actin polymerization. The Rac-specific GEF (guanine-nucleotide exchange factor) Tiam1 (T-lymphoma invasion and **metastasis** 1) regulates migration, cell-matrix and cell-cell adhesion by modulating the actin cytoskeleton through the GTPase, Rac1. Using yeast two-hybrid screening and biochemical assays, we found that Tiam1 interacts with the p21-Arc [Arp (actin-related protein) complex] subunit of the Arp2/3 complex. Association occurred through the N-terminal pleckstrin homology domain and the adjacent coiled-coil region of Tiam1. As a result, Tiam1 co-localizes with the Arp2/3 complex at sites of actin polymerization, such as epithelial cell-cell contacts and membrane ruffles. Deletion of the p21-Arc-binding domain in Tiam1 impairs its subcellular localization and capacity to activate Rac1, suggesting that binding to the Arp2/3 complex is important for the function of Tiam1. Indeed, blocking Arp2/3 activation with a WASP (Wiskott-Aldrich syndrome protein) inhibitor leads to subcellular relocalization of Tiam1 and decreased Rac activation. Conversely, functionally active Tiam1, but not a GEF-deficient mutant, promotes activation of the Arp2/3 complex and its association with cytoskeletal components, indicating that Tiam1 and Arp2/3 are mutually dependent for their correct localization and signalling. Our data suggests a model in which the Arp2/3 complex acts as a scaffold to localize Tiam1, and thereby Rac activity, which are both required for activation of the Arp2/3 complex and further Arp2/3 recruitment. This 'self-amplifying' signalling module involving Tiam1, Rac and the Arp2/3 complex could thus drive actin polymerization at specific sites in cells that are required for dynamic morphological changes.
11. Inhibition of Rac1 promotes BMP-2-induced osteoblastic differentiation. Small G proteins of the Rho family are pivotal regulators of several signaling networks. The Ras homolog family (Rho) and one of its targets, Rho-associated protein kinase (ROCK), participate in a wide variety of biological processes, including bone formation. A previous study has demonstrated that the ROCK inhibitor Y-27632 enhanced bone formation induced by recombinant human bone morphogenetic protein-2 (BMP-2) in vivo and in vitro. However, the effect of other Rho family members, such as Ras-related C3 botulinum toxin substrate 1 (Rac1) and cell division cycle 42 (Cdc42), on bone formation remains unknown. In this study, we investigated whether Rac1 also participates in BMP-2-induced osteogenesis. Expression of a dominant-negative mutant of Rac1 enhanced BMP-2-induced osteoblastic differentiation in C2C12 cells, whereas a constitutively active mutant of Rac1 attenuated that effect. Knockdown of T-lymphoma invasion and **metastasis** 1 (Tiam1), a Rac-specific guanine nucleotide exchange factor, enhanced BMP-2-induced alkaline phosphatase activity. Further, we demonstrated that BMP-2 stimulated Rac1 activity. These results indicate that the activation of Rac1 attenuates osteoblastic differentiation in C2C12 cells.
12. Tyr724 phosphorylation of ELMO1 by Src is involved in cell spreading and migration via Rac1 activation. The complex of Dock180/ELMO1 that functions as a bipartite guanine nucleotide exchange factor for Rac is essential for diverse physiological and pathological processes of cells such as cell migration, phagocytosis, and invasion of cancer cells. Among the Src-family tyrosine kinases (SFKs), it has been reported that Hck directly phosphorylates ELMO1, regulating phagocytosis by promoting activation of Rac1; however, the involvement of other SFKs in ELMO1 phosphorylation has remained unknown. Here, we identified novel tyrosine (Y) residues of ELMO1 phosphorylated by SFKs, and examined the effects on Rac1 activity, cell adhesion, spreading, and cell motility on extracellular matrix (ECM).In this study, we unveiled that Src and Fyn can induce tyrosine phosphorylation of ELMO1 in in vivo and in vitro phosphorylation assays. Mutational analyses identified both Y720 and Y724 residues of ELMO1 as Src-mediated phosphorylation sites, preferentially on Y724. Single substitution of Y724 to Phe abrogated Rac1 activation triggered by Src. To elucidate the biological function of pY724, we established NIH3T3 cells stably expressing wild-type ELMO1 or its Y724F mutant together with Dock180. Among them, Y724-deficient cells exhibited a depletion of Rac1 activity with diminished phosphorylation of ELMO1 even upon the ECM-stimulation. It is noteworthy that NIH3T3 cells with ELMO1 Y724F were strikingly defective to promote cell spreading on fibronectin-coated dish, concomitantly exhibiting immature assemblies of actin stress fibers and focal adhesions. Eventually, ELMO1 Y724F significantly impaired cell migration.These results define that Src-mediated Y724 phosphorylation in ELMO1 plays a critical role for cell spreading via activation of Rac1, leading to promotion of cell migration. As the overexpression and/or hyperactivation of Src have been shown in a wide variety of human cancers, Src-mediated phosphorylation of Y724 in ELMO1 may regulate cancer cell adhesion to the ECM, invasion into surrounding tissues, and subsequent distant **metastasis**.
13. The interaction between caveolin-1 and Rho-GTPases promotes **metastasis** by controlling the expression of alpha5-integrin and the activation of Src, Ras and Erk. Proteins containing a caveolin-binding domain (CBD), such as the Rho-GTPases, can interact with caveolin-1 (Cav1) through its caveolin scaffold domain. Rho-GTPases are important regulators of p130(Cas), which is crucial for both normal cell migration and Src kinase-mediated **metastasis** of cancer cells. However, although Rho-GTPases (particularly RhoC) and Cav1 have been linked to cancer progression and **metastasis**, the underlying molecular mechanisms are largely unknown. To investigate the function of Cav1-Rho-GTPase interaction in **metastasis**, we disrupted Cav1-Rho-GTPase binding in melanoma and mammary epithelial tumor cells by overexpressing CBD, and examined the loss-of-function of RhoC in metastatic cancer cells. Cancer cells overexpressing CBD or lacking RhoC had reduced p130(Cas) phosphorylation and Rac1 activation, resulting in an inhibition of migration and invasion in vitro. The activity of Src and the activation of its downstream targets FAK, Pyk2, Ras and extracellular signal-regulated kinase (Erk)1/2 were also impaired. A reduction in α5-integrin expression, which is required for binding to fibronectin and thus cell migration and survival, was observed in CBD-expressing cells and cells lacking RhoC. As a result of these defects, CBD-expressing melanoma cells had a reduced ability to metastasize in recipient mice, and impaired extravasation and survival in secondary sites in chicken embryos. Our data indicate that interaction between Cav1 and Rho-GTPases (most likely RhoC but not RhoA) promotes **metastasi**s by stimulating α5-integrin expression and regulating the Src-dependent activation of p130(Cas)/Rac1, FAK/Pyk2 and Ras/Erk1/2 signaling cascades.
14. Hypoxia-induced IL-18 increases hypoxia-inducible factor-1alpha expression through a Rac1-dependent NF-kappaB pathway. Interleukin-18 (IL-18) plays pivotal roles in linking inflammatory immune responses and tumor progression and **metastasis**, yet the manner in which this occurs remains to be sufficiently clarified. Here we report that hypoxia induces the transcription and secretion of IL-18, which subsequently induces the expression of hypoxia-inducible factor-1alpha (HIF-1alpha). Mechanistically, IL-18 induces HIF-1alpha through the activity of the GTPase Rac1, which inducibly associates with the IL-18 receptor beta (IL-18Rbeta) subunit, via a PI3K-AKT-NF-kappaB-dependent pathway. Importantly, the knockdown of the IL-18Rbeta subunit inhibited IL-18-driven tumor cell **metastasis**. Collectively, these findings demonstrate a feed-forward pathway in HIF-1alpha-mediated tumor progression, in which the induction of IL-18 by hypoxia or inflammatory cells augments the expression of both HIF-1alpha and tumor cell **metastasis**.

#### GeneRIFs

1. Rack1/PI3K/Rac1 signaling pathway may play a crucial role in malignant biological behaviors of mouse hepatocarcinoma cells with lymphatic **metastasis** potential
2. Data indicate that interaction between Cav1 and Rho-GTPases (most likely RhoC but not RhoA) promotes **metastasis** by stimulating alpha5-integrin expression and regulating the Src-dependent activation of Ras/Erk1/2 signaling cascades.
3. These findings demonstrate a feed-forward pathway in HIF-1alpha-mediated tumor progression, in which the induction of IL-18 by hypoxia or inflammatory cells augments the expression of both HIF-1alpha and tumor cell **metastasis**.
4. a novel cascade triggered by c-Met and mediated by RAC-1 and reactive oxygen species, linked to **metastasis**

### [Cc]ell [Ii]nvasion

#### PubMed

1. Stat3 promotes directional cell migration by regulating Rac1 activity via its activator betaPIX. Stat3 is a member of the signal transducer and activator of transcription family, which is important for cytokine signaling as well as for a number of cellular processes including cell proliferation, anti-apoptosis and immune responses. In recent years, evidence has emerged suggesting that Stat3 also participates in **cell invasion** and motility. However, how Stat3 regulates these processes remains poorly understood. Here, we find that loss of Stat3 expression in mouse embryonic fibroblasts leads to an elevation of Rac1 activity, which promotes a random mode of migration by reducing directional persistence and formation of actin stress fibers. Through rescue experiments, we demonstrate that Stat3 can regulate the activation of Rac1 to mediate persistent directional migration and that this function is not dependent on Stat3 transcriptional activity. We find that Stat3 binds to betaPIX, a Rac1 activator, and that this interaction could represent a mechanism by which cytoplasmic Stat3 regulates Rac1 activity to modulate the organization of actin cytoskeleton and directional migration.

---

## Jun

- NCBI Gene
- GeneRIF
- Pubmed

| Search Term | Rank | Fields |
| --- | --- | --- |
| [Mm]igration | 6 | Process(1); PubMed(43); GeneRIFs(4) |
| ECM | 7 | PubMed(3) |
| [Ee]xtracellular [Mm]atrix | 8 | PubMed(14); GeneRIFs(1) |
| [Ii]nvasive | 10 | PubMed(11) |
| [Mm]etastasis | 12 | PubMed(7); GeneRIFs(1) |
| [Cc]ell [Ii]nvasion | 13 | PubMed(3); GeneRIFs(1) |

  
hide/show details for Jun

### [Mm]igration

#### Process

1. positive regulation of epithelial cell **migration**

#### PubMed

1. The goya mouse mutant reveals distinct newly identified roles for MAP3K1 in the development and survival of cochlear sensory hair cells. Mitogen-activated protein kinase, MAP3K1, plays an important role in a number of cellular processes, including epithelial **migration** during eye organogenesis. In addition, studies in keratinocytes indicate that MAP3K1 signalling through JNK is important for actin stress fibre formation and cell **migration**. However, MAP3K1 can also act independently of JNK in the regulation of cell proliferation and apoptosis. We have identified a mouse mutant, goya, which exhibits the eyes-open-at-birth and microphthalmia phenotypes. In addition, these mice also have hearing loss. The goya mice carry a splice site mutation in the Map3k1 gene. We show that goya and kinase-deficient Map3k1 homozygotes initially develop supernumerary cochlear outer hair cells (OHCs) that subsequently degenerate, and a progressive profound hearing loss is observed by 9 weeks of age. Heterozygote mice also develop supernumerary OHCs, but no cellular degeneration or hearing loss is observed. MAP3K1 is expressed in a number of inner-ear cell types, including outer and inner hair cells, stria vascularis and spiral ganglion. Investigation of targets downstream of MAP3K1 identified an increase in p38 phosphorylation (Thr180/Tyr182) in multiple cochlear tissues. We also show that the extra OHCs do not arise from aberrant control of proliferation via p27KIP1. The identification of the goya mutant reveals a signalling molecule involved with hair-cell development and survival. Mammalian hair cells do not have the ability to regenerate after damage, which can lead to irreversible sensorineural hearing loss. Given the observed goya phenotype, and the many diverse cellular processes that MAP3K1 is known to act upon, further investigation of this model might help to elaborate upon the mechanisms underlying sensory hair cell specification, and pathways important for their survival. In addition, MAP3K1 is revealed as a new candidate gene for human sensorineural hearing loss.
2. Targeted deletion of the mitogen-activated protein kinase kinase 4 gene in the nervous system causes severe brain developmental defects and premature death. The c-Jun NH2-terminal protein kinase (JNK) is a mitogen-activated protein kinase (MAPK) involved in the regulation of various physiological processes. Its activity is increased upon phosphorylation by the MAPK kinases MKK4 and MKK7. The early embryonic death of mice lacking an mkk4 or mkk7 gene has provided genetic evidence that MKK4 and MKK7 have nonredundant functions in vivo. To elucidate the physiological role of MKK4, we generated a novel mouse model in which the mkk4 gene could be specifically deleted in the brain. At birth, the mutant mice were indistinguishable from their control littermates, but they stopped growing a few days later and died prematurely, displaying severe neurological defects. Decreased JNK activity in the absence of MKK4 correlated with impaired phosphorylation of a subset of physiologically relevant JNK substrates and with altered gene expression. These defects resulted in the misalignment of the Purkinje cells in the cerebellum and delayed radial **migration** in the cerebral cortex. Together, our data demonstrate for the first time that MKK4 is an essential activator of JNK required for the normal development of the brain.
3. Disruption of c-Jun reduces cellular **migration** and invasion through inhibition of c-Src and hyperactivation of ROCK II kinase. The spread of metastatic tumors to different organs is associated with poor prognosis. The metastatic process requires **migration** and cellular invasion. The protooncogene c-jun encodes the founding member of the activator protein-1 family and is required for cellular proliferation and DNA synthesis in response to oncogenic signals and plays an essential role in chemical carcinogenesis. The role of c-Jun in cellular invasion remains to be defined. Genetic deletion of c-Jun in transgenic mice is embryonic lethal; therefore, transgenic mice encoding a c-Jun gene flanked by LoxP sites (c-jun(f/f)) were used. c-jun gene deletion reduced c-Src expression, hyperactivated ROCK II signaling, and reduced cellular polarity, **migration**, and invasiveness. c-Jun increased c-Src mRNA abundance and c-Src promoter activity involving an AP-1 site in the c-Src promoter. Transduction of c-jun(-/-) cells with either c-Jun or c-Src retroviral expression systems restored the defective cellular **migration** of c-jun(-/-) cells. As c-Src is a critical component of pathways regulating proliferation, survival, and metastasis, the induction of c-Src abundance, by c-Jun, provides a novel mechanism of cooperative signaling in cellular invasion.
4. Grhl3 and Lmo4 play coordinate roles in epidermal **migration**. In addition to its role in formation of the epidermal barrier, the mammalian transcription factor Grainy head-like 3 (Grhl3) is also essential for neural tube closure and wound repair, processes that are dependent in part on epidermal **migration**. Here, we demonstrate that the LIM-only domain protein, LMO4 serves as a functional partner of GRHL3 in its established roles, and define a new cooperative role for these factors in another developmental epidermal **migration** event, eyelid fusion. GRHL3 and LMO4 interact biochemically and genetically, with mutant mice exhibiting fully penetrant exencephaly, thoraco-lumbo-sacral spina bifida, defective skin barrier formation, and a co-incident eyes-open-at-birth (EOB) phenotype, which is not observed in the original individual null lines. The two genes are co-expressed in the surface ectoderm of the migrating eyelid root, and electron microscopy of Grhl3/Lmo4-null eyes reveals a failure in epithelial extension and a lack of peridermal clump formation at the eyelid margins. Accumulation of actin fibers is also absent in the circumference of these eyelids, and ERK1/2 phosphorylation is lost in the epidermis and eyelids of Grhl3(-/-)/Lmo4(-/-) embryos. Keratinocytes from mutant mice fail to "heal" in in vitro scratch assays, consistent with a general epidermal migratory defect that is dependent on ERK activation and actin cable formation.
5. Inactivation of PI3Kδ induces vascular injury and promotes aneurysm development by upregulating the AP-1/MMP-12 pathway in macrophages. An aneurysm is an inflammatory vascular condition. Phosphatidylinositol 3-kinases δ is highly expressed in leukocytes, and play a key role in innate immunity. However, the link between phosphatidylinositol 3-kinases δ and aneurysm development has not yet been elucidated.Carotid ligation unexpectedly induced characteristic aneurysm formation beneath the ligation point in p110δ(D910A/D910A) mice (n=25; P<0.001 versus wild-type). Besides, p110δ inactivation exacerbated CaCl2-induced abdominal aortic aneurysms development. A reverse transcription polymerase chain reaction microarray revealed significant extracellular matrix components degradation and matrix metalloproteinases (MMPs) upregulation in the abdominal aorta of p110δ(D910A/D910A) mice. Similarly, the expression of both collagen I and IV was significantly decreased (n=10; P<0.05 versus wild-type) in carotid artery. Western blot assay confirmed that MMP-12 was significantly upregulated in arteries of p110δ(D910A/D910A) mice (n=10; P<0.01 versus wild-type). In vitro, p110δ inactivation marked increase peritoneal macrophages recruitment and synergistically enhance tumor necrosis factor-α-induced recruitment. A specific phosphatidylinositol 3-kinases δ inhibitor (IC87114) or genetic p110δ inactivation upregulated MMP-12 expression and c-Jun phosphorylation (n=6; P<0.05 versus wild-type macrophages). IC87114 also increased activator protein-1 DNA-binding activity (n=6; P<0.001 versus control) and enhanced the effect of tumor necrosis factor-α on activator protein-1-binding activity (n=5; P<0.01 versus tumor necrosis factor-α treatment groups). Knockdown of c-Jun suppressed the effect of the IC87114 and tumor necrosis factor-α on MMP-12 mRNA expression (n=5 in each group; P<0.01 versus scrRNA treatment groups).Our findings demonstrate that p110δ inactivation leads to extracellular matrix degradation in vessels and promotes aneurysm development by induci**ng macrop**hages migration and upregulating the activator protein-1/MMP-12 pathway in macrophages.
6. Fbxo45 forms a novel ubiquitin ligase complex and is required for neuronal development. Fbxo45 is an F-box protein that is restricted to the nervous system. Unlike other F-box proteins, Fbxo45 was found not to form an SCF complex as a result of an amino acid substitution in the consensus sequence for Cul1 binding. Proteomics analysis revealed that Fbxo45 specifically associates with PAM (protein associated with Myc), a RING finger-type ubiquitin ligase. Mice deficient in Fbxo45 were generated and found to die soon after birth as a result of respiratory distress. Fbxo45(-)(/)(-) embryos show abnormal innervation of the diaphragm, impaired synapse formation at neuromuscular junctions, and aberrant development of axon fiber tracts in the brain. Similar defects are also observed in mice lacking Phr1 (mouse ortholog of PAM), suggesting that Fbxo45 and Phr1 function in the same pathway. In addition, neuronal **migration** was impaired in Fbxo45(-)(/)(-) mice. These results suggest that Fbxo45 forms a novel Fbxo45-PAM ubiquitin ligase complex that plays an important role in neural development.
7. Inactivation of PI3Kδ induces vascular injury and promotes aneurysm development by upregulating the AP-1/MMP-12 pathway in macrophages. An aneurysm is an inflammatory vascular condition. Phosphatidylinositol 3-kinases δ is highly expressed in leukocytes, and play a key role in innate immunity. However, the link between phosphatidylinositol 3-kinases δ and aneurysm development has not yet been elucidated.Carotid ligation unexpectedly induced characteristic aneurysm formation beneath the ligation point in p110δ(D910A/D910A) mice (n=25; P<0.001 versus wild-type). Besides, p110δ inactivation exacerbated CaCl2-induced abdominal aortic aneurysms development. A reverse transcription polymerase chain reaction microarray revealed significant extracellular matrix components degradation and matrix metalloproteinases (MMPs) upregulation in the abdominal aorta of p110δ(D910A/D910A) mice. Similarly, the expression of both collagen I and IV was significantly decreased (n=10; P<0.05 versus wild-type) in carotid artery. Western blot assay confirmed that MMP-12 was significantly upregulated in arteries of p110δ(D910A/D910A) mice (n=10; P<0.01 versus wild-type). In vitro, p110δ inactivation marked increase peritoneal macrophages recruitment and synergistically enhance tumor necrosis factor-α-induced recruitment. A specific phosphatidylinositol 3-kinases δ inhibitor (IC87114) or genetic p110δ inactivation upregulated MMP-12 expression and c-Jun phosphorylation (n=6; P<0.05 versus wild-type macrophages). IC87114 also increased activator protein-1 DNA-binding activity (n=6; P<0.001 versus control) and enhanced the effect of tumor necrosis factor-α on activator protein-1-binding activity (n=5; P<0.01 versus tumor necrosis factor-α treatment groups). Knockdown of c-Jun suppressed the effect of the IC87114 and tumor necrosis factor-α on MMP-12 mRNA expression (n=5 in each group; P<0.01 versus scrRNA treatment groups).Our findings demonstrate that p110δ inactivation leads to extracellular matrix degradation in vessels and promotes aneurysm development by induci**ng macrop**hages migration and upregulating the activator protein-1/MMP-12 pathway in macrophages.
8. Disruption of c-Jun reduces cellular **migration** and invasion through inhibition of c-Src and hyperactivation of ROCK II kinase. The spread of metastatic tumors to different organs is associated with poor prognosis. The metastatic process requires **migration** and cellular invasion. The protooncogene c-jun encodes the founding member of the activator protein-1 family and is required for cellular proliferation and DNA synthesis in response to oncogenic signals and plays an essential role in chemical carcinogenesis. The role of c-Jun in cellular invasion remains to be defined. Genetic deletion of c-Jun in transgenic mice is embryonic lethal; therefore, transgenic mice encoding a c-Jun gene flanked by LoxP sites (c-jun(f/f)) were used. c-jun gene deletion reduced c-Src expression, hyperactivated ROCK II signaling, and reduced cellular polarity, **migration**, and invasiveness. c-Jun increased c-Src mRNA abundance and c-Src promoter activity involving an AP-1 site in the c-Src promoter. Transduction of c-jun(-/-) cells with either c-Jun or c-Src retroviral expression systems restored the defective cellular **migration** of c-jun(-/-) cells. As c-Src is a critical component of pathways regulating proliferation, survival, and metastasis, the induction of c-Src abundance, by c-Jun, provides a novel mechanism of cooperative signaling in cellular invasion.
9. Changes in protooncogene expression correlated with general and sex-specific differentiation in murine primordial germ cells. Primordial germ cells (PGCs) of the mouse undergo key developmental transitions during embryonic days 12-15. On day 12 they complete **migration** into the gonads. They cease mitotic proliferation on day 13 and subsequently enter sex-specific pathways of development. The molecular mechanisms controlling these transitions are poorly understood, yet they are crucial to production of normal gametes later in life. We have used the polymerase chain reaction (PCR) to directly compare levels of expression of several protooncogenes proposed to be involved in control of cell proliferation and differentiation in proliferating and differentiating PCGs of both sexes over a 4 day time course. We report here that mRNA levels for nuclear protooncogenes c-myc, c-fos, and c-jun increase dramatically in both sexes from little or no detectable expression on day 12 to high expression on days 13-15. We observe c-kit message on day 12 in combined PGCs of both sexes, in female but not male PGCs on day 13, and in both sexes on day 14, c-kit mRNA is undetectable on day 15 in either sex, c-mos is not expressed at detectable levels on day 12 in either sex, but increases gradually in female PGCs to very high levels on day 15. In male PGCs, c-mos is expressed at high levels on days 13-15. Our results are consistent with a role for protooncogenes c-myc, c-fos and c-jun in mediating the initial differentiation of PGCs of both sexes that occurs upon colonization of the gonad. Because c-kit and c-mos are expressed differentially in male and female day 13-15 germ cells, they may play roles in initiating or mediating progress along the sex-specific pathways of development that PGCs embark upon at this time.
10. The F-box protein Fbw7 is required for cerebellar development. The F-box protein Fbw7 (also known as Fbxw7, hCdc4 and Sel-10) functions as a substrate recognition component of a SCF-type E3 ubiquitin ligase. SCF(Fbw7) facilitates polyubiquitination and subsequent degradation of various proteins such as Notch, cyclin E, c-Myc and c-Jun. Fbw7 is highly expressed in the nervous system and controls neural stem cell differentiation and apoptosis via Notch and c-Jun during embryonic development (Hoeck et al., 2010). Fbw7 deletion in the neural lineage is perinatal lethal and thus prohibits studying the role of Fbw7 in the adult nervous system. fbw7 mRNA is highly expressed in the postnatal brain and to gain insights into the function of Fbw7 in postnatal neurogenesis we analysed Fbw7 function in the cerebellum. We generated conditional Fbw7-knockout mice (fbw7(∆Cb)) by inactivating Fbw7 specifically in the cerebellar anlage. This resulted in decreased cerebellar size, reduced Purkinje cell number and defects in axonal arborisation. Moreover, Fbw7-deficient cerebella showed supranumeral fissures and aberrant progenitor cel**l migrati**on. Protein levels of the Fbw7 substrates Notch1 and N-terminally phosphorylated c-Jun were upregulated in fbw7(∆Cb) mice. Concomitant deletion of c-Jun, and also the junAA knock-in mutation which specifically abrogates c-Jun N-terminal phosphorylation, rescued Purkinje cell numbers and arborisation in the fbw7(∆Cb) background. Taken together these data demonstrate that Fbw7 is essential during cerebellar development, and identify N-terminally phosphorylated c-Jun as an important substrate of SCF(Fbw7) during neurogenesis.
11. AP-1 (Fra-1/c-Jun)-mediated induction of expression of matrix metalloproteinase-2 is required for 15S-hydroxyeicosatetraenoic acid-induced angiogenesis. To understand the involvement of matrix metalloproteinases (MMPs) in 15(S)-hydroxyeicosatetraenoic acid (15(S)-HETE)-induced angiogenesis, we have studied the role of MMP-2. 15(S)-HETE induced MMP-2 expression and activity in a time-dependent manner in human dermal microvascular endothelial cells (HDMVECs). Inhibition of MMP-2 activity or depletion of its levels attenuated 15(S)-HETE-induced HDMVEC **migration**, tube formation, and Matrigel plug angiogenesis. 15(S)-HETE also induced Fra-1 and c-Jun expression in a Rac1-MEK1-JNK1-dependent manner. In addition, 15(S)-HETE-induced MMP-2 expression and activity were mediated by Rac1-MEK1-JNK1-dependent activation of AP-1 (Fra-1/c-Jun). Cloning and site-directed mutagenesis of MMP-2 promoter revealed that AP-1 site proximal to the transcriptional start site is required for 15(S)-HETE-induced MMP-2 expression, and Fra-1 and c-Jun are the essential components of AP-1 that bind to MMP-2 promoter in response to 15(S)-HETE. Hind limb ischemia led to an increase in MEK1 and JNK1 activation and Fra-1, c-Jun, and MMP-2 expression resulting in enhanced neovascularization and recovery of blood perfusion in wild-type mice as compared with 12/15-Lox(-/-) mice. Together, these results provide the first direct evidence for a role of 12/15-Lox-12/15(S)-HETE axis in the regulation of ischemia-induced angiogenesis.
12. Anxa5 mediates the in vitro malignant behaviours of murine hepatocarcinoma Hca-F cells with high lymph node metastasis potential preferentially via ERK2/p-ERK2/c-Jun/p-c-Jun(Ser73) and E-cadherin. Annexin A5 (Anxa5) is associated with the progression of some cancers, while its role and regulation mechanism in tumor lymphatic metastasis is rarely reported. This study aims to investigate the influence of Anxa5 knockdown on the malignant behaviours of murine hepatocarcinoma Hca-F cell line with high lymph node metastatic (LNM) potential and the underlying regulation mechanism.RNA interfering was performed to silence Anxa5 in Hca-F. Monoclonal shRNA-Anxa5- Hca-F cells were obtained via G418 screening by limited dilution method. Quantitative real-time RT-PCR (qRT-PCR) and Western blotting (WB) were applied to measure Anxa5 expression levels. CCK-8, Boyden transwell-chamber and in situ LN adhesion assays were performed to explore the effects of Anxa5 on the proliferation, **migration**, invasion and adhesion capacities of Hca-F. WB and qRT-PCR were used to detect the level changes of key molecules in corresponding signal pathways.We obtained two monoclonal shRNA-Anxa5-transfected Hca-F cell lines with stable knockdowns of Anxa5. Anxa5 knockdown resulted in significantly reduced proliferation, **migration**, invasion and in situ LN adhesion potentials of Hca-F in proportion to its knockdown extent. Anxa5 downregulation enhanced E-cadherin levels in Hca-F. Moreover, Anxa5 affected Hca-F behaviours specifically via ERK2/p-ERK2/c-Jun/p-c-Jun(Ser73) instead of p38MAPK/c-Jun, Jnk/c-Jun and AKT/c-Jun pathways.Anxa5 mediates the in vitro malignant behaviours of murine hepatocarcinoma Hca-F cells via ERK2/c-Jun/p-c-Jun(Ser73) and ERK2/E-cadherin pathways. It is an important molecule in metastasis (especially LNM) and a potential therapeutic target for hepatocarcinoma.
13. Decursin and decursinol inhibit VEGF-induced angiogenesis by blocking the activation of extracellular signal-regulated kinase and c-Jun N-terminal kinase. The root of Angelica gigas Nakai contains two major coumarins, which have been previously identified as decursin and decursinol. Decursin has been demonstrated to exhibit potent anti-cancer activity both in vitro and in vivo. In this study, we found that decursin and decursinol at non-cytotoxic doses inhibited the VEGF-induced proliferation, **migration**, and capillary-tube formation of HUVECs. Moreover, decursin and decursinol suppressed microvessel formation on chorioallantoic membranes in fertilized eggs and into mouse Matrigel plugs. The oral administration of decursin and decursinol also reduced VEGF-induced angiogenesis in Matrigel. Furthermore, decursin and decursinol reduced the phosphorylation of ERK and JNK, but not p38 MAPK, in VEGF-stimulated HUVECs. Taken together, our results reveal that decursin and decursinol inhibit VEGF-induced angiogenesis by reducing the activation of ERK and JNK in HUVECs, and possess potent in vivo anti-angiogenic activity, coupled with the advantage of oral dosing. Thus, these compounds may have the potential for the treatment of cancers dependent on VEGF-induced vascularization.
14. The LIM protein, Limd1, regulates AP-1 activation through an interaction with Traf6 to influence osteoclast development. Increasingly a number of proteins important in the regulation of bone osteoclast development have been shown primarily influence osteoclastogenesis under conditions of physiologic or pathologic stress. Why basal osteoclastogenesis is normal and how these proteins regulate stress osteoclastogenic responses, as opposed to basal osteoclastogenesis, is unclear. LIM proteins of the Ajuba/Zyxin family localize to cellular sites of cell adhesion where they contribute to the regulation of cell adhesion and **migration**, translocate into the nucleus where they can affect cell fate, but are also found in the cytoplasm where their function is largely unknown. We show that one member of this LIM protein family, Limd1, is uniquely up-regulated during osteoclast differentiation and interacts with Traf6, a critical cytosolic regulator of RANK-L-regulated osteoclast development. Limd1 positively affects the capacity of Traf6 to activate AP-1, and Limd1(-/-) osteoclast precursor cells are defective in the activation of AP-1 and thus induction of NFAT2. Limd1(-/-) mice, although having normal basal bone osteoclast numbers and bone density, are resistant to physiological and pathologic osteoclastogenic stimuli. These results implicate Limd1 as a potentially important regulator of osteoclast development under conditions of stress.
15. Arsenic-induced alterations in embryonic transcription factor gene expression: implications for abnormal neural development. We examined the morphological and molecular consequences of acute in utero exposure to teratogenic concentrations of arsenate. The treatment produced a dose-related increase in neural tube defects, along with a significant alteration in the pattern of gene expression for several transcription factors (creb, Hox 3.1, Pax3, and Emx-1) that were examined using in situ transcription and antisense RNA amplification procedures. On gestational day 9:0, there was a significant delay in the embryos progression through neural tube closure, accompanied by a significant downregulation of Hox 3.1 expression and a significant upregulation of Pax3, Emx-1, and creb. As both Hox 3.1 and Pax3 serve to regulate N-CAM expression, it is possible that abnormalities associated with N-CAM may compromise neural crest cell **migration** and normal neural tube closure.
16. Podoplanin is regulated by AP-1 and promotes platelet aggregation and cell **migration** in osteosarcoma. Podoplanin is a type-I transmembrane sialomucin-like protein, which is expressed in a wide range of cell types and is involved in platelet aggregation and tumor metastasis. Here, we investigated the function, regulation, and expression of podoplanin in osteosarcoma. Podoplanin expression was observed in three osteosarcoma cell lines (MG-63, HOS, and U-2 OS) with platelet aggregation-inducing ability, which was blocked by podoplanin small-interfering RNA or a neutralizing antibody. Overexpression of podoplanin in nonmetastatic Dunn osteosarcoma cells promoted cell **migration** without attenuating cell proliferation. Both podoplanin and TGF-β1 were up-regulated by c-Fos induction in MC3T3-E1 osteoblastic cells, and were highly expressed in c-Fos transgenic mouse osteosarcomas and c-Fos-transformed osteosarcoma cell lines. Immunohistochemistry of human osteosarcoma tissue microarrays (n = 133) showed staining of tumor cells embedded in an excess of irregular neoplastic bone matrix in 100% of tumors undergoing so-called "normalization/maturation." Podoplanin was also expressed in osteosarcoma subtypes, with 65% of osteoblastic, 100% of chondroblastic, and 79% of fibroblastic tumors. CD44 and pERM immunohistochemistry showed coexpression with podoplanin in both mouse and human osteosarcoma. Podoplanin expression was significantly higher in metastatic osteosarcomas (n = 6) than in primary osteosarcomas (n = 10). Our data suggest that podoplanin, which is not expressed in normal osteoblasts but in osteocytes, is aberrantly expressed in transformed osteoblasts and in osteosarcoma, and is under AP-1 transcriptional control. Thus podoplanin is a candidate molecule for therapeutic targeting.
17. c-Jun is essential for organization of the epidermal leading edge. The **migration** of epithelial layers requires specific and coordinated organization of the cells at the leading edge of the sheet. Mice that are conditionally deleted for the c-jun protooncogene in epidermis are born at expected frequencies, but with open eyes and with defects in epidermal wound healing. Keratinocytes lacking c-Jun are unable to migrate or elongate properly in culture at the border of scratch assays. Histological analyses in vitro and in vivo demonstrate an inability to activate EGF receptor at the leading edge of wounds, and we demonstrate that this can be rescued by supplementation with conditioned medium or the EGF receptor ligand HB-EGF. Lack of c-Jun prevents EGF-induced expression of HB-EGF, indicating that c-jun controls formation of the epidermal leading edge through its control of an EGF receptor autocrine loop.
18. Epithelial sheet movement requires the cooperation of c-Jun and MAP3K1. Epithelial sheet movement is an essential morphogenetic process during mouse embryonic eyelid closure in which Mitogen-Activated Protein 3 Kinase 1 (MAP3K1) and c-Jun play a critical role. Here we show that MAP3K1 associates with the cytoskeleton, activates Jun N-terminal kinase (JNK) and actin polymerization, and promotes the eyelid inferior epithelial cell elongation and epithelium protrusion. Following epithelium protrusion, c-Jun begins to express and acts to promote ERK phosphorylation and **migration** of the protruding epithelial cells. Homozygous deletion of either gene causes defective eyelid closure, but non-allelic non-complementation does not occur between Map3k1 and c-Jun and the double heterozygotes have normal eyelid closure. Results from this study suggest that MAP3K1 and c-Jun signal through distinct temporal-spatial pathways and that productive epithelium movement for eyelid closure requires the consecutive action of MAP3K1-dependent cytoskeleton reorganization followed by c-Jun-mediated **migration**.
19. Stem cell factor induces AP-1-dependent mast cell IL-6 production via MAPK kinase 3 activity. Mast cells are critical immune effectors abundant in tissues interfacing with the environment and have major roles in allergen-induced inflammation and host responses to infection. SCF is a regulator of mast cell function and growth. However, the critical mechanisms in SCF-directed events remain incompletely defined. Here, we have investigated the role of MKK3 in mast cell SCF signaling-dependent functions by using BMMCs from MKK3-deficient mice. MKK3 was phosphorylated rapidly and persistently following SCF-induced activation and contributed to mast cell proliferation but not survival or **migration** in response to SCF. Analysis of SCF-induced mast cell mediator secretion demonstrated that IL-6 production is specifically dependent on MKK3 signals, both independently and in concert with IgE. Analysis of SCF-induced signaling showed that sustained p38 phosphorylation was impaired in MKK3-deficient mast cells, where as early JNK and IκBα activation were enhanced. Notably, SCF-inducible expression and activation of c-Jun, a component of the AP-1 transcription factor, was significantly dependent on MKK3. Accordingly, AP-1 DNA-binding activity and interaction with the IL6 gene promoter was markedly impaired in MKK3-deficient mast cells, whereas transcription factors of the Egr family, NF-κB, and NFAT retained near-full activity. These results designate MKK3 as a novel, positive regulator of SCF-induced mast cell proliferation and a critical signaling protein for AP-1-dependent IL-6 production.
20. A novel allele of Alx4 results in reduced Fgf10 expression and failure of eyelid fusion in mice. Normal fusion of developing eyelids requires coordination of inductive signals from the eyelid mesenchyme with **migration** of the periderm cell layer and constriction of the eyelids across the eye. Failure of this process results in an eyelids open at birth (EOB) phenotype in mice. We have identified a novel spontaneous allele of Alx4 that displays EOB, in addition to polydactyly and cranial malformations. Alx4 is expressed in the eyelid mesenchyme prior to and during eyelid fusion in a domain overlapping the expression of genes that also play a role in normal eyelid development. We show that Alx4 mutant mice have reduced expression of Fgf10, a key factor expressed in the mesenchyme that is required for initiation of eyelid fusion by the periderm. This is accompanied by a reduced number of periderm cells expressing phosphorylated c-Jun, consistent with the incomplete ablation of Fgf10 expression. Together, these data demonstrate that eyelid fusion in mice requires the expression of Alx4, accompanied by the loss of normal expression of essential components of the eyelid fusion pathway.
21. Blockade of the RhoA-JNK-c-Jun-MMP2 cascade by atorvastatin reduces osteosarcoma cell invasion. Osteosarcoma is characterized by a high malignant and metastatic potential, which points to the need for new therapeutic strategies to prevent cell metastasis. In this study, we show that statin-induced HMG-CoA reductase inhibition reduces cell **migration** and invasion in human and murine osteosarcoma cells, independently of the genotype. The statin-induced reduction of cell **migration** and invasion was independent of induction of apoptosis and was geranylgeranylpyrophosphate-dependent. The statin reduced matrix metalloproteinase (MMP) 2, 9, and 14 and TIMP2 expression or activity in invading cells. Forced expression of MMP2 and MMP14 overcame the inhibitory effect of the statin on cell invasion, suggesting a role for these MMPs in invasive potential. We also investigated the mechanisms involved in the reduced MMP2 activity and cell invasion. Inhibition of JNK, but not ERK1/2 signaling, reduced MMP2 activity. Pharmacological or constitutive activation of JNK overcame the reduced MMP2 activity and cell invasion induced by the statin. The statin decreased JNK phosphorylation and c-Jun nuclear translocation, suggesting that HMG-CoA reductase inhibition targets the JNK-c-Jun signaling pathway. We showed that mevalonate or geranylgeranylpyrophosphate treatment prevented the statin-induced reduction in JNK phosphorylation, MMP2 activity, and cell invasion. Forced expression of a constitutively active form of RhoA increased JNK phosphorylation and overcame the inhibitory effect of atorvastatin on MMP2 activity and cell invasion. The data establish a link between RhoA, JNK, c-Jun, and MMP2 activity that is functionally involved in the reduction in osteosarcoma cell invasion by the statin. This suggests a novel strategy targeting RhoA-JNK-c-Jun signaling to reduce osteosarcoma cell tumorigenesis.
22. The c-Jun-induced transformation process involves complex regulation of tenascin-C expression. In cooperation with an activated ras oncogene, the site-dependent AP-1 transcription factor c-Jun transforms primary rat embryo fibroblasts (REF). Although signal transduction pathways leading to activation of c-Jun proteins have been extensively studied, little is known about c-Jun cellular targets. We identified c-Jun-upregulated cDNA clones homologous to the tenascin-C gene by differential screening of a cDNA library from REF. This tightly regulated gene encodes a rare extracellular matrix protein involved in cell attachment and **migration** and in the control of cell growth. Transient overexpression of c-Jun induced tenascin-C expression in primary REF and in FR3T3, an established fibroblast cell line. Surprisingly, tenascin-C synthesis was repressed after stable transformation by c-Jun compared to that in the nontransformed parental cells. As assessed by using the tenascin-C (-220 to +79) promoter fragment cloned in a reporter construct, the c-Jun-induced transient activation is mediated by two binding sites: one GCN4/AP-1-like site, at position -146, and one NF-kappaB site, at position -210. Furthermore, as demonstrated by gel shift experiments and cotransfections of the reporter plasmid and expression vectors encoding the p65 subunit of NF-kappaB and c-Jun, the two transcription factors bind and synergistically transactivate the tenascin-C promoter. We previously described two other extracellular matrix proteins, SPARC and thrombospondin-1, as c-Jun targets. Thus, our results strongly suggest that the regulation of the extracellular matrix composition plays a central role in c-Jun-induced transformation.
23. c-Jun promotes cell **migration** and drives expression of the motility factor ENPP2 in soft tissue sarcomas. Genomic amplification of the c-Jun proto-oncogene has been identified in ∼30% of dedifferentiated liposarcomas (DDLPS), but the functional contribution of c-Jun to the progression of DDLPS remains poorly understood. In previous work we showed that knock-down of c-Jun by RNA interference impaired the in vitro proliferation and in vivo growth of a DDLPS cell line (LP6) with genomic amplification of the c-Jun locus. Here, we used gene expression analysis and functional studies in a broad panel of cell lines to further define the role of c-Jun in DDLPS and other soft tissue sarcomas. We show that c-Jun knock-down impairs transition through the G1 phase of the cell cycle in multiple DDLPS cell lines. We also found that high levels of c-Jun expression are both necessary and sufficient to promote DDLPS cel**l migrati**on and invasion in vitro. Our data suggest that high levels of c-Jun enhance motility in part by driving the expression of ENPP2/Autotaxin. c-Jun over-expression has minimal effects on in vitro proliferation but substantially enhances the in vivo growth of weakly tumourigenic DDLPS cell lines. Finally, we provide evidence that c-Jun genomic amplification and over-expression may have similar functional consequences in other types of soft tissue sarcoma. Our data suggest a model in which relatively low levels of c-Jun are sufficient for in vitro proliferation, but high levels of c-Jun enhance invasiveness and capacity for in vivo tumour growth. These observations provide an explanation for the selective advantage provided by c-Jun genomic amplification in vivo and suggest that sarcomas with elevated c-Jun levels are likely to have a particularly high malignant potential. Data from exon array and RNA-Seq experiments have been deposited in the GEO database (Accession No. GSE57531).
24. Differential expression of fos and jun family members during murine postnatal intestinal development. We have examined the patterns of expression of members of the fos and jun families of transcription factors during murine postnatal intestinal development. Northern analysis showed increases in fosB, c-fos, and junB mRNA levels in both proximal jejunum and colon. These increases coincide with weaning a period of major changes in intestinal cell proliferation and **migration**. Administration of dexamethasone to suckling mice resulted in transient induction of c-fos mRNA levels in all gut segments. Thus, modifications in expression of certain members of the fos and jun families in vivo correlate with major maturational changes during murine intestinal development and support a role for these transcription factors in the regulation of intestinal functions.
25. Keratinocyte **migration** in the developing eyelid requires LIMK2. In vitro studies have identified LIMK2 as a key downstream effector of Rho GTPase-induced changes in cytoskeletal organization. LIMK2 is phosphorylated and activated by Rho associated coiled-coil kinases (ROCKs) in response to a variety of growth factors. The biochemical targets of LIMK2 belong to a family of actin binding proteins that are potent modulators of actin assembly and disassembly. Although numerous studies have suggested that LIMK2 regulates cell morphology and motility, evidence supportive of these functions in vivo has remained elusive. In this study, a knockout mouse was created that abolished LIMK2 biochemical activity resulting in a profound inhibition of epithelial sheet **migration** during eyelid development. In the absence of LIMK2, nascent eyelid keratinocytes differentiate and acquire a pre-migratory phenotype but the leading cells fail to nucleate filamentous actin and remain immobile causing an eyes open at birth (EOB) phenotype. The failed nucleation of actin was associated with significant reductions in phosphorylated cofilin, a major LIMK2 biochemical substrate and potent modulator of actin dynamics. These results demonstrate that LIMK2 activity is required for keratinocyte **migration** in the developing eyelid.
26. Polysaccharide isolated from the liquid culture broth of Inonotus obliquus suppresses invasion of B16-F10 melanoma cells via AKT/NF-κB signaling pathway. A number of polysaccharides exhibit pharmacological activities. Polysaccharides derived from Inonotus obliquus (PLIO) appear to have various potential pharmacological properties, including anti‑tumor activity. However, the molecular mechanisms underlying these properties remain to be elucidated. The present study investigated the anti‑metastatic potential of PLIO and the underlying signaling pathways in B16‑F10 murine melanoma cells using the MTT colorimetric assay, **in vitro** migration and invasion assays, and flow cytometric and western blot analyses. PLIO inhibited the invasion of B16‑F10 cells and suppressed the expression of matrix metalloproteinases. PLIO treatment inhibited nuclear factor‑κB (NF‑κB) nuclear translocation in B16‑F10 cells. In addition, PLIO treatment inhibited the phosphorylation of c-Jun N‑terminal kinases and AKT. These results suggest that PLIO may suppress the invasion of highly metastatic melanoma cells via inhibition of the AKT/NF-κB signaling pathways.
27. Somatic excision demonstrates that c-Jun induces cellular **migration** and invasion through induction of stem cell factor. Cancer cells arise through sequential acquisition of mutations in tumor suppressors and oncogenes. c-Jun, a critical component of the AP-1 complex, is frequently overexpressed in diverse tumor types and has been implicated in promoting cellular proliferation, **migration**, and angiogenesis. Functional analysis of candidate genetic targets using germ line deletion in murine models can be compromised through compensatory mechanisms. As germ line deletion of c-jun induces embryonic lethality, somatic deletion of the c-jun gene was conducted using floxed c-jun (c-jun(f/f)) conditional knockout mice. c-jun-deleted cells showed increased cellular adhesion, stress fiber formation, and reduced cellular **migration**. The reduced migratory velocity and migratory directionality was rescued by either c-Jun reintroduction or addition of secreted factors from wild-type cells. An unbiased analysis of cytokines and growth factors, differentially expressed and showing loss of secretion upon c-jun deletion, identified stem cell factor (SCF) as a c-Jun target gene. Immunoneutralizing antibody to SCF reduced **migration** of wild-type cells. SCF addition rescued the defect in cellular adhesion, cellular velocity, directional **migration**, transwell **migration**, and cellular invasion of c-jun(-/-) cells. c-Jun induced SCF protein, mRNA, and promoter activity. Induction of the SCF promoter required the c-Jun DNA-binding domain. c-Jun bound to the SCF promoter in chromatin immunoprecipitation assays. Mutation of the c-Jun binding site abolished c-Jun-mediated induction of the SCF promoter. These studies demonstrate an essential role of c-Jun in cellular **migration** through induction of SCF.
28. DCX, a new mediator of the JNK pathway. Mutations in the X-linked gene DCX result in lissencephaly in males, and abnormal neuronal positioning in females, suggesting a role for this gene product during neuronal **migration**. In spite of several known protein interactions, the involvement of DCX in a signaling pathway is still elusive. Here we demonstrate that DCX is a substrate of JNK and interacts with both c-Jun N-terminal kinase (JNK) and JNK interacting protein (JIP). The localization of this signaling module in the developing brain suggests its functionality in migrating neurons. The localization of DCX at neurite tips is determined by its interaction with JIP and by the interaction of the latter with kinesin. DCX is phosphorylated by JNK in growth cones. DCX mutated in sites phosphorylated by JNK affected neurite outgrowth, and the velocity and relative pause time of migrating neurons. We hypothesize that during neuronal **migration**, there is a need to regulate molecular motors that are working in the cell in opposite directions: kinesin (a plus-end directed molecular motor) versus dynein (a minus-end directed molecular motor).
29. Regulation of lipopolysaccharide-induced lung inflammation by plasminogen activator Inhibitor-1 through a JNK-mediated pathway. The neutrophil is of undoubted importance in lung inflammation after exposure to LPS. We have shown recently that systemic inhibition of JNK decreased neutrophil recruitment to the lung after exposure to LPS, although the mechanisms underlying this inhibition are incompletely understood. As plasminogen activator inhibitor-1 (PAI-1) accentuates cell **migration**, with JNK activation recently shown to up-regulate PAI-1 expression, this suggested that systemic JNK inhibition may down-regulate LPS-induced pulmonary neutrophil recruitment through a decrease in PAI-1 expression. We show in this study that exposure of mice to aerosolized LPS increased PAI-1 expression in the lung and alveolar compartment, which was decreased by pretreatment with the JNK inhibitor SP600125. Exogenous, intratracheally administered PAI-1 prevented the inhibition of pulmonary neutrophil recruitment in the setting of systemic JNK inhibition, thereby suggesting a role for PAI-1 in the JNK-mediated pathway regulating LPS-induced neutrophil recruitment. In addition, PAI-1(-/-) mice had a decrease in neutrophil recruitment to the alveolar compartment after exposure to LPS, compared with wild-type controls, further suggesting a role for PAI-1 in LPS-induced lung inflammation. An increase in the intravascular level of KC is a likely mechanism for the inhibition of pulmonary neutrophil recruitment after LPS exposure in the setting of decreased PAI-1 expression, as systemic KC levels after exposure to LPS were increased in PAI-1-deficient mice and in mice pretreated with SP600125, with augmentation of intravascular KC levels inhibiting neutrophil recruitment to the lung after exposure to LPS.
30. Stress-activated protein kinase MKK7 regulates axon elongation in the developing cerebral cortex. The c-Jun NH(2)-terminal protein kinase (JNK), which belongs to the mitogen-activated protein kinase family, plays important roles in a broad range of physiological processes. JNK is controlled by two upstream regulators, mitogen-activated protein kinase kinase (MKK) 7 and MKK4. To elucidate the physiological functions of MKK7, we used Nestin-Cre to generate a novel mouse model in which the mkk7 gene was specifically deleted in the nervous system (Mkk7(flox/flox) Nestin-Cre mice). These mice were indistinguishable from their control littermates in gross appearance during embryogenesis but died immediately after birth without breathing. Histological examination showed that the mutants had severe defects in brain development, including enlarged ventricles, reduced striatum, and minimal axon tracts. Electron microscopy revealed abnormal accumulations of filamentous structures and autophagic vacuoles in Mkk7(flox/flox) Nestin-Cre brain. Further analysis showed that MKK7 deletion decreased numbers of TAG-1-expressing axons and delayed neuronal **migration** in the cerebrum. Neuronal differentiation was not altered. In utero electroporation studies showed that contralateral projection of axons by layer 2/3 neurons was impaired in the absence of MKK7. Moreover, MKK7 regulated axon elongation in a cell-autonomous manner in vivo, a finding confirmed in vitro. Finally, phosphorylation levels of JNK substrates, including c-Jun, neurofilament heavy chain, microtubule-associated protein 1B, and doublecortin, were reduced in Mkk7(flox/flox) Nestin-Cre brain. Our findings demonstrate that the phenotype of Mkk7(flox/flox) Nestin-Cre mice differs substantially from that of Mkk4(flox/flox) Nestin-Cre mice, and establish that MKK7-mediated regulation of JNK is uniquely critical for both axon elongation and radial **migration** in the developing brain.
31. A role for MEK kinase 1 in TGF-beta/activin-induced epithelium movement and embryonic eyelid closure. MEKK1-deficient mice show an eye open at birth phenotype caused by impairment in embryonic eyelid closure. MEK kinase 1 (MEKK1) is highly expressed in the growing tip of the eyelid epithelium, which displays loose cell-cell contacts and prominent F-actin fibers in wild-type mice, but compact cell contacts, lack of polymerized actin and a concomitant impairment in c-Jun N-terminal phosphorylation in MEKK1-deficient mice. In cultured keratinocytes, MEKK1 is essential for JNK activation by TGF-beta and activin, but not by TGF-alpha. MEKK1-driven JNK activation is required for actin stress fiber formation, c-Jun phosphorylation and cell **migration**. However, MEKK1 ablation does not impair other TGF-beta/activin functions, such as nuclear translocation of Smad4. These results establish a specific role for the MEKK1-JNK cascade in transmission of TGF-beta and activin signals that control epithelial cell movement, providing the mechanistic basis for the regulation of eyelid closure by MEKK1. This study also suggests that the signaling mechanisms that control eyelid closure in mammals and dorsal closure in Drosophila are evolutionarily conserved.
32. Spry1 and Spry2 are necessary for eyelid closure. Sproutys (Sprys) are downstream targets and negative feedback regulators of the FGF-Ras-ERK signaling pathway. Our previous studies have shown that Spry1 and Spry2, through negative modulation of FGF-ERK signaling, allow lens vesicle separation from the overlying ectoderm and regulate corneal epithelial proliferation. Here we show that Spry1 and Spry2 are necessary for eyelid closure. Murine palpebral conjunctival epithelial cells that differentiate as inner eyelids and adjacent mesenchymal cells express Spry1 and Spry2 prior to eyelid closure. Conditional deletion of both Spry1 and Spry2, but not either one alone, in the ocular surface epithelial cells result in the "EOB" (eyes open at birth) phenotype suggesting redundant roles for these proteins during eyelid closure. Spry mutant eyelids show increased proliferation of conjunctival epithelial cells with concomitant induction of FGF targets, Erm, Pea3 and Dusp6 and elevated ERK phosphorylation. Peridermal cells at the leading edge of Spry-mutant eyelids showed reduced c-Jun, but not ERK, phosphorylation, reduced F-actin polymerization and reduced motility in vitro. Spry mutant eyelids also showed disruptions in epithelial mesenchymal interactions reflected in the enhanced mesenchymal Spry1 and Spry4 expression, disaggregation of BMP4-positive mesenchymal cells and loss of Shh in the eyelid epithelium. Spry mutant eyelids also showed increased Wnt signaling and reduced expression of Foxc1 and Foxc2, two transcription factors previously shown to be necessary for eyelid closure. Collectively, our results show that conjunctival epithelial Spry1 and Spry2 redundantly promote eyelid closure by (a) stimulating ERK-independent, c-Jun-mediated peridermal **migration**, (b) suppressing conjunctival epithelial proliferation through FGF-ERK signaling, (c) mediating conjunctival epithelial-mesenchymal interactions and (d) maintaining expression of Foxc1 and Foxc2.
33. Regulation of the transcriptional activation of CTRP3 in chondrocytes by c-Jun. Although C1qTNF-related protein 3 (CTRP3) is highly expressed in cartilage and is involved in the proliferation and **migration** of chondrocytes, the molecular mechanism governing the transcriptional regulation of CTRP3 in chondrocytes is largely unknown. Here, we demonstrated the mechanism of transcriptional activation of the CTRP3 gene in chondrogenic cells. We observed that c-Jun stimulated CTRP3 promoter activity in ATDC5 cells. Deletion analysis of the CTRP3 promoter showed that the c-Jun-responsive region is localized at the position between -359 and -176. Mutation of the AP-1 binding site in this region eliminated c-Jun-mediated CTRP3 promoter activity. A promoter enzyme immunoassay and a chromatin immunoprecipitation assay showed that c-Jun binds directly to the AP-1 binding site at the region -184/-177 of the mouse CTRP3 promoter. Thus, these results demonstrated that c-Jun is a cis-acting element for CTRP3 regulation in chondrogenic cells.
34. Hepatocyte **migration** during liver development requires Prox1. Several genes are required during the early phases of liver specification, proliferation and differentiation. Here we report that Prox1 is required for hepatocyte **migration**. Loss of Prox1 leads to formation of a smaller liver with a reduced population of clustered hepatocytes surrounded by a laminin-rich basal membrane.
35. Glial origin of mesenchymal stem cells in a tooth model system. Mesenchymal stem cells occupy niches in stromal tissues where they provide sources of cells for specialized mesenchymal derivatives during growth and repair. The origins of mesenchymal stem cells have been the subject of considerable discussion, and current consensus holds that perivascular cells form mesenchymal stem cells in most tissues. The continuously growing mouse incisor tooth offers an excellent model to address the origin of mesenchymal stem cells. These stem cells dwell in a niche at the tooth apex where they produce a variety of differentiated derivatives. Cells constituting the tooth are mostly derived from two embryonic sources: neural crest ectomesenchyme and ectodermal epithelium. It has been thought for decades that the dental mesenchymal stem cells giving rise to pulp cells and odontoblasts derive from neural crest cells after their **migration** in the early head and formation of ectomesenchymal tissue. Here we show that a significant population of mesenchymal stem cells during development, self-renewal and repair of a tooth are derived from peripheral nerve-associated glia. Glial cells generate multipotent mesenchymal stem cells that produce pulp cells and odontoblasts. By combining a clonal colour-coding technique with tracing of peripheral glia, we provide new insights into the dynamics of tooth organogenesis and growth.
36. AP-1-controlled hepatocyte growth factor activation promotes keratinocyte **migration** via CEACAM1 and urokinase plasminogen activator/urokinase plasminogen receptor. Keratinocyte **migration** is essential for the rapid closure of the epidermis in the process of wound healing. Mesenchymal cell-derived hepatocyte growth factor (HGF) is a central regulator of this process. However, the molecular mechanisms and relevant genes that facilitate this cellular response are still poorly defined. We used heterologous cocultures combining primary human keratinocytes and genetically modified murine fibroblasts to identify key factors mediating HGF-induced epidermal cell **migration**. The absence of c-Jun activity in fibroblasts completely abolished the expression of HGF in these cells and consequently altered the behavior of keratinocytes. Time-resolved expression series of keratinocytes stimulated with HGF disclosed target genes regulating HGF-dependent motility. In addition to well-established HGF-dependent wound healing-associated genes, carcinoembryogenic antigen-related cell adhesion molecule (CEACAM)-1 and the urokinase plasminogen activator (uPA)/uPA-receptor (uPAR) pathway were identified as possible mediators in HGF-induced keratinocyte **migration**. The functional relevance of CEACAM-1 and uPA/uPAR on epidermal cell motility was demonstrated using the HaCaT cell culture model. In conclusion, the distinct spatiotemporal regulation of genes by HGF is essential for proper epidermal cell **migration** in cutaneous wound healing.
37. Inhibition of AP-1 transcriptional activity blocks the **migration**, invasion, and experimental metastasis of murine osteosarcoma. A well-characterized murine osteosarcoma model for metastasis and invasion was used in this study to determine the role of AP-1 in the progression of this disease. We analyzed K12 and K7M2 cells, two clonally related murine osteosarcoma cell lines that have been characterized as low metastatic or high metastatic, respectively, for AP-1 components and activity. AP-1 DNA binding was similar between the two cell lines; however AP-1 transcriptional activity was enhanced by 3- to 5-fold in K7M2 cells relative to that in K12 cells. The AP-1 complexes in K12 and K7M2 cells was composed primarily of cJun, JunD, FosB, Fra1, and Fra2, with the contribution of individual components in the complex varying between the two cell lines. In addition, an increase in phosphorylated cJun, JNK activity, and phosphorylated ERK1/2 was associated with the more metastatic osteosarcoma phenotype. The significance of AP-1 activation was confirmed by conditional expression of TAM67, a dominant negative mutant of cJun. Under conditions where TAM67 inhibited AP-1 activity in K7M2 cells, **migration** and invasion potential was significantly blocked. Tam67 expression in aggressive osteosarcoma cells decreased long-term in vivo experimental metastasis and increased survival of mice. This study shows that differences in metastatic activity can be due to AP-1 activation. The inhibition of AP-1 activity may serve as a therapeutic tool in the management of osteosarcoma.
38. Histone H3 lysine 36 methyltransferase Hypb/Setd2 is required for embryonic vascular remodeling. HYPB is a human histone H3 lysine 36 (H3K36)-specific methyltransferase and acts as the ortholog of yeast Set2. This study explored the physiological function of mammalian HYPB using knockout mice. Homozygous disruption of Hypb impaired H3K36 trimethylation but not mono- or dimethylation, and resulted in embryonic lethality at E10.5-E11.5. Severe vascular defects were observed in the Hypb(-/-) embryo, yolk sac, and placenta. The abnormally dilated capillaries in mutant embryos and yolk sacs could not be remodeled into large blood vessels or intricate networks, and the aberrantly rounded mesodermal cells exhibited weakened interaction with endothelial cells. The embryonic vessels failed to invade the labyrinthine layer of placenta, which impaired the embryonic-maternal vascular connection. These defects could not be rescued by wild-type tetraploid blastocysts, excluding the possibility that they were caused by the extraembryonic tissues. Consistent with these phenotypes, gene expression profiling in wild-type and Hypb(-/-) yolk sacs revealed that the Hypb disruption altered the expression of some genes involved in vascular remodeling. At the cellular level, Hypb(-/-) embryonic stem cell-derived embryonic bodies, as well as in vitro-cultured human endothelial cells with siRNA-mediated suppression of HYPB, showed obvious defects in cell **migration** and invasion during vessel formation, suggesting an intrinsic role of Hypb in vascular development. Taken together, these results indicate that Hypb is required for embryonic vascular remodeling and provide a tool to study the function of H3K36 methylation in vasculogenesis/angiogenesis.
39. The c-Jun N-terminal kinase activator dual leucine zipper kinase regulates axon growth and neuronal **migration** in the developing cerebral cortex. Mammalian corticogenesis substantially depends on **migration** and axon projection of newborn neurons that are coordinated by a yet unidentified molecular mechanism. Dual leucine zipper kinase (DLK) induces activation of c-Jun N-terminal kinase (JNK), a molecule that regulates morphogenesis in various organisms. We show here, using gene targeting in mice, that DLK is indispensable for establishing axon tracts, especially those originating from neocortical pyramidal neurons of the cerebrum. Direct and quantitative analysis of radial **migration** of pyramidal neurons using slice culture and a time-lapse imaging system revealed that acceleration around the subplate was affected by DLK gene disruption and by administration of a JNK inhibitor. Phosphorylation of JNK substrates, including c-Jun and doublecortin, and of JNK itself at the activation loop were partially affected in brains of DLK-deficient mouse embryos. These data suggest that DLK plays a significant role in the coordinated regulation of radial **migration** and axon projection by modulating JNK activity.
40. c-Jun is required for TGF-β-mediated cellular **migratio**n via nuclear Ca²⁺ signaling. Tumor progression involves the acquisition of invasiveness through a basement membrane. The c-jun proto-oncogene is overexpressed in human tumors and has been identified at the leading edge of human breast tumors. TGF-β plays a bifunctional role in tumorigenesis and cell**ular migr**ation. Although c-Jun and the activator protein 1 (AP-1) complex have been implicated in human cancer, the molecular mechanisms governing cell**ular migr**ation via c-Jun and the role of c-Jun in TGF-β signaling remains poorly understood. Here, we analyze TGF-β mediated ce**llular mi**gration in mouse embryo fibroblasts using floxed c-jun transgenic mice. We compared the c-jun wild type with the c-jun knockout cells through the use of Cre recombinase. Herein, TGF-β stimulated c**ellular m**igration and intracellular calcium release requiring endogenous c-Jun. TGF-β mediated Ca(2+) release was independent of extracellular calcium and was suppressed by both U73122 and neomycin, pharmacological inhibitors of the breakdown of PIP(2) into IP(3). Unlike TGF-β-mediated Ca(2+) release, which was c-Jun dependent, ATP mediated Ca(2+) release was c-Jun independent. These studies identify a novel pathway by which TGF-β regulate**s cellula**r migration and Ca(2+) release via endogenous c-Jun.
41. JUN siRNA regulates matrix metalloproteinase-2 expression, microvascular endothelial growth and retinal neovascularisation. Transcription factors link changes in the extracellular environment with alterations in gene expression. As such, these molecules serve as attractive targets for intervention in pathological settings. Since JUN has been linked with microvascular disease in humans, we hypothesised that small interfering RNA (siRNA) targeting this immediate-early gene may be useful agents that suppress endothelial growth and neovascularisation. Here we show that Jun siRNA inhibits Jun mRNA and protein expression in murine microvascular endothelial cells, blocks cell proliferation and suppresses **migration** in a scratch-wound assay. It also inhibits three-dimensional tubular formation on basement membrane extracts and reduces angiogenesis in mice bearing Matrigel plugs as subcutaneous implants. Single intravitreal administration of Jun siRNA reduces neovascularisation in a murine model of proliferative retinopathy, and suppresses endothelial JUN and matrix metalloproteinase-2 (MMP-2) immunoreactivity in retinal vessels, data supported by its repression of MMP-2 expression and gelatinolytic activity in vitro. Co-administration of TGFbeta with the siRNA reverses this neovascular inhibitory effect, which is in turn abrogated by cis-9-octadecenoyl-N-hydroxylamide, consistent with the involvement of a metalloproteinase such as MMP-2. Thus, JUN siRNA can serve as a specific inhibitor of aberrant endothelial and neovascular growth.
42. JNK signalling modulates intestinal homeostasis and tumourigenesis in mice. Wnt signalling is a crucial signalling pathway controlling intestinal homeostasis and cancer. We show here that the JNK MAP kinase pathway and one of its most important substrates, the AP-1 transcription factor c-Jun, modulates Wnt signalling strength in the intestine. Transgenic gut-specific augmentation of JNK signalling stimulated progenitor cell proliferation and **migration**, resulting in increased villus length. In the crypt, c-Jun protein was highly expressed in progenitor cells and the absence of c-Jun resulted in decreased proliferation and villus length. In addition to several known c-Jun/AP-1 target genes, expression of Wnt target genes Axin2 and Lgr5 were stimulated by JNK activation, suggesting a cross talk of JNK to Wnt signalling. Expression of the Wnt pathway component TCF4 was controlled by JNK activity, and chromatin immunoprecipitation and reporter assays identified tcf4 as a direct c-Jun target gene. Consequently, increased JNK activity accelerated tumourigenesis in a model of colorectal carcinogenesis. As c-jun is a direct target of the TCF4/beta-catenin complex, the control of tcf4 expression by JNK/c-Jun leads to a positive feedback loop that connects JNK and Wnt signalling. This mechanism regulates the physiological function of progenitor cells and oncogenic transformation.
43. Excess biglycan causes eyelid malformation by perturbing muscle development and TGF-alpha signaling. Tissue morphogenesis during development is regulated by growth factors and cytokines, and is characterized by constant remodeling of extracellular matrix (ECM) in response to signaling molecules, for example, growth factors, cytokines, and so forth. Proteoglycans that bind growth factors are potential regulators of tissue morphogenesis during embryonic development. In this study, we showed that transgenic mice overexpressing biglycan under the keratocan promoter exhibited exposure keratitis and premature eye opening from noninfectious eyelid ulceration due to perturbation of eyelid muscle formation and the failure of meibomian gland formation. In addition, in vitro analysis revealed that biglycan binds to TGF-alpha, thus interrupting EGFR signaling pathways essential for mesenchymal cell **migration** induced by eyelid epithelium. The defects of TGF-alpha signaling by excess biglycan were further augmented by the interruption of the autocrine or paracrine loop of the EGFR signaling pathway of HB-EGF expression elicited by TGF-alpha. These results are consistent with the notion that under physiological conditions, biglycan secreted by mesenchymal cells serves as a regulatory molecule for the formation of a TGF-alpha gradient serving as a morphogen of eyelid morphogenesis.

#### GeneRIFs

1. Pik3cd inactivation leads to extracellular matrix degradation in vessels and promotes aneurysm development by inducing macrophage **migration** and upregulating the activator protein-1/MMP-12 pathway in macrophages.
2. Podoplanin is regulated by AP-1 and promotes platelet aggregation and cell **migration** in osteosarcoma
3. These studies demonstrate an essential role of c-Jun in cellular **migration** through induction of SCF.
4. show that in murine microvascular endothelial cells, Jun siRNA inhibits Jun mRNA and protein expression, matrix metalloproteinase-2 expression, blocks cell proliferation and suppresses **migration**.

### ECM

#### PubMed

1. Insulin-mediated activation of activator protein-1 through the mitogen-activated protein kinase pathway stimulates collagenase-1 gene transcription in the MES 13 mesangial cell line. The initial stages of diabetic nephropathy are characterized, in part, by expansion of the mesangial matrix and thickening of the glomerular basement membrane which are caused by increased extracellular matrix (**ECM**) protein synthesis and reduced degradation, a consequence of decreased matrix metalloproteinase (MMP) activity. These changes have been largely attributed to the effects of hyperglycemia such that the potential contribution of impaired insulin action to alterations in the **ECM** have not been studied in detail. We have shown here that insulin stimulates collagenase-1 fusion gene transcription in the MES 13 mesangial-derived cell line. Multiple collagenase-1 promoter elements are required for the full stimulatory effect of insulin but the action of insulin appears to be mediated through an activator protein-1 (AP-1) motif. Thus, mutation of this AP-1 motif abolishes insulin-stimulated collagenase fusion gene transcription and, in isolation, this AP-1 motif can mediate a stimulatory effect of insulin on the expression of a heterologous fusion gene. This suggested that the other collagenase-1 promoter elements that are required for the full stimulatory effect of insulin probably bind accessory factors that enhance the effect of insulin mediated through the AP-1 motif. In MES 13 cells, the AP-1 motif is bound by Fra-1, Fra-2, Jun B and Jun D. Stimulation of collagenase-1 fusion gene transcription by insulin requires activation of the mitogen-activated protein kinase (MEK) pathway since inhibition of MEK-1 and -2 blocks this effect. The potential significance of these observations with respect to a role for insulin in the pathophysiology of diabetic glomerulosclerosis is discussed.
2. MEK kinase 1 regulates c-Jun phosphorylation in the control of corneal morphogenesis. To study the in vivo role of MEK kinase 1 (MEKK1) in corneal development.Wild type and Mekk1DeltaKD/DeltaKD mice eye tissues were examined by staining with hematoxylin and eosin for morphogenesis and Masson's trichrome for extracellular matrix (**ECM**) deposition. The cells expressing **ECM** gene transcripts of Collagen I, Keratocan, and Lumican in corneal stroma were identified by in situ hybridization and the level of Collagen I mRNA in the developing cornea was quantified by real-time RT-PCR. Immunohistochemistry staining was employed to study the expression and N-terminal phosphorylation of c-Jun and the expression of epithelium differentiation markers and intercellular structural proteins of the corneal epithelium.Mekk1DeltaKD/DeltaKD mice exhibited the "eye open at birth" phenotype (EOB), and developed eye defects and severe pathology secondary to impaired eyelid formation. The corneal stroma of Mekk1DeltaKD/DeltaKD fetuses, although exhibiting normal morphology, thickness, and keratocyte proliferation, showed reduced extracellular matrix (**ECM**) accumulation, corresponding to a decrease in transcription of Lumican, Keratocan, and Collagen I. Immunohistochemistry studies demonstrated that MEKK1 ablation caused a remarkable reduction in the expression of occludin and zonula occluden protein-1 (ZO-1), components of tight junction, but had no effect on the expression of E-cadherin and beta-catenin for adherens junctions, desmoplakin and desmoglein for desmosomes and cytokeratins 12 and 14 for cornea-type epithelial differentiation in the developing cornea. c-Jun was abundantly expressed in the developing corneal epithelium and its phosphorylation was considerably reduced in Mekk1DeltaKD/DeltaKD fetuses.In addition to its role in eyelid morphogenesis, MEKK1 is crucial for corneal development such that its ablation caused a reduction of **ECM** deposition in corneal stroma and disturbance of tight junctions in corneal epithelium. c-Jun phosphorylation in corneal epithelium is a downstream event of the MEKK1 pathway, likely contributing to corneal development and function. Altogether, MEKK1 plays a major role in ocular surface morphogenesis and its ablation leads to damage and various eye manifestations at postnatal stages.
3. Excess biglycan causes eyelid malformation by perturbing muscle development and TGF-alpha signaling. Tissue morphogenesis during development is regulated by growth factors and cytokines, and is characterized by constant remodeling of extracellular matrix (**ECM**) in response to signaling molecules, for example, growth factors, cytokines, and so forth. Proteoglycans that bind growth factors are potential regulators of tissue morphogenesis during embryonic development. In this study, we showed that transgenic mice overexpressing biglycan under the keratocan promoter exhibited exposure keratitis and premature eye opening from noninfectious eyelid ulceration due to perturbation of eyelid muscle formation and the failure of meibomian gland formation. In addition, in vitro analysis revealed that biglycan binds to TGF-alpha, thus interrupting EGFR signaling pathways essential for mesenchymal cell migration induced by eyelid epithelium. The defects of TGF-alpha signaling by excess biglycan were further augmented by the interruption of the autocrine or paracrine loop of the EGFR signaling pathway of HB-EGF expression elicited by TGF-alpha. These results are consistent with the notion that under physiological conditions, biglycan secreted by mesenchymal cells serves as a regulatory molecule for the formation of a TGF-alpha gradient serving as a morphogen of eyelid morphogenesis.

### [Ee]xtracellular [Mm]atrix

#### PubMed

1. Transcriptome analysis in prenatal IGF1-deficient mice identifies molecular pathways and target genes involved in distal lung differentiation. Insulin-like Growth Factor 1 (IGF1) is a multifunctional regulator of somatic growth and development throughout evolution. IGF1 signaling through IGF type 1 receptor (IGF1R) controls cell proliferation, survival and differentiation in multiple cell types. IGF1 deficiency in mice disrupts lung morphogenesis, causing altered prenatal pulmonary alveologenesis. Nevertheless, little is known about the cellular and molecular basis of IGF1 activity during lung development.Prenatal Igf1(-/-) mutant mice with a C57Bl/6J genetic background displayed severe disproportional lung hypoplasia, leading to lethal neonatal respiratory distress. Immuno-histological analysis of their lungs showed a thickened mesenchyme, alterations in **extracellular matrix** deposition, thinner smooth muscles and dilated blood vessels, which indicated immature and delayed distal pulmonary organogenesis. Transcriptomic analysis of Igf1(-/-) E18.5 lungs using RNA microarrays identified deregulated genes related to vascularization, morphogenesis and cellular growth, and to MAP-kinase, Wnt and cell-adhesion pathways. Up-regulation of immunity-related genes was verified by an increase in inflammatory markers. Increased expression of Nfib and reduced expression of Klf2, Egr1 and Ctgf regulatory proteins as well as activation of ERK2 MAP-kinase were corroborated by Western blot. Among IGF-system genes only IGFBP2 revealed a reduction in mRNA expression in mutant lungs. Immuno-staining patterns for IGF1R and IGF2, similar in both genotypes, correlated to alterations found in specific cell compartments of Igf1(-/-) lungs. IGF1 addition to Igf1(-/-) embryonic lungs cultured ex vivo increased airway septa remodeling and distal epithelium maturation, processes accompanied by up-regulation of Nfib and Klf2 transcription factors and Cyr61 matricellular protein.We demonstrated the functional tissue specific implication of IGF1 on fetal lung development in mice. Results revealed novel target genes and gene networks mediators of IGF1 action on pulmonary cellular proliferation, differentiation, adhesion and immunity, and on vascular and distal epithelium maturation during prenatal lung development.
2. Inactivation of PI3Kδ induces vascular injury and promotes aneurysm development by upregulating the AP-1/MMP-12 pathway in macrophages. An aneurysm is an inflammatory vascular condition. Phosphatidylinositol 3-kinases δ is highly expressed in leukocytes, and play a key role in innate immunity. However, the link between phosphatidylinositol 3-kinases δ and aneurysm development has not yet been elucidated.Carotid ligation unexpectedly induced characteristic aneurysm formation beneath the ligation point in p110δ(D910A/D910A) mice (n=25; P<0.001 versus wild-type). Besides, p110δ inactivation exacerbated CaCl2-induced abdominal aortic aneurysms development. A reverse transcription polymerase chain reaction microarray revealed signifi**cant extracellular m**atrix components degradation and matrix metalloproteinases (MMPs) upregulation in the abdominal aorta of p110δ(D910A/D910A) mice. Similarly, the expression of both collagen I and IV was significantly decreased (n=10; P<0.05 versus wild-type) in carotid artery. Western blot assay confirmed that MMP-12 was significantly upregulated in arteries of p110δ(D910A/D910A) mice (n=10; P<0.01 versus wild-type). In vitro, p110δ inactivation marked increase peritoneal macrophages recruitment and synergistically enhance tumor necrosis factor-α-induced recruitment. A specific phosphatidylinositol 3-kinases δ inhibitor (IC87114) or genetic p110δ inactivation upregulated MMP-12 expression and c-Jun phosphorylation (n=6; P<0.05 versus wild-type macrophages). IC87114 also increased activator protein-1 DNA-binding activity (n=6; P<0.001 versus control) and enhanced the effect of tumor necrosis factor-α on activator protein-1-binding activity (n=5; P<0.01 versus tumor necrosis factor-α treatment groups). Knockdown of c-Jun suppressed the effect of the IC87114 and tumor necrosis factor-α on MMP-12 mRNA expression (n=5 in each group; P<0.01 versus scrRNA treatment groups).Our findings demonstrate that p110δ inactiv**ation leads to extra**cellular matrix degradation in vessels and promotes aneurysm development by inducing macrophages migration and upregulating the activator protein-1/MMP-12 pathway in macrophages.
3. Phosphorylation of c-Fos by members of the p38 MAPK family. Role in the AP-1 response to UV light. Exposure to sources of UV radiation, such as sunlight, induces a number of cellular alterations that are highly dependent on its ability to affect gene expression. Among them, the rapid activation of genes coding for two subfamilies of proto-oncoproteins, Fos and Jun, which constitute the AP-1 transcription factor, plays a key role in the subsequent regulation of expression of genes involved in DNA repair, cell proliferation, cell cycle arrest, death by apoptosis, and tissue and **extracellular matrix** remodeling proteases. Besides being regulated at the transcriptional level, Jun and Fos transcriptional activities are also regulated by phosphorylation as a result of the activation of intracellular signaling cascades. In this regard, the phosphorylation of c-Jun by UV-induced JNK has been readily documented, whereas a role for Fos proteins in UV-mediated responses and the identification of Fos-activating kinases has remained elusive. Here we identify p38 MAPKs as proteins that can associate with c-Fos and phosphorylate its transactivation domain both in vitro and in vivo. This phosphorylation is transduced into changes in its transcriptional ability as p38-activated c-Fos enhances AP1-driven gene expression. Our findings indicate that as a consequence of the activation of stress pathways induced by UV light, endogenous c-Fos becomes a substrate of p38 MAPKs and, for the first time, provide evidence that support a critical role for p38 MAPKs in mediating stress-induced c-Fos phosphorylation and gene transcription activation. Using a specific pharmacological inhibitor for p38alpha and -beta, we found that most likely these two isoforms mediate UV-induced c-Fos phosphorylation in vivo. Thus, these newly described pathways act concomitantly with the activation of c-Jun by JNK/MAPKs, thereby contributing to the complexity of AP1-driven gene transcription regulation.
4. Inactivation of PI3Kδ induces vascular injury and promotes aneurysm development by upregulating the AP-1/MMP-12 pathway in macrophages. An aneurysm is an inflammatory vascular condition. Phosphatidylinositol 3-kinases δ is highly expressed in leukocytes, and play a key role in innate immunity. However, the link between phosphatidylinositol 3-kinases δ and aneurysm development has not yet been elucidated.Carotid ligation unexpectedly induced characteristic aneurysm formation beneath the ligation point in p110δ(D910A/D910A) mice (n=25; P<0.001 versus wild-type). Besides, p110δ inactivation exacerbated CaCl2-induced abdominal aortic aneurysms development. A reverse transcription polymerase chain reaction microarray revealed signifi**cant extracellular m**atrix components degradation and matrix metalloproteinases (MMPs) upregulation in the abdominal aorta of p110δ(D910A/D910A) mice. Similarly, the expression of both collagen I and IV was significantly decreased (n=10; P<0.05 versus wild-type) in carotid artery. Western blot assay confirmed that MMP-12 was significantly upregulated in arteries of p110δ(D910A/D910A) mice (n=10; P<0.01 versus wild-type). In vitro, p110δ inactivation marked increase peritoneal macrophages recruitment and synergistically enhance tumor necrosis factor-α-induced recruitment. A specific phosphatidylinositol 3-kinases δ inhibitor (IC87114) or genetic p110δ inactivation upregulated MMP-12 expression and c-Jun phosphorylation (n=6; P<0.05 versus wild-type macrophages). IC87114 also increased activator protein-1 DNA-binding activity (n=6; P<0.001 versus control) and enhanced the effect of tumor necrosis factor-α on activator protein-1-binding activity (n=5; P<0.01 versus tumor necrosis factor-α treatment groups). Knockdown of c-Jun suppressed the effect of the IC87114 and tumor necrosis factor-α on MMP-12 mRNA expression (n=5 in each group; P<0.01 versus scrRNA treatment groups).Our findings demonstrate that p110δ inactiv**ation leads to extra**cellular matrix degradation in vessels and promotes aneurysm development by inducing macrophages migration and upregulating the activator protein-1/MMP-12 pathway in macrophages.
5. Phosphorylation of c-Fos by members of the p38 MAPK family. Role in the AP-1 response to UV light. Exposure to sources of UV radiation, such as sunlight, induces a number of cellular alterations that are highly dependent on its ability to affect gene expression. Among them, the rapid activation of genes coding for two subfamilies of proto-oncoproteins, Fos and Jun, which constitute the AP-1 transcription factor, plays a key role in the subsequent regulation of expression of genes involved in DNA repair, cell proliferation, cell cycle arrest, death by apoptosis, and tissue and **extracellular matrix** remodeling proteases. Besides being regulated at the transcriptional level, Jun and Fos transcriptional activities are also regulated by phosphorylation as a result of the activation of intracellular signaling cascades. In this regard, the phosphorylation of c-Jun by UV-induced JNK has been readily documented, whereas a role for Fos proteins in UV-mediated responses and the identification of Fos-activating kinases has remained elusive. Here we identify p38 MAPKs as proteins that can associate with c-Fos and phosphorylate its transactivation domain both in vitro and in vivo. This phosphorylation is transduced into changes in its transcriptional ability as p38-activated c-Fos enhances AP1-driven gene expression. Our findings indicate that as a consequence of the activation of stress pathways induced by UV light, endogenous c-Fos becomes a substrate of p38 MAPKs and, for the first time, provide evidence that support a critical role for p38 MAPKs in mediating stress-induced c-Fos phosphorylation and gene transcription activation. Using a specific pharmacological inhibitor for p38alpha and -beta, we found that most likely these two isoforms mediate UV-induced c-Fos phosphorylation in vivo. Thus, these newly described pathways act concomitantly with the activation of c-Jun by JNK/MAPKs, thereby contributing to the complexity of AP1-driven gene transcription regulation.
6. Role of AP-1 and RE-1 binding sites in matrix metalloproteinase-2 transcriptional regulation in skeletal muscle atrophy. Previous work has suggested that an **extracellular matrix** degrading enzyme-matrix metalloproteinase-2 (MMP-2) plays an important role in the development of muscle atrophy. However, the transcriptional regulation mechanism of MMP-2 in skeletal muscle atrophy remains largely unknown. Using transgenic MMP-2 promoter reporter mice, we have demonstrated that AP-1 and RE-1 binding sites in the MMP-2 promoter region, coupled with increased binding of Fra-1, Fra-2 and AP-2, play a critical role in MMP-2 transcriptional regulation in muscle atrophy. Novel information gained from this study has improved our understanding of in vivo transcriptional regulation of MMP-2 in skeletal muscle atrophy.
7. Jun-regulated genes promote interaction of diffuse large B-cell lymphoma with the microenvironment. Diffuse large B-cell lymphoma (DLBCL) is an aggressive disease with a high proliferation rate. However, the molecular and genetic features that drive the aggressive clinical behavior of DLBCL are not fully defined. Here, we have demonstrated that activated Jun signaling is a frequent event in DLBCL that promotes dissemination of malignant cells. Downregulation of Jun dramatically reduces lymphoma cell adhesion to **extracellular matrix** proteins, subcutaneous tumor size in nude mice, and invasive behavior, including bone marrow infiltration and interaction with bone marrow stromal cells. Furthermore, using a combination of RNA interference and gene expression profiling, we identified Jun target genes that are associated with disseminated lymphoma. Among them, ITGAV, FoxC1, and CX3CR1 are significantly enriched in patients with 2 or more extranodal sites. Our results point to activated Jun signaling as a major driver of the aggressive phenotype of DLBCL.
8. Insulin-mediated activation of activator protein-1 through the mitogen-activated protein kinase pathway stimulates collagenase-1 gene transcription in the MES 13 mesangial cell line. The initial stages of diabetic nephropathy are characterized, in part, by expansion of the mesangial matrix and thickening of the glomerular basement membrane which are caused by increased **extracellular matrix** (ECM) protein synthesis and reduced degradation, a consequence of decreased matrix metalloproteinase (MMP) activity. These changes have been largely attributed to the effects of hyperglycemia such that the potential contribution of impaired insulin action to alterations in the ECM have not been studied in detail. We have shown here that insulin stimulates collagenase-1 fusion gene transcription in the MES 13 mesangial-derived cell line. Multiple collagenase-1 promoter elements are required for the full stimulatory effect of insulin but the action of insulin appears to be mediated through an activator protein-1 (AP-1) motif. Thus, mutation of this AP-1 motif abolishes insulin-stimulated collagenase fusion gene transcription and, in isolation, this AP-1 motif can mediate a stimulatory effect of insulin on the expression of a heterologous fusion gene. This suggested that the other collagenase-1 promoter elements that are required for the full stimulatory effect of insulin probably bind accessory factors that enhance the effect of insulin mediated through the AP-1 motif. In MES 13 cells, the AP-1 motif is bound by Fra-1, Fra-2, Jun B and Jun D. Stimulation of collagenase-1 fusion gene transcription by insulin requires activation of the mitogen-activated protein kinase (MEK) pathway since inhibition of MEK-1 and -2 blocks this effect. The potential significance of these observations with respect to a role for insulin in the pathophysiology of diabetic glomerulosclerosis is discussed.
9. Transcriptional regulation of dentin matrix protein 1 (DMP1) by AP-1 (c-fos/c-jun) factors. Dentin matrix protein 1 (DMP1) is an **extracellular matrix** phosphoprotein whose expression is precisely controlled temporally and spatially during the differentiation of the odontoblasts. Odontoblasts are postmitotic cells that differentiate to mature polarized cells and are responsible for the synthesis of a calcified dentin matrix. Although the DMP1 promoter has been isolated from the mouse and the rat, little is actually known about their control by specific transcription factors. Analysis of the rat DMP1 promoter has identified several regulatory transcription factors. These factors include AP-1 family members, SP1 and ETS. Therefore the transcription of DMP1 may be controlled positively or negatively by a variety of stimuli resulting in a developmentally regulated gene expression. This study demonstrates the role of c-fos and c-jun on the transcriptional regulation of DMP1 gene.
10. The c-Jun-induced transformation process involves complex regulation of tenascin-C expression. In cooperation with an activated ras oncogene, the site-dependent AP-1 transcription factor c-Jun transforms primary rat embryo fibroblasts (REF). Although signal transduction pathways leading to activation of c-Jun proteins have been extensively studied, little is known about c-Jun cellular targets. We identified c-Jun-upregulated cDNA clones homologous to the tenascin-C gene by differential screening of a cDNA library from REF. This tightly regulated gene encodes a rare **extracellular matrix** protein involved in cell attachment and migration and in the control of cell growth. Transient overexpression of c-Jun induced tenascin-C expression in primary REF and in FR3T3, an established fibroblast cell line. Surprisingly, tenascin-C synthesis was repressed after stable transformation by c-Jun compared to that in the nontransformed parental cells. As assessed by using the tenascin-C (-220 to +79) promoter fragment cloned in a reporter construct, the c-Jun-induced transient activation is mediated by two binding sites: one GCN4/AP-1-like site, at position -146, and one NF-kappaB site, at position -210. Furthermore, as demonstrated by gel shift experiments and cotransfections of the reporter plasmid and expression vectors encoding the p65 subunit of NF-kappaB and c-Jun, the two transcription factors bind and synergistically transactivate the tenascin-C promoter. We previously described two other **extracellular matrix** proteins, SPARC and thrombospondin-1, as c-Jun targets. Thus, our results strongly suggest that the regulation of the **extracellular matrix** composition plays a central role in c-Jun-induced transformation.
11. Altered gene expression in neurons during programmed cell death: identification of c-jun as necessary for neuronal apoptosis. We have examined the hypothesis that neuronal programmed cell death requires a genetic program; we used a model wherein rat sympathetic neurons maintained in vitro are deprived of NGF and subsequently undergo apoptosis. To evaluate gene expression potentially necessary for this process, we used a PCR-based technique and in situ hybridization; patterns of general gene repression and selective gene induction were identified in NGF-deprived neurons. A temporal cascade of induced genes included "immediate early genes," which were remarkable in that their induction occurred hours after the initial stimulus of NGF removal and the synthesis of some required ongoing protein synthesis. The cascade also included the cell cycle gene c-myb and the genes encoding the **extracellular matrix** proteases transin and collagenase. Concurrent in situ hybridization and nuclear staining revealed that while c-jun was induced in most neurons, c-fos induction was restricted to neurons undergoing chromatin condensation, a hallmark of apoptosis. To evaluate the functional role of the proteins encoded by these genes, neutralizing antibodies were injected into neurons. Antibodies specific for either c-Jun or the Fos family (c-Fos, Fos B, Fra-1, and Fra-2) protected NGF-deprived neurons from apoptosis, whereas antibodies specific for Jun B, Jun D, or three nonimmune antibody preparations had no protective effect. Because these induced genes encode proteins ranging from a transcription factor necessary for death to proteases likely involved in tissue remodeling concurrent with death, these data may outline a genetic program responsible for neuronal programmed cell death.
12. MEK kinase 1 regulates c-Jun phosphorylation in the control of corneal morphogenesis. To study the in vivo role of MEK kinase 1 (MEKK1) in corneal development.Wild type and Mekk1DeltaKD/DeltaKD mice eye tissues were examined by staining with hematoxylin and eosin for morphogenesis and Masson's trichrome for **extracellular matrix** (ECM) deposition. The cells expressing ECM gene transcripts of Collagen I, Keratocan, and Lumican in corneal stroma were identified by in situ hybridization and the level of Collagen I mRNA in the developing cornea was quantified by real-time RT-PCR. Immunohistochemistry staining was employed to study the expression and N-terminal phosphorylation of c-Jun and the expression of epithelium differentiation markers and intercellular structural proteins of the corneal epithelium.Mekk1DeltaKD/DeltaKD mice exhibited the "eye open at birth" phenotype (EOB), and developed eye defects and severe pathology secondary to impaired eyelid formation. The corneal stroma of Mekk1DeltaKD/DeltaKD fetuses, although exhibiting normal morphology, thickness, and keratocyte proliferation, showed reduced **extracellular matrix** (ECM) accumulation, corresponding to a decrease in transcription of Lumican, Keratocan, and Collagen I. Immunohistochemistry studies demonstrated that MEKK1 ablation caused a remarkable reduction in the expression of occludin and zonula occluden protein-1 (ZO-1), components of tight junction, but had no effect on the expression of E-cadherin and beta-catenin for adherens junctions, desmoplakin and desmoglein for desmosomes and cytokeratins 12 and 14 for cornea-type epithelial differentiation in the developing cornea. c-Jun was abundantly expressed in the developing corneal epithelium and its phosphorylation was considerably reduced in Mekk1DeltaKD/DeltaKD fetuses.In addition to its role in eyelid morphogenesis, MEKK1 is crucial for corneal development such that its ablation caused a reduction of ECM deposition in corneal stroma and disturbance of tight junctions in corneal epithelium. c-Jun phosphorylation in corneal epithelium is a downstream event of the MEKK1 pathway, likely contributing to corneal development and function. Altogether, MEKK1 plays a major role in ocular surface morphogenesis and its ablation leads to damage and various eye manifestations at postnatal stages.
13. Excess biglycan causes eyelid malformation by perturbing muscle development and TGF-alpha signaling. Tissue morphogenesis during development is regulated by growth factors and cytokines, and is characterized by constant remodeling of **extracellular matrix** (ECM) in response to signaling molecules, for example, growth factors, cytokines, and so forth. Proteoglycans that bind growth factors are potential regulators of tissue morphogenesis during embryonic development. In this study, we showed that transgenic mice overexpressing biglycan under the keratocan promoter exhibited exposure keratitis and premature eye opening from noninfectious eyelid ulceration due to perturbation of eyelid muscle formation and the failure of meibomian gland formation. In addition, in vitro analysis revealed that biglycan binds to TGF-alpha, thus interrupting EGFR signaling pathways essential for mesenchymal cell migration induced by eyelid epithelium. The defects of TGF-alpha signaling by excess biglycan were further augmented by the interruption of the autocrine or paracrine loop of the EGFR signaling pathway of HB-EGF expression elicited by TGF-alpha. These results are consistent with the notion that under physiological conditions, biglycan secreted by mesenchymal cells serves as a regulatory molecule for the formation of a TGF-alpha gradient serving as a morphogen of eyelid morphogenesis.
14. The AP-1 transcription factor c-Jun prevents stress-imposed maladaptive remodeling of the heart. Systemic hypertension increases cardiac workload and subsequently induces signaling networks in heart that underlie myocyte growth (hypertrophic response) through expansion of sarcomeres with the aim to increase contractility. However, conditions of increased workload can induce both adaptive and maladaptive growth of heart muscle. Previous studies implicate two members of the AP-1 transcription factor family, junD and fra-1, in regulation of heart growth during hypertrophic response. In this study, we investigate the function of the AP-1 transcription factors, c-jun and c-fos, in heart growth. Using pressure overload-induced cardiac hypertrophy in mice and targeted deletion of Jun or Fos in cardiomyocytes, we show that c-jun is required for adaptive cardiac hypertrophy, while c-fos is dispensable in this context. c-jun promotes expression of sarcomere proteins and suppresses expression of **extracellular matrix** proteins. Capacity of cardiac muscle to contract depends on organization of principal thick and thin filaments, myosin and actin, within the sarcomere. In line with decreased expression of sarcomere-associated proteins, Jun-deficient cardiomyocytes present disarrangement of filaments in sarcomeres and actin cytoskeleton disorganization. Moreover, Jun-deficient hearts subjected to pressure overload display pronounced fibrosis and increased myocyte apoptosis finally resulting in dilated cardiomyopathy. In conclusion, c-jun but not c-fos is required to induce a transcriptional program aimed at adapting heart growth upon increased workload.

#### GeneRIFs

1. Pik3cd inactivation leads to **extracellular matrix** degradation in vessels and promotes aneurysm development by inducing macrophage migration and upregulating the activator protein-1/MMP-12 pathway in macrophages.

### [Ii]nvasive

#### PubMed

1. Disruption of c-Jun reduces cellular migration and invasion through inhibition of c-Src and hyperactivation of ROCK II kinase. The spread of metastatic tumors to different organs is associated with poor prognosis. The metastatic process requires migration and cellular invasion. The protooncogene c-jun encodes the founding member of the activator protein-1 family and is required for cellular proliferation and DNA synthesis in response to oncogenic signals and plays an essential role in chemical carcinogenesis. The role of c-Jun in cellular invasion remains to be defined. Genetic deletion of c-Jun in transgenic mice is embryonic lethal; therefore, transgenic mice encoding a c-Jun gene flanked by LoxP sites (c-jun(f/f)) were used. c-jun gene deletion reduced c-Src expression, hyperactivated ROCK II signaling, and reduced cellular polarity, migration, and **invasive**ness. c-Jun increased c-Src mRNA abundance and c-Src promoter activity involving an AP-1 site in the c-Src promoter. Transduction of c-jun(-/-) cells with either c-Jun or c-Src retroviral expression systems restored the defective cellular migration of c-jun(-/-) cells. As c-Src is a critical component of pathways regulating proliferation, survival, and metastasis, the induction of c-Src abundance, by c-Jun, provides a novel mechanism of cooperative signaling in cellular invasion.
2. Disruption of c-Jun reduces cellular migration and invasion through inhibition of c-Src and hyperactivation of ROCK II kinase. The spread of metastatic tumors to different organs is associated with poor prognosis. The metastatic process requires migration and cellular invasion. The protooncogene c-jun encodes the founding member of the activator protein-1 family and is required for cellular proliferation and DNA synthesis in response to oncogenic signals and plays an essential role in chemical carcinogenesis. The role of c-Jun in cellular invasion remains to be defined. Genetic deletion of c-Jun in transgenic mice is embryonic lethal; therefore, transgenic mice encoding a c-Jun gene flanked by LoxP sites (c-jun(f/f)) were used. c-jun gene deletion reduced c-Src expression, hyperactivated ROCK II signaling, and reduced cellular polarity, migration, and **invasive**ness. c-Jun increased c-Src mRNA abundance and c-Src promoter activity involving an AP-1 site in the c-Src promoter. Transduction of c-jun(-/-) cells with either c-Jun or c-Src retroviral expression systems restored the defective cellular migration of c-jun(-/-) cells. As c-Src is a critical component of pathways regulating proliferation, survival, and metastasis, the induction of c-Src abundance, by c-Jun, provides a novel mechanism of cooperative signaling in cellular invasion.
3. Loss of JUNB/AP-1 promotes **invasive** prostate cancer. Prostate cancer is a frequent cause of male death in the Western world. Relatively few genetic alterations have been identified, likely owing to disease heterogeneity. Here, we show that the transcription factor JUNB/AP-1 limits prostate cancer progression. JUNB expression is increased in low-grade prostate cancer compared with normal human prostate, but downregulated in high-grade samples and further decreased in all metastatic samples. To model the hypothesis that this downregulation is functionally significant, we genetically inactivated Junb in the prostate epithelium of mice. When combined with Pten (phosphatase and tensin homologue) loss, double-mutant mice were prone to **invasive** cancer development. Importantly, **invasive** tumours also developed when Junb and Pten were inactivated in a small cell population of the adult anterior prostate by topical Cre recombinase delivery. The resulting tumours displayed strong histological similarity with human prostate cancer. Loss of JunB expression led to increased proliferation and decreased senescence, likely owing to decreased p16(Ink4a) and p21(CIP1) in epithelial cells. Furthermore, the tumour stroma was altered with increased osteopontin and S100 calcium-binding protein A8/9 expression, which correlated with poor prognoses in patients. These data demonstrate that JUNB/AP-1 cooperates with PTEN signalling as barriers to **invasive** prostate cancer, whose concomitant genetic or epigenetic suppression induce malignant progression.
4. Jun-regulated genes promote interaction of diffuse large B-cell lymphoma with the microenvironment. Diffuse large B-cell lymphoma (DLBCL) is an aggressive disease with a high proliferation rate. However, the molecular and genetic features that drive the aggressive clinical behavior of DLBCL are not fully defined. Here, we have demonstrated that activated Jun signaling is a frequent event in DLBCL that promotes dissemination of malignant cells. Downregulation of Jun dramatically reduces lymphoma cell adhesion to extracellular matrix proteins, subcutaneous tumor size in nude mice, and **invasive** behavior, including bone marrow infiltration and interaction with bone marrow stromal cells. Furthermore, using a combination of RNA interference and gene expression profiling, we identified Jun target genes that are associated with disseminated lymphoma. Among them, ITGAV, FoxC1, and CX3CR1 are significantly enriched in patients with 2 or more extranodal sites. Our results point to activated Jun signaling as a major driver of the aggressive phenotype of DLBCL.
5. Squamous cell carcinoma growth in mice and in culture is regulated by c-Jun and its control of matrix metalloproteinase-2 and -9 expression. Squamous cell carcinoma (SCC) is an **invasive** malignancy of epidermal keratinocytes. Surgical excision is currently the main treatment; however, this can cause scarring and disfigurement. There is accordingly, an acute need for alternative strategies to treat SCC. The transcription factor c-Jun is expressed in human SCC and another common form of **invasive** skin cancer, basal cell carcinoma together with the mitogenic marker-proliferating cell nuclear antigen. Here, we have employed DNAzymes (catalytic DNA molecules) targeting c-Jun (Dz13) to inhibit c-Jun expression in SCC cells. Dz13 inhibits SCC proliferation and suppresses solid SCC tumor growth and tumor angiogenesis in severe combined immunodeficient mice. We further demonstrate that Dz13 inhibits c-Jun, together with matrix metalloproteinase (MMP)-2 and MMP-9 expression in the tumors, consistent with DNAzyme inhibition of MMP-2 and MMP-9 gelatinolytic activity by zymography. Dz13 also suppressed the expression of vascular endothelial growth factor and fibroblast growth factor-2 in the tumors. These findings demonstrate that c-Jun regulates SCC growth and suggest that DNAzymes targeting this transcription factor may potentially be useful as inhibitors of cutaneous carcinoma.
6. Blockade of the RhoA-JNK-c-Jun-MMP2 cascade by atorvastatin reduces osteosarcoma cell invasion. Osteosarcoma is characterized by a high malignant and metastatic potential, which points to the need for new therapeutic strategies to prevent cell metastasis. In this study, we show that statin-induced HMG-CoA reductase inhibition reduces cell migration and invasion in human and murine osteosarcoma cells, independently of the genotype. The statin-induced reduction of cell migration and invasion was independent of induction of apoptosis and was geranylgeranylpyrophosphate-dependent. The statin reduced matrix metalloproteinase (MMP) 2, 9, and 14 and TIMP2 expression or activity in invading cells. Forced expression of MMP2 and MMP14 overcame the inhibitory effect of the statin on cell invasion, suggesting a role for these MMPs in **invasive** potential. We also investigated the mechanisms involved in the reduced MMP2 activity and cell invasion. Inhibition of JNK, but not ERK1/2 signaling, reduced MMP2 activity. Pharmacological or constitutive activation of JNK overcame the reduced MMP2 activity and cell invasion induced by the statin. The statin decreased JNK phosphorylation and c-Jun nuclear translocation, suggesting that HMG-CoA reductase inhibition targets the JNK-c-Jun signaling pathway. We showed that mevalonate or geranylgeranylpyrophosphate treatment prevented the statin-induced reduction in JNK phosphorylation, MMP2 activity, and cell invasion. Forced expression of a constitutively active form of RhoA increased JNK phosphorylation and overcame the inhibitory effect of atorvastatin on MMP2 activity and cell invasion. The data establish a link between RhoA, JNK, c-Jun, and MMP2 activity that is functionally involved in the reduction in osteosarcoma cell invasion by the statin. This suggests a novel strategy targeting RhoA-JNK-c-Jun signaling to reduce osteosarcoma cell tumorigenesis.
7. c-Jun promotes cell migration and drives expression of the motility factor ENPP2 in soft tissue sarcomas. Genomic amplification of the c-Jun proto-oncogene has been identified in ∼30% of dedifferentiated liposarcomas (DDLPS), but the functional contribution of c-Jun to the progression of DDLPS remains poorly understood. In previous work we showed that knock-down of c-Jun by RNA interference impaired the in vitro proliferation and in vivo growth of a DDLPS cell line (LP6) with genomic amplification of the c-Jun locus. Here, we used gene expression analysis and functional studies in a broad panel of cell lines to further define the role of c-Jun in DDLPS and other soft tissue sarcomas. We show that c-Jun knock-down impairs transition through the G1 phase of the cell cycle in multiple DDLPS cell lines. We also found that high levels of c-Jun expression are both necessary and sufficient to promote DDLPS cell migration and invasion in vitro. Our data suggest that high levels of c-Jun enhance motility in part by driving the expression of ENPP2/Autotaxin. c-Jun over-expression has minimal effects on in vitro proliferation but substantially enhances the in vivo growth of weakly tumourigenic DDLPS cell lines. Finally, we provide evidence that c-Jun genomic amplification and over-expression may have similar functional consequences in other types of soft tissue sarcoma. Our data suggest a model in which relatively low levels of c-Jun are sufficient for in vitro proliferation, but high levels of c-Jun enhanc**e invasi**veness and capacity for in vivo tumour growth. These observations provide an explanation for the selective advantage provided by c-Jun genomic amplification in vivo and suggest that sarcomas with elevated c-Jun levels are likely to have a particularly high malignant potential. Data from exon array and RNA-Seq experiments have been deposited in the GEO database (Accession No. GSE57531).
8. Reversion of the Jun-induced oncogenic phenotype by enhanced synthesis of sialosyllactosylceramide (GM3 ganglioside). In the mouse fibroblast cell line C3H 10T1/2 and the chicken fibroblast cell line DF1, the ganglioside GM3 is the major glycosphingolipid component of the plasma membrane. Expression of the viral oncoprotein Jun (v-Jun) induces transformed cell clones with greatly reduced levels of GM3 and GM3 synthase (lactosylceramide alpha2,3-sialyltransferase) mRNA in both 10T1/2 and DF1 cell cultures. Compared with nontransformed controls, v-Jun transfectants show enhanced ability of anchorage-independent growth, and their growth rates as adherent cells are increased. When the mouse GM3 synthase gene is transfected with the pcDNA vector into v-Jun-transformed 10T1/2 cells, the levels of GM3 synthase and corresponding mRNA are restored to those of control cells. Reexpression of GM3 correlates with a reduced ability of the cells to form colonies in nutrient agar. Similarly, when the newly cloned chicken GM3 synthase gene is transfected into v-Jun-transformed DF1 with the pcDNA vector, the GM3 synthase level is restored to that of control cells, and the ability of the cells to form agar colonies is reduced. The levels of GM3 in the cell also affect membrane microdomains. The complex of GM3 with tetraspanin CD9 and integrin alpha5beta1 inhibits motility and **invasive**ness. The amounts of this complex are greatly reduced in transformed cells. Expression of GM3 and consequent reversion of the transformed phenotype results in increased levels of that microdomain complex.
9. Dynamic regulation of c-Jun N-terminal kinase activity in mouse brain by environmental stimuli. Activation of the recently identified c-Jun N-terminal kinases (JNKs) typically results in programmed cell death (apoptosis) in neurons and other cell types grown in culture. However, the effects of JNK activation in the central nervous system in vivo are unknown. At baseline, JNK activity in mice was on average 17-fold higher in brain than in peripheral organs, whereas JNK protein levels were similar. In brain, JNK was expressed primarily in neurons. Restraining mice or allowing them to explore a novel environment rapidly increased JNK activity 3- to 15-fold in various brain regions, but these manipulations did not increase brain activity of the extracellular signal-regulated kinase. Because non**invasive** environmental stimuli that do not induce neurodegeneration elicited prominent increases in JNK activity in the brain, we conclude that acute activation of the JNK cascade in central nervous system neurons does not induce neuronal apoptosis in vivo. In contrast, the high baseline activity of JNK in the brain and the activation of the JNK cascade by environmental stimuli suggest that this kinase may play an important physiological role in neuronal function.
10. DYRK2 priming phosphorylation of c-Jun and c-Myc modulates cell cycle progression in human cancer cells. Dysregulation of the G(1)/S transition in the cell cycle contributes to tumor development. The oncogenic transcription factors c-Jun and c-Myc are indispensable regulators at this transition, and their aberrant expression is associated with many malignancies. Degradation of c-Jun/c-Myc is a critical process for the G(1)/S transition, which is initiated upon phosphorylation by glycogen synthase kinase 3 β (GSK3β). However, a specific kinase or kinases responsible for priming phosphorylation events that precede this GSK3β modification has not been definitively identified. Here, we found that the dual-specificity tyrosine phosphorylation-regulated kinase DYRK2 functions as a priming kinase of c-Jun and c-Myc. Knockdown of DYRK2 in human cancer cells shortened the G(1) phase and accelerated cell proliferation due to escape of c-Jun and c-Myc from ubiquitination-mediated degradation. In concert with these results, silencing DYRK2 increased cell proliferation in human cancer cells, and this promotion was completely impeded by codeprivation of c-Jun or c-Myc in vivo. We also found marked attenuation of DYRK2 expression in multiple human tumor samples. Downregulation of DYRK2 correlated with high levels of unphosphorylated c-Jun and c-Myc and, importantly, wi**th invas**iveness of human breast cancers. These results reveal that DYRK2 regulates tumor progression through modulation of c-Jun and c-Myc.
11. c-Jun is required for TGF-β-mediated cellular migration via nuclear Ca²⁺ signaling. Tumor progression involves the acquisition **of inva**siveness through a basement membrane. The c-jun proto-oncogene is overexpressed in human tumors and has been identified at the leading edge of human breast tumors. TGF-β plays a bifunctional role in tumorigenesis and cellular migration. Although c-Jun and the activator protein 1 (AP-1) complex have been implicated in human cancer, the molecular mechanisms governing cellular migration via c-Jun and the role of c-Jun in TGF-β signaling remains poorly understood. Here, we analyze TGF-β mediated cellular migration in mouse embryo fibroblasts using floxed c-jun transgenic mice. We compared the c-jun wild type with the c-jun knockout cells through the use of Cre recombinase. Herein, TGF-β stimulated cellular migration and intracellular calcium release requiring endogenous c-Jun. TGF-β mediated Ca(2+) release was independent of extracellular calcium and was suppressed by both U73122 and neomycin, pharmacological inhibitors of the breakdown of PIP(2) into IP(3). Unlike TGF-β-mediated Ca(2+) release, which was c-Jun dependent, ATP mediated Ca(2+) release was c-Jun independent. These studies identify a novel pathway by which TGF-β regulates cellular migration and Ca(2+) release via endogenous c-Jun.

### [Mm]etastasis

#### PubMed

1. Disruption of c-Jun reduces cellular migration and invasion through inhibition of c-Src and hyperactivation of ROCK II kinase. The spread of metastatic tumors to different organs is associated with poor prognosis. The metastatic process requires migration and cellular invasion. The protooncogene c-jun encodes the founding member of the activator protein-1 family and is required for cellular proliferation and DNA synthesis in response to oncogenic signals and plays an essential role in chemical carcinogenesis. The role of c-Jun in cellular invasion remains to be defined. Genetic deletion of c-Jun in transgenic mice is embryonic lethal; therefore, transgenic mice encoding a c-Jun gene flanked by LoxP sites (c-jun(f/f)) were used. c-jun gene deletion reduced c-Src expression, hyperactivated ROCK II signaling, and reduced cellular polarity, migration, and invasiveness. c-Jun increased c-Src mRNA abundance and c-Src promoter activity involving an AP-1 site in the c-Src promoter. Transduction of c-jun(-/-) cells with either c-Jun or c-Src retroviral expression systems restored the defective cellular migration of c-jun(-/-) cells. As c-Src is a critical component of pathways regulating proliferation, survival, and **metastasis**, the induction of c-Src abundance, by c-Jun, provides a novel mechanism of cooperative signaling in cellular invasion.
2. Disruption of c-Jun reduces cellular migration and invasion through inhibition of c-Src and hyperactivation of ROCK II kinase. The spread of metastatic tumors to different organs is associated with poor prognosis. The metastatic process requires migration and cellular invasion. The protooncogene c-jun encodes the founding member of the activator protein-1 family and is required for cellular proliferation and DNA synthesis in response to oncogenic signals and plays an essential role in chemical carcinogenesis. The role of c-Jun in cellular invasion remains to be defined. Genetic deletion of c-Jun in transgenic mice is embryonic lethal; therefore, transgenic mice encoding a c-Jun gene flanked by LoxP sites (c-jun(f/f)) were used. c-jun gene deletion reduced c-Src expression, hyperactivated ROCK II signaling, and reduced cellular polarity, migration, and invasiveness. c-Jun increased c-Src mRNA abundance and c-Src promoter activity involving an AP-1 site in the c-Src promoter. Transduction of c-jun(-/-) cells with either c-Jun or c-Src retroviral expression systems restored the defective cellular migration of c-jun(-/-) cells. As c-Src is a critical component of pathways regulating proliferation, survival, and **metastasis**, the induction of c-Src abundance, by c-Jun, provides a novel mechanism of cooperative signaling in cellular invasion.
3. Anxa5 mediates the in vitro malignant behaviours of murine hepatocarcinoma Hca-F cells with high lymph node **metastasis** potential preferentially via ERK2/p-ERK2/c-Jun/p-c-Jun(Ser73) and E-cadherin. Annexin A5 (Anxa5) is associated with the progression of some cancers, while its role and regulation mechanism in tumor lymphatic **metastasis** is rarely reported. This study aims to investigate the influence of Anxa5 knockdown on the malignant behaviours of murine hepatocarcinoma Hca-F cell line with high lymph node metastatic (LNM) potential and the underlying regulation mechanism.RNA interfering was performed to silence Anxa5 in Hca-F. Monoclonal shRNA-Anxa5- Hca-F cells were obtained via G418 screening by limited dilution method. Quantitative real-time RT-PCR (qRT-PCR) and Western blotting (WB) were applied to measure Anxa5 expression levels. CCK-8, Boyden transwell-chamber and in situ LN adhesion assays were performed to explore the effects of Anxa5 on the proliferation, migration, invasion and adhesion capacities of Hca-F. WB and qRT-PCR were used to detect the level changes of key molecules in corresponding signal pathways.We obtained two monoclonal shRNA-Anxa5-transfected Hca-F cell lines with stable knockdowns of Anxa5. Anxa5 knockdown resulted in significantly reduced proliferation, migration, invasion and in situ LN adhesion potentials of Hca-F in proportion to its knockdown extent. Anxa5 downregulation enhanced E-cadherin levels in Hca-F. Moreover, Anxa5 affected Hca-F behaviours specifically via ERK2/p-ERK2/c-Jun/p-c-Jun(Ser73) instead of p38MAPK/c-Jun, Jnk/c-Jun and AKT/c-Jun pathways.Anxa5 mediates the in vitro malignant behaviours of murine hepatocarcinoma Hca-F cells via ERK2/c-Jun/p-c-Jun(Ser73) and ERK2/E-cadherin pathways. It is an important molecule in **metastasis** (especially LNM) and a potential therapeutic target for hepatocarcinoma.
4. Podoplanin is regulated by AP-1 and promotes platelet aggregation and cell migration in osteosarcoma. Podoplanin is a type-I transmembrane sialomucin-like protein, which is expressed in a wide range of cell types and is involved in platelet aggregation and tumor **metastasis**. Here, we investigated the function, regulation, and expression of podoplanin in osteosarcoma. Podoplanin expression was observed in three osteosarcoma cell lines (MG-63, HOS, and U-2 OS) with platelet aggregation-inducing ability, which was blocked by podoplanin small-interfering RNA or a neutralizing antibody. Overexpression of podoplanin in nonmetastatic Dunn osteosarcoma cells promoted cell migration without attenuating cell proliferation. Both podoplanin and TGF-β1 were up-regulated by c-Fos induction in MC3T3-E1 osteoblastic cells, and were highly expressed in c-Fos transgenic mouse osteosarcomas and c-Fos-transformed osteosarcoma cell lines. Immunohistochemistry of human osteosarcoma tissue microarrays (n = 133) showed staining of tumor cells embedded in an excess of irregular neoplastic bone matrix in 100% of tumors undergoing so-called "normalization/maturation." Podoplanin was also expressed in osteosarcoma subtypes, with 65% of osteoblastic, 100% of chondroblastic, and 79% of fibroblastic tumors. CD44 and pERM immunohistochemistry showed coexpression with podoplanin in both mouse and human osteosarcoma. Podoplanin expression was significantly higher in metastatic osteosarcomas (n = 6) than in primary osteosarcomas (n = 10). Our data suggest that podoplanin, which is not expressed in normal osteoblasts but in osteocytes, is aberrantly expressed in transformed osteoblasts and in osteosarcoma, and is under AP-1 transcriptional control. Thus podoplanin is a candidate molecule for therapeutic targeting.
5. Downregulation of c-jun results in apoptosis-mediated anti-osteosarcoma activity in an orthotopic model. c-jun has been found to be upregulated in a variety of cancers including osteosarcoma. DNAzymes are oligonucleotides capable of specific downregulation of target genes. c-jun knockdown-mediated apoptosis in osteosarcoma cells involved caspases-1, -2 and -8, but not the Fas/FasL pathway. A c-jun DNAzyme, encapsulated within a novel cationic multilamellar vesicle liposome, inhibited the growth and **metastasis** of osteosarcoma in an orthotopic spontaneously **metastasis**ing model of the disease. The 60 nm DDAB:DOPE liposome was formulated using ethanol injection/extrusion. Clinically, downregulation of c-jun may proffer an improved treatment outcome for these tumours originating in bone.
6. Blockade of the RhoA-JNK-c-Jun-MMP2 cascade by atorvastatin reduces osteosarcoma cell invasion. Osteosarcoma is characterized by a high malignant and metastatic potential, which points to the need for new therapeutic strategies to prevent cell **metastasis**. In this study, we show that statin-induced HMG-CoA reductase inhibition reduces cell migration and invasion in human and murine osteosarcoma cells, independently of the genotype. The statin-induced reduction of cell migration and invasion was independent of induction of apoptosis and was geranylgeranylpyrophosphate-dependent. The statin reduced matrix metalloproteinase (MMP) 2, 9, and 14 and TIMP2 expression or activity in invading cells. Forced expression of MMP2 and MMP14 overcame the inhibitory effect of the statin on cell invasion, suggesting a role for these MMPs in invasive potential. We also investigated the mechanisms involved in the reduced MMP2 activity and cell invasion. Inhibition of JNK, but not ERK1/2 signaling, reduced MMP2 activity. Pharmacological or constitutive activation of JNK overcame the reduced MMP2 activity and cell invasion induced by the statin. The statin decreased JNK phosphorylation and c-Jun nuclear translocation, suggesting that HMG-CoA reductase inhibition targets the JNK-c-Jun signaling pathway. We showed that mevalonate or geranylgeranylpyrophosphate treatment prevented the statin-induced reduction in JNK phosphorylation, MMP2 activity, and cell invasion. Forced expression of a constitutively active form of RhoA increased JNK phosphorylation and overcame the inhibitory effect of atorvastatin on MMP2 activity and cell invasion. The data establish a link between RhoA, JNK, c-Jun, and MMP2 activity that is functionally involved in the reduction in osteosarcoma cell invasion by the statin. This suggests a novel strategy targeting RhoA-JNK-c-Jun signaling to reduce osteosarcoma cell tumorigenesis.
7. Inhibition of AP-1 transcriptional activity blocks the migration, invasion, and experimental **metastasis** of murine osteosarcoma. A well-characterized murine osteosarcoma model for **metastasis** and invasion was used in this study to determine the role of AP-1 in the progression of this disease. We analyzed K12 and K7M2 cells, two clonally related murine osteosarcoma cell lines that have been characterized as low metastatic or high metastatic, respectively, for AP-1 components and activity. AP-1 DNA binding was similar between the two cell lines; however AP-1 transcriptional activity was enhanced by 3- to 5-fold in K7M2 cells relative to that in K12 cells. The AP-1 complexes in K12 and K7M2 cells was composed primarily of cJun, JunD, FosB, Fra1, and Fra2, with the contribution of individual components in the complex varying between the two cell lines. In addition, an increase in phosphorylated cJun, JNK activity, and phosphorylated ERK1/2 was associated with the more metastatic osteosarcoma phenotype. The significance of AP-1 activation was confirmed by conditional expression of TAM67, a dominant negative mutant of cJun. Under conditions where TAM67 inhibited AP-1 activity in K7M2 cells, migration and invasion potential was significantly blocked. Tam67 expression in aggressive osteosarcoma cells decreased long-term in vivo experimental **metastasis** and increased survival of mice. This study shows that differences in metastatic activity can be due to AP-1 activation. The inhibition of AP-1 activity may serve as a therapeutic tool in the management of osteosarcoma.

#### GeneRIFs

1. As c-Src is a critical component of pathways regulating proliferation, survival, and **metastasis**, the induction of c-Src abundance, by c-Jun, provides a novel mechanism of cooperative signaling in cellular invasion.

### [Cc]ell [Ii]nvasion

#### PubMed

1. v-jun oncogene suppresses both phorbol ester-induced **cell invasion** and stromelysin gene expression in a mouse papilloma cell line. The viral jun (v-jun) oncogene encodes a transcription factor that can participate in the transactivation of genes through the AP-1 complex. Evidence indicates that the ability of v-jun to transform cells and stimulate transcription depends on the cell type. We have asked whether expression of the v-jun gene in benign tumor forming mouse keratinocytes that already express an activated c-rasHa oncogene would cause malignant progression. Our results showed that the v-jun transfection did not result in malignant progression; instead, we made the unexpected observation that the ability of these cells to invade reconstituted basement membrane matrix (in vitro) in response to the phorbol ester, 12-O-tetradecanoylphorbol-13-acetate, was suppressed. This phenomenon could, in part, be explained by the suppression of the induction by phorbol ester of expression of the metalloproteinase, stromelysin (transin). Of interest was the finding that 12-O-tetradecanoylphorbol-13-acetate induction of other cellular genes known to be regulated by AP-1 was not inhibited in the benign tumor cells expressing v-jun.
2. Blockade of the RhoA-JNK-c-Jun-MMP2 cascade by atorvastatin reduces osteosarcoma **cell invasion**. Osteosarcoma is characterized by a high malignant and metastatic potential, which points to the need for new therapeutic strategies to prevent cell metastasis. In this study, we show that statin-induced HMG-CoA reductase inhibition reduces cell migration and invasion in human and murine osteosarcoma cells, independently of the genotype. The statin-induced reduction of cell migration and invasion was independent of induction of apoptosis and was geranylgeranylpyrophosphate-dependent. The statin reduced matrix metalloproteinase (MMP) 2, 9, and 14 and TIMP2 expression or activity in invading cells. Forced expression of MMP2 and MMP14 overcame the inhibitory effect of the statin on **cell invasion**, suggesting a role for these MMPs in invasive potential. We also investigated the mechanisms involved in the reduced MMP2 activity and **cell invasion**. Inhibition of JNK, but not ERK1/2 signaling, reduced MMP2 activity. Pharmacological or constitutive activation of JNK overcame the reduced MMP2 activity and **cell invasion** induced by the statin. The statin decreased JNK phosphorylation and c-Jun nuclear translocation, suggesting that HMG-CoA reductase inhibition targets the JNK-c-Jun signaling pathway. We showed that mevalonate or geranylgeranylpyrophosphate treatment prevented the statin-induced reduction in JNK phosphorylation, MMP2 activity, and **cell invasion**. Forced expression of a constitutively active form of RhoA increased JNK phosphorylation and overcame the inhibitory effect of atorvastatin on MMP2 activity and **cell invasion**. The data establish a link between RhoA, JNK, c-Jun, and MMP2 activity that is functionally involved in the reduction in osteosarcoma **cell invasion** by the statin. This suggests a novel strategy targeting RhoA-JNK-c-Jun signaling to reduce osteosarcoma cell tumorigenesis.
3. The FGF-BMP signaling axis regulates outflow tract valve primordium formation by promoting cushion neural crest cell differentiation. Heart valves develop from precursor structures called cardiac cushions, an endothelial-lined cardiac jelly that resides in the inner side of the heart tube. The cushions are then invaded by cells from different sources, undergo a series of complicated and poorly understood remodeling processes, and give rise to valves. Disruption of the fibroblast growth factor (FGF) signaling axis impairs morphogenesis of the outflow tract (OFT). Yet, whether FGF signaling regulates OFT valve formation is unknown.To study how OFT valve formation is regulated and how aberrant cell signaling causes valve defects.By using mouse genetic manipulation, cell lineage tracing, ex vivo heart culture, and molecular biology approaches, we demonstrated that FGF signaling in the OFT myocardium upregulated Bmp4 expression, which then enhanced smooth muscle differentiation of neural crest cells (NCCs) in the cushion. FGF signaling also promoted OFT myocardial **cell invasion** to the cushion. Disrupting FGF signaling interrupted cushion remodeling with reduced NCCs differentiation into smooth muscle and less cardiomyocyte invasion and resulted in malformed OFT valves.The results demonstrate a novel mechanism by which the FGF-BMP signaling axis regulates formation of OFT valve primordia by controlling smooth muscle differentiation of cushion NCCs.

#### GeneRIFs

1. a link between RhoA, JNK, c-Jun, and MMP2 activity that is functionally involved in the reduction in osteosarcoma **cell invasion** by the statin. This suggests a novel strategy targeting RhoA-JNK-c-Jun signaling to reduce osteosarcoma cell tumorigenesis.

---

## Ppm1f

- NCBI Gene
- GeneRIF
- Pubmed

| Search Term | Rank | Fields |
| --- | --- | --- |
| [Mm]igration | 6 | Process(1) |
| [Ii]nvasive | 10 | PubMed(1); GeneRIFs(1) |

  
hide/show details for Ppm1f

### [Mm]igration

#### Process

1. positive regulation of epithelial cell **migration**

### [Ii]nvasive

#### PubMed

1. The POPX2 phosphatase regulates cancer cell motility and **invasive**ness. Rho GTP ases play major roles in the regulation of actin cytoskeleton, cell movement and cell cycle. PAK, one of the effector kinases of these small GTP ases, has long been associated with different types of cancer. Therefore, it is likely that deregulation of PAK activity or expression may contribute to the development of cancer. POP X2, a PP 2C serine/threonine phosphatase, is known to dephosphorylate PAK and downregulate its activity. We find that POPX2 is expressed in a wide variety of tumour cell lines, the levels being highest in the more **invasive** MDA-MB-231 and lowest in the non-**invasive** MCF7 breast cancer lines. We show that silencing of POPX2 reduces the amount of stress fibers and focal adhesions in both cells lines. Interestingly, POPX2 deficiency dramatically reduces cell motility and **invasive**ness in MDA-MB-231 cells, and cell motility in MCF7 cells. Conversely, overexpression of POP X2 in MDA-MB-231 and MCF7 cells increased their motility. The silencing of POP X2 also inhibits the expression of beta1 integrin implying that POP X2 may modulate cell attachment to the extra-cellular matrix, as reflected in diminished initial colonization of POPX2 knockdown cells in nude mice. Based on these results, we propose a mechanism by which POP X2 regulates the **invasive** behavior of the cells.

#### GeneRIFs

1. The POPX2 phosphatase regulates cancer cell motility and **invasive**ness.

---

## Pip5k1a

- NCBI Gene
- GeneRIF
- Pubmed

| Search Term | Rank | Fields |
| --- | --- | --- |
| [Mm]igration | 6 | Process(1); PubMed(2) |

  
hide/show details for Pip5k1a

### [Mm]igration

#### Process

1. fibroblast **migration**

#### PubMed

1. The LIM protein Ajuba regulates phosphatidylinositol 4,5-bisphosphate levels in migrating cells through an interaction with and activation of PIPKI alpha. The phosphoinositide phosphatidylinositol 4,5-bisphosphate [PI(4,5)P2] regulates the activity of many actin-binding proteins and as such is an important modulator of cytoskeleton organization during cell **migration**, for example. In migrating cells actin remodeling is tightly regulated and localized; therefore, how the PI(4,5)P2 level is spatially and temporally regulated is crucial to understanding how it controls cell **migration**. Here we show that the LIM protein Ajuba contributes to the cellular regulation of PI(4,5)P2 levels by interacting with and activating the enzymatic activity of the PI(4)P 5-kinase (PIPKIalpha), the predominant enzyme in the synthesis of PI(4,5)P2, in a **migration** stimulus-regulated manner. In migrating primary mouse embryonic fibroblasts (MEFs) from Ajuba(-/-) mice the level of PI(4,5)P2 was decreased with a corresponding increase in the level of the substrate PI(4)P. Reintroduction of Ajuba into these cells normalized PI(4,5)P2 levels. Localization of PI(4,5)P2 synthesis and PIPKIalpha in the leading lamellipodia and membrane ruffles, respectively, of migrating Ajuba(-/-) MEFs was impaired. In vitro, Ajuba dramatically activated the enzymatic activity of PIPKIalpha while inhibiting the activity of PIPKIIbeta. Thus, in addition to its effects upon Rac activity Ajuba can also influence cell **migration** through regulation of PI(4,5)P2 synthesis through direct activation of PIPKIalpha enzyme activity.
2. Focal adhesion kinase is required for neural crest cell morphogenesis during mouse cardiovascular development. Neural crest cells (NCCs) participate in the remodeling of the cardiac outflow tract and pharyngeal arch arteries during cardiovascular development. Focal adhesion kinase (FAK) mediates signal transduction by integrin and growth factor receptors, each of which is important for normal cardiovascular development. To investigate the role of FAK in NCC morphogenesis, we deleted it in murine NCCs using Wnt1cre, yielding craniofacial and cardiovascular malformations resembling those observed in individuals with DiGeorge syndrome. In these mice, we observed normal cardiac NCC **migration** but reduced differentiation into smooth muscle within the aortic arch arteries and impaired cardiac outflow tract rotation, which resulted in a dextroposed aortic root. Moreover, within the conotruncal cushions, Fak-deficient NCCs formed a less organized mesenchyme, with reduced expression of perlecan and semaphorin 3C, and exhibited disorganized F-actin stress fibers within the aorticopulmonary septum. Additionally, absence of Fak resulted in reduced in vivo phosphorylation of Crkl and Erk1/2, components of a signaling pathway essential for NCC development. Consistent with this, both TGF-beta and FGF induced FAK and Crkl phosphorylation in control but not Fak-deficient NCCs in vitro. Our results indicate that FAK plays an essential role in cardiac outflow tract development by promoting the activation of molecules such as Crkl and Erk1/2.

---

## Chac1

- NCBI Gene
- GeneRIF
- Pubmed

| Search Term | Rank | Fields |
| --- | --- | --- |
| [Ee]xtracellular [Mm]atrix | 8 | PubMed(1) |

  
hide/show details for Chac1

### [Ee]xtracellular [Mm]atrix

#### PubMed

1. Global defects in collagen secretion in a Mia3/TANGO1 knockout mouse. Melanoma inhibitory activity member 3 (MIA3/TANGO1) [corrected] is an evolutionarily conserved endoplasmic reticulum resident transmembrane protein. Recent in vitro studies have shown that it is required for the loading of collagen VII, but not collagen I, into COPII-coated transport vesicles. In this paper, we show that mice lacking Mia3 are defective for the secretion of numerous collagens, including collagens I, II, III, IV, VII, and IX, from chondrocytes, fibroblasts, endothelial cells, and mural cells. Collagen deposition by these cell types is abnormal, and **extracellular matrix** composition is compromised. These changes are associated with intracellular accumulation of collagen and the induction of a strong unfolded protein response, primarily within the developing skeleton. Chondrocyte maturation and bone mineralization are severely compromised in Mia3-null embryos, leading to dwarfism and neonatal lethality. Thus, Mia3's role in protein secretion is much broader than previously realized, and it may, in fact, be required for the efficient secretion of all collagen molecules in higher organisms.

---

## Ptpn21

- NCBI Gene
- GeneRIF
- Pubmed

| Search Term | Rank | Fields |
| --- | --- | --- |
| [Mm]igration | 6 | PubMed(1) |

  
hide/show details for Ptpn21

### [Mm]igration

#### PubMed

1. Protein-tyrosine phosphatase PTPD1 regulates focal adhesion kinase autophosphorylation and cell **migration**. PTPD1 is a cytosolic nonreceptor tyrosine phosphatase and a positive regulator of the Src-epidermal growth factor transduction pathway. We show that PTPD1 localizes along actin filaments and at adhesion plaques. PTPD1 forms a stable complex via distinct molecular modules with actin, Src tyrosine kinase, and focal adhesion kinase (FAK), a scaffold protein kinase enriched at adhesion plaques. Overexpression of PTPD1 promoted cell scattering and **migration**, short hairpin RNA-mediated silencing of endogenous PTPD1, or expression of PTPD1 mutants lacking either catalytic activity (PTPD1(C1108S)) or the FERM domain (PTPD1(Delta1-325)) significantly reduced cell motility. PTPD1 and Src catalytic activities were both required for epidermal growth factor-induced FAK autophosphorylation at its active site and for downstream propagation of ERK1/2 signaling. Our findings demonstrate that PTPD1 is a component of a multivalent scaffold complex nucleated by FAK at specific intracellular sites. By modulating Src-FAK signaling at adhesion sites, PTPD1 promotes the cytoskeleton events that induce cell adhesion and **migration**.

---

## Myl6

- NCBI Gene
- GeneRIF
- Pubmed

| Search Term | Rank | Fields |
| --- | --- | --- |
| [Mm]igration | 6 | PubMed(1) |
| [Ee]xtracellular [Mm]atrix | 8 | Component(1) |
| [Mm]etastasis | 12 | PubMed(1) |

  
hide/show details for Myl6

### [Mm]igration

#### PubMed

1. Vangl2-regulated polarisation of second heart field-derived cells is required for outflow tract lengthening during cardiac development. Planar cell polarity (PCP) is the mechanism by which cells orient themselves in the plane of an epithelium or during directed cell **migration**, and is regulated by a highly conserved signalling pathway. Mutations in the PCP gene Vangl2, as well as in other key components of the pathway, cause a spectrum of cardiac outflow tract defects. However, it is unclear why cells within the mesodermal heart tissue require PCP signalling. Using a new conditionally floxed allele we show that Vangl2 is required solely within the second heart field (SHF) to direct normal outflow tract lengthening, a process that is required for septation and normal alignment of the aorta and pulmonary trunk with the ventricular chambers. Analysis of a range of markers of polarised epithelial tissues showed that in the normal heart, undifferentiated SHF cells move from the dorsal pericardial wall into the distal outflow tract where they acquire an epithelial phenotype, before moving proximally where they differentiate into cardiomyocytes. Thus there is a transition zone in the distal outflow tract where SHF cells become more polarised, turn off progenitor markers and start to differentiate to cardiomyocytes. Membrane-bound Vangl2 marks the proximal extent of this transition zone and in the absence of Vangl2, the SHF-derived cells are abnormally polarised and disorganised. The consequent thickening, rather than lengthening, of the outflow wall leads to a shortened outflow tract. Premature down regulation of the SHF-progenitor marker Isl1 in the mutants, and accompanied premature differentiation to cardiomyocytes, suggests that the organisation of the cells within the transition zone is important for maintaining the undifferentiated phenotype. Thus, Vangl2-regulated polarisation and subsequent acquisition of an epithelial phenotype is essential to lengthen the tubular outflow vessel, a process that is essential for on-going cardiac morphogenesis.

### [Ee]xtracellular [Mm]atrix

#### Component

1. **extracellular matrix**

### [Mm]etastasis

#### PubMed

1. Lack of periostin leads to suppression of Notch1 signaling and calcific aortic valve disease. The Postn gene encodes protein periostin. During embryonic development, it is highly expressed in the outflow tract (OFT) endocardial cushions of the developing heart, which give rise to several structures of the mature heart including the aortic valve. Periostin was previously implicated in osteoblast differentiation, cancer **metastasis**, and tooth and bone development, but its role in cardiac OFT development is unclear. To elucidate the role that periostin plays in the developing heart we analyzed cardiac OFT phenotype in mice after deletion of the Postn gene. We found that lack of periostin in the embryonic OFT leads to ectopic expression of the proosteogenic growth factor pleiotrophin (Ptn) and overexpression of delta-like 1 homolog (Dlk1), a negative regulator of Notch1, in the distal (prevalvular) cushions of the OFT. This resulted in suppression of Notch1 signaling, strong induction of the central transcriptional regulator of osteoblast cell fate Runx2, upregulation of osteopontin and osteocalcin expression, and subsequent calcification of the aortic valve. Our data suggest that periostin represses a default osteogenic program in the OFT cushion mesenchyme and promotes differentiation along a fibrogenic lineage. Lack of periostin causes derepression of the osteogenic potential of OFT mesenchymal cells, calcium deposition, and calcific aortic valve disease. These results establish periostin as a key regulator of OFT endocardial cushion mesenchymal cell fate during embryonic development.

---

## Myl1

- NCBI Gene
- GeneRIF
- Pubmed

| Search Term | Rank | Fields |
| --- | --- | --- |
| [Mm]igration | 6 | PubMed(1) |

  
hide/show details for Myl1

### [Mm]igration

#### PubMed

1. Src Family Kinases (SFK) Mediate Angiotensin II-Induced Myosin Light Chain Phosphorylation and Hypertension. Angiotensin (Ang) II is the major bioactive peptide of the renin-angiotensin system (RAS); it contributes to the pathogenesis of hypertension by inducing vascular contraction and adverse remodeling, thus elevated peripheral resistance. Ang II also activates Src family kinases (SFK) in the vascular system, which has been implicated in cell proliferation and **migration**. However, the role of SFK in Ang II-induced hypertension is largely unknown. In this study, we found that administration of a SFK inhibitor SU6656 markedly lowered the level of systemic BP in Ang II-treated mice, which was associated with an attenuated phosphorylation of the smooth-muscle myosin-light-chain (MLC) in the mesenteric resistant arteries. In the cultured human coronary artery smooth muscle cells (SMCs), pretreatment with SU6656 blocked Ang II-induced MLC phosphorylation and contraction. These results for the first time demonstrate that SFK directly regulate vascular contractile machinery to influence BP. Thus our study provides an additional mechanistic link between Ang II and vasoconstriction via SFK-enhanced MLC phosphorylation in SMCs, and suggests that targeted inhibition of Src may provide a new therapeutic opportunity in the treatment of hypertension.

---

## Ano1

- NCBI Gene
- GeneRIF
- Pubmed

| Search Term | Rank | Fields |
| --- | --- | --- |
| [Mm]igration | 6 | PubMed(2); GeneRIFs(1) |

  
hide/show details for Ano1

### [Mm]igration

#### PubMed

1. The role of TMEM16A (ANO1) and TMEM16F (ANO6) in cell **migration**. Members of the TMEM16 family have recently been described as Ca(2+)-activated Cl(-) channels. They have been implicated in cancer and appear to be associated with poor patient prognosis. Here, we investigate the role of TMEM16 channels in cell **migration**, which is largely unknown. We focused on TMEM16A and TMEM16F channels that have the highest expression of TMEM16 channels in Ehrlich Lettre ascites (ELA) cells. Due to the lack of specific pharmacological modulators, we employed a miRNA approach and stably knocked down the expression of TMEM16A and TMEM16F channels, respectively. **Migration** analysis shows that TMEM16A KD clones are affected in their directional **migration**, whereas TMEM16F KD clones show a 40 % reduced rate of cell **migration**. Moreover, TMEM16A KD clones have a smaller projected cell area, and they are rounder than TMEM16F KD clones. The morphological changes are linearly correlated with the directionality of cells. TMEM16A and TMEM16F, thus, have an important function in cell **migration**-TMEM16A in directional **migration**, TMEM16F in determination of the speed of **migration**. We conclude that TMEM16A and TMEM16F channels have a distinct impact on the steering and motor mechanisms of migrating ELA cells.
2. Anoctamin 1 dysregulation alters bronchial epithelial repair in cystic fibrosis. Cystic fibrosis (CF) airway epithelium is constantly subjected to injury events due to chronic infection and inflammation. Moreover, abnormalities in CF airway epithelium repair have been described and contribute to the lung function decline seen in CF patients. In the last past years, it has been proposed that anoctamin 1 (ANO1), a Ca(2+)-activated Cl(-) channel, might offset the CFTR deficiency but this protein has not been characterized in CF airways. Interestingly, recent evidence indicates a role for ANO1 in cell proliferation and tumor growth. Our aims were to study non-CF and CF bronchial epithelial repair and to determine whether ANO1 is involved in airway epithelial repair. Here, we showed, with human bronchial epithelial cell lines and primary cells, that both cell proliferation and **migration** during epithelial repair are delayed in CF compared to non-CF cells. We then demonstrated that ANO1 Cl(-) channel activity was significantly decreased in CF versus non-CF cells. To explain this decreased Cl(-) channel activity in CF context, we compared ANO1 expression in non-CF vs. CF bronchial epithelial cell lines and primary cells, in lung explants from wild-type vs. F508del mice and non-CF vs. CF patients. In all these models, ANO1 expression was markedly lower in CF compared to non-CF. Finally, we established that ANO1 inhibition or overexpression was associated respectively with decreases and increases in cell proliferation and **migration**. In summary, our study demonstrates involvement of ANO1 decreased activity and expression in abnormal CF airway epithelial repair and suggests that ANO1 correction may improve this process.

#### GeneRIFs

1. findings show TMEM16A and TMEM16F have an important function in cell **migration**-TMEM16A in directional **migration**, TMEM16F in determination of speed of **migration**; conclude that TMEM16A and TMEM16F channels have a distinct impact on steering and motor mechanisms of migrating ELA cells

---

## Adamts9

- NCBI Gene
- GeneRIF
- Pubmed

| Search Term | Rank | Fields |
| --- | --- | --- |
| ECM | 7 | PubMed(1) |

  
hide/show details for Adamts9

### ECM

#### PubMed

1. Characterization of ADAMTS-9 and ADAMTS-20 as a distinct ADAMTS subfamily related to Caenorhabditis elegans GON-1. We demonstrate that in humans, two metalloproteases, ADAMTS-9 (1935 amino acids) and ADAMTS-20 (1911 amino acids) are orthologs of GON-1, an ADAMTS protease required for gonadal morphogenesis in Caenorhabditis elegans. ADAMTS-9 and ADAMTS-20 have an identical modular structure, are distinct in possessing 15 TSRs and a unique C-terminal domain, and have a similar gene structure, suggesting that they comprise a new subfamily of human ADAMTS proteases. ADAMTS20 is very sparingly expressed, although it is detectable in epithelial cells of the breast and lung. However, ADAMTS9 is highly expressed in embryonic and adult tissues, and therefore we characterized the ADAMTS-9 protein further. Although the ADAMTS-9 zymogen has many proprotein convertase processing sites, pulse-chase analysis, site-directed mutagenesis, and amino acid sequencing demonstrated that maturation to the active form occurs by selective proprotein convertase (e.g. furin) cleavage of the Arg(287)-Phe(288) bond. Although lacking a transmembrane sequence, ADAMTS-9 is retained near the cell surface as well as in the **ECM** of transiently transfected COS-1 and 293 cells. COS-1 cells transfected with ADAMTS9 (but not vector-transfected cells) proteolytically cleaved bovine versican and aggrecan core proteins at the Glu(441)-Ala(442) bond of versican V1 and the Glu(1771)-Ala(1772) bond of aggrecan, respectively. In contrast, the ADAMTS-9 catalytic domain alone was neither localized to the cell surface nor able to confer these proteolytic activities on cells, demonstrating that the ancillary domains of ADAMTS-9, including the TSRs, are required both for specific extracellular localization and for its versicanase and aggrecanase activities.

---

## Adamts9

- NCBI Gene
- GeneRIF
- Pubmed

| Search Term | Rank | Fields |
| --- | --- | --- |
| [Ff]ilopodia | 1 | PubMed(1) |
| [Mm]igration | 6 | PubMed(2) |
| ECM | 7 | PubMed(9) |
| [Ee]xtracellular [Mm]atrix | 8 | PubMed(11); Component(1); GeneRIFs(1) |

  
hide/show details for Adamts9

### [Ff]ilopodia

#### PubMed

1. ADAMTS9 is a cell-autonomously acting, anti-angiogenic metalloprotease expressed by microvascular endothelial cells. The metalloprotease ADAMTS9 participates in melanoblast development and is a tumor suppressor in esophageal and nasopharyngeal cancer. ADAMTS9 null mice die before gastrulation, but, ADAMTS9+/- mice were initially thought to be normal. However, when congenic with the C57Bl/6 strain, 80% of ADAMTS9+/- mice developed spontaneous corneal neovascularization. beta-Galactosidase staining enabled by a lacZ cassette targeted to the ADAMTS9 locus showed that capillary endothelial cells (ECs) in embryonic and adult tissues and in capillaries growing into heterotopic tumors expressed ADAMTS9. Heterotopic B.16-F10 melanomas elicited greater vascular induction in ADAMTS9+/- mice than in wild-type littermates, suggesting a potential inhibitory role in tumor angiogenesis. Treatment of cultured human microvascular ECs with ADAMTS9 small-interfering RNA resulted in enhanced **filopodia**l extension, decreased cell adhesion, increased cell migration, and enhanced formation of tube-like structures on Matrigel. Conversely, overexpression of catalytically active, but not inactive, ADAMTS9 in ECs led to fewer tube-like structures, demonstrating that the proteolytic activity of ADAMTS9 was essential. However, unlike the related metalloprotease ADAMTS1, which exerts anti-angiogenic effects by cleavage of thrombospondins and sequestration of vascular endothelial growth factor165, ADAMTS9 neither cleaved thrombospondins 1 and 2, nor bound vascular endothelial growth factor165. Taken together, these data identify ADAMTS9 as a novel, constitutive, endogenous angiogenesis inhibitor that operates cell-autonomously in ECs via molecular mechanisms that are distinct from those used by ADAMTS1.

### [Mm]igration

#### PubMed

1. The secreted metalloprotease ADAMTS20 is required for melanoblast survival. ADAMTS20 (Adisintegrin-like and metalloprotease domain with thrombospondin type-1 motifs) is a member of a family of secreted metalloproteases that can process a variety of extracellular matrix (ECM) components and secreted molecules. Adamts20 mutations in belted (bt) mice cause white spotting of the dorsal and ventral torso, indicative of defective neural crest (NC)-derived melanoblast development. The expression pattern of Adamts20 in dermal mesenchymal cells adjacent to migrating melanoblasts led us to initially propose that Adamts20 regulated melanoblast **migration**. However, using a Dct-LacZ transgene to track melanoblast development, we determined that melanoblasts were distributed normally in whole mount E12.5 bt/bt embryos, but were specifically reduced in the trunk of E13.5 bt/bt embryos due to a seven-fold higher rate of apoptosis. The melanoblast defect was exacerbated in newborn skin and embryos from bt/bt animals that were also haploinsufficient for Adamts9, a close homolog of Adamts20, indicating that these metalloproteases functionally overlap in melanoblast development. We identified two potential mechanisms by which Adamts20 may regulate melanoblast survival. First, skin explant cultures demonstrated that Adamts20 was required for melanoblasts to respond to soluble Kit ligand (sKitl). In support of this requirement, bt/bt;Kit(tm1Alf)/+ and bt/bt;Kitl(Sl)/+ mice exhibited synergistically increased spotting. Second, ADAMTS20 cleaved the aggregating proteoglycan versican in vitro and was necessary for versican processing in vivo, raising the possibility that versican can participate in melanoblast development. These findings reveal previously unrecognized roles for Adamts proteases in cell survival and in mediating Kit signaling during melanoblast colonization of the skin. Our results have implications not only for understanding mechanisms of NC-derived melanoblast development but also provide insights on novel biological functions of secreted metalloproteases.
2. ADAMTS9 is a cell-autonomously acting, anti-angiogenic metalloprotease expressed by microvascular endothelial cells. The metalloprotease ADAMTS9 participates in melanoblast development and is a tumor suppressor in esophageal and nasopharyngeal cancer. ADAMTS9 null mice die before gastrulation, but, ADAMTS9+/- mice were initially thought to be normal. However, when congenic with the C57Bl/6 strain, 80% of ADAMTS9+/- mice developed spontaneous corneal neovascularization. beta-Galactosidase staining enabled by a lacZ cassette targeted to the ADAMTS9 locus showed that capillary endothelial cells (ECs) in embryonic and adult tissues and in capillaries growing into heterotopic tumors expressed ADAMTS9. Heterotopic B.16-F10 melanomas elicited greater vascular induction in ADAMTS9+/- mice than in wild-type littermates, suggesting a potential inhibitory role in tumor angiogenesis. Treatment of cultured human microvascular ECs with ADAMTS9 small-interfering RNA resulted in enhanced filopodial extension, decreased cell adhesion, increased cell **migration**, and enhanced formation of tube-like structures on Matrigel. Conversely, overexpression of catalytically active, but not inactive, ADAMTS9 in ECs led to fewer tube-like structures, demonstrating that the proteolytic activity of ADAMTS9 was essential. However, unlike the related metalloprotease ADAMTS1, which exerts anti-angiogenic effects by cleavage of thrombospondins and sequestration of vascular endothelial growth factor165, ADAMTS9 neither cleaved thrombospondins 1 and 2, nor bound vascular endothelial growth factor165. Taken together, these data identify ADAMTS9 as a novel, constitutive, endogenous angiogenesis inhibitor that operates cell-autonomously in ECs via molecular mechanisms that are distinct from those used by ADAMTS1.

### ECM

#### PubMed

1. ADAMTS9-Mediated Extracellular Matrix Dynamics Regulates Umbilical Cord Vascular Smooth Muscle Differentiation and Rotation. Despite the significance for fetal nourishment in mammals, mechanisms of umbilical cord vascular growth remain poorly understood. Here, the secreted metalloprotease ADAMTS9 is shown to be necessary for murine umbilical cord vascular development. Restricting it to the cell surface using a gene trap allele, Adamts9(Gt), impaired umbilical vessel elongation and radial growth via reduced versican proteolysis and accumulation of extracellular matrix (**ECM**). Both Adamts9(Gt) and conditional Adamts9 deletion revealed that ADAMTS9 produced by mesenchymal cells acted non-autonomously to regulate smooth muscle cell (SMC) proliferation, differentiation, and orthogonal reorientation during growth of the umbilical vasculature. In Adamts9(Gt/Gt), we observed interference with PDGFRβ signaling via the mitogen-activated protein kinase/extracellular signal-regulated kinase (MAPK/ERK) pathway, which regulates cytoskeletal dynamics during SMC rotation. In addition, we observed disrupted Shh signaling and perturbed orientation of the mesenchymal primary cilium. Thus, **EC**M dynamics is a major influence on umbilical vascular SMC fate, with ADAMTS9 acting as its principal mediator.
2. Characterization of ADAMTS-9 and ADAMTS-20 as a distinct ADAMTS subfamily related to Caenorhabditis elegans GON-1. We demonstrate that in humans, two metalloproteases, ADAMTS-9 (1935 amino acids) and ADAMTS-20 (1911 amino acids) are orthologs of GON-1, an ADAMTS protease required for gonadal morphogenesis in Caenorhabditis elegans. ADAMTS-9 and ADAMTS-20 have an identical modular structure, are distinct in possessing 15 TSRs and a unique C-terminal domain, and have a similar gene structure, suggesting that they comprise a new subfamily of human ADAMTS proteases. ADAMTS20 is very sparingly expressed, although it is detectable in epithelial cells of the breast and lung. However, ADAMTS9 is highly expressed in embryonic and adult tissues, and therefore we characterized the ADAMTS-9 protein further. Although the ADAMTS-9 zymogen has many proprotein convertase processing sites, pulse-chase analysis, site-directed mutagenesis, and amino acid sequencing demonstrated that maturation to the active form occurs by selective proprotein convertase (e.g. furin) cleavage of the Arg(287)-Phe(288) bond. Although lacking a transmembrane sequence, ADAMTS-9 is retained near the cell surface as well as in the **ECM** of transiently transfected COS-1 and 293 cells. COS-1 cells transfected with ADAMTS9 (but not vector-transfected cells) proteolytically cleaved bovine versican and aggrecan core proteins at the Glu(441)-Ala(442) bond of versican V1 and the Glu(1771)-Ala(1772) bond of aggrecan, respectively. In contrast, the ADAMTS-9 catalytic domain alone was neither localized to the cell surface nor able to confer these proteolytic activities on cells, demonstrating that the ancillary domains of ADAMTS-9, including the TSRs, are required both for specific extracellular localization and for its versicanase and aggrecanase activities.
3. ADAMTS9-Mediated Extracellular Matrix Dynamics Regulates Umbilical Cord Vascular Smooth Muscle Differentiation and Rotation. Despite the significance for fetal nourishment in mammals, mechanisms of umbilical cord vascular growth remain poorly understood. Here, the secreted metalloprotease ADAMTS9 is shown to be necessary for murine umbilical cord vascular development. Restricting it to the cell surface using a gene trap allele, Adamts9(Gt), impaired umbilical vessel elongation and radial growth via reduced versican proteolysis and accumulation of extracellular matrix (**ECM**). Both Adamts9(Gt) and conditional Adamts9 deletion revealed that ADAMTS9 produced by mesenchymal cells acted non-autonomously to regulate smooth muscle cell (SMC) proliferation, differentiation, and orthogonal reorientation during growth of the umbilical vasculature. In Adamts9(Gt/Gt), we observed interference with PDGFRβ signaling via the mitogen-activated protein kinase/extracellular signal-regulated kinase (MAPK/ERK) pathway, which regulates cytoskeletal dynamics during SMC rotation. In addition, we observed disrupted Shh signaling and perturbed orientation of the mesenchymal primary cilium. Thus, **EC**M dynamics is a major influence on umbilical vascular SMC fate, with ADAMTS9 acting as its principal mediator.
4. Characterization of ADAMTS-9 and ADAMTS-20 as a distinct ADAMTS subfamily related to Caenorhabditis elegans GON-1. We demonstrate that in humans, two metalloproteases, ADAMTS-9 (1935 amino acids) and ADAMTS-20 (1911 amino acids) are orthologs of GON-1, an ADAMTS protease required for gonadal morphogenesis in Caenorhabditis elegans. ADAMTS-9 and ADAMTS-20 have an identical modular structure, are distinct in possessing 15 TSRs and a unique C-terminal domain, and have a similar gene structure, suggesting that they comprise a new subfamily of human ADAMTS proteases. ADAMTS20 is very sparingly expressed, although it is detectable in epithelial cells of the breast and lung. However, ADAMTS9 is highly expressed in embryonic and adult tissues, and therefore we characterized the ADAMTS-9 protein further. Although the ADAMTS-9 zymogen has many proprotein convertase processing sites, pulse-chase analysis, site-directed mutagenesis, and amino acid sequencing demonstrated that maturation to the active form occurs by selective proprotein convertase (e.g. furin) cleavage of the Arg(287)-Phe(288) bond. Although lacking a transmembrane sequence, ADAMTS-9 is retained near the cell surface as well as in the **ECM** of transiently transfected COS-1 and 293 cells. COS-1 cells transfected with ADAMTS9 (but not vector-transfected cells) proteolytically cleaved bovine versican and aggrecan core proteins at the Glu(441)-Ala(442) bond of versican V1 and the Glu(1771)-Ala(1772) bond of aggrecan, respectively. In contrast, the ADAMTS-9 catalytic domain alone was neither localized to the cell surface nor able to confer these proteolytic activities on cells, demonstrating that the ancillary domains of ADAMTS-9, including the TSRs, are required both for specific extracellular localization and for its versicanase and aggrecanase activities.
5. The secreted metalloprotease ADAMTS20 is required for melanoblast survival. ADAMTS20 (Adisintegrin-like and metalloprotease domain with thrombospondin type-1 motifs) is a member of a family of secreted metalloproteases that can process a variety of extracellular matrix (**ECM**) components and secreted molecules. Adamts20 mutations in belted (bt) mice cause white spotting of the dorsal and ventral torso, indicative of defective neural crest (NC)-derived melanoblast development. The expression pattern of Adamts20 in dermal mesenchymal cells adjacent to migrating melanoblasts led us to initially propose that Adamts20 regulated melanoblast migration. However, using a Dct-LacZ transgene to track melanoblast development, we determined that melanoblasts were distributed normally in whole mount E12.5 bt/bt embryos, but were specifically reduced in the trunk of E13.5 bt/bt embryos due to a seven-fold higher rate of apoptosis. The melanoblast defect was exacerbated in newborn skin and embryos from bt/bt animals that were also haploinsufficient for Adamts9, a close homolog of Adamts20, indicating that these metalloproteases functionally overlap in melanoblast development. We identified two potential mechanisms by which Adamts20 may regulate melanoblast survival. First, skin explant cultures demonstrated that Adamts20 was required for melanoblasts to respond to soluble Kit ligand (sKitl). In support of this requirement, bt/bt;Kit(tm1Alf)/+ and bt/bt;Kitl(Sl)/+ mice exhibited synergistically increased spotting. Second, ADAMTS20 cleaved the aggregating proteoglycan versican in vitro and was necessary for versican processing in vivo, raising the possibility that versican can participate in melanoblast development. These findings reveal previously unrecognized roles for Adamts proteases in cell survival and in mediating Kit signaling during melanoblast colonization of the skin. Our results have implications not only for understanding mechanisms of NC-derived melanoblast development but also provide insights on novel biological functions of secreted metalloproteases.
6. O-fucosylation of thrombospondin type 1 repeats restricts epithelial to mesenchymal transition (EMT) and maintains epiblast pluripotency during mouse gastrulation. Thrombospondin type 1 repeat (TSR) superfamily members regulate diverse biological activities ranging from cell motility to inhibition of angiogenesis. In this study, we verified that mouse protein O-fucosyltransferase-2 (POFUT2) specifically adds O-fucose to TSRs. Using two Pofut2 gene-trap lines, we demonstrated that O-fucosylation of TSRs was essential for restricting epithelial to mesenchymal transition in the primitive streak, correct patterning of mesoderm, and localization of the definitive endoderm. Although Pofut2 mutant embryos established anterior/posterior polarity, they underwent extensive mesoderm differentiation at the expense of maintaining epiblast pluripotency. Moreover, mesoderm differentiation was biased towards the vascular endothelial cell lineage. Localization of Foxa2 and Cer1 expressing cells within the interior of Pofut2 mutant embryos suggested that POFUT2 activity was also required for the displacement of the primitive endoderm by definitive endoderm. Notably, Nodal, BMP4, Fgf8, and Wnt3 expression were markedly elevated and expanded in Pofut2 mutants, providing evidence that O-fucose modification of TSRs was essential for modulation of growth factor signaling during gastrulation. The ability of Pofut2 mutant embryos to form teratomas comprised of tissues from all three germ layer origins suggested that defects in Pofut2 mutant embryos resulted from abnormalities in the extracellular environment. This prediction is consistent with the observation that POFUT2 targets are constitutive components of the extracellular matrix (**ECM**) or associate with the **ECM**. For this reason, the Pofut2 mutants represent a valuable tool for studying the role of O-fucosylation in **ECM** synthesis and remodeling, and will be a valuable model to study how post-translational modification of **ECM** components regulates the formation of tissue boundaries, cell movements, and signaling.
7. ADAMTS metalloproteases generate active versican fragments that regulate interdigital web regression. We show that combinatorial mouse alleles for the secreted metalloproteases Adamts5, Adamts20 (bt), and Adamts9 result in fully penetrant soft-tissue syndactyly. Interdigital webs in Adamts5(-/-);bt/bt mice had reduced apoptosis and decreased cleavage of the proteoglycan versican; however, the BMP-FGF axis, which regulates interdigital apoptosis was unaffected. BMP4 induced apoptosis, but without concomitant versican proteolysis. Haploinsufficiency of either Vcan or Fbln1, a cofactor for versican processing by ADAMTS5, led to highly penetrant syndactyly in bt mice, suggesting that cleaved versican was essential for web regression. The local application of an aminoterminal versican fragment corresponding to ADAMTS-processed versican, induced cell death in Adamts5(-/-);bt/bt webs. Thus, ADAMTS proteases cooperatively maintain versican proteolysis above a required threshold to create a permissive environment for apoptosis. The data highlight the developmental significance of proteolytic action on the **ECM**, not only as a clearance mechanism, but also as a means to generate bioactive versican fragments.
8. Cooperation of two ADAMTS metalloproteases in closure of the mouse palate identifies a requirement for versican proteolysis in regulating palatal mesenchyme proliferation. We have identified a role for two evolutionarily related, secreted metalloproteases of the ADAMTS family, ADAMTS20 and ADAMTS9, in palatogenesis. Adamts20 mutations cause the mouse white-spotting mutant belted (bt), whereas Adamts9 is essential for survival beyond 7.5 days gestation (E7.5). Functional overlap of Adamts9 with Adamts20 was identified using Adamts9(+/-);bt/bt mice, which have a fully penetrant cleft palate. Palate closure was delayed, although eventually completed, in both Adamts9(+/-);bt/+ and bt/bt mice, demonstrating cooperation of these genes. Adamts20 is expressed in palatal mesenchyme, whereas Adamts9 is expressed exclusively in palate microvascular endothelium. Palatal shelves isolated from Adamts9(+/-);bt/bt mice fused in culture, suggesting an intact epithelial TGFβ3 signaling pathway. Cleft palate resulted from a temporally specific delay in palatal shelf elevation and growth towards the midline. Mesenchyme of Adamts9(+/-);bt/bt palatal shelves had reduced cell proliferation, a lower cell density and decreased processing of versican (VCAN), an extracellular matrix **(EC**M) proteoglycan and ADAMTS9/20 substrate, from E13.5 to E14.5. Vcan haploinsufficiency led to greater penetrance of cleft palate in bt mice, with a similar defect in palatal shelf extension as Adamts9(+/-);bt/bt mice. Cell density was normal in bt/bt;Vcan(hdf)(/+) mice, consistent with reduced total intact versican in **EC**M, but impaired proliferation persisted in palate mesenchyme, suggesting that ADAMTS-cleaved versican is required for cell proliferation. These findings support a model in which cooperative versican proteolysis by ADAMTS9 in vascular endothelium and by ADAMTS20 in palate mesenchyme drives palatal shelf sculpting and extension.
9. Proteolytic cleavage of versican during cardiac cushion morphogenesis. The proteoglycan versican is essential to the formation of endocardial cushion mesenchyme by epithelial-mesenchymal transformation (EMT). A potentially important factor in the regulation of versican activity during cushion EMT is proteolysis by ADAMTS metalloproteinases. Using antibodies to the DPEAAE neoepitope created by ADAMTS proteolysis of versican, we detected the amino terminal 70-kDa versican cleavage fragment in cardiac cushions. Initially (i.e., 9.5 days post coitum [dpc]), the fragment is associated with endocardial cells undergoing EMT and with newly derived mesenchymal cells. ADAMTS-1 and its cofactor fibulin-1 were also associated with these cells. As cushions become increasingly populated with mesenchymal cells (10.5-12.5 dpc), the fragment remains asymmetrically distributed compared with the pattern of total versican. Highest levels of the fragment are present in regions immediately subjacent to the endocardium characterized as having densely packed, rounded cells, lacking cellular protrusions. With further development (i.e., 12.5-14.5 dpc), the pattern of fragment distribution within cushions broadens to include the **ECM** surrounding loosely packed mesenchymal cells in the cushion core. Together, the findings reveal that versican proteolysis leading to the production of the 70-kDa fragment is integral to the formation and differentiation of endocardial cushion mesenchyme.

### [Ee]xtracellular [Mm]atrix

#### PubMed

1. ADAMTS9-Mediated **Extracellular Matrix** Dynamics Regulates Umbilical Cord Vascular Smooth Muscle Differentiation and Rotation. Despite the significance for fetal nourishment in mammals, mechanisms of umbilical cord vascular growth remain poorly understood. Here, the secreted metalloprotease ADAMTS9 is shown to be necessary for murine umbilical cord vascular development. Restricting it to the cell surface using a gene trap allele, Adamts9(Gt), impaired umbilical vessel elongation and radial growth via reduced versican proteolysis and accumulation of **extracellular matrix** (ECM). Both Adamts9(Gt) and conditional Adamts9 deletion revealed that ADAMTS9 produced by mesenchymal cells acted non-autonomously to regulate smooth muscle cell (SMC) proliferation, differentiation, and orthogonal reorientation during growth of the umbilical vasculature. In Adamts9(Gt/Gt), we observed interference with PDGFRβ signaling via the mitogen-activated protein kinase/extracellular signal-regulated kinase (MAPK/ERK) pathway, which regulates cytoskeletal dynamics during SMC rotation. In addition, we observed disrupted Shh signaling and perturbed orientation of the mesenchymal primary cilium. Thus, ECM dynamics is a major influence on umbilical vascular SMC fate, with ADAMTS9 acting as its principal mediator.
2. ADAMTS9-Mediated **Extracellular Matrix** Dynamics Regulates Umbilical Cord Vascular Smooth Muscle Differentiation and Rotation. Despite the significance for fetal nourishment in mammals, mechanisms of umbilical cord vascular growth remain poorly understood. Here, the secreted metalloprotease ADAMTS9 is shown to be necessary for murine umbilical cord vascular development. Restricting it to the cell surface using a gene trap allele, Adamts9(Gt), impaired umbilical vessel elongation and radial growth via reduced versican proteolysis and accumulation of **extracellular matrix** (ECM). Both Adamts9(Gt) and conditional Adamts9 deletion revealed that ADAMTS9 produced by mesenchymal cells acted non-autonomously to regulate smooth muscle cell (SMC) proliferation, differentiation, and orthogonal reorientation during growth of the umbilical vasculature. In Adamts9(Gt/Gt), we observed interference with PDGFRβ signaling via the mitogen-activated protein kinase/extracellular signal-regulated kinase (MAPK/ERK) pathway, which regulates cytoskeletal dynamics during SMC rotation. In addition, we observed disrupted Shh signaling and perturbed orientation of the mesenchymal primary cilium. Thus, ECM dynamics is a major influence on umbilical vascular SMC fate, with ADAMTS9 acting as its principal mediator.
3. The secreted metalloprotease ADAMTS20 is required for melanoblast survival. ADAMTS20 (Adisintegrin-like and metalloprotease domain with thrombospondin type-1 motifs) is a member of a family of secreted metalloproteases that can process a variety of **extracellular matrix** (ECM) components and secreted molecules. Adamts20 mutations in belted (bt) mice cause white spotting of the dorsal and ventral torso, indicative of defective neural crest (NC)-derived melanoblast development. The expression pattern of Adamts20 in dermal mesenchymal cells adjacent to migrating melanoblasts led us to initially propose that Adamts20 regulated melanoblast migration. However, using a Dct-LacZ transgene to track melanoblast development, we determined that melanoblasts were distributed normally in whole mount E12.5 bt/bt embryos, but were specifically reduced in the trunk of E13.5 bt/bt embryos due to a seven-fold higher rate of apoptosis. The melanoblast defect was exacerbated in newborn skin and embryos from bt/bt animals that were also haploinsufficient for Adamts9, a close homolog of Adamts20, indicating that these metalloproteases functionally overlap in melanoblast development. We identified two potential mechanisms by which Adamts20 may regulate melanoblast survival. First, skin explant cultures demonstrated that Adamts20 was required for melanoblasts to respond to soluble Kit ligand (sKitl). In support of this requirement, bt/bt;Kit(tm1Alf)/+ and bt/bt;Kitl(Sl)/+ mice exhibited synergistically increased spotting. Second, ADAMTS20 cleaved the aggregating proteoglycan versican in vitro and was necessary for versican processing in vivo, raising the possibility that versican can participate in melanoblast development. These findings reveal previously unrecognized roles for Adamts proteases in cell survival and in mediating Kit signaling during melanoblast colonization of the skin. Our results have implications not only for understanding mechanisms of NC-derived melanoblast development but also provide insights on novel biological functions of secreted metalloproteases.
4. Fibulin-1 is required during cardiac ventricular morphogenesis for versican cleavage, suppression of ErbB2 and Erk1/2 activation, and to attenuate trabecular cardiomyocyte proliferation. Trabeculation is an integral component of cardiac ventricular morphogenesis and is dependent on the matrix metalloproteinase, ADAMTS1. A substrate of ADAMTS1 is the proteoglycan versican which is expressed in the developing ventricle and which has been implicated in trabeculation. Fibulin-1 is a versican and ADAMTS1-binding **extracellular matrix** protein required for ventricular morphogenesis. Here we investigated the involvement of fibulin-1 in ADAMTS1-mediated cleavage of versican in vitro, and the involvement of fibulin-1 in versican cleavage in ventricular morphogenesis.We show that fibulin-1 is a cofactor for ADAMTS1-dependent in vitro cleavage of versican V1, yielding a 70-kDa amino-terminal fragment. Furthermore, fibulin-1-deficiency in mice was found to cause a significant reduction (>90%) in ventricular levels of the 70-kDa versican V1 cleavage product and a 2-fold increase in trabecular cardiomyocyte proliferation. Decreased versican V1 cleavage and augmented trabecular cardiomyocyte proliferation in fibulin-1 null hearts is accompanied by increased ventricular activation of ErbB2 and Erk1/2. By contrast, versican deficiency was found to lead to decreased cardiomyocyte proliferation and reduced ventricular trabeculation.We conclude that fibulin-1 regulates versican-dependent events in ventricular morphogenesis by promoting ADAMTS1 cleavage of versican leading to suppression of trabecular cardiomyocyte proliferation mediated by the ErbB2-Map kinase pathway.
5. O-fucosylation of thrombospondin type 1 repeats restricts epithelial to mesenchymal transition (EMT) and maintains epiblast pluripotency during mouse gastrulation. Thrombospondin type 1 repeat (TSR) superfamily members regulate diverse biological activities ranging from cell motility to inhibition of angiogenesis. In this study, we verified that mouse protein O-fucosyltransferase-2 (POFUT2) specifically adds O-fucose to TSRs. Using two Pofut2 gene-trap lines, we demonstrated that O-fucosylation of TSRs was essential for restricting epithelial to mesenchymal transition in the primitive streak, correct patterning of mesoderm, and localization of the definitive endoderm. Although Pofut2 mutant embryos established anterior/posterior polarity, they underwent extensive mesoderm differentiation at the expense of maintaining epiblast pluripotency. Moreover, mesoderm differentiation was biased towards the vascular endothelial cell lineage. Localization of Foxa2 and Cer1 expressing cells within the interior of Pofut2 mutant embryos suggested that POFUT2 activity was also required for the displacement of the primitive endoderm by definitive endoderm. Notably, Nodal, BMP4, Fgf8, and Wnt3 expression were markedly elevated and expanded in Pofut2 mutants, providing evidence that O-fucose modification of TSRs was essential for modulation of growth factor signaling during gastrulation. The ability of Pofut2 mutant embryos to form teratomas comprised of tissues from all three germ layer origins suggested that defects in Pofut2 mutant embryos resulted from abnormalities in the extracellular environment. This prediction is consistent with the observation that POFUT2 targets are constitutive components of the **extracellular matrix** (ECM) or associate with the ECM. For this reason, the Pofut2 mutants represent a valuable tool for studying the role of O-fucosylation in ECM synthesis and remodeling, and will be a valuable model to study how post-translational modification of ECM components regulates the formation of tissue boundaries, cell movements, and signaling.
6. Reduced versican cleavage due to Adamts9 haploinsufficiency is associated with cardiac and aortic anomalies. Here, we demonstrate that ADAMTS9, a highly conserved versican-degrading protease, is required for correct cardiovascular development and adult homeostasis. Analysis of Adamts9(+/LacZ) adult mice revealed anomalies in the aortic wall, valvulosinus and valve leaflets. Abnormal myocardial projections and 'spongy' myocardium consistent with non-compaction of the left ventricle were also found in Adamts9(+/LacZ) mice. During development, Adamts9 was expressed in derivatives of the Secondary Heart Field, vascular smooth muscle cells in the arterial wall, mesenchymal cells of the valves, and non-myocardial cells of the ventricles, but expression also continued in the adult heart and ascending aorta. Thus, the adult cardiovascular anomalies found in Adamts9(+/LacZ) hearts could result from subtle developmental alterations in **extracellular matrix** remodeling or defects in adult homeostasis. The valvular and aortic anomalies of Adamts9(+/LacZ) hearts were associated with accumulation of versican and a decrease in cleaved versican relative to WT littermates. These data suggest a potentially important role for ADAMTS9 cleavage of versican, or other, as yet undefined substrates in development and allostasis of cardiovascular **extracellular matrix**. In addition, these studies identify ADAMTS9 as a potential candidate gene for congenital cardiac anomalies. Mouse models of ADAMTS9 deficiency may be useful to study myxomatous valve degeneration.
7. Genetic and biochemical evidence that gastrulation defects in Pofut2 mutants result from defects in ADAMTS9 secretion. Protein O-fucosyltransferase 2 (POFUT2) adds O-linked fucose to Thrombospondin Type 1 Repeats (TSR) in 49 potential target proteins. Nearly half the POFUT2 targets belong to the A Disintegrin and Metalloprotease with ThromboSpondin type-1 motifs (ADAMTS) or ADAMTS-like family of proteins. Both the mouse Pofut2 RST434 gene trap allele and the Adamts9 knockout were reported to result in early embryonic lethality, suggesting that defects in Pofut2 mutant embryos could result from loss of O-fucosylation on ADAMTS9. To address this question, we compared the Pofut2 and Adamts9 knockout phenotypes and used Cre-mediated deletion of Pofut2 and Adamts9 to dissect the tissue-specific role of O-fucosylated ADAMTS9 during gastrulation. Disruption of Pofut2 using the knockout (LoxP) or gene trap (RST434) allele, as well as deletion of Adamts9, resulted in disorganized epithelia (epiblast, extraembryonic ectoderm, and visceral endoderm) and blocked mesoderm formation during gastrulation. The similarity between Pofut2 and Adamts9 mutants suggested that disruption of ADAMTS9 function could be responsible for the gastrulation defects observed in Pofut2 mutants. Consistent with this prediction, CRISPR/Cas9 knockout of POFUT2 in HEK293T cells blocked secretion of ADAMTS9. We determined that Adamts9 was dynamically expressed during mouse gastrulation by trophoblast giant cells, parietal endoderm, the most proximal visceral endoderm adjacent to the ectoplacental cone, extraembryonic mesoderm, and anterior primitive streak. Conditional deletion of either Pofut2 or Adamts9 in the epiblast rescues the gastrulation defects, and identified a new role for O-fucosylated ADAMTS9 during morphogenesis of the amnion and axial mesendoderm. Combined, these results suggested that loss of ADAMTS9 function in the extra embryonic tissue is responsible for gastrulation defects in the Pofut2 knockout. We hypothesize that loss of ADAMTS9 function in the most proximal visceral endoderm leads to slippage of the visceral endoderm and altered characteristics of the extraembryonic ectoderm. Consequently, loss of input from the extraembryonic ectoderm and/or compression of the epiblast by Reichert's membrane blocks gastrulation. In the future, the Pofut2 and Adamts9 knockouts will be valuable tools for understanding how local changes in the properties of the **extracellular matrix** influence the organization of tissues during mammalian development.
8. Cooperation of two ADAMTS metalloproteases in closure of the mouse palate identifies a requirement for versican proteolysis in regulating palatal mesenchyme proliferation. We have identified a role for two evolutionarily related, secreted metalloproteases of the ADAMTS family, ADAMTS20 and ADAMTS9, in palatogenesis. Adamts20 mutations cause the mouse white-spotting mutant belted (bt), whereas Adamts9 is essential for survival beyond 7.5 days gestation (E7.5). Functional overlap of Adamts9 with Adamts20 was identified using Adamts9(+/-);bt/bt mice, which have a fully penetrant cleft palate. Palate closure was delayed, although eventually completed, in both Adamts9(+/-);bt/+ and bt/bt mice, demonstrating cooperation of these genes. Adamts20 is expressed in palatal mesenchyme, whereas Adamts9 is expressed exclusively in palate microvascular endothelium. Palatal shelves isolated from Adamts9(+/-);bt/bt mice fused in culture, suggesting an intact epithelial TGFβ3 signaling pathway. Cleft palate resulted from a temporally specific delay in palatal shelf elevation and growth towards the midline. Mesenchyme of Adamts9(+/-);bt/bt palatal shelves had reduced cell proliferation, a lower cell density and decreased processing of versican (VCAN), an **extracellular matri**x (ECM) proteoglycan and ADAMTS9/20 substrate, from E13.5 to E14.5. Vcan haploinsufficiency led to greater penetrance of cleft palate in bt mice, with a similar defect in palatal shelf extension as Adamts9(+/-);bt/bt mice. Cell density was normal in bt/bt;Vcan(hdf)(/+) mice, consistent with reduced total intact versican in ECM, but impaired proliferation persisted in palate mesenchyme, suggesting that ADAMTS-cleaved versican is required for cell proliferation. These findings support a model in which cooperative versican proteolysis by ADAMTS9 in vascular endothelium and by ADAMTS20 in palate mesenchyme drives palatal shelf sculpting and extension.
9. ADAMTS1, ADAMTS5, ADAMTS9 and aggrecanase-generated proteoglycan fragments are induced following spinal cord injury in mouse. ADAMTS (a disintegrin and metalloproteinase with thrombospondin motifs) proteinases are involved in a variety of biological processes such as angiogenesis, cancer and arthritis. ADAMTSs appears to be responsible for the cleavage of proteoglycans in several tissues including brain and cartilage. Chondroitin sulfate proteoglycans (CSPGs) maintains the integrity of the brain **extracellular matrix** and major inhibitory contributors for glial scar and neural plasticity. The activity of aggrecanases in the central nervous system (CNS) has been reported. ADAMTSs are an enzyme degrading CSPGs in the brain. However, there is a little knowledge regarding ADAMTSs in the CNS. We investigated the expression levels of ADAMTSs mRNAs by RT-PCR after spinal cord injury in mouse. Transcripts encoding 4 of the 19 known ADAMTSs were evaluated in the mouse spinal cord following injury. ADAMTS1, -5 and -9 expression levels were found to be upregulated. No change was observed in ADAMTS4 expression. By means of immunohistochemistry, ADAMTSs were detected in the astrocytes implying its cellular source in SCI. Western blot analyses indicated that aggrecanase-generated proteoglycan fragments are produced after SCI.
10. Endothelial deletion of ADAM17 in mice results in defective remodeling of the semilunar valves and cardiac dysfunction in adults. Global inactivation of the metalloproteinase ADAM17 during mouse development results in perinatal lethality and abnormalities of the heart, including late embryonic cardiomegaly and thickened semilunar and atrioventricular valves. These defects have been attributed in part to a lack of ADAM17-mediated processing of HB-EGF, as absence of soluble HB-EGF results in similar phenotypes. Because valvular mesenchymal cells are largely derived from cardiac endothelial cells, we generated mice with a floxed Adam17 allele and crossed these animals with Tie2-Cre transgenics to focus on the role of endothelial ADAM17 in valvulogenesis. We find that although hearts from late-stage embryos with ablation of endothelial ADAM17 appear normal, an increase in valve size and cell number is evident, but only in the semilunar cusps. Unlike Hbegf(-/-) valves, ADAM17-null semilunar valves do not differ from controls in acute cell proliferation at embryonic day 14.5 (E14.5), suggesting compensatory processing of HB-EGF. However, levels of the proteoglycan versican are significantly reduced in mutant hearts early in valve remodeling (E12.5). After birth, aortic valve cusps from mutants are not only hyperplastic but also show expansion of the glycosaminoglycan-rich component, with the majority of adults exhibiting aberrant compartmentalization of versican and increased deposition of collagen. The inability of mutant outflow valve precursors to transition into fully mature cusps is associated with decreased postnatal viability, progressive cardiomegaly, and systolic dysfunction. Together, our data indicate that ADAM17 is required in valvular endothelial cells for regulating cell content as well as **extracellular matrix** composition and organization in semilunar valve remodeling and homeostasis.
11. Insights on ADAMTS proteases and ADAMTS-like proteins from mammalian genetics. The mammalian ADAMTS superfamily comprises 19 secreted metalloproteinases and 7 ADAMTS-like proteins, each the product of a distinct gene. Thus far, all appear to be relevant to **extracellular matrix** function or to cell-matrix interactions. Most ADAMTS functions first emerged from analysis of spontaneous human and animal mutations and genetically engineered animals. The clinical manifestations of Mendelian disorders resulting from mutations in ADAMTS2, ADAMTS10, ADAMTS13, ADAMTS17, ADAMTSL2 and ADAMTSL4 identified essential roles for each gene, but also suggested potential cooperative functions of ADAMTS proteins. These observations were extended by analysis of spontaneous animal mutations, such as in bovine ADAMTS2, canine ADAMTS10, ADAMTS17 and ADAMTSL2 and mouse ADAMTS20. These human and animal disorders are recessive and their manifestations appear to result from a loss-of-function mechanism. Genome-wide analyses have determined an association of some ADAMTS loci such as ADAMTS9 and ADAMTS7, with specific traits and acquired disorders. Analysis of genetically engineered rodent mutations, now achieved for over half the superfamily, has provided novel biological insights and animal models for the respective human genetic disorders and suggested potential candidate genes for related human phenotypes. Engineered mouse mutants have been interbred to generate combinatorial mutants, uncovering cooperative functions of ADAMTS proteins in morphogenesis. Specific genetic models have provided crucial insights on mechanisms of osteoarthritis (OA), a common adult-onset degenerative condition. Engineered mutants will facilitate interpretation of exome variants identified in isolated birth defects and rare genetic conditions, as well as in genome-wide screens for trait and disease associations. Mammalian forward and reverse genetics, together with genome-wide analysis, together constitute a powerful force for revealing the functions of ADAMTS proteins in physiological pathways and health disorders. Their continuing use, together with genome-editing technology and the ability to generate stem cells from mutants, presents numerous opportunities for advancing basic knowledge, human disease pathways and therapy.

#### Component

1. proteinaceous **extracellular matrix**

#### GeneRIFs

1. **Extracellular matrix** dynamics is a major influence on umbilical vascular SMC fate, with ADAMTS9 acting as its principal mediator.

---

## Adamts20

- NCBI Gene
- GeneRIF
- Pubmed

| Search Term | Rank | Fields |
| --- | --- | --- |
| [Mm]igration | 6 | PubMed(4); GeneRIFs(1) |
| ECM | 7 | PubMed(10) |
| [Ee]xtracellular [Mm]atrix | 8 | Process(1); PubMed(8); Component(2) |

  
hide/show details for Adamts20

### [Mm]igration

#### PubMed

1. The secreted metalloprotease ADAMTS20 is required for melanoblast survival. ADAMTS20 (Adisintegrin-like and metalloprotease domain with thrombospondin type-1 motifs) is a member of a family of secreted metalloproteases that can process a variety of extracellular matrix (ECM) components and secreted molecules. Adamts20 mutations in belted (bt) mice cause white spotting of the dorsal and ventral torso, indicative of defective neural crest (NC)-derived melanoblast development. The expression pattern of Adamts20 in dermal mesenchymal cells adjacent to migrating melanoblasts led us to initially propose that Adamts20 regulated melanoblast **migration**. However, using a Dct-LacZ transgene to track melanoblast development, we determined that melanoblasts were distributed normally in whole mount E12.5 bt/bt embryos, but were specifically reduced in the trunk of E13.5 bt/bt embryos due to a seven-fold higher rate of apoptosis. The melanoblast defect was exacerbated in newborn skin and embryos from bt/bt animals that were also haploinsufficient for Adamts9, a close homolog of Adamts20, indicating that these metalloproteases functionally overlap in melanoblast development. We identified two potential mechanisms by which Adamts20 may regulate melanoblast survival. First, skin explant cultures demonstrated that Adamts20 was required for melanoblasts to respond to soluble Kit ligand (sKitl). In support of this requirement, bt/bt;Kit(tm1Alf)/+ and bt/bt;Kitl(Sl)/+ mice exhibited synergistically increased spotting. Second, ADAMTS20 cleaved the aggregating proteoglycan versican in vitro and was necessary for versican processing in vivo, raising the possibility that versican can participate in melanoblast development. These findings reveal previously unrecognized roles for Adamts proteases in cell survival and in mediating Kit signaling during melanoblast colonization of the skin. Our results have implications not only for understanding mechanisms of NC-derived melanoblast development but also provide insights on novel biological functions of secreted metalloproteases.
2. The secreted metalloprotease ADAMTS20 is required for melanoblast survival. ADAMTS20 (Adisintegrin-like and metalloprotease domain with thrombospondin type-1 motifs) is a member of a family of secreted metalloproteases that can process a variety of extracellular matrix (ECM) components and secreted molecules. Adamts20 mutations in belted (bt) mice cause white spotting of the dorsal and ventral torso, indicative of defective neural crest (NC)-derived melanoblast development. The expression pattern of Adamts20 in dermal mesenchymal cells adjacent to migrating melanoblasts led us to initially propose that Adamts20 regulated melanoblast **migration**. However, using a Dct-LacZ transgene to track melanoblast development, we determined that melanoblasts were distributed normally in whole mount E12.5 bt/bt embryos, but were specifically reduced in the trunk of E13.5 bt/bt embryos due to a seven-fold higher rate of apoptosis. The melanoblast defect was exacerbated in newborn skin and embryos from bt/bt animals that were also haploinsufficient for Adamts9, a close homolog of Adamts20, indicating that these metalloproteases functionally overlap in melanoblast development. We identified two potential mechanisms by which Adamts20 may regulate melanoblast survival. First, skin explant cultures demonstrated that Adamts20 was required for melanoblasts to respond to soluble Kit ligand (sKitl). In support of this requirement, bt/bt;Kit(tm1Alf)/+ and bt/bt;Kitl(Sl)/+ mice exhibited synergistically increased spotting. Second, ADAMTS20 cleaved the aggregating proteoglycan versican in vitro and was necessary for versican processing in vivo, raising the possibility that versican can participate in melanoblast development. These findings reveal previously unrecognized roles for Adamts proteases in cell survival and in mediating Kit signaling during melanoblast colonization of the skin. Our results have implications not only for understanding mechanisms of NC-derived melanoblast development but also provide insights on novel biological functions of secreted metalloproteases.
3. Cytoarchitectonic abnormalities in hippocampal formation and cerebellum of dreher mutant mouse. The laminated structures in the hippocampal formation and cerebellum of homozygous dreher mice were compared to their littermates and to C57BL/6J mice in Nissl- and myelin-stained preparations. In the dreher dentate gyrus, ectopic granule cells were situated in the molecular layer, and frequently there was either partial or complete absence of the infrapyramidal limb of the granule cell layer. In the dreher hippocampus, the cells of the pyramidal cell layer in area CA3 formed widely dispersed arrangements, and there were ectopically situated pyramidal cells in the stratum radiatum and stratum oriens. In the dreher cerebellum, 3 abnormal patterns were observed: (1) disruptions of foliation with normal cytoarchitectonic structure, (2) foliation with a mixture of normal laminated structure and abnormal laminated structure, and (3) almost complete absence of the cerebellum. In abnormal folia exhibiting the second or third pattern, islands consisting of agglomerations of both granule cells and Purkinje cells or just granule cells were observed. The neuronal heterotopias and cytoarchitectonic disorganization observed in the present study are apparently secondary to disruption of cell proliferation and neuronal **migration** produced directly or indirectly by the dreher mutation. In addition, the fact that the phenotypic abnormalities in homozygous dreher mice produces different abnormal morphologies in different specimens may be useful for analyzing the development of the hippocampal formation and cerebellum.
4. A defect in a novel ADAMTS family member is the cause of the belted white-spotting mutation. Several features of the pigment defect in belted (bt) mutant mice suggest that it occurs as a result of a defect in melanocyte development that is unique from those described for other classical white-spotting mutations. We report here that bt mice carry mutations in Adamts20, a novel member of the ADAMTS family of secreted metalloproteases. Adamts20 shows a highly dynamic pattern of expression in the developing embryo that generally precedes the appearance of melanoblasts in the same region, and is not expressed in the migrating cells themselves. Adamts20 shows remarkable homology with GON-1, an ADAMTS family protease required for distal tip cell **migration** in C. elegans. Our results suggest that the role of ADAMTS proteases in the regulation of cell **migration** has been conserved in mammalian development.

#### GeneRIFs

1. Results suggest that the role of ADAMTS proteases, including ADAMTS20, in the regulation of cell **migration** has been conserved in mammalian development.

### ECM

#### PubMed

1. ADAMTS9-Mediated Extracellular Matrix Dynamics Regulates Umbilical Cord Vascular Smooth Muscle Differentiation and Rotation. Despite the significance for fetal nourishment in mammals, mechanisms of umbilical cord vascular growth remain poorly understood. Here, the secreted metalloprotease ADAMTS9 is shown to be necessary for murine umbilical cord vascular development. Restricting it to the cell surface using a gene trap allele, Adamts9(Gt), impaired umbilical vessel elongation and radial growth via reduced versican proteolysis and accumulation of extracellular matrix (**ECM**). Both Adamts9(Gt) and conditional Adamts9 deletion revealed that ADAMTS9 produced by mesenchymal cells acted non-autonomously to regulate smooth muscle cell (SMC) proliferation, differentiation, and orthogonal reorientation during growth of the umbilical vasculature. In Adamts9(Gt/Gt), we observed interference with PDGFRβ signaling via the mitogen-activated protein kinase/extracellular signal-regulated kinase (MAPK/ERK) pathway, which regulates cytoskeletal dynamics during SMC rotation. In addition, we observed disrupted Shh signaling and perturbed orientation of the mesenchymal primary cilium. Thus, **EC**M dynamics is a major influence on umbilical vascular SMC fate, with ADAMTS9 acting as its principal mediator.
2. The secreted metalloprotease ADAMTS20 is required for melanoblast survival. ADAMTS20 (Adisintegrin-like and metalloprotease domain with thrombospondin type-1 motifs) is a member of a family of secreted metalloproteases that can process a variety of extracellular matrix (**ECM**) components and secreted molecules. Adamts20 mutations in belted (bt) mice cause white spotting of the dorsal and ventral torso, indicative of defective neural crest (NC)-derived melanoblast development. The expression pattern of Adamts20 in dermal mesenchymal cells adjacent to migrating melanoblasts led us to initially propose that Adamts20 regulated melanoblast migration. However, using a Dct-LacZ transgene to track melanoblast development, we determined that melanoblasts were distributed normally in whole mount E12.5 bt/bt embryos, but were specifically reduced in the trunk of E13.5 bt/bt embryos due to a seven-fold higher rate of apoptosis. The melanoblast defect was exacerbated in newborn skin and embryos from bt/bt animals that were also haploinsufficient for Adamts9, a close homolog of Adamts20, indicating that these metalloproteases functionally overlap in melanoblast development. We identified two potential mechanisms by which Adamts20 may regulate melanoblast survival. First, skin explant cultures demonstrated that Adamts20 was required for melanoblasts to respond to soluble Kit ligand (sKitl). In support of this requirement, bt/bt;Kit(tm1Alf)/+ and bt/bt;Kitl(Sl)/+ mice exhibited synergistically increased spotting. Second, ADAMTS20 cleaved the aggregating proteoglycan versican in vitro and was necessary for versican processing in vivo, raising the possibility that versican can participate in melanoblast development. These findings reveal previously unrecognized roles for Adamts proteases in cell survival and in mediating Kit signaling during melanoblast colonization of the skin. Our results have implications not only for understanding mechanisms of NC-derived melanoblast development but also provide insights on novel biological functions of secreted metalloproteases.
3. Cooperation of two ADAMTS metalloproteases in closure of the mouse palate identifies a requirement for versican proteolysis in regulating palatal mesenchyme proliferation. We have identified a role for two evolutionarily related, secreted metalloproteases of the ADAMTS family, ADAMTS20 and ADAMTS9, in palatogenesis. Adamts20 mutations cause the mouse white-spotting mutant belted (bt), whereas Adamts9 is essential for survival beyond 7.5 days gestation (E7.5). Functional overlap of Adamts9 with Adamts20 was identified using Adamts9(+/-);bt/bt mice, which have a fully penetrant cleft palate. Palate closure was delayed, although eventually completed, in both Adamts9(+/-);bt/+ and bt/bt mice, demonstrating cooperation of these genes. Adamts20 is expressed in palatal mesenchyme, whereas Adamts9 is expressed exclusively in palate microvascular endothelium. Palatal shelves isolated from Adamts9(+/-);bt/bt mice fused in culture, suggesting an intact epithelial TGFβ3 signaling pathway. Cleft palate resulted from a temporally specific delay in palatal shelf elevation and growth towards the midline. Mesenchyme of Adamts9(+/-);bt/bt palatal shelves had reduced cell proliferation, a lower cell density and decreased processing of versican (VCAN), an extracellular matrix **(EC**M) proteoglycan and ADAMTS9/20 substrate, from E13.5 to E14.5. Vcan haploinsufficiency led to greater penetrance of cleft palate in bt mice, with a similar defect in palatal shelf extension as Adamts9(+/-);bt/bt mice. Cell density was normal in bt/bt;Vcan(hdf)(/+) mice, consistent with reduced total intact versican in **EC**M, but impaired proliferation persisted in palate mesenchyme, suggesting that ADAMTS-cleaved versican is required for cell proliferation. These findings support a model in which cooperative versican proteolysis by ADAMTS9 in vascular endothelium and by ADAMTS20 in palate mesenchyme drives palatal shelf sculpting and extension.
4. O-fucosylation of thrombospondin type 1 repeats restricts epithelial to mesenchymal transition (EMT) and maintains epiblast pluripotency during mouse gastrulation. Thrombospondin type 1 repeat (TSR) superfamily members regulate diverse biological activities ranging from cell motility to inhibition of angiogenesis. In this study, we verified that mouse protein O-fucosyltransferase-2 (POFUT2) specifically adds O-fucose to TSRs. Using two Pofut2 gene-trap lines, we demonstrated that O-fucosylation of TSRs was essential for restricting epithelial to mesenchymal transition in the primitive streak, correct patterning of mesoderm, and localization of the definitive endoderm. Although Pofut2 mutant embryos established anterior/posterior polarity, they underwent extensive mesoderm differentiation at the expense of maintaining epiblast pluripotency. Moreover, mesoderm differentiation was biased towards the vascular endothelial cell lineage. Localization of Foxa2 and Cer1 expressing cells within the interior of Pofut2 mutant embryos suggested that POFUT2 activity was also required for the displacement of the primitive endoderm by definitive endoderm. Notably, Nodal, BMP4, Fgf8, and Wnt3 expression were markedly elevated and expanded in Pofut2 mutants, providing evidence that O-fucose modification of TSRs was essential for modulation of growth factor signaling during gastrulation. The ability of Pofut2 mutant embryos to form teratomas comprised of tissues from all three germ layer origins suggested that defects in Pofut2 mutant embryos resulted from abnormalities in the extracellular environment. This prediction is consistent with the observation that POFUT2 targets are constitutive components of the extracellular matrix (**ECM**) or associate with the **ECM**. For this reason, the Pofut2 mutants represent a valuable tool for studying the role of O-fucosylation in **ECM** synthesis and remodeling, and will be a valuable model to study how post-translational modification of **ECM** components regulates the formation of tissue boundaries, cell movements, and signaling.
5. ADAMTS metalloproteases generate active versican fragments that regulate interdigital web regression. We show that combinatorial mouse alleles for the secreted metalloproteases Adamts5, Adamts20 (bt), and Adamts9 result in fully penetrant soft-tissue syndactyly. Interdigital webs in Adamts5(-/-);bt/bt mice had reduced apoptosis and decreased cleavage of the proteoglycan versican; however, the BMP-FGF axis, which regulates interdigital apoptosis was unaffected. BMP4 induced apoptosis, but without concomitant versican proteolysis. Haploinsufficiency of either Vcan or Fbln1, a cofactor for versican processing by ADAMTS5, led to highly penetrant syndactyly in bt mice, suggesting that cleaved versican was essential for web regression. The local application of an aminoterminal versican fragment corresponding to ADAMTS-processed versican, induced cell death in Adamts5(-/-);bt/bt webs. Thus, ADAMTS proteases cooperatively maintain versican proteolysis above a required threshold to create a permissive environment for apoptosis. The data highlight the developmental significance of proteolytic action on the **ECM**, not only as a clearance mechanism, but also as a means to generate bioactive versican fragments.
6. Characterization of ADAMTS-9 and ADAMTS-20 as a distinct ADAMTS subfamily related to Caenorhabditis elegans GON-1. We demonstrate that in humans, two metalloproteases, ADAMTS-9 (1935 amino acids) and ADAMTS-20 (1911 amino acids) are orthologs of GON-1, an ADAMTS protease required for gonadal morphogenesis in Caenorhabditis elegans. ADAMTS-9 and ADAMTS-20 have an identical modular structure, are distinct in possessing 15 TSRs and a unique C-terminal domain, and have a similar gene structure, suggesting that they comprise a new subfamily of human ADAMTS proteases. ADAMTS20 is very sparingly expressed, although it is detectable in epithelial cells of the breast and lung. However, ADAMTS9 is highly expressed in embryonic and adult tissues, and therefore we characterized the ADAMTS-9 protein further. Although the ADAMTS-9 zymogen has many proprotein convertase processing sites, pulse-chase analysis, site-directed mutagenesis, and amino acid sequencing demonstrated that maturation to the active form occurs by selective proprotein convertase (e.g. furin) cleavage of the Arg(287)-Phe(288) bond. Although lacking a transmembrane sequence, ADAMTS-9 is retained near the cell surface as well as in the **ECM** of transiently transfected COS-1 and 293 cells. COS-1 cells transfected with ADAMTS9 (but not vector-transfected cells) proteolytically cleaved bovine versican and aggrecan core proteins at the Glu(441)-Ala(442) bond of versican V1 and the Glu(1771)-Ala(1772) bond of aggrecan, respectively. In contrast, the ADAMTS-9 catalytic domain alone was neither localized to the cell surface nor able to confer these proteolytic activities on cells, demonstrating that the ancillary domains of ADAMTS-9, including the TSRs, are required both for specific extracellular localization and for its versicanase and aggrecanase activities.
7. Cooperation of two ADAMTS metalloproteases in closure of the mouse palate identifies a requirement for versican proteolysis in regulating palatal mesenchyme proliferation. We have identified a role for two evolutionarily related, secreted metalloproteases of the ADAMTS family, ADAMTS20 and ADAMTS9, in palatogenesis. Adamts20 mutations cause the mouse white-spotting mutant belted (bt), whereas Adamts9 is essential for survival beyond 7.5 days gestation (E7.5). Functional overlap of Adamts9 with Adamts20 was identified using Adamts9(+/-);bt/bt mice, which have a fully penetrant cleft palate. Palate closure was delayed, although eventually completed, in both Adamts9(+/-);bt/+ and bt/bt mice, demonstrating cooperation of these genes. Adamts20 is expressed in palatal mesenchyme, whereas Adamts9 is expressed exclusively in palate microvascular endothelium. Palatal shelves isolated from Adamts9(+/-);bt/bt mice fused in culture, suggesting an intact epithelial TGFβ3 signaling pathway. Cleft palate resulted from a temporally specific delay in palatal shelf elevation and growth towards the midline. Mesenchyme of Adamts9(+/-);bt/bt palatal shelves had reduced cell proliferation, a lower cell density and decreased processing of versican (VCAN), an extracellular matrix **(EC**M) proteoglycan and ADAMTS9/20 substrate, from E13.5 to E14.5. Vcan haploinsufficiency led to greater penetrance of cleft palate in bt mice, with a similar defect in palatal shelf extension as Adamts9(+/-);bt/bt mice. Cell density was normal in bt/bt;Vcan(hdf)(/+) mice, consistent with reduced total intact versican in **EC**M, but impaired proliferation persisted in palate mesenchyme, suggesting that ADAMTS-cleaved versican is required for cell proliferation. These findings support a model in which cooperative versican proteolysis by ADAMTS9 in vascular endothelium and by ADAMTS20 in palate mesenchyme drives palatal shelf sculpting and extension.
8. ADAMTS metalloproteases generate active versican fragments that regulate interdigital web regression. We show that combinatorial mouse alleles for the secreted metalloproteases Adamts5, Adamts20 (bt), and Adamts9 result in fully penetrant soft-tissue syndactyly. Interdigital webs in Adamts5(-/-);bt/bt mice had reduced apoptosis and decreased cleavage of the proteoglycan versican; however, the BMP-FGF axis, which regulates interdigital apoptosis was unaffected. BMP4 induced apoptosis, but without concomitant versican proteolysis. Haploinsufficiency of either Vcan or Fbln1, a cofactor for versican processing by ADAMTS5, led to highly penetrant syndactyly in bt mice, suggesting that cleaved versican was essential for web regression. The local application of an aminoterminal versican fragment corresponding to ADAMTS-processed versican, induced cell death in Adamts5(-/-);bt/bt webs. Thus, ADAMTS proteases cooperatively maintain versican proteolysis above a required threshold to create a permissive environment for apoptosis. The data highlight the developmental significance of proteolytic action on the **ECM**, not only as a clearance mechanism, but also as a means to generate bioactive versican fragments.
9. The secreted metalloprotease ADAMTS20 is required for melanoblast survival. ADAMTS20 (Adisintegrin-like and metalloprotease domain with thrombospondin type-1 motifs) is a member of a family of secreted metalloproteases that can process a variety of extracellular matrix (**ECM**) components and secreted molecules. Adamts20 mutations in belted (bt) mice cause white spotting of the dorsal and ventral torso, indicative of defective neural crest (NC)-derived melanoblast development. The expression pattern of Adamts20 in dermal mesenchymal cells adjacent to migrating melanoblasts led us to initially propose that Adamts20 regulated melanoblast migration. However, using a Dct-LacZ transgene to track melanoblast development, we determined that melanoblasts were distributed normally in whole mount E12.5 bt/bt embryos, but were specifically reduced in the trunk of E13.5 bt/bt embryos due to a seven-fold higher rate of apoptosis. The melanoblast defect was exacerbated in newborn skin and embryos from bt/bt animals that were also haploinsufficient for Adamts9, a close homolog of Adamts20, indicating that these metalloproteases functionally overlap in melanoblast development. We identified two potential mechanisms by which Adamts20 may regulate melanoblast survival. First, skin explant cultures demonstrated that Adamts20 was required for melanoblasts to respond to soluble Kit ligand (sKitl). In support of this requirement, bt/bt;Kit(tm1Alf)/+ and bt/bt;Kitl(Sl)/+ mice exhibited synergistically increased spotting. Second, ADAMTS20 cleaved the aggregating proteoglycan versican in vitro and was necessary for versican processing in vivo, raising the possibility that versican can participate in melanoblast development. These findings reveal previously unrecognized roles for Adamts proteases in cell survival and in mediating Kit signaling during melanoblast colonization of the skin. Our results have implications not only for understanding mechanisms of NC-derived melanoblast development but also provide insights on novel biological functions of secreted metalloproteases.
10. Characterization of ADAMTS-9 and ADAMTS-20 as a distinct ADAMTS subfamily related to Caenorhabditis elegans GON-1. We demonstrate that in humans, two metalloproteases, ADAMTS-9 (1935 amino acids) and ADAMTS-20 (1911 amino acids) are orthologs of GON-1, an ADAMTS protease required for gonadal morphogenesis in Caenorhabditis elegans. ADAMTS-9 and ADAMTS-20 have an identical modular structure, are distinct in possessing 15 TSRs and a unique C-terminal domain, and have a similar gene structure, suggesting that they comprise a new subfamily of human ADAMTS proteases. ADAMTS20 is very sparingly expressed, although it is detectable in epithelial cells of the breast and lung. However, ADAMTS9 is highly expressed in embryonic and adult tissues, and therefore we characterized the ADAMTS-9 protein further. Although the ADAMTS-9 zymogen has many proprotein convertase processing sites, pulse-chase analysis, site-directed mutagenesis, and amino acid sequencing demonstrated that maturation to the active form occurs by selective proprotein convertase (e.g. furin) cleavage of the Arg(287)-Phe(288) bond. Although lacking a transmembrane sequence, ADAMTS-9 is retained near the cell surface as well as in the **ECM** of transiently transfected COS-1 and 293 cells. COS-1 cells transfected with ADAMTS9 (but not vector-transfected cells) proteolytically cleaved bovine versican and aggrecan core proteins at the Glu(441)-Ala(442) bond of versican V1 and the Glu(1771)-Ala(1772) bond of aggrecan, respectively. In contrast, the ADAMTS-9 catalytic domain alone was neither localized to the cell surface nor able to confer these proteolytic activities on cells, demonstrating that the ancillary domains of ADAMTS-9, including the TSRs, are required both for specific extracellular localization and for its versicanase and aggrecanase activities.

### [Ee]xtracellular [Mm]atrix

#### Process

1. **extracellular matrix** organization

#### PubMed

1. Fibulin-1 is required during cardiac ventricular morphogenesis for versican cleavage, suppression of ErbB2 and Erk1/2 activation, and to attenuate trabecular cardiomyocyte proliferation. Trabeculation is an integral component of cardiac ventricular morphogenesis and is dependent on the matrix metalloproteinase, ADAMTS1. A substrate of ADAMTS1 is the proteoglycan versican which is expressed in the developing ventricle and which has been implicated in trabeculation. Fibulin-1 is a versican and ADAMTS1-binding **extracellular matrix** protein required for ventricular morphogenesis. Here we investigated the involvement of fibulin-1 in ADAMTS1-mediated cleavage of versican in vitro, and the involvement of fibulin-1 in versican cleavage in ventricular morphogenesis.We show that fibulin-1 is a cofactor for ADAMTS1-dependent in vitro cleavage of versican V1, yielding a 70-kDa amino-terminal fragment. Furthermore, fibulin-1-deficiency in mice was found to cause a significant reduction (>90%) in ventricular levels of the 70-kDa versican V1 cleavage product and a 2-fold increase in trabecular cardiomyocyte proliferation. Decreased versican V1 cleavage and augmented trabecular cardiomyocyte proliferation in fibulin-1 null hearts is accompanied by increased ventricular activation of ErbB2 and Erk1/2. By contrast, versican deficiency was found to lead to decreased cardiomyocyte proliferation and reduced ventricular trabeculation.We conclude that fibulin-1 regulates versican-dependent events in ventricular morphogenesis by promoting ADAMTS1 cleavage of versican leading to suppression of trabecular cardiomyocyte proliferation mediated by the ErbB2-Map kinase pathway.
2. Insights on ADAMTS proteases and ADAMTS-like proteins from mammalian genetics. The mammalian ADAMTS superfamily comprises 19 secreted metalloproteinases and 7 ADAMTS-like proteins, each the product of a distinct gene. Thus far, all appear to be relevant to **extracellular matrix** function or to cell-matrix interactions. Most ADAMTS functions first emerged from analysis of spontaneous human and animal mutations and genetically engineered animals. The clinical manifestations of Mendelian disorders resulting from mutations in ADAMTS2, ADAMTS10, ADAMTS13, ADAMTS17, ADAMTSL2 and ADAMTSL4 identified essential roles for each gene, but also suggested potential cooperative functions of ADAMTS proteins. These observations were extended by analysis of spontaneous animal mutations, such as in bovine ADAMTS2, canine ADAMTS10, ADAMTS17 and ADAMTSL2 and mouse ADAMTS20. These human and animal disorders are recessive and their manifestations appear to result from a loss-of-function mechanism. Genome-wide analyses have determined an association of some ADAMTS loci such as ADAMTS9 and ADAMTS7, with specific traits and acquired disorders. Analysis of genetically engineered rodent mutations, now achieved for over half the superfamily, has provided novel biological insights and animal models for the respective human genetic disorders and suggested potential candidate genes for related human phenotypes. Engineered mouse mutants have been interbred to generate combinatorial mutants, uncovering cooperative functions of ADAMTS proteins in morphogenesis. Specific genetic models have provided crucial insights on mechanisms of osteoarthritis (OA), a common adult-onset degenerative condition. Engineered mutants will facilitate interpretation of exome variants identified in isolated birth defects and rare genetic conditions, as well as in genome-wide screens for trait and disease associations. Mammalian forward and reverse genetics, together with genome-wide analysis, together constitute a powerful force for revealing the functions of ADAMTS proteins in physiological pathways and health disorders. Their continuing use, together with genome-editing technology and the ability to generate stem cells from mutants, presents numerous opportunities for advancing basic knowledge, human disease pathways and therapy.
3. ADAMTS9-Mediated **Extracellular Matrix** Dynamics Regulates Umbilical Cord Vascular Smooth Muscle Differentiation and Rotation. Despite the significance for fetal nourishment in mammals, mechanisms of umbilical cord vascular growth remain poorly understood. Here, the secreted metalloprotease ADAMTS9 is shown to be necessary for murine umbilical cord vascular development. Restricting it to the cell surface using a gene trap allele, Adamts9(Gt), impaired umbilical vessel elongation and radial growth via reduced versican proteolysis and accumulation of **extracellular matrix** (ECM). Both Adamts9(Gt) and conditional Adamts9 deletion revealed that ADAMTS9 produced by mesenchymal cells acted non-autonomously to regulate smooth muscle cell (SMC) proliferation, differentiation, and orthogonal reorientation during growth of the umbilical vasculature. In Adamts9(Gt/Gt), we observed interference with PDGFRβ signaling via the mitogen-activated protein kinase/extracellular signal-regulated kinase (MAPK/ERK) pathway, which regulates cytoskeletal dynamics during SMC rotation. In addition, we observed disrupted Shh signaling and perturbed orientation of the mesenchymal primary cilium. Thus, ECM dynamics is a major influence on umbilical vascular SMC fate, with ADAMTS9 acting as its principal mediator.
4. The secreted metalloprotease ADAMTS20 is required for melanoblast survival. ADAMTS20 (Adisintegrin-like and metalloprotease domain with thrombospondin type-1 motifs) is a member of a family of secreted metalloproteases that can process a variety of **extracellular matrix** (ECM) components and secreted molecules. Adamts20 mutations in belted (bt) mice cause white spotting of the dorsal and ventral torso, indicative of defective neural crest (NC)-derived melanoblast development. The expression pattern of Adamts20 in dermal mesenchymal cells adjacent to migrating melanoblasts led us to initially propose that Adamts20 regulated melanoblast migration. However, using a Dct-LacZ transgene to track melanoblast development, we determined that melanoblasts were distributed normally in whole mount E12.5 bt/bt embryos, but were specifically reduced in the trunk of E13.5 bt/bt embryos due to a seven-fold higher rate of apoptosis. The melanoblast defect was exacerbated in newborn skin and embryos from bt/bt animals that were also haploinsufficient for Adamts9, a close homolog of Adamts20, indicating that these metalloproteases functionally overlap in melanoblast development. We identified two potential mechanisms by which Adamts20 may regulate melanoblast survival. First, skin explant cultures demonstrated that Adamts20 was required for melanoblasts to respond to soluble Kit ligand (sKitl). In support of this requirement, bt/bt;Kit(tm1Alf)/+ and bt/bt;Kitl(Sl)/+ mice exhibited synergistically increased spotting. Second, ADAMTS20 cleaved the aggregating proteoglycan versican in vitro and was necessary for versican processing in vivo, raising the possibility that versican can participate in melanoblast development. These findings reveal previously unrecognized roles for Adamts proteases in cell survival and in mediating Kit signaling during melanoblast colonization of the skin. Our results have implications not only for understanding mechanisms of NC-derived melanoblast development but also provide insights on novel biological functions of secreted metalloproteases.
5. Cooperation of two ADAMTS metalloproteases in closure of the mouse palate identifies a requirement for versican proteolysis in regulating palatal mesenchyme proliferation. We have identified a role for two evolutionarily related, secreted metalloproteases of the ADAMTS family, ADAMTS20 and ADAMTS9, in palatogenesis. Adamts20 mutations cause the mouse white-spotting mutant belted (bt), whereas Adamts9 is essential for survival beyond 7.5 days gestation (E7.5). Functional overlap of Adamts9 with Adamts20 was identified using Adamts9(+/-);bt/bt mice, which have a fully penetrant cleft palate. Palate closure was delayed, although eventually completed, in both Adamts9(+/-);bt/+ and bt/bt mice, demonstrating cooperation of these genes. Adamts20 is expressed in palatal mesenchyme, whereas Adamts9 is expressed exclusively in palate microvascular endothelium. Palatal shelves isolated from Adamts9(+/-);bt/bt mice fused in culture, suggesting an intact epithelial TGFβ3 signaling pathway. Cleft palate resulted from a temporally specific delay in palatal shelf elevation and growth towards the midline. Mesenchyme of Adamts9(+/-);bt/bt palatal shelves had reduced cell proliferation, a lower cell density and decreased processing of versican (VCAN), an **extracellular matri**x (ECM) proteoglycan and ADAMTS9/20 substrate, from E13.5 to E14.5. Vcan haploinsufficiency led to greater penetrance of cleft palate in bt mice, with a similar defect in palatal shelf extension as Adamts9(+/-);bt/bt mice. Cell density was normal in bt/bt;Vcan(hdf)(/+) mice, consistent with reduced total intact versican in ECM, but impaired proliferation persisted in palate mesenchyme, suggesting that ADAMTS-cleaved versican is required for cell proliferation. These findings support a model in which cooperative versican proteolysis by ADAMTS9 in vascular endothelium and by ADAMTS20 in palate mesenchyme drives palatal shelf sculpting and extension.
6. O-fucosylation of thrombospondin type 1 repeats restricts epithelial to mesenchymal transition (EMT) and maintains epiblast pluripotency during mouse gastrulation. Thrombospondin type 1 repeat (TSR) superfamily members regulate diverse biological activities ranging from cell motility to inhibition of angiogenesis. In this study, we verified that mouse protein O-fucosyltransferase-2 (POFUT2) specifically adds O-fucose to TSRs. Using two Pofut2 gene-trap lines, we demonstrated that O-fucosylation of TSRs was essential for restricting epithelial to mesenchymal transition in the primitive streak, correct patterning of mesoderm, and localization of the definitive endoderm. Although Pofut2 mutant embryos established anterior/posterior polarity, they underwent extensive mesoderm differentiation at the expense of maintaining epiblast pluripotency. Moreover, mesoderm differentiation was biased towards the vascular endothelial cell lineage. Localization of Foxa2 and Cer1 expressing cells within the interior of Pofut2 mutant embryos suggested that POFUT2 activity was also required for the displacement of the primitive endoderm by definitive endoderm. Notably, Nodal, BMP4, Fgf8, and Wnt3 expression were markedly elevated and expanded in Pofut2 mutants, providing evidence that O-fucose modification of TSRs was essential for modulation of growth factor signaling during gastrulation. The ability of Pofut2 mutant embryos to form teratomas comprised of tissues from all three germ layer origins suggested that defects in Pofut2 mutant embryos resulted from abnormalities in the extracellular environment. This prediction is consistent with the observation that POFUT2 targets are constitutive components of the **extracellular matrix** (ECM) or associate with the ECM. For this reason, the Pofut2 mutants represent a valuable tool for studying the role of O-fucosylation in ECM synthesis and remodeling, and will be a valuable model to study how post-translational modification of ECM components regulates the formation of tissue boundaries, cell movements, and signaling.
7. Cooperation of two ADAMTS metalloproteases in closure of the mouse palate identifies a requirement for versican proteolysis in regulating palatal mesenchyme proliferation. We have identified a role for two evolutionarily related, secreted metalloproteases of the ADAMTS family, ADAMTS20 and ADAMTS9, in palatogenesis. Adamts20 mutations cause the mouse white-spotting mutant belted (bt), whereas Adamts9 is essential for survival beyond 7.5 days gestation (E7.5). Functional overlap of Adamts9 with Adamts20 was identified using Adamts9(+/-);bt/bt mice, which have a fully penetrant cleft palate. Palate closure was delayed, although eventually completed, in both Adamts9(+/-);bt/+ and bt/bt mice, demonstrating cooperation of these genes. Adamts20 is expressed in palatal mesenchyme, whereas Adamts9 is expressed exclusively in palate microvascular endothelium. Palatal shelves isolated from Adamts9(+/-);bt/bt mice fused in culture, suggesting an intact epithelial TGFβ3 signaling pathway. Cleft palate resulted from a temporally specific delay in palatal shelf elevation and growth towards the midline. Mesenchyme of Adamts9(+/-);bt/bt palatal shelves had reduced cell proliferation, a lower cell density and decreased processing of versican (VCAN), an **extracellular matri**x (ECM) proteoglycan and ADAMTS9/20 substrate, from E13.5 to E14.5. Vcan haploinsufficiency led to greater penetrance of cleft palate in bt mice, with a similar defect in palatal shelf extension as Adamts9(+/-);bt/bt mice. Cell density was normal in bt/bt;Vcan(hdf)(/+) mice, consistent with reduced total intact versican in ECM, but impaired proliferation persisted in palate mesenchyme, suggesting that ADAMTS-cleaved versican is required for cell proliferation. These findings support a model in which cooperative versican proteolysis by ADAMTS9 in vascular endothelium and by ADAMTS20 in palate mesenchyme drives palatal shelf sculpting and extension.
8. The secreted metalloprotease ADAMTS20 is required for melanoblast survival. ADAMTS20 (Adisintegrin-like and metalloprotease domain with thrombospondin type-1 motifs) is a member of a family of secreted metalloproteases that can process a variety of **extracellular matrix** (ECM) components and secreted molecules. Adamts20 mutations in belted (bt) mice cause white spotting of the dorsal and ventral torso, indicative of defective neural crest (NC)-derived melanoblast development. The expression pattern of Adamts20 in dermal mesenchymal cells adjacent to migrating melanoblasts led us to initially propose that Adamts20 regulated melanoblast migration. However, using a Dct-LacZ transgene to track melanoblast development, we determined that melanoblasts were distributed normally in whole mount E12.5 bt/bt embryos, but were specifically reduced in the trunk of E13.5 bt/bt embryos due to a seven-fold higher rate of apoptosis. The melanoblast defect was exacerbated in newborn skin and embryos from bt/bt animals that were also haploinsufficient for Adamts9, a close homolog of Adamts20, indicating that these metalloproteases functionally overlap in melanoblast development. We identified two potential mechanisms by which Adamts20 may regulate melanoblast survival. First, skin explant cultures demonstrated that Adamts20 was required for melanoblasts to respond to soluble Kit ligand (sKitl). In support of this requirement, bt/bt;Kit(tm1Alf)/+ and bt/bt;Kitl(Sl)/+ mice exhibited synergistically increased spotting. Second, ADAMTS20 cleaved the aggregating proteoglycan versican in vitro and was necessary for versican processing in vivo, raising the possibility that versican can participate in melanoblast development. These findings reveal previously unrecognized roles for Adamts proteases in cell survival and in mediating Kit signaling during melanoblast colonization of the skin. Our results have implications not only for understanding mechanisms of NC-derived melanoblast development but also provide insights on novel biological functions of secreted metalloproteases.

#### Component

1. **extracellular matrix**
2. proteinaceous **extracellular matrix**

---

## Abcc1

- NCBI Gene
- GeneRIF
- Pubmed

| Search Term | Rank | Fields |
| --- | --- | --- |
| [Mm]igration | 6 | Process(1); PubMed(3) |

  
hide/show details for Abcc1

### [Mm]igration

#### Process

1. positive regulation of cell **migration**

#### PubMed

1. The leukotriene C(4) transporter MRP1 regulates CCL19 (MIP-3beta, ELC)-dependent mobilization of dendritic cells to lymph nodes. Adaptive immune responses begin after antigen-bearing dendritic cells (DCs) traffic from peripheral tissues to lymph nodes. Here, we show that DC **migration** from skin to lymph nodes utilizes the leukotriene C(4) (LTC(4)) transporter multidrug resistance-associated protein 1 (MRP1). DC mobilization from the epidermis and trafficking into lymphatic vessels was greatly reduced in MRP1(-/-) mice, but **migration** was restored by exogenous cysteinyl leukotrienes LTC(4) or LTD(4). In vitro, these cysteinyl leukotrienes promoted optimal chemotaxis to the chemokine CCL19, but not to other related chemokines. Antagonism of CCL19 in vivo prevented DC **migration** out of the epidermis. Thus, MRP-1 regulates DC **migration** to lymph nodes, apparently by transporting LTC(4), which in turn promotes chemotaxis to CCL19 and mobilization of DCs from the epidermis.
2. ABCC multidrug transporters in childhood neuroblastoma: clinical and biological effects independent of cytotoxic drug efflux. Although the prognostic value of the ATP-binding cassette, subfamily C (ABCC) transporters in childhood neuroblastoma is usually attributed to their role in cytotoxic drug efflux, certain observations have suggested that these multidrug transporters might contribute to the malignant phenotype independent of cytotoxic drug efflux.A v-myc myelocytomatosis viral related oncogene, neuroblastoma derived (MYCN)-driven transgenic mouse neuroblastoma model was crossed with an Abcc1-deficient mouse strain (658 hMYCN(1/-), 205 hMYCN(+/1) mice) or, alternatively, treated with the ABCC1 inhibitor, Reversan (n = 20). ABCC genes were suppressed using short interfering RNA or overexpressed by stable transfection in neuroblastoma cell lines BE(2)-C, SH-EP, and SH-SY5Y, which were then assessed for wound closure ability, clonogenic capacity, morphological differentiation, and cell growth. Real-time quantitative polymerase chain reaction was used to examine the clinical significance of ABCC family gene expression in a large prospectively accrued cohort of patients (n = 209) with primary neuroblastomas. Kaplan-Meier survival analysis and Cox regression were used to test for associations with event-free and overall survival. Except where noted, all statistical tests were two-sided.Inhibition of ABCC1 statistically significantly inhibited neuroblastoma development in hMYCN transgenic mice (mean age for palpable tumor: treated mice, 47.2 days; control mice, 41.9 days; hazard ratio [HR] = 9.3, 95% confidence interval [CI] = 2.65 to 32; P < .001). Suppression of ABCC1 in vitro inhibited wound closure (P < .001) and clonogenicity (P = .006); suppression of ABCC4 enhanced morphological differentiation (P < .001) and inhibited cell growth (P < .001). Analysis of 209 neuroblastoma patient tumors revealed that, in contrast with ABCC1 and ABCC4, low rather than high ABCC3 expression was associated with reduced event-free survival (HR of recurrence or death = 2.4, 95% CI = 1.4 to 4.2; P = .001), with 23 of 53 patients with low ABCC3 expression experiencing recurrence or death compared with 31 of 155 patients with high ABCC3. Moreover, overexpression of ABCC3 in vitro inhibited neuroblastoma cell **migration** (P < .001) and clonogenicity (P = .03). The combined expression of ABCC1, ABCC3, and ABCC4 was associated with patients having an adverse event, such that of the 12 patients with the "poor prognosis" expression pattern, 10 experienced recurrence or death (HR of recurrence or death = 12.3, 95% CI = 6 to 27; P < .001).ABCC transporters can affect neuroblastoma biology independently of their role in chemotherapeutic drug efflux, enhancing their potential as targets for therapeutic intervention.
3. Modulation of dendritic cell trafficking to and from the airways. We investigated the fate of latex (LX) particles that were introduced into mice intranasally. Macrophages acquired the vast majority of particles and outnumbered LX particle-bearing airway dendritic cells (DCs) by at least two orders of magnitude. Yet alveolar macrophages were refractory to **migration** to the draining lymph node (DLN), and all transport to the DLN could be ascribed to the few LX(+) airway DCs. Upon macrophage depletion, markedly greater numbers of DCs were recruited into the alveolar space. Consequently, the number of DCs that carried particles to the DLN was boosted by 20-fold. Thus, a so far overlooked aspect of macrophage-mediated suppression of airway DC function stems from the modulation of DC recruitment into the airway. This increase in DC recruitment permitted the development of a robust assay to quantify the subsequent **migration** of DCs to the DLN. Therefore, we determined whether lung DCs use the same molecules that skin DCs use during **migration** to DLNs. Like skin DCs, lung DCs used CCR7 ligands and CCR8 for e**migration** to DLN, but the leukotriene C(4) transporter multidrug resistance-related protein 1 did not mediate lung DC **migration** as it does in skin, indicating that pathways governing DC **migration** from different tissues partially differ in molecular regulation.

---

## Actb

- NCBI Gene
- GeneRIF
- Pubmed

| Search Term | Rank | Fields |
| --- | --- | --- |
| [Ff]ilopodia | 1 | PubMed(2) |
| [Pp]odosome(s)? | 5 | PubMed(2); Component(1) |
| [Mm]igration | 6 | PubMed(20); GeneRIFs(4) |
| ECM | 7 | PubMed(1) |
| [Ee]xtracellular [Mm]atrix | 8 | PubMed(3) |
| [Ii]nvasive | 10 | PubMed(1) |
| [Mm]etastasis | 12 | PubMed(2) |

  
hide/show details for Actb

### [Ff]ilopodia

#### PubMed

1. Serum response factor is required for sprouting angiogenesis and vascular integrity. Serum response factor (SRF) is a transcription factor that controls the expression of cytoskeletal proteins and immediate early genes in different cell types. Here, we found that SRF expression is restricted to endothelial cells (ECs) of small vessels such as capillaries in the mouse embryo. EC-specific Srf deletion led to aneurysms and hemorrhages from 11.5 days of mouse development (E11.5) and lethality at E14.5. Mutant embryos presented a reduced capillary density and defects in EC migration, with fewer numbers of **filopodia** in tip cells and ECs showing defects in actin polymerization and intercellular junctions. We show that SRF is essential for the expression of VE-cadherin and beta-actin in ECs both in vivo and in vitro. Moreover, knockdown of SRF in ECs impaired VEGF- and FGF-induced in vitro angiogenesis. Taken together, our results demonstrate that SRF plays an important role in sprouting angiogenesis and small vessel integrity in the mouse embryo.
2. Optogenetic control of PIP3: PIP3 is sufficient to induce the actin-based active part of growth cones and is regulated via endocytosis. Phosphatidylinositol-3,4,5-trisphosphate (PIP3) is highly regulated in a spatiotemporal manner and plays multiple roles in individual cells. However, the local dynamics and primary functions of PIP3 in developing neurons remain unclear because of a lack of techniques for manipulating PIP3 spatiotemporally. We addressed this issue by combining optogenetic control and observation of endogenous PIP3 signaling. Endogenous PIP3 was abundant in actin-rich structures such as growth cones and "waves", and PIP3-rich plasma membranes moved actively within growth cones. To study the role of PIP3 in developing neurons, we developed a PI3K photoswitch that can induce production of PIP3 at specific locations upon blue light exposure. We succeeded in producing PIP3 locally in mouse hippocampal neurons. Local PIP3 elevation at neurite tips did not induce neurite elongation, but it was sufficient to induce the formation of **filopodia** and lamellipodia. Interestingly, ectopic PIP3 elevation alone activated membranes to form actin-based structures whose behavior was similar to that of growth-cone-like "waves". We also found that endocytosis regulates effective PIP3 concentration at plasma membranes. These results revealed the local dynamics and primary functions of PIP3, providing fundamental information about PIP3 signaling in neurons.

### [Pp]odosome(s)?

#### PubMed

1. The transient appearance of zipper-like actin superstructures during the fusion of osteoclasts. Multinucleated osteoclasts are responsible for bone resorption. Hypermultinucleated osteoclasts are often observed in some bone-related diseases such as Paget's disease and cherubism. The cellular mechanics controlling the size of osteoclasts is poorly understood. We introduced EGFP-actin into RAW 264.7 cells to monitor actin dynamics during osteoclast differentiation. Before their terminal differentiation into osteoclasts, syncytia displayed two main types of actin assembly, **podosome** clusters and clusters of zipper-like structures. The zipper-like structures morphologically resembled the adhesion zippers found at the initial stage of cell-cell adhesion in keratinocytes. In the zipper-like structure, Arp3 and cortactin overlapped with the distribution of dense F-actin, whereas integrin β3, paxillin and vinculin were localized to the periphery of the structure. The structure was negative for WGA-lectin staining and biotin labeling. The zipper-like structure broke down and transformed into a large actin ring, called a **podosom**e belt. Syncytia containing clusters of zipper-like structures had more nuclei than those with **podosom**e clusters. Differentiated osteoclasts with a **podosom**e belt also formed the zipper-like structure at the cell contact site during cell fusion. The breakdown of the cell contact site resulted in the fusion of the **podosom**e belts following plasma membrane fusion. Additionally, osteoclasts in mouse calvariae formed the zipper-like structure in the sealing zone. Therefore, we propose that the zipper-like actin superstructures might be involved in cell-cell interaction to achieve efficient multinucleation of osteoclasts. Understanding of the zipper-like structure might lead to selective therapeutics for bone diseases caused by hypermultinucleated osteoclasts.
2. TRPM7, a novel regulator of actomyosin contractility and cell adhesion. Actomyosin contractility regulates various cell biological processes including cytokinesis, adhesion and migration. While in lower eukaryotes, alpha-kinases control actomyosin relaxation, a similar role for mammalian alpha-kinases has yet to be established. Here, we examined whether TRPM7, a cation channel fused to an alpha-kinase, can affect actomyosin function. We demonstrate that activation of TRPM7 by bradykinin leads to a Ca(2+)- and kinase-dependent interaction with the actomyosin cytoskeleton. Moreover, TRPM7 phosphorylates the myosin IIA heavy chain. Accordingly, low overexpression of TRPM7 increases intracellular Ca2+ levels accompanied by cell spreading, adhesion and the formation of focal adhesions. Activation of TRPM7 induces the transformation of these focal adhesions into **podosomes** by a kinase-dependent mechanism, an effect that can be mimicked by pharmacological inhibition of myosin II. Collectively, our results demonstrate that regulation of cell adhesion by TRPM7 is the combined effect of kinase-dependent and -independent pathways on actomyosin contractility.

#### Component

1. **podosome**

### [Mm]igration

#### PubMed

1. Serum response factor is required for sprouting angiogenesis and vascular integrity. Serum response factor (SRF) is a transcription factor that controls the expression of cytoskeletal proteins and immediate early genes in different cell types. Here, we found that SRF expression is restricted to endothelial cells (ECs) of small vessels such as capillaries in the mouse embryo. EC-specific Srf deletion led to aneurysms and hemorrhages from 11.5 days of mouse development (E11.5) and lethality at E14.5. Mutant embryos presented a reduced capillary density and defects in EC **migration**, with fewer numbers of filopodia in tip cells and ECs showing defects in actin polymerization and intercellular junctions. We show that SRF is essential for the expression of VE-cadherin and beta-actin in ECs both in vivo and in vitro. Moreover, knockdown of SRF in ECs impaired VEGF- and FGF-induced in vitro angiogenesis. Taken together, our results demonstrate that SRF plays an important role in sprouting angiogenesis and small vessel integrity in the mouse embryo.
2. Consequences of lack of beta 1 integrin gene expression in mice. beta 1 integrins are cell-surface receptors that mediate cell-cell and cell-matrix interactions. We have generated a null mutation in the gene for the beta 1 integrin subunit in mice and embryonic stem (ES) cells. Heterozygous mice are indistinguishable from normal littermates. Homozygous null embryos develop normally to the blastocyst stage, implant, and invade the uterine basement membrane but die shortly thereafter. Using beta 1 integrin-deficient ES cells we have established chimeric embryos and adult mice. Analysis of the chimeric embryos demonstrated the presence of beta 1 integrin-deficient cells in all germ layers indicating that beta 1-null cells can differentiate and migrate in a context of normal tissue. When evaluated at embryonic day 9.5 (E9.5), embryos with a beta 1-null cell contribution below 25% were developing normally, whereas embryos with a contribution above this threshold were distorted and showed abnormal morphogenesis. In adult chimeric mice beta 1 integrin-deficient cells failed to colonize liver and spleen but were found in all other tissues analyzed at levels from 2%-25%. Immunostaining of chimeric mice showed that in cardiac muscle, there were small, scattered patches of myocytes that were beta 1-null. In contrast, many myotubes showed some beta 1-null contribution as a result of fusion between wild-type and mutant myoblasts to form mixed myotubes. The adult chimeric brain contained beta 1-null cells in all regions analyzed. Also, tissues derived from the neural crest contained beta 1 integrin-deficient cells indicating that **migration** of neuronal cells as well as neural crest cells can occur in the absence of beta 1 integrins.
3. Planning your every move: the role of β-actin and its post-transcriptional regulation in cell motility. Cell motility is a tightly regulated process that involves the polymerization of actin subunits. The formation of actin filaments is controlled through a variety of protein factors that accelerate or perturb the polymerization process. As is the case for most biological events, cell movement is also controlled at the level of gene expression. Growing research explains how the β-actin isoform of actin is particularly regulated through post-transcriptional events. This includes the discovery of multiple sites in the 3' untranslated region of β-actin mRNA to which RNA-binding proteins can associate. The control such proteins have on β-actin expression, and as a result, c**ell migra**tion, continues to develop, and presents a thorough process that involves guiding an mRNA out of the nucleus, to a specific cytosolic destination, and then controlling the translation and decay of this message. In this review we will provide an overview on the recent progress regarding the mechanisms by which actin polymerization modulates cell movement and invasion and we will discuss the importance of post-transcriptional regulatory events in β-actin mediated effects on these processes.
4. p27(Kip1) is a microtubule-associated protein that promotes microtubule polymerization during neuron **migration**. The **migration** of cortical interneurons is characterized by extensive morphological changes that result from successive cycles of nucleokinesis and neurite branching. Their molecular bases remain elusive, and the present work describes how p27(Kip1) controls cell-cycle-unrelated signaling pathways to regulate these morphological remodelings. Live imaging reveals that interneurons lacking p27(Kip1) show delayed tangential **migration** resulting from defects in both nucleokinesis and dynamic branching of the leading process. At the molecular level, p27(Kip1) is a microtubule-associated protein that promotes polymerization of microtubules in extending neurites, thereby contributing to tangential **migration**. Furthermore, we show that p27(Kip1) controls actomyosin contractions that drive both forward translocation of the nucleus and growth cone splitting. Thus, p27(Kip1) cell-autonomously controls nucleokinesis and neurite branching by regulating both actin and microtubule cytoskeletons.
5. The reeler gene encodes a protein with an EGF-like motif expressed by pioneer neurons. We have identified a strong candidate cDNA for the mouse reeler gene. This 5 kb transcript encodes a 99.4 kD protein consisting of 881 amino acids and possessing two EGF-like motifs. We assayed two independent mutant alleles--'Jackson reeler', which has a deletion of the entire gene, and 'Orleans reeler' which exhibits a 220 bp deletion in the open reading frame, including the second EGF-like motif and resulting in a frame shift. In situ hybridization reveals that the transcript is detected exclusively in the pioneer neurons which guide neuronal cell **migration** along the radial array. Our findings offer an explanation for how the reeler mutant phenotype causes a disturbance of the complex architecture of the neuronal network.
6. Measurements of spatiotemporal changes in G-actin concentration reveal its effect on stimulus-induced actin assembly and lamellipodium extension. To understand the intracellular role of G-actin concentration in stimulus-induced actin assembly and lamellipodium extension during cell **migration**, we developed a novel technique for quantifying spatiotemporal changes in G-actin concentration in live cells, consisting of sequential measurements of fluorescent decay after photoactivation (FDAP) of Dronpa-labeled actin. Cytoplasmic G-actin concentrations decreased by ∼40% immediately after cell stimulation and thereafter the cell area extended. The extent of stimulus-induced G-actin loss and cell extension correlated linearly with G-actin concentration in unstimulated cells, even at concentrations much higher than the critical concentration of actin filaments, indicating that cytoplasmic G-actin concentration is a critical parameter for determining the extent of stimulus-induced G-actin assembly and cell extension. Multipoint FDAP analysis revealed that G-actin concentration in lamellipodia was comparable to that in the cell body. We also assessed the cellular concentrations of free G-actin, profilin- and thymosin-β4-bound G-actin, and free barbed and pointed ends of actin filaments by model fitting of jasplakinolide-induced temporal changes in G-actin concentration.
7. In murine 3T3 fibroblasts, different second messenger pathways resulting in the induction of NO synthase II (iNOS) converge in the activation of transcription factor NF-kappaB. Transcription factor NF-kappaB is essential for the induction of nitric oxide synthase (NOS) II (iNOS) by bacterial lipopolysaccharide in murine macrophages (Xie, Q. W., Kashiwabara, Y., and Nathan, C. (1994) J. Biol. Chem. 269, 4705-4708). In 3T3 fibroblasts, agents other than cytokines are efficacious inducers of NOS II expression. In addition to cytokines such as interferon-gamma or tumor necrosis factor-alpha, protein kinase C-stimulating agents such as tetradecanoylphorbol-13-acetate, or cyclic AMP-elevating agents such as forskolin and 8-bromo-cAMP markedly increased NOS II mRNA (measured by Sl nuclease and RNase protection analyses), NOS II protein (determined by Western blotting), and NOS activity (measured by chemiluminescence detection of NO2-). Transforming growth factor-beta1 (which is an inhibitor of NOS II induction in other cell types) potentiated NOS II mRNA expression produced by all inducing agents listed, whereas dexamethasone, pyrrolidine dithiocarbamate and 3,4-dichloroisocoumarin (inhibitors of NF-kappaB activation) suppressed NOS II mRNA induction in response to all stimulants. In electrophoretic mobility shift assays, nuclear protein extracts from 3T3 cells stimulated with any of the inducing agents significantly slowed the **migration** of an NF-kappaB-binding oligonucleotide, whereas nuclear extracts from untreated control cells did not. These experiments indicate that NF-kappaB is the key control element for the induction of NOS II in response to at least three different second messenger pathways in 3T3 cells.
8. Silencing of the Tropomyosin-1 gene by DNA methylation alters tumor suppressor function of TGF-beta. Loss of actin stress fibers has been associated with cell transformation and metastasis. TGF-beta induction of stress fibers in epithelial cells requires high molecular weight tropomyosins encoded by TPM1 and TPM2 genes. Here, we investigated the mechanism underlying the failure of TGF-beta to induce stress fibers and inhibit cell **migration** in metastatic cells. RT-PCR analysis in carcinoma cell lines revealed a significant reduction in TPM1 transcripts in metastatic MDA-MB-231, MDA-MB-435 and SW620 cell lines. Treatment of these cells with demethylating agent 5-aza-2'-deoxycytidine (5-aza-dC) increased mRNA levels of TPM1 with no effect on TPM2. Importantly, 5-aza-dC treatment of MDA-MB-231 cells restored TGF-beta induction of TPM1 and formation of stress fibers. Forced expression of TPM1 by using Tet-Off system increased stress fibers in MDA-MB-231 cells and reduced cell **migration**. A potential CpG island spanning the TPM1 proximal promoter, exon 1, and the beginning of intron 1 was identified. Bisulfite sequencing showed significant cytosine methylation in metastatic cell lines that correlated with a reduced expression of TPM1. Together these results suggest that epigenetic suppression of TPM1 may alter TGF-beta tumor suppressor function and contribute to metastatic properties of tumor cells.
9. Interaction of the Coxsackie and adenovirus receptor (CAR) with the cytoskeleton: binding to actin. The Coxsackie and adenovirus receptor (CAR) is a cell adhesion molecule that is highly expressed in the developing brain. CAR is enriched in growth cone particles (GCP) after subcellular fractionation. In GCP, we identified actin as an interaction partner of the cytoplasmic domain of CAR. In vivo, actin and CAR co-immunoprecipitate and co-localize. In vitro, the binding is direct, with a K(d) of approximately 2.6 microM, and leads to actin bundling. We previously demonstrated that CAR interacts with microtubules. These data suggest a role for CAR in processes requiring dynamic reorganization of the cytoskeleton such as neurite outgrowth and cell **migration**.
10. Beta-actin is required for proper mouse neural crest ontogeny. The mouse genome consists of six functional actin genes of which the expression patterns are temporally and spatially regulated during development and in the adult organism. Deletion of beta-actin in mouse is lethal during embryonic development, although there is compensatory expression of other actin isoforms. This suggests different isoform specific functions and, more in particular, an important function for beta-actin during early mammalian development. We here report a role for beta-actin during neural crest ontogeny. Although beta-actin null neural crest cells show expression of neural crest markers, less cells delaminate and their **migration** arrests shortly after. These phenotypes were associated with elevated apoptosis levels in neural crest cells, whereas proliferation levels were unchanged. Specifically the pre-migratory neural crest cells displayed higher levels of apoptosis, suggesting increased apoptosis in the neural tube accounts for the decreased amount of migrating neural crest cells seen in the beta-actin null embryos. These cells additionally displayed a lack of membrane bound N-cadherin and dramatic decrease in cadherin-11 expression which was more pronounced in the pre-migratory neural crest population, potentially indicating linkage between the cadherin-11 expression and apoptosis. By inhibiting ROCK ex vivo, the knockout neural crest cells regained migratory capacity and cadherin-11 expression was upregulated. We conclude that the presence of beta-actin is vital for survival, specifically of pre-migratory neural crest cells, their proper e**migration** from the neural tube and their subsequent **migration**. Furthermore, the absence of beta-actin affects cadherin-11 and N-cadherin function, which could partly be alleviated by ROCK inhibition, situating the Rho-ROCK signaling in a feedback loop with cadherin-11.
11. Beta- and gamma-cytoplasmic actins are required for meiosis in mouse oocytes. In mammals, female meiosis consists of two asymmetric cell divisions, which generate a large haploid oocyte and two small polar bodies. Asymmetric partitioning of the cytoplasm results from **migration** of the meiotic spindle toward the cortex and requires actin filaments. However, the subcellular localization and the role of the existing two cytoplasmic actin (CYA) isoforms, beta and gamma, have not been characterized. We show that beta- and gamma-CYA are differentially distributed in the maturing oocyte from late metaphase I as well as in preimplantation embryos. Gamma-CYA is preferentially enriched in oocyte cortices and is absent from all cell-cell contact areas from metaphase II until the blastocyst stage. Beta-CYA is enriched in contractile structures, at cytokinesis, at cell-cell contacts, and around the forming blastocoel. Alteration of beta- or gamma-CYA function by isoform-specific antibody microinjection suggests that gamma-CYA holds a major and specific role in the establishment and/or maintenance of asymmetry in meiosis I and in the maintenance of overall cortical integrity. In contrast, beta- and gamma-CYA, together, appear to participate in the formation and the cortical anchorage of the second meiotic spindle in waiting for fertilization. Finally, differences in gamma-CYA expression are amongst the earliest markers of cell fate determination in development.
12. Cells lacking β-actin are genetically reprogrammed and maintain conditional migratory capacity. Vertebrate nonmuscle cells express two actin isoforms: cytoplasmic β- and γ-actin. Because of the presence and localized translation of β-actin at the leading edge, this isoform is generally accepted to specifically generate protrusive forces for c**ell migra**tion. Recent evidence also implicates β-actin in gene regulation. **Cell migr**ation without β-actin has remained unstudied until recently and it is unclear whether other actin isoforms can compensate for this cytoplasmic function and/or for its nuclear role. Primary mouse embryonic fibroblasts lacking β-actin display compensatory expression of other actin isoforms. Consistent with this preservation of polymerization capacity, β-actin knockout cells have unchanged lamellipodial protrusion rates despite a **severe m**igration defect. To solve this paradox we applied quantitative proteomics revealing a broad genetic reprogramming of β-actin knockout cells. This also explains why reintroducing β-actin in knockout cells does not restore the affe**cted cell** migration. Pathway analysis suggested increased Rho-ROCK signaling, consistent with observed phenotypic changes. We therefore developed and tested a model explaining the phenotypes in β-actin knockout cells based on increased Rho-ROCK signaling and increased TGFβ production resulting in increased adhesion and contractility in the knockout cells. Inhibiting ROCK or myos**in restor**es migration of β-actin knockout cells indicating that other actins compensate for β-actin in this process. Consequently, isoactins act redundantly in providing propulsive fo**rces for** cell migration, but β-actin has a unique nuclear function, regulating expression on transcriptional and post-translational levels, thereby preventing myogenic differentiation.
13. Cell dynamics in fetal intestinal epithelium: implications for intestinal growth and morphogenesis. The cellular mechanisms that drive growth and remodeling of the early intestinal epithelium are poorly understood. Current dogma suggests that the murine fetal intestinal epithelium is stratified, that villi are formed by an epithelial remodeling process involving the de novo formation of apical surface at secondary lumina, and that radial intercalation of the stratified cells constitutes a major intestinal lengthening mechanism. Here, we investigate cell polarity, cell cycle dynamics and cell shape in the fetal murine intestine between E12.5 and E14.5. We show that, contrary to previous assumptions, this epithelium is pseudostratified. Furthermore, epithelial nuclei exhibit interkinetic nuclear **migration**, a process wherein nuclei move in concert with the cell cycle, from the basal side (where DNA is synthesized) to the apical surface (where mitosis takes place); such nuclear movements were previously misinterpreted as the radial intercalation of cells. We further demonstrate that growth of epithelial girth between E12.5 and E14.5 is driven by microtubule- and actinomyosin-dependent apicobasal elongation, rather than by progressive epithelial stratification as was previously thought. Finally, we show that the actin-binding protein Shroom3 is crucial for the maintenance of the single-layered pseudostratified epithelium. In mice lacking Shroom3, the epithelium is disorganized and temporarily stratified during villus emergence. These results favor an alternative model of intestinal morphogenesis in which the epithelium remains single layered and apicobasally polarized throughout early intestinal development.
14. β-Actin mRNA compartmentalization enhances focal adhesion stability and directs cell **migratio**n. Directed cell motility is at the basis of biological phenomena such as development, wound healing, and metastasis. It has been shown that substrate attachments mediate motility by coupling the cell's cytoskeleton with force generation. However, it has been unclear how the persistence of cell directionality is facilitated. We show that mRNA localization plays an important role in this process, but the mechanism of action is still unknown. In this study, we show that the zipcode-binding protein 1 transports β-actin mRNA to the focal adhesion compartment, where it dwells for minutes, suggesting a means for associating its localization with motility through the formation of stable connections between adhesions and newly synthesized actin filaments. In order to demonstrate this, we developed an approach for assessing the functional consequences of β-actin mRNA and protein localization by tethering the mRNA to a specific location-in this case, the focal adhesion complex. This approach will have a significant impact on cell biology because it is now possible to forcibly direct any mRNA and its cognate protein to specific locations in the cell. This will reveal the importance of localized protein translation on various cellular processes.
15. β-Actin specifically controls cell growth, **migratio**n, and the G-actin pool. Ubiquitously expressed β-actin and γ-actin isoforms play critical roles in most cellular processes; however, their unique contributions are not well understood. We generated whole-body β-actin-knockout (Actb(-/-)) mice and demonstrated that β-actin is required for early embryonic development. Lethality of Actb(-/-) embryos correlated with severe growth impairment **and migr**ation defects in β-actin-knockout primary mouse embryonic fibroblasts (MEFs) that were not observed in γ-actin-null **MEFs. Mi**gration defects were associated with reduced membrane protrusion dynamics and increased focal adhesions. We also iden**tified mi**gration defects upon conditional ablation of β-actin in highly motile T cells. Of great interest, ablation of β-actin altered the ratio of globular actin (G-actin) to filamentous actin in MEFs, with corresponding changes in expression of genes that regulate the cell cycle and motility. These data support an essential role for β-actin in regula**ting cell** migration and gene expression through control of the cellular G-actin pool.
16. Specific interaction of KIF11 with ZBP1 regulates the transport of β-actin mRNA and cell motility. ZBP1-modulated localization of β-actin mRNA enables a cell to establish polarity and structural asymmetry. Although the mechanism of β-actin mRNA localization has been well established, the underlying mechanism of how a specific molecular motor contributes to the transport of the ZBP1 (also known as IGF2BP1) complex in non-neuronal cells remains elusive. In this study, we report the isolation and identification of KIF11, a microtubule motor, which physically interacts with ZBP1 and is a component of β-actin messenger ribonucleoprotein particles (mRNPs). We show that KIF11 colocalizes with the β-actin mRNA, and the ability of KIF11 to transport β-actin mRNA is dependent on ZBP1. We characterize the corresponding regions of ZBP1 and KIF11 that mediate the interaction of the two proteins in vitro and in vivo. Disruption of the in vivo interaction of KIF11 with ZBP1 delocalizes β-actin mRNA and affect**s cell mi**gration. Our study reveals a molecular mechanism by which a particular microtubule motor mediates the transport of an mRNP through direct interaction with an mRNA-binding protein.
17. TRPM7, a novel regulator of actomyosin contractility and cell adhesion. Actomyosin contractility regulates various cell biological processes including cytokinesis, adhesion and **migration**. While in lower eukaryotes, alpha-kinases control actomyosin relaxation, a similar role for mammalian alpha-kinases has yet to be established. Here, we examined whether TRPM7, a cation channel fused to an alpha-kinase, can affect actomyosin function. We demonstrate that activation of TRPM7 by bradykinin leads to a Ca(2+)- and kinase-dependent interaction with the actomyosin cytoskeleton. Moreover, TRPM7 phosphorylates the myosin IIA heavy chain. Accordingly, low overexpression of TRPM7 increases intracellular Ca2+ levels accompanied by cell spreading, adhesion and the formation of focal adhesions. Activation of TRPM7 induces the transformation of these focal adhesions into podosomes by a kinase-dependent mechanism, an effect that can be mimicked by pharmacological inhibition of myosin II. Collectively, our results demonstrate that regulation of cell adhesion by TRPM7 is the combined effect of kinase-dependent and -independent pathways on actomyosin contractility.
18. Developmental expression of MMP-9 (gelatinase B) mRNA in mouse embryos. Considerable remodeling of the extracellular matrix as well as cellular **migration** takes place during embryogenesis. Since the metalloproteinase MMP-9 is implicated in these functions in cancer cells, we studied the patterns of expression of MMP-9 mRNA during the development of post-implantation mouse embryos. MMP-9 mRNA was detected using the ribonuclease protection assay in poly A+RNA from 13 to 17 day embryos, but not at 11 days. In order to localize these transcripts, in situ hybridization was performed on sections of murine embryos from 7.5 to 15 days of gestation. At the time of implantation, MMP-9 mRNA was localized to the invading trophoblast cells. Strong signals were also seen in the yolk sac. No signal for MMP-9 mRNA was seen by in situ hybridization in the embryo until day 11 when detectable reaction was seen in the central nervous system. By day 15 strong signals were seen in the liver, in the developing bronchial epithelium of the lungs and in the primordial alveoli, in the epithelium of the thyroid gland, in the thymus, in the endochondrial plates of the bone, and in neural cells. The liver from day 15 embryos contained gelatinase activity at 105 kDa consistent with MMP-9. Thus, MMP-9 expression appears to be expressed in specific organs in a precise temporal sequence during development.
19. Partial deficiency of sphingosine-1-phosphate lyase confers protection in experimental autoimmune encephalomyelitis. Sphingosine-1-phosphate (S1P) regulates the egress of T cells from lymphoid organs; levels of S1P in the tissues are controlled by S1P lyase (Sgpl1). Hence, Sgpl1 offers a target to block T cell-dependent inflammatory processes. However, the involvement of Sgpl1 in models of disease has not been fully elucidated yet, since Sgpl1 KO mice have a short life-span.We generated inducible Sgpl1 KO mice featuring partial reduction of Sgpl1 activity and analyzed them with respect to sphingolipid levels, T-cell distribution, and response in models of inflammation.The partially Sgpl1 deficient mice are viable but feature profound reduction of peripheral T cells, similar to the constitutive KO mice. While thymic T cell development in these mice appears normal, mature T cells are retained in thymus and lymph nodes, leading to reduced T cell numbers in spleen and blood, with a skewing towards increased proportions of memory T cells and T regulatory cells. The therapeutic relevance of Sgpl1 is demonstrated by the fact that the inducible KO mice are protected in experimental autoimmune encephalomyelitis (EAE). T cell im**migration** into the CNS was found to be profoundly reduced. Since S1P levels in the brain of the animals are unchanged, we conclude that protection in EAE is due to the peripheral effect on T cells, leading to reduced CNS im**migration**, rather than on local effects in the CNS.The data suggest Sgpl1 as a novel therapeutic target for the treatment of multiple sclerosis.
20. Multiple origins of Cajal-Retzius cells at the borders of the developing pallium. Cajal-Retzius cells are critical in cortical lamination, but very little is known about their origin and development. The homeodomain transcription factor Dbx1 is expressed in restricted progenitor domains of the developing pallium: the ventral pallium (VP) and the septum. Using genetic tracing and ablation experiments in mice, we show that two subpopulations of Reelin(+) Cajal-Retzius cells are generated from Dbx1-expressing progenitors. VP- and septum-derived Reelin(+) neurons differ in their onset of appearance, **migration** routes, destination and expression of molecular markers. Together with reported data supporting the generation of Reelin(+) cells in the cortical hem, our results show that Cajal-Retzius cells are generated at least at three focal sites at the borders of the developing pallium and are redistributed by tangential **migration**. Our data also strongly suggest that distinct Cajal-Retzius subtypes exist and that their presence in different territories of the developing cortex might contribute to region-specific properties.

#### GeneRIFs

1. Disruption of the in vivo interaction of KIF11 with ZBP1 delocalizes beta-actin mRNA and affects cell **migration**.
2. Absence of beta-actin severely hampers formation of the peripheral nervous system, due to defective neural crest **migration**.
3. Data suggest that in beta-actin knockout cells other actins compensate for beta-actin in cell **migration** process.
4. These data support an essential role for beta-actin in regulating cell **migration** and gene expression through control of the cellular G-actin pool

### ECM

#### PubMed

1. Depletion of nuclear actin is a key mediator of quiescence in epithelial cells. Functional differentiation is orchestrated by precise growth-regulatory controls conveyed by the tissue microenvironment. Cues from laminin 111 (LN1) lower transcription and suppress mammary epithelial cell growth in culture, but how LN1 induces quiescence is unknown. Recent literature points to involvement of nuclear β-actin in transcriptional regulation. Here, we show that quiescence induced by growth factor withdrawal, or LN1 addition, rapidly decreases nuclear β-actin. LN1, but not other extracellular matrix **(E**CM) molecules, decreases the levels of nuclear β-actin and destabilizes RNA polymerase (RNA Pol) II and III binding to transcription sites, leading to a dramatic drop in transcription and DNA synthesis. Constitutive overexpression of globular β-actin in the nucleus reverses the effect of LN1 on transcription and RNA Pol II association and prevents the cells from becoming quiescent in the presence of LN1. The physiological relevance of our findings was verified by identifying a clear spatial separation of LN1 and β-actin in developing mammary end buds. These data indicate a novel role for nuclear β-actin in growth arrest of epithelial cells and underscore the importance of the integrity of the basement membrane in homeostasis.

### [Ee]xtracellular [Mm]atrix

#### PubMed

1. Serum response factor is required for cell contact maintenance but dispensable for proliferation in visceral yolk sac endothelium. Endothelial-specific knockout of the transcription factor serum response factor (SRF) results in embryonic lethality by mid-gestation. The associated phenotype exhibits vascular failure in embryos as well as visceral yolk sac (VYS) tissues. Previous data suggest that this vascular failure is caused by alterations in cell-cell and cell-matrix contacts. In the current study, we sought to more carefully address the role of SRF in endothelial function and cell contact interactions in VYS tissues.Tie2-Cre recombinase-mediated knockout of SRF expression resulted in loss of detectable SRF from VYS mesoderm by E12.5. This loss was accompanied by decreased expression of smooth muscle alpha-actin as well as vascular endothelial cadherin and claudin 5, endothelial-specific components of adherens and tight junctions, respectively. Focal adhesion (FA) integrins alpha5 and beta1 were largely unchanged in contrast to loss of the FA-associated molecule vinculin. The integrin binding partner fibronectin-1 was also profoundly decreased in the **extracellular matrix**, indicating another aspect of impaired adhesive function and integrin signaling. Additionally, cells in SRF-null VYS mesoderm failed to reduce proliferation, suggesting not only that integrin-mediated contact inhibition is impaired but also that SRF protein is not required for proliferation in these cells.Our data support a model in which SRF is critical in maintaining functional cell-cell and cell-matrix adhesion in endothelial cells. Furthermore, we provide evidence that supports a model in which loss of SRF protein results in a sustained proliferation defect due in part to failed integrin signaling.
2. Depletion of nuclear actin is a key mediator of quiescence in epithelial cells. Functional differentiation is orchestrated by precise growth-regulatory controls conveyed by the tissue microenvironment. Cues from laminin 111 (LN1) lower transcription and suppress mammary epithelial cell growth in culture, but how LN1 induces quiescence is unknown. Recent literature points to involvement of nuclear β-actin in transcriptional regulation. Here, we show that quiescence induced by growth factor withdrawal, or LN1 addition, rapidly decreases nuclear β-actin. LN1, but not othe**r extracellular matr**ix (ECM) molecules, decreases the levels of nuclear β-actin and destabilizes RNA polymerase (RNA Pol) II and III binding to transcription sites, leading to a dramatic drop in transcription and DNA synthesis. Constitutive overexpression of globular β-actin in the nucleus reverses the effect of LN1 on transcription and RNA Pol II association and prevents the cells from becoming quiescent in the presence of LN1. The physiological relevance of our findings was verified by identifying a clear spatial separation of LN1 and β-actin in developing mammary end buds. These data indicate a novel role for nuclear β-actin in growth arrest of epithelial cells and underscore the importance of the integrity of the basement membrane in homeostasis.
3. Developmental expression of MMP-9 (gelatinase B) mRNA in mouse embryos. Considerable remodeling of the **extracellular matrix** as well as cellular migration takes place during embryogenesis. Since the metalloproteinase MMP-9 is implicated in these functions in cancer cells, we studied the patterns of expression of MMP-9 mRNA during the development of post-implantation mouse embryos. MMP-9 mRNA was detected using the ribonuclease protection assay in poly A+RNA from 13 to 17 day embryos, but not at 11 days. In order to localize these transcripts, in situ hybridization was performed on sections of murine embryos from 7.5 to 15 days of gestation. At the time of implantation, MMP-9 mRNA was localized to the invading trophoblast cells. Strong signals were also seen in the yolk sac. No signal for MMP-9 mRNA was seen by in situ hybridization in the embryo until day 11 when detectable reaction was seen in the central nervous system. By day 15 strong signals were seen in the liver, in the developing bronchial epithelium of the lungs and in the primordial alveoli, in the epithelium of the thyroid gland, in the thymus, in the endochondrial plates of the bone, and in neural cells. The liver from day 15 embryos contained gelatinase activity at 105 kDa consistent with MMP-9. Thus, MMP-9 expression appears to be expressed in specific organs in a precise temporal sequence during development.

### [Ii]nvasive

#### PubMed

1. 'Designer' tumors in mice. We have developed and tested successfully a general method based on Cre-mediated recombination that can be used for ubiquitous or tissue-specific expression of protein products, including tumor-inducing oncoproteins. Depending on the specificity of a chosen promoter driving cre expression, tumors develop by design in bitransgenic mouse progeny derived by crossing Cre-producing mice with partners carrying a dormant oncogenic transgene (targeted into the 3' noncoding region of the cytoplasmic beta-actin locus) that becomes functional after excision of a 'floxed' DNA segment. To provide proof-of-principle, we have used as models transgenes encoding the polyomavirus middle T antigen (PVMT) and the T antigens of the SV40 early region (SVER). Cre-dependent activation of widespread SVER expression resulted in hyperplasias or **invasive** tumors affecting particular visceral smooth muscles, whereas Cre-dependent, mammary gland-specific expression of PVMT-induced adenocarcinomas, according to plan. Unexpectedly, we also encountered spontaneous (Cre-independent) oncogene expression occurring as a rare event, which simulates the initiation of sporadic tumors and leads to PVMT-induced hemangiomas and mammary carcinomas or SVER-induced disseminated sarcomas, thus, revealing particular tissue susceptibilities to the actions of these oncoproteins.

### [Mm]etastasis

#### PubMed

1. Silencing of the Tropomyosin-1 gene by DNA methylation alters tumor suppressor function of TGF-beta. Loss of actin stress fibers has been associated with cell transformation and **metastasis**. TGF-beta induction of stress fibers in epithelial cells requires high molecular weight tropomyosins encoded by TPM1 and TPM2 genes. Here, we investigated the mechanism underlying the failure of TGF-beta to induce stress fibers and inhibit cell migration in metastatic cells. RT-PCR analysis in carcinoma cell lines revealed a significant reduction in TPM1 transcripts in metastatic MDA-MB-231, MDA-MB-435 and SW620 cell lines. Treatment of these cells with demethylating agent 5-aza-2'-deoxycytidine (5-aza-dC) increased mRNA levels of TPM1 with no effect on TPM2. Importantly, 5-aza-dC treatment of MDA-MB-231 cells restored TGF-beta induction of TPM1 and formation of stress fibers. Forced expression of TPM1 by using Tet-Off system increased stress fibers in MDA-MB-231 cells and reduced cell migration. A potential CpG island spanning the TPM1 proximal promoter, exon 1, and the beginning of intron 1 was identified. Bisulfite sequencing showed significant cytosine methylation in metastatic cell lines that correlated with a reduced expression of TPM1. Together these results suggest that epigenetic suppression of TPM1 may alter TGF-beta tumor suppressor function and contribute to metastatic properties of tumor cells.
2. β-Actin mRNA compartmentalization enhances focal adhesion stability and directs cell migration. Directed cell motility is at the basis of biological phenomena such as development, wound healing, and **metastasi**s. It has been shown that substrate attachments mediate motility by coupling the cell's cytoskeleton with force generation. However, it has been unclear how the persistence of cell directionality is facilitated. We show that mRNA localization plays an important role in this process, but the mechanism of action is still unknown. In this study, we show that the zipcode-binding protein 1 transports β-actin mRNA to the focal adhesion compartment, where it dwells for minutes, suggesting a means for associating its localization with motility through the formation of stable connections between adhesions and newly synthesized actin filaments. In order to demonstrate this, we developed an approach for assessing the functional consequences of β-actin mRNA and protein localization by tethering the mRNA to a specific location-in this case, the focal adhesion complex. This approach will have a significant impact on cell biology because it is now possible to forcibly direct any mRNA and its cognate protein to specific locations in the cell. This will reveal the importance of localized protein translation on various cellular processes.

---

## Sarm1

- NCBI Gene
- GeneRIF
- Pubmed

| Search Term | Rank | Fields |
| --- | --- | --- |
| [Ff]ilopodia | 1 | PubMed(1) |

  
hide/show details for Sarm1

### [Ff]ilopodia

#### PubMed

1. Sarm1, a negative regulator of innate immunity, interacts with syndecan-2 and regulates neuronal morphology. Dendritic arborization is a critical neuronal differentiation process. Here, we demonstrate that syndecan-2 (Sdc2), a synaptic heparan sulfate proteoglycan that triggers dendritic **filopodia** and spine formation, regulates dendritic arborization in cultured hippocampal neurons. This process is controlled by sterile α and TIR motif-containing 1 protein (Sarm1), a negative regulator of Toll-like receptor 3 (TLR3) in innate immunity signaling. We show that Sarm1 interacts with and receives signal from Sdc2 and controls dendritic arborization through the MKK4-JNK pathway. In Sarm1 knockdown mice, dendritic arbors of neurons were less complex than those of wild-type littermates. In addition to acting downstream of Sdc2, Sarm1 is expressed earlier than Sdc2, which suggests that it has multiple roles in neuronal morphogenesis. Specifically, it is required for proper initiation and elongation of dendrites, axonal outgrowth, and neuronal polarization. These functions likely involve Sarm1-mediated regulation of microtubule stability, as Sarm1 influenced tubulin acetylation. This study thus reveals the molecular mechanism underlying the action of Sarm1 in neuronal morphogenesis.

---

## Actg1

- NCBI Gene
- GeneRIF
- Pubmed

| Search Term | Rank | Fields |
| --- | --- | --- |
| [Ff]ilopodia | 1 | PubMed(1) |
| [Pp]odosome(s)? | 5 | PubMed(1) |
| [Mm]igration | 6 | PubMed(9); GeneRIFs(1) |
| ECM | 7 | PubMed(1) |
| [Ee]xtracellular [Mm]atrix | 8 | PubMed(1); Component(1) |

  
hide/show details for Actg1

### [Ff]ilopodia

#### PubMed

1. Serum response factor is required for sprouting angiogenesis and vascular integrity. Serum response factor (SRF) is a transcription factor that controls the expression of cytoskeletal proteins and immediate early genes in different cell types. Here, we found that SRF expression is restricted to endothelial cells (ECs) of small vessels such as capillaries in the mouse embryo. EC-specific Srf deletion led to aneurysms and hemorrhages from 11.5 days of mouse development (E11.5) and lethality at E14.5. Mutant embryos presented a reduced capillary density and defects in EC migration, with fewer numbers of **filopodia** in tip cells and ECs showing defects in actin polymerization and intercellular junctions. We show that SRF is essential for the expression of VE-cadherin and beta-actin in ECs both in vivo and in vitro. Moreover, knockdown of SRF in ECs impaired VEGF- and FGF-induced in vitro angiogenesis. Taken together, our results demonstrate that SRF plays an important role in sprouting angiogenesis and small vessel integrity in the mouse embryo.

### [Pp]odosome(s)?

#### PubMed

1. A congenital activating mutant of WASp causes altered plasma membrane topography and adhesion under flow in lymphocytes. Leukocytes rely on dynamic actin-dependent changes in cell shape to pass through blood vessels, which is fundamental to immune surveillance. Wiskott-Aldrich Syndrome protein (WASp) is a hematopoietic cell-restricted cytoskeletal regulator important for modulating cell shape through Arp2/3-mediated actin polymerization. A recently identified WASp(I294T) mutation was shown to render WASp constitutively active in vivo, causing increased filamentous (F)-actin polymerization, high **podosome** turnover in macrophages, and myelodysplasia. The aim of this study was to determine the effect of WASp(I294T) expression in lymphocytes. Here, we report that lymphocytes isolated from a patient with WASp(I294T), and in a cellular model of WASp(I294T), displayed abnormal microvillar architecture, associated with an increase in total cellular F-actin. Microvillus function was additionally altered as lymphocytes bearing the WASp(I294T) mutation failed to roll normally on L-selectin ligand under flow. This was not because of defects in L-selectin expression, shedding, cytoskeletal anchorage, or membranal positioning; however, under static conditions of adhesion, WASp(I294T)-expressing lymphocytes exhibited altered dynamic interaction with L-selectin ligand, with a significantly reduced rate of adhesion turnover. Together, our results demonstrate that WASp(I294T) significantly affects lymphocyte membrane topography and L-selectin-dependent adhesion, which may be linked to defective hematopoiesis and leukocyte function in affected patients.

### [Mm]igration

#### PubMed

1. Beta- and gamma-cytoplasmic actins are required for meiosis in mouse oocytes. In mammals, female meiosis consists of two asymmetric cell divisions, which generate a large haploid oocyte and two small polar bodies. Asymmetric partitioning of the cytoplasm results from **migration** of the meiotic spindle toward the cortex and requires actin filaments. However, the subcellular localization and the role of the existing two cytoplasmic actin (CYA) isoforms, beta and gamma, have not been characterized. We show that beta- and gamma-CYA are differentially distributed in the maturing oocyte from late metaphase I as well as in preimplantation embryos. Gamma-CYA is preferentially enriched in oocyte cortices and is absent from all cell-cell contact areas from metaphase II until the blastocyst stage. Beta-CYA is enriched in contractile structures, at cytokinesis, at cell-cell contacts, and around the forming blastocoel. Alteration of beta- or gamma-CYA function by isoform-specific antibody microinjection suggests that gamma-CYA holds a major and specific role in the establishment and/or maintenance of asymmetry in meiosis I and in the maintenance of overall cortical integrity. In contrast, beta- and gamma-CYA, together, appear to participate in the formation and the cortical anchorage of the second meiotic spindle in waiting for fertilization. Finally, differences in gamma-CYA expression are amongst the earliest markers of cell fate determination in development.
2. Serum response factor is required for sprouting angiogenesis and vascular integrity. Serum response factor (SRF) is a transcription factor that controls the expression of cytoskeletal proteins and immediate early genes in different cell types. Here, we found that SRF expression is restricted to endothelial cells (ECs) of small vessels such as capillaries in the mouse embryo. EC-specific Srf deletion led to aneurysms and hemorrhages from 11.5 days of mouse development (E11.5) and lethality at E14.5. Mutant embryos presented a reduced capillary density and defects in EC **migration**, with fewer numbers of filopodia in tip cells and ECs showing defects in actin polymerization and intercellular junctions. We show that SRF is essential for the expression of VE-cadherin and beta-actin in ECs both in vivo and in vitro. Moreover, knockdown of SRF in ECs impaired VEGF- and FGF-induced in vitro angiogenesis. Taken together, our results demonstrate that SRF plays an important role in sprouting angiogenesis and small vessel integrity in the mouse embryo.
3. β-Actin specifically controls cell growth, **migratio**n, and the G-actin pool. Ubiquitously expressed β-actin and γ-actin isoforms play critical roles in most cellular processes; however, their unique contributions are not well understood. We generated whole-body β-actin-knockout (Actb(-/-)) mice and demonstrated that β-actin is required for early embryonic development. Lethality of Actb(-/-) embryos correlated with severe growth impairment **and migr**ation defects in β-actin-knockout primary mouse embryonic fibroblasts (MEFs) that were not observed in γ-actin-null **MEFs. Mi**gration defects were associated with reduced membrane protrusion dynamics and increased focal adhesions. We also iden**tified mi**gration defects upon conditional ablation of β-actin in highly motile T cells. Of great interest, ablation of β-actin altered the ratio of globular actin (G-actin) to filamentous actin in MEFs, with corresponding changes in expression of genes that regulate the cell cycle and motility. These data support an essential role for β-actin in regula**ting cell** migration and gene expression through control of the cellular G-actin pool.
4. Measurements of spatiotemporal changes in G-actin concentration reveal its effect on stimulus-induced actin assembly and lamellipodium extension. To understand the intracellular role of G-actin concentration in stimulus-induced actin assembly and lamellipodium extension during cell **migration**, we developed a novel technique for quantifying spatiotemporal changes in G-actin concentration in live cells, consisting of sequential measurements of fluorescent decay after photoactivation (FDAP) of Dronpa-labeled actin. Cytoplasmic G-actin concentrations decreased by ∼40% immediately after cell stimulation and thereafter the cell area extended. The extent of stimulus-induced G-actin loss and cell extension correlated linearly with G-actin concentration in unstimulated cells, even at concentrations much higher than the critical concentration of actin filaments, indicating that cytoplasmic G-actin concentration is a critical parameter for determining the extent of stimulus-induced G-actin assembly and cell extension. Multipoint FDAP analysis revealed that G-actin concentration in lamellipodia was comparable to that in the cell body. We also assessed the cellular concentrations of free G-actin, profilin- and thymosin-β4-bound G-actin, and free barbed and pointed ends of actin filaments by model fitting of jasplakinolide-induced temporal changes in G-actin concentration.
5. Beta- and gamma-cytoplasmic actins are required for meiosis in mouse oocytes. In mammals, female meiosis consists of two asymmetric cell divisions, which generate a large haploid oocyte and two small polar bodies. Asymmetric partitioning of the cytoplasm results from **migration** of the meiotic spindle toward the cortex and requires actin filaments. However, the subcellular localization and the role of the existing two cytoplasmic actin (CYA) isoforms, beta and gamma, have not been characterized. We show that beta- and gamma-CYA are differentially distributed in the maturing oocyte from late metaphase I as well as in preimplantation embryos. Gamma-CYA is preferentially enriched in oocyte cortices and is absent from all cell-cell contact areas from metaphase II until the blastocyst stage. Beta-CYA is enriched in contractile structures, at cytokinesis, at cell-cell contacts, and around the forming blastocoel. Alteration of beta- or gamma-CYA function by isoform-specific antibody microinjection suggests that gamma-CYA holds a major and specific role in the establishment and/or maintenance of asymmetry in meiosis I and in the maintenance of overall cortical integrity. In contrast, beta- and gamma-CYA, together, appear to participate in the formation and the cortical anchorage of the second meiotic spindle in waiting for fertilization. Finally, differences in gamma-CYA expression are amongst the earliest markers of cell fate determination in development.
6. β-Actin specifically controls cell growth, **migratio**n, and the G-actin pool. Ubiquitously expressed β-actin and γ-actin isoforms play critical roles in most cellular processes; however, their unique contributions are not well understood. We generated whole-body β-actin-knockout (Actb(-/-)) mice and demonstrated that β-actin is required for early embryonic development. Lethality of Actb(-/-) embryos correlated with severe growth impairment **and migr**ation defects in β-actin-knockout primary mouse embryonic fibroblasts (MEFs) that were not observed in γ-actin-null **MEFs. Mi**gration defects were associated with reduced membrane protrusion dynamics and increased focal adhesions. We also iden**tified mi**gration defects upon conditional ablation of β-actin in highly motile T cells. Of great interest, ablation of β-actin altered the ratio of globular actin (G-actin) to filamentous actin in MEFs, with corresponding changes in expression of genes that regulate the cell cycle and motility. These data support an essential role for β-actin in regula**ting cell** migration and gene expression through control of the cellular G-actin pool.
7. The Sphingosine-1-phospate receptor 1 mediates S1P action during cardiac development. Sphingosine-1-phosophate (S1P) is a biologically active sphingolipid metabolite that influences cellular events including differentiation, proliferation, and **migration**. S1P acts through five distinct cell surface receptors designated S1P1-5R, with S1P1R having the highest expression level in the developing heart. S1P1R is critical for vascular maturation, with its loss leading to embryonic death by E14.5; however, its function during early cardiac development is not well known. Our previous studies demonstrated that altered S1P levels adversely affects atrioventricular (AV) canal development in vitro, with reduced levels leading to cell death and elevated levels inhibiting cell **migration** and endothelial to mesenchymal cell transformation (EMT).We determined, by real-time PCR analysis, that S1P1R was expressed at least 10-fold higher than other S1P receptors in the developing heart. Immunohistochemical analysis revealed S1P1R protein expression in both endothelial and myocardial cells in the developing atrium and ventricle. Using AV canal cultures, we observed that treatment with either FTY720 (an S1P1,3,4,5R agonist) or KRP203 (an S1P1R-specific agonist) caused similar effects on AV canal cultures as S1P treatment, including induction of cell rounding, inhibition of cell **migration**, and inhibition of EMT. In vivo, morphological analysis of embryonic hearts at E10.5 revealed that S1P1R-/- hearts were malformed with reduced myocardial tissue. In addition to reduced myocardial tissue, E12.5 S1P1R-/- hearts had disrupted morphology of the heart wall and trabeculae, with thickened and disorganized outer compact layer and reduced fibronectin (FN) deposition compared to S1P1R+/+ littermates. The reduced myocardium was accompanied by a decrease in cell proliferation but not an increase in apoptosis.These data indicate that S1P1R is the primary mediator of S1P action in AV canal cultures and that loss of S1P1R expression in vivo leads to malformed embryonic hearts, in part due to reduced fibronectin expression and reduced cell proliferation.
8. Delayed embryonic development and impaired cell growth and survival in Actg1 null mice. Actins are among the most highly expressed proteins in eukaryotes and play a central role in nearly all aspects of cell biology. While the intricate process of development undoubtedly requires a properly regulated actin cytoskeleton, little is known about the contributions of different actin isoforms during embryogenesis. Of the six actin isoforms, only the two cytoplasmic actins, beta(cyto)- and gamma(cyto)-actin, are ubiquitously expressed. We found that gamma(cyto)-actin null (Actg1(-/-)) mice were fully viable during embryonic development, but most died within 48 h of birth due to respiratory failure and cannibalization by the parents. While no morphogenetic defects were identified, Actg1(-/-) mice exhibited stunted growth during embryonic and postnatal development as well as delayed cardiac outflow tract formation that resolved by birth. Using primary mouse embryonic fibroblasts, we confirm that gamma(cyto)-actin is not required for cell **migration**. The Actg1(-/-) cells, however, exhibited growth impairment and reduced cell viability, defects which perhaps contribute to the stunted growth and developmental delays observed in Actg1(-/-) embryos. Since the total amount of actin protein was maintained in Actg1(-/-) cells, our data suggests a distinct requirement for gamma(cyto)-actin in cell growth and survival.
9. Potential target genes of EMX2 include Odz/Ten-M and other gene families with implications for cortical patterning. EMX2 and PAX6 are expressed by cortical progenitors and specify area patterning. We used representational difference analysis (RDA) to compare expressed RNAs from wild type and Emx2-/- cortex and identified 41 unique clones. Using secondary screening by in situ hybridization, we selected five genes for further analysis, Cdk4, Cofilin1, Crmp1, ME2, and Odz4, involved in neuronal proliferation, differentiation, **migration**, and axon guidance. Each exhibits differential expression in wild type cortex. Odz4 is one of four members of a vertebrate gene family homologous to the Drosophila pair-rule patterning gene, Odd Oz (Odz), a transmembrane receptor. We show that Odz genes are expressed in complementary patterns in cortex, as well as in nuclei-specific patterns in thalamus that relates to their area-unique cortical expression. In addition, each of the genes analyzed shows different expression patterns in wild type cortex, Emx2, and Pax6 mutant cortex, consistent with potential roles in area patterning. These findings identify potential targets of EMX2 that might account for its function and the defects in Emx2-/- cortex, and suggest that the Odz family of transmembrane proteins influences cortical area patterning downstream to EMX2 and PAX6.

#### GeneRIFs

1. These data support an essential role for beta-actin in regulating cell **migration** and gene expression through control of the cellular G-actin pool

### ECM

#### PubMed

1. Small leucine-rich proteoglycans exhibit unique spatiotemporal expression profiles during cardiac valve development. Small Leucine Rich Proteoglycans (SLRPs) play a role in collagen fiber formation and also function as signaling molecules. Given the importance of collagen synthesis to the cardiovascular extracellular matrix (**ECM**), we examined the spatiotemporal expression of SLRPs, not previously investigated in the murine heart.Cardiac expression using antibodies specific for biglycan (BGN), decorin (DCN), fibromodulin (FMOD), and lumican (LUM) revealed distinct patterns among the SLRPs in mesenchymal-derived tissues. DCN showed the most intense localization within the developing valve cusps, while LUM was evident primarily in the hinge region of postnatal cardiac valves. BGN, DCN, and FMOD were immunolocalized to regions where cardiac valves anchor into adjacent tissues. Medial (BGN) and adventitial (BGN, DCN, FMOD and LUM) layers of the pulmonary and aortic arteries also showed intense staining of SLRPs but this spatiotemporal expression varied with developmental age.The unique expression patterns of SLRPs suggest they have adapted to specialized roles in the cardiovascular **ECM**. SLRP expression patterns overlap with areas where TGFβ signaling is critical to the developing heart. Therefore, we speculate that SLRPs may not only be required to facilitate collagen fiber formation but may also regulate TGFβ signaling in the murine heart.

### [Ee]xtracellular [Mm]atrix

#### PubMed

1. Small leucine-rich proteoglycans exhibit unique spatiotemporal expression profiles during cardiac valve development. Small Leucine Rich Proteoglycans (SLRPs) play a role in collagen fiber formation and also function as signaling molecules. Given the importance of collagen synthesis to the cardiovascular **extracellular matrix** (ECM), we examined the spatiotemporal expression of SLRPs, not previously investigated in the murine heart.Cardiac expression using antibodies specific for biglycan (BGN), decorin (DCN), fibromodulin (FMOD), and lumican (LUM) revealed distinct patterns among the SLRPs in mesenchymal-derived tissues. DCN showed the most intense localization within the developing valve cusps, while LUM was evident primarily in the hinge region of postnatal cardiac valves. BGN, DCN, and FMOD were immunolocalized to regions where cardiac valves anchor into adjacent tissues. Medial (BGN) and adventitial (BGN, DCN, FMOD and LUM) layers of the pulmonary and aortic arteries also showed intense staining of SLRPs but this spatiotemporal expression varied with developmental age.The unique expression patterns of SLRPs suggest they have adapted to specialized roles in the cardiovascular ECM. SLRP expression patterns overlap with areas where TGFβ signaling is critical to the developing heart. Therefore, we speculate that SLRPs may not only be required to facilitate collagen fiber formation but may also regulate TGFβ signaling in the murine heart.

#### Component

1. **extracellular matrix**

---

## Timp3

- NCBI Gene
- GeneRIF
- Pubmed

| Search Term | Rank | Fields |
| --- | --- | --- |
| [Mm]igration | 6 | PubMed(9) |
| ECM | 7 | PubMed(23) |
| [Ee]xtracellular [Mm]atrix | 8 | PubMed(42); Component(2); GeneRIFs(1); Conserved Domains(1) |
| [Ii]nvasive | 10 | PubMed(5) |
| [Mm]etastasis | 12 | PubMed(4) |
| [Cc]ell [Ii]nvasion | 13 | PubMed(2) |

  
hide/show details for Timp3

### [Mm]igration

#### PubMed

1. GPR124, an orphan G protein-coupled receptor, is required for CNS-specific vascularization and establishment of the blood-brain barrier. Every organ in the body requires blood vessels for efficient delivery of oxygen and nutrients, but independent vascular beds are highly specialized to meet the individual needs of specific organs. The vasculature of the brain is tightly sealed, with blood-brain barrier (BBB) properties developing coincident with neural vascularization. G protein-coupled receptor 124 (GPR124) (tumor endothelial marker 5, TEM5), an orphan member of the adhesion family of G protein-coupled receptors, was previously identified on the basis of its overexpression in tumor vasculature. Here, we show that global deletion or endothelial-specific deletion of GPR124 in mice results in embryonic lethality associated with abnormal angiogenesis of the forebrain and spinal cord. Expression of GPR124 was found to be required for invasion and **migration** of blood vessels into neuroepithelium, establishment of BBB properties, and expansion of the cerebral cortex. Thus, GPR124 is an important regulator of neurovasculature development and a potential drug target for cerebrovascular diseases.
2. Association of TIMP-2 with extracellular matrix exposed to mechanical stress and its co-distribution with periostin during mouse mandible development. Matrix remodeling is regulated by matrix metalloproteinases (MMPs) and tissue inhibitors of metalloproteinases (TIMPs). Periostin, originally identified in a mouse osteoblastic library, plays a role in cell adhesion and **migration** and in mechanical stress-induced matrix remodeling. In this study, we analyzed and compared the distribution patterns of TIMP-2 and periostin during mouse mandible development. Immunohistochemical staining for TIMP-2 and periostin was carried out on serial cryosections obtained from mice at embryonic days 13-16, postnatal day 2 (P2), P35, and 12 weeks of age. TIMP-2 and periostin exhibited a strikingly similar protein distribution during mandible development. From bud to early bell stages of molars, TIMP-2 and periostin were highly expressed on the lingual and anterior sides of the basement membrane and on the adjacent jaw mesenchyme. In pre- and postnatal incisors, the basement membrane of the apical loop and dental follicle was immunostained for TIMP-2 and periostin. At postnatal stages, TIMP-2 and periostin were prominently confined to the extracellular matrix (ECM) of gingival tissues, periodontal ligaments, and tendons (all recipients of mechanical strain). However, periostin was solely detected in the lower portion of the inner root sheath of hair follicles. Gingiva of P2 cultured in anti-TIMP-2 antibody-conditioned medium showed markedly reduced staining of periostin. We suggest that TIMP-2 and periostin are co-distributed on ECM exposed to mechanical forces and coordinately function as ECM modulators.
3. Matrix metalloproteinases regulate morphogenesis, **migration** and remodeling of epithelium, tongue skeletal muscle and cartilage in the mandibular arch. We have investigated the role of proteinases in the developmental program of bone, cartilage, tongue muscle and epithelial differentiation and remodeling in the mandibular arch during murine embryogenesis. Expression of matrix metalloproteinases (MMPs) and their tissue inhibitors (TIMPs) was tissue-specific with little or no expression in the epithelium of tooth buds, tongue or oral cavity. Gelatinase A mRNA transcripts were strongly expressed in the perichondrium of Meckel's cartilage and mesenchymal areas of embryonic day 13-15 mandibles, whereas gelatinase B, collagenase-3, TIMP-1 and TIMP-2 mRNA were found primarily in the ossifying areas of the mandibles. The skeletal muscle of the tongue expressed stromelysin-3, TIMP-2 and TIMP-3 mRNA while stromelysin-3, TIMP-2 and gelatinase A were seen in the overlying connective tissue layer. Gelatinase A, gelatinase B, stromelysin-1, urokinase, TIMP-1 and TIMP-2 mRNA and protein activities were also detected in cultured mandibular explants. Culture of day 10 mandibular explants with a hydroxamic acid metalloproteinase inhibitor, but not with inhibitors of metalloendopeptidases (thiorphan and phosphoramidon), serine proteinases (aprotinin), cysteine proteinases (leupeptin) and urokinase (amiloride), altered mandibular morphogenesis dramatically. Development of the tongue (glossogenesis) and cartilage, but not bone or teeth was affected. Formation of the oral sulcus and fusion of the two epithelia of the medial sulcus were inhibited, and number and **migration** of myoblasts decreased. The resulting 'tongue-tied phenotype' indicates that MMPs are involved in epithelial morphogenesis and the **migration** of myoblasts to the region of the tongue. Development of the anterior segment of Meckel's cartilage was also inhibited and proteoglycan content of the cartilage was reduced by inhibiting MMPs. Our data suggest that matrix metalloproteinases play a pivotal role in the morphogenesis of structures derived from epithelium (oral sulcus), cranial paraxial mesoderm (tongue) and cranial neural crest (Meckel's cartilage).
4. Endothelial cell lumen and vascular guidance tunnel formation requires MT1-MMP-dependent proteolysis in 3-dimensional collagen matrices. Here we show that endothelial cells (EC) require matrix type 1-metalloproteinase (MT1-MMP) for the formation of lumens and tube networks in 3-dimensional (3D) collagen matrices. A fundamental consequence of EC lumen formation is the generation of vascular guidance tunnels within collagen matrices through an MT1-MMP-dependent proteolytic process. Vascular guidance tunnels represent a conduit for EC motility within these spaces (a newly remodeled 2D matrix surface) to both assemble and remodel tube structures. Interestingly, it appears that twice as many tunnel spaces are created than are occupied by tube networks after several days of culture. After tunnel formation, these spaces represent a 2D migratory surface within 3D collagen matrices allowing for EC **migration** in an MMP-independent fashion. Blockade of EC lumenogenesis using inhibitors that interfere with the process (eg, integrin, MMP, PKC, Src) completely abrogates the formation of vascular guidance tunnels. Thus, the MT1-MMP-dependent proteolytic process that creates tunnel spaces is directly and functionally coupled to the signaling mechanisms required for EC lumen and tube network formation. In summary, a fundamental and previously unrecognized purpose of EC tube morphogenesis is to create networks of matrix conduits that are necessary for EC **migration** and tube remodeling events critical to blood vessel assembly.
5. Identification of FGF10 targets in the embryonic lung epithelium during bud morphogenesis. Genetic studies implicate Fgf10-Fgfr2 signaling as a critical regulator of bud morphogenesis in the embryo. However, little is known about the transcriptional targets of Fgf10 during this process. Here we identified global changes in gene expression in lung epithelial explants undergoing FGF10-mediated budding in the absence of other growth factors and mesenchyme. Targets were confirmed by their localization at sites where endogenous Fgf10 signaling is active in embryonic lungs and by demonstrating their induction in intact lungs in response to local application of FGF10 protein. We show that the initial stages of budding are characterized by marked up-regulation of genes associated with cell rearrangement and cell **migration**, inflammatory process, and lipid metabolism but not cell proliferation. We also found that some genes implicated in tumor invasion and metastatic behavior are epithelial targets of Fgf10 in the lung and other developing organs that depend on Fgf10-Fgfr2 signaling to properly form. Our approach identifies Fgf10 targets that are common to multiple biological processes and provides insights into potential mechanisms by which Fgf signaling regulates epithelial cell behavior.
6. Matrix metalloproteinases regulate mesonephric cell **migration** in developing XY gonads which correlates with the inhibition of tissue inhibitor of metalloproteinase-3 by Sry. In the mouse, the sex determining gene Sry, on the Y chromosome, controls testis differentiation during embryogenesis. Following Sry expression, indifferent XY gonads increase their size relative to XX gonads and form cord-like structures with the adjacent mesonephros, providing XY gonad somatic cells. This mesonephric cell **migration** is known to depend on Sry, but the molecular mechanism of mesonephric cell **migration** remains unknown. In this study, it was shown that cells expressing Sry induced proliferation of mesonephric cells migrating into male gonads, and inhibited expression of the tissue inhibitor of metalloproteinases (TIMP)-3 gene, which is the endogenous inhibitor of matrix metalloproteinases (MMP). In addition, the mesonephric cell **migration** was blocked by a chemically synthesized inhibitor of MMP in a gonad/mesonephros organ co-culture system with enhanced green fluorescent protein transgenic embryos. The findings indicate that MMP may play a critical role in mesonephric cell **migration**, and the function of MMP may be regulated by a Sry-TIMP-3 cascade. These findings are an important clue for the elucidation of testicular formation in developing gonads.
7. Expression of matrix metalloproteinases during murine chorioallantoic placenta maturation. A large body of experimental evidence supports the participation of two groups of extracellular proteases, matrix metalloproteinases (MMPs), and plasminogen activators/plasmin, in tissue remodeling in physiological and pathological invasion. In the late mouse placenta, several tissue remodeling and cell invasion processes take place. Spongiotrophoblast **migration** into maternal decidua, as well as decidual extracellular matrix remodeling require the coordinated action of extracellular proteolytic enzymes. Via Northern and in situ hybridization, we have analyzed the spatio-temporal expression patterns of members of the MMP family (stromelysin-3, gelatinases A and B), as well as their inhibitors TIMP-1, -2 and -3 in late murine placenta (days 10.5 to 18.5 of gestation). Gelatinase activity in placental extracts was assessed by substrate zymography. Gelatinase A and stromelysin-3 were found to be prominently expressed in decidual tissue; shortly after midpregnancy, the decidual expression patterns of gelatinase A and stromelysin-3 became overlapping with each other, as well as with the expression domain of TIMP-2. On the other hand, gelatinase B transcripts were expressed only by trophoblast giant cells at day 10.5, and were downregulated at later stages. TIMP-1 and TIMP-3 transcripts were detected in decidual periphery at day 10.5, while later the expression was restricted to the endometrial stroma and spongiotrophoblasts, respectively. The areas of stromelysin-3 expression were the same (giant trophoblasts) or adjacent (decidua) to those where urokinase (uPA) transcripts were detected, suggesting a possible cooperation between these proteinases in placental remodeling. We generated mice doubly deficient for stromelysin-3 and uPA, and report here that these mice are viable and fertile. Furthermore, these animals do not manifest obvious placental abnormalities, thereby suggesting the existence of compensatory/redundant mechanisms involving other proteolytic enzymes. Our findings document the participation of MMPs and their inhibitors in the process of late murine placenta maturation, and warrant the characterization of other members of the MMP family, like membrane type-MMPs, in this process.
8. S156C mutation in tissue inhibitor of metalloproteinases-3 induces increased angiogenesis. Tissue Inhibitor of metalloproteinases-3 (TIMP-3) is a potent matrix-bound angiogenesis inhibitor. Mutations in TIMP-3 cause Sorsby Fundus Dystrophy, a dominant inherited, early onset macular degenerative disease, with choroidal neovascularization causing a loss of vision in the majority of patients. Here we report that expression of S156C TIMP-3 mutation in endothelial cells results in an abnormal localization of the protein, increased glycosylation, decreased matrix metalloproteinase inhibitory activity, and increased vascular endothelial growth factor (VEGF) binding with a consequent increase in VEGF-dependent **migration** and tube formation. These enhanced signaling events appear to be mediated as a consequence of a post-transcriptionally regulated increase in the expression of membrane-associated VEGFR-2 in endothelial cells of Timp-3(156/156) mutant mice as well as in human Sorsby fundus dystrophy eyes. Understanding the mechanism(s) by which mutant TIMP-3 can induce abnormal neovascularization provides important insight into the pathophysiology of a number of diseases with increased angiogenesis.
9. Overexpression of delta-like 4 induces arterialization and attenuates vessel formation in developing mouse embryos. The importance of Notch signaling pathway in the regulation of vascular development and angiogenesis is suggested by the expression of Notch receptors and ligands in vascular endothelial cells (ECs) and the observed vascular phenotypes in mutants of Notch receptors or ligands, especially Dll4. DLL4 is specifically expressed in arterial ECs during development, and haplo-insufficiency is embryonically lethal in mice. To address the role of Dll4 in vascular development, we produced mDll4 conditionally overexpressed transgenic mice that were crossed with constitutive recombinase cre lines. Double transgenic embryos displayed grossly enlarged dorsal aortae (DA) and died before embryonic day 10.5 (E10.5), showing a variable degree of premature arteriovenous fusion. Veins displayed ectopic expression of arterial markers. Other defects included reduced vascular sprouting, EC proliferation, and **migration**. mDll4 overexpression also inhibited VEGF signaling and increased fibronectin accumulation around the vessels. In vitro and in vivo studies of DLL4-FL (Dll4-full-length) in ECs recapitulate many of the mDll4 transgenics findings, including decreased tube formation, reduced vascular branching, fewer vessels, increased pericyte recruitment, and increased fibronectin expression. These results establish the role of Dll4 in arterial identity determination, and regulation of angiogenesis subject to dose and location.

### ECM

#### PubMed

1. TIMP3 is the primary TIMP to regulate agonist-induced vascular remodelling and hypertension. Hypertension is accompanied by structural remodelling of vascular extracellular matrix (**ECM**). Tissue inhibitor of metalloproteinases (TIMPs) inhibits matrix metalloproteinases (MMPs) that degrade the matrix structural proteins. In response to a hypertensive stimulus, the balance between MMPs and TIMPs is altered. We examined the role of TIMPs in agonist-induced hypertension.We subjected TIMP-knockout mice to angiotensin II (Ang II) infusion, and found that Ang-II-induced hypertension in TIMP1(-/-), TIMP2(-/-), and TIMP4(-/-) mice was comparable to wild-type (WT) mice, but significantly suppressed in TIMP3(-/-) mice. Ex vivo pressure myography analyses on carotid and mesenteric arteries revealed that Ang-II-infused TIMP3(-/-) arteries were more distensible with impaired elastic recoil compared with the WT group. The acute response to vasoconstriction and vasodilation was intact in TIMP3(-/-) mesenteric and carotid arteries. Mesenteric arteries from TIMP3(-/-)-Ang II mice exhibited a reduced media-to-lumen ratio, suppressed collagen and elastin levels, elevated elastase and gelatinase proteolytic activities compared with WT-Ang II. TIMP3(-/-)-Ang II carotid arteries also showed adverse structural remodelling. Treatment of mice with doxycycline, a matrix metalloproteinase inhibitor, improved matrix integrity in mesenteric and carotid arteries in TIMP3(-/-)-Ang II and differentially regulated elastin and collagen levels in WT-Ang II vs. TIMP3(-/-)-Ang II.Our study demonstrates a critical role for TIMP3, among all TIMPs, is preserving arterial **ECM** in response to Ang II. It is critical to acknowledge that the suppressed Ang-II-induced hypertension in TIMP3(-/-) mice is not a protective mechanism but owing to adverse remodelling in arterial matrix.
2. The role of TIMPs in regulation of extracellular matrix proteolysis. Tissue inhibitors of metalloproteinases (TIMPs), which inhibit matrix metalloproteinases (MMPs) as well as the closely related, a disintegrin and metalloproteinases (ADAMs) and ADAMs with thrombospondin motifs (ADAMTSs), were traditionally thought to control extracellular matrix (**ECM**) proteolysis through direct inhibition of MMP-dependent **ECM** proteolysis. This classical role for TIMPs suggests that increased TIMP levels results in **ECM** accumulation (or fibrosis), whereas loss of TIMPs leads to enhanced matrix proteolysis. Mice lacking TIMP family members have provided support for such a role; however, studies with these TIMP deficient mice have also demonstrated that loss of TIMPs can often be associated with an accumulation of **ECM**. Collectively, these studies suggest that the divergent roles of TIMPs in matrix accumulation and proteolysis, which together can be referred to as **ECM** turnover, are dependent on the TIMP, specific tissue, and local tissue environment (i.e. health vs. injury/disease). Ultimately, these combined factors dictate the specific metalloproteinases being regulated by a given TIMP, and it is likely the diversity of metalloproteinases and their physiological substrates that determines whether TIMPs inhibit matrix proteolysis or accumulation. In this review, we discuss the evidence for the dichotomous roles of TIMPs in **ECM** turnover highlighting some of the common findings between different TIMP family members. Importantly, while we now have a better understanding of the role of TIMPs in regulating **ECM** turnover, much remains to be determined. Data on the specific metalloproteinases inhibited by different TIMPs in vivo remains limited and must be the focus of future studies.
3. Loss of Timp3 gene leads to abdominal aortic aneurysm formation in response to angiotensin II. Aortic aneurysm is dilation of the aorta primarily due to degradation of the aortic wall extracellular matrix (**ECM**). Tissue inhibitors of metalloproteinases (TIMPs) inhibit matrix metalloproteinases (MMPs), the proteases that degrade the **ECM**. Timp3 is the only **ECM**-bound Timp, and its levels are altered in the aorta from patients with abdominal aortic aneurysm (AAA). We investigated the causal role of Timp3 in AAA formation. Infusion of angiotensin II (Ang II) using micro-osmotic (Alzet) pumps in Timp3(-/-) male mice, but not in wild type control mice, led to adverse remodeling of the abdominal aorta, reduced collagen and elastin proteins but not mRNA, and elevated proteolytic activities, suggesting excess protein degradation within 2 weeks that led to formation of AAA by 4 weeks. Intriguingly, despite early up-regulation of MMP2 in Timp3(-/-)Ang II aortas, additional deletion of Mmp2 in these mice (Timp3(-/-)/Mmp2(-/-)) resulted in exacerbated AAA, compromised survival due to aortic rupture, and inflammation in the abdominal aorta. Reconstitution of WT bone marrow in Timp3(-/-)/Mmp2(-/-) mice reduced inflammation and prevented AAA in these animals following Ang II infusion. Treatment with a broad spectrum MMP inhibitor (PD166793) prevented the Ang II-induced AAA in Timp3(-/-) and Timp3(-/-)/Mmp2(-/-) mice. Our study demonstrates that the regulatory function of TIMP3 is critical in preventing adverse vascular remodeling and AAA. Hence, replenishing TIMP3, a physiological inhibitor of a number of metalloproteinases, could serve as a therapeutic approach in limiting AAA development or expansion.
4. TGF-beta3-induced palatogenesis requires matrix metalloproteinases. Cleft lip and palate syndromes are among the most common congenital malformations in humans. Mammalian palatogenesis is a complex process involving highly regulated interactions between epithelial and mesenchymal cells of the palate to permit correct positioning of the palatal shelves, the remodeling of the extracellular matrix (**ECM**), and subsequent fusion of the palatal shelves. Here we show that several matrix metalloproteinases (MMPs), including a cell membrane-associated MMP (MT1-MMP) and tissue inhibitor of metalloproteinase-2 (TIMP-2) were highly expressed by the medial edge epithelium (MEE). MMP-13 was expressed both in MEE and in adjacent mesenchyme, whereas gelatinase A (MMP-2) was expressed by mesenchymal cells neighboring the MEE. Transforming growth factor (TGF)-beta3-deficient mice, which suffer from clefting of the secondary palate, showed complete absence of TIMP-2 in the midline and expressed significantly lower levels of MMP-13 and slightly reduced levels of MMP-2. In concordance with these findings, MMP-13 expression was strongly induced by TGF-beta3 in palatal fibroblasts. Finally, palatal shelves from prefusion wild-type mouse embryos cultured in the presence of a synthetic inhibitor of MMPs or excess of TIMP-2 failed to fuse and MEE cells did not transdifferentiate, phenocopying the defect of the TGF-beta3-deficient mice. Our observations indicate for the first time that the proteolytic degradation of the **ECM** by MMPs is a necessary step for palatal fusion.
5. A null mutation for tissue inhibitor of metalloproteinases-3 (Timp-3) impairs murine bronchiole branching morphogenesis. Tissue inhibitors of metalloproteinases (TIMPs) regulate extracellular matrix (**ECM**) degradation by matrix metalloproteinases (MMPs). We have examined the role of TIMP-3 on **ECM** homeostasis and bronchiole branching morphogenesis during murine embryogenesis. Employing an in vitro organ culture system, we found decreased bronchiolar branching in null lungs when compared with wild type (WT) counterparts after 2 days in culture. When a synthetic inhibitor of MMPs at low dose was added to the culture system, branching was augmented regardless of genotype. Gelatin and in situ zymography revealed that null lungs exhibited enhanced activation of MMPs throughout lung development. We analysed the impact of increased MMP activity on a number of **ECM** molecules by Western blot analysis, but found that only fibronectin abundance was consistently reduced in the null lungs throughout development. To confirm that our observed defect in culture was not simply a developmental delay in the null lung, we examined null and WT lungs from newborn pups. Here, we found not only a reduced number of bronchioles in the null, but also that the bronchiole tubes were dilated compared with controls and that alveologenesis was attenuated. We propose that the deletion of TIMP-3 disrupts the exquisite TIMP/MMP balance required for proper focal **ECM** proteolysis, which leads to correct bronchiole branching morphogenesis in the developing mouse lung.
6. Tissue inhibitor of metalloproteinases 3 regulates extracellular matrix--cell signaling during bronchiole branching morphogenesis. Tissue inhibitors of metalloproteinases (TIMPs) regulate extracellular matrix (**ECM**) degradation by matrix metalloproteinases (MMPs) throughout embryogenesis. We examined lungs from TIMP3 null mice and found decreased bronchiole branching, enhanced activity of MMPs and enhanced fibronectin degradation throughout lung development compared to controls. Activation of focal adhesion kinase (FAK) was also reduced from embryonic days 12.5 through 14.5 in TIMP3 null lungs. Treatment with a synthetic MMP inhibitor, GM6001, in utero enhanced the branching pattern in both wild type and null lungs accompanied by a restoration of fibronectin localization, signaling through FAK and epithelial cell proliferation in null lungs. Direct down-regulation of FAK abundance in WT lung organ culture by siRNA targeting resulted in reduced bronchiole branching, phenocopying the TIMP3 defect. We propose that enhanced MMP activity in the absence of TIMP3 interferes with focal **ECM** proteolysis, perturbing the intracellular signaling necessary for correct pattern formation of the bronchiole tree during bronchiole branching morphogenesis. Thus, TIMP3 can indirectly regulate epithelial cell proliferation via MMP inhibitory activity. While others have demonstrated this function for MMPs, and there is in vitro evidence that TIMP3 controls proliferation, to our knowledge this is the first evidence of TIMP3 regulating proliferation in vivo.
7. Absence of tissue inhibitor of metalloproteinases 3 disrupts alveologenesis in the mouse. Tissue inhibitors of metalloproteinases (TIMPs) regulate extracellular matrix (**ECM**) degradation by matrix metalloproteinases (MMPs) throughout lung development. We examined lungs from TIMP3 null mice and found significant air space enlargement compared with wild type (WT) animals during a time course spanning early alveologenesis (post-partum days 1, 5, 9 and 14). Trichrome staining revealed a similar pattern of collagen distribution in the walls of nascent alveoli; however, the alveolar walls of TIMP3 mutant mice appeared to be thinner than controls. Assessment of MMP2 and MMP9 activities by gelatin zymography demonstrated a significant elevation in the active form of MMP2 at post-partum days 1 and 5. Treatment of null pregnant dams with a broad spectrum synthetic metalloproteinase inhibitor, GM6001, on embryonic day 16.5 enhanced the formation of primitive alveoli during the saccular stage of lung development as evidenced by a partial, but significant, rescue of alveolar size in post-partum day 1 animals. We propose that increased MMP activity in the absence of TIMP3 enhances **ECM** proteolysis, upsetting proper formation of primitive alveolar septa during the saccular stage of alveologenesis. Therefore, TIMP3 indirectly regulates alveolar formation in the mouse. To our knowledge, ours is the first study to demonstrate that in utero manipulation of the TIMP/MMP proteolytic axis, to specifically inhibit proteolysis, significantly affects lung development.
8. Temporospatial distribution of matrix metalloproteinase and tissue inhibitors of matrix metalloproteinases during murine secondary palate morphogenesis. Extracellular matrix (**ECM**) molecules are known to play a pivotal role in the morphogenesis of the secondary palate. The maintenance and degradation of the **ECM** is mediated in part by the matrix metalloproteinases (MMPs) and their endogenous inhibitors TIMPs. MMPs and TIMPs have previously been shown to be developmentally regulated within the palatal shelf during secondary palate morphogenesis. This study was conducted to examine the temporospatial distribution of these enzymes and their inhibitors within the palatal shelves using immunofluorescent localization to determine if specific changes occur in their distribution concomitant with events in palatal shelf formation and reorientation. Frontal sections through the posterior palatal shelves at gestational day (gd) 12, 13 and 14 were immunofluorescently stained for MMPs 2, 3, 9, and 13 and TIMPs 1, 2, and 3 using standard protocols and commercially available antibodies. The results demonstrated that MMPs and TIMPs were already present within the palatal shelf mesenchyme 30 h prior to reorientation and closure and that their expression within the shelf mesenchyme increased as the shelves remodeled, then decreased with closure and fusion. Increased distribution of MMPs and TIMPs within specific regions of the palatal mesenchyme and palatal epithelial basement membrane preceded decreases previously observed within these areas for their substrates, fibronectin, collagen III and collagen I. In addition, MMP-3 and TIMP-3 were immunolocalized to regions of the palatal epithelium that undergo reorganization concomitant with reorientation. The results of this study indicate that MMPs and TIMPs are developmentally regulated during palatal shelf morphogenesis and that their distribution correlates with the distribution of the **ECM** components of the palatal shelf they regulate. These results provide support for the idea that temporospatially controlled interactions between MMPs and their substrates may be pivotal in modulating events in palatal morphogenesis.
9. Association of TIMP-2 with extracellular matrix exposed to mechanical stress and its co-distribution with periostin during mouse mandible development. Matrix remodeling is regulated by matrix metalloproteinases (MMPs) and tissue inhibitors of metalloproteinases (TIMPs). Periostin, originally identified in a mouse osteoblastic library, plays a role in cell adhesion and migration and in mechanical stress-induced matrix remodeling. In this study, we analyzed and compared the distribution patterns of TIMP-2 and periostin during mouse mandible development. Immunohistochemical staining for TIMP-2 and periostin was carried out on serial cryosections obtained from mice at embryonic days 13-16, postnatal day 2 (P2), P35, and 12 weeks of age. TIMP-2 and periostin exhibited a strikingly similar protein distribution during mandible development. From bud to early bell stages of molars, TIMP-2 and periostin were highly expressed on the lingual and anterior sides of the basement membrane and on the adjacent jaw mesenchyme. In pre- and postnatal incisors, the basement membrane of the apical loop and dental follicle was immunostained for TIMP-2 and periostin. At postnatal stages, TIMP-2 and periostin were prominently confined to the extracellular matrix (**ECM**) of gingival tissues, periodontal ligaments, and tendons (all recipients of mechanical strain). However, periostin was solely detected in the lower portion of the inner root sheath of hair follicles. Gingiva of P2 cultured in anti-TIMP-2 antibody-conditioned medium showed markedly reduced staining of periostin. We suggest that TIMP-2 and periostin are co-distributed on **ECM** exposed to mechanical forces and coordinately function as **ECM** modulators.
10. Regulation of tissue inhibitor of metalloproteinases-3 gene expression by transforming growth factor-beta and dexamethasone in bovine and human articular chondrocytes. Physiological and pathological degradation of cartilage extracellular matrix (**ECM**) is regulated by the balance between tissue inhibitors of metalloproteinases (TIMPs) and matrix metalloproteinases (MMPs). We examined the potential of chondrocytes from normal bovine or human osteoarthritic (OA) cartilage to express RNA for the new inhibitor TIMP-3 and studied its regulation by an inducer of matrix synthesis, transforming growth factor-beta (TGF-beta). Freshly released chondrocytes constitutively expressed three transcripts of TIMP-3 that are induced by serum factors. In primary cultures of chondrocytes, one of these factors, TGF-beta, increased TIMP-3 mRNA in a dose-dependent fashion that required de novo protein synthesis and transcription. TGF-beta did not alter stability of the TIMP-3 transcripts in RNA decay time-courses, suggesting a transcriptional control. Nuclear run-on assays confirmed increased rate of TIMP-3 gene transcription by TGF-beta. An antiinflammatory glucocorticoid, dexamethasone, inhibited the basal, and suppressed partially the TGF-beta-inducible, TIMP-3 expression in primary bovine and human chondrocytes. DNA sequencing of bovine TIMP-3 cDNA revealed an open reading frame of a 211-amino-acid protein containing signal peptide and 12 conserved cysteines. The encoded protein differed from human TIMP-3 at four positions. The constitutive expression and evolutionary conservation of TIMP-3 imply its important function. TIMP-3 induction by TGF-beta suggests the role of this factor and TIMP-3 in cartilage remodeling with important implications for arthritis.
11. Timp-1, -2 and -3 show coexpression with gelatinases A and B during mouse tooth morphogenesis. Matrix metalloproteinases (MMPs) have been implicated in tissue remodelling and in regulation of cell-matrix interactions during organ development. The activity of MMPs is regulated by members of the TIMP (tissue inhibitors of metalloproteinase) family. We analyzed by in situ hybridization the expression of gelatinase A (MMP-2) and gelatinase B (MMP-9) as well as Timp-1, -2 and -3 during different stages of mouse tooth development. Gene expression was generally found in mesenchymal tissues except for Timp-3, which also was found in dental epithelial cells. During early tooth development, gelatinase A and Timp-2 were widely expressed in the branchial arch, while gelatinase B and Timp-1 and Timp-3 expression showed clear association with epithelial morphogenesis and was restricted to the mesenchyme at the tip of the growing tooth bud. Gelatinase A and Timp-1 showed transient expression in secretory odontoblasts at the time of basement membrane degradation, while Timp-2 expression continued throughout the dental papilla. At the time of tooth eruption, Timp-3 was expressed in most dental epithelial cells except secretory ameloblasts, and gelatinase B was intensely expressed in osteoclasts in the jaw bone. The exact co-localization of gelatinase A and Timp-1 in secretory odontoblasts, and the correlation between gelatinase B and Timp-3 during bone resorption may indicate interaction of the proteins during degradation of the basement membrane and in the control of **ECM** turnover in connection with tooth eruption.
12. Molecular dissection of TIMP3 mutation S156C associated with Sorsby fundus dystrophy. Sorsby fundus dystrophy (SFD) is an autosomal dominant macular degeneration of late onset. A key feature of the disease is the thickening of Bruch's membrane, an **ECM** structure located between the RPE and the choroid. SFD is caused by mutations in the gene encoding the **ECM**-associated tissue inhibitor of metalloproteases-3 (TIMP3). We have recently generated two Timp3 gene-targeted mouse lines, one deficient for the murine gene (Timp3-/-) and one carrying an SFD-related S156C mutation. Based on extracts and cell cultures derived from tissues of these animals we now evaluated TIMP3 functionality and its contribution to SFD. We show that the activity levels of TIMP3 target proteases including TACE, ADAMTS4/5 and aggrecan-cleaving MMPs are similar in Timp3S156/+ and Timp3S156C/S156C mice when compared to controls. In Timp3-/- mice, a significant enhancement of enzyme activity was observed for TACE but not for ADAMTS4/5 and MMPs indicating a compensatory effect of other inhibitors regulating the latter two groups of proteases. Fibrin bead assays show that angiogenesis in Timp3S156/+ and Timp3S156C/S156C mice is not altered whereas increased formation of capillary tubes was observed in Timp3-/- animals over controls. Rescue experiments using recombinant proteins demonstrate that the inhibitory activities of TIMP3 towards TACE and aggrecan-cleaving MMPs as well as the anti-angiogenic properties of TIMP3 are not impaired by SFD mutation S156C. We finally demonstrate that wild-type and S156C-TIMP3 proteins block the binding of VEGF to its receptor VEGFR2 to a similar extent. Taken together, this study shows that S156C-TIMP3 retains its known functional properties suggesting that causes other than an imbalance in protease or angiogenic activities represent the primary molecular defect underlying SFD.
13. Timp-3 deficiency impairs cognitive function in mice. Extracellular matrix (**ECM**) degradation is performed primarily by matrix metalloproteinases (MMPs). MMPs have recently been shown to regulate synaptic activity in the hippocampus and to affect memory and learning. The tissue inhibitor of metalloproteinase (Timp) is an endogenous factor that controls MMP activity by binding to the catalytic site of MMPs. At present, four Timp isotypes have been reported (Timp-1 through Timp-4) with 35-50% amino-acid sequence homology. Timp-3 is a unique member of Timp proteins in that it is bound to the **ECM**. In this study, we used the passive avoidance test, active avoidance test, and water maze test to examine the cognitive function in Timp-3 knockout (KO) mice. Habituation was evaluated using the open-field test. The water maze test showed that Timp-3 KO mice exhibit deterioration in cognitive function compared with wild-type (WT) mice. The open-field test showed decreased habituation of Timp-3 KO mice. Immunostaining of brain slices revealed the expression of Timp-3 in the hippocampus. In situ zymography of the hippocampus showed increased gelatinolytic activity in Timp-3 KO mice compared with WT mice. These results present the first evidence of Timp-3 involvement in cognitive function and hippocampal MMP activity in mice. Moreover, our findings suggest a novel therapeutic target to be explored for improvement of cognitive function in humans.
14. Early activation of matrix metalloproteinases underlies the exacerbated systolic and diastolic dysfunction in mice lacking TIMP3 following myocardial infarction. Extracellular matrix (**ECM**) remodeling is a critical aspect of cardiac remodeling following myocardial infarction. Tissue inhibitors of metalloproteinases (TIMPs) are physiological inhibitors of matrix metalloproteinases (MMPs) that degrade the **ECM** proteins. TIMP3 is highly expressed in the heart, and is markedly downregulated in patients with ischemic cardiomyopathy. We therefore examined the time- and region-dependent role of TIMP3 in the cardiac response to myocardial infarction (MI). TIMP3(-/-) and wild-type (WT) mice were subjected to MI by ligation of the left anterior descending artery. TIMP3(-/-)-MI mice exhibited a significantly compromised rate of survival compared with WT-MI mice, primarily due to increased left ventricular (LV) rupture, greater infarct expansion, exacerbated LV dilation, and greater systolic and diastolic dysfunction. Second harmonic generation imaging of unfixed and unstained hearts revealed greater collagen disarray and reduced density in the TIMP3(-/-) infarct myocardium compared with the WT group. Gelatinolytic and collagenolytic activities increased in TIMP3(-/-) compared with WT hearts at 1 day post-MI but not at 3 days or 1 wk post-MI. Neutrophil infiltration and inflammatory MMPs were significantly increased in the infarct and peri-infarct regions of TIMP3(-/-)-MI hearts. Treatment of TIMP3(-/-) mice with a broad-spectrum MMP inhibitor (PD-166793) for 2 days before and 2 days after MI markedly improved post-MI infarct expansion, LV rupture incident, LV dilation, and systolic dysfunction in these mice up to 1 wk post-MI. Our data demonstrate that the initial rise in proteolytic activities early post-MI is a triggering factor for subsequent LV adverse remodeling, LV rupture, and dilated cardiomyopathy. Hence, timing of treatments to improve cardiac response to MI may be critical in producing favorable outcome.
15. ATXN1 protein family and CIC regulate extracellular matrix remodeling and lung alveolarization. Although expansion of CAG repeats in ATAXIN1 (ATXN1) causes Spinocerebellar ataxia type 1, the functions of ATXN1 and ATAXIN1-Like (ATXN1L) remain poorly understood. To investigate the function of these proteins, we generated and characterized Atxn1L(-/-) and Atxn1(-/-); Atxn1L(-/-) mice. Atxn1L(-/-) mice have hydrocephalus, omphalocele, and lung alveolarization defects. These phenotypes are more penetrant and severe in Atxn1(-/-); Atxn1L(-/-) mice, suggesting that ATXN1 and ATXN1L are functionally redundant. Upon pursuing the molecular mechanism, we discovered that several Matrix metalloproteinase (Mmp) genes are overexpressed and that the transcriptional repressor Capicua (CIC) is destabilized in Atxn1L(-/-) lungs. Consistent with this, Cic deficiency causes lung alveolarization defect. Loss of either ATXN1L or CIC derepresses Etv4, an activator for Mmp genes, thereby mediating MMP9 overexpression. These findings demonstrate a critical role of ATXN1/ATXN1L-CIC complexes in extracellular matrix **(EC**M) remodeling during development and their potential roles in pathogenesis of disorders affecting **EC**M remodeling.
16. Gene encoding a novel murine tissue inhibitor of metalloproteinases (TIMP), TIMP-3, is expressed in developing mouse epithelia, cartilage, and muscle, and is located on mouse chromosome 10. Remodeling of the extracellular matrix (**ECM**) is an essential component of normal development and is also involved in the pathogenesis of arthritis and the spread of cancer. The matrix metalloproteinases and their natural inhibitors, the tissue inhibitors of metalloproteinases (TIMPs), play an important role in this context. We have isolated mouse cDNA clones encoding a novel member of the TIMP family, designated TIMP-3. We have assigned the Timp-3 locus to the [C1-D1] region of mouse chromosome 10 using both genetic and cytogenetic methods. The conceptual translation product of the Timp-3 cDNA shows a high degree of similarity with ChIMP-3, a recently cloned chicken metalloproteinase inhibitor, as well as significant structural similarity with the amino acid sequences of the previously isolated members of this family, TIMP-1 and TIMP-2. The pattern of expression of Timp-3 in the developing mouse embryo is distinct from that previously reported for Timp-1. Timp-3 is expressed in cartilage and skeletal muscle, in myocardium, in the skin, oral and nasal epithelium, in the newborn mouse liver, in the epithelium of some tubular structures such as the developing bronchial tree, oesophagus, colon, urogenital sinus, bile duct, in the kidney, salivary glands, and in the choroid plexus of the brain. The patterns of Timp-3 expression in surface epithelia and in the epithelial lining of many tubular organs suggests that TIMP-3 may be involved in regulating **ECM** remodeling during the folding of epithelia and during the formation, branching, and expansion of epithelial tubes. In the mouse placenta, expression is seen in the trophoblast, raising the possibility that TIMP-3 may be involved in regulating trophoblastic invasion of the uterus. We propose a role for TIMP-3 in musculoskeletal and cardiac development, in the morphogenesis of certain epithelial structures, and placental implantation.
17. Spontaneous air space enlargement in the lungs of mice lacking tissue inhibitor of metalloproteinases-3 (TIMP-3). Tissue inhibitors of metalloproteinases regulate **ECM** degradation by matrix metalloproteinases (MMPs). We have developed a mouse line deficient for tissue inhibitor of metalloproteinases-3 (TIMP-3), the only TIMP known to reside within the **ECM**. Homozygous Timp-3-null animals develop spontaneous air space enlargement in the lung that is evident at 2 weeks after birth and progresses with age of the animal. As early as 13 months of age animals become moribund. Lung function, measured by carbon monoxide uptake, is impaired in aged null animals. Lungs from aged null animals have reduced abundance of collagen, enhanced degradation of collagen in the peribronchiolar space, and disorganization of collagen fibrils in the alveolar interstitium, but no increase in inflammatory cell infiltration or evidence of fibrosis in comparison with controls. Using in situ zymography, we show that lungs from aged null animals have heightened MMP activity over wild-type and heterozygotic animals. Finally, TIMP-3-null fibroblast cultures demonstrate enhanced destruction of **ECM** molecules in vitro. We propose that the deletion of TIMP-3 results in a shift of the TIMP/MMP balance in the lung to favor **ECM** degradation, culminating in incapacitating illness and a shorter life span.
18. Differential expression of matrix metalloproteinases and their inhibitors in human and mouse lung development. Lung development is a highly orchestrated process characterized by timed expression and activation of growth factor and protease/antiprotease systems. This interplay is essential in regulating vasculogenesis, alveolarization, and epithelial to mesenchymal transition during lung development. Alterations in the proteolytic/antiproteolytic balance of the lung have been associated with several respiratory diseases characterized by changes in the lung extracellular matrix (**ECM**). Here, we characterized the expression pattern of matrix metalloproteases (MMP) and their inhibitors, the tissue inhibitors of metalloproteases (TIMP), in human and mouse lung development. Using MMP/TIMP expression arrays, RT-PCR, Western Blotting, and ELISA analyses, we demonstrate that fetal human lung is characterized by a dominant proteolytic profile with high MMP-2 and little TIMP-3 expression. Adult human lung, in contrast, exhibits a more anti-proteolytic profile with decreased MMP-2 and increased TIMP-3 expression. MMP-14, MMP-20, TIMP-1, and TIMP-2 were constitutively expressed, irrespective of the developmental stage. Similar results were obtained using mouse lungs of different developmental stages, with the addition that in mouse lung, TIMP-2 and TIMP-3 were upregulated as lung development progressed. Exposure of neonatal mice to chronic hypoxia (10% O2), a stimulus that leads to an arrest of lung development, resulted in upregulation of MMP-2 with a concomitant downregulation of TIMP-2. These results provide a comprehensive analysis of MMP and TIMP expression during human and mouse lung development. MMP-2, TIMP-2, and TIMP-3 may be key regulatory enzymes during lung development, possibly through their complex action on **ECM** components, membrane receptor ectodomain shedding, and growth factor bioactivity.
19. Matrix metalloproteinases and oral cancer. For tumours to invade and metastasise, neoplastic cells must be capable of degrading the extracellular matrix (**ECM**), and accessing blood vessels and lymphatics. This process is mediated in the pericellular environment and is a highly controlled cascade of events utilising the same mechanisms that normal cells use for migrating through tissue barriers, for example, in development and wound healing. Proteolytic enzymes from several families, including matrix metalloproteinases (MMPs), are involved in **ECM** remodelling. Increased production of these enzymes has been associated with the invasive and/or metastatic phenotype in many tumours. Several MMPs have been shown to play a role in the invasion and metastasis of oral carcinoma, and it is increasingly apparent that tumour cells, as well as producing endogenous MMP, are capable of utilising MMP produced by tumour stromal cells, indicating an active role for stroma in tumour invasion. It is not clear whether a particular invasive system is favoured by oral carcinoma, but it is likely that further understanding of the interactions between carcinoma and stromal cells will provide an opportunity to refine the therapeutic interventions that are currently being tested.
20. Combination of tumor necrosis factor-alpha ablation and matrix metalloproteinase inhibition prevents heart failure after pressure overload in tissue inhibitor of metalloproteinase-3 knock-out mice. Cytokine and extracellular matrix (**ECM**) homeostasis are distinct systems that are each dysregulated in heart failure. Here we show that tissue inhibitor of metalloproteinase (TIMP)-3 is a critical regulator of both systems in a mouse model of left ventricular (LV) dilation and dysfunction. Timp-3(-/-) mice develop precipitous LV dilation and dysfunction reminiscent of dilated cardiomyopathy (DCM), culminating in early onset of heart failure by 6 weeks, compared with wild-type aortic-banding (AB). Timp-3 deficiency resulted in increased TNFalpha converting enzyme (TACE) activity within 6 hours after AB leading to enhanced tumor necrosis factor-alpha (TNFalpha) processing. In addition, TNFalpha production increased in timp-3(-/-)-AB myocardium. A significant elevation in gelatinase and collagenase activities was observed 1 week after AB, with localized **ECM** degradation in timp-3(-/-)-AB myocardium. Timp-3(-/-)/tnfalpha(-/-) mice were generated and subjected to AB for comparative analyses with timp-3(-/-)-AB mice. This revealed the critical role of TNFalpha in the early phase of LV remodeling, de novo expression of Matrix metalloproteinases (MMP)-8 in the absence of TNFalpha, and highlighted the importance of interstitial collagenases (MMP-2, MMP-13, and MT1-MMP) for cardiac **ECM** degradation. Ablation of TNFalpha, or limiting MMP activity with a synthetic MMP inhibitor (PD166793), each partially attenuated LV dilation and cardiac dysfunction in timp-3(-/-)-AB mice. Notably, combining TNFalpha ablation with MMP inhibition completely rescued heart disease in timp-3(-/-)-AB mice. This study provides a basis for anti-TNFalpha and MMP inhibitor combination therapy in heart disease.
21. Loss of TIMP3 selectively exacerbates diabetic nephropathy. Diabetic nephropathy is the most common cause of end-stage renal disease. Polymorphism in the tissue inhibitor of metalloproteinase-3 (TIMP3) gene, and the **ECM**-bound inhibitor of matrix metalloproteinases (MMPs), has been linked to diabetic nephropathy in humans. To elucidate the mechanism, we generated double mutant mice in which the TIMP3 gene was deleted in the genetic diabetic Akita mouse background. The aggravation of diabetic injury occurred in the absence of worsening of hypertension or hyperglycemia. In fact, myocardial TIMP3 levels were not affected in Akita hearts, and cardiac diastolic and systolic function remained unchanged in the double mutant mice. However, TIMP3 levels increased in Akita kidneys and deletion of TIMP3 exacerbated the diabetic renal injury in the Akita mouse, characterized by increased albuminuria, mesangial matrix expansion, and kidney hypertrophy. The progression of diabetic renal injury was accompanied by the upregulation of fibrotic and inflammatory markers, increased production of reactive oxygen species and NADPH oxidase activity, and elevated activity of TNF-α-converting enzyme (TACE) in the TIMP3(-/-)/Akita kidneys. Moreover, while the elevated phospho-Akt (S473 and T308) and phospho-ERK1/2 in the Akita mice was not detected in the TIMP3(-/-)/Akita kidneys, PKCβ1 (but not PKCα) was markedly elevated in the double mutant kidneys. Our data provide definitive evidence for a critical and selective role of TIMP3 in diabetic renal injury consistent with gene expression findings from human diabetic kidneys.
22. Regulation of TIMP3 in diabetic nephropathy: a role for microRNAs. Diabetic nephropathy (DN) is the major cause of chronic kidney disease in developed countries and contributes significantly to increased morbidity and mortality among diabetic patients. Morphologically, DN is characterized by tubulo-interstitial fibrosis, thickening of the glomerular basement membrane and mesangial expansion mainly due to accumulation of extracellular matrix (**ECM**). **ECM** turnover is regulated by metalloproteinases and tissue inhibitors of metalloproteinases (TIMPs) activities. In diabetic conditions, TIMP3 expression in kidney is strongly reduced, but the causes of this reduction are still unknown. The aim of this study was to elucidate at least one of these mechanisms which relies on differential expression of TIMP3-targeting microRNAs (miRs) in a hyperglycemic environment either in vitro (MES13 cell line) or in vivo (mouse kidney and human biopsies). Among the TIMP3-targeting miRs, miR-21 and miR-221 were significantly upregulated in kidneys from diabetic mice compared to control littermates, and in a mesangial cell line grown in high glucose conditions. In human samples, only miR-21 expression was increased in kidney biopsies from diabetic patients compared to healthy controls. The expression of miR-217, which targets TIMP3 indirectly through downregulation of SirT1, was also increased in diabetic kidney and MES13 cell line. In agreement with these result, SirT1 expression was reduced in mouse and human diabetic kidneys as well as in MES13 mesangial cell line. TIMP3 deficiency has recently emerged as a hallmark of DN in mouse and human. In this study, we demonstrated that this reduction is due, at least in part, to increased expression of certain TIMP3-targeting miRs in diabetic kidneys compared to healthy controls. Unveiling the post-transcriptional mechanisms responsible for TIMP3 downregulation in hyperglycemic conditions may orient toward the use of this protein as a possible therapeutic target in DN.
23. Tissue inhibitor of metalloproteinases-3 (TIMP-3) is an extracellular matrix-associated protein with a distinctive pattern of expression in mouse cells and tissues. We have isolated cDNA clones corresponding to a novel mouse metalloproteinase inhibitor. Five overlapping cDNA clones contain most of the information for a prominent 4.5-kilobase transcript that was detected in RNA from mouse fibroblasts and adult tissues. Sequence analysis revealed an open reading frame (ORF) for a protein of 212 amino acids that is 80% identical to chicken inhibitor of metalloproteinases-3 (ChIMP-3). The 3'-untranslated sequence also showed remarkable conservation with the chicken gene. The ORF directed the expression of a 24-kDa protein in COS-1 cells that localized to the extracellular matrix (**ECM**). On the basis of these similarities we propose to identify the new gene as murine tissue inhibitor of metalloproteinases-3 (TIMP-3). Mouse C3H 10T1/2 fibroblasts produced a 24-kDa metalloproteinase inhibitor that also localized to the **ECM** and was recognized by a polyclonal antibody to ChIMP-3. Like TIMP-1, TIMP-3 was highly inducible in mouse C3H 10T1/2 fibroblasts by phorbol ester (PMA), epidermal growth factor (EGF), and transforming growth factor-beta 1, but nuclear run-on assays showed that the on/off transcription kinetics were faster for TIMP-3 than TIMP-1. A major difference in vitro was the stimulation of expression of TIMP-3 by dexamethasone which inhibits EGF- and PMA-induced TIMP-1 transcription. Also, TIMP-3 showed a distinctive pattern of expression in adult tissues with abundant transcripts detected in kidney, lung, and brain but only low levels detected in bone, a prominent location of TIMP-1 transcripts. We propose that TIMP-3 functions in a tissue-specific fashion as part of an acute response to remodeling stimuli.

### [Ee]xtracellular [Mm]atrix

#### PubMed

1. **Extracellular matrix** secretion by cardiac fibroblasts: role of microRNA-29b and microRNA-30c. MicroRNAs (miRNAs), in particular miR-29b and miR-30c, have been implicated as important regulators of cardiac fibrosis.To perform a proteomics comparison of miRNA effects on **extracellular matrix** secretion by cardiac fibroblasts.Mouse cardiac fibroblasts were transfected with pre-/anti-miR of miR-29b and miR-30c, and their conditioned medium was analyzed by mass spectrometry. miR-29b targeted a cadre of proteins involved in fibrosis, including multiple collagens, matrix metalloproteinases, and leukemia inhibitory factor, insulin-like growth factor 1, and pentraxin 3, 3 predicted targets of miR-29b. miR-29b also attenuated the cardiac fibroblast response to transforming growth factor-β. In contrast, miR-30c had little effect on **extracellular matri**x production but opposite effects regarding leukemia inhibitory factor and insulin-like growth factor 1. Both miRNAs indirectly affected cardiac myocytes. On transfection with pre-miR-29b, the conditioned medium of cardiac fibroblasts lost its ability to support adhesion of rat ventricular myocytes and led to a significant reduction of cardiac myocyte proteins (α-actinin, cardiac myosin-binding protein C, and cardiac troponin I). Similarly, cardiomyocytes derived from mouse embryonic stem cells atrophied under pre-miR-29 conditioned medium, whereas pre-miR-30c conditioned medium had a prohypertrophic effect. Levels of miR-29a, miR-29c, and miR-30c, but not miR-29b, were significantly reduced in a mouse model of pathological but not physiological hypertrophy. Treatment with antagomiRs to miR-29b induced excess fibrosis after aortic constriction without overt deterioration in cardiac function.Our proteomic analysis revealed novel molecular targets of miRNAs that are linked to a fibrogenic cardiac phenotype. Such comprehensive screening methods are essential to define the concerted actions of miRNAs in cardiovascular disease.
2. TIMP3 is the primary TIMP to regulate agonist-induced vascular remodelling and hypertension. Hypertension is accompanied by structural remodelling of vascular **extracellular matrix** (ECM). Tissue inhibitor of metalloproteinases (TIMPs) inhibits matrix metalloproteinases (MMPs) that degrade the matrix structural proteins. In response to a hypertensive stimulus, the balance between MMPs and TIMPs is altered. We examined the role of TIMPs in agonist-induced hypertension.We subjected TIMP-knockout mice to angiotensin II (Ang II) infusion, and found that Ang-II-induced hypertension in TIMP1(-/-), TIMP2(-/-), and TIMP4(-/-) mice was comparable to wild-type (WT) mice, but significantly suppressed in TIMP3(-/-) mice. Ex vivo pressure myography analyses on carotid and mesenteric arteries revealed that Ang-II-infused TIMP3(-/-) arteries were more distensible with impaired elastic recoil compared with the WT group. The acute response to vasoconstriction and vasodilation was intact in TIMP3(-/-) mesenteric and carotid arteries. Mesenteric arteries from TIMP3(-/-)-Ang II mice exhibited a reduced media-to-lumen ratio, suppressed collagen and elastin levels, elevated elastase and gelatinase proteolytic activities compared with WT-Ang II. TIMP3(-/-)-Ang II carotid arteries also showed adverse structural remodelling. Treatment of mice with doxycycline, a matrix metalloproteinase inhibitor, improved matrix integrity in mesenteric and carotid arteries in TIMP3(-/-)-Ang II and differentially regulated elastin and collagen levels in WT-Ang II vs. TIMP3(-/-)-Ang II.Our study demonstrates a critical role for TIMP3, among all TIMPs, is preserving arterial ECM in response to Ang II. It is critical to acknowledge that the suppressed Ang-II-induced hypertension in TIMP3(-/-) mice is not a protective mechanism but owing to adverse remodelling in arterial matrix.
3. Scleraxis is required for cell lineage differentiation and **extracellular matrix** remodeling during murine heart valve formation in vivo. Heart valve structures, derived from mesenchyme precursor cells, are composed of differentiated cell types and **extracellular matrix** arranged to facilitate valve function. Scleraxis (scx) is a transcription factor required for tendon cell differentiation and matrix organization. This study identified high levels of scx expression in remodeling heart valve structures at embryonic day 15.5 through postnatal stages using scx-GFP reporter mice and determined the in vivo function using mice null for scx. Scx(-/-) mice display significantly thickened heart valve structures from embryonic day 17.5, and valves from mutant mice show alterations in valve precursor cell differentiation and matrix organization. This is indicated by decreased expression of the tendon-related collagen type XIV, increased expression of cartilage-associated genes including sox9, as well as persistent expression of mesenchyme cell markers including msx1 and snai1. In addition, ultrastructure analysis reveals disarray of **extracellular matrix** and collagen fiber organization within the valve leaflet. Thickened valve structures and increased expression of matrix remodeling genes characteristic of human heart valve disease are observed in juvenile scx(-/-) mice. In addition, excessive collagen deposition in annular structures within the atrioventricular junction is observed. Collectively, our studies have identified an in vivo requirement for scx during valvulogenesis and demonstrate its role in cell lineage differentiation and matrix distribution in remodeling valve structures.
4. The role of TIMPs in regulation of **extracellular matrix** proteolysis. Tissue inhibitors of metalloproteinases (TIMPs), which inhibit matrix metalloproteinases (MMPs) as well as the closely related, a disintegrin and metalloproteinases (ADAMs) and ADAMs with thrombospondin motifs (ADAMTSs), were traditionally thought to control **extracellular matrix** (ECM) proteolysis through direct inhibition of MMP-dependent ECM proteolysis. This classical role for TIMPs suggests that increased TIMP levels results in ECM accumulation (or fibrosis), whereas loss of TIMPs leads to enhanced matrix proteolysis. Mice lacking TIMP family members have provided support for such a role; however, studies with these TIMP deficient mice have also demonstrated that loss of TIMPs can often be associated with an accumulation of ECM. Collectively, these studies suggest that the divergent roles of TIMPs in matrix accumulation and proteolysis, which together can be referred to as ECM turnover, are dependent on the TIMP, specific tissue, and local tissue environment (i.e. health vs. injury/disease). Ultimately, these combined factors dictate the specific metalloproteinases being regulated by a given TIMP, and it is likely the diversity of metalloproteinases and their physiological substrates that determines whether TIMPs inhibit matrix proteolysis or accumulation. In this review, we discuss the evidence for the dichotomous roles of TIMPs in ECM turnover highlighting some of the common findings between different TIMP family members. Importantly, while we now have a better understanding of the role of TIMPs in regulating ECM turnover, much remains to be determined. Data on the specific metalloproteinases inhibited by different TIMPs in vivo remains limited and must be the focus of future studies.
5. Loss of Timp3 gene leads to abdominal aortic aneurysm formation in response to angiotensin II. Aortic aneurysm is dilation of the aorta primarily due to degradation of the aortic wall **extracellular matrix** (ECM). Tissue inhibitors of metalloproteinases (TIMPs) inhibit matrix metalloproteinases (MMPs), the proteases that degrade the ECM. Timp3 is the only ECM-bound Timp, and its levels are altered in the aorta from patients with abdominal aortic aneurysm (AAA). We investigated the causal role of Timp3 in AAA formation. Infusion of angiotensin II (Ang II) using micro-osmotic (Alzet) pumps in Timp3(-/-) male mice, but not in wild type control mice, led to adverse remodeling of the abdominal aorta, reduced collagen and elastin proteins but not mRNA, and elevated proteolytic activities, suggesting excess protein degradation within 2 weeks that led to formation of AAA by 4 weeks. Intriguingly, despite early up-regulation of MMP2 in Timp3(-/-)Ang II aortas, additional deletion of Mmp2 in these mice (Timp3(-/-)/Mmp2(-/-)) resulted in exacerbated AAA, compromised survival due to aortic rupture, and inflammation in the abdominal aorta. Reconstitution of WT bone marrow in Timp3(-/-)/Mmp2(-/-) mice reduced inflammation and prevented AAA in these animals following Ang II infusion. Treatment with a broad spectrum MMP inhibitor (PD166793) prevented the Ang II-induced AAA in Timp3(-/-) and Timp3(-/-)/Mmp2(-/-) mice. Our study demonstrates that the regulatory function of TIMP3 is critical in preventing adverse vascular remodeling and AAA. Hence, replenishing TIMP3, a physiological inhibitor of a number of metalloproteinases, could serve as a therapeutic approach in limiting AAA development or expansion.
6. TGF-beta3-induced palatogenesis requires matrix metalloproteinases. Cleft lip and palate syndromes are among the most common congenital malformations in humans. Mammalian palatogenesis is a complex process involving highly regulated interactions between epithelial and mesenchymal cells of the palate to permit correct positioning of the palatal shelves, the remodeling of the **extracellular matrix** (ECM), and subsequent fusion of the palatal shelves. Here we show that several matrix metalloproteinases (MMPs), including a cell membrane-associated MMP (MT1-MMP) and tissue inhibitor of metalloproteinase-2 (TIMP-2) were highly expressed by the medial edge epithelium (MEE). MMP-13 was expressed both in MEE and in adjacent mesenchyme, whereas gelatinase A (MMP-2) was expressed by mesenchymal cells neighboring the MEE. Transforming growth factor (TGF)-beta3-deficient mice, which suffer from clefting of the secondary palate, showed complete absence of TIMP-2 in the midline and expressed significantly lower levels of MMP-13 and slightly reduced levels of MMP-2. In concordance with these findings, MMP-13 expression was strongly induced by TGF-beta3 in palatal fibroblasts. Finally, palatal shelves from prefusion wild-type mouse embryos cultured in the presence of a synthetic inhibitor of MMPs or excess of TIMP-2 failed to fuse and MEE cells did not transdifferentiate, phenocopying the defect of the TGF-beta3-deficient mice. Our observations indicate for the first time that the proteolytic degradation of the ECM by MMPs is a necessary step for palatal fusion.
7. A null mutation for tissue inhibitor of metalloproteinases-3 (Timp-3) impairs murine bronchiole branching morphogenesis. Tissue inhibitors of metalloproteinases (TIMPs) regulate **extracellular matrix** (ECM) degradation by matrix metalloproteinases (MMPs). We have examined the role of TIMP-3 on ECM homeostasis and bronchiole branching morphogenesis during murine embryogenesis. Employing an in vitro organ culture system, we found decreased bronchiolar branching in null lungs when compared with wild type (WT) counterparts after 2 days in culture. When a synthetic inhibitor of MMPs at low dose was added to the culture system, branching was augmented regardless of genotype. Gelatin and in situ zymography revealed that null lungs exhibited enhanced activation of MMPs throughout lung development. We analysed the impact of increased MMP activity on a number of ECM molecules by Western blot analysis, but found that only fibronectin abundance was consistently reduced in the null lungs throughout development. To confirm that our observed defect in culture was not simply a developmental delay in the null lung, we examined null and WT lungs from newborn pups. Here, we found not only a reduced number of bronchioles in the null, but also that the bronchiole tubes were dilated compared with controls and that alveologenesis was attenuated. We propose that the deletion of TIMP-3 disrupts the exquisite TIMP/MMP balance required for proper focal ECM proteolysis, which leads to correct bronchiole branching morphogenesis in the developing mouse lung.
8. Tissue inhibitor of metalloproteinases 3 regulates **extracellular matrix**--cell signaling during bronchiole branching morphogenesis. Tissue inhibitors of metalloproteinases (TIMPs) regulate **extracellular matrix** (ECM) degradation by matrix metalloproteinases (MMPs) throughout embryogenesis. We examined lungs from TIMP3 null mice and found decreased bronchiole branching, enhanced activity of MMPs and enhanced fibronectin degradation throughout lung development compared to controls. Activation of focal adhesion kinase (FAK) was also reduced from embryonic days 12.5 through 14.5 in TIMP3 null lungs. Treatment with a synthetic MMP inhibitor, GM6001, in utero enhanced the branching pattern in both wild type and null lungs accompanied by a restoration of fibronectin localization, signaling through FAK and epithelial cell proliferation in null lungs. Direct down-regulation of FAK abundance in WT lung organ culture by siRNA targeting resulted in reduced bronchiole branching, phenocopying the TIMP3 defect. We propose that enhanced MMP activity in the absence of TIMP3 interferes with focal ECM proteolysis, perturbing the intracellular signaling necessary for correct pattern formation of the bronchiole tree during bronchiole branching morphogenesis. Thus, TIMP3 can indirectly regulate epithelial cell proliferation via MMP inhibitory activity. While others have demonstrated this function for MMPs, and there is in vitro evidence that TIMP3 controls proliferation, to our knowledge this is the first evidence of TIMP3 regulating proliferation in vivo.
9. Tissue inhibitor of metalloproteinase-3 knockout mice exhibit enhanced energy expenditure through thermogenesis. Tissue inhibitors of metalloproteinases (TIMPs) regulate matrix metalloproteinase activity and maintain **extracellular matrix** homeostasis. Although TIMP-3 has multiple functions (e.g., apoptosis, inhibition of VEGF binding to VEGF receptor, and inhibition of TNFα converting enzyme), its roles in thermogenesis and metabolism, which influence energy expenditure and can lead to the development of metabolic disorders when dysregulated, are poorly understood. This study aimed to determine whether TIMP-3 is implicated in metabolism by analyzing TIMP-3 knockout (KO) mice. TIMP-3 KO mice had higher body temperature, oxygen consumption, and carbon dioxide production than wild-type (WT) mice, although there were no differences in food intake and locomotor activity. These results suggest that metabolism is enhanced in TIMP-3 KO mice. Real-time PCR analysis showed that the expression of PPAR-δ, UCP-2, NRF-1 and NRF-2 in soleus muscle, and PGC-1α and UCP-2 in gastrocnemius muscle, was higher in TIMP-3 KO mice than in WT mice, suggesting that TIMP-3 deficiency may increase mitochondrial activity. When exposed to cold for 8 hours to induce thermogenesis, TIMP-3 KO mice had a higher body temperature than WT mice. In the treadmill test, oxygen consumption and carbon dioxide production were higher in TIMP-3 KO mice both before and after starting exercise, and the difference was more pronounced after starting exercise. Our findings suggest that TIMP-3 KO mice exhibit enhanced metabolism, as reflected by a higher body temperature than WT mice, possibly due to increased mitochondrial activity. Given that TIMP-3 deficiency increases energy expenditure, TIMP-3 may present a novel therapeutic target for preventing metabolic disorders.
10. Loss of the Timp gene family is sufficient for the acquisition of the CAF-like cell state. Cancer-associated fibroblasts (CAFs) drive tumour progression, but the emergence of this cell state is poorly understood. A broad spectrum of metalloproteinases, controlled by the Timp gene family, influence the tumour microenvironment in human cancers. Here, we generate quadruple TIMP knockout (TIMPless) fibroblasts to unleash metalloproteinase activity within the tumour-stromal compartment and show that complete Timp loss is sufficient for the acquisition of hallmark CAF functions. Exosomes produced by TIMPless fibroblasts induce cancer cell motility and cancer stem cell markers. The proteome of these exosomes is enriched in **extracellular matrix** proteins and the metalloproteinase ADAM10. Exosomal ADAM10 increases aldehyde dehydrogenase expression in breast cancer cells through Notch receptor activation and enhances motility through the GTPase RhoA. Moreover, ADAM10 knockdown in TIMPless fibroblasts abrogates their CAF function. Importantly, human CAFs secrete ADAM10-rich exosomes that promote cell motility and activate RhoA and Notch signalling in cancer cells. Thus, Timps suppress cancer stroma where activated-fibroblast-secreted exosomes impact tumour progression.
11. Murine tissue inhibitor of metalloproteinases-4 (Timp-4): cDNA isolation and expression in adult mouse tissues. We have isolated cDNA clones corresponding to a new member of the murine tissue inhibitor of metalloproteinase (TIMP) family, designated Timp-4. The nucleotide sequence predicts a protein of 22,609 Da that contains the characteristic 12 cysteine TIMP signature. TIMP-4 is more closely related to TIMP-2 and TIMP-3 than to TIMP-1 (48%, 45% and 38% identity, respectively). Analysis of Timp-4 mRNA expression in adult mouse tissues indicated a 1.2 kb transcript in brain, heart, ovary and skeletal muscle. This pattern of expression distinguishes Timp-4 from other Timps, suggesting that the TIMP-4 protein may be an important tissue-specific regulator of **extracellular matrix** remodelling.
12. Down-regulation of tissue inhibitor of metalloproteinase-3 (TIMP-3) expression is necessary for adipocyte differentiation. Matrix metalloproteinase activity is essential for proper **extracellular matrix** remodeling that takes place during adipose tissue formation. Four tissue inhibitors of matrix metalloproteinases (TIMPs) regulate their activity. However, the role of TIMPs in adipocyte differentiation is poorly understood. We found that the expression of all TIMPs was modified during adipocyte differentiation, but that of TIMP-3 was distinguished by its extreme down-regulation. TIMP-3 expression was closely linked to the differentiation process. Indeed, it remained low during the adipocyte differentiation but increased when cell differentiation was prevented. We identified the transcription factor Sp1 as being responsible for the regulation of TIMP-3 expression during adipocyte differentiation. Overexpression of TIMP-3 reduced adipocyte differentiation, underlining its active role in this process. TIMP-3 overexpression decreased the expression of the early and obligate key inductors of adipogenesis Krüppel-like factor 4 (Klf4), early growth response 2 (Egr2/Krox20), and CAAT/enhancer-binding protein beta (C/EBPbeta). Our results indicate that during preadipocyte differentiation, the Sp1-dependent decrease in TIMP-3 expression is required for the successful implementation of the adipocyte differentiation program.
13. Differential regulation of TIMP-1, -2, and -3 mRNA and protein expressions during mouse incisor development. Tissue inhibitors of metalloproteinases (TIMPs) possess multiple functions, in addition to their matrix metalloproteinase (MMP) inhibitory activity. The continuously growing incisor of mouse possesses a stem cell compartment at the apical end of the epithelium (the apical loop) and thus provides an excellent tool to analyze the mechanisms of organogenesis and cytodifferentiation. To understand the functions of TIMPs in tooth development, we have analyzed the gene expression and protein localization of TIMP-1, -2, and -3 during mouse incisor development, from embryonic day 13 (E13) to postnatal day 3 (P3). TIMP-1 was present on the basement membrane during early developmental stages. At P2, TIMP-1 was strongly detected along the apical loop, transiently disappeared from the basement membrane in the cytodifferentiation zone, and later reappeared at the distal end of functional ameloblasts. Expression of TIMP-2 protein was restricted to the outer part of the apical loop throughout the examined stages. At P2, TIMP-2 was present on the basement membrane at the outer part of the apical loop. The dental follicle also expressed Timp-2, and the corresponding protein was abundant within the **extracellular matrix**. Timp-3 mRNA was highly expressed in the mesenchyme surrounding the apical loop. During matrix formation, Timp-3 was expressed by subodontoblasts, and the protein was detected in this layer and between odontoblasts. Distinct temporal and spatial expression patterns of TIMPs suggest divergent functions of these factors in incisor organogenesis.
14. Absence of tissue inhibitor of metalloproteinases 3 disrupts alveologenesis in the mouse. Tissue inhibitors of metalloproteinases (TIMPs) regulate **extracellular matrix** (ECM) degradation by matrix metalloproteinases (MMPs) throughout lung development. We examined lungs from TIMP3 null mice and found significant air space enlargement compared with wild type (WT) animals during a time course spanning early alveologenesis (post-partum days 1, 5, 9 and 14). Trichrome staining revealed a similar pattern of collagen distribution in the walls of nascent alveoli; however, the alveolar walls of TIMP3 mutant mice appeared to be thinner than controls. Assessment of MMP2 and MMP9 activities by gelatin zymography demonstrated a significant elevation in the active form of MMP2 at post-partum days 1 and 5. Treatment of null pregnant dams with a broad spectrum synthetic metalloproteinase inhibitor, GM6001, on embryonic day 16.5 enhanced the formation of primitive alveoli during the saccular stage of lung development as evidenced by a partial, but significant, rescue of alveolar size in post-partum day 1 animals. We propose that increased MMP activity in the absence of TIMP3 enhances ECM proteolysis, upsetting proper formation of primitive alveolar septa during the saccular stage of alveologenesis. Therefore, TIMP3 indirectly regulates alveolar formation in the mouse. To our knowledge, ours is the first study to demonstrate that in utero manipulation of the TIMP/MMP proteolytic axis, to specifically inhibit proteolysis, significantly affects lung development.
15. Increased neovascularization in mice lacking tissue inhibitor of metalloproteinases-3. Tissue inhibitor of metalloproteinases-3 (TIMP-3) is a matrix-bound inhibitor of matrix metalloproteinases (MMPs). The authors have previously determined a novel function of TIMP-3 to inhibit vascular endothelial growth factor (VEGF)-mediated angiogenesis. Here, the authors examined the in vivo angiogenic phenotype of ocular vessels in mice deficient in TIMP-3.VEGF-mediated corneal neovascularization and laser-induced choroidal neovascularization (CNV) were examined in TIMP-3-null mice. The effects of the absence of TIMP-3 on the phosphorylation status of the VEGF-receptor-2 (VEGFR-2) and the downstream signaling pathways were evaluated biochemically. In addition, the activation state of MMPs in the retina of TIMP-3-deficient mice was examined by in situ zymography.The results of these studies determine an accentuation of pathologic VEGF-mediated angiogenesis in the cornea and laser-induced CNV in mice lacking TIMP-3. In the absence of the MMP inhibitor, pathophysiological changes were observed in the choroidal vasculature concomitantly with an increase in gelatinolytic activity. These results suggest that an imbalance of **extracellular matrix** homeostasis, together with a loss of an angiogenesis inhibitor, can prime vascular beds to be more responsive to an angiogenic stimulus.In light of the recent studies suggesting that genetic variants near TIMP-3 influence susceptibility to age-related macular degeneration, these results imply that TIMP-3 may regulate the development of the choroidal vasculature and is a likely contributor to increased susceptibility to choroidal neovascularization.
16. Abnormal vessel formation in the choroid of mice lacking tissue inhibitor of metalloprotease-3. Tissue inhibitor of metalloprotease (TIMP)-3 is an inhibitor of matrix metalloprotease (MMP) and regulates angiogenesis. In the eye, TIMP3 is tightly associated with Bruch's membrane. In this study, the authors analyzed mice lacking TIMP3 for retinal abnormalities.Mice with targeted disruption of the Timp3 gene were generated (Timp3(-/-)) and bred into C57/Bl6 and CD1 backgrounds. Eyes were analyzed by light and electron microscopy. Vasculature was examined by scanning laser ophthalmoscopy, corrosion casts, and whole mount preparations. MMP activity was assessed by in situ zymography, angiogenic potential was evaluated by tube formation, and aortic ring assays and signaling pathways were studied by immunoblotting.TIMP3-deficient mice develop abnormal vessels with dilated capillaries throughout the choroid. Enhanced MMP activity in the choroid region of Timp3(-/-) eyes was detected when compared with controls. Timp3(-/-)-derived tissue showed an increased angiogenic activity over wild-type, an effect that could specifically be inhibited by recombinant TIMP3. Moreover, the antiangiogenic property of TIMP3 was demonstrated to reside within the C-terminal domain. When VEGFR2 inhibitor was added to Timp3(-/-) aortic explants, endothelial sprout formation was markedly reduced, which provided evidence for an unbalanced VEGF-mediated angiogenesis in Timp3(-/-) animals. Finally, angiogenic signaling pathways are activated in Timp3(-/-)-derived cells.These findings suggest that the distinct choroidal phenotype in mice lacking TIMP3 may be the result of a local disruption of **extracellular matrix** and angiogenic homeostasis, and they support an important role of TIMP3 in the regulation of choroidal vascularization.
17. Tissue inhibitor of metalloproteinase-3 plays important roles in the kidney following unilateral ureteral obstruction. Tissue inhibitor of metalloproteinase-3 (Timp-3), an inhibitor of matrix-degrading enzymes, is an important molecule for maintenance of the **extracellular matrix**. In this study, we generated Timp-3-deficient mice and used them to examine the effect of Timp-3-deficiency on blood pressure and to investigate the role of Timp-3 in the kidney following unilateral ureteral obstruction. The blood pressure and heart rate of Timp-3-deficient mice were not significantly different from those of wild-type mice. On the other hand, the obstructed kidneys of Timp-3-deficient mice developed more severe hydronephrosis than those of wild-type animals. Matrix metalloproteinase activities assessed by in situ zymography and transforming growth factor-beta expression were elevated in Timp-3-deficient mice. The renal tissues were thinner and the ratio of renal medulla to cortex was significantly lower in the obstructed Timp-3-deficient kidneys. These findings indicate that Timp-3-deficiency does not substantially affect the blood pressure in mice, and that Timp-3 plays an important role in the maintenance of renal macrostructure after unilateral ureteral obstruction.
18. Temporospatial distribution of matrix metalloproteinase and tissue inhibitors of matrix metalloproteinases during murine secondary palate morphogenesis. **Extracellular matrix** (ECM) molecules are known to play a pivotal role in the morphogenesis of the secondary palate. The maintenance and degradation of the ECM is mediated in part by the matrix metalloproteinases (MMPs) and their endogenous inhibitors TIMPs. MMPs and TIMPs have previously been shown to be developmentally regulated within the palatal shelf during secondary palate morphogenesis. This study was conducted to examine the temporospatial distribution of these enzymes and their inhibitors within the palatal shelves using immunofluorescent localization to determine if specific changes occur in their distribution concomitant with events in palatal shelf formation and reorientation. Frontal sections through the posterior palatal shelves at gestational day (gd) 12, 13 and 14 were immunofluorescently stained for MMPs 2, 3, 9, and 13 and TIMPs 1, 2, and 3 using standard protocols and commercially available antibodies. The results demonstrated that MMPs and TIMPs were already present within the palatal shelf mesenchyme 30 h prior to reorientation and closure and that their expression within the shelf mesenchyme increased as the shelves remodeled, then decreased with closure and fusion. Increased distribution of MMPs and TIMPs within specific regions of the palatal mesenchyme and palatal epithelial basement membrane preceded decreases previously observed within these areas for their substrates, fibronectin, collagen III and collagen I. In addition, MMP-3 and TIMP-3 were immunolocalized to regions of the palatal epithelium that undergo reorganization concomitant with reorientation. The results of this study indicate that MMPs and TIMPs are developmentally regulated during palatal shelf morphogenesis and that their distribution correlates with the distribution of the ECM components of the palatal shelf they regulate. These results provide support for the idea that temporospatially controlled interactions between MMPs and their substrates may be pivotal in modulating events in palatal morphogenesis.
19. Association of TIMP-2 with **extracellular matrix** exposed to mechanical stress and its co-distribution with periostin during mouse mandible development. Matrix remodeling is regulated by matrix metalloproteinases (MMPs) and tissue inhibitors of metalloproteinases (TIMPs). Periostin, originally identified in a mouse osteoblastic library, plays a role in cell adhesion and migration and in mechanical stress-induced matrix remodeling. In this study, we analyzed and compared the distribution patterns of TIMP-2 and periostin during mouse mandible development. Immunohistochemical staining for TIMP-2 and periostin was carried out on serial cryosections obtained from mice at embryonic days 13-16, postnatal day 2 (P2), P35, and 12 weeks of age. TIMP-2 and periostin exhibited a strikingly similar protein distribution during mandible development. From bud to early bell stages of molars, TIMP-2 and periostin were highly expressed on the lingual and anterior sides of the basement membrane and on the adjacent jaw mesenchyme. In pre- and postnatal incisors, the basement membrane of the apical loop and dental follicle was immunostained for TIMP-2 and periostin. At postnatal stages, TIMP-2 and periostin were prominently confined to the **extracellular matrix** (ECM) of gingival tissues, periodontal ligaments, and tendons (all recipients of mechanical strain). However, periostin was solely detected in the lower portion of the inner root sheath of hair follicles. Gingiva of P2 cultured in anti-TIMP-2 antibody-conditioned medium showed markedly reduced staining of periostin. We suggest that TIMP-2 and periostin are co-distributed on ECM exposed to mechanical forces and coordinately function as ECM modulators.
20. Regulation of tissue inhibitor of metalloproteinases-3 gene expression by transforming growth factor-beta and dexamethasone in bovine and human articular chondrocytes. Physiological and pathological degradation of cartilage **extracellular matrix** (ECM) is regulated by the balance between tissue inhibitors of metalloproteinases (TIMPs) and matrix metalloproteinases (MMPs). We examined the potential of chondrocytes from normal bovine or human osteoarthritic (OA) cartilage to express RNA for the new inhibitor TIMP-3 and studied its regulation by an inducer of matrix synthesis, transforming growth factor-beta (TGF-beta). Freshly released chondrocytes constitutively expressed three transcripts of TIMP-3 that are induced by serum factors. In primary cultures of chondrocytes, one of these factors, TGF-beta, increased TIMP-3 mRNA in a dose-dependent fashion that required de novo protein synthesis and transcription. TGF-beta did not alter stability of the TIMP-3 transcripts in RNA decay time-courses, suggesting a transcriptional control. Nuclear run-on assays confirmed increased rate of TIMP-3 gene transcription by TGF-beta. An antiinflammatory glucocorticoid, dexamethasone, inhibited the basal, and suppressed partially the TGF-beta-inducible, TIMP-3 expression in primary bovine and human chondrocytes. DNA sequencing of bovine TIMP-3 cDNA revealed an open reading frame of a 211-amino-acid protein containing signal peptide and 12 conserved cysteines. The encoded protein differed from human TIMP-3 at four positions. The constitutive expression and evolutionary conservation of TIMP-3 imply its important function. TIMP-3 induction by TGF-beta suggests the role of this factor and TIMP-3 in cartilage remodeling with important implications for arthritis.
21. Biglycan and decorin differentially regulate signaling in the fetal membranes. Preterm birth is the leading cause of newborn mortality in the United States and about one third of cases are caused by preterm premature rupture of fetal membranes, a complication that is frequently observed in patients with Ehlers-Danlos Syndrome. Notably, a subtype of Ehlers-Danlos Syndrome is caused by expression of abnormal biglycan and decorin proteoglycans. As compound deficiency of these two small leucine-rich proteoglycans is a model of preterm birth, we investigated the fetal membranes of Bgn(-/-); Dcn(-/-) double-null and single-null mice. Our results showed that biglycan signaling supported fetal membrane remodeling during early gestation in the absence of concomitant changes in TGFβ levels. In late gestation, biglycan signaling acted in a TGFβ-dependent manner to aid in membrane stabilization. In contrast, decorin signaling supported fetal membrane remodeling at early stages of gestation in a TGFβ-dependent manner, and fetal membrane stabilization at later stages of gestation without changes in TGFβ levels. Furthermore, exogenous soluble decorin was capable of rescuing the TGFβ signaling pathway in fetal membrane mesenchymal cells. Collectively, these findings provide novel targets for manipulation of fetal memb**rane extracellular m**atrix stability and could represent novel targets for research on preventive strategies for preterm premature rupture of fetal membranes.
22. Reducing Timp3 or vitronectin ameliorates disease manifestations in CADASIL mice. CADASIL is a genetic paradigm of cerebral small vessel disease caused by NOTCH3 mutations that stereotypically lead to the extracellular deposition of NOTCH3 ectodomain (Notch3(ECD) ) on the vessels. TIMP3 and vitronectin are 2 **extracellular matrix** proteins that abnormally accumulate in Notch3(ECD) -containing deposits on brain vessels of mice and patients with CADASIL. Herein, we investigated whether increased levels of TIMP3 and vitronectin are responsible for aspects of CADASIL disease phenotypes.Timp3 and vitronectin expression were genetically reduced in TgNotch3(R169C) mice, a well-established preclinical model of CADASIL. A mouse overexpressing human TIMP3 (TgBAC-TIMP3) was developed. Disease-related phenotypes, including cerebral blood flow (CBF) deficits, white matter lesions, and Notch3(ECD) deposition, were evaluated between 6 and 20 months of age.CBF responses to neural activity (functional hyperemia), topical application of vasodilators, and decreases in blood pressure (CBF autoregulation) were similarly reduced in TgNotch3(R169C) and TgBAC-TIMP3 mice, and myogenic responses of brain arteries were likewise attenuated. These defects were rescued in TgNotch3(R169C) mice by haploinsufficiency of Timp3, although the number of white matter lesions was unaffected. In contrast, haploinsufficiency or loss of vitronectin in TgNotch3(R169C) mice ameliorated white matter lesions, although CBF responses were unchanged. Amelioration of cerebrovascular reactivity or white matter lesions in these mice was not associated with reduced Notch3(ECD) deposition in brain vessels.Elevated levels of TIMP3 and vitronectin, acting downstream of Notch3(ECD) deposition, play a role in CADASIL, producing divergent influences on early CBF deficits and later white matter lesions.
23. Timp-3 deficiency impairs cognitive function in mice. **Extracellular matrix** (ECM) degradation is performed primarily by matrix metalloproteinases (MMPs). MMPs have recently been shown to regulate synaptic activity in the hippocampus and to affect memory and learning. The tissue inhibitor of metalloproteinase (Timp) is an endogenous factor that controls MMP activity by binding to the catalytic site of MMPs. At present, four Timp isotypes have been reported (Timp-1 through Timp-4) with 35-50% amino-acid sequence homology. Timp-3 is a unique member of Timp proteins in that it is bound to the ECM. In this study, we used the passive avoidance test, active avoidance test, and water maze test to examine the cognitive function in Timp-3 knockout (KO) mice. Habituation was evaluated using the open-field test. The water maze test showed that Timp-3 KO mice exhibit deterioration in cognitive function compared with wild-type (WT) mice. The open-field test showed decreased habituation of Timp-3 KO mice. Immunostaining of brain slices revealed the expression of Timp-3 in the hippocampus. In situ zymography of the hippocampus showed increased gelatinolytic activity in Timp-3 KO mice compared with WT mice. These results present the first evidence of Timp-3 involvement in cognitive function and hippocampal MMP activity in mice. Moreover, our findings suggest a novel therapeutic target to be explored for improvement of cognitive function in humans.
24. The gene structure of tissue inhibitor of metalloproteinases (TIMP)-3 and its inhibitory activities define the distinct TIMP gene family. Matrix metalloproteinases (MMPs) and tissue inhibitors of metalloproteinases (TIMPs) play a critical role in **extracellular matrix** homeostasis. We have previously cloned human and mouse TIMP-3 cDNAs and mapped their chromosomal loci (Apte, S. S., Mattei, M-G., and Olsen, B. R. (1994) Genomics 19, 86-90; Apte, S. S., Hayashi, K., Seldin, M. F., Mattei, M-G., Hayashi, M., and Olsen, B. R. (1994) Dev. Dynam. 200, 177-197); the identification of TIMP3 mutations in Sorsby's fundus dystrophy has underscored the functional importance of TIMP-3. We now report that TIMP-3 is encoded by five exons spanning over 30 kilobase pairs of mouse genomic DNA. In the attribution of protein domains to specific exons, as well as exon structures, the Timp-3 and Timp-1 genes are similar, confirming the common evolutionary origin of the TIMPs and defining a distinct gene family. We have expressed human and mouse TIMP-3 in mouse NSO myeloma cells. In each case, an N-glycosylated 27-kDa protein was generated, that, like TIMP-1 and TIMP-2, inhibited collagenase-1, stromelysin-1, and gelatinases A and B. TIMP-3 and TIMP-1 inhibition were quantitatively similar, implying that all TIMPs are equally efficient in MMP inhibition. Instead, differential regulation of the TIMP genes or divergent C-terminal protein sequences may underlie distinct biological functions for each TIMP.
25. Early activation of matrix metalloproteinases underlies the exacerbated systolic and diastolic dysfunction in mice lacking TIMP3 following myocardial infarction. **Extracellular matrix** (ECM) remodeling is a critical aspect of cardiac remodeling following myocardial infarction. Tissue inhibitors of metalloproteinases (TIMPs) are physiological inhibitors of matrix metalloproteinases (MMPs) that degrade the ECM proteins. TIMP3 is highly expressed in the heart, and is markedly downregulated in patients with ischemic cardiomyopathy. We therefore examined the time- and region-dependent role of TIMP3 in the cardiac response to myocardial infarction (MI). TIMP3(-/-) and wild-type (WT) mice were subjected to MI by ligation of the left anterior descending artery. TIMP3(-/-)-MI mice exhibited a significantly compromised rate of survival compared with WT-MI mice, primarily due to increased left ventricular (LV) rupture, greater infarct expansion, exacerbated LV dilation, and greater systolic and diastolic dysfunction. Second harmonic generation imaging of unfixed and unstained hearts revealed greater collagen disarray and reduced density in the TIMP3(-/-) infarct myocardium compared with the WT group. Gelatinolytic and collagenolytic activities increased in TIMP3(-/-) compared with WT hearts at 1 day post-MI but not at 3 days or 1 wk post-MI. Neutrophil infiltration and inflammatory MMPs were significantly increased in the infarct and peri-infarct regions of TIMP3(-/-)-MI hearts. Treatment of TIMP3(-/-) mice with a broad-spectrum MMP inhibitor (PD-166793) for 2 days before and 2 days after MI markedly improved post-MI infarct expansion, LV rupture incident, LV dilation, and systolic dysfunction in these mice up to 1 wk post-MI. Our data demonstrate that the initial rise in proteolytic activities early post-MI is a triggering factor for subsequent LV adverse remodeling, LV rupture, and dilated cardiomyopathy. Hence, timing of treatments to improve cardiac response to MI may be critical in producing favorable outcome.
26. ATXN1 protein family and CIC regulate **extracellular matrix** remodeling and lung alveolarization. Although expansion of CAG repeats in ATAXIN1 (ATXN1) causes Spinocerebellar ataxia type 1, the functions of ATXN1 and ATAXIN1-Like (ATXN1L) remain poorly understood. To investigate the function of these proteins, we generated and characterized Atxn1L(-/-) and Atxn1(-/-); Atxn1L(-/-) mice. Atxn1L(-/-) mice have hydrocephalus, omphalocele, and lung alveolarization defects. These phenotypes are more penetrant and severe in Atxn1(-/-); Atxn1L(-/-) mice, suggesting that ATXN1 and ATXN1L are functionally redundant. Upon pursuing the molecular mechanism, we discovered that several Matrix metalloproteinase (Mmp) genes are overexpressed and that the transcriptional repressor Capicua (CIC) is destabilized in Atxn1L(-/-) lungs. Consistent with this, Cic deficiency causes lung alveolarization defect. Loss of either ATXN1L or CIC derepresses Etv4, an activator for Mmp genes, thereby mediating MMP9 overexpression. These findings demonstrate a critical role of ATXN1/ATXN1L-CIC complexes in **extracellular matri**x (ECM) remodeling during development and their potential roles in pathogenesis of disorders affecting ECM remodeling.
27. Expression and function of matrix metalloproteinases and their inhibitors at the maternal-embryonic boundary during mouse embryo implantation. Gelatinase B, a matrix metalloproteinase (MMP) of high specific activity, is highly expressed and activated by mouse blastocysts in culture, and inhibition of this enzyme activity inhibits lysis of **extracellular matrix** (Behrendtsen, O., Alexander, C. M. and Werb, Z. (1992) Development 114, 447-456). Because gelatinase B expression is linked to invasive potential, we studied the expression of gelatinase B mRNA and protein in vivo, in implanting trophoblast giant cells, and found that it was expressed and activated during colonization of the maternal decidua. mRNAs for several other MMPs (stromelysin-1, stromelysin-3 and gelatinase A) and MMP inhibitors (TIMP-1 and TIMP-2) were expressed in the undifferentiated stroma toward the outside of the decidua, and TIMP-3 mRNA was expressed in primary and some mature decidual cells during their differentiation. Both mRNA and TIMP-3 protein were present at high concentrations transiently, and declined from 6.5 days post coitum onward, as the cells underwent apoptosis during the main period of gelatinase B expression and ectoplacental growth and expansion. To assess the function of MMPs during implantation and decidual development, we either injected a peptide hydroxamate MMP inhibitor into normal mice or studied transgenic mice overexpressing TIMP-1. In both cases, decidual length and overall size were reduced, and the embryo was displaced mesometrially. Embryo orientation was less strictly regulated in inhibitor-treated deciduae than in control deciduae. Morphogenesis and development of oil-induced deciduomas were also slowed in the presence of the inhibitor. We conclude that administration of MMP inhibitors retards decidual remodeling and growth, and we suggest that the MMPs expressed in precursor stromal cells promote their differentiation and expansion.
28. Metalloproteinases promote plaque rupture and myocardial infarction: A persuasive concept waiting for clinical translation. Atherosclerotic plaque rupture provokes most myocardial infarctions. Matrix metalloproteinases (MMPs) have counteracting roles in intimal thickening, which stabilizes plaques, on the one hand and **extracellular matrix** destruction that leads to plaque rupture on the other. This review briefly summarizes the key points supporting the involvement of individual MMPs in provoking plaque rupture and discusses the barriers that stand in the way of clinical translation, which can be itemised as follows: structural and functional complexity of the MMP family; lack of adequate preclinical models partly owing to different expression patterns of MMPs and TIMPs in mouse and human macrophages; the need to target individual MMPs selectively; the difficulties in establishing causality in human studies; and the requirement for surrogate markers of efficacy. Overcoming these barriers would open the way to new treatments that could have a major impact on cardiovascular mortality worldwide.
29. Gene encoding a novel murine tissue inhibitor of metalloproteinases (TIMP), TIMP-3, is expressed in developing mouse epithelia, cartilage, and muscle, and is located on mouse chromosome 10. Remodeling of the **extracellular matrix** (ECM) is an essential component of normal development and is also involved in the pathogenesis of arthritis and the spread of cancer. The matrix metalloproteinases and their natural inhibitors, the tissue inhibitors of metalloproteinases (TIMPs), play an important role in this context. We have isolated mouse cDNA clones encoding a novel member of the TIMP family, designated TIMP-3. We have assigned the Timp-3 locus to the [C1-D1] region of mouse chromosome 10 using both genetic and cytogenetic methods. The conceptual translation product of the Timp-3 cDNA shows a high degree of similarity with ChIMP-3, a recently cloned chicken metalloproteinase inhibitor, as well as significant structural similarity with the amino acid sequences of the previously isolated members of this family, TIMP-1 and TIMP-2. The pattern of expression of Timp-3 in the developing mouse embryo is distinct from that previously reported for Timp-1. Timp-3 is expressed in cartilage and skeletal muscle, in myocardium, in the skin, oral and nasal epithelium, in the newborn mouse liver, in the epithelium of some tubular structures such as the developing bronchial tree, oesophagus, colon, urogenital sinus, bile duct, in the kidney, salivary glands, and in the choroid plexus of the brain. The patterns of Timp-3 expression in surface epithelia and in the epithelial lining of many tubular organs suggests that TIMP-3 may be involved in regulating ECM remodeling during the folding of epithelia and during the formation, branching, and expansion of epithelial tubes. In the mouse placenta, expression is seen in the trophoblast, raising the possibility that TIMP-3 may be involved in regulating trophoblastic invasion of the uterus. We propose a role for TIMP-3 in musculoskeletal and cardiac development, in the morphogenesis of certain epithelial structures, and placental implantation.
30. Mechanistic insights into a TIMP3-sensitive pathway constitutively engaged in the regulation of cerebral hemodynamics. Cerebral small vessel disease (SVD) is a leading cause of stroke and dementia. CADASIL, an inherited SVD, alters cerebral artery function, compromising blood flow to the working brain. TIMP3 (tissue inhibitor of metalloproteinase 3) accumulation in the vascular **extracellular matrix** in CADASIL is a key contributor to cerebrovascular dysfunction. However, the linkage between elevated TIMP3 and compromised cerebral blood flow (CBF) remains unknown. Here, we show that TIMP3 acts through inhibition of the metalloprotease ADAM17 and HB-EGF to regulate cerebral arterial tone and blood flow responses. In a clinically relevant CADASIL mouse model, we show that exogenous ADAM17 or HB-EGF restores cerebral arterial tone and blood flow responses, and identify upregulated voltage-dependent potassium channel (KV) number in cerebral arterial myocytes as a heretofore-unrecognized downstream effector of TIMP3-induced deficits. These results support the concept that the balance of TIMP3 and ADAM17 activity modulates CBF through regulation of myocyte KV channel number.
31. Differential expression of matrix metalloproteinases and their inhibitors in human and mouse lung development. Lung development is a highly orchestrated process characterized by timed expression and activation of growth factor and protease/antiprotease systems. This interplay is essential in regulating vasculogenesis, alveolarization, and epithelial to mesenchymal transition during lung development. Alterations in the proteolytic/antiproteolytic balance of the lung have been associated with several respiratory diseases characterized by changes in the lung **extracellular matrix** (ECM). Here, we characterized the expression pattern of matrix metalloproteases (MMP) and their inhibitors, the tissue inhibitors of metalloproteases (TIMP), in human and mouse lung development. Using MMP/TIMP expression arrays, RT-PCR, Western Blotting, and ELISA analyses, we demonstrate that fetal human lung is characterized by a dominant proteolytic profile with high MMP-2 and little TIMP-3 expression. Adult human lung, in contrast, exhibits a more anti-proteolytic profile with decreased MMP-2 and increased TIMP-3 expression. MMP-14, MMP-20, TIMP-1, and TIMP-2 were constitutively expressed, irrespective of the developmental stage. Similar results were obtained using mouse lungs of different developmental stages, with the addition that in mouse lung, TIMP-2 and TIMP-3 were upregulated as lung development progressed. Exposure of neonatal mice to chronic hypoxia (10% O2), a stimulus that leads to an arrest of lung development, resulted in upregulation of MMP-2 with a concomitant downregulation of TIMP-2. These results provide a comprehensive analysis of MMP and TIMP expression during human and mouse lung development. MMP-2, TIMP-2, and TIMP-3 may be key regulatory enzymes during lung development, possibly through their complex action on ECM components, membrane receptor ectodomain shedding, and growth factor bioactivity.
32. Expression of the type IV collagenase system during mouse kidney development and tubule segmentation. Type IV collagenases matrix metalloproteinase-2 (MMP2) and MMP9 and their related proteins, MT1-MMP, tissue inhibitor of metalloproteinases 1 (TIMP1), TIMP2, and TIMP3, are expressed during kidney morphogenesis and nephrogenesis, but the renal ontogeny of these proteins is only partially known, and their persistence in the adult remains controversial. Their expression was analyzed from early metanephric stages to adulthood by Western blot semiquantitative analysis; laser confocal microscopy of whole-mount kidneys; and a two-step immunoperoxidase labeling procedure using specific markers of proximal tubule (megalin), ascending limb of Henle's loop (Tamm Horsfall protein), and collecting duct (Dolichos biflorus agglutinin lectin). By Western blot, all antigens were detected at day 11.5, peaked at day 16.5, and persisted in the adult at lower levels, although MMP2 was less modulated. All antigens were expressed in metanephric mesenchyme at embryonic day 11.5 and became concentrated in neural cell adhesion molecule-positive-induced mesenchymal cells at day 12.5. Only MT1-MMP and to a lesser extent MMP2 were detected in the ureter bud. At day 16.5, all antigens predominated in the cytoplasm of the proximal tubule, except TIMP1, which was mostly expressed in the ascending limb of Henle's loop and distal tubule. During tubule segmentation, components of the type IV collagenase system showed both spatial and temporal regulation. The distribution of gelatinases was not strictly superimposable to that of their natural inhibitors TIMP, especially for MMP9 and TIMP1. All components persisted in specific segments of the adult renal tubule, where MMP9, MMP2, and MT1-MMP showed an apical expression, suggesting that substrates for these enzymes should be in the tubule lumen or in the apical cell domain and not in the **extracellular matrix**. These results suggest that a regulated balance of gelatinase activity is required during kidney organogenesis and that gelatinases continue to play a role in adult renal tubule physiology.
33. Loss of TIMP3 enhances interstitial nephritis and fibrosis. The balance of matrix metalloproteinases (MMPs) and tissue inhibitors of matrix metalloproteinases (TIMPs) determines the integrity of the **extracellular matrix**. TIMP3 is the most highly expressed tissue inhibitor of metalloproteinase (TIMP) in the kidney, but its function in renal disease is incompletely understood. In this study, TIMP3-/- mice demonstrated an age-dependent chronic tubulointerstitial fibrosis. After unilateral ureteral obstruction (UUO), young TIMP3-/- mice exhibited increased renal injury (tubular atrophy, cortical and medullary thinning, and vascular damage) compared with wild-type mice. In addition, TIMP3-/- mice had greater interstitial fibrosis; increased synthesis and deposition of type I collagen; increased activation of fibroblasts; enhanced apoptosis; and greater activation of MMP2, but not MMP9, after UUO. TIMP3 deficiency also led to accelerated processing of TNFalpha, demonstrated by significantly higher TACE activity and greater soluble TNFalpha levels by 3 d after UUO. The additional deletion of TNFalpha markedly reduced inflammation, apoptosis, and induction of a number of MMPs. Moreover, inhibition of MMPs in TIMP3-/-/TNFalpha-/- mice further abrogated postobstructive injury and prevented tubulointerestitial fibrosis. In humans, TIMP3 expression increased in the renal arteries and proximal tubules of subjects with diabetic nephropathy or chronic allograft nephropathy. Taken together, these results provide evidence that TIMP3 is an important mediator of kidney injury, and regulating its activity may have therapeutic benefit for patients with kidney disease.
34. Enforced expression of tissue inhibitor of matrix metalloproteinase-3 affects functional capillary morphogenesis and inhibits tumor growth in a murine tumor model. Homeostasis of the **extracellular matrix** is a delicate balance between degradation and remodeling, the balance being maintained by the interaction of activated matrix metalloproteinases (MMPs) and specific tissue inhibitors of matrix metalloproteinases (TIMPs). Up-regulation of MMP activity, favoring proteolytic degradation of the basement membrane and **extracellular matrix**, has been linked to tumor growth and metastasis, as well as tumor-associated angiogenesis, whereas inhibition of MMP activity appears to restrict these processes. We have used retroviral-mediated gene delivery to effect sustained autocrine expression of TIMP-3 in murine neuroblastoma and melanoma tumor cells in order to further examine the ability of TIMPs to inhibit angiogenesis in vivo. Growth of both histologic types of gene-modified tumor cells in severe combined immunodeficiency (SCID) mice was significantly restricted when compared with controls. Grossly, these tumors were small and had few feeding vessels. Histologic evaluation revealed that although tumors overexpressing TIMP-3 had an increased number of CD31(+) endothelial cells, these endothelial cells had not formed functional tubules, as evidenced by decreased vessel continuity and minimal pericyte recruitment. This effect appears to be mediated, in part, by decreased expression of vascular endothelial (VE)-cadherin by endothelial cells in the presence of TIMP-3 as seen both in an in vitro assay and in TIMP-3-overexpressing tumors. Taken together, these results demonstrate that overexpression of TIMP-3 can inhibit angiogenesis and associated tumor growth, and that the antiangiogenic effects of TIMP-3 appear to be mediated through the inhibition of functional capillary morphogenesis.
35. Matrix metalloproteinases and oral cancer. For tumours to invade and metastasise, neoplastic cells must be capable of degrading the **extracellular matrix** (ECM), and accessing blood vessels and lymphatics. This process is mediated in the pericellular environment and is a highly controlled cascade of events utilising the same mechanisms that normal cells use for migrating through tissue barriers, for example, in development and wound healing. Proteolytic enzymes from several families, including matrix metalloproteinases (MMPs), are involved in ECM remodelling. Increased production of these enzymes has been associated with the invasive and/or metastatic phenotype in many tumours. Several MMPs have been shown to play a role in the invasion and metastasis of oral carcinoma, and it is increasingly apparent that tumour cells, as well as producing endogenous MMP, are capable of utilising MMP produced by tumour stromal cells, indicating an active role for stroma in tumour invasion. It is not clear whether a particular invasive system is favoured by oral carcinoma, but it is likely that further understanding of the interactions between carcinoma and stromal cells will provide an opportunity to refine the therapeutic interventions that are currently being tested.
36. Expression of matrix metalloproteinases and tissue inhibitors of metalloproteinases in the mouse uterus during the peri-implantation period. The attachment of the blastocyst to the uterine luminal epithelium and the subsequent invasion by trophoblast cells through the stroma and deciduum occur in a highly regulated manner by remodeling of the **extracellular matrix**. We investigated the temporal and spatial expression of mRNAs for four matrix metalloproteinases (MMPs; MMP-2 [gelatinase A], MMP-3 [stromelysin 1], MMPs; MMP-2 [gelatinase B], and MMP-13 [collagenase 3]) and tissue inhibitors of metalloproteinases (TIMPs; TIMP-1, TIMP-2, and TIMP-3) in the mouse uterus from days 1 to 8 of pregnancy. Northern blot analyses showed the transcripts for MMP-2, MMP-3, RNA on these days. However, MMP-13 mRNA was not detected in the uterus, and only weak signals for MMP-3 mRNA were detected in the myometrium. Striking expression was observed with MMP-2 mRNA in the subepithelial stroma on days 3-5. With the progression of decidualization on day 6, signals were primarily in the secondary decidual zone. On day 8, MMP-2 mRNA was localized at the site of placenta formation in the mesometrial pole. Signals for MMP-9 mRNA were first detected in a small population of stromal cells exclusively at the site of implantation on day 5 at the antimesometrial pole. However, the most pronounced expressed was noted in trophoblast giant cells on day 8. TIMP-1 mRNA was present in the myometrium on day 1. On days 2-5, modest signals were detected in the stroma, and on days 6 and 8, they were in the secondary decidual zone. Localization of TIMP-2 mRNA was similar to that of TIMP-1 except it was restricted to the stroma on day 1. The regulation of TIMP-3 was more pronounced. While a gradual increase in signals was observed in stromal cells from days 1 to 4, strong signals were detected in antimesometrial stromal cells at the sites of blastocyst attachment on day 5. On days 6 and 7, even stronger signals were present in the primary decidual zone surrounding the embryo, and on day 8 signals were localized primarily in the mesometrial decidual bed. These results suggest that MMP-2 may participate in the early phase of decidualization and neovascularization required for placentation. The restricted MMP-9 expression in stromal cells on day 5 and in trophoblast giant cells on day 8, coupled with the expression of TIMP-3 in the stroma surrounding the embryo, suggests that a fine balance between MMP-9 and TIMP-3 may regulate trophoblast invasion in the uterus.
37. Combination of tumor necrosis factor-alpha ablation and matrix metalloproteinase inhibition prevents heart failure after pressure overload in tissue inhibitor of metalloproteinase-3 knock-out mice. Cytokine and **extracellular matrix** (ECM) homeostasis are distinct systems that are each dysregulated in heart failure. Here we show that tissue inhibitor of metalloproteinase (TIMP)-3 is a critical regulator of both systems in a mouse model of left ventricular (LV) dilation and dysfunction. Timp-3(-/-) mice develop precipitous LV dilation and dysfunction reminiscent of dilated cardiomyopathy (DCM), culminating in early onset of heart failure by 6 weeks, compared with wild-type aortic-banding (AB). Timp-3 deficiency resulted in increased TNFalpha converting enzyme (TACE) activity within 6 hours after AB leading to enhanced tumor necrosis factor-alpha (TNFalpha) processing. In addition, TNFalpha production increased in timp-3(-/-)-AB myocardium. A significant elevation in gelatinase and collagenase activities was observed 1 week after AB, with localized ECM degradation in timp-3(-/-)-AB myocardium. Timp-3(-/-)/tnfalpha(-/-) mice were generated and subjected to AB for comparative analyses with timp-3(-/-)-AB mice. This revealed the critical role of TNFalpha in the early phase of LV remodeling, de novo expression of Matrix metalloproteinases (MMP)-8 in the absence of TNFalpha, and highlighted the importance of interstitial collagenases (MMP-2, MMP-13, and MT1-MMP) for cardiac ECM degradation. Ablation of TNFalpha, or limiting MMP activity with a synthetic MMP inhibitor (PD166793), each partially attenuated LV dilation and cardiac dysfunction in timp-3(-/-)-AB mice. Notably, combining TNFalpha ablation with MMP inhibition completely rescued heart disease in timp-3(-/-)-AB mice. This study provides a basis for anti-TNFalpha and MMP inhibitor combination therapy in heart disease.
38. TIMP-3 recruits quiescent hematopoietic stem cells into active cell cycle and expands multipotent progenitor pool. Regulating transition of hematopoietic stem cells (HSCs) between quiescent and cycling states is critical for maintaining homeostasis of blood cell production. The cycling states of HSCs are regulated by the extracellular factors such as cytokines and **extracellular matrix**; however, the molecular circuitry for such regulation remains elusive. Here we show that tissue inhibitor of metalloproteinase-3 (TIMP-3), an endogenous regulator of metalloproteinases, stimulates HSC proliferation by recruiting quiescent HSCs into the cell cycle. Myelosuppression induced TIMP-3 in the bone marrow before hematopoietic recovery. Interestingly, TIMP-3 enhanced proliferation of HSCs and promoted expansion of multipotent progenitors, which was achieved by stimulating cell-cycle entry of quiescent HSCs without compensating their long-term repopulating activity. Surprisingly, this effect did not require metalloproteinase inhibitory activity of TIMP-3 and was possibly mediated through a direct inhibition of angiopoietin-1 signaling, a critical mediator for HSC quiescence. Furthermore, bone marrow recovery from myelosuppression was accelerated by over-expression of TIMP-3, and in turn, impaired in TIMP-3-deficient animals. These results suggest that TIMP-3 may act as a molecular cue in response to myelosuppression for recruiting dormant HSCs into active cell cycle and may be clinically useful for facilitating hematopoietic recovery after chemotherapy or ex vivo expansion of HSCs.
39. Steroidal regulation of uterine edema and tissue inhibitors of metalloproteinase (TIMP)-3 messenger RNA expression is altered in TIMP-1-deficient mice. Tissue inhibitors of metalloproteinases (TIMPs) are expressed within the uteri of virtually all species where they are postulated to control **extracellular matrix** turnover, cellular apoptosis, and proliferation. The objective of the current study was to examine the steroidal regulation of uterine TIMP expression and to determine the potential role of the TIMP-1 gene product in this regulation. To accomplish these goals, ovariectomized female TIMP-1 wild-type and null mice were treated with estradiol, progesterone, or estradiol and progesterone and killed at various times after steroid administration. Estradiol induced a significant reduction in uterine TIMP-3 expression in wild-type mice at 8 and 24 h post-steroid administration, but the ability of this steroid to decrease TIMP-3 expression was impaired in the uteri of TIMP-1 null mice. Further, estrogen-induced uterine wet-weight gain/edema was enhanced in the TIMP-1 null mice, and the antiestrogen compound ICI 182780 or progesterone could only partially block this estrogenic effect. It is concluded from this study that steroidal modulation of uterine TIMP-3 expression and regulation of wet-weight gain/edema are altered in TIMP-1 null mice. These observations suggest that steroids induce uterine TIMP-1 expression and, in turn, that TIMP-1 influences TIMP-3 mRNA expression and uterine edema.
40. Expression of matrix metalloproteinases during murine chorioallantoic placenta maturation. A large body of experimental evidence supports the participation of two groups of extracellular proteases, matrix metalloproteinases (MMPs), and plasminogen activators/plasmin, in tissue remodeling in physiological and pathological invasion. In the late mouse placenta, several tissue remodeling and cell invasion processes take place. Spongiotrophoblast migration into maternal decidua, as well as decidual **extracellular matrix** remodeling require the coordinated action of extracellular proteolytic enzymes. Via Northern and in situ hybridization, we have analyzed the spatio-temporal expression patterns of members of the MMP family (stromelysin-3, gelatinases A and B), as well as their inhibitors TIMP-1, -2 and -3 in late murine placenta (days 10.5 to 18.5 of gestation). Gelatinase activity in placental extracts was assessed by substrate zymography. Gelatinase A and stromelysin-3 were found to be prominently expressed in decidual tissue; shortly after midpregnancy, the decidual expression patterns of gelatinase A and stromelysin-3 became overlapping with each other, as well as with the expression domain of TIMP-2. On the other hand, gelatinase B transcripts were expressed only by trophoblast giant cells at day 10.5, and were downregulated at later stages. TIMP-1 and TIMP-3 transcripts were detected in decidual periphery at day 10.5, while later the expression was restricted to the endometrial stroma and spongiotrophoblasts, respectively. The areas of stromelysin-3 expression were the same (giant trophoblasts) or adjacent (decidua) to those where urokinase (uPA) transcripts were detected, suggesting a possible cooperation between these proteinases in placental remodeling. We generated mice doubly deficient for stromelysin-3 and uPA, and report here that these mice are viable and fertile. Furthermore, these animals do not manifest obvious placental abnormalities, thereby suggesting the existence of compensatory/redundant mechanisms involving other proteolytic enzymes. Our findings document the participation of MMPs and their inhibitors in the process of late murine placenta maturation, and warrant the characterization of other members of the MMP family, like membrane type-MMPs, in this process.
41. Regulation of TIMP3 in diabetic nephropathy: a role for microRNAs. Diabetic nephropathy (DN) is the major cause of chronic kidney disease in developed countries and contributes significantly to increased morbidity and mortality among diabetic patients. Morphologically, DN is characterized by tubulo-interstitial fibrosis, thickening of the glomerular basement membrane and mesangial expansion mainly due to accumulation of **extracellular matrix** (ECM). ECM turnover is regulated by metalloproteinases and tissue inhibitors of metalloproteinases (TIMPs) activities. In diabetic conditions, TIMP3 expression in kidney is strongly reduced, but the causes of this reduction are still unknown. The aim of this study was to elucidate at least one of these mechanisms which relies on differential expression of TIMP3-targeting microRNAs (miRs) in a hyperglycemic environment either in vitro (MES13 cell line) or in vivo (mouse kidney and human biopsies). Among the TIMP3-targeting miRs, miR-21 and miR-221 were significantly upregulated in kidneys from diabetic mice compared to control littermates, and in a mesangial cell line grown in high glucose conditions. In human samples, only miR-21 expression was increased in kidney biopsies from diabetic patients compared to healthy controls. The expression of miR-217, which targets TIMP3 indirectly through downregulation of SirT1, was also increased in diabetic kidney and MES13 cell line. In agreement with these result, SirT1 expression was reduced in mouse and human diabetic kidneys as well as in MES13 mesangial cell line. TIMP3 deficiency has recently emerged as a hallmark of DN in mouse and human. In this study, we demonstrated that this reduction is due, at least in part, to increased expression of certain TIMP3-targeting miRs in diabetic kidneys compared to healthy controls. Unveiling the post-transcriptional mechanisms responsible for TIMP3 downregulation in hyperglycemic conditions may orient toward the use of this protein as a possible therapeutic target in DN.
42. Tissue inhibitor of metalloproteinases-3 (TIMP-3) is an **extracellular matrix**-associated protein with a distinctive pattern of expression in mouse cells and tissues. We have isolated cDNA clones corresponding to a novel mouse metalloproteinase inhibitor. Five overlapping cDNA clones contain most of the information for a prominent 4.5-kilobase transcript that was detected in RNA from mouse fibroblasts and adult tissues. Sequence analysis revealed an open reading frame (ORF) for a protein of 212 amino acids that is 80% identical to chicken inhibitor of metalloproteinases-3 (ChIMP-3). The 3'-untranslated sequence also showed remarkable conservation with the chicken gene. The ORF directed the expression of a 24-kDa protein in COS-1 cells that localized to the **extracellular matrix** (ECM). On the basis of these similarities we propose to identify the new gene as murine tissue inhibitor of metalloproteinases-3 (TIMP-3). Mouse C3H 10T1/2 fibroblasts produced a 24-kDa metalloproteinase inhibitor that also localized to the ECM and was recognized by a polyclonal antibody to ChIMP-3. Like TIMP-1, TIMP-3 was highly inducible in mouse C3H 10T1/2 fibroblasts by phorbol ester (PMA), epidermal growth factor (EGF), and transforming growth factor-beta 1, but nuclear run-on assays showed that the on/off transcription kinetics were faster for TIMP-3 than TIMP-1. A major difference in vitro was the stimulation of expression of TIMP-3 by dexamethasone which inhibits EGF- and PMA-induced TIMP-1 transcription. Also, TIMP-3 showed a distinctive pattern of expression in adult tissues with abundant transcripts detected in kidney, lung, and brain but only low levels detected in bone, a prominent location of TIMP-1 transcripts. We propose that TIMP-3 functions in a tissue-specific fashion as part of an acute response to remodeling stimuli.

#### Component

1. **extracellular matrix**
2. proteinaceous **extracellular matrix**

#### GeneRIFs

1. Distinct choroidal phenotype in mice lacking TIMP3 may be result of local disruption of **extracellular matrix** and angiogenic homeostasis. Important role of TIMP3 in regulation of choroidal vascularization.

#### Conserved Domains

1. cd03585: NTR\_TIMP; NTR domain, TIMP subfamily; TIMPs, or tissue inibitors of metalloproteases, are essential regulators of **extracellular matrix** turnover and remodeling. They form complexes with matrix metalloproteases (MMPs) and inactivate them irreversibly by ...

### [Ii]nvasive

#### PubMed

1. Expression and function of matrix metalloproteinases and their inhibitors at the maternal-embryonic boundary during mouse embryo implantation. Gelatinase B, a matrix metalloproteinase (MMP) of high specific activity, is highly expressed and activated by mouse blastocysts in culture, and inhibition of this enzyme activity inhibits lysis of extracellular matrix (Behrendtsen, O., Alexander, C. M. and Werb, Z. (1992) Development 114, 447-456). Because gelatinase B expression is linked to **invasive** potential, we studied the expression of gelatinase B mRNA and protein in vivo, in implanting trophoblast giant cells, and found that it was expressed and activated during colonization of the maternal decidua. mRNAs for several other MMPs (stromelysin-1, stromelysin-3 and gelatinase A) and MMP inhibitors (TIMP-1 and TIMP-2) were expressed in the undifferentiated stroma toward the outside of the decidua, and TIMP-3 mRNA was expressed in primary and some mature decidual cells during their differentiation. Both mRNA and TIMP-3 protein were present at high concentrations transiently, and declined from 6.5 days post coitum onward, as the cells underwent apoptosis during the main period of gelatinase B expression and ectoplacental growth and expansion. To assess the function of MMPs during implantation and decidual development, we either injected a peptide hydroxamate MMP inhibitor into normal mice or studied transgenic mice overexpressing TIMP-1. In both cases, decidual length and overall size were reduced, and the embryo was displaced mesometrially. Embryo orientation was less strictly regulated in inhibitor-treated deciduae than in control deciduae. Morphogenesis and development of oil-induced deciduomas were also slowed in the presence of the inhibitor. We conclude that administration of MMP inhibitors retards decidual remodeling and growth, and we suggest that the MMPs expressed in precursor stromal cells promote their differentiation and expansion.
2. Proteinase expression in early mouse embryos is regulated by leukaemia inhibitory factor and epidermal growth factor. Several proteinases from different multigene families have been implicated in the uterine invasion required for establishment of pregnancy in some mammals. In this study, the expression of matrix metalloproteinase gelatinase B (MMP-9), urokinase-type plasminogen activator (uPA) and their inhibitors was investigated during early mouse embryo development. Transcripts for tissue inhibitors of metalloproteinases (TIMP-1,-2,-3) and uPA receptor were detected throughout pre- and peri-implantation development whilst MMP-9 and uPA mRNAs were first detected in peri-implantation blastocysts associated with the **invasive** phase of implantation. Through use of in situ hybridization, it was shown that MMP-9 transcripts were strongly expressed in the network of trophoblast giant cells at the periphery of implanting 7.5 day embryos and TIMP-3 transcripts were strongly expressed in the decidua immediately adjacent to the implanting embryo. uPA transcripts were preferentially expressed in the ectoplacental cone and its derivatives. Because these proteinases are regulated by growth factors and cytokines in other tissues, the effect of leukaemia inhibitory factor (LIF) and epidermal growth factor (EGF) on their activity was investigated. Both LIF and EGF, like the proteinases, have been implicated in peri-implantation development. Blastocysts collected on day 4 of pregnancy were cultured 2 days in TCM 199 + 10% fetal bovine serum to allow outgrowth followed by 24 hour culture in defined media containing either LIF or EGF. Conditioned media were assayed for uPA activity by a chromogenic assay and MMP activity by gelatin zymography. Both LIF and EGF stimulated uPA and MMP-9 activity in blastocyst outgrowths after 3 days of culture (day 7). Proteinase activity was assayed again at the 5th to 6th day of culture (day 9 to 10). EGF was found to have no effect whereas LIF decreased production of both proteinases. These results demonstrate that proteinase activity in early embryos can be regulated by growth factors and cytokines during the implantation process and, in particular, they demonstrate the possible involvement of LIF in establishment of the correct temporal programme of proteinase expression.
3. Matrix metalloproteinases and oral cancer. For tumours to invade and metastasise, neoplastic cells must be capable of degrading the extracellular matrix (ECM), and accessing blood vessels and lymphatics. This process is mediated in the pericellular environment and is a highly controlled cascade of events utilising the same mechanisms that normal cells use for migrating through tissue barriers, for example, in development and wound healing. Proteolytic enzymes from several families, including matrix metalloproteinases (MMPs), are involved in ECM remodelling. Increased production of these enzymes has been associated with the **invasive** and/or metastatic phenotype in many tumours. Several MMPs have been shown to play a role in the invasion and metastasis of oral carcinoma, and it is increasingly apparent that tumour cells, as well as producing endogenous MMP, are capable of utilising MMP produced by tumour stromal cells, indicating an active role for stroma in tumour invasion. It is not clear whether a particular **invasive** system is favoured by oral carcinoma, but it is likely that further understanding of the interactions between carcinoma and stromal cells will provide an opportunity to refine the therapeutic interventions that are currently being tested.
4. Subtilisin proprotein convertase-6 expression in the mouse uterus during implantation and artificially induced decidualization. During implantation, a balance of factors regulates the **invasive** properties of the embryo and the anti-**invasive** properties of uterine decidua. Although antiproteinases such as the metalloproteinase inhibitor TIMP-3 are thought to play critical roles in preventing the overaggressive invasion of trophoblasts, the mechanism of antiproteinase regulation is unknown. Recently, the prohormone convertase SPC-6 has been found to be co-expressed in embryo-proximal decidua in association with TIMP-3. As members of this serine proteinase family are known to activate latent TGFbeta family members which regulate decidual TIMP-3 levels, we sought to characterize the expression of SPC-6 during pregnancy and artificial decidualization. In this study, we demonstrate that the zone of SPC-6 gene expression exhibits a great degree of temporal and spatial overlap with TIMP-3 gene expression in uterine decidua from E5.5 through to E8.5. Like TIMP-3, we demonstrate that SPC-6 expression is induced during the decidual cell response using an in vivo model of artificial decidualization. Both the secreted and membrane bound forms of SPC-6 are expressed throughout the period of decidualization, suggesting that SPC-6 may play multiple roles during this developmental period. This is confirmed by our observation of the movement of SPC-6 expression to the presumptive placental region, as TIMP-3 expression regresses at the implantation site.
5. Tissue inhibitor of metalloproteinases-3 is the major metalloproteinase inhibitor in the decidualizing murine uterus. Embryo implantation in the mouse is a highly orchestrated process, a key aspect of which is the invasion of trophoblast cells of the blastocyst into the maternal uterine endometrium. Invasion is facilitated via proteinases expressed by trophoblast cells and balanced by expression of inhibitors of proteinases in the maternal decidua. The predominant proteinase expressed by trophectodermal derivatives of the implanting mouse embryo is matrix metalloproteinase-9 (MMP-9; gelatinase B). Using in situ hybridization, transcripts for MMP-9 were detected in trophoblast cells of the embryo from the earliest stage of decidual formation (day 6.0) examined. MMP-9 transcripts were localized to trophoblast giant cells at the periphery of the embryo at the egg cylinder stage (day 7.0). By the neural-fold stage (day 8.5), expression was restricted to giant cells adjacent to the maternal side of the developing placenta, and by day 9.5 few MMP-9-positive cells remained. The major tissue inhibitor of metalloproteinases (TIMP) produced during this period was TIMP-3. Transcripts encoding TIMP-3 were detected from day 6.0-7.0 in the maternal decidua immediately adjacent to embryonic cells expressing MMP-9. The intensity of TIMP-3 expression in later-stage embryos declined in parallel with MMP-9 expression. Maternal TIMP-3 expression also occurred in the absence of embryonic MMP-9 expression in decidual reactions induced by parthenogenetic embryos (where MMP-9 positive cells were not detected) or in oil-induced deciduomas. These results support the hypothesis that MMP-9 is an important mediator of cellular **invasive**ness during embryo implantation, and that TIMP-3 serves as a regulator within the uterus to restrict invasion to the site of implantation.

### [Mm]etastasis

#### PubMed

1. Timp3 loss accelerates tumour invasion and increases prostate inflammation in a mouse model of prostate cancer. Altered expression and activity of proteases is implicated in inflammation and cancer progression. An important negative regulator of protease activity is TIMP3 (tissue inhibitor of metalloproteinase 3). TIMP3 expression is lacking in many cancers including advanced prostate cancer, and this may facilitate invasion and **metastasis** by allowing unrestrained protease activity.To investigate the role of TIMP3 in prostate cancer progression, we crossed TIMP3-deficient mice (Timp3(-/-)) to mice with prostate-specific deletion of the tumor suppressor Pten (Pten(-/-)), a well-established mouse model of prostate cancer. Tumor growth and progression were compared between Pten(-/-), Timp3(-/-) and control (Pten(-/-), Timp3(+/+)) mice at 16 weeks of age by histopathology and markers of proliferation, vascularity, and tumor invasion. Metalloproteinase activity within the tumors was assessed by gelatin zymography. Inflammatory infiltrates were assessed by immunohistochemistry for macrophages and lymphocytes whereas expression of cytokines and other inflammatory mediators was assessed by quantitative real time PCR and multiplex ELISA.Increased tumor growth, proliferation index, increased microvascular density, and invasion was observed in Pten(-/-), Timp3(-/-) prostate tumors compared to Pten(-/-), Timp3(+/+) tumors. Tumor cell invasion in Pten(-/-), Timp3(-/-) mice was associated with increased expression of matrix metalloprotease (MMP)-9 and activation of MMP-2. There was markedly increased inflammatory cell infiltration into the TIMP3-deficient prostate tumors along with increased expression of monocyte chemoattractant protein-1, cyclooxygenase-2, TNF-α, and interleukin-1β; all of which are implicated in inflammation and cancer.This study provides important insights into the role of altered protease activity in promoting prostate cancer invasion and implicates prostate inflammation as an important promoting factor in prostate cancer progression.
2. Enhanced metastatic dissemination to multiple organs by melanoma and lymphoma cells in timp-3-/- mice. Identifying versatile inhibitors of **metastasis** that operate in multiple sites against distinct cancer cell types is important for designing novel therapeutics for **metastasis**. We show that multiple tissues of timp-3-/- mice are more susceptible to metastatic colonization. Overall, a 5-14-fold increase in liver and kidney colonization occurred by EL-4 lymphoma cells, and a twofold increase upon targeting B16F10 melanoma cells to the bone or lung of timp-3-/- mice. There was a general lack of macrophage or neutrophil localization to metastases in the liver, kidney and lung, and of osteoclasts to bone in both genotypes. Analysis of lung showed that proliferation or angiogenesis were unaltered within the metastatic colonies. Lung-trap assays revealed that initial tumor cell trapping was similar in the lung vasculature of timp-3-/- and wild-type mice. However, more tumor cells were found in timp-3-/- lungs at 48 and 96 h after tumor cell injection indicating more efficient extravasation and initial proliferation. Activation of pro-MMP-2 was greater in timp-3-/- lungs at these time points. These data demonstrate TIMP-3 functions to inhibit metastatic dissemination of diverse cancer cells to multiple organs. TIMP-3 regulates MMP-2 activation to limit tumor cell extravasation and subsequent colonization of the lung, without augmenting inflammatory cell response.
3. Enforced expression of tissue inhibitor of matrix metalloproteinase-3 affects functional capillary morphogenesis and inhibits tumor growth in a murine tumor model. Homeostasis of the extracellular matrix is a delicate balance between degradation and remodeling, the balance being maintained by the interaction of activated matrix metalloproteinases (MMPs) and specific tissue inhibitors of matrix metalloproteinases (TIMPs). Up-regulation of MMP activity, favoring proteolytic degradation of the basement membrane and extracellular matrix, has been linked to tumor growth and **metastasis**, as well as tumor-associated angiogenesis, whereas inhibition of MMP activity appears to restrict these processes. We have used retroviral-mediated gene delivery to effect sustained autocrine expression of TIMP-3 in murine neuroblastoma and melanoma tumor cells in order to further examine the ability of TIMPs to inhibit angiogenesis in vivo. Growth of both histologic types of gene-modified tumor cells in severe combined immunodeficiency (SCID) mice was significantly restricted when compared with controls. Grossly, these tumors were small and had few feeding vessels. Histologic evaluation revealed that although tumors overexpressing TIMP-3 had an increased number of CD31(+) endothelial cells, these endothelial cells had not formed functional tubules, as evidenced by decreased vessel continuity and minimal pericyte recruitment. This effect appears to be mediated, in part, by decreased expression of vascular endothelial (VE)-cadherin by endothelial cells in the presence of TIMP-3 as seen both in an in vitro assay and in TIMP-3-overexpressing tumors. Taken together, these results demonstrate that overexpression of TIMP-3 can inhibit angiogenesis and associated tumor growth, and that the antiangiogenic effects of TIMP-3 appear to be mediated through the inhibition of functional capillary morphogenesis.
4. Matrix metalloproteinases and oral cancer. For tumours to invade and **metastasis**e, neoplastic cells must be capable of degrading the extracellular matrix (ECM), and accessing blood vessels and lymphatics. This process is mediated in the pericellular environment and is a highly controlled cascade of events utilising the same mechanisms that normal cells use for migrating through tissue barriers, for example, in development and wound healing. Proteolytic enzymes from several families, including matrix metalloproteinases (MMPs), are involved in ECM remodelling. Increased production of these enzymes has been associated with the invasive and/or metastatic phenotype in many tumours. Several MMPs have been shown to play a role in the invasion and **metastasis** of oral carcinoma, and it is increasingly apparent that tumour cells, as well as producing endogenous MMP, are capable of utilising MMP produced by tumour stromal cells, indicating an active role for stroma in tumour invasion. It is not clear whether a particular invasive system is favoured by oral carcinoma, but it is likely that further understanding of the interactions between carcinoma and stromal cells will provide an opportunity to refine the therapeutic interventions that are currently being tested.

### [Cc]ell [Ii]nvasion

#### PubMed

1. Timp3 loss accelerates tumour invasion and increases prostate inflammation in a mouse model of prostate cancer. Altered expression and activity of proteases is implicated in inflammation and cancer progression. An important negative regulator of protease activity is TIMP3 (tissue inhibitor of metalloproteinase 3). TIMP3 expression is lacking in many cancers including advanced prostate cancer, and this may facilitate invasion and metastasis by allowing unrestrained protease activity.To investigate the role of TIMP3 in prostate cancer progression, we crossed TIMP3-deficient mice (Timp3(-/-)) to mice with prostate-specific deletion of the tumor suppressor Pten (Pten(-/-)), a well-established mouse model of prostate cancer. Tumor growth and progression were compared between Pten(-/-), Timp3(-/-) and control (Pten(-/-), Timp3(+/+)) mice at 16 weeks of age by histopathology and markers of proliferation, vascularity, and tumor invasion. Metalloproteinase activity within the tumors was assessed by gelatin zymography. Inflammatory infiltrates were assessed by immunohistochemistry for macrophages and lymphocytes whereas expression of cytokines and other inflammatory mediators was assessed by quantitative real time PCR and multiplex ELISA.Increased tumor growth, proliferation index, increased microvascular density, and invasion was observed in Pten(-/-), Timp3(-/-) prostate tumors compared to Pten(-/-), Timp3(+/+) tumors. Tumor **cell invasion** in Pten(-/-), Timp3(-/-) mice was associated with increased expression of matrix metalloprotease (MMP)-9 and activation of MMP-2. There was markedly increased inflammatory cell infiltration into the TIMP3-deficient prostate tumors along with increased expression of monocyte chemoattractant protein-1, cyclooxygenase-2, TNF-α, and interleukin-1β; all of which are implicated in inflammation and cancer.This study provides important insights into the role of altered protease activity in promoting prostate cancer invasion and implicates prostate inflammation as an important promoting factor in prostate cancer progression.
2. Expression of matrix metalloproteinases during murine chorioallantoic placenta maturation. A large body of experimental evidence supports the participation of two groups of extracellular proteases, matrix metalloproteinases (MMPs), and plasminogen activators/plasmin, in tissue remodeling in physiological and pathological invasion. In the late mouse placenta, several tissue remodeling and **cell invasion** processes take place. Spongiotrophoblast migration into maternal decidua, as well as decidual extracellular matrix remodeling require the coordinated action of extracellular proteolytic enzymes. Via Northern and in situ hybridization, we have analyzed the spatio-temporal expression patterns of members of the MMP family (stromelysin-3, gelatinases A and B), as well as their inhibitors TIMP-1, -2 and -3 in late murine placenta (days 10.5 to 18.5 of gestation). Gelatinase activity in placental extracts was assessed by substrate zymography. Gelatinase A and stromelysin-3 were found to be prominently expressed in decidual tissue; shortly after midpregnancy, the decidual expression patterns of gelatinase A and stromelysin-3 became overlapping with each other, as well as with the expression domain of TIMP-2. On the other hand, gelatinase B transcripts were expressed only by trophoblast giant cells at day 10.5, and were downregulated at later stages. TIMP-1 and TIMP-3 transcripts were detected in decidual periphery at day 10.5, while later the expression was restricted to the endometrial stroma and spongiotrophoblasts, respectively. The areas of stromelysin-3 expression were the same (giant trophoblasts) or adjacent (decidua) to those where urokinase (uPA) transcripts were detected, suggesting a possible cooperation between these proteinases in placental remodeling. We generated mice doubly deficient for stromelysin-3 and uPA, and report here that these mice are viable and fertile. Furthermore, these animals do not manifest obvious placental abnormalities, thereby suggesting the existence of compensatory/redundant mechanisms involving other proteolytic enzymes. Our findings document the participation of MMPs and their inhibitors in the process of late murine placenta maturation, and warrant the characterization of other members of the MMP family, like membrane type-MMPs, in this process.

---

## Trib2

- NCBI Gene
- GeneRIF
- Pubmed

| Search Term | Rank | Fields |
| --- | --- | --- |
| [Mm]igration | 6 | PubMed(1) |

  
hide/show details for Trib2

### [Mm]igration

#### PubMed

1. Survival factor withdrawal-induced apoptosis of TF-1 cells involves a TRB2-Mcl-1 axis-dependent pathway. Tribbles, an atypical protein kinase superfamily member, coordinates cell proliferation, **migration**, and morphogenesis during the development of Drosophila and Xenopus embryos. Although Tribbles are highly conserved throughout evolution, the physiological functions of mammalian Tribbles family remain largely unclear. Here we report that human TRB2 is a pro-apoptotic molecule that induces apoptosis of cells mainly of the hematopoietic origin. TRB2 mRNA is selectively induced by removal of granulocyte macrophage colony-stimulating factor (GM-CSF) or interleukin-2 from human erythroleukemia-derived TF-1 cell line or activated primary CD4(+) T cells, respectively. It is, however, not induced by many other treatments that trigger apoptosis of these two cell types. Overexpression of TRB2 activates many apoptotic events observed in GM-CSF-deprived TF-1 cells, including loss of mitochondrial membrane potential, Mcl-1 cleavage/degradation, and activation of Bax and a number of caspases. Specific knockdown of TRB2 significantly suppresses GM-CSF deprivation-induced apoptosis and all apoptotic events mentioned above. Finally, we demonstrate that TRB2-induced cleavage and degradation of Mcl-1 are mediated via a caspase-dependent but proteasome-independent mechanism, and overexpression of Mcl-1 or its upstream activator Akt can markedly overcome the apoptogenic effect of TRB2. Altogether, these results suggest that the TRB2-Mcl-1 axis plays an important role in survival factor withdrawal-induced apoptosis of TF-1 cells.

---

## Ddc

- NCBI Gene
- GeneRIF
- Pubmed

| Search Term | Rank | Fields |
| --- | --- | --- |
| [Mm]igration | 6 | PubMed(1) |

  
hide/show details for Ddc

### [Mm]igration

#### PubMed

1. Excitatory neurons of the proprioceptive, interoceptive, and arousal hindbrain networks share a developmental requirement for Math1. Hindbrain networks important for sensation and arousal contain diverse neuronal populations with distinct projections, yet share specific characteristics such as neurotransmitter expression. The relationship between the function of these neurons, their developmental origin, and the timing of their **migration** remains unclear. Mice lacking the proneural transcription factor Math1 (Atoh1) lose neurons essential for hearing, balance, and unconscious proprioception. By using a new, inducible Math1(Cre\*PR) allele, we found that Math1 is also required for the conscious proprioceptive system, including excitatory projection neurons of the dorsal column nuclei and for vital components of the interoceptive system, such as Barrington's nucleus, that is closely associated with arousal. In addition to specific networks, Math1 lineages shared specific neurotransmitter expression, including glutamate, acetylcholine, somatostatin, corticotropin releasing hormone, and nitric oxide. These findings identify twenty novel Math1 lineages and indicate that the Math1 network functions partly as an interface for conscious (early-born) and unconscious (late-born) proprioceptive inputs to the cortex and cerebellum, respectively. In addition, these data provide previously unsuspected genetic and developmental links between proprioception, interoception, hearing, and arousal.

---

## Cyth1

- NCBI Gene
- GeneRIF
- Pubmed

| Search Term | Rank | Fields |
| --- | --- | --- |
| [Mm]igration | 6 | PubMed(3); GeneRIFs(2) |

  
hide/show details for Cyth1

### [Mm]igration

#### PubMed

1. Cytohesin-1 controls the activation of RhoA and modulates integrin-dependent adhesion and **migration** of dendritic cells. Adhesion and motility of mammalian leukocytes are essential requirements for innate and adaptive immune defense mechanisms. We show here that the guanine nucleotide exchange factor cytohesin-1, which had previously been demonstrated to be an important component of beta-2 integrin activation in lymphocytes, regulates the activation of the small GTPase RhoA in primary dendritic cells (DCs). Cytohesin-1 and RhoA are both required for the induction of chemokine-dependent conformational changes of the integrin beta-2 subunit of DCs during adhesion under physiological flow conditions. Furthermore, use of RNAi in murine bone marrow DCs (BM-DCs) revealed that interference with cytohesin-1 signaling impairs **migration** of wild-type dendritic cells in complex 3D environments and in vivo. This phenotype was not observed in the complete absence of integrins. We thus demonstrate an essential role of cytohesin-1/RhoA during ameboid **migration** in the presence of integrins and further suggest that DCs without integrins switch to a different **migration** mode.
2. Cytohesin-1 controls the activation of RhoA and modulates integrin-dependent adhesion and **migration** of dendritic cells. Adhesion and motility of mammalian leukocytes are essential requirements for innate and adaptive immune defense mechanisms. We show here that the guanine nucleotide exchange factor cytohesin-1, which had previously been demonstrated to be an important component of beta-2 integrin activation in lymphocytes, regulates the activation of the small GTPase RhoA in primary dendritic cells (DCs). Cytohesin-1 and RhoA are both required for the induction of chemokine-dependent conformational changes of the integrin beta-2 subunit of DCs during adhesion under physiological flow conditions. Furthermore, use of RNAi in murine bone marrow DCs (BM-DCs) revealed that interference with cytohesin-1 signaling impairs **migration** of wild-type dendritic cells in complex 3D environments and in vivo. This phenotype was not observed in the complete absence of integrins. We thus demonstrate an essential role of cytohesin-1/RhoA during ameboid **migration** in the presence of integrins and further suggest that DCs without integrins switch to a different **migration** mode.
3. Signaling through Arf6 guanine-nucleotide exchange factor cytohesin-1 regulates **migration** in Schwann cells. During development of the peripheral nervous system (PNS), Schwann cells migrate along neuronal axons before initiating myelination of the axons. While intercellular signals controlling **migration**, between Schwann cells and peripheral neurons, are established, how their intracellular transduction of the signals into Schwann cells still remains to be clarified. Here, we show that cytohesin-1, a guanine-nucleotide exchange factor (GEF), and the effector Arf6 are required for **migration** of primary Schwann cells. Knockdown of cytohesin-1 or Arf6 in Schwann cells, as well as treatment with the chemical cytohesin inhibitor SecinH3 or knockout of cytohesin-1, inhibits peripheral neuronal conditioned medium-mediated **migration**. Similar effects are also observed following stimulation with each of growth factors contained in a conditioned medium, suggesting that cytohesin-1 plays a role in transducing soluble ligand signals from neurons. Reintroduction of small interfering (si)RNA-resistant cytohesin-1 into Schwann cells reverses blunted **migration** in the siRNA-transfected Schwann cells, illustrating the importance of cytohesin-1 in **migration**. On the other hand, introduction of cytohesin-1 that harbors the Tyr-382 mutation, which is an amino acid that is important for its activation, failed to reverse the reduction in primary Schwann cell **migration**. These results suggest that signaling through cytohesin-1 is required for Schwann cell **migration**, revealing a novel mechanism for Schwann cell **migration**.

#### GeneRIFs

1. Cytohesin-1 is required for Schwann cell **migration** and that phosphorylation of cytohesin-1 at the Tyr-382 position is important for **migration**.
2. demonstrate an essential role of cytohesin-1/RhoA during ameboid **migration** in the presence of integrins

---

## Ero1lb

- NCBI Gene
- GeneRIF
- Pubmed

| Search Term | Rank | Fields |
| --- | --- | --- |
| [Ee]xtracellular [Mm]atrix | 8 | Process(1); PubMed(1); GeneRIFs(1) |

  
hide/show details for Ero1lb

### [Ee]xtracellular [Mm]atrix

#### Process

1. **extracellular matrix** organization

#### PubMed

1. Endoplasmic reticulum thiol oxidase deficiency leads to ascorbic acid depletion and noncanonical scurvy in mice. Endoplasmic reticulum (ER) thiol oxidases initiate a disulfide relay to oxidatively fold secreted proteins. We found that combined loss-of-function mutations in genes encoding the ER thiol oxidases ERO1α, ERO1β, and PRDX4 compromised th**e extracellular matr**ix in mice and interfered with the intracellular maturation of procollagen. These severe abnormalities were associated with an unexpectedly modest delay in disulfide bond formation in secreted proteins but a profound, 5-fold lower procollagen 4-hydroxyproline content and enhanced cysteinyl sulfenic acid modification of ER proteins. Tissue ascorbic acid content was lower in mutant mice, and ascorbic acid supplementation improved procollagen maturation and lowered sulfenic acid content in vivo. In vitro, the presence of a sulfenic acid donor accelerated the oxidative inactivation of ascorbate by an H(2)O(2)-generating system. Compromised ER disulfide relay thus exposes protein thiols to competing oxidation to sulfenic acid, resulting in depletion of ascorbic acid, impaired procollagen proline 4-hydroxylation, and a noncanonical form of scurvy.

#### GeneRIFs

1. combined loss-of-function mutations in genes encoding the ER thiol oxidases ERO1alpha, ERO1beta, and PRDX4 compromised the **extracellular matrix** and interfered with the intracellular maturation of procollagen

---

## Ero1l

- NCBI Gene
- GeneRIF
- Pubmed

| Search Term | Rank | Fields |
| --- | --- | --- |
| [Ee]xtracellular [Mm]atrix | 8 | Process(1); PubMed(2); GeneRIFs(1) |

  
hide/show details for Ero1l

### [Ee]xtracellular [Mm]atrix

#### Process

1. **extracellular matrix** organization

#### PubMed

1. Endoplasmic reticulum thiol oxidase deficiency leads to ascorbic acid depletion and noncanonical scurvy in mice. Endoplasmic reticulum (ER) thiol oxidases initiate a disulfide relay to oxidatively fold secreted proteins. We found that combined loss-of-function mutations in genes encoding the ER thiol oxidases ERO1α, ERO1β, and PRDX4 compromised th**e extracellular matr**ix in mice and interfered with the intracellular maturation of procollagen. These severe abnormalities were associated with an unexpectedly modest delay in disulfide bond formation in secreted proteins but a profound, 5-fold lower procollagen 4-hydroxyproline content and enhanced cysteinyl sulfenic acid modification of ER proteins. Tissue ascorbic acid content was lower in mutant mice, and ascorbic acid supplementation improved procollagen maturation and lowered sulfenic acid content in vivo. In vitro, the presence of a sulfenic acid donor accelerated the oxidative inactivation of ascorbate by an H(2)O(2)-generating system. Compromised ER disulfide relay thus exposes protein thiols to competing oxidation to sulfenic acid, resulting in depletion of ascorbic acid, impaired procollagen proline 4-hydroxylation, and a noncanonical form of scurvy.
2. Endoplasmic reticulum thiol oxidase deficiency leads to ascorbic acid depletion and noncanonical scurvy in mice. Endoplasmic reticulum (ER) thiol oxidases initiate a disulfide relay to oxidatively fold secreted proteins. We found that combined loss-of-function mutations in genes encoding the ER thiol oxidases ERO1α, ERO1β, and PRDX4 compromised th**e extracellular matr**ix in mice and interfered with the intracellular maturation of procollagen. These severe abnormalities were associated with an unexpectedly modest delay in disulfide bond formation in secreted proteins but a profound, 5-fold lower procollagen 4-hydroxyproline content and enhanced cysteinyl sulfenic acid modification of ER proteins. Tissue ascorbic acid content was lower in mutant mice, and ascorbic acid supplementation improved procollagen maturation and lowered sulfenic acid content in vivo. In vitro, the presence of a sulfenic acid donor accelerated the oxidative inactivation of ascorbate by an H(2)O(2)-generating system. Compromised ER disulfide relay thus exposes protein thiols to competing oxidation to sulfenic acid, resulting in depletion of ascorbic acid, impaired procollagen proline 4-hydroxylation, and a noncanonical form of scurvy.

#### GeneRIFs

1. combined loss-of-function mutations in genes encoding the ER thiol oxidases ERO1alpha, ERO1beta, and PRDX4 compromised the **extracellular matrix** and interfered with the intracellular maturation of procollagen

---

## Magi2

- NCBI Gene
- GeneRIF
- Pubmed

| Search Term | Rank | Fields |
| --- | --- | --- |
| [Mm]igration | 6 | Process(1); PubMed(1) |
| [Ii]nvasive | 10 | PubMed(1) |

  
hide/show details for Magi2

### [Mm]igration

#### Process

1. negative regulation of cell **migration**

#### PubMed

1. Delta proteins and MAGI proteins: an interaction of Notch ligands with intracellular scaffolding molecules and its significance for zebrafish development. Delta proteins activate Notch through a binding reaction that depends on their extracellular domains; but the intracellular (C-terminal) domains of the Deltas also have significant functions. All classes of vertebrates possess a subset of Delta proteins with a conserved ATEV\* motif at their C termini. These ATEV Deltas include Delta1 and Delta4 in mammals and DeltaD and DeltaC in the zebrafish. We show that these Deltas associate with the membrane-associated scaffolding proteins MAGI1, MAGI2 and MAGI3, through a direct interaction between the C termini of the Deltas and a specific PDZ domain (PDZ4) of the MAGIs. In cultured cells and in subsets of cells in the intact zebrafish embryo, DeltaD and MAGI1 are co-localized at the plasma membrane. The interaction and the co-localization can be abolished by injection of a morpholino that blocks the mRNA splicing reaction that gives DeltaD its terminal valine, on which the interaction depends. Embryos treated in this way appear normal with respect to some known functions of DeltaD as a Notch ligand, including the control of somite segmentation, neurogenesis, and hypochord formation. They do, however, show an anomalous distribution of Rohon-Beard neurons in the dorsal neural tube, suggesting that the Delta-MAGI interaction may play some part in the control of neuron **migration**.

### [Ii]nvasive

#### PubMed

1. Optimising experimental design for high-throughput phenotyping in mice: a case study. To further the functional annotation of the mammalian genome, the Sanger Mouse Genetics Programme aims to generate and characterise knockout mice in a high-throughput manner. Annually, approximately 200 lines of knockout mice will be characterised using a standardised battery of phenotyping tests covering key disease indications ranging from obesity to sensory acuity. From these findings secondary centres will select putative mutants of interest for more in-depth, confirmatory experiments. Optimising experimental design and data analysis is essential to maximise output using the resources with greatest efficiency, thereby attaining our biological objective of understanding the role of genes in normal development and disease. This study uses the example of the non**invasive** blood pressure test to demonstrate how statistical investigation is important for generating meaningful, reliable results and assessing the design for the defined research objectives. The analysis adjusts for the multiple-testing problem by applying the false discovery rate, which controls the number of false calls within those highlighted as significant. A variance analysis finds that the variation between mice dominates this assay. These variance measures were used to examine the interplay between days, readings, and number of mice on power, the ability to detect change. If an experiment is underpowered, we cannot conclude whether failure to detect a biological difference arises from low power or lack of a distinct phenotype, hence the mice are subjected to testing without gain. Consequently, in confirmatory studies, a power analysis along with the 3Rs can provide justification to increase the number of mice used.

---

## Inpp5f

- NCBI Gene
- GeneRIF
- Pubmed

| Search Term | Rank | Fields |
| --- | --- | --- |
| [Mm]igration | 6 | PubMed(1) |

  
hide/show details for Inpp5f

### [Mm]igration

#### PubMed

1. Spatiotemporal control of phosphatidylinositol 4-phosphate by Sac2 regulates endocytic recycling. It is well established that the spatial- and temporal-restricted generation and turnover of phosphoinositides (PIs) by a cascade of PI-metabolizing enzymes is a key regulatory mechanism in the endocytic pathway. Here, we demonstrate that the Sac1 domain-containing protein Sac2 is a PI 4-phosphatase that specifically hydrolyzes phosphatidylinositol 4-phosphate in vitro. We further show that Sac2 colocalizes with early endosomal markers and is recruited to transferrin (Tfn)-containing vesicles during endocytic recycling. Exogenous expression of the catalytically inactive mutant Sac2C458S resulted in altered cellular distribution of Tfn receptors and delayed Tfn recycling. Furthermore, genomic ablation of Sac2 caused a similar perturbation on Tfn and integrin recycling as well as defects in cell **migration**. Structural characterization of Sac2 revealed a unique pleckstrin-like homology Sac2 domain conserved in all Sac2 orthologues. Collectively, our findings provide evidence for the tight regulation of PIs by Sac2 in the endocytic recycling pathway.

---

## Pou3f3

- NCBI Gene
- GeneRIF
- Pubmed

| Search Term | Rank | Fields |
| --- | --- | --- |
| [Mm]igration | 6 | Process(1); PubMed(14); GeneRIFs(1) |

  
hide/show details for Pou3f3

### [Mm]igration

#### Process

1. cerebral cortex radially oriented cell **migration**

#### PubMed

1. Rac1 controls the formation of midline commissures and the competency of tangential **migration** in ventral telencephalic neurons. Previous studies using dominant-mutant constructs have implicated Rac1 GTPase in neuritogenesis and neuronal **migration**. However, overexpression of dominant mutants generally blocks Rho-GTPase activity; thus, it may not reveal the specific or physiological functions of Rac1. To address this issue, we have applied a conditional gene-targeting strategy, using Foxg1-Cre mice to delete Rac1 in the ventricular zone (VZ) of telencephalon and Dlx5/6-Cre-IRES (internal ribosomal entry site)-EGFP (enhanced green fluorescent protein) (Dlx5/6-CIE) in the subventricular zone (SVZ) of ventral telencephalon, respectively. Surprisingly, the deletion of Rac1 in VZ progenitors did not prevent axonal outgrowth of telencephalic neurons. However, the anterior commissure was absent, and the corpus callosal as well as hippocampal commissural axons failed to cross the midline in Rac1/Foxg1-Cre knock-out embryos. The thalamocortical and corticothalamic axons also showed defasciculation or projection defects. These results suggest that Rac1 controls axon guidance rather than neuritogenesis. In addition, although Rac1/Foxg1-Cre knock-out embryos showed delayed radial **migration** of cortical projection neurons and severe impairment of tangential **migration** by the ventral telencephalon-derived interneurons, deletion of Rac1 in the SVZ by Dlx5/6-CIE mice produced no discernible defects in tangential **migration**. These contrasting effects of Rac1 deletion on tangential **migration** suggest that Rac1 is dispensable for cellular motility per se during neuronal **migration**. Together, these results underscore the challenge of deciphering the biological functions of Rac1, and Rho-GTPases in general, during mammalian brain development. Moreover, they indicate that Rac1 has a critical role in axon guidance and in acquisition of migratory competency during differentiation of the progenitors for the ventral telencephalon-derived interneurons.
2. High affinity binding of Dab1 to Reelin receptors promotes normal positioning of upper layer cortical plate neurons. The positions of neurons in the neocortex, hippocampus, cerebellum and various other laminated brain regions are regulated by a signaling pathway initiated by the secreted protein Reelin and requiring the intracellular adaptor protein Dab1. Dab1 and the Reelin receptors VLDLR and ApoER2 are expressed by neurons whose **migration**s are coordinated by Reelin. In vitro, Dab1 binds with high affinity to the cytoplasmic tails of VLDLR and ApoER2 via its PTB domain. To test the importance of Dab1 binding to VLDLR and ApoER2, we replaced the Dab1 gene with a cDNA cassette encoding a point mutant allele, Dab1(F158V). This mutation strongly decreases Dab1 binding in vitro to peptides containing the ApoER2 or VLDLR cytoplasmic regions. Surprisingly, Dab1(F158V/F158V) homozygotes have no discernable phenotype. However, Dab1(F158V/-) hemizygous animals have a subtle phenotype in which late-generated cortical plate neurons migrate excessively into the marginal zone. Early cortical plate neurons, subplate neurons, hippocampal pyramidal cells and cerebellar Purkinje cells are positioned normally. Thus Dab(F158V) is a weak loss-of-function (hypomorphic) allele that has no detectable effect when homozygous. The phenotype of Dab1(F158V/-) hemizygotes shows that late cortical plate neurons of layers 2-3 require efficient Reelin-Dab1 signaling to prevent them entering the marginal zone. The Dab1(F158V) allele adds to a series of Dab1 alleles that demonstrates cell type-specific variation in the Reelin-Dab1 pathway.
3. Ephrin signalling controls brain size by regulating apoptosis of neural progenitors. Mechanisms controlling brain size include the regulation of neural progenitor cell proliferation, differentiation, survival and **migration**. Here we show that ephrin-A/EphA receptor signalling plays a key role in controlling the size of the mouse cerebral cortex by regulating cortical progenitor cell apoptosis. In vivo gain of EphA receptor function, achieved through ectopic expression of ephrin-A5 in early cortical progenitors expressing EphA7, caused a transient wave of neural progenitor cell apoptosis, resulting in premature depletion of progenitors and a subsequent dramatic decrease in cortical size. In vitro treatment with soluble ephrin-A ligands similarly induced the rapid death of cultured dissociated cortical progenitors in a caspase-3-dependent manner, thereby confirming a direct effect of ephrin/Eph signalling on apoptotic cascades. Conversely, in vivo loss of EphA function, achieved through EphA7 gene disruption, caused a reduction in apoptosis occurring normally in forebrain neural progenitors, resulting in an increase in cortical size and, in extreme cases, exencephalic forebrain overgrowth. Together, these results identify ephrin/Eph signalling as a physiological trigger for apoptosis that can alter brain size and shape by regulating the number of neural progenitors.
4. Transcriptional regulation of cortical neuron **migration** by POU domain factors. The identification of pathways mediated by the kinase Cdk5 and the ligand reelin has provided a conceptual framework for exploring the molecular mechanisms underlying proper lamination of the developing mammalian cerebral cortex. In this report, we identify a component of the regulation of Cdk5-mediated cortical lamination by genetic analysis of the roles of the class III POU domain transcription factors, Brn-1 and Brn-2, expressed during the development of the forebrain and coexpressed in most layer II-V cortical neurons. Brn-1 and Brn-2 appear to critically control the initiation of radial **migration**, redundantly regulating the cell-autonomous expression of the p35 and p39 regulatory subunits of Cdk5 in migrating cortical neurons, with Brn-1(-/-)/Brn-2(-/-) mice exhibiting cortical inversion.
5. The adenomatous polyposis coli protein is an essential regulator of radial glial polarity and construction of the cerebral cortex. Radial glia are highly polarized cells that serve as neuronal progenitors and as scaffolds for neuronal **migration** during construction of the cerebral cortex. How radial glial cells establish and maintain their morphological polarity is unknown. Using conditional gene targeting in mice, we demonstrate that adenomatous polyposis coli (APC) serves an essential function in the maintenance of polarized radial glial scaffold during brain development. In the absence of APC, radial glial cells lose their polarity and responsiveness to the extracellular polarity maintenance cues, such as neuregulin-1. Elimination of APC further leads to marked instability of the radial glial microtubule cytoskeleton. The resultant changes in radial glial function and loss of APC in radial glial progeny lead to defective generation and **migration** of cortical neurons, severely disrupted cortical layer formation, and aberrant axonal tract development. Thus, APC is an essential regulator of radial glial polarity and is critical for the construction of cerebral cortex in mammals.
6. Ephrin Bs are essential components of the Reelin pathway to regulate neuronal **migration**. Coordinated **migration** of neurons in the developing and adult brain is essential for its proper function. The secreted glycoprotein Reelin (also known as RELN) guides **migration** of neurons by binding to two lipoprotein receptors, the very-low-density lipoprotein receptor (VLDLR) and apolipoprotein E receptor 2 (ApoER2, also known as LRP8). Loss of Reelin function in humans results in the severe developmental disorder lissencephaly and it has also been associated with other neurological disorders such as epilepsy, schizophrenia and Alzheimer's disease. The molecular mechanisms by which Reelin activates its receptors and controls cellular functions are largely unknown. Here we show that the neuronal guidance cues ephrin B proteins are essential for Reelin signalling during the development of laminated structures in the brain. We show that ephrin Bs genetically interact with Reelin. Notably, compound mouse mutants (Reln(+/-); Efnb3(-/-) or Reln(+/-); Efnb2(-/-)) and triple ephrin B1, B2, B3 knockouts show neuronal **migration** defects that recapitulate the ones observed in the neocortex, hippocampus and cerebellum of the reeler mouse. Mechanistically, we show that Reelin binds to the extracellular domain of ephrin Bs, which associate at the membrane with VLDLR and ApoER2 in neurons. Clustering of ephrin Bs leads to the recruitment and phosphorylation of Dab1 which is necessary for Reelin signalling. Conversely, loss of function of ephrin Bs severely impairs Reelin-induced Dab1 phosphorylation. Importantly, activation of ephrin Bs can rescue the reeler neuronal **migration** defects in the absence of Reelin protein. Together, our results identify ephrin Bs as essential components of the Reelin receptor/signalling pathway to control neuronal **migration** during the development of the nervous system.
7. Brn-1 and Brn-2 share crucial roles in the production and positioning of mouse neocortical neurons. Formation of highly organized neocortical structure depends on the production and correct placement of the appropriate number and types of neurons. POU homeodomain proteins Brn-1 and Brn-2 are coexpressed in the developing neocortex, both in the late precursor cells and in the migrating neurons. Here we show that double disruption of both Brn-1 and Brn-2 genes in mice leads to abnormal formation of the neocortex with dramatically reduced production of layer IV-II neurons and defective **migration** of neurons unable to express mDab1. These data indicate that Brn-1 and Brn-2 share roles in the production and positioning of neocortical neuron development.
8. Phosphorylation of SCG10/stathmin-2 determines multipolar stage exit and neuronal **migration** rate. Cell **migration** is the consequence of the sum of positive and negative regulatory mechanisms. Although appropriate **migration** of neurons is a principal feature of brain development, the negative regulatory mechanisms remain obscure. We found that JNK1 was highly active in developing cortex and that selective inhibition of JNK in the cytoplasm markedly increased both the frequency of exit from the multipolar stage and radial **migration** rate and ultimately led to an ill-defined cellular organization. Moreover, regulation of multipolar-stage exit and radial **migration** in Jnk1(-/-) (also known as Mapk8) mice, resulted from consequential changes in phosphorylation of the microtubule regulator SCG10 (also called stathmin-2). Expression of an SCG10 mutant that mimics the JNK1-phosphorylated form restored normal **migration** in the brains of Jnk1(-/-) mouse embryos. These findings indicate that the phosphorylation of SCG10 by JNK1 is a fundamental mechanism that governs the transition from the multipolar stage and the rate of neuronal cell movement during cortical development.
9. POU-III transcription factors (Brn1, Brn2, and Oct6) influence neurogenesis, molecular identity, and migratory destination of upper-layer cells of the cerebral cortex. The upper layers (II-IV) are the most prominent distinguishing feature of mammalian neocortex compared with avian or reptilian dorsal cortex, and are vastly expanded in primates. Although the time-dependent embryonic generation of upper-layer cells is genetically instructed within their parental progenitors, mechanisms governing cell-intrinsic fate transitions remain obscure. POU-homeodomain transcription factors Pou3f3 and Pou3f2 (Brn1 and Brn2) are known to label postmitotic upper-layer cells, and are redundantly required for their production. We find that the onset of Pou3f3/2 expression actually occurs in ventricular zone (VZ) progenitors, and that Pou3f3/2 subsequently label neural progeny switching from deep-layer Ctip2(+) identity to Satb2(+) upper-layer fate as they migrate to proper superficial positions. By using an Engrailed dominant-negative repressor, we show that sustained neurogenesis after the deep- to upper-layer transition requires the proneual action of Pou3fs in VZ progenitors. Conversely, single-gene overexpression of any Pou3f in early neural progenitors is sufficient to specify the precocious birth of Satb2(+) daughter neurons that extend axons to the contralateral hemisphere, as well as exhibit robust pia-directed **migration** that is characteristic of upper-layer cells. Finally, we demonstrate that Pou3fs influence multiple stages of neurogenesis by suppressing Notch effector Hes5, and promoting the expression of proneural transcription factors Tbr2 and Tbr1.
10. LPIAT1 regulates arachidonic acid content in phosphatidylinositol and is required for cortical lamination in mice. Dietary arachidonic acid (AA) has roles in growth, neuronal development, and cognitive function in infants. AA is remarkably enriched in phosphatidylinositol (PI), an important constituent of biological membranes in mammals; however, the physiological significance of AA-containing PI remains unknown. In an RNA interference-based genetic screen using Caenorhabditis elegans, we recently cloned mboa-7 as an acyltransferase that selectively incorporates AA into PI. Here we show that lysophosphatidylinositol acyltransferase 1 (LPIAT1, also known as MBOAT7), the closest mammalian homologue, plays a crucial role in brain development in mice. Lpiat1(-/-) mice show almost no LPIAT activity with arachidonoyl-CoA as an acyl donor and show reduced AA contents in PI and PI phosphates. Lpiat1(-/-) mice die within a month and show atrophy of the cerebral cortex and hippocampus. Immunohistochemical analysis reveals disordered cortical lamination and delayed neuronal **migration** in the cortex of E18.5 Lpiat1(-/-) mice. LPIAT1 deficiency also causes disordered neuronal processes in the cortex and reduced neurite outgrowth in vitro. Taken together, these results demonstrate that AA-containing PI/PI phosphates play an important role in normal cortical lamination during brain development in mice.
11. Absence of Fyn and Src causes a reeler-like phenotype. Nonreceptor protein tyrosine kinases of the Src family regulate the survival, proliferation, differentiation, and motility of many cell types, but their roles in brain development are unclear. Biochemical and in vitro experiments implicate Src and Fyn in the Reelin-dependent tyrosine phosphorylation of Dab1, which controls the positioning of radially migrating neurons in many brain regions. However, genetic evidence that either Src or Fyn mediates Reelin-dependent **migration**s in vivo has been lacking. Here, we report that, although Src is dispensable and although the absence of Fyn causes an intermediate phenotype, the combined absence of Src and Fyn almost abolishes tyrosine phosphorylation of Dab1 and causes defects in the fetal cortex and cerebellum very similar to those of dab1 mutants of the same age. Neurogenesis is not detectably affected, but the layering of neurons in the cortex is inverted, and the formation of the Purkinje plate is impaired. This implies that Src and Fyn are needed for Reelin-dependent events during brain development.
12. Proneural bHLH and Brn proteins coregulate a neurogenic program through cooperative binding to a conserved DNA motif. Proneural proteins play a central role in vertebrate neurogenesis, but little is known of the genes that they regulate and of the factors that interact with proneural proteins to activate a neurogenic program. Here, we demonstrate that the proneural protein Mash1 and the POU proteins Brn1 and Brn2 interact on the promoter of the Notch ligand Delta1 and synergistically activate Delta1 transcription, a key step in neurogenesis. Overexpression experiments in vivo indicate that Brn2, like Mash1, regulates additional aspects of neurogenesis, including the division of progenitors and the differentiation and **migration** of neurons. We identify by in silico screening a number of additional candidate target genes, which are recognized by Mash1 and Brn proteins through a DNA-binding motif similar to that found in the Delta1 gene and present a broad range of activities. We thus propose that Mash1 synergizes with Brn factors to regulate multiple steps of neurogenesis.
13. Mitotic spindle regulation by Nde1 controls cerebral cortical size. Ablation of the LIS1-interacting protein Nde1 (formerly mNudE) in mouse produces a small brain (microcephaly), with the most dramatic reduction affecting the cerebral cortex. While cortical lamination is mostly preserved, the mutant cortex has fewer neurons and very thin superficial cortical layers (II to IV). BrdU birthdating revealed retarded and modestly disorganized neuronal **migration**; however, more dramatic defects on mitotic progression, mitotic orientation, and mitotic chromosome localization in cortical progenitors were observed in Nde1 mutant embryos. The small cerebral cortex seems to reflect both reduced progenitor cell division and altered neuronal cell fates. In vitro analysis demonstrated that Nde1 is essential for centrosome duplication and mitotic spindle assembly. Our data show that mitotic spindle function and orientation are essential for normal development of mammalian cerebral cortex.
14. MACF1 regulates the **migration** of pyramidal neurons via microtubule dynamics and GSK-3 signaling. Neuronal **migration** and subsequent differentiation play critical roles for establishing functional neural circuitry in the developing brain. However, the molecular mechanisms that regulate these processes are poorly understood. Here, we show that microtubule actin crosslinking factor 1 (MACF1) determines neuronal positioning by regulating microtubule dynamics and mediating GSK-3 signaling during brain development. First, using MACF1 floxed allele mice and in utero gene manipulation, we find that MACF1 deletion suppresses **migration** of cortical pyramidal neurons and results in aberrant neuronal positioning in the developing brain. The cell autonomous deficit in **migration** is associated with abnormal dynamics of leading processes and centrosomes. Furthermore, microtubule stability is severely damaged in neurons lacking MACF1, resulting in abnormal microtubule dynamics. Finally, MACF1 interacts with and mediates GSK-3 signaling in developing neurons. Our findings establish a cellular mechanism underlying neuronal **migration** and provide insights into the regulation of cytoskeleton dynamics in developing neurons.

#### GeneRIFs

1. Brn-1 and Brn-2 appear to critically control the initiation of radial **migration**, redundantly regulating the cell-autonomous expression of the p35 and p39 regulatory subunits of Cdk5 in migrating cortical neurons

---

## Pou3f2

- NCBI Gene
- GeneRIF
- Pubmed

| Search Term | Rank | Fields |
| --- | --- | --- |
| [Mm]igration | 6 | Process(1); PubMed(27); GeneRIFs(2) |
| [Ii]nvasive | 10 | PubMed(1) |
| [Mm]etastasize | 11 | PubMed(1) |
| [Mm]etastasis | 12 | PubMed(1) |

  
hide/show details for Pou3f2

### [Mm]igration

#### Process

1. cerebral cortex radially oriented cell **migration**

#### PubMed

1. PTB deficiency causes the loss of adherens junctions in the dorsal telencephalon and leads to lethal hydrocephalus. Polypyrimidine tract-binding protein (PTB) is a well-characterized RNA-binding protein and known to be preferentially expressed in neural stem cells (NSCs) in the central nervous system; however, its role in NSCs in the developing brain remains unclear. To explore the role of PTB in embryonic NSCs in vivo, Nestin-Cre-mediated conditional Ptb knockout mice were generated for this study. In the mutant forebrain, despite the depletion of PTB protein, neither abnormal neurogenesis nor flagrant morphological abnormalities were observed at embryonic day 14.5 (E14.5). Nevertheless, by 10 weeks, nearly all mutant mice succumbed to hydrocephalus (HC), which was caused by a lack of the ependymal cell layer in the dorsal cortex. Upon further analysis, a gradual loss of adherens junctions (AJs) was observed in the ventricular zone (VZ) of the dorsal telencephalon in the mutant brains, beginning at E14.5. In the AJs-deficient VZ, impaired interkinetic nuclear **migration** and precocious differentiation of NSCs were observed after E14.5. These findings demonstrated that PTB depletion in the dorsal telencephalon is causally involved in the development of HC and that PTB is important for the maintenance of AJs in the NSCs of the dorsal telencephalon.
2. Transcriptional regulation of cortical neuron **migration** by POU domain factors. The identification of pathways mediated by the kinase Cdk5 and the ligand reelin has provided a conceptual framework for exploring the molecular mechanisms underlying proper lamination of the developing mammalian cerebral cortex. In this report, we identify a component of the regulation of Cdk5-mediated cortical lamination by genetic analysis of the roles of the class III POU domain transcription factors, Brn-1 and Brn-2, expressed during the development of the forebrain and coexpressed in most layer II-V cortical neurons. Brn-1 and Brn-2 appear to critically control the initiation of radial **migration**, redundantly regulating the cell-autonomous expression of the p35 and p39 regulatory subunits of Cdk5 in migrating cortical neurons, with Brn-1(-/-)/Brn-2(-/-) mice exhibiting cortical inversion.
3. Stress-activated protein kinase MKK7 regulates axon elongation in the developing cerebral cortex. The c-Jun NH(2)-terminal protein kinase (JNK), which belongs to the mitogen-activated protein kinase family, plays important roles in a broad range of physiological processes. JNK is controlled by two upstream regulators, mitogen-activated protein kinase kinase (MKK) 7 and MKK4. To elucidate the physiological functions of MKK7, we used Nestin-Cre to generate a novel mouse model in which the mkk7 gene was specifically deleted in the nervous system (Mkk7(flox/flox) Nestin-Cre mice). These mice were indistinguishable from their control littermates in gross appearance during embryogenesis but died immediately after birth without breathing. Histological examination showed that the mutants had severe defects in brain development, including enlarged ventricles, reduced striatum, and minimal axon tracts. Electron microscopy revealed abnormal accumulations of filamentous structures and autophagic vacuoles in Mkk7(flox/flox) Nestin-Cre brain. Further analysis showed that MKK7 deletion decreased numbers of TAG-1-expressing axons and delayed neuronal **migration** in the cerebrum. Neuronal differentiation was not altered. In utero electroporation studies showed that contralateral projection of axons by layer 2/3 neurons was impaired in the absence of MKK7. Moreover, MKK7 regulated axon elongation in a cell-autonomous manner in vivo, a finding confirmed in vitro. Finally, phosphorylation levels of JNK substrates, including c-Jun, neurofilament heavy chain, microtubule-associated protein 1B, and doublecortin, were reduced in Mkk7(flox/flox) Nestin-Cre brain. Our findings demonstrate that the phenotype of Mkk7(flox/flox) Nestin-Cre mice differs substantially from that of Mkk4(flox/flox) Nestin-Cre mice, and establish that MKK7-mediated regulation of JNK is uniquely critical for both axon elongation and radial **migration** in the developing brain.
4. Brn-1 and Brn-2 share crucial roles in the production and positioning of mouse neocortical neurons. Formation of highly organized neocortical structure depends on the production and correct placement of the appropriate number and types of neurons. POU homeodomain proteins Brn-1 and Brn-2 are coexpressed in the developing neocortex, both in the late precursor cells and in the migrating neurons. Here we show that double disruption of both Brn-1 and Brn-2 genes in mice leads to abnormal formation of the neocortex with dramatically reduced production of layer IV-II neurons and defective **migration** of neurons unable to express mDab1. These data indicate that Brn-1 and Brn-2 share roles in the production and positioning of neocortical neuron development.
5. POU-III transcription factors (Brn1, Brn2, and Oct6) influence neurogenesis, molecular identity, and migratory destination of upper-layer cells of the cerebral cortex. The upper layers (II-IV) are the most prominent distinguishing feature of mammalian neocortex compared with avian or reptilian dorsal cortex, and are vastly expanded in primates. Although the time-dependent embryonic generation of upper-layer cells is genetically instructed within their parental progenitors, mechanisms governing cell-intrinsic fate transitions remain obscure. POU-homeodomain transcription factors Pou3f3 and Pou3f2 (Brn1 and Brn2) are known to label postmitotic upper-layer cells, and are redundantly required for their production. We find that the onset of Pou3f3/2 expression actually occurs in ventricular zone (VZ) progenitors, and that Pou3f3/2 subsequently label neural progeny switching from deep-layer Ctip2(+) identity to Satb2(+) upper-layer fate as they migrate to proper superficial positions. By using an Engrailed dominant-negative repressor, we show that sustained neurogenesis after the deep- to upper-layer transition requires the proneual action of Pou3fs in VZ progenitors. Conversely, single-gene overexpression of any Pou3f in early neural progenitors is sufficient to specify the precocious birth of Satb2(+) daughter neurons that extend axons to the contralateral hemisphere, as well as exhibit robust pia-directed **migration** that is characteristic of upper-layer cells. Finally, we demonstrate that Pou3fs influence multiple stages of neurogenesis by suppressing Notch effector Hes5, and promoting the expression of proneural transcription factors Tbr2 and Tbr1.
6. Proneural bHLH and Brn proteins coregulate a neurogenic program through cooperative binding to a conserved DNA motif. Proneural proteins play a central role in vertebrate neurogenesis, but little is known of the genes that they regulate and of the factors that interact with proneural proteins to activate a neurogenic program. Here, we demonstrate that the proneural protein Mash1 and the POU proteins Brn1 and Brn2 interact on the promoter of the Notch ligand Delta1 and synergistically activate Delta1 transcription, a key step in neurogenesis. Overexpression experiments in vivo indicate that Brn2, like Mash1, regulates additional aspects of neurogenesis, including the division of progenitors and the differentiation and **migration** of neurons. We identify by in silico screening a number of additional candidate target genes, which are recognized by Mash1 and Brn proteins through a DNA-binding motif similar to that found in the Delta1 gene and present a broad range of activities. We thus propose that Mash1 synergizes with Brn factors to regulate multiple steps of neurogenesis.
7. POU-III transcription factors (Brn1, Brn2, and Oct6) influence neurogenesis, molecular identity, and migratory destination of upper-layer cells of the cerebral cortex. The upper layers (II-IV) are the most prominent distinguishing feature of mammalian neocortex compared with avian or reptilian dorsal cortex, and are vastly expanded in primates. Although the time-dependent embryonic generation of upper-layer cells is genetically instructed within their parental progenitors, mechanisms governing cell-intrinsic fate transitions remain obscure. POU-homeodomain transcription factors Pou3f3 and Pou3f2 (Brn1 and Brn2) are known to label postmitotic upper-layer cells, and are redundantly required for their production. We find that the onset of Pou3f3/2 expression actually occurs in ventricular zone (VZ) progenitors, and that Pou3f3/2 subsequently label neural progeny switching from deep-layer Ctip2(+) identity to Satb2(+) upper-layer fate as they migrate to proper superficial positions. By using an Engrailed dominant-negative repressor, we show that sustained neurogenesis after the deep- to upper-layer transition requires the proneual action of Pou3fs in VZ progenitors. Conversely, single-gene overexpression of any Pou3f in early neural progenitors is sufficient to specify the precocious birth of Satb2(+) daughter neurons that extend axons to the contralateral hemisphere, as well as exhibit robust pia-directed **migration** that is characteristic of upper-layer cells. Finally, we demonstrate that Pou3fs influence multiple stages of neurogenesis by suppressing Notch effector Hes5, and promoting the expression of proneural transcription factors Tbr2 and Tbr1.
8. Brn-1 and Brn-2 share crucial roles in the production and positioning of mouse neocortical neurons. Formation of highly organized neocortical structure depends on the production and correct placement of the appropriate number and types of neurons. POU homeodomain proteins Brn-1 and Brn-2 are coexpressed in the developing neocortex, both in the late precursor cells and in the migrating neurons. Here we show that double disruption of both Brn-1 and Brn-2 genes in mice leads to abnormal formation of the neocortex with dramatically reduced production of layer IV-II neurons and defective **migration** of neurons unable to express mDab1. These data indicate that Brn-1 and Brn-2 share roles in the production and positioning of neocortical neuron development.
9. Transcriptional regulation of cortical neuron **migration** by POU domain factors. The identification of pathways mediated by the kinase Cdk5 and the ligand reelin has provided a conceptual framework for exploring the molecular mechanisms underlying proper lamination of the developing mammalian cerebral cortex. In this report, we identify a component of the regulation of Cdk5-mediated cortical lamination by genetic analysis of the roles of the class III POU domain transcription factors, Brn-1 and Brn-2, expressed during the development of the forebrain and coexpressed in most layer II-V cortical neurons. Brn-1 and Brn-2 appear to critically control the initiation of radial **migration**, redundantly regulating the cell-autonomous expression of the p35 and p39 regulatory subunits of Cdk5 in migrating cortical neurons, with Brn-1(-/-)/Brn-2(-/-) mice exhibiting cortical inversion.
10. Fezl is required for the birth and specification of corticospinal motor neurons. The molecular mechanisms controlling the differentiation of neural progenitors into distinct subtypes of neurons during neocortical development are unknown. Here, we report that Fezl is required for the specification of corticospinal motor neurons and other subcerebral projection neurons, which are absent from Fezl null mutant neocortex. There is neither an increase in cell death in Fezl(-/-) cortex nor abnormalities in **migration**, indicating that the absence of subcerebral projection neurons is due to a failure in fate specification. In striking contrast, other neuronal populations in the same and other cortical layers are born normally. Overexpression of Fezl results in excess production of subcerebral projection neurons and arrested **migration** of these neurons in the germinal zone. These data indicate that Fezl plays a central role in the specification of corticospinal motor neurons and other subcerebral projection neurons, controlling early decisions regarding lineage-specific differentiation from neural progenitors.
11. Cortical **migration** defects in mice expressing A-RAF from the B-RAF locus. We have previously shown that mice lacking the protein kinase B-RAF have defects in both neural and endothelial cell lineages and die around embryonic day 12 (E12). To delineate the function of B-RAF in the brain, B-RAF KIN/KIN mice lacking B-RAF and expressing A-RAF under the control of the B-RAF locus were created. B-RAF KIN/KIN embryos displayed no vascular defects, no endothelial and neuronal apoptosis, or gross developmental abnormalities, and a significant proportion of these animals survived for up to 8 weeks. Cell proliferation in the neocortex was reduced from E14.5 onwards. Newborn cortical neurons were impaired in their **migration** toward the cortical plate, causing a depletion of Brn-2-expressing pyramidal neurons in layers II, III, and V of the postnatal cortex. Our data reveal that B-RAF is an important mediator of neuronal survival, **migration**, and dendrite formation and that A-RAF cannot fully compensate for these functions.
12. Common partner Smad-independent canonical bone morphogenetic protein signaling in the specification process of the anterior rhombic lip during cerebellum development. Bone morphogenetic protein (BMP) signaling is critical for cerebellum development. However, the details of receptor regulated-Smad (R-Smad) and common partner Smad (Co-Smad, or Smad4) involvement are unclear. Here, we report that cerebellum-specific double conditional inactivation of Smad1 and Smad5 (Smad1/5) results in cerebellar hypoplasia, reduced granule cell numbers, and disorganized Purkinje neuron **migration** during embryonic development. However, single conditional inactivation of either Smad1 or Smad5 did not result in cerebellar abnormalities. Surprisingly, conditional inactivation of Smad4, which is considered to be the central mediator of canonical BMP-Smad signaling, resulted only in very mild cerebellar defects. Conditional inactivation of Smad1/5 led to developmental defects in the anterior rhombic lip (ARL), as shown by reduced cell proliferation and loss of Pax6 and Atoh1 expression. These defects subsequently caused the loss of the nuclear transitory zone and a region of the deep cerebellar nuclei. The normal maturation of the remaining granule cell precursors in the external granular layer (EGL) suggests Smad1/5 signaling is required for the specification process in ARL but not for the subsequent EGL development. Our results demonstrate functional redundancy for Smad1 and Smad5 but functional discrepancy between Smad1/5 and Smad4 during cerebellum development.
13. Patterns of neurogenesis and amplitude of Reelin expression are essential for making a mammalian-type cortex. The mammalian neocortex is characterized as a six-layered laminar structure, in which distinct types of pyramidal neurons are distributed coordinately during embryogenesis. In contrast, no other vertebrate class possesses a brain region that is strictly analogous to the neocortical structure. Although it is widely accepted that the pallium, a dorsal forebrain region, is specified in all vertebrate species, little is known of the differential mechanisms underlying laminated or non-laminated structures in the pallium. Here we show that differences in patterns of neuronal specification and **migration** provide the pallial architectonic diversity. We compared the neurogenesis in mammalian and avian pallium, focusing on subtype-specific gene expression, and found that the avian pallium generates distinct types of neurons in a spatially restricted manner. Furthermore, expression of Reelin gene is hardly detected in the developing avian pallium, and an experimental increase in Reelin-positive cells in the avian pallium modified radial fiber organization, which resulted in dramatic changes in the morphology of migrating neurons. Our results demonstrate that distinct mechanisms govern the patterns of neuronal specification in mammalian and avian pallial development, and that Reelin-dependent neuronal **migration** plays a critical role in mammalian type corticogenesis. These lines of evidence shed light on the developmental programs underlying the evolution of the mammalian specific laminated cortex.
14. Crucial roles of the Arp2/3 complex during mammalian corticogenesis. The polarity and organization of radial glial cells (RGCs), which serve as both stem cells and scaffolds for neuronal **migration**, are crucial for cortical development. However, the cytoskeletal mechanisms that drive radial glial outgrowth and maintain RGC polarity remain poorly understood. Here, we show that the Arp2/3 complex - the unique actin nucleator that produces branched actin networks - plays essential roles in RGC polarity and morphogenesis. Disruption of the Arp2/3 complex in murine RGCs retards process outgrowth toward the basal surface and impairs apical polarity and adherens junctions. Whereas the former is correlated with an abnormal actin-based leading edge, the latter is consistent with blockage in membrane trafficking. These defects result in altered cell fate, disrupted cortical lamination and abnormal angiogenesis. In addition, we present evidence that the Arp2/3 complex is a cell-autonomous regulator of neuronal **migration**. Our data suggest that Arp2/3-mediated actin assembly might be particularly important for neuronal cell motility in a soft or poorly adhesive matrix environment.
15. 14-3-3ε and ζ regulate neurogenesis and differentiation of neuronal progenitor cells in the developing brain. During brain development, neural progenitor cells proliferate and differentiate into neural precursors. These neural precursors migrate along the radial glial processes and localize at their final destination in the cortex. Numerous reports have revealed that 14-3-3 proteins are involved in many neuronal activities, although their functions in neurogenesis remain unclear. Here, using 14-3-3ε/ζ double knock-out mice, we found that 14-3-3 proteins are important for proliferation and differentiation of neural progenitor cells in the cortex, resulting in neuro**nal migra**tion defects and seizures. 14-3-3 deficiency resulted in the increase of δ-catenin and the decrease of β-catenin and αN-catenin. 14-3-3 proteins regulated neuronal differentiation into neurons via direct interactions with phosphorylated δ-catenin to promote F-actin formation through a catenin/Rho GTPase/Limk1/cofilin signaling pathway. Conversely, n**euronal m**igration defects seen in the double knock-out mice were restored by phosphomimic Ndel1 mutants, but not δ-catenin. Our findings provide new evidence that 14-3-3 proteins play important roles in neurogenesis and **neuronal** migration via the regulation of distinct signaling cascades.
16. Predominant expression of Brn-2 in the postmitotic neurons of the developing mouse neocortex. The expression of Brn-2, a central nervous systems (CNS)-specific POU domain transcription factor, in the developing mouse neocortex was examined with an anti-Brn-2 antibody. Brn-2 protein was first detected in CNS on embryonic day (E) 11.5, and remained strong until E15.5. From E11.5 to postnatal day (P) 0, a high level of Brn-2 expression was observed in the subventricular zone, the intermediate zone, and the outer layer of the neocortex, but not in the ventricular zone. In the double-staining experiments, most of the Brn-2 positive cells were also positive for NCAM-H, an adhesion molecule specific to post-mitotic neurons. Furthermore, BrdU-labeling experiments demonstrated the presence of Brn-2 protein exclusively in postmitotic cells. These results indicated that, in the developing neocortex, Brn-2 expression is up-regulated after the final cell division. Therefore, this transcription factor may be involved in the **migration** and/or maturation process of the immature neuronal cells.
17. Expression of liver X receptor beta is essential for formation of superficial cortical layers and **migration** of later-born neurons. Liver X receptor (LXR) beta regulates cholesterol levels in the brain and is essential for maintenance of motor neurons in the spinal cord and dopaminergic neurons in the substantia nigra. Here, we have examined the expression pattern of LXRbeta protein in the cerebral cortex and looked for defects in cortical development in LXRbeta knockout (LXRbeta(-/-)) mice. LXRbeta protein was widely expressed in the mouse brain at later embryonic stages, and the expression pattern in the cerebral cortex was developmentally regulated. In normal postnatal mice, LXRbeta was localized mainly in the upper layers of the cerebral cortex. In LXRbeta(-/-) mice layers II and III were thinner with fewer neurons. Layer I was slightly thicker, whereas layers IV-VI were essentially normal. Consistent with this finding, Brn2 and NeuN expression were decreased in the upper layers in the LXRbeta(-/-) neonatal cortex. The number of S-phase progenitor cells in the cortex between embryonic day (E) 12.5 to E16.5, was similar in WT and LXRbeta(-/-) littermates but BrdU birth dating revealed that late-generated neurons labeled by BrdU injections administered at E14.5 or E16.5, and destined to cortical layers II/III, were disorganized and failed to migrate. The defect in **migration** appears to be caused by a reduction in the number of vertical processes emanating from the radial glia. These processes are the architectural guides for later-born migrating neurons. Taken together, these findings suggest that LXRbeta expression in the cerebral cortex is involved in cortex lamination and is essential for the **migration** of late-generated neocortical neurons.
18. Liver X receptor beta and thyroid hormone receptor alpha in brain cortical layering. In the past year, two members of the nuclear receptor family, liver X receptor beta (LXRbeta) and thyroid hormone receptor alpha (TRalpha), have been found to be essential for correct **migration** of neurons in the developing cortex in mouse embryos. TRalpha and LXRbeta bind to identical response elements on DNA and sometimes regulate the same genes. The reason for the **migration** defect in the LXRbeta(-/-) mouse and the possibility that TRalpha may be involved are the subjects of the present study. At E15.5, expression of reelin and VLDLR was similar but expression of apolipoprotein E receptor 2 (ApoER2) (the reelin receptor) was much lower in LXRbeta(-/-) than in WT mice. Knockout of ApoER2 is known to lead to abnormal cortical lamination. Surprisingly, by postnatal day 14 (P14), no morphological abnormalities were detectable in the cortex of LXRbeta(-/-) mice and ApoER2 expression was much stronger than in WT controls. Thus, a postnatal mechanism leads to increase in ApoER2 expression by P14. TRalpha also regulates ApoER2. In both WT and LXRbeta(-/-) mice, expression of TRalpha was high at postnatal day 2. By P14 it was reduced to low levels in WT mice but was still abundantly expressed in the cortex of LXRbeta(-/-) mice. Based on the present data we hypothesize that reduction in the level of ApoER2 is the reason for the retarded **migration** of later-born neurons in LXRbeta(-/-) mice but that as thyroid hormone (TH) increases after birth the neurons do find their correct place in the cortex.
19. Development of the deep cerebellar nuclei: transcription factors and cell **migration** from the rhombic lip. The deep cerebellar nuclei (DCN) are the main output centers of the cerebellum, but little is known about their development. Using transcription factors as cell type-specific markers, we found that DCN neurons in mice are produced in the rhombic lip and migrate rostrally in a subpial stream to the nuclear transitory zone (NTZ). The rhombic lip-derived cells express transcription factors Pax6, Tbr2, and Tbr1 sequentially as they enter the NTZ. A subset of rhombic lip-derived cells also express reelin, a key regulator of Purkinje cell **migration**s. In organotypic slice cultures, the rhombic lip was necessary and sufficient to produce cells that migrate in the subpial stream, enter the NTZ, and express Pax6, Tbr2, Tbr1, and reelin. In later stages of development, the subpial stream is replaced by the external granular layer, and the NTZ organizes into distinct DCN nuclei. Tbr1 expression persists to adulthood in a subset of medial DCN projection neurons. In reeler mutant mice, which have a severe cerebellar malformation, rhombic lip-derived cells migrated to the NTZ, despite reelin deficiency. Studies in Tbr1 mutant mice suggested that Tbr1 plays a role in DCN morphogenesis but is not required for reelin expression, glutamatergic differentiation, or the initial formation of efferent axon pathways. Our findings reveal underlying similarities in the transcriptional programs for glutamatergic neuron production in the DCN and the cerebral cortex, and they support a model of cerebellar neurogenesis in which glutamatergic and GABAergic neurons are produced from separate progenitor compartments.
20. Phosphorylation of BRN2 modulates its interaction with the Pax3 promoter to control melanocyte **migration** and proliferation. MITF-M and PAX3 are proteins central to the establishment and transformation of the melanocyte lineage. They control various cellular mechanisms, including **migration** and proliferation. BRN2 is a POU domain transcription factor expressed in melanoma cell lines and is involved in proliferation and invasion, at least in part by regulating the expression of MITF-M and PAX3. The T361 and S362 residues of BRN2, both in the POU domain, are conserved throughout the POU protein family and are targets for phosphorylation, but their roles in vivo remain unknown. To examine the role of this phosphorylation, we generated mutant BRN2 in which these two residues were replaced with alanines (BRN2TS→BRN2AA). When expressed in melanocytes in vitro or in the melanocyte lineage in transgenic mice, BRN2TS induced proliferation and represse**d migrati**on, whereas BRN2AA repressed both proliferation an**d migrati**on. BRN2TS and BRN2AA bound and repressed the MITF-M promoter, whereas PAX3 transcription was induced by BRN2TS but repressed by BRN2AA. Expression of the BRN2AA transgene in a Mitf heterozygous background and in a Pax3 mutant background enhanced the coat color phenotype. Our findings show that melanocyt**e migrati**on and proliferation are controlled both through the regulation of PAX3 by nonphosphorylated BRN2 and through the regulation of MITF-M by the overall BRN2 level.
21. BRN2 is a transcriptional repressor of CDH13 (T-cadherin) in melanoma cells. T-cadherin (cadherin 13, H-cadherin, gene name CDH13) has been proposed to act as a tumor-suppressor gene as its expression is significantly diminished in several types of carcinomas, including melanomas. Allelic loss and promoter hypermethylation have been proposed as mechanisms for silencing of CDH13. However, they do not account for loss of T-cadherin expression in all carcinomas, and other genetic or epigenetic alterations can be presumed. The present study investigated transcriptional regulation of CDH13 in melanoma. Bioinformatical analysis pointed to the presence of known BRN2 (also known as POU3F2 and N-Oct-3)-binding motifs in the CDH13 promoter sequence. We found an inverse correlation between BRN2 and T-cadherin protein and transcript expression. Reporter gene analysis and electrophoretic mobility shift assays in melanoma cells demonstrated that CDH13 is a direct target of BRN2 and that BRN2 is a functional transcriptional repressor of CDH13 promoter activity. The regulatory binding element of BRN2 was located -219 bp of the CDH13 promoter proximal to the start codon and was identified as 5'-CATGCAAAA-3'. Ectopic expression of BRN2 in BRN2-negative/T-cadherin-positive melanoma cells resulted in suppression of CDH13 promoter activity, whereas BRN2 knockdown in BRN2-positive/T-cadherin-negative melanoma cells resulted in re-expression of T-cadherin transcripts and protein. Transcriptional repression of CDH13 by BRN2 may participate in malignant transformation of melanoma by increasing invasion an**d migrati**on potentials of melanoma cells. The study has identified CDH13 as a novel direct BRN2 transcriptional target gene and has advanced knowledge of mechanisms underlying loss of T-cadherin expression in melanoma.
22. SDCCAG8 regulates pericentriolar material recruitment and neuronal **migration** in the developing cortex. Mutations of SDCCAG8 are associated with nephronophthisis and Bardet-Biedl syndrome, as well as schizophrenia; however, the function of SDCCAG8 remains largely unknown. Here, we show that SDCCAG8 regulates centrosomal accumulation of pericentriolar material and neuronal polarization and **migration** in the developing mouse cortex. Sdccag8 expression is selectively elevated in newborn neurons prior to their commencement of radial locomotion, and suppression of this expression by short-hairpin RNAs or a loss-of-function allele impairs centrosomal recruitment of γ-tubulin and pericentrin, interferes with microtubule organization, decouples the centrosome and the nucleus, and disrupts neuronal **migratio**n. Moreover, SDCCAG8 interacts and cotraffics with pericentriolar material 1 (PCM1), a centriolar satellite protein crucial for targeting proteins to the centrosome. Expression of SDCCAG8 carrying a human mutation causes neuronal **migratio**n defects. These results reveal a critical role for SDCCAG8 in controlling centrosomal properties and function, and provide insights into the basis of neurological defects linked to SDCCAG8 mutations.
23. Progressive impairment of developing neuroendocrine cell lineages in the hypothalamus of mice lacking the Orthopedia gene. Development of the neuroendocrine hypothalamus is characterized by a precise series of morphogenetic milestones culminating in terminal differentiation of neurosecretory cell lineages. The homeobox-containing gene Orthopedia (Otp) is expressed in neurons giving rise to the paraventricular (PVN), supraoptic (SON), anterior periventricular (aPV), and arcuate (ARN) nuclei throughout their development. Homozygous Otp(-/-) mice die soon after birth and display progressive impairment of crucial neuroendocrine developmental events such as reduced cell proliferation, abnormal cell **migration**, and failure in terminal differentiation of the parvocellular and magnocellular neurons of the aPV, PVN, SON, and ARN. Moreover, our data provide evidence that Otp and Sim1, a bHLH-PAS transcription factor that directs terminal differentiation of the PVN, SON, and aPV, act in parallel and are both required to maintain Brn2 expression which, in turn, is required for neuronal cell lineages secreting oxytocin (OT), arginine vasopressin (AVP), and corticotropin-releasing hormone (CRH).
24. Nova2 regulates neuronal **migration** through an RNA switch in disabled-1 signaling. Neuronal **migration** leads to a highly organized laminar structure in the mammalian brain, and its misregulation causes lissencephaly and behavioral and cognitive defects. Reelin signaling, which is mediated in part by a key adaptor, disabled-1 (Dab1), plays a critical but incompletely understood role in this process. We found that the neuron-specific RNA-binding protein Nova2 regulates neuronal **migration** in late-generated cortical and Purkinje neurons. An unbiased HITS-CLIP and exon junction array search for Nova-dependent reelin-pathway RNAs at E14.5 revealed only one candidate-an alternatively spliced isoform of Dab1 (Dab1.7bc). In utero electroporation demonstrated that Dab1.7bc was sufficient to induce neuronal **migration** defects in wild-type mice and exacerbate defects when Dab1 levels were reduced, whereas Dab1 overexpression mitigates defects in Nova2 null mice. Thus, Nova2 regulates an RNA switch controlling the ability of Dab1 to mediate neuronal responsiveness to reelin signaling and neuronal **migration**, suggesting new links between splicing regulation, brain disease, and development.
25. Disc1 point mutations in mice affect development of the cerebral cortex. Disrupted-in-Schizophrenia 1 (DISC1) is a strong candidate gene for schizophrenia and other mental disorders. DISC1 regulates neurodevelopmental processes including neurogenesis, neuronal **migration**, neurite outgrowth, and neurotransmitter signaling. Abnormal neuronal morphology and cortical architecture are seen in human postmortem brain from patients with schizophrenia. However, the etiology and development of these histological abnormalities remain unclear. We analyzed the histology of two Disc1 mutant mice with point mutations (Q31L and L100P) and found a relative reduction in neuron number, decreased neurogenesis, and altered neuron distribution compared to wild-type littermates. Frontal cortical neurons have shorter dendrites and decreased surface area and spine density. Overall, the histology of Disc1 mutant mouse cortex is reminiscent of the findings in schizophrenia. These results provide further evidence that Disc1 participates in cortical development, including neurogenesis and neuron **migration**.
26. MicroRNA-9 regulates neurogenesis in mouse telencephalon by targeting multiple transcription factors. microRNA-9-2 and microRNA-9-3 double-mutant mice demonstrate that microRNA-9 (miR-9) controls neural progenitor proliferation and differentiation in the developing telencephalon by regulating the expression of multiple transcription factors. As suggested by our previous study, the Foxg1 expression was elevated, and the production of Cajal-Retzius cells and early-born neurons was suppressed in the miR-9-2/3 double-mutant pallium. At embryonic day 16.5 (E16.5), however, the Foxg1 expression was no longer elevated. The expression of an AU-rich RNA-binding protein Elavl2 increased at E16.5, Elav2 associated with Foxg1 3' untranslated region (UTR), and it countered the Foxg1 suppression by miR-9. Later, progenitor proliferation was reduced in the miR-9-2/3 double-mutant pallium with the decrease in Nr2e1 and Pax6 expression and the increase in Meis2 expression. The analyses suggest that microRNA-9 indirectly inhibits Pax6 expression by suppressing Meis2 expression. In contrast, together with Elavl1 and Msi1, microRNA-9 targets Nr2e1 mRNA 3' UTR to enhance the expression. Concomitantly, cortical layers were reduced, each cortical projection was malformed, and the tangential **migration** of interneurons into the pallium was impaired in the miR-9-2/3 double mutants. miR-9 also targets Gsh2 3' UTR, and Gsh2, as well as Foxg1, expression was elevated in the miR-9-2/3 double-mutant subpallium. The subpallium progenitor proliferation was enhanced, and the development of basal ganglia including striatum and globus pallidus was suppressed. Pallial/subpallial boundary shifted dorsally, and the ventral pallium was lost. Corridor was malformed, and thalamocortical and corticofugal axons were misrouted in the miR-9-2/3 double mutants.
27. Bcl11a (Ctip1) Controls **Migration** of Cortical Projection Neurons through Regulation of Sema3c. During neocortical development, neurons undergo polarization, oriented **migration**, and layer-type-specific differentiation. The transcriptional programs underlying these processes are not completely understood. Here, we show that the transcription factor Bcl11a regulates polarity and **migration** of upper layer neurons. Bcl11a-deficient late-born neurons fail to correctly switch from multipolar to bipolar morphology, resulting in impaired radial **migration**. We show that the expression of Sema3c is increased in migrating Bcl11a-deficient neurons and that Bcl11a is a direct negative regulator of Sema3c transcription. In vivo gain-of-function and rescue experiments demonstrate that Sema3c is a major downstream effector of Bcl11a required for the cell polarity switch and for the **migration** of upper layer neurons. Our data uncover a novel Bcl11a/Sema3c-dependent regulatory pathway used by migrating cortical neurons.

#### GeneRIFs

1. Melanocyte **migration** and proliferation are controlled both through the regulation of Pax3 by nonphosphorylated Brn2 and through the regulation of MITF-M by the overall BRN2 level.
2. Brn-1 and Brn-2 appear to critically control the initiation of radial **migration**, redundantly regulating the cell-autonomous expression of the p35 and p39 regulatory subunits of Cdk5 in migrating cortical neurons

### [Ii]nvasive

#### PubMed

1. Intravital imaging reveals transient changes in pigment production and Brn2 expression during metastatic melanoma dissemination. How melanoma acquire a metastatic phenotype is a key issue. One possible mechanism is that metastasis is driven by microenvironment-induced switching between non**invasive** and **invasive** states. However, whether switching is a reversible or hierarchical process is not known and is difficult to assess by comparison of primary and metastatic tumors. We address this issue in a model of melanoma metastasis using a novel intravital imaging method for melanosomes combined with a reporter construct in which the Brn-2 promoter drives green fluorescent protein (GFP) expression. A subpopulation of cells containing little or no pigment and high levels of Brn2::GFP expression are motile in the primary tumor and enter the vasculature. Significantly, the less differentiated state of motile and intravasated cells is not maintained at secondary sites, implying switching between states as melanoma cells metastasize. We show that melanoma cells can switch in both directions between high- and low-pigment states. However, switching from Brn2::GFP high to low was greatly favored over the reverse direction. Microarray analysis of high- and low-pigment populations revealed that transforming growth factor (TGF)beta2 was up-regulated in the poorly pigmented cells. Furthermore, TGFbeta signaling induced hypopigmentation and increased cell motility. Thus, a subset of less differentiated cells exits the primary tumor but subsequently give rise to metastases that include a range of more differentiated and pigment-producing cells. These data show reversible phenotype switching during melanoma metastasis.

### [Mm]etastasize

#### PubMed

1. Intravital imaging reveals transient changes in pigment production and Brn2 expression during metastatic melanoma dissemination. How melanoma acquire a metastatic phenotype is a key issue. One possible mechanism is that metastasis is driven by microenvironment-induced switching between noninvasive and invasive states. However, whether switching is a reversible or hierarchical process is not known and is difficult to assess by comparison of primary and metastatic tumors. We address this issue in a model of melanoma metastasis using a novel intravital imaging method for melanosomes combined with a reporter construct in which the Brn-2 promoter drives green fluorescent protein (GFP) expression. A subpopulation of cells containing little or no pigment and high levels of Brn2::GFP expression are motile in the primary tumor and enter the vasculature. Significantly, the less differentiated state of motile and intravasated cells is not maintained at secondary sites, implying switching between states as melanoma cells **metastasize**. We show that melanoma cells can switch in both directions between high- and low-pigment states. However, switching from Brn2::GFP high to low was greatly favored over the reverse direction. Microarray analysis of high- and low-pigment populations revealed that transforming growth factor (TGF)beta2 was up-regulated in the poorly pigmented cells. Furthermore, TGFbeta signaling induced hypopigmentation and increased cell motility. Thus, a subset of less differentiated cells exits the primary tumor but subsequently give rise to metastases that include a range of more differentiated and pigment-producing cells. These data show reversible phenotype switching during melanoma metastasis.

### [Mm]etastasis

#### PubMed

1. Intravital imaging reveals transient changes in pigment production and Brn2 expression during metastatic melanoma dissemination. How melanoma acquire a metastatic phenotype is a key issue. One possible mechanism is that **metastasis** is driven by microenvironment-induced switching between noninvasive and invasive states. However, whether switching is a reversible or hierarchical process is not known and is difficult to assess by comparison of primary and metastatic tumors. We address this issue in a model of melanoma **metastasis** using a novel intravital imaging method for melanosomes combined with a reporter construct in which the Brn-2 promoter drives green fluorescent protein (GFP) expression. A subpopulation of cells containing little or no pigment and high levels of Brn2::GFP expression are motile in the primary tumor and enter the vasculature. Significantly, the less differentiated state of motile and intravasated cells is not maintained at secondary sites, implying switching between states as melanoma cells metastasize. We show that melanoma cells can switch in both directions between high- and low-pigment states. However, switching from Brn2::GFP high to low was greatly favored over the reverse direction. Microarray analysis of high- and low-pigment populations revealed that transforming growth factor (TGF)beta2 was up-regulated in the poorly pigmented cells. Furthermore, TGFbeta signaling induced hypopigmentation and increased cell motility. Thus, a subset of less differentiated cells exits the primary tumor but subsequently give rise to metastases that include a range of more differentiated and pigment-producing cells. These data show reversible phenotype switching during melanoma **metastasis**.

---

## Pou3f4

- NCBI Gene
- GeneRIF
- Pubmed

| Search Term | Rank | Fields |
| --- | --- | --- |
| [Mm]igration | 6 | PubMed(4) |

  
hide/show details for Pou3f4

### [Mm]igration

#### PubMed

1. Brn-1 and Brn-2 share crucial roles in the production and positioning of mouse neocortical neurons. Formation of highly organized neocortical structure depends on the production and correct placement of the appropriate number and types of neurons. POU homeodomain proteins Brn-1 and Brn-2 are coexpressed in the developing neocortex, both in the late precursor cells and in the migrating neurons. Here we show that double disruption of both Brn-1 and Brn-2 genes in mice leads to abnormal formation of the neocortex with dramatically reduced production of layer IV-II neurons and defective **migration** of neurons unable to express mDab1. These data indicate that Brn-1 and Brn-2 share roles in the production and positioning of neocortical neuron development.
2. Proneural bHLH and Brn proteins coregulate a neurogenic program through cooperative binding to a conserved DNA motif. Proneural proteins play a central role in vertebrate neurogenesis, but little is known of the genes that they regulate and of the factors that interact with proneural proteins to activate a neurogenic program. Here, we demonstrate that the proneural protein Mash1 and the POU proteins Brn1 and Brn2 interact on the promoter of the Notch ligand Delta1 and synergistically activate Delta1 transcription, a key step in neurogenesis. Overexpression experiments in vivo indicate that Brn2, like Mash1, regulates additional aspects of neurogenesis, including the division of progenitors and the differentiation and **migration** of neurons. We identify by in silico screening a number of additional candidate target genes, which are recognized by Mash1 and Brn proteins through a DNA-binding motif similar to that found in the Delta1 gene and present a broad range of activities. We thus propose that Mash1 synergizes with Brn factors to regulate multiple steps of neurogenesis.
3. Pou3f4-mediated regulation of ephrin-b2 controls temporal bone development in the mouse. The temporal bone encases conductive and sensorineural elements of the ear. Mutations of POU3F4 are associated with unique temporal bone abnormalities and X-linked mixed deafness (DFNX2/DFN3). However, the target genes and developmental processes controlled by POU3F4 transcription factor activity have remained largely uncharacterized. Ephrin-B2 (Efnb2) is a signaling molecule with well-documented effects on cell adhesion, proliferation, and **migration**. Our analyses of targeted mouse mutants revealed that Efnb2 loss-of-function phenocopies temporal bone abnormalities of Pou3f4 hemizygous null neonates: qualitatively identical malformations of the stapes, styloid process, internal auditory canal, and cochlear capsule were present in both mutants. Using failed/insufficient separation of the stapes and styloid process as a quantitative trait, we found that single gene Efnb2 loss-of-function and compound Pou3f4/Efnb2 loss-of-function caused a more severe phenotype than single gene Pou3f4 loss-of-function. Pou3f4 and Efnb2 gene expression domains overlapped at the site of impending stapes-styloid process separation and at subcapsular mesenchyme surrounding the cochlea; at both these sites, Efnb2 expression was attenuated in Pou3f4 hemizygous null mutants relative to control. Results of immunoprecipitation experiments using chromatin isolated from nascent middle ear mesenchyme supported the hypothesis of a physical association between Pou3f4 and specific non-coding sequence of Efnb2. We propose that Efnb2 is a target of Pou3f4 transcription factor activity and an effector of mesenchymal patterning during temporal bone development.
4. Neuronal **migration** and ventral subtype identity in the telencephalon depend on SOX1. Little is known about the molecular mechanisms and intrinsic factors that are responsible for the emergence of neuronal subtype identity. Several transcription factors that are expressed mainly in precursors of the ventral telencephalon have been shown to control neuronal specification, but it has been unclear whether subtype identity is also specified in these precursors, or if this happens in postmitotic neurons, and whether it involves the same or different factors. SOX1, an HMG box transcription factor, is expressed widely in neural precursors along with the two other SOXB1 subfamily members, SOX2 and SOX3, and all three have been implicated in neurogenesis. SOX1 is also uniquely expressed at a high level in the majority of telencephalic neurons that constitute the ventral striatum (VS). These neurons are missing in Sox1-null mutant mice. In the present study, we have addressed the requirement for SOX1 at a cellular level, revealing both the nature and timing of the defect. By generating a novel Sox1-null allele expressing beta-galactosidase, we found that the VS precursors and their early neuronal differentiation are unaffected in the absence of SOX1, but the prospective neurons fail to migrate to their appropriate position. Furthermore, the **migration** of non-Sox1-expressing VS neurons (such as those expressing Pax6) was also affected in the absence of SOX1, suggesting that Sox1-expressing neurons play a role in structuring the area of the VS. To test whether SOX1 is required in postmitotic cells for the emergence of VS neuronal identity, we generated mice in which Sox1 expression was directed to all ventral telencephalic precursors, but to only a very few VS neurons. These mice again lacked most of the VS, indicating that SOX1 expression in precursors is not sufficient for VS development. Conversely, the few neurons in which Sox1 expression was maintained were able to migrate to the VS. In conclusion, Sox1 expression in precursors is not sufficient for VS neuronal identity and **migration**, but this is accomplished in postmitotic cells, which require the continued presence of SOX1. Our data also suggest that other SOXB1 members showing expression in specific neuronal populations are likely to play continuous roles from the establishment of precursors to their final differentiation.

---

## Tubb3

- NCBI Gene
- GeneRIF
- Pubmed

| Search Term | Rank | Fields |
| --- | --- | --- |
| [Ff]ilopodia | 1 | PubMed(2) |
| [Ff]ilopodium | 2 | PubMed(1) |
| [Mm]igration | 6 | PubMed(239); GeneRIFs(1) |
| ECM | 7 | PubMed(7) |
| [Ee]xtracellular [Mm]atrix | 8 | PubMed(15) |
| [Ii]nvasive | 10 | PubMed(2) |
| [Mm]etastasis | 12 | PubMed(1) |

  
hide/show details for Tubb3

### [Ff]ilopodia

#### PubMed

1. Capzb2 interacts with beta-tubulin to regulate growth cone morphology and neurite outgrowth. Capping protein (CP) is a heterodimer that regulates actin assembly by binding to the barbed end of F-actin. In cultured nonneuronal cells, each CP subunit plays a critical role in the organization and dynamics of lamellipodia and **filopodia**. Mutations in either alpha or beta CP subunit result in retinal degeneration in Drosophila. However, the function of CP subunits in mammalian neurons remains unclear. Here, we investigate the role of the beta CP subunit expressed in the brain, Capzb2, in growth cone morphology and neurite outgrowth. We found that silencing Capzb2 in hippocampal neurons resulted in short neurites and misshapen growth cones in which microtubules overgrew into the periphery and completely overlapped with F-actin. In searching for the mechanisms underlying these cytoskeletal abnormalities, we identified beta-tubulin as a novel binding partner of Capzb2 and demonstrated that Capzb2 decreases the rate and the extent of tubulin polymerization in vitro. We mapped the region of Capzb2 that was required for the subunit to interact with beta-tubulin and inhibit microtubule polymerization. A mutant Capzb2 lacking this region was able to bind F-actin and form a CP heterodimer with alpha2-subunit. However, this mutant was unable to rescue the growth cone and neurite outgrowth phenotypes caused by Capzb2 knockdown. Together, these data suggest that Capzb2 plays an important role in growth cone formation and neurite outgrowth and that the underlying mechanism may involve direct interaction between Capzb2 and microtubules.
2. Ena/VASP Is Required for neuritogenesis in the developing cortex. Mammalian cortical development involves neuronal migration and neuritogenesis; this latter process forms the structural precursors to axons and dendrites. Elucidating the pathways that regulate the cytoskeleton to drive these processes is fundamental to our understanding of cortical development. Here we show that loss of all three murine Ena/VASP proteins, a family of actin regulatory proteins, causes neuronal ectopias, alters intralayer positioning in the cortical plate, and, surprisingly, blocks axon fiber tract formation during corticogenesis. Cortical fiber tract defects in the absence of Ena/VASP arise from a failure in neurite initiation, a prerequisite for axon formation. Neurite initiation defects in Ena/VASP-deficient neurons are preceded by a failure to form bundled actin filaments and **filopodia**. These findings provide insight into the regulation of neurite formation and the role of the actin cytoskeleton during cortical development.

### [Ff]ilopodium

#### PubMed

1. The Oxygen Sensor PHD2 Controls Dendritic Spines and Synapses via Modification of Filamin A. Neuronal function is highly sensitive to changes in oxygen levels, but how hypoxia affects dendritic spine formation and synaptogenesis is unknown. Here we report that hypoxia, chemical inhibition of the oxygen-sensing prolyl hydroxylase domain proteins (PHDs), and silencing of Phd2 induce immature **filopodium**-like dendritic protrusions, promote spine regression, reduce synaptic density, and decrease the frequency of spontaneous action potentials independently of HIF signaling. We identified the actin cross-linker filamin A (FLNA) as a target of PHD2 mediating these effects. In normoxia, PHD2 hydroxylates the proline residues P2309 and P2316 in FLNA, leading to von Hippel-Lindau (VHL)-mediated ubiquitination and proteasomal degradation. In hypoxia, PHD2 inactivation rapidly upregulates FLNA protein levels because of blockage of its proteasomal degradation. FLNA upregulation induces more immature spines, whereas Flna silencing rescues the immature spine phenotype induced by PHD2 inhibition.

### [Mm]igration

#### PubMed

1. Balancing neural crest cell intrinsic processes with those of the microenvironment in Tcof1 haploinsufficient mice enables complete enteric nervous system formation. The enteric nervous system (ENS) comprises a complex neuronal network that regulates peristalsis of the gut wall and secretions into the lumen. The ENS is formed from a multipotent progenitor cell population called the neural crest, which is derived from the neuroepithelium. Neural crest cells (NCCs) migrate over incredible distances to colonize the entire length of the gut and during their **migration** they must survive, proliferate and ultimately differentiate. The absence of an ENS from variable lengths of the colon results in Hirschsprung's disease (HSCR) or colonic aganglionosis. Mutations in about 12 different genes have been identified in HSCR patients but the complex pattern of inheritance and variable penetrance suggests that additional genes or modifiers must be involved in the etiology and pathogenesis of this disease. We discovered that Tcof1 haploinsufficiency in mice models many of the early features of HSCR. Neuroepithelial apoptosis diminished the size of the neural stem cell pool resulting in reduced NCC numbers and their delayed **migration** along the gut from E10.5 to E14.5. Surprisingly however, we observe continued and complete colonization of the entire colon throughout E14.5-E18.5, a period in which the gut is considered to be non- or less-permissive to NCC. Thus, we reveal for the first time that reduced NCC progenitor numbers and delayed **migration** do not unequivocally equate with a predisposition for the pathogenesis of HSCR. In fact, these deficiencies can be overcome by balancing NCC intrinsic processes of proliferation and differentiation with extrinsic influences of the gut microenvironment.
2. Close homolog of L1 modulates area-specific neuronal positioning and dendrite orientation in the cerebral cortex. We show that the neural cell recognition molecule Close Homolog of L1 (CHL1) is required for neuronal positioning and dendritic growth of pyramidal neurons in the posterior region of the developing mouse neocortex. CHL1 was expressed in pyramidal neurons in a high-caudal to low-rostral gradient within the developing cortex. Deep layer pyramidal neurons of CHL1-minus mice were shifted to lower laminar positions in the visual and somatosensory cortex and developed misoriented, often inverted apical dendrites. Impaired **migration** of CHL1-minus cortical neurons was suggested by strikingly slower rates of radial **migration** in cortical slices, failure to potentiate integrin-dependent haptotactic cell **migration** in vitro, and accumulation of migratory cells in the intermediate and ventricular/subventricular zones in vivo. The restriction of CHL1 expression and effects of its deletion in posterior neocortical areas suggests that CHL1 may regulate area-specific neuronal connectivity and, by extension, function in the visual and somatosensory cortex.
3. Crucial roles of the Arp2/3 complex during mammalian corticogenesis. The polarity and organization of radial glial cells (RGCs), which serve as both stem cells and scaffolds for neuronal **migration**, are crucial for cortical development. However, the cytoskeletal mechanisms that drive radial glial outgrowth and maintain RGC polarity remain poorly understood. Here, we show that the Arp2/3 complex - the unique actin nucleator that produces branched actin networks - plays essential roles in RGC polarity and morphogenesis. Disruption of the Arp2/3 complex in murine RGCs retards process outgrowth toward the basal surface and impairs apical polarity and adherens junctions. Whereas the former is correlated with an abnormal actin-based leading edge, the latter is consistent with blockage in membrane trafficking. These defects result in altered cell fate, disrupted cortical lamination and abnormal angiogenesis. In addition, we present evidence that the Arp2/3 complex is a cell-autonomous regulator of neuronal **migration**. Our data suggest that Arp2/3-mediated actin assembly might be particularly important for neuronal cell motility in a soft or poorly adhesive matrix environment.
4. N-cadherin and β1-integrins cooperate during the development of the enteric nervous system. Cell adhesion controls various embryonic morphogenetic processes, including the development of the enteric nervous system (ENS). Ablation of β1-integrin (β1-/-) expression in enteric neural crest cells (ENCC) in mice leads to major alterations in the ENS structure caused by reduc**ed migrat**ion and increased aggregation properties of ENCC during gut colonization, which gives rise to a Hirschsprung's disease-like phenotype. In the present study, we examined the role of N-cadherin in ENS development and the interplay with β1 integrins during this process. The Ht-PA-Cre mouse model was used to target gene disruption of N-cadherin and β1 integrin in migratory NCC and to produce single- and double-conditional mutants for these two types of adhesion receptors. Double mutation of N-cadherin and β1 integrin led to embryonic lethality with severe defects in ENS development. N-cadherin-null (Ncad-/-) ENCC exhibited a delayed colonization in the developing gut at E12.5, although this was to a lesser extent than in β1-/- mutants. This delay of Ncad-/**- ENCC mi**gration was recovered at later stages of development. The double Ncad-/-; β1-/- mutant ENCC failed to colonize the distal part of the gut and there was more severe aganglionosis in the proximal hindgut than in the single mutants for N-cadherin or β1-integrin. This was due to an altered speed of locomotion and directionality in the gut wall. The abnormal aggregation defect of ENCC and the disorganized ganglia network in the β1-/- mutant was not observed in the double mutant. This indicates that N-cadherin enhances the effect of the β1-integrin mutation and demonstrates cooperation between these two adhesion receptors during ENS ontogenesis. In conclusion, our data reveal that N-cadherin is not essential for ENS development but it does modulate the mod**es of ENC**C migration and acts in concert with β1-integrin to control the proper development of the ENS.
5. Liver X receptor beta and thyroid hormone receptor alpha in brain cortical layering. In the past year, two members of the nuclear receptor family, liver X receptor beta (LXRbeta) and thyroid hormone receptor alpha (TRalpha), have been found to be essential for correct **migration** of neurons in the developing cortex in mouse embryos. TRalpha and LXRbeta bind to identical response elements on DNA and sometimes regulate the same genes. The reason for the **migration** defect in the LXRbeta(-/-) mouse and the possibility that TRalpha may be involved are the subjects of the present study. At E15.5, expression of reelin and VLDLR was similar but expression of apolipoprotein E receptor 2 (ApoER2) (the reelin receptor) was much lower in LXRbeta(-/-) than in WT mice. Knockout of ApoER2 is known to lead to abnormal cortical lamination. Surprisingly, by postnatal day 14 (P14), no morphological abnormalities were detectable in the cortex of LXRbeta(-/-) mice and ApoER2 expression was much stronger than in WT controls. Thus, a postnatal mechanism leads to increase in ApoER2 expression by P14. TRalpha also regulates ApoER2. In both WT and LXRbeta(-/-) mice, expression of TRalpha was high at postnatal day 2. By P14 it was reduced to low levels in WT mice but was still abundantly expressed in the cortex of LXRbeta(-/-) mice. Based on the present data we hypothesize that reduction in the level of ApoER2 is the reason for the retarded **migration** of later-born neurons in LXRbeta(-/-) mice but that as thyroid hormone (TH) increases after birth the neurons do find their correct place in the cortex.
6. Phosphorylation of SCG10/stathmin-2 determines multipolar stage exit and neuronal **migration** rate. Cell **migration** is the consequence of the sum of positive and negative regulatory mechanisms. Although appropriate **migration** of neurons is a principal feature of brain development, the negative regulatory mechanisms remain obscure. We found that JNK1 was highly active in developing cortex and that selective inhibition of JNK in the cytoplasm markedly increased both the frequency of exit from the multipolar stage and radial **migration** rate and ultimately led to an ill-defined cellular organization. Moreover, regulation of multipolar-stage exit and radial **migration** in Jnk1(-/-) (also known as Mapk8) mice, resulted from consequential changes in phosphorylation of the microtubule regulator SCG10 (also called stathmin-2). Expression of an SCG10 mutant that mimics the JNK1-phosphorylated form restored normal **migration** in the brains of Jnk1(-/-) mouse embryos. These findings indicate that the phosphorylation of SCG10 by JNK1 is a fundamental mechanism that governs the transition from the multipolar stage and the rate of neuronal cell movement during cortical development.
7. Integrin alpha4beta1 (VLA-4) expression and activity in retinal and peripheral neurons. The integrin alpha4beta1 fulfills important roles in inflammation and hematopoesis, but its functions in neurons are not well understood. Here we show that the alpha4 subunit is expressed on mouse retinal ganglion cells (RGCs) and undifferentiated retinal neuroblasts during the period of axon extension and **migration**. To determine if alpha4 integrins expressed by retinal neurons were active, neurons were cultured on known alpha4 ligands in vitro. Recombinant soluble vascular cell adhesion molecule 1 (rsVCAM-1), fibronectin, and osteopontin (OPN) induced neurite outgrowth that was diminished by function blocking antibodies specific for alpha4. Neurite outgrowth on OPN was also blocked by antibodies to the integrin beta1 subunit, implicating the alpha4beta1 heterodimer as one integrin receptor mediating outgrowth on OPN. OPN immunoreactivity was detected in the RGC fiber layer and optic nerve, suggesting that it may act as an alpha4 ligand in vivo. Neurons from chick lumbar sympathetic ganglia, chick dorsal root ganglia, and mouse superior cervical ganglia also extended neurites on rsVCAM-1, suggesting that integrin alpha4beta1 may play a role in the development of multiple neuronal cell types.
8. Ephrin signalling controls brain size by regulating apoptosis of neural progenitors. Mechanisms controlling brain size include the regulation of neural progenitor cell proliferation, differentiation, survival and **migration**. Here we show that ephrin-A/EphA receptor signalling plays a key role in controlling the size of the mouse cerebral cortex by regulating cortical progenitor cell apoptosis. In vivo gain of EphA receptor function, achieved through ectopic expression of ephrin-A5 in early cortical progenitors expressing EphA7, caused a transient wave of neural progenitor cell apoptosis, resulting in premature depletion of progenitors and a subsequent dramatic decrease in cortical size. In vitro treatment with soluble ephrin-A ligands similarly induced the rapid death of cultured dissociated cortical progenitors in a caspase-3-dependent manner, thereby confirming a direct effect of ephrin/Eph signalling on apoptotic cascades. Conversely, in vivo loss of EphA function, achieved through EphA7 gene disruption, caused a reduction in apoptosis occurring normally in forebrain neural progenitors, resulting in an increase in cortical size and, in extreme cases, exencephalic forebrain overgrowth. Together, these results identify ephrin/Eph signalling as a physiological trigger for apoptosis that can alter brain size and shape by regulating the number of neural progenitors.
9. Neuronal **migration** and ventral subtype identity in the telencephalon depend on SOX1. Little is known about the molecular mechanisms and intrinsic factors that are responsible for the emergence of neuronal subtype identity. Several transcription factors that are expressed mainly in precursors of the ventral telencephalon have been shown to control neuronal specification, but it has been unclear whether subtype identity is also specified in these precursors, or if this happens in postmitotic neurons, and whether it involves the same or different factors. SOX1, an HMG box transcription factor, is expressed widely in neural precursors along with the two other SOXB1 subfamily members, SOX2 and SOX3, and all three have been implicated in neurogenesis. SOX1 is also uniquely expressed at a high level in the majority of telencephalic neurons that constitute the ventral striatum (VS). These neurons are missing in Sox1-null mutant mice. In the present study, we have addressed the requirement for SOX1 at a cellular level, revealing both the nature and timing of the defect. By generating a novel Sox1-null allele expressing beta-galactosidase, we found that the VS precursors and their early neuronal differentiation are unaffected in the absence of SOX1, but the prospective neurons fail to migrate to their appropriate position. Furthermore, the **migration** of non-Sox1-expressing VS neurons (such as those expressing Pax6) was also affected in the absence of SOX1, suggesting that Sox1-expressing neurons play a role in structuring the area of the VS. To test whether SOX1 is required in postmitotic cells for the emergence of VS neuronal identity, we generated mice in which Sox1 expression was directed to all ventral telencephalic precursors, but to only a very few VS neurons. These mice again lacked most of the VS, indicating that SOX1 expression in precursors is not sufficient for VS development. Conversely, the few neurons in which Sox1 expression was maintained were able to migrate to the VS. In conclusion, Sox1 expression in precursors is not sufficient for VS neuronal identity and **migration**, but this is accomplished in postmitotic cells, which require the continued presence of SOX1. Our data also suggest that other SOXB1 members showing expression in specific neuronal populations are likely to play continuous roles from the establishment of precursors to their final differentiation.
10. MAPK-upstream protein kinase (MUK) regulates the radial **migration** of immature neurons in telencephalon of mouse embryo. The radial **migration** of differentiating neurons provides an essential step in the generation of laminated neocortex, although its molecular mechanism is not fully understood. We show that the protein levels of a JNK activator kinase, MUK/DLK/ZPK, and JNK activity increase potently and temporally in newly generated neurons in developing mouse telencephalon during radial **migration**. The ectopic expression of MUK/DLK/ZPK in neural precursor cells in utero impairs radial **migration**, whereas it allows these cells to leave the ventricular zone and differentiate into neural cells. The MUK/DLK/ZPK protein is associated with dotted structures that are frequently located along microtubules and with Golgi apparatus in cultured embryonic cortical cells. In COS-1 cells, MUK/DLK/ZPK overexpression impairs the radial organization of microtubules without massive depolymerization. These results suggest that MUK/DLK/ZPK and JNK regulate radial cell **migration** via microtubule-based events.
11. Common partner Smad-independent canonical bone morphogenetic protein signaling in the specification process of the anterior rhombic lip during cerebellum development. Bone morphogenetic protein (BMP) signaling is critical for cerebellum development. However, the details of receptor regulated-Smad (R-Smad) and common partner Smad (Co-Smad, or Smad4) involvement are unclear. Here, we report that cerebellum-specific double conditional inactivation of Smad1 and Smad5 (Smad1/5) results in cerebellar hypoplasia, reduced granule cell numbers, and disorganized Purkinje neuron **migration** during embryonic development. However, single conditional inactivation of either Smad1 or Smad5 did not result in cerebellar abnormalities. Surprisingly, conditional inactivation of Smad4, which is considered to be the central mediator of canonical BMP-Smad signaling, resulted only in very mild cerebellar defects. Conditional inactivation of Smad1/5 led to developmental defects in the anterior rhombic lip (ARL), as shown by reduced cell proliferation and loss of Pax6 and Atoh1 expression. These defects subsequently caused the loss of the nuclear transitory zone and a region of the deep cerebellar nuclei. The normal maturation of the remaining granule cell precursors in the external granular layer (EGL) suggests Smad1/5 signaling is required for the specification process in ARL but not for the subsequent EGL development. Our results demonstrate functional redundancy for Smad1 and Smad5 but functional discrepancy between Smad1/5 and Smad4 during cerebellum development.
12. MEKK4 signaling regulates filamin expression and neuronal **migration**. Periventricular heterotopia (PVH) is a congenital malformation of human cerebral cortex frequently associated with Filamin-A (FLN-A) mutations but the pathogenetic mechanisms remain unclear. Here, we show that the MEKK4 (MAP3K4) pathway is involved in Fln-A regulation and PVH formation. MEKK4(-/-) mice developed PVH associated with breaches in the neuroependymal lining which were largely comprised of neurons that failed to reach the cortical plate. RNA interference (RNAi) targeting MEKK4 also impaired neuronal **migration**. Expression of Fln was elevated in MEKK4(-/-) forebrain, most notably near sites of failed neuronal **migration**. Importantly, recombinant MKK4 protein precipitated a complex containing MEKK4 and Fln-A, and MKK4 mediated signaling between MEKK4 and Fln-A, suggesting that MKK4 may bridge these molecules during development. Finally, we showed that wild-type FLN-A overexpression inhibited neuronal **migration**. Collectively, our results demonstrate a link between MEKK4 and Fln-A that impacts neuronal **migration** initiation and provides insight into the pathogenesis of human PVH.
13. Beta-actin is required for proper mouse neural crest ontogeny. The mouse genome consists of six functional actin genes of which the expression patterns are temporally and spatially regulated during development and in the adult organism. Deletion of beta-actin in mouse is lethal during embryonic development, although there is compensatory expression of other actin isoforms. This suggests different isoform specific functions and, more in particular, an important function for beta-actin during early mammalian development. We here report a role for beta-actin during neural crest ontogeny. Although beta-actin null neural crest cells show expression of neural crest markers, less cells delaminate and their **migration** arrests shortly after. These phenotypes were associated with elevated apoptosis levels in neural crest cells, whereas proliferation levels were unchanged. Specifically the pre-migratory neural crest cells displayed higher levels of apoptosis, suggesting increased apoptosis in the neural tube accounts for the decreased amount of migrating neural crest cells seen in the beta-actin null embryos. These cells additionally displayed a lack of membrane bound N-cadherin and dramatic decrease in cadherin-11 expression which was more pronounced in the pre-migratory neural crest population, potentially indicating linkage between the cadherin-11 expression and apoptosis. By inhibiting ROCK ex vivo, the knockout neural crest cells regained migratory capacity and cadherin-11 expression was upregulated. We conclude that the presence of beta-actin is vital for survival, specifically of pre-migratory neural crest cells, their proper e**migration** from the neural tube and their subsequent **migration**. Furthermore, the absence of beta-actin affects cadherin-11 and N-cadherin function, which could partly be alleviated by ROCK inhibition, situating the Rho-ROCK signaling in a feedback loop with cadherin-11.
14. Expression of liver X receptor beta is essential for formation of superficial cortical layers and **migration** of later-born neurons. Liver X receptor (LXR) beta regulates cholesterol levels in the brain and is essential for maintenance of motor neurons in the spinal cord and dopaminergic neurons in the substantia nigra. Here, we have examined the expression pattern of LXRbeta protein in the cerebral cortex and looked for defects in cortical development in LXRbeta knockout (LXRbeta(-/-)) mice. LXRbeta protein was widely expressed in the mouse brain at later embryonic stages, and the expression pattern in the cerebral cortex was developmentally regulated. In normal postnatal mice, LXRbeta was localized mainly in the upper layers of the cerebral cortex. In LXRbeta(-/-) mice layers II and III were thinner with fewer neurons. Layer I was slightly thicker, whereas layers IV-VI were essentially normal. Consistent with this finding, Brn2 and NeuN expression were decreased in the upper layers in the LXRbeta(-/-) neonatal cortex. The number of S-phase progenitor cells in the cortex between embryonic day (E) 12.5 to E16.5, was similar in WT and LXRbeta(-/-) littermates but BrdU birth dating revealed that late-generated neurons labeled by BrdU injections administered at E14.5 or E16.5, and destined to cortical layers II/III, were disorganized and failed to migrate. The defect in **migration** appears to be caused by a reduction in the number of vertical processes emanating from the radial glia. These processes are the architectural guides for later-born migrating neurons. Taken together, these findings suggest that LXRbeta expression in the cerebral cortex is involved in cortex lamination and is essential for the **migration** of late-generated neocortical neurons.
15. DCC is specifically required for the survival of retinal ganglion and displaced amacrine cells in the developing mouse retina. Netrin-1 and DCC are well known for their roles in neurite growth, axonal guidance, and neuronal **migration**. Recently, a number of studies showed that DCC is involved in the induction of apoptosis, and this proapoptotic activity can be blocked in the presence of Netrin-1. However, here, we found that DCC is required for the survival of two types of neurons selectively in the developing mouse retina where DCC is abundantly expressed. Our results showed that the DCC(-/-) retina displayed a reduced ganglion cell layer with relatively normal neuroblastic layer. Immunostaining assays revealed that in DCC(-/-) mice, initial neurogenesis within retina was unchanged while the numbers of differentiated retinal ganglion cells and displaced amacrine cells in ganglion cell layer were greatly reduced due to increased apoptosis. By contrast, other neuronal types including horizontal cells, bipolar cells, amacrine cells, photoreceptors, and Müller cells appeared normal in DCC mutant retinas. Moreover, DCC(kanga) mice that lack the intracellular P3 domain of DCC receptor displayed the same defects as DCC(-/-) mice. Thus, our findings suggest that DCC is a key regulator for the survival of specific types of neurons during retinal development and that DCC-P3 domain is essential for this developing event.
16. PTB deficiency causes the loss of adherens junctions in the dorsal telencephalon and leads to lethal hydrocephalus. Polypyrimidine tract-binding protein (PTB) is a well-characterized RNA-binding protein and known to be preferentially expressed in neural stem cells (NSCs) in the central nervous system; however, its role in NSCs in the developing brain remains unclear. To explore the role of PTB in embryonic NSCs in vivo, Nestin-Cre-mediated conditional Ptb knockout mice were generated for this study. In the mutant forebrain, despite the depletion of PTB protein, neither abnormal neurogenesis nor flagrant morphological abnormalities were observed at embryonic day 14.5 (E14.5). Nevertheless, by 10 weeks, nearly all mutant mice succumbed to hydrocephalus (HC), which was caused by a lack of the ependymal cell layer in the dorsal cortex. Upon further analysis, a gradual loss of adherens junctions (AJs) was observed in the ventricular zone (VZ) of the dorsal telencephalon in the mutant brains, beginning at E14.5. In the AJs-deficient VZ, impaired interkinetic nuclear **migration** and precocious differentiation of NSCs were observed after E14.5. These findings demonstrated that PTB depletion in the dorsal telencephalon is causally involved in the development of HC and that PTB is important for the maintenance of AJs in the NSCs of the dorsal telencephalon.
17. p27(Kip1) is a microtubule-associated protein that promotes microtubule polymerization during neuron **migration**. The **migration** of cortical interneurons is characterized by extensive morphological changes that result from successive cycles of nucleokinesis and neurite branching. Their molecular bases remain elusive, and the present work describes how p27(Kip1) controls cell-cycle-unrelated signaling pathways to regulate these morphological remodelings. Live imaging reveals that interneurons lacking p27(Kip1) show delayed tangential **migration** resulting from defects in both nucleokinesis and dynamic branching of the leading process. At the molecular level, p27(Kip1) is a microtubule-associated protein that promotes polymerization of microtubules in extending neurites, thereby contributing to tangential **migration**. Furthermore, we show that p27(Kip1) controls actomyosin contractions that drive both forward translocation of the nucleus and growth cone splitting. Thus, p27(Kip1) cell-autonomously controls nucleokinesis and neurite branching by regulating both actin and microtubule cytoskeletons.
18. Stress-activated protein kinase MKK7 regulates axon elongation in the developing cerebral cortex. The c-Jun NH(2)-terminal protein kinase (JNK), which belongs to the mitogen-activated protein kinase family, plays important roles in a broad range of physiological processes. JNK is controlled by two upstream regulators, mitogen-activated protein kinase kinase (MKK) 7 and MKK4. To elucidate the physiological functions of MKK7, we used Nestin-Cre to generate a novel mouse model in which the mkk7 gene was specifically deleted in the nervous system (Mkk7(flox/flox) Nestin-Cre mice). These mice were indistinguishable from their control littermates in gross appearance during embryogenesis but died immediately after birth without breathing. Histological examination showed that the mutants had severe defects in brain development, including enlarged ventricles, reduced striatum, and minimal axon tracts. Electron microscopy revealed abnormal accumulations of filamentous structures and autophagic vacuoles in Mkk7(flox/flox) Nestin-Cre brain. Further analysis showed that MKK7 deletion decreased numbers of TAG-1-expressing axons and delayed neuronal **migration** in the cerebrum. Neuronal differentiation was not altered. In utero electroporation studies showed that contralateral projection of axons by layer 2/3 neurons was impaired in the absence of MKK7. Moreover, MKK7 regulated axon elongation in a cell-autonomous manner in vivo, a finding confirmed in vitro. Finally, phosphorylation levels of JNK substrates, including c-Jun, neurofilament heavy chain, microtubule-associated protein 1B, and doublecortin, were reduced in Mkk7(flox/flox) Nestin-Cre brain. Our findings demonstrate that the phenotype of Mkk7(flox/flox) Nestin-Cre mice differs substantially from that of Mkk4(flox/flox) Nestin-Cre mice, and establish that MKK7-mediated regulation of JNK is uniquely critical for both axon elongation and radial **migration** in the developing brain.
19. beta1 integrin maintains integrity of the embryonic neocortical stem cell niche. During embryogenesis, the neural stem cells (NSC) of the developing cerebral cortex are located in the ventricular zone (VZ) lining the cerebral ventricles. They exhibit apical and basal processes that contact the ventricular surface and the pial basement membrane, respectively. This unique architecture is important for VZ physical integrity and fate determination of NSC daughter cells. In addition, the shorter apical process is critical for interkinetic nuclear **migration** (INM), which enables VZ cell mitoses at the ventricular surface. Despite their importance, the mechanisms required for NSC adhesion to the ventricle are poorly understood. We have shown previously that one class of candidate adhesion molecules, laminins, are present in the ventricular region and that their integrin receptors are expressed by NSC. However, prior studies only demonstrate a role for their interaction in the attachment of the basal process to the overlying pial basement membrane. Here we use antibody-blocking and genetic experiments to reveal an additional and novel requirement for laminin/integrin interactions in apical process adhesion and NSC regulation. Transient abrogation of integrin binding and signalling using blocking antibodies to specifically target the ventricular region in utero results in abnormal INM and alterations in the orientation of NSC divisions. We found that these defects were also observed in laminin alpha2 deficient mice. More detailed analyses using a multidisciplinary approach to analyse stem cell behaviour by expression of fluorescent transgenes and multiphoton time-lapse imaging revealed that the transient embryonic disruption of laminin/integrin signalling at the VZ surface resulted in apical process detachment from the ventricular surface, dystrophic radial glia fibers, and substantial layering defects in the postnatal neocortex. Collectively, these data reveal novel roles for the laminin/integrin interaction in anchoring embryonic NSCs to the ventricular surface and maintaining the physical integrity of the neocortical niche, with even transient perturbations resulting in long-lasting cortical defects.
20. MicroRNA-9 regulates neurogenesis in mouse telencephalon by targeting multiple transcription factors. microRNA-9-2 and microRNA-9-3 double-mutant mice demonstrate that microRNA-9 (miR-9) controls neural progenitor proliferation and differentiation in the developing telencephalon by regulating the expression of multiple transcription factors. As suggested by our previous study, the Foxg1 expression was elevated, and the production of Cajal-Retzius cells and early-born neurons was suppressed in the miR-9-2/3 double-mutant pallium. At embryonic day 16.5 (E16.5), however, the Foxg1 expression was no longer elevated. The expression of an AU-rich RNA-binding protein Elavl2 increased at E16.5, Elav2 associated with Foxg1 3' untranslated region (UTR), and it countered the Foxg1 suppression by miR-9. Later, progenitor proliferation was reduced in the miR-9-2/3 double-mutant pallium with the decrease in Nr2e1 and Pax6 expression and the increase in Meis2 expression. The analyses suggest that microRNA-9 indirectly inhibits Pax6 expression by suppressing Meis2 expression. In contrast, together with Elavl1 and Msi1, microRNA-9 targets Nr2e1 mRNA 3' UTR to enhance the expression. Concomitantly, cortical layers were reduced, each cortical projection was malformed, and the tangential **migration** of interneurons into the pallium was impaired in the miR-9-2/3 double mutants. miR-9 also targets Gsh2 3' UTR, and Gsh2, as well as Foxg1, expression was elevated in the miR-9-2/3 double-mutant subpallium. The subpallium progenitor proliferation was enhanced, and the development of basal ganglia including striatum and globus pallidus was suppressed. Pallial/subpallial boundary shifted dorsally, and the ventral pallium was lost. Corridor was malformed, and thalamocortical and corticofugal axons were misrouted in the miR-9-2/3 double mutants.
21. Glial origin of mesenchymal stem cells in a tooth model system. Mesenchymal stem cells occupy niches in stromal tissues where they provide sources of cells for specialized mesenchymal derivatives during growth and repair. The origins of mesenchymal stem cells have been the subject of considerable discussion, and current consensus holds that perivascular cells form mesenchymal stem cells in most tissues. The continuously growing mouse incisor tooth offers an excellent model to address the origin of mesenchymal stem cells. These stem cells dwell in a niche at the tooth apex where they produce a variety of differentiated derivatives. Cells constituting the tooth are mostly derived from two embryonic sources: neural crest ectomesenchyme and ectodermal epithelium. It has been thought for decades that the dental mesenchymal stem cells giving rise to pulp cells and odontoblasts derive from neural crest cells after their **migration** in the early head and formation of ectomesenchymal tissue. Here we show that a significant population of mesenchymal stem cells during development, self-renewal and repair of a tooth are derived from peripheral nerve-associated glia. Glial cells generate multipotent mesenchymal stem cells that produce pulp cells and odontoblasts. By combining a clonal colour-coding technique with tracing of peripheral glia, we provide new insights into the dynamics of tooth organogenesis and growth.
22. LPIAT1 regulates arachidonic acid content in phosphatidylinositol and is required for cortical lamination in mice. Dietary arachidonic acid (AA) has roles in growth, neuronal development, and cognitive function in infants. AA is remarkably enriched in phosphatidylinositol (PI), an important constituent of biological membranes in mammals; however, the physiological significance of AA-containing PI remains unknown. In an RNA interference-based genetic screen using Caenorhabditis elegans, we recently cloned mboa-7 as an acyltransferase that selectively incorporates AA into PI. Here we show that lysophosphatidylinositol acyltransferase 1 (LPIAT1, also known as MBOAT7), the closest mammalian homologue, plays a crucial role in brain development in mice. Lpiat1(-/-) mice show almost no LPIAT activity with arachidonoyl-CoA as an acyl donor and show reduced AA contents in PI and PI phosphates. Lpiat1(-/-) mice die within a month and show atrophy of the cerebral cortex and hippocampus. Immunohistochemical analysis reveals disordered cortical lamination and delayed neuronal **migration** in the cortex of E18.5 Lpiat1(-/-) mice. LPIAT1 deficiency also causes disordered neuronal processes in the cortex and reduced neurite outgrowth in vitro. Taken together, these results demonstrate that AA-containing PI/PI phosphates play an important role in normal cortical lamination during brain development in mice.
23. Protein 600 is a microtubule/endoplasmic reticulum-associated protein in CNS neurons. There is an increasing body of literature pointing to cytoskeletal proteins as spatial organizers and interactors of organelles. In this study, we identified protein 600 (p600) as a novel microtubule-associated protein (MAP) developmentally regulated in neurons. p600 exhibits the unique feature to interact with the endoplasmic reticulum (ER). Silencing of p600 by RNA interference (RNAi) destabilizes neuronal processes in young primary neurons undergoing neurite extension and containing scarce staining of the ER marker Bip. Furthermore, in utero electroporation of p600 RNAi alters neuronal **migration**, a process that depends on synergistic actions of microtubule dynamics and ER functions. p600-depleted migrating neurons display thin, crooked, and "zigzag" leading process with very few ER membranes. Thus, p600 constitutes the only known MAP to associate with the ER in neurons, and this interaction may impact on multiple cellular processes ranging from neuronal development to neuronal maturation and plasticity.
24. Arginylation-dependent neural crest cell **migration** is essential for mouse development. Coordinated cell **migration** during development is crucial for morphogenesis and largely relies on cells of the neural crest lineage that migrate over long distances to give rise to organs and tissues throughout the body. Recent studies of protein arginylation implicated this poorly understood posttranslational modification in the functioning of actin cytoskeleton and in cell **migration** in culture. Knockout of arginyltransferase (Ate1) in mice leads to embryonic lethality and severe heart defects that are reminiscent of cell **migration**-dependent phenotypes seen in other mouse models. To test the hypothesis that arginylation regulates cell **migration** during morphogenesis, we produced Wnt1-Cre Ate1 conditional knockout mice (Wnt1-Ate1), with Ate1 deletion in the neural crest cells driven by Wnt1 promoter. Wnt1-Ate1 mice die at birth and in the first 2-3 weeks after birth with severe breathing problems and with growth and behavioral retardation. Wnt1-Ate1 pups have prominent defects, including short palate and altered opening to the nasopharynx, and cranial defects that likely contribute to the abnormal breathing and early death. Analysis of neural crest cell movement patterns in situ and cell motility in culture shows an overall delay in the **migration** of Ate1 knockout cells that is likely regulated by intracellular mechanisms rather than extracellular signaling events. Taken together, our data suggest that arginylation plays a general role in the **migration** of the neural crest cells in development by regulating the molecular machinery that underlies cell **migration** through tissues and organs during morphogenesis.
25. Sox10 and Itgb1 interaction in enteric neural crest cell **migration**. SOX10 involvement in syndromic form of Hirschsprung disease (intestinal aganglionosis, HSCR) in humans as well as developmental defects in animal models highlight the importance of this transcription factor in control of the pool of enteric progenitors and their differentiation. Here, we characterized the role of SOX10 in cell **migration** and its interactions with β1-integrins. To this end, we crossed the Sox10(lacZ/+) mice with the conditional Ht-PA::Cre; beta1(neo/+) and beta1(fl/fl) mice and compared the phenotype of embryos of different genotypes during enteric nervous system (ENS) development. The Sox10(lacZ/+); Ht-PA::Cre; beta1(neo/fl) double mutant embryos presented with increased intestinal aganglionosis length and more severe neuronal network disorganization compared to single mutants. These defects, detected by E11.5, are not compensated after birth, showing that a coordinated and balanced interaction between these two genes is required for normal ENS development. Use of video-microscopy revealed that defects observed result from reduced **migratio**n speed and altered directionality of enteric neural crest cells. Expression of β1-integrins upon SOX10 overexpression or in Sox10(lacZ/+) mice was also analyzed. The modulation of SOX10 expression altered β1-integrins, suggesting that SOX10 levels are critical for proper expression and function of this adhesion molecule. Together with previous studies, our results strongly indicate that SOX10 mediates ENCC adhesion a**nd migrat**ion, and contribute to the understanding of the molecular and cellular basis of ENS defects observed both in mutant mouse models and in patients carrying SOX10 mutations.
26. 14-3-3ε and ζ regulate neurogenesis and differentiation of neuronal progenitor cells in the developing brain. During brain development, neural progenitor cells proliferate and differentiate into neural precursors. These neural precursors migrate along the radial glial processes and localize at their final destination in the cortex. Numerous reports have revealed that 14-3-3 proteins are involved in many neuronal activities, although their functions in neurogenesis remain unclear. Here, using 14-3-3ε/ζ double knock-out mice, we found that 14-3-3 proteins are important for proliferation and differentiation of neural progenitor cells in the cortex, resulting in neuro**nal migra**tion defects and seizures. 14-3-3 deficiency resulted in the increase of δ-catenin and the decrease of β-catenin and αN-catenin. 14-3-3 proteins regulated neuronal differentiation into neurons via direct interactions with phosphorylated δ-catenin to promote F-actin formation through a catenin/Rho GTPase/Limk1/cofilin signaling pathway. Conversely, n**euronal m**igration defects seen in the double knock-out mice were restored by phosphomimic Ndel1 mutants, but not δ-catenin. Our findings provide new evidence that 14-3-3 proteins play important roles in neurogenesis and **neuronal** migration via the regulation of distinct signaling cascades.
27. Cortical **migration** defects in mice expressing A-RAF from the B-RAF locus. We have previously shown that mice lacking the protein kinase B-RAF have defects in both neural and endothelial cell lineages and die around embryonic day 12 (E12). To delineate the function of B-RAF in the brain, B-RAF KIN/KIN mice lacking B-RAF and expressing A-RAF under the control of the B-RAF locus were created. B-RAF KIN/KIN embryos displayed no vascular defects, no endothelial and neuronal apoptosis, or gross developmental abnormalities, and a significant proportion of these animals survived for up to 8 weeks. Cell proliferation in the neocortex was reduced from E14.5 onwards. Newborn cortical neurons were impaired in their **migration** toward the cortical plate, causing a depletion of Brn-2-expressing pyramidal neurons in layers II, III, and V of the postnatal cortex. Our data reveal that B-RAF is an important mediator of neuronal survival, **migration**, and dendrite formation and that A-RAF cannot fully compensate for these functions.
28. Upregulation of neurovascular communication through filamin abrogation promotes ectopic periventricular neurogenesis. Neuronal fate-restricted intermediate progenitors (IPs) are derived from the multipotent radial glia (RGs) and serve as the direct precursors for cerebral cortical neurons, but factors that control their neurogenic plasticity remain elusive. Here we report that IPs' neuron production is enhanced by abrogating filamin function, leading to the generation of periventricular neurons independent of normal neocortical neurogenesis and neuronal **migration**. Loss of Flna in neural progenitor cells (NPCs) led RGs to undergo changes resembling epithelial-mesenchymal transition (EMT) along with exuberant angiogenesis that together changed the microenvironment and increased neurogenesis of IPs. We show that by collaborating with β-arrestin, Flna maintains the homeostatic signaling between the vasculature and NPCs, and loss of this function results in escalated Vegfa and Igf2 signaling, which exacerbates both EMT and angiogenesis to further potentiate IPs' neurogenesis. These results suggest that the neurogenic potential of IPs may be boosted in vivo by manipulating Flna-mediated neurovascular communication.
29. The c-Jun N-terminal kinase activator dual leucine zipper kinase regulates axon growth and neuronal **migration** in the developing cerebral cortex. Mammalian corticogenesis substantially depends on **migration** and axon projection of newborn neurons that are coordinated by a yet unidentified molecular mechanism. Dual leucine zipper kinase (DLK) induces activation of c-Jun N-terminal kinase (JNK), a molecule that regulates morphogenesis in various organisms. We show here, using gene targeting in mice, that DLK is indispensable for establishing axon tracts, especially those originating from neocortical pyramidal neurons of the cerebrum. Direct and quantitative analysis of radial **migration** of pyramidal neurons using slice culture and a time-lapse imaging system revealed that acceleration around the subplate was affected by DLK gene disruption and by administration of a JNK inhibitor. Phosphorylation of JNK substrates, including c-Jun and doublecortin, and of JNK itself at the activation loop were partially affected in brains of DLK-deficient mouse embryos. These data suggest that DLK plays a significant role in the coordinated regulation of radial **migration** and axon projection by modulating JNK activity.
30. Bcl11a (Ctip1) Controls **Migration** of Cortical Projection Neurons through Regulation of Sema3c. During neocortical development, neurons undergo polarization, oriented **migration**, and layer-type-specific differentiation. The transcriptional programs underlying these processes are not completely understood. Here, we show that the transcription factor Bcl11a regulates polarity and **migration** of upper layer neurons. Bcl11a-deficient late-born neurons fail to correctly switch from multipolar to bipolar morphology, resulting in impaired radial **migration**. We show that the expression of Sema3c is increased in migrating Bcl11a-deficient neurons and that Bcl11a is a direct negative regulator of Sema3c transcription. In vivo gain-of-function and rescue experiments demonstrate that Sema3c is a major downstream effector of Bcl11a required for the cell polarity switch and for the **migration** of upper layer neurons. Our data uncover a novel Bcl11a/Sema3c-dependent regulatory pathway used by migrating cortical neurons.
31. Absence of Fyn and Src causes a reeler-like phenotype. Nonreceptor protein tyrosine kinases of the Src family regulate the survival, proliferation, differentiation, and motility of many cell types, but their roles in brain development are unclear. Biochemical and in vitro experiments implicate Src and Fyn in the Reelin-dependent tyrosine phosphorylation of Dab1, which controls the positioning of radially migrating neurons in many brain regions. However, genetic evidence that either Src or Fyn mediates Reelin-dependent **migration**s in vivo has been lacking. Here, we report that, although Src is dispensable and although the absence of Fyn causes an intermediate phenotype, the combined absence of Src and Fyn almost abolishes tyrosine phosphorylation of Dab1 and causes defects in the fetal cortex and cerebellum very similar to those of dab1 mutants of the same age. Neurogenesis is not detectably affected, but the layering of neurons in the cortex is inverted, and the formation of the Purkinje plate is impaired. This implies that Src and Fyn are needed for Reelin-dependent events during brain development.
32. STAT5A/B activity is required in the developing forebrain and spinal cord. Formation of the CNS requires the coordination and integration of processes such as cell proliferation, neuronal differentiation, neuronal **migration**, axon tract formation and synaptogenesis, all of which must occur at precise times and places during development. Although growth factors are known to play a role in regulating many of these processes, very little is known of the signaling events immediately downstream of ligand-receptor interactions in the developing CNS. Here we present evidence that STAT5, an important mediator of cytokine signaling, is required for some aspects of CNS development. We show that phosphorylated and hence activated forms of STAT5 (pSTAT5) are expressed in a temporally restricted manner in a subset of early-born telencephalic neurons and axons. Accordingly, Stat5 mutants have reduced numbers of interneurons in the cortical marginal zone, suggestive of **migration** defects. Moreover, corticofugal axons develop aberrantly in Stat5 mutants, indicative of a role for pSTAT5 in axon guidance. Notably, pSTAT5 is also expressed in commissural axons in the embryonic spinal cord, where it is also required for their guidance. Taken together, we provide the first evidence that STAT5 is a key effector molecule in the developing mammalian CNS.
33. Cranial nerve development requires co-ordinated Shh and canonical Wnt signaling. Cranial nerves govern sensory and motor information exchange between the brain and tissues of the head and neck. The cranial nerves are derived from two specialized populations of cells, cranial neural crest cells and ectodermal placode cells. Defects in either cell type can result in cranial nerve developmental defects. Although several signaling pathways are known to regulate cranial nerve formation our understanding of how intercellular signaling between neural crest cells and placode cells is coordinated during cranial ganglia morphogenesis is poorly understood. Sonic Hedgehog (Shh) signaling is one key pathway that regulates multiple aspects of craniofacial development, but whether it co-ordinates cranial neural crest cell and placodal cell interactions during cranial ganglia formation remains unclear. In this study we examined a new Patched1 (Ptch1) loss-of-function mouse mutant and characterized the role of Ptch1 in regulating Shh signaling during cranial ganglia development. Ptch1(Wig/ Wig) mutants exhibit elevated Shh signaling in concert with disorganization of the trigeminal and facial nerves. Importantly, we discovered that enhanced Shh signaling suppressed canonical Wnt signaling in the cranial nerve region. This critically affected the survival and **migration** of cranial neural crest cells and the development of placodal cells as well as the integration between neural crest and placodes. Collectively, our findings highlight a novel and critical role for Shh signaling in cranial nerve development via the cross regulation of canonical Wnt signaling.
34. Functions of a jumonji-cyclin D1 pathway in the coordination of cell cycle exit and **migration** during neurogenesis in the mouse hindbrain. During development of the mouse central nervous system (CNS), most neural progenitor cells proliferate in the ventricular zone (VZ). In many regions of the CNS, neural progenitor cells give rise to postmitotic neurons that initiate neuronal differentiation and migrate out of the VZ to the mantle zone (MZ). Thereafter, they remain in a quiescent state. Here, we found many ectopic mitotic cells and cell clusters expressing neural progenitor or proneural marker genes in the MZ of the hindbrain of jumonji (jmj) mutant embryos. When we examined the expression of cyclin D1, which is repressed by jmj in the repression of cardiac myocyte proliferation, we found many ectopic clusters expressing both cyclin D1 and Musashi 1 in the MZ of mutant embryos. jmj is mainly expressed in the cyclin D1 negative region in the hindbrain, and cyclin D1 expression in the VZ was upregulated in jmj mutant mice. In jmj and cyclin D1 double mutant mice, the ectopic mitosis and formation of the abnormal clusters in the MZ were rescued. These results suggest that a jmj-cyclin D1 pathway is required for the precise coordination of cell cycle exit and **migration** during neurogenesis in the mouse hindbrain.
35. Endocannabinoid signaling controls pyramidal cell specification and long-range axon patterning. Endocannabinoids (eCBs) have recently been identified as axon guidance cues shaping the connectivity of local GABAergic interneurons in the developing cerebrum. However, eCB functions during pyramidal cell specification and establishment of long-range axonal connections are unknown. Here, we show that eCB signaling is operational in subcortical proliferative zones from embryonic day 12 in the mouse telencephalon and controls the proliferation of pyramidal cell progenitors and radial **migration** of immature pyramidal cells. When layer patterning is accomplished, developing pyramidal cells rely on eCB signaling to initiate the elongation and fasciculation of their long-range axons. Accordingly, CB(1) cannabinoid receptor (CB(1)R) null and pyramidal cell-specific conditional mutant (CB(1)R(f/f,NEX-Cre)) mice develop deficits in neuronal progenitor proliferation and axon fasciculation. Likewise, axonal pathfinding becomes impaired after in utero pharmacological blockade of CB(1)Rs. Overall, eCBs are fundamental developmental cues controlling pyramidal cell development during corticogenesis.
36. Nervous-tissue-specific elimination of microtubule-actin crosslinking factor 1a results in multiple developmental defects in the mouse brain. The microtubule-actin crosslinking factor 1 (MACF1) is a ubiquitous cytoskeletal linker protein with multiple spliced isoforms expressed in different tissues. The MACF1a isoform contains microtubule and actin-binding regions and is expressed at high levels in the nervous system. Macf1-/- mice are early embryonic lethal and hence the role of MACF1 in the nervous system could not be determined. We have specifically knocked out MACF1a in the developing mouse nervous system using Cre/loxP technology. Mutant mice died within 24-36h after birth of apparent respiratory distress. Their brains displayed a disorganized cerebral cortex with a mixed layer structure, heterotopia in the pyramidal layer of the hippocampus, disorganized thalamocortical and corticofugal fibers, and aplastic anterior and hippocampal commissures. Embryonic neurons showed a defect in traversing the cortical plate. Our data suggest a critical role for MACF1 in neuronal **migration** that is dependent on its ability to interact with both microfilaments and microtubules.
37. Inactivation of aPKClambda results in the loss of adherens junctions in neuroepithelial cells without affecting neurogenesis in mouse neocortex. In developing mammalian telencephalon, the loss of adherens junctions and cell cycle exit represent crucial steps in the differentiation of neuroepithelial cells into neurons, but the relationship between these cellular events remains obscure. Atypical protein kinase C (aPKC) is known to contribute to junction formation in epithelial cells and to cell fate determination for Drosophila neuroblasts. To elucidate the functions of aPKClambda, one out of two aPKC members, in mouse neocortical neurogenesis, a Nestin-Cre mediated conditional gene targeting system was employed. In conditional aPKClambda knockout mice, neuroepithelial cells of the neocortical region lost aPKClambda protein at embryonic day 15 and demonstrated a loss of adherens junctions, retraction of apical processes and impaired interkinetic nuclear **migration** that resulted in disordered neuroepithelial tissue architecture. These results are evidence that aPKClambda is indispensable for the maintenance of adherens junctions and may function in the regulation of adherens junction integrity upon differentiation of neuroepithelial cells into neurons. In spite of the loss of adherens junctions in the neuroepithelium of conditional aPKClambda knockout mice, neurons were produced at a normal rate. Therefore, we concluded that, at least in the later stages of neurogenesis, regulation of cell cycle exit is independent of adherens junctions.
38. Expression and subcellular location of alpha-synuclein during mouse-embryonic development. Alpha-synuclein (alpha-SYN) is one of the major components of intracellular fibrillary aggregates in the brains of a subset of neurodegenerative disorders. Although alpha-SYN expression has been found in developing mouse brain, a detailed distribution during mouse-embryonic development has not been made. Here we describe the expression pattern of alpha-SYN during the development of mice from E9.5 to P0 by immunohistochemistry (IHC). As a result, alpha-SYN was detected as early as E9.5. During the embryonic stages, alpha-SYN was dynamically expressed in several regions of the brain. In the neocortex, expression was detected in the marginal zone (MZ) in the early stages and was later condensed in the MZ and in the subplate (SP); in the cerebellum, expression was initially detected in the deep cerebellar nuclei (DCN) and was later condensed in the Purkinje cells. These spatio-temporal expression patterns matched the neuronal migratory pathways and the formation of the synapse connections. Additionally, alpha-SYN was detected in the sensory systems, including the nasal mucosa, the optic cup, the sensory ganglia, and their dominating nerve fibers. Furthermore, the nuclear location of alpha-SYN protein was found in developing neurons in the early stages, and later it was mostly found in the non-nuclear compartments. This finding was further confirmed by Western blot analysis. These results suggest that alpha-SYN may be involved not only in the **migration** of neurons and in the synaptogenesis of the central nervous system (CNS) but also in the establishment of the sensory systems. The nuclear location of alpha-SYN may hint at an important function in these events.
39. BMP signaling regulates murine enteric nervous system precursor **migration**, neurite fasciculation, and patterning via altered Ncam1 polysialic acid addition. The enteric nervous system (ENS) forms from migrating neural crest-derived precursors that differentiate into neurons and glia, aggregate into ganglion cell clusters, and extend neuronal processes to form a complex interacting network that controls many aspects of intestinal function. Bone morphogenetic proteins (BMPs) have diverse roles in development and influence the differentiation, proliferation, and survival of ENS precursors. We hypothesized that BMP signaling might also be important for the ENS precursor **migration**, ganglion cell aggregation, and neurite fasciculation necessary to form the enteric nervous system. We now demonstrate that BMP signaling restricts murine ENS precursors to the outer bowel wall during **migration**. In addition, blocking BMP signaling causes faster colonization of the murine colon, reduces ganglion cell aggregation, and reduces neurite fasciculation. BMP signaling also influences patterns of neurite extension within the developing bowel wall. These effects on ENS precursor **migration** and neurite fasciculation appear to be mediated at least in part by increased polysialic acid addition to neural cell adhesion molecule (Ncam1) in response to BMP. Removing PSA enzymatically reverses the BMP effects on ENS precursor **migration** and neurite fasciculation. These studies demonstrate several novel roles for BMP signaling and highlight new functions for sialyltransferases in the developing ENS.
40. The zinc finger transcription factor RP58 negatively regulates Rnd2 for the control of neuronal **migration** during cerebral cortical development. The zinc finger transcription factor RP58 (also known as ZNF238) regulates neurogenesis of the mouse neocortex and cerebellum (Okado et al. 2009; Xiang et al. 2011; Baubet et al. 2012; Ohtaka-Maruyama et al. 2013), but its mechanism of action remains unclear. In this study, we report a cell-autonomous function for RP58 during the differentiation of embryonic cortical projection neurons via its activities as a transcriptional repressor. Disruption of RP58 expression alters the differentiation of immature neurons and impairs their **migration** and positioning within the mouse cerebral cortex. Loss of RP58 within the embryonic cortex also leads to elevated mRNA for Rnd2, a member of the Rnd family of atypical RhoA-like GTPase proteins important for cortical neuron **migration** (Heng et al. 2008). Mechanistically, RP58 represses transcription of Rnd2 via binding to a 3'-regulatory enhancer in a sequence-specific fashion. Using reporter assays, we found that RP58 repression of Rnd2 is competed by proneural basic helix-loop-helix transcriptional activators. Finally, our rescue experiments revealed that negative regulation of Rnd2 by RP58 was important for cortical cell **migration** in vivo. Taken together, these studies demonstrate that RP58 is a key player in the transcriptional control of cell **migration** in the developing cerebral cortex.
41. FoxP2 protein levels regulate cell morphology changes and **migration** patterns in the vertebrate developing telencephalon. In the mammalian telencephalon, part of the progenitor cells transition from multipolar to bipolar morphology as they invade the mantle zone. This associates with changing patterns of radial **migration**. However, the molecules implicated in these morphology transitions are not well known. In the present work, we analyzed the function of FoxP2 protein in this process during telencephalic development in vertebrates. We analyzed the expression of FoxP2 protein and its relation with cell morphology and migratory patterns in mouse and chicken developing striatum. We observed FoxP2 protein expressed in a gradient from the subventricular zone to the mantle layer in mice embryos. In the FoxP2 low domain cells showed multipolar **migration**. In the striatal mantle layer where FoxP2 protein expression is higher, cells showed locomoting **migration** and bipolar morphology. In contrast, FoxP2 showed a high and homogenous expression pattern in chicken striatum, thus bipolar morphology predominated. Elevation of FoxP2 in the striatal subventricular zone by in utero electroporation promoted bipolar morphology and impaired multipolar radial **migration**. In mouse cerebral cortex we obtained similar results. FoxP2 promotes transition from multipolar to bipolar morphology by means of gradiental expression in mouse striatum and cortex. Together these results indicate a role of FoxP2 differential expression in cell morphology control of the vertebrate telencephalon.
42. Transcription factor Sox11 is essential for both embryonic and adult neurogenesis. Neurogenesis requires neural progenitor cell (NPC) proliferation, neuronal **migration**, and differentiation. During embryonic development, neurons are generated in specific areas of the developing neuroepithelium and migrate to their appropriate positions. In the adult brain, neurogenesis continues in the subgranular zone (SGZ) of the hippocampal dentate gyrus and the subventricular zone (SVZ) of the lateral ventricle. Although neurogenesis is fundamental to brain development and function, our understanding of the molecular mechanisms that regulate neurogenesis is still limited.In this study, we generated a Sox11 floxed allele and a Sox11 null allele in mice using the Cre-loxP technology. We first analyzed the role of the transcription factor Sox11 in embryonic neurogenesis using Sox11 null embryos. We also examined the role of Sox11 in adult hippocampal neurogenesis using Sox11 conditional knockout mice in which Sox11 is specifically deleted in adult NPCs. Sox11 null embryos developed small and disorganized brains, accompanied by transient proliferation deficits in NPCs. Deletion of Sox11 in adult NPCs blunted proliferation in the SGZ. Using functional genomics, we identified potential downstream target genes of Sox11.Taken together, our work provides evidence that Sox11 is required for both embryonic and adult neurogenesis, and identifies potential downstream target genes.
43. Ena/VASP Is Required for neuritogenesis in the developing cortex. Mammalian cortical development involves neuronal **migration** and neuritogenesis; this latter process forms the structural precursors to axons and dendrites. Elucidating the pathways that regulate the cytoskeleton to drive these processes is fundamental to our understanding of cortical development. Here we show that loss of all three murine Ena/VASP proteins, a family of actin regulatory proteins, causes neuronal ectopias, alters intralayer positioning in the cortical plate, and, surprisingly, blocks axon fiber tract formation during corticogenesis. Cortical fiber tract defects in the absence of Ena/VASP arise from a failure in neurite initiation, a prerequisite for axon formation. Neurite initiation defects in Ena/VASP-deficient neurons are preceded by a failure to form bundled actin filaments and filopodia. These findings provide insight into the regulation of neurite formation and the role of the actin cytoskeleton during cortical development.
44. Characterization of neogenin-expressing neural progenitor populations and migrating neuroblasts in the embryonic mouse forebrain. Many studies have demonstrated a role for netrin-1-deleted in colorectal cancer (DCC) interactions in both axon guidance and neuronal **migration**. Neogenin, a member of the DCC receptor family, has recently been shown to be a chemorepulsive axon guidance receptor for the repulsive guidance molecule (RGM) family of guidance cues [Rajagopalan S, Deitinghoff L, Davis D, Conrad S, Skutella T, Chedotal A, Mueller B, Strittmatter S (2004) Neogenin mediates the action of repulsive guidance molecule. Nat Cell Biol 6:755-762]. Here we show that neogenin is present on neural progenitors, including neurogenic radial glia, in the embryonic mouse forebrain suggesting that neogenin expression is a hallmark of neural progenitor populations. Neogenin-positive progenitors were isolated from embryonic day 14.5 forebrain using flow cytometry and cultured as neurospheres. Neogenin-positive progenitors gave rise to neurospheres displaying a high proliferative and neurogenic potential. In contrast, neogenin-negative forebrain cells did not produce long-term neurosphere cultures and did not possess a significant neurogenic potential. These observations argue strongly for a role for neogenin in neural progenitor biology. In addition, we also observed neogenin on parvalbumin- and calbindin-positive interneuron neuroblasts that were migrating through the medial and lateral ganglionic eminences, suggesting a role for neogenin in tangential **migration**. Therefore, neogenin may be a multi-functional receptor regulating both progenitor activity and neuroblast **migration** in the embryonic forebrain.
45. RhoA and Cdc42 are required in pre-migratory progenitors of the medial ganglionic eminence ventricular zone for proper cortical interneuron **migration**. Cortical interneurons arise from the ganglionic eminences in the ventral telencephalon and migrate tangentially to the cortex. Although RhoA and Cdc42, members of the Rho family of small GTPases, have been implicated in regulating neuronal **migration**, their respective roles in the tangential **migration** of cortical interneurons remain unknown. Here we show that loss of RhoA and Cdc42 in the ventricular zone (VZ) of the medial ganglionic eminence (MGE) using Olig2-Cre mice causes moderate or severe defects in the **migration** of cortical interneurons, respectively. Furthermore, RhoA- or Cdc42-deleted MGE cells exhibit impaired **migration** in vitro. To determine whether RhoA and Cdc42 directly regulate the motility of cortical interneurons during **migration**, we deleted RhoA and Cdc42 in the subventricular zone (SVZ), where more fate-restricted progenitors are located within the ganglionic eminences, using Dlx5/6-Cre-ires-EGFP (Dlx5/6-CIE) mice. Deletion of either gene within the SVZ does not cause any obvious defects in cortical interneuron **migration**, indicating that cell motility is not dependent upon RhoA or Cdc42. These findings provide genetic evidence that RhoA and Cdc42 are required in progenitors of the MGE in the VZ, but not the SVZ, for proper cortical interneuron **migration**.
46. Peripheral nerve-derived CXCL12 and VEGF-A regulate the patterning of arterial vessel branching in developing limb skin. In developing limb skin, peripheral nerves provide a spatial template that controls the branching pattern and differentiation of arteries. Our previous studies indicate that nerve-derived VEGF-A is required for arterial differentiation but not for nerve-vessel alignment. In this study, we demonstrate that nerve-vessel alignment depends on the activity of Cxcl12-Cxcr4 chemokine signaling. Genetic inactivation of Cxcl12-Cxcr4 signaling perturbs nerve-vessel alignment and abolishes arteriogenesis. Further in vitro assays allow us to uncouple nerve-vessel alignment and arteriogenesis, revealing that nerve-derived Cxcl12 stimulates endothelial cell **migration**, whereas nerve-derived VEGF-A is responsible for arterial differentiation. These findings suggest a coordinated sequential action in which nerve Cxcl12 functions over a distance to recruit vessels to align with nerves, and subsequent arterial differentiation presumably requires a local action of nerve VEGF-A in the nerve-associated vessels.
47. G protein-coupled receptor 56 and collagen III, a receptor-ligand pair, regulates cortical development and lamination. GPR56, an orphan G protein-coupled receptor (GPCR) from the family of adhesion GPCRs, plays an indispensable role in cortical development and lamination. Mutations in the GPR56 gene cause a malformed cerebral cortex in both humans and mice that resembles cobblestone lissencephaly, which is characterized by over**migration** of neurons beyond the pial basement membrane. However, the molecular mechanisms through which GPR56 regulates cortical development remain elusive due to the unknown status of its ligand. Here we identify collagen, type III, alpha-1 (gene symbol Col3a1) as the ligand of GPR56 through an in vitro biotinylation/proteomics approach. Further studies demonstrated that Col3a1 null mutant mice exhibit over**migration** of neurons beyond the pial basement membrane and a cobblestone-like cortical malformation similar to the phenotype seen in Gpr56 null mutant mice. Functional studies suggest that the interaction of collagen III with its receptor GPR56 inhibits neural **migration** in vitro. As for intracellular signaling, GPR56 couples to the Gα(12/13) family of G proteins and activates RhoA pathway upon ligand binding. Thus, collagen III regulates the proper lamination of the cerebral cortex by acting as the major ligand of GPR56 in the developing brain.
48. Involvement of the myelin-associated inhibitor Nogo-A in early cortical development and neuronal maturation. Nogo-A is a myelin-associated protein expressed by neurons and myelinating mature oligodendrocytes in the central nervous system. Although most research has focused on the participation of Nogo-A in the prevention of axonal regeneration and plasticity in the adult, little attention has been paid to the putative functions of Nogo-A during embryonic development. Here we examined the general pattern and cell-specific distribution of Nogo-A in the prenatal mouse telencephalon. In addition, we studied the development of the major axon tracts and radial and tangential **migration** in Nogo-A/B/C knockout mice. The pattern of Nogo-A showed distinct distribution in radial glia and postmitotic neurons, in which it is particularly enriched in developing axons. Similarly, Nogo-A was enriched at the leading process of tangentially migrating interneurons but not detectable in radial migrating neurons. Although a low level of Nogo-A appears to be on the surface of many cortical neurons, most proteins have intracellular localization. In Nogo-deficient background, neurons displayed early polarization and increased branching in vitro, probably reflecting a cell-intrinsic role of Nogo proteins in branching reduction, and early tangential **migration** was delayed. On the basis of these observations, we propose that Nogo proteins, particularly Nogo-A, are involved in multiple processes during cortical development.
49. Identification of a novel brain-specific and Reelin-regulated gene that encodes a protein colocalized with synapsin. We carried out a screening of genes that are differentially expressed in normal mice and reeler mutants and are characterized by abnormal neuronal **migration** and neurite deployment due to defective Reelin signalling. A novel gene, provisionally named C61, was overexpressed in Reelin-deficient embryonic mouse brain RNA. C61 encodes a 3.7 kb mRNA that is brain specific and developmentally regulated, with predominant expression in differentiating neurons. The predicted protein is 664 amino acids long, and contains LAG1 and Ezrin/Radixin/Moesin-Myosin-Filament motifs, suggesting that it may function as an intracellular adaptor. From E14.5 to birth, C61 was highly expressed in all neuronal differentiation fields, with the highest signal in the telencephalic cortical plate and mitral cells in the olfactory bulb. When expressed as a GFP fusion protein in transfected non-neuronal cells and primary neurons, this protein localizes, respectively, to the nuclear membrane or axonal outgrowths, indicating a function in axonal traffic or signalling.
50. Regulation of neural progenitor cell motility by ceramide and potential implications for mouse brain development. We provide evidence that the sphingolipid ceramide, in addition to its pro-apoptotic function, regulates neural progenitor (NP) motility in vitro and brain development in vivo. Ceramide (N-palmitoyl d-erythro sphingosine and N-oleoyl d-erythro sphingosine) and the ceramide analog N-oleoyl serinol (S18) stimulate **migration** of NPs in scratch (wounding) **migration** assays. Sphingolipid depletion by inhibition of de novo ceramide biosynthesis, or ceramide inactivation using an anti-ceramide antibody, obliterates NP motility, which is restored by ceramide or S18. These results suggest that ceramide is crucial for NP motility. Wounding of the NP monolayer activates neutral sphingomyelinase indicating that ceramide is generated from sphingomyelin. In membrane processes, ceramide is co-distributed with its binding partner atypical protein kinase C zeta/lambda (aPKC), and Cdc42, alpha/beta-tubulin, and beta-catenin, three proteins involved in aPKC-dependent regulation of cell polarity and motility. Sphingolipid depletion by myriocin prevents membrane translocation of aPKC and Cdc42, which is restored by ceramide or S18. These results suggest that ceramide-mediated membrane association of aPKC/Cdc42 is important for NP motility. In vivo, sphingolipid depletion leads to ectopic localization of mitotic or post-mitotic neural cells in the embryonic brain, while S18 restores the normal brain organization. In summary, our study provides novel evidence that ceramide is critical for NP motility and polarity in vitro and in vivo.
51. Spatiotemporal mapping of vascularization and innervation in the fetal murine intestine. In mice, the intestinal tube develops from the splanchopleure before embryonic day 9.5. Subsequent patterning of nerves and blood vessels is critical for normal digestive function. A hierarchical branching vascular network allows for efficient nutrient absorption, while the complex enteric nervous system regulates intestinal motility as well as secretion, absorption, and blood flow. Despite the well-recognized significance of these systems, the precise mechanisms by which they develop have not been clearly established in mammals.Using a novel whole-mount immunohistochemical protocol, we visualize the pattern of intestinal neurovascular development in mice between embryonic day 10.5 and birth. In particular, we focus on the development and remodeling of the enteric vascular plexus, the **migration** and organization of enteric neural crest-derived cells, and the integration of peripheral sympathetic nerves with the enteric nervous system. These correlative data lead us to hypothesize a functional interaction between migrating neural crest-derived cells and endothelial cells of the primary capillary plexus, as well as a subsequent interaction between developing peripheral autonomic nerves and differentiated neural crest-derived cells.These studies provide useful anatomical data for continuing investigations on the functional mechanisms underlying intestinal organogenesis.
52. Tcof1 acts as a modifier of Pax3 during enteric nervous system development and in the pathogenesis of colonic aganglionosis. Hirschsprung disease (HSCR) is a human congenital disorder, defined by the absence of ganglia from variable lengths of the colon. These ganglia comprise the enteric nervous system (ENS) and are derived from migratory neural crest cells (NCCs). The inheritance of HSCR is complex, often non-Mendelian and characterized by variable penetrance. Although extensive research has identified many key players in the pathogenesis of Hirschsprung disease, a large number of cases remain genetically undefined. Therefore, additional unidentified genes or modifiers must contribute to the etiology and pathogenesis of Hirschsprung disease. We have discovered that Tcof1 may be one such modifier. Haploinsufficiency of Tcof1 in mice results in a reduction of vagal NCCs and their delayed **migration** along the length of the gut during early development. This alone, however, is not sufficient to cause colonic aganglionosis as alterations in the balance of NCC proliferation and differentiation ensures NCC colonize the entire length of the gut of Tcof1(+/-) mice by E18.5. In contrast, Tcof1 haploinsufficiency is able to sensitize Pax3(+/-) mice to colonic aganglionosis. Although, Pax3 heterozygous mice do not show ENS defects, compound Pax3;Tcof1 heterozygous mice exhibit cumulative apoptosis which severely reduces the NCC population that migrates into the foregut. In addition, the proliferative capacity of these NCC is also diminished. Taken together with the opposing effects of Pax3 and Tcof1 on NCC differentiation, the synergistic haploinsufficiency of Tcof1 and Pax3 results in colonic aganglionosis in mice and may contribute to the pathogenesis of Hirschsprung disease.
53. Neural crest boundary cap cells constitute a source of neuronal and glial cells of the PNS. Boundary cap (BC) cells are neural crest derivatives that form clusters at the surface of the neural tube, at entry and exit points of peripheral nerve roots. Using various knock-in alleles of the mouse gene Egr2 (also known as Krox20), the expression of which, in trunk regions, is initially restricted to BC cells, we were able to trace BC cell progeny during development and analyze their fate. Trunk BC-derived cells migrated along peripheral axons and colonized spinal nerve roots and dorsal root ganglia (DRG). All Schwann cell precursors occupying the dorsal roots were derived from BC cells. In the DRG, BC-derived cells were the progenitors of both neurons, mainly nociceptive afferents, and satellite cells. These data indicate that BC cells constitute a source of peripheral nervous system (PNS) components that, after the major neural crest ventrolateral migratory stream, feeds a secondary wave of **migration** to the PNS.
54. A mutation in the pericentrin gene causes abnormal interneuron **migration** to the olfactory bulb in mice. Precise control of neuronal **migration** is essential for proper function of the brain. Taking a forward genetic screen, we isolated a mutant mouse with defects in interneuron **migration**. By genetic mapping, we identified a frame shift mutation in the pericentrin (Pcnt) gene. The Pcnt gene encodes a large centrosomal coiled-coil protein that has been implicated in schizophrenia. Recently, frame shift and premature termination mutations in the pericentrin (PCNT) gene were identified in individuals with Seckel syndrome and microcephalic osteodysplastic primordial dwarfism (MOPD II), both of which are characterized by greatly reduced body and brain sizes. The mouse Pcnt mutant shares features with the human syndromes in its overall growth retardation and reduced brain size. We found that dorsal lateral ganglionic eminence (dLGE)-derived olfactory bulb interneurons are severely affected and distributed abnormally in the rostral forebrain in the mutant. Furthermore, mutant interneurons exhibit abnormal **migration** behavior and RNA interference knockdown of Pcnt impairs cell **migration** along the rostal migratory stream (RMS) into the olfactory bulb. These findings indicate that pericentrin is required for proper **migration** of olfactory bulb interneurons and provide a developmental basis for association of pericentrin function with interneuron defects in human schizophrenia.
55. Nogo-a regulates neural precursor **migration** in the embryonic mouse cortex. Although Nogo-A has been intensively studied for its inhibitory effect on axonal regeneration in the adult central nervous system, little is known about its function during brain development. In the embryonic mouse cortex, Nogo-A is expressed by radial precursor/glial cells and by tangentially migrating as well as postmigratory neurons. We studied radially migrating neuroblasts in wild-type and Nogo-A knockout (KO) mouse embryos. In vitro analysis showed that Nogo-A and its receptor components NgR, Lingo-1, TROY, and p75 are expressed in cells emigrating from embryonic forebrain-derived neurospheres. Live imaging revealed an increased cell motility when Nogo-A was knocked out or blocked with antibodies. Antibodies blocking NgR or Lingo-1 showed the same motility-enhancing effect supporting a direct role of surface Nogo-A on **migration**. Bromodeoxyuridine (BrdU) labeling of embryonic day (E)15.5 embryos demonstrated that Nogo-A influences the radial **migration** of neuronal precursors. At E17.5, the normal transient accumulation of radially migrating precursors within the subventricular zone was not detectable in the Nogo-A KO mouse cortex. At E19, **migration** to the upper cortical layers was disturbed. These findings suggest that Nogo-A and its receptor complex play a role in the interplay of adhesive and repulsive cell interactions in radial **migration** during cortical development.
56. A collagen VI-dependent pathogenic mechanism for Hirschsprung's disease. Hirschsprung's disease (HSCR) is a severe congenital anomaly of the enteric nervous system (ENS) characterized by functional intestinal obstruction due to a lack of intrinsic innervation in the distal bowel. Distal innervation deficiency results from incomplete colonization of the bowel by enteric neural crest cells (eNCCs), the ENS precursors. Here, we report the generation of a mouse model for HSCR--named Holstein--that contains an untargeted transgenic insertion upstream of the collagen-6α4 (Col6a4) gene. This insertion induces eNCC-specific upregulation of Col6a4 expression that increases total collagen VI protein levels in the extracellular matrix (ECM) surrounding both the developing and the postnatal ENS. Increased collagen VI levels during development mainly result in slower **migratio**n of eNCCs. This appears to be due to the fact that collagen VI is a poor substratum for supporting eNCC **migratio**n and can even interfere with the **migratio**n-promoting effects of fibronectin. Importantly, for a majority of patients in a HSCR cohort, the myenteric ganglia from the ganglionated region are also specifically surrounded by abundant collagen VI microfibrils, an outcome accentuated by Down syndrome. Collectively, our data thus unveil a clinically relevant pathogenic mechanism for HSCR that involves cell-autonomous changes in ECM composition surrounding eNCCs. Moreover, as COL6A1 and COL6A2 are on human Chr.21q, this mechanism is highly relevant to the predisposition of patients with Down syndrome to HSCR.
57. Both Myosin-10 isoforms are required for radial neuronal **migration** in the developing cerebral cortex. During embryonic development of the mammalian cerebral cortex, postmitotic cortical neurons migrate radially from the ventricular zone to the cortical plate. Proper **migration** involves the correct orientation of migrating neurons and the transition from a multipolar to a mature bipolar morphology. Herein, we report that the 2 isoforms of Myosin-10 (Myo10) play distinct roles in the regulation of radial **migration** in the mouse cortex. We show that the full-length Myo10 (fMyo10) isoform is located in deeper layers of the cortex and is involved in establishing proper **migration** orientation. We also demonstrate that fMyo10-dependent orientation of radial **migration** is mediated at least in part by the netrin-1 receptor deleted in colorectal cancer. Moreover, we show that the headless Myo10 (hMyo10) isoform is required for the transition from multipolar to bipolar morphologies in the intermediate zone. Our study reveals divergent functions for the 2 Myo10 isoforms in controlling both the direction of **migration** and neuronal morphogenesis during radial cortical neuronal **migration**.
58. Expression of glial progenitor markers p75NTR and S100 protein in the developing mouse parathyroid gland. Drosophila glial cells missing (Drosophila Gcm) is a transcription factor that is required for the differentiation of glial cells. Gcm2, a mouse homologue of Drosophila Gcm, is a master regulatory gene of parathyroid development and is expressed in the parathyroid rudiment. We have found that the mouse parathyroid exhibits the glial progenitor markers, p75(NTR) and S100 protein, during fetal development. At embryonic day 11.5 (E11.5), a bulge of the parathyroid rudiment is formed in the cranial part of the third pharyngeal pouch. The rudiment exhibits immunoreactivity for p75(NTR) and S100 protein, in addition to secretory protein 1/chromogranin A. While the thymus rudiment, which arises from the caudal part of the third pharyngeal pouch, is moving downwards, the parathyroid is attached to the top of thymus. The parathyroid comes into contact with the thyroid lobe at E13.5 and then separates from the thymus. The parathyroid maintains intense immunoreactivity for p75(NTR) and S100 protein during the **migration** and development in the thyroid lobe. The co-localization of p75(NTR) and S100 in the developing parathyroid cells has been confirmed by confocal microscopy. Other glial markers, viz. glial fibrillary acidic protein, Sox10, vimentin and nestin, are not expressed in the parathyroid at any stage of development. The neural progenitor markers, neurofilament 160 and TuJ1, are also absent from the parathyroid. Taken together, we suggest that Gcm2 supplies only some glial progenitor characteristics to the parathyroid rudiment.
59. Zfp423 controls proliferation and differentiation of neural precursors in cerebellar vermis formation. Neural stem cells and progenitors in the developing brain must choose between proliferation with renewal and differentiation. Defects in navigating this choice can result in malformations or cancers, but the genetic mechanisms that shape this choice are not fully understood. We show by positional cloning that the 30-zinc finger transcription factor Zfp423 (OAZ) is required for patterning the development of neuronal and glial precursors in the developing brain, particularly in midline structures. Mutation of Zfp423 results in loss of the corpus callosum, reduction of hippocampus, and a malformation of the cerebellum reminiscent of human Dandy-Walker patients. Within the cerebellum, Zfp423 is expressed in both ventricular and external germinal zones. Loss of Zfp423 results in diminished proliferation by granule cell precursors in the external germinal layer, especially near the midline, and abnormal differentiation and **migration** of ventricular zone-derived neurons and Bergmann glia.
60. Guidance of trunk neural crest **migration** requires neuropilin 2/semaphorin 3F signaling. In vertebrate embryos, neural crest cells migrate only through the anterior half of each somite while avoiding the posterior half. We demonstrate that neural crest cells express the receptor neuropilin 2 (Npn2), while its repulsive ligand semaphorin 3F (Sema3f) is restricted to the posterior-half somite. In Npn2 and Sema3f mutant mice, neural crest cells lose their segmental **migration** pattern and instead migrate as a uniform sheet, although somite polarity itself remains unchanged. Furthermore, Npn2 is cell autonomously required for neural crest cells to avoid Sema3f in vitro. These data show that Npn2/Sema3f signaling guides neural crest **migration** through the somite. Interestingly, neural crest cells still condense into segmentally arranged dorsal root ganglia in Npn2 nulls, suggesting that segmental neural crest **migration** and segmentation of the peripheral nervous system are separable processes.
61. Enteric nervous system progenitors are coordinately controlled by the G protein-coupled receptor EDNRB and the receptor tyrosine kinase RET. The enteric nervous system (ENS) in vertebrates is derived mainly from vagal neural crest cells that enter the foregut and colonize the entire wall of the gastrointestinal tract. Failure to completely colonize the gut results in the absence of enteric ganglia (Hirschsprung's disease). Two signaling systems mediated by RET and EDNRB have been identified as critical players in enteric neurogenesis. We demonstrate that interaction between these signaling pathways controls ENS development throughout the intestine. Activation of EDNRB specifically enhances the effect of RET signaling on the proliferation of uncommitted ENS progenitors. In addition, we reveal novel antagonistic roles of these pathways on the **migration** of ENS progenitors. Protein kinase A is a key component of the molecular mechanisms that integrate signaling by the two receptors. Our data provide strong evidence that the coordinate and balanced interaction between receptor tyrosine kinases and G protein-coupled receptors controls the development of the nervous system in mammals.
62. Ephrin B1 is expressed on neuroepithelial cells in correlation with neocortical neurogenesis. To identify molecules involved in neurogenesis, we have raised monoclonal antibodies against embryonic day 12.5 mouse telencephalon. One antibody, monoclonal antibody 25H11, stains predominantly the ventricular zone of the anterior and lateral telencephalon. Purification of the 25H11 antigen, a 47 kDa integral membrane protein, from approximately 2500 mouse telencephali reveals its identity with ephrin B1. Ephrin B1 appears at the onset of neocortical neurogenesis, being first expressed in neuron-generating neuroepithelial cells and rapidly thereafter in virtually all neuroepithelial cells. Expression of ephrin B1 persists through the period of neocortical neurogenesis and is downregulated thereafter. Ephrin B1 is present on the ventricular as well as basolateral plasma membrane of neuroepithelial cells and exhibits an ventricular-high to pial-low gradient across the ventricular zone. Expression of ephrin B1 is also detected on radial glial cells, extending all the way to their pial endfeet, and on neurons in the mantle/intermediate zone but not in the cortical plate. Our results suggest that ephrin B1, presumably via ephrin-Eph receptor signaling, has a role in neurogenesis. Given the ventricular-to-pial gradient of ephrin B1 on the neuroepithelial cell surface and its known role in cell **migration** in other systems mediated by its repulsive properties, we propose that ephrin B1 may be involved in the **migration** of newborn neurons out from the ventricular zone toward the neocortex.
63. Cell **migration** along the lateral cortical stream to the developing basal telencephalic limbic system. During embryogenesis, the lateral cortical stream (LCS) emerges from the corticostriatal border (CSB), the boundary between the developing cerebral cortex and striatum. The LCS is comprised of a mix of pallial- and subpallial-derived neural progenitor cells that migrate to the developing structures of the basal telencephalon, most notably the piriform cortex and amygdala. Using a combination of in vitro and in vivo approaches, we analyzed the timing, composition, migratory modes, origin, and requirement of the homeodomain-containing transcription factor Gsh2 (genomic screened homeobox 2) in the development of this prominent migratory stream. We reveal that Pax6 (paired box gene 6)-positive pallial-derived and Dlx2 (distal-less homeobox 2)-positive subpallial-derived subpopulations of LCS cells are generated in distinct temporal windows during embryogenesis. Furthermore, our data indicate the CSB border not only is comprised of separate populations of pallial- and subpallial-derived progenitors that contribute to the LCS but also a subpopulation of cells coexpressing Pax6 and Dlx2. Moreover, despite migrating along a route outlined by a cascade of radial glia, the Dlx2-positive population appears to migrate primarily in an apparent chain-like manner, with LCS migratory cells being generated locally at the CSB with little contribution from other subpallial structures such as the medial, lateral, or caudal ganglionic eminences. We further demonstrate that the generation of the LCS is dependent on the homeodomain-containing gene Gsh2, revealing a novel requirement for Gsh2 in telencephalic development.
64. Stereotyped fetal brain disorganization is induced by hypoxia and requires lysophosphatidic acid receptor 1 (LPA1) signaling. Fetal hypoxia is a common risk factor that has been associated with a range of CNS disorders including epilepsy, schizophrenia, and autism. Cellular and molecular mechanisms through which hypoxia may damage the developing brain are incompletely understood but are likely to involve disruption of the laminar organization of the cerebral cortex. Lysophosphatidic acid (LPA) is a bioactive lipid capable of cortical influences via one or more of six cognate G protein-coupled receptors, LPA(1-6), several of which are enriched in fetal neural progenitor cells (NPCs). Here we report that fetal hypoxia induces cortical disruption via increased LPA(1) signaling involving stereotyped effects on NPCs: N-cadherin disruption, displacement of mitotic NPCs, and impaired neuronal **migration**, as assessed both ex vivo and in vivo. Importantly, genetic removal or pharmacological inhibition of LPA(1) prevented the occurrence of these hypoxia-induced phenomena. Hypoxia resulted in overactivation of LPA(1) through selective inhibition of G protein-coupled receptor kinase 2 expression and activation of downstream pathways including G(αi) and Ras-related C3 botulinum toxin substrate 1. These data identify stereotyped and selective hypoxia-induced cerebral cortical disruption requiring LPA(1) signaling, inhibition of which can reduce or prevent disease-associated sequelae, and may take us closer to therapeutic treatment of fetal hypoxia-induced CNS disorders and possibly other forms of hypoxic injury.
65. Serum response factor is required for cortical axon growth but is dispensable for neurogenesis and neocortical lamination. Previous studies have shown that neuron-specific deletion of serum response factor (SRF) results in deficits in tangential cell **migration**, guidance-dependent circuit assembly, activity-dependent gene expression, and synaptic plasticity in the hippocampus. Furthermore, SRF deletion in mouse embryonic stem cells causes cell death in vitro. However, the requirement of SRF for early neuronal development including neural stem cell homeostasis, neurogenesis, and axonal innervations remains unknown. Here, we report that SRF is critical for development of major axonal tracts in the forebrain. Conditional mutant mice lacking SRF in neural progenitor cells (Srf-Nestin-cKO) exhibit striking deficits in cortical axonal projections including corticostriatal, corticospinal, and corticothalamic tracts, and they show a variable loss of the corpus callosum. Neurogenesis and interneuron specification occur normally in the absence of SRF and the deficits in axonal projections were not due to a decrease or loss in cell numbers. Radial **migration** of neurons and neocortical lamination were also not affected. No aberrant cell death was observed during development, whereas there was an increase in the number of proliferative cells in the ventricular zone from embryonic day 14 to day 18. Similar axonal tract deficits were also observed in mutant mice lacking SRF in the developing excitatory neurons of neocortex and hippocampus (Srf-NEX-cKO). Together, these findings suggest distinct roles for SRF during neuronal development; SRF is specifically required in a cell-autonomous manner for axonal tract development but is dispensable for cell survival, neurogenesis, neocortical lamination, and neuronal differentiation.
66. The protocadherin gene Celsr3 is required for interneuron **migration** in the mouse forebrain. Interneurons are extremely diverse in the mammalian brain and provide an essential balance for functional neural circuitry. The vast majority of murine cortical interneurons are generated in the subpallium and migrate tangentially over a long distance to acquire their final positions. By using a mouse line with a deletion of the Celsr3 (Flamingo, or Fmi1) gene and a knock-in of the green fluorescent protein reporter, we find that Celsr3, a member of the nonclustered protocadherin (Pcdh) family, is predominantly expressed in the cortical interneurons in adults and in the interneuron germinal zones in embryos. We show that Celsr3 is crucial for interneuron **migration** in the developing mouse forebrain. Specifically, in Celsr3 knockout mice, calretinin-positive interneurons are reduced in the developing neocortex, accumulated in the corticostriatal boundary, and increased in the striatum. Moreover, the laminar distribution of cortical calbindin-positive cells is altered. Finally, we found that expression patterns of NRG1 (neuregulin-1) and its receptor ErbB4, which are essential for interneuron **migration**, are changed in Celsr3 mutants. These results demonstrate that the protocadherin Celsr3 gene is essential for both tangential and radial interneuron **migration**s in a class-specific manner.
67. Tbr1 regulates differentiation of the preplate and layer 6. During corticogenesis, early-born neurons of the preplate and layer 6 are important for guiding subsequent neuronal **migration**s and axonal projections. Tbr1 is a putative transcription factor that is highly expressed in glutamatergic early-born cortical neurons. In Tbr1-deficient mice, these early-born neurons had molecular and functional defects. Cajal-Retzius cells expressed decreased levels of Reelin, resulting in a reeler-like cortical **migration** disorder. Impaired subplate differentiation was associated with ectopic projection of thalamocortical fibers into the basal telencephalon. Layer 6 defects contributed to errors in the thalamocortical, corticothalamic, and callosal projections. These results show that Tbr1 is a common genetic determinant for the differentiation of early-born glutamatergic neocortical neurons and provide insights into the functions of these neurons as regulators of cortical development.
68. Expression of Disabled 1 suppresses astroglial differentiation in neural stem cells. Disabled 1 (Dab1), a cytoplasmic adaptor protein expressed predominantly in the CNS, transduces a Reelin-initiated signaling that controls neuronal **migration** and positioning during brain development. To determine the role of Dab1 in neural stem cell (NSC) differentiation, we established a culture of neurospheres derived from the embryonic forebrain of the Dab1(-/-) mice, yotari. Differentiating Dab1(-/-) neurospheres exhibited a higher expression of GFAP, an astrocytic marker, at the expense of neuronal markers. Under Dab1-deficient condition, the expression of NeuroD, a transcription factor for neuronal differentiation, was decreased and the JAK-STAT pathway was evidently increased during differentiation of NSC, suggesting the possible involvement of Dab1 in astrocyte differentiation via JAK-STAT pathway. Notably, expression of neural and glial markers and the level of JAK-STAT signaling molecules were not changed in differentiating NSC by Reelin treatment, indicating that differentiation of NSC is Reelin-independent. Immunohistochemical analyses showed a decrease in the number of neurons and an increase in the number of GFAP-positive cells in developing yotari brains. Our results suggest that Dab1 participates in the differentiation of NSCs into a specific cell lineage, thereby maintaining a balance between neurogenesis and gliogenesis.
69. A radial glia-specific role of RhoA in double cortex formation. The positioning of neurons in the cerebral cortex is of crucial importance for its function as highlighted by the severe consequences of **migration**al disorders in patients. Here we show that genetic deletion of the small GTPase RhoA in the developing cerebral cortex results in two **migration**al disorders: subcortical band heterotopia (SBH), a heterotopic cortex underlying the normotopic cortex, and cobblestone lissencephaly, in which neurons protrude beyond layer I at the pial surface of the brain. Surprisingly, RhoA(-/-) neurons migrated normally when transplanted into wild-type cerebral cortex, whereas the converse was not the case. Alterations in the radial glia scaffold are demonstrated to cause these **migration**al defects through destabilization of both the actin and the microtubules cytoskeleton. These data not only demonstrate that RhoA is largely dispensable for **migration** in neurons but also showed that defects in radial glial cells, rather than neurons, can be sufficient to produce SBH.
70. Cxcr4 regulation of interneuron **migration** is disrupted in 22q11.2 deletion syndrome. Interneurons are thought to be a primary pathogenic target for several behavioral disorders that arise during development, including schizophrenia and autism. It is not known, however, whether genetic lesions associated with these diseases disrupt established molecular mechanisms of interneuron development. We found that diminished 22q11.2 gene dosage-the primary genetic lesion in 22q11.2 deletion syndrome (22q11.2 DS)-specifically compromises the distribution of early-generated parvalbumin-expressing interneurons in the Large Deletion (LgDel) 22q11.2DS mouse model. This change reflects cell-autonomous disruption of interneuron **migration** caused by altered expression of the cytokine C-X-C chemokine receptor type 4 (Cxcr4), an established regulator of this process. Cxcr4 is specifically reduced in LgDel migrating interneurons, and genetic analysis confirms that diminished Cxcr4 alters interneuron **migration** in LgDel mice. Thus, diminished 22q11.2 gene dosage disrupts cortical circuit development by modifying a critical molecular signaling pathway via Cxcr4 that regulates cortical interneuron **migration** and placement.
71. Slit2 and Robo3 modulate the **migration** of GnRH-secreting neurons. Gonadotropin-releasing hormone (GnRH) neurons are born in the nasal placode and migrate along olfactory and vomeronasal axons to reach the forebrain and settle in the hypothalamus, where they control reproduction. The molecular cues that guide their **migration** have not been fully identified, but are thought to control either cell movement directly or the patterning of their axonal substrates. Using genetically altered mouse models we show that the **migration** of GnRH neurons is directly modulated by Slit2 and Robo3, members of the axon guidance Slit ligand and Robo receptor families. Mice lacking Slit2 or Robo3 have a reduced number of GnRH neurons in the forebrain, but a normal complement of their supporting axons, pointing to a direct role for these molecules in GnRH neuron **migration**.
72. The SH2 domain protein Shep1 regulates the in vivo signaling function of the scaffolding protein Cas. The members of the p130Cas (Cas) family are important scaffolding proteins that orchestrate cell adhesion, **migration** and invasiveness downstream of integrin adhesion receptors and receptor tyrosine kinases by recruiting enzymes and structural molecules. Shep1, BCAR3/AND-34 and NSP1 define a recently identified family of SH2 domain-containing proteins that constitutively bind Cas proteins through a Cdc25-type nucleotide exchange factor-like domain. To gain insight into the functional interplay between Shep1 and Cas in vivo, we have inactivated the Shep1 gene in the mouse through Cre-mediated deletion of the exon encoding the SH2 domain. Analysis of Cas tyrosine phosphorylation in the brains of newborn mice, where Shep1 is highly expressed, revealed a strong decrease in Cas substrate domain phosphorylation in knockout compared to wild-type brains. Src family kinases bind to Cas via their SH3 and SH2 domains, which contributes to their activation, and phosphorylate multiple tyrosines in the Cas substrate domain. These tyrosine-phosphorylated motifs represent docking sites for the Crk adaptor, linking Cas to the downstream Rac1 and Rap1 GTPases to regulate cell adhesion and actin cytoskeleton organization. Accordingly, we detected lower Cas-Crk association and lower phosphorylation of the Src activation loop in Shep1 knockout brains compared to wild-type. Conversely, Shep1 transfection in COS cells increases Cas tyrosine phosphorylation. The SH2 domain is likely critical for the effects of Shep1 on Cas and Src signaling because the knockout mice express Shep1 fragments that lack the amino-terminal region including the SH2 domain, presumably due to aberrant translation from internal ATG codons. These fragments retain the ability to increase Cas levels in transfected cells, similar to full-length Shep1. However, they do not affect Cas phosphorylation on their own or in the presence of co-transfected full-length Shep1. They also do not show dominant negative effects on the activity of full-length Shep1 in vivo because the heterozygous mice, which express the fragments, have a normal life span. This is in contrast to the homozygous knockout mice, most of which die soon after birth. These data demonstrate that Shep1 plays a critical role in the in vivo regulation of Src activity and Cas downstream signaling through Crk, and suggest that the SH2 domain of Shep1 is critical for these effects.
73. The cyclin-dependent kinase inhibitor p57Kip2 regulates cell cycle exit, differentiation, and **migration** of embryonic cerebral cortical precursors. Mounting evidence indicates cyclin-dependent kinase (CDK) inhibitors (CKIs) of the Cip/Kip family, including p57(Kip2) and p27(Kip1), control not only cell cycle exit but also corticogenesis. Nevertheless, distinct activities of p57(Kip2) remain poorly defined. Using in vivo and culture approaches, we show p57(Kip2) overexpression at E14.5-15.5 elicits precursor cell cycle exit, promotes transition from proliferation to neuronal differentiation, and enhances process outgrowth, while opposite effects occur in p57(Kip2)-deficient precursors. Studies at later ages indicate p57(Kip2) overexpression also induces precocious glial differentiation, suggesting stage-dependent effects. In embryonic cortex, p57(Kip2) overexpression advances cell radial **migration** and alters postnatal laminar positioning. While both CKIs induce differentiation, p57(Kip2) was twice as effective as p27(Kip1) in inducing neuronal differentiation and was not permissive to astrogliogenic effects of ciliary neurotrophic factor, suggesting that the CKIs differentially modulate cell fate decisions. At molecular levels, although highly conserved N-terminal regions of both CKIs elicit cycle withdrawal and differentiation, the C-terminal region of p57(Kip2) alone inhibits in vivo **migration**. Furthermore, p57(Kip2) effects on neurogenesis and gliogenesis require the N-terminal cyclin/CDK binding/inhibitory domains, while previous p27(Kip1) studies report cell cycle-independent functions. These observations suggest p57(Kip2) coordinates multiple stages of corticogenesis and exhibits distinct and common activities compared with related family member p27(Kip1).
74. Six3 is required for ependymal cell maturation. Ependymal cells are part of the neurogenic niche in the adult subventricular zone of the lateral ventricles, where they regulate neurogenesis and neuroblast **migration**. Ependymal cells are generated from radial glia cells during embryonic brain development and acquire their final characteristics postnatally. The homeobox gene Six3 is expressed in ependymal cells during the formation of the lateral wall of the lateral ventricles in the brain. Here, we show that Six3 is necessary for ependymal cell maturation during postnatal stages of brain development. In its absence, ependymal cells fail to suppress radial glia characteristics, resulting in a defective lateral wall, abnormal neuroblast **migration** and differentiation, and hydrocephaly.
75. The transcriptional repressor RP58 is crucial for cell-division patterning and neuronal survival in the developing cortex. The neocortex and the hippocampus comprise several specific layers containing distinct neurons that originate from progenitors at specific development times, under the control of an adequate cell-division patterning mechanism. Although many molecules are known to regulate this cell-division patterning process, its details are not well understood. Here, we show that, in the developing cerebral cortex, the RP58 transcription repressor protein was expressed both in postmitotic glutamatergic projection neurons and in their progenitor cells, but not in GABAergic interneurons. Targeted deletion of the RP58 gene led to dysplasia of the neocortex and of the hippocampus, reduction of the number of mature cortical neurons, and defects of laminar organization, which reflect abnormal neuronal **migration** within the cortical plate. We demonstrate an impairment of the cell-division patterning during the late embryonic stage and an enhancement of apoptosis of the postmitotic neurons in the RP58-deficient cortex. These results suggest that RP58 controls cell division of progenitor cells and regulates the survival of postmitotic cortical neurons.
76. NFIX Regulates Proliferation and **Migration** Within the Murine SVZ Neurogenic Niche. Transcription factors of the nuclear factor one (NFI) family play a pivotal role in the development of the nervous system. One member, NFIX, regulates the development of the neocortex, hippocampus, and cerebellum. Postnatal Nfix(-/-) mice also display abnormalities within the subventricular zone (SVZ) lining the lateral ventricles, a region of the brain comprising a neurogenic niche that provides ongoing neurogenesis throughout life. Specifically, Nfix(-/-) mice exhibit more PAX6-expressing progenitor cells within the SVZ. However, the mechanism underlying the development of this phenotype remains undefined. Here, we reveal that NFIX contributes to multiple facets of SVZ development. Postnatal Nfix(-/-) mice exhibit increased levels of proliferation within the SVZ, both in vivo and in vitro as assessed by a neurosphere assay. Furthermore, we show that the **migration** of SVZ-derived neuroblasts to the olfactory bulb is impaired, and that the olfactory bulbs of postnatal Nfix(-/-) mice are smaller. We also demonstrate that gliogenesis within the rostral migratory stream is delayed in the absence of Nfix, and reveal that Gdnf (glial-derived neurotrophic factor), a known attractant for SVZ-derived neuroblasts, is a target for transcriptional activation by NFIX. Collectively, these findings suggest that NFIX regulates both proliferation and **migration** during the development of the SVZ neurogenic niche.
77. EphB-ephrin-B2 interactions are required for thymus **migration** during organogenesis. Thymus organogenesis requires coordinated interactions of multiple cell types, including neural crest (NC) cells, to orchestrate the formation, separation, and subsequent **migration** of the developing thymus from the third pharyngeal pouch to the thoracic cavity. The molecular mechanisms driving these processes are unclear; however, NC-derived mesenchyme has been shown to play an important role. Here, we show that, in the absence of ephrin-B2 expression on thymic NC-derived mesenchyme, the thymus remains in the cervical area instead of migrating into the thoracic cavity. Analysis of individual NC-derived thymic mesenchymal cells shows that, in the absence of ephrin-B2, their motility is impaired as a result of defective EphB receptor signaling. This implies a NC-derived cell-specific role of EphB-ephrin-B2 interactions in the collective **migration** of the thymic rudiment during organogenesis.
78. Netrin1 is required for neural and glial precursor **migration**s into the olfactory bulb. Netrin1 (NTN1) deficiency in mouse brain causes defects in axon guidance and cell **migration** during embryonic development. Here we show that NTN1 is required for olfactory bulb (OB) development at late embryogenesis and at early postnatal stages to facilitate the accumulation of proper numbers of granular and glomerular neuron subtypes and oligodendrocytes into the OB. In addition to the analysis of Ntn1-/- mice we made tissue and neurosphere cultures to clarify the role of NTN1 in the anterior forebrain. We propose that a subset of neural progenitors/precursors requires NTN1 to efficiently enter the rostral migratory stream to migrate into the OB. The analysis of postnatal Ntn1-/- OBs revealed a reduction of specific types of interneurons which have been shown to originate from particular subregions of the lateral ventricle walls. Based on Ntn1 expression in ventral parts of the ventricle walls, we observed a decrease in the mainly ventrally derived type II interneurons that express calcium-binding proteins calretinin and calbindin. Instead, no change in the numbers of dorsally derived tyrosine hydroxylase expressing interneurons was detected. In addition to the specific reduction of type II interneurons, our results indicate that NTN1 is required for oligodendroglial **migration** into the OB. Furthermore, we characterised the Ntn1 expressing subpopulation of neurosphere-forming cells from embryonic and adult brain as multipotent and self-renewing. However, NTN1 is dispensable for the proliferation of neurosphere forming progenitor cells and for their differentiation.
79. The Rb/E2F pathway modulates neurogenesis through direct regulation of the Dlx1/Dlx2 bigene cluster. During brain morphogenesis, the mechanisms through which the cell cycle machinery integrates with differentiation signals remain elusive. Here we show that the Rb/E2F pathway regulates key aspects of differentiation and **migration** through direct control of the Dlx1 and Dlx2 homeodomain proteins, required for interneuron specification. Rb deficiency results in a dramatic reduction of Dlx1 and Dlx2 gene expression manifested by loss of interneuron subtypes and severe **migration** defects in the mouse brain. The Rb/E2F pathway modulates Dlx1/Dlx2 regulation through direct interaction with a Dlx forebrain-specific enhancer, I12b, and the Dlx1/Dlx2 proximal promoter regions, through repressor E2F sites both in vitro and in vivo. In the absence of Rb, we demonstrate that repressor E2Fs inhibit Dlx transcription at the Dlx1/Dlx2 promoters and Dlx1/2-I12b enhancer to suppress differentiation. Our findings support a model whereby the cell cycle machinery not only controls cell division but also modulates neuronal differentiation and **migration** through direct regulation of the Dlx1/Dlx2 bigene cluster during embryonic development.
80. Zac1 Regulates the Differentiation and **Migration** of Neocortical Neurons via Pac1. Imprinted genes are dosage sensitive, and their dysregulated expression is linked to disorders of growth and proliferation, including fetal and postnatal growth restriction. Common sequelae of growth disorders include neurodevelopmental defects, some of which are indirectly related to placental insufficiency. However, several growth-associated imprinted genes are also expressed in the embryonic CNS, in which their aberrant expression may more directly affect neurodevelopment. To test whether growth-associated genes influence neural lineage progression, we focused on the maternally imprinted gene Zac1. In humans, either loss or gain of ZAC1 expression is associated with reduced growth rates and intellectual disability. To test whether increased Zac1 expression directly perturbs neurodevelopment, we misexpressed Zac1 in murine neocortical progenitors. The effects were striking: Zac1 delayed the transition of apical radial glial cells to basal intermediate neuronal progenitors and postponed their subsequent differentiation into neurons. Zac1 misexpression also blocked neuronal **migration**, with Zac1-overexpressing neurons pausing more frequently and forming fewer neurite branches during the period when locomoting neurons undergo dynamic morphological transitions. Similar, albeit less striking, neuronal **migration** and morphological defects were observed on Zac1 knockdown, indicating that Zac1 levels must be regulated precisely. Finally, Zac1 controlled neuronal **migration** by regulating Pac1 transcription, a receptor for the neuropeptide pituitary adenylate cyclase-activating polypeptide (PACAP). Pac1 and Zac1 loss- and gain-of-function presented as phenocopies, and overexpression of Pac1 rescued the Zac1 knockdown neuronal **migration** phenotype. Thus, dysregulated Zac1 expression has striking consequences on neocortical development, suggesting that misexpression of this transcription factor in the brain in certain growth disorders may contribute to neurocognitive deficits. Significance statement: Altered expression of imprinted genes is linked to cognitive dysfunction and neuropsychological disorders, such as Angelman and Prader-Willi syndromes, and autism spectrum disorder. Mouse models have also revealed the importance of imprinting for brain development, with chimeras generated with parthenogenetic (two maternal chromosomes) or androgenetic (two paternal chromosomes) cells displaying altered brain sizes and cellular defects. Despite these striking phenotypes, only a handful of imprinted genes are known or suspected to regulate brain development (e.g., Dlk1, Peg3, Ube3a, necdin, and Grb10). Herein we show that the maternally imprinted gene Zac1 is a critical regulator of neocortical development. Our studies are relevant because loss of 6q24 maternal imprinting in humans results in elevated ZAC1 expression, which has been associated with neurocognitive defects.
81. Phactr4 regulates directional **migration** of enteric neural crest through PP1, integrin signaling, and cofilin activity. Hirschsprung disease (HSCR) is caused by a reduction of enteric neural crest cells (ENCCs) in the gut and gastrointestinal blockage. Knowledge of the genetics underlying HSCR is incomplete, particularly genes that control cellular behaviors of ENCC **migration**. Here we report a novel regulator of ENCC **migration** in mice. Disruption of the Phactr4 gene causes an embryonic gastrointestinal defect due to colon hypoganglionosis, which resembles human HSCR. Time-lapse imaging of ENCCs within the embryonic gut demonstrates a collective cell **migration** defect. Mutant ENCCs show undirected cellular protrusions and disrupted directional and chain **migration**. Phactr4 acts cell-autonomously in ENCCs and colocalizes with integrin and cofilin at cell protrusions. Mechanistically, we show that Phactr4 negatively regulates integrin signaling through the RHO/ROCK pathway and coordinates protein phosphatase 1 (PP1) with cofilin activity to regulate cytoskeletal dynamics. Strikingly, lamellipodia formation and in vivo ENCC chain **migration** defects are rescued by inhibition of ROCK or integrin function. Our results demonstrate a previously unknown pathway in ENCC collective **migration** in vivo and provide new candidate genes for human genetic studies of HSCR.
82. RET signaling is essential for **migration**, axonal growth and axon guidance of developing sympathetic neurons. Sympathetic axons use blood vessels as an intermediate path to reach their final target tissues. The initial contact between differentiating sympathetic neurons and blood vessels occurs following the primary sympathetic chain formation, where precursors of sympathetic neurons migrate and project axons along or toward blood vessels. We demonstrate that, in Ret-deficient mice, neuronal precursors throughout the entire sympathetic nervous system fail to migrate and project axons properly. These primary deficits lead to mis-routing of sympathetic nerve trunks and accelerated cell death of sympathetic neurons later in development. Artemin is expressed in blood vessels during periods of early sympathetic differentiation, and can promote and attract axonal growth of the sympathetic ganglion in vitro. This analysis identifies RET and artemin as central regulators of early sympathetic innervation.
83. Gene deletion mutants reveal a role for semaphorin receptors of the plexin-B family in mechanisms underlying corticogenesis. Semaphorins and their receptors, plexins, are emerging as key regulators of various aspects of neural and nonneural development. Semaphorin 4D (Sema4D) and B-type plexins demonstrate distinct expression patterns over critical time windows during the development of the murine neocortex. Here, analysis of mice genetically lacking plexin-B1 or plexin-B2 revealed the significance of Sema4D-plexin-B signaling in cortical development. Deficiency of plexin-B2 resulted in abnormal cortical layering and defective **migration** and differentiation of several subtypes of cortical neurons, including Cajal-Retzius cells, GABAergic interneurons, and principal cells in vivo. In contrast, a lack of plexin-B1 did not impact on cortical development in vivo. In various ex vivo assays on embryonic forebrain, Sema4D enhanced the radial and tangential **migration** of developing neurons in a plexin-B2-dependent manner. These results suggest that Sema4D-plexin-B2 interactions regulate mechanisms underlying cell specification, differentiation, and **migration** during corticogenesis.
84. Scratch2 modulates neurogenesis and cell **migration** through antagonism of bHLH proteins in the developing neocortex. Scratch genes (Scrt) are neural-specific zinc-finger transcription factors (TFs) with an unknown function in the developing brain. Here, we show that, in addition to the reported expression of mammalian Scrt2 in postmitotic differentiating and mature neurons in the developing and early postnatal brain, Scrt2 is also localized in subsets of mitotic and neurogenic radial glial (RGP) and intermediate (IP) progenitors, as well as in their descendants-postmitotic IPs and differentiating neurons at the border subventricular/intermediate zone. Conditional activation of transgenic Scrt2 in cortical progenitors in mice promotes neuronal differentiation by favoring the direct mode of neurogenesis of RGPs at the onset of neurogenesis, at the expense of IP generation. Neuronal amplification via indirect IP neurogenesis is thereby extenuated, leading to a mild postnatal reduction of cortical thickness. Forced in vivo overexpression of Scrt2 suppressed the generation of IPs from RGPs and caused a delay in the radial **migration** of upper layer neurons toward the cortical plate. Mechanistically, our results indicate that Scrt2 negatively regulates the transcriptional activation of the basic helix loop helix TFs Ngn2/NeuroD1 on E-box containing common target genes, including Rnd2, a well-known major effector for **migration**al defects in developing cortex. Altogether, these findings reveal a modulatory role of Scrt2 protein in cortical neurogenesis and neuronal **migration**.
85. Temporal regulation of EphA4 in astroglia during murine retinal and optic nerve development. Eph receptors and their ephrin ligands play important roles in many aspects of visual system development. In this study, we characterized the spatial and temporal expression pattern of EphA4 in astrocyte precursor cell (APC) and astrocyte populations in the murine retina and optic nerve. EphA4 is expressed by immotile optic disc astrocyte precursor cells (ODAPS), but EphA4 is downregulated as these cells migrate into the retina. Surprisingly, mature astrocytes in the adult retina re-express EphA4. Within the optic nerve, EphA4 is expressed in specialized astrocytes that form a meshwork at the optic nerve head (ONH). Our in vitro and in vivo data indicate that EphA4 is dispensable for retinal ganglion cell (RGC) axon growth and projections through the chiasm. While optic stalk structure, APC proliferation and **migration**, retinal vascularization, and oligodendrocyte **migration** appear normal in EphA4 mutants, the expression of EphA4 in APCs and in the astrocyte meshwork at the ONH has implications for optic nerve pathologies.
86. The survival promoting peptide Y-P30 promotes cellular **migration**. Y-P30, the 30 amino acid N-terminal peptide of the dermcidin gene, has been found to promote neuronal survival and differentiation. Its early presence in development and import to the fetal brain led to the hypothesis that Y-P30 has an influence on proliferation, differentiation and **migration**. Neurospheres derived from neural stem cells isolated from E13 mouse cortex and striatal ganglionic eminences were treated with Y-P30, however, the proportion of progenitors, neurons and astrocytes generated in differentiation assays was not altered. A short Y-P30 treatment of undifferentiated striatal and cortical neurospheres failed to alter the proportion of BrdU-positive cells. A longer treatment reduced the percentage of BrdU-positive cells and GABA-immunoreactive neurons only in striatal spheres. The presence of Y-P30 enhanced **migration** of T24 human bladder carcinoma cells in a wound-healing assay in vitro. Further, Y-P30 enhanced **migration** of T24 cells, rat primary cortical astrocytes and PC12 cells in chemotactic Boyden chamber assays. Together, these findings suggest that a major function of Y-P30 is to promote **migration** of neural and non-neural cell types.
87. Olfactory ensheathing glia are required for embryonic olfactory axon targeting and the **migration** of gonadotropin-releasing hormone neurons. Kallmann's syndrome is caused by the failure of olfactory axons and gonadotropin-releasing hormone (GnRH) neurons to enter the embryonic forebrain, resulting in anosmia and sterility. Sox10 mutations have been associated with Kallmann's syndrome phenotypes, but their effect on olfactory system development is unknown. We recently showed that Sox10 is expressed by neural crest-derived olfactory ensheathing cells (OECs). Here, we demonstrate that in homozygous Sox10(lacZ/lacZ) mouse embryos, OEC differentiation is disrupted; olfactory axons accumulate in the ventromedial olfactory nerve layer and fewer olfactory receptor neurons express the maturation marker OMP (most likely owing to the failure of axonal targeting). Furthermore, GnRH neurons clump together in the periphery and a smaller proportion enters the forebrain. Our data suggest that human Sox10 mutations cause Kallmann's syndrome by disrupting the differentiation of OECs, which promote embryonic olfactory axon targeting and hence olfactory receptor neuron maturation, and GnRH neuron **migration** to the forebrain.
88. Tangentially migrating transient glutamatergic neurons control neurogenesis and maintenance of cerebral cortical progenitor pools. The relative contribution of intrinsic and extrinsic cues in the regulation of cortical neurogenesis remains a crucial challenge in developmental neurobiology. We previously reported that a transient population of glutamatergic neurons, the cortical plate (CP) transient neurons, migrates from the ventral pallium (VP) over long distances and participate in neocortical development. Here, we show that the genetic ablation of this population leads to a reduction in the number of cortical neurons especially fated to superficial layers. These defects result from precocious neurogenesis followed by a depletion of the progenitor pools. Notably, these changes progress from caudolateral to rostrodorsal pallial territories between E12.5 and E14.5 along the expected trajectory of the ablated cells. Conversely, we describe enhanced proliferation resulting in an increase in the number of cortical neurons in the Gsx2 mutants which present an expansion of the VP and a higher number of CP transient neurons migrating into the pallium. Our findings indicate that these neurons act to maintain the proliferative state of neocortical progenitors and delay differentiation during their **migration** from extraneocortical regions and, thus, participate in the extrinsic control of cortical neuronal numbers.
89. Mice lacking sister chromatid cohesion protein PDS5B exhibit developmental abnormalities reminiscent of Cornelia de Lange syndrome. PDS5B is a sister chromatid cohesion protein that is crucial for faithful segregation of duplicated chromosomes in lower organisms. Mutations in cohesion proteins are associated with the developmental disorder Cornelia de Lange syndrome (CdLS) in humans. To delineate the physiological roles of PDS5B in mammals, we generated mice lacking PDS5B (APRIN). Pds5B-deficient mice died shortly after birth. They exhibited multiple congenital anomalies, including heart defects, cleft palate, fusion of the ribs, short limbs, distal colon aganglionosis, abnormal **migration** and axonal projections of sympathetic neurons, and germ cell depletion, many of which are similar to abnormalities found in humans with CdLS. Unexpectedly, we found no cohesion defects in Pds5B(-/-) cells and detected high PDS5B expression in post-mitotic neurons in the brain. These results, along with the developmental anomalies of Pds5B(-/-) mice, the presence of a DNA-binding domain in PDS5B in vertebrates and its nucleolar localization, suggest that PDS5B and the cohesin complex have important functions beyond their role in chromosomal dynamics.
90. Premutation CGG-repeat expansion of the Fmr1 gene impairs mouse neocortical development. Fragile X-associated tremor/ataxia syndrome (FXTAS) is a late adult-onset neurodegenerative disorder caused by a premutation CGG-trinucleotide repeat expansion (55-200 CGG repeats) within the 5'-untranslated region of the FMR1 gene. Although FXTAS generally affects premutation carriers over 50 years of age, cognitive and psychological symptoms can appear in carriers during childhood, suggesting that the FMR1 premutation affects brain function early in life. Recent work with cultured hippocampal neurons from a premutation (Fmr1 CGG knock-in) mouse model revealed impaired development of early postnatal neurons, consistent with the developmental clinical involvement of premutation carriers. In the current work, we show that the presence of premutation CGG-repeat expansions in the mouse Fmr1 gene alters embryonic neocortical development. Specifically, embryonic premutation mice display **migration** defects in the neocortex and altered expression of neuronal lineage markers. The current data demonstrate that premutation alleles of the Fmr1 gene are associated with defects in developmental programs operating during prenatal stages of brain formation and provide further evidence that the FMR1 premutation has a neurodevelopmental component.
91. GATA3 and NeuroD distinguish auditory and vestibular neurons during development of the mammalian inner ear. The function of the zinc finger transcription factor GATA3 was studied in a newly established, conditionally immortal cell line derived to represent auditory sensory neuroblasts migrating from the mouse otic vesicle at embryonic day E10.5. The cell line, US/VOT-33, expressed GATA3, the bHLH transcription factor NeuroD and the POU-domain transcription factor Brn3a, as do auditory neuroblasts in vivo. When GATA3 was knocked down reversibly with antisense oligonucleotides, NeuroD was reversibly down-regulated. Auditory and vestibular neurons form from neuroblasts that express NeuroD and that migrate from the antero-ventral, otic epithelium at E9.5-10.5. On the medial side, neuroblasts and epithelial cells express GATA3 but on the lateral side they do not. At E13.5 most auditory neurons express GATA3 but no longer express NeuroD, whereas vestibular neurons express NeuroD but not GATA3. Neuroblasts expressing NeuroD and GATA3 were located in the ventral, otic epithelium, the adjacent mesenchyme and the developing auditory ganglion. The results suggest that auditory and vestibular neurons arise from different, otic epithelial domains and that they gain their identity prior to **migration**. In auditory neuroblasts, NeuroD appears to be dependent on the expression of GATA3.
92. Gastrointestinal defects of the Gas1 mutant involve dysregulated Hedgehog and Ret signaling. The gastrointestinal (GI) tract defines the digestive system and is composed of the stomach, intestine and colon. Among the major cell types lining radially along the GI tract are the epithelium, mucosa, smooth muscles and enteric neurons. The Hedgehog (Hh) pathway has been implicated in directing various aspects of the developing GI tract, notably the mucosa and smooth muscle growth, and enteric neuron patterning, while the Ret signaling pathway is selectively required for enteric neuron **migration**, proliferation, and differentiation. The growth arrest specific gene 1 (Gas1) encodes a GPI-anchored membrane protein known to bind to Sonic Hh (Shh), Indian Hh (Ihh), and Ret. However, its role in the GI tract has not been examined. Here we show that the Gas1 mutant GI tract, compared to the control, is shorter, has thinner smooth muscles, and contains more enteric progenitors that are abnormally distributed. These phenotypes are similar to those of the Shh mutant, supporting that Gas1 mediates most of the Shh activity in the GI tract. Because Gas1 has been shown to inhibit Ret signaling elicited by Glial cell line-derived neurotrophic factor (Gdnf), we explored whether Gas1 mutant enteric neurons displayed any alteration of Ret signaling levels. Indeed, isolated mutant enteric progenitors not only showed increased levels of phospho-Ret and its downstream effectors, phospho-Akt and phospho-Erk, but also displayed altered responses to Gdnf and Shh. We therefore conclude that phenotypes observed in the Gas1 mutant are due to a combination of reduced Hh signaling and increased Ret signaling.
93. Cholecystokinin modulates **migration** of gonadotropin-releasing hormone-1 neurons. Expression of the brain-gut peptide cholecystokinin (CCK) in the developing olfactory-gonadotropin-releasing hormone-1 (GnRH-1) neuroendocrine systems was characterized, and the function of CCK in these systems was analyzed both in vivo and in vitro. We present novel data demonstrating that CCK transcript and protein are expressed in sensory cells in the developing olfactory epithelium and vomeronasal organ, with both ligand and receptors (CCK-1R and CCK-2R) found on olfactory axons throughout prenatal development. In addition, migrating GnRH-1 neurons in nasal regions express CCK-1R but not CCK-2R receptors. The role of CCK in olfactory-GnRH-1 system development was evaluated using nasal explants, after assessing that the in vivo expression of both CCK and CCK receptors was mimicked in this in vitro model. Exogenous application of CCK (10(-7) m) reduced both olfactory axon outgrowth and **migration** of GnRH-1 cells. This inhibition was mediated by CCK-1R receptors. Moreover, CCK-1R but not CCK-2R antagonism caused a shift in the location of GnRH-1 neurons, increasing the distance that the cells migrated. GnRH-1 neuronal **migration** in mice carrying a genetic deletion of either CCK-1R or CCK-2R receptor genes was also analyzed. At embryonic day 14.5, the total number of GnRH-1 cells was identical in wild-type and mutant mice; however, the number of GnRH-1 neurons within forebrain was significantly greater in CCK-1R-/- embryos, consistent with an accelerated migratory process. These results indicate that CCK provides an inhibitory influence on GnRH-1 neuronal **migration**, contributing to the appropriate entrance of these neuroendocrine cells into the brain, and thus represent the first report of a developmental role for CCK.
94. The newly identified **migration** inhibitory protein regulates the radial **migration** in the developing neocortex. Neuronal **migration** is a crucial process in the organization of the developing cerebral cortex. Although a number of positive regulatory mechanisms of radial **migration** have been identified, negative cell-autonomous mechanisms have yet to be fully described. Here we report a newly identified **Migration** Inhibitory Protein (MINP, formerly known as 2900011O08Rik) that negatively regulates radial **migration**. MINP mRNA was specifically detected in the central and peripheral nervous system, and especially enriched in the cerebral cortex. MINP immunoreactivity co-localized with the neuronal marker Tuj1 and was detected in the cytoplasm of post-mitotic neurons. To elucidate the function of MINP in the developing brain, we performed in utero electroporation of MINP siRNA, MINP shRNA, or MINP-overexpressing vectors into mouse cortices and carried out in vivo **migration** assays. Whereas knockdown of MINP did not alter neuronal morphology, the radial **migration** was found accelerated by MINP knockdown, and reduced by MINP overexpression. This **migration** phenotype was also confirmed in vitro, indicating that MINP regulates neuronal **migration** in a cell-autonomous fashion. Furthermore, downregulation of MINP affected microtubule stability by interacting with tubulin that is a potential mechanism involved in the regulation of neuronal **migration**.
95. Developmental distribution of coxsackie virus and adenovirus receptor localized in the nervous system. Mouse coxsackie virus and adenovirus receptor (mCAR), which was isolated from the nerve growth cone-enriched fraction of newborn mouse brains, is a member of immunoglobulin-super family, and functions as a homophilic adhesion molecule. We observed the expression of mCAR in embryos to adult tissues by means of immunohistochemical analysis with a peptide antibody. mCAR expression was first detected in the embryonic ectoderm in the uterus on embryonic day 6.5 (E6.5). Then it was strongly expressed in the neuroepithelium of the neural tube, the developing brain and the spinal cord from E8.5 to postnatal day 7 (P7), in the cranial motor nerves from E9.5 to E11.5, and in the optic nerve from E13.5 to P7, which agrees with periods of their respective morphogenetic peaks. This expression of mCAR decreased postnatally and was absent in adult tissues. We found that mCAR occurred in a few proliferating cells of the hippocampal dentate gyrus, the subventricular zone (SVZ) of the lateral ventricles, and the rostral migratory stream (RMS) over P21. These observations demonstrate that mCAR was expressed characteristically in the immature neuroepithelium including progenitor cells or radial cells derived from the neural tube and in immature cells in a selected germinal zone of the mature brain. Based on our findings, we propose that mCAR is involved in **migration** and fasciculation during a restricted period as an adhesion molecule.
96. The transcriptional control of trunk neural crest induction, survival, and delamination. Trunk neural crest cells are generated at the border between the neural plate and nonneural ectoderm, where they initiate a distinct program of gene expression, undergo an epithelial-mesenchymal transition (EMT), and delaminate from the neuroepithelium. Here, we provide evidence that members of three families of transcription induce these properties in premigratory neural crest cells. Sox9 acts to provide the competence for neural crest cells to undergo an EMT and is required for trunk neural crest survival. In the absence of Sox9, cells apoptose prior to or shortly after delamination. Slug/Snail, in the presence of Sox9, is sufficient to induce an EMT in neural epithelial cells, while FoxD3 regulates the expression of cell-cell adhesion molecules required for neural crest **migration**. Together, the data suggest a model in which a combination of transcription factors regulates the acquisition of the diverse properties of neural crest cells.
97. Scratch regulates neuronal **migration** onset via an epithelial-mesenchymal transition-like mechanism. During neocortical development, the neuroepithelial or neural precursor cells that commit to neuronal fate need to delaminate and start **migration** toward the pial surface. However, the mechanism that couples neuronal fate commitment to detachment from the neuroepithelium remains largely unknown. Here we show that Scratch1 and Scratch2, members of the Snail superfamily of transcription factors, are expressed upon neuronal fate commitment under the control of proneural genes and promote apical process detachment and radial **migration** in the developing mouse neocortex. Scratch-induced delamination from the apical surface was mediated by transcriptional repression of the adhesion molecule E-cadherin. These findings suggest that Scratch proteins constitute a molecular link between neuronal fate commitment and the onset of neuronal **migration**. On the basis of their similarity to proteins involved in the epithelial-mesenchymal transition (EMT), we propose that Scratch proteins mediate the conversion of neuroepithelial cells to migrating neurons or intermediate neuronal progenitors through an EMT-related mechanism.
98. Arl13b-regulated cilia activities are essential for polarized radial glial scaffold formation. The construction of cerebral cortex begins with the formation of radial glia. Once formed, polarized radial glial cells divide either symmetrically or asymmetrically to balance appropriate production of progenitor cells and neurons. Following birth, neurons use the processes of radial glia as scaffolding for oriented **migration**. Radial glia therefore provide an instructive structural matrix to coordinate the generation and placement of distinct groups of cortical neurons in the developing cerebral cortex. We found that Arl13b, a cilia-enriched small GTPase that is mutated in Joubert syndrome, was critical for the initial formation of the polarized radial progenitor scaffold. Using developmental stage-specific deletion of Arl13b in mouse cortical progenitors, we found that early neuroepithelial deletion of ciliary Arl13b led to a reversal of the apical-basal polarity of radial progenitors and aberrant neuronal placement. Arl13b modulated ciliary signaling necessary for radial glial polarity. Our findings indicate that Arl13b signaling in primary cilia is crucial for the initial formation of a polarized radial glial scaffold and suggest that disruption of this process may contribute to aberrant neurodevelopment and brain abnormalities in Joubert syndrome-related ciliopathies.
99. Murine neocortical histogenesis is perturbed by prenatal exposure to low doses of Bisphenol A. Bisphenol A (BPA) has been shown to disrupt thyroid hormone function. We therefore studied whether prenatal exposure to low-doses of BPA affects the morphology and the expression of some genes related to brain development in the murine fetal neocortex. Pregnant mice were injected subcutaneously with 20 microg/kg of BPA daily from embryonic day 0 (E0). Control animals received vehicle alone. For evaluating cell proliferation, neuronal differentiation and **migration**, bromodeoxyuridine (BrdU) was injected intraperitoneally into pregnant mice with various regimens and the brains were processed for immunohistochemistry. The total RNA was extracted from the embryonic telencephalon at various embryonic stages. The BrdU-labeled cells examined 1 hour after BrdU injection showed no differences between the BPA-treated and control groups (n = 10, each), which indicated that the proliferation of precursor cells was not affected. The BrdU-labeled cells, analysed 2 days after BrdU injection, were decreased in the ventricular zone of BPA-treated mice at E14.5 and E16.5, whereas they were increased in the cortical plate at E14.5 as compared with those in control mice (n = 10, each). Furthermore, the expression of Math3, Ngn2, Hes1, LICAM, and THRalpha was significantly upregulated at E14.5 in the BPA-treated group. These results suggested that BPA might disrupt normal neocortical development by accelerating neuronal differentiation/**migration**.
100. Reelin signaling directly affects radial glia morphology and biochemical maturation. Radial glial cells are characterized, besides their astroglial properties, by long radial processes extending from the ventricular zone to the pial surface, a crucial feature for the radial **migration** of neurons. The molecular signals that regulate this characteristic morphology, however, are largely unknown. We show an important role of the secreted molecule reelin for the establishment of radial glia processes. We describe a significant reduction in ventricular zone cells with long radial processes in the absence of reelin in the cortex of reeler mutant mice. These defects were correlated to a decrease in the content of brain lipid-binding protein (Blbp) and were detected exclusively in the cerebral cortex, but not in the basal ganglia of reeler mice. Conversely, reelin addition in vitro increased the Blbp content and process extension of radial glia from the cortex, but not the basal ganglia. Isolation of radial glia by fluorescent-activated cell sorting showed that these effects are due to direct signaling of reelin to radial glial cells. We could further demonstrate that this signaling requires Dab1, as the increase in Blbp upon reelin addition failed to occur in Dab1-/- mice. Taken together, these results unravel a novel role of reelin signaling to radial glial cells that is crucial for the regulation of their Blbp content and characteristic morphology in a region-specific manner.
101. Nuclear factor one B regulates neural stem cell differentiation and axonal projection of corticofugal neurons. During development of the cerebral cortex, neural stem cells divide to expand the progenitor pool and generate basal progenitors, outer radial glia, and cortical neurons. As these newly born neurons differentiate, they must properly migrate toward their final destination in the cortical plate, project axons to appropriate targets, and develop dendrites. However, a complete understanding of the precise genetic mechanisms regulating these steps is lacking. Here we show that a member of the nuclear factor one (NFI) family of transcription factors, NFIB, is essential for many of these processes in mice. We performed a detailed analysis of NFIB expression during cortical development, and investigated defects in cortical neurogenesis, neuronal **migration**, and differentiation in NfiB(-/-) brains. We found that NFIB is strongly expressed in radial glia and corticofugal neurons throughout cortical development. However, in NfiB(-/-) cortices, radial glia failed to generate outer radial glia, subsequently resulting in a loss of late basal progenitors. In addition, corticofugal neurons showed a severe loss of axonal projections, whereas late-born cortical neurons displayed defects in **migration** and ectopically expressed the early-born neuronal marker CTIP2. Furthermore, gene expression analysis, by RNA sequencing, revealed a misexpression of genes that regulate the cell cycle, neuronal differentiation and **migration** in NfiB(-/-) brains. Together these results demonstrate the critical functions of NFIB in regulating cortical development.
102. Human TUBB3 mutations perturb microtubule dynamics, kinesin interactions, and axon guidance. We report that eight heterozygous missense mutations in TUBB3, encoding the neuron-specific beta-tubulin isotype III, result in a spectrum of human nervous system disorders that we now call the TUBB3 syndromes. Each mutation causes the ocular motility disorder CFEOM3, whereas some also result in intellectual and behavioral impairments, facial paralysis, and/or later-onset axonal sensorimotor polyneuropathy. Neuroimaging reveals a spectrum of abnormalities including hypoplasia of oculomotor nerves and dysgenesis of the corpus callosum, anterior commissure, and corticospinal tracts. A knock-in disease mouse model reveals axon guidance defects without evidence of cortical cell **migration** abnormalities. We show that the disease-associated mutations can impair tubulin heterodimer formation in vitro, although folded mutant heterodimers can still polymerize into microtubules. Modeling each mutation in yeast tubulin demonstrates that all alter dynamic instability whereas a subset disrupts the interaction of microtubules with kinesin motors. These findings demonstrate that normal TUBB3 is required for axon guidance and maintenance in mammals.
103. Phosphorylation of Neurogenin2 specifies the **migration** properties and the dendritic morphology of pyramidal neurons in the neocortex. The molecular mechanisms specifying the dendritic morphology of different neuronal subtypes are poorly understood. Here we demonstrate that the bHLH transcription factor Neurogenin2 (Ngn2) is both necessary and sufficient for specifying the dendritic morphology of pyramidal neurons in vivo by specifying the polarity of its leading process during the initiation of radial **migration**. The ability of Ngn2 to promote a polarized leading process outgrowth requires the phosphorylation of a single tyrosine residue at position 241, an event that is neither involved in Ngn2 direct transactivation properties nor its proneural function. Interestingly, the **migration** defect observed in the Ngn2 knockout mouse and in progenitors expressing the Ngn2(Y241F) mutation can be rescued by inhibiting the activity of the small-GTPase RhoA in cortical progenitors. Our results demonstrate that Ngn2 coordinates the acquisition of the radial **migration** properties and the unipolar dendritic morphology characterizing pyramidal neurons through molecular mechanisms distinct from those mediating its proneural activity.
104. Multiple dose-dependent effects of Lis1 on cerebral cortical development. Humans with heterozygous inactivating mutations of the Lis1 gene display type I lissencephaly, a severe form of cortical dysplasia hypothesized to result from abnormal neuronal **migration**. Previously we reported the construction of an allelic series of the Lis1 gene in mice to analyze the effects of graded reduction of LIS1 protein on the pathogenesis of this disorder and demonstrated a cell autonomous defect in neuronal **migration** (Hirotsune et al., 1998). Here we report the systematic examination of the consequences of dosage reduction of LIS1 on neocortical development using wild-type, null heterozygous (45% LIS1 protein), and compound null/hypomorphic (35% LIS1 protein) mice. The development of the preplate, Cajal-Retzius cells, and the radial glial scaffold appeared unaffected by LIS1 levels. However, a dose-dependent morphologic change in disorganization of the subplate was noted. LIS1 dose-dependent defects in neuronal **migration** were found in vivo and in vitro. The position and number of mitotic cells in the ventricular zone were more abnormal as LIS1 levels decreased, suggesting defects in interkinetic nuclear **migration** and neuroblast proliferation. LIS1 dose-dependent progressive thinning of the cortex and ventricular zone occurred by programmed cell death. Thus, in addition to its requirement for cell autonomous neuronal **migration**, LIS1 influences the generation and survival of cortical ventricular zone neuroblasts. These studies reveal the importance of LIS1 levels in orderly cerebral cortical morphogenesis and suggest new insights into the pathogenesis of type I lissencephaly.
105. The role of the transcription factor Rbpj in the development of dorsal root ganglia. The dorsal root ganglion (DRG) is composed of well-characterized populations of sensory neurons and glia derived from a common pool of neural crest stem cells (NCCs), and is a good system to study the mechanisms of neurogenesis and gliogenesis. Notch signaling is known to play important roles in DRG development, but the full scope of Notch functions in mammalian DRG development remains poorly understood.In the present study, we used Wnt1-Cre to conditionally inactivate the transcription factor Rbpj, a critical integrator of activation signals from all Notch receptors, in NCCs and their derived cells. Deletion of Rbpj caused the up-regulation of NeuroD1 and precocious neurogenesis in DRG early development but led to an eventual deficit of sensory neurons at later stages, due to reduced cell proliferation and abnormal cell death. In addition, gliogenesis was delayed initially, but a near-complete loss of glia was observed finally in Rbpj-deficient DRG. Furthermore, we found P75 and Sox10, which are normally expressed exclusively in neuronal and glial progenitors of the DRG after the NCCs have completed their **migration**, were co-expressed in many cells of the DRG of Rbpj conditional knock-out mice.Our data indicate that Rbpj-mediated canonical Notch signaling inhibits DRG neuronal differentiation, possibly by regulating NeuroD1 expression, and is required for DRG gliogenesis in vivo.
106. Ongoing roles of Phox2 homeodomain transcription factors during neuronal differentiation. Transcriptional determinants of neuronal identity often stay expressed after their downstream genetic program is launched. Whether this maintenance of expression plays a role is for the most part unknown. Here, we address this question for the paralogous paired-like homeobox genes Phox2a and Phox2b, which specify several classes of visceral neurons at the progenitor stage in the central and peripheral nervous systems. By temporally controlled inactivation of Phox2b, we find that the gene, which is required in ventral neural progenitors of the hindbrain for the production of branchio-visceral motoneuronal precursors, is also required in these post-mitotic precursors to maintain their molecular signature - including downstream transcription factors - and allow their tangential **migration** and the histogenesis of the corresponding nuclei. Similarly, maintenance of noradrenergic differentiation during embryogenesis requires ongoing expression of Phox2b in sympathetic ganglia, and of Phox2a in the main noradrenergic center, the locus coeruleus. These data illustrate cases where the neuronal differentiation program does not unfold as a transcriptional `cascade' whereby downstream events are irreversibly triggered by an upstream regulator, but instead require continuous transcriptional input from it.
107. The lysine acetyltransferase activator Brpf1 governs dentate gyrus development through neural stem cells and progenitors. Lysine acetylation has recently emerged as an important post-translational modification in diverse organisms, but relatively little is known about its roles in mammalian development and stem cells. Bromodomain- and PHD finger-containing protein 1 (BRPF1) is a multidomain histone binder and a master activator of three lysine acetyltransferases, MOZ, MORF and HBO1, which are also known as KAT6A, KAT6B and KAT7, respectively. While the MOZ and MORF genes are rearranged in leukemia, the MORF gene is also mutated in prostate and other cancers and in four genetic disorders with intellectual disability. Here we show that forebrain-specific inactivation of the mouse Brpf1 gene causes hypoplasia in the dentate gyrus, including underdevelopment of the suprapyramidal blade and complete loss of the infrapyramidal blade. We trace the developmental origin to compromised Sox2+ neural stem cells and Tbr2+ intermediate neuronal progenitors. We further demonstrate that Brpf1 loss deregulates neuronal **migration**, cell cycle progression and transcriptional control, thereby causing abnormal morphogenesis of the hippocampus. These results link histone binding and acetylation control to hippocampus development and identify an important epigenetic regulator for patterning the dentate gyrus, a brain structure critical for learning, memory and adult neurogenesis.
108. Ion channel expression in the developing enteric nervous system. The enteric nervous system arises from neural crest-derived cells (ENCCs) that migrate caudally along the embryonic gut. The expression of ion channels by ENCCs in embryonic mice was investigated using a PCR-based array, RT-PCR and immunohistochemistry. Many ion channels, including chloride, calcium, potassium and sodium channels were already expressed by ENCCs at E11.5. There was an increase in the expression of numerous ion channel genes between E11.5 and E14.5, which coincides with ENCC **migration** and the first extension of neurites by enteric neurons. Previous studies have shown that a variety of ion channels regulates neurite extension and **migration** of many cell types. Pharmacological inhibition of a range of chloride or calcium channels had no effect on ENCC **migration** in cultured explants or neuritogenesis in vitro. The non-selective potassium channel inhibitors, TEA and 4-AP, retarded ENCC **migration** and neuritogenesis, but only at concentrations that also resulted in cell death. In summary, a large range of ion channels is expressed while ENCCs are colonizing the gut, but we found no evidence that ENCC **migration** or neuritogenesis requires chloride, calcium or potassium channel activity. Many of the ion channels are likely to be involved in the development of electrical excitability of enteric neurons.
109. BMP signaling regulates sympathetic nervous system development through Smad4-dependent and -independent pathways. Induction of the sympathetic nervous system (SNS) from its neural crest (NC) precursors is dependent on BMP signaling from the dorsal aorta. To determine the roles of BMP signaling and the pathways involved in SNS development, we conditionally knocked out components of the BMP pathways. To determine if BMP signaling is a cell-autonomous requirement of SNS development, the Alk3 (BMP receptor IA) was deleted in the NC lineage. The loss of Alk3 does not prevent NC cell **migration**, but the cells die immediately after reaching the dorsal aorta. The paired homeodomain factor Phox2b, known to be essential for survival of SNS precursors, is downregulated, suggesting that Phox2b is a target of BMP signaling. To determine if Alk3 signals through the canonical BMP pathway, Smad4 was deleted in the NC lineage. Loss of Smad4 does not affect neurogenesis and ganglia formation; however, proliferation and noradrenergic differentiation are reduced. Analysis of transcription factors regulating SNS development shows that the basic helix-loop-helix factor Ascl1 is downregulated by loss of Smad4 and that Ascl1 regulates SNS proliferation but not noradrenergic differentiation. To determine if the BMP-activated Tak1 (Map3k7) pathway plays a role in SNS development, Tak1 was deleted in the NC lineage. We show that Tak1 is not involved in SNS development. Taken together, our results suggest multiple roles for BMP signaling during SNS development. The Smad4-independent pathway acts through the activation of Phox2b to regulate survival of SNS precursors, whereas the Smad4-dependent pathway controls noradrenergic differentiation and regulates proliferation by maintaining Ascl1 expression.
110. CoREST/LSD1 control the development of pyramidal cortical neurons. The development of a neuron from a precursor cell comprises a complex set of steps ranging from regulation of the proliferative cycle through the acquisition of distinct morphology and functionality. How these processes are orchestrated is largely unknown. Using in utero manipulation of gene expression in the mouse embryonic cerebral cortex, we found that the transition between multipolar and bipolar stages of newborn cortical pyramidal neurons is markedly delayed by depletion of CoREST, a corepressor component of chromatin remodeling complexes. This profoundly affects the onset of their radial **migration**. The loss of CoREST function also perturbs the dynamics of neuronal precursor cell populations, transiently increasing the fraction of cells remaining in progenitor states, but not the acquisition of the neuronal glutamatergic fate of pyramidal cells. The function of CoREST in these processes appears to be independent of its best-known interactor, the RE-1 silencer of transcription/neural restrictive silencing factor, and requires the histone demethylase LSD1. This reveals the importance of epigenetic control in the execution of neural development programs, specifically in the cerebral cortex.
111. Presenilin 1 in **migration** and morphogenesis in the central nervous system. Morphogenesis of the central nervous system relies in large part upon the correct **migration** of neuronal cells from birthplace to final position. Two general modes of **migration** govern CNS morphogenesis: radial, which is mostly glia-guided and topologically relatively simple; and tangential, which often involves complex movement of neurons in more than one direction. We describe the consequences of loss of function of presenilin 1 on these fundamental processes. Previous studies of the central nervous system in presenilin 1 homozygote mutant embryos identified a premature neuronal differentiation that is transient and localized, with cortical dysplasia at later stages. We document widespread effects on CNS morphogenesis that appear strongly linked to defective neuronal **migration**. Loss of presenilin 1 function perturbs both radial and tangential **migration** in cerebral cortex, and several tangential migratory pathways in the brainstem. The inability of cells to execute their migratory trajectories affects cortical lamination, formation of the facial branchiomotor nucleus, the spread of cerebellar granule cell precursors to form the external granule layer and development of the pontine nuclei. Finally, overall morphogenesis of the mid-hindbrain region is abnormal, resulting in incomplete midline fusion of the cerebellum and overgrowth of the caudal midbrain. These observations indicate that in the absence of presenilin 1 function, the ability of a cell to move can be severely impaired regardless of its mode of **migration**, and, at a grosser level, brain morphogenesis is perturbed. Our results demonstrate that presenilin 1 plays a much more important role in brain development than has been assumed, consistent with a pleiotropic involvement of this molecule in cellular signaling.
112. Impairment of radial glial scaffold-dependent neuronal **migration** and formation of double cortex by genetic ablation of afadin. Studies of human brain malformations, such as lissencephaly and double cortex, have revealed the importance of neuronal **migration** during cortical development. Afadin, a membrane scaffolding protein, regulates the formation of adherens junctions (AJs) and cell **migration** to form and maintain tissue structures. Here, we report that mice with dorsal telencephalon-specific ablation of afadin gene exhibited defects similar to human double cortex, in which the heterotopic cortex was located underneath the normotopic cortex. The normotopic cortex of the mutant mice was arranged in the pattern similar to the cortex of the control mice, while the heterotopic cortex was disorganized. As seen in human patients, double cortex in the mutant mice was formed by impaired neuronal **migration** during cortical development. Genetic ablation of afadin in the embryonic cerebral cortex disrupted AJs of radial glial cells, likely resulting in the retraction of the apical endfeet from the ventricular surface and the dispersion of radial glial cells from the ventricular zone to the subventricular and intermediate zones. These results indicate that afadin is required for the maintenance of AJs of radial glial cells and that the disruption of AJs might cause an abnormal radial scaffold for neuronal **migration**. In contrast, the proliferation or differentiation of radial glial cells was not significantly affected. Taken together, these findings indicate that afadin is required for the maintenance of the radial glial scaffold for neuronal **migration** and that the genetic ablation of afadin leads to the formation of double cortex.
113. Prokineticin-1 modulates proliferation and differentiation of enteric neural crest cells. Prokineticins (Prok-1 and Prok-2) belong to a newly identified AVIT protein family. They are involved in variety of activities in various tissues, including smooth muscle contraction of the gastrointestinal tract and promoting proliferation of endothelial cells derived from adrenal gland. Importantly, they also act as the survival factors to modulate growth and survival of neurons and hematopoietic stem cells. In this study we demonstrated that Prok-1 (but not Prok-2) protein is expressed in the mucosa and mesenchyme of the mouse embryonic gut during enteric nervous system development. Its receptor, PK-R1 is expressed in the enteric neural crest cells (NCCs). To elucidate the physiological role(s) of Prok-1 in NCCs, we isolated the NCCs from the mouse embryonic gut (E11.5) and cultured them in the form of neurospheres. In an in vitro NCC culture, Prok-1 was able to activate both Akt and MAPK pathways and induce the proliferation and differentiation (but not **migration**) of NCCs via PK-R1. Knock-down of PK-R1 using siRNA resulted in a complete abolishment of Prok-1 induced proliferation. Taken together, it is the first report demonstrating that Prok-1 acts as a gut mucosa/mesenchyme-derived factor and maintains proliferation and differentiation of enteric NCCs.
114. Heparan sulfate proteoglycan syndecan-3 is a novel receptor for GDNF, neurturin, and artemin. Glial cell line-derived neurotrophic factor (GDNF) family ligands (GFLs) are potent survival factors for dopaminergic neurons and motoneurons with therapeutic potential for Parkinson's disease. Soluble GFLs bind to a ligand-specific glycosylphosphatidylinositol-anchored coreceptor (GDNF family receptor α) and signal through the receptor tyrosine kinase RET. In this paper, we show that all immobilized matrix-bound GFLs, except persephin, use a fundamentally different receptor. They interact with syndecan-3, a transmembrane heparan sulfate (HS) proteoglycan, by binding to its HS chains with high affinity. GFL-syndecan-3 interaction mediates both cell spreading and neurite outgrowth with the involvement of Src kinase activation. GDNF promotes **migratio**n of cortical neurons in a syndecan-3-dependent manner, and in agreement, mice lacking syndecan-3 or GDNF have a reduced number of cortical γ-aminobutyric acid-releasing neurons, suggesting a central role for the two molecules in cortical development. Collectively, syndecan-3 may directly transduce GFL signals or serve as a coreceptor, presenting GFLs to the signaling receptor RET.
115. Midline radial glia translocation and corpus callosum formation require FGF signaling. Midline astroglia in the cerebral cortex develop earlier than other astrocytes through mechanisms that are still unknown. We show that radial glia in dorsomedial cortex retract their apical endfeet at midneurogenesis and translocate to the overlaying pia, forming the indusium griseum. These cells require the fibroblast growth factor receptor 1 (Fgfr1) gene for their precocious somal translocation to the dorsal midline, as demonstrated by inactivating the Fgfr1 gene in radial glial cells and by RNAi knockdown of Fgfr1 in vivo. Dysfunctional astroglial **migration** underlies the callosal dysgenesis in conditional Fgfr1 knockout mice, suggesting that precise targeting of astroglia to the cortex has unexpected roles in axon guidance. FGF signaling is sufficient to induce somal translocation of radial glial cells throughout the cortex; furthermore, the targeting of astroglia to dorsolateral cortex requires FGFr2 signaling after neurogenesis. Hence, FGFs have an important role in the transition from radial glia to astrocytes by stimulating somal translocation of radial glial cells.
116. RTP801/REDD1 regulates the timing of cortical neurogenesis and neuron **migration**. The generation, differentiation, and **migration** of newborn neurons are critical features of normal brain development that are subject to both extracellular and intracellular regulation. However, the means of such control are only partially understood. Here, we show that expression of RTP801/REDD1, an inhibitor of mTOR (mammalian target of rapamycin) activation, is regulated during neuronal differentiation and that RTP801 functions to influence the timing of both neurogenesis and neuron **migration**. RTP801 levels are high in embryonic cortical neuroprogenitors, diminished in newborn neurons, and low in mature neurons. Knockdown of RTP801 in vitro and in vivo accelerates cell cycle exit by neuroprogenitors and their differentiation into neurons. It also disrupts **migration** of rat newborn neurons to the cortical plate and results in the ectopic localization of mature neurons. On the other hand, RTP801 overexpression delays neuronal differentiation. These findings suggest that endogenous RTP801 plays an essential role in temporal control of cortical development and in cortical patterning.
117. Novel embryonic neuronal **migration** and proliferation defects in Dcx mutant mice are exacerbated by Lis1 reduction. Heterozygous LIS1 mutations and males with loss of the X-linked DCX result in lissencephaly, a neuronal **migration** defect. LIS1 regulates nuclear translocation and mitotic division of neural progenitor cells, while the role of DCX in cortical development remains poorly understood. Here, we uncovered novel neuronal **migration** and proliferation defects in the Dcx mutant embryonic brains. Although cortical organization was fairly well preserved, Dcx(ko/Y) neurons displayed defective **migration** velocities similar to Lis1(+/ko) neurons when characterized by time-lapse video-microscopy of embryonic cortical slices. Dcx(ko/Y) migrating neurons displayed novel multidirectional movements with abnormal morphology and increased branching. Surprisingly, Dcx(ko/Y) radial glial cells displayed spindle orientation abnormalities similar to Lis1(+/ko) cells that in turn lead to moderate proliferation defects both in vivo and in vitro. We found functional genetic interaction of the two genes, with the combined effects of Lis1 haploinsufficiency and Dcx knock-out leading to more severe neuronal **migration** and proliferation phenotypes in the Lis1(+/ko);Dcx(ko/Y) male double mutant compared with the single mutants, resulting in cortical disorganization and depletion of the progenitor pool. Thus, we provide definitive evidence for a critical role for Dcx in neuronal **migration** and neurogenesis, as well as for the in vivo genetic interaction of the two genes most commonly involved in human neuronal **migration** defects.
118. Abnormal development of the cerebral cortex and cerebellum in the setting of lamin B2 deficiency. Nuclear lamins are components of the nuclear lamina, a structural scaffolding for the cell nucleus. Defects in lamins A and C cause an array of human diseases, including muscular dystrophy, lipodystrophy, and progeria, but no diseases have been linked to the loss of lamins B1 or B2. To explore the functional relevance of lamin B2, we generated lamin B2-deficient mice and found that they have severe brain abnormalities resembling lissencephaly, with abnormal layering of neurons in the cerebral cortex and cerebellum. This neuronal layering abnormality is due to defective neuronal **migration**, a process that is dependent on the organized movement of the nucleus within the cell. These studies establish an essential function for lamin B2 in neuronal **migration** and brain development.
119. Dpy19l1, a multi-transmembrane protein, regulates the radial **migration** of glutamatergic neurons in the developing cerebral cortex. During corticogenesis, the regulation of neuronal **migration** is crucial for the functional organization of the neocortex. Glutamatergic neurons are major excitatory components of the mammalian neocortex. In order to elucidate the specific molecular mechanisms underlying their development, we used single-cell microarray analysis to screen for mouse genes that are highly expressed in developing glutamatergic neurons. We identified dpy-19-like 1 (Dpy19l1), a homolog of C. elegans dpy-19, which encodes a putative multi-transmembrane protein shown to regulate directed **migration** of Q neuroblasts in C. elegans. At embryonic stages Dpy19l1 is highly expressed in glutamatergic neurons in the mouse cerebral cortex, whereas in the subpallium, where GABAergic neurons are generated, expression was below detectable levels. Downregulation of Dpy19l1 mediated by shRNA resulted in defective radial **migration** of glutamatergic neurons in vivo, which was restored by the expression of shRNA-insensitive Dpy19l1. Many Dpy19l1-knockdown cells were aberrantly arrested in the intermediate zone and the deep layer and, additionally, some extended single long processes towards the pial surface. Furthermore, we observed defective radial **migration** of bipolar cells in Dpy19l1-knockdown brains. Despite these **migration** defects, these cells correctly expressed Cux1, which is a marker for upper layer neurons, suggesting that Dpy19l1 knockdown results in **migration** defects but does not affect cell type specification. These results indicate that Dpy19l1 is required for the proper radial **migration** of glutamatergic neurons, and suggest an evolutionarily conserved role for the Dpy19 family in neuronal **migration**.
120. Basement membrane attachment is dispensable for radial glial cell fate and for proliferation, but affects positioning of neuronal subtypes. Radial glial cells have been shown to act as neuronal precursors in the developing cortex and to maintain their radial processes attached to the basement membrane (BM) during cell division. Here, we examined a potential role of direct signalling from the BM to radial glial cells in three mouse mutants where radial glia attachment to the BM is disrupted. This is the case if the nidogen-binding site of the laminin gamma1 chain is mutated, in the absence of alpha6 integrin or of perlecan, an essential BM component. Surprisingly, cortical radial glial cells lacking contact to the BM were not affected in their proliferation, interkinetic nuclear **migration**, orientation of cell division and neurogenesis. Only a small subset of precursors was located ectopically within the cortical parenchyma. Notably, however, neuronal subtype composition was severely disturbed at late developmental stages (E18) in the cortex of the laminin gamma1III4-/- mice. Thus, although BM attachment seems dispensable for precursor cells, an intact BM is required for adequate neuronal composition of the cerebral cortex.
121. Doublecortin is required in mice for lamination of the hippocampus but not the neocortex. Doublecortin (DCX) is a microtubule-associated protein that is required for normal neocortical and hippocampal development in humans. Mutations in the X-linked human DCX gene cause gross neocortical disorganization (lissencephaly or "smooth brain") in hemizygous males, whereas heterozygous females show a mosaic phenotype with a normal cortex as well as a second band of misplaced (heterotopic) neurons beneath the cortex ("double cortex syndrome"). We created a mouse carrying a targeted mutation in the Dcx gene. Hemizygous male Dcx mice show severe postnatal lethality; the few that survive to adulthood are variably fertile. Dcx mutant mice show neocortical lamination that is largely indistinguishable from wild type and show normal patterns of neocortical neurogenesis and neuronal **migration**. In contrast, the hippocampus of both heterozygous females and hemizygous males shows disrupted lamination that is most severe in the CA3 region. Behavioral tests show defects in context and cued conditioned fear tests, suggesting that deficits in hippocampal learning accompany the abnormal cytoarchitecture.
122. SPARC-like 1 (SC1) is a diversely expressed and developmentally regulated matricellular protein that does not compensate for the absence of SPARC in the CNS. SPARC-like 1 (SC1) is a member of the SPARC family of matricellular proteins that has been implicated in the regulation of processes such as cell **migration**, proliferation, and differentiation. Here we show that SC1 exhibits remarkably diverse and dynamic expression in the developing and adult nervous system. During development, SC1 localizes to radial glia and pial-derived structures, including the vasculature, choroid plexus, and pial membranes. SC1 is not downregulated in postnatal development, but its expression shifts to distinct time windows in subtypes of glia and neurons, including astrocytes, large projection neurons, Bergmann glia, Schwann cells, and ganglionic satellite cells. In addition, SC1 expression levels and patterns are not altered in the SPARC null mouse, suggesting that SC1 does not compensate for the absence of SPARC. We conclude that SC1 and SPARC may share significant homology, but are likely to have distinct but complementary roles in nervous system development.
123. Determination of the migratory capacity of embryonic cortical cells lacking the transcription factor Pax-6. The cerebral cortex forms by the orderly **migration** and subsequent differentiation of neuronal precursors generated in the proliferative ventricular zone. We studied the role of the transcription factor Pax-6, which is expressed in the ventricular zone, in cortical development. Embryos homozygous for a mutation of Pax-6 (Small eye; Sey) had abnormalities suggesting defective **migration** of late-born cortical precursors. When late-born Sey/Sey precursors were transplanted into wild-type embryonic rat cortex, they showed similar integrative, **migration**al and differentiative abilities to those of transplanted wild-type mouse precursors. These results suggest that postmitotic cortical cells do not need Pax-6 to acquire the capacity to migrate and differentiate, but that Pax-6 generates a cortical environment that permits later-born precursors to express their full developmental potential.
124. Wnt signaling mutants have decreased dentate granule cell production and radial glial scaffolding abnormalities. LRP6 mutant mice have generalized defects in the Wnt/beta-catenin signaling pathway because of the crucial function of LRP6 as a Wnt signaling co-receptor (Pinson et al., 2000). We examined the hippocampal phenotype of single LRP6 mutant mice as well as LRP6/Lef1 double mutant mice. LRP6 mutants had reduced production of dentate granule neurons and abnormalities of the radial glial scaffolding in the forming dentate gyrus. These defects were more severe with the addition of a single Lef1 null allele to an LRP6 null background. Pyramidal cell fields were unaffected in the LRP6, Lef1, or double mutants. The dentate defects were accompanied by decreased numbers of mitotic precursors in the migratory pathway to the dentate and in the displaced proliferative zone in the dentate itself. At earlier gestational ages, there was a reduction in the number of dentate granule cell progenitors in the dentate ventricular zone before the e**migration** of the earliest differentiated granule neurons and precursors to form the dentate anlage.
125. The X-linked intellectual disability protein PHF6 associates with the PAF1 complex and regulates neuronal **migration** in the mammalian brain. Intellectual disability is a prevalent disorder that remains incurable. Mutations of the X-linked protein PHF6 cause the intellectual disability disorder Börjeson-Forssman-Lehmann syndrome (BFLS). However, the biological role of PHF6 relevant to BFLS pathogenesis has remained unknown. We report that knockdown of PHF6 profoundly impairs neurona**l migrati**on in the mouse cerebral cortex in vivo, leading to the formation of white matter heterotopias displaying neuronal hyperexcitability. We find that PHF6 physically associates with the PAF1 transcription elongation complex, and inhibition of PAF1 phenocopies the PHF6 knockdown-induc**ed migrat**ion phenotype in vivo. We also identify Neuroglycan C/Chondroitin sulfate proteoglycan 5 (NGC/CSPG5), a potential schizophrenia susceptibility gene, as a critical downstream target of PHF6 in the control of neuro**nal migra**tion. These findings define PHF6, PAF1, and NGC/CSPG5 as components of a cell-intrinsic transcriptional pathway that orchestrates neuro**nal migra**tion in the brain, with important implications for the pathogenesis of developmental disorders of cognition.
126. Control of patterns of corneal innervation by Pax6. Corneal nerves play essential roles in maintaining the ocular surface through provision of neurotrophic support, but genetic control of corneal innervation is poorly understood. The possibility of a neurotrophic failure in ocular surface disease associated with heterozygosity at the Pax6 locus (aniridia-related keratopathy [ARK]) was investigated.Patterns of corneal innervation were studied during development and aging in mice with different Pax6 dosages and in chimeras. Immunohistochemistry and ELISA-based assays were used to determine the molecular basis of defects seen in Pax6 mutants, and wound healing assays were performed.In adults, the Pax6(+/-) epithelium was less densely innervated than the wild-type epithelium, and radial projection of epithelial nerves was disrupted. Neurotrophic support of the corneal epithelium appeared normal. Directed nerve projection correlated with patterns of epithelial cell **migration** in adult wild-types, but innervation defects observed in Pax6(+/-) mice were not fully corrected in wound healing or chimeric models where directed epithelial **migration** was restored.Pax6 dosage nonautonomously controls robust directed radial projection of corneal neurons, and the guidance cues for growth cone guidance are not solely dependent on directed epithelial **migration**. There is little evidence that ARK represents neurotrophic keratitis.
127. Twist1 directly regulates genes that promote cell proliferation and **migration** in developing heart valves. Twist1, a basic helix-loop-helix transcription factor, is expressed in mesenchymal precursor populations during embryogenesis and in metastatic cancer cells. In the developing heart, Twist1 is highly expressed in endocardial cushion (ECC) valve mesenchymal cells and is down regulated during valve differentiation and remodeling. Previous studies demonstrated that Twist1 promotes cell proliferation, **migration**, and expression of primitive extracellular matrix (ECM) molecules in ECC mesenchymal cells. Furthermore, Twist1 expression is induced in human pediatric and adult diseased heart valves. However, the Twist1 downstream target genes that mediate increased cell proliferation and **migration** during early heart valve development remain largely unknown. Candidate gene and global gene profiling approaches were used to identify transcriptional targets of Twist1 during heart valve development. Candidate target genes were analyzed for evolutionarily conserved regions (ECRs) containing E-box consensus sequences that are potential Twist1 binding sites. ECRs containing conserved E-box sequences were identified for Twist1 responsive genes Tbx20, Cdh11, Sema3C, Rab39b, and Gadd45a. Twist1 binding to these sequences in vivo was determined by chromatin immunoprecipitation (ChIP) assays, and binding was detected in ECCs but not late stage remodeling valves. In addition identified Twist1 target genes are highly expressed in ECCs and have reduced expression during heart valve remodeling in vivo, which is consistent with the expression pattern of Twist1. Together these analyses identify multiple new genes involved in cell proliferation and **migration** that are differentially expressed in the developing heart valves, are responsive to Twist1 transcriptional function, and contain Twist1-responsive regulatory sequences.
128. Tissue specific characterisation of Lim-kinase 1 expression during mouse embryogenesis. The Lim-kinase (LIMK) proteins are important for the regulation of the actin cytoskeleton, in particular the control of actin nucleation and depolymerisation via regulation of cofilin, and hence may control a large number of processes during development, including cell tensegrity, **migration**, cell cycling, and axon guidance. LIMK1/LIMK2 knockouts disrupt spinal cord morphogenesis and synapse formation but other tissues and developmental processes that require LIMK are yet to be fully determined. To identify tissues and cell-types that may require LIMK, we characterised the pattern of LIMK1 protein during mouse embryogenesis. We showed that LIMK1 displays an expression pattern that is temporally dynamic and tissue-specific. In several tissues LIMK1 is detected in cell-types that also express Wilms' tumour protein 1 and that undergo transitions between epithelial and mesenchymal states, including the pleura, epicardium, kidney nephrons, and gonads. LIMK1 was also found in a subset of cells in the dorsal retina, and in mesenchymal cells surrounding the peripheral nerves. This detailed study of the spatial and temporal expression of LIMK1 shows that LIMK1 expression is more dynamic than previously reported, in particular at sites of tissue-tissue interactions guiding multiple developmental processes.
129. Hes genes and neurogenin regulate non-neural versus neural fate specification in the dorsal telencephalic midline. The choroid plexus in the brain is unique because it is a non-neural secretory tissue. It secretes the cerebrospinal fluid and functions as a blood-brain barrier, but the precise mechanism of specification of this non-neural tissue has not yet been determined. Using mouse embryos and lineage-tracing analysis, we found that the prospective choroid plexus region initially gives rise to Cajal-Retzius cells, specialized neurons that guide neuronal **migration**. Inactivation of the bHLH repressor genes Hes1, Hes3 and Hes5 upregulated expression of the proneural gene neurogenin 2 (Ngn2) and prematurely depleted Bmp-expressing progenitor cells, leading to enhanced formation of Cajal-Retzius cells and complete loss of choroid plexus epithelial cells. Overexpression of Ngn2 had similar effects. These data indicate that Hes genes promote specification of the fate of choroid plexus epithelial cells rather than the fate of Cajal-Retzius cells by antagonizing Ngn2 in the dorsal telencephalic midline region, and thus this study has identified a novel role for bHLH genes in the process of deciding which cells will have a non-neural versus a neural fate.
130. Cdk5 phosphorylation of ErbB4 is required for tangential **migration** of cortical interneurons. Interneuron dysfunction in humans is often associated with neurological and psychiatric disorders, such as epilepsy, schizophrenia, and autism. Some of these disorders are believed to emerge during brain formation, at the time of interneuron specification, **migration**, and synapse formation. Here, using a mouse model and a host of histological and molecular biological techniques, we report that the signaling molecule cyclin-dependent kinase 5 (Cdk5), and its activator p35, control the tangential **migration** of interneurons toward and within the cerebral cortex by modulating the critical neurodevelopmental signaling pathway, ErbB4/phosphatidylinositol 3-kinase, that has been repeatedly linked to schizophrenia. This finding identifies Cdk5 as a crucial signaling factor in cortical interneuron development in mammals.
131. Onecut transcription factors are required for the second phase of development of the A13 dopaminergic nucleus in the mouse. The A13 dopaminergic nucleus belongs to the incerto-hypothalamic area. It is thought to exert autonomous roles by integrating sensory input to autonomic, neuroendocrine, and motor output. Although its early development has been well characterized, the factors that contribute to later steps of its formation remain unknown. Transcription factors of the Onecut family have been detected in the A13 nucleus, raising the question of possible roles of these factors during A13 development. Using a combination of immunofluorescence analyses on sections and after whole-mount labeling followed by 3D reconstructions, we further characterized the second phase of development of the A13 nucleus in the mouse, described the distribution of the Onecut proteins throughout A13 development, and analyzed the phenotype of this nucleus in single or compound mutant embryos for the Onecut factors. Here we show that A13 development can be divided into two successive phases. First, during radial **migration** toward the pial surface the A13 cells differentiate into dopaminergic neurons. Second, these cells gather in the vicinity of the third ventricle. Onecut factors are dynamically and differentially expressed in the A13 nucleus during these two phases of development. In Onecut mutant embryos, the A13 neurons differentiate normally but scatter in the diencephalon and fail to properly gather close to the third ventricle. Hence, Onecut factors are markers of the A13 nucleus throughout embryonic development. They are dispensable for the first phase of A13 development but are required for the second phase of development and for maintenance of this nucleus.
132. CXC chemokine receptor 7 (CXCR7) affects the **migration** of GnRH neurons by regulating CXCL12 availability. Gonadotropin-releasing hormone (GnRH) neurons are neuroendocrine cells, located in the hypothalamus, that play an essential role in mammalian reproduction. These neurons originate in the nasal placode and migrate during embryonic development, in association with olfactory/vomeronasal nerves, first in the nose, then through the cribriform plate to enter the forebrain, before settling in the hypothalamus. One of the molecules required for their early **migration** in the nose is the chemokine CXCL12, which is expressed in the embryonic nasal mesenchyme in an increasing ventral to dorsal gradient, presumably guiding GnRH neurons toward the forebrain. Mice lacking CXCR4, the receptor for CXCL12, exhibit defective GnRH cell movement and a significant reduction in their number, suggesting that CXCL12/CXCR4 signaling is important in the **migration** and survival of these neurons. Here, we investigated the role of the more recently identified second CXCL12 receptor, CXCR7, in GnRH neuron development. We demonstrate that CXCR7 is expressed along the migratory path of GnRH neurons in the nasal cavity and, although not expressed by GnRH neurons, it affects their **migration** as indicated by the ectopic accumulation of these cells in the nasal compartment in CXCR7(-/-) mice. Absence of CXCR7 caused abnormal accumulation of CXCL12-RFP at CXCR4-positive sites in the nasal area of CXCL12-RFP-transgenic mice and excessive CXCL12-dependent intracellular clustering of CXCR4 in GnRH neurons, suggesting internalization. These findings imply that CXCR7 regulates CXCL12 availability by acting as a scavenger along the migratory path of GnRH neurons and, thus, influences the **migration** of these cells in a noncell-autonomous manner.
133. Defective postnatal neurogenesis and disorganization of the rostral migratory stream in absence of the Vax1 homeobox gene. The subventricular zone (SVZ) is one of the sources of adult neural stem cells (ANSCs) in the mouse brain. Precursor cells proliferate in the SVZ and migrate through the rostral migratory stream (RMS) to the olfactory bulb (OB), where they differentiate into granule and periglomerular cells. Few transcription factors are known to be responsible for regulating NSC proliferation, **migration**, and differentiation processes; even fewer have been found to be responsible for the organization of the SVZ and RMS. For this reason, we studied the ventral anterior homeobox (Vax1) gene in NSC proliferation and in SVZ organization. We found that Vax1 is strongly expressed in the SVZ and in the RMS and that, in the absence of Vax1, embryonic precursor cells proliferate 100 times more than wild-type controls, in vitro. The SVZ of Vax1(-/-) brains is hyperplastic and mostly disorganized, and the RMS is missing, causing a failure of precursor cell **migration** to the OBs, which as a result are severely hypoplastic. Moreover, we found that Vax1 is essential for the correct differentiation of ependyma and astrocytes. Together, these data indicate that Vax1 is a potent regulator of SVZ organization and NSC proliferation, with important consequences on postnatal neurogenesis.
134. Mash1 regulates the development of C cells in mouse thyroid glands. In mammals, the ultimobranchial body derived from the fourth pharyngeal pouch gives rise to thyroid C cells. The C cells of newborn mice are immunoreactive for calcitonin, calcitonin gene-related peptide (CGRP), protein gene product (PGP) 9.5 and NeuroD, and transiently exhibit the neuronal markers TuJ1 and somatostatin during fetal development. The basic helix-loop-helix (bHLH) transcription factor Mash1 plays a role in the differentiation of autonomic neurons. We show that in wild-type mouse embryos, Mash1 is expressed in the ultimobranchial body at embryonic day (E) 12.5, when the body is located close to the great arch arteries. It is also expressed in the ultimobanchial body fused with the thyroid lobe at E 13.5. Targeted disruption of Mash1 resulted in the absence of C cells in the mouse thyroid glands, since cells displaying the C-cell markers and expressing NeuroD were not detected during fetal development or at birth. The failure of C-cell formation in the null mutant thyroids was also confirmed by electron microscopy. While the formation and **migration** of the ultimobranchial body were not affected in the Mash1 null mutants, at E 12.5-E 13.5 both the ultimobranchial body located close to the arteries and the organ populating the thyroid lobe exhibited a marked increase in apoptotic cell numbers. Thus, in the mutant mice, the ultimobranchial body fails to complete its differentiation program and finally dies. These results indicate that Mash1 enhances survival of the C-cell progenitors by inhibiting apoptosis.
135. Enteric nervous system specific deletion of Foxd3 disrupts glial cell differentiation and activates compensatory enteric progenitors. The enteric nervous system (ENS) arises from the coordinated **migration**, expansion and differentiation of vagal and sacral neural crest progenitor cells. During development, vagal neural crest cells enter the foregut and migrate in a rostro-to-caudal direction, colonizing the entire gastrointestinal tract and generating the majority of the ENS. Sacral neural crest contributes to a subset of enteric ganglia in the hindgut, colonizing the colon in a caudal-to-rostral wave. During this process, enteric neural crest-derived progenitors (ENPs) self-renew and begin expressing markers of neural and glial lineages as they populate the intestine. Our earlier work demonstrated that the transcription factor Foxd3 is required early in neural crest-derived progenitors for self-renewal, multipotency and establishment of multiple neural crest-derived cells and structures including the ENS. Here, we describe Foxd3 expression within the fetal and postnatal intestine: Foxd3 was strongly expressed in ENPs as they colonize the gastrointestinal tract and was progressively restricted to enteric glial cells. Using a novel Ednrb-iCre transgene to delete Foxd3 after vagal neural crest cells migrate into the midgut, we demonstrated a late temporal requirement for Foxd3 during ENS development. Lineage labeling of Ednrb-iCre expressing cells in Foxd3 mutant embryos revealed a reduction of ENPs throughout the gut and loss of Ednrb-iCre lineage cells in the distal colon. Although mutant mice were viable, defects in patterning and distribution of ENPs were associated with reduced proliferation and severe reduction of glial cells derived from the Ednrb-iCre lineage. Analyses of ENS-lineage and differentiation in mutant embryos suggested activation of a compensatory population of Foxd3-positive ENPs that did not express the Ednrb-iCre transgene. Our findings highlight the crucial roles played by Foxd3 during ENS development including progenitor proliferation, neural patterning, and glial differentiation and may help delineate distinct molecular programs controlling vagal versus sacral neural crest development.
136. Emergence of the sensory nervous system as defined by Foxs1 expression. Peripheral sensory neurons are derived from two distinct structures, the ectodermal placodes and the neural crest. Here, we establish the forkhead family transcription factor Foxs1 as an early sensory neuronal marker. Early embryonic Foxs1 expression was present in all the sensory nervous system regardless of cellular origin, but was not found in other placode and neural crest-derived cell types. Foxs1 expression was turned on in the sensory neuron precursors of the trunk. Consistently, expression of Sox10, that is present in undifferentiated multipotent neural crest cells (NCCs), was mutually exclusive to Foxs1. Acquirement of Foxs1 expression was used to study the emergence of the dorsal root ganglion (DRG) sensory neurons. Migrating pioneering Foxs1 expressing NCCs were found at the anterior dorsal somitic lip at the 18-somite stage. These cells showed limited proliferation and migrated to form a cluster in the ventral aspect of the coalescing ganglion, surrounded by Foxs1(-)/Sox10(+) migrating NCCs retaining a high rate of proliferation. Sensory neurogenesis of the Foxs1(-)/Sox10(+) precursors occurred within the condensed DRG starting with neurogenin-1 (Ngn1) and Brn3a expression. These data define a sequential emergence of neuronal precursors of the sensory nervous system with different molecular characteristics, starting during **migration** and continuing well after DRG condensation.
137. α2-chimaerin controls neuronal **migratio**n and functioning of the cerebral cortex through CRMP-2. Disrupted cortical neuronal **migratio**n is associated with epileptic seizures and developmental delay. However, the molecular mechanism by which disruptions of early cortical development result in neurological symptoms is poorly understood. Here we report α2-chimaerin as a key regulator of cortical neurona**l migrati**on and function. In utero suppression of α2-chimaerin arrested neuron**al migrat**ion at the multipolar stage, leading to accumulation of ectopic neurons in the subcortical region. Mice with su**ch migrat**ion defects showed an imbalance between excitation and inhibition in local cortical circuitry and greater susceptibility to convulsant-induced seizures. We further show that α2-chimaerin regulates bipolar transition and neuro**nal migra**tion through modulating the activity of CRMP-2, a microtubule-associated protein. These findings establish a new α2-chimaerin-dependent mechanism underlying neur**onal migr**ation and proper functioning of the cerebral cortex and provide insights into the pathogenesis of seizure-related neurodevelopmental disorders.
138. Sox11 regulates survival and axonal growth of embryonic sensory neurons. Sensory neurons transduce various stimuli including temperature, pain, and touch from the periphery to the central nervous system. Sensory neuron development is governed by a combination of extracellular cues and specific gene expression. We demonstrated that the transcription factor Sox11 was highly expressed in the developing sensory neurons. To test the function of Sox11, we used a knockin mouse model where the entire coding region of Sox11 was replaced by a LacZ reporter. The ablation of Sox11 caused severe reduction in sensory neuron survival in the trigeminal and dorsal root ganglia, although it did not affect **migration** of neural crest cells or acquisition of major sensory neuron subtypes. We further demonstrated that ablating Sox11 caused an arrest of axonal outgrowth in vivo and in vitro. This defect could not be fully rescued by blocking cell death. Our data suggest that Sox11 is a key regulator of sensory neuron development.
139. Receptor protein tyrosine phosphatases are expressed by cycling retinal progenitor cells and involved in neuronal development of mouse retina. Receptor protein tyrosine phosphatases (RPTPs) appear to coordinate many aspects of neural development, including cell proliferation, **migration** and differentiation. Here we investigated potential roles of RPTPs in the developing mouse retina. Using a degenerate oligonucleotide-based reverse transcription polymerase chain reaction approach, we identified 11 different RPTPs in the retina at embryonic stage 13 (E13). Subsequently, the expression patterns of RPTPkappa, RPTPJ, RPTPRR, RPTPsigma, RPTPepsilon and RPTPgamma in the retina from embryonic stages to adult were analyzed in detail using quantitative real-time-PCR, in situ hybridization, immunohistochemistry and Western blotting. At E13, all six RPTPs are expressed in actively cycling retinal progenitor cells and postmitotic newborn retinal neurons. With ongoing maturation, RPTPkappa, RPTPJ, RPTPRR, RPTPsigma, RPTPepsilon and RPTPgamma display a different spatiotemporal regulation of mRNAs and proteins in the pre- and postnatal retina. Finally, in adulthood these six RPTPs localize to distinct cellular compartments of multiple retinal neurons. Additional studies in RPTPgamma(-/-) and RPTPbeta/zeta(-/-) (also known as PTPRZ1, RPTPbeta or RPTPzeta) mice at postnatal stage P1 reveal no apparent differences in retinal laminar organization or in the expression pattern of specific retinal cell-type markers when compared with wild type. However, in RPTPbeta/zeta(-/-) retinas, immunoreactivity of vimentin, a marker of Müller glial cells, is selectively reduced and the morphology of vimentin-immunoreactive radial processes of Müller cells is considerably disturbed. Our results suggest distinct roles of RPTPs in cell proliferation and establishing phenotypes of different retinal cells during retinogenesis as well as later in the maintenance of mature retina.
140. Akirin2 is essential for the formation of the cerebral cortex. The proper spatial and temporal regulation of dorsal telencephalic progenitor behavior is a prerequisite for the formation of the highly-organized, six-layered cerebral cortex. Premature differentiation of cells, disruption of cell cycle timing, excessive apoptosis, and/or incorrect neuronal **migration** signals can have devastating effects, resulting in a number of neurodevelopmental disorders involving microcephaly and/or lissencephaly. Though genes encoding many key players in cortical development have been identified, our understanding remains incomplete. We show that the gene encoding Akirin2, a small nuclear protein, is expressed in the embryonic telencephalon. Converging evidence indicates that Akirin2 acts as a bridge between transcription factors (including Twist and NF-κB proteins) and the BAF (SWI/SNF) chromatin remodeling machinery to regulate patterns of gene expression. Constitutive knockout of Akirin2 is early embryonic lethal in mice, while restricted loss in B cells led to disrupted proliferation and cell survival.We generated cortex-restricted Akirin2 knockouts by crossing mice harboring a floxed Akirin2 allele with the Emx1-Cre transgenic line and assessed the resulting embryos using in situ hybridization, EdU labeling, and immunohistochemistry.The vast majority of Akirin2 mutants do not survive past birth, and exhibit extreme microcephaly, with little dorsal telencephalic tissue and no recognizable cortex. This is primarily due to massive cell death of early cortical progenitors, which begins at embryonic day (E)10, shortly after Emx1-Cre is active. Immunostaining and cell cycle analysis using EdU labeling indicate that Akirin2-null progenitors fail to proliferate normally, produce fewer neurons, and undergo extensive apoptosis. All of the neurons that are generated in Akirin2 mutants also undergo apoptosis by E12. In situ hybridization for Wnt3a and Wnt-responsive genes suggest defective formation and/or function of the cortical hem in Akirin2 null mice. Furthermore, the apical ventricular surface becomes disrupted, and Sox2-positive progenitors are found to "spill" into the lateral ventricle.Our data demonstrate a previously-unsuspected role for Akirin2 in early cortical development and, given its known nuclear roles, suggest that it may act to regulate gene expression patterns critical for early progenitor cell behavior and cortical neuron production.
141. Semaphorin 4D regulates gonadotropin hormone-releasing hormone-1 neuronal **migration** through PlexinB1-Met complex. In mammals, reproduction is dependent on specific neurons secreting the neuropeptide gonadotropin hormone-releasing hormone-1 (GnRH-1). These cells originate during embryonic development in the olfactory placode and migrate into the forebrain, where they become integral members of the hypothalamic-pituitary-gonadal axis. This migratory process is regulated by a wide range of guidance cues, which allow GnRH-1 cells to travel over long distances to reach their appropriate destinations. The Semaphorin4D (Sema4D) receptor, PlexinB1, is highly expressed in the developing olfactory placode, but its function in this context is still unknown. Here, we demonstrate that PlexinB1-deficient mice exhibit a migratory defect of GnRH-1 neurons, resulting in reduction of this cell population in the adult brain. Moreover, Sema4D promotes directional **migration** in GnRH-1 cells by coupling PlexinB1 with activation of the Met tyrosine kinase (hepatocyte growth factor receptor). This work identifies a function for PlexinB1 during brain development and provides evidence that Sema4D controls **migration** of GnRH-1 neurons.
142. ASPP2 binds Par-3 and controls the polarity and proliferation of neural progenitors during CNS development. Cell polarity plays a key role in the development of the central nervous system (CNS). Interestingly, disruption of cell polarity is seen in many cancers. ASPP2 is a haplo-insufficient tumor suppressor and an activator of the p53 family. In this study, we show that ASPP2 controls the polarity and proliferation of neural progenitors in vivo, leading to the formation of neuroblastic rosettes that resemble primitive neuroepithelial tumors. Consistent with its role in cell polarity, ASPP2 influences interkinetic nuclear **migration** and lamination during CNS development. Mechanistically, ASPP2 maintains the integrity of tight/adherens junctions. ASPP2 binds Par-3 and controls its apical/junctional localization without affecting its expression or Par-3/aPKC lambda binding. The junctional localization of ASPP2 and Par-3 is interdependent, suggesting that they are prime targets for each other. These results identify ASPP2 as a regulator of Par-3, which plays a key role in controlling cell proliferation, polarity, and tissue organization during CNS development.
143. Neural precursor death is central to the pathogenesis of intestinal aganglionosis in Ret hypomorphic mice. The RET tyrosine kinase is required for the **migration**, proliferation, and survival of the enteric neural crest-derived cells (ENCCs) that form the enteric nervous system (ENS). Hypomorphic RET alleles cause intestinal aganglionosis [Hirschsprung disease (HSCR)], in which delayed **migration** and successive nonapoptotic ENCC death are considered to be major contributory factors. The significance of ENCC death in intestinal aganglionosis, however, has remained unclear. We show that elevated expression of Bcl-xL inhibits ENCC death in both Ret-null and hypomorphic states. However, the rescued Ret-null mice showed ENS malfunction with reduced nitric oxide synthase expression in colonic neurons, revealing the requirement of RET for neuronal differentiation. In contrast, the inhibition of cell death allows morphologically and functionally normal ENS formation in Ret hypomorphic mice. These results indicate that ENCC death is a principal cause of intestinal aganglionosis in a Ret hypomorphic state, and suggest that the inhibition of cell death is a route to the prevention of HSCR.
144. Elongator controls the **migration** and differentiation of cortical neurons through acetylation of alpha-tubulin. The generation of cortical projection neurons relies on the coordination of radial **migration** with branching. Here, we report that the multisubunit histone acetyltransferase Elongator complex, which contributes to transcript elongation, also regulates the maturation of projection neurons. Indeed, silencing of its scaffold (Elp1) or catalytic subunit (Elp3) cell-autonomously delays the **migration** and impairs the branching of projection neurons. Strikingly, neurons defective in Elongator show reduced levels of acetylated alpha-tubulin. Reduction of alpha-tubulin acetylation via expression of a nonacetylatable alpha-tubulin mutant leads to comparable defects in cortical neurons and suggests that alpha-tubulin is a target of Elp3. This is further supported by the demonstration that Elp3 promotes acetylation and counteracts HDAC6-mediated deacetylation of this substrate in vitro. Our results uncover alpha-tubulin as a target of the Elongator complex and suggest that a tight regulation of its acetylation underlies the maturation of cortical projection neurons.
145. Regulation of apoptosis and neurite extension by FKBP38 is required for neural tube formation in the mouse. FKBP38 (also known as FKBP8) is a transmembrane chaperone protein that inhibits apoptosis by recruiting the anti-apoptotic proteins Bcl-2 and Bcl-x(L) to mitochondria. We have now generated mice harboring a loss-of-function mutation in Fkbp38. The Fkbp38(-/-) mice die soon after birth manifesting defects in neural tube closure in the thoraco-lumbar-sacral region (spina bifida) as well as skeletal defects including scoliosis, rib deformities, club foot and curled tail. The neuroepithelium is disorganized and that formation of dorsal root ganglia is defective in Fkbp38(-/-) embryos, likely as a result of an increased frequency of apoptosis and aberrant **migration** of neuronal cells. Furthermore, the extension of nerve fibers in the spinal cord is abnormal in the mutant embryos. To explore the mechanisms underlying these characteristics, we screened for proteins that interact with FKBP38 in the yeast two-hybrid system and thereby identified protrudin, a protein that promotes process formation by regulating membrane trafficking. Protrudin was found to be hyperphosphorylated in the brain of Fkbp38(-/-) mice, suggesting that FKBP38 regulates protrudin-dependent membrane recycling and neurite outgrowth. Together, our findings suggest that FKBP38 is required for neuroectodermal organization during neural tube formation as a result of its anti-apoptotic activity and regulation of neurite extension.
146. Role of Pax6 in development of the cerebellar system. Post-mitotic neurons generated at the rhombic lip undertake long distance **migration** to widely dispersed destinations, giving rise to cerebellar granule cells and the precerebellar nuclei. Here we show that Pax6, a key regulator in CNS and eye development, is strongly expressed in rhombic lip and in cells migrating away from it. Development of some structures derived from these cells is severely affected in Pax6-null Small eye (Pax6(Sey)/Pax6(Sey)) embryos. Cell proliferation and initial differentiation seem unaffected, but cell **migration** and neurite extension are disrupted in mutant embryos. Three of the five precerebellar nuclei fail to form correctly. In the cerebellum the pre-migratory granule cell sub-layer and fissures are absent. Some granule cells are found in ectopic positions in the inferior colliculus which may result from the complete absence of Unc5h3 expression in Pax6(Sey)/Pax6(Sey) granule cells. Our results suggest that Pax6 plays a strong role during hindbrain **migration** processes and at least part of its activity is mediated through regulation of the netrin receptor Unc5h3.
147. GPR56 functions together with α3β1 integrin in regulating cerebral cortical development. Loss of function mutations in GPR56, which encodes a G protein-coupled receptor, cause a specific human brain malformation called bilateral frontoparietal polymicrogyria (BFPP). Studies from BFPP postmortem brain tissue and Gpr56 knockout mice have previously showed that GPR56 deletion leads to breaches in the pial basement membrane (BM) and neuronal ectopias during cerebral cortical development. Since α3β1 integrin also plays a role in pial BM assembly and maintenance, we evaluated whether it functions together with GPR56 in regulating the same developmental process. We reveal that loss of α3 integrin enhances the cortical phenotype associated with Gpr56 deletion, and that neuronal **overmigr**ation through a breached pial BM occurs earlier in double knockout than in Gpr56 single knockout mice. These observations provide compelling evidence of the synergism of GPR56 and α3β1 integrin in regulating the development of cerebral cortex.
148. Tangential neuronal **migration** controls axon guidance: a role for neuregulin-1 in thalamocortical axon navigation. Neuronal **migration** and axon guidance constitute fundamental processes in brain development that are generally studied independently. Although both share common mechanisms of cell biology and biochemistry, little is known about their coordinated integration in the formation of neural circuits. Here we show that the development of the thalamocortical projection, one of the most prominent tracts in the mammalian brain, depends on the early tangential **migration** of a population of neurons derived from the ventral telencephalon. This tangential **migration** contributes to the establishment of a permissive corridor that is essential for thalamocortical axon pathfinding. Our results also demonstrate that in this process two different products of the Neuregulin-1 gene, CRD-NRG1 and Ig-NRG1, mediate the guidance of thalamocortical axons. These results show that neuronal tangential **migration** constitutes a novel mechanism to control the timely arrangement of guidance cues required for axonal tract formation in the mammalian brain.
149. Mice lacking the transcription factor SHOX2 display impaired cerebellar development and deficits in motor coordination. Purkinje cells of the developing cerebellum secrete the morphogen sonic hedgehog (SHH), which is required to maintain the proliferative state of granule cell precursors (GCPs) prior to their differentiation and **migration** to form the internal granule layer (IGL). Despite a wealth of knowledge regarding the function of SHH during cerebellar development, the upstream regulators of Shh expression during this process remain largely unknown. Here we report that the murine short stature homeobox 2 (Shox2) gene is required for normal Shh expression in dorsal-residing Purkinje cells. Using two different Cre drivers, we show that elimination of Shox2 in the brain results in developmental defects in the inferior colliculus and cerebellum. Specifically, loss of Shox2 in the cerebellum results in precocious differentiation and **migration** of GCPs from the external granule layer (EGL) to the IGL. This correlates with premature bone morphogenetic protein 4 (Bmp4) expression in granule cells of the dorsal cerebellum. The size of the neonatal cerebellum is reduced in Shox2-mutant animals, which is consistent with a reduction in the number of GCPs present in the EGL, and could account for the smaller vermis and thinner IGL present in adult Shox2mutants. Shox2-mutant mice also display reduced exploratory activity, altered gait and impaired motor coordination. Our findings are the first to show a role for Shox2 in brain development. We provide evidence that Shox2 plays an important role during cerebellar development, perhaps to maintain the proper balance of Shh and Bmp expression levels in the dorsal vermis, and demonstrate that in the absence of Shox2, mice display both cerebellar impairments and deficits in motor coordination, ultimately highlighting the importance of Shox2 in the cerebellum.
150. The Rho-GTPase cdc42 regulates neural progenitor fate at the apical surface. Stem cell persistence into adulthood requires self-renewal from early developmental stages. In the developing mouse brain, only apical progenitors located at the ventricle are self-renewing, whereas basal progenitors gradually deplete. However, nothing is known about the mechanisms regulating the fundamental difference between these progenitors. Here we show that the conditional deletion of the small Rho-GTPase cdc42 at different stages of neurogenesis in mouse telencephalon results in an immediate increase in basal mitoses. Whereas cdc42-deficient progenitors have normal cell cycle length, orientation of cell division and basement membrane contact, the apical location of the Par complex and adherens junctions are gradually lost, leading to an increasing failure of apically directed interkinetic nuclear **migration**. These cells then undergo mitoses at basal positions and acquire the fate of basal progenitors. Thus, cdc42 has a crucial role at the apical pole of progenitors, thereby regulating the position of mitoses and cell fate.
151. Unipolar brush cells of the cerebellum are produced in the rhombic lip and migrate through developing white matter. Unipolar brush cells (UBCs) are glutamatergic interneurons in the cerebellar cortex and dorsal cochlear nucleus. We studied the development of UBCs, using transcription factor Tbr2/Eomes as a marker for UBCs and their progenitors in embryonic and postnatal mouse cerebellum. Tbr2+ UBCs appeared to migrate out of the upper rhombic lip via two cellular streams: a dorsal pathway into developing cerebellar white matter, where the migrating cells dispersed widely before entering the internal granular layer, and a rostral pathway along the cerebellar ventricular zone toward the brainstem. Ablation of the rhombic lip in organotypic slice cultures substantially reduced the production of Tbr2+ UBCs. In coculture experiments, Tbr2+ UBCs migrated from rhombic lip explants directly into the developing white matter of adjacent cerebellar slices. The origin of Tbr2+ UBCs was confirmed by colocalization with beta-galactosidase expressed from the Math1 locus, a molecular marker of rhombic lip lineages. Moreover, the production of Tbr2+ UBCs was Math1 dependent, as Tbr2+ UBCs were severely reduced in Math1-null cerebellum. In reeler mutant mice, Tbr2+ UBCs accumulated near the rhombic lip, consistent with impaired **migration** through developing white matter. Our results suggest that UBCs arise from the rhombic lip and migrate via novel pathways to their final destinations in the cerebellum and dorsal cochlear nucleus. Our findings support a model of cerebellar neurogenesis, in which glutamatergic and GABAergic neurons are produced from separate progenitor pools located mainly in the rhombic lip and the cerebellar ventricular zone, respectively.
152. Pax6 regulates regional development and neuronal **migration** in the cerebral cortex. Mutations in the Pax6 gene disrupt telencephalic development, resulting in a thin cortical plate, expansion of proliferative layers, and the absence of the olfactory bulb. The primary defect in the neuronal cell population of the developing cerebral cortex was analysed by using mouse chimeras containing a mixture of wild-type and Pax6-deficient cells. The chimeric analysis shows that Pax6 influences cellular activity throughout corticogenesis. At early stages, Pax6-deficient and wildtype cells segregate into exclusive patches, indicating an inability of different cell genotypes to interact. At later stages, cells are sorted further based on telencephalic domains. Pax6-deficient cells are specifically reduced in the mediocaudal domain of the dorsal telencephalon, indicating a role in regionalization. In addition, Pax6 regulates the process of radial **migration** of neuronal precursors. Loss of Pax6 particularly affects movement of neuronal precursors at the subventricular zone/intermediate zone boundary at a transitional migratory phase essential for entry into the intermediate zone. We suggest that the primary role of Pax6 is the continual regulation of cell surface properties responsible for both cellular identity and radial **migration**, defects of which cause regional cell sorting and abnormalities of **migration** in chimeras.
153. Beta tubulin isoforms are not interchangeable for rescuing impaired radial **migration** due to Tubb3 knockdown. Over the last years, the critical role of cytoskeletal proteins in cortical development including neuronal **migration** as well as in neuronal morphology has been well established. Inputs from genetic studies were provided through the identification of several mutated genes encoding either proteins associated with microtubules (DCX, LIS1, KIF2A, KIF5C, DYNC1H1) or tubulin subunits (TUBA1A, TUBB2B, TUBB5 and TUBG1), in malformations of cortical development (MCD). We also reported the identification of missense mutations in TUBB3, the postmitotic neuronal specific tubulin, in six different families presenting either polymicrogyria or gyral disorganization in combination with cerebellar and basal ganglial abnormalities. Here, we investigate further the association between TUBB3 mutations and MCDs by analyzing the consequences of Tubb3 knockdown on cortical development in mice. Using the in utero-electroporation approach, we demonstrate that Tubb3 knockdown leads to delayed bipolar morphology and radial **migration** with evidence, suggesting that the neuronal arrest is a transient phenomenon overcome after birth. Silenced blocked cells display a round-shape and decreased number of processes and a delay in the acquisition of the bipolar morphology. Also, more Tbr2 positive cells are observed, although less cells express the proliferation marker Ki67, suggesting that Tubb3 inactivation might have an indirect effect on intermediate progenitor proliferation. Furthermore, we show by rescue experiments the non-interchangeability of other beta-tubulins which are unable to rescue the phenotype. Our study highlights the critical and specific role of Tubb3 on the stereotyped morphological changes and polarization processes that are required for initiating radial **migration** to the cortical plate.
154. Neurogenin 2 controls cortical neuron **migration** through regulation of Rnd2. Motility is a universal property of newly generated neurons. How cell **migration** is coordinately regulated with other aspects of neuron production is not well understood. Here we show that the proneural protein neurogenin 2 (Neurog2), which controls neurogenesis in the embryonic cerebral cortex, directly induces the expression of the small GTP-binding protein Rnd2 (ref. 3) in newly generated mouse cortical neurons before they initiate **migration**. Rnd2 silencing leads to a defect in radial **migration** of cortical neurons similar to that observed when the Neurog2 gene is deleted. Remarkably, restoring Rnd2 expression in Neurog2-mutant neurons is sufficient to rescue their ability to migrate. Our results identify Rnd2 as a novel essential regulator of neuronal **migration** in the cerebral cortex and demonstrate that Rnd2 is a major effector of Neurog2 function in the promotion of **migration**. Thus, a proneural protein controls the complex cellular behaviour of cell **migration** through a remarkably direct pathway involving the transcriptional activation of a small GTP-binding protein.
155. Dlx5 and Dlx6 regulate the development of parvalbumin-expressing cortical interneurons. Dlx5 and Dlx6 homeobox genes are expressed in developing and mature cortical interneurons. Simultaneous deletion of Dlx5 and 6 results in exencephaly of the anterior brain; despite this defect, prenatal basal ganglia differentiation appeared largely intact, while tangential **migration** of Lhx6(+) and Mafb(+) interneurons to the cortex was reduced and disordered. The **migration** deficits were associated with reduced CXCR4 expression. Transplantation of mutant immature interneurons into a wild-type brain demonstrated that loss of either Dlx5 or Dlx5&6 preferentially reduced the number of mature parvalbumin(+) interneurons; those parvalbumin(+) interneurons that were present had increased dendritic branching. Dlx5/6(+/-) mice, which appear normal histologically, show spontaneous electrographic seizures and reduced power of gamma oscillations. Thus, Dlx5&6 appeared to be required for development and function of somal innervating (parvalbumin(+)) neocortical interneurons. This contrasts with Dlx1, whose function is required for dendrite innervating (calretinin(+), somatostatin(+), and neuropeptide Y(+)) interneurons (Cobos et al., 2005).
156. FRS2 alpha 2F/2F mice lack carotid body and exhibit abnormalities of the superior cervical sympathetic ganglion and carotid sinus nerve. The docking protein FRS2 alpha is an important mediator of fibroblast growth factor (FGF)-induced signal transduction, and functions by linking FGF receptors (FGFRs) to a variety of intracellular signaling pathways. We show that the carotid body is absent in FRS2 alpha(2F/2F) mice, in which the Shp2-binding sites of FRS2 alpha are disrupted. We also show that the carotid body rudiment is not formed in the wall of the third arch artery in mutant embryos. In wild-type mice, the superior cervical ganglion of the sympathetic trunk connects to the carotid body in the carotid bifurcation region, and extends thick nerve bundles into the carotid body. In FRS2 alpha(2F/2F) mice, the superior cervical ganglion was present in the lower cervical region as an elongated feature, but failed to undergo cranio-ventral **migration**. In addition, few neuronal processes extended from the ganglion into the carotid bifurcation region. The number of carotid sinus nerve fibers that reached the carotid bifurcation region was markedly decreased, and baroreceptor fibers belonging to the glossopharyngeal nerve were absent from the basal part of the internal carotid artery in FRS2 alpha(2F/2F) mutant mice. In some of the mutant mice (5 out of 14), baroreceptors and some glomus cells were distributed in the wall of the common carotid artery, onto which the sympathetic ganglion abutted. We propose that the sympathetic ganglion provides glomus cell precursors into the third arch artery derivative in the presence of sensory fibers of the glossopharyngeal nerve.
157. Fyn tyrosine kinase is a critical regulator of disabled-1 during brain development. Disabled-1 (Dab1) is an intracellular adaptor protein that regulates **migration**s of various classes of neurons during mammalian brain development. Dab1 function depends on its tyrosine phosphorylation, which is stimulated by Reelin, an extracellular signaling molecule. Reelin increases the stoichiometry of Dab1 phosphorylation and downregulates Dab1 protein levels. Reelin binds to various cell surface receptors, including two members of the low-density lipoprotein receptor family that also bind to Dab1. Mutations in Dab1, its phosphorylation sites, Reelin, or the Reelin receptors cause a common phenotype. However, the molecular mechanism whereby Reelin regulates Dab1 tyrosine phosphorylation is poorly understood.We found that Reelin-induced Dab1 tyrosine phosphorylation in neuron cultures is inhibited by acute treatment with pharmacological inhibitors of Src family, but not Abl family, kinases. In addition, Reelin stimulates Src family kinases by a mechanism involving Dab1. We analyzed the Dab1 protein level and tyrosine phosphorylation stoichiometry by using brain samples and cultured neurons that were obtained from mouse embryos carrying mutations in Src family tyrosine kinases. We found that fyn is required for proper Dab1 levels and phosphorylation in vivo and in vitro. When fyn copy number is reduced, src, but not yes, becomes important, reflecting a partial redundancy between fyn and src.Reelin activates Fyn to phosphorylate and downregulate Dab1 during brain development. The results were unexpected because Fyn deficiency does not cause the same developmental phenotype as Dab1 or Reelin deficiency. This suggests additional complexity in the Reelin signaling pathway.
158. N-cofilin is associated with neuronal **migration** disorders and cell cycle control in the cerebral cortex. Many neuronal disorders such as lissencephaly, epilepsy, and schizophrenia are caused by the abnormal **migration** of neurons in the developing brain. The role of the actin cytoskeleton in neuronal **migration** disorders has in large part remained elusive. Here we show that the F-actin depolymerizing factor n-cofilin controls cell **migration** and cell cycle progression in the cerebral cortex. Loss of n-cofilin impairs radial **migration**, resulting in the lack of intermediate cortical layers. Neuronal progenitors in the ventricular zone show increased cell cycle exit and exaggerated neuronal differentiation, leading to the depletion of the neuronal progenitor pool. These results demonstrate that mutations affecting regulators of the actin cytoskeleton contribute to the pathology of cortex development.
159. Effects of different regions of the developing gut on the **migration** of enteric neural crest-derived cells: a role for Sema3A, but not Sema3F. The enteric nervous system arises from vagal (caudal hindbrain) and sacral level neural crest-derived cells that migrate into and along the developing gut. Data from previous studies have suggested that (i) there may be gradients along the gut that induce the caudally directed **migration** of vagal enteric neural precursors (ENPs), (ii) exposure to the caecum might alter the migratory ability of vagal ENPs and (iii) Sema3A might regulate the entry into the hindgut of ENPs derived from sacral neural crest. Using co-cultures we show that there is no detectable gradient of chemoattractive molecules along the pre-caecal gut that specifically promotes the caudally directed **migration** of vagal ENPs, although vagal ENPs migrate faster caudally than rostrally along explants of hindgut. Exposure to the caecum did not alter the rate at which ENPs colonized explants of hindgut, but it did alter the ability of ENPs to colonize the midgut. The co-cultures also revealed that there is localized expression of a repulsive cue in the distal hindgut, which might delay the entry of sacral ENPs. We show that Sema3A is expressed by the hindgut mesenchyme and its receptor, neuropilin-1, is expressed by migrating ENPs. Furthermore, there is premature entry of sacral ENPs and extrinsic axons into the distal hindgut of fetal mice lacking Sema3A. These data show that Sema3A expressed by the distal hindgut regulates the entry of sacral ENPs and extrinsic axons into the hindgut. ENPs did not express neuropilin-2 and there was no detectable change in the timetable by which ENPs colonize the gut in mice lacking neuropilin-2.
160. A phenotype-driven ENU mutagenesis screen identifies novel alleles with functional roles in early mouse craniofacial development. Proper craniofacial development begins during gastrulation and requires the coordinated integration of each germ layer tissue (ectoderm, mesoderm, and endoderm) and its derivatives in concert with the precise regulation of cell proliferation, **migration**, and differentiation. Neural crest cells, which are derived from ectoderm, are a migratory progenitor cell population that generates most of the cartilage, bone, and connective tissue of the head and face. Neural crest cell development is regulated by a combination of intrinsic cell autonomous signals acquired during their formation, balanced with extrinsic signals from tissues with which the neural crest cells interact during their **migration** and differentiation. Although craniofacial anomalies are typically attributed to defects in neural crest cell development, the cause may be intrinsic or extrinsic. Therefore, we performed a phenotype-driven ENU mutagenesis screen in mice with the aim of identifying novel alleles in an unbiased manner, that are critically required for early craniofacial development. Here we describe 10 new mutant lines, which exhibit phenotypes affecting frontonasal and pharyngeal arch patterning, neural and vascular development as well as sensory organ morphogenesis. Interestingly, our data imply that neural crest cells and endothelial cells may employ similar developmental programs and be interdependent during early embryogenesis, which collectively is critical for normal craniofacial morphogenesis. Furthermore our novel mutants that model human conditions such as exencephaly, craniorachischisis, DiGeorge, and Velocardiofacial sydnromes could be very useful in furthering our understanding of the complexities of specific human diseases.
161. Stromal cell-derived factor-1 (chemokine C-X-C motif ligand 12) and chemokine C-X-C motif receptor 4 are required for **migration** of gonadotropin-releasing hormone neurons to the forebrain. Gonadotropin-releasing hormone (GnRH) neurons migrate from the vomeronasal organ (VNO) in the nasal compartment to the basal forebrain in mice, beginning on embryonic day 11 (E11). These neurons use vomeronasal axons as guides to migrate through the nasal mesenchyme. Most GnRH neurons then migrate along the caudal branch of the vomeronasal nerve to reach the hypothalamus. We show here that stromal cell-derived factor-1 [SDF-1, also known as chemokine C-X-C motif ligand 12 (CXCL12)] is expressed in the embryonic nasal mesenchyme from as early as E10 in an increasing rostral to caudal gradient that is most intense at the border of the nasal mesenchyme and the telencephalon. Chemokine C-X-C motif receptor 4 (CXCR4), the receptor for SDF-1, is expressed by neurons in the olfactory epithelium and VNO. Cells derived from these sensory epithelia, including migrating GnRH neurons and ensheathing glial precursors of the migrating mass (MM), also express CXCR4, suggesting that they may use SDF-1 as a chemokine. In support of this, most GnRH neurons of CXCR4-/- mice fail to exit the VNO at E13, and comparatively few GnRH neurons reach the forebrain. There is also a significant decrease in the total number of GnRH neurons in CXCR4-/- mice and an increase in cell death within the VNO relative to controls. The MM is smaller in CXCR4-/- mice, suggesting that some MM cells also require SDF-1/CXCR4 function for **migration** and survival.
162. Different timings of Dicer deletion affect neurogenesis and gliogenesis in the developing mouse central nervous system. MicroRNAs, processed by the RNAase III enzyme Dicer, are approximately 22 nucleotide endogenous noncoding small RNAs. The function of Dicer in the mouse central nervous system (CNS) development is not well understood. Here, we show that specifically deleting Dicer expression in the CNS and in the cerebral cortex using two Cre lines results in reduced progenitor numbers, abnormal neuronal differentiation, and thinner cortical wall. Incomplete Dicer deletion during early embryonic stages contributes to normal development of early-born neurons in the cortex and motor neurons in the spinal cord. However, at late embryonic stages when Dicer is completely ablated in the CNS, the **migration** of late-born neurons in the cortex and oligodendrocyte precursor expansion and differentiation in the spinal cord are greatly affected. Our studies of different timings of Dicer deletion demonstrate the importance of the Dicer-mediated microRNA pathway in regulating distinct phases of neurogenesis and gliogenesis during the CNS development.
163. Functional redundancy of NSCL-1 and NeuroD during development of the petrosal and vestibulocochlear ganglia. To study the role of different members of the bHLH gene family for sensory organ development we have generated NSCL-1 and NeuroD compound-mutant mice. Double homozygous animals were characterized by a more severe reduction of the petrosal and vestibulocochlear ganglia than NeuroD-knockout mice. The more severe reduction of the petrosal and vestibulocochlear ganglia in double-knockout mice indicates overlapping functions of the two genes during neuronal development. Interestingly, we also found that the two genes are jointly regulated by thyroid hormone during sensory hair cell development. We further present a detailed expression analysis of NSCL-1 and NSCL-2 during sensory neuron development. NSCL-1 expression was detected in all developing cranial ganglia including the petrosal and vestibulocochlear ganglion, in inner and outer hair cells of the organ of Corti and in hair cells of the vestibular system. Expression domains in other sensory structures include the retina, Merkel cells of the developing skin and sensory cells of the tongue. The expression of NSCL-2 was restricted to developing cranial ganglia, the retina and the vestibular nerve. Both NSCL-1 and NSCL-2 genes are active only in postmitotic neurons, indicating a role for neuronal cell **migration** and/or differentiation within the sensory system.
164. Shp2-dependent ERK signaling is essential for induction of Bergmann glia and foliation of the cerebellum. Folding of the cortex and the persistence of radial glia (RG)-like cells called Bergmann glia (BG) are hallmarks of the mammalian cerebellum. Similar to basal RG in the embryonic neocortex, BG maintain only basal processes and continuously express neural stem cell markers. Past studies had focused on the function of BG in granule cell **migration** and how granule cell progenitors (GCP) regulate cerebellar foliation. The molecular control of BG generation and its role in cerebellar foliation are less understood. Here, we have analyzed the function of the protein tyrosine phosphatase Shp2 in mice by deleting its gene Ptpn11 in the entire cerebellum or selectively in the GCP lineage. Deleting Ptpn11 in the entire cerebellum by En1-cre blocks transformation of RG into BG but preserves other major cerebellar cell types. In the absence of BG, inward invagination of GCP persists but is uncoupled from the folding of the Purkinje cell layer and the basement membrane, leading to disorganized lamination and an absence of cerebellar folia. In contrast, removing Ptpn11 in the GCP lineage by Atoh1-cre has no effect on cerebellar development, indicating that Shp2 is not cell autonomously required in GCP. Furthermore, we demonstrate that Ptpn11 interacts with Fgf8 and is essential for ERK activation in RG and nascent BG. Finally, expressing constitutively active MEK1 rescues BG formation and cerebellar foliation in Shp2-deficient cerebella. Our results demonstrate an essential role of Shp2 in BG specification via fibroblast growth factor/extracellular signal-regulated protein kinase signaling, and reveal a crucial function of BG in organizing cerebellar foliation.
165. ERBB3-mediated regulation of Bergmann glia proliferation in cerebellar lamination. Cortical lamination is crucial for the assembly of cerebellar circuitry. In this process, granule neurons (GNs) migrate along Bergmann glia (BG), which are specialized astroglial cells, from the external granule layer to the internal granule layer. However, the molecular mechanisms underlying BG development are not well understood. Here, we show that GFAP::Cre;Erbb3(F/F) mice, which lack Erbb3 in both radial glia and neurons, exhibit impairments in balance and motor coordination. Cerebellar lamination is aberrant, with misplaced Purkinje neurons and GN clusters. These phenotypes were not observed in Math1::CreER(T2);Erbb3(F/F) mice, where the Erbb3 gene was deleted in GNs, suggesting involvement of non-neuronal Erbb3 in cerebellar lamination. Mechanistic studies indicate that ERBB3 is crucial for the proliferation of BG, which are required for GN **migration**. These observations identify a crucial role for ERBB3 in cerebellar lamination and reveal a novel mechanism that regulates BG development.
166. LMO4 functions as a co-activator of neurogenin 2 in the developing cortex. The proneural protein neurogenin 2 (NGN2) is a key transcription factor in regulating both neurogenesis and neuronal radial **migration** in the embryonic cerebral cortex. However, the co-factors that support the action of NGN2 in the cortex remain unclear. Here, we show that the LIM-only protein LMO4 functions as a novel co-factor of NGN2 in the developing cortex. LMO4 and its binding partner nuclear LIM interactor (NLI/LDB1/CLIM2) interact with NGN2 simultaneously, forming a multi-protein transcription complex. This complex is recruited to the E-box containing enhancers of NGN2-target genes, which regulate various aspects of cortical development, and activates NGN2-mediated transcription. Correspondingly, analysis of Lmo4-null embryos shows that the loss of LMO4 leads to impairments of neuronal differentiation in the cortex. In addition, expression of LMO4 facilitates NGN2-mediated radial **migration** of cortical neurons in the embryonic cortex. Our results indicate that LMO4 promotes the acquisition of cortical neuronal identities by forming a complex with NGN2 and subsequently activating NGN2-dependent gene expression.
167. Frizzled-3 is required for the development of major fiber tracts in the rostral CNS. Many ligand/receptor families are known to contribute to axonal growth and targeting. Thus far, there have been no reports implicating Wnts and Frizzleds in this process, despite their large numbers and widespread expression within the CNS. In this study, we show that targeted deletion of the mouse fz3 gene leads to severe defects in several major axon tracts within the forebrain. In particular, fz3(-/-) mice show a complete loss of the thalamocortical, corticothalamic, and nigrostriatal tracts and of the anterior commissure, and they show a variable loss of the corpus callosum. Peripheral nerve fibers and major axon tracts in the more caudal regions of the CNS are mostly or completely unaffected. Cell proliferation in the ventricular zone and cell **migration** to the developing cortex proceed normally until at least embryonic day 14. Extensive cell death in the fz3(-/-) striatum occurs late in gestation, perhaps secondary to the nearly complete absence of long-range connections. In contrast, there is little cell death, as assayed by terminal deoxynucleotidyl transferase-mediated biotinylated UTP nick end labeling, in the cortex. These data provide the first link between Frizzled signaling and axonal development.
168. Plexin-B2, but not Plexin-B1, critically modulates neuronal **migration** and patterning of the developing nervous system in vivo. Semaphorins and their receptors, plexins, have emerged as important cellular cues regulating key developmental processes. B-type plexins directly regulate the actin cytoskeleton in a variety of cell types. Recently, B-type plexins have been shown to be expressed in striking patterns in the nervous system over critical developmental windows. However, in contrast to the well characterized plexin-A family, the functional role of plexin-B proteins in neural development and organogenesis in vertebrates in vivo is not known. Here, we have elucidated the functional contribution of the two neuronally expressed plexin-B proteins, Plexin-B1 or Plexin-B2, toward the development of the peripheral nervous system and the CNS by generating and analyzing constitutive knock-out mice. The development of the nervous system was found to be normal in mice lacking Plexin-B1, whereas mice lacking Plexin-B2 demonstrated defects in closure of the neural tube and a conspicuous disorganization of the embryonic brain. After analyzing mutant mice, which bypassed neural tube defects, we observed a key requirement for Plexin-B2 in proliferation and **migration** of granule cell precursors in the developing dentate gyrus, olfactory bulb, and cerebellum. Furthermore, we identified semaphorin 4C as a high-affinity ligand for Plexin-B2 in binding and functional assays. Semaphorin 4C stimulated activation of ErbB-2 and RhoA via Plexin-B2 and enhanced proliferation and **migration** of granule cell precursors. Semaphorin 4C-induced proliferation of ventricular zone neuroblasts was abrogated in mice lacking Plexin-B2. These genetic and functional analyses reveal a key requirement for Plexin-B2, but not Plexin-B1, in patterning of the vertebrate nervous system in vivo.
169. Requirement for downregulation of kreisler during late patterning of the hindbrain. Pattern formation in the hindbrain is governed by a segmentation process that provides the basis for the organisation of cranial motor nerves. A cascade of transcriptional activators, including the bZIP transcription factor encoded by the kreisler gene controls this segmentation process. In kreisler mutants, r5 fails to form and this correlates with abnormalities in the neuroanatomical organisation of the hindbrain. Studies of Hox gene regulation suggest that kreisler may regulate the identity as well as the formation of r5, but such a role cannot be detected in kreisler mutants since r5 is absent. To gain further insights into the function of kreisler we have generated transgenic mice in which kreisler is ectopically expressed in r3 and for an extended period in r5. In these transgenic mice, the Fgf3, Krox20, Hoxa3 and Hoxb3 genes have ectopic or prolonged expression domains in r3, indicating that it acquires molecular characteristics of r5. Prolonged kreisler expression subsequently causes morphological alterations of r3/r5 that are due to an inhibition of neuronal differentiation and **migration** from the ventricular zone to form the mantle layer. We find that these alterations in r5 correlate with an arrest of facial branchiomotor neurone **migration** from r4 into the caudal hindbrain, which is possibly due to the deficiency in the mantle layer through which they normally migrate. We propose that the requirement for the downregulation of segmental kreisler expression prior to neuronal differentiation reflects the stage-specific roles of this gene and its targets.
170. Cadherin 2/4 signaling via PTP1B and catenins is crucial for nucleokinesis during radial neuronal **migration** in the neocortex. Cadherins are crucial for the radial **migration** of excitatory projection neurons into the developing neocortical wall. However, the specific cadherins and the signaling pathways that regulate radial **migration** are not well understood. Here, we show that cadherin 2 (CDH2) and CDH4 cooperate to regulate radial **migration** in mouse brain via the protein tyrosine phosphatase 1B (PTP1B) and α- and β-catenins. Surprisingly, perturbation of cadherin-mediated signaling does not affect the formation and extension of leading processes of migrating neocortical neurons. Instead, movement of the cell body and nucleus (nucleokinesis) is disrupted. This defect is partially rescued by overexpression of LIS1, a microtubule-associated protein that has previously been shown to regulate nucleokinesis. Taken together, our findings indicate that cadherin-mediated signaling to the cytoskeleton is crucial for nucleokinesis of neocortical projection neurons during their radia**l migrati**on.
171. Role of heat-shock factor 2 in cerebral cortex formation and as a regulator of p35 expression. Heat-shock factors (HSFs) are associated with multiple developmental processes, but their mechanisms of action in these processes remain largely enigmatic. Hsf2-null mice display gametogenesis defects and brain abnormalities characterized by enlarged ventricles. Here, we show that Hsf2-/- cerebral cortex displays mispositioning of neurons of superficial layers. HSF2 deficiency resulted in a reduced number of radial glia fibers, the architectural guides for migrating neurons, and of Cajal-Retzius cells, which secrete the positioning signal Reelin. Therefore, we focused on the radial **migration** signaling pathways. The levels of Reelin and Dab1 tyrosine phosphorylation were reduced, suggesting that the Reelin cascade is affected in Hsf2-/- cortices. The expression of p35, an activator of cyclin-dependent kinase 5 (Cdk5), essential for radial **migration**, was dependent on the amount of HSF2 in gain- and loss-of-function systems. p39, another Cdk5 activator, displayed reduced mRNA levels in Hsf2-/- cortices, which, together with the lowered p35 levels, decreased Cdk5 activity. We demonstrate in vivo binding of HSF2 to the p35 promoter and thereby identify p35 as the first target gene for HSF2 in cortical development. In conclusion, HSF2 affects cellular populations that assist in radial **migration** and directly regulates the expression of p35, a crucial actor of radial neuronal **migration**.
172. Macrophage **migration** inhibitory factor acts as a neurotrophin in the developing inner ear. This study is the first to demonstrate that macrophage **migration** inhibitory factor (MIF), an immune system 'inflammatory' cytokine that is released by the developing otocyst, plays a role in regulating early innervation of the mouse and chick inner ear. We demonstrate that MIF is a major bioactive component of the previously uncharacterized otocyst-derived factor, which directs initial neurite outgrowth from the statoacoustic ganglion (SAG) to the developing inner ear. Recombinant MIF acts as a neurotrophin in promoting both SAG directional neurite outgrowth and neuronal survival and is expressed in both the developing and mature inner ear of chick and mouse. A MIF receptor, CD74, is found on both embryonic SAG neurons and adult mouse spiral ganglion neurons. Mif knockout mice are hearing impaired and demonstrate altered innervation to the organ of Corti, as well as fewer sensory hair cells. Furthermore, mouse embryonic stem cells become neuron-like when exposed to picomolar levels of MIF, suggesting the general importance of this cytokine in neural development.
173. Adenomatous polyposis coli is required for early events in the normal growth and differentiation of the developing cerebral cortex. Adenomatous polyposis coli (Apc) is a large multifunctional protein known to be important for Wnt/beta-catenin signalling, cytoskeletal dynamics, and cell polarity. In the developing cerebral cortex, Apc is expressed in proliferating cells and its expression increases as cells migrate to the cortical plate. We examined the consequences of loss of Apc function for the early development of the cerebral cortex.We used Emx1(Cre) to inactivate Apc specifically in proliferating cerebral cortical cells and their descendents starting from embryonic day 9.5. We observed reduction in the size of the mutant cerebral cortex, disruption to its organisation, and changes in the molecular identity of its cells. Loss of Apc leads to a decrease in the size of the proliferative pool, disrupted interkinetic nuclear **migration**, and increased apoptosis. beta-Catenin, pericentrin, and N-cadherin proteins no longer adopt their normal high concentration at the apical surface of the cerebral cortical ventricular zone, indicating that cell polarity is disrupted. Consistent with enhanced Wnt/beta-catenin signalling resulting from loss of Apc we found increased levels of TCF/LEF-dependent transcription and expression of endogenous Wnt/beta-catenin target genes (Axin2 (conductin), Lef1, and c-myc) in the mutant cerebral cortex. In the Apc mutant cerebral cortex the expression of transcription factors Foxg1, Pax6, Tbr1, and Tbr2 is drastically reduced compared to normal and many cells ectopically express Pax3, Wnt1, and Wt1 (but not Wnt2b, Wnt8b, Ptc, Gli1, Mash1, Olig2, or Islet1). This indicates that loss of Apc function causes cerebral cortical cells to lose their normal identity and redirect to fates normally found in more posterior-dorsal regions of the central nervous system.Apc is required for multiple aspects of early cerebral cortical development, including the regulation of cell number, interkinetic nuclear **migration**, cell polarity, and cell type specification.
174. Cell **migration** from the ganglionic eminences is required for the development of hippocampal GABAergic interneurons. GABAergic interneurons have major roles in hippocampal function and dysfunction. Here we provide evidence that, in mice, virtually all of these cells originate from progenitors in the basal telencephalon. Immature interneurons tangentially migrate from the basal telencephalon through the neocortex to take up their final positions in the hippocampus. Disrupting differentiation in the embryonic basal telencephalon (lateral and medial ganglionic eminences) through loss of Dlx1/2 homeobox function blocks the **migration** of virtually all GABAergic interneurons to the hippocampus. On the other hand, disrupting specification of the medial ganglionic eminence through loss of Nkx2.1 homeobox function depletes the hippocampus of a distinct subset of hippocampal interneurons. Loss of hippocampal interneurons does not appear to have major effects on the early development of hippocampal projection neurons nor on the pathfinding of afferrent tracts.
175. Robo1 modulates proliferation and neurogenesis in the developing neocortex. The elaborate cytoarchitecture of the mammalian neocortex requires the timely production of its constituent pyramidal neurons and interneurons and their disposition in appropriate layers. Numerous chemotropic factors present in the forebrain throughout cortical development play important roles in the orchestration of these events. The Roundabout (Robo) family of receptors and their ligands, the Slit proteins, are expressed in the developing forebrain, and are known to play important roles in the generation and **migration** of cortical interneurons. However, few studies have investigated their function(s) in the development of pyramidal cells. Here, we observed expression of Robo1 and Slit genes (Slit1, Slit2) in cells lining the telencephalic ventricles, and found significant increases in progenitor cells (basal and apical) at embryonic day (E)12.5 and E14.5 in the developing cortex of Robo1(-/-), Slit1(-/-), and Slit1(-/-)/Slit2(-/-), but not in mice lacking the other Robo or Slit genes. Using layer-specific markers, we found that both early- and late-born pyramidal neuron populations were significantly increased in the cortices of Robo1(-/-) mice at the end of corticogenesis (E18.5). The excess number of cortical pyramidal neurons generated prenatally appears to die in early postnatal life. The observed increase in pyramidal neurons was due to prolonged proliferative activity of their progenitors and not due to changes in cell cycle events. This finding, confirmed by in utero electroporation with Robo1 short hairpin RNA (shRNA) or control constructs into progenitors along the ventricular zone as well as in dissociated cortical cell cultures, points to a novel role for Robo1 in regulating the proliferation and generation of pyramidal neurons.
176. An Fgf8 mouse mutant phenocopies human 22q11 deletion syndrome. Deletion of chromosome 22q11, the most common microdeletion detected in humans, is associated with a life-threatening array of birth defects. Although 90% of affected individuals share the same three megabase deletion, their phenotype is highly variable and includes craniofacial and cardiovascular anomalies, hypoplasia or aplasia of the thymus with associated deficiency of T cells, hypocalcemia with hypoplasia or aplasia of the parathyroids, and a variety of central nervous system abnormalities. Because ablation of neural crest in chicks produces many features of the deletion 22q11 syndrome, it has been proposed that haploinsufficiency in this region impacts neural crest function during cardiac and pharyngeal arch development. Few factors required for **migration**, survival, proliferation and subsequent differentiation of pharyngeal arch neural crest and mesoderm-derived mesenchyme into their respective cardiovascular, musculoskeletal, and glandular derivatives have been identified. However, the importance of epithelial-mesenchymal interactions and pharyngeal endoderm function is becoming increasingly clear. Fibroblast growth factor 8 is a signaling molecule expressed in the ectoderm and endoderm of the developing pharyngeal arches and known to play an important role in survival and patterning of first arch tissues. We demonstrate a dosage-sensitive requirement for FGF8 during development of pharyngeal arch, pharyngeal pouch and neural crest-derived tissues. We show that FGF8 deficient embryos have lethal malformations of the cardiac outflow tract, great vessels and heart due, at least in part, to failure to form the fourth pharyngeal arch arteries, altered expression of Fgf10 in the pharyngeal mesenchyme, and abnormal apoptosis in pharyngeal and cardiac neural crest. The Fgf8 mutants described herein display the complete array of cardiovascular, glandular and craniofacial phenotypes seen in human deletion 22q11 syndromes. This represents the first single gene disruption outside the typically deleted region of human chromosome 22 to fully recapitulate the deletion 22q11 phenotype. FGF8 may operate directly in molecular pathways affected by deletions in 22q11 or function in parallel pathways required for normal development of pharyngeal arch and neural crest-derived tissues. In either case, Fgf8 may function as a modifier of the 22q11 deletion and contribute to the phenotypic variability of this syndrome.
177. Directional neuronal **migration** is impaired in mice lacking adenomatous polyposis coli 2. Adenomatous polyposis coli 2 (APC2) is a family member of APC and mainly expressed in the nervous system. We previously reported that APC2 plays a critical role in axonal projection through the regulation of microtubule stability. Here, we show that a lack of Apc2 induces severe laminary defects in some regions of the mouse brain, including the cerebral cortex and cerebellum. In vivo BrdU labeling and immunohistochemical analyses with specific markers revealed that the laminary abnormalities are a result of dysregulated neuronal **migration** by a cell-autonomous mechanism. Using total internal reflection fluorescent microscopy, we found that APC2 is distributed along actin fibers as well as microtubules. Cerebellar granule cells in dissociated cultures and in vivo showed that BDNF-stimulated directional **migration** is impaired in Apc2-deficient neurons. We revealed that this impairment stems from the dysregulations of Rho family GTPase activation and TrkB localization, which disrupts the formation of BDNF-stimulated F-actin at the leading edge. Thus, APC2 is an essential mediator of the cytoskeletal regulation at leading edges in response to extracellular signals.
178. Absence of the vagus nerve in the stomach of Tbx1-/- mutant mice. Tbx1 is a member of the Tbox family of binding domain transcription factors. TBX1 maps within the region of chromosome 22q11 deleted in humans with DiGeorge syndrome (DGS), a common genetic disorder characterized by numerous physical manifestations including craniofacial and cardiac anomalies. Mice with homozygous null mutations in Tbx1 phenocopy this disorder and have defects including abnormal cranial ganglia formation and cardiac neural crest cell **migration**. These defects prompted us to investigate whether extrinsic vagus nerve or intrinsic enteric nervous system abnormalities are prevalent in the gastrointestinal tract of Tbx1 mutant mice.We used in situ hybridization for Ret, and immunohistochemical staining for neurofilament, HuC/D and βIII-tubulin to study cranial ganglia, vagus nerve, and enteric nervous system development in Tbx1 mutant and control mice.In Tbx1(-/-) embryos, cranial ganglia of the glossopharyngeal (IXth) and vagus (Xth) nerves were malformed and abnormally fused. In the gastrointestinal tract, the vagus nerves adjacent to the esophagus were severely hypoplastic and they did not extend beyond the gastro-esophageal junction nor project branches within the stomach wall, as was observed in Tbx1(+/+) mice.Although cranial ganglia morphology appeared normal in Tbx1(+/-) mice, these animals had a spectrum of stomach vagus innervation defects ranging from mild to severe. In all Tbx1 genotypes, the intrinsic enteric nervous system developed normally. The deficit in vagal innervation of the stomach in mice mutant for a gene implicated in DGS raises the possibility that similar defects may underlie a number of as yet unidentified/unreported congenital disorders affecting gastrointestinal function.
179. NDUFV2 regulates neuronal **migration** in the developing cerebral cortex through modulation of the multipolar-bipolar transition. Abnormalities during brain development are tightly linked several psychiatric disorders. Mutations in NADH dehydrogenase ubiquinone flavoprotein 2 (NDUFV2) are responsible for schizophrenia, bipolar disorder and Parkinson׳s disease. However, the function of NDUFV2 during brain development remains unclear. Here we reported that ndufv2 is expressed in the developing cerebral cortex. In utero suppression of ndufv2 arrested neuronal **migratio**n, leading to accumulation of ectopic neurons in the intermediate zone. ndufv2 inhibition did not affect radial glia scaffold, progenitor cells or neurons survival. However, the loss of ndufv2 impairs neuronal multipolar-bipolar transition in vivo and polarization in vitro. Moreover, ndufv2 affected actin cytoskeleton and tubulin stabilization in cortical neurons. Overall, our findings establish a new NDUFV2 dependent mechanism underlying neuronal **migratio**n and psychiatric disorders.
180. Familial dysautonomia model reveals Ikbkap deletion causes apoptosis of Pax3+ progenitors and peripheral neurons. Familial dysautonomia (FD) is a devastating developmental and progressive peripheral neuropathy caused by a mutation in the gene inhibitor of kappa B kinase complex-associated protein (IKBKAP). To identify the cellular and molecular mechanisms that cause FD, we generated mice in which Ikbkap expression is ablated in the peripheral nervous system and identify the steps in peripheral nervous system development that are Ikbkap-dependent. We show that Ikbkap is not required for trunk neural crest **migration** or pathfinding, nor for the formation of dorsal root or sympathetic ganglia, or the adrenal medulla. Instead, Ikbkap is essential for the second wave of neurogenesis during which the majority of tropomyosin-related kinase A (TrkA(+)) nociceptors and thermoreceptors arise. In its absence, approximately half the normal complement of TrkA(+) neurons are lost, which we show is partly due to p53-mediated premature differentiation and death of mitotically-active progenitors that express the paired-box gene Pax3 and give rise to the majority of TrkA(+) neurons. By the end of sensory development, the number of TrkC neurons is significantly increased, which may result from an increase in Runx3(+) cells. Furthermore, our data demonstrate that TrkA(+) (but not TrkC(+)) sensory and sympathetic neurons undergo exacerbated Caspase 3-mediated programmed cell death in the absence of Ikbkap and that this death is not due to a reduction in nerve growth factor synthesis. In summary, these data suggest that FD does not result from a failure in trunk neural crest **migration**, but rather from a critical function for Ikbkap in TrkA progenitors and TrkA(+) neurons.
181. Retinaldehyde dehydrogenase enzymes regulate colon enteric nervous system structure and function. The enteric nervous system (ENS) forms from the neural crest-derived precursors that colonize the bowel before differentiating into a network of neurons and glia that control intestinal function. Retinoids are essential for normal ENS development, but the role of retinoic acid (RA) metabolism in development remains incompletely understood. Because RA is produced locally in the tissues where it acts by stimulating RAR and RXR receptors, RA signaling during development is absolutely dependent on the rate of RA synthesis and degradation. RA is produced by three different enzymes called retinaldehyde dehydrogenases (RALDH1, RALDH2 and RALDH3) that are all expressed in the developing bowel. To determine the relative importance of these enzymes for ENS development, we analyzed whole mount preparations of adult (8-12-week old) myenteric and submucosal plexus stained with NADPH diaphorase (neurons and neurites), anti-TuJ1 (neurons and neurites), anti-HuC/HuD (neurons), and anti-S100β (glia) in an allelic series of mice with mutations in Raldh1, Raldh2, and Raldh3. We found that Raldh1-/-, Raldh2+/-, Raldh3+/- (R1(KO)R2(Het)R3(Het)) mutant mice had a reduced colon myenteric neuron density, reduced colon myenteric neuron to glia ratio, reduced colon submucosal neuron density, and increased colon myenteric fibers per neuron when compared to the wild type (WT; Raldh1WT, Raldh2WT, Raldh3WT) mice. These defects are unlikely to be due to defective ENS precursor **migratio**n since R1(KO)R2(Het)R3(KO) mice had increased enteric neuron progenitor **migratio**n into the distal colon compared to WT during development. RALDH mutant mice also have reduced contractility in the colon compared to WT mice. These data suggest that RALDH1, RALDH2 and RALDH3 each contribute to ENS development and function.
182. Abnormal neocortical development in mice lacking cGMP-dependent protein kinase I. Cyclic GMP-dependent protein kinase type I (cGKI) is a key signaling intermediate important for synaptic potentiation in the hippocampus and cerebellum, but its expression and function in cortical development have not been elucidated. The expression of cGKI in the developing mouse neocortex was evaluated by immunofluorescence labeling, and effect of cGKI deletion on cortical development was studied in adult cGKI knockout mice. cGKI was expressed at highest levels at embryonic stages in young neurons and radial glial fibers, corresponding to the major period of radial **migration** and laminar development of pyramidal neurons (embryonic day E13.5-E14.5), declining upon maturation (E17.5-postnatal day P28). The cerebral cortex of homozygous null mutant mice lacking cGKI exhibited heterotopic collections of neurons in the upper cortical layers and abnormal invaginations of layer I, in accord with a neuronal **migration** or positioning defect. Some cGKI mutant mice displayed defects in midline development resulting in partial fusion of cerebral hemispheres with adjacent neuronal heterotopias. Apical dendrites of cortical pyramidal neurons were misoriented in the cerebral cortex of cGKI null mutants, as shown in reporter mice expressing yellow fluorescent protein in layer V pyramidal neurons and by Golgi impregnation. These results demonstrate a role for cGKI signaling in cortical development related to neuronal **migration**/positioning that is important for dendritic orientation and connectivity.
183. Wnt5a is a transcriptional target of Dlx homeogenes and promotes differentiation of interneuron progenitors in vitro and in vivo. During brain development, neurogenesis, **migration**, and differentiation of neural progenitor cells are regulated by an interplay between intrinsic genetic programs and extrinsic cues. The Dlx homeogene transcription factors have been proposed to directly control the genesis and maturation of GABAergic interneurons of the olfactory bulb (OB), subpallium, and cortex. Here we provide evidence that Dlx genes promote differentiation of olfactory interneurons via the signaling molecule Wnt5a. Dlx2 and Dlx5 interact with homeodomain binding sequences within the Wnt5a locus and activate its transcription. Exogenously provided Wnt5a promotes GABAergic differentiation in dissociated OB neurons and in organ-type brain cultures. Finally, we show that the Dlx-mutant environment is unfavorable for GABA differentiation, in vivo and in vitro. We conclude that Dlx genes favor interneuron differentiation also in a non-cell-autonomous fashion, via expression of Wnt5a.
184. GFR alpha3, a component of the artemin receptor, is required for **migration** and survival of the superior cervical ganglion. GFR alpha3 is a component of the receptor for the neurotrophic factor artemin. The role of GFR alpha3 in nervous system development was examined by generating mice in which the Gfr alpha3 gene was disrupted. The Gfr alpha3-/- mice exhibited severe defects in the superior cervical ganglion (SCG), whereas other ganglia appeared normal. SCG precursor cells in the mutant embryos failed to migrate to the correct position, and they subsequently failed to innervate the target organs. In wild-type embryos, Gfr alpha3 was expressed in migrating SCG precursors, and artemin was expressed in and near the SCG. After birth, SCG neurons in the mutant mice underwent progressive cell death. These observations suggest that GFR alpha3-mediated signaling is required both for the rostral **migration** of SCG precursors and for the survival of mature SCG neurons.
185. Mouse B-type lamins are required for proper organogenesis but not by embryonic stem cells. B-type lamins, the major components of the nuclear lamina, are believed to be essential for cell proliferation and survival. We found that mouse embryonic stem cells (ESCs) do not need any lamins for self-renewal and pluripotency. Although genome-wide lamin-B binding profiles correlate with reduced gene expression, such binding is not directly required for gene silencing in ESCs or trophectoderm cells. However, B-type lamins are required for proper organogenesis. Defects in spindle orientation in neural progenitor cells and **migration** of neurons probably cause brain disorganizations found in lamin-B null mice. Thus, our studies not only disprove several prevailing views of lamin-Bs but also establish a foundation for redefining the function of the nuclear lamina in the context of tissue building and homeostasis.
186. Cyclin-dependent kinase 5 is essential for neuronal cell cycle arrest and differentiation. Cyclin-dependent kinase 5 (Cdk5) is a serine/threonine kinase with significant homology to cell cycle-related Cdks but is not believed to be active in a typical cell cycle. In Cdk5-deficient embryos and Cdk5 chimeras, **migration** and survival of postmitotic neurons is compromised in a cell-autonomous manner. In the present study, we show that loss of Cdk5 leads to both failure of neuronal differentiation and loss of cell cycle control. Using specific cytoskeletal proteins as indices of neuronal differentiation, we find that Cdk5-deficient neurons are significantly arrested or delayed in their developmental program both in vivo and in vitro. For example, immunocytochemistry of embryonic day 16 (E16) cortex reveals that the expression of microtubule-associated protein 2c (Map-2c), a marker of mature neurons, is nearly absent in Cdk5(-/-) cells that have migrated to the cortical plate while these same cells continue to express nestin. Similarly, in vitro, Map-2-positive cells are rare in cultures from E16 Cdk5(-/-) embryos. Cell cycle control is also deficient in Cdk5(-/-) cells. In vivo, neurons engaged in cell cycle activities are found in the cortical plate, and, in vitro, class III beta-tubulin-positive cells continue to label with bromodeoxyuridine even after 5 d of incubation. Transfection of a wild-type Cdk5 construct reveals that cell cycle control can be regained in Cdk5(-/-) cells by overexpression of Cdk5. These data indicate that Cdk5 is necessary for both neuronal differentiation and cell cycle inhibition.
187. Ccm3, a gene associated with cerebral cavernous malformations, is required for neuronal **migration**. Loss of function of cerebral cavernous malformation 3 (CCM3) results in an autosomal dominant cerebrovascular disorder. Here, we uncover a developmental role for CCM3 in regulating neuronal **migration** in the neocortex. Using cell type-specific gene inactivation in mice, we show that CCM3 has both cell autonomous and cell non-autonomous functions in neural progenitors and is specifically required in radial glia and newly born pyramidal neurons migrating through the subventricular zone, but not in those migrating through the cortical plate. Loss of CCM3 function leads to RhoA activation, alterations in the actin and microtubule cytoskeleton affecting neuronal morphology, and abnormalities in laminar positioning of primarily late-born neurons, indicating CCM3 involvement in radial glia-dependent locomotion and possible interaction with the Cdk5/RhoA pathway. Thus, we identify a novel cytoplasmic regulator of neuronal **migration** and demonstrate that its inactivation in radial glia progenitors and nascent neurons produces severe malformations of cortical development.
188. Neuropilin 1 signaling guides neural crest cells to coordinate pathway choice with cell specification. Neural crest cells (NCCs) are highly motile embryonic stem cells that delaminate from the neuroectoderm early during vertebrate embryogenesis and differentiate at defined target sites into various essential cell types. To reach their targets, NCCs follow 1 of 3 sequential pathways that correlate with NCC fate. The firstborn NCCs travel ventrally alongside intersomitic blood vessels to form sympathetic neuronal progenitors near the dorsal aorta, while the lastborn NCCs migrate superficially beneath the epidermis to give rise to melanocytes. Yet, most NCCs enter the somites to form the intermediate wave that gives rise to sympathetic and sensory neurons. Here we show that the repulsive guidance cue SEMA3A and its receptor neuropilin 1 (NRP1) are essential to direct the intermediate wave NCC precursors of peripheral neurons from a default pathway alongside intersomitic blood vessels into the anterior sclerotome. Thus, loss of function for either gene caused excessive intersomitic NCC **migration**, and this led to ectopic neuronal differentiation along both the anteroposterior and dorsoventral axes of the trunk. The choice of migratory pathway did not affect the specification of NCCs, as they retained their commitment to differentiate into sympathetic or sensory neurons, even when they migrated on an ectopic dorsolateral path that is normally taken by melanocyte precursors. We conclude that NRP1 signaling coordinates pathway choice with NCC fate and therefore confines neuronal differentiation to appropriate locations.
189. Loss-of-function mutations in SOX10 cause Kallmann syndrome with deafness. Transcription factor SOX10 plays a role in the maintenance of progenitor cell multipotency, lineage specification, and cell differentiation and is a major actor in the development of the neural crest. It has been implicated in Waardenburg syndrome (WS), a rare disorder characterized by the association between pigmentation abnormalities and deafness, but SOX10 mutations cause a variable phenotype that spreads over the initial limits of the syndrome definition. On the basis of recent findings of olfactory-bulb agenesis in WS individuals, we suspected SOX10 was also involved in Kallmann syndrome (KS). KS is defined by the association between anosmia and hypogonadotropic hypogonadism due to incomplete **migration** of neuroendocrine gonadotropin-releasing hormone (GnRH) cells along the olfactory, vomeronasal, and terminal nerves. Mutations in any of the nine genes identified to date account for only 30% of the KS cases. KS can be either isolated or associated with a variety of other symptoms, including deafness. This study reports SOX10 loss-of-function mutations in approximately one-third of KS individuals with deafness, indicating a substantial involvement in this clinical condition. Study of SOX10-null mutant mice revealed a developmental role of SOX10 in a subpopulation of glial cells called olfactory ensheathing cells. These mice indeed showed an almost complete absence of these cells along the olfactory nerve pathway, as well as defasciculation and misrouting of the nerve fibers, impaired **migration** of GnRH cells, and disorganization of the olfactory nerve layer of the olfactory bulbs.
190. ABBA regulates plasma-membrane and actin dynamics to promote radial glia extension. Radial glia play key roles in neuronal **migration**, axon guidance, and neurogenesis during development of the central nervous system. However, the molecular mechanisms regulating growth and morphology of these extended cells are unknown. We show that ABBA, a novel member of the IRSp53-MIM protein family, is enriched in different types of radial glia. ABBA binds ATP-actin monomers with high affinity and deforms PtdIns(4,5)P(2)-rich membranes in vitro through its WH2 and IM domains, respectively. In radial-glia-like C6-R cells, ABBA localises to the interface between the actin cytoskeleton and plasma membrane, and its depletion by RNAi led to defects in lamellipodial dynamics and process extension. Together, this study identifies ABBA as a novel regulator of actin and plasma membrane dynamics in radial glial cells, and provides evidence that membrane binding and deformation activity is critical for the cellular functions of IRSp53-MIM-ABBA family proteins.
191. Abnormalities in neural crest cell **migration** in laminin alpha5 mutant mice. Although numerous in vitro experiments suggest that extracellular matrix molecules like laminin can influence neural crest **migration**, little is known about their function in the embryo. Here, we show that laminin alpha5, a gene up-regulated during neural crest induction, is localized in regions of newly formed cranial and trunk neural folds and adjacent neural crest migratory pathways in a manner largely conserved between chick and mouse. In laminin alpha5 mutant mice, neural crest migratory streams appear expanded in width compared to wild type. Conversely, neural folds exposed to laminin alpha5 in vitro show a reduction by half in the number of migratory neural crest cells. During gangliogenesis, laminin alpha5 mutants exhibit defects in condensing cranial sensory and trunk sympathetic ganglia. However, ganglia apparently recover at later stages. These data suggest that the laminin alpha5 subunit functions as a cue that restricts neural crest cells, focusing their migratory pathways and condensation into ganglia. Thus, it is required for proper **migration** and timely differentiation of some neural crest populations.
192. Allocation of paraventricular and supraoptic neurons requires Sim1 function: a role for a Sim1 downstream gene PlexinC1. SIM1 is a transcription factor essential for the developmental expression of the endocrine hormone genes, e.g. vasopressin (Vp) and oxytocin (Ot), in the paraventricular nucleus (PVN) and supraoptic nucleus (SON) of the hypothalamus. Mice mutant for Sim1 lack structural PVN and SON, attributed in previous studies to the death of the PVN/SON progenitor cells. Here, we use a tau-LacZ knock-in allele at the Sim1 locus to trace Sim1 mutant cells and show that they are generated normally and survive to birth, contrasting to the previous proposal. Mutant cells adopt neuronal characteristics and maintain their PVN/SON identity as they continue to express PVN/SON progenitor markers. However, they occupy an ectopic position between the normal PVN and SON, indicating a defect in neuronal **migration**. To explore candidate molecular cues that contribute to PVN/SON neuronal **migration**, we focused on the Plexin family of genes. We found that PlexinA1 is expressed in regions surrounding the PVN and SON, whereas PlexinC1 is expressed within the PVN and SON. PlexinA1 expression becomes up-regulated in Sim1 mutant cells, whereas PlexinC1 expression is down-regulated. Finally, the PlexinC1 mutant has a selective defect in partitioning the VP and OT neurons coherently into the PVN and SON. Together, our results uncover a transcriptional regulation of neuronal **migration** cues initiated by Sim1 that contribute to the organization of the PVN and SON.
193. Disruption of neural progenitors along the ventricular and subventricular zones in periventricular heterotopia. Periventricular heterotopia (PH) is a disorder characterized by neuronal nodules, ectopically positioned along the lateral ventricles of the cerebral cortex. Mutations in either of two human genes, Filamin A (FLNA) or ADP-ribosylation factor guanine exchange factor 2 (ARFGEF2), cause PH (Fox et al. in 'Mutations in filamin 1 prevent **migration** of cerebral cortical neurons in human periventricular heterotopia'. Neuron, 21, 1315-1325, 1998; Sheen et al. in 'Mutations in ARFGEF2 implicate vesicle trafficking in neural progenitor proliferation and **migration** in the human cerebral cortex'. Nat. Genet., 36, 69-76, 2004). Recent studies have shown that mutations in mitogen-activated protein kinase kinase kinase-4 (Mekk4), an indirect interactor with FlnA, also lead to periventricular nodule formation in mice (Sarkisian et al. in 'MEKK4 signaling regulates filamin expression and neuronal **migration**'. Neuron, 52, 789-801, 2006). Here we show that neurons in post-mortem human PH brains migrated appropriately into the cortex, that periventricular nodules were primarily composed of later-born neurons, and that the neuroependyma was disrupted in all PH cases. As studied in the mouse, loss of FlnA or Big2 function in neural precursors impaired neuronal **migration** from the germinal zone, disrupted cell adhesion and compromised neuroepithelial integrity. Finally, the hydrocephalus with hop gait (hyh) mouse, which harbors a mutation in Napa [encoding N-ethylmaleimide-sensitive factor attachment protein alpha (alpha-SNAP)], also develops a progressive denudation of the neuroepithelium, leading to periventricular nodule formation. Previous studies have shown that Arfgef2 and Napa direct vesicle trafficking and fusion, whereas FlnA associates dynamically with the Golgi membranes during budding and trafficking of transport vesicles. Our current findings suggest that PH formation arises from a final common pathway involving disruption of vesicle trafficking, leading to impaired cell adhesion and loss of neuroependymal integrity.
194. A dynamic code of dorsal neural tube genes regulates the segregation between neurogenic and melanogenic neural crest cells. Understanding when and how multipotent progenitors segregate into diverse fates is a key question during embryonic development. The neural crest (NC) is an exemplary model system with which to investigate the dynamics of progenitor cell specification, as it generates a multitude of derivatives. Based on 'in ovo' lineage analysis, we previously suggested an early fate restriction of premigratory trunk NC to generate neural versus melanogenic fates, yet the timing of fate segregation and the underlying mechanisms remained unknown. Analysis of progenitors expressing a Foxd3 reporter reveals that prospective melanoblasts downregulate Foxd3 and have already segregated from neural lineages before e**migration**. When this downregulation is prevented, late-emigrating avian precursors fail to upregulate the melanogenic markers Mitf and MC/1 and the guidance receptor Ednrb2, generating instead glial cells that express P0 and Fabp. In this context, Foxd3 lies downstream of Snail2 and Sox9, constituting a minimal network upstream of Mitf and Ednrb2 to link melanogenic specification with **migration**. Consistent with the gain-of-function data in avians, loss of Foxd3 function in mouse NC results in ectopic melanogenesis in the dorsal tube and sensory ganglia. Altogether, Foxd3 is part of a dynamically expressed gene network that is necessary and sufficient to regulate fate decisions in premigratory NC. Their timely downregulation in the dorsal neural tube is thus necessary for the switch between neural and melanocytic phases of NC development.
195. Integrity of developing spinal motor columns is regulated by neural crest derivatives at motor exit points. Spinal motor neurons must extend their axons into the periphery through motor exit points (MEPs), but their cell bodies remain within spinal motor columns. It is not known how this partitioning is established in development. We show here that motor neuron somata are confined to the CNS by interactions with a neural crest subpopulation, boundary cap (BC) cells that prefigure the sites of spinal MEPs. Elimination of BC cells by surgical or targeted genetic ablation does not perturb motor axon outgrowth but results in motor neuron somata migrating out of the spinal cord by translocating along their axons. Heterologous neural crest grafts in crest-ablated embryos stop motor neuron e**migration**. Thus, before the formation of a mature transitional zone at the MEP, BC cells maintain a cell-tight boundary that allows motor axons to cross but blocks neuron **migration**.
196. SPARC is expressed by macroglia and microglia in the developing and mature nervous system. SPARC (secreted protein, acidic and rich in cysteine) is a matricellular protein that is highly expressed during development, tissue remodeling, and repair. SPARC produced by olfactory ensheathing cells (OECs) can promote axon sprouting in vitro and in vivo. Here, we show that in the developing nervous system of the mouse, SPARC is expressed by radial glia, blood vessels, and other pial-derived structures during embryogenesis and postnatal development. The rostral migratory stream contains SPARC that becomes progressively restricted to the SVZ in adulthood. In the adult CNS, SPARC is enriched in specialized radial glial derivatives (Müller and Bergmann glia), microglia, and brainstem astrocytes. The peripheral glia, Schwann cells, and OECs express SPARC throughout development and in maturity, although it appears to be down-regulated with maturation. These data suggest that SPARC may be expressed by glia in a spatiotemporal manner consistent with a role in cell **migratio**n, neurogenesis, synaptic plasticity, and angiogenesis.
197. Neuropilin receptors guide distinct phases of sensory and motor neuronal segmentation. The segmented trunk peripheral nervous system is generated by ventrally migrating neural crest cells that exclusively invade the anterior sclerotome and differentiate into metameric dorsal root and sympathetic ganglia. Meanwhile, ventral spinal motor axons also project through the somites in a segmental fashion. How peripheral nervous system segmentation is generated is unknown. We previously showed that neuropilin 2 (Nrp2)/semaphorin 3F (Sema3F) signaling is required for segmental neural crest **migration**, but not for metameric dorsal root gangliogenesis. We now expand these results to show that Nrp2 patterns initial motor axon outgrowth as well. Later, Nrp1/Sema3A signaling is essential for segmental dorsal root gangliogenesis and motor axonal fasciculation into ventral roots. Strikingly, Nrp/Sema signaling is not required for sympathetic ganglia segmentation. These data show that Nrp2 and Nrp1 work together to produce segmentation of sensory and motor nerves, and that dorsal peripheral nervous system metamerism is generated in a stepwise, Nrp-dependent process.
198. Expression of Kv1.1, a Shaker-like potassium channel, is temporally regulated in embryonic neurons and glia. Kv1.1, a Shaker-like voltage-gated potassium channel, is strongly expressed in a variety of neurons in adult rodents, in which it appears to be involved in regulating neuronal excitability. Here we show that Kv1.1 is also expressed during embryonic development in the mouse, exhibiting two transient peaks of expression around embryonic day 9.5 (E9.5) and E14.5. Using both in situ hybridization and immunocytochemistry, we have identified several cell types and tissues that express Kv1.1 RNA and protein. At E9.5, Kv1.1 RNA and protein are detected transiently in non-neuronal cells in several regions of the early CNS, including rhombomeres 3 and 5 and ventricular zones in the mesencephalon and diencephalon. At E14.5, several cell types in both the CNS and peripheral nervous system express Kv1.1, including neuronal cells (sensory ganglia and outer aspect of cerebral hemispheres) and glial cells (radial glia, satellite cells, and Schwann cell precursors). These data show that Kv1.1 is expressed transiently in a variety of neuronal and non-neuronal cells during restricted periods of embryonic development. Although the functional roles of Kv1.1 in development are not understood, the cell-specific localization and timing of expression suggest this channel may play a role in several developmental processes, including proliferation, **migration**, or cell-cell adhesion.
199. Expression of MUK/DLK/ZPK, an activator of the JNK pathway, in the nervous systems of the developing mouse embryo. C-Jun N-terminal kinase (JNK) is implicated in regulating the various cellular events during neural development that include differentiation, apoptosis and **migration**. MUK/DLK/ZPK is a MAP kinase kinase kinase (MAPKKK) enzyme that activates JNK via MAP kinase kinases (MAPKK) such as MKK7. We show here that the expression of MUK/DLK/ZPK protein in the developing mouse embryo is almost totally specific for the neural tissues, including central, peripheral, and autonomic nervous systems. The only obvious exception is the liver, in which the protein is temporally expressed at around E11. The expression becomes obvious in the neurons of the brain and neural crest tissues at embryonic day 10 (E10) after neuron production is initiated. By E14, MUK/DLK/ZPK proteins are found in various neural tissues including the brain, spinal cord, sensory ganglia (such as trigeminal and dorsal root ganglia), and the sympathetic and visceral nerves. The localization of MUK/DLK/ZPK protein in neural cells almost consistently overlapped that of betaIII-tubulin, a neuron specific tubulin isoform, and both proteins were more concentrated in axons than in cell bodies and dendrites. The intensely overlapping localization of betaIII-tubulin and MUK/DLK/ZPK indicated that this protein kinase is tightly associated with the microtubules of neurons.
200. Loss of the Prader-Willi syndrome protein necdin causes defective **migration**, axonal outgrowth, and survival of embryonic sympathetic neurons. Prader-Willi syndrome is a neurodevelopmental disorder marked by abnormalities in feeding, drinking, thermoregulation, intestinal motility, and reproduction, suggesting disruption of the autonomic nervous system. Necdin, one of several proteins genetically inactivated in individuals with Prader-Willi syndrome, is important for the differentiation of central and sensory neurons. We now show that formation, **migration**, and survival of sympathetic superior cervical ganglion neurons are impaired in Ndn-null embryos. We observed reduced innervation of superior cervical ganglion target organs, including the submandibular gland, parotid gland, and nasal mucosa. While the formation of other sympathetic chain ganglia is unaffected, axonal extension is impaired throughout the sympathetic nervous system. These results demonstrate a novel role for necdin in cellular **migration**, in addition to its roles in survival and axon outgrowth. Furthermore, reduced sympathetic function provides a plausible explanation for deficiencies of salivary gland function in individuals with congenital necdin deficiency consequent to Prader-Willi syndrome.
201. Myocardin-related transcription factors regulate the Cdk5/Pctaire1 kinase cascade to control neurite outgrowth, neuronal **migration** and brain development. Numerous motile cell functions depend on signaling from the cytoskeleton to the nucleus. Myocardin-related transcription factors (MRTFs) translocate to the nucleus in response to actin polymerization and cooperate with serum response factor (Srf) to regulate the expression of genes encoding actin and other components of the cytoskeleton. Here, we show that MRTF-A (Mkl1) and MRTF-B (Mkl2) redundantly control neuronal **migration** and neurite outgrowth during mouse brain development. Conditional deletion of the genes encoding these Srf coactivators disrupts the formation of multiple brain structures, reflecting a failure in neuronal actin polymerization and cytoskeletal assembly. These abnormalities were accompanied by dysregulation of the actin-severing protein gelsolin and Pctaire1 (Cdk16) kinase, which cooperates with Cdk5 to initiate a kinase cascade that governs cytoskeletal rearrangements essential for neuron **migration** and neurite outgrowth. Thus, the MRTF/Srf partnership interlinks two key signaling pathways that control actin treadmilling and neuronal maturation, thereby fulfilling a regulatory loop that couples cytoskeletal dynamics to nuclear gene transcription during brain development.
202. Cerebellar defects in Pdss2 conditional knockout mice during embryonic development and in adulthood. PDSS2 is a gene that encodes one of the two subunits of trans-prenyl diphosphate synthase that is essential for ubiquinone biosynthesis. It is known that mutations in PDSS2 can cause primary ubiquinone deficiency in humans and a similar disease in mice. Cerebellum is the most often affected organ in ubiquinone deficiency, and cerebellar atrophy has been diagnosed in many infants with this disease. In this study, two Pdss2 conditional knockout mouse lines directed by Pax2-cre and Pcp2-cre were generated to investigate the effect of ubiquinone deficiency on cerebellum during embryonic development and in adulthood, respectively. The Pdss2(f/-); Pax2-cre mouse recapitulates some symptoms of ubiquinone deficiency in infants, including severe cerebellum hypoplasia and lipid accumulation in skeletal muscles at birth. During early cerebellum development (E12.5-14.5), Pdss2 knockout initially causes the delay of radial glial cell growth and neuron progenitor **migration**, so the growth of mutant cerebellum is retarded. During later development (E15.5-P0), increased ectopic apoptosis of neuroblasts and impaired cell proliferation result in the progression of cerebellum hypoplasia in the mutant. Thus, the mutant cerebellum contains fewer neurons at birth, and the cells are disorganized. The developmental defect of mutant cerebellum does not result from reduced Fgf8 expression before E12.5. Electron microscopy reveals mitochondrial defects and increased autophagic-like vacuolization that may arise in response to abnormal mitochondria in the mutant cerebellum. Nevertheless, the mutant mice die soon after birth probably due to cleft palate and micrognathia, which may result from Pdss2 knockout caused by ectopic Pax2-cre expression in the first branchial arch. On the other hand, the Pdss2(f/-); Pcp2-cre mouse is healthy at birth but gradually loses cerebellar Purkinje cells and develops ataxia-like symptoms at 9.5 months; thus this conditional knockout mouse may serve as a model for ubiquinone deficiency in adult patients. In conclusion, this study provides two mouse models of Pdss2 based ubiquinone deficiency. During cerebellum development, Pdss2 knockout results in severe cerebellum hypoplasia by impairing cell **migration** and eliciting ectopic apoptosis, whereas Pdss2 knockout in Purkinje cells at postnatal stages leads to the development of cerebellar ataxia.
203. Cajal-Retzius cells instruct neuronal **migration** by coincidence signaling between secreted and contact-dependent guidance cues. Cajal-Retzius (CR) cells are a transient cell population of the CNS that is critical for brain development. In the neocortex, CR cells secrete reelin to instruct the radial **migration** of projection neurons. It has remained unexplored, however, whether CR cells provide additional molecular cues important for brain development. Here, we show that CR cells express the immunoglobulin-like adhesion molecule nectin1, whereas neocortical projection neurons express its preferred binding partner, nectin3. We demonstrate that nectin1- and nectin3-mediated interactions between CR cells and migrating neurons are critical for radial **migration**. Furthermore, reelin signaling to Rap1 promotes neuronal Cdh2 function via nectin3 and afadin, thus directing the broadly expressed homophilic cell adhesion molecule Cdh2 toward mediating heterotypic cell-cell interactions between neurons and CR cells. Our findings identify nectins and afadin as components of the reelin signaling pathway and demonstrate that coincidence signaling between CR cell-derived secreted and short-range guidance cues direct neuronal **migration**.
204. GPR56 regulates pial basement membrane integrity and cortical lamination. GPR56 is a member of the family of adhesion G-protein-coupled receptors that have a large extracellular region containing a GPS (G-protein proteolytic site) domain. Loss-of-function mutations in the GPR56 gene cause a specific human brain malformation called bilateral frontoparietal polymicrogyria (BFPP). BFPP is a radiological diagnosis and its histopathology remains unclear. This study demonstrates that loss of the mouse Gpr56 gene leads to neuronal ectopia in the cerebral cortex, a cobblestone-like cortical malformation. There are four crucial events in the development of cobblestone cortex, namely defective pial basement membrane (BM), abnormal anchorage of radial glial endfeet, mislocalized Cajal-Retzius cells, and neuronal over**migration**. By detailed time course analysis, we reveal that the leading causal events are likely the breaches in the pial BM. We show further that GPR56 is present in abundance in radial glial endfeet. Furthermore, a putative ligand of GPR56 is localized in the marginal zone or overlying extracellular matrix. These observations provide compelling evidence that GPR56 functions in regulating pial BM integrity during cortical development.
205. Retinoic acid regulates murine enteric nervous system precursor proliferation, enhances neuronal precursor differentiation, and reduces neurite growth in vitro. Enteric nervous system (ENS) precursors undergo a complex process of cell **migration**, proliferation, and differentiation to form an integrated network of neurons and glia within the bowel wall. Although retinoids regulate ENS development, molecular and cellular mechanisms of retinoid effects on the ENS are not well understood. We hypothesized that retinoids might directly affect ENS precursor differentiation and proliferation, and tested that hypothesis using immunoselected fetal ENS precursors in primary culture. We now demonstrate that all retinoid receptors and many retinoid biosynthetic enzymes are present in the fetal bowel at about the time that migrating ENS precursors reach the distal bowel. We further demonstrate that retinoic acid (RA) enhances proliferation of subsets of ENS precursors in a time-dependent fashion and increases neuronal differentiation. Surprisingly, however, enteric neurons that develop in retinoid deficient media have dramatically longer neurites than those exposed to RA. This difference in neurite growth correlates with increased RhoA protein at the neurite tip, decreased Smurf1 (a protein that targets RhoA for degradation), and dramatically decreased Smurf1 mRNA in response to RA. Collectively these data demonstrate diverse effects of RA on ENS precursor development and suggest that altered fetal retinoid availability or metabolism could contribute to intestinal motility disorders.
206. Mutations in the β-tubulin gene TUBB5 cause microcephaly with structural brain abnormalities. The formation of the mammalian cortex requires the generation, **migratio**n, and differentiation of neurons. The vital role that the microtubule cytoskeleton plays in these cellular processes is reflected by the discovery that mutations in various tubulin isotypes cause different neurodevelopmental diseases, including lissencephaly (TUBA1A), polymicrogyria (TUBA1A, TUBB2B, TUBB3), and an ocular motility disorder (TUBB3). Here, we show that Tubb5 is expressed in neurogenic progenitors in the mouse and that its depletion in vivo perturbs the cell cycle of progenitors and alters the position of migrating neurons. We report the occurrence of three microcephalic patients with structural brain abnormalities harboring de novo mutations in TUBB5 (M299V, V353I, and E401K). These mutant proteins, which affect the chaperone-dependent assembly of tubulin heterodimers in different ways, disrupt neurogenic division and/or **migratio**n in vivo. Our results provide insight into the functional repertoire of the tubulin gene family, specifically implicating TUBB5 in embryonic neurogenesis and microcephaly.
207. Premature expression of KCC2 in embryonic mice perturbs neural development by an ion transport-independent mechanism. During neuronal maturation, the neuron-specific K-Cl co-transporter KCC2 lowers the intracellular chloride and thereby renders GABAergic transmission hyperpolarizing. Independently of its role as a co-transporter, KCC2 plays a crucial role in the maturation of dendritic spines, most probably via an interaction with the cytoskeleton-associated protein 4.1N. In this study, we show that neural-specific overexpression of KCC2 impairs the development of the neural tube- and neural crest-related structures in mouse embryos. At early stages (E9.5-11.5), the transgenic embryos had a thinner neural tube and abnormal body curvature. They displayed a reduced neuronal differentiation and altered neural crest cell pattern. At later stages (E11.5-15.5), the transgenic embryos had smaller brain structures and a distinctive cleft palate. Similar results were obtained using overexpression of a transport-inactive N-terminal-deleted variant of KCC2, implying that the effects were not dependent on KCC2's role as a K-Cl co-transporter. Interestingly, the neural tube of transgenic embryos had an aberrant cytoplasmic distribution of 4.1N and actin. This was corroborated in a neural stem cell line with ectopic expression of KCC2. Embryo phenotype and cell morphology were unaffected by a mutated variant of KCC2 which is unable to bind 4.1N. These results point to a role of KCC2 in neuronal differentiation and **migration** during early development mediated by its direct structural interactions with the neuronal cytoskeleton.
208. Disrupted-in-Schizophrenia-1 expression is regulated by beta-site amyloid precursor protein cleaving enzyme-1-neuregulin cascade. Neuregulin-1 (NRG1) and Disrupted-in-Schizophrenia-1 (DISC1) are promising susceptibility factors for schizophrenia. Both are multifunctional proteins with roles in a variety of neurodevelopmental processes, including progenitor cell proliferation, **migration**, and differentiation. Here, we provide evidence linking these factors together in a single pathway, which is mediated by ErbB receptors and PI3K/Akt. We show that signaling by NRG1 and NRG2, but not NRG3, increase expression of an isoform of DISC1 in vitro. Receptors ErbB2 and ErbB3, but not ErbB4, are responsible for transducing this effect, and PI3K/Akt signaling is also required. In NRG1 knockout mice, this DISC1 isoform is selectively reduced during neurodevelopment. Furthermore, a similar decrease in DISC1 expression is seen in beta-site amyloid precursor protein cleaving enzyme-1 (BACE1) knockout mice, in which NRG1/Akt signaling is reportedly impaired. In contrast to neuronal DISC1 that was reported and characterized, expression of DISC1 in other types of cells in the brain has not been addressed. Here we demonstrate that DISC1, like NRG and ErbB proteins, is expressed in neurons, astrocytes, oligodendrocytes, microglia, and radial progenitors. These findings may connect NRG1, ErbBs, Akt, and DISC1 in a common pathway, which may regulate neurodevelopment and contribute to susceptibility to schizophrenia.
209. Dosage effects of cohesin regulatory factor PDS5 on mammalian development: implications for cohesinopathies. Cornelia de Lange syndrome (CdLS), a disorder caused by mutations in cohesion proteins, is characterized by multisystem developmental abnormalities. PDS5, a cohesion protein, is important for proper chromosome segregation in lower organisms and has two homologues in vertebrates (PDS5A and PDS5B). Pds5B mutant mice have developmental abnormalities resembling CdLS; however the role of Pds5A in mammals and the association of PDS5 proteins with CdLS are unknown. To delineate genetic interactions between Pds5A and Pds5B and explore mechanisms underlying phenotypic variability, we generated Pds5A-deficient mice. Curiously, these mice exhibit multiple abnormalities that were previously observed in Pds5B-deficient mice, including cleft palate, skeletal patterning defects, growth retardation, congenital heart defects and delayed **migration** of enteric neuron precursors. They also frequently display renal agenesis, an abnormality not observed in Pds5B(-/-) mice. While Pds5A(-/-) and Pds5B(-/-) mice die at birth, embryos harboring 3 mutant Pds5 alleles die between E11.5 and E12.5 most likely of heart failure, indicating that total Pds5 gene dosage is critical for normal development. In addition, characterization of these compound homozygous-heterozygous mice revealed a severe abnormality in lens formation that does not occur in either Pds5A(-/-) or Pds5B(-/-) mice. We further identified a functional missense mutation (R1292Q) in the PDS5B DNA-binding domain in a familial case of CdLS, in which affected individuals also develop megacolon. This study shows that PDS5A and PDS5B functions other than those involving chromosomal dynamics are important for normal development, highlights the sensitivity of key developmental processes on PDS5 signaling, and provides mechanistic insights into how PDS5 mutations may lead to CdLS.
210. Distinct cortical **migration**s from the medial and lateral ganglionic eminences. Recent evidence suggests that projection neurons and interneurons of the cerebral cortex are generally derived from distinct proliferative zones. Cortical projection neurons originate from the cortical ventricular zone (VZ), and then migrate radially into the cortical mantle, whereas most cortical interneurons originate from the basal telencephalon and migrate tangentially into the developing cortex. Previous studies using methods that label both proliferative and postmitotic cells have found that cortical interneurons migrate from two major subdivisions of the developing basal telencephalon: the medial and lateral ganglionic eminences (MGE and LGE). Since these studies labeled cells by methods that do not distinguish between the proliferating cells and those that may have originated elsewhere, we have studied the contribution of the MGE and LGE to cortical interneurons using fate mapping and genetic methods. Transplantation of BrdU-labeled MGE or LGE neuroepithelium into the basal telencephalon of unlabeled telencephalic slices enabled us to follow the fate of neurons derived from each of these primordia. We have determined that early in neurogenesis GABA-expressing cells from the MGE tangentially migrate into the cerebral cortex, primarily via the intermediate zone, whereas cells from the LGE do not. Later in neurogenesis, LGE-derived cells also migrate into the cortex, although this **migration** occurs primarily through the subventricular zone. Some of these LGE-derived cells invade the cortical plate and express GABA, while others remain within the cortical proliferative zone and appear to become mitotically active late in gestation. In addition, by comparing the phenotypes of mouse mutants with differential effects on MGE and LGE **migration**, we provide evidence that the MGE and LGE may give rise to different subtypes of cortical interneurons.
211. Impaired cell cycle control of neuronal precursor cells in the neocortical primordium of presenilin-1-deficient mice. Recent studies have implicated presenilin-1 (PS-1) in the processing of the amyloid precursor protein and Notch-1. We show that PS-1 has biological effects on differentiation and cell cycle control of neuronal precursor cells in vivo using PS-1-deficient mice. The expression of Class III beta-tubulin was upregulated throughout the neocortical primordia of PS-1-deficient E14 embryos, especially on the ventricular surface. The increased speed of **migration** of the immature neurons from the ventricular zone outward in the PS-1-deficient neocortical primordia was indicated by an in vivo bromodeoxyuridine (BrdU)-labeling assay and a DiI-labeling assay in slice culture. Furthermore, we investigated the cell cycle of neuronal precursor cells in the neocortical ventricular zone using an in vivo cumulative BrdU-labeling assay. The length of the cell cycle in the neocortical precursor cells of wild-type mice was 11.4 hr whereas that of the PS-1-deficient mice was 15.4 hr. Among all phases of the cell cycle, S-phase exhibited the most prominent change in length, increasing from 2.4 hr in the wild-type mice to 7.4 hr in the mutant mice. The distribution of beta-catenin was specifically affected in the ventricular zone of the PS-1-deficient mice. These findings suggest that PS-1 is involved in the differentiation and the cell cycle control of neuronal precursor cells in the ventricular proliferating zone of the neocortical primordium.
212. Disruption of segmental neural crest **migration** and ephrin expression in delta-1 null mice. Neural crest cells migrate segmentally through the rostral half of each trunk somite due to inhibitory influences of ephrins and other molecules present in the caudal-half of somites. To examine the potential role of Notch/Delta signaling in establishing the segmental distribution of ephrins, we examined neural crest **migration** and ephrin expression in Delta-1 mutant mice. Using Sox-10 as a marker, we noted that neural crest cells moved through both rostral and caudal halves of the somites in mutants, consistent with the finding that ephrinB2 levels are significantly reduced in the caudal-half somites. Later, mutant embryos had aberrantly fused and/or reduced dorsal root and sympathetic ganglia, with a marked diminution in peripheral glia. These results show that Delta-1 is essential for proper **migration** and differentiation of neural crest cells. Interestingly, absence of Delta-1 leads to diminution of both neurons and glia in peripheral ganglia, suggesting a general depletion of the ganglion precursor pool in mutant mice.
213. Neural RNA-binding protein Musashi1 controls midline crossing of precerebellar neurons through posttranscriptional regulation of Robo3/Rig-1 expression. Precisely regulated spatiotemporal gene expression is essential for the establishment of neural circuits. In contrast to the increasing evidence for transcriptional regulation of axon guidance cues and receptors, the role of posttranscriptional regulation in axon guidance, especially in vivo, remains poorly characterized. Here, we demonstrate that the expression of Slit receptor Robo3/Rig-1, which plays crucial roles in axonal midline crossing, is regulated by a neural RNA-binding protein Musashi1 (Msi1). Msi1 binds to Robo3 mRNA through RNA recognition motifs and increases the protein level of Robo3 without affecting its mRNA level. In Msi1-deficient precerebellar neurons, Robo3 protein, but not its mRNA, is dramatically reduced. Moreover, similar to defects in Robo3-deficient mice, axonal midline crossing and neuronal **migration** of precerebellar neurons are severely impaired in Msi1-deficient mice. Together, these findings indicate that Msi1-mediated posttranscriptional regulation of Robo3 controls midline crossing of precerebellar neurons.
214. The SRC homology 2 domain protein Shep1 plays an important role in the penetration of olfactory sensory axons into the forebrain. Shep1 is a multidomain signaling protein that forms a complex with Cas, a key scaffolding component of integrin signaling pathways, to promote the **migration** of non-neuronal cells. However, the physiological function of Shep1 in the nervous system remains unknown. Interestingly, we found that Shep1 and Cas are both concentrated in the axons of developing olfactory sensory neurons (OSNs). These neurons extend their axons from the olfactory epithelium to the olfactory bulb located at the anterior tip of the forebrain. However, in developing Shep1 knock-out mice, we did not detect penetration of OSN axons across the pial basement membrane surrounding the olfactory bulb, suggesting that Shep1 function is important for the establishment of OSN connections with the olfactory bulb. Interestingly, we observed reduced levels of Cas tyrosine phosphorylation in OSN axons of Shep1 knock-out mice, suggesting compromised Cas signaling function. Indeed, when embedded in a three-dimensional gel of basement membrane proteins, explants from Shep1 knock-out olfactory epithelium extend neuronal processes less efficiently than explants from control epithelium. Furthermore, ectopic expression of Shep1 in non-neuronal cells promotes cell **migration** through a collagen gel. Later in development, loss of Shep1 function also causes a marked reduction in olfactory bulb size and disruption of bulb lamination, which may be primarily attributable to the defective innervation. The greatly reduced OSN connections and hypoplasia of the olfactory bulb, likely resulting in anosmia, are reminiscent of the symptoms of Kallmann syndrome, a human developmental disease that can be caused by mutations in a growing number of genes.
215. GDNF is required for neural colonization of the pancreas. The mammalian pancreas is densely innervated by both the sympathetic and parasympathetic nervous systems, which control exocrine and endocrine secretion. During embryonic development, neural crest cells migrating in a rostrocaudal direction populate the gut, giving rise to neural progenitor cells. Recent studies in mice have shown that neural crest cells enter the pancreatic epithelium at E11.5. However, the cues that guide the **migration** of neural progenitors into the pancreas are poorly defined. In this study we identify glial cell line-derived neurotrophic factor (GDNF) as a key player in this process. GDNF displays a dynamic expression pattern during embryonic development that parallels the chronology of **migration** and differentiation of neural crest derivatives in the pancreas. Conditional inactivation of Gdnf in the pancreatic epithelium results in a dramatic loss of neuronal and glial cells and in reduced parasympathetic innervation in the pancreas. Importantly, the innervation of other regions of the gut remains unaffected. Analysis of Gdnf mutant mouse embryos and ex vivo experiments indicate that GDNF produced in the pancreas acts as a neurotrophic factor for gut-resident neural progenitor cells. Our data further show that exogenous GDNF promotes the proliferation of pancreatic progenitor cells in organ culture. In summary, our results point to GDNF as crucial for the development of the intrinsic innervation of the pancreas.
216. Peripheral contributions to olfactory bulb cell populations (**migration**s towards the olfactory bulb). The olfactory system represents one of the most suitable models to study interactions between the peripheral and central nervous systems. The developing olfactory epithelium (olfactory placode and pit) gives rise to several cell populations that migrate towards the telencephalic vesicle. One of these cell populations, called the Migratory Mass (MM), accompanies the first emerging olfactory axons from the olfactory placode, but the fate of these cells and their contribution to the Olfactory Bulb (OB) populations has not been properly addressed. To asses this issue we performed ultrasound-guided in utero retroviral injections at embryonic day (E) 11 revealing the MM as an early source of Olfactory Ensheathing Cells in later postnatal stages. Employing a wide number of antibodies to identify the nature of the infected cells we described that those cells generated within the MM at E11 belong to different cell populations both in the mesenchyma, where they envelop olfactory axons and express the most common glial markers, and in the olfactory bulb, where they are restricted to the Olfactory Nerve and Glomerular layers. Thus, the data reveal the existence of a novel progenitor class within the MM, potentially derived from the olfactory placode which gives rise to different neural cell population including some CNS neurons, glia and olfactory ensheathing cells.
217. Functional dicer is necessary for appropriate specification of radial glia during early development of mouse telencephalon. Early telencephalic development involves transformation of neuroepithelial stem cells into radial glia, which are themselves neuronal progenitors, around the time when the tissue begins to generate postmitotic neurons. To achieve this transformation, radial precursors express a specific combination of proteins. We investigate the hypothesis that micro RNAs regulate the ability of the early telencephalic progenitors to establish radial glia. We ablate functional Dicer, which is required for the generation of mature micro RNAs, by conditionally mutating the Dicer1 gene in the early embryonic telencephalon and analyse the molecular specification of radial glia as well as their progeny, namely postmitotic neurons and basal progenitors. Conditional mutation of Dicer1 from the telencephalon at around embryonic day 8 does not prevent morphological development of radial glia, but their expression of Nestin, Sox9, and ErbB2 is abnormally low. The population of basal progenitors, which are generated by the radial glia, is disorganised and expanded in Dicer1⁻/⁻ dorsal telencephalon. While the proportion of cells expressing markers of postmitotic neurons is unchanged, their laminar organisation in the telencephalic wall is disrupted suggesting a defect in radial glial gui**ded migra**tion. We found that the laminar disruption could not be accounted for by a reduction of the population of Cajal Retzius neurons. Together, our data suggest novel roles for micro RNAs during early development of progenitor cells in the embryonic telencephalon.
218. Homeodomain interacting protein kinase 2 regulates postnatal development of enteric dopaminergic neurons and glia via BMP signaling. Trophic factor signaling is important for the **migration**, differentiation, and survival of enteric neurons during development. The mechanisms that regulate the maturation of enteric neurons in postnatal life, however, are poorly understood. Here, we show that transcriptional cofactor HIPK2 (homeodomain interacting protein kinase 2) is required for the maturation of enteric neurons and for regulating gliogenesis during postnatal development. Mice lacking HIPK2 display a spectrum of gastrointestinal (GI) phenotypes, including distention of colon and slowed GI transit time. Although loss of HIPK2 does not affect the enteric neurons in prenatal development, a progressive loss of enteric neurons occurs during postnatal life in Hipk2(-/-) mutant mice that preferentially affects the dopaminergic population of neurons in the caudal region of the intestine. The mechanism by which HIPK2 regulates postnatal enteric neuron development appears to involve the response of enteric neurons to bone morphogenetic proteins (BMPs). Specifically, compared to wild type mice, a larger proportion of enteric neurons in Hipk2(-/-) mutants have an abnormally high level of phosphorylated Smad1/5/8. Consistent with the ability of BMP signaling to promote gliogenesis, Hipk2(-/-) mutants show a significant increase in glia in the enteric nervous system. In addition, numbers of autophagosomes are increased in enteric neurons in Hipk2(-/-) mutants, and synaptic maturation is arrested. These results reveal a new role for HIPK2 as an important transcriptional cofactor that regulates the BMP signaling pathway in the maintenance of enteric neurons and glia, and further suggest that HIPK2 and its associated signaling mechanisms may be therapeutically altered to promote postnatal neuronal maturation.
219. Composition of the migratory mass during development of the olfactory nerve. The embryonic development of the olfactory nerve includes the differentiation of cells within the olfactory placode, **migration** of cells into the mesenchyme from the placode, and extension of axons by the olfactory sensory neurons (OSNs). The coalition of both placode-derived migratory cells and OSN axons within the mesenchyme is collectively termed the "migratory mass." Here we address the sequence and coordination of the events that give rise to the migratory mass. Using neuronal and developmental markers, we show subpopulations of neurons emerging from the placode by embryonic day (E)10, a time at which the migratory mass is largely cellular and only a few isolated OSN axons are seen, prior to the first appearance of OSN axon fascicles at E11. These neurons also precede the emergence of the gonadotropin-releasing hormone neurons and ensheathing glia which are also resident in the mesenchyme as part of the migratory mass beginning at about E11. The data reported here begin to establish a spatiotemporal framework for the **migration** of molecularly heterogeneous placode-derived cells in the mesenchyme. The precocious e**migration** of the early arriving neurons in the mesenchyme suggests they may serve as "guidepost cells" that contribute to the establishment of a scaffold for the extension and coalescence of the OSN axons.
220. Fate of mesencephalic AHD2-expressing dopamine progenitor cells in NURR1 mutant mice. The orphan nuclear receptor NURR1 was previously demonstrated to be required for the generation of mesencephalic dopamine (DA) cells. However, even in the absence of NURR1, which is normally expressed as cells become postmitotic, neuronal differentiation is induced and expression of several genes detected in developing dopamine cells appears normal during early stages of development. These include the homeobox transcription factors engrailed and Ptx-3 as well as aldehyde dehydrogenase 2, here defined as the earliest marker identified in developing DA cells, expressed already in mitotic DA progenitors. We have used the expression of these dopaminergic markers, retrograde axonal tracing, and apoptosis analyses to study the fate of the DA progenitor cells in the absence of NURR1. We conclude that NURR1 plays a critical role in the maturation, **migration**, striatal target area innervation, and survival of differentiating mesencephalic DA cells.
221. Vascularisation is not necessary for gut colonisation by enteric neural crest cells. The vasculature and nervous system share striking similarities in their networked, tree-like architecture and in the way they are super-imposed in mature organs. It has previously been suggested that the intestinal microvasculature network directs the **migration** of enteric neural crest cells (ENCC) along the gut to promote the formation of the enteric nervous system (ENS). To investigate the inter-relationship of migrating ENCC, ENS formation and gut vascular development we combined fate-mapping of ENCC with immunolabelling and intravascular dye injection to visualise nascent blood vessel networks. We found that the enteric and vascular networks initially had very distinct patterns of development. In the foregut, ENCC migrated through areas devoid of established vascular networks. In vessel-rich areas, such as the midgut and hindgut, the distribution of migrating ENCC did not support the idea that these cells followed a pre-established vascular network. Moreover, when gut vascular development was impaired, either genetically in Vegfa(120/120) or Tie2-Cre;Nrp1(fl/-) mice or using an in vitro Wnt1-Cre;Rosa26(Yfp/+) mouse model of ENS development, ENCC still colonised the entire length of the gut, including the terminal hindgut. These results demonstrate that blood vessel networks are not necessary to guide migrating ENCC during ENS development. Conversely, in miRet(51) mice, which lack ENS in the hindgut, the vascular network in this region appeared to be normal suggesting that in early development both networks form independently of each other.
222. Developmental dynamics of PAFAH1B subunits during mouse brain development. Platelet-activating factor (PAF) mediates an array of biological processes in the mammalian central nervous system as a bioactive lipid messenger in synaptic function and dysfunction (plasticity, memory, and neurodegeneration). The intracellular enzyme that deacetylates the PAF (PAFAH1B) is composed of a tetramer of two catalytic subunits, ALPHA1 (PAFAH1B3) and ALPHA2 (PAFAH1B2), and a regulatory dimer of LIS1 (PAFAH1B1). We have investigated the mouse PAFAH1B subunit genes during brain development in normal mice and in mice with a hypomorphic allele for Lis1 (Lis1/sLis1; Cahana et al. [2001] Proc Natl Acad Sci U S A 98:6429-6434). We have analyzed quantitatively (by means of real-time polymerase chain reaction) and qualitatively (by in situ hybridization techniques) the amounts and expression patterns of their transcription in developing and postnatal brain, focusing mainly on differences in two laminated encephalic regions, the forebrain (telencephalon) and hindbrain (cerebellum) separately. The results revealed significant differences in cDNA content between these two brain subdivisions but, more importantly, between the LIS1 complex subunits. In addition, we found significant spatial differences in gene expression patterns. Comparison of results obtained with Lis1/sLis1 analysis also revealed significant temporal and spatial differences in Alpha1 and Lis1 expression levels. Thus, small changes in the amount of the Lis1 gene may differentially regulate expression of Alpha1 and Alpha2, depending on the brain region, which suggests different roles for each LIS1 complex subunit during neural differentiation and neural **migration**.
223. Tangential **migration** and proliferation of intermediate progenitors of GABAergic neurons in the mouse telencephalon. In the embryonic neocortex, neuronal precursors are generated in the ventricular zone (VZ) and accumulate in the cortical plate. Recently, the subventricular zone (SVZ) of the embryonic neocortex was recognized as an additional neurogenic site for both principal excitatory neurons and GABAergic inhibitory neurons. To gain insight into the neurogenesis of GABAergic neurons in the SVZ, we investigated the characteristics of intermediate progenitors of GABAergic neurons (IPGNs) in mouse neocortex by immunohistochemistry, immunocytochemistry, single-cell RT-PCR and single-cell array analysis. IPGNs were identified by their expression of some neuronal and cell cycle markers. Moreover, we investigated the origins of the neocortical IPGNs by Cre-loxP fate mapping in transgenic mice and the transduction of part of the telencephalic VZ by Cre-reporter plasmids, and found them in the medial and lateral ganglionic eminence. Therefore, they must migrate tangentially within the telencephalon to reach the neocortex. Cell-lineage analysis by simple-retrovirus transduction revealed that the neocortical IPGNs self-renew and give rise to a small number of neocortical GABAergic neurons and to a large number of granule and periglomerular cells in the olfactory bulb. IPGNs are maintained in the neocortex and may act as progenitors for adult neurogenesis.
224. The lack of Emx2 causes impairment of Reelin signaling and defects of neuronal **migration** in the developing cerebral cortex. Neocorticogenesis in mice homozygous for an Emx2 null allele is the topic of this article. The development of both main components of neocortex, primordial plexiform layer derivatives and cortical plate, was analyzed, paying special attention to radial **migration** of neurons forming the cortical plate. The products of the Reelin gene, normally playing a key role in orchestrating radial **migration** of these neurons, display normal distribution at the beginning of the cortical neuronogenesis but are absent in the neocortical marginal zone of the mutant mice at the time when the cortical plate is laid down. As a consequence, the development of radial glia is impaired, and neurons making up the cortical plate display abnormal **migration** patterns. In addition, restricted defects along the rostrocaudal and the mediolateral axes are present in the subplate, suggesting an Emx2-specific role in priming the proper development of this layer.
225. Mcl-1 is a key regulator of apoptosis during CNS development and after DNA damage. Despite the importance of Mcl-1, an anti-apoptotic Bcl-2 family member, in the regulation of apoptosis, little is known regarding its role in nervous system development and injury-induced neuronal cell death. Because germline deletion of Mcl-1 results in peri-implantation lethality, we address the function of Mcl-1 in the nervous system using two different conditional Mcl-1 mouse mutants in the developing nervous system. Here, we show for the first time that Mcl-1 is required for neuronal development. Neural precursors within the ventricular zone and newly committed neurons in the cortical plate express high levels of Mcl-1 throughout cortical neurogenesis. Loss of Mcl-1 in neuronal progenitors results in widespread apoptosis. Double labeling with active caspase 3 and Tuj1 reveals that newly committed Mcl1 deficient neurons undergo apoptosis as they commence **migration** away from the ventricular zone. Examination of neural progenitor differentiation in vitro demonstrated that cell death in the absence of Mcl1 is cell autonomous. Although conditional deletion of Mcl-1 in cultured neurons does not trigger apoptosis, loss of Mcl-1 sensitizes neurons to an acute DNA damaging insult. Indeed, the rapid reduction of Mcl-1 mRNA and protein levels are early events after DNA damage in neurons, and maintaining high Mcl-1 levels can protect neurons against death. Together, our results are the first to demonstrate the requirement of Mcl-1, an anti-apoptotic Bcl-2 family protein, for cortical neurogenesis and the survival of neurons after DNA damage.
226. VEGF signalling controls GnRH neuron survival via NRP1 independently of KDR and blood vessels. Gonadotropin-releasing hormone (GnRH) neurons are neuroendocrine cells that are born in the nasal placode during embryonic development and migrate through the nose and forebrain to the hypothalamus, where they regulate reproduction. Many molecular pathways that guide their **migration** have been identified, but little is known about the factors that control the survival of the migrating GnRH neurons as they negotiate different environments. We previously reported that the class 3 semaphorin SEMA3A signals through its neuropilin receptors, NRP1 and NRP2, to organise the axons that guide migrating GnRH neurons from their birthplace into the brain. By combining analysis of genetically altered mice with in vitro models, we show here that the alternative neuropilin ligand VEGF164 promotes the survival of migrating GnRH neurons by co-activating the ERK and AKT signalling pathways through NRP1. We also demonstrate that survival signalling relies on neuronal, but not endothelial, NRP1 expression and that it occurs independently of KDR, the main VEGF receptor in blood vessels. Therefore, VEGF164 provides survival signals directly to developing GnRH neurons, independently of its role in blood vessels. Finally, we show that the VEGF164-mediated neuronal survival and SEMA3A-mediated axon guidance cooperate to ensure that migrating GnRH neurons reach the brain. Thus, the loss of both neuropilin ligands leads to an almost complete failure to establish the GnRH neuron system.
227. Doublecortin is a microtubule-associated protein and is expressed widely by migrating neurons. Doublecortin (DCX) is required for normal **migration** of neurons into the cerebral cortex, since mutations in the human gene cause a disruption of cortical neuronal **migration**. To date, little is known about the distribution of DCX protein or its function. Here, we demonstrate that DCX is expressed in migrating neurons throughout the central and peripheral nervous system during embryonic and postnatal development. DCX protein localization overlaps with microtubules in cultured primary cortical neurons, and this overlapping expression is disrupted by microtubule depolymerization. DCX coassembles with brain microtubules, and recombinant DCX stimulates the polymerization of purified tubulin. Finally, overexpression of DCX in heterologous cells leads to a dramatic microtubule phenotype that is resistant to depolymerization. Therefore, DCX likely directs neuronal **migration** by regulating the organization and stability of microtubules.
228. Expression of stathmin and SCG10 proteins in the olfactory neurogenesis during development and after lesion in the adulthood. Stathmin and SCG10 belong to a family of phosphoproteins associated to cell proliferation and differentiation. In the present study, we have analyzed immunocytochemically the distribution of these proteins during neurogenesis in the mouse olfactory system, from midgestation to adulthood. Data show that already at embryonic day 12, stathmin and SCG10 immunoreactivities were present in the olfactory and vomeronasal neurons, and their number increased greatly, colocalizing with neuronal specific tubulin, a marker of immature neurons. Later on up to adulthood, the distribution of stathmin and SCG10 became progressively restricted to a few immature receptor and chemosensory neurons. Significantly, in the olfactory epithelium, stathmin was seen in immature neurons and also in basal cells representing precursors of neuronal elements. Interestingly, before birth stathmin and SCG10 immunopositive cells were seen outside the olfactory epithelium, seemingly migrating toward the olfactory bulb. After regeneration in the adult following peripheral lesion of the olfactory epithelium, stathmin and SCG10 were again strongly expressed and generally colocalized with neuronal specific tubulin immunoreactivity. Overall these results indicate that stathmin and SCG10 are expressed in immature olfactory neurons as well as in the migrating cells generated from the olfactory epithelium, supporting the role of these proteins in neurogenesis and cell **migration**.
229. DOCK7 interacts with TACC3 to regulate interkinetic nuclear **migration** and cortical neurogenesis. Neurogenesis in the developing neocortex relies on the ability of radial glial progenitor cells (RGCs) to switch from proliferative to differentiative neuron-generating divisions, but the molecular mechanisms that control this switch in a correct temporal manner are not well understood. Here, we show that DOCK7, a member of the DOCK180 family of proteins, regulates RGC proliferation versus differentiation. Silencing of DOCK7 in RGCs of developing mouse embryos impedes neuronal differentiation and maintains cells as cycling progenitors. In contrast, DOCK7 overexpression promotes RGC differentiation to basal progenitors and neurons. We further present evidence that DOCK7 influences neurogenesis by controlling apically directed interkinetic nuclear **migration** of RGCs. DOCK7 exerts its effects by antagonizing the microtubule growth-promoting function of the centrosome-associated protein TACC3. Thus, DOCK7 interaction with TACC3 controls interkinetic nuclear **migration** and the genesis of neurons from RGCs during cortical development.
230. IGF-I promotes neuronal **migration** and positioning in the olfactory bulb and the exit of neuroblasts from the subventricular zone. While insulin-like growth factor-I (IGF-I) supports neuronal and glial differentiation in the CNS, it is largely unknown whether IGF-I also influences neuronal **migration** and positioning. We show here that the pattern of olfactory bulb (OB) layering is altered in Igf-I (-/-) mice. In these animals, Tbr1(+)-glutamatergic neurons are misplaced in the mitral cell layer (ML) and the external plexiform layer (EPL). In addition, there are fewer interneurons in the glomerular layer and the EPL of the Igf-I (-/-) mice, and fewer newborn neurons are incorporated into the OB from the forebrain subventricular zone (SVZ). Indeed, neuroblasts accumulate in the postnatal/adult SVZ of Igf-I (-/-) mice. Significantly, the positioning of Tbr1(+)-cells in a primitive ML is stimulated by IGF-I in cultured embryonic OB slices, an effect that is partially repressed by the phosphoinositide 3-kinase (PI3K) inhibitor. In OB cell cultures, IGF-I increases the phosphorylation of disabled1 (P-Dab1), an adaptor protein that is a target of Src family kinases (SFK) in the reelin signalling pathway, whereas reduced P-Dab1 levels were found in Igf-I (-/-) mice. Neuroblast **migration** from the rostral migratory stream (RMS) explants of postnatal Igf-I (-/-) was similar to that from Igf-I (+/+) explants. However, cell **migration** was significantly enhanced by IGF-I added to the explants, an effect that was repressed by PI3K and SFK inhibitors. These findings suggest that IGF-I promotes neuronal positioning in the OB and support a role for IGF-I in stimulating neuroblast exit from the SVZ into the RMS, thereby promoting the incorporation of newly formed neurons into the OB.
231. Emx1-lineage progenitors differentially contribute to neural diversity in the striatum and amygdala. In the developing mammalian basal telencephalon, neural progenitors from the subpallium generate the majority of inhibitory medium spiny neurons (MSNs) in the striatum, while both pallial- and subpallial-derived progenitors contribute to excitatory and inhibitory neuronal diversity in the amygdala. Using a combination of approaches, including genetic fate mapping, cell birth dating, cell **migration** assays, and electrophysiology, we find that cells derived from the Emx1 lineage contribute to two distinct neuronal populations in the mature basal forebrain: inhibitory MSNs in the striatum and functionally distinct subclasses of excitatory neurons in the amygdala. Our cell birth-dating studies reveal that these two populations are born at different times during early neurogenesis, with the amygdala population born before the MSNs. In the striatum, Emx1-lineage neurons represent a unique subpopulation of MSNs: they are disproportionately localized to the dorsal striatum, are found in dopamine receiving, reelin-positive patches, and are born throughout striatal neurogenesis. In addition, our data suggest that a subpopulation of these Emx1-lineage cells originate in the pallium and subsequently migrate to the developing striatum and amygdala. Our intersectional fate-mapping analysis further reveals that Emx1-lineage cells that coexpress Dlx exclusively generate MSNs but do not contribute to the excitatory neurons in the amygdala. Thus, both the timing of neurogenesis and differential combinatorial gene expression appear to be key determinants of striatal versus amygdala fate decisions of Emx1-lineage cells.
232. Hand2 is necessary for terminal differentiation of enteric neurons from crest-derived precursors but not for their **migration** into the gut or for formation of glia. Hand genes encode basic helix-loop-helix transcription factors that are expressed in the developing gut, where their function is unknown. We now report that enteric Hand2 expression is limited to crest-derived cells, whereas Hand1 expression is restricted to muscle and interstitial cells of Cajal. Hand2 is developmentally regulated and is intranuclear in precursors but cytoplasmic in neurons. Neurons develop in explants from wild-type but not Hand2(-/-) bowel, although, in both, crest-derived cells are present and glia arise. Similarly, small interfering RNA (siRNA) silencing of Hand2 in enteric crest-derived cells prevents neuronal development. Terminally differentiated enteric neurons do not develop after conditional inactivation of Hand2 in migrating crest-derived cells; nevertheless, conditional Hand2 inactivation does not prevent precursors from expressing early neural markers. We suggest that enteric neuronal development occurs in stages and that Hand2 expression is required for terminal differentiation but not for precursors to enter the neuronal lineage.
233. Zic1 and Zic3 regulate medial forebrain development through expansion of neuronal progenitors. The medial telencephalon is a source of neurons that follow distinct tangential trajectories of **migration** to various structures such as the cerebral cortex, striatum, and olfactory bulb. In the present study, we characterized the forebrain anomalies in Zic1/Zic3 compound mutant mice. Zic1 and Zic3 were strongly expressed in the medial structures, including the septum, medial cerebral cortex, and choroid plexus. Mice homozygous for the Zic1 mutant allele together with the null Zic3 allele showed medial forebrain defects, which were not obvious in either Zic1 or Zic3 single mutants. Absence of both Zic1 and Zic3 caused hypoplasia of the hippocampus, septum, and olfactory bulb. Analysis of the cell cycle revealed that the cell cycle exit rate was increased in the septa of double mutants. Misexpression of Zic3 in the ventricular layer of the cerebral cortex inhibited neuronal differentiation. These results indicated that both Zic1 and Zic3 function in maintaining neural precursor cells in an undifferentiated state. The functions of these genes may be essential to increasing neural cell numbers regionally in the medial telencephalon and to proper mediolateral patterning of the telencephalon.
234. p75 is important for axon growth and schwann cell **migration** during development. Mice lacking the low-affinity neurotrophin receptor p75 have multiple peripheral neural deficits. Here we examined the developmental nature of these deficiencies. Peripheral axons in p75 -/- embryos were severely stunted and poorly arborized from embryonic day 11.5 (E11.5) to E14.5. In vitro, neurite outgrowth from the dorsal root ganglia was significantly decreased in the p75 -/- embryos at E12.5, suggesting that stunted axonal growth in the embryo may result in part from defects in neurite elongation. Additionally, Schwann cell marker S100beta immunoreactivity was decreased or absent along the growing axons of the ophthalmic branch from the trigeminal ganglia in p75 -/- embryos. Electron microscopy studies of the axons of the trigeminal ganglion at E13.5 revealed that in the p75 mutant embryo, nerve bundles were highly impaired and that coverage of the growing axons by Schwann cell cytoplasm was substantially reduced. In vitro, Schwann cell **migration** from the dorsal root ganglia was significantly decreased in the p75 -/- embryos at E12.5, suggesting that the lack of S100beta staining and Schwann cell coverage in the p75 mutant results from a deficit in Schwann cell **migration**. These results provide evidence that p75 is important in the developing embryo for regulating axon growth and arborization and for Schwann cell **migration**.
235. Essential role for the SLK protein kinase in embryogenesis and placental tissue development. Over the past decade, the Ste20-like kinase SLK, has been implicated in several signaling processes. SLK repression has been shown to impair cell cycle kinetics and inhibit FAK-mediated cell **migration**. Here, using a gene trapped allele, we have generated mice expressing a truncated form of the SLK kinase.Our results show that an SLK-LacZ fusion protein is expressed in embryonic stem cells and in embryos throughout development. We find that the SLK-LacZ fusion protein is less efficient at phosphorylating substrates resulting in reduced cell proliferation within the embryos and angiogenic defects in the placentae of the homozygous mutant animals at embryonic day (E) 12.5. This results in marked developmental defects and apoptotic lesions in the embryos by E14.5.Homozygotes expressing the SLK-LacZ fusion protein present with an embryonic lethal phenotype occurring between E12.5 and E14.5. Overall, we demonstrate a requirement for SLK kinase activity in the developing embryo and placenta.
236. Orphan G protein-coupled receptor GPR56 regulates neural progenitor cell **migration** via a G alpha 12/13 and Rho pathway. In the developing forebrain, the **migration** and positioning of neural progenitor cells (NPCs) are regulated coordinately by various molecules. Mutation of these molecules, therefore, causes cortical malformation. GPR56 has been reported as a cortical malformation-related gene that is mutated in patients with bilateral frontoparietal polymicrogyria. GPR56 encodes an orphan G protein-coupled receptor, and its mutations reduce the cell surface expression. It has also been reported that the expression level of GPR56 is involved in cancer cell adhesion and metastasis. However, it remains to be clarified how GPR56 functions in brain development and which signaling pathways are activated by GPR56. In this study, we showed that GPR56 is highly expressed in NPCs and has the ability to inhibit NPC **migration**. We found that GPR56 coupled with Galpha(12/13) and induced Rho-dependent activation of the transcription mediated through a serum-responsive element and NF-kappaB-responsive element and actin fiber reorganization. The transcriptional activation and actin reorganization were inhibited by an RGS domain of the p115 Rho-specific guanine nucleotide exchange factor (p115 RhoGEF RGS) and dominant negative form of Rho. Moreover, we have demonstrated that a functional anti-GPR56 antibody, which has an agonistic activity, inhibited NPC **migration**. This inhibition was attenuated by p115 RhoGEF RGS, C3 exoenzyme, and GPR56 knockdown. These results indicate that GPR56 participates in the regulation of NPC movement through the Galpha(12/13) and Rho signaling pathway, suggesting its important role in the development of the central nervous system.
237. MPP3 is required for maintenance of the apical junctional complex, neuronal **migration**, and stratification in the developing cortex. During mammalian cortical development, division of progenitor cells occurs at the apical ventricular zone. Apical complex proteins and adherens junctions regulate the different modes of division. Here, we have identified the membrane-associated guanylate kinase protein membrane palmitoylated protein 3 (MPP3) as an essential protein for the maintenance of these complexes. MPP3 localizes at the apical membrane in which it shows partial colocalization with adherens junction proteins and apical proteins. We generated Mpp3 conditional knock-out mice and specifically ablated Mpp3 expression in cortical progenitor cells. Conditional deletion of Mpp3 during cortical development resulted in a gradual loss of the apical complex proteins and disrupted adherens junctions. Although there is cellular disorganization in the ventricular zone, gross morphology of the cortex was unaffected during loss of MPP3. However, in the ventricular zone, removal of MPP3 resulted in randomization of spindle orientation and ectopically localized mitotic cells. Loss of MPP3 in the developing cortex resulted in delayed **migration** of progenitor cells, whereas the rate of cell division and exit from the cell cycle was not affected. This resulted in defects in cortical stratification and ectopically localized layer II-IV pyramidal neurons and interneurons. These data show that MPP3 is required for maintenance of the apical protein complex and adherens junctions and for stratification and proper **migration** of neurons during the development of the cortex.
238. Mice lacking the homologue of the human 22q11.2 gene CRKL phenocopy neurocristopathies of DiGeorge syndrome. Heterozygous deletions within human chromosome 22q11 are the genetic basis of DiGeorge/velocardiofacial syndrome (DGS/VCFS), the most common deletion syndrome (1 in 4,000 live births) in humans. CRKL maps within the common deletion region for DGS/VCFS (ref. 2) and encodes an SH2-SH3-SH3 adapter protein closely related to the Crk gene products. Here we report that mice homozygous for a targeted null mutation at the CrkL locus (gene symbol Crkol for mice) exhibit defects in multiple cranial and cardiac neural crest derivatives including the cranial ganglia, aortic arch arteries, cardiac outflow tract, thymus, parathyroid glands and craniofacial structures. We show that the **migration** and early expansion of neural crest cells is unaffected in Crkol-/- embryos. These results therefore indicate an essential stage- and tissue-specific role for Crkol in the function, differentiation, and/or survival of neural crest cells during development. The similarity between the Crkol-/- phenotype and the clinical manifestations of DGS/VCFS implicate defects in CRKL-mediated signaling pathways as part of the molecular mechanism underlying this syndrome.
239. Changes in Sef levels influence auditory brainstem development and function. During development of the CNS, secreted morphogens of the fibroblast growth factor (FGF) family have multiple effects on cell division, **migration**, and survival depending on where, when, and how much FGF signal is received. The consequences of misregulating the FGF pathway were studied in a mouse with decreased levels of the FGF antagonist Sef. To uncover effects in the nervous system, we focused on the auditory system, which is accessible to physiological analysis. We found that the mitogen-activated protein kinase pathway is active in the rhombic lip, a germinal zone that generates diverse types of neurons, including the cochlear nucleus complex of the auditory system. Sef is expressed immediately adjacent to the rhombic lip, overlapping with FGF15 and FGFR1, which is also present in the lip itself. This pattern suggests that Sef may normally function in non-rhombic lip cells and prevent them from responding to FGF ligand in the vicinity. Consistent with this idea, overexpression of Sef in chicks decreased the size of the auditory nuclei. Cochlear nucleus defects were also apparent in mice with reduced levels of Sef, with 13% exhibiting grossly dysmorphic cochlear nuclei and 26% showing decreased amounts of GFAP in the cochlear nucleus. Additional evidence for cochlear nucleus defects was obtained by electrophysiological analysis of Sef mutant mice, which have normal auditory thresholds but abnormal auditory brainstem responses. These results show both increases and decreases in Sef levels affect the assembly and function of the auditory brainstem.

#### GeneRIFs

1. Study shows the critical and specific role of Tubb3 on the stereotyped morphological changes and polarization processes that are required for initiating radial **migration** to the cortical plate.

### ECM

#### PubMed

1. Patterns of laminins and integrins in the embryonic ventricular zone of the CNS. The extracellular matrix (**ECM**) provides both a physical framework and a microenvironment that supplies instructive signals from the earliest stages of multicellular development. As a first step toward understanding the role of the **ECM** in regulating the behavior of neural stem cells (NSCs), here we show the localization of laminins, a heterotrimeric family of **ECM** molecules expressed in many different stem cell microenvironments, and their corresponding receptors in the embryonic murine ventricular zone (VZ) within which the NSCs undergo symmetrical and asymmetrical divisions required for cortical development. In addition to the presence of laminins containing both the alpha2 and alpha4 chains, we find distinct patterns of **ECM** receptor expression in the VZ and in the overlying cortex. Neural stem cells derived from the VZ express high levels of the integrin laminin receptor alpha6beta1. At developmental stages at which NSCs undergo asymmetrical divisions, integrin beta1 was unevenly distributed in some mitotic pairs at the ventricular wall. These results suggest a significant role in the regulation of NSC fate for laminin/integrin signaling within the microenvironment of the VZ and provide a framework for future molecular and cellular analyses of the role of the **ECM** in neural development.
2. MBTPS1/SKI-1/S1P proprotein convertase is required for **ECM** signaling and axial elongation during somitogenesis and vertebral development†. Caudal regression syndrome (sacral agenesis), which impairs development of the caudal region of the body, occurs with a frequency of about 2 live births per 100 000 newborns although this incidence rises to 1 in 350 infants born to mothers with gestational diabetes. The lower back and limbs can be affected as well as the genitourinary and gastrointestinal tracts. The axial skeleton is formed during embryogenesis through the process of somitogenesis in which the paraxial mesoderm periodically segments into bilateral tissue blocks, called somites. Somites are the precursors of vertebrae and associated muscle, tendons and dorsal dermis. Vertebral anomalies in caudal regression syndrome may arise through perturbation of somitogenesis or, alternatively, could result from defective bone formation and patterning. We discovered that MBTPS1/SKI-1/S1P, which proteolytically activates a class of transmembrane transcription factors, plays a critical role in somitogenesis and the pathogenesis of lumbar/sacral vertebral anomalies. Conditional deletion of Mbtps1 yields a viable mouse with misshapen, fused and reduced number of lumbar and sacral vertebrae, under-developed hind limb bones and a kinky, shortened tail. We show that Mbtps1 is required to (i) maintain the Fgf8 'wavefront' in the presomitic mesoderm that underpins axial elongation, (ii) sustain the Lfng oscillatory 'clock' activity that governs the periodicity of somite formation and (iii) preserve the composition and character of the somitic extracellular matrix containing fibronectin, fibrillin2 and laminin. Based on this spinal phenotype and known functions of MBTPS1, we reason that loss-of-function mutations in Mbtps1 may cause the etiology of caudal regression syndrome.
3. A collagen VI-dependent pathogenic mechanism for Hirschsprung's disease. Hirschsprung's disease (HSCR) is a severe congenital anomaly of the enteric nervous system (ENS) characterized by functional intestinal obstruction due to a lack of intrinsic innervation in the distal bowel. Distal innervation deficiency results from incomplete colonization of the bowel by enteric neural crest cells (eNCCs), the ENS precursors. Here, we report the generation of a mouse model for HSCR--named Holstein--that contains an untargeted transgenic insertion upstream of the collagen-6α4 (Col6a4) gene. This insertion induces eNCC-specific upregulation of Col6a4 expression that increases total collagen VI protein levels in the extracellular matrix **(EC**M) surrounding both the developing and the postnatal ENS. Increased collagen VI levels during development mainly result in slower migration of eNCCs. This appears to be due to the fact that collagen VI is a poor substratum for supporting eNCC migration and can even interfere with the migration-promoting effects of fibronectin. Importantly, for a majority of patients in a HSCR cohort, the myenteric ganglia from the ganglionated region are also specifically surrounded by abundant collagen VI microfibrils, an outcome accentuated by Down syndrome. Collectively, our data thus unveil a clinically relevant pathogenic mechanism for HSCR that involves cell-autonomous changes in **EC**M composition surrounding eNCCs. Moreover, as COL6A1 and COL6A2 are on human Chr.21q, this mechanism is highly relevant to the predisposition of patients with Down syndrome to HSCR.
4. Tenascin C regulates proliferation and differentiation processes during embryonic retinogenesis and modulates the de-differentiation capacity of Müller glia by influencing growth factor responsiveness and the extracellular matrix compartment. The retina represents an ideal model system for studying developmental processes during morphogenesis. The knowledge of the precise regulation and combination of genetic pre-dispositions and environmental circumstances enables the understanding of pathologies and the subsequent development or/and improvement of therapeutic strategies. This study focused on the functional analysis of the extracellular matrix **(EC**M) molecule Tenascin C (Tnc) in the retinal stem/progenitor cell environment. In this perspective, a Tnc(-/-) mouse was examined for potential alterations in proliferation and differentiation programs by using immunohistochemistry, RT-PCR analysis and bioassays. It could be shown that both cycling G2-phase cells and early post-mitotic neurons were significantly increased in the retina due to Tnc-deficiency. Further investigations suggested that Tnc regulates these processes via the Wnt-signaling cascade. Therapeutic approaches in the treatment of degenerative diseases often integrate cell-replacement strategies. Retinal Müller glia cells represent the glia of the retina and are described to possess the ability to re-enter the cell cycle and generate neurons in response to injury. In this study, the de-differentiation was induced by FGF2. It was found out that Tnc influences the de-differentiation behavior of adherent Müller glia in vitro. Moreover, it was interesting to investigate the effect of the absence of Tnc on the composition of other components of t**he** ECM. A special focus lay on the expression of a specifically sulfated carbohydrate motif on chondroitin sulfate glycosaminoglycan chains, which can be detected with the mAb 473HD. It was possible to note a significant increase of this particular chondroitin sulfate in the Tnc-deficie**nt** ECM.
5. Twist1 directly regulates genes that promote cell proliferation and migration in developing heart valves. Twist1, a basic helix-loop-helix transcription factor, is expressed in mesenchymal precursor populations during embryogenesis and in metastatic cancer cells. In the developing heart, Twist1 is highly expressed in endocardial cushion (ECC) valve mesenchymal cells and is down regulated during valve differentiation and remodeling. Previous studies demonstrated that Twist1 promotes cell proliferation, migration, and expression of primitive extracellular matrix (**ECM**) molecules in ECC mesenchymal cells. Furthermore, Twist1 expression is induced in human pediatric and adult diseased heart valves. However, the Twist1 downstream target genes that mediate increased cell proliferation and migration during early heart valve development remain largely unknown. Candidate gene and global gene profiling approaches were used to identify transcriptional targets of Twist1 during heart valve development. Candidate target genes were analyzed for evolutionarily conserved regions (ECRs) containing E-box consensus sequences that are potential Twist1 binding sites. ECRs containing conserved E-box sequences were identified for Twist1 responsive genes Tbx20, Cdh11, Sema3C, Rab39b, and Gadd45a. Twist1 binding to these sequences in vivo was determined by chromatin immunoprecipitation (ChIP) assays, and binding was detected in ECCs but not late stage remodeling valves. In addition identified Twist1 target genes are highly expressed in ECCs and have reduced expression during heart valve remodeling in vivo, which is consistent with the expression pattern of Twist1. Together these analyses identify multiple new genes involved in cell proliferation and migration that are differentially expressed in the developing heart valves, are responsive to Twist1 transcriptional function, and contain Twist1-responsive regulatory sequences.
6. Outgrowing olfactory axons contain the Reelin receptor VLDLR and navigate through the Reelin-rich cribriform mesenchyme. Chemosensory neurons in the olfactory epithelium (OE) project axonal processes to the olfactory bulb (OB) of the brain. During embryonic stages, on their trajectory to the OB, the outgrowing axons traverse the so-called cribriform mesenchyme, which is located between the OE and the OB. The molecular cues guiding these axons through the cribriform mesenchyme are largely unknown. To identify molecules influencing the axonal trajectory in the murine cribriform mesenchyme, we performed microarray analyses focusing on extracellular matrix (**ECM**) proteins present in this tissue. Thereby, the **ECM** protein Reelin turned out to be an interesting candidate. Reelin was found to be expressed by numerous cells in the cribriform mesenchyme during the embryonic stages when the first axons navigate from the OE to the OB. These cells were closely associated with olfactory axons and apparently lack glial and neuronal markers. In the mesenchyme underlying the OE, localization of the Reelin protein was not confined to the Reelin-expressing cells, but it was also observed to be widely distributed in the **ECM**-most prominently in regions traversed by olfactory axons. Importantly, these axons were found to be endowed with the Reelin receptor very-low-density lipoprotein receptor (VLDLR). Finally, Reelin expression was also detectable in neuronal cells of the OB, which are contacted by VLDLR-positive olfactory axons. In summary, the results of the present study suggest that a Reelin/VLDLR signaling pathway might contribute to the formation of olfactory projections to the OB and the establishment of initial contacts between the incoming axons and neurons in the OB.
7. Thrombospondin-4 and matrix three-dimensionality in axon outgrowth and adhesion in the developing retina. Thrombospondin-4 (TSP-4), a large pentameric glycoprotein of the extracellular matrix, has been described as a neurite outgrowth-promoting molecule. However, the means by which TSP-4 promotes neurite outgrowth in the developing eye is unclear. Here we show that TSP-4 is present at the appropriate time in development and displays a localization pattern within the developing mouse retina consistent with a role in retinal ganglion cell (RGC) neurite outgrowth. Furthermore, results indicate that while TSP-4 alone does not support adhesion or neurite extension, it enhances the ability of laminins to promote adhesion and neurite outgrowth of embryonic retinal cells. The mechanism of enhancement is, in part, based on the ability of TSP-4 to enhance the three-dimensionality and/or clustering of laminins within the substrate matrix. These results support a model where TSP-4 acts as an organizer of adhesive and axon outgrowth-promoting molecules in the **ECM** to optimize retinal ganglion cell responses.

### [Ee]xtracellular [Mm]atrix

#### PubMed

1. Patterns of laminins and integrins in the embryonic ventricular zone of the CNS. The **extracellular matrix** (ECM) provides both a physical framework and a microenvironment that supplies instructive signals from the earliest stages of multicellular development. As a first step toward understanding the role of the ECM in regulating the behavior of neural stem cells (NSCs), here we show the localization of laminins, a heterotrimeric family of ECM molecules expressed in many different stem cell microenvironments, and their corresponding receptors in the embryonic murine ventricular zone (VZ) within which the NSCs undergo symmetrical and asymmetrical divisions required for cortical development. In addition to the presence of laminins containing both the alpha2 and alpha4 chains, we find distinct patterns of ECM receptor expression in the VZ and in the overlying cortex. Neural stem cells derived from the VZ express high levels of the integrin laminin receptor alpha6beta1. At developmental stages at which NSCs undergo asymmetrical divisions, integrin beta1 was unevenly distributed in some mitotic pairs at the ventricular wall. These results suggest a significant role in the regulation of NSC fate for laminin/integrin signaling within the microenvironment of the VZ and provide a framework for future molecular and cellular analyses of the role of the ECM in neural development.
2. The **extracellular matrix** glycoprotein tenascin-R regulates neurogenesis during development and in the adult dentate gyrus of mice. Abnormal generation of inhibitory neurons that synthesize γ-aminobutyric acid (GABAergic) is characteristic of neuropsychological disorders. We provide evidence that the **extracellular matri**x molecule tenascin-R (TNR) - which is predominantly expressed by a subpopulation of interneurons - plays a role in the generation of GABAergic and granule neurons in the murine dentate gyrus by regulating fate determination of neural stem or progenitor cells (NSCs). During development, absence of TNR in constitutively TNR-deficient (TNR(-/-)) mice results in increased numbers of dentate gyrus GABAergic neurons, decreased expression of its receptor β1 integrin, increased activation of p38 MAPK and increased expression of the GABAergic specification gene Ascl1. Postnatally, increased GABAergic input to adult hippocampal NSCs in TNR(-/-) mice is associated not only with increased numbers of GABAergic and, particularly, parvalbumin-immunoreactive neurons, as seen during development, but also with increased numbers of granule neurons, thus contributing to the increased differentiation of NSCs into granule cells. These findings indicate the importance of TNR in the regulation of hippocampal neurogenesis and suggest that TNR acts through distinct direct and indirect mechanisms during development and in the adult.
3. MBTPS1/SKI-1/S1P proprotein convertase is required for ECM signaling and axial elongation during somitogenesis and vertebral development†. Caudal regression syndrome (sacral agenesis), which impairs development of the caudal region of the body, occurs with a frequency of about 2 live births per 100 000 newborns although this incidence rises to 1 in 350 infants born to mothers with gestational diabetes. The lower back and limbs can be affected as well as the genitourinary and gastrointestinal tracts. The axial skeleton is formed during embryogenesis through the process of somitogenesis in which the paraxial mesoderm periodically segments into bilateral tissue blocks, called somites. Somites are the precursors of vertebrae and associated muscle, tendons and dorsal dermis. Vertebral anomalies in caudal regression syndrome may arise through perturbation of somitogenesis or, alternatively, could result from defective bone formation and patterning. We discovered that MBTPS1/SKI-1/S1P, which proteolytically activates a class of transmembrane transcription factors, plays a critical role in somitogenesis and the pathogenesis of lumbar/sacral vertebral anomalies. Conditional deletion of Mbtps1 yields a viable mouse with misshapen, fused and reduced number of lumbar and sacral vertebrae, under-developed hind limb bones and a kinky, shortened tail. We show that Mbtps1 is required to (i) maintain the Fgf8 'wavefront' in the presomitic mesoderm that underpins axial elongation, (ii) sustain the Lfng oscillatory 'clock' activity that governs the periodicity of somite formation and (iii) preserve the composition and character of the somiti**c extracellular matr**ix containing fibronectin, fibrillin2 and laminin. Based on this spinal phenotype and known functions of MBTPS1, we reason that loss-of-function mutations in Mbtps1 may cause the etiology of caudal regression syndrome.
4. Heparan sulfate deficiency in periocular mesenchyme causes microphthalmia and ciliary body dysgenesis. The heparan sulfate (HS) is a component of proteoglycans in the **extracellular matrix** and on cell surfaces, modulating developmental processes. The aim of this study is to investigate whether the defect of HS in the periocular mesenchyme impairs ocular morphogenesis. First, using Protein 0-Cre transgenic mice, we ablated Ext1, which encodes an indispensable enzyme for HS synthesis, in the developing periocular mesenchyme. The expression of Ext1 messenger RNA (mRNA) and HS were observed by RT-PCR and immunohistochemistry, respectively. The phenotypes in the mutant were evaluated by light microscopy and immunohistochemistry for cellular makers. Second, the distribution of the mutant periocular mesenchymal cells was tracked using a Rosa26 Cre-reporter gene. No mutant embryos (Protein 0-Cre;Ext1(flox/flox)) were identified after embryonic day 14.5 (E14.5). RT-PCR showed that an intense band amplified from Ext1 was observed in cDNAs from the control periocular mesenchymal cells at E13.5; however, the band for Ext1 was hardly detectable in cDNA from the mutant embryo, indicating that the mRNA was missing in the mutant periocular mesenchyme at E13.5. The HS expression was disrupted in the periocular mesenchyme of the mutant ocular tissues. The HS deficiency resulted in microphthalmia with reduced axial lengths, lens diameters, and vitreous sizes compared with the littermate eyes. The mutant embryos showed agenesis of the anterior chamber, where cells expressing Cre recombinase were distributed. Moreover, the mutants showed phenotypic alterations in the neural ectoderm including dysgenesis of the presumptive ciliary body and agenesis of the optic nerve head. These findings demonstrate that HS in the periocular mesenchyme plays a critical role in normal ocular morphogenesis, indicating reciprocal interactions between the periocular mesenchyme and the neural ectoderm.
5. A collagen VI-dependent pathogenic mechanism for Hirschsprung's disease. Hirschsprung's disease (HSCR) is a severe congenital anomaly of the enteric nervous system (ENS) characterized by functional intestinal obstruction due to a lack of intrinsic innervation in the distal bowel. Distal innervation deficiency results from incomplete colonization of the bowel by enteric neural crest cells (eNCCs), the ENS precursors. Here, we report the generation of a mouse model for HSCR--named Holstein--that contains an untargeted transgenic insertion upstream of the collagen-6α4 (Col6a4) gene. This insertion induces eNCC-specific upregulation of Col6a4 expression that increases total collagen VI protein levels in the **extracellular matri**x (ECM) surrounding both the developing and the postnatal ENS. Increased collagen VI levels during development mainly result in slower migration of eNCCs. This appears to be due to the fact that collagen VI is a poor substratum for supporting eNCC migration and can even interfere with the migration-promoting effects of fibronectin. Importantly, for a majority of patients in a HSCR cohort, the myenteric ganglia from the ganglionated region are also specifically surrounded by abundant collagen VI microfibrils, an outcome accentuated by Down syndrome. Collectively, our data thus unveil a clinically relevant pathogenic mechanism for HSCR that involves cell-autonomous changes in ECM composition surrounding eNCCs. Moreover, as COL6A1 and COL6A2 are on human Chr.21q, this mechanism is highly relevant to the predisposition of patients with Down syndrome to HSCR.
6. Perlecan maintains the integrity of cartilage and some basement membranes. Perlecan is a heparan sulfate proteoglycan that is expressed in all basement membranes (BMs), in cartilage, and several other mesenchymal tissues during development. Perlecan binds growth factors and interacts with various **extracellular matrix** proteins and cell adhesion molecules. Homozygous mice with a null mutation in the perlecan gene exhibit normal formation of BMs. However, BMs deteriorate in regions with increased mechanical stress such as the contracting myocardium and the expanding brain vesicles showing that perlecan is crucial for maintaining BM integrity. As a consequence, small clefts are formed in the cardiac muscle leading to blood leakage into the pericardial cavity and an arrest of heart function. The defects in the BM separating the brain from the adjacent mesenchyme caused invasion of brain tissue into the overlaying ectoderm leading to abnormal expansion of neuroepithelium, neuronal ectopias, and exencephaly. Finally, homozygotes developed a severe defect in cartilage, a tissue that lacks BMs. The chondrodysplasia is characterized by a reduction of the fibrillar collagen network, shortened collagen fibers, and elevated expression of cartilage **extracellular matrix** genes, suggesting that perlecan protects cartilage **extracellular matrix** from degradation.
7. Tenascin C regulates proliferation and differentiation processes during embryonic retinogenesis and modulates the de-differentiation capacity of Müller glia by influencing growth factor responsiveness and the **extracellular matri**x compartment. The retina represents an ideal model system for studying developmental processes during morphogenesis. The knowledge of the precise regulation and combination of genetic pre-dispositions and environmental circumstances enables the understanding of pathologies and the subsequent development or/and improvement of therapeutic strategies. This study focused on the functional analysis of the **extracellular matri**x (ECM) molecule Tenascin C (Tnc) in the retinal stem/progenitor cell environment. In this perspective, a Tnc(-/-) mouse was examined for potential alterations in proliferation and differentiation programs by using immunohistochemistry, RT-PCR analysis and bioassays. It could be shown that both cycling G2-phase cells and early post-mitotic neurons were significantly increased in the retina due to Tnc-deficiency. Further investigations suggested that Tnc regulates these processes via the Wnt-signaling cascade. Therapeutic approaches in the treatment of degenerative diseases often integrate cell-replacement strategies. Retinal Müller glia cells represent the glia of the retina and are described to possess the ability to re-enter the cell cycle and generate neurons in response to injury. In this study, the de-differentiation was induced by FGF2. It was found out that Tnc influences the de-differentiation behavior of adherent Müller glia in vitro. Moreover, it was interesting to investigate the effect of the absence of Tnc on the composition of other components of the ECM. A special focus lay on the expression of a specifically sulfated carbohydrate motif on chondroitin sulfate glycosaminoglycan chains, which can be detected with the mAb 473HD. It was possible to note a significant increase of this particular chondroitin sulfate in the Tnc-deficient ECM.
8. p190 RhoGAP is the principal Src substrate in brain and regulates axon outgrowth, guidance and fasciculation. The Src tyrosine kinases have been implicated in several aspects of neural development and nervous system function; however, their relevant substrates in brain and their mechanism of action in neurons remain to be established clearly. Here we identify the potent Rho regulatory protein, p190 RhoGAP (GTPase-activating protein), as the principal Src substrate detected in the developing and mature nervous system. We also find that mice lacking functional p190 RhoGAP exhibit defects in axon guidance and fasciculation. p190 RhoGAP is co-enriched with F-actin in the distal tips of axons, and overexpressing p190 RhoGAP in neuroblastoma cells promotes extensive neurite outgrowth, indicating that p190 RhoGAP may be an important regulator of Rho-mediated actin reorganization in neuronal growth cones. p190 RhoGAP transduces signals downstream of cell-surface adhesion molecules, and we find that p190-RhoGAP-mediated neurite outgrowth is promoted by the **extracellular matrix** protein laminin. Together with the fact that mice lacking neural adhesion molecules or Src kinases also exhibit defects in axon outgrowth, guidance and fasciculation, our results suggest that p190 RhoGAP mediates a Src-dependent adhesion signal for neuritogenesis to the actin cytoskeleton through the Rho GTPase.
9. Vascular endothelial growth factor coordinates islet innervation via vascular scaffolding. Neurovascular alignment is a common anatomical feature of organs, but the mechanisms leading to this arrangement are incompletely understood. Here, we show that vascular endothelial growth factor (VEGF) signaling profoundly affects both vascularization and innervation of the pancreatic islet. In mature islets, nerves are closely associated with capillaries, but the islet vascularization process during embryonic organogenesis significantly precedes islet innervation. Although a simple neuronal meshwork interconnects the developing islet clusters as they begin to form at E14.5, the substantial ingrowth of nerve fibers into islets occurs postnatally, when islet vascularization is already complete. Using genetic mouse models, we demonstrate that VEGF regulates islet innervation indirectly through its effects on intra-islet endothelial cells. Our data indicate that formation of a VEGF-directed, intra-islet vascular plexus is required for development of islet innervation, and that VEGF-induced islet hypervascularization leads to increased nerve fiber ingrowth. Transcriptome analysis of hypervascularized islets revealed an increased expression of **extracellular matrix** components and axon guidance molecules, with these transcripts being enriched in the islet-derived endothelial cell population. We propose a mechanism for coordinated neurovascular development within pancreatic islets, in which endocrine cell-derived VEGF directs the patterning of intra-islet capillaries during embryogenesis, forming a scaffold for the postnatal ingrowth of essential autonomic nerve fibers.
10. Twist1 directly regulates genes that promote cell proliferation and migration in developing heart valves. Twist1, a basic helix-loop-helix transcription factor, is expressed in mesenchymal precursor populations during embryogenesis and in metastatic cancer cells. In the developing heart, Twist1 is highly expressed in endocardial cushion (ECC) valve mesenchymal cells and is down regulated during valve differentiation and remodeling. Previous studies demonstrated that Twist1 promotes cell proliferation, migration, and expression of primitive **extracellular matrix** (ECM) molecules in ECC mesenchymal cells. Furthermore, Twist1 expression is induced in human pediatric and adult diseased heart valves. However, the Twist1 downstream target genes that mediate increased cell proliferation and migration during early heart valve development remain largely unknown. Candidate gene and global gene profiling approaches were used to identify transcriptional targets of Twist1 during heart valve development. Candidate target genes were analyzed for evolutionarily conserved regions (ECRs) containing E-box consensus sequences that are potential Twist1 binding sites. ECRs containing conserved E-box sequences were identified for Twist1 responsive genes Tbx20, Cdh11, Sema3C, Rab39b, and Gadd45a. Twist1 binding to these sequences in vivo was determined by chromatin immunoprecipitation (ChIP) assays, and binding was detected in ECCs but not late stage remodeling valves. In addition identified Twist1 target genes are highly expressed in ECCs and have reduced expression during heart valve remodeling in vivo, which is consistent with the expression pattern of Twist1. Together these analyses identify multiple new genes involved in cell proliferation and migration that are differentially expressed in the developing heart valves, are responsive to Twist1 transcriptional function, and contain Twist1-responsive regulatory sequences.
11. Outgrowing olfactory axons contain the Reelin receptor VLDLR and navigate through the Reelin-rich cribriform mesenchyme. Chemosensory neurons in the olfactory epithelium (OE) project axonal processes to the olfactory bulb (OB) of the brain. During embryonic stages, on their trajectory to the OB, the outgrowing axons traverse the so-called cribriform mesenchyme, which is located between the OE and the OB. The molecular cues guiding these axons through the cribriform mesenchyme are largely unknown. To identify molecules influencing the axonal trajectory in the murine cribriform mesenchyme, we performed microarray analyses focusing on **extracellular matrix** (ECM) proteins present in this tissue. Thereby, the ECM protein Reelin turned out to be an interesting candidate. Reelin was found to be expressed by numerous cells in the cribriform mesenchyme during the embryonic stages when the first axons navigate from the OE to the OB. These cells were closely associated with olfactory axons and apparently lack glial and neuronal markers. In the mesenchyme underlying the OE, localization of the Reelin protein was not confined to the Reelin-expressing cells, but it was also observed to be widely distributed in the ECM-most prominently in regions traversed by olfactory axons. Importantly, these axons were found to be endowed with the Reelin receptor very-low-density lipoprotein receptor (VLDLR). Finally, Reelin expression was also detectable in neuronal cells of the OB, which are contacted by VLDLR-positive olfactory axons. In summary, the results of the present study suggest that a Reelin/VLDLR signaling pathway might contribute to the formation of olfactory projections to the OB and the establishment of initial contacts between the incoming axons and neurons in the OB.
12. Thrombospondin-4 and matrix three-dimensionality in axon outgrowth and adhesion in the developing retina. Thrombospondin-4 (TSP-4), a large pentameric glycoprotein of the **extracellular matrix**, has been described as a neurite outgrowth-promoting molecule. However, the means by which TSP-4 promotes neurite outgrowth in the developing eye is unclear. Here we show that TSP-4 is present at the appropriate time in development and displays a localization pattern within the developing mouse retina consistent with a role in retinal ganglion cell (RGC) neurite outgrowth. Furthermore, results indicate that while TSP-4 alone does not support adhesion or neurite extension, it enhances the ability of laminins to promote adhesion and neurite outgrowth of embryonic retinal cells. The mechanism of enhancement is, in part, based on the ability of TSP-4 to enhance the three-dimensionality and/or clustering of laminins within the substrate matrix. These results support a model where TSP-4 acts as an organizer of adhesive and axon outgrowth-promoting molecules in the ECM to optimize retinal ganglion cell responses.
13. Abnormalities in neural crest cell migration in laminin alpha5 mutant mice. Although numerous in vitro experiments suggest that **extracellular matrix** molecules like laminin can influence neural crest migration, little is known about their function in the embryo. Here, we show that laminin alpha5, a gene up-regulated during neural crest induction, is localized in regions of newly formed cranial and trunk neural folds and adjacent neural crest migratory pathways in a manner largely conserved between chick and mouse. In laminin alpha5 mutant mice, neural crest migratory streams appear expanded in width compared to wild type. Conversely, neural folds exposed to laminin alpha5 in vitro show a reduction by half in the number of migratory neural crest cells. During gangliogenesis, laminin alpha5 mutants exhibit defects in condensing cranial sensory and trunk sympathetic ganglia. However, ganglia apparently recover at later stages. These data suggest that the laminin alpha5 subunit functions as a cue that restricts neural crest cells, focusing their migratory pathways and condensation into ganglia. Thus, it is required for proper migration and timely differentiation of some neural crest populations.
14. The **extracellular matrix** protein WARP is a novel component of a distinct subset of basement membranes. WARP is a recently described member of the von Willebrand factor A domain superfamily of **extracellular matrix** proteins, and is encoded by the Vwa1 gene. We have previously shown that WARP is a multimeric component of the chondrocyte pericellular matrix in articular cartilage and intervertebral disc, where it interacts with the basement membrane heparan sulfate proteoglycan perlecan. However, the tissue-specific expression of WARP in non-cartilaginous tissues and its localization in the **extracellular matrix** of other perlecan-containing tissues have not been analyzed in detail. To visualize WARP-expressing cells, we generated a reporter gene knock-in mouse by targeted replacement of the Vwa1 gene with beta-galactosidase. Analysis of reporter gene expression and WARP protein localization by immunostaining demonstrates that WARP is a component of a limited number of distinct basement membranes. WARP is expressed in the vasculature of neural tissues and in basement membrane structures of the peripheral nervous system. Furthermore, WARP is also expressed in the apical ectodermal ridge of developing limb buds, and in skeletal and cardiac muscle. These findings are the first evidence for WARP expression in non-cartilaginous tissues, and the identification of WARP as a component of a limited range of specialized basement membranes provides further evidence for the heterogeneous composition of basement membranes between different tissues.
15. GPR56 regulates pial basement membrane integrity and cortical lamination. GPR56 is a member of the family of adhesion G-protein-coupled receptors that have a large extracellular region containing a GPS (G-protein proteolytic site) domain. Loss-of-function mutations in the GPR56 gene cause a specific human brain malformation called bilateral frontoparietal polymicrogyria (BFPP). BFPP is a radiological diagnosis and its histopathology remains unclear. This study demonstrates that loss of the mouse Gpr56 gene leads to neuronal ectopia in the cerebral cortex, a cobblestone-like cortical malformation. There are four crucial events in the development of cobblestone cortex, namely defective pial basement membrane (BM), abnormal anchorage of radial glial endfeet, mislocalized Cajal-Retzius cells, and neuronal overmigration. By detailed time course analysis, we reveal that the leading causal events are likely the breaches in the pial BM. We show further that GPR56 is present in abundance in radial glial endfeet. Furthermore, a putative ligand of GPR56 is localized in the marginal zone or overlying **extracellular matrix**. These observations provide compelling evidence that GPR56 functions in regulating pial BM integrity during cortical development.

### [Ii]nvasive

#### PubMed

1. Regeneration of axons in injured spinal cord by activation of bone morphogenetic protein/Smad1 signaling pathway in adult neurons. Axon growth potential is highest in young neurons but diminishes with age, thus becoming a significant obstacle to axonal regeneration after injury in maturity. The mechanism for the decline is incompletely understood, and no effective clinical treatment is available to rekindle innate growth capability. Here, we show that Smad1-dependent bone morphogenetic protein (BMP) signaling is developmentally regulated and governs axonal growth in dorsal root ganglion (DRG) neurons. Down-regulation of the pathway contributes to the age-related decline of the axon growth potential. Reactivating Smad1 selectively in adult DRG neurons results in sensory axon regeneration in a mouse model of spinal cord injury (SCI). Smad1 signaling can be effectively manipulated by an adeno-associated virus (AAV) vector encoding BMP4 delivered by a clinically applicable and minimally **invasive** technique, an approach devoid of unwanted abnormalities in mechanosensation or pain perception. Importantly, transected axons are able to regenerate even when the AAV treatment is delivered after SCI, thus mimicking a clinically relevant scenario. Together, our results identify a therapeutic target to promote axonal regeneration after SCI.
2. The SH2 domain protein Shep1 regulates the in vivo signaling function of the scaffolding protein Cas. The members of the p130Cas (Cas) family are important scaffolding proteins that orchestrate cell adhesion, migration and **invasive**ness downstream of integrin adhesion receptors and receptor tyrosine kinases by recruiting enzymes and structural molecules. Shep1, BCAR3/AND-34 and NSP1 define a recently identified family of SH2 domain-containing proteins that constitutively bind Cas proteins through a Cdc25-type nucleotide exchange factor-like domain. To gain insight into the functional interplay between Shep1 and Cas in vivo, we have inactivated the Shep1 gene in the mouse through Cre-mediated deletion of the exon encoding the SH2 domain. Analysis of Cas tyrosine phosphorylation in the brains of newborn mice, where Shep1 is highly expressed, revealed a strong decrease in Cas substrate domain phosphorylation in knockout compared to wild-type brains. Src family kinases bind to Cas via their SH3 and SH2 domains, which contributes to their activation, and phosphorylate multiple tyrosines in the Cas substrate domain. These tyrosine-phosphorylated motifs represent docking sites for the Crk adaptor, linking Cas to the downstream Rac1 and Rap1 GTPases to regulate cell adhesion and actin cytoskeleton organization. Accordingly, we detected lower Cas-Crk association and lower phosphorylation of the Src activation loop in Shep1 knockout brains compared to wild-type. Conversely, Shep1 transfection in COS cells increases Cas tyrosine phosphorylation. The SH2 domain is likely critical for the effects of Shep1 on Cas and Src signaling because the knockout mice express Shep1 fragments that lack the amino-terminal region including the SH2 domain, presumably due to aberrant translation from internal ATG codons. These fragments retain the ability to increase Cas levels in transfected cells, similar to full-length Shep1. However, they do not affect Cas phosphorylation on their own or in the presence of co-transfected full-length Shep1. They also do not show dominant negative effects on the activity of full-length Shep1 in vivo because the heterozygous mice, which express the fragments, have a normal life span. This is in contrast to the homozygous knockout mice, most of which die soon after birth. These data demonstrate that Shep1 plays a critical role in the in vivo regulation of Src activity and Cas downstream signaling through Crk, and suggest that the SH2 domain of Shep1 is critical for these effects.

### [Mm]etastasis

#### PubMed

1. Orphan G protein-coupled receptor GPR56 regulates neural progenitor cell migration via a G alpha 12/13 and Rho pathway. In the developing forebrain, the migration and positioning of neural progenitor cells (NPCs) are regulated coordinately by various molecules. Mutation of these molecules, therefore, causes cortical malformation. GPR56 has been reported as a cortical malformation-related gene that is mutated in patients with bilateral frontoparietal polymicrogyria. GPR56 encodes an orphan G protein-coupled receptor, and its mutations reduce the cell surface expression. It has also been reported that the expression level of GPR56 is involved in cancer cell adhesion and **metastasis**. However, it remains to be clarified how GPR56 functions in brain development and which signaling pathways are activated by GPR56. In this study, we showed that GPR56 is highly expressed in NPCs and has the ability to inhibit NPC migration. We found that GPR56 coupled with Galpha(12/13) and induced Rho-dependent activation of the transcription mediated through a serum-responsive element and NF-kappaB-responsive element and actin fiber reorganization. The transcriptional activation and actin reorganization were inhibited by an RGS domain of the p115 Rho-specific guanine nucleotide exchange factor (p115 RhoGEF RGS) and dominant negative form of Rho. Moreover, we have demonstrated that a functional anti-GPR56 antibody, which has an agonistic activity, inhibited NPC migration. This inhibition was attenuated by p115 RhoGEF RGS, C3 exoenzyme, and GPR56 knockdown. These results indicate that GPR56 participates in the regulation of NPC movement through the Galpha(12/13) and Rho signaling pathway, suggesting its important role in the development of the central nervous system.

---

## Cd109

- NCBI Gene
- GeneRIF
- Pubmed

| Search Term | Rank | Fields |
| --- | --- | --- |
| ECM | 7 | PubMed(1) |
| [Ee]xtracellular [Mm]atrix | 8 | PubMed(5); GeneRIFs(1) |

  
hide/show details for Cd109

### ECM

#### PubMed

1. CD109 overexpression ameliorates skin fibrosis in a mouse model of bleomycin-induced scleroderma. Transforming growth factor β (TGFβ) is a profibrotic cytokine, and its aberrant function is implicated in several types of fibrotic pathologies including scleroderma (systemic sclerosis [SSc]). Multiple lines of evidence show that increased TGFβ signaling contributes to progressive fibrosis in SSc by promoting fibroblast activation, excessive extracellular matri**x (**ECM) deposition, and dermal thickening. We have previously identified CD109 as a TGFβ coreceptor and have shown that it antagonizes TGFβ signaling and TGFβ-in**duc**ed ECM expression in vitro in human keratinocytes and fibroblasts. The aim of the present study was to examine the ability of CD109 to prevent skin fibrosis in a mouse model of bleomycin-induced SSc.Transgenic mice overexpressing CD109 in the epidermis and their wild-type (WT) littermates were injected with bleomycin in phosphate buffered saline (PBS) or with PBS alone every other day for 21 days or 28 days. Dermal thickness and collagen deposition were determined histologically using Masson's trichrome and picrosirius red staining. In addition, collagen and fibronectin content was analyzed using Western blotting, and activation of TGFβ signaling was examined by determining phospho-Smad2 and phospho-Smad3 levels using Western blotting and immunohistochemistry.Transgenic mice overexpressing CD109 in the epidermis showed resistance to bleomycin-induced skin fibrosis, as evidenced by a significant decrease in dermal thickness, collagen crosslinking, collagen and fibronectin content, and phospho-Smad2/3 levels, as compared to their WT littermates.Our findings suggest that CD109 inhibits TGFβ signaling and fibrotic responses in experimental murine scleroderma. They also suggest that CD109 regulates dermal-epidermal interactions to decrease extracellular matrix synthesis in the dermis. Thus, CD109 is a potential molecular target for therapeutic intervention in scleroderma.

### [Ee]xtracellular [Mm]atrix

#### PubMed

1. **Extracellular matrix** secretion by cardiac fibroblasts: role of microRNA-29b and microRNA-30c. MicroRNAs (miRNAs), in particular miR-29b and miR-30c, have been implicated as important regulators of cardiac fibrosis.To perform a proteomics comparison of miRNA effects on **extracellular matrix** secretion by cardiac fibroblasts.Mouse cardiac fibroblasts were transfected with pre-/anti-miR of miR-29b and miR-30c, and their conditioned medium was analyzed by mass spectrometry. miR-29b targeted a cadre of proteins involved in fibrosis, including multiple collagens, matrix metalloproteinases, and leukemia inhibitory factor, insulin-like growth factor 1, and pentraxin 3, 3 predicted targets of miR-29b. miR-29b also attenuated the cardiac fibroblast response to transforming growth factor-β. In contrast, miR-30c had little effect on **extracellular matri**x production but opposite effects regarding leukemia inhibitory factor and insulin-like growth factor 1. Both miRNAs indirectly affected cardiac myocytes. On transfection with pre-miR-29b, the conditioned medium of cardiac fibroblasts lost its ability to support adhesion of rat ventricular myocytes and led to a significant reduction of cardiac myocyte proteins (α-actinin, cardiac myosin-binding protein C, and cardiac troponin I). Similarly, cardiomyocytes derived from mouse embryonic stem cells atrophied under pre-miR-29 conditioned medium, whereas pre-miR-30c conditioned medium had a prohypertrophic effect. Levels of miR-29a, miR-29c, and miR-30c, but not miR-29b, were significantly reduced in a mouse model of pathological but not physiological hypertrophy. Treatment with antagomiRs to miR-29b induced excess fibrosis after aortic constriction without overt deterioration in cardiac function.Our proteomic analysis revealed novel molecular targets of miRNAs that are linked to a fibrogenic cardiac phenotype. Such comprehensive screening methods are essential to define the concerted actions of miRNAs in cardiovascular disease.
2. Overexpression of CD109 in the Epidermis Differentially Regulates ALK1 Versus ALK5 Signaling and Modulates **Extracellular Matrix** Synthesis in the Skin. Transforming growth factor-β (TGF-β) is a multifunctional growth factor involved in many physiological processes including wound healing and inflammation. Excessive TGF-β signaling in the skin has been implicated in fibrotic skin disorders such as keloids and scleroderma. We previously identified CD109 as a TGF-β co-receptor and inhibitor of TGF-β signaling and have shown that transgenic mice overexpressing CD109 in the epidermis display decreased scarring. In certain cell types, in addition to the canonical type I receptor, ALK5, which activates Smad2/3, TGF-β can signal through another type I receptor, ALK1, which activates Smad1/5. Here we demonstrate that ALK1 is expressed and co-localizes with CD109 in mouse keratinocytes and that mice overexpressing CD109 in the epidermis display enhanced ALK1-Smad1/5 signaling but decreased ALK5-Smad2/3 signaling, TGF-β expressio**n, and extracellular** matrix production in the skin when compared with wild-type littermates. Furthermore, treatment with conditioned media from isolated keratinocytes or epidermal explants from CD109 transgenic mouse skin leads to a decre**ase in extracellular** matrix production in mouse skin fibroblasts. Taken together, our findings suggest that CD109 differentially regulates TGF-β-induced ALK1-Smad1/5 versus ALK5-Smad2/3 pathways, leading to de**creased extracellula**r matrix production in the skin and that epidermal CD109 expression regulates dermal function through a paracrine mechanism.
3. Transgenic mice overexpressing CD109 in the epidermis display decreased inflammation and granulation tissue and improved collagen architecture during wound healing. Transforming growth factor-β (TGF-β) is a multifunctional growth factor involved in all aspects of wound healing. TGF-β accelerates wound healing, but an excess of its presence at the wound site has been implicated in pathological scar formation. Our group has recently identified CD109, a glycophosphatidylinositol-anchored protein, as a novel TGF-β coreceptor and inhibitor of TGF-β signaling in vitro. To determine the effects of CD109 in vivo on wound healing, we generated transgenic mice overexpressing CD109 in the epidermis. In excisional wounds, we show that CD109 transgenic mice display markedly reduced macrophage and neutrophil recruitment, granulation tissue area, and decreased Smad2 and Smad3 phosphorylation, whereas wound closure remains unaffected as compared with wild-type littermates. Futhermore, we demonstrate that the expression of the proinflammatory cytokines interleukin-1α and monocyte chemoattractant protein-1**, and extracellular** matrix components is markedly decreased during wound healing in CD109 transgenic mice. In incisional wounds, CD109 transgenic mice show improved dermal architecture, whereas the tensile strength of the wound remains unchanged. Taken together, our findings demonstrate that CD109 overexpression in the epidermis reduces inflammation and granulation tissue area and improves collagen organization in vivo.
4. CD109, a novel TGF-β antagonist, decreases fibrotic responses in a hypoxic wound model. Excessive **extracellular matri**x deposition that occurs in many fibrotic skin disorders such as hypertrophic scarring and scleroderma is often associated with hypoxia. CD109 is a novel TGF-β co-receptor and TGF-β antagonist shown to inhibit TGF-β-indu**ced extracellular ma**trix protein production in vitro. We examined whether CD109 is able to regul**ate extracellular ma**trix deposition under low oxygen tension in vivo using transgenic mice overexpressing CD109 in the epidermis. By creating dorsal bipedicle skin flaps with centrally located excisional wounds in these mice and their wild-type littermates, we generated a novel murine hypoxic wound model. Mice were sacrificed on 7 or 14 days post-wounding, and tissues were harvested for histological and biochemical analysis. Hypoxic wounds in both transgenic and wild-type mice showed increased levels of HIF-1α and delayed wound closure, validating this model in mice. Hypoxic wounds in CD109 transgenic mice demonstrated decreased collagen type 1 and fibronectin expression, and reduced dermal thickness on day 7 post-wounding as compared to those in wild-type mice and to non-hypoxic control wounds. These results suggest that CD109 decre**ases extracellular m**atrix production and fibrotic responses during hypoxic wound healing. Manipulating CD109 levels may have potential therapeutic value for the treatment of fibrotic skin disorders associated with poor oxygen delivery.
5. CD109 overexpression ameliorates skin fibrosis in a mouse model of bleomycin-induced scleroderma. Transforming growth factor β (TGFβ) is a profibrotic cytokine, and its aberrant function is implicated in several types of fibrotic pathologies including scleroderma (systemic sclerosis [SSc]). Multiple lines of evidence show that increased TGFβ signaling contributes to progressive fibrosis in SSc by promoting fibroblast activation, excessi**ve extracellular mat**rix (ECM) deposition, and dermal thickening. We have previously identified CD109 as a TGFβ coreceptor and have shown that it antagonizes TGFβ signaling and TGFβ-induced ECM expression in vitro in human keratinocytes and fibroblasts. The aim of the present study was to examine the ability of CD109 to prevent skin fibrosis in a mouse model of bleomycin-induced SSc.Transgenic mice overexpressing CD109 in the epidermis and their wild-type (WT) littermates were injected with bleomycin in phosphate buffered saline (PBS) or with PBS alone every other day for 21 days or 28 days. Dermal thickness and collagen deposition were determined histologically using Masson's trichrome and picrosirius red staining. In addition, collagen and fibronectin content was analyzed using Western blotting, and activation of TGFβ signaling was examined by determining phospho-Smad2 and phospho-Smad3 levels using Western blotting and immunohistochemistry.Transgenic mice overexpressing CD109 in the epidermis showed resistance to bleomycin-induced skin fibrosis, as evidenced by a significant decrease in dermal thickness, collagen crosslinking, collagen and fibronectin content, and phospho-Smad2/3 levels, as compared to their WT littermates.Our findings suggest that CD109 inhibits TGFβ signaling and fibrotic responses in experimental murine scleroderma. They also suggest that CD109 regulates dermal-epidermal interactions to d**ecrease extracellula**r matrix synthesis in the dermis. Thus, CD109 is a potential molecular target for therapeutic intervention in scleroderma.

#### GeneRIFs

1. CD109 decreases **extracellular matrix** production and fibrotic responses during hypoxic wound healing

---

## Kcnj12

- NCBI Gene
- GeneRIF
- Pubmed

| Search Term | Rank | Fields |
| --- | --- | --- |
| [Ii]nvasive | 10 | PubMed(1) |

  
hide/show details for Kcnj12

### [Ii]nvasive

#### PubMed

1. In vivo and in vitro investigations of heterozygous nebulin knock-out mice disclose a mild skeletal muscle phenotype. Nemaline myopathy is the most common congenital skeletal muscle disease, and mutations in the nebulin gene account for 50% of all cases. Recent studies suggest that the disease severity might be related to the nebulin expression levels. Considering that mutations in the nebulin gene are typically recessive, one would expect that a single functional nebulin allele would maintain nebulin protein expression which would result in preserved skeletal muscle function. We investigated skeletal muscle function of heterozygous nebulin knock-out (i.e., nebulin(+/-)) mice using a multidisciplinary approach including protein and gene expression analysis and combined in vivo and in vitro force measurements. Skeletal muscle anatomy and energy metabolism were studied strictly non-**invasive**ly using magnetic resonance imaging and 31P-magnetic resonance spectroscopy. Maximal force production was reduced by around 16% in isolated muscle of nebulin(+/-) mice while in vivo force generating capacity was preserved. Muscle weakness was associated with a shift toward a slower proteomic phenotype, but was not related to nebulin protein deficiency or to an impaired energy metabolism. Further studies would be warranted in order to determine the mechanisms leading to a mild skeletal muscle phenotype resulting from the expression of a single nebulin allele.

---

## Anxa7

- NCBI Gene
- GeneRIF
- Pubmed

| Search Term | Rank | Fields |
| --- | --- | --- |
| [Mm]igration | 6 | PubMed(6); GeneRIFs(2) |
| [Mm]etastasize | 11 | PubMed(1) |
| [Mm]etastasis | 12 | PubMed(8); GeneRIFs(6) |

  
hide/show details for Anxa7

### [Mm]igration

#### PubMed

1. Clic1 plays a role in mouse hepatocarcinoma via modulating Annexin A7 and Gelsolin in vitro and in vivo. Clic1 is a member of the family of chloride intracellular ion channels. Previous studies suggest that Clic1 is involved in **migration** and invasion of the lymphatic metastasis in hepatocarcinoma, however, the mechanism is not fully understood. In the present study, we observed Clic1 is abundant in cytoplasm, higher expression in Hca-F cell than Hca-P cell, and we showed that downregulation of Clic1 by RNA interference was able to markedly enhance the expression of tumor metastasis genes Annexin A7 and Gelsolin in vitro, and downregulation of Annexin A7 and Gelsolin also enhanced the expression of Clic1 in vitro and in vivo. Our results provide novel insight that Clic1 have a role in **migration** and invasion in hepatocarcinoma maybe via modulating the expression of Annexin A7 and Gelsolin, and provide novel insight into the mechanisms of Clic1 for hepatocarcinoma treatment.
2. Guanine nucleotide-binding protein subunit beta-2-like 1, a new Annexin A7 interacting protein. We report for the first time that Guanine nucleotide-binding protein subunit beta-2-like 1 (RACK1) formed a complex with Annexin A7. Hca-F and Hca-P are a pair of syngeneic mouse hepatocarcinoma cell lines established and maintained in our laboratory. Our previous study showed that both Annexin A7 and RACK1 were expressed higher in Hca-F (lymph node metastasis >70%) than Hca-P (lymph node metastasis <30%). Suppression of Annexin A7 expression in Hca-F cells induced decreased **migration** and invasion ability. In this study, knockdown of RACK1 by RNA interference (RNAi) had the same impact on metastasis potential of Hca-F cells as Annexin A7 down-regulation. Furthermore, by co-immunoprecipitation and double immunofluorescence confocal imaging, we found that RACK1 was in complex with Annexin A7 in control cells, but not in the RACK1-down-regulated cells, indicating the abolishment of RACK1-Annexin A7 interaction in Hca-F cells by RACK1 RNAi. Taken together, these results suggest that RACK1-Annexin A7 interaction may be one of the means by which RACK1 and Annexin A7 influence the metastasis potential of mouse hepatocarcinoma cells in vitro.
3. Annexin A7 and its binding protein galectin-3 influence mouse hepatocellular carcinoma cell line in vitro. Lymph node metastasis is recognized as an important mode of liver cancer metastasis. Our previous study has built two hepatocarcinoma cell lines, Hca-F with high (75%) and Hca-P with low (25%) incidences of lymph node metastasis, and has indicated that annexin A7 is an important factor in the lymphatic metastasis of tumors. There is evidence that galectin-3 is the binding protein of annexin A7 and works in protein complexes. Our current study shows that both annexin A7 and galectin-3 express higher in Hca-F than Hca-P. Annexin A7 was successfully down-regulated in Hca-P by RNA interference, and this resulted in concomitant reduction of galactin 3 expression in annexin A7 down regulated compared to the control and N-control cells. Using CCK-8 assay, the expression level of annexin A7 and galectin-3 were found to have correlation with the proliferation ability; Transwell assay showed annexin A7 and galectin-3 are involved in cell **migration** and invasion regulation in mouse hepatocellular carcinoma cell lines, immunofluorescence assay indicate annexin A7 and galectin-3 were co-located annexin A7 and galectin-3 played roles in DNA damage and cell proliferation cycle checkpoint arrest pathway. Those phenomena indicated that annexin A7 influences lymphatic metastasis of tumors by interacting with galectin-3 through the regulation of tumor cell proliferation, attachment, **migration** and invasion.
4. Ezrin expression is altered in mice lymphatic metastatic hepatocellular carcinoma and subcellular fractions upon Annexin 7 modulation in-vitro. Ezrin and Annexin seven (A7) have been suggested to be involved in several roles in cancers metastasis. However, the role of Ezrin and the effect of A7 on Ezrin expression in lymphatic metastatic hepatocellular carcinoma (LNM-HCC) have not been extensively explored yet. This study reports expression of Ezrin in high lymphatic metastasis (Hca-F >70%) and low metastatic metastasis (Hca-P <30%) HCC cell lines, and the effect of A7 on Ezrin expression. Real-Time PCR, Western blot, Subcellular fractionation, Immunocytochemistry and Immunofluorescence were used to investigate Ezrin expression in addition to **migration** and invasion behaviors of A7 up-regulated Hca-F cells, A7 down-regulated Hca-P and in their respective negative control (NC) cells. Ezrin expression was higher in high LNM-HCC than low LNM-HCC (p=0.0046). Cell fractionation analysis reveals that Ezrin was highly present in the cytoplasm, nucleus and cytoskeleton of NC-Hca-F cells. However, Ezrin was highly observed in the cell membrane, nucleus and cytoskeleton of NC-Hca-P cells. A7 up-regulation in Hca-F suppressed Ezrin expression (p=0.0248), but increase the **migration** and invasion, whereas Ezrin was mainly located in the cytoplasm and nucleus fractions. Down-regulation of A7 in Hca-P cells, enhanced Ezrin expression (p<0.0001) in the cytoplasm and nucleus fractions, and suppressed **migration** and invasion. In conclusion, Ezrin may play a role in LNM-HCC and might be inversely associated with A7 expression. The subcellular localization of Ezrin and A7 was varied according to the metastatic levels. Ezrin may thus be a potential diagnostic and/or prognostic biomarker for HCC.
5. Inhibition of annexin A7 gene and protein induces the apotosis and decreases the invasion, **migration** of the hepatocarcinoma cell line. Our previous studies have shown that annexin A7 (ANXA7) gives different expressions in the mouse hepatocarcinoma cell lines with low or high lymphatic metastatic potential in both gene and protein levels. In this study, whether by using RNA interference (RNAi) technique downregulating ANXA7 in the gene level or by using antibody against ANXA7 in the protein level, the depressed expression of ANXA7 could induce apotosis and decrease the invasion, **migration** capacities of the Hca-P cell, a hepatocarcinoma cell line with low lymphatic metastatic potential in vitro. The results indicate that ANXA7 is an important factor in tumors with the lymphatic metastasis.
6. Annexin A7 gene is an important factor in the lymphatic metastasis of tumors. In the tumor malignancy progression, lymph node metastasis (LNM) is recognized as an important factor. In this study, RNA interference (RNAi) was employed to down-regulate ANXA7 gene in Hca-F cells, a hepatocarcinoma cell line with high LNM rate. There was no significant effect on cell proliferation ability, but cell division, motility, and invasion abilities were markedly inhibited. By contrast, up-regulating the expression of ANXA7 gene in Hca-P cells with lower LNM rate, cell **migration** ability was improved and the percentage of cells in S phase was significantly decreased in vitro. Here, we reported that the expression of Ech1, GSN and JNK1 genes, which were relevant to tumor lymphatic metastasis, had been inhibited due to down-regulation ANXA7 gene and promoted due to up-regulation ANXA7 gene by western blot analysis. These results indicated that ANXA7 is a critical factor in the development of lymphatic metastasis in hepatocarcinoma progression.

#### GeneRIFs

1. Clic1 have a role in **migration** and invasion in hepatocarcinoma maybe via modulating the expression of Annexin A7 and Gelsolin.
2. Annexin A7 and galectin-3 are involved in cell **migration** and invasion regulation in mouse hepatocellular carcinoma cell lines.

### [Mm]etastasize

#### PubMed

1. Annexin A7 suppresses lymph node metastasis of hepatocarcinoma cells in a mouse model. Hepatocellular carcinoma (HCC) is one of the leading causes of cancer death in China. This study investigated the effects of Annexin A7 (ANXA7) on the inhibition of HCC lymph node metastasis in a mouse model.The stable knockup and knockdown of Annexin A7-expressing HCC cells using Annexin A7 cDNA and shRNA vectors, respectively, were injected into a mouse footpad to establish primary and metastatic tumors in mice. On the 14th, 21st, and 28th days after HCC cells inoculation, the mice were sacrificed for inspection of primary and secondary tumors and immunohistochemistry of Annexin A7 expression.The lymph node metastasis rate of the FANXA7-control group was 77%, and the lymph node metastasis rate of the FANXA7-down group was 100% (p<0.05). In contrast, the lymph node metastasis rate of the PANXA7-up group was 0% and that of the PANXA7-control group was 36% (p<0.05). Furthermore, immunohistochemistry experiments revealed that the subcellular localization of Annexin A7 protein in both primary and lymph node-**metastasize**d tumors was mainly in the cytosol. In addition, the expression of the 47 kDa and 51 kDa isoforms of Annexin A7 protein changed during tumor progression.This study indicated that Annexin A7 expression was able to inhibit HCC lymph node metastasis, whereas knockdown of Annexin A7 expression significantly induced HCC metastasis to local lymph nodes.

### [Mm]etastasis

#### PubMed

1. In vivo and in vitro effect of hepatocarcinoma lymph node **metastasis** by upregulation of Annexin A7 and relevant mechanisms. We unveiled the association of Annexin A7 with vascular endothelial growth factor-C (VEGF-C) and the effect of upregulation of Annexin A7 in Hca-F and Hca-P cells on inhibiting hepatocarcinoma (HCC) lymph node **metastasis** (LNM) in vitro and in vivo. A total of 200 inbred 615 mice were randomly divided into four equal groups inoculated with Hca-F, Hca-P, FAnxa7-upregulated, and PAnxa7-upregulated cells, respectively. The primary tumor, popliteal, inguinal, and iliac lymph nodes were prepared for immunohistochemical (IHC) staining, real-time quantitative polymerase chain reaction (qRT-PCR) analysis, Western blot, and hematoxylin-eosin (H&E) staining. There was over 50 % increase both in the number of FAnxa7-upregulated and PAnxa7-upregulated cells migrated through the filter compared to their controls (FAnxa7-control, Hca-F and PAnxa7-control, Hca-P). However, no significant differences were noted in invasion ability between them (all P > 0.05). Tumor lymph vessels were significantly reduced in FAnxa7-upregulated and PAnxa7-upregulated tumors when compared with Hca-F and Hca-P tumors (all P < 0.05). Blood vessel density did not differ significantly between FAnxa7-upregulated and PAnxa7-upregulated tumors and Hca-F and Hca-P tumors. Enzyme-linked immunosorbent assay (ELISA) for VEGF-C showed that upregulating Annexin A7 decreased VEGF-C secretion in FAnxa7-upregulated and PAnxa7-upregulated cells (P < 0.05). The IHC staining result showed that the level of serum Annexin A7 was found to be statistically higher in all experimental groups than that in the control group (P < 0.05). The present results indicated that alterations in serum Annexin A7 expression may be of prognostic relevance **in HCC lym**phatic metastasis.
2. Clic1 plays a role in mouse hepatocarcinoma via modulating Annexin A7 and Gelsolin in vitro and in vivo. Clic1 is a member of the family of chloride intracellular ion channels. Previous studies suggest that Clic1 is involved in migration and invasion of the lymphatic **metastasis** in hepatocarcinoma, however, the mechanism is not fully understood. In the present study, we observed Clic1 is abundant in cytoplasm, higher expression in Hca-F cell than Hca-P cell, and we showed that downregulation of Clic1 by RNA interference was able to markedly enhance the expression of tumor **metastasis** genes Annexin A7 and Gelsolin in vitro, and downregulation of Annexin A7 and Gelsolin also enhanced the expression of Clic1 in vitro and in vivo. Our results provide novel insight that Clic1 have a role in migration and invasion in hepatocarcinoma maybe via modulating the expression of Annexin A7 and Gelsolin, and provide novel insight into the mechanisms of Clic1 for hepatocarcinoma treatment.
3. Guanine nucleotide-binding protein subunit beta-2-like 1, a new Annexin A7 interacting protein. We report for the first time that Guanine nucleotide-binding protein subunit beta-2-like 1 (RACK1) formed a complex with Annexin A7. Hca-F and Hca-P are a pair of syngeneic mouse hepatocarcinoma cell lines established and maintained in our laboratory. Our previous study showed that both Annexin A7 and RACK1 were expressed higher in Hca-F (lymph node **metastasis** >70%) than Hca-P (lymph node **metastasis** <30%). Suppression of Annexin A7 expression in Hca-F cells induced decreased migration and invasion ability. In this study, knockdown of RACK1 by RNA interference (RNAi) had the same impact on **metastasis** potential of Hca-F cells as Annexin A7 down-regulation. Furthermore, by co-immunoprecipitation and double immunofluorescence confocal imaging, we found that RACK1 was in complex with Annexin A7 in control cells, but not in the RACK1-down-regulated cells, indicating the abolishment of RACK1-Annexin A7 interaction in Hca-F cells by RACK1 RNAi. Taken together, these results suggest that RACK1-Annexin A7 interaction may be one of the means by which RACK1 and Annexin A7 influence the **metastasis** potential of mouse hepatocarcinoma cells in vitro.
4. Annexin A7 suppresses lymph node **metastasis** of hepatocarcinoma cells in a mouse model. Hepatocellular carcinoma (HCC) is one of the leading causes of cancer death in China. This study investigated the effects of Annexin A7 (ANXA7) on the inhibition of HCC lymph node **metastasis** in a mouse model.The stable knockup and knockdown of Annexin A7-expressing HCC cells using Annexin A7 cDNA and shRNA vectors, respectively, were injected into a mouse footpad to establish primary and metastatic tumors in mice. On the 14th, 21st, and 28th days after HCC cells inoculation, the mice were sacrificed for inspection of primary and secondary tumors and immunohistochemistry of Annexin A7 expression.The lymph node **metastasis** rate of the FANXA7-control group was 77%, and the lymph node **metastasis** rate of the FANXA7-down group was 100% (p<0.05). In contrast, the lymph node **metastasis** rate of the PANXA7-up group was 0% and that of the PANXA7-control group was 36% (p<0.05). Furthermore, immunohistochemistry experiments revealed that the subcellular localization of Annexin A7 protein in both primary and lymph node-metastasized tumors was mainly in the cytosol. In addition, the expression of the 47 kDa and 51 kDa isoforms of Annexin A7 protein changed during tumor progression.This study indicated that Annexin A7 expression was able to inhibit HCC lymph node **metastasis**, whereas knockdown of Annexin A7 expression significantly induced HCC **metastasis** to local lymph nodes.
5. Annexin A7 and its binding protein galectin-3 influence mouse hepatocellular carcinoma cell line in vitro. Lymph node **metastasis** is recognized as an important mode of liver cancer **metastasis**. Our previous study has built two hepatocarcinoma cell lines, Hca-F with high (75%) and Hca-P with low (25%) incidences of lymph node **metastasis**, and has indicated that annexin A7 is an important factor in the lymphatic **metastasis** of tumors. There is evidence that galectin-3 is the binding protein of annexin A7 and works in protein complexes. Our current study shows that both annexin A7 and galectin-3 express higher in Hca-F than Hca-P. Annexin A7 was successfully down-regulated in Hca-P by RNA interference, and this resulted in concomitant reduction of galactin 3 expression in annexin A7 down regulated compared to the control and N-control cells. Using CCK-8 assay, the expression level of annexin A7 and galectin-3 were found to have correlation with the proliferation ability; Transwell assay showed annexin A7 and galectin-3 are involved in cell migration and invasion regulation in mouse hepatocellular carcinoma cell lines, immunofluorescence assay indicate annexin A7 and galectin-3 were co-located annexin A7 and galectin-3 played roles in DNA damage and cell proliferation cycle checkpoint arrest pathway. Those phenomena indicated that annexin A7 influences lymphatic **metastasis** of tumors by interacting with galectin-3 through the regulation of tumor cell proliferation, attachment, migration and invasion.
6. Ezrin expression is altered in mice lymphatic metastatic hepatocellular carcinoma and subcellular fractions upon Annexin 7 modulation in-vitro. Ezrin and Annexin seven (A7) have been suggested to be involved in several roles in cancers **metastasis**. However, the role of Ezrin and the effect of A7 on Ezrin expression in lymphatic metastatic hepatocellular carcinoma (LNM-HCC) have not been extensively explored yet. This study reports expression of Ezrin in high lymphatic **metastasis** (Hca-F >70%) and low metastatic **metastasis** (Hca-P <30%) HCC cell lines, and the effect of A7 on Ezrin expression. Real-Time PCR, Western blot, Subcellular fractionation, Immunocytochemistry and Immunofluorescence were used to investigate Ezrin expression in addition to migration and invasion behaviors of A7 up-regulated Hca-F cells, A7 down-regulated Hca-P and in their respective negative control (NC) cells. Ezrin expression was higher in high LNM-HCC than low LNM-HCC (p=0.0046). Cell fractionation analysis reveals that Ezrin was highly present in the cytoplasm, nucleus and cytoskeleton of NC-Hca-F cells. However, Ezrin was highly observed in the cell membrane, nucleus and cytoskeleton of NC-Hca-P cells. A7 up-regulation in Hca-F suppressed Ezrin expression (p=0.0248), but increase the migration and invasion, whereas Ezrin was mainly located in the cytoplasm and nucleus fractions. Down-regulation of A7 in Hca-P cells, enhanced Ezrin expression (p<0.0001) in the cytoplasm and nucleus fractions, and suppressed migration and invasion. In conclusion, Ezrin may play a role in LNM-HCC and might be inversely associated with A7 expression. The subcellular localization of Ezrin and A7 was varied according to the metastatic levels. Ezrin may thus be a potential diagnostic and/or prognostic biomarker for HCC.
7. Inhibition of annexin A7 gene and protein induces the apotosis and decreases the invasion, migration of the hepatocarcinoma cell line. Our previous studies have shown that annexin A7 (ANXA7) gives different expressions in the mouse hepatocarcinoma cell lines with low or high lymphatic metastatic potential in both gene and protein levels. In this study, whether by using RNA interference (RNAi) technique downregulating ANXA7 in the gene level or by using antibody against ANXA7 in the protein level, the depressed expression of ANXA7 could induce apotosis and decrease the invasion, migration capacities of the Hca-P cell, a hepatocarcinoma cell line with low lymphatic metastatic potential in vitro. The results indicate that ANXA7 is an important factor in tumors with the lymphatic **metastasis**.
8. Annexin A7 gene is an important factor in the lymphatic **metastasis** of tumors. In the tumor malignancy progression, lymph node **metastasis** (LNM) is recognized as an important factor. In this study, RNA interference (RNAi) was employed to down-regulate ANXA7 gene in Hca-F cells, a hepatocarcinoma cell line with high LNM rate. There was no significant effect on cell proliferation ability, but cell division, motility, and invasion abilities were markedly inhibited. By contrast, up-regulating the expression of ANXA7 gene in Hca-P cells with lower LNM rate, cell migration ability was improved and the percentage of cells in S phase was significantly decreased in vitro. Here, we reported that the expression of Ech1, GSN and JNK1 genes, which were relevant to tumor lymphatic **metastasis**, had been inhibited due to down-regulation ANXA7 gene and promoted due to up-regulation ANXA7 gene by western blot analysis. These results indicated that ANXA7 is a critical factor in the development of lymphatic **metastasis** in hepatocarcinoma progression.

#### GeneRIFs

1. alterations in serum Annexin A7 expression may be of prognostic relevance in hepatocarcinoma lymphatic **metastasis**
2. Ezrin may play a role in regulating lymphatic **metastasis** in hepatocellular carcinoma and might be inversely associated with A7 expression.
3. ANXA7 is an important factor in tumors with the lymphatic **metastasis**.
4. Annexin A7 expression was able to inhibit HCC lymph node **metastasis**, whereas knockdown of Annexin A7 expression significantly induced HCC **metastasis** to local lymph nodes.
5. Results suggest that RACK1-Annexin A7 interaction may be one of the means by which RACK1 and Annexin A7 influence the **metastasis** potential of mouse hepatocarcinoma cells in vitro.
6. ANXA7 is a critical factor in the development of lymphatic **metastasis** in hepatocarcinoma progression

---

## Prdx6

- NCBI Gene
- GeneRIF
- Pubmed

| Search Term | Rank | Fields |
| --- | --- | --- |
| [Mm]igration | 6 | PubMed(1) |
| [Mm]etastasis | 12 | PubMed(1); GeneRIFs(1) |

  
hide/show details for Prdx6

### [Mm]igration

#### PubMed

1. Calcium prevents tumorigenesis in a mouse model of colorectal cancer. Calcium has been proposed as a mediator of the chemoprevention of colorectal cancer (CRC), but the comprehensive mechanism underlying this preventive effect is not yet clear. Hence, we conducted this study to evaluate the possible roles and mechanisms of calcium-mediated prevention of CRC induced by 1,2-dimethylhydrazine (DMH) in mice.For gene expression analysis, 6 non-tumor colorectal tissues of mice from the DMH + Calcium group and 3 samples each from the DMH and control groups were hybridized on a 4×44 K Agilent whole genome oligo microarray, and selected genes were validated by real-time polymerase chain reaction (PCR). Functional analysis of the microarray data was performed using KEGG and Gene Ontology (GO) analyses. Hub genes were identified using Pathway Studio software.The tumor incidence rates in the DMH and DMH + Calcium groups were 90% and 40%, respectively. Microarray gene expression analysis showed that S100a9, Defa20, Mmp10, Mmp7, Ptgs2, and Ang2 were among the most downregulated genes, whereas Per3, Tef, Rnf152, and Prdx6 were significantly upregulated in the DMH + Calcium group compared with the DMH group. Functional analysis showed that the Wnt, cell cycle, and arachidonic acid pathways were significantly downregulated in the DMH + Calcium group, and that the GO terms related to cell differentiation, cell cycle, proliferation, cell death, adhesion, and cell **migratio**n were significantly affected. Forkhead box M1 (FoxM1) and nuclear factor kappa-B (NF-κB) were considered as potent hub genes.In the DMH-induced CRC mouse model, comprehensive mechanisms were involved with complex gene expression alterations encompassing many altered pathways and GO terms. However, how calcium regulates these events remains to be studied.

### [Mm]etastasis

#### PubMed

1. [Effects of yiqi chutan recipe on tumor growth, survival time and expressions of PRDX-1 and PRDX-6 in Lewis lung carcinoma model mice with pi-deficiency syndrome]. To investigate the effects of Yiqi Chutan Recipe (YCR, a Chinese herbal prescription for invigorating qi and removing phlegm) on the growth and **metastasis** of tumor, survival time, and the expressions of peroxiredoxin (PRDX-1 and PRDX-6) in tumor tissue of C57BL/6J mice bearing Lewis lung carcinoma.Lewis lung carcinoma cells were transplanted to 90 C57BL/6J mice receiving preconditioning for inducing Pi-deficiency syndrome and divided into three groups treated respectively with saline, high dose YCR (3.0 g/kg) and low dose YCR (1.0 g/kg) once a day via gastric infusion. Besides, a group of 30 healthy mice simply received tumor cell transplantation was set up for controls. Ten mice selected from each group were sacrificed 21 days later, the size, weight and lung **metastasis** foci of tumor in mice were measured, and expressions of PRDX-1 and PRDX-6 in tumor tissue were detected using immunohistochemical method. The survival time of the remained 20 mice in each groups was observed.Tumor size, weight and the numbers of lung metastatic foci were (1.14 +/- 0.30) cm3, (0.83 +/- 0.26) g, (6.20 +/- 2.53) foci in the high dose YCR treated group, which were significantly lower than those in the control group [(2.83 +/- 0.35) cm3, (2.08 +/- 0.28) g, and (8.60 +/- 1.84) foci] respectively, also lower than those in the saline treated group [(2.29 +/- 0.49) cm3, (1.67 +/- 0.33) g and (8.70 +/- 2.00) foci]. The median survival time in the three groups, in above order, were 29.00 +/- 0.89 days, 22.00 +/- 0.75 days and 21.00 +/- 0.53 days; the average survival time in them 29.60 +/- 0.53 days, 22.90 +/- 0.50 days and 20.95 +/- 0.44 days; the PRDX-1 expression were 0.15 +/- 0.03, 0.52 +/- 0.07 and 0.61 +/- 0.09; and the PRDX-6 expression were 0.12 +/- 0.02, 0.43 +/- 0.06 and 0.56 +/- 0.07, all showed significant difference in comparing the indices in the high dose treated group with those in the control group and in the saline treated group (P < 0.05 or P < 0.01). The tumor growth inhibition rate was 50.30% in the high dose YCR group with life prolongation rate of 41.29%, all better than those in the low dose YCR treated group (P < 0.05).YCR can remarkably inhibit the growth and **metastasis** of Lewis lung carcinoma in mice with Pi-asthenia syndrome, prolong their survival period, and its mechanism is possibly related to the reduction of over expressed PRDX-1 and PRDX-6.

#### GeneRIFs

1. Yiqi chutan recipe can inhibit the growth and **metastasis** of Lewis lung carcinoma and reduce expression of PRDX1 and PRDX6.

---

## Fap

- NCBI Gene
- GeneRIF
- Pubmed

| Search Term | Rank | Fields |
| --- | --- | --- |
| [Ii]nvadopodium | 4 | Component(1) |
| [Mm]igration | 6 | Process(1); PubMed(2) |
| ECM | 7 | PubMed(3); GeneRIFs(1); Summary(1) |
| [Ee]xtracellular [Mm]atrix | 8 | Process(2); PubMed(5); Summary(1) |
| [Ii]nvasive | 10 | PubMed(4); GeneRIFs(1) |
| [Mm]etastasis | 12 | PubMed(2) |
| [Cc]ell [Ii]nvasion | 13 | PubMed(1) |

  
hide/show details for Fap

### [Ii]nvadopodium

#### Component

1. **invadopodium** membrane

### [Mm]igration

#### Process

1. endothelial cell **migration**

#### PubMed

1. Regulation of mesenchymal-to-epithelial transition by PARAXIS during somitogenesis. Dynamic alterations in cell shape, **migration**, and adhesion play a central role in tissue morphogenesis during embryonic development and congenital disease. The mesenchymal-to-epithelial transition that occurs during vertebrate somitogenesis is required for proper patterning of the axial musculoskeletal system. Somitic MET is initiated in the presomitic mesoderm by PARAXIS-dependent changes in cell adhesion, cell polarity, and the composition of the extracellular matrix. However, the target genes downstream of the transcription factor PARAXIS remain poorly described.A genome-wide comparison of gene expression in the anterior presomitic mesoderm and newly formed somites of Paraxis(-/-) embryos resulted in a set of deregulated genes enriched for factors associated with extracellular matrix and cytoskeletal organization and cell-cell and cell-ECM adhesion. The greatest change in expression was seen in fibroblast activation protein alpha (Fap), encoding a dipeptidyl peptidase capable of increasing fibronectin and collagen fiber organization in extracellular matrix. Further, downstream genes in the Wnt and Notch signaling pathways were downregulated, predicting that PARAXIS participates in positive feedback loops in both pathways.These data demonstrate that PARAXIS initiates and stabilizes somite epithelialization by integrating signals from multiple pathways to control the reorganization of the ECM, cytoskeleton, and adhesion junctions during MET.
2. Elevation of seprase expression and promotion of an invasive phenotype by collagenous matrices in ovarian tumor cells. Tumor cells do not constitutively exhibit invasive activity, but rather, can be transiently induced to adhere and form lesions. We report here that the expression of seprase, a dominant EDTA-resistant gelatinase in malignant tumors, is dependent on tumor cell exposure to type I collagen gel (TICg). The induced seprase expression of ovarian tumor cells influences their collagen contraction and invasion capability. Importantly, tumor cells with reduced seprase expression, due to manipulation by RNA interference, showed a reduction of TICg contraction in the gel contractility assay, inhibition of tumor cell invasion through TICg as shown by a transwell **migration** assay and inhibition of peritoneal membrane tumor lesion in a mouse model. In addition, mAb C27, an antibody against beta1 integrin, which blocks cellular avidity to TICg, can induce seprase RNA expression and promote the invasive phenotype and metastatic potential of ovarian tumor cells. Thus, collagenous matrices in the tumor cell niche induce the expression of seprase and initiate tumor invasion and metastatic cascades.

### ECM

#### PubMed

1. Fibroblast activation protein-α promotes tumor growth and invasion of breast cancer cells through non-enzymatic functions. Fibroblast activation protein-α (FAP) is a cell surface, serine protease of the post-prolyl peptidase family that is expressed in human breast cancer but not in normal tissues. Previously, we showed that FAP expression increased tumor growth rates in a mouse model of human breast cancer. Here the role of the proteolytic activities of FAP in promoting tumor growth, matrix degradation and invasion was investigated. Mammary fat pads of female SCID mice were inoculated with breast cancer cells that express FAP and the mice treated with normal saline or Val-boroPro (talabostat); Glu-boroPro (PT-630); or 1-[[(3-hydroxy-1-adamantyl)amino]acetyl]-2-cyano-(S)-pyrrolidine (LAF-237) that inhibit prolyl peptidases. Other mice were injected with breast cancer cells expressing a catalytically inactive mutant of FAP and did not receive inhibitor treatment. PT-630 and LAF-237 did not slow growth of tumors produced by any of the three cell lines expressing FAP. Talabostat slightly decreased the growth rates of the FAP-expressing tumors but because PT-630 and LAF-237 did not, the growth retardation was likely not related to the inhibition of FAP or the related post-prolyl peptidase dipeptidyl peptidase IV. Breast cancer cells expressing a catalytically inactive mutant of FAP (FAP(S624A)) also produced tumors that grew rapidly. In vitro studies revealed that cells expressing wild type FAP or FAP(S624A) degrade extracellular matrix **(E**CM) more extensively, accumulate higher levels of matrix metalloproteinase-9 (MMP-9) in conditioned medium, are more invasive in type I collagen gels, and have altered signaling compared to control transfectants that do not express FAP and form slow growing tumors. We conclude that the proteolytic activity of FAP participates in matrix degradation, but other functions of the protein stimulate increased tumor growth.
2. FAP-overexpressing fibroblasts produce an extracellular matrix that enhances invasive velocity and directionality of pancreatic cancer cells. Alterations towards a permissive stromal microenvironment provide important cues for tumor growth, invasion, and metastasis. In this study, Fibroblast activation protein (FAP), a serine protease selectively produced by tumor-associated fibroblasts in over 90% of epithelial tumors, was used as a platform for studying tumor-stromal interactions. We tested the hypothesis that FAP enzymatic activity locally modifies stromal **ECM** (extracellular matrix) components thus facilitating the formation of a permissive microenvironment promoting tumor invasion in human pancreatic cancer.We generated a tetracycline-inducible FAP overexpressing fibroblastic cell line to synthesize an in vivo-like 3-dimensional (3D) matrix system which was utilized as a stromal landscape for studying matrix-induced cancer cell behaviors. A FAP-dependent topographical and compositional alteration of the **ECM** was characterized by measuring the relative orientation angles of fibronectin fibers and by Western blot analyses. The role of FAP in the matrix-induced permissive tumor behavior was assessed in Panc-1 cells in assorted matrices by time-lapse acquisition assays. Also, FAP+ matrix-induced regulatory molecules in cancer cells were determined by Western blot analyses.We observed that FAP remodels the **ECM** through modulating protein levels, as well as through increasing levels of fibronectin and collagen fiber organization. FAP-dependent architectural/compositional alterations of the **ECM** promote tumor invasion along characteristic parallel fiber orientations, as demonstrated by enhanced directionality and velocity of pancreatic cancer cells on FAP+ matrices. This phenotype can be reversed by inhibition of FAP enzymatic activity during matrix production resulting in the disorganization of the **ECM** and impeded tumor invasion. We also report that the FAP+ matrix-induced tumor invasion phenotype is β1-integrin/FAK mediated.Cancer cell invasiveness can be affected by alterations in the tumor microenvironment. Disruption of FAP activity and β1-integrins may abrogate the invasive capabilities of pancreatic and other tumors by disrupting the FAP-directed organization of stroma**l E**CM and blocking β1-integrin dependent cell-matrix interactions. This provides a novel preclinical rationale for therapeutics aimed at interfering with the architectural organization of tumor-associat**ed** ECM. Better understanding of the stromal influences that fuel progressive tumorigenic behaviors may allow the effective future use of targeted therapeutics aimed at disrupting specific tumor-stromal interactions.
3. Regulation of mesenchymal-to-epithelial transition by PARAXIS during somitogenesis. Dynamic alterations in cell shape, migration, and adhesion play a central role in tissue morphogenesis during embryonic development and congenital disease. The mesenchymal-to-epithelial transition that occurs during vertebrate somitogenesis is required for proper patterning of the axial musculoskeletal system. Somitic MET is initiated in the presomitic mesoderm by PARAXIS-dependent changes in cell adhesion, cell polarity, and the composition of the extracellular matrix. However, the target genes downstream of the transcription factor PARAXIS remain poorly described.A genome-wide comparison of gene expression in the anterior presomitic mesoderm and newly formed somites of Paraxis(-/-) embryos resulted in a set of deregulated genes enriched for factors associated with extracellular matrix and cytoskeletal organization and cell-cell and cell-**ECM** adhesion. The greatest change in expression was seen in fibroblast activation protein alpha (Fap), encoding a dipeptidyl peptidase capable of increasing fibronectin and collagen fiber organization in extracellular matrix. Further, downstream genes in the Wnt and Notch signaling pathways were downregulated, predicting that PARAXIS participates in positive feedback loops in both pathways.These data demonstrate that PARAXIS initiates and stabilizes somite epithelialization by integrating signals from multiple pathways to control the reorganization of the **ECM**, cytoskeleton, and adhesion junctions during MET.

#### GeneRIFs

1. In NIH 3T3 cells overexpressing recombinant mouse FAP, FAP enzymatic activity during matrix production is important for the topographical organization of the **ECM** fibers.

#### Summary

1. This gene belongs to the serine protease family. The encoded protein is an inducible cell-surface bound glycoprotein specifically expressed in tumor-associated fibroblasts and pericytes of epithelial tumors and has protease and gelatinase activity. The protein plays a role in remodeling of the extracellular matrix (**ECM**) and may affect tumorigenesis and tissue repair. Alternately spliced transcript variants of this gene are described in the literature (PMID 9139873), but the full-length sequence of these variants is not available. [provided by RefSeq, Apr 2013]

### [Ee]xtracellular [Mm]atrix

#### Process

1. negative regulation of **extracellular matrix** disassembly
2. negative regulation of **extracellular matrix** organization

#### PubMed

1. Fibroblast activation protein-α promotes tumor growth and invasion of breast cancer cells through non-enzymatic functions. Fibroblast activation protein-α (FAP) is a cell surface, serine protease of the post-prolyl peptidase family that is expressed in human breast cancer but not in normal tissues. Previously, we showed that FAP expression increased tumor growth rates in a mouse model of human breast cancer. Here the role of the proteolytic activities of FAP in promoting tumor growth, matrix degradation and invasion was investigated. Mammary fat pads of female SCID mice were inoculated with breast cancer cells that express FAP and the mice treated with normal saline or Val-boroPro (talabostat); Glu-boroPro (PT-630); or 1-[[(3-hydroxy-1-adamantyl)amino]acetyl]-2-cyano-(S)-pyrrolidine (LAF-237) that inhibit prolyl peptidases. Other mice were injected with breast cancer cells expressing a catalytically inactive mutant of FAP and did not receive inhibitor treatment. PT-630 and LAF-237 did not slow growth of tumors produced by any of the three cell lines expressing FAP. Talabostat slightly decreased the growth rates of the FAP-expressing tumors but because PT-630 and LAF-237 did not, the growth retardation was likely not related to the inhibition of FAP or the related post-prolyl peptidase dipeptidyl peptidase IV. Breast cancer cells expressing a catalytically inactive mutant of FAP (FAP(S624A)) also produced tumors that grew rapidly. In vitro studies revealed that cells expressing wild type FAP or FAP(S624A) degrad**e extracellular matr**ix (ECM) more extensively, accumulate higher levels of matrix metalloproteinase-9 (MMP-9) in conditioned medium, are more invasive in type I collagen gels, and have altered signaling compared to control transfectants that do not express FAP and form slow growing tumors. We conclude that the proteolytic activity of FAP participates in matrix degradation, but other functions of the protein stimulate increased tumor growth.
2. FAP-overexpressing fibroblasts produce an **extracellular matrix** that enhances invasive velocity and directionality of pancreatic cancer cells. Alterations towards a permissive stromal microenvironment provide important cues for tumor growth, invasion, and metastasis. In this study, Fibroblast activation protein (FAP), a serine protease selectively produced by tumor-associated fibroblasts in over 90% of epithelial tumors, was used as a platform for studying tumor-stromal interactions. We tested the hypothesis that FAP enzymatic activity locally modifies stromal ECM (**extracellular matrix**) components thus facilitating the formation of a permissive microenvironment promoting tumor invasion in human pancreatic cancer.We generated a tetracycline-inducible FAP overexpressing fibroblastic cell line to synthesize an in vivo-like 3-dimensional (3D) matrix system which was utilized as a stromal landscape for studying matrix-induced cancer cell behaviors. A FAP-dependent topographical and compositional alteration of the ECM was characterized by measuring the relative orientation angles of fibronectin fibers and by Western blot analyses. The role of FAP in the matrix-induced permissive tumor behavior was assessed in Panc-1 cells in assorted matrices by time-lapse acquisition assays. Also, FAP+ matrix-induced regulatory molecules in cancer cells were determined by Western blot analyses.We observed that FAP remodels the ECM through modulating protein levels, as well as through increasing levels of fibronectin and collagen fiber organization. FAP-dependent architectural/compositional alterations of the ECM promote tumor invasion along characteristic parallel fiber orientations, as demonstrated by enhanced directionality and velocity of pancreatic cancer cells on FAP+ matrices. This phenotype can be reversed by inhibition of FAP enzymatic activity during matrix production resulting in the disorganization of the ECM and impeded tumor invasion. We also report that the FAP+ matrix-induced tumor invasion phenotype is β1-integrin/FAK mediated.Cancer cell invasiveness can be affected by alterations in the tumor microenvironment. Disruption of FAP activity and β1-integrins may abrogate the invasive capabilities of pancreatic and other tumors by disrupting the FAP-directed organization of stromal ECM and blocking β1-integrin dependent cell-matrix interactions. This provides a novel preclinical rationale for therapeutics aimed at interfering with the architectural organization of tumor-associated ECM. Better understanding of the stromal influences that fuel progressive tumorigenic behaviors may allow the effective future use of targeted therapeutics aimed at disrupting specific tumor-stromal interactions.
3. Regulation of mesenchymal-to-epithelial transition by PARAXIS during somitogenesis. Dynamic alterations in cell shape, migration, and adhesion play a central role in tissue morphogenesis during embryonic development and congenital disease. The mesenchymal-to-epithelial transition that occurs during vertebrate somitogenesis is required for proper patterning of the axial musculoskeletal system. Somitic MET is initiated in the presomitic mesoderm by PARAXIS-dependent changes in cell adhesion, cell polarity, and the composition of the **extracellular matrix**. However, the target genes downstream of the transcription factor PARAXIS remain poorly described.A genome-wide comparison of gene expression in the anterior presomitic mesoderm and newly formed somites of Paraxis(-/-) embryos resulted in a set of deregulated genes enriched for factors associated with **extracellular matrix** and cytoskeletal organization and cell-cell and cell-ECM adhesion. The greatest change in expression was seen in fibroblast activation protein alpha (Fap), encoding a dipeptidyl peptidase capable of increasing fibronectin and collagen fiber organization in **extracellular matrix**. Further, downstream genes in the Wnt and Notch signaling pathways were downregulated, predicting that PARAXIS participates in positive feedback loops in both pathways.These data demonstrate that PARAXIS initiates and stabilizes somite epithelialization by integrating signals from multiple pathways to control the reorganization of the ECM, cytoskeleton, and adhesion junctions during MET.
4. Fibroblast Activation Protein (FAP) Accelerates Collagen Degradation and Clearance from Lungs in Mice. Idiopathic pulmonary fibrosis is a disease characterized by progressive, unrelenting lung scarring, with death from respiratory failure within 2-4 years unless lung transplantation is performed. New effective therapies are clearly needed. Fibroblast activation protein (FAP) is a cell surface-associated serine protease up-regulated in the lungs of patients with idiopathic pulmonary fibrosis as well as in wound healing and cancer. We postulate that FAP is not only a marker of disease but influences the development of pulmonary fibrosis after lung injury. In two different models of pulmonary fibrosis, intratracheal bleomycin instillation and thoracic irradiation, we find increased mortality and increased lung fibrosis in FAP-deficient mice compared with wild-type mice. Lung **extracellular matrix** analysis reveals accumulation of intermediate-sized collagen fragments in FAP-deficient mouse lungs, consistent within vitrostudies showing that FAP mediates ordered proteolytic processing of matrix metalloproteinase (MMP)-derived collagen cleavage products. FAP-mediated collagen processing leads to increased collagen internalization without altering expression of the endocytic collagen receptor, Endo180. Pharmacologic FAP inhibition decreases collagen internalization as expected. Conversely, restoration of FAP expression in the lungs of FAP-deficient mice decreases lung hydroxyproline content after intratracheal bleomycin to levels comparable with that of wild-type controls. Our findings indicate that FAP participates directly, in concert with MMPs, in collagen catabolism and clearance and is an important factor in resolving scar after injury and restoring lung homeostasis. Our study identifies FAP as a novel endogenous regulator of fibrosis and is the first to show FAP's protective effects in the lung.
5. Expression of the fibroblast activation protein during mouse embryo development. Human Fibroblast Activation Protein (FAP), a member of the serine prolyl oligopeptidase family, is a type II cell surface glycoprotein that acts as a dual-specificity dipeptidyl-peptidase (DPP) and collagenase in vitro. Its restricted expression pattern in embryonic mesenchyme, in wound healing and in reactive stromal fibroblasts of epithelial cancers, has suggested a role for the FAP protease in **extracellular matrix** degradation or growth factor activation in sites of tissue remodeling. The FAP homologue in Xenopus laevis has been reported to be induced in the thyroid hormone-induced tail resorption program during tadpole metamorphosis supporting a role for FAP in tissue remodeling processes during embryonic development. However, Fap-deficient mice show no overt developmental defects and are viable. To study the expression of FAP during mouse embryogenesis, a second Fap-deficient mouse strain expressing beta-Galactosidase under the control of the Fap promoter was generated by homologous recombination (Fap-/- lacZ mice). FAP deficiency was confirmed by the absence of FAP-specific dipeptidyl-peptidase activity in detergent-soluble extracts isolated from 17.5 d.p.c. Fap-/- lacZ embryos. We report that Fap-/- lacZ mice express beta-Galactosidase at regions of active tissue remodeling during embryogenesis including somites and perichondrial mesenchyme from cartilage primordia.

#### Summary

1. This gene belongs to the serine protease family. The encoded protein is an inducible cell-surface bound glycoprotein specifically expressed in tumor-associated fibroblasts and pericytes of epithelial tumors and has protease and gelatinase activity. The protein plays a role in remodeling of the **extracellular matrix** (ECM) and may affect tumorigenesis and tissue repair. Alternately spliced transcript variants of this gene are described in the literature (PMID 9139873), but the full-length sequence of these variants is not available. [provided by RefSeq, Apr 2013]

### [Ii]nvasive

#### PubMed

1. Fibroblast activation protein-α promotes tumor growth and invasion of breast cancer cells through non-enzymatic functions. Fibroblast activation protein-α (FAP) is a cell surface, serine protease of the post-prolyl peptidase family that is expressed in human breast cancer but not in normal tissues. Previously, we showed that FAP expression increased tumor growth rates in a mouse model of human breast cancer. Here the role of the proteolytic activities of FAP in promoting tumor growth, matrix degradation and invasion was investigated. Mammary fat pads of female SCID mice were inoculated with breast cancer cells that express FAP and the mice treated with normal saline or Val-boroPro (talabostat); Glu-boroPro (PT-630); or 1-[[(3-hydroxy-1-adamantyl)amino]acetyl]-2-cyano-(S)-pyrrolidine (LAF-237) that inhibit prolyl peptidases. Other mice were injected with breast cancer cells expressing a catalytically inactive mutant of FAP and did not receive inhibitor treatment. PT-630 and LAF-237 did not slow growth of tumors produced by any of the three cell lines expressing FAP. Talabostat slightly decreased the growth rates of the FAP-expressing tumors but because PT-630 and LAF-237 did not, the growth retardation was likely not related to the inhibition of FAP or the related post-prolyl peptidase dipeptidyl peptidase IV. Breast cancer cells expressing a catalytically inactive mutant of FAP (FAP(S624A)) also produced tumors that grew rapidly. In vitro studies revealed that cells expressing wild type FAP or FAP(S624A) degrade extracellular matrix (ECM) more extensively, accumulate higher levels of matrix metalloproteinase-9 (MMP-9) in conditioned medium, are mor**e invasi**ve in type I collagen gels, and have altered signaling compared to control transfectants that do not express FAP and form slow growing tumors. We conclude that the proteolytic activity of FAP participates in matrix degradation, but other functions of the protein stimulate increased tumor growth.
2. FAP-overexpressing fibroblasts produce an extracellular matrix that enhances **invasive** velocity and directionality of pancreatic cancer cells. Alterations towards a permissive stromal microenvironment provide important cues for tumor growth, invasion, and metastasis. In this study, Fibroblast activation protein (FAP), a serine protease selectively produced by tumor-associated fibroblasts in over 90% of epithelial tumors, was used as a platform for studying tumor-stromal interactions. We tested the hypothesis that FAP enzymatic activity locally modifies stromal ECM (extracellular matrix) components thus facilitating the formation of a permissive microenvironment promoting tumor invasion in human pancreatic cancer.We generated a tetracycline-inducible FAP overexpressing fibroblastic cell line to synthesize an in vivo-like 3-dimensional (3D) matrix system which was utilized as a stromal landscape for studying matrix-induced cancer cell behaviors. A FAP-dependent topographical and compositional alteration of the ECM was characterized by measuring the relative orientation angles of fibronectin fibers and by Western blot analyses. The role of FAP in the matrix-induced permissive tumor behavior was assessed in Panc-1 cells in assorted matrices by time-lapse acquisition assays. Also, FAP+ matrix-induced regulatory molecules in cancer cells were determined by Western blot analyses.We observed that FAP remodels the ECM through modulating protein levels, as well as through increasing levels of fibronectin and collagen fiber organization. FAP-dependent architectural/compositional alterations of the ECM promote tumor invasion along characteristic parallel fiber orientations, as demonstrated by enhanced directionality and velocity of pancreatic cancer cells on FAP+ matrices. This phenotype can be reversed by inhibition of FAP enzymatic activity during matrix production resulting in the disorganization of the ECM and impeded tumor invasion. We also report that the FAP+ matrix-induced tumor invasion phenotype is β1-integrin/FAK mediated.Cancer cell **invasiv**eness can be affected by alterations in the tumor microenvironment. Disruption of FAP activity and β1-integrins may abrogate th**e invasi**ve capabilities of pancreatic and other tumors by disrupting the FAP-directed organization of stromal ECM and blocking β1-integrin dependent cell-matrix interactions. This provides a novel preclinical rationale for therapeutics aimed at interfering with the architectural organization of tumor-associated ECM. Better understanding of the stromal influences that fuel progressive tumorigenic behaviors may allow the effective future use of targeted therapeutics aimed at disrupting specific tumor-stromal interactions.
3. Luciferase expression is driven by the promoter of fibroblast activation protein-α in murine pulmonary fibrosis. We used optical imaging of live animals and transgenic technology to develop a pulmonary fibrosis model in mice that can non**-invasiv**ely and in real-time trace the pulmonary fibrosis process.Fibroblast activation protein-α (FAPα) is selectively expressed in fibrotic foci of human pulmonary fibrosis. It is not expressed in normal tissue. We confirmed that FAPα is upregulated in fibroblasts of murine pulmonary fibrosis. Moreover, TGF-β1, a central pathological mediator of fibrotic diseases, could promote FAPα expression in mouse embryonic fibroblasts. Luciferase reporter assays showed that 5.4 kb FAPα promoter response activities to TGF-β1 was stronger than of the 2.1 kb promoter. We generated a transgenic mouse line expressing firefly luciferase under the control of the 5.4 kb FAPα gene promoter (FAPα-p-luc). After experimentally inducing murine pulmonary fibrosis, there luminescence appeared in the chests and excised lungs of FAPα-p-luc mice. The intensity of luminescence became stronger with the exacerbation of pulmonary fibrosis.Fluorescence intensity reflects the degree of pulmonary fibrosis in FAPα-p-luc mice. and this mouse model may be used to investigate molecular mechanisms and drug screening of pulmonary fibrosis.
4. Elevation of seprase expression and promotion of an **invasive** phenotype by collagenous matrices in ovarian tumor cells. Tumor cells do not constitutively exhibit **invasive** activity, but rather, can be transiently induced to adhere and form lesions. We report here that the expression of seprase, a dominant EDTA-resistant gelatinase in malignant tumors, is dependent on tumor cell exposure to type I collagen gel (TICg). The induced seprase expression of ovarian tumor cells influences their collagen contraction and invasion capability. Importantly, tumor cells with reduced seprase expression, due to manipulation by RNA interference, showed a reduction of TICg contraction in the gel contractility assay, inhibition of tumor cell invasion through TICg as shown by a transwell migration assay and inhibition of peritoneal membrane tumor lesion in a mouse model. In addition, mAb C27, an antibody against beta1 integrin, which blocks cellular avidity to TICg, can induce seprase RNA expression and promote the **invasive** phenotype and metastatic potential of ovarian tumor cells. Thus, collagenous matrices in the tumor cell niche induce the expression of seprase and initiate tumor invasion and metastatic cascades.

#### GeneRIFs

1. seprase may have a role in promotion of an **invasive** phenotype by collagenous matrices in ovarian tumor cells

### [Mm]etastasis

#### PubMed

1. FAP-overexpressing fibroblasts produce an extracellular matrix that enhances invasive velocity and directionality of pancreatic cancer cells. Alterations towards a permissive stromal microenvironment provide important cues for tumor growth, invasion, and **metastasis**. In this study, Fibroblast activation protein (FAP), a serine protease selectively produced by tumor-associated fibroblasts in over 90% of epithelial tumors, was used as a platform for studying tumor-stromal interactions. We tested the hypothesis that FAP enzymatic activity locally modifies stromal ECM (extracellular matrix) components thus facilitating the formation of a permissive microenvironment promoting tumor invasion in human pancreatic cancer.We generated a tetracycline-inducible FAP overexpressing fibroblastic cell line to synthesize an in vivo-like 3-dimensional (3D) matrix system which was utilized as a stromal landscape for studying matrix-induced cancer cell behaviors. A FAP-dependent topographical and compositional alteration of the ECM was characterized by measuring the relative orientation angles of fibronectin fibers and by Western blot analyses. The role of FAP in the matrix-induced permissive tumor behavior was assessed in Panc-1 cells in assorted matrices by time-lapse acquisition assays. Also, FAP+ matrix-induced regulatory molecules in cancer cells were determined by Western blot analyses.We observed that FAP remodels the ECM through modulating protein levels, as well as through increasing levels of fibronectin and collagen fiber organization. FAP-dependent architectural/compositional alterations of the ECM promote tumor invasion along characteristic parallel fiber orientations, as demonstrated by enhanced directionality and velocity of pancreatic cancer cells on FAP+ matrices. This phenotype can be reversed by inhibition of FAP enzymatic activity during matrix production resulting in the disorganization of the ECM and impeded tumor invasion. We also report that the FAP+ matrix-induced tumor invasion phenotype is β1-integrin/FAK mediated.Cancer cell invasiveness can be affected by alterations in the tumor microenvironment. Disruption of FAP activity and β1-integrins may abrogate the invasive capabilities of pancreatic and other tumors by disrupting the FAP-directed organization of stromal ECM and blocking β1-integrin dependent cell-matrix interactions. This provides a novel preclinical rationale for therapeutics aimed at interfering with the architectural organization of tumor-associated ECM. Better understanding of the stromal influences that fuel progressive tumorigenic behaviors may allow the effective future use of targeted therapeutics aimed at disrupting specific tumor-stromal interactions.
2. Targeting fibroblast activation protein inhibits tumor stromagenesis and growth in mice. Membrane-bound proteases have recently emerged as critical mediators of tumorigenesis, angiogenesis, and **metastasis**. However, the mechanisms by which they regulate these processes remain unknown. As the cell surface serine protease fibroblast activation protein (FAP) is selectively expressed on tumor-associated fibroblasts and pericytes in epithelial tumors, we set out to investigate the role of FAP in mouse models of epithelial-derived solid tumors. In this study, we demonstrate that genetic deletion and pharmacologic inhibition of FAP inhibited tumor growth in both an endogenous mouse model of lung cancer driven by the K-rasG12D mutant and a mouse model of colon cancer, in which CT26 mouse colon cancer cells were transplanted into immune competent syngeneic mice. Interestingly, growth of only the K-rasG12D-driven lung tumors was also attenuated by inhibition of the closely related protease dipeptidyl peptidase IV (DPPIV). Our results indicate that FAP depletion inhibits tumor cell proliferation indirectly, increases accumulation of collagen, decreases myofibroblast content, and decreases blood vessel density in tumors. These data provide proof of principle that targeting stromal cell-mediated modifications of the tumor microenvironment may be an effective approach to treating epithelial-derived solid tumors.

### [Cc]ell [Ii]nvasion

#### PubMed

1. Elevation of seprase expression and promotion of an invasive phenotype by collagenous matrices in ovarian tumor cells. Tumor cells do not constitutively exhibit invasive activity, but rather, can be transiently induced to adhere and form lesions. We report here that the expression of seprase, a dominant EDTA-resistant gelatinase in malignant tumors, is dependent on tumor cell exposure to type I collagen gel (TICg). The induced seprase expression of ovarian tumor cells influences their collagen contraction and invasion capability. Importantly, tumor cells with reduced seprase expression, due to manipulation by RNA interference, showed a reduction of TICg contraction in the gel contractility assay, inhibition of tumor **cell invasion** through TICg as shown by a transwell migration assay and inhibition of peritoneal membrane tumor lesion in a mouse model. In addition, mAb C27, an antibody against beta1 integrin, which blocks cellular avidity to TICg, can induce seprase RNA expression and promote the invasive phenotype and metastatic potential of ovarian tumor cells. Thus, collagenous matrices in the tumor cell niche induce the expression of seprase and initiate tumor invasion and metastatic cascades.

---

## Flt1

- NCBI Gene
- GeneRIF
- Pubmed

| Search Term | Rank | Fields |
| --- | --- | --- |
| [Ff]ilopodia | 1 | PubMed(3) |
| [Mm]igration | 6 | Process(4); PubMed(32); GeneRIFs(2) |
| [Ee]xtracellular [Mm]atrix | 8 | PubMed(2) |
| [Ii]nvasive | 10 | PubMed(2) |
| [Mm]etastasis | 12 | PubMed(12); GeneRIFs(2) |
| [Cc]ell [Ii]nvasion | 13 | PubMed(1) |

  
hide/show details for Flt1

### [Ff]ilopodia

#### PubMed

1. Serum response factor is required for sprouting angiogenesis and vascular integrity. Serum response factor (SRF) is a transcription factor that controls the expression of cytoskeletal proteins and immediate early genes in different cell types. Here, we found that SRF expression is restricted to endothelial cells (ECs) of small vessels such as capillaries in the mouse embryo. EC-specific Srf deletion led to aneurysms and hemorrhages from 11.5 days of mouse development (E11.5) and lethality at E14.5. Mutant embryos presented a reduced capillary density and defects in EC migration, with fewer numbers of **filopodia** in tip cells and ECs showing defects in actin polymerization and intercellular junctions. We show that SRF is essential for the expression of VE-cadherin and beta-actin in ECs both in vivo and in vitro. Moreover, knockdown of SRF in ECs impaired VEGF- and FGF-induced in vitro angiogenesis. Taken together, our results demonstrate that SRF plays an important role in sprouting angiogenesis and small vessel integrity in the mouse embryo.
2. Flt1 acts as a negative regulator of tip cell formation and branching morphogenesis in the zebrafish embryo. Endothelial tip cells guide angiogenic sprouts by exploring the local environment for guidance cues such as vascular endothelial growth factor (VegfA). Here we present Flt1 (Vegf receptor 1) loss- and gain-of-function data in zebrafish showing that Flt1 regulates tip cell formation and arterial branching morphogenesis. Zebrafish embryos expressed soluble Flt1 (sFlt1) and membrane-bound Flt1 (mFlt1). In Tg(flt1(BAC):yfp) × Tg(kdrl:ras-cherry)(s916) embryos, flt1:yfp was expressed in tip, stalk and base cells of segmental artery sprouts and overlapped with kdrl:cherry expression in these domains. flt1 morphants showed increased tip cell numbers, enhanced angiogenic behavior and hyperbranching of segmental artery sprouts. The additional arterial branches developed into functional vessels carrying blood flow. In support of a functional role for the extracellular VEGF-binding domain of Flt1, overexpression of sflt1 or mflt1 rescued aberrant branching in flt1 morphants, and overexpression of sflt1 or mflt1 in controls resulted in short arterial sprouts with reduced numbers of **filopodi**a. flt1 morphants showed reduced expression of Notch receptors and of the Notch downstream target efnb2a, and ectopic expression of flt4 in arteries, consistent with loss of Notch signaling. Conditional overexpression of the notch1a intracellular cleaved domain in flt1 morphants restored segmental artery patterning. The developing nervous system of the trunk contributed to the distribution of Flt1, and the loss of flt1 affected neurons. Thus, Flt1 acts in a Notch-dependent manner as a negative regulator of tip cell differentiation and branching. Flt1 distribution may be fine-tuned, involving interactions with the developing nervous system.
3. The Notch ligand Delta-like 4 negatively regulates endothelial tip cell formation and vessel branching. Delta-like 4 (Dll4) is a transmembrane ligand for Notch receptors that is expressed in arterial blood vessels and sprouting endothelial cells. Here we show that Dll4 regulates vessel branching during development by inhibiting endothelial tip cell formation. Heterozygous deletion of dll4 or pharmacological inhibition of Notch signaling using gamma-secretase inhibitor revealed a striking vascular phenotype, with greatly increased numbers of **filopodia**-extending endothelial tip cells and increased expression of tip cell marker genes compared with controls. **Filopodia** extension in dll4(+/-) retinal vessels required the vascular growth factor VEGF and was inhibited when VEGF signaling was blocked. Although VEGF expression was not significantly altered in dll4(+/-) retinas, dll4(+/-) vessels showed increased expression of VEGF receptor 2 and decreased expression of VEGF receptor 1 compared with wild-type, suggesting they could be more responsive to VEGF stimulation. In addition, expression of dll4 in wild-type tip cells was itself decreased when VEGF signaling was blocked, indicating that dll4 may act downstream of VEGF as a "brake" on VEGF-mediated angiogenic sprouting. Taken together, these data reveal Dll4 as a negative regulator of vascular sprouting and vessel branching that is required for normal vascular network formation during development.

### [Mm]igration

#### Process

1. cell **migration**
2. cell **migration**
3. cell **migration**
4. positive regulation of cell **migration**

#### PubMed

1. Serum response factor is required for sprouting angiogenesis and vascular integrity. Serum response factor (SRF) is a transcription factor that controls the expression of cytoskeletal proteins and immediate early genes in different cell types. Here, we found that SRF expression is restricted to endothelial cells (ECs) of small vessels such as capillaries in the mouse embryo. EC-specific Srf deletion led to aneurysms and hemorrhages from 11.5 days of mouse development (E11.5) and lethality at E14.5. Mutant embryos presented a reduced capillary density and defects in EC **migration**, with fewer numbers of filopodia in tip cells and ECs showing defects in actin polymerization and intercellular junctions. We show that SRF is essential for the expression of VE-cadherin and beta-actin in ECs both in vivo and in vitro. Moreover, knockdown of SRF in ECs impaired VEGF- and FGF-induced in vitro angiogenesis. Taken together, our results demonstrate that SRF plays an important role in sprouting angiogenesis and small vessel integrity in the mouse embryo.
2. Lack of pericytes leads to endothelial hyperplasia and abnormal vascular morphogenesis. The association of pericytes (PCs) to newly formed blood vessels has been suggested to regulate endothelial cell (EC) proliferation, survival, **migration**, differentiation, and vascular branching. Here, we addressed these issues using PDGF-B-- and PDGF receptor-beta (PDGFR-beta)--deficient mice as in vivo models of brain angiogenesis in the absence of PCs. Quantitative morphological analysis showed that these mutants have normal microvessel density, length, and number of branch points. However, absence of PCs correlates with endothelial hyperplasia, increased capillary diameter, abnormal EC shape and ultrastructure, changed cellular distribution of certain junctional proteins, and morphological signs of increased transendothelial permeability. Brain endothelial hyperplasia was observed already at embryonic day (E) 11.5 and persisted throughout development. From E 13.5, vascular endothelial growth factor-A (VEGF-A) and other genes responsive to metabolic stress became upregulated, suggesting that the abnormal microvessel architecture has systemic metabolic consequences. VEGF-A upregulation correlated temporally with the occurrence of vascular abnormalities in the placenta and dilation of the heart. Thus, although PC deficiency appears to have direct effects on EC number before E 13.5, the subsequent increased VEGF-A levels may further abrogate microvessel architecture, promote vascular permeability, and contribute to formation of the edematous phenotype observed in late gestation PDGF-B and PDGFR-beta knock out embryos.
3. GPR124, an orphan G protein-coupled receptor, is required for CNS-specific vascularization and establishment of the blood-brain barrier. Every organ in the body requires blood vessels for efficient delivery of oxygen and nutrients, but independent vascular beds are highly specialized to meet the individual needs of specific organs. The vasculature of the brain is tightly sealed, with blood-brain barrier (BBB) properties developing coincident with neural vascularization. G protein-coupled receptor 124 (GPR124) (tumor endothelial marker 5, TEM5), an orphan member of the adhesion family of G protein-coupled receptors, was previously identified on the basis of its overexpression in tumor vasculature. Here, we show that global deletion or endothelial-specific deletion of GPR124 in mice results in embryonic lethality associated with abnormal angiogenesis of the forebrain and spinal cord. Expression of GPR124 was found to be required for invasion and **migration** of blood vessels into neuroepithelium, establishment of BBB properties, and expansion of the cerebral cortex. Thus, GPR124 is an important regulator of neurovasculature development and a potential drug target for cerebrovascular diseases.
4. Vascular endothelial growth factor A (VEGF-A) is involved in guidance of VEGF receptor-positive cells to the anterior portion of early embryos. The hemangioblast in the mesoderm gives rise to both angioblasts and hematopoietic stem cells. The movement of hemangioblast precursor cells in the fetal trunk is a critical event in early embryogenesis. Vascular endothelial growth factor (VEGF) signaling is likely involved in this **migration** given the partial disturbance of VEGF receptor (VEGFR)-positive cell accumulation and **migration** in VEGFR2 null mice or mice with a truncated VEGFR1. However, it is not clear how the VEGF system regulates this **migration** or its direction. We show here that the expression of VEGF-A is dominant in the anterior portion of the embryo, whereas VEGFR1 and VEGFR2 are expressed in the posterior portion of the embryo. An inhibitor of VEGFR kinase blocked the **migration** of VEGFR-positive cells in a whole-embryo culture system. In addition, VEGFR-positive cells migrated toward a VEGFR1- or VEGFR2-specific ligand in vitro. Furthermore, VEGFR-positive cells derived from wild-type or VEGFR2(+/-) mice moved rapidly anteriorly, whereas cells derived from VEGFR2(+/-) mice carrying a truncated VEGFR1 [VEGFR1(TM-TK)(-/-)] migrated little when injected into wild-type mice. These results suggest that the VEGF-A protein concentrated in the anterior region plays an important role in the guidance of VEGFR-positive cells from the posterior portion to the head region by interacting with VEGFR in the mouse embryo.
5. Identification and characterization of VEGF-A-responsive neutrophils expressing CD49d, VEGFR1, and CXCR4 in mice and humans. Vascular endothelial growth factor A (VEGF-A) is upregulated during hypoxia and is the major regulator of angiogenesis. VEGF-A expression has also been found to recruit myeloid cells to ischemic tissues where they contribute to angiogenesis. This study investigates the mechanisms underlying neutrophil recruitment to VEGF-A as well as the characteristics of these neutrophils. A previously undefined circulating subset of neutrophils shown to be CD49d(+)VEGFR1(high)CXCR4(high) was identified in mice and humans. By using chimeric mice with impaired VEGF receptor 1 (VEGFR1) or VEGFR2 signaling (Flt-1tk(-/-), tsad(-/-)), we found that parallel activation of VEGFR1 on neutrophils and VEGFR2 on endothelial cells was required for VEGF-A-induced recruitment of circulating neutrophils to tissue. Intravital microscopy of mouse microcirculation revealed that neutrophil recruitment by VEGF-A versus by the chemokine macrophage inflammatory protein 2 (MIP-2 [CXCL2]) involved the same steps of the recruitment cascade but that an additional neutrophil integrin (eg, VLA-4 [CD49d/CD29]) played a crucial role in neutrophil crawling and e**migration** to VEGF-A. Isolated CD49d(+) neutrophils featured increased chemokinesis but not chemotaxis compared with CD49d(-) neutrophils in the presence of VEGF-A. Finally, by targeting the integrin α4 subunit (CD49d) in a transplantation-based angiogenesis model that used avascular pancreatic islets transplanted to striated muscle, we demonstrated that inhibiting the recruitment of circulating proangiogenic neutrophils to hypoxic tissue impairs vessel neoformation. Thus, angiogenesis can be modulated by targeting cell-surface receptors specifically involved in VEGF-A-dependent recruitment of proangiogenic neutrophils without compromising recruitment of the neutrophil population involved in the immune response to pathogens.
6. Expression of vascular endothelial growth factor receptor 1 in bone marrow-derived mesenchymal cells is dependent on hypoxia-inducible factor 1. Bone marrow-derived cells are recruited to sites of ischemia, where they promote tissue vascularization. This response is dependent upon the expression of vascular endothelial growth factor (VEGF) receptor 1 (VEGFR1), which mediates cell **migration** in response to VEGF or placental growth factor (PLGF). In this study, we found that exposure of cultured mouse bone marrow-derived mesenchymal stromal cells (MSC) to hypoxia or an adenovirus encoding a constitutively active form of hypoxia-inducible factor 1 (HIF-1) induced VEGFR1 mRNA and protein expression and promoted ex vivo **migration** in response to VEGF or PLGF. MSC in which HIF-1 activity was inhibited by a dominant negative or RNA interference approach expressed markedly reduced levels of VEGFR1 and failed to migrate or activate AKT in response to VEGF or PLGF. Thus, loss-of-function and gain-of-function approaches demonstrated that HIF-1 activity is necessary and sufficient for basal and hypoxia-induced VEGFR1 expression in bone marrow-derived MSC.
7. Glutaredoxin-1 up-regulation induces soluble vascular endothelial growth factor receptor 1, attenuating post-ischemia limb revascularization. Glutaredoxin-1 (Glrx) is a cytosolic enzyme that regulates diverse cellular function by removal of GSH adducts from S-glutathionylated proteins including signaling molecules and transcription factors. Glrx is up-regulated during inflammation and diabetes, and Glrx overexpression inhibits VEGF-induced EC **migration**. The aim was to investigate the role of up-regulated Glrx in EC angiogenic capacities and in vivo revascularization in the setting of hind limb ischemia. Glrx-overexpressing EC from Glrx transgenic (TG) mice showed impaired **migration** and network formation and secreted higher levels of soluble VEGF receptor 1 (sFlt), an antagonizing factor to VEGF. After hind limb ischemia surgery Glrx TG mice demonstrated impaired blood flow recovery, associated with lower capillary density and poorer limb motor function compared with wild type littermates. There were also higher levels of anti-angiogenic sFlt expression in the muscle and plasma of Glrx TG mice after surgery. Noncanonical Wnt5a is known to induce sFlt. Wnt5a was highly expressed in ischemic muscles and EC from Glrx TG mice, and exogenous Wnt5a induced sFlt expression and inhibited network formation in human microvascular EC. Adenoviral Glrx-induced sFlt in EC was inhibited by a competitive Wnt5a inhibitor. Furthermore, Glrx overexpression removed GSH adducts on p65 in ischemic muscle and EC and enhanced NF-κB activity, which was responsible for Wnt5a-sFlt induction. Taken together, up-regulated Glrx induces sFlt in EC via NF-κB-dependent Wnt5a, resulting in attenuated revascularization in hind limb ischemia. The Glrx-induced sFlt explains part of the mechanism of redox-regulated VEGF signaling.
8. Robo1 regulates semaphorin signaling to guide the **migration** of cortical interneurons through the ventral forebrain. Cortical interneurons, generated predominantly in the medial ganglionic eminence, migrate around and avoid the developing striatum in the subpallium en route to the cortex. This is attributable to the chemorepulsive cues of class 3 semaphorins expressed in the striatal mantle and acting through neuropilin (Nrp1 and Nrp2) receptors expressed in these cells. Cortical interneurons also express Robo receptors, and we show here that in mice lacking Robo1, but not Robo2, these cells migrate aberrantly through the striatum. In vitro experiments demonstrated that interneurons lacking Robo1 function are significantly less responsive to the effects of semaphorins. Failure to respond to semaphorin appears to be attributable to a reduction in Nrp1 and PlexinA1 receptors within these cells. Biochemical studies further demonstrated that Robo1 binds directly to Nrp1, but not to semaphorins, and this interaction is mediated by a region contained within its first two Ig domains. Thus, we show for the first time that Robo1 interacts with Nrp1 to modulate semaphorin signaling in the developing forebrain and direct the **migration** of interneurons through the subpallium and into the cortex.
9. Autoantibody from women with preeclampsia induces soluble Fms-like tyrosine kinase-1 production via angiotensin type 1 receptor and calcineurin/nuclear factor of activated T-cells signaling. Preeclampsia is a pregnancy-specific hypertensive syndrome that causes substantial maternal and fetal morbidity and mortality. Recent evidence indicates that maternal endothelial dysfunction in preeclampsia results from increased soluble Fms-like tyrosine kinase-1 (sFlt-1), a circulating antiangiogenic protein. Factors responsible for excessive production of sFlt-1 in preeclampsia have not been identified. We tested the hypothesis that angiotensin II type 1 (AT(1)) receptor activating autoantibodies, which occur in women with preeclampsia, contribute to increased production of sFlt-1. IgG from women with preeclampsia stimulates the synthesis and secretion of sFlt-1 via AT(1) receptor activation in pregnant mice, human placental villous explants, and human trophoblast cells. Using FK506 or short-interfering RNA targeted to the calcineurin catalytic subunit mRNA, we determined that calcineurin/nuclear factor of activated T-cells signaling functions downstream of the AT(1) receptor to induce sFlt-1 synthesis and secretion by AT(1)-receptor activating autoantibodies. AT(1)-receptor activating autoantibody-induced sFlt-1 secretion resulted in inhibition of endothelial cell **migration** and capillary tube formation in vitro. Overall, our studies demonstrate that an autoantibody from women with preeclampsia induces sFlt-1 production via angiotensin receptor activation and downstream calcineurin/nuclear factor of activated T-cells signaling. These autoantibodies represent potentially important targets for diagnosis and therapeutic intervention.
10. Heterozygous expression of the oncogenic Pik3ca(H1047R) mutation during murine development results in fatal embryonic and extraembryonic defects. The phosphoinositide 3-kinase (PI3K)/AKT signalling pathway regulates many cellular functions including proliferation, **migration**, survival and protein synthesis. Somatic mutations in PIK3CA, the gene encoding the p110α catalytic subunit of PI3K enzyme, are commonly associated with many human cancers as well as recently being implicated in human overgrowth syndromes. However, it is not clear if such mutations can be inherited through the germline. We have used a novel mouse model with Cre recombinase (Cre)-conditional knock-in of the common H1047R mutation into the endogenous Pik3ca gene. Heterozygous expression of the Pik3ca(H1047R) mutation in the developing mouse embryo resulted in failed 'turning' of the embryo and disrupted vascular remodelling within the embryonic and extraembryonic tissues, leading to lethality prior to E10. As vascular endothelial growth factor A (VEGF-A) signalling was disrupted in these embryos, we used Cre under the control of the Tie2 promoter to target the Pik3ca(H1047R) mutation specifically to endothelial cells. In these embryos turning occurred normally but the vascular remodelling defects and embryonic lethality remained, likely as a result of endothelial hyperproliferation. Our results confirm the lethality associated with heterozygous expression of the Pik3ca(H1047R) mutation during development and likely explain the lack of inherited germline PIK3CA mutations in humans.
11. Essential role of Gab1 for signaling by the c-Met receptor in vivo. The docking protein Gab1 binds phosphorylated c-Met receptor tyrosine kinase directly and mediates signals of c-Met in cell culture. Gab1 is phosphorylated by c-Met and by other receptor and nonreceptor tyrosine kinases. Here, we report the functional analysis of Gab1 by targeted mutagenesis in the mouse, and compare the phenotypes of the Gab1 and c-Met mutations. Gab1 is essential for several steps in development: **migration** of myogenic precursor cells into the limb anlage is impaired in Gab1-/- embryos. As a consequence, extensor muscle groups of the forelimbs are virtually absent, and the flexor muscles reach less far. Fewer hindlimb muscles exist, which are smaller and disorganized. Muscles in the diaphragm, which also originate from migratory precursors, are missing. Moreover, Gab1-/- embryos die in a broad time window between E13.5 and E18.5, and display reduced liver size and placental defects. The labyrinth layer, but not the spongiotrophoblast layer, of the placenta is severely reduced, resulting in impaired communication between maternal and fetal circulation. Thus, extensive similarities between the phenotypes of c-Met and HGF/SF mutant mice exist, and the muscle **migration** phenotype is even more pronounced in Gab1-/-:c-Met+/- embryos. This is genetic evidence that Gab1 is essential for c-Met signaling in vivo. Analogy exists to signal transmission by insulin receptors, which require IRS1 and IRS2 as specific docking proteins.
12. Identification of Flk-1 target genes in vasculogenesis: Pim-1 is required for endothelial and mural cell differentiation in vitro. The tyrosine kinase receptor fetal liver kinase 1 (Flk-1) plays a crucial role in vasculogenesis and angiogenesis, but its target genes remain elusive. Comparing Flk-1(+/+) with Flk-1(-/-) embryonic stem (ES) cells, we identified transcripts regulated by the vascular endothelial growth factor A (VEGF-A)/Flk-1 pathway at an early stage of their differentiation to endothelial and mural precursors. Further analysis of a number of these genes (Nm23-M1, Nm23-M2, Slug, Set, pp32, Cbp, Ship-1, Btk, and Pim-1) showed that their products were transiently up-regulated in vivo in endothelial cells (ECs) during angiogenesis of the ovary, and their mRNA was rapidly induced in vitro by VEGF-A in human umbilical cord vein endothelial cells (HUVECs). Functional analysis by RNA interference (RNAi) in ES cells induced to differentiate demonstrated that Pim-1 is required for their differentiation into ECs and smooth muscle cells (SMCs). In HUVECs, RNAi showed that Pim-1 is required in ECs for VEGF-A-dependent proliferation and **migration**. The identification of Flk-1 target genes should help in elucidating the molecular pathways that govern the vasculogenesis and angiogenesis processes.
13. Bovine papular stomatitis virus encodes a functionally distinct VEGF that binds both VEGFR-1 and VEGFR-2. Bovine papular stomatitis virus (BPSV), a member of the genus Parapoxvirus, causes proliferative dermatitis in cattle and humans. Other species of the genus cause similar lesions, the nature of which has been attributed, at least in part, to a viral-encoded vascular endothelial growth factor (VEGF) that induces vascularization and dermal oedema through VEGF receptor-2 (VEGFR-2). The results of this study showed that BPSV strain V660 encodes a novel VEGF and that the predicted BPSV protein showed only 33-52% amino acid identity to VEGFs encoded by the other species of the genus. BPSV VEGF showed higher identity to mammalian VEGF-A (51%) than the other parapoxviral VEGFs (31-46%). Assays of the purified BPSV VEGF (BPSVV660VEGF) demonstrated that it was also functionally more similar to VEGF-A, as it showed significant binding to VEGFR-1 and induced monocyte **migration**. Like VEGF-A and the other viral VEGFs, BPSVV660VEGF bound VEGFR-2 with high affinity. Sequence analysis and structural modelling of BPSVV660VEGF revealed specific residues, outside the known receptor-binding face, that are predicted either to influence VEGF structure or to mediate binding directly to the VEGFRs. These results indicate that BPSVV660VEGF is a biologically active member of the VEGF family and that, via its interaction with VEGFR-2, it is likely to contribute to the proliferative and highly vascularized nature of BPSV lesions. This is also the first example of a viral VEGF acting via VEGFR-1 and influencing haematopoietic cell function. These data suggest that BPSVV660VEGF is an evolutionary and functional intermediate between VEGF-A and the other parapoxviral VEGFs.
14. Comparative structure-function analysis of VEGFR-1 and VEGFR-2: What have we learned from chimeric systems? Activation of vascular endothelial growth factor receptor-1 and -2 (VEGFR-1 and VEGFR-2) plays a critical role in vasculogenesis and angiogenesis. However, the mechanism by which activation of VEGFRs elicits these cellular events is not fully understood. We recently constructed chimeric receptors containing the extracellular domain of human CSF-1R/c-fms fused with the entire transmembrane and cytoplasmic domains of murine VEGFR-1 and VEGFR-2. Selective activation of chimeric VEGFR-2, but not chimeric VEGFR-1, stimulated endothelial cell growth, **migration**, and differentiation. Stimulation of cells coexpressing chimeric VEGFR-1 and VEGFR-2 suppressed VEGFR-2-mediated endothelial cell growth. Site-directed mutagenesis demonstrated that tyrosines 799 and 1173 are required for VEGFR-2-mediated endothelial cell growth and activation of PI3 kinase. Further site-directed mutagenesis demonstrated that tyrosine 1212, located in the carboxyl tail of VEGFR-2, is required for the ligand-dependent autophosphorylation of the receptor and its ability to activate signaling proteins. Collectively, our results suggest that activation of VEGFR-1 and VEGFR-2 differentially regulates endothelial cell function and angiogenesis. Second, activation of VEGFR-2 is associated with many endothelial cell functions, including cell proliferation, **migration**, and differentiation. Third, activation of PI3 kinase by VEGFR-2 regulates endothelial cell proliferation.
15. SnoN facilitates ALK1-Smad1/5 signaling during embryonic angiogenesis. In endothelial cells, two type I receptors of the transforming growth factor β (TGF-β) family, ALK1 and ALK5, coordinate to regulate embryonic angiogenesis in response to BMP9/10 and TGF-β. Whereas TGF-β binds to and activates ALK5, leading to Smad2/3 phosphorylation and inhibition of endothelial cell proliferation **and migra**tion, BMP9/10 and TGF-β also bind to ALK1, resulting in the activation of Smad1/5. SnoN is a negative regulator of ALK5 signaling through the binding and repression of Smad2/3. Here we uncover a positive role of SnoN in enhancing Smad1/5 activation in endothelial cells to promote angiogenesis. Upon ligand binding, SnoN directly bound to ALK1 on the plasma membrane and facilitated the interaction between ALK1 and Smad1/5, enhancing Smad1/5 phosphorylation. Disruption of this SnoN-Smad interaction impaired Smad1/5 activation and up-regulated Smad2/3 activity. This resulted in defective angiogenesis and arteriovenous malformations, leading to embryonic lethality at E12.5. Thus, SnoN is essential for TGF-β/BMP9-dependent biological processes by its ability to both positively and negatively modulate the activities of Smad-dependent pathways.
16. Distinct requirements for Gab1 in Met and EGF receptor signaling in vivo. Gab1 is a multiadaptor protein that has been shown to be required for multiple processes in embryonic development and oncogenic transformation. Gab1 functions by amplifying signal transduction downstream of various receptor tyrosine kinases through recruitment of multiple signaling effectors, including phosphatidylinositol 3-kinase and Shp2. Until now, the functional significance of individual interactions in vivo was not known. Here we have generated knockin mice that carry point mutations in either the P13K or Shp2 binding sites of Gab1. We show that different effector interactions with Gab1 play distinct biological roles downstream of Gab1 during the development of different organs. Recruitment of phosphatidylinositol 3-kinase by Gab1 is essential for EGF receptor-mediated embryonic eyelid closure and keratinocyte **migration**, and the Gab1-Shp2 interaction is crucial for Met receptor-directed placental development and muscle progenitor cell **migration** to the limbs. Furthermore, we investigate the dual association of Gab1 with the Met receptor. By analyzing knockin mice with mutations in the Grb2 or Met binding site of Gab1, we show that the requirements for Gab1 recruitment to Met varies in different biological contexts. Either the direct or the indirect interaction of Gab1 with Met is sufficient for Met-dependent muscle precursor cell **migration**, whereas both modes of interaction are required and neither is sufficient for placenta development, liver growth, and palatal shelf closure. These data demonstrate that Gab1 induces different biological responses through the recruitment of distinct effectors and that different modes of recruitment for Gab1 are required in different organs.
17. VEGFR-1 regulates adult olfactory bulb neurogenesis and **migration** of neural progenitors in the rostral migratory stream in vivo. The generation of new neurons in the olfactory bulb (OB) persists into adulthood and is a multistep process that includes proliferation, fate choice, **migration**, survival, and differentiation. Neural precursor cells destined to form olfactory interneurons arise in the subventricular zone (SVZ) and migrate along the rostral migratory stream (RMS) to the OB. Recently, some factors classically known from their effects on the vascular system have been found to influence different steps of adult neurogenesis. In the present study, we report a modulatory function for the vascular endothelial growth factor receptor-1 (VEGFR-1) in adult olfactory neurogenesis. We identified expression of VEGFR-1 in GFAP-positive cells within regions involved in neurogenesis of the adult mouse brain. To determine functions for VEGFR-1 in adult neurogenesis, we compared neural progenitor cell proliferation, **migration**, and differentiation from wild-type and VEGFR-1 signaling-deficient mice (Flt-1TK(-/-) mice). Our data show that VEGFR-1 signaling is involved in the regulation of proliferation of neuronal progenitor cells within the SVZ, **migration** along the RMS, and in neuronal differentiation and anatomical composition of interneuron subtypes within the OB. RMS **migration** in Flt-1TK(-/-) mice was altered mainly as a result of increased levels of its ligand VEGF-A, which results in an increased phosphorylation of VEGFR-2 in neuronal progenitor cells within the SVZ and the RMS. This study reveals that proper RMS **migration** is dependent on endogenous VEGF-A protein.
18. The PTEN/PI3K pathway governs normal vascular development and tumor angiogenesis. PTEN is an important tumor suppressor gene. Hereditary mutation of PTEN causes tumor-susceptibility diseases such as Cowden disease. We used the Cre-loxP system to generate an endothelial cell-specific mutation of Pten (Tie2CrePten) in mice. Tie2CrePten(flox/+) mice displayed enhanced tumorigenesis due to an increase in angiogenesis driven by vascular growth factors. This effect was partially dependent on the PI3K subunits p85alpha and p110gamma. In vitro, Tie2CrePten(flox/+) endothelial cells showed enhanced proliferation/**migration**. Tie2CrePten(flox/flox) mice died before embryonic day 11.5 (E11.5) due to bleeding and cardiac failure caused by impaired recruitment of pericytes and vascular smooth muscle cells to blood vessels, and of cardiomyocytes to the endocardium. These phenotypes depend strongly on p110gamma rather than on p85alpha and were associated with decreased expression of Ang-1, VCAM-1, connexin 40, and ephrinB2 but increased expression of Ang-2, VEGF-A, VEGFR1, and VEGFR2. Pten is thus indispensable for normal cardiovascular morphogenesis and post-natal angiogenesis, including tumor angiogenesis.
19. Vascular endothelial growth factor a signaling in the podocyte-endothelial compartment is required for mesangial cell **migration** and survival. The glomerular filtration barrier separates the blood from the urinary space and consists of two major cell types: podocytes and fenestrated endothelial cells. Mesangial cells sit between the capillary loops and provide structural support. Proliferation and loss of mesangial cells both are central findings in a number of renal diseases, including diabetic nephropathy and mesangiolysis, respectively. Using cell-specific gene targeting, it was shown previously that vascular endothelial growth factor A (VEGF-A) production by podocytes is required for glomerular endothelial cell **migration**, differentiation, and survival. For further investigation of the effect of gene dose and VEGF-A knockdown within the glomerulus, mice that carry one hypomorphic VEGF-A allele and one podocyte-specific null VEGF-A allele (VEGFhypo/loxP,Neph-Cre+/-) were generated; in these mice, the "allelic dose" of VEGF-A is intermediate between glomerular-specific heterozygous and null states. VEGFhypo/loxP,Neph-Cre+/- mice die at 3 wk of age from renal failure. Although endothelial cell defects are observed, striking loss of mesangial cells occurs postnatally. In addition, differentiated mesangial cells cannot be found in glomeruli of podocyte-specific null VEGF-A mice (VEGFloxP/loxP,Cre+/-). Together, these results demonstrate a key role for VEGF-A production in the podocyte for mesangial cell survival and differentiation.
20. Herbal compound farnesiferol C exerts antiangiogenic and antitumor activity and targets multiple aspects of VEGFR1 (Flt1) or VEGFR2 (Flk1) signaling cascades. Farnesiferol C (FC) is one of the major compounds isolated from Ferula assafoetida, an Asian herbal spice used for cancer treatment as a folk remedy. Here, we examined the hypothesis that novel antiangiogenic activities of FC contribute to anticancer efficacy. In human umbilical vein endothelial cells (HUVEC), exposure to the 10 to 40 mumol/L concentration range of FC inhibited vascular endothelial growth factor (VEGF)-induced cell proliferation, **migration**, invasion, tube formation, and the expression of matrix metalloproteinase-2. In addition, FC inhibited the angiogenic sprouting of VEGF-treated rat aorta in an ex vivo model. Furthermore, FC inhibited the in vivo growth of mouse Lewis lung cancer allograft model by 60% (P < 0.001) at a daily i.p. dosage of 1 mg/kg body weight without any negative effect on the weight of the host mice. Immunohistochemistry staining showed decreased microvessel density (CD34) and proliferative index (Ki-67) without affecting the apoptotic (terminal deoxynucleotidyl transferase-mediated dUTP nick end labeling) index. Mechanistically, FC decreased the binding of VEGF to VEGFR1/Flt-1, but not to VEGFR2/KDR/Flk-1. In terms of early signaling, FC exerted a rapid inhibitory action (examined within 10 minutes) on VEGF-induced autophosphorylation of VEGFR1 without affecting that of VEGFR2. Nevertheless, FC decreased the phosphorylation of most of the kinases downstream of VEGFR2: focal adhesion kinase, Src, extracellular signal-regulated kinase 1/2, p38 mitogen-activated protein kinase, and c-jun-NH(2)-kinase without affecting AKT. Computer simulation suggests that FC may inhibit Src or focal adhesion kinase protein activities directly through its docking to their ATP-binding sites. Taken together, the multitargeting actions of FC, particularly VEGFR1 inhibition, may make it a novel drug candidate to complement current VEGF/VEGFR2-targeting antiangiogenic modalities for cancer.
21. Flt-1, but not Flk-1 mediates hyperpermeability through activation of the PI3-K/Akt pathway. Vascular endothelial growth factor (VEGF), a potent mediator of endothelial proliferation and **migration**, has an important role also in brain edema formation during hypoxia and ischemia. VEGF binds to the tyrosine kinase receptors Flt-1 and Flk-1. Yet, their relative importance for hypoxia-induced hyperpermeability is not well understood. We used an in vitro blood-brain barrier (BBB) model consisting of porcine brain microvascular endothelial cells (BMEC) to determine the role of Flt-1 in VEGF-induced endothelial cell (EC) barrier dysfunction. Soluble Flt-1 abolished hypoxia/VEGF-induced hyperpermeability. Furthermore, selective antisense oligonucleotides to Flt-1, but not to Flk-1, inhibited hypoxia-induced permeability changes. Consistent with these data, addition of the receptor-specific homolog placenta-derived growth factor, which binds Flt-1 but not Flk-1, increased endothelial permeability to the same extent as VEGF, whereas adding VEGF-E, a viral VEGF molecule from the orf virus family activating Flk-1 and neuropilin-1, but not Flt-1, did not show any effect. Using the carcinoma submandibular gland cell line (CSG), only expressing Flt-1, it was demonstrated that activation of Flt-1 is sufficient to induce hyperpermeability by hypoxia and VEGF. Hyperpermeability, induced by hypoxia/VEGF, depends on activation of phosphatidylinositol 3-kinase/Akt (PI3-K/Akt), nitric oxide synthase (NOS) and protein kinase G (PKG). The activation of the PI3-K/Akt pathway by hypoxia was confirmed using an in vivo mice hypoxia model. These results demonstrate that hypoxia/VEGF-induced hyperpermeability can be mediated by activation of Flt-1 independently on the presence of Flk-1 and indicate a central role for activation of the PI3-K/Akt pathway, followed by induction of NOS and PKG activity.
22. Early endocardial morphogenesis requires Scl/Tal1. The primitive heart tube is composed of an outer myocardial and an inner endocardial layer that will give rise to the cardiac valves and septa. Specification and differentiation of these two cell layers are among the earliest events in heart development, but the embryonic origins and genetic regulation of early endocardial development remain largely undefined. We have analyzed early endocardial development in the zebrafish using time-lapse confocal microscopy and show that the endocardium seems to originate from a region in the lateral plate mesoderm that will give rise to hematopoietic cells of the primitive myeloid lineage. Endocardial precursors appear to rapidly migrate to the site of heart tube formation, where they arrive prior to the bilateral myocardial primordia. Analysis of a newly discovered zebrafish Scl/Tal1 mutant showed an additional and previously undescribed role of this transcription factor during the development of the endocardium. In Scl/Tal1 mutant embryos, endocardial precursors are specified, but **migration** is severely defective and endocardial cells aggregate at the ventricular pole of the heart. We further show that the initial fusion of the bilateral myocardial precursor populations occurs independently of the endocardium and tal1 function. Our results suggest early separation of the two components of the primitive heart tube and imply Scl/Tal1 as an indispensable component of the molecular hierarchy that controls endocardium morphogenesis.
23. Loss of Notch signalling induced by Dll4 causes arterial calibre reduction by increasing endothelial cell response to angiogenic stimuli. In the vascular system, Notch receptors and ligands are expressed mainly on arteries, with Delta-like 4 (Dll4) being the only ligand known to be expressed early during the development of arterial endothelial cells and capillaries. Dll4 null embryos die very early in development with severely reduced arterial calibre and lumen and loss of arterial cell identity.The current detailed analysis of these mutants shows that the arterial defect precedes the initiation of blood flow and that the arterial Dll4-/- endothelial cells proliferate and migrate more actively. Dll4-/- mutants reveal a defective basement membrane around the forming aorta and increased endothelial cell **migration** from the dorsal aorta to peripheral regions, which constitute the main causes of arterial lumen reduction in these embryos. The increased proliferation and **migration** of Dll4-/- endothelial cells was found to coincide with increased expression of the receptors VEGFR-2 and Robo4 and with downregulation of the TGF-beta accessory receptor Endoglin.Together, these results strongly suggest that Notch signalling can increase arterial stability and calibre by decreasing the response of arterial endothelial cells to local gradients of pro-angiogenic factors like VEGF.
24. Flt-1 lacking the tyrosine kinase domain is sufficient for normal development and angiogenesis in mice. Receptor tyrosine kinases Flt-1 and Flk-1/KDR, and their ligand, the vascular endothelial growth factor (VEGF), were shown to be essential for angiogenesis in the mouse embryo by gene targeting. Flk-1/KDR null mutant mice exhibited impaired endothelial and hematopoietic cell development. On the other hand, Flt-1 null mutation resulted in early embryonic death at embryonic day 8.5, showing disorganization of blood vessels, such as overgrowth of endothelial cells. Flt-1 differs from Flk-1 in that it displays a higher affinity for VEGF but lower kinase activity, suggesting the importance of its extracellular domain. To examine the biological role of Flt-1 in embryonic development and vascular formation, we deleted the kinase domain without affecting the ligand binding region. Flt-1 tyrosine kinase-deficient homozygous mice (flt-1(TK-/-)) developed normal vessels and survived. However, VEGF-induced macrophage **migration** was strongly suppressed in flt-1(TK-/-) mice. These results indicate that Flt-1 without tyrosine kinase domain is sufficient to allow embryonic development with normal angiogenesis, and that a receptor tyrosine kinase plays a main biological role as a ligand-binding molecule.
25. VEGFR1-positive macrophages facilitate liver repair and sinusoidal reconstruction after hepatic ischemia/reperfusion injury. Liver repair after acute liver injury is characterized by hepatocyte proliferation, removal of necrotic tissue, and restoration of hepatocellular and hepatic microvascular architecture. Macrophage recruitment is essential for liver tissue repair and recovery from injury; however, the underlying mechanisms are unclear. Signaling through vascular endothelial growth factor receptor 1 (VEGFR1) is suggested to play a role in macrophage **migration** and angiogenesis. The aim of the present study was to examine the role of VEGFR1 in liver repair and sinusoidal reconstruction after hepatic ischemia/reperfusion (I/R). VEGFR1 tyrosine kinase knockout mice (VEGFR1 TK-/- mice) and wild-type (WT) mice were subjected to hepatic warm I/R, and the processes of liver repair and sinusoidal reconstruction were examined. Compared with WT mice, VEGFR1 TK-/- mice exhibited delayed liver repair after hepatic I/R. VEGFR1-expressing macrophages recruited to the injured liver showed reduced expression of epidermal growth factor (EGF). VEGFR1 TK-/- mice also showed evidence of sustained sinusoidal functional and structural damage, and reduced expression of pro-angiogenic factors. Treatment of VEGFR1 TK-/- mice with EGF attenuated hepatoceullar and sinusoidal injury during hepatic I/R. VEGFR1 TK-/- bone marrow (BM) chimeric mice showed impaired liver repair and sinusoidal reconstruction, and reduced recruitment of VEGFR1-expressing macrophages to the injured liver. VEGFR1-macrophages recruited to the liver during hepatic I/R contribute to liver repair and sinusoidal reconstruction. VEGFR1 activation is a potential therapeutic strategy for promoting liver repair and sinusoidal restoration after acute liver injury.
26. Distinct roles of vascular endothelial growth factor receptor-1- and receptor-2-mediated signaling in T cell priming and Th17 polarization to lipopolysaccharide-containing allergens in the lung. Vascular endothelial growth factor (VEGF) is a key mediator in the development of airway immune dysfunction to inhaled allergens. However, the exact role of its receptors-mediated signaling is controversial. In this study, we evaluated the role of VEGF receptor (VEGFR)-1- and VEGFR-2-mediated signaling in T cell priming and polarization in the context of inhalation of LPS-containing allergens. A murine asthma model of mixed Th1 and Th17 cell responses was generated using intranasal sensitization with LPS-containing allergens. Pharmacologic intervention was performed during sensitization. In vivo production of VEGF and Th1- and Th17-polarizing cytokines (IL-12p70 and IL-6, respectively) were upregulated by airway exposure to LPS. Pharmacological intervention with a VEGFR-2-neutralizing Ab (anti-Flk1 mAb) abolished the production of IL-6 (but not IL-12p70) and the subsequent development of allergen-specific Th17 cell response. On the other hand, blocking VEGFR-1 signaling with a VEGFR-1 antagonist (anti-Flt1 hexapeptide) did not affect the production of IL-12p70 and IL-6. However, blocking VEGFR-1 signaling resulted in T cell tolerance rather than priming, mainly by inhibiting the maturation of lung dendritic cells, and their **migration** into lung-draining lymph nodes. These results suggest that T cell priming to LPS-containing allergens depends on VEGFR-1-mediated signaling, and the subsequent Th17 polarization depends on VEGFR-2 signaling.
27. Endothelial progenitor cells induce transplant arteriosclerosis via VEGFR-1/2 activity. Acute rejection (AR) after organ transplantation results in transplant arteriosclerosis (TA). Endothelial progenitor cells (EPCs) are involved in tissue repair and blood vessel formation but are suspected to be a cause of TA.In this study, we introduced a syngeneic and allogeneic abdominal aortic transplant model with C57BL/6 and BALB/c mice. Syngeneic and allogeneic grafts were histopathologically analyzed after transplantation. Bone marrow-derived EPCs were injected into transplant model animals to observe their distribution and temporal concentration changes. Changes of vascular endothelial growth factor receptor 1 (VEGFR-1), phosphorylated VEGFR-1 (pVEGFR-1), VEGFR-2, pVEGFR-2, protein kinase B (Akt), pAkt, extracellular signal-regulated kinase 1 (Erk1), pErk1 levels in EPCs upon VEGF165 and the VEGFR inhibitor Vandetanib exposure were analyzed in vitro with western blotting.In the allogeneic transplant group, two weeks after transplantation, formations of new intima layers could be observed, and its proliferation gradually increased to four and six weeks post-transplantation (p < 0.05), accompanied by significant arterial stenoses. Exogenous EPCs mainly localized to the damaged sites of the transplant arteries. In vivo, Vandetanib caused a significant dose dependent decrease of transplant hyperplasia (p < 0.05) and inhibited VEGF related prolifera**tion, mig**ration and adhesion of EPCs.Vandetanib treatment can reduce arteriosclerosis induced by abdominal aorta transplantation by blocking VEGFRs in EPCs.
28. The VEGF receptor flt-1 (VEGFR-1) is a positive modulator of vascular sprout formation and branching morphogenesis. Sprouting angiogenesis is critical to blood vessel formation, but the cellular and molecular controls of this process are poorly understood. We used time-lapse imaging of green fluorescent protein (GFP)-expressing vessels derived from stem cells to analyze dynamic aspects of vascular sprout formation and to determine how the vascular endothelial growth factor (VEGF) receptor flt-1 affects sprouting. Surprisingly, loss of flt-1 led to decreased sprout formation and **migration**, which resulted in reduced vascular branching. This phenotype was also seen in vivo, as flt-1(-/-) embryos had defective sprouting from the dorsal aorta. We previously showed that loss of flt-1 increases the rate of endothelial cell division. However, the timing of division versus morphogenetic effects suggested that these phenotypes were not causally linked, and in fact mitoses were prevalent in the sprout field of both wild-type and flt-1(-/-) mutant vessels. Rather, rescue of the branching defect by a soluble flt-1 (sflt-1) transgene supports a model whereby flt-1 normally positively regulates sprout formation by production of sflt-1, a soluble form of the receptor that antagonizes VEGF signaling. Thus precise levels of bioactive VEGF-A and perhaps spatial localization of the VEGF signal are likely modulated by flt-1 to ensure proper sprout formation during blood vessel formation.
29. Increased hemangioblast commitment, not vascular disorganization, is the primary defect in flt-1 knock-out mice. We previously demonstrated the essential role of the flt-1 gene in regulating the development of the cardiovascular system. While the inactivation of the flt-1 gene leads to a very severe disorganization of the vascular system, the primary defect at the cellular level was unknown. Here we report a surprising finding that it is an increase in the number of endothelial progenitors that leads to the vascular disorganization in flt-1(-/-) mice. At the early primitive streak stage (prior to the formation of blood islands), hemangioblasts are formed much more abundantly in flt-1(-/-) embryos. This increase is primarily due to an alteration in cell fate determination among mesenchymal cells, rather than to increased proliferation, **migration** or reduced apoptosis of flt-1(-/-) hemangioblasts. We further show that the increased population density of hemangioblasts is responsible for the observed vascular disorganization, based on the following observations: (1) both flt-1(-/-) and flt-1(+/+) endothelial cells formed normal vascular channels in chimaeric embryos; (2) wild-type endothelial cells formed abnormal vascular channels when their population density was significantly increased; and (3) in the absence of wild-type endothelial cells, flt-1(-/-) endothelial cells alone could form normal vascular channels when sufficiently diluted in a developing embryo. These results define the primary defect in flt-1(-/-) embryos at the cellular level and demonstrate the importance of population density of progenitor cells in pattern formation.
30. Edg-1, the G protein-coupled receptor for sphingosine-1-phosphate, is essential for vascular maturation. Sphingolipid signaling pathways have been implicated in many critical cellular events. Sphingosine-1-phosphate (SPP), a sphingolipid metabolite found in high concentrations in platelets and blood, stimulates members of the endothelial differentiation gene (Edg) family of G protein-coupled receptors and triggers diverse effects, including cell growth, survival, **migration**, and morphogenesis. To determine the in vivo functions of the SPP/Edg signaling pathway, we disrupted the Edg1 gene in mice. Edg1(-/-) mice exhibited embryonic hemorrhage leading to intrauterine death between E12.5 and E14.5. Vasculogenesis and angiogenesis appeared normal in the mutant embryos. However, vascular maturation was incomplete due to a deficiency of vascular smooth muscle cells/pericytes. We also show that Edg-1 mediates an SPP-induced **migration** response that is defective in mutant cells due to an inability to activate the small GTPase, Rac. Our data reveal Edg-1 to be the first G protein-coupled receptor required for blood vessel formation and show that sphingolipid signaling is essential during mammalian development.
31. Vascular endothelial growth factor receptor-1 signaling promotes mobilization of macrophage lineage cells from bone marrow and stimulates solid tumor growth. Vascular endothelial growth factor and its receptors, including Flt-1 and Flk-1, are involved in angiogenesis under physiologic and pathologic conditions. Recently, Flt-1-expressing cells were reported to contribute to the intracranial growth of glioma cells. However, the role of Flt-1 signaling in solid tumor growth in s.c. tissue has not been elucidated. To investigate how Flt-1 signaling is involved in the proliferation of solid tumors, we implanted tumor cells into wild-type (Wt) and Flt-1 tyrosine kinase (TK)-deficient (Flt-1 TK(-/-)) mice. Growth of HSML and B16 but not Lewis lung carcinoma cell in s.c. tissue was significantly decreased in Flt-1 TK(-/-) mice. Angiogenesis in HSML and B16 tumors was remarkably reduced in Flt-1 TK(-/-) mice. Moreover, the infiltration of macrophage lineage cells into HSML and B16 tumors was clearly suppressed in Flt-1 TK(-/-) mice. Pericyte marker(+) cells were also reduced in Flt-1 TK(-/-) mice. However, in the border area of tumor, angiogenesis and the infiltration of macrophage lineage cell were basically similar between Wt and Flt-1 TK(-/-) mice. In bone marrow (BM) transplantation experiments, tumor angiogenesis, infiltration of macrophage lineage cells, and tumor growth were significantly suppressed in Wt/Flt-1 TK(-/-) mice implanted with Flt-1 TK(-/-) BM cells compared with those implanted with Wt BM cells. We conclude that Flt-1 signaling is involved in the function of BM-derived cell, such as the **migration** of macrophages into cancerous tissues, and significantly contributes to angiogenesis and tumor progression.
32. Identification of Thymosin β4 as an effector of Hand1-mediated vascular development. The bHLH transcription factor Hand1 (Heart and neural crest-derived transcript-1) has a fundamental role in cardiovascular development; however, the molecular mechanisms have not been elucidated. In this paper we identify Thymosin β4 (Tβ4/Tmsb4x), which encodes an actin monomer-binding protein implicated in ce**ll migrat**ion and angiogenesis, as a direct target of Hand1. We demonstrate that Hand1 binds an upstream regulatory region proximal to the promoter of Tβ4 at consensus Thing1 and E-Box sites and identify both activation and repression of Tβ4 by Hand1, through direct binding within either non-canonical or canonical E-boxes, providing new insight into gene regulation by bHLH transcription factors. Hand1-mediated activation of Tβ4 is essential for yolk sac vasculogenesis and embryonic survival, and administration of synthetic TB4 partially rescues yolk sac capillary plexus formation in Hand1-null embryos. Thus, we identify an in vivo downstream target of Hand1 and reveal impaired yolk sac vasculogenesis as a primary cause of early embryonic lethality following loss of this critical bHLH factor.

#### GeneRIFs

1. VEGFR-1 is a negative regulator of adult olfactory neurogenesis and **migration**, acting predominantly via a paracrine mechanism.
2. assays of purified bovine papular stomatitis virus VEGF (BPSVV660VEGF) demonstrated that it showed significant binding to VEGFR-1 and induced monocyte **migration**

### [Ee]xtracellular [Mm]atrix

#### PubMed

1. Thymosin beta 4 is dispensable for murine cardiac development and function. Thymosin beta 4 (Tβ4) is a 43-amino acid factor encoded by an X-linked gene. Recent studies have suggested that Tβ4 is a key factor in cardiac development, growth, disease, epicardial integrity, and blood vessel formation. Cardiac-specific short hairpin (sh)RNA knockdown of tβ4 has been reported to result in embryonic lethality at E14.5-16.5, with severe cardiac and angiogenic defects. However, this shRNA tβ4-knockdown model did not completely abrogate Tβ4 expression. To completely ablate Tβ4 and to rule out the possibility of off-target effects associated with shRNA gene silencing, further studies of global or cardiac-specific knockouts are critical.We examined the role of Tβ4 in developing and adult heart through global and cardiac specific tβ4-knockout mouse models.Global tβ4-knockout mice were born at mendelian ratios and exhibited normal heart and blood vessel formation. Furthermore, in adult global tβ4-knockout mice, cardiac function, capillary density, expression of key cardiac fetal and angiogenic genes, epicardial marker expres**sion, and extracellu**lar matrix deposition were indistinguishable from that of controls. Tissue-specific tβ4-deficient mice, generated by crossing tβ4-floxed mice to Nkx2.5-Cre and αMHC-Cre, were also found to have no phenotype.We conclude that Tβ4 is dispensable for embryonic viability, heart development, coronary vessel development, and adult myocardial function.
2. Forkhead box transcription factor FoxC1 preserves corneal transparency by regulating vascular growth. Normal vision requires the precise control of vascular growth to maintain corneal transparency. Here we provide evidence for a unique mechanism by which the Forkhead box transcription factor FoxC1 regulates corneal vascular development. Murine Foxc1 is essential for development of the ocular anterior segment, and in humans, mutations have been identified in Axenfeld-Rieger syndrome, a disorder characterized by anterior segment dysgenesis. We show that FOXC1 mutations also lead to corneal angiogenesis, and that mice homozygous for either a global (Foxc1(-/-)) or neural crest (NC)-specific (NC-Foxc1(-/-)) null mutation display excessive growth of corneal blood and lymphatic vessels. This is associated with disorganization of the **extracellular matrix** and increased expression of multiple matrix metalloproteinases. Heterozygous mutants (Foxc1(+/-) and NC-Foxc1(+/-)) exhibit milder phenotypes, such as disrupted limbal vasculature. Moreover, environmental exposure to corneal injury significantly increases growth of both blood and lymphatic vessels in both Foxc1(+/-) and NC-Foxc1(+/-) mice compared with controls. Notably, this amplification of the angiogenic response is abolished by inhibition of VEGF receptor 2. Collectively, these findings identify a role for FoxC1 in inhibiting corneal angiogenesis, thereby maintaining corneal transparency by regulating VEGF signaling.

### [Ii]nvasive

#### PubMed

1. The imprinted Phlda2 gene regulates extraembryonic energy stores. An important difference between placental mammals and marsupials is the maturity of the fetus at birth. Placental mammals achieved this maturity by developing a complex and **invasive** placenta to support and prolong internal development. The exact genomic modifications that facilitated the evolution of this complex structure are unknown, but the emergence of genomic imprinting within mammalian lineages suggests a role for gene dosage. Here we show that a maximally altered placental structure is achieved by a single extra dose of the imprinted Phlda2 gene characterized by a dramatically reduced junctional zone and a decrease in stored glycogen. In addition, glycogen cells do not migrate into the maternal decidua in a timely fashion, but instead, Tpbpa-positive cells progressively mislocalize into the labyrinth. These defects are linked to a progressive restriction of embryonic growth from embryonic day 16.5. This work has identified a critical role for the imprinted Phlda2 gene in regulating glycogen storage in the eutherian placenta and implies that imprinting has provided a mechanism to boost nutrient supply to the fetus late in gestation, when the fetus is placing the highest demands on maternal resources, to enhance growth.
2. Multiparity increases trophoblast invasion and vascular endothelial growth factor expression at the maternal-fetal interface in mice. To analyze immunomodulating effects related to parity status, we studied trophoblast invasion grade, placental expression and systemic concentration of VEGF and its receptor Flt-1 in normal fertile (CBA/JxBALB/c) mice and abortion-prone (CBA/JxDBA/2) H-2(d)xH-2(k) mice. BALB/c or DBA/2 mated CBA/J females were, respectively, divided into the following groups: primiparous young (3.0+/-0.5 months old); primiparous old (8.5+/-0.5 months old) and multiparous old (8.5+/-0.5 months old, with 4 pregnancies). Immunohistochemical analysis of term placentae from both multiparous groups revealed various layers of **invasive** trophoblast tissue, identified as cytokeratin+/vimentin- cells, in contrast to the single layer detected in the placentae of primiparous animals, indicating that multiparity increases trophoblast invasion regardless of the success of the pregnancy outcome. **Invasive** trophoblast tissue from primiparous CBA/JxDBA/2 placentae showed diminished VEGF expression in comparison with the normal fertile group, while both multiparous groups demonstrated high expression of VEGF in the **invasive** trophoblast tissue. Placental expression of Flt-1 was similar in all groups. However, the primiparous CBA/JxBALB/c group showed the highest plasma concentration of sFlt-1 at term, while both multiparous groups demonstrated low circulating levels. No differences in circulating VEGF levels were observed among the groups. These results demonstrate an increase in trophoblast invasion tissue and expression of VEGF in the maternal-fetal interface in multiparous mice compared to primiparous mice. Moreover, the placenta appears to be able to regulate the circulating levels of VEGF by releasing sFlt-1.

### [Mm]etastasis

#### PubMed

1. Chemotherapy enhances **metastasis** formation via VEGFR-1-expressing endothelial cells. Recent studies suggest that chemotherapy, in addition to its cytotoxic effects on tumor cells, can induce a cascade of host events to support tumor growth and spread. Here, we used an experimental pulmonary **metastasis** model to investigate the role of this host response in **metastasis** formation. Mice were pretreated with chemotherapy and after clearance of the drugs from circulation, tumor cells were administered intravenously to study potential "protumorigenic" host effects of chemotherapy. Pretreatment with the commonly used chemotherapeutic agents cisplatin and paclitaxel significantly enhanced lung **metastasis** in this model. This corresponded to enhanced adhesion of tumor cells to an endothelial cell monolayer that had been pretreated with chemotherapy in vitro. Interestingly, chemotherapy exposure enhanced the expression of VEGF receptor 1 (VEGFR-1) on endothelial cells both in vitro and in vivo. Administration of antibodies targeting VEGFR-1 reversed the early retention of tumor cells in the lungs, thereby preventing the formation of chemotherapy-induced pulmonary metastases. The data indicate that chemotherapy-induced expression of VEGFR-1 on endothelial cells can create an environment favorable to tumor cell homing. Inhibition of VEGFR-1 function may therefore be used to counteract chemotherapy-induced retention of tumor cells within the metastatic niche, providing a novel level of tumor control in chemotherapy.
2. VEGFR1 activity modulates myeloid cell infiltration in growing lung metastases but is not required for spontaneous **metastasis** formation. The role of vascular endothelial growth factor receptor 1 (VEGFR1/Flt1) in tumor **metastasis** remains incompletely characterized. Recent reports suggested that blocking VEGFR1 activity or the interaction with its ligands (VEGF and PlGF) has anti-tumor effects. Moreover, several studies showed that VEGFR1 mediates tumor progression to distant **metastasis**. All these effects may be exerted indirectly by recruitment of bone marrow-derived cells (BMDCs), such as myeloid cells. We investigated the role of VEGFR1 activity in BMDCs during the pre-metastatic phase, i.e., prior to metastatic nodule formation in mice after surgical removal of the primary tumor. Using pharmacologic blockade or genetic deletion of the tyrosine kinase domain of VEGFR1, we demonstrate that VEGFR1 activity is not required for the infiltration of de novo myeloid BMDCs in the pre-metastatic lungs in two tumor models and in two mouse models. Moreover, in line with emerging clinical observations, we show that blockade of VEGFR1 activity neither prevents nor changes the rate of spontaneous **metastasis** formation after primary tumor removal. Prevention of **metastasis** will require further identification and exploration of cellular and molecular pathways that mediate the priming of the metastatic soil.
3. VEGFR-1 mediates endothelial differentiation and formation of blood vessels in a murine model of infantile hemangioma. Vascular endothelial growth factor receptor-1 (VEGFR-1) is a member of the VEGFR family, and binds to VEGF-A, VEGF-B, and placental growth factor. VEGFR-1 contributes to tumor growth and **metastasis**, but its role in the pathological formation of blood vessels is still poorly understood. Herein, we used infantile hemangioma (IH), the most common tumor of infancy, as a means to study VEGFR-1 activation in pathological vasculogenesis. IH arises from stem cells (HemSCs) that can form the three most prominent cell types in the tumor: endothelial cells, pericytes, and adipocytes. HemSCs can recapitulate the IH life cycle when injected in immuncompromised mice, and are targeted by corticosteroids, the traditional treatment for IH. We report here that VEGF-A or VEGF-B induces VEGFR-1-mediated ERK1/2 phosphorylation in HemSCs and promotes differentiation of HemSCs to endothelial cells. Studies of VEGFR-2 phosphorylation status and down-regulation of neuropilin-1 in the HemSCs demonstrate that VEGFR-2 and NRP1 are not needed for VEGF-A- or VEGF-B-induced ERK1/2 activation. U0216-mediated blockade of ERK1/2 phosphorylation or shRNA-mediated suppression of VEGFR-1 prevents HemSC-to-EC differentiation. Furthermore, the down-regulation of VEGFR-1 in the HemSCs results in decreased formation of blood vessels in vivo and reduced ERK1/2 activation. Thus, our study reveals a critical role for VEGFR-1 in the HemSC-to-EC differentiation that underpins pathological vasculogenesis in IH.
4. PlGF blockade does not inhibit angiogenesis during primary tumor growth. It has been recently reported that treatment with an anti-placenta growth factor (PlGF) antibody inhibits **metastasis** and primary tumor growth. Here we show that, although anti-PlGF treatment inhibited wound healing, extravasation of B16F10 cells, and growth of a tumor engineered to overexpress the PlGF receptor (VEGFR-1), neutralization of PlGF using four novel blocking antibodies had no significant effect on tumor angiogenesis in 15 models. Also, genetic ablation of the tyrosine kinase domain of VEGFR-1 in the host did not result in growth inhibition of the anti-VEGF-A sensitive or resistant tumors tested. Furthermore, combination of anti-PlGF with anti-VEGF-A antibodies did not result in greater antitumor efficacy than anti-VEGF-A monotherapy. In conclusion, our data argue against an important role of PlGF during primary tumor growth in most models and suggest that clinical evaluation of anti-PlGF antibodies may be challenging.
5. FLT1 signaling in **metastasis**-associated macrophages activates an inflammatory signature that promotes breast cancer **metastasis**. Although the link between inflammation and cancer initiation is well established, its role in metastatic diseases, the primary cause of cancer deaths, has been poorly explored. Our previous studies identified a population of **metastasis**-associated macrophages (MAMs) recruited to the lung that promote tumor cell seeding and growth. Here we show that FMS-like tyrosine kinase 1 (Flt1, also known as VEGFR1) labels a subset of macrophages in human breast cancers that are significantly enriched in metastatic sites. In mouse models of breast cancer pulmonary **metastasis**, MAMs uniquely express FLT1. Using several genetic models, we show that macrophage FLT1 signaling is critical for **metastasis**. FLT1 inhibition does not affect MAM recruitment to metastatic lesions but regulates a set of inflammatory response genes, including colony-stimulating factor 1 (CSF1), a central regulator of macrophage biology. Using a gain-of-function approach, we show that CSF1-mediated autocrine signaling in MAMs is downstream of FLT1 and can restore the tumor-promoting activity of FLT1-inhibited MAMs. Thus, CSF1 is epistatic to FLT1, establishing a link between FLT1 and inflammatory responses within breast tumor metastases. Importantly, FLT1 inhibition reduces tumor metastatic efficiency even after initial seeding, suggesting that these pathways represent therapeutic targets in metastatic disease.
6. Flt-1 signaling in macrophages promotes glioma growth in vivo. Several lines of evidence indicate that Flt-1, a fms-like tyrosine kinase receptor, which binds to vascular endothelial growth factor (VEGF)-A, VEGF-B, and PlGF, is a positive regulator of angiogenesis in the context of tumor growth and **metastasis**. However, the molecular basis of its action is still not clear. Besides endothelial cells, Flt-1 is also expressed by other different cell types, including myeloid hematopoeitic cells (monocytes and macrophages). To examine the functions of Flt-1 expressed by bone marrow-derived myeloid cells in supporting tumor growth and angiogenesis, Flt-1 tyrosine kinase-deficient (Flt-1 TK-/-) bone marrow cells were transplanted into lethally irradiated syngeneic recipients. After hematopoietic reconstitution, we orthotopically implanted syngeneic wild-type glioma cells or glioma cells overexpressing either VEGF(164) or PlGF-2. Loss of Flt-1 signaling in bone marrow-derived myeloid cells led to a significant decrease in tumor volume and vascularization in gliomas. VEGF but not PlGF overexpressed by glioma cells restored the tumor growth rate in Flt-1 TK-/- bone marrow chimera. VEGF and PlGF overexpression by tumor cells induced an accumulation of bone marrow-derived myeloid cells into tumor tissue. This infiltration was decreased in tumors grown in Flt-1 TK-/- bone marrow chimeras. When investigating chemokines and growth factors involved in myeloid cell recruitment, we determined elevated SDF-1/CXCL12 levels in VEGF- and PlGF-overexpressing tumors. Collectively, these results suggest that Flt-1 signaling in myeloid cells is essential to amplify the angiogenic response and to promote glioma growth.
7. VEGFR1-positive haematopoietic bone marrow progenitors initiate the pre-metastatic niche. The cellular and molecular mechanisms by which a tumour cell undergoes **metastasis** to a predetermined location are largely unknown. Here we demonstrate that bone marrow-derived haematopoietic progenitor cells that express vascular endothelial growth factor receptor 1 (VEGFR1; also known as Flt1) home to tumour-specific pre-metastatic sites and form cellular clusters before the arrival of tumour cells. Preventing VEGFR1 function using antibodies or by the removal of VEGFR1(+) cells from the bone marrow of wild-type mice abrogates the formation of these pre-metastatic clusters and prevents tumour **metastasis**, whereas reconstitution with selected Id3 (inhibitor of differentiation 3)-competent VEGFR1+ cells establishes cluster formation and tumour **metastasis** in Id3 knockout mice. We also show that VEGFR1+ cells express VLA-4 (also known as integrin alpha4beta1), and that tumour-specific growth factors upregulate fibronectin--a VLA-4 ligand--in resident fibroblasts, providing a permissive niche for incoming tumour cells. Conditioned media obtained from distinct tumour types with unique patterns of metastatic spread redirected fibronectin expression and cluster formation, thereby transforming the metastatic profile. These findings demonstrate a requirement for VEGFR1+ haematopoietic progenitors in the regulation of **metastasis**, and suggest that expression patterns of fibronectin and VEGFR1+VLA-4+ clusters dictate organ-specific tumour spread.
8. Effect of vegf gene knockdown on growth of the murine sarcoma cell line MS-K. The murine sarcoma cell line MS-K was previously established as a Ki-ras-positive cell line. Inoculation of this cell line under the flank of C3H/HeN mice results in the growth of large tumors with well-developed blood vessels within day 30 of transplantation without any **metastasis** because MS-K cells produce vascular endothelial growth factor (VEGF). To elucidate the role of VEGF in tumor formation in vivo, stable vegf-knockdown-MS-K clones were obtained using plasmid-based knockdown vectors. Interestingly, tumorigenesis was completely suppressed in a vegf-A-knockdown-MS-K clone [designated MS-K (A-KD)]. Proliferation and colony formation capacity of the MS-K (A-KD) cells in a semi-solid medium under low serum conditions was significantly lower than that of control MS-K (SCR) cells; however, the expression of vegf-receptor 1 (vegf-r-1) was not changed. Addition of the recombinant VEGF-A(165) partially restored the colony formation capacity of MS-K (A-KD) cells and caused the phosphorylation of VEGF-r-1 (Flt-1) in MS-K (Normal) cells. Furthermore, tumorigenicity of the vegf-r-1-knockdown-MS-K clone [designated MS-K (R1-KD)] had obviously delayed or strongly suppressed compared with the MS-K (Normal). These results indicate that Vascular endothelial growth factor-A, produced from MS-K, acts as a growth factor for MS-K cells itself and supports tumor formation in vivo by inducing the blood vessel formation.
9. Hypoxia-inducible factor-2α regulates GM-CSF-derived soluble vascular endothelial growth factor receptor 1 production from macrophages and inhibits tumor growth and angiogenesis. Macrophage secretion of vascular endothelial growth factor (VEGF) in response to the hypoxic tumor microenvironment contributes to tumor growth, angiogenesis, and **metastasi**s. We have recently demonstrated that macrophages stimulated with GM-CSF at low O(2) secrete high levels of a soluble form of the VEGF receptor 1 (sVEGFR-1), which neutralizes VEGF and inhibits its biological activity. Using small interfering RNA targeting to deplete hypoxia-inducible factor (HIF)-1α or HIF-2α in murine macrophages, we found that macrophage production of sVEGFR-1 in response to low O(2) was dependent on HIF-2α, whereas HIF-1α specifically regulated VEGF production. In our current report, we evaluated the growth of B16F10 malignant melanoma in mice with a monocyte/macrophage-selective deletion of HIF-1α or HIF-2α (HIF-1α(flox/flox)- or HIF-2α(flox/+)/LysMcre mice). GM-CSF treatment increased intratumoral VEGF and sVEGFR-1 in control mice, an effect that was associated with a decrease in microvessel density. GM-CSF treatment of HIF-1α(flox/flox)/LysMcre mice induced sVEGFR-1 but not VEGF, resulting in an overall greater reduction in tumor growth and angiogenesis compared with control mice. In addition, real-time PCR for melanoma-specific genes revealed a significantly reduced presence of lung micrometastases in HIF-1α(flox/flox)/LysMcre mice treated with GM-CSF. Conversely, GM-CSF treatment induced VEGF but not sVEGFR-1 in HIF-2α(flox/+)/LysMcre mice, and, correspondingly, GM-CSF did not decrease tumor growth, angiogenes**is, or lun**g metastasis in these mice. This study reveals opposing roles for the HIFs in the regulation of angiogenesis by tumor-associated macrophages and suggests that administration of GM-CSF might be an effective means of inducing sVEGFR-1 and inhibiting tumor growth and angiogenesis in patients with melanoma.
10. c-Myc is essential for vasculogenesis and angiogenesis during development and tumor progression. c-Myc promotes cell growth and transformation by ill-defined mechanisms. c-myc(-/-) mice die by embryonic day 10.5 (E10.5) with defects in growth and in cardiac and neural development. Here we report that the lethality of c-myc(-/-) embryos is also associated with profound defects in vasculogenesis and primitive erythropoiesis. Furthermore, c-myc(-/-) embryonic stem (ES) and yolk sac cells are compromised in their differentiative and growth potential. These defects are intrinsic to c-Myc, and are in part associated with a requirement for c-Myc for the expression of vascular endothelial growth factor (VEGF), as VEGF can partially rescue these defects. However, c-Myc is also required for the proper expression of other angiogenic factors in ES and yolk sac cells, including angiopoietin-2, and the angiogenic inhibitors thrombospondin-1 and angiopoietin-1. Finally, c-myc(-/-) ES cells are dramatically impaired in their ability to form tumors in immune-compromised mice, and the small tumors that sometimes develop are poorly vascularized. Therefore, c-Myc function is also necessary for the angiogenic switch that is indispensable for the progression and **metastasis** of tumors. These findings support the model wherein c-Myc promotes cell growth and transformation, as well as vascular and hematopoietic development, by functioning as a master regulator of angiogenic factors.
11. Anti-PlGF inhibits growth of VEGF(R)-inhibitor-resistant tumors without affecting healthy vessels. Novel antiangiogenic strategies with complementary mechanisms are needed to maximize efficacy and minimize resistance to current angiogenesis inhibitors. We explored the therapeutic potential and mechanisms of alphaPlGF, an antibody against placental growth factor (PlGF), a VEGF homolog, which regulates the angiogenic switch in disease, but not in health. alphaPlGF inhibited growth and **metastasis** of various tumors, including those resistant to VEGF(R) inhibitors (VEGF(R)Is), and enhanced the efficacy of chemotherapy and VEGF(R)Is. alphaPlGF inhibited angiogenesis, lymphangiogenesis, and tumor cell motility. Distinct from VEGF(R)Is, alphaPlGF prevented infiltration of angiogenic macrophages and severe tumor hypoxia, and thus, did not switch on the angiogenic rescue program responsible for resistance to VEGF(R)Is. Moreover, it did not cause or enhance VEGF(R)I-related side effects. The efficacy and safety of alphaPlGF, its pleiotropic and complementary mechanism to VEGF(R)Is, and the negligible induction of an angiogenic rescue program suggest that alphaPlGF may constitute a novel approach for cancer treatment.
12. miR-200 Inhibits lung adenocarcinoma cell invasion and **metastasis** by targeting Flt1/VEGFR1. The microRNA-200 (miR-200) family is part of a gene expression signature that predicts poor prognosis in lung cancer patients. In a mouse model of K-ras/p53-mutant lung adenocarcinoma, miR-200 levels are suppressed in **metastasis**-prone tumor cells, and forced miR-200 expression inhibits tumor growth and **metastasis**, but the miR-200 target genes that drive lung tumorigenesis have not been fully elucidated. Here, we scanned the genome for putative miR-200 binding sites and found them in the 3'-untranslated region (3'-UTR) of 35 genes that are amplified in human cancer. Mining of a database of resected human lung adenocarcinomas revealed that the levels of one of these genes, Flt1/VEGFR1, correlate inversely with duration of survival. Forced miR-200 expression suppressed Flt1 levels in **metastasis**-prone lung adenocarcinoma cells derived from K-ras/p53-mutant mice, and negatively regulated the Flt1 3'-UTR in reporter assays. Cancer-associated fibroblasts (CAFs) isolated from murine lung adenocarcinomas secreted abundant VEGF and enhanced tumor cell invasion in coculture studies. CAF-induced tumor cell invasion was abrogated by VEGF neutralization or Flt1 knockdown in tumor cells. Flt1 knockdown decreased the growth and **metastasis** of tumor cells in syngeneic mice. We conclude that miR-200 suppresses lung tumorigenesis by targeting Flt1.

#### GeneRIFs

1. VEGFR1 activity modulates myeloid cell infiltration in growing lung metastases but is not required for spontaneous **metastasis** formation
2. findings demonstrate a requirement for VEGFR1+ haematopoietic progenitors in the regulation of **metastasis**, and suggest that expression patterns of fibronectin and VEGFR1+VLA-4+ clusters dictate organ-specific tumour spread

### [Cc]ell [Ii]nvasion

#### PubMed

1. miR-200 Inhibits lung adenocarcinoma **cell invasion** and metastasis by targeting Flt1/VEGFR1. The microRNA-200 (miR-200) family is part of a gene expression signature that predicts poor prognosis in lung cancer patients. In a mouse model of K-ras/p53-mutant lung adenocarcinoma, miR-200 levels are suppressed in metastasis-prone tumor cells, and forced miR-200 expression inhibits tumor growth and metastasis, but the miR-200 target genes that drive lung tumorigenesis have not been fully elucidated. Here, we scanned the genome for putative miR-200 binding sites and found them in the 3'-untranslated region (3'-UTR) of 35 genes that are amplified in human cancer. Mining of a database of resected human lung adenocarcinomas revealed that the levels of one of these genes, Flt1/VEGFR1, correlate inversely with duration of survival. Forced miR-200 expression suppressed Flt1 levels in metastasis-prone lung adenocarcinoma cells derived from K-ras/p53-mutant mice, and negatively regulated the Flt1 3'-UTR in reporter assays. Cancer-associated fibroblasts (CAFs) isolated from murine lung adenocarcinomas secreted abundant VEGF and enhanced tumor **cell invasion** in coculture studies. CAF-induced tumor **cell invasion** was abrogated by VEGF neutralization or Flt1 knockdown in tumor cells. Flt1 knockdown decreased the growth and metastasis of tumor cells in syngeneic mice. We conclude that miR-200 suppresses lung tumorigenesis by targeting Flt1.

---

## Vamp2

- NCBI Gene
- GeneRIF
- Pubmed

| Search Term | Rank | Fields |
| --- | --- | --- |
| [Mm]igration | 6 | PubMed(4) |
| ECM | 7 | PubMed(1) |
| [Ii]nvasive | 10 | PubMed(1) |

  
hide/show details for Vamp2

### [Mm]igration

#### PubMed

1. **Migration** Speed of Cajal-Retzius Cells Modulated by Vesicular Trafficking Controls the Size of Higher-Order Cortical Areas. In the neocortex, higher-order areas are essential to integrate sensory-motor information and have expanded in size during evolution. How higher-order areas are specified, however, remains largely unknown. Here, we show that the **migration** and distribution of early-born neurons, the Cajal-Retzius cells (CRs), controls the size of higher-order areas in the mouse somatosensory, auditory, and visual cortex. Using live imaging, genetics, and in silico modeling, we show that subtype-specific differences in the onset, speed, and directionality of CR **migration** determine their differential invasion of the developing cortical surface. CR **migration** speed is cell autonomously modulated by vesicle-associated membrane protein 3 (VAMP3), a classically non-neuronal mediator of endosomal recycling. Increasing CR **migration** speed alters their distribution in the developing cerebral cortex and leads to an expansion of postnatal higher-order areas and congruent rewiring of thalamo-cortical input. Our findings thus identify novel roles for neuronal **migration** and VAMP3-dependent vesicular trafficking in cortical wiring.
2. Syntaxin 1 is required for DCC/Netrin-1-dependent chemoattraction of migrating neurons from the lower rhombic lip. Directed cell **migration** and axonal guidance are essential steps in neural development that share many molecular mechanisms. The guidance of developing axons and migrating neurons is likely to depend on the precise control of plasmalemma turnover in selected regions of leading edges and growth cones, respectively. Previous results provided evidence of a signaling mechanism that couples chemotropic deleted in colorectal cancer (DCC)/Netrin-1 axonal guidance and exocytosis through Syntaxin1(Sytx1)/TI-VAMP SNARE proteins. Here we studied whether Netrin-1-dependent neuronal **migration** relies on a similar SNARE mechanism. We show that migrating neurons in the lower rhombic lip (LRL) express several SNARE proteins, and that DCC co-associates with Sytx1 and TI-VAMP in these cells. We also demonstrate that cleavage of Sytx1 by botulinum toxin C1 (BoNT/C1) abolishes Netrin-1-dependent chemoattraction of migrating neurons, and that interference of Sytx1 functions with shRNAs or Sytx1-dominant negatives disrupts Netrin-1-dependent chemoattraction of LRL neurons. These findings indicate that a Sytx1/DCC interaction is required for Netrin-1 guidance of migrating neurons, thereby highlighting a relationship between guidance signaling and SNARE proteins that regulate membrane turnover.
3. Genetic interactions between doublecortin and doublecortin-like kinase in neuronal **migration** and axon outgrowth. Although mutations in the human doublecortin gene (DCX) cause profound defects in cortical neuronal **migration**, a genetic deletion of Dcx in mice produces a milder defect. A second locus, doublecortin-like kinase (Dclk), encodes a protein with similar "doublecortin domains" and microtubule stabilization properties that may compensate for Dcx. Here, we generate a mouse with a Dclk mutation that causes no obvious **migration**al abnormalities but show that mice mutant for both Dcx and Dclk demonstrate perinatal lethality, disorganized neocortical layering, and profound hippocampal cytoarchitectural disorganization. Surprisingly, Dcx(-/y);Dclk(-/-) mutants have widespread axonal defects, affecting the corpus callosum, anterior commissure, subcortical fiber tracts, and internal capsule. Dcx/Dclk-deficient dissociated neurons show abnormal axon outgrowth and dendritic structure, with defects in axonal transport of synaptic vesicle proteins. Dcx and Dclk may directly or indirectly regulate microtubule-based vesicle transport, a process critical to both neuronal **migration** and axon outgrowth.
4. A signaling mechanism coupling netrin-1/deleted in colorectal cancer chemoattraction to SNARE-mediated exocytosis in axonal growth cones. Directed cell **migration** and axonal guidance are essential steps in neural development. Both processes are controlled by specific guidance cues that activate the signaling cascades that ultimately control cytoskeletal dynamics. Another essential step in **migration** and axonal guidance is the regulation of plasmalemma turnover and exocytosis in leading edges and growth cones. However, the cross talk mechanisms linking guidance receptors and membrane exocytosis are not understood. Netrin-1 is a chemoattractive cue required for the formation of commissural pathways. Here, we show that the Netrin-1 receptor deleted in colorectal cancer (DCC) forms a protein complex with the t-SNARE (target SNARE) protein Syntaxin-1 (Sytx1). This interaction is Netrin-1 dependent both in vitro and in vivo, and requires specific Sytx1 and DCC domains. Blockade of Sytx1 function by using botulinum toxins abolished Netrin-1-dependent chemoattraction of axons in mouse neuronal cultures. Similar loss-of-function experiments in the chicken spinal cord in vivo using dominant-negative Sytx1 constructs or RNAi led to defects in commissural axon pathfinding reminiscent to those described in Netrin-1 and DCC loss-of-function models. We also show that Netrin-1 elicits exocytosis at growth cones in a Sytx1-dependent manner. Moreover, we demonstrate that the Sytx1/DCC complex associates with the v-SNARE (vesicle SNARE) tetanus neurotoxin-insensitive vesicle-associated membrane protein (TI-VAMP) and that knockdown of TI-VAMP in the commissural pathway in the spinal cord results in aberrant axonal guidance phenotypes. Our data provide evidence of a new signaling mechanism that couples chemotropic Netrin-1/DCC axonal guidance and Sytx1/TI-VAMP SNARE proteins regulating membrane turnover and exocytosis.

### ECM

#### PubMed

1. α3(V) collagen is critical for glucose homeostasis in mice due to effects in pancreatic islets and peripheral tissues. Collagen V, broadly expressed as α1(V)2 α2(V) heterotrimers that regulate collagen fibril geometry and strength, also occurs in some tissues, such as white adipose tissue (WAT), pancreatic islets, and skeletal muscle, as the poorly characterized α1(V) α2(V) α3(V) heterotrimer. Here, we investigate the role of α3(V) collagen chains by generating mice with a null allele of the α3(V) gene Col5a3 (Col5a3–/– mice). Female Col5a3–/– mice had reduced dermal fat and were resistant to high-fat diet–induced weight gain. Male and female mutant mice were glucose intolerant, insulin-resistant, and hyperglycemic, and these metabolic defects worsened with age. Col5a3–/– mice demonstrated decreased numbers of pancreatic islets, which were more susceptible to streptozotocin-induced apoptosis, and islets isolated from mutant mice displayed blunted glucose-stimulated insulin secretion. Moreover, Col5a3–/– WAT and skeletal muscle were defective in glucose uptake and mobilization of intracellular GLUT4 glucose transporter to the plasma membrane in response to insulin. Our results underscore the emerging **vie**w of the importance of ECM to the microenvironments that inform proper development/functioning of specialized cells, such as adipocytes, β cells, and skeletal muscle.

### [Ii]nvasive

#### PubMed

1. Genetically-directed, cell type-specific sparse labeling for the analysis of neuronal morphology. In mammals, genetically-directed cell labeling technologies have not yet been applied to the morphologic analysis of neurons with very large and complex arbors, an application that requires extremely sparse labeling and that is only rendered practical by limiting the labeled population to one or a few predetermined neuronal subtypes.In the present study we have addressed this application by using CreER technology to non-**invasive**ly label very small numbers of neurons so that their morphologies can be fully visualized. Four lines of IRES-CreER knock-in mice were constructed to permit labeling selectively in cholinergic or catecholaminergic neurons [choline acetyltransferase (ChAT)-IRES-CreER or tyrosine hydroxylase (TH)-IRES-CreER], predominantly in projection neurons [neurofilament light chain (NFL)-IRES-CreER], or broadly in neurons and some glia [vesicle-associated membrane protein2 (VAMP2)-IRES-CreER]. When crossed to the Z/AP reporter and exposed to 4-hydroxytamoxifen in the early postnatal period, the number of neurons expressing the human placental alkaline phosphatase reporter can be reproducibly lowered to fewer than 50 per brain. Sparse Cre-mediated recombination in ChAT-IRES-CreER;Z/AP mice shows the full axonal and dendritic arbors of individual forebrain cholinergic neurons, the first time that the complete morphologies of these very large neurons have been revealed in any species.Sparse genetically-directed, cell type-specific neuronal labeling with IRES-creER lines should prove useful for studying a wide variety of questions in neuronal development and disease.

---

## Igf1r

- NCBI Gene
- GeneRIF
- Pubmed

| Search Term | Rank | Fields |
| --- | --- | --- |
| [Mm]igration | 6 | Process(1); PubMed(14); GeneRIFs(2) |
| [Ee]xtracellular [Mm]atrix | 8 | PubMed(6) |
| [Ii]nvasive | 10 | PubMed(6) |
| [Mm]etastasis | 12 | PubMed(6); GeneRIFs(2) |
| [Cc]ell [Ii]nvasion | 13 | PubMed(1); GeneRIFs(1) |

  
hide/show details for Igf1r

### [Mm]igration

#### Process

1. positive regulation of cell **migration**

#### PubMed

1. Upregulation of neurovascular communication through filamin abrogation promotes ectopic periventricular neurogenesis. Neuronal fate-restricted intermediate progenitors (IPs) are derived from the multipotent radial glia (RGs) and serve as the direct precursors for cerebral cortical neurons, but factors that control their neurogenic plasticity remain elusive. Here we report that IPs' neuron production is enhanced by abrogating filamin function, leading to the generation of periventricular neurons independent of normal neocortical neurogenesis and neuronal **migration**. Loss of Flna in neural progenitor cells (NPCs) led RGs to undergo changes resembling epithelial-mesenchymal transition (EMT) along with exuberant angiogenesis that together changed the microenvironment and increased neurogenesis of IPs. We show that by collaborating with β-arrestin, Flna maintains the homeostatic signaling between the vasculature and NPCs, and loss of this function results in escalated Vegfa and Igf2 signaling, which exacerbates both EMT and angiogenesis to further potentiate IPs' neurogenesis. These results suggest that the neurogenic potential of IPs may be boosted in vivo by manipulating Flna-mediated neurovascular communication.
2. Protein tyrosine phosphatase-alpha complexes with the IGF-I receptor and undergoes IGF-I-stimulated tyrosine phosphorylation that mediates cell **migration**. Protein tyrosine phosphatase-alpha (PTPalpha) is a widely expressed receptor-type phosphatase that functions in multiple signaling systems. The actions of PTPalpha can be regulated by its phosphorylation on serine and tyrosine residues, although little is known about the conditions that promote PTPalpha phosphorylation. In this study, we tested the ability of several extracellular factors to stimulate PTPalpha tyrosine phosphorylation. The growth factors IGF-I and acidic FGF induced the highest increase in PTPalpha phosphorylation at tyrosine 789, followed by PMA and lysophosphatidic acid, while EGF had little effect. Further investigation of IGF-I-induced PTPalpha tyrosine phosphorylation demonstrated that this occurs through a novel Src family kinase-independent mechanism that does not require focal adhesion kinase, phosphatidylinositol 3-kinase, or MEK. We also show that PTPalpha physically interacts with the IGF-I receptor. In contrast to IGF-I-induced PTPalpha phosphorylation, this association does not require IGF-I. The interaction of PTPalpha and the IGF-I receptor is independent of PTPalpha catalytic activity, and expression of exogenous PTPalpha does not promote IGF-I receptor tyrosine dephosphorylation, indicating that PTPalpha does not act as an IGF-I receptor phosphatase. However, PTPalpha mediates IGF-I signaling, because IGF-I-stimulated fibroblast **migration** was reduced by approximately 50% in cells lacking PTPalpha or in cells with mutant PTPalpha lacking the tyrosine 789 phosphorylation site. Our results suggest that PTPalpha tyrosine phosphorylation can occur in response to diverse stimuli and can be mediated by various tyrosine kinases. In the case of IGF-I, we propose that IGF-I-induced tyrosine 789 phosphorylation of PTPalpha, possibly catalyzed by the PTPalpha-associated IGF-I receptor tyrosine kinase, is required for efficient cell **migration** in response to this growth factor.
3. Dichotomy of decorin activity on the insulin-like growth factor-I system. The stromal-specific proteoglycan decorin has emerged in recent years as a critical regulator of tumor initiation and progression. Decorin regulates the biology of various types of cancer by modulating the activity of several receptor tyrosine kinases coordinating growth, survival, **migration**, and angiogenesis. Decorin binds to surface receptors for epidermal growth factor and hepatocyte growth factor with high affinity, and negatively regulates their activity and signaling via robust internalization and eventual degradation. The insulin-like growth factor (IGF)-I system plays a critical role in the regulation of cell growth both in vivo and in vitro. The IGF-I receptor (IGF-IR) is also essential for cellular transformation, owing to its ability to enhance cell proliferation and protect cancer cells from apoptosis. Recent data have pointed to a role of decorin in regulating the IGF-I system in both nontransformed and transformed cells. Significantly, there is a surprising dichotomy in the mechanism of decorin action on IGF-IR signaling, which differs considerably between physiological and pathological cellular models. In this review, we summarize the current knowledge on decorin regulation of the IGF-I system in normal and transformed cells, and discuss possible decorin-based therapeutic approaches to target IGF-IR-driven tumors.
4. Novel role of the IGF-1 receptor in endothelial function and repair: studies in endothelium-targeted IGF-1 receptor transgenic mice. We recently demonstrated that reducing IGF-1 receptor (IGF-1R) numbers in the endothelium enhances nitric oxide (NO) bioavailability and endothelial cell insulin sensitivity. In the present report, we aimed to examine the effect of increasing IGF-1R on endothelial cell function and repair. To examine the effect of increasing IGF-1R in the endothelium, we generated mice overexpressing human IGF-1R in the endothelium (human IGF-1R endothelium-overexpressing mice [hIGFREO]) under direction of the Tie2 promoter enhancer. hIGFREO aorta had reduced basal NO bioavailability (percent constriction to N(G)-monomethyl-l-arginine [mean (SEM) wild type 106% (30%); hIGFREO 48% (10%)]; P < 0.05). Endothelial cells from hIGFREO had reduced insulin-stimulated endothelial NO synthase activation (mean [SEM] wild type 170% [25%], hIGFREO 58% [3%]; P = 0.04) and insulin-stimulated NO release (mean [SEM] wild type 4,500 AU [1,000], hIGFREO 1,500 AU [700]; P < 0.05). hIGFREO mice had enhanced endothelium regeneration after denuding arterial injury (mean [SEM] percent recovered area, wild type 57% [2%], hIGFREO 47% [5%]; P < 0.05) and enhanced endothelial cell **migration** in vitro. The IGF-1R, although reducing NO bioavailability, enhances in situ endothelium regeneration. Manipulating IGF-1R in the endothelium may be a useful strategy to treat disorders of vascular growth and repair.
5. Focal adhesion kinase (FAK) activates and stabilizes IGF-1 receptor. Recent studies have shown a direct association between IGF-1R and FAK, two important mediators of cell growth, survival and **migration**. However, the mechanism by which FAK affects IGF-1R function remains unknown. This study investigates the potential role of FAK in mediating activation and stability of IGF-1R. Autophosphorylation and phosphorylation capacities of wild type and mutant IGF-1R were studied. Surprisingly, we found that the mutant IGF-1R lacking the three core tyrosine residues in the activation-loop can be phosphorylated although it is unable to undergo autophosphorylation, suggesting that another kinase possesses the ability to phosphorylate IGF-1R. By using wild type MEFs and FAK-/- MEFs we could demonstrate that FAK mediates activation-loop independent phosphorylation, as well as Akt and ERK activation. Furthermore, the stability of IGF-1R was decreased upon FAK siRNA or inactivation. Taken together, our data suggest a role for FAK in phosphorylation, signaling and stability of the IGF-1R.
6. MesP1: a novel basic helix-loop-helix protein expressed in the nascent mesodermal cells during mouse gastrulation. A subtractive hybridization strategy was used to isolate putative genes involved in the development of mouse primordial germ cells (PGC). Complimentary DNA was amplified on RNA isolated from the base of the allantois where PGC are located in the 7.5 days post coitum (dpc) mouse embryo. It was then subtracted by hybridization with cDNA amplified on RNA of the anterior region where PGC are absent. A novel gene thus isolated is designated as Mesp1 and encodes a possible transcription factor MesP1 containing a basic helix-loop-helix motif. Its earliest expression was observed at the onset of gastrulation, as early as 6.5 dpc, in the nascent mesodermal cells that first ingressed at the end of the primitive streak. These expressing cells in the lateral and extraembryonic mesoderm showed a wing-shaped distribution. Its initial expression was soon down-regulated at 7.5 dpc before the completion of gastrulation, except at the proximal end of the primitive streak which included the extraembryonic mesoderm and the base of allantois. At 8 dpc, the expression at the base of the allantois moved laterally. This distribution between 7.0 and 8.0 dpc was similar to that of PGC detected by the alkaline phosphatase activity. However, the expression of Mesp1 was down-regulated thereafter, when PGC entered in the **migration** stage. After birth, Mesp1 expression was detected only in mature testes, but in a different isoform from that expressed in the embryo. Mesp1 was mapped to the mid region of chromosome 7, near the mesodermal deficiency gene (mesd). However, a Southern hybridization study clearly showed that Mesp1 was distinctly different from mesd. The amino acid sequence and its expression pattern suggest that MesP1 plays an important role in the development of the nascent mesoderm including PGC.
7. Inhibition of type I insulin-like growth factor receptor signaling attenuates the development of breast cancer brain metastasis. Brain metastasis is a common cause of mortality in cancer patients, yet potential therapeutic targets remain largely unknown. The type I insulin-like growth factor receptor (IGF-IR) is known to play a role in the progression of breast cancer and is currently being investigated in the clinical setting for various types of cancer. The present study demonstrates that IGF-IR is constitutively autophosphorylated in brain-seeking breast cancer sublines. Knockdown of IGF-IR results in a decrease of phospho-AKT and phospho-p70s6k, as well as decreased **migration** and invasion of MDA-MB-231Br brain-seeking cells. In addition, transient ablation of IGFBP3, which is overexpressed in brain-seeking cells, blocks IGF-IR activation. Using an in vivo experimental brain metastasis model, we show that IGF-IR knockdown brain-seeking cells have reduced potential to establish brain metastases. Finally, we demonstrate that the malignancy of brain-seeking cells is attenuated by pharmacological inhibition with picropodophyllin, an IGF-IR-specific tyrosine kinase inhibitor. Together, our data suggest that the IGF-IR is an important mediator of brain metastasis and its ablation delays the onset of brain metastases in our model system.
8. Insulin-like growth factor-I receptor blockade improves outcome in mouse model of lung injury. The insulin-like growth factor-I (IGF-I) pathway is an important determinant of survival and proliferation in many cells. However, little is known about the role of the IGF-I pathway in lung injury. We previously showed elevated levels of IGF-I in bronchoalveolar lavage fluid from patients with acute respiratory distress syndrome. Furthermore, immunodepletion of IGF from acute respiratory distress syndrome bronchoalveolar lavage increased fibroblast apoptosis.We examined the effect of blockade of type 1 IGF tyrosine kinase receptor (IGF-IR) in a murine model of bleomycin-induced lung injury and fibrosis.Mice were treated with a monoclonal antibody against the IGF-I receptor (A12) or vehicle after intratracheal bleomycin instillation.Mice treated with A12 antibody had significantly improved survival after bleomycin injury compared with control mice. Both groups of mice had a similar degree of fibrosis on days 7 and 14, but by Day 28 the A12-treated group had significantly less fibrosis. Delayed treatment with A12 also resulted in decreased fibrosis. A12-treated mice had significantly decreased apoptotic cells on Day 28 compared with control mice. We confirmed that A12 treatment induced mouse lung fibroblast apoptosis in vitro. In addition, IGF-I increased lung fibroblast **migration**. The primary pathway activated by IGF-I in lung fibroblasts was the insulin receptor substrate-2/phosphatidylinositol 3-kinase/Akt axis.IGF-I regulated survival and **migration** of fibrogenic cells in the lung. Blockade of the IGF pathway increased fibroblast apoptosis and subsequent resolution of pulmonary fibrosis. Thus, IGF-IR may be a potential target for treatment of lung injury and fibrosis.
9. Insulin/insulin-like growth factor I hybrid receptors have different biological characteristics depending on the insulin receptor isoform involved. The insulin receptor (IR) and the insulin-like growth factor I receptor (IGF-IR) have a highly homologous structure, but different biological effects. Insulin and IGF-I half-receptors can heterodimerize, leading to the formation of insulin/IGF-I hybrid receptors (Hybrid-Rs) that bind IGF-I with high affinity. As the IR exists in two isoforms (IR-A and IR-B), we evaluated whether the assembly of the IGF-IR with either IR-A or IR-B moieties may differently affect Hybrid-R signaling and biological role. Three different models were studied: (a) 3T3-like mouse fibroblasts with a disrupted IGF-IR gene (R(-) cells) cotransfected with the human IGF-IR and with either the IR-A or IR-B cDNA; (b) a panel of human cell lines variably expressing the two IR isoforms; and (c) HepG2 human hepatoblastoma cells predominantly expressing either IR-A or IR-B, depending on their differentiation state. We found that Hybrid-Rs containing IR-A (Hybrid-Rs(A)) bound to and were activated by IGF-I, IGF-II, and insulin. By binding to Hybrid-Rs(A), insulin activated the IGF-I half-receptor beta-subunit and the IGF-IR-specific substrate CrkII. In contrast, Hybrid-Rs(B) bound to and were activated with high affinity by IGF-I, with low affinity by IGF-II, and insignificantly by insulin. As a consequence, cell proliferation and **migration** in response to both insulin and IGFs were more effectively stimulated in Hybrid-R(A)-containing cells than in Hybrid-R(B)-containing cells. The relative abundance of IR isoforms therefore affects IGF system activation through Hybrid-Rs, with important consequences for tissue-specific responses to both insulin and IGFs.
10. Selective tyrosine kinase inhibition of insulin-like growth factor-1 receptor inhibits human and mouse breast cancer-induced bone cell activity, bone remodeling, and osteolysis. Insulin-like growth factor 1 (IGF-1) plays an important role in both bone metabolism and breast cancer. In this study, we investigated the effects of the novel IGF-1 receptor tyrosine kinase inhibitor cis-3-[3-(4-methyl-piperazin-l-yl)-cyclobutyl]-1-(2-phenyl-quinolin-7-yl)-imidazo[1,5-a]pyrazin-8-ylamine (PQIP) on osteolytic bone disease associated with breast cancer. Human MDA-MB-231 and mouse 4T1 breast cancer cells enhanced osteoclast formation in receptor activator of NF-κB ligand (RANKL) and macrophage colony-stimulating factor (M-CSF) stimulated bone marrow cultures, and these effects were significantly inhibited by PQIP. Functional studies in osteoclasts showed that PQIP inhibited both IGF-1 and conditioned medium-induced osteoclast formation by preventing phosphatidylinositol 3-kinase (PI3K)/protein kinase B (Akt) activation without interfering with RANKL or M-CSF signaling. Treatment of osteoblasts with PQIP significantly inhibited the increase in RANKL/osteoprotegerin (OPG) ratio by IGF-1 and conditioned medium and totally prevented conditioned medium-induced osteoclast formation in osteoblast-bone marrow (BM) cell cocultures, thereby suggesting an inhibitory effect on osteoblast-osteoclast coupling. PQIP also inhibited IGF-1-induced osteoblast differentiation, spreading, **migratio**n, and bone nodule formation. Treatment with PQIP significantly reduced MDA-MB-231 conditioned medium-induced osteolytic bone loss in a mouse calvarial organ culture system ex vivo and in adult mice in vivo. Moreover, once daily oral administration of PQIP significantly decreased trabecular bone loss and reduced the size of osteolytic bone lesions following 4T1 intratibial injection in mice. Quantitative histomorphometry showed a significant reduction in bone resorption and formation indices, indicative of a reduced rate of cancer-associated bone turnover. We conclude that inhibition of IGF-1 receptor tyrosine kinase activity by PQIP suppresses breast cancer-induced bone turnover and osteolysis. Therefore, PQIP, and its novel derivatives that are currently in advanced clinical development for the treatment of a number of solid tumors, may be of value in the treatment of osteolytic bone disease associated with breast cancer.
11. Leukocyte antigen-related deficiency enhances insulin-like growth factor-1 signaling in vascular smooth muscle cells and promotes neointima formation in response to vascular injury. Increase in the expression of leukocyte antigen-related (LAR) protein causes insulin resistance, an important contributor to atherosclerosis. However, the function of LAR in atherosclerosis is not known. To address whether LAR is important in the response of vascular cells to atherogenic stimuli, we investigated cell proliferation, **migration**, and insulin-like growth factor-1 receptor (IGF-1R) signaling in wild-type and LAR(-/-) mouse vascular smooth muscle cells (VSMC) treated with IGF-1. Absence of LAR significantly enhanced proliferation and **migration** of VSMC compared with wild-type cells after IGF-1 treatment. U0126 and LY249002, specific inhibitors of MAPK/ERK kinase (MEK) and phosphoinositide 3-kinase, respectively, inhibited IGF-1-induced DNA synthesis and **migration** in both wild-type and LAR(-/-) VSMC. IGF-1 markedly enhanced IGF-1R phosphorylation in both wild-type and LAR(-/-) VSMC, but the phosphorylation was 90% higher in knock-out cells compared with wild-type cells. Absence of LAR enhanced phosphorylation of insulin receptor substrate-1 and insulin receptor substrate-1-associated phosphoinositide 3-kinase activity in VSMC treated with IGF-1. IGF-1-induced phosphorylation of ERK1/2 also increased significantly in LAR(-/-) VSMC compared with wild-type cells. Furthermore, LAR directly binds to IGF-1R in glutathione S-transferase-LAR pull-down and IGF-1R immunoprecipitation experiments and recombinant LAR dephosphorylates IGF-1R in vitro. Neointima formation in response to arterial injury and IGF-1R phosphorylation in neointima increased significantly in LAR(-/-) mice compared with wild-type mice. A significant decrease in body weight, fasting insulin, and IGF-1 levels were observed in LAR(-/-) mice compared with wild-type mice. Together, these data indicate that LAR regulates IGF-1R signaling in VSMC and dysregulation of this phosphatase may lead to VSMC hyperplasia.
12. Impaired Shc, Ras, and MAPK activation but normal Akt activation in FL5.12 cells expressing an insulin-like growth factor I receptor mutated at tyrosines 1250 and 1251. The Y1250F/Y1251F mutant of the insulin-like growth factor I receptor (IGF-IR) has tyrosines 1250 and 1251 mutated to phenylalanines and is deficient in IGF-I-mediated suppression of apoptosis in FL5.12 lymphocytic cells. To address the mechanism of loss of function in this mutant we investigated signaling responses in FL5.12 cells overexpressing either a wild-type (WT) or Y1250F/Y1251F (mutant) IGF-IR. Cells expressing the mutant receptor were deficient in IGF-I-induced phosphorylation of the JNK pathway and had decreased ERK and p38 phosphorylation. IGF-I induced phosphorylation of Akt was comparable in WT and mutant expressing cells. The decreased activation of the mitogen-activated protein kinase (MAPK) pathways was accompanied by greatly decreased Ras activation in response to IGF-I. Although phosphorylation of Gab2 was similar in WT and mutant cell lines, phosphorylation of Shc on Tyr(313) in response to IGF-I was decreased in cells expressing the mutant receptor, as was recruitment of Grb2 and Ship to Shc. However, phosphorylation of Shc on Tyr(239), the Src phosphorylation site, was normal. A role for JNK in the survival of FL5.12 cells was supported by the observation that the JNK inhibitor SP600125 suppressed IGF-I-mediated protection from apoptosis. Altogether these data demonstrate that phosphorylation of Shc, and assembly of the Shc complex necessary for activation of Ras and the MAPK pathways are deficient in cells expressing the Y1250F/Y1251F mutant IGF-IR. This would explain the loss of IGF-I-mediated survival in FL5.12 cells expressing this mutant and may also explain why this mutant IGF-IR is deficient in functions associated with cellular transformation and cell **migration** in fibroblasts and epithelial tumor cells.
13. Protective role of insulin-like growth factor-1 receptor in endothelial cells against unilateral ureteral obstruction-induced renal fibrosis. Insulin-like growth factor-1 receptor (IGF-1R) can regulate vascular homeostasis and endothelial function. We studied the role of IGF-1R in oxidative stress-induced endothelial dysfunction. Unilateral ureteral obstruction (UUO) was performed in wild-type (WT) mice and mice with endothelial cell (EC)-specific IGF-1R knockout (KO). After UUO in endothelial IGF-1R KO mice, endothelial barrier dysfunction was more severe than in WT mice, as seen by increased inflammatory cell infiltration and vascular endothelial (VE)-cadherin phosphorylation. UUO in endothelial IGF-1R KO mice increased interstitial fibroblast accumulation and enhanced extracellular protein deposition as compared with the WT mice. Endothelial barrier function measured by transendothelial **migration** in response to hydrogen peroxide (H2O2) was impaired in ECs. Silencing IGF-1R enhanced the influence of H2O2 in disrupting the VE-protein tyrosine phosphatase/VE-cadherin interaction. Overexpression of IGF-1R suppressed H2O2-induced endothelial barrier dysfunction. Furthermore, by using the piggyBac transposon system, we expressed IGF-1R in VE cells in mice. The expression of IGF-1R in ECs also suppressed the inflammatory cell infiltration and renal fibrosis induced by UUO. IGF-1R KO in the VE-cadherin lineage of bone marrow cells had no significant effect on the UUO-induced fibrosis, as compared with control mice. Our results indicate that IGF-1R in the endothelium maintains the endothelial barrier function by stabilization of the VE-protein tyrosine phosphatase/VE-cadherin complex. Decreased expression of IGF-1R impairs endothelial function and increases the fibrosis of kidney disease.
14. Expression of insulin receptor (IR) A and B isoforms, IGF-IR, and IR/IGF-IR hybrid receptors in vascular smooth muscle cells and their role in cell **migration** in atherosclerosis. Abnormal proliferation and **migration** of vascular smooth muscle cells (VSMCs) is a major contributor to the development of atherosclerotic process. In a previous work, we demonstrated that the insulin receptor isoform A (IRA) and its association with the insulin-like growth factor-I receptor (IGF-IR) confer a proliferative advantage to VSMCs. However, the role of IR and IGF-IR in VSMC **migration** remains poorly understood.Wound healing assays were performed in VSMCs bearing IR (IRLoxP(+/+) VSMCs), or not (IR(-/-) VSMCs), expressing IRA (IRA VSMCs) or expressing IRB (IRB VSMCs). To study the role of IR isoforms and IGF-IR in experimental atherosclerosis, we used ApoE(-/-) mice at 8, 12, 18 and 24 weeks of age. Finally, we analyzed the mRNA expression of total IR, IRB isoform, IGF-IR and IGFs by qRT-PCR in the medial layer of human aortas.IGF-I strongly induced **migratio**n of the four cell lines through IGF-IR. In contrast, insulin and IGF-II only caused a significant increase of IRA VSMC **migratio**n which might be favored by the formation of IRA/IGF-IR receptors. Additionally, a specific IGF-IR inhibitor, picropodophyllin, completely abolished insulin- and IGF-II-induced **migratio**n in IRB, but not in IRA VSMCs. A significant increase of IRA and IGF-IR, and VSMC **migratio**n were observed in fibrous plaques from 24-week-old ApoE(-/-) mice. Finally, we observed a marked increase of IGF-IR, IGF-I and IGF-II in media from fatty streaks as compared with both healthy aortas and fibrolipidic lesions, favoring the ability of medial VSMCs to migrate into the intima.Our data suggest that overexpression of IGF-IR or IRA isoform, as homodimers or as part of IRA/IGF-IR hybrid receptors, confers a stronger migratory capability to VSMCs as might occur in early stages of atherosclerotic process.

#### GeneRIFs

1. Protein tyrosine phosphatase-alpha complexes with the IGF-I receptor and undergoes IGF-I-stimulated tyrosine phosphorylation that mediates cell **migration**.
2. IGF-I regulated survival and **migration** of fibrogenic cells in the lung. Blockade of the IGF pathway increased fibroblast apoptosis and subsequent resolution of pulmonary fibrosis.

### [Ee]xtracellular [Mm]atrix

#### PubMed

1. Transcriptome analysis in prenatal IGF1-deficient mice identifies molecular pathways and target genes involved in distal lung differentiation. Insulin-like Growth Factor 1 (IGF1) is a multifunctional regulator of somatic growth and development throughout evolution. IGF1 signaling through IGF type 1 receptor (IGF1R) controls cell proliferation, survival and differentiation in multiple cell types. IGF1 deficiency in mice disrupts lung morphogenesis, causing altered prenatal pulmonary alveologenesis. Nevertheless, little is known about the cellular and molecular basis of IGF1 activity during lung development.Prenatal Igf1(-/-) mutant mice with a C57Bl/6J genetic background displayed severe disproportional lung hypoplasia, leading to lethal neonatal respiratory distress. Immuno-histological analysis of their lungs showed a thickened mesenchyme, alterations in **extracellular matrix** deposition, thinner smooth muscles and dilated blood vessels, which indicated immature and delayed distal pulmonary organogenesis. Transcriptomic analysis of Igf1(-/-) E18.5 lungs using RNA microarrays identified deregulated genes related to vascularization, morphogenesis and cellular growth, and to MAP-kinase, Wnt and cell-adhesion pathways. Up-regulation of immunity-related genes was verified by an increase in inflammatory markers. Increased expression of Nfib and reduced expression of Klf2, Egr1 and Ctgf regulatory proteins as well as activation of ERK2 MAP-kinase were corroborated by Western blot. Among IGF-system genes only IGFBP2 revealed a reduction in mRNA expression in mutant lungs. Immuno-staining patterns for IGF1R and IGF2, similar in both genotypes, correlated to alterations found in specific cell compartments of Igf1(-/-) lungs. IGF1 addition to Igf1(-/-) embryonic lungs cultured ex vivo increased airway septa remodeling and distal epithelium maturation, processes accompanied by up-regulation of Nfib and Klf2 transcription factors and Cyr61 matricellular protein.We demonstrated the functional tissue specific implication of IGF1 on fetal lung development in mice. Results revealed novel target genes and gene networks mediators of IGF1 action on pulmonary cellular proliferation, differentiation, adhesion and immunity, and on vascular and distal epithelium maturation during prenatal lung development.
2. Reduced expression of insulin-like growth factor 1 receptor leads to accelerated intervertebral disc degeneration in mice. Insulin-like growth factor 1 (IGF-1) and its receptor (insulin-like growth factor 1 receptor, IGF1R) can regulate the **extracellular matrix** synthesis and play a crucial role in maintaining the normal functions of the intervertebral disc (IVD). The objective of this study was to investigate whether there would be accelerated IVD degeneration (IVDD) in IGF1R+/- mice. Three IGF1R+/- male mice and three wild-type male mice were sacrificed respectively at 6, 12, and 18 weeks after birth. Six lumbar disc samples were harvested from each mouse, with a total of 54 disc samples taken from each genotype. Histomorphological analysis for the IVD was performed to assess the degenerative extent according to the classification system proposed by Boos et al. Quantitative real-time PCR and semi-quantitative histologic scoring (HScore) for immunohistochemical staining were used to evaluate the expression level of type-II collagen, aggrecan and matrix metallopeptidase 13 (MMP-13). Histomorphological analysis for the discs revealed significantly less amounts of proteoglycan and type-II collagen, and significantly higher total degenerative score in IGF1R+/- mice than in wild-type mice. Real-time PCR showed that the mRNA expressions of type-II collagen and aggrecan in the discs were significantly lower, while MMP-13 was significantly higher in IGF1R+/- mice than in wild-type mice. The results of HScore analysis were similar to those obtained from the quantitative real-time PCR. Taken together, our study indicates that reduced expression of IGF1R would lead to accelerated degeneration of IVD. IGF1R+/- mice could be regarded as a good animal model to study IVD degeneration (IVDD), and studies on the IVD of IGF1R+/- mice could provide further insight into the pathogenesis of IVDD.
3. Type 1 insulin-like growth factor regulates MT1-MMP synthesis and tumor invasion via PI 3-kinase/Akt signaling. The membrane type 1 matrix metalloproteinase (MT1-MMP) has been identified as a major activator of MMP-2 - a process involving the formation of a trimolecular complex with TIMP-2. We previously identified the IGF-I receptor as a positive regulator of MMP-2 synthesis. Here, we investigated the role of IGF-IR in the regulation of MT1-MMP. Highly invasive Lewis lung carcinoma subline H-59 cells express MT1-MMP and utilize it to activate their major **extracellular matrix** degrading proteinase-MMP-2. These cells were transiently transfected with a plasmid vector expressing a luciferase reporter gene downstream of the mouse MT1-MMP promoter. IGF-I treatment increased luciferase activity in the transfected cells by up to 10-fold and augmented endogenous MT1-MMP mRNA and protein synthesis by up to 2-3-fold, relative to controls. MT1-MMP induction and invasion were blocked by the PI 3-kinase inhibitors LY294002 and wortmannin and by rapamycin, but not by the MEK inhibitor PD98059. Overexpression of a dominant negative Akt mutant or of the tumor suppressor phosphatase and tensin homologue, PTEN, in these cells also caused a significant reduction in MT1-MMP expression and invasion. The results demonstrate that IGF-IR controls tumor cell invasion by coordinately regulating MMP-2 expression and its MT1-MMP-mediated activation and identify PI 3-kinase/Akt/mTOR signaling as critical to this regulation.
4. Cloning of cDNA for the alpha subunit of mouse insulin-like growth factor I receptor and the role of the receptor in metanephric development. Various growth factors influence mammalian development by binding to specific cell surface receptors. These interactions are followed by a series of intracellular transductional events leading to a wide variety of biological effects. To establish the role of insulin-like growth factor I receptor (IGF-IR) in renal development, cDNA for the alpha subunit of the mouse IGF-IR was isolated, characterized, and used in expression studies and antisense experiments in a metanephric organ culture system. A 989-bp insert, encoding the signal peptide and 299 amino acids, isolated from a newborn mouse kidney cDNA library had 99% and 91% homology with the nucleotide sequences encoding the rat and the human IGF-IR, respectively. An approximately 11-kb message was readily detected by Northern blot analysis of RNA from the developing kidney at day 13 of gestation, and it declined during the subsequent embryonal and neonatal periods. In situ hybridization revealed high levels of message over the ureteric bud and its branches. A lower level of message was seen in the neonatal kidney, confined mainly to the tubules. Antisense oligodeoxynucleotide-treated metanephric kidneys were reduced in size and had a decreased population of nephrons with marked disorganization of ureteric bud branches. Immunofluorescence studies indicated an arrest of IGF-IR translation after antisense exposure. Immunoprecipitation studies showed a marked decrease in the biosynthesis of various **extracellular matrix** proteins that serve as regulators of morphogenesis. These studies suggest that the nucleotide sequence encoding the alpha subunit of mouse IGF-IR is highly conserved and that the receptor might play an essential role in the organogenesis of the kidney.
5. Secretion of Annexin II via activation of insulin receptor and insulin-like growth factor receptor. Annexin II is secreted into the extracellular environment, where, via interactions with specific proteases and **extracellular matrix** proteins, it participates in plasminogen activation, cell adhesion, and tumor metastasis and invasion. However, mechanisms regulating annexin II transport across the cellular membrane are unknown. In this study, we used coimmunoprecipitation to show that Annexin-II was bound to insulin and insulin-like growth factor-1 (IGF-1) receptors in PC12 cells and NIH-3T3 cells overexpressing insulin (NIH-3T3(IR)) or IGF-1 receptor (NIH-3T3(IGF-1R)). Stimulation of insulin and IGF-1 receptors by insulin caused a temporary dissociation of annexin II from these receptors, which was accompanied by an increased amount of extracellular annexin II detected in the media of PC12, NIH-3T3(IR), and NIH-3T3(IGF-1R) cells but not in that of untransfected NIH-3T3 cells. Activation of a different growth factor receptor, the platelet-derived growth factor receptor, did not produce such results. Tyrphostin AG1024, a tyrosine kinase inhibitor of insulin and IGF-1 receptor, was shown to inhibit annexin II secretion along with reduced receptor phosphorylation. Inhibitors of a few downstream signaling enzymes including phosphatidylinositol 3-kinase, pp60c-Src, and protein kinase C had no effect on insulin-induced annexin II secretion, suggesting a possible direct link between receptor activation and annexin II secretion. Immunocytochemistry revealed that insulin also induced transport of the membrane-bound form of annexin II to the outside layer of the cell membrane and appeared to promote cell aggregation. These results suggest that the insulin receptor and its signaling pathways may participate in molecular mechanisms mediating annexin II secretion.
6. The direct binding of insulin-like growth factor-1 (IGF-1) to integrin alphavbeta3 is involved in IGF-1 signaling. It has been proposed that ligand occupancy of integrin alphavbeta3 with **extracellular matrix** ligands (e.g. vitronectin) plays a critical role in insulin-like growth factor-1 (IGF-1) signaling. We found that expression of alphavbeta3 enhanced IGF-1-induced proliferation of Chinese hamster ovary cells in serum-free conditions (in the absence of vitronectin). We hypothesized that the direct integrin binding to IGF-1 may play a role in IGF-1 signaling. We demonstrated that alphavbeta3 specifically and directly bound to IGF-1 in cell adhesion, enzyme-linked immunosorbent assay-type binding, and surface plasmon resonance studies. We localized the amino acid residues of IGF-1 that are critical for integrin binding by docking simulation and mutagenesis. We found that mutating two Arg residues at positions 36 and 37 in the C-domain of IGF-1 to Glu (the R36E/R37E mutation) effectively reduced integrin binding. Interestingly, although the mutant still bound to IGF1R, it was defective in inducing IGF1R phosphorylation, AKT and ERK1/2 activation, and cell proliferation. Furthermore wild type IGF-1 mediated co-precipitation of alphavbeta3 and IGF1R, whereas the R36E/R37E mutant did not, suggesting that IGF-1 mediates the interaction between alphavbeta3 and IGF1R. These results suggest that the direct binding to IGF-1 to integrin alphavbeta3 plays a role in IGF-1 signaling through ternary complex formation (alphavbeta3-IGF-IGF1R), and integrin-IGF-1 interaction is a novel target for drug discovery.

### [Ii]nvasive

#### PubMed

1. The imprinted Phlda2 gene regulates extraembryonic energy stores. An important difference between placental mammals and marsupials is the maturity of the fetus at birth. Placental mammals achieved this maturity by developing a complex and **invasive** placenta to support and prolong internal development. The exact genomic modifications that facilitated the evolution of this complex structure are unknown, but the emergence of genomic imprinting within mammalian lineages suggests a role for gene dosage. Here we show that a maximally altered placental structure is achieved by a single extra dose of the imprinted Phlda2 gene characterized by a dramatically reduced junctional zone and a decrease in stored glycogen. In addition, glycogen cells do not migrate into the maternal decidua in a timely fashion, but instead, Tpbpa-positive cells progressively mislocalize into the labyrinth. These defects are linked to a progressive restriction of embryonic growth from embryonic day 16.5. This work has identified a critical role for the imprinted Phlda2 gene in regulating glycogen storage in the eutherian placenta and implies that imprinting has provided a mechanism to boost nutrient supply to the fetus late in gestation, when the fetus is placing the highest demands on maternal resources, to enhance growth.
2. Repression of malignant tumor progression upon pharmacologic IGF1R blockade in a mouse model of insulinoma. NVP-AEW541, a specific ATP-competitive inhibitor of the insulin-like growth factor-1 receptor (IGF1R) tyrosine kinase, has been reported to interfere with tumor growth in various tumor transplantation models. We have assessed the efficacy of NVP-AEW541 in repressing tumor growth and tumor progression in the Rip1Tag2 transgenic mouse model of pancreatic β-cell carcinogenesis. In addition, we have tested NVP-AEW541 in Rip1Tag2;RipIGF1R double-transgenic mice which show accelerated tumor growth and increased tumor malignancy compared with Rip1Tag2 single-transgenic mice. Previously, we have shown that high levels of IGF-2, a high-affinity ligand for IGF1R, are required for Rip1Tag2 tumor cell survival and tumor growth. Unexpectedly, treatment of Rip1Tag2 mice with NVP-AEW541 in prevention and intervention trials neither did affect tumor growth nor tumor cell proliferation and apoptosis. Yet, it significantly repressed progression to tumor malignancy, that is, the rate of the transition from differentiated adenoma to **invasiv**e carcinoma. Treatment of Rip1Tag2;RipIGF1R double-transgenic mice resulted in moderately reduced tumor volumes and increased rates of tumor cell apoptosis. Sustained expression of IGF-2 and of the IGF-2-binding form of insulin receptor (IR-A) in tumor cells suggests a compensatory role of IR-A upon IGF1R blockade. The results indicate that inhibition of IGF1R alone is not sufficient to efficiently block insulinoma growth and imply an overlapping role of IGF1R and insulin receptor in executing mitogenic and survival stimuli elicited by IGF-2. The reduction of tumor invasion upon IGF1R blockade on the other hand indicates a critical function of IGF1R signaling for the acquisition of a malignant phenotype.
3. Igf1r as a therapeutic target in a mouse model of basal-like breast cancer. Considering the strong association between dysregulated insulin-like growth factor (IGF) signaling and various human cancers, we have used an expedient combination of genetic analysis and pharmacological treatment to evaluate the potential of the type 1 IGF receptor (Igf1r) for targeted anticancer therapy in a mouse model of mammary tumorigenesis. In this particular strain of genetically modified animals, histopathologically heterogeneous **invasive** carcinomas exhibiting up-regulation of the Igf1r gene developed extremely rapidly by mammary gland-specific overexpression of constitutively active oncogenic Kras\* (mutant Kras(G12D)). Immunophenotyping data and expression profiling analyses showed that, except for a minor luminal component, these mouse tumors resembled basal-like human breast cancers. This is a group of aggressive tumors of poor prognosis for which there is no targeted therapy currently available, and it includes a subtype correlating with KRAS locus amplification. Conditional ablation of Igf1r in the mouse mammary epithelium increased the latency of Kras\*-induced tumors very significantly (approximately 11-fold in comparison with the intact model), whereas treatment of tumor-bearing animals by administration of picropodophyllin (PPP), a specific Igf1r inhibitor, resulted in a dramatic decrease in tumor mass of the main forms of basal-like carcinomas. PPP also was effective against xenografts of the human basal-like cancer cell line MDA-MB-231, which carries a KRAS(G13D) mutation.
4. Type 1 insulin-like growth factor regulates MT1-MMP synthesis and tumor invasion via PI 3-kinase/Akt signaling. The membrane type 1 matrix metalloproteinase (MT1-MMP) has been identified as a major activator of MMP-2 - a process involving the formation of a trimolecular complex with TIMP-2. We previously identified the IGF-I receptor as a positive regulator of MMP-2 synthesis. Here, we investigated the role of IGF-IR in the regulation of MT1-MMP. Highly **invasive** Lewis lung carcinoma subline H-59 cells express MT1-MMP and utilize it to activate their major extracellular matrix degrading proteinase-MMP-2. These cells were transiently transfected with a plasmid vector expressing a luciferase reporter gene downstream of the mouse MT1-MMP promoter. IGF-I treatment increased luciferase activity in the transfected cells by up to 10-fold and augmented endogenous MT1-MMP mRNA and protein synthesis by up to 2-3-fold, relative to controls. MT1-MMP induction and invasion were blocked by the PI 3-kinase inhibitors LY294002 and wortmannin and by rapamycin, but not by the MEK inhibitor PD98059. Overexpression of a dominant negative Akt mutant or of the tumor suppressor phosphatase and tensin homologue, PTEN, in these cells also caused a significant reduction in MT1-MMP expression and invasion. The results demonstrate that IGF-IR controls tumor cell invasion by coordinately regulating MMP-2 expression and its MT1-MMP-mediated activation and identify PI 3-kinase/Akt/mTOR signaling as critical to this regulation.
5. Elevated levels of IGF-1 receptor convey **invasive** and metastatic capability in a mouse model of pancreatic islet tumorigenesis. In a prototypical model of multistage tumorigenesis involving pancreatic islets in RIP1-Tag2 transgenic mice, activation of insulin-like growth factor II (IGF-II) was previously shown to serve as a survival factor that inhibited apoptosis. Now IGF-1R, the receptor tyrosine kinase for IGF-II, has been found to be variably upregulated, first uniformly in dysplastic and angiogenic progenitors and then focally at the margins and in **invasive** regions of carcinomas. When the levels of IGF-1R were forcibly elevated throughout islet tumorigenesis, progression was accelerated at all stages in the pathway, although apoptosis was not differentially suppressed. Notably, encapsulated tumors were absent; instead, **invasive** carcinomas with downregulated E-cadherin were prevalent, and the majority of mice had local lymph node metastasis.
6. Role of calcineurin, hnRNPA2 and Akt in mitochondrial respiratory stress-mediated transcription activation of nuclear gene targets. Pathophysiological conditions causing mitochondrial dysfunction and altered transmembrane potential (psim) initiate a mitochondrial respiratory stress response, also known as mitochondrial retrograde response, in a variety of mammalian cells. An increase in the cytosolic Ca2+ [Ca2+]c as part of this signaling cascade activates Ca2+ responsive phosphatase, calcineurin (Cn). Activation of IGF1R accompanied by increased glycolysis, **invasive**ness, and resistance to apoptosis is a phenotypic hallmark of C2C12 skeletal muscle cells subjected to this stress. The signaling is associated with activation and increased nuclear translocation of a number of transcription factors including a novel NFkappaB (cRel:p50) pathway, NFAT, CREB and C/EBPdelta. This culminates in the upregulation of a number of nuclear genes including Cathepsin L, RyR1, Glut4 and Akt1. We observed that stress regulated transcription activation of nuclear genes involves a cooperative interplay between NFkappaB (cRel:p50), C/EBPdelta, CREB, and NFAT. Our results show that the functional synergy of these factors requires the stress-activated heterogeneous nuclear ribonucleoprotein, hnRNPA2 as a transcriptional coactivator. We report here that mitochondrial stress leads to induced expression and activation of serine threonine kinase Akt1. Interestingly, we observe that Akt1 phosphorylates hnRNPA2 under mitochondrial stress conditions, which is a crucial step for the recruitment of this coactivator to the stress target promoters and culmination in mitochondrial stress-mediated transcription activation of target genes. We propose that mitochondrial stress plays an important role in tumor progression and emergence of **invasive** phenotypes.

### [Mm]etastasis

#### PubMed

1. Inhibition of type I insulin-like growth factor receptor signaling attenuates the development of breast cancer brain **metastasis**. Brain **metastasis** is a common cause of mortality in cancer patients, yet potential therapeutic targets remain largely unknown. The type I insulin-like growth factor receptor (IGF-IR) is known to play a role in the progression of breast cancer and is currently being investigated in the clinical setting for various types of cancer. The present study demonstrates that IGF-IR is constitutively autophosphorylated in brain-seeking breast cancer sublines. Knockdown of IGF-IR results in a decrease of phospho-AKT and phospho-p70s6k, as well as decreased migration and invasion of MDA-MB-231Br brain-seeking cells. In addition, transient ablation of IGFBP3, which is overexpressed in brain-seeking cells, blocks IGF-IR activation. Using an in vivo experimental brain **metastasis** model, we show that IGF-IR knockdown brain-seeking cells have reduced potential to establish brain metastases. Finally, we demonstrate that the malignancy of brain-seeking cells is attenuated by pharmacological inhibition with picropodophyllin, an IGF-IR-specific tyrosine kinase inhibitor. Together, our data suggest that the IGF-IR is an important mediator of brain **metastasis** and its ablation delays the onset of brain metastases in our model system.
2. Vascular endothelial growth factor C expression and lymph node **metastasis** are regulated by the type I insulin-like growth factor receptor. Vascular endothelial growth factor (VEGF)-C is a lymphangiogenic factor implicated in lymphatic **metastasis**. In this study, we investigated the role of the type I insulin-like growth factor receptor (IGF-IR) in the regulation of VEGF-C expression. We used Lewis lung carcinoma subline M-27 cells transfected with human IGF-IR cDNA. These cells, but not the wild-type cells, expressed VEGF-C mRNA, produced a M(r) 58,000 VEGF-C precursor protein, and secreted a M(r) 29,000 processed form in response to IGF-I. In vivo, they acquired a lymph node metastasizing potential. VEGF-C induction was abolished in cells expressing an IGF-IR with tyrosine-phenylalanine substitutions in the kinase domain, but not in the COOH-terminal domain. The induction was phosphatidylinositol 3'-kinase dependent and, to a lesser extent, mitogen-activated protein kinase signaling dependent, as determined by the use of the respective inhibitors LY294002 (84.6% reduction) and PD98059 (38% reduction). The results identify the IGF-IR as a positive regulator of VEGF-C expression and implicate it in the control of lymphatic **metastasis**.
3. Components of the hematopoietic compartments in tumor stroma and tumor-bearing mice. Solid tumors are composed of cancerous cells and non-cancerous stroma. A better understanding of the tumor stroma could lead to new therapeutic applications. However, the exact compositions and functions of the tumor stroma are still largely unknown. Here, using a Lewis lung carcinoma implantation mouse model, we examined the hematopoietic compartments in tumor stroma and tumor-bearing mice. Different lineages of differentiated hematopoietic cells existed in tumor stroma with the percentage of myeloid cells increasing and the percentage of lymphoid and erythroid cells decreasing over time. Using bone marrow reconstitution analysis, we showed that the tumor stroma also contained functional hematopoietic stem cells. All hematopoietic cells in the tumor stroma originated from bone marrow. In the bone marrow and peripheral blood of tumor-bearing mice, myeloid populations increased and lymphoid and erythroid populations decreased and numbers of hematopoietic stem cells markedly increased with time. To investigate the function of hematopoietic cells in tumor stroma, we co-implanted various types of hematopoietic cells with cancer cells. We found that total hematopoietic cells in the tumor stroma promoted tumor development. Furthermore, the growth of the primary implanted Lewis lung carcinomas and their **metastasis** were significantly decreased in mice reconstituted with IGF type I receptor-deficient hematopoietic stem cells, indicating that IGF signaling in the hematopoietic tumor stroma supports tumor outgrowth. These results reveal that hematopoietic cells in the tumor stroma regulate tumor development and that tumor progression significantly alters the host hematopoietic compartment.
4. Secretion of Annexin II via activation of insulin receptor and insulin-like growth factor receptor. Annexin II is secreted into the extracellular environment, where, via interactions with specific proteases and extracellular matrix proteins, it participates in plasminogen activation, cell adhesion, and tumor **metastasis** and invasion. However, mechanisms regulating annexin II transport across the cellular membrane are unknown. In this study, we used coimmunoprecipitation to show that Annexin-II was bound to insulin and insulin-like growth factor-1 (IGF-1) receptors in PC12 cells and NIH-3T3 cells overexpressing insulin (NIH-3T3(IR)) or IGF-1 receptor (NIH-3T3(IGF-1R)). Stimulation of insulin and IGF-1 receptors by insulin caused a temporary dissociation of annexin II from these receptors, which was accompanied by an increased amount of extracellular annexin II detected in the media of PC12, NIH-3T3(IR), and NIH-3T3(IGF-1R) cells but not in that of untransfected NIH-3T3 cells. Activation of a different growth factor receptor, the platelet-derived growth factor receptor, did not produce such results. Tyrphostin AG1024, a tyrosine kinase inhibitor of insulin and IGF-1 receptor, was shown to inhibit annexin II secretion along with reduced receptor phosphorylation. Inhibitors of a few downstream signaling enzymes including phosphatidylinositol 3-kinase, pp60c-Src, and protein kinase C had no effect on insulin-induced annexin II secretion, suggesting a possible direct link between receptor activation and annexin II secretion. Immunocytochemistry revealed that insulin also induced transport of the membrane-bound form of annexin II to the outside layer of the cell membrane and appeared to promote cell aggregation. These results suggest that the insulin receptor and its signaling pathways may participate in molecular mechanisms mediating annexin II secretion.
5. Elevated levels of IGF-1 receptor convey invasive and metastatic capability in a mouse model of pancreatic islet tumorigenesis. In a prototypical model of multistage tumorigenesis involving pancreatic islets in RIP1-Tag2 transgenic mice, activation of insulin-like growth factor II (IGF-II) was previously shown to serve as a survival factor that inhibited apoptosis. Now IGF-1R, the receptor tyrosine kinase for IGF-II, has been found to be variably upregulated, first uniformly in dysplastic and angiogenic progenitors and then focally at the margins and in invasive regions of carcinomas. When the levels of IGF-1R were forcibly elevated throughout islet tumorigenesis, progression was accelerated at all stages in the pathway, although apoptosis was not differentially suppressed. Notably, encapsulated tumors were absent; instead, invasive carcinomas with downregulated E-cadherin were prevalent, and the majority of mice had local lymph node **metastasis**.
6. Selective inhibition of proprotein convertases represses the metastatic potential of human colorectal tumor cells. The proprotein convertases (PCs) are implicated in the activation of various precursor proteins that play an important role in tumor cell **metastasis**. Here, we report their involvement in the regulation of the metastatic potential of colorectal tumor cells. PC function in the human and murine colon carcinoma cell lines HT-29 and CT-26, respectively, was inhibited using siRNA targeting the PCs furin, PACE4, PC5, and PC7 or by overexpression of the general PC inhibitor alpha1-antitrypsin Portland (alpha1-PDX). We found that overexpression of alpha1-PDX and knockdown of furin expression inhibited processing of IGF-1 receptor and its subsequent activation by IGF-1 to induce IRS-1 and Akt phosphorylation, all important in colon carcinoma **metastasis**. These data suggest that the PC furin is a major IGF-1 receptor convertase. Expression of alpha1-PDX reduced the production of TNF-alpha and IL-1alpha by human colon carcinoma cells, and incubation of murine liver endothelial cells with conditioned media derived from these cells failed to induce tumor cell adhesion to activated murine endothelial cells, a critical step in metastatic invasion. Furthermore, colon carcinoma cells in which PC activity was inhibited by overexpression of alpha1-PDX when injected into the portal vein of mice showed a significantly reduced ability to form liver metastases. This suggests that inhibition of PCs is a potentially promising strategy for the prevention of colorectal liver **metastasis**.

#### GeneRIFs

1. Using an in vivo experimental brain **metastasis** model, the study shows that Igf1r knockdown brain-seeking cells have reduced potential to establish brain metastases.
2. Results identify the IGF-IR as a positive regulator of VEGF-C expression and implicate it in the control of lymphatic **metastasis**.

### [Cc]ell [Ii]nvasion

#### PubMed

1. Type 1 insulin-like growth factor regulates MT1-MMP synthesis and tumor invasion via PI 3-kinase/Akt signaling. The membrane type 1 matrix metalloproteinase (MT1-MMP) has been identified as a major activator of MMP-2 - a process involving the formation of a trimolecular complex with TIMP-2. We previously identified the IGF-I receptor as a positive regulator of MMP-2 synthesis. Here, we investigated the role of IGF-IR in the regulation of MT1-MMP. Highly invasive Lewis lung carcinoma subline H-59 cells express MT1-MMP and utilize it to activate their major extracellular matrix degrading proteinase-MMP-2. These cells were transiently transfected with a plasmid vector expressing a luciferase reporter gene downstream of the mouse MT1-MMP promoter. IGF-I treatment increased luciferase activity in the transfected cells by up to 10-fold and augmented endogenous MT1-MMP mRNA and protein synthesis by up to 2-3-fold, relative to controls. MT1-MMP induction and invasion were blocked by the PI 3-kinase inhibitors LY294002 and wortmannin and by rapamycin, but not by the MEK inhibitor PD98059. Overexpression of a dominant negative Akt mutant or of the tumor suppressor phosphatase and tensin homologue, PTEN, in these cells also caused a significant reduction in MT1-MMP expression and invasion. The results demonstrate that IGF-IR controls tumor **cell invasion** by coordinately regulating MMP-2 expression and its MT1-MMP-mediated activation and identify PI 3-kinase/Akt/mTOR signaling as critical to this regulation.

#### GeneRIFs

1. IGF-IR controls tumor **cell invasion** by coordinately regulating MMP-2 expression and its MT1-MMP-mediated activation and identify PI 3-kinase/Akt/mTOR signaling as critical to this regulation.

---

## Casp7

- NCBI Gene
- GeneRIF
- Pubmed

| Search Term | Rank | Fields |
| --- | --- | --- |
| [Mm]igration | 6 | PubMed(1) |
| ECM | 7 | PubMed(2) |
| [Ee]xtracellular [Mm]atrix | 8 | PubMed(1) |

  
hide/show details for Casp7

### [Mm]igration

#### PubMed

1. Fine-tuning nucleophosmin in macrophage differentiation and activation. M-CSF-driven differentiation of peripheral blood monocytes is one of the sources of tissue macrophages. In humans and mice, the differentiation process involves the activation of caspases that cleave a limited number of proteins. One of these proteins is nucleophosmin (NPM1), a multifunctional and ubiquitous protein. Here, we show that caspases activated in monocytes exposed to M-CSF cleave NPM1 at D213 to generate a 30-kDa N-terminal fragment. The protein is further cleaved into a 20-kDa fragment, which involves cathepsin B. NPM1 fragments contribute to the limited motility, **migration**, and phagocytosis capabilities of resting macrophages. Their activation with lipopolysaccharides inhibits proteolytic processes and restores expression of the full-length protein that negatively regulates the transcription of genes encoding inflammatory cytokines (eg, NPM1 is recruited with NF-κB on the MCP1 gene promoter to decrease its transcription). In mice with heterozygous npm gene deletion, cytokine production in response to lipopolysaccharides, including CXCL1 (KC), MCP1, and MIP2, is dramatically enhanced. These results indicate a dual function of NPM1 in M-CSF-differentiated macrophages. Proteolysis of the protein participates in the establishment of a mature macrophage phenotype. In response to inflammatory stimuli, the full-length protein negatively regulates inflammatory cytokine production.

### ECM

#### PubMed

1. Role and Association of Inflammatory and Apoptotic Caspases in Renal Tubulointerstitial Fibrosis. Caspases, an evolutionary conserved family of aspartate-specific cystein proteases, play pivotal roles in apoptotic and inflammatory signaling. Thus far, 14 mammalian caspases are identified and categorized into 3 distinct sub-types: inflammatory caspases, apoptotic initiator and apoptotic executioner. Caspase-1 is an inflammatory caspase, while caspase-7 belongs to apoptotic executioner. The roles and association of these two distinct types of caspases in renal tubulointerstitial fibrosis (TIF) have not been well recognized.Caspase-1 inhibitor Z-YVAD-FMK and caspase-7 siRNA were used in tubular epithelial cell line NRK-52E (TECs) to test their effects on transforming growth factor-beta1 (TGF-β1) stimulation. In vivo, Unilateral ureteral obstruction (UUO) animal model was employed in wild-type (WT) and caspase-1 knock out (KO) (caspase-1-/-) mice.In current study, we found that caspase-7 was obviously activated in cultured TECs stimulated by TGF-β1 and in UUO model of WT mice. While in UUO model of caspase-1 KO mice, the increased caspase-7 activation was suppressed significantly along with reduced trans-differentiation and minimized extracellular matrix **(E**CM) accumulation, as demonstrated by western blot, Masson trichrome staining and immunohistochemistry. In addition, pharmacological inhibition of caspase-1 dampened caspase-7 activation and TECs' transdifferentiation induced by TGF-β1 exposure, which was consistent with in vivo study. Notably, caspase-7 gene knock down by specific siRNA abrogated TGF-β1 driven TECs' trans-differentiation and redu**ced** ECM accumulation.Our study associated inflammatory and apoptotic caspases in TIF for the first time and we further confirmed that caspase-1 activation is an upstream event of apoptotic caspase-7 induction in TIF triggered by UUO and in TECs mediated by TGF-β1 induced transdifferentiation.
2. Caspase-9, caspase-3 and caspase-7 have distinct roles during intrinsic apoptosis. Apoptosis is a form of programmed cell death that is regulated by the Bcl-2 family and caspase family of proteins. The caspase cascade responsible for executing cell death following cytochrome c release is well described; however the distinct roles of caspases-9, -3 and -7 during this process are not completely defined.Here we demonstrate several unique functions for each of these caspases during cell death. Specific inhibition of caspase-9 allows for efficient release of cytochrome c, but blocks changes in mitochondrial morphology and ROS production. We show that caspase-9 can cleave Bid into tBid at amino acid 59 and that this cleavage of Bid is required for ROS production following serum withdrawal. We also demonstrate that caspase-3-deficient MEFs are less sensitive to intrinsic cell death stimulation, yet have higher ROS production. In contrast, caspase-7-deficient MEFs are not resistance to intrinsic cell death, but remain attached to the **ECM**.Taken together, these data suggest that caspase-9 is required for mitochondrial morphological changes and ROS production by cleaving and activating Bid into tBid. After activation by caspase-9, caspase-3 inhibits ROS production and is required for efficient execution of apoptosis, while effector caspase-7 is required for apoptotic cell detachment.

### [Ee]xtracellular [Mm]atrix

#### PubMed

1. Role and Association of Inflammatory and Apoptotic Caspases in Renal Tubulointerstitial Fibrosis. Caspases, an evolutionary conserved family of aspartate-specific cystein proteases, play pivotal roles in apoptotic and inflammatory signaling. Thus far, 14 mammalian caspases are identified and categorized into 3 distinct sub-types: inflammatory caspases, apoptotic initiator and apoptotic executioner. Caspase-1 is an inflammatory caspase, while caspase-7 belongs to apoptotic executioner. The roles and association of these two distinct types of caspases in renal tubulointerstitial fibrosis (TIF) have not been well recognized.Caspase-1 inhibitor Z-YVAD-FMK and caspase-7 siRNA were used in tubular epithelial cell line NRK-52E (TECs) to test their effects on transforming growth factor-beta1 (TGF-β1) stimulation. In vivo, Unilateral ureteral obstruction (UUO) animal model was employed in wild-type (WT) and caspase-1 knock out (KO) (caspase-1-/-) mice.In current study, we found that caspase-7 was obviously activated in cultured TECs stimulated by TGF-β1 and in UUO model of WT mice. While in UUO model of caspase-1 KO mice, the increased caspase-7 activation was suppressed significantly along with reduced trans-differentiation and minimize**d extracellular matr**ix (ECM) accumulation, as demonstrated by western blot, Masson trichrome staining and immunohistochemistry. In addition, pharmacological inhibition of caspase-1 dampened caspase-7 activation and TECs' transdifferentiation induced by TGF-β1 exposure, which was consistent with in vivo study. Notably, caspase-7 gene knock down by specific siRNA abrogated TGF-β1 driven TECs' trans-differentiation and reduced ECM accumulation.Our study associated inflammatory and apoptotic caspases in TIF for the first time and we further confirmed that caspase-1 activation is an upstream event of apoptotic caspase-7 induction in TIF triggered by UUO and in TECs mediated by TGF-β1 induced transdifferentiation.

---

## Rab11fip4

- NCBI Gene
- GeneRIF
- Pubmed

| Search Term | Rank | Fields |
| --- | --- | --- |
| [Mm]igration | 6 | PubMed(1) |
| [Ee]xtracellular [Mm]atrix | 8 | PubMed(1) |

  
hide/show details for Rab11fip4

### [Mm]igration

#### PubMed

1. ADP Ribosylation Factor 6 Regulates Neuronal **Migration** in the Developing Cerebral Cortex through FIP3/Arfophilin-1-dependent Endosomal Trafficking of N-cadherin. During neural development, endosomal trafficking controls cell shape and motility through the polarized transport of membrane proteins related to cell-cell and cell-extracellular matrix interactions. ADP ribosylation factor 6 (Arf6) is a critical small GTPase that regulates membrane trafficking between the plasma membrane and endosomes. We herein demonstrated that the knockdown of endogenous Arf6 in mouse cerebral cortices led to impaired neuronal **migration** in the intermediate zone and cytoplasmic retention of N-cadherin and syntaxin12 in migrating neurons. Rescue experiments with separation-of-function Arf6 mutants identified Rab11 family-interacting protein 3 (FIP3)/Arfophilin-1, a dual effector for Arf6 and Rab11, as a downstream effector of Arf6 in migrating neurons. The knockdown of FIP3 led to impaired neuronal **migration** in the intermediate zone and cytoplasmic retention of N-cadherin in migrating neurons, similar to that of Arf6, which could be rescued by the coexpression of wild-type FIP3 but not FIP3 mutants lacking the binding site for Arf6 or Rab11. These results suggest that Arf6 regulates cortical neuronal **migration** in the intermediate zone through the FIP3-dependent endosomal trafficking.

### [Ee]xtracellular [Mm]atrix

#### PubMed

1. ADP Ribosylation Factor 6 Regulates Neuronal Migration in the Developing Cerebral Cortex through FIP3/Arfophilin-1-dependent Endosomal Trafficking of N-cadherin. During neural development, endosomal trafficking controls cell shape and motility through the polarized transport of membrane proteins related to cell-cell and cell-**extracellular matrix** interactions. ADP ribosylation factor 6 (Arf6) is a critical small GTPase that regulates membrane trafficking between the plasma membrane and endosomes. We herein demonstrated that the knockdown of endogenous Arf6 in mouse cerebral cortices led to impaired neuronal migration in the intermediate zone and cytoplasmic retention of N-cadherin and syntaxin12 in migrating neurons. Rescue experiments with separation-of-function Arf6 mutants identified Rab11 family-interacting protein 3 (FIP3)/Arfophilin-1, a dual effector for Arf6 and Rab11, as a downstream effector of Arf6 in migrating neurons. The knockdown of FIP3 led to impaired neuronal migration in the intermediate zone and cytoplasmic retention of N-cadherin in migrating neurons, similar to that of Arf6, which could be rescued by the coexpression of wild-type FIP3 but not FIP3 mutants lacking the binding site for Arf6 or Rab11. These results suggest that Arf6 regulates cortical neuronal migration in the intermediate zone through the FIP3-dependent endosomal trafficking.

---

## Grina

- NCBI Gene
- GeneRIF
- Pubmed

| Search Term | Rank | Fields |
| --- | --- | --- |
| [Mm]igration | 6 | PubMed(1) |

  
hide/show details for Grina

### [Mm]igration

#### PubMed

1. Identification of molecules preferentially expressed beneath the marginal zone in the developing cerebral cortex. During cerebral cortical development, the majority of excitatory neurons are born near the ventricle and migrate radially toward the marginal zone (MZ). Since the cells invariably stop migrating beneath the MZ, neurons are aligned in an "inside-out" manner in the cortical plate (CP); that is, the early-born and late-born neurons are ultimately positioned in the deep and superficial layers, respectively. Since dramatic morphological changes occur in cells beneath the MZ, several events critical for proper neuronal maturation and layer formation must take place. In this study, we screened for molecules strongly expressed beneath the MZ, and identified 28 genes that are preferentially expressed in the upper half of the mouse CP on both embryonic day (E) 16.5 and E18.5. Expression analyses in reeler and yotari mice, in which neurons terminate **migration** throughout the CP, suggested that these genes were indeed related to the events beneath the MZ rather than unrelatedly induced by the structures near the brain surface. Pathway analyses suggested calcium signaling to have an important role in cells beneath the MZ. The gene list presented here will be useful for clarifying the molecular mechanisms that control cortical development.

---

## Kcnn2

- NCBI Gene
- GeneRIF
- Pubmed

| Search Term | Rank | Fields |
| --- | --- | --- |
| [Mm]igration | 6 | PubMed(2); GeneRIFs(1) |

  
hide/show details for Kcnn2

### [Mm]igration

#### PubMed

1. Ion channel expression in the developing enteric nervous system. The enteric nervous system arises from neural crest-derived cells (ENCCs) that migrate caudally along the embryonic gut. The expression of ion channels by ENCCs in embryonic mice was investigated using a PCR-based array, RT-PCR and immunohistochemistry. Many ion channels, including chloride, calcium, potassium and sodium channels were already expressed by ENCCs at E11.5. There was an increase in the expression of numerous ion channel genes between E11.5 and E14.5, which coincides with ENCC **migration** and the first extension of neurites by enteric neurons. Previous studies have shown that a variety of ion channels regulates neurite extension and **migration** of many cell types. Pharmacological inhibition of a range of chloride or calcium channels had no effect on ENCC **migration** in cultured explants or neuritogenesis in vitro. The non-selective potassium channel inhibitors, TEA and 4-AP, retarded ENCC **migration** and neuritogenesis, but only at concentrations that also resulted in cell death. In summary, a large range of ion channels is expressed while ENCCs are colonizing the gut, but we found no evidence that ENCC **migration** or neuritogenesis requires chloride, calcium or potassium channel activity. Many of the ion channels are likely to be involved in the development of electrical excitability of enteric neurons.
2. Knockdown of the small conductance Ca(2+) -activated K(+) channels is potently cytotoxic in breast cancer cell lines. Small conductance calcium-activated potassium (KCa 2.x) channels have a widely accepted canonical function in regulating cellular excitability. In this study, we address a potential non-canonical function of KCa 2.x channels in breast cancer cell survival, using in vitro models.The expression of all KCa 2.x channel isoforms was initially probed using RT-PCR, Western blotting and microarray analysis in five widely studied breast cancer cell lines. In order to assess the effect of pharmacological blockade and siRNA-mediated knockdown of KCa 2.x channels on these cell lines, we utilized MTS proliferation assays and also followed the corresponding expression of apoptotic markers.All of the breast cancer cell lines, regardless of their lineage or endocrine responsiveness, were highly sensitive to KCa 2.x channel blockade. UCL1684 caused cytotoxicity, with LD50 values in the low nanomolar range, in all cell lines. The role of KCa 2.x channels was confirmed using pharmacological inhibition and siRNA-mediated knockdown. This reduced cell viability and also reduced expression of Bcl-2 but increased expression of active caspase-7 and caspase-9. Complementary to these results, a variety of cell lines can be protected from apoptosis induced by staurosporine using the KCa 2.x channel activator CyPPA.In addition to a well-established role for KCa 2.x channels in **migration**, blockade of these channels was potently cytotoxic in breast cancer cell lines, pointing to modulation of KCa 2.x channels as a potential therapeutic approach to breast cancer.

#### GeneRIFs

1. In addition to a well-established role for KCa2.x channels in **migration**, blockade of these channels was potently cytotoxic in breast cancer cell lines.

---

## Sirt7

- NCBI Gene
- GeneRIF
- Pubmed

| Search Term | Rank | Fields |
| --- | --- | --- |
| [Mm]etastasis | 12 | PubMed(1) |

  
hide/show details for Sirt7

### [Mm]etastasis

#### PubMed

1. Sirt7 Contributes to Myocardial Tissue Repair by Maintaining Transforming Growth Factor-β Signaling Pathway. Sirt7, 1 of the 7 members of the mammalian sirtuin family, promotes oncogenic transformation. Tumor growth and **metastasi**s require fibrotic and angiogenic responses. Here, we investigated the role of Sirt7 in cardiovascular tissue repair process.In wild-type mice, Sirt7 expression increased in response to acute cardiovascular injury, including myocardial infarction and hind-limb ischemia, particularly at the active wound healing site. Compared with wild-type mice, homozygous Sirt7-deficient (Sirt7(-/-)) mice showed susceptibility to cardiac rupture after myocardial infarction, delayed blood flow recovery after hind-limb ischemia, and impaired wound healing after skin injury. Histological analysis showed reduced fibrosis, fibroblast differentiation, and inflammatory cell infiltration in the border zone of infarction in Sirt7(-/-) mice. In vitro, Sirt7(-/-) mouse-derived or Sirt7 siRNA-treated cardiac fibroblasts showed reduced transforming growth factor-β signal activation and low expression levels of fibrosis-related genes compared with wild-type mice-derived or control siRNA-treated cells. These changes were accompanied by reduction in transforming growth factor receptor I protein. Loss of Sirt7 activated autophagy in cardiac fibroblasts. Transforming growth factor-β receptor I downregulation induced by loss of Sirt7 was blocked by autophagy inhibitor, and interaction of Sirt7 with protein interacting with protein kinase-Cα was involved in this process.Sirt7 maintains transforming growth factor receptor I by modulating autophagy and is involved in the tissue repair process.

---

## Cntn1

- NCBI Gene
- GeneRIF
- Pubmed

| Search Term | Rank | Fields |
| --- | --- | --- |
| [Mm]igration | 6 | PubMed(4) |
| [Ee]xtracellular [Mm]atrix | 8 | PubMed(3) |

  
hide/show details for Cntn1

### [Mm]igration

#### PubMed

1. A combination of chain and neurophilic **migration** involving the adhesion molecule TAG-1 in the caudal medulla. Neuronal populations destined to form several precerebellar nuclei are generated by the rhombic lip in the caudal hindbrain. These immature neurons gather into the olivary and the superficial migratory streams and migrate tangentially around the hindbrain to reach their final position. We focus on the cells of the superficial stream that migrate ventrally, cross the midline and form the lateral reticular (LRN) and external cuneate (ECN) nuclei. The cells of the superficial steam are preceded by long leading processes; in the dorsal neural tube, they migrate in close apposition to each other and form distinct chains, whereas they disperse and follow Tuj-1 immunoreactive axons on reaching the ventral hindbrain. This suggests that, in the superficial stream, neuronal **migration** combines both homotypic and heterotypic mechanisms. We also show that the adhesion molecule TAG-1 is expressed by the migrating cells. Blocking TAG-1 function results in alterations in the superficial **migration**, indicating that TAG-1 is involved in the superficial **migration**. Other members of the immunoglobulin superfamily and known ligands of TAG-1 are also expressed in the region of the **migration** but are not involved in the **migration**. These findings provide evidence that the TAG-1 protein is involved as a contact-dependent signal guiding not only axonal outgrowth but also cell **migration**.
2. Functional reciprocity between Na+ channel Nav1.6 and beta1 subunits in the coordinated regulation of excitability and neurite outgrowth. Voltage-gated Na(+) channel (VGSC) beta1 subunits regulate cell-cell adhesion and channel activity in vitro. We previously showed that beta1 promotes neurite outgrowth in cerebellar granule neurons (CGNs) via homophilic cell adhesion, fyn kinase, and contactin. Here we demonstrate that beta1-mediated neurite outgrowth requires Na(+) current (I(Na)) mediated by Na(v)1.6. In addition, beta1 is required for high-frequency action potential firing. Transient I(Na) is unchanged in Scn1b (beta1) null CGNs; however, the resurgent I(Na), thought to underlie high-frequency firing in Na(v)1.6-expressing cerebellar neurons, is reduced. The proportion of axon initial segments (AIS) expressing Na(v)1.6 is reduced in Scn1b null cerebellar neurons. In place of Na(v)1.6 at the AIS, we observed an increase in Na(v)1.1, whereas Na(v)1.2 was unchanged. This indicates that beta1 is required for normal localization of Na(v)1.6 at the AIS during the postnatal developmental switch to Na(v)1.6-mediated high-frequency firing. In agreement with this, beta1 is normally expressed with alpha subunits at the AIS of P14 CGNs. We propose reciprocity of function between beta1 and Na(v)1.6 such that beta1-mediated neurite outgrowth requires Na(v)1.6-mediated I(Na), and Na(v)1.6 localization and consequent high-frequency firing require beta1. We conclude that VGSC subunits function in macromolecular signaling complexes regulating both neuronal excitability and **migration** during cerebellar development.
3. Developmental abnormalities of neuronal structure and function in prenatal mice lacking the prader-willi syndrome gene necdin. Necdin (Ndn) is one of a cluster of genes deleted in the neurodevelopmental disorder Prader-Willi syndrome (PWS). Ndntm2Stw mutant mice die shortly after birth because of abnormal respiratory rhythmogenesis generated by a key medullary nucleus, the pre-Bötzinger complex (preBötC). Here, we address two fundamental issues relevant to its pathogenesis. First, we performed a detailed anatomical study of the developing medulla to determine whether there were defects within the preBötC or synaptic inputs that regulate respiratory rhythmogenesis. Second, in vitro studies determined if the unstable respiratory rhythm in Ndntm2Stw mice could be normalized by neuromodulators. Anatomical defects in Ndntm2Stw mice included defasciculation and irregular projections of axonal tracts, aberrant neuron**al migrat**ion, and a major defect in the cytoarchitecture of the cuneate/gracile nuclei, including dystrophic axons. Exogenous application of neuromodulators alleviated the long periods of slow respiratory rhythms and apnea, but some instability of rhythmogenesis persisted. We conclude that deficiencies in the neuromodulatory drive necessary for preBötC function contribute to respiratory dysfunction of Ndntm2Stw mice. These abnormalities are part of a more widespread deficit in neuro**nal migra**tion and the extension, arborization, and fasciculation of axons during early stages of central nervous system development that may account for respiratory, sensory, motor, and behavioral problems associated with PWS.
4. Transgenic mice expressing F3/contactin from the TAG-1 promoter exhibit developmentally regulated changes in the differentiation of cerebellar neurons. F3/contactin (CNTN1) and TAG-1 (CNTN2) are closely related axonal glycoproteins that are differentially regulated during development. In the cerebellar cortex TAG-1 is expressed first as granule cell progenitors differentiate in the premigratory zone of the external germinal layer. However, as these cells begin radial **migration**, TAG-1 is replaced by F3/contactin. To address the significance of this differential regulation, we have generated transgenic mice in which F3/contactin expression is driven by TAG-1 gene regulatory sequences, which results in premature expression of F3/contactin in granule cells. These animals (TAG/F3 mice) display a developmentally regulated cerebellar phenotype in which the size of the cerebellum is markedly reduced during the first two postnatal weeks but subsequently recovers. This is due in part to a reduction in the number of granule cells, most evident in the external germinal layer at postnatal day 3 and in the inner granular layer between postnatal days 8 and 11. The reduction in granule cell number is accompanied by a decrease in precursor granule cell proliferation at postnatal day 3, followed by an increase in the number of cycling cells at postnatal day 8. In the same developmental window the size of the molecular layer is markedly reduced and Purkinje cell dendrites fail to elaborate normally. These data are consistent with a model in which deployment of F3/contactin on granule cells affects proliferation and differentiation of these neurons as well as the differentiation of their synaptic partners, the Purkinje cells. Together, these findings indicate that precise spatio-temporal regulation of TAG-1 and F3/contactin expression is critical for normal cerebellar morphogenesis.

### [Ee]xtracellular [Mm]atrix

#### PubMed

1. Differential expression of phosphacan/RPTPbeta isoforms in the developing mouse visual system. The chondroitin sulfate proteoglycan DSD-1-PG/phosphacan represents one of four splice variants of receptor-protein-tyrosine-phosphatase-beta/zeta (RPTPbeta/zeta). This receptor is expressed by glial cells and occurs in two isoforms, RPTPbeta(long) and RPTPbeta(short). The secreted forms phosphacan and phosphacan short isoform (PSI) bind to **extracellular matrix** and adhesion molecules and might mediate astroglial effects on neuronal differentiation. Phosphacan and RPTPbeta(long) both carry the DSD-1 epitope, a glycosaminoglycan modification that is involved in stimulating neurite outgrowth of embryonic rat mesencephalic and hippocampal neurons in a polycationic environment. Additionally, phosphacan inhibits neurite outgrowth of embryonic DRG neurons in the presence of laminin. In the light of these functional properties we examined the expression patterns of the DSD-1 epitope and phosphacan isoforms in the developing mouse visual system. During retinal development the DSD-1 epitope appears around embryonic day (E)13, peaks around postnatal day (P)6, and is downregulated from P9 to adolescence. By comparison, the phosphacan core protein is first detectable at E12, reaches maximal levels around P14, and persists, although at lower levels, to adulthood. The DSD-1 epitope is restricted to the nerve fiber and the inner plexiform layers. In contrast, the phosphacan core protein immunoreactivity extends from the nerve fiber layer to the outer plexiform layer. The level of expression of the phosphacan/RPTPbeta gene was investigated by reverse-transcriptase polymerase chain reaction. These experiments suggest that there is a shift in the expression patterns of the different phosphacan/RPTPbeta isoforms during late embryonic and postnatal development. In situ hybridization experiments support the conclusion that at least one of the phosphacan/RPTPbeta isoforms in the retina is expressed by neurons.
2. Phosphacan short isoform, a novel non-proteoglycan variant of phosphacan/receptor protein tyrosine phosphatase-beta, interacts with neuronal receptors and promotes neurite outgrowth. Phosphacan, one of the principal proteoglycans in the **extracellular matrix** of the central nervous system, is implicated in neuron-glia interactions associated with neuronal differentiation and myelination. We report here the identification of a novel truncated form of phosphacan, phosphacan short isoform (PSI), that corresponds to the N-terminal carbonic anhydrase- and fibronectin type III-like domains and half of the spacer region. The novel cDNA transcript was isolated by screening of a neonatal brain cDNA expression library using a polyclonal antibody raised against phosphacan. Expression of this transcript in vivo was confirmed by Northern blot hybridization. Analysis of brain protein extracts reveals the presence of a 90-kDa glycosylated protein in the phosphate-buffered saline-insoluble 100000 x g fraction that reacts with antisera against both phosphacan and a recombinant PSI protein and that has the predicted N-terminal sequence. This protein is post-translationally modified with oligosaccharides, including the HNK-1 epitope, but, unlike phosphacan, it is not a proteoglycan. The expression of the PSI protein varies during central nervous system development in a fashion similar to that observed for phosphacan, being first detected around embryonic day 16 and then showing a dramatic increase in expression to plateau around the second week post-natal. Both the native and recombinant PSI protein can interact with the Ig cell adhesion molecules, F3/contactin and L1, and in neurite outgrowth assays, the PSI protein can promote outgrowth of cortical neurons when used as a coated substrate. Hence, the identification of this novel isoform of phosphacan/receptor protein tyrosine phosphatase-beta provides a new component in cell-cell and cell-**extracellular matrix** signaling events in which these proteins have been implicated.
3. The F3/11 cell adhesion molecule mediates the repulsion of neurons by the **extracellular matrix** glycoprotein J1-160/180. The oligodendrocyte-derived **extracellular matrix** protein J1-160/180 displays repellent substrate properties toward neurons. In a search for neuronal ligands mediating the response to J1-160/180, we have identified the F3/11 cell surface protein, a glyco-phosphatidylinositol-anchored member of the immunoglobulin superfamily. F3/11 mediates the initial recognition between a J1-160/180 substrate and cerebellar neurons or F3-transfected CHO cells. In cerebellar neurons, the F3/11-J1-160/180 interaction induces a repulsion consisting of the loss of substrate adhesion with time in culture and inhibition of neurite outgrowth. Antibody blocking experiments show that the avoidance response of neurites at J1-160/180 substrate borders is also mediated by F3/11. Active cell-cell and cell-substrate repulsion is considered a major mechanism governing the extent and directionality of axonal growth, but the ligand-receptor interactions involved have remained unknown. Our results show that F3/11 mediates the neuronal response to the repellent molecule J1-160/180 and may thus be involved in signal transduction leading to cell repulsion.

---

## Mmp14

- NCBI Gene
- GeneRIF
- Pubmed

| Search Term | Rank | Fields |
| --- | --- | --- |
| [Ii]nvadopodia | 3 | PubMed(1) |
| [Pp]odosome(s)? | 5 | PubMed(2); GeneRIFs(1) |
| [Mm]igration | 6 | Process(3); PubMed(29); GeneRIFs(7) |
| ECM | 7 | PubMed(26); GeneRIFs(3) |
| [Ee]xtracellular [Mm]atrix | 8 | Component(1); PubMed(66); Conserved Domains(1); GeneRIFs(3); Summary(1) |
| [Ii]nvasive | 10 | PubMed(20); GeneRIFs(4) |
| [Mm]etastasize | 11 | PubMed(1) |
| [Mm]etastasis | 12 | PubMed(12) |
| [Cc]ell [Ii]nvasion | 13 | PubMed(9); GeneRIFs(1) |

  
hide/show details for Mmp14

### [Ii]nvadopodia

#### PubMed

1. Dendritic cell podosomes are protrusive and invade the extracellular matrix using metalloproteinase MMP-14. Podosomes are spot-like actin-rich structures formed at the ventral surface of monocytic and haematopoietic cells. Podosomes degrade extracellular matrix and are proposed to be involved in cell migration. A key question is whether podosomes form protrusions similar to the **invadopodia** of cancer cells. We characterised podosomes of immature dendritic cells using electron microscopy combined with both conventional and novel high-resolution structured illumination light microscopy. Dendritic cell podosomes are composed of actin foci surrounded by a specialised ring region that is rich in material containing paxillin. We found that podosomes were preferential sites for protrusion into polycarbonate filters impregnated with crosslinked gelatin, degrading up to 2 micrometers of matrix in 24 hours. Podosome-associated uptake of colloidal gold-labelled gelatin matrix appeared to occur via large phagosome-like structures or narrow tubular invaginations. The motor protein myosin-II was excluded from ring or core regions but was concentrated around them and the myosin-II inhibitor Blebbistatin reduced the length of podosome protrusions. Finally, we found that degradation, protrusion and endocytosis in this system are dependent on the matrix metalloproteinase MMP-14. We propose that podosomes mediate migration of dendritic cells through tissues by means of myosin-II-dependent protrusion coupled to MMP-14-dependent degradation and endocytosis.

### [Pp]odosome(s)?

#### PubMed

1. Dendritic cell **podosomes** are protrusive and invade the extracellular matrix using metalloproteinase MMP-14. **Podosomes** are spot-like actin-rich structures formed at the ventral surface of monocytic and haematopoietic cells. **Podosomes** degrade extracellular matrix and are proposed to be involved in cell migration. A key question is whether **podosomes** form protrusions similar to the invadopodia of cancer cells. We characterised **podosomes** of immature dendritic cells using electron microscopy combined with both conventional and novel high-resolution structured illumination light microscopy. Dendritic cell **podosomes** are composed of actin foci surrounded by a specialised ring region that is rich in material containing paxillin. We found that **podosomes** were preferential sites for protrusion into polycarbonate filters impregnated with crosslinked gelatin, degrading up to 2 micrometers of matrix in 24 hours. **Podosome**-associated uptake of colloidal gold-labelled gelatin matrix appeared to occur via large phagosome-like structures or narrow tubular invaginations. The motor protein myosin-II was excluded from ring or core regions but was concentrated around them and the myosin-II inhibitor Blebbistatin reduced the length of **podosome** protrusions. Finally, we found that degradation, protrusion and endocytosis in this system are dependent on the matrix metalloproteinase MMP-14. We propose that **podosomes** mediate migration of dendritic cells through tissues by means of myosin-II-dependent protrusion coupled to MMP-14-dependent degradation and endocytosis.
2. TLR ligand-induced **podosome** disassembly in dendritic cells is ADAM17 dependent. Toll-like receptor (TLR) signaling induces a rapid reorganization of the actin cytoskeleton in cultured mouse dendritic cells (DC), leading to enhanced antigen endocytosis and a concomitant loss of filamentous actin-rich **podosomes**. We show that as **podosomes** are lost, TLR signaling induces prominent focal contacts and a transient reduction in DC migratory capacity in vitro. We further show that **podosomes** in mouse DC are foci of pronounced gelatinase activity, dependent on the enzyme membrane type I matrix metalloprotease (MT1-MMP), and that DC transiently lose the ability to degrade the extracellular matrix after TLR signaling. Surprisingly, MMP inhibitors block TLR signaling-induced **podosome** disassembly, although stimulated endocytosis is unaffected, which demonstrates that the two phenomena are not obligatorily coupled. **Podosome** disassembly caused by TLR signaling occurs normally in DC lacking MT1-MMP, and instead requires the tumor necrosis factor alpha-converting enzyme ADAM17 (a disintegrin and metalloprotease 17), which demonstrates a novel role for this "sheddase" in regulating an actin-based structure.

#### GeneRIFs

1. **Podosomes** mediate migration of dendritic cells through tissues by means of myosin-II-dependent protrusion coupled to MMP-14-dependent degradation and endocytosis.

### [Mm]igration

#### Process

1. cell **migration**
2. positive regulation of cell **migration**
3. positive regulation of macrophage **migration**

#### PubMed

1. MT1-MMP is required for myeloid cell fusion via regulation of Rac1 signaling. Cell fusion is essential for fertilization, myotube formation, and inflammation. Macrophages fuse under various circumstances, but the molecular signals involved in the distinct steps of their fusion are not fully characterized. Using null mice and derived cells, we show that the protease MT1-MMP is necessary for macrophage fusion during osteoclast and giant-cell formation in vitro and in vivo. Specifically, MT1-MMP is required for lamellipodia formation and for proper cell morphology and motility of bone marrow myeloid progenitors prior to membrane fusion. These functions of MT1-MMP do not depend on MT1-MMP catalytic activity or downstream pro-MMP-2 activation. Instead, MT1-MMP null cells show a decreased Rac1 activity and reduced membrane targeting of Rac1 and the adaptor protein p130Cas. Retroviral rescue experiments and protein binding assays delineate a signaling pathway in which MT1-MMP, via its cytosolic tail, contributes to macrophage **migration** and fusion by regulating Rac1 activity through an association with p130Cas.
2. MT1-MMP is required for myeloid cell fusion via regulation of Rac1 signaling. Cell fusion is essential for fertilization, myotube formation, and inflammation. Macrophages fuse under various circumstances, but the molecular signals involved in the distinct steps of their fusion are not fully characterized. Using null mice and derived cells, we show that the protease MT1-MMP is necessary for macrophage fusion during osteoclast and giant-cell formation in vitro and in vivo. Specifically, MT1-MMP is required for lamellipodia formation and for proper cell morphology and motility of bone marrow myeloid progenitors prior to membrane fusion. These functions of MT1-MMP do not depend on MT1-MMP catalytic activity or downstream pro-MMP-2 activation. Instead, MT1-MMP null cells show a decreased Rac1 activity and reduced membrane targeting of Rac1 and the adaptor protein p130Cas. Retroviral rescue experiments and protein binding assays delineate a signaling pathway in which MT1-MMP, via its cytosolic tail, contributes to macrophage **migration** and fusion by regulating Rac1 activity through an association with p130Cas.
3. A novel and selective membrane type-1 matrix metalloproteinase (MT1-MMP) inhibitor reduces cancer cell motility and tumor growth. Matrix metalloproteinases (MMPs), and especially membrane type-1 matrix metalloproteinase (MT1-MMP, MMP-14), play a role in cancer progression and can have a prognostic value. Various synthetic broad-spectrum MMP inhibitors have been developed but have had little success in cancer patient treatment owing to side effects. Until recently, selective targeting of specific MMPs has not been possible due to lack of specific inhibitors. Here we have developed a selective MT1-MMP peptide-inhibitor GACFSIAHECGA, which did not affect the activities of many other MMPs including MMP-1, -2, -3, -7, -8, -9, -10, -11, -12, -13, -15, -17 or -20. In a fluorescent peptide cleavage assay it displayed an IC(50) value of 150 microM. The peptide effectively inhibited the **migration** and invasion of cancer cell lines in vitro. Furthermore, in vivo the peptide reduced the growth of tongue carcinoma xenografts and prolonged the survival of mice. Overall these results suggest that selective MT1-MMP inhibitors may have utility as anticancer agents.
4. Pulmonary fibroblasts mobilize the membrane-tethered matrix metalloprotease, MT1-MMP, to destructively remodel and invade interstitial type I collagen barriers. In acute and chronic lung disease, widespread disruption of tissue architecture underlies compromised pulmonary function. Pulmonary fibroblasts have been implicated as critical effectors of tissue-destructive extracellular matrix (ECM) remodeling by mobilizing a spectrum of proteolytic enzymes. Although efforts to date have focused on the catabolism of type I collagen, the predominant component of the lung interstitial matrix, the key collagenolytic enzymes employed by pulmonary fibroblasts remain unidentified. Herein, membrane type-1 matrix metalloprotease (MT1-MMP) is identified as the dominant and direct-acting protease responsible for the type I collagenolytic activity mediated by both mouse and human pulmonary fibroblasts. Furthermore, MT1-MMP is shown to be essential for pulmonary fibroblast **migration** within three-dimensional (3-D) hydrogels of cross-linked type I collagen that recapitulate ECM barriers encountered in the in vivo environment. Together, these findings demonstrate that MT1-MMP serves as a key effector of type I collagenolytic activity in pulmonary fibroblasts and earmark this pericellular collagenase as a potential target for therapeutic intervention.
5. Dendritic cell podosomes are protrusive and invade the extracellular matrix using metalloproteinase MMP-14. Podosomes are spot-like actin-rich structures formed at the ventral surface of monocytic and haematopoietic cells. Podosomes degrade extracellular matrix and are proposed to be involved in cell **migration**. A key question is whether podosomes form protrusions similar to the invadopodia of cancer cells. We characterised podosomes of immature dendritic cells using electron microscopy combined with both conventional and novel high-resolution structured illumination light microscopy. Dendritic cell podosomes are composed of actin foci surrounded by a specialised ring region that is rich in material containing paxillin. We found that podosomes were preferential sites for protrusion into polycarbonate filters impregnated with crosslinked gelatin, degrading up to 2 micrometers of matrix in 24 hours. Podosome-associated uptake of colloidal gold-labelled gelatin matrix appeared to occur via large phagosome-like structures or narrow tubular invaginations. The motor protein myosin-II was excluded from ring or core regions but was concentrated around them and the myosin-II inhibitor Blebbistatin reduced the length of podosome protrusions. Finally, we found that degradation, protrusion and endocytosis in this system are dependent on the matrix metalloproteinase MMP-14. We propose that podosomes mediate **migration** of dendritic cells through tissues by means of myosin-II-dependent protrusion coupled to MMP-14-dependent degradation and endocytosis.
6. MMP-2 null mice exhibit an early onset and severe experimental autoimmune encephalomyelitis due to an increase in MMP-9 expression and activity. Matrix metalloproteinase-2 (MMP-2; gelatinase A) is known to degrade a broad range of extracellular matrix components and chemokines, and has important roles in the processes of cell **migration**, invasion, and involution during development, as well as during tumor growth and metastasis and in inflammation and repair. To better elucidate the roles of this matrix metalloproteinase in the development and progression of experimental autoimmune encephalomyelitis, we used MMP-2-deficient (KO) mice. Surprisingly, we found that MMP-2 KO mice exhibited an earlier onset and more severe disease than did their wild-type (WT) counterparts. WT mice engrafted with MMP-2 KO bone marrow exhibited a similar earlier onset and more severe clinical disease score than WT mice engrafted with WT bone marrow. Lymphocytes derived from MMP-2 KO mice exhibited increased trans**migration** through endothelial cell monolayers as well as through collagen type IV and laminin-coated BD BIOCOAT inserts, which correlated with a 3-fold increase in expression of MMP-9 and was abrogated by inhibition of MMP activity. We demonstrated a correlation between expression levels of MMP-9 and MT1-MMP expression and suggest a signaling pathway involving tethering of MMP-2 to MT1-MMP as a modulator of MMP-9 expression. Last, we discuss other possible MMP-2-mediated mechanisms which may contribute to the observed phenotype.
7. Proteolytic processing of laminin-5 by MT1-MMP in tissues and its effects on epithelial cell morphology. The extracellular matrix macromolecule laminin-5 (Ln-5) is converted by matrix metalloproteinases (MMP) MT1-MMP and MMP-2 into a **migration**-promoting substrate in vitro. We now report that cleavage of Ln-5 by MT1-MMP occurs in vivo and affects epithelial tissue organization and probably Ln-5 turnover. In MT1-MMP knockout (KO) mice, the kidneys showed increased levels of total Ln-5 gamma2 subunit, but significantly reduced amounts of gamma2', an amino-terminal truncated proteolytic form of gamma2. The kidney tubular epithelia of KO animals were poorly differentiated, a phenotype reminiscent of human congenital mixed hypoplastic/dysplastic disorders. To establish a better link between Ln-5 proteolytic cleavage and epithelial morphology, MT1-MMP expression was reconstituted by transfection of MT1-MMP into a Ln-5 positive, MT1-MMP deficient epithelial cell line. MT1-MMP transfectants demonstrated increased levels of processed Ln-5 gamma2 chain and enhanced spreading on Ln-5, but not fibronectin. Recombinant MT1-MMP cleaved gamma2 constructs in vitro at a known in vivo gamma2 gamma2' processing site. These results strongly indicate that Ln-5 is a physiological substrate of MT1-MMP in vivo. Proteolytic processing of gamma2 subunit by MT1-MMP may influence Ln-5 turnover in epithelial basement membranes and affect epithelial morphogenesis.
8. Msx1 and Msx2 function together in the regulation of primordial germ cell **migration** in the mouse. Primordial germ cells (PGCs) are a highly migratory cell population that gives rise to eggs and sperm. Much is known about PGC specification, but less about the processes that control PGC **migration**. In this study, we document a deficiency in PGC development in embryos carrying global homozygous null mutations in Msx1 and Msx2, both immediate downstream effectors of Bmp signaling pathway. We show that Msx1(-/-);Msx2(-/-) mutant embryos have defects in PGC **migration** as well as a reduced number of PGCs. These phenotypes are also evident in a Mesp1-Cre-mediated mesoderm-specific mutant line of Msx1 and Msx2. Since PGCs are not marked in Mesp1-lineage tracing, our results suggest that Msx1 and Msx2 function cell non-autonomously in directing PGC **migration**. Consistent with this hypothesis, we noted an upregulation of fibronectin, well known as a mediator of cell **migration**, in tissues through which PGCs migrate. We also noted a reduction in the expression of Wnt5a and an increase in the expression in Bmp4 in such tissues in Msx1(-/-);Msx2(-/-) mutants, both known effectors of PGC development.
9. Angiotensin II stimulates cardiac fibroblast **migration** via the differential regulation of matrixins and RECK. Sustained induction and activation of matrixins (matrix metalloproteinases or MMPs), and the destruction and deposition of extracellular matrix (ECM), are the hallmarks of cardiac fibrosis. The reversion-inducing-cysteine-rich protein with Kazal motifs (RECK) is a unique membrane-anchored endogenous MMP regulator. We hypothesized that elevated angiotensin II (Ang II), which is associated with fibrosis in the heart, differentially regulates MMPs and RECK both in vivo and in vitro. Continuous infusion of Ang II into male C57Bl/6 mice for 2weeks resulted in cardiac fibrosis, with increased expressions of MMPs 2, 7, 9 and 14, and of collagens Ia1 and IIIa1. The expression of RECK, however, was markedly suppressed. These effects were inhibited by co-treatment with the Ang II type 1 receptor (AT1) antagonist losartan. In vitro, Ang II suppressed RECK expression in adult mouse cardiac fibroblasts (CF) via AT1/Nox4-dependent ERK/Sp1 activation, but induced MMPs 2, 14 and 9 via NF-κB, AP-1 and/or Sp1 activation. Further, while forced expression of RECK inhibits, its knockdown potentiates Ang II-induced CF **migratio**n. Notably, RECK overexpression reduced Ang II-induced MMPs 2, 9 and 14 activation, but enhanced collagens Ia1 and IIIa1 expression and soluble collagen release. These results demonstrate for the first time that Ang II suppresses RECK, but induces MMPs both in vivo and in vitro, and RECK overexpression blunts Ang II-induced MMP activation and CF **migratio**n in vitro. Strategies that upregulate RECK expression in vivo have the potential to attenuate sustained MMP expression, and blunt fibrosis and adverse remodeling in hypertensive heart diseases.
10. Taspase1-dependent TFIIA cleavage coordinates head morphogenesis by limiting Cdkn2a locus transcription. Head morphogenesis requires complex signal relays to enable precisely coordinated proliferation, **migration**, and patterning. Here, we demonstrate that, during mouse head formation, taspase1-mediated (TASP1-mediated) cleavage of the general transcription factor TFIIA ensures proper coordination of rapid cell proliferation and morphogenesis by maintaining limited transcription of the negative cell cycle regulators p16Ink4a and p19Arf from the Cdkn2a locus. In mice, loss of TASP1 function led to catastrophic craniofacial malformations that were associated with inadequate cell proliferation. Compound deficiency of Cdkn2a, especially p16Ink4a deficiency, markedly reduced the craniofacial anomalies of TASP1-deficent mice. Furthermore, evaluation of mice expressing noncleavable TASP1 targets revealed that TFIIA is the principal TASP1 substrate that orchestrates craniofacial morphogenesis. ChIP analyses determined that noncleaved TFIIA accumulates at the p16Ink4a and p19Arf promoters to drive transcription of these negative regulators. In summary, our study elucidates a regulatory circuit comprising proteolysis, transcription, and proliferation that is pivotal for construction of the mammalian head.
11. Cytoplasmic tail of MT1-MMP regulates macrophage motility independently from its protease activity. Membrane type-1 matrix metalloproteinase (MT1-MMP) is a proinvasive protease that regulates various cellular functions as evidenced by myriad defects in different types of cells and tissues in MT1-MMP-deficient (MT1(-/-)) mice. Here we demonstrate that MT1(-/-) mice exhibit fewer infiltrating macrophages into sites of inflammation. MT1(-/-)macrophages exhibited a reduced ability to invade reconstituted basement membrane (Matrigel) and invasion by wild type (WT) macrophages was inhibited by a synthetic MMP inhibitor (BB94) to a level similar to that of MT1(-/-) cells. The rate of **migration** of MT1(-/-) macrophages was also low compared to that of the WT cells and re-expression of MT1-MMP in MT1(-/-) macrophages reconstituted their migratory activity. Unexpectedly, however, BB94 did not inhibit the **migration** of WT macrophages. The **migration**-boosting activity of MT1-MMP is retained in a mutant that lacks most of the extracellular portion including the catalytic and hemopexin-like domains. In contrast, deletion of the cytoplasmic (CP) tail abolished the activity completely. Thus, we have demonstrated that MT1-MMP regulates macrophages via its invasion-promoting protease activity as well as its CP-dependent non-proteolytic activity to boost cell **migration**.
12. Distinctive functions of membrane type 1 matrix-metalloprotease (MT1-MMP or MMP-14) in lung and submandibular gland development are independent of its role in pro-MMP-2 activation. Membrane type 1-matrix metalloprotease (MT1-MMP or MMP-14) is a major activator of pro-MMP-2 and is essential for skeletal development. We show here that it is required for branching morphogenesis of the submandibular gland but not the lung. Instead, in the lung, it is essential for postnatal development of alveolar septae. Lung development in Mmp14-/- mice is arrested at the prealveolar stage with compensatory hyperinflation of immature saccules. Mmp2-/- mice lacked comparable defects in the lung and submandibular gland, suggesting that MT1-MMP acts via mechanisms independent of pro-MMP-2 activation. Since the developmental defects in the lung are first manifest around the time of initial vascularization (E16.5), we investigated the behavior of pulmonary endothelial cells from Mmp14+/+ and Mmp14-/- mice. Endothelial cells from lungs of 1-week-old Mmp14-/- mice show reduced **migration** and formation of three-dimensional structures on Matrigel. Since pulmonary septal development requires capillary growth, the underlying mechanism of pulmonary hypoplasia in Mmp14-/- mice may be defective angiogenesis, supporting a model in which angiogenesis is a critical rate-limiting step for acquisition of pulmonary parenchymal mass.
13. Functional interplay between endothelial nitric oxide synthase and membrane type 1 matrix metalloproteinase in migrating endothelial cells. Nitric oxide (NO) is essential for vascular homeostasis and is also a critical modulator of angiogenesis; however, the molecular mechanisms of NO action during angiogenesis remain elusive. We have investigated the potential relationship between NO and membrane type 1-matrix metalloproteinase (MT1-MMP) during endothelial **migration** and capillary tube formation. Endothelial NO synthase (eNOS) colocalizes with MT1-MMP at motility-associated structures in migratory human endothelial cells (ECs); moreover, NO is produced at these structures and is released into the medium during EC **migration**. We have therefore addressed 2 questions: (1) the putative regulation of MT1-MMP by NO in migratory ECs; and (2) the requirement for MT1-MMP in NO-induced EC **migration** and tube formation. NO upregulates MT1-MMP membrane clustering on migratory human ECs, and this is accompanied by increased degradation of type I collagen substrate. MT1-MMP membrane expression and localization are impaired in lung ECs from eNOS-deficient mice, and these cells also show impaired **migration** and tube formation in vitro. Inhibition of MT1-MMP with a neutralizing antibody impairs NOinduced tube formation by human ECs, and NO-induced endothelial **migration** and tube formation are impaired in lung ECs from mice deficient in MT1-MMP. MT1-MMP thus appears to be a key molecular effector of NO during the EC **migration** and angiogenic processes, and is a potential therapeutic target for NO-associated vascular disorders.
14. Membrane localization of membrane type 1 matrix metalloproteinase by CD44 regulates the activation of pro-matrix metalloproteinase 9 in osteoclasts. CD44, MT1-MMP, and MMP9 are implicated in the **migration** of osteoclast and bone resorption. This study was designed to determine the functional relationship between CD44 and MT1-MMP in the activation of pro-MMP9. We used osteoclasts isolated from wild-type and CD44-null mice. Results showed that MT1-MMP is present in multiple forms with a molecular mass ~63, 55, and 45 kDa in the membrane of wild-type osteoclasts. CD44-null osteoclasts demonstrated a 55 kDa active MT1-MMP form in the membrane and conditioned medium. It failed to activate pro-MMP9 because TIMP2 binds and inhibits this MT1-MMP (~55 kDa) in CD44-null osteoclasts. The role of MT1-MMP in the activation of pro-MMP9, CD44 expression, **and migra**tion was confirmed by knockdown of MT1-MMP in wild-type osteoclasts. Although knockdown of MMP9 suppressed osteocl**ast migra**tion, it had no effects on MT1-MMP activity or CD44 expression. These results suggest that CD44 and MT1-MMP are directly or indirectly involved in the regulation of pro-MMP9 activation. Surface expression of CD44, membrane localization of MT1-MMP, and activation of pro-MMP9 are the necessary sequence of events in osteocl**ast migra**tion.
15. A concerted HIF-1α/MT1-MMP signalling axis regulates the expression of the 3BP2 adaptor protein in hypoxic mesenchymal stromal cells. Increased plasticity, migratory and immunosuppressive abilities characterize mesenchymal stromal cells (MSC) which enable them to be active participants in the development of hypoxic solid tumours. Our understanding of the oncogenic adaptation of MSC to hypoxia however lacks the identification and characterization of specific biomarkers. In this study, we assessed the hypoxic regulation of 3BP2/SH3BP2 (Abl SH3-binding protein 2), an immune response adaptor/scaffold protein which regulates leukocyte differentiation and motility. Gene silencing of 3BP2 abrogated MSC **migratio**n in response to hypoxic cues and generation of MSC stably expressing the transcription factor hypoxia inducible factor 1alpha (HIF-1α) resulted in increased endogenous 3BP2 expression as well as cel**l migrati**on. Analysis of the 3BP2 promoter sequence revealed only one potential HIF-1α binding site within the human but none in the murine sequence. An alternate early signalling cascade that regulated 3BP2 expression was found to involve membrane type-1 matrix metalloproteinase (MT1-MMP) transcriptional regulation which gene silencing abrogated 3BP2 expression in response to hypoxia. Collectively, we provide evidence for a concerted HIF-1α/MT1-MMP signalling axis that explains the induction of adaptor protein 3BP2 and which may link protein binding partners together and stimulate oncogenic **MSC migra**tion. These mechanistic observations support the potential for malignant transformation of MSC within hypoxic tumour stroma and may contribute to evasion of the immune system by a tumour.
16. Effect of MT1-MMP deficiency and overexpression in corneal keratocytes on vascular endothelial cell **migration** and proliferation. To determine the effect of keratocyte-derived MT1-MMP on calf pulmonary artery endothelial cell (CPAE) proliferation and **migration**.Keratocyte lines were generated from MT1-MMP knockout (KO) and wild type (WT) mice. WT keratocytes were transfected with WT or mutant MT1-MMP DNAs (DeltaTC or E240A). The effect of keratocyte-conditioned media on CPAE proliferation and **migration** was assayed.KO keratocyte conditioned media resulted in the greatest increase of CPAE cell proliferation (190.5+/-6.0%; p<0.01). WT keratocyte conditioned media showed higher CPAE proliferation (155.4+/-3.6%) than WT/MT1-MMP-transfected keratocytes (119.7+/-2.2%; p<0.001). **Migration** assays confirmed these findings.Keratocyte-derived MT1-MMP has anti-angiogenic effects in CPAE cells.
17. Matrix metalloproteinases 2 and 9 are dispensable for pancreatic islet formation and function in vivo. Pancreatic islet formation is a highly regulated process that is initiated at the end of gestation in rodents. Endocrine precursor cells first form within the epithelium of duct-like structures and then delaminate from the epithelium, migrate, and cluster during the early stages of islet formation. The molecular mechanisms that regulate endocrine cell **migration** and islet formation are not well understood. Cell culture studies suggest that matrix metalloproteinases (MMPs) 2 and 9 are required for islet formation. To address whether MMP2 and MMP9 function are essential for endocrine cell **migration** and islet formation in vivo, we analyzed pancreas development in MMP2/MMP9 double-deficient mice. Our results show that islet architecture and function are unperturbed in these knockout mice, demonstrating that both MMP2 and MMP9 functions are dispensable for islet formation and endocrine cell differentiation. Our studies also show that a number of other MMPs are expressed at the time islet formation is initiated. This observation suggests that other MMPs may substitute for MMP2 and MMP9 loss in pancreatic tissue. However, islet formation is unaffected in transgenic mice with modified tissue inhibitor of metalloproteinase-1 (TIMP1) levels, suggesting that MMP activity may contribute little to islet morphogenesis in vivo.
18. Membrane-type MMPs enable extracellular matrix permissiveness and mesenchymal cell proliferation during embryogenesis. Peri-cellular remodeling of mesenchymal extracellular matrices is considered a prerequisite for cell proliferation, motility and development. Here we demonstrate that membrane-type 3 MMP, MT3-MMP, is expressed in mesenchymal tissues of the skeleton and in peri-skeletal soft connective tissue. Consistent with this localization, MT3-MMP-deficient mice display growth inhibition tied to a decreased viability of mesenchymal cells in skeletal tissues. We document that MT3-MMP works as a major collagenolytic enzyme, enabling cartilage and bone cells to cleave high-density fibrillar collagen and modulate their resident matrix to make it permissive for proliferation and **migration**. Collectively, these data uncover a novel extracellular matrix remodeling mechanism required for proper function of mesenchymal cells. The physiological significance of MT3-MMP is highlighted in mice double deficient for MT1-MMP and MT3-MMP. Double deficiency transcends the combined effects of the individual single deficiencies and leads to severe embryonic defects in palatogenesis and bone formation incompatible with life. These defects are directly tied to loss of indispensable collagenolytic activities required in collagen-rich mesenchymal tissues for extracellular matrix remodeling and cell proliferation during embryogenesis.
19. MT1-MMP-mediated basement membrane remodeling modulates renal development. Extracellular matrix (ECM) remodeling regulates multiple cellular functions required for normal development and tissue repair. Matrix metalloproteinases (MMPs) are key mediators of this process and membrane targeted MMPs (MT-MMPs) in particular have been shown to be important in normal development of specific organs. In this study we investigated the role of MT1-MMP in kidney development. We demonstrate that loss of MT1-MMP leads to a renal phenotype characterized by a moderate decrease in ureteric bud branching morphogenesis and a severe proliferation defect. The kidneys of MT1-MMP-null mice have increased deposition of collagen IV, laminins, perlecan, and nidogen and the phenotype is independent of the MT-1MMP target, MMP-2. Utilizing in vitro systems we demonstrated that MTI-MMP proteolytic activity is required for renal tubule cells to proliferate in three dimensional matrices and to migrate on collagen IV and laminins. Together these data suggest an important role for MT1-MMP in kidney development, which is mediated by its ability to regulate cell proliferation and **migration** by proteolytically cleaving kidney basement membrane components.
20. Monocyte/macrophage MMP-14 modulates cell infiltration and T-cell attraction in contact dermatitis but not in murine wound healing. Monocyte infiltration and subsequent differentiation into macrophages has been shown to be crucial during inflammation. Metalloproteinases are key enzymes in these processes, but the role of MMP-14 remains largely unknown. To address this question, we generated animals with conditional ablation of MMP-14 in the monocyte/macrophage lineage. The knockout (KO) animals (LysM-Cre(+)MMP-14(fl/fl)) were healthy and fertile, and neither skin architecture nor differentiation was altered from the wild type (WT). Full-thickness wounds were induced, and careful analysis of wound closure, granulation tissue formation, and angiogenesis revealed no differences between genotypes. The inflammatory response, monocyte influx, differentiation, and lymphocyte infiltration was also similar in KO and WT animals. Ear swelling after croton oil application was similar in the KO and WT animals. Interestingly, the number of monocytes and macrophages, as well as of T cells, was significantly reduced in KO animals, compared with WT animals. Similarly, both P-selectin and proinflammatory cytokine levels were markedly reduced in KO animals. In vitro, the migratory capacity of isolated KO macrophages was significantly impaired on fibronectin, a substrate of MMP-14. These data point to a role of MMP-14 during transendothelial **migration** of monocytes and T-cell attraction.
21. Lumican effects in the control of tumour progression and their links with metalloproteinases and integrins. Lumican is a member of the small leucine-rich proteoglycan family. It is present in numerous extracellular matrices of different tissues, such as muscle, cartilage, and cornea. In skin, lumican is present as a glycoprotein. It plays a critical role in collagen fibrillogenesis, as shown by knocking out of its gene in mice. A direct link between lumican expression and melanoma progression and metastasis has been demonstrated. Lumican was shown to impede tumour cell **migration** and invasion by directly interacting with the α2β1 integrin. In addition, an active sequence of the lumican core protein, called lumcorin, was identified as being responsible for inhibition of melanoma cel**l migrati**on. Lumican was also shown to exert angiostatic properties by downregulating the proteolytic activity associated with endothelial cell membranes, particularly matrix metalloproteinase (MMP)-14 and MMP-9. Globally, lumican appears to be a potent agent for inhibiting tumour progression rather than tumorigenesis. However, progressive changes in proteoglycans occur in the tumour environment. The complexity and diversity of proteoglycan structure might be responsible for a variety of functions that regulate cell behaviour. Through their core protein and their glycosaminoglycan chains, proteoglycans can interact with growth factors and chemokines. These interactions affect cell signalling, motility, adhesion, growth, and apoptosis. This review summarizes recent data concerning lumican control of tumour progression in different cancers, with a particular focus on its interactions with MMPs and integrins. Its potential therapeutic implications are discussed.
22. MT1-MMP plays a critical role in hematopoiesis by regulating HIF-mediated chemokine/cytokine gene transcription within niche cells. HSC fate decisions are regulated by cell-intrinsic and cell-extrinsic cues. The latter cues are derived from the BM niche. Membrane-type 1 matrix metalloproteinase (MT1-MMP), which is best known for its proteolytic role in pericellular matrix remodeling, is highly expressed in HSCs and stromal/niche cells. We found that, in MT1-MMP(-/-) mice, in addition to a stem cell defect, the transcription and release of kit ligand (KitL), stromal cell-derived factor-1 (SDF-1/CXCL12), erythropoietin (Epo), and IL-7 was impaired, resulting in a trilineage hematopoietic differentiation block, while addition of exogenous KitL and SDF-1 restored hematopoiesis. Further mechanistic studies revealed that MT1-MMP activates the hypoxia-inducible factor-1 (HIF-1) pathway via factor inhibiting HIF-1 (FIH-1) within niche cells, thereby inducing the transcription of HIF-responsive genes, which induce terminal hematopoietic differentiation. Thus, MT1-MMP in niche cells regulates postnatal hematopoiesis, by modulating hematopoietic HIF-dependent niche factors that are critical for terminal differentiation and **migration**.
23. Inhibition of membrane type-1 matrix metalloproteinase by cancer drugs interferes with the homing of diabetogenic T cells into the pancreas. We have discovered that clinically tested inhibitors of matrix metalloproteinases can control the functional activity of T cell membrane type-1 matrix metalloproteinase (MT1-MMP) and the onset of disease in a rodent model of type 1 diabetes in non-obese diabetic mice. We determined that MT1-MMP proteolysis of the T cell surface CD44 adhesion receptor affects the homing of T cells into the pancreas. We also determined that both the induction of the intrinsic T cell MT1-MMP activity and the shedding of cellular CD44 follow the adhesion of insulin-specific, CD8-positive, Kd-restricted T cells to the matrix. Conversely, inhibition of these events by AG3340 (a potent hydroxamate inhibitor that was widely used in clinical trials in cancer patents) impedes the trans**migration** of diabetogenic T cells into the pancreas and protects non-obese diabetic mice from diabetes onset. Overall, our studies have divulged a previously unknown function of MT1-MMP and identified a promising novel drug target in type I diabetes.
24. Complementary roles of intracellular and pericellular collagen degradation pathways in vivo. Collagen degradation is essential for cell **migration**, proliferation, and differentiation. Two key turnover pathways have been described for collagen: intracellular cathepsin-mediated degradation and pericellular collagenase-mediated degradation. However, the functional relationship between these two pathways is unclear and even controversial. Here we show that intracellular and pericellular collagen turnover pathways have complementary roles in vivo. Individual deficits in intracellular collagen degradation (urokinase plasminogen activator receptor-associated protein/Endo180 ablation) or pericellular collagen degradation (membrane type 1-matrix metalloproteinase ablation) were compatible with development and survival. Their combined deficits, however, synergized to cause postnatal death by severely impairing bone formation. Interestingly, this was mechanistically linked to the proliferative failure and poor survival of cartilage- and bone-forming cells within their collagen-rich microenvironment. These findings have important implications for the use of pharmacological inhibitors of collagenase activity to prevent connective tissue destruction in a variety of diseases.
25. Induction of a MT1-MMP and MT2-MMP-dependent basement membrane trans**migration** program in cancer cells by Snail1. The ability of carcinoma cells arising at primary sites to cross their underlying basement membrane (BM), a specialized form of extracellular matrix that subtends all epithelial cells, and to access the host vasculature are central features of the malignant phenotype. The initiation of the invasive phenotype has been linked to the aberrant expression of zinc-finger transcriptional repressors, like Snail1, which act by triggering an epithelial-mesenchymal cell-like transformation (EMT-like) via the regulation of largely undefined, downstream effectors. Herein, we find that Snail1 induces cancer cells to (i) degrade and perforate BM barriers, (ii) initiate angiogenesis, and (iii) and intravasate vascular networks in vivo via a matrix metalloproteinase (MMP)-dependent process. Unexpectedly, the complete Snail1 invasion program can be recapitulated by expressing directly either of the membrane-anchored metalloproteinases, MT1-MMP or MT2-MMP. The pro-invasive, angiogenic, and metastatic activities of MT1-MMP and MT2-MMP are unique relative to all other metalloproteinase family members and cannot be mimicked in vivo by the secreted MMPs, MMP-1, MMP-2, MMP-3, MMP-7, MMP-9, or MMP-13. Further, siRNA-specific silencing of MT1-MMP and MT2-MMP ablates completely the ability of Snail1 to drive cancer cell BM invasion, induce angiogenesis, or trigger intravasation. Taken together, these data demonstrate that MT1-MMP and MT2-MMP cooperatively function as direct-acting, pro-invasive factors that confer Snail1-triggered cells with the key activities most frequently linked to morbidity and mortality in cancer.
26. Functional roles of MMP14 and MMP15 in early postnatal mammary gland development. During late embryogenesis, mammary epithelial cells initiate **migration** programs that drive ductal invasion into the surrounding adipose-rich mesenchyme. Currently, branching morphogenesis is thought to depend on the mobilization of the membrane-anchored matrix metalloproteinases MMP14 (MT1-MMP) and MMP15 (MT2-MMP), which drive epithelial cell invasion by remodeling the extracellular matrix and triggering associated signaling cascades. However, the roles that these proteinases play during mammary gland development in vivo remain undefined. Here, we characterize the impact of global Mmp14 and Mmp15 targeting on early postnatal mammary gland development in mice. Unexpectedly, both Mmp14(-/-) and Mmp15(-/-) mammary glands retain the ability to generate intact ductal networks. Although neither proteinase is required for branching morphogenesis, transcriptome profiling reveals a key role for MMP14 and MMP15 in regulating mammary gland adipocyte differentiation. Whereas MMP14 promotes the generation of white fat depots crucial for energy storage, MMP15 differentially controls the formation of thermogenic brown fat. Taken together, these data not only indicate that current paradigms relevant to proteinase-dependent morphogenesis need be revisited, but also identify new roles for the enzymes in regulating adipocyte fate determination in the developing mammary gland.
27. Role of metalloproteinases at the onset of liver development. At the onset of liver development, the hepatic precursor cells, namely, the hepatoblasts, derive from the ventral foregut endoderm and form a bud surrounded by a basement membrane (BM). To initiate liver growth, the hepatoblasts migrate across the BM and invade the neighboring septum transversum mesenchyme. In the present study, carried out in the mouse embryo, we searched for effectors involved in this process and we examined the role of matrix metalloproteinases (MMPs). We found expression of a broad range of MMPs, among which MMP-2 was predominantly expressed in the septum transversum and MMP-14 in the hepatoblasts. Using a new liver explant culture system we showed that inhibition of MMP activity represses **migration** of the hepatoblasts. We conclude that MMPs are required to initiate expansion of the liver during development and that our culture system provides a new model to study hepatoblast **migration**.
28. Ovarian cancer cell detachment and multicellular aggregate formation are regulated by membrane type 1 matrix metalloproteinase: a potential role in I.p. metastatic dissemination. An early event in the metastasis of epithelial ovarian carcinoma is shedding of cells from the primary tumor into the peritoneal cavity followed by diffuse i.p. seeding of secondary lesions. Anchorage-independent metastatic cells are present as both single cells and multicellular aggregates (MCA), the latter of which adhere to and disaggregate on human mesothelial cell monolayers, subsequently forming invasive foci. Although this unique metastatic mechanism presents a distinct set of therapeutic challenges, factors that regulate MCA formation and dissemination have not been extensively evaluated. Proteolytic activity is important at multiple stages in i.p. metastasis, catalyzing **migration** through the mesothelial monolayer and invasion of the collagen-rich submesothelial matrix to anchor secondary lesions, and acquisition of membrane type 1 matrix metalloproteinase (MT1-MMP; MMP-14) expression promotes a collagen-invasive phenotype in ovarian carcinoma. MT1-MMP is regulated posttranslationally through multiple mechanisms including phosphorylation of its cytoplasmic tail, and the current data using ovarian cancer cells expressing wild-type, phosphomimetic (T567E-MT1-MMP), and phosphodefective (T567A-MT1-MMP) MT1-MMP show that MT1-MMP promotes MCA formation. Confluent T567E-MT1-MMP-expressing cells exhibit rapid detachment kinetics, spontaneous release as cell-cell adherent sheets concomitant with MT1-MMP-catalyzed alpha(3) integrin ectodomain shedding, and robust MCA formation. Expansive growth within three-dimensional collagen gels is also MT1-MMP dependent, with T567E-MT1-MMP-expressing cells exhibiting multiple collagen invasive foci. Analysis of human ovarian tumors shows elevated MT1-MMP in metastases relative to paired primary tumors. These data suggest that MT1-MMP activity may be key to ovarian carcinoma metastatic success by promoting both formation and dissemination of MCAs.
29. Diminished matrix metalloproteinase 2 (MMP-2) in ectomesenchyme-derived tissues of the Patch mutant mouse: regulation of MMP-2 by PDGF and effects on mesenchymal cell **migration**. Platelet-derived growth factors (PDGF) regulate cell proliferation, survival, morphology, and **migration**, as well as deposition and turnover of the extracellular matrix. Important roles for the A form of PDGF (PDGF-A) during connective tissue morphogenesis have been highlighted by the murine Patch mutation, which includes a deletion of the alpha subunit of the PDGF receptor. Homozygous (Ph/Ph) embryos exhibit multiple connective tissue defects including cleft face (involving the first branchial arch and frontonasal processes), incomplete heart septation, and heart valve abnormalities before they die in utero. Analyses of the cell biology underlying the defects in Ph/Ph embryos have revealed a deficit in a matrix metalloproteinase (MMP-2) and one of its activators (MT-MMP) that are likely to be involved in cell **migration** and tissue remodeling, two processes necessary for normal cardiac and craniofacial development. Morphogenesis of these structures requires infiltration of ectomesenchymal precursors and their subsequent deposition and remodeling of extracellular matrix components. First branchial arch and heart tissue from E10.5 embryos were examined by gelatin zymography and RT-PCR in order to characterize the expression of MMPs in these tissues. Of the MMPs examined, only MMP-2 and one of its activators, MT-MMP, were expressed in the first arch and heart at this stage of development. Tissues from Ph/Ph embryos exhibited a significant decrease in both MMP-2 and MT-MMP compared to tissues from normal embryos of the same developmental stage. In order to assess whether this decrease affects the motile activity of mesenchymal cells, cell **migration** from Ph/Ph branchial arch explants was compared to **migration** from normal arch tissue and found to be significantly less. In addition, the migratory ability of branchial arch cells from normal explants could be reduced in a similar manner using a specific MMP inhibitor. Although it is still unclear whether the MMP-2 reduction is a direct result of the absence of response of Ph/Ph cells to PDGF-A treatment of normal branchial arch cells in vitro with recombinant PDGF-AA significantly upregulated MMP-2 protein. Together, these results suggest that PDGF-A regulates MMP-2 expression and activation during normal development and that faulty proteinase expression may be at least partially responsible for the developmental defects exhibited by Ph/Ph embryos.

#### GeneRIFs

1. These data point to a role of MMP-14 during transendothelial **migration** of monocytes and T-cell attraction.
2. MT1-MMP in niche cells regulates postnatal hematopoiesis, by modulating hematopoietic hypoxia-inducible factor-1 - dependent niche factors that are critical for terminal differentiation and **migration**.
3. Data provide evidence for a concerted HIF-1alpha/MT1-MMP signalling axis that explains the induction of adaptor protein 3BP2 and which may link protein binding partners together and stimulate oncogenic MSC **migration**.
4. important role for MT1-MMP in kidney development, which is mediated by its ability to regulate cell proliferation and **migration** by proteolytically cleaving kidney basement membrane components
5. Podosomes mediate **migration** of dendritic cells through tissues by means of myosin-II-dependent protrusion coupled to MMP-14-dependent degradation and endocytosis.
6. The MT1-MMP regulates macrophages via its invasion-promoting protease activity as well as its cytoplasmic-dependent non-proteolytic activity to boost cell **migration**.
7. Retroviral rescue experiments and protein binding assays delineate a signaling pathway in which MT1-MMP, via its cytosolic tail, contributes to macrophage **migration** and fusion by regulating Rac1 activity through an association with p130Cas.

### ECM

#### PubMed

1. ATXN1 protein family and CIC regulate extracellular matrix remodeling and lung alveolarization. Although expansion of CAG repeats in ATAXIN1 (ATXN1) causes Spinocerebellar ataxia type 1, the functions of ATXN1 and ATAXIN1-Like (ATXN1L) remain poorly understood. To investigate the function of these proteins, we generated and characterized Atxn1L(-/-) and Atxn1(-/-); Atxn1L(-/-) mice. Atxn1L(-/-) mice have hydrocephalus, omphalocele, and lung alveolarization defects. These phenotypes are more penetrant and severe in Atxn1(-/-); Atxn1L(-/-) mice, suggesting that ATXN1 and ATXN1L are functionally redundant. Upon pursuing the molecular mechanism, we discovered that several Matrix metalloproteinase (Mmp) genes are overexpressed and that the transcriptional repressor Capicua (CIC) is destabilized in Atxn1L(-/-) lungs. Consistent with this, Cic deficiency causes lung alveolarization defect. Loss of either ATXN1L or CIC derepresses Etv4, an activator for Mmp genes, thereby mediating MMP9 overexpression. These findings demonstrate a critical role of ATXN1/ATXN1L-CIC complexes in extracellular matrix **(EC**M) remodeling during development and their potential roles in pathogenesis of disorders affecting **EC**M remodeling.
2. MT1-MMP-dependent control of skeletal stem cell commitment via a β1-integrin/YAP/TAZ signaling axis. In vitro, topographical and biophysical cues arising from the extracellular matrix **(EC**M) direct skeletal stem cell (SSC) commitment and differentiation. However, the mechanisms by which the SSC**-EC**M interface is regulated and the outcome of such interactions on stem cell fate in vivo remain unknown. Here we demonstrate that conditional deletion of the membrane-anchored metalloproteinase MT1-MMP (Mmp14) in mesenchymal progenitors, but not in committed osteoblasts, redirects SSC fate decisions from osteogenesis to adipo- and chondrogenesis. By effecting **EC**M remodeling, MT1-MMP regulates stem cell shape, thereby activating a β1-integrin/RhoGTPase signaling cascade and triggering the nuclear localization of the transcriptional coactivators YAP and TAZ, which serve to control SSC lineage commitment. These data identify a critical MT1-MMP/integrin/YAP/TAZ axis operative in the stem cell niche that oversees SSC fate determination.
3. Differential expression of matrix metalloproteinases and their inhibitors in human and mouse lung development. Lung development is a highly orchestrated process characterized by timed expression and activation of growth factor and protease/antiprotease systems. This interplay is essential in regulating vasculogenesis, alveolarization, and epithelial to mesenchymal transition during lung development. Alterations in the proteolytic/antiproteolytic balance of the lung have been associated with several respiratory diseases characterized by changes in the lung extracellular matrix (**ECM**). Here, we characterized the expression pattern of matrix metalloproteases (MMP) and their inhibitors, the tissue inhibitors of metalloproteases (TIMP), in human and mouse lung development. Using MMP/TIMP expression arrays, RT-PCR, Western Blotting, and ELISA analyses, we demonstrate that fetal human lung is characterized by a dominant proteolytic profile with high MMP-2 and little TIMP-3 expression. Adult human lung, in contrast, exhibits a more anti-proteolytic profile with decreased MMP-2 and increased TIMP-3 expression. MMP-14, MMP-20, TIMP-1, and TIMP-2 were constitutively expressed, irrespective of the developmental stage. Similar results were obtained using mouse lungs of different developmental stages, with the addition that in mouse lung, TIMP-2 and TIMP-3 were upregulated as lung development progressed. Exposure of neonatal mice to chronic hypoxia (10% O2), a stimulus that leads to an arrest of lung development, resulted in upregulation of MMP-2 with a concomitant downregulation of TIMP-2. These results provide a comprehensive analysis of MMP and TIMP expression during human and mouse lung development. MMP-2, TIMP-2, and TIMP-3 may be key regulatory enzymes during lung development, possibly through their complex action on **ECM** components, membrane receptor ectodomain shedding, and growth factor bioactivity.
4. TGF-beta3-induced palatogenesis requires matrix metalloproteinases. Cleft lip and palate syndromes are among the most common congenital malformations in humans. Mammalian palatogenesis is a complex process involving highly regulated interactions between epithelial and mesenchymal cells of the palate to permit correct positioning of the palatal shelves, the remodeling of the extracellular matrix (**ECM**), and subsequent fusion of the palatal shelves. Here we show that several matrix metalloproteinases (MMPs), including a cell membrane-associated MMP (MT1-MMP) and tissue inhibitor of metalloproteinase-2 (TIMP-2) were highly expressed by the medial edge epithelium (MEE). MMP-13 was expressed both in MEE and in adjacent mesenchyme, whereas gelatinase A (MMP-2) was expressed by mesenchymal cells neighboring the MEE. Transforming growth factor (TGF)-beta3-deficient mice, which suffer from clefting of the secondary palate, showed complete absence of TIMP-2 in the midline and expressed significantly lower levels of MMP-13 and slightly reduced levels of MMP-2. In concordance with these findings, MMP-13 expression was strongly induced by TGF-beta3 in palatal fibroblasts. Finally, palatal shelves from prefusion wild-type mouse embryos cultured in the presence of a synthetic inhibitor of MMPs or excess of TIMP-2 failed to fuse and MEE cells did not transdifferentiate, phenocopying the defect of the TGF-beta3-deficient mice. Our observations indicate for the first time that the proteolytic degradation of the **ECM** by MMPs is a necessary step for palatal fusion.
5. Transmembrane/cytoplasmic, rather than catalytic, domains of Mmp14 signal to MAPK activation and mammary branching morphogenesis via binding to integrin β1. Epithelial cell invasion through the extracellular matrix **(EC**M) is a crucial step in branching morphogenesis. The mechanisms by which the mammary epithelium integrates cues from the **EC**M with intracellular signaling in order to coordinate invasion through the stroma to make the mammary tree are poorly understood. Because the cell membrane-bound matrix metalloproteinase Mmp14 is known to play a key role in cancer cell invasion, we hypothesized that it could also be centrally involved in integrating signals for mammary epithelial cells (MECs) to navigate the collagen 1 (CL-1)-rich stroma of the mammary gland. Expression studies in nulliparous mice that carry a NLS-lacZ transgene downstream of the Mmp14 promoter revealed that Mmp14 is expressed in MECs at the tips of the branches. Using both mammary organoids and 3D organotypic cultures, we show that MMP activity is necessary for invasion through dense CL-1 (3 mg/ml) gels, but dispensable for MEC branching in sparse CL-1 (1 mg/ml) gels. Surprisingly, however, Mmp14 without its catalytic activity was still necessary for branching. Silencing Mmp14 prevented cell invasion through CL-1 and disrupted branching altogether; it also reduced integrin β1 (Itgb1) levels and attenuated MAPK signaling, disrupting Itgb1-dependent invasion/branching within CL-1 gels. FRET imaging revealed that Mmp14 associates directly with Itgb1. We identified a domain of Mmp14 that is required for modulating the levels of Itgb1, MEC signaling and the rate of invasion within CL-1. These results shed light on hitherto undescribed non-proteolytic activities of Mmp14 that are necessary for the Itgb1-dependent biochemical and mechanical signals that regulate branching in the mammary epithelium.
6. MT1-MMP-dependent control of skeletal stem cell commitment via a β1-integrin/YAP/TAZ signaling axis. In vitro, topographical and biophysical cues arising from the extracellular matrix **(EC**M) direct skeletal stem cell (SSC) commitment and differentiation. However, the mechanisms by which the SSC**-EC**M interface is regulated and the outcome of such interactions on stem cell fate in vivo remain unknown. Here we demonstrate that conditional deletion of the membrane-anchored metalloproteinase MT1-MMP (Mmp14) in mesenchymal progenitors, but not in committed osteoblasts, redirects SSC fate decisions from osteogenesis to adipo- and chondrogenesis. By effecting **EC**M remodeling, MT1-MMP regulates stem cell shape, thereby activating a β1-integrin/RhoGTPase signaling cascade and triggering the nuclear localization of the transcriptional coactivators YAP and TAZ, which serve to control SSC lineage commitment. These data identify a critical MT1-MMP/integrin/YAP/TAZ axis operative in the stem cell niche that oversees SSC fate determination.
7. Transmembrane/cytoplasmic, rather than catalytic, domains of Mmp14 signal to MAPK activation and mammary branching morphogenesis via binding to integrin β1. Epithelial cell invasion through the extracellular matrix **(EC**M) is a crucial step in branching morphogenesis. The mechanisms by which the mammary epithelium integrates cues from the **EC**M with intracellular signaling in order to coordinate invasion through the stroma to make the mammary tree are poorly understood. Because the cell membrane-bound matrix metalloproteinase Mmp14 is known to play a key role in cancer cell invasion, we hypothesized that it could also be centrally involved in integrating signals for mammary epithelial cells (MECs) to navigate the collagen 1 (CL-1)-rich stroma of the mammary gland. Expression studies in nulliparous mice that carry a NLS-lacZ transgene downstream of the Mmp14 promoter revealed that Mmp14 is expressed in MECs at the tips of the branches. Using both mammary organoids and 3D organotypic cultures, we show that MMP activity is necessary for invasion through dense CL-1 (3 mg/ml) gels, but dispensable for MEC branching in sparse CL-1 (1 mg/ml) gels. Surprisingly, however, Mmp14 without its catalytic activity was still necessary for branching. Silencing Mmp14 prevented cell invasion through CL-1 and disrupted branching altogether; it also reduced integrin β1 (Itgb1) levels and attenuated MAPK signaling, disrupting Itgb1-dependent invasion/branching within CL-1 gels. FRET imaging revealed that Mmp14 associates directly with Itgb1. We identified a domain of Mmp14 that is required for modulating the levels of Itgb1, MEC signaling and the rate of invasion within CL-1. These results shed light on hitherto undescribed non-proteolytic activities of Mmp14 that are necessary for the Itgb1-dependent biochemical and mechanical signals that regulate branching in the mammary epithelium.
8. Pulmonary fibroblasts mobilize the membrane-tethered matrix metalloprotease, MT1-MMP, to destructively remodel and invade interstitial type I collagen barriers. In acute and chronic lung disease, widespread disruption of tissue architecture underlies compromised pulmonary function. Pulmonary fibroblasts have been implicated as critical effectors of tissue-destructive extracellular matrix (**ECM**) remodeling by mobilizing a spectrum of proteolytic enzymes. Although efforts to date have focused on the catabolism of type I collagen, the predominant component of the lung interstitial matrix, the key collagenolytic enzymes employed by pulmonary fibroblasts remain unidentified. Herein, membrane type-1 matrix metalloprotease (MT1-MMP) is identified as the dominant and direct-acting protease responsible for the type I collagenolytic activity mediated by both mouse and human pulmonary fibroblasts. Furthermore, MT1-MMP is shown to be essential for pulmonary fibroblast migration within three-dimensional (3-D) hydrogels of cross-linked type I collagen that recapitulate **ECM** barriers encountered in the in vivo environment. Together, these findings demonstrate that MT1-MMP serves as a key effector of type I collagenolytic activity in pulmonary fibroblasts and earmark this pericellular collagenase as a potential target for therapeutic intervention.
9. Angiotensin II-induced MMP-2 activity and MMP-14 and basigin protein expression are mediated via the angiotensin II receptor type 1-mitogen-activated protein kinase 1 pathway in retinal pigment epithelium: implications for age-related macular degeneration. Accumulation of various lipid-rich extracellular matrix (**ECM**) deposits under the retinal pigment epithelium (RPE) has been observed in eyes with age-related macular degeneration (AMD). RPE-derived matrix metalloproteinase (MMP)-2, MMP-14, and basigin (BSG) are major enzymes involved in the maintenance of **ECM** turnover. Hypertension (HTN) is a systemic risk factor for AMD. It has previously been reported that angiotensin II (Ang II), one of the most important hormones associated with HTN, increases MMP-2 activity and its key regulator, MMP-14, in RPE, inducing breakdown of the RPE basement membrane, which may lead to progression of sub-RPE deposits. Ang II exerts most of its actions by activating the mitogen-activated protein kinase (MAPK) signaling pathway. Herein is explored the MAPK signaling pathway as a potential key intracellular modulator of Ang II-induced increase in MMP-2 activity and MMP-14 and BSG protein expression. It was observed that Ang II stimulates phosphorylation of extracellular signal-regulated kinase (ERK) and p38 MAPK in RPE cells and ERK/p38 and Jun N-terminal kinase (JNK) in mice. These effects were mediated by Ang II type 1 receptors. Blockade of ERK or p38 MAPK abrogated the increase in MMP-2 activity and MMP-14 and BSG proteins in ARPE-19 cells. A better understanding of the molecular events by which Ang II induces **ECM** dysregulation is of critical importance to further define its contribution to the progression of sub-RPE deposits in AMD patients with HTN.
10. TIMP2 deficiency accelerates adverse post-myocardial infarction remodeling because of enhanced MT1-MMP activity despite lack of MMP2 activation. Myocardial infarction (MI) results in remodeling of the myocardium and the extracellular matrix (**ECM**). Tissue inhibitors of metalloproteinases (TIMPs) are critical regulators of **ECM** integrity via inhibiting matrix metalloproteinases (MMPs). TIMP2 is highly expressed in the heart and is the only TIMP that, in addition to inhibiting MMPs, is required for cell surface activation of pro-MMP2. Hence, it is difficult to predict the function of TIMP2 as protective (MMP-inhibiting) or harmful (MMP-activating) in heart disease.We examined the role of TIMP2 in the cardiac response to MI.MI was induced in 11- to 12-week-old male TIMP2(-/-) and age-matched wild-type mice. Cardiac function was monitored by echocardiography at 1 and 4 weeks post-MI. **ECM** fibrillar structure was visualized using second harmonic generation and multiphoton imaging of unfixed/unstained hearts. Molecular analyses were performed at 3 days and 1 week post-MI on flash-frozen infarct, periinfarct, and noninfarct tissue. Membrane type 1 (MT1)-MMP levels and activity were measured in membrane protein fractions. TIMP2(-/-)-MI mice exhibited a 25% greater infarct expansion, markedly exacerbated left ventricular dilation (by 12%) and dysfunction (by 30%), and more severe inflammation compared to wild-type MI mice. Adverse **ECM** remodeling was detected by reduced density and enhanced disarray of fibrillar collagen in TIMP2(-/-)-MI compared to wild-type MI hearts. TIMP2 deficiency completely abrogated MMP2 activation but markedly increased collagenase activity, particularly MT1-MMP activity post-MI.The MMP-inhibitory function of TIMP2 is a key determinant of post-MI myocardial remodeling primarily because of its inhibitory action on MT1-MMP. TIMP2 replenishment in diseased myocardium could provide a potential therapy in reducing or preventing disease progression.
11. MT1-MMP down-regulates the glucose 6-phosphate transporter expression in marrow stromal cells: a molecular link between pro-MMP-2 activation, chemotaxis, and cell survival. Bone marrow-derived stromal cells (BMSC) are avidly recruited by experimental vascularizing tumors, which implies that they must respond to tumor-derived growth factor cues. In fact, BMSC chemotaxis and cell survival are regulated, in part, by the membrane type-1 matrix metalloproteinase (MT1-MMP), an MMP also involved in pro-MMP-2 activation and in degradation of the extracellular matrix (**ECM**). Given that impaired chemotaxis was recently observed in bone marrow cells isolated from a glucose 6-phosphate transporter-deficient (G6PT-/-) mouse model, we sought to investigate the potential MT1-MMP/G6PT signaling axis in BMSC. We show that MT1-MMP-mediated activation of pro-MMP-2 by concanavalin A (ConA) correlated with an increase in the sub-G1 cell cycle phase as well as with cell necrosis, indicative of a decrease in BMSC survival. BMSC isolated from Egr-1-/- mouse or MT1-MMP gene silencing in BMSC with small interfering RNA (siMT1-MMP) antagonized both the ConA-mediated activation of pro-MMP-2 and the induction of cell necrosis. Overexpression of recombinant full-length MT1-MMP triggered necrosis and this was signaled through the cytoplasmic domain of MT1-MMP. ConA inhibited both the gene and protein expression of G6PT, while overexpression of recombinant G6PT inhibited MT1-MMP-mediated pro-MMP-2 activation but could not rescue BMSC from ConA-induced cell necrosis. Cell chemotaxis in response to the tumorigenic growth factor sphingosine 1-phosphate was significantly abrogated in siMT1-MMP BMSC and in chlorogenic acid-treated BMSC. Altogether, we provide evidence for an MT1-MMP/G6PT signaling axis that regulates BMSC survival, **ECM** degradation, and mobilization. This may lead to optimized clinical applications that use BMSC as a platform for the systemic delivery of therapeutic or anti-cancer recombinant proteins in vivo.
12. 3-phosphoinositide-dependent protein kinase-1 (PDK1) promotes invasion and activation of matrix metalloproteinases. Metastasis is a major cause of morbidity and mortality in breast cancer with tumor cell invasion playing a crucial role in the metastatic process. PDK1 is a key molecule that couples PI3K to cell proliferation and survival signals in response to growth factor receptor activation, and is oncogenic when expressed in mouse mammary epithelial cells. We now present evidence showing that PDK1-expressing cells exhibit enhanced anchorage-dependent and -independent cell growth and are highly invasive when grown on Matrigel. These properties correlate with induction of MMP-2 activity, increased MT1-MMP expression and a unique gene expression profile.Invasion assays in Matrigel, MMP-2 zymogram analysis, gene microarray analysis and mammary isografts were used to characterize the invasive and proliferative function of cells expressing PDK1. Tissue microarray analysis of human breast cancers was used to measure PDK1 expression in invasive tumors by IHC.Enhanced invasion on Matrigel in PDK1-expressing cells was accompanied by increased MMP-2 activity resulting from stabilization against proteasomal degradation. Increased MMP-2 activity was accompanied by elevated levels of MT1-MMP, which is involved in generating active MMP-2. Gene microarray analysis identified increased expression of the **ECM**-associated genes decorin and type I procollagen, whose gene products are substrates of MT1-MMP. Mammary fat pad isografts of PDK1-expressing cells produced invasive adenocarcinomas. Tissue microarray analysis of human invasive breast cancer indicated that PDK1pSer241 was strongly expressed in 90% of samples.These results indicate that PDK1 serves as an important effector of mammary epithelial cell growth and invasion in the transformed phenotype. PDK1 mediates its effect in part by MT1-MMP induction, which in turn activates MMP-2 and modulates the **ECM** proteins decorin and collagen. The presence of increased PDK1 expression in the majority of invasive breast cancers suggests its importance in the metastatic process.
13. Dysregulation of TGF-beta1 receptor activation leads to abnormal lung development and emphysema-like phenotype in core fucose-deficient mice. The core fucosylation (alpha1,6-fucosylation) of glycoproteins is widely distributed in mammalian tissues, and is altered under pathological conditions. To investigate physiological functions of the core fucose, we generated alpha1,6-fucosyltransferase (Fut8)-null mice and found that disruption of Fut8 induces severe growth retardation and death during postnatal development. Histopathological analysis revealed that Fut8(-/-) mice showed emphysema-like changes in the lung, verified by a physiological compliance analysis. Biochemical studies indicated that lungs from Fut8(-/-) mice exhibit a marked overexpression of matrix metalloproteinases (MMPs), such as MMP-12 and MMP-13, highly associated with lung-destructive phenotypes, and a down-regulation of extracellular matrix (**ECM**) proteins such as elastin, as well as retarded alveolar epithelia cell differentiation. These changes should be consistent with a deficiency in TGF-beta1 signaling, a pleiotropic factor that controls **ECM** homeostasis by down-regulating MMP expression and inducing **ECM** protein components. In fact, Fut8(-/-) mice have a marked dysregulation of TGF-beta1 receptor activation and signaling, as assessed by TGF-beta1 binding assays and Smad2 phosphorylation analysis. We also show that these TGF-beta1 receptor defects found in Fut8(-/-) cells can be rescued by reintroducing Fut8 into Fut8(-/-) cells. Furthermore, exogenous TGF-beta1 potentially rescued emphysema-like phenotype and concomitantly reduced MMP expression in Fut8(-/-) lung. We propose that the lack of core fucosylation of TGF-beta1 receptors is crucial for a developmental and progressive/destructive emphysema, suggesting that perturbation of this function could underlie certain cases of human emphysema.
14. Angiotensin II stimulates cardiac fibroblast migration via the differential regulation of matrixins and RECK. Sustained induction and activation of matrixins (matrix metalloproteinases or MMPs), and the destruction and deposition of extracellular matrix (**ECM**), are the hallmarks of cardiac fibrosis. The reversion-inducing-cysteine-rich protein with Kazal motifs (RECK) is a unique membrane-anchored endogenous MMP regulator. We hypothesized that elevated angiotensin II (Ang II), which is associated with fibrosis in the heart, differentially regulates MMPs and RECK both in vivo and in vitro. Continuous infusion of Ang II into male C57Bl/6 mice for 2weeks resulted in cardiac fibrosis, with increased expressions of MMPs 2, 7, 9 and 14, and of collagens Ia1 and IIIa1. The expression of RECK, however, was markedly suppressed. These effects were inhibited by co-treatment with the Ang II type 1 receptor (AT1) antagonist losartan. In vitro, Ang II suppressed RECK expression in adult mouse cardiac fibroblasts (CF) via AT1/Nox4-dependent ERK/Sp1 activation, but induced MMPs 2, 14 and 9 via NF-κB, AP-1 and/or Sp1 activation. Further, while forced expression of RECK inhibits, its knockdown potentiates Ang II-induced CF migration. Notably, RECK overexpression reduced Ang II-induced MMPs 2, 9 and 14 activation, but enhanced collagens Ia1 and IIIa1 expression and soluble collagen release. These results demonstrate for the first time that Ang II suppresses RECK, but induces MMPs both in vivo and in vitro, and RECK overexpression blunts Ang II-induced MMP activation and CF migration in vitro. Strategies that upregulate RECK expression in vivo have the potential to attenuate sustained MMP expression, and blunt fibrosis and adverse remodeling in hypertensive heart diseases.
15. Loss of MT1-MMP causes cell senescence and nuclear defects which can be reversed by retinoic acid. MT1-MMP (MMP14) is a collagenolytic enzyme located at the cell surface and implicated in extracellular matrix (**ECM**) remodeling. Mmp14(-/-) mice present dwarfism, bone abnormalities, and premature death. We demonstrate herein that the loss of MT1-MMP also causes cardiac defects and severe metabolic changes, and alters the cytoskeleton and the nuclear lamina structure. Moreover, the absence of MT1-MMP induces a senescent phenotype characterized by up-regulation of p16(INK4a) and p21(CIP1/WAF) (1), increased activity of senescence-associated β-galactosidase, generation of a senescence-associated secretory phenotype, and somatotroph axis alterations. Consistent with the role of retinoic acid signaling in nuclear lamina stabilization, treatment of Mmp14(-/-) mice with all-trans retinoic acid reversed the nuclear lamina alterations, partially rescued the cell senescence phenotypes, ameliorated the pathological defects in bone, skin, and heart, and extended their life span. These results demonstrate that nuclear architecture and cell senescence can be modulated by a membrane protease, in a process involving the **EC**M as a key regulator of nuclear stiffness under cell stress conditions.
16. MT1-MMP-dependent remodeling of cardiac extracellular matrix structure and function following myocardial infarction. The myocardial extracellular matrix (**ECM**), an interwoven meshwork of proteins, glycoproteins, proteoglycans, and glycosaminoglycans that is dominated by polymeric fibrils of type I collagen, serves as the mechanical scaffold on which myocytes are arrayed for coordinated and synergistic force transduction. Following ischemic injury, cardiac **ECM** remodeling is initiated via localized proteolysis, the bulk of which has been assigned to matrix metalloproteinase (MMP) family members. Nevertheless, the key effector(s) of myocardial type I collagenolysis both in vitro and in vivo have remained unidentified. In this study, using cardiac explants from mice deficient in each of the major type I collagenolytic MMPs, including MMP-13, MMP-8, MMP-2, MMP-9, or MT1-MMP, we identify the membrane-anchored MMP, MT1-MMP, as the dominant collagenase that is operative within myocardial tissues in vitro. Extending these observations to an in vivo setting, mice heterozygous for an MT1-MMP-null allele display a distinct survival advantage and retain myocardial function relative to wild-type littermates in an experimental model of myocardial infarction, effects associated with preservation of the myocardial type I collagen network as a consequence of the decreased collagenolytic potential of cardiac fibroblasts. This study identifies MT1-MMP as a key MMP responsible for effecting postinfarction cardiac **ECM** remodeling and cardiac dysfunction.
17. MT1-MMP regulates the PI3Kδ·Mi-2/NuRD-dependent control of macrophage immune function. Macrophages play critical roles in events ranging from host defense to obesity and cancer, where they infiltrate affected tissues and orchestrate immune responses in tandem with the remodeling of the extracellular matrix **(E**CM). Despite the dual roles played by macrophages in inflammation, the functions of macrophage-derived proteinases are typically relegated to tissue-invasive or -degradative events. Here we report that the membrane-tethered matrix metalloenzyme MT1-MMP not only serves as a**n E**CM-directed proteinase, but unexpectedly controls inflammatory gene responses wherein MT1-MMP(-/-) macrophages mount exaggerated chemokine and cytokine responses to immune stimuli both in vitro and in vivo. MT1-MMP modulates inflammatory responses in a protease-independent fashion in tandem with its trafficking to the nuclear compartment, where it triggers the expression and activation of a phosphoinositide 3-kinase δ (PI3Kδ)/Akt/GSK3β signaling cascade. In turn, MT1-MMP-dependent PI3Kδ activation regulates the immunoregulatory Mi-2/NuRD nucleosome remodeling complex that is responsible for controlling macrophage immune response. These findings identify a novel role for nuclear MT1-MMP as a previously unsuspected transactivator of signaling networks central to macrophage immune responses.
18. MT1-MMP-mediated basement membrane remodeling modulates renal development. Extracellular matrix (**ECM**) remodeling regulates multiple cellular functions required for normal development and tissue repair. Matrix metalloproteinases (MMPs) are key mediators of this process and membrane targeted MMPs (MT-MMPs) in particular have been shown to be important in normal development of specific organs. In this study we investigated the role of MT1-MMP in kidney development. We demonstrate that loss of MT1-MMP leads to a renal phenotype characterized by a moderate decrease in ureteric bud branching morphogenesis and a severe proliferation defect. The kidneys of MT1-MMP-null mice have increased deposition of collagen IV, laminins, perlecan, and nidogen and the phenotype is independent of the MT-1MMP target, MMP-2. Utilizing in vitro systems we demonstrated that MTI-MMP proteolytic activity is required for renal tubule cells to proliferate in three dimensional matrices and to migrate on collagen IV and laminins. Together these data suggest an important role for MT1-MMP in kidney development, which is mediated by its ability to regulate cell proliferation and migration by proteolytically cleaving kidney basement membrane components.
19. A pericellular collagenase directs the 3-dimensional development of white adipose tissue. White adipose tissue (WAT) serves as the primary energy depot in the body by storing fat. During development, fat cell precursors (i.e., preadipocytes) undergo a hypertrophic response as they mature into lipid-laden adipocytes. However, the mechanisms that regulate adipocyte size and mass remain undefined. Herein, we demonstrate that the membrane-anchored metalloproteinase, MT1-MMP, coordinates adipocyte differentiation in vivo. In the absence of the protease, WAT development is aborted, leaving tissues populated by mini-adipocytes which render null mice lipodystrophic. While MT1-MMP preadipocytes display a cell autonomous defect in vivo, null progenitors retain the ability to differentiate into functional adipocytes during 2-dimensional (2-D) culture. By contrast, within the context of the 3-dimensional (3-D) **ECM**, normal adipocyte maturation requires a burst in MT1-MMP-mediated proteolysis that modulates pericellular collagen rigidity in a fashion that controls adipogenesis. Hence, MT1-MMP acts as a 3-D-specific adipogenic factor that directs the dynamic adipocyte-**ECM** interactions critical to WAT development.
20. Matrix remodeling by MMPs during wound repair. Repair following injury involves a range of processes - such as re-epithelialization, scar formation, angiogenesis, inflammation, and more - that function, often together, to restore tissue architecture. MMPs carry out diverse roles in all of these activities. In this article, we discuss how specific MMPs act on **ECM** during two critical repair processes: re-epithelialization and resolution of scar tissue. For wound closure, we discuss how two MMPs - MMP1 in human epidermis and MMP7 in mucosal epithelia - facilitate re-epithelialization by cleaving different **ECM** or **ECM**-associated proteins to affect similar integrin:matrix adhesion. In scars and fibrotic tissues, we discuss that a variety of MMPs carry out a diverse range of activities that can either promote or limit **ECM** deposition. However, few of these MMP-driven activities have been demonstrated to be due a direct action on **ECM**.
21. Matrix metalloproteinases in tumor invasion. Controlled degradation of extracellular matrix (**ECM**) is essential for the growth, invasion, and metastasis of malignant tumors, and for tumor-induced angiogenesis. Matrix metalloproteinases (MMPs) are a family of zinc-dependent neutral endopeptidases collectively capable of degrading essentially all **ECM** components and they apparently play an important role in all these aspects of tumor development. Furthermore, recent evidence suggests that MMPs also play a role in tumor cell survival. In this review, we discuss the current concept concerning the role of MMPs and their inhibitors in tumor invasion, as a basis for prognosis and targeted therapeutic intervention in cancer.
22. Multifunctional roles of MT1-MMP in myofiber formation and morphostatic maintenance of skeletal muscle. Sequential activation of muscle-specific transcription factors is the critical basis for myogenic differentiation. However, the complexity of this process does not exclude the possibility that other molecules and systems are regulatory as well. We observed that myogenic differentiation proceeded through three distinct stages of proliferation, elongation and fusion, which are distinguishable by their cellular morphologies and gene expression patterns of proliferation- and differentiation-specific markers. Treatment of the differentiating myoblasts with inhibitors of matrix metalloproteinases (MMPs) revealed that MMP activity at the elongation stage is a critical prerequisite to complete the successive myoblast cell fusion. The MMP regulated the myogenic differentiation independently from the genetic program that governs expression of the myogenic genes. Membrane-type 1 matrix metalloproteinase (MT1-MMP) was identified as a major contributor to this checkpoint for morphological differentiation and degraded fibronectin, a possible inhibitory factor for myogenic cell fusion. A MT1-MMP deficiency caused similar myogenic impediments forming smaller myofibers in situ. Additionally, the mutant mice demonstrated some central nucleation of the myofibers typically found in muscular dystrophy and MT1-MMP was found to cleave laminin-2/4 in the basement membrane. Thus, MT1-MMP is a new multilateral regulator for muscle differentiation and maintenance through processing of stage-specific distinct **ECM** substrates.
23. Adipogenic histone mark regulation by matrix metalloproteinase 14 in collagen-rich microenvironments. Adipogenesis is directed by both transcriptional network and posttranslational modification of chromatin structure. Although adipogenesis in vivo proceeds in collagen-rich extracellular matrix (**ECM**) environments, the impact of **ECM** proteins and their modifying enzymes on the epigenetic regulation of adipogenesis has been largely unknown. We aimed to define the role of fibrillar type I collagen and its modifying enzymes in regulating adipogenic chromatin signatures and gene regulation in the in vivo-like settings. Adipogenic cocktail induces a robust increase in the level of protranscriptional acetylated histone H3 at lysine 9 (H3K9ac) within 24 h. When cultured atop fibrillar type I collagen gel, however, H3K9ac levels in differentiating 3T3-L1 cells are substantially reduced. The suppression of adipogenic histone mark in differentiating 3T3-L1 cells is type I collagen density dependent and released by heat denaturing of the subjacent collagen substratum, pointing to the critical role played by the triple-helical structure of type I collagen. By probing adipogenic collagenolysis with a series of proteinase inhibitors, matrix metalloproteinase (MMP) family members are found to be responsible for adipogenic collagenolysis. At the same time, MMP inhibitor specifically blocked the adipogenic induction of H3K9ac. By targeting individual MMP using small interfering RNA oligos, MMP14 was identified as the major adipogenic MMP critical for H3K9 acetylation. Consistently, MMP14-null adipose tissues display diminished protranscriptional histone mark H3K9ac while maintaining repressive histone mark tri-methylated histone H3 at lysine 9 (H3K9me3). Taken together, MMP14-dependent collagenolysis plays the major role in regulating adipogenic histone marks by releasing the epigenetic constraints imposed by fibrillar type I collagen.
24. Tumor cell traffic through the extracellular matrix is controlled by the membrane-anchored collagenase MT1-MMP. As cancer cells traverse collagen-rich extracellular matrix (**ECM**) barriers and intravasate, they adopt a fibroblast-like phenotype and engage undefined proteolytic cascades that mediate invasive activity. Herein, we find that fibroblasts and cancer cells express an indistinguishable pericellular collagenolytic activity that allows them to traverse the **ECM**. Using fibroblasts isolated from gene-targeted mice, a matrix metalloproteinase (MMP)-dependent activity is identified that drives invasion independently of plasminogen, the gelatinase A/TIMP-2 axis, gelatinase B, collagenase-3, collagenase-2, or stromelysin-1. In contrast, deleting or suppressing expression of the membrane-tethered MMP, MT1-MMP, in fibroblasts or tumor cells results in a loss of collagenolytic and invasive activity in vitro or in vivo. Thus, MT1-MMP serves as the major cell-associated proteinase necessary to confer normal or neoplastic cells with invasive activity.
25. Mammary Branching Morphogenesis Requires Reciprocal Signaling by Heparanase and MMP-14. The development of the mammary gland involves formation of a branched arboreal structure resulting from the penetration and proliferation of epithelial cells into the fat pad. The mammary cells invade by remodeling their surrounding extracellular matrix (**ECM**), which are rich in proteins, and glycans such as heparan sulfate proteoglycans (HSPGs). There is increasing literature on how the interaction between signaling by **ECM** and matrix metalloproteinases (MMPs) is relevant to morphogenetic and physiological contexts. Here we sought to understand how heparanase, the sole mammalian heparan sulfate-degrading endoglycosidase may regulate mammary gland development. We found a robust localization of heparanase within growing end buds during branching in vivo. Using three-dimensional (3D) organotypic cultures, we showed that heparanase expression and activity are required for mammary epithelial invasion/branching within dense collagen I gels. Morphometric analysis of glands from both heparanase-overexpressing and knockout mice showed a direct correlation between degree of branching and the heparanase levels, confirming our 3D organotypic culture observations. Finally, we uncovered a reciprocal association between levels of heparanase and MMP14, a membrane-bound MMP, shedding further light on how branching occurs within developing mammary glands.
26. Interleukin-6 enhances matrix metalloproteinase-14 expression via the RAF-mitogen-activated protein kinase kinase-extracellular signal-regulated kinase 1/2-activator protein-1 pathway. 1. Interleukin (IL)-6 is a pivotal cytokine that regulates extracellular matrix (**ECM**) metabolism by increasing collagen degradation via activation of matrix metalloproteinases (MMPs), such as MMP-14. In the present study, we investigated the role of IL-6 in atherosclerotic plaque and signalling pathways in apolipoprotein E-deficient (ApoE(-/-)) mice. 2. Twenty-five male ApoE(-/-) mice were fed a high-fat diet and atherosclerotic lesions in the right common carotid artery were induced by perivascular placement of a constrictive collar. Immunohistochemical analysis detected expression of IL-6 and MMP-14 in atherosclerotic lesions of the right common carotid artery. 3. On silencing activator protein (AP)-1 expression with a specific small interfering RNA, 75% of the IL-6-induced increase in MMP-14 expression was abolished through the RAF-mitogen-activated protein kinase kinase-extracellular signal-regulated kinase 1/2-AP-1 pathway. 4. These findings suggest a novel molecular pathway for inflammation-associated **ECM** dysregulation, which may account for atherosclerotic plaque rupture.

#### GeneRIFs

1. Hence, MT1-MMP acts as a 3-D-specific adipogenic factor that directs the dynamic adipocyte-**ECM** interactions critical to WAT development.
2. there is an MT1-MMP/G6PT signaling axis that regulates BMSC survival, **ECM** degradation, and mobilization
3. PDK1 mediates mammary epithelial cell growth and invasion in part by MT1-MMP induction, which in turn activates MMP-2 and modulates the **ECM** proteins decorin and collagen

### [Ee]xtracellular [Mm]atrix

#### Component

1. **extracellular matrix**

#### PubMed

1. ATXN1 protein family and CIC regulate **extracellular matrix** remodeling and lung alveolarization. Although expansion of CAG repeats in ATAXIN1 (ATXN1) causes Spinocerebellar ataxia type 1, the functions of ATXN1 and ATAXIN1-Like (ATXN1L) remain poorly understood. To investigate the function of these proteins, we generated and characterized Atxn1L(-/-) and Atxn1(-/-); Atxn1L(-/-) mice. Atxn1L(-/-) mice have hydrocephalus, omphalocele, and lung alveolarization defects. These phenotypes are more penetrant and severe in Atxn1(-/-); Atxn1L(-/-) mice, suggesting that ATXN1 and ATXN1L are functionally redundant. Upon pursuing the molecular mechanism, we discovered that several Matrix metalloproteinase (Mmp) genes are overexpressed and that the transcriptional repressor Capicua (CIC) is destabilized in Atxn1L(-/-) lungs. Consistent with this, Cic deficiency causes lung alveolarization defect. Loss of either ATXN1L or CIC derepresses Etv4, an activator for Mmp genes, thereby mediating MMP9 overexpression. These findings demonstrate a critical role of ATXN1/ATXN1L-CIC complexes in **extracellular matri**x (ECM) remodeling during development and their potential roles in pathogenesis of disorders affecting ECM remodeling.
2. MT1-MMP-dependent control of skeletal stem cell commitment via a β1-integrin/YAP/TAZ signaling axis. In vitro, topographical and biophysical cues arising from the **extracellular matri**x (ECM) direct skeletal stem cell (SSC) commitment and differentiation. However, the mechanisms by which the SSC-ECM interface is regulated and the outcome of such interactions on stem cell fate in vivo remain unknown. Here we demonstrate that conditional deletion of the membrane-anchored metalloproteinase MT1-MMP (Mmp14) in mesenchymal progenitors, but not in committed osteoblasts, redirects SSC fate decisions from osteogenesis to adipo- and chondrogenesis. By effecting ECM remodeling, MT1-MMP regulates stem cell shape, thereby activating a β1-integrin/RhoGTPase signaling cascade and triggering the nuclear localization of the transcriptional coactivators YAP and TAZ, which serve to control SSC lineage commitment. These data identify a critical MT1-MMP/integrin/YAP/TAZ axis operative in the stem cell niche that oversees SSC fate determination.
3. Forkhead box transcription factor FoxC1 preserves corneal transparency by regulating vascular growth. Normal vision requires the precise control of vascular growth to maintain corneal transparency. Here we provide evidence for a unique mechanism by which the Forkhead box transcription factor FoxC1 regulates corneal vascular development. Murine Foxc1 is essential for development of the ocular anterior segment, and in humans, mutations have been identified in Axenfeld-Rieger syndrome, a disorder characterized by anterior segment dysgenesis. We show that FOXC1 mutations also lead to corneal angiogenesis, and that mice homozygous for either a global (Foxc1(-/-)) or neural crest (NC)-specific (NC-Foxc1(-/-)) null mutation display excessive growth of corneal blood and lymphatic vessels. This is associated with disorganization of the **extracellular matrix** and increased expression of multiple matrix metalloproteinases. Heterozygous mutants (Foxc1(+/-) and NC-Foxc1(+/-)) exhibit milder phenotypes, such as disrupted limbal vasculature. Moreover, environmental exposure to corneal injury significantly increases growth of both blood and lymphatic vessels in both Foxc1(+/-) and NC-Foxc1(+/-) mice compared with controls. Notably, this amplification of the angiogenic response is abolished by inhibition of VEGF receptor 2. Collectively, these findings identify a role for FoxC1 in inhibiting corneal angiogenesis, thereby maintaining corneal transparency by regulating VEGF signaling.
4. Differential expression of matrix metalloproteinases and their inhibitors in human and mouse lung development. Lung development is a highly orchestrated process characterized by timed expression and activation of growth factor and protease/antiprotease systems. This interplay is essential in regulating vasculogenesis, alveolarization, and epithelial to mesenchymal transition during lung development. Alterations in the proteolytic/antiproteolytic balance of the lung have been associated with several respiratory diseases characterized by changes in the lung **extracellular matrix** (ECM). Here, we characterized the expression pattern of matrix metalloproteases (MMP) and their inhibitors, the tissue inhibitors of metalloproteases (TIMP), in human and mouse lung development. Using MMP/TIMP expression arrays, RT-PCR, Western Blotting, and ELISA analyses, we demonstrate that fetal human lung is characterized by a dominant proteolytic profile with high MMP-2 and little TIMP-3 expression. Adult human lung, in contrast, exhibits a more anti-proteolytic profile with decreased MMP-2 and increased TIMP-3 expression. MMP-14, MMP-20, TIMP-1, and TIMP-2 were constitutively expressed, irrespective of the developmental stage. Similar results were obtained using mouse lungs of different developmental stages, with the addition that in mouse lung, TIMP-2 and TIMP-3 were upregulated as lung development progressed. Exposure of neonatal mice to chronic hypoxia (10% O2), a stimulus that leads to an arrest of lung development, resulted in upregulation of MMP-2 with a concomitant downregulation of TIMP-2. These results provide a comprehensive analysis of MMP and TIMP expression during human and mouse lung development. MMP-2, TIMP-2, and TIMP-3 may be key regulatory enzymes during lung development, possibly through their complex action on ECM components, membrane receptor ectodomain shedding, and growth factor bioactivity.
5. Expression of the type IV collagenase system during mouse kidney development and tubule segmentation. Type IV collagenases matrix metalloproteinase-2 (MMP2) and MMP9 and their related proteins, MT1-MMP, tissue inhibitor of metalloproteinases 1 (TIMP1), TIMP2, and TIMP3, are expressed during kidney morphogenesis and nephrogenesis, but the renal ontogeny of these proteins is only partially known, and their persistence in the adult remains controversial. Their expression was analyzed from early metanephric stages to adulthood by Western blot semiquantitative analysis; laser confocal microscopy of whole-mount kidneys; and a two-step immunoperoxidase labeling procedure using specific markers of proximal tubule (megalin), ascending limb of Henle's loop (Tamm Horsfall protein), and collecting duct (Dolichos biflorus agglutinin lectin). By Western blot, all antigens were detected at day 11.5, peaked at day 16.5, and persisted in the adult at lower levels, although MMP2 was less modulated. All antigens were expressed in metanephric mesenchyme at embryonic day 11.5 and became concentrated in neural cell adhesion molecule-positive-induced mesenchymal cells at day 12.5. Only MT1-MMP and to a lesser extent MMP2 were detected in the ureter bud. At day 16.5, all antigens predominated in the cytoplasm of the proximal tubule, except TIMP1, which was mostly expressed in the ascending limb of Henle's loop and distal tubule. During tubule segmentation, components of the type IV collagenase system showed both spatial and temporal regulation. The distribution of gelatinases was not strictly superimposable to that of their natural inhibitors TIMP, especially for MMP9 and TIMP1. All components persisted in specific segments of the adult renal tubule, where MMP9, MMP2, and MT1-MMP showed an apical expression, suggesting that substrates for these enzymes should be in the tubule lumen or in the apical cell domain and not in the **extracellular matrix**. These results suggest that a regulated balance of gelatinase activity is required during kidney organogenesis and that gelatinases continue to play a role in adult renal tubule physiology.
6. TGF-beta3-induced palatogenesis requires matrix metalloproteinases. Cleft lip and palate syndromes are among the most common congenital malformations in humans. Mammalian palatogenesis is a complex process involving highly regulated interactions between epithelial and mesenchymal cells of the palate to permit correct positioning of the palatal shelves, the remodeling of the **extracellular matrix** (ECM), and subsequent fusion of the palatal shelves. Here we show that several matrix metalloproteinases (MMPs), including a cell membrane-associated MMP (MT1-MMP) and tissue inhibitor of metalloproteinase-2 (TIMP-2) were highly expressed by the medial edge epithelium (MEE). MMP-13 was expressed both in MEE and in adjacent mesenchyme, whereas gelatinase A (MMP-2) was expressed by mesenchymal cells neighboring the MEE. Transforming growth factor (TGF)-beta3-deficient mice, which suffer from clefting of the secondary palate, showed complete absence of TIMP-2 in the midline and expressed significantly lower levels of MMP-13 and slightly reduced levels of MMP-2. In concordance with these findings, MMP-13 expression was strongly induced by TGF-beta3 in palatal fibroblasts. Finally, palatal shelves from prefusion wild-type mouse embryos cultured in the presence of a synthetic inhibitor of MMPs or excess of TIMP-2 failed to fuse and MEE cells did not transdifferentiate, phenocopying the defect of the TGF-beta3-deficient mice. Our observations indicate for the first time that the proteolytic degradation of the ECM by MMPs is a necessary step for palatal fusion.
7. Transmembrane/cytoplasmic, rather than catalytic, domains of Mmp14 signal to MAPK activation and mammary branching morphogenesis via binding to integrin β1. Epithelial cell invasion through the **extracellular matri**x (ECM) is a crucial step in branching morphogenesis. The mechanisms by which the mammary epithelium integrates cues from the ECM with intracellular signaling in order to coordinate invasion through the stroma to make the mammary tree are poorly understood. Because the cell membrane-bound matrix metalloproteinase Mmp14 is known to play a key role in cancer cell invasion, we hypothesized that it could also be centrally involved in integrating signals for mammary epithelial cells (MECs) to navigate the collagen 1 (CL-1)-rich stroma of the mammary gland. Expression studies in nulliparous mice that carry a NLS-lacZ transgene downstream of the Mmp14 promoter revealed that Mmp14 is expressed in MECs at the tips of the branches. Using both mammary organoids and 3D organotypic cultures, we show that MMP activity is necessary for invasion through dense CL-1 (3 mg/ml) gels, but dispensable for MEC branching in sparse CL-1 (1 mg/ml) gels. Surprisingly, however, Mmp14 without its catalytic activity was still necessary for branching. Silencing Mmp14 prevented cell invasion through CL-1 and disrupted branching altogether; it also reduced integrin β1 (Itgb1) levels and attenuated MAPK signaling, disrupting Itgb1-dependent invasion/branching within CL-1 gels. FRET imaging revealed that Mmp14 associates directly with Itgb1. We identified a domain of Mmp14 that is required for modulating the levels of Itgb1, MEC signaling and the rate of invasion within CL-1. These results shed light on hitherto undescribed non-proteolytic activities of Mmp14 that are necessary for the Itgb1-dependent biochemical and mechanical signals that regulate branching in the mammary epithelium.
8. Metalloproteinases promote plaque rupture and myocardial infarction: A persuasive concept waiting for clinical translation. Atherosclerotic plaque rupture provokes most myocardial infarctions. Matrix metalloproteinases (MMPs) have counteracting roles in intimal thickening, which stabilizes plaques, on the one hand and **extracellular matrix** destruction that leads to plaque rupture on the other. This review briefly summarizes the key points supporting the involvement of individual MMPs in provoking plaque rupture and discusses the barriers that stand in the way of clinical translation, which can be itemised as follows: structural and functional complexity of the MMP family; lack of adequate preclinical models partly owing to different expression patterns of MMPs and TIMPs in mouse and human macrophages; the need to target individual MMPs selectively; the difficulties in establishing causality in human studies; and the requirement for surrogate markers of efficacy. Overcoming these barriers would open the way to new treatments that could have a major impact on cardiovascular mortality worldwide.
9. Type 1 insulin-like growth factor regulates MT1-MMP synthesis and tumor invasion via PI 3-kinase/Akt signaling. The membrane type 1 matrix metalloproteinase (MT1-MMP) has been identified as a major activator of MMP-2 - a process involving the formation of a trimolecular complex with TIMP-2. We previously identified the IGF-I receptor as a positive regulator of MMP-2 synthesis. Here, we investigated the role of IGF-IR in the regulation of MT1-MMP. Highly invasive Lewis lung carcinoma subline H-59 cells express MT1-MMP and utilize it to activate their major **extracellular matrix** degrading proteinase-MMP-2. These cells were transiently transfected with a plasmid vector expressing a luciferase reporter gene downstream of the mouse MT1-MMP promoter. IGF-I treatment increased luciferase activity in the transfected cells by up to 10-fold and augmented endogenous MT1-MMP mRNA and protein synthesis by up to 2-3-fold, relative to controls. MT1-MMP induction and invasion were blocked by the PI 3-kinase inhibitors LY294002 and wortmannin and by rapamycin, but not by the MEK inhibitor PD98059. Overexpression of a dominant negative Akt mutant or of the tumor suppressor phosphatase and tensin homologue, PTEN, in these cells also caused a significant reduction in MT1-MMP expression and invasion. The results demonstrate that IGF-IR controls tumor cell invasion by coordinately regulating MMP-2 expression and its MT1-MMP-mediated activation and identify PI 3-kinase/Akt/mTOR signaling as critical to this regulation.
10. MT1-MMP-dependent control of skeletal stem cell commitment via a β1-integrin/YAP/TAZ signaling axis. In vitro, topographical and biophysical cues arising from the **extracellular matri**x (ECM) direct skeletal stem cell (SSC) commitment and differentiation. However, the mechanisms by which the SSC-ECM interface is regulated and the outcome of such interactions on stem cell fate in vivo remain unknown. Here we demonstrate that conditional deletion of the membrane-anchored metalloproteinase MT1-MMP (Mmp14) in mesenchymal progenitors, but not in committed osteoblasts, redirects SSC fate decisions from osteogenesis to adipo- and chondrogenesis. By effecting ECM remodeling, MT1-MMP regulates stem cell shape, thereby activating a β1-integrin/RhoGTPase signaling cascade and triggering the nuclear localization of the transcriptional coactivators YAP and TAZ, which serve to control SSC lineage commitment. These data identify a critical MT1-MMP/integrin/YAP/TAZ axis operative in the stem cell niche that oversees SSC fate determination.
11. Transmembrane/cytoplasmic, rather than catalytic, domains of Mmp14 signal to MAPK activation and mammary branching morphogenesis via binding to integrin β1. Epithelial cell invasion through the **extracellular matri**x (ECM) is a crucial step in branching morphogenesis. The mechanisms by which the mammary epithelium integrates cues from the ECM with intracellular signaling in order to coordinate invasion through the stroma to make the mammary tree are poorly understood. Because the cell membrane-bound matrix metalloproteinase Mmp14 is known to play a key role in cancer cell invasion, we hypothesized that it could also be centrally involved in integrating signals for mammary epithelial cells (MECs) to navigate the collagen 1 (CL-1)-rich stroma of the mammary gland. Expression studies in nulliparous mice that carry a NLS-lacZ transgene downstream of the Mmp14 promoter revealed that Mmp14 is expressed in MECs at the tips of the branches. Using both mammary organoids and 3D organotypic cultures, we show that MMP activity is necessary for invasion through dense CL-1 (3 mg/ml) gels, but dispensable for MEC branching in sparse CL-1 (1 mg/ml) gels. Surprisingly, however, Mmp14 without its catalytic activity was still necessary for branching. Silencing Mmp14 prevented cell invasion through CL-1 and disrupted branching altogether; it also reduced integrin β1 (Itgb1) levels and attenuated MAPK signaling, disrupting Itgb1-dependent invasion/branching within CL-1 gels. FRET imaging revealed that Mmp14 associates directly with Itgb1. We identified a domain of Mmp14 that is required for modulating the levels of Itgb1, MEC signaling and the rate of invasion within CL-1. These results shed light on hitherto undescribed non-proteolytic activities of Mmp14 that are necessary for the Itgb1-dependent biochemical and mechanical signals that regulate branching in the mammary epithelium.
12. Type 1 insulin-like growth factor regulates MT1-MMP synthesis and tumor invasion via PI 3-kinase/Akt signaling. The membrane type 1 matrix metalloproteinase (MT1-MMP) has been identified as a major activator of MMP-2 - a process involving the formation of a trimolecular complex with TIMP-2. We previously identified the IGF-I receptor as a positive regulator of MMP-2 synthesis. Here, we investigated the role of IGF-IR in the regulation of MT1-MMP. Highly invasive Lewis lung carcinoma subline H-59 cells express MT1-MMP and utilize it to activate their major **extracellular matrix** degrading proteinase-MMP-2. These cells were transiently transfected with a plasmid vector expressing a luciferase reporter gene downstream of the mouse MT1-MMP promoter. IGF-I treatment increased luciferase activity in the transfected cells by up to 10-fold and augmented endogenous MT1-MMP mRNA and protein synthesis by up to 2-3-fold, relative to controls. MT1-MMP induction and invasion were blocked by the PI 3-kinase inhibitors LY294002 and wortmannin and by rapamycin, but not by the MEK inhibitor PD98059. Overexpression of a dominant negative Akt mutant or of the tumor suppressor phosphatase and tensin homologue, PTEN, in these cells also caused a significant reduction in MT1-MMP expression and invasion. The results demonstrate that IGF-IR controls tumor cell invasion by coordinately regulating MMP-2 expression and its MT1-MMP-mediated activation and identify PI 3-kinase/Akt/mTOR signaling as critical to this regulation.
13. Targeting a single function of the multifunctional matrix metalloprotease MT1-MMP: impact on lymphangiogenesis. The group of matrix metalloproteases (MMPs) is responsible for multiple processes of **extracellular matrix** remodeling in the healthy body but also for matrix and tissue destruction during cancer invasion and metastasis. The understanding of the contributions from each individual MMP, both in healthy and pathological events, has been complicated by the lack of specific inhibitors and the fact that some of the potent MMPs are multifunctional enzymes. These factors have also hampered the setup of therapeutic strategies targeting MMP activity. A tempting target is the membrane-associated MT1-MMP, which has well-documented importance in matrix degradation but which takes part in more than one pathway in this regard. In this report, we describe the selective targeting of a single function of this enzyme by means of a specific monoclonal antibody against MT1-MMP, raised in an MT1-MMP knock-out mouse. The antibody blocks the enzyme ability to activate proMMP-2 without interfering with the collagenolytic function or the general proteolytic activity of MT1-MMP. Using this antibody, we have shown that the MT1-MMP-catalyzed activation of proMMP-2 is involved in the outgrowth of cultured lymphatic endothelial cells in a collagen matrix in vitro, as well as in lymphatic vessel sprouting assayed ex vivo. This is the first example of the complete inactivation of a single function of a multifunctional MMP and the use of this strategy to pursue its role.
14. Pulmonary fibroblasts mobilize the membrane-tethered matrix metalloprotease, MT1-MMP, to destructively remodel and invade interstitial type I collagen barriers. In acute and chronic lung disease, widespread disruption of tissue architecture underlies compromised pulmonary function. Pulmonary fibroblasts have been implicated as critical effectors of tissue-destructive **extracellular matrix** (ECM) remodeling by mobilizing a spectrum of proteolytic enzymes. Although efforts to date have focused on the catabolism of type I collagen, the predominant component of the lung interstitial matrix, the key collagenolytic enzymes employed by pulmonary fibroblasts remain unidentified. Herein, membrane type-1 matrix metalloprotease (MT1-MMP) is identified as the dominant and direct-acting protease responsible for the type I collagenolytic activity mediated by both mouse and human pulmonary fibroblasts. Furthermore, MT1-MMP is shown to be essential for pulmonary fibroblast migration within three-dimensional (3-D) hydrogels of cross-linked type I collagen that recapitulate ECM barriers encountered in the in vivo environment. Together, these findings demonstrate that MT1-MMP serves as a key effector of type I collagenolytic activity in pulmonary fibroblasts and earmark this pericellular collagenase as a potential target for therapeutic intervention.
15. Genetic dissection of proteolytic and non-proteolytic contributions of MT1-MMP to macrophage invasion. MT1-MMP/MMP-14 is a major invasion-promoting membrane protease expressed in macrophages. In addition to its proteolytic activity that degrades the **extracellular matrix**, MT1-MMP also boosts ATP production in cells in a manner independent of its proteolytic activity. It remains unclear to what extent the proteolytic and energy-boosting activities of MT1-MMP contribute to macrophage invasion. Recently, we demonstrated that the cytoplasmic tail of MT1-MMP makes use of APBA3/Mint3 to activate HIF-1 and thereby boosts glycolysis for ATP production. Here, we used Apba3(-/-) macrophages to dissect the contribution of the proteolytic and the energy-boosting activities of MT1-MMP. The proteolytic activity of MT1-MMP was not affected by the lack of APBA3 in macrophages. Apba3(-/-) and Mmp14(-/-) macrophages exhibited a 55% reduction of ATP levels compared to wild-type (WT) cells and the rate of motility of the mutant cells was accordingly reduced. In contrast, matrigel invasion by Mmp14(-/-) and Apba3(-/-) macrophages was reduced to 24% and 55.4%, respectively, of the level observed in WT cells. These results represent the first attempt to dissect the contribution of the two invasion-promoting activities of MT1-MMP to macrophage invasion.
16. Dendritic cell podosomes are protrusive and invade the **extracellular matrix** using metalloproteinase MMP-14. Podosomes are spot-like actin-rich structures formed at the ventral surface of monocytic and haematopoietic cells. Podosomes degrade **extracellular matrix** and are proposed to be involved in cell migration. A key question is whether podosomes form protrusions similar to the invadopodia of cancer cells. We characterised podosomes of immature dendritic cells using electron microscopy combined with both conventional and novel high-resolution structured illumination light microscopy. Dendritic cell podosomes are composed of actin foci surrounded by a specialised ring region that is rich in material containing paxillin. We found that podosomes were preferential sites for protrusion into polycarbonate filters impregnated with crosslinked gelatin, degrading up to 2 micrometers of matrix in 24 hours. Podosome-associated uptake of colloidal gold-labelled gelatin matrix appeared to occur via large phagosome-like structures or narrow tubular invaginations. The motor protein myosin-II was excluded from ring or core regions but was concentrated around them and the myosin-II inhibitor Blebbistatin reduced the length of podosome protrusions. Finally, we found that degradation, protrusion and endocytosis in this system are dependent on the matrix metalloproteinase MMP-14. We propose that podosomes mediate migration of dendritic cells through tissues by means of myosin-II-dependent protrusion coupled to MMP-14-dependent degradation and endocytosis.
17. Alterations in membrane type-1 matrix metalloproteinase abundance after the induction of thoracic aortic aneurysm in a murine model. Thoracic aortic aneurysms (TAAs) develop as a result of dysregulated **extracellular matrix** remodeling mediated by several matrix metalloproteinases (MMPs). Membrane type-1 MMP (MT1-MMP) is the prototypical member of a unique family of membrane-bound MMPs, possessing multiple substrates and functions. The present study tested the hypothesis that MT1-MMP expression, abundance, and activity would be elevated during TAA development and that this protease is produced primarily by mesenchymal cells within the thoracic aorta. Descending thoracic aortas were harvested from C57BL/6J mice at multiple time points (2, 4, 8, and 16 wk, n = 15 each) post-TAA induction (0.5M CaCl(2), 15 min) and compared with reference controls (n = 15). The expression and abundance of MT1-MMP, MMP-2, and tissue inhibitor of metalloproteinase (TIMP)-2 were assessed by quantitative PCR and immunoblot analysis. MT1-MMP activity was determined by fluorescent peptide assay. MT1-MMP was localized within the aortic wall by immunohistochemistry. MT1-MMP abundance and localization in live animals (8 wk post-TAA induction vs. control) was determined by micro-ultrasound imaging with an MT1-MMP-targeted microbubble contrast agent. Aortic diameter was increased 172 +/- 7% at 16 wk post-TAA induction (P < 0.05). MT1-MMP and MMP-2 mRNA levels were elevated at 2 wk post-TAA induction (P < 0.05). MT1-MMP protein abundance increased progressively to a maximum of 178 +/- 26% at 16 wk post-TAA induction, whereas MMP-2 and TIMP-2 peaked at 2 wk post-TAA induction (526 +/- 93% and 376 +/- 48%, respectively, P < 0.05). MT1-MMP colocalized with fibroblasts, and MT1-MMP-targeted contrast binding was elevated in 8-wk TAA-induced mice versus control mice (217 +/- 53% vs. 81 +/- 8%, P < 0.05). In conclusion, these novel results suggest that MT1-MMP plays a dynamic multifunctional role in TAA development and, therefore, may provide a significant target for therapeutic strategies.
18. Angiotensin II-induced MMP-2 activity and MMP-14 and basigin protein expression are mediated via the angiotensin II receptor type 1-mitogen-activated protein kinase 1 pathway in retinal pigment epithelium: implications for age-related macular degeneration. Accumulation of various lipid-rich **extracellular matrix** (ECM) deposits under the retinal pigment epithelium (RPE) has been observed in eyes with age-related macular degeneration (AMD). RPE-derived matrix metalloproteinase (MMP)-2, MMP-14, and basigin (BSG) are major enzymes involved in the maintenance of ECM turnover. Hypertension (HTN) is a systemic risk factor for AMD. It has previously been reported that angiotensin II (Ang II), one of the most important hormones associated with HTN, increases MMP-2 activity and its key regulator, MMP-14, in RPE, inducing breakdown of the RPE basement membrane, which may lead to progression of sub-RPE deposits. Ang II exerts most of its actions by activating the mitogen-activated protein kinase (MAPK) signaling pathway. Herein is explored the MAPK signaling pathway as a potential key intracellular modulator of Ang II-induced increase in MMP-2 activity and MMP-14 and BSG protein expression. It was observed that Ang II stimulates phosphorylation of extracellular signal-regulated kinase (ERK) and p38 MAPK in RPE cells and ERK/p38 and Jun N-terminal kinase (JNK) in mice. These effects were mediated by Ang II type 1 receptors. Blockade of ERK or p38 MAPK abrogated the increase in MMP-2 activity and MMP-14 and BSG proteins in ARPE-19 cells. A better understanding of the molecular events by which Ang II induces ECM dysregulation is of critical importance to further define its contribution to the progression of sub-RPE deposits in AMD patients with HTN.
19. Matrix metalloproteinase 14 is required for fibrous tissue expansion. Type I collagen-containing fibrils are major structural components of the **extracellular matrix** of vertebrate tissues, especially tendon, but how they are formed is not fully understood. MMP14 is a potent pericellular collagenase that can cleave type I collagen in vitro. In this study, we show that tendon development is arrested in Scleraxis-Cre::Mmp14 lox/lox mice that are unable to release collagen fibrils from plasma membrane fibripositors. In contrast to its role in collagen turnover in adult tissue, MMP14 promotes embryonic tissue formation by releasing collagen fibrils from the cell surface. Notably, the tendons grow to normal size and collagen fibril release from fibripositors occurs in Col-r/r mice that have a mutated collagen-I that is uncleavable by MMPs. Furthermore, fibronectin (not collagen-I) accumulates in the tendons of Mmp14-null mice. We propose a model for cell-regulated collagen fibril assembly during tendon development in which MMP14 cleaves a molecular bridge tethering collagen fibrils to the plasma membrane of fibripositors.
20. Loss of epidermal MMP-14 expression interferes with angiogenesis but not with re-epithelialization. Synthesis and activation of matrix metalloproteinases during wound healing are important for remodeling the **extracellular matrix** and modulating various cellular functions. The membrane-type 1 matrix metalloproteinase (MMP-14) has been shown to play a key role during these processes. To analyze the function of epidermal-derived MMP-14 during skin repair we generated mice lacking MMP-14 expression in the epidermis (MMP-14(ep-/-)). These mice displayed overall normal skin morphology and epidermal differentiation patterns. Wound repair in MMP-14(ep-/-) followed the same kinetics as in wild type mice (MMP-14(ep+/+)), and infiltration of neutrophils, leukocytes, and macrophages into the wound site was comparable. Microscopic analysis showed no altered re-epithelialization in the absence of epidermal MMP-14. Furthermore, epidermal differentiation at the end of the repair process and scar formation was normal. However, at day 14 post wounding, sustained angiogenesis was observed in MMP-14(ep-/-) mice in contrast to control mice. Interestingly, decreased levels of endostatin were detected in wound lysates of MMP-14(ep-/-) mice as well as in cultured keratinocytes. Taken together, these data indicate that MMP-14 expression in keratinocytes is dispensable for skin homeostasis and repair, but plays a crucial role in the epidermal-dermal crosstalk leading to modulation of vessel density.
21. Secreted versus membrane-anchored collagenases: relative roles in fibroblast-dependent collagenolysis and invasion. Fibroblasts degrade type I collagen, the major extracellular protein found in mammals, during events ranging from bulk tissue resorption to invasion through the three-dimensional **extracellular matrix**. Current evidence suggests that type I collagenolysis is mediated by secreted as well as membrane-anchored members of the matrix metalloproteinase (MMP) gene family. However, the roles played by these multiple and possibly redundant, degradative systems during fibroblast-mediated matrix remodeling is undefined. Herein, we use fibroblasts isolated from Mmp13(-/-), Mmp8(-/-), Mmp2(-/-), Mmp9(-/-), Mmp14(-/-) and Mmp16(-/-) mice to define the functional roles for secreted and membrane-anchored collagenases during collagen-resorptive versus collagen-invasive events. In the presence of a functional plasminogen activator-plasminogen axis, secreted collagenases arm cells with a redundant collagenolytic potential that allows fibroblasts harboring single deficiencies for either MMP-13, MMP-8, MMP-2, or MMP-9 to continue to degrade collagen comparably to wild-type fibroblasts. Likewise, Mmp14(-/-) or Mmp16(-/-) fibroblasts retain near-normal collagenolytic activity in the presence of plasminogen via the mobilization of secreted collagenases, but only Mmp14 (MT1-MMP) plays a required role in the collagenolytic processes that support fibroblast invasive activity. Furthermore, by artificially tethering a secreted collagenase to the surface of Mmp14(-/-) fibroblasts, we demonstrate that localized pericellular collagenolytic activity differentiates the collagen-invasive phenotype from bulk collagen degradation. Hence, whereas secreted collagenases arm fibroblasts with potent matrix-resorptive activity, only MT1-MMP confers the focal collagenolytic activity necessary for supporting the tissue-invasive phenotype.
22. TIMP2 deficiency accelerates adverse post-myocardial infarction remodeling because of enhanced MT1-MMP activity despite lack of MMP2 activation. Myocardial infarction (MI) results in remodeling of the myocardium and the **extracellular matrix** (ECM). Tissue inhibitors of metalloproteinases (TIMPs) are critical regulators of ECM integrity via inhibiting matrix metalloproteinases (MMPs). TIMP2 is highly expressed in the heart and is the only TIMP that, in addition to inhibiting MMPs, is required for cell surface activation of pro-MMP2. Hence, it is difficult to predict the function of TIMP2 as protective (MMP-inhibiting) or harmful (MMP-activating) in heart disease.We examined the role of TIMP2 in the cardiac response to MI.MI was induced in 11- to 12-week-old male TIMP2(-/-) and age-matched wild-type mice. Cardiac function was monitored by echocardiography at 1 and 4 weeks post-MI. ECM fibrillar structure was visualized using second harmonic generation and multiphoton imaging of unfixed/unstained hearts. Molecular analyses were performed at 3 days and 1 week post-MI on flash-frozen infarct, periinfarct, and noninfarct tissue. Membrane type 1 (MT1)-MMP levels and activity were measured in membrane protein fractions. TIMP2(-/-)-MI mice exhibited a 25% greater infarct expansion, markedly exacerbated left ventricular dilation (by 12%) and dysfunction (by 30%), and more severe inflammation compared to wild-type MI mice. Adverse ECM remodeling was detected by reduced density and enhanced disarray of fibrillar collagen in TIMP2(-/-)-MI compared to wild-type MI hearts. TIMP2 deficiency completely abrogated MMP2 activation but markedly increased collagenase activity, particularly MT1-MMP activity post-MI.The MMP-inhibitory function of TIMP2 is a key determinant of post-MI myocardial remodeling primarily because of its inhibitory action on MT1-MMP. TIMP2 replenishment in diseased myocardium could provide a potential therapy in reducing or preventing disease progression.
23. Overexpression of membrane-type matrix metalloproteinase-1 gene induces mammary gland abnormalities and adenocarcinoma in transgenic mice. To investigate the role of membrane-type matrix metalloproteinase-1 (MT1-MMP) in mammary gland development and tumorigenesis, transgenic mice overexpressing MT1-MMP in mammary gland under the control of the mouse mammary tumor virus long terminal repeat-promoter were generated. The mouse mammary tumor virus/MT1-MMP transgenic mice displayed abnormalities in 82% of female mammary glands. The abnormalities were verified as lymphocytic infiltration, fibrosis, hyperplasia, alveolar structure disruption, dysplasia, and adenocarcinoma. Northern and reverse transcription-PCR analyses demonstrated that MT1-MMP mRNA was overexpressed in mammary glands exhibiting abnormalities. Western blot analysis and immunohistochemical studies have revealed that the protein expression level was also increased in these glands. In addition, the beta-casein gene as a functional epithelial cell marker was poorly expressed in the mammary glands of transgenic mice exhibiting abnormalities. Gelatin zymography showed significantly increased MMP-2 activation in these mammary glands. These results showed that overexpression of MT1-MMP induced remodeling of the **extracellular matrix** and tumor formation in the mammary glands of transgenic mice. Therefore, we suggest that overexpression of MT1-MMP may play a key role in development and tumorigenesis in mammary glands.
24. TLR ligand-induced podosome disassembly in dendritic cells is ADAM17 dependent. Toll-like receptor (TLR) signaling induces a rapid reorganization of the actin cytoskeleton in cultured mouse dendritic cells (DC), leading to enhanced antigen endocytosis and a concomitant loss of filamentous actin-rich podosomes. We show that as podosomes are lost, TLR signaling induces prominent focal contacts and a transient reduction in DC migratory capacity in vitro. We further show that podosomes in mouse DC are foci of pronounced gelatinase activity, dependent on the enzyme membrane type I matrix metalloprotease (MT1-MMP), and that DC transiently lose the ability to degrade the **extracellular matrix** after TLR signaling. Surprisingly, MMP inhibitors block TLR signaling-induced podosome disassembly, although stimulated endocytosis is unaffected, which demonstrates that the two phenomena are not obligatorily coupled. Podosome disassembly caused by TLR signaling occurs normally in DC lacking MT1-MMP, and instead requires the tumor necrosis factor alpha-converting enzyme ADAM17 (a disintegrin and metalloprotease 17), which demonstrates a novel role for this "sheddase" in regulating an actin-based structure.
25. MMP-2 null mice exhibit an early onset and severe experimental autoimmune encephalomyelitis due to an increase in MMP-9 expression and activity. Matrix metalloproteinase-2 (MMP-2; gelatinase A) is known to degrade a broad range of **extracellular matrix** components and chemokines, and has important roles in the processes of cell migration, invasion, and involution during development, as well as during tumor growth and metastasis and in inflammation and repair. To better elucidate the roles of this matrix metalloproteinase in the development and progression of experimental autoimmune encephalomyelitis, we used MMP-2-deficient (KO) mice. Surprisingly, we found that MMP-2 KO mice exhibited an earlier onset and more severe disease than did their wild-type (WT) counterparts. WT mice engrafted with MMP-2 KO bone marrow exhibited a similar earlier onset and more severe clinical disease score than WT mice engrafted with WT bone marrow. Lymphocytes derived from MMP-2 KO mice exhibited increased transmigration through endothelial cell monolayers as well as through collagen type IV and laminin-coated BD BIOCOAT inserts, which correlated with a 3-fold increase in expression of MMP-9 and was abrogated by inhibition of MMP activity. We demonstrated a correlation between expression levels of MMP-9 and MT1-MMP expression and suggest a signaling pathway involving tethering of MMP-2 to MT1-MMP as a modulator of MMP-9 expression. Last, we discuss other possible MMP-2-mediated mechanisms which may contribute to the observed phenotype.
26. Matrix metalloproteinase induction in the tumor stroma does not depend on CD147 expression in murine B16 melanoma. It was conclusively demonstrated that the cell surface glycoprotein CD147 on tumor cells mediates induction of matrix metalloproteinases (MMPs) by stromal cells in humans. However, for murine models such evidence remains elusive.To address the impact of CD147 on MMP expression in the murine B16 melanoma model, we consequently stably knocked down CD147 expression in two B16 sublines. The CD147 knockdown remained stable under in vivo conditions as confirmed by immunohistochemistry. However, no differences in MMP-2, MMP-9 and MT1-MMP expression by stromal and tumor cells were detectable in CD147+ and CD147- tumors. Since the tumor microenvironment is a complex system, involving several cell types, the **extracellular matrix** and plethora soluble factors, we subsequently studied the role of murine CD147 in vitro. Coculture of melanoma cells with different fibroblast cell lines demonstrated that neither CD147+ nor CD147- B16 tumor cells altered the expression of MMP-2 or MMP-9 by the fibroblasts, although we could confirm the susceptibility of these fibroblasts for MMP induction.At least for the murine B16 melanoma model, CD147 expression on tumor cells seems not to be crucial for MMP-2, MMP-9 and MT1-MMP induction on tumor-associated stromal cells.
27. Temporal expression and activation of matrix metalloproteinases-2, -9, and membrane type 1-matrix metalloproteinase following acute hindlimb ischemia. Matrix metalloproteinase (MMP) activity is essential for remodeling of ischemic tissue. The murine hindlimb ischemia model exhibits tissue remodeling including revascularization in part due to angiogenesis. MMP-2 and -9 are type IV collagenases necessary for basement membrane degradation as a part of **extracellular matrix** remodeling and angiogenesis. Polymorphonuclear leukocytes (PMNs) contain MMP-9, and in the presence of membrane type 1 (MT1)-MMP, are able to activate proMMP-2 in vitro. Activation of MMP-2 and -9 may be essential in ischemic limbs both for tissue remodeling and revascularization via angiogenesis. We hypothesized that MMP-2 and -9 would be activated following acute hindlimb ischemia (HI), and this activation would be temporally related to PMN infiltration.HI was achieved by unilateral femoral artery ligation in 20 FVB/N mice. Five mice underwent sham operation without hindlimb ischemia. Gastrocnemius muscle was harvested from both hindlimbs at 1, 3, 14, and 30 days following ligation and assayed for MMP-2, -9 (gelatin zymography), and MT1-MMP (Western blotting). MMP-2 and -9 expression and activation were analyzed by gelatin zymography and quantified by densitometry with NIH Image Analysis software. Neutrophils per high power field were counted. The results were expressed as a ratio of ischemic to nonischemic limbs and compared at each time point using ANOVA.Zymographic analysis revealed a 212% increase in active MMP-2 3 days postligation (P <.05). Active MMP-9 reached its maximum level (800% over baseline) on postoperative day 3 and continued to be elevated on day 14 (737% over baseline) (P <.05). The increase in active MMP-2 and -9 levels paralleled PMN infiltration that also peaked 3 days postligation (1184% over baseline) (P <.05). PMN count, MMP-2, and -9 all returned to baseline levels by postoperative Day 30. MT1-MMP was present in tissue samples from all time points as confirmed by Western blot.Limb ischemia causes an early activation of MMP-2 and -9 in temporal relation to PMN infiltration. HI may prime PMNs, leading to their sequestration in ischemic tissue. Primed PMNs, along with constitutively expressed MT1-MMP, may activate MMPs-2 and -9 and enable tissue remodeling essential for limb revascularization and angiogenesis.
28. MT1-MMP down-regulates the glucose 6-phosphate transporter expression in marrow stromal cells: a molecular link between pro-MMP-2 activation, chemotaxis, and cell survival. Bone marrow-derived stromal cells (BMSC) are avidly recruited by experimental vascularizing tumors, which implies that they must respond to tumor-derived growth factor cues. In fact, BMSC chemotaxis and cell survival are regulated, in part, by the membrane type-1 matrix metalloproteinase (MT1-MMP), an MMP also involved in pro-MMP-2 activation and in degradation of the **extracellular matrix** (ECM). Given that impaired chemotaxis was recently observed in bone marrow cells isolated from a glucose 6-phosphate transporter-deficient (G6PT-/-) mouse model, we sought to investigate the potential MT1-MMP/G6PT signaling axis in BMSC. We show that MT1-MMP-mediated activation of pro-MMP-2 by concanavalin A (ConA) correlated with an increase in the sub-G1 cell cycle phase as well as with cell necrosis, indicative of a decrease in BMSC survival. BMSC isolated from Egr-1-/- mouse or MT1-MMP gene silencing in BMSC with small interfering RNA (siMT1-MMP) antagonized both the ConA-mediated activation of pro-MMP-2 and the induction of cell necrosis. Overexpression of recombinant full-length MT1-MMP triggered necrosis and this was signaled through the cytoplasmic domain of MT1-MMP. ConA inhibited both the gene and protein expression of G6PT, while overexpression of recombinant G6PT inhibited MT1-MMP-mediated pro-MMP-2 activation but could not rescue BMSC from ConA-induced cell necrosis. Cell chemotaxis in response to the tumorigenic growth factor sphingosine 1-phosphate was significantly abrogated in siMT1-MMP BMSC and in chlorogenic acid-treated BMSC. Altogether, we provide evidence for an MT1-MMP/G6PT signaling axis that regulates BMSC survival, ECM degradation, and mobilization. This may lead to optimized clinical applications that use BMSC as a platform for the systemic delivery of therapeutic or anti-cancer recombinant proteins in vivo.
29. MT1-MMP modulates bFGF-induced VEGF-A expression in corneal fibroblasts. The cornea is physiologically avascular. Following a corneal injury, wound healing often proceeds without neovascularization (NV); however, corneal NV may be induced during wound healing in certain inflammatory, infectious, degenerative, and traumatic states. Such states disrupt the physiologic balance between pro-angiogenic and antiangiogenic mediators, favoring angiogenesis. Contributors to such states are matrix metalloproteinases (MMPs), which are key factors in both **extracellular matrix** remodeling and angiogenesis. Similarly, vascular endothelial growth factor A (VEGF-A) and basic fibroblast growth factor (bFGF) exert pro-angiogenic effects. Here, we elaborate on the facilitative role of MMPs-specifically Membrane Type 1 MMP (MT1-MMP, MMP14)-in corneal NV. Additionally, we provide new insight into the signaling relating to MT1-MMP, Ras, and ERK in the bFGF-induced VEGF-A expression pathways within the corneal fibroblasts.
30. The membrane-anchored MMP inhibitor RECK is a key regulator of **extracellular matrix** integrity and angiogenesis. Matrix metalloproteinases (MMPs) are essential for proper **extracellular matrix** remodeling. We previously found that a membrane-anchored glycoprotein, RECK, negatively regulates MMP-9 and inhibits tumor invasion and metastasis. Here we show that RECK regulates two other MMPs, MMP-2 and MT1-MMP, known to be involved in cancer progression, that mice lacking a functional RECK gene die around E10.5 with defects in collagen fibrils, the basal lamina, and vascular development, and that this phenotype is partially suppressed by MMP-2 null mutation. Also, vascular sprouting is dramatically suppressed in tumors derived from RECK-expressing fibrosarcoma cells grown in nude mice. These results support a role for RECK in the regulation of MMP-2 in vivo and implicate RECK downregulation in tumor angiogenesis.
31. Membrane-type 1 matrix metalloproteinase is required for normal alveolar development. Matrix metalloproteinases (MMPs) are expressed during lung development, but their role may be limited, as mice deficient in MMP-3, 7, 9, or 12 develop a normal adult lung. Because membrane-type 1 matrix metalloproteinase (MT1-MMP) is expressed in the developing lung epithelium, we examined the lung structure of MT1-MMP-deficient (-/-) mice. Branching morphogenesis was normal, but alveolar development was abnormal in the MT1-MMP-/- lungs with 40% less alveolar surface area at 1 month (P < 0.01). MT1-MMP-/- airways and alveoli had an abnormal ultrastructural appearance, but epithelial cell differentiation markers were distributed similarly in both strains. There was no evidence of excess **extracellular matrix** deposition or inflammation at the time points examined. In contrast, by adulthood MMP-2-/- mice had normal alveolar size and structure, indicating normal alveolar development was not dependent on the ability of MT1-MMP to activate pro-MMP-2. These data indicate that MT1-MMP is required for normal lung development.
32. MT1-MMP-dependent neovessel formation within the confines of the three-dimensional **extracellular matrix**. During angiogenesis, endothelial cells initiate a tissue-invasive program within an interstitial matrix comprised largely of type I collagen. **Extracellular matrix**-degradative enzymes, including the matrix metalloproteinases (MMPs) MMP-2 and MMP-9, are thought to play key roles in angiogenesis by binding to docking sites on the cell surface after activation by plasmin- and/or membrane-type (MT) 1-MMP-dependent processes. To identify proteinases critical to neovessel formation, an ex vivo model of angiogenesis has been established wherein tissue explants from gene-targeted mice are embedded within a three-dimensional, type I collagen matrix. Unexpectedly, neither MMP-2, MMP-9, their cognate cell-surface receptors (i.e., beta3 integrin and CD44), nor plasminogen are essential for collagenolytic activity, endothelial cell invasion, or neovessel formation. Instead, the membrane-anchored MMP, MT1-MMP, confers endothelial cells with the ability to express invasive and tubulogenic activity in a collagen-rich milieu, in vitro or in vivo, where it plays an indispensable role in driving neovessel formation.
33. Proteolytic processing of laminin-5 by MT1-MMP in tissues and its effects on epithelial cell morphology. The **extracellular matrix** macromolecule laminin-5 (Ln-5) is converted by matrix metalloproteinases (MMP) MT1-MMP and MMP-2 into a migration-promoting substrate in vitro. We now report that cleavage of Ln-5 by MT1-MMP occurs in vivo and affects epithelial tissue organization and probably Ln-5 turnover. In MT1-MMP knockout (KO) mice, the kidneys showed increased levels of total Ln-5 gamma2 subunit, but significantly reduced amounts of gamma2', an amino-terminal truncated proteolytic form of gamma2. The kidney tubular epithelia of KO animals were poorly differentiated, a phenotype reminiscent of human congenital mixed hypoplastic/dysplastic disorders. To establish a better link between Ln-5 proteolytic cleavage and epithelial morphology, MT1-MMP expression was reconstituted by transfection of MT1-MMP into a Ln-5 positive, MT1-MMP deficient epithelial cell line. MT1-MMP transfectants demonstrated increased levels of processed Ln-5 gamma2 chain and enhanced spreading on Ln-5, but not fibronectin. Recombinant MT1-MMP cleaved gamma2 constructs in vitro at a known in vivo gamma2 gamma2' processing site. These results strongly indicate that Ln-5 is a physiological substrate of MT1-MMP in vivo. Proteolytic processing of gamma2 subunit by MT1-MMP may influence Ln-5 turnover in epithelial basement membranes and affect epithelial morphogenesis.
34. Functional interactions between matrix metalloproteinases and glycosaminoglycans. Similar to most proteinases, matrix metalloproteinases (MMP) do not recognize a consensus cleavage site. Thus, it is not surprising that, in a defined in vitro reaction, most MMPs can act on a wide range of proteins, including many **extracellular matri**x proteins. However, the findings obtained from in vivo studies with genetic models have demonstrated that individual MMPs act on just a few extracellular protein substrates, typically not matrix proteins. The limited, precise functions of an MMP imply that mechanisms have evolved to control the specificity of proteinase:substrate interactions. We discuss the possibility that interactions with the glycosaminoglycan chains of proteoglycans may function as allosteric regulators or accessory factors directing MMP catalysis to specific substrates. We propose that understanding how the activity of specific MMPs is confined to discreet compartments and targeted to defined substrates via interactions with other macromolecules may provide a means of blocking potentially deleterious MMP-mediated processes at the same time as sparing any beneficial functions.
35. Dimerization of endogenous MT1-MMP is a regulatory step in the activation of the 72-kDa gelatinase MMP-2 on fibroblasts and fibrosarcoma cells. The secreted gelatinase matrix metalloprotease-2 (MMP-2) and the membrane-anchored matrix metalloprotease MT1-MMP (MMP-14), are central players in pericellular proteolysis in **extracellular matrix** degradation. In addition to possessing a direct collagenolytic and gelatinolytic activity, these enzymes take part in a cascade pathway in which MT1-MMP activates the MMP-2 proenzyme. This reaction occurs in an interplay with the matrix metalloprotease inhibitor, TIMP-2, and the proposed mechanism involves two molecules of MT1-MMP in complex with one TIMP-2 molecule. We provide positive evidence that proMMP-2 activation is governed by dimerization of MT1-MMP on the surface of fibroblasts and fibrosarcoma cells. Even in the absence of transfection and overexpression, dimerization of MT1-MMP markedly stimulated the formation of active MMP-2 products. The effect demonstrated here was brought about by a monoclonal antibody that binds specifically to MT1-MMP as shown by immunofluorescence experiments. The antibody has no effect on the catalytic activity. The effect on proMMP-2 activation involves MT1-MMP dimerization because it requires the divalent monoclonal antibody, with no effect obtained with monovalent Fab fragments. Since only a negligible level of proMMP-2 activation was obtained with MT1-MMP-expressing cells in the absence of dimerization, our results identify the dimerization event as a critical level of proteolytic cascade regulation.
36. MT1-MMP modulates the mechanosensitivity of osteocytes. Membrane-type matrix metalloproteinase-1 (MT1-MMP) is expressed by mechanosensitive osteocytes and affects bone mass. The extracellular domain of MT1-MMP is connected to **extracellular matrix**, while its intracellular domain is a strong modulator of cell signaling. In theory MT1-MMP could thus transduce mechanical stimuli into a chemical response. We hypothesized that MT1-MMP plays a role in the osteocyte response to mechanical stimuli. MT1-MMP-positive and knockdown (siRNA) MLO-Y4 osteocytes were mechanically stimulated with a pulsating fluid flow (PFF). Focal adhesions were visualized by paxillin immunostaining. Osteocyte number, number of empty lacunae, and osteocyte morphology were measured in long bones of MT1-MMP(+/+) and MT1-MMP(-/-) mice. PFF decreased MT1-MMP mRNA and protein expression in MLO-Y4 osteocytes, suggesting that mechanical loading may affect pericellular matrix remodeling by osteocytes. MT1-MMP knockdown enhanced NO production and c-jun and c-fos mRNA expression in response to PFF, concomitantly with an increased number and size of focal adhesions, indicating that MT1-MMP knockdown osteocytes have an increased sensitivity to mechanical loading. Osteocytes in MT1-MMP(-/-) bone were more elongated and followed the principle loading direction, suggesting that they might sense mechanical loading. This was supported by a lower number of empty lacunae in MT1-MMP(-/-) bone, as osteocytes lacking mechanical stimuli tend to undergo apoptosis. In conclusion, mechanical stimulation decreased MT1-MMP expression by MLO-Y4 osteocytes, and MT1-MMP knockdown increased the osteocyte response to mechanical stimulation, demonstrating a novel and unexpected role for MT1-MMP in mechanosensing.
37. MT-MMP, the cell surface activator of proMMP-2 (pro-gelatinase A), is expressed with its substrate in mouse tissue during embryogenesis. Matrix metalloproteinases (MMPs), which degrade the components of the **extracellular matrix**, are key enzymes involved in the tissue remodeling of multicellular organisms. Since MMPs are secreted as inactive zymogens (pro-MMPs), they have to be activated to function. We identified a membrane-type MMP (MT-MMP) that activated proMMP-2 (pro-gelatinase A = 72 kDa type IV pro-collagenase) and described its expression on the invasive tumor cell surface. In this study we further examined the expression and role of MT-MMP in the activation of proMMP-2 during mouse embryogenesis. Northern blotting demonstrated that MT-MMP expression was increased together with that of MMP-2 and its inhibitor gene, TIMP-2, in embryos depending upon the number of days after gestation, and decreased with maturation after birth. In situ hybridization and immunohistochemistry localized MT-MMP mRNA and protein in the cells of ossifying tissues where both MMP-2 and TIMP-2 were expressed. Activated MMP-2 was detected by gelatin zymography in the lysates prepared from the micro dissected tissues that expressed the three genes. The activation rate of proMMP-2 was proportional to the expression of MMP-2 and MT-MMP. These results indicated that proMMP-2 activation through its activator, MT-MMP, is a physiological system used by organisms to initiate tissue remodeling on the cell surface.
38. Dysregulation of TGF-beta1 receptor activation leads to abnormal lung development and emphysema-like phenotype in core fucose-deficient mice. The core fucosylation (alpha1,6-fucosylation) of glycoproteins is widely distributed in mammalian tissues, and is altered under pathological conditions. To investigate physiological functions of the core fucose, we generated alpha1,6-fucosyltransferase (Fut8)-null mice and found that disruption of Fut8 induces severe growth retardation and death during postnatal development. Histopathological analysis revealed that Fut8(-/-) mice showed emphysema-like changes in the lung, verified by a physiological compliance analysis. Biochemical studies indicated that lungs from Fut8(-/-) mice exhibit a marked overexpression of matrix metalloproteinases (MMPs), such as MMP-12 and MMP-13, highly associated with lung-destructive phenotypes, and a down-regulation of **extracellular matrix** (ECM) proteins such as elastin, as well as retarded alveolar epithelia cell differentiation. These changes should be consistent with a deficiency in TGF-beta1 signaling, a pleiotropic factor that controls ECM homeostasis by down-regulating MMP expression and inducing ECM protein components. In fact, Fut8(-/-) mice have a marked dysregulation of TGF-beta1 receptor activation and signaling, as assessed by TGF-beta1 binding assays and Smad2 phosphorylation analysis. We also show that these TGF-beta1 receptor defects found in Fut8(-/-) cells can be rescued by reintroducing Fut8 into Fut8(-/-) cells. Furthermore, exogenous TGF-beta1 potentially rescued emphysema-like phenotype and concomitantly reduced MMP expression in Fut8(-/-) lung. We propose that the lack of core fucosylation of TGF-beta1 receptors is crucial for a developmental and progressive/destructive emphysema, suggesting that perturbation of this function could underlie certain cases of human emphysema.
39. Angiotensin II stimulates cardiac fibroblast migration via the differential regulation of matrixins and RECK. Sustained induction and activation of matrixins (matrix metalloproteinases or MMPs), and the destruction and deposition of **extracellular matrix** (ECM), are the hallmarks of cardiac fibrosis. The reversion-inducing-cysteine-rich protein with Kazal motifs (RECK) is a unique membrane-anchored endogenous MMP regulator. We hypothesized that elevated angiotensin II (Ang II), which is associated with fibrosis in the heart, differentially regulates MMPs and RECK both in vivo and in vitro. Continuous infusion of Ang II into male C57Bl/6 mice for 2weeks resulted in cardiac fibrosis, with increased expressions of MMPs 2, 7, 9 and 14, and of collagens Ia1 and IIIa1. The expression of RECK, however, was markedly suppressed. These effects were inhibited by co-treatment with the Ang II type 1 receptor (AT1) antagonist losartan. In vitro, Ang II suppressed RECK expression in adult mouse cardiac fibroblasts (CF) via AT1/Nox4-dependent ERK/Sp1 activation, but induced MMPs 2, 14 and 9 via NF-κB, AP-1 and/or Sp1 activation. Further, while forced expression of RECK inhibits, its knockdown potentiates Ang II-induced CF migration. Notably, RECK overexpression reduced Ang II-induced MMPs 2, 9 and 14 activation, but enhanced collagens Ia1 and IIIa1 expression and soluble collagen release. These results demonstrate for the first time that Ang II suppresses RECK, but induces MMPs both in vivo and in vitro, and RECK overexpression blunts Ang II-induced MMP activation and CF migration in vitro. Strategies that upregulate RECK expression in vivo have the potential to attenuate sustained MMP expression, and blunt fibrosis and adverse remodeling in hypertensive heart diseases.
40. Loss of MT1-MMP causes cell senescence and nuclear defects which can be reversed by retinoic acid. MT1-MMP (MMP14) is a collagenolytic enzyme located at the cell surface and implicated in **extracellular matrix** (ECM) remodeling. Mmp14(-/-) mice present dwarfism, bone abnormalities, and premature death. We demonstrate herein that the loss of MT1-MMP also causes cardiac defects and severe metabolic changes, and alters the cytoskeleton and the nuclear lamina structure. Moreover, the absence of MT1-MMP induces a senescent phenotype characterized by up-regulation of p16(INK4a) and p21(CIP1/WAF) (1), increased activity of senescence-associated β-galactosidase, generation of a senescence-associated secretory phenotype, and somatotroph axis alterations. Consistent with the role of retinoic acid signaling in nuclear lamina stabilization, treatment of Mmp14(-/-) mice with all-trans retinoic acid reversed the nuclear lamina alterations, partially rescued the cell senescence phenotypes, ameliorated the pathological defects in bone, skin, and heart, and extended their life span. These results demonstrate that nuclear architecture and cell senescence can be modulated by a membrane protease, in a process involving the ECM as a key regulator of nuclear stiffness under cell stress conditions.
41. Cloning of murine membrane-type-1-matrix metalloproteinase (MT-1-MMP) and its metanephric developmental regulation with respect to MMP-2 and its inhibitor. **Extracellular matrix** macromolecules regulate morphogenesis of embryonic organs, and are developmentally regulated. Their expression and turnover is regulated by matrix metalloproteinases (MMPs). Recently, an epithelial cell "membrane" associated metalloproteinase (MT-1-MMP) has been identified that acts as an activator of a "secreted" MMP-2, and is produced by mesenchymal fibroblasts. The activity of MMP-2 is inhibited by a "soluble" tissue inhibitor of MMP-2, TIMP-2. The role of MT-1-MMP in renal development is unknown.MT-1-MMP was cloned from embryonic mouse kidney cDNA library, and its spatio-temporal distribution during development in the context of MMP-2 and tissue inhibitor of metalloproteinase-2 (TIMP-2) was studied.The cloned MT-1-MMP exhibited approximately 86% nucleotide sequence homology with human MT-1-MMP, and had a catalytic domain and a zinc binding site preceded by a RRKR furin recognition motif. A approximately 4.5 Kb MT-1-MMP mRNA transcript was detected, and its expression was developmentally regulated. A parallel developmental regulation of MMP-2 mRNA expression was also observed. TIMP-2 expression was also developmentally regulated, but lagged behind MT-1-MMP and MMP-2. By in situ hybridization, MT-1-MMP mRNA was seen to be confined to ureteric bud epithelia, and was absent in the mesenchyme, while MMP-2 was confined to the mesenchyme. MT-1-MMP protein expression was seen on ureteric bud epithelia, induced mesenchyme and nascent nephrons, and it was highest during mid gestation. Similar spatio-temporal expressions of MMP-2 and TIMP-2 proteins were observed.mRNAs of MT-MMP-1 and MMP-2 are expressed in the respective epithelial and mesenchymal compartments, while their proteins are co-expressed in the epithelia suggest that MT-1-MMP and MMP-2, in conjunction with TIMP-2, may be involved in paracrine/juxtacrine epithelial:mesenchymal interactions during metanephrogenesis.
42. Matrix metalloproteinase-14 mediates formation of bile ducts and hepatic maturation of fetal hepatic progenitor cells. Fetal hepatic stem/progenitor cells, called hepatoblasts, play central roles in liver development; however, the molecular mechanisms regulating the phenotype of these cells have not been completely elucidated. Matrix metalloproteinase (MMP)-14 is a type I transmembrane proteinase regulating pericellular proteolysis of the **extracellular matrix** and is essential for the activation of several MMPs and cytokines. However, the physiological functions of MMP-14 in liver development are unknown. Here we describe a functional role for MMP-14 in hepatic and biliary differentiation of mouse hepatoblasts. MMP-14 was upregulated in cells around the portal vein in perinatal stage liver. Formation of bile duct-like structures in MMP-14-deficient livers was significantly delayed compared with wild-type livers in vivo. In vitro biliary differentiation assays showed that formation of cholangiocytic cysts derived from MMP-14-deficient hepatoblasts was completely impaired, and that overexpression of MMP-14 in hepatoblasts promoted the formation of bile duct-like cysts. In contrast, the expression of molecules associated with metabolic functions in hepatocytes, including hepatic nuclear factor 4α and tryptophan 2,3-dioxygenase, were significantly increased in MMP-14-deficient livers. Expression of the epidermal growth factor receptor and phosphorylation of mitogen-activated protein kinases were significantly upregulated in MMP-14-deficient livers. We demonstrate that MMP-14-mediated signaling in fetal hepatic progenitor cells promotes biliary luminal formation around the portal vein and negatively controls the maturation of hepatocytes.
43. Extracellular collagenases and the endocytic receptor, urokinase plasminogen activator receptor-associated protein/Endo180, cooperate in fibroblast-mediated collagen degradation. The collagens of the **extracellular matrix** are the most abundant structural proteins in the mammalian body. In tissue remodeling and in the invasive growth of malignant tumors, collagens constitute an important barrier, and consequently, the turnover of collagen is a rate-limiting process in these events. A recently discovered turnover route with importance for tumor growth involves intracellular collagen degradation and is governed by the collagen receptor, urokinase plasminogen activator receptor-associated protein (uPARAP or Endo180). The interplay between this mechanism and extracellular collagenolysis is not known. In this report, we demonstrate the existence of a new, composite collagen breakdown pathway. Thus, fibroblast-mediated collagen degradation proceeds preferentially as a sequential mechanism in which extracellular collagenolysis is followed by uPARAP/Endo180-mediated endocytosis of large collagen fragments. First, we show that collagen that has been pre-cleaved by a mammalian collagenase is taken up much more efficiently than intact, native collagen by uPARAP/Endo180-positive cells. Second, we demonstrate that this preference is governed by the acquisition of a gelatin-like structure by the collagen, occurring upon collagenase-mediated cleavage under native conditions. Third, we demonstrate that the growth of uPARAP/Endo180-deficient fibroblasts on a native collagen matrix leads to substantial extracellular accumulation of well defined collagen fragments, whereas, wild-type fibroblasts possess the ability to direct an organized and complete degradation sequence comprising both the initial cleavage, the endocytic uptake, and the intracellular breakdown of collagen.
44. Detection of gelatinolytic activity in developing basement membranes of the mouse embryo head by combining sensitive in situ zymography with immunolabeling. Genetic evidence indicates that the major gelatinases MMP-2 and MMP-9 are involved in mammalian craniofacial development. Since these matrix metalloproteinases are secreted as proenzymes that require activation, their tissue distribution does not necessarily reflect the sites of enzymatic activity. Information regarding the spatial and temporal expression of gelatinolytic activity in the head of the mammalian embryo is sparse. Sensitive in situ zymography with dye-quenched gelatin (DQ-gelatin) has been introduced recently; gelatinolytic activity results in a local increase in fluorescence. Using frontal sections of wild-type mouse embryo heads from embryonic day 14.5-15.5, we optimized and validated a simple double-labeling in situ technique for combining DQ-gelatin zymography with immunofluorescence staining. MMP inhibitors were tested to confirm the specificity of the reaction in situ, and results were compared to standard SDS-gel zymography of tissue extracts. Double-labeling was used to show the spatial relationship in situ between gelatinolytic activity and immunostaining for gelatinases MMP-2 and MMP-9, collagenase 3 (MMP-13) and MT1-MMP (MMP-14), a major activator of pro-gelatinases. Strong gelatinolytic activity, which partially overlapped with MMP proteins, was confirmed for Meckel's cartilage and developing mandibular bone. In addition, we combined in situ zymography with immunostaining for **extracellular matrix** proteins that are potential gelatinase substrates. Interestingly, gelatinolytic activity colocalized precisely with laminin-positive basement membranes at specific sites around growing epithelia in the developing mouse head, such as the ducts of salivary glands or the epithelial fold between tongue and lower jaw region. Thus, this sensitive method allows to associate, with high spatial resolution, gelatinolytic activity with epithelial morphogenesis in the embryo.
45. Mechanistic relationship between membrane type-1 matrix metalloproteinase and the myocardial response to pressure overload. Although matrix metalloproteinases (MMPs) were initially thought to result primarily in **extracellular matrix** degradation, certain MMP types, such as membrane type-1 (MT1) MMP, may also be involved in profibrotic cascades through hydrolysis of latency-associated transforming growth factor-binding protein (LTBP-1) and activation of transforming growth factor-dependent profibrotic signaling. The present study tested the hypothesis that MT1-MMP plays a direct role in the matrix remodeling response to a left ventricular (LV) pressure overload (PO) stimulus.Wild-type (WT) and transgenic mice with cardiac-restricted MT1-MMP overexpression or MT1-MMP reduced expression underwent PO for 4 weeks. PO resulted in a 57% increase in LV mass (no change in LV end diastolic volume, resulting in an increase in the LV mass/volume ratio consistent with concentric remodeling), a 60% increase in MT1-MMP-mediated LTBP-1 hydrolysis and a 190% increase in collagen content in WT mice. Although LV mass was similar among WT, MT1-MMP overexpression, and MT1-MMP reduced expression after PO, significant differences in LV function, MT1-MMP-mediated LTBP-1 hydrolysis, and collagen content occurred. PO in MT1-MMP overexpression increased LTBP-1 hydrolysis (18%), collagen content (60%), and left atrial dimension (19%; indicative of LV diastolic dysfunction) when compared with WT. PO in MT1-MMP reduced expression reduced left atrial dimension (19%), LTBP-1 hydrolysis (40%), and collagen content (32%) when compared with both WT.Despite an equivalent PO stimulus and magnitude of LV myocardial growth, altering MT1-MMP levels caused specific matrix-dependent changes in remodeling, thereby demonstrating a mechanistic role in the development of the maladaptive remodeling and myocardial fibrotic response to PO.
46. Galectin-3 is a downstream regulator of matrix metalloproteinase-9 function during endochondral bone formation. Endochondral bone formation is characterized by the progressive replacement of a cartilage anlagen by bone at the growth plate with a tight balance between the rates of chondrocyte proliferation, differentiation, and cell death. Deficiency of matrix metalloproteinase-9 (MMP-9) leads to an accumulation of late hypertrophic chondrocytes. We found that galectin-3, an in vitro substrate of MMP-9, accumulates in the late hypertrophic chondrocytes and their surrounding **extracellular matrix** in the expanded hypertrophic cartilage zone. Treatment of wild-type embryonic metatarsals in culture with full-length galectin-3, but not galectin-3 cleaved by MMP-9, mimicked the embryonic phenotype of Mmp-9 null mice, with an increased hypertrophic zone and decreased osteoclast recruitment. These results indicate that extracellular galectin-3 could be an endogenous substrate of MMP-9 that acts downstream to regulate hypertrophic chondrocyte death and osteoclast recruitment during endochondral bone formation. Thus, the disruption of growth plate homeostasis in Mmp-9 null mice links galectin-3 and MMP-9 in the regulation of the clearance of late chondrocytes through regulation of their terminal differentiation.
47. MT1-MMP inactivates ADAM9 to regulate FGFR2 signaling and calvarial osteogenesis. MMP14 encodes a membrane-tethered metalloproteinase MT1-MMP, capable of remodeling the **extracellular matrix** and modulating receptors on the cell surface. Loss of MT1-MMP results in craniofacial abnormalities. Here we show that MT1-MMP forms a complex with FGFR2 and ADAM9 in osteoblasts and proteolytically inactivates ADAM9, hence protecting FGFR2 from ADAM9-mediated ectodomain shedding on the cell surface. In Mmp14-/- osteoblasts, FGF-induced proliferation and downstream signaling are specifically compromised, in conjunction with ADAM9 upregulation and FGFR2 shedding. The retarded parietal growth in Mmp14-/- embryos starts at 15.5 dpc, attributable to the impaired FGFR2 signaling due to increased shedding mediated by ADAM9. Adam9 depletion completely rescues the defective FGFR2 signaling and largely restores calvarial bone growth in Mmp14-/- embryos. These data reveal a regulatory paradigm for FGRF2 signaling and identify MT1-MMP as a critical negative modulator of ADAM9 activity to maintain FGFR2 signaling in calvarial osteogenesis.
48. Multiple essential MT1-MMP functions in tooth root formation, dentinogenesis, and tooth eruption. Membrane-type matrix metalloproteinase 1 (MT1-MMP) is a transmembrane zinc-endopeptidase that breaks down **extracellular matrix** components, including several collagens, during tissue development and physiological remodeling. MT1-MMP-deficient mice (MT1-MMP(-/-)) feature severe defects in connective tissues, such as impaired growth, osteopenia, fibrosis, and conspicuous loss of molar tooth eruption and root formation. In order to define the functions of MT1-MMP during root formation and tooth eruption, we analyzed the development of teeth and surrounding tissues in the absence of MT1-MMP. In situ hybridization showed that MT1-MMP was widely expressed in cells associated with teeth and surrounding connective tissues during development. Multiple defects in dentoalveolar tissues were associated with loss of MT1-MMP. Root formation was inhibited by defective structure and function of Hertwig's epithelial root sheath (HERS). However, no defect was found in creation of the eruption pathway, suggesting that tooth eruption was hampered by lack of alveolar bone modeling/remodeling coincident with reduced periodontal ligament (PDL) formation and integration with the alveolar bone. Additionally, we identified a significant defect in dentin formation and mineralization associated with the loss of MT1-MMP. To segregate these multiple defects and trace their cellular origin, conditional ablation of MT1-MMP was performed in epithelia and mesenchyme. Mice featuring selective loss of MT1-MMP activity in the epithelium were indistinguishable from wild type mice, and importantly, featured a normal HERS structure and molar eruption. In contrast, selective knock-out of MT1-MMP in Osterix-expressing mesenchymal cells, including osteoblasts and odontoblasts, recapitulated major defects from the global knock-out including altered HERS structure, short roots, defective dentin formation and mineralization, and reduced alveolar bone formation, although molars were able to erupt. These data indicate that MT1-MMP activity in the dental mesenchyme, and not in epithelial-derived HERS, is essential for proper tooth root formation and eruption. In summary, our studies point to an indispensable role for MT1-MMP-mediated matrix remodeling in tooth eruption through effects on bone formation, soft tissue remodeling and organization of the follicle/PDL region.
49. MT1-MMP-dependent remodeling of cardiac **extracellular matrix** structure and function following myocardial infarction. The myocardial **extracellular matrix** (ECM), an interwoven meshwork of proteins, glycoproteins, proteoglycans, and glycosaminoglycans that is dominated by polymeric fibrils of type I collagen, serves as the mechanical scaffold on which myocytes are arrayed for coordinated and synergistic force transduction. Following ischemic injury, cardiac ECM remodeling is initiated via localized proteolysis, the bulk of which has been assigned to matrix metalloproteinase (MMP) family members. Nevertheless, the key effector(s) of myocardial type I collagenolysis both in vitro and in vivo have remained unidentified. In this study, using cardiac explants from mice deficient in each of the major type I collagenolytic MMPs, including MMP-13, MMP-8, MMP-2, MMP-9, or MT1-MMP, we identify the membrane-anchored MMP, MT1-MMP, as the dominant collagenase that is operative within myocardial tissues in vitro. Extending these observations to an in vivo setting, mice heterozygous for an MT1-MMP-null allele display a distinct survival advantage and retain myocardial function relative to wild-type littermates in an experimental model of myocardial infarction, effects associated with preservation of the myocardial type I collagen network as a consequence of the decreased collagenolytic potential of cardiac fibroblasts. This study identifies MT1-MMP as a key MMP responsible for effecting postinfarction cardiac ECM remodeling and cardiac dysfunction.
50. MT1-MMP-deficient mice develop dwarfism, osteopenia, arthritis, and connective tissue disease due to inadequate collagen turnover. MT1-MMP is a membrane-bound matrix metalloproteinase (MT-MMP) capable of mediating pericellular proteolysis of **extracellular matrix** components. MT1-MMP is therefore thought to be an important molecular tool for cellular remodeling of the surrounding matrix. To establish the biological role of this membrane proteinase we generated MT1-MMP-deficient mice by gene targeting. MT1-MMP deficiency causes craniofacial dysmorphism, arthritis, osteopenia, dwarfism, and fibrosis of soft tissues due to ablation of a collagenolytic activity that is essential for modeling of skeletal and extraskeletal connective tissues. Our findings demonstrate the pivotal function of MT1-MMP in connective tissue metabolism, and illustrate that modeling of the soft connective tissue matrix by resident cells is essential for the development and maintenance of the hard tissues of the skeleton.
51. MT1-MMP regulates the PI3Kδ·Mi-2/NuRD-dependent control of macrophage immune function. Macrophages play critical roles in events ranging from host defense to obesity and cancer, where they infiltrate affected tissues and orchestrate immune responses in tandem with the remodeling of th**e extracellular matr**ix (ECM). Despite the dual roles played by macrophages in inflammation, the functions of macrophage-derived proteinases are typically relegated to tissue-invasive or -degradative events. Here we report that the membrane-tethered matrix metalloenzyme MT1-MMP not only serves as an ECM-directed proteinase, but unexpectedly controls inflammatory gene responses wherein MT1-MMP(-/-) macrophages mount exaggerated chemokine and cytokine responses to immune stimuli both in vitro and in vivo. MT1-MMP modulates inflammatory responses in a protease-independent fashion in tandem with its trafficking to the nuclear compartment, where it triggers the expression and activation of a phosphoinositide 3-kinase δ (PI3Kδ)/Akt/GSK3β signaling cascade. In turn, MT1-MMP-dependent PI3Kδ activation regulates the immunoregulatory Mi-2/NuRD nucleosome remodeling complex that is responsible for controlling macrophage immune response. These findings identify a novel role for nuclear MT1-MMP as a previously unsuspected transactivator of signaling networks central to macrophage immune responses.
52. Membrane-type MMPs enable **extracellular matrix** permissiveness and mesenchymal cell proliferation during embryogenesis. Peri-cellular remodeling of mesenchymal extracellular matrices is considered a prerequisite for cell proliferation, motility and development. Here we demonstrate that membrane-type 3 MMP, MT3-MMP, is expressed in mesenchymal tissues of the skeleton and in peri-skeletal soft connective tissue. Consistent with this localization, MT3-MMP-deficient mice display growth inhibition tied to a decreased viability of mesenchymal cells in skeletal tissues. We document that MT3-MMP works as a major collagenolytic enzyme, enabling cartilage and bone cells to cleave high-density fibrillar collagen and modulate their resident matrix to make it permissive for proliferation and migration. Collectively, these data uncover a novel **extracellular matrix** remodeling mechanism required for proper function of mesenchymal cells. The physiological significance of MT3-MMP is highlighted in mice double deficient for MT1-MMP and MT3-MMP. Double deficiency transcends the combined effects of the individual single deficiencies and leads to severe embryonic defects in palatogenesis and bone formation incompatible with life. These defects are directly tied to loss of indispensable collagenolytic activities required in collagen-rich mesenchymal tissues for **extracellular matrix** remodeling and cell proliferation during embryogenesis.
53. MT1-MMP-mediated basement membrane remodeling modulates renal development. **Extracellular matrix** (ECM) remodeling regulates multiple cellular functions required for normal development and tissue repair. Matrix metalloproteinases (MMPs) are key mediators of this process and membrane targeted MMPs (MT-MMPs) in particular have been shown to be important in normal development of specific organs. In this study we investigated the role of MT1-MMP in kidney development. We demonstrate that loss of MT1-MMP leads to a renal phenotype characterized by a moderate decrease in ureteric bud branching morphogenesis and a severe proliferation defect. The kidneys of MT1-MMP-null mice have increased deposition of collagen IV, laminins, perlecan, and nidogen and the phenotype is independent of the MT-1MMP target, MMP-2. Utilizing in vitro systems we demonstrated that MTI-MMP proteolytic activity is required for renal tubule cells to proliferate in three dimensional matrices and to migrate on collagen IV and laminins. Together these data suggest an important role for MT1-MMP in kidney development, which is mediated by its ability to regulate cell proliferation and migration by proteolytically cleaving kidney basement membrane components.
54. MT1-matrix metalloproteinase directs arterial wall invasion and neointima formation by vascular smooth muscle cells. During pathologic vessel remodeling, vascular smooth muscle cells (VSMCs) embedded within the collagen-rich matrix of the artery wall mobilize uncharacterized proteolytic systems to infiltrate the subendothelial space and generate neointimal lesions. Although the VSMC-derived serine proteinases, plasminogen activator and plasminogen, the cysteine proteinases, cathepsins L, S, and K, and the matrix metalloproteinases MMP-2 and MMP-9 have each been linked to pathologic matrix-remodeling states in vitro and in vivo, the role that these or other proteinases play in allowing VSMCs to negotiate the three-dimensional (3-D) cross-linked **extracellular matrix** of the arterial wall remains undefined. Herein, we demonstrate that VSMCs proteolytically remodel and invade collagenous barriers independently of plasmin, cathepsins L, S, or K, MMP-2, or MMP-9. Instead, we identify the membrane-anchored matrix metalloproteinase, MT1-MMP, as the key pericellular collagenolysin that controls the ability of VSMCs to degrade and infiltrate 3-D barriers of interstitial collagen, including the arterial wall. Furthermore, genetic deletion of the proteinase affords mice with a protected status against neointimal hyperplasia and lumen narrowing in vivo. These studies suggest that therapeutic interventions designed to target MT1-MMP could prove beneficial in a range of human vascular disease states associated with the destructive remodeling of the vessel wall **extracellular matrix**.
55. Induction of a MT1-MMP and MT2-MMP-dependent basement membrane transmigration program in cancer cells by Snail1. The ability of carcinoma cells arising at primary sites to cross their underlying basement membrane (BM), a specialized form of **extracellular matrix** that subtends all epithelial cells, and to access the host vasculature are central features of the malignant phenotype. The initiation of the invasive phenotype has been linked to the aberrant expression of zinc-finger transcriptional repressors, like Snail1, which act by triggering an epithelial-mesenchymal cell-like transformation (EMT-like) via the regulation of largely undefined, downstream effectors. Herein, we find that Snail1 induces cancer cells to (i) degrade and perforate BM barriers, (ii) initiate angiogenesis, and (iii) and intravasate vascular networks in vivo via a matrix metalloproteinase (MMP)-dependent process. Unexpectedly, the complete Snail1 invasion program can be recapitulated by expressing directly either of the membrane-anchored metalloproteinases, MT1-MMP or MT2-MMP. The pro-invasive, angiogenic, and metastatic activities of MT1-MMP and MT2-MMP are unique relative to all other metalloproteinase family members and cannot be mimicked in vivo by the secreted MMPs, MMP-1, MMP-2, MMP-3, MMP-7, MMP-9, or MMP-13. Further, siRNA-specific silencing of MT1-MMP and MT2-MMP ablates completely the ability of Snail1 to drive cancer cell BM invasion, induce angiogenesis, or trigger intravasation. Taken together, these data demonstrate that MT1-MMP and MT2-MMP cooperatively function as direct-acting, pro-invasive factors that confer Snail1-triggered cells with the key activities most frequently linked to morbidity and mortality in cancer.
56. Functional roles of MMP14 and MMP15 in early postnatal mammary gland development. During late embryogenesis, mammary epithelial cells initiate migration programs that drive ductal invasion into the surrounding adipose-rich mesenchyme. Currently, branching morphogenesis is thought to depend on the mobilization of the membrane-anchored matrix metalloproteinases MMP14 (MT1-MMP) and MMP15 (MT2-MMP), which drive epithelial cell invasion by remodeling the **extracellular matrix** and triggering associated signaling cascades. However, the roles that these proteinases play during mammary gland development in vivo remain undefined. Here, we characterize the impact of global Mmp14 and Mmp15 targeting on early postnatal mammary gland development in mice. Unexpectedly, both Mmp14(-/-) and Mmp15(-/-) mammary glands retain the ability to generate intact ductal networks. Although neither proteinase is required for branching morphogenesis, transcriptome profiling reveals a key role for MMP14 and MMP15 in regulating mammary gland adipocyte differentiation. Whereas MMP14 promotes the generation of white fat depots crucial for energy storage, MMP15 differentially controls the formation of thermogenic brown fat. Taken together, these data not only indicate that current paradigms relevant to proteinase-dependent morphogenesis need be revisited, but also identify new roles for the enzymes in regulating adipocyte fate determination in the developing mammary gland.
57. Matrix metalloproteinases in tumor invasion. Controlled degradation of **extracellular matrix** (ECM) is essential for the growth, invasion, and metastasis of malignant tumors, and for tumor-induced angiogenesis. Matrix metalloproteinases (MMPs) are a family of zinc-dependent neutral endopeptidases collectively capable of degrading essentially all ECM components and they apparently play an important role in all these aspects of tumor development. Furthermore, recent evidence suggests that MMPs also play a role in tumor cell survival. In this review, we discuss the current concept concerning the role of MMPs and their inhibitors in tumor invasion, as a basis for prognosis and targeted therapeutic intervention in cancer.
58. Involvement of matrix metalloproteinases in the adipose conversion of 3T3-L1 preadipocytes. When mouse 3T3-L1 preadipocytes are induced to differentiate into adipocytes, they change from an extended fibroblast-like morphology to a rounded one. This change most likely occurs through **extracellular matrix** remodelling, a process known to be mediated in part by matrix metalloproteinases (MMPs). In this study, we have shown by semi-quantitative reverse transcriptase-PCR, zymographic and immunoblot analysis that MMP-2, MMP-9 and membrane type 1 (MT1)-MMP are regulated during adipose conversion. To assess the importance of MMPs for adipocytic differentiation we have used MMP-specific inhibitors as well as neutralizing antibodies. Treatment of 3T3-L1 preadipocytes with the broad MMP inhibitor Ilomastat or the more restricted MMP-2 Inhibitor I prevented their differentiation into adipocytes in a dose-dependent manner, as evidenced by absence of triglyceride accumulation. Inhibitor treatment prevented the fibronectin-network degradation, as well as the induction of the genes for peroxisome-proliferator-activated receptor gamma and adipsin, two adipocyte phenotype markers. Inhibitor treatment was effective when applied during the early stages of adipocytic conversion, whereas inhibitor treatment during later stages had little effect. Inhibitor treatment did not inhibit clonal mitotic expansion; nor did it affect the expression pattern of the adipogenic transcription factor CCAAT/enhancer-binding protein beta (C/EBPbeta) or its nuclear translocation. It did, however, markedly reduce C/EBPbeta DNA-binding capacity. Taken together, these results suggest that MMPs, and notably MMP-2 and MMP-9, may be necessary mediators of adipocytic differentiation of 3T3-L1 cells.
59. Adipogenic histone mark regulation by matrix metalloproteinase 14 in collagen-rich microenvironments. Adipogenesis is directed by both transcriptional network and posttranslational modification of chromatin structure. Although adipogenesis in vivo proceeds in collagen-rich **extracellular matrix** (ECM) environments, the impact of ECM proteins and their modifying enzymes on the epigenetic regulation of adipogenesis has been largely unknown. We aimed to define the role of fibrillar type I collagen and its modifying enzymes in regulating adipogenic chromatin signatures and gene regulation in the in vivo-like settings. Adipogenic cocktail induces a robust increase in the level of protranscriptional acetylated histone H3 at lysine 9 (H3K9ac) within 24 h. When cultured atop fibrillar type I collagen gel, however, H3K9ac levels in differentiating 3T3-L1 cells are substantially reduced. The suppression of adipogenic histone mark in differentiating 3T3-L1 cells is type I collagen density dependent and released by heat denaturing of the subjacent collagen substratum, pointing to the critical role played by the triple-helical structure of type I collagen. By probing adipogenic collagenolysis with a series of proteinase inhibitors, matrix metalloproteinase (MMP) family members are found to be responsible for adipogenic collagenolysis. At the same time, MMP inhibitor specifically blocked the adipogenic induction of H3K9ac. By targeting individual MMP using small interfering RNA oligos, MMP14 was identified as the major adipogenic MMP critical for H3K9 acetylation. Consistently, MMP14-null adipose tissues display diminished protranscriptional histone mark H3K9ac while maintaining repressive histone mark tri-methylated histone H3 at lysine 9 (H3K9me3). Taken together, MMP14-dependent collagenolysis plays the major role in regulating adipogenic histone marks by releasing the epigenetic constraints imposed by fibrillar type I collagen.
60. Tumor cell traffic through the **extracellular matrix** is controlled by the membrane-anchored collagenase MT1-MMP. As cancer cells traverse collagen-rich **extracellular matrix** (ECM) barriers and intravasate, they adopt a fibroblast-like phenotype and engage undefined proteolytic cascades that mediate invasive activity. Herein, we find that fibroblasts and cancer cells express an indistinguishable pericellular collagenolytic activity that allows them to traverse the ECM. Using fibroblasts isolated from gene-targeted mice, a matrix metalloproteinase (MMP)-dependent activity is identified that drives invasion independently of plasminogen, the gelatinase A/TIMP-2 axis, gelatinase B, collagenase-3, collagenase-2, or stromelysin-1. In contrast, deleting or suppressing expression of the membrane-tethered MMP, MT1-MMP, in fibroblasts or tumor cells results in a loss of collagenolytic and invasive activity in vitro or in vivo. Thus, MT1-MMP serves as the major cell-associated proteinase necessary to confer normal or neoplastic cells with invasive activity.
61. Mammary Branching Morphogenesis Requires Reciprocal Signaling by Heparanase and MMP-14. The development of the mammary gland involves formation of a branched arboreal structure resulting from the penetration and proliferation of epithelial cells into the fat pad. The mammary cells invade by remodeling their surrounding **extracellular matrix** (ECM), which are rich in proteins, and glycans such as heparan sulfate proteoglycans (HSPGs). There is increasing literature on how the interaction between signaling by ECM and matrix metalloproteinases (MMPs) is relevant to morphogenetic and physiological contexts. Here we sought to understand how heparanase, the sole mammalian heparan sulfate-degrading endoglycosidase may regulate mammary gland development. We found a robust localization of heparanase within growing end buds during branching in vivo. Using three-dimensional (3D) organotypic cultures, we showed that heparanase expression and activity are required for mammary epithelial invasion/branching within dense collagen I gels. Morphometric analysis of glands from both heparanase-overexpressing and knockout mice showed a direct correlation between degree of branching and the heparanase levels, confirming our 3D organotypic culture observations. Finally, we uncovered a reciprocal association between levels of heparanase and MMP14, a membrane-bound MMP, shedding further light on how branching occurs within developing mammary glands.
62. Mutations in two matrix metalloproteinase genes, MMP-2 and MT1-MMP, are synthetic lethal in mice. The matrix metalloproteinase (MMP) family (approximately 25 members in mammals) has been implicated in **extracellular matrix** remodeling associated with embryonic development, cancer formation and progression, and various other physiological and pathological events. Inactivating mutations in individual matrix metalloproteinase genes in mice described so far, however, are nonlethal at least up to the first few weeks after birth, suggesting functional redundancy among MMP family members. Here, we report that mice lacking two MMPs, MMP-2 (nonmembrane type) and MT1-MMP (membrane type), die immediately after birth with respiratory failure, abnormal blood vessels, and immature muscle fibers reminiscent of central core disease. In the absence of MMP-2 and MT1-MMP, myoblast fusion in vitro is also significantly retarded. These findings suggest functional overlap in mice between the two MMPs with distinct molecular natures.
63. Interleukin-6 enhances matrix metalloproteinase-14 expression via the RAF-mitogen-activated protein kinase kinase-extracellular signal-regulated kinase 1/2-activator protein-1 pathway. 1. Interleukin (IL)-6 is a pivotal cytokine that regulates **extracellular matrix** (ECM) metabolism by increasing collagen degradation via activation of matrix metalloproteinases (MMPs), such as MMP-14. In the present study, we investigated the role of IL-6 in atherosclerotic plaque and signalling pathways in apolipoprotein E-deficient (ApoE(-/-)) mice. 2. Twenty-five male ApoE(-/-) mice were fed a high-fat diet and atherosclerotic lesions in the right common carotid artery were induced by perivascular placement of a constrictive collar. Immunohistochemical analysis detected expression of IL-6 and MMP-14 in atherosclerotic lesions of the right common carotid artery. 3. On silencing activator protein (AP)-1 expression with a specific small interfering RNA, 75% of the IL-6-induced increase in MMP-14 expression was abolished through the RAF-mitogen-activated protein kinase kinase-extracellular signal-regulated kinase 1/2-AP-1 pathway. 4. These findings suggest a novel molecular pathway for inflammation-associated ECM dysregulation, which may account for atherosclerotic plaque rupture.
64. A matrix metalloproteinase expressed on the surface of invasive tumour cells. Gelatinase A (type-IV collagenase; M(r) 72,000) is produced by tumour stroma cells and is believed to be crucial for their invasion and metastasis, acting by degrading **extracellular matrix** macro-molecules such as type IV collagen. An inactive precursor of gelatinase A (pro-gelatinase A) is secreted and activated in invasive tumour tissue as a result of proteolysis which is mediated by a fraction of tumour cell membrane that is sensitive to metalloproteinase inhibitors. Here we report the cloning of the complementary DNA encoding a new matrix metalloproteinase with a potential transmembrane domain. Expression of the gene product on the cell surface induces specific activation of pro-gelatinase A in vitro and enhances cellular invasion of the reconstituted basement membrane. Tumour cells of invasive lung carcinomas, which contain activated forms of gelatinase A, were found to express the transcript and the gene product. The new metalloproteinase may thus trigger invasion by tumour cells by activating pro-gelatinase A on the tumour cell surface.
65. Diminished matrix metalloproteinase 2 (MMP-2) in ectomesenchyme-derived tissues of the Patch mutant mouse: regulation of MMP-2 by PDGF and effects on mesenchymal cell migration. Platelet-derived growth factors (PDGF) regulate cell proliferation, survival, morphology, and migration, as well as deposition and turnover of the **extracellular matrix**. Important roles for the A form of PDGF (PDGF-A) during connective tissue morphogenesis have been highlighted by the murine Patch mutation, which includes a deletion of the alpha subunit of the PDGF receptor. Homozygous (Ph/Ph) embryos exhibit multiple connective tissue defects including cleft face (involving the first branchial arch and frontonasal processes), incomplete heart septation, and heart valve abnormalities before they die in utero. Analyses of the cell biology underlying the defects in Ph/Ph embryos have revealed a deficit in a matrix metalloproteinase (MMP-2) and one of its activators (MT-MMP) that are likely to be involved in cell migration and tissue remodeling, two processes necessary for normal cardiac and craniofacial development. Morphogenesis of these structures requires infiltration of ectomesenchymal precursors and their subsequent deposition and remodeling of **extracellular matrix** components. First branchial arch and heart tissue from E10.5 embryos were examined by gelatin zymography and RT-PCR in order to characterize the expression of MMPs in these tissues. Of the MMPs examined, only MMP-2 and one of its activators, MT-MMP, were expressed in the first arch and heart at this stage of development. Tissues from Ph/Ph embryos exhibited a significant decrease in both MMP-2 and MT-MMP compared to tissues from normal embryos of the same developmental stage. In order to assess whether this decrease affects the motile activity of mesenchymal cells, cell migration from Ph/Ph branchial arch explants was compared to migration from normal arch tissue and found to be significantly less. In addition, the migratory ability of branchial arch cells from normal explants could be reduced in a similar manner using a specific MMP inhibitor. Although it is still unclear whether the MMP-2 reduction is a direct result of the absence of response of Ph/Ph cells to PDGF-A treatment of normal branchial arch cells in vitro with recombinant PDGF-AA significantly upregulated MMP-2 protein. Together, these results suggest that PDGF-A regulates MMP-2 expression and activation during normal development and that faulty proteinase expression may be at least partially responsible for the developmental defects exhibited by Ph/Ph embryos.
66. Expression pattern of four membrane-type matrix metalloproteinases in the normal and diseased mouse mammary gland. Both mammary gland development and mammary carcinogenesis involve extensive remodeling of the mammary gland **extracellular matrix**. The expression of four membrane-type matrix metalloproteinases (MT-MMPs) with matrix remodeling potential in development and tumorigenesis was evaluated by in-situ hybridization on mouse mammary gland sections. MT1-MMP and MT3-MMP were found in the mammary stroma mainly around epithelial structures in both developing and mature mammary gland. In contrast, MT2-MMP was found exclusively in the mammary epithelium. Lactating gland expressed none of the examined MT-MMPs. Mammary gland tumors expressed MT1-MMP, MT2-MMP, and MT3-MMP while MT4-MMP was not expressed in any developmental or cancerous stage analyzed here. Our results suggest that MT1-MMP, MT2-MMP, and MT3-MMP may be involved in remodeling of both the normal and diseased mammary gland either directly or indirectly by activation of other MMPs.

#### Conserved Domains

1. cd04278: ZnMc\_MMP; Zinc-dependent metalloprotease, matrix metalloproteinase (MMP) sub-family. MMPs are responsible for a great deal of pericellular proteolysis of **extracellular matrix** and cell surface molecules, playing crucial roles in morphogenesis, cell fate ...

#### GeneRIFs

1. MT1-MMP controls stem cell commitment by remodeling the 3D **extracellular matrix**.
2. This study identifies MT1-MMP as a key matrix metalloproteinase responsible for effecting postinfarction cardiac **extracellular matrix** remodeling and cardiac dysfunction.
3. MT1-MMP is a new multilateral regulator for muscle differentiation and maintenance through processing of stage-specific distinct **extracellular matrix** substrates.

#### Summary

1. This gene encodes a member of the matrix metalloproteinase family of **extracellular matrix**-degrading enzymes that are involved in tissue remodeling, wound repair, progression of atherosclerosis and tumor invasion. The encoded preproprotein undergoes proteolytic processing to generate a mature, zinc-dependent endopeptidase enzyme. Mice lacking the encoded protein exhibit craniofacial dysmorphism, arthritis, osteopenia, dwarfism, and fibrosis of soft tissues. [provided by RefSeq, Feb 2016]

### [Ii]nvasive

#### PubMed

1. **Invasive** potential of melanoma cells correlates with the expression of MT1-MMP and regulated by modulating its association with motility receptors via N-glycosylation on the receptors. Matrix remodeling and invasion of basement membrane are the major determinants of malignant progression. Matrix degrading enzymes play a pivotal role in this process and have been shown to be regulated at multiple levels. Using high metastatic B16F10 and its **invasive** variant B16BL6 cells, we previously demonstrated that the expression of β1,6 branched N-oligosaccharides promotes cellular adhesion on different matrix components which in turn induces secretion of MMP9. The present investigations report that although the two cell lines do not differ in the expression of uPAR, expression of MT1-MMP is significantly higher on B16BL6 cells. Analysis of the transcripts of tissue inhibitors of matrix metalloproteinases (TIMPs) showed that expression of both TIMP1 and TIMP2 correlates negatively with the **invasiv**e potential of cells. CD44 and β1 integrin, the two important receptors involved in motility, were identified to carry β1,6 branched N-oligosaccharides in **an invas**ive potential dependent manner. However, their glycosylation status did not appear to influence their surface expression. Although glycosylation on CD44 had no effect, that on β1 integrin significantly affected association of β1 integrin with MT1-MMP. The results thus demonstrate that the cancer cells use multiple mechanisms for degradation of matrix in a controlled manner to couple it with movement for effective invasion.
2. Type 1 insulin-like growth factor regulates MT1-MMP synthesis and tumor invasion via PI 3-kinase/Akt signaling. The membrane type 1 matrix metalloproteinase (MT1-MMP) has been identified as a major activator of MMP-2 - a process involving the formation of a trimolecular complex with TIMP-2. We previously identified the IGF-I receptor as a positive regulator of MMP-2 synthesis. Here, we investigated the role of IGF-IR in the regulation of MT1-MMP. Highly **invasive** Lewis lung carcinoma subline H-59 cells express MT1-MMP and utilize it to activate their major extracellular matrix degrading proteinase-MMP-2. These cells were transiently transfected with a plasmid vector expressing a luciferase reporter gene downstream of the mouse MT1-MMP promoter. IGF-I treatment increased luciferase activity in the transfected cells by up to 10-fold and augmented endogenous MT1-MMP mRNA and protein synthesis by up to 2-3-fold, relative to controls. MT1-MMP induction and invasion were blocked by the PI 3-kinase inhibitors LY294002 and wortmannin and by rapamycin, but not by the MEK inhibitor PD98059. Overexpression of a dominant negative Akt mutant or of the tumor suppressor phosphatase and tensin homologue, PTEN, in these cells also caused a significant reduction in MT1-MMP expression and invasion. The results demonstrate that IGF-IR controls tumor cell invasion by coordinately regulating MMP-2 expression and its MT1-MMP-mediated activation and identify PI 3-kinase/Akt/mTOR signaling as critical to this regulation.
3. **Invasive** potential of melanoma cells correlates with the expression of MT1-MMP and regulated by modulating its association with motility receptors via N-glycosylation on the receptors. Matrix remodeling and invasion of basement membrane are the major determinants of malignant progression. Matrix degrading enzymes play a pivotal role in this process and have been shown to be regulated at multiple levels. Using high metastatic B16F10 and its **invasive** variant B16BL6 cells, we previously demonstrated that the expression of β1,6 branched N-oligosaccharides promotes cellular adhesion on different matrix components which in turn induces secretion of MMP9. The present investigations report that although the two cell lines do not differ in the expression of uPAR, expression of MT1-MMP is significantly higher on B16BL6 cells. Analysis of the transcripts of tissue inhibitors of matrix metalloproteinases (TIMPs) showed that expression of both TIMP1 and TIMP2 correlates negatively with the **invasiv**e potential of cells. CD44 and β1 integrin, the two important receptors involved in motility, were identified to carry β1,6 branched N-oligosaccharides in **an invas**ive potential dependent manner. However, their glycosylation status did not appear to influence their surface expression. Although glycosylation on CD44 had no effect, that on β1 integrin significantly affected association of β1 integrin with MT1-MMP. The results thus demonstrate that the cancer cells use multiple mechanisms for degradation of matrix in a controlled manner to couple it with movement for effective invasion.
4. Type 1 insulin-like growth factor regulates MT1-MMP synthesis and tumor invasion via PI 3-kinase/Akt signaling. The membrane type 1 matrix metalloproteinase (MT1-MMP) has been identified as a major activator of MMP-2 - a process involving the formation of a trimolecular complex with TIMP-2. We previously identified the IGF-I receptor as a positive regulator of MMP-2 synthesis. Here, we investigated the role of IGF-IR in the regulation of MT1-MMP. Highly **invasive** Lewis lung carcinoma subline H-59 cells express MT1-MMP and utilize it to activate their major extracellular matrix degrading proteinase-MMP-2. These cells were transiently transfected with a plasmid vector expressing a luciferase reporter gene downstream of the mouse MT1-MMP promoter. IGF-I treatment increased luciferase activity in the transfected cells by up to 10-fold and augmented endogenous MT1-MMP mRNA and protein synthesis by up to 2-3-fold, relative to controls. MT1-MMP induction and invasion were blocked by the PI 3-kinase inhibitors LY294002 and wortmannin and by rapamycin, but not by the MEK inhibitor PD98059. Overexpression of a dominant negative Akt mutant or of the tumor suppressor phosphatase and tensin homologue, PTEN, in these cells also caused a significant reduction in MT1-MMP expression and invasion. The results demonstrate that IGF-IR controls tumor cell invasion by coordinately regulating MMP-2 expression and its MT1-MMP-mediated activation and identify PI 3-kinase/Akt/mTOR signaling as critical to this regulation.
5. Secreted versus membrane-anchored collagenases: relative roles in fibroblast-dependent collagenolysis and invasion. Fibroblasts degrade type I collagen, the major extracellular protein found in mammals, during events ranging from bulk tissue resorption to invasion through the three-dimensional extracellular matrix. Current evidence suggests that type I collagenolysis is mediated by secreted as well as membrane-anchored members of the matrix metalloproteinase (MMP) gene family. However, the roles played by these multiple and possibly redundant, degradative systems during fibroblast-mediated matrix remodeling is undefined. Herein, we use fibroblasts isolated from Mmp13(-/-), Mmp8(-/-), Mmp2(-/-), Mmp9(-/-), Mmp14(-/-) and Mmp16(-/-) mice to define the functional roles for secreted and membrane-anchored collagenases during collagen-resorptive versus collagen-**invasive** events. In the presence of a functional plasminogen activator-plasminogen axis, secreted collagenases arm cells with a redundant collagenolytic potential that allows fibroblasts harboring single deficiencies for either MMP-13, MMP-8, MMP-2, or MMP-9 to continue to degrade collagen comparably to wild-type fibroblasts. Likewise, Mmp14(-/-) or Mmp16(-/-) fibroblasts retain near-normal collagenolytic activity in the presence of plasminogen via the mobilization of secreted collagenases, but only Mmp14 (MT1-MMP) plays a required role in the collagenolytic processes that support fibroblast **invasive** activity. Furthermore, by artificially tethering a secreted collagenase to the surface of Mmp14(-/-) fibroblasts, we demonstrate that localized pericellular collagenolytic activity differentiates the collagen-**invasive** phenotype from bulk collagen degradation. Hence, whereas secreted collagenases arm fibroblasts with potent matrix-resorptive activity, only MT1-MMP confers the focal collagenolytic activity necessary for supporting the tissue-**invasive** phenotype.
6. MT1-MMP promotes vascular smooth muscle dedifferentiation through LRP1 processing. At sites of vessel-wall injury, vascular smooth muscle cells (VSMCs) can dedifferentiate to express an **invasive** and proliferative phenotype, which contributes to the development of neointimal lesions and vascular disorders. Herein, we demonstrate that the loss of the VSMC differentiated phenotype, as the repression of contractile-protein expression, is correlated with a dramatic upregulation of the membrane-anchored matrix metalloproteinase MT1-MMP (also known as MMP14 and membrane-type 1 matrix metalloproteinase). Matrix metalloproteinase (MMP) inhibitors or MT1-MMP deficiency led to attenuated VSMC dedifferentiation, whereas the phenotypic switch was re-engaged following the restoration of MT1-MMP activity in MT1-MMP(-/-) cells. MT1-MMP-dependent dedifferentiation was mediated by the PDGF-BB-PDGFRbeta pathway in parallel with the proteolytic processing of the multifunctional LDL receptor-related protein LRP1 and the dynamic internalization of a PDGFRbeta-beta3-integrin-MT1-MMP-LRP1 multi-component complex. Importantly, LRP1 silencing allowed the PDGF-BB-induced dedifferentiation program to proceed in the absence of MT1-MMP activity, supporting the role of unprocessed LRP1 as a gatekeeper of VSMC differentiation. Hence, MT1-MMP and LRP1 serve as a new effector-target-molecule axis that controls the PDGF-BB-PDGFRbeta-dependent VSMC phenotype and function.
7. 3-phosphoinositide-dependent protein kinase-1 (PDK1) promotes invasion and activation of matrix metalloproteinases. Metastasis is a major cause of morbidity and mortality in breast cancer with tumor cell invasion playing a crucial role in the metastatic process. PDK1 is a key molecule that couples PI3K to cell proliferation and survival signals in response to growth factor receptor activation, and is oncogenic when expressed in mouse mammary epithelial cells. We now present evidence showing that PDK1-expressing cells exhibit enhanced anchorage-dependent and -independent cell growth and are highly **invasive** when grown on Matrigel. These properties correlate with induction of MMP-2 activity, increased MT1-MMP expression and a unique gene expression profile.Invasion assays in Matrigel, MMP-2 zymogram analysis, gene microarray analysis and mammary isografts were used to characterize the **invasive** and proliferative function of cells expressing PDK1. Tissue microarray analysis of human breast cancers was used to measure PDK1 expression in **invasive** tumors by IHC.Enhanced invasion on Matrigel in PDK1-expressing cells was accompanied by increased MMP-2 activity resulting from stabilization against proteasomal degradation. Increased MMP-2 activity was accompanied by elevated levels of MT1-MMP, which is involved in generating active MMP-2. Gene microarray analysis identified increased expression of the ECM-associated genes decorin and type I procollagen, whose gene products are substrates of MT1-MMP. Mammary fat pad isografts of PDK1-expressing cells produced **invasive** adenocarcinomas. Tissue microarray analysis of human **invasive** breast cancer indicated that PDK1pSer241 was strongly expressed in 90% of samples.These results indicate that PDK1 serves as an important effector of mammary epithelial cell growth and invasion in the transformed phenotype. PDK1 mediates its effect in part by MT1-MMP induction, which in turn activates MMP-2 and modulates the ECM proteins decorin and collagen. The presence of increased PDK1 expression in the majority of **invasive** breast cancers suggests its importance in the metastatic process.
8. Posttranslational regulation of membrane type 1-matrix metalloproteinase (MT1-MMP) in mouse PTEN null prostate cancer cells: Enhanced surface expression and differential O-glycosylation of MT1-MMP. Membrane type 1 (MT1)-matrix metalloproteinase (MT1-MMP) is a membrane-tethered MMP that has been shown to play a key role in promoting cancer cell invasion. MT1-MMP is highly expressed in bone metastasis of prostate cancer (PC) patients and promotes intraosseous tumor growth of PC cells in mice. The majority of metastatic prostate cancers harbor loss-of-function mutations or deletions of the tumor suppressor PTEN (phosphatase and tensin homologue deleted on chromosome ten). However, the role of PTEN inactivation in MT1-MMP expression in PC cells has not been examined. In this study, prostate epithelial cell lines derived from mice that are either heterozygous (PTEN(+/-)) or homozygous (PTEN(-/-)) for PTEN deletion or harboring a wild-type PTEN (PTEN(+/+)) were used to investigate the expression of MT1-MMP. We found that biallelic loss of PTEN is associated with posttranslational regulation of MT1-MMP protein in mouse PC cells. PTEN(-/-) PC cells display higher levels of MT1-MMP at the cell surface when compared to PTEN(+/+) and PTEN(+/-) cells and consequently exhibited enhanced migratory and collagen-**invasive** activities. MT1-MMP displayed by PTEN(-/-) cells is differentially O-glycosylated and exhibits a slow rate of turnover. MT1-MMP expression in PTEN(-/-) cells is under control of the PI3K/AKT signaling pathway, as determined using pharmacological inhibitors. Interestingly, rapamycin, an mTOR inhibitor, upregulates MT1-MMP expression in PTEN(+/+) cells via PI3K activity. Collectively, these data in a mouse prostate cell system uncover for the first time a novel and complex relationship between PTEN loss-mediated PI3K/AKT activation and posttranslational regulation of MT1-MMP, which may play a role in PC progression.
9. MT1-MMP-dependent neovessel formation within the confines of the three-dimensional extracellular matrix. During angiogenesis, endothelial cells initiate a tissue-**invasive** program within an interstitial matrix comprised largely of type I collagen. Extracellular matrix-degradative enzymes, including the matrix metalloproteinases (MMPs) MMP-2 and MMP-9, are thought to play key roles in angiogenesis by binding to docking sites on the cell surface after activation by plasmin- and/or membrane-type (MT) 1-MMP-dependent processes. To identify proteinases critical to neovessel formation, an ex vivo model of angiogenesis has been established wherein tissue explants from gene-targeted mice are embedded within a three-dimensional, type I collagen matrix. Unexpectedly, neither MMP-2, MMP-9, their cognate cell-surface receptors (i.e., beta3 integrin and CD44), nor plasminogen are essential for collagenolytic activity, endothelial cell invasion, or neovessel formation. Instead, the membrane-anchored MMP, MT1-MMP, confers endothelial cells with the ability to express **invasive** and tubulogenic activity in a collagen-rich milieu, in vitro or in vivo, where it plays an indispensable role in driving neovessel formation.
10. MT-MMP, the cell surface activator of proMMP-2 (pro-gelatinase A), is expressed with its substrate in mouse tissue during embryogenesis. Matrix metalloproteinases (MMPs), which degrade the components of the extracellular matrix, are key enzymes involved in the tissue remodeling of multicellular organisms. Since MMPs are secreted as inactive zymogens (pro-MMPs), they have to be activated to function. We identified a membrane-type MMP (MT-MMP) that activated proMMP-2 (pro-gelatinase A = 72 kDa type IV pro-collagenase) and described its expression on the **invasive** tumor cell surface. In this study we further examined the expression and role of MT-MMP in the activation of proMMP-2 during mouse embryogenesis. Northern blotting demonstrated that MT-MMP expression was increased together with that of MMP-2 and its inhibitor gene, TIMP-2, in embryos depending upon the number of days after gestation, and decreased with maturation after birth. In situ hybridization and immunohistochemistry localized MT-MMP mRNA and protein in the cells of ossifying tissues where both MMP-2 and TIMP-2 were expressed. Activated MMP-2 was detected by gelatin zymography in the lysates prepared from the micro dissected tissues that expressed the three genes. The activation rate of proMMP-2 was proportional to the expression of MMP-2 and MT-MMP. These results indicated that proMMP-2 activation through its activator, MT-MMP, is a physiological system used by organisms to initiate tissue remodeling on the cell surface.
11. Cytoplasmic tail of MT1-MMP regulates macrophage motility independently from its protease activity. Membrane type-1 matrix metalloproteinase (MT1-MMP) is a pro**invasive** protease that regulates various cellular functions as evidenced by myriad defects in different types of cells and tissues in MT1-MMP-deficient (MT1(-/-)) mice. Here we demonstrate that MT1(-/-) mice exhibit fewer infiltrating macrophages into sites of inflammation. MT1(-/-)macrophages exhibited a reduced ability to invade reconstituted basement membrane (Matrigel) and invasion by wild type (WT) macrophages was inhibited by a synthetic MMP inhibitor (BB94) to a level similar to that of MT1(-/-) cells. The rate of migration of MT1(-/-) macrophages was also low compared to that of the WT cells and re-expression of MT1-MMP in MT1(-/-) macrophages reconstituted their migratory activity. Unexpectedly, however, BB94 did not inhibit the migration of WT macrophages. The migration-boosting activity of MT1-MMP is retained in a mutant that lacks most of the extracellular portion including the catalytic and hemopexin-like domains. In contrast, deletion of the cytoplasmic (CP) tail abolished the activity completely. Thus, we have demonstrated that MT1-MMP regulates macrophages via its invasion-promoting protease activity as well as its CP-dependent non-proteolytic activity to boost cell migration.
12. Extracellular collagenases and the endocytic receptor, urokinase plasminogen activator receptor-associated protein/Endo180, cooperate in fibroblast-mediated collagen degradation. The collagens of the extracellular matrix are the most abundant structural proteins in the mammalian body. In tissue remodeling and in the **invasive** growth of malignant tumors, collagens constitute an important barrier, and consequently, the turnover of collagen is a rate-limiting process in these events. A recently discovered turnover route with importance for tumor growth involves intracellular collagen degradation and is governed by the collagen receptor, urokinase plasminogen activator receptor-associated protein (uPARAP or Endo180). The interplay between this mechanism and extracellular collagenolysis is not known. In this report, we demonstrate the existence of a new, composite collagen breakdown pathway. Thus, fibroblast-mediated collagen degradation proceeds preferentially as a sequential mechanism in which extracellular collagenolysis is followed by uPARAP/Endo180-mediated endocytosis of large collagen fragments. First, we show that collagen that has been pre-cleaved by a mammalian collagenase is taken up much more efficiently than intact, native collagen by uPARAP/Endo180-positive cells. Second, we demonstrate that this preference is governed by the acquisition of a gelatin-like structure by the collagen, occurring upon collagenase-mediated cleavage under native conditions. Third, we demonstrate that the growth of uPARAP/Endo180-deficient fibroblasts on a native collagen matrix leads to substantial extracellular accumulation of well defined collagen fragments, whereas, wild-type fibroblasts possess the ability to direct an organized and complete degradation sequence comprising both the initial cleavage, the endocytic uptake, and the intracellular breakdown of collagen.
13. MicroRNA-24 regulates macrophage behavior and retards atherosclerosis. Our recent studies have highlighted membrane type-1 matrix metalloproteinase (MMP)-14 as a selective marker for an **invasive** subset of macrophages potentially related to atherosclerotic plaque progression. Moreover, colony stimulating factors (CSF) may exert divergent effects on macrophage MMP expression, possibly through microRNAs. We, therefore, aim to identify and test the pathophysiological role of microRNAs, which modulate macrophage MMP-14 expression in atherosclerotic plaque progression.Compared with macrophage CSF-differentiated macrophages, granulocyte/macrophage CSF-matured macrophages exhibited reduced MMP-14 mRNA levels but increased protein expression and activity, which resulted in heightened macrophage invasion. MicroRNA-24, identified to target MMP-14, was accordingly increased in macrophage CSF compared with granulocyte/macrophage CSF macrophages. Silencing microRNA-24 in macrophage CSF macrophages significantly increased MMP-14 expression and enhanced their **invasive** capacity, mimicking granulocyte/macrophage CSF macrophages, and suggesting that granulocyte/macrophage CSF modulates MMP-14 protein expression and subsequent macrophage invasion in a microRNA-24-dependent manner. In human coronary atherosclerotic plaques, increased MMP-14 protein expression in foam cell macrophages was associated with lesions exhibiting histological characteristics associated with an unstable phenotype. Furthermore, microRNA-24 expression in these atherosclerotic plaques was inversely related to MMP-14 protein expression. Moreover, stable plaques contained higher microRNA-24 levels than unstable plaques, and microRNA-24 colocalized with foam cell macrophages that exhibited low MMP-14 protein expression. Finally, in atherosclerotic mice (apolipoprotein E-deficient), microRNA-24 inhibition increased plaque size and macrophage MMP-14 expression.Taken together, our data demonstrates that downregulation of microRNA-24 promotes an **invasive** macrophage subset and plays a novel regulatory role in MMP-14 proteolytic activity and, therefore, plaque stability, highlighting its therapeutic potential.
14. MT1-MMP regulates the PI3Kδ·Mi-2/NuRD-dependent control of macrophage immune function. Macrophages play critical roles in events ranging from host defense to obesity and cancer, where they infiltrate affected tissues and orchestrate immune responses in tandem with the remodeling of the extracellular matrix (ECM). Despite the dual roles played by macrophages in inflammation, the functions of macrophage-derived proteinases are typically relegated to tissu**e-invasi**ve or -degradative events. Here we report that the membrane-tethered matrix metalloenzyme MT1-MMP not only serves as an ECM-directed proteinase, but unexpectedly controls inflammatory gene responses wherein MT1-MMP(-/-) macrophages mount exaggerated chemokine and cytokine responses to immune stimuli both in vitro and in vivo. MT1-MMP modulates inflammatory responses in a protease-independent fashion in tandem with its trafficking to the nuclear compartment, where it triggers the expression and activation of a phosphoinositide 3-kinase δ (PI3Kδ)/Akt/GSK3β signaling cascade. In turn, MT1-MMP-dependent PI3Kδ activation regulates the immunoregulatory Mi-2/NuRD nucleosome remodeling complex that is responsible for controlling macrophage immune response. These findings identify a novel role for nuclear MT1-MMP as a previously unsuspected transactivator of signaling networks central to macrophage immune responses.
15. The metalloproteinase MT1-MMP is required for normal development and maintenance of osteocyte processes in bone. The osteocyte is the terminally differentiated state of the osteogenic mesenchymal progenitor immobilized in the bone matrix. Despite their numerical prominence, little is known about osteocytes and their formation. Osteocytes are physically separated in the bone matrix but seemingly compensate for their seclusion from other cells by maintaining an elaborate network of cell processes through which they interact with other osteocytes and bone-lining cells at the periosteal and endosteal surfaces of the bone. This highly organized architecture suggests that osteocytes make an active contribution to the structure and maintenance of their environment rather than passively submitting to random embedding during bone growth or repair. The most abundant matrix protein in the osteocyte environment is type-I collagen and we demonstrate here that, in the mouse, osteocyte phenotype and the formation of osteocyte processes is highly dependent on continuous cleavage of type-I collagen. This collagenolytic activity and formation of osteocyte processes is dependent on matrix metalloproteinase activity. Specifically, a deficiency of membrane type-1 matrix metalloproteinase leads to disruption of collagen cleavage in osteocytes and ultimately to the loss of formation of osteocyte processes. Osteocytogenesis is thus an active **invasive** process requiring cleavage of collagen for maintenance of the osteocyte phenotype.
16. Induction of a MT1-MMP and MT2-MMP-dependent basement membrane transmigration program in cancer cells by Snail1. The ability of carcinoma cells arising at primary sites to cross their underlying basement membrane (BM), a specialized form of extracellular matrix that subtends all epithelial cells, and to access the host vasculature are central features of the malignant phenotype. The initiation of the **invasive** phenotype has been linked to the aberrant expression of zinc-finger transcriptional repressors, like Snail1, which act by triggering an epithelial-mesenchymal cell-like transformation (EMT-like) via the regulation of largely undefined, downstream effectors. Herein, we find that Snail1 induces cancer cells to (i) degrade and perforate BM barriers, (ii) initiate angiogenesis, and (iii) and intravasate vascular networks in vivo via a matrix metalloproteinase (MMP)-dependent process. Unexpectedly, the complete Snail1 invasion program can be recapitulated by expressing directly either of the membrane-anchored metalloproteinases, MT1-MMP or MT2-MMP. The pro-**invasive**, angiogenic, and metastatic activities of MT1-MMP and MT2-MMP are unique relative to all other metalloproteinase family members and cannot be mimicked in vivo by the secreted MMPs, MMP-1, MMP-2, MMP-3, MMP-7, MMP-9, or MMP-13. Further, siRNA-specific silencing of MT1-MMP and MT2-MMP ablates completely the ability of Snail1 to drive cancer cell BM invasion, induce angiogenesis, or trigger intravasation. Taken together, these data demonstrate that MT1-MMP and MT2-MMP cooperatively function as direct-acting, pro-**invasive** factors that confer Snail1-triggered cells with the key activities most frequently linked to morbidity and mortality in cancer.
17. Tumor cell traffic through the extracellular matrix is controlled by the membrane-anchored collagenase MT1-MMP. As cancer cells traverse collagen-rich extracellular matrix (ECM) barriers and intravasate, they adopt a fibroblast-like phenotype and engage undefined proteolytic cascades that mediate **invasive** activity. Herein, we find that fibroblasts and cancer cells express an indistinguishable pericellular collagenolytic activity that allows them to traverse the ECM. Using fibroblasts isolated from gene-targeted mice, a matrix metalloproteinase (MMP)-dependent activity is identified that drives invasion independently of plasminogen, the gelatinase A/TIMP-2 axis, gelatinase B, collagenase-3, collagenase-2, or stromelysin-1. In contrast, deleting or suppressing expression of the membrane-tethered MMP, MT1-MMP, in fibroblasts or tumor cells results in a loss of collagenolytic and **invasive** activity in vitro or in vivo. Thus, MT1-MMP serves as the major cell-associated proteinase necessary to confer normal or neoplastic cells with **invasive** activity.
18. Ovarian cancer cell detachment and multicellular aggregate formation are regulated by membrane type 1 matrix metalloproteinase: a potential role in I.p. metastatic dissemination. An early event in the metastasis of epithelial ovarian carcinoma is shedding of cells from the primary tumor into the peritoneal cavity followed by diffuse i.p. seeding of secondary lesions. Anchorage-independent metastatic cells are present as both single cells and multicellular aggregates (MCA), the latter of which adhere to and disaggregate on human mesothelial cell monolayers, subsequently forming **invasive** foci. Although this unique metastatic mechanism presents a distinct set of therapeutic challenges, factors that regulate MCA formation and dissemination have not been extensively evaluated. Proteolytic activity is important at multiple stages in i.p. metastasis, catalyzing migration through the mesothelial monolayer and invasion of the collagen-rich submesothelial matrix to anchor secondary lesions, and acquisition of membrane type 1 matrix metalloproteinase (MT1-MMP; MMP-14) expression promotes a collagen-**invasive** phenotype in ovarian carcinoma. MT1-MMP is regulated posttranslationally through multiple mechanisms including phosphorylation of its cytoplasmic tail, and the current data using ovarian cancer cells expressing wild-type, phosphomimetic (T567E-MT1-MMP), and phosphodefective (T567A-MT1-MMP) MT1-MMP show that MT1-MMP promotes MCA formation. Confluent T567E-MT1-MMP-expressing cells exhibit rapid detachment kinetics, spontaneous release as cell-cell adherent sheets concomitant with MT1-MMP-catalyzed alpha(3) integrin ectodomain shedding, and robust MCA formation. Expansive growth within three-dimensional collagen gels is also MT1-MMP dependent, with T567E-MT1-MMP-expressing cells exhibiting multiple collagen **invasive** foci. Analysis of human ovarian tumors shows elevated MT1-MMP in metastases relative to paired primary tumors. These data suggest that MT1-MMP activity may be key to ovarian carcinoma metastatic success by promoting both formation and dissemination of MCAs.
19. Matrix metalloproteinases (MMPs) regulate fibrin-**invasive** activity via MT1-MMP-dependent and -independent processes. Cross-linked fibrin is deposited in tissues surrounding wounds, inflammatory sites, or tumors and serves not only as a supporting substratum for trafficking cells, but also as a structural barrier to invasion. While the plasminogen activator-plasminogen axis provides cells with a powerful fibrinolytic system, plasminogen-deleted animals use alternate proteolytic processes that allow fibrin invasion to proceed normally. Using fibroblasts recovered from wild-type or gene-deleted mice, invasion of three-dimensional fibrin gels proceeded in a matrix metalloproteinase (MMP)-dependent fashion. Consistent with earlier studies supporting a singular role for the membrane-anchored MMP, MT1-MMP, in fibrin-**invasive** events, fibroblasts from MT1-MMP-null mice displayed an early defect in invasion. However, MT1-MMP-deleted fibroblasts circumvented this early deficiency and exhibited compensatory fibrin-**invasive** activity. The MT1-MMP-independent process was sensitive to MMP inhibitors that target membrane-anchored MMPs, and further studies identified MT2-MMP and MT3-MMP, but not MT4-MMP, as alternate pro-**invasive** factors. Given the widespread distribution of MT1-, 2-, and 3-MMP in normal and neoplastic cells, these data identify a subset of membrane-anchored MMPs that operate in an autonomous fashion to drive fibrin-**invasive** activity.
20. A matrix metalloproteinase expressed on the surface of **invasive** tumour cells. Gelatinase A (type-IV collagenase; M(r) 72,000) is produced by tumour stroma cells and is believed to be crucial for their invasion and metastasis, acting by degrading extracellular matrix macro-molecules such as type IV collagen. An inactive precursor of gelatinase A (pro-gelatinase A) is secreted and activated in **invasive** tumour tissue as a result of proteolysis which is mediated by a fraction of tumour cell membrane that is sensitive to metalloproteinase inhibitors. Here we report the cloning of the complementary DNA encoding a new matrix metalloproteinase with a potential transmembrane domain. Expression of the gene product on the cell surface induces specific activation of pro-gelatinase A in vitro and enhances cellular invasion of the reconstituted basement membrane. Tumour cells of **invasive** lung carcinomas, which contain activated forms of gelatinase A, were found to express the transcript and the gene product. The new metalloproteinase may thus trigger invasion by tumour cells by activating pro-gelatinase A on the tumour cell surface.

#### GeneRIFs

1. of the transcripts of tissue inhibitors of matrix metalloproteinases (TIMPs) showed that expression of both TIMP1 and TIMP2 correlates negatively with the **invasive** potential of cells.
2. MT1-MMP confers endothelial cells with the ability to express **invasive** and tubulogenic activity in a collagen-rich milieu, in vitro or in vivo, where it plays an indispensable role in driving neovessel formation.
3. MT1-MMP serves as the major cell-associated proteinase necessary to confer normal or neoplastic cells with **invasive** activity.
4. Matrix metalloproteinases (MMPs) regulate fibrin-**invasive** activity via MT1-MMP-dependent and -independent processes.

### [Mm]etastasize

#### PubMed

1. MT1-MMP is required for efficient tumor dissemination in experimental metastatic disease. Membrane-type I matrix metalloproteinase (MT1-MMP) is associated with multiple forms of cancer including mammary cancer. To directly evaluate the significance of MT1-MMP expression in tumor progression and metastasis using a genetically induced cancer model, we crossed MT1-MMP-deficient mice to MMTV-polyoma virus middle T-antigen (PyMT) mice. Expression of PyMT in the MT1-MMP-deficient background consistently resulted in hyperplasia of the mammary gland as seen in wild-type PyMT littermates. Following orthotopic transplantation of PyMT+ glands into the cleared mammary fat pad of syngeneic recipient mice, MT1-MMP-deficient tumors were palpable earlier than wild-type tumors. Moreover, MT1-MMP-deficient tumors grew to the experimental end point size quicker than control tumors, but demonstrated markedly reduced ability to **metastasize** to the lungs of recipient mice. Accordingly, MT1-MMP-deficient mice displayed an overall reduction in metastasis count of 50%. MT1-MMP was expressed solely in the stroma of PyMT-induced tumors and those metastatic nodules that formed in the lungs were devoid of MT1-MMP expression. Stromal fibroblasts isolated from MT1-MMP-deficient tumors did not degrade type I collagen suggesting that efficient dissemination of tumor cells is dependent on stromal cell remodeling of the tumor environment. The data demonstrate directly that MT1-MMP-mediated proteolysis by stromal cells is important in the metastatic process.

### [Mm]etastasis

#### PubMed

1. Targeting a single function of the multifunctional matrix metalloprotease MT1-MMP: impact on lymphangiogenesis. The group of matrix metalloproteases (MMPs) is responsible for multiple processes of extracellular matrix remodeling in the healthy body but also for matrix and tissue destruction during cancer invasion and **metastasis**. The understanding of the contributions from each individual MMP, both in healthy and pathological events, has been complicated by the lack of specific inhibitors and the fact that some of the potent MMPs are multifunctional enzymes. These factors have also hampered the setup of therapeutic strategies targeting MMP activity. A tempting target is the membrane-associated MT1-MMP, which has well-documented importance in matrix degradation but which takes part in more than one pathway in this regard. In this report, we describe the selective targeting of a single function of this enzyme by means of a specific monoclonal antibody against MT1-MMP, raised in an MT1-MMP knock-out mouse. The antibody blocks the enzyme ability to activate proMMP-2 without interfering with the collagenolytic function or the general proteolytic activity of MT1-MMP. Using this antibody, we have shown that the MT1-MMP-catalyzed activation of proMMP-2 is involved in the outgrowth of cultured lymphatic endothelial cells in a collagen matrix in vitro, as well as in lymphatic vessel sprouting assayed ex vivo. This is the first example of the complete inactivation of a single function of a multifunctional MMP and the use of this strategy to pursue its role.
2. MT1-MMP is required for efficient tumor dissemination in experimental metastatic disease. Membrane-type I matrix metalloproteinase (MT1-MMP) is associated with multiple forms of cancer including mammary cancer. To directly evaluate the significance of MT1-MMP expression in tumor progression and **metastasis** using a genetically induced cancer model, we crossed MT1-MMP-deficient mice to MMTV-polyoma virus middle T-antigen (PyMT) mice. Expression of PyMT in the MT1-MMP-deficient background consistently resulted in hyperplasia of the mammary gland as seen in wild-type PyMT littermates. Following orthotopic transplantation of PyMT+ glands into the cleared mammary fat pad of syngeneic recipient mice, MT1-MMP-deficient tumors were palpable earlier than wild-type tumors. Moreover, MT1-MMP-deficient tumors grew to the experimental end point size quicker than control tumors, but demonstrated markedly reduced ability to metastasize to the lungs of recipient mice. Accordingly, MT1-MMP-deficient mice displayed an overall reduction in **metastasis** count of 50%. MT1-MMP was expressed solely in the stroma of PyMT-induced tumors and those metastatic nodules that formed in the lungs were devoid of MT1-MMP expression. Stromal fibroblasts isolated from MT1-MMP-deficient tumors did not degrade type I collagen suggesting that efficient dissemination of tumor cells is dependent on stromal cell remodeling of the tumor environment. The data demonstrate directly that MT1-MMP-mediated proteolysis by stromal cells is important in the metastatic process.
3. MMP-2 null mice exhibit an early onset and severe experimental autoimmune encephalomyelitis due to an increase in MMP-9 expression and activity. Matrix metalloproteinase-2 (MMP-2; gelatinase A) is known to degrade a broad range of extracellular matrix components and chemokines, and has important roles in the processes of cell migration, invasion, and involution during development, as well as during tumor growth and **metastasis** and in inflammation and repair. To better elucidate the roles of this matrix metalloproteinase in the development and progression of experimental autoimmune encephalomyelitis, we used MMP-2-deficient (KO) mice. Surprisingly, we found that MMP-2 KO mice exhibited an earlier onset and more severe disease than did their wild-type (WT) counterparts. WT mice engrafted with MMP-2 KO bone marrow exhibited a similar earlier onset and more severe clinical disease score than WT mice engrafted with WT bone marrow. Lymphocytes derived from MMP-2 KO mice exhibited increased transmigration through endothelial cell monolayers as well as through collagen type IV and laminin-coated BD BIOCOAT inserts, which correlated with a 3-fold increase in expression of MMP-9 and was abrogated by inhibition of MMP activity. We demonstrated a correlation between expression levels of MMP-9 and MT1-MMP expression and suggest a signaling pathway involving tethering of MMP-2 to MT1-MMP as a modulator of MMP-9 expression. Last, we discuss other possible MMP-2-mediated mechanisms which may contribute to the observed phenotype.
4. 3-phosphoinositide-dependent protein kinase-1 (PDK1) promotes invasion and activation of matrix metalloproteinases. **Metastasis** is a major cause of morbidity and mortality in breast cancer with tumor cell invasion playing a crucial role in the metastatic process. PDK1 is a key molecule that couples PI3K to cell proliferation and survival signals in response to growth factor receptor activation, and is oncogenic when expressed in mouse mammary epithelial cells. We now present evidence showing that PDK1-expressing cells exhibit enhanced anchorage-dependent and -independent cell growth and are highly invasive when grown on Matrigel. These properties correlate with induction of MMP-2 activity, increased MT1-MMP expression and a unique gene expression profile.Invasion assays in Matrigel, MMP-2 zymogram analysis, gene microarray analysis and mammary isografts were used to characterize the invasive and proliferative function of cells expressing PDK1. Tissue microarray analysis of human breast cancers was used to measure PDK1 expression in invasive tumors by IHC.Enhanced invasion on Matrigel in PDK1-expressing cells was accompanied by increased MMP-2 activity resulting from stabilization against proteasomal degradation. Increased MMP-2 activity was accompanied by elevated levels of MT1-MMP, which is involved in generating active MMP-2. Gene microarray analysis identified increased expression of the ECM-associated genes decorin and type I procollagen, whose gene products are substrates of MT1-MMP. Mammary fat pad isografts of PDK1-expressing cells produced invasive adenocarcinomas. Tissue microarray analysis of human invasive breast cancer indicated that PDK1pSer241 was strongly expressed in 90% of samples.These results indicate that PDK1 serves as an important effector of mammary epithelial cell growth and invasion in the transformed phenotype. PDK1 mediates its effect in part by MT1-MMP induction, which in turn activates MMP-2 and modulates the ECM proteins decorin and collagen. The presence of increased PDK1 expression in the majority of invasive breast cancers suggests its importance in the metastatic process.
5. The membrane-anchored MMP inhibitor RECK is a key regulator of extracellular matrix integrity and angiogenesis. Matrix metalloproteinases (MMPs) are essential for proper extracellular matrix remodeling. We previously found that a membrane-anchored glycoprotein, RECK, negatively regulates MMP-9 and inhibits tumor invasion and **metastasis**. Here we show that RECK regulates two other MMPs, MMP-2 and MT1-MMP, known to be involved in cancer progression, that mice lacking a functional RECK gene die around E10.5 with defects in collagen fibrils, the basal lamina, and vascular development, and that this phenotype is partially suppressed by MMP-2 null mutation. Also, vascular sprouting is dramatically suppressed in tumors derived from RECK-expressing fibrosarcoma cells grown in nude mice. These results support a role for RECK in the regulation of MMP-2 in vivo and implicate RECK downregulation in tumor angiogenesis.
6. Posttranslational regulation of membrane type 1-matrix metalloproteinase (MT1-MMP) in mouse PTEN null prostate cancer cells: Enhanced surface expression and differential O-glycosylation of MT1-MMP. Membrane type 1 (MT1)-matrix metalloproteinase (MT1-MMP) is a membrane-tethered MMP that has been shown to play a key role in promoting cancer cell invasion. MT1-MMP is highly expressed in bone **metastasis** of prostate cancer (PC) patients and promotes intraosseous tumor growth of PC cells in mice. The majority of metastatic prostate cancers harbor loss-of-function mutations or deletions of the tumor suppressor PTEN (phosphatase and tensin homologue deleted on chromosome ten). However, the role of PTEN inactivation in MT1-MMP expression in PC cells has not been examined. In this study, prostate epithelial cell lines derived from mice that are either heterozygous (PTEN(+/-)) or homozygous (PTEN(-/-)) for PTEN deletion or harboring a wild-type PTEN (PTEN(+/+)) were used to investigate the expression of MT1-MMP. We found that biallelic loss of PTEN is associated with posttranslational regulation of MT1-MMP protein in mouse PC cells. PTEN(-/-) PC cells display higher levels of MT1-MMP at the cell surface when compared to PTEN(+/+) and PTEN(+/-) cells and consequently exhibited enhanced migratory and collagen-invasive activities. MT1-MMP displayed by PTEN(-/-) cells is differentially O-glycosylated and exhibits a slow rate of turnover. MT1-MMP expression in PTEN(-/-) cells is under control of the PI3K/AKT signaling pathway, as determined using pharmacological inhibitors. Interestingly, rapamycin, an mTOR inhibitor, upregulates MT1-MMP expression in PTEN(+/+) cells via PI3K activity. Collectively, these data in a mouse prostate cell system uncover for the first time a novel and complex relationship between PTEN loss-mediated PI3K/AKT activation and posttranslational regulation of MT1-MMP, which may play a role in PC progression.
7. Interference with the complement system by tumor cell membrane type-1 matrix metalloproteinase plays a significant role in promoting **metastasis** in mice. Neoplasms have developed strategies to protect themselves against the complement-mediated host immunity. Invasion- and **metastasis**-promoting membrane type-1 (MT1) matrix metalloproteinase (MMP) is strongly associated with many metastatic cancer types. The relative importance of the individual functions of MT1-MMP in **metastasis** was, however, unknown. We have now determined that the expression of murine MT1-MMP in murine melanoma B16F1 cells strongly increased the number of metastatic loci in the lungs of syngeneic C57BL/6 mice. In contrast, MT1-MMP did not affect the number of metastatic loci in complement-deficient C57BL/6-C3-/- mice. Our results indicated, for the first time, that the anticomplement activity of MT1-MMP played a significant role in promoting **metastasis** in vivo and determined the relative importance of the anticomplement activity in the total metastatic effect of this multifunctional proteolytic enzyme. We believe that our results shed additional light on the functions of MT1-MMP in cancer and clearly make this protease a promising drug target in metastatic malignancies.
8. Lumican effects in the control of tumour progression and their links with metalloproteinases and integrins. Lumican is a member of the small leucine-rich proteoglycan family. It is present in numerous extracellular matrices of different tissues, such as muscle, cartilage, and cornea. In skin, lumican is present as a glycoprotein. It plays a critical role in collagen fibrillogenesis, as shown by knocking out of its gene in mice. A direct link between lumican expression and melanoma progression and **metastasis** has been demonstrated. Lumican was shown to impede tumour cell migration and invasion by directly interacting with the α2β1 integrin. In addition, an active sequence of the lumican core protein, called lumcorin, was identified as being responsible for inhibition of melanoma cell migration. Lumican was also shown to exert angiostatic properties by downregulating the proteolytic activity associated with endothelial cell membranes, particularly matrix metalloproteinase (MMP)-14 and MMP-9. Globally, lumican appears to be a potent agent for inhibiting tumour progression rather than tumorigenesis. However, progressive changes in proteoglycans occur in the tumour environment. The complexity and diversity of proteoglycan structure might be responsible for a variety of functions that regulate cell behaviour. Through their core protein and their glycosaminoglycan chains, proteoglycans can interact with growth factors and chemokines. These interactions affect cell signalling, motility, adhesion, growth, and apoptosis. This review summarizes recent data concerning lumican control of tumour progression in different cancers, with a particular focus on its interactions with MMPs and integrins. Its potential therapeutic implications are discussed.
9. Matrix metalloproteinases in tumor invasion. Controlled degradation of extracellular matrix (ECM) is essential for the growth, invasion, and **metastasis** of malignant tumors, and for tumor-induced angiogenesis. Matrix metalloproteinases (MMPs) are a family of zinc-dependent neutral endopeptidases collectively capable of degrading essentially all ECM components and they apparently play an important role in all these aspects of tumor development. Furthermore, recent evidence suggests that MMPs also play a role in tumor cell survival. In this review, we discuss the current concept concerning the role of MMPs and their inhibitors in tumor invasion, as a basis for prognosis and targeted therapeutic intervention in cancer.
10. Ovarian cancer cell detachment and multicellular aggregate formation are regulated by membrane type 1 matrix metalloproteinase: a potential role in I.p. metastatic dissemination. An early event in the **metastasis** of epithelial ovarian carcinoma is shedding of cells from the primary tumor into the peritoneal cavity followed by diffuse i.p. seeding of secondary lesions. Anchorage-independent metastatic cells are present as both single cells and multicellular aggregates (MCA), the latter of which adhere to and disaggregate on human mesothelial cell monolayers, subsequently forming invasive foci. Although this unique metastatic mechanism presents a distinct set of therapeutic challenges, factors that regulate MCA formation and dissemination have not been extensively evaluated. Proteolytic activity is important at multiple stages in i.p. **metastasis**, catalyzing migration through the mesothelial monolayer and invasion of the collagen-rich submesothelial matrix to anchor secondary lesions, and acquisition of membrane type 1 matrix metalloproteinase (MT1-MMP; MMP-14) expression promotes a collagen-invasive phenotype in ovarian carcinoma. MT1-MMP is regulated posttranslationally through multiple mechanisms including phosphorylation of its cytoplasmic tail, and the current data using ovarian cancer cells expressing wild-type, phosphomimetic (T567E-MT1-MMP), and phosphodefective (T567A-MT1-MMP) MT1-MMP show that MT1-MMP promotes MCA formation. Confluent T567E-MT1-MMP-expressing cells exhibit rapid detachment kinetics, spontaneous release as cell-cell adherent sheets concomitant with MT1-MMP-catalyzed alpha(3) integrin ectodomain shedding, and robust MCA formation. Expansive growth within three-dimensional collagen gels is also MT1-MMP dependent, with T567E-MT1-MMP-expressing cells exhibiting multiple collagen invasive foci. Analysis of human ovarian tumors shows elevated MT1-MMP in metastases relative to paired primary tumors. These data suggest that MT1-MMP activity may be key to ovarian carcinoma metastatic success by promoting both formation and dissemination of MCAs.
11. MT1-MMP sheds LYVE-1 on lymphatic endothelial cells and suppresses VEGF-C production to inhibit lymphangiogenesis. Lymphangiogensis is involved in various pathological conditions, such as arthritis and cancer **metastasis**. Although many factors have been identified to stimulate lymphatic vessel growth, little is known about lymphangiogenesis inhibitors. Here we report that membrane type 1-matrix metalloproteinase (MT1-MMP) is an endogenous suppressor of lymphatic vessel growth. MT1-MMP-deficient mice exhibit spontaneous corneal lymphangiogenesis without concomitant changes in angiogenesis. Mice lacking MT1-MMP in either lymphatic endothelial cells or macrophages recapitulate corneal lymphangiogenic phenotypes observed in Mmp14(-/-) mice, suggesting that the spontaneous lymphangiogenesis is both lymphatic endothelial cells autonomous and macrophage associated. Mechanistically, MT1-MMP directly cleaves LYVE-1 on lymphatic endothelial cells to inhibit LYVE-1-mediated lymphangiogenic responses. In addition, MT1-MMP-mediated PI3Kδ signalling restrains the production of VEGF-C from prolymphangiogenic macrophages through repressing the activation of NF-κB signalling. Thus, we identify MT1-MMP as an endogenous inhibitor of physiological lymphangiogenesis.
12. A matrix metalloproteinase expressed on the surface of invasive tumour cells. Gelatinase A (type-IV collagenase; M(r) 72,000) is produced by tumour stroma cells and is believed to be crucial for their invasion and **metastasis**, acting by degrading extracellular matrix macro-molecules such as type IV collagen. An inactive precursor of gelatinase A (pro-gelatinase A) is secreted and activated in invasive tumour tissue as a result of proteolysis which is mediated by a fraction of tumour cell membrane that is sensitive to metalloproteinase inhibitors. Here we report the cloning of the complementary DNA encoding a new matrix metalloproteinase with a potential transmembrane domain. Expression of the gene product on the cell surface induces specific activation of pro-gelatinase A in vitro and enhances cellular invasion of the reconstituted basement membrane. Tumour cells of invasive lung carcinomas, which contain activated forms of gelatinase A, were found to express the transcript and the gene product. The new metalloproteinase may thus trigger invasion by tumour cells by activating pro-gelatinase A on the tumour cell surface.

### [Cc]ell [Ii]nvasion

#### PubMed

1. Transmembrane/cytoplasmic, rather than catalytic, domains of Mmp14 signal to MAPK activation and mammary branching morphogenesis via binding to integrin β1. Epithelial **cell invasio**n through the extracellular matrix (ECM) is a crucial step in branching morphogenesis. The mechanisms by which the mammary epithelium integrates cues from the ECM with intracellular signaling in order to coordinate invasion through the stroma to make the mammary tree are poorly understood. Because the cell membrane-bound matrix metalloproteinase Mmp14 is known to play a key role in cancer **cell invasio**n, we hypothesized that it could also be centrally involved in integrating signals for mammary epithelial cells (MECs) to navigate the collagen 1 (CL-1)-rich stroma of the mammary gland. Expression studies in nulliparous mice that carry a NLS-lacZ transgene downstream of the Mmp14 promoter revealed that Mmp14 is expressed in MECs at the tips of the branches. Using both mammary organoids and 3D organotypic cultures, we show that MMP activity is necessary for invasion through dense CL-1 (3 mg/ml) gels, but dispensable for MEC branching in sparse CL-1 (1 mg/ml) gels. Surprisingly, however, Mmp14 without its catalytic activity was still necessary for branching. Silencing Mmp14 prevented **cell invasio**n through CL-1 and disrupted branching altogether; it also reduced integrin β1 (Itgb1) levels and attenuated MAPK signaling, disrupting Itgb1-dependent invasion/branching within CL-1 gels. FRET imaging revealed that Mmp14 associates directly with Itgb1. We identified a domain of Mmp14 that is required for modulating the levels of Itgb1, MEC signaling and the rate of invasion within CL-1. These results shed light on hitherto undescribed non-proteolytic activities of Mmp14 that are necessary for the Itgb1-dependent biochemical and mechanical signals that regulate branching in the mammary epithelium.
2. Type 1 insulin-like growth factor regulates MT1-MMP synthesis and tumor invasion via PI 3-kinase/Akt signaling. The membrane type 1 matrix metalloproteinase (MT1-MMP) has been identified as a major activator of MMP-2 - a process involving the formation of a trimolecular complex with TIMP-2. We previously identified the IGF-I receptor as a positive regulator of MMP-2 synthesis. Here, we investigated the role of IGF-IR in the regulation of MT1-MMP. Highly invasive Lewis lung carcinoma subline H-59 cells express MT1-MMP and utilize it to activate their major extracellular matrix degrading proteinase-MMP-2. These cells were transiently transfected with a plasmid vector expressing a luciferase reporter gene downstream of the mouse MT1-MMP promoter. IGF-I treatment increased luciferase activity in the transfected cells by up to 10-fold and augmented endogenous MT1-MMP mRNA and protein synthesis by up to 2-3-fold, relative to controls. MT1-MMP induction and invasion were blocked by the PI 3-kinase inhibitors LY294002 and wortmannin and by rapamycin, but not by the MEK inhibitor PD98059. Overexpression of a dominant negative Akt mutant or of the tumor suppressor phosphatase and tensin homologue, PTEN, in these cells also caused a significant reduction in MT1-MMP expression and invasion. The results demonstrate that IGF-IR controls tumor **cell invasion** by coordinately regulating MMP-2 expression and its MT1-MMP-mediated activation and identify PI 3-kinase/Akt/mTOR signaling as critical to this regulation.
3. Transmembrane/cytoplasmic, rather than catalytic, domains of Mmp14 signal to MAPK activation and mammary branching morphogenesis via binding to integrin β1. Epithelial **cell invasio**n through the extracellular matrix (ECM) is a crucial step in branching morphogenesis. The mechanisms by which the mammary epithelium integrates cues from the ECM with intracellular signaling in order to coordinate invasion through the stroma to make the mammary tree are poorly understood. Because the cell membrane-bound matrix metalloproteinase Mmp14 is known to play a key role in cancer **cell invasio**n, we hypothesized that it could also be centrally involved in integrating signals for mammary epithelial cells (MECs) to navigate the collagen 1 (CL-1)-rich stroma of the mammary gland. Expression studies in nulliparous mice that carry a NLS-lacZ transgene downstream of the Mmp14 promoter revealed that Mmp14 is expressed in MECs at the tips of the branches. Using both mammary organoids and 3D organotypic cultures, we show that MMP activity is necessary for invasion through dense CL-1 (3 mg/ml) gels, but dispensable for MEC branching in sparse CL-1 (1 mg/ml) gels. Surprisingly, however, Mmp14 without its catalytic activity was still necessary for branching. Silencing Mmp14 prevented **cell invasio**n through CL-1 and disrupted branching altogether; it also reduced integrin β1 (Itgb1) levels and attenuated MAPK signaling, disrupting Itgb1-dependent invasion/branching within CL-1 gels. FRET imaging revealed that Mmp14 associates directly with Itgb1. We identified a domain of Mmp14 that is required for modulating the levels of Itgb1, MEC signaling and the rate of invasion within CL-1. These results shed light on hitherto undescribed non-proteolytic activities of Mmp14 that are necessary for the Itgb1-dependent biochemical and mechanical signals that regulate branching in the mammary epithelium.
4. Type 1 insulin-like growth factor regulates MT1-MMP synthesis and tumor invasion via PI 3-kinase/Akt signaling. The membrane type 1 matrix metalloproteinase (MT1-MMP) has been identified as a major activator of MMP-2 - a process involving the formation of a trimolecular complex with TIMP-2. We previously identified the IGF-I receptor as a positive regulator of MMP-2 synthesis. Here, we investigated the role of IGF-IR in the regulation of MT1-MMP. Highly invasive Lewis lung carcinoma subline H-59 cells express MT1-MMP and utilize it to activate their major extracellular matrix degrading proteinase-MMP-2. These cells were transiently transfected with a plasmid vector expressing a luciferase reporter gene downstream of the mouse MT1-MMP promoter. IGF-I treatment increased luciferase activity in the transfected cells by up to 10-fold and augmented endogenous MT1-MMP mRNA and protein synthesis by up to 2-3-fold, relative to controls. MT1-MMP induction and invasion were blocked by the PI 3-kinase inhibitors LY294002 and wortmannin and by rapamycin, but not by the MEK inhibitor PD98059. Overexpression of a dominant negative Akt mutant or of the tumor suppressor phosphatase and tensin homologue, PTEN, in these cells also caused a significant reduction in MT1-MMP expression and invasion. The results demonstrate that IGF-IR controls tumor **cell invasion** by coordinately regulating MMP-2 expression and its MT1-MMP-mediated activation and identify PI 3-kinase/Akt/mTOR signaling as critical to this regulation.
5. 3-phosphoinositide-dependent protein kinase-1 (PDK1) promotes invasion and activation of matrix metalloproteinases. Metastasis is a major cause of morbidity and mortality in breast cancer with tumor **cell invasion** playing a crucial role in the metastatic process. PDK1 is a key molecule that couples PI3K to cell proliferation and survival signals in response to growth factor receptor activation, and is oncogenic when expressed in mouse mammary epithelial cells. We now present evidence showing that PDK1-expressing cells exhibit enhanced anchorage-dependent and -independent cell growth and are highly invasive when grown on Matrigel. These properties correlate with induction of MMP-2 activity, increased MT1-MMP expression and a unique gene expression profile.Invasion assays in Matrigel, MMP-2 zymogram analysis, gene microarray analysis and mammary isografts were used to characterize the invasive and proliferative function of cells expressing PDK1. Tissue microarray analysis of human breast cancers was used to measure PDK1 expression in invasive tumors by IHC.Enhanced invasion on Matrigel in PDK1-expressing cells was accompanied by increased MMP-2 activity resulting from stabilization against proteasomal degradation. Increased MMP-2 activity was accompanied by elevated levels of MT1-MMP, which is involved in generating active MMP-2. Gene microarray analysis identified increased expression of the ECM-associated genes decorin and type I procollagen, whose gene products are substrates of MT1-MMP. Mammary fat pad isografts of PDK1-expressing cells produced invasive adenocarcinomas. Tissue microarray analysis of human invasive breast cancer indicated that PDK1pSer241 was strongly expressed in 90% of samples.These results indicate that PDK1 serves as an important effector of mammary epithelial cell growth and invasion in the transformed phenotype. PDK1 mediates its effect in part by MT1-MMP induction, which in turn activates MMP-2 and modulates the ECM proteins decorin and collagen. The presence of increased PDK1 expression in the majority of invasive breast cancers suggests its importance in the metastatic process.
6. Posttranslational regulation of membrane type 1-matrix metalloproteinase (MT1-MMP) in mouse PTEN null prostate cancer cells: Enhanced surface expression and differential O-glycosylation of MT1-MMP. Membrane type 1 (MT1)-matrix metalloproteinase (MT1-MMP) is a membrane-tethered MMP that has been shown to play a key role in promoting cancer **cell invasion**. MT1-MMP is highly expressed in bone metastasis of prostate cancer (PC) patients and promotes intraosseous tumor growth of PC cells in mice. The majority of metastatic prostate cancers harbor loss-of-function mutations or deletions of the tumor suppressor PTEN (phosphatase and tensin homologue deleted on chromosome ten). However, the role of PTEN inactivation in MT1-MMP expression in PC cells has not been examined. In this study, prostate epithelial cell lines derived from mice that are either heterozygous (PTEN(+/-)) or homozygous (PTEN(-/-)) for PTEN deletion or harboring a wild-type PTEN (PTEN(+/+)) were used to investigate the expression of MT1-MMP. We found that biallelic loss of PTEN is associated with posttranslational regulation of MT1-MMP protein in mouse PC cells. PTEN(-/-) PC cells display higher levels of MT1-MMP at the cell surface when compared to PTEN(+/+) and PTEN(+/-) cells and consequently exhibited enhanced migratory and collagen-invasive activities. MT1-MMP displayed by PTEN(-/-) cells is differentially O-glycosylated and exhibits a slow rate of turnover. MT1-MMP expression in PTEN(-/-) cells is under control of the PI3K/AKT signaling pathway, as determined using pharmacological inhibitors. Interestingly, rapamycin, an mTOR inhibitor, upregulates MT1-MMP expression in PTEN(+/+) cells via PI3K activity. Collectively, these data in a mouse prostate cell system uncover for the first time a novel and complex relationship between PTEN loss-mediated PI3K/AKT activation and posttranslational regulation of MT1-MMP, which may play a role in PC progression.
7. MT1-MMP-dependent neovessel formation within the confines of the three-dimensional extracellular matrix. During angiogenesis, endothelial cells initiate a tissue-invasive program within an interstitial matrix comprised largely of type I collagen. Extracellular matrix-degradative enzymes, including the matrix metalloproteinases (MMPs) MMP-2 and MMP-9, are thought to play key roles in angiogenesis by binding to docking sites on the cell surface after activation by plasmin- and/or membrane-type (MT) 1-MMP-dependent processes. To identify proteinases critical to neovessel formation, an ex vivo model of angiogenesis has been established wherein tissue explants from gene-targeted mice are embedded within a three-dimensional, type I collagen matrix. Unexpectedly, neither MMP-2, MMP-9, their cognate cell-surface receptors (i.e., beta3 integrin and CD44), nor plasminogen are essential for collagenolytic activity, endothelial **cell invasion**, or neovessel formation. Instead, the membrane-anchored MMP, MT1-MMP, confers endothelial cells with the ability to express invasive and tubulogenic activity in a collagen-rich milieu, in vitro or in vivo, where it plays an indispensable role in driving neovessel formation.
8. Stage-specific secretion of HMGB1 in cartilage regulates endochondral ossification. High mobility group box 1 protein (HMGB1) is a chromatin protein that has a dual function as a nuclear factor and as an extracellular factor. Extracellular HMGB1 released by damaged cells acts as a chemoattractant, as well as a proinflammatory cytokine, suggesting that HMGB1 is tightly connected to the process of tissue organization. However, the role of HMGB1 in bone and cartilage that undergo remodeling during embryogenesis, tissue repair, and disease is largely unknown. We show here that the stage-specific secretion of HMGB1 in cartilage regulates endochondral ossification. We analyzed the skeletal development of Hmgb1(-/-) mice during embryogenesis and found that endochondral ossification is significantly impaired due to the delay of cartilage invasion by osteoclasts, osteoblasts, and blood vessels. Immunohistochemical analysis revealed that HMGB1 protein accumulated in the cytosol of hypertrophic chondrocytes at growth plates, and its extracellular release from the chondrocytes was verified by organ culture. Furthermore, we demonstrated that the chondrocyte-secreted HMGB1 functions as a chemoattractant for osteoclasts and osteoblasts, as well as for endothelial cells, further supporting the conclusion that Hmgb1(-/-) mice are defective in **cell invasion**. Collectively, these findings suggest that HMGB1 released from differentiating chondrocytes acts, at least in part, as a regulator of endochondral ossification during osteogenesis.
9. Functional roles of MMP14 and MMP15 in early postnatal mammary gland development. During late embryogenesis, mammary epithelial cells initiate migration programs that drive ductal invasion into the surrounding adipose-rich mesenchyme. Currently, branching morphogenesis is thought to depend on the mobilization of the membrane-anchored matrix metalloproteinases MMP14 (MT1-MMP) and MMP15 (MT2-MMP), which drive epithelial **cell invasion** by remodeling the extracellular matrix and triggering associated signaling cascades. However, the roles that these proteinases play during mammary gland development in vivo remain undefined. Here, we characterize the impact of global Mmp14 and Mmp15 targeting on early postnatal mammary gland development in mice. Unexpectedly, both Mmp14(-/-) and Mmp15(-/-) mammary glands retain the ability to generate intact ductal networks. Although neither proteinase is required for branching morphogenesis, transcriptome profiling reveals a key role for MMP14 and MMP15 in regulating mammary gland adipocyte differentiation. Whereas MMP14 promotes the generation of white fat depots crucial for energy storage, MMP15 differentially controls the formation of thermogenic brown fat. Taken together, these data not only indicate that current paradigms relevant to proteinase-dependent morphogenesis need be revisited, but also identify new roles for the enzymes in regulating adipocyte fate determination in the developing mammary gland.

#### GeneRIFs

1. IGF-IR controls tumor **cell invasion** by coordinately regulating MMP-2 expression and its MT1-MMP-mediated activation and identify PI 3-kinase/Akt/mTOR signaling as critical to this regulation.

---

## Mmp24

- NCBI Gene
- GeneRIF
- Pubmed

| Search Term | Rank | Fields |
| --- | --- | --- |
| [Ff]ilopodia | 1 | PubMed(1) |
| [Mm]igration | 6 | PubMed(3) |
| ECM | 7 | PubMed(3); GeneRIFs(1) |
| [Ee]xtracellular [Mm]atrix | 8 | PubMed(6); Component(2); Conserved Domains(1); Summary(1) |
| [Mm]etastasis | 12 | PubMed(1) |

  
hide/show details for Mmp24

### [Ff]ilopodia

#### PubMed

1. Membrane localization of membrane type 5 matrix metalloproteinase by AMPA receptor binding protein and cleavage of cadherins. Matrix metalloproteinases (MMPs) have been proposed to remodel the extracellular environment of neurons. Here, we report that the metalloproteinase membrane-type 5 MMP (MT5-MMP) binds to AMPA receptor binding protein (ABP) and GRIP (glutamate receptor interaction protein), two related postsynaptic density (PSD) PDZ (postsynaptic density-95/Discs large/zona occludens-1) domain proteins that target AMPA receptors to synapses. The MT5-MMP C terminus binds ABP PDZ5 and the two proteins coimmunoprecipitated and colocalized in heterologous cells and neurons. MT5-MMP localized in **filopodia** at the tips of growth cones in young [2-5 d in vitro (DIV)] cultured embryonic hippocampal neurons, and at synapses in mature (21 DIV) neurons. Its enrichment in synaptosomes also indicated a synaptic localization in the mature brain. Deletion of the PDZ binding site impaired membrane trafficking of MT5-MMP, whereas exogenous ABP splice forms that are associated either with the plasma membrane or with the cytosol, respectively, colocalized with MT5-MMP in synaptic spines or recruited MT5-MMP to intracellular compartments. We show that endogenous MT5-MMP is found in cultured neurons and brain lysates in a proenzyme form that is activated by furin and degraded by auto-proteolysis. We also identify cadherins as MT5-MMP substrates. These results suggest that ABP directs MT5-MMP proteolytic activity to growth cones and synaptic sites in neurons, where it may regulate axon pathfinding or synapse remodeling through proteolysis of cadherins or other ECM or cell adhesion molecules.

### [Mm]igration

#### PubMed

1. Matrix metalloproteinases 2 and 9 are dispensable for pancreatic islet formation and function in vivo. Pancreatic islet formation is a highly regulated process that is initiated at the end of gestation in rodents. Endocrine precursor cells first form within the epithelium of duct-like structures and then delaminate from the epithelium, migrate, and cluster during the early stages of islet formation. The molecular mechanisms that regulate endocrine cell **migration** and islet formation are not well understood. Cell culture studies suggest that matrix metalloproteinases (MMPs) 2 and 9 are required for islet formation. To address whether MMP2 and MMP9 function are essential for endocrine cell **migration** and islet formation in vivo, we analyzed pancreas development in MMP2/MMP9 double-deficient mice. Our results show that islet architecture and function are unperturbed in these knockout mice, demonstrating that both MMP2 and MMP9 functions are dispensable for islet formation and endocrine cell differentiation. Our studies also show that a number of other MMPs are expressed at the time islet formation is initiated. This observation suggests that other MMPs may substitute for MMP2 and MMP9 loss in pancreatic tissue. However, islet formation is unaffected in transgenic mice with modified tissue inhibitor of metalloproteinase-1 (TIMP1) levels, suggesting that MMP activity may contribute little to islet morphogenesis in vivo.
2. Targeted deletion of Sox10 by Wnt1-cre defects neuronal **migration** and projection in the mouse inner ear. Sensory nerves of the brainstem are mostly composed of placode-derived neurons, neural crest-derived neurons and neural crest-derived Schwann cells. This mixed origin of cells has made it difficult to dissect interdependence for fiber guidance. Inner ear-derived neurons are known to connect to the brain after delayed loss of Schwann cells in ErbB2 mutants. However, the ErbB2 mutant related alterations in the ear and the brain compound interpretation of the data. We present here a new model to evaluate exclusively the effect of Schwann cell loss on inner ear innervation. Conditional deletion of the neural crest specific transcription factor, Sox10, using the rhombic lip/neural crest specific Wnt1-cre driver spares Sox10 expression in the ear. We confirm that neural crest-derived cells provide a stop signal for migrating spiral ganglion neurons. In the absence of Schwann cells, spiral ganglion neurons migrate into the center of the cochlea and even out of the ear toward the brain. Spiral ganglion neuron afferent processes reach the organ of Corti, but many afferent fibers bypass the organ of Corti to enter the lateral wall of the cochlea. In contrast to this peripheral disorganization, the central projection to cochlear nuclei is normal. Compared to ErbB2 mutants, conditional Sox10 mutants have limited cell death in spiral ganglion neurons, indicating that the absence of Schwann cells alone contributes little to the embryonic survival of neurons. These data suggest that neural crest-derived cells are dispensable for all central and some peripheral targeting of inner ear neurons. However, Schwann cells provide a stop signal for migratory spiral ganglion neurons and facilitate proper targeting of the organ of Corti by spiral ganglion afferents.
3. Mint-3 regulates the retrieval of the internalized membrane-type matrix metalloproteinase, MT5-MMP, to the plasma membrane by binding to its carboxyl end motif EWV. Membrane type matrix metalloproteinases (MT-MMPs) play a critical role in promoting cell growth and **migration** within the extracellular matrix by trafficking to specialized areas. Here we show that the carboxyl EWV motif of MT5-MMP serves as a retrieval signal for internalized MT5-MMP by interacting with Mint-3, a protein with two type III PDZ domains. Deletion of the EWV signal impairs the recycling of MT5-MMP without affecting its internalization, leading to decreased activity on the cell surface. A yeast two-hybrid screening identified Mint-3 as the EWV-binding protein. Mint-3 stimulates MT5-MMP activity when expressed at low levels in an EWV-dependent fashion, but inhibits its activity at higher levels independent of the EWV motif. siRNA-mediated knockdown of endogenous Mint-3 decreased MT5-MMP activity. Furthermore, Mint-3 significantly increased the level of MT5-MMP on the cell surface without affecting its synthesis and internalization. Therefore, Mints may be the adaptor proteins that regulate the trafficking of MT-MMPs.

### ECM

#### PubMed

1. Matrix metalloproteinases in tumor invasion. Controlled degradation of extracellular matrix (**ECM**) is essential for the growth, invasion, and metastasis of malignant tumors, and for tumor-induced angiogenesis. Matrix metalloproteinases (MMPs) are a family of zinc-dependent neutral endopeptidases collectively capable of degrading essentially all **ECM** components and they apparently play an important role in all these aspects of tumor development. Furthermore, recent evidence suggests that MMPs also play a role in tumor cell survival. In this review, we discuss the current concept concerning the role of MMPs and their inhibitors in tumor invasion, as a basis for prognosis and targeted therapeutic intervention in cancer.
2. Membrane localization of membrane type 5 matrix metalloproteinase by AMPA receptor binding protein and cleavage of cadherins. Matrix metalloproteinases (MMPs) have been proposed to remodel the extracellular environment of neurons. Here, we report that the metalloproteinase membrane-type 5 MMP (MT5-MMP) binds to AMPA receptor binding protein (ABP) and GRIP (glutamate receptor interaction protein), two related postsynaptic density (PSD) PDZ (postsynaptic density-95/Discs large/zona occludens-1) domain proteins that target AMPA receptors to synapses. The MT5-MMP C terminus binds ABP PDZ5 and the two proteins coimmunoprecipitated and colocalized in heterologous cells and neurons. MT5-MMP localized in filopodia at the tips of growth cones in young [2-5 d in vitro (DIV)] cultured embryonic hippocampal neurons, and at synapses in mature (21 DIV) neurons. Its enrichment in synaptosomes also indicated a synaptic localization in the mature brain. Deletion of the PDZ binding site impaired membrane trafficking of MT5-MMP, whereas exogenous ABP splice forms that are associated either with the plasma membrane or with the cytosol, respectively, colocalized with MT5-MMP in synaptic spines or recruited MT5-MMP to intracellular compartments. We show that endogenous MT5-MMP is found in cultured neurons and brain lysates in a proenzyme form that is activated by furin and degraded by auto-proteolysis. We also identify cadherins as MT5-MMP substrates. These results suggest that ABP directs MT5-MMP proteolytic activity to growth cones and synaptic sites in neurons, where it may regulate axon pathfinding or synapse remodeling through proteolysis of cadherins or other **ECM** or cell adhesion molecules.
3. Expression, purification and characterization of recombinant mouse MT5-MMP protein products. We have recently identified the fifth member of the membrane-type matrix metalloproteinase subfamily, MT5-MMP/MMP24, which is expressed in a brain specific manner (Duanqing Pei (1999) J. Biol. Chem. 274, 8925-8932). To further characterize its enzymic properties, an expression construct was engineered to produce MT5-MMP as a soluble and active form by truncating its transmembrane domain. Stable expression cell lines were subsequently established from MDCK cells transfected with this construct. Unfortunately, purification of MT5-MMP from the culture media in large quantity proves to be difficult initially due to its rapid turnover via a mechanism which can be inhibited by a broad spectrum metalloproteinase inhibitor, BB94. Thus, BB94 was included in the cell culture medium and throughout the purification process except the final step of chromatography to protect MT5-MMP from destruction. Purified to homogeneity and free of the synthetic inhibitor, MT5-MMP can activate progelatinase A efficiently in a TIMP2 sensitive fashion. A preliminary screen for its potential substrates among extracellular matrix components identified the proteoglycans as the preferred substrates for MT5-MMP. Furthermore, it is determined that the stability of purified MT5-MMP is temperature dependent with rapid destruction at 37 degrees C, but being relatively stable at temperatures 4 degrees C or lower. These observations establish MT5-MMP as a proteoglycanase with a short half-life at body temperature, which may be critical for tightly controlled turnover of **ECM** components such as those in the brain.

#### GeneRIFs

1. These results suggest that ABP directs MT5-MMP proteolytic activity to growth cones and synaptic sites in neurons, where it may regulate axon pathfinding or synapse remodeling through proteolysis of cadherins or other **ECM** or cell adhesion molecules.

### [Ee]xtracellular [Mm]atrix

#### PubMed

1. Functional interactions between matrix metalloproteinases and glycosaminoglycans. Similar to most proteinases, matrix metalloproteinases (MMP) do not recognize a consensus cleavage site. Thus, it is not surprising that, in a defined in vitro reaction, most MMPs can act on a wide range of proteins, including many **extracellular matri**x proteins. However, the findings obtained from in vivo studies with genetic models have demonstrated that individual MMPs act on just a few extracellular protein substrates, typically not matrix proteins. The limited, precise functions of an MMP imply that mechanisms have evolved to control the specificity of proteinase:substrate interactions. We discuss the possibility that interactions with the glycosaminoglycan chains of proteoglycans may function as allosteric regulators or accessory factors directing MMP catalysis to specific substrates. We propose that understanding how the activity of specific MMPs is confined to discreet compartments and targeted to defined substrates via interactions with other macromolecules may provide a means of blocking potentially deleterious MMP-mediated processes at the same time as sparing any beneficial functions.
2. Matrix metalloproteinases in tumor invasion. Controlled degradation of **extracellular matrix** (ECM) is essential for the growth, invasion, and metastasis of malignant tumors, and for tumor-induced angiogenesis. Matrix metalloproteinases (MMPs) are a family of zinc-dependent neutral endopeptidases collectively capable of degrading essentially all ECM components and they apparently play an important role in all these aspects of tumor development. Furthermore, recent evidence suggests that MMPs also play a role in tumor cell survival. In this review, we discuss the current concept concerning the role of MMPs and their inhibitors in tumor invasion, as a basis for prognosis and targeted therapeutic intervention in cancer.
3. Absence of mechanical allodynia and Abeta-fiber sprouting after sciatic nerve injury in mice lacking membrane-type 5 matrix metalloproteinase. Matrix metalloproteinases (MMPs) are a family of endopeptidases that degrade **extracellular matrix** components. Membrane-type 5 MMP (MT5-MMP/MMP-24) was identified as neuron-specific, and is believed to contribute to neuronal circuit formation and plasticity. To elucidate its function in vivo, we have generated mice lacking MT5-MMP by gene targeting. MT5-MMP-deficient mice were born without obvious morphological abnormalities. No apparent histological defects were observed in the nervous system either. However, MT5-MMP-deficient mice did not develop neuropathic pain with mechanical allodynia after sciatic nerve injury, though responses to acute noxious stimuli were normal. Neuropathic pain induced by peripheral nerve lesions is known to accompany structural reorganization of the nervous system. Intraneural injection of cholera toxin B subunit, a transganglionic tracer, into the injured sciatic nerve of wild-type mice revealed that the myelinated Abeta-fiber primary afferents sprouted from laminae III-VI of the dorsal horn of the spinal cord and invaded lamina II. However, no such sprouting and invasion of Abeta-fibers were observed in MT5-MMP-deficient mice. These findings suggest that MT5-MMP is essential for the development of mechanical allodynia and plays an important role in neuronal plasticity in this mouse model.
4. Expression, purification and characterization of recombinant mouse MT5-MMP protein products. We have recently identified the fifth member of the membrane-type matrix metalloproteinase subfamily, MT5-MMP/MMP24, which is expressed in a brain specific manner (Duanqing Pei (1999) J. Biol. Chem. 274, 8925-8932). To further characterize its enzymic properties, an expression construct was engineered to produce MT5-MMP as a soluble and active form by truncating its transmembrane domain. Stable expression cell lines were subsequently established from MDCK cells transfected with this construct. Unfortunately, purification of MT5-MMP from the culture media in large quantity proves to be difficult initially due to its rapid turnover via a mechanism which can be inhibited by a broad spectrum metalloproteinase inhibitor, BB94. Thus, BB94 was included in the cell culture medium and throughout the purification process except the final step of chromatography to protect MT5-MMP from destruction. Purified to homogeneity and free of the synthetic inhibitor, MT5-MMP can activate progelatinase A efficiently in a TIMP2 sensitive fashion. A preliminary screen for its potential substrates among **extracellular matrix** components identified the proteoglycans as the preferred substrates for MT5-MMP. Furthermore, it is determined that the stability of purified MT5-MMP is temperature dependent with rapid destruction at 37 degrees C, but being relatively stable at temperatures 4 degrees C or lower. These observations establish MT5-MMP as a proteoglycanase with a short half-life at body temperature, which may be critical for tightly controlled turnover of ECM components such as those in the brain.
5. Identification and characterization of the fifth membrane-type matrix metalloproteinase MT5-MMP. A new member of the membrane-type matrix metalloproteinase (MT-MMP) subfamily tentatively named MT5-MMP was isolated from mouse brain cDNA library. It is predicted to contain (i) a candidate signal sequence, (ii) a propeptide region with the highly conserved PRCGVPD sequence, (iii) a potential furin recognition motif RRRRNKR, (iv) a zinc-binding catalytic domain, (v) a hemopexin-like domain, (vi) a 24-residue hydrophobic domain as a potential transmembrane domain, and (vii) a short cytosolic domain. Reverse transcriptase-polymerase chain reaction analysis of its transcripts indicates that MT5-MMP is expressed in a brain-specific manner consistent with the origin of its EST clone from cerebellum. It is also highly expressed during embryonic development at stages day 11 and 15. Like other MT-MMPs, MT5-MMP specifically activates progelatinase A when co-expressed in Madin-Darby canine kidney cells. Its ability to activate progelatinase A is dependent on its proteolytic activity since a mutation converting Glu to Ala in the zinc binding motif HE255LGH renders MT5-MMP inactive against progelatinase A. In contrast to other MT-MMPs, MT5-MMP tends to shed from cell surface as soluble proteinases, thus offering flexibility as both a cell bound and soluble proteinase for **extracellular matrix** remodeling processes. Taken together, these properties serve to distinguish MT5-MMP as a versatile MT-MMP playing an important role in **extracellular matrix** remodeling events in the brain and during embryonic development.
6. Mint-3 regulates the retrieval of the internalized membrane-type matrix metalloproteinase, MT5-MMP, to the plasma membrane by binding to its carboxyl end motif EWV. Membrane type matrix metalloproteinases (MT-MMPs) play a critical role in promoting cell growth and migration within the **extracellular matrix** by trafficking to specialized areas. Here we show that the carboxyl EWV motif of MT5-MMP serves as a retrieval signal for internalized MT5-MMP by interacting with Mint-3, a protein with two type III PDZ domains. Deletion of the EWV signal impairs the recycling of MT5-MMP without affecting its internalization, leading to decreased activity on the cell surface. A yeast two-hybrid screening identified Mint-3 as the EWV-binding protein. Mint-3 stimulates MT5-MMP activity when expressed at low levels in an EWV-dependent fashion, but inhibits its activity at higher levels independent of the EWV motif. siRNA-mediated knockdown of endogenous Mint-3 decreased MT5-MMP activity. Furthermore, Mint-3 significantly increased the level of MT5-MMP on the cell surface without affecting its synthesis and internalization. Therefore, Mints may be the adaptor proteins that regulate the trafficking of MT-MMPs.

#### Component

1. **extracellular matrix**
2. proteinaceous **extracellular matrix**

#### Conserved Domains

1. cd04278: ZnMc\_MMP; Zinc-dependent metalloprotease, matrix metalloproteinase (MMP) sub-family. MMPs are responsible for a great deal of pericellular proteolysis of **extracellular matrix** and cell surface molecules, playing crucial roles in morphogenesis, cell fate ...

#### Summary

1. This gene encodes a member of the matrix metalloproteinase family of **extracellular matrix**-degrading enzymes that are involved in tissue remodeling, wound repair, progression of atherosclerosis and tumor invasion. The encoded preproprotein undergoes proteolytic processing to generate a mature, zinc-dependent endopeptidase enzyme. Mice lacking the encoded protein do not develop neuropathic pain with mechanical allodynia after sciatic nerve injury, display enhanced sensitivity to noxious thermal stimuli under basal conditions, and develop hyperalgesia during inflammation. [provided by RefSeq, Feb 2016]

### [Mm]etastasis

#### PubMed

1. Matrix metalloproteinases in tumor invasion. Controlled degradation of extracellular matrix (ECM) is essential for the growth, invasion, and **metastasis** of malignant tumors, and for tumor-induced angiogenesis. Matrix metalloproteinases (MMPs) are a family of zinc-dependent neutral endopeptidases collectively capable of degrading essentially all ECM components and they apparently play an important role in all these aspects of tumor development. Furthermore, recent evidence suggests that MMPs also play a role in tumor cell survival. In this review, we discuss the current concept concerning the role of MMPs and their inhibitors in tumor invasion, as a basis for prognosis and targeted therapeutic intervention in cancer.

---

## Insc

- NCBI Gene
- GeneRIF
- Pubmed

| Search Term | Rank | Fields |
| --- | --- | --- |
| [Mm]igration | 6 | PubMed(2); GeneRIFs(1) |
| [Ee]xtracellular [Mm]atrix | 8 | PubMed(1) |

  
hide/show details for Insc

### [Mm]igration

#### PubMed

1. Arid3b is essential for second heart field cell deployment and heart patterning. Arid3b, a member of the conserved ARID family of transcription factors, is essential for mouse embryonic development but its precise roles are poorly understood. Here, we show that Arid3b is expressed in the myocardium of the tubular heart and in second heart field progenitors. Arid3b-deficient embryos show cardiac abnormalities, including a notable shortening of the poles, absence of myocardial differentiation and altered patterning of the atrioventricular canal, which also lacks epithelial-to-mesenchymal transition. Proliferation and death of progenitors as well as early patterning of the heart appear normal. However, DiI labelling of second heart field progenitors revealed a defect in the addition of cells to the heart. RNA microarray analysis uncovered a set of differentially expressed genes in Arid3b-deficient tissues, including Bhlhb2, a regulator of cardiomyocyte differentiation, and Lims2, a gene involved in cell **migration**. Arid3b is thus required for heart development by regulating the motility and differentiation of heart progenitors. These findings identify Arid3b as a candidate gene involved in the aetiology of human congenital malformations.
2. The cell polarity protein mInsc regulates neutrophil chemotaxis via a noncanonical G protein signaling pathway. Successful chemotaxis requires not only increased motility but also sustained directionality. Here, we show that, during neutrophil chemotaxis via receptors coupled with the Gi family of heterotrimeric G proteins, directional movement is regulated by mInsc, a mammalian protein distantly related to the Drosophila polarity-organizer Inscuteable. The GDP-bound, Gβγ-free Gαi subunit accumulates at the front of chemotaxing neutrophils to recruit mInsc-complexed with the Par3-aPKC evolutionarily conserved polarity complex-via LGN/AGS3 that simultaneously binds to Gαi-GDP and mInsc. Both mInsc-deficient and aPKC-blocked neutrophils exhibit a normal motile activity but migrate in an undirected manner. mInsc deficiency prevents neutrophils from efficiently stabilizing pseudopods at the leading edge; the stability is restored by wild-type mInsc, but not by a mutant protein defective in binding to LGN/AGS3. Thus, mInsc controls directio**nal migra**tion via noncanonical G protein signaling, in which Gβγ-free Gαi-GDP, a product from Gαi-GTP released after receptor activation, plays a central role.

#### GeneRIFs

1. Insc controls directional **migration** via noncanonical G protein signaling, in which Gbetagamma-free Galphai-GDP, a product from Galphai-GTP released after receptor activation, plays a central role.

### [Ee]xtracellular [Mm]atrix

#### PubMed

1. Asymmetric cell divisions promote stratification and differentiation of mammalian skin. The epidermis is a stratified squamous epithelium forming the barrier that excludes harmful microbes and retains body fluids. To perform these functions, proliferative basal cells in the innermost layer periodically detach from an underlying basement membrane of **extracellular matrix**, move outward and eventually die. Once suprabasal, cells stop dividing and enter a differentiation programme to form the barrier. The mechanism of stratification is poorly understood. Although studies in vitro have led to the view that stratification occurs through the delamination and subsequent movement of epidermal cells, most culture conditions favour keratinocytes that lack the polarity and cuboidal morphology of basal keratinocytes in tissue. These features could be important in considering an alternative mechanism, that stratification occurs through asymmetric cell divisions in which the mitotic spindle orients perpendicularly to the basement membrane. Here we show that basal epidermal cells use their polarity to divide asymmetrically, generating a committed suprabasal cell and a proliferative basal cell. We further demonstrate that integrins and cadherins are essential for the apical localization of atypical protein kinase C, the Par3-LGN-Inscuteable complex and NuMA-dynactin to align the spindle.

---

## Rela

- NCBI Gene
- GeneRIF
- Pubmed

| Search Term | Rank | Fields |
| --- | --- | --- |
| [Mm]igration | 6 | PubMed(14); GeneRIFs(2) |
| ECM | 7 | PubMed(3) |
| [Ee]xtracellular [Mm]atrix | 8 | PubMed(7) |
| [Ii]nvasive | 10 | PubMed(2); GeneRIFs(1) |
| [Mm]etastasize | 11 | PubMed(1) |
| [Mm]etastasis | 12 | PubMed(5) |
| [Cc]ell [Ii]nvasion | 13 | PubMed(1) |

  
hide/show details for Rela

### [Mm]igration

#### PubMed

1. TAK1 kinase signaling regulates embryonic angiogenesis by modulating endothelial cell survival and **migration**. TGF-β activated kinase 1 (TAK1) is a mediator of various cytokine signaling pathways. Germline deficiency of Tak1 causes multiple abnormalities, including dilated blood vessels at midgestation. However, the mechanisms by which TAK1 regulates vessel formation have not been elucidated. TAK1 binding proteins 1 and 2 (TAB1 and TAB2) are activators of TAK1, but their roles in embryonic TAK1 signaling have not been determined. In the present study, we characterized mouse embryos harboring endothelial-specific deletions of Tak1, Tab1, or Tab2 and found that endothelial TAK1 and TAB2, but not TAB1, were critically involved in vascular formation. TAK1 deficiency in endothelial cells caused increased cell death and vessel regression at embryonic day 10.5 (E10.5). Deletion of TNF signaling largely rescued endothelial cell death in TAK1-deficient embryos at E10.5. However, embryos deficient in both TAK1 and TNF signaling still exhibited dilated capillary networks at E12.5. TAB2 deficiency caused reduced TAK1 activity, resulting in abnormal capillary blood vessels, similar to the compound deficiency of TAK1 and TNF signaling. Ablation of either TAK1 or TAB2 impaired cell **migratio**n and tube formation. Our results show that endothelial TAK1 signaling is important for 2 biologic processes in angiogenesis: inhibiting TNF-dependent endothelial cell death and promoting TNF-independent angiogenic cell **migratio**n.
2. Osteopontin induces nuclear factor kappa B-mediated promatrix metalloproteinase-2 activation through I kappa B alpha /IKK signaling pathways, and curcumin (diferulolylmethane) down-regulates these pathways. We have recently reported that osteopontin (OPN) stimulates tumor growth and activation of promatrix metalloproteinase-2 (pro-MMP-2) through nuclear factor kappa B (NF kappa B)-mediated induction of membrane type 1 matrix metalloproteinase (MT1-MMP) in murine melanoma cells (Philip, S., Bulbule, A., and Kundu, G. C. (2001) J. Biol. Chem. 276, 44926-44935). However, the molecular mechanism by which OPN activates NF kappa B and regulates pro-MMP-2 activation in murine melanoma (B16F10) cells is not well defined. We also investigated the mechanism of action of curcumin (diferulolylmethane) on OPN-induced NF kappa B-mediated activation of pro-MMP-2 in B16F10 cells. Here we report that OPN induces phosphorylation and degradation of the inhibitor of nuclear factor kappa B (I kappa B alpha) by inducing the activity of I kappa B kinase (IKK) in these cells. OPN also induces the nuclear accumulation of NF kappa B p65, NF kappa B-DNA binding, and transactivation. However, curcumin a known anti-inflammatory and anticarcinogenic agent suppressed OPN-induced I kappa B alpha phosphorylation and degradation by inhibiting the IKK activity. Moreover, our data revealed that curcumin inhibited the OPN-induced translocation of p65, NF kappa B-DNA binding, and NF kappa B transcriptional activity. The OPN-induced pro-MMP-2 activation and MT1-MMP expression were also drastically reduced by curcumin. Curcumin also inhibited OPN-induced cell proliferation, cell **migration**, extracellular matrix invasion, and synergistically induced apoptotic morphology with OPN in these cells. Most importantly, curcumin suppressed the OPN-induced tumor growth in nude mice, and the levels of pro-MMP-2 expression and activation in OPN-induced tumor were inhibited by curcumin. To our knowledge, this is the first report that OPN induces NF kappa B activity through phosphorylation and degradation of I kappa B alpha by activating IKK that ultimately triggers the activation of pro-MMP-2 and further demonstrates that curcumin potently suppresses OPN-induced cell **migration**, tumor growth, and NF kappa B-mediated pro-MMP-2 activation by blocking the IKK/I kappa B alpha signaling pathways.
3. ΔNp63 versatilely regulates a Broad NF-κB gene program and promotes squamous epithelial proliferation**, migrati**on, and inflammation. Head and neck squamous cell carcinoma (HNSCC) and many other epithelial malignancies exhibit increased proliferation, invasion, and inflammation, concomitant with aberrant nuclear activation of TP53 and NF-κB family members ΔNp63, cRel, and RelA. However, the mechanisms of cross-talk by which these transcription factors coordinate gene expression and the malignant phenotype remain elusive. In this study, we showed that ΔNp63 regulates a cohort of genes involved in cell growth, survival, adhesion, and inflammation, which substantially overlaps with the NF-κB transcriptome. ΔNp63 with cRel and/or RelA are recruited to form novel binding complexes on p63 or NF-κB/Rel sites of multitarget gene promoters. Overexpressed ΔNp63- or TNF-α-induced NF-κB and inflammatory cytokine interleukin-8 (IL-8) reporter activation depended on RelA/cRel regulatory binding sites. Depletion of RelA or ΔNp63 by small interfering RNA (siRNA) significantly inhibited NF-κB-specific, or TNF-α-induced IL-8 reporter activation. ΔNp63 siRNA significantly inhibited proliferation, **survival**, and migration by HNSCC cells in vitro. Consistent with these data, an increase in nuclear ΔNp63, accompanied by increased proliferation (Ki-67) and adhesion (β4 integrin) markers, and induced inflammatory cell infiltration was observed throughout HNSCC specimens, when compared with the basilar pattern of protein expression and minimal inflammation seen in nonmalignant mucosa. Furthermore, overexpression of ΔNp63α in squamous epithelial cells in transgenic mice leads to increased suprabasilar cRel, Ki-67, and cytokine expression, together with epidermal hyperplasia and diffuse inflammation, similar to HNSCC. Our study reveals ΔNp63 as a master transcription factor that, in coordination with NF-κB/Rels, orchestrates a broad gene program promoting epidermal hyperplasia, inflammation, and the malignant phenotype of HNSCC.
4. Possible mechanism of the anti-inflammatory activity of ruscogenin: role of intercellular adhesion molecule-1 and nuclear factor-kappaB. Ruscogenin (RUS), first isolated from Ruscus aculeatus, also a major steroidal sapogenin of traditional Chinese herb Radix Ophiopogon japonicus, has been found to exert significant anti-inflammatory and anti-thrombotic activities. Our previous studies suggested that ruscogenin remarkably inhibited adhesion of leukocytes to a human umbilical vein endothelial cell line (ECV304) injured by tumor necrosis factor-alpha (TNF-alpha) in a concentration-dependent manner. Yet the underlying mechanisms remain unclear. In this study, the in vivo effects of ruscogenin on leukocyte **migration** and celiac prostaglandin E(2) (PGE(2)) level induced by zymosan A were studied in mice. Furthermore, the effects of ruscogenin on TNF-alpha-induced intercellular adhesion molecule-1 (ICAM-1) expression and nuclear factor-kappaB (NF-kappaB) activation were also investigated under consideration of their key roles in leukocyte recruitment. The results showed that ruscogenin significantly suppressed zymosan A-evoked peritoneal total leukocyte **migration** in mice in a dose-dependent manner, while it had no obvious effect on PGE(2) content in peritoneal exudant. Ruscogenin also inhibited TNF-alpha-induced over expression of ICAM-1 both at the mRNA and protein levels and suppressed NF-kappaB activation considerably by decreasing NF-kappaB p65 translocation and DNA binding activity. These findings provide some new insights that may explain the possible molecular mechanism of ruscogenin and Radix Ophiopogon japonicus for the inhibition of endothelial responses to cytokines during inflammatory and vascular disorders.
5. Targeted mutation of TNF receptor I rescues the RelA-deficient mouse and reveals a critical role for NF-kappa B in leukocyte recruitment. NF-kappaB binding sites are present in the promoter regions of many acute phase and inflammatory response genes, suggesting that NF-kappaB plays an important role in the initiation of innate immune responses. However, targeted mutations of the various NF-kappaB family members have yet to identify members responsible for this critical role. RelA-deficient mice die on embryonic day 15 from TNF-alpha-induced liver degeneration. To investigate the importance of RelA in innate immunity, we genetically suppressed this embryonic lethality by breeding the RelA deficiency onto a TNFR type 1 (TNFR1)-deficient background. TNFR1/RelA-deficient mice were born healthy, but were susceptible to bacterial infections and bacteremia and died within a few weeks after birth. Hemopoiesis was intact in TNFR1/RelA-deficient newborns, but neutrophil e**migration** to alveoli during LPS-induced pneumonia was severely reduced relative to that in wild-type or TNFR1-deficient mice. In contrast, radiation chimeras reconstituted with RelA or TNFR1/RelA-deficient hemopoietic cells were healthy and demonstrated no defect in neutrophil e**migration** during LPS-induced pneumonia. Analysis of RNA harvested from the lungs of mice 4 h after LPS insufflation revealed that the induction of several genes important for neutrophil recruitment to the lung was significantly reduced in TNFR1/RelA-deficient mice relative to that in wild-type or TNFR1-deficient mice. These results suggest that TNFR1-independent activation of RelA is essential in cells of nonhemopoietic origin during the initiation of an innate immune response.
6. Epithelial-mesenchymal transition induced by TNF-α requires NF-κB-mediated transcriptional upregulation of Twist1. Proinflammatory cytokines produced in the tumor microenvironment facilitate tumor development and metastatic progression. In particular, TNF-α promotes cancer invasion and angiogenesis associated with epithelial-mesenchymal transition (EMT); however, the mechanisms underlying its induction of EMT in cancer cells remain unclear. Here we show that EMT and cancer stemness properties induced by chronic treatment with TNF-α are mediated by the upregulation of the transcriptional repressor Twist1. Exposure to TNF-α rapidly induced Twist1 mRNA and protein expression in normal breast epithelial and breast cancer cells. Both IKK-β and NF-κB p65 were required for TNF-α-induced expression of Twist1, suggesting the involvement of canonical NF-κB signaling. In support of this likelihood, we defined a functional NF-κB-binding site in the Twist1 promoter, and overexpression of p65 was sufficient to induce transcriptional upregulation of Twist1 along with EMT in mammary epithelial cells. Conversely, suppressing Twist1 expression abrogated p65-ind**uced cell** migration, invasion, EMT, and stemness properties, establishing that Twist1 is required for NF-κB to induce these aggressive phenotypes in breast cancer cells. Taken together, our results establish a signaling axis through which the tumor microenvironment elicits Twist1 expression to promote cancer metastasis. We suggest that targeting NF-κB-mediated Twist1 upregulation may offer an effective a therapeutic strategy for breast cancer treatment.
7. Functions and regulation of NF-kappaB RelA during pneumococcal pneumonia. Eradication of bacteria in the lower respiratory tract depends on the coordinated expression of proinflammatory cytokines and consequent neutrophilic inflammation. To determine the roles of the NF-kappaB subunit RelA in facilitating these events, we infected RelA-deficient mice (generated on a TNFR1-deficient background) with Streptococcus pneumoniae. RelA deficiency decreased cytokine expression, alveolar neutrophil e**migration**, and lung bacterial killing. S. pneumoniae killing was also diminished in the lungs of mice expressing a dominant-negative form of IkappaBalpha in airway epithelial cells, implicating this cell type as an important locus of NF-kappaB activation during pneumonia. To study mechanisms of epithelial RelA activation, we stimulated a murine alveolar epithelial cell line (MLE-15) with bronchoalveolar lavage fluid (BALF) harvested from mice infected with S. pneumoniae. Pneumonic BALF, but not S. pneumoniae, induced degradation of IkappaBalpha and IkappaBbeta and rapid nuclear accumulation of RelA. Moreover, BALF-induced RelA activity was completely abolished following combined but not individual neutralization of TNF and IL-1 signaling, suggesting either cytokine is sufficient and necessary for alveolar epithelial RelA activation during pneumonia. Our results demonstrate that RelA is essential for the host defense response to pneumococcus in the lungs and that RelA in airway epithelial cells is primarily activated by TNF and IL-1.
8. Pinellia ternata lectin exerts a pro-inflammatory effect on macrophages by inducing the release of pro-inflammatory cytokines, the activation of the nuclear factor-κB signaling pathway and the overproduction of reactive oxygen species. Pinellia ternata (PT) is a widely used traditional Chinese medicine. The raw material has a throat-irritating toxicity that is associated with the PT lectin (PTL). PTL is a monocot lectin isolated from the tubers of PT, which exhibits mouse peritoneal acute inflammatory effects in vivo. The present study aimed to investigate the pro-inflammatory effect of PTL on macrophages. PTL (50 µg/ml)‑stimulated macrophages enhanced the chemotactic activity of neutrophils. PTL (50, 100, 200 and 400 µg/ml) significantly elevated the production of cytokines [tumor necrosis factor‑α (TNF-α) , interleukin (IL)‑1β and IL‑6]. PTL (25, 50 and 100 µg/ml) induced intracellular reactive oxygen species (ROS) overproduction. PTL also caused transfer of p65 from the macrophage cytoplasm to the nucleus and activated the nuclear factor‑κB (NF‑κB) signaling pathway. Scanning electron microscope images revealed severe cell swelling and membrane integrity defection of macrophages following PTL (100 µg/ml) stimulation, which was also associated with inflammation. PTL had pro‑inflammatory activity, **involvin**g induced neutrophil migration, cytokine release, ROS overproduction and the activation of the NF-κB signaling pathway, which was associated with the activation of macrophages.
9. An NF-κB--EphrinA5-Dependent Communication between NG2(+) Interstitial Cells and Myoblasts Promotes Muscle Growth in Neonates. Skeletal muscle growth immediately following birth is critical for proper body posture and locomotion. However, compared with embryogenesis and adulthood, the processes regulating the maturation of neonatal muscles is considerably less clear. Studies in the 1960s predicted that neonatal muscle growth results from nuclear accretion of myoblasts preferentially at the tips of myofibers. Remarkably, little information has been added since then to resolve how myoblasts migrate to the ends of fibers. Here, we provide insight into this process by revealing a unique NF-κB-dependent communication between NG2(+) interstitial cells and myoblasts. NF-κB in NG2(+) cells promotes myobla**st migrat**ion to the tips of myofibers through cell-cell contact. This occurs through expression of ephrinA5 from NG2(+) cells, which we further deduce is an NF-κB target gene. Together, these results suggest that NF-κB plays an important role in the development of newborn muscles to ensure proper myob**last migr**ation for fiber growth.
10. The role of RelA (p65) threonine 505 phosphorylation in the regulation of cell growth, survival, and **migration**. The NF-κB family of transcription factors is a well-established regulator of the immune and inflammatory responses and also plays a key role in other cellular processes, including cell death, proliferation, and **migratio**n. Conserved residues in the trans-activation domain of RelA, which can be posttranslationally modified, regulate divergent NF-κB functions in response to different cellular stimuli. Using rela(-/-) mouse embryonic fibroblasts reconstituted with RelA, we find that mutation of the threonine 505 (T505) phospho site to alanine has wide-ranging effects on NF-κB function. These include previously described effects on chemotherapeutic drug-induced apoptosis, as well as new roles for this modification in autophagy, cell proliferation, a**nd migrat**ion. This last effect was associated with alterations in the actin cytoskeleton and expression of cellul**ar migrat**ion-associated genes such as WAVE3 and α-actinin 4. We also define a new component of cisplatin-induced, RelA T505-dependent apoptosis, involving induction of NOXA gene expression, an effect explained at least in part through induction of the p53 homologue, p73. Therefore, in contrast to other RelA phosphorylation events, which positively regulate NF-κB function, we identified RelA T505 phosphorylation as a negative regulator of its ability to induce diverse cellular processes such as apoptosis, autophagy, proliferation, **and migr**ation.
11. Synaptic activation of NF-kappa B by glutamate in cerebellar granule neurons in vitro. Neuronal proliferation, **migration**, and differentiation are regulated by the sequential expression of particular genes at specific stages of development. Such processes rely on differential gene expression modulated through second-messenger systems. Early postnatal mouse cerebellar granule cells migrate into the internal granular layer and acquire differentiated properties. The neurotransmitter glutamate has been shown to play an important role in this developmental process. We show here by immunohistochemistry that the RelA subunit of the transcription factor NF-kappa B is present in several areas of the mouse brain. Moreover, immunofluorescence microscopy and electrophoretic mobility-shift assay demonstrate that in cerebellar granule cell cultures derived from 3- to 7-day-old mice, glutamate specifically activates the transcription factor NF-kappa B, as shown by binding of nuclear extract proteins to a synthetic oligonucleotide reproducing the kappa B site of human immunodeficiency virus. The use of different antagonists of the glutamate recpetors indicates that the effect of glutamate occurs mainly via N-methyl-D-aspartate (NMDA)-receptor activation, possibly as a result of an increase in intracellular Ca2+. The synaptic specificity of the effect is strongly suggested by the observation that glutamate failed to activate NF-kappa B in astrocytes, while cytokines, such as interleukin 1 alpha and tumor necrosis factor alpha, did so. The effect of glutamate appears to be developmentally regulated. Indeed, NF-kappa B is found in an inducible form in the cytoplasm of neurons of 3- to 7-day-old mice but is constitutively activated in the nuclei of neurons derived from older pups (8-10 days postnatal). Overall, these observations suggest the existence of a new pathway of trans-synaptic regulation of gene expression.
12. Members of the NF-kappaB family expressed in zones of active neurogenesis in the postnatal and adult mouse brain. The Rel/NF-kappaB family of transcription factors is implicated in cell proliferation, cell death, cell **migration** and cell interactions. Here, we examined by immunohistochemistry the expression pattern of various members of this family during postnatal telencephalon development and during adulthood, and we used neuronal and glial markers to identify the cells types where they are expressed. Distinct Rel/NF-kappaB proteins are highly expressed postnatally in the subventricular zone and in the rostral migratory stream. In particular, Rel A and p50 are expressed in radial glial cells, in migrating neuron precursors and in a population belonging to the astrocytic lineage. Rel B, on the other hand, is only expressed in migrating neuron precursors, whereas c-Rel is present in a few cells located at the edges of the rostral migratory stream. The expression of Rel A and p50 persists into adulthood, particularly in subventricular zone astrocyte-like cells and in migrating neuron precursors, respectively. The selective expression of NF-kappaB members in the postnatal subventricular zone and rostral migratory stream and their persistence into adulthood in regions of ongoing neurogenesis suggests possible mechanisms linking NF-kappaB expression with cell proliferation and **migration**. Their presence in actively proliferating progenitor cells, detected by BrdU staining, further suggests that NF-kappaB may be part of a signaling pathway that is important for neurogenesis.
13. ROS-NFkappaB mediates TGF-beta1-induced expression of urokinase-type plasminogen activator, matrix metalloproteinase-9 and cell invasion. TGF-beta1 has been postulated as a pro-oncogenic factor in the late step of the tumoral progression. In transformed cells, TGF-beta1 enhances the capacity to degrade the extracellular matrix, cell invasiveness and epithelial-mesenchymal transition, which are crucial steps for metastasis. Urokinase-type plasminogen activator (uPA) and matrix metalloproteinase-9 (MMP-9) are critical components in cell **migration** and invasion induced by TGF-beta1, however, the exact mechanism by which TGF-beta1 regulates uPA and MMP-9 is not well elucidated so far. In the present study, we analyzed the role of ROS-NFkappaB, signal as mediator in the cell malignity enhancement by TGF-beta1. We found that TGF-beta1 activates NFkappaB, through Rac1-NOXs-ROS-dependent mechanism. Our results shows that TGF-beta1 stimulation of uPA and MMP-9 expression involve NOXs-dependent ROS and NFkappaB, activation, demonstrated by using DPI, NOXs inhibitor, ROS scavenger N-acetylcysteine and SN50, an NFkb inhibitor. Furthermore, we found that the inhibition of ROS and NFkappaB, abrogates TGF-beta1 stimulation of EMT, cell motility and invasion. Thus, ROS-NFkappaB acts as the crucial signal in TGF-beta1-induced uPA and MMP-9 expression thereby mediating the enhancement of cellular malignity by TGF-beta1.
14. **Migration** of cells in a social context. In multicellular organisms and complex ecosystems, cells migrate in a social context. Whereas this is essential for the basic processes of life, the influence of neighboring cells on the individual remains poorly understood. Previous work on isolated cells has observed a stereotypical migratory behavior characterized by short-time directional persistence with long-time random movement. We discovered a much richer dynamic in the social context, with significant variations in directionality, displacement, and speed, which are all modulated by local cell density. We developed a mathematical model based on the experimentally identified "cellular traffic rules" and basic physics that revealed that these emergent behaviors are caused by the interplay of single-cell properties and intercellular interactions, the latter being dominated by a pseudopod formation bias mediated by secreted chemicals and pseudopod collapse following collisions. The model demonstrates how aspects of complex biology can be explained by simple rules of physics and constitutes a rapid test bed for future studies of collective **migration** of individual cells.

#### GeneRIFs

1. we identified RelA T505 phosphorylation as a negative regulator of its ability to induce diverse cellular processes such as apoptosis, autophagy, proliferation, and **migration**
2. curcumin potently suppresses osteopontin-induced cell **migration**, tumor growth, and NF kappa B-mediated pro-MMP-2 activation by blocking the IKK/I kappa B alpha signaling pathways

### ECM

#### PubMed

1. TNF-alpha suppressed TGF-beta-induced CTGF expression by switching the binding preference of p300 from Smad4 to p65. TGF-beta regulates diverse biologic effects including cell growth, cell death or apoptosis, cell differentiation, and extracellular matrix (**ECM**) synthesis. Connective tissue growth factor (CTGF), induced by TGF-beta has been reported to mediate stimulatory action of TGF-beta-induced **ECM**. Although TNF-alpha was reported to suppress theTGF-beta-induced CTGF gene expression, the molecular mechanism is not well clarified. In this study, we found the inhibitory effect of TNF-alpha on TGF-beta-induced CTGF expression in WT but not p65-/-MEF cells. TNF-alpha neither induced Smad7 expression nor affected TGF-beta-induced Smad2 phosphorylation and nuclear translocation. We demonstrated that p300 physically associated with p65 rather than Smad4 in the presence of both TNF-alpha and TGF-beta. Moreover, the TGF-beta-induced binding of p300 and acetylated H4, but not Smad4 to the CTGF promoter was disturbed by TNF-alpha treatment. Overall, our data showed that suppression of TNF-alpha on TGF-beta-induced CTGF expression is due to the competition of p300 by p65 and Smad4.
2. NF-kappa B activation mediates the cross-talk between extracellular matrix and interferon-gamma (IFN-gamma) leading to enhanced monokine induced by IFN-gamma (MIG) expression in macrophages. In intact tissue, the extracellular matrix (**ECM**) provides support and helps maintain homeostasis but is considered biologically inert. In the setting of inflammation, not only is the **ECM** the target of inflammation, but its breakdown products modulate the magnitude and quality of an immune response. Fragments of the **ECM** component hyaluronan (HA) induce macrophage expression of chemokines, cytokines, and growth factors as well greatly enhance IFN-gamma-induced MIG expression. In this report, we demonstrate that the synergistic induction of MIG by HA and IFN-gamma occurs at the level of transcription via NF-kappaB. Using electrophoretic mobility shift assays and reporter assays, we have identified two NF-kappaB sites proximal to the IFN-gamma-responsive element-1 (gammaRE-1) that mediate this effect. Interestingly, our experiments also revealed a critical role for NF-kappaB in mediating IFN-gamma-induced MIG expression independent of HA. These data emphasize the ability of "degraded self" to activate/modify immune responses through the NF-kappaB pathway.
3. Astragaloside IV from Astragalus membranaceus ameliorates renal interstitial fibrosis by inhibiting inflammation via TLR4/NF-кB in vivo and in vitro. Renal fibrosis is characterized by infiltration of inflammatory cells, activation and proliferation of fibroblasts, and accumulation of extracellular matrix **(EC**M). Astragalus membranaceus (AM) is traditional Chinese medicine and has a range of pharmacological effects. Astragaloside IV (As IV) is the main compound of AM and has anti-inflammation activities. Whether As IV ameliorates renal interstitial fibrosis by inhibiting inflammation remains unknown. Accordingly, this study investigated the ameliorating effect of As IV on renal fibrosis. Renal fibrosis was induced in vivo using the unilateral ureteral obstruction (UUO) model. UUO mice were administered intragastrically with As IV (20 and 40mg/kg/day). After a week, **EC**M including fibronectin and collagen I was examined by Immunohistochemistry and Western blot, inflammatory cells (CD68 and CD3) were detected by Immunohistochemistry, the release of inflammatory cytokines (tumor necrosis factor-α and interleukin-1β) was inspected by polymerase chain reaction, and signaling pathway was determined by Western blot. In vitro, 100ng/ml lipopolysaccharide (LPS) stimulated epithelial cells to construct the inflammatory model; these cells were treated by As IV (10 and 20μM) with or without TAK-242 (1μM) for 48h. The released inflammatory cytokines were assayed by enzyme-linked immunosorbent assay, and signaling pathway was evaluated by Western blot. As IV decreased accumulatio**n o**f ECM and infiltration of inflammatory cells in UUO-induced renal fibrosis. Furthermore, As IV markedly attenuated pro-inflammatory cytokines in UUO mouse and LPS-induced epithelial cells. As IV also inhibited the TLR4 and nuclear factor (NF)-кB signaling pathway in vivo and vitro. These results demonstrate that As IV protects against the progression of renal fibrosis by inhibiting inflammation via the TLR4/NF-кB signaling pathway.

### [Ee]xtracellular [Mm]atrix

#### PubMed

1. Osteopontin induces nuclear factor kappa B-mediated promatrix metalloproteinase-2 activation through I kappa B alpha /IKK signaling pathways, and curcumin (diferulolylmethane) down-regulates these pathways. We have recently reported that osteopontin (OPN) stimulates tumor growth and activation of promatrix metalloproteinase-2 (pro-MMP-2) through nuclear factor kappa B (NF kappa B)-mediated induction of membrane type 1 matrix metalloproteinase (MT1-MMP) in murine melanoma cells (Philip, S., Bulbule, A., and Kundu, G. C. (2001) J. Biol. Chem. 276, 44926-44935). However, the molecular mechanism by which OPN activates NF kappa B and regulates pro-MMP-2 activation in murine melanoma (B16F10) cells is not well defined. We also investigated the mechanism of action of curcumin (diferulolylmethane) on OPN-induced NF kappa B-mediated activation of pro-MMP-2 in B16F10 cells. Here we report that OPN induces phosphorylation and degradation of the inhibitor of nuclear factor kappa B (I kappa B alpha) by inducing the activity of I kappa B kinase (IKK) in these cells. OPN also induces the nuclear accumulation of NF kappa B p65, NF kappa B-DNA binding, and transactivation. However, curcumin a known anti-inflammatory and anticarcinogenic agent suppressed OPN-induced I kappa B alpha phosphorylation and degradation by inhibiting the IKK activity. Moreover, our data revealed that curcumin inhibited the OPN-induced translocation of p65, NF kappa B-DNA binding, and NF kappa B transcriptional activity. The OPN-induced pro-MMP-2 activation and MT1-MMP expression were also drastically reduced by curcumin. Curcumin also inhibited OPN-induced cell proliferation, cell migration, **extracellular matrix** invasion, and synergistically induced apoptotic morphology with OPN in these cells. Most importantly, curcumin suppressed the OPN-induced tumor growth in nude mice, and the levels of pro-MMP-2 expression and activation in OPN-induced tumor were inhibited by curcumin. To our knowledge, this is the first report that OPN induces NF kappa B activity through phosphorylation and degradation of I kappa B alpha by activating IKK that ultimately triggers the activation of pro-MMP-2 and further demonstrates that curcumin potently suppresses OPN-induced cell migration, tumor growth, and NF kappa B-mediated pro-MMP-2 activation by blocking the IKK/I kappa B alpha signaling pathways.
2. Relaxin induces matrix-metalloproteinases-9 and -13 via RXFP1: induction of MMP-9 involves the PI3K, ERK, Akt and PKC-ζ pathways. We determined the precise role of relaxin family peptide (RXFP) receptors-1 and -2 in the regulation of MMP-9 and -13 by relaxin, and delineated the signaling cascade that contributes to relaxin's modulation of MMP-9 in fibrocartilaginous cells. Relaxin treatment of cells in which RXFP1 was silenced resulted in diminished induction of MMP-9 and -13 by relaxin, whereas overexpression of RXFP1 potentiated the relaxin-induced expression of these proteinases. Suppression or overexpression of RXFP2 resulted in no changes in the relaxin-induced MMP-9 and -13. Studies using chemical inhibitors and siRNAs to signaling molecules showed that PI3K, Akt, ERK and PKC-ζ and the transcription factors Elk-1, c-fos and, to a lesser extent, NF-κB are involved in relaxin's induction of MMP-9. Our findings provide the first characterization of signaling cascade involved in the regulation of any MMP by relaxin and offer mechanistic insights on how relaxin likely mediat**es extracellular mat**rix turnover.
3. TNF-alpha suppressed TGF-beta-induced CTGF expression by switching the binding preference of p300 from Smad4 to p65. TGF-beta regulates diverse biologic effects including cell growth, cell death or apoptosis, cell differentiation, and **extracellular matrix** (ECM) synthesis. Connective tissue growth factor (CTGF), induced by TGF-beta has been reported to mediate stimulatory action of TGF-beta-induced ECM. Although TNF-alpha was reported to suppress theTGF-beta-induced CTGF gene expression, the molecular mechanism is not well clarified. In this study, we found the inhibitory effect of TNF-alpha on TGF-beta-induced CTGF expression in WT but not p65-/-MEF cells. TNF-alpha neither induced Smad7 expression nor affected TGF-beta-induced Smad2 phosphorylation and nuclear translocation. We demonstrated that p300 physically associated with p65 rather than Smad4 in the presence of both TNF-alpha and TGF-beta. Moreover, the TGF-beta-induced binding of p300 and acetylated H4, but not Smad4 to the CTGF promoter was disturbed by TNF-alpha treatment. Overall, our data showed that suppression of TNF-alpha on TGF-beta-induced CTGF expression is due to the competition of p300 by p65 and Smad4.
4. NF-kappa B activation mediates the cross-talk between **extracellular matrix** and interferon-gamma (IFN-gamma) leading to enhanced monokine induced by IFN-gamma (MIG) expression in macrophages. In intact tissue, the **extracellular matrix** (ECM) provides support and helps maintain homeostasis but is considered biologically inert. In the setting of inflammation, not only is the ECM the target of inflammation, but its breakdown products modulate the magnitude and quality of an immune response. Fragments of the ECM component hyaluronan (HA) induce macrophage expression of chemokines, cytokines, and growth factors as well greatly enhance IFN-gamma-induced MIG expression. In this report, we demonstrate that the synergistic induction of MIG by HA and IFN-gamma occurs at the level of transcription via NF-kappaB. Using electrophoretic mobility shift assays and reporter assays, we have identified two NF-kappaB sites proximal to the IFN-gamma-responsive element-1 (gammaRE-1) that mediate this effect. Interestingly, our experiments also revealed a critical role for NF-kappaB in mediating IFN-gamma-induced MIG expression independent of HA. These data emphasize the ability of "degraded self" to activate/modify immune responses through the NF-kappaB pathway.
5. ROS-NFkappaB mediates TGF-beta1-induced expression of urokinase-type plasminogen activator, matrix metalloproteinase-9 and cell invasion. TGF-beta1 has been postulated as a pro-oncogenic factor in the late step of the tumoral progression. In transformed cells, TGF-beta1 enhances the capacity to degrade the **extracellular matrix**, cell invasiveness and epithelial-mesenchymal transition, which are crucial steps for metastasis. Urokinase-type plasminogen activator (uPA) and matrix metalloproteinase-9 (MMP-9) are critical components in cell migration and invasion induced by TGF-beta1, however, the exact mechanism by which TGF-beta1 regulates uPA and MMP-9 is not well elucidated so far. In the present study, we analyzed the role of ROS-NFkappaB, signal as mediator in the cell malignity enhancement by TGF-beta1. We found that TGF-beta1 activates NFkappaB, through Rac1-NOXs-ROS-dependent mechanism. Our results shows that TGF-beta1 stimulation of uPA and MMP-9 expression involve NOXs-dependent ROS and NFkappaB, activation, demonstrated by using DPI, NOXs inhibitor, ROS scavenger N-acetylcysteine and SN50, an NFkb inhibitor. Furthermore, we found that the inhibition of ROS and NFkappaB, abrogates TGF-beta1 stimulation of EMT, cell motility and invasion. Thus, ROS-NFkappaB acts as the crucial signal in TGF-beta1-induced uPA and MMP-9 expression thereby mediating the enhancement of cellular malignity by TGF-beta1.
6. Activation of NF-kappaB by fluid shear stress, but not TNF-alpha, requires focal adhesion kinase in osteoblasts. When bone is mechanically loaded fluid shear stress (FSS) is generated as a result of the movement of interstitial fluid across the membranes of osteoblasts and osteocytes. This external mechanical loading stimulates changes in the activity of cytoplasmic signaling molecules and alters gene expression in bone cells. This process, referred to as mechanotransduction, is vital for maintaining bone health in vivo by regulating the balance between bone formation and bone resorption. This current study focuses on the role of focal adhesions, sites of integrin-mediated cellular attachment to the **extracellular matrix**, and their proposed function as mechanosensors in bone cells. We examined the role of a key component of focal adhesions and of mechanotransduction, focal adhesion kinase (FAK) in regulation of FSS- and tumor necrosis factor-alpha (TNF-alpha)-induced activation of nuclear factor-kappa B (NF-kappaB) signaling in osteoblasts. Immortalized FAK(+/+) and FAK(-)(/)(-) osteoblasts were exposed to periods of oscillatory fluid shear stress (OFF) and NF-kappaB activation was analyzed. We determined that FAK is required for OFF-induced nuclear translocation and activation of NF-kappaB in osteoblasts. In addition we found that OFF-induced phosphorylation of the IkappaB kinases (IKKalpha/beta) in both FAK(+/+) and FAK(-/-) osteoblasts, but only FAK(+/+) osteoblasts demonstrated the resulting degradation of NF-kappaB inhibitors IkappaBalpha and IkappaBbeta. OFF did not induce the degradation of IkappaBepsilon or the processing of p105 in either FAK(+/+) and FAK(-/-) osteoblasts. To compare the role of FAK in mediating OFF-induced mechanotransduction to the well characterized activation of NF-kappaB by inflammatory cytokines, we exposed FAK(+/+) and FAK(-/-) osteoblasts to TNF-alpha. Interestingly, FAK was not required for TNF-alpha induced NF-kappaB activation in osteoblasts. In addition we determined that TNF-alpha treatment did not induce the degradation of IkappaBbeta as did OFF. These data indicate a novel relationship between FAK and NF-kappaB activation in osteoblast mechanotransduction and demonstrates that the mechanism of FSS-induced NF-kappaB activation in osteoblasts differs from the well characterized TNF-alpha-induced activation.
7. Astragaloside IV from Astragalus membranaceus ameliorates renal interstitial fibrosis by inhibiting inflammation via TLR4/NF-кB in vivo and in vitro. Renal fibrosis is characterized by infiltration of inflammatory cells, activation and proliferation of fibroblasts, and accumulation of **extracellular matri**x (ECM). Astragalus membranaceus (AM) is traditional Chinese medicine and has a range of pharmacological effects. Astragaloside IV (As IV) is the main compound of AM and has anti-inflammation activities. Whether As IV ameliorates renal interstitial fibrosis by inhibiting inflammation remains unknown. Accordingly, this study investigated the ameliorating effect of As IV on renal fibrosis. Renal fibrosis was induced in vivo using the unilateral ureteral obstruction (UUO) model. UUO mice were administered intragastrically with As IV (20 and 40mg/kg/day). After a week, ECM including fibronectin and collagen I was examined by Immunohistochemistry and Western blot, inflammatory cells (CD68 and CD3) were detected by Immunohistochemistry, the release of inflammatory cytokines (tumor necrosis factor-α and interleukin-1β) was inspected by polymerase chain reaction, and signaling pathway was determined by Western blot. In vitro, 100ng/ml lipopolysaccharide (LPS) stimulated epithelial cells to construct the inflammatory model; these cells were treated by As IV (10 and 20μM) with or without TAK-242 (1μM) for 48h. The released inflammatory cytokines were assayed by enzyme-linked immunosorbent assay, and signaling pathway was evaluated by Western blot. As IV decreased accumulation of ECM and infiltration of inflammatory cells in UUO-induced renal fibrosis. Furthermore, As IV markedly attenuated pro-inflammatory cytokines in UUO mouse and LPS-induced epithelial cells. As IV also inhibited the TLR4 and nuclear factor (NF)-кB signaling pathway in vivo and vitro. These results demonstrate that As IV protects against the progression of renal fibrosis by inhibiting inflammation via the TLR4/NF-кB signaling pathway.

### [Ii]nvasive

#### PubMed

1. Down-regulation of NF kappa B activation is an effective therapeutic modality in acquired platinum-resistant bladder cancer. No previous study has addressed the efficacy of NF-κB blockade when bladder tumors develop acquired resistance toward CDDP-treatments. We investigated the changes in NF-κB activation and therapeutic impact of NF-κB blockade by the novel NF-κB inhibitor dehydroxymethyl derivative of epoxyquinomicin (DHMEQ) in CDDP-resistant bladder cancer cells.Two hu**man inva**sive bladder cancer cell lines, T24 and T24PR, were used. The T24PR cell line was newly established as an acquired platinum-resistant subline by culturing in CDDP-containing medium for 6 months. Expression of intranuclear p65 protein in the fractionated two cell lines was determined by Western blotting analysis. DNA-binding activity of NF-κB was detected by electrophoretic mobility shift assay. The cytotoxic effects and induction of apoptosis were analyzed in vivo and in vitro.Intranuclear expression and DNA-binding activity of p65 were strongly enhanced in T24PR cells compared with those of T24 cells, and both were significantly suppressed by DHMEQ. Lowered cell viability and strong induction of apoptosis were observed by treatment with DHMEQ alone in these chemo-resistant cells compared with parent cells. As T24PR cells did not show dramatic cross-resistance to paclitaxel in the in vitro study, we next examined whether the combination of DHMEQ with paclitaxel could enhance the therapeutic effect of the paclitaxel treatment in T24PR tumors. Using mouse xenograft models, the mean volume of tumors treated with the combination of DHMEQ (2 mg/kg) and paclitaxel (10 mg/kg) was significantly smaller than those treated with paclitaxel alone (p < 0.05), and the reduction of tumor volume in mice treated with DHMEQ in combination with paclitaxel and paclitaxel alone as compared to vehicle control was 66.9% and 17.0%, respectively.There was a distinct change in the activation level of NF-κB between T24 and T24PR cells, suggesting strong nuclear localization of NF-κB could be a promising target after developing acquired platinum-resistance in bladder cancer.
2. ROS-NFkappaB mediates TGF-beta1-induced expression of urokinase-type plasminogen activator, matrix metalloproteinase-9 and cell invasion. TGF-beta1 has been postulated as a pro-oncogenic factor in the late step of the tumoral progression. In transformed cells, TGF-beta1 enhances the capacity to degrade the extracellular matrix, cell **invasive**ness and epithelial-mesenchymal transition, which are crucial steps for metastasis. Urokinase-type plasminogen activator (uPA) and matrix metalloproteinase-9 (MMP-9) are critical components in cell migration and invasion induced by TGF-beta1, however, the exact mechanism by which TGF-beta1 regulates uPA and MMP-9 is not well elucidated so far. In the present study, we analyzed the role of ROS-NFkappaB, signal as mediator in the cell malignity enhancement by TGF-beta1. We found that TGF-beta1 activates NFkappaB, through Rac1-NOXs-ROS-dependent mechanism. Our results shows that TGF-beta1 stimulation of uPA and MMP-9 expression involve NOXs-dependent ROS and NFkappaB, activation, demonstrated by using DPI, NOXs inhibitor, ROS scavenger N-acetylcysteine and SN50, an NFkb inhibitor. Furthermore, we found that the inhibition of ROS and NFkappaB, abrogates TGF-beta1 stimulation of EMT, cell motility and invasion. Thus, ROS-NFkappaB acts as the crucial signal in TGF-beta1-induced uPA and MMP-9 expression thereby mediating the enhancement of cellular malignity by TGF-beta1.

#### GeneRIFs

1. ROS-NFkappaB acts as the crucial signal in TGF-beta1-induced uPA and MMP-9 expression thereby mediating the enhancement of cell **invasive**ness by TGF-beta1.

### [Mm]etastasize

#### PubMed

1. Tumor angiogenesis is caused by single melanoma cells in a manner dependent on reactive oxygen species and NF-κB. Melanomas have a high angiogenic potential, but respond poorly to medical treatment and **metastasiz**e very early. To understand the early events in tumor angiogenesis, animal models with high tumor resolution and blood vessel resolution are required, which provide the opportunity to test the ability of small molecule inhibitors to modulate the angiogenic tumor program. We have established a transgenic melanoma angiogenesis model in the small laboratory fish species Japanese medaka. Here, pigment cells are transformed by an oncogenic receptor tyrosine kinase in fish expressing GFP throughout their vasculature. We show that angiogenesis occurs in a reactive oxygen species (ROS)- and NF-κB-dependent, but hypoxia-independent manner. Intriguingly, we observed that blood vessel sprouting is induced even by single transformed pigment cells. The oncogenic receptor as well as human melanoma cells harboring other oncogenes caused the production of pro-angiogenic factors, most prominently angiogenin, through NF-κB signaling. Inhibiting NF-κB prevented tumor angiogenesis and led to the regression of existing tumor blood vessels. In conclusion, our high-resolution medaka melanoma model discloses that ROS and NF-κB signaling from single tumor cells causes hypoxia-independent angiogenesis, thus, demonstrating that the intrinsic malignant tumor cell features are sufficient to initiate and maintain a pro-angiogenic signaling threshold.

### [Mm]etastasis

#### PubMed

1. Sipa1 is a candidate for underlying the **metastasis** efficiency modifier locus Mtes1. We previously identified loci in the mouse genome that substantially influence the metastatic efficiency of mammary tumors. Here, we present data supporting the idea that the signal transduction molecule, Sipa1, is a candidate for underlying the **metastasis** efficiency modifier locus Mtes1. Analysis of candidate genes identified a nonsynonymous amino acid polymorphism in Sipa1 that affects the Sipa1 Rap-GAP function. Spontaneous **metastasis** assays using cells ectopically expressing Sipa1 or cells with knocked-down Sipa1 expression showed that metastatic capacity was correlated with cellular Sipa1 levels. We examined human expression data and found that they were consistent with the idea that Sipa1 concentration has a role in **metastasis**. Taken together, these data suggest that the Sipa1 polymorphism is one of the genetic polymorphisms underlying the Mtes1 locus. This report is also the first demonstration, to our knowledge, of a constitutional genetic polymorphism affecting tumor **metastasis**.
2. Epithelial-mesenchymal transition induced by TNF-α requires NF-κB-mediated transcriptional upregulation of Twist1. Proinflammatory cytokines produced in the tumor microenvironment facilitate tumor development and metastatic progression. In particular, TNF-α promotes cancer invasion and angiogenesis associated with epithelial-mesenchymal transition (EMT); however, the mechanisms underlying its induction of EMT in cancer cells remain unclear. Here we show that EMT and cancer stemness properties induced by chronic treatment with TNF-α are mediated by the upregulation of the transcriptional repressor Twist1. Exposure to TNF-α rapidly induced Twist1 mRNA and protein expression in normal breast epithelial and breast cancer cells. Both IKK-β and NF-κB p65 were required for TNF-α-induced expression of Twist1, suggesting the involvement of canonical NF-κB signaling. In support of this likelihood, we defined a functional NF-κB-binding site in the Twist1 promoter, and overexpression of p65 was sufficient to induce transcriptional upregulation of Twist1 along with EMT in mammary epithelial cells. Conversely, suppressing Twist1 expression abrogated p65-induced cell migration, invasion, EMT, and stemness properties, establishing that Twist1 is required for NF-κB to induce these aggressive phenotypes in breast cancer cells. Taken together, our results establish a signaling axis through which the tumor microenvironment elicits Twist1 expression to prom**ote cancer** metastasis. We suggest that targeting NF-κB-mediated Twist1 upregulation may offer an effective a therapeutic strategy for breast cancer treatment.
3. Identification of gene function and functional pathways by systemic plasmid-based ribozyme targeting in adult mice. To date, functional genomic studies have been confined to either cell-based assays or germline mutations, using transgenic or knockout animals. However, these approaches are often unable either to recapitulate complex biologic phenotypes, such as tumor **metastasis**, or to identify the specific genes and functional pathways that produce serious diseases in adult animals. Although the transcription factor NF-kappaB transactivates many **metastasis**-related genes in cells, the precise genes and functional-pathways through which NF-kappaB regulates **metastasis** in tumor-bearing hosts are poorly understood. Here, we show that the systemic delivery of plasmid-based ribozymes targeting NF-kappaB in adult, tumor-bearing mice suppressed NF-kappaB expression in metastatic melanoma cells, as well as in normal cell types, and significantly reduced metastatic spread. Plasmid-based ribozymes suppressed target-gene expression with sequence specificity not achievable by using synthetic oligonucleotide-based approaches. NF-kappaB seemed to regulate tumor **metastasis** through invasion-related, rather than angiogenesis-, cell-cycle- or apoptosis-related pathways in tumor-bearing mice. Furthermore, ribozymes targeting either of the NF-kappaB-regulated genes, integrin beta(3) or PECAM-1 (a ligand-receptor pair linked to cell adhesion), reduced tumor **metastasis** at a level comparable to NF-kappaB. These studies demonstrate the utility of gene targeting by means of systemic, plasmid-based ribozymes to dissect out the functional genomics of complex biologic phenotypes, including tumor **metastasis**.
4. ROS-NFkappaB mediates TGF-beta1-induced expression of urokinase-type plasminogen activator, matrix metalloproteinase-9 and cell invasion. TGF-beta1 has been postulated as a pro-oncogenic factor in the late step of the tumoral progression. In transformed cells, TGF-beta1 enhances the capacity to degrade the extracellular matrix, cell invasiveness and epithelial-mesenchymal transition, which are crucial steps for **metastasis**. Urokinase-type plasminogen activator (uPA) and matrix metalloproteinase-9 (MMP-9) are critical components in cell migration and invasion induced by TGF-beta1, however, the exact mechanism by which TGF-beta1 regulates uPA and MMP-9 is not well elucidated so far. In the present study, we analyzed the role of ROS-NFkappaB, signal as mediator in the cell malignity enhancement by TGF-beta1. We found that TGF-beta1 activates NFkappaB, through Rac1-NOXs-ROS-dependent mechanism. Our results shows that TGF-beta1 stimulation of uPA and MMP-9 expression involve NOXs-dependent ROS and NFkappaB, activation, demonstrated by using DPI, NOXs inhibitor, ROS scavenger N-acetylcysteine and SN50, an NFkb inhibitor. Furthermore, we found that the inhibition of ROS and NFkappaB, abrogates TGF-beta1 stimulation of EMT, cell motility and invasion. Thus, ROS-NFkappaB acts as the crucial signal in TGF-beta1-induced uPA and MMP-9 expression thereby mediating the enhancement of cellular malignity by TGF-beta1.
5. Norcantharidin facilitates LPS-mediated immune responses by up-regulation of AKT/NF-κB signaling in macrophages. Norcantharidin (NCTD), a demethylated analog of cantharidin, is a common used clinical drug to inhibit proliferation and **metastasi**s of cancer cells. But the role of NCTD in modulating immune responses remains unknown. Here, we investigated the function and mechanism of NCTD in regulation of TLR4 associated immune response in macrophages. We evaluated the influence of NCTD on host defense against invaded pathogens by acute peritonitis mouse model, ELISA, Q-PCR, nitrite quantification, phagocytosis assay and gelatin zymography assay. Our data showed that the survival and the serum concentrations of IL-6 and TNF-α were all enhanced by NCTD significantly in peritonitis mouse model. Accordingly, LPS-induced cytokine, nitric oxide and MMP-9 production as well as the phagocytosis of bacteria were all up-regulated by NCTD in a dose dependent manner in both RAW264.7 cells and bone marrow-derived macrophages (BMMs). Then we further analyzed TLR4 associated signaling pathway by Western blot, Immunofluorescence and EMSA in the presence or absence of LPS. The phosphorylation of AKT and p65 at serine 536 but not serine 468 was enhanced obviously by NCTD in a dose dependent manner, whereas the degradation of IκBα was little effected. Consequently, the nuclear translocation and DNA binding ability of NF-κB was also increased by NCTD obviously in RAW264.7 cells. Our results demonstrated that NCTD could facilitate LPS-mediated immune response through promoting the phosphorylation of AKT/p65 and transcriptional activity of NF-κB, thus reprofiling the traditional anti-tumor drug NCTD as a novel immune regulator in promoting host defense against bacterial infection.

### [Cc]ell [Ii]nvasion

#### PubMed

1. ROS-NFkappaB mediates TGF-beta1-induced expression of urokinase-type plasminogen activator, matrix metalloproteinase-9 and **cell invasion**. TGF-beta1 has been postulated as a pro-oncogenic factor in the late step of the tumoral progression. In transformed cells, TGF-beta1 enhances the capacity to degrade the extracellular matrix, cell invasiveness and epithelial-mesenchymal transition, which are crucial steps for metastasis. Urokinase-type plasminogen activator (uPA) and matrix metalloproteinase-9 (MMP-9) are critical components in cell migration and invasion induced by TGF-beta1, however, the exact mechanism by which TGF-beta1 regulates uPA and MMP-9 is not well elucidated so far. In the present study, we analyzed the role of ROS-NFkappaB, signal as mediator in the cell malignity enhancement by TGF-beta1. We found that TGF-beta1 activates NFkappaB, through Rac1-NOXs-ROS-dependent mechanism. Our results shows that TGF-beta1 stimulation of uPA and MMP-9 expression involve NOXs-dependent ROS and NFkappaB, activation, demonstrated by using DPI, NOXs inhibitor, ROS scavenger N-acetylcysteine and SN50, an NFkb inhibitor. Furthermore, we found that the inhibition of ROS and NFkappaB, abrogates TGF-beta1 stimulation of EMT, cell motility and invasion. Thus, ROS-NFkappaB acts as the crucial signal in TGF-beta1-induced uPA and MMP-9 expression thereby mediating the enhancement of cellular malignity by TGF-beta1.

---

## Creb3l2

- NCBI Gene
- GeneRIF
- Pubmed

| Search Term | Rank | Fields |
| --- | --- | --- |
| ECM | 7 | PubMed(1) |
| [Ee]xtracellular [Mm]atrix | 8 | PubMed(2) |

  
hide/show details for Creb3l2

### ECM

#### PubMed

1. The endoplasmic reticulum stress transducer BBF2H7 suppresses apoptosis by activating the ATF5-MCL1 pathway in growth plate cartilage. BBF2H7 (box B-binding factor 2 human homolog on chromosome 7) is a basic leucine zipper transmembrane transcription factor that belongs to the cyclic AMP-responsive element-binding protein (CREB)/activating transcription factor (ATF) family. This novel endoplasmic reticulum (ER) stress transducer is localized in the ER and is cleaved in its transmembrane region in response to ER stress. BBF2H7 has been shown to be expressed in proliferating chondrocytes in cartilage during the development of long bones. The target of BBF2H7 is Sec23a, one of the coat protein complex II components. Bbf2h7-deficient (Bbf2h7(-/-)) mice exhibit severe chondrodysplasia, with expansion of the rough ER in proliferating chondrocytes caused by impaired secretion of extracellular matrix (**ECM**) proteins. We observed a decrease in the number of proliferating chondrocytes in the cartilage of Bbf2h7(-/-) mice. TUNEL staining of the cartilage showed that apoptosis was promoted in Bbf2h7(-/-) chondrocytes. Atf5 (activating transcription factor 5), another member of the CREB/ATF family and an antiapoptotic factor, was also found to be a target of BBF2H7 in chondrocytes. ATF5 activated the transcription of Mcl1 (myeloid cell leukemia sequence 1), which belongs to the antiapoptotic B-cell leukemia/lymphoma 2 family, to suppress apoptosis. Finally, we found that the BBF2H7-ATF5-MCL1 pathway specifically suppressed ER stress-induced apoptosis in chondrocytes. Taken together, our findings indicate that BBF2H7 is activated in response to ER stress caused by synthesis of abundant **ECM** proteins and plays crucial roles as a bifunctional regulator to accelerate **ECM** protein secretion and suppress ER stress-induced apoptosis by activating the ATF5-MCL1 pathway during chondrogenesis.

### [Ee]xtracellular [Mm]atrix

#### PubMed

1. Regulation of endoplasmic reticulum stress response by a BBF2H7-mediated Sec23a pathway is essential for chondrogenesis. Many tissues have a specific signal transduction system for endoplasmic reticulum (ER) dysfunction; however, the mechanisms underlying the ER stress response in cartilage remain unclear. BBF2H7 (BBF2 human homologue on chromosome 7), an ER-resident basic leucine zipper transcription factor, is activated in response to ER stress and is highly expressed in chondrocytes. In this study, we generated Bbf2h7(-/-) mice to assess the in vivo function of BBF2H7. The mice showed severe chondrodysplasia and died by suffocation shortly after birth because of an immature chest cavity. The cartilage showed a lack of typical columnar structure in the proliferating zone and a decrease in the size of the hypertrophic zone, resulting in a significant reduction of **extracellular matrix** proteins. Interestingly, proliferating chondrocytes showed abnormally expanded ER, containing aggregated type II collagen (Col2) and cartilage oligomeric matrix protein (COMP). We identified Sec23a, which encodes a coat protein complex II component responsible for protein transport from the ER to the Golgi, as a target of BBF2H7, which directly bound to a CRE-like sequence in the promoter region of Sec23a to activate its transcription. When Sec23a was introduced to Bbf2h7(-/-) chondrocytes, the impaired transport and secretion of cartilage matrix proteins was totally restored, indicating that by activating protein secretion the BBF2H7-Sec23a pathway has a crucial role in chondrogenesis. Our findings provide a new link by which ER stress is converted to signalling for the activation of ER-to-Golgi trafficking.
2. The endoplasmic reticulum stress transducer BBF2H7 suppresses apoptosis by activating the ATF5-MCL1 pathway in growth plate cartilage. BBF2H7 (box B-binding factor 2 human homolog on chromosome 7) is a basic leucine zipper transmembrane transcription factor that belongs to the cyclic AMP-responsive element-binding protein (CREB)/activating transcription factor (ATF) family. This novel endoplasmic reticulum (ER) stress transducer is localized in the ER and is cleaved in its transmembrane region in response to ER stress. BBF2H7 has been shown to be expressed in proliferating chondrocytes in cartilage during the development of long bones. The target of BBF2H7 is Sec23a, one of the coat protein complex II components. Bbf2h7-deficient (Bbf2h7(-/-)) mice exhibit severe chondrodysplasia, with expansion of the rough ER in proliferating chondrocytes caused by impaired secretion of **extracellular matrix** (ECM) proteins. We observed a decrease in the number of proliferating chondrocytes in the cartilage of Bbf2h7(-/-) mice. TUNEL staining of the cartilage showed that apoptosis was promoted in Bbf2h7(-/-) chondrocytes. Atf5 (activating transcription factor 5), another member of the CREB/ATF family and an antiapoptotic factor, was also found to be a target of BBF2H7 in chondrocytes. ATF5 activated the transcription of Mcl1 (myeloid cell leukemia sequence 1), which belongs to the antiapoptotic B-cell leukemia/lymphoma 2 family, to suppress apoptosis. Finally, we found that the BBF2H7-ATF5-MCL1 pathway specifically suppressed ER stress-induced apoptosis in chondrocytes. Taken together, our findings indicate that BBF2H7 is activated in response to ER stress caused by synthesis of abundant ECM proteins and plays crucial roles as a bifunctional regulator to accelerate ECM protein secretion and suppress ER stress-induced apoptosis by activating the ATF5-MCL1 pathway during chondrogenesis.

---

## Hes1

- NCBI Gene
- GeneRIF
- Pubmed

| Search Term | Rank | Fields |
| --- | --- | --- |
| [Ff]ilopodia | 1 | PubMed(2) |
| [Mm]igration | 6 | Process(1); PubMed(22) |
| ECM | 7 | PubMed(2) |
| [Ee]xtracellular [Mm]atrix | 8 | PubMed(3) |
| [Ii]nvasive | 10 | PubMed(1) |
| [Mm]etastasis | 12 | PubMed(2) |

  
hide/show details for Hes1

### [Ff]ilopodia

#### PubMed

1. Dynamic interactions between intermediate neurogenic progenitors and radial glia in embryonic mouse neocortex: potential role in Dll1-Notch signaling. The mammalian neocortical progenitor cell niche is composed of a diverse repertoire of neuroepithelial cells, radial glia (RG), and intermediate neurogenic progenitors (INPs). Previously, live-cell imaging experiments have proved crucial in identifying these distinct progenitor populations, especially INPs, which amplify neural output by undergoing additional rounds of proliferation before differentiating into new neurons. INPs also provide feedback to the RG pool by serving as a source of Delta-like 1 (Dll1), a key ligand for activating Notch signaling in neighboring cells, a well-known mechanism for maintaining RG identity. While much is known about Dll1-Notch signaling at the molecular level, little is known about how this cell-cell contact dependent feedback is transmitted at the cellular level. To investigate how RG and INPs might interact to convey Notch signals, we used high-resolution live-cell multiphoton microscopy (MPM) to directly observe cellular interactions and dynamics, in conjunction with Notch-pathway specific reporters in the neocortical neural stem cell niche in organotypic brain slices from embryonic mice. We found that INPs and RG interact via dynamic and transient elongate processes, some apparently long-range (extending from the subventricular zone to the ventricular zone), and some short-range (**filopodia**-like). Gene expression profiling of RG and INPs revealed further progenitor cell diversification, including different subpopulations of Hes1+ and/or Hes5+ RG, and Dll1+ and/or Dll3+ INPs. Thus, the embryonic progenitor niche includes a network of dynamic cell-cell interactions, using different combinations of Notch signaling molecules to maintain and likely diversify progenitor pools.
2. NRP1 Regulates CDC42 Activation to Promote **Filopodia** Formation in Endothelial Tip Cells. Sprouting blood vessels are led by **filopodia**-studded endothelial tip cells that respond to angiogenic signals. Mosaic lineage tracing previously revealed that NRP1 is essential for tip cell function, although its mechanistic role in tip cells remains poorly defined. Here, we show that NRP1 is dispensable for genetic tip cell identity. Instead, we find that NRP1 is essential to form the **filopodia**l bursts that distinguish tip cells morphologically from neighboring stalk cells, because it enables the extracellular matrix (ECM)-induced activation of CDC42, a key regulator of **filopodia** formation. Accordingly, NRP1 knockdown and pharmacological CDC42 inhibition similarly impaired **filopodia** formation in vitro and in developing zebrafish in vivo. During mouse retinal angiogenesis, CDC42 inhibition impaired tip cell and vascular network formation, causing defects that resembled those due to loss of ECM-induced, but not VEGF-induced, NRP1 signaling. We conclude that NRP1 enables ECM-induced **filopodia** formation for tip cell function during sprouting angiogenesis.

### [Mm]igration

#### Process

1. cell **migration**

#### PubMed

1. NFIX Regulates Proliferation and **Migration** Within the Murine SVZ Neurogenic Niche. Transcription factors of the nuclear factor one (NFI) family play a pivotal role in the development of the nervous system. One member, NFIX, regulates the development of the neocortex, hippocampus, and cerebellum. Postnatal Nfix(-/-) mice also display abnormalities within the subventricular zone (SVZ) lining the lateral ventricles, a region of the brain comprising a neurogenic niche that provides ongoing neurogenesis throughout life. Specifically, Nfix(-/-) mice exhibit more PAX6-expressing progenitor cells within the SVZ. However, the mechanism underlying the development of this phenotype remains undefined. Here, we reveal that NFIX contributes to multiple facets of SVZ development. Postnatal Nfix(-/-) mice exhibit increased levels of proliferation within the SVZ, both in vivo and in vitro as assessed by a neurosphere assay. Furthermore, we show that the **migration** of SVZ-derived neuroblasts to the olfactory bulb is impaired, and that the olfactory bulbs of postnatal Nfix(-/-) mice are smaller. We also demonstrate that gliogenesis within the rostral migratory stream is delayed in the absence of Nfix, and reveal that Gdnf (glial-derived neurotrophic factor), a known attractant for SVZ-derived neuroblasts, is a target for transcriptional activation by NFIX. Collectively, these findings suggest that NFIX regulates both proliferation and **migration** during the development of the SVZ neurogenic niche.
2. Murine neocortical histogenesis is perturbed by prenatal exposure to low doses of Bisphenol A. Bisphenol A (BPA) has been shown to disrupt thyroid hormone function. We therefore studied whether prenatal exposure to low-doses of BPA affects the morphology and the expression of some genes related to brain development in the murine fetal neocortex. Pregnant mice were injected subcutaneously with 20 microg/kg of BPA daily from embryonic day 0 (E0). Control animals received vehicle alone. For evaluating cell proliferation, neuronal differentiation and **migration**, bromodeoxyuridine (BrdU) was injected intraperitoneally into pregnant mice with various regimens and the brains were processed for immunohistochemistry. The total RNA was extracted from the embryonic telencephalon at various embryonic stages. The BrdU-labeled cells examined 1 hour after BrdU injection showed no differences between the BPA-treated and control groups (n = 10, each), which indicated that the proliferation of precursor cells was not affected. The BrdU-labeled cells, analysed 2 days after BrdU injection, were decreased in the ventricular zone of BPA-treated mice at E14.5 and E16.5, whereas they were increased in the cortical plate at E14.5 as compared with those in control mice (n = 10, each). Furthermore, the expression of Math3, Ngn2, Hes1, LICAM, and THRalpha was significantly upregulated at E14.5 in the BPA-treated group. These results suggested that BPA might disrupt normal neocortical development by accelerating neuronal differentiation/**migration**.
3. Nuclear factor one B regulates neural stem cell differentiation and axonal projection of corticofugal neurons. During development of the cerebral cortex, neural stem cells divide to expand the progenitor pool and generate basal progenitors, outer radial glia, and cortical neurons. As these newly born neurons differentiate, they must properly migrate toward their final destination in the cortical plate, project axons to appropriate targets, and develop dendrites. However, a complete understanding of the precise genetic mechanisms regulating these steps is lacking. Here we show that a member of the nuclear factor one (NFI) family of transcription factors, NFIB, is essential for many of these processes in mice. We performed a detailed analysis of NFIB expression during cortical development, and investigated defects in cortical neurogenesis, neuronal **migration**, and differentiation in NfiB(-/-) brains. We found that NFIB is strongly expressed in radial glia and corticofugal neurons throughout cortical development. However, in NfiB(-/-) cortices, radial glia failed to generate outer radial glia, subsequently resulting in a loss of late basal progenitors. In addition, corticofugal neurons showed a severe loss of axonal projections, whereas late-born cortical neurons displayed defects in **migration** and ectopically expressed the early-born neuronal marker CTIP2. Furthermore, gene expression analysis, by RNA sequencing, revealed a misexpression of genes that regulate the cell cycle, neuronal differentiation and **migration** in NfiB(-/-) brains. Together these results demonstrate the critical functions of NFIB in regulating cortical development.
4. Hes genes and neurogenin regulate non-neural versus neural fate specification in the dorsal telencephalic midline. The choroid plexus in the brain is unique because it is a non-neural secretory tissue. It secretes the cerebrospinal fluid and functions as a blood-brain barrier, but the precise mechanism of specification of this non-neural tissue has not yet been determined. Using mouse embryos and lineage-tracing analysis, we found that the prospective choroid plexus region initially gives rise to Cajal-Retzius cells, specialized neurons that guide neuronal **migration**. Inactivation of the bHLH repressor genes Hes1, Hes3 and Hes5 upregulated expression of the proneural gene neurogenin 2 (Ngn2) and prematurely depleted Bmp-expressing progenitor cells, leading to enhanced formation of Cajal-Retzius cells and complete loss of choroid plexus epithelial cells. Overexpression of Ngn2 had similar effects. These data indicate that Hes genes promote specification of the fate of choroid plexus epithelial cells rather than the fate of Cajal-Retzius cells by antagonizing Ngn2 in the dorsal telencephalic midline region, and thus this study has identified a novel role for bHLH genes in the process of deciding which cells will have a non-neural versus a neural fate.
5. SnoN facilitates ALK1-Smad1/5 signaling during embryonic angiogenesis. In endothelial cells, two type I receptors of the transforming growth factor β (TGF-β) family, ALK1 and ALK5, coordinate to regulate embryonic angiogenesis in response to BMP9/10 and TGF-β. Whereas TGF-β binds to and activates ALK5, leading to Smad2/3 phosphorylation and inhibition of endothelial cell proliferation **and migra**tion, BMP9/10 and TGF-β also bind to ALK1, resulting in the activation of Smad1/5. SnoN is a negative regulator of ALK5 signaling through the binding and repression of Smad2/3. Here we uncover a positive role of SnoN in enhancing Smad1/5 activation in endothelial cells to promote angiogenesis. Upon ligand binding, SnoN directly bound to ALK1 on the plasma membrane and facilitated the interaction between ALK1 and Smad1/5, enhancing Smad1/5 phosphorylation. Disruption of this SnoN-Smad interaction impaired Smad1/5 activation and up-regulated Smad2/3 activity. This resulted in defective angiogenesis and arteriovenous malformations, leading to embryonic lethality at E12.5. Thus, SnoN is essential for TGF-β/BMP9-dependent biological processes by its ability to both positively and negatively modulate the activities of Smad-dependent pathways.
6. Shp2-dependent ERK signaling is essential for induction of Bergmann glia and foliation of the cerebellum. Folding of the cortex and the persistence of radial glia (RG)-like cells called Bergmann glia (BG) are hallmarks of the mammalian cerebellum. Similar to basal RG in the embryonic neocortex, BG maintain only basal processes and continuously express neural stem cell markers. Past studies had focused on the function of BG in granule cell **migration** and how granule cell progenitors (GCP) regulate cerebellar foliation. The molecular control of BG generation and its role in cerebellar foliation are less understood. Here, we have analyzed the function of the protein tyrosine phosphatase Shp2 in mice by deleting its gene Ptpn11 in the entire cerebellum or selectively in the GCP lineage. Deleting Ptpn11 in the entire cerebellum by En1-cre blocks transformation of RG into BG but preserves other major cerebellar cell types. In the absence of BG, inward invagination of GCP persists but is uncoupled from the folding of the Purkinje cell layer and the basement membrane, leading to disorganized lamination and an absence of cerebellar folia. In contrast, removing Ptpn11 in the GCP lineage by Atoh1-cre has no effect on cerebellar development, indicating that Shp2 is not cell autonomously required in GCP. Furthermore, we demonstrate that Ptpn11 interacts with Fgf8 and is essential for ERK activation in RG and nascent BG. Finally, expressing constitutively active MEK1 rescues BG formation and cerebellar foliation in Shp2-deficient cerebella. Our results demonstrate an essential role of Shp2 in BG specification via fibroblast growth factor/extracellular signal-regulated protein kinase signaling, and reveal a crucial function of BG in organizing cerebellar foliation.
7. Prox1 ablation in hepatic progenitors causes defective hepatocyte specification and increases biliary cell commitment. The liver has multiple functions that preserve homeostasis. Liver diseases are debilitating, costly and often result in death. Elucidating the developmental mechanisms that establish the liver's architecture or generate the cellular diversity of this organ should help advance the prevention, diagnosis and treatment of hepatic diseases. We previously reported that **migration** of early hepatic precursors away from the gut epithelium requires the activity of the homeobox gene Prox1. Here, we show that Prox1 is a novel regulator of cell differentiation and morphogenesis during hepatogenesis. Prox1 ablation in bipotent hepatoblasts dramatically reduced the expression of multiple hepatocyte genes and led to very defective hepatocyte morphogenesis. As a result, abnormal epithelial structures expressing hepatocyte and cholangiocyte markers or resembling ectopic bile ducts developed in the Prox1-deficient liver parenchyma. By contrast, excessive commitment of hepatoblasts into cholangiocytes, premature intrahepatic bile duct morphogenesis, and biliary hyperplasia occurred in periportal areas of Prox1-deficient livers. Together, these abnormalities indicate that Prox1 activity is necessary to correctly allocate cell fates in liver precursors. These results increase our understanding of differentiation anomalies in pathological conditions and will contribute to improving stem cell protocols in which differentiation is directed towards hepatocytes and cholangiocytes.
8. Premature differentiation and aberrant movement of pituitary cells lacking both Hes1 and Prop1. In the pituitary, the transition from proliferating progenitor cell into differentiated hormone producing cell is carefully regulated in a time-dependent and spatially-restricted manner. We report that two targets of Notch signaling, Hes1 and Prop1, are needed to maintain progenitors within Rathke's pouch and for the restriction of differentiated cells to the ventral pituitary. We observed ACTH and alphaGSU producing cells that had prematurely differentiated within Rathke's pouch along with correlated ectopic expression of Mash1 only when both Prop1 and Hes1 were lost. We also discovered that downregulation of N-cadherin expression in cells as they transition from Rathke's pouch to the anterior lobe appears to be essential for their movement. In the Prop1 mutant, cells are trapped in Rathke's pouch and N-cadherin expression remains high. Also, Slug, a marker of epithelial-to-mesenchymal transition, is absent in the dorsal anterior lobe. When Hes1 is lost in the Prop1 mutant, N-cadherin is downregulated and cells are able to exit Rathke's pouch but have lost their **migration**al cues and form ectopic foci surrounding Rathke's pouch. Our data reveal important overlapping functions of Hes1 and Prop1 in cell differentiation and movement that are critical for pituitary organogenesis.
9. Hes1 is required for the development of pharyngeal organs and survival of neural crest-derived mesenchymal cells in pharyngeal arches. Hes genes are required to maintain diverse progenitor cell populations during embryonic development. Loss of Hes1 results in a spectrum of malformations of pharyngeal endoderm-derived organs, including the ultimobranchial body (progenitor of C cells), parathyroid, thymus and thyroid glands, together with highly penetrant C-cell aplasia (81%) and parathyroid aplasia (28%). The hypoplastic parathyroid and thymus are mostly located around the pharyngeal cavity, even at embryonic day (E) 15.5 to E18.5, indicating the failure of **migration** of the organs. To clarify the relationship between these phenotypes and neural crest cells, we examine fate mapping of neural crest cells colonized in pharyngeal arches in Hes1 null mutants by using the Wnt1-Cre/R26R reporter system. In null mutants, the number of neural crest cells labeled by X-gal staining is markedly decreased in the pharyngeal mesenchyme at E12.5 when the primordia of the thymus, parathyroid and ultimobranchial body migrate toward their destinations. Furthermore, phospho-Histone-H3-positive proliferating cells are reduced in number in the pharyngeal mesenchyme at this stage. Our data indicate that the development of pharyngeal organs and survival of neural-crest-derived mesenchyme in pharyngeal arches are critically dependent on Hes1. We propose that the defective survival of neural-crest-derived mesenchymal cells in pharyngeal arches directly or indirectly leads to deficiencies of pharyngeal organs.
10. Fibroblast growth factor-2 increases the expression of neurogenic genes and promotes the **migration** and differentiation of neurons derived from transplanted neural stem/progenitor cells. The capacity of neural stem cells (NSC) to generate different types of neurons and glia depends on the action of intrinsic determinants and extracellular signals. Here, we isolated adult olfactory bulb stem cells (aOBSC) that express nestin, RC2 and Sox2, and that have the capacity to generate neurons possessing mature features in culture and in vivo. The differentiation of aOBSC into neurons and glia, as well as their genetic profile, was compared to that of embryonic OBSC (eOBSC) and ganglionic eminence stem cells (GESC). While these eOBSC express neurogenin (Ngn) 1 and 2, two telencephalic dorsal markers, GESC only express Ngn2. Adult OBSC express either little or no detectable Ngn1 and 2, and they produced significantly fewer neurons in culture than eOBSC. By contrast, Dlx2 transcripts (a telencephalic ventral marker) were only clearly detected in GESC. When transplanted into the early postnatal P5-P7 OB, each of the three populations gave rise to cells with a distinct pattern of neuronal **migration** and/or dendritic arborization. Overall, these findings suggest that cultured NSC partially maintain their regional and temporal specification. Notably, significant neuronal **migration** and differentiation were only observed in vivo when the NSC were briefly exposed to fibroblast growth factor-2 (FGF-2) before grafting, a treatment that enhanced the neurogenin expression. Hence, the **migration** and maturation of neurons derived from transplanted NSC can be promoted by upregulating neurogenic gene expression with FGF-2.
11. The Notch effector gene Hes1 regulates **migration** of hypothalamic neurons, neuropeptide content and axon targeting to the pituitary. Proper development of the hypothalamic-pituitary axis requires precise neuronal signaling to establish a network that regulates homeostasis. The developing hypothalamus and pituitary utilize similar signaling pathways for differentiation in embryonic development. The Notch signaling effector gene Hes1 is present in the developing hypothalamus and pituitary and is required for proper formation of the pituitary, which contains axons of arginine vasopressin (AVP) neurons from the hypothalamic paraventricular nucleus (PVN) and supraoptic nucleus (SON). We hypothesized that Hes1 is necessary for the generation, placement and projection of AVP neurons. We found that Hes1 null mice show no significant difference in cell proliferation or death in the developing diencephalon at embryonic day 10.5 (e10.5) or e11.5. By e16.5, AVP cell bodies are formed in the SON and PVN, but are abnormally placed, suggesting that Hes1 may be necessary for the **migration** of AVP neurons. GAD67 immunoreactivity is ectopically expressed in Hes1 null mice, which may contribute to cell body misplacement. Additionally, at e18.5 Hes1 null mice show continued misplacement of AVP cell bodies in the PVN and SON and additionally exhibit abnormal axonal projection. Using mass spectrometry to characterize peptide content, we found that Hes1 null pituitaries have aberrant somatostatin (SS) peptide, which correlates with abnormal SS cells in the pituitary and misplaced SS axon tracts at e18.5. Our results indicate that Notch signaling facilitates the **migration** and guidance of hypothalamic neurons, as well as neuropeptide content.
12. Notch signaling coordinates the patterning of striatal compartments. Numerous lines of evidence suggest that Notch signaling plays a pivotal role in controlling the production of neurons from progenitor cells. However, most experiments have relied on gain-of-function approaches because perturbation of Notch signaling results in death prior to the onset of neurogenesis. Here, we examine the requirement for Notch signaling in the development of the striatum through the analysis of different single and compound Notch1 conditional and Notch3 null mutants. We find that normal development of the striatum depends on the presence of appropriate Notch signals in progenitors during a critical window of embryonic development. Early removal of Notch1 prior to neurogenesis alters early-born patch neurons but not late-born matrix neurons in the striatum. We further show that the late-born striatal neurons in these mutants are spared as a result of functional compensation by Notch3. Notably, however, the removal of Notch signaling subsequent to cells leaving the germinal zone has no obvious effect on striatal organization and patterning. These results indicate that Notch signaling is required in neural progenitor cells to control cell fate in the striatum, but is dispensable during subsequent phases of neuronal **migration** and differentiation.
13. Keratin 79 identifies a novel population of migratory epithelial cells that initiates hair canal morphogenesis and regeneration. The formation of epithelial tubes underlies the development of diverse organs. In the skin, hair follicles resemble tube-like structures with lumens that are generated through poorly understood cellular rearrangements. Here, we show that creation of the hair follicle lumen is mediated by early outward movement of keratinocytes from within the cores of developing hair buds. These migratory keratinocytes express keratin 79 (K79) and stream out of the hair germ and into the epidermis prior to lumen formation in the embryo. Remarkably, this process is recapitulated during hair regeneration in the adult mouse, when K79(+) cells migrate out of the reactivated secondary hair germ prior to formation of a new hair canal. During homeostasis, K79(+) cells line the hair follicle infundibulum, a domain we show to be multilayered, biochemically distinct and maintained by Lrig1(+) stem cell-derived progeny. Upward movement of these cells sustains the infundibulum, while perturbation of this domain during acne progression is often accompanied by loss of K79. Our findings uncover previously unappreciated long-distance cell movements throughout the life cycle of the hair follicle, and suggest a novel mechanism by which the follicle generates its hollow core through outward cell **migration**.
14. Impaired angiogenesis and altered Notch signaling in mice overexpressing endothelial Egfl7. Epidermal growth factor-like domain 7 (Egfl7) is important for regulating tubulogenesis in zebrafish, but its role in mammals remains unresolved. We show here that endothelial overexpression of Egfl7 in transgenic mice leads to partial lethality, hemorrhaging, and altered cardiac morphogenesis. These defects are accompanied by abnormal vascular patterning and remodeling in both the embryonic and postnatal vasculature. Egfl7 overexpression in the neonatal retina results in a hyperangiogenic response, and EGFL7 knockdown in human primary endothelial cells suppresses endothelial cell proliferation, sprouting, and **migration**. These phenotypes are reminiscent of Notch inhibition. In addition, our results show that EGFL7 and endothelial-specific NOTCH physically interact in vivo and strongly suggest that Egfl7 antagonizes Notch in both the postnatal retina and in primary endothelial cells. Specifically, Egfl7 inhibits Notch reporter activity and down-regulates the level of Notch target genes when overexpressed. In conclusion, we have uncovered a critical role for Egfl7 in vascular development and have shown that some of these functions are mediated through modulation of Notch signaling.
15. A role for smoothened during murine lens and cornea development. Various studies suggest that Hedgehog (Hh) signalling plays roles in human and zebrafish ocular development. Recent studies (Kerr et al., Invest Ophthalmol Vis Sci. 2012; 53, 3316-30) showed that conditionally activating Hh signals promotes murine lens epithelial cell proliferation and disrupts fibre differentiation. In this study we examined the expression of the Hh pathway and the requirement for the Smoothened gene in murine lens development. Expression of Hh pathway components in developing lens was examined by RT-PCR, immunofluorescence and in situ hybridisation. The requirement of Smo in lens development was determined by conditional loss-of-function mutations, using LeCre and MLR10 Cre transgenic mice. The phenotype of mutant mice was examined by immunofluorescence for various markers of cell cycle, lens and cornea differentiation. Hh pathway components (Ptch1, Smo, Gli2, Gli3) were detected in lens epithelium from E12.5. Gli2 was particularly localised to mitotic nuclei and, at E13.5, Gli3 exhibited a shift from cytosol to nucleus, suggesting distinct roles for these transcription factors. Conditional deletion of Smo, from ∼E12.5 (MLR10 Cre) did not affect ocular development, whereas deletion from ∼E9.5 (LeCre) resulted in lens and corneal defects from E14.5. Mutant lenses were smaller and showed normal expression of p57Kip2, c-Maf, E-cadherin and Pax6, reduced expression of FoxE3 and Ptch1 and decreased nuclear Hes1. There was normal G1-S phase but decreased G2-M phase transition at E16.5 and epithelial cell death from E14.5-E16.5. Mutant corneas were thicker due to aberr**ant migra**tion of Nrp2+ cells from the extraocular mesenchyme, resulting in delayed corneal endothelial but normal epithelial differentiation. These results indicate the Hh pathway is required during a discrete period (E9.5-E12.5) in lens development to regulate lens epithelial cell proliferation, survival and FoxE3 expression. Defective corneal development occurs secondary to defects in lens and appears to be due to defect**ive migra**tion of peri-ocular Nrp2+ neural crest/mesenchymal cells.
16. Genetic deletion of Rnd3 results in aqueductal stenosis leading to hydrocephalus through up-regulation of Notch signaling. Rho family guanosine triphosphatase (GTPase) 3 (Rnd3), a member of the small Rho GTPase family, is involved in the regulation of cell actin cytoskeleton dynamics, cell **migration**, and proliferation through the Rho kinase-dependent signaling pathway. We report a role of Rnd3 in the pathogenesis of hydrocephalus disorder. Mice with Rnd3 genetic deletion developed severe obstructive hydrocephalus with enlargement of the lateral and third ventricles, but not of the fourth ventricles. The cerebral aqueducts in Rnd3-null mice were partially or completely blocked by the overgrowth of ependymal epithelia. We examined the molecular mechanism contributing to this Rnd3-deficiency-mediated hydrocephalus and found that Rnd3 is a regulator of Notch signaling. Rnd3 deficiency, through either gene deletion or siRNA knockdown, resulted in a decrease in Notch intracellular domain (NICD) protein degradation. However, there was no correlated change in mRNA change, which in turn led to an increase in NICD protein levels. Immunoprecipitation analysis demonstrated that Rnd3 and NICD physically interacted, and that down-regulation of Rnd3 attenuated NICD protein ubiquitination. This eventually enhanced Notch signaling activity and promoted aberrant growth of aqueduct ependymal cells, resulting in aqueduct stenosis and the development of congenital hydrocephalus. Inhibition of Notch activity rescued the hydrocephalus disorder in the mutant animals.
17. Key basic helix-loop-helix transcription factor genes Hes1 and Ngn2 are regulated by Pax3 during mouse embryonic development. Pax3 is expressed early during embryonic development in spatially restricted domains including limb muscle, neural crest, and neural tube. Pax3 functions at the nodal point in melanocyte stem cell differentiation, cardiogenesis and neurogenesis. Additionally Pax3 has been implicated in **migration** and differentiation of precursor cell populations. Currently there are questions about how Pax3 regulates these diverse functions. In this study we found that in the absence of functional Pax3, as in Splotch embryos, the neural crest cells undergo premature neurogenesis, as evidenced by increased Brn3a positive staining in neural tube explants, in comparison with wild-type. Premature neurogenesis in the absence of functional Pax3 may be due to a change in the regulation of basic helix-loop-helix transcription factors implicated in proliferation and differentiation. Using promoter-luciferase activity measurements in transient co-transfection experiments and electro-mobility shift assays, we show that Pax3 regulates Hairy and enhancer of split homolog-1 (Hes1) and Neurogenin2 (Ngn2) by directly binding to their promoters. Chromatin immunoprecipitation assays confirmed that Pax3 bound to cis-regulatory elements within Hes1 and Ngn2 promoters. These observations suggest that Pax3 regulates Hes1 and Ngn2 and imply that it may couple **migration** with neural stem cell maintenance and neurogenesis.
18. Overexpression of lunatic fringe does not affect epithelial cell differentiation in the developing mouse lung. The Notch/Notch-ligand pathway regulates cell fate decisions and patterning in various tissues. Several of its components are expressed in the developing lung, suggesting that this pathway is important for airway cellular patterning. Fringe proteins, which modulate Notch signaling, are crucial for defining morphogenic borders in several organs. Their role in controlling cellular differentiation along anterior-posterior axis of the airways is unknown. Herein, we report the temporal-spatial expression patterns of Lunatic fringe (Lfng) and Notch-regulated basic helix-loop-helix factors, Hes1 and Mash-1, during murine lung development. Lfng was only expressed during early development in epithelial cells lining the larger airways. Those epithelial cells also expressed Hes1, but at later gestation Hes1 expression was confined to epithelium lining the terminal bronchioles. Mash-1 displayed a very characteristic expression pattern. It followed neural crest **migration** in the early lung, whereas at later stages Mash-1 was expressed in lung neuroendocrine cells. To clarify whether Lfng influences airway cell differentiation, Lfng was overexpressed in distal epithelial cells of the developing mouse lung. Overexpression of Lfng did not affect spatial or temporal expression of Hes1 and Mash-1. Neuroendocrine CGRP and protein gene product 9.5 expression was not altered by Lfng overexpression. Expression of proximal ciliated (beta-tubulin IV), nonciliated (CCSP), and distal epithelial cell (SP-C, T1alpha) markers also was not influenced by Lfng excess. Overexpression of Lfng had no effect on mesenchymal cell marker (alpha-sma, vWF, PECAM-1) expression. Collectively, the data suggest that Lunatic fringe does not play a significant role in determining cell fate in fetal airway epithelium.
19. Primary cilia maintain corneal epithelial homeostasis by regulation of the Notch signaling pathway. Primary cilia have been linked to signaling pathways involved in cell proliferation, cell motility and cell polarity. Defects in ciliary function result in developmental abnormalities and multiple ciliopathies. Patients affected by severe ciliopathies, such as Meckel syndrome, present several ocular surface disease conditions of unclear pathogenesis. Here, we show that primary cilia are predominantly present on basal cells of the mouse corneal epithelium (CE) throughout development and in the adult. Conditional ablation of cilia in the CE leads to an increase in proliferation and vertical **migration** of basal corneal epithelial cells (CECs). A consequent increase in cell density of suprabasal layers results in a thicker than normal CE. Surprisingly, in cilia-deficient CE, cilia-mediated signaling pathways, including Hh and Wnt pathways, were not affected but the intensity of Notch signaling was severely diminished. Although Notch1 and Notch2 receptors were expressed normally, nuclear Notch1 intracellular domain (N1ICD) expression was severely reduced. Postnatal development analysis revealed that in cilia-deficient CECs downregulation of the Notch pathway precedes cell proliferation defects. Thus, we have uncovered a function of the primary cilium in maintaining homeostasis of the CE by balancing proliferation and vertical **migration** of basal CECs through modulation of Notch signaling.
20. Reelin sets the pace of neocortical neurogenesis. **Migration** of neurons during cortical development is often assumed to rely on purely post-proliferative reelin signaling. However, Notch signaling, long known to regulate neural precursor formation and maintenance, is required for the effects of reelin on neuronal **migration**. Here, we show that reelin gain-of-function causes a higher expression of Notch target genes in radial glia and accelerates the production of both neurons and intermediate progenitor cells. Converse alterations correlate with reelin loss-of-function, consistent with reelin controlling Notch signaling during neurogenesis. Ectopic expression of reelin in isolated clones of progenitors causes a severe reduction in neuronal differentiation. In mosaic cell cultures, reelin-primed progenitor cells respond to wild-type cells by further decreasing neuronal differentiation, consistent with an increased sensitivity to lateral inhibition. These results indicate that reelin and Notch signaling cooperate to set the pace of neocortical neurogenesis, a prerequisite for proper neuronal **migration** and cortical layering.
21. Presenilin-1 regulates neuronal differentiation during neurogenesis. Mutations in Presenilin-1 (PS1) are a major cause of familial Alzheimer's disease. Our previous studies showed that PS1 is required for murine neural development. Here we report that lack of PS1 leads to premature differentiation of neural progenitor cells, indicating a role for PS1 in a cell fate decision between postmitotic neurons and neural progenitor cells. Neural proliferation and apoptotic cell death during neurogenesis are unaltered in PS1(-/-) mice, suggesting that the reduction in the neural progenitor cells observed in the PS1(-/-) brain is due to premature differentiation of progenitor cells, rather than to increased apoptotic cell death or decreased cell proliferation. In addition, the premature neuronal differentiation in the PS1(-/-) brain is associated with aberrant neuronal **migration** and disorganization of the laminar architecture of the developing cerebral hemisphere. In the ventricular zone of PS1(-/-) mice, expression of the Notch1 downstream effector gene Hes5 is reduced and expression of the Notch1 ligand Dll1 is elevated, whereas expression of Notch1 is unchanged. The level of Dll1 transcripts is also increased in the presomitic mesoderm of PS1(-/-) embryos, while the level of Notch1 transcripts is unchanged, in contrast to a previous report (Wong et al., 1997, Nature 387, 288-292). These results provide direct evidence that PS1 controls neuronal differentiation in association with the downregulation of Notch signalling during neurogenesis.
22. Crumbs3 is essential for proper epithelial development and viability. First identified in Drosophila, the Crumbs (Crb) proteins are important in epithelial polarity, apical membrane formation, and tight junction (TJ) assembly. The conserved Crb intracellular region includes a FERM (band 4.1/ezrin/radixin/moesin) binding domain (FBD) whose mammalian binding partners are not well understood and a PDZ binding motif that interacts with mammalian Pals1 (protein associated with lin seven) (also known as MPP5). Pals1 binds Patj (Pals1-associated tight-junction protein), a multi-PDZ-domain protein that associates with many tight junction proteins. The Crb complex also binds the conserved Par3/Par6/atypical protein kinase C (aPKC) polarity cassette that restricts **migration** of basolateral proteins through phosphorylation. Here, we describe a Crb3 knockout mouse that demonstrates extensive defects in epithelial morphogenesis. The mice die shortly after birth, with cystic kidneys and proteinaceous debris throughout the lungs. The intestines display villus fusion, apical membrane blebs, and disrupted microvilli. These intestinal defects phenocopy those of Ezrin knockout mice, and we demonstrate an interaction between Crumbs3 and ezrin. Taken together, our data indicate that Crumbs3 is crucial for epithelial morphogenesis and plays a role in linking the apical membrane to the underlying ezrin-containing cytoskeleton.

### ECM

#### PubMed

1. β1-integrin controls cell fate specification in early lens development. Integrins are heterodimeric cell surface molecules that mediate cell-extracellular matrix **(EC**M) adhesion, **EC**M assembly, and regulation of both **EC**M and growth factor induced signaling. However, the developmental context of these diverse functions is not clear. Loss of β1-integrin from the lens vesicle (mouse E10.5) results in abnormal exit of anterior lens epithelial cells (LECs) from the cell cycle and their aberrant elongation toward the presumptive cornea by E12.5. These cells lose expression of LEC markers and initiate expression of the Maf (also known as c-Maf) and Prox1 transcription factors as well as other lens fiber cell markers. β1-integrin null LECs also upregulate the ERK, AKT and Smad1/5/8 phosphorylation indicative of BMP and FGF signaling. By E14.5, β1-integrin null lenses have undergone a complete conversion of all lens epithelial cells into fiber cells. These data suggest that shortly after lens vesicle closure, β1-integrin blocks inappropriate differentiation of the lens epithelium into fibers, potentially by inhibiting BMP and/or FGF receptor activation. Thus, β1-integrin has an important role in fine-tuning the response of the early lens to the gradient of growth factors that regulate lens fiber cell differentiation.
2. NRP1 Regulates CDC42 Activation to Promote Filopodia Formation in Endothelial Tip Cells. Sprouting blood vessels are led by filopodia-studded endothelial tip cells that respond to angiogenic signals. Mosaic lineage tracing previously revealed that NRP1 is essential for tip cell function, although its mechanistic role in tip cells remains poorly defined. Here, we show that NRP1 is dispensable for genetic tip cell identity. Instead, we find that NRP1 is essential to form the filopodial bursts that distinguish tip cells morphologically from neighboring stalk cells, because it enables the extracellular matrix (**ECM**)-induced activation of CDC42, a key regulator of filopodia formation. Accordingly, NRP1 knockdown and pharmacological CDC42 inhibition similarly impaired filopodia formation in vitro and in developing zebrafish in vivo. During mouse retinal angiogenesis, CDC42 inhibition impaired tip cell and vascular network formation, causing defects that resembled those due to loss of **ECM**-induced, but not VEGF-induced, NRP1 signaling. We conclude that NRP1 enables **ECM**-induced filopodia formation for tip cell function during sprouting angiogenesis.

### [Ee]xtracellular [Mm]atrix

#### PubMed

1. β1-integrin controls cell fate specification in early lens development. Integrins are heterodimeric cell surface molecules that mediate cell**-extracellular matri**x (ECM) adhesion, ECM assembly, and regulation of both ECM and growth factor induced signaling. However, the developmental context of these diverse functions is not clear. Loss of β1-integrin from the lens vesicle (mouse E10.5) results in abnormal exit of anterior lens epithelial cells (LECs) from the cell cycle and their aberrant elongation toward the presumptive cornea by E12.5. These cells lose expression of LEC markers and initiate expression of the Maf (also known as c-Maf) and Prox1 transcription factors as well as other lens fiber cell markers. β1-integrin null LECs also upregulate the ERK, AKT and Smad1/5/8 phosphorylation indicative of BMP and FGF signaling. By E14.5, β1-integrin null lenses have undergone a complete conversion of all lens epithelial cells into fiber cells. These data suggest that shortly after lens vesicle closure, β1-integrin blocks inappropriate differentiation of the lens epithelium into fibers, potentially by inhibiting BMP and/or FGF receptor activation. Thus, β1-integrin has an important role in fine-tuning the response of the early lens to the gradient of growth factors that regulate lens fiber cell differentiation.
2. NRP1 Regulates CDC42 Activation to Promote Filopodia Formation in Endothelial Tip Cells. Sprouting blood vessels are led by filopodia-studded endothelial tip cells that respond to angiogenic signals. Mosaic lineage tracing previously revealed that NRP1 is essential for tip cell function, although its mechanistic role in tip cells remains poorly defined. Here, we show that NRP1 is dispensable for genetic tip cell identity. Instead, we find that NRP1 is essential to form the filopodial bursts that distinguish tip cells morphologically from neighboring stalk cells, because it enables the **extracellular matrix** (ECM)-induced activation of CDC42, a key regulator of filopodia formation. Accordingly, NRP1 knockdown and pharmacological CDC42 inhibition similarly impaired filopodia formation in vitro and in developing zebrafish in vivo. During mouse retinal angiogenesis, CDC42 inhibition impaired tip cell and vascular network formation, causing defects that resembled those due to loss of ECM-induced, but not VEGF-induced, NRP1 signaling. We conclude that NRP1 enables ECM-induced filopodia formation for tip cell function during sprouting angiogenesis.
3. Transcription factors SOX4 and SOX9 cooperatively control development of bile ducts. In developing liver, cholangiocytes derive from the hepatoblasts and organize to form the bile ducts. Earlier work has shown that the SRY-related High Mobility Group box transcription factor 9 (SOX9) is transiently required for bile duct development, raising the question of the potential involvement of other SOX family members in biliary morphogenesis. Here we identify SOX4 as a new regulator of cholangiocyte development. Liver-specific inactivation of SOX4, combined or not with inactivation of SOX9, affects cholangiocyte differentiation, apico-basal polarity and bile duct formation. Both factors cooperate to control the expression of mediators of the Transforming Growth Factor-β, Notch, and Hippo-Yap signaling pathways, which are required for normal development of the bile ducts. In addition, SOX4 and SOX9 control formation of primary cilia, which are known signaling regulators. The two factors also stimulate secretion of laminin α5, a**n extracellular matr**ix component promoting bile duct maturation. We conclude that SOX4 is a new regulator of liver development and that it exerts a pleiotropic control on bile duct development in cooperation with SOX9.

### [Ii]nvasive

#### PubMed

1. APC haploinsufficiency coupled with p53 loss sufficiently induces mucinous cystic neoplasms and **invasive** pancreatic carcinoma in mice. Adenomatous polyposis coli (APC), a tumor-suppressor gene critically involved in familial adenomatous polyposis, is integral in Wnt/β-catenin signaling and is implicated in the development of sporadic tumors of the distal gastrointestinal tract including pancreatic cancer (PC). Here we report for the first time that functional APC is required for the growth and maintenance of pancreatic islets and maturation. Subsequently, a non-Kras mutation-induced premalignancy mouse model was developed; in this model, APC haploinsufficiency coupled with p53 deletion resulted in the development of a distinct type of pancreatic premalignant precursors, mucinous cystic neoplasms (MCNs), exhibiting pathomechanisms identical to those observed in human MCNs, including accumulation of cystic fluid secreted by neoplastic and ovarian-like stromal cells, with 100% penetrance and the presence of hepatic and gastric metastases in >30% of the mice. The major clinical implications of this study suggest targeting the Wnt signaling pathway as a novel strategy for managing MCN.

### [Mm]etastasis

#### PubMed

1. Lack of periostin leads to suppression of Notch1 signaling and calcific aortic valve disease. The Postn gene encodes protein periostin. During embryonic development, it is highly expressed in the outflow tract (OFT) endocardial cushions of the developing heart, which give rise to several structures of the mature heart including the aortic valve. Periostin was previously implicated in osteoblast differentiation, cancer **metastasis**, and tooth and bone development, but its role in cardiac OFT development is unclear. To elucidate the role that periostin plays in the developing heart we analyzed cardiac OFT phenotype in mice after deletion of the Postn gene. We found that lack of periostin in the embryonic OFT leads to ectopic expression of the proosteogenic growth factor pleiotrophin (Ptn) and overexpression of delta-like 1 homolog (Dlk1), a negative regulator of Notch1, in the distal (prevalvular) cushions of the OFT. This resulted in suppression of Notch1 signaling, strong induction of the central transcriptional regulator of osteoblast cell fate Runx2, upregulation of osteopontin and osteocalcin expression, and subsequent calcification of the aortic valve. Our data suggest that periostin represses a default osteogenic program in the OFT cushion mesenchyme and promotes differentiation along a fibrogenic lineage. Lack of periostin causes derepression of the osteogenic potential of OFT mesenchymal cells, calcium deposition, and calcific aortic valve disease. These results establish periostin as a key regulator of OFT endocardial cushion mesenchymal cell fate during embryonic development.
2. Enhancer-bound LDB1 regulates a corticotrope promoter-pausing repression program. Substantial evidence supports the hypothesis that enhancers are critical regulators of cell-type determination, orchestrating both positive and negative transcriptional programs; however, the basic mechanisms by which enhancers orchestrate interactions with cognate promoters during activation and repression events remain incompletely understood. Here we report the required actions of LIM domain-binding protein 1 (LDB1)/cofactor of LIM homeodomain protein 2/nuclear LIM interactor, interacting with the enhancer-binding protein achaete-scute complex homolog 1, to mediate looping to target gene promoters and target gene regulation in corticotrope cells. LDB1-mediated enhancer:promoter looping appears to be required for both activation and repression of these target genes. Although LDB1-dependent activated genes are regulated at the level of transcriptional initiation, the LDB1-dependent repressed transcription units appear to be regulated primarily at the level of promoter pausing, with LDB1 regulating recruitment of **metastasis**-associated 1 family, member 2, a component of the nucleosome remodeling deacetylase complex, on these negative enhancers, required for the repressive enhancer function. These results indicate that LDB1-dependent looping events can deliver repressive cargo to cognate promoters to mediate promoter pausing events in a pituitary cell type.

---

## Abcg1

- NCBI Gene
- GeneRIF
- Pubmed

| Search Term | Rank | Fields |
| --- | --- | --- |
| [Mm]igration | 6 | PubMed(3); GeneRIFs(1) |
| [Ee]xtracellular [Mm]atrix | 8 | PubMed(1) |

  
hide/show details for Abcg1

### [Mm]igration

#### PubMed

1. Deletion of ABCA1 and ABCG1 impairs macrophage **migration** because of increased Rac1 signaling. Reduced plasma cholesterol and increased high-density lipoprotein (HDL) levels promote regression of atherosclerosis, in a process characterized by lipid unloading and e**migration** of macrophages from lesions. In contrast free cholesterol loading of macrophages leads to imbalanced Rac1/Rho activities and impaired chemotaxis.To study the role of HDL and the ATP-binding cassette transporters ABCA1 and ABCG1 in modulating the chemotaxis of macrophages.Abca1(-/-)Abcg1(-/-) mouse macrophages displayed profoundly impaired chemotaxis both in a Transwell chamber assay and in the peritoneal cavity of wild-type (WT) mice. HDL reversed impaired chemotaxis in free cholesterol-loaded WT macrophages but was without effect in Abca1(-/-)Abcg1(-/-) cells, whereas cyclodextrin was effective in both. Abca1(-/-)Abcg1(-/-) macrophages had markedly increased Rac1 activity and increased association of Rac1 with the plasma membrane (PM). Their defective chemotaxis was reversed by a Rac1 inhibitor. To gain a better understanding of the role of transporters in PM cholesterol movement, we measured transbilayer PM sterol distribution. In WT macrophages, the majority of cholesterol was located on the inner leaflet, whereas on upregulation of transporters by liver X receptor activation, PM sterol was shifted to the outer leaflet, where it could be removed by HDL. Abca1(-/-)Abcg1(-/-) macrophages showed increased PM sterol content and defective redistribution of sterol to the outer leaflet.Deletion of ABCA1 and ABCG1 causes an increased cholesterol content on the inner leaflet of the PM, associated with increased Rac1 PM localization, activation, and impairment of **migration**. ABCA1 and ABCG1 facilitate macrophage chemotaxis by promoting PM transbilayer cholesterol movement and may contribute to the ability of HDL to promote regression of atherosclerosis.
2. Deletion of ABCA1 and ABCG1 impairs macrophage **migration** because of increased Rac1 signaling. Reduced plasma cholesterol and increased high-density lipoprotein (HDL) levels promote regression of atherosclerosis, in a process characterized by lipid unloading and e**migration** of macrophages from lesions. In contrast free cholesterol loading of macrophages leads to imbalanced Rac1/Rho activities and impaired chemotaxis.To study the role of HDL and the ATP-binding cassette transporters ABCA1 and ABCG1 in modulating the chemotaxis of macrophages.Abca1(-/-)Abcg1(-/-) mouse macrophages displayed profoundly impaired chemotaxis both in a Transwell chamber assay and in the peritoneal cavity of wild-type (WT) mice. HDL reversed impaired chemotaxis in free cholesterol-loaded WT macrophages but was without effect in Abca1(-/-)Abcg1(-/-) cells, whereas cyclodextrin was effective in both. Abca1(-/-)Abcg1(-/-) macrophages had markedly increased Rac1 activity and increased association of Rac1 with the plasma membrane (PM). Their defective chemotaxis was reversed by a Rac1 inhibitor. To gain a better understanding of the role of transporters in PM cholesterol movement, we measured transbilayer PM sterol distribution. In WT macrophages, the majority of cholesterol was located on the inner leaflet, whereas on upregulation of transporters by liver X receptor activation, PM sterol was shifted to the outer leaflet, where it could be removed by HDL. Abca1(-/-)Abcg1(-/-) macrophages showed increased PM sterol content and defective redistribution of sterol to the outer leaflet.Deletion of ABCA1 and ABCG1 causes an increased cholesterol content on the inner leaflet of the PM, associated with increased Rac1 PM localization, activation, and impairment of **migration**. ABCA1 and ABCG1 facilitate macrophage chemotaxis by promoting PM transbilayer cholesterol movement and may contribute to the ability of HDL to promote regression of atherosclerosis.
3. Control of angiogenesis by AIBP-mediated cholesterol efflux. Cholesterol is a structural component of the cell and is indispensable for normal cellular function, although its excess often leads to abnormal proliferation, **migration**, inflammatory responses and/or cell death. To prevent cholesterol overload, ATP-binding cassette (ABC) transporters mediate cholesterol efflux from the cells to apolipoprotein A-I (apoA-I) and the apoA-I-containing high-density lipoprotein (HDL). Maintaining efficient cholesterol efflux is essential for normal cellular function. However, the role of cholesterol efflux in angiogenesis and the identity of its local regulators are poorly understood. Here we show that apoA-I binding protein (AIBP) accelerates cholesterol efflux from endothelial cells to HDL and thereby regulates angiogenesis. AIBP- and HDL-mediated cholesterol depletion reduces lipid rafts, interferes with VEGFR2 (also known as KDR) dimerization and signalling and inhibits vascular endothelial growth factor-induced angiogenesis in vitro and mouse aortic neovascularization ex vivo. Notably, Aibp, a zebrafish homologue of human AIBP, regulates the membrane lipid order in embryonic zebrafish vasculature and functions as a non-cell-autonomous regulator of angiogenesis. aibp knockdown results in dysregulated sprouting/branching angiogenesis, whereas forced Aibp expression inhibits angiogenesis. Dysregulated angiogenesis is phenocopied in Abca1 (also known as Abca1a) Abcg1-deficient embryos, and cholesterol levels are increased in Aibp-deficient and Abca1 Abcg1-deficient embryos. Our findings demonstrate that secreted AIBP positively regulates cholesterol efflux from endothelial cells and that effective cholesterol efflux is critical for proper angiogenesis.

#### GeneRIFs

1. Deletion of ABCA1 and ABCG1 impairs macrophage **migration** because of increased Rac1 signaling.

### [Ee]xtracellular [Mm]atrix

#### PubMed

1. ABCG1-mediated generation of extracellular cholesterol microdomains. Previous studies have demonstrated that the ATP-binding cassette transporters (ABC)A1 and ABCG1 function in many aspects of cholesterol efflux from macrophages. In this current study, we continued our investigation of extracellular cholesterol microdomains that form during enrichment of macrophages with cholesterol. Human monocyte-derived macrophages and mouse bone marrow-derived macrophages, differentiated with macrophage colony-stimulating factor (M-CSF) or granulocyte macrophage colony-stimulation factor (GM-CSF), were incubated with acetylated LDL (AcLDL) to allow for cholesterol enrichment and processing. We utilized an anti-cholesterol microdomain monoclonal antibody to reveal pools of unesterified cholesterol, which were found both in the **extracellular matrix** and associated with the cell surface, that we show function in reverse cholesterol transport. Coincubation of AcLDL with 50 μg/ml apoA-I eliminated all extracellular and cell surface-associated cholesterol microdomains, while coincubation with the same concentration of HDL only removed **extracellular matri**x-associated cholesterol microdomains. Only at an HDL concentration of 200 µg/ml did HDL eliminate the cholesterol microdomains that were cell-surface associated. The deposition of cholesterol microdomains was inhibited by probucol, but it was increased by the liver X receptor (LXR) agonist TO901317, which upregulates ABCA1 and ABCG1. Extracellular cholesterol microdomains did not develop when ABCG1-deficient mouse bone marrow-derived macrophages were enriched with cholesterol. Our findings show that generation of extracellular cholesterol microdomains is mediated by ABCG1 and that reverse cholesterol transport occurs not only at the cell surface but also within the extracellular space.

---

## Nfkbia

- NCBI Gene
- GeneRIF
- Pubmed

| Search Term | Rank | Fields |
| --- | --- | --- |
| [Mm]igration | 6 | PubMed(2) |
| ECM | 7 | PubMed(1) |
| [Ee]xtracellular [Mm]atrix | 8 | PubMed(3) |
| [Ii]nvasive | 10 | PubMed(2) |
| [Mm]etastasis | 12 | PubMed(1) |

  
hide/show details for Nfkbia

### [Mm]igration

#### PubMed

1. Cardiac-specific abrogation of NF- kappa B activation in mice by transdominant expression of a mutant I kappa B alpha. Nuclear factor-kappaB (NF-kappa B) is a pleiotropic oxidant-sensitive transcription factor that is present in the cytosol in an inactive form complexed to an inhibitory kappaB (I kappa B) monomer. Various stimuli, including ischemia, hypoxia, free radicals, cytokines, and lipopolysaccharide (LPS), activate NF-kappa B by inducing phosphorylation of I kappa B. Phosphorylation of serine residues at positions 32 and 36 is critical for ubiquitination and degradation of I kappa B alpha with consequent **migration** of NF-kappa B to the nucleus. Although NF-kappa B is thought to contribute to numerous pathophysiologic processes, definitive assessment of its role has been hindered by the inability to achieve specific inhibition in vivo. Pharmacologic inhibitors of NF-kappa B are available, but their utility for in vivo studies is limited by their relative lack of specificity. Targeted ablation of genes encoding NF-kappa B subunits has not been productive in this regard because of fetal lethality in the case of p65 and functional redundancy in the Rel family of proteins. To overcome these limitations, we have created a viable transgenic mouse that expresses a phosphorylation-resistant mutant of I kappa B alpha (I kappa B alpha(S32A,S36A)) under the direction of a cardiac-specific promoter. Several transgenic lines were obtained with copy numbers ranging from one to seven. The mice exhibit normal cardiac morphology and histology. Total myocardial I kappa B alpha protein level is elevated 3.5- to 6.5-fold with a concomitant 50-60% decrease in the level of I kappa B beta. Importantly, expression of I kappa B(S32A,S36A) results in complete abrogation of myocardial NF-kappa B activation in response to tumor necrosis factor- alpha (TNF-alpha) and LPS stimulation. Thus, novel transgenic mice have been created that make it possible to achieve cardiac-specific and selective inhibition of NF-kappa B in vivo. These transgenic mice should be useful in studies of various cardiac pathophysiological phenomena that involve NF-kappa B activation, including ischemic preconditioning, heart failure, septic shock, acute coronary syndromes, cardiac allograft rejection, and apoptosis.
2. Functions of IkappaB proteins in inflammatory responses to Escherichia coli LPS in mouse lungs. Acute inflammation induced by intrapulmonary LPS requires nuclear factor (NF)-kappaB RelA. This study elucidates the effects of intrapulmonary LPS on IkappaB proteins, endogenous inhibitors of RelA, and the effects of deficiency of IkappaB-beta. IkappaB-alpha, IkappaB-beta, and IkappaB-epsilon each complexed with RelA in uninfected murine lungs. Intratracheal instillation of LPS induced the degradation of IkappaB-alpha and IkappaB-beta, as measured by the loss of immunoreactive proteins in non-nuclear fractions. Degradation was apparent by 2 h and sustained through 6 h. In contrast, net IkappaB-epsilon content increased over this period. The small amounts of IkappaB-alpha and IkappaB-beta that were detected in nuclear fractions from the lungs also decreased over this time frame, whereas intranuclear NF-kappaB content (including both RelA and p50) increased. The hypophosphorylated form of IkappaB-beta, which facilitates transcription induced by NF-kappaB, was not detected. Neutrophil recruitment and edema accumulation did not differ between wild type mice and gene-targeted mice deficient in IkappaB-beta, suggesting that IkappaB-beta is not specifically required for these responses. Altogether, these data suggest that RelA is liberated during LPS-induced pulmonary inflammation by the regulated degradation of both IkappaB-alpha and IkappaB-beta. In the absence of IkappaB-beta, IkappaB-alpha or other inhibitory proteins can regulate NF-kappaB functions essential to acute neutrophil e**migration** in the lungs.

### ECM

#### PubMed

1. Hyaluronan fragments activate an NF-kappa B/I-kappa B alpha autoregulatory loop in murine macrophages. Macrophages play an important role in the acute tissue inflammatory response through the release of cytokines and growth factors in response to stimuli such as lipopolysaccharide (LPS). Macrophage inflammatory effector functions are also influenced by interactions with the extracellular matrix (**ECM**). Such macrophage-**ECM** interactions may be important in regulating chronic inflammatory responses. Recent evidence has suggested that hyaluronan (HA), a glycosaminoglycan (GAG) component of **ECM** can induce inflammatory gene expression in murine macrophages. HA exists in its native form as a large polymer, but is found as smaller fragments under inflammatory conditions. The NF-kappa B/I-kappa B transcriptional regulatory system has been shown to be a critical component of the host inflammatory response. We examined the effects of high molecular weight HA and lower molecular weight HA fragments on NF-kappa B activation in mouse macrophages. Only the smaller HA fragments were found to activate NF-kappa B DNA binding activity. After HA stimulation, I-kappa B alpha mRNA was induced and I-kappa B alpha protein levels, which initially decreased, were restored. The induction of I-kappa Balpha expression was not observed for other GAGs. The time course of I-kappa B alpha protein regeneration in response to HA fragments was consistent with an autoregulatory mechanism. In support of this mechanism, in vitro translated murine I-kappa B alpha inhibited HA fragment-induced NF-kappa B DNA binding activity. The NF-kappa B DNA binding complex in HA-stimulated extracts was found to contain p50 and p65 subunits. Activation of the NF-kappa B/I-kappa B system in macrophages by **ECM** fragments may be an important mechanism for propagating the tissue inflammatory response.

### [Ee]xtracellular [Mm]atrix

#### PubMed

1. Fibromodulin suppresses nuclear factor-kappaB activity by inducing the delayed degradation of IKBA via a JNK-dependent pathway coupled to fibroblast apoptosis. Fibulin-5 (FBLN5) belongs to the Fibulin family of secreted **extracellular matrix** proteins, and our laboratory first established FBLN5 as a novel target for TGF-β in fibroblasts and endothelial cells. To better understand the pathophysiology of FBLN5, we carried out microarray analysis to identify fibroblast genes whose expressions were regulated by FBLN5 and TGF-β. In doing so, we identified fibromodulin (Fmod) as a novel target gene of FBLN5, and we validated the differential expression of Fmod and 12 other FBLN5-regulated genes by semi-quantitative real time PCR. Fmod belongs to the small leucine-rich family of proteoglycans, which are important constituents of mammalian extracellular matrices. Interestingly, parental 3T3-L1 fibroblasts displayed high levels of nuclear factor-κB (NF-κB) activity, although those engineered to express Fmod constitutively exhibited significantly reduced NF-κB activity, suggesting that Fmod functions to inhibit NF-κB signaling. By monitoring alterations in the activation of NF-κB and the degradation of its inhibitor, IκBα, we demonstrate for the first time that Fmod contributes to the constitutive degradation of IκBα protein in 3T3-L1 fibroblasts. Mechanistically, we observed Fmod to delay the degradation of IκBα by promoting the following: (i) activation of c-Jun N-terminal kinase; (ii) inhibition of calpain and casein kinase 2 activity; and (iii) induction of fibroblast apoptosis. Taken together, our study identified a novel function for Fmod in directing extracellular signaling, particularly the regulation of NF-κB activity and cell survival.
2. Hyaluronan fragments activate an NF-kappa B/I-kappa B alpha autoregulatory loop in murine macrophages. Macrophages play an important role in the acute tissue inflammatory response through the release of cytokines and growth factors in response to stimuli such as lipopolysaccharide (LPS). Macrophage inflammatory effector functions are also influenced by interactions with the **extracellular matrix** (ECM). Such macrophage-ECM interactions may be important in regulating chronic inflammatory responses. Recent evidence has suggested that hyaluronan (HA), a glycosaminoglycan (GAG) component of ECM can induce inflammatory gene expression in murine macrophages. HA exists in its native form as a large polymer, but is found as smaller fragments under inflammatory conditions. The NF-kappa B/I-kappa B transcriptional regulatory system has been shown to be a critical component of the host inflammatory response. We examined the effects of high molecular weight HA and lower molecular weight HA fragments on NF-kappa B activation in mouse macrophages. Only the smaller HA fragments were found to activate NF-kappa B DNA binding activity. After HA stimulation, I-kappa B alpha mRNA was induced and I-kappa B alpha protein levels, which initially decreased, were restored. The induction of I-kappa Balpha expression was not observed for other GAGs. The time course of I-kappa B alpha protein regeneration in response to HA fragments was consistent with an autoregulatory mechanism. In support of this mechanism, in vitro translated murine I-kappa B alpha inhibited HA fragment-induced NF-kappa B DNA binding activity. The NF-kappa B DNA binding complex in HA-stimulated extracts was found to contain p50 and p65 subunits. Activation of the NF-kappa B/I-kappa B system in macrophages by ECM fragments may be an important mechanism for propagating the tissue inflammatory response.
3. Nuclear factor-kappaB (NF-kappaB) regulates proliferation and branching in mouse mammary epithelium. The nuclear factor-kappaB (NF-kappaB) family of transcription factors has been shown to regulate proliferation in several cell types. Although recent studies have demonstrated aberrant expression or activity of NF-kappaB in human breast cancer cell lines and tumors, little is known regarding the precise role of NF-kappaB in normal proliferation and development of the mammary epithelium. We investigated the function of NF-kappaB during murine early postnatal mammary gland development by observing the consequences of increased NF-kappaB activity in mouse mammary epithelium lacking the gene encoding IkappaBalpha, a major inhibitor of NF-kappaB. Mammary tissue containing epithelium from inhibitor kappaBalpha (IkappaBalpha)-deficient female donors was transplanted into the gland-free mammary stroma of wild-type mice, resulting in an increase in lateral ductal branching and pervasive intraductal hyperplasia. A two- to threefold increase in epithelial cell number was observed in IkappaBalpha-deficient epithelium compared with controls. Epithelial cell proliferation was strikingly increased in IkappaBalpha-deficient epithelium, and no alteration in apoptosis was detected. The **extracellular matrix** adjacent to IkappaBalpha-deficient epithelium was reduced. Consistent with in vivo data, a fourfold increase in epithelial branching was also observed in purified IkappaBalpha-deficient primary epithelial cells in three-dimensional culture. These data demonstrate that NF-kappaB positively regulates mammary epithelial proliferation, branching, and functions in maintenance of normal epithelial architecture during early postnatal development.

### [Ii]nvasive

#### PubMed

1. Role of calcineurin, hnRNPA2 and Akt in mitochondrial respiratory stress-mediated transcription activation of nuclear gene targets. Pathophysiological conditions causing mitochondrial dysfunction and altered transmembrane potential (psim) initiate a mitochondrial respiratory stress response, also known as mitochondrial retrograde response, in a variety of mammalian cells. An increase in the cytosolic Ca2+ [Ca2+]c as part of this signaling cascade activates Ca2+ responsive phosphatase, calcineurin (Cn). Activation of IGF1R accompanied by increased glycolysis, **invasive**ness, and resistance to apoptosis is a phenotypic hallmark of C2C12 skeletal muscle cells subjected to this stress. The signaling is associated with activation and increased nuclear translocation of a number of transcription factors including a novel NFkappaB (cRel:p50) pathway, NFAT, CREB and C/EBPdelta. This culminates in the upregulation of a number of nuclear genes including Cathepsin L, RyR1, Glut4 and Akt1. We observed that stress regulated transcription activation of nuclear genes involves a cooperative interplay between NFkappaB (cRel:p50), C/EBPdelta, CREB, and NFAT. Our results show that the functional synergy of these factors requires the stress-activated heterogeneous nuclear ribonucleoprotein, hnRNPA2 as a transcriptional coactivator. We report here that mitochondrial stress leads to induced expression and activation of serine threonine kinase Akt1. Interestingly, we observe that Akt1 phosphorylates hnRNPA2 under mitochondrial stress conditions, which is a crucial step for the recruitment of this coactivator to the stress target promoters and culmination in mitochondrial stress-mediated transcription activation of target genes. We propose that mitochondrial stress plays an important role in tumor progression and emergence of **invasive** phenotypes.
2. Links between alpha-catenin, NF-kappaB, and squamous cell carcinoma in skin. Cancers display a diverse set of cellular defects, which are thought to be elicited by multiple genetic mutations. In this study, we show that when a single adherens junction protein, alpha-catenin, is removed by conditional targeting, the entire skin epidermis systematically transforms to a hyperproliferative, **invasive** tissue replete with inflammation. Transcriptional profiling and biochemical analyses reveal that alpha-catenin ablation is accompanied by activation of NF-kappaB and its proinflammatory target genes, along with genes involved in proliferation, wound healing, angiogenesis, and metastasis. Many of these alterations occur in vitro and in the embryo, and thus seem at least partly to be intrinsic to the loss of alpha-catenin. We show that reductions in alpha-catenin, activation of NF-kappaB, and inflammation are common features of human squamous cell carcinomas of the skin.

### [Mm]etastasis

#### PubMed

1. Links between alpha-catenin, NF-kappaB, and squamous cell carcinoma in skin. Cancers display a diverse set of cellular defects, which are thought to be elicited by multiple genetic mutations. In this study, we show that when a single adherens junction protein, alpha-catenin, is removed by conditional targeting, the entire skin epidermis systematically transforms to a hyperproliferative, invasive tissue replete with inflammation. Transcriptional profiling and biochemical analyses reveal that alpha-catenin ablation is accompanied by activation of NF-kappaB and its proinflammatory target genes, along with genes involved in proliferation, wound healing, angiogenesis, and **metastasis**. Many of these alterations occur in vitro and in the embryo, and thus seem at least partly to be intrinsic to the loss of alpha-catenin. We show that reductions in alpha-catenin, activation of NF-kappaB, and inflammation are common features of human squamous cell carcinomas of the skin.

---

## Irak4

- NCBI Gene
- GeneRIF
- Pubmed

| Search Term | Rank | Fields |
| --- | --- | --- |
| [Mm]igration | 6 | Process(1); PubMed(2); GeneRIFs(1) |
| [Ee]xtracellular [Mm]atrix | 8 | PubMed(1) |

  
hide/show details for Irak4

### [Mm]igration

#### Process

1. neutrophil **migration**

#### PubMed

1. The critical role of kinase activity of interleukin-1 receptor-associated kinase 4 in animal models of joint inflammation. We have previously reported that the kinase activity of interleukin-1 receptor-associated kinase 4 (IRAK-4) is important for Toll-like receptor and interleukin-1 receptor signaling in vitro. Using mice devoid of IRAK-4 kinase activity (IRAK-4 KD mice), we undertook this study to determine the importance of IRAK-4 kinase function in complex disease models of joint inflammation.IRAK-4 KD mice were subjected to serum transfer-induced (K/BxN) arthritis, and **migration** of transferred spleen lymphocytes into joints and cartilage and bone degradation were assessed. T cell response in vivo was tested in antigen-induced arthritis (AIA) by measuring the T cell-dependent antigen-specific IgG production and frequency of antigen-specific T cells in the spleen and lymph nodes. T cell allogeneic response was tested in vitro by mixed lymphocyte reaction (MLR).Lipopolysaccharide-induced local neutrophil influx into subcutaneous air pouches was impaired in IRAK-4 KD mice. These mice were also protected from inflammation in the K/BxN and AIA models, as shown by reduced swelling of joints. Histologic analysis of joints of K/BxN serum-injected mice revealed that bone erosion, osteoclast formation, and cartilage matrix proteoglycan loss were reduced in IRAK-4 KD mice. Assessment of T cell response by MLR, by frequency of antigen-specific clones, and by production of antigen-specific IgG did not reveal substantial differences between IRAK-4 KD and wild-type mice.These results demonstrate that IRAK-4 is a key component for the development of proarthritis inflammation, but that it is not crucial for T cell activation. Therefore, the kinase function of IRAK-4 appears to be an attractive therapeutic target in chronic inflammation.
2. Innate immune interleukin-1 receptor-associated kinase 4 exacerbates viral myocarditis by reducing CCR5(+) CD11b(+) monocyte **migration** and impairing interferon production. Viral myocarditis follows a fatal course in ≈30% of patients. Interleukin-1 receptor-associated kinase 4 (IRAK4), a major nodal signal transducer in innate immunity, can play a pivotal role in host inflammatory response. We sought to determine how IRAK4 modulates inflammation and outcome in a mouse model of viral myocarditis.Myocarditis was induced after intraperitoneal inoculation of coxsackievirus B3 into C57Bl/6 IRAK4-deficient mice and their littermate controls. Mortality and viral proliferation were markedly reduced in IRAK4(-/-) mice compared with their IRAK4(+/+) littermates. Disease resistance of IRAK4(-/-) mice paralleled increased amounts of protective heart-infiltrating CCR5(+) monocytes/macrophages and enhanced interferon-α and interferon-γ production 2 days after infection. Competitive bone marrow chimera demonstrated that intact IRAK4 function inhibited heart-speci**fic migra**tion of bone marrow-derived CCR5(+) cells. Mechanistically, lack of IRAK4 resulted in interferon regulatory factor 5 homodimerization via reduced melanoma differentiation-associated protein 5 degradation and enhanced Stat1 and Stat5 phosphorylation. Consequently, antiviral interferon-α and interferon-γ production, as well as CCR5(+) cell recruitment, increased, whereas the overall proinflammatory response was drastically reduced in the absence of IRAK4.Innate immunity signal transducer IRAK4 exacerbates viral myocarditis through inhibition of interferon production and reduced mobilization of protective CCR5(+) monocytes/macrophages to the heart. The combination of IRAK4 inhibitors and antiviral adjuvants may become an attractive therapeutic approach against viral myocarditis in the future.

#### GeneRIFs

1. Intact IRAK4 function inhibited heart-specific **migration** of bone-marrow-derived CCR5(+) cells.

### [Ee]xtracellular [Mm]atrix

#### PubMed

1. Bleomycin-induced pulmonary fibrosis susceptibility genes in AcB/BcA recombinant congenic mice. The genetic basis of susceptibility to pulmonary fibrosis is largely unknown. Initially, in this study, loci regulating the response of bleomycin-induced pulmonary fibrosis were mapped using a set of recombinant congenic strains bred from pulmonary fibrosis-resistant A/J and susceptible C57BL/6J (B6) mice. Linkage was identified (logarithm of the odds score = 4.9) on chromosome 9, and other suggestive loci were detected. The putative loci included alleles from both the B6 and A/J strains as increasing the fibrosis response of congenic mice. Gene expression analysis with microarrays revealed 3,304 genes or expressed sequence tags to be differentially expressed (P < 0.01) in lung tissue between bleomycin-treated B6 and A/J mice, and 246 of these genes mapped to potential susceptibility loci. Pulmonary genes differentially expressed between bleomycin-treated B6 and A/J mice included those of heparin binding and **extracellular matrix** deposition pathways. A review of available genomic sequences revealed 809 (43% of total) genes in the linkage intervals to have variations predicted to alter the encoded proteins or their regulation, 68 (8.4%) of which were also differentially expressed. Genomic approaches were combined to produce a set of candidate genes that may influence susceptibility to bleomycin-induced pulmonary fibrosis in the A/J:B6 mouse model.

---

## Foxg1

- NCBI Gene
- GeneRIF
- Pubmed

| Search Term | Rank | Fields |
| --- | --- | --- |
| [Mm]igration | 6 | Process(1); PubMed(33); GeneRIFs(1) |
| [Ee]xtracellular [Mm]atrix | 8 | PubMed(1) |
| [Cc]ell [Ii]nvasion | 13 | PubMed(1) |

  
hide/show details for Foxg1

### [Mm]igration

#### Process

1. pyramidal neuron **migration**

#### PubMed

1. MicroRNA-9 regulates neurogenesis in mouse telencephalon by targeting multiple transcription factors. microRNA-9-2 and microRNA-9-3 double-mutant mice demonstrate that microRNA-9 (miR-9) controls neural progenitor proliferation and differentiation in the developing telencephalon by regulating the expression of multiple transcription factors. As suggested by our previous study, the Foxg1 expression was elevated, and the production of Cajal-Retzius cells and early-born neurons was suppressed in the miR-9-2/3 double-mutant pallium. At embryonic day 16.5 (E16.5), however, the Foxg1 expression was no longer elevated. The expression of an AU-rich RNA-binding protein Elavl2 increased at E16.5, Elav2 associated with Foxg1 3' untranslated region (UTR), and it countered the Foxg1 suppression by miR-9. Later, progenitor proliferation was reduced in the miR-9-2/3 double-mutant pallium with the decrease in Nr2e1 and Pax6 expression and the increase in Meis2 expression. The analyses suggest that microRNA-9 indirectly inhibits Pax6 expression by suppressing Meis2 expression. In contrast, together with Elavl1 and Msi1, microRNA-9 targets Nr2e1 mRNA 3' UTR to enhance the expression. Concomitantly, cortical layers were reduced, each cortical projection was malformed, and the tangential **migration** of interneurons into the pallium was impaired in the miR-9-2/3 double mutants. miR-9 also targets Gsh2 3' UTR, and Gsh2, as well as Foxg1, expression was elevated in the miR-9-2/3 double-mutant subpallium. The subpallium progenitor proliferation was enhanced, and the development of basal ganglia including striatum and globus pallidus was suppressed. Pallial/subpallial boundary shifted dorsally, and the ventral pallium was lost. Corridor was malformed, and thalamocortical and corticofugal axons were misrouted in the miR-9-2/3 double mutants.
2. The Rb/E2F pathway modulates neurogenesis through direct regulation of the Dlx1/Dlx2 bigene cluster. During brain morphogenesis, the mechanisms through which the cell cycle machinery integrates with differentiation signals remain elusive. Here we show that the Rb/E2F pathway regulates key aspects of differentiation and **migration** through direct control of the Dlx1 and Dlx2 homeodomain proteins, required for interneuron specification. Rb deficiency results in a dramatic reduction of Dlx1 and Dlx2 gene expression manifested by loss of interneuron subtypes and severe **migration** defects in the mouse brain. The Rb/E2F pathway modulates Dlx1/Dlx2 regulation through direct interaction with a Dlx forebrain-specific enhancer, I12b, and the Dlx1/Dlx2 proximal promoter regions, through repressor E2F sites both in vitro and in vivo. In the absence of Rb, we demonstrate that repressor E2Fs inhibit Dlx transcription at the Dlx1/Dlx2 promoters and Dlx1/2-I12b enhancer to suppress differentiation. Our findings support a model whereby the cell cycle machinery not only controls cell division but also modulates neuronal differentiation and **migration** through direct regulation of the Dlx1/Dlx2 bigene cluster during embryonic development.
3. Arl13b-regulated cilia activities are essential for polarized radial glial scaffold formation. The construction of cerebral cortex begins with the formation of radial glia. Once formed, polarized radial glial cells divide either symmetrically or asymmetrically to balance appropriate production of progenitor cells and neurons. Following birth, neurons use the processes of radial glia as scaffolding for oriented **migration**. Radial glia therefore provide an instructive structural matrix to coordinate the generation and placement of distinct groups of cortical neurons in the developing cerebral cortex. We found that Arl13b, a cilia-enriched small GTPase that is mutated in Joubert syndrome, was critical for the initial formation of the polarized radial progenitor scaffold. Using developmental stage-specific deletion of Arl13b in mouse cortical progenitors, we found that early neuroepithelial deletion of ciliary Arl13b led to a reversal of the apical-basal polarity of radial progenitors and aberrant neuronal placement. Arl13b modulated ciliary signaling necessary for radial glial polarity. Our findings indicate that Arl13b signaling in primary cilia is crucial for the initial formation of a polarized radial glial scaffold and suggest that disruption of this process may contribute to aberrant neurodevelopment and brain abnormalities in Joubert syndrome-related ciliopathies.
4. Notch signaling coordinates the patterning of striatal compartments. Numerous lines of evidence suggest that Notch signaling plays a pivotal role in controlling the production of neurons from progenitor cells. However, most experiments have relied on gain-of-function approaches because perturbation of Notch signaling results in death prior to the onset of neurogenesis. Here, we examine the requirement for Notch signaling in the development of the striatum through the analysis of different single and compound Notch1 conditional and Notch3 null mutants. We find that normal development of the striatum depends on the presence of appropriate Notch signals in progenitors during a critical window of embryonic development. Early removal of Notch1 prior to neurogenesis alters early-born patch neurons but not late-born matrix neurons in the striatum. We further show that the late-born striatal neurons in these mutants are spared as a result of functional compensation by Notch3. Notably, however, the removal of Notch signaling subsequent to cells leaving the germinal zone has no obvious effect on striatal organization and patterning. These results indicate that Notch signaling is required in neural progenitor cells to control cell fate in the striatum, but is dispensable during subsequent phases of neuronal **migration** and differentiation.
5. The lysine acetyltransferase activator Brpf1 governs dentate gyrus development through neural stem cells and progenitors. Lysine acetylation has recently emerged as an important post-translational modification in diverse organisms, but relatively little is known about its roles in mammalian development and stem cells. Bromodomain- and PHD finger-containing protein 1 (BRPF1) is a multidomain histone binder and a master activator of three lysine acetyltransferases, MOZ, MORF and HBO1, which are also known as KAT6A, KAT6B and KAT7, respectively. While the MOZ and MORF genes are rearranged in leukemia, the MORF gene is also mutated in prostate and other cancers and in four genetic disorders with intellectual disability. Here we show that forebrain-specific inactivation of the mouse Brpf1 gene causes hypoplasia in the dentate gyrus, including underdevelopment of the suprapyramidal blade and complete loss of the infrapyramidal blade. We trace the developmental origin to compromised Sox2+ neural stem cells and Tbr2+ intermediate neuronal progenitors. We further demonstrate that Brpf1 loss deregulates neuronal **migration**, cell cycle progression and transcriptional control, thereby causing abnormal morphogenesis of the hippocampus. These results link histone binding and acetylation control to hippocampus development and identify an important epigenetic regulator for patterning the dentate gyrus, a brain structure critical for learning, memory and adult neurogenesis.
6. Rac1 controls the formation of midline commissures and the competency of tangential **migration** in ventral telencephalic neurons. Previous studies using dominant-mutant constructs have implicated Rac1 GTPase in neuritogenesis and neuronal **migration**. However, overexpression of dominant mutants generally blocks Rho-GTPase activity; thus, it may not reveal the specific or physiological functions of Rac1. To address this issue, we have applied a conditional gene-targeting strategy, using Foxg1-Cre mice to delete Rac1 in the ventricular zone (VZ) of telencephalon and Dlx5/6-Cre-IRES (internal ribosomal entry site)-EGFP (enhanced green fluorescent protein) (Dlx5/6-CIE) in the subventricular zone (SVZ) of ventral telencephalon, respectively. Surprisingly, the deletion of Rac1 in VZ progenitors did not prevent axonal outgrowth of telencephalic neurons. However, the anterior commissure was absent, and the corpus callosal as well as hippocampal commissural axons failed to cross the midline in Rac1/Foxg1-Cre knock-out embryos. The thalamocortical and corticothalamic axons also showed defasciculation or projection defects. These results suggest that Rac1 controls axon guidance rather than neuritogenesis. In addition, although Rac1/Foxg1-Cre knock-out embryos showed delayed radial **migration** of cortical projection neurons and severe impairment of tangential **migration** by the ventral telencephalon-derived interneurons, deletion of Rac1 in the SVZ by Dlx5/6-CIE mice produced no discernible defects in tangential **migration**. These contrasting effects of Rac1 deletion on tangential **migration** suggest that Rac1 is dispensable for cellular motility per se during neuronal **migration**. Together, these results underscore the challenge of deciphering the biological functions of Rac1, and Rho-GTPases in general, during mammalian brain development. Moreover, they indicate that Rac1 has a critical role in axon guidance and in acquisition of migratory competency during differentiation of the progenitors for the ventral telencephalon-derived interneurons.
7. Hes genes and neurogenin regulate non-neural versus neural fate specification in the dorsal telencephalic midline. The choroid plexus in the brain is unique because it is a non-neural secretory tissue. It secretes the cerebrospinal fluid and functions as a blood-brain barrier, but the precise mechanism of specification of this non-neural tissue has not yet been determined. Using mouse embryos and lineage-tracing analysis, we found that the prospective choroid plexus region initially gives rise to Cajal-Retzius cells, specialized neurons that guide neuronal **migration**. Inactivation of the bHLH repressor genes Hes1, Hes3 and Hes5 upregulated expression of the proneural gene neurogenin 2 (Ngn2) and prematurely depleted Bmp-expressing progenitor cells, leading to enhanced formation of Cajal-Retzius cells and complete loss of choroid plexus epithelial cells. Overexpression of Ngn2 had similar effects. These data indicate that Hes genes promote specification of the fate of choroid plexus epithelial cells rather than the fate of Cajal-Retzius cells by antagonizing Ngn2 in the dorsal telencephalic midline region, and thus this study has identified a novel role for bHLH genes in the process of deciding which cells will have a non-neural versus a neural fate.
8. Tangential neuronal **migration** controls axon guidance: a role for neuregulin-1 in thalamocortical axon navigation. Neuronal **migration** and axon guidance constitute fundamental processes in brain development that are generally studied independently. Although both share common mechanisms of cell biology and biochemistry, little is known about their coordinated integration in the formation of neural circuits. Here we show that the development of the thalamocortical projection, one of the most prominent tracts in the mammalian brain, depends on the early tangential **migration** of a population of neurons derived from the ventral telencephalon. This tangential **migration** contributes to the establishment of a permissive corridor that is essential for thalamocortical axon pathfinding. Our results also demonstrate that in this process two different products of the Neuregulin-1 gene, CRD-NRG1 and Ig-NRG1, mediate the guidance of thalamocortical axons. These results show that neuronal tangential **migration** constitutes a novel mechanism to control the timely arrangement of guidance cues required for axonal tract formation in the mammalian brain.
9. Mcl-1 is a key regulator of apoptosis during CNS development and after DNA damage. Despite the importance of Mcl-1, an anti-apoptotic Bcl-2 family member, in the regulation of apoptosis, little is known regarding its role in nervous system development and injury-induced neuronal cell death. Because germline deletion of Mcl-1 results in peri-implantation lethality, we address the function of Mcl-1 in the nervous system using two different conditional Mcl-1 mouse mutants in the developing nervous system. Here, we show for the first time that Mcl-1 is required for neuronal development. Neural precursors within the ventricular zone and newly committed neurons in the cortical plate express high levels of Mcl-1 throughout cortical neurogenesis. Loss of Mcl-1 in neuronal progenitors results in widespread apoptosis. Double labeling with active caspase 3 and Tuj1 reveals that newly committed Mcl1 deficient neurons undergo apoptosis as they commence **migration** away from the ventricular zone. Examination of neural progenitor differentiation in vitro demonstrated that cell death in the absence of Mcl1 is cell autonomous. Although conditional deletion of Mcl-1 in cultured neurons does not trigger apoptosis, loss of Mcl-1 sensitizes neurons to an acute DNA damaging insult. Indeed, the rapid reduction of Mcl-1 mRNA and protein levels are early events after DNA damage in neurons, and maintaining high Mcl-1 levels can protect neurons against death. Together, our results are the first to demonstrate the requirement of Mcl-1, an anti-apoptotic Bcl-2 family protein, for cortical neurogenesis and the survival of neurons after DNA damage.
10. Adenomatous polyposis coli is required for early events in the normal growth and differentiation of the developing cerebral cortex. Adenomatous polyposis coli (Apc) is a large multifunctional protein known to be important for Wnt/beta-catenin signalling, cytoskeletal dynamics, and cell polarity. In the developing cerebral cortex, Apc is expressed in proliferating cells and its expression increases as cells migrate to the cortical plate. We examined the consequences of loss of Apc function for the early development of the cerebral cortex.We used Emx1(Cre) to inactivate Apc specifically in proliferating cerebral cortical cells and their descendents starting from embryonic day 9.5. We observed reduction in the size of the mutant cerebral cortex, disruption to its organisation, and changes in the molecular identity of its cells. Loss of Apc leads to a decrease in the size of the proliferative pool, disrupted interkinetic nuclear **migration**, and increased apoptosis. beta-Catenin, pericentrin, and N-cadherin proteins no longer adopt their normal high concentration at the apical surface of the cerebral cortical ventricular zone, indicating that cell polarity is disrupted. Consistent with enhanced Wnt/beta-catenin signalling resulting from loss of Apc we found increased levels of TCF/LEF-dependent transcription and expression of endogenous Wnt/beta-catenin target genes (Axin2 (conductin), Lef1, and c-myc) in the mutant cerebral cortex. In the Apc mutant cerebral cortex the expression of transcription factors Foxg1, Pax6, Tbr1, and Tbr2 is drastically reduced compared to normal and many cells ectopically express Pax3, Wnt1, and Wt1 (but not Wnt2b, Wnt8b, Ptc, Gli1, Mash1, Olig2, or Islet1). This indicates that loss of Apc function causes cerebral cortical cells to lose their normal identity and redirect to fates normally found in more posterior-dorsal regions of the central nervous system.Apc is required for multiple aspects of early cerebral cortical development, including the regulation of cell number, interkinetic nuclear **migration**, cell polarity, and cell type specification.
11. Functional dicer is necessary for appropriate specification of radial glia during early development of mouse telencephalon. Early telencephalic development involves transformation of neuroepithelial stem cells into radial glia, which are themselves neuronal progenitors, around the time when the tissue begins to generate postmitotic neurons. To achieve this transformation, radial precursors express a specific combination of proteins. We investigate the hypothesis that micro RNAs regulate the ability of the early telencephalic progenitors to establish radial glia. We ablate functional Dicer, which is required for the generation of mature micro RNAs, by conditionally mutating the Dicer1 gene in the early embryonic telencephalon and analyse the molecular specification of radial glia as well as their progeny, namely postmitotic neurons and basal progenitors. Conditional mutation of Dicer1 from the telencephalon at around embryonic day 8 does not prevent morphological development of radial glia, but their expression of Nestin, Sox9, and ErbB2 is abnormally low. The population of basal progenitors, which are generated by the radial glia, is disorganised and expanded in Dicer1⁻/⁻ dorsal telencephalon. While the proportion of cells expressing markers of postmitotic neurons is unchanged, their laminar organisation in the telencephalic wall is disrupted suggesting a defect in radial glial gui**ded migra**tion. We found that the laminar disruption could not be accounted for by a reduction of the population of Cajal Retzius neurons. Together, our data suggest novel roles for micro RNAs during early development of progenitor cells in the embryonic telencephalon.
12. Rac1 plays an essential role in axon growth and guidance and in neuronal survival in the central and peripheral nervous systems. Rac1 is a critical regulator of cytoskeletal dynamics in multiple cell types. In the nervous system, it has been implicated in the control of cell proliferation, neuronal **migration**, and axon development.To systematically investigate the role of Rac1 in axon growth and guidance in the developing nervous system, we have examined the phenotypes associated with deleting Rac1 in the embryonic mouse forebrain, in cranial and spinal motor neurons, in cranial sensory and dorsal root ganglion neurons, and in the retina. We observe a widespread requirement for Rac1 in axon growth and guidance and a cell-autonomous defect in axon growth in Rac1 (-/-) motor neurons in culture. Neuronal death, presumably a secondary consequence of the axon growth and/or guidance defects, was observed in multiple locations. Following deletion of Rac1 in the forebrain, thalamocortical axons were misrouted inferiorly, with the majority projecting to the contralateral thalamus and a minority projecting ipsilaterally to the ventral cortex, a pattern of misrouting that is indistinguishable from the pattern previously observed in Frizzled3 (-/-) and Celsr3 (-/-) forebrains. In the limbs, motor-neuron-specific deletion of Rac1 produced a distinctive stalling of axons within the dorsal nerve of the hindlimb but a much milder loss of axons in the ventral hindlimb and forelimb nerves, a pattern that is virtually identical to the one previously observed in Frizzled3 (-/-) limbs.The similarities in axon growth and guidance phenotypes caused by Rac1, Frizzled3, and Celsr3 loss-of-function mutations suggest a mechanistic connection between tissue polarity/planar cell polarity signaling and Rac1-dependent cytoskeletal regulation.
13. A conserved Pbx-Wnt-p63-Irf6 regulatory module controls face morphogenesis by promoting epithelial apoptosis. Morphogenesis of mammalian facial processes requires coordination of cellular proliferation, **migration**, and apoptosis to develop intricate features. Cleft lip and/or palate (CL/P), the most frequent human craniofacial birth defect, can be caused by perturbation of any of these programs. Mutations of WNT, P63, and IRF6 yield CL/P in humans and mice; however, how these genes are regulated remains elusive. We generated mouse lines lacking Pbx genes in cephalic ectoderm and demonstrated that they exhibit fully penetrant CL/P and perturbed Wnt signaling. We also characterized a midfacial regulatory element that Pbx proteins bind to control the expression of Wnt9b-Wnt3, which in turn regulates p63. Altogether, we establish a Pbx-dependent Wnt-p63-Irf6 regulatory module in midfacial ectoderm that is conserved within mammals. Dysregulation of this network leads to localized suppression of midfacial apoptosis and CL/P. Ectopic Wnt ectodermal expression in Pbx mutants rescues the clefting, opening avenues for tissue repair.
14. Twist function is required for the morphogenesis of the cephalic neural tube and the differentiation of the cranial neural crest cells in the mouse embryo. Loss of Twist function in the cranial mesenchyme of the mouse embryo causes failure of closure of the cephalic neural tube and malformation of the branchial arches. In the Twist(-/-) embryo, the expression of molecular markers that signify dorsal forebrain tissues is either absent or reduced, but those associated with ventral tissues display expanded domains of expression. Dorsoventral organization of the mid- and hindbrain and the anterior-posterior pattern of the neural tube are not affected. In the Twist(-/-) embryo, neural crest cells stray from the subectodermal migratory path and the late-migrating subpopulation invades the cell-free zone separating streams of cells going to the first and second branchial arches. Cell transplantation studies reveal that Twist activity is required in the cranial mesenchyme for directing the **migration** of the neural crest cells, as well as in the neural crest cells within the first branchial arch to achieve correct localization. Twist is also required for the proper differentiation of the first arch tissues into bone, muscle, and teeth.
15. MicroRNA targeting of CoREST controls polarization of migrating cortical neurons. The **migration** of cortical projection neurons is a multistep process characterized by dynamic cell shape remodeling. The molecular basis of these changes remains elusive, and the present work describes how microRNAs (miRNAs) control neuronal polarization during radial **migration**. We show that miR-22 and miR-124 are expressed in the cortical wall where they target components of the CoREST/REST transcriptional repressor complex, thereby regulating doublecortin transcription in migrating neurons. This molecular pathway underlies radial **migration** by promoting dynamic multipolar-bipolar cell conversion at early phases of **migration**, and later stabilization of cell polarity to support locomotion on radial glia fibers. Thus, our work emphasizes key roles of some miRNAs that control radial **migration** during cerebral corticogenesis.
16. Brain lipid-binding protein is a direct target of Notch signaling in radial glial cells. Radial glia function during CNS development both as neural progenitors and as a scaffolding supporting neuronal **migration**. To elucidate pathways involved in these functions, we mapped in vivo the promoter for Blbp, a radial glial gene. We show here that a binding site for the Notch effector CBF1 is essential for all Blbp transcription in radial glia, and that BLBP expression is significantly reduced in the forebrains of mice lacking the Notch1 and Notch3 receptors. These results identify Blbp as the first predominantly CNS-specific Notch target gene and suggest that it mediates some aspects of Notch signaling in radial glia.
17. Neuronal death resulting from targeted disruption of the Snf2 protein ATRX is mediated by p53. ATRX, a chromatin remodeling protein of the Snf2 family, participates in diverse cellular functions including regulation of gene expression and chromosome alignment during mitosis and meiosis. Mutations in the human gene cause alpha thalassemia mental retardation, X-linked (ATR-X) syndrome, a rare disorder characterized by severe cognitive deficits, microcephaly and epileptic seizures. Conditional inactivation of the Atrx gene in the mouse forebrain leads to neonatal lethality and defective neurogenesis manifested by increased cell death and reduced cellularity in the developing neocortex and hippocampus. Here, we show that Atrx-null forebrains do not generate dentate granule cells due to a reduction in precursor cell number and abnormal **migration** of differentiating granule cells. In addition, fewer GABA-producing interneurons are generated that migrate from the ventral telencephalon to the cortex and hippocampus. Staining for cleaved caspase 3 demonstrated increased apoptosis in both the hippocampal hem and basal telencephalon concurrent with p53 pathway activation. Elimination of the tumor suppressor protein p53 in double knock-out mice rescued cell death in the embryonic telencephalon but only partially ameliorated the Atrx-null phenotypes at birth. Together, these findings show that ATRX deficiency leads to p53-dependent neuronal apoptosis which is responsible for some but not all of the phenotypic consequences of ATRX deficiency in the forebrain.
18. Dual origins of the mammalian accessory olfactory bulb revealed by an evolutionarily conserved migratory stream. The accessory olfactory bulb (AOB) is a critical olfactory structure that has been implicated in mediating social behavior. It receives input from the vomeronasal organ and projects to targets in the amygdaloid complex. Its anterior and posterior components (aAOB and pAOB) display molecular, connectional and functional segregation in processing reproductive and defensive and aggressive behaviors, respectively. We observed a dichotomy in the development of the projection neurons of the aAOB and pAOB in mice. We found that they had distinct sites of origin and that different regulatory molecules were required for their specification and **migration**. aAOB neurons arose locally in the rostral telencephalon, similar to main olfactory bulb neurons. In contrast, pAOB neurons arose caudally, from the neuroepithelium of the diencephalic-telencephalic boundary, from which they migrated rostrally to reach their destination. This unusual origin and **migration** is conserved in Xenopus, providing an insight into the origin of a key component of this system in evolution.
19. Unique requirement for Rb/E2F3 in neuronal **migration**: evidence for cell cycle-independent functions. The cell cycle regulatory retinoblastoma (Rb) protein is a key regulator of neural precursor proliferation; however, its role has been expanded to include a novel cell-autonomous role in mediating neuronal **migration**. We sought to determine the Rb-interacting factors that mediate both the cell cycle and **migration** defects. E2F1 and E2F3 are likely Rb-interacting candidates that we have shown to be deregulated in the absence of Rb. Using mice with compound null mutations of Rb and E2F1 or E2F3, we asked to what extent either E2F1 or E2F3 interacts with Rb in neurogenesis. Here, we report that E2F1 and E2F3 are both functionally relevant targets in neural precursor proliferation, cell cycle exit, and laminar patterning. Each also partially mediates the Rb requirement for neuronal survival. Neuronal **migration**, however, is specifically mediated through E2F3, beyond its role in cell cycle regulation. This study not only outlines overlapping and distinct functions for E2Fs in neurogenesis but also is the first to establish a physiologically relevant role for the Rb/E2F pathway beyond cell cycle regulation in vivo.
20. Neural deletion of Tgfbr2 impairs angiogenesis through an altered secretome. Simultaneous generation of neural cells and that of the nutrient-supplying vasculature during brain development is called neurovascular coupling. We report on a transgenic mouse with impaired transforming growth factor β (TGFβ)-signalling in forebrain-derived neural cells using a Foxg1-cre knock-in to drive the conditional knock-out of the Tgfbr2. Although the expression of FOXG1 is assigned to neural progenitors and neurons of the telencephalon, Foxg1(cre/+);Tgfbr2(flox/flox) (Tgfbr2-cKO) mutants displayed intracerebral haemorrhage. Blood vessels exhibited an atypical, clustered appearance were less in number and displayed reduced branching. Vascular endothelial growth factor (VEGF) A, insulin-like growth factor (IGF) 1, IGF2, TGFβ, inhibitor of DNA binding (ID) 1, thrombospondin (THBS) 2, and a disintegrin and metalloproteinase with thrombospondin motifs (ADAMTS) 1 were altered in either expression levels or tissue distribution. Accordingly, human umbilical vein endothelial cells (HUVEC) displayed branching defects after stimulation with conditioned medium (CM) that was derived from primary neural cultures of the ventral and dorsal telencephalon of Tgfbr2-cKO. Supplementing CM of Tgfbr2-cKO with VEGFA rescued these defects, but application of TGFβ aggravated them. HUVEC showed redu**ced migra**tion towards CM of mutants compared with controls. Supplementing the CM with growth factors VEGFA, fibroblast growth factor (FGF) 2 and IGF1 partially restored HU**VEC migra**tion. In contrast, TGFβ supplementation further impa**ired migr**ation of HUVEC. We observed differences along the dorso-ventral axis of the telencephalon with regard to the impact of these factors on the phenotype. Together these data establish a TGFBR2-dependent molecular crosstalk between neural and endothelial cells during brain vessel development. These findings will be useful to further elucidate neurovascular interaction in general and to understand pathologies of the blood vessel system such as intracerebral haemorrhages, hereditary haemorrhagic telangiectasia, Alzheimeŕs disease, cerebral amyloid angiopathy or tumour biology.
21. Neural progenitor cells orchestrate microglia **migration** and positioning into the developing cortex. Microglia are observed in the early developing forebrain and contribute to the regulation of neurogenesis through still unravelled mechanisms. In the developing cerebral cortex, microglia cluster in the ventricular/subventricular zone (VZ/SVZ), a region containing Cxcl12-expressing basal progenitors (BPs). Here we show that the ablation of BP as well as genetic loss of Cxcl12 affect microglia recruitment into the SVZ. Ectopic Cxcl12 expression or pharmacological blockage of CxcR4 further supports that Cxcl12/CxcR4 signalling is involved in microglial recruitment during cortical development. Furthermore, we found that cell death in the developing forebrain triggers microglial proliferation and that this is mediated by the release of macrophage **migration** inhibitory factor (MIF). Finally, we show that the depletion of microglia in mice lacking receptor for colony-stimulating factor-1 (Csf-1R) reduces BPs into the cerebral cortex.
22. Dynamic FoxG1 expression coordinates the integration of multipolar pyramidal neuron precursors into the cortical plate. Pyramidal cells of the cerebral cortex are born in the ventricular zone and migrate through the intermediate zone to enter into the cortical plate. In the intermediate zone, these migrating precursors move tangentially and initiate the extension of their axons by transiently adopting a characteristic multipolar morphology. We observe that expression of the forkhead transcription factor FoxG1 is dynamically regulated during this transitional period. By utilizing conditional genetic strategies, we show that the downregulation of FoxG1 at the beginning of the multipolar cell phase induces Unc5D expression, the timing of which ultimately determines the laminar identity of pyramidal neurons. In addition, we demonstrate that the re-expression of FoxG1 is required for cells to transit out of the multipolar cell phase and to enter into the cortical plate. Thus, the dynamic expression of FoxG1 during **migration** within the intermediate zone is essential for the proper assembly of the cerebral cortex.
23. Ectodermal Wnt controls nasal pit morphogenesis through modulation of the BMP/FGF/JNK signaling axis. Mutations of WNT3, WNT5A, WNT9B, and WNT11 genes are associated with orofacial birth defects, including nonsyndromic cleft lip with cleft palate in humans. However, the source of Wnt ligands and their signaling effects on the orofacial morphogenetic process remain elusive.Using Foxg1-Cre to impair Wnt secretion through the inactivation of Gpr177/mWls, we investigate the relevant regulation of Wnt production and signaling in nasal-facial development. Ectodermal ablation of Gpr177 leads to severe facial deformities resulting from dramatically reduced cell proliferation and increased cell death due to a combined loss of WNT, FGF and BMP signaling in the developing facial prominence. In the invaginating nasal pit, the Gpr177 disruption also causes a detrimental effect on **migration** of the olfactory epithelial cells into the mesenchymal region. The blockage of Wnt secretion apparently impairs the olfactory epithelial cells through modulation of JNK signaling.Our study thus suggests the head ectoderm, including the facial ectoderm and the neuroectoderm, as the source of canonical as well as noncanonical Wnt ligands during early development of the nasal-facial prominence. Both β-catenin-dependent and -independent signaling pathways are required for proper development of these morphogenetic processes.
24. Endothelin-2 signaling in the neural retina promotes the endothelial tip cell state and inhibits angiogenesis. Endothelin signaling is required for neural crest **migration** and homeostatic regulation of blood pressure. Here, we report that constitutive overexpression of Endothelin-2 (Edn2) in the mouse retina perturbs vascular development by inhibiting endothelial cell **migration** across the retinal surface and subsequent endothelial cell invasion into the retina. Developing endothelial cells exist in one of two states: tip cells at the growing front and stalk cells in the vascular plexus behind the front. This division of endothelial cell states is one of the central organizing principles of angiogenesis. In the developing retina, Edn2 overexpression leads to overproduction of endothelial tip cells by both morphologic and molecular criteria. Spatially localized overexpression of Edn2 produces a correspondingly localized endothelial response. Edn2 overexpression in the early embryo inhibits vascular development at midgestation, but Edn2 overexpression in developing skin and brain has no discernible effect on vascular structure. Inhibition of retinal angiogenesis by Edn2 requires expression of Endothelin receptor A but not Endothelin receptor B in the neural retina. Taken together, these observations imply that the neural retina responds to Edn2 by synthesizing one or more factors that promote the endothelial tip cell state and inhibit angiogenesis. The response to Edn2 is sufficiently potent that it overrides the activities of other homeostatic regulators of angiogenesis, such as Vegf.
25. Cubilin, a high affinity receptor for fibroblast growth factor 8, is required for cell survival in the developing vertebrate head. Cubilin (Cubn) is a multiligand endocytic receptor critical for the intestinal absorption of vitamin B12 and renal protein reabsorption. During mouse development, Cubn is expressed in both embryonic and extra-embryonic tissues, and Cubn gene inactivation results in early embryo lethality most likely due to the impairment of the function of extra-embryonic Cubn. Here, we focus on the developmental role of Cubn expressed in the embryonic head. We report that Cubn is a novel, interspecies-conserved Fgf receptor. Epiblast-specific inactivation of Cubn in the mouse embryo as well as Cubn silencing in the anterior head of frog or the cephalic neural crest of chick embryos show that Cubn is required during early somite stages to convey survival signals in the developing vertebrate head. Surface plasmon resonance analysis reveals that fibroblast growth factor 8 (Fgf8), a key mediator of cell survival, **migration**, proliferation, and patterning in the developing head, is a high affinity ligand for Cubn. Cell uptake studies show that binding to Cubn is necessary for the phosphorylation of the Fgf signaling mediators MAPK and Smad1. Although Cubn may not form stable ternary complexes with Fgf receptors (FgfRs), it acts together with and/or is necessary for optimal FgfR activity. We propose that plasma membrane binding of Fgf8, and most likely of the Fgf8 family members Fgf17 and Fgf18, to Cubn improves Fgf ligand endocytosis and availability to FgfRs, thus modulating Fgf signaling activity.
26. Dbx1-expressing cells are necessary for the survival of the mammalian anterior neural and craniofacial structures. Development of the vertebrate forebrain and craniofacial structures are intimately linked processes, the coordinated growth of these tissues being required to ensure normal head formation. In this study, we identify five small subsets of progenitors expressing the transcription factor dbx1 in the cephalic region of developing mouse embryos at E8.5. Using genetic tracing we show that dbx1-expressing cells and their progeny have a modest contribution to the forebrain and face tissues. However, their genetic ablation triggers extensive and non cell-autonomous apoptosis as well as a decrease in proliferation in surrounding tissues, resulting in the progressive loss of most of the forebrain and frontonasal structures. Targeted ablation of the different subsets reveals that the very first dbx1-expressing progenitors are critically required for the survival of anterior neural tissues, the production and/or **migration** of cephalic neural crest cells and, ultimately, forebrain formation. In addition, we find that the other subsets, generated at slightly later stages, each play a specific function during head development and that their coordinated activity is required for accurate craniofacial morphogenesis. Our results demonstrate that dbx1-expressing cells have a unique function during head development, notably by controlling cell survival in a non cell-autonomous manner.
27. Impaired Interneuron Development after Foxg1 Disruption. Interneurons play pivotal roles in the modulation of cortical function; however, the mechanisms that control interneuron development remain unclear. This study aimed to explore a new role for Foxg1 in interneuron development. By crossing Foxg1fl/fl mice with a Dlx5/6-Cre line, we determined that conditional disruption of Foxg1 in the subpallium results in defects in interneuron development. In developing interneurons, the expression levels of several receptors, including roundabout-1, Eph receptor A4, and C-X-C motif receptor 4/7, were strongly downregulated, which led to **migration** defects after Foxg1 ablation. The transcription factors Dlx1/2 and Mash1, which have been reported to be involved in interneuron development, were significantly upregulated at the mRNA levels. Foxg1 mutant cells developed shorter neurites and fewer branches and displayed severe **migration** defects in vitro. Notably, Prox1, which is a transcription factor that functions as a key regulator in the development of excitatory neurons, was also dramatically upregulated at both the mRNA and protein levels, suggesting that Prox1 is also important for interneuron development. Our work demonstrates that Foxg1 may act as a critical upstream regulator of Dlx1/2, Mash1, and Prox1 to control interneuron development. These findings will further our understanding of the molecular mechanisms of interneuron development.
28. The homeobox gene Hex is required in definitive endodermal tissues for normal forebrain, liver and thyroid formation. The homeobox gene Hex is expressed in the anterior visceral endoderm (AVE) and rostral definitive endoderm of early mouse embryos. Later, Hex transcripts are detected in liver, thyroid and endothelial precursor cells. A null mutation was introduced into the Hex locus by homologous recombination in embryonic stem cells. Hex mutant embryos exhibit varying degrees of anterior truncation as well as liver and thyroid dysplasia. The liver diverticulum is formed but **migration** of hepatocytes into the septum transversum fails to occur. Development of the thyroid is arrested at the thyroid bud stage at 9.5 dpc. Brain defects are restricted to the rostral forebrain and have a caudal limit at the zona limitans intrathalamica, the boundary between dorsal and ventral thalamus. Analysis of Hex(-/-) mutants at early stages shows that the prospective forebrain ectoderm is correctly induced and patterned at 7.5 days post coitum (dpc), but subsequently fails to develop. AVE markers are expressed and correctly positioned but development of rostral definitive endoderm is greatly disturbed in Hex(-/-) embryos. Chimeric embryos composed of Hex(-/-) cells developing within a wild-type visceral endoderm show forebrain defects indicating that Hex is required in the definitive endoderm. All together, these results demonstrate that Hex function is essential in definitive endoderm for normal development of the forebrain, liver and thyroid gland.
29. Foxg1 has an essential role in postnatal development of the dentate gyrus. Foxg1, formerly BF-1, is expressed continuously in the postnatal and adult hippocampal dentate gyrus (DG). This transcription factor (TF) is thought to be involved in Rett syndrome, which is characterized by reduced hippocampus size, indicating its important role in hippocampal development. Due to the perinatal death of Foxg1(-/-) mice, the function of Foxg1 in postnatal DG neurogenesis remains to be explored. Here, we describe the generation of a Foxg1(fl/fl) mouse line. Foxg1 was conditionally ablated from the DG during prenatal and postnatal development by crossing this line with a Frizzled9-CreER(TM) line and inducing recombination with tamoxifen. In this study, we first show that disruption of Foxg1 results in the loss of the subgranular zone and a severely disrupted secondary radial glial scaffold, leading to the impaired **migration** of granule cells. Moreover, detailed analysis reveals that Foxg1 may be necessary for the maintenance of the DG progenitor pool and that the lack of Foxg1 promotes both gliogenesis and neurogenesis. We additionally show that Foxg1 may be required for the survival and maturation of postmitotic neurons and that Foxg1 may be involved in Reelin signaling in regulating postnatal DG development. Last, prenatal deletion of Foxg1 suggests that it is rarely involved in the **migration** of primordial granule cells. In summary, we report that Foxg1 is critical for DG formation, especially during early postnatal stage.
30. Tbr2-positive intermediate (basal) neuronal progenitors safeguard cerebral cortex expansion by controlling amplification of pallial glutamatergic neurons and attraction of subpallial GABAergic interneurons. Little is known about how, during its formidable expansion in development and evolution, the cerebral cortex is able to maintain the correct balance between excitatory and inhibitory neurons. In fact, while the former are born within the cortical primordium, the latter originate outward in the ventral pallium. Therefore, it remains to be addressed how these two neuronal populations might coordinate their relative amounts in order to build a functional cortical network. Here, we show that Tbr2-positive cortical intermediate (basal) neuronal progenitors (INPs) dictate the migratory route and control the amount of subpallial GABAergic interneurons in the subventricular zone (SVZ) through a non-cell-autonomous mechanism. In fact, Tbr2 interneuron attractive activity is moderated by Cxcl12 chemokine signaling, whose forced expression in the Tbr2 mutants can rescue, to some extent, SVZ cell **migration**. We thus propose that INPs are able to control simultaneously the increase of glutamatergic and GABAergic neuronal pools, thereby creating a simple way to intrinsically balance their relative accumulation.
31. Short- and long-range attraction of cortical GABAergic interneurons by neuregulin-1. Most cortical interneurons arise from the subcortical telencephalon, but the molecules that control their **migration** remain largely unidentified. Here, we show that different isoforms of Neuregulin-1 are expressed in the developing cortex and in the route that migrating interneurons follow toward the cortex, whereas a population of the migrating interneurons express ErbB4, a receptor for Neuregulin-1. The different isoforms of Neuregulin-1 act as short- and long-range attractants for migrating interneurons, and perturbing ErbB4 function in vitro decreases the number of interneurons that tangentially migrate to the cortex. In vivo, loss of Neuregulin-1/ErbB4 signaling causes an alteration in the tangential **migration** of cortical interneurons and a reduction in the number of GABAergic interneurons in the postnatal cortex. These observations provide evidence that Neuregulin-1 and its ErbB4 receptor directly control neuronal **migration** in the nervous system.
32. A transgenic marker mouse line labels Cajal-Retzius cells from the cortical hem and thalamocortical axons. Cajal-Retzius (CR) cells are among the earliest born cortical neurons and are required for normal cortical development in rodents and humans; however, their embryonic origin has been controversial. Recent genetic lineage studies and direct visualization of **migration** of CR cells have demonstrated multiple germinative sources for CR cells on the edges of the developing pallium. We generated transgenic mice using 5' untranslated regions of the Frizzled10 gene in order to mark the cortical hem (the most caudomedial edge of the telencephalic neuroepithelium) and found that these mice faithfully reproduce the previously described expression pattern of Frizzled10 mRNA in the cortical hem, dorsal thalamus and dorsal neural tube. In the cortical hem, expression of LacZ mRNA was confined to the ventricular zone and perdurance of LacZ protein served as lineage marker for CR cells derived from the hem during embryonic life. When these marker mice were crossed with FoxG1 (BF1) mutants, they confirmed the previous finding that in these mice the cortical hem is expanded leading to increased production of CR cells from the medial wall of the cortex.
33. Rb/E2F regulates expression of neogenin during neuronal **migration**. The Rb/E2F pathway has long been appreciated for its role in regulating cell cycle progression. Emerging evidence indicates that it also influences physiological events beyond regulation of the cell cycle. We have previously described a requirement for Rb/E2F mediating neuronal **migration**; however, the molecular mechanisms remain unknown, making this an ideal system to identify Rb/E2F-mediated atypical gene regulation in vivo. Here, we report that Rb regulates the expression of neogenin, a gene encoding a receptor involved in cell **migration** and axon guidance. Rb is capable of repressing E2F-mediated neogenin expression while E2F3 occupies a region containing E2F consensus sites on the neogenin promoter in native chromatin. Absence of Rb results in aberrant neuronal **migration** and adhesion in response to netrin-1, a known ligand for neogenin. Increased expression of neogenin through ex vivo electroporation results in impaired neuronal **migration** similar to that detected in forebrain-specific Rb deficiency. These findings show direct regulation of neogenin by the Rb/E2F pathway and demonstrate that regulation of neogenin expression is required for neural precursor **migration**. These studies identify a novel mechanism through which Rb regulates transcription of a gene beyond the classical E2F targets to regulate events distinct from cell cycle progression.

#### GeneRIFs

1. the dynamic expression of FoxG1 during **migration** within the intermediate zone is essential for the proper assembly of the cerebral cortex.

### [Ee]xtracellular [Mm]atrix

#### PubMed

1. Exosomal MicroRNA Transport from Salivary Mesenchyme Regulates Epithelial Progenitor Expansion during Organogenesis. Epithelial-mesenchymal interactions involve fundamental communication between tissues during organogenesis and are primarily regulated by growth factors and **extracellular matrix**. It is unclear whether RNA-containing exosomes are mobile genetic signals regulating epithelial-mesenchymal interactions. Here we identify that exosomes loaded with mesenchyme-specific mature microRNA contribute mobile genetic signals from mesenchyme to epithelium. The mature mesenchymal miR-133b-3p, loaded into exosomes, was transported from mesenchyme to the salivary epithelium, which did not express primary miR-133b-3p. Knockdown of miR-133b-3p in culture decreased endbud morphogenesis, reduced proliferation of epithelial KIT(+) progenitors, and increased expression of a target gene, Disco-interacting protein 2 homolog B (Dip2b). DIP2B, which is involved in DNA methylation, was localized with 5-methylcytosine in the prophase nucleus of a subset of KIT(+) progenitors during mitosis. In summary, exosomal transport of miR-133b-3p from mesenchyme to epithelium decreases DIP2B, which may function as an epigenetic regulator of genes responsible for KIT(+) progenitor expansion during organogenesis.

### [Cc]ell [Ii]nvasion

#### PubMed

1. Endothelin-2 signaling in the neural retina promotes the endothelial tip cell state and inhibits angiogenesis. Endothelin signaling is required for neural crest migration and homeostatic regulation of blood pressure. Here, we report that constitutive overexpression of Endothelin-2 (Edn2) in the mouse retina perturbs vascular development by inhibiting endothelial cell migration across the retinal surface and subsequent endothelial **cell invasion** into the retina. Developing endothelial cells exist in one of two states: tip cells at the growing front and stalk cells in the vascular plexus behind the front. This division of endothelial cell states is one of the central organizing principles of angiogenesis. In the developing retina, Edn2 overexpression leads to overproduction of endothelial tip cells by both morphologic and molecular criteria. Spatially localized overexpression of Edn2 produces a correspondingly localized endothelial response. Edn2 overexpression in the early embryo inhibits vascular development at midgestation, but Edn2 overexpression in developing skin and brain has no discernible effect on vascular structure. Inhibition of retinal angiogenesis by Edn2 requires expression of Endothelin receptor A but not Endothelin receptor B in the neural retina. Taken together, these observations imply that the neural retina responds to Edn2 by synthesizing one or more factors that promote the endothelial tip cell state and inhibit angiogenesis. The response to Edn2 is sufficiently potent that it overrides the activities of other homeostatic regulators of angiogenesis, such as Vegf.

---

## Adam12

- NCBI Gene
- GeneRIF
- Pubmed

| Search Term | Rank | Fields |
| --- | --- | --- |
| [Mm]igration | 6 | PubMed(4); Summary(1) |
| ECM | 7 | PubMed(3) |
| [Ee]xtracellular [Mm]atrix | 8 | PubMed(6) |
| [Ii]nvasive | 10 | PubMed(1) |
| [Mm]etastasis | 12 | PubMed(1) |

  
hide/show details for Adam12

### [Mm]igration

#### PubMed

1. Erbb2 up-regulation of ADAM12 expression accelerates skin cancer progression. Solar ultraviolet (UV) radiation can cause severe damage to the skin and is the primary cause of most skin cancer. UV radiation causes DNA damage leading to mutations and also activates the Erbb2/HER2 receptor through indirect mechanisms involving reactive oxygen species. We hypothesized that Erbb2 activation accelerates the malignant progression of UV-induced skin cancer. Following the induction of benign squamous papillomas by UV exposure of v-ras(Ha) transgenic Tg.AC mice, mice were treated topically with the Erbb2 inhibitor AG825 and tumor progression monitored. AG825 treatment reduced tumor volume, increased tumor regression, and delayed the development of malignant squamous cell carcinoma (SCC). Progression to malignancy was associated with increased Erbb2 and ADAM12 (A Disintegin And Metalloproteinase 12) transcripts and protein, while inhibition of Erbb2 blocked the increase in ADAM12 message upon malignant progression. Similarly, human SCC and SCC cell lines had increased ADAM12 protein and transcripts when compared to normal controls. To determine whether Erbb2 up-regulation of ADAM12 contributed to malignant progression of skin cancer, Erbb2 expression was modulated in cultured SCC cells using forced over-expression or siRNA targeting, demonstrating up-regulation of ADAM12 by Erbb2. Furthermore, ADAM12 transfection or siRNA targeting revealed that ADAM12 increased both the **migration** and invasion of cutaneous SCC cells. Collectively, these results suggest Erbb2 up-regulation of ADAM12 as a novel mechanism contributing to the malignant progression of UV-induced skin cancer. Inhibition of Erbb2/HER2 reduced tumor burden, increased tumor regression, and delayed the progression of benign skin tumors to malignant SCC in UV-exposed mice. Inhibition of Erbb2 suppressed the increase in metalloproteinase ADAM12 expression in skin tumors, which in turn increased **migration** and tumor cell invasiveness.
2. Evidence for an interaction of the metalloprotease-disintegrin tumour necrosis factor alpha convertase (TACE) with mitotic arrest deficient 2 (MAD2), and of the metalloprotease-disintegrin MDC9 with a novel MAD2-related protein, MAD2beta. Metalloprotease-disintegrins are a family of transmembrane glycoproteins that have a role in fertilization, sperm **migration**, myoblast fusion, neural development and ectodomain shedding. In the present study we used the yeast two-hybrid system to search for proteins that interact with the cytoplasmic domain of two metalloprotease-disintegrins, tumour necrosis factor alpha convertase (TACE; ADAM17) and MDC9 (ADAM9; meltrin gamma). We have identified mitotic arrest deficient 2 (MAD2) as a binding partner of the TACE cytoplasmic domain, and a novel MAD2-related protein, MAD2beta, as a binding partner of the MDC9 cytoplasmic domain. MAD2beta has 23% sequence identity with MAD2, which is a component of the spindle assembly (or mitotic) checkpoint mechanism. Northern blot analysis of human tissues indicates that MAD2beta mRNA is expressed ubiquitously. The interaction of the TACE and MDC9 cytoplasmic domains with their binding partners has been confirmed biochemically. The independent identification of MAD2 and MAD2beta as potential interacting partners of distinct metalloprotease-disintegrins raises the possibility of a link between metalloprotease-disintegrins and the cell cycle, or of functions for MAD2 and MAD2beta that are not related to cell cycle control.
3. Selective modulation of integrin-mediated cell **migration** by distinct ADAM family members. A disintegrin and a metalloprotease (ADAM) family members have been implicated in many biological processes. Although it is recognized that recombinant ADAM disintegrin domains can interact with integrins, little is known about ADAM-integrin interactions in cellular context. Here, we tested whether ADAMs can selectively regulate integrin-mediated cell **migration**. ADAMs were expressed in Chinese hamster ovary cells that express defined integrins (alpha4beta1, alpha5beta1, or both), and cell **migration** on full-length fibronectin or on its alpha4beta1 or alpha5beta1 binding fragments was studied. We found that ADAMs inhibit integrin-mediated cell **migration** in patterns dictated by the integrin binding profiles of their isolated disintegrin domains. ADAM12 inhibited cell **migration** mediated by the alpha4beta1 but not the alpha5beta1 integrin. ADAM17 had the reciprocal effect; it inhibited alpha5beta1- but not alpha4beta1-mediated cell **migration**. ADAM19 and ADAM33 inhibited **migration** mediated by both alpha4beta1 and alpha5beta1 integrins. A point mutation in the ADAM12 disintegrin loop partially reduced the inhibitory effect of ADAM12 on cell **migration** on the alpha4beta1 binding fragment of fibronectin, whereas mutations that block metalloprotease activity had no effect. Our results indicate that distinct ADAMs can modulate cell **migration** mediated by specific integrins in a pattern dictated, at least in part, by their disintegrin domains.
4. Breast cancer-associated mutations in metalloprotease disintegrin ADAM12 interfere with the intracellular trafficking and processing of the protein. ADAM12 has recently emerged as a Candidate Cancer Gene in a comprehensive genetic analysis of human breast cancers. Three somatic mutations in ADAM12 were observed at significant frequencies in breast cancers: D301H, G479E and L792F. The first 2 of these mutations involve highly conserved residues in ADAM12, and our computational sequence analysis confirms that they may be cancer-related. We show that the corresponding mutations in mouse ADAM12 inhibit the proteolytic processing and activation of ADAM12 in NIH3T3, COS-7, CHO-K1 cells and in MCF-7 breast cancer cells. The D/H and G/E ADAM12 mutants exert a dominant-negative effect on the processing of the wild-type ADAM12. Immunofluorescence analysis and cell surface biotinylation experiments demonstrate that the D/H and G/E mutants are retained inside the cell and are not transported to the cell surface. Consequently, the D/H and G/E mutants, unlike the wild-type ADAM12, are not capable of shedding Delta-like l, a ligand for Notch receptor, at the cell surface, or of stimulating cell **migration**. Our results suggest that the breast cancer-associated mutations interfere with the intracellular trafficking of ADAM12 and result in loss of the functional ADAM12 at the cell surface.

#### Summary

1. This gene encodes a member of a disintegrin and metalloprotease (ADAM) family of endoproteases that play important roles in various biological processes including cell signaling, adhesion and **migration**. The encoded preproprotein undergoes proteolytic processing to generate a mature, functional protein that localizes to the cell surface. About a third of the mice lacking the encoded protein die before weaning. Overexpression of the encoded protein in a mouse model of Duchenne muscular dystrophy alleviates the muscle pathology by preventing cell necrosis and inflammation. [provided by RefSeq, May 2016]

### ECM

#### PubMed

1. Insulin-Like Growth Factor-I Increases Laminin, Integrin Subunits and Metalloprotease ADAM12 in Mouse Myoblasts. The extracellular matrix (**ECM**) is considered a part of the myogenesis signaling mechanism. we hypothesized that insulin-like growth factor-I (IGF-I) modifies **ECM** during differentiation of mouse C2C12 cells. The myogenic effect of IGF-I (30 nmol/l) was manifested by increased myogenin and myosin heavy chain (MyHC) levels as well as fusion index (2.6 times over control) on the 3rd day of differentiation. IGF-I markedly augmented laminin, but not fibronectin. Cellular contents of integrin α3, α5 and β1 during 3-day differentiation increased in the presence of IGF-I. Treatment with IGF-I increased the expression of the long form of metalloprotease ADAM12 (100 kDa) in myocytes. In conclusion: i) IGF-I caused an increase of laminin, integrin α3 and β1 in C2C12 myogenic cells that can be secondary to stimulation of myogenesis; ii) IGF-I augmented integrin α5 and ADAM12 levels, suggesting a role of this growth factor in determination of the pool of reserve cells during myogenesis.
2. Distinct fibroblast lineages determine dermal architecture in skin development and repair. Fibroblasts are the major mesenchymal cell type in connective tissue and deposit the collagen and elastic fibres of the extracellular matrix (**ECM**). Even within a single tissue, fibroblasts exhibit considerable functional diversity, but it is not known whether this reflects the existence of a differentiation hierarchy or is a response to different environmental factors. Here we show, using transplantation assays and lineage tracing in mice, that the fibroblasts of skin connective tissue arise from two distinct lineages. One forms the upper dermis, including the dermal papilla that regulates hair growth and the arrector pili muscle, which controls piloerection. The other forms the lower dermis, including the reticular fibroblasts that synthesize the bulk of the fibrillar **ECM**, and the preadipocytes and adipocytes of the hypodermis. The upper lineage is required for hair follicle formation. In wounded adult skin, the initial wave of dermal repair is mediated by the lower lineage and upper dermal fibroblasts are recruited only during re-epithelialization. Epidermal β-catenin activation stimulates the expansion of the upper dermal lineage, rendering wounds permissive for hair follicle formation. Our findings explain why wounding is linked to formation of **EC**M-rich scar tissue that lacks hair follicles. They also form a platform for discovering fibroblast lineages in other tissues and for examining fibroblast changes in ageing and disease.
3. Tumor necrosis factor-α alters integrins and metalloprotease ADAM12 levels and signaling in differentiating myoblasts. The extracellular matrix **(EC**M) is important in the regulation of myogenesis. We hypothesized that tumor necrosis factor-α (TNF-α) modifi**es** ECM during differentiation of mouse C2C12 myoblasts. Exogenous TNF-α (1 ng/ml) stimulated myoblast fusion on the 3rd day (by 160% vs control) but not on the 5th day of myogenesis. The level of integrin α5 was significantly augmented by TNF-α during 5 day-differentiation; however, integrin β1 was higher than control only on the 3rd day of cytokine treatment. Both the abundance of integrin α5 bound to actin and the level of integrin β1 complexed with integrin α5 increased in the presence of TNF-α, especially on the 3rd day of differentiation. Similarly, the stimulatory effects of TNF-α on integrin α3, metalloprotease ADAM12 and kinases related to integrins, FAK and ILK, were limited to the 3rd day of differentiation. We concluded that TNF-α-induc**ed** changes in ECM components in differentiating myogenic cells, i.e. i) increased expression of integrin α5, β1, α3, and metalloprotease ADAM12, ii) enhanced formation of α5β1 integrin receptors and interaction of integrin α5-cytoskeleton, and iii) increased expression of kinases associated with integrin signaling, FAK and ILK, were temporarily associated with the onset of myocyte fusion.

### [Ee]xtracellular [Mm]atrix

#### PubMed

1. **Extracellular matrix** secretion by cardiac fibroblasts: role of microRNA-29b and microRNA-30c. MicroRNAs (miRNAs), in particular miR-29b and miR-30c, have been implicated as important regulators of cardiac fibrosis.To perform a proteomics comparison of miRNA effects on **extracellular matrix** secretion by cardiac fibroblasts.Mouse cardiac fibroblasts were transfected with pre-/anti-miR of miR-29b and miR-30c, and their conditioned medium was analyzed by mass spectrometry. miR-29b targeted a cadre of proteins involved in fibrosis, including multiple collagens, matrix metalloproteinases, and leukemia inhibitory factor, insulin-like growth factor 1, and pentraxin 3, 3 predicted targets of miR-29b. miR-29b also attenuated the cardiac fibroblast response to transforming growth factor-β. In contrast, miR-30c had little effect on **extracellular matri**x production but opposite effects regarding leukemia inhibitory factor and insulin-like growth factor 1. Both miRNAs indirectly affected cardiac myocytes. On transfection with pre-miR-29b, the conditioned medium of cardiac fibroblasts lost its ability to support adhesion of rat ventricular myocytes and led to a significant reduction of cardiac myocyte proteins (α-actinin, cardiac myosin-binding protein C, and cardiac troponin I). Similarly, cardiomyocytes derived from mouse embryonic stem cells atrophied under pre-miR-29 conditioned medium, whereas pre-miR-30c conditioned medium had a prohypertrophic effect. Levels of miR-29a, miR-29c, and miR-30c, but not miR-29b, were significantly reduced in a mouse model of pathological but not physiological hypertrophy. Treatment with antagomiRs to miR-29b induced excess fibrosis after aortic constriction without overt deterioration in cardiac function.Our proteomic analysis revealed novel molecular targets of miRNAs that are linked to a fibrogenic cardiac phenotype. Such comprehensive screening methods are essential to define the concerted actions of miRNAs in cardiovascular disease.
2. Insulin-Like Growth Factor-I Increases Laminin, Integrin Subunits and Metalloprotease ADAM12 in Mouse Myoblasts. The **extracellular matrix** (ECM) is considered a part of the myogenesis signaling mechanism. we hypothesized that insulin-like growth factor-I (IGF-I) modifies ECM during differentiation of mouse C2C12 cells. The myogenic effect of IGF-I (30 nmol/l) was manifested by increased myogenin and myosin heavy chain (MyHC) levels as well as fusion index (2.6 times over control) on the 3rd day of differentiation. IGF-I markedly augmented laminin, but not fibronectin. Cellular contents of integrin α3, α5 and β1 during 3-day differentiation increased in the presence of IGF-I. Treatment with IGF-I increased the expression of the long form of metalloprotease ADAM12 (100 kDa) in myocytes. In conclusion: i) IGF-I caused an increase of laminin, integrin α3 and β1 in C2C12 myogenic cells that can be secondary to stimulation of myogenesis; ii) IGF-I augmented integrin α5 and ADAM12 levels, suggesting a role of this growth factor in determination of the pool of reserve cells during myogenesis.
3. Distinct fibroblast lineages determine dermal architecture in skin development and repair. Fibroblasts are the major mesenchymal cell type in connective tissue and deposit the collagen and elastic fibres of the **extracellular matrix** (ECM). Even within a single tissue, fibroblasts exhibit considerable functional diversity, but it is not known whether this reflects the existence of a differentiation hierarchy or is a response to different environmental factors. Here we show, using transplantation assays and lineage tracing in mice, that the fibroblasts of skin connective tissue arise from two distinct lineages. One forms the upper dermis, including the dermal papilla that regulates hair growth and the arrector pili muscle, which controls piloerection. The other forms the lower dermis, including the reticular fibroblasts that synthesize the bulk of the fibrillar ECM, and the preadipocytes and adipocytes of the hypodermis. The upper lineage is required for hair follicle formation. In wounded adult skin, the initial wave of dermal repair is mediated by the lower lineage and upper dermal fibroblasts are recruited only during re-epithelialization. Epidermal β-catenin activation stimulates the expansion of the upper dermal lineage, rendering wounds permissive for hair follicle formation. Our findings explain why wounding is linked to formation of ECM-rich scar tissue that lacks hair follicles. They also form a platform for discovering fibroblast lineages in other tissues and for examining fibroblast changes in ageing and disease.
4. ADAM12 induces actin cytoskeleton and **extracellular matrix** reorganization during early adipocyte differentiation by regulating beta1 integrin function. Changes in cell shape are a morphological hallmark of differentiation. In this study we report that the expression of ADAM12, a disintegrin and metalloprotease, dramatically affects cell morphology in preadipocytes, changing them from a flattened, fibroblastic appearance to a more rounded shape. We showed that the highest levels of ADAM12 mRNA were detected in preadipocytes at the critical stage when preadipocytes become permissive for adipogenic differentiation. Furthermore, as assessed by immunostaining, ADAM12 was transiently expressed at the cell surface concomitant with the reduced activity of beta1 integrin. Co-immunoprecipitation studies indicated the formation of ADAM12/beta1 integrin complexes in these preadipocytes. Overexpression of ADAM12 at the cell surface of 3T3-L1 preadipocytes achieved by transient transfection or retroviral transduction led to the disappearance of the extensive network of actin stress fibers that are characteristic of these cells, and its reorganization into a cortical network located beneath the cell membrane. The cells became more rounded, exhibited fewer vinculin-positive focal adhesions, and adhered less efficiently to fibronectin in attachment assays. Moreover, ADAM12-expressing cells were more prone to apoptosis, which could be prevented by treating the cells with beta1-activating antibodies. A reduced and re-organized fibronectin-rich **extracellular matrix** accompanied these changes. In addition, beta1 integrin was more readily extracted with Triton X-100 from cells overexpressing ADAM12 than from control cells. Collectively, these results show that surface expression of ADAM12 impairs the function of beta1 integrins and, consequently, alters the organization of the actin cytoskeleton and **extracellular matrix**. These events may be necessary for early adipocyte differentiation.
5. Tumor necrosis factor-α alters integrins and metalloprotease ADAM12 levels and signaling in differentiating myoblasts. The **extracellular matri**x (ECM) is important in the regulation of myogenesis. We hypothesized that tumor necrosis factor-α (TNF-α) modifies ECM during differentiation of mouse C2C12 myoblasts. Exogenous TNF-α (1 ng/ml) stimulated myoblast fusion on the 3rd day (by 160% vs control) but not on the 5th day of myogenesis. The level of integrin α5 was significantly augmented by TNF-α during 5 day-differentiation; however, integrin β1 was higher than control only on the 3rd day of cytokine treatment. Both the abundance of integrin α5 bound to actin and the level of integrin β1 complexed with integrin α5 increased in the presence of TNF-α, especially on the 3rd day of differentiation. Similarly, the stimulatory effects of TNF-α on integrin α3, metalloprotease ADAM12 and kinases related to integrins, FAK and ILK, were limited to the 3rd day of differentiation. We concluded that TNF-α-induced changes in ECM components in differentiating myogenic cells, i.e. i) increased expression of integrin α5, β1, α3, and metalloprotease ADAM12, ii) enhanced formation of α5β1 integrin receptors and interaction of integrin α5-cytoskeleton, and iii) increased expression of kinases associated with integrin signaling, FAK and ILK, were temporarily associated with the onset of myocyte fusion.
6. ADAM12-S stimulates bone growth in transgenic mice by modulating chondrocyte proliferation and maturation. ADAM12-S transgenic mice exhibit a pronounced increase in the length of bones, such as femur, tibia, and vertebrae. The effect of ADAM12-S on longitudinal bone growth involves the modulation of chondrocyte proliferation and maturation, likely through proteolytic activities and altered cell-**extracellular matrix** interactions in the growth plate.The disintegrin and metalloprotease ADAM12 is expressed in both osteoblasts and osteoclasts, suggesting a regulatory role of ADAM12 in bone. However, thus far, no in vivo function of ADAM12 in the skeleton has been reported.Transgenic mice expressing the secreted form of human ADAM12, ADAM12-S, or a truncated metalloprotease-deficient form of ADAM12-S in the circulation were used to study the effects of ADAM12 on the skeleton. In addition, murine chondrocyte cultures were used to study the effect of ADAM12-S on cell-**extracellular matrix** interactions.ADAM12-S transgenic mice exhibit increased longitudinal bone growth. The increased bone length is progressive and age dependent, with a maximum increase of 17% seen in the femur from 6-month-old transgenic mice. The effect is gene dose dependent, being more pronounced in mice expressing higher levels of the transgene than in a lower-expressing line. Histological analysis revealed no alterations in the growth plate organization, but mean growth plate width was increased. Both the cellular incorporation of bromodeoxyuridine and the width of the collagen type X-positive hypertrophic zone were increased in the growth plate of ADAM12-S transgenic mice. Importantly, mice expressing a truncated form of ADAM12-S that lacked the pro- and metalloprotease domains showed no alterations in bone length, suggesting that protease activity is required for the ADAM12-S effect. In vitro studies showed that ADAM12-S inhibits chondrocyte adhesion to fibronectin and collagen type II.ADAM12-S stimulates bone growth in mice by modulating chondrocyte proliferation and maturation through mechanisms probably involving both metalloprotease and adhesion activities.

### [Ii]nvasive

#### PubMed

1. Erbb2 up-regulation of ADAM12 expression accelerates skin cancer progression. Solar ultraviolet (UV) radiation can cause severe damage to the skin and is the primary cause of most skin cancer. UV radiation causes DNA damage leading to mutations and also activates the Erbb2/HER2 receptor through indirect mechanisms involving reactive oxygen species. We hypothesized that Erbb2 activation accelerates the malignant progression of UV-induced skin cancer. Following the induction of benign squamous papillomas by UV exposure of v-ras(Ha) transgenic Tg.AC mice, mice were treated topically with the Erbb2 inhibitor AG825 and tumor progression monitored. AG825 treatment reduced tumor volume, increased tumor regression, and delayed the development of malignant squamous cell carcinoma (SCC). Progression to malignancy was associated with increased Erbb2 and ADAM12 (A Disintegin And Metalloproteinase 12) transcripts and protein, while inhibition of Erbb2 blocked the increase in ADAM12 message upon malignant progression. Similarly, human SCC and SCC cell lines had increased ADAM12 protein and transcripts when compared to normal controls. To determine whether Erbb2 up-regulation of ADAM12 contributed to malignant progression of skin cancer, Erbb2 expression was modulated in cultured SCC cells using forced over-expression or siRNA targeting, demonstrating up-regulation of ADAM12 by Erbb2. Furthermore, ADAM12 transfection or siRNA targeting revealed that ADAM12 increased both the migration and invasion of cutaneous SCC cells. Collectively, these results suggest Erbb2 up-regulation of ADAM12 as a novel mechanism contributing to the malignant progression of UV-induced skin cancer. Inhibition of Erbb2/HER2 reduced tumor burden, increased tumor regression, and delayed the progression of benign skin tumors to malignant SCC in UV-exposed mice. Inhibition of Erbb2 suppressed the increase in metalloproteinase ADAM12 expression in skin tumors, which in turn increased migration and tumor cell **invasive**ness.

### [Mm]etastasis

#### PubMed

1. ADAM12-cleaved ephrin-A1 contributes to lung **metastasis**. Eph receptor tyrosine kinases and their ephrin ligands have been implicated in neuronal development and neovascularization. Overexpression of ephrin-A1 has been implicated in tumor progression and poor prognosis. However, the mechanisms are not clear. Here, we report a role of the Eph/ephrin system in a cell adhesion mechanism. Clustered erythropoietin-producing hepatocellular receptor A1 (EphA1)/ephrin-A1 complexes on the plasma membrane did not undergo endocytosis, and the cell remained adherent to one another. The cell-cell contacts were maintained in an Eph tyrosine kinase activity-independent manner even in the absence of E-cadherin. EphA1 and ephrin-A1 co-localized in pulmonary endothelial cells, and regulated vascular permeability and **metastasis** in the lungs. We identified ADAM12 (A disintegrin and metalloproteinase 12) as an EphA1-binding partner by yeast two-hybrid screening and found that ADAM12 enhanced ephrin-A1 cleavage in response to transforming growth factor-β1 in primary tumors. Released soluble ephrin-A1 in the serum deteriorated the EphA1/ephrin-A1-mediated cell adhesion in the lungs in an endocrine manner, causing lung hyperpermeability that facilitated tumor cell entry into the lungs. Depletion of soluble ephrin-A1 by its neutralizing antibody significantly inhibited lung **metastasi**s.

---

## Egfl8

- NCBI Gene
- GeneRIF
- Pubmed

| Search Term | Rank | Fields |
| --- | --- | --- |
| [Mm]igration | 6 | PubMed(1) |

  
hide/show details for Egfl8

### [Mm]igration

#### PubMed

1. Regulatory role of mouse epidermal growth factor-like protein 8 in thymic epithelial cells. Unlike epidermal growth factor-like protein 7 (EGFL7), which is a secreted protein implicated in the regulation of blood vessel formation and cell **migration**, little is known about the physiological function of EGFL8. Thymic epithelial cells (TECs) play a pivotal role in T-cell development by regulating cellular interactions and expression of growth factors, cytokines, and chemokines. In order to investigate the functional role of EGFL8 in TECs, we transfected TECs with an EGFL8-expressing vector to overexpress EGFL8 protein and with an EGFL8 siRNA to knockdown EGFL8 expression. EGFL8-silenced TECs showed significant increase in the number of adherent thymocytes by enhancing the expression of intercellular adhesion molecule-1 (ICAM-1), while the overexpression of EGFL8 inhibited the adherence of TECs to thymocytes by suppressing ICAM-1 expression. Furthermore, in vitro co-culture study revealed that knockdown of EGFL8 facilitated the maturation of thymocytes to CD4(+) and CD8(+) single-positive populations. These regulatory effects of EGFL8 in T-cell development were further confirmed by the results that knockdown of EGFL8 enhanced the expression of genes involved in thymopoiesis, such as interleukin-7 (IL-7), granulocyte/macrophage-colony stimulating factor (GM-CSF), and thymus-expressed chemokine (TECK). Our data show that EGFL8 exerts inhibitory effects on TECs and thymocytes, suggesting that EGFL8 acts as a negative regulatory molecule in the development of T cells in the mouse thymus.

---

## Arhgef6

- NCBI Gene
- GeneRIF
- Pubmed

| Search Term | Rank | Fields |
| --- | --- | --- |
| [Mm]igration | 6 | PubMed(4); GeneRIFs(1) |
| ECM | 7 | PubMed(1) |
| [Ee]xtracellular [Mm]atrix | 8 | PubMed(1) |

  
hide/show details for Arhgef6

### [Mm]igration

#### PubMed

1. Regulation of mesenchymal-to-epithelial transition by PARAXIS during somitogenesis. Dynamic alterations in cell shape, **migration**, and adhesion play a central role in tissue morphogenesis during embryonic development and congenital disease. The mesenchymal-to-epithelial transition that occurs during vertebrate somitogenesis is required for proper patterning of the axial musculoskeletal system. Somitic MET is initiated in the presomitic mesoderm by PARAXIS-dependent changes in cell adhesion, cell polarity, and the composition of the extracellular matrix. However, the target genes downstream of the transcription factor PARAXIS remain poorly described.A genome-wide comparison of gene expression in the anterior presomitic mesoderm and newly formed somites of Paraxis(-/-) embryos resulted in a set of deregulated genes enriched for factors associated with extracellular matrix and cytoskeletal organization and cell-cell and cell-ECM adhesion. The greatest change in expression was seen in fibroblast activation protein alpha (Fap), encoding a dipeptidyl peptidase capable of increasing fibronectin and collagen fiber organization in extracellular matrix. Further, downstream genes in the Wnt and Notch signaling pathways were downregulated, predicting that PARAXIS participates in positive feedback loops in both pathways.These data demonstrate that PARAXIS initiates and stabilizes somite epithelialization by integrating signals from multiple pathways to control the reorganization of the ECM, cytoskeleton, and adhesion junctions during MET.
2. Genes, plasticity and mental retardation. Functional and structural plasticity is a fundamental property of the brain involved in diverse processes ranging from brain construction and repair to storage of experiences during lifetime. Our current understanding of different forms of brain plasticity mechanisms has advanced tremendously in the last decades, benefiting from studies of development and memory storage in adulthood and from investigations of diverse diseased conditions. In this review, we focus on the role of mental retardation (MR) genes and show how this developing area of research can enrich our knowledge of the cellular and molecular mechanisms of brain plasticity and cognitive functions, and of the dysfunctional mechanisms underlying MR. We describe two main groups of MR genes; those leading to dysfunctional neurodevelopmental programs and brain malformations, and those which rely on alterations in molecular mechanisms underlying synaptic organization and plasticity. We first explore the role of MR genes in key mechanisms of neurogenesis and neuronal **migration** during development and in the adult, such as actin and microtubule-cytoskeletal dynamics and signal transduction. We then define the contribution of MR genes to forms of activity-dependent synaptic modifications, such as those involved in molecular organization of the synapse, intracellular signaling regulating gene programs and neuronal cytoskeleton to control network remodeling. We trace the characteristics of MR genes playing key roles in many forms of brain plasticity mechanisms, and highlight specific MR genes that endorse distinct roles in different cell types or brain regions, and at various times of a brain lifetime.
3. αPIX RhoGEF supports positive selection by restraining **migratio**n and promoting arrest of thymocytes. Thymocytes mature in a series of stages by migrating through specific areas of the thymus and interacting with other cells to receive the necessary developmental signals; however, little is known about the molecular mechanisms governing this **migratio**n. We report that murine thymocytes with a knockout mutation in α-PAK (p21-activated kinase)-interacting exchange factor (PIX; Arhgef6), an activator of Rho GTPases, showed greatly increased motility and altered morphology in two-dimensiona**l migrati**on on ICAM-1. αPIX was also required for efficient positive selection, but not negative selection, of thymocytes. TCR signaling was normal in αPix(-) thymocytes, indicating that the effects of αPIX on positive selection are largely independent of TCR signaling. αPix(-) thymocytes also paused less d**uring mig**ration in the thymic cortex, interacted less with ICAM-1 coated beads, and could overcome TCR stop signals, consistent with defective scanning behavior. These results identify αPIX as a regulator of thy**mocyte mi**gration and subsequent arrest that is linked to positive selection.
4. AlphaPIX Rho GTPase guanine nucleotide exchange factor regulates lymphocyte functions and antigen receptor signaling. AlphaPIX is a Rho GTPase guanine nucleotide exchange factor domain-containing signaling protein that associates with other proteins involved in cytoskeletal-membrane complexes. It has been shown that PIX proteins play roles in some immune cells, including neutrophils and T cells. In this study, we report the immune system phenotype of alphaPIX knockout mice. We extended alphaPIX expression experiments and found that whereas alphaPIX was specific to immune cells, its homolog betaPIX was expressed in a wider range of cells. Mice lacking alphaPIX had reduced numbers of mature lymphocytes and defective immune responses. Antigen receptor-directed proliferation of alphaPIX(-) T and B cells was also reduced, but basal **migration** was enhanced. Accompanying these defects, formation of T-cell-B-cell conjugates and recruitment of PAK and Lfa-1 integrin to the immune synapse were impaired in the absence of alphaPIX. Proximal antigen receptor signaling was largely unaffected, with the exception of reduced phosphorylation of PAK and expression of GIT2 in both T cells and B cells. These results reveal specific roles for alphaPIX in the immune system and suggest that redundancy with betaPIX precludes a more severe immune phenotype.

#### GeneRIFs

1. Results indicate Arhgef6 (alphaPIX) as a regulator of thymocyte **migration** and subsequent arrest that is linked to positive selection.

### ECM

#### PubMed

1. Regulation of mesenchymal-to-epithelial transition by PARAXIS during somitogenesis. Dynamic alterations in cell shape, migration, and adhesion play a central role in tissue morphogenesis during embryonic development and congenital disease. The mesenchymal-to-epithelial transition that occurs during vertebrate somitogenesis is required for proper patterning of the axial musculoskeletal system. Somitic MET is initiated in the presomitic mesoderm by PARAXIS-dependent changes in cell adhesion, cell polarity, and the composition of the extracellular matrix. However, the target genes downstream of the transcription factor PARAXIS remain poorly described.A genome-wide comparison of gene expression in the anterior presomitic mesoderm and newly formed somites of Paraxis(-/-) embryos resulted in a set of deregulated genes enriched for factors associated with extracellular matrix and cytoskeletal organization and cell-cell and cell-**ECM** adhesion. The greatest change in expression was seen in fibroblast activation protein alpha (Fap), encoding a dipeptidyl peptidase capable of increasing fibronectin and collagen fiber organization in extracellular matrix. Further, downstream genes in the Wnt and Notch signaling pathways were downregulated, predicting that PARAXIS participates in positive feedback loops in both pathways.These data demonstrate that PARAXIS initiates and stabilizes somite epithelialization by integrating signals from multiple pathways to control the reorganization of the **ECM**, cytoskeleton, and adhesion junctions during MET.

### [Ee]xtracellular [Mm]atrix

#### PubMed

1. Regulation of mesenchymal-to-epithelial transition by PARAXIS during somitogenesis. Dynamic alterations in cell shape, migration, and adhesion play a central role in tissue morphogenesis during embryonic development and congenital disease. The mesenchymal-to-epithelial transition that occurs during vertebrate somitogenesis is required for proper patterning of the axial musculoskeletal system. Somitic MET is initiated in the presomitic mesoderm by PARAXIS-dependent changes in cell adhesion, cell polarity, and the composition of the **extracellular matrix**. However, the target genes downstream of the transcription factor PARAXIS remain poorly described.A genome-wide comparison of gene expression in the anterior presomitic mesoderm and newly formed somites of Paraxis(-/-) embryos resulted in a set of deregulated genes enriched for factors associated with **extracellular matrix** and cytoskeletal organization and cell-cell and cell-ECM adhesion. The greatest change in expression was seen in fibroblast activation protein alpha (Fap), encoding a dipeptidyl peptidase capable of increasing fibronectin and collagen fiber organization in **extracellular matrix**. Further, downstream genes in the Wnt and Notch signaling pathways were downregulated, predicting that PARAXIS participates in positive feedback loops in both pathways.These data demonstrate that PARAXIS initiates and stabilizes somite epithelialization by integrating signals from multiple pathways to control the reorganization of the ECM, cytoskeleton, and adhesion junctions during MET.

---

## Arhgef7

- NCBI Gene
- GeneRIF
- Pubmed

| Search Term | Rank | Fields |
| --- | --- | --- |
| [Ff]ilopodium | 2 | PubMed(1) |
| [Mm]igration | 6 | Process(2); PubMed(11); GeneRIFs(3) |
| [Ee]xtracellular [Mm]atrix | 8 | PubMed(2) |
| [Ii]nvasive | 10 | PubMed(1); GeneRIFs(1) |
| [Mm]etastasis | 12 | PubMed(1) |
| [Cc]ell [Ii]nvasion | 13 | PubMed(2) |

  
hide/show details for Arhgef7

### [Ff]ilopodium

#### PubMed

1. An integrin-linked machinery of cytoskeletal regulation that enables experimental tumor initiation and metastatic colonization. Recently extravasated metastatic cancer cells use the Rif/mDia2 actin-nucleating/polymerizing machinery in order to extend integrin β1-containing, **filopodiu**m-like protrusions (FLPs), which enable them to interact productively with the surrounding extracellular matrix; this process governs the initial proliferation of these cancer cells. Here, we identify the signaling pathway governing FLP lifetime, which involves integrin-linked kinase (ILK) and β-parvin, two integrin:actin-bridging proteins that block cofilin-mediated actin-filament severing. Notably, the combined actions of Rif/mDia2 and ILK/β-parvin/cofilin pathways on FLPs are required not only for metastatic outgrowth but also for primary tumor formation following experimental implantation. This provides one mechanistic explanation for how the epithelial-mesenchymal transition (EMT) program imparts tumor-initiating powers to carcinoma cells, since it enhances FLP formation through the activation of ILK/β-parvin/cofilin pathway.

### [Mm]igration

#### Process

1. astrocyte cell **migration**
2. positive regulation of fibroblast **migration**

#### PubMed

1. Stat3 promotes directional cell **migration** by regulating Rac1 activity via its activator betaPIX. Stat3 is a member of the signal transducer and activator of transcription family, which is important for cytokine signaling as well as for a number of cellular processes including cell proliferation, anti-apoptosis and immune responses. In recent years, evidence has emerged suggesting that Stat3 also participates in cell invasion and motility. However, how Stat3 regulates these processes remains poorly understood. Here, we find that loss of Stat3 expression in mouse embryonic fibroblasts leads to an elevation of Rac1 activity, which promotes a random mode of **migration** by reducing directional persistence and formation of actin stress fibers. Through rescue experiments, we demonstrate that Stat3 can regulate the activation of Rac1 to mediate persistent directional **migration** and that this function is not dependent on Stat3 transcriptional activity. We find that Stat3 binds to betaPIX, a Rac1 activator, and that this interaction could represent a mechanism by which cytoplasmic Stat3 regulates Rac1 activity to modulate the organization of actin cytoskeleton and directional **migration**.
2. Stat3 promotes directional cell **migration** by regulating Rac1 activity via its activator betaPIX. Stat3 is a member of the signal transducer and activator of transcription family, which is important for cytokine signaling as well as for a number of cellular processes including cell proliferation, anti-apoptosis and immune responses. In recent years, evidence has emerged suggesting that Stat3 also participates in cell invasion and motility. However, how Stat3 regulates these processes remains poorly understood. Here, we find that loss of Stat3 expression in mouse embryonic fibroblasts leads to an elevation of Rac1 activity, which promotes a random mode of **migration** by reducing directional persistence and formation of actin stress fibers. Through rescue experiments, we demonstrate that Stat3 can regulate the activation of Rac1 to mediate persistent directional **migration** and that this function is not dependent on Stat3 transcriptional activity. We find that Stat3 binds to betaPIX, a Rac1 activator, and that this interaction could represent a mechanism by which cytoplasmic Stat3 regulates Rac1 activity to modulate the organization of actin cytoskeleton and directional **migration**.
3. Phosphorylation of the cool-1/beta-Pix protein serves as a regulatory signal for the **migration** and invasive activity of Src-transformed cells. Previously we showed that Cool-1 (Cloned out of library-1)/beta-Pix (Pak-interactive exchange factor) is phosphorylated at a specific tyrosine residue (Tyr-442) in a Src-dependent manner and serves as a dual function guanine nucleotide exchange factor (GEF)/signaling-effector for Cdc42 that is essential for transformation by Src. Here, we show that knocking-down Cool-1 or overexpressing a Cool-1 mutant that contains substitutions within its Dbl homology domain and is defective for GEF activity, inhibits Src-promoted cell **migration**. Similarly, the expression of a Cool-1 mutant containing a tyrosine to phenylalanine substitution at position 442, making it incapable of being phosphorylated in response to serum, epidermal growth factor (EGF), or Src, also causes a significant inhibition of the **migration** and invasive activity of cells expressing oncogenic Src. We further demonstrate that the phosphorylation of Cool-1 at Tyr-442 weakens its ability to bind to one of its primary interaction-partners, Cat-1 (Cool-associated tyrosine phosphosubstrate-1)/Git-1 (G protein-coupled receptor kinase-interactor-1), thus making Cat more accessible for binding to paxillin. This enables cells to alternate between states where they contain large numbers of focal complexes (i.e. conditions favoring Cool-1-Cat interactions) versus reduced numbers of focal complexes (conditions favoring Cat-paxillin interactions). Overall, these findings show that the phosphorylation-dephosphorylation cycle of Cool-1 at Tyr-442 can serve as a key regulatory signal for focal complex assembly-disassembly, and consequently, for the **migration** and invasive activity of Src-transformed cells.
4. β-Pix directs collective **migratio**n of anterior visceral endoderm cells in the early mouse embryo. Collective epithelial **migratio**n is important throughout embryonic development. The underlying mechanisms are poorly understood but likely involve spatially localized activation of Rho GTPases. We previously reported that Rac1 is essential for generating the protrusive activity that drives the collective **migratio**n of anterior visceral endoderm (AVE) cells in the early mouse embryo. To identify potential regulators of Rac1, we first performed an RNAi screen of Rho family exchange factors (guanine nucleotide exchange factor [GEF]) in an in vitro collective epithelial **migratio**n assay and identified β-Pix. Genetic deletion of β-Pix in mice disrupts collective A**VE migrat**ion, while high-resolution live imaging revealed that this is associated with randomly directed protrusive activity. We conclude that β-Pix controls the spatial localization of Rac1 activity to drive collective **AVE migra**tion at a critical stage in mouse development.
5. Molecular cloning of neuronally expressed mouse betaPix isoforms. Pix, a Pak-interacting exchange factor, is known to be involved in the regulation of Cdc42/Rac GTPases and Pak kinase activity. In this study, we cloned the cDNAs encoding two betaPix isoforms from mouse brain cDNA library. Both of the cloned genes, designated betaPix-b and betaPix-c (GenBank Accession Nos. AF247654 and AF247655, respectively), have a novel insert region consisting of 59 amino acid residues. In betaPix-c, 75 amino acid residues are deleted in the proline-rich region at the carboxyl-terminus of betaPix. In situ hybridization studies with insert region-specific probe in rat embryo show that insert region-containing isoforms are expressed mainly in the central nervous system. Moreover, temporal expression pattern of isoforms is correlated with the active neurogenesis period in the cerebral cortex and cerebellum. These results strongly suggest that betaPix isoforms may play important roles in the cellular events required for brain development such as neuronal **migration**.
6. P-cadherin promotes collective cell **migration** via a Cdc42-mediated increase in mechanical forces. Collective cell **migration** (CCM) is essential for organism development, wound healing, and metastatic transition, the primary cause of cancer-related death, and it involves cell-cell adhesion molecules of the cadherin family. Increased P-cadherin expression levels are correlated with tumor aggressiveness in carcinoma and aggressive sarcoma; however, how P-cadherin promotes tumor malignancy remains unknown. Here, using integrated cell biology and biophysical approaches, we determined that P-cadherin specifically induces polarization and CCM through an increase in the strength and anisotropy of mechanical forces. We show that this mechanical regulation is mediated by the P-cadherin/β-PIX/Cdc42 axis; P-cadherin specifically activates Cdc42 through β-PIX, which is specifically recruited at cell-cell contacts upon CCM. This mechanism of cell polarization an**d migrati**on is absent in cells expressing E- or R-cadherin. Thus, we identify a specific role of P-cadherin through β-PIX-mediated Cdc42 activation in the regulation of cell polarity and force anisotropy that drives CCM.
7. Arhgef7 promotes activation of the Hippo pathway core kinase Lats. The Hippo pathway regulates tissue growth and organ size, and inactivation contributes to cancer. Signals flow through Mst/Lats kinases, which phosphorylate and promote cytoplasmic localization of the transcriptional regulators Yap and Taz to inhibit transcription. Here, we identify the multidomain-containing guanine nucleotide exchange factor (GEF) Arhgef7, or βPix, as a positive Hippo pathway regulator. We show that βPix, which localizes to the cytoplasm, binds both Lats and Yap/Taz and thereby promotes Lats-mediated phosphorylation of Yap/Taz in a GEF-independent manner. βPix is required downstream of both cell density sensing and actin cytoskeletal rearrangements, and we demonstrate that loss of βPix expression in normal mammary epithelial cells strongly reduces Yap/Taz phosphorylation, promotes nuclear localization and increases target gene expression. Conversely, increased expression of βPIX in breast cancer cell lines re-couples the Hippo kinase cassette to Yap/Taz, promoting localization of Yap/Taz to the cytoplasm and inhibiting **cell migr**ation and proliferation. These studies thus define βPix as a key component that links the Hippo kinase cassette to Yap/Taz in response to multiple upstream Hippo pathway activators.
8. Analysis of the myosin-II-responsive focal adhesion proteome reveals a role for β-Pix in negative regulation of focal adhesion maturation. Focal adhesions undergo myosin-II-mediated maturation wherein they grow and change composition to modulate integrin signalling for cell **migratio**n, growth and differentiation. To determine how focal adhesion composition is affected by myosin II activity, we performed proteomic analysis of isolated focal adhesions and compared protein abundance in focal adhesions from cells with and without myosin II inhibition. We identified 905 focal adhesion proteins, 459 of which changed in abundance with myosin II inhibition, defining the myosin-II-responsive focal adhesion proteome. The abundance of 73% of the proteins in the myosin-II-responsive focal adhesion proteome was enhanced by contractility, including proteins involved in Rho-mediated focal adhesion maturation and endocytosis- and calpain-dependent focal adhesion disassembly. During myosin II inhibition, 27% of proteins in the myosin-II-responsive focal adhesion proteome, including proteins involved in Rac-mediated lamellipodial protrusion, were enriched in focal adhesions, establishing that focal adhesion protein recruitment is also negatively regulated by contractility. We focused on the Rac guanine nucleotide exchange factor β-Pix, documenting its role in the negative regulation of focal adhesion maturation and the promotion of lamellipodial protrusion and focal adhesion turnover to drive cel**l migrati**on.
9. Sorting nexin 27 protein regulates trafficking of a p21-activated kinase (PAK) interacting exchange factor (β-Pix)-G protein-coupled receptor kinase interacting protein (GIT) complex via a PDZ domain interaction. Sorting nexin 27 (SNX27) is a 62-kDa protein localized to early endosomes and known to regulate the intracellular trafficking of ion channels and receptors. In addition to a PX domain, SNX27 is the only sorting family member that contains a PDZ domain. To identify novel SNX27-PDZ binding partners, we performed a proteomic screen in mouse principal kidney cortical collecting duct cells using a GST-SNX27 fusion construct as bait. We found that β-Pix (p21-activated kinase-interactive exchange factor), a guanine nucleotide exchange factor for the Rho family of small GTPases known to regulate cell motility directly interacted with SNX27. The association of β-Pix and SNX27 is specific for β-Pix isoforms terminating in the type-1 PDZ binding motif (ETNL). In the same screen we also identified Git1/2 as a potential SNX27 interacting protein. The interaction between SNX27 and Git1/2 is indirect and mediated by β-Pix. Furthermore, we show recruitment of the β-Pix·Git complex to endosomal sites in a SNX27-dependent manner. Fi**nally, mi**gration assays revealed that depletion of SNX27 from HeLa and mouse principal kidney cortical collecting duct cells significantly decreases cell motility. We propose a model by which SNX27 regulates trafficking of β-Pix to focal adhesions and thereby influences cell motility.
10. p85 beta-PIX is required for cell motility through phosphorylations of focal adhesion kinase and p38 MAP kinase. Lysophosphatidic acid (LPA) mediates diverse biological responses, including cell **migration**, through the activation of G-protein-coupled receptors. Recently, we have shown that LPA stimulates p21-activated kinase (PAK) that is critical for focal adhesion kinase (FAK) phosphorylation and cell motility. Here, we provide the direct evidence that p85 beta-PIX is required for cell motility of NIH-3T3 cells by LPA through FAK and p38 MAP kinase phosphorylations. LPA induced p85 beta-PIX binding to FAK in NIH-3T3 cells that was inhibited by pretreatment of the cells with phosphoinositide 3-kinase inhibitor, LY294002. Furthermore, the similar inhibition of the complex formation was also observed, when the cells were transfected with either p85 beta-PIX mutant that cannot bind GIT or dominant negative mutants of Rac1 (N17Rac1) and PAK (PAK-PID). Transfection of the cells with specific p85 beta-PIX siRNA led to drastic inhibition of LPA-induced FAK phosphorylation, peripheral redistribution of p85 beta-PIX with FAK and GIT1, and cell motility. p85 beta-PIX was also required for p38 MAP kinase phosphorylation induced by LPA. Finally, dominant negative mutant of Rho (N19Rho)-transfected cells did not affect PAK activation, while the cells stably transfected with p85 beta-PIX siRNA or N17Rac1 showed the reduction of LPA-induced PAK activation. Taken together, the present data suggest that p85 beta-PIX, located downstream of Rac1, is a key regulator for the activations of FAK or p38 MAP kinase and plays a pivotal role in focal complex formation and cell motility induced by LPA.
11. N-α-acetyltransferase 10 protein suppresses cancer cell metastasis by binding PIX proteins and inhibiting Cdc42/Rac1 activity. N-α-acetyltransferase 10 protein, Naa10p, is an N-acetyltransferase known to be involved in cell cycle control. We found that Naa10p was expressed lower in varieties of malignancies with lymph node metastasis compared with non-lymph node metastasis. Higher Naa10p expression correlates the survival of lung cancer patients. Naa10p significantly suppresse**d migrati**on, tumor growth, and metastasis independent of its enzymatic activity. Instead, Naa10p binds to the GIT-binding domain of PIX, thereby preventing the formation of the GIT-PIX-Paxillin complex, resulting in reduced intrinsic Cdc42/Rac1 activity and decreased cel**l migrati**on. Forced expression of PIX in Naa10-transfected tumor cells restored th**e migrati**on and metastasis ability. We suggest that Naa10p functions as a tumor metastasis suppressor by disrupting the migratory complex, PIX-GIT- Paxillin, in cancer cells.

#### GeneRIFs

1. beta-Pix controls the spatial localization of Rac1 activity to drive collective AVE **migration** at a critical stage in mouse development.
2. Stat3 binds to betaPIX, a Rac1 activator,and regulates Rac1 activity to modulate the organization of actin cytoskeleton and directional **migration**
3. phosphorylation-dephosphorylation cycle of Cool-1 at Tyr-442 can serve as a key regulatory signal for focal complex assembly-disassembly, and consequently, for the **migration** and invasive activity of Src-transformed

### [Ee]xtracellular [Mm]atrix

#### PubMed

1. An integrin-linked machinery of cytoskeletal regulation that enables experimental tumor initiation and metastatic colonization. Recently extravasated metastatic cancer cells use the Rif/mDia2 actin-nucleating/polymerizing machinery in order to extend integrin β1-containing, filopodium-like protrusions (FLPs), which enable them to interact productively with the surrounding **extracellular matri**x; this process governs the initial proliferation of these cancer cells. Here, we identify the signaling pathway governing FLP lifetime, which involves integrin-linked kinase (ILK) and β-parvin, two integrin:actin-bridging proteins that block cofilin-mediated actin-filament severing. Notably, the combined actions of Rif/mDia2 and ILK/β-parvin/cofilin pathways on FLPs are required not only for metastatic outgrowth but also for primary tumor formation following experimental implantation. This provides one mechanistic explanation for how the epithelial-mesenchymal transition (EMT) program imparts tumor-initiating powers to carcinoma cells, since it enhances FLP formation through the activation of ILK/β-parvin/cofilin pathway.
2. FAK potentiates Rac1 activation and localization to matrix adhesion sites: a role for betaPIX. FAK, a cytoplasmic protein tyrosine kinase, is activated and localized to focal adhesions upon cell attachment to **extracellular matrix**. FAK null cells spread poorly and exhibit altered focal adhesion turnover. Rac1 is a member of the Rho-family GTPases that promotes membrane ruffling, leading edge extension, and cell spreading. We investigated the activation and subcellular location of Rac1 in FAK null and FAK reexpressing fibroblasts. FAK reexpressers had a more robust pattern of Rac1 activation after cell adhesion to fibronectin than the FAK null cells. Translocation of Rac1 to focal adhesions was observed in FAK reexpressers, but seldom in FAK null cells. Experiments with constitutively active L61Rac1 and dominant negative N17Rac1 indicated that the activation state of Rac1 regulated its localization to focal adhesions. We demonstrated that FAK tyrosine-phosphorylated betaPIX and thereby increased its binding to Rac1. In addition, betaPIX facilitated the targeting of activated Rac1 to focal adhesions and the efficiency of cell spreading. These data indicate that FAK has a role in the activation and focal adhesion translocation of Rac1 through the tyrosine phosphorylation of betaPIX.

### [Ii]nvasive

#### PubMed

1. Phosphorylation of the cool-1/beta-Pix protein serves as a regulatory signal for the migration and **invasive** activity of Src-transformed cells. Previously we showed that Cool-1 (Cloned out of library-1)/beta-Pix (Pak-interactive exchange factor) is phosphorylated at a specific tyrosine residue (Tyr-442) in a Src-dependent manner and serves as a dual function guanine nucleotide exchange factor (GEF)/signaling-effector for Cdc42 that is essential for transformation by Src. Here, we show that knocking-down Cool-1 or overexpressing a Cool-1 mutant that contains substitutions within its Dbl homology domain and is defective for GEF activity, inhibits Src-promoted cell migration. Similarly, the expression of a Cool-1 mutant containing a tyrosine to phenylalanine substitution at position 442, making it incapable of being phosphorylated in response to serum, epidermal growth factor (EGF), or Src, also causes a significant inhibition of the migration and **invasive** activity of cells expressing oncogenic Src. We further demonstrate that the phosphorylation of Cool-1 at Tyr-442 weakens its ability to bind to one of its primary interaction-partners, Cat-1 (Cool-associated tyrosine phosphosubstrate-1)/Git-1 (G protein-coupled receptor kinase-interactor-1), thus making Cat more accessible for binding to paxillin. This enables cells to alternate between states where they contain large numbers of focal complexes (i.e. conditions favoring Cool-1-Cat interactions) versus reduced numbers of focal complexes (conditions favoring Cat-paxillin interactions). Overall, these findings show that the phosphorylation-dephosphorylation cycle of Cool-1 at Tyr-442 can serve as a key regulatory signal for focal complex assembly-disassembly, and consequently, for the migration and **invasive** activity of Src-transformed cells.

#### GeneRIFs

1. phosphorylation-dephosphorylation cycle of Cool-1 at Tyr-442 can serve as a key regulatory signal for focal complex assembly-disassembly, and consequently, for the migration and **invasive** activity of Src-transformed

### [Mm]etastasis

#### PubMed

1. N-α-acetyltransferase 10 protein suppresses cancer cell **metastasi**s by binding PIX proteins and inhibiting Cdc42/Rac1 activity. N-α-acetyltransferase 10 protein, Naa10p, is an N-acetyltransferase known to be involved in cell cycle control. We found that Naa10p was expressed lower in varieties of malignancies with lymph nod**e metastas**is compared with non-lymph nod**e metastas**is. Higher Naa10p expression correlates the survival of lung cancer patients. Naa10p significantly suppressed migration, tumor growth, an**d metastas**is independent of its enzymatic activity. Instead, Naa10p binds to the GIT-binding domain of PIX, thereby preventing the formation of the GIT-PIX-Paxillin complex, resulting in reduced intrinsic Cdc42/Rac1 activity and decreased cell migration. Forced expression of PIX in Naa10-transfected tumor cells restored the migration an**d metastas**is ability. We suggest that Naa10p functions as a tumo**r metastas**is suppressor by disrupting the migratory complex, PIX-GIT- Paxillin, in cancer cells.

### [Cc]ell [Ii]nvasion

#### PubMed

1. Stat3 promotes directional cell migration by regulating Rac1 activity via its activator betaPIX. Stat3 is a member of the signal transducer and activator of transcription family, which is important for cytokine signaling as well as for a number of cellular processes including cell proliferation, anti-apoptosis and immune responses. In recent years, evidence has emerged suggesting that Stat3 also participates in **cell invasion** and motility. However, how Stat3 regulates these processes remains poorly understood. Here, we find that loss of Stat3 expression in mouse embryonic fibroblasts leads to an elevation of Rac1 activity, which promotes a random mode of migration by reducing directional persistence and formation of actin stress fibers. Through rescue experiments, we demonstrate that Stat3 can regulate the activation of Rac1 to mediate persistent directional migration and that this function is not dependent on Stat3 transcriptional activity. We find that Stat3 binds to betaPIX, a Rac1 activator, and that this interaction could represent a mechanism by which cytoplasmic Stat3 regulates Rac1 activity to modulate the organization of actin cytoskeleton and directional migration.
2. Stat3 promotes directional cell migration by regulating Rac1 activity via its activator betaPIX. Stat3 is a member of the signal transducer and activator of transcription family, which is important for cytokine signaling as well as for a number of cellular processes including cell proliferation, anti-apoptosis and immune responses. In recent years, evidence has emerged suggesting that Stat3 also participates in **cell invasion** and motility. However, how Stat3 regulates these processes remains poorly understood. Here, we find that loss of Stat3 expression in mouse embryonic fibroblasts leads to an elevation of Rac1 activity, which promotes a random mode of migration by reducing directional persistence and formation of actin stress fibers. Through rescue experiments, we demonstrate that Stat3 can regulate the activation of Rac1 to mediate persistent directional migration and that this function is not dependent on Stat3 transcriptional activity. We find that Stat3 binds to betaPIX, a Rac1 activator, and that this interaction could represent a mechanism by which cytoplasmic Stat3 regulates Rac1 activity to modulate the organization of actin cytoskeleton and directional migration.

---

## Bri3

- NCBI Gene
- GeneRIF
- Pubmed

| Search Term | Rank | Fields |
| --- | --- | --- |
| ECM | 7 | PubMed(1) |
| [Ee]xtracellular [Mm]atrix | 8 | PubMed(1) |

  
hide/show details for Bri3

### ECM

#### PubMed

1. Nell1-deficient mice have reduced expression of extracellular matrix proteins causing cranial and vertebral defects. The mammalian Nell1 gene encodes a protein kinase C-beta1 (PKC-beta1) binding protein that belongs to a new class of cell-signaling molecules controlling cell growth and differentiation. Over-expression of Nell1 in the developing cranial sutures in both human and mouse induces craniosynostosis, the premature fusion of the growing cranial bone fronts. Here, we report the generation, positional cloning and characterization of Nell1(6R), a recessive, neonatal-lethal point mutation in the mouse Nell1 gene, induced by N-ethyl-N-nitrosourea. Nell1(6R) has a T-->A base change that converts a codon for cysteine into a premature stop codon [Cys(502)Ter], resulting in severe truncation of the predicted protein product and marked reduction in steady-state levels of the transcript. In addition to the expected alteration of cranial morphology, Nell1(6R) mutants manifest skeletal defects in the vertebral column and ribcage, revealing a hitherto undefined role for Nell1 in signal transduction in endochondral ossification. Real-time quantitative reverse transcription-PCR assays of 219 genes showed an association between the loss of Nell1 function and reduced expression of genes for extracellular matrix (**ECM**) proteins critical for chondrogenesis and osteogenesis. Several affected genes are involved in the human cartilage disorder Ehlers-Danlos Syndrome and other disorders associated with spinal curvature anomalies. Nell1(6R) mutant mice are a new tool for elucidating basic mechanisms in osteoblast and chrondrocyte differentiation in the developing skull and vertebral column and understanding how perturbations in the production of **ECM** proteins can lead to anomalies in these structures.

### [Ee]xtracellular [Mm]atrix

#### PubMed

1. Nell1-deficient mice have reduced expression of **extracellular matrix** proteins causing cranial and vertebral defects. The mammalian Nell1 gene encodes a protein kinase C-beta1 (PKC-beta1) binding protein that belongs to a new class of cell-signaling molecules controlling cell growth and differentiation. Over-expression of Nell1 in the developing cranial sutures in both human and mouse induces craniosynostosis, the premature fusion of the growing cranial bone fronts. Here, we report the generation, positional cloning and characterization of Nell1(6R), a recessive, neonatal-lethal point mutation in the mouse Nell1 gene, induced by N-ethyl-N-nitrosourea. Nell1(6R) has a T-->A base change that converts a codon for cysteine into a premature stop codon [Cys(502)Ter], resulting in severe truncation of the predicted protein product and marked reduction in steady-state levels of the transcript. In addition to the expected alteration of cranial morphology, Nell1(6R) mutants manifest skeletal defects in the vertebral column and ribcage, revealing a hitherto undefined role for Nell1 in signal transduction in endochondral ossification. Real-time quantitative reverse transcription-PCR assays of 219 genes showed an association between the loss of Nell1 function and reduced expression of genes for **extracellular matrix** (ECM) proteins critical for chondrogenesis and osteogenesis. Several affected genes are involved in the human cartilage disorder Ehlers-Danlos Syndrome and other disorders associated with spinal curvature anomalies. Nell1(6R) mutant mice are a new tool for elucidating basic mechanisms in osteoblast and chrondrocyte differentiation in the developing skull and vertebral column and understanding how perturbations in the production of ECM proteins can lead to anomalies in these structures.

---

## Etv6

- NCBI Gene
- GeneRIF
- Pubmed

| Search Term | Rank | Fields |
| --- | --- | --- |
| [Mm]igration | 6 | PubMed(1) |

  
hide/show details for Etv6

### [Mm]igration

#### PubMed

1. Cytoskeletal regulatory gene expression and migratory properties of B-cell progenitors are affected by the ETV6-RUNX1 rearrangement. Although the ETV6-RUNX1 fusion is a frequent initiating event in childhood leukemia, its role in leukemogenesis is only partly understood. The main impact of the fusion itself is to generate and sustain a clone of clinically silent preleukemic B-cell progenitors (BCP). Additional oncogenic hits, occurring even several years later, are required for overt disease. The understanding of the features and interactions of ETV6-RUNX1-positive cells during this "latency" period may explain how these silent cells can persist and whether they could be prone to additional genetic changes. In this study, two in vitro murine models were used to investigate whether ETV6-RUNX1 alters the cellular adhesion and **migration** properties of BCP. ETV6-RUNX1-expressing cells showed a significant defect in the chemotactic response to CXCL12, caused by a block in CXCR4 signaling, as demonstrated by inhibition of CXCL12-associated calcium flux and lack of ERK phosphorylation. Moreover, the induction of ETV6-RUNX1 caused changes in the expression of cell-surface adhesion molecules. The expression of genes regulating the cytoskeleton was also affected, resulting in a block of CDC42 signaling. The abnormalities described here could alter the interaction of ETV6-RUNX1 preleukemic BCP with the microenvironment and contribute to the pathogenesis of the disease.Alterations in the expression of cytoskeletal regulatory genes and **migration** properties of BCP represent early events in the evolution of the disease, from the preleukemic phase to the clinical onset, and suggest new strategies for effective eradication of leukemia.

---

## Keap1

- NCBI Gene
- GeneRIF
- Pubmed

| Search Term | Rank | Fields |
| --- | --- | --- |
| [Mm]igration | 6 | PubMed(2); GeneRIFs(1) |
| [Ii]nvasive | 10 | PubMed(3) |
| [Mm]etastasis | 12 | PubMed(2) |

  
hide/show details for Keap1

### [Mm]igration

#### PubMed

1. The subcellular localization and activity of cortactin is regulated by acetylation and interaction with Keap1. Cortactin is an F-actin-binding protein that localizes to the cell cortex, where the actin remodeling that is required for cell **migration** occurs. We found that cortactin shuttled between the cytoplasm and the nucleus under basal conditions. We identified Kelch-like ECH-associated protein 1 (Keap1), a cytosolic protein that is involved in oxidant stress responses, as a binding partner of cortactin that promoted the cortical localization of cortactin and cell **migration**. The ability of cortactin to promote cell **migration** is regulated by various posttranslational modifications, including acetylation. We showed that the acetylated form of cortactin was mainly localized to the nucleus and that acetylation of cortactin decreased cell **migration** by inhibiting the binding of cortactin to Keap1. Our findings reveal that Keap1 regulates cell **migration** by affecting the subcellular localization and activity of cortactin independently of its role in oxidant stress responses.
2. Subcellular localization and cytoplasmic complex status of endogenous Keap1. Keap1 acts as a sensor for oxidative/electrophilic stress, an adaptor for Cullin-3-based ubiquitin ligase, and a regulator of Nrf2 activity through the interaction with Nrf2 Neh2 domain. However, the mechanism(s) of Nrf2 **migration** into the nucleus in response to stress remains largely unknown due to the lack of a reliable antibody for the detection of endogenous Keap1 molecule. Here, we report the generation of a new monoclonal antibody for the detection of endogenous Keap1 molecules. Immunocytochemical analysis of mouse embryonic fibroblasts with the antibody revealed that under normal, unstressed condition, Keap1 is localized primarily in the cytoplasm with minimal amount in the nucleus and endoplasmic reticulum. This subcellular localization profile of Keap1 appears unchanged after treatment of cells with diethyl maleate, an electrophile, and/or Leptomycin B, a nuclear export inhibitor. Subcellular fractionation analysis of mouse liver cells showed similar results. No substantial change in the subcellular distribution profile could be observed in cells isolated from butylated hydroxyanisole-treated mice. Analyses of sucrose density gradient centrifugation of mouse liver cells indicated that Keap1 appears to form multiprotein complexes in the cytoplasm. These results demonstrate that endogenous Keap1 remains mostly in the cytoplasm, and electrophiles promote nuclear accumulation of Nrf2 without altering the subcellular localization of Keap1.

#### GeneRIFs

1. findings reveal that Keap1 regulates cell **migration** by affecting the subcellular localization and activity of cortactin independently of its role in oxidant stress responses.

### [Ii]nvasive

#### PubMed

1. Role of KEAP1/NRF2 and TP53 Mutations in Lung Squamous Cell Carcinoma Development and Radiation Resistance. Lung squamous cell carcinoma (LSCC) pathogenesis remains incompletely understood, and biomarkers predicting treatment response remain lacking. Here, we describe novel murine LSCC models driven by loss of Trp53 and Keap1, both of which are frequently mutated in human LSCCs. Homozygous inactivation of Keap1 or Trp53 promoted airway basal stem cell (ABSC) self-renewal, suggesting that mutations in these genes lead to expansion of mutant stem cell clones. Deletion of Trp53 and Keap1 in ABSCs, but not more differentiated tracheal cells, produced tumors recapitulating histologic and molecular features of human LSCCs, indicating that they represent the likely cell of origin in this model. Deletion of Keap1 promoted tumor aggressiveness, metastasis, and resistance to oxidative stress and radiotherapy (RT). KEAP1/NRF2 mutation status predicted risk of local recurrence after RT in patients with non-small lung cancer (NSCLC) and could be non**invasive**ly identified in circulating tumor DNA. Thus, KEAP1/NRF2 mutations could serve as predictive biomarkers for personalization of therapeutic strategies for NSCLCs.We developed an LSCC mouse model involving Trp53 and Keap1, which are frequently mutated in human LSCCs. In this model, ABSCs are the cell of origin of these tumors. KEAP1/NRF2 mutations increase radioresistance and predict local tumor recurrence in radiotherapy patients. Our findings are of potential clinical relevance and could lead to personalized treatment strategies for tumors with KEAP1/NRF2 mutations. Cancer Discov; 7(1); 86-101. ©2016 AACR.This article is highlighted in the In This Issue feature, p. 1.
2. Dissection of the role of p62/Sqstm1 in activation of Nrf2 during xenophagy. Upon infection of a cell by Salmonella, p62/Sqstm1 assembles on the microbes; simultaneously, p62/Sqstm1 is phosphorylated at Ser351, leading to inactivation of Keap1, which is responsible for degrading Nrf2. Thus, cytoprotective Nrf2 targets are induced at the same time that autophagosomes entrap the microbes (xenophagy). However, the detailed role of p62/Sqstm1 during xenophagy has remained unclear. Here we show that translocation of p62/Sqstm1 to **invasive** Salmonella precedes Ser351 phosphorylation. Furthermore, in addition to Ser351 phosphorylation, oligomerization of p62/Sqstm1 is also required for localization of Keap1 onto microbes, which is followed by Nrf2 activation. Our data reveal the sequential dynamics of p62/Sqstm1 in response to bacterial infection.
3. Activation of liver X receptor improves viability of adipose-derived mesenchymal stem cells to attenuate myocardial ischemia injury through TLR4/NF-κB and Keap-1/Nrf-2 signaling pathways. Clinical application of cellular therapy for cardiac regeneration is significantly hampered by the low retention of engrafted cells, mainly attributable to the poor microenvironment dominated by inflammation and oxidative stress in the host's infarcted myocardium. This study aims at investigating whether liver X receptor (LXR) agonist T0901317 will improve survival of adipose-derived mesenchymal stem cells (AD-MSCs) after transplantation into infarcted hearts.No**ninvasiv**e in vivo bioluminescence imaging and histological staining showed that LXR agonist T0901317 improved the retention and survival of intramyocardially injected AD-MSCs. Moreover, combined therapy of LXR agonist and AD-MSCs inhibited host cardiomyocyte apoptosis, reduced fibrosis, and improved cardiac function, while it concomitantly decreased inflammatory cytokines (e.g., tumor necrosis factor-α and interleukin-6) and increased growth factor (e.g., vascular endothelial growth factor and basic fibroblast growth factor) expression in infarct myocardium. To reveal possible mechanisms, AD-MSCs were subjected to hypoxia/serum deprivation (H/SD) injury to simulate ischemic conditions in vivo. The LXR agonist (10(-7) mM) improved AD-MSC survival under H/SD condition. Western blot revealed that the LXR agonist reduced TLR4, TRAF-6, and MyD88 protein expression, inhibited IκBα phosphorylation and NF-κB-p65 nuclear translocation, which resulted in accelerated Keap-1 protein degradation, enhanced Nrf-2 nuclear translocation, and increased HO-1 protein expression.LXR agonist can enhance the functional survival of transplanted AD-MSCs in infarcted myocardium, at least partially, via modulation of the TLR4/NF-κB and Keap-1/Nrf-2 signaling pathways. Moreover, combined therapy of LXR agonist and AD-MSCs has a synergetic effect on cardiac repair and functional improvement after infarction.

### [Mm]etastasis

#### PubMed

1. Nrf2-deficiency creates a responsive microenvironment for **metastasis** to the lung. The Nrf2 transcription factor is crucial for regulating the cellular defense against various carcinogens. However, relationship between host Nrf2 and cancer **metastasis** remains unexplored. To address this issue, we examined susceptibility of Nrf2-deficient mice to pulmonary cancer **metastasis** following implantation of the mouse Lewis lung carcinoma (3LL) cell line. Nrf2-deficient mice reproducibly exhibited a higher number of pulmonary metastatic nodules than wild-type mice did. The lung and bone marrow (BM) of cancer-bearing Nrf2-deficient mice contained increased numbers of inflammatory cells, including myeloid-derived suppressor cells (MDSCs), a potent population of immunosuppressive cells. MDSCs can attenuate CD8(+) T-cell immunity through modification of the T-cell receptor complex exploiting reactive oxygen species (ROS). MDSCs of Nrf2-deficient mice retained elevated levels of ROS relative to wild-type mice. BM transplantation experiments revealed functional disturbance in the hematopoietic and immune systems of Nrf2-deficient mice. Wild-type recipient mice with Nrf2-deficient BM cells showed increased levels of lung **metastasis** after cancer cell inoculation. These mice exhibited high-level accumulation of ROS in MDSCs, which showed very good coincidence to the decrease of splenic CD8(+) T-cells. In contrast, Keap1-knockdown mutant mice harboring high-level Nrf2 expression displayed increased resistance against the cancer cell **metastasis** to the lung, accompanied by a decrease in ROS in the MDSCs fraction. Our results thus reveal a novel function for Nrf2 in the prevention of cancer **metastasis**, presumably by its ability to preserve the redox balance in the hematopoietic and immune systems.
2. Role of KEAP1/NRF2 and TP53 Mutations in Lung Squamous Cell Carcinoma Development and Radiation Resistance. Lung squamous cell carcinoma (LSCC) pathogenesis remains incompletely understood, and biomarkers predicting treatment response remain lacking. Here, we describe novel murine LSCC models driven by loss of Trp53 and Keap1, both of which are frequently mutated in human LSCCs. Homozygous inactivation of Keap1 or Trp53 promoted airway basal stem cell (ABSC) self-renewal, suggesting that mutations in these genes lead to expansion of mutant stem cell clones. Deletion of Trp53 and Keap1 in ABSCs, but not more differentiated tracheal cells, produced tumors recapitulating histologic and molecular features of human LSCCs, indicating that they represent the likely cell of origin in this model. Deletion of Keap1 promoted tumor aggressiveness, **metastasis**, and resistance to oxidative stress and radiotherapy (RT). KEAP1/NRF2 mutation status predicted risk of local recurrence after RT in patients with non-small lung cancer (NSCLC) and could be noninvasively identified in circulating tumor DNA. Thus, KEAP1/NRF2 mutations could serve as predictive biomarkers for personalization of therapeutic strategies for NSCLCs.We developed an LSCC mouse model involving Trp53 and Keap1, which are frequently mutated in human LSCCs. In this model, ABSCs are the cell of origin of these tumors. KEAP1/NRF2 mutations increase radioresistance and predict local tumor recurrence in radiotherapy patients. Our findings are of potential clinical relevance and could lead to personalized treatment strategies for tumors with KEAP1/NRF2 mutations. Cancer Discov; 7(1); 86-101. ©2016 AACR.This article is highlighted in the In This Issue feature, p. 1.

---

## Bag3

- NCBI Gene
- GeneRIF
- Pubmed

| Search Term | Rank | Fields |
| --- | --- | --- |
| [Ff]ilopodia | 1 | PubMed(1) |
| [Mm]igration | 6 | PubMed(2) |
| [Mm]etastasis | 12 | PubMed(2) |

  
hide/show details for Bag3

### [Ff]ilopodia

#### PubMed

1. BAG3 regulates motility and adhesion of epithelial cancer cells. BAG3 protein binds to and regulates Hsp70 chaperone activity. The BAG3 protein contains a WW domain and a proline-rich region with SH3-binding motifs, suggesting that it may interact with proteins relevant to signal transduction, recruiting Hsp70 to signaling complexes and altering cell responses. BAG3 overexpression has been observed in human cancers. We show here that homozygous BAG3-deficient mouse embryonic fibroblasts (MEF) exhibit delayed formation of **filopodia** and focal adhesion complexes when freshly plated. BAG3-deficient MEFs show reduced cell motility in culture. We observed that endogenous BAG3 protein is highly expressed in many human epithelial cancer cell lines, especially adenocarcinomas. Gene transfer-mediated overexpression of BAG3 increased motility of Cos7 cell and several human cancer cell lines, including breast cancer MCF7 and prostate cancer DU145 and ALVA31 cell lines. Conversely, reduction of BAG3 protein by RNA interference (RNAi) decreased cell motility in four of four epithelial tumor lines tested. We observed an influence of BAG3 on cell adhesion in culture. In Cos7 kidney epithelial cells, BAG3 protein partially colocalizes with actin at the leading edge of migrating cells, wherein active actin polymerization and nucleation occur. RNAi-mediated reductions in BAG3 expression were associated with decreased Rac1 activity, suggesting a role for BAG3 in regulating this small GTPase involved in actin-cytoskeleton dynamics. In mice, RNAi-mediated reductions in BAG3 in a human tumor xenograft suppressed invasion and metastasis in vivo. Thus, the high levels of BAG3 protein seen in some epithelial cancer cell lines may be relevant to mechanisms of tumor invasion and metastasis.

### [Mm]igration

#### PubMed

1. Deletion of the bis gene results in a marked increase in the production of corticosterone that is associated with thymic atrophy in mice. Bis (Bag3) is known to be involved in cell survival, **migration**, the regulating of chaperones, and protein quality control. We reported recently on the production of bis gene-deleted mice, which show early lethality within 3 wk after birth with a phenotype showing severe malnutrition and shrinkage of the thymus. In this report, we provide evidence to show that an intrinsic problem of adrenal gland is the the primary cause for the severe atrophy of the thymus in bis(-/-) mice. The bis(-/-) mice show significantly higher levels of corticosterone, but CRH and ACTH levels were considerably lower than those of wild littermates. The transcription of steroidogenic enzymes was increased in the adrenal glands of bis(-/-) mice, accompanied by an increase in the thickness of the zona reticularis. An analysis of thymus tissue from bis(-/-) mice revealed that the severe atrophy of the thymus is due to the specific loss of immature double-positive (CD4(+)CD8(+)) cortical thymocytes by apoptosis, as evidenced by immunohistochemical examination and flow cytometric analysis, which were restored by injection of an inhibitor of glucocorticoid synthesis. In vitro cultures of thymocytes with increasing doses of dexamethasone exhibited a similar degree of apoptosis between wild and bis(-/-) thymocytes. The corticosterone levels from fasted wild littermates were one-half those of bis(-/-) mice, although serum glucose levels were similar. Thus, the deletion of the bis gene resulted in the intrinsic defect in the adrenal gland, leading to a marked increase in glucocorticoid levels, probably upon starvation stress, which accounts for the massive apoptosis of the thymus.
2. Myosin heavy chain is stabilized by BCL-2 interacting cell death suppressor (BIS) in skeletal muscle. BCL-2 interacting cell death suppressor (BIS), which is ubiquitously expressed, has important roles in various cellular processes, such as apoptosis, the cellular stress response, **migration** and invasion and protein quality control. In particular, BIS is highly expressed in skeletal and cardiac muscles, and BIS gene mutations result in human myopathy. In this study, we show that mRNA and protein levels of BIS were markedly increased during skeletal myogenesis in C2C12 cells and mouse satellite cells. BIS knockdown did not prevent the early stage of skeletal myogenesis, but did induce muscle atrophy and a decrease in the diameter of myotubes. BIS knockdown significantly suppressed the expression level of myosin heavy chain (MyHC) without changing the expression levels of myogenic marker proteins, such as Mgn, Cav-3 and MG53. In addition, BIS endogenously interacted with MyHC, and BIS knockdown induced MyHC ubiquitination and degradation. From these data, we conclude that molecular association of MyHC and BIS is necessary for MyHC stabilization in skeletal muscle.

### [Mm]etastasis

#### PubMed

1. BAG3 promotes pancreatic ductal adenocarcinoma growth by activating stromal macrophages. The incidence and death rate of pancreatic ductal adenocarcinoma (PDAC) have increased in recent years, therefore the identification of novel targets for treatment is extremely important. Interactions between cancer and stromal cells are critically involved in tumour formation and development of **metastasis**. Here we report that PDAC cells secrete BAG3, which binds and activates macrophages, inducing their activation and the secretion of PDAC supporting factors. We also identify IFITM-2 as a BAG3 receptor and show that it signals through PI3K and the p38 MAPK pathways. Finally, we show that the use of an anti-BAG3 antibody results in reduced tumour growth and prevents **metastasis** formation in three different mouse models. In conclusion, we identify a paracrine loop involved in PDAC growth and metastatic spreading, and show that an anti-BAG3 antibody has therapeutic potential.
2. BAG3 regulates motility and adhesion of epithelial cancer cells. BAG3 protein binds to and regulates Hsp70 chaperone activity. The BAG3 protein contains a WW domain and a proline-rich region with SH3-binding motifs, suggesting that it may interact with proteins relevant to signal transduction, recruiting Hsp70 to signaling complexes and altering cell responses. BAG3 overexpression has been observed in human cancers. We show here that homozygous BAG3-deficient mouse embryonic fibroblasts (MEF) exhibit delayed formation of filopodia and focal adhesion complexes when freshly plated. BAG3-deficient MEFs show reduced cell motility in culture. We observed that endogenous BAG3 protein is highly expressed in many human epithelial cancer cell lines, especially adenocarcinomas. Gene transfer-mediated overexpression of BAG3 increased motility of Cos7 cell and several human cancer cell lines, including breast cancer MCF7 and prostate cancer DU145 and ALVA31 cell lines. Conversely, reduction of BAG3 protein by RNA interference (RNAi) decreased cell motility in four of four epithelial tumor lines tested. We observed an influence of BAG3 on cell adhesion in culture. In Cos7 kidney epithelial cells, BAG3 protein partially colocalizes with actin at the leading edge of migrating cells, wherein active actin polymerization and nucleation occur. RNAi-mediated reductions in BAG3 expression were associated with decreased Rac1 activity, suggesting a role for BAG3 in regulating this small GTPase involved in actin-cytoskeleton dynamics. In mice, RNAi-mediated reductions in BAG3 in a human tumor xenograft suppressed invasion and **metastasis** in vivo. Thus, the high levels of BAG3 protein seen in some epithelial cancer cell lines may be relevant to mechanisms of tumor invasion and **metastasis**.

---

## Serpini1

- NCBI Gene
- GeneRIF
- Pubmed

| Search Term | Rank | Fields |
| --- | --- | --- |
| [Mm]igration | 6 | PubMed(2) |
| [Ee]xtracellular [Mm]atrix | 8 | PubMed(1) |
| [Ii]nvasive | 10 | PubMed(1) |
| [Mm]etastasis | 12 | PubMed(2); GeneRIFs(1) |

  
hide/show details for Serpini1

### [Mm]igration

#### PubMed

1. Expression of neuroserpin, an inhibitor of tissue plasminogen activator, in the developing and adult nervous system of the mouse. Neuroserpin is a serine protease inhibitor of the serpin family that has been identified as an axonally secreted glycoprotein in neuronal cultures of chicken dorsal root ganglia. To obtain an indication for possible functions of neuroserpin, we analyzed its expression in the developing and the adult CNS of the mouse. In the adult CNS, neuroserpin was most strongly expressed in the neocortex, the hippocampal formation, the olfactory bulb, and the amygdala. In contrast, most thalamic nuclei, the caudate putamen, and the cerebellar granule cells were devoid of neuroserpin mRNA. During embryonic development, neuroserpin mRNA was not detectable in neuroepithelia, but it was expressed in the differentiating fields of most CNS regions concurrent with their appearance. In the cerebellum, the granule cells and a subgroup of Purkinje cells were neuroserpin-positive during postnatal development. As a further step toward the elucidation of neuroserpin function, we performed a study to identify potential target proteases. In vitro, neuroserpin formed SDS-stable complexes and inhibited the amidolytic activity of tissue plasminogen activator, urokinase, and plasmin. In contrast, no complex formation with or inhibition of thrombin was found. Expression pattern and inhibitory specificity implicate neuroserpin as a candidate regulator of plasminogen activators, which have been suggested to participate in the modulation or reorganization of synaptic connections in the adult. During development, neuroserpin may attenuate extracellular proteolysis related to processes such as neuronal **migration**, axogenesis, or the formation of mature synaptic connections.
2. Anti-Mullerian-hormone-dependent regulation of the brain serine-protease inhibitor neuroserpin. The balance between tissue-type plasminogen activator (tPA) and one of its inhibitors, neuroserpin, has crucial roles in the central nervous system, including the control of neuronal **migration**, neuronal plasticity and neuronal death. In the present study, we demonstrate that the activation of the transforming growth factor-beta (TGFbeta)-related BMPR-IB (also known as BMPR1B and Alk6)- and Smad5-dependent signalling pathways controls neuroserpin transcription. Accordingly, we demonstrate for the first time that anti-Mullerian hormone (AMH), a member of the TGFbeta family, promotes the expression of neuroserpin in cultured neurons but not in astrocytes. The relevance of these findings is confirmed by the presence of both AMH and AMH type-II receptor (AMHR-II) in brain tissues, and is supported by the observation of reduced levels of neuroserpin in the brain of AMHR-II-deficient mice. Interestingly, as previously demonstrated for neuroserpin, AMH protects neurons against N-methyl-D-aspartate (NMDA)-mediated excitotoxicity both in vitro and in vivo. This study demonstrates the existence of an AMH-dependent signalling pathway in the brain leading to an overexpression of the serine-protease inhibitor, neuroserpin, and neuronal survival.

### [Ee]xtracellular [Mm]atrix

#### PubMed

1. **Extracellular matrix** secretion by cardiac fibroblasts: role of microRNA-29b and microRNA-30c. MicroRNAs (miRNAs), in particular miR-29b and miR-30c, have been implicated as important regulators of cardiac fibrosis.To perform a proteomics comparison of miRNA effects on **extracellular matrix** secretion by cardiac fibroblasts.Mouse cardiac fibroblasts were transfected with pre-/anti-miR of miR-29b and miR-30c, and their conditioned medium was analyzed by mass spectrometry. miR-29b targeted a cadre of proteins involved in fibrosis, including multiple collagens, matrix metalloproteinases, and leukemia inhibitory factor, insulin-like growth factor 1, and pentraxin 3, 3 predicted targets of miR-29b. miR-29b also attenuated the cardiac fibroblast response to transforming growth factor-β. In contrast, miR-30c had little effect on **extracellular matri**x production but opposite effects regarding leukemia inhibitory factor and insulin-like growth factor 1. Both miRNAs indirectly affected cardiac myocytes. On transfection with pre-miR-29b, the conditioned medium of cardiac fibroblasts lost its ability to support adhesion of rat ventricular myocytes and led to a significant reduction of cardiac myocyte proteins (α-actinin, cardiac myosin-binding protein C, and cardiac troponin I). Similarly, cardiomyocytes derived from mouse embryonic stem cells atrophied under pre-miR-29 conditioned medium, whereas pre-miR-30c conditioned medium had a prohypertrophic effect. Levels of miR-29a, miR-29c, and miR-30c, but not miR-29b, were significantly reduced in a mouse model of pathological but not physiological hypertrophy. Treatment with antagomiRs to miR-29b induced excess fibrosis after aortic constriction without overt deterioration in cardiac function.Our proteomic analysis revealed novel molecular targets of miRNAs that are linked to a fibrogenic cardiac phenotype. Such comprehensive screening methods are essential to define the concerted actions of miRNAs in cardiovascular disease.

### [Ii]nvasive

#### PubMed

1. In vivo and in vitro investigations of heterozygous nebulin knock-out mice disclose a mild skeletal muscle phenotype. Nemaline myopathy is the most common congenital skeletal muscle disease, and mutations in the nebulin gene account for 50% of all cases. Recent studies suggest that the disease severity might be related to the nebulin expression levels. Considering that mutations in the nebulin gene are typically recessive, one would expect that a single functional nebulin allele would maintain nebulin protein expression which would result in preserved skeletal muscle function. We investigated skeletal muscle function of heterozygous nebulin knock-out (i.e., nebulin(+/-)) mice using a multidisciplinary approach including protein and gene expression analysis and combined in vivo and in vitro force measurements. Skeletal muscle anatomy and energy metabolism were studied strictly non-**invasive**ly using magnetic resonance imaging and 31P-magnetic resonance spectroscopy. Maximal force production was reduced by around 16% in isolated muscle of nebulin(+/-) mice while in vivo force generating capacity was preserved. Muscle weakness was associated with a shift toward a slower proteomic phenotype, but was not related to nebulin protein deficiency or to an impaired energy metabolism. Further studies would be warranted in order to determine the mechanisms leading to a mild skeletal muscle phenotype resulting from the expression of a single nebulin allele.

### [Mm]etastasis

#### PubMed

1. Serpins promote cancer cell survival and vascular co-option in brain **metastasis**. Brain **metastasis** is an ominous complication of cancer, yet most cancer cells that infiltrate the brain die of unknown causes. Here, we identify plasmin from the reactive brain stroma as a defense against metastatic invasion, and plasminogen activator (PA) inhibitory serpins in cancer cells as a shield against this defense. Plasmin suppresses brain **metastasis** in two ways: by converting membrane-bound astrocytic FasL into a paracrine death signal for cancer cells, and by inactivating the axon pathfinding molecule L1CAM, which metastatic cells express for spreading along brain capillaries and for metastatic outgrowth. Brain metastatic cells from lung cancer and breast cancer express high levels of anti-PA serpins, including neuroserpin and serpin B2, to prevent plasmin generation and its **metastasis**-suppressive effects. By protecting cancer cells from death signals and fostering vascular co-option, anti-PA serpins provide a unifying mechanism for the initiation of brain **metastasis** in lung and breast cancers.
2. Update of the human and mouse SERPIN gene superfamily. The serpin family comprises a structurally similar, yet functionally diverse, set of proteins. Named originally for their function as serine proteinase inhibitors, many of its members are not inhibitors but rather chaperones, involved in storage, transport, and other roles. Serpins are found in genomes of all kingdoms, with 36 human protein-coding genes and five pseudogenes. The mouse has 60 Serpin functional genes, many of which are orthologous to human SERPIN genes and some of which have expanded into multiple paralogous genes. Serpins are found in tissues throughout the body; whereas most are extracellular, there is a class of intracellular serpins. Serpins appear to have roles in inflammation, immune function, tumorigenesis, blood clotting, dementia, and cancer **metastasis**. Further characterization of these proteins will likely reveal potential biomarkers and therapeutic targets for disease.

#### GeneRIFs

1. Brain metastatic cells from lung cancer and breast cancer express high levels of anti-plasminogen activator (PA) serpins, including neuroserpin and serpin B2, to prevent plasmin generation and its **metastasis**-suppressive effects.

---

## Slc9a3r1

- NCBI Gene
- GeneRIF
- Pubmed

| Search Term | Rank | Fields |
| --- | --- | --- |
| [Mm]igration | 6 | Process(1); PubMed(2); GeneRIFs(1) |

  
hide/show details for Slc9a3r1

### [Mm]igration

#### Process

1. negative regulation of cell **migration**

#### PubMed

1. PTEN tumor suppressor associates with NHERF proteins to attenuate PDGF receptor signaling. PTEN, a tumor suppressor frequently inactivated in many human cancers, directly antagonizes the activity of phosphatidylinositol-3-OH kinase (PI3K) by dephosphorylating phosphoinositides. We show here that PTEN interacts directly with the NHERF1 and NHERF2 (Na+/H+ exchanger regulatory factor) homologous adaptor proteins through the PDZ motif of PTEN and the PDZ1 domain of NHERF1 or both PDZ domains of NHERF2. NHERFs were shown to interact directly with platelet-derived growth factor receptor (PDGFR), and we demonstrate the assembly of a ternary complex between PTEN, NHERFs and PDGFR. The activation of the PI3K pathway after PDGFR stimulation was prolonged in NHERF1(-/-) mouse embryonic fibroblasts as compared to wild-type cells, consistent with defective PTEN recruitment to PDGFR in the absence of NHERF1. Depletion of NHERF2 by small interfering RNA similarly increased PI3K signaling. Phenotypically, the loss of NHERF1 enhanced the PDGF-induced cytoskeletal rearrangements and chemotactic **migration** of the cells. These data indicate that, in normal cells, NHERF proteins recruit PTEN to PDGFR to restrict the activation of the PI3K.
2. EBP50 promotes focal adhesion turnover and vascular smooth muscle cells **migration**. The ezrin-radixin-moesin-binding phosphoprotein 50 (EBP50) is a PDZ-containing scaffolding protein that regulates a variety of physiological functions. In the vasculature, EBP50 promotes neointima formation following arterial injury. In this study the role of EBP50 on vascular smooth muscle cell (VSMC) **migration** was characterized. The spreading and motility of primary VSMC isolated from EBP50 knockout (KO) mice were significantly reduced compared to wild-type (WT) cells. EBP50-null VSMC had fewer and larger focal adhesions than wild-type cells. Assembly and disassembly of focal adhesion-assessed by live-cell total internal reflection fluorescence imaging-in response to epidermal growth factor (EGF) were significantly reduced in KO cells. Immunoprecipitation experiments showed that EBP50 interacts with EGF receptor via the PDZ2 domain and with focal adhesion kinase (FAK) via the C-terminal ERM domain. EBP50 promoted the formation of a complex containing both EGF receptor and FAK. Phosphorylation of Tyr-925 of FAK in response to EGF was significantly reduced in KO cell compared to WT cells. The residence time of FAK in focal adhesions-determined by fluorescence recovery after photobleaching-was increased in WT cells. Collectively, these studies indicate that EBP50, by scaffolding EGF receptor and FAK, facilitates activation of FAK, focal adhesion turnover, and **migration** of VSMC.

#### GeneRIFs

1. EBP50 promotes focal adhesion turnover and vascular smooth muscle cells **migration**.

---

## Slc9a3r2

- NCBI Gene
- GeneRIF
- Pubmed

| Search Term | Rank | Fields |
| --- | --- | --- |
| [Mm]igration | 6 | PubMed(1) |

  
hide/show details for Slc9a3r2

### [Mm]igration

#### PubMed

1. PTEN tumor suppressor associates with NHERF proteins to attenuate PDGF receptor signaling. PTEN, a tumor suppressor frequently inactivated in many human cancers, directly antagonizes the activity of phosphatidylinositol-3-OH kinase (PI3K) by dephosphorylating phosphoinositides. We show here that PTEN interacts directly with the NHERF1 and NHERF2 (Na+/H+ exchanger regulatory factor) homologous adaptor proteins through the PDZ motif of PTEN and the PDZ1 domain of NHERF1 or both PDZ domains of NHERF2. NHERFs were shown to interact directly with platelet-derived growth factor receptor (PDGFR), and we demonstrate the assembly of a ternary complex between PTEN, NHERFs and PDGFR. The activation of the PI3K pathway after PDGFR stimulation was prolonged in NHERF1(-/-) mouse embryonic fibroblasts as compared to wild-type cells, consistent with defective PTEN recruitment to PDGFR in the absence of NHERF1. Depletion of NHERF2 by small interfering RNA similarly increased PI3K signaling. Phenotypically, the loss of NHERF1 enhanced the PDGF-induced cytoskeletal rearrangements and chemotactic **migration** of the cells. These data indicate that, in normal cells, NHERF proteins recruit PTEN to PDGFR to restrict the activation of the PI3K.

---

## Rrad

- NCBI Gene
- GeneRIF
- Pubmed

| Search Term | Rank | Fields |
| --- | --- | --- |
| ECM | 7 | PubMed(1) |
| [Ee]xtracellular [Mm]atrix | 8 | PubMed(1) |

  
hide/show details for Rrad

### ECM

#### PubMed

1. Rad GTPase inhibits cardiac fibrosis through connective tissue growth factor. Our previous studies documented that Rad (Ras associated with diabetes), a member of the RGK (Rad, Gem, and Kir) family of Ras-related small G protein, is significantly decreased in human failing hearts and plays an important role in attenuating cardiac hypertrophy. The goal of this study is to identify the effect of Rad on cardiac fibrosis and the underlying mechanisms.Rad knockout (KO) mice showed more severe cardiac fibrosis compared with wild-type littermate controls as detected by Sirius Red staining. Western blot analyses demonstrated that the expression of connective tissue growth factor (CTGF), a key mediator of fibrosis, increased dramatically in Rad KO mice. Overexpression of Rad in cultured neonatal cardiomyocytes suppressed both basal and transforming growth factor-β1-induced CTGF expression. Elevated CTGF expression was observed in cardiomyocytes when Rad was reduced by RNA interference. Moreover, cardiac fibroblasts produced greater extracellular matrix **(EC**M) when stimulated with conditioned medium from Rad-knockdown cardiomyocytes. **EC**M production was completely abolished by adding a CTGF-neutralizing antibody into the medium. CCAAT/enhancer-binding protein δ (C/EBP-δ) was demonstrated to activate CTGF in cardiomyocytes. Chromatin immunoprecipitation assay and co-immunoprecipitation further demonstrated that Rad inhibited the binding of C/EBP-δ to the CTGF promoter via direct interaction with C/EBP-δ.Our data reveal that Rad deficiency can lead to cardiac fibrosis. Rad inhibits CTGF expression through binding with C/EBP-δ, thus regul**ati**ng ECM production in the heart. This study suggests a potential link between decreased Rad levels and increased cardiac fibrosis in human failing hearts.

### [Ee]xtracellular [Mm]atrix

#### PubMed

1. Rad GTPase inhibits cardiac fibrosis through connective tissue growth factor. Our previous studies documented that Rad (Ras associated with diabetes), a member of the RGK (Rad, Gem, and Kir) family of Ras-related small G protein, is significantly decreased in human failing hearts and plays an important role in attenuating cardiac hypertrophy. The goal of this study is to identify the effect of Rad on cardiac fibrosis and the underlying mechanisms.Rad knockout (KO) mice showed more severe cardiac fibrosis compared with wild-type littermate controls as detected by Sirius Red staining. Western blot analyses demonstrated that the expression of connective tissue growth factor (CTGF), a key mediator of fibrosis, increased dramatically in Rad KO mice. Overexpression of Rad in cultured neonatal cardiomyocytes suppressed both basal and transforming growth factor-β1-induced CTGF expression. Elevated CTGF expression was observed in cardiomyocytes when Rad was reduced by RNA interference. Moreover, cardiac fibroblasts produced greater **extracellular matri**x (ECM) when stimulated with conditioned medium from Rad-knockdown cardiomyocytes. ECM production was completely abolished by adding a CTGF-neutralizing antibody into the medium. CCAAT/enhancer-binding protein δ (C/EBP-δ) was demonstrated to activate CTGF in cardiomyocytes. Chromatin immunoprecipitation assay and co-immunoprecipitation further demonstrated that Rad inhibited the binding of C/EBP-δ to the CTGF promoter via direct interaction with C/EBP-δ.Our data reveal that Rad deficiency can lead to cardiac fibrosis. Rad inhibits CTGF expression through binding with C/EBP-δ, thus regulating ECM production in the heart. This study suggests a potential link between decreased Rad levels and increased cardiac fibrosis in human failing hearts.

---

## Anks1

- NCBI Gene
- GeneRIF
- Pubmed

| Search Term | Rank | Fields |
| --- | --- | --- |
| [Mm]igration | 6 | Process(1); PubMed(2) |

  
hide/show details for Anks1

### [Mm]igration

#### Process

1. substrate-dependent cell **migration**

#### PubMed

1. The SAM domains of Anks family proteins are critically involved in modulating the degradation of EphA receptors. We recently reported that the phosphotyrosine-binding (PTB) domain of Anks family proteins binds to EphA8, thereby positively regulating EphA8-mediated signaling pathways. In the current study, we identified a potential role for the SAM domains of Anks family proteins in EphA signaling. We found that SAM domains of Anks family proteins directly bind to ubiquitin, suggesting that Anks proteins regulate the degradation of ubiquitinated EphA receptors. Consistent with the role of Cbl ubiquitin ligases in the degradation of Eph receptors, our results revealed that the ubiquitin ligase c-Cbl induced the ubiquitination and degradation of EphA8 upon ligand binding. Ubiquitinated EphA8 also bound to the SAM domains of Odin, a member of the Anks family proteins. More importantly, the overexpression of wild-type Odin protected EphA8 and EphA2 from undergoing degradation following ligand stimulation and promoted EphA-mediated inhibition of cell **migration**. In contrast, a SAM domain deletion mutant of Odin strongly impaired the function of endogenous Odin, suggesting that the mutant functions in a dominant-negative manner. An analysis of Odin-deficient primary embryonic fibroblasts indicated that Odin levels play a critical role in regulating the stability of EphA2 in response to ligand stimulation. Taken together, our studies suggest that the SAM domains of Anks family proteins play a pivotal role in enhancing the stability of EphA receptors by modulating the ubiquitination process.
2. Identification of phosphotyrosine binding domain-containing proteins as novel downstream targets of the EphA8 signaling function. Eph receptors and ephrins have been implicated in a variety of cellular processes, including morphology and motility, because of their ability to modulate intricate signaling networks. Here we show that the phosphotyrosine binding (PTB) domain-containing proteins AIDA-1b and Odin are tightly associated with the EphA8 receptor in response to ligand stimulation. Both AIDA-1b and Odin belong to the ankyrin repeat and sterile alpha motif domain-containing (Anks) protein family. The PTB domain of Anks family proteins is crucial for their association with the juxtamembrane domain of EphA8, whereas EphA8 tyrosine kinase activity is not required for this protein-protein interaction. In addition, we found that Odin is a more physiologically relevant partner of EphA8 in mammalian cells. Interestingly, overexpression of the Odin PTB domain alone attenuated EphA8-mediated inhibition of cell **migration** in HEK293 cells, suggesting that it acts as a dominant-negative mutant of the endogenous Odin protein. More importantly, small interfering RNA-mediated Odin silencing significantly diminished ephrinA5-induced EphA8 signaling effects, which inhibit cell **migration** in HEK293 cells and retract growing neurites of Neuro2a cells. Taken together, our findings support a possible function for Anks family proteins as scaffolding proteins of the EphA8 signaling pathway.

---

## Klf6

- NCBI Gene
- GeneRIF
- Pubmed

| Search Term | Rank | Fields |
| --- | --- | --- |
| [Mm]igration | 6 | PubMed(3) |
| [Ee]xtracellular [Mm]atrix | 8 | PubMed(1); GeneRIFs(1) |
| [Mm]etastasize | 11 | PubMed(1) |
| [Mm]etastasis | 12 | PubMed(2); GeneRIFs(1) |

  
hide/show details for Klf6

### [Mm]igration

#### PubMed

1. Serum response factor is required for cortical axon growth but is dispensable for neurogenesis and neocortical lamination. Previous studies have shown that neuron-specific deletion of serum response factor (SRF) results in deficits in tangential cell **migration**, guidance-dependent circuit assembly, activity-dependent gene expression, and synaptic plasticity in the hippocampus. Furthermore, SRF deletion in mouse embryonic stem cells causes cell death in vitro. However, the requirement of SRF for early neuronal development including neural stem cell homeostasis, neurogenesis, and axonal innervations remains unknown. Here, we report that SRF is critical for development of major axonal tracts in the forebrain. Conditional mutant mice lacking SRF in neural progenitor cells (Srf-Nestin-cKO) exhibit striking deficits in cortical axonal projections including corticostriatal, corticospinal, and corticothalamic tracts, and they show a variable loss of the corpus callosum. Neurogenesis and interneuron specification occur normally in the absence of SRF and the deficits in axonal projections were not due to a decrease or loss in cell numbers. Radial **migration** of neurons and neocortical lamination were also not affected. No aberrant cell death was observed during development, whereas there was an increase in the number of proliferative cells in the ventricular zone from embryonic day 14 to day 18. Similar axonal tract deficits were also observed in mutant mice lacking SRF in the developing excitatory neurons of neocortex and hippocampus (Srf-NEX-cKO). Together, these findings suggest distinct roles for SRF during neuronal development; SRF is specifically required in a cell-autonomous manner for axonal tract development but is dispensable for cell survival, neurogenesis, neocortical lamination, and neuronal differentiation.
2. Relationships between hematopoiesis and hepatogenesis in the midtrimester fetal liver characterized by dynamic transcriptomic and proteomic profiles. In fetal hematopoietic organs, the switch from hematopoiesis is hypothesized to be a critical time point for organogenesis, but it is not yet evidenced. The transient coexistence of hematopoiesis will be useful to understand the development of fetal liver (FL) around this time and its relationship to hematopoiesis. Here, the temporal and the comparative transcriptomic and proteomic profiles were observed during the critical time points corresponding to the initiation (E11.5), peak (E14.5), recession (E15.5), and disappearance (3 ddp) of mouse FL hematopoiesis. We found that E11.5-E14.5 corresponds to a FL hematopoietic expansion phase with distinct molecular features, including the expression of new transcription factors, many of which are novel KRAB (Kruppel-associated box)-containing zinc finger proteins. This time period is also characterized by extensive depression of some liver functions, especially catabolism/utilization, immune and defense, classical complement cascades, and intrinsic blood coagulation. Instead, the other liver functions increased, such as xenobiotic and sterol metabolism, synthesis of carbohydrate and glycan, the alternate and lectin complement cascades and extrinsic blood coagulation, and etc. Strikingly, all of the liver functions were significantly increased at E14.5-E15.5 and thereafter, and the depression of the key pathways attributes to build the hematopoietic microenvironment. These findings signal hematopoiesis e**migration** is the key to open the door of liver maturation.
3. A novel KLF6-Rho GTPase axis regulates hepatocellular carcinoma cell **migration** and dissemination. The presence of invasion into the extra-hepatic portion of the portal vein or the development of distant metastases renders hepatocellular carcinoma (HCC) patients ineligible for the only potential curative options for this malignancy-tumor resection or organ transplantation. Gene expression profiling of murine HCC cell lines identified KLF6 as a potential regulator of HCC cell **migration**. KLF6 knockdown increases cell **migration**, consistent with the correlation between decreased KLF6 mRNA levels and the presence of vascular invasion in human HCC. Concordantly, single-copy deletion of Klf6 in a HCC mouse model results in increased tumor formation, increased metastasis to the lungs and decreased survival, indicating that KLF6 suppresses both HCC development and metastasis. By combining gene expression profiling and chromatin immunoprecipitation coupled to deep sequencing, we identified novel transcriptional targets of KLF6 in HCC cells including VAV3, a known activator of the RAC1 small GTPase. Indeed, RAC1 activity is increased in KLF6-knockdown cells in a VAV3-dependent manner, and knockdown of either RAC1 or VAV3 impairs HCC cell **migration**. Together, our data demonstrate a novel function for KLF6 in constraining HCC dissemination through the regulation of a VAV3-RAC1 signaling axis.

### [Ee]xtracellular [Mm]atrix

#### PubMed

1. Modulation of cardiac fibrosis by Krüppel-like factor 6 through transcriptional control of thrombospondin 4 in cardiomyocytes. Krüppel-like factors (KLFs) are a family of transcription factors which play important roles in the heart under pathological and developmental conditions. We previously identified and cloned Klf6 whose homozygous mutation in mice results in embryonic lethality suggesting a role in cardiovascular development. Effects of KLF6 on pathological regulation of the heart were investigated in the present study.Mice heterozygous for Klf6 resulted in significantly diminished levels of cardiac fibrosis in response to angiotensin II infusion. Intriguingly, a similar phenotype was seen in cardiomyocyte-specific Klf6 knockout mice, but not in cardiac fibroblast-specific knockout mice. Microarray analysis revealed increased levels of th**e extracellular matr**ix factor, thrombospondin 4 (TSP4), in the Klf6-ablated heart. Mechanistically, KLF6 directly suppressed Tsp4 expression levels, and cardiac TSP4 regulated the activation of cardiac fibroblasts to regulate cardiac fibrosis.Our present studies on the cardiac function of KLF6 show a new mechanism whereby cardiomyocytes regulate cardiac fibrosis through transcriptional control of th**e extracellular matr**ix factor, TSP4, which, in turn, modulates activation of cardiac fibroblasts.

#### GeneRIFs

1. present studies on the cardiac function of KLF6 show a new mechanism whereby cardiomyocytes regulate cardiac fibrosis through transcriptional control of the **extracellular matrix** factor thrombospondin 4

### [Mm]etastasize

#### PubMed

1. KLF6-SV1 overexpression accelerates human and mouse prostate cancer progression and metastasis. Metastatic prostate cancer (PCa) is one of the leading causes of death from cancer in men. The molecular mechanisms underlying the transition from localized tumor to hormone-refractory metastatic PCa remain largely unknown, and their identification is key for predicting prognosis and targeted therapy. Here we demonstrated that increased expression of a splice variant of the Kruppel-like factor 6 (KLF6) tumor suppressor gene, known as KLF6-SV1, in tumors from men after prostatectomy predicted markedly poorer survival and disease recurrence profiles. Analysis of tumor samples revealed that KLF6-SV1 levels were specifically upregulated in hormone-refractory metastatic PCa. In 2 complementary mouse models of metastatic PCa, KLF6-SV1-overexpressing PCa cells were shown by in vivo and ex vivo bioluminescent imaging to **metastasize** more rapidly and to disseminate to lymph nodes, bone, and brain more often. Interestingly, while KLF6-SV1 overexpression increased metastasis, it did not affect localized tumor growth. KLF6-SV1 inhibition using RNAi induced spontaneous apoptosis in cultured PCa cell lines and suppressed tumor growth in mice. Together, these findings demonstrate that KLF6-SV1 expression levels in PCa tumors at the time of diagnosis can predict the metastatic behavior of the tumor; thus, KLF-SV1 may represent a novel therapeutic target.

### [Mm]etastasis

#### PubMed

1. A novel KLF6-Rho GTPase axis regulates hepatocellular carcinoma cell migration and dissemination. The presence of invasion into the extra-hepatic portion of the portal vein or the development of distant metastases renders hepatocellular carcinoma (HCC) patients ineligible for the only potential curative options for this malignancy-tumor resection or organ transplantation. Gene expression profiling of murine HCC cell lines identified KLF6 as a potential regulator of HCC cell migration. KLF6 knockdown increases cell migration, consistent with the correlation between decreased KLF6 mRNA levels and the presence of vascular invasion in human HCC. Concordantly, single-copy deletion of Klf6 in a HCC mouse model results in increased tumor formation, increased **metastasis** to the lungs and decreased survival, indicating that KLF6 suppresses both HCC development and **metastasis**. By combining gene expression profiling and chromatin immunoprecipitation coupled to deep sequencing, we identified novel transcriptional targets of KLF6 in HCC cells including VAV3, a known activator of the RAC1 small GTPase. Indeed, RAC1 activity is increased in KLF6-knockdown cells in a VAV3-dependent manner, and knockdown of either RAC1 or VAV3 impairs HCC cell migration. Together, our data demonstrate a novel function for KLF6 in constraining HCC dissemination through the regulation of a VAV3-RAC1 signaling axis.
2. KLF6-SV1 overexpression accelerates human and mouse prostate cancer progression and **metastasis**. Metastatic prostate cancer (PCa) is one of the leading causes of death from cancer in men. The molecular mechanisms underlying the transition from localized tumor to hormone-refractory metastatic PCa remain largely unknown, and their identification is key for predicting prognosis and targeted therapy. Here we demonstrated that increased expression of a splice variant of the Kruppel-like factor 6 (KLF6) tumor suppressor gene, known as KLF6-SV1, in tumors from men after prostatectomy predicted markedly poorer survival and disease recurrence profiles. Analysis of tumor samples revealed that KLF6-SV1 levels were specifically upregulated in hormone-refractory metastatic PCa. In 2 complementary mouse models of metastatic PCa, KLF6-SV1-overexpressing PCa cells were shown by in vivo and ex vivo bioluminescent imaging to metastasize more rapidly and to disseminate to lymph nodes, bone, and brain more often. Interestingly, while KLF6-SV1 overexpression increased **metastasis**, it did not affect localized tumor growth. KLF6-SV1 inhibition using RNAi induced spontaneous apoptosis in cultured PCa cell lines and suppressed tumor growth in mice. Together, these findings demonstrate that KLF6-SV1 expression levels in PCa tumors at the time of diagnosis can predict the metastatic behavior of the tumor; thus, KLF-SV1 may represent a novel therapeutic target.

#### GeneRIFs

1. Klf6 overexpression accelerates mouse prostate cancer progression and **metastasis**.

---

## Nova2

- NCBI Gene
- GeneRIF
- Pubmed

| Search Term | Rank | Fields |
| --- | --- | --- |
| [Mm]igration | 6 | PubMed(3); GeneRIFs(1) |

  
hide/show details for Nova2

### [Mm]igration

#### PubMed

1. Nova2 regulates neuronal **migration** through an RNA switch in disabled-1 signaling. Neuronal **migration** leads to a highly organized laminar structure in the mammalian brain, and its misregulation causes lissencephaly and behavioral and cognitive defects. Reelin signaling, which is mediated in part by a key adaptor, disabled-1 (Dab1), plays a critical but incompletely understood role in this process. We found that the neuron-specific RNA-binding protein Nova2 regulates neuronal **migration** in late-generated cortical and Purkinje neurons. An unbiased HITS-CLIP and exon junction array search for Nova-dependent reelin-pathway RNAs at E14.5 revealed only one candidate-an alternatively spliced isoform of Dab1 (Dab1.7bc). In utero electroporation demonstrated that Dab1.7bc was sufficient to induce neuronal **migration** defects in wild-type mice and exacerbate defects when Dab1 levels were reduced, whereas Dab1 overexpression mitigates defects in Nova2 null mice. Thus, Nova2 regulates an RNA switch controlling the ability of Dab1 to mediate neuronal responsiveness to reelin signaling and neuronal **migration**, suggesting new links between splicing regulation, brain disease, and development.
2. Nova2 regulates neuronal **migration** through an RNA switch in disabled-1 signaling. Neuronal **migration** leads to a highly organized laminar structure in the mammalian brain, and its misregulation causes lissencephaly and behavioral and cognitive defects. Reelin signaling, which is mediated in part by a key adaptor, disabled-1 (Dab1), plays a critical but incompletely understood role in this process. We found that the neuron-specific RNA-binding protein Nova2 regulates neuronal **migration** in late-generated cortical and Purkinje neurons. An unbiased HITS-CLIP and exon junction array search for Nova-dependent reelin-pathway RNAs at E14.5 revealed only one candidate-an alternatively spliced isoform of Dab1 (Dab1.7bc). In utero electroporation demonstrated that Dab1.7bc was sufficient to induce neuronal **migration** defects in wild-type mice and exacerbate defects when Dab1 levels were reduced, whereas Dab1 overexpression mitigates defects in Nova2 null mice. Thus, Nova2 regulates an RNA switch controlling the ability of Dab1 to mediate neuronal responsiveness to reelin signaling and neuronal **migration**, suggesting new links between splicing regulation, brain disease, and development.
3. NOVA regulates Dcc alternative splicing during neuronal **migration** and axon guidance in the spinal cord. RNA-binding proteins (RBPs) control multiple aspects of post-transcriptional gene regulation and function during various biological processes in the nervous system. To further reveal the functional significance of RBPs during neural development, we carried out an in vivo RNAi screen in the dorsal spinal cord interneurons, including the commissural neurons. We found that the NOVA family of RBPs play a key role in neuronal **migration**, axon outgrowth, and axon guidance. Interestingly, Nova mutants display similar defects as the knockout of the Dcc transmembrane receptor. We show here that Nova deficiency disrupts the alternative splicing of Dcc, and that restoring Dcc splicing in Nova knockouts is able to rescue the defects. Together, our results demonstrate that the production of DCC splice variants controlled by NOVA has a crucial function during many stages of commissural neuron development.

#### GeneRIFs

1. Nova2 regulates an RNA switch controlling the ability of Dab1 to mediate neuronal responsiveness to reelin signaling and neuronal **migration**, suggesting new links between splicing regulation, brain disease, and development.

---

## Nova1

- NCBI Gene
- GeneRIF
- Pubmed

| Search Term | Rank | Fields |
| --- | --- | --- |
| [Mm]igration | 6 | PubMed(2) |

  
hide/show details for Nova1

### [Mm]igration

#### PubMed

1. NOVA regulates Dcc alternative splicing during neuronal **migration** and axon guidance in the spinal cord. RNA-binding proteins (RBPs) control multiple aspects of post-transcriptional gene regulation and function during various biological processes in the nervous system. To further reveal the functional significance of RBPs during neural development, we carried out an in vivo RNAi screen in the dorsal spinal cord interneurons, including the commissural neurons. We found that the NOVA family of RBPs play a key role in neuronal **migration**, axon outgrowth, and axon guidance. Interestingly, Nova mutants display similar defects as the knockout of the Dcc transmembrane receptor. We show here that Nova deficiency disrupts the alternative splicing of Dcc, and that restoring Dcc splicing in Nova knockouts is able to rescue the defects. Together, our results demonstrate that the production of DCC splice variants controlled by NOVA has a crucial function during many stages of commissural neuron development.
2. Nova2 regulates neuronal **migration** through an RNA switch in disabled-1 signaling. Neuronal **migration** leads to a highly organized laminar structure in the mammalian brain, and its misregulation causes lissencephaly and behavioral and cognitive defects. Reelin signaling, which is mediated in part by a key adaptor, disabled-1 (Dab1), plays a critical but incompletely understood role in this process. We found that the neuron-specific RNA-binding protein Nova2 regulates neuronal **migration** in late-generated cortical and Purkinje neurons. An unbiased HITS-CLIP and exon junction array search for Nova-dependent reelin-pathway RNAs at E14.5 revealed only one candidate-an alternatively spliced isoform of Dab1 (Dab1.7bc). In utero electroporation demonstrated that Dab1.7bc was sufficient to induce neuronal **migration** defects in wild-type mice and exacerbate defects when Dab1 levels were reduced, whereas Dab1 overexpression mitigates defects in Nova2 null mice. Thus, Nova2 regulates an RNA switch controlling the ability of Dab1 to mediate neuronal responsiveness to reelin signaling and neuronal **migration**, suggesting new links between splicing regulation, brain disease, and development.

---

## Apc

- NCBI Gene
- GeneRIF
- Pubmed

| Search Term | Rank | Fields |
| --- | --- | --- |
| [Mm]igration | 6 | Process(6); PubMed(65); GeneRIFs(5) |
| ECM | 7 | PubMed(1) |
| [Ee]xtracellular [Mm]atrix | 8 | PubMed(13) |
| [Ii]nvasive | 10 | PubMed(78); GeneRIFs(3) |
| [Mm]etastasize | 11 | PubMed(6) |
| [Mm]etastasis | 12 | PubMed(45) |
| [Cc]ell [Ii]nvasion | 13 | PubMed(3) |

  
hide/show details for Apc

### [Mm]igration

#### Process

1. cell **migration**
2. cell **migration**
3. positive regulation of cell **migration**
4. positive regulation of cell **migration**
5. regulation of cell **migration**
6. regulation of cell **migration**

#### PubMed

1. The adenomatous polyposis coli protein is an essential regulator of radial glial polarity and construction of the cerebral cortex. Radial glia are highly polarized cells that serve as neuronal progenitors and as scaffolds for neuronal **migration** during construction of the cerebral cortex. How radial glial cells establish and maintain their morphological polarity is unknown. Using conditional gene targeting in mice, we demonstrate that adenomatous polyposis coli (APC) serves an essential function in the maintenance of polarized radial glial scaffold during brain development. In the absence of APC, radial glial cells lose their polarity and responsiveness to the extracellular polarity maintenance cues, such as neuregulin-1. Elimination of APC further leads to marked instability of the radial glial microtubule cytoskeleton. The resultant changes in radial glial function and loss of APC in radial glial progeny lead to defective generation and **migration** of cortical neurons, severely disrupted cortical layer formation, and aberrant axonal tract development. Thus, APC is an essential regulator of radial glial polarity and is critical for the construction of cerebral cortex in mammals.
2. Adenomatous polyposis coli is required for early events in the normal growth and differentiation of the developing cerebral cortex. Adenomatous polyposis coli (Apc) is a large multifunctional protein known to be important for Wnt/beta-catenin signalling, cytoskeletal dynamics, and cell polarity. In the developing cerebral cortex, Apc is expressed in proliferating cells and its expression increases as cells migrate to the cortical plate. We examined the consequences of loss of Apc function for the early development of the cerebral cortex.We used Emx1(Cre) to inactivate Apc specifically in proliferating cerebral cortical cells and their descendents starting from embryonic day 9.5. We observed reduction in the size of the mutant cerebral cortex, disruption to its organisation, and changes in the molecular identity of its cells. Loss of Apc leads to a decrease in the size of the proliferative pool, disrupted interkinetic nuclear **migration**, and increased apoptosis. beta-Catenin, pericentrin, and N-cadherin proteins no longer adopt their normal high concentration at the apical surface of the cerebral cortical ventricular zone, indicating that cell polarity is disrupted. Consistent with enhanced Wnt/beta-catenin signalling resulting from loss of Apc we found increased levels of TCF/LEF-dependent transcription and expression of endogenous Wnt/beta-catenin target genes (Axin2 (conductin), Lef1, and c-myc) in the mutant cerebral cortex. In the Apc mutant cerebral cortex the expression of transcription factors Foxg1, Pax6, Tbr1, and Tbr2 is drastically reduced compared to normal and many cells ectopically express Pax3, Wnt1, and Wt1 (but not Wnt2b, Wnt8b, Ptc, Gli1, Mash1, Olig2, or Islet1). This indicates that loss of Apc function causes cerebral cortical cells to lose their normal identity and redirect to fates normally found in more posterior-dorsal regions of the central nervous system.Apc is required for multiple aspects of early cerebral cortical development, including the regulation of cell number, interkinetic nuclear **migration**, cell polarity, and cell type specification.
3. Adenomatous polyposis coli is required for early events in the normal growth and differentiation of the developing cerebral cortex. Adenomatous polyposis coli (Apc) is a large multifunctional protein known to be important for Wnt/beta-catenin signalling, cytoskeletal dynamics, and cell polarity. In the developing cerebral cortex, Apc is expressed in proliferating cells and its expression increases as cells migrate to the cortical plate. We examined the consequences of loss of Apc function for the early development of the cerebral cortex.We used Emx1(Cre) to inactivate Apc specifically in proliferating cerebral cortical cells and their descendents starting from embryonic day 9.5. We observed reduction in the size of the mutant cerebral cortex, disruption to its organisation, and changes in the molecular identity of its cells. Loss of Apc leads to a decrease in the size of the proliferative pool, disrupted interkinetic nuclear **migration**, and increased apoptosis. beta-Catenin, pericentrin, and N-cadherin proteins no longer adopt their normal high concentration at the apical surface of the cerebral cortical ventricular zone, indicating that cell polarity is disrupted. Consistent with enhanced Wnt/beta-catenin signalling resulting from loss of Apc we found increased levels of TCF/LEF-dependent transcription and expression of endogenous Wnt/beta-catenin target genes (Axin2 (conductin), Lef1, and c-myc) in the mutant cerebral cortex. In the Apc mutant cerebral cortex the expression of transcription factors Foxg1, Pax6, Tbr1, and Tbr2 is drastically reduced compared to normal and many cells ectopically express Pax3, Wnt1, and Wt1 (but not Wnt2b, Wnt8b, Ptc, Gli1, Mash1, Olig2, or Islet1). This indicates that loss of Apc function causes cerebral cortical cells to lose their normal identity and redirect to fates normally found in more posterior-dorsal regions of the central nervous system.Apc is required for multiple aspects of early cerebral cortical development, including the regulation of cell number, interkinetic nuclear **migration**, cell polarity, and cell type specification.
4. The adenomatous polyposis coli protein is an essential regulator of radial glial polarity and construction of the cerebral cortex. Radial glia are highly polarized cells that serve as neuronal progenitors and as scaffolds for neuronal **migration** during construction of the cerebral cortex. How radial glial cells establish and maintain their morphological polarity is unknown. Using conditional gene targeting in mice, we demonstrate that adenomatous polyposis coli (APC) serves an essential function in the maintenance of polarized radial glial scaffold during brain development. In the absence of APC, radial glial cells lose their polarity and responsiveness to the extracellular polarity maintenance cues, such as neuregulin-1. Elimination of APC further leads to marked instability of the radial glial microtubule cytoskeleton. The resultant changes in radial glial function and loss of APC in radial glial progeny lead to defective generation and **migration** of cortical neurons, severely disrupted cortical layer formation, and aberrant axonal tract development. Thus, APC is an essential regulator of radial glial polarity and is critical for the construction of cerebral cortex in mammals.
5. The C-terminus of Apc does not influence intestinal adenoma development or progression. Adenomatous polyposis coli (APC ) mutations are found in most colorectal tumours. These mutations are almost always protein-truncating, deleting both central domains that regulate Wnt signalling and C-terminal domains that interact with the cytoskeleton. The importance of Wnt dysregulation for colorectal tumourigenesis is well characterized. It is, however, unclear whether loss of C-terminal functions contributes to tumourigenesis, although this protein region has been implicated in cellular processes--including polarity, **migration**, mitosis, and chromosomal instability (CIN)—that have been postulated as critical for the development and progression of intestinal tumours. Since almost all APC mutations in human patients disrupt both central and C-terminal regions, we created a mouse model to test the role of the C-terminus of APC in intestinal tumourigenesis. This mouse (Apc(ΔSAMP)) carries an internal deletion within Apc that dysregulates Wnt by removing the beta-catenin-binding and SAMP repeats, but leaves the C-terminus intact. We compared Apc(ΔSAMP) mice with Apc(1322T) animals. The latter allele represented the most commonly found human APC mutation and was identical to Apc(ΔSAMP) except for absence of the entire C-terminus. Apc(ΔSAMP) mice developed numerous intestinal adenomas indistinguishable in number, location, and dysplasia from those seen in Apc(1322T) mice. No carcinomas were found in Apc(ΔSAMP) or Apc(1322T) animals. While similar disruption of the Wnt signalling pathway was observed in tumours from both mice, no evidence of differential C-terminus functions (such a**s cell mi**gration, CIN, or localization of APC and EB1) was seen. We conclude that the C-terminus of APC does not influence intestinal adenoma development or progression.
6. Colorectal cancer in mice genetically deficient in the mucin Muc2. The gastrointestinal tract is lined by a layer of mucus comprised of highly glycosylated proteins called mucins. To evaluate the importance of mucin in intestinal carcinogenesis, we constructed mice genetically deficient in Muc2, the most abundant secreted gastrointestinal mucin. Muc2-/- mice displayed aberrant intestinal crypt morphology and altered cell maturation and **migration**. Most notably, the mice frequently developed adenomas in the small intestine that progressed to invasive adenocarcinoma, as well as rectal tumors. Thus, Muc2 is involved in the suppression of colorectal cancer.
7. HEF1, a novel target of Wnt signaling, promotes colonic cell **migration** and cancer progression. Misregulation of the canonical Wnt/β-catenin pathway and aberrant activation of Wnt signaling target genes are common in colorectal cancer (CRC) and contribute to cancer progression. Altered expression of human enhancer of filamentation 1 (HEF1; also known as NEDD9 or Cas-L) has been implicated in progression of melanoma, breast, and CRC. However, the regulation of HEF1 and the role of HEF1 in CRC tumorigenesis are not fully understood. We here identify HEF1 as a novel Wnt signaling target. The expression of HEF1 was upregulated by Wnt-3a, β-catenin, and Dvl2 in a dose-dependent manner, and was suppressed following β-catenin downregulation by shRNA. In addition, elevated HEF1 mRNA and protein levels were observed in CRC cell lines and primary tumor tissues, as well as in the colon and adenoma polyps of Apc(Min/+) mice. Moreover, HEF1 levels in human colorectal tumor tissues increased with the tumor grade. Chromatin immunoprecipitation (ChIP) assays and promoter analyses revealed three functional T-cell factor (TCF)-binding sites in the promoter of HEF1 responsible for HEF1 induction by Wnt signaling. Ectopic expression of HEF1 increased cell proliferation and colony formation, while downregulation of HEF1 in SW480 cells by shRNA had the opposite effects and inhibited the xenograft tumor growth. Furthermore, overexpression of HEF1 in SW480 cells promoted ce**ll migrat**ion and invasion. Together, our results determined a novel role of HEF1 as a mediator of the canonical Wnt/β-catenin signaling pathway for cell proliferati**on, migra**tion, and tumorigenesis, as well as an important player in colorectal tumorigenesis and progression. HEF1 may represent an attractive candidate for drug targeting in CRC.
8. Adenomatous polyposis coli (APC) plays multiple roles in the intestinal and colorectal epithelia. The adenomatous polyposis coli (APC) gene is mutated in familial adenomatous polyposis and in most sporadic colorectal tumors. During both embryonic and postnatal periods, APC is widely expressed in a variety of tissues, including the brain and gastrointestinal tract. The APC gene product (APC) is a large multidomain protein consisting of 2843 amino acids. APC downregulates the Wnt signaling pathway through its binding to beta-catenin and Axin. Most mutated APC proteins in colorectal tumors lack the beta-catenin-binding regions and fail to inhibit Wnt signaling, leading to the overproliferation of tumor cells. Several mouse models (APC580D, APCDelta716, APC1309, APCMin, APC1638T) have been established to investigate carcinogenesis caused by APC mutations. APC also binds to APC-stimulated guanine nucleotide exchange factor, the kinesin superfamily-associated protein 3, IQGAP1, microtubules, EB1, and discs large (DLG). APC has both nuclear localization signals and nuclear export signals in its molecule, suggesting its occasional nuclear localization and export of beta-catenin from the nucleus. APC is highly expressed in the intestinal and colorectal epithelia and may be involved in homeostasis of the enterocyte renewal phenomena, in which proliferation, **migration**, differentiation, and apoptosis are highly regulated both temporally and spatially. Through the many binding proteins mentioned, APC can exert multiple functions involved in epithelial homeostasis.
9. Localized gene action controlling intestinal neoplasia in mice. Mice heterozygous for the ApcMin (Min) mutation develop adenomas throughout the intestinal tract. Apc is believed to be involved in cell **migration**, adhesion, and polarity. Adenoma multiplicity and growth rate are modulated by an unlinked modifier locus, Mom1. The secretory phospholipase Pla2g2a is a candidate for Mom1. Here, we investigate the range of action of Apc and Mom1. Analysis of chimeric Min mice indicates that the actions of both Apc and Mom1 are localized within the cell lineage that gives rise to intestinal tumors.
10. Macrophage **migration** inhibitory factor promotes intestinal tumorigenesis. The cytokine macrophage **migration** inhibitory factor (MIF) is expressed throughout the human gastrointestinal tract. Recently, protumorigenic activity of MIF has been described in several cancer models. Therefore, we investigated the expression and function of MIF during the early stages of intestinal tumorigenesis.MIF messenger RNA, protein, and tautomerase activity were measured in normal intestinal mucosa and adenomas from patients with sporadic colorectal adenomas and in the adenomatous polyposis coli (Apc)Min/+ mouse model of intestinal tumorigenesis. MIF function was investigated by using VACO-235 human colorectal adenoma cells in vitro and by testing the effect of genetic deletion of Mif on ApcMin/+ mouse intestinal tumorigenesis.MIF expression and tautomerase activity were increased in human and ApcMin/+ mouse intestinal adenomas compared with adjacent normal mucosa. Up-regulation of MIF occurred mainly in epithelial cells (associated with an increasing grade of dysplasia), but also in stromal plasma cells. Exogenous MIF inhibited apoptosis and promoted anchorage-independent growth of VACO-235 cells (maximal at 100 ng/mL). Homozygous deletion of Mif was associated with a reduction in the number and size of ApcMin/+ mouse adenomas (P = .025 for the difference in large [>7-mm] tumors) and decreased angiogenesis (43% decrease in mean tumor microvessel density), but there was no alteration in epithelial cell apoptosis or proliferation.MIF expression is increased in sporadic human colorectal adenomas, and exogenous MIF drives tumorigenic behavior of epithelial cells in vitro. Mif also promotes intestinal tumorigenesis (predominantly via angiogenesis) in the ApcMin/+ mouse. Therefore, MIF is a potential colorectal cancer chemoprevention target.
11. Differential CD74 (major histocompatibility complex Class II invariant chain) expression in mouse and human intestinal adenomas. CD74 (major histocompatibility complex (MHC) Class II invariant chain) has recently been identified as the cell-surface receptor for the pro-tumorigenic cytokine macrophage **migration** inhibitory factor (MIF). Therefore, we investigated CD74 gene expression in intestinal adenomas in Apc(Min/+) mice and humans. CD74 mRNA (p31 and p41 splice variants) and immunoreactive CD74 protein levels were significantly lower in small intestinal and colonic Apc(Min/+) mouse adenomas compared with histologically normal mucosa. These findings were mirrored by a reduction in MHC Class II expression and Class II trans-activator type IV transcripts. Conversely, CD74 protein levels were actually increased in dysplastic epithelial cells in 47/55 (85%) human colorectal adenomas, with CD74 and MIF protein levels together predicting increasing dysplasia in individual adenomas (P=0.003). Down-regulation of CD74 during Apc(Min/+) mouse intestinal tumorigenesis does not model increased CD74 expression at the early, benign stages of human colorectal carcinogenesis. Epithelial cell CD74 represents a valid target for anti-CRC therapy.
12. IQ-domain GTPase-activating protein 1 regulates beta-catenin at membrane ruffles and its role in macropinocytosis of N-cadherin and adenomatous polyposis coli. Beta-catenin is an integral component of E-cadherin dependent cell-cell junctions. Here we show that beta-catenin co-localizes with IQ-domain GTPase-activating protein 1 (IQGAP1), adenomatous polyposis coli (APC), and N-cadherin at actin-positive membrane ruffles in NIH 3T3 fibroblasts. We used deletion mapping to identify the membrane ruffle-targeting region of beta-catenin, localizing it to amino acids 47-217, which overlap the IQGAP1 binding site. Knockdown by small interference RNA (siRNA) revealed IQGAP1-dependent membrane targeting of beta-catenin, APC, and N-cadherin. Transient overexpression of IQGAP1 or N-cadherin increased beta-catenin at membrane ruffles. IQGAP1/APC regulates cell **migration**, and using a wound healing assay we demonstrate that siRNA-mediated loss of beta-catenin also caused a modest reduction in the rate of cell **migration**. More significantly, we discovered that beta-catenin is internalized by Arf6-dependent macropinocytosis near sites of membrane ruffling. The beta-catenin macropinosomes co-stained for APC, N-cadherin, and to a lesser extent IQGAP1, and internalization of each binding partner was abrogated by siRNA-dependent knockdown of beta-catenin. In addition, beta-catenin macropinosomes co-localized with the lysosomal marker, lysosome associated membrane protein 1, consistent with their recycling by the late endosomal machinery. Our findings expand on current knowledge of beta-catenin function. We propose that in motile cells beta-catenin is recruited by IQGAP1 and N-cadherin to active membrane ruffles, wherein beta-catenin mediates the internalization and possible recycling of the membrane-associated proteins N-cadherin and APC.
13. A urokinase-type plasminogen activator deficiency diminishes the frequency of intestinal adenomas in ApcMin/+ mice. The interaction of urokinase-type plasminogen activator (uPA) and its receptor, uPAR, on cell surfaces facilitates the generation of cell-bound plasmin, thus allowing cells to establish a proteolytic front that enables their **migration** through protein barriers. This complex also activates cell signalling pathways that influence cell functions. Clinical studies have identified uPA as an indicator of poor overall survival in patients with colorectal cancer. In the current study, a mouse model of colon cancer, Apc(Min/+), with an additional deficiency of uPA (Apc(Min/+)/Plau-/-) was used to determine the effects of uPA on tumour initiation and growth. Utilizing this model, it was found that the number of tumours was diminished in these mice relative to Apc(Min/+) mice, which correlated with the decreased leukocyte infiltration in the tumours. However, tumour growth was not impeded in Apc(Min/+)/Plau-/- mice, and proliferation and tumour vascularization were, in fact, enhanced in Apc(Min/+)/Plau-/- mice. These latter effects are consistent with a mechanism involving up-regulation of COX-2 expression and Akt pathway activation in Apc(Min/+)/Plau-/- mice. The results from this study suggest that uPA plays dual and opposing roles in regulating lesion development: one early, during the transition from normal epithelia to dysplastic lesions, and another later during tumour growth.
14. Investigation of Schwann cells at neoplastic cell sites before the onset of cancer invasion. In neural invasion (NI), cancer cells are classically assumed to actively invade nerves and to cause local recurrence and pain. However, the opposite possibility, that nerves may reach cancer cells even in their preinvasive stage and thereby promote cancer spread, has not yet been genuinely considered. The present study analyzes the reaction of Schwann cells of peripheral nerves to carcinogenesis in pancreatic cancer and colon cancer.Two novel 3D **migration** and Schwann cell outgrowth assays were developed to monitor the timing and the specificity of Schwann cell **migration** and cancer invasion toward peripheral neurons through digital-time-lapse microscopy and after blockade of nerve growth factor (NGF) signalling via siRNA or a small-molecule inhibitor of the p75(NTR) receptor. The frequency and emergence of the Schwann cell markers Sox10, S100, ALDH1L1, and glial-fibrillary-acidic-protein (GFAP) around cancer precursor lesions were studied in human and conditional murine pancreatic and colon cancer specimens using multiple immunolabeling.Schwann cells migrated toward pancreatic and colon cancer cells, but not toward benign cells, before the onset of cancer **migration** toward peripheral neurons. This chemoattraction was inhibited after blockade of p75(NTR)-signaling on Schwann and pancreatic cancer cells. Schwann cells were specifically detected around murine and human pancreatic intraepithelial neoplasias (PanINs) (mean percent of murine PanINs surrounded by Schwann cells = 78.9%, 95% CI = 70.9 to 86.8%, and mean percent of human PanINs surrounded by Schwann cells = 52.5%, 95% CI = 14.7 to 90.4%; human: n = 44, murine: n = 14) and intestinal adenomas (mean percent of murine adenomas surrounded by Schwann cells = 64.2%, 95% CI = 28.6 to 99.8%, and mean percent of human adenomas surrounded by Schwann cells = 17.2%, 95% CI = -126.9 to 161.4; human: n = 36, murine: n = 12). The Schwann cell presence in this premalignant stage was associated with the frequency of NI in the malignant phase.Schwann cells have particular and specific affinity to cancer cells. Emergence of Schwann cells in the premalignant phase of pancreatic and colon cancer implies that, in contrast with the traditional assumption, nerves-and not cancer cells-migrate first during NI.
15. Deficiency of SPARC suppresses intestinal tumorigenesis in APCMin/+ mice. SPARC (secreted protein acidic, rich in cysteine) is a matricellular protein that has been found to be activated in a number of human cancers. More recently, it has been shown to be upregulated in human gastric and colorectal cancer. We therefore wished to address the functional importance of SPARC upregulation to intestinal tumorigenesis in vivo.SPARC upregulation was determined in intestinal adenomas of tumour-prone Apc(Min/+) mice at both the RNA and the protein level. To determine the functional importance of SPARC for intestinal tumorigenesis we then intercrossed Sparc knockout mice with Apc(Min/+) mice (n = 20). Intestinal enterocyte **migration** was examined using bromodeoxyuridine labelling studies.Levels of murine Sparc and several related proteins were upregulated in adenomas arising in Apc(Min/+) mice. A deficiency of Sparc strongly suppressed adenoma formation in Apc(Min/+) mice (p>or=0.0001). Importantly, a deficiency of Sparc also accelerated enterocyte **migration** (p = 0.01), as perturbed slow epithelial **migration** may underpin adenoma formation in the intestine.These data implicate Sparc in both cell **migration** and tumour formation, and identify Sparc as a potential therapeutic target for colorectal cancer.
16. Inhibition of CXCR2 profoundly suppresses inflammation-driven and spontaneous tumorigenesis. The chemokine receptor CXCR2 is a key mediator of neutrophil **migration** that also plays a role in tumor development. However, CXCR2 influences tumors through multiple mechanisms and might promote or inhibit tumor development depending on context. Here, we used several mouse models of spontaneous and inflammation-driven neoplasia to define indispensable roles for CXCR2 in benign and malignant tumors. CXCR2-activating chemokines were part of the secretome of cultured primary benign intestinal adenomas (ApcMin/+) and highly expressed by all tumors in all models. CXCR2 deficiency profoundly suppressed inflammation-driven tumorigenesis in skin and intestine as well as spontaneous adenocarcinoma formation in a model of invasive intestinal adenocarcinoma (AhCreER;Apcfl/+;Ptenfl/fl mice). Pepducin-mediated CXCR2 inhibition reduced tumorigenesis in ApcMin/+ mice. Ly6G+ neutrophils were the dominant source of CXCR2 in blood, and CXCR2 deficiency attenuated neutrophil recruitment. Moreover, systemic Ly6G+ cell depletion purged CXCR2-dependent tumor-associated leukocytes, suppressed established skin tumor growth and colitis-associated tumorigenesis, and reduced ApcMin/+ adenoma formation. CXCR2 is thus a potent protumorigenic chemokine receptor that directs recruitment of tumor-promoting leukocytes into tissues during tumor-inducing and tumor-driven inflammation. Similar leukocyte populations were also found in human intestinal adenomas, which suggests that CXCR2 antagonists may have therapeutic and prophylactic potential in the treatment of cancer.
17. Transcription factors Sox5 and Sox6 exert direct and indirect influences on oligodendroglial **migration** in spinal cord and forebrain. Transcription factors of the SoxD protein family have previously been shown to prevent precocious specification and terminal differentiation of oligodendrocyte progenitor cells in the developing spinal cord. Using mice with specific deletion of the SoxD proteins Sox5 and Sox6 in the central nervous system, we now show that SoxD proteins additionally influence **migration** of oligodendrocyte progenitors in the spinal cord as well as in the forebrain. In mutant mice, e**migration** of oligodendrocyte progenitors from the ventricular zone and colonization of the mantle zone are significantly delayed probably because of reduced expression of Pdgf receptor alpha and decreased responsiveness toward Pdgf-A as a main migratory cue. In addition to this direct cell-autonomous effect on Pdgf receptor alpha expression, SoxD proteins furthermore promote oligodendroglial **migration** by keeping the cells in an undifferentiated state and preventing a premature loss of their migratory capacity. This indirect effect becomes particularly important during late embryonic and early postnatal phases of oligodendroglial development. Finally, we show that Sox5 and Sox6 cooperate with Sox9 and Sox10 to activate Pdgf receptor alpha expression and thereby maintain oligodendrocyte progenitors in the immature state. This contrasts with their behavior on myelin genes where they antagonize the function of SoxE proteins. It argues that SoxD proteins can function either as repressors or as co-activators of SoxE proteins thereby modulating their function in a stage-specific manner.
18. ERK5 signalling rescues intestinal epithelial turnover and tumour cell proliferation upon ERK1/2 abrogation. The ERK1/2 MAPK signalling module integrates extracellular cues that induce proliferation and differentiation of epithelial lineages, and is an established oncogenic driver, particularly in the intestine. However, the interrelation of the ERK1/2 module relative to other signalling pathways in intestinal epithelial cells and colorectal cancer (CRC) is unclear. Here we show that loss of Erk1/2 in intestinal epithelial cells results in defects in nutrient absorption, epithelial cell **migration** and secretory cell differentiation. However, intestinal epithelial cell proliferation is not impeded, implying compensatory mechanisms. Genetic deletion of Erk1/2 or pharmacological targeting of MEK1/2 results in supraphysiological activity of the ERK5 pathway. Furthermore, targeting both pathways causes a more effective suppression of cell proliferation in murine intestinal organoids and human CRC lines. These results suggest that ERK5 provides a common bypass route in intestinal epithelial cells, which rescues cell proliferation upon abrogation of ERK1/2 signalling, with therapeutic implications in CRC.
19. Treg-cell depletion promotes chemokine production and accumulation of CXCR3(+) conventional T cells in intestinal tumors. Colorectal cancer (CRC) is one of the most prevalent tumor types worldwide and tumor-infiltrating T cells are crucial for anti-tumor immunity. We previously demonstrated that Treg cells from CRC patients inhibit transendothelial **migration** of conventional T cells. However, it remains unclear if local Treg cells affect lymphocyte **migration** into colonic tumors. By breeding APC(Min/+) mice with depletion of regulatory T cells mice, expressing the diphtheria toxin receptor under the control of the FoxP3 promoter, we were able to selectively deplete Treg cells in tumor-bearing mice, and investigate the impact of these cells on the infiltration of conventional T cells into intestinal tumors. Short-term Treg-cell depletion led to a substantial increase in the frequencies of T cells in the tumors, attributed by both increased infiltration and proliferation of T cells in the Treg-cell-depleted tumors. We also demonstrate a selective increase of the chemokines CXCL9 and CXCL10 in Treg-cell-depleted tumors, which were accompanied by accumulation of CXCR3(+) T cells, and increased IFN-γ mRNA expression. In conclusion, Treg-cell depletion increases the accumulation of conventional T cells in intestinal tumors, and targeting Treg cells could be a possible anti-tumor immunotherapy, which not only affects T-cell effector functions, but also their recruitment to tumors.
20. A critical role for the regulated wnt-myc pathway in naive T cell survival. Wnt signaling is involved in T cell development, activation, and differentiation. However, the role for Wnt signaling in mature naive T cells has not been investigated. In this article, we report that activation of Wnt signaling in T cell lineages by deletion of the Apc (adenomatous polyposis coli) gene causes spontaneous T cell activation and severe T cell lymphopenia. The lymphopenia is the result of rapid apoptosis of newly exported, mature T cells in the periphery and is not due to defects in thymocyte development or e**migration**. Using chimera mice consisting of both wild-type and Apc-deficient T cells, we found that loss of naive T cells is due to T cell intrinsic dysregulation of Wnt signaling. Because Apc deletion causes overexpression of the Wnt target gene cMyc, we generated mice with combined deletion of the cMyc gene. Because combined deletion of cMyc and Apc attenuated T cell loss, cMyc overexpression is partially responsible for spontaneous T cell apoptosis and lymphopenia. Cumulatively, our data reveal a missing link between Wnt signaling and survival of naive T cells.
21. The absence of LPA2 attenuates tumor formation in an experimental model of colitis-associated cancer. Chronic inflammation is a risk factor for colon cancer (CC). Lysophosphatidic acid (LPA), a naturally produced phospholipid, mediates multiple effects that are vital to disease process, including inflammation and cancer. The expression of LPA receptor 2 (LPA2) is up-regulated in several types of cancer, including ovarian and colon cancer, but the importance of LPA and LPA2 in the development and progression of CC is unclear. In this study, we sought to determine whether LPA and LPA2 regulate the progression of CC in vivo.We examined the potential role of LPA in CC progression by administering LPA to mice heterozygous for the adenomatous polyposis coli (Apc) allele. We determined the loss of LPA2 function in tumorigenesis in the colon by treating mice with genetic deletion of LPA2 (LPA2-/-) with azoxymethane and dextran sulfate sodium.We found that LPA increased tumor incidence in Apc(min/+) mice. LPA2-/- mice showed reduced mucosal damage and fewer tumors than wild-type (WT) mice. Reduced epithelial cell proliferation and decreases in beta-catenin, Krüppel-like factor 5, and cyclooxygenase-2 expression were observed in LPA2-/- mice. Unlike WT mice, induction of monocyte chemoattractant protein-1 and macrophage **migratio**n inhibitory factor was significantly attenuated in LPA2-/- mice with reduced infiltration by macrophages.These results show that LPA is capable of promoting tumorigenesis in the colon. The absence of LPA2 attenuates several effects that contribute to cancer progression in vivo, and, hence, the current study identifies LPA2 as an important modulator of CC.
22. PHLPP is a negative regulator of RAF1, which reduces colorectal cancer cell motility and prevents tumor progression in mice. Hyperactivation of the RAS-RAF signaling pathway in colorectal tumors is associated with metastasis and poor outcomes of patients. Little is known about how RAS-RAF signaling is turned off once activated. We investigated how the pH domain and leucine-rich repeat protein phosphatases (PHLPPs) control RAS-RAF signaling and colorectal cancer (CRC) development.We used co-immunoprecipitation assays to identify substrates of PHLPP1 and PHLPP2. We studied phosphorylation of RAF1 in CRC cells that express exogenous PHLPP1 or PHLPP2, or lentiviral-based small hairpin RNAs against their transcripts; we measured effects on cell motility, **migration**, and invasion in vitro. Tumor progression and survival were analyzed in Phlpp1(-/-) Apc(Min) and Apc(Min)/Phlpp1(-/-) mice. Microarray datasets of colorectal tumor and nontumor tissues were analyzed for PHLPP gene expression.PHLPP1 and 2 were found to dephosphorylate RAF1 at S338, inhibiting its kinase activity in vitro and in CRC cells. In cells, knockdown of PHLPP1 or PHLPP2 increased the amplitude and duration of RAF-MEK-ERK signaling downstream of epidermal growth factor receptor and KRAS, whereas overexpression had the opposite effect. In addition, knockdown of PHLPP1 or PHLPP2 caused CRC cells to express markers of the epithelial-mesenchymal transition, and increased cel**l migrati**on and invasion. Apc(Min)/Phlpp1(-/-) mice had decreased survival and developed larger intestinal and colon tumors compared to Apc(Min) mice. Whereas Apc(Min) mice developed mostly low-grade adenomas, 20% of the tumors that developed in Apc(Min)/Phlpp1(-/-) mice were invasive adenocarcinomas. Normal villi and adenomas of Apc(Min)/Phlpp1(-/-) mice had significantly fewer apoptotic cells than Apc(Min) mice. Human CRC patient microarray data revealed that the expression of PHLPP1 or PHLPP2 is positively correlated with CDH1.PHLPP1 and PHLPP2 dephosphorylate RAF1 to reduce its signaling, increase the invasive and migratory activities of CRC cells, and activate the epithelial-mesenchymal transition. In Apc(Min) mice, loss of PHLPP1 promotes tumor progression.
23. miR-23a promotes the transition from indolent to invasive colorectal cancer. Colorectal cancer is a classic example of a tumor that progresses through multiple distinct stages in its evolution. To understand the mechanisms regulating the transition from indolent to invasive disease, we profiled somatic copy number alterations in noninvasive adenomas and invasive adenocarcinomas from Apc and DNA mismatch repair (MMR) mutant mouse models. We identified a recurrent amplicon on mouse chromosome 8 that encodes microRNA (miRNA) 23a and -27a (miR). miR-23a and -27a levels are upregulated in mouse intestinal adenocarcinomas, primary tumors from patients with stage I/II colorectal cancers, as well as in human colorectal cancer cell lines and cancer stem cells. Functionally, miR-23a promotes the **migration** and invasion of colorectal cancer cells and stem cells, whereas miR-27a primarily promotes proliferation. We computationally and experimentally validated that metastasis suppressor 1 (MTSS1) is a direct miR-23a target and similarly validated that the ubiquitin ligase FBXW7 is a direct miR-27a target. Analyses of computationally predicted target genes in microarray data sets of patients with colorectal cancers are consistent with a role for miR-23a, but not miR-27a, specifically in invasive colorectal cancers.
24. Suppression of colon cancer metastasis by Aes through inhibition of Notch signaling. Metastasis is responsible for most cancer deaths. Here, we show that Aes (or Grg5) gene functions as an endogenous metastasis suppressor. Expression of Aes was decreased in liver metastases compared with primary colon tumors in both mice and humans. Aes inhibited Notch signaling by converting active Rbpj transcription complexes into repression complexes on insoluble nuclear matrix. In tumor cells, Notch signaling was triggered by ligands on adjoining blood vessels, and stimulated transendothelial **migration**. Genetic depletion of Aes in Apc(Δ716) intestinal polyposis mice caused marked tumor invasion and intravasation that were suppressed by Notch signaling inhibition. These results suggest that inhibition of Notch signaling can be a promising strategy for prevention and treatment of colon cancer metastasis.
25. Adenomatous polyposis coli protein nucleates actin assembly and synergizes with the formin mDia1. The tumor suppressor protein adenomatous polyposis coli (APC) regulates cell protrusion and cell **migration**, processes that require the coordinated regulation of actin and microtubule dynamics. APC localizes in vivo to microtubule plus ends and actin-rich cortical protrusions, and has well-documented direct effects on microtubule dynamics. However, its potential effects on actin dynamics have remained elusive. Here, we show that the C-terminal "basic" domain of APC (APC-B) potently nucleates the formation of actin filaments in vitro and stimulates actin assembly in cells. Nucleation is achieved by a mechanism involving APC-B dimerization and recruitment of multiple actin monomers. Further, APC-B nucleation activity is synergistic with its in vivo binding partner, the formin mDia1. Together, APC-B and mDia1 overcome a dual cellular barrier to actin assembly imposed by profilin and capping protein. These observations define a new function for APC and support an emerging view of collaboration between distinct actin assembly-promoting factors with complementary activities.
26. Crypt-restricted proliferation and commitment to the Paneth cell lineage following Apc loss in the mouse intestine. Loss of Apc appears to be one of the major events initiating colorectal cancer. However, the first events responsible for this initiation process are not well defined and the ways in which different epithelial cell types respond to Apc loss are unknown. We used a conditional gene-ablation approach in transgenic mice expressing tamoxifen-dependent Cre recombinase all along the crypt-villus axis to analyze the immediate effects of Apc loss in the small intestinal epithelium, both in the stem-cell compartment and in postmitotic epithelial cells. Within 4 days, Apc loss induced a dramatic enlargement of the crypt compartment associated with intense cell proliferation, apoptosis and impairment of cell **migration**. This result confirms the gatekeeper role of Apc in the intestinal epithelium in vivo. Although Apc deletion activated beta-catenin signaling in the villi, we observed neither proliferation nor morphological change in this compartment. This highlights the dramatic difference in the responses of immature and differentiated epithelial cells to aberrant beta-catenin signaling. These distinct biological responses were confirmed by molecular analyses, revealing that Myc and cyclin D1, two canonical beta-catenin target genes, were induced in distinct compartments. We also showed that Apc is a crucial determinant of cell fate in the murine intestinal epithelium. Apc loss perturbs differentiation along the enterocyte, goblet and enteroendocrine lineages, and promotes commitment to the Paneth cell lineage through beta-catenin/Tcf4-mediated transcriptional control of specific markers of Paneth cells, the cryptdin/defensin genes.
27. Transient inhibition of the ERK pathway prevents cerebellar developmental defects and improves long-term motor functions in murine models of neurofibromatosis type 1. Individuals with neurofibromatosis type 1 (NF1) frequently exhibit cognitive and motor impairments and characteristics of autism. The cerebellum plays a critical role in motor control, cognition, and social interaction, suggesting that cerebellar defects likely contribute to NF1-associated neurodevelopmental disorders. Here we show that Nf1 inactivation during early, but not late stages of cerebellar development, disrupts neuronal lamination, which is partially caused by overproduction of glia and subsequent disruption of the Bergmann glia (BG) scaffold. Specific Nf1 inactivation in glutamatergic neuronal precursors causes premature differentiation of granule cell (GC) precursors and ectopic production of unipolar brush cells (UBCs), indirectly disrupting neuronal **migration**. Transient MEK inhibition during a neonatal window prevents cerebellar developmental defects and improves long-term motor performance of Nf1-deficient mice. This study reveals essential roles of Nf1 in GC/UBC **migration** by generating correct numbers of glia and controlling GC/UBC fate-specification/differentiation, identifying a therapeutic prevention strategy for multiple NF1-associcated developmental abnormalities.
28. Loss of Apc in vivo immediately perturbs Wnt signaling, differentiation, and **migration**. Although Apc is well characterized as a tumor-suppressor gene in the intestine, the precise mechanism of this suppression remains to be defined. Using a novel inducible Ahcre transgenic line in conjunction with a loxP-flanked Apc allele we, show that loss of Apc acutely activates Wnt signaling through the nuclear accumulation of beta-catenin. Coincidentally, it perturbs differentiation, **migration**, proliferation, and apoptosis, such that Apc-deficient cells maintain a "crypt progenitor-like" phenotype. Critically, for the first time we confirm a series of Wnt target molecules in an in vivo setting and also identify a series of new candidate targets within the same setting.
29. Dissociation of EphB2 signaling pathways mediating progenitor cell proliferation and tumor suppression. Signaling proteins driving the proliferation of stem and progenitor cells are often encoded by proto-oncogenes. EphB receptors represent a rare exception; they promote cell proliferation in the intestinal epithelium and function as tumor suppressors by controlling cell **migration** and inhibiting invasive growth. We show that cell **migration** and proliferation are controlled independently by the receptor EphB2. EphB2 regulated cell positioning is kinase-independent and mediated via phosphatidylinositol 3-kinase, whereas EphB2 tyrosine kinase activity regulates cell proliferation through an Abl-cyclin D1 pathway. Cyclin D1 regulation becomes uncoupled from EphB signaling during the progression from adenoma to colon carcinoma in humans, allowing continued proliferation with invasive growth. The dissociation of EphB2 signaling pathways enables the selective inhibition of the mitogenic effect without affecting the tumor suppressor function and identifies a pharmacological strategy to suppress adenoma growth.
30. Matrix metalloproteinases and genetic mouse models in cancer research: a mini-review. Carcinogenesis is a multistep and also a multifactorial process that involves agents like genetic and environmental factors. Matrix metalloproteinases (MMPs) are major proteolytic enzymes which are involved in cancer cell **migration**, invasion, and metastasis. Genetic variations in genes encoding the MMPs were shown in human studies to influence cancer risk and phenotypic features of a tumor. The complex role of MMPs seems to be important in the mechanism of carcinogenesis, but it is not well recognized. Rodent studies concentrated particularly on the better understanding of the biological functions of the MMPs and their impact on the pathological process, also through the modification of Mmp genes. This review presents current knowledge and the existing evidence on the importance of selected MMPs in genetic mouse models of cancer and human genetic association studies. Further, this work can be useful for scientists studying the role of the genetic impact of MMPs in carcinogenesis.
31. CXCL1 induced by prostaglandin E2 promotes angiogenesis in colorectal cancer. Chronic inflammation is a well-known risk factor for cancer. Proinflammatory mediators such as prostaglandin E2 (PGE2) promote colorectal tumor growth by stimulating angiogenesis, cell invasion, and cell growth, and inhibiting apoptosis. Molecules that regulate tumor-associated angiogenesis provide promising therapeutic targets for treatment of colorectal cancer (CRC) as indicated by the recent development of the novel anti-angiogenic agent bevacizumab (Avastin). However, use of this drug only prolongs survival by several months, highlighting the importance of finding more effective treatment regimens. We report here that PGE2 induces expression of CXCL1 (growth-regulated oncogene alpha), a pro-angiogenic chemokine, in human CRC cells. More importantly, CXCL1 released from carcinoma cells induces microvascular endothelial cell **migration** and tube formation in vitro. Furthermore, PGE2 promotes tumor growth in vivo by induction of CXCL1 expression, which results in increased tumor microvessel formation. These results have potential clinical significance because we found that CXCL1 expression correlates with PGE2 levels in human CRCs. Collectively, our findings show for the first time that CXCL1 is regulated by PGE2 and indicate that CXCL1 inhibitors should be evaluated further as potential anti-angiogenic agents for treatment of CRC.
32. The role of P-glycoprotein in intestinal tumorigenesis: disruption of mdr1a suppresses polyp formation in Apc(Min/+) mice. P-glycoprotein (P-gp) mediates the active transport of various substrates including xenobiotics, and it thus has a protective function in various cell types and tissues/organs including the intestinal epithelium. However, whether or not P-gp plays a positive role in the intestinal tumorigenesis is unclear. We have introduced disrupted alleles of the murine P-gp gene, mdr1a, into Apc(Min/+) mice to evaluate whether P-gp plays any role in intestinal carcinogenesis. Spontaneously occurring DNA damage was significantly increased in both the small and large intestine of mdr1a(-/-), Apc(Min/+) mice compared with mdr1a(+/+), Apc(Min/+) mice. Furthermore, we observed active proliferation and rapid **migration**/disappearance of enterocytes in the intestine of the compound mice deficient in mdr1a. Finally, we found that the number of polyps and cancers was markedly decreased in mdr1a(-/-), Apc(Min/+) mice (P=0.0016). P-gp thus appears to play a positive role during intestinal tumorigenesis.
33. Gadd45a regulates matrix metalloproteinases by suppressing DeltaNp63alpha and beta-catenin via p38 MAP kinase and APC complex activation. The p53-regulated growth arrest and DNA damage-inducible gene product Gadd45a has been recently identified as a key factor protecting the epidermis against ultraviolet radiation (UVR)-induced skin tumors by activating p53 via the stress mitogen-activated protein kinase (MAPK) signaling pathway. Herein we identify Gadd45a as an important negative regulator of two oncogenes commonly over-expressed in epithelial tumors: the p53 homologue DeltaNp63alpha and beta-catenin. DeltaNp63alpha is one of the several p63 isoforms and is the predominant species expressed in basal epidermal keratinocytes. DeltaNp63alpha lacks the N-terminal transactivation domain and behaves as a dominant-negative factor blocking expression of several p53-effector genes. DeltaNp63alpha also associates with and blocks activation of the adenomatous polyposis coli (APC) destruction complex that targets free cytoplasmic beta-catenin for degradation. While most beta-catenin protein is localized to the cell membrane and is involved in cell-cell adhesion, accumulation of free cytoplasmic beta-catenin will translocate into the nucleus where it functions in a bipartite transcription factor complex, whose targets include invasion and metastasis promoting endopeptidases, matrix metalloproteinases (MMP). We show that Gadd45a not only directly associates with two components of the APC complex, namely protein phosphatase 2A (PP2A) and glycogen synthase kinase 3beta (GSK3beta) but also promotes GSK3beta dephosphorylation at Ser9, which is essential for GSK3beta activation, and resultant activation of the APC destruction complex. We demonstrate that lack of Gadd45a not only prevents DeltaNp63alpha suppression and GSK3beta dephosphorylation but also prevents free cytoplasmic beta-catenin degradation after UV irradiation. The inability of Gadd45a-null keratinocytes to suppress beta-catenin may contribute to the resulting observation of increased MMP expression and activity along with significantly faster keratinocyte **migration** in Matrigel in vitro and accelerated wound closure in vivo. Furthermore, epidermal keratinocytes treated with p38 MAPK inhibitors, both in vivo and in vitro, behave very similarly to Gadd45a-null keratinocytes after UVR. Similarly, Trp53-null mice are unable to attenuate DeltaNp63alpha expression in epidermal keratinocytes after such stress. These findings demonstrate a dependence on Gadd45a-mediated p38 MAPK and p53 activation for proper modulation of DeltaNp63alpha, GSK3beta, and beta-catenin after irradiation. Taken together, our results indicate that Gadd45a is able to repress DeltaNp63alpha, beta-catenin, and consequently MMP expression by two means: by maintaining UVR-induced p38 MAPK and p53 activation and also by associating with the APC complex. This implicates Gadd45a in the negative regulation of cell **migration**, and invasion.
34. MYC-nick promotes cell **migration** by inducing fascin expression and Cdc42 activation. MYC-nick is a cytoplasmic, transcriptionally inactive member of the MYC oncoprotein family, generated by a proteolytic cleavage of full-length MYC. MYC-nick promotes **migration** and survival of cells in response to chemotherapeutic agents or withdrawal of glucose. Here we report that MYC-nick is abundant in colonic and intestinal tumors derived from mouse models with mutations in the Wnt, TGF-β, and PI3K pathways. Moreover, MYC-nick is elevated in colon cancer cells deleted for FBWX7, which encodes the major E3 ligase of full-length MYC frequently mutated in colorectal cancers. MYC-nick promotes the **migratio**n of colon cancer cells assayed in 3D cultures or grown as xenografts in a zebrafish metastasis model. MYC-nick accelerates **migratio**n by activating the Rho GTPase Cdc42 and inducing fascin expression. MYC-nick, fascin, and Cdc42 are frequently up-regulated in cells present at the invasive front of human colorectal tumors, suggesting a coordinated role for these proteins in tumor **migratio**n.
35. Loss of functional Fas ligand enhances intestinal tumorigenesis in the Min mouse model. Fas ligand (FasL/CD95L), a member of the tumor necrosis factor family, interacts with a specific receptor Fas, ultimately leading to cell death. Tumor expression of FasL has been proposed to aid in immune evasion through a "Fas counterattack" mechanism but has also been described as a proinflammatory factor. Here, we tested the role of FasL in a mouse model of spontaneous tumor development. We used the Min mouse in which multiple benign polyps develop in the intestine due to a mutation in the Apc tumor suppressor gene. Mutant mice deficient in functional FasL, termed gld/gld, were crossed to Min mice to generate tumor-prone animals lacking functional FasL. Comparison of FasL-deficient versus proficient Min mice revealed a significant increase in polyp number in the gld/gld mice. We next assessed immune cell infiltration into adenomas. There was no difference in the number of either lymphocytes or macrophages; however, the number of tumor-infiltrating neutrophils was 3-fold lower in the gld/gld specimens compared with controls. Neutrophil **migration** in vitro was stimulated by wild-type but not mutant FasL. In a nontumor-bearing colitis model in vivo, neutrophil recruitment to the intestine was also reduced in gld/gld mice. Although the Fas counterattack hypothesis suggests that the absence of FasL would result in increased immune-mediated tumor elimination, the opposite is true in the Min model with lack of functional FasL associated with reduced neutrophil influx and increased tumor development. Thus, the proinflammatory rather than counterattack role of tumor FasL is more relevant.
36. Myc deletion rescues Apc deficiency in the small intestine. The APC gene encodes the adenomatous polyposis coli tumour suppressor protein, germline mutation of which characterizes familial adenomatous polyposis (FAP), an autosomal intestinal cancer syndrome. Inactivation of APC is also recognized as the key early event in the development of sporadic colorectal cancers, and its loss results in constitutive activity of the beta-catenin-Tcf4 transcription complex. The proto-oncogene c-MYC has been identified as a target of the Wnt pathway in colorectal cancer cells in vitro, in normal crypts in vivo and in intestinal epithelial cells acutely transformed on in vivo deletion of the APC gene; however, the significance of this is unclear. Therefore, to elucidate the role Myc has in the intestine after Apc loss, we have simultaneously deleted both Apc and Myc in the adult murine small intestine. Here we show that loss of Myc rescued the phenotypes of perturbed differentiation, **migration**, proliferation and apoptosis, which occur on deletion of Apc. Remarkably, this rescue occurred in the presence of high levels of nuclear beta-catenin. Array analysis revealed that Myc is required for the majority of Wnt target gene activation following Apc loss. These data establish Myc as the critical mediator of the early stages of neoplasia following Apc loss.
37. Novel roles for MLH3 deficiency and TLE6-like amplification in DNA mismatch repair-deficient gastrointestinal tumorigenesis and progression. DNA mismatch repair suppresses gastrointestinal tumorgenesis. Four mammalian E. coli MutL homologues heterodimerize to form three distinct complexes: MLH1/PMS2, MLH1/MLH3, and MLH1/PMS1. To understand the mechanistic contributions of MLH3 and PMS2 in gastrointestinal tumor suppression, we generated Mlh3(-/-);Apc(1638N) and Mlh3(-/-);Pms2(-/-);Apc(1638N) (MPA) mice. Mlh3 nullizygosity significantly increased Apc frameshift mutations and tumor multiplicity. Combined Mlh3;Pms2 nullizygosity further increased Apc base-substitution mutations. The spectrum of MPA tumor mutations was distinct from that observed in Mlh1(-/-);Apc(1638N) mice, implicating the first potential role for MLH1/PMS1 in tumor suppression. Because Mlh3;Pms2 deficiency also increased gastrointestinal tumor progression, we used array-CGH to identify a recurrent tumor amplicon. This amplicon contained a previously uncharacterized Transducin enhancer of Split (Tle) family gene, Tle6-like. Expression of Tle6-like, or the similar human TLE6D splice isoform in colon cancer cells increased cell proliferation, colony-formation, cell **migration**, and xenograft tumorgenicity. Tle6-like;TLE6D directly interact with the gastrointestinal tumor suppressor RUNX3 and antagonize RUNX3 target transactivation. TLE6D is recurrently overexpressed in human colorectal cancers and TLE6D expression correlates with RUNX3 expression. Collectively, these findings provide important insights into the molecular mechanisms of individual MutL homologue tumor suppression and demonstrate an association between TLE mediated antagonism of RUNX3 and accelerated human colorectal cancer progression.
38. Morphological and molecular processes of polyp formation in Apc(delta716) knockout mice. Mutations in the human adenomatous polyposis coli (APC) gene are responsible for not only familial adenomatous polyposis but also many sporadic cancers of the digestive tract. Using homologous recombination in embryonic stem cells, we recently constructed Apc gene knockout mice that contained a truncation mutation at codon 716 (Apc(delta716)). The heterozygous mice developed numerous intestinal polyps. All microadenomas dissected from nascent polyps had already lost the wild-type allele, indicating the loss of heterozygosity (M. Oshima et al., Proc. Natl. Acad. Sci. USA, 92: 4482-4486, 1995). We also demonstrated that cyclooxygenase 2 is induced in the polyps at an early stage and plays a key role in polyp development (M. Oshima et al., Cell 87: 803-809, 1996). We have analyzed the process of polyp development in these mice both at morphological and molecular levels. A small intestinal microadenoma is initiated as an outpocketing pouch in a single crypt and develops into the inner (lacteal) side of a neighboring villus forming a double-layer nascent polyp. The microadenoma then enlarges and gets folded inside the villus. When it fills the intravillous space, it expands downward and extends into adjoining villi, rather than rupturing into the intestinal lumen. During this course of development, the basement membrane remains intact, and the labeling index of the microadenoma cells is similar to that of the normal crypt epithelium. As in the crypt cells, neither transforming growth factor beta1 nor its receptor type II is expressed in the microadenoma cells. No hot spot mutations in the K-ras gene are found in the microadenoma tissue during these early stages of polyp development. Essentially, the same results have been obtained for the colonic polyps as well. These results suggest that early adenomas in the Apc(delta716) polyps are very similar to the normal proliferating cells of the crypt except for the lack of directed **migration** along the crypt-villus axis.
39. Oligodendrocyte precursors migrate along vasculature in the developing nervous system. Oligodendrocytes myelinate axons in the central nervous system and develop from oligodendrocyte precursor cells (OPCs) that must first migrate extensively during brain and spinal cord development. We show that OPCs require the vasculature as a physical substrate for **migration**. We observed that OPCs of the embryonic mouse brain and spinal cord, as well as the human cortex, emerge from progenitor domains and associate with the abluminal endothelial surface of nearby blood vessels. Migrating OPCs crawl along and jump between vessels. OPC **migration** in vivo was disrupted in mice with defective vascular architecture but was normal in mice lacking pericytes. Thus, physical interactions with the vascular endothelium are required for OPC **migration**. We identify Wnt-Cxcr4 (chemokine receptor 4) signaling in regulation of OPC-endothelial interactions and propose that this signaling coordinates OPC **migration** with differentiation.
40. A critical role for Apc in hematopoietic stem and progenitor cell survival. The adenomatous polyposis coli (Apc) tumor suppressor is involved in the initiation and progression of colorectal cancer via regulation of the Wnt signaling cascade. In addition, Apc plays an important role in multiple cellular functions, including cell **migration** and adhesion, spindle assembly, and chromosome segregation. However, its role during adult hematopoiesis is unknown. We show that conditional inactivation of Apc in vivo dramatically increases apoptosis and enhances cell cycle entry of hematopoietic stem cells (HSCs)/ hematopoietic progenitor cells (HPCs), leading to their rapid disappearance and bone marrow failure. The defect in HSCs/HPCs caused by Apc ablation is cell autonomous. In addition, we found that loss of Apc leads to exhaustion of the myeloid progenitor pool (common myeloid progenitor, granulocyte-monocyte progenitor, and megakaryocyte-erythroid progenitor), as well as the lymphoid-primed multipotent progenitor pool. Down-regulation of the genes encoding Cdkn1a, Cdkn1b, and Mcl1 occurs after acute Apc excision in candidate HSC populations. Together, our data demonstrate that Apc is essential for HSC and HPC maintenance and survival.
41. Genotype-phenotype correlation in murine Apc mutation: differences in enterocyte **migration** and response to sulindac. The adenomatous polyposis coli (APC) gene product mediates coordinated cell growth in the intestinal mucosa. In humans, germ-line mutations of APC are associated with colorectal carcinogenesis, a process that varies in severity depending on the length of the protein resulting from the mutant allele. In a previous study of the C57BL/6J-Min/+ (Min/+) mouse, we found that the protein fragment resulting from truncation at codon 850 of murine Apc was associated with changes in enterocyte **migration**, proliferation, apoptosis, and beta-catenin expression. This effect was reversed upon treatment of Min/+ mice with the chemopreventive drug sulindac sulfide. In this study, we measured enterocyte **migration** in the Apc1638N mouse, an animal with an Apc mutation that yields no detectable APC protein. We found no difference in enterocyte **migration**, proliferation, apoptosis, or beta-catenin levels in the Apc1638N mouse when compared to wild-type littermates bearing two normal Apc alleles. Furthermore, administration of sulindac sulfide to Apc1638N mice did not alter enterocyte **migration**. These observations suggest that a dominant negative effect altering cell **migration** is exerted by the truncated APC protein present in the Min/+ mouse. These data also suggest that the effectiveness of chemopreventive agents in preventing Apc-related tumor formation may depend on which type of mutation is present.
42. Amer2 protein interacts with EB1 protein and adenomatous polyposis coli (APC) and controls microtubule stability and cell **migration**. EB1 is key factor in the organization of the microtubule cytoskeleton by binding to the plus-ends of microtubules and serving as a platform for a number of interacting proteins (termed +TIPs) that control microtubule dynamics. Together with its direct binding partner adenomatous polyposis coli (APC), EB1 can stabilize microtubules. Here, we show that Amer2 (APC membrane recruitment 2), a previously identified membrane-associated APC-binding protein, is a direct interaction partner of EB1 and acts as regulator of microtubule stability together with EB1. Amer2 binds to EB1 via specific (S/T)xIP motifs and recruits it to the plasma membrane. Coexpression of Amer2 and EB1 generates stabilized microtubules at the plasma membrane, whereas knockdown of Amer2 leads to destabilization of microtubules. Knockdown of Amer2, APC, or EB1 reduces cell **migration**, and morpholino-mediated down-regulation of Xenopus Amer2 blocks convergent extension cell movements, suggesting that the Amer2-EB1-APC complex regulates cell **migration** by altering microtubule stability.
43. Apc gene mutation is associated with a dominant-negative effect upon intestinal cell **migration**. Apc-associated intestinal tumor formation appears to require functional loss of both Apc alleles. Apc has, therefore, been classified as a tumor suppressor gene. Loss of APC protein function results in increased intracellular beta-catenin, a molecule important to both cell-cell adhesion and regulation of cellular growth. In mice bearing a germ-line Apc mutation, we found that enterocyte beta-catenin expression was also increased in histologically normal intestinal mucosa. Enterocyte crypt-villus **migration** was decreased by 25%, and treatment of Min/+ animals with sulindac sulfide normalized both beta-catenin expression and enterocyte **migration**. Our data suggest that alterations in enterocyte **migration** occur in cells bearing a single mutant Apc allele, and that sulindac sulfide may normalize enterocyte growth in these cells.
44. EB1 and APC bind to mDia to stabilize microtubules downstream of Rho and promote cell **migration**. Lysophosphatidic acid (LPA) stimulates Rho GTPase and its effector, the formin mDia, to capture and stabilize microtubules in fibroblasts. We investigated whether mammalian EB1 and adenomatous polyposis coli (APC) function downstream of Rho-mDia in microtubule stabilization. A carboxy-terminal APC-binding fragment of EB1 (EB1-C) functioned as a dominant-negative inhibitor of microtubule stabilization induced by LPA or active mDia. Knockdown of EB1 with small interfering RNAs also prevented microtubule stabilization. Expression of either full-length EB1 or APC, but not an APC-binding mutant of EB1, was sufficient to stabilize microtubules. Binding and localization studies showed that EB1, APC and mDia may form a complex at stable microtubule ends. Furthermore, EB1-C, but not an APC-binding mutant, inhibited fibroblast **migration** in an in vitro wounding assay. These results show an evolutionarily conserved pathway for microtubule capture, and suggest that mDia functions as a scaffold protein for EB1 and APC to stabilize microtubules and promote cell **migration**.
45. Distinct biological roles for the akt family in mammary tumor progression. The phosphatidylinositol 3' kinase/Akt pathway is frequently dysregulated in cancer, which can have unfavorable consequences in terms of cell proliferation, survival, metabolism, and **migration**. Increasing evidence suggests that Akt1, Akt2, and Akt3 play unique roles in breast cancer initiation and progression. We have recently shown that in contrast to Akt1, which accelerates mammary tumor induction in transgenic mice, Akt2 promotes metastasis of tumor cells without affecting the latency of tumor development. Despite the distinct phenotypic outputs resulting from Akt1 or Akt2 activation, very little is known about the mode by which such unique functions originate from these highly related kinases. Here we discuss potential mechanisms contributing to the differing functional specificity of Akt1 and Akt2 with respect to **migration**, invasion, and metastasis.
46. Altered SK3/KCa2.3-mediated **migration** in adenomatous polyposis coli (Apc) mutated mouse colon epithelial cells. Lost of adenomatous polyposis coli gene (Apc) disturbs the **migration** of intestinal epithelial cells but the mechanisms have not been fully characterized. Since we have demonstrated that SK3/KCa2.3 channel promotes cancer cell **migration**, we hypothesized that Apc mutation may affect SK3/KCa2.3 channel-mediated colon epithelial cell motility. We report evidence that SK3/KCa2.3 channel promotes colon epithelial cells motility. Following Apc mutation SK3/KCa2.3 expression is largely reduced leading to a suppression of the SK3/KCa2.3 channel mediated-cell **migration**. Our findings reveal a previously unknown function of the SK3/KCa2.3 channel in epithelial colonic cells, and suggest that Apc is a powerful regulator SK3/KCa2.3 channel.
47. Altered dynamics of intestinal cell maturation in Apc1638N/+ mice. Novel imaging of active transcription sites in interphase nuclei of intestinal epithelial cells in situ showed that key genes associated with Wnt and Notch signaling were dynamically regulated as the cells underwent normal maturation during their **migration** along the mouse crypt-villus axis (CVA). However, oscillating patterns of activation of these genes were displaced along this axis in the histologically normal intestinal mucosa of Apc(1638N/+) mice before tumor development. Gene expression profiling then showed that the normal reprogramming of cells along the CVA was dampened in the Apc(1638N/+) mice, with an overrepresentation of c-myc target genes among those loci affected in the mutant mice. Moreover, in the Apc(1638N/+) mice, there was a perturbed pattern of expression of lineage-specific markers along the CVA consistent with transcription site repression of the Math1 gene, and genes encoding enzymes of every step of the tricarboxylic acid cycle were downregulated in the crypt of Apc(1638N/+) mice compared with WT, but not in the villus. These changes may alter energy metabolism and generate a pseudohypoxic state, suggested by elevated expression of Hif1alpha and its target genes. Thus, although intestinal tumors develop in Apc(1638N/+) mice on focal loss or inactivation of the WT allele, our results show that in the Apc(1638N/+) mouse, inheritance of only a single WT Apc allele perturbs the dynamic and complex reprogramming underlying normal cell maturation, which links epithelial function and homeostasis with architectural organization of the intestine.
48. Dietary-induced ERbeta upregulation counteracts intestinal neoplasia development in intact male ApcMin/+ mice. Most sporadic colorectal cancers (CRCs) develop through the adenoma-carcinoma sequence pathway and are initiated by adenomatous polyposis coli (APC) gene mutations. Estrogen receptor beta (ERbeta) is recognized to progressively reduce its expression in adenomatous and carcinomatous tissues in humans. Moreover, ERbeta deficiency enhances small intestinal tumorigenesis in rodents. In the Apc(Min/+) mouse model, we evaluated intestinal polyp development and ERbeta expression plus other biological parameters influencing tumor growth (epithelial cell proliferation, apoptosis and **migration**) following the addition of a combination of the ERbeta-selective agonist silymarin (SIL) and/or lignin (LIG) to a high-fat/low-fiber diet. Forty-five Apc(Min/+) mice were divided in four groups: animals fed on the tumorigenic high-fat/low-fiber diet, the tumorigenic diet supplemented with SIL (0.02%) or purified LIG (6.24%) or SIL (0.005%) + LIG (6.24%). In these animals, we assessed polyp number and volume and their degree of dysplasia together with ERbeta messenger RNA (mRNA) and protein levels and epithelial cell proliferation, **migration** and apoptosis. The latter group of parameters was evaluated in normal and adenomatous mucosa and the results compared with those found in wild-type (WT) mice fed on the control diet. The addition of SIL or LIG to the diet and even more the specific combination of the two significantly counteracted intestinal tumorigenesis and increased ERbeta mRNA and protein levels. Cell proliferation and apoptosis were rebalanced and cell **migration** accelerated, restoring values similar to those observed in WT animals. Our results further support a protective effect of ERbeta in CRC suggesting the use of the combination of SIL-LIG as a potential approach against CRC development.
49. Modulation of tumor formation and intestinal cell **migration** by estrogens in the Apc(Min/+) mouse model of colorectal cancer. Epidemiological studies suggest that post-menopausal hormone replacement therapy (HRT) reduces colorectal cancer (CRC) incidence. Phytoestrogens, including the soy isoflavone genistein and coumestrol, are used by many women as alternatives to HRT. Previous studies showed that ovariectomy induced a 77% increase in intestinal adenoma number in the C57BL/6J-Min/+ (Min/+) mouse, an animal model of adenomatous polyposis coli (APC)-associated CRC. Replacement of estradiol (E(2)) in ovariectomized Min/+ mice reduced tumor number to baseline and up-regulated the expression of estrogen receptor beta (ERbeta). We hypothesized that the phytoestrogens genistein and coumestrol would inhibit intestinal tumorigenesis in ovariectomized Min/+ mice. Min/+ and Apc(+/+) (WT) mice were ovariectomized and assigned to either a control diet or treatment with E(2), genistein or coumestrol. Treatment of ovariectomized Min/+ (Min/+ OX) mice with genistein resulted in a non-significant reduction in tumor number. Min/+ OX mice treated with coumestrol had significantly fewer tumors than untreated Min/+ OX controls and the same number of tumors as non-ovariectomized Min/+ mice. Bromodeoxyuridine **migration** assays also demonstrated that treatment with E(2) or coumestrol improved enterocyte **migration** rate. Immunoprecipitation and immunohistochemistry analyses showed that impaired association of the adherens junction proteins E-cadherin and beta-catenin in Min/+ mice was improved by treatment with either E(2) or coumestrol. Immunoblot analyses also showed that expression of ERbeta was elevated in enterocytes of Min/+ OX mice treated with E(2) or coumestrol as compared with those of untreated Min/+ OX mice. In conclusion, both coumestrol and E(2) prevent intestinal tumorigenesis and ameliorate enterocyte **migration** and intercellular adhesion in the Apc(Min/+) mouse model of CRC.
50. Tumorigenic fragments of APC cause dominant defects in directional cell **migration** in multiple model systems. Nonsense mutations that result in the expression of truncated, N-terminal, fragments of the adenomatous polyposis coli (APC) tumour suppressor protein are found in most sporadic and some hereditary colorectal cancers. These mutations can cause tumorigenesis by eliminating β-catenin-binding sites from APC, which leads to upregulation of β-catenin and thereby results in the induction of oncogenes such as MYC. Here we show that, in three distinct experimental model systems, expression of an N-terminal fragment of APC (N-APC) results in loss of directionality, but not speed, of cell motility independently of changes in β-catenin regulation. We developed a system to culture and fluorescently label live pieces of gut tissue to record high-resolution three-dimensional time-lapse movies of cells in situ. This revealed an unexpected complexity of normal gut ce**ll migrat**ion, a key process in gut epithelial maintenance, with cells moving with spatial and temporal discontinuity. Quantitative comparison of gut tissue from wild-type mice and APC heterozygotes (APC(Min/+); multiple intestinal neoplasia model) demonstrated that cells in precancerous epithelia lack directional preference when moving along the crypt-villus axis. This effect was reproduced in diverse experimental systems: in developing chicken embryos, mesoderm cells expressing N-APC failed to migrate normally; in amoeboid Dictyostelium, which lack endogenous APC, expressing an N-APC fragment maintained cell motility, but the cells failed to perform directional chemotaxis; and multicellular Dictyostelium slug aggregates similarly failed to perform phototaxis. We propose that N-terminal fragments of APC represent a gain-of-function mutation that causes cells within tissue to fail to migrate directionally in response to relevant guidance cues. Consistent with this idea, crypts in histologically normal tissues of APC(Min/+) intestines are overpopulated with cells, suggesting that a lack **of migrat**ion might cause cell accumulation in a precancerous state.
51. Genetic enhancement of the Lis1+/- phenotype by a heterozygous mutation in the adenomatous polyposis coli gene. Hemizygous Lis1 mutations cause type 1 lissencephaly, a neuronal **migration** disorder in humans. The Lis1+/- mouse is a model for lissencephaly; mice exhibit neuronal **migration** defects but are viable and fertile. On an inbred genetic background, 20% of Lis1+/- mice develop hydrocephalus and die prematurely. Lis1 functions with the microtubule motor cytoplasmic dynein. Because dynactin, a dynein regulator, interacts with end-binding protein 1 (EB1) and beta-catenin, two known binding partners of the adenomatous polyposis coli (APC) protein, we looked for a genetic interaction between Lis1 and APC. Mice with a heterozygous truncating mutation in APC (Min mutation) do not exhibit neuronal **migration** defects or develop hydrocephalus. However, the presence of the APC mutation increases the **migration** deficit and the incidence of hydrocephalus in Lis1+/- animals. Lis1 and dynein distribution is altered in cells derived from Min mice, and both Lis1 and dynein interact with the C terminus of APC in vitro. Together, our findings point to a previously unknown interaction between APC and Lis1 during mammalian brain development.
52. Prickle1 mutation causes planar cell polarity and directional cell **migration** defects associated with cardiac outflow tract anomalies and other structural birth defects. Planar cell polarity (PCP) is controlled by a conserved pathway that regulates directional cell behavior. Here, we show that mutant mice harboring a newly described mutation termed Beetlejuice (Bj) in Prickle1 (Pk1), a PCP component, exhibit developmental phenotypes involving cell polarity defects, including skeletal, cochlear and congenital cardiac anomalies. Bj mutants die neonatally with cardiac outflow tract (OFT) malalignment. This is associated with OFT shortening due to loss of polarized cell orientation and failure of second heart field cell intercalation mediating OFT lengthening. OFT myocardialization was disrupted with cardiomyocytes failing to align with the direction of cell invasion into the outflow cushions. The expression of genes mediating Wnt signaling was altered. Also noted were shortened but widened bile ducts and disruption in canonical Wnt signaling. Using an in vitro wound closure assay, we showed Bj mutant fibroblasts cannot establish polarized cell morphology or engage in directional cell **migration**, and their actin cytoskeleton failed to align with the direction of wound closure. Unexpectedly, Pk1 mutants exhibited primary and motile cilia defects. Given Bj mutant phenotypes are reminiscent of ciliopathies, these findings suggest Pk1 may also regulate ciliogenesis. Together these findings show Pk1 plays an essential role in regulating cell polarity and directional cell **migration** during development.
53. 15-Hydroxyprostaglandin dehydrogenase is down-regulated in colorectal cancer. Prostaglandin E2 (PGE2) can stimulate tumor progression by modulating several proneoplastic pathways, including proliferation, angiogenesis, cell **migration**, invasion, and apoptosis. Although steady-state tissue levels of PGE2 stem from relative rates of biosynthesis and breakdown, most reports examining PGE2 have focused solely on the cyclooxygenase-dependent formation of this bioactive lipid. Enzymatic degradation of PGE2 involves the NAD+-dependent 15-hydroxyprostaglandin dehydrogenase (15-PGDH). The present study examined a range of normal tissues in the human and mouse and found high levels of 15-PGDH in the large intestine. By contrast, the expression of 15-PGDH is decreased in several colorectal carcinoma cell lines and in other human malignancies such as breast and lung carcinomas. Consistent with these findings, we observe diminished 15-Pgdh expression in ApcMin+/- mouse adenomas. Enzymatic activity of 15-PGDH correlates with expression levels and the genetic disruption of 15-Pgdh completely blocks production of the urinary PGE2 metabolite. Finally, 15-PGDH expression and activity are significantly down-regulated in human colorectal carcinomas relative to matched normal tissue. In summary, these results suggest a novel tumor suppressive role for 15-PGDH due to loss of expression during colorectal tumor progression.
54. The APC network regulates the removal of mutated cells from colonic crypts. Self-renewal is essential for multicellular organisms but carries the risk of somatic mutations that can lead to cancer, which is particularly critical for rapidly renewing tissues in a highly mutagenic environment such as the intestinal epithelium. Using computational modeling and in vivo experimentation, we have analyzed how adenomatous polyposis coli (APC) mutations and β-catenin aberrations affect the maintenance of mutant cells in colonic crypts. The increasing abundance of APC along the crypt axis forms a gradient of cellular adhesion that causes more proliferative cells to accelerate their movement toward the top of the crypt, where they are shed into the lumen. Thus, the normal crypt can efficiently eliminate β-catenin mutant cells, whereas APC mutations favor retention. Together, the molecular design of the APC/β-catenin signaling network integrates cell proliferation a**nd migrat**ion dynamics to translate intracellular signal processing and protein gradients along the crypt into intercellular interactions and whole-crypt physiological or pathological behavior.
55. Membrane localization of adenomatous polyposis coli protein at cellular protrusions: targeting sequences and regulation by beta-catenin. Adenomatous polyposis coli protein (APC) translocates to, and stabilizes, the plus-ends of microtubules. In microtubule-dependent cellular protrusions, APC frequently accumulates in peripheral clusters at the basal membrane. APC targeting to membrane clusters is important for cell **migration**, but the localization mechanism is poorly understood. In this study, we performed deletion mapping and defined a minimal sequence (amino acids 1-2226) that efficiently targets APC to membrane clusters. This sequence lacks DLG-1 and EB1 binding sites, suggesting that these partners are not absolutely required for APC membrane targeting. A series of APC sequences were transiently expressed in cells and compared for their ability to compete endogenous APC at the membrane; potent inhibition of endogenous APC targeting was elicited by the Armadillo- (binds KAP3A, B56alpha, and ASEF) and beta-catenin-binding domains. The Armadillo domain was predicted to inhibit APC membrane localization through sequestration of the kinesin-KAP3A complex. The role of beta-catenin in APC membrane localization was unexpected but affirmed by overexpressing the APC binding sequence of beta-catenin, which similarly reduced APC membrane staining. Furthermore, we used RNA interference to show that loss of beta-catenin reduced APC at membrane clusters in migrating cells. In addition, we report that transiently expressed APC-yellow fluorescent protein co-localized with beta-catenin, KAP3A, EB1, and DLG-1 at membrane clusters, but only beta-catenin stimulated APC anchorage at the membrane. Our findings identify beta-catenin as a regulator of APC targeting to membrane clusters and link these two proteins to cell **migration**.
56. Lack of adenomatous polyposis coli protein correlates with a decrease in cell **migration** and overall changes in microtubule stability. Most sporadic colorectal tumors carry truncation mutations in the adenomatous polyposis coli (APC) gene. The APC protein is involved in many processes that govern gut tissue. In addition to its involvement in the regulation of beta-catenin, APC is a cytoskeletal regulator with direct and indirect effects on microtubules. Cancer-related truncation mutations lack direct and indirect binding sites for microtubules in APC, suggesting that loss of this function contributes to defects in APC-mutant cells. In this study, we show that loss of APC results in disappearance of cellular protrusions and decreased cell **migration**. These changes are accompanied by a decrease in overall microtubule stability and also by a decrease in posttranslationally modified microtubules in the cell periphery particularly the migrating edge. Consistent with the ability of APC to affect cell shape, the overexpression of APC in cells can induce cellular protrusions. These data demonstrate that cell **migration** and microtubule stability are linked to APC status, thereby revealing a weakness in APC-deficient cells with potential therapeutic implications.
57. Essential requirement for β-arrestin2 in mouse intestinal tumors with elevated Wnt signaling. β-Arrestins (Arrb) participate in the regulation of multiple signaling pathways, including Wnt/β-catenin, the major actor in human colorectal cancer initiation. To better understand the roles of Arrb in intestinal tumorigenesis, a reverse genetic approach (Arrb(-/-)) and in vivo siRNA treatment were used in Apc(Δ14/+) mice. Mice with Arrb2 depletion (knockout and siRNA) developed only 33% of the tumors detected in their Arrb2-WT littermates, whereas Arrb1 depletion remained without significant effect. These remaining tumors grow normally and are essentially Arrb2-independent. Unsupervised hierarchical clustering analysis showed that they clustered with 25% of Apc(Δ14/+);Arrb2(+/+) tumors. Genes overexpressed in this subset reflect a high interaction with the immune system, whereas those overexpressed in Arrb2-dependent tumors are predominantly involved in Wnt signaling, cell adhes**ion, migr**ation, and extracellular matrix remodeling. The involvement of Arrb2 in intestinal tumor development via the regulation of the Wnt pathway is supported by ex vivo and in vitro experiments using either tumors from Apc(Δ14/+) mice or murine Apc(Min/+) cells. Indeed, Arrb2 siRNAs decreased the expression of Wnt target genes in cells isolated from 12 of 18 tumors from Apc(Δ14/+) mice. In Apc(Min/+) cells, Arrb2 siRNAs completely reversed the increased Wnt activity and colony formation in soft agar induced by Apc siRNA treatment, whereas they did not affect these parameters in basal conditions or in cells expressing constitutively active β-catenin. We demonstrate that Arrb2 is essential for the initiation and growth of intestinal tumors displaying elevated Wnt pathway activity and identify a previously unsuspected molecular heterogeneity among tumors induced by truncating Apc mutations.
58. Intestinal tumor progression is promoted by decreased apoptosis and dysregulated Wnt signaling in Ceacam1-/- mice. The carcinoembryonic antigen cell adhesion molecule 1 (CEACAM1) is downregulated in colonic and intestinal hyperplastic lesions as well as in other cancers, where it functions as a tumor suppressor. To investigate the functions of CEACAM1 in the normal intestine and in intestinal tumors, we generated a compound knockout mouse model and examined both Ceacam1(-/-) and Apc(1638N/+):Ceacam1(-/-) mice. Ceacam1(-/-) intestinal cells exhibited a significant decrease in apoptosis, with no change in proliferation or **migration**, however. Compound Apc(1638N/+):Ceacam1(-/-) mice demonstrated an increase in intestinal tumor multiplicity and tumor progression. Increases in intussusceptions and desmoid lesions were also observed. We have shown that CEACAM1-L associates with beta-catenin by co-immunoprecipitation and colocalization in CEACAM1-L-transfected CT26 and CT51 mouse colon carcinoma cells. Ceacam1(-/-) enterocytes displayed decreased glycogen synthase kinase 3-beta activity with corresponding nuclear localization of beta-catenin. Increased T-cell factor/Lef transcriptional activity was observed in CEACAM1-null CT51 colonic cells and in Caco2 colon cancer cells in which CEACAM1 was downregulated. A significant increased expression in c-Myc and cyclin D1 targets of the Wnt signaling pathway was also revealed in the Ceacam1(-/-) intestine. CEACAM1 therefore actively participates in Wnt signaling in intestinal cells and its downregulation in intestinal tissue contributes to malignancy by augmenting tumor multiplicity and progression.
59. APC/β-catenin-rich complexes at membrane protrusions regulate mammary tumor cell **migratio**n and mesenchymal morphology. The APC tumor suppressor is mutated or downregulated in many tumor types, and is prominently localized to punctate clusters at protrusion tips in migratory cells, such as in astrocytes where it has been implicated in directed cell motility. Although APC loss is considered an initiating event in colorectal cancer, for example, it is less clear what role APC plays in tumor cell motility and whether loss of APC might be an important promoter of tumor progression in addition to initiation.The localization of APC and β-catenin was analyzed in multiple cell lines, including non-transformed epithelial lines treated with a proteasome inhibitor or TGFβ to induce an epithelial-to-mesenchymal transition (EMT), as well as several breast cancer lines, by immunofluorescence. APC expression was knocked down in 4T07 mammary tumor cells using lentiviral-mediated delivery of APC-specific short-hairpin (sh) RNAs, and assessed using quantitative (q) reverse-transcriptase (RT)-PCR and western blotting. Tumor cell motility was analyzed by performing wound-filling assays, and morphology via immunofluorescence (IF) and phase-contrast microscopy. Additionally, proliferation was measured using BrdU incorporation, and TCF reporter assays were performed to determine β-catenin/TCF-mediated transcriptional activity.APC/β-catenin-rich complexes were observed at protrusion ends of migratory epithelial cells treated with a proteasome inhibitor or when EMT has been induced and in tumor cells with a mesenchymal, spindle-like morphology. 4T07 tumor cells with reduced APC levels were significantly less motile and had a more rounded morphology; yet, they did not differ significantly in proliferation or β-catenin/TCF transcriptional activity. Furthermore, we found that APC/β-catenin-rich complexes at protrusion ends were dependent upon an intact microtubule cytoskeleton.These findings indicate that membrane protrusions with APC/β-catenin-containing puncta control the migratory potential and mesenchymal morphology of mammary tumor cells and suggest that APC loss during later stages of tumor progression might impact tumor cell dissemination or colonization.
60. Differential regulation of microtubule severing by APC underlies distinct patterns of projection neuron and interneuron **migration**. Coordinated **migration** of distinct classes of neurons to appropriate positions leads to the formation of functional neuronal circuitry in the cerebral cortex. The two major classes of cortical neurons, interneurons and projection neurons, utilize distinctly different modes (radial versus tangential) and routes of **migration** to arrive at their final positions in the cerebral cortex. Here, we show that adenomatous polyposis coli (APC) modulates microtubule (MT) severing in interneurons to facilitate tangential mode of interneuron **migration**, but not the glial-guided, radial **migration** of projection neurons. APC regulates the stability and activity of the MT-severing protein p60-katanin in interneurons to promote the rapid remodeling of neuronal processes necessary for interneuron **migration**. These findings reveal how severing and restructuring of MTs facilitate distinct modes of neuronal **migration** necessary for laminar organization of neurons in the developing cerebral cortex.
61. Cell type-specific expression of adenomatous polyposis coli in lung development, injury, and repair. Adenomatous polyposis coli (Apc) is critical for Wnt signaling and cell **migration**. The current study examined Apc expression during lung development, injury, and repair. Apc was first detectable in smooth muscle layers in early lung morphogenesis, and was highly expressed in ciliated and neuroendocrine cells in the advanced stages. No Apc immunoreactivity was detected in Clara or basal cells, which function as stem/progenitor cell in adult lung. In ciliated cells, Apc is associated mainly with apical cytoplasmic domain. In response to naphthalene-induced injury, Apc(positive) cells underwent squamous metaplasia, accompanied by changes in Apc subcellular distribution. In conclusion, both spatial and temporal expression of Apc is dynamically regulated during lung development and injury repair. Differential expression of Apc in progenitor vs. nonprogenitor cells suggests a functional role in cell-type specification. Subcellular localization changes of Apc in response to naphthalene injury suggest a role in cell shape and cell **migration**.
62. Deficiency of the LIM-only protein FHL2 reduces intestinal tumorigenesis in Apc mutant mice. The four and a half LIM-only protein 2 (FHL2) is capable of shuttling between focal adhesion and nucleus where it signals through direct interaction with a number of proteins including beta-catenin. Although FHL2 activation has been found in various human cancers, evidence of its functional contribution to carcinogenesis has been lacking.Here we have investigated the role of FHL2 in intestinal tumorigenesis in which activation of the Wnt pathway by mutations in the adenomatous polyposis coli gene (Apc) or in beta-catenin constitutes the primary transforming event. In this murine model, introduction of a biallelic deletion of FHL2 into mutant Apc(Delta14/+) mice substantially reduces the number of intestinal adenomas but not tumor growth, suggesting a role of FHL2 in the initial steps of tumorigenesis. In the lesions, Wnt signalling is not affected by FHL2 deficiency, remaining constitutively active. Nevertheless, loss of FHL2 activity is associated with increased epithelial cell **migration** in intestinal epithelium, which might allow to eliminate more efficiently deleterious cells and reduce the risk of tumorigenesis. This finding may provide a mechanistic basis for tumor suppression by FHL2 deficiency. In human colorectal carcinoma but not in low-grade dysplasia, we detected up-regulation and enhanced nuclear localization of FHL2, indicating the activation of FHL2 during the development of malignancy.Our data demonstrate that FHL2 represents a critical factor in intestinal tumorigenesis.
63. Wnt5a promotes human colon cancer cell **migration** and invasion but does not augment intestinal tumorigenesis in Apc1638N mice. Whereas aberrant activation of canonical Wnt/β-catenin signaling underlies the majority of colorectal cancer cases, the contribution of non-canonical Wnt signaling is unclear. As enhanced expression of the most extensively studied non-canonical Wnt ligand WNT5A is observed in various diseases including colon cancer, WNT5A is gaining attention nowadays. Numerous in vitro studies suggest modulating capacities of WNT5A on proliferation, differentiation, **migratio**n and invasion, affecting tumor and non-mutant cells. However, a possible contribution of WNT5A to colorectal cancer remains to be elucidated. We have analyzed WNT5A expression in colorectal cancer profiling data sets, altered WNT5A expression in colon cancer cells and used our inducible Wnt5a transgenic mouse model to gain more insight into the role of WNT5A in intestinal cancer. We observed that increased WNT5A expression is associated with poor prognosis of colorectal cancer patients. WNT5A knockdown in human colon cancer cells caused reduced directional **migratio**n, deregulated focal adhesion site formation and reduced invasion, whereas Wnt5a administration promoted the directional **migratio**n of colon cancer cells. Despite these observed protumorigenic activities of WNT5A, the induction of Wnt5a expression in intestinal tumors of Apc1638N mice was not sufficient to augment malignancy or metastasis by itself. In conclusion, WNT5A promotes adhesion sites to form in a focal fashion and promotes the directional **migratio**n and invasion of colon cancer cells. Although these activities appear insufficient by themselves to augment malignancy or metastasis in Apc1638N mice, they might explain the poor colon cancer prognosis associated with enhanced WNT5A expression.
64. APC is an RNA-binding protein, and its interactome provides a link to neural development and microtubule assembly. Adenomatous polyposis coli (APC) is a microtubule plus-end scaffolding protein important in biology and disease. APC is implicated in RNA localization, although the mechanisms and functional significance remain unclear. We show APC is an RNA-binding protein and identify an RNA interactome by HITS-CLIP. Targets were highly enriched for APC-related functions, including microtubule organization, cell motility, cancer, and neurologic disease. Among the targets is β2B-tubulin, known to be required in human neuron and axon **migratio**n. We show β2B-tubulin is synthesized in axons and localizes preferentially to dynamic microtubules in the growth cone periphery. APC binds the β2B-tubulin 3' UTR; experiments interfering with this interaction reduced β2B-tubulin mRNA axonal localization and expression, depleted dynamic microtubules and the growth cone periphery, and impaired neu**ron migra**tion. These results identify APC as a platform binding functionally related protein and RNA networks, and suggest a self-organizing model for the microtubule to localize synthesis of its own subunits.
65. Natural sphingadienes inhibit Akt-dependent signaling and prevent intestinal tumorigenesis. Sphingolipid metabolites regulate cell proliferation, **migration**, and stress responses. Alterations in sphingolipid metabolism have been proposed to contribute to carcinogenesis, cancer progression, and drug resistance. We identified a family of natural sphingolipids called sphingadienes and investigated their effects in colon cancer. We find that sphingadienes induce colon cancer cell death in vitro and prevent intestinal tumorigenesis in vivo. Sphingadienes exert their influence by blocking Akt translocation from the cytosol to the membrane, thereby inhibiting protein translation and promoting apoptosis and autophagy. Sphingadienes are orally available, are slowly metabolized through the sphingolipid degradative pathway, and show limited short-term toxicity. Thus, sphingadienes represent a new class of therapeutic and/or chemopreventive agents that blocks Akt signaling in neoplastic and preneoplastic cells.

#### GeneRIFs

1. adenomatous polyposis coli (APC) modulates microtubule (MT) severing in interneurons to facilitate tangential mode of interneuron **migration**, but not the glial-guided, radial **migration** of projection neurons
2. Study shows APC is an RNA-binding protein and identify an RNA interactome; among the targets is beta2B-tubulin, known to be required in human neuron and axon **migration**; results identify APC as a platform binding functionally related protein and RNA networks, and suggest a self-organizing model for the microtubule to localize synthesis of its own subunits.
3. the Amer2-EB1-APC complex regulates cell **migration** by altering microtubule stability.
4. beta-catenin is a regulator of APC targeting to membrane clusters; both proteins have roles in cell **migration**
5. results show an evolutionarily conserved pathway for microtubule capture, and suggest that mDia functions as a scaffold protein for EB1 and APC to stabilize microtubules and promote cell **migration**.

### ECM

#### PubMed

1. Estrogen receptor α or β loss in the colon of Min/+ mice promotes crypt expansion and impairs TGFβ and HNF3β signaling. Adenomatous polyposis coli (APC)-regulated Wnt and transforming growth factor-β (TGFβ) signaling cooperate in the intestine to maintain normal enterocyte functions. Human clinical trials showed that estrogen [17β-estradiol (E2)], the ligand of nuclear receptors estrogen receptor α (ERα) and ERβ, inhibited colorectal cancer (CRC) in women. Consistent with this finding, we reported that E2, ERα and ERβ suppressed intestinal tumorigenesis in the C57BL/6J-Min/+ (Min/+) mouse, a CRC model. Here, we extended our results with further comparisons of colon and small intestine from intact female Apc (+/+) (WT), Min/+ and ER-deficient Min/+ mice. In the colon of ER-deficient Min/+ mice, ER loss reduced TGFβ signaling in crypt base cells as evidenced by minimal expression of the effectors Smad 2, 3 and 4 in these strains. We also found reduced expression of Indian hedgehog (Ihh), bone morphogenetic protein 4 and hepatocyte nuclear factor 3β or FoxA2, factors needed for paracrine signaling between enterocytes and mesenchyme. In proximal colon, ER loss produced a >10-fold increased incidence of crypt fission, a marker for wound healing and tumor promotion. These data, combined with our previous work detailing the specific roles of E2, ERα and ERβ in the colon, suggest that ER activity helps to maintain the intestinal stem cell (ISC) microenvironment by modulating epithelial-stromal crosstalk in ways that regulate cytokine, Wnt and Ihh availability in the extrac**ell**ular matrix (ECM).

### [Ee]xtracellular [Mm]atrix

#### PubMed

1. Integrin signalling regulates YAP and TAZ to control skin homeostasis. The skin is a squamous epithelium that is continuously renewed by a population of basal layer stem/progenitor cells and can heal wounds. Here, we show that the transcription regulators YAP and TAZ localise to the nucleus in the basal layer of skin and are elevated upon wound healing. Skin-specific deletion of both YAP and TAZ in adult mice slows proliferation of basal layer cells, leads to hair loss and impairs regeneration after wounding. Contact with the basal **extracellular matrix** and consequent integrin-Src signalling is a key determinant of the nuclear localisation of YAP/TAZ in basal layer cells and in skin tumours. Contact with the basement membrane is lost in differentiating daughter cells, where YAP and TAZ become mostly cytoplasmic. In other types of squamous epithelia and squamous cell carcinomas, a similar control mechanism is present. By contrast, columnar epithelia differentiate an apical domain that recruits CRB3, Merlin (also known as NF2), KIBRA (also known as WWC1) and SAV1 to induce Hippo signalling and retain YAP/TAZ in the cytoplasm despite contact with the basal layer **extracellular matrix**. When columnar epithelial tumours lose their apical domain and become invasive, YAP/TAZ becomes nuclear and tumour growth becomes sensitive to the Src inhibitor Dasatinib.
2. Lgr4-mediated Wnt/β-catenin signaling in peritubular myoid cells is essential for spermatogenesis. Peritubular myoid cells (PMCs) are myofibroblast-like cells that surround the seminiferous tubules and play essential roles in male fertility. How these cells modulate spermatogenesis and the signaling pathways that are involved are largely unknown. Here we report that Lgr4 is selectively expressed in mouse PMCs in the testes, and loss of Lgr4 leads to germ cells arresting at meiosis I and then undergoing apoptosis. In PMCs of Lgr4 mutant mice, the expression of androgen receptor, alpha-smooth muscle actin and **extracellular matri**x proteins was dramatically reduced. Malfunctioning PMCs further affected Sertoli cell nuclear localization and functional protein expression in Lgr4(-/-) mice. In addition, Wnt/β-catenin signaling was activated in wild-type PMCs but attenuated in those of Lgr4(-/-) mice. When Wnt/β-catenin signaling was reactivated by crossing with Apc(min/+) mice or by Gsk3β inhibitor treatment, the Lgr4 deficiency phenotype in testis was partially rescued. Together, these data demonstrate that Lgr4 signaling through Wnt/β-catenin regulates PMCs and is essential for spermatogenesis.
3. Matrix metalloproteinase activity modulates tumor size, cell motility, and cell invasiveness in murine aggressive fibromatosis. Matrix metalloproteinases (MMPs) and tissue inhibitors of MMPs (TIMPs) regulate the degradation of **extracellular matrix** components and play important roles in the progression of select neoplastic processes. The locally invasive soft tissue tumor, aggressive fibromatosis (also called desmoid tumor), is caused by mutations resulting in beta-catenin-mediated T-cell factor (tcf)-dependent transcriptional activity. Because beta-catenin can regulate MMP expression, we investigated the expression of several MMPs and TIMPs in aggressive fibromatosis tumors that develop in Apc+/Apc1638N mice. Mmp-3 and Timp-1 were differentially regulated (5-fold and 0.5-fold, respectively) in tumors compared with normal fibrous tissue. Conditioned media from tumor cells showed an increased ability to degrade collagen, and inhibition of MMPs using GM6001 decreased the ability of the tumor cells to invade through Matrigel. Both the treatment of Apc/Apc1638N mice with GM6001 or crossing with a transgenic mouse that overexpresses Timp-1 resulted in a significant reduction in tumor volume. Surprisingly, overexpression of Timp-1 also resulted in a 50% increase in tumor number. Although TIMP-1 can induce growth stimulatory effects in some cell types, we found no difference in proliferation or apoptosis rate in cells from tumors that developed in the Timp-1-transgenic mice compared with mice that did not express the Timp-1 transgene, suggesting that TIMP-1 promotes aggressive fibromatosis tumor formation through an alternate mechanism. These data suggest that MMPs play a crucial role in regulating the invasiveness of mesenchymal cells and in modulating aggressive fibromatosis tumor progression. Because this is a locally invasive tumor, MMP inhibition could slow tumor growth and may prove to be an effective adjuvant therapy.
4. Cancer stemness in Apc- vs. Apc/KRAS-driven intestinal tumorigenesis. Constitutive activation of the Wnt pathway leads to adenoma formation, an obligatory step towards intestinal cancer. In view of the established role of Wnt in regulating stemness, we attempted the isolation of cancer stem cells (CSCs) from Apc- and Apc/KRAS-mutant intestinal tumours. Whereas CSCs are present in Apc/KRAS tumours, they appear to be very rare (<10(-6)) in the Apc-mutant adenomas. In contrast, the Lin(-)CD24(hi)CD29(+) subpopulation of adenocarcinoma cells appear to be enriched in CSCs with increased levels of active β-catenin. Expression profiling analysis of the CSC-enriched subpopulation confirmed their enhanced Wnt activity and revealed additional differential expression of other signalling pathways, growth factor binding proteins, and **extracellular matri**x components. As expected, genes characteristic of the Paneth cell lineage (e.g. defensins) are co-expressed together with stem cell genes (e.g. Lgr5) within the CSC-enriched subpopulation. This is of interest as it may indicate a cancer stem cell niche role for tumor-derived Paneth-like cells, similar to their role in supporting Lgr5(+) stem cells in the normal intestinal crypt. Overall, our results indicate that oncogenic KRAS activation in Apc-driven tumours results in the expansion of the CSCs compartment by increasing ®-catenin intracellular stabilization.
5. Estrogen receptor α or β loss in the colon of Min/+ mice promotes crypt expansion and impairs TGFβ and HNF3β signaling. Adenomatous polyposis coli (APC)-regulated Wnt and transforming growth factor-β (TGFβ) signaling cooperate in the intestine to maintain normal enterocyte functions. Human clinical trials showed that estrogen [17β-estradiol (E2)], the ligand of nuclear receptors estrogen receptor α (ERα) and ERβ, inhibited colorectal cancer (CRC) in women. Consistent with this finding, we reported that E2, ERα and ERβ suppressed intestinal tumorigenesis in the C57BL/6J-Min/+ (Min/+) mouse, a CRC model. Here, we extended our results with further comparisons of colon and small intestine from intact female Apc (+/+) (WT), Min/+ and ER-deficient Min/+ mice. In the colon of ER-deficient Min/+ mice, ER loss reduced TGFβ signaling in crypt base cells as evidenced by minimal expression of the effectors Smad 2, 3 and 4 in these strains. We also found reduced expression of Indian hedgehog (Ihh), bone morphogenetic protein 4 and hepatocyte nuclear factor 3β or FoxA2, factors needed for paracrine signaling between enterocytes and mesenchyme. In proximal colon, ER loss produced a >10-fold increased incidence of crypt fission, a marker for wound healing and tumor promotion. These data, combined with our previous work detailing the specific roles of E2, ERα and ERβ in the colon, suggest that ER activity helps to maintain the intestinal stem cell (ISC) microenvironment by modulating epithelial-stromal crosstalk in ways that regulate cytokine, Wnt and Ihh avai**lability in the extr**acellular matrix (ECM).
6. The receptor tyrosine kinase EPHB4 has tumor suppressor activities in intestinal tumorigenesis. Colorectal cancer is the second cause of cancer-related death in the western world, and although the genetic and molecular mechanisms involved in the initiation and progression of these tumors are among the best characterized, there are significant gaps in our understanding of this disease. The role of EPHB signaling in colorectal cancer has only recently been realized. Here, we use animal models to investigate the role of EphB4 in intestinal tumorigenesis. Modulation of EPHB4 levels in colon cancer cell lines resulted in significant differences in tumor growth in a xenograft model, with low levels of EPHB4 associated with faster growth. In addition, using a genetic model of intestinal tumorigenesis where adenomatous polyposis coli (Apc) mutations lead to initiation of the tumorigenic process (Apc(min) mice), we show that inactivation of a single allele of EphB4 results in higher proliferation in both the normal epithelium and intestinal tumors, significantly larger tumors in the small intestine, and a 10-fold increase in the number of tumors in the large intestine. This was associated with a 25% reduction in the lifespan of Apc(min) mice (P < 0.0001). Gene expression analysis showed that EphB4 mutations result in a profound transcriptional reprogramming, affecting genes involved in cell proliferation, remodeling of the **extracellular matrix**, and cell attachment to the basement membrane among other functional groups of genes. Importantly, in agreement with the expression profiling experiments, using an in vitro assay, we show that loss of EPHB4 in colon cancer cells results in a significantly increased potential to invade through a complex **extracellular matrix**. Collectively, these results indicate that EphB4 has tumor suppressor activities and that regulation of cell proliferation, **extracellular matrix** remodeling, and invasive potential are important mechanisms of tumor suppression.
7. Curcumin enhances dasatinib-induced inhibition of growth and transformation of colon cancer cells. Colorectal cancer is the third most common form of malignancy, behind prostate and lung cancers. Despite recent advances in medicine, mortality from colorectal cancer remains high, highlighting the need for improved therapies. Numerous studies have demonstrated increased activation of EGFR and its family members (EGFRs), IGF-1R as well as c-Src in colorectal cancer. The current study was undertaken to examine the effectiveness of combination therapy of dasatinib (BMS-354825; Bristol-Myers Squibb), a highly specific inhibitor of Src family kinases (SFK) and a nontoxic dietary agent; curcumin (diferuloylmethane), in colorectal cancer in in vitro and in vivo experimental models. For the latter, we utilized C57BL/6 APC(Min+/-) mice. Initial in vitro studies revealed synergistic interactions between the two agents. Additionally, we have observed that combination treatment causes a much greater inhibition of the following metastatic processes than either agent alone: (i) colony formation, (ii) invasion through **extracellular matrix** and (iii) tubule formation by endothelial cells. Dasatinib affects the cell adhesion phenotype of colon cancer HCT-116 cells whereas the combination therapy enhances this effect to a greater extent. Preclinical investigation revealed that the combination therapy to be highly effective causing an over 95% regression of intestinal adenomas in Apc(Min+/-) mice, which could be attributed to decreased proliferation and increased apoptosis. In conclusion, our data suggest that combination treatment of dasatinib and curcumin could be a potential therapeutic strategy for colorectal cancer.
8. Loss of cathepsin L activity promotes claudin-1 overexpression and intestinal neoplasia. Intestinal epithelial integrity and polarity are maintained by cohesive interactions between cells via the formation of tight junctions. Irregularities in tight junctions have only recently been found to be associated with the initiation and progression of intestinal neoplasia. The claudin family of proteins is integral to the structure and function of the tight junction but little is known of the molecular events that regulate the expression of these components. The present report identifies cathepsin L, classically a lysosomal cysteine protease, as being induced during intestinal epithelial cell polarization and differentiation. Inhibition of intracellular cathepsin L activity results in the accumulation of disorganized cell layers and a decline in the expression of differentiation markers in cultured intestinal epithelial cells. This coincides with a rapid up-regulation of claudin-1 protein accumulation. Mutant mice defective in cathepsin L activity (furless) display an elevated level of intestinal claudin-1 and claudin-2 expression. Loss of cathepsin L activity leads to a marked increase in tumor multiplicity in the intestine of Apc(Min) mice. Given the traditionally viewed biological role of cathepsin L in the processing of lysosomal content as well as in pathological **extracellular matrix** remodeling, the results here demonstrate an as yet unsuspected intracellular role for this protease in normal intestinal epithelial polarization and initiation of neoplasia.
9. Thrombospondin 1--a regulator of adenoma growth and carcinoma progression in the APC(Min/+) mouse model. Thrombospondin 1 (TSP-1) is a multifunctional **extracellular matrix** protein that is an endogenous regulator of tumor angiogenesis. The effects of TSP-1 on adenoma formation and development into cancerous lesions has been evaluated in the Min(/+) (multiple intestinal neoplasia) mouse model. These mice develop multiple adenomas in the small intestine due to a mutation in the homologous APC (adenomatous polyposis coli) gene. As in its human counterpart, these adenomas may progress to carcinomas. Intestines of APC(Min/+) mice were dissected and histologic evaluation of adenomas was then conducted. Significant increases in vascularization and proliferation were observed in adenomatous, as compared with normal, mucosa. TSP-1 immunostaining revealed significant decreases in the number and intensity of positive cells in adenomas, as compared with normal mucosa. TSP-1 scores were inversely correlated with vascularity and proliferation rate. Cross breeding of mice homozygous for a deletion of the TSP-1 gene (TSP-1(-/-)) with mice heterozygous for the APC gene mutation (APC(Min/+)), resulted in animals that showed a significant increase in adenoma number and diameter. Also, histopathological examination of these adenomas showed accelerated dysplasic changes, carcinoma in situ and early invasion, compared with their APC(Min/+) littermates. Moreover, a significant decrease of TUNEL-positive cells was observed in intestinal adenomas of TSP-1(-/-)/APC(Min/+) mice. This study reports the first in vivo impact of TSP-1 during early stages of tumor initiation and development in an intestinal carcinogenesis model and demonstrates that TSP-1 affects both angiogenesis and tumor cell apoptosis.
10. IL-33 activates tumor stroma to promote intestinal polyposis. Tumor epithelial cells develop within a microenvironment consisting of **extracellular matrix**, growth factors, and cytokines produced by nonepithelial stromal cells. In response to paracrine signals from tumor epithelia, stromal cells modify the microenvironment to promote tumor growth and metastasis. Here, we identify interleukin 33 (IL-33) as a regulator of tumor stromal cell activation and mediator of intestinal polyposis. In human colorectal cancer, IL-33 expression was induced in the tumor epithelium of adenomas and carcinomas, and expression of the IL-33 receptor, IL1RL1 (also referred to as IL1-R4 or ST2), localized predominantly to the stroma of adenoma and both the stroma and epithelium of carcinoma. Genetic and antibody abrogation of responsiveness to IL-33 in the Apc(Min/+) mouse model of intestinal tumorigenesis inhibited proliferation, induced apoptosis, and suppressed angiogenesis in adenomatous polyps, which reduced both tumor number and size. Similar to human adenomas, IL-33 expression localized to tumor epithelial cells and expression of IL1RL1 associated with two stromal cell types, subepithelial myofibroblasts and mast cells, in Apc(Min/+) polyps. In vitro, IL-33 stimulation of human subepithelial myofibroblasts induced the expression of **extracellular matrix** components and growth factors associated with intestinal tumor progression. IL-33 deficiency reduced mast cell accumulation in Apc(Min/+) polyps and suppressed the expression of mast cell-derived proteases and cytokines known to promote polyposis. Based on these findings, we propose that IL-33 derived from the tumor epithelium promotes polyposis through the coordinated activation of stromal cells and the formation of a protumorigenic microenvironment.
11. Matrix metalloproteinase 7 is required for tumor formation, but dispensable for invasion and fibrosis in SMAD4-deficient intestinal adenocarcinomas. Expression of matrix metalloproteinase 7 (MMP7) is increased in the human colorectal carcinomas, and correlates with malignant progression. However, its contribution to colon cancer pathogenesis is not understood thoroughly. To investigate the roles of MMP7 in colon cancer progression, we introduced an Mmp7 knockout mutation into the cis-Apc/Smad4 mutant mouse, a model of invasive colon cancer in which SMAD4-dependent TGF-beta family signaling is inactivated. We demonstrate here that lack of MMP7 reduces the number and size of tumors in the cis-Apc/Smad4 mice. On the other hand, MMP7-deficiency does not affect the depth of tumor invasion, number of stromal fibroblasts or levels of **extracellular matrix** components in the tumors. These results indicate that MMP7 is required for tumor formation, but not for the invasion or fibrosis of the colon cancer whose SMAD4-dependent TGF-beta family signaling is blocked.
12. Essential requirement for β-arrestin2 in mouse intestinal tumors with elevated Wnt signaling. β-Arrestins (Arrb) participate in the regulation of multiple signaling pathways, including Wnt/β-catenin, the major actor in human colorectal cancer initiation. To better understand the roles of Arrb in intestinal tumorigenesis, a reverse genetic approach (Arrb(-/-)) and in vivo siRNA treatment were used in Apc(Δ14/+) mice. Mice with Arrb2 depletion (knockout and siRNA) developed only 33% of the tumors detected in their Arrb2-WT littermates, whereas Arrb1 depletion remained without significant effect. These remaining tumors grow normally and are essentially Arrb2-independent. Unsupervised hierarchical clustering analysis showed that they clustered with 25% of Apc(Δ14/+);Arrb2(+/+) tumors. Genes overexpressed in this subset reflect a high interaction with the immune system, whereas those overexpressed in Arrb2-dependent tumors are predominantly involved in Wnt signaling, cell adhesion, migration, **and extracellular m**atrix remodeling. The involvement of Arrb2 in intestinal tumor development via the regulation of the Wnt pathway is supported by ex vivo and in vitro experiments using either tumors from Apc(Δ14/+) mice or murine Apc(Min/+) cells. Indeed, Arrb2 siRNAs decreased the expression of Wnt target genes in cells isolated from 12 of 18 tumors from Apc(Δ14/+) mice. In Apc(Min/+) cells, Arrb2 siRNAs completely reversed the increased Wnt activity and colony formation in soft agar induced by Apc siRNA treatment, whereas they did not affect these parameters in basal conditions or in cells expressing constitutively active β-catenin. We demonstrate that Arrb2 is essential for the initiation and growth of intestinal tumors displaying elevated Wnt pathway activity and identify a previously unsuspected molecular heterogeneity among tumors induced by truncating Apc mutations.
13. Intestinal tumorigenesis is suppressed in mice lacking the metalloproteinase matrilysin. Matrix metalloproteinases (MMPs) classically have been implicated in basement membrane destruction associated with late-stage tumor cell invasion and metastasis. However, recent studies have demonstrated that one MMP family member, matrilysin, is expressed in a high percentage of early-stage human colorectal tumors. We analyzed matrilysin expression in benign intestinal tumors from mice heterozygous for the ApcMin allele (Min/+) and found that the mRNA was induced in the majority (88%) of these adenomas. Protein was detected in the tumor cells, where, surprisingly, it was predominantly immunolocalized to the lumenal surface of dysplastic glands rather than the basement membrane or **extracellular matrix**. To address the role of matrilysin in Min intestinal tumorigenesis, we generated Min/+ mice deficient in this MMP by gene targeting and homologous recombination. The absence of matrilysin resulted in a reduction in mean tumor multiplicity in Min/+ animals of approximately 60% and a significant decrease in the average tumor diameter. Based on these findings, we conclude that matrilysin is a suppressor of the Min phenotype, possibly by functioning in a capacity independent of matrix degradation. These results argue for the use of MMP inhibitors in the treatment and prevention of early-stage colon cancer.

### [Ii]nvasive

#### PubMed

1. APC haploinsufficiency coupled with p53 loss sufficiently induces mucinous cystic neoplasms and **invasive** pancreatic carcinoma in mice. Adenomatous polyposis coli (APC), a tumor-suppressor gene critically involved in familial adenomatous polyposis, is integral in Wnt/β-catenin signaling and is implicated in the development of sporadic tumors of the distal gastrointestinal tract including pancreatic cancer (PC). Here we report for the first time that functional APC is required for the growth and maintenance of pancreatic islets and maturation. Subsequently, a non-Kras mutation-induced premalignancy mouse model was developed; in this model, APC haploinsufficiency coupled with p53 deletion resulted in the development of a distinct type of pancreatic premalignant precursors, mucinous cystic neoplasms (MCNs), exhibiting pathomechanisms identical to those observed in human MCNs, including accumulation of cystic fluid secreted by neoplastic and ovarian-like stromal cells, with 100% penetrance and the presence of hepatic and gastric metastases in >30% of the mice. The major clinical implications of this study suggest targeting the Wnt signaling pathway as a novel strategy for managing MCN.
2. The adenomatous polyposis coli-associated exchange factors Asef and Asef2 are required for adenoma formation in Apc(Min/+)mice. Sporadic and familial colorectal tumours usually harbour biallelic adenomatous polyposis coli (APC)-associated mutations that result in constitutive activation of Wnt signalling. Furthermore, APC activates Asef and Asef2, which are guanine-nucleotide exchange factors specific for Rac1 and Cdc42. Here, we show that Asef and Asef2 expression is aberrantly enhanced in intestinal adenomas and tumours. We also show that deficiency of either Asef or Asef2 significantly reduces the number and size of adenomas in Apc(Min/+) mice, which are heterozygous for an APC mutation and spontaneously develop adenomas in the intestine. We observed that the APC-Asef/Asef2 complex induces c-Jun amino-terminal kinase-mediated transactivation of matrix metalloproteinase 9, and is required for the **invasive** activity of colorectal tumour cells. Furthermore, we show that Asef and Asef2 are required for tumour angiogenesis. These results suggest that Asef and Asef2 have a crucial role in intestinal adenoma formation and tumour progression, and might be promising molecular targets for the treatment of colorectal tumours.
3. Integrin signalling regulates YAP and TAZ to control skin homeostasis. The skin is a squamous epithelium that is continuously renewed by a population of basal layer stem/progenitor cells and can heal wounds. Here, we show that the transcription regulators YAP and TAZ localise to the nucleus in the basal layer of skin and are elevated upon wound healing. Skin-specific deletion of both YAP and TAZ in adult mice slows proliferation of basal layer cells, leads to hair loss and impairs regeneration after wounding. Contact with the basal extracellular matrix and consequent integrin-Src signalling is a key determinant of the nuclear localisation of YAP/TAZ in basal layer cells and in skin tumours. Contact with the basement membrane is lost in differentiating daughter cells, where YAP and TAZ become mostly cytoplasmic. In other types of squamous epithelia and squamous cell carcinomas, a similar control mechanism is present. By contrast, columnar epithelia differentiate an apical domain that recruits CRB3, Merlin (also known as NF2), KIBRA (also known as WWC1) and SAV1 to induce Hippo signalling and retain YAP/TAZ in the cytoplasm despite contact with the basal layer extracellular matrix. When columnar epithelial tumours lose their apical domain and become **invasive**, YAP/TAZ becomes nuclear and tumour growth becomes sensitive to the Src inhibitor Dasatinib.
4. Inducible loss of one Apc allele in Lrig1-expressing progenitor cells results in multiple distal colonic tumors with features of familial adenomatous polyposis. Individuals with familial adenomatous polyposis (FAP) harbor a germline mutation in adenomatous polyposis coli (APC). The major clinical manifestation is development of multiple colonic tumors at a young age due to stochastic loss of the remaining APC allele. Extracolonic features, including periampullary tumors, gastric abnormalities, and congenital hypertrophy of the retinal pigment epithelium, may occur. The objective of this study was to develop a mouse model that simulates these features of FAP. We combined our Lrig1-CreERT2/+ mice with Apcfl/+ mice, eliminated one copy of Apc in leucine-rich repeats and immunoglobulin-like domains protein 1 (Lrig1)-positive (Lrig1(+)) progenitor cells with tamoxifen injection, and monitored tumor formation in the colon by colonoscopy and PET. Initial loss of one Apc allele in Lrig1(+) cells results in a predictable pattern of preneoplastic changes, culminating in multiple distal colonic tumors within 50 days of induction, as well as the extracolonic manifestations of FAP mentioned above. We show that tumor formation can be monitored by non**invasive** PET imaging. This inducible stem cell-driven model recapitulates features of FAP and offers a tractable platform on which therapeutic interventions can be monitored over time by colonoscopy and non**invasive** imaging.
5. A senescence-inflammatory switch from cancer-inhibitory to cancer-promoting mechanism. Senescence, perceived as a cancer barrier, is paradoxically associated with inflammation, which promotes tumorigenesis. Here, we characterize a distinct low-grade inflammatory process in stressed epithelium that is related to para-inflammation; this process either represses or promotes tumorigenesis, depending on p53 activity. Csnk1a1 (CKIα) downregulation induces a senescence-associated inflammatory response (SIR) with growth arrest in colorectal tumors, which loses its growth control capacity in the absence of p53 and instead, accelerates growth and **invasiv**eness. Corresponding processes occur in CKIα-deleted intestinal organoids, assuming tumorigenic transformation properties ex vivo, upon p53 loss. Treatment of organoids and mice with anti-inflammatory agents suppresses the SIR and prevents p53-deficient organoid transformation and mouse carcinogenesis. SIR/para-inflammation suppression may therefore constitute a key mechanism in the anticarcinogenic effects of nonsteroidal anti-inflammatory drugs.
6. A new mouse model for evaluating the immunotherapy of human colorectal cancer. A new murine model of human colorectal cancer was generated by crossing human carcinoembryonic antigen (CEA) transgenic mice (H-2K(b)) with adenomatous polyposis coli (Apc1638N) knockout mice (H-2K(b)). The resulting hybrid mice developed gastrointestinal polyps in 6-8 months that progressed to **invasive** carcinomas with a similar pattern of dysplasia and CEA expression as observed in human colorectal cancer. These animals exhibited incomplete or partial tolerance to CEA as evidenced by delayed growth of CEA-expressing tumors and the inability to inhibit CEA-specific CTL responses. These results have important implications for understanding the role of CEA-specific immunity in human colon cancer patients and suggest that vaccine strategies targeting CEA may be feasible. This model provides a powerful system for evaluating antigen-specific tumor immunity against spontaneous tumors arising in an orthotopic location and permits evaluation of therapeutic vaccine strategies for human colorectal cancer.
7. Colorectal cancer in mice genetically deficient in the mucin Muc2. The gastrointestinal tract is lined by a layer of mucus comprised of highly glycosylated proteins called mucins. To evaluate the importance of mucin in intestinal carcinogenesis, we constructed mice genetically deficient in Muc2, the most abundant secreted gastrointestinal mucin. Muc2-/- mice displayed aberrant intestinal crypt morphology and altered cell maturation and migration. Most notably, the mice frequently developed adenomas in the small intestine that progressed to **invasive** adenocarcinoma, as well as rectal tumors. Thus, Muc2 is involved in the suppression of colorectal cancer.
8. No effects of Smad2 (madh2) null mutation on malignant progression of intestinal polyps in Apc(delta716) knockout mice. The loss of heterozygosity (LOH) in human chromosome 18q21 is found at high frequencies in advanced pancreatic and colorectal cancers. Several candidate tumor suppressor genes, such as SMAD2, SMAD4, and DCC, are located in this region. The homologues of these genes in the mouse are also clustered on chromosome 18. Mutations in the adenomatous polyposis coli (APC) gene are responsible for familial adenomatous polyposis, and we earlier constructed a mouse model for familial adenomatous polyposis, Apc(delta716). Although human APC is located on chromosome 5q, mouse Apc is on chromosome 18, 30 cM proximal to the Dcc-Smad4-Smad2 locus. Taking advantage of this fact, we constructed previously a cis-compound Apc(delta716) Smad4 mutant, the intestinal polyps of which progress to very **invasive** adenocarcinomas. To determine whether Smad2 mutations play similar roles in malignant progression, here we constructed compound mutant mice carrying Apc and Smad2 knockouts in the cis configuration. In contrast to the cis-compound Apc(delta716) Smad4 heterozygotes, the polyps in the cis-compound Apc(delta716) Smad2 heterozygotes showed no difference in the number, size, or histopathology from the polyps in the simple Apc(delta716) heterozygotes. These results suggest that, on human chromosome 18q21, the SMAD4 LOH plays a more significant role, and SMAD2 LOH is insufficient to cause malignant progression of colonic polyps.
9. Sonic hedgehog acts as a negative regulator of {beta}-catenin signaling in the adult tongue epithelium. Wnt/beta-catenin signaling has been implicated in taste papilla development; however, its role in epithelial maintenance and tumor progression in the adult tongue remains elusive. We show Wnt/beta-catenin pathway activation in reporter mice and by nuclear beta-catenin staining in the epithelium and taste papilla of adult mouse and human tongues. beta-Catenin activation in APC(min/+) mice, which carry a mutation in adenomatous poliposis coli (APC), up-regulates Sonic hedgehog (Shh) and Jagged-2 (JAG2) in the tongue epithelium without formation of squamous cell carcinoma (SCC). We demonstrate that Shh suppresses beta-catenin transcriptional activity in a signaling-dependent manner in vitro and in vivo. A similar regulation and function was observed for JAG2, suggesting that both pathways negatively regulate beta-catenin, thereby preventing SCC formation in the tongue. This was supported by reduced nuclear beta-catenin in the tongue epithelium of Patched(+/-) mice, exhibiting dominant active Shh signaling. At the **invasive** front of human tongue cancer, nuclear beta-catenin and Shh were increased, suggesting their participation in tumor progression. Interestingly, Shh but not JAG2 was able to reduce beta-catenin signaling in SCC cells, arguing for a partial loss of negative feedback on beta-catenin transcription in tongue cancer. We show for the first time that the putative Wnt/beta-catenin targets Shh and JAG2 control beta-catenin signaling in the adult tongue epithelium, a function that is partially lost in lingual SCC.
10. Estrogen receptors alpha and beta are inhibitory modifiers of Apc-dependent tumorigenesis in the proximal colon of Min/+ mice. Estrogen replacement therapy in postmenopausal women is associated with a reduction in colorectal cancer risk, potentially via interactions between 17beta-estradiol (E(2)) and the estrogen receptors (ER) alpha and beta. To study the role of E(2) in intestinal tumor inhibition, we separately crossed C57BL/6J-Min/+ (Min/+) mice with Eralpha(+/-) and Erbeta(+/-) mice to generate ER-deficient Min/+ progeny. We found an increased incidence of visible colon tumors and dysplastic microadenomas in ER-deficient Min/+ relative to Er(+/+)Min/+ controls. Small intestinal tumor numbers were unaffected. **Invasive** carcinomas were found only in Eralpha(+/-)Min/+ mice, suggesting that ERalpha plays additional non-cell autonomous roles that limit tumor progression. Histologic analyses of ER-deficient Min/+ colons, as well as colons from ovariectomized Min/+ mice (OvxMin/+) and E(2)-treated OvxMin/+ mice (OvxMin/+ +E(2)), revealed significant differences in crypt architecture, enterocyte proliferation, and goblet cell differentiation relative to Min/+ and Er(+/+)Apc(+/+) (wild-type) controls. The expression of ERalpha and ERbeta was regionally compartmentalized along the colonic crypt axis, suggesting functional antagonism. Our results indicate that ERalpha and ERbeta are inhibitory modifiers of Apc-dependent colon tumorigenesis. As a result, loss of E(2) and ER signaling in postmenopausal women may contribute to colorectal cancer development.
11. alpha2HS-glycoprotein, an antagonist of transforming growth factor beta in vivo, inhibits intestinal tumor progression. Transforming growth factor (TGF)-beta1 is associated with tumor progression and resistance to chemotherapy in established cancers, as well as host immune suppression. Here, we show that the serum glycoprotein alpha2-HS-glycoprotein (AHSG) blocks TGF-beta1 binding to cell surface receptors, suppresses TGF-beta signal transduction, and inhibits TGF-beta-induced epithelial-mesenchymal transition, suggesting that AHSG may play a role in tumor progression. In 66 consecutive sporadic human colorectal cancer specimens, we observed a 3-fold depletion of ASHG in tumor compared with normal tissue, whereas levels of other abundant plasma proteins, albumin and transferrin, were equivalent. Using the Multiple intestinal neoplasia/+ (Min/+) mouse model of intestinal tumorigenesis, we found twice as many intestinal polyps overall, twice as many large polyps (>3 mm diameter), and more progression to **invasive** adenocarcinoma in Min/+ Ahsg-/- mice than in littermates expressing Ahsg. Phosphorylated Smad2 was more abundant in the intestinal mucosa and tumors of Min/+ mice lacking Ahsg, demonstrating increased TGF-beta signaling in vivo. Furthermore, TGF-beta-mediated suppression of immune cell function was exaggerated in Ahsg-/- animals, as shown by inhibition of macrophage activation and reduction in 12-O-tetradecanoylphorbol 13-acetate-induced cutaneous inflammation. Reconstitution of Ahsg-/- mice with bovine Ahsg suppressed endogenous TGF-beta-dependent signaling to wild-type levels, suggesting that therapeutic enhancement of AHSG levels may benefit patients whose tumors are driven by TGF-beta.
12. LIN28 cooperates with WNT signaling to drive **invasive** intestinal and colorectal adenocarcinoma in mice and humans. Colorectal cancer (CRC) remains a major contributor to cancer-related mortality. LIN28A and LIN28B are highly related RNA-binding protein paralogs that regulate biogenesis of let-7 microRNAs and influence development, metabolism, tissue regeneration, and oncogenesis. Here we demonstrate that overexpression of either LIN28 paralog cooperates with the Wnt pathway to promote **invasive** intestinal adenocarcinoma in murine models. When LIN28 alone is induced genetically, half of the resulting tumors harbor Ctnnb1 (β-catenin) mutation. When overexpressed in Apc(Min/+) mice, LIN28 accelerates tumor formation and enhances proliferation and **invasiv**eness. In conditional genetic models, enforced expression of a LIN28-resistant form of the let-7 microRNA reduces LIN28-induced tumor burden, while silencing of LIN28 expression reduces tumor volume and increases tumor differentiation, indicating that LIN28 contributes to tumor maintenance. We detected aberrant expression of LIN28A and/or LIN28B in 38% of a large series of human CRC samples (n = 595), where LIN28 expression levels were associated with **invasiv**e tumor growth. Our late-stage CRC murine models and analysis of primary human tumors demonstrate prominent roles for both LIN28 paralogs in promoting CRC growth and progression and implicate the LIN28/let-7 pathway as a therapeutic target.
13. Tumorigenesis in the multiple intestinal neoplasia mouse: redundancy of negative regulators and specificity of modifiers. The interaction between mutations in the tumor-suppressor genes Apc and p53 was studied in congenic mouse strains to minimize the influence of polymorphic modifiers. The multiplicity and **invasive**ness of intestinal adenomas of Apc(Min/+) (Min) mice was enhanced by deficiency for p53. In addition, the occurrence of desmoid fibromas was strongly enhanced by p53 deficiency. The genetic modifier Mom1 and the pharmacological agents piroxicam and difluoromethylornithine each reduced intestinal adenoma multiplicity in the absence of p53 function. Mom1 showed no influence on the development of desmoid fibromas, whereas the combination of piroxicam and difluoromethylornithine exerted a moderate effect. The ensemble of tumor suppressors and modifiers of a neoplastic process can be usefully analyzed in respect to tissue specificity and synergy.
14. Oncogenic transformation of diverse gastrointestinal tissues in primary organoid culture. The application of primary organoid cultures containing epithelial and mesenchymal elements to cancer modeling holds promise for combining the accurate multilineage differentiation and physiology of in vivo systems with the facile in vitro manipulation of transformed cell lines. Here we used a single air-liquid interface culture method without modification to engineer oncogenic mutations into primary epithelial and mesenchymal organoids from mouse colon, stomach and pancreas. Pancreatic and gastric organoids exhibited dysplasia as a result of expression of Kras carrying the G12D mutation (Kras(G12D)), p53 loss or both and readily generated adenocarcinoma after in vivo transplantation. In contrast, primary colon organoids required combinatorial Apc, p53, Kras(G12D) and Smad4 mutations for progressive transformation to **invasive** adenocarcinoma-like histology in vitro and tumorigenicity in vivo, recapitulating multi-hit models of colorectal cancer (CRC), as compared to the more promiscuous transformation of small intestinal organoids. Colon organoid culture functionally validated the microRNA miR-483 as a dominant driver oncogene at the IGF2 (insulin-like growth factor-2) 11p15.5 CRC amplicon, inducing dysplasia in vitro and tumorigenicity in vivo. These studies demonstrate the general utility of a highly tractable primary organoid system for cancer modeling and driver oncogene validation in diverse gastrointestinal tissues.
15. Heterozygous disruption of the PTEN promotes intestinal neoplasia in APCmin/+ mouse: roles of osteopontin. The persistent activation of the phosphatidylinositol 3-kinase (PI3K)/Akt pathway is oncogenic and involved in colorectal neoplasia. Mutations of both regulatory subunit and catalytic subunit of PI3K have been demonstrated in colon cancers. In the present study, we show that heterozygous disruption of the phosphatase and tensin homolog (PTEN) tumor suppressor gene promoted tumor progression in APC(min/+) mice. Number and size of intestinal tumors were significantly increased in mice bearing both adenomatous polyposis coli (APC) and PTEN mutations. While APC(min/+)PTEN(+/+) mice developed adenomas, **invasive** carcinomas developed in APC(min/+)PTEN(+/-) mice. Large tumors often resulted in intestinal intussusception and early death of APC(min/+)PTEN(+/-) mice. Targeted array revealed that osteopontin (OPN) was the leading gene whose expression was strongly induced by deficiency of PTEN. In colon cancer cells, gain-of-function mutation of PI3K robustly increased levels of OPN and treatment with OPN reduced growth factor deprivation-induced programmed cell death. Moreover, OPN expression was strongly increased in Ras-induced transformation of intestinal epithelial cells in a PI3K-dependent manner. Inhibition of OPN expression by specific small interfering RNA reduced uncontrolled growth and **invasive**ness of Ras-transformed intestinal epithelial cells. Thus, our results suggest that the PI3K pathway promotes the transformation of intestinal adenoma to adenocarcinoma. OPN, a downstream effector of PI3K, protects transformed intestinal epithelial cells from programmed cell death and stimulates their anchorage-independent growth.
16. Long-lived Min mice develop advanced intestinal cancers through a genetically conservative pathway. C57BL/6J mice carrying the Min allele of Adenomatous polyposis coli (Apc) develop numerous adenomas along the entire length of the intestine and consequently die at an early age. This short lifespan would prevent the accumulation of somatic genetic mutations or epigenetic alterations necessary for tumor progression. To overcome this limitation, we generated F(1) Apc(Min/+) hybrids by crossing C57BR/cdcJ and SWR/J females to C57BL/6J Apc(Min/+) males. These hybrids developed few intestinal tumors and often lived longer than 1 year. Many of the tumors (24-87%) were **invasive** adenocarcinomas, in which neoplastic tissue penetrated through the muscle wall into the mesentery. In a few cases (3%), lesions metastasized by extension to regional lymph nodes. The development of these familial cancers does not require chromosomal gains or losses, a high level of microsatellite instability, or the presence of Helicobacter. To test whether genetic instability might accelerate tumor progression, we generated Apc(Min/+) mice homozygous for the hypomorphic allele of the Nijmegen breakage syndrome gene (Nbs1(DeltaB)) and also treated Apc(Min/+) mice with a strong somatic mutagen. These imposed genetic instabilities did not reduce the time required for cancers to form nor increase the percentage of cancers nor drive progression to the point of distant metastasis. In summary, we have found that the Apc(Min/+) mouse model for familial intestinal cancer can develop frequent **invasive** cancers in the absence of overt genomic instability. Possible factors that promote invasion include age-dependent epigenetic changes, conservative somatic recombination, or direct effects of alleles in the F(1) hybrid genetic background.
17. Matrix metalloproteinase activity modulates tumor size, cell motility, and cell **invasive**ness in murine aggressive fibromatosis. Matrix metalloproteinases (MMPs) and tissue inhibitors of MMPs (TIMPs) regulate the degradation of extracellular matrix components and play important roles in the progression of select neoplastic processes. The locally **invasive** soft tissue tumor, aggressive fibromatosis (also called desmoid tumor), is caused by mutations resulting in beta-catenin-mediated T-cell factor (tcf)-dependent transcriptional activity. Because beta-catenin can regulate MMP expression, we investigated the expression of several MMPs and TIMPs in aggressive fibromatosis tumors that develop in Apc+/Apc1638N mice. Mmp-3 and Timp-1 were differentially regulated (5-fold and 0.5-fold, respectively) in tumors compared with normal fibrous tissue. Conditioned media from tumor cells showed an increased ability to degrade collagen, and inhibition of MMPs using GM6001 decreased the ability of the tumor cells to invade through Matrigel. Both the treatment of Apc/Apc1638N mice with GM6001 or crossing with a transgenic mouse that overexpresses Timp-1 resulted in a significant reduction in tumor volume. Surprisingly, overexpression of Timp-1 also resulted in a 50% increase in tumor number. Although TIMP-1 can induce growth stimulatory effects in some cell types, we found no difference in proliferation or apoptosis rate in cells from tumors that developed in the Timp-1-transgenic mice compared with mice that did not express the Timp-1 transgene, suggesting that TIMP-1 promotes aggressive fibromatosis tumor formation through an alternate mechanism. These data suggest that MMPs play a crucial role in regulating the **invasive**ness of mesenchymal cells and in modulating aggressive fibromatosis tumor progression. Because this is a locally **invasive** tumor, MMP inhibition could slow tumor growth and may prove to be an effective adjuvant therapy.
18. Alpha-catenin is essential in intestinal adenoma formation. A loss-of-function mutation in the APC gene initiates colorectal carcinogenesis. Although the molecular mechanism of tumor initiation is complex, several modifier genes have been identified using mouse models, including the ApcMin mouse. Among the familial adenomatous polyposis mouse lines carrying a truncation mutation at codon 580 in Apc (Apc580D), one line (line19-Apc(580D/+)) showed a remarkably reduced incidence of intestinal adenomas (<5% compared with other lines). Extensive genetic analysis identified a deletion in the alpha-catenin (Ctnna1) gene as the cause of this suppression. Notably, the suppression only occurred when the Ctnna1 deletion was in cis-configuration with the Apc580D mutation. In all adenomas generated in line19-Apc(580D/+), somatic recombination between the Apc and Ctnna1 loci retained the wild-type Ctnna1 allele. These data strongly indicate that simultaneous inactivation of alpha-catenin and Apc during tumor initiation suppresses adenoma formation in line19-Apc(580D/+), suggesting that alpha-catenin plays an essential role in the initiation of intestinal adenomas. Although accumulating evidence obtained from human colon tumors with **invasive** or metastatic potential has established a tumor-suppressive role for alpha-catenin in late-stage tumorigenesis, the role of alpha-catenin in the initiation of intestinal tumorigenesis is not well documented, especially compared with that of beta-catenin. A mouse model used in this study focused on the early stage of tumor initiation and clearly indicated an essential role for alpha-catenin. Thus, alpha-catenin has dual roles in intestinal tumorigenesis, a supporting role in tumor initiation, and a suppressive role in tumor progression.
19. The cyclooxygenase-2-mediated prostaglandin signaling is causally related to epithelial carcinogenesis. Epidemiologic, pharmacologic, clinical, and experimental studies document the importance of prostaglandin (PG) signaling in cancer development, including non-melanoma skin cancer lesions in humans and mice. First of all, enzymes involved in PG biosynthesis, such as cyclooxygenase (COX)-2 and/or membrane prostaglandin E synthase (mPGES)-1, were found to be overexpressed in a wide range of premalignant and malignant epithelial tumors, including those of the skin, breast, esophagus, stomach, colorectum, pancreas, and bladder. On the other hand, 15-hydroxy-prostaglandin dehydrogenase (15-PGDH), which is involved in the degradation pathway of PG including PGE(2,) thus counteracting the activities of COX-2 and PGES, was found to be downregulated in human epithelial tumors, indicating a tumor suppressor activity of this enzyme. Most remarkably, genetic studies showed that mice, which are deficient in COX-2 and/or PGES are resistant to the development of cancer of skin, colon, and stomach. In contrast, the forced overexpression of COX-2 in proliferative compartments of simple or stratified epithelia such as skin epidermis, urinary bladder, mammary gland, and pancreas results in spontaneous hyperplasia and dysplasia in transgenic mice. In skin, the pathological changes are found to be due to an abnormal process of terminal differentiation, while in other tissues, hyperproliferation seems to be the main contributor to the pre-**invasive** neoplasms. Moreover, the COX-2 transgenic mouse lines are sensitized for cancer development.
20. Apc Restoration Promotes Cellular Differentiation and Reestablishes Crypt Homeostasis in Colorectal Cancer. The adenomatous polyposis coli (APC) tumor suppressor is mutated in the vast majority of human colorectal cancers (CRC) and leads to deregulated Wnt signaling. To determine whether Apc disruption is required for tumor maintenance, we developed a mouse model of CRC whereby Apc can be conditionally suppressed using a doxycycline-regulated shRNA. Apc suppression produces adenomas in both the small intestine and colon that, in the presence of Kras and p53 mutations, can progress to **invasive** carcinoma. In established tumors, Apc restoration drives rapid and widespread tumor-cell differentiation and sustained regression without relapse. Tumor regression is accompanied by the re-establishment of normal crypt-villus homeostasis, such that once aberrantly proliferating cells reacquire self-renewal and multi-lineage differentiation capability. Our study reveals that CRC cells can revert to functioning normal cells given appropriate signals and provide compelling in vivo validation of the Wnt pathway as a therapeutic target for treatment of CRC.
21. Development of gastric carcinoma from intestinal metaplasia in Cdx2-transgenic mice. In the progression of chronic gastritis, gastric mucosal cells deviate from the normal pathway of gastric differentiation to an intestinal phenotype. Many epidemiologic studies have found an association between the formation of intestinal metaplasia and the development of gastric carcinoma. However, there is no direct evidence that shows intestinal metaplasia is a precursor lesion of gastric carcinoma, to date. We periodically examined the intestinal metaplastic mucosa of Cdx2-transgenic mice we have previously generated. Gastric polyps developed from intestinal metaplastic mucosa in all stomachs of Cdx2-transgenic mice examined. These gastric polyps consisted of intestinal-type adenocarcinoma that invaded the submucosa and muscularis propria and occasionally spread into the subserosa. p53 and APC gene mutations were recognized in the adenocarcinomas. The participation of APC and p53 gene mutations in gastric carcinogenesis from the intestinal metaplasia was verified by the Cdx2-transgenic mice, carrying Apc(Min) mutation or p53 deficiency, that developed gastric polyps much earlier than Cdx2 alone. We successfully showed that long-term intestinal metaplasia induces **invasive** gastric carcinoma. These results indicate that intestinal metaplasia itself plays a significant role in the genesis and progression of gastric carcinoma.
22. The Min (multiple intestinal neoplasia) mutation: its effect on gut epithelial cell differentiation and interaction with a modifier system. Min is a fully penetrant dominant mutation that leads to the development of multiple intestinal adenomas throughout the duodenal-to-colonic axis. Min/+ C57BL6/J mice have an average life-span of 120 d. Multi-label immunocytochemical studies of these lesions demonstrate patches of differentiated enterocytes, and scattered enteroendocrine, goblet and Paneth cells. Expression of endogenous marker genes within these differentiated cells can be directly correlated with the position occupied by the adenoma along the duodenal-to-colonic axis and mirrors the regional differentiation of the normal gut epithelium. The presence of multiple lineages in adenomas together with their retention of spatial information suggests that tumorigenesis in Min/+ mice may be initiated in a multipotent stem cell normally located at the base of intestinal crypts. To study the time-dependent properties of these tumors, genetic conditions were sought in which Min/+ animals could survive for up to 300 d. Min is fully penetrant in hybrids with either AKR/J or MA/MyJ. However, the hybrids demonstrate a reduction in the number of intestinal adenomas. Preliminary backcross analysis is consistent with a single major modifier locus unlinked to Min in both the AKR/J and MA/MyJ strains. The increased lifespan of the hybrid animals is also associated with the development of **invasive** tumors. New tumors do not arise continuously over the lifespan of these animals; instead all adenomas appear to be established by 100 d of age or sooner. These studies indicate that the Min/+ mouse is a powerful model system for analyzing the mechanisms that establish and maintain a balance between proliferation and differentiation in the continuously renewing gut epithelium and for an assessment of the multi-step hypothesis of intestinal neoplasia.
23. Smad3 deficiency promotes tumorigenesis in the distal colon of ApcMin/+ mice. Colorectal cancer, one of the most common human malignancies in the Western world, is often subdivided based on tumor location in either the distal or proximal colon. Several mouse models have been developed to study human colorectal cancer, but few display this clear distinction between the two colonic locations. By crossing Apc(Min/+) and Smad3 mutant mice, we showed that combined activation of the Wnt pathway and attenuation of the transforming growth factor-beta (TGF-beta) pathway causes high multiplicity and rapid onset of **invasive** tumorigenesis almost exclusively in the distal colon, closely mimicking the familial adenomatous polyposis (FAP) disease and consisting with distinct colorectal cancer etiologies based on tumor location. Transcriptional profiling revealed higher expression of several TGF-beta activators in the normal distal mucosa than in proximal mucosa, suggesting a stronger reliance on TGF-beta-mediated growth control in the distal than in the proximal colon. Apc(Min/+)Smad3(-/-) mice provide an alternative model to Apc(Min/+) mice to study FAP and distal sporadic colorectal cancer. This model will be useful in dissecting mechanistic and etiologic differences between proximal and distal colonic cancer, whereas the confinement of tumorigenesis to the distal colon offers unique advantages in monitoring tumor progression by in vivo imaging.
24. Investigation of Schwann cells at neoplastic cell sites before the onset of cancer invasion. In neural invasion (NI), cancer cells are classically assumed to actively invade nerves and to cause local recurrence and pain. However, the opposite possibility, that nerves may reach cancer cells even in their pre**invasive** stage and thereby promote cancer spread, has not yet been genuinely considered. The present study analyzes the reaction of Schwann cells of peripheral nerves to carcinogenesis in pancreatic cancer and colon cancer.Two novel 3D migration and Schwann cell outgrowth assays were developed to monitor the timing and the specificity of Schwann cell migration and cancer invasion toward peripheral neurons through digital-time-lapse microscopy and after blockade of nerve growth factor (NGF) signalling via siRNA or a small-molecule inhibitor of the p75(NTR) receptor. The frequency and emergence of the Schwann cell markers Sox10, S100, ALDH1L1, and glial-fibrillary-acidic-protein (GFAP) around cancer precursor lesions were studied in human and conditional murine pancreatic and colon cancer specimens using multiple immunolabeling.Schwann cells migrated toward pancreatic and colon cancer cells, but not toward benign cells, before the onset of cancer migration toward peripheral neurons. This chemoattraction was inhibited after blockade of p75(NTR)-signaling on Schwann and pancreatic cancer cells. Schwann cells were specifically detected around murine and human pancreatic intraepithelial neoplasias (PanINs) (mean percent of murine PanINs surrounded by Schwann cells = 78.9%, 95% CI = 70.9 to 86.8%, and mean percent of human PanINs surrounded by Schwann cells = 52.5%, 95% CI = 14.7 to 90.4%; human: n = 44, murine: n = 14) and intestinal adenomas (mean percent of murine adenomas surrounded by Schwann cells = 64.2%, 95% CI = 28.6 to 99.8%, and mean percent of human adenomas surrounded by Schwann cells = 17.2%, 95% CI = -126.9 to 161.4; human: n = 36, murine: n = 12). The Schwann cell presence in this premalignant stage was associated with the frequency of NI in the malignant phase.Schwann cells have particular and specific affinity to cancer cells. Emergence of Schwann cells in the premalignant phase of pancreatic and colon cancer implies that, in contrast with the traditional assumption, nerves-and not cancer cells-migrate first during NI.
25. Inhibition of CXCR2 profoundly suppresses inflammation-driven and spontaneous tumorigenesis. The chemokine receptor CXCR2 is a key mediator of neutrophil migration that also plays a role in tumor development. However, CXCR2 influences tumors through multiple mechanisms and might promote or inhibit tumor development depending on context. Here, we used several mouse models of spontaneous and inflammation-driven neoplasia to define indispensable roles for CXCR2 in benign and malignant tumors. CXCR2-activating chemokines were part of the secretome of cultured primary benign intestinal adenomas (ApcMin/+) and highly expressed by all tumors in all models. CXCR2 deficiency profoundly suppressed inflammation-driven tumorigenesis in skin and intestine as well as spontaneous adenocarcinoma formation in a model of **invasive** intestinal adenocarcinoma (AhCreER;Apcfl/+;Ptenfl/fl mice). Pepducin-mediated CXCR2 inhibition reduced tumorigenesis in ApcMin/+ mice. Ly6G+ neutrophils were the dominant source of CXCR2 in blood, and CXCR2 deficiency attenuated neutrophil recruitment. Moreover, systemic Ly6G+ cell depletion purged CXCR2-dependent tumor-associated leukocytes, suppressed established skin tumor growth and colitis-associated tumorigenesis, and reduced ApcMin/+ adenoma formation. CXCR2 is thus a potent protumorigenic chemokine receptor that directs recruitment of tumor-promoting leukocytes into tissues during tumor-inducing and tumor-driven inflammation. Similar leukocyte populations were also found in human intestinal adenomas, which suggests that CXCR2 antagonists may have therapeutic and prophylactic potential in the treatment of cancer.
26. The receptor tyrosine kinase EPHB4 has tumor suppressor activities in intestinal tumorigenesis. Colorectal cancer is the second cause of cancer-related death in the western world, and although the genetic and molecular mechanisms involved in the initiation and progression of these tumors are among the best characterized, there are significant gaps in our understanding of this disease. The role of EPHB signaling in colorectal cancer has only recently been realized. Here, we use animal models to investigate the role of EphB4 in intestinal tumorigenesis. Modulation of EPHB4 levels in colon cancer cell lines resulted in significant differences in tumor growth in a xenograft model, with low levels of EPHB4 associated with faster growth. In addition, using a genetic model of intestinal tumorigenesis where adenomatous polyposis coli (Apc) mutations lead to initiation of the tumorigenic process (Apc(min) mice), we show that inactivation of a single allele of EphB4 results in higher proliferation in both the normal epithelium and intestinal tumors, significantly larger tumors in the small intestine, and a 10-fold increase in the number of tumors in the large intestine. This was associated with a 25% reduction in the lifespan of Apc(min) mice (P < 0.0001). Gene expression analysis showed that EphB4 mutations result in a profound transcriptional reprogramming, affecting genes involved in cell proliferation, remodeling of the extracellular matrix, and cell attachment to the basement membrane among other functional groups of genes. Importantly, in agreement with the expression profiling experiments, using an in vitro assay, we show that loss of EPHB4 in colon cancer cells results in a significantly increased potential to invade through a complex extracellular matrix. Collectively, these results indicate that EphB4 has tumor suppressor activities and that regulation of cell proliferation, extracellular matrix remodeling, and **invasive** potential are important mechanisms of tumor suppression.
27. Compound disruption of smad2 accelerates malignant progression of intestinal tumors in apc knockout mice. Smad2 is a receptor-regulated Smad that is activated specifically by transforming growth factor beta and activin signaling. We disrupted the mouse Smad2 gene by gene targeting. Homozygous Smad2 mutant mice died around E8.5 with impaired visceral endoderm function and deficiency of mesoderm formation. Heterozygotes were fertile and had no apparent abnormality up to at least 1 1/2 year of age. To examine the role of Smad2 inactivation in the process of carcinogenesis, we prepared compound heterozygous mice, which carry both Apc and Smad2 mutations on the same chromosome in the cis-configuration. Compound inactivation of Smad2 in heterozygous Apc mutant mice did not change the total number of intestinal tumors but increased sudden death from intestinal obstruction caused by extremely large tumors. Furthermore, histological examination revealed that Apc/Smad2 cis-compound heterozygotes developed multiple **invasive** cancers that had never been observed in Apc single heterozygotes. These results indicate that loss of Smad2 does not initiate tumorigenesis by itself but accelerates malignant progression of tumors to **invasive** cancer in the late stages of carcinogenesis.
28. β-catenin tyrosine 654 phosphorylation increases Wnt signalling and intestinal tumorigenesis. Objective Deregulation of the Wnt signalling pathway by mutations in the Apc or β-catenin genes underlies colorectal carcinogenesis. As a result, β-catenin stabilises, translocates to the nucleus, and activates gene transcription. Intestinal tumours show a heterogeneous pattern of nuclear β-catenin, with the highest levels observed at the invasion front. Activation of receptor tyrosine kinases in these tumour areas by growth factors expressed by surrounding stromal cells phosphorylate β-catenin at tyrosine residues, which is thought to increase β-catenin nuclear translocation and t**umour in**vasiveness. This study investigates the relevance of β-catenin tyrosine phosphorylation for Wnt signalling and intestinal tumorigenesis in vivo. Design A conditional knock-in mouse model was generated into which the phospho-mimicking Y654E modification in the endogenous β-catenin gene was introduced. Results This study provided in vivo evidence that β-catenin(E654) is characterised by reduced affinity for cadherins, increased signalling and strongly increased phosphorylation at serine 675 by protein kinase A (PKA). In addition, homozygosity for the β-catenin(E654) targeted allele caused embryonic lethality, whereas heterozygosity predisposed to intestinal tumour development, and strongly enhanced Apc-driven intestinal tumour initiation associated with increased nuclear accumulation of βcatenin. Surprisingly, the expression of β-catenin(E654) did not affect histological grade or in**duce tum**our invasiveness. Conclusions A thus far unknown mechanism was uncovered in which Y654 phosphorylation of β-catenin facilitates additional phosphorylation at serine 675 by PKA. In addition, in contrast to the current belief that β-catenin Y654 phosphorylation increases tumour progress**ion to a** more invasive phenotype, these results show that it rather increases tumour initiation by enhancing Wnt signalling.
29. PHLPP is a negative regulator of RAF1, which reduces colorectal cancer cell motility and prevents tumor progression in mice. Hyperactivation of the RAS-RAF signaling pathway in colorectal tumors is associated with metastasis and poor outcomes of patients. Little is known about how RAS-RAF signaling is turned off once activated. We investigated how the pH domain and leucine-rich repeat protein phosphatases (PHLPPs) control RAS-RAF signaling and colorectal cancer (CRC) development.We used co-immunoprecipitation assays to identify substrates of PHLPP1 and PHLPP2. We studied phosphorylation of RAF1 in CRC cells that express exogenous PHLPP1 or PHLPP2, or lentiviral-based small hairpin RNAs against their transcripts; we measured effects on cell motility, migration, and invasion in vitro. Tumor progression and survival were analyzed in Phlpp1(-/-) Apc(Min) and Apc(Min)/Phlpp1(-/-) mice. Microarray datasets of colorectal tumor and nontumor tissues were analyzed for PHLPP gene expression.PHLPP1 and 2 were found to dephosphorylate RAF1 at S338, inhibiting its kinase activity in vitro and in CRC cells. In cells, knockdown of PHLPP1 or PHLPP2 increased the amplitude and duration of RAF-MEK-ERK signaling downstream of epidermal growth factor receptor and KRAS, whereas overexpression had the opposite effect. In addition, knockdown of PHLPP1 or PHLPP2 caused CRC cells to express markers of the epithelial-mesenchymal transition, and increased cell migration and invasion. Apc(Min)/Phlpp1(-/-) mice had decreased survival and developed larger intestinal and colon tumors compared to Apc(Min) mice. Whereas Apc(Min) mice developed mostly low-grade adenomas, 20% of the tumors that developed in Apc(Min)/Phlpp1(-/-) mice wer**e invasi**ve adenocarcinomas. Normal villi and adenomas of Apc(Min)/Phlpp1(-/-) mice had significantly fewer apoptotic cells than Apc(Min) mice. Human CRC patient microarray data revealed that the expression of PHLPP1 or PHLPP2 is positively correlated with CDH1.PHLPP1 and PHLPP2 dephosphorylate RAF1 to reduce its signaling, increase th**e invasi**ve and migratory activities of CRC cells, and activate the epithelial-mesenchymal transition. In Apc(Min) mice, loss of PHLPP1 promotes tumor progression.
30. miR-23a promotes the transition from indolent to **invasive** colorectal cancer. Colorectal cancer is a classic example of a tumor that progresses through multiple distinct stages in its evolution. To understand the mechanisms regulating the transition from indolent to **invasive** disease, we profiled somatic copy number alterations in non**invasive** adenomas and **invasive** adenocarcinomas from Apc and DNA mismatch repair (MMR) mutant mouse models. We identified a recurrent amplicon on mouse chromosome 8 that encodes microRNA (miRNA) 23a and -27a (miR). miR-23a and -27a levels are upregulated in mouse intestinal adenocarcinomas, primary tumors from patients with stage I/II colorectal cancers, as well as in human colorectal cancer cell lines and cancer stem cells. Functionally, miR-23a promotes the migration and invasion of colorectal cancer cells and stem cells, whereas miR-27a primarily promotes proliferation. We computationally and experimentally validated that metastasis suppressor 1 (MTSS1) is a direct miR-23a target and similarly validated that the ubiquitin ligase FBXW7 is a direct miR-27a target. Analyses of computationally predicted target genes in microarray data sets of patients with colorectal cancers are consistent with a role for miR-23a, but not miR-27a, specifically in **invasive** colorectal cancers.
31. Targeted expression of oncogenic K-ras in intestinal epithelium causes spontaneous tumorigenesis in mice. Ras oncoproteins are mutated in about 50% of human colorectal cancers, but their precise role in tumor initiation or progression is still unclear.This study presents transgenic mice that express K-ras(V12G), the most frequent oncogenic mutation in human tumors, under control of the murine villin promoter in epithelial cells of the large and small intestine.More than 80% of the transgenic animals displayed single or multiple intestinal lesions, ranging from aberrant crypt foci (ACF) to **invasive** adenocarcinomas. Expression of K-ras(V12G) caused activation of the MAP kinase cascade, and the tumors were frequently characterized by deregulated cellular proliferation. Unexpectedly, we obtained no evidence of inactivating mutations of the tumor suppressor gene Apc, the "gatekeeper" in colonic epithelial proliferation. However, spontaneous mutation of the tumor-suppressor gene p53, a frequent feature in the human disease, was found in 3 of 7 tumors that were tested.This animal model recapitulates the stages of tumor progression as well as a part of the genetic alterations found in human colorectal cancer. Furthermore, it indicates that activation of K-ras in concert with mutations in p53 may constitute a route to digestive tumor formation and growth, underlining the fact that the pathway to intestinal cancer is not necessarily a single road.
32. Fas/CD95 deficiency in ApcMin/+ mice increases intestinal tumor burden. Fas, a member of the tumor necrosis family, is responsible for initiating the apoptotic pathway when bound to its ligand, Fas-L. Defects in the Fas-mediated apoptotic pathway have been reported in colorectal cancer.In the present study, a variant of the Apc(Min/+) mouse, a model for the human condition, Familial Adenomatous Polyposis (FAP), was generated with an additional deficiency of Fas (Apc(Min/+)/Fas(lpr)) by cross-breeding Apc(Min/+) mice with Fas deficient (Fas(lpr)) mice. One of the main limitations of the Apc(Min/+) mouse model is that it only develops benign polyps. However, Apc(Min/+)/Fas(lpr) mice presented with a dramatic increase in tumor burden relative to Apc(Min/+) mice and **invasive** lesions at advanced ages. Proliferation and apoptosis markers revealed an increase in cellular proliferation, but negligible changes in apoptosis, while p53 increased at early ages. Fas-L was lower in Apc(Min/+)/Fas(lpr) mice relative to Apc(Min/+) cohorts, which resulted in enhanced inflammation.This study demonstrated that imposition of a Fas deletion in an Apc(Min/+) background results in a more aggressive phenotype of the Apc(Min/+) mouse model, with more rapid development of **invasive** intestinal tumors and a decrease in Fas-L levels.
33. Dietary modulation of carcinoma development in a mouse model for human familial adenomatous polyposis. Familial adenomatous polyposis (FAP) is caused by a dominant mutation in the adenomatous polyposis coli (APC) gene. Individuals with FAP progressively develop adenomas and carcinomas of the colon and rectum. We developed a mouse model for this disorder by genetically modifying the Apc gene. The resulting mice Apc1638 progressively develop neoplasms in the colon and remainder of the gastrointestinal tract. In this study when Apc1638 mice were fed a Western-style diet, they developed an increased incidence of the end point of carcinomas and number of **invasive** tumors. The findings therefore demonstrated dietary modulation of carcinoma incidence in mice with a targeted mutation providing a model for the study of gene-environment interactions in cancer.
34. PIK3CA and APC mutations are synergistic in the development of intestinal cancers. Human colorectal cancers are known to possess multiple mutations, though how these mutations interact in tumor development and progression has not been fully investigated. We have previously described the FCPIK3ca\* murine colon cancer model, which expresses a constitutively activated phosphoinositide-3 kinase (PI3K) in the intestinal epithelium. The expression of this dominantly active form of PI3K results in hyperplasia and **invasive** mucinous adenocarcinomas. These cancers form via a non-canonical mechanism of tumor initiation that is mediated through activation of PI3K and not through aberrations in WNT signaling. Since the Adenomatous Polyposis Coli (APC) gene is mutated in the majority of human colon cancers and often occurs simultaneously with PIK3CA mutations, we sought to better understand the interaction between APC and PIK3CA mutations in the mammalian intestine. In this study, we have generated mice in which the expression of a constitutively active PI3K and the loss of APC occur simultaneously in the distal small intestine and colon. Here, we demonstrate that expression of a dominant active PI3K synergizes with loss of APC activity resulting in a dramatic change in tumor multiplicity, size, morphology and **invasive**ness. Activation of the PI3K pathway is not able to directly activate WNT signaling through the nuclear localization of CTNNB1 (β-catenin) in the absence of aberrant WNT signaling. Alterations at the transcriptional level, including increased CCND1, may be the etiology of synergy between these activated pathways.
35. Total abdominal 18F-FDG uptake reflects intestinal adenoma burden in Apc mutant mice. Apc mutant (Apc(Min)) mice develop multiple adenomas in their intestines and are widely used to study colorectal carcinogenesis and chemopreventive approaches. Molecular imaging of intestinal adenomas could potentially provide non**invasive** longitudinal evaluation of these lesions in living mice. Therefore, the aim of this study was to investigate the role of (18)F-FDG PET in the Apc(Min) mouse model.Apc(Min) mice (n = 8) fed a purified diet were imaged serially after injection of (18)F-FDG at age 9 and 12 wk using a small-animal PET scanner. Abdominal uptake of the tracer was quantified. After dissection, intestines were imaged separately, and intestinal tracer uptake was quantified. Tracer distribution was compared with results from microscopic examination regarding adenoma number and size. Thereafter, findings were validated serially in 20 Apc(Min) mice aged 6, 8, 10, and 12 wk that received standard chow to increase adenoma numbers. In vivo abdominal (18)F-FDG uptake was correlated with microscopy results.Microscopic examination showed that the mice developed 25-35 intestinal adenomas at age 12 wk. Ex vivo (18)F-FDG PET of the dissected intestines visualized all large adenomas and most small adenomas. Ex vivo total intestinal (18)F-FDG uptake correlated with in vivo total abdominal uptake and with the number of large adenomas at age 9 and 12 wk. At 12 wk, there was a clear correlation between in vivo abdominal tracer uptake and the number of large adenomas but not the total number of lesions.Intestinal adenomas in Apc(Min) mice are metabolically active lesions that take up (18)F-FDG. Abdominal (18)F-FDG uptake at age 12 wk serves as a readout modality for large intestinal adenomas.
36. Pathogenic intestinal bacteria enhance prostate cancer development via systemic activation of immune cells in mice. A role for microbes has been suspected in prostate cancer but difficult to confirm in human patients. We show here that a gastrointestinal (GI) tract bacterial infection is sufficient to enhance prostate intraepithelial neoplasia (PIN) and micro**invasive** carcinoma in a mouse model. We found that animals with a genetic predilection for dysregulation of wnt signaling, Apc (Min/+) mutant mice, were significantly susceptible to prostate cancer in an inflammation-dependent manner following infection with Helicobacter hepaticus. Further, early neoplasia observed in infected Apc (Min/+) mice was transmissible to uninfected mice by intraperitoneal injection of mesenteric lymph node (MLN) cells alone from H. hepaticus-infected mutant mice. Transmissibility of neoplasia was preventable by prior neutralization of inflammation using anti-TNF-α antibody in infected MLN donor mice. Taken together, these data confirm that systemic inflammation triggered by GI tract bacteria plays a pivotal role in tumorigenesis of the prostate gland.
37. Dissociation of EphB2 signaling pathways mediating progenitor cell proliferation and tumor suppression. Signaling proteins driving the proliferation of stem and progenitor cells are often encoded by proto-oncogenes. EphB receptors represent a rare exception; they promote cell proliferation in the intestinal epithelium and function as tumor suppressors by controlling cell migration and inhibiting **invasive** growth. We show that cell migration and proliferation are controlled independently by the receptor EphB2. EphB2 regulated cell positioning is kinase-independent and mediated via phosphatidylinositol 3-kinase, whereas EphB2 tyrosine kinase activity regulates cell proliferation through an Abl-cyclin D1 pathway. Cyclin D1 regulation becomes uncoupled from EphB signaling during the progression from adenoma to colon carcinoma in humans, allowing continued proliferation with **invasive** growth. The dissociation of EphB2 signaling pathways enables the selective inhibition of the mitogenic effect without affecting the tumor suppressor function and identifies a pharmacological strategy to suppress adenoma growth.
38. Genetic deletion of receptor for hyaluronan-mediated motility (Rhamm) attenuates the formation of aggressive fibromatosis (desmoid tumor). Aggressive fibromatosis (desmoid tumor) is a locally **invasive** soft tissue neoplasm associated with mutations resulting in beta-catenin-mediated transcriptional activation. This tumor is composed of cells with histological and molecular characteristics common to proliferating mesenchymal cells of dermal wounds. Using immunohistochemistry and RT-PCR, we show that Rhamm, a protein with an important role in wound healing and neoplastic progression, is also expressed at high levels in aggressive fibromatosis. A mouse harboring a targeted deletion in Rhamm was generated, resulting in viable Rhamm-/- animals. Rhamm-/- mice were crossed with Apc/Apc1638N mice, which harbor a targeted mutation in the Apc gene predisposing animals to gastrointestinal and aggressive fibromatosis tumors. Rhamm deficiency significantly decreased the number of aggressive fibromatosis tumors formed, but did not alter the number of gastrointestinal polyps. Cell culture studies show that Rhamm regulates cell proliferation in both fibroblasts and fibromatosis cells under conditions of low density, but not high density. These results suggest that Rhamm regulates proliferation of cells with sparse cell-cell contacts, such as occurs in aggressive fibromatosis; provides the first genetic evidence implicating Rhamm in tumor pathology; and suggest Rhamm blockade as a potential therapeutic target for this otherwise difficult-to-treat neoplasm.
39. Cyclooxygenase-two (COX-2) modulates proliferation in aggressive fibromatosis (desmoid tumor). Aggressive fibromatosis is a locally **invasive** soft tissue lesion. Seventy-five per cent of cases harbor a somatic mutation in either the APC or beta-catenin genes, resulting in beta-catenin protein stabilization. Cyclooxygenase-2 (COX-2) is an enzyme involved in prostaglandin synthesis that modulates the formation of colonic neoplasia, especially in cases due to mutations resulting in beta-catenin stabilization. Human aggressive fibromatoses and lesions from the Apc+/Apc1638N mouse (a murine model for Apc-driven fibromatosis) demonstrated elevated COX-2 levels. COX-2 blockade either by the selective agent DFU or by non-selective COX blocking agents results in reduced proliferation in human tumor cell cultures. Breeding mice with Cox-2-/- mice resulted in no difference in number of aggressive fibromatoses formed, but in a smaller tumor size, while there was a decrease in number of GI lesions by 50%. Mice fed various COX blocking agents also showed a decline in tumor size. COX-2 expression was regulated by tcf-dependent transcription in this lesion. COX-2 partially regulates proliferation due to beta-catenin stabilization in aggressive fibromatosis. Although COX blockade alone does not cause tumor regression, this data suggests that it may have a role as an adjuvant therapy to slow tumor growth in this lesion.
40. Proteomic profiling of a mouse model of acute intestinal Apc deletion leads to identification of potential novel biomarkers of human colorectal cancer (CRC). Colorectal cancer (CRC) is the fourth most common cause of cancer-related death worldwide. Accurate non-**invasive** screening for CRC would greatly enhance a population's health. Adenomatous polyposis coli (Apc) gene mutations commonly occur in human colorectal adenomas and carcinomas, leading to Wnt signalling pathway activation. Acute conditional transgenic deletion of Apc in murine intestinal epithelium (AhCre(+)Apc(fl)(/)(fl)) causes phenotypic changes similar to those found during colorectal tumourigenesis. This study comprised a proteomic analysis of murine small intestinal epithelial cells following acute Apc deletion to identify proteins that show altered expression during human colorectal carcinogenesis, thus identifying proteins that may prove clinically useful as blood/serum biomarkers of colorectal neoplasia. Eighty-one proteins showed significantly increased expression following iTRAQ analysis, and validation of nine of these by Ingenuity Pathaway Analysis showed they could be detected in blood or serum. Expression was assessed in AhCre(+)Apc(fl)(/)(fl) small intestinal epithelium by immunohistochemistry, western blot and quantitative real-time PCR; increased nucelolin concentrations were also detected in the serum of AhCre(+)Apc(fl)(/)(fl) and Apc(Min)(/)(+) mice by ELISA. Six proteins; heat shock 60kDa protein 1, Nucleolin, Prohibitin, Cytokeratin 18, Ribosomal protein L6 and DEAD (Asp-Glu-Ala-Asp) box polypeptide 5,were selected for further investigation. Increased expression of 4 of these was confirmed in human CRC by qPCR. In conclusion, several novel candidate biomarkers have been identified from analysis of transgenic mice in which the Apc gene was deleted in the intestinal epithelium that also showed increased expression in human CRC. Some of these warrant further investigation as potential serum-based biomarkers of human CRC.
41. Differing effects of endogenous and synthetic inhibitors of metalloproteinases on intestinal tumorigenesis. Matrix metalloproteinase (MMP) activity has been associated with tumor invasion and metastasis in many different tumor types, but recent studies also support a role for these enzymes in earlier stages of the tumor progression continuum. Specifically, the expression pattern of MMPs in benign human and mouse gastrointestinal tumors suggests that they may function in the development or growth of non-**invasive** tumors. To address the contribution of MMP activity to the development of intestinal adenomas, we administered the synthetic MMP inhibitor batimastat and expressed the tissue inhibitor of metalloproteinases-1 (TIMP-1) in the gastrointestinal tract of Min mice, which spontaneously develop pre-malignant small and large intestinal tumors. Batimastat administration resulted in a 48% decrease in the number of Min tumors. This reduction in tumor number is similar to that observed in mice lacking the metalloproteinase matrilysin, and demonstrates the therapeutic and chemopreventive potential of MMP inhibitors for pre-malignant intestinal tumors. In contrast, forced TIMP-1 expression in transgenic mice had no effect or, in one line, unexpectedly augmented Min tumor multiplicity by 32%. This observation supports an in vivo tumor-promoting activity of TIMP-1 that could be related to the growth stimulatory effects of TIMP that have been documented in vitro. Taken together, these 2 approaches of modulating MMP activity in Min mice support a critical function of MMPs in Min tumorigenesis, underscore the importance of an MMP/inhibitor balance in maintaining tissue homeostasis and demonstrate that endogenous MMP inhibitors can have complex effects in particular cellular contexts.
42. Tumor progression in Apc(1638N) mice with Exo1 and Fen1 deficiencies. Flap endonuclease 1 (Fen1) and exonuclease 1 (Exo1) have sequence homology and similar nuclease capabilities. Both function in multiple pathways of DNA metabolism, but appear to have distinct in vivo nucleic acid substrates, and therefore distinct metabolic roles. When combined with Apc(1638N), Fen1 promotes tumor progression. Because of functional similarity to Fen1, and because Exo1 is involved in DNA mismatch repair (MMR) by interaction with Msh2 and Mlh1, genes that cause hereditary nonpolyposis colorectal cancer (HNPCC), we investigated the possibility that Exo1 might also act as a modifier to Apc(1638N). We present evidence that mice with combined mutations in Apc(1638N) and Exo1 and Apc(1638N), Exo1 and Fen1 genes show moderate increased tumor incidence and multiplicity in comparison to Apc(1638N) siblings, implying a low penetrance role for Exo1 in early gastrointestinal (GI) tumorigenesis. Despite a decrease in median survival (10 months) in Apc(1638N) Exo1 mice, their tumors do not progress any more rapidly than those of Apc(1638N). Instead these animals die from infections that are the result of impaired immune response. Apc(1638N) Exo1 Fen1 mice survive longer (18 months), and therefore appear relatively immune competent. They die of **invasive** GI tumors that display microsatellite instability (MSI). Our results show that Exo1 has a modest tumor suppressor function.
43. MYC-nick promotes cell migration by inducing fascin expression and Cdc42 activation. MYC-nick is a cytoplasmic, transcriptionally inactive member of the MYC oncoprotein family, generated by a proteolytic cleavage of full-length MYC. MYC-nick promotes migration and survival of cells in response to chemotherapeutic agents or withdrawal of glucose. Here we report that MYC-nick is abundant in colonic and intestinal tumors derived from mouse models with mutations in the Wnt, TGF-β, and PI3K pathways. Moreover, MYC-nick is elevated in colon cancer cells deleted for FBWX7, which encodes the major E3 ligase of full-length MYC frequently mutated in colorectal cancers. MYC-nick promotes the migration of colon cancer cells assayed in 3D cultures or grown as xenografts in a zebrafish metastasis model. MYC-nick accelerates migration by activating the Rho GTPase Cdc42 and inducing fascin expression. MYC-nick, fascin, and Cdc42 are frequently up-regulated in cells present at the **invasiv**e front of human colorectal tumors, suggesting a coordinated role for these proteins in tumor migration.
44. β-catenin/TCF4 complex induces the epithelial-to-mesenchymal transition (EMT)-activator ZEB1 to regulate tumor **invasiv**eness. In most carcinomas, invasion of malignant cells into surrounding tissues involves their molecular reprogramming as part of an epithelial-to-mesenchymal transition (EMT). Mutation of the APC gene in most colorectal carcinomas (CRCs) contributes to the nuclear translocation of the oncoprotein β-catenin that upon binding to T-cell and lymphoid enhancer (TCF-LEF) factors triggers an EMT and a p**roinvasi**ve gene expression profile. A key inducer of EMT is the ZEB1 transcription factor whose expression promotes tumorigenesis and metastasis in carcinomas. As inhibitor of the epithelial phenotype, ZEB1 is never present in the epithelium of normal colon or the tumor center of CRCs where β-catenin remains membranous. We show here that ZEB1 is expressed by epithelial cells in intestinal tumors from human patients (familial adenomatous polyposis) and mouse models (APC(Min/+)) with germline mutations of APC that result in nuclear accumulation of β-catenin. However, ZEB1 is not expressed in the epithelium of hereditary forms of CRCs that carry wild-type APC and where β-catenin is excluded from the nucleus (Lynch syndrome). We found that β-catenin/TCF4 binds directly to the ZEB1 promoter and activates its transcription. Knockdown of β-catenin and TCF4 in APC-mutated CRC cells inhibited endogenous ZEB1, whereas forced translocation of β-catenin to the nucleus in APC-wild-type CRC cells induced de novo expression of ZEB1. Upregulation of MT1-MMP and LAMC2 by β-catenin/TCF4 has been l**inked to** invasiveness in CRCs, and we show here that both proteins are activated by ZEB1 coexpressing with it in primary colorectal tumors with mutated APC. These results set ZEB1 as an effector of β-catenin/TCF4 signaling in EMT and tumor progression.
45. Signal transducer and activator of transcription 3 (STAT3) protein suppresses adenoma-to-carcinoma transition in Apcmin/+ mice via regulation of Snail-1 (SNAI) protein stability. STAT3 was recently reported to suppress tumor invasion in Apc(min)(/+) mice. We investigated the mechanisms by which STAT3 inhibits intestinal epithelial tumors using Apc(min)(/+)/Stat3(IEC-KO) mice (intestinal epithelial cell (IEC)-specific deletion of STAT3 in the Apc(min)(/+) background) to determine the role of STAT3 in carcinogenesis in vivo as well as colorectal cancer cell lines in vitro. To inhibit invasion of IEC tumors, STAT3 functions as a molecular adaptor rather than a transcription factor. Accordingly, the tumors in Apc(min)(/+)/Stat3(IEC-KO) mice undergo adenoma-to-carcinoma transition and acquire an **invasive** phenotype. Similarly, STAT3 knockdown in a colorectal cell line enhances IEC invasion. We demonstrate that STAT3 down-regulates SNAI (Snail-1) expression levels and hence suppresses epithelial-mesenchymal transition of colorectal cancer cells. Mechanistically, STAT3 facilitates glycogen synthase kinase (GSK) 3β-mediated degradation of SNAI by regulating phosphorylation of GSK3β. Our data identified a new role for STAT3 in the adenoma-to-carcinoma sequence of intestinal tumors.
46. DCC constrains tumour progression via its dependence receptor activity. The role of deleted in colorectal carcinoma (DCC) as a tumour suppressor has been a matter of debate for the past 15 years. DCC gene expression is lost or markedly reduced in the majority of advanced colorectal cancers and, by functioning as a dependence receptor, DCC has been shown to induce apoptosis unless engaged by its ligand, netrin-1 (ref. 2). However, so far no animal model has supported the view that the DCC loss-of-function is causally implicated as predisposing to aggressive cancer development. To investigate the role of DCC-induced apoptosis in the control of tumour progression, here we created a mouse model in which the pro-apoptotic activity of DCC is genetically silenced. Although the loss of DCC-induced apoptosis in this mouse model is not associated with a major disorganization of the intestines, it leads to spontaneous intestinal neoplasia at a relatively low frequency. Loss of DCC-induced apoptosis is also associated with an increase in the number and aggressiveness of intestinal tumours in a predisposing APC mutant context, resulting in the development of highly **invasive** adenocarcinomas. These results demonstrate that DCC functions as a tumour suppressor via its ability to trigger tumour cell apoptosis.
47. Modulation of NKG2D ligand expression and metastasis in tumors by spironolactone via RXRγ activation. Tumor metastasis and lack of NKG2D ligand (NKG2DL) expression are associated with poor prognosis in patients with colon cancer. Here, we found that spironolactone (SPIR), an FDA-approved diuretic drug with a long-term safety profile, can up-regulate NKG2DL expression in multiple colon cancer cell lines by activating the ATM-Chk2-mediated checkpoint pathway, which in turn enhances tumor elimination by natural killer cells. SPIR can also up-regulate the expression of metastasis-suppressor genes TIMP2 and TIMP3, thereby reducing tumor cell **invasiv**eness. Although SPIR is an aldosterone antagonist, its antitumor effects are independent of the mineralocorticoid receptor pathway. By screening the human nuclear hormone receptor siRNA library, we identified retinoid X receptor γ (RXRγ) instead as being indispensable for the antitumor functions of SPIR. Collectively, our results strongly support the use of SPIR or other RXRγ agonists with minimal side effects for colon cancer prevention and therapy.
48. Matrix metalloproteinase 7 is required for tumor formation, but dispensable for invasion and fibrosis in SMAD4-deficient intestinal adenocarcinomas. Expression of matrix metalloproteinase 7 (MMP7) is increased in the human colorectal carcinomas, and correlates with malignant progression. However, its contribution to colon cancer pathogenesis is not understood thoroughly. To investigate the roles of MMP7 in colon cancer progression, we introduced an Mmp7 knockout mutation into the cis-Apc/Smad4 mutant mouse, a model of **invasive** colon cancer in which SMAD4-dependent TGF-beta family signaling is inactivated. We demonstrate here that lack of MMP7 reduces the number and size of tumors in the cis-Apc/Smad4 mice. On the other hand, MMP7-deficiency does not affect the depth of tumor invasion, number of stromal fibroblasts or levels of extracellular matrix components in the tumors. These results indicate that MMP7 is required for tumor formation, but not for the invasion or fibrosis of the colon cancer whose SMAD4-dependent TGF-beta family signaling is blocked.
49. Aggressive fibromatosis (desmoid tumor) is derived from mesenchymal progenitor cells. The cellular origins from which most tumors arise are poorly defined, especially in mesenchymal neoplasms. Aggressive fibromatosis, also known as desmoid tumor, is a locally **invasive** soft tissue tumor that has mesenchymal characteristics. We found that aggressive fibromatosis tumors express genes and cell surface markers characteristic of mesenchymal stem cells (MSC). In mice that are genetically predisposed to develop aggressive fibromatosis tumors (Apc(wt/1638N)), we found that the number of tumors formed was proportional to the number of MSCs present. Sca-1(-/-) mice, which develop fewer MSCs, were crossed with Apc(wt/1638N) mice. Doubly mutant mice deficient in Sca-1 developed substantially fewer aggressive fibromatosis tumors than wild-type (WT) littermates, but Sca-1 deficiency had no effect on the formation of epithelial-derived intestinal polyps. MSCs isolated from Apc(wt/1638N) mice (or mice expressing a stabilized form of β-catenin) induced aberrant cellular growth reminiscent of aggressive fibromatosis tumors after engraftment to immunocompromised mice, but WT cells and mature fibroblasts from the same animals did not. Taken together, our findings indicate that aggressive fibromatosis is derived from MSCs, and that β-catenin supports tumorigenesis by maintaining mesenchymal progenitor cells in a less differentiated state. Protecting this progenitor cell population might prevent tumor formation in patients harboring a germline APC mutation, where fibromatosis is currently the leading cause of mortality.
50. TGF-beta receptor inactivation and mutant Kras induce intestinal neoplasms in mice via a beta-catenin-independent pathway. During colorectal cancer pathogenesis, mutations and epigenetic events cause neoplastic behavior in epithelial cells by deregulating the Wnt, Ras-Raf-extracellular signal-regulated kinase (ERK), and transforming growth factor (TGF)-beta-signaling pathways, among others. The TGF-beta-signaling pathway is often inactivated in colon cancer cells by mutations in the gene encoding the TGF-beta receptor TGFBR2. The RAS-RAF-ERK pathway is frequently up-regulated in colon cancer via mutational activation of KRAS or BRAF. We assessed how these pathways interact in vivo and affect formation of colorectal tumors.We analyzed intestinal tumors that arose in mice that express an oncogenic (active) form of Kras and that have Tgfbr2 inactivations-2 common molecular events observed in human colorectal tumors. LSL-KrasG12D mice were crossed with Villin-Cre;Tgfbr2E2flx/E2flx mice, which do not express Tgfbr2 in the intestinal epithelium.Neither inactivation of Tgfbr2 nor expression of oncogenic Kras alone was sufficient to induce formation of intestinal neoplasms. Histologic abnormalities arose in mice that expressed Kras, but only the combination of Tgfbr2 inactivation and Kras activation led to intestinal neoplasms and metastases. The cancers arose via a beta-catenin-independent mechanism; the epidermal growth factor-signaling pathway was also activated. Cells in the resulting tumors proliferated at higher rates, expressed decreased levels of p15, and expressed increased levels of cyclin D1 and cdk4, compared with control cells.A combination of inactivation of the TGF-beta-signaling pathway and expression of oncogenic Kras leads to formation of **invasive** intestinal neoplasms through a beta-catenin-independent pathway; these adenocarcinomas have the capacity to metastasize.
51. Physiological expression of the PI3K-activating mutation Pik3ca(H1047R) combines with Apc loss to promote development of **invasive** intestinal adenocarcinomas in mice. PIK3CA, the gene encoding the p110α catalytic subunit of PI3K (phosphoinositide 3-kinase), is mutated in approximately 20% of sporadic CRCs (colorectal cancers), but the role of these mutations in the pathogenesis of CRC remains unclear. In the present study we used a novel mouse model to investigate the role of the Pik3caH1047R mutation, the most common PIK3CA mutation in CRC, during the development and progression of intestinal cancer. Our results demonstrate that Pik3caH1047R, when expressed at physiological levels, is insufficient to initiate intestinal tumorigenesis; however, in the context of Apc (adenomatous polyposis coli) loss, which is observed in 80% of CRCs and by itself results in benign intestinal adenomas, the Pik3caH1047R mutation promotes the development of highly aggressive and **invasiv**e adenocarcinomas in both the small and large intestines. The results of the present study show that an activating Pik3ca mutation can act in tandem with Apc loss to drive the progression of gastrointestinal cancer and thus this disease may be susceptible to therapeutic targeting using PI3K pathway inhibitors.
52. Prostate cancer induced by loss of Apc is restrained by TGFβ signaling. Recent work with mouse models of prostate cancer (CaP) has shown that inactivation of TGFβ signaling in prostate epithelium can cooperate with deletion of the Pten tumor suppressor to drive locally aggressive cancer and metastatic disease. Here, we show that inactivating the TGFβ pathway by deleting the gene encoding the TGFβ type II receptor (Tgfbr2) in combination with a deletion of the Apc tumor suppressor gene specifically in mouse prostate epithelium, results in the rapid onset **of inva**sive CaP. Micro-metastases were observed in the lymph nodes and lungs of a proportion of the double mutant mice, whereas no metastases were observed in Apc single mutant mice. Prostate-specific Apc;Tgfbr2 mutants had a lower frequency of metastasis and survived significantly longer than Pten;Tgfbr2 double mutants. However, all Apc;Tgfbr2 mutants develo**ped inva**sive cancer by 30 weeks of age, wher**eas inva**sive cancer was rarely observed in Apc single mutant animals, even by one year of age. Further comparison of the Pten and Apc models of CaP revealed additional differences, including adenosquamous carcinoma in the Apc;Tgfbr2 mutants that was not seen in the Pten model, and a lack of robust induction of the TGFβ pathway in Apc null prostate. In addition to causing high-grade prostate intra-epithelial neoplasia (HGPIN), deletion of either Pten or Apc induced senescence in affected prostate ducts, and this restraint was overcome by loss of Tgfbr2. In summary, this work demonstrates that TGFβ signaling restrains the progression of CaP induced by different tumor suppressor mutations, suggesting that TGFβ signaling exerts a general tumor suppressive effect in prostate.
53. Ephrin-A1 promotes the malignant progression of intestinal tumors in Apc(min/+) mice. The ephrin-A1 and EphA receptors are frequently highly expressed in different human cancers, suggesting that they may promote tumor development and progression. We generated transgenic mice carrying Fabpl(4xat-132) ephrin-A1, which express ephrin-A1 in the intestinal epithelial cells. Those mice were then mated with Apc(min/+) mice to produce the compound mice, which overexpress ephrin-A1 in the intestinal tumors of Apc(min/+) mice. We compared the number, size and histopathological features of the intestinal tumors in the Fabpl(4xat-132) ephrin-A1/Apc(min/+) compound mice with those of the Apc(min/+) mice. The compound mice showed an increased number of intestinal tumors, significantly in the large intestine, and developed more **invasive** tumors. Among the 20 mice of each type examined, 5 Apc(min/+) mice developed 5 **invasive** tumors, 1 **invasive** tumor in each mouse, in the proximal or middle portions of the small intestine. On the other hand, 14 out of 20 compound mice developed 29 **invasive** tumors and 16 of them were in the distal small intestine and the large intestine, where transgenic ephrin-A1 was highly expressed. These results suggested that the increased expression of ephrin-A1 accelerated the malignant progression of the intestinal adenoma to **invasive** tumors.
54. Loss of Apc allows phenotypic manifestation of the transforming properties of an endogenous K-ras oncogene in vivo. Oncogenic mutations in the K-ras gene occur in approximately 50% of human colorectal cancers. However, the precise role that K-ras oncogenes play in tumor formation is still unclear. To address this issue, we have conditionally expressed an oncogenic K-ras(V12) allele in the small intestine of adult mice either alone or in the context of Apc deficiency. We found that expression of K-ras(V12) does not affect normal intestinal homeostasis or the immediate phenotypes associated with Apc deficiency. Mechanistically we failed to find activation of the Raf/MEK/ERK pathway, which may be a consequence of the up-regulation of a number of negative feedback loops. However, K-ras(V12) expression accelerates intestinal tumorigenesis and confers **invasive** properties after Apc loss over the long term. In renal epithelium, expression of the oncogenic K-ras(V12) allele in the absence of Apc induces the rapid development of renal carcinoma. These tumors, unlike those of intestinal origin, display activation of the Raf/MEK/ERK and Akt signaling pathways. Taken together, these data indicate that normal intestinal and kidney epithelium are resistant to malignant transformation by an endogenous K-ras oncogene. However, activation of K-ras(V12) after Apc loss results in increased tumorigenesis with distinct kinetics. Whereas the effect of K-ras oncogenes in the intestine can been observed only after long latencies, they result in rapid carcinogenesis in the kidney epithelium. These data imply a window of opportunity for anti-K-ras therapies after tumor initiation in preventing tumor growth and invasion.
55. Spontaneous initiation, promotion and progression of colorectal cancer in the novel A/J Min/+ mouse. The C57BL/6J multiple intestinal neoplasia (Min/+) mouse is a widely used murine model for familial adenomatous polyposis, a hereditary form of human colorectal cancer. However, it is a questionable model partly because the vast majority of tumors arise in the small intestine, and partly because the fraction of tumors that progress to **invasive** carcinomas is minuscule. A/J mice are typically more susceptible to carcinogen-induced colorectal cancer than C57BL/6J mice. To investigate whether the novel Min/+ mouse on the A/J genetic background could be a better model for colorectal cancer, we examined the spontaneous intestinal tumorigenesis in 81 A/J Min/+ mice ranging in age from 4 to 60 weeks. The A/J Min/+ mouse exhibited a dramatic increase in number of colonic lesions when compared to what has been reported for the conventional Min/+ mouse; however, an increase in small intestinal lesions did not occur. In addition, this novel mouse model displayed a continual development of colonic lesions highlighted by the transition from early lesions (flat ACF) to tumors over time. In mice older than 40 weeks, 13 colonic (95% CI: 8.7-16.3) and 21 small intestinal (95% CI: 18.6-24.3) tumors were recorded. Notably, a considerable proportion of those lesions progressed to carcinomas in both the colon (21%) and small intestine (51%). These findings more closely reflect aspects of human colorectal carcinogenesis. In conclusion, the novel A/J Min/+ mouse may be a relevant model for initiation, promotion and progression of colorectal cancer.
56. Elevated Coexpression of KITENIN and the ErbB4 CYT-2 Isoform Promotes the Transition from Colon Adenoma to Carcinoma Following APC loss. The molecular events in the malignant progression of colon adenoma after loss of adenomatous polyposis coli (APC) are not fully understood. KITENIN (KAI1 C-terminal interacting tetraspanin) increases the **invasive**ness of colorectal cancer cells, and we identified a novel EGFR-independent oncogenic signal of EGF that works under coexpressed KITENIN and ErbB4. Here we tested whether elevated KITENIN and ErbB4 contribute to further progression of intestinal adenoma following APC loss.The intestinal tissues of villin-KITENIN transgenic mice in which villin-driven KITENIN expression induces increased c-Jun expression exhibit mild epithelial cell proliferation but no epithelial lineage changes compared with those of nontransgenic mice. Among the four ErbB4 isoforms, JM-a/CYT-2 and JM-b/CYT-2 exhibited the highest AP-1 activity when cells coexpressing KITENIN and each isoform were stimulated by EGF. Interestingly, predominant overexpression of the ErB4-CYT-2 mRNA as well as increased EGFR expression were observed in intestinal adenoma of APC(min/+) mice, which makes the microenvironment of activated EGF signaling. When we crossed villin-KITENIN mice with APC(min/+) mice, intestinal tumor tissues in the crossed mice showed the characteristics of early-stage invading adenocarcinoma. In patients with colorectal cancer, ErbB4-CYT-2 mRNA expression was significantly greater in tumor tissues than in normal adjacent tissues, but no significant differences in tumor tissue expression were found between different colorectal cancer stages. Furthermore, the mRNA expression of KITENIN and that of ErbB4-CYT-2 were positively correlated in human colorectal cancer tissue.Elevated coexpression of KITENIN and ErbB4-CYT-2 promotes the transition of colon adenoma to adenocarcinoma within an APC loss-associated tumor microenvironment.
57. Myeloid translocation genes differentially regulate colorectal cancer programs. Myeloid translocation genes (MTGs), originally identified as chromosomal translocations in acute myelogenous leukemia, are transcriptional corepressors that regulate hematopoietic stem cell programs. Analysis of The Cancer Genome Atlas (TCGA) database revealed that MTGs were mutated in epithelial malignancy and suggested that loss of function might promote tumorigenesis. Genetic deletion of MTGR1 and MTG16 in the mouse has revealed unexpected and unique roles within the intestinal epithelium. Mtgr1(-/-) mice have progressive depletion of all intestinal secretory cells, and Mtg16(-/-) mice have a decrease in goblet cells. Furthermore, both Mtgr1(-/-) and Mtg16(-/-) mice have increased intestinal epithelial cell proliferation. We thus hypothesized that loss of MTGR1 or MTG16 would modify Apc(1638/+)-dependent intestinal tumorigenesis. Mtgr1(-/-) mice, but not Mtg16(-/-) mice, had a 10-fold increase in tumor multiplicity. This was associated with more advanced dysplasia, including progression to **invasive** adenocarcinoma, and augmented intratumoral proliferation. Analysis of chromatin immunoprecipitation sequencing data sets for MTGR1 and MTG16 targets indicated that MTGR1 can regulate Wnt and Notch signaling. In support of this, immunohistochemistry and gene expression analysis revealed that both Wnt and Notch signaling pathways were hyperactive in Mtgr1(-/-) tumors. Furthermore, in human colorectal cancer (CRC) samples MTGR1 was downregulated at both the transcript and protein level. Overall our data indicates that MTGR1 has a context-dependent effect on intestinal tumorigenesis.
58. CKIα ablation highlights a critical role for p53 in **invasiv**eness control. The mature gut renews continuously and rapidly throughout adult life, often in a damage-inflicting micro-environment. The major driving force for self-renewal of the intestinal epithelium is the Wnt-mediated signalling pathway, and Wnt signalling is frequently hyperactivated in colorectal cancer. Here we show that casein kinase Iα (CKIα), a component of the β-catenin-destruction complex, is a critical regulator of the Wnt signalling pathway. Inducing the ablation of Csnk1a1 (the gene encoding CKIα) in the gut triggers massive Wnt activation, surprisingly without causing tumorigenesis. CKIα-deficient epithelium shows many of the features of human colorectal tumours in addition to Wnt activation, in particular the induction of the DNA damage response and cellular senescence, both of which are thought to provide a barrier against malignant transformation. The epithelial DNA damage response in mice is accompanied by substantial activation of p53, suggesting that the p53 pathway may counteract the pro-tumorigenic effects of Wnt hyperactivation. Notably, the transition from benign adenom**as to in**vasive colorectal cancer in humans is typically linked to p53 inactivation, underscoring the importance of p53 as a safeguard against malignant progression; however, the mechanism of p53-mediated tumour suppression is unknown. We show that the maintenance of intestinal homeostasis in CKIα-deficient gut requires p53-mediated growth control, because the combined ablation of Csnk1a1 and either p53 or its target gene p21 (also known as Waf1, Cip1, Sdi1 and Cdkn1a) triggered high-grade dysplasia with extensive proliferation. Unexpectedly, these ablations also induced non-proliferating cells to invade the villous lamina propria rapidly, pro**ducing i**nvasive carcinomas throughout the small bowel. Furthermore, in p53-deficient gut, loss of heterozygosity of the gene encoding CKIα caused a **highly** invasive carcinoma, indicating that CKIα functions as a tumour suppressor when p53 is inactivated. We identified a set of genes (the p53-su**ppressed** invasiveness signature, PSIS) that is activated by the loss of both p53 and CKIα and which probably accounts for the brisk ind**uction o**f invasiveness. PSIS transcription and tumour invasion were suppressed by p21, independently of cell cycle control. Restraining tissue invasion through suppressing PSIS expression is thus a novel tumour-suppressor function of wild-type p53.
59. Mice expressing activated PI3K rapidly develop advanced colon cancer. Aberrations in the phosphoinositide 3-kinase (PI3K) signaling pathway play a key role in the pathogenesis of numerous cancers by altering cellular growth, metabolism, proliferation, and apoptosis. Mutations in the catalytic domain of PI3K that generate a dominantly active kinase are commonly found in human colorectal cancers and have been thought to drive tumor progression but not initiation. However, the effects of constitutively activated PI3K upon the intestinal mucosa have not been previously studied in animal models. Here, we show that the expression of a dominantly active form of the PI3K protein in the mouse intestine results in hyperplasia and advanced neoplasia. Mice expressing constitutively active PI3K in the epithelial cells of the distal small bowel and colon rapidly developed **invasive** adenocarcinomas in the colon that spread into the mesentery and adjacent organs. The histologic characteristics of these tumors were strikingly similar to **invasive** mucinous colon cancers in humans. Interestingly, these tumors formed without a benign polypoid intermediary, consistent with the lack of aberrant WNT signaling observed. Together, our findings indicate a noncanonical mechanism of colon tumor initiation that is mediated through activation of PI3K. This unique model has the potential to further our understanding of human disease and facilitate the development of therapeutics through pharmacologic screening and biomarker identification.
60. Colon Tumors with the Simultaneous Induction of Driver Mutations in APC, KRAS, and PIK3CA Still Progress through the Adenoma-to-carcinoma Sequence. Human colorectal cancers often possess multiple mutations, including three to six driver mutations per tumor. The timing of when these mutations occur during tumor development and progression continues to be debated. More advanced lesions carry a greater number of driver mutations, indicating that colon tumors might progress from adenomas to carcinomas through the stepwise accumulation of mutations following tumor initiation. However, mutations that have been implicated in tumor progression have been identified in normal-appearing epithelial cells of the colon, leaving the possibility that these mutations might be present before the initiation of tumorigenesis. We utilized mouse models of colon cancer to investigate whether tumorigenesis still occurs through the adenoma-to-carcinoma sequence when multiple mutations are present at the time of tumor initiation. To create a model in which tumors could concomitantly possess mutations in Apc, Kras, and Pik3ca, we developed a novel minimally **invasive** technique to administer an adenovirus expressing Cre recombinase to a focal region of the colon. Here, we demonstrate that the presence of these additional driver mutations at the time of tumor initiation results in increased tumor multiplicity and an increased rate of progression to **invasive** adenocarcinomas. These cancers can even metastasize to retroperitoneal lymph nodes or the liver. However, despite having as many as three concomitant driver mutations at the time of initiation, these tumors still proceed through the adenoma-to-carcinoma sequence.
61. Defining the role of APC in the mitotic spindle checkpoint in vivo: APC-deficient cells are resistant to Taxol. Mutations in the adenomatous polyposis coli (APC) tumour suppressor are the key initiating event of colorectal cancer. Although the control of WNT signalling is well established as a central tumour-suppressive function, the significance of APC in regulating chromosome instability is less well established. In this study, we test whether APC-deficient cells have a functional spindle assembly checkpoint (SAC) in vivo by examining the response of these cells to Taxol and Vinorelbine. We also show for the first time that APC deficiency compromises the arrest response to Taxol in vivo. This effect is independent of the role that APC has in WNT signalling. At higher levels of Taxol, APC-deficient cells arrest as efficiently as wild-type cells. Importantly, this dose of Taxol strongly suppresses intestinal tumourigenesis in models of benign (APC(Min/+) mouse) and **invasive** (AhCreER(+)APC(fl/+)PTEN(fl/fl)) cancer. In contrast to intestinal enterocytes with a general SAC defect because of Bub1 (budding uninhibited by benzimidazole 1) deletion, APC-deficient enterocytes arrest equivalently to wild type when treated with Vinorelbine. This suggests that the failed arrest in response to Taxol is because of a specific defect in microtubule stabilization following Taxol treatment rather than a general role of the APC protein in the mitotic spindle checkpoint. In summary, this study clarifies the role of APC as a mitotic spindle checkpoint protein in vivo and shows that APC-deficient cells have a compromised response to Taxol.
62. Cyclin D1 expression in the intestinal mucosa and tumors of Apc1638N mice. Altered expression of cyclin D1 contributes to the development of several types of cancer, including colorectal cancer. This study examined cyclin D1 expression in 32 intestinal tumors in different stages of tumorigenesis in Apc1638N mice, a mouse model for human familial adenomatous polyposis (FAP). Three morphological patterns of expression of cyclin D1 in intestinal epithelial cells were found: nuclear, punctate-cytoplasmic and fine granular cytoplasmic. The nuclear pattern of cyclin D1 was detected in all of the tumors, including adenomas (n = 18) and adenocarcinomas (n = 14); this pattern was found predominantly in the tubular region of the tumors and in flat mucosa adjacent to a subset of the tumors (67% of adenomas and 57% of carcinomas). The punctate-cytoplasmic pattern of cyclin D1 expression was found in all adenocarcinomas and a majority of adenomas (80%), mainly in **invasive** and villous areas of the tumors; it was not found in normal flat adjacent mucosa suggesting that this pattern and altered cytoplasmic/nuclear expression were associated with tumor progression. Fine cytoplasmic granules were located in normal duodenum in the basal portion of the crypts and in colon in epithelial cells at the surface of the colonic crypts; in both duodenum and colon the number of cells with fine cytoplasmic granules significantly increased after feeding a Western-style diet. These altered patterns of expression of cyclin D1 may provide useful biomarkers of abnormal cell development for studies of tumorigenesis and the effects of chemopreventive agents.
63. Animal models of human prostate cancer: the consensus report of the New York meeting of the Mouse Models of Human Cancers Consortium Prostate Pathology Committee. Animal models, particularly mouse models, play a central role in the study of the etiology, prevention, and treatment of human prostate cancer. While tissue culture models are extremely useful in understanding the biology of prostate cancer, they cannot recapitulate the complex cellular interactions within the tumor microenvironment that play a key role in cancer initiation and progression. The National Cancer Institute (NCI) Mouse Models of Human Cancers Consortium convened a group of human and veterinary pathologists to review the current animal models of prostate cancer and make recommendations about the pathologic analysis of these models. More than 40 different models with 439 samples were reviewed, including genetically engineered mouse models, xenograft, rat, and canine models. Numerous relevant models have been developed over the past 15 years, and each approach has strengths and weaknesses. Analysis of multiple genetically engineered models has shown that reactive stroma formation is present in all the models developing **invasive** carcinomas. In addition, numerous models with multiple genetic alterations display aggressive phenotypes characterized by sarcomatoid carcinomas and metastases, which is presumably a histologic manifestation of epithelial-mesenchymal transition. The significant progress in development of improved models of prostate cancer has already accelerated our understanding of the complex biology of prostate cancer and promises to enhance development of new approaches to prevention, detection, and treatment of this common malignancy.
64. Expression of CD44 in Apc and Tcf mutant mice implies regulation by the WNT pathway. Overexpression of cell surface glycoproteins of the CD44 family is an early event in the colorectal adenoma-carcinoma sequence. This suggests a link with disruption of APC tumor suppressor protein-mediated regulation of beta-catenin/Tcf-4 signaling, which is crucial in initiating tumorigenesis. To explore this hypothesis, we analyzed CD44 expression in the intestinal mucosa of mice and humans with genetic defects in either APC or Tcf-4, leading to constitutive activation or blockade of the beta-catenin/Tcf-4 pathway, respectively. We show that CD44 expression in the non-neoplastic intestinal mucosa of Apc mutant mice is confined to the crypt epithelium but that CD44 is strongly overexpressed in adenomas as well as in **invasive** carcinomas. This overexpression includes the standard part of the CD44 (CD44s) as well as variant exons (CD44v). Interestingly, deregulated CD44 expression is already present in aberrant crypt foci with dysplasia (ACFs), the earliest detectable lesions of colorectal neoplasia. Like ACFs of Apc-mutant mice, ACFs of familial adenomatous polyposis (FAP) patients also overexpress CD44. In sharp contrast, Tcf-4 mutant mice show a complete absence of CD44 in the epithelium of the small intestine. This loss of CD44 concurs with loss of stem cell characteristics, shared with adenoma cells. Our results indicate that CD44 expression is part of a genetic program controlled by the beta-catenin/Tcf-4 signaling pathway and suggest a role for CD44 in the generation and turnover of epithelial cells.
65. An EphB-Abl signaling pathway is associated with intestinal tumor initiation and growth. EphB receptors regulate the proliferation and positioning of intestinal stem and progenitor cells. In addition, they can act as tumor promoters for adenoma development but suppress progression to **invasive** carcinoma. We used imatinib to abrogate Abl kinase activity in Apc(Min/+) mice and in mice with LGR5(+) stem cells that were genetically engineered to develop adenomatous polyposis coli. Imatinib treatment inhibited the tumor-promoting effects of EphB signaling without attenuating EphB-mediated tumor suppression, demonstrating a role for EphB signaling in the initiation of intestinal tumors. The imatinib treatment regimen extended the life span of Apc(Min/+) mice and reduced cell proliferation in cultured slices of adenomas from patients with familial adenomatous polyposis. These findings connect the EphB signaling pathway to the regulation of intestinal adenoma initiation via Abl kinase. Our findings may have clinical implications for pharmacological therapy against adenoma formation and cancer progression in patients predisposed to develop colorectal cancer.
66. PTEN loss and KRAS activation leads to the formation of serrated adenomas and metastatic carcinoma in the mouse intestine. Mutation or loss of the genes PTEN and KRAS have been implicated in human colorectal cancer (CRC), and have been shown to co-occur despite both playing a role in the PI3' kinase (PI3'K) pathway. We investigated the role of these genes in intestinal tumour progression in vivo, using genetically engineered mouse models, with the aim of generating more representative models of human CRC. Intestinal-specific deletion of Pten and activation of an oncogenic allele of Kras was induced in wild-type (WT) mice and mice with a predisposition to adenoma development (Apc(fl/+) ). The animals were euthanized when they became symptomatic of a high tumour burden. Histopathological examination of the tissues was carried out, and immunohistochemistry used to characterize signalling pathway activation. Mutation of Pten and Kras resulted in a significant life-span reduction of mice predisposed to adenomas. **Invasive** adenocarcinoma was observed in these animals, with evidence of activation of the PI3'K pathway but no metastasis. However, mutation of Pten and Kras in WT animals not predisposed to adenomas led to perturbed homeostasis of the intestinal epithelium and the development of hyperplastic polyps, dysplastic sessile serrated adenomas and metastasizing adenocarcinomas with serrated features. These studies demonstrate synergism between Pten and Kras mutations in intestinal tumour progression, in an autochthonous and immunocompetent murine model, with potential application to preclinical drug testing. In particular, they show that Pten and Kras mutations alone predispose mice to the spectrum of serrated lesions that reflect the serrated pathway of CRC progression in humans.
67. IFN-{beta} signaling positively regulates tumorigenesis in aggressive fibromatosis, potentially by modulating mesenchymal progenitors. Aggressive fibromatosis (also called desmoid tumor) is a benign, locally **invasive**, soft tissue tumor composed of cells with mesenchymal characteristics. These tumors are characterized by increased levels of beta-catenin-mediated T-cell factor (TCF)-dependent transcriptional activation. We found that type 1 IFN signaling is activated in human and murine aggressive fibromatosis tumors and that the expression of associated response genes is regulated by beta-catenin. When mice deficient for the type 1 IFN receptor (Ifnar1-/-) were crossed with mice predisposed to developing aggressive fibromatosis tumors (Apc/Apc1638N), a significant decrease in aggressive fibromatosis tumor formation was observed compared with littermate controls, showing a novel role for type 1 IFN signaling in promoting tumor formation. Type 1 IFN activation inhibits cell proliferation but does not alter cell apoptosis or the level of beta-catenin-mediated TCF-dependent transcriptional activation in aggressive fibromatosis cell cultures. Thus, these changes cannot explain our in vivo results. Intriguingly, Ifnar1-/- mice have smaller numbers of mesenchymal progenitor cells compared with littermate controls, and treatment of aggressive fibromatosis cell cultures with IFN increases the proportion of cells that exclude Hoechst dye and sort to the side population, raising the possibility that type 1 IFN signaling regulates the number of precursor cells present that drive aggressive fibromatosis tumor formation and maintenance. This study identified a novel role for IFN type 1 signaling as a positive regulator of neoplasia and suggests that IFN treatment is a less than optimal therapy for this tumor type.
68. SMAD4-deficient intestinal tumors recruit CCR1+ myeloid cells that promote invasion. Inactivation of TGF-beta family signaling is implicated in colorectal tumor progression. Using cis-Apc(+/Delta716) Smad4(+/-) mutant mice (referred to as cis-Apc/Smad4), a model of **invasive** colorectal cancer in which TGF-beta family signaling is blocked, we show here that a new type of immature myeloid cell (iMC) is recruited from the bone marrow to the tumor invasion front. These CD34(+) iMCs express the matrix metalloproteinases MMP9 and MMP2 and the CC-chemokine receptor 1 (CCR1) and migrate toward the CCR1 ligand CCL9. In adenocarcinomas, expression of CCL9 is increased in the tumor epithelium. By deleting Ccr1 in the background of the cis-Apc/Smad4 mutant, we further show that lack of CCR1 prevents accumulation of CD34(+) iMCs at the invasion front and suppresses tumor invasion. These results indicate that loss of transforming growth factor-beta family signaling in tumor epithelium causes accumulation of iMCs that promote tumor invasion.
69. Administration of an unconjugated bile acid increases duodenal tumors in a murine model of familial adenomatous polyposis. Intestinal carcinogenesis involves the successive accumulation of multiple genetic defects until cellular transformation to an **invasive** phenotype occurs. This process is modulated by many epigenetic factors. Unconjugated bile acids are tumor promoters whose presence in intestinal tissues is regulated by dietary factors. We studied the role of the unconjugated bile acid, chenodeoxycholate, in an animal model of familial adenomatous polyposis. Mice susceptible to intestinal tumors as a result of a germline mutation in Apc (Min/+ mice) were given a 10 week dietary treatment with 0.5% chenodeoxycholate. Following this, the mice were examined to determine tumor number, enterocyte proliferation, apoptosis and beta-catenin expression. Intestinal tissue prostaglandin E2 (PGE2) levels were also assessed. Administration of chenodeoxycholate in the diet increased duodenal tumor number in Min/+ mice. Promotion of duodenal tumor formation was accompanied by increased beta-catenin expression in duodenal cells, as well as increased PGE2 in duodenal tissue. These data suggest that unconjugated bile acids contribute to periampullary tumor formation in the setting of an Apc mutation.
70. Plasminogen activator inhibitor-1 (PAI-1) modifies the formation of aggressive fibromatosis (desmoid tumor). Aggressive fibromatosis is a mesenchymal neoplasm associated with mutations, resulting in beta-catenin-mediated transcriptional activation. We found that plasminogen activator inhibitor-1 (PAI-1) was upregulated fourfold in aggressive fibromatosis. We investigated the ability of beta-catenin to regulate a PAI-1 reporter, and found that PAI-1 is an indirect target. To determine the role of PAI-1 in vivo, a mouse containing a targeted deletion in Pai-1 was crossed with a mouse that develops aggressive fibromatosis and gastrointestinal tumors (Apc/Apc1638N mouse). Pai-1 deficiency reduced the number of aggressive fibromatosis tumors formed, but not the number of gastrointestinal tumors. Deficiency of Pai-1 reduced tumor cell proliferation and motility rate. Although PAI-1 can alter cell motility by competing for a common binding site on vitronectin, blocking this site did not alter the motility rate. The number of cells moving through matrigel (invasion rate) did not change with Pai-1 deficiency, but because of the low motility rate the invasion index (invasion rate/motility) was increased in Pai-1-deficient cells. This suggests a proteolytic effect for PAI-1 regulating cell **invasive**ness. Our study found that, although PAI-1 has cellular effects that could inhibit or enhance tumor growth, on balance, it acts as a tumor enhancer in aggressive fibromatosis.
71. Gastric and duodenal polyps in Smad4 (Dpc4) knockout mice. The SMAD4 (DPC4) gene was initially isolated as a candidate tumor suppressor from the convergent site of homozygous deletions on 18q in a panel of pancreatic carcinoma cell lines. It encodes a common cytoplasmic signaling molecule shared by the transforming growth factor-beta, activin, and bone morphogenic pathways. We recently inactivated its mouse homologue Smad4 and demonstrated its role in the malignant progression of benign adenomas to **invasive** adenocarcinomas by analyzing mice with Apc and Smad4 compound mutations. Although simple Smad4 homozygotes were embryonically lethal, the heterozygotes were fertile and appeared normal up to the age of 1 year. Upon further investigation, however, they have developed inflammatory polyps in the glandular stomach and duodenum. By PCR genotyping and immunohistochemical staining, the wild-type Smad4 allele has been lost in the polyp epithelial cells, ie., loss of heterozygosity. On the other hand, we have not found any mutations in such genes as K-Ras, H-Ras, N-Ras, p53, or PTEN. Histologically, the polyps are similar to human juvenile polyps showing moderate stromal cell proliferation and infiltrations by eosinophils and plasma cells. In addition, foci of adenocarcinoma with signet ring cells are also found. These results are consistent with a recent report that germ-line SMAD4 mutations are found in a subset of familial juvenile polyposis.
72. Colon-specific tumorigenesis in mice driven by Cre-mediated inactivation of Apc and activation of mutant Kras. Several genetically engineered mouse (GEM) models of colorectal cancer have been developed and are a mainstay in our efforts to identify means of preventing and treating this disease. Many of these models involve a germline disruption of the adenomatous polyposis coli (Apc) tumor suppressor gene and share the limitation that the great preponderance of tumors appear in the small rather than large intestine. In recent years efforts have been made to increase the similarity of these models to human sporadic colorectal cancer by disrupting Apc in a tissue-specific fashion using the Cre-Lox system so that the genetic aberrations are confined to the colonic epithelium. These models have shown great promise but reproducible and high penetrance colon-specific tumorigenesis has not yet been achieved without **invasive** techniques to introduce the Cre enzyme. We therefore sought to create a new model with high penetrance colon-specific tumorigenesis but without the need for exogenous Cre administration. We utilized existing mice possessing a conditional knock out for the Apc gene and a latent activated Kras allele and crossed them with mice expressing Cre recombinase solely in the large intestine. Using this approach we generated mice that developed 1-9 colonic adenomas per mouse (average 4.3) but without any tumors in the small intestine or cecum. No **invasive** tumors were observed. Despite the apparent lack of invasion, the geographical correctness, complete penetrance and intermediate tumor burden make this model a promising addition to our toolkit for the study of colorectal cancer treatment and prevention.
73. Suppressive effect of global DNA hypomethylation on gastric carcinogenesis. Global DNA hypomethylation and concomitant site-specific gene hypermethylation are among the most common molecular alterations in human neoplasia. Although site-specific DNA hypermethylation has been shown to be associated with the development of various tumors accompanied by transcriptional silencing of target genes, the functional significance of global DNA hypomethylation in tumorigenesis remains unclear. Previous studies have revealed that a genetic reduction of the DNA methylation levels leads to opposing effects on tumor development, depending on the tumor cell type and the stage of tumorigenesis. In the present study, we investigated the effect of DNA hypomethylation on gastric carcinogenesis in mice. The genetic reduction of DNA methylation levels suppressed the incidence, number and size of gastric tumors in two different mouse models for gastric tumorigenesis: the N-methyl-N-nitrosourea-induced model and the Apc(Min/+) mouse model that spontaneously develops gastric tumors with aging. Histological analyses revealed DNA hypomethylation to completely inhibit the development of **invasive** gastric tumors. These findings indicate that the reduction of DNA methylation levels suppresses gastric carcinogenesis and suggest that DNA methylation is closely associated with gastric tumorigenesis.
74. Cyclooxygenase 2- and prostaglandin E(2) receptor EP(2)-dependent angiogenesis in Apc(Delta716) mouse intestinal polyps. To investigate angiogenesis during intestinal polyp development, we determined the microvessel density (MVD) in polyps of Apc knockout (Apc(Delta716)) mice, a model for human familial adenomatous polyposis. We scored MVD also in several compound mutants carrying Apc(Delta716), namely, mice with an additional mutation in Smad4, in which the polyps progress into **invasive** adenocarcinomas; mice with a cyclooxygenase (COX)-2 gene (Ptgs2) mutation, in which adenoma growth is suppressed; and mice with prostaglandin E(2) EP receptor gene mutations. In both simple Apc(Delta716) and compound Apc(Delta716) Smad4 mutants, MVD increased in a polyp size-dependent manner only in the polyps expanded beyond a threshold of about 1 mm in diameter. These results indicate that tumor angiogenesis is stimulated only after tumors grow to a certain size, and this angiogenic switch is common to both benign adenomas and malignant adenocarcinomas. In Apc(Delta716) polyposis attenuated by the COX-2 gene mutation, in contrast, MVD did not increase even in polyps larger than 1 mm. The same phenomenon was observed in the compound mutant mice with Apc(Delta716) and the EP(2) receptor gene mutations, but not in other EP compound mutants. We also immunohistochemically studied COX-2 and angiogenic factors, vascular endothelial growth factor and basic fibroblast growth factor. Interestingly, expression of these proteins was also increased in polyps larger than 1 mm. These results suggest that, in both benign and malignant mouse intestinal tumors, stromal expression of COX-2 results in elevated prostaglandin E(2) levels that stimulate cell surface receptor EP(2), followed by induction of vascular endothelial growth factor that causes tumor angiogenesis.
75. Deficiency of caveolin-1 in Apc(min/+) mice promotes colorectal tumorigenesis. Caveolin-1 (Cav1), a scaffold protein of membrane caveolae and coactivator of peroxisome proliferator-activated receptor gamma (PPARg), inhibits oncogenic signaling through Ras and wingless. However, the in vivo role of Cav1 in colorectal cancer (CRC) remained unknown. To test whether loss of Cav1 accelerates tumorigenesis, we generated a novel mouse model of CRC by crossing C57BL/6 Apc(min/+) with B6129 Cav1 knockout (Cav1-/-) mice. Apc(min/+) Cav1-/- mice developed large, micro**invasive** and vascularized intraepithelial adenocarcinomas in the distal colon and rectum with higher incidence than Apc(min/+) Cav1+/- and Apc(min/+) Cav1+/+ littermates. Intratumoral gene signatures related to Ras and wingless signaling were elevated, nuclear localization of PPARg protein and expression of PPARg-target genes were reduced independently of Cav1. The PPARg-agonist rosiglitazone prevented tumor formation in mice irrespectively of the Cav1 status and upregulated expression of the Ras-inhibitory protein docking protein-1. Thus, codeficiency of Cav1 and adenomatous polyposis coli facilitated formation of CRC, and activation of PPARg may offer novel strategies for treatment of CRC.
76. Differential expression of matrilysin and cyclooxygenase-2 in intestinal and colorectal neoplasms. Both the matrix metalloproteinase matrilysin and the prostaglandin H synthase cyclooxygenase-2 (Cox-2), are thought to play key roles in colorectal carcinogenesis. These enzymes are overexpressed in 85-90% of human colorectal cancers. Furthermore, mice carrying an adenomatous polyposis coli germline mutation that are also nullizygous for either matrilysin or Cox-2 display a significant reduction in tumor multiplicity. To determine if there is a direct link between matrilysin and Cox-2, their expression was characterized in two mouse models of intestinal carcinogenesis and in human colorectal tumor samples. Both matrilysin and Cox-2 expression was increased in the mouse models and in the human colorectal cancers; however, immunohistochemistry and in situ hybridization indicated that their localization within the tumors was different. In the mouse models, Cox-2 was expressed in the superficial stroma, whereas matrilysin expression was localized exclusively to the neoplastic epithelium. In contrast, in human colorectal cancers, both Cox-2 and matrilysin were expressed in the neoplastic epithelium. Although over 80% of the specimens expressed both matrilysin and Cox-2, the levels and localization of matrilysin and Cox-2 expression were distinct. Cox-2 expression was strongest in well-differentiated areas, and matrilysin immunostaining was strongest in the more dysplastic and **invasive** regions of the tumor. These results indicate that these two important modulators of colorectal tumorigenesis are differentially expressed and imply that the therapeutic benefit may be improved by combination therapy utilizing selective Cox-2 and matrilysin inhibitors.
77. Interleukin-11 is the dominant IL-6 family cytokine during gastrointestinal tumorigenesis and can be targeted therapeutically. Among the cytokines linked to inflammation-associated cancer, interleukin (IL)-6 drives many of the cancer "hallmarks" through downstream activation of the gp130/STAT3 signaling pathway. However, we show that the related cytokine IL-11 has a stronger correlation with elevated STAT3 activation in human gastrointestinal cancers. Using genetic mouse models, we reveal that IL-11 has a more prominent role compared to IL-6 during the progression of sporadic and inflammation-associated colon and gastric cancers. Accordingly, in these models and in human tumor cell line xenograft models, pharmacologic inhibition of IL-11 signaling alleviated STAT3 activation, suppressed tumor cell proliferation, and reduced the **invasive** capacity and growth of tumors. Our results identify IL-11 signaling as a potential therapeutic target for the treatment of gastrointestinal cancers.
78. Loss of liver E-cadherin induces sclerosing cholangitis and promotes carcinogenesis. E-cadherin is an important adhesion molecule whose loss is associated with progression and poor prognosis of liver cancer. However, it is unclear whether the loss of E-cadherin is a real culprit or a bystander in liver cancer progression. In addition, the precise role of E-cadherin in maintaining liver homeostasis is also still unknown, especially in vivo. Here we demonstrate that liver-specific E-cadherin knockout mice develop spontaneous periportal inflammation via an impaired intrahepatic biliary network, as well as periductal fibrosis, which resembles primary sclerosing cholangitis. Inducible gene knockout studies identified E-cadherin loss in biliary epithelial cells as a causal factor of cholangitis induction. Furthermore, a few of the E-cadherin knockout mice developed spontaneous liver cancer. When knockout of E-cadherin is combined with Ras activation or chemical carcinogen administration, E-cadherin knockout mice display markedly accelerated carcinogenesis and an **invasive** phenotype associated with epithelial-mesenchymal transition, up-regulation of stem cell markers, and elevated ERK activation. Also in human hepatocellular carcinoma, E-cadherin loss correlates with increased expression of mesenchymal and stem cell markers, and silencing of E-cadherin in hepatocellular carcinoma cell lines causes epithelial-mesenchymal transition and increased **invasive**ness, suggesting that E-cadherin loss can be a causal factor of these phenotypes. Thus, E-cadherin plays critical roles in maintaining homeostasis and suppressing carcinogenesis in the liver.

#### GeneRIFs

1. Apc suppression produces adenomas in both the small intestine and colon that, in the presence of Kras and p53 mutations, can progress to **invasive** carcinoma.
2. Expression of a dominant active PI3K synergizes with loss of APC activity resulting in a dramatic change in tumor multiplicity, size, morphology and **invasive**ness.
3. Data show that that the Apc(Min/+) mouse model for familial intestinal cancer can develop frequent **invasive** cancers in the absence of overt genomic instability.

### [Mm]etastasize

#### PubMed

1. Inactivation of conditional Rb by Villin-Cre leads to aggressive tumors outside the gastrointestinal tract. We have crossed mice carrying the conditional Rb(tm2Brn) allele with a constitutive Villin-Cre transgenic mouse. The Villin promoter in these animals is highly expressed in adult intestine and kidney proximal tubules and is expressed in the gut and nephros anlagen during embryogenesis. We report here that these mice develop tumors between 12 and 17 months old outside the gastrointestinal (GI) tract. A high penetrance of pituitary tumors and medullar carcinoma of the thyroid is observed with a lower incidence of hyperplasia of pulmonary neuroendocrine cells and aggressive liver, bile duct, stomach, oral cavity tumors, and lipomas. Rb rearrangement due to ectopic Villin promoter activity in neural crest or neural crest stem cells during embryogenesis is most likely responsible for the medullar carcinoma of the thyroid phenotype. The aggressive nature of the medullar carcinoma of the thyroid and its ability to **metastasize** to unusual sites make the model suitable for the study of tumor progression and mechanism of metastasis. Observed sites of metastasis include the stomach, small intestine, liver, lung, kidney, pancreas, spleen, bone marrow, salivary gland, fat, lymph nodes, and dorsal root ganglion. Because the Villin promoter is highly active throughout the GI and in the nephros anlagen during development, we find that Rb inactivation is not sufficient to initiate tumorigenesis in the GI or kidneys in mice.
2. Long-lived Min mice develop advanced intestinal cancers through a genetically conservative pathway. C57BL/6J mice carrying the Min allele of Adenomatous polyposis coli (Apc) develop numerous adenomas along the entire length of the intestine and consequently die at an early age. This short lifespan would prevent the accumulation of somatic genetic mutations or epigenetic alterations necessary for tumor progression. To overcome this limitation, we generated F(1) Apc(Min/+) hybrids by crossing C57BR/cdcJ and SWR/J females to C57BL/6J Apc(Min/+) males. These hybrids developed few intestinal tumors and often lived longer than 1 year. Many of the tumors (24-87%) were invasive adenocarcinomas, in which neoplastic tissue penetrated through the muscle wall into the mesentery. In a few cases (3%), lesions **metastasize**d by extension to regional lymph nodes. The development of these familial cancers does not require chromosomal gains or losses, a high level of microsatellite instability, or the presence of Helicobacter. To test whether genetic instability might accelerate tumor progression, we generated Apc(Min/+) mice homozygous for the hypomorphic allele of the Nijmegen breakage syndrome gene (Nbs1(DeltaB)) and also treated Apc(Min/+) mice with a strong somatic mutagen. These imposed genetic instabilities did not reduce the time required for cancers to form nor increase the percentage of cancers nor drive progression to the point of distant metastasis. In summary, we have found that the Apc(Min/+) mouse model for familial intestinal cancer can develop frequent invasive cancers in the absence of overt genomic instability. Possible factors that promote invasion include age-dependent epigenetic changes, conservative somatic recombination, or direct effects of alleles in the F(1) hybrid genetic background.
3. Smad3 mutant mice develop metastatic colorectal cancer. TGFbeta-related growth factors have been implicated in a variety of developmental and physiological processes in organisms ranging from nematodes to mammals. TGFbeta transduces its signal to the interior of the cell via Smad2, Smad3, and Smad4. We report the cloning and targeted disruption of the mouse Smad3 gene. Smad3 mutant mice are viable and fertile. Between 4 and 6 months of age, the Smad3 mutant mice become moribund with colorectal adenocarcinomas. The neoplasms penetrate through the intestinal wall and **metastasize** to lymph nodes. These results directly implicate TGFbeta signaling in the pathogenesis of colorectal cancer and provide a compelling animal model for the study of human colorectal cancer.
4. TGF-beta receptor inactivation and mutant Kras induce intestinal neoplasms in mice via a beta-catenin-independent pathway. During colorectal cancer pathogenesis, mutations and epigenetic events cause neoplastic behavior in epithelial cells by deregulating the Wnt, Ras-Raf-extracellular signal-regulated kinase (ERK), and transforming growth factor (TGF)-beta-signaling pathways, among others. The TGF-beta-signaling pathway is often inactivated in colon cancer cells by mutations in the gene encoding the TGF-beta receptor TGFBR2. The RAS-RAF-ERK pathway is frequently up-regulated in colon cancer via mutational activation of KRAS or BRAF. We assessed how these pathways interact in vivo and affect formation of colorectal tumors.We analyzed intestinal tumors that arose in mice that express an oncogenic (active) form of Kras and that have Tgfbr2 inactivations-2 common molecular events observed in human colorectal tumors. LSL-KrasG12D mice were crossed with Villin-Cre;Tgfbr2E2flx/E2flx mice, which do not express Tgfbr2 in the intestinal epithelium.Neither inactivation of Tgfbr2 nor expression of oncogenic Kras alone was sufficient to induce formation of intestinal neoplasms. Histologic abnormalities arose in mice that expressed Kras, but only the combination of Tgfbr2 inactivation and Kras activation led to intestinal neoplasms and metastases. The cancers arose via a beta-catenin-independent mechanism; the epidermal growth factor-signaling pathway was also activated. Cells in the resulting tumors proliferated at higher rates, expressed decreased levels of p15, and expressed increased levels of cyclin D1 and cdk4, compared with control cells.A combination of inactivation of the TGF-beta-signaling pathway and expression of oncogenic Kras leads to formation of invasive intestinal neoplasms through a beta-catenin-independent pathway; these adenocarcinomas have the capacity to **metastasize**.
5. Colon Tumors with the Simultaneous Induction of Driver Mutations in APC, KRAS, and PIK3CA Still Progress through the Adenoma-to-carcinoma Sequence. Human colorectal cancers often possess multiple mutations, including three to six driver mutations per tumor. The timing of when these mutations occur during tumor development and progression continues to be debated. More advanced lesions carry a greater number of driver mutations, indicating that colon tumors might progress from adenomas to carcinomas through the stepwise accumulation of mutations following tumor initiation. However, mutations that have been implicated in tumor progression have been identified in normal-appearing epithelial cells of the colon, leaving the possibility that these mutations might be present before the initiation of tumorigenesis. We utilized mouse models of colon cancer to investigate whether tumorigenesis still occurs through the adenoma-to-carcinoma sequence when multiple mutations are present at the time of tumor initiation. To create a model in which tumors could concomitantly possess mutations in Apc, Kras, and Pik3ca, we developed a novel minimally invasive technique to administer an adenovirus expressing Cre recombinase to a focal region of the colon. Here, we demonstrate that the presence of these additional driver mutations at the time of tumor initiation results in increased tumor multiplicity and an increased rate of progression to invasive adenocarcinomas. These cancers can even **metastasize** to retroperitoneal lymph nodes or the liver. However, despite having as many as three concomitant driver mutations at the time of initiation, these tumors still proceed through the adenoma-to-carcinoma sequence.
6. Lymph node-independent liver metastasis in a model of metastatic colorectal cancer. Deciphering metastatic routes is critically important as metastasis is a primary cause of cancer mortality. In colorectal cancer (CRC), it is unknown whether liver metastases derive from cancer cells that first colonize intestinal lymph nodes, or whether such metastases can form without prior lymph node involvement. A lack of relevant metastatic CRC models has precluded investigations into metastatic routes. Here we describe a metastatic CRC mouse model and show that liver metastases can manifest without a lymph node metastatic intermediary. Colorectal tumours transplanted onto the colonic mucosa invade and **metastasize** to specific target organs including the intestinal lymph nodes, liver and lungs. Importantly, this metastatic pattern differs from that observed following caecum implantation, which invariably involves peritoneal carcinomatosis. Anti-angiogenesis inhibits liver metastasis, yet anti-lymphangiogenesis does not impact liver metastasis despite abrogating lymph node metastasis. Our data demonstrate direct hematogenous spread as a dissemination route that contributes to CRC liver malignancy.

### [Mm]etastasis

#### PubMed

1. Cancer stemness in Wnt-driven mammary tumorigenesis. Wnt signaling plays a central role in mammary stem cell (MaSC) homeostasis and in breast cancer. In particular, epigenetic alterations at different members of the Wnt pathway have been identified among triple-negative, basal-like breast cancers. Previously, we developed a mouse model for metaplastic breast adenocarcinoma, a subtype of triple-negative breast cancer, by targeting a hypomorphic mutations in the endogenous Apc gene (Apc (1572T/+)). Here, by employing the CD24 and CD29 cell surface antigens, we have identified a subpopulation of mammary cancer stem cells (MaCSCs) from Apc (1572T/+) capable of self-renewal and differentiation both in vivo and in vitro. Moreover, immunohistochemical analysis of micro- and macrolung metastases and preliminary intravenous transplantation assays suggest that the MaCSCs underlie **metastasis** at distant organ sites. Expression profiling of the normal and tumor cell subpopulations encompassing MaSCs and CSCs revealed that the normal stem cell compartment is more similar to tumor cells than to their own differentiated progenies. Accordingly, Wnt signaling appears to be active in both the normal and cancer stem cell compartments, although at different levels. By comparing normal with cancer mouse mammary compartments, we identified a MaCSC gene signature able to predict outcome in breast cancer in man. Overall, our data indicate that constitutive Wnt signaling activation affects self-renewal and differentiation of MaSCs leading to metaplasia and basal-like adenocarcinomas.
2. The tumor suppressor CDX2 opposes pro-metastatic biomechanical modifications of colon cancer cells through organization of the actin cytoskeleton. The vast majority of cancer deaths are caused by the formation of metastases rather than the primary tumor itself. Despite this clinical importance, the molecular and cellular events that support the dissemination of cancer cells are not yet fully unraveled. We have previously shown that CDX2, a homeotic transcription factor essential for gut development, acts as a colon-specific tumor suppressor and opposes **metastasis**. Here, using a combination of biochemical, biophysical, and immunofluorescence techniques, we further investigated the mechanisms promoted by CDX2 that might antagonize tumor cell dissemination. We found that CDX2 expression regulates the transcription of RHO GEFs, thereby activating RHO signaling cascades that lead to reorganization of the actin cytoskeleton and enhanced adherent junctions. Accordingly, we observed by atomic force microscopy (AFM) that colon cancer cells expressing CDX2 are less deformable, a feature that has been shown to correlate with poor metastatic potential. Thus, this study illustrates how the loss of expression of a transcription factor during colon cancer progression modifies the biomechanical characteristics of tumor cells and hence facilitates invasion and **metastasis**.
3. Fusion between Intestinal epithelial cells and macrophages in a cancer context results in nuclear reprogramming. The most deadly phase in cancer progression is attributed to the inappropriate acquisition of molecular machinery leading to metastatic transformation and spread of disease to distant organs. Although it is appreciated that **metastasis** involves epithelial-mesenchymal interplay, the underlying mechanism defining this process is poorly understood. Specifically, how cancer cells evade immune surveillance and gain the ability to navigate the circulatory system remains a focus. One possible mechanism underlying metastatic conversion is fusion between blood-derived immune cells and cancer cells. While this notion is a century old, in vivo evidence that cell fusion occurs within tumors and imparts genetic or physiologic changes remains controversial. We have previously demonstrated in vivo cell fusion between blood cells and intestinal epithelial cells in an injury setting. Here, we hypothesize that immune cells, such as macrophages, fuse with tumor cells imparting metastatic capabilities by transferring their cellular identity. We used parabiosis to introduce fluorescent-labeled bone marrow-derived cells to mice with intestinal tumors, finding that fusion between circulating blood-derived cells and tumor epithelium occurs during the natural course of tumorigenesis. Moreover, we identify the macrophage as a key cellular partner for this process. Interestingly, cell fusion hybrids retain a transcriptome identity characteristic of both parental derivatives, while also expressing a unique subset of transcripts. Our data supports the novel possibility that tumorigenic cell fusion may impart physical behavior attributed to migratory macrophages, including navigation of circulation and immune evasion. As such, cell fusion may represent a promising novel mechanism underlying the metastatic conversion of cancer cells.
4. Protein kinase C and cancer: what we know and what we do not. Since their discovery in the late 1970s, protein kinase C (PKC) isozymes represent one of the most extensively studied signaling kinases. PKCs signal through multiple pathways and control the expression of genes relevant for cell cycle progression, tumorigenesis and metastatic dissemination. Despite the vast amount of information concerning the mechanisms that control PKC activation and function in cellular models, the relevance of individual PKC isozymes in the progression of human cancer is still a matter of controversy. Although the expression of PKC isozymes is altered in multiple cancer types, the causal relationship between such changes and the initiation and progression of the disease remains poorly defined. Animal models developed in the last years helped to better understand the involvement of individual PKCs in various cancer types and in the context of specific oncogenic alterations. Unraveling the enormous complexity in the mechanisms by which PKC isozymes have an impact on tumorigenesis and **metastasis** is key for reassessing their potential as pharmacological targets for cancer treatment.
5. Role of oncogenic K-Ras in cancer stem cell activation by aberrant Wnt/β-catenin signaling. Adenomatous polyposis coli (APC) loss-of-function mutations and K-Ras gain-of-function mutations are common abnormalities that occur during the initiation and intermediate adenoma stages of colorectal tumorigenesis, respectively. However, little is known about the role these mutations play in cancer stem cells (CSCs) associated with colorectal cancer (CRC) tumorigenesis.We analyzed tissue from CRC patients (n = 49) to determine whether K-Ras mutations contributed to CSC activation during colorectal tumorigenesis. DLD-1-K-Ras-WT and DLD-1-K-Ras-MT cells were cultured and evaluated for their ability to differentiate, form spheroids in vitro, and form tumors in vivo. Interaction between APC and K-Ras mutations in colorectal tumorigenesis was evaluated using APC (Min/+)/K-Ras (LA2) mice and DLD-1-K-Ras-WT and DLD-1-K-Ras-MT cell xenografts. (n = 4) Group differences were determined by Student t test. All statistical tests were two-sided.The sphere-forming capability of DLD-1-K-Ras-MT cells was statistically significantly higher than that of DLD-1-K-Ras-WT cells (DLD-1-K-Ras-MT mean = 86.661 pixel, 95% confidence interval [CI] = 81.701 to 91.621 pixel; DLD-1-K-Ras-WT mean = 42.367 pixel, 95% CI = 36.467 to 48.267 pixel; P = .003). Moreover, both the size and weight of tumors from DLD-1-K-Ras-MT xenografts were markedly increased compared with tumors from DLD-1-K-Ras-WT cells. Expression of the CSC markers CD44, CD133, and CD166 was induced in intestinal tumors from APC (Min/+)/K-Ras (LA2)mice, but not K-Ras (LA2) mice, indicating that APC mutation is required for CSC activation by oncogenic K-Ras mutation.K-Ras mutation activates CSCs, contributing to colorectal tumorigenesis and **metastasi**s in CRC cells harboring APC mutations. Initial activation of β-catenin by APC loss and further enhancement through K-Ras mutation induces CD44, CD133, and CD166 expression.
6. Inactivation of conditional Rb by Villin-Cre leads to aggressive tumors outside the gastrointestinal tract. We have crossed mice carrying the conditional Rb(tm2Brn) allele with a constitutive Villin-Cre transgenic mouse. The Villin promoter in these animals is highly expressed in adult intestine and kidney proximal tubules and is expressed in the gut and nephros anlagen during embryogenesis. We report here that these mice develop tumors between 12 and 17 months old outside the gastrointestinal (GI) tract. A high penetrance of pituitary tumors and medullar carcinoma of the thyroid is observed with a lower incidence of hyperplasia of pulmonary neuroendocrine cells and aggressive liver, bile duct, stomach, oral cavity tumors, and lipomas. Rb rearrangement due to ectopic Villin promoter activity in neural crest or neural crest stem cells during embryogenesis is most likely responsible for the medullar carcinoma of the thyroid phenotype. The aggressive nature of the medullar carcinoma of the thyroid and its ability to metastasize to unusual sites make the model suitable for the study of tumor progression and mechanism of **metastasis**. Observed sites of **metastasis** include the stomach, small intestine, liver, lung, kidney, pancreas, spleen, bone marrow, salivary gland, fat, lymph nodes, and dorsal root ganglion. Because the Villin promoter is highly active throughout the GI and in the nephros anlagen during development, we find that Rb inactivation is not sufficient to initiate tumorigenesis in the GI or kidneys in mice.
7. CXCR2-expressing myeloid-derived suppressor cells are essential to promote colitis-associated tumorigenesis. A large body of evidence indicates that chronic inflammation is one of several key risk factors for cancer initiation, progression, and **metastasis**. However, the underlying mechanisms responsible for the contribution of inflammation and inflammatory mediators to cancer remain elusive. Here, we present genetic evidence that loss of CXCR2 dramatically suppresses chronic colonic inflammation and colitis-associated tumorigenesis through inhibiting infiltration of myeloid-derived suppressor cells (MDSCs) into colonic mucosa and tumors in a mouse model of colitis-associated cancer. CXCR2 ligands were elevated in inflamed colonic mucosa and tumors and induced MDSC chemotaxis. Adoptive transfer of wild-type MDSCs into Cxcr2(-/-) mice restored AOM/DSS-induced tumor progression. MDSCs accelerated tumor growth by inhibiting CD8(+) T cell cytotoxic activity.
8. Long-lived Min mice develop advanced intestinal cancers through a genetically conservative pathway. C57BL/6J mice carrying the Min allele of Adenomatous polyposis coli (Apc) develop numerous adenomas along the entire length of the intestine and consequently die at an early age. This short lifespan would prevent the accumulation of somatic genetic mutations or epigenetic alterations necessary for tumor progression. To overcome this limitation, we generated F(1) Apc(Min/+) hybrids by crossing C57BR/cdcJ and SWR/J females to C57BL/6J Apc(Min/+) males. These hybrids developed few intestinal tumors and often lived longer than 1 year. Many of the tumors (24-87%) were invasive adenocarcinomas, in which neoplastic tissue penetrated through the muscle wall into the mesentery. In a few cases (3%), lesions metastasized by extension to regional lymph nodes. The development of these familial cancers does not require chromosomal gains or losses, a high level of microsatellite instability, or the presence of Helicobacter. To test whether genetic instability might accelerate tumor progression, we generated Apc(Min/+) mice homozygous for the hypomorphic allele of the Nijmegen breakage syndrome gene (Nbs1(DeltaB)) and also treated Apc(Min/+) mice with a strong somatic mutagen. These imposed genetic instabilities did not reduce the time required for cancers to form nor increase the percentage of cancers nor drive progression to the point of distant **metastasis**. In summary, we have found that the Apc(Min/+) mouse model for familial intestinal cancer can develop frequent invasive cancers in the absence of overt genomic instability. Possible factors that promote invasion include age-dependent epigenetic changes, conservative somatic recombination, or direct effects of alleles in the F(1) hybrid genetic background.
9. Dual inhibition of epidermal growth factor and insulin-like 1 growth factor receptors reduce intestinal adenoma burden in the Apc(min/+) mouse. Identification of early molecular pathway changes may be useful as biomarkers for tumour response/resistance prediction, and here we provide direct in vivo proof of this concept. The type 1 insulin-like growth factor receptor (IGF1R) has been implicated in various aspects of adenoma development and **metastasis**. We show here that, in murine intestinal adenomas acutely exposed to a small molecular inhibitor of EGFR (gefitinib), there is concurrent suppression of EGFR downstream signalling and induction of IGF signalling. We therefore tested the hypothesis that blockade of EGFR signalling was being tempered by compensatory activation of the IGF pathway by examining the effect of chronic suppression of IGF1R using AZ12253801, a small molecular tyrosine kinase inhibitor of IGF1R.Male Apc(min/+) mice with an intestinal tumour burden were exposed to a single dose of an inhibitor against EGFR (gefitinib), IGF1R (AZ12253801), 0.5% Tween 80 or combined EGFR/IGF1R inhibitor and culled 4 h post dosing. Tumour tissue was analysed to detect the early molecular pathways induced and anti-tumour phenotypic changes. Cohorts of male Apc(min/+) mice (n=15-17) were subsequently treated with gefitinib for a period of 8 weeks and subsequently exposed to single (either gefitinib or AZ12253801) or combined (gefitinib and AZ12253801) therapy. We also included a vehicle-treated cohort, which was never exposed to gefitinib and became symptomatic of the disease by day 150.Both single treatments delayed the onset of disease symptoms. Combined dosing with gefitinib and AZ12253801 similarly delayed the onset of symptoms, and at 200 days suppressed small intestinal tumourigenesis more effectively than either treatment alone (median small intestinal adenoma volume (47 mm(3) (comb) vs 248 mm(3) (AZ12253801), P=0.0003 and 47 mm(3) (comb) vs 123 mm(3) (gefitinib), P=0.0042, Mann-Whitney (two-sided) test).Our data provide evidence in support of the use of combinatorial therapy, and establishes the need to further define the precise benefit in vivo.
10. Development of a mouse model for sporadic and metastatic colon tumors and its use in assessing drug treatment. Most genetically engineered mouse (GEM) models for colon cancer are based on tissuewide or germline gene modification, resulting in tumors predominantly of the small intestine. Several of these models involve modification of the adenomatous polyposis coli (Apc) gene and are excellent models for familial cancer predisposition syndromes. We have developed a stochastic somatic mutation model for sporadic colon cancer that presents with isolated primary tumors in the distal colon and recapitulates the entire adenoma-carcinoma-**metastasis** axis seen in human colon cancer. Using this model, we have analyzed tumors that are either solely mutant in the Apc gene or in combination with another colon cancer-associated mutant gene, the Kras G12D allele. Because of the restricted location in the distal colon, the natural history of the tumors can be analyzed by serial colonoscopy. As the mammalian target of rapamycin (mTOR) pathway is a critical component of the complex signaling network in colon cancer, we used this model to assess the efficacy of mTOR blockade through rapamycin treatment of mice with established tumors. After treatment, Apc mutant tumors were more than 80% smaller than control tumors. However, tumors that possessed both Apc and Kras mutations did not respond to rapamycin treatment. These studies suggest that mTOR inhibitors should be further explored as potential colorectal cancer therapies in patients whose tumors do not have activating mutations in KRAS.
11. PHLPP is a negative regulator of RAF1, which reduces colorectal cancer cell motility and prevents tumor progression in mice. Hyperactivation of the RAS-RAF signaling pathway in colorectal tumors is associated with **metastasis** and poor outcomes of patients. Little is known about how RAS-RAF signaling is turned off once activated. We investigated how the pH domain and leucine-rich repeat protein phosphatases (PHLPPs) control RAS-RAF signaling and colorectal cancer (CRC) development.We used co-immunoprecipitation assays to identify substrates of PHLPP1 and PHLPP2. We studied phosphorylation of RAF1 in CRC cells that express exogenous PHLPP1 or PHLPP2, or lentiviral-based small hairpin RNAs against their transcripts; we measured effects on cell motility, migration, and invasion in vitro. Tumor progression and survival were analyzed in Phlpp1(-/-) Apc(Min) and Apc(Min)/Phlpp1(-/-) mice. Microarray datasets of colorectal tumor and nontumor tissues were analyzed for PHLPP gene expression.PHLPP1 and 2 were found to dephosphorylate RAF1 at S338, inhibiting its kinase activity in vitro and in CRC cells. In cells, knockdown of PHLPP1 or PHLPP2 increased the amplitude and duration of RAF-MEK-ERK signaling downstream of epidermal growth factor receptor and KRAS, whereas overexpression had the opposite effect. In addition, knockdown of PHLPP1 or PHLPP2 caused CRC cells to express markers of the epithelial-mesenchymal transition, and increased cell migration and invasion. Apc(Min)/Phlpp1(-/-) mice had decreased survival and developed larger intestinal and colon tumors compared to Apc(Min) mice. Whereas Apc(Min) mice developed mostly low-grade adenomas, 20% of the tumors that developed in Apc(Min)/Phlpp1(-/-) mice were invasive adenocarcinomas. Normal villi and adenomas of Apc(Min)/Phlpp1(-/-) mice had significantly fewer apoptotic cells than Apc(Min) mice. Human CRC patient microarray data revealed that the expression of PHLPP1 or PHLPP2 is positively correlated with CDH1.PHLPP1 and PHLPP2 dephosphorylate RAF1 to reduce its signaling, increase the invasive and migratory activities of CRC cells, and activate the epithelial-mesenchymal transition. In Apc(Min) mice, loss of PHLPP1 promotes tumor progression.
12. miR-23a promotes the transition from indolent to invasive colorectal cancer. Colorectal cancer is a classic example of a tumor that progresses through multiple distinct stages in its evolution. To understand the mechanisms regulating the transition from indolent to invasive disease, we profiled somatic copy number alterations in noninvasive adenomas and invasive adenocarcinomas from Apc and DNA mismatch repair (MMR) mutant mouse models. We identified a recurrent amplicon on mouse chromosome 8 that encodes microRNA (miRNA) 23a and -27a (miR). miR-23a and -27a levels are upregulated in mouse intestinal adenocarcinomas, primary tumors from patients with stage I/II colorectal cancers, as well as in human colorectal cancer cell lines and cancer stem cells. Functionally, miR-23a promotes the migration and invasion of colorectal cancer cells and stem cells, whereas miR-27a primarily promotes proliferation. We computationally and experimentally validated that **metastasis** suppressor 1 (MTSS1) is a direct miR-23a target and similarly validated that the ubiquitin ligase FBXW7 is a direct miR-27a target. Analyses of computationally predicted target genes in microarray data sets of patients with colorectal cancers are consistent with a role for miR-23a, but not miR-27a, specifically in invasive colorectal cancers.
13. Promotion of colorectal cancer invasion and **metastasis** through activation of NOTCH-DAB1-ABL-RHOGEF protein TRIO. We have recently identified a **metastasis** suppressor gene for colorectal cancer: AES/Aes, which encodes an endogenous inhibitor of NOTCH signaling. When Aes is knocked out in the adenomatous epithelium of intestinal polyposis mice, their tumors become malignant, showing marked submucosal invasion and intravasation. Here, we show that one of the genes induced by NOTCH signaling in colorectal cancer is DAB1/Dab1. Genetic depletion of DAB1 suppresses cancer invasion and **metastasis** in the NOTCH signaling-activated mice. DAB1 is phosphorylated by ABL tyrosine kinase, which activates ABL reciprocally. Consistently, inhibition of ABL suppresses cancer invasion in mice. Furthermore, we show that one of the targets of ABL is the RAC/RHOGEF protein TRIO, and that phosphorylation at its Tyr residue 2681 (pY2681) causes RHO activation in colorectal cancer cells. Its unphosphorylatable mutation TRIO Y2681F reduces RHOGEF activity and inhibits invasion of colorectal cancer cells. Importantly, TRIO pY2681 correlates with significantly poorer prognosis of patients with colorectal cancer after surgery.These results indicate that TRIO pY2681 is one of the downstream effectors of NOTCH signaling activation in colorectal cancer, and can be a prognostic marker, helping to determine the therapeutic modality of patients with colorectal cancer.
14. Cross-species regulatory network analysis identifies a synergistic interaction between FOXM1 and CENPF that drives prostate cancer malignancy. To identify regulatory drivers of prostate cancer malignancy, we have assembled genome-wide regulatory networks (interactomes) for human and mouse prostate cancer from expression profiles of human tumors and of genetically engineered mouse models, respectively. Cross-species computational analysis of these interactomes has identified FOXM1 and CENPF as synergistic master regulators of prostate cancer malignancy. Experimental validation shows that FOXM1 and CENPF function synergistically to promote tumor growth by coordinated regulation of target gene expression and activation of key signaling pathways associated with prostate cancer malignancy. Furthermore, co-expression of FOXM1 and CENPF is a robust prognostic indicator of poor survival and **metastasis**. Thus, genome-wide cross-species interrogation of regulatory networks represents a valuable strategy to identify causal mechanisms of human cancer.
15. Suppression of colon cancer **metastasis** by Aes through inhibition of Notch signaling. **Metastasis** is responsible for most cancer deaths. Here, we show that Aes (or Grg5) gene functions as an endogenous **metastasis** suppressor. Expression of Aes was decreased in liver metastases compared with primary colon tumors in both mice and humans. Aes inhibited Notch signaling by converting active Rbpj transcription complexes into repression complexes on insoluble nuclear matrix. In tumor cells, Notch signaling was triggered by ligands on adjoining blood vessels, and stimulated transendothelial migration. Genetic depletion of Aes in Apc(Δ716) intestinal polyposis mice caused marked tumor invasion and intravasation that were suppressed by Notch signaling inhibition. These results suggest that inhibition of Notch signaling can be a promising strategy for prevention and treatment of colon cancer **metastasi**s.
16. Deficiency of phospholipase A2 group 7 decreases intestinal polyposis and colon tumorigenesis in Apc(Min/+) mice. Platelet-activating factor (PAF) is a naturally occurring phospholipid that mediates diverse effects such as physiological and pathological inflammation, immunosuppression, and cancer. Several lines of evidence support both positive and negative roles for PAF in carcinogenesis. PAF stimulates cell growth, oncogenic transformation, and **metastasis**, but can also limit proliferation and induce apoptosis. The biological context and microenvironment seem to define whether PAF has pro- or anticarcinogenic effects. To investigate the role of exacerbated PAF signaling in colon cancer, we conducted cell-based and in vivo studies using genetically engineered mice lacking expression of phospholipase A2 group 7 (PLA2G7), an enzyme that specifically metabolizes PAF and structurally related glycerophospholipids. Absence of Pla2g7 robustly decreased intestinal polyposis and colon tumor formation in Apc(Min)(/+) mice, suggesting an antitumorigenic role for PAF in settings characterized by aberrant function of the tumor suppressor Adenomatous polyposis coli (Apc). In colonic epithelial cells, exposure to a PAF analog led to dephosphorylation of Akt at serine-473 and induction of apoptosis. The mechanism of this response involved formation of a complex between β-arrestin 1 and the Akt phosphatase PHLPP2, and activation of the intrinsic pathway of apoptosis. Our results suggest that strategies based on inhibiting PLA2G7 activity or increasing PAF-mediated signaling hold promise for the treatment of intestinal malignancies that harbor mutations in APC.
17. Lineage-specific T-cell responses to cancer mucosa antigen oppose systemic metastases without mucosal inflammatory disease. Cancer mucosa antigens are emerging as a new category of self-antigens expressed normally in immunologically privileged mucosal compartments and universally by their derivative tumors. These antigens leverage the established immunologic partitioning of systemic and mucosal compartments, limiting tolerance opposing systemic antitumor efficacy. An unresolved issue surrounding self-antigens as immunotherapeutic targets is autoimmunity following systemic immunization. In the context of cancer mucosa antigens, immune effectors to self-antigens risk amplifying mucosal inflammatory disease promoting carcinogenesis. Here, we examined the relationship between immunotherapy for systemic colon cancer metastases targeting the intestinal cancer mucosa antigen guanylyl cyclase C (GCC) and its effect on inflammatory bowel disease and carcinogenesis in mice. Immunization with GCC-expressing viral vectors opposed nascent tumor growth in mouse models of pulmonary **metastasis**, reflecting systemic lineage-specific tolerance characterized by CD8(+), but not CD4(+), T-cell or antibody responses. Responses protecting against systemic metastases spared intestinal epithelium from autoimmunity, and systemic GCC immunity did not amplify chemically induced inflammatory bowel disease. Moreover, GCC immunization failed to promote intestinal carcinogenesis induced by germ-line mutations or chronic inflammation. The established role of CD8(+) T cells in antitumor efficacy, but CD4(+) T cells in autoimmunity, suggests that lineage-specific responses to GCC are particularly advantageous to protect against systemic metastases without mucosal inflammation. These observations support the utility of GCC-targeted immunotherapy in patients at risk for systemic metastases, including those with inflammatory bowel disease, hereditary colorectal cancer syndromes, and sporadic colorectal cancer.
18. Matrix metalloproteinases and genetic mouse models in cancer research: a mini-review. Carcinogenesis is a multistep and also a multifactorial process that involves agents like genetic and environmental factors. Matrix metalloproteinases (MMPs) are major proteolytic enzymes which are involved in cancer cell migration, invasion, and **metastasis**. Genetic variations in genes encoding the MMPs were shown in human studies to influence cancer risk and phenotypic features of a tumor. The complex role of MMPs seems to be important in the mechanism of carcinogenesis, but it is not well recognized. Rodent studies concentrated particularly on the better understanding of the biological functions of the MMPs and their impact on the pathological process, also through the modification of Mmp genes. This review presents current knowledge and the existing evidence on the importance of selected MMPs in genetic mouse models of cancer and human genetic association studies. Further, this work can be useful for scientists studying the role of the genetic impact of MMPs in carcinogenesis.
19. IL-33 activates tumor stroma to promote intestinal polyposis. Tumor epithelial cells develop within a microenvironment consisting of extracellular matrix, growth factors, and cytokines produced by nonepithelial stromal cells. In response to paracrine signals from tumor epithelia, stromal cells modify the microenvironment to promote tumor growth and **metastasis**. Here, we identify interleukin 33 (IL-33) as a regulator of tumor stromal cell activation and mediator of intestinal polyposis. In human colorectal cancer, IL-33 expression was induced in the tumor epithelium of adenomas and carcinomas, and expression of the IL-33 receptor, IL1RL1 (also referred to as IL1-R4 or ST2), localized predominantly to the stroma of adenoma and both the stroma and epithelium of carcinoma. Genetic and antibody abrogation of responsiveness to IL-33 in the Apc(Min/+) mouse model of intestinal tumorigenesis inhibited proliferation, induced apoptosis, and suppressed angiogenesis in adenomatous polyps, which reduced both tumor number and size. Similar to human adenomas, IL-33 expression localized to tumor epithelial cells and expression of IL1RL1 associated with two stromal cell types, subepithelial myofibroblasts and mast cells, in Apc(Min/+) polyps. In vitro, IL-33 stimulation of human subepithelial myofibroblasts induced the expression of extracellular matrix components and growth factors associated with intestinal tumor progression. IL-33 deficiency reduced mast cell accumulation in Apc(Min/+) polyps and suppressed the expression of mast cell-derived proteases and cytokines known to promote polyposis. Based on these findings, we propose that IL-33 derived from the tumor epithelium promotes polyposis through the coordinated activation of stromal cells and the formation of a protumorigenic microenvironment.
20. Gadd45a regulates matrix metalloproteinases by suppressing DeltaNp63alpha and beta-catenin via p38 MAP kinase and APC complex activation. The p53-regulated growth arrest and DNA damage-inducible gene product Gadd45a has been recently identified as a key factor protecting the epidermis against ultraviolet radiation (UVR)-induced skin tumors by activating p53 via the stress mitogen-activated protein kinase (MAPK) signaling pathway. Herein we identify Gadd45a as an important negative regulator of two oncogenes commonly over-expressed in epithelial tumors: the p53 homologue DeltaNp63alpha and beta-catenin. DeltaNp63alpha is one of the several p63 isoforms and is the predominant species expressed in basal epidermal keratinocytes. DeltaNp63alpha lacks the N-terminal transactivation domain and behaves as a dominant-negative factor blocking expression of several p53-effector genes. DeltaNp63alpha also associates with and blocks activation of the adenomatous polyposis coli (APC) destruction complex that targets free cytoplasmic beta-catenin for degradation. While most beta-catenin protein is localized to the cell membrane and is involved in cell-cell adhesion, accumulation of free cytoplasmic beta-catenin will translocate into the nucleus where it functions in a bipartite transcription factor complex, whose targets include invasion and **metastasis** promoting endopeptidases, matrix metalloproteinases (MMP). We show that Gadd45a not only directly associates with two components of the APC complex, namely protein phosphatase 2A (PP2A) and glycogen synthase kinase 3beta (GSK3beta) but also promotes GSK3beta dephosphorylation at Ser9, which is essential for GSK3beta activation, and resultant activation of the APC destruction complex. We demonstrate that lack of Gadd45a not only prevents DeltaNp63alpha suppression and GSK3beta dephosphorylation but also prevents free cytoplasmic beta-catenin degradation after UV irradiation. The inability of Gadd45a-null keratinocytes to suppress beta-catenin may contribute to the resulting observation of increased MMP expression and activity along with significantly faster keratinocyte migration in Matrigel in vitro and accelerated wound closure in vivo. Furthermore, epidermal keratinocytes treated with p38 MAPK inhibitors, both in vivo and in vitro, behave very similarly to Gadd45a-null keratinocytes after UVR. Similarly, Trp53-null mice are unable to attenuate DeltaNp63alpha expression in epidermal keratinocytes after such stress. These findings demonstrate a dependence on Gadd45a-mediated p38 MAPK and p53 activation for proper modulation of DeltaNp63alpha, GSK3beta, and beta-catenin after irradiation. Taken together, our results indicate that Gadd45a is able to repress DeltaNp63alpha, beta-catenin, and consequently MMP expression by two means: by maintaining UVR-induced p38 MAPK and p53 activation and also by associating with the APC complex. This implicates Gadd45a in the negative regulation of cell migration, and invasion.
21. Absence of the CD44 gene prevents sarcoma **metastasis**. To test the role of the CD44 gene in tumorigenesis, mice with the min mutation of the APC gene or with the tm1 mutation of the p53 gene were crossed with CD44 knockout mice. The absence of CD44 gene products did not affect tumor incidence or survival; however, mice with disruption of the CD44 gene showed virtually aborted **metastasis** formation of osteosarcomas. This is in agreement with the role attributed to CD44 variants in the spread of cancer. Therefore, CD44 gene products are not essential for tumor incidence and growth but are important in regulating **metastasis** formation.
22. Differing effects of endogenous and synthetic inhibitors of metalloproteinases on intestinal tumorigenesis. Matrix metalloproteinase (MMP) activity has been associated with tumor invasion and **metastasis** in many different tumor types, but recent studies also support a role for these enzymes in earlier stages of the tumor progression continuum. Specifically, the expression pattern of MMPs in benign human and mouse gastrointestinal tumors suggests that they may function in the development or growth of non-invasive tumors. To address the contribution of MMP activity to the development of intestinal adenomas, we administered the synthetic MMP inhibitor batimastat and expressed the tissue inhibitor of metalloproteinases-1 (TIMP-1) in the gastrointestinal tract of Min mice, which spontaneously develop pre-malignant small and large intestinal tumors. Batimastat administration resulted in a 48% decrease in the number of Min tumors. This reduction in tumor number is similar to that observed in mice lacking the metalloproteinase matrilysin, and demonstrates the therapeutic and chemopreventive potential of MMP inhibitors for pre-malignant intestinal tumors. In contrast, forced TIMP-1 expression in transgenic mice had no effect or, in one line, unexpectedly augmented Min tumor multiplicity by 32%. This observation supports an in vivo tumor-promoting activity of TIMP-1 that could be related to the growth stimulatory effects of TIMP that have been documented in vitro. Taken together, these 2 approaches of modulating MMP activity in Min mice support a critical function of MMPs in Min tumorigenesis, underscore the importance of an MMP/inhibitor balance in maintaining tissue homeostasis and demonstrate that endogenous MMP inhibitors can have complex effects in particular cellular contexts.
23. Review of colorectal cancer and its metastases in rodent models: comparative aspects with those in humans. Colorectal cancer (CRC) remains one of the most common cancer forms developing in industrialized countries, and its incidence appears to be rising. Studies of human population groups provide insufficient information about carcinogenesis, pathogenesis, and treatment of CRC. To study these phenomena in detail, a number of animal models of human CRC have been developed. The hypothetical ideal animal model should mimic the human disease in terms of morphology, biochemical alterations, and biological behavior. No existing model replicates the disease as an entity, but available models approximate many of the characteristics of human colonic carcinogenesis and **metastasis**. So far few comparative evaluations of the various animal models of CRC have been made.Animal studies cannot replace human clinical trials, but they can be used as a pre-screening tool, so that human trials become more directed, with greater chances of success. The orthotopic transplantation of colon cancer cells into the cecum of syngeneic animals or intraportal inoculation appears to resemble the human metastatic disease most closely, providing a model for study of the treatment of metastases. Which model(s) to choose depends on the goal(s) of the experiment(s). The review published here can provide help in selecting the most optimal CRC model(s) for a certain purpose and in preventing unnecessary duplication of animal experimentation.
24. MYC-nick promotes cell migration by inducing fascin expression and Cdc42 activation. MYC-nick is a cytoplasmic, transcriptionally inactive member of the MYC oncoprotein family, generated by a proteolytic cleavage of full-length MYC. MYC-nick promotes migration and survival of cells in response to chemotherapeutic agents or withdrawal of glucose. Here we report that MYC-nick is abundant in colonic and intestinal tumors derived from mouse models with mutations in the Wnt, TGF-β, and PI3K pathways. Moreover, MYC-nick is elevated in colon cancer cells deleted for FBWX7, which encodes the major E3 ligase of full-length MYC frequently mutated in colorectal cancers. MYC-nick promotes the migration of colon cancer cells assayed in 3D cultures or grown as xenografts in a zebrafish **metastasi**s model. MYC-nick accelerates migration by activating the Rho GTPase Cdc42 and inducing fascin expression. MYC-nick, fascin, and Cdc42 are frequently up-regulated in cells present at the invasive front of human colorectal tumors, suggesting a coordinated role for these proteins in tumor migration.
25. β-catenin/TCF4 complex induces the epithelial-to-mesenchymal transition (EMT)-activator ZEB1 to regulate tumor invasiveness. In most carcinomas, invasion of malignant cells into surrounding tissues involves their molecular reprogramming as part of an epithelial-to-mesenchymal transition (EMT). Mutation of the APC gene in most colorectal carcinomas (CRCs) contributes to the nuclear translocation of the oncoprotein β-catenin that upon binding to T-cell and lymphoid enhancer (TCF-LEF) factors triggers an EMT and a proinvasive gene expression profile. A key inducer of EMT is the ZEB1 transcription factor whose expression promotes tumorigenesis an**d metastas**is in carcinomas. As inhibitor of the epithelial phenotype, ZEB1 is never present in the epithelium of normal colon or the tumor center of CRCs where β-catenin remains membranous. We show here that ZEB1 is expressed by epithelial cells in intestinal tumors from human patients (familial adenomatous polyposis) and mouse models (APC(Min/+)) with germline mutations of APC that result in nuclear accumulation of β-catenin. However, ZEB1 is not expressed in the epithelium of hereditary forms of CRCs that carry wild-type APC and where β-catenin is excluded from the nucleus (Lynch syndrome). We found that β-catenin/TCF4 binds directly to the ZEB1 promoter and activates its transcription. Knockdown of β-catenin and TCF4 in APC-mutated CRC cells inhibited endogenous ZEB1, whereas forced translocation of β-catenin to the nucleus in APC-wild-type CRC cells induced de novo expression of ZEB1. Upregulation of MT1-MMP and LAMC2 by β-catenin/TCF4 has been linked to invasiveness in CRCs, and we show here that both proteins are activated by ZEB1 coexpressing with it in primary colorectal tumors with mutated APC. These results set ZEB1 as an effector of β-catenin/TCF4 signaling in EMT and tumor progression.
26. CCR6, the sole receptor for the chemokine CCL20, promotes spontaneous intestinal tumorigenesis. Interactions between the inflammatory chemokine CCL20 and its receptor CCR6 have been associated with colorectal cancer growth and **metastasis**, however, a causal role for CCL20 signaling through CCR6 in promoting intestinal carcinogenesis has not been demonstrated in vivo. In this study, we aimed to determine the role of CCL20-CCR6 interactions in spontaneous intestinal tumorigenesis. CCR6-deficient mice were crossed with mice heterozygous for a mutation in the adenomatous polyposis coli (APC) gene (APCMIN/+ mice) to generate APCMIN/+ mice with CCR6 knocked out (CCR6KO-APCMIN/+ mice). CCR6KO-APCMIN/+ mice had diminished spontaneous intestinal tumorigenesis. CCR6KO-APCMIN/+ also had normal sized spleens as compared to the enlarged spleens found in APCMIN/+ mice. Decreased macrophage infiltration into intestinal adenomas and non-tumor epithelium was observed in CCR6KO-APCMIN/+ as compared to APCMIN/+ mice. CCL20 signaling through CCR6 caused increased production of CCL20 by colorectal cancer cell lines. Furthermore, CCL20 had a direct mitogenic effect on colorectal cancer cells. Thus, interactions between CCL20 and CCR6 promote intestinal carcinogenesis. Our results suggest that the intestinal tumorigenesis driven by CCL20-CCR6 interactions may be driven by macrophage recruitment into the intestine as well as proliferation of neoplastic epithelial cells. This interaction could be targeted for the treatment or prevention of malignancy.
27. Modulation of NKG2D ligand expression and **metastasis** in tumors by spironolactone via RXRγ activation. Tumor **metastasi**s and lack of NKG2D ligand (NKG2DL) expression are associated with poor prognosis in patients with colon cancer. Here, we found that spironolactone (SPIR), an FDA-approved diuretic drug with a long-term safety profile, can up-regulate NKG2DL expression in multiple colon cancer cell lines by activating the ATM-Chk2-mediated checkpoint pathway, which in turn enhances tumor elimination by natural killer cells. SPIR can also up-regulate the expression of **metastasi**s-suppressor genes TIMP2 and TIMP3, thereby reducing tumor cell invasiveness. Although SPIR is an aldosterone antagonist, its antitumor effects are independent of the mineralocorticoid receptor pathway. By screening the human nuclear hormone receptor siRNA library, we identified retinoid X receptor γ (RXRγ) instead as being indispensable for the antitumor functions of SPIR. Collectively, our results strongly support the use of SPIR or other RXRγ agonists with minimal side effects for colon cancer prevention and therapy.
28. HOXA5 Counteracts Stem Cell Traits by Inhibiting Wnt Signaling in Colorectal Cancer. Hierarchical organization of tissues relies on stem cells, which either self-renew or produce committed progenitors predestined for lineage differentiation. Here we identify HOXA5 as an important repressor of intestinal stem cell fate in vivo and identify a reciprocal feedback between HOXA5 and Wnt signaling. HOXA5 is suppressed by the Wnt pathway to maintain stemness and becomes active only outside the intestinal crypt where it inhibits Wnt signaling to enforce differentiation. In colon cancer, HOXA5 is downregulated, and its re-expression induces loss of the cancer stem cell phenotype, preventing tumor progression and **metastasis**. Tumor regression by HOXA5 induction can be triggered by retinoids, which represent tangible means to treat colon cancer by eliminating cancer stem cells.
29. The proprotein convertase PC5/6 is protective against intestinal tumorigenesis: in vivo mouse model. The secretory basic amino acid-specific proprotein convertases (PCs) have often been associated with cancer/**metastasis**. By controlling the cleavage of cancer-associated proteins, PCs play key roles in multiple steps of cancer development. Most analyses of the implication of PCs in cancer/**metastasis** relied on the use of in vitro overexpression systems or inhibitors that can affect more than one PC. Aside from the role of furin in salivary gland tumorigenesis, no other in vivo genetic model of PC-knockout was reported in relation to cancer development.Since PC5/6 is highly expressed in the small intestine, the present study examined its in vivo role in intestinal tumorigenesis. Analysis of human intestinal tumors at various stages showed a systematic down-regulation of PC5/6 expression. Since gene inactivation of PC5/6 leads to lethality at birth, we generated mice lacking PC5/6 in enterocytes and analyzed the impact of the presence or absence of this PC in the mouse ApcMin/+ model that develops numerous adenocarcinomas along the intestinal tract. This resulted in viable mice with almost no expression of PC5/6 in small intestine, but with no overt phenotype. The data showed that by themselves ApcMin/+ tumors express lower levels of PC5/6 mRNA, and that the lack of PC5/6 in enterocytes results in a significantly higher tumor number in the duodenum, with a similar trend in other intestinal segments. Finally, the absence of PC5/6 is also associated with a premature mortality of ApcMin/+ mice.Overall, these data suggest that intestinal PC5/6 is protective towards tumorigenesis, especially in mouse duodenum, and possibly in human colon.
30. Distinct biological roles for the akt family in mammary tumor progression. The phosphatidylinositol 3' kinase/Akt pathway is frequently dysregulated in cancer, which can have unfavorable consequences in terms of cell proliferation, survival, metabolism, and migration. Increasing evidence suggests that Akt1, Akt2, and Akt3 play unique roles in breast cancer initiation and progression. We have recently shown that in contrast to Akt1, which accelerates mammary tumor induction in transgenic mice, Akt2 promotes **metastasis** of tumor cells without affecting the latency of tumor development. Despite the distinct phenotypic outputs resulting from Akt1 or Akt2 activation, very little is known about the mode by which such unique functions originate from these highly related kinases. Here we discuss potential mechanisms contributing to the differing functional specificity of Akt1 and Akt2 with respect to migration, invasion, and **metastasis**.
31. Prostate cancer induced by loss of Apc is restrained by TGFβ signaling. Recent work with mouse models of prostate cancer (CaP) has shown that inactivation of TGFβ signaling in prostate epithelium can cooperate with deletion of the Pten tumor suppressor to drive locally aggressive cancer and metastatic disease. Here, we show that inactivating the TGFβ pathway by deleting the gene encoding the TGFβ type II receptor (Tgfbr2) in combination with a deletion of the Apc tumor suppressor gene specifically in mouse prostate epithelium, results in the rapid onset of invasive CaP. Micro-metastases were observed in the lymph nodes and lungs of a proportion of the double mutant mice, whereas no metastases were observed in Apc single mutant mice. Prostate-specific Apc;Tgfbr2 mutants had a lower frequency **of metast**asis and survived significantly longer than Pten;Tgfbr2 double mutants. However, all Apc;Tgfbr2 mutants developed invasive cancer by 30 weeks of age, whereas invasive cancer was rarely observed in Apc single mutant animals, even by one year of age. Further comparison of the Pten and Apc models of CaP revealed additional differences, including adenosquamous carcinoma in the Apc;Tgfbr2 mutants that was not seen in the Pten model, and a lack of robust induction of the TGFβ pathway in Apc null prostate. In addition to causing high-grade prostate intra-epithelial neoplasia (HGPIN), deletion of either Pten or Apc induced senescence in affected prostate ducts, and this restraint was overcome by loss of Tgfbr2. In summary, this work demonstrates that TGFβ signaling restrains the progression of CaP induced by different tumor suppressor mutations, suggesting that TGFβ signaling exerts a general tumor suppressive effect in prostate.
32. RHOA inactivation enhances Wnt signalling and promotes colorectal cancer. Activation of the small GTPase RHOA has strong oncogenic effects in many tumour types, although its role in colorectal cancer remains unclear. Here we show that RHOA inactivation contributes to colorectal cancer progression/**metastasis**, largely through the activation of Wnt/β-catenin signalling. RhoA inactivation in the murine intestine accelerates the tumorigenic process and in human colon cancer cells leads to the redistribution of β-catenin from the membrane to the nucleus and enhanced Wnt/β-catenin signalling, resulting in increased proliferation, invasion and de-differentiation. In mice, RHOA inactivation contributes to colon canc**er metasta**sis and reduced RHOA levels were observed at metastatic sites compared with primary human colon tumours. Therefore, we have identified a new mechanism of activation of Wnt/β-catenin signalling and characterized the role of RHOA as a novel tumour suppressor in colorectal cancer. These results constitute a shift from the current paradigm and demonstrate that RHO GTPases can suppress tumour progression **and metast**asis.
33. PTEN loss and KRAS activation leads to the formation of serrated adenomas and metastatic carcinoma in the mouse intestine. Mutation or loss of the genes PTEN and KRAS have been implicated in human colorectal cancer (CRC), and have been shown to co-occur despite both playing a role in the PI3' kinase (PI3'K) pathway. We investigated the role of these genes in intestinal tumour progression in vivo, using genetically engineered mouse models, with the aim of generating more representative models of human CRC. Intestinal-specific deletion of Pten and activation of an oncogenic allele of Kras was induced in wild-type (WT) mice and mice with a predisposition to adenoma development (Apc(fl/+) ). The animals were euthanized when they became symptomatic of a high tumour burden. Histopathological examination of the tissues was carried out, and immunohistochemistry used to characterize signalling pathway activation. Mutation of Pten and Kras resulted in a significant life-span reduction of mice predisposed to adenomas. Invasive adenocarcinoma was observed in these animals, with evidence of activation of the PI3'K pathway but no **metastasis**. However, mutation of Pten and Kras in WT animals not predisposed to adenomas led to perturbed homeostasis of the intestinal epithelium and the development of hyperplastic polyps, dysplastic sessile serrated adenomas and metastasizing adenocarcinomas with serrated features. These studies demonstrate synergism between Pten and Kras mutations in intestinal tumour progression, in an autochthonous and immunocompetent murine model, with potential application to preclinical drug testing. In particular, they show that Pten and Kras mutations alone predispose mice to the spectrum of serrated lesions that reflect the serrated pathway of CRC progression in humans.
34. Prostaglandin E2 regulates the nuclear receptor NR4A2 in colorectal cancer. Many lines of research implicate cyclooxygenase 2-derived prostaglandins in tumor growth and **metastasis**. More specifically, we have shown that prostaglandin E2 (PGE2) promotes cell proliferation and invasion through transactivation of the epidermal growth factor receptor, initiates immune evasion through induction of decay accelerating factor, and transactivates peroxisome proliferator-activated receptor delta, leading to increased polyp size and multiplicity. We continue to identify novel PGE2 target genes in colorectal carcinoma cells and report here that an immediate early gene, nuclear factor NR4A2 (Nurr1), is induced by PGE2 that in turn regulates cell death. Originally described as a critical dopaminergic neuron growth factor receptor, NR4A2 expression is rapidly but transiently induced by PGE2 in a cAMP/protein kinase A-dependent manner. NR4A2 binds to the cognate NBRE response element and enhances transcription of a reporter construct in colorectal carcinoma cells. Furthermore, NR4A2 expression is elevated in Apc-/+ mouse adenomas and its levels were further increased following PGE2 treatment. Human colorectal cancers relative to matched normal mucosa showed increased NR4A2 expression. Although not previously described in epithelial tissues, NR4A2 protein localizes to proliferating crypts of Apc-/+ mouse intestine. Finally, functional studies reveal that PGE2-mediated protection from apoptosis is completely inhibited by a dominant-negative NR4A2 construct. Building on previous reports from our group on the peroxisome proliferator-activated receptor family of nuclear receptors, these most recent data suggest that NR4A2, a member of another family of nuclear receptors can stimulate progression of colorectal cancer downstream from cyclooxygenase 2-derived PGE2.
35. Insights into cancer from transgenic mouse models. The generation of mice designed to overexpress activated forms of oncogenes or carrying targeted mutations in tumour suppressor genes, has allowed scientists to causally link the function of these genes with specific tumour processes, such as proliferation, apoptosis, angiogenesis or **metastasis**. In addition, these mice have been interbred to assess the extent of cooperativity between different genetic lesions in disease progression, leading to a greater understanding of the multi-stage nature of tumourigenesis. The effect of genetic mutations is often influenced by the genetic background of the mouse and by analysing strain-dependent phenotypes, modifier loci have been identified. Although genetic mutations in mouse and humans do not always lead to the same tumour spectrum, the underlying molecular mechanisms are frequently relevant to both species. Furthermore, new technical approaches creating conditional mouse mutants which develop tumours in a tissue-specific manner, will allow the effect of mutation of certain genes to be studied in specific tissues, free from the fatal effects of the mutation in other clinically less relevant tissues. Several exising mouse strains have already been used to develop and test new therapies and conditional mutagenesis will undoubtedly increase the potential use of transgenic mice in understanding and treating cancer.
36. A Sleeping Beauty transposon-mediated screen identifies murine susceptibility genes for adenomatous polyposis coli (Apc)-dependent intestinal tumorigenesis. It is proposed that a progressive series of mutations and epigenetic events leads to human colorectal cancer (CRC) and **metastasis**. Furthermore, data from resequencing of the coding regions of human CRC suggests that a relatively large number of mutations occur in individual human CRC, most at low frequency. The functional role of these low-frequency mutations in CRC, and specifically how they may cooperate with high-frequency mutations, is not well understood. One of the most common rate-limiting mutations in human CRC occurs in the adenomatous polyposis coli (APC) gene. To identify mutations that cooperate with mutant APC, we performed a forward genetic screen in mice carrying a mutant allele of Apc (Apc(Min)) using Sleeping Beauty (SB) transposon-mediated mutagenesis. Apc(Min) SB-mutagenized mice developed three times as many polyps as mice with the Apc(Min) allele alone. Analysis of transposon common insertion sites (CIS) identified the Apc locus as a major target of SB-induced mutagenesis, suggesting that SB insertions provide an efficient route to biallelic Apc inactivation. We also identified an additional 32 CIS genes/loci that may represent modifiers of the Apc(Min) phenotype. Five CIS genes tested for their role in proliferation caused a significant change in cell viability when message levels were reduced in human CRC cells. These findings demonstrate the utility of using transposon mutagenesis to identify low-frequency and cooperating cancer genes; this approach will aid in the development of combinatorial therapies targeting this deadly disease.
37. Inactivation of Apc in the mouse prostate causes prostate carcinoma. Alterations of the Wnt/beta-catenin signaling pathway are positively associated with the development and progression of human cancer, including carcinoma of the prostate. To determine the role of activated Wnt/beta-catenin signaling in mouse prostate carcinogenesis, we created a mouse prostate tumor model using probasin-Cre-mediated deletion of Apc. Prostate tumors induced by the deletion of Apc have elevated levels of beta-catenin protein and are highly proliferative. Tumor formation is fully penetrant and follows a consistent pattern of progression. Hyperplasia is observed as early as 4.5 weeks of age, and adenocarcinoma is observed by 7 months. Continued tumor growth usually necessitated sacrifice between 12 and 15 months of age. Despite the high proliferation rate, we have not observed **metastasis** of these tumors to the lymph nodes or other organs. Surgical castration of 6-week-old mice inhibited tumor formation, and castration of mice with more advanced tumors resulted in the partial regression of specific prostate glands. However, significant areas of carcinoma remained 2 months postcastration, suggesting that tumors induced by Apc loss of function are capable of growth under conditions of androgen depletion. We conclude that the prostate-specific deletion of Apc and the increased expression of beta-catenin associated with prostate carcinoma suggests a role for beta-catenin in prostate cancer and offers an appropriate animal model to investigate the interaction of Wnt signaling with other genetic and epigenetic signals in prostate carcinogenesis.
38. Oncogenic β-catenin triggers an inflammatory response that determines the aggressiveness of hepatocellular carcinoma in mice. Hepatocellular carcinoma (HCC) is the third leading cause of cancer-related death worldwide. Its pathogenesis is frequently linked to liver inflammation. Gain-of-function mutations in the gene encoding β-catenin are frequent genetic modifications found in human HCCs. Thus, we investigated whether inflammation was a component of β-catenin-induced tumorigenesis using genetically modified mouse models that recapitulated the stages of initiation and progression of this tumoral process. Oncogenic β-catenin signaling was found to induce an inflammatory program in hepatocytes that involved direct transcriptional control by β-catenin and activation of the NF-κB pathway. This led to a specific inflammatory response, the intensity of which determined the degree of tumor aggressiveness. The chemokine-like chemotactic factor leukocyte cell-derived chemotaxin 2 (LECT2) and invariant NKT (iNKT) cells were identified as key interconnected effectors of liver β-catenin-induced inflammation. In genetic deletion models lacking the gene encoding LECT2 or iNKT cells, hepatic β-catenin signaling triggered the formation of highly malignant HCCs wi**th lung me**tastasis. Thus, our results identify inflammation as a key player in β-catenin-induced liver tumorigenesis. We provide strong evidence that, by activating pro- and antiinflammatory mediators, β-catenin signaling produces an inflammatory microenvironment that has an impact on tumoral development. Our data are consistent with the fact that most β-catenin-activated HCCs are of better prognosis.
39. Roles of arrest-defective protein 1(225) and hypoxia-inducible factor 1alpha in tumor growth and **metastasis**. Vascular endothelial growth factor A (VEGFA), a critical mediator of tumor angiogenesis, is a well-characterized target of hypoxia-inducible factor 1 (HIF-1). Murine arrest-defective protein 1A (mARD1A(225)) acetylates HIF-1alpha, triggering its degradation, and thus may play a role in decreased expression of VEGFA.We generated Apc(Min/+)/mARD1A(225) transgenic mice and quantified growth of intestinal polyps. Human gastric MKN74 and murine melanoma B16F10 cells overexpressing mARD1A(225) were injected into mice, and tumor growth and **metastasis** were measured. VEGFA expression and microvessel density in tumors were assessed using immunohistochemistry. To evaluate the role of mARD1A(225) acetylation of Lys532 in HIF-1alpha, we injected B16F10-mARD1A(225) cell lines stably expressing mutant HIF-1alpha/K532R into mice and measured **metastasis**. All statistical tests were two-sided, and P values less than .05 were considered statistically significant.Apc(Min/+)/mARD1A(225) transgenic mice (n = 25) had statistically significantly fewer intestinal polyps than Apc(Min/+) mice (n = 21) (number of intestinal polyps per mouse: Apc(Min/+) mice vs Apc(Min/+)/mARD1A(225) transgenic mice, mean = 83.4 vs 38.0 polyps, difference = 45.4 polyps, 95% confidence interval [CI] = 41.8 to 48.6; P < .001). The growth and metastases of transplanted tumors were also statistically significantly reduced in mice injected with mARD1A(225)-overexpressing cells than in mice injected with control cells (P < .01). Moreover, overexpression of mARD1A(225) decreased VEGFA expression and microvessel density in tumor xenografts (P < .04) and Apc(Min/+) intestinal polyps (P = .001). Mutation of lysine 532 of HIF-1alpha in B16F10-mARD1A(225) cells prevented HIF-1alpha degradation and inhibited the antimetastatic effect of mARD1A(225) (P < .001).mARD1A(225) may be a novel upstream target that blocks VEGFA expression and tumor-related angiogenesis.
40. Mutated K-ras(Asp12) promotes tumourigenesis in Apc(Min) mice more in the large than the small intestines, with synergistic effects between K-ras and Wnt pathways. Summary K-ras mutations are found in 40-50% of human colorectal adenomas and carcinomas, but their functional contribution remains incompletely understood. Here, we show that a conditional mutant K-ras mouse model (K-ras(Asp12)/Cre), with transient intestinal Cre activation by beta-Naphthoflavone (beta-NF) treatment, displayed transgene recombination and K-ras(Asp12) expression in the murine intestines, but developed few intestinal adenomas over 2 years. However, when crossed with Apc(Min/+) mice, the K-ras(Asp12)/Cre/Apc(Min/+) offspring showed acceleration of intestinal tumourigenesis with significantly changed average lifespan (P < 0.05) decreased to 18.4 +/- 5.4 weeks from 20.9 +/- 4.7 weeks (control Apc(Min/+) mice). The numbers of adenomas in the small intestine and large intestine were significantly (P < 0.01) increased by 1.5-fold and 5.7-fold, respectively, in K-ras(Asp12)/Cre/Apc(Min/+) mice compared with Apc(Min/+) mice, with the more marked increase in adenoma prevalence in the large intestine. To explore possible mechanisms for K-ras(Asp12) and Apc(Min) co-operation, the Mitogen-activated protein kinase (Mapk), Akt and Wnt signalling pathways, including selected target gene expression levels, were evaluated in normal large intestine and large intestinal tumours. K-ras(Asp12) increased activation of Mapk and Akt signalling pathway targets phospho-extracellular signal-regulated kinase (pErk) and pAkt, and increased relative expression levels of Wnt pathway targets vascular endothelial growth factor (VEGF), gastrin, cyclo-oxygenase 2 (Cox2) and T-cell lymphoma invasion and **metastasis** 1 (Tiam1) in K-ras(Asp12)/Cre/Apc(Min/+) adenomas compared with that of Apc(Min/+) adenomas, although other Wnt signalling pathway target genes such as Peroxisome proliferator-activated receptor delta (PPARd), matrix metalloproteinase 7 (MMP7), protein phosphatase 1 alpha (PP1A) and c-myc remained unchanged. In conclusion, intestinal expression of K-ras(Asp12) promotes mutant Apc-initiated intestinal adenoma formation in vivo more in the large intestine than the small intestine, with evidence of synergistic co-operation between mutant K-ras and Apc involving increased expression of some Wnt-pathway target genes.
41. IMP1 promotes tumor growth, dissemination and a tumor-initiating cell phenotype in colorectal cancer cell xenografts. Igf2 mRNA binding protein 1 (IMP1, CRD-BP, ZBP-1) is a messenger RNA binding protein that we have shown previously to regulate colorectal cancer (CRC) cell growth in vitro. Furthermore, increased IMP1 expression correlates with enhanced **metastasis** and poor prognosis in CRC patients. In the current study, we sought to elucidate IMP1-mediated functions in CRC pathogenesis in vivo. Using CRC cell xenografts, we demonstrate that IMP1 overexpression promotes xenograft tumor growth and dissemination into the blood. Furthermore, intestine-specific knockdown of Imp1 dramatically reduces tumor number in the Apc (Min/+) mouse model of intestinal tumorigenesis. In addition, IMP1 knockdown xenografts exhibit a reduced number of tumor cells entering the circulation, suggesting that IMP1 may directly modulate this early metastatic event. We further demonstrate that IMP1 overexpression decreases E-cadherin expression, promotes survival of single tumor cell-derived colonospheres and promotes enrichment and maintenance of a population of CD24+CD44+ cells, signifying that IMP1 overexpressing cells display evidence of loss of epithelial identity and enhancement of a tumor-initiating cell phenotype. Taken together, these findings implicate IMP1 as a modulator of tumor growth and provide evidence for a novel role of IMP1 in early events in CRC **metastasis**.
42. Wnt5a promotes human colon cancer cell migration and invasion but does not augment intestinal tumorigenesis in Apc1638N mice. Whereas aberrant activation of canonical Wnt/β-catenin signaling underlies the majority of colorectal cancer cases, the contribution of non-canonical Wnt signaling is unclear. As enhanced expression of the most extensively studied non-canonical Wnt ligand WNT5A is observed in various diseases including colon cancer, WNT5A is gaining attention nowadays. Numerous in vitro studies suggest modulating capacities of WNT5A on proliferation, differentiation, migration and invasion, affecting tumor and non-mutant cells. However, a possible contribution of WNT5A to colorectal cancer remains to be elucidated. We have analyzed WNT5A expression in colorectal cancer profiling data sets, altered WNT5A expression in colon cancer cells and used our inducible Wnt5a transgenic mouse model to gain more insight into the role of WNT5A in intestinal cancer. We observed that increased WNT5A expression is associated with poor prognosis of colorectal cancer patients. WNT5A knockdown in human colon cancer cells caused reduced directional migration, deregulated focal adhesion site formation and reduced invasion, whereas Wnt5a administration promoted the directional migration of colon cancer cells. Despite these observed protumorigenic activities of WNT5A, the induction of Wnt5a expression in intestinal tumors of Apc1638N mice was not sufficient to augment malignancy or **metastasi**s by itself. In conclusion, WNT5A promotes adhesion sites to form in a focal fashion and promotes the directional migration and invasion of colon cancer cells. Although these activities appear insufficient by themselves to augment malignancy or **metastasi**s in Apc1638N mice, they might explain the poor colon cancer prognosis associated with enhanced WNT5A expression.
43. Lymph node-independent liver **metastasis** in a model of metastatic colorectal cancer. Deciphering metastatic routes is critically important as **metastasis** is a primary cause of cancer mortality. In colorectal cancer (CRC), it is unknown whether liver metastases derive from cancer cells that first colonize intestinal lymph nodes, or whether such metastases can form without prior lymph node involvement. A lack of relevant metastatic CRC models has precluded investigations into metastatic routes. Here we describe a metastatic CRC mouse model and show that liver metastases can manifest without a lymph node metastatic intermediary. Colorectal tumours transplanted onto the colonic mucosa invade and metastasize to specific target organs including the intestinal lymph nodes, liver and lungs. Importantly, this metastatic pattern differs from that observed following caecum implantation, which invariably involves peritoneal carcinomatosis. Anti-angiogenesis inhibits liver **metastasis**, yet anti-lymphangiogenesis does not impact liver **metastasis** despite abrogating lymph node **metastasis**. Our data demonstrate direct hematogenous spread as a dissemination route that contributes to CRC liver malignancy.
44. Intestinal tumorigenesis is suppressed in mice lacking the metalloproteinase matrilysin. Matrix metalloproteinases (MMPs) classically have been implicated in basement membrane destruction associated with late-stage tumor cell invasion and **metastasis**. However, recent studies have demonstrated that one MMP family member, matrilysin, is expressed in a high percentage of early-stage human colorectal tumors. We analyzed matrilysin expression in benign intestinal tumors from mice heterozygous for the ApcMin allele (Min/+) and found that the mRNA was induced in the majority (88%) of these adenomas. Protein was detected in the tumor cells, where, surprisingly, it was predominantly immunolocalized to the lumenal surface of dysplastic glands rather than the basement membrane or extracellular matrix. To address the role of matrilysin in Min intestinal tumorigenesis, we generated Min/+ mice deficient in this MMP by gene targeting and homologous recombination. The absence of matrilysin resulted in a reduction in mean tumor multiplicity in Min/+ animals of approximately 60% and a significant decrease in the average tumor diameter. Based on these findings, we conclude that matrilysin is a suppressor of the Min phenotype, possibly by functioning in a capacity independent of matrix degradation. These results argue for the use of MMP inhibitors in the treatment and prevention of early-stage colon cancer.
45. Type I to type II ovarian carcinoma progression: mutant Trp53 or Pik3ca confers a more aggressive tumor phenotype in a mouse model of ovarian cancer. A dualistic pathway model of ovarian carcinoma (OvCA) pathogenesis has been proposed: type I OvCAs are low grade, genetically stable, and relatively more indolent than type II OvCAs, most of which are high-grade serous carcinomas. Endometrioid OvCA (EOC) is a prototypical type I tumor, often harboring mutations that affect the Wnt and phosphatidylinositol 3-kinase/AKT/mammalian target of rapamycin signaling pathways. Molecular and histopathologic analyses indicate type I and II OvCAs share overlapping features, and a subset of EOCs may undergo type I→type II progression accompanied by acquisition of somatic TP53 or PIK3CA mutations. We used a murine model of EOC initiated by conditional inactivation of the Apc and Pten tumor suppressor genes to investigate mutant Trp53 or Pik3ca alleles as key drivers of type I→type II OvCA progression. In the mouse EOC model, the presence of somatic Trp53 or Pik3ca mutations resulted in shortened survival and more widespr**ead metast**asis. Activation of mutant Pik3ca alone had no demonstrable effect on the ovarian surface epithelium but resulted in papillary hyperplasia when coupled with Pten inactivation. Our findings indicate that the adverse prognosis associated with TP53 and PIK3CA mutations in human cancers can be functionally replicated in mouse models of type I→type II OvCA progression. Moreover, the models should represent a robust platform for assessment of the contributions of Trp53 or Pik3ca defects in the response of EOCs to conventional and targeted drugs.

### [Cc]ell [Ii]nvasion

#### PubMed

1. CXCL1 induced by prostaglandin E2 promotes angiogenesis in colorectal cancer. Chronic inflammation is a well-known risk factor for cancer. Proinflammatory mediators such as prostaglandin E2 (PGE2) promote colorectal tumor growth by stimulating angiogenesis, **cell invasion**, and cell growth, and inhibiting apoptosis. Molecules that regulate tumor-associated angiogenesis provide promising therapeutic targets for treatment of colorectal cancer (CRC) as indicated by the recent development of the novel anti-angiogenic agent bevacizumab (Avastin). However, use of this drug only prolongs survival by several months, highlighting the importance of finding more effective treatment regimens. We report here that PGE2 induces expression of CXCL1 (growth-regulated oncogene alpha), a pro-angiogenic chemokine, in human CRC cells. More importantly, CXCL1 released from carcinoma cells induces microvascular endothelial cell migration and tube formation in vitro. Furthermore, PGE2 promotes tumor growth in vivo by induction of CXCL1 expression, which results in increased tumor microvessel formation. These results have potential clinical significance because we found that CXCL1 expression correlates with PGE2 levels in human CRCs. Collectively, our findings show for the first time that CXCL1 is regulated by PGE2 and indicate that CXCL1 inhibitors should be evaluated further as potential anti-angiogenic agents for treatment of CRC.
2. Prickle1 mutation causes planar cell polarity and directional cell migration defects associated with cardiac outflow tract anomalies and other structural birth defects. Planar cell polarity (PCP) is controlled by a conserved pathway that regulates directional cell behavior. Here, we show that mutant mice harboring a newly described mutation termed Beetlejuice (Bj) in Prickle1 (Pk1), a PCP component, exhibit developmental phenotypes involving cell polarity defects, including skeletal, cochlear and congenital cardiac anomalies. Bj mutants die neonatally with cardiac outflow tract (OFT) malalignment. This is associated with OFT shortening due to loss of polarized cell orientation and failure of second heart field cell intercalation mediating OFT lengthening. OFT myocardialization was disrupted with cardiomyocytes failing to align with the direction of **cell invasion** into the outflow cushions. The expression of genes mediating Wnt signaling was altered. Also noted were shortened but widened bile ducts and disruption in canonical Wnt signaling. Using an in vitro wound closure assay, we showed Bj mutant fibroblasts cannot establish polarized cell morphology or engage in directional cell migration, and their actin cytoskeleton failed to align with the direction of wound closure. Unexpectedly, Pk1 mutants exhibited primary and motile cilia defects. Given Bj mutant phenotypes are reminiscent of ciliopathies, these findings suggest Pk1 may also regulate ciliogenesis. Together these findings show Pk1 plays an essential role in regulating cell polarity and directional cell migration during development.
3. Intestinal tumorigenesis is suppressed in mice lacking the metalloproteinase matrilysin. Matrix metalloproteinases (MMPs) classically have been implicated in basement membrane destruction associated with late-stage tumor **cell invasion** and metastasis. However, recent studies have demonstrated that one MMP family member, matrilysin, is expressed in a high percentage of early-stage human colorectal tumors. We analyzed matrilysin expression in benign intestinal tumors from mice heterozygous for the ApcMin allele (Min/+) and found that the mRNA was induced in the majority (88%) of these adenomas. Protein was detected in the tumor cells, where, surprisingly, it was predominantly immunolocalized to the lumenal surface of dysplastic glands rather than the basement membrane or extracellular matrix. To address the role of matrilysin in Min intestinal tumorigenesis, we generated Min/+ mice deficient in this MMP by gene targeting and homologous recombination. The absence of matrilysin resulted in a reduction in mean tumor multiplicity in Min/+ animals of approximately 60% and a significant decrease in the average tumor diameter. Based on these findings, we conclude that matrilysin is a suppressor of the Min phenotype, possibly by functioning in a capacity independent of matrix degradation. These results argue for the use of MMP inhibitors in the treatment and prevention of early-stage colon cancer.

---

## Apc2

- NCBI Gene
- GeneRIF
- Pubmed

| Search Term | Rank | Fields |
| --- | --- | --- |
| [Mm]igration | 6 | PubMed(4) |

  
hide/show details for Apc2

### [Mm]igration

#### PubMed

1. Directional neuronal **migration** is impaired in mice lacking adenomatous polyposis coli 2. Adenomatous polyposis coli 2 (APC2) is a family member of APC and mainly expressed in the nervous system. We previously reported that APC2 plays a critical role in axonal projection through the regulation of microtubule stability. Here, we show that a lack of Apc2 induces severe laminary defects in some regions of the mouse brain, including the cerebral cortex and cerebellum. In vivo BrdU labeling and immunohistochemical analyses with specific markers revealed that the laminary abnormalities are a result of dysregulated neuronal **migration** by a cell-autonomous mechanism. Using total internal reflection fluorescent microscopy, we found that APC2 is distributed along actin fibers as well as microtubules. Cerebellar granule cells in dissociated cultures and in vivo showed that BDNF-stimulated directional **migration** is impaired in Apc2-deficient neurons. We revealed that this impairment stems from the dysregulations of Rho family GTPase activation and TrkB localization, which disrupts the formation of BDNF-stimulated F-actin at the leading edge. Thus, APC2 is an essential mediator of the cytoskeletal regulation at leading edges in response to extracellular signals.
2. Directional neuronal **migration** is impaired in mice lacking adenomatous polyposis coli 2. Adenomatous polyposis coli 2 (APC2) is a family member of APC and mainly expressed in the nervous system. We previously reported that APC2 plays a critical role in axonal projection through the regulation of microtubule stability. Here, we show that a lack of Apc2 induces severe laminary defects in some regions of the mouse brain, including the cerebral cortex and cerebellum. In vivo BrdU labeling and immunohistochemical analyses with specific markers revealed that the laminary abnormalities are a result of dysregulated neuronal **migration** by a cell-autonomous mechanism. Using total internal reflection fluorescent microscopy, we found that APC2 is distributed along actin fibers as well as microtubules. Cerebellar granule cells in dissociated cultures and in vivo showed that BDNF-stimulated directional **migration** is impaired in Apc2-deficient neurons. We revealed that this impairment stems from the dysregulations of Rho family GTPase activation and TrkB localization, which disrupts the formation of BDNF-stimulated F-actin at the leading edge. Thus, APC2 is an essential mediator of the cytoskeletal regulation at leading edges in response to extracellular signals.
3. NeuroD1 reprograms chromatin and transcription factor landscapes to induce the neuronal program. Cell fate specification relies on the action of critical transcription factors that become available at distinct stages of embryonic development. One such factor is NeuroD1, which is essential for eliciting the neuronal development program and possesses the ability to reprogram other cell types into neurons. Given this capacity, it is important to understand its targets and the mechanism underlying neuronal specification. Here, we show that NeuroD1 directly binds regulatory elements of neuronal genes that are developmentally silenced by epigenetic mechanisms. This targeting is sufficient to initiate events that confer transcriptional competence, including reprogramming of transcription factor landscape, conversion of heterochromatin to euchromatin, and increased chromatin accessibility, indicating potential pioneer factor ability of NeuroD1. The transcriptional induction of neuronal fate genes is maintained via epigenetic memory despite a transient NeuroD1 induction during neurogenesis. NeuroD1 also induces genes involved in the epithelial-to-mesenchymal transition, thereby promoting neuronal **migration**. Our study not only reveals the NeuroD1-dependent gene regulatory program driving neurogenesis but also increases our understanding of how cell fate specification during development involves a concerted action of transcription factors and epigenetic mechanisms.
4. Loss-of-Function Mutation in APC2 Causes Sotos Syndrome Features. Sotos syndrome, characterized by intellectual disability and characteristic facial features, is caused by haploinsufficiency in the NSD1 gene. We conducted an etiological study on two siblings with Sotos features without mutations in NSD1 and detected a homozygous frameshift mutation in the APC2 gene by whole-exome sequencing, which resulted in the loss of function of cytoskeletal regulation in neurons. Apc2-deficient (Apc2(-/-)) mice exhibited impaired learning and memory abilities along with an abnormal head shape. Endogenous Apc2 expression was downregulated by the knockdown of Nsd1, indicating that APC2 is a downstream effector of NSD1 in neurons. Nsd1 knockdown in embryonic mouse brains impaired the **migration** and laminar positioning of cortical neurons, as observed in Apc2(-/-) mice, and this defect was rescued by the forced expression of Apc2. Thus, APC2 is a crucial target of NSD1, which provides an explanation for the intellectual disability associated with Sotos syndrome.

---

## Ninj1

- NCBI Gene
- GeneRIF
- Pubmed

| Search Term | Rank | Fields |
| --- | --- | --- |
| [Ff]ilopodia | 1 | PubMed(1) |
| [Mm]igration | 6 | PubMed(3) |

  
hide/show details for Ninj1

### [Ff]ilopodia

#### PubMed

1. Ninjurin1 enhances the basal motility and transendothelial migration of immune cells by inducing protrusive membrane dynamics. Ninjurin1 is involved in the pathogenesis of experimental autoimmune encephalomyelitis, an animal model of multiple sclerosis, by mediating leukocyte extravasation, a process that depends on homotypic binding. However, the precise regulatory mechanisms of Ninjurin1 during inflammation are largely undefined. We therefore examined the pro-migratory function of Ninjurin1 and its regulatory mechanisms in macrophages. Interestingly, Ninjurin1-deficient bone marrow-derived macrophages exhibited reduced membrane protrusion formation and dynamics, resulting in the impairment of cell motility. Furthermore, exogenous Ninjurin1 was distributed at the membrane of **filopodia**l structures in Raw264.7 macrophage cells. In Raw264.7 cells, RNA interference of Ninjurin1 reduced the number of **filopodia**l projections, whereas overexpression of Ninjurin1 facilitated their formation and thus promoted cell motility. Ninjurin1-induced **filopodia**l protrusion formation required the activation of Rac1. In Raw264.7 cells penetrating an MBEC4 endothelial cell monolayer, Ninjurin1 was localized to the membrane of protrusions and promoted their formation, suggesting that Ninjurin1-induced protrusive activity contributed to transendothelial migration. Taking these data together, we conclude that Ninjurin1 enhances macrophage motility and consequent extravasation of immune cells through the regulation of protrusive membrane dynamics. We expect these findings to provide insight into the understanding of immune responses mediated by Ninjurin1.

### [Mm]igration

#### PubMed

1. Contribution of Ninjurin1 to Toll-like receptor 4 signaling and systemic inflammation. Nerve injury-induced protein (Ninjurin [Ninj]) 1 is an adhesion molecule originally identified in Schwann cells after nerve injury, whereas it is also expressed in leukocytes, epithelium, endothelium, and various organs, and is induced under inflammatory conditions. Its contribution to inflammation was so far restricted to the nervous system and exclusively attributed to its role during leukocyte **migration**. We hypothesized a proinflammatory role for Ninj1 also outside the nervous system. To elucidate its impact during inflammation, we analyzed expression levels and its contribution to inflammation in septic mice and studied its effect on inflammatory signaling in vitro. The effect on inflammation was analyzed by genetic (only in vitro) and pharmacologic repression in septic mice (cecal ligation and puncture) and cell culture, respectively. Repression of Ninj1 by an inhibitory peptide or small interfering RNA attenuated LPS-triggered inflammation in macrophages and endothelial cells by modulating p38 phosphorylation and activator protein-1 activation. Inhibition of Ninj1 in septic mice reduced systemic and pulmonary inflammation as well as organ damage, and ameliorated survival after 24 hours. Ninj1 is elevated under inflammatory conditions and contributes to inflammation not only by mediating leukocyte **migration**, but also by modulating Toll-like receptor 4-dependent expression of inflammatory mediators. We assume that, owing to both mechanisms, inhibition reduces systemic inflammation and organ damage in septic mice. Our data contribute to a better understanding of the complex inflammatory mechanisms and add a novel therapeutic target for inflammatory conditions such as sepsis.
2. Ninjurin1 enhances the basal motility and transendothelial **migration** of immune cells by inducing protrusive membrane dynamics. Ninjurin1 is involved in the pathogenesis of experimental autoimmune encephalomyelitis, an animal model of multiple sclerosis, by mediating leukocyte extravasation, a process that depends on homotypic binding. However, the precise regulatory mechanisms of Ninjurin1 during inflammation are largely undefined. We therefore examined the pro-migratory function of Ninjurin1 and its regulatory mechanisms in macrophages. Interestingly, Ninjurin1-deficient bone marrow-derived macrophages exhibited reduced membrane protrusion formation and dynamics, resulting in the impairment of cell motility. Furthermore, exogenous Ninjurin1 was distributed at the membrane of filopodial structures in Raw264.7 macrophage cells. In Raw264.7 cells, RNA interference of Ninjurin1 reduced the number of filopodial projections, whereas overexpression of Ninjurin1 facilitated their formation and thus promoted cell motility. Ninjurin1-induced filopodial protrusion formation required the activation of Rac1. In Raw264.7 cells penetrating an MBEC4 endothelial cell monolayer, Ninjurin1 was localized to the membrane of protrusions and promoted their formation, suggesting that Ninjurin1-induced protrusive activity contributed to transendothelial **migration**. Taking these data together, we conclude that Ninjurin1 enhances macrophage motility and consequent extravasation of immune cells through the regulation of protrusive membrane dynamics. We expect these findings to provide insight into the understanding of immune responses mediated by Ninjurin1.
3. Ninjurin1 deficiency attenuates susceptibility of experimental autoimmune encephalomyelitis in mice. Ninjurin1 is a homotypic adhesion molecule that contributes to leukocyte trafficking in experimental autoimmune encephalomyelitis (EAE), an animal model of multiple sclerosis. However, in vivo gene deficiency animal studies have not yet been done. Here, we constructed Ninjurin1 knock-out (KO) mice and investigated the role of Ninjurin1 on leukocyte trafficking under inflammation conditions such as EAE and endotoxin-induced uveitis. Ninjurin1 KO mice attenuated EAE susceptibility by reducing leukocyte recruitment into the injury regions of the spinal cord and showed less adhesion of leukocytes on inflamed retinal vessels in endotoxin-induced uveitis mice. Moreover, the administration of a custom-made antibody (Ab26-37) targeting the Ninjurin1 binding domain ameliorated the EAE symptoms, showing the contribution of its adhesion activity to leukocyte trafficking. In addition, we addressed the transendothelial **migration** (TEM) activity of bone marrow-derived macrophages and Raw264.7 cells according to the expression level of Ninjurin1. TEM activity was decreased in Ninjurin1 KO bone marrow-derived macrophages and siNinj1 Raw264.7 cells. Consistent with this, GFP-tagged mNinj1-overexpressing Raw264.7 cells increased their TEM activity. Taken together, we have clarified the contribution of Ninjurin1 to leukocyte trafficking in vivo and delineated its direct functions to TEM, emphasizing Ninjurin1 as a beneficial therapeutic target against inflammatory diseases such as multiple sclerosis.

---

## Fli1

- NCBI Gene
- GeneRIF
- Pubmed

| Search Term | Rank | Fields |
| --- | --- | --- |
| [Mm]igration | 6 | PubMed(4) |
| ECM | 7 | PubMed(1) |
| [Ee]xtracellular [Mm]atrix | 8 | PubMed(1) |

  
hide/show details for Fli1

### [Mm]igration

#### PubMed

1. Genomic cloning of mouse MIF (macrophage inhibitory factor) and genetic mapping of the human and mouse expressed gene and nine mouse pseudogenes. The single functional mouse gene for MIF (macrophage **migration** inhibitory factor) has been cloned from a P1 library, and its exon/intron structure determined and shown to resemble that of the human gene. The gene was mapped to chromosome 10 using two multilocus crosses between laboratory strains and either Mus musculus musculus or Mus spretus. Nine additional loci containing related sequences, apparently all processed pseudogenes, were also mapped to chromosomes 1, 2, 3, 7, 8, 9, 12, 17, and 19. While most of these pseudogenes were found in inbred mice and M. spretus, some are species specific. This suggests that there have been active phases of pseudogene formation in Mus both before and after the separation of musculus and spretus. The human genome contains no pseudogenes; we assigned the human gene to chromosome 19, consistent with the location of mouse and human functional genes for MIF in a region of conserved linkage.
2. Redundant role of protein kinase C delta and epsilon during mouse embryonic development. Protein Kinase C delta and epsilon are mediators of important cellular events, such as cell proliferation, **migration** or apoptosis. The formation of blood vessels, i.e., vasculo- and angiogenesis, is a process where these isoforms have also been shown to participate. However, mice deficient in either Protein Kinase C delta or epsilon are viable and therefore their individual contribution to the formation of the vasculature appeared so far dispensable. In this study, we show that double null mutation of Protein Kinase C delta and epsilon causes embryonic lethality at approximately E9.5. At this stage, whole mount staining of the endothelial marker CD31 in double null embryos revealed defective blood vessel formation. Moreover, culture of double deficient mouse allantois showed impaired endothelial cell organization, and analyses of double deficient embryo sections showed dilated vessels, decreased endothelial-specific adherent junctions, and decreased contact of endothelial cells with mural cells. Protein kinase C delta and epsilon also appeared essential for vascular smooth muscle cell differentiation, since α-smooth muscle actin, a classical marker for vascular smooth muscle cells, was almost undetectable in double deficient embryonic aorta at E9.5. Subsequent qPCR analyses showed decreased VE-cadherin, Vegfr2, Cd31, Cdh2, Ets1, and Fli-1, among other angiogenesis related transcripts in double deficient embryos. Taken together, these data suggest for the first time an in vivo redundant role between members of the novel Protein Kinase C subfamily that allows for mutual compensation during mouse embryonic development, with vasculogenesis/angiogenesis as an obvious common function of these two Protein Kinase Cs. Protein Kinase C delta and epsilon might therefore be useful targets for inhibiting vasculo- and/or angiogenesis.
3. The Friend leukaemia virus integration 1 (Fli-1) transcription factor affects lupus nephritis development by regulating inflammatory cell infiltration into the kidney. The transcription factor Friend leukaemia virus integration 1 (Fli-1) is implicated in the pathogenesis of systemic lupus erythematosus in both human patients and murine models of lupus. Murphy Roths large (MRL)/lpr mice and New Zealand mixed (NZM)2410 mice, murine models of lupus, with decreased expression of Fli-1 had significantly prolonged survival and reduced nephritis. Lupus nephritis is a major cause of mortality and morbidity in patients, and inflammatory cell infiltration plays a key role in the development of the disease. To study how the expression of Fli-1 affects the infiltration of inflammatory cells into the kidneys, we generated congenic enhanced green fluorescent protein (GFP) transgenic MRL/lpr mice. A significantly increased number of GFP-expressing inflammatory cells infiltrated the kidneys of wild-type MRL/lpr mice compared to Fli-1 heterozygous (Fli-1(+/-)) MRL/lpr mice after injection of GFP(+) cells. Expression of inflammatory chemokine mRNA, including chemokine (C-C motif) ligand (CCL)2, CCL3, CCL4 and CCL5, was significantly lower in the kidneys from Fli-1(+/-) MRL/lpr mice compared to wild-type littermates. Numbers of infiltrated cells into the kidneys correlate with expression levels of CCL2, CCL4 and CCL5, but not the titres of anti-dsDNA autoantibodies in these mice. Significantly increased inflammatory cells from wild-type MRL/lpr mice infiltrated into kidneys compared to the cells from Fli-1(+/-) MRL/lpr mice. The chemotaxis of inflammatory cells from Fli-1(+/-) MRL/lpr mice towards each chemokine was decreased significantly compared to inflammatory cells from wild-type MRL/lpr mice in the transwell **migration** assay in vitro. Our results indicate that Fli-1 affects lupus nephritis development by regulating the expression of chemokines in the kidney and the **migration** of inflammatory cells.
4. The Ets family member Erg gene is expressed in mesodermal tissues and neural crests at fundamental steps during mouse embryogenesis. The Erg gene belongs to the Ets family encoding a class of transcription factors. To gain new insight on the in vivo functional specificity of the Erg gene within the wide Ets family, we used in situ hybridization to determine its expression pattern during murine embryogenesis. We found that the Erg gene expression predominates in mesodermal tissues, including the endothelial, precartilaginous and urogenital areas. A specific Erg gene expression was also identified in migrating neural crest cells. A comparison with Fli-1, the most closely Erg-related gene, revealed that both gene expressions partially overlap, suggesting that they may contribute to related functions in these tissues. Like other Ets family genes, Erg seems involved in several fundamental developmental steps in murine embryogenesis, including epithelio-mesenchymal transition, cell **migration**, settlement and differentiation.

### ECM

#### PubMed

1. Fli1 is a negative regulator of estrogen receptor α in dermal fibroblasts. Estrogen is an important regulator of dermal fibroblast functions, including extracellular matrix **(EC**M) synthesis. Estrogen mediates its effects through estrogen receptors (ERs), ERα and ERβ; however, regulation of ERs in dermal fibroblasts remains poorly understood. Friend leukemia integration factor 1 (Fli1), a member of the Ets transcription factor family, has been shown to play a pivotal role in regulation of t**he** ECM genes in dermal fibroblasts. The aim of this study was to examine a possible interaction between Fli1 and estrogen pathways, focusing on ERα. We show that treatment of human dermal fibroblasts with transforming growth factor-β (TGF-β) increases ERα protein and mRNA levels. Similarly, ERα expression was increased in response to small interfering RNA (siRNA)-mediated depletion of Fli1, suggesting that Fli1 is a mediator of the TGF-β effects on ERα expression. Accordingly, we showed that Fli1 binds to the most proximal region of the ERα promoter, and dissociates from the promoter upon TGF-β treatment. An inverse correlation between Fli1 and ERα expression levels was confirmed in cultured skin fibroblasts obtained from Fli1(+/-) mice and in the skin of Fli1(+/-) mice in vivo. This study supports a role of Fli1 as a negative regulator of the ERα gene in dermal fibroblasts.

### [Ee]xtracellular [Mm]atrix

#### PubMed

1. Fli1 is a negative regulator of estrogen receptor α in dermal fibroblasts. Estrogen is an important regulator of dermal fibroblast functions, including **extracellular matri**x (ECM) synthesis. Estrogen mediates its effects through estrogen receptors (ERs), ERα and ERβ; however, regulation of ERs in dermal fibroblasts remains poorly understood. Friend leukemia integration factor 1 (Fli1), a member of the Ets transcription factor family, has been shown to play a pivotal role in regulation of the ECM genes in dermal fibroblasts. The aim of this study was to examine a possible interaction between Fli1 and estrogen pathways, focusing on ERα. We show that treatment of human dermal fibroblasts with transforming growth factor-β (TGF-β) increases ERα protein and mRNA levels. Similarly, ERα expression was increased in response to small interfering RNA (siRNA)-mediated depletion of Fli1, suggesting that Fli1 is a mediator of the TGF-β effects on ERα expression. Accordingly, we showed that Fli1 binds to the most proximal region of the ERα promoter, and dissociates from the promoter upon TGF-β treatment. An inverse correlation between Fli1 and ERα expression levels was confirmed in cultured skin fibroblasts obtained from Fli1(+/-) mice and in the skin of Fli1(+/-) mice in vivo. This study supports a role of Fli1 as a negative regulator of the ERα gene in dermal fibroblasts.

---

## Nfkb1

- NCBI Gene
- GeneRIF
- Pubmed

| Search Term | Rank | Fields |
| --- | --- | --- |
| [Mm]igration | 6 | PubMed(45); GeneRIFs(4) |
| ECM | 7 | PubMed(6) |
| [Ee]xtracellular [Mm]atrix | 8 | PubMed(10) |
| [Ii]nvasive | 10 | PubMed(19); GeneRIFs(1) |
| [Mm]etastasis | 12 | PubMed(24); GeneRIFs(7) |

  
hide/show details for Nfkb1

### [Mm]igration

#### PubMed

1. ΔNp63 versatilely regulates a Broad NF-κB gene program and promotes squamous epithelial proliferation**, migrati**on, and inflammation. Head and neck squamous cell carcinoma (HNSCC) and many other epithelial malignancies exhibit increased proliferation, invasion, and inflammation, concomitant with aberrant nuclear activation of TP53 and NF-κB family members ΔNp63, cRel, and RelA. However, the mechanisms of cross-talk by which these transcription factors coordinate gene expression and the malignant phenotype remain elusive. In this study, we showed that ΔNp63 regulates a cohort of genes involved in cell growth, survival, adhesion, and inflammation, which substantially overlaps with the NF-κB transcriptome. ΔNp63 with cRel and/or RelA are recruited to form novel binding complexes on p63 or NF-κB/Rel sites of multitarget gene promoters. Overexpressed ΔNp63- or TNF-α-induced NF-κB and inflammatory cytokine interleukin-8 (IL-8) reporter activation depended on RelA/cRel regulatory binding sites. Depletion of RelA or ΔNp63 by small interfering RNA (siRNA) significantly inhibited NF-κB-specific, or TNF-α-induced IL-8 reporter activation. ΔNp63 siRNA significantly inhibited proliferation, **survival**, and migration by HNSCC cells in vitro. Consistent with these data, an increase in nuclear ΔNp63, accompanied by increased proliferation (Ki-67) and adhesion (β4 integrin) markers, and induced inflammatory cell infiltration was observed throughout HNSCC specimens, when compared with the basilar pattern of protein expression and minimal inflammation seen in nonmalignant mucosa. Furthermore, overexpression of ΔNp63α in squamous epithelial cells in transgenic mice leads to increased suprabasilar cRel, Ki-67, and cytokine expression, together with epidermal hyperplasia and diffuse inflammation, similar to HNSCC. Our study reveals ΔNp63 as a master transcription factor that, in coordination with NF-κB/Rels, orchestrates a broad gene program promoting epidermal hyperplasia, inflammation, and the malignant phenotype of HNSCC.
2. SASH1 is a scaffold molecule in endothelial TLR4 signaling. Recognition of microbial products by TLRs is critical for mediating innate immune responses to invading pathogens. In this study, we identify a novel scaffold protein in TLR4 signaling called SAM and SH3 domain containing protein 1 (SASH1). Sash1 is expressed across all microvascular beds and functions as a scaffold molecule to independently bind TRAF6, TAK1, IκB kinase α, and IκB kinase β. This interaction fosters ubiquitination of TRAF6 and TAK1 and promotes LPS-induced NF-κB, JNK, and p38 activation, culminating in increased production of proinflammatory cytokines and increased LPS-induced endothe**lial migr**ation. Our findings suggest that SASH1 acts to assemble a signaling complex downstream of TLR4 to activate early endothelial responses to receptor activation.
3. Synaptic activation of NF-kappa B by glutamate in cerebellar granule neurons in vitro. Neuronal proliferation, **migration**, and differentiation are regulated by the sequential expression of particular genes at specific stages of development. Such processes rely on differential gene expression modulated through second-messenger systems. Early postnatal mouse cerebellar granule cells migrate into the internal granular layer and acquire differentiated properties. The neurotransmitter glutamate has been shown to play an important role in this developmental process. We show here by immunohistochemistry that the RelA subunit of the transcription factor NF-kappa B is present in several areas of the mouse brain. Moreover, immunofluorescence microscopy and electrophoretic mobility-shift assay demonstrate that in cerebellar granule cell cultures derived from 3- to 7-day-old mice, glutamate specifically activates the transcription factor NF-kappa B, as shown by binding of nuclear extract proteins to a synthetic oligonucleotide reproducing the kappa B site of human immunodeficiency virus. The use of different antagonists of the glutamate recpetors indicates that the effect of glutamate occurs mainly via N-methyl-D-aspartate (NMDA)-receptor activation, possibly as a result of an increase in intracellular Ca2+. The synaptic specificity of the effect is strongly suggested by the observation that glutamate failed to activate NF-kappa B in astrocytes, while cytokines, such as interleukin 1 alpha and tumor necrosis factor alpha, did so. The effect of glutamate appears to be developmentally regulated. Indeed, NF-kappa B is found in an inducible form in the cytoplasm of neurons of 3- to 7-day-old mice but is constitutively activated in the nuclei of neurons derived from older pups (8-10 days postnatal). Overall, these observations suggest the existence of a new pathway of trans-synaptic regulation of gene expression.
4. An NF-κB--EphrinA5-Dependent Communication between NG2(+) Interstitial Cells and Myoblasts Promotes Muscle Growth in Neonates. Skeletal muscle growth immediately following birth is critical for proper body posture and locomotion. However, compared with embryogenesis and adulthood, the processes regulating the maturation of neonatal muscles is considerably less clear. Studies in the 1960s predicted that neonatal muscle growth results from nuclear accretion of myoblasts preferentially at the tips of myofibers. Remarkably, little information has been added since then to resolve how myoblasts migrate to the ends of fibers. Here, we provide insight into this process by revealing a unique NF-κB-dependent communication between NG2(+) interstitial cells and myoblasts. NF-κB in NG2(+) cells promotes myobla**st migrat**ion to the tips of myofibers through cell-cell contact. This occurs through expression of ephrinA5 from NG2(+) cells, which we further deduce is an NF-κB target gene. Together, these results suggest that NF-κB plays an important role in the development of newborn muscles to ensure proper myob**last migr**ation for fiber growth.
5. Members of the NF-kappaB family expressed in zones of active neurogenesis in the postnatal and adult mouse brain. The Rel/NF-kappaB family of transcription factors is implicated in cell proliferation, cell death, cell **migration** and cell interactions. Here, we examined by immunohistochemistry the expression pattern of various members of this family during postnatal telencephalon development and during adulthood, and we used neuronal and glial markers to identify the cells types where they are expressed. Distinct Rel/NF-kappaB proteins are highly expressed postnatally in the subventricular zone and in the rostral migratory stream. In particular, Rel A and p50 are expressed in radial glial cells, in migrating neuron precursors and in a population belonging to the astrocytic lineage. Rel B, on the other hand, is only expressed in migrating neuron precursors, whereas c-Rel is present in a few cells located at the edges of the rostral migratory stream. The expression of Rel A and p50 persists into adulthood, particularly in subventricular zone astrocyte-like cells and in migrating neuron precursors, respectively. The selective expression of NF-kappaB members in the postnatal subventricular zone and rostral migratory stream and their persistence into adulthood in regions of ongoing neurogenesis suggests possible mechanisms linking NF-kappaB expression with cell proliferation and **migration**. Their presence in actively proliferating progenitor cells, detected by BrdU staining, further suggests that NF-kappaB may be part of a signaling pathway that is important for neurogenesis.
6. An NF-κB--EphrinA5-Dependent Communication between NG2(+) Interstitial Cells and Myoblasts Promotes Muscle Growth in Neonates. Skeletal muscle growth immediately following birth is critical for proper body posture and locomotion. However, compared with embryogenesis and adulthood, the processes regulating the maturation of neonatal muscles is considerably less clear. Studies in the 1960s predicted that neonatal muscle growth results from nuclear accretion of myoblasts preferentially at the tips of myofibers. Remarkably, little information has been added since then to resolve how myoblasts migrate to the ends of fibers. Here, we provide insight into this process by revealing a unique NF-κB-dependent communication between NG2(+) interstitial cells and myoblasts. NF-κB in NG2(+) cells promotes myobla**st migrat**ion to the tips of myofibers through cell-cell contact. This occurs through expression of ephrinA5 from NG2(+) cells, which we further deduce is an NF-κB target gene. Together, these results suggest that NF-κB plays an important role in the development of newborn muscles to ensure proper myob**last migr**ation for fiber growth.
7. ΔNp63 versatilely regulates a Broad NF-κB gene program and promotes squamous epithelial proliferation**, migrati**on, and inflammation. Head and neck squamous cell carcinoma (HNSCC) and many other epithelial malignancies exhibit increased proliferation, invasion, and inflammation, concomitant with aberrant nuclear activation of TP53 and NF-κB family members ΔNp63, cRel, and RelA. However, the mechanisms of cross-talk by which these transcription factors coordinate gene expression and the malignant phenotype remain elusive. In this study, we showed that ΔNp63 regulates a cohort of genes involved in cell growth, survival, adhesion, and inflammation, which substantially overlaps with the NF-κB transcriptome. ΔNp63 with cRel and/or RelA are recruited to form novel binding complexes on p63 or NF-κB/Rel sites of multitarget gene promoters. Overexpressed ΔNp63- or TNF-α-induced NF-κB and inflammatory cytokine interleukin-8 (IL-8) reporter activation depended on RelA/cRel regulatory binding sites. Depletion of RelA or ΔNp63 by small interfering RNA (siRNA) significantly inhibited NF-κB-specific, or TNF-α-induced IL-8 reporter activation. ΔNp63 siRNA significantly inhibited proliferation, **survival**, and migration by HNSCC cells in vitro. Consistent with these data, an increase in nuclear ΔNp63, accompanied by increased proliferation (Ki-67) and adhesion (β4 integrin) markers, and induced inflammatory cell infiltration was observed throughout HNSCC specimens, when compared with the basilar pattern of protein expression and minimal inflammation seen in nonmalignant mucosa. Furthermore, overexpression of ΔNp63α in squamous epithelial cells in transgenic mice leads to increased suprabasilar cRel, Ki-67, and cytokine expression, together with epidermal hyperplasia and diffuse inflammation, similar to HNSCC. Our study reveals ΔNp63 as a master transcription factor that, in coordination with NF-κB/Rels, orchestrates a broad gene program promoting epidermal hyperplasia, inflammation, and the malignant phenotype of HNSCC.
8. Members of the NF-kappaB family expressed in zones of active neurogenesis in the postnatal and adult mouse brain. The Rel/NF-kappaB family of transcription factors is implicated in cell proliferation, cell death, cell **migration** and cell interactions. Here, we examined by immunohistochemistry the expression pattern of various members of this family during postnatal telencephalon development and during adulthood, and we used neuronal and glial markers to identify the cells types where they are expressed. Distinct Rel/NF-kappaB proteins are highly expressed postnatally in the subventricular zone and in the rostral migratory stream. In particular, Rel A and p50 are expressed in radial glial cells, in migrating neuron precursors and in a population belonging to the astrocytic lineage. Rel B, on the other hand, is only expressed in migrating neuron precursors, whereas c-Rel is present in a few cells located at the edges of the rostral migratory stream. The expression of Rel A and p50 persists into adulthood, particularly in subventricular zone astrocyte-like cells and in migrating neuron precursors, respectively. The selective expression of NF-kappaB members in the postnatal subventricular zone and rostral migratory stream and their persistence into adulthood in regions of ongoing neurogenesis suggests possible mechanisms linking NF-kappaB expression with cell proliferation and **migration**. Their presence in actively proliferating progenitor cells, detected by BrdU staining, further suggests that NF-kappaB may be part of a signaling pathway that is important for neurogenesis.
9. The effects of protein malnutrition on the TNF-RI and NF-κB expression via the TNF-α signaling pathway. Malnutrition is a nutritional condition that can affect many aspects of the immunological response, including by decreasing cel**l migrati**on and stimulating phagocytosis; the bactericidal response; changes in reactive oxygen and nitrogen species production; and the production of proinflammatory cytokines, such as tumor necrosis factor alpha (TNF-α). This cytokine is primarily produced by macrophages and is associated with a wide range of biological activities, including inflammatory processes, growth, differentiation, and apoptosis. TNF-α acts through the activation of TNF receptors, and mainly receptor I (TNF-RI), which is responsible for most of the effects of TNF-α. This activation triggers a series of intracellular events that result in the activation of the transcription factor NF-κB. In this study, we evaluated the expression of the transcription factor NF-κB, mediated by TNF-α through TNF-RI, in a protein malnutrition (PM) model. Adult male BALB/c mice were submitted to PM, and after loss of approximately 20% of their body weight, their peritoneal macrophages were collected and cultivated with or without TNF-α. The expression of TNF-RI and proteins in its signaling pathway (TRADD, TRAF, RIP, IKK, IKB-α, pIKB-α, NF-κB, and pNF-κB) were evaluated, as well as cytokine production (IL-1α, IL-1β, IL-6, and IL-12). The compiled results highlight that the malnourished animals presented anemia, leukopenia, and decreased peritoneal cellularity. TNF-RI expression was reduced in the malnourished animals, and NF-κB phosphorylation was also reduced, in association with reduced production of IL-1β and IL-12. In this study, we observed aspects related to the innate immune response, and the outcome data allowed us to conclude that nutritional status interferes with the macrophage activation and the response capabilities of these cells.
10. Ethyl pyruvate, an anti-inflammatory agent, inhibits tumor angiogenesis through inhibition of the NF-κB signaling pathway. Ethyl pyruvate (EP), simple derivative of pyruvate, has been shown to have anti-inflammatory properties. Here, we demonstrate EP's strong anti-angiogenic activity. EP inhibited in vivo angiogenesis in the mouse Matrigel-plug assay and tumor growth in the mouse Lewis lung carcinoma model. EP also interfered with the angiogenic cascade, including growth, invasion, **migratio**n, and tube formation. Activation of NF-κB by vascular endothelial cell growth factor was reduced by EP. Taken together, we suggest that EP may have potential as a new multi-functional drug candidate for anti-angiogenesis and cancer therapy.
11. Changes in mitochondrial DNA alter expression of nuclear encoded genes associated with tumorigenesis. We previously reported the presence of a mtDNA mutation hotspot in UV-induced premalignant and malignant skin tumors in hairless mice. We have modeled this change (9821insA) in murine cybrid cells and demonstrated that this alteration in mtDNA associated with mtBALB haplotype can alter the biochemical characteristics of cybrids and subsequently can contribute to significant changes in their behavioral capabilities. This study shows that changes in mtDNA can produce differences in expression levels of specific nuclear-encoded genes, which are capable of triggering the phenotypes such as seen in malignant cells. From a potential list of differentially expressed genes discovered by microarray analysis, we selected MMP-9 and Col1a1 for further studies. Real-time PCR confirmed up-regulation of MMP-9 and down-regulation of Col1a1 in cybrids harboring the mtDNA associated with the skin tumors. These cybrids also showed significantly increased **migration** and invasion abilities compared to wild type. The non-specific MMP inhibitor, GM6001, was able to inhibit migratory and invasive abilities of the 9821insA cybrids confirming a critical role of MMPs in cellular motility. Nuclear factor-κB (NF-κB) is a key transcription factor for production of MMPs. An inhibitor of NF-κB activation, Bay 11-7082, was able to inhibit the expression of MMP-9 and ultimately decrea**se migrat**ion and invasion of mutant cybrids containing 9821insA. These studies confirm a role of NF-κB in the regulation of MMP-9 expression and through this regulation modulates the migratory and invasive capabilities of cybrids with mutant mtDNA. Enhan**ced migra**tion and invasion abilities caused by up-regulated MMP-9 may contribute to the tumorigenic phenotypic characteristics of mutant cybrids.
12. Activation of epidermal growth factor receptor is required for NTHi-induced NF-κB-dependent inflammation. Inflammation is a hallmark of many serious human diseases. Nontypeable Haemophilus influenzae (NTHi) is an important human pathogen causing respiratory tract infections in both adults and children. NTHi infections are characterized by inflammation, which is mainly mediated by nuclear transcription factor-kappa B (NF-κB)-dependent production of proinflammatory mediators. Epidermal growth factor receptor (EGFR) has been shown to play important roles in regulating diverse biological processes, including cell growth, differentiation, apoptosis, adhesion, an**d migrati**on. Its role in regulating NF-κB activation and inflammation, however, remains largely unknown.In the present study, we demonstrate that EGFR plays a vital role in NTHi-induced NF-κB activation and the subsequent induction of proinflammatory mediators in human middle ear epithelial cells and other cell types. Importantly, we found that AG1478, a specific tyrosine kinase inhibitor of EGFR potently inhibited NTHi-induced inflammatory responses in the middle ears and lungs of mice in vivo. Moreover, we found that MKK3/6-p38 and PI3K/Akt signaling pathways are required for mediating EGFR-dependent NF-κB activation and inflammatory responses by NTHi.Here, we provide direct evidence that EGFR plays a critical role in mediating NTHi-induced NF-κB activation and inflammation in vitro and in vivo. Given that EGFR inhibitors have been approved in clinical use for the treatment of cancers, current studies will not only provide novel insights into the molecular mechanisms underlying the regulation of inflammation, but may also lead to the development of novel therapeutic strategies for the treatment of respiratory inflammatory diseases and other inflammatory diseases.
13. The **migration** of neural progenitor cell mediated by SDF-1 is NF-κB/HIF-1α dependent upon hypoxia. Stromal cell-derived factor 1 (SDF-1) is critical for neural progenitor cell (NPC**) migrati**on after ischemia for nerve repair, but how hypoxic induction of SDF-1 is regulated has not been fully addressed. Here, we examined the regulation of SDF-1 hypoxic induction by the transcription factors nuclear factor-κB (NF-κB) and hypoxic inducible factor 1α (HIF-1α) in astrocytes.Stromal cell-derived factor-1 in astrocyte-conditioned medium (ACM) collected from hypoxic astrocytes induced a time- and dose-dependent increase i**n NPC mig**ration using chemotaxis assay. The increase i**n NPC mig**ration correlated with increased SDF-1 production in astrocytes by real-time PCR and ELISA assays. Astrocytes produced SDF-1 time-dependently upon 3% O(2) treatment, which was associated with increased levels of NF-κB and HIF-1α using Western blot analysis. Anti-HIF-1α compound, 3-(5'-hydroxymethyl-2'-furyl)-1-benzylindazole (YC-1) and NF-κB inhibitor pyrrolidine dithiocarbamate (PDTC), decreased hypoxic induction of SDF-1, and PDTC pretreatment cancelled HIF-1α expression as well**, thus NP**C migration induced by ACM was decreased accordingly. Moreover, lentiviurs siRNA for NF-κB p65 abrogated induction of HIF-1α and SDF-1 under hypoxia in astrocytes.Hypoxic induction of SDF-1 is reliant upon NF-κB and HIF-1α. There is a cross-talk between HIF-1α and NF-κB, both HIF-1α and SDF-1 are downstream targets of NF-κB in hypoxia condition.
14. Phosphorylation of transglutaminase 2 (TG2) at serine-216 has a role in TG2 mediated activation of nuclear factor-kappa B and in the downregulation of PTEN. Transglutaminase 2 (TG2) and its phosphorylation have been consistently found to be upregulated in a number of cancer cell types. At the molecular level, TG2 has been associated with the activation of nuclear factor-kappa B (NF-κB), protein kinase B (PKB/Akt) and in the downregulation of phosphatase and tensin homologue deleted on chromosome 10 (PTEN). However, the underlying mechanism involved is not known. We have reported that protein kinase A (PKA) induced phosphorylation of TG2 at serine-216 (Ser(216)) regulates TG2 function and facilitates protein-protein interaction. However, the role of TG2 phosphorylation in the modulation of NF-κB, Akt and PTEN is not explored.In this study we have investigated the effect of TG2 phosphorylation on NF-κB, Akt and PTEN using embryonic fibroblasts derived from TG2 null mice (MEF(tg2-/-)) overexpressing native TG2 or mutant-TG2 (m-TG2) lacking Ser(216) phosphorylation site with and without dibutyryl cyclic-AMP (db-cAMP) stimulation. Functional consequences on cell cycle and cell motility were determined by fluorescence activated cell sorting (FACS) analysis and ce**ll migrat**ion assay respectively.PKA activation in TG2 overexpressing MEF(tg2-/-) cells resulted in an increased activation of NF-κB and Akt phosphorylation in comparison to empty vector transfected control cells as determined by the reporter-gene assay and immunoblot analysis respectively. These effects were not observed in MEF(tg2-/-) cells overexpressing m-TG2. Similarly, a significant downregulation of PTEN at both, the mRNA and protein levels were found in cells overexpressing TG2 in comparison to empty vector control and m-TG2 transfected cells. Furthermore, Akt activation correlated with the simultaneous activation of NF-κB and a decrease in PTEN suggesting that the facilitatory effect of TG2 on Akt activation occurs in a PTEN-dependent manner. Similar results were found with MCF-7 and T-47D breast cancer cells overexpressing TG2 and m-TG2 further supporting the role of TG2 phosphorylation in NF-κB activation and in the downregulation of PTEN.Collectively, these data suggest that phosphorylation of TG2 at Ser(216) plays a role in TG2 mediated activation of NF-κB, Akt and in the downregulation of PTEN. Blocking TG2 phosphorylation may provide a novel strategy to attenuate NF-κB activation and downregulation of PTEN in TG2 overexpressing cancers.
15. DDR2 inhibition reduces **migration** and invasion of murine metastatic melanoma cells by suppressing MMP2/9 expression through ERK/NF-κB pathway. Metastatic melanoma is one of the most deadly and evasive cancers. Collagen I in the extracellular matrix promotes the **migratio**n and invasion of tumor cells through the production of matrix metalloproteinase (MMP) 2 and 9. Discoidin domain receptor (DDR) 2 is a collagen receptor that is implicated in several cancer types including breast and prostate cancers. However, the role of DDR2 in the **migratio**n and invasion of murine melanoma cells is less studied. In the present study, we investigated the effects and underlying mechanisms of DDR2 in **migratio**n and invasion of B16BL6 melanoma cells in response to collagen I. Results demonstrated that DDR2 is expressed and is phosphorylated by collagen I in the cells. Upon down-regulation of DDR2 using small-interfering RNA (siRNA) approach, both of the cell migratory and invasive phenotypes were significantly attenuated when compared with the control cells. This effect was mediated via suppression of MMP2/9 upon DDR2 inhibition. Furthermore, inhibition of DDR2 by specific siRNA markedly reduced the activation of extracellular regulated kinase (ERK) 1 and 2 and nuclear factor of kappa B (NF-κB) in the cells when compared with the control cells. Overall, these data demonstrated that DDR2 siRNA-mediated suppression of ERK1/2 and NF-κB could down-regulate the expressions of MMP2/9 in response to collagen I to reduce the migratory and invasive phenotypes of the cells.
16. Apoptosis induced by lipid-associated membrane proteins from Mycoplasma penetrans is mediated by nuclear factor kappaB activation in mouse macrophage. Mycoplasma penetrans was shown to be involved in alteration of several eukaryotical cells functions and a causative agent in urogenital infectious diseases. Lipid-associated membrane proteins (LAMPs) may be responsible for the pathogenicity of some mycoplamas. In this study, we investigated whether M. penetrans LAMPs have pathogenic potential by inducing apoptosis in mouse macrophages. As analyzed by annexin-V - fluorescein isothiocyanate staining, significant early- and late-stage apoptosis was induced in M. penetrans LAMPs-challenged mouse macrophages. And agarose gel electrophoresis of the DNA of M. penetrans LAMPs-challenged cells revealed a ladder-like pattern of **migration** of DNA indicative of apoptosis. The possible molecular mechanisms responsible for the induction of apoptosis were also investigated by characterizing the activation of nuclear transcription factor kappaB (NFkappaB). NFkappaB was activated and translocated into the nucleus in mouse macrophages stimulated by M. penetrans LAMPs. The activation of NFkappaB and M. penetrans LAMPs-induced apoptosis in mouse macrophages was partially inhibited by the NFkappaB-specific inhibitor pyrrolidine dithiocarbamate. Thus, this study demonstrates that M. penetrans LAMPs may be an important etiological factor owing to their ability to induce apoptosis in mouse macrophages, which is probably mediated through the activation of NFkappaB.
17. Synergistic effect of IL-2, IL-12, and IL-18 on thymocyte apoptosis and Th1/Th2 cytokine expression. In the periphery, IL-18 synergistically induces the expression of the Th1 cytokine IFN-gamma in the presence of IL-12 and the Th2 cytokines IL-5 and IL-13 in the presence of IL-2. Although the expression of these cytokines has been described in the thymus, their role in thymic development and function remains uncertain. We report here that freshly isolated thymocytes from C57BL/6 and BALB/c mice stimulated in vitro with IL-2-plus-IL-18 or IL-12-plus-IL-18 produce large amounts of IFN-gamma and IL-13. Analysis of the thymic subsets, CD4(-)CD8(-) (DN), CD4(+)CD8(+), CD4(+)CD8(-), and CD4(-)CD8(+) revealed that IL-18 in combination with IL-2 or IL-12 induces IFN-gamma and IL-13 preferentially from DN cells. Moreover, DN2 and DN3 thymocytes contained more IFN-gamma(+) cells than cells in the later stage of maturation. Additionally, IL-18 in combination with IL-2 induces CCR4 (Th2-associated) and CCR5 (Th1-associated) gene expression. In contrast, IL-18-plus-IL-12 specifically induced CCR5 expression. The IL-2-plus-IL-18 or IL-12-plus-IL-18 effect on IFN-gamma and IL-13 expression is dependent on Stat4 and NF-kappaB but independent of Stat6, T-bet, or NFAT. Furthermore, IL-12-plus-IL-18 induces significant thymocyte apoptosis when expressed in vivo or in vitro, and this effect is exacerbated in the absence of IFN-gamma. IL-12-plus-IL-18-stimulated thymocytes can also induce IA-IE expression on cortical and medullary thymic epithelial cells in an IFN-gamma-dependent manner. Thus, the combination of IL-2, IL-12, and IL-18 can induce phenotypic and functional changes in thymocytes that may alter **migration**, differentiation, and cell death of immature T cells inside the thymus and potentially affect the Th1/Th2 bias in peripheral immune compartments.
18. The I kappa B/NF-kappa B system: a key determinant of mucosalinflammation and protection. The ubiquitous transcription factor NF-kappa B is a central regulator of the transcriptional activation of a number of genes involved in cell adhesion, immune and proinflammatory responses, apoptosis, differentiation, and growth. Induction of these genes in intestinal epithelial cells (IECs) by activated NF-kappa B profoundly influences mucosal inflammation and repair. NF-kappa B activation requires the removal of I kappa B from NF-kappa B by inducible proteolysis, which liberates this transcription factor for **migration** to the nucleus, where it binds to kappa B-regulatory elements and induces transcription. I kappa B alpha degradation is incomplete and delayed in IECs, resulting in buffered responses to luminal stimuli. The stimulatory environment partially determines whether the effect of NF-kappa B is protective or deleterious for the host. kappa B-dependent proinflammatory gene expression, particularly chemokines, major histocompatibility complex class II antigens, and adhesion molecules may be extremely important in early protective responses to mucosal pathogens but, when dysregulated, could lead to the development of chronic inflammation, as seen in inflammatory bowel diseases. The key role of NF-kappa B in regulating expression of a number of proinflammatory genes makes this protein an attractive target for selective therapeutic intervention.
19. Phospholipase D1 has a pivotal role in interleukin-1β-driven chronic autoimmune arthritis through regulation of NF-κB, hypoxia-inducible factor 1α, and FoxO3a. Interleukin-1β (IL-1β) is a potent proinflammatory and immunoregulatory cytokine playing an important role in the progression of rheumatoid arthritis (RA). However, the signaling network of IL-1β in synoviocytes from RA patients is still poorly understood. Here, we show for the first time that phospholipase D1 (PLD1), but not PLD2, is selectively upregulated in IL-1β-stimulated synoviocytes, as well as synovium, from RA patients. IL-1β enhanced the binding of NF-κB and ATF-2 to the PLD1 promoter, thereby enhancing PLD1 expression. PLD1 inhibition abolished the IL-1β-induced expression of proinflammatory mediators and angiogenic factors by suppressing the binding of NF-κB or hypoxia-inducible factor 1α to the promoter of its target genes, as well as IL-1β-induced prol**iferation** or migration. However, suppression of PLD1 activity promoted cell cycle arrest via transactivation of FoxO3a. Furthermore, PLD1 inhibitor significantly suppressed joint inflammation and destruction in IL-1 receptor antagonist-deficient (IL-1Ra(-/-)) mice, a model of spontaneous arthritis. Taken together, these results suggest that the abnormal upregulation of PLD1 may contribute to the pathogenesis of IL-1β-induced chronic arthritis and that a selective PLD1 inhibitor might provide a potential therapeutic molecule for the treatment of chronic inflammatory autoimmune disorders.
20. Mouse aorta smooth muscle cells differentiate into lymphoid tissue organizer-like cells on combined tumor necrosis factor receptor-1/lymphotoxin beta-receptor NF-kappaB signaling. Mouse aorta smooth muscle cells (SMC) express tumor necrosis factor receptor superfamily member 1A (TNFR-1) and lymphotoxin beta-receptor (LTbetaR). Circumstantial evidence has linked the SMC LTbetaR to tertiary lymphoid organogenesis in hyperlipidemic mice. Here, we explored TNFR-1 and LTbetaR signaling in cultured SMC.TNFR-1 signaling activated the classical RelA NF-kappaB pathway, whereas LTbetaR signaling activated the classical RelA and alternative RelB NF-kappaB pathways, and both signaling pathways synergized to enhance p100 inhibitor processing to the p52 subunit of NF-kappaB. Microarrays showed that simultaneous TNFR-1/LTbetaR activation resulted in elevated mRNA encoding leukocyte homeostatic chemokines CCL2, CCL5, CXCL1, and CX3CL1. Importantly, SMC acquired features of lymphoid tissue organizers, which control tertiary lymphoid organogenesis in autoimmune diseases through hyperinduction of CCL7, CCL9, CXCL13, CCL19, CXCL16, vascular cell adhesion molecule-1, and intercellular adhesion molecule-1. TNFR-1/LTbetaR cross-talk resulted in augmented secretion of lymphorganogenic chemokine proteins. Supernatants of TNFR-1/LTbetaR-activated SMC markedly supported **migration** of splenic T cells, B cells, and macrophages/dendritic cells. Experiments with ltbr(-/-) SMC indicated that LTbetaR-RelB activation was obligatory to generate the lymphoid tissue organizer phenotype.SMC may participate in the formation of tertiary lymphoid tissue in atherosclerosis by upregulation of lymphorganogenic chemokines involved in T-lymphocyte, B-lymphocyte, and macrophage/dendritic cell attraction.
21. Angiopoietin-1/Tie2 signaling pathway inhibits lipopolysaccharide-induced activation of RAW264.7 macrophage cells. Angiopoietin-1 (Ang1) is a ligand for the endothelial-specific tyrosine kinase receptor Tie2 and has been shown to play an essential role in embryonic vasculature development. There have been many studies about the anti-inflammatory effects of Ang1, most of which focus on endothelium cells. In the present study, we explore the role of Ang1-Tie2 signaling in the activation of macrophages upon lipopolysaccharide (LPS) stimulation. We found that Tie2 receptor is expressed on macrophages and Ang1 could inhibit LPS-induced activation of macrophages, as evidenced by cell **migration** and TNF-alpha production, specifically through Tie2 receptor. We further investigated the mechanism and found that Ang1-Tie2 could block LPS-induced activation of NF-kappaB which has been shown to be necessary for macrophage activation with LPS treatment. Thus, we described, for the first time, the role of Ang1-Tie2 signaling in macrophage activation and the possible mechanisms in response to immune stimulation.
22. CTGF promotes inflammatory cell infiltration of the renal interstitium by activating NF-kappaB. Connective tissue growth factor (CTGF) is an important profibrotic factor in kidney diseases. Blockade of endogenous CTGF ameliorates experimental renal damage and inhibits synthesis of extracellular matrix in cultured renal cells. CTGF regulates several cellular responses, including adhesion, **migration**, proliferation, and synthesis of proinflammatory factors. Here, we investigated whether CTGF participates in the inflammatory process in the kidney by evaluating the nuclear factor-kappa B (NF-kappaB) pathway, a key signaling system that controls inflammation and immune responses. Systemic administration of CTGF to mice for 24 h induced marked infiltration of inflammatory cells in the renal interstitium (T lymphocytes and monocytes/macrophages) and led to elevated renal NF-kappaB activity. Administration of CTGF increased renal expression of chemokines (MCP-1 and RANTES) and cytokines (INF-gamma, IL-6, and IL-4) that recruit immune cells and promote inflammation. Treatment with a NF-kappaB inhibitor, parthenolide, inhibited CTGF-induced renal inflammatory responses, including the up-regulation of chemokines and cytokines. In cultured murine tubuloepithelial cells, CTGF rapidly activated the NF-kappaB pathway and the cascade of mitogen-activated protein kinases, demonstrating crosstalk between these signaling pathways. CTGF, via mitogen-activated protein kinase and NF-kappaB activation, increased proinflammatory gene expression. These data show that in addition to its profibrotic properties, CTGF contributes to the recruitment of inflammatory cells in the kidney by activating the NF-kappaB pathway.
23. Role for nuclear factor-kappaB in augmented lung injury because of interaction between hyperoxia and high stretch ventilation. High-tidal-volume mechanical ventilation and hyperoxia used in patients with acute lung injury (ALI) can induce alveolar coagulopathy and fibrin depositions within the airways. Hyperoxia has been shown to increase ventilator-induced lung injury (VILI), but the mechanisms that regulate interaction between high-tidal-volume mechanical ventilation and hyperoxia are unclear. We hypothesized that mechanical stretch with hyperoxia synergistically augmented neutrophil infiltration and production of plasminogen activator inhibitor-1 (PAI-1) via the nuclear factor-kappaB (NF-kappaB) pathway. C57BL/6 mice (n=5 per group) were exposed to high-tidal-volume (30 mL/kg) or low-tidal-volume (6 mL/kg) mechanical ventilation with room air or hyperoxia for 1 to 5h after 2-microg/g NF-kappaB inhibitor (SN-50) administration. Nonventilated mice with room air or hyperoxia served as control groups. Evans blue dye, myeloperoxidase, electrophoretic mobility shifting of nuclear protein, and inflammatory cytokine were measured. The expression of tumor necrosis factor-alpha (TNF-alpha) and PAI-1 were studied by immunohistochemistry. The addition of hyperoxia to high-tidal-volume ventilation-augmented lung injury, as demonstrated by increased microvascular leak, neutrophil **migration** into the lung, TNF-alpha and active PAI-1 production, DNA binding activity of NF-kappaB, and NF-kappaB activation. No statistically significant increase of neutrophil infiltration and inflammatory cytokine production was found in the mice ventilated at 6 mL/kg using hyperoxia. Hyperoxia-induced augmentation of VILI was attenuated in mice with pharmacologic inhibition of NF-kappaB activity by SN-50. We conclude that hyperoxia increased high-tidal-volume-induced cytokine production and neutrophil influx through activation of the NF-kappaB pathway.
24. Suppression of NF-kappaB activity by NDRG2 expression attenuates the invasive potential of highly malignant tumor cells. Downregulation of the N-myc downstream-regulated gene 2 (NDRG2) gene is involved in the progression of aggressive forms of cancer, along with the poor prognosis of cancer patients. In the current study, we examined the effect of NDRG2 expression on the metastatic potential of HT1080 human fibrosarcoma and B16F10 murine melanoma cells in both in vitro and in vivo systems. In gelatin zymography, NDRG2 expression remarkably suppressed the matrix metalloproteinase (MMP)-9 activity and slightly inhibited MMP-2 activity of both cell lines. Tumor **migration** and invasion in vitro were significantly reduced by NDRG2 expression, and NDRG2 inhibited tumor cell proliferation in an anchorage-independent semisolid agar assay. Specifically, we found that NDRG2 affects invasion through suppression of nuclear factor kappa B (NF-kappaB) activity. In animal experiments, subcutaneously injected B16F10-NDRG2 cells showed delayed tumor growth compared with B16F10-mock cells. Furthermore, severe metastasis from primary tumor mass into the draining lymph nodes was observed after injection of B16F10-mock cells, but not with B16F10-NDRG2 cells. Pulmonary metastasis after intravenous injection of B16F10 cells was also reduced by NDRG2 expression. Intra- and peritumoral angiogenesis that is critical for the tumor growth and metastasis was clearly found in tumors after injection with B16F10-mock cells, whereas it was impaired in tumors after injection with B16F10-NDRG2 cells. Collectively, our data show that NDRG2 expression significantly suppresses tumor invasion by inhibiting MMP activities, which are regulated through the NF-kappaB signaling. Moreover, results from animal experiments provide evidence for the regulatory role of the NDRG2 gene in metastatic tumors.
25. Exendin-4 ameliorates oxidized-LDL-induced inhibition of macrophage **migration** in vitro via the NF-κB pathway. To investigate the effects of the glucagon-like peptide-1 (GLP-1) receptor agonist exendin-4 on oxidized low-density lipoprotein (ox-LDL)-induced inhibition of macrophage **migratio**n and the mechanisms underlying the effects of exendin-4.Primary peritoneal macrophages were extracted from the peritoneal cavity of mice treated with 3% thioglycollate (2 mL, ip). **Migratio**n of the macrophages was examined using a cell **migratio**n assay. Macrophage **migratio**n-related factors including leptin-like ox-LDL receptor (LOX-1), cyclooxygenase 2 (COX-2), tumor necrosis factor (TNF)-α, interleukin-1 (IL-1)β, matrix metalloproteinase-2 (MMP-2), intercellular adhesion molecule (ICAM)-1 and macropha**ge migrat**ion inhibitory factor (MIF) were measured using semi-quantitative RT-PCR. Expression of MIF and ICAM-1 proteins was examined with ELISA. Gelatin zymography was used to evaluate the activity of MMP-9. Activation of the NF-κB pathway was determined by confocal laser scanning microscopy.Treatment of the macrophages with ox-LDL (50 μg/mL) markedly suppressed the macrop**hage migr**ation. Furthermore, ox-LDL treatment substantially increased the expression of the macrop**hage migr**ation-related factors, the activity of MMP-9 and the translocation of the NF-κB p65 subunit. These effects of ox-LDL were significantly ameliorated by pretreatment with the specific NF-κB inhibitor ammonium pyrrolidine dithiocarbamate (100 μmol/L). These effects of ox-LDL were also significantly ameliorated by pretreatment with exendin-4 (25 and 50 nmol/L).Exendin-4 ameliorates the inhibition of ox-LDL on mac**rophage m**igration in vitro, via suppressing ox-LDL-induced expression of ICAM-1 and MIF, which is probably mediated by the NF-κB pathway.
26. Inflammation induces lymphangiogenesis through up-regulation of VEGFR-3 mediated by NF-kappaB and Prox1. The concept of inflammation-induced lymphangiogenesis (ie, formation of new lymphatic vessels) has long been recognized, but the molecular mechanisms remained largely unknown. The 2 primary mediators of lymphangiogenesis are vascular endothelial growth factor receptor-3 (VEGFR-3) and Prox1. The key factors that regulate inflammation-induced transcription are members of the nuclear factor-kappaB (NF-kappaB) family; however, the role of NF-kappaB in regulation of lymphatic-specific genes has not been defined. Here, we identified VEGFR-3 and Prox1 as downstream targets of the NF-kappaB pathway. In vivo time-course analysis of inflammation-induced lymphangiogenesis showed activation of NF-kappaB followed by sequential up-regulation of Prox1 and VEGFR-3 that preceded lymphangiogenesis by 4 and 2 days, respectively. Activation of NF-kappaB by inflammatory stimuli also elevated Prox1 and VEGFR-3 expression in cultured lymphatic endothelial cells, resulting in increased proliferation and **migration**. We also show that Prox1 synergizes with the p50 of NF-kappaB to control VEGFR-3 expression. Collectively, our findings suggest that induction of the NF-kappaB pathway by inflammatory stimuli activates Prox1, and both NF-kappaB and Prox1 activate the VEGFR-3 promoter leading to increased receptor expression in lymphatic endothelial cells. This, in turn, enhances the responsiveness of preexisting lymphatic endothelium to VEGFR-3 binding factors, VEGF-C and VEGF-D, ultimately resulting in robust lymphangiogenesis.
27. Role of the adipocyte-specific NF-κB activity in the regulation of IP-10 and T cell **migratio**n. Infiltration of immune cells into adipose tissue plays a central role in the pathophysiology of obesity-associated low-grade inflammation. The aim of this study was to analyze the role of adipocyte NF-κB signaling in the regulation of the chemokine/adipokine interferon-γ-induced protein 10 kDa (IP-10) and adipocyte-mediated T ce**ll migrat**ion. Therefore, the regulation of IP-10 was investigated in adipose tissue of male C57BL/6J mice, primary human and 3T3-L1 preadipocytes/adipocytes. To specifically block the NF-κB pathway, 3T3-L1 cells stably overexpressing a transdominant mutant of IκBα were generated, and the chemical NF-κB inhibitor Bay117082 was used. Adipocyte-mediated **T cell mi**gration was assesse**d by a mi**gration assay. It could be shown that IP-10 expression was higher in mature adipocytes compared with preadipocytes. Induced IP-10 expression and secretion were completely blocked by an NF-κB inhibitor in 3T3-L1 and primary human adipocytes. Stable overexpression of a transdominant mutant of IκBα in 3T3-L1 adipocytes led to an inhibition of basal and stimulated IP-10 expression and secretio**n. T cell** migration was induced by 3T3-L1 adipocyte-conditioned medium, and both basal and induc**ed T cell** migration was strongly inhibited by stable overexpression of a transdominant IκBα mutant. In addition, with the use of an anti-IP-10 antibody, a significant decrease of adipocyte-ind**uced T ce**ll migration was shown. In conclusion, in this study, we could demonstrate that the NF-κB pathway is essential for the regulation of IP-10 in 3T3-L1 and primary human adipocytes. Adipocytes rather than preadipocytes contribute to NF-κB-dependent IP-10 expression and secretion. Furthermore, NF-κB-dependent factors and especially IP-10 represent novel signals from adipocytes to **induce T** cell migration.
28. NFκB activation by modified vaccinia virus as a novel strategy to enhance neutrophil **migratio**n and HIV-specific T-cell responses. Neutrophils are antigen-transporting cells that generate vaccinia virus (VACV)-specific T-cell responses, yet how VACV modulates neutrophil recruitment and its significance in the immune response are unknown. We generated an attenuated VACV strain that expresses HIV-1 clade C antigens but lacks three specific viral genes (A52R, K7R, and B15R). We found that these genes act together to inhibit the NFκB signaling pathway. Triple ablation in modified virus restored NFκB function in macrophages. After virus infection of mice, NFκB pathway activation led to expression of several cytokines/chemokines that increased **the migra**tion of neutrophil populations (Nα and Nβ) to the infection site. Nβ cells displayed features of antigen-presenting cells and activated virus-specific CD8 T cells. Enhanced neutrophil trafficking to the infection site correlated with an increased T-cell response to HIV vector-delivered antigens. These results identify a mechanism for poxvirus-induced immune response and alternatives for vaccine vector design.
29. Epidermal growth factor (EGF)-induced corneal epithelial wound healing through nuclear factor κB subtype-regulated CCCTC binding factor (CTCF) activation. Epidermal growth factor (EGF) plays an important role in corneal epithelial **migratio**n and proliferation to improve the wound healing process. This study aimed to understand the role of NFκB in EGF-induced corneal epithelial wound healing through regulation of CTCF activity, which plays important roles in cell motility an**d migrati**on to promote wound healing. The effect of NFκB p50 on corneal epithelial wound healing was investigated by comparing the eyes of wild-type and p50 knockout mice. We found that there was a significant retardation in corneal epithelial wound healing in the corneas of p50 knockout mice. Wound closure rates were measured in human corneal epithelial cells transfected with an NFκB activation-sensitive CTCF expression construct to demonstrate the effect of human CTCF expression under the control of EGF-induced NFκB activation on wound healing. EGF stimulation activated NFκB, which directly triggered the expression of the exogenous human CTCF in transfected cells and, subsequently, promoted human corneal epithelial cell moti**lity, mig**ration, and wound healing. Overexpression of CTCF in corneal epithelial cells and mouse corneas significantly enhanced the wound healing process. Furthermore, the effect of overexpressing NFκB p50 in corneal epithelial cells on the promotion of wound healing was abolished by knockdown of CTCF with CTCF-specific shRNA. Thus, a direct regulatory relationship between EGF-induced NFκB p50 and CTCF activation affecting corneal epithelial wound healing has been established, indicating that CTCF is, indeed, a NFκB p50-targeted and effective gene product in the core transcriptional network downstream from the growth factor-induced NFκB signaling pathway.
30. TNF-α mediates mitochondrial uncoupling and enhances ROS-dependent cell **migratio**n via NF-κB activation in liver cells. Development of hepatocellular carcinoma (HCC) is accompanied by a continuous increase in reactive oxygen species (ROS) levels. To investigate the primary source of ROS in liver cells, we used tumor necrosis factor-alpha (TNF-α) as stimulus. Applying inhibitors against the respiratory chain complexes, we identified mitochondria as primary source of ROS production. TNF-α altered mitochondrial integrity by mimicking a mild uncoupling effect in liver cells, as indicated by a 40% reduction in membrane potential and ATP depletion (35%). TNF-α-induced ROS production activated NF-κB 3.5-fold and subsequently enh**anced mig**ration up to 12.7-fold. This study identifies complex I and complex III of the mitochondrial respiratory chain as point of release of ROS upon TNF-α stimulation of liver cells, which enhance**s cell mi**gration by activating NF-κB signalling.
31. Developmental endothelial locus-1 inhibits MIF production through suppression of NF-κB in macrophages. Macrophage **migratio**n inhibitory factor (MIF) is a proinflammatory cytokine that regulates leukocyte recruitment, thereby playing a pivotal role in the regulation of innate and adaptive immunity and tumor progression. Elevated levels of MIF are associated with numerous inflammatory disorders and cancers. To determine whether developmental endothelial locus-1 (Del-1) regulated MIF, RAW264.7 macrophages were treated with Del-1 and assessed using ELISA. The results showed that MIF was downregulated in macrophages by Del-1, an endogenous anti-inflammatory protein that was previously shown to limit leukocyte adhesion and **migratio**n. Treatment of RAW264.7 macrophages with Del-1 inhibited constitutive and lipopolysaccharide (LPS)-induced MIF secretion. Recombinant Del-1 protein attenuated the phosphorylation of IκBα induced by a relatively low concentration of LPS in THP-1 monocytes, but did not inhibit IκBα phosphorylation in response to a relatively high concentration of LPS. Concomitantly, translocation of NF-κB to the nucleus was inhibited by Del-1 in LPS-activated macrophages. In addition, conditioned medium harvested from cells transfected with a Del-1 expression plasmid suppressed NF-κB activation in response to relatively low concentrations of TNF-α, albeit not the activation that was induced by a relatively high concentration of TNF-α. On the other hand, although Del-1 enhanced the macrophage expression of p53, a known negative regulator of MIF production, MIF production was not significantly affected by the level of p53 in mouse bone marrow-derived macrophages. These findings suggested that Del-1 controls NF-κB-activated MIF production in macrophages, and the potential application of Del-1 to therapeutic modalities for chronic inflammation-associated cancers.
32. Modulation of matrix metalloproteinase-9 activity by hyaluronan is dependent on NF-kappaB activity in lymphoma cell lines with dissimilar invasive behavior. Expression and activity of matrix metalloproteinase-9 (MMP-9) as well as its relationship with hyaluronan (HA) and NF-kappaB activity were analyzed in two murine lymphoma cell lines with dissimilar **migration** and invasive behavior. MMP activity was evaluated by zymograms in supernatants, membrane extracts of tumor cells, and in the organs invaded by these cells. The more aggressive LBLa cell line showed MMP-9 activity in vitro, which increased after HA treatment and was blocked by anti-CD44 mAb. Such activity was not found in the less aggressive LBLc. MMP-9 and MMP-2 activity was found in organs invaded by both cell lines, although differential MMP-9 activity was observed in lung infiltrated only by LBLa cell line. NF-kappaB activation was evaluated to determine whether differential activity of MMP-9 was dependent on downstream signaling pathway, showing higher NF-kappaB activity in the more aggressive LBLa cell line. Our results showed that MMP-9 activity modulated by HA through NF-kappaB signaling pathway may be involved in the aggressive behavior of LBLa.
33. Late stages of T cell maturation in the thymus involve NF-κB and tonic type I interferon signaling. Positive selection occurs in the thymic cortex, but critical maturation events occur later in the medulla. Here we defined the precise stage at which T cells acquired competence to proliferate and emigrate. Transcriptome analysis of late gene changes suggested roles for the transcription factor NF-κB and interferon signaling. Mice lacking the inhibitor of NF-κB (IκB) kinase (IKK) kinase TAK1 underwent normal positive selection but exhibited a specific block in functional maturation. NF-κB signaling provided protection from death mediated by the cytokine TNF and was required for proliferation **and emigr**ation. The interferon signature was independent of NF-κB; however, thymocytes deficient in the interferon-α (IFN-α) receptor IFN-αR showed reduced expression of the transcription factor STAT1 and phenotypic abnormality but were able to proliferate. Thus, both NF-κB and tonic interferon signals are involved in the final maturation of thymocytes into naive T cells.
34. Inhibition of the classical NF-kappaB pathway prevents osteoclast bone-resorbing activity. The classical NF-kappaB pathway plays an important role in osteoclast formation and differentiation; however, the role of NF-kappaB in osteoclast bone-resorbing activity is not well understood. To elucidate whether NF-kappaB is important for osteoclast bone-resorbing activity, we used a selective peptide inhibitor of the classical NF-kappaB pathway named the NBD peptide. Osteoclasts were generated using bone marrow macrophages in the presence of M-CSF and RANKL. The NBD peptide dose-dependently blocked the bone-resorbing activity of osteoclasts by reducing area, volume (p < 0.001) and depths (p < 0.05) of pits. The reduced resorption by the peptide was due to reduced osteoclast bone-resorbing activity, but not reduced differentiation as the number of osteoclasts was similar in all groups. The peptide inhibited bone resorption by reducing TRAP activity, disrupting actin rings and preventing osteoclast **migration**. Gene expressions of a panel of bone resorption markers were significantly reduced. The NBD peptide dose-dependently reduced the RANKL-induced c-Src kinase activity, which is important for actin ring formation and osteoclast bone resorption. Therefore, these data suggest that the classical NF-kappaB pathway plays a pivotal role in osteoclast bone-resorbing activity.
35. Nuclear **migration** of NF-kappa B correlates with TNF-alpha mRNA accumulation. Previous work on the transcriptional regulation of the mouse TNF-alpha promoter had indicated a major role for the NF-kappa B transcription factor in the induction of the gene by endotoxin. However, similar studies using the human promoter failed to establish a role for this factor. We measured the nuclear **migration** of NF-kappa B and the accumulation of TNF-alpha mRNA in murine T lymphoblasts and bone marrow-derived macrophages (BMDM) under different activation conditions, seeking to establish a correlation. Activation of NF-kappa B and accumulation of TNF-alpha mRNA correlated semiquantitatively under the following conditions: (1) inhibition of NF-kappa B by dithiocarbamates; (2) induction of TNF synthesis by taxol; (3) partial induction of TNF-alpha mRNA by various inducers in macrophages from lpsd mice; and (4) inhibition of NF-kappa B activation by a protease inhibitor. However, inhibition of TNF-alpha mRNA accumulation by cAMP inducers had no effect on NF-kappa B induction. We conclude that NF-kappa B translocation is necessary, but not sufficient for the transcriptional induction of the TNF-alpha gene by LPS in macrophages or by phorbol ester and ionomycin in T lymphocytes.
36. The compromise of macrophage functions by hyperoxia is attenuated by ethacrynic acid via inhibition of NF-κB-mediated release of high-mobility group box-1. The prolonged exposure to hyperoxia can compromise macrophage functions and contribute to the development of ventilator-associated pneumonia. High levels of extracellular high-mobility group box-1 (HMGB1) in the airways of mice exposed to hyperoxia can directly cause macrophage dysfunction. Hence, inhibition of the release of nuclear HMGB1 into the extracellular milieu may help to maintain macrophage functions under hyperoxic conditions. The present study investigates whether ethacrynic acid (EA) affects hyperoxia-induced HMGB1 release from macrophages and improves their functions. Macrophage-like RAW 264.7 cells and bone marrow-derived macrophages were exposed to different concentrations of EA for 24 hours in the presence of 95% O2. EA significantly decreased the accumulation of extracellular HMGB1 in cultured media. Importantly, the phagocytic activity and **migratio**n capability of macrophages were significantly enhanced in EA-treated cells. Interestingly, hyperoxia-induced NF-κB activation was also inhibited in these cells. To determine whether NF-κB plays a role in hyperoxia-induced HMGB1 release, BAY 11-7082, an inhibitor of NF-κB activation, was used. Similar to EA, BAY 11-7082 significantly inhibited the accumulation of extracellular HMGB1 and improved hyperoxia-compromised macroph**age migra**tion and phagocytic activity. Furthermore, 24-hour hyperoxic exposure of macrophages caused hyperacetylation of HMGB1 and its subsequent cytoplasmic translocation and release, which were inhibited by EA and BAY 11-7082. Together, these results suggest that EA enhances hyperoxia-compromised macrophage functions by inhibiting HMGB1 hyperacetylation and its release from macrophages, possibly through attenuation of the NF-κB activation. Therefore, the activation of NF-κB could be one of the underlying mechanisms that mediate hyperoxia-compromised macrophage functions.
37. Down-regulation of MIF by NFκB under hypoxia accelerated neuronal loss during stroke. Neuronal apoptosis is one of the major causes of poststroke neurological deficits. Inflammation during the acute phase of stroke results in nuclear translocation of NFκB in affected cells in the infarct area. Macrophag**e migrati**on inhibitory factor (MIF) promotes cardiomyocyte survival in mice following heart ischemia. However, the role of MIF during stroke remains limited. In this study, we showed that MIF expression is down-regulated by 0.75 ± 0.10-fold of the control in the infarct area in the mouse brains. Two functional cis-acing NFκB response elements were identified in the human MIF promoter. Dual activation of hypoxia and NFκB signaling resulted in significant reduction of MIF promoter activity to 0.86 ± 0.01-fold of the control. Furthermore, MIF reduced caspase-3 activation and protected neurons from oxidative stress- and in vitro ischemia/reperfusion-induced apoptosis. H2O2 significantly induced cell death with 12.81 ± 0.58-fold increase of TUNEL-positive cells, and overexpression of MIF blocked the H2O2-induced cell death. Disruption of the MIF gene in MIF-knockout mice resulted in caspase-3 activation, neuronal loss, and increased infarct development during stroke in vivo. The infarct volume was increased from 6.51 ± 0.74% in the wild-type mice to 9.07 ± 0.66% in the MIF-knockout mice. Our study demonstrates that MIF exerts a neuronal protective effect and that down-regulation of MIF by NFκB-mediated signaling under hypoxia accelerates neuronal loss during stroke. Our results suggest that MIF is an important molecule for preserving a longer time window for stroke treatment, and strategies to maintain MIF expression at physiological level could have beneficial effects for stroke patients.
38. Silencing of ST6GalNAc I suppresses the proliferation, **migration** and invasion of hepatocarcinoma cells through PI3K/AKT/NF-κB pathway. ST6GalNAc I is the major Sialyl-Tn antigen (STn) synthase that is highly correlated with tumor invasion and metastasis. However, the roles and molecular mechanisms by which ST6GalNAc I mediates the malignant phenotypes of hepatocarcinoma cells still remain poorly unknown. In this study, we investigated the expression of STn and ST6GalNAc I in mouse hepatocarcinoma cell lines Hca-F, Hca-P, and Hepa1-6, which have different metastatic potential, as compared with normal mouse liver cell line IAR-20. The results showed that the expression of ST6GalNAc I and STn in Hca-F and Hca-P cells was much higher than that in Hepa1-6 and IAR20 cells. Knockdown of ST6GalNAc I by shRNA in Hca-F cells significantly decreased the expression of STn and inhibited the growth of tumor cells in vitro and in vivo. This reduction of ST6GalNAc I expression also led to the decreased **migratio**n and invasion of Hca-F cells. Furthermore, we found that ST6GalNAc I knockdown inhibited the expression levels of PI3k, p-Akt473, p-Akt308, NF-κB, and their downstream molecules. Together, our results suggest a role of ST6GalNAc I in promoting the growth and invasion of hepatocarcinoma cells through regulating PI3K/AKT signaling, and ST6GalNAc I might be a promising marker for the prognosis and therapy of hepatocarcinoma.
39. Poly(ADP-ribose) polymerase inhibition down-regulates expression of metastasis-related genes in CT26 colon carcinoma cells. The current study was designed to test the hypothesis that inhibition of poly(ADP-ribose) polymerase in colorectal cancer mediates down-regulation of metastasis-related gene expression through the regulation of nuclear factor-kappaB (NF-kappaB) activity.Mouse colon carcinoma cells (CT26) were treated with and without the PARP inhibitor 5-aminoisoquinolin-1(2H)-one hydrochloride (5-AIQ). We investigated adhesion, **migration** and invasion of differently treated CT26 cells. In addition, the expression levels of PARP, NF-kappaB, integrin beta(1), MMP-9 and MMP-2 as well as the activities of NF-kappaB, MMP-9 and MMP-2 were determined by Western blot, electrophoretic mobility gel shift assay and zymography, respectively.Inhibition ofPARP attenuated the adhesion of CT26 cells to the extracellular matrix and their **migration** and invasion through Matrigel. In addition, the results of Western blot showed that the expression levels of PARP, NF-kappaB, integrin beta(1), MMP-9 and MMP-2 were reduced in 5-AIQ-treated CT26 cells; the activities of NF-kappaB, MMP-9 and MMP-2 were also suppressed.Inhibition of PARP down-regulates the expression of metastasis-related genes in mouse colon carcinoma cells. This could be, at least in part, through the regulation of NF-kappaB activity, but the precise mechanisms of action remain to be elucidated.
40. CXCR4 expression on pathogenic T cells facilitates their bone marrow infiltration in a mouse model of aplastic anemia. Aplastic anemia (AA) is a disease characterized by T-cell-mediated destruction of bone marrow (BM) hematopoietic stem and progenitor cells. Physiologically, T cells migrate to the BM in response to chemokines, such as SDF-1α, the ligand for CXCR4. However, how T cells traffic to the BM in AA is poorly understood. CXCR4 is aberrantly expressed in immune-mediated diseases and its regulation by nuclear factor-κB (NF-κB) in cancer models is well documented. In this study, we show that CXCR4 is highly expressed on BM-infiltrating CD4(+) and CD8(+) T cells in a mouse model of AA. Inhibiting CXCR4 in AA mice, using CXCR4(-/-) splenocytes or AMD3100, significantly reduced BM infiltration of T cells. We also report that NF-κB occupancy at the CXCR4 promoter is enhanced in BM-infiltrating CD8(+) T cells of AA mice. Moreover, inhibiting NF-κB signaling in AA mice using Bay11 or dehydroxymethylepoxyquinomicin, or transferring p50(-/-) splenocytes, decreased CXCR4 expression on CD8(+) T cells, significantly reduced BM infiltration of T cells, and strongly attenuated disease symptoms. Remarkably, therapeutic administration of Bay11 significantly extended survival of AA mice. Overall, we demonstrate that CXCR4 medi**ates migr**ation of pathogenic T cells to the BM in AA mice, and inhibiting NF-κB signaling may represent a novel therapeutic approach to treating AA.
41. BRAF activated non-coding RNA (BANCR) promoting gastric cancer cells proliferation via regulation of NF-κB1. Long non-coding RNA, BANCR, has been demonstrated to contribute to the proliferation and **migratio**n of tumors. However, its molecular mechanism underlying gastric cancer is still unknown. In present study, we investigated whether BANCR was involved in the development of gastric cancer cells via regulation of NF-κB1.Human gastric cancer tissues were isolated as well as human gastric cell lines MGC803 and BGC823 were cultured to investigate the role of BANCR in gastric cancer.BANCR expression was significantly up-regulated in gastric tumor tissues and gastric cell lines. Down-regulation of BANCR inhibited gastric cancer cell growth and promoted cell apoptosis, and it also contributed to a significant decrease of NF-κB1 (P50/105) expression and 3'UTR of NF-κB1 activity. Overexpression of NF-κB1 reversed the effect of BANCR on cancer cell growth and apoptosis. MiroRNA-9 (miR-9) targeted NF-κB1, and miR-9 inhibitor also reversed the effects of BANCR on gastric cancer cell growth and apoptosis.BANCR was highly expressed both in gastric tumor tissues and in cancer cells. NF-κB1 and miR-9 were involved in the role of BANCR in gastric cancer cell growth and apoptosis.
42. Targeted deletion of the inhibitory NF-kappaB p50 subunit in bone marrow-derived cells improves collateral growth after arterial occlusion. Adaptive collateral artery growth (arteriogenesis) is an important mechanism to maintain tissue perfusion upon arterial obstruction. Leucocytes and inflammatory mediators play a crucial role in this process. Depletion of the nuclear factor kappa B (NF-κB) p50 subunit modulates inflammatory processes in cardiovascular disease. We hypothesized that NF-κB p50 is a regulator of the inflammatory response after arterial occlusion and subsequent collateral perfusion.Unilateral femoral artery ligation was performed in NF-κB p50-/- and wild-type (Wt, B6/129PF2) mice. Seven days after arterial occlusion, tissue perfusion restoration was significantly enhanced in NF-κB p50-/- mice compared with Wt mice (42.9 ± 3.9 vs. 32.0 ± 2.6% perfusion recovery, P = 0.04). Transplantation of NF-κB p50-/- bone marrow (bm) into Wt mice and vice versa showed that the effect of p50 subunit depletion can be predominantly attributed to the bone marrow-derived circulating cells (NF-κB p50-/- bm in Wt mice 42.1 ± 1.5%, Wt bm in NF-κB p50-/- mice 35.4±1.5% perfusion recovery). Histological analyses revealed a more elaborate extravasation of monocytes in hindlimb tissue of NF-κB p50-/- mice. Chemotaxis assays confirmed th**e increas**ed migration ability of NF-κB p50-/- monocytes, which may be due to an observed increased integrin expression. Upon stimulation of blood from NF-κB p50-/- and Wt mice more interleukin-6 was produced, confirming the pro-inflammatory phenotype in absence of the p50 subunit.Depletion of the NF-κB p50 subunit enhances collateral artery growth. Its absence in circulating cells improves tissue perfusion restoration after femoral artery ligation by increasing macrophage influx into the growing collateral vessels.
43. NF-kappaB activation, dependent on acetylation/deacetylation, contributes to HIF-1 activity and **migration** of bone metastatic breast carcinoma cells. Here, we show that NF-kappaB-HIF-1 interaction contributed to breast cancer metastatic capacity by means of an incomplete epithelial/mesenchymal transition and influencing **migration**, as shown in 1833 (human) and 4T1 (mouse) metastatic cells after different stimuli. The 1833 and the transforming growth factor-beta1-exposed 4T1 cells showed both epithelial (E-cadherins) and mesenchymal (N-cadherins and vimentin) markers, and common mechanisms contributed to the retention of certain epithelial characteristics and the control of **migration**. The complex NF-kappaB-HIF-1 reciprocal regulation and the enhanced c-Jun expression played a functional role in exacerbating the invasiveness of 1833 cells after p50/p65 transfection and of 4T1 cells exposed to transforming growth factor-beta1. Twist expression seemed to exert a permissive role also regulating epithelial/mesenchymal transition markers. After c-Src wild-type (Srcwt) transfection, c-Src-signal transducer overexpression in 1833 cells increased HIF-1 transactivating activity and invasiveness, and changed E-cadherin/N-cadherin ratio versus mesenchymal phenotype. The transcription factor pattern and the motile phenotype of metastatic 1833 cells were influenced by p65-lysine acetylation and HDAC-dependent epigenetic mechanisms, which positively regulated basal NF-kappaB and HIF-1 activities. However, HDAC3 acted as a corepressor of NF-kappaB activity in parental MDA-MB231 cells, thus explaining many differences from the derived 1833 clone, including reduced HIF-1alpha and c-Jun expression. Invasiveness was differently affected by HDAC knockdown in 1833 and MDA-MB231 cells. We suggest that acetylation/deacetylation are critical in establishing the bone-metastatic gene signature of 1833 cells by regulating the activity of NF-kappaB and HIF-1, and further clarify the epigenetic control of transcription factor network in the motile phenotype of 1833 cells.
44. Sequestration of PDLIM2 in the cytoplasm of monocytic/macrophage cells is associated with adhesion and increased nuclear activity of NF-kappaB. PDLIM2 (Mystique/SLIM) is a postsynaptic density-95/discs large/zonula occludens-1-Lin-11, Isl-1, Mec-3 (PDZ-LIM) domain protein expressed in the nucleus of T lymphocytes, where it promotes degradation of the p65 subunit of NF-kappaB. It is also expressed at the cytoskeleton in epithelial cells, where it is essential for cell **migration**. It is not known whether PDLIM2 function at the nucleus and cytoskeleton is linked and whether PDLIM2 subcellular location is regulated in hematopoietic cells. To investigate this, we used the human monocytic leukemia cell line THP-1 that can differentiate into adherent macrophages and the adherent murine macrophage cell line RAW264.7. PMA-induced differentiation of THP-1 cells resulted in increased accumulation of PDLIM2. In differentiated cells, PDLIM2 exhibited retarded mobility indicative of serine phosphorylation, which could be reversed by phosphatases and by inhibition of protein kinase C or ERK kinases. In nondifferentiated THP-1 cells, PDLIM2 was located predominantly in the nucleus, whereas in differentiated cells, PDLIM2 was located predominantly in the cytoplasm. Suppression of PDLIM2 expression in THP-1 and RAW 264.7 cells resulted in decreased adhesion, increased NF-kappaB transcription reporter activity, and increased LPS-induced TNF-alpha production. Overexpression of PDLIM2 in THP-1 cells enhanced cell adhesion. Overall, these findings indicate that PDLIM2 sequestration in the cytoplasm is associated with cell adhesion and increased nuclear activity of NF-kappaB p65. The data suggest that sequestration of PDLIM2 at the cytoskeleton regulates its nuclear function.
45. Cyp1B1 expression promotes angiogenesis by suppressing NF-κB activity. Nuclear factor-κB (NF-κB) is a master regulator of genes that control a large number of cellular processes, including angiogenesis and inflammation. We recently demonstrated that cytochrome P-450 1B1 (Cyp1B1) deficiency in endothelial cells (EC) and pericytes (PC) results in increased oxidative stress, alterations **in migrat**ion, attenuation of capillary morphogenesis, sustained activation of NF-κB, and increased expression of thrombospondin-2 (TSP2), an endogenous inhibitor of angiogenesis. On the basis of a growing body of evidence that phenethyl isothiocyanate (PEITC) and pyrrolidine dithiocarbamate (PDTC) function as antioxidants and suppressors of NF-κB activation, we investigated their potential ability to restore a normal phenotype in Cyp1B1-deficient (cyp1b1(-/-)) vascular cells. PEITC and PDTC inhibited NF-κB activity and expression in cyp1b1(-/-) EC and PC. We also observed restorati**on of mig**ration and capillary morphogenesis of cyp1b1(-/-) EC and decreased cellular oxidative stress in cyp1b1(-/-) EC and PC without restoration to normal TSP2 levels. In addition, expression of a dominant-negative inhibitor κBα, a suppressor of NF-κB activation, decreased NF-κB activity without affecting TSP2 expression in these cells. In contrast, knockdown of TSP2 expression resulted in attenuation of NF-κB activity in cyp1b1(-/-) vascular cells. Furthermore, expression of TSP2 in wild-type (cyp1b1(+/+)) cells resulted in increased NF-κB activity. Together, our results demonstrate an important role for TSP2 in modulation of NF-κB activity and attenuation of angiogenesis. Thus Cyp1B1 expression in vascular cells plays an important role in the regulation of vascular homeostasis through modulation of the cellular reductive state, TSP2 expression, and NF-κB activation.

#### GeneRIFs

1. NF-kappaB in NG2(+) cells promotes myoblast **migration** to the tips of myofibers through cell-cell contact through expression of ephrinA5 from NG2(+) cells
2. NFkappaB pathway activation led to expression of several cytokines/chemokines that increased the **migration** of neutrophil populations (Nalpha and Nbeta) to the infection site.
3. Data indicate that tumor necrosis factor-alpha (TNF-alpha)-induced reactive oxygen species (ROS) production activates NF-kappaB and enhances liver cell **migration**.
4. NF-kappaB-HIF-1 interaction contributed to breast cancer metastatic capacity by means of an incomplete epithelial/mesenchymal transition and influencing **migration**.

### ECM

#### PubMed

1. NF-kappa B activation mediates the cross-talk between extracellular matrix and interferon-gamma (IFN-gamma) leading to enhanced monokine induced by IFN-gamma (MIG) expression in macrophages. In intact tissue, the extracellular matrix (**ECM**) provides support and helps maintain homeostasis but is considered biologically inert. In the setting of inflammation, not only is the **ECM** the target of inflammation, but its breakdown products modulate the magnitude and quality of an immune response. Fragments of the **ECM** component hyaluronan (HA) induce macrophage expression of chemokines, cytokines, and growth factors as well greatly enhance IFN-gamma-induced MIG expression. In this report, we demonstrate that the synergistic induction of MIG by HA and IFN-gamma occurs at the level of transcription via NF-kappaB. Using electrophoretic mobility shift assays and reporter assays, we have identified two NF-kappaB sites proximal to the IFN-gamma-responsive element-1 (gammaRE-1) that mediate this effect. Interestingly, our experiments also revealed a critical role for NF-kappaB in mediating IFN-gamma-induced MIG expression independent of HA. These data emphasize the ability of "degraded self" to activate/modify immune responses through the NF-kappaB pathway.
2. Hyaluronan fragments activate an NF-kappa B/I-kappa B alpha autoregulatory loop in murine macrophages. Macrophages play an important role in the acute tissue inflammatory response through the release of cytokines and growth factors in response to stimuli such as lipopolysaccharide (LPS). Macrophage inflammatory effector functions are also influenced by interactions with the extracellular matrix (**ECM**). Such macrophage-**ECM** interactions may be important in regulating chronic inflammatory responses. Recent evidence has suggested that hyaluronan (HA), a glycosaminoglycan (GAG) component of **ECM** can induce inflammatory gene expression in murine macrophages. HA exists in its native form as a large polymer, but is found as smaller fragments under inflammatory conditions. The NF-kappa B/I-kappa B transcriptional regulatory system has been shown to be a critical component of the host inflammatory response. We examined the effects of high molecular weight HA and lower molecular weight HA fragments on NF-kappa B activation in mouse macrophages. Only the smaller HA fragments were found to activate NF-kappa B DNA binding activity. After HA stimulation, I-kappa B alpha mRNA was induced and I-kappa B alpha protein levels, which initially decreased, were restored. The induction of I-kappa Balpha expression was not observed for other GAGs. The time course of I-kappa B alpha protein regeneration in response to HA fragments was consistent with an autoregulatory mechanism. In support of this mechanism, in vitro translated murine I-kappa B alpha inhibited HA fragment-induced NF-kappa B DNA binding activity. The NF-kappa B DNA binding complex in HA-stimulated extracts was found to contain p50 and p65 subunits. Activation of the NF-kappa B/I-kappa B system in macrophages by **ECM** fragments may be an important mechanism for propagating the tissue inflammatory response.
3. NF-kappa B activation mediates the cross-talk between extracellular matrix and interferon-gamma (IFN-gamma) leading to enhanced monokine induced by IFN-gamma (MIG) expression in macrophages. In intact tissue, the extracellular matrix (**ECM**) provides support and helps maintain homeostasis but is considered biologically inert. In the setting of inflammation, not only is the **ECM** the target of inflammation, but its breakdown products modulate the magnitude and quality of an immune response. Fragments of the **ECM** component hyaluronan (HA) induce macrophage expression of chemokines, cytokines, and growth factors as well greatly enhance IFN-gamma-induced MIG expression. In this report, we demonstrate that the synergistic induction of MIG by HA and IFN-gamma occurs at the level of transcription via NF-kappaB. Using electrophoretic mobility shift assays and reporter assays, we have identified two NF-kappaB sites proximal to the IFN-gamma-responsive element-1 (gammaRE-1) that mediate this effect. Interestingly, our experiments also revealed a critical role for NF-kappaB in mediating IFN-gamma-induced MIG expression independent of HA. These data emphasize the ability of "degraded self" to activate/modify immune responses through the NF-kappaB pathway.
4. Bit-1 mediates integrin-dependent cell survival through activation of the NFkappaB pathway. Loss of properly regulated cell death and cell survival pathways can contribute to the development of cancer and cancer metastasis. Cell survival signals are modulated by many different receptors, including integrins. Bit-1 is an effector of anoikis (cell death due to loss of attachment) in suspended cells. The anoikis function of Bit-1 can be counteracted by integrin-mediated cell attachment. Here, we explored integrin regulation of Bit-1 in adherent cells. We show that knockdown of endogenous Bit-1 in adherent cells decreased cell survival and re-expression of Bit-1 abrogated this effect. Furthermore, reduction of Bit-1 promoted both staurosporine and serum-deprivation induced apoptosis. Indeed knockdown of Bit-1 in these cells led to increased apoptosis as determined by caspase-3 activation and positive TUNEL staining. Bit-1 expression protected cells from apoptosis by increasing phospho-IκB levels and subsequently bcl-2 gene transcription. Protection from apoptosis under serum-free conditions correlated with bcl-2 transcription and Bcl-2 protein expression. Finally, Bit-1-mediated regulation of bcl-2 was dependent on focal adhesion kinase, PI3K, and AKT. Thus, we have elucidated an integrin-controlled pathway in which Bit-1 is, in part, responsible for the survival effects of cell**-EC**M interactions.
5. Novel curcumin analogue UBS109 potently stimulates osteoblastogenesis and suppresses osteoclastogenesis: involvement in Smad activation and NF-κB inhibition. Bone homeostasis is maintained through a balance between osteoblastic bone formation and osteoclastic bone resorption. Bone loss is induced due to decreased osteoblastic bone formation and increased osteoclastic bone resorption with various pathologic states. Osteoporosis with its accompanying decrease in bone mass is widely recognized as a major public health problem. Pharmacologic and functional food factors may play a role in the prevention of bone loss with aging. This study was undertaken to determine the effect of curcumin analogues (curcumin, EF31, **EC**MN909, and UBS109), which were newly synthesized, on osteoblastogenesis and osteoclastogenesis in vitro. Among these compounds, UBS109 had a unique stimulatory effect on osteoblastic differentiation and mineralization. UBS109 stimulated both basal and bone morphogenic protein-2 (BMP2)-increased Smad-luciferase activity, the Smad signaling of which is related to osteoblastogenesis. Such an effect was not seen with other compounds. Moreover, UBS109 potently suppressed tumor necrosis factor-α (TNF-α)-increased osteoblastic nuclear factor kappa B (NF-κB)-luciferase activity. In addition, EF**31,** ECMN909, and UBS109 had a suppressive effect on osteoclastogenesis as compared with that of curcum**in.** ECMN909 and UBS109 potently inhibited the receptor activator of NF-κB (RANK) ligand (RANKL)-increased preosteoclastic NF-κB-luciferase activity, in which NF-κB signaling plays a pivotal role in osteoclastogenesis. In the present study, curcumin analogue UBS109 was found to have a stimulating effect on osteoblastogenesis and a suppressive effect on osteoclastogenesis in vitro, suggesting an anabolic effect of the compound on bone mass.
6. Astragaloside IV suppresses transforming growth factor-β1 induced fibrosis of cultured mouse renal fibroblasts via inhibition of the MAPK and NF-κB signaling pathways. Renal fibrosis, a progressive process characterized by the accumulation of extracellular matrix **(E**CM) leading to organ dysfunction, is a characteristic of chronic kidney diseases. Among fibrogenic factors known to regulate the renal fibrotic process, transforming growth factor-β (TGF-β) plays a central role. In the present study, we examined the effect of Astragaloside IV (AS-IV), a component of the traditional Chinese medicinal plant Astragalus membranaceus, on the processes associated with renal fibrosis in cultured mouse renal fibroblasts treated with TGF-β1. RT-PCR, western blotting, immunofluorescence staining and collagen assays showed that AS-IV suppressed TGF-β1 induced fibroblast proliferation, transdifferentiation**, a**nd ECM production in a dose-dependent manner. Examination of the underlying mechanisms showed that the effect of AS-IV on the inhibition of fibroblast differentiatio**n a**nd ECM formation were mediated by its modulation of the activity of the MAPK and NF-κB signaling pathways. Taken together, our results indicate that AS-IV alleviates renal interstitial fibrosis via a mechanism involving the MAPK and NF-κB signaling pathways and demonstrate the therapeutic potential of AS-IV for the treatment of chronic kidney diseases.

### [Ee]xtracellular [Mm]atrix

#### PubMed

1. NF-kappa B activation mediates the cross-talk between **extracellular matrix** and interferon-gamma (IFN-gamma) leading to enhanced monokine induced by IFN-gamma (MIG) expression in macrophages. In intact tissue, the **extracellular matrix** (ECM) provides support and helps maintain homeostasis but is considered biologically inert. In the setting of inflammation, not only is the ECM the target of inflammation, but its breakdown products modulate the magnitude and quality of an immune response. Fragments of the ECM component hyaluronan (HA) induce macrophage expression of chemokines, cytokines, and growth factors as well greatly enhance IFN-gamma-induced MIG expression. In this report, we demonstrate that the synergistic induction of MIG by HA and IFN-gamma occurs at the level of transcription via NF-kappaB. Using electrophoretic mobility shift assays and reporter assays, we have identified two NF-kappaB sites proximal to the IFN-gamma-responsive element-1 (gammaRE-1) that mediate this effect. Interestingly, our experiments also revealed a critical role for NF-kappaB in mediating IFN-gamma-induced MIG expression independent of HA. These data emphasize the ability of "degraded self" to activate/modify immune responses through the NF-kappaB pathway.
2. Hyaluronan fragments activate an NF-kappa B/I-kappa B alpha autoregulatory loop in murine macrophages. Macrophages play an important role in the acute tissue inflammatory response through the release of cytokines and growth factors in response to stimuli such as lipopolysaccharide (LPS). Macrophage inflammatory effector functions are also influenced by interactions with the **extracellular matrix** (ECM). Such macrophage-ECM interactions may be important in regulating chronic inflammatory responses. Recent evidence has suggested that hyaluronan (HA), a glycosaminoglycan (GAG) component of ECM can induce inflammatory gene expression in murine macrophages. HA exists in its native form as a large polymer, but is found as smaller fragments under inflammatory conditions. The NF-kappa B/I-kappa B transcriptional regulatory system has been shown to be a critical component of the host inflammatory response. We examined the effects of high molecular weight HA and lower molecular weight HA fragments on NF-kappa B activation in mouse macrophages. Only the smaller HA fragments were found to activate NF-kappa B DNA binding activity. After HA stimulation, I-kappa B alpha mRNA was induced and I-kappa B alpha protein levels, which initially decreased, were restored. The induction of I-kappa Balpha expression was not observed for other GAGs. The time course of I-kappa B alpha protein regeneration in response to HA fragments was consistent with an autoregulatory mechanism. In support of this mechanism, in vitro translated murine I-kappa B alpha inhibited HA fragment-induced NF-kappa B DNA binding activity. The NF-kappa B DNA binding complex in HA-stimulated extracts was found to contain p50 and p65 subunits. Activation of the NF-kappa B/I-kappa B system in macrophages by ECM fragments may be an important mechanism for propagating the tissue inflammatory response.
3. NF-kappa B activation mediates the cross-talk between **extracellular matrix** and interferon-gamma (IFN-gamma) leading to enhanced monokine induced by IFN-gamma (MIG) expression in macrophages. In intact tissue, the **extracellular matrix** (ECM) provides support and helps maintain homeostasis but is considered biologically inert. In the setting of inflammation, not only is the ECM the target of inflammation, but its breakdown products modulate the magnitude and quality of an immune response. Fragments of the ECM component hyaluronan (HA) induce macrophage expression of chemokines, cytokines, and growth factors as well greatly enhance IFN-gamma-induced MIG expression. In this report, we demonstrate that the synergistic induction of MIG by HA and IFN-gamma occurs at the level of transcription via NF-kappaB. Using electrophoretic mobility shift assays and reporter assays, we have identified two NF-kappaB sites proximal to the IFN-gamma-responsive element-1 (gammaRE-1) that mediate this effect. Interestingly, our experiments also revealed a critical role for NF-kappaB in mediating IFN-gamma-induced MIG expression independent of HA. These data emphasize the ability of "degraded self" to activate/modify immune responses through the NF-kappaB pathway.
4. DDR2 inhibition reduces migration and invasion of murine metastatic melanoma cells by suppressing MMP2/9 expression through ERK/NF-κB pathway. Metastatic melanoma is one of the most deadly and evasive cancers. Collagen I in the **extracellular matri**x promotes the migration and invasion of tumor cells through the production of matrix metalloproteinase (MMP) 2 and 9. Discoidin domain receptor (DDR) 2 is a collagen receptor that is implicated in several cancer types including breast and prostate cancers. However, the role of DDR2 in the migration and invasion of murine melanoma cells is less studied. In the present study, we investigated the effects and underlying mechanisms of DDR2 in migration and invasion of B16BL6 melanoma cells in response to collagen I. Results demonstrated that DDR2 is expressed and is phosphorylated by collagen I in the cells. Upon down-regulation of DDR2 using small-interfering RNA (siRNA) approach, both of the cell migratory and invasive phenotypes were significantly attenuated when compared with the control cells. This effect was mediated via suppression of MMP2/9 upon DDR2 inhibition. Furthermore, inhibition of DDR2 by specific siRNA markedly reduced the activation of extracellular regulated kinase (ERK) 1 and 2 and nuclear factor of kappa B (NF-κB) in the cells when compared with the control cells. Overall, these data demonstrated that DDR2 siRNA-mediated suppression of ERK1/2 and NF-κB could down-regulate the expressions of MMP2/9 in response to collagen I to reduce the migratory and invasive phenotypes of the cells.
5. Involvement of the NF-kappa B/matrix metalloproteinase pathway in cardiac fibrosis of mice lacking guanylyl cyclase/natriuretic peptide receptor A. Mice carrying a targeted disruption of the Npr1 gene (coding for guanylyl cyclase/natriuretic peptide receptor A (NPRA)) exhibit increased blood pressure, cardiac hypertrophy, and congestive heart failure, similar to untreated human hypertensive patients. The objective of this study was to determine whether permanent ablation of NPRA signaling in mice alters the expression of matrix metalloproteinase (MMP)-2 and MMP-9 and pro-inflammatory mediators such as tumor necrosis factor-alpha (TNF-alpha), leading to myocardial collagen remodeling. Here, we report that expression levels of the MMP-2 and MMP-9 genes were increased by 3-5-fold and that the expression of the TNF-alpha gene was enhanced by 8-fold in Npr1 homozygous null mutant (Npr1-/-) mouse hearts compared with wild-type (Npr1+/+) control mouse hearts. Myocardial fibrosis, total collagen, and the collagen type I/III ratio (p < 0.01) were dramatically increased in adult Npr1-/- mice compared with age-matched wild-type counterparts. Hypertrophic marker genes, including the beta-myosin heavy chain and transforming growth factor-beta1, were significantly up-regulated (3-5-fold) in both young and adult Npr1-/- mouse hearts. NF-kappa B binding activity in ventricular tissues was enhanced by 4-fold with increased translocation of the p65 subunit from the cytoplasmic to nuclear fraction in Npr1-/- mice. Our results show that reduced NPRA signaling activates MMP, transforming growth factor-beta1, and TNF-alpha expression in Npr1-/- mouse hearts. The findings of this study demonstrate that disruption of NPRA/cGMP signaling promotes hypertrophic growth and **extracellular matrix** remodeling, leading to the development of cardiac hypertrophy, myocardial fibrosis, and congestive heart failure.
6. CTGF promotes inflammatory cell infiltration of the renal interstitium by activating NF-kappaB. Connective tissue growth factor (CTGF) is an important profibrotic factor in kidney diseases. Blockade of endogenous CTGF ameliorates experimental renal damage and inhibits synthesis of **extracellular matrix** in cultured renal cells. CTGF regulates several cellular responses, including adhesion, migration, proliferation, and synthesis of proinflammatory factors. Here, we investigated whether CTGF participates in the inflammatory process in the kidney by evaluating the nuclear factor-kappa B (NF-kappaB) pathway, a key signaling system that controls inflammation and immune responses. Systemic administration of CTGF to mice for 24 h induced marked infiltration of inflammatory cells in the renal interstitium (T lymphocytes and monocytes/macrophages) and led to elevated renal NF-kappaB activity. Administration of CTGF increased renal expression of chemokines (MCP-1 and RANTES) and cytokines (INF-gamma, IL-6, and IL-4) that recruit immune cells and promote inflammation. Treatment with a NF-kappaB inhibitor, parthenolide, inhibited CTGF-induced renal inflammatory responses, including the up-regulation of chemokines and cytokines. In cultured murine tubuloepithelial cells, CTGF rapidly activated the NF-kappaB pathway and the cascade of mitogen-activated protein kinases, demonstrating crosstalk between these signaling pathways. CTGF, via mitogen-activated protein kinase and NF-kappaB activation, increased proinflammatory gene expression. These data show that in addition to its profibrotic properties, CTGF contributes to the recruitment of inflammatory cells in the kidney by activating the NF-kappaB pathway.
7. Fibroblast expression of an IκB dominant-negative transgene attenuates renal fibrosis. It is not clear whether interstitial fibroblasts or tubular epithelial cells are primarily responsible for the profibrotic effects of NF-κB activation during renal fibrogenesis. Here, we crossed mice carrying a conditional IκB dominant-negative transgene (IκBdN) with mice transgenic for cell-specific FSP1.Cre (FSP1(+) fibroblasts) or γGT.Cre (proximal tubular epithelia) and challenged all progeny with unilateral ureteral obstruction. We determined NF-κB activation by nuclear localization of phosphorylated p65 ((p)p65) in renal tissues after 7 days. We observed inhibition of NF-κB activation in interstitial cells and tubular epithelia in obstructed kidneys of FSP1.Cre;IκBdN and γGT.Cre;IκBdN mice, respectively, compared with IκBdN controls (P < 0.05). Dep**osition of extracell**ular matrix, however, was significantly lower in the obstructed kidneys of FSP1.Cre;IκBdN mice but not in γGT.Cre;IκBdN mice (P < 0.05). In addition, levels of mRNA encoding the profibrotic PAI-1, fibronectin-EIIIA, and type I (α1) procollagen were significantly lower in obstructed kidneys of FSP1.Cre;IκBdN mice compared with γGT.Cre;IκBdN mice (P < 0.05). Taken together, these data support a profibrotic role for fibroblasts, but not proximal tubular epithelial cells, in modulating NF-κB activation during renal fibrogenesis.
8. The interactions between pristine graphene and macrophages and the production of cytokines/chemokines via TLR- and NF-κB-related signaling pathways. Graphene may have attractive properties for some biomedical applications, but its potential adverse biological effects, in particular, possible modulation of immune responses, require further investigation. Macrophages are one of the most important effector cells of the innate immune system, and play pivotal roles in the response to graphene exposure. We have previously reported that exposure of macrophages to high concentrations of graphene triggers cell death via MAPK- and TGF-related pathways. However, little is known about the influence of exposure to low concentrations of graphene on the function of macrophages. In the present investigation, we demonstrate the biological effects of sub-cytotoxic concentrations of commercial pristine graphene on both primary murine macrophages and immortalized macrophages. Graphene significantly stimulates the secretion of Th1/Th2 cytokines including IL-1α, IL-6, IL-10, TNF-α and GM-CSF as well as chemokines such as MCP-1, MIP-1α, MIP-1β and RANTES, probably by activating TLR-mediated and NF-κB-dependent transcription. Furthermore, these graphene-induced factors alter the morphology of naïve macrophages by remodeling their actin assembly, decreasing their ability to adhere **to the extracellular** matrix, and attenuating their phagocytosis. This negative feedback of the immune response of macrophages by graphene-induced factors may play an important role in the prevention of their over-activation after graphene exposure. These findings shed light on the interaction of graphene and macrophages in vitro. Further research is needed to systematically assess the biological responses of graphene, both to improve its safety and to contribute to the design of new biological applications.
9. Poly(ADP-ribose) polymerase inhibition down-regulates expression of metastasis-related genes in CT26 colon carcinoma cells. The current study was designed to test the hypothesis that inhibition of poly(ADP-ribose) polymerase in colorectal cancer mediates down-regulation of metastasis-related gene expression through the regulation of nuclear factor-kappaB (NF-kappaB) activity.Mouse colon carcinoma cells (CT26) were treated with and without the PARP inhibitor 5-aminoisoquinolin-1(2H)-one hydrochloride (5-AIQ). We investigated adhesion, migration and invasion of differently treated CT26 cells. In addition, the expression levels of PARP, NF-kappaB, integrin beta(1), MMP-9 and MMP-2 as well as the activities of NF-kappaB, MMP-9 and MMP-2 were determined by Western blot, electrophoretic mobility gel shift assay and zymography, respectively.Inhibition ofPARP attenuated the adhesion of CT26 cells to the **extracellular matrix** and their migration and invasion through Matrigel. In addition, the results of Western blot showed that the expression levels of PARP, NF-kappaB, integrin beta(1), MMP-9 and MMP-2 were reduced in 5-AIQ-treated CT26 cells; the activities of NF-kappaB, MMP-9 and MMP-2 were also suppressed.Inhibition of PARP down-regulates the expression of metastasis-related genes in mouse colon carcinoma cells. This could be, at least in part, through the regulation of NF-kappaB activity, but the precise mechanisms of action remain to be elucidated.
10. Astragaloside IV suppresses transforming growth factor-β1 induced fibrosis of cultured mouse renal fibroblasts via inhibition of the MAPK and NF-κB signaling pathways. Renal fibrosis, a progressive process characterized by the accumulation o**f extracellular matr**ix (ECM) leading to organ dysfunction, is a characteristic of chronic kidney diseases. Among fibrogenic factors known to regulate the renal fibrotic process, transforming growth factor-β (TGF-β) plays a central role. In the present study, we examined the effect of Astragaloside IV (AS-IV), a component of the traditional Chinese medicinal plant Astragalus membranaceus, on the processes associated with renal fibrosis in cultured mouse renal fibroblasts treated with TGF-β1. RT-PCR, western blotting, immunofluorescence staining and collagen assays showed that AS-IV suppressed TGF-β1 induced fibroblast proliferation, transdifferentiation, and ECM production in a dose-dependent manner. Examination of the underlying mechanisms showed that the effect of AS-IV on the inhibition of fibroblast differentiation and ECM formation were mediated by its modulation of the activity of the MAPK and NF-κB signaling pathways. Taken together, our results indicate that AS-IV alleviates renal interstitial fibrosis via a mechanism involving the MAPK and NF-κB signaling pathways and demonstrate the therapeutic potential of AS-IV for the treatment of chronic kidney diseases.

### [Ii]nvasive

#### PubMed

1. Links between alpha-catenin, NF-kappaB, and squamous cell carcinoma in skin. Cancers display a diverse set of cellular defects, which are thought to be elicited by multiple genetic mutations. In this study, we show that when a single adherens junction protein, alpha-catenin, is removed by conditional targeting, the entire skin epidermis systematically transforms to a hyperproliferative, **invasive** tissue replete with inflammation. Transcriptional profiling and biochemical analyses reveal that alpha-catenin ablation is accompanied by activation of NF-kappaB and its proinflammatory target genes, along with genes involved in proliferation, wound healing, angiogenesis, and metastasis. Many of these alterations occur in vitro and in the embryo, and thus seem at least partly to be intrinsic to the loss of alpha-catenin. We show that reductions in alpha-catenin, activation of NF-kappaB, and inflammation are common features of human squamous cell carcinomas of the skin.
2. Synthetic cationic peptide IDR-1002 provides protection against bacterial infections through chemokine induction and enhanced leukocyte recruitment. With the rapid rise in the incidence of multidrug resistant infections, there is substantial interest in host defense peptides as templates for production of new antimicrobial therapeutics. Natural peptides are multifunctional mediators of the innate immune response, with some direct antimicrobial activity and diverse immunomodulatory properties. We have previously developed an innate defense regulator (IDR) 1, with protective activity against bacterial infection mediated entirely through its effects on the immunity of the host, as a novel approach to anti-infective therapy. In this study, an immunomodulatory peptide IDR-1002 was selected from a library of bactenecin derivatives based on its substantially more potent ability to induce chemokines in human PBMCs. The enhanced chemokine induction activity of the peptide in vitro correlated with stronger protective activity in vivo in the Staphylococcus aureus-**invasive** infection model, with a >5-fold reduction in the protective dose in direct comparison with IDR-1. IDR-1002 also afforded protection against the Gram-negative bacterial pathogen Escherichia coli. Chemokine induction by IDR-1002 was found to be mediated through a Gi-coupled receptor and the PI3K, NF-kappaB, and MAPK signaling pathways. The protective activity of the peptide was associated with in vivo augmentation of chemokine production and recruitment of neutrophils and monocytes to the site of infection. These results highlight the importance of the chemokine induction activity of host defense peptides and demonstrate that the optimization of the ex vivo chemokine-induction properties of peptides is a promising method for the rational development of immunomodulatory IDR peptides with enhanced anti-infective activity.
3. Synthetic cationic peptide IDR-1002 provides protection against bacterial infections through chemokine induction and enhanced leukocyte recruitment. With the rapid rise in the incidence of multidrug resistant infections, there is substantial interest in host defense peptides as templates for production of new antimicrobial therapeutics. Natural peptides are multifunctional mediators of the innate immune response, with some direct antimicrobial activity and diverse immunomodulatory properties. We have previously developed an innate defense regulator (IDR) 1, with protective activity against bacterial infection mediated entirely through its effects on the immunity of the host, as a novel approach to anti-infective therapy. In this study, an immunomodulatory peptide IDR-1002 was selected from a library of bactenecin derivatives based on its substantially more potent ability to induce chemokines in human PBMCs. The enhanced chemokine induction activity of the peptide in vitro correlated with stronger protective activity in vivo in the Staphylococcus aureus-**invasive** infection model, with a >5-fold reduction in the protective dose in direct comparison with IDR-1. IDR-1002 also afforded protection against the Gram-negative bacterial pathogen Escherichia coli. Chemokine induction by IDR-1002 was found to be mediated through a Gi-coupled receptor and the PI3K, NF-kappaB, and MAPK signaling pathways. The protective activity of the peptide was associated with in vivo augmentation of chemokine production and recruitment of neutrophils and monocytes to the site of infection. These results highlight the importance of the chemokine induction activity of host defense peptides and demonstrate that the optimization of the ex vivo chemokine-induction properties of peptides is a promising method for the rational development of immunomodulatory IDR peptides with enhanced anti-infective activity.
4. Changes in mitochondrial DNA alter expression of nuclear encoded genes associated with tumorigenesis. We previously reported the presence of a mtDNA mutation hotspot in UV-induced premalignant and malignant skin tumors in hairless mice. We have modeled this change (9821insA) in murine cybrid cells and demonstrated that this alteration in mtDNA associated with mtBALB haplotype can alter the biochemical characteristics of cybrids and subsequently can contribute to significant changes in their behavioral capabilities. This study shows that changes in mtDNA can produce differences in expression levels of specific nuclear-encoded genes, which are capable of triggering the phenotypes such as seen in malignant cells. From a potential list of differentially expressed genes discovered by microarray analysis, we selected MMP-9 and Col1a1 for further studies. Real-time PCR confirmed up-regulation of MMP-9 and down-regulation of Col1a1 in cybrids harboring the mtDNA associated with the skin tumors. These cybrids also showed significantly increased migration and invasion abilities compared to wild type. The non-specific MMP inhibitor, GM6001, was able to inhibit migratory and **invasive** abilities of the 9821insA cybrids confirming a critical role of MMPs in cellular motility. Nuclear factor-κB (NF-κB) is a key transcription factor for production of MMPs. An inhibitor of NF-κB activation, Bay 11-7082, was able to inhibit the expression of MMP-9 and ultimately decrease migration and invasion of mutant cybrids containing 9821insA. These studies confirm a role of NF-κB in the regulation of MMP-9 expression and through this regulation modulates the migratory **and inva**sive capabilities of cybrids with mutant mtDNA. Enhanced migration and invasion abilities caused by up-regulated MMP-9 may contribute to the tumorigenic phenotypic characteristics of mutant cybrids.
5. Modulation of NFkappaB activity and E-cadherin by the type III transforming growth factor beta receptor regulates cell growth and motility. Transforming growth factor beta is growth-inhibitory in non-transformed epithelial cells but becomes growth-promoting during tumorigenesis. The role of the type I and II receptors in tumorigenesis has been extensively studied, but the role of the ubiquitously expressed type III receptor (TbetaRIII) remains elusive. We developed short hairpin RNAs directed against TbetaRIII to investigate the role of this receptor in breast cancer tumorigenesis. Nontumorigenic NMuMG mouse cells stably expressing short hairpin RNA specific to mouse TbetaRIII (NM-kd) demonstrated increased cell growth, motility, and invasion as compared with control cells expressing shRNA to human TbetaRIII (NM-con). Reconstitution of TbetaRIII expression with rat TbetaRIII abrogated the increased growth and motility seen in the NM-kd cells. In addition, the NM-kd cells exhibited marked reduction in the expression of the adherens junction protein, E-cadherin. This loss of E-cadherin was due to increased NFkappaB activity that, in turn, resulted in increased expression of the transcriptional repressors of E-cadherin such as Snail, Slug, Twist, and Sip1. Finally, NMuMG cells in which TbetaRIII had been knocked down formed **invasive** tumors in athymic nude mice, whereas the control cells did not. These data indicate that TbetaRIII acts as a tumor suppressor in nontumorigenic mammary epithelial cells at least in part by inhibiting NFkappaB-mediated repression of E-cadherin.
6. NF-kappaB and not the MAPK signaling pathway regulates GADD45beta expression during acute inflammation. The GADD45 (growth arrest and DNA damage-inducible) family of genes is involved in the regulation of cell cycle progression and apoptosis. To study signaling pathways affecting GADD45beta expression and to examine systematically in vivo the GADD45beta expression in tissues following various toxic stresses, we created a transgenic mouse by fusing the GADD45beta promoter to firefly luciferase (Gadd45beta-luc). In vivo GADD45beta expression was assessed by measuring the luciferase activity in the Gadd45beta-luc transgenic mouse using a non-**invasive** imaging system (IVIS Imaging System, Xenogen Corporation). We found that a number of agents that induce oxidative stress, such as sodium arsenite, CCl4, lipopolysaccharide (LPS), or tumor necrosis factor-alpha, are able to induce luciferase expression throughout the entire animal. In liver, spleen, lung, intestine, kidney, and heart, we observed an induction of luciferase activity after LPS treatment, which correlates with an increase of GADD45beta mRNA in these tissues. Processes that induce DNA damage activate the NF-kappaB signaling pathway. Several inhibitors of the NF-kappaB signaling pathway, including dexamethasone, thalidomide, and a proteasome inhibitor, bortezomib, showed inhibitory effects on LPS-induced GADD45beta expression as indicated by a decrease of the luciferase activity. Northern blot analysis confirmed a broad inhibitory effect of bortezomib on LPS-induced GADD45beta mRNA expression in spleen, lung, and intestine. In liver of bortezomib-treated mice, we observed a reverse correlation between the luciferase activity and the GADD45beta mRNA level. We speculate that such a discrepancy could be due to severe liver toxicity caused by bortezomib and LPS co-treatment. MAPK inhibitors had transient and inconsistent effects on LPS-induced luciferase expression. Our data are consistent with the notion that NF-kappaB, but not the MAPK signaling pathways, is involved in the in vivo regulation of GADD45beta expression. Thus, NF-kappaB signaling involves induction of GADD45beta expression, which supports the proposed role of GADD45beta in protecting cells against DNA damaged under various stress conditions.
7. DDR2 inhibition reduces migration and invasion of murine metastatic melanoma cells by suppressing MMP2/9 expression through ERK/NF-κB pathway. Metastatic melanoma is one of the most deadly and evasive cancers. Collagen I in the extracellular matrix promotes the migration and invasion of tumor cells through the production of matrix metalloproteinase (MMP) 2 and 9. Discoidin domain receptor (DDR) 2 is a collagen receptor that is implicated in several cancer types including breast and prostate cancers. However, the role of DDR2 in the migration and invasion of murine melanoma cells is less studied. In the present study, we investigated the effects and underlying mechanisms of DDR2 in migration and invasion of B16BL6 melanoma cells in response to collagen I. Results demonstrated that DDR2 is expressed and is phosphorylated by collagen I in the cells. Upon down-regulation of DDR2 using small-interfering RNA (siRNA) approach, both of the cell migratory and **invasiv**e phenotypes were significantly attenuated when compared with the control cells. This effect was mediated via suppression of MMP2/9 upon DDR2 inhibition. Furthermore, inhibition of DDR2 by specific siRNA markedly reduced the activation of extracellular regulated kinase (ERK) 1 and 2 and nuclear factor of kappa B (NF-κB) in the cells when compared with the control cells. Overall, these data demonstrated that DDR2 siRNA-mediated suppression of ERK1/2 and NF-κB could down-regulate the expressions of MMP2/9 in response to collagen I to reduce the migratory a**nd invas**ive phenotypes of the cells.
8. Simultaneous inactivation of Par-4 and PTEN in vivo leads to synergistic NF-kappaB activation and **invasive** prostate carcinoma. Prostate cancer is one of the most common neoplasias in men. The tumor suppressor Par-4 is an important negative regulator of the canonical NF-kappaB pathway and is highly expressed in prostate. Here we show that Par-4 expression is lost in a high percentage of human prostate carcinomas, and this occurs in association with phosphatase and tensin homolog deleted from chromosome 10 (PTEN) loss. Par-4 null mice, similar to PTEN-heterozygous mice, only develop benign prostate lesions, but, importantly, concomitant Par-4 ablation and PTEN-heterozygosity lead to **invasive** prostate carcinoma in mice. This strong tumorigenic cooperation is anticipated in the preneoplastic prostate epithelium by an additive increase in Akt activation and a synergistic stimulation of NF-kappaB. These results establish the cooperation between Par-4 and PTEN as relevant for the development of prostate cancer and implicate the NF-kappaB pathway as a critical event in prostate tumorigenesis.
9. Suppression of NF-kappaB activity by NDRG2 expression attenuates the **invasive** potential of highly malignant tumor cells. Downregulation of the N-myc downstream-regulated gene 2 (NDRG2) gene is involved in the progression of aggressive forms of cancer, along with the poor prognosis of cancer patients. In the current study, we examined the effect of NDRG2 expression on the metastatic potential of HT1080 human fibrosarcoma and B16F10 murine melanoma cells in both in vitro and in vivo systems. In gelatin zymography, NDRG2 expression remarkably suppressed the matrix metalloproteinase (MMP)-9 activity and slightly inhibited MMP-2 activity of both cell lines. Tumor migration and invasion in vitro were significantly reduced by NDRG2 expression, and NDRG2 inhibited tumor cell proliferation in an anchorage-independent semisolid agar assay. Specifically, we found that NDRG2 affects invasion through suppression of nuclear factor kappa B (NF-kappaB) activity. In animal experiments, subcutaneously injected B16F10-NDRG2 cells showed delayed tumor growth compared with B16F10-mock cells. Furthermore, severe metastasis from primary tumor mass into the draining lymph nodes was observed after injection of B16F10-mock cells, but not with B16F10-NDRG2 cells. Pulmonary metastasis after intravenous injection of B16F10 cells was also reduced by NDRG2 expression. Intra- and peritumoral angiogenesis that is critical for the tumor growth and metastasis was clearly found in tumors after injection with B16F10-mock cells, whereas it was impaired in tumors after injection with B16F10-NDRG2 cells. Collectively, our data show that NDRG2 expression significantly suppresses tumor invasion by inhibiting MMP activities, which are regulated through the NF-kappaB signaling. Moreover, results from animal experiments provide evidence for the regulatory role of the NDRG2 gene in metastatic tumors.
10. Chronic exposure to 12-O-tetradecanoylphorbol-13-acetate represses sod2 induction in vivo: the negative role of p50. It is well documented that the tumor promoter 12-O-tetradecanoylphorbol-13-acetate (TPA) can activate manganese superoxide dismutase (MnSOD) expression. However, it is unclear how repeated exposure to TPA following a single application of tumor initiator 7,12-dimethylbenz-(a)-anthracene causes tumor development. We generated transgenic mice expressing human MnSOD promoter- and enhancer-driven luciferase reporter gene and used a non-**invasive** imaging system to investigate the effects of TPA on MnSOD expression in vivo. Our data indicate that TPA initially activates MnSOD expression, but this positive effect declines after repeated applications. Changes in MnSOD expression in vivo were verified by measuring MnSOD mRNA and protein levels. Using chromatin immunoprecipitation coupled to Western analysis of the transcription factors known to be essential for the constitutive and TPA-induced transcription of MnSOD, we found that TPA treatment leads to both activation and inactivation of MnSOD gene transcription. During the activation phase, the levels of p50, p65, specificity protein 1 (Sp1) and nucleophosmin (NPM) increase after TPA treatments. Sustained treatments with TPA lead to further increase of p50 but not p65, Sp1 or NPM, suggesting that excess p50 may have inhibitory effects leading to the suppression of MnSOD. Alteration of p50 levels by expressing p50 cDNA or p50 small interfering RNA in mouse epithelial (JB6) cells confirms that p50 is inhibitory to MnSOD transcription. These findings identify p50 as having a negative effect on MnSOD induction upon repeated applications of TPA and provide an insight into a cause for the reduction of MnSOD expression during early stages of skin carcinogenesis.
11. Distinct roles for the NF-kappaB1 (p50) and c-Rel transcription factors in inflammatory arthritis. Rheumatoid arthritis (RA) is a complex disease, with contributions from systemic autoimmunity and local inflammation. Persistent synovial joint inflammation and **invasive** synovial pannus tissue lead to joint destruction. RA is characterized by the production of inflammatory mediators, many of which are regulated by the Rel/NF-kappaB transcription factors. Although an attractive target for therapeutic intervention in inflammatory diseases, Rel/NF-kappaB is involved in normal physiology, thus global inhibition could be harmful. An alternate approach is to identify and target the Rel/NF-kappaB subunits critical for components of disease. To assess this, mice with null mutations in c-rel or nfkb1 were used to examine directly the roles of c-Rel and p50 in models of acute and chronic inflammatory arthritis. We found c-Rel-deficient mice were resistant to collagen-induced arthritis but had a normal response in an acute, destructive arthritis model (methylated BSA/IL-1 induced arthritis) suggesting c-Rel is required for systemic but not local joint disease. In contrast, p50-deficient mice were refractory to induction of both the chronic and acute arthritis models, showing this subunit is essential for local joint inflammation and destruction. Our data suggest Rel/NF-kappaB subunits play distinct roles in the pathogenesis of inflammatory arthritis and may provide a rationale for more specific therapeutic blockade of Rel/NF-kappaB in RA.
12. Modulation of matrix metalloproteinase-9 activity by hyaluronan is dependent on NF-kappaB activity in lymphoma cell lines with dissimilar **invasive** behavior. Expression and activity of matrix metalloproteinase-9 (MMP-9) as well as its relationship with hyaluronan (HA) and NF-kappaB activity were analyzed in two murine lymphoma cell lines with dissimilar migration and **invasive** behavior. MMP activity was evaluated by zymograms in supernatants, membrane extracts of tumor cells, and in the organs invaded by these cells. The more aggressive LBLa cell line showed MMP-9 activity in vitro, which increased after HA treatment and was blocked by anti-CD44 mAb. Such activity was not found in the less aggressive LBLc. MMP-9 and MMP-2 activity was found in organs invaded by both cell lines, although differential MMP-9 activity was observed in lung infiltrated only by LBLa cell line. NF-kappaB activation was evaluated to determine whether differential activity of MMP-9 was dependent on downstream signaling pathway, showing higher NF-kappaB activity in the more aggressive LBLa cell line. Our results showed that MMP-9 activity modulated by HA through NF-kappaB signaling pathway may be involved in the aggressive behavior of LBLa.
13. Combined stimulation of Toll-like receptor 5 and NOD1 strongly potentiates activity of NF-κB, resulting in enhanced innate immune reactions and resistance to Salmonella enterica serovar Typhimurium infection. Pathogen recognition receptors (PRRs) are essential components of host innate immune systems that detect specific conserved pathogen-associated molecular patterns (PAMPs) presented by microorganisms. Members of two families of PRRs, transmembrane Toll-like receptors (TLRs 1, 2, 4, 5, and 6) and cytosolic NOD receptors (NOD1 and NOD2), are stimulated upon recognition of various bacterial PAMPs. Such stimulation leads to induction of a number of immune defense reactions, mainly triggered via activation of the transcription factor NF-κB. While coordination of responses initiated via different PRRs sensing multiple PAMPS present during an infection makes clear biological sense for the host, such interactions have not been fully characterized. Here, we demonstrate that combined stimulation of NOD1 and TLR5 (as well as other NOD and TLR family members) strongly potentiates activity of NF-κB and induces enhanced levels of innate immune reactions (e.g., cytokine production) both in vitro and in vivo. Moreover, we show that an increased level of NF-κB activity plays a critical role in formation of downstream responses. In live mice, synergy between these receptors resulting in potentiation of NF-κB activity was organ specific, being most prominent in the gastrointestinal tract. Coordinated activity of NOD1 and TLR5 significantly increased protection of mice against e**nteroinv**asive Salmonella infection. Obtained results suggest that cooperation of NOD and TLR receptors is important for effective responses to microbial infection in vivo.
14. Priming by lipopolysaccharide exaggerates acute lung injury and mortality in responses to peptidoglycan through up-regulation of Toll-like receptor-2 expression in mice. **Invasive** infection mixed with Gram-positive and Gram-negative bacteria often results in severe sepsis and septic shock, the prognosis of which is extremely poor and the mortality is high. Here, we hypothesized that lipopolysaccharide (LPS) from Gram-negative bacteria may exert a priming effect on the innate immune response to peptidoglycan (PepG) from Gram-positive bacteria and if so, examined the molecular mechanism of this priming. We found that mice who underwent intratracheal instillation with PepG (5 mg/kg) following prior administration of LPS (5 mg/kg) had a marked decline in survival as compared with the animals given each bacterial cell wall component alone. Furthermore, blood gas exchange impairment and pulmonary vascular hyperpermeability were greatly enhanced in mice given PepG after LPS stimulation, indicating a severe development of acute lung injury. LPS significantly up-regulated the expression levels of Toll-like receptor (TLR)-2 mRNA and protein in mouse lungs. Translocation of TLR-2 to the membranes was also increased by LPS stimulation. This was supported by immunohistochemical examination showing that TLR-2 expression was changed from the cytoplasm to the luminal surface of bronchiolar epithelial cells following LPS stimulation. We also demonstrated an LPS-induced increase in TLR-2 mRNA expression in type-II pneumocytes by reverse transcription-polymerase chain reaction following laser-assisted microdissection. In vivo transfection of nuclear factor-kappaB (NF-kappaB) oligonucleotides strongly prevented the up-regulation of TLR-2 after LPS stimulation at pulmonary cellular and tissue levels. We conclude that the priming effect of LPS on PepG-induced lung injury and death is preceded by NF-kappaB-mediated up-regulation of TLR-2.
15. Elevation of intracellular cyclic AMP inhibits NF-kappaB-mediated thymosin beta4 expression in melanoma cells. Thymosin beta4 (Tbeta4) is a major actin-sequestering protein that has been implicated in the growth, survival, motility, and metastasis of certain tumors and is considered an indicator for malignant progression. Therefore, identifying compounds that can downregulate Tbeta4 expression is very important for the development of anti-cancer chemotherapies. In this study, we investigated the effects of elevated cAMP on Tbeta4 expression and the metastatic potential of murine B16 melanoma cells. In addition, we also dissected the mechanism underlying cAMP-mediated Tbeta4 suppression. We found that treatment with the cAMP-inducing compounds alpha-MSH (alpha-melanocyte stimulating hormone) and IBMX (3-isobutyl-1-methylxanthine) significantly suppressed Tbeta4 expression and regulated EMT-associated genes through the suppression of NF-kappaB activation in B16F10 cells. Along with decreased Tbeta4 expression, the in vitro **invasive**ness and anchorage-independent growth in a semi-solid agar of these cells were also inhibited. In animal experiments, the metastatic potential of the alpha-MSH- or IBMX-treated B16F10 melanoma cells was decreased compared to untreated control cells. Collectively, our data demonstrate that elevated intracellular cAMP significantly suppresses Tbeta4 expression and reduces MMP-9 activity, which leads to decreased metastatic potential. Moreover, suppression of NF-kappaB activation by alpha-MSH or IBMX is critical for inhibiting Tbeta4 expression.
16. Protease-activated Receptor-2 (PAR-2)-mediated Nf-κB Activation Suppresses Inflammation-associated Tumor Suppressor MicroRNAs in Oral Squamous Cell Carcinoma. Oral cancer is the sixth most common cause of death from cancer with an estimated 400,000 deaths worldwide and a low (50%) 5-year survival rate. The most common form of oral cancer is oral squamous cell carcinoma (OSCC). OSCC is highly inflammatory and **invasiv**e, and the degree of inflammation correlates with tumor aggressiveness. The G protein-coupled receptor protease-activated receptor-2 (PAR-2) plays a key role in inflammation. PAR-2 is activated via proteolytic cleavage by trypsin-like serine proteases, including kallikrein-5 (KLK5), or by treatment with activating peptides. PAR-2 activation induces G protein-α-mediated signaling, mobilizing intracellular calcium and Nf-κB signaling, leading to the increased expression of pro-inflammatory mRNAs. Little is known, however, about PAR-2 regulation of inflammation-related microRNAs. Here, we assess PAR-2 expression and function in OSCC cell lines and tissues. Stimulation of PAR-2 activates Nf-κB signaling, resulting in RelA nuclear translocation and enhanced expression of pro-inflammatory mRNAs. Concomitantly, suppression of the anti-inflammatory tumor suppressor microRNAs let-7d, miR-23b, and miR-200c was observed following PAR-2 stimulation. Analysis of orthotopic oral tumors generated by cells with reduced KLK5 expression showed smaller, less aggressive lesions with reduced inflammatory infiltrate relative to tumors generated by KLK5-expressing control cells. Together, these data support a model wherein KLK5-mediated PAR-2 activation regulates the expression of inflammation-associated mRNAs and microRNAs, thereby modulating progression of oral tumors.
17. Toll-like receptor 7 regulates pancreatic carcinogenesis in mice and humans. Pancreatic ductal adenocarcinoma is an aggressive cancer that interacts with stromal cells to produce a highly inflammatory tumor microenvironment that promotes tumor growth and **invasive**ness. The precise interplay between tumor and stroma remains poorly understood. TLRs mediate interactions between environmental stimuli and innate immunity and trigger proinflammatory signaling cascades. Our finding that TLR7 expression is upregulated in both epithelial and stromal compartments in human and murine pancreatic cancer led us to postulate that carcinogenesis is dependent on TLR7 signaling. In a mouse model of pancreatic cancer, TLR7 ligation vigorously accelerated tumor progression and induced loss of expression of PTEN, p16, and cyclin D1 and upregulation of p21, p27, p53, c-Myc, SHPTP1, TGF-β, PPARγ, and cyclin B1. Furthermore, TLR7 ligation induced STAT3 activation and interfaced with Notch as well as canonical NF-κB and MAP kinase pathways, but downregulated expression of Notch target genes. Moreover, blockade of TLR7 protected against carcinogenesis. Since pancreatic tumorigenesis requires stromal expansion, we proposed that TLR7 ligation modulates pancreatic cancer by driving stromal inflammation. Accordingly, we found that mice lacking TLR7 exclusively within their inflammatory cells were protected from neoplasia. These data suggest that targeting TLR7 holds promise for treatment of human pancreatic cancer.
18. The p50 subunit of NF-kappaB is critical for in vivo clearance of the non**invasive** enteric pathogen Citrobacter rodentium. Citrobacter rodentium, a natural mouse pathogen, belongs to the family of extracellular enteric pathogens that includes enteropathogenic Escherichia coli (EPEC) and enterohemorrhagic E. coli (EHEC). C. rodentium shares many virulence factors with EPEC and EHEC and relies on attaching-and-effacing lesion formation for colonization and infection of the gut. In vivo, C. rodentium infection is characterized by increased epithelial cell proliferation, mucosal thickening, and a TH1-type immune response, but with protective immunity believed to be mediated by serum immunoglobulin G (IgG). In this work, we characterize the immune response and pathology of mice lacking the p50 subunit of the transcription factor nuclear factor kappa B (NF-kappaB) during C. rodentium infection. We show that p50(-/-) mice are unable to clear C. rodentium infection. Furthermore, these animals show a reduced influx of immune cells into infected colonic tissue and greater levels of mucosal hyperplasia and the cytokines tumor necrosis factor alpha and gamma interferon. Surprisingly, despite being unable to eliminate infection, p50(-/-) mice showed markedly higher levels of anti-Citrobacter IgG and IgM, suggesting that antibody alone is not responsible for bacterial clearance. These data also demonstrate that non-NF-kappaB-dependent defenses are insufficient to control C. rodentium infection, and hence, the NF-kappaB p50 subunit is critical for defense against this non**invasive** pathogen.
19. NF-kappaB activation, dependent on acetylation/deacetylation, contributes to HIF-1 activity and migration of bone metastatic breast carcinoma cells. Here, we show that NF-kappaB-HIF-1 interaction contributed to breast cancer metastatic capacity by means of an incomplete epithelial/mesenchymal transition and influencing migration, as shown in 1833 (human) and 4T1 (mouse) metastatic cells after different stimuli. The 1833 and the transforming growth factor-beta1-exposed 4T1 cells showed both epithelial (E-cadherins) and mesenchymal (N-cadherins and vimentin) markers, and common mechanisms contributed to the retention of certain epithelial characteristics and the control of migration. The complex NF-kappaB-HIF-1 reciprocal regulation and the enhanced c-Jun expression played a functional role in exacerbating the **invasive**ness of 1833 cells after p50/p65 transfection and of 4T1 cells exposed to transforming growth factor-beta1. Twist expression seemed to exert a permissive role also regulating epithelial/mesenchymal transition markers. After c-Src wild-type (Srcwt) transfection, c-Src-signal transducer overexpression in 1833 cells increased HIF-1 transactivating activity and **invasive**ness, and changed E-cadherin/N-cadherin ratio versus mesenchymal phenotype. The transcription factor pattern and the motile phenotype of metastatic 1833 cells were influenced by p65-lysine acetylation and HDAC-dependent epigenetic mechanisms, which positively regulated basal NF-kappaB and HIF-1 activities. However, HDAC3 acted as a corepressor of NF-kappaB activity in parental MDA-MB231 cells, thus explaining many differences from the derived 1833 clone, including reduced HIF-1alpha and c-Jun expression. **Invasive**ness was differently affected by HDAC knockdown in 1833 and MDA-MB231 cells. We suggest that acetylation/deacetylation are critical in establishing the bone-metastatic gene signature of 1833 cells by regulating the activity of NF-kappaB and HIF-1, and further clarify the epigenetic control of transcription factor network in the motile phenotype of 1833 cells.

#### GeneRIFs

1. NF-kappaB p50 subunit is critical for defense against this non**invasive** pathogen

### [Mm]etastasis

#### PubMed

1. Links between alpha-catenin, NF-kappaB, and squamous cell carcinoma in skin. Cancers display a diverse set of cellular defects, which are thought to be elicited by multiple genetic mutations. In this study, we show that when a single adherens junction protein, alpha-catenin, is removed by conditional targeting, the entire skin epidermis systematically transforms to a hyperproliferative, invasive tissue replete with inflammation. Transcriptional profiling and biochemical analyses reveal that alpha-catenin ablation is accompanied by activation of NF-kappaB and its proinflammatory target genes, along with genes involved in proliferation, wound healing, angiogenesis, and **metastasis**. Many of these alterations occur in vitro and in the embryo, and thus seem at least partly to be intrinsic to the loss of alpha-catenin. We show that reductions in alpha-catenin, activation of NF-kappaB, and inflammation are common features of human squamous cell carcinomas of the skin.
2. Identification of gene function and functional pathways by systemic plasmid-based ribozyme targeting in adult mice. To date, functional genomic studies have been confined to either cell-based assays or germline mutations, using transgenic or knockout animals. However, these approaches are often unable either to recapitulate complex biologic phenotypes, such as tumor **metastasis**, or to identify the specific genes and functional pathways that produce serious diseases in adult animals. Although the transcription factor NF-kappaB transactivates many **metastasis**-related genes in cells, the precise genes and functional-pathways through which NF-kappaB regulates **metastasis** in tumor-bearing hosts are poorly understood. Here, we show that the systemic delivery of plasmid-based ribozymes targeting NF-kappaB in adult, tumor-bearing mice suppressed NF-kappaB expression in metastatic melanoma cells, as well as in normal cell types, and significantly reduced metastatic spread. Plasmid-based ribozymes suppressed target-gene expression with sequence specificity not achievable by using synthetic oligonucleotide-based approaches. NF-kappaB seemed to regulate tumor **metastasis** through invasion-related, rather than angiogenesis-, cell-cycle- or apoptosis-related pathways in tumor-bearing mice. Furthermore, ribozymes targeting either of the NF-kappaB-regulated genes, integrin beta(3) or PECAM-1 (a ligand-receptor pair linked to cell adhesion), reduced tumor **metastasis** at a level comparable to NF-kappaB. These studies demonstrate the utility of gene targeting by means of systemic, plasmid-based ribozymes to dissect out the functional genomics of complex biologic phenotypes, including tumor **metastasis**.
3. Hypoxia-induced IL-18 increases hypoxia-inducible factor-1alpha expression through a Rac1-dependent NF-kappaB pathway. Interleukin-18 (IL-18) plays pivotal roles in linking inflammatory immune responses and tumor progression and **metastasis**, yet the manner in which this occurs remains to be sufficiently clarified. Here we report that hypoxia induces the transcription and secretion of IL-18, which subsequently induces the expression of hypoxia-inducible factor-1alpha (HIF-1alpha). Mechanistically, IL-18 induces HIF-1alpha through the activity of the GTPase Rac1, which inducibly associates with the IL-18 receptor beta (IL-18Rbeta) subunit, via a PI3K-AKT-NF-kappaB-dependent pathway. Importantly, the knockdown of the IL-18Rbeta subunit inhibited IL-18-driven tumor cell **metastasis**. Collectively, these findings demonstrate a feed-forward pathway in HIF-1alpha-mediated tumor progression, in which the induction of IL-18 by hypoxia or inflammatory cells augments the expression of both HIF-1alpha and tumor cell **metastasis**.
4. Hypoxia-induced IL-18 increases hypoxia-inducible factor-1alpha expression through a Rac1-dependent NF-kappaB pathway. Interleukin-18 (IL-18) plays pivotal roles in linking inflammatory immune responses and tumor progression and **metastasis**, yet the manner in which this occurs remains to be sufficiently clarified. Here we report that hypoxia induces the transcription and secretion of IL-18, which subsequently induces the expression of hypoxia-inducible factor-1alpha (HIF-1alpha). Mechanistically, IL-18 induces HIF-1alpha through the activity of the GTPase Rac1, which inducibly associates with the IL-18 receptor beta (IL-18Rbeta) subunit, via a PI3K-AKT-NF-kappaB-dependent pathway. Importantly, the knockdown of the IL-18Rbeta subunit inhibited IL-18-driven tumor cell **metastasis**. Collectively, these findings demonstrate a feed-forward pathway in HIF-1alpha-mediated tumor progression, in which the induction of IL-18 by hypoxia or inflammatory cells augments the expression of both HIF-1alpha and tumor cell **metastasis**.
5. Identification of gene function and functional pathways by systemic plasmid-based ribozyme targeting in adult mice. To date, functional genomic studies have been confined to either cell-based assays or germline mutations, using transgenic or knockout animals. However, these approaches are often unable either to recapitulate complex biologic phenotypes, such as tumor **metastasis**, or to identify the specific genes and functional pathways that produce serious diseases in adult animals. Although the transcription factor NF-kappaB transactivates many **metastasis**-related genes in cells, the precise genes and functional-pathways through which NF-kappaB regulates **metastasis** in tumor-bearing hosts are poorly understood. Here, we show that the systemic delivery of plasmid-based ribozymes targeting NF-kappaB in adult, tumor-bearing mice suppressed NF-kappaB expression in metastatic melanoma cells, as well as in normal cell types, and significantly reduced metastatic spread. Plasmid-based ribozymes suppressed target-gene expression with sequence specificity not achievable by using synthetic oligonucleotide-based approaches. NF-kappaB seemed to regulate tumor **metastasis** through invasion-related, rather than angiogenesis-, cell-cycle- or apoptosis-related pathways in tumor-bearing mice. Furthermore, ribozymes targeting either of the NF-kappaB-regulated genes, integrin beta(3) or PECAM-1 (a ligand-receptor pair linked to cell adhesion), reduced tumor **metastasis** at a level comparable to NF-kappaB. These studies demonstrate the utility of gene targeting by means of systemic, plasmid-based ribozymes to dissect out the functional genomics of complex biologic phenotypes, including tumor **metastasis**.
6. Nucling, a novel apoptosis-associated protein, controls mammary gland involution by regulating NF-κB and STAT3. Postpartum mammary gland involution is the physiological process by which the lactating gland returns to its pre-pregnant state. In rodent models, the microenvironment of mammary gland involution is sufficient to induce enhanced tumor cell growth, local invasion, and **metastasi**s. Therefore, a deeper understanding of the physiological regulation of involution may provide in-depth information on breast cancer therapy. We herein identified Nucling as an important regulator of involution of the mammary gland. A knock-out mouse model was generated and revealed that postpartum involution were impaired in mice lacking Nucling. Involution is normally associated with an increase in the activation of NF-κB and STAT3, which is required for the organized regulation of involution, and was observed in WT glands, but not in the absence of Nucling. Furthermore, the loss of Nucling led to the suppression of Calpain-1, IL-6, and C/EBPδ factors, which are known to be essential for normal involution. The number of M2 macrophages, which are crucial for epithelial cell death and adipocyte repopulation after weaning, was also reduced in Nucling-KO glands. Taken together, the results of the present study demonstrated that Nucling played an important role in mammary gland involution by regulating NF-κB and STAT3 signaling pathways.
7. Bit-1 mediates integrin-dependent cell survival through activation of the NFkappaB pathway. Loss of properly regulated cell death and cell survival pathways can contribute to the development of cancer and cancer **metastasis**. Cell survival signals are modulated by many different receptors, including integrins. Bit-1 is an effector of anoikis (cell death due to loss of attachment) in suspended cells. The anoikis function of Bit-1 can be counteracted by integrin-mediated cell attachment. Here, we explored integrin regulation of Bit-1 in adherent cells. We show that knockdown of endogenous Bit-1 in adherent cells decreased cell survival and re-expression of Bit-1 abrogated this effect. Furthermore, reduction of Bit-1 promoted both staurosporine and serum-deprivation induced apoptosis. Indeed knockdown of Bit-1 in these cells led to increased apoptosis as determined by caspase-3 activation and positive TUNEL staining. Bit-1 expression protected cells from apoptosis by increasing phospho-IκB levels and subsequently bcl-2 gene transcription. Protection from apoptosis under serum-free conditions correlated with bcl-2 transcription and Bcl-2 protein expression. Finally, Bit-1-mediated regulation of bcl-2 was dependent on focal adhesion kinase, PI3K, and AKT. Thus, we have elucidated an integrin-controlled pathway in which Bit-1 is, in part, responsible for the survival effects of cell-ECM interactions.
8. Activated K-ras and INK4a/Arf deficiency cooperate during the development of pancreatic cancer by activation of Notch and NF-κB signaling pathways. Pancreatic ductal adenocarcinoma (PDAC) is the fourth leading cause of cancer-related death in the United States, suggesting that novel strategies for the prevention and treatment of PDAC are urgently needed. K-ras mutations are observed in >90% of pancreatic cancer, suggesting its role in the initiation and early developmental stages of PDAC. In order to gain mechanistic insight as to the role of mutated K-ras, several mouse models have been developed by targeting a conditionally mutated K-ras(G12D) for recapitulating PDAC. A significant co-operativity has been shown in tumor development and **metastasi**s in a compound mouse model with activated K-ras and Ink4a/Arf deficiency. However, the molecular mechanism(s) by which K-ras and Ink4a/Arf deficiency contribute to PDAC has not been fully elucidated.To assess the molecular mechanism(s) that are involved in the development of PDAC in the compound transgenic mice with activated K-ras and Ink4a/Arf deficiency, we used multiple methods, such as Real-time RT-PCR, western blotting assay, immunohistochemistry, MTT assay, invasion, EMSA and ELISA. We found that the deletion of Ink4a/Arf in K-ras(G12D) expressing mice leads to PDAC, which is in part mediated through the activation of Notch and NF-κB signaling pathways. Moreover, we found down-regulation of miR-200 family, which could also play important roles in tumor development and progression of PDAC in the compound transgenic mice.Our results suggest that the activation of Notch and NF-κB together with the loss of miR-200 family is mechanistically linked with the development and progression of PDAC in the compound K-ras(G12D) and Ink4a/Arf deficient transgenic mice.
9. Targeting nuclear factor-κB suppresses the negative effect of toll-like receptor 4 signaling on ant**imetastasi**s therapy based on targeting αvβ3. The targeting of αvβ3 is a promising therapeutic strategy for suppressing t**umor metas**tasis. However, it is unclear whether the therapeutic efficacy could be influence**d by metas**tasis-promoting factor(s) in vivo. Here we report that Toll-like receptor 4 (TLR4) ligand released from damaged tumor cells or bacteria had a negative effect on the therapeutic effect of a recombinant CBD-HepII polypeptide of fibronectin (CH50) that suppresses t**umor metas**tasis by targeting αvβ3. The TLR4 ligand could antagonize the inhibitory effect of CH50 on tumor cell adhesion and invasion by promoting the expression and activity of αvβ3 in tumor cells. The TLR4 ligand also reduced **the antim**etastasis effect of CH50 by promoting tumor cell survival in circulation. Moreover, TLR4 ligands released by tumor cells in circulation could increase the survival and proliferation capacity of tumor cells after extravasation, resulting in the formation of more metastatic nodules. The effect of TLR4 signaling was mainly mediated by nuclear factor-κB (NF-κB). Inhibiting NF-κB could abrogate the negative effect of TLR4 ligand, and augment the inhibitory effect of CH**50 on tumo**r metastasis. Consistently, the combination of NF-κB inhibitor and CH50 significant**ly inhibit**ed metastasis of tumor cells in vivo and prolonged the survival of mice. The findings in this study suggest that the combination of NF-κB inhibitor and αvβ3 antagonist would be a novel therapeutic option for the prev**ention of** tumor metastasis.
10. Parthenolide, a natural inhibitor of Nuclear Factor-kappaB, inhibits lung colonization of murine osteosarcoma cells. The transcription factor nuclear factor-kappaB (NF-kappaB) regulates the expression of several genes important for tumor **metastasis** and is constitutively active in the highly metastatic murine osteosarcoma cell line LM8. Parthenolide, a sesquiterpene lactone, was reported to inhibit the DNA binding of NF-kappaB. The purpose of this study is to investigate the usefulness of parthenolide as target for antimetastatic therapies.We examined the effect of parthenolide on **metastasis**-associated phenotypes in vitro and in murine experimental lung **metastasis** models by s.c. and i.v. inoculation of LM8 cells.We found that parthenolide strongly induced apoptosis and inhibited cell proliferation and the expression of vascular endothelial growth factor in vitro. In the in vivo **metastasis** models, parthenolide treatment suppressed lung **metastasis** when treatment was initiated concurrently with s.c. or i.v. inoculation of tumor cells, whereas lung **metastasis** was not reduced when parthenolide was given after the homing of tumor cells. The growth of s.c. tumors that developed at the inoculation site was not suppressed by parthenolide. We also found that the genetic inhibition of NF-kappaB activity by expressing mutant IkappaBalpha suppressed lung **metastasis** in vivo but not s.c. tumor growth. This supports our notion that the **metastasis**-preventing effect of parthenolide is mediated at least in part by inhibition of NF-kappaB activity.These findings suggested that NF-kappaB is a potential molecular target for designing specific prophylactic interventions against distant **metastasis** and that parthenolide is a hopeful candidate for an antimetastatic drug.
11. Suppression of NF-kappaB activity by NDRG2 expression attenuates the invasive potential of highly malignant tumor cells. Downregulation of the N-myc downstream-regulated gene 2 (NDRG2) gene is involved in the progression of aggressive forms of cancer, along with the poor prognosis of cancer patients. In the current study, we examined the effect of NDRG2 expression on the metastatic potential of HT1080 human fibrosarcoma and B16F10 murine melanoma cells in both in vitro and in vivo systems. In gelatin zymography, NDRG2 expression remarkably suppressed the matrix metalloproteinase (MMP)-9 activity and slightly inhibited MMP-2 activity of both cell lines. Tumor migration and invasion in vitro were significantly reduced by NDRG2 expression, and NDRG2 inhibited tumor cell proliferation in an anchorage-independent semisolid agar assay. Specifically, we found that NDRG2 affects invasion through suppression of nuclear factor kappa B (NF-kappaB) activity. In animal experiments, subcutaneously injected B16F10-NDRG2 cells showed delayed tumor growth compared with B16F10-mock cells. Furthermore, severe **metastasis** from primary tumor mass into the draining lymph nodes was observed after injection of B16F10-mock cells, but not with B16F10-NDRG2 cells. Pulmonary **metastasis** after intravenous injection of B16F10 cells was also reduced by NDRG2 expression. Intra- and peritumoral angiogenesis that is critical for the tumor growth and **metastasis** was clearly found in tumors after injection with B16F10-mock cells, whereas it was impaired in tumors after injection with B16F10-NDRG2 cells. Collectively, our data show that NDRG2 expression significantly suppresses tumor invasion by inhibiting MMP activities, which are regulated through the NF-kappaB signaling. Moreover, results from animal experiments provide evidence for the regulatory role of the NDRG2 gene in metastatic tumors.
12. Platelet-activating factor enhances tumour **metastasis** via the reactive oxygen species-dependent protein kinase casein kinase 2-mediated nuclear factor-κB activation. Platelet-activating factor (PAF) promotes tumour **metastasi**s via activation of the transcription factor nuclear factor-κB (NF-κB). We here investigated the role of the protein kinase CK2 (formerly Casein Kinase 2 or II) in PAF-induced NF-κB activation and tum**our metast**asis, given that PAF has been reported to increase CK2 activity, and that CK2 plays a key role in NF-κB activation. PAF increased CK2 activity, phosphorylation and protein expression in vivo as well as in vitro. CK2 inhibitors inhibited the PAF-mediated NF-κB activation and expression of NF-κB-dependent pro-inflammatory cytokines and anti-apoptotic factors. Pre-treatment with the antioxidant N-Acetyl-L-Cysteine (NAC) resulted in a significant inhibition in PAF-induced enhancement of CK2 activity, phosphorylation and protein expression in vivo as well as in vitro. H2 O2 and known reactive oxygen species inducers, lipopolysaccharide (LPS) and tumour necrosis factor-α (TNF-α) enhanced CK2 activity, phosphorylation and protein expression, which was again inhibited by antioxidant. PAF, LPS and TNF-α induced increased CK2 activity, phosphorylationand protein expression, which were inhibited by p38 inhibitor. PAF, LPS or TNF-α increased **pulmonary** metastasis of B16F10, which was inhibited by antioxidants, CK2 inhibitor and p38 inhibitor. Our data suggest that (i) reactive oxygen species activate CK2 via p38, which, in turn, induces NF-κB activation, and (ii) PAF, LPS and TNF-α increase pulm**onary tumo**ur metastasis via the induction of the reactive oxygen species (ROS)/p38/CK2/NF-κB pathway.
13. Subcellular localization of apurinic endonuclease 1 promotes lung tumor aggressiveness via NF-kappaB activation. Apurinic endonuclease 1 (Ape1) is not only involved in base excision repair, but also activates some transcriptional factors through its redox activity. However, which subcellular localization of Ape1 is involved in the activation of transcriptional factor remains unclear. We first observed that Cox-2 expression was associated with cytoplasmic Ape1 expression in lung tumors and cancer cell lines. We thus hypothesize that nuclear factor (NF)-kappaB is activated by cytoplasmic Ape1 to cause Cox-2 expression. Herein, we generated cytoplasmic and nuclear Ape1 in Ape1-knockdown lung cancer cells by exogenous expression of Ape1 containing various deletions and/or mutations of the nuclear localization sequence. It was observed that cytoplasmic Ape1, but not nuclear Ape1, induced Cox-2 expression through NF-kappaB activation. NF-kappaB activation by cytoplasmic Ape1 was diminished by the Ape1 redox activity inhibitor resveratrol. Cells expressing cytoplasmic Ape1 exhibited tumor progression and **metastasis** in vitro and in vivo as xenografts, but cells expressing nuclear Ape1 did not. Patients with tumors containing elevated cytoplasmic Ape1 had a poor prognosis and a 3.722-fold risk of tumor recurrence and/or **metastasis**. Cytoplasmic Ape1 could therefore enhance lung tumor malignancy through NF-kappaB activation, suggesting that combination of cisplatin and specific redox inhibitor could improve chemotherapeutic response in patients with tumors containing elevated cytoplasmic Ape1.
14. Ikappa B kinasebeta/nuclear factor-kappaB activation controls the development of liver **metastasis** by way of interleukin-6 expression. Nuclear factor kappaB (NF-kappaB) plays an important role in the regulation of innate immune responses, apoptosis, inflammation, and oncogenesis. NF-kappaB activation in the liver was observed after intrasplenic administration of a lung carcinoma cell line, LLC, which induces liver **metastasis**. To explore the role of Ikappa B kinase beta (IKKbeta), which is the critical kinase of the IKK complex, and NF-kappaB activation in **metastasis**, we injected LLC cells into hepatocyte-specific IKKbeta knockout mice (Ikkbeta(Deltahep)), whole-liver knockout (Ikkbeta(DeltaL+H)) mice, and control (Ikkbeta(F/F)) mice. Ikkbeta(DeltaL+H) mice developed liver **metastasis** with significantly lower liver weights and fewer metastatic foci compared to Ikkbeta(Deltahep) and Ikkbeta(F/F) mice. Furthermore, intrasplenic LLC injection induced the messenger RNA (mRNA) expression of interleukin (IL)-6 and IL-1beta in Ikkbeta(F/F) mice, whereas these genes were less expressed in Ikkbeta(DeltaL+H) mice. IL-6(-/-) mice and treatment with anti-IL-6 receptor antibody showed a lesser degree of metastatic tumor, indicating that IL-6 is associated with liver **metastasis**.Collectively, these observations suggest that IKKbeta/NF-kappaB activation controls the development of liver **metastasis** by way of IL-6 expression and is a potential target for the development of antimetastatic drugs.
15. Hydrogen peroxide-mediated nuclear factor kappaB activation in both liver and tumor cells during initial stages of hepatic **metastasis**. Various factors involved in tumor **metastasis** are regulated by the transcription factor nuclear factor kappaB (NF-kappaB). Because NF-kappaB activation may contribute to establishment of hepatic **metastasis**, its activation in liver cells and tumor cells was separately evaluated in a mouse model of hepatic **metastasis**. pNF-kappaB-Luc, a firefly luciferase-expressing plasmid DNA depending on the NF-kappaB activity, was injected into the tail vein of mice by the hydrodynamics-based procedure, a well-established method for gene transfer to BALB/c male mouse liver. The luciferase activity in the liver was significantly increased by an intraportal inoculation of murine adenocarcinoma colon26 cells, but not of peritoneal macrophages, suggesting that the NF-kappaB in liver cells is activated when tumor cells enter the hepatic circulation. Then, colon26 cells stably transfected with pNF-kappaB-Luc were inoculated. The firefly luciferase activity, an indicator of NF-kappaB activity in tumor cells, was significantly increased when colon26/NFkappaB-Luc cells were inoculated into the portal vein of BALB/c male mice. The NF-kappaB activation in both liver and tumor cells was significantly inhibited by injection of catalase derivatives, which have been reported to inhibit hepatic **metastasis** of tumor cells. These findings indicate for the first time that NF-kappaB, a key agent regulating the expression of various molecules involved in tumor **metastasis**, is activated in both liver and tumor cells during the initial stages of tumor **metastasis** through a hydrogen peroxide mediated pathway. Thus, the removal of hydrogen peroxide will be a promising approach to treating hepatic **metastasis**.
16. Regulation of anoikis by deleted in breast cancer-1 (DBC1) through NF-κB. Anoikis-resistance of tumor cells is critical for anchorage-independent growth and **metastasi**s. The inflammatory-response transcription factor NF-κB contributes to anoikis-resistance and tumor progression through mechanisms that are understood incompletely. Deleted in breast cancer-1 (DBC1) protein (KIAA1967) is over-expressed in several tumor types, and correlates with a poorer prognosis in some cases. We report here that DBC1 suppressed anoikis in normal epithelial and breast cancer cell lines. DBC1 interacted with IKK-β, stimulating its kinase activity, promoting NF-κB transcriptional activity through the phosphorylation of relA serine-536 and enhancing the expression of the NF-κB target genes, c-FLIP and bcl-xl. Our results indicate that DBC1 is an important co-factor for the control of the IKK-β-NF-κB signaling pathway that regulates anoikis.
17. Breast tumor cell TACE-shed MCSF promotes pro-angiogenic macrophages through NF-κB signaling. Most deaths associated with breast cancer, the most common malignancy in women, are caused by **metastasi**s. Tumor associated macrophages significantly contribute to breast cancer progression and development of **metastasi**s through the promotion of angiogenesis which involves a central regulator of macrophage functions: nuclear factor kappa-light-chain-enhancer of activated B cells (NF-κB). Macrophages are activated by macrophage colony stimulating factor (MCSF) and chemokine (C-C motif) ligand 2 (CCL2) to secrete angiogenic factors including vascular endothelial growth factor (VEGF). The release of MCSF from tumor cells is mediated by ectodomain shedding through tumor necrosis factor alpha converting enzyme activation (TACE). Here we determined whether tumor cells TACE-shed MCSF promotes angiogenesis through activation of the NF-κB pathway in macrophages and the subsequent release of VEGF. These interactions were modeled in vitro using a panel of mammary cells mimicking the breast cancer progression from normal murine mammary gland cells to metastatic 4T1 cells along with J774 macrophages, all derived from BALB/c mice. TACE and MCSF expressions were higher in metastatic cells compared to epithelial cells (p < 0.05). Tumor conditioned medias activated the expression of VEGF by macrophages through stimulation of the NF-κB pathway and resulting macrophage secretions that promoted high levels of endothelial cell tubes. Furthermore, the combinations of CCL2, also highly expressed by tumor cells, and MCSF promoted pro-angiogenic macrophages. These results highlight the key role of tumor cell TACE-shed MCSF and secreted CCL2 in stimulating pro-angiogenic macrophages.
18. Inhibitor of growth-4 promotes IkappaB promoter activation to suppress NF-kappaB signaling and innate immunity. Ing4 is a member of the inhibitor of growth (ING) family of chromatin-modifying proteins. Biochemical experiments indicate that Ing4 is a subunit of the HB01-JADE-hEAF6 histone acetyltransferase complex responsible for most nucleosomal histone H4 acetylation in eukaryotes, and transfection studies suggest that Ing4 may regulate a wide variety of cellular processes, including DNA repair, apoptosis, cell-cycle regulation, **metastasis**, angiogenesis, and tumor suppression. However, in vivo evidence for a physiological role for Ing4 in cell-growth regulation is lacking. We have generated Ing4-deficient mice to explore the role of Ing4 in development, tumorigenesis, and in NF-kappaB signaling. Ing4-null mice develop normally and are viable. Although mice deficient for Ing4 fail to form spontaneous tumors, they are hypersensitive to LPS treatment and display elevated cytokine responses. Macrophages isolated from Ing4-null mice have increased levels of nuclear p65/RelA protein, resulting in increased RelA binding to NF-kappaB target promoters and up-regulation of cytokine gene expression. However, increased promoter occupancy by RelA in LPS-stimulated, Ing4-null cells does not always correlate with increased NF-kappaB target-gene expression, as RelA activation of a subset of cytokine promoters also requires Ing4 for proper histone H4 acetylation. Furthermore, activation of the IkappaB alpha promoter by RelA is also Ing4-dependent, and LPS-stimulated, Ing4-null cells have reduced levels of IkappaB alpha promoter H4 acetylation and IkappaB gene expression. Thus, Ing4 negatively regulates the cytokine-mediated inflammatory response in mice by facilitating NF-kappaB activation of IkappaB promoters, thereby suppressing nuclear RelA levels and the activation of select NF-kappaB target cytokines.
19. Prolyl hydroxylase-3 is down-regulated in colorectal cancer cells and inhibits IKKbeta independent of hydroxylase activity. Prolyl hydroxylase (PHD) hydroxylates hypoxia inducible factor (HIF) alpha, leading to HIFalpha degradation. The PHD family comprises PHD1, PHD2, and PHD3. The enzymatic activity of PHDs is O(2)-dependent, so PHDs are believed to be oxygen sensors as well as tumor suppressors. However, the expression pattern of PHDs in colorectal cancer and the correlation between their expression level and tumorigenesis is unclear.We determined the expression of PHDs in 60 human primary colorectal carcinoma tissues, paired with normal colorectal tissues. PHD3 expression levels were knocked down using small interfering RNA (siRNA); cells were analyzed by immunoblotting, immunoprecipitation, and histochemical analyses. In vivo tumor growth was analyzed in nu/nu mice.Expression of PHD3 is decreased in colorectal cancer tissues. Decreased expression of PHD3 is associated with higher tumor grade and **metastasis**. PHD3 inhibits phosphorylation of inhibitor of kappaB (IkappaB) kinase (IKK) beta and activation of (NF) kappaB, independent of its hydroxylase activity. PHD3 associates with IKKbeta but does not target it for destruction; instead, PHD3 blocks the interaction between IKKbeta and Hsp90 that is required for phosphorylation of IKKbeta. Knockdown of PHD3 increased resistance of colorectal cancer cells to the effects of tumor necrosis factor-alpha and tumorigenesis.PHD3 appears to be a tumor suppressor in colorectal cancer cells that inhibits IKKbeta/NF-kappaB signaling, independent of its hydroxylase activity. Activation of NF-kappaB has been observed in colon cancer. Determination of PHD3 status could aid targeted therapy selection for patients with colorectal tumors that have increased NF-kappaB activity.
20. Singleminded-2s (Sim2s) promotes delayed involution of the mouse mammary gland through suppression of Stat3 and NFκB. Postlactational involution of the mammary gland provides a unique model to study breast cancer susceptibility and **metastasi**s. We have shown that the short isoform of Singleminded-2s (Sim2s), a basic helix loop helix/PAS transcription factor, plays a role in promoting lactogenic differentiation, as well as maintaining mammary epithelial differentiation and malignancy. Sim2s is dynamically expressed during mammary gland development, with expression peaking during lactation, and decreasing in early involution. To determine the role of SIM2S in involution, we used transgenic mice expressing SIM2S under the mouse mammary tumor virus-Sim2s promoter. Overexpression of Sim2s in the mouse mammary gland resulted in delayed involution, indicated by a lower proportion of cleaved caspase-3-positive cells and slower reestablishment of the mammary fat pad. Immunohistochemical and quantitative RNA analysis showed a decrease in apoptotic markers and inflammatory response genes, and an increase in antiapoptotic genes, which were accompanied by inhibition of signal transducer and activator of transcription 3 activity. Microarray analysis confirmed that genes in the signal transducer and activator of transcription 3 signaling pathway were repressed by SIM2S expression, along with nuclear factor-κB and other key pathways involved in mammary gland development. Multiparous mouse mammary tumor virus-Sim2s females displayed a more differentiated phenotype compared with wild-type controls, characterized by enhanced β-casein expression and alveolar structures. Together, these results suggest a role for SIM2S in the normal involuting gland and identify potential downstream pathways regulated by SIM2S.
21. Elevation of intracellular cyclic AMP inhibits NF-kappaB-mediated thymosin beta4 expression in melanoma cells. Thymosin beta4 (Tbeta4) is a major actin-sequestering protein that has been implicated in the growth, survival, motility, and **metastasis** of certain tumors and is considered an indicator for malignant progression. Therefore, identifying compounds that can downregulate Tbeta4 expression is very important for the development of anti-cancer chemotherapies. In this study, we investigated the effects of elevated cAMP on Tbeta4 expression and the metastatic potential of murine B16 melanoma cells. In addition, we also dissected the mechanism underlying cAMP-mediated Tbeta4 suppression. We found that treatment with the cAMP-inducing compounds alpha-MSH (alpha-melanocyte stimulating hormone) and IBMX (3-isobutyl-1-methylxanthine) significantly suppressed Tbeta4 expression and regulated EMT-associated genes through the suppression of NF-kappaB activation in B16F10 cells. Along with decreased Tbeta4 expression, the in vitro invasiveness and anchorage-independent growth in a semi-solid agar of these cells were also inhibited. In animal experiments, the metastatic potential of the alpha-MSH- or IBMX-treated B16F10 melanoma cells was decreased compared to untreated control cells. Collectively, our data demonstrate that elevated intracellular cAMP significantly suppresses Tbeta4 expression and reduces MMP-9 activity, which leads to decreased metastatic potential. Moreover, suppression of NF-kappaB activation by alpha-MSH or IBMX is critical for inhibiting Tbeta4 expression.
22. Silencing of ST6GalNAc I suppresses the proliferation, migration and invasion of hepatocarcinoma cells through PI3K/AKT/NF-κB pathway. ST6GalNAc I is the major Sialyl-Tn antigen (STn) synthase that is highly correlated with tumor invasion and **metastasi**s. However, the roles and molecular mechanisms by which ST6GalNAc I mediates the malignant phenotypes of hepatocarcinoma cells still remain poorly unknown. In this study, we investigated the expression of STn and ST6GalNAc I in mouse hepatocarcinoma cell lines Hca-F, Hca-P, and Hepa1-6, which have different metastatic potential, as compared with normal mouse liver cell line IAR-20. The results showed that the expression of ST6GalNAc I and STn in Hca-F and Hca-P cells was much higher than that in Hepa1-6 and IAR20 cells. Knockdown of ST6GalNAc I by shRNA in Hca-F cells significantly decreased the expression of STn and inhibited the growth of tumor cells in vitro and in vivo. This reduction of ST6GalNAc I expression also led to the decreased migration and invasion of Hca-F cells. Furthermore, we found that ST6GalNAc I knockdown inhibited the expression levels of PI3k, p-Akt473, p-Akt308, NF-κB, and their downstream molecules. Together, our results suggest a role of ST6GalNAc I in promoting the growth and invasion of hepatocarcinoma cells through regulating PI3K/AKT signaling, and ST6GalNAc I might be a promising marker for the prognosis and therapy of hepatocarcinoma.
23. Poly(ADP-ribose) polymerase inhibition down-regulates expression of **metastasis**-related genes in CT26 colon carcinoma cells. The current study was designed to test the hypothesis that inhibition of poly(ADP-ribose) polymerase in colorectal cancer mediates down-regulation of **metastasis**-related gene expression through the regulation of nuclear factor-kappaB (NF-kappaB) activity.Mouse colon carcinoma cells (CT26) were treated with and without the PARP inhibitor 5-aminoisoquinolin-1(2H)-one hydrochloride (5-AIQ). We investigated adhesion, migration and invasion of differently treated CT26 cells. In addition, the expression levels of PARP, NF-kappaB, integrin beta(1), MMP-9 and MMP-2 as well as the activities of NF-kappaB, MMP-9 and MMP-2 were determined by Western blot, electrophoretic mobility gel shift assay and zymography, respectively.Inhibition ofPARP attenuated the adhesion of CT26 cells to the extracellular matrix and their migration and invasion through Matrigel. In addition, the results of Western blot showed that the expression levels of PARP, NF-kappaB, integrin beta(1), MMP-9 and MMP-2 were reduced in 5-AIQ-treated CT26 cells; the activities of NF-kappaB, MMP-9 and MMP-2 were also suppressed.Inhibition of PARP down-regulates the expression of **metastasis**-related genes in mouse colon carcinoma cells. This could be, at least in part, through the regulation of NF-kappaB activity, but the precise mechanisms of action remain to be elucidated.
24. Regulation of interleukin-8 expression in melanoma-stimulated neutrophil inflammatory response. Inflammation facilitates tumor progression including **metastasis**. Interleukin-8 (IL-8) is a chemokine that regulates polymorphonuclear neutrophil (PMN) mobilization and activity and we hypothesize that this cytokine influences tumor behavior. We have demonstrated that IL-8 is crucial for PMN-mediated melanoma extravasation under flow conditions. In addition, IL-8 is up-regulated in PMNs upon co-culturing with melanoma cells. Melanoma cells induce IkappaB-alpha degradation in PMNs indicating that NF-kappaB signaling is active in PMNs. Furthermore, the production of IL-8 in PMNs is NF-kappaB dependent. We have further identified that interleukin-6 (IL-6) and interleukin-1beta (IL-1beta) from PMN-melanoma co-cultures synergistically contribute to IkappaB-alpha degradation and IL-8 synthesis in PMNs. Taken together, these findings show that melanoma cells induce PMNs to secrete IL-8 through activation of NF-kappaB and suggest a model in which this interaction promotes a microenvironment that is favorable for **metastasis**.

#### GeneRIFs

1. we investigated whether Platelet-activating factor could induce NF-kappaB activation and tumour **metastasis** via enhancing CK2 activity.
2. Data indicate that the effect of TLR4 signaling was mainly mediated by nuclear factor-kappaB (NF-kappaB), and suggest that the combination of NF-kappaB inhibitor and alphavbeta3 antagonist would be a novel therapeutic option for the prevention of tumor **metastasis**.
3. Ikappa B kinasebeta/nuclear factor-kappaB activation controls the development of liver **metastasis** by way of interleukin-6 expression.
4. Inhibition of PARP down-regulates the expression of **metastasis**-related genes in mouse colon carcinoma cells. This could be, at least in part, through the regulation of NF-kappaB activity.
5. These findings demonstrate a feed-forward pathway in HIF-1alpha-mediated tumor progression, in which the induction of IL-18 by hypoxia or inflammatory cells augments the expression of both HIF-1alpha and tumor cell **metastasis**.
6. These findings indicate for the first time that NF-kappaB, a key agent regulating the expression of various molecules involved in tumor **metastasis**, is activated in both liver and tumor cells
7. these findings show that melanoma cells induce PMNs to secrete IL-8 through activation of NF-kappaB and suggest a model in which this interaction promotes a microenvironment that is favorable for **metastasis**.

---

## Nfkb2

- NCBI Gene
- GeneRIF
- Pubmed

| Search Term | Rank | Fields |
| --- | --- | --- |
| [Mm]igration | 6 | PubMed(5) |
| [Ee]xtracellular [Mm]atrix | 8 | Process(1) |

  
hide/show details for Nfkb2

### [Mm]igration

#### PubMed

1. Inhibitor of NF-kappa B kinases alpha and beta are both essential for high mobility group box 1-mediated chemotaxis [corrected]. Inhibitor of NF-kappaB kinases beta (IKKbeta) and alpha (IKKalpha) activate distinct NF-kappaB signaling modules. The IKKbeta/canonical NF-kappaB pathway rapidly responds to stress-like conditions, whereas the IKKalpha/noncanonical pathway controls adaptive immunity. Moreover, IKKalpha can attenuate IKKbeta-initiated inflammatory responses. High mobility group box 1 (HMGB1), a chromatin protein, is an extracellular signal of tissue damage-attracting cells in inflammation, tissue regeneration, and scar formation. We show that IKKalpha and IKKbeta are each critically important for HMGB1-elicited chemotaxis of fibroblasts, macrophages, and neutrophils in vitro and neutrophils in vivo. By time-lapse microscopy we dissected different parameters of the HMGB1 **migration** response and found that IKKalpha and IKKbeta are each essential to polarize cells toward HMGB1 and that each kinase also differentially affects cellular velocity in a time-dependent manner. In addition, HMGB1 modestly induces noncanonical IKKalpha-dependent p52 nuclear translocation and p52/RelB target gene expression. Akin to IKKalpha and IKKbeta, p52 and RelB are also required for HMGB1 chemotaxis, and p52 is essential for cellular orientation toward an HMGB1 gradient. RAGE, a ubiquitously expressed HMGB1 receptor, is required for HMGB1 chemotaxis. Moreover, IKKbeta, but not IKKalpha, is required for HMGB1 to induce RAGE mRNA, suggesting that RAGE is at least one IKKbeta target involved in HMGB1 **migration** responses, and in accord with these results enforced RAGE expression rescues the HMGB1 **migration** defect of IKKbeta, but not IKKalpha, null cells. Thus, proinflammatory HMGB1 chemotactic responses mechanistically require the differential collaboration of both IKK-dependent NF-kappaB signaling pathways.
2. Constitutive alternative NF-kappaB signaling promotes marginal zone B-cell development but disrupts the marginal sinus and induces HEV-like structures in the spleen. Nuclear factor-kappaB (NF-kappaB) plays a crucial role in B-cell and lymphoid organ development. Here, we studied the consequences of constitutive, signal-independent activation of the alternative NF-kappaB pathway for the splenic marginal zone (MZ). In contrast to nfkb2(-/-) mice, which lack both p100 and p52, mice that lack only the inhibitory p100 precursor but still express the p52 subunit of NF-kappaB2 (p100(-/-)) had markedly elevated MZ B-cell numbers. Both cell-intrinsic mechanisms and increased stromal expression of vascular cell adhesion molecule-1 (VCAM-1) contributed to the accumulation of MZ B cells in p100(-/-) spleens. While **migration** of p100(-/-) MZ B cells toward the lysophospholipid sphingosine-1 phosphate (S1P) was not affected, CXCL13-stimulated chemotaxis was impaired, correlating with reduced **migration** of MZ B cells into follicles in response to lipopolysaccharide (LPS). Strikingly, p100 deficiency resulted in the absence of a normal marginal sinus, strongly induced expression of mucosal addressin cell adhesion molecule-1 (MAdCAM-1) and glycosylated cell adhesion molecule-1 (GlyCAM-1), and the formation of nonfunctional ectopic high endothelial venule (HEV)-like structures in the red pulp. Thus, constitutive activation of the alternative NF-kappaB pathway favors MZ B-cell development and accumulation but leads to a disorganized spleen microarchitecture.
3. The IKKα-dependent NF-κB p52/RelB noncanonical pathway is essential to sustain a CXCL12 autocrine loop in cells migrating in response to HMGB1. HMGB1 is a chromatin architectural protein that is released by dead or damaged cells at sites of tissue injury. Extracellular HMGB1 functions as a proinflammatory cytokine and chemoattractant for immune effector and progenitor cells. Previously, we have shown that the inhibitor of NF-κB kinase (IKK)β- and IKKα-dependent NF-κB signaling pathways are simultaneously required for **cell mig**ration to HMGB1. The IKKβ-dependent canonical pathway is needed to maintain expression of receptor for advanced glycation end products, the ubiquitously expressed receptor for HMGB1, but the target of the IKKα non-canonical pathway was not known. In this study, we show that the IKKα-dependent p52/RelB noncanonical pathway is critical to sustain CXCL12/SDF1 production in order for cells to migrate toward HMGB1. Using both mouse bone marrow-derived macrophages and mouse embryo fibroblasts (MEFs), it was observed that neutralization of CXCL12 by a CXCL12 mAb completely eliminated chemotaxis to HMGB1. In addition, t**he HMGB1** migration defect of IKKα KO and p52 KO cells could be rescued by adding recombinant CXCL12 to cells. Moreover, p52 KO MEFs stably transduced with a GFP retroviral vector that enforces physiologic expression of CXCL12 also showed ne**ar normal** migration toward HMGB1. Finally, both AMD3100, a specific antagonist of CXCL12's G protein-coupled receptor CXCR4, and an anti-CXCR4 Ab blocked HMGB1 chemotactic responses. These results indicate that HMGB1-CXCL12 interplay dr**ives cell** migration toward HMGB1 by engaging receptors of both chemoattractants. This novel requirement for a second receptor-ligand pair enhances our understanding of the molecular mechanisms regulating HMGB1-dependent cell recruitment to sites of tissue injury.
4. Dendritic cells require the NF-kappaB2 pathway for cross-presentation of soluble antigens. NF-kappaB-inducing kinase (NIK) is responsible for activation of the non-canonical p100 processing pathway of NF-kappaB activation. This kinase has been shown to be critical for activation of this pathway after signaling through several TNF family members including CD40. The functional importance of this pathway in CD40 and TLR-induced dendritic cell (DC) differentiation was studied in vivo in the alymphoplasia (Aly) mouse. The Aly mouse expresses a mutant NIK molecule that prohibits the induction of the non-canonical pathway. We show that while MHC class II presentation and in vivo **migration** of Aly DCs is intact, these cells are unable to cross-prime CD8+ T cells to exogenous Ag. Gene expression array analysis of DCs matured in vivo indicates multiple defects in Ag processing pathways after maturation and provide a global view of the genes that are regulated by the NF-kappaB2 pathway in DCs. These experiments indicate a possible role for NIK in mediating cross-priming of soluble Ag. In addition, our findings explain the profound immune unresponsiveness of the Aly mouse.
5. p100 Deficiency is insufficient for full activation of the alternative NF-κB pathway: TNF cooperates with p52-RelB in target gene transcription. Constitutive activation of the alternative NF-κB pathway leads to marginal zone B cell expansion and disorganized spleen microarchitecture. Furthermore, uncontrolled alternative NF-κB signaling may result in the development and progression of cancer. Here, we focused on the question how does the constitutive alternative NF-κB signaling exert its effects in these malignant processes.To explore the consequences of unrestricted alternative NF-κB activation on genome-wide transcription, we compared gene expression profiles of wild-type and NF-κB2/p100-deficient (p100(-/-)) primary mouse embryonic fibroblasts (MEFs) and spleens. Microarray experiments revealed only 73 differentially regulated genes in p100(-/-) vs. wild-type MEFs. Chromatin immunoprecipitation (ChIP) assays showed in p100(-/-) MEFs direct binding of p52 and RelB to the promoter of the Enpp2 gene encoding ENPP2/Autotaxin, a protein with an important role in lymphocyte homing and **cell mig**ration. Gene ontology analysis revealed upregulation of genes with anti-apoptotic/proliferative activity (Enpp2/Atx, Serpina3g, Traf1, Rrad), chemotactic/locomotory activity (Enpp2/Atx, Ccl8), and lymphocyte homing activity (Enpp2/Atx, Cd34). Most importantly, biochemical and gene expression analyses of MEFs and spleen, respectively, indicated a marked crosstalk between classical and alternative NF-κB pathways.Our results show that p100 deficiency alone was insufficient for full induction of genes regulated by the alternative NF-κB pathway. Moreover, alternative NF-κB signaling strongly synergized both in vitro and in vivo with classical NF-κB activation, thereby extending the number of genes under the control of the p100 inhibitor of the alternative NF-κB signaling pathway.

### [Ee]xtracellular [Mm]atrix

#### Process

1. **extracellular matrix** organization

---

## Arap3

- NCBI Gene
- GeneRIF
- Pubmed

| Search Term | Rank | Fields |
| --- | --- | --- |
| [Mm]igration | 6 | Process(1); PubMed(2) |
| ECM | 7 | PubMed(1) |
| [Ee]xtracellular [Mm]atrix | 8 | PubMed(1) |

  
hide/show details for Arap3

### [Mm]igration

#### Process

1. negative regulation of cell **migration**

#### PubMed

1. Arap3 is dysregulated in a mouse model of hypotrichosis-lymphedema-telangiectasia and regulates lymphatic vascular development. Mutations in SOX18, VEGFC and Vascular Endothelial Growth Factor 3 underlie the hereditary lymphatic disorders hypotrichosis-lymphedema-telangiectasia (HLT), Milroy-like lymphedema and Milroy disease, respectively. Genes responsible for hereditary lymphedema are key regulators of lymphatic vascular development in the embryo. To identify novel modulators of lymphangiogenesis, we used a mouse model of HLT (Ragged Opossum) and performed gene expression profiling of aberrant dermal lymphatic vessels. Expression studies and functional analysis in zebrafish and mice revealed one candidate, ArfGAP with RhoGAP domain, Ankyrin repeat and PH domain 3 (ARAP3), which is down-regulated in HLT mouse lymphatic vessels and necessary for lymphatic vascular development in mice and zebrafish. We position this known regulator of cell behaviour during **migration** as a mediator of the cellular response to Vegfc signalling in lymphatic endothelial cells in vitro and in vivo. Our data refine common mechanisms that are likely to contribute during both development and the pathogenesis of lymphatic vascular disorders.
2. Nck adapters are involved in the formation of dorsal ruffles, cell **migration**, and Rho signaling downstream of the platelet-derived growth factor beta receptor. The SH3 and SH2 domain-containing adapter proteins Nck1 and Nck2 are known to function downstream of activated tyrosine kinase receptors, such as the platelet-derived growth factor (PDGF) receptors. The SH2 domain of Nck1 binds to phosphorylated tyrosine residue 751 in PDGFbeta receptor and has been suggested to have a role in the PDGF-induced mobilization of the actin filament system. Because Tyr-751 is a site for additional receptor interactors, it has been difficult to discriminate the signaling from Nck from signaling via other molecules. For this reason we have used mouse embryonic fibroblasts derived from mice in which the genes for Nck1 and Nck2 have been inactivated by gene targeting (knock-out (KO) cells). The mutant cells had a reduced ability to form edge ruffles in response to PDGF, and the presence of Nck was obligatory for the formation of dorsal ruffles. In addition, the KO cells had a reduced chemotactic and migratory potential. Importantly, KO cells had reduced cell attachment properties and a reduced ability to form focal adhesions in response to serum stimulation. Moreover, signaling involving the Rho GTPases was defective in KO cells. In summary, our observations suggest that the Nck adapters are needed for signaling to Rho GTPases and actin dynamics downstream of the PDGFbeta receptor.

### ECM

#### PubMed

1. ARAP3 is transiently tyrosine phosphorylated in cells attaching to fibronectin and inhibits cell spreading in a RhoGAP-dependent manner. ARAP3 is a GTPase activating protein (GAP) for Rho and Arf GTPases that is implicated in phosphoinositide 3-kinase (PI 3-kinase) signalling pathways controlling lamellipodia formation and actin stress fibre assembly. We have identified ARAP3 as a phosphorylated target of protein tyrosine kinases. In cells, ARAP3 was tyrosine phosphorylated when co-expressed with Src-family kinases (SFKs), upon stimulation with growth factors and during adhesion to the extracellular matrix (**ECM**) substrate fibronectin. Adhesion-induced phosphorylation of ARAP3 was suppressed by selective inhibitors of Src-family kinases and PI 3-kinase and by a Src dominant interfering mutant. Inducible expression of ARAP3 in HEK293 epithelial cells resulted in increased cell rounding, membrane process formation and cell clustering on **ECM** substrates. In contrast, ARAP3 dramatically slowed the kinetics of cell spreading on fibronectin but had no effect on cell adhesion. These effects of ARAP3 required a functional Rho GAP domain and were associated with reduced cellular levels of active RhoA and Rac1 but did not require the sterile alpha motif (SAM) or Arf GAP domains. Mutation of two phosphorylation sites, Y1399 and Y1404, enhanced some ARAP3 activities, suggesting that ARAP3 may be negatively regulated by phosphorylation on these tyrosine residues. These results implicate ARAP3 in integrin-mediated tyrosine kinase signalling pathways controlling Rho GTPases and cell spreading.

### [Ee]xtracellular [Mm]atrix

#### PubMed

1. ARAP3 is transiently tyrosine phosphorylated in cells attaching to fibronectin and inhibits cell spreading in a RhoGAP-dependent manner. ARAP3 is a GTPase activating protein (GAP) for Rho and Arf GTPases that is implicated in phosphoinositide 3-kinase (PI 3-kinase) signalling pathways controlling lamellipodia formation and actin stress fibre assembly. We have identified ARAP3 as a phosphorylated target of protein tyrosine kinases. In cells, ARAP3 was tyrosine phosphorylated when co-expressed with Src-family kinases (SFKs), upon stimulation with growth factors and during adhesion to the **extracellular matrix** (ECM) substrate fibronectin. Adhesion-induced phosphorylation of ARAP3 was suppressed by selective inhibitors of Src-family kinases and PI 3-kinase and by a Src dominant interfering mutant. Inducible expression of ARAP3 in HEK293 epithelial cells resulted in increased cell rounding, membrane process formation and cell clustering on ECM substrates. In contrast, ARAP3 dramatically slowed the kinetics of cell spreading on fibronectin but had no effect on cell adhesion. These effects of ARAP3 required a functional Rho GAP domain and were associated with reduced cellular levels of active RhoA and Rac1 but did not require the sterile alpha motif (SAM) or Arf GAP domains. Mutation of two phosphorylation sites, Y1399 and Y1404, enhanced some ARAP3 activities, suggesting that ARAP3 may be negatively regulated by phosphorylation on these tyrosine residues. These results implicate ARAP3 in integrin-mediated tyrosine kinase signalling pathways controlling Rho GTPases and cell spreading.

---

## Socs4

- NCBI Gene
- GeneRIF
- Pubmed

| Search Term | Rank | Fields |
| --- | --- | --- |
| [Mm]igration | 6 | PubMed(1) |

  
hide/show details for Socs4

### [Mm]igration

#### PubMed

1. Rbx2 regulates neuronal **migration** through different cullin 5-RING ligase adaptors. Morphogenesis requires the proper **migration** and positioning of different cell types in the embryo. Much more is known about how cells start and guide their **migration**s than about how they stop when they reach their destinations. Here we provide evidence that Rbx2, a subunit of the Cullin 5-RING E3 ubiquitin ligase (CRL5) complex, stops neocortical projection neurons at their target layers. Rbx2 mutation causes neocortical and cerebellar ectopias dependent on Dab1, a key signaling protein in the Reelin pathway. SOCS7, a CRL5 substrate adaptor protein, is also required for neocortical layering. SOCS7-CRL5 complexes stimulate the ubiquitylation and turnover of Dab1. SOCS7 is upregulated during projection neuron **migration**, and unscheduled SOCS7 expression stops **migration** prematurely. Cerebellar development requires Rbx2 but not SOCS7, pointing to the importance of other CRL5 adaptors. Our results suggest that CRL5 adaptor expression is spatiotemporally regulated to modulate Reelin signaling and ensure normal neuron positioning in the developing brain.

---

## Socs5

- NCBI Gene
- GeneRIF
- Pubmed

| Search Term | Rank | Fields |
| --- | --- | --- |
| [Mm]igration | 6 | PubMed(1) |

  
hide/show details for Socs5

### [Mm]igration

#### PubMed

1. Rbx2 regulates neuronal **migration** through different cullin 5-RING ligase adaptors. Morphogenesis requires the proper **migration** and positioning of different cell types in the embryo. Much more is known about how cells start and guide their **migration**s than about how they stop when they reach their destinations. Here we provide evidence that Rbx2, a subunit of the Cullin 5-RING E3 ubiquitin ligase (CRL5) complex, stops neocortical projection neurons at their target layers. Rbx2 mutation causes neocortical and cerebellar ectopias dependent on Dab1, a key signaling protein in the Reelin pathway. SOCS7, a CRL5 substrate adaptor protein, is also required for neocortical layering. SOCS7-CRL5 complexes stimulate the ubiquitylation and turnover of Dab1. SOCS7 is upregulated during projection neuron **migration**, and unscheduled SOCS7 expression stops **migration** prematurely. Cerebellar development requires Rbx2 but not SOCS7, pointing to the importance of other CRL5 adaptors. Our results suggest that CRL5 adaptor expression is spatiotemporally regulated to modulate Reelin signaling and ensure normal neuron positioning in the developing brain.

---

## Gadd45g

- NCBI Gene
- GeneRIF
- Pubmed

| Search Term | Rank | Fields |
| --- | --- | --- |
| [Mm]igration | 6 | PubMed(3) |
| [Mm]etastasis | 12 | PubMed(1); GeneRIFs(1) |

  
hide/show details for Gadd45g

### [Mm]igration

#### PubMed

1. Potential target genes of EMX2 include Odz/Ten-M and other gene families with implications for cortical patterning. EMX2 and PAX6 are expressed by cortical progenitors and specify area patterning. We used representational difference analysis (RDA) to compare expressed RNAs from wild type and Emx2-/- cortex and identified 41 unique clones. Using secondary screening by in situ hybridization, we selected five genes for further analysis, Cdk4, Cofilin1, Crmp1, ME2, and Odz4, involved in neuronal proliferation, differentiation, **migration**, and axon guidance. Each exhibits differential expression in wild type cortex. Odz4 is one of four members of a vertebrate gene family homologous to the Drosophila pair-rule patterning gene, Odd Oz (Odz), a transmembrane receptor. We show that Odz genes are expressed in complementary patterns in cortex, as well as in nuclei-specific patterns in thalamus that relates to their area-unique cortical expression. In addition, each of the genes analyzed shows different expression patterns in wild type cortex, Emx2, and Pax6 mutant cortex, consistent with potential roles in area patterning. These findings identify potential targets of EMX2 that might account for its function and the defects in Emx2-/- cortex, and suggest that the Odz family of transmembrane proteins influences cortical area patterning downstream to EMX2 and PAX6.
2. Paired box 5 is a frequently methylated lung cancer tumour suppressor gene interfering β-catenin signalling and GADD45G expression. Recent studies suggest that paired box 5 (PAX5) is down-regulated in multiple tumours through its promoter methylation. However, the role of PAX5 in non-small cell lung cancer (NSCLC) pathogenesis remains unclear. The aim of this study is to examine PAX5 expression, its methylation status, biological functions and related molecular mechanism in NSCLC. We found that PAX5 was widely expressed in normal adult tissues but silenced or down-regulated in 88% (7/8) of NSCLC cell lines. PAX5 expression level was significantly lower in NSCLC than that in adjacent non-cancerous tissues (P = 0.0201). PAX5 down-regulation was closely associated with its promoter hypermethylation status and PAX5 expression could be restored by demethylation treatment. Frequent PAX5 promoter methylation in primary tumours (70%) was correlated with lung tumour histological types (P = 0.006). Ectopic expression of PAX5 in silenced lung cancer cell lines (A549 and H1975) inhibited their colony formation and cell viability, arrested cell cycle at G2 phase and suppressed cell **migratio**n/invasion as well as tumorigenicity in nude mice. Restoration of PAX5 expression resulted in the down-regulation of β-catenin and up-regulation of tissue inhibitors of metalloproteinase 2, GADD45G in lung tumour cells. In summary, PAX5 was found to be an epigenetically inactivated tumour suppressor that inhibits NSCLC cell proliferation and metastasis, through down-regulating the β-catenin pathway and up-regulating GADD45G expression.
3. Neurogenin2-d4Venus and Gadd45g-d4Venus transgenic mice: visualizing mitotic and migratory behaviors of cells committed to the neuronal lineage in the developing mammalian brain. To achieve highly sensitive and comprehensive assessment of the morphology and dynamics of cells committed to the neuronal lineage in mammalian brain primordia, we generated two transgenic mouse lines expressing a destabilized (d4) Venus controlled by regulatory elements of the Neurogenin2 (Neurog2) or Gadd45g gene. In mid-embryonic neocortical walls, expression of Neurog2-d4Venus mostly overlapped with that of Neurog2 protein, with a slightly (1 h) delayed onset. Although Neurog2-d4Venus and Gadd45g-d4Venus mice exhibited very similar labeling patterns in the ventricular zone (VZ), in Gadd45g-d4Venus mice cells could be visualized in more basal areas containing fully differentiated neurons, where Neurog2-d4Venus fluorescence was absent. Time-lapse monitoring revealed that most d4Venus(+) cells in the VZ had processes extending to the apical surface; many of these cells eventually retracted their apical process and migrated basally to the subventricular zone, where neurons, as well as the intermediate neurogenic progenitors that undergo terminal neuron-producing division, could be live-monitored by d4Venus fluorescence. Some d4Venus(+) VZ cells instead underwent nuclear **migratio**n to the apical surface, where they divided to generate two d4Venus(+) daughter cells, suggesting that the symmetric terminal division that gives rise to neuron pairs at the apical surface can be reliably live-monitored. Similar lineage-committed cells were observed in other developing neural regions including retina, spinal cord, and cerebellum, as well as in regions of the peripheral nervous system such as dorsal root ganglia. These mouse lines will be useful for elucidating the cellular and molecular mechanisms underlying development of the mammalian nervous system.

### [Mm]etastasis

#### PubMed

1. Paired box 5 is a frequently methylated lung cancer tumour suppressor gene interfering β-catenin signalling and GADD45G expression. Recent studies suggest that paired box 5 (PAX5) is down-regulated in multiple tumours through its promoter methylation. However, the role of PAX5 in non-small cell lung cancer (NSCLC) pathogenesis remains unclear. The aim of this study is to examine PAX5 expression, its methylation status, biological functions and related molecular mechanism in NSCLC. We found that PAX5 was widely expressed in normal adult tissues but silenced or down-regulated in 88% (7/8) of NSCLC cell lines. PAX5 expression level was significantly lower in NSCLC than that in adjacent non-cancerous tissues (P = 0.0201). PAX5 down-regulation was closely associated with its promoter hypermethylation status and PAX5 expression could be restored by demethylation treatment. Frequent PAX5 promoter methylation in primary tumours (70%) was correlated with lung tumour histological types (P = 0.006). Ectopic expression of PAX5 in silenced lung cancer cell lines (A549 and H1975) inhibited their colony formation and cell viability, arrested cell cycle at G2 phase and suppressed cell migration/invasion as well as tumorigenicity in nude mice. Restoration of PAX5 expression resulted in the down-regulation of β-catenin and up-regulation of tissue inhibitors of metalloproteinase 2, GADD45G in lung tumour cells. In summary, PAX5 was found to be an epigenetically inactivated tumour suppressor that inhibits NSCLC cell proliferation an**d metastas**is, through down-regulating the β-catenin pathway and up-regulating GADD45G expression.

#### GeneRIFs

1. PAX5 was found to be an epigenetically inactivated tumor suppressor that inhibited non-small-cell lung proliferation and **metastasis**, through down-regulating the beta-catenin pathway and up-regulating GADD45G expression.

---

## Plpp1

- NCBI Gene
- GeneRIF
- Pubmed

| Search Term | Rank | Fields |
| --- | --- | --- |
| [Mm]igration | 6 | PubMed(4) |
| [Mm]etastasis | 12 | PubMed(2) |

  
hide/show details for Plpp1

### [Mm]igration

#### PubMed

1. Lipid phosphate phosphatases dimerise, but this interaction is not required for in vivo activity. Lipid phosphate phosphatases (LPPs) are integral membrane proteins believed to dephosphorylate bioactive lipid messengers, so modifying or attenuating their activities. Wunen, a Drosophila LPP homologue, has been shown to play a pivotal role in primordial germ cell (PGC) **migration** and survival during embryogenesis. It has been hypothesised that LPPs may form oligomeric complexes, and may even function as hexamers. We were interested in exploring this possibility, to confirm whether LPPs can oligomerise, and if they do, whether oligomerisation is required for either in vitro or in vivo activity.We present evidence that Wunen dimerises, that these associations require the last thirty-five C-terminal amino-acids and depend upon the presence of an intact catalytic site. Expression of a truncated, monomeric form of Wunen in Drosophila embryos results in perturbation of germ cell **migration** and germ cell loss, as observed for full-length Wunen. We also observed that murine LPP-1 and human LPP-3 can also form associations, but do not form interactions with Wunen or each other. Furthermore, Wunen does not form dimers with its closely related counterpart Wunen-2. Finally we discovered that addition of a trimeric myc tag to the C-terminus of Wunen does not prevent dimerisation or in vitro activity, but does prevent activity in vivo.LPPs do form complexes, but these do not seem to be specifically required for activity either in vitro or in vivo. Since neither dimerisation nor the C-terminus seem to be involved in substrate recognition, they may instead confer structural or functional stability through dimerisation. The results indicate that the associations we see are highly specific and occur only between monomers of the same protein.
2. Decreased peritoneal ovarian cancer growth in mice lacking expression of lipid phosphate phosphohydrolase 1. Lysophosphatidic acid (LPA) is a bioactive lipid that enhances ovarian cancer cell proliferation, **migration** and invasion in vitro and stimulates peritoneal metastasis in vivo. LPA is generated through the action of autotaxin or phospholipases, and degradation begins with lipid phosphate phosphohydrolase (LPP)-dependent removal of the phosphate. While the effects of LPA on ovarian cancer progression are clear, the effects of LPA metabolism within the tumor microenvironment on peritoneal metastasis have not been reported. We examined the contribution of lipid phosphatase activity to ovarian cancer peritoneal metastasis using mice deficient in LPP1 expression. Homozygous deletion of LPP1 (LPP1 KO) results in elevated levels and decreased turnover of LPA in vivo. Within 2 weeks of intraperitoneal injection of syngeneic mouse ovarian cancer cells, we observed enhanced tumor seeding in the LPP1 KO mice compared to wild type. However, tumor growth plateaued in the LPP1 KO mice by 3 weeks while tumors continued to grow in wild type mice. The decreased tumor burden was accompanied by increased apoptosis and no change in proliferation or angiogenesis. Tumor growth was restored and apoptosis reversed with exogenous administration of LPA. Together, these observations demonstrate that the elevated levels of LPA per se in LPP1 KO mice do not inhibit tumor growth. Rather, the data support the notion that either elevated LPA concentration or altered LPA metabolism affects other growth-promoting contributions of the tumor microenvironment.
3. Lipid phosphate phosphatase-1 expression in cancer cells attenuates tumor growth and metastasis in mice. Lipid phosphate phosphatase-1 (LPP1) degrades lysophosphatidate (LPA) and attenuates receptor-mediated signaling. LPP1 expression is low in many cancer cells and tumors compared with normal tissues. It was hypothesized from studies with cultured cells that increasing LPP1 activity would decrease tumor growth and metastasis. This hypothesis has never been tested in vivo. To do this, we inducibly expressed LPP1 or a catalytically inactive mutant in cancer cells. Expressing active LPP1 increased extracellular LPA degradation by 5-fold. It also decreased the stimulation of Ca(2+) transients by LPA, a nondephosphorylatable LPA1/2 receptor agonist and a protease-activated receptor-1 peptide. The latter results demonstrate that LPP1 has effects downstream of receptor activation. Decreased Ca(2+) mobilization and Rho activation contributed to the effects of LPP1 in attenuating the LPA-induced **migration** of MDA-MB-231 breast cancer cells and their growth in 3D culture. Increasing LPP1 expression in breast and thyroid cancer cells decreased tumor growth and the metastasis by up to 80% compared with expression of inactive LPP1 or green fluorescent protein in syngeneic and xenograft mouse models. The present work demonstrates for the first time that increasing the LPP1 activity in three lines of aggressive cancer cells decreases their abilities to produce tumors and metastases in mice.
4. Fly and mammalian lipid phosphate phosphatase isoforms differ in activity both in vitro and in vivo. Wunen (Wun), a homologue of a lipid phosphate phosphatase (LPP), has a crucial function in the **migration** and survival of primordial germ cells (PGCs) during Drosophila embryogenesis. Past work has indicated that the LPP isoforms may show functional redundancy in certain systems, and that they have broad-range lipid phosphatase activities in vitro, with little apparent specificity between them. We show here that there are marked differences in biochemical activity between fly Wun and mammalian LPPs, with Wun having a narrower activity range than has been reported for the mammalian LPPs. Furthermore, although it is active on a range of substrates in vitro, mouse Lpp1 has no activity on an endogenous Drosophila germ-cell-specific factor in vivo. Conversely, human LPP3 is active, resulting in aberrant **migration** and PGC death. These results show an absolute difference in bioactivity among LPP isoforms for the first time in a model organism and may point towards an underlying signalling system that is conserved between flies and humans.

### [Mm]etastasis

#### PubMed

1. Decreased peritoneal ovarian cancer growth in mice lacking expression of lipid phosphate phosphohydrolase 1. Lysophosphatidic acid (LPA) is a bioactive lipid that enhances ovarian cancer cell proliferation, migration and invasion in vitro and stimulates peritoneal **metastasis** in vivo. LPA is generated through the action of autotaxin or phospholipases, and degradation begins with lipid phosphate phosphohydrolase (LPP)-dependent removal of the phosphate. While the effects of LPA on ovarian cancer progression are clear, the effects of LPA metabolism within the tumor microenvironment on peritoneal **metastasis** have not been reported. We examined the contribution of lipid phosphatase activity to ovarian cancer peritoneal **metastasis** using mice deficient in LPP1 expression. Homozygous deletion of LPP1 (LPP1 KO) results in elevated levels and decreased turnover of LPA in vivo. Within 2 weeks of intraperitoneal injection of syngeneic mouse ovarian cancer cells, we observed enhanced tumor seeding in the LPP1 KO mice compared to wild type. However, tumor growth plateaued in the LPP1 KO mice by 3 weeks while tumors continued to grow in wild type mice. The decreased tumor burden was accompanied by increased apoptosis and no change in proliferation or angiogenesis. Tumor growth was restored and apoptosis reversed with exogenous administration of LPA. Together, these observations demonstrate that the elevated levels of LPA per se in LPP1 KO mice do not inhibit tumor growth. Rather, the data support the notion that either elevated LPA concentration or altered LPA metabolism affects other growth-promoting contributions of the tumor microenvironment.
2. Lipid phosphate phosphatase-1 expression in cancer cells attenuates tumor growth and **metastasis** in mice. Lipid phosphate phosphatase-1 (LPP1) degrades lysophosphatidate (LPA) and attenuates receptor-mediated signaling. LPP1 expression is low in many cancer cells and tumors compared with normal tissues. It was hypothesized from studies with cultured cells that increasing LPP1 activity would decrease tumor growth and **metastasis**. This hypothesis has never been tested in vivo. To do this, we inducibly expressed LPP1 or a catalytically inactive mutant in cancer cells. Expressing active LPP1 increased extracellular LPA degradation by 5-fold. It also decreased the stimulation of Ca(2+) transients by LPA, a nondephosphorylatable LPA1/2 receptor agonist and a protease-activated receptor-1 peptide. The latter results demonstrate that LPP1 has effects downstream of receptor activation. Decreased Ca(2+) mobilization and Rho activation contributed to the effects of LPP1 in attenuating the LPA-induced migration of MDA-MB-231 breast cancer cells and their growth in 3D culture. Increasing LPP1 expression in breast and thyroid cancer cells decreased tumor growth and the **metastasis** by up to 80% compared with expression of inactive LPP1 or green fluorescent protein in syngeneic and xenograft mouse models. The present work demonstrates for the first time that increasing the LPP1 activity in three lines of aggressive cancer cells decreases their abilities to produce tumors and metastases in mice.

---

## Itga4

- NCBI Gene
- GeneRIF
- Pubmed

| Search Term | Rank | Fields |
| --- | --- | --- |
| [Mm]igration | 6 | Process(5); PubMed(58); GeneRIFs(8) |
| ECM | 7 | PubMed(2) |
| [Ee]xtracellular [Mm]atrix | 8 | PubMed(22) |
| [Ii]nvasive | 10 | PubMed(2) |
| [Mm]etastasize | 11 | PubMed(1) |
| [Mm]etastasis | 12 | PubMed(15); GeneRIFs(3) |
| [Cc]ell [Ii]nvasion | 13 | PubMed(1) |

  
hide/show details for Itga4

### [Mm]igration

#### Process

1. T cell **migration**
2. cell **migration**
3. cell-matrix adhesion involved in ameboidal cell **migration**
4. positive regulation of T cell **migration**
5. positive regulation of leukocyte **migration**

#### PubMed

1. The role of chemokine activation of Rac GTPases in hematopoietic stem cell marrow homing, retention, and peripheral mobilization. Signaling downstream from the chemokine receptor CXCR4, the tyrosine kinase receptor c-kit and beta1-integrins has been shown to be crucial in the regulation of **migration**, homing, and engraftment of hematopoietic stem cells and progenitors. Each of these receptors signal through Rac-type Rho guanosine triphosphatases (GTPases). Rac GTPases play a major role in the organization of the actin cytoskeleton and also in the control of gene expression and the activation of proliferation and survival pathways. Here we review the specific roles of the members of the Rac subfamily of the Rho GTPase family in regulating the intracellular signaling of hematopoietic cells responsible for regulation of homing, marrow retention, and peripheral mobilization.
2. **Migration** of encephalitogenic CD8 T cells into the central nervous system is dependent on the α4β1-integrin. Although CD8 T cells are key players in neuroinflammation, little is known about their trafficking cues into the central nervous system (CNS). We used a murine model of CNS autoimmunity to define the molecules involved in cytotoxic CD8 T-cel**l migrati**on into the CNS. Using a panel of mAbs, we here show that the α4β1-integrin is essential for CD8 T-cell interaction with CNS endothelium. We also investigated which α4β1-integrin ligands expressed by endothelial cells are implicated. The blockade of VCAM-1 did not protect against autoimmune encephalomyelitis, and only partly decreased the CD8(+) T-cell infiltration into the CNS. In addition, inhibition of junctional adhesion molecule-B expressed by CNS endothelial cells also decreases CD8 T-cell infiltration. CD8 T cells may use additional and possibly unidentified adhesion molecules to gain access to the CNS.
3. Adhesion molecules involved in macrophage responses to Wallerian degeneration in the murine peripheral nervous system. When a peripheral nerve is damaged the severed axon undergoes Wallerian degeneration. The distal nerve is infiltrated by large numbers of monocyte-derived macrophages which participate in the phagocytosis of degenerating myelin. In other tissues, adhesion molecules play a crucial role in leukocyte recruitment during inflammation. Blood-borne cells enter damaged tissue by interacting with adhesion molecules expressed on activated endothelium. Having crossed the endothelium, leukocytes must adhere and migrate within the tissue. We investigated the adhesion molecules involved in both stages of the macrophage response to transection of one sciatic nerve of BALB/c mice. By injecting monoclonal antibodies in vivo, before and after peripheral nerve injury, we showed that intercellular adhesion molecule-1 (ICAM-1) and integrins alpha4beta1 (VLA-4) and alphaMbeta2 (type 3 complement receptor) are unlikely to be involved in the transendothelial **migration** of monocytes responding to peripheral nerve degeneration. We also studied the adhesion of macrophages within the endoneurium, using an in vitro adhesion assay. Macrophages showed much greater levels of adhesion to cryostat sections of transected nerves than to control nerves. This increased adhesion was partially inhibited by antibodies to the beta1-integrin chain, and more strongly inhibited by the extracellular matrix molecules fibronectin and collagen. Adhesion was unaffected by laminin-1 and by antibodies to other adhesion molecules, including alpha4beta1- and alpha5beta1-integrins. Thus we conclude that monocyte entry into a degenerating peripheral nerve is independent of alphaLbeta2/alphaMbeta2-ICAM-1 or alpha4beta1/VCAM-1 interactions, and that adhesion within the endoneurium is mediated in part by a beta1-integrin other than alpha4beta1 or alpha5beta1.
4. Dual functions of [alpha]4[beta]1 integrin in epicardial development: initial **migration** and long-term attachment. The epicardium of the mammalian heart arises from progenitor cells outside the developing heart. The epicardial progenitor (EPP) cells migrate onto the heart through a cyst-mediated mechanism in which the progenitors are released from the tissue of origin as cysts; the cysts float in the fluid of the pericardial cavity and attach to the naked myocardial surface of the heart, and cells in the cysts then migrate out to form an epithelial sheet. In this paper, we show that the gene encoding the alpha4 subunit of alpha4beta1 integrin (alpha4beta1) is essential for this migratory process. We have generated a knockin mutation in mice replacing the alpha4 integrin gene with the lacZ reporter gene, placing lacZ under the control of the alpha4 integrin promoter. We show that in homozygous mutant embryos, the **migration** of EPP progenitor cells is impaired due to inefficient budding of the cysts and a failure of the cells in the cysts to migrate on the heart. This study provides direct genetic evidence for essential roles for alpha4beta1 integrin-mediated cell adhesion in the **migration** of progenitor cells to form the epicardium, in addition to a previous finding that alpha4beta1 is essential for maintaining the epicardium (Yang, J.T., H. Rayburn, and R.O. Hynes. 1995. Development. 121:549-560).
5. Beta1 integrin expression on endothelial cells is required for angiogenesis but not for vasculogenesis. Integrins are a family of cell adhesion receptors that are involved in cell-matrix and cell-cell communications. They facilitate cell proliferation, **migration**, and survival. Using the Cre-Lox system, we deleted beta1 integrin on Tie2-positive (Tie2-cre beta1 Int (fl/fl)) vascular endothelial cells. Deletion of beta1 integrin on vascular endothelial cells results in embryonic lethality. Blood vessel defects are encountered in the Tie2-Cre beta1 Int (fl/fl) embryos at embryonic age (E9.5), and embryos die before reaching E10.5. The embryos exhibit growth retardation and both histological evaluation and PECAM-1 staining of E9.5 embryos revealed defects in angiogenic sprouting and vascular branching morphogenesis. Large and medium-size vessel formation is not affected in these embryos. Angiogenic defects were observed in several regions of the embryo and yolk sacs. These results indicate that beta1 integrin expression on vascular endothelial cells is crucial for embryonic angiogenesis but dispensable for vasculogenesis.
6. VCAM-1 and VLA-4 modulate dendritic cell IL-12p40 production in experimental visceral leishmaniasis. Vascular cell adhesion molecule-1 (VCAM-1) interacts with its major ligand very late antigen-4 (VLA-4) to mediate cell adhesion and transendothelial **migration** of leukocytes. We report an important role for VCAM-1/VLA-4 interactions in the generation of immune responses during experimental visceral leishmaniasis caused by Leishmania donovani. Our studies demonstrate that these molecules play no direct role in the recruitment of leukocytes to the infected liver, but instead contribute to IL-12p40-production by splenic CD8(+) dendritic cells (DC). Blockade of VCAM-1/VLA-4 interactions using whole antibody or anti-VCAM-1 Fab' fragments reduced IL-12p40 mRNA accumulation by splenic DC 5 hours after L. donovani infection. This was associated with reduced anti-parasitic CD4(+) T cell activation in the spleen and lowered hepatic IFNgamma, TNF and nitric oxide production by 14 days post infection. Importantly, these effects were associated with enhanced parasite growth in the liver in studies with either anti-VCAM-1 or anti-VLA-4 antibodies. These data indicate a role for VCAM-1 and VLA-4 in DC activation during infectious disease.
7. Integrin alpha4beta1 signaling is required for lymphangiogenesis and tumor metastasis. Recent studies have shown that lymphangiogenesis or the growth of lymphatic vessels at the periphery of tumors promotes tumor metastasis to lymph nodes. We show here that the fibronectin-binding integrin alpha4beta1 and its ligand fibronectin are novel functional markers of proliferative lymphatic endothelium. Tumors and lymphangiogenic growth factors, such as vascular endothelial growth factor-C (VEGF-C) and VEGF-A, induce lymphatic vessel expression of integrin alpha4beta1. Integrin alpha4beta1 then promotes growth factor and tumor-induced lymphangiogenesis, as genetic loss of integrin alpha4beta1 expression in Tie2Cre+ alpha4(loxp/loxp) mice or genetic loss of alpha4 signaling in alpha4Y991A knock-in mice blocks growth factor and tumor-induced lymphangiogenesis, as well as tumor metastasis to lymph nodes. In addition, antagonists of integrin alpha4beta1 suppress lymphangiogenesis and tumor metastasis. Our studies show that integrin alpha4beta1 and the signals it transduces regulate the adhesion, **migration**, invasion, and survival of proliferating lymphatic endothelial cells. As suppression of alpha4beta1 expression, signal transduction, or function in tumor lymphatic endothelium not only inhibits tumor lymphangiogenesis but also prevents metastatic disease, these results show that integrin alpha4beta1-mediated tumor lymphangiogenesis promotes metastasis and is a useful target for the suppression of metastatic disease.
8. Defective blood vessel development and pericyte/pvSMC distribution in alpha 4 integrin-deficient mouse embryos. Blood vessel development is in part regulated by pericytes/presumptive vascular smooth muscle cells (PC/pvSMCs). Here, we demonstrate that interactions between PC/pvSMCs and extracellular matrix play a critical role in this event. We show that the cranial vessels in alpha4 integrin-deficient mouse embryos at the stage of vessel remodeling are increased in diameter. This defect is accompanied by a failure of PC/pvSMCs, which normally express alpha4beta1 integrin, to spread uniformly along the vessels. We also find that fibronectin but not VCAM-1 is localized in the cranial vessels at this stage. Furthermore, cultured alpha4 integrin-null PC/pvSMCs plated on fibronectin display a delay in initiating **migration**, a reduction in **migration** speed, and a decrease in directional persistence in response to a polarized force of shear flow. These results suggest that specific motile activities of PC/pvSMCs regulated by mechanical signals imposed by the interstitial extracellular matrix may also be required in vivo for the distribution and function of the PC/pvSMCs during blood vessel development.
9. Relationships between hematopoiesis and hepatogenesis in the midtrimester fetal liver characterized by dynamic transcriptomic and proteomic profiles. In fetal hematopoietic organs, the switch from hematopoiesis is hypothesized to be a critical time point for organogenesis, but it is not yet evidenced. The transient coexistence of hematopoiesis will be useful to understand the development of fetal liver (FL) around this time and its relationship to hematopoiesis. Here, the temporal and the comparative transcriptomic and proteomic profiles were observed during the critical time points corresponding to the initiation (E11.5), peak (E14.5), recession (E15.5), and disappearance (3 ddp) of mouse FL hematopoiesis. We found that E11.5-E14.5 corresponds to a FL hematopoietic expansion phase with distinct molecular features, including the expression of new transcription factors, many of which are novel KRAB (Kruppel-associated box)-containing zinc finger proteins. This time period is also characterized by extensive depression of some liver functions, especially catabolism/utilization, immune and defense, classical complement cascades, and intrinsic blood coagulation. Instead, the other liver functions increased, such as xenobiotic and sterol metabolism, synthesis of carbohydrate and glycan, the alternate and lectin complement cascades and extrinsic blood coagulation, and etc. Strikingly, all of the liver functions were significantly increased at E14.5-E15.5 and thereafter, and the depression of the key pathways attributes to build the hematopoietic microenvironment. These findings signal hematopoiesis e**migration** is the key to open the door of liver maturation.
10. Phagocytic cells of the thymic reticulum interact with thymocytes via extracellular matrix ligands and receptors. We previously showed that, in the context of thymic epithelial cells, thymocyte **migration** is partially controlled by extracellular matrix (ECM)-mediated interactions. Herein we evaluated whether these interactions could be involved in cell **migration** related events in the context of non-epithelial cells of the thymic microenvironment, the phagocytic cells of the thymic reticulum (PTR). We first showed, by immunocytochemistry, cytofluorometry, and RT-PCR, that PTR produce ECM components, including fibronectin and laminin, and express the corresponding integrin-type receptors, VLA-4, VLA-5, and VLA-6. Thymocytes adhere onto PTR monolayers, with immature CD4(+)CD8(+) cells being predominant. Importantly, such an adhesion is partially mediated by ECM ligands and receptors, since it was impaired by anti-ECM or anti-ECM receptor antibodies. Conjointly, our data reveal that the ECM-dependence for thymocyte adhesion onto the thymic microenvironment is not restricted to the epithelial cells, being also seen when they encounter non-epithelial phagocytic cells.
11. Enhanced stem cell **migration** mediated by VCAM-1/VLA-4 interaction improves cardiac function in virus-induced dilated cardiomyopathy. Endogenous circulation of bone marrow-derived cells (BMCs) was observed in patients with dilated cardiomyopathy (DCM) who showed cardiac upregulation of Vascular Cell Adhesion Protein-1 (VCAM-1). However, the underlying pathophysiology is currently unknown. Thus, we aimed to analyze circulation, **migration** and G-CSF-based mobilization of BMCs in a murine model of virus-induced DCM. Mice with coxsackievirus B3 (CVB3) induced DCM and healthy controls were analyzed regarding their myocardial homing factors by PCR. To determine cardiac VCAM-1 expression ELISA and immunohistochemistry were applied. Flow cytometry was performed to analyze BMCs. Cardiac diameters and function were evaluated by echocardiography before and 4 weeks after G-CSF treatment. In murine CVB3-induced DCM an increase of BMCs in peripheral blood and a decrease of BMCs in bone marrow was observed. We found an enhanced **migration** of Very Late Antigen-4 (VLA-4⁺) BMCs to the diseased heart overexpressing VCAM-1 and higher numbers of CD45⁻CD34⁻Sca-1⁺ and CD45⁻CD34⁻c-kit⁺ cells. Mobilization of BMCs by **G-CSF boo**sted migration along the VCAM-1/VLA-4 axis and reduced apoptosis of cardiomyocytes. Significant improvement of cardiac function was detected by echocardiography in G-CSF-treated mice. Blocking VCAM-1 by a neutralizing antibody reduced the G-CSF-dependent effects **on stem** cell migration and cardiac function. This is the first study showing that in virus-induced DCM VCAM-1/VLA-4 interaction is crucial for recruitment of circulating BMCs leading to beneficial anti-apoptotic effects resulting in improved cardiac function after G-CSF-induced mobilization.
12. Essential role of VLA-4/VCAM-1 pathway in the establishment of CD8+ T-cell-mediated Trypanosoma cruzi-elicited meningoencephalitis. Central nervous system (CNS) damage can occur during Trypanosoma cruzi infection, especially in immunosuppressed patients. The enhanced susceptibility of C3H/He mice to CD8-mediated acute meningoencephalitis is associated with higher up-regulation of vascular cell adhesion molecule-1 (VCAM-1) on CNS vascular endothelia than in the less susceptible C57BL/6. Further, in vitro adhesion of activated peripheral blood cells to CNS blood vessels was abrogated by anti-VLA-4 antibodies that also inhibited cell **migration** into the CNS of T. cruzi-infected mice. Lastly, the reactivation of meningoencephalitis in immunosuppressed chronically infected mice was associated with VCAM-1 up-regulation. Therefore, we hypothesize that VLA-4/VCAM-1 pathway plays a pivotal role in the establishment of T. cruzi-elicited encephalitis.
13. Integrin alpha4beta1 (VLA-4) expression and activity in retinal and peripheral neurons. The integrin alpha4beta1 fulfills important roles in inflammation and hematopoesis, but its functions in neurons are not well understood. Here we show that the alpha4 subunit is expressed on mouse retinal ganglion cells (RGCs) and undifferentiated retinal neuroblasts during the period of axon extension and **migration**. To determine if alpha4 integrins expressed by retinal neurons were active, neurons were cultured on known alpha4 ligands in vitro. Recombinant soluble vascular cell adhesion molecule 1 (rsVCAM-1), fibronectin, and osteopontin (OPN) induced neurite outgrowth that was diminished by function blocking antibodies specific for alpha4. Neurite outgrowth on OPN was also blocked by antibodies to the integrin beta1 subunit, implicating the alpha4beta1 heterodimer as one integrin receptor mediating outgrowth on OPN. OPN immunoreactivity was detected in the RGC fiber layer and optic nerve, suggesting that it may act as an alpha4 ligand in vivo. Neurons from chick lumbar sympathetic ganglia, chick dorsal root ganglia, and mouse superior cervical ganglia also extended neurites on rsVCAM-1, suggesting that integrin alpha4beta1 may play a role in the development of multiple neuronal cell types.
14. The integrins Mac-1 and alpha4beta1 perform crucial roles in neutrophil and T cell recruitment to lungs during Streptococcus pneumoniae infection. Neutrophils and T cells play an important role in host protection against pulmonary infection caused by Streptococcus pneumoniae. However, the role of the integrins in recruitment of these cells to infected lungs is not well understood. In this study we used the twin approaches of mAb blockade and gene-deficient mice to investigate the relative impact of specific integrins on cellular recruitment and bacterial loads following pneumococcal infection. We find that both Mac-1 (CD11b/CD18) and α(4)β(1) (CD49d/CD29) integrins, but surprisingly not LFA-1 (CD11a/CD18), contribute to two aspects of the response. In terms of recruitment from the circulation into lungs, neutrophils depend on Mac-1 and α(4)β(1), whereas the T cells are entirely dependent on α(4)β(1). Second, immunohistochemistry results indicate that adhesion also plays a role within infected lung tissue itself. There is widespread expression of ICAM-1 within lung tissue. Use of ICAM-1(-/-) mice revealed that neutrophils make use of this Mac-1 ligand, not for lung entry o**r for mig**ration within lung tissue, but for combating the pneumococcal infection. In contrast to ICAM-1, there is restricted and constitutive expression of the α(4)β(1) ligand, VCAM-1, on the bronchioles, allowing direct access of the leukocytes to the airways via this integrin at an early stage of pneumococcal infection. Therefore, integrins Mac-1 and α(4)β(1) have a pivotal role in prevention of pneumococcal outgrowth during disease both in regulating neutrophil and T cell recruitment into infected lungs and by influencing their behavior within the lung tissue itself.
15. Enhanced trafficking to the pancreatic lymph nodes and auto-antigen presentation capacity distinguishes peritoneal B lymphocytes in non-obese diabetic mice. NOD.Igmicro ( null ) mice lacking mature B cells are highly resistant to diabetes and display poor CD4 T cell responses to autoantigens. Nevertheless, the degree to which different B cell subsets contribute to diabetes in NOD mice remains unresolved. Due to their role in the recognition of microbial and autoantigens, peritoneal B cell characteristics were examined in NOD mice to see if they differ developmentally, phenotypically or functionally in aspects relevant to diabetogenesis.The population dynamics, activation state, migratory behaviour and antigen presentation function were investigated in NOD peritoneal B cells.NOD peritoneal B cells were found to express abnormally high levels of co-stimulatory molecules (CD40, CD86 and CD69). In contrast, the expression of L-selectin and integrin alpha4beta1 was markedly reduced in NOD mice compared with BALB/c and C57BL/6 mice. The number of B cells in the peritoneum was lower in NOD than in control mice throughout development; **migration** of B cells from the peritoneum to the pancreatic lymph nodes in NOD mice was enhanced tenfold. NOD B cells showed no chemotactic response to sphingosine-1-phosphate, which normally acts to retain B cells in the peritoneum. Peritoneal B cells of NOD mice also presented insulin autoantigen to CD4 T cells, inducing T cell proliferation.NOD peritoneal B cells are hyperactivated, migrate to the pancreatic lymph nodes and are capable of driving insulin-specific CD4 T cell activation. These characteristics could make them important for inducing or amplifying T cell responses against islet-antigens.
16. **Migration** of encephalitogenic CD8 T cells into the central nervous system is dependent on the α4β1-integrin. Although CD8 T cells are key players in neuroinflammation, little is known about their trafficking cues into the central nervous system (CNS). We used a murine model of CNS autoimmunity to define the molecules involved in cytotoxic CD8 T-cel**l migrati**on into the CNS. Using a panel of mAbs, we here show that the α4β1-integrin is essential for CD8 T-cell interaction with CNS endothelium. We also investigated which α4β1-integrin ligands expressed by endothelial cells are implicated. The blockade of VCAM-1 did not protect against autoimmune encephalomyelitis, and only partly decreased the CD8(+) T-cell infiltration into the CNS. In addition, inhibition of junctional adhesion molecule-B expressed by CNS endothelial cells also decreases CD8 T-cell infiltration. CD8 T cells may use additional and possibly unidentified adhesion molecules to gain access to the CNS.
17. Enhanced stem cell **migration** mediated by VCAM-1/VLA-4 interaction improves cardiac function in virus-induced dilated cardiomyopathy. Endogenous circulation of bone marrow-derived cells (BMCs) was observed in patients with dilated cardiomyopathy (DCM) who showed cardiac upregulation of Vascular Cell Adhesion Protein-1 (VCAM-1). However, the underlying pathophysiology is currently unknown. Thus, we aimed to analyze circulation, **migration** and G-CSF-based mobilization of BMCs in a murine model of virus-induced DCM. Mice with coxsackievirus B3 (CVB3) induced DCM and healthy controls were analyzed regarding their myocardial homing factors by PCR. To determine cardiac VCAM-1 expression ELISA and immunohistochemistry were applied. Flow cytometry was performed to analyze BMCs. Cardiac diameters and function were evaluated by echocardiography before and 4 weeks after G-CSF treatment. In murine CVB3-induced DCM an increase of BMCs in peripheral blood and a decrease of BMCs in bone marrow was observed. We found an enhanced **migration** of Very Late Antigen-4 (VLA-4⁺) BMCs to the diseased heart overexpressing VCAM-1 and higher numbers of CD45⁻CD34⁻Sca-1⁺ and CD45⁻CD34⁻c-kit⁺ cells. Mobilization of BMCs by **G-CSF boo**sted migration along the VCAM-1/VLA-4 axis and reduced apoptosis of cardiomyocytes. Significant improvement of cardiac function was detected by echocardiography in G-CSF-treated mice. Blocking VCAM-1 by a neutralizing antibody reduced the G-CSF-dependent effects **on stem** cell migration and cardiac function. This is the first study showing that in virus-induced DCM VCAM-1/VLA-4 interaction is crucial for recruitment of circulating BMCs leading to beneficial anti-apoptotic effects resulting in improved cardiac function after G-CSF-induced mobilization.
18. The integrins Mac-1 and alpha4beta1 perform crucial roles in neutrophil and T cell recruitment to lungs during Streptococcus pneumoniae infection. Neutrophils and T cells play an important role in host protection against pulmonary infection caused by Streptococcus pneumoniae. However, the role of the integrins in recruitment of these cells to infected lungs is not well understood. In this study we used the twin approaches of mAb blockade and gene-deficient mice to investigate the relative impact of specific integrins on cellular recruitment and bacterial loads following pneumococcal infection. We find that both Mac-1 (CD11b/CD18) and α(4)β(1) (CD49d/CD29) integrins, but surprisingly not LFA-1 (CD11a/CD18), contribute to two aspects of the response. In terms of recruitment from the circulation into lungs, neutrophils depend on Mac-1 and α(4)β(1), whereas the T cells are entirely dependent on α(4)β(1). Second, immunohistochemistry results indicate that adhesion also plays a role within infected lung tissue itself. There is widespread expression of ICAM-1 within lung tissue. Use of ICAM-1(-/-) mice revealed that neutrophils make use of this Mac-1 ligand, not for lung entry o**r for mig**ration within lung tissue, but for combating the pneumococcal infection. In contrast to ICAM-1, there is restricted and constitutive expression of the α(4)β(1) ligand, VCAM-1, on the bronchioles, allowing direct access of the leukocytes to the airways via this integrin at an early stage of pneumococcal infection. Therefore, integrins Mac-1 and α(4)β(1) have a pivotal role in prevention of pneumococcal outgrowth during disease both in regulating neutrophil and T cell recruitment into infected lungs and by influencing their behavior within the lung tissue itself.
19. Integrin alpha4beta1 signaling is required for lymphangiogenesis and tumor metastasis. Recent studies have shown that lymphangiogenesis or the growth of lymphatic vessels at the periphery of tumors promotes tumor metastasis to lymph nodes. We show here that the fibronectin-binding integrin alpha4beta1 and its ligand fibronectin are novel functional markers of proliferative lymphatic endothelium. Tumors and lymphangiogenic growth factors, such as vascular endothelial growth factor-C (VEGF-C) and VEGF-A, induce lymphatic vessel expression of integrin alpha4beta1. Integrin alpha4beta1 then promotes growth factor and tumor-induced lymphangiogenesis, as genetic loss of integrin alpha4beta1 expression in Tie2Cre+ alpha4(loxp/loxp) mice or genetic loss of alpha4 signaling in alpha4Y991A knock-in mice blocks growth factor and tumor-induced lymphangiogenesis, as well as tumor metastasis to lymph nodes. In addition, antagonists of integrin alpha4beta1 suppress lymphangiogenesis and tumor metastasis. Our studies show that integrin alpha4beta1 and the signals it transduces regulate the adhesion, **migration**, invasion, and survival of proliferating lymphatic endothelial cells. As suppression of alpha4beta1 expression, signal transduction, or function in tumor lymphatic endothelium not only inhibits tumor lymphangiogenesis but also prevents metastatic disease, these results show that integrin alpha4beta1-mediated tumor lymphangiogenesis promotes metastasis and is a useful target for the suppression of metastatic disease.
20. VCAM-1 and VLA-4 modulate dendritic cell IL-12p40 production in experimental visceral leishmaniasis. Vascular cell adhesion molecule-1 (VCAM-1) interacts with its major ligand very late antigen-4 (VLA-4) to mediate cell adhesion and transendothelial **migration** of leukocytes. We report an important role for VCAM-1/VLA-4 interactions in the generation of immune responses during experimental visceral leishmaniasis caused by Leishmania donovani. Our studies demonstrate that these molecules play no direct role in the recruitment of leukocytes to the infected liver, but instead contribute to IL-12p40-production by splenic CD8(+) dendritic cells (DC). Blockade of VCAM-1/VLA-4 interactions using whole antibody or anti-VCAM-1 Fab' fragments reduced IL-12p40 mRNA accumulation by splenic DC 5 hours after L. donovani infection. This was associated with reduced anti-parasitic CD4(+) T cell activation in the spleen and lowered hepatic IFNgamma, TNF and nitric oxide production by 14 days post infection. Importantly, these effects were associated with enhanced parasite growth in the liver in studies with either anti-VCAM-1 or anti-VLA-4 antibodies. These data indicate a role for VCAM-1 and VLA-4 in DC activation during infectious disease.
21. Integrin alpha4beta1 (VLA-4) expression and activity in retinal and peripheral neurons. The integrin alpha4beta1 fulfills important roles in inflammation and hematopoesis, but its functions in neurons are not well understood. Here we show that the alpha4 subunit is expressed on mouse retinal ganglion cells (RGCs) and undifferentiated retinal neuroblasts during the period of axon extension and **migration**. To determine if alpha4 integrins expressed by retinal neurons were active, neurons were cultured on known alpha4 ligands in vitro. Recombinant soluble vascular cell adhesion molecule 1 (rsVCAM-1), fibronectin, and osteopontin (OPN) induced neurite outgrowth that was diminished by function blocking antibodies specific for alpha4. Neurite outgrowth on OPN was also blocked by antibodies to the integrin beta1 subunit, implicating the alpha4beta1 heterodimer as one integrin receptor mediating outgrowth on OPN. OPN immunoreactivity was detected in the RGC fiber layer and optic nerve, suggesting that it may act as an alpha4 ligand in vivo. Neurons from chick lumbar sympathetic ganglia, chick dorsal root ganglia, and mouse superior cervical ganglia also extended neurites on rsVCAM-1, suggesting that integrin alpha4beta1 may play a role in the development of multiple neuronal cell types.
22. Dual functions of [alpha]4[beta]1 integrin in epicardial development: initial **migration** and long-term attachment. The epicardium of the mammalian heart arises from progenitor cells outside the developing heart. The epicardial progenitor (EPP) cells migrate onto the heart through a cyst-mediated mechanism in which the progenitors are released from the tissue of origin as cysts; the cysts float in the fluid of the pericardial cavity and attach to the naked myocardial surface of the heart, and cells in the cysts then migrate out to form an epithelial sheet. In this paper, we show that the gene encoding the alpha4 subunit of alpha4beta1 integrin (alpha4beta1) is essential for this migratory process. We have generated a knockin mutation in mice replacing the alpha4 integrin gene with the lacZ reporter gene, placing lacZ under the control of the alpha4 integrin promoter. We show that in homozygous mutant embryos, the **migration** of EPP progenitor cells is impaired due to inefficient budding of the cysts and a failure of the cells in the cysts to migrate on the heart. This study provides direct genetic evidence for essential roles for alpha4beta1 integrin-mediated cell adhesion in the **migration** of progenitor cells to form the epicardium, in addition to a previous finding that alpha4beta1 is essential for maintaining the epicardium (Yang, J.T., H. Rayburn, and R.O. Hynes. 1995. Development. 121:549-560).
23. Essential role of VLA-4/VCAM-1 pathway in the establishment of CD8+ T-cell-mediated Trypanosoma cruzi-elicited meningoencephalitis. Central nervous system (CNS) damage can occur during Trypanosoma cruzi infection, especially in immunosuppressed patients. The enhanced susceptibility of C3H/He mice to CD8-mediated acute meningoencephalitis is associated with higher up-regulation of vascular cell adhesion molecule-1 (VCAM-1) on CNS vascular endothelia than in the less susceptible C57BL/6. Further, in vitro adhesion of activated peripheral blood cells to CNS blood vessels was abrogated by anti-VLA-4 antibodies that also inhibited cell **migration** into the CNS of T. cruzi-infected mice. Lastly, the reactivation of meningoencephalitis in immunosuppressed chronically infected mice was associated with VCAM-1 up-regulation. Therefore, we hypothesize that VLA-4/VCAM-1 pathway plays a pivotal role in the establishment of T. cruzi-elicited encephalitis.
24. Defective blood vessel development and pericyte/pvSMC distribution in alpha 4 integrin-deficient mouse embryos. Blood vessel development is in part regulated by pericytes/presumptive vascular smooth muscle cells (PC/pvSMCs). Here, we demonstrate that interactions between PC/pvSMCs and extracellular matrix play a critical role in this event. We show that the cranial vessels in alpha4 integrin-deficient mouse embryos at the stage of vessel remodeling are increased in diameter. This defect is accompanied by a failure of PC/pvSMCs, which normally express alpha4beta1 integrin, to spread uniformly along the vessels. We also find that fibronectin but not VCAM-1 is localized in the cranial vessels at this stage. Furthermore, cultured alpha4 integrin-null PC/pvSMCs plated on fibronectin display a delay in initiating **migration**, a reduction in **migration** speed, and a decrease in directional persistence in response to a polarized force of shear flow. These results suggest that specific motile activities of PC/pvSMCs regulated by mechanical signals imposed by the interstitial extracellular matrix may also be required in vivo for the distribution and function of the PC/pvSMCs during blood vessel development.
25. Abl-interactor-1 (Abi1) has a role in cardiovascular and placental development and is a binding partner of the alpha4 integrin. Dynamic signals linking the actin cytoskeleton and cell adhesion receptors are essential for morphogenesis during development and normal tissue homeostasis. Abi1 is a central regulator of actin polymerization through interactions with multiple protein complexes. However, the in vivo role of Abi1 remains to be defined. The α4 integrin adhesion receptor is associated with enhanced protrusive activity and regulation of directional cell **migratio**n. Among integrin subunits, α4 exhibits unique properties in that it predominantly accumulates at the leading edge of migrating cells; however, the pathways that link the actin-regulatory machinery to α4 at the leading edge have remained elusive. We generated Abi1 KO mice and found that loss of Abi1 phenocopies KO of α4. Mice lacking Abi1 or α4 exhibit midgestational lethality with abnormalities in placental and cardiovascular development. Notably, purified Abi1 protein binds directly to the α4 cytoplasmic tail and endogenous Abi1 colocalizes with phosphorylated α4 at the leading edge of spreading cells. Moreover, Abi1-deficient cells expressing α4 have impaired cell spreading, which is rescued by WT Abi1 but not an Abi1 mutant lacking the α4-binding site. These data reveal a direct link between the α4 integrin and actin polymerization and uncover a role for Abi1 in the regulation of morphogenesis in vivo. The Abi1-α4 interaction establishes a mechanistic paradigm for signaling between adhesion events and enhanced actin polymerization at the earliest stages of protrusion.
26. Association between α4 integrin cytoplasmic tail and non-muscle myosin IIA regulates cell **migratio**n. α4β1 integrin regulates ce**ll migrat**ion via cytoplasmic interactions. Here, we report an association between the cytoplasmic tail of α4 integrin (α4 tail) and non-muscle myosin IIA (MIIA), demonstrated by co-immunoprecipitation of the MIIA heavy chain (HC) with anti-α4-integrin antibodies and pull-down of MIIA-HC with recombinant α4 tail from cell lysates. The association between the α4 tail and MIIA does not require paxillin binding or phosphorylation at Ser988 in the α4 tail. We found that substituting Glu982 in the α4 tail with alanine (E982A) disrupts the α4-MIIA association without interfering with the paxillin binding or Ser988 phosphorylation. By comparing stably transfected CHO cells, we show that the E982A mutation reduces the ability of α4β1 integrin to mediate cell spreading and to promote front-back polarization. In addition, we show that E982A impairs shear-**flow-indu**ced migration of the α4-integrin-expressing CHO cells by r**educing t**heir migration speed and directional persistence. The E982A mutation also leads to defects in the organization of MIIA filament bundles. Furthermore, when cells are plated on fibronectin and simulated with shear flow, α4β1 integrin forms filament-like patterns that co-align with MIIA filament bundles. These results provide a new mechanism for linking integrins to the actomyosin cytoskeleton and for **regulatin**g cell migration by integrins and non-muscle myosin II.
27. Mab21l2 is essential for embryonic heart and liver development. During mouse embryogenesis, proper formation of the heart and liver is especially important and is crucial for embryonic viability. In this study, we showed that Mab21l2 was expressed in the trabecular and compact myocardium, and that deletion of Mab21l2 resulted in a reduction of the trabecular myocardium and thinning of the compact myocardium. Mab21l2-deficient embryonic hearts had decreased expression of genes that regulate cell proliferation and apoptosis of cardiomyocytes. These results show that Mab21l2 functions during heart development by regulating the expression of such genes. Mab21l2 was also expressed in the septum transversum mesenchyme (STM). Epicardial progenitor cells are localized to the anterior surface of the STM (proepicardium), and proepicardial cells migrate onto the surface of the heart and form the epicardium, which plays an important role in heart development. The rest of the STM is essential for the growth and survival of hepatoblasts, which are bipotential progenitors for hepatocytes and cholangiocytes. Proepicardial cells in Mab21l2-deficient embryos had defects in cell proliferation, which led to a small proepicardium, in which α4 integrin expression, which is essential for the **migratio**n of proepicardial cells, was down-regulated, suggesting that defects occurred in its **migratio**n. In Mab21l2-deficient embryos, epicardial formation was defective, suggesting that Mab21l2 plays important roles in epicardial formation through the regulation of the cell proliferation of proepicardial cells and the migratory process of proepicardial cells. Mab21l2-deficient embryos also exhibited hypoplasia of the STM surrounding hepatoblasts and decreased hepatoblast proliferation with a resultant loss of defective morphogenesis of the liver. These findings demonstrate that Mab21l2 plays a crucial role in both heart and liver development through STM formation.
28. β7 integrins are required to give rise to intestinal mononuclear phagocytes with tolerogenic potential. While pro-inflammatory monocyte trafficking to the intestine has been partially characterised, the molecules required for **migratio**n of tolerogenic mononuclear phagocytes (dendritic cells (DC) and macrophages) are unknown. We hypothesised that the gut-homing receptor integrin α4β7 is required for this process.We used a T cell-mediated colitis model to study the role of α4β7 in the innate immune compartment. We then performed competitive bone marrow (BM) reconstitution experiments to assess the requirement of α4β7 in the generation of intestinal retinoic acid (RA)-producing CD11c(hi) DC (ALDE(+)DC) and CD64 macrophages. Using mixed BM chimeras we also asked whether α4β7 is required to give rise to tolerogenic mononuclear phagocytes.Lack of β7 integrins in the innate immune compartment (β7(-/-)RAG2(-/-) mice) markedly accelerated T cell-mediated colitis, which was correlated with lower numbers and frequencies of ALDE(+)DC in mesenteric lymph nodes. Consistent with a role of α4β7 in the generation of intestinal mononuclear phagocytes, BM cells from β7(-/-) mice poorly reconstituted small intestine ALDE(+)DC and Mφ when compared to their wild type counterparts. In addition, mice lacking β7 integrins in the CD11c(hi) compartment showed decreased ability to induce Foxp3(+) T(REG) and IL-10-producing T cells.Mice lacking β7 integrins in the innate immune compartment are more susceptible to intestinal inflammation, which is correlated with a requirement of β7 integrins to reconstitute gut mononuclear phagocytes with tolerogenic potential.
29. α4β7 Integrin (LPAM-1) is upregulated at atherosclerotic lesions and is involved in atherosclerosis progression. Integrin activation and lymphocyt**e migrati**on to the vascular intima is a key event in early atherosclerosis. α4β7 integrin (LPAM-1) and its ligand, mucosal addressin cell adhesion molecule (MAdCAM-1) are known to play an important role in homing of activated lymphocytes to gut-associated lymphoid tissues. However, it is unclear whether α4β7 integrin is involved in the pathogenesis of atherosclerosis.The expressions of α4β7 integrin and its ligands in atherosclerosis plaques from 12 week high fat diet (HFD) fed ApoE(-/-) and C57BL/6 mice were examined using immunofluorescent and immunohistochemical assays, respectively. We also generated ApoE/β7 double deficient mice and compared atherosclerotic lesion development in β7(+/+)ApoE(-/-) and β7(-/-)ApoE(-/-) mice that were fed with HFD for 12 weeks.We found an upregulation of α4β7 integrin and its ligands VCAM-1 and MAdCAM-1 at atherosclerosis plaques in Apolipoprotein E deficient (ApoE(-/-)) mice fed with HFD for 12 weeks. Over the 12 week HFD period, peripheral blood lymphocyte (PBL) expression of α4β7 integrin increased in parallel with aortic lesion size. A removal of α4β7 integrin by genetic deletion of the β7 chain in the ApoE(-/-) mouse resulted in a markedly decreased 12 week-HFD atherosclerotic plaque area. β7(-/-) ApoE(-/-) macrophages showed reduced acetylated and native LDL uptake and phagocytic activity, revealing possible roles for α4β7 at two distinct stages of macrophage dysfunction during atherogenesis. Finally, a reduced activity of integrin downstream signalling components focal adhesion kinase (FAK) and MAPK/ERK1/2 in macrophage indicates their possible engagement during α4β7 integrin signalling in atherosclerosis.Together our results reveal a critical role of α4β7 in diet-induced atherosclerosis in mouse.
30. Expanding roles for alpha 4 integrin and its ligands in development. Interaction of alpha 4 integrins with vascular cell adhesion molecule-1 (VCAM-1) is classically important for immune function. However, we found recently that these receptors have a second role, in embryogenesis, where they mediate cell-cell interactions that are important for skeletal muscle differentiation. Here, we present evidence of an expanding role for these receptors in murine development. alpha 4 and VCAM-1 were found at embryonic sites of hematopoiesis, suggesting a role for these receptors during embryogenesis that parallels their hematopoietic function in adult bone marrow. During angiogenesis in the lung, alpha 4 and VCAM-1 were found on mesenchyme that gives rise to vascular endothelium and smooth muscle. alpha 4 persisted on the smooth muscle and the endothelium of newly forming vessels where it colocalized with its extracellular matrix ligand, fibronectin (FN). These patterns suggest several roles for alpha 4 integrins and their ligands in angiogenesis. alpha 4 was also found on neural crest derivatives where it colocalized with FN. alpha 4 was expressed selectively on cells in the dorsal root ganglia: it was apparent along ventral projections, but absent from dorsal projections, suggesting that alpha 4 integrins could be involved in defining neuronal fates. Although VCAM-1 was not expressed on most neural crest derivatives, it was found in the neural crest-derived outflow tract of the embryonic heart, where it colocalized with alpha 4. These results imply that alpha 4 integrins and their ligands could be important for **migration** or differentiation of neural crest. alpha 4 was also expressed on embryonic retina and FN was found on inductive mesenchyme surrounding the eye, suggesting a role for these proteins in eye development. Finally, based on their patterns of expression, we conclude that VCAM-1 only participates in a subset of interactions involving alpha 4 integrins, whereas FN appears to be the more general ligand.
31. An integrin alpha4beta7•IgG heterodimeric chimera binds to MAdCAM-1 on high endothelial venules in gut-associated lymphoid tissue. Lymphocyte homing is regulated by a multistep process mediated by sequential adhesive interactions between circulating lymphocytes and high endothelial venules (HEVs). In gut-associated lymphoid tissue (GALT), the initial interactive step, "tethering and rolling," is partly mediated by integrin α4β7 expressed on GALT-homing lymphocytes and its ligand MAdCAM-1, which is exclusively expressed on HEVs in GALT. To probe functional MAdCAM-1 in tissue sections, we developed a soluble integrin α4β7 heterodimeric IgG chimera by joining the extracellular region of mouse integrin α4 and β7 subunits to a human IgG Fc domain. Western blot analysis revealed that co-transfection of HEK 293T cells with expression vectors encoding integrin α4•IgG and β7•IgG results in the formation of α4β7•IgG heterodimeric chimeras. This complex preferentially binds to CHO cells expressing MAdCAM-1 and, to a lesser extent, to cells expressing VCAM-1, but not to cells expressing ICAM-1. Moreover, α4β7•IgG specifically binds to HEVs in GALT in situ in a divalent cation-dependent fashion and inhibits lymphocyte binding to HEVs in GALT. These findings indicate that α4β7•IgG can be used as a probe for functional MAdCAM-1 expressed on HEVs in GALT and could potentially serve as an anti-inflammatory drug inhibiting **GALT-spe**cific lymphocyte migration.
32. Integrin α4 Enhances Metastasis and May Be Associated with Poor Prognosis in MYCN-low Neuroblastoma. High-risk neuroblastoma is associated with an overall survival rate of 30-50%. Neuroblastoma-expressed cell adhesion receptors of the integrin family impact cell adhesion, **migratio**n, proliferation and survival. Integrin α4 is essential for neural crest cell motility during development, is highly expressed on leukocytes, and is critical for transendothelia**l migrati**on. Thus, cancer cells that express this receptor may exhibit increased metastatic potential. We show that α4 expression in human and murine neuroblastoma cell lines selectively enhances in vitro interaction with the alternatively spliced connecting segment 1 of fibronectin, as well as vascular cell adhesion molecule-1 and increas**es migrat**ion. Integrin α4 expression enhanced experimental metastasis in a syngeneic tumor model, reconstituting a pattern of organ involvement similar to that seen in patients. Accordingly, antagonism of integrin α4 blocked metastasis, suggesting adhesive function of the integrin is required. However, adhesive function was not sufficient, as mutants of integrin α4 that conserved the matrix-adhesive and promigratory function in vitro were compromised in their metastatic capacity in vivo. Clinically, integrin α4 is more frequently expressed in non-MYNC amplified tumors, and is selectively associated with poor prognosis in this subset of disease. These results reveal an unexpected role for integrin α4 in neuroblastoma dissemination and identify α4 as a potential prognostic indicator and therapeutic target.
33. Inhibition of alpha(4) integrin mediated adhesion was involved in the reduction of B16-F10 melanoma cells lung colonization in C57BL/6 mice treated with gambogic acid. Gambogic acid, the major active ingredient of gamboge, has been shown to exhibit anti-cancer activity both in vivo and in vitro. However, its potential activity in tumor metastasis remains unclear. In the present study, we found that gambogic acid strongly inhibited the adhesion of highly metastatic mouse melanoma B16-F10 cells in vitro. Gambogic acid also exhibited significant anti-metastasis activity on the development of in vivo artificial metastases (i.e. following tail vein i.v. injection of the B16-F10 melanoma tumor cells in C57BL/6 mice). Flow cytometric analysis and Western blot showed that gambogic acid inhibited the cell surface expression of alpha(4) integrin, while exhibited negligible effects on the expression of alpha(5) and beta(1) integrin. Thus we concluded for the first time that gambogic acid could inhibit the adhesion and **migration** of B16-F10 cells in vitro and in vivo, in which down-regulation of alpha(4) integrin expression was involved.
34. The alpha4 subunit of integrin is important for neural crest cell **migration**. We identify the alpha4 subunit of integrin as a predominant integrin expressed by neural crest cells in both avian and murine embryos. Using degenerate primers, we obtained a PCR fragment of the chick integrin alpha4 subunit that was subsequently used to clone the full-length subunit with a predicted amino acid sequence 60% identical to human and mouse alpha4 subunits. In situ hybridization demonstrates that chick integrin alpha4 mRNA is expressed at high levels by migrating neural crest cells and neural crest-derived ganglia at both cranial and trunk levels. An antibody against the murine alpha4 subunit revealed similar distribution patterns in mouse to chick. In addition to neural crest cells, the integrin alpha4 subunit was later observed on the muscle masses of the limb, the apical ectodermal ridge, and the developing liver. To examine the functional role of the integrin alpha4 subunit in neural crest cell **migration**, we used an explant preparation that allows visualization of neural crest cells in their normal environment with or without perturbing reagents. In the presence of a blocking antibody against the mouse integrin alpha4 subunit, there was a profound abrogation of neural crest cell **migration** at trunk and hindbrain levels. Both the numbers of migrating neural crest cells and the total distance traversed were markedly reduced. Similarly, avian embryos injected with synthetic peptides that contain the integrin alpha4 binding site in fibronectin displayed abnormal neural crest cell **migration**. Our results suggest that the integrin alpha4 subunit is important for normal neural crest cell **migration** and may be one of the primary alpha subunits used for neural crest cell **migration** in vivo. Furthermore, the integrin alpha4 subunit represents a useful neural crest marker in the mouse.
35. Absence of alpha 4 but not beta 2 integrins restrains development of chronic allergic asthma using mouse genetic models. Chronic asthma is characterized by ongoing recruitment of inflammatory cells and airway hyperresponsiveness leading to structural airway remodeling. Although alpha 4 beta 1 and beta2 integrins regulate leukocyte **migration** in inflammatory diseases and play decisive roles in acute asthma, their role has not been explored under the chronic asthma setting. To extend our earlier studies with alpha 4(Delta/Delta) and beta2(-/-) mice, which showed that both alpha 4 and beta2 integrins have nonredundant regulatory roles in acute ovalbumin (OVA)-induced asthma, we explored to what extent these molecular pathways control development of structural airway remodeling in chronic asthma.Control, alpha 4(Delta/Delta), and beta2(-/-) mouse groups, sensitized by intraperitoneal OVA as allergen, received intratracheal OVA periodically over days 8 to 55 to induce a chronic asthma phenotype. Post-OVA assessment of inflammation and pulmonary function (airway hyperresponsiveness), together with airway modeling measured by goblet cell metaplasia, collagen content of lung, and transforming growth factor beta1 expression in lung homogenates, were evaluated.In contrast to control and beta2(-/-) mice, alpha 4(Delta/Delta) mice failed to develop and maintain the composite chronic asthma phenotype evaluated as mentioned and subepithelial collagen content was comparable to baseline. These data indicate that beta2 integrins, although required for inflammatory **migration** in acute asthma, are dispensable for structural remodeling in chronic asthma.alpha 4 integrins appear to have a regulatory role in directing transforming growth factor beta-induced collagen deposition and structural alterations in lung architecture likely through interactions of Th2 cells, eosinophils, or mast cells with endothelium, resident airway cells, and/or extracellular matrix.
36. Genetic perturbation of the putative cytoplasmic membrane-proximal salt bridge aberrantly activates alpha(4) integrins. alpha(4) integrins play a pivotal role in leukocyte **migration** and tissue-specific homing. The ability of integrins to bind ligand is dynamically regulated by activation-dependent conformational changes triggered in the cytoplasmic domain. An NMR solution structure defined a putative membrane-proximal salt bridge between the alpha(IIb)beta(3) integrin cytoplasmic tails, which restrains integrins in their low-affinity state. However, the physiological importance of this salt bridge in alpha(4) integrin regulation remains to be elucidated. To address this question, we disrupted the salt bridge in murine germ line by mutating the conserved cytoplasmic arginine R(GFFKR) in alpha(4) integrins. In lymphocytes from knock-in mice (alpha(4)-R/A(GFFKR)), alpha(4)beta(1) and alpha(4)beta(7) integrins exhibited constitutively up-regulated ligand binding. However, trans**migration** of these cells across VCAM-1 and MAdCAM-1 substrates, or across endothelial monolayers, was reduced. Perturbed detachment of the tail appeared to cause the reduced cell **migration** of alpha(4)-R/A(GFFKR) lymphocytes. In vivo, alpha(4)-R/A(GFFKR) cells exhibited increased firm adhesion to Peyer patch venules but reduced homing to the gut. Our results demonstrate that the membrane-proximal salt bridge plays a critical role in supporting proper alpha(4) integrin adhesive dynamics. Loss of this interaction destabilizes the nonadhesive conformation, and thereby perturbs the properly balanced cycles of adhesion and deadhesion required for efficient cell **migration**.
37. Genetic evidence for convergence of c-Kit- and alpha4 integrin-mediated signals on class IA PI-3kinase and the Rac pathway in regulating integrin-directed **migration** in mast cells. Mast cells play a critical role in host defense against a number of pathogens. Increased mast cell infiltration has been described in allergic asthma, in rheumatoid arthritis, and during helminthes infection. Despite the importance of mast cells in allergic disease and defense against infection, little is known about the mechanisms by which mast cells migrate to various tissues under steady state conditions or during infection or inflammation. Here, we show that activation of c-Kit by its ligand, stem cell factor (SCF), cooperates with alpha4 integrin in inducing directed **migration** of mast cells on fibronectin. A reduction in **migration** and activation of a small G protein, Rac, was observed in mast cells derived from class IA phosphoinositide-3 kinase (PI-3kinase)-deficient mice in response to SCF stimulation and in mast cells expressing the dominant-negative Rac (RacN17), as well as in mast cells deficient in the hematopoietic-specific small G protein, Rac2. In addition, a PI-3kinase inhibitor inhibited alpha4- as well as SCF-induced **migration** in a dose-dependent fashion. In contrast, a mitogen-activated protein kinase (MAPK) inhibitor had little effect. Consistent with the pharmacologic results, abrogating the binding of the p85alpha subunit of class IA PI-3kinase to c-Kit also resulted in inhibition of SCF-induced **migration** on fibronectin. These genetic and biochemical data demonstrate that both c-Kit and alpha4 integrin signaling are linked to class IA PI-3kinase and Rac pathways and regulate integrin-directed (haptotactic) **migration** in mast cells.
38. Blockade of alpha4 integrin signaling ameliorates the metabolic consequences of high-fat diet-induced obesity. Many prevalent diseases of advanced societies, such as obesity-induced type 2 diabetes, are linked to indolent mononuclear cell-dependent inflammation. We previously proposed that blockade of alpha4 integrin signaling can inhibit inflammation while limiting mechanism-based toxicities of loss of alpha4 function. Thus, we hypothesized that mice bearing an alpha4(Y991A) mutation, which blocks signaling, would be protected from development of high-fat diet-induced insulin resistance.Six- to eight-week-old wild-type and alpha4(Y991A) C57Bl/6 male mice were placed on either a high-fat diet that derived 60% calories from lipids or a chow diet. Metabolic testing was performed after 16-22 weeks of diet.Alpha4(Y991A) mice were protected from development of high-fat diet-induced insulin resistance. This protection was conferred on wild-type mice by alpha4(Y991A) bone marrow transplantation. In the reverse experiment, wild-type bone marrow renders high-fat diet-fed alpha4(Y991A) acceptor animals insulin resistant. Furthermore, fat-fed alpha4(Y991A) mice showed a dramatic reduction of monocyte/macrophages in adipose tissue. This reduction was due to reduced monocyte/macrophage **migration** rather than reduced monocyte chemoattractant protein-1 production.Alpha4 integrins contribute to the development of HFD-induced insulin resistance by mediating the trafficking of monocytes into adipose tissue; hence, blockade of alpha4 integrin signaling can prevent the development of obesity-induced insulin resistance.
39. IgA ASC accumulation to the lactating mammary gland is dependent on VCAM-1 and alpha4 integrins. The homing and **migration** of IgA antibody secreting cells (ASC) to the lactating mammary gland is essential to the passive transfer of immunity from mother to nursing neonate. Antibody secreting cells, located within the lactating mammary gland, produce high levels of antigen-specific IgA antibodies. These antibodies, which are subsequently transferred to the nursing neonate via breast milk, provide passive immune protection against antigens previously encountered by the mother to the nursing infant. The efficient homing and accumulation of lymphocytes is highly dependent on cellular adhesion molecules expressed on the vascular endothelium and their integrin ligands. Vasculature within the lactating mammary gland is known to express several adhesion molecules, including VCAM-1 and MAdCAM-1. However, the role of these molecules in vivo has not been previously described. Here we show that alpha4 integrins and VCAM-1 play essential roles in mediating the accumulation of IgA ASCs to the lactating mammary gland. Conversely, neither MAdCAM-1 nor its major ligand alpha4beta7 are required for efficient IgA ASC accumulation to this tissue.
40. CD44, alpha(4) integrin, and fucoidin receptor-mediated phagocytosis of apoptotic leukocytes. Various types of phagocytes mediate the clearance of apoptotic cells. We previously reported that human and murine high endothelial venule (HEV) cells ingest apoptotic cells. In this report, we examined endothelial cell fucoidin receptor-mediated phagocytosis using a murine endothelial cell model mHEV. mHEV cell recognition of apoptotic leukocytes was blocked by fucoidin but not by other phagocytic receptor inhibitors such as mannose, fucose, N-acetylglucosamine, phosphatidylserine (PS), or blocking anti-PS receptor antibodies. Thus, the mHEV cells used fucoidin receptors for recognition and phagocytosis of apoptotic leukocytes. The fucoidin receptor-mediated endothelial cell phagocytosis was specific for apoptotic leukocytes, as necrotic cells were not ingested. This is in contrast to macrophages, which ingest apoptotic and necrotic cells. Endothelial cell phagocytosis of apoptotic cells did not alter viable lymphocyte **migration** across these endothelial cells. Antibody blocking of CD44 and alpha4 integrin on the apoptotic leukocyte inhibited this endothelial cell phagocytosis, suggesting a novel function for these adhesion molecules in the removal of apoptotic targets. The removal of apoptotic leukocytes by endothelial cells may protect the microvasculature, thus ensuring that viable lymphocytes can successfully migrate into tissues.
41. Distinct signaling mechanisms regulate **migration** in unconfined versus confined spaces. Using a microchannel assay, we demonstrate that cells adopt distinct signaling strategies to modulate cell **migration** in different physical microenvironments. We studied α4β1 integrin-mediated signaling, which regulates cel**l migrati**on pertinent to embryonic development, leukocyte trafficking, and melanoma invasion. We show that α4β1 integrin promotes c**ell migra**tion through both unconfined and confined spaces. However, unlike unconfined (**2D) migra**tion, which depends on enhanced Rac1 activity achieved by preventing α4/paxillin binding, conf**ined migr**ation requires myosin II-driven contractility, which is increased when Rac1 is inhibited by α4/paxillin binding. This Rac1-myosin II cross talk mechanism also con**trols mig**ration of fibroblast-like cells lacking α4β1 integrin, in which Rac1 and myosin II modulate unconfined and c**onfined m**igration, respectively. We further demonstrate the distinct roles of myosin II isoforms, MIIA and MIIB, which are primarily required for confined and unc**onfined m**igration, respectively. This work provides a paradigm for the plasticity of cells migrating through different physical microenvironments.
42. Unique and redundant roles of alpha4 and beta2 integrins in kinetics of recruitment of lymphoid vs myeloid cell subsets to the inflamed peritoneum revealed by studies of genetically deficient mice. Leukocyte recruitment to inflammatory sites is a prominent feature of acute and chronic inflammation. Instrumental in this process is the coordinated upregulation of leukocyte integrins (among which alpha4beta1 and beta2 integrins are major players) and their cognate receptors in inflamed tissues. To avoid the ambiguity of previous short-term antibody-based studies and to allow for long-term observation, we used genetically deficient mice to compare roles of alpha4 and beta2 integrins in leukocyte trafficking.Aseptic peritonitis was induced in alpha4 or beta2 integrin-deficient (conditional and conventional knockouts, respectively) and control mice, and recruitment of major leukocyte subsets to the inflamed peritoneum was followed for up to 4 days.Despite normal chemokine levels in the peritoneum and adequate numbers, optimal recruitment of myeloid cells was impaired in both alpha4- and beta2-deficient mice. Furthermore, clearance of recruited neutrophils and macrophages was delayed in these mice. Lymphocyte **migration** to the peritoneum in the absence of alpha4 integrins was drastically decreased, both at steady state and during inflammation, a finding consistent with impaired lymphocyte in vitro adhesion and signaling. By contrast, in the absence of beta2 integrins, defects in lymphocyte recruitment were only evident when peritonitis was established.Our data with concurrent use of genetic models of integrin deficiency reveal nonredundant functions of alpha4 integrins in lymphocyte **migration** to the peritoneum and further refine specific roles of alpha4 and beta2 integrins concerning trafficking and clearance of other leukocyte subsets at homeostasis and during inflammation.
43. Integrin receptors are required for cell survival and proliferation during development of the peripheral glial lineage. Proliferation and survival of Schwann cells are important for nerve development and for disease processes in peripheral nerves. We have analyzed embryos lacking alpha4- or alpha5-integrins and show here that these integrins contribute to the control of glial cell numbers. To overcome early embryonic lethality an explant and grafting system that allows the study of isolated glial progenitor cells both in vitro and in vivo was used. Schwann cells differentiate in the absence of alpha5 but their numbers and the proliferation rate of early progenitor cells are reduced, suggesting that alpha5 is essential for normal proliferation. Survival, rather than proliferation, is compromised in alpha4-deficient explants. Conditional immortalization allowed further characterization and revealed that alpha4 contributes to survival in a cell-density-dependent fashion. In addition, transplants into chicken embryos were used to analyze in vivo cell **migration** and showed that cell death occurs mainly in highly motile, individually migrating cells. The cell death patterns in vitro and in vivo argue that alpha4-integrins play a role in survival during cell **migration**. Neural crest **migration** has been suggested to require these integrins; however, no defects in **migration** were observed in the absence of alpha4 or alpha5. We conclude that integrins can complement growth factors in the control of glial cell numbers.
44. Alpha4beta7 integrin/MAdCAM-1 adhesion pathway is crucial for B cell **migration** into pancreatic lymph nodes in nonobese diabetic mice. Although B cells are crucial antigen-presenting cells in the initiation of T cell autoimmunity to islet beta cell autoantigens in type 1 diabetes (T1D), adhesion molecules that control **migration** of B cells into pancreatic lymph nodes (PanLN) in the nonobese diabetic (NOD) mouse model of human T1D have not been defined. In this study, we found that B cells from PanLN of 3-4-week-old female NOD mice expressed high levels of alpha(4) integrin and LFA-1 and intermediate levels of beta(7) integrin; half of B cells were L-selectin(high). In short-term in vivo lymphocyte **migration** assays, B cells migrated from the bloodstream into PanLN more efficiently than into peripheral LNs. Moreover, antibodies to mucosal addressin cell adhesion molecule 1 (MAdCAM-1) and alpha(4)beta(7) integrin inhibited >90% of B cell **migration** into PanLN. In contrast, antibodies to peripheral node addressin, L-selectin or LFA-1 partially inhibited B cell **migration** into PanLN. Furthermore, one intraperitoneal injection of anti-MAdCAM-1 antibody into 3-week-old NOD mice significantly inhibited entry of B cells into PanLN for at least 2 weeks. Taken together, these results indicate that the alpha(4)beta(7) integrin/MAdCAM-1 adhesion pathway plays a predominant role in **migration** of B cells into PanLN in NOD mice. Thus, specific blockage of alpha(4)beta(7) integrin/MAdCAM-1 adhesion pathway-mediated B cell **migration** may be a potential treatment for T1D.
45. Blocking the alpha 4 integrin-paxillin interaction selectively impairs mononuclear leukocyte recruitment to an inflammatory site. Antagonists to alpha4 integrin show promise for several autoimmune and inflammatory diseases but may exhibit mechanism-based toxicities. We tested the capacity of blockade of alpha4 integrin signaling to perturb functions involved in inflammation, while limiting potential adverse effects. We generated and characterized mice bearing a Y991A mutation in alpha4 integrin [alpha4(Y991A) mice], which blocks paxillin binding and inhibits alpha4 integrin signals that support leukocyte **migration**. In contrast to the embryonic-lethal phenotype of alpha4 integrin-null mice, mice bearing the alpha4(Y991A) mutation were viable and fertile; however, they exhibited defective recruitment of mononuclear leukocytes into thioglycollate-induced peritonitis. Alpha4 integrins are essential for definitive hematopoiesis; however, the alpha4(Y991A) mice had intact lymphohematopoiesis and, with the exception of reduced Peyer's patches, normal architecture and cellularity of secondary lymphoid tissues. We conclude that interference with alpha4 integrin signaling can selectively impair mononuclear leukocyte recruitment to sites of inflammation while sparing vital functions of alpha4 integrins in development and hematopoiesis.
46. Runx1 modulates adult hair follicle stem cell emergence and maintenance from distinct embryonic skin compartments. Runx1 controls hematopoietic stem cell emergence and hair follicle stem cell (HFSC) activation and proliferation in adult skin. Here we use lineage tracing and mouse genetic manipulation to address the role of Runx1 in the embryonic development of HFSCs. We find Runx1 is expressed in distinct classes of embryonic skin precursors for short-term HF progenitors, adult HFSCs, and mesenchymal progenitors. Runx1 acts in the embryonic epithelium for timely emergence of adult HFSCs and short-term progenitors, but is dispensable for both of them. In contrast, Runx1 is strictly needed in the embryonic mesenchyme for proper adult HFSC differentiation and long-term skin integrity. Our data implicate Runx1 in epithelial cell adhesion and **migration** and in regulation of paracrine epithelial-mesenchymal cross talk. The latter involves Lef1 and Wnt signaling modulation in opposing directions from two distinct skin compartments. Thus, a master regulator of hematopoiesis also controls HFSC emergence and maintenance via modulation of bidirectional cross talking between nascent stem cells and their niche.
47. Aberrant activation of integrin alpha4beta7 suppresses lymphocyte **migration** to the gut. Integrin adhesion molecules mediate lymphocyte **migration** and homing to normal and inflamed tissues. While the ligand-binding activity of integrins is known to be modulated by conformational changes, little is known about how the appropriate balance of integrin adhesiveness is maintained in order to optimize the migratory capacity of lymphocytes in vivo. In this study we examined the regulation of the gut homing receptor alpha4beta7 integrin by manipulating at the germline level an integrin regulatory domain known as adjacent to metal ion-dependent adhesion site (ADMIDAS). ADMIDAS normally serves to raise the activation threshold of alpha4beta7, thereby stabilizing it in the default nonadhesive state. Lymphocytes from knockin beta7 (D146A) mice, which harbor a disrupted ADMIDAS, not only expressed an alpha4beta7 integrin that persistently adhered to mucosal addressin cell adhesion molecule-1 (MAdCAM-1), but also exhibited perturbed cell **migration** along MAdCAM-1 substrates resulting from improper de-adhesion of the lymphocyte trailing edge. In vivo, aberrantly activated alpha4beta7 enhanced adhesion to Peyer's patch venules, but suppressed lymphocyte homing to the gut, diminishing the capacity of T cells to induce colitis. Our results underscore the importance of a proper balance in the adhesion and de-adhesion of the alpha4beta7 integrin, both for lymphocyte trafficking to the gut and for colitis progression.
48. Ex vivo expansion of hematopoietic progenitor cells is associated with downregulation of alpha4 integrin- and CXCR4-mediated engraftment in NOD/SCID beta2-microglobulin-null mice. Several studies indicate that ex vivo cytokine-supported expansion induces defective hematopoietic stem cell engraftment. We investigated the role of alpha4 integrin, alpha5 integrin and CXCR4 in engraftment of unmanipulated and cytokine-treated human cord blood CD34(+) cells.Uncultured or expanded CD34(+) cells were infused in NOD/SCID-beta(2)microglobulin-null mice. The function of alpha4, and alpha5 integrins and CXCR4 was assessed by incubating cells with specific neutralizing antibodies, prior to transplant. The activation state of alpha4 integrin was further tested by adhesion and **migration** assays.Neutralization of either alpha4 integrin or CXCR4 abolished engraftment of uncultured CD34(+) cells at 6 week spost-transplant, while alpha5 integrin neutralization had no significant effect. However, after short-term ex vivo culture, blocking alpha4 integrin or CXCR4 did not affect repopulating activity whereas neutralization of alpha5 integrin inhibited engraftment. Using soluble vascular cell adhesion molecule-1 binding assays, we observed that alpha4 integrin affinity in fresh CD34(+) cells was low and susceptible to stimulation while in cultured CD34(+) cells, it was high and insensitive to further activation. In addition, stromal cell-derived factor-1 stimulated **migration** across vascular cell adhesion molecule-1 in fresh CD34(+) cells but not in cultured CD34(+) cells.Our data show that ex vivo culture of hematopoietic progenitor cells is associated with downregulation of both alpha4 integrin- and CXCR4-mediated engraftment. Further investigations suggest that this is caused by supraphysiological increase of alpha4 integrin affinity, which impairs directional **migration** across vascular cell adhesion molecule-1 in response to stromal cell-derived factor-1. Such changes may underlie the engraftment defect of cytokine-stimulated CD34(+) cells.
49. In vivo CD44-CD49d complex formation in autoimmune disease has consequences on T cell activation and apoptosis resistance. CD44 is involved in leukocyte **migration** and activation and has recently been reported to contribute to leukocyte extravasation by associating with CD49d. We explored whether similar changes in CD44 activity are seen in vivo using murine alopecia areata (AA) as a chronic, organ-related autoimmune disease model system. Expression of the activated, hyaluronan-binding form of CD44, and of CD49d, was elevated in draining lymph node cells (LNC) of AA-affected mice as compared to control mice. LNC of AA mice displayed increased motility, proliferative activity and apoptosis resistance, which were equally well inhibited by anti-CD44 and anti-CD49d. The latter is the sequelae of the association between CD44 and CD49d that is seen in activated lymphocytes. Significantly, due to CD44-CD49d complex formation, CD44 gains access to focal adhesion kinase and CD49d gains access to CD44-associated lck and ezrin, such that downstream kinases become activated via CD44 or CD49d engagement. Thus, by their association, CD44 and CD49d mutually avail themselves of the partner's signaling pathways and the ligand binding of each one triggers signaling pathways of both. This strongly influences the lymphocytes' activation state and function.
50. [LFA-1 and VLA-4 involved in vasoendothelial adhesion and transendothelial **migration** of human high proliferative potential endothelial progenitor cells]. To investigate whether lymphocyte function-associated antigen 1 (LFA-1) and very late antigen 4 (VLA-4) are involved in vasoendothelial adhesion and transendothelial **migration** of high proliferative potential endothelial progenitor cells (HPP-EPCs), flow cytometry was used to analyze the expression of integrin beta1 and beta2, the expression of intercellular adhesion molecule (ICAM-1, 2) and vascular cell adhesion molecule (VCAM-1) in mouse bone marrow endothelial cells (mBMECs). The adhesion and trans**migration** through endothelial cells of the HPP-EPCs blocked by functional grade neutralizing antibodies of VLA-4 and LFA-1 were studied in vitro. The results revealed that HPP-EPCs were positive for CD11a and CD49d in HPP-EPCs. The expression of ICAM-1and VCAM-1 of mBMECs increased after activated by IL-1beta and TNF-alpha. The results of adhesion in vitro revealed that the numbers of the adhered and migrated cells in the CD11a antibody group, in the CD49d antibody group and in the combinational antibody group were less than those in the isotype control antibody group. Furthermore, the number of adhered and migrated cells in the combinational antibody group was less than that in the CD11a or the CD49d antibody group (p < 0.05). It is concluded that both LFA-1 and VLA-4 are involved in vasoendothelial adhesion and transendothelial **migration** of HPP-EPCs.
51. Single-cell analysis of murine long-term hematopoietic stem cells reveals distinct patterns of gene expression during fetal **migration**. Long-term hematopoietic stem cells (LT-HSCs) migrate from the fetal liver (FL) to the fetal bone marrow (FBM) during development. Various adhesion and chemotactic receptor genes have been implicated in the **migration** of adult LT-HSCs. However, their role in the **migration** of fetal LT-HSCs is not clearly understood due, in part, to the rare number of these cells in fetal tissues, which preclude classical gene expression analysis. The aim of this study is to characterize the expression of **migration** related genes in fetal LT-HSC across different anatomical locations during development.We isolated fetal LT-HSC from different developmental stages, as well as different anatomical locations, and performed single-cell multiplex RT-qPCR and flow cytometry analysis of eight molecules involved in adult LT-HSC **migration**. Our results show that the gene expression of the chemokine receptor Cxcr4 in LT-HSC varies across developmental microenvironments and times, while the cadherin Cdh2 (Ncad) and the calcium receptor Casr show higher gene expression and variability only in FBM at 17.5 days post coitum (dpc). The cadherin Cdh5 (Vecad) maintains high expression variability only during fetal development, while the integrin subunit Itga5 (α5) increases its variability after 14.5 dpc. The integrin subunits Itga4 (α4) and Itgal (Lfa1), as well as the selectin ligand Selplg (Psgl1), did not show differences in their expression in single LT-HSCs irrespective of the developmental times or anatomical microenvironments studied.Our data demonstrate that the expression pattern of phenotypically identical, single LT-HSCs fluctuates as a function of developmental stage and anatomical microenvironment. This is the first exhaustive gene expression comparison o**f migrati**on-related molecules in fetal tissues across developmental times, enhancing the understanding of LT-HS**C migrati**on fate decisions during development.
52. EMILIN1-α4/α9 integrin interaction inhibits dermal fibroblast and keratinocyte proliferation. EMILIN1 promotes α4β1 integrin-dependent cell adhesion **and migra**tion and reduces pro-transforming growth factor-β processing. A knockout mouse model was used to unravel EMILIN1 functions in skin where the protein was abundantly expressed in the dermal stroma and where EMILIN1-positive fibrils reached the basal keratinocyte layer. Loss of EMILIN1 caused dermal and epidermal hyperproliferation and accelerated wound closure. We identified the direct engagement of EMILIN1 to α4β1 and α9β1 integrins as the mechanism underlying the homeostatic role exerted by EMILIN1. The lack of EMILIN1-α4/α9 integrin interaction was accompanied by activation of PI3K/Akt and Erk1/2 pathways as a result of the reduction of PTEN. The down-regulation of PTEN empowered Erk1/2 phosphorylation that in turn inhibited Smad2 signaling by phosphorylation of residues Ser245/250/255. These results highlight the important regulatory role of an extracellular matrix component in skin proliferation. In addition, EMILIN1 is identified as a novel ligand for keratinocyte α9β1 integrin, suggesting prospective roles for this receptor-ligand pair in skin homeostasis.
53. Alpha4 and beta2 integrins have nonredundant roles for asthma development, but for optimal allergen sensitization only alpha4 is critical. Recruitment of effector cell subsets to inflammatory lung, together with airway resident cells responsive to secreted products, play pivotal roles in developing and maintaining asthma. Differential use of adhesion molecules dictates the recruitment patterns of specific cell subsets, yet a clear understanding of the distinctive adhesive molecular pathways guiding them to lung is lacking. To provide further insight into the role of alpha4beta1/VCAM-1 pathway and to compare this to the role of beta2 integrin in the development of acute asthma phenotype, we used genetically deficient mice, in contrast to previous studies with anti-functional antibodies yielding ambiguous results.Allergen-dependent airway inflammation and hyperresponsiveness was induced in conditional alpha4(Delta/Delta), VCAM-1(-/-), and beta2(-/-) mice. Cytology, immunocytochemistry, cytokine and immunoglobulin measurements, and cell type accumulation in lung, BAL fluid, plasma, and hemopoietic tissues were carried out.Asthma phenotype was totally abrogated in alpha4- or beta2-deficient mice. Adoptive transfer of sensitized alpha4(Delta/Delta) CD4(+) cells into challenged normal mice failed to induce asthma, whereas alpha4(+/+) CD4(+) cells were able to induce asthma in challenged alpha4(Delta/Delta) mice. Parallel studies with beta2(-/-) or VCAM-1(-/-) mice uncovered novel mechanistic insights in primary sensitization and into redundant or unique functional roles of these adhesion pathways in allergic asthma.The lack of alpha4 integrin not only impedes the **migration** of all white cell subsets to lung and airways, but also prevents upregulation of vascular cell adhesion molecule-1 (VCAM-1) in inflamed lung vasculature and, unlike beta2, attenuates optimal sensitization and ovalbumin-specific IgE production in vivo. As VCAM-1 deficiency did not protect mice from asthma, interactions of alpha4beta1(+) or alpha4beta7(+) cells with other ligands are suggested.
54. Alpha4 integrins are type I cAMP-dependent protein kinase-anchoring proteins. A-kinase anchoring proteins (AKAPs) control the localization and substrate specificity of cAMP-dependent protein kinase (PKA), tetramers of regulatory (PKA-R) and catalytic (PKA-C) subunits, by binding to PKA-R subunits. Most mammalian AKAPs bind Type II PKA through PKA-RII (ref. 2), whereas dual specificity AKAPs bind both PKA-RI and PKA-RII (ref. 3). Inhibition of PKA-AKAP interactions modulates PKA signalling. Localized PKA activation in pseudopodia of migrating cells phosphorylates alpha4 integrins to provide spatial cues governing cell motility. Here, we report that the alpha4 cytoplasmic domain is a Type I PKA-specific AKAP that is distinct from canonical AKAPs in two ways: the alpha4 interaction requires the PKA holoenzyme, and is insensitive to amphipathic peptides that disrupt most PKA-AKAP interactions. We exploited type-specific PKA anchoring peptides to create genetically encoded baits that sequester specific PKA isoforms to the mitochondria and found that mislocalization of Type I, but not Type II, PKA disrupts alpha4 phosphorylation and markedly inhibits the velocity and directional persistence of cell **migration**.
55. Osteopontin mediates macrophage chemotaxis via α4 and α9 integrins and survival via the α4 integrin. Osteopontin (OPN) is highly expressed by macrophages and plays a key role in the pathology of several chronic inflammatory diseases including atherosclerosis and the foreign body reaction. However, the molecular mechanism behind OPN regulation of macrophage functions is not well understood. OPN is a secreted molecule and interacts with several integrins via two domains: the RGD sequence binding to α(v) -containing integrins, and the SLAYGLR sequence binding to α(4) β(1), α(4) β(7), and α(9) β(1) integrins. Here we determined the role of OPN in macrophage survival, chemotaxis, and activation state. For survival studies, OPN treated-bone marrow derived macrophages (BMDMs) were challenged with growth factor withdrawal and neutralizing integrin antibodies. We found that survival in BMDMs is mediated primarily through the α(4) integrin. In chemotaxis studies, we obs**erved tha**t migration to OPN was blocked by neutralizing α(4) and α(9) integrin antibodies. Further, OPN did not affect macrophage activation as measured by IL-12 production. Finally, the relative contributions of the RGD and the SLAYGLR functional domains of OPN to leukocyte recruitment were evaluated in an in vivo model. We generated chimeric mice expressing mutated forms of OPN in myeloid-derived leukocytes, and found that the SLAYGLR functional domain of OPN, but not the RGD, mediates macrophage accumulation in response to thioglycollate-elicited peritonitis. Collectively, these data indicate that α(4) and α(9) integrins interacting with OPN via the SLAYGLR domain play a key role in macrophage biology **by regul**ating migration, survival, and accumulation.
56. Alpha4 integrins regulate the proliferation/differentiation balance of multilineage hematopoietic progenitors in vivo. We investigated roles of alpha4 integrins during hematopoiesis using mutant and chimeric mice. Yolk sac erythropoiesis and **migration** of hematopoietic progenitors to fetal liver, spleen, and bone marrow can occur without alpha4 integrins. Although terminal differentiation of these progenitors is possible without alpha4 integrins, these receptors are essential to maintain normal hematopoiesis in fetal liver, spleen, and bone marrow microenvironments. Moreover, alpha4-deficient erythroid progenitors and pre-B cells neither transmigrate beneath the stroma nor expand-properly in vitro. In contrast, alpha4-null cells migrate and differentiate efficiently into T lymphocytes within the thymus. In summary, alpha4 integrins are essential for normal development of all hematopoietic lineages in fetal liver, bone marrow, and spleen, likely by regulating the proliferation/differentiation balance of hematopoietic progenitors.
57. A small molecule that inhibits the interaction of paxillin and alpha 4 integrin inhibits accumulation of mononuclear leukocytes at a site of inflammation. Extracellular antagonists of alpha 4 integrin are an effective therapy for several autoimmune and inflammatory diseases; however, these agents that directly block ligand binding may exhibit mechanism-based toxicities. Inhibition of alpha 4 integrin signaling by mutations of alpha 4 that block paxillin binding inhibits inflammation while limiting mechanism-based toxicities. Here, we test a pharmacological approach by identifying small molecules that inhibit the alpha 4 integrin-paxillin interaction. By screening a large (approximately 40,000-compound) chemical library, we identified a noncytotoxic inhibitor of this interaction that impaired integrin alpha 4-mediated but not alpha L beta 2-mediated Jurkat T cell **migration**. The identified compound had no effect on alpha 4-mediated **migration** in cells bearing the alpha 4(Y991A) mutation that disrupts the alpha 4-paxillin interaction, establishing the specificity of its action. Administration of this compound to mice led to impaired recruitment of mononuclear leukocytes to a site of inflammation in vivo, whereas an isomer that does not inhibit the alpha 4-paxillin interaction had no effect on alpha 4-mediated cell **migration**, cell spreading, or recruitment of leukocytes to an inflammatory site. Thus, a small molecule inhibitor that interferes with alpha 4 integrin signaling reduces alpha 4-mediated T cell **migration** in vivo, thus providing proof of principle for inhibition of alpha 4 integrin signaling as a target for the pharmacological reduction of inflammation.
58. Genetic aspects and microenvironment affect expression of CD18 and VLA-4 in experimental tuberculosis. Control of infection by Mycobacterium tuberculosis is dependent on macrophage activation and efficient **migration** of effector T-cell populations. Lymphocyte differentiation is associated with changes in cell surface phenotype and alterations in the migratory pattern of these cells. In this study, we investigated the expression of adhesion receptors involved in activation and **migration** process in experimental tuberculosis. We observed that susceptible BALB/c mice infected with virulent M. tuberculosis by intraperitoneal route presented downmodulation of very late antigen 4 (VLA-4) and unchanged levels of CD18 and CD44hi on peritoneal lymphocytes. On the other hand, lymphocytes from resistant C57BL/6 mice infected by the same route showed unchanged levels of VLA-4 and upregulation of CD18 and CD44hi. However, when BALB/c mice were infected by intratracheal route, lung lymphocytes presented a different pattern of CD18, CD44hi and VLA-4 expression from that observed on peritoneal cells, characterized by unchanged levels of VLA-4 and upregulation of CD18 and CD44hi- coincidentally the same phenotype found on peritoneal cells from C57BL/6. These results suggest that susceptibility and resistance to M. tuberculosis infection, depending on the experimental model, are related to the expression of CD18, CD44hi and VLA-4. Moreover, the microenvironment at the site of infection seems to differentially regulate the expression of these receptors. Thus, the up- or downmodulation of these adhesion receptors is probably associated with differential recruitment of T cells at the site of infection, which may or may not mediate protection in experimental tuberculosis.

#### GeneRIFs

1. Mobilization of bone marrow-derived cells by G-CSF boosted **migration** along the VCAM-1/VLA-4 axis and reduced apoptosis of cardiomyocytes.
2. alpha(4) and alpha(9) integrins interacting with OPN via the SLAYGLR domain play a key role in macrophage biology by regulating **migration**, survival, and accumulation.
3. role in **migration** of B cells into pancreatic lymph nodes in NOD mice
4. Both LFA-1 and VLA-4 are involved in vasoendothelial adhesion and transendothelial **migration** of high proliferative potential endothelial progenitor cells.
5. Gambogic acid could inhibit the adhesion and **migration** of B16-F10 cells in vitro and in vivo, and down-regulation of alpha(4) integrin expression was involved.
6. alpha4 subunit is expressed on mouse retinal ganglion cells (RGCs) and undifferentiated retinal neuroblasts during the period of axon extension and **migration**.
7. genetic and biochemical data demonstrate that both c-Kit and alpha4 integrin signaling are linked to class IA phosphatidylinositol 3-kinase and Rac pathways and regulate integrin-directed **migration** in mast cells
8. Dual functions of [alpha]4[beta]1 integrin in epicardial development: initial **migration** and long-term attachment.

### ECM

#### PubMed

1. Phagocytic cells of the thymic reticulum interact with thymocytes via extracellular matrix ligands and receptors. We previously showed that, in the context of thymic epithelial cells, thymocyte migration is partially controlled by extracellular matrix (**ECM**)-mediated interactions. Herein we evaluated whether these interactions could be involved in cell migration related events in the context of non-epithelial cells of the thymic microenvironment, the phagocytic cells of the thymic reticulum (PTR). We first showed, by immunocytochemistry, cytofluorometry, and RT-PCR, that PTR produce **ECM** components, including fibronectin and laminin, and express the corresponding integrin-type receptors, VLA-4, VLA-5, and VLA-6. Thymocytes adhere onto PTR monolayers, with immature CD4(+)CD8(+) cells being predominant. Importantly, such an adhesion is partially mediated by **ECM** ligands and receptors, since it was impaired by anti-**ECM** or anti-**ECM** receptor antibodies. Conjointly, our data reveal that the **ECM**-dependence for thymocyte adhesion onto the thymic microenvironment is not restricted to the epithelial cells, being also seen when they encounter non-epithelial phagocytic cells.
2. Cell adhesion events mediated by alpha 4 integrins are essential in placental and cardiac development. alpha 4 integrins are cell surface receptors that mediate cell-extracellular matrix (**ECM**) and cell-cell adhesions by interacting with fibronectin (FN) and vascular cell adhesion molecule 1 (VCAM-1), respectively. We have generated a null mutation in the gene for the alpha 4 integrin subunit. Homozygous null embryos express no alpha 4 integrins and show two unexpected defects, both of which lead to embryonic lethality. The first defect is failure of fusion of the allantois with the chorion during placentation. The second is in the development of the epicardium and coronary vessels leading to cardiac hemorrhage. Both processes clearly involve alpha 4 integrin interactions that were previously unsuspected. alpha 4 integrin and VCAM-1 are expressed at the sites of these interactions. These results raise the possibility of abortifacients targeting alpha 4 integrins, and raise serious questions about potential side effects of drugs currently being designed to block alpha 4 integrin functions in inflammation.

### [Ee]xtracellular [Mm]atrix

#### PubMed

1. Integrin expression patterns during early limb muscle development in the mouse. Cell-**extracellular matrix** interactions play crucial roles in limb muscle development but practically nothing is known on what integrins are involved before the differentiation of muscle precursor cells (MPCs) in the limb muscle masses. In this study we determine the expression patterns of integrins during early forelimb muscle development in the mouse. alpha6beta1 integrin is downregulated in the lateral dermomyotome when delamination of MPCs occurs. In late E9.5 embryos, alpha1beta1 and alpha5beta1 are expressed in a pattern very similar to pax3, which marks MPCs migrating to the limb bud. After myf5 upregulation in the limb bud, alpha1beta1 and alpha5beta1 expression is maintained and the alpha4beta1 integrin starts being expressed.
2. PAR3 is essential for cyst-mediated epicardial development by establishing apical cortical domains. Epithelial cysts are one of the fundamental architectures for mammalian organogenesis. Although in vitro studies using cultured epithelial cells have revealed proteins required for cyst formation, the mechanisms that orchestrate the functions of these proteins in vivo remain to be clarified. We show that the targeted disruption of the mouse Par3 gene results in midgestational embryonic lethality with defective epicardial development. The epicardium is mainly derived from epicardial cysts and essential for cardiomyocyte proliferation during cardiac morphogenesis. PAR3-deficient epicardial progenitor (EPP) cells do not form cell cysts and show defects in the establishment of apical cortical domains, but not in basolateral domains. In PAR3-deficient EPP cells, the localizations of aPKC, PAR6beta and ezrin to the apical cortical domains are disturbed. By contrast, ZO1 and alpha4/beta1 integrins normally localize to cell-cell junctions and basal domains, respectively. Our observations indicate that EPP cell cyst formation requires PAR3 to interpret the polarity cues from cell-cell and cell-**extracellular matrix** interactions so that each EPP cell establishes apical cortical domains. These results also provide a clear example of the proper organization of epithelial tissues through the regulation of individual cell polarity.
3. The mouse KRAB zinc-finger protein CHATO is required in embryonic-derived tissues to control yolk sac and placenta morphogenesis. Yolk sac and placenta are required to sustain embryonic development in mammals, yet our understanding of the genes and processes that control morphogenesis of these extraembryonic tissues is still limited. The chato mutation disrupts ZFP568, a Krüppel-Associated-Box (KRAB) domain Zinc finger protein, and causes a unique set of extraembryonic malformations, including ruffling of the yolk sac membrane, defective extraembryonic mesoderm morphogenesis and vasculogenesis, failure to close the ectoplacental cavity, and incomplete placental development. Phenotypic analysis of chato embryos indicated that ZFP568 does not control proliferation or differentiation of extraembryonic lineages but rather regulates the morphogenetic events that shape extraembryonic tissues. Analysis of chimeric embryos showed that Zfp568 function is required in embryonic-derived lineages, including the extraembryonic mesoderm. Depleting Zfp568 affects the ability of extraembryonic mesoderm cells to migrate. However, explanted Zfp568 mutant cells could migrate properly when plated on appropriate **extracellular matri**x conditions. We show that expression of Fibronectin and Indian Hedgehog are reduced in chato mutant yolk sacs. These data suggest that ZFP568 controls the production of secreted factors required to promote morphogenesis of extraembryonic tissues. Our results support previously undescribed roles of the extraembryonic mesoderm in yolk sac morphogenesis and in the closure of the ectoplacental cavity and identify a novel role of ZFP568 in the development of extraembryonic tissues.
4. Adhesion molecules involved in macrophage responses to Wallerian degeneration in the murine peripheral nervous system. When a peripheral nerve is damaged the severed axon undergoes Wallerian degeneration. The distal nerve is infiltrated by large numbers of monocyte-derived macrophages which participate in the phagocytosis of degenerating myelin. In other tissues, adhesion molecules play a crucial role in leukocyte recruitment during inflammation. Blood-borne cells enter damaged tissue by interacting with adhesion molecules expressed on activated endothelium. Having crossed the endothelium, leukocytes must adhere and migrate within the tissue. We investigated the adhesion molecules involved in both stages of the macrophage response to transection of one sciatic nerve of BALB/c mice. By injecting monoclonal antibodies in vivo, before and after peripheral nerve injury, we showed that intercellular adhesion molecule-1 (ICAM-1) and integrins alpha4beta1 (VLA-4) and alphaMbeta2 (type 3 complement receptor) are unlikely to be involved in the transendothelial migration of monocytes responding to peripheral nerve degeneration. We also studied the adhesion of macrophages within the endoneurium, using an in vitro adhesion assay. Macrophages showed much greater levels of adhesion to cryostat sections of transected nerves than to control nerves. This increased adhesion was partially inhibited by antibodies to the beta1-integrin chain, and more strongly inhibited by the **extracellular matrix** molecules fibronectin and collagen. Adhesion was unaffected by laminin-1 and by antibodies to other adhesion molecules, including alpha4beta1- and alpha5beta1-integrins. Thus we conclude that monocyte entry into a degenerating peripheral nerve is independent of alphaLbeta2/alphaMbeta2-ICAM-1 or alpha4beta1/VCAM-1 interactions, and that adhesion within the endoneurium is mediated in part by a beta1-integrin other than alpha4beta1 or alpha5beta1.
5. Chemerin contributes to inflammation by promoting macrophage adhesion to VCAM-1 and fibronectin through clustering of VLA-4 and VLA-5. Chemerin is a potent macrophage chemoattractant protein. We used murine peritoneal exudate cells (PECs) in adhesion, flow cytometry, and confocal microscopy assays to test the hypothesis that chemerin can also contribute to inflammation by promoting macrophage adhesion. Chemerin stimulated the adhesion of PECs to the **extracellular matrix** protein fibronectin and to the adhesion molecule VCAM-1 within a minute, with an EC(50) of 322 and 196 pM, respectively. Experiments using pertussis toxin and PECs from ChemR23(-/-) mice demonstrated that chemerin stimulated the adhesion of macrophages via the Gi protein-coupled receptor ChemR23. Blocking Abs against integrin subunits revealed that 89% of chemerin-stimulated adhesion to fibronectin was dependent on increased avidity of the integrin VLA-5 (alpha(5)beta(1)) and that 88% of adhesion to VCAM-1 was dependent on increased avidity of VLA-4 (alpha(4)beta(1)). Although chemerin was unable to induce an increase in integrin affinity as judged by the binding of soluble ligand, experiments using confocal microscopy revealed an increase in valency resulting from integrin clustering as the mechanism responsible for chemerin-stimulated macrophage adhesion. PI3K, Akt, and p38 were identified as key signaling mediators in chemerin-stimulated adhesion. The finding that chemerin can rapidly stimulate macrophage adhesion to **extracellular matrix** proteins and adhesion molecules, taken together with its ability to promote chemotaxis, suggests a novel role for chemerin in the recruitment and retention of macrophages at sites of inflammation.
6. Requirement of alpha(4)beta(1) and alpha(5)beta(1) integrin expression in bone-marrow-derived progenitor cells in preventing endotoxin-induced lung vascular injury and edema in mice. The goal of this study was to determine the role of integrin-mediated adhesion of bone-marrow-derived progenitor cells (BMPCs) as a requirement for the endothelial barrier protection in a lung injury model. C57BL mice were used as the source for BMPCs, which were characterized as CD34(+) and fetal liver kinase-1 (Flk1)(+) and also an expression of a repertoire of integrins. We used a mouse model of bacterial lipopolysaccharide (LPS)-induced lung vascular injury and edema formation to test the effects of BMPC integrin expression in preventing endothelial barrier injury. Adhesion of BMPCs to purified **extracellular matrix** proteins induced focal adhesion kinase (Fak) phosphorylation and formation of branching point structures in a alpha(4) and alpha(5) integrin-dependent manner. BMPCs expressing red fluorescent protein (RFP) were administered via the retro-orbital venous route in mice treated intraperitonially with LPS (7.5 mg/kg body weight). We observed increased retention of RFP-labeled Flk1(+) and CD34(+) BMPCs for up to 8 weeks in mice injured with LPS. BMPC transplantation increased survival by 50% (at 72-96 hours after LPS) and reduced lung vascular injury and extravascular water content induced by LPS. However, blocking with anti-alpha(4) or anti-alpha(5) integrin antibody or shRNA-mediated silencing of alpha(4) or alpha(5) integrins in donor BMPCs failed to prevent the vascular injury or edema formation and mortality. Thus, alpha(4) and alpha(5) integrin-dependent adhesion of BMPCs in lung tissue plays a critical role in preventing lung vascular injury and increasing survival in a mouse model of LPS-induced acute lung injury.
7. Defective blood vessel development and pericyte/pvSMC distribution in alpha 4 integrin-deficient mouse embryos. Blood vessel development is in part regulated by pericytes/presumptive vascular smooth muscle cells (PC/pvSMCs). Here, we demonstrate that interactions between PC/pvSMCs and **extracellular matrix** play a critical role in this event. We show that the cranial vessels in alpha4 integrin-deficient mouse embryos at the stage of vessel remodeling are increased in diameter. This defect is accompanied by a failure of PC/pvSMCs, which normally express alpha4beta1 integrin, to spread uniformly along the vessels. We also find that fibronectin but not VCAM-1 is localized in the cranial vessels at this stage. Furthermore, cultured alpha4 integrin-null PC/pvSMCs plated on fibronectin display a delay in initiating migration, a reduction in migration speed, and a decrease in directional persistence in response to a polarized force of shear flow. These results suggest that specific motile activities of PC/pvSMCs regulated by mechanical signals imposed by the interstitial **extracellular matrix** may also be required in vivo for the distribution and function of the PC/pvSMCs during blood vessel development.
8. Phagocytic cells of the thymic reticulum interact with thymocytes via **extracellular matrix** ligands and receptors. We previously showed that, in the context of thymic epithelial cells, thymocyte migration is partially controlled by **extracellular matrix** (ECM)-mediated interactions. Herein we evaluated whether these interactions could be involved in cell migration related events in the context of non-epithelial cells of the thymic microenvironment, the phagocytic cells of the thymic reticulum (PTR). We first showed, by immunocytochemistry, cytofluorometry, and RT-PCR, that PTR produce ECM components, including fibronectin and laminin, and express the corresponding integrin-type receptors, VLA-4, VLA-5, and VLA-6. Thymocytes adhere onto PTR monolayers, with immature CD4(+)CD8(+) cells being predominant. Importantly, such an adhesion is partially mediated by ECM ligands and receptors, since it was impaired by anti-ECM or anti-ECM receptor antibodies. Conjointly, our data reveal that the ECM-dependence for thymocyte adhesion onto the thymic microenvironment is not restricted to the epithelial cells, being also seen when they encounter non-epithelial phagocytic cells.
9. Chemerin contributes to inflammation by promoting macrophage adhesion to VCAM-1 and fibronectin through clustering of VLA-4 and VLA-5. Chemerin is a potent macrophage chemoattractant protein. We used murine peritoneal exudate cells (PECs) in adhesion, flow cytometry, and confocal microscopy assays to test the hypothesis that chemerin can also contribute to inflammation by promoting macrophage adhesion. Chemerin stimulated the adhesion of PECs to the **extracellular matrix** protein fibronectin and to the adhesion molecule VCAM-1 within a minute, with an EC(50) of 322 and 196 pM, respectively. Experiments using pertussis toxin and PECs from ChemR23(-/-) mice demonstrated that chemerin stimulated the adhesion of macrophages via the Gi protein-coupled receptor ChemR23. Blocking Abs against integrin subunits revealed that 89% of chemerin-stimulated adhesion to fibronectin was dependent on increased avidity of the integrin VLA-5 (alpha(5)beta(1)) and that 88% of adhesion to VCAM-1 was dependent on increased avidity of VLA-4 (alpha(4)beta(1)). Although chemerin was unable to induce an increase in integrin affinity as judged by the binding of soluble ligand, experiments using confocal microscopy revealed an increase in valency resulting from integrin clustering as the mechanism responsible for chemerin-stimulated macrophage adhesion. PI3K, Akt, and p38 were identified as key signaling mediators in chemerin-stimulated adhesion. The finding that chemerin can rapidly stimulate macrophage adhesion to **extracellular matrix** proteins and adhesion molecules, taken together with its ability to promote chemotaxis, suggests a novel role for chemerin in the recruitment and retention of macrophages at sites of inflammation.
10. Requirement of alpha(4)beta(1) and alpha(5)beta(1) integrin expression in bone-marrow-derived progenitor cells in preventing endotoxin-induced lung vascular injury and edema in mice. The goal of this study was to determine the role of integrin-mediated adhesion of bone-marrow-derived progenitor cells (BMPCs) as a requirement for the endothelial barrier protection in a lung injury model. C57BL mice were used as the source for BMPCs, which were characterized as CD34(+) and fetal liver kinase-1 (Flk1)(+) and also an expression of a repertoire of integrins. We used a mouse model of bacterial lipopolysaccharide (LPS)-induced lung vascular injury and edema formation to test the effects of BMPC integrin expression in preventing endothelial barrier injury. Adhesion of BMPCs to purified **extracellular matrix** proteins induced focal adhesion kinase (Fak) phosphorylation and formation of branching point structures in a alpha(4) and alpha(5) integrin-dependent manner. BMPCs expressing red fluorescent protein (RFP) were administered via the retro-orbital venous route in mice treated intraperitonially with LPS (7.5 mg/kg body weight). We observed increased retention of RFP-labeled Flk1(+) and CD34(+) BMPCs for up to 8 weeks in mice injured with LPS. BMPC transplantation increased survival by 50% (at 72-96 hours after LPS) and reduced lung vascular injury and extravascular water content induced by LPS. However, blocking with anti-alpha(4) or anti-alpha(5) integrin antibody or shRNA-mediated silencing of alpha(4) or alpha(5) integrins in donor BMPCs failed to prevent the vascular injury or edema formation and mortality. Thus, alpha(4) and alpha(5) integrin-dependent adhesion of BMPCs in lung tissue plays a critical role in preventing lung vascular injury and increasing survival in a mouse model of LPS-induced acute lung injury.
11. Defective blood vessel development and pericyte/pvSMC distribution in alpha 4 integrin-deficient mouse embryos. Blood vessel development is in part regulated by pericytes/presumptive vascular smooth muscle cells (PC/pvSMCs). Here, we demonstrate that interactions between PC/pvSMCs and **extracellular matrix** play a critical role in this event. We show that the cranial vessels in alpha4 integrin-deficient mouse embryos at the stage of vessel remodeling are increased in diameter. This defect is accompanied by a failure of PC/pvSMCs, which normally express alpha4beta1 integrin, to spread uniformly along the vessels. We also find that fibronectin but not VCAM-1 is localized in the cranial vessels at this stage. Furthermore, cultured alpha4 integrin-null PC/pvSMCs plated on fibronectin display a delay in initiating migration, a reduction in migration speed, and a decrease in directional persistence in response to a polarized force of shear flow. These results suggest that specific motile activities of PC/pvSMCs regulated by mechanical signals imposed by the interstitial **extracellular matrix** may also be required in vivo for the distribution and function of the PC/pvSMCs during blood vessel development.
12. Expanding roles for alpha 4 integrin and its ligands in development. Interaction of alpha 4 integrins with vascular cell adhesion molecule-1 (VCAM-1) is classically important for immune function. However, we found recently that these receptors have a second role, in embryogenesis, where they mediate cell-cell interactions that are important for skeletal muscle differentiation. Here, we present evidence of an expanding role for these receptors in murine development. alpha 4 and VCAM-1 were found at embryonic sites of hematopoiesis, suggesting a role for these receptors during embryogenesis that parallels their hematopoietic function in adult bone marrow. During angiogenesis in the lung, alpha 4 and VCAM-1 were found on mesenchyme that gives rise to vascular endothelium and smooth muscle. alpha 4 persisted on the smooth muscle and the endothelium of newly forming vessels where it colocalized with its **extracellular matrix** ligand, fibronectin (FN). These patterns suggest several roles for alpha 4 integrins and their ligands in angiogenesis. alpha 4 was also found on neural crest derivatives where it colocalized with FN. alpha 4 was expressed selectively on cells in the dorsal root ganglia: it was apparent along ventral projections, but absent from dorsal projections, suggesting that alpha 4 integrins could be involved in defining neuronal fates. Although VCAM-1 was not expressed on most neural crest derivatives, it was found in the neural crest-derived outflow tract of the embryonic heart, where it colocalized with alpha 4. These results imply that alpha 4 integrins and their ligands could be important for migration or differentiation of neural crest. alpha 4 was also expressed on embryonic retina and FN was found on inductive mesenchyme surrounding the eye, suggesting a role for these proteins in eye development. Finally, based on their patterns of expression, we conclude that VCAM-1 only participates in a subset of interactions involving alpha 4 integrins, whereas FN appears to be the more general ligand.
13. Cell adhesion events mediated by alpha 4 integrins are essential in placental and cardiac development. alpha 4 integrins are cell surface receptors that mediate cell-**extracellular matrix** (ECM) and cell-cell adhesions by interacting with fibronectin (FN) and vascular cell adhesion molecule 1 (VCAM-1), respectively. We have generated a null mutation in the gene for the alpha 4 integrin subunit. Homozygous null embryos express no alpha 4 integrins and show two unexpected defects, both of which lead to embryonic lethality. The first defect is failure of fusion of the allantois with the chorion during placentation. The second is in the development of the epicardium and coronary vessels leading to cardiac hemorrhage. Both processes clearly involve alpha 4 integrin interactions that were previously unsuspected. alpha 4 integrin and VCAM-1 are expressed at the sites of these interactions. These results raise the possibility of abortifacients targeting alpha 4 integrins, and raise serious questions about potential side effects of drugs currently being designed to block alpha 4 integrin functions in inflammation.
14. Absence of alpha 4 but not beta 2 integrins restrains development of chronic allergic asthma using mouse genetic models. Chronic asthma is characterized by ongoing recruitment of inflammatory cells and airway hyperresponsiveness leading to structural airway remodeling. Although alpha 4 beta 1 and beta2 integrins regulate leukocyte migration in inflammatory diseases and play decisive roles in acute asthma, their role has not been explored under the chronic asthma setting. To extend our earlier studies with alpha 4(Delta/Delta) and beta2(-/-) mice, which showed that both alpha 4 and beta2 integrins have nonredundant regulatory roles in acute ovalbumin (OVA)-induced asthma, we explored to what extent these molecular pathways control development of structural airway remodeling in chronic asthma.Control, alpha 4(Delta/Delta), and beta2(-/-) mouse groups, sensitized by intraperitoneal OVA as allergen, received intratracheal OVA periodically over days 8 to 55 to induce a chronic asthma phenotype. Post-OVA assessment of inflammation and pulmonary function (airway hyperresponsiveness), together with airway modeling measured by goblet cell metaplasia, collagen content of lung, and transforming growth factor beta1 expression in lung homogenates, were evaluated.In contrast to control and beta2(-/-) mice, alpha 4(Delta/Delta) mice failed to develop and maintain the composite chronic asthma phenotype evaluated as mentioned and subepithelial collagen content was comparable to baseline. These data indicate that beta2 integrins, although required for inflammatory migration in acute asthma, are dispensable for structural remodeling in chronic asthma.alpha 4 integrins appear to have a regulatory role in directing transforming growth factor beta-induced collagen deposition and structural alterations in lung architecture likely through interactions of Th2 cells, eosinophils, or mast cells with endothelium, resident airway cells, and/or **extracellular matrix**.
15. Rac2 controls tumor growth, metastasis and M1-M2 macrophage differentiation in vivo. Although it is well-established that the macrophage M1 to M2 transition plays a role in tumor progression, the molecular basis for this process remains incompletely understood. Herein, we demonstrate that the small GTPase, Rac2 controls macrophage M1 to M2 differentiation and the metastatic phenotype in vivo. Using a genetic approach, combined with syngeneic and orthotopic tumor models we demonstrate that Rac2-/- mice display a marked defect in tumor growth, angiogenesis and metastasis. Microarray, RT-PCR and metabolomic analysis on bone marrow derived macrophages isolated from the Rac2-/- mice identify an important role for Rac2 in M2 macrophage differentiation. Furthermore, we define a novel molecular mechanism by which signals transmitted from the **extracellular matrix** via the α4β1 integrin and MCSF receptor lead to the activation of Rac2 and potentially regulate macrophage M2 differentiation. Collectively, our findings demonstrate a macrophage autonomous process by which the Rac2 GTPase is activated downstream of the α4β1 integrin and the MCSF receptor to control tumor growth, metastasis and macrophage differentiation into the M2 phenotype. Finally, using gene expression and metabolomic data from our Rac2-/- model, and information related to M1-M2 macrophage differentiation curated from the literature we executed a systems biologic analysis of hierarchical protein-protein interaction networks in an effort to develop an iterative interactome map which will predict additional mechanisms by which Rac2 may coordinately control macrophage M1 to M2 differentiation and metastasis.
16. Expression of laminin alpha1, alpha2, alpha4, and alpha5 chains, fibronectin, and tenascin-C in skeletal muscle of dystrophic 129ReJ dy/dy mice. The dy/dy mouse is an animal model for human merosin-negative congenital muscular dystrophy (CMD), which has been reported to have reduced or no expression of the basement membrane protein laminin alpha2. We here investigate various myogenic and nonmyogenic tissues of mature dy/dy and control 129ReJ mice histologically and for laminin alpha2 expression. In addition, expression patterns of laminin alpha1, alpha2, alpha4, and alpha5 chains, the interstitial proteins fibronectin and tenascin-C, and the adhesion molecules VCAM-1, ICAM-1, and alpha4 integrin were characterized in skeletal muscle of 1- and 7-day and mature (>6 weeks old) dy/dy and control 129ReJ mice. The laminin alpha2 chain remained detectable in myogenic tissues of dy/dy mice by immunofluorescence using two different monoclonal antibodies and by Northern blot analysis. However, laminin alpha2 expression was significantly reduced or not detectable in nonmyogenic tissues of dy/dy mice, including skin, lung, kidney, brain, thymus, and eye. Focal lesions were observed in mature skeletal muscle only, characterized by necrotic tissue, isolated VCAM-1- and ICAM-1-positive cells indicative of inflammatory processes, and regenerating muscle fibers surrounded by intense tenascin-C and fibronectin expression. In contrast to studies on human CMD muscle, laminin alpha1 was not detectable in either dy/dy or control skeletal muscle using immunofluorescence or Northern blot analysis. Immunofluorescence localized laminin alpha4 to basement membranes of blood vessels, the endoneurium of the intramuscular nerves, and the neuromuscular junction in skeletal muscle of 1- and 7-day-old dy/dy and control mice. In mature muscle, laminin alpha4 expression shifted to the perineurium of intramuscular nerves in both dy/dy and control mice. Furthermore, strong upregulation of laminin alpha4 in the basement membranes of blood vessels, the perineurium of intramuscular nerves, and of isolated regenerating muscle fibers in the dy/dy mice was apparent. Investigation of 1-day-old animals revealed expression of laminin alpha5 in skeletal muscle fiber basement membranes of dy/dy but not control animals. This difference between dy/dy and control animals was no longer apparent at 7 days after birth, indicating a temporary shift in expression pattern of laminin alpha5 in dy/dy animals. Analysis of the **extracellular matrix** components of 1- and 7-day-old dy/dy and control skeletal muscle revealed an early onset of the dystrophy, even before histopathological features of the disease were evident. Our data confirm the absence of laminin alpha1 chain in myogenic tissues of both dy/dy and control mice and suggest compensation for reduced laminin alpha2 in dy/dy skeletal muscle by laminin alpha4 and, in early development, also laminin alpha5. These results have significant ramifications in the diagnosis of human merosin-negative CMD.
17. The genomic structure of the murine alpha 4 integrin gene. The VLA-4 (alpha 4 beta 1) integrin is a leukocyte glycoprotein involved in both cell-**extracellular matrix** and cell-cell interactions. We report here the cloning of the murine alpha 4 gene whose protein product is antigenically related to the human VLA-4 alpha chain. The alpha 4 m gene is about 75 kb long and consists of 28 exons, ranging in size from 46 bp (exon 13) to 437 bp (exon 1). The introns varied from 79 bp (intron 8) to more than 17 kb (intron 2). Three mRNA transcripts from this alpha 4 m gene can be visualized on Northern blot. After cloning the 3' untranslated region (3' UTR), four polyadenylation sites could be identified, presumably responsible for the presence of three to four transcripts of the alpha 4 gene, differing substantially in length.
18. The novel α4B murine α4 integrin protein splicing variant inhibits α4 protein-dependent cell adhesion. Integrins affect the motility of multiple cell types to control cell survival, growth, or differentiation, which are mediated by cell-cell and ce**ll-extracellular mat**rix interactions. We reported previously that the α9 integrin splicing variant, SFα9, promotes WT α9 integrin-dependent adhesion. In this study, we introduced a new murine α4 integrin splicing variant, α4B, which has a novel short cytoplasmic tail. In inflamed tissues, the expression of α4B, as well as WT α4 integrin, was up-regulated. Cells expressing α4B specifically bound to VCAM-1 but not other α4 integrin ligands, such as fibronectin CS1 or osteopontin. The binding of cells expressing WT α4 integrin to α4 integrin ligands is inhibited by coexpression of α4B. Knockdown of α4B in metastatic melanoma cell lines results in a significant increase in lung metastasis. Expression levels of WT α4 integrin are unaltered by α4B, with α4B acting as a regulatory subunit for WT α4 integrin by a dominant-negative effect or inhibiting α4 integrin activation.
19. Integrin trafficking regulates adhesion to fibronectin during differentiation of mouse peri-implantation blastocysts. Trophoblast cells of the peri-implantation blastocyst differentiate from a polarized epithelium, the trophectoderm, into invasive cells having an apical surface occupied by integrins that mediate adhesion to the **extracellular matrix**. Blastocyst differentiation was assessed during serum-free culture using a fibronectin binding assay with intact mouse blastocysts. Fibronectin binding activity became elevated during a 24-h "window" after approximately 72 h of culture. Blastocyst differentiation was unaffected by transcriptional inhibition with alpha-amanitin, however, exposure of cavitating morulae to the drug significantly delayed the onset of maximal fibronectin-binding activity. Inhibition of de novo protein synthesis with cycloheximide delayed development only when added during the first 24 h of blastocyst culture, indicating that proteins required for adhesion to fibronectin were synthesized at least 24 h before blastocyst differentiation was completed. Since blastocyst differentiation did not appear to be regulated temporally by gene expression, the possible role of protein trafficking was investigated using the inhibitor, brefeldin A. Brefeldin A caused a reversible, dose-dependent decrease in fibronectin-binding activity when added to the culture medium between 48 and 72 h of culture. During the period of brefeldin A sensitivity, alpha 5 beta 1 integrin, a major fibronectin receptor, translocated to the apical surface of trophoblast cells, as determined by immunohistochemistry and confocal microscopy. Mouse blastocysts expressed other integrins that recognize the central cell-binding domain of fibronectin, including the alpha v integrins and alpha llb beta 3, but not alpha4 which recognizes the lllCS site. Trafficking of alpha 5 beta 1, and possibly other integrins, to the apical surface of trophoblast cells appears to be a critical step in the differentiation of the mouse blastocyst to an invasive phenotype.
20. EMILIN1-α4/α9 integrin interaction inhibits dermal fibroblast and keratinocyte proliferation. EMILIN1 promotes α4β1 integrin-dependent cell adhesion and migration and reduces pro-transforming growth factor-β processing. A knockout mouse model was used to unravel EMILIN1 functions in skin where the protein was abundantly expressed in the dermal stroma and where EMILIN1-positive fibrils reached the basal keratinocyte layer. Loss of EMILIN1 caused dermal and epidermal hyperproliferation and accelerated wound closure. We identified the direct engagement of EMILIN1 to α4β1 and α9β1 integrins as the mechanism underlying the homeostatic role exerted by EMILIN1. The lack of EMILIN1-α4/α9 integrin interaction was accompanied by activation of PI3K/Akt and Erk1/2 pathways as a result of the reduction of PTEN. The down-regulation of PTEN empowered Erk1/2 phosphorylation that in turn inhibited Smad2 signaling by phosphorylation of residues Ser245/250/255. These results highlight the important regulatory **role of an extracell**ular matrix component in skin proliferation. In addition, EMILIN1 is identified as a novel ligand for keratinocyte α9β1 integrin, suggesting prospective roles for this receptor-ligand pair in skin homeostasis.
21. Integrin repertoire on myogenic cells changes during the course of primary myogenesis in the mouse. Cells interact with the **extracellular matrix** through receptors, most commonly of the integrin family. We (Cachaco et al. [2003] Development 130:1659-1671) and others (Schwander et al. [2003] Dev. Cell 4:673-685) have demonstrated a role for beta1 integrins in mouse primary myogenesis. However, it is unclear what alpha subunits pair with beta1 during this process in vivo. Here, we determined alpha subunit expression patterns at embryonic day (E) 11.5-E14.5. Differentiated myotomal myocytes express all alpha subunits studied. As the muscle masses form both in trunk (E12.5) and limbs (E11.5-E12.5), laminin receptors alpha6beta1 and alpha7beta1 are undetectable, and an assembled laminin matrix is absent. Instead alpha1beta1, alpha4beta1, alpha5beta1, and an alpha v-containing integrin are expressed and unassembled laminin and fibronectin are abundant around myogenic cells. At E13.5-E14.5, alpha6beta1 and alpha7beta1 are expressed, and a laminin matrix forms around individual myotubes. Thus, myogenic cells change their integrin expression pattern during the course of primary myogenesis in the mouse, suggesting different roles for fibronectin- and laminin-containing matrices in this process.
22. Integrin-alpha9 is required for fibronectin matrix assembly during lymphatic valve morphogenesis. Dysfunction of lymphatic valves underlies human lymphedema, yet the process of valve morphogenesis is poorly understood. Here, we show that during embryogenesis, lymphatic valve leaflet formation is initiated by upregulation of integrin-alpha9 expression and deposition of its ligand fibronectin-EIIIA (FN-EIIIA) in the **extracellular matrix**. Endothelial cell-specific deletion of Itga9 (encoding integrin-alpha9) in mouse embryos results in the development of rudimentary valve leaflets characterized by disorganized FN matrix, short cusps, and retrograde lymphatic flow. Similar morphological and functional defects are observed in mice lacking the EIIIA domain of FN. Mechanistically, we demonstrate that in primary human lymphatic endothelial cells, the integrin-alpha9-EIIIA interaction directly regulates FN fibril assembly, which is essential for the formation of the **extracellular matrix** core of valve leaflets. Our findings reveal an important role for integrin-alpha9 signaling during lymphatic valve morphogenesis and implicate it as a candidate gene for primary lymphedema caused by valve defects.

### [Ii]nvasive

#### PubMed

1. Integrin trafficking regulates adhesion to fibronectin during differentiation of mouse peri-implantation blastocysts. Trophoblast cells of the peri-implantation blastocyst differentiate from a polarized epithelium, the trophectoderm, into **invasive** cells having an apical surface occupied by integrins that mediate adhesion to the extracellular matrix. Blastocyst differentiation was assessed during serum-free culture using a fibronectin binding assay with intact mouse blastocysts. Fibronectin binding activity became elevated during a 24-h "window" after approximately 72 h of culture. Blastocyst differentiation was unaffected by transcriptional inhibition with alpha-amanitin, however, exposure of cavitating morulae to the drug significantly delayed the onset of maximal fibronectin-binding activity. Inhibition of de novo protein synthesis with cycloheximide delayed development only when added during the first 24 h of blastocyst culture, indicating that proteins required for adhesion to fibronectin were synthesized at least 24 h before blastocyst differentiation was completed. Since blastocyst differentiation did not appear to be regulated temporally by gene expression, the possible role of protein trafficking was investigated using the inhibitor, brefeldin A. Brefeldin A caused a reversible, dose-dependent decrease in fibronectin-binding activity when added to the culture medium between 48 and 72 h of culture. During the period of brefeldin A sensitivity, alpha 5 beta 1 integrin, a major fibronectin receptor, translocated to the apical surface of trophoblast cells, as determined by immunohistochemistry and confocal microscopy. Mouse blastocysts expressed other integrins that recognize the central cell-binding domain of fibronectin, including the alpha v integrins and alpha llb beta 3, but not alpha4 which recognizes the lllCS site. Trafficking of alpha 5 beta 1, and possibly other integrins, to the apical surface of trophoblast cells appears to be a critical step in the differentiation of the mouse blastocyst to an **invasive** phenotype.
2. Expression of the integrin alpha 4 beta 1 on melanoma cells can inhibit the **invasive** stage of metastasis formation. Among a series of adhesion molecules, expression of integrin alpha 4 beta 1 showed a unique inverse correlation with the **invasive** potential of B16 melanoma cell lines. When an alpha 4 cDNA was introduced into an alpha 4-beta 1+ highly **invasive** melanoma line, alpha 4 beta 1 heterodimers were expressed on the surface. Matrigel invasion by the alpha 4+ beta 1+ cells was reduced. Pulmonary metastasis was also suppressed when the transfectants were placed subcutaneously, but not when injected intravenously. Expression of alpha 4 beta 1 promoted homotypic intercellular adhesion. The homotypic adhesion was abrogated, and the alpha 4+ beta 1+ (less **invasive** cell lines) increased matrigel invasion following the anti-alpha 4 MAb treatment. These results suggest that integrin alpha 4 beta 1 could play a role in controlling melanoma cell metastasis at the **invasive** stage.

### [Mm]etastasize

#### PubMed

1. Constitutive expression of the alpha4 integrin correlates with tumorigenicity and lymph node metastasis of the B16 murine melanoma. The lymphatic system plays a critical role in melanoma metastasis, and yet, virtually no information exists regarding the cellular and molecular mechanisms that take place between melanoma cells and the lymphatic vasculature. Here, we generated B16-F1 melanoma cells that expressed high (B16alpha(4)+) and negligible (B16alpha(4)-) levels of alpha(4) integrin to determine how the expression of alpha(4) integrins affects tumor cell interactions with lymphatic endothelial cells in vitro and how it impacts lymphatic metastasis in vivo. We found a direct correlation between alpha(4) integrin expression on B16-F1 melanoma cells and their ability to form adhesive interactions with monolayers of lymphatic endothelial cells. Adhesion of B16-F1 melanoma cells to lymphatic endothelial cells was mediated by the melanoma cell alpha(4) integrin binding to its counterreceptor, vascular cell adhesion molecule 1 (VCAM-1), that was constitutively expressed on the lymphatic endothelial cells. VCAM-1 was also expressed on the tumor-associated lymphatic vessels of B16-F1 and B16alpha(4)+ tumors growing in the subcutaneous space of C57BL/6J mice. B16-F1 tumors **metastasize**d to lymph nodes in 30% of mice, whereas B16alpha(4)+ tumors generated lymph node metastases in 80% of mice. B16-F1 melanoma cells that were deficient in alpha(4) integrins (B16alpha(4)-) were nontumorigenic. Collectively, these data show that the alpha(4) integrin expressed by melanoma cells contributes to tumorigenesis and may also facilitate metastasis to regional lymph nodes by promoting stable adhesion of melanoma cells to the lymphatic vasculature.

### [Mm]etastasis

#### PubMed

1. Bone homing of mesenchymal stem cells by ectopic alpha 4 integrin expression. The pluripotent nature of mesenchymal stem cells (MSC) widens their potential for tissue regeneration and as vehicles for cell therapy in molecular medicine. Although the MSC are relatively easier to obtain and propagate in culture, a major impediment remains in their engraftment to target tissues on autologous transfer. We report here that transient, ectopic expression of alpha4 integrin (CD49d) on MSC greatly increases bone homing in an immunocompetent mouse model. Heterodimerization of the alpha4 integrin with endogenous beta1 integrin (CD29) was confirmed to influence this targeting. In addition to retaining their stem cell property, the engrafted MSC were also found to form osteoblasts and osteocytes in the growth plate of recipient mouse limb bones (femur/tibia) in vivo. These findings provide evidence for a novel strategy to achieve bone homing of genetically engineered MSC, which may broadly benefit in targeted therapies for osteopenic bone defects and cancer bone **metastasis**.
2. Melanoma cell adhesion can be blocked by heparin in vitro: suggestion of VLA-4 as a novel target for antimetastatic approaches. The clinical benefit of heparin in cancer patients to prolong survival can be attributed to non-anticoagulant mechanisms. Since adhesion molecules are crucially involved in tumour cell **metastasis**, their inhibition offers an attractive approach for interfering with the metastatic cascade. Heparin is known to attenuate **metastasis** in a selectin-dependent manner and possesses a variety of additional effects that are thought to influence tumour cell dissemination, proliferation, and angiogenesis. We investigated the adhesion behaviour of B16F10 melanoma cells in vitro regarding selectin- and VLA-4/VCAM-1-mediated binding to get an insight into underlying mechanisms of melanoma cell **metastasis**. We show that B16F10 cells display binding ability to P- and L-selectin as well as to isolated platelets. In contrast, B16F10 cells did not adhere to immobilized P-selectin under flow. This contributes to recent findings that elucidate a major role of platelet P-selectin for microemboli formation and thus, facilitating **metastasis**. In contrast, B16F10 cells adhered to endothelial cells under flow, which could partly be inhibited by a function-blocking anti-VCAM-1 mAb. To emphasize VCAM-1 function, we analyzed cell adhesion at immobilized VCAM-1 and observed an integrin dependency. Inhibition experiments reveal that heparin influences VLA-4-mediated binding pathways. By a combination of different techniques we prove that the site of heparin action is rather VLA-4 than VCAM-1. To our knowledge, this is the first time that heparin is shown to interfere with the VLA-4/VCAM-1 interaction leading to the suggestion of a novel heparin target. Our results may contribute to the understanding of how heparin exerts its anti-metastatic activity.
3. Integrin alpha4beta1 signaling is required for lymphangiogenesis and tumor **metastasis**. Recent studies have shown that lymphangiogenesis or the growth of lymphatic vessels at the periphery of tumors promotes tumor **metastasis** to lymph nodes. We show here that the fibronectin-binding integrin alpha4beta1 and its ligand fibronectin are novel functional markers of proliferative lymphatic endothelium. Tumors and lymphangiogenic growth factors, such as vascular endothelial growth factor-C (VEGF-C) and VEGF-A, induce lymphatic vessel expression of integrin alpha4beta1. Integrin alpha4beta1 then promotes growth factor and tumor-induced lymphangiogenesis, as genetic loss of integrin alpha4beta1 expression in Tie2Cre+ alpha4(loxp/loxp) mice or genetic loss of alpha4 signaling in alpha4Y991A knock-in mice blocks growth factor and tumor-induced lymphangiogenesis, as well as tumor **metastasis** to lymph nodes. In addition, antagonists of integrin alpha4beta1 suppress lymphangiogenesis and tumor **metastasis**. Our studies show that integrin alpha4beta1 and the signals it transduces regulate the adhesion, migration, invasion, and survival of proliferating lymphatic endothelial cells. As suppression of alpha4beta1 expression, signal transduction, or function in tumor lymphatic endothelium not only inhibits tumor lymphangiogenesis but also prevents metastatic disease, these results show that integrin alpha4beta1-mediated tumor lymphangiogenesis promotes **metastasis** and is a useful target for the suppression of metastatic disease.
4. Integrin alpha4beta1 signaling is required for lymphangiogenesis and tumor **metastasis**. Recent studies have shown that lymphangiogenesis or the growth of lymphatic vessels at the periphery of tumors promotes tumor **metastasis** to lymph nodes. We show here that the fibronectin-binding integrin alpha4beta1 and its ligand fibronectin are novel functional markers of proliferative lymphatic endothelium. Tumors and lymphangiogenic growth factors, such as vascular endothelial growth factor-C (VEGF-C) and VEGF-A, induce lymphatic vessel expression of integrin alpha4beta1. Integrin alpha4beta1 then promotes growth factor and tumor-induced lymphangiogenesis, as genetic loss of integrin alpha4beta1 expression in Tie2Cre+ alpha4(loxp/loxp) mice or genetic loss of alpha4 signaling in alpha4Y991A knock-in mice blocks growth factor and tumor-induced lymphangiogenesis, as well as tumor **metastasis** to lymph nodes. In addition, antagonists of integrin alpha4beta1 suppress lymphangiogenesis and tumor **metastasis**. Our studies show that integrin alpha4beta1 and the signals it transduces regulate the adhesion, migration, invasion, and survival of proliferating lymphatic endothelial cells. As suppression of alpha4beta1 expression, signal transduction, or function in tumor lymphatic endothelium not only inhibits tumor lymphangiogenesis but also prevents metastatic disease, these results show that integrin alpha4beta1-mediated tumor lymphangiogenesis promotes **metastasis** and is a useful target for the suppression of metastatic disease.
5. Melanoma cell adhesion can be blocked by heparin in vitro: suggestion of VLA-4 as a novel target for antimetastatic approaches. The clinical benefit of heparin in cancer patients to prolong survival can be attributed to non-anticoagulant mechanisms. Since adhesion molecules are crucially involved in tumour cell **metastasis**, their inhibition offers an attractive approach for interfering with the metastatic cascade. Heparin is known to attenuate **metastasis** in a selectin-dependent manner and possesses a variety of additional effects that are thought to influence tumour cell dissemination, proliferation, and angiogenesis. We investigated the adhesion behaviour of B16F10 melanoma cells in vitro regarding selectin- and VLA-4/VCAM-1-mediated binding to get an insight into underlying mechanisms of melanoma cell **metastasis**. We show that B16F10 cells display binding ability to P- and L-selectin as well as to isolated platelets. In contrast, B16F10 cells did not adhere to immobilized P-selectin under flow. This contributes to recent findings that elucidate a major role of platelet P-selectin for microemboli formation and thus, facilitating **metastasis**. In contrast, B16F10 cells adhered to endothelial cells under flow, which could partly be inhibited by a function-blocking anti-VCAM-1 mAb. To emphasize VCAM-1 function, we analyzed cell adhesion at immobilized VCAM-1 and observed an integrin dependency. Inhibition experiments reveal that heparin influences VLA-4-mediated binding pathways. By a combination of different techniques we prove that the site of heparin action is rather VLA-4 than VCAM-1. To our knowledge, this is the first time that heparin is shown to interfere with the VLA-4/VCAM-1 interaction leading to the suggestion of a novel heparin target. Our results may contribute to the understanding of how heparin exerts its anti-metastatic activity.
6. Bone homing of mesenchymal stem cells by ectopic alpha 4 integrin expression. The pluripotent nature of mesenchymal stem cells (MSC) widens their potential for tissue regeneration and as vehicles for cell therapy in molecular medicine. Although the MSC are relatively easier to obtain and propagate in culture, a major impediment remains in their engraftment to target tissues on autologous transfer. We report here that transient, ectopic expression of alpha4 integrin (CD49d) on MSC greatly increases bone homing in an immunocompetent mouse model. Heterodimerization of the alpha4 integrin with endogenous beta1 integrin (CD29) was confirmed to influence this targeting. In addition to retaining their stem cell property, the engrafted MSC were also found to form osteoblasts and osteocytes in the growth plate of recipient mouse limb bones (femur/tibia) in vivo. These findings provide evidence for a novel strategy to achieve bone homing of genetically engineered MSC, which may broadly benefit in targeted therapies for osteopenic bone defects and cancer bone **metastasis**.
7. Constitutive expression of the alpha4 integrin correlates with tumorigenicity and lymph node **metastasis** of the B16 murine melanoma. The lymphatic system plays a critical role in melanoma **metastasis**, and yet, virtually no information exists regarding the cellular and molecular mechanisms that take place between melanoma cells and the lymphatic vasculature. Here, we generated B16-F1 melanoma cells that expressed high (B16alpha(4)+) and negligible (B16alpha(4)-) levels of alpha(4) integrin to determine how the expression of alpha(4) integrins affects tumor cell interactions with lymphatic endothelial cells in vitro and how it impacts lymphatic **metastasis** in vivo. We found a direct correlation between alpha(4) integrin expression on B16-F1 melanoma cells and their ability to form adhesive interactions with monolayers of lymphatic endothelial cells. Adhesion of B16-F1 melanoma cells to lymphatic endothelial cells was mediated by the melanoma cell alpha(4) integrin binding to its counterreceptor, vascular cell adhesion molecule 1 (VCAM-1), that was constitutively expressed on the lymphatic endothelial cells. VCAM-1 was also expressed on the tumor-associated lymphatic vessels of B16-F1 and B16alpha(4)+ tumors growing in the subcutaneous space of C57BL/6J mice. B16-F1 tumors metastasized to lymph nodes in 30% of mice, whereas B16alpha(4)+ tumors generated lymph node metastases in 80% of mice. B16-F1 melanoma cells that were deficient in alpha(4) integrins (B16alpha(4)-) were nontumorigenic. Collectively, these data show that the alpha(4) integrin expressed by melanoma cells contributes to tumorigenesis and may also facilitate **metastasis** to regional lymph nodes by promoting stable adhesion of melanoma cells to the lymphatic vasculature.
8. PI3Kα activates integrin α4β1 to establish a metastatic niche in lymph nodes. Lymph nodes are initial sites of tum**or metasta**sis, yet whether the lymph node microenvironment actively promotes tum**or metasta**sis remains unknown. We show here that VEGF-C/PI3Kα-driven remodeling of lymph nodes promotes tu**mor metast**asis by activating integrin α4β1 on lymph node lymphatic endothelium. Activated integrin α4β1 promotes expansion of the lymphatic endothelium in lymph nodes and serves as an adhesive ligand that captures vascular cell adhesion molecule 1 (VCAM-1)(+) metastatic tumor cells, thereby promoting lym**ph node me**tastasis. Experimental induction of α4β1 expression in lymph nodes is sufficient to promote tumor cell adhesion to lymphatic endothelium and l**ymph node** metastasis in vivo, whereas genetic or pharmacological blockade of integrin α4β1 or VCAM-1 inhibits it. As lymph node metastases accurately predict poor disease outcome, and integrin α4β1 is a biomarker of lymphatic endothelium in tumor-draining lymph nodes from animals and patients, these results indicate that targeting integrin α4β1 or VCAM to inhibit the interactions of tumor cells with the lymph node microenvironment may be an effective strategy to **suppress** tumor metastasis.
9. Integrin α4 Enhances **Metastasi**s and May Be Associated with Poor Prognosis in MYCN-low Neuroblastoma. High-risk neuroblastoma is associated with an overall survival rate of 30-50%. Neuroblastoma-expressed cell adhesion receptors of the integrin family impact cell adhesion, migration, proliferation and survival. Integrin α4 is essential for neural crest cell motility during development, is highly expressed on leukocytes, and is critical for transendothelial migration. Thus, cancer cells that express this receptor may exhibit increased metastatic potential. We show that α4 expression in human and murine neuroblastoma cell lines selectively enhances in vitro interaction with the alternatively spliced connecting segment 1 of fibronectin, as well as vascular cell adhesion molecule-1 and increases migration. Integrin α4 expression enhanced experimen**tal metast**asis in a syngeneic tumor model, reconstituting a pattern of organ involvement similar to that seen in patients. Accordingly, antagonism of integrin α4 blo**cked metas**tasis, suggesting adhesive function of the integrin is required. However, adhesive function was not sufficient, as mutants of integrin α4 that conserved the matrix-adhesive and promigratory function in vitro were compromised in their metastatic capacity in vivo. Clinically, integrin α4 is more frequently expressed in non-MYNC amplified tumors, and is selectively associated with poor prognosis in this subset of disease. These results reveal an unexpected role for integrin α4 in neuroblastoma dissemination and identify α4 as a potential prognostic indicator and therapeutic target.
10. Inhibition of alpha(4) integrin mediated adhesion was involved in the reduction of B16-F10 melanoma cells lung colonization in C57BL/6 mice treated with gambogic acid. Gambogic acid, the major active ingredient of gamboge, has been shown to exhibit anti-cancer activity both in vivo and in vitro. However, its potential activity in tumor **metastasis** remains unclear. In the present study, we found that gambogic acid strongly inhibited the adhesion of highly metastatic mouse melanoma B16-F10 cells in vitro. Gambogic acid also exhibited significant anti-**metastasis** activity on the development of in vivo artificial metastases (i.e. following tail vein i.v. injection of the B16-F10 melanoma tumor cells in C57BL/6 mice). Flow cytometric analysis and Western blot showed that gambogic acid inhibited the cell surface expression of alpha(4) integrin, while exhibited negligible effects on the expression of alpha(5) and beta(1) integrin. Thus we concluded for the first time that gambogic acid could inhibit the adhesion and migration of B16-F10 cells in vitro and in vivo, in which down-regulation of alpha(4) integrin expression was involved.
11. Receptor tyrosine kinases and TLR/IL1Rs unexpectedly activate myeloid cell PI3kγ, a single convergent point promoting tumor inflammation and progression. Tumor inflammation promotes angiogenesis, immunosuppression, and tumor growth, but the mechanisms controlling inflammatory cell recruitment to tumors are not well understood. We found that a range of chemoattractants activating G protein-coupled receptors (GPCRs), receptor tyrosine kinases (RTKs) and Toll-like/IL-1 receptors (TLR/IL1Rs) unexpectedly initiate tumor inflammation by activating the PI3-kinase isoform p110γ in Gr1+CD11b+ myeloid cells. Whereas GPCRs activate p110γ in a Ras/p101-dependent manner, RTKs and TLR/IL1Rs directly activate p110γ in a Ras/p87-dependent manner. Once activated, p110γ promotes inside-out activation of a single integrin, α4β1, causing myeloid cell invasion into tumors. Pharmacological or genetic blockade of p110γ suppressed inflammation, grow**th, and me**tastasis of implanted and spontaneous tumors, revealing an important therapeutic target in oncology.
12. Rac2 controls tumor growth, **metastasis** and M1-M2 macrophage differentiation in vivo. Although it is well-established that the macrophage M1 to M2 transition plays a role in tumor progression, the molecular basis for this process remains incompletely understood. Herein, we demonstrate that the small GTPase, Rac2 controls macrophage M1 to M2 differentiation and the metastatic phenotype in vivo. Using a genetic approach, combined with syngeneic and orthotopic tumor models we demonstrate that Rac2-/- mice display a marked defect in tumor growth, angiogenesis and **metastasis**. Microarray, RT-PCR and metabolomic analysis on bone marrow derived macrophages isolated from the Rac2-/- mice identify an important role for Rac2 in M2 macrophage differentiation. Furthermore, we define a novel molecular mechanism by which signals transmitted from the extracellular matrix via the α4β1 integrin and MCSF receptor lead to the activation of Rac2 and potentially regulate macrophage M2 differentiation. Collectively, our findings demonstrate a macrophage autonomous process by which the Rac2 GTPase is activated downstream of the α4β1 integrin and the MCSF receptor to control tumor grow**th, metast**asis and macrophage differentiation into the M2 phenotype. Finally, using gene expression and metabolomic data from our Rac2-/- model, and information related to M1-M2 macrophage differentiation curated from the literature we executed a systems biologic analysis of hierarchical protein-protein interaction networks in an effort to develop an iterative interactome map which will predict additional mechanisms by which Rac2 may coordinately control macrophage M1 to M2 differentiation **and metast**asis.
13. PI3-kinase γ promotes Rap1a-mediated activation of myeloid cell integrin α4β1, leading to tumor inflammation and growth. Tumor inflammation, the recruitment of myeloid lineage cells into the tumor microenvironment, promotes angiogenesis, immunosuppression a**nd metasta**sis. CD11b+Gr1lo monocytic lineage cells and CD11b+Gr1hi granulocytic lineage cells are recruited from the circulation by tumor-derived chemoattractants, which stimulate PI3-kinase γ (PI3Kγ)-mediated integrin α4 activation and extravasation. We show here that PI3Kγ activates PLCγ, leading to RasGrp/CalDAG-GEF-I&II mediated, Rap1a-dependent activation of integrin α4β1, extravasation of monocytes and granulocytes, and inflammation-associated tumor progression. Genetic depletion of PLCγ, CalDAG-GEFI or II, Rap1a, or the Rap1 effector RIAM was sufficient to prevent integrin α4 activation by chemoattractants or activated PI3Kγ (p110γCAAX), while activated Rap (RapV12) promoted constitutive integrin activation and cell adhesion that could only be blocked by inhibition of RIAM or integrin α4β1. Similar to blockade of PI3Kγ or integrin α4β1, blockade of Rap1a suppressed both the recruitment of monocytes and granulocytes to tumors and tumor progression. These results demonstrate critical roles for a PI3Kγ-Rap1a-dependent pathway in integrin activation during tumor inflammation and suggest novel avenues for cancer therapy.
14. The novel α4B murine α4 integrin protein splicing variant inhibits α4 protein-dependent cell adhesion. Integrins affect the motility of multiple cell types to control cell survival, growth, or differentiation, which are mediated by cell-cell and cell-extracellular matrix interactions. We reported previously that the α9 integrin splicing variant, SFα9, promotes WT α9 integrin-dependent adhesion. In this study, we introduced a new murine α4 integrin splicing variant, α4B, which has a novel short cytoplasmic tail. In inflamed tissues, the expression of α4B, as well as WT α4 integrin, was up-regulated. Cells expressing α4B specifically bound to VCAM-1 but not other α4 integrin ligands, such as fibronectin CS1 or osteopontin. The binding of cells expressing WT α4 integrin to α4 integrin ligands is inhibited by coexpression of α4B. Knockdown of α4B in metastatic melanoma cell lines results in a significant i**ncrease in** lung metastasis. Expression levels of WT α4 integrin are unaltered by α4B, with α4B acting as a regulatory subunit for WT α4 integrin by a dominant-negative effect or inhibiting α4 integrin activation.
15. Expression of the integrin alpha 4 beta 1 on melanoma cells can inhibit the invasive stage of **metastasis** formation. Among a series of adhesion molecules, expression of integrin alpha 4 beta 1 showed a unique inverse correlation with the invasive potential of B16 melanoma cell lines. When an alpha 4 cDNA was introduced into an alpha 4-beta 1+ highly invasive melanoma line, alpha 4 beta 1 heterodimers were expressed on the surface. Matrigel invasion by the alpha 4+ beta 1+ cells was reduced. Pulmonary **metastasis** was also suppressed when the transfectants were placed subcutaneously, but not when injected intravenously. Expression of alpha 4 beta 1 promoted homotypic intercellular adhesion. The homotypic adhesion was abrogated, and the alpha 4+ beta 1+ (less invasive cell lines) increased matrigel invasion following the anti-alpha 4 MAb treatment. These results suggest that integrin alpha 4 beta 1 could play a role in controlling melanoma cell **metastasis** at the invasive stage.

#### GeneRIFs

1. ITGA4 expression enhances **metastasis** in MYCN low neuroblastoma.
2. alpha4beta1-mediated tumor lymphangiogenesis promotes **metastasis**
3. alpha(4) integrin expressed by melanoma cells contributes to tumorigenesis and may also facilitate **metastasis** to regional lymph nodes

### [Cc]ell [Ii]nvasion

#### PubMed

1. Receptor tyrosine kinases and TLR/IL1Rs unexpectedly activate myeloid cell PI3kγ, a single convergent point promoting tumor inflammation and progression. Tumor inflammation promotes angiogenesis, immunosuppression, and tumor growth, but the mechanisms controlling inflammatory cell recruitment to tumors are not well understood. We found that a range of chemoattractants activating G protein-coupled receptors (GPCRs), receptor tyrosine kinases (RTKs) and Toll-like/IL-1 receptors (TLR/IL1Rs) unexpectedly initiate tumor inflammation by activating the PI3-kinase isoform p110γ in Gr1+CD11b+ myeloid cells. Whereas GPCRs activate p110γ in a Ras/p101-dependent manner, RTKs and TLR/IL1Rs directly activate p110γ in a Ras/p87-dependent manner. Once activated, p110γ promotes inside-out activation of a single integrin, α4β1, causing m**yeloid cell i**nvasion into tumors. Pharmacological or genetic blockade of p110γ suppressed inflammation, growth, and metastasis of implanted and spontaneous tumors, revealing an important therapeutic target in oncology.

---

## Dusp10

- NCBI Gene
- GeneRIF
- Pubmed

| Search Term | Rank | Fields |
| --- | --- | --- |
| [Mm]igration | 6 | PubMed(1) |

  
hide/show details for Dusp10

### [Mm]igration

#### PubMed

1. DUSP10 regulates intestinal epithelial cell growth and colorectal tumorigenesis. Dual specificity phosphatase 10 (DUSP10), also known as MAP kinase phosphatase 5 (MKP5), negatively regulates the activation of MAP kinases. Genetic polymorphisms and aberrant expression of this gene are associated with colorectal cancer (CRC) in humans. However, the role of DUSP10 in intestinal epithelial tumorigenesis is not clear. Here, we showed that DUSP10 knockout (KO) mice had increased intestinal epithelial cell (IEC) proliferation and **migration** and developed less severe colitis than wild-type (WT) mice in response to dextran sodium sulphate (DSS) treatment, which is associated with increased ERK1/2 activation and Krüppel-like factor 5 (KLF5) expression in IEC. In line with increased IEC proliferation, DUSP10 KO mice developed more colon tumours with increased severity compared with WT mice in response to administration of DSS and azoxymethane (AOM). Furthermore, survival analysis of CRC patients demonstrated that high DUSP10 expression in tumours was associated with significant improvement in survival probability. Overexpression of DUSP10 in Caco-2 and RCM-1 cells inhibited cell proliferation. Our study showed that DUSP10 negatively regulates IEC growth and acts as a suppressor for CRC. Therefore, it could be targeted for the development of therapies for colitis and CRC.

---

## Relb

- NCBI Gene
- GeneRIF
- Pubmed

| Search Term | Rank | Fields |
| --- | --- | --- |
| [Mm]igration | 6 | PubMed(9); GeneRIFs(1) |

  
hide/show details for Relb

### [Mm]igration

#### PubMed

1. p100 Deficiency is insufficient for full activation of the alternative NF-κB pathway: TNF cooperates with p52-RelB in target gene transcription. Constitutive activation of the alternative NF-κB pathway leads to marginal zone B cell expansion and disorganized spleen microarchitecture. Furthermore, uncontrolled alternative NF-κB signaling may result in the development and progression of cancer. Here, we focused on the question how does the constitutive alternative NF-κB signaling exert its effects in these malignant processes.To explore the consequences of unrestricted alternative NF-κB activation on genome-wide transcription, we compared gene expression profiles of wild-type and NF-κB2/p100-deficient (p100(-/-)) primary mouse embryonic fibroblasts (MEFs) and spleens. Microarray experiments revealed only 73 differentially regulated genes in p100(-/-) vs. wild-type MEFs. Chromatin immunoprecipitation (ChIP) assays showed in p100(-/-) MEFs direct binding of p52 and RelB to the promoter of the Enpp2 gene encoding ENPP2/Autotaxin, a protein with an important role in lymphocyte homing and **cell mig**ration. Gene ontology analysis revealed upregulation of genes with anti-apoptotic/proliferative activity (Enpp2/Atx, Serpina3g, Traf1, Rrad), chemotactic/locomotory activity (Enpp2/Atx, Ccl8), and lymphocyte homing activity (Enpp2/Atx, Cd34). Most importantly, biochemical and gene expression analyses of MEFs and spleen, respectively, indicated a marked crosstalk between classical and alternative NF-κB pathways.Our results show that p100 deficiency alone was insufficient for full induction of genes regulated by the alternative NF-κB pathway. Moreover, alternative NF-κB signaling strongly synergized both in vitro and in vivo with classical NF-κB activation, thereby extending the number of genes under the control of the p100 inhibitor of the alternative NF-κB signaling pathway.
2. Constitutive alternative NF-kappaB signaling promotes marginal zone B-cell development but disrupts the marginal sinus and induces HEV-like structures in the spleen. Nuclear factor-kappaB (NF-kappaB) plays a crucial role in B-cell and lymphoid organ development. Here, we studied the consequences of constitutive, signal-independent activation of the alternative NF-kappaB pathway for the splenic marginal zone (MZ). In contrast to nfkb2(-/-) mice, which lack both p100 and p52, mice that lack only the inhibitory p100 precursor but still express the p52 subunit of NF-kappaB2 (p100(-/-)) had markedly elevated MZ B-cell numbers. Both cell-intrinsic mechanisms and increased stromal expression of vascular cell adhesion molecule-1 (VCAM-1) contributed to the accumulation of MZ B cells in p100(-/-) spleens. While **migration** of p100(-/-) MZ B cells toward the lysophospholipid sphingosine-1 phosphate (S1P) was not affected, CXCL13-stimulated chemotaxis was impaired, correlating with reduced **migration** of MZ B cells into follicles in response to lipopolysaccharide (LPS). Strikingly, p100 deficiency resulted in the absence of a normal marginal sinus, strongly induced expression of mucosal addressin cell adhesion molecule-1 (MAdCAM-1) and glycosylated cell adhesion molecule-1 (GlyCAM-1), and the formation of nonfunctional ectopic high endothelial venule (HEV)-like structures in the red pulp. Thus, constitutive activation of the alternative NF-kappaB pathway favors MZ B-cell development and accumulation but leads to a disorganized spleen microarchitecture.
3. The IKKα-dependent NF-κB p52/RelB noncanonical pathway is essential to sustain a CXCL12 autocrine loop in cells migrating in response to HMGB1. HMGB1 is a chromatin architectural protein that is released by dead or damaged cells at sites of tissue injury. Extracellular HMGB1 functions as a proinflammatory cytokine and chemoattractant for immune effector and progenitor cells. Previously, we have shown that the inhibitor of NF-κB kinase (IKK)β- and IKKα-dependent NF-κB signaling pathways are simultaneously required for **cell mig**ration to HMGB1. The IKKβ-dependent canonical pathway is needed to maintain expression of receptor for advanced glycation end products, the ubiquitously expressed receptor for HMGB1, but the target of the IKKα non-canonical pathway was not known. In this study, we show that the IKKα-dependent p52/RelB noncanonical pathway is critical to sustain CXCL12/SDF1 production in order for cells to migrate toward HMGB1. Using both mouse bone marrow-derived macrophages and mouse embryo fibroblasts (MEFs), it was observed that neutralization of CXCL12 by a CXCL12 mAb completely eliminated chemotaxis to HMGB1. In addition, t**he HMGB1** migration defect of IKKα KO and p52 KO cells could be rescued by adding recombinant CXCL12 to cells. Moreover, p52 KO MEFs stably transduced with a GFP retroviral vector that enforces physiologic expression of CXCL12 also showed ne**ar normal** migration toward HMGB1. Finally, both AMD3100, a specific antagonist of CXCL12's G protein-coupled receptor CXCR4, and an anti-CXCR4 Ab blocked HMGB1 chemotactic responses. These results indicate that HMGB1-CXCL12 interplay dr**ives cell** migration toward HMGB1 by engaging receptors of both chemoattractants. This novel requirement for a second receptor-ligand pair enhances our understanding of the molecular mechanisms regulating HMGB1-dependent cell recruitment to sites of tissue injury.
4. p100 Deficiency is insufficient for full activation of the alternative NF-κB pathway: TNF cooperates with p52-RelB in target gene transcription. Constitutive activation of the alternative NF-κB pathway leads to marginal zone B cell expansion and disorganized spleen microarchitecture. Furthermore, uncontrolled alternative NF-κB signaling may result in the development and progression of cancer. Here, we focused on the question how does the constitutive alternative NF-κB signaling exert its effects in these malignant processes.To explore the consequences of unrestricted alternative NF-κB activation on genome-wide transcription, we compared gene expression profiles of wild-type and NF-κB2/p100-deficient (p100(-/-)) primary mouse embryonic fibroblasts (MEFs) and spleens. Microarray experiments revealed only 73 differentially regulated genes in p100(-/-) vs. wild-type MEFs. Chromatin immunoprecipitation (ChIP) assays showed in p100(-/-) MEFs direct binding of p52 and RelB to the promoter of the Enpp2 gene encoding ENPP2/Autotaxin, a protein with an important role in lymphocyte homing and **cell mig**ration. Gene ontology analysis revealed upregulation of genes with anti-apoptotic/proliferative activity (Enpp2/Atx, Serpina3g, Traf1, Rrad), chemotactic/locomotory activity (Enpp2/Atx, Ccl8), and lymphocyte homing activity (Enpp2/Atx, Cd34). Most importantly, biochemical and gene expression analyses of MEFs and spleen, respectively, indicated a marked crosstalk between classical and alternative NF-κB pathways.Our results show that p100 deficiency alone was insufficient for full induction of genes regulated by the alternative NF-κB pathway. Moreover, alternative NF-κB signaling strongly synergized both in vitro and in vivo with classical NF-κB activation, thereby extending the number of genes under the control of the p100 inhibitor of the alternative NF-κB signaling pathway.
5. The IKKα-dependent NF-κB p52/RelB noncanonical pathway is essential to sustain a CXCL12 autocrine loop in cells migrating in response to HMGB1. HMGB1 is a chromatin architectural protein that is released by dead or damaged cells at sites of tissue injury. Extracellular HMGB1 functions as a proinflammatory cytokine and chemoattractant for immune effector and progenitor cells. Previously, we have shown that the inhibitor of NF-κB kinase (IKK)β- and IKKα-dependent NF-κB signaling pathways are simultaneously required for **cell mig**ration to HMGB1. The IKKβ-dependent canonical pathway is needed to maintain expression of receptor for advanced glycation end products, the ubiquitously expressed receptor for HMGB1, but the target of the IKKα non-canonical pathway was not known. In this study, we show that the IKKα-dependent p52/RelB noncanonical pathway is critical to sustain CXCL12/SDF1 production in order for cells to migrate toward HMGB1. Using both mouse bone marrow-derived macrophages and mouse embryo fibroblasts (MEFs), it was observed that neutralization of CXCL12 by a CXCL12 mAb completely eliminated chemotaxis to HMGB1. In addition, t**he HMGB1** migration defect of IKKα KO and p52 KO cells could be rescued by adding recombinant CXCL12 to cells. Moreover, p52 KO MEFs stably transduced with a GFP retroviral vector that enforces physiologic expression of CXCL12 also showed ne**ar normal** migration toward HMGB1. Finally, both AMD3100, a specific antagonist of CXCL12's G protein-coupled receptor CXCR4, and an anti-CXCR4 Ab blocked HMGB1 chemotactic responses. These results indicate that HMGB1-CXCL12 interplay dr**ives cell** migration toward HMGB1 by engaging receptors of both chemoattractants. This novel requirement for a second receptor-ligand pair enhances our understanding of the molecular mechanisms regulating HMGB1-dependent cell recruitment to sites of tissue injury.
6. Lung-targeted overexpression of the NF-κB member RelB inhibits cigarette smoke-induced inflammation. Acute lung inflammation can be caused by a variety of respirable agents, including cigarette smoke. Long-term cigarette smoke exposure can cause chronic obstructive pulmonary disease (COPD), a serious illness that affects >10 million Americans. Cigarette smoke is a known inducer of inflammation and is responsible for approximately 90% of all COPD cases. RelB, a member of the NF-κB family, attenuates cigarette smoke-induced inflammatory mediator production in mouse lung fibroblasts in vitro. We hypothesized that overexpression of RelB in the airways of mice would dampen acute smoke-induced pulmonary inflammation. Mice received a recombinant adenovirus encoding RelB by intranasal aspiration to induce transient RelB overexpression in the lungs and were subsequently exposed to mainstream cigarette smoke. Markers of inflammation were analyzed after smoke exposure. Neutrophil infiltration, normally increased by smoke exposure, was significantly and potently decreased after RelB overexpression. Cigarette smoke-induced proinflammatory cytokine and chemokine production, cyclooxygenase-2 expression, and prostaglandin E(2) production were also significantly decreased in the context of RelB overexpression. The expression of intercellular adhesion molecule 1, an NF-κB-dependent protein, was decreased, indicating a potential mechanism through which RelB can regulate inflammatory ce**ll migrat**ion. Therefore, increased expression and/or activation of RelB could be a novel therapeutic strategy against acute lung inflammation caused by respirable agents and possibly against chronic injury, such as COPD.
7. Aryl hydrocarbon receptor (AhR) attenuation of subchronic cigarette smoke-induced pulmonary neutrophilia is associated with retention of nuclear RelB and suppression of intercellular adhesion molecule-1 (ICAM-1). Cigarette smoke is associated with chronic and enhanced pulmonary inflammation characterized by increased cytokine production and leukocyte recruitment to the lung. Although the aryl hydrocarbon receptor (AhR) is well-known to mediate toxic effects of manmade environmental contaminants, the AhR has emerged as a suppressor of acute cigarette smoke-induced neutrophilia by a mechanism involving the NF-κB protein RelB. Yet individuals who smoke often smoke for many years and vary in their cigarette consumption. As there is currently no information on the AhR prevention of lung inflammation, including neutrophilia, due to varied and prolonged exposure regimes, we exposed control and AhR(-/-) mice to cigarette smoke for 2 weeks (subchronic exposure) utilizing low and high exposure protocols and evaluated pulmonary inflammation. Subchronic cigarette smoke exposure significantly increased pulmonary neutrophilia dose-dependently in AhR(-/-) mice. Surprisingly, there was no difference between smoke-exposed AhR(+/-) and AhR(-/-) mice in the expression of cytokines associated with neutrophil recruitment. Expression of pulmonary intercellular adhesion molecule-1 (ICAM-1), an adhesion molecule involved in neutrophil **migratio**n and retention, was higher in pulmonary endothelial cells from AhR(-/-) mice. Although total lung RelB expression was increased by cigarette smoke, nuclear RelB was significantly lower in subchronically exposed AhR(-/-) mice. Inhibition of AhR activity by CH-223191 in endothelial cells potentiated ICAM-1 expression and prevented RelB nuclear translocation but had no effect on neutrophil adhesion. These data support that genetic absence of the AhR contributes to heightened pulmonary neutrophilia in response to ongoing cigarette smoke exposure. Interindividual variations in AhR expression may enhance the susceptibility to cigarette smoke-induced diseases.
8. IKK phosphorylates RelB to modulate its promoter specificity and promote fibroblast **migration** downstream of TNF receptors. TNFα is a potent cytokine that plays a critical role in numerous cellular processes, particularly immune and inflammatory responses, programmed cell death, angiogenesis, and cell **migratio**n. Thus, understanding the molecular mechanisms that mediate TNFα-induced cellular responses is a crucial issue. It is generally accepted that global DNA binding activity of the NF-κB avian reticuloendotheliosis viral (v-rel) oncogene related B (RelB) subunit is not induced upon TNFα treatment in fibroblasts, despite its TNFα-induced nuclear accumulation. Here, we demonstrate that RelB plays a critical role in promoting fibrob**last migr**ation upon prolonged TNFα treatment. We identified the two kinases IκB kinase α (IKKα) and IκB kinase β (IKKβ) as RelB interacting partners whose activation by TNFα promotes RelB phosphorylation at serine 472. Once phosphorylated on serine 472, nuclear RelB dissociates from its interaction with the inhibitory protein IκBα and binds to the promot**er of cri**tical migration-associated genes, such as the matrix metallopeptidase 3 (MMP3). Further, we show that RelB serine 472 phosphorylation status controls MMP3 exp**ression a**nd promigration activity downstream of TNF receptors. Our findings provide new insights into the regulation of RelB activity and reveal a novel link between selective NF-κB target gene expression and cellular response in response to TNFα.
9. Developmental pathway of CD4+CD8- medullary thymocytes during mouse ontogeny and its defect in Aire-/- mice. The newly generated single-positive (SP) thymocytes undergo further maturation in the thymic medulla before their e**migration** to the periphery. The present study was undertaken to validate a developmental program we proposed for CD4SP medullary thymocytes and to explore the mechanisms regulating this process. During mouse ontogeny, the emergence of different subsets of CD4SP thymocytes followed a strict temporal order from SP1 to SP4. Parallel to the transition in surface phenotype, a steady increase in function was observed. As further evidence, purified SP1 cells were able to sequentially give rise to SP2, SP3, and SP4 cells in intrathymic adoptive transfer and in culture. Notably, the development of CD4SP cells in the medulla seemed to be critically dependent on a functionally intact medullary epithelial cell compartment because Relb and Aire deficiency were found to cause severe blockage at the transition from SP3 to SP4. Taken together, this work establishes an ontogenetically and functionally relevant maturation program for CD4SP thymocytes. Precise dissection of this program should facilitate further inquiry into the molecular mechanisms governing normal thymocyte development and its disturbance in pathological conditions.

#### GeneRIFs

1. RelB serine 472 phosphorylation status controls MMP3 expression and pro**migration** activity downstream of TNF receptors.

---

## Sv2a

- NCBI Gene
- GeneRIF
- Pubmed

| Search Term | Rank | Fields |
| --- | --- | --- |
| [Mm]igration | 6 | PubMed(2) |

  
hide/show details for Sv2a

### [Mm]igration

#### PubMed

1. **Migration** and synaptogenesis of cone photoreceptors in the developing mouse retina. Mouse retinal photoreceptor cell generation and morphogenesis take place in a well-characterized temporal sequence. Both rod and cone photoreceptor differentiation and synaptogenesis occur postnatally, but the relative timing of these events has been difficult to document due to the paucity of cell-specific markers. We have found that antibodies to neuron-specific enolase (NSE) preferentially label a subpopulation of photoreceptors in the outer nuclear layer (ONL) of the mouse retina in addition to labeling ganglion, amacrine, bipolar, and horizontal cells within the inner layers of the retina. The appearance of NSE immunoreactivity in the different classes of retinal neurons during development showed a close temporal relationship to the onset of expression of the synaptic vesicle-associated protein SV2 and clearly preceded the sequential development of synaptic connections in both inner and outer synaptic layers. The NSE-immunoreactive photoreceptors were identified as cones by dual labeling of their inner segments with the lectin peanut agglutinin or by colabeling with antisera to cone photopigments. Axonal extensions of NSE-labeled cone cells were shown to interact with those of differentiating horizontal cells as early as postnatal day 3 (P3). Colocalization of NSE with SV2 indicated that cone cells began to make synaptic contacts with horizontal cell processes several days prior to the development of rod synaptic terminals. Between P4 and P11, cone photoreceptor cell nuclei were observed to be scattered at various levels throughout the ONL and thus appeared to have become displaced from their previous position directly beneath the outer limiting membrane (OLM). By P12, the cone nuclei had migrated sclerad once again and were now observed to be neatly aligned adjacent to the OLM. In the rd mouse mutant, this migratory process was delayed, so that, at P12, positioning of the cone cell nuclei within the ONL was still quite irregular. Thus, we have identified a late migratory phase for cone photoreceptors during the second week after birth that correlates with the timing of maturation of the rod synaptic terminals just prior to eye opening. The types of cues used by maturing cone cells for their eventual sclerad location remain to be elucidated.
2. Reelin deficiency causes specific defects in the molecular composition of the synapses in the adult brain. The extracellular protein Reelin regulates radial neuronal **migration** in the embryonic brain, promotes dendrite outgrowth in the developing postnatal forebrain, and strengthens synaptic transmission in the adult brain. Heterozygous reeler mice expressing reduced levels of Reelin are grossly normal but exhibit behavioral and physiological abnormalities. We previously demonstrated that dendritic spine density is reduced in the developing hippocampus of these mice. In this study, we investigated the consequence of Reelin deficiency on synapse formation in adult heterozygous reeler mice using imaging and biochemical approaches. Using a reeler colony that expresses yellow fluorescent protein in selected neurons, we analyzed spine density in hippocampal area CA1 by confocal microscopy and found modest abnormalities in heterozygous reeler mice. However, biochemical analysis of synaptic composition revealed specific postsynaptic defects in scaffolding proteins, neurotransmitter receptors, and signaling proteins. Using whole brain homogenates and purified pre- and postsynaptic fractions, we found that the defects were localized to the postsynaptic compartment of heterozygous reeler synapses. Decreased levels of postsynaptic density-95 (PSD-95), the N-methyl d-aspartate (NMDA) receptor subunits NR2A and NR2B, and the phosphatase PTEN were found specifically in the postsynaptic density fraction obtained from these mice. Furthermore, we found that PSD-95, NR2A, and PTEN interact with each other at the synapse. Finally, we show that levels of NR2A are reduced in conditional Pten knock out mice, demonstrating that the PTEN phosphatase regulates NMDA receptor expression at the synapse in vivo. These studies may provide insights into the etiology of cognitive disorders associated with deficiencies in Reelin signaling and PTEN dysfunction.

---

## Phldb1

- NCBI Gene
- GeneRIF
- Pubmed

| Search Term | Rank | Fields |
| --- | --- | --- |
| [Mm]igration | 6 | PubMed(1) |

  
hide/show details for Phldb1

### [Mm]igration

#### PubMed

1. A collection of cDNAs enriched in upper cortical layers of the embryonic mouse brain. In an attempt to elucidate the molecular basis of neuronal **migration** and corticogenesis, we performed subtractive hybridization of mRNAs from the upper cortical layers (layer I and upper cortical plate) against mRNAs from the remaining cerebral cortex at E15-E16. We obtained a collection of subtracted cDNA clones and analyzed their 3' UTR sequences, 47% of which correspond to EST sequences, and may represent novel products. Among the cloned sequences, we identified gene products that have not been reported in brain or in the cerebral cortex before. We examined the expression pattern of 39 subtracted clones, which was enriched in the upper layers of the cerebral cortex at embryonic stages. The expression of most clones is developmentally regulated, and especially high in embryonic and early postnatal stages. Four of the unknown clones were studied in more detail and identified as a new member of the tetraspanin superfamily, a putative RNA binding protein, a specific product of the adult dentate gyrus and a protein containing a beta-catenin repeat. We thus cloned a collection of subtracted cDNAs coding for protein products that may be involved in the development of the cerebral cortex.

---

## Stom

- NCBI Gene
- GeneRIF
- Pubmed

| Search Term | Rank | Fields |
| --- | --- | --- |
| [Ee]xtracellular [Mm]atrix | 8 | PubMed(1) |

  
hide/show details for Stom

### [Ee]xtracellular [Mm]atrix

#### PubMed

1. Stomatin, a MEC-2 like protein, is expressed by mammalian sensory neurons. The molecular mechanism whereby vertebrate primary sensory neurons convert mechanical energy at their receptive fields into action potentials is unknown. In recent years, genetic screens for touch insensitive mutants of the nematode worm Caenorhabditis elegans have led to the identification of several genes required for mechanical sensitivity. A model has been proposed in which a mechanically gated ion channel is connected both to the **extracellular matrix** and to the cytoskeleton. Displacement of the membrane is proposed to produce a shearing force that pulls the channel open. MEC-2 is thought to play an important role in this complex by linking the ion channel to the cytoskeleton. MEC-2 is highly homologous to a vertebrate protein called stomatin. Stomatin was first isolated from erythrocytes where it is a major integral membrane protein. To date, however, no data on neuronal expression of stomatin in the peripheral nervous system (PNS) or central nervous system (CNS) is available. Here, we have used RT-PCR, in situ hybridization, Northern blotting, and immunocytochemistry to demonstrate that stomatin is expressed by all sensory neurons in mouse dorsal root ganglia. Indirect immunofluorescence together with transfection of cultured adult sensory neurons with epitope-tagged stomatin show that stomatin is localized in spots on somatic and axonal membranes. During development, stomatin begins to be expressed by sensory neurons only as target innervation occurs. The onset of expression of stomatin thus coincides with the onset of functional mechanical sensitivity. Together, our data suggest that stomatin, like the C. elegans MEC-2 gene, is expressed in an appropriate temporal and spatial manner to participate in a putative vertebrate mechanotransduction complex.

---

## Zeb1

- NCBI Gene
- GeneRIF
- Pubmed

| Search Term | Rank | Fields |
| --- | --- | --- |
| [Mm]igration | 6 | PubMed(13); GeneRIFs(2) |
| ECM | 7 | PubMed(1) |
| [Ee]xtracellular [Mm]atrix | 8 | PubMed(2) |
| [Ii]nvasive | 10 | PubMed(5); GeneRIFs(1) |
| [Mm]etastasis | 12 | PubMed(15); GeneRIFs(1) |
| [Cc]ell [Ii]nvasion | 13 | PubMed(2) |

  
hide/show details for Zeb1

### [Mm]igration

#### PubMed

1. Rap1 GTPase is required for mouse lens epithelial maintenance and morphogenesis. Rap1, a Ras-like small GTPase, plays a crucial role in cell-matrix adhesive interactions, cell-cell junction formation, cell polarity and **migration**. The role of Rap1 in vertebrate organ development and tissue architecture, however, remains elusive. We addressed this question in a mouse lens model system using a conditional gene targeting approach. While individual germline deficiency of either Rap1a or Rap1b did not cause overt defects in mouse lens, conditional double deficiency (Rap1 cKO) prior to lens placode formation led to an ocular phenotype including microphthalmia and lens opacification in embryonic mice. The embryonic Rap1 cKO mouse lens exhibited striking defects including loss of E-cadherin- and ZO-1-based cell-cell junctions, disruption of paxillin and β1-integrin-based cell adhesive interactions along with abnormalities in cell shape and apical-basal polarity of epithelium. These epithelial changes were accompanied by increased levels of α-smooth muscle actin, vimentin and N-cadherin, and expression of transcriptional suppressors of E-cadherin (Snai1, Slug and Zeb2), and a mesenchymal metabolic protein (Dihydropyrimidine dehydrogenase). Additionally, while lens differentiation was not overtly affected, increased apoptosis and dysregulated cell cycle progression were noted in epithelium and fibers in Rap1 cKO mice. Collectively these observations uncover a requirement for Rap1 in maintenance of lens epithelial phenotype and morphogenesis.
2. Loss of Sip1 leads to **migration** defects and retention of ectodermal markers during lens development. SIP1 encodes a DNA-binding transcription factor that regulates multiple developmental processes, as highlighted by the pleiotropic defects observed in Mowat-Wilson syndrome, which results from mutations in this gene. Further, in adults, dysregulated SIP1 expression has been implicated in both cancer and fibrotic diseases, where it functionally links TGFβ signaling to the loss of epithelial cell characteristics and gene expression. In the ocular lens, an epithelial tissue important for vision, Sip1 is co-expressed with epithelial markers, such as E-cadherin, and is required for the complete separation of the lens vesicle from the head ectoderm during early ocular morphogenesis. However, the function of Sip1 after early lens morphogenesis is still unknown. Here, we conditionally deleted Sip1 from the developing mouse lens shortly after lens vesicle closure, leading to defects in coordinated fiber cell tip **migratio**n, defective suture formation, and cataract. Interestingly, RNA-Sequencing analysis on Sip1 knockout lenses identified 190 differentially expressed genes, all of which are distinct from previously described Sip1 target genes. Furthermore, 34% of the genes with increased expression in the Sip1 knockout lenses are normally downregulated as the lens transitions from the lens vesicle to early lens, while 49% of the genes with decreased expression in the Sip1 knockout lenses are normally upregulated during early lens development. Overall, these data imply that Sip1 plays a major role in reprogramming the lens vesicle away from a surface ectoderm cell fate towards that necessary for the development of a transparent lens and demonstrate that Sip1 regulates distinctly different sets of genes in different cellular contexts.
3. The miR-200 family inhibits epithelial-mesenchymal transition and cancer cell **migration** by direct targeting of E-cadherin transcriptional repressors ZEB1 and ZEB2. MicroRNAs are small non-coding RNA molecules that can regulate gene expression by interacting with multiple mRNAs and inducing either translation suppression or degradation of mRNA. Recently, several miRNAs were identified as either promoters or suppressors of metastasis. However, it is unclear in which step(s) of the multistep metastatic cascade these miRNAs play a defined functional role. To study the functional importance of miRNAs in epithelial-mesenchymal transition (EMT), a process thought to initiate metastasis by enhancing the motility of tumor cells, we used a well established in vitro EMT assay: transforming growth factor-beta-induced EMT in NMuMG murine mammary epithelial cells. We found that members of the miR-200 family, organized as two clusters in the genome, were repressed during EMT. Overexpression of each miRNA individually or as clusters in NMuMG cells hindered EMT by enhancing E-cadherin expression through direct targeting of ZEB1 and ZEB2, which encode transcriptional repressors of E-cadherin. In the 4TO7 mouse carcinoma cell line, which expresses low levels of endogenous E-cadherin and displays a mesenchymal phenotype, ectopic expression of the miR-200 family miRNAs significantly increased E-cadherin expression and altered cell morphology to an epithelial phenotype. Furthermore, ectopic expression of each miR-200 miRNA cluster significantly reduced the in vitro motility of 4TO7 cells in **migration** assays. These results suggested that loss of expression of the miR-200 family members may play a critical role in the repression of E-cadherin by ZEB1 and ZEB2 during EMT, thereby enhancing **migration** and invasion during cancer progression.
4. Porphyromonas gingivalis initiates a mesenchymal-like transition through ZEB1 in gingival epithelial cells. The oral anaerobe Porphyromonas gingivalis is associated with the development of cancers including oral squamous cell carcinoma (OSCC). Here, we show that infection of gingival epithelial cells with P. gingivalis induces expression and nuclear localization of the ZEB1 transcription factor, which controls epithelial-mesenchymal transition. P. gingivalis also caused an increase in ZEB1 expression as a dual species community with Fusobacterium nucleatum or Streptococcus gordonii. Increased ZEB1 expression was associated with elevated ZEB1 promoter activity and did not require suppression of the miR-200 family of microRNAs. P. gingivalis strains lacking the FimA fimbrial protein were attenuated in their ability to induce ZEB1 expression. ZEB1 levels correlated with an increase in expression of mesenchymal markers, including vimentin and MMP-9, and with enh**anced mig**ration of epithelial cells into matrigel. Knockdown of ZEB1 with siRNA prevented the P. gingivalis-induced increase in mesenchymal markers and epitheli**al cell m**igration. Oral infection of mice by P. gingivalis increased ZEB1 levels in gingival tissues, and intracellular P. gingivalis were detected by antibody staining in biopsy samples from OSCC. These findings indicate that FimA-driven ZEB1 expression could provide a mechanistic basis for a P. gingivalis contribution to OSCC.
5. Transcriptional mechanisms link epithelial plasticity to adhesion and differentiation of epidermal progenitor cells. During epithelial tissue morphogenesis, developmental progenitor cells undergo dynamic adhesive and cytoskeletal remodeling to trigger proliferation and **migration**. Transcriptional mechanisms that restrict such a mild form of epithelial plasticity to maintain lineage-restricted differentiation in committed epithelial tissues are poorly understood. Here, we report that simultaneous ablation of transcriptional repressor-encoding Ovol1 and Ovol2 results in expansion and blocked terminal differentiation of embryonic epidermal progenitor cells. Conversely, mice overexpressing Ovol2 in their skin epithelia exhibit precocious differentiation accompanied by smaller progenitor cell compartments. We show that Ovol1/Ovol2-deficient epidermal cells fail to undertake α-catenin-driven actin cytoskeletal reorganization and adhesive maturation and exhibit changes that resemble epithelial-to-mesenchymal transition (EMT). Remarkably, these alterations and defective terminal differentiation are reversed upon depletion of EMT-promoting transcriptional factor Zeb1. Collectively, our findings reveal Ovol-Zeb1-α-catenin sequential repression and highlight Ovol1 and Ovol2 as gatekeepers of epithelial adhesion and differentiation by inhibiting progenitor-like traits and epithelial plasticity.
6. Decrease of ZEB1 expression inhibits the B16F10 cancer stem-like properties. Increasing evidence supports that cancer stem cells (CSCs) are responsible for driving tumor initiation and maintenance. Zinc-finger E-box binding homeobox 1 (ZEB1) is a transcription factor for regulating tumor progression, and contributes to maintenance of CSC-like properties. The goal of the present study is to investigate the effect of decreasing ZEB1 expression on the B16F10 CSC-like properties. The recombinant shRNA targeting ZEB1 were transfected into melanoma B16F10 cells, and shZEB1-CD133(+)CD44(+) CSCs were isolated from the stable transfected cells using the magnetic-associated cell sorting method. The shZEB1-CD133(+)CD44(+) CSC-like properties were systematically analyzed. The results show the B16F10 shZEB1-CD133(+)CD44(+) CSCs significantly decreased the ability of clonogenicity, cellular proliferation, **migration**, and invasion. Importantly, tumorigenicity and tumor lung metastasis was significantly inhibited in B16F10 shZEB1-CD133(+)CD44(+) CSCs compared with B16F10 scramble-CD133(+)CD44(+) CSCs. The decrease of ZEB1 expression markedly resulted in down-regulation of vimentin and N-cadherin expression as well as up-regulation of E-cadherin expression in tumor tissues from the mice injected with B16F10 shZEB1-CD44(+)CD133(+) CSCs. These findings contribute to understanding the maintenance of B16F10 CD133(+)CD44(+) CSC-like properties that was closely associated with ZEB1 expression. ZEB1 may serve as a new therapeutic target for treatment of malignant melanoma.
7. C-reactive protein inhibits expression of N-cadherin and ZEB-1 in murine colon adenocarcinoma. Epithelial-to-mesenchymal transition (EMT) is thought to play a key role in cancer cell invasion and metastasis. We previously demonstrated that cancer cell **migration** is inhibited by C-reactive protein (CRP), which is widely used as a biomarker of inflammation, though its functions are not fully understood. In the present study, we evaluated the effect of CRP on cancer cell **migration** and expression of mesenchymal and epithelial markers of EMT and of related transcription factors. MCA-38 murine colon adenocarcinoma cells were subcutaneously inoculated into the backs of C57BL/6 mice, which also received 1 μg of recombinant mouse CRP or vehicle (phosphate-buffered saline) subcutaneously every 3 days for 4 weeks. Thereafter, the mice were sacrificed for evaluation using quantitative real-time polymerase chain reaction (PCR) and immunohistochemistry. There was no statistical difference in tumor size between the control and CRP groups, but CRP dose-dependently inhibited MCA-38 cell **migratio**n. PCR analysis confirmed that CRP suppresses expression of N-cadherin (p < 0.01), a mesenchymal marker of EMT, and ZEB-1, an EMT-related transcription factor (p < 0.01). These findings suggest that CRP inhibits EMT in a MCA-38 tumor-bearing mouse model. CRP may thus be a potentially useful tool for preventing cancer progression through suppression of EMT.
8. miR-200b regulates cell **migration** via Zeb family during mouse palate development. Palate development requires coordinating proper cellular and molecular events in palatogenesis, including the epithelial-mesenchymal transition (EMT), apoptosis, cell proliferation, and cell **migration**. Zeb1 and Zeb2 regulate epithelial cadherin (E-cadherin) and EMT during organogenesis. While microRNA 200b (miR-200b) is known to be a negative regulator of Zeb1 and Zeb2 in cancer progression, its regulatory effects on Zeb1 and Zeb2 in palatogenesis have not yet been clarified. The aim of this study is to investigate the relationship between the regulators of palatal development, specifically, miR-200b and the Zeb family. Expression of both Zeb1 and Zeb2 was detected in the mesenchyme of the mouse palate, while miR-200b was expressed in the medial edge epithelium. After contact with the palatal shelves, miR-200b was expressed in the palatal epithelial lining and epithelial island around the fusion region but not in the palatal mesenchyme. The function of miR-200b was examined by overexpression via a lentiviral vector in the palatal shelves. Ectopic expression of miR-200b resulted in suppression of the Zeb family, upregulation of E-cadherin, and changes in cell **migration** and palatal fusion. These results suggest that miR-200b plays crucial roles in cell **migration** and palatal fusion by regulating Zeb1 and Zeb2 as a noncoding RNA during palate development.
9. Zeb1 Is a Potential Regulator of Six2 in the Proliferation, Apoptosis and **Migration** of Metanephric Mesenchyme Cells. Nephron progenitor cells surround around the ureteric bud tips (UB) and inductively interact with the UB to originate nephrons, the basic units of renal function. This process is determined by the internal balance between self-renewal and consumption of the nephron progenitor cells, which is depending on the complicated regulation networks. It has been reported that Zeb1 regulates the proliferation of mesenchymal cells in mouse embryos. However, the role of Zeb1 in nephrons generation is not clear, especially in metanephric mesenchyme (MM). Here, we detected cell proliferation, apoptosis and **migration** in MM cells by EdU assay, flow cytometry assay and wound healing assay, respectively. Meanwhile, Western and RT-PCR were used to measure the expression level of Zeb1 and Six2 in MM cells and developing kidney. Besides, the dual-luciferase assay was conducted to study the molecular relationship between Zeb1 and Six2. We found that knock-down of Zeb1 decreased cell proliferation, **migration** and promoted cell apoptosis in MM cells and Zeb1 overexpression leaded to the opposite data. Western-blot and RT-PCR results showed that knock-down of Zeb1 decreased the expression of Six2 in MM cells and Zeb1 overexpression contributed to the opposite results. Similarly, Zeb1 promoted Six2 promoter reporter activity in luciferase assays. However, double knock-down of Zeb1 and Six2 did not enhance the apoptosis of MM cells compared with control cells. Nevertheless, double silence of Zeb1 and Six2 repressed cell proliferation. In addition, we also found that Zeb1 and Six2 had an identical pattern in distinct developing phases of embryonic kidney. These results indicated that there may exist a complicated regulation network between Six2 and Zeb1. Together, we demonstrate Zeb1 promotes proliferation and apoptosis and inhibits the **migration** of MM cells, in association with Six2.
10. TMPRSS2/ERG promotes epithelial to mesenchymal transition through the ZEB1/ZEB2 axis in a prostate cancer model. Prostate cancer is the most common non-dermatologic malignancy in men in the Western world. Recently, a frequent chromosomal aberration fusing androgen regulated TMPRSS2 promoter and the ERG gene (TMPRSS2/ERG) was discovered in prostate cancer. Several studies demonstrated cooperation between TMPRSS2/ERG and other defective pathways in cancer progression. However, the unveiling of more specific pathways in which TMPRSS2/ERG takes part, requires further investigation. Using immortalized prostate epithelial cells we were able to show that TMPRSS2/ERG over-expressing cells undergo an Epithelial to Mesenchymal Transition (EMT), manifested by acquisition of mesenchymal morphology and markers as well as **migration** and invasion capabilities. These findings were corroborated in vivo, where the control cells gave rise to discrete nodules while the TMPRSS2/ERG-expressing cells formed malignant tumors, which expressed EMT markers. To further investigate the general transcription scheme induced by TMPRSS2/ERG, cells were subjected to a microarray analysis that revealed a distinct EMT expression program, including up-regulation of the EMT facilitators, ZEB1 and ZEB2, and down-regulation of the epithelial marker CDH1(E-Cadherin). A chromatin immunoprecipitation assay revealed direct binding of TMPRSS2/ERG to the promoter of ZEB1 but not ZEB2. However, TMPRSS2/ERG was able to bind the promoters of the ZEB2 modulators, IL1R2 and SPINT1. This set of experiments further illuminates the mechanism by which the TMPRSS2/ERG fusion affects prostate cancer progression and might assist in targeting TMPRSS2/ERG and its downstream targets in future drug design efforts.
11. Identification of a ZEB2-MITF-ZEB1 transcriptional network that controls melanogenesis and melanoma progression. Deregulation of signaling pathways that control differentiation, expansion and **migration** of neural crest-derived melanoblasts during normal development contributes also to melanoma progression and metastasis. Although several epithelial-to-mesenchymal (EMT) transcription factors, such as zinc finger E-box binding protein 1 (ZEB1) and ZEB2, have been implicated in neural crest cell biology, little is known about their role in melanocyte homeostasis and melanoma. Here we show that mice lacking Zeb2 in the melanocyte lineage exhibit a melanoblast **migration** defect and, unexpectedly, a severe melanocyte differentiation defect. Loss of Zeb2 in the melanocyte lineage results in a downregulation of the Microphthalmia-associated transcription factor (Mitf) and melanocyte differentiation markers concomitant with an upregulation of Zeb1. We identify a transcriptional signaling network in which the EMT transcription factor ZEB2 regulates MITF levels to control melanocyte differentiation. Moreover, our data are also relevant for human melanomagenesis as loss of ZEB2 expression is associated with reduced patient survival.
12. ZEB1 Promotes invasiveness of colorectal carcinoma cells through the opposing regulation of uPA and PAI-1. Carcinoma cells enhance their invasive capacity through dedifferentiation and dissolution of intercellular adhesions. A key activator of this process is the ZEB1 transcription factor, which is induced in invading cancer cells by canonical Wnt signaling (β-catenin/TCF4). Tumor invasiveness also entails proteolytic remodeling of the peritumoral stroma. This study aimed to investigate the potential regulation by ZEB1 of the plasminogen proteolytic system constituted by the urokinase plasminogen activator (uPA), and its inhibitor, plasminogen activator inhibitor-1 (PAI-1).Through multiple experimental approaches, colorectal carcinoma (CRC) cell lines and samples from human primary CRC and ZEB1 (-/-) mice were used to examine ZEB1-mediated regulation of uPA and PAI-1 at the protein, mRNA, and transcriptional level.ZEB1 regulates uPA and PAI-1 in opposite directions: induces uPA and inhibits PAI-1. In vivo expression of uPA depends on ZEB1 as it is severely reduced in the developing intestine of ZEB1 null (-/-) mice. Optimal induction of uPA by Wnt signaling requires ZEB1 expression. ZEB1 binds to the uPA promoter and activates its transcription through a mechanism implicating the histone acetyltransferase p300. In contrast, inhibition of PAI-1 by ZEB1 does not involve transcriptional repression but rather downregulation of mRNA stability. ZEB1-mediated tumor cell **migratio**n and invasion depend on its induction of uPA. ZEB1 coexpresses with uPA in cancer cells at the invasive front of CRCs.ZEB1 promotes tumor invasiveness not only via induction in cancer cells of a motile dedifferentiated phenotype but also by differential regulation of genes involved in stroma remodeling.
13. Maternal DNA Methylation Regulates Early Trophoblast Development. Critical roles for DNA methylation in embryonic development are well established, but less is known about its roles during trophoblast development, the extraembryonic lineage that gives rise to the placenta. We dissected the role of DNA methylation in trophoblast development by performing mRNA and DNA methylation profiling of Dnmt3a/3b mutants. We find that oocyte-derived methylation plays a major role in regulating trophoblast development but that imprinting of the key placental regulator Ascl2 is only partially responsible for these effects. We have identified several methylation-regulated genes associated with trophoblast differentiation that are involved in cell adhesion and **migration**, potentially affecting trophoblast invasion. Specifically, trophoblast-specific DNA methylation is linked to the silencing of Scml2, a Polycomb Repressive Complex 1 protein that drives loss of cell adhesion in methylation-deficient trophoblast. Our results reveal that maternal DNA methylation controls multiple differentiation-related and physiological processes in trophoblast via both imprinting-dependent and -independent mechanisms.

#### GeneRIFs

1. Zeb1 promotes proliferation and apoptosis and inhibits the **migration** of metanephric mesenchyme cells, in association with Six2.
2. loss of expression of the miR-200 family members may play a critical role in the repression of E-cadherin by ZEB1 and ZEB2 during EMT, thereby enhancing **migration** and invasion during cancer progression.

### ECM

#### PubMed

1. Regulation of collagen type I in vascular smooth muscle cells by competition between Nkx2.5 and deltaEF1/ZEB1. A major component of the vessel wall of large arteries and veins is the extracellular matrix (**ECM**), which consists of collagens, elastin, and proteoglycans. Collagen type I is one of the most abundant of the **ECM** proteins. We have previously shown that the pro-collagen type I alpha 2 gene contains an enhancer which confers tissue-specific expression in the majority of collagen-producing cells, including blood vessels. In this paper, we delineate a specific vascular smooth muscle cell (vSMC) element: a 100-bp sequence around -16.6 kb upstream of the transcription start site that regulates collagen expression exclusively in vSMCs. Furthermore, we show that the expression is activated through the binding of the homeodomain protein Nkx2.5, which is further potentiated in the presence of GATA6. In contrast, this element was repressed by the binding of the zinc-finger protein deltaEF1/ZEB1. We propose a model of regulation where the activating transcription factor Nkx2.5 and the repressor deltaEF1/ZEB1 compete for an overlapping DNA binding site. This element is important in understanding the molecular mechanisms of vessel remodeling and is a potential target for intervention in vascular diseases where there is excessive deposition of collagen in the vessel wall.

### [Ee]xtracellular [Mm]atrix

#### PubMed

1. Regulation of collagen type I in vascular smooth muscle cells by competition between Nkx2.5 and deltaEF1/ZEB1. A major component of the vessel wall of large arteries and veins is the **extracellular matrix** (ECM), which consists of collagens, elastin, and proteoglycans. Collagen type I is one of the most abundant of the ECM proteins. We have previously shown that the pro-collagen type I alpha 2 gene contains an enhancer which confers tissue-specific expression in the majority of collagen-producing cells, including blood vessels. In this paper, we delineate a specific vascular smooth muscle cell (vSMC) element: a 100-bp sequence around -16.6 kb upstream of the transcription start site that regulates collagen expression exclusively in vSMCs. Furthermore, we show that the expression is activated through the binding of the homeodomain protein Nkx2.5, which is further potentiated in the presence of GATA6. In contrast, this element was repressed by the binding of the zinc-finger protein deltaEF1/ZEB1. We propose a model of regulation where the activating transcription factor Nkx2.5 and the repressor deltaEF1/ZEB1 compete for an overlapping DNA binding site. This element is important in understanding the molecular mechanisms of vessel remodeling and is a potential target for intervention in vascular diseases where there is excessive deposition of collagen in the vessel wall.
2. MicroRNA-192 in diabetic kidney glomeruli and its function in TGF-beta-induced collagen expression via inhibition of E-box repressors. Key features of diabetic nephropathy (DN) include the accumulation of **extracellular matrix** proteins such as collagen 1-alpha 1 and -2 (Col1a1 and -2). Transforming growth factor beta1 (TGF-beta), a key regulator of these **extracellular matrix** genes, is increased in mesangial cells (MC) in DN. By microarray profiling, we noted that TGF-beta increased Col1a2 mRNA in mouse MC (MMC) but also decreased mRNA levels of an E-box repressor, deltaEF1. TGF-beta treatment or short hairpin RNAs targeting deltaEF1 increased enhancer activity of upstream E-box elements in the Col1a2 gene. TGF-beta also decreased the expression of Smad-interacting protein 1 (SIP1), another E-box repressor similar to deltaEF1. Interestingly, we noted that SIP1 is a target of microRNA-192 (miR-192), a key miR highly expressed in the kidney. miR-192 levels also were increased by TGF-beta in MMC. TGF-beta treatment or transfection with miR-192 decreased endogenous SIP1 expression as well as reporter activity of a SIP1 3' UTR-containing luciferase construct in MMC. Conversely, a miR-192 inhibitor enhanced the luciferase activity, confirming SIP1 to be a miR-192 target. Furthermore, miR-192 synergized with deltaEF1 short hairpin RNAs to increase Col1a2 E-box-luc activity. Importantly, the in vivo relevance was noted by the observation that miR-192 levels were enhanced significantly in glomeruli isolated from streptozotocin-injected diabetic mice as well as diabetic db/db mice relative to corresponding nondiabetic controls, in parallel with increased TGF-beta and Col1a2 levels. These results uncover a role for miRs in the kidney and DN in controlling TGF-beta-induced Col1a2 expression by down-regulating E-box repressors.

### [Ii]nvasive

#### PubMed

1. ZEB1 sensitizes lung adenocarcinoma to metastasis suppression by PI3K antagonism. Epithelial tumor cells that have undergone epithelial-to-mesenchymal transition (EMT) are typically prone to metastasis and drug resistance and contribute to a poor clinical outcome. The transcription factor ZEB1 is a known driver of EMT, and mediators of ZEB1 represent potential therapeutic targets for metastasis suppression. Here, we have shown that phosphatidylinositol 3-kinase-targeted (PI3K-targeted) therapy suppresses metastasis in a mouse model of Kras/Tp53-mutant lung adenocarcinoma that develops metastatic disease due to high expression of ZEB1. In lung adenocarcinoma cells from Kras/Tp53-mutant animals and human lung cancer cell lines, ZEB1 activated PI3K by derepressing miR-200 targets, including amphiregulin (AREG), betacellulin (BTC), and the transcription factor GATA6, which stimulated an EGFR/ERBB2 autocrine loop. Additionally, ZEB1-dependent derepression of the miR-200 and miR-183 target friend of GATA 2 (FOG2) enhanced GATA3-induced expression of the p110α catalytic subunit of PI3K. Knockdown of FOG2, p110α, and RHEB ameliorate**d invasi**ve and metastatic propensities of tumor cells. Surprisingly, FOG2 was not required for mesenchymal differentiation, suggesting that mesenchymal differentiation and invasion are distinct and separable processes. Together, these results indicate that ZEB1 sensitizes lung adenocarcinoma cells to metastasis suppression by PI3K-targeted therapy and suggest that treatments to selectively modify the metastatic behavior of mesenchymal tumor cells are feasible and may be of clinical value.
2. Down-regulation of TCF8 is involved in the leukemogenesis of adult T-cell leukemia/lymphoma. Adult T-cell leukemia/lymphoma (ATLL) is caused by latent human T-lymphotropic virus-1 (HTLV-1) infection. To clarify the molecular mechanism underlying leukemogenesis after viral infection, we precisely mapped 605 chromosomal breakpoints in 61 ATLL cases by spectral karyotyping and identified frequent chromosomal breakpoints in 10p11, 14q11, and 14q32. Single nucleotide polymorphism (SNP) array-comparative genomic hybridization (CGH), genetic, and expression analyses of the genes mapped within a common breakpoint cluster region in 10p11.2 revealed that in ATLL cells, transcription factor 8 (TCF8) was frequently disrupted by several mechanisms, including mainly epigenetic dysregulation. TCF8 mutant mice frequently developed **invasive** CD4(+) T-cell lymphomas in the thymus or in ascitic fluid in vivo. Down-regulation of TCF8 expression in ATLL cells in vitro was associated with resistance to transforming growth factor beta1 (TGF-beta1), a well-known characteristic of ATLL cells, suggesting that escape from TGF-beta1-mediated growth inhibition is important in the pathogenesis of ATLL. These findings indicate that TCF8 has a tumor suppressor role in ATLL.
3. Sequential inductions of the ZEB1 transcription factor caused by mutation of Rb and then Ras proteins are required for tumor initiation and progression. Rb1 restricts cell cycle progression, and it imposes cell contact inhibition to suppress tumor outgrowth. It also triggers oncogene-induced senescence to block Ras mutation. Loss of the Rb1 pathway, which is a hallmark of cancer cells, then provides a permissive environment for Ras mutation, and Ras is sufficient for **invasive** tumor formation in Rb1 family mutant mouse embryo fibroblasts (MEFs). These results demonstrate that sequential mutation of the Rb1 and Ras pathways comprises a tumor initiation axis. Both Rb1 and Ras regulate expression of the transcription factor ZEB1, thereby linking tumor initiation to the subsequent invasion and metastasis, which is induced by ZEB1. ZEB1 acts in a negative feedback loop to block expression of miR-200, which is thought to facilitate tumor invasion and metastasis. However, ZEB1 also represses cyclin-dependent kinase (cdk) inhibitors to control the cell cycle; its mutation in MEFs leads to induction of these inhibitors and premature senescence. Here, we provide evidence for two sequential inductions of ZEB1 during Ras transformation of MEFs. Rb1 constitutively represses cdk inhibitors, and induction of ZEB1 when the Rb1 pathway is lost is required to maintain this repression, allowing for the classic immortalization and loss of cell contact inhibition seen when the Rb1 pathway is lost. In vivo, we show that this induction of ZEB1 is required for Ras-initiated tumor formation. ZEB1 is then further induced by Ras, beyond the level seen with Rb1 mutation, and this Ras superinduction is required to reach a threshold of ZEB1 sufficient for repression of miR-200 and tumor invasion.
4. ZEB1 Promotes **invasive**ness of colorectal carcinoma cells through the opposing regulation of uPA and PAI-1. Carcinoma cells enhance their **invasive** capacity through dedifferentiation and dissolution of intercellular adhesions. A key activator of this process is the ZEB1 transcription factor, which is induced in invading cancer cells by canonical Wnt signaling (β-catenin/TCF4). Tumor **invasiv**eness also entails proteolytic remodeling of the peritumoral stroma. This study aimed to investigate the potential regulation by ZEB1 of the plasminogen proteolytic system constituted by the urokinase plasminogen activator (uPA), and its inhibitor, plasminogen activator inhibitor-1 (PAI-1).Through multiple experimental approaches, colorectal carcinoma (CRC) cell lines and samples from human primary CRC and ZEB1 (-/-) mice were used to examine ZEB1-mediated regulation of uPA and PAI-1 at the protein, mRNA, and transcriptional level.ZEB1 regulates uPA and PAI-1 in opposite directions: induces uPA and inhibits PAI-1. In vivo expression of uPA depends on ZEB1 as it is severely reduced in the developing intestine of ZEB1 null (-/-) mice. Optimal induction of uPA by Wnt signaling requires ZEB1 expression. ZEB1 binds to the uPA promoter and activates its transcription through a mechanism implicating the histone acetyltransferase p300. In contrast, inhibition of PAI-1 by ZEB1 does not involve transcriptional repression but rather downregulation of mRNA stability. ZEB1-mediated tumor cell migration and invasion depend on its induction of uPA. ZEB1 coexpresses with uPA in cancer cells at the **invasiv**e front of CRCs.ZEB1 promotes tumor **invasiv**eness not only via induction in cancer cells of a motile dedifferentiated phenotype but also by differential regulation of genes involved in stroma remodeling.
5. ASPP2 controls epithelial plasticity and inhibits metastasis through β-catenin-dependent regulation of ZEB1. Epithelial to mesenchymal transition (EMT), and the reverse mesenchymal to epithelial transition (MET), are known examples of epithelial plasticity that are important in kidney development and cancer metastasis. Here we identify ASPP2, a haploinsufficient tumour suppressor, p53 activator and PAR3 binding partner, as a molecular switch of MET and EMT. ASPP2 contributes to MET in mouse kidney in vivo. Mechanistically, ASPP2 induces MET through its PAR3-binding amino-terminus, independently of p53 binding. ASPP2 prevents β-catenin from transactivating ZEB1, directly by forming an ASPP2-β-catenin-E-cadherin ternary complex and indirectly by inhibiting β-catenin's N-terminal phosphorylation to stabilize the β-catenin-E-cadherin complex. ASPP2 limits the **pro-inv**asive property of oncogenic RAS and inhibits tumour metastasis in vivo. Reduced ASPP2 expression results in EMT, and is associated with poor survival in hepatocellular carcinoma and breast cancer patients. Hence, ASPP2 is a key regulator of epithelial plasticity that connects cell polarity to the suppression of WNT signalling, EMT and tumour metastasis.

#### GeneRIFs

1. ZEB1 Promotes **invasive**ness of colorectal carcinoma cells through the opposing regulation of uPA and PAI-1.

### [Mm]etastasis

#### PubMed

1. ZEB1 sensitizes lung adenocarcinoma to **metastasis** suppression by PI3K antagonism. Epithelial tumor cells that have undergone epithelial-to-mesenchymal transition (EMT) are typically prone to **metastasis** and drug resistance and contribute to a poor clinical outcome. The transcription factor ZEB1 is a known driver of EMT, and mediators of ZEB1 represent potential therapeutic targets for **metastasis** suppression. Here, we have shown that phosphatidylinositol 3-kinase-targeted (PI3K-targeted) therapy suppresses **metastasis** in a mouse model of Kras/Tp53-mutant lung adenocarcinoma that develops metastatic disease due to high expression of ZEB1. In lung adenocarcinoma cells from Kras/Tp53-mutant animals and human lung cancer cell lines, ZEB1 activated PI3K by derepressing miR-200 targets, including amphiregulin (AREG), betacellulin (BTC), and the transcription factor GATA6, which stimulated an EGFR/ERBB2 autocrine loop. Additionally, ZEB1-dependent derepression of the miR-200 and miR-183 target friend of GATA 2 (FOG2) enhanced GATA3-induced expression of the p110α catalytic subunit of PI3K. Knockdown of FOG2, p110α, and RHEB ameliorated invasive and metastatic propensities of tumor cells. Surprisingly, FOG2 was not required for mesenchymal differentiation, suggesting that mesenchymal differentiation and invasion are distinct and separable processes. Together, these results indicate that ZEB1 sensitizes lung adenocarcinoma cells t**o metastas**is suppression by PI3K-targeted therapy and suggest that treatments to selectively modify the metastatic behavior of mesenchymal tumor cells are feasible and may be of clinical value.
2. The ZEB1 transcription factor acts in a negative feedback loop with miR200 downstream of Ras and Rb1 to regulate Bmi1 expression. Ras mutations are frequent in cancer cells where they drive proliferation and resistance to apoptosis. However in primary cells, mutant Ras instead can cause oncogene-induced senescence, a tumor suppressor function linked to repression of the polycomb factor Bmi1, which normally regulates cell cycle inhibitory cyclin-dependent kinase inhibitors (cdki). It is unclear how Ras causes repression of Bmi1 in primary cells to suppress tumor formation while inducing the gene in cancer cells to drive tumor progression. Ras also induces the EMT transcription factor ZEB1 to trigger tumor invasion and **metastasis**. Beyond its well-documented role in EMT, ZEB1 is important for maintaining repression of cdki. Indeed, heterozygous mutation of ZEB1 is sufficient for elevated cdki expression, leading to premature senescence of primary cells. A similar phenotype is evident with Bmi1 mutation. We show that activation of Rb1 in response to mutant Ras causes dominant repression of ZEB1 in primary cells, but loss of the Rb1 pathway is a hallmark of cancer cells and in the absence of such Rb1 repression Ras induces ZEB1 in cancer cells. ZEB1 represses miR-200 in the context of a mutual repression loop. Because miR-200 represses Bmi1, induction of ZEB1 leads to induction of Bmi1. Rb1 pathway status then dictates the opposing effects of mutant Ras on the ZEB1-miR-200 loop in primary versus cancer cells. This loop not only triggers EMT, surprisingly we show it acts downstream of Ras to regulate Bmi1 expression and thus the critical decision between oncogene-induced senescence and tumor initiation.
3. The miR-200 family inhibits epithelial-mesenchymal transition and cancer cell migration by direct targeting of E-cadherin transcriptional repressors ZEB1 and ZEB2. MicroRNAs are small non-coding RNA molecules that can regulate gene expression by interacting with multiple mRNAs and inducing either translation suppression or degradation of mRNA. Recently, several miRNAs were identified as either promoters or suppressors of **metastasis**. However, it is unclear in which step(s) of the multistep metastatic cascade these miRNAs play a defined functional role. To study the functional importance of miRNAs in epithelial-mesenchymal transition (EMT), a process thought to initiate **metastasis** by enhancing the motility of tumor cells, we used a well established in vitro EMT assay: transforming growth factor-beta-induced EMT in NMuMG murine mammary epithelial cells. We found that members of the miR-200 family, organized as two clusters in the genome, were repressed during EMT. Overexpression of each miRNA individually or as clusters in NMuMG cells hindered EMT by enhancing E-cadherin expression through direct targeting of ZEB1 and ZEB2, which encode transcriptional repressors of E-cadherin. In the 4TO7 mouse carcinoma cell line, which expresses low levels of endogenous E-cadherin and displays a mesenchymal phenotype, ectopic expression of the miR-200 family miRNAs significantly increased E-cadherin expression and altered cell morphology to an epithelial phenotype. Furthermore, ectopic expression of each miR-200 miRNA cluster significantly reduced the in vitro motility of 4TO7 cells in migration assays. These results suggested that loss of expression of the miR-200 family members may play a critical role in the repression of E-cadherin by ZEB1 and ZEB2 during EMT, thereby enhancing migration and invasion during cancer progression.
4. Different thresholds of ZEB1 are required for Ras-mediated tumour initiation and **metastasis**. Ras pathway mutation is frequent in carcinomas where it induces expression of the transcriptional repressor ZEB1. Although ZEB1 is classically linked to epithelial-mesenchymal transition and tumour **metastasis**, it has an emerging second role in generation of cancer-initiating cells. Here we show that Ras induction of ZEB1 is required for tumour initiation in a lung cancer model, and we link this function to repression Pten, whose loss is critical for emergence of cancer-initiating cells. These two roles for ZEB1 in tumour progression can be distinguished by their requirement for different levels of ZEB1. A lower threshold of ZEB1 is sufficient for cancer initiation, whereas further induction is necessary for tumour **metastasis**.
5. The EMT-activator ZEB1 promotes tumorigenicity by repressing stemness-inhibiting microRNAs. Invasion and **metastasis** of carcinomas is promoted by the activation of the embryonic 'epithelial to mesenchymal transition' (EMT) program, which triggers cellular mobility and subsequent dissemination of tumour cells. We recently showed that the EMT-activator ZEB1 (zinc finger E-box binding homeobox 1) is a crucial promoter of **metastasis** and demonstrated that ZEB1 inhibits expression of the microRNA-200 (miR-200) family, whose members are strong inducers of epithelial differentiation. Here, we report that ZEB1 not only promotes tumour cell dissemination, but is also necessary for the tumour-initiating capacity of pancreatic and colorectal cancer cells. We show that ZEB1 represses expression of stemness-inhibiting miR-203 and that candidate targets of miR-200 family members are also stem cell factors, such as Sox2 and Klf4. Moreover, miR-200c, miR-203 and miR-183 cooperate to suppress expression of stem cell factors in cancer cells and mouse embryonic stem (ES) cells, as demonstrated for the polycomb repressor Bmi1. We propose that ZEB1 links EMT-activation and stemness-maintenance by suppressing stemness-inhibiting microRNAs (miRNAs) and thereby is a promoter of mobile, migrating cancer stem cells. Thus, targeting the ZEB1-miR-200 feedback loop might form the basis of a promising treatment for fatal tumours, such as pancreatic cancer.
6. A double-negative feedback loop between ZEB1-SIP1 and the microRNA-200 family regulates epithelial-mesenchymal transition. Epithelial to mesenchymal transition occurs during embryologic development to allow tissue remodeling and is proposed to be a key step in the **metastasis** of epithelial-derived tumors. The miR-200 family of microRNAs plays a major role in specifying the epithelial phenotype by preventing expression of the transcription repressors, ZEB1/deltaEF1 and SIP1/ZEB2. We show here that miR-200a, miR-200b, and the related miR-429 are all encoded on a 7.5-kb polycistronic primary miRNA (pri-miR) transcript. We show that the promoter for the pri-miR is located within a 300-bp segment located 4 kb upstream of miR-200b. This promoter region is sufficient to confer expression in epithelial cells and is repressed in mesenchymal cells by ZEB1 and SIP1 through their binding to a conserved pair of ZEB-type E-box elements located proximal to the transcription start site. These findings establish a double-negative feedback loop controlling ZEB1-SIP1 and miR-200 family expression that regulates cellular phenotype and has direct relevance to the role of these factors in tumor progression.
7. Decrease of ZEB1 expression inhibits the B16F10 cancer stem-like properties. Increasing evidence supports that cancer stem cells (CSCs) are responsible for driving tumor initiation and maintenance. Zinc-finger E-box binding homeobox 1 (ZEB1) is a transcription factor for regulating tumor progression, and contributes to maintenance of CSC-like properties. The goal of the present study is to investigate the effect of decreasing ZEB1 expression on the B16F10 CSC-like properties. The recombinant shRNA targeting ZEB1 were transfected into melanoma B16F10 cells, and shZEB1-CD133(+)CD44(+) CSCs were isolated from the stable transfected cells using the magnetic-associated cell sorting method. The shZEB1-CD133(+)CD44(+) CSC-like properties were systematically analyzed. The results show the B16F10 shZEB1-CD133(+)CD44(+) CSCs significantly decreased the ability of clonogenicity, cellular proliferation, migration, and invasion. Importantly, tumorigenicity and tumor lung **metastasis** was significantly inhibited in B16F10 shZEB1-CD133(+)CD44(+) CSCs compared with B16F10 scramble-CD133(+)CD44(+) CSCs. The decrease of ZEB1 expression markedly resulted in down-regulation of vimentin and N-cadherin expression as well as up-regulation of E-cadherin expression in tumor tissues from the mice injected with B16F10 shZEB1-CD44(+)CD133(+) CSCs. These findings contribute to understanding the maintenance of B16F10 CD133(+)CD44(+) CSC-like properties that was closely associated with ZEB1 expression. ZEB1 may serve as a new therapeutic target for treatment of malignant melanoma.
8. C-reactive protein inhibits expression of N-cadherin and ZEB-1 in murine colon adenocarcinoma. Epithelial-to-mesenchymal transition (EMT) is thought to play a key role in cancer cell invasion and **metastasis**. We previously demonstrated that cancer cell migration is inhibited by C-reactive protein (CRP), which is widely used as a biomarker of inflammation, though its functions are not fully understood. In the present study, we evaluated the effect of CRP on cancer cell migration and expression of mesenchymal and epithelial markers of EMT and of related transcription factors. MCA-38 murine colon adenocarcinoma cells were subcutaneously inoculated into the backs of C57BL/6 mice, which also received 1 μg of recombinant mouse CRP or vehicle (phosphate-buffered saline) subcutaneously every 3 days for 4 weeks. Thereafter, the mice were sacrificed for evaluation using quantitative real-time polymerase chain reaction (PCR) and immunohistochemistry. There was no statistical difference in tumor size between the control and CRP groups, but CRP dose-dependently inhibited MCA-38 cell migration. PCR analysis confirmed that CRP suppresses expression of N-cadherin (p < 0.01), a mesenchymal marker of EMT, and ZEB-1, an EMT-related transcription factor (p < 0.01). These findings suggest that CRP inhibits EMT in a MCA-38 tumor-bearing mouse model. CRP may thus be a potentially useful tool for preventing cancer progression through suppression of EMT.
9. The ZEB1/miR-200 feedback loop controls Notch signalling in cancer cells. Notch signalling is important for development and tissue homeostasis and activated in many human cancers. Nevertheless, mutations in Notch pathway components are rare in solid tumours. ZEB1 is an activator of an epithelial-mesenchymal transition (EMT) and has crucial roles in tumour progression towards **metastasis**. ZEB1 and miR-200 family members repress expression of each other in a reciprocal feedback loop. Since miR-200 members target stem cell factors, ZEB1 indirectly induces stemness maintenance and associated drug resistance. Here, we link ZEB1 and its cancer promoting properties to Notch activation. We show that miR-200 members target Notch pathway components, such as Jagged1 (Jag1) and the mastermind-like coactivators Maml2 and Maml3, thereby mediating enhanced Notch activation by ZEB1. We further detected a coordinated upregulation of Jag1 and ZEB1, associated with reduced miR-200 expression in two aggressive types of human cancer, pancreatic adenocarcinoma and basal type of breast cancer. These findings explain increased Notch signalling in some types of cancers, where mutations in Notch pathway genes are rare. Moreover, they indicate an additional way how ZEB1 exerts its tumour progressing functions.
10. **Metastasis** is regulated via microRNA-200/ZEB1 axis control of tumour cell PD-L1 expression and intratumoral immunosuppression. Immunosuppression of tumour-infiltrating lymphocytes (TIL) is a common feature of advanced cancer, but its biological basis has remained obscure. We demonstrate here a molecular link between epithelial-to-mesenchymal transition (EMT) and CD8(+) TIL immunosuppression, two key drivers of cancer progression. We show that microRNA-200 (miR-200), a cell-autonomous suppressor of EMT and **metastasis**, targets PD-L1. Moreover, ZEB1, an EMT activator and transcriptional repressor of miR-200, relieves miR-200 repression of PD-L1 on tumour cells, leading to CD8(+) T-cell immunosuppression and **metastasis**. These findings are supported by robust correlations between the EMT score, miR-200 levels and PD-L1 expression in multiple human lung cancer datasets. In addition to revealing a link between EMT and T-cell dysfunction, these findings also show that ZEB1 promotes **metastasis** through a heretofore unappreciated cell non-autonomous mechanism, and suggest that subgroups of patients in whom malignant progression is driven by EMT activators may respond to treatment with PD-L1 antagonists.
11. Identification of a ZEB2-MITF-ZEB1 transcriptional network that controls melanogenesis and melanoma progression. Deregulation of signaling pathways that control differentiation, expansion and migration of neural crest-derived melanoblasts during normal development contributes also to melanoma progression and **metastasis**. Although several epithelial-to-mesenchymal (EMT) transcription factors, such as zinc finger E-box binding protein 1 (ZEB1) and ZEB2, have been implicated in neural crest cell biology, little is known about their role in melanocyte homeostasis and melanoma. Here we show that mice lacking Zeb2 in the melanocyte lineage exhibit a melanoblast migration defect and, unexpectedly, a severe melanocyte differentiation defect. Loss of Zeb2 in the melanocyte lineage results in a downregulation of the Microphthalmia-associated transcription factor (Mitf) and melanocyte differentiation markers concomitant with an upregulation of Zeb1. We identify a transcriptional signaling network in which the EMT transcription factor ZEB2 regulates MITF levels to control melanocyte differentiation. Moreover, our data are also relevant for human melanomagenesis as loss of ZEB2 expression is associated with reduced patient survival.
12. Zeb1 mutant mice as a model of posterior corneal dystrophy. The zinc finger transcription factor Zeb1 binds to E-box-like sequences and is important for maintaining repression of epithelial specification genes in vivo. Overexpression of Zeb1 in cancer triggers epithelial-mesenchymal transition, which facilitates **metastasis**. The mutation of ZEB1 in humans is linked to posterior polymorphous corneal dystrophy (PPCD), in which an epithelial transition of the corneal endothelium is associated with abnormal endothelial proliferation. The purpose of this study is to determine whether Zeb1 null or heterozygous mice may provide an animal model for PPCD.Corneal morphology, protein and mRNA expression, and cell proliferation were compared in wild-type and Zeb1 gene knockout mice by immunostaining, real-time PCR, and BrdU incorporation. mRNA expression in isolated embryo fibroblasts derived from wild-type, Zeb1 heterozygous, and null mice was analyzed by real-time PCR RESULTS: Zeb1 null mice late in gestation show ectopic expression of epithelial genes in the corneal endothelium and keratocytes, including the basement membrane component COL4A3, which is ectopically expressed by the corneal endothelium in PPCD. These embryos also show abnormal corneal endothelial and keratocyte proliferation, corneal thickening, and corneolenticular and iridocorneal adhesions. Adult Zeb1 heterozygous mice exhibit these same corneal defects. The ectopic expression of epithelial genes extended to embryonic fibroblasts derived from Zeb1 heterozygous and null mice, suggesting that Zeb1 may have a more general role in the suppression of an epithelial phenotype.The authors conclude that Zeb1 heterozygous and null mice show features of PPCD and thus should provide an animal model for genetic dissection of pathways contributing to the disease.
13. Sequential inductions of the ZEB1 transcription factor caused by mutation of Rb and then Ras proteins are required for tumor initiation and progression. Rb1 restricts cell cycle progression, and it imposes cell contact inhibition to suppress tumor outgrowth. It also triggers oncogene-induced senescence to block Ras mutation. Loss of the Rb1 pathway, which is a hallmark of cancer cells, then provides a permissive environment for Ras mutation, and Ras is sufficient for invasive tumor formation in Rb1 family mutant mouse embryo fibroblasts (MEFs). These results demonstrate that sequential mutation of the Rb1 and Ras pathways comprises a tumor initiation axis. Both Rb1 and Ras regulate expression of the transcription factor ZEB1, thereby linking tumor initiation to the subsequent invasion and **metastasis**, which is induced by ZEB1. ZEB1 acts in a negative feedback loop to block expression of miR-200, which is thought to facilitate tumor invasion and **metastasis**. However, ZEB1 also represses cyclin-dependent kinase (cdk) inhibitors to control the cell cycle; its mutation in MEFs leads to induction of these inhibitors and premature senescence. Here, we provide evidence for two sequential inductions of ZEB1 during Ras transformation of MEFs. Rb1 constitutively represses cdk inhibitors, and induction of ZEB1 when the Rb1 pathway is lost is required to maintain this repression, allowing for the classic immortalization and loss of cell contact inhibition seen when the Rb1 pathway is lost. In vivo, we show that this induction of ZEB1 is required for Ras-initiated tumor formation. ZEB1 is then further induced by Ras, beyond the level seen with Rb1 mutation, and this Ras superinduction is required to reach a threshold of ZEB1 sufficient for repression of miR-200 and tumor invasion.
14. ASPP2 controls epithelial plasticity and inhibits **metastasis** through β-catenin-dependent regulation of ZEB1. Epithelial to mesenchymal transition (EMT), and the reverse mesenchymal to epithelial transition (MET), are known examples of epithelial plasticity that are important in kidney development and cancer **metastasi**s. Here we identify ASPP2, a haploinsufficient tumour suppressor, p53 activator and PAR3 binding partner, as a molecular switch of MET and EMT. ASPP2 contributes to MET in mouse kidney in vivo. Mechanistically, ASPP2 induces MET through its PAR3-binding amino-terminus, independently of p53 binding. ASPP2 prevents β-catenin from transactivating ZEB1, directly by forming an ASPP2-β-catenin-E-cadherin ternary complex and indirectly by inhibiting β-catenin's N-terminal phosphorylation to stabilize the β-catenin-E-cadherin complex. ASPP2 limits the pro-invasive property of oncogenic RAS and inhibits tu**mour metas**tasis in vivo. Reduced ASPP2 expression results in EMT, and is associated with poor survival in hepatocellular carcinoma and breast cancer patients. Hence, ASPP2 is a key regulator of epithelial plasticity that connects cell polarity to the suppression of WNT signalling, EMT and tu**mour metas**tasis.
15. Zeb1 links epithelial-mesenchymal transition and cellular senescence. Overexpression of zinc finger E-box binding homeobox transcription factor 1 (Zeb1) in cancer leads to epithelial-to-mesenchymal transition (EMT) and increased **metastasis**. As opposed to overexpression, we show that mutation of Zeb1 in mice causes a mesenchymal-epithelial transition in gene expression characterized by ectopic expression of epithelial genes such as E-cadherin and loss of expression of mesenchymal genes such as vimentin. In contrast to rapid proliferation in cancer cells where Zeb1 is overexpressed, this mesenchymal-epithelial transition in mutant mice is associated with diminished proliferation of progenitor cells at sites of developmental defects, including the forming palate, skeleton and CNS. Zeb1 dosage-dependent deregulation of epithelial and mesenchymal genes extends to mouse embryonic fibroblasts (MEFs), and mutant MEFs also display diminished replicative capacity in culture, leading to premature senescence. Replicative senescence in MEFs is classically triggered by products of the Ink4a (Cdkn2a) gene. However, this Ink4a pathway is not activated during senescence of Zeb1 mutant MEFs. Instead, there is ectopic expression of two other cell cycle inhibitory cyclin-dependent kinase inhibitors, p15Ink4b (Cdkn2b) and p21Cdkn1a (Cdkn1a). We demonstrate that this ectopic expression of p15Ink4b extends in vivo to sites of diminished progenitor cell proliferation and developmental defects in Zeb1-null mice.

#### GeneRIFs

1. Ras induction of ZEB1 is required for tumour initiation in a lung adenocarcinoma model via Pten repression. A lower threshold of ZEB1 is sufficient for cancer initiation, whereas further induction is necessary for tumour **metastasis**.

### [Cc]ell [Ii]nvasion

#### PubMed

1. C-reactive protein inhibits expression of N-cadherin and ZEB-1 in murine colon adenocarcinoma. Epithelial-to-mesenchymal transition (EMT) is thought to play a key role in cancer **cell invasion** and metastasis. We previously demonstrated that cancer cell migration is inhibited by C-reactive protein (CRP), which is widely used as a biomarker of inflammation, though its functions are not fully understood. In the present study, we evaluated the effect of CRP on cancer cell migration and expression of mesenchymal and epithelial markers of EMT and of related transcription factors. MCA-38 murine colon adenocarcinoma cells were subcutaneously inoculated into the backs of C57BL/6 mice, which also received 1 μg of recombinant mouse CRP or vehicle (phosphate-buffered saline) subcutaneously every 3 days for 4 weeks. Thereafter, the mice were sacrificed for evaluation using quantitative real-time polymerase chain reaction (PCR) and immunohistochemistry. There was no statistical difference in tumor size between the control and CRP groups, but CRP dose-dependently inhibited MCA-38 cell migration. PCR analysis confirmed that CRP suppresses expression of N-cadherin (p < 0.01), a mesenchymal marker of EMT, and ZEB-1, an EMT-related transcription factor (p < 0.01). These findings suggest that CRP inhibits EMT in a MCA-38 tumor-bearing mouse model. CRP may thus be a potentially useful tool for preventing cancer progression through suppression of EMT.
2. Integral role of transcription factor 8 in the negative regulation of tumor angiogenesis. Angiogenesis is involved in various physiologic and pathological conditions, including tumor growth, and is tightly regulated by the orchestration of proangiogenic and antiangiogenic factors. Inhibition of vascular endothelial growth factor (VEGF), the best-established antiangiogenic treatment in cancer, has shown some effectiveness; however, the identification of novel regulators, whose function is independent of VEGF, is required to achieve better outcomes. Here, we show that transcription factor 8 (TCF8) is up-regulated in endothelial cells during angiogenesis, acting as a negative regulator. Furthermore, TCF8 is specifically expressed in the endothelium of tumor vessels. Tcf8-heterozygous knockout mice are more permissive than wild-type mice to the formation of tumor blood vessels in s.c. implanted melanoma, which seems to contribute to the more aggressive growth and the lung metastases of the tumor in mutant mice. Suppression of TCF8 facilitates angiogenesis in both in vitro and ex vivo models, and displays comprehensive cellular phenotypes, including enhanced **cell invasion**, impaired cell adhesion, and increased cell monolayer permeability due to, at least partly, MMP1 overexpression, attenuation of focal adhesion formation, and insufficient VE-cadherin recruitment, respectively. Taken together, our findings define a novel, integral role for TCF8 in the regulation of pathologic angiogenesis, and propose TCF8 as a target for therapeutic intervention in cancer.

---

## Zeb2

- NCBI Gene
- GeneRIF
- Pubmed

| Search Term | Rank | Fields |
| --- | --- | --- |
| [Mm]igration | 6 | Process(2); PubMed(20); GeneRIFs(2) |
| [Ee]xtracellular [Mm]atrix | 8 | PubMed(2) |
| [Mm]etastasize | 11 | PubMed(1) |
| [Mm]etastasis | 12 | PubMed(7) |
| [Cc]ell [Ii]nvasion | 13 | PubMed(1) |

  
hide/show details for Zeb2

### [Mm]igration

#### Process

1. melanocyte **migration**
2. neural crest cell **migration**

#### PubMed

1. Loss of Sip1 leads to **migration** defects and retention of ectodermal markers during lens development. SIP1 encodes a DNA-binding transcription factor that regulates multiple developmental processes, as highlighted by the pleiotropic defects observed in Mowat-Wilson syndrome, which results from mutations in this gene. Further, in adults, dysregulated SIP1 expression has been implicated in both cancer and fibrotic diseases, where it functionally links TGFβ signaling to the loss of epithelial cell characteristics and gene expression. In the ocular lens, an epithelial tissue important for vision, Sip1 is co-expressed with epithelial markers, such as E-cadherin, and is required for the complete separation of the lens vesicle from the head ectoderm during early ocular morphogenesis. However, the function of Sip1 after early lens morphogenesis is still unknown. Here, we conditionally deleted Sip1 from the developing mouse lens shortly after lens vesicle closure, leading to defects in coordinated fiber cell tip **migratio**n, defective suture formation, and cataract. Interestingly, RNA-Sequencing analysis on Sip1 knockout lenses identified 190 differentially expressed genes, all of which are distinct from previously described Sip1 target genes. Furthermore, 34% of the genes with increased expression in the Sip1 knockout lenses are normally downregulated as the lens transitions from the lens vesicle to early lens, while 49% of the genes with decreased expression in the Sip1 knockout lenses are normally upregulated during early lens development. Overall, these data imply that Sip1 plays a major role in reprogramming the lens vesicle away from a surface ectoderm cell fate towards that necessary for the development of a transparent lens and demonstrate that Sip1 regulates distinctly different sets of genes in different cellular contexts.
2. Fezl is required for the birth and specification of corticospinal motor neurons. The molecular mechanisms controlling the differentiation of neural progenitors into distinct subtypes of neurons during neocortical development are unknown. Here, we report that Fezl is required for the specification of corticospinal motor neurons and other subcerebral projection neurons, which are absent from Fezl null mutant neocortex. There is neither an increase in cell death in Fezl(-/-) cortex nor abnormalities in **migration**, indicating that the absence of subcerebral projection neurons is due to a failure in fate specification. In striking contrast, other neuronal populations in the same and other cortical layers are born normally. Overexpression of Fezl results in excess production of subcerebral projection neurons and arrested **migration** of these neurons in the germinal zone. These data indicate that Fezl plays a central role in the specification of corticospinal motor neurons and other subcerebral projection neurons, controlling early decisions regarding lineage-specific differentiation from neural progenitors.
3. The miR-200 family inhibits epithelial-mesenchymal transition and cancer cell **migration** by direct targeting of E-cadherin transcriptional repressors ZEB1 and ZEB2. MicroRNAs are small non-coding RNA molecules that can regulate gene expression by interacting with multiple mRNAs and inducing either translation suppression or degradation of mRNA. Recently, several miRNAs were identified as either promoters or suppressors of metastasis. However, it is unclear in which step(s) of the multistep metastatic cascade these miRNAs play a defined functional role. To study the functional importance of miRNAs in epithelial-mesenchymal transition (EMT), a process thought to initiate metastasis by enhancing the motility of tumor cells, we used a well established in vitro EMT assay: transforming growth factor-beta-induced EMT in NMuMG murine mammary epithelial cells. We found that members of the miR-200 family, organized as two clusters in the genome, were repressed during EMT. Overexpression of each miRNA individually or as clusters in NMuMG cells hindered EMT by enhancing E-cadherin expression through direct targeting of ZEB1 and ZEB2, which encode transcriptional repressors of E-cadherin. In the 4TO7 mouse carcinoma cell line, which expresses low levels of endogenous E-cadherin and displays a mesenchymal phenotype, ectopic expression of the miR-200 family miRNAs significantly increased E-cadherin expression and altered cell morphology to an epithelial phenotype. Furthermore, ectopic expression of each miR-200 miRNA cluster significantly reduced the in vitro motility of 4TO7 cells in **migration** assays. These results suggested that loss of expression of the miR-200 family members may play a critical role in the repression of E-cadherin by ZEB1 and ZEB2 during EMT, thereby enhancing **migration** and invasion during cancer progression.
4. miR-200b regulates cell **migration** via Zeb family during mouse palate development. Palate development requires coordinating proper cellular and molecular events in palatogenesis, including the epithelial-mesenchymal transition (EMT), apoptosis, cell proliferation, and cell **migration**. Zeb1 and Zeb2 regulate epithelial cadherin (E-cadherin) and EMT during organogenesis. While microRNA 200b (miR-200b) is known to be a negative regulator of Zeb1 and Zeb2 in cancer progression, its regulatory effects on Zeb1 and Zeb2 in palatogenesis have not yet been clarified. The aim of this study is to investigate the relationship between the regulators of palatal development, specifically, miR-200b and the Zeb family. Expression of both Zeb1 and Zeb2 was detected in the mesenchyme of the mouse palate, while miR-200b was expressed in the medial edge epithelium. After contact with the palatal shelves, miR-200b was expressed in the palatal epithelial lining and epithelial island around the fusion region but not in the palatal mesenchyme. The function of miR-200b was examined by overexpression via a lentiviral vector in the palatal shelves. Ectopic expression of miR-200b resulted in suppression of the Zeb family, upregulation of E-cadherin, and changes in cell **migration** and palatal fusion. These results suggest that miR-200b plays crucial roles in cell **migration** and palatal fusion by regulating Zeb1 and Zeb2 as a noncoding RNA during palate development.
5. TMPRSS2/ERG promotes epithelial to mesenchymal transition through the ZEB1/ZEB2 axis in a prostate cancer model. Prostate cancer is the most common non-dermatologic malignancy in men in the Western world. Recently, a frequent chromosomal aberration fusing androgen regulated TMPRSS2 promoter and the ERG gene (TMPRSS2/ERG) was discovered in prostate cancer. Several studies demonstrated cooperation between TMPRSS2/ERG and other defective pathways in cancer progression. However, the unveiling of more specific pathways in which TMPRSS2/ERG takes part, requires further investigation. Using immortalized prostate epithelial cells we were able to show that TMPRSS2/ERG over-expressing cells undergo an Epithelial to Mesenchymal Transition (EMT), manifested by acquisition of mesenchymal morphology and markers as well as **migration** and invasion capabilities. These findings were corroborated in vivo, where the control cells gave rise to discrete nodules while the TMPRSS2/ERG-expressing cells formed malignant tumors, which expressed EMT markers. To further investigate the general transcription scheme induced by TMPRSS2/ERG, cells were subjected to a microarray analysis that revealed a distinct EMT expression program, including up-regulation of the EMT facilitators, ZEB1 and ZEB2, and down-regulation of the epithelial marker CDH1(E-Cadherin). A chromatin immunoprecipitation assay revealed direct binding of TMPRSS2/ERG to the promoter of ZEB1 but not ZEB2. However, TMPRSS2/ERG was able to bind the promoters of the ZEB2 modulators, IL1R2 and SPINT1. This set of experiments further illuminates the mechanism by which the TMPRSS2/ERG fusion affects prostate cancer progression and might assist in targeting TMPRSS2/ERG and its downstream targets in future drug design efforts.
6. Identification of a ZEB2-MITF-ZEB1 transcriptional network that controls melanogenesis and melanoma progression. Deregulation of signaling pathways that control differentiation, expansion and **migration** of neural crest-derived melanoblasts during normal development contributes also to melanoma progression and metastasis. Although several epithelial-to-mesenchymal (EMT) transcription factors, such as zinc finger E-box binding protein 1 (ZEB1) and ZEB2, have been implicated in neural crest cell biology, little is known about their role in melanocyte homeostasis and melanoma. Here we show that mice lacking Zeb2 in the melanocyte lineage exhibit a melanoblast **migration** defect and, unexpectedly, a severe melanocyte differentiation defect. Loss of Zeb2 in the melanocyte lineage results in a downregulation of the Microphthalmia-associated transcription factor (Mitf) and melanocyte differentiation markers concomitant with an upregulation of Zeb1. We identify a transcriptional signaling network in which the EMT transcription factor ZEB2 regulates MITF levels to control melanocyte differentiation. Moreover, our data are also relevant for human melanomagenesis as loss of ZEB2 expression is associated with reduced patient survival.
7. Rap1 GTPase is required for mouse lens epithelial maintenance and morphogenesis. Rap1, a Ras-like small GTPase, plays a crucial role in cell-matrix adhesive interactions, cell-cell junction formation, cell polarity and **migration**. The role of Rap1 in vertebrate organ development and tissue architecture, however, remains elusive. We addressed this question in a mouse lens model system using a conditional gene targeting approach. While individual germline deficiency of either Rap1a or Rap1b did not cause overt defects in mouse lens, conditional double deficiency (Rap1 cKO) prior to lens placode formation led to an ocular phenotype including microphthalmia and lens opacification in embryonic mice. The embryonic Rap1 cKO mouse lens exhibited striking defects including loss of E-cadherin- and ZO-1-based cell-cell junctions, disruption of paxillin and β1-integrin-based cell adhesive interactions along with abnormalities in cell shape and apical-basal polarity of epithelium. These epithelial changes were accompanied by increased levels of α-smooth muscle actin, vimentin and N-cadherin, and expression of transcriptional suppressors of E-cadherin (Snai1, Slug and Zeb2), and a mesenchymal metabolic protein (Dihydropyrimidine dehydrogenase). Additionally, while lens differentiation was not overtly affected, increased apoptosis and dysregulated cell cycle progression were noted in epithelium and fibers in Rap1 cKO mice. Collectively these observations uncover a requirement for Rap1 in maintenance of lens epithelial phenotype and morphogenesis.
8. Maternal DNA Methylation Regulates Early Trophoblast Development. Critical roles for DNA methylation in embryonic development are well established, but less is known about its roles during trophoblast development, the extraembryonic lineage that gives rise to the placenta. We dissected the role of DNA methylation in trophoblast development by performing mRNA and DNA methylation profiling of Dnmt3a/3b mutants. We find that oocyte-derived methylation plays a major role in regulating trophoblast development but that imprinting of the key placental regulator Ascl2 is only partially responsible for these effects. We have identified several methylation-regulated genes associated with trophoblast differentiation that are involved in cell adhesion and **migration**, potentially affecting trophoblast invasion. Specifically, trophoblast-specific DNA methylation is linked to the silencing of Scml2, a Polycomb Repressive Complex 1 protein that drives loss of cell adhesion in methylation-deficient trophoblast. Our results reveal that maternal DNA methylation controls multiple differentiation-related and physiological processes in trophoblast via both imprinting-dependent and -independent mechanisms.
9. Mouse models of Hirschsprung disease and other developmental disorders of the enteric nervous system: Old and new players. Hirschsprung disease (HSCR, intestinal aganglionosis) is a multigenic disorder with variable penetrance and severity that has a general population incidence of 1/5000 live births. Studies using animal models have contributed to our understanding of the developmental origins of HSCR and the genetic complexity of this disease. This review summarizes recent progress in understanding control of enteric nervous system (ENS) development through analyses in mouse models. An overview of signaling pathways that have long been known to control the **migration**, proliferation and differentiation of enteric neural progenitors into and along the developing gut is provided as a framework for the latest information on factors that influence enteric ganglia formation and maintenance. Newly identified genes and additional factors beyond discrete genes that contribute to ENS pathology including regulatory sequences, miRNAs and environmental factors are also introduced. Finally, because HSCR has become a paradigm for complex oligogenic diseases with non-Mendelian inheritance, the importance of gene interactions, modifier genes, and initial studies on genetic background effects are outlined.
10. Identification of a ZEB2-MITF-ZEB1 transcriptional network that controls melanogenesis and melanoma progression. Deregulation of signaling pathways that control differentiation, expansion and **migration** of neural crest-derived melanoblasts during normal development contributes also to melanoma progression and metastasis. Although several epithelial-to-mesenchymal (EMT) transcription factors, such as zinc finger E-box binding protein 1 (ZEB1) and ZEB2, have been implicated in neural crest cell biology, little is known about their role in melanocyte homeostasis and melanoma. Here we show that mice lacking Zeb2 in the melanocyte lineage exhibit a melanoblast **migration** defect and, unexpectedly, a severe melanocyte differentiation defect. Loss of Zeb2 in the melanocyte lineage results in a downregulation of the Microphthalmia-associated transcription factor (Mitf) and melanocyte differentiation markers concomitant with an upregulation of Zeb1. We identify a transcriptional signaling network in which the EMT transcription factor ZEB2 regulates MITF levels to control melanocyte differentiation. Moreover, our data are also relevant for human melanomagenesis as loss of ZEB2 expression is associated with reduced patient survival.
11. Loss of Sip1 leads to **migration** defects and retention of ectodermal markers during lens development. SIP1 encodes a DNA-binding transcription factor that regulates multiple developmental processes, as highlighted by the pleiotropic defects observed in Mowat-Wilson syndrome, which results from mutations in this gene. Further, in adults, dysregulated SIP1 expression has been implicated in both cancer and fibrotic diseases, where it functionally links TGFβ signaling to the loss of epithelial cell characteristics and gene expression. In the ocular lens, an epithelial tissue important for vision, Sip1 is co-expressed with epithelial markers, such as E-cadherin, and is required for the complete separation of the lens vesicle from the head ectoderm during early ocular morphogenesis. However, the function of Sip1 after early lens morphogenesis is still unknown. Here, we conditionally deleted Sip1 from the developing mouse lens shortly after lens vesicle closure, leading to defects in coordinated fiber cell tip **migratio**n, defective suture formation, and cataract. Interestingly, RNA-Sequencing analysis on Sip1 knockout lenses identified 190 differentially expressed genes, all of which are distinct from previously described Sip1 target genes. Furthermore, 34% of the genes with increased expression in the Sip1 knockout lenses are normally downregulated as the lens transitions from the lens vesicle to early lens, while 49% of the genes with decreased expression in the Sip1 knockout lenses are normally upregulated during early lens development. Overall, these data imply that Sip1 plays a major role in reprogramming the lens vesicle away from a surface ectoderm cell fate towards that necessary for the development of a transparent lens and demonstrate that Sip1 regulates distinctly different sets of genes in different cellular contexts.
12. miR-200b regulates cell **migration** via Zeb family during mouse palate development. Palate development requires coordinating proper cellular and molecular events in palatogenesis, including the epithelial-mesenchymal transition (EMT), apoptosis, cell proliferation, and cell **migration**. Zeb1 and Zeb2 regulate epithelial cadherin (E-cadherin) and EMT during organogenesis. While microRNA 200b (miR-200b) is known to be a negative regulator of Zeb1 and Zeb2 in cancer progression, its regulatory effects on Zeb1 and Zeb2 in palatogenesis have not yet been clarified. The aim of this study is to investigate the relationship between the regulators of palatal development, specifically, miR-200b and the Zeb family. Expression of both Zeb1 and Zeb2 was detected in the mesenchyme of the mouse palate, while miR-200b was expressed in the medial edge epithelium. After contact with the palatal shelves, miR-200b was expressed in the palatal epithelial lining and epithelial island around the fusion region but not in the palatal mesenchyme. The function of miR-200b was examined by overexpression via a lentiviral vector in the palatal shelves. Ectopic expression of miR-200b resulted in suppression of the Zeb family, upregulation of E-cadherin, and changes in cell **migration** and palatal fusion. These results suggest that miR-200b plays crucial roles in cell **migration** and palatal fusion by regulating Zeb1 and Zeb2 as a noncoding RNA during palate development.
13. TMPRSS2/ERG promotes epithelial to mesenchymal transition through the ZEB1/ZEB2 axis in a prostate cancer model. Prostate cancer is the most common non-dermatologic malignancy in men in the Western world. Recently, a frequent chromosomal aberration fusing androgen regulated TMPRSS2 promoter and the ERG gene (TMPRSS2/ERG) was discovered in prostate cancer. Several studies demonstrated cooperation between TMPRSS2/ERG and other defective pathways in cancer progression. However, the unveiling of more specific pathways in which TMPRSS2/ERG takes part, requires further investigation. Using immortalized prostate epithelial cells we were able to show that TMPRSS2/ERG over-expressing cells undergo an Epithelial to Mesenchymal Transition (EMT), manifested by acquisition of mesenchymal morphology and markers as well as **migration** and invasion capabilities. These findings were corroborated in vivo, where the control cells gave rise to discrete nodules while the TMPRSS2/ERG-expressing cells formed malignant tumors, which expressed EMT markers. To further investigate the general transcription scheme induced by TMPRSS2/ERG, cells were subjected to a microarray analysis that revealed a distinct EMT expression program, including up-regulation of the EMT facilitators, ZEB1 and ZEB2, and down-regulation of the epithelial marker CDH1(E-Cadherin). A chromatin immunoprecipitation assay revealed direct binding of TMPRSS2/ERG to the promoter of ZEB1 but not ZEB2. However, TMPRSS2/ERG was able to bind the promoters of the ZEB2 modulators, IL1R2 and SPINT1. This set of experiments further illuminates the mechanism by which the TMPRSS2/ERG fusion affects prostate cancer progression and might assist in targeting TMPRSS2/ERG and its downstream targets in future drug design efforts.
14. The miR-200 family inhibits epithelial-mesenchymal transition and cancer cell **migration** by direct targeting of E-cadherin transcriptional repressors ZEB1 and ZEB2. MicroRNAs are small non-coding RNA molecules that can regulate gene expression by interacting with multiple mRNAs and inducing either translation suppression or degradation of mRNA. Recently, several miRNAs were identified as either promoters or suppressors of metastasis. However, it is unclear in which step(s) of the multistep metastatic cascade these miRNAs play a defined functional role. To study the functional importance of miRNAs in epithelial-mesenchymal transition (EMT), a process thought to initiate metastasis by enhancing the motility of tumor cells, we used a well established in vitro EMT assay: transforming growth factor-beta-induced EMT in NMuMG murine mammary epithelial cells. We found that members of the miR-200 family, organized as two clusters in the genome, were repressed during EMT. Overexpression of each miRNA individually or as clusters in NMuMG cells hindered EMT by enhancing E-cadherin expression through direct targeting of ZEB1 and ZEB2, which encode transcriptional repressors of E-cadherin. In the 4TO7 mouse carcinoma cell line, which expresses low levels of endogenous E-cadherin and displays a mesenchymal phenotype, ectopic expression of the miR-200 family miRNAs significantly increased E-cadherin expression and altered cell morphology to an epithelial phenotype. Furthermore, ectopic expression of each miR-200 miRNA cluster significantly reduced the in vitro motility of 4TO7 cells in **migration** assays. These results suggested that loss of expression of the miR-200 family members may play a critical role in the repression of E-cadherin by ZEB1 and ZEB2 during EMT, thereby enhancing **migration** and invasion during cancer progression.
15. Aebp2 as an epigenetic regulator for neural crest cells. Aebp2 is a potential targeting protein for the mammalian Polycomb Repression Complex 2 (PRC2). We generated a mutant mouse line disrupting the transcription of Aebp2 to investigate its in vivo roles. Aebp2-mutant homozygotes were embryonic lethal while heterozygotes survived to adulthood with fertility. In developing mouse embryos, Aebp2 is expressed mainly within cells of neural crest origin. In addition, many heterozygotes display a set of phenotypes, enlarged colon and hypopigmentation, similar to those observed in human patients with Hirschsprung's disease and Waardenburg syndrome. These phenotypes are usually caused by the absence of the neural crest-derived ganglia in hindguts and melanocytes. ChIP analyses demonstrated that the majority of the genes involved in the **migration** and development process of neural crest cells are downstream target genes of AEBP2 and PRC2. Furthermore, expression analyses confirmed that some of these genes are indeed affected in the Aebp2 heterozygotes. Taken together, these results suggest that Aebp2 may regulate the **migration** and development of the neural crest cells through the PRC2-mediated epigenetic mechanism.
16. A novel mode of tangential **migration** of cortical projection neurons. Projection neurons of the developing cerebral cortex are generated in the cerebral ventricular zone and subsequently move to the developing cortical plate via radial **migration**. Conversely, most inhibitory interneurons originate in the ganglionic eminences and enter the developing cortical plate by tangential **migration**. Using immunohistochemical analysis together with tracer labeling experiments in organotypic brain slices, we show that a portion of cortical projection neurons migrates tangentially over long distances. Lineage analysis revealed that these neurons are derived from Emx1+ cortical progenitors and express the transcription factor Satb2 but do not express GABA or Olig1. In vitro and in vivo analysis of reeler mutant brains demonstrated that although reeler mutation does not influence tangential **migration** of interneurons, it affects the tangential **migration** of cortical projection neurons.
17. Directed **migration** of cortical interneurons depends on the cell-autonomous action of Sip1. GABAergic interneurons mainly originate in the medial ganglionic eminence (MGE) of the embryonic ventral telencephalon (VT) and migrate tangentially to the cortex, guided by membrane-bound and secreted factors. We found that Sip1 (Zfhx1b, Zeb2), a transcription factor enriched in migrating cortical interneurons, is required for their proper differentiation and correct guidance. The majority of Sip1 knockout interneurons fail to migrate to the neocortex and stall in the VT. RNA sequencing reveals that Sip1 knockout interneurons do not acquire a fully mature cortical interneuron identity and contain increased levels of the repulsive receptor Unc5b. Focal electroporation of Unc5b-encoding vectors in the MGE of wild-type brain slices disturbs **migration** to the neocortex, whereas reducing Unc5b levels in Sip1 knockout slices and brains rescues the **migration** defect. Our results reveal that Sip1, through tuning of Unc5b levels, is essential for cortical interneuron guidance.
18. PROP1 triggers epithelial-mesenchymal transition-like process in pituitary stem cells. Mutations in PROP1 are the most common cause of hypopituitarism in humans; therefore, unraveling its mechanism of action is highly relevant from a therapeutic perspective. Our current understanding of the role of PROP1 in the pituitary gland is limited to the repression and activation of the pituitary transcription factor genes Hesx1 and Pou1f1, respectively. To elucidate the comprehensive PROP1-dependent gene regulatory network, we conducted genome-wide analysis of PROP1 DNA binding and effects on gene expression in mutant mice, mouse isolated stem cells and engineered mouse cell lines. We determined that PROP1 is essential for stimulating stem cells to undergo an epithelial to mesenchymal transition-like process necessary for cell **migration** and differentiation. Genomic profiling reveals that PROP1 binds to genes expressed in epithelial cells like Claudin 23, and to EMT inducer genes like Zeb2, Notch2 and Gli2. Zeb2 activation appears to be a key step in the EMT process. Our findings identify PROP1 as a central transcriptional component of pituitary stem cell differentiation.
19. Reduced endothelin converting enzyme-1 and endothelin-3 mRNA in the developing bowel of male mice may increase expressivity and penetrance of Hirschsprung disease-like distal intestinal aganglionosis. Hirschsprung disease (distal intestinal aganglionosis, HSCR) is a multigenic disorder with incomplete penetrance, variable expressivity, and a strong male gender bias. Recent studies demonstrated that these genetic patterns arise because gene interactions determine whether enteric nervous system (ENS) precursors successfully proliferate and migrate into the distal bowel. We now demonstrate that male gender bias in the extent of distal intestinal aganglionosis occurs in mice with Ret dominant-negative mutations (RetDN) that mimic human HSCR. We hypothesized that male gender bias could result from reduced expression of a gene already known to be essential for ENS development. Using quantitative real-time polymerase chain reaction (PCR) we demonstrated reduced levels of endothelin converting enzyme-1 and endothelin-3 mRNA in the male mouse bowel at the time that ENS precursors migrate into the colon. Other HSCR-associated genes are expressed at comparable levels in male and female mice. Testosterone and Mullerian inhibiting substance had no deleterious effect on ENS precursor development, but adding EDN3 peptide to E11.5 male RetDN heterozygous mouse gut explants in organ culture significantly increased the rate of ENS precursor **migration** through the bowel.
20. Downregulation of Sphingosine 1-Phosphate Receptor 1 Promotes the Switch from Tangential to Radial **Migration** in the OB. Neuroblast **migration** is a highly orchestrated process that ensures the proper integration of newborn neurons into complex neuronal circuits. In the postnatal rodent brain, neuroblasts migrate long distances from the subependymal zone of the lateral ventricles to the olfactory bulb (OB) within the rostral migratory stream (RMS). They first migrate tangentially in close contact to each other and later radially as single cells until they reach their final destination in the OB. Sphingosine 1-phosphate (S1P) is a bioactive lipid that interacts with cell-surface receptors to exert different cellular responses. Although well studied in other systems and a target for the treatment of multiple sclerosis, little is known about S1P in the postnatal brain. Here, we report that the S1P receptor 1 (S1P1) is expressed in neuroblasts migrating in the RMS. Using in vivo and in vitro gain- and loss-of-function approaches in both wild-type and transgenic mice, we found that the activation of S1P1 by its natural ligand S1P, acting as a paracrine signal, contributes to maintain neuroblasts attached to each other while they migrate in chains within the RMS. Once in the OB, neuroblasts cease to express S1P1, which results in cell detachment and initiation of radial **migration**, likely via downregulation of NCAM1 and β1 integrin. Our results reveal a novel physiological function for S1P1 in the postnatal brain, directing the path followed by newborn neurons in the neurogenic niche.The function of each neuron is highly determined by the position it occupies within a neuronal circuit. Frequently, newborn neurons must travel long distances from their birthplace to their predetermined final location and, to do so, they use different modes of **migratio**n. In this study, we identify the sphingosine 1-phosphate (S1P) receptor 1 (S1P1) as one of the key players that govern the switch from tangential to radial **migratio**n of postnatally generated neuroblasts in the olfactory bulb. Of interest is the evidence that the ligand, S1P, is provided by nearby astrocytes. Finally, we also propose adhesion molecules that act downstream of S1P1 and initiate the transition from tangential chain **migratio**n to individual radial **migratio**n outside of the stream.

#### GeneRIFs

1. This study identify the sphingosine 1-phosphate (S1P) receptor 1 (S1P1) as one of the key players that govern the switch from tangential to radial **migration** of postnatally generated neuroblasts in the olfactory bulb.
2. loss of expression of the miR-200 family members may play a critical role in the repression of E-cadherin by ZEB1 and ZEB2 during EMT, thereby enhancing **migration** and invasion during cancer progression.

### [Ee]xtracellular [Mm]atrix

#### PubMed

1. MicroRNA-192 in diabetic kidney glomeruli and its function in TGF-beta-induced collagen expression via inhibition of E-box repressors. Key features of diabetic nephropathy (DN) include the accumulation of **extracellular matrix** proteins such as collagen 1-alpha 1 and -2 (Col1a1 and -2). Transforming growth factor beta1 (TGF-beta), a key regulator of these **extracellular matrix** genes, is increased in mesangial cells (MC) in DN. By microarray profiling, we noted that TGF-beta increased Col1a2 mRNA in mouse MC (MMC) but also decreased mRNA levels of an E-box repressor, deltaEF1. TGF-beta treatment or short hairpin RNAs targeting deltaEF1 increased enhancer activity of upstream E-box elements in the Col1a2 gene. TGF-beta also decreased the expression of Smad-interacting protein 1 (SIP1), another E-box repressor similar to deltaEF1. Interestingly, we noted that SIP1 is a target of microRNA-192 (miR-192), a key miR highly expressed in the kidney. miR-192 levels also were increased by TGF-beta in MMC. TGF-beta treatment or transfection with miR-192 decreased endogenous SIP1 expression as well as reporter activity of a SIP1 3' UTR-containing luciferase construct in MMC. Conversely, a miR-192 inhibitor enhanced the luciferase activity, confirming SIP1 to be a miR-192 target. Furthermore, miR-192 synergized with deltaEF1 short hairpin RNAs to increase Col1a2 E-box-luc activity. Importantly, the in vivo relevance was noted by the observation that miR-192 levels were enhanced significantly in glomeruli isolated from streptozotocin-injected diabetic mice as well as diabetic db/db mice relative to corresponding nondiabetic controls, in parallel with increased TGF-beta and Col1a2 levels. These results uncover a role for miRs in the kidney and DN in controlling TGF-beta-induced Col1a2 expression by down-regulating E-box repressors.
2. MicroRNA-192 in diabetic kidney glomeruli and its function in TGF-beta-induced collagen expression via inhibition of E-box repressors. Key features of diabetic nephropathy (DN) include the accumulation of **extracellular matrix** proteins such as collagen 1-alpha 1 and -2 (Col1a1 and -2). Transforming growth factor beta1 (TGF-beta), a key regulator of these **extracellular matrix** genes, is increased in mesangial cells (MC) in DN. By microarray profiling, we noted that TGF-beta increased Col1a2 mRNA in mouse MC (MMC) but also decreased mRNA levels of an E-box repressor, deltaEF1. TGF-beta treatment or short hairpin RNAs targeting deltaEF1 increased enhancer activity of upstream E-box elements in the Col1a2 gene. TGF-beta also decreased the expression of Smad-interacting protein 1 (SIP1), another E-box repressor similar to deltaEF1. Interestingly, we noted that SIP1 is a target of microRNA-192 (miR-192), a key miR highly expressed in the kidney. miR-192 levels also were increased by TGF-beta in MMC. TGF-beta treatment or transfection with miR-192 decreased endogenous SIP1 expression as well as reporter activity of a SIP1 3' UTR-containing luciferase construct in MMC. Conversely, a miR-192 inhibitor enhanced the luciferase activity, confirming SIP1 to be a miR-192 target. Furthermore, miR-192 synergized with deltaEF1 short hairpin RNAs to increase Col1a2 E-box-luc activity. Importantly, the in vivo relevance was noted by the observation that miR-192 levels were enhanced significantly in glomeruli isolated from streptozotocin-injected diabetic mice as well as diabetic db/db mice relative to corresponding nondiabetic controls, in parallel with increased TGF-beta and Col1a2 levels. These results uncover a role for miRs in the kidney and DN in controlling TGF-beta-induced Col1a2 expression by down-regulating E-box repressors.

### [Mm]etastasize

#### PubMed

1. miR-200 enhances mouse breast cancer cell colonization to form distant metastases. The development of metastases involves the dissociation of cells from the primary tumor to penetrate the basement membrane, invade and then exit the vasculature to seed, and colonize distant tissues. The last step, establishment of macroscopic tumors at distant sites, is the least well understood. Four isogenic mouse breast cancer cell lines (67NR, 168FARN, 4TO7, and 4T1) that differ in their ability to **metastasize** when implanted into the mammary fat pad are used to model the steps of metastasis. Only 4T1 forms macroscopic lung and liver metastases. Because some miRNAs are dysregulated in cancer and affect cellular transformation, tumor formation, and metastasis, we examined whether changes in miRNA expression might explain the differences in metastasis of these cells.miRNA expression was analyzed by miRNA microarray and quantitative RT-PCR in isogenic mouse breast cancer cells with distinct metastatic capabilities. 4T1 cells that form macroscopic metastases had elevated expression of miR-200 family miRNAs compared to related cells that invade distant tissues, but are unable to colonize. Moreover, over-expressing miR-200 in 4TO7 cells enabled them to **metastasize** to lung and liver. These findings are surprising since the miR-200 family was previously shown to promote epithelial characteristics by inhibiting the transcriptional repressor Zeb2 and thereby enhancing E-cadherin expression. We confirmed these findings in these cells. The most metastatic 4T1 cells acquired epithelial properties (high expression of E-cadherin and cytokeratin-18) compared to the less metastatic cells.Expression of miR-200, which promotes a mesenchymal to epithelial cell transition (MET) by inhibiting Zeb2 expression, unexpectedly enhances macroscopic metastases in mouse breast cancer cell lines. These results suggest that for some tumors, tumor colonization at metastatic sites might be enhanced by MET. Therefore the epithelial nature of a tumor does not predict metastatic outcome.

### [Mm]etastasis

#### PubMed

1. The miR-200 family inhibits epithelial-mesenchymal transition and cancer cell migration by direct targeting of E-cadherin transcriptional repressors ZEB1 and ZEB2. MicroRNAs are small non-coding RNA molecules that can regulate gene expression by interacting with multiple mRNAs and inducing either translation suppression or degradation of mRNA. Recently, several miRNAs were identified as either promoters or suppressors of **metastasis**. However, it is unclear in which step(s) of the multistep metastatic cascade these miRNAs play a defined functional role. To study the functional importance of miRNAs in epithelial-mesenchymal transition (EMT), a process thought to initiate **metastasis** by enhancing the motility of tumor cells, we used a well established in vitro EMT assay: transforming growth factor-beta-induced EMT in NMuMG murine mammary epithelial cells. We found that members of the miR-200 family, organized as two clusters in the genome, were repressed during EMT. Overexpression of each miRNA individually or as clusters in NMuMG cells hindered EMT by enhancing E-cadherin expression through direct targeting of ZEB1 and ZEB2, which encode transcriptional repressors of E-cadherin. In the 4TO7 mouse carcinoma cell line, which expresses low levels of endogenous E-cadherin and displays a mesenchymal phenotype, ectopic expression of the miR-200 family miRNAs significantly increased E-cadherin expression and altered cell morphology to an epithelial phenotype. Furthermore, ectopic expression of each miR-200 miRNA cluster significantly reduced the in vitro motility of 4TO7 cells in migration assays. These results suggested that loss of expression of the miR-200 family members may play a critical role in the repression of E-cadherin by ZEB1 and ZEB2 during EMT, thereby enhancing migration and invasion during cancer progression.
2. A double-negative feedback loop between ZEB1-SIP1 and the microRNA-200 family regulates epithelial-mesenchymal transition. Epithelial to mesenchymal transition occurs during embryologic development to allow tissue remodeling and is proposed to be a key step in the **metastasis** of epithelial-derived tumors. The miR-200 family of microRNAs plays a major role in specifying the epithelial phenotype by preventing expression of the transcription repressors, ZEB1/deltaEF1 and SIP1/ZEB2. We show here that miR-200a, miR-200b, and the related miR-429 are all encoded on a 7.5-kb polycistronic primary miRNA (pri-miR) transcript. We show that the promoter for the pri-miR is located within a 300-bp segment located 4 kb upstream of miR-200b. This promoter region is sufficient to confer expression in epithelial cells and is repressed in mesenchymal cells by ZEB1 and SIP1 through their binding to a conserved pair of ZEB-type E-box elements located proximal to the transcription start site. These findings establish a double-negative feedback loop controlling ZEB1-SIP1 and miR-200 family expression that regulates cellular phenotype and has direct relevance to the role of these factors in tumor progression.
3. Identification of a ZEB2-MITF-ZEB1 transcriptional network that controls melanogenesis and melanoma progression. Deregulation of signaling pathways that control differentiation, expansion and migration of neural crest-derived melanoblasts during normal development contributes also to melanoma progression and **metastasis**. Although several epithelial-to-mesenchymal (EMT) transcription factors, such as zinc finger E-box binding protein 1 (ZEB1) and ZEB2, have been implicated in neural crest cell biology, little is known about their role in melanocyte homeostasis and melanoma. Here we show that mice lacking Zeb2 in the melanocyte lineage exhibit a melanoblast migration defect and, unexpectedly, a severe melanocyte differentiation defect. Loss of Zeb2 in the melanocyte lineage results in a downregulation of the Microphthalmia-associated transcription factor (Mitf) and melanocyte differentiation markers concomitant with an upregulation of Zeb1. We identify a transcriptional signaling network in which the EMT transcription factor ZEB2 regulates MITF levels to control melanocyte differentiation. Moreover, our data are also relevant for human melanomagenesis as loss of ZEB2 expression is associated with reduced patient survival.
4. Identification of a ZEB2-MITF-ZEB1 transcriptional network that controls melanogenesis and melanoma progression. Deregulation of signaling pathways that control differentiation, expansion and migration of neural crest-derived melanoblasts during normal development contributes also to melanoma progression and **metastasis**. Although several epithelial-to-mesenchymal (EMT) transcription factors, such as zinc finger E-box binding protein 1 (ZEB1) and ZEB2, have been implicated in neural crest cell biology, little is known about their role in melanocyte homeostasis and melanoma. Here we show that mice lacking Zeb2 in the melanocyte lineage exhibit a melanoblast migration defect and, unexpectedly, a severe melanocyte differentiation defect. Loss of Zeb2 in the melanocyte lineage results in a downregulation of the Microphthalmia-associated transcription factor (Mitf) and melanocyte differentiation markers concomitant with an upregulation of Zeb1. We identify a transcriptional signaling network in which the EMT transcription factor ZEB2 regulates MITF levels to control melanocyte differentiation. Moreover, our data are also relevant for human melanomagenesis as loss of ZEB2 expression is associated with reduced patient survival.
5. The miR-200 family inhibits epithelial-mesenchymal transition and cancer cell migration by direct targeting of E-cadherin transcriptional repressors ZEB1 and ZEB2. MicroRNAs are small non-coding RNA molecules that can regulate gene expression by interacting with multiple mRNAs and inducing either translation suppression or degradation of mRNA. Recently, several miRNAs were identified as either promoters or suppressors of **metastasis**. However, it is unclear in which step(s) of the multistep metastatic cascade these miRNAs play a defined functional role. To study the functional importance of miRNAs in epithelial-mesenchymal transition (EMT), a process thought to initiate **metastasis** by enhancing the motility of tumor cells, we used a well established in vitro EMT assay: transforming growth factor-beta-induced EMT in NMuMG murine mammary epithelial cells. We found that members of the miR-200 family, organized as two clusters in the genome, were repressed during EMT. Overexpression of each miRNA individually or as clusters in NMuMG cells hindered EMT by enhancing E-cadherin expression through direct targeting of ZEB1 and ZEB2, which encode transcriptional repressors of E-cadherin. In the 4TO7 mouse carcinoma cell line, which expresses low levels of endogenous E-cadherin and displays a mesenchymal phenotype, ectopic expression of the miR-200 family miRNAs significantly increased E-cadherin expression and altered cell morphology to an epithelial phenotype. Furthermore, ectopic expression of each miR-200 miRNA cluster significantly reduced the in vitro motility of 4TO7 cells in migration assays. These results suggested that loss of expression of the miR-200 family members may play a critical role in the repression of E-cadherin by ZEB1 and ZEB2 during EMT, thereby enhancing migration and invasion during cancer progression.
6. Transcriptional repressor ZEB2 promotes terminal differentiation of CD8+ effector and memory T cell populations during infection. ZEB2 is a multi-zinc-finger transcription factor known to play a significant role in early neurogenesis and in epithelial-mesenchymal transition-dependent tumor **metastasis**. Although the function of ZEB2 in T lymphocytes is unknown, activity of the closely related family member ZEB1 has been implicated in lymphocyte development. Here, we find that ZEB2 expression is up-regulated by activated T cells, specifically in the KLRG1(hi) effector CD8(+) T cell subset. Loss of ZEB2 expression results in a significant loss of antigen-specific CD8(+) T cells after primary and secondary infection with a severe impairment in the generation of the KLRG1(hi) effector memory cell population. We show that ZEB2, which can bind DNA at tandem, consensus E-box sites, regulates gene expression of several E-protein targets and may directly repress Il7r and Il2 in CD8(+) T cells responding to infection. Furthermore, we find that T-bet binds to highly conserved T-box sites in the Zeb2 gene and that T-bet and ZEB2 regulate similar gene expression programs in effector T cells, suggesting that T-bet acts upstream and through regulation of ZEB2. Collectively, we place ZEB2 in a larger transcriptional network that is responsible for the balance between terminal differentiation and formation of memory CD8(+) T cells.
7. miR-200 enhances mouse breast cancer cell colonization to form distant metastases. The development of metastases involves the dissociation of cells from the primary tumor to penetrate the basement membrane, invade and then exit the vasculature to seed, and colonize distant tissues. The last step, establishment of macroscopic tumors at distant sites, is the least well understood. Four isogenic mouse breast cancer cell lines (67NR, 168FARN, 4TO7, and 4T1) that differ in their ability to metastasize when implanted into the mammary fat pad are used to model the steps of **metastasis**. Only 4T1 forms macroscopic lung and liver metastases. Because some miRNAs are dysregulated in cancer and affect cellular transformation, tumor formation, and **metastasis**, we examined whether changes in miRNA expression might explain the differences in **metastasis** of these cells.miRNA expression was analyzed by miRNA microarray and quantitative RT-PCR in isogenic mouse breast cancer cells with distinct metastatic capabilities. 4T1 cells that form macroscopic metastases had elevated expression of miR-200 family miRNAs compared to related cells that invade distant tissues, but are unable to colonize. Moreover, over-expressing miR-200 in 4TO7 cells enabled them to metastasize to lung and liver. These findings are surprising since the miR-200 family was previously shown to promote epithelial characteristics by inhibiting the transcriptional repressor Zeb2 and thereby enhancing E-cadherin expression. We confirmed these findings in these cells. The most metastatic 4T1 cells acquired epithelial properties (high expression of E-cadherin and cytokeratin-18) compared to the less metastatic cells.Expression of miR-200, which promotes a mesenchymal to epithelial cell transition (MET) by inhibiting Zeb2 expression, unexpectedly enhances macroscopic metastases in mouse breast cancer cell lines. These results suggest that for some tumors, tumor colonization at metastatic sites might be enhanced by MET. Therefore the epithelial nature of a tumor does not predict metastatic outcome.

### [Cc]ell [Ii]nvasion

#### PubMed

1. miR-200 promotes the mesenchymal to epithelial transition by suppressing multiple members of the Zeb2 and Snail1 transcriptional repressor complexes. The miR-200 family promotes the epithelial state by suppressing the Zeb1/Zeb2 epithelial gene transcriptional repressors. To identify other miR-200-regulated genes, we isolated mRNAs bound to transfected biotinylated miR-200c in mouse breast cancer cells. In all, 520 mRNAs were significantly enriched in miR-200c binding at least twofold. Putative miR-200-regulated genes included Zeb2, enriched 3.5-fold in the pull down. However, Zeb2 knockdown does not fully recapitulate miR-200c overexpression, suggesting that regulating other miR-200 targets contributes to miR-200's enhancement of epithelial gene expression. Candidate genes were highly enriched for miR-200c seed pairing in their 3'UTR and coding sequence and for genes that were downregulated by miR-200c overexpression. Epidermal growth factor receptor and downstream MAPK signaling pathways were the most enriched pathways. Genes whose products mediate transforming growth factor (TGF)-β signaling were also significantly overrepresented, and miR-200 counteracted the suppressive effects of TGF-β and bone morphogenic protein 2 (BMP-2) on epithelial gene expression. miR-200c regulated the 3'UTRs of 12 of 14 putative miR-200c-binding mRNAs tested. The extent of mRNA binding to miR-200c strongly correlated with gene suppression. Twelve targets of miR-200c (Crtap, Fhod1, Smad2, Map3k1, Tob1, Ywhag/14-3-3γ, Ywhab/14-3-3β, Smad5, Zfp36, Xbp1, Mapk12, Snail1) were experimentally validated by identifying their 3'UTR miR-200 recognition elements. Smad2 and Smad5 form a complex with Zeb2 and Ywhab/14-3-3β and Ywhag/14-3-3γ form a complex with Snail1. These complexes that repress transcription assemble on epithelial gene promoters. miR-200 overexpression induced RNA polymerase II localization and reduced Zeb2 and Snail1 binding to epithelial gene promoters. Expression of miR-200-resistant Smad5 modestly, but significantly, reduced epithelial gene induction by miR-200. miR-200 expression and Zeb2 knockdown are known to in**hibit cell in**vasion in in vitro assays. Knockdown of each of three novel miR-200 target genes identified here, Smad5, Ywhag and Crtap, also profoundly suppr**essed cell in**vasion. Thus, miR-200 suppresses TGF-β/BMP signaling, promotes epithelial gene expression and supp**resses cell i**nvasion by regulating a network of genes.

---

## Birc3

- NCBI Gene
- GeneRIF
- Pubmed

| Search Term | Rank | Fields |
| --- | --- | --- |
| [Ff]ilopodia | 1 | PubMed(1) |
| [Mm]etastasis | 12 | PubMed(1) |

  
hide/show details for Birc3

### [Ff]ilopodia

#### PubMed

1. cIAP1 regulates TNF-mediated cdc42 activation and **filopodia** formation. Tumour necrosis factor-α (TNF) is a cytokine endowed with multiple functions, depending on the cellular and environmental context. TNF receptor engagement induces the formation of a multimolecular complex including the TNFR-associated factor TRAF2, the receptor-interaction protein kinase RIP1 and the cellular inhibitor of apoptosis cIAP1, the latter being essential for NF-κB activation. Here, we show that cIAP1 also regulates TNF-induced actin cytoskeleton reorganization through a cdc42-dependent, NF-κB-independent pathway. Deletion of cIAP1 prevents TNF-induc**ed filopo**dia and cdc42 activation. The expression of cIAP1 or its E3-ubiquitin ligase-defective mutant restores the ability of cIAP1(-/-) MEFs to produ**ce filopo**dia, whereas a cIAP1 mutant unable to bind TRAF2 does not. Accordingly, the silencing of TRAF2 inhibits TNF-mediat**ed filopo**dia formation, whereas silencing of RIP1 does not. cIAP1 directly binds cdc42 and promotes its RhoGDIα-mediated stabilization. TNF decreases cIAP1-cdc42 interaction, suggesting that TNF-induced recruitment of cIAP1/TRAF2 to the receptor releases cdc42, which in turn triggers actin remodeling. cIAP1 also regulates cdc42 activation in response to EGF and HRas-V12 expression. A downregulation of cIAP1 altered the cell polarization, the cell adhesion to endothelial cells and cell intercalation, which are cdc42-dependent processes. Finally, we demonstrated that the deletion of cIAP1 regulated the HRas-V12-mediated transformation process, including anchorage-dependent cell growth, tumour growth in a xenograft model and the development of experimental metastasis in the lung.

### [Mm]etastasis

#### PubMed

1. cIAP1 regulates TNF-mediated cdc42 activation and filopodia formation. Tumour necrosis factor-α (TNF) is a cytokine endowed with multiple functions, depending on the cellular and environmental context. TNF receptor engagement induces the formation of a multimolecular complex including the TNFR-associated factor TRAF2, the receptor-interaction protein kinase RIP1 and the cellular inhibitor of apoptosis cIAP1, the latter being essential for NF-κB activation. Here, we show that cIAP1 also regulates TNF-induced actin cytoskeleton reorganization through a cdc42-dependent, NF-κB-independent pathway. Deletion of cIAP1 prevents TNF-induced filopodia and cdc42 activation. The expression of cIAP1 or its E3-ubiquitin ligase-defective mutant restores the ability of cIAP1(-/-) MEFs to produce filopodia, whereas a cIAP1 mutant unable to bind TRAF2 does not. Accordingly, the silencing of TRAF2 inhibits TNF-mediated filopodia formation, whereas silencing of RIP1 does not. cIAP1 directly binds cdc42 and promotes its RhoGDIα-mediated stabilization. TNF decreases cIAP1-cdc42 interaction, suggesting that TNF-induced recruitment of cIAP1/TRAF2 to the receptor releases cdc42, which in turn triggers actin remodeling. cIAP1 also regulates cdc42 activation in response to EGF and HRas-V12 expression. A downregulation of cIAP1 altered the cell polarization, the cell adhesion to endothelial cells and cell intercalation, which are cdc42-dependent processes. Finally, we demonstrated that the deletion of cIAP1 regulated the HRas-V12-mediated transformation process, including anchorage-dependent cell growth, tumour growth in a xenograft model and the development of experimen**tal metast**asis in the lung.

---

## Xiap

- NCBI Gene
- GeneRIF
- Pubmed

| Search Term | Rank | Fields |
| --- | --- | --- |
| [Mm]igration | 6 | PubMed(1) |
| ECM | 7 | PubMed(1) |
| [Ee]xtracellular [Mm]atrix | 8 | PubMed(2) |
| [Ii]nvasive | 10 | PubMed(1) |
| [Mm]etastasis | 12 | PubMed(2); GeneRIFs(1) |
| [Cc]ell [Ii]nvasion | 13 | PubMed(1) |

  
hide/show details for Xiap

### [Mm]igration

#### PubMed

1. IAPs regulate the plasticity of cell **migration** by directly targeting Rac1 for degradation. Inhibitors of apoptosis proteins (IAPs) are a highly conserved class of multifunctional proteins. Rac1 is a well-studied Rho GTPase that controls numerous basic cellular processes. While the regulation of nucleotide binding to Rac1 is well understood, the molecular mechanisms controlling Rac1 degradation are not known. Here, we demonstrate X-linked IAP (XIAP) and cellular IAP1 (c-IAP1) directly bind to Rac1 in a nucleotide-independent manner to promote its polyubiquitination at Lys147 and proteasomal degradation. These IAPs are also required for degradation of Rac1 upon CNF1 toxin treatment or RhoGDI depletion. Consistently, downregulation of XIAP or c-IAP1 by various strategies led to an increase in Rac1 protein levels in primary and tumour cells, leading to an elongated morphology and enhanced cell **migration**. Further, XIAP counteracts Rac1-dependent cellular polarization in the developing zebrafish hindbrain and promotes the delamination of neurons from the normal tissue architecture. These observations unveil an evolutionarily conserved role of IAPs in controlling Rac1 stability thereby regulating the plasticity of cell **migration** and morphogenesis.

### ECM

#### PubMed

1. ras Oncogene triggers up-regulation of cIAP2 and XIAP in intestinal epithelial cells: epidermal growth factor receptor-dependent and -independent mechanisms of ras-induced transformation. Detachment of normal epithelial cells from the extracellular matrix (**ECM**) triggers apoptosis, a phenomenon called anoikis. Conversely, carcinomas (cancers of epithelial origin) represent three-dimensional disorganized multicellular masses in which cells are deprived of adhesion to the **ECM** but remain viable. Resistance of cancer cells to anoikis is thought to be critical for tumor progression. However, the knowledge about molecular mechanisms of this type of resistance remains limited. Herein we report that ras oncogene, an established inhibitor of anoikis, triggers a significant upregulation of anti-apoptotic proteins cIAP2 and XIAP in intestinal epithelial cells. We also observed that the effect of ras on cIAP2 requires ras-induced autocrine production of transforming growth factor alpha (TGF-alpha), a ligand for epidermal growth factor receptor, whereas ras-triggered up-regulation of XIAP is TGF-alpha-independent. Moreover, overexpression of either cIAP2 or XIAP in nonmalignant intestinal epithelial cell was found to block anoikis. In addition, an established IAP antagonist Smac or Smac-derived cell-permeable peptide suppressed ras-induced anoikis resistance and subsequent anchorage-independent growth of ras-transformed cells. We conclude that ras-induced overexpression of cIAP2 and XIAP significantly contributes to the ability of ras-transformed intestinal epithelial cells to survive in the absence of adhesion to the **ECM** and grow in a three-dimensional manner.

### [Ee]xtracellular [Mm]atrix

#### PubMed

1. Role for X-linked Inhibitor of apoptosis protein upstream of mitochondrial permeabilization. Apoptosis is controlled by a signaling equilibrium between prosurvival and proapoptotic pathways, such that unwanted apoptosis is avoided, but when required it occurs rapidly and efficiently. Many apoptosis regulators display dual roles, depending upon whether a cell has received an apoptotic stimulus or not. Here, we identify a novel and unexpected function for X-linked inhibitor of apoptosis (XIAP) that occurs when apoptosis is triggered under physiological conditions. We show that in response to loss of survival signals provided by cell adhesion, endogenous XIAP translocates from the cytosol into a mitochondrial 400-kDa complex and that this occurs very early in the apoptosis process. Membrane-associated XIAP induces mitochondrial outer membrane permeabilization leading to cytochrome c and Smac release, which is dependent on Bax and Bak. Thus, although XIAP suppresses apoptosis in healthy cells, our data indicate that XIAP may contribute to it in response to a proapoptotic signal such as loss of **extracellular matrix**-dependent survival signaling. We suggest that, as with Bcl-2 family proteins, more diverse functions for XIAP exist than previously identified. Moreover, switching the function of proteins from anti- to proapoptotic forms may be a common theme in the efficient execution of cell death.
2. ras Oncogene triggers up-regulation of cIAP2 and XIAP in intestinal epithelial cells: epidermal growth factor receptor-dependent and -independent mechanisms of ras-induced transformation. Detachment of normal epithelial cells from the **extracellular matrix** (ECM) triggers apoptosis, a phenomenon called anoikis. Conversely, carcinomas (cancers of epithelial origin) represent three-dimensional disorganized multicellular masses in which cells are deprived of adhesion to the ECM but remain viable. Resistance of cancer cells to anoikis is thought to be critical for tumor progression. However, the knowledge about molecular mechanisms of this type of resistance remains limited. Herein we report that ras oncogene, an established inhibitor of anoikis, triggers a significant upregulation of anti-apoptotic proteins cIAP2 and XIAP in intestinal epithelial cells. We also observed that the effect of ras on cIAP2 requires ras-induced autocrine production of transforming growth factor alpha (TGF-alpha), a ligand for epidermal growth factor receptor, whereas ras-triggered up-regulation of XIAP is TGF-alpha-independent. Moreover, overexpression of either cIAP2 or XIAP in nonmalignant intestinal epithelial cell was found to block anoikis. In addition, an established IAP antagonist Smac or Smac-derived cell-permeable peptide suppressed ras-induced anoikis resistance and subsequent anchorage-independent growth of ras-transformed cells. We conclude that ras-induced overexpression of cIAP2 and XIAP significantly contributes to the ability of ras-transformed intestinal epithelial cells to survive in the absence of adhesion to the ECM and grow in a three-dimensional manner.

### [Ii]nvasive

#### PubMed

1. BID-dependent release of mitochondrial SMAC dampens XIAP-mediated immunity against Shigella. The X-linked inhibitor of apoptosis protein (XIAP) is a potent caspase inhibitor, best known for its anti-apoptotic function in cancer. During apoptosis, XIAP is antagonized by SMAC, which is released from the mitochondria upon caspase-mediated activation of BID. Recent studies suggest that XIAP is involved in immune signaling. Here, we explore XIAP as an important mediator of an immune response against the entero**invasive** bacterium Shigella flexneri, both in vitro and in vivo. Our data demonstrate for the first time that Shigella evades the XIAP-mediated immune response by inducing the BID-dependent release of SMAC from the mitochondria. Unlike apoptotic stimuli, Shigella activates the calpain-dependent cleavage of BID to trigger the release of SMAC, which antagonizes the inflammatory action of XIAP without inducing apoptosis. Our results demonstrate how the cellular death machinery can be subverted by an **invasive** pathogen to ensure bacterial colonization.

### [Mm]etastasis

#### PubMed

1. IAP regulation of **metastasis**. Inhibitor-of-Apoptosis (IAP) proteins contribute to tumor progression, but the requirements of this pathway are not understood. Here, we show that intermolecular cooperation between XIAP and survivin stimulates tumor cell invasion and promotes **metastasis**. This pathway is independent of IAP inhibition of cell death. Instead, a survivin-XIAP complex activates NF-kappaB, which in turn leads to increased fibronectin gene expression, signaling by beta1 integrins, and activation of cell motility kinases FAK and Src. Therefore, IAPs are direct **metastasis** genes, and their antagonists could provide antimetastatic therapies in patients with cancer.
2. Ceramide targets xIAP and cIAP1 to sensitize metastatic colon and breast cancer cells to apoptosis induction to suppress tumor progression. Ceramide is a bioeffector that mediates various cellular processes, including apoptosis. However, the mechanism underlying ceramide function in apoptosis is apparently cell type-dependent and is not well-understood. We aimed at identifying molecular targets of ceramide in metastatic human colon and breast cancer cells, and determining the efficacy of ceramide analog in suppression of colon and breast cancer **metastasis**.The activity of and mechanism underlying ceramide as a cytotoxic agent, and as a sensitizer for Fas-mediated apoptosis was analyzed in human cell lines established from primary or metastatic colon and breast cancers. The efficacy of ceramide analog LCL85 in suppression of **metastasis** was examined in preclinical mouse tumor models.Exposure of human colon carcinoma cells to ceramide analog LCL85 results in apoptosis in a dose-dependent manner. Interestingly, a sublethal dose of LCL85 increased C16 ceramide content and overcame tumor cell resistance to Fas-mediated apoptosis. Subsequently, treatment of tumor cells with exogenous C16 ceramide resulted in increased tumor cell sensitivity to Fas-mediated apoptosis. LCL85 resembles Smac mimetic BV6 in sensitization of colon carcinoma cells to Fas-mediated apoptosis by inducing proteasomal degradation of cIAP1 and xIAP proteins. LCL85 also decreased xIAP1 and cIAP1 protein levels and sensitized metastatic human breast cancer cells to Fas-mediated apoptosis. Silencing xIAP and cIAP1 with specific siRNAs significantly increased the metastatic human colon carcinoma cell sensitivity to Fas-mediated apoptosis, suggesting that IAP proteins mediate apoptosis resistance in metastatic human colon carcinoma cells and ceramide induces IAP protein degradation to sensitize the tumor cells to apoptosis induction. Consistent with its apoptosis sensitization activity, subtoxic doses of LCL85 suppressed colon carcinoma cell metastatic potential in an experimental lung **metastasis** mouse model, as well as breast cancer growth and spontaneous lung **metastasis** in an orthotopic breast cancer mouse model.We have identified xIAP and cIAP1 as molecular targets of ceramide and determined that ceramide analog LCL85 is an effective sensitizer in overcoming resistance of human cell lines established from metastatic colon and breast cancers to apoptosis induction to suppress **metastasis** in vivo.

#### GeneRIFs

1. Identify xIAP and cIAP1 as molecular targets of ceramide and show ceramide analog LCL85 is an effective sensitizer in overcoming resistance of metastatic colon and breast cancers to apoptosis induction to suppress **metastasis** in vivo.

### [Cc]ell [Ii]nvasion

#### PubMed

1. IAP regulation of metastasis. Inhibitor-of-Apoptosis (IAP) proteins contribute to tumor progression, but the requirements of this pathway are not understood. Here, we show that intermolecular cooperation between XIAP and survivin stimulates tumor **cell invasion** and promotes metastasis. This pathway is independent of IAP inhibition of cell death. Instead, a survivin-XIAP complex activates NF-kappaB, which in turn leads to increased fibronectin gene expression, signaling by beta1 integrins, and activation of cell motility kinases FAK and Src. Therefore, IAPs are direct metastasis genes, and their antagonists could provide antimetastatic therapies in patients with cancer.

---

## Birc2

- NCBI Gene
- GeneRIF
- Pubmed

| Search Term | Rank | Fields |
| --- | --- | --- |
| [Mm]igration | 6 | PubMed(1) |
| [Ii]nvasive | 10 | PubMed(1) |
| [Mm]etastasis | 12 | PubMed(2); GeneRIFs(1) |

  
hide/show details for Birc2

### [Mm]igration

#### PubMed

1. IAPs regulate the plasticity of cell **migration** by directly targeting Rac1 for degradation. Inhibitors of apoptosis proteins (IAPs) are a highly conserved class of multifunctional proteins. Rac1 is a well-studied Rho GTPase that controls numerous basic cellular processes. While the regulation of nucleotide binding to Rac1 is well understood, the molecular mechanisms controlling Rac1 degradation are not known. Here, we demonstrate X-linked IAP (XIAP) and cellular IAP1 (c-IAP1) directly bind to Rac1 in a nucleotide-independent manner to promote its polyubiquitination at Lys147 and proteasomal degradation. These IAPs are also required for degradation of Rac1 upon CNF1 toxin treatment or RhoGDI depletion. Consistently, downregulation of XIAP or c-IAP1 by various strategies led to an increase in Rac1 protein levels in primary and tumour cells, leading to an elongated morphology and enhanced cell **migration**. Further, XIAP counteracts Rac1-dependent cellular polarization in the developing zebrafish hindbrain and promotes the delamination of neurons from the normal tissue architecture. These observations unveil an evolutionarily conserved role of IAPs in controlling Rac1 stability thereby regulating the plasticity of cell **migration** and morphogenesis.

### [Ii]nvasive

#### PubMed

1. cIAP-1 controls innate immunity to C. pneumoniae pulmonary infection. The resistance of epithelial cells infected with Chlamydophila pneumoniae for apoptosis has been attributed to the induced expression and increased stability of anti-apoptotic proteins called inhibitor of apoptosis proteins (IAPs). The significance of cellular inhibitor of apoptosis protein-1 (cIAP-1) in C. pneumoniae pulmonary infection and innate immune response was investigated in cIAP-1 knockout (KO) mice using a novel non-**invasive** intra-tracheal infection method. In contrast to wildtype, cIAP-1 knockout mice failed to clear the infection from their lungs. Wildtype mice responded to infection with a strong inflammatory response in the lung. In contrast, the recruitment of macrophages was reduced in cIAP-1 KO mice compared to wildtype mice. The concentration of Interferon gamma (IFN-gamma) was increased whereas that of Tumor Necrosis Factor (TNF-alpha) was reduced in the lungs of infected cIAP-1 KO mice compared to infected wildtype mice. Ex vivo experiments on mouse peritoneal macrophages and splenocytes revealed that cIAP-1 is required for innate immune responses of these cells. Our findings thus suggest a new immunoregulatory role of cIAP-1 in the course of bacterial infection.

### [Mm]etastasis

#### PubMed

1. Ceramide targets xIAP and cIAP1 to sensitize metastatic colon and breast cancer cells to apoptosis induction to suppress tumor progression. Ceramide is a bioeffector that mediates various cellular processes, including apoptosis. However, the mechanism underlying ceramide function in apoptosis is apparently cell type-dependent and is not well-understood. We aimed at identifying molecular targets of ceramide in metastatic human colon and breast cancer cells, and determining the efficacy of ceramide analog in suppression of colon and breast cancer **metastasis**.The activity of and mechanism underlying ceramide as a cytotoxic agent, and as a sensitizer for Fas-mediated apoptosis was analyzed in human cell lines established from primary or metastatic colon and breast cancers. The efficacy of ceramide analog LCL85 in suppression of **metastasis** was examined in preclinical mouse tumor models.Exposure of human colon carcinoma cells to ceramide analog LCL85 results in apoptosis in a dose-dependent manner. Interestingly, a sublethal dose of LCL85 increased C16 ceramide content and overcame tumor cell resistance to Fas-mediated apoptosis. Subsequently, treatment of tumor cells with exogenous C16 ceramide resulted in increased tumor cell sensitivity to Fas-mediated apoptosis. LCL85 resembles Smac mimetic BV6 in sensitization of colon carcinoma cells to Fas-mediated apoptosis by inducing proteasomal degradation of cIAP1 and xIAP proteins. LCL85 also decreased xIAP1 and cIAP1 protein levels and sensitized metastatic human breast cancer cells to Fas-mediated apoptosis. Silencing xIAP and cIAP1 with specific siRNAs significantly increased the metastatic human colon carcinoma cell sensitivity to Fas-mediated apoptosis, suggesting that IAP proteins mediate apoptosis resistance in metastatic human colon carcinoma cells and ceramide induces IAP protein degradation to sensitize the tumor cells to apoptosis induction. Consistent with its apoptosis sensitization activity, subtoxic doses of LCL85 suppressed colon carcinoma cell metastatic potential in an experimental lung **metastasis** mouse model, as well as breast cancer growth and spontaneous lung **metastasis** in an orthotopic breast cancer mouse model.We have identified xIAP and cIAP1 as molecular targets of ceramide and determined that ceramide analog LCL85 is an effective sensitizer in overcoming resistance of human cell lines established from metastatic colon and breast cancers to apoptosis induction to suppress **metastasis** in vivo.
2. Ceramide targets xIAP and cIAP1 to sensitize metastatic colon and breast cancer cells to apoptosis induction to suppress tumor progression. Ceramide is a bioeffector that mediates various cellular processes, including apoptosis. However, the mechanism underlying ceramide function in apoptosis is apparently cell type-dependent and is not well-understood. We aimed at identifying molecular targets of ceramide in metastatic human colon and breast cancer cells, and determining the efficacy of ceramide analog in suppression of colon and breast cancer **metastasis**.The activity of and mechanism underlying ceramide as a cytotoxic agent, and as a sensitizer for Fas-mediated apoptosis was analyzed in human cell lines established from primary or metastatic colon and breast cancers. The efficacy of ceramide analog LCL85 in suppression of **metastasis** was examined in preclinical mouse tumor models.Exposure of human colon carcinoma cells to ceramide analog LCL85 results in apoptosis in a dose-dependent manner. Interestingly, a sublethal dose of LCL85 increased C16 ceramide content and overcame tumor cell resistance to Fas-mediated apoptosis. Subsequently, treatment of tumor cells with exogenous C16 ceramide resulted in increased tumor cell sensitivity to Fas-mediated apoptosis. LCL85 resembles Smac mimetic BV6 in sensitization of colon carcinoma cells to Fas-mediated apoptosis by inducing proteasomal degradation of cIAP1 and xIAP proteins. LCL85 also decreased xIAP1 and cIAP1 protein levels and sensitized metastatic human breast cancer cells to Fas-mediated apoptosis. Silencing xIAP and cIAP1 with specific siRNAs significantly increased the metastatic human colon carcinoma cell sensitivity to Fas-mediated apoptosis, suggesting that IAP proteins mediate apoptosis resistance in metastatic human colon carcinoma cells and ceramide induces IAP protein degradation to sensitize the tumor cells to apoptosis induction. Consistent with its apoptosis sensitization activity, subtoxic doses of LCL85 suppressed colon carcinoma cell metastatic potential in an experimental lung **metastasis** mouse model, as well as breast cancer growth and spontaneous lung **metastasis** in an orthotopic breast cancer mouse model.We have identified xIAP and cIAP1 as molecular targets of ceramide and determined that ceramide analog LCL85 is an effective sensitizer in overcoming resistance of human cell lines established from metastatic colon and breast cancers to apoptosis induction to suppress **metastasis** in vivo.

#### GeneRIFs

1. Identify xIAP and cIAP1 as molecular targets of ceramide and show ceramide analog LCL85 is an effective sensitizer in overcoming resistance of metastatic colon and breast cancers to apoptosis induction to suppress **metastasis** in vivo.

---

## Fmn2

- NCBI Gene
- GeneRIF
- Pubmed

| Search Term | Rank | Fields |
| --- | --- | --- |
| [Ff]ilopodia | 1 | PubMed(1) |
| [Mm]igration | 6 | PubMed(4); GeneRIFs(1) |

  
hide/show details for Fmn2

### [Ff]ilopodia

#### PubMed

1. Formin 2 regulates the stabilization of **filopodia**l tip adhesions in growth cones and affects neuronal outgrowth and pathfinding in vivo. Growth cone **filopodia** are actin-based mechanosensory structures that are essential for chemoreception and the generation of contractile forces necessary for directional motility. However, little is known about the influence of **filopodia**l actin structures on substrate adhesion and **filopodia**l contractility. Formin 2 (Fmn2) localizes along **filopodia**l actin bundles and its depletion does not affect **filopodia** initiation or elongation. However, Fmn2 activity is required for **filopodia**l tip adhesion maturation and the ability of **filopodia** to generate traction forces. Dysregulation of **filopodia** in Fmn2-depleted neurons leads to compromised growth cone motility. Additionally, in mouse fibroblasts, Fmn2 regulates ventral stress fiber assembly and affects the stability of focal adhesions. In the developing chick spinal cord, Fmn2 activity is required cell-autonomously for the outgrowth and pathfinding of spinal commissural neurons. Our results reveal an unanticipated function for Fmn2 in neural development. Fmn2 regulates structurally diverse bundled actin structures, parallel **filopodia**l bundles in growth cones and anti-parallel stress fibers in fibroblasts, in turn modulating the stability of substrate adhesions. We propose Fmn2 as a mediator of actin bundle integrity, enabling efficient force transmission to the adhesion sites.

### [Mm]igration

#### PubMed

1. PtdIns(3,4,5)P3 is constitutively synthesized and required for spindle translocation during meiosis in mouse oocytes. Prior to ovulation, mammalian oocytes complete their first meiotic division and arrest at metaphase II. During this marked asymmetric cell division, the meiotic spindle moves dramatically from the center of the oocyte to the cortex to facilitate segregation of half of its chromosomal content into the diminutive first polar body. Recent investigations have documented crucial roles for filamentous actin (F-actin) in meiotic spindle translocation. However, the identity of the upstream regulators responsible for these carefully orchestrated movements has remained elusive. Utilizing fluorescently tagged probes and time-lapse confocal microscopy, we document that phosphatidylinositol 3,4,5-trisphosphate [PtdIns(3,4,5)P3] is constitutively synthesized with spatial and temporal dynamics similar to that of F-actin and Formin 2 (Fmn2). Blockage of PtdIns(3,4,5)P3 synthesis by LY294002, a specific inhibitor of phosphoinositide 3-kinase (PI3K), disrupts cytoplasmic F-actin organization and meiotic spindle **migration** to the cortex. F-actin nucleator Fmn2 and Rho GTPase Cdc42 play roles in mediating the effect of PtdIns(3,4,5)P3 on F-actin assembly. Moreover, the spatial and temporal dynamics of PtdIns(3,4,5)P3 is impaired by depletion of MATER or Filia, two oocyte proteins encoded by maternal effect genes. Thus, PtdIns(3,4,5)P3 is synthesized during meiotic maturation and acts upstream of Cdc42 and Fmn2, but downstream of MATER/Filia proteins to regulate the F-actin organization and spindle translocation to the cortex during mouse oocyte meiosis.
2. Sequential actin-based pushing forces drive meiosis I chromosome **migration** and symmetry breaking in oocytes. Polar body extrusion during oocyte maturation is critically dependent on asymmetric positioning of the meiotic spindle, which is established through **migration** of the meiosis I (MI) spindle/chromosomes from the oocyte interior to a subcortical location. In this study, we show that MI chromosome **migration** is biphasic and driven by consecutive actin-based pushing forces regulated by two actin nucleators, Fmn2, a formin family protein, and the Arp2/3 complex. Fmn2 was recruited to endoplasmic reticulum structures surrounding the MI spindle, where it nucleated actin filaments to initiate an initially slow and poorly directed motion of the spindle away from the cell center. A fast and highly directed second **migration** phase was driven by actin-mediated cytoplasmic streaming and occurred as the chromosomes reach a sufficient proximity to the cortex to activate the Arp2/3 complex. We propose that decisive symmetry breaking in mouse oocytes results from Fmn2-mediated perturbation of spindle position and the positive feedback loop between chromosome signal-induced Arp2/3 activation and Arp2/3-orchestrated cytoplasmic streaming that transports the chromosomes.
3. Spindle positioning in mouse oocytes relies on a dynamic meshwork of actin filaments. Female meiosis in higher organisms consists of highly asymmetric divisions, which retain most maternal stores in the oocyte for embryo development. Asymmetric partitioning of the cytoplasm results from the spindle's "off-center" positioning, which, in mouse oocytes, depends mainly on actin filaments [1, 2]. This is a unique situation compared to most systems, in which spindle positioning requires interactions between astral microtubules and cortical actin filaments [3]. Formin 2, a straight-actin-filament nucleator, is required for the first meiotic spindle **migration** to the cortex and cytokinesis in mouse oocytes [4, 5]. Although the requirement for actin filaments in the control of spindle positioning is well established in this model, no one has been able to detect them in the cytoplasm [6]. Through the expression of an F-actin-specific probe and live confocal microscopy, we show the presence of a cytoplasmic actin meshwork, organized by Formin 2, that controls spindle **migration**. In late meiosis I, these filaments organize into a spindle-like F-actin structure, which is connected to the cortex. At anaphase, global reorganization of this meshwork allows polar-body extrusion. In addition, using actin-YFP, our FRAP analysis confirms the presence of a highly dynamic cytoplasmic actin meshwork that is tightly regulated in time and space.
4. Symmetry breaking in mouse oocytes requires transient F-actin meshwork destabilization. Female meiotic divisions are extremely asymmetric, giving rise to a large oocyte and small degenerating polar bodies, keeping the maternal stores for further embryo development. This asymmetry is achieved via off-center positioning of the division spindle. Mouse oocytes have developed a formin-2-dependent actin-based spindle positioning mechanism that allows the meiotic spindle to migrate towards the closest cortex. Using spinning disk microscopy and FRAP analysis, we studied the changes in the organization of the cytoplasmic F-actin meshwork during the first meiotic division. It is very dense in prophase I, undergoes a significant density drop upon meiosis resumption and reforms progressively later on. This meshwork remodeling correlates with endogenous formin 2 regulation. High formin 2 levels at meiosis I entry induce meshwork maintenance, leading to equal forces being exerted on the chromosomes, preventing spindle **migration**. Hence, the meshwork density drop at meiosis resumption is germane to the symmetry-breaking event required for successful asymmetric meiotic divisions.

#### GeneRIFs

1. High formin 2 levels at meiosis I entry induce meshwork maintenance, leading to equal forces being exerted on the chromosomes, preventing spindle **migration**.

---

## Gab2

- NCBI Gene
- GeneRIF
- Pubmed

| Search Term | Rank | Fields |
| --- | --- | --- |
| [Mm]igration | 6 | Process(2); PubMed(5); GeneRIFs(2) |
| [Ii]nvasive | 10 | PubMed(1) |
| [Mm]etastasis | 12 | PubMed(1) |

  
hide/show details for Gab2

### [Mm]igration

#### Process

1. cell **migration**
2. positive regulation of epithelial cell **migration**

#### PubMed

1. Docking protein Gab1 is an essential component of postnatal angiogenesis after ischemia via HGF/c-met signaling. Grb2-associated binder (Gab) docking proteins, consisting of Gab1, Gab2, and Gab3, have crucial roles in growth factor-dependent signaling. Various proangiogenic growth factors regulate angiogenesis and endothelial function. However, the roles of Gab proteins in angiogenesis remain elusive.To elucidate the role of Gab proteins in postnatal angiogenesis.Endothelium-specific Gab1 knockout (Gab1ECKO) mice were viable and showed no obvious defects in vascular development. Therefore, we analyzed a hindlimb ischemia (HLI) model of control, Gab1ECKO, or conventional Gab2 knockout (Gab2KO) mice. Intriguingly, impaired blood flow recovery and necrosis in the operated limb was observed in all of Gab1ECKO, but not in control or Gab2KO mice. Among several proangiogenic growth factors, hepatocyte growth factor (HGF) induced the most prominent tyrosine phosphorylation of Gab1 and subsequent complex formation of Gab1 with SHP2 (Src homology-2-containing protein tyrosine phosphatase 2) and phosphatidylinositol 3-kinase subunit p85 in human endothelial cells (ECs). Gab1-SHP2 complex was required for HGF-induced **migration** and proliferation of ECs via extracellular signal-regulated kinase (ERK)1/2 pathway and for HGF-induced stabilization of ECs via ERK5. In contrast, Gab1-p85 complex regulated activation of AKT and contributed partially to **migration** of ECs after HGF stimulation. Microarray analysis demonstrated that HGF upregulated angiogenesis-related genes such as KLF2 (Krüppel-like factor 2) and Egr1 (early growth response 1) via Gab1-SHP2 complex in human ECs. In Gab1ECKO mice, gene transfer of vascular endothelial growth factor, but not HGF, improved blood flow recovery and ameliorated limb necrosis after HLI.Gab1 is essential for postnatal angiogenesis after ischemia via HGF/c-Met signaling.
2. Gab2 phosphorylation by RSK inhibits Shp2 recruitment and cell motility. The scaffolding adapter protein Gab2 (Grb2-associated binder) participates in the signaling response evoked by various growth factors and cytokines. Gab2 is overexpressed in several human malignancies, including breast cancer, and was shown to promote mammary epithelial cell **migration**. The role of Gab2 in the activation of different signaling pathways is well documented, but less is known regarding the feedback mechanisms responsible for its inactivation. We now demonstrate that activation of the Ras/mitogen-activated protein kinase (MAPK) pathway promotes Gab2 phosphorylation on basic consensus motifs. More specifically, we show that RSK (p90 ribosomal S6 kinase) phosphorylates Gab2 on three conserved residues, both in vivo and in vitro. Mutation of these phosphorylation sites does not alter Gab2 binding to Grb2, but instead, we show that Gab2 phosphorylation inhibits the recruitment of the tyrosine phosphatase Shp2 in response to growth factors. Expression of an unphosphorylatable Gab2 mutant in mammary epithelial cells promotes an invasion-like phenotype and increases cell motility. Taken together, these results suggest that RSK is part of a negative-feedback loop that restricts Gab2-dependent epithelial cell motility. On the basis of the widespread role of Gab2 in receptor signaling, these findings also suggest that RSK plays a regulatory function in diverse receptor systems.
3. Non-redundant roles of the Gab1 and Gab2 scaffolding adapters in VEGF-mediated signalling, **migration**, and survival of endothelial cells. Gab1 was previously described as a positive modulator of Akt, Src, ERK1/2, endothelial cell **migration**, and capillary formation in response to vascular endothelial growth factor (VEGF). However, its involvement in endothelial cell survival, as well as the potential contribution of the other family member Gab2 to signalling and biological responses remained unknown. Here, we show that Gab2 is tyrosine phosphorylated in a Grb2-dependent manner downstream of activated VEGF receptor-2 (VEGFR2), and that it associates with signalling proteins including PI3K and SHP2, but apparently not with the receptor. Similarly to Gab1, over-expression of Gab2 induces endothelial cell **migration** in response to VEGF, whereas its depletion using siRNAs results in its reduction. Importantly, depletion of both Gab1 and Gab2 leads to an even greater inhibition of VEGF-induced cell **migration**. However, contrary to what has been reported for Gab1, the silencing of Gab2 results in increased Src, Akt and ERK1/2 activation, slightly reduced p38 phosphorylation, and up-regulation of Gab1 protein levels. Accordingly, re-expression of Gab2 in Gab2-/- fibroblasts leads to opposite results, suggesting that the modulation of both Gab2 and Gab1 expression in these conditions might contribute to the impaired signalling observed. Consistent with their opposite roles on Akt, the depletion of Gab1, but not of Gab2, results in reduced FOXO1 phosphorylation and VEGF-mediated endothelial cell survival. Mutation of VEGFR2 Y801 and Y1214, which abrogates the phosphorylation of Gab1, also correlates with inhibition of Akt. Altogether, these results underscore the non-redundant and essential roles of Gab1 and Gab2 in endothelial cells, and suggest major contributions of these proteins during in vivo angiogenesis.
4. Role of Gab2 in mammary tumorigenesis and metastasis. Overexpression of the adaptor/scaffolding protein Gab2 has been detected in primary human breast cancer cells and cell lines, although its functional significance in breast carcinogenesis is not fully understood. Here, we show a requirement for Gab2 in promoting mammary tumor metastasis. Although Gab2 expression levels were elevated in mammary tumors induced by the Neu (ErbB-2) oncogene, homozygous deletion of Gab2 in mice had only a modest effect on the initiation of Neu-induced mammary tumors. Notably, ablation of Gab2 severely suppressed lung metastasis. Gab2-deficient cancer cells displayed normal Akt activities, and their proliferative rate in vitro was similar to control cells. However, Gab2(-/-) cancer cells exhibited decreased **migration** and impaired Erk activation, and the defects were rescued by re-introduction of Gab2 into Gab2(-/-) cells. These findings suggest that although Gab2 overexpression may confer growth advantage to tumor cells, the functional requirement for Gab2 in mammary tumor initiation/growth may be dispensable, and that Gab2 may have a prominent role in promoting mammary tumor metastasis.
5. Role of the docking protein Gab2 in beta(1)-integrin signaling pathway-mediated hematopoietic cell adhesion and **migration**. Gab2, a newly identified pleckstrin homology domain-containing docking protein, is a major binding protein of SHP-2 tyrosine phosphatase in interleukin (IL)-3-stimulated hematopoietic cells. Its signaling mechanism remains largely unknown. We report here an important regulatory role for Gab2 in beta(1) integrin signaling pathway that mediates hematopoietic cell adhesion and **migration**. Cross-linking of the beta(1) integrin on Ba/F3 cells induced rapid tyrosine phosphorylation of Gab2 and its association with Syk kinase, SHP-2 phosphatase, and the p85 subunit of phosphatidylinositol (PI)-3 kinase. In addition, Gab2 was also constitutively associated with SHP-1 phosphatase via its C-terminal Src homology 2 domain. Overexpression of the pleckstrin homology domain or a mutant Gab2 molecule lacking SHP-2 binding sites resulted in significant reductions in Ba/F3 cell adhesion and **migration**. Biochemical analyses revealed that enforced expression of Gab2 mutant molecules dramatically reduced beta(1)-integrin ligation-triggered PI3 kinase activation, whereas Erk kinase activation remained unaltered. Furthermore, transduction of primary hematopoietic progenitor cells from viable motheaten mice with these mutant Gab2 molecules also significantly ameliorated their enhanced **migration** capacity associated with the SHP1 gene mutation. Taken together, these results suggest an important signaling role for Gab2 in regulating hematopoietic cell adhesion and **migration**.

#### GeneRIFs

1. Gab2(-/-) mammary tumor cancer cells exhibited decreased **migration** and impaired Erk activation.
2. role in beta1-integrin signaling pathway hematooietic stem cell adhesion and **migration**

### [Ii]nvasive

#### PubMed

1. A role for the scaffolding adapter GAB2 in breast cancer. The scaffolding adapter GAB2 maps to a region (11q13-14) commonly amplified in human breast cancer, and is overexpressed in breast cancer cell lines and primary tumors, but its functional role in mammary carcinogenesis has remained unexplored. We found that overexpression of GAB2 (Grb2-associated binding protein 2) increases proliferation of MCF10A mammary cells in three-dimensional culture. Coexpression of GAB2 with antiapoptotic oncogenes causes lumenal filling, whereas coexpression with Neu (also known as ErbB2 and HER2) results in an **invasive** phenotype. These effects of GAB2 are mediated by hyperactivation of the Shp2-Erk pathway. Furthermore, overexpression of Gab2 potentiates, whereas deficiency of Gab2 ameliorates, Neu-evoked breast carcinogenesis in mice. Finally, GAB2 is amplified in some GAB2-overexpressing human breast tumors. Our data suggest that GAB2 may be a key gene within an 11q13 amplicon in human breast cancer and propose a role for overexpression of GAB2 in mammary carcinogenesis. Agents that target GAB2 or GAB2-dependent pathways may be useful for treating breast tumors that overexpress GAB2 or HER2 or both.

### [Mm]etastasis

#### PubMed

1. Role of Gab2 in mammary tumorigenesis and **metastasis**. Overexpression of the adaptor/scaffolding protein Gab2 has been detected in primary human breast cancer cells and cell lines, although its functional significance in breast carcinogenesis is not fully understood. Here, we show a requirement for Gab2 in promoting mammary tumor **metastasis**. Although Gab2 expression levels were elevated in mammary tumors induced by the Neu (ErbB-2) oncogene, homozygous deletion of Gab2 in mice had only a modest effect on the initiation of Neu-induced mammary tumors. Notably, ablation of Gab2 severely suppressed lung **metastasis**. Gab2-deficient cancer cells displayed normal Akt activities, and their proliferative rate in vitro was similar to control cells. However, Gab2(-/-) cancer cells exhibited decreased migration and impaired Erk activation, and the defects were rescued by re-introduction of Gab2 into Gab2(-/-) cells. These findings suggest that although Gab2 overexpression may confer growth advantage to tumor cells, the functional requirement for Gab2 in mammary tumor initiation/growth may be dispensable, and that Gab2 may have a prominent role in promoting mammary tumor **metastasis**.

---

## Gab1

- NCBI Gene
- GeneRIF
- Pubmed

| Search Term | Rank | Fields |
| --- | --- | --- |
| [Ff]ilopodia | 1 | PubMed(1) |
| [Mm]igration | 6 | Process(1); PubMed(9) |
| [Mm]etastasis | 12 | PubMed(1) |

  
hide/show details for Gab1

### [Ff]ilopodia

#### PubMed

1. Gab1 mediates hepatocyte growth factor-stimulated mitogenicity and morphogenesis in multipotent myeloid cells. Hepatocyte growth factor (HGF)-stimulated mitogenesis, motogenesis and morphogenesis in various cell types begins with activation of the Met receptor tyrosine kinase and the recruitment of intracellular adaptors and kinase substrates. The adapter protein Gab1 is a critical effector and substrate of activated Met, mediating morphogenesis, among other activities, in epithelial cells. To define its role downstream of Met in hematopoietic cells, Gab1 was expressed in the HGF-responsive, Gab1-negative murine myeloid cell line 32D. Interestingly, the adhesion and motility of Gab1-expressing cells were significantly greater than parental cells, independent of growth factor treatment. Downstream of activated Met, Gab1 expression was specifically associated with rapid Shp-2 recruitment and activation, increased mitogenic potency, suppression of GATA-1 expression and concomitant upregulation of GATA-2 transcription. In addition to enhanced proliferation, continuous culture of Gab1-expressing 32D cells in HGF resulted in cell attachment, **filopodia** extension and phenotypic changes suggestive of monocytic differentiation. Our results suggest that in myeloid cells, Gab1 is likely to enhance HGF mitogenicity by coupling Met to Shp-2 and GATA-2 expression, thereby potentially contributing to normal myeloid differentiation as well as oncogenic transformation.

### [Mm]igration

#### Process

1. regulation of cell **migration**

#### PubMed

1. Essential role of Gab1 for signaling by the c-Met receptor in vivo. The docking protein Gab1 binds phosphorylated c-Met receptor tyrosine kinase directly and mediates signals of c-Met in cell culture. Gab1 is phosphorylated by c-Met and by other receptor and nonreceptor tyrosine kinases. Here, we report the functional analysis of Gab1 by targeted mutagenesis in the mouse, and compare the phenotypes of the Gab1 and c-Met mutations. Gab1 is essential for several steps in development: **migration** of myogenic precursor cells into the limb anlage is impaired in Gab1-/- embryos. As a consequence, extensor muscle groups of the forelimbs are virtually absent, and the flexor muscles reach less far. Fewer hindlimb muscles exist, which are smaller and disorganized. Muscles in the diaphragm, which also originate from migratory precursors, are missing. Moreover, Gab1-/- embryos die in a broad time window between E13.5 and E18.5, and display reduced liver size and placental defects. The labyrinth layer, but not the spongiotrophoblast layer, of the placenta is severely reduced, resulting in impaired communication between maternal and fetal circulation. Thus, extensive similarities between the phenotypes of c-Met and HGF/SF mutant mice exist, and the muscle **migration** phenotype is even more pronounced in Gab1-/-:c-Met+/- embryos. This is genetic evidence that Gab1 is essential for c-Met signaling in vivo. Analogy exists to signal transmission by insulin receptors, which require IRS1 and IRS2 as specific docking proteins.
2. Distinct requirements for Gab1 in Met and EGF receptor signaling in vivo. Gab1 is a multiadaptor protein that has been shown to be required for multiple processes in embryonic development and oncogenic transformation. Gab1 functions by amplifying signal transduction downstream of various receptor tyrosine kinases through recruitment of multiple signaling effectors, including phosphatidylinositol 3-kinase and Shp2. Until now, the functional significance of individual interactions in vivo was not known. Here we have generated knockin mice that carry point mutations in either the P13K or Shp2 binding sites of Gab1. We show that different effector interactions with Gab1 play distinct biological roles downstream of Gab1 during the development of different organs. Recruitment of phosphatidylinositol 3-kinase by Gab1 is essential for EGF receptor-mediated embryonic eyelid closure and keratinocyte **migration**, and the Gab1-Shp2 interaction is crucial for Met receptor-directed placental development and muscle progenitor cell **migration** to the limbs. Furthermore, we investigate the dual association of Gab1 with the Met receptor. By analyzing knockin mice with mutations in the Grb2 or Met binding site of Gab1, we show that the requirements for Gab1 recruitment to Met varies in different biological contexts. Either the direct or the indirect interaction of Gab1 with Met is sufficient for Met-dependent muscle precursor cell **migration**, whereas both modes of interaction are required and neither is sufficient for placenta development, liver growth, and palatal shelf closure. These data demonstrate that Gab1 induces different biological responses through the recruitment of distinct effectors and that different modes of recruitment for Gab1 are required in different organs.
3. Docking protein Gab1 is an essential component of postnatal angiogenesis after ischemia via HGF/c-met signaling. Grb2-associated binder (Gab) docking proteins, consisting of Gab1, Gab2, and Gab3, have crucial roles in growth factor-dependent signaling. Various proangiogenic growth factors regulate angiogenesis and endothelial function. However, the roles of Gab proteins in angiogenesis remain elusive.To elucidate the role of Gab proteins in postnatal angiogenesis.Endothelium-specific Gab1 knockout (Gab1ECKO) mice were viable and showed no obvious defects in vascular development. Therefore, we analyzed a hindlimb ischemia (HLI) model of control, Gab1ECKO, or conventional Gab2 knockout (Gab2KO) mice. Intriguingly, impaired blood flow recovery and necrosis in the operated limb was observed in all of Gab1ECKO, but not in control or Gab2KO mice. Among several proangiogenic growth factors, hepatocyte growth factor (HGF) induced the most prominent tyrosine phosphorylation of Gab1 and subsequent complex formation of Gab1 with SHP2 (Src homology-2-containing protein tyrosine phosphatase 2) and phosphatidylinositol 3-kinase subunit p85 in human endothelial cells (ECs). Gab1-SHP2 complex was required for HGF-induced **migration** and proliferation of ECs via extracellular signal-regulated kinase (ERK)1/2 pathway and for HGF-induced stabilization of ECs via ERK5. In contrast, Gab1-p85 complex regulated activation of AKT and contributed partially to **migration** of ECs after HGF stimulation. Microarray analysis demonstrated that HGF upregulated angiogenesis-related genes such as KLF2 (Krüppel-like factor 2) and Egr1 (early growth response 1) via Gab1-SHP2 complex in human ECs. In Gab1ECKO mice, gene transfer of vascular endothelial growth factor, but not HGF, improved blood flow recovery and ameliorated limb necrosis after HLI.Gab1 is essential for postnatal angiogenesis after ischemia via HGF/c-Met signaling.
4. Non-redundant roles of the Gab1 and Gab2 scaffolding adapters in VEGF-mediated signalling, **migration**, and survival of endothelial cells. Gab1 was previously described as a positive modulator of Akt, Src, ERK1/2, endothelial cell **migration**, and capillary formation in response to vascular endothelial growth factor (VEGF). However, its involvement in endothelial cell survival, as well as the potential contribution of the other family member Gab2 to signalling and biological responses remained unknown. Here, we show that Gab2 is tyrosine phosphorylated in a Grb2-dependent manner downstream of activated VEGF receptor-2 (VEGFR2), and that it associates with signalling proteins including PI3K and SHP2, but apparently not with the receptor. Similarly to Gab1, over-expression of Gab2 induces endothelial cell **migration** in response to VEGF, whereas its depletion using siRNAs results in its reduction. Importantly, depletion of both Gab1 and Gab2 leads to an even greater inhibition of VEGF-induced cell **migration**. However, contrary to what has been reported for Gab1, the silencing of Gab2 results in increased Src, Akt and ERK1/2 activation, slightly reduced p38 phosphorylation, and up-regulation of Gab1 protein levels. Accordingly, re-expression of Gab2 in Gab2-/- fibroblasts leads to opposite results, suggesting that the modulation of both Gab2 and Gab1 expression in these conditions might contribute to the impaired signalling observed. Consistent with their opposite roles on Akt, the depletion of Gab1, but not of Gab2, results in reduced FOXO1 phosphorylation and VEGF-mediated endothelial cell survival. Mutation of VEGFR2 Y801 and Y1214, which abrogates the phosphorylation of Gab1, also correlates with inhibition of Akt. Altogether, these results underscore the non-redundant and essential roles of Gab1 and Gab2 in endothelial cells, and suggest major contributions of these proteins during in vivo angiogenesis.
5. Non-redundant roles of the Gab1 and Gab2 scaffolding adapters in VEGF-mediated signalling, **migration**, and survival of endothelial cells. Gab1 was previously described as a positive modulator of Akt, Src, ERK1/2, endothelial cell **migration**, and capillary formation in response to vascular endothelial growth factor (VEGF). However, its involvement in endothelial cell survival, as well as the potential contribution of the other family member Gab2 to signalling and biological responses remained unknown. Here, we show that Gab2 is tyrosine phosphorylated in a Grb2-dependent manner downstream of activated VEGF receptor-2 (VEGFR2), and that it associates with signalling proteins including PI3K and SHP2, but apparently not with the receptor. Similarly to Gab1, over-expression of Gab2 induces endothelial cell **migration** in response to VEGF, whereas its depletion using siRNAs results in its reduction. Importantly, depletion of both Gab1 and Gab2 leads to an even greater inhibition of VEGF-induced cell **migration**. However, contrary to what has been reported for Gab1, the silencing of Gab2 results in increased Src, Akt and ERK1/2 activation, slightly reduced p38 phosphorylation, and up-regulation of Gab1 protein levels. Accordingly, re-expression of Gab2 in Gab2-/- fibroblasts leads to opposite results, suggesting that the modulation of both Gab2 and Gab1 expression in these conditions might contribute to the impaired signalling observed. Consistent with their opposite roles on Akt, the depletion of Gab1, but not of Gab2, results in reduced FOXO1 phosphorylation and VEGF-mediated endothelial cell survival. Mutation of VEGFR2 Y801 and Y1214, which abrogates the phosphorylation of Gab1, also correlates with inhibition of Akt. Altogether, these results underscore the non-redundant and essential roles of Gab1 and Gab2 in endothelial cells, and suggest major contributions of these proteins during in vivo angiogenesis.
6. Distinct requirements for Gab1 in Met and EGF receptor signaling in vivo. Gab1 is a multiadaptor protein that has been shown to be required for multiple processes in embryonic development and oncogenic transformation. Gab1 functions by amplifying signal transduction downstream of various receptor tyrosine kinases through recruitment of multiple signaling effectors, including phosphatidylinositol 3-kinase and Shp2. Until now, the functional significance of individual interactions in vivo was not known. Here we have generated knockin mice that carry point mutations in either the P13K or Shp2 binding sites of Gab1. We show that different effector interactions with Gab1 play distinct biological roles downstream of Gab1 during the development of different organs. Recruitment of phosphatidylinositol 3-kinase by Gab1 is essential for EGF receptor-mediated embryonic eyelid closure and keratinocyte **migration**, and the Gab1-Shp2 interaction is crucial for Met receptor-directed placental development and muscle progenitor cell **migration** to the limbs. Furthermore, we investigate the dual association of Gab1 with the Met receptor. By analyzing knockin mice with mutations in the Grb2 or Met binding site of Gab1, we show that the requirements for Gab1 recruitment to Met varies in different biological contexts. Either the direct or the indirect interaction of Gab1 with Met is sufficient for Met-dependent muscle precursor cell **migration**, whereas both modes of interaction are required and neither is sufficient for placenta development, liver growth, and palatal shelf closure. These data demonstrate that Gab1 induces different biological responses through the recruitment of distinct effectors and that different modes of recruitment for Gab1 are required in different organs.
7. The Gab1 scaffold regulates RTK-dependent dorsal ruffle formation through the adaptor Nck. The polarised distribution of signals downstream from receptor tyrosine kinases (RTKs) regulates fundamental cellular processes that control cell **migration**, growth and morphogenesis. It is poorly understood how RTKs are involved in the localised signalling and actin remodelling required for these processes. Here, we show that the Gab1 scaffold is essential for the formation of a class of polarised actin microdomain, namely dorsal ruffles, downstream from the Met, EGF and PDGF RTKs. Gab1 associates constitutively with the actin-nucleating factor N-WASP. Following RTK activation, Gab1 recruits Nck, an activator of N-WASP, into a signalling complex localised to dorsal ruffles. Formation of dorsal ruffles requires interaction between Gab1 and Nck, and also requires functional N-WASP. Epithelial cells expressing Gab1DeltaNck (Y407F) exhibit decreased Met-dependent Rac activation, fail to induce dorsal ruffles, and have impaired cell **migration** and epithelial remodelling. These data show that a Gab1-Nck signalling complex interacts with several RTKs to promote polarised actin remodelling and downstream biological responses.
8. CXCR4 and Gab1 cooperate to control the development of migrating muscle progenitor cells. Long-range migrating progenitor cells generate hypaxial muscle, for instance the muscle of the limbs, hypoglossal cord, and diaphragm. We show here that migrating muscle progenitors express the chemokine receptor CXCR4. The corresponding ligand, SDF1, is expressed in limb and branchial arch mesenchyme; i.e., along the routes and at the targets of the migratory cells. Ectopic application of SDF1 in the chick limb attracts muscle progenitor cells. In CXCR4 mutant mice, the number of muscle progenitors that colonize the anlage of the tongue and the dorsal limb was reduced. Changes in the distribution of the muscle progenitor cells were accompanied by increased apoptosis, indicating that CXCR4 signals provide not only attractive cues but also control survival. Gab1 encodes an adaptor protein that transduces signals elicited by tyrosine kinase receptors, for instance the c-Met receptor, and plays a role in the **migration** of muscle progenitor cells. We found that CXCR4 and Gab1 interact genetically. For instance, muscle progenitors do not reach the anlage of the tongue in CXCR4;Gab1 double mutants; this target is colonized in either of the single mutants. Our analysis reveals a role of SDF1/CXCR4 signaling in the development of migrating muscle progenitors and shows that a threshold number of progenitor cells is required to generate muscle of appropriate size.
9. Endothelial Grb2-associated binder 1 is crucial for postnatal angiogenesis. Grb2-associated binder 1 (Gab1), a scaffolding adaptor protein, plays an important role in transmitting key signals that control cell growth, differentiation, and function from multiple tyrosine kinase receptors. The study was designed to investigate the role of endothelial Gab1 in angiogenesis and its underlying molecular mechanisms.Using Cre-Lox recombination technology, we generated endothelial-specific Gab1 knockout (Gab1-ecKO) mice. Gab1-ecKO mice are viable and showed no obvious developmental defects in the vascular system. To analyze the role of Gab1 in postnatal angiogenesis, we used hindlimb ischemia and Matrigel plug models. We found that loss of endothelial Gab1 in mice dramatically impaired postnatal angiogenesis. Gab1-ecKO mice had impaired ischemia-initiated blood flow recovery, exhibited reduced angiogenesis, and were associated with marked limb necrosis. We further observed significant endothelial cell (EC) death in the ischemic hindlimb of Gab1-ecKO mice. Matrigel plug assay showed that hepatocyte growth factor (HGF)-mediated angiogenesis was inhibited in Gab1-ecKO mice. In vitro studies showed that Gab1 was required for HGF-induced EC **migration**, tube formation, and microvessel sprouting. Mechanistically, HGF stimulated Gab1 tyrosine phosphorylation in ECs, leading to activation of extracellular regulated MAP kinase 1/2 and Akt, which are angiogenic and survival signaling.Gab1 is essential for postnatal angiogenesis through mediating angiogenic and survival signaling.

### [Mm]etastasis

#### PubMed

1. Epidermal growth factor receptor-dependent activation of Gab1 is involved in ErbB-2-mediated mammary tumor progression. Activation of the epidermal growth factor receptor (EGFR) family is thought to play an important role in mammary tumorigenesis and **metastasis**. The potent transforming activity of the EGFR family is due to their ability to heterodimerize with each other in response to a number of mitogenic ligands. The formation of EGFR and ErbB-2 heterodimers has been recently implicated as an important factor in the induction of sporadic human breast cancers. To directly assess whether the catalytic activity of EGFR is required for ErbB-2 induction of mammary tumors, we have interbred transgenic mice expressing ErbB-2 oncogene under the transcriptional control of the mouse mammary tumor virus (MMTV) promoter/enhancer to a naturally occurring mouse mutant carrying a catalytically impaired EGFR (waved-2 mice). Although the female transgenic mice possessing mutant EGFR developed mammary tumors, the tumors occurred only after a delayed latency period, and were fewer in number. The impaired tumor phenotype was further correlated with debilitated phosphorylation of the Gab1 multisubstrate adapter. These observations provide evidence that efficient ErbB-2-induced mammary tumor progression requires EGFR-dependent activation of Gab1.

---

## Hspd1

- NCBI Gene
- GeneRIF
- Pubmed

| Search Term | Rank | Fields |
| --- | --- | --- |
| [Mm]igration | 6 | PubMed(1); GeneRIFs(1) |
| [Ee]xtracellular [Mm]atrix | 8 | Component(1) |

  
hide/show details for Hspd1

### [Mm]igration

#### PubMed

1. Heat shock protein 60 stimulates the **migration** of vascular smooth muscle cells via Toll-like receptor 4 and ERK MAPK activation. Accumulating evidence indicates that heat shock protein (HSP) 60 is strongly associated with the pathology of atherosclerosis (AS). However, the precise mechanisms by which HSP60 promotes atherosclerosis remain unclear. In the present study, we found that HSP60 mRNA and protein expression levels in the thoracic aorta are enhanced not only in a mouse model of AS but also in high-fat diet (HFD) mice. HSP60 expression and secretion was activated by platelet-derived growth factor-BB (PDGF-BB) and interleukin (IL)-8 in both human umbilical vein endothelial cells (HUVECs) and vascular smooth muscle cells (VSMCs). HSP60 was found to induce VSMC **migration**, and exposure to HSP60 activated ERK MAPK signaling. U0126, an inhibitor of ERK, reduced VSMC **migration**. The HSP60-stimulated VSMCs were found to express TLR4 mRNA but not TLR2 mRNA. Knockdown of TLR4 by siRNA reduced HSP60-induced VSMC **migration** and HSP60-induced ERK activation. Finally, HSP60 induced IL-8 secretion in VSMCs. Together these results suggest that HSP60 is involved in the stimulation of VSMC **migration**, via TLR4 and ERK MAPK activation. Meanwhile, activation of HSP60 is one of the most powerful methods of sending a 'danger signal' to the immune system to generate IL-8, which assists in the management of an infection or disease.

#### GeneRIFs

1. Heat shock protein 60 stimulates the **migration** of vascular smooth muscle cells via Toll-like receptor 4 and ERK MAPK activation

### [Ee]xtracellular [Mm]atrix

#### Component

1. **extracellular matrix**

---

## Dab2ip

- NCBI Gene
- GeneRIF
- Pubmed

| Search Term | Rank | Fields |
| --- | --- | --- |
| [Mm]igration | 6 | Process(5); PubMed(6); GeneRIFs(2) |
| [Ii]nvasive | 10 | PubMed(1) |
| [Mm]etastasis | 12 | PubMed(3); GeneRIFs(1) |

  
hide/show details for Dab2ip

### [Mm]igration

#### Process

1. cell motility involved in cerebral cortex radial glia guided **migration**
2. negative regulation of endothelial cell **migration**
3. negative regulation of endothelial cell **migration**
4. negative regulation of epithelial cell **migration**
5. positive regulation of neuron **migration**

#### PubMed

1. AIP1 prevents graft arteriosclerosis by inhibiting interferon-γ-dependent smooth muscle cell proliferation and intimal expansion. ASK1-interacting protein-1 (AIP1), a Ras GTPase-activating protein family member, is highly expressed in endothelial cells and vascular smooth musccells (VSMCs). The role of AIP1 in VSMCs and VSMC proliferative disease is not known.We used mouse graft arteriosclerosis models characterized by VSMC accumulation and intimal expansion to determine the function of AIP1.In a single minor histocompatibility antigen (male to female)-dependent aorta transplantation model, AIP1 deletion in the graft augmented neointima formation, an effect reversed in AIP1/interferon-γ receptor (IFN-γR) doubly-deficient aorta donors. In a syngeneic aortic transplantation model in which wild-type or AIP1-knockout mouse aortas were transplanted into IFN-γR-deficient recipients and in which neointima formation was induced by intravenous administration of an adenovirus that encoded a mouse IFN-γ transgene, donor grafts from AIP1-knockout mice enhanced IFN-γ-induced VSMC proliferation and neointima formation. Mechanistically, knockout or knockdown of AIP1 in VSMCs significantly enhanced IFN-γ-induced JAK-STAT signaling and IFN-γ-depende**nt VSMC m**igration and proliferation, 2 critical steps in neointima formation. Furthermore, AIP1 specifically bound to JAK2 and inhibited its activity.AIP1 functions as a negative regulator in IFN-γ-induced intimal formation, in part by downregulating IFN-γ-JAK2-STAT1/3-dependent migratory and proliferative signaling in VSMCs.
2. AIP1-mediated actin disassembly is required for postnatal germ cell **migration** and spermatogonial stem cell niche establishment. In mammals, spermatogonial stem cells (SSCs) arise from early germ cells called gonocytes, which are derived from primordial germ cells during embryogenesis and remain quiescent until birth. After birth, these germ cells migrate from the center of testicular cord, through Sertoli cells, and toward the basement membrane to form the SSC pool and establish the SSC niche architecture. However, molecular mechanisms underlying germ cell **migration** and niche establishment are largely unknown. Here, we show that the actin disassembly factor actin interacting protein 1 (AIP1) is required in both germ cells and Sertoli cells to regulate this process. Germ cell-specific or Sertoli cell-specific deletion of Aip1 gene each led to significant defects in germ cell **migration** after postnatal day 4 or 5, accompanied by elevated levels of actin filaments (F-actin) in the affected cells. Furthermore, our data demonstrated that interaction between germ cells and Sertoli cells, likely through E-cadherin-mediated cell adhesion, is critical for germ cells' **migration** toward the basement membrane. At last, Aip1 deletion in Sertoli cells decreased SSC self-renewal, increased spermatogonial differentiation, but did not affect the expression and secretion levels of growth factors, suggesting that the disruption of SSC function results from architectural changes in the postnatal niche.
3. Dab2IP Regulates Neuronal Positioning, Rap1 Activity and Integrin Signaling in the Developing Cortex. Dab2IP (DOC-2/DAB2 interacting protein) is a GTPase-activating protein which is involved in various aspects of brain development in addition to its roles in tumor formation and apoptosis in other systems. In this study, we carefully examined the expression profile of Dab2IP and investigated its physiological role during brain development using a Dab2IP-knockdown (KD) mouse model created by retroviral insertion of a LacZ-encoding gene-trapping cassette. LacZ staining revealed that Dab2IP is expressed in the ventricular zone as well as the cortical plate and the intermediate zone. Immunohistochemical analysis showed that Dab2IP protein is localized in the leading process and proximal cytoplasmic regions of migrating neurons in the intermediate zone. Bromodeoxyuridine birth dating experiments in combination with immunohistochemical analysis using layer-specific markers showed that Dab2IP is important for proper positioning of a subset of layer II-IV neurons in the developing cortex. Notably, neuronal **migration** was not completely disrupted in the cerebral cortex of Dab2IP-KD mice and disruption of **migration** was not strictly layer specific. Previously, we found that Dab2IP regulates multipolar transition in cortical neurons. Others have shown that Rap1 regulates the transition from multipolar to bipolar morphology in migrating postmitotic neurons through N-cadherin signaling and somal translocation in the superficial layer of the cortical plate through integrin signaling. Therefore, we examined whether Rap1 and integrin signaling were affected in Dab2IP-KD brains. We found that Dab2IP-KD resulted in higher levels of activated Rap1 and integrin in the developing cortex. Taken together, our results suggest that Dab2IP plays an important role in the **migration** and positioning of a subpopulation of later-born (layers II-IV) neurons, likely through the regulation of Rap1 and integrin signaling.
4. Dab2ip regulates neuronal **migration** and neurite outgrowth in the developing neocortex. Dab2ip (DOC-2/DAB2 interacting protein) is a member of the Ras GTPase-activating protein (GAP) family that has been previously shown to function as a tumor suppressor in several systems. Dab2ip is also highly expressed in the brain where it interacts with Dab1, a key mediator of the Reelin pathway that controls several aspects of brain development and function. We found that Dab2ip is highly expressed in the developing cerebral cortex, but that mutations in the Reelin signaling pathway do not affect its expression. To determine whether Dab2ip plays a role in brain development, we knocked down or over expressed it in neuronal progenitor cells of the embryonic mouse neocortex using in utero electroporation. Dab2ip down-regulation severely disrupts neuronal **migration**, affecting preferentially late-born principal cortical neurons. Dab2ip overexpression also leads to **migration** defects. Structure-function experiments in vivo further show that both PH and GRD domains of Dab2ip are important for neuronal **migration**. A detailed analysis of transfected neurons reveals that Dab2ip down- or up-regulation disrupts the transition from a multipolar to a bipolar neuronal morphology in the intermediate zone. Knock down of Dab2ip in neurons ex-vivo indicates that this protein is necessary for proper neurite development and for the expression of several major neuronal microtubule associated proteins (MAPs), which are important for neurite growth and stabilization. Thus, our study identifies, for the first time, a critical role for Dab2ip in mammalian cortical development and begins to reveal molecular mechanisms that underlie this function.
5. Expression of mouse Dab2ip transcript variants and gene methylation during brain development. Dab2ip (DOC-2/DAB2 interacting protein) is a RasGAP protein which shows a growth-inhibitory effect in human prostate cancer cell lines. Recent studies have shown that Dab2ip also plays an important role in regulating dendrite development and neuronal **migration** during brain development. In this study, we provide a more complete description of the mouse Dab2ip (mDab2ip) gene locus and examined DNA methylation and expression of Dab2ip during cerebellar development. Analysis of cDNA sequences in public databases revealed a total of 20 possible exons for mDab2ip gene, spanning over 172kb. Using Cap Analysis of Gene Expression (CAGE) data available through FANTOM5 project, we deduced five different transcription start sites for mDab2ip. Here, we characterized three different mDab2ip transcript variants beginning with exon 1. These transcripts varied by the presence or absence of exons 3 and 5, which encode a putative nuclear localization signal and the N-terminal region of a PH-domain, respectively. The 5' region of the mDab2ip gene contains three putative CpG islands (CpG131, CpG54, and CpG85). Interestingly, CpG54 and CpG85 are localized on exons 3 and 5. Bisulfate DNA sequencing showed that methylation level of CpG54 remained constant whereas methylation of CpG85 increased during cerebellar development. Real-time PCR analysis showed that the proportion of PH-domain containing mDab2ip transcripts increased during cerebellar development, in correlation with the increase in CpG85 methylation. These data suggest that site-specific methylation of mDab2ip gene during cerebellar development may play a role in inclusion of exon 5, resulting in a Dab2ip transcript variant that encodes a full pleckstrin homology (PH) domain.
6. AIP1 functions as an endogenous inhibitor of VEGFR2-mediated signaling and inflammatory angiogenesis in mice. ASK1-interacting protein-1 (AIP1), a recently identified member of the Ras GTPase-activating protein family, is highly expressed in vascular ECs and regulates EC apoptosis in vitro. However, its function in vivo has not been established. To study this, we generated AIP1-deficient mice (KO mice). Although these mice showed no obvious defects in vascular development, they exhibited dramatically enhanced angiogenesis in 2 models of inflammatory angiogenesis. In one of these models, the enhanced angiogenesis observed in the KO mice was associated with increased VEGF-VEGFR2 signaling. Consistent with this, VEGF-induced ear, cornea, and retina neovascularization were greatly augmented in KO mice and the enhanced retinal angiogenesis was markedly diminished by overexpression of AIP1. In vitro, VEGF-induced EC **migration** was inhibited by AIP1 overexpression, whereas it was augmented by both AIP1 knockout and knockdown, with the enhanced EC **migration** caused by AIP1 knockdown being associated with increased VEGFR2 signaling. We present mechanistic data that suggest AIP1 is recruited to the VEGFR2-PI3K complex, binding to both VEGFR2 and PI3K p85, at a late phase of the VEGF response, and that this leads to inhibition of VEGFR2 signaling. Taken together, our data demonstrate that AIP1 functions as an endogenous inhibitor in VEGFR2-mediated adaptive angiogenesis in mice.

#### GeneRIFs

1. Germ cell-specific or Sertoli cell-specific deletion of Aip1 gene each led to significant defects in germ cell **migration** after postnatal day 4 or 5, accompanied by elevated levels of actin filaments (F-actin) in the affected cells.
2. results suggest that Dab2IP plays an important role in the **migration** and positioning of a subpopulation of later-born (layers II-IV) neurons, likely through the regulation of Rap1 and integrin signaling.

### [Ii]nvasive

#### PubMed

1. Mutant p53 reprograms TNF signaling in cancer cells through interaction with the tumor suppressor DAB2IP. Inflammation is a significant factor in cancer development, and a molecular understanding of the parameters dictating the impact of inflammation on cancers could significantly improve treatment. The tumor suppressor p53 is frequently mutated in cancer, and p53 missense mutants (mutp53) can acquire oncogenic properties. We report that cancer cells with mutp53 respond to inflammatory cytokines increasing their **invasive** behavior. Notably, this action is coupled to expression of chemokines that can expose the tumor to host immunity, potentially affecting response to therapy. Mechanistically, mutp53 fuels NF-κB activation while it dampens activation of ASK1/JNK by TNFα, and this action depends on mutp53 binding and inhibiting the tumor suppressor DAB2IP in the cytoplasm. Interfering with such interaction reduced aggressiveness of cancer cells in xenografts. This interaction is an unexplored mechanism by which mutant p53 can influence tumor evolution, with implications for our understanding of the complex role of inflammation in cancer.

### [Mm]etastasis

#### PubMed

1. AIP1 Expression in Tumor Niche Suppresses Tumor Progression and **Metastasis**. Studies from tumor cells suggest that tumor-suppressor AIP1 inhibits epithelial-mesenchymal transition (EMT). However, the role of AIP1 in the tumor microenvironment has not been examined. We show that a global or vascular endothelial cell (EC)-specific deletion of the AIP1 gene in mice augments tumor growth and **metastasis** in melanoma and breast cancer models. AIP1-deficient vascular environment not only enhances tumor neovascularization and increases premetastatic niche formation, but also secretes tumor EMT-promoting factors. These effects from AIP1 loss are associated with increased VEGFR2 signaling in the vascular EC and could be abrogated by systemic administration of VEGFR2 kinase inhibitors. Mechanistically, AIP1 blocks VEGFR2-dependent signaling by directly binding to the phosphotyrosine residues within the activation loop of VEGFR2. Our data reveal that AIP1, by inhibiting VEGFR2-dependent signaling in tumor niche, suppresses tumor EMT switch, tumor angiogenesis, and tumor premetastatic niche formation to limit tumor growth and **metastasis**.
2. Role of DAB2IP in modulating epithelial-to-mesenchymal transition and prostate cancer **metastasis**. A single nucleotide polymorphism in the DAB2IP gene is associated with risk of aggressive prostate cancer (PCa), and loss of DAB2IP expression is frequently detected in metastatic PCa. However, the functional role of DAB2IP in PCa remains unknown. Here, we show that the loss of DAB2IP expression initiates epithelial-to-mesenchymal transition (EMT), which is visualized by repression of E-cadherin and up-regulation of vimentin in both human normal prostate epithelial and prostate carcinoma cells as well as in clinical prostate-cancer specimens. Conversely, restoring DAB2IP in metastatic PCa cells reversed EMT. In DAB2IP knockout mice, prostate epithelial cells exhibited elevated mesenchymal markers, which is characteristic of EMT. Using a human prostate xenograft-mouse model, we observed that knocking down endogenous DAB2IP in human carcinoma cells led to the development of multiple lymph node and distant organ metastases. Moreover, we showed that DAB2IP functions as a scaffold protein in regulating EMT by modulating nuclear beta-catenin/T-cell factor activity. These results show the mechanism of DAB2IP in EMT and suggest that assessment of DAB2IP may provide a prognostic biomarker and potential therapeutic target for PCa **metastasis**.
3. An oncogene-tumor suppressor cascade drives metastatic prostate cancer by coordinately activating Ras and nuclear factor-kappaB. **Metastasis** is responsible for the majority of prostate cancer-related deaths; however, little is known about the molecular mechanisms that underlie this process. Here we identify an oncogene-tumor suppressor cascade that promotes prostate cancer growth and **metastasis** by coordinately activating the small GTPase Ras and nuclear factor-kappaB (NF-kappaB). Specifically, we show that loss of the Ras GTPase-activating protein (RasGAP) gene DAB2IP induces metastatic prostate cancer in an orthotopic mouse tumor model. Notably, DAB2IP functions as a signaling scaffold that coordinately regulates Ras and NF-kappaB through distinct domains to promote tumor growth and **metastasis**, respectively. DAB2IP is suppressed in human prostate cancer, where its expression inversely correlates with tumor grade and predicts prognosis. Moreover, we report that epigenetic silencing of DAB2IP is a key mechanism by which the polycomb-group protein histone-lysine N-methyltransferase EZH2 activates Ras and NF-kappaB and triggers **metastasis**. These studies define the mechanism by which two major pathways can be simultaneously activated in metastatic prostate cancer and establish EZH2 as a driver of **metastasis**.

#### GeneRIFs

1. Our data reveal that AIP1, by inhibiting VEGFR2-dependent signaling in tumor niche, suppresses tumor EMT switch, tumor angiogenesis, and tumor premetastatic niche formation to limit tumor growth and **metastasis**.

---

## Rasal2

- NCBI Gene
- GeneRIF
- Pubmed

| Search Term | Rank | Fields |
| --- | --- | --- |
| [Mm]igration | 6 | PubMed(1) |
| [Mm]etastasis | 12 | PubMed(2); GeneRIFs(1) |

  
hide/show details for Rasal2

### [Mm]igration

#### PubMed

1. RASAL2 promotes lung cancer metastasis through epithelial-mesenchymal transition. It was reported that genetically-engineered RASAL2 knockout mice are prone to development of several sporadic tumor, including lung adenocarcinoma. However, a causative relationship between RASAL2 deficiency and lung adenocarcinoma development still remains unknown. In the present study, RASAL2 level was determined in patients with lung adenocarcinoma and control subjects in an attempt to explore its potential clinical diagnostic and prognostic value. Low RASAL2 expression levels were found in 71% (37 of 52) of lung adenocarcinoma, which were correlated with lymph node metastasis in lung adenocarcinoma. Moreover, Low RASAL2 expression levels were correlated with reduced overall survival (OS) in lung adenocarcinoma. We find that inactivation of RASAL2 promotes lung cancer cell **migration** through the induction of epithelial mesenchymal transition (EMT) and promoted lung metastasis in nude mice. Our results suggest that the down-regulation of RASAL2 promotes metastatic progression of lung adenocarcinoma, hence it could serve as a potential target for the development of lung cancer therapies.

### [Mm]etastasis

#### PubMed

1. The RasGAP gene, RASAL2, is a tumor and **metastasis** suppressor. RAS genes are commonly mutated in cancer; however, RAS mutations are rare in breast cancer, despite frequent hyperactivation of Ras and ERK. Here, we report that the RasGAP gene, RASAL2, functions as a tumor and **metastasis** suppressor. RASAL2 is mutated or suppressed in human breast cancer, and RASAL2 ablation promotes tumor growth, progression, and **metastasis** in mouse models. In human breast cancer, RASAL2 loss is associated with metastatic disease; low RASAL2 levels correlate with recurrence of luminal B tumors; and RASAL2 ablation promotes **metastasis** of luminal mouse tumors. Additional data reveal a broader role for RASAL2 inactivation in other tumor types. These studies highlight the expanding role of RasGAPs and reveal an alternative mechanism of activating Ras in cancer.
2. RASAL2 promotes lung cancer **metastasis** through epithelial-mesenchymal transition. It was reported that genetically-engineered RASAL2 knockout mice are prone to development of several sporadic tumor, including lung adenocarcinoma. However, a causative relationship between RASAL2 deficiency and lung adenocarcinoma development still remains unknown. In the present study, RASAL2 level was determined in patients with lung adenocarcinoma and control subjects in an attempt to explore its potential clinical diagnostic and prognostic value. Low RASAL2 expression levels were found in 71% (37 of 52) of lung adenocarcinoma, which were correlated with lymph node **metastasis** in lung adenocarcinoma. Moreover, Low RASAL2 expression levels were correlated with reduced overall survival (OS) in lung adenocarcinoma. We find that inactivation of RASAL2 promotes lung cancer cell migration through the induction of epithelial mesenchymal transition (EMT) and promoted lung **metastasis** in nude mice. Our results suggest that the down-regulation of RASAL2 promotes metastatic progression of lung adenocarcinoma, hence it could serve as a potential target for the development of lung cancer therapies.

#### GeneRIFs

1. The RasGAP gene, RASAL2, is a tumor and **metastasis** suppressor

---

## Dlc1

- NCBI Gene
- GeneRIF
- Pubmed

| Search Term | Rank | Fields |
| --- | --- | --- |
| [Mm]igration | 6 | Process(1); PubMed(3); GeneRIFs(1) |
| [Ii]nvasive | 10 | PubMed(2) |
| [Mm]etastasis | 12 | PubMed(3); GeneRIFs(1) |

  
hide/show details for Dlc1

### [Mm]igration

#### Process

1. negative regulation of cell **migration**

#### PubMed

1. The role of Dlc1 isoform 2 in K-Ras2(G12D) induced thymic cancer. The Deleted in liver cancer one (Dlc1) tumor suppressor gene encodes a RhoGTPase activating protein (RhoGAP). The Dlc1 gene has multiple transcriptional isoforms and we have previously established a mouse strain containing a gene trap (gt) insertion, which specifically reduces the expression of the 6.1 kb isoform (isoform 2). This gene trapped allele when homozygous results in embryonic lethality and the heterozygous gene trapped mice do not show an increased incidence of cancers, suggesting that cooperating oncogenic changes may be required for transformation. In the present work, we have studied the in vivo cooperation between oncogenic K-Ras2 and Dlc1 genes in tumourigenesis. We have observed an increase in invasive thymic cancers, including both thymomas and lymphomas, resulting in significantly shortened life spans in mice heterozygous for the gt Dlc1 allele and an inducible LSL-K-Ras2(G12D) allele compared with the LSL-K-Ras2(G12D) only mice. The heterozygous mice showed a high degree of metastasis in the lung. We have found tumour specific selective hypermethylation of the Dlc1 isoform 2 promoter and reduction of the corresponding protein expression in thymic lymphoma (TL) and thymic epithelial carcinoma (TEC) derived from the thymic tumours. The Dlc1 deficient thymic lymphoma cell lines exhibited increased trans-endothelial cell **migration**. TEC cell lines also exhibited increased stress fiber formation and Rho activity. Introduction of the three Dlc1 isoforms tagged with GFP into these cells resulted in different morphological changes. These results suggest that loss of expression of only isoform 2 may be sufficient for the development of thymic tumors and metastasis.
2. Identification and characterization of Dlc1 isoforms in the mouse and study of the biological function of a single gene trapped isoform. The Dlc1 (deleted in liver cancer 1) tumour suppressor gene codes for a RhoGTPase activating protein that is found inactivated in many tumour types. Several transcriptional isoforms have been described but the functional significance and tissue distribution of each form is presently poorly understood. Also, differences in the number of isoforms and splice variants reported still exist between different mammalian species. In order to better understand the number and function of the different variants of the Dlc1 gene in the mouse, we have carried out a detailed analysis. Extensive 3' RACE experiments were carried out in order to identify all possible Dlc1 isoforms and splice variants in the mouse. In addition, we have generated a gene trapped mouse that targets one of these isoforms in order to study its biological function. The effect of this gene trap insertion on the splicing of other isoforms has also been studied.In addition to the known 6.1 and 6.2 Kb transcripts of Dlc1, our study revealed the existence of a novel 7.6 Kb transcriptional isoform in the mouse, which corresponds to the human 7.4 Kb (KIAA1723) cDNA transcript. A gene trapped embryonic cell line, with an insertion between Exon 1 and 2 of the 6.1 Kb transcriptional isoform, was used to generate a transgenic mouse. This line showed a significant reduction in the expression of the trapped isoform. However, reduced expression of the other isoforms was not seen. Mice heterozygous for the gene trapped allele were phenotypically normal, but homozygous mutant embryos did not survive beyond 10.5 days post coitum. Dlc1gt/gt embryos showed defects in the brain, heart, and placental blood vessels. Cultured serum-free mouse embryo cells from Dlc1 deficient embryos had elevated RhoA activity and displayed alterations in the organization of actin filaments and focal adhesions. The Dlc1 deficient cells also exhibited increased wound closure in an in vitro scratch assay.The mouse has three major transcriptional isoforms of the Dlc1 gene that are differentially expressed in various tissues. A mouse with exon 1 of the 6.1 Kb transcript gt resulted in hypomorphic expression of Dlc1 protein and an embryonic lethal phenotype in the homozygous condition, which indicates that this isoform plays a major role in mouse development. The Dlc1 deficient cells showed altered cytoskeleton structure, increased RhoA activity and cellular **migration**.
3. Tensin1 positively regulates RhoA activity through its interaction with DLC1. DLC1 is a RhoGAP-containing tumor suppressor and many of DLC1's functions are absolutely dependent on its RhoGAP activity. Through its RhoGAP domain, DLC1 inhibits the activity of RhoA GTPase, which regulates actin cytoskeleton networks and dis/assembly of focal adhesions. Tensin1 (TNS1) is a focal adhesion molecule that links the actin cytoskeleton to integrins and forms signaling complexes through its multiple binding domains. Here, we report that TNS1 enhances RhoA activity in a DLC1-dependent manner. This is accomplished by binding to DLC1 through TNS1's C2, SH2, and PTB domains. Point mutations at these three sites disrupt TNS1's interaction with DLC1 as well as its effect on RhoA activity. The biological relevance of this TNS1-DLC1-RhoA signaling axis is investigated in TNS1 knockout (KO) cells and mice. Endothelial cells isolated from TNS1 KO mice or those silenced with TNS1 siRNA show significant reduction in proliferation, **migration**, and tube formation activities. Concomitantly, the RhoA activity is down-regulated in TNS1 KO cells and this reduction is restored by further silencing of DLC1. Furthermore, the angiogenic process is compromised in TNS1 KO mice. These studies demonstrate that TNS1 binds to DLC1 and fine-tunes its RhoGAP activity toward RhoA and that the TNS1-DLC1-RhoA signaling axis is critical in regulating cellular functions that lead to angiogenesis.

#### GeneRIFs

1. The Dlc1 deficient cells showed altered cytoskeleton structure, increased RhoA activity and cellular **migration**.

### [Ii]nvasive

#### PubMed

1. In vivo and in vitro investigations of heterozygous nebulin knock-out mice disclose a mild skeletal muscle phenotype. Nemaline myopathy is the most common congenital skeletal muscle disease, and mutations in the nebulin gene account for 50% of all cases. Recent studies suggest that the disease severity might be related to the nebulin expression levels. Considering that mutations in the nebulin gene are typically recessive, one would expect that a single functional nebulin allele would maintain nebulin protein expression which would result in preserved skeletal muscle function. We investigated skeletal muscle function of heterozygous nebulin knock-out (i.e., nebulin(+/-)) mice using a multidisciplinary approach including protein and gene expression analysis and combined in vivo and in vitro force measurements. Skeletal muscle anatomy and energy metabolism were studied strictly non-**invasive**ly using magnetic resonance imaging and 31P-magnetic resonance spectroscopy. Maximal force production was reduced by around 16% in isolated muscle of nebulin(+/-) mice while in vivo force generating capacity was preserved. Muscle weakness was associated with a shift toward a slower proteomic phenotype, but was not related to nebulin protein deficiency or to an impaired energy metabolism. Further studies would be warranted in order to determine the mechanisms leading to a mild skeletal muscle phenotype resulting from the expression of a single nebulin allele.
2. The role of Dlc1 isoform 2 in K-Ras2(G12D) induced thymic cancer. The Deleted in liver cancer one (Dlc1) tumor suppressor gene encodes a RhoGTPase activating protein (RhoGAP). The Dlc1 gene has multiple transcriptional isoforms and we have previously established a mouse strain containing a gene trap (gt) insertion, which specifically reduces the expression of the 6.1 kb isoform (isoform 2). This gene trapped allele when homozygous results in embryonic lethality and the heterozygous gene trapped mice do not show an increased incidence of cancers, suggesting that cooperating oncogenic changes may be required for transformation. In the present work, we have studied the in vivo cooperation between oncogenic K-Ras2 and Dlc1 genes in tumourigenesis. We have observed an increase in **invasive** thymic cancers, including both thymomas and lymphomas, resulting in significantly shortened life spans in mice heterozygous for the gt Dlc1 allele and an inducible LSL-K-Ras2(G12D) allele compared with the LSL-K-Ras2(G12D) only mice. The heterozygous mice showed a high degree of metastasis in the lung. We have found tumour specific selective hypermethylation of the Dlc1 isoform 2 promoter and reduction of the corresponding protein expression in thymic lymphoma (TL) and thymic epithelial carcinoma (TEC) derived from the thymic tumours. The Dlc1 deficient thymic lymphoma cell lines exhibited increased trans-endothelial cell migration. TEC cell lines also exhibited increased stress fiber formation and Rho activity. Introduction of the three Dlc1 isoforms tagged with GFP into these cells resulted in different morphological changes. These results suggest that loss of expression of only isoform 2 may be sufficient for the development of thymic tumors and metastasis.

### [Mm]etastasis

#### PubMed

1. PKA-induced dimerization of the RhoGAP DLC1 promotes its inhibition of tumorigenesis and **metastasis**. Deleted in Liver Cancer 1 (DLC1) is a tumour suppressor that encodes a RhoGTPase-activating protein (RhoGAP) and is frequently inactivated in many human cancers. The RhoGAP activity of DLC1 against Rho signalling is well documented and is strongly associated with the tumour suppressor functions of DLC1. However, the mechanism by which the RhoGAP activity of DLC1 is regulated remains obscure. Here, we report that phosphorylation of DLC1 at Ser549 by cyclic AMP-dependent protein kinase A contributes to enhanced RhoGAP activity and promotes the activation of DLC1, which suppresses hepatoma cell growth, motility and **metastasis** in both in vitro and in vivo models. Intriguingly, we found that Ser549 phosphorylation induces the dimerization of DLC1 and that inducible dimerization of DLC1 can rescue the tumour suppressive and RhoGAP activities of DLC1 containing a Ser549 deletion. Our study establishes a novel regulatory mechanism for DLC1 RhoGAP activity via dimerization induced by protein kinase A signalling.
2. DLC1-dependent parathyroid hormone-like hormone inhibition suppresses breast cancer bone **metastasis**. Bone **metastasis** is a frequent complication of breast cancer that is often accelerated by TGF-β signaling; however, little is known about how the TGF-β pathway is regulated during bon**e metastas**is. Here we report that deleted in liver cancer 1 (DLC1) is an important regulator of TGF-β responses and osteolyt**ic metasta**sis of breast cancer cells. In murine models, breast cancer cells lacking DLC1 expression exhibited enhanced capabilities of bo**ne metasta**sis. Knockdown of DLC1 in cancer cells promoted bo**ne metasta**sis, leading to manifested osteolysis and accelerated death in mice, while DLC1 overexpression suppressed bo**ne metasta**sis. Activation of Rho-ROCK signaling in the absence of DLC1 mediated SMAD3 linker region phosphorylation and TGF-β-induced expression of parathyroid hormone-like hormone (PTHLH), leading to osteoclast maturation for osteolytic colonization. Furthermore, pharmacological inhibition of Rho-ROCK effectively reduced PTHLH production and breast cancer b**one metast**asis in vitro and in vivo. Evaluation of clinical breast tumor samples revealed that reduced DLC1 expression was linked to elevated PTHLH expression and organ-speci**fic metast**asis to bone. Overall, our findings define a stroma-dependent paradigm of Rho signaling in cancer and implicate Rho-TGF-β crosstalk in osteolytic **bone metas**tasis.
3. The role of Dlc1 isoform 2 in K-Ras2(G12D) induced thymic cancer. The Deleted in liver cancer one (Dlc1) tumor suppressor gene encodes a RhoGTPase activating protein (RhoGAP). The Dlc1 gene has multiple transcriptional isoforms and we have previously established a mouse strain containing a gene trap (gt) insertion, which specifically reduces the expression of the 6.1 kb isoform (isoform 2). This gene trapped allele when homozygous results in embryonic lethality and the heterozygous gene trapped mice do not show an increased incidence of cancers, suggesting that cooperating oncogenic changes may be required for transformation. In the present work, we have studied the in vivo cooperation between oncogenic K-Ras2 and Dlc1 genes in tumourigenesis. We have observed an increase in invasive thymic cancers, including both thymomas and lymphomas, resulting in significantly shortened life spans in mice heterozygous for the gt Dlc1 allele and an inducible LSL-K-Ras2(G12D) allele compared with the LSL-K-Ras2(G12D) only mice. The heterozygous mice showed a high degree of **metastasis** in the lung. We have found tumour specific selective hypermethylation of the Dlc1 isoform 2 promoter and reduction of the corresponding protein expression in thymic lymphoma (TL) and thymic epithelial carcinoma (TEC) derived from the thymic tumours. The Dlc1 deficient thymic lymphoma cell lines exhibited increased trans-endothelial cell migration. TEC cell lines also exhibited increased stress fiber formation and Rho activity. Introduction of the three Dlc1 isoforms tagged with GFP into these cells resulted in different morphological changes. These results suggest that loss of expression of only isoform 2 may be sufficient for the development of thymic tumors and **metastasis**.

#### GeneRIFs

1. Loss of expression of only Dlc1 isoform 2 may be sufficient for the development of thymic tumors and **metastasis**.

---

## Lyst

- NCBI Gene
- GeneRIF
- Pubmed

| Search Term | Rank | Fields |
| --- | --- | --- |
| [Mm]igration | 6 | PubMed(2) |
| [Ee]xtracellular [Mm]atrix | 8 | PubMed(1) |
| [Ii]nvasive | 10 | PubMed(1) |
| [Mm]etastasis | 12 | PubMed(7) |

  
hide/show details for Lyst

### [Mm]igration

#### PubMed

1. Milky spots promote ovarian cancer metastatic colonization of peritoneal adipose in experimental models. The goal of controlling ovarian cancer metastasis formation has elicited considerable interest in identifying the tissue microenvironments involved in cancer cell colonization of the omentum. Omental adipose is a site of prodigious metastasis in both ovarian cancer models and clinical disease. This tissue is unusual for its milky spots, comprised of immune cells, stromal cells, and structural elements surrounding glomerulus-like capillary beds. The present study shows the novel finding that milky spots and adipocytes play distinct and complementary roles in omental metastatic colonization. In vivo assays showed that ID8, CaOV3, HeyA8, and SKOV3ip.1 cancer cells preferentially lodge and grow within omental and splenoportal fat, which contain milky spots, rather than in peritoneal fat depots. Similarly, medium conditioned by milky spot-containing adipose tissue caused 75% more cell **migratio**n than did medium conditioned by milky spot-deficient adipose. Studies with immunodeficient mice showed that the mouse genetic background does not alter omental milky spot number and size, nor does it affect ovarian cancer colonization. Finally, consistent with the role of lipids as an energy source for cancer cell growth, in vivo time-course studies revealed an inverse relationship between metastatic burden and omental adipocyte content. Our findings support a two-step model in which both milky spots and adipose have specific roles in colonization of the omentum by ovarian cancer cells.
2. Cleavage of the CD11b extracellular domain by the leukocyte serprocidins is critical for neutrophil detachment during chemotaxis. The β(2)-integrin CD11b/CD18 mediates the firm adhesion of neutrophils (PMNs) to epithelial monolayers, a key step in PMN transepithelial **migratio**n. To complete the tran**smigratio**n process, adherent PMNs must detach from epithelial monolayer surfaces to move forward. The mechanism that governs the detachment of adherent PMNs, however, is not clear. Here, we present evidence that cleavage of the CD11b extracellular domain containing the ligand-binding I-domain by 3 structural and functional related serine proteases (elastase, proteinase-3 and cathepsin G) serves as a novel mechanism for PMN detachment after the initial cell adhesion. Kinetic studies showed that the cleavage of CD11b is positively correlated with PMN detachment and subsequent tran**smigratio**n. Moreover, the results demonstrated that elastase, proteinase-3 and cathepsin G all cleaved the purified, functionally active form of CD11b in a pattern similar to the CD11b shedding that occurs during PMN tran**smigratio**n. Their cleavage sites on purified CD11b were located at (761)Thr-Ala(762) (elastase/proteinase-3) and (760)Phe-Thr(761) (cathepsin G), respectively. CD11b cleavage and PMN detachment and chemotaxis, were impaired in elastase/cathepsin G-deficient Beige mice; this defect could be restored by the addition of extracellular elastase. By illustrating CD11b shedding by elastase, proteinase-3 and cathepsin G as a novel mechanism for PMN detachment, our study provides novel therapeutic targets for controlling inflammation.

### [Ee]xtracellular [Mm]atrix

#### PubMed

1. Positional cloning of the Chediak-Higashi syndrome gene: genetic mapping of the beige locus on mouse chromosome 13. Chediak-Higashi syndrome (CHS) is a systemic disorder of human and mouse (beige, bg) that is characterized by aberrant intracellular protein kinesis and lysosomal trafficking. Affected individuals exhibit a severe primary immune deficiency that principally affects the function of granulocytes and cytolytic lymphocytes and partial oculocutaneous albinism, platelet dysfunction, and neurodegeneration. Chediak-Higashi syndrome is inherited as an autosomal recessive Mendelian trait in human and mouse and maps on proximal mouse Chromosome 13.To clone positionally the defective gene in CHS, we have generated a large number of backcross mice who segregate for beige. Genomic DNA from these mice was genotyped for 26 genetic markers known to map on proximal mouse Chromosome 13.By segregation analysis, bg was localized to a 0.24 centiMorgan interval and was shown to cosegregate with 6 genetic markers (Nid, Estm9, D13Mit56, D13Mit162, D13Mit237, and D13Mit240). Two of these loci, Nid and Estm9, are genes and represent candidates for bg. Nidogen (Nid) encodes an **extracellular matrix** protein that is a component of basement membranes. Estm9 is a sequence that is transcribed ubiquitously in mouse embryos and encodes a protein of unknown function. Mutation analysis of Nid and Estm9 was undertaken in 6 bg alleles; no differences were observed between bg and coisogenic controls by analysis of Northern blots, Southern blots, or by quantitative reverse transcription and polymerase chain reaction.These studies indicate that a genomic rearrangement affecting Nid or Estm9 does not underlie bg. The bg locus has been localized on mouse Chromosome 13 with sufficient precision to enable rapid cloning of the bg non-recombinant interval and eventual identification of the gene for Chediak-Higashi syndrome among sequences within the interval.

### [Ii]nvasive

#### PubMed

1. Differentiating inbred mouse strains from each other and those with single gene mutations using hair proteomics. Mutant laboratory mice with distinctive hair phenotypes are useful for identifying genes responsible for hair diseases. The work presented here demonstrates that shotgun proteomic profiling can distinguish hair shafts from different inbred mouse strains. For this purpose, analyzing the total hair shaft provided better discrimination than analyzing the isolated solubilized and particulate (cross-linked) fractions. Over 100 proteins exhibited significant differences among the 11 strains and 5 mutant stocks across the wide spectrum of strains surveyed. Effects on the profile of single gene mutations causing hair shaft defects were profound. Since the hair shaft provides a discrete sampling of the species proteome, with constituents serving important functions in epidermal appendages and throughout the body, this work provides a foundation for non-**invasive** diagnosis of genetic diseases of hair and perhaps other tissues.

### [Mm]etastasis

#### PubMed

1. Exercise and in vivo natural cytotoxicity against tumour cells of varying metastatic capacity. Identification of modifiable risk factors which contribute to tumour **metastasis** is an important goal of cancer prevention research. This study was designed to evaluate the role of moderate exercise conditioning on the in vivo retention of radiolabelled H-ras-transformed fibroblasts (CIRAS 1 and CIRAS 3 cell lines) in mice with (C3H/He-bg2J/+) and without (C3H/HeJ) the beige (bg) mutation which produces impaired natural killer (NK) cell function. Mice were randomly assigned to treadmill training (20 m/min, 30 min/day, 5 times/week) or remained sedentary for 9 weeks. C3H beige mice had significantly higher retention in the lungs of CIRAS 1 tumour cells (P < 0.0001) than genotypically normal C3H/HeJ mice. There was a significant main effect of exercise conditioning (P < 0.05) with trained mice (irrespective of genotype) having lower tumour retention (44 +/- 3%) than sedentary controls (53 +/- 3%). Comparing the in vivo retention of CIRAS 1 and CIRAS 3 in the lungs of genotypically normal mice, we observed higher retention of CIRAS 1 (48 +/- 2%, compared with 18 +/- 2% for CIRAS 3; P < 0.0001). There was a significant interaction effect of exercise and tumour type (P < 0.05); trained mice had a lower retention for CIRAS 1 than sedentary controls (43 +/- 3%, compared with 53 +/- 3%), while training status had no effect on CIRAS 3 retention. There were no significant differences in final tumour multiplicity in the lung as a function of exercise condition. The data suggest that exercise conditioning reduces some measures of in vivo burden but only for the less aggressive tumour variant (CIRAS 1); further, innate immune mechanisms other than NK cells are likely to be involved in this modest protective effect.
2. Role of natural killer cells on engraftment of human lymphoid cells and on **metastasis** of human T-lymphoblastoid leukemia cells in C57BL/6J-scid mice and in C57BL/6J-scid bg mice. The severe combined immunodeficiency (scid) mutation was backcrossed onto the C57BL/6J strain background in order to study the role of natural killer (NK) cells in rejection of normal and malignant human lymphohematopoietic cells. C57BL/6J-scid/scid mice showed severe loss of mature T and B cells accompanied by increased percentages of NK1.1+ cells and myeloid cells. Although little or no serum immunoglobulin was detectable prior to 2 months of age, all mice tested had circulating immunoglobulin by 7.5 months of age. C57BL/6J-scid/scid mice had markedly elevated levels of both hemolytic complement activity and NK cell activity compared with C57BL/6J - (+/+) controls. Weekly injections with anti-NK1.1 antibody resulted in elimination of NK cell activity in C57BL/6J-scid/scid mice throughout 8 weeks of treatment. Although human CEM-C7 T lymphoblastoid tumor cells grew slowly in unmanipulated C57BL/6J-scid/scid mice, anti-NK1.1 treatment resulted in increased growth accompanied by **metastasis** of human lymphoma cells to the brain, liver, and kidney. In contrast to T lymphoblastoid tumor cells, nonmalignant human peripheral blood mononuclear cells engrafted at low levels in anti-NK1.1-treated as well as in unmanipulated C57BL/6-scid/scid mice. Backcrossing of the beige (bgJ) mutation onto the C57BL/6-scid/scid genetic stock caused decreased NK cell activity accompanied by granulocyte defects. C57BL/6-scid/scid bgJ)/bgJ) mice showed **metastasis** of human CEM-C7 cells to the brain and other organs but supported only low levels of engraftment with human peripheral blood mononuclear cells. These results demonstrate that NK cells, in the absence of an adaptive immune system, function in resistance to **metastasis** of human lymphomas and suggest that innate immune factors in addition to NK cell function mediate resistance to engraftment of normal human peripheral blood leukocytes.
3. Role of NK cells in tumour growth and **metastasis** in beige mice. Although natural killer (NK) cells are thought to give the host a spontaneous resistance against tumours and have been postulated to act in vivo as surveillor cells, definitive data in support of these hypotheses has not been obtained. Recently the beige (bg) mouse, a morphological homologue of the human Chediak-Higashi (CH) syndrome, was shown to be deficient in NK activity. Specifically, spleen cells of bg mice were demonstrated to be incapable of in vitro natural cytotoxicity against tumour cells. We report here that a tumour line, modified to be sensitive to NK cytotoxicity by in vitro culture, demonstrated in vivo an increased growth rate, faster induction time and an increased metastatic capability in bg compared to control mice. This was not found with a tumour line insensitive to NK activity (without in vitro culture). In vivo activation of NK cells in bg and control mice resulted in a decrease in tumour growth rate and metastatic frequency. These results demonstrate that NK cells have an important function in the host's control of tumour growth and **metastasis**.
4. Evaluation of three lines of immunodeficient mice for the study of spontaneous metastatic tumors. Various immunodeficient animals have been used as transplantation recipients for studying the growth of human tumors. We have been assessing the value of immunodeficiencies for the study of naturally arising tumors, using a model system of transgenic mice that spontaneously develop cancer of the pancreas as a result of elastase promoter-driven expression of the large tumor antigen gene of simian virus 40. We previously reported the establishment of transgenic mice that carried the SCID and/or beige mutations, eliminating B- and T-cell function and reducing lytic NK cell activity, respectively. In SCID beige animals, **metastasis** rates and target organs for metastases were similar to those observed in humans with pancreatic cancer. We describe here analysis of subsequent more highly inbred generations of these mice. The data show that inbreeding has almost negated the value of these immunodeficiencies for enhancing disease progression, and we observe high rates of **metastasis** even in immunocompetent animals. The data suggest that SCID and beige immunodeficiencies may not always have the same value for the modeling of spontaneous tumors as they do for the study of xenografts.
5. Milky spots promote ovarian cancer metastatic colonization of peritoneal adipose in experimental models. The goal of controlling ovarian cancer **metastasis** formation has elicited considerable interest in identifying the tissue microenvironments involved in cancer cell colonization of the omentum. Omental adipose is a site of prodigious **metastasis** in both ovarian cancer models and clinical disease. This tissue is unusual for its milky spots, comprised of immune cells, stromal cells, and structural elements surrounding glomerulus-like capillary beds. The present study shows the novel finding that milky spots and adipocytes play distinct and complementary roles in omental metastatic colonization. In vivo assays showed that ID8, CaOV3, HeyA8, and SKOV3ip.1 cancer cells preferentially lodge and grow within omental and splenoportal fat, which contain milky spots, rather than in peritoneal fat depots. Similarly, medium conditioned by milky spot-containing adipose tissue caused 75% more cell migration than did medium conditioned by milky spot-deficient adipose. Studies with immunodeficient mice showed that the mouse genetic background does not alter omental milky spot number and size, nor does it affect ovarian cancer colonization. Finally, consistent with the role of lipids as an energy source for cancer cell growth, in vivo time-course studies revealed an inverse relationship between metastatic burden and omental adipocyte content. Our findings support a two-step model in which both milky spots and adipose have specific roles in colonization of the omentum by ovarian cancer cells.
6. Enhanced human tumor cell transplantability in a new congenic immunodeficient mouse; KSN-BNX. We introduced two mutant genes (beige; bg that induces the deficiency of natural killer (NK) activity and xid that decreases the production of immunoglobulin) into KSN nude mice with high reproductive performances. We produced KSN bg/bg(nu/nu) (KSN-bg), KSN-xid/xid(nu/nn) (KSN-xid), KSN xid/xid,bg/bg(nu/nu) (KSN-BNX) and KSN-nu/+ (KS) mice by back-cross (cross-intercross method). All strains showed as high a reproductivity rate as the parental KSN mice. KSN-xid and KSN-BNX mice had a reduced percentage of B220 positive cells in the spleens compared to KSN and KSN-bg mice, but they showed increased percentages of Thy-1 and asialo GM1 positive cells. The serum immunoglobulin concentrations of KSN-BNX were as low as KSN-xid. Both KSN-bg and KSN-BNX mice showed deficient NK activity in spleens, whereas KSN-xid mice showed an elevated NK activity. Compared to nude mice, the growth of both human tumor cell TCO-1 and BxPc-3 transplanted subcutaneously was enhanced in KSN-BNX mice. However Panc-1 cells that was rejected in nude mice was not accepted in KSN-BNX mice. Liver **metastasis** of human pancreatic tumor cells; Capan-1, BxPc-3 and MIAPaCa-2 were studied. No significant difference was observed in the percentage of **metastasis** formed mice between nude and KSN-BNX mice.
7. Mechanisms of the antimetastatic effect in the liver and of the hepatocyte injury induced by alpha-galactosylceramide in mice. The role of mouse liver NK1.1 Ag(+) T (NKT) cells in the antitumor effect of alpha-galactosylceramide (alpha-GalCer) has been unclear. We now show that, whereas alpha-GalCer increased the serum IFN-gamma concentration and alanine aminotransferase activity in NK cell-depleted C57BL/6 (B6) mice and B6-beige/beige mice similarly to its effects in control B6 mice, its enhancement of the antitumor cytotoxicity of liver mononuclear cells (MNCs) was abrogated. Depletion of both NK and NKT cells in B6 mice reduced all these effects of alpha-GALCER: Injection of Abs to IFN-gamma also inhibited the alpha-GalCer-induced increase in antitumor cytotoxicity of MNCS: alpha-GalCer induced the expression of Fas ligand on NKT cells in the liver of B6 mice. Whereas alpha-GalCer did not increase serum alanine aminotransferase activity in B6-lpr/lpr mice and B6-gld/gld mice, it increased the antitumor cytotoxicity of liver MNCS: The alpha-GalCer-induced increase in survival rate apparent in B6 mice injected intrasplenically with B16 tumor cells was abrogated in beige/beige mice, NK cell-depleted B6 mice, and B6 mice treated with Abs to IFN-gamma. Depletion of CD8(+) T cells did not affect the alpha-GalCer-induced antitumor cytotoxicity of liver MNCs but reduced the effect of alpha-GalCer on the survival of B6 mice. Thus, IFN-gamma produced by alpha-GalCer-activated NKT cells increases both the innate antitumor cytotoxicity of NK cells and the adaptive antitumor response of CD8(+) T cells, with consequent inhibition of tumor **metastasis** to the liver. Moreover, NKT cells mediate alpha-GalCer-induced hepatocyte injury through Fas-Fas ligand signaling.

---

## Mapre1

- NCBI Gene
- GeneRIF
- Pubmed

| Search Term | Rank | Fields |
| --- | --- | --- |
| [Ff]ilopodia | 1 | PubMed(2); GeneRIFs(1) |
| [Pp]odosome(s)? | 5 | PubMed(1) |
| [Mm]igration | 6 | PubMed(11); GeneRIFs(3) |

  
hide/show details for Mapre1

### [Ff]ilopodia

#### PubMed

1. Visualization of microtubule growth in living platelets reveals a dynamic marginal band with multiple microtubules. The marginal band of microtubules maintains the discoid shape of resting blood platelets. Although studies of platelet microtubule coil structure conclude that it is composed of a single microtubule, no investigations of its dynamics exist. In contrast to previous studies, permeabilized platelets incubated with GTP-rhodamine-tubulin revealed tubulin incorporation at 7.9 (+/- 1.9) points throughout the coil, and anti-EB1 antibodies stained 8.7 (+/- 2.0) sites, indicative of multiple free microtubules. To pursue this result, we expressed the microtubule plus-end marker EB3-GFP in megakaryocytes and examined its behavior in living platelets released from these cells. Time-lapse microscopy of EB3-GFP in resting platelets revealed multiple assembly sites within the coil and a bidirectional pattern of assembly. Consistent with these findings, tyrosinated tubulin, a marker of newly assembled microtubules, localized to resting platelet microtubule coils. These results suggest that the resting platelet marginal band contains multiple highly dynamic microtubules of mixed polarity. Analysis of microtubule coil diameters in newly formed resting platelets indicates that microtubule coil shrinkage occurs with aging. In addition, activated EB3-GFP-expressing platelets exhibited a dramatic increase in polymerizing microtubules, which travel outward and into **filopodia**. Thus, the dynamic microtubules associated with the marginal band likely function during both resting and activated platelet states.
2. Migration and actin protrusion in melanoma cells are regulated by EB1 protein. Remodeling of actin and microtubule cytoskeletons is thought to be coupled; however, the interplay between these two systems is not fully understood. We show a microtubule end-binding protein, EB1, is required for formation of polarize morphology and motility of melanoma cells. EB1 depletion decreased lamellipodia protrusion, and resulted in loss of opposed protruding and retracting cell edges. Lamellipodia attenuation correlated with mis-localization of **filopodia** throughout the cell and decreased Arp3 localization. EB1-depleted cells displayed less persistent migration and reduced velocity in single-cell motility experiments. We propose EB1 coordinates melanoma cell migration through regulating the balance between lamellipodial and **filopodia**l protrusion.

#### GeneRIFs

1. EB1 coordinates melanoma cell migration through regulating the balance between lamellipodial and **filopodia**l protrusion.

### [Pp]odosome(s)?

#### PubMed

1. Microtubule dynamic instability controls **podosome** patterning in osteoclasts through EB1, cortactin, and Src. In osteoclasts (OCs) **podosomes** are organized in a belt, a feature critical for bone resorption. Although microtubules (MTs) promote the formation and stability of the belt, the MT and/or **podosome** molecules that mediate the interaction of the two systems are not identified. Because the growing "plus" ends of MTs point toward the **podosome** belt, plus-end tracking proteins (+TIPs) might regulate **podosome** patterning. Among the +TIPs, EB1 increased as OCs matured and was enriched in the **podosome** belt, and EB1-positive MTs targeted **podosomes**. Suppression of MT dynamic instability, displacement of EB1 from MT ends, or EB1 depletion resulted in the loss of the **podosome** belt. We identified cortactin as an Src-dependent interacting partner of EB1. Cortactin-deficient OCs presented a defective MT targeting to, and patterning of, **podosomes** and reduced bone resorption. Suppression of MT dynamic instability or EB1 depletion increased cortactin phosphorylation, decreasing its acetylation and affecting its interaction with EB1. Thus, dynamic MTs and **podosomes** interact to control bone resorption.

### [Mm]igration

#### PubMed

1. Membrane localization of adenomatous polyposis coli protein at cellular protrusions: targeting sequences and regulation by beta-catenin. Adenomatous polyposis coli protein (APC) translocates to, and stabilizes, the plus-ends of microtubules. In microtubule-dependent cellular protrusions, APC frequently accumulates in peripheral clusters at the basal membrane. APC targeting to membrane clusters is important for cell **migration**, but the localization mechanism is poorly understood. In this study, we performed deletion mapping and defined a minimal sequence (amino acids 1-2226) that efficiently targets APC to membrane clusters. This sequence lacks DLG-1 and EB1 binding sites, suggesting that these partners are not absolutely required for APC membrane targeting. A series of APC sequences were transiently expressed in cells and compared for their ability to compete endogenous APC at the membrane; potent inhibition of endogenous APC targeting was elicited by the Armadillo- (binds KAP3A, B56alpha, and ASEF) and beta-catenin-binding domains. The Armadillo domain was predicted to inhibit APC membrane localization through sequestration of the kinesin-KAP3A complex. The role of beta-catenin in APC membrane localization was unexpected but affirmed by overexpressing the APC binding sequence of beta-catenin, which similarly reduced APC membrane staining. Furthermore, we used RNA interference to show that loss of beta-catenin reduced APC at membrane clusters in migrating cells. In addition, we report that transiently expressed APC-yellow fluorescent protein co-localized with beta-catenin, KAP3A, EB1, and DLG-1 at membrane clusters, but only beta-catenin stimulated APC anchorage at the membrane. Our findings identify beta-catenin as a regulator of APC targeting to membrane clusters and link these two proteins to cell **migration**.
2. ACF7 regulates cytoskeletal-focal adhesion dynamics and **migration** and has ATPase activity. Coordinated interactions between microtubule (MT) and actin cytoskeletons are involved in many polarized cellular processes. Spectraplakins are enormous (>500 kDa) proteins able to bind both MTs and actin filaments (F-actin) directly. To elucidate the physiological significance and functions of mammalian spectraplakin ACF7, we've conditionally targeted it in skin epidermis. Intriguingly, ACF7 deficiency compromises the targeting of microtubules along F-actin to focal adhesions (FAs), stabilizes FA-actin networks, and impairs epidermal **migration**. Exploring underlying mechanisms, we show that ACF7's binding domains for F-actin, MTs, and MT plus-end proteins are not sufficient to rescue the defects in FA-cytoskeletal dynamics and **migration** functions of ACF7 null keratinocytes. We've uncovered an intrinsic actin-regulated ATPase domain in ACF7 and demonstrate that it is both functional and essential for these roles. Our findings provide insight into the functions of this important cytoskeletal crosslinking protein in regulating dynamic interactions between MTs and F-actin to sustain directional cell movement.
3. Amer2 protein interacts with EB1 protein and adenomatous polyposis coli (APC) and controls microtubule stability and cell **migration**. EB1 is key factor in the organization of the microtubule cytoskeleton by binding to the plus-ends of microtubules and serving as a platform for a number of interacting proteins (termed +TIPs) that control microtubule dynamics. Together with its direct binding partner adenomatous polyposis coli (APC), EB1 can stabilize microtubules. Here, we show that Amer2 (APC membrane recruitment 2), a previously identified membrane-associated APC-binding protein, is a direct interaction partner of EB1 and acts as regulator of microtubule stability together with EB1. Amer2 binds to EB1 via specific (S/T)xIP motifs and recruits it to the plasma membrane. Coexpression of Amer2 and EB1 generates stabilized microtubules at the plasma membrane, whereas knockdown of Amer2 leads to destabilization of microtubules. Knockdown of Amer2, APC, or EB1 reduces cell **migration**, and morpholino-mediated down-regulation of Xenopus Amer2 blocks convergent extension cell movements, suggesting that the Amer2-EB1-APC complex regulates cell **migration** by altering microtubule stability.
4. EB1 and APC bind to mDia to stabilize microtubules downstream of Rho and promote cell **migration**. Lysophosphatidic acid (LPA) stimulates Rho GTPase and its effector, the formin mDia, to capture and stabilize microtubules in fibroblasts. We investigated whether mammalian EB1 and adenomatous polyposis coli (APC) function downstream of Rho-mDia in microtubule stabilization. A carboxy-terminal APC-binding fragment of EB1 (EB1-C) functioned as a dominant-negative inhibitor of microtubule stabilization induced by LPA or active mDia. Knockdown of EB1 with small interfering RNAs also prevented microtubule stabilization. Expression of either full-length EB1 or APC, but not an APC-binding mutant of EB1, was sufficient to stabilize microtubules. Binding and localization studies showed that EB1, APC and mDia may form a complex at stable microtubule ends. Furthermore, EB1-C, but not an APC-binding mutant, inhibited fibroblast **migration** in an in vitro wounding assay. These results show an evolutionarily conserved pathway for microtubule capture, and suggest that mDia functions as a scaffold protein for EB1 and APC to stabilize microtubules and promote cell **migration**.
5. Amer2 protein interacts with EB1 protein and adenomatous polyposis coli (APC) and controls microtubule stability and cell **migration**. EB1 is key factor in the organization of the microtubule cytoskeleton by binding to the plus-ends of microtubules and serving as a platform for a number of interacting proteins (termed +TIPs) that control microtubule dynamics. Together with its direct binding partner adenomatous polyposis coli (APC), EB1 can stabilize microtubules. Here, we show that Amer2 (APC membrane recruitment 2), a previously identified membrane-associated APC-binding protein, is a direct interaction partner of EB1 and acts as regulator of microtubule stability together with EB1. Amer2 binds to EB1 via specific (S/T)xIP motifs and recruits it to the plasma membrane. Coexpression of Amer2 and EB1 generates stabilized microtubules at the plasma membrane, whereas knockdown of Amer2 leads to destabilization of microtubules. Knockdown of Amer2, APC, or EB1 reduces cell **migration**, and morpholino-mediated down-regulation of Xenopus Amer2 blocks convergent extension cell movements, suggesting that the Amer2-EB1-APC complex regulates cell **migration** by altering microtubule stability.
6. EB1 and APC bind to mDia to stabilize microtubules downstream of Rho and promote cell **migration**. Lysophosphatidic acid (LPA) stimulates Rho GTPase and its effector, the formin mDia, to capture and stabilize microtubules in fibroblasts. We investigated whether mammalian EB1 and adenomatous polyposis coli (APC) function downstream of Rho-mDia in microtubule stabilization. A carboxy-terminal APC-binding fragment of EB1 (EB1-C) functioned as a dominant-negative inhibitor of microtubule stabilization induced by LPA or active mDia. Knockdown of EB1 with small interfering RNAs also prevented microtubule stabilization. Expression of either full-length EB1 or APC, but not an APC-binding mutant of EB1, was sufficient to stabilize microtubules. Binding and localization studies showed that EB1, APC and mDia may form a complex at stable microtubule ends. Furthermore, EB1-C, but not an APC-binding mutant, inhibited fibroblast **migration** in an in vitro wounding assay. These results show an evolutionarily conserved pathway for microtubule capture, and suggest that mDia functions as a scaffold protein for EB1 and APC to stabilize microtubules and promote cell **migration**.
7. Bim1p/Yeb1p mediates the Kar9p-dependent cortical attachment of cytoplasmic microtubules. In Saccharomyces cerevisiae, positioning of the mitotic spindle depends on the interaction of cytoplasmic microtubules with the cell cortex. In this process, cortical Kar9p in the bud acts as a link between the actin and microtubule cytoskeletons. To identify Kar9p-interacting proteins, a two-hybrid screen was conducted with the use of full-length Kar9p as bait, and three genes were identified: BIM1, STU2, and KAR9 itself. STU2 encodes a component of the spindle pole body. Bim1p is the yeast homologue of the human microtubule-binding protein EB1, which is a binding partner to the adenomatous polyposis coli protein involved in colon cancer. Eighty-nine amino acids within the third quarter of Bim1p was sufficient to confer interaction with Kar9p. The two-hybrid interactions were confirmed with the use of coimmunoprecipitation experiments. Genetic analysis placed Bim1p in the Kar9p pathway for nuclear **migration**. Bim1p was not required for Kar9p's cortical or spindle pole body localization. However, deletion of BIM1 eliminated Kar9p localization along cytoplasmic microtubules. Furthermore, in the bim1 mutants, the cytoplasmic microtubules no longer intersected the cortical dot of Green Fluorescent Protein-Kar9p. These experiments demonstrate that the interaction of cytoplasmic microtubules with the Kar9p cortical attachment site requires the microtubule-binding protein Bim1p.
8. Microtubule plus-end binding protein EB1 is necessary for muscle cell differentiation, elongation and fusion. During muscle differentiation, microtubule stability, nucleation and orientation all undergo profound changes, which are simultaneous with and possibly necessary for the elongation and fusion of muscle cells. We do not yet understand these events, but they present similarities with the polarized **migration** of fibroblasts, in which EB1 is necessary for microtubule stabilization. However, it was recently reported that EB3, not EB1, is involved in muscle cell elongation and fusion, and that neither of these two proteins influences microtubule stabilization. To re-examine the role of EB1, we have generated C2 cell lines permanently expressing EB1-targeted shRNAs. In these lines, EB1 is specifically knocked down by more than 90% before any differentiation-related changes can take place. We find that differentiation (assessed by myogenin expression), elongation and fusion are prevented. In addition, two early events that normally precede differentiation - microtubule stabilization and the accumulation of cadherin and beta-catenin on the plasma membrane - are inhibited. Re-expression of EB1 as EB1-GFP restores all aspects of normal differentiation, whereas overexpression of EB3-GFP restores elongation but not fusion. We conclude that EB1 is necessary for the early stages of muscle differentiation.
9. **Migration** and actin protrusion in melanoma cells are regulated by EB1 protein. Remodeling of actin and microtubule cytoskeletons is thought to be coupled; however, the interplay between these two systems is not fully understood. We show a microtubule end-binding protein, EB1, is required for formation of polarize morphology and motility of melanoma cells. EB1 depletion decreased lamellipodia protrusion, and resulted in loss of opposed protruding and retracting cell edges. Lamellipodia attenuation correlated with mis-localization of filopodia throughout the cell and decreased Arp3 localization. EB1-depleted cells displayed less persistent **migration** and reduced velocity in single-cell motility experiments. We propose EB1 coordinates melanoma cell **migration** through regulating the balance between lamellipodial and filopodial protrusion.
10. The microtubule-associated protein EB1 maintains cell polarity through activation of protein kinase C. The plus-ends of microtubules target the cell cortex to modulate actin protrusion dynamics and polarity, but little is known of the molecular mechanism that couples the interaction. EB1 protein associates with the plus-ends of microtubules, placing EB1 in an ideal spatial position to mediate microtubule-actin cross talk. The objective of the current study was to further understand intracellular signaling involved in EB1-dependent cell polarity and motility. B16F10 mouse melanoma cells were depleted of EB1 protein using short hair-pin RNA interference. Correlative live cell-immunofluorescence microscopy was performed to determine localization of WAVE2 and IQGAP1 to protruding versus retracting edges. EB1 knock down caused poor subcellular separation of WAVE2 and IQGAP1, and overall decreased localization. Activation of PKC corrected defects in WAVE2 and IQGAP1 localization, cell spreading and cell shape to levels observed in control cells, but did not correct defects in cell **migration**. Consistent with these findings, decreased PKC phosphorylation was observed in EB1 knock down cells. These findings support a model where EB1 protein links microtubules to actin protrusion and cell polarity through signaling pathways involving PKC.
11. SMRT analysis of MTOC and nuclear positioning reveals the role of EB1 and LIC1 in single-cell polarization. In several migratory cells, the microtubule-organizing center (MTOC) is repositioned between the leading edge and nucleus, creating a polarized morphology. Although our understanding of polarization has progressed as a result of various scratch-wound and cell **migration** studies, variations in culture conditions required for such assays have prevented a unified understanding of the intricacies of MTOC and nucleus positioning that result in cell polarization. Here, we employ a new SMRT (for sparse, monolayer, round, triangular) analysis that uses a universal coordinate system based on cell centroid to examine the pathways regulating MTOC and nuclear positions in cells plated in a variety of conditions. We find that MTOC and nucleus positioning are crucially and independently affected by cell shape and confluence; MTOC off-centering correlates with the polarization of single cells; acto-myosin contractility and microtubule dynamics are required for single-cell polarization; and end binding protein 1 and light intermediate chain 1, but not Par3 and light intermediate chain 2, are required for single-cell polarization and directional cell motility. Using various cellular geometries and conditions, we implement a systematic and reproducible approach to identify regulators of MTOC and nucleus positioning that depend on extracellular guidance cues.

#### GeneRIFs

1. the Amer2-EB1-APC complex regulates cell **migration** by altering microtubule stability.
2. EB1 coordinates melanoma cell **migration** through regulating the balance between lamellipodial and filopodial protrusion.
3. results show an evolutionarily conserved pathway for microtubule capture, and suggest that mDia functions as a scaffold protein for EB1 and APC to stabilize microtubules and promote cell **migration**.

---

## Slc6a5

- NCBI Gene
- GeneRIF
- Pubmed

| Search Term | Rank | Fields |
| --- | --- | --- |
| [Mm]igration | 6 | PubMed(1) |

  
hide/show details for Slc6a5

### [Mm]igration

#### PubMed

1. Excitatory neurons of the proprioceptive, interoceptive, and arousal hindbrain networks share a developmental requirement for Math1. Hindbrain networks important for sensation and arousal contain diverse neuronal populations with distinct projections, yet share specific characteristics such as neurotransmitter expression. The relationship between the function of these neurons, their developmental origin, and the timing of their **migration** remains unclear. Mice lacking the proneural transcription factor Math1 (Atoh1) lose neurons essential for hearing, balance, and unconscious proprioception. By using a new, inducible Math1(Cre\*PR) allele, we found that Math1 is also required for the conscious proprioceptive system, including excitatory projection neurons of the dorsal column nuclei and for vital components of the interoceptive system, such as Barrington's nucleus, that is closely associated with arousal. In addition to specific networks, Math1 lineages shared specific neurotransmitter expression, including glutamate, acetylcholine, somatostatin, corticotropin releasing hormone, and nitric oxide. These findings identify twenty novel Math1 lineages and indicate that the Math1 network functions partly as an interface for conscious (early-born) and unconscious (late-born) proprioceptive inputs to the cortex and cerebellum, respectively. In addition, these data provide previously unsuspected genetic and developmental links between proprioception, interoception, hearing, and arousal.

---

## Gnal

- NCBI Gene
- GeneRIF
- Pubmed

| Search Term | Rank | Fields |
| --- | --- | --- |
| [Mm]igration | 6 | PubMed(1) |

  
hide/show details for Gnal

### [Mm]igration

#### PubMed

1. Lhx2-dependent specification of olfactory sensory neurons is required for successful integration of olfactory, vomeronasal, and GnRH neurons. Inactivation of the LIM-homeodomain 2 gene (Lhx2) results in a severe defect in specification of olfactory sensory neurons (OSNs). However, the ramifications of lack of Lhx2-dependent OSN specification for formation of the primary olfactory pathway have not been addressed, since mutant mice die in utero. We have analyzed prenatal and postnatal consequences of conditionally inactivating Lhx2 selectively in OSNs. A cell-autonomous effect is that OSN axons cannot innervate their target, the olfactory bulb. Moreover, the lack of Lhx2 in OSNs causes unpredicted, non-cell-autonomous phenotypes. First, the olfactory bulb shows pronounced hypoplasia in adults, and the data suggest that innervation by correctly specified OSNs is necessary for adult bulb size and organization. Second, absence of an olfactory nerve in the conditional mutant reveals that the vomeronasal nerve is dependent on olfactory nerve formation. Third, the lack of a proper vomeronasal nerve prevents **migration** of gonadotropin-releasing hormone (GnRH) cells the whole distance to their final positions in the hypothalamus during embryo development. As adults, the conditional mutants do not pass puberty, and these findings support the view of an exclusive nasal origin of GnRH neurons in the mouse. Thus, Lhx2 in OSNs is required for functional development of three separate systems.

---

## Hspa1a

- NCBI Gene
- GeneRIF
- Pubmed

| Search Term | Rank | Fields |
| --- | --- | --- |
| [Mm]igration | 6 | PubMed(1) |
| [Ee]xtracellular [Mm]atrix | 8 | PubMed(2) |
| [Ii]nvasive | 10 | PubMed(1) |

  
hide/show details for Hspa1a

### [Mm]igration

#### PubMed

1. Early transcriptional activation of the hsp70.1 gene by osmotic stress in one-cell embryos of the mouse. In fertilized mouse eggs, de novo transcription of embryonic genes is first observed during the S phase of the one-cell stage. This transcription, however, is mostly limited to the male pronucleus and possibly uncoupled from translation, making the functional meaning obscure. We found that one-cell mouse embryos respond to the osmotic shock of in vitro isolation with **migration** of HSF1, the canonical stress activator of mammalian heat shock genes, to pronuclei and by transient transcription of the hsp70.1, but not hsp70.3 and hsp90, heat shock genes. Isolated growing dictyate oocytes also display a nuclear HSF1 localization, but, in contrast with embryos, they transcribe both hsp70.1 and hsp70.3 genes only after heat shock. Intranuclear injection of double-stranded oligodeoxyribonucleotides containing HSE, GAGA box or GC box consensus sequences, and antibodies raised to transcription factors HSF1, HSF2, Drosophila melanogaster GAGA factor, or Sp1 demonstrated that hsp70.1 transcription depends on HSF1 in both oocytes and embryos and that Sp1 is dispensable in oocytes and inhibitory in the embryos. Hsp70.1 thus represents the first endogenous gene so far identified to be physiologically activated and tightly regulated after fertilization in mammals.

### [Ee]xtracellular [Mm]atrix

#### PubMed

1. The 70 kDa heat shock protein protects against experimental traumatic brain injury. Traumatic brain injury (TBI) causes disruption of the blood brain barrier (BBB) leading to hemorrhage which can complicate an already catastrophic illness. Matrix metalloproteinases (MMPs) involved in the breakdown of the **extracellular matrix** may lead to brain hemorrhage. We explore the contribution of the 70 kDa heat shock protein (Hsp70) to outcome and brain hemorrhage in a model of TBI. Male, wildtype (Wt), Hsp70 knockout (Ko) and transgenic (Tg) mice were subjected to TBI using controlled cortical impact (CCI). Motor function, brain hemorrhage and lesion size were assessed at 3, 7 and 14 days. Brains were evaluated for the effects of Hsp70 on MMPs. In Hsp70 Tg mice, CCI led to smaller brain lesions, decreased hemorrhage and reduced expression and activation of MMPs compared to Wt. CCI also significantly decreased right-biased swings and corner turns in the Hsp70 Tg mice. Conversely, Hsp70 Ko mice had significantly increased lesion size, worsened brain hemorrhage and increased expression and activation of MMPs with worsened behavioral outcomes compared to Wt. Hsp70 is protective in experimental TBI. To our knowledge, this is the direct demonstration of brain protection by Hsp70 in a TBI model. Our data demonstrate a new mechanism linking TBI-induced hemorrhage and neuronal injury to the suppression of MMPs by Hsp70, and support the development of Hsp70 enhancing strategies for the treatment of TBI.
2. Recombination in the class III region of the mouse major histocompatibility complex. The sites of meiotic recombination in the class II region of the mouse major histocompatibility complex (MHC) are clustered at hotspots. To search for hotspots in the class III region, we mapped recombinational breakpoints of 79 Ab:H2-D recombinants with 11 DNA markers; these included Tnx, the gene for an **extracellular matrix** protein, tenascin X, the Notch-related Int3 gene, and a microsatellite marker, D17Mit13, none of which had previously been mapped precisely. The results gave the gene order of Eb-61.1-Int3-Tnx-Cyp21/C4-Bf-Hsp68c-D17Mit13+ ++-Tnfa/Tnfb-D. The crossover sites in 40 of the 79 recombinants were confined within the Eb/Int3:Tnx/Cyp21 interval. The result demonstrated that an unequal distribution of recombination is a general feature of the mouse MHC, suggesting the presence of a recombinational hotspot within the Int3:Tnx interval.

### [Ii]nvasive

#### PubMed

1. Inducible heat shock protein 70 prevents multifocal flat dysplastic lesions and **invasive** tumors in an inflammatory model of colon cancer. Heat shock protein 70 (Hsp70) regulates protein biosynthesis and refolding of denatured proteins. Since Hsp70 participates in recovery from stress injury, we examined the effect of Hsp70 genetic deletion in the azoxymethane (AOM)/dextran sulfate sodium (DSS) model of inflammation and colon cancer.Hsp70 mutant mice (Hsp70.1(-/-)/70.3(-/-)) and wild-type (WT) littermates received AOM and three cycles of DSS and were killed 24 weeks later. Tumors were graded for histology and immunostained for p53, adenomatous polyposis coli, beta-catenin, cyclooxygenase-2 (Cox-2) and inducible nitric oxide synthase (iNOS) and sequenced for p53 mutations.Elevated adenomas developed in 4/10 WT mice with no dysplasia in adjacent mucosa. In contrast, 7/8 Hsp70 knock out (KO) mice developed chronic mucosal inflammation and multifocal areas of flat dysplasia and 4/8 progressed to **invasive** carcinomas arising in a background of flat dysplastic mucosa. These differences in the incidence of flat dysplasia and **invasive** cancers were significant (P < 0.05). Nuclear p53 was stronger in Hsp70 KO tumors compared with WT tumors, and sequencing confirmed p53 mutations in 2/5 tumors from Hsp70(-/-) versus 0/5 in WT mice. In Hsp70 WT tumors, beta-catenin was predominantly nuclear, compared with membranous beta-catenin in Hsp70(-/-) tumors, suggesting that Hsp70 regulates beta-catenin in colonic tumorigenesis. Cox-2 and iNOS levels were increased in tumors from Hsp70(-/-) mice compared with Hsp70 WT tumors.Hsp70-deleted mice treated with AOM/DSS develop flat **invasive** colonic tumors that mimic many histological and molecular features of ulcerative colitis colon cancer. This model will be useful to dissect the role of Hsp70 in inflammatory bowel disease colon cancer.

---

## Per3

- NCBI Gene
- GeneRIF
- Pubmed

| Search Term | Rank | Fields |
| --- | --- | --- |
| [Mm]igration | 6 | PubMed(2) |
| [Ee]xtracellular [Mm]atrix | 8 | PubMed(1) |

  
hide/show details for Per3

### [Mm]igration

#### PubMed

1. Calcium prevents tumorigenesis in a mouse model of colorectal cancer. Calcium has been proposed as a mediator of the chemoprevention of colorectal cancer (CRC), but the comprehensive mechanism underlying this preventive effect is not yet clear. Hence, we conducted this study to evaluate the possible roles and mechanisms of calcium-mediated prevention of CRC induced by 1,2-dimethylhydrazine (DMH) in mice.For gene expression analysis, 6 non-tumor colorectal tissues of mice from the DMH + Calcium group and 3 samples each from the DMH and control groups were hybridized on a 4×44 K Agilent whole genome oligo microarray, and selected genes were validated by real-time polymerase chain reaction (PCR). Functional analysis of the microarray data was performed using KEGG and Gene Ontology (GO) analyses. Hub genes were identified using Pathway Studio software.The tumor incidence rates in the DMH and DMH + Calcium groups were 90% and 40%, respectively. Microarray gene expression analysis showed that S100a9, Defa20, Mmp10, Mmp7, Ptgs2, and Ang2 were among the most downregulated genes, whereas Per3, Tef, Rnf152, and Prdx6 were significantly upregulated in the DMH + Calcium group compared with the DMH group. Functional analysis showed that the Wnt, cell cycle, and arachidonic acid pathways were significantly downregulated in the DMH + Calcium group, and that the GO terms related to cell differentiation, cell cycle, proliferation, cell death, adhesion, and cell **migratio**n were significantly affected. Forkhead box M1 (FoxM1) and nuclear factor kappa-B (NF-κB) were considered as potent hub genes.In the DMH-induced CRC mouse model, comprehensive mechanisms were involved with complex gene expression alterations encompassing many altered pathways and GO terms. However, how calcium regulates these events remains to be studied.
2. Calcium prevents tumorigenesis in a mouse model of colorectal cancer. Calcium has been proposed as a mediator of the chemoprevention of colorectal cancer (CRC), but the comprehensive mechanism underlying this preventive effect is not yet clear. Hence, we conducted this study to evaluate the possible roles and mechanisms of calcium-mediated prevention of CRC induced by 1,2-dimethylhydrazine (DMH) in mice.For gene expression analysis, 6 non-tumor colorectal tissues of mice from the DMH + Calcium group and 3 samples each from the DMH and control groups were hybridized on a 4×44 K Agilent whole genome oligo microarray, and selected genes were validated by real-time polymerase chain reaction (PCR). Functional analysis of the microarray data was performed using KEGG and Gene Ontology (GO) analyses. Hub genes were identified using Pathway Studio software.The tumor incidence rates in the DMH and DMH + Calcium groups were 90% and 40%, respectively. Microarray gene expression analysis showed that S100a9, Defa20, Mmp10, Mmp7, Ptgs2, and Ang2 were among the most downregulated genes, whereas Per3, Tef, Rnf152, and Prdx6 were significantly upregulated in the DMH + Calcium group compared with the DMH group. Functional analysis showed that the Wnt, cell cycle, and arachidonic acid pathways were significantly downregulated in the DMH + Calcium group, and that the GO terms related to cell differentiation, cell cycle, proliferation, cell death, adhesion, and cell **migratio**n were significantly affected. Forkhead box M1 (FoxM1) and nuclear factor kappa-B (NF-κB) were considered as potent hub genes.In the DMH-induced CRC mouse model, comprehensive mechanisms were involved with complex gene expression alterations encompassing many altered pathways and GO terms. However, how calcium regulates these events remains to be studied.

### [Ee]xtracellular [Mm]atrix

#### PubMed

1. Matrix metalloproteinase 2 and 9 dysfunction underlie vascular stiffness in circadian clock mutant mice. To determine if elasticity in blood vessels is compromised in circadian clock-mutant mice (Bmal1-knockout [KO] and Per-triple KO) and if matrix metalloproteinases (MMPs) might confer these changes in compliance.High-resolution ultrasonography in vivo revealed impaired remodeling and increased pulse-wave velocity in the arteries of Bmal1-KO and Per-triple KO mice. In addition, compliance of remodeled arteries and naïve pressurized arterioles ex vivo from Bmal1-KO and Per-triple KO mice was reduced, consistent with stiffening of the vascular bed. The observed vascular stiffness was coincident with dysregulation of MMP-2 and MMP-9 in Bmal1-KO mice. Furthermore, inhibition of MMPs improved indexes of pathological remodeling in wild-type mice, but the effect was abolished in Bmal1-KO mice.Circadian clock dysfunction contributes to hardening of arteries, which may involve impaired control of the **extracellular matri**x composition.

---

## Myc

- NCBI Gene
- GeneRIF
- Pubmed

| Search Term | Rank | Fields |
| --- | --- | --- |
| [Ff]ilopodia | 1 | PubMed(1) |
| [Mm]igration | 6 | Process(1); PubMed(22); GeneRIFs(2) |
| ECM | 7 | PubMed(1) |
| [Ee]xtracellular [Mm]atrix | 8 | PubMed(7) |
| [Ii]nvasive | 10 | PubMed(26); GeneRIFs(1) |
| [Mm]etastasize | 11 | PubMed(2) |
| [Mm]etastasis | 12 | PubMed(19); GeneRIFs(4) |
| [Cc]ell [Ii]nvasion | 13 | PubMed(1) |

  
hide/show details for Myc

### [Ff]ilopodia

#### PubMed

1. Stress-induced cleavage of Myc promotes cancer cell survival. Evasion of apoptosis is critical in Myc-induced tumor progression. Here we report that cancer cells evade death under stress by activating calpain-mediated proteolysis of Myc. This generates Myc-nick, a cytoplasmic, transcriptionally inactive cleavage product of Myc. We found conversion of Myc into Myc-nick in cell lines and tissues derived from multiple cancers. In colon cancer, the production of Myc-nick is enhanced under stress conditions such as hypoxia and nutrient deprivation. Under these conditions, ectopic expression of Myc-nick promotes anchorage-independent growth and cell survival at least in part by promoting autophagy. Myc-nick also delays colon cancer cell death after treatment with chemotherapeutic drugs such as etoposide, cisplatin, and imatinib. Furthermore, colon cancer cells expressing a cleavage-resistant form of Myc undergo extensive apoptosis but are rescued by overexpression of Myc-nick. We also found that ectopic expression of Myc-nick results in the induction of the actin-bundling protein fascin, formation of **filopodia**, and increased cell motility-all mediators of tumor metastasis. Myc-nick-induced survival, autophagy, and motility require Myc box II (MBII), a region of Myc-nick that recruits acetyltransferases that in turn modify cytoplasmic proteins, including α-tubulin and ATG3. Our results suggest that Myc-nick-induced survival and motility contribute to colon cancer progression and metastasis.

### [Mm]igration

#### Process

1. positive regulation of smooth muscle cell **migration**

#### PubMed

1. Changes in protooncogene expression correlated with general and sex-specific differentiation in murine primordial germ cells. Primordial germ cells (PGCs) of the mouse undergo key developmental transitions during embryonic days 12-15. On day 12 they complete **migration** into the gonads. They cease mitotic proliferation on day 13 and subsequently enter sex-specific pathways of development. The molecular mechanisms controlling these transitions are poorly understood, yet they are crucial to production of normal gametes later in life. We have used the polymerase chain reaction (PCR) to directly compare levels of expression of several protooncogenes proposed to be involved in control of cell proliferation and differentiation in proliferating and differentiating PCGs of both sexes over a 4 day time course. We report here that mRNA levels for nuclear protooncogenes c-myc, c-fos, and c-jun increase dramatically in both sexes from little or no detectable expression on day 12 to high expression on days 13-15. We observe c-kit message on day 12 in combined PGCs of both sexes, in female but not male PGCs on day 13, and in both sexes on day 14, c-kit mRNA is undetectable on day 15 in either sex, c-mos is not expressed at detectable levels on day 12 in either sex, but increases gradually in female PGCs to very high levels on day 15. In male PGCs, c-mos is expressed at high levels on days 13-15. Our results are consistent with a role for protooncogenes c-myc, c-fos and c-jun in mediating the initial differentiation of PGCs of both sexes that occurs upon colonization of the gonad. Because c-kit and c-mos are expressed differentially in male and female day 13-15 germ cells, they may play roles in initiating or mediating progress along the sex-specific pathways of development that PGCs embark upon at this time.
2. A critical role for the regulated wnt-myc pathway in naive T cell survival. Wnt signaling is involved in T cell development, activation, and differentiation. However, the role for Wnt signaling in mature naive T cells has not been investigated. In this article, we report that activation of Wnt signaling in T cell lineages by deletion of the Apc (adenomatous polyposis coli) gene causes spontaneous T cell activation and severe T cell lymphopenia. The lymphopenia is the result of rapid apoptosis of newly exported, mature T cells in the periphery and is not due to defects in thymocyte development or e**migration**. Using chimera mice consisting of both wild-type and Apc-deficient T cells, we found that loss of naive T cells is due to T cell intrinsic dysregulation of Wnt signaling. Because Apc deletion causes overexpression of the Wnt target gene cMyc, we generated mice with combined deletion of the cMyc gene. Because combined deletion of cMyc and Apc attenuated T cell loss, cMyc overexpression is partially responsible for spontaneous T cell apoptosis and lymphopenia. Cumulatively, our data reveal a missing link between Wnt signaling and survival of naive T cells.
3. Rac1 regulates pancreatic islet morphogenesis. Pancreatic islets of Langerhans originate from endocrine progenitors within the pancreatic ductal epithelium. Concomitant with differentiation of these progenitors into hormone-producing cells such cells delaminate, aggregate and migrate away from the ductal epithelium. The cellular and molecular mechanisms regulating islet cell delamination and cell **migration** are poorly understood. Extensive biochemical and cell biological studies using cultured cells demonstrated that Rac1, a member of the Rho family of small GTPases, acts as a key regulator of cell **migration**.To address the functional role of Rac1 in islet morphogenesis, we generated transgenic mice expressing dominant negative Rac1 under regulation of the Rat Insulin Promoter. Blocking Rac1 function in beta cells inhibited their **migration** away from the ductal epithelium in vivo. Consistently, transgenic islet cell spreading was compromised in vitro. We also show that the EGF-receptor ligand betacellulin induced actin remodelling and cell spreading in wild-type islets, but not in transgenic islets. Finally, we demonstrate that cell-cell contact E-cadherin increased as a consequence of blocking Rac1 activity.Our data support a model where Rac1 signalling controls islet cell **migration** by modulating E-cadherin-mediated cell-cell adhesion. Furthermore, in vitro experiments show that betacellulin stimulated islet cell spreading and actin remodelling is compromised in transgenic islets, suggesting that betacellulin may act as a regulator of Rac1 activity and islet **migration** in vivo. Our results further emphasize Rac1 as a key regulator of cell **migration** and cell adhesion during tissue and organ morphogenesis.
4. Myc deletion rescues Apc deficiency in the small intestine. The APC gene encodes the adenomatous polyposis coli tumour suppressor protein, germline mutation of which characterizes familial adenomatous polyposis (FAP), an autosomal intestinal cancer syndrome. Inactivation of APC is also recognized as the key early event in the development of sporadic colorectal cancers, and its loss results in constitutive activity of the beta-catenin-Tcf4 transcription complex. The proto-oncogene c-MYC has been identified as a target of the Wnt pathway in colorectal cancer cells in vitro, in normal crypts in vivo and in intestinal epithelial cells acutely transformed on in vivo deletion of the APC gene; however, the significance of this is unclear. Therefore, to elucidate the role Myc has in the intestine after Apc loss, we have simultaneously deleted both Apc and Myc in the adult murine small intestine. Here we show that loss of Myc rescued the phenotypes of perturbed differentiation, **migration**, proliferation and apoptosis, which occur on deletion of Apc. Remarkably, this rescue occurred in the presence of high levels of nuclear beta-catenin. Array analysis revealed that Myc is required for the majority of Wnt target gene activation following Apc loss. These data establish Myc as the critical mediator of the early stages of neoplasia following Apc loss.
5. Colorectal cancer in mice genetically deficient in the mucin Muc2. The gastrointestinal tract is lined by a layer of mucus comprised of highly glycosylated proteins called mucins. To evaluate the importance of mucin in intestinal carcinogenesis, we constructed mice genetically deficient in Muc2, the most abundant secreted gastrointestinal mucin. Muc2-/- mice displayed aberrant intestinal crypt morphology and altered cell maturation and **migration**. Most notably, the mice frequently developed adenomas in the small intestine that progressed to invasive adenocarcinoma, as well as rectal tumors. Thus, Muc2 is involved in the suppression of colorectal cancer.
6. Adenomatous polyposis coli is required for early events in the normal growth and differentiation of the developing cerebral cortex. Adenomatous polyposis coli (Apc) is a large multifunctional protein known to be important for Wnt/beta-catenin signalling, cytoskeletal dynamics, and cell polarity. In the developing cerebral cortex, Apc is expressed in proliferating cells and its expression increases as cells migrate to the cortical plate. We examined the consequences of loss of Apc function for the early development of the cerebral cortex.We used Emx1(Cre) to inactivate Apc specifically in proliferating cerebral cortical cells and their descendents starting from embryonic day 9.5. We observed reduction in the size of the mutant cerebral cortex, disruption to its organisation, and changes in the molecular identity of its cells. Loss of Apc leads to a decrease in the size of the proliferative pool, disrupted interkinetic nuclear **migration**, and increased apoptosis. beta-Catenin, pericentrin, and N-cadherin proteins no longer adopt their normal high concentration at the apical surface of the cerebral cortical ventricular zone, indicating that cell polarity is disrupted. Consistent with enhanced Wnt/beta-catenin signalling resulting from loss of Apc we found increased levels of TCF/LEF-dependent transcription and expression of endogenous Wnt/beta-catenin target genes (Axin2 (conductin), Lef1, and c-myc) in the mutant cerebral cortex. In the Apc mutant cerebral cortex the expression of transcription factors Foxg1, Pax6, Tbr1, and Tbr2 is drastically reduced compared to normal and many cells ectopically express Pax3, Wnt1, and Wt1 (but not Wnt2b, Wnt8b, Ptc, Gli1, Mash1, Olig2, or Islet1). This indicates that loss of Apc function causes cerebral cortical cells to lose their normal identity and redirect to fates normally found in more posterior-dorsal regions of the central nervous system.Apc is required for multiple aspects of early cerebral cortical development, including the regulation of cell number, interkinetic nuclear **migration**, cell polarity, and cell type specification.
7. Myc deletion rescues Apc deficiency in the small intestine. The APC gene encodes the adenomatous polyposis coli tumour suppressor protein, germline mutation of which characterizes familial adenomatous polyposis (FAP), an autosomal intestinal cancer syndrome. Inactivation of APC is also recognized as the key early event in the development of sporadic colorectal cancers, and its loss results in constitutive activity of the beta-catenin-Tcf4 transcription complex. The proto-oncogene c-MYC has been identified as a target of the Wnt pathway in colorectal cancer cells in vitro, in normal crypts in vivo and in intestinal epithelial cells acutely transformed on in vivo deletion of the APC gene; however, the significance of this is unclear. Therefore, to elucidate the role Myc has in the intestine after Apc loss, we have simultaneously deleted both Apc and Myc in the adult murine small intestine. Here we show that loss of Myc rescued the phenotypes of perturbed differentiation, **migration**, proliferation and apoptosis, which occur on deletion of Apc. Remarkably, this rescue occurred in the presence of high levels of nuclear beta-catenin. Array analysis revealed that Myc is required for the majority of Wnt target gene activation following Apc loss. These data establish Myc as the critical mediator of the early stages of neoplasia following Apc loss.
8. c-myc and N-myc promote active stem cell metabolism and cycling as architects of the developing brain. myc genes are associated with a wide variety of human cancers including most types of nervous system tumors. While the mechanisms by which myc overexpression causes tumorigenesis are multifaceted and have yet to be clearly elucidated, they are at least in part related to endogenous myc function in normal cells. Knockout (KO) of either c-myc or N-myc genes in neural stem and precursor cells (NSC) driven by nestin-cre impairs mouse brain growth and mutation of N-myc also causes microcephaly in humans in Feingold Syndrome. To further define myc function in NSC and nervous system development, we created a double KO (DKO) for c- and N-myc using nestin-cre. The DKO mice display profoundly impaired overall brain growth associated with decreased cell cycling and **migration** of NSC, which are strikingly decreased in number. The DKO brain also exhibits specific changes in gene expression including downregulation of genes involved in protein and nucleotide metabolism, mitosis, and chromatin structure as well as upregulation of genes associated with differentiation. Together these data support a model of nervous system tumorigenesis in which excess myc aberrantly locks in a developmentally active chromatin state characterized by overactive cell cycling, and metabolism as well as blocked differentiation.
9. c-Myc is essential for urokinase plasminogen activator expression on hypoxia-induced vascular smooth muscle cells. The purpose of this study was to investigate whether c-Myc regulates expression of urokinase plasminogen activator (uPA) on hypoxia-induced vascular smooth muscle cells (VSMCs).VSMCs were isolated from thoracic aorta of wild-type (WT), tissue plasminogen activator (tPA) and uPA-deficient mice. Gene and protein expression levels were examined by reverse-transcription PCR and Western blotting, respectively. c-Myc and uPA transcriptional activity were determined by luciferase analysis. Zymography analysis was used to test the activity of matrix metalloproteinases (MMPs), tPA, and uPA.Hypoxia significantly promoted WT and tPA-/- VSMC **migration** and invasion. However, uPA-/- severely decreased hypoxia-induced VSMC **migration** and invasion. Hypoxia increased uPA and MMP-2 activity, while uPA-/- decreased hypoxia-induced MMP-2 activity. c-Myc expression and transcriptional activity were increased in response to hypoxia, and silenced c-Myc abolished hypoxia-induced uPA and MMP-2 activity. In addition, hypoxia-induced Bcl2 expression and Bcl2 binding to c-Myc led to enhanced c-Myc-mediated uPA and MMP-2 activity in response to hypoxia.The results show that c-Myc was essential for hypoxia-induced uPA expression and activity, resulting in VSMC **migration** and invasion. In addition, Bcl2 enhanced the c-Myc-mediated uPA/MMP-2 pathway.
10. Requirement for the homeobox gene Hb9 in the consolidation of motor neuron identity. The homeobox gene Hb9, like its close relative MNR2, is expressed selectively by motor neurons (MNs) in the developing vertebrate CNS. In embryonic chick spinal cord, the ectopic expression of MNR2 or Hb9 is sufficient to trigger MN differentiation and to repress the differentiation of an adjacent population of V2 interneurons. Here, we provide genetic evidence that Hb9 has an essential role in MN differentiation. In mice lacking Hb9 function, MNs are generated on schedule and in normal numbers but transiently acquire molecular features of V2 interneurons. The aberrant specification of MN identity is associated with defects in the **migration** of MNs, the emergence of the subtype identities of MNs, and the projection of motor axons. These findings show that HB9 has an essential function in consolidating the identity of postmitotic MNs.
11. Astroglial c-Myc overexpression predisposes mice to primary malignant gliomas. Malignant astrocytomas are common human primary brain tumors that result from neoplastic transformation of astroglia or their progenitors. Here we show that deregulation of the c-Myc pathway in developing astroglia predisposes mice to malignant astrocytomas within 2-3 weeks of age. The genetically engineered murine (GEM) gliomas harbor a molecular signature resembling that of human primary glioblastoma multiforme, including up-regulation of epidermal growth factor receptor and Mdm2. The GEM gliomas seem to originate in an abnormal population of glial fibrillary acidic protein-expressing cells in the ventricular zone and, analogous to human glioblastomas, exhibit molecular and morphological heterogeneity. Levels of connexin 43 in the majority of the tumors are unaltered from normal tissue, indicating that GEM tumors have retained the capacity to establish syncytial networks. In line with this, individual glioma foci are composed of a mixture of actively proliferating cells expressing c-Myc and proliferating cell nuclear antigen and less dividing bystander cells that express glial fibrillary acidic protein and the broad complex tramtrack bric-a-brac/poxvirus and zinc finger domain protein HOF. A subset of the transgenic mice harbored, in addition to brain tumors, vestigial cerebellums in which granule cell **migration** and radial Bergman glial cell differentiation were disturbed. These observations argue for a window of vulnerability during astrocyte development where c-Myc overexpression is sufficient to trigger the neoplastic process, presumably by inducing the sustained growth of early astroglial cells. This is in contrast to most other transgenic studies in which c-Myc overexpression requires co-operating transgenes for rapid tumor induction.
12. Deregulated expression of c-Myc depletes epidermal stem cells. The beta-catenin/TCF signaling pathway is essential for the maintenance of epithelial stem cells in the small intestine. c-Myc a downstream target of beta-catenin/TCF (ref. 2), can induce differentiation of epidermal stem cells in vitro. To determine the role of c-Myc in epidermal stem cells in vivo, we have targeted expression of human MYC2 to the hair follicles and the basal layer of mouse epidermis using a keratin 14 vector (K14.MYC2). Adult K14.MYC2 mice gradually lose their hair and develop spontaneous ulcerated lesions due to a severe impairment in wound healing; their keratinocytes show impaired **migration** in response to wounding. The expression of beta1 integrin, which is preferentially expressed in epidermal stem cells is unusually low in the epidermis of K14.MYC2 mice. Label-retaining analysis to identify epidermal stem cells reveals a 75% reduction in the number of stem cells in 3-month-old K14.MYC2 mice, compared with wildtype mice. We conclude that deregulated expression of c-Myc in stem cells reduces beta1 integrin expression, which is essential to both keratinocyte **migration** and stem cell maintenance.
13. XIAP-associated factor 1 (XAF1) suppresses angiogenesis in mouse endothelial cells. XIAP-associated factor 1 (XAF1) is a tumor suppressor gene, but its role in angiogenesis is unknown. We investigated whether XAF1 has any antiangiogenesis effect.MS1 (a mouse endothelial cell line) was infected with an adenoviral vector ZD55-XAF1. Controls were uninfected or infected with ZD55-EGFP. Wound healing assay and tube formation assay were used to assess angiogenesis. Cell proliferation was detected by WST-1 assay, and apoptosis was detected by TUNEL and APOPercentagetrade mark assays.ZD55-XAF1 significantly upregulated XAF1, which was associated with decreased cell proliferation, **migration** and tube formation of MS1 cells. Ectopic overexpression of XAF1 induced apoptosis in MS1 and also sensitized cells to 5-fluorouracil-induced apoptosis. A significant decrease in the expression of Tie-1, Ang-1, Ang-2 and c-Myc was observed.Our data suggest that XAF1 possesses a potential antiangiogenesis effect. Suppressed expression of Tie-1, Ang-1, Ang-2 and c-Myc may be mechanistically responsible for the observed antiangiogenesis effect.
14. Loss of the Max-interacting protein Mnt in mice results in decreased viability, defective embryonic growth and craniofacial defects: relevance to Miller-Dieker syndrome. The Mnt gene encodes a Mad-family bHLH transcription factor located on human 17p13.3. Mnt is one of 20 genes deleted in a heterozygous fashion in Miller-Dieker syndrome (MDS), a contiguous gene syndrome that consists of severe neuronal **migration** defects and craniofacial dysmorphic features. Mnt can inhibit Myc-dependent cell transformation and is hypothesized to counterbalance the effects of c-Myc on growth and proliferation in vivo by competing with Myc for binding to Max and by repressing target genes activated by Myc : Max heterodimers. Unlike the related Mad family members, Mnt is expressed ubiquitously and Mnt/Max heterodimers are found in proliferating cells that contain Myc/Max heterodimers, suggesting a unique role for Mnt during proliferation. To examine the role of Mnt in vivo, we produced mice with null (Mnt(KO)) and loxP-flanked conditional knock-out (Mnt(CKO)) alleles of Mnt. Virtually all Mnt(KO/KO) mutants in a mixed (129S6 x NIH Black Swiss) or inbred (129S6) genetic background died perinatally. Mnt-deficient embryos exhibited small size throughout development and showed reduced levels of c-Myc and N-Myc. In addition, 37% of the mixed background mutants displayed cleft palate as well as retardation of skull development, a phenotype not observed in the inbred mutants. These results demonstrate an important role for Mnt in embryonic development and survival, and suggest that Mnt may play a role in the craniofacial defects displayed by MDS patients.
15. Decorin antagonizes Met receptor activity and down-regulates {beta}-catenin and Myc levels. A theme emerging during the past few years is that members of the small leucine-rich proteoglycan gene family affect cell growth by interacting with multiple receptor tyrosine kinases (RTKs), mostly by a physical down-regulation of the receptors, thereby depriving tumor cells of pro-survival signals. Decorin binds and down-regulates several RTKs, including Met, the receptor for hepatocyte growth factor. Here we demonstrate that decorin blocks several biological activities mediated by the Met signaling axis, including cell scatter, evasion, and **migration**. These effects were mediated by a profound down-regulation of noncanonical β-catenin levels. In addition, Myc, a downstream target of β-catenin, was markedly down-regulated by decorin, whereas phosphorylation of Myc at threonine 58 was markedly induced. The latter is known to destabilize Myc and target it for proteasomal degradation. We also discovered that systemic delivery of decorin using three distinct tumor xenograft models caused down-regulation of Met and a concurrent suppression of β-catenin and Myc levels. We found that decorin protein core labeled with the near infrared dye IR800 specifically targeted the tumor cells expressing Met. Even 68-h post-injection, decorin was found to reside within the tumor xenografts with little or no binding to other tissues. Collectively, our results indicate a role for a secreted proteoglycan in suppressing the expression of key oncogenic factors required for tumor progression.
16. Nitric oxide prevents aortic neointimal hyperplasia by controlling macrophage polarization. Nitric oxide synthase 3 (NOS3) prevents neointima hyperplasia by still unknown mechanisms. To demonstrate the significance of endothelial nitric oxide in the polarization of infiltrated macrophages through the expression of matrix metalloproteinase (MMP)-13 in neointima formation.After aortic endothelial denudation, NOS3 null mice show elevated neointima formation, detecting increased mobilization of LSK (lineage-negative [Lin]-stem-cell antigen 1 [SCA1]+KIT+) progenitor cells, and high ratios of M1 (proinflammatory) to M2 (resolving) macrophages, accompanied by high expression of interleukin-5, interleukin-6, MCP-1 (monocyte chemoattractant protein), VEGF (vascular endothelial growth factor), GM-CSF (granulocyte-macrophage colony stimulating factor), interleukin-1β, and interferon-γ. In conditional c-Myc knockout mice, in which M2 polarization is defective, denuded aortas showed extensive wall thickening as well. Conditioned medium from NOS3-deficient endothelium induced extensive repolarization of M2 macrophages to an M1 phenotype, and vascular smooth muscle cells proliferated and migrated faster in conditioned medium from M1 macrophages. Among the different proteins participating in cel**l migrati**on, MMP-13 was preferentially expressed by M1 macrophages. M1-mediated vascular smooth muscle cel**l migrati**on was inhibited when macrophages were isolated from MMP-13-deficient mice, whereas exogenous administration of MMP-13 to vascular smooth muscle cell fully restore**d migrati**on. Excess vessel wall thickening in mice lacking NOS3 was partially reversed by simultaneous deletion of MMP-13, indicating that NOS3 prevents neointimal hyperplasia by preventing MMP-13 activity. An excess of M1-polarized macrophages that coexpress MMP-13 was also detected in human carotid samples from endarterectomized patients.These findings indicate that at least M1 macrophage-mediated expression of MMP-13 in NOS3 null mice induces neointima formation after vascular injury, suggesting that MMP-13 may represent a new promising target in vascular disease.
17. A new variant 15; 16 translocation in mouse plasmacytoma leads to the juxtaposition of c-myc and immunoglobulin lambda. Mouse plasmacytomas (MPCs) induced by pristane oil, or by a combination of pristane oil and Abelson virus, carry one of two chromosomal translocations. The typical 12; 15 translocation leads to the juxtaposition of c-myc and immunoglobulin heavy-chain sequences, whereas the 6; 15 translocation links the kappa light-chain locus with the pvt-1 (plasmacytoma variant translocation) locus, located at least 75kb 3' of c-myc [Cory, S., Graham, M., Webb, E., Corcoran, L. & Adams, J. (1985). EMBO J., 4, 675-681]. Unlike the human Burkitt's lymphoma-associated translocation, the lambda/myc juxtaposed variant translocation has not been found previously in MPCs. Using unconventional MPC induction systems in which the tumor precursor cell was induced to proliferate in a secondary host, we have recently identified a 15; 16 translocation in six of the derived MPCs [Wiener, F., Silva, S., Sugiyama, H., Babonits, M. & Klein, G. (1990). Genes Chromosomes Cancer, 2, 36-43]. Chromosome 16 harbors the lambda light-chain gene. To explore whether the 15; 16 translocation represents the lambda/myc juxtaposition, we have mapped the breakpoints on chromosomes 15 and 16 by pulsed-field gel electrophoresis (PFGE). The pvt-1 region was mapped to approximately 220 kb 3' of c-myc. The breakpoint on chromosome 15 in ABPC-Ch-163-10, one of the six 15; 16 translocation-carrying MPCs, was situated approximately 80 kb 3' of c-myc and 140 kb 5' of pvt-1b, the major breakpoint cluster region of the previously analysed 6; 15 variant MPCs. The breakpoint on chromosome 16 was found to cut between the V1 and C3 regions of the lambda locus. Co-**migration** experiments showed that the C3 and the myc gene were juxtaposed head to tail on the 15; 16 translocation chromosome. On the reciprocal product V1 was juxtaposed to pvt-1.
18. Diet-induced obesity promotes murine gastric cancer growth through a nampt/sirt1/c-myc positive feedback loop. Obesity increases the risk of gastric cancer and may promote its growth, as was recently demonstrated by our novel in vivo mouse model. However, the underlying mechanisms of this correlation remain unclear. The purpose of this study was to investigate the precise effects of obesity on gastric cancer growth and to elucidate the potential molecular mechanisms. Diet-induced obese mice were insulin-resistant, glucose-intolerant and had high serum visfatin concentration. In the subcutaneous mouse model, tumors were more aggressive in diet-induced obese mice compared with lean mice. Tumor weights showed a significant positive correlation with mouse body weights, as well as serum insulin and visfatin concentrations. Immunohistochemical staining showed that the expression levels of iNampt, Sirt1 and c-MYC proteins were upregulated in the subcutaneous tumors from obese mice compared to those from lean animals. Furthermore, obesity not only prompted significantly murine forestomach carcinoma cell **migratio**n, proliferation, but also affected cellular apoptosis and cell cycle by endocrine mechanisms. These were associated with increased expression of the pro-survival nampt/sirt1/c-myc positive feedback loop confirmed by RT-PCR and western blotting. These results suggested that diet-induced obesity could promote murine gastric cancer growth by upregulating the expression of the nampt, sirt1 and c-myc genes.
19. MicroRNA-34a modulates c-Myc transcriptional complexes to suppress malignancy in human prostate cancer cells. MicroRNA-34a (miR-34a), a potent mediator of tumor suppressor p53, has been reported to function as a tumor suppressor and miR-34a was found to be downregulated in prostate cancer tissues. We studied the functional effects of miR-34a on c-Myc transcriptional complexes in PC-3 prostate cancer cells. Transfection of miR-34a into PC-3 cells strongly inhibited in vitro cell proliferation, cell invasion and promoted apoptosis. Transfection of miR-34a into PC-3 cells also significantly inhibited in vivo xenograft tumor growth in nude mice. miR-34a downregulated expression of c-Myc oncogene by targeting its 3' UTR as shown by luciferase reporter assays. miR-34a was found to repress RhoA, a regulator of cell **migration** and invasion, by suppressing c-Myc-Skp2-Miz1 transcriptional complex that activates RhoA. Overexpression of c-Myc reversed miR-34a suppression of RhoA expression, suggesting that miR-34a inhibits invasion by suppressing RhoA through c-Myc. miR-34a was also found to repress c-Myc-pTEFB transcription elongation complex, indicating one of the mechanisms by which miR-34a has profound effects on cellular function. This is the first report to document that miR-34a suppresses assembly and function of the c-Myc-Skp2-Miz1 complex that activates RhoA and the c-Myc-pTEFB complex that elongates transcription of various genes, suggesting a novel role of miR-34a in the regulation of transcription by c-Myc complex.
20. Modulation of cellular **migration** and survival by c-Myc through the downregulation of urokinase (uPA) and uPA receptor. It has been proposed that c-Myc proapoptotic activity accounts for most of its restraint of tumor formation. We established a telomerase-immortalized human epithelial cell line expressing an activatable c-Myc protein. We found that c-Myc activation induces, in addition to increased sensitivity to apoptosis, reductions in cell motility and invasiveness. Transcriptome analysis revealed that urokinase (uPA) and uPA receptor (uPAR) were strongly downregulated by c-Myc. Evidence is provided that the repression of uPA and uPAR may account for most of the antimigratory and proapoptotic activities of c-Myc. c-Myc is known to cooperate with Ras in cellular transformation. We therefore investigated if this cooperation could converge in the control of uPA/uPAR expression. We found that Ras is able to block the effects of c-Myc activation on apoptosis and cellular motility but not on cell invasiveness. Accordingly, the activation of c-Myc in the context of Ras expression had only minor influence on uPAR expression but still had a profound repressive effect on uPA expression. Thus, the differential regulation of uPA and uPAR by c-Myc and Ras correlates with the effects of these two oncoproteins on cell motility, invasiveness, and survival. In conclusion, we have discovered a novel link between c-Myc and uPA/uPAR. We propose that reductions of cell motility and invasiveness could contribute to the inhibition of tumorigenesis by c-Myc and that the regulation of uPA and uPAR expression may be a component of the ability of c-Myc to reduce motility and invasiveness.
21. AZT exerts its antitumoral effect by telomeric and non-telomeric effects in a mammary adenocarcinoma model. Limitless replicative potential is one of the hallmarks of cancer that is mainly due to the activity of telomerase. This holoenzyme maintains telomere length, adding TTAGGG repetitions at the end of chromosomes in each cell division. In addition to this function, there are extratelomeric roles of telomerase that are involved in cancer promoting events. It has been demonstrated that TERT, the catalytic component of telomerase, acts as a transcriptional modulator in many signaling pathways. Taking into account this evidence and our experience on the study of azidothymidine (AZT) as an inhibitor of telomerase activity, the present study analyzes the effect of AZT on some telomeric and extratelomeric activities. To carry out the present study, we evaluated the transcription of genes that are modulated by the Wnt/β-catenin pathway, such as c-Myc and cyclin-D1 (Cyc-D1) and cell processes related with their expression, such as, proliferation, modifications of the actin cytoskeleton, cell **migratio**n and cell cycle in a mammary carcinoma cell line (F3II). Results obtained after treatment with AZT (600 µM) for 15 passages confirmed the inhibitory effect on telomerase. Regarding extratelomeric activities, our results showed a decrease of 64, 38 and 25% in the transcription of c-Myc, Cyc-D1 and TERT, respectively (p<0.05) after AZT treatment. Furthermore, we found an effect on c**ell migra**tion, reaching an inhibition of 48% (p<0.05) and a significant passage-dependent increase on cell doubling time during treatment. Finally, we evaluated the effect on cell cycle, obtaining a decline in G0/G1 in AZT-treated cells. These results allow us to postulate that AZT is not only an inhibitor of telomerase activity, but also a potential modulator of extratelomeric processes involved in cancer promotion.
22. c-myc in the hematopoietic lineage is crucial for its angiogenic function in the mouse embryo. The c-myc proto-oncogene, which is crucial for the progression of many human cancers, has been implicated in key cellular processes in diverse cell types, including endothelial cells that line the blood vessels and are critical for angiogenesis. The de novo differentiation of endothelial cells is known as vasculogenesis, whereas the growth of new blood vessels from pre-existing vessels is known as angiogenesis. To ascertain the function of c-myc in vascular development, we deleted c-myc in selected cell lineages. Embryos lacking c-myc in endothelial and hematopoietic lineages phenocopied those lacking c-myc in the entire embryo proper. At embryonic day (E) 10.5, both mutant embryos were grossly normal, had initiated primitive hematopoiesis, and both survived until E11.5-12.5, longer than the complete null. However, they progressively developed defective hematopoiesis and angiogenesis. The majority of embryos lacking c-myc specifically in hematopoietic cells phenocopied those lacking c-myc in endothelial and hematopoietic lineages, with impaired definitive hematopoiesis as well as angiogenic remodeling. c-myc is required for embryonic hematopoietic stem cell differentiation, through a cell-autonomous mechanism. Surprisingly, c-myc is not required for vasculogenesis in the embryo. c-myc deletion in endothelial cells does not abrogate endothelial proliferation, survival, **migration** or capillary formation. Embryos lacking c-myc in a majority of endothelial cells can survive beyond E12.5. Our findings reveal that hematopoiesis is a major function of c-myc in embryos and support the notion that c-myc functions in selected cell lineages rather than in a ubiquitous manner in mammalian development.

#### GeneRIFs

1. Regarding extratelomeric activities, our results showed a decrease of 64, 38 and 25% in the transcription of c-Myc, Cyc-D1 and TERT, respectively (p<0.05) after AZT treatment. Furthermore, we found an effect on cell **migration**, reaching an inhibition of 48% (p<0.05) and a significant passage-dependent increase on cell doubling time during treatment.
2. The Myc 3' Wnt responsive DNA element controls intestinal cell proliferation and **migration**.

### ECM

#### PubMed

1. Long form of latent TGF-beta binding protein 1 (Ltbp1L) is essential for cardiac outflow tract septation and remodeling. Latent TGF-beta binding protein 1 (LTBP1) is a member of the LTBP/fibrillin family of extracellular proteins. Due to the usage of different promoters, LTBP1 exists in two major forms, long (L) and short (S), each expressed in a temporally and spatially unique fashion. Both LTBP1 molecules covalently interact with latent TGF-beta and regulate its function, presumably via interaction with the extracellular matrix (**ECM**). To explore the in vivo role of Ltbp1 in mouse development, at the time when only the L isoform is expressed, we mutated the Ltbp1L locus by gene targeting. Ltbp1L-null animals die shortly after birth from defects in heart development, consisting of the improper septation of the cardiac outflow tract (OFT) and remodeling of the associated vessels. These cardiac anomalies present as persistent truncus arteriosus (PTA) and interrupted aortic arch (IAA), which are associated with the faulty function of cardiac neural crest cells (CNCCs). The lack of Ltbp1L in the **ECM** of the septating OFT and associated vessels results in altered gene expression and function of CNCCs and decreased Tgf-beta activity in the OFT. This phenotype reveals a crucial role for Ltbp1L and matrix as extracellular regulators of Tgf-beta activity in heart organogenesis.

### [Ee]xtracellular [Mm]atrix

#### PubMed

1. Integrin signalling regulates YAP and TAZ to control skin homeostasis. The skin is a squamous epithelium that is continuously renewed by a population of basal layer stem/progenitor cells and can heal wounds. Here, we show that the transcription regulators YAP and TAZ localise to the nucleus in the basal layer of skin and are elevated upon wound healing. Skin-specific deletion of both YAP and TAZ in adult mice slows proliferation of basal layer cells, leads to hair loss and impairs regeneration after wounding. Contact with the basal **extracellular matrix** and consequent integrin-Src signalling is a key determinant of the nuclear localisation of YAP/TAZ in basal layer cells and in skin tumours. Contact with the basement membrane is lost in differentiating daughter cells, where YAP and TAZ become mostly cytoplasmic. In other types of squamous epithelia and squamous cell carcinomas, a similar control mechanism is present. By contrast, columnar epithelia differentiate an apical domain that recruits CRB3, Merlin (also known as NF2), KIBRA (also known as WWC1) and SAV1 to induce Hippo signalling and retain YAP/TAZ in the cytoplasm despite contact with the basal layer **extracellular matrix**. When columnar epithelial tumours lose their apical domain and become invasive, YAP/TAZ becomes nuclear and tumour growth becomes sensitive to the Src inhibitor Dasatinib.
2. The Myc-dependent angiogenic switch in tumors is mediated by interleukin 1beta. Although induction of blood vessel growth is acknowledged as a pivotal requirement for the evolution of macroscopic tumors, the events that trigger onset of tumor angiogenesis remain largely obscure. The pervasive Myc oncoprotein is itself a potent inducer of angiogenesis in a wide range of tissues. We have used a reversibly switchable mouse transgenic model of Myc-dependent beta-cell carcinogenesis to delineate the kinetics and causal sequence of angiogenic processes following acute Myc activation. We show that onset of endothelial cell proliferation is induced shortly after Myc-induced cell cycle entry of beta cells. Endothelial cell proliferation is not indirectly induced by local tissue hypoxia but instead via a diffusible angiogenic signal produced by Myc-expressing beta cells. This signal triggers the release of pre-existing, sequestered VEGF from the islet **extracellular matrix**, that then homes to the endothelial compartment where it induces endothelial cell proliferation. Myc activation in beta cells rapidly induces expression and release of the proinflammatory cytokine interleukin 1beta (IL-1beta). We show that IL-1beta is the principal effector downstream of Myc responsible for triggering rapid onset of islet angiogenesis. Together, our data delineate a complete pathway in vivo by which the highly pleiotropic Myc oncoproteins elicits coexpansion of the vascular compartment during tumorigenic progression.
3. Sox9 plays multiple roles in the lung epithelium during branching morphogenesis. Lung branching morphogenesis is a highly orchestrated process that gives rise to the complex network of gas-exchanging units in the adult lung. Intricate regulation of signaling pathways, transcription factors, and epithelial-mesenchymal cross-talk are critical to ensuring branching morphogenesis occurs properly. Here, we describe a role for the transcription factor Sox9 during lung branching morphogenesis. Sox9 is expressed at the distal tips of the branching epithelium in a highly dynamic manner as branching occurs and is down-regulated starting at embryonic day 16.5, concurrent with the onset of terminal differentiation of type 1 and type 2 alveolar cells. Using epithelial-specific genetic loss- and gain-of-function approaches, our results demonstrate that Sox9 controls multiple aspects of lung branching. Fine regulation of Sox9 levels is required to balance proliferation and differentiation of epithelial tip progenitor cells, and loss of Sox9 leads to direct and indirect cellular defects including **extracellular matrix** defects, cytoskeletal disorganization, and aberrant epithelial movement. Our evidence shows that unlike other endoderm-derived epithelial tissues, such as the intestine, Wnt/β-catenin signaling does not regulate Sox9 expression in the lung. We conclude that Sox9 collectively promotes proper branching morphogenesis by controlling the balance between proliferation and differentiation and regulating the **extracellular matri**x.
4. Dwarfism and impaired gut development in insulin-like growth factor II mRNA-binding protein 1-deficient mice. Insulin-like growth factor II mRNA-binding protein 1 (IMP1) belongs to a family of RNA-binding proteins implicated in mRNA localization, turnover, and translational control. Mouse IMP1 is expressed during early development, and an increase in expression occurs around embryonic day 12.5 (E12.5). To characterize the physiological role of IMP1, we generated IMP1-deficient mice carrying a gene trap insertion in the Imp1 gene. Imp1(-/-) mice were on average 40% smaller than wild-type and heterozygous sex-matched littermates. Growth retardation was apparent from E17.5 and remained permanent into adult life. Moreover, Imp1(-/-) mice exhibited high perinatal mortality, and only 50% were alive 3 days after birth. In contrast to most other organs, intestinal epithelial cells continue to express IMP1 postnatally, and Imp1(-/-) mice exhibited impaired development of the intestine, with small and misshapen villi and twisted colon crypts. Analysis of target mRNAs and global expression profiling at E12.5 indicated that Igf2 translation was downregulated, whereas the postnatal intestine showed reduced expression of transcripts encoding **extracellular matrix** components, such as galectin- 1, lumican, tenascin-C, procollagen transcripts, and the Hsp47 procollagen chaperone. Taken together, the results demonstrate that IMP1 is essential for normal growth and development. Moreover, IMP1 may facilitate intestinal morphogenesis via regulation of **extracellular matrix** formation.
5. The mouse Col2a-1 gene is highly conserved and is linked to Int-1 on chromosome 15. Type II collagen is the major **extracellular matrix** component of cartilage and correct expression of the alpha 1(II) collagen gene is important for vertebrate skeletal development. In order to provide the basis for studying the control of type II collagen gene expression in embryogenesis and in mouse models of human connective tissue disease, the complete mouse Col2-a1 gene has been isolated in a single cosmid clone, cosMco1.2, and partially characterized. The gene is approximately 30 kb and is highly conserved in exon/intron structure and nucleotide and amino acid sequence (greater than 80% homology) when compared with the human, rat, bovine and chicken equivalents. A high degree of conservation was also found in the 5' flanking region of the rat, human and mouse alpha 1(II) collagen genes, including the presence of several G + C and C + T rich, direct repeat motifs. The sites of transcription start, termination codon and polyadenylation have also been identified. Unlike chicken, bovine and human, where polyA attachment is at a single site, for the mouse Col2a-1 gene two polyadenylation sites are utilized. Col2a-1 has also been localized by interspecies backcross analysis to the central portion of mouse Chromosome (Chr) 15, approximately 8 centiMorgans (cM) proximal of Int-1 and 18 cM distal of Myc. Col2a-1 is therefore included in a linkage group which is conserved on human Chr 12q.
6. Long form of latent TGF-beta binding protein 1 (Ltbp1L) is essential for cardiac outflow tract septation and remodeling. Latent TGF-beta binding protein 1 (LTBP1) is a member of the LTBP/fibrillin family of extracellular proteins. Due to the usage of different promoters, LTBP1 exists in two major forms, long (L) and short (S), each expressed in a temporally and spatially unique fashion. Both LTBP1 molecules covalently interact with latent TGF-beta and regulate its function, presumably via interaction with the **extracellular matrix** (ECM). To explore the in vivo role of Ltbp1 in mouse development, at the time when only the L isoform is expressed, we mutated the Ltbp1L locus by gene targeting. Ltbp1L-null animals die shortly after birth from defects in heart development, consisting of the improper septation of the cardiac outflow tract (OFT) and remodeling of the associated vessels. These cardiac anomalies present as persistent truncus arteriosus (PTA) and interrupted aortic arch (IAA), which are associated with the faulty function of cardiac neural crest cells (CNCCs). The lack of Ltbp1L in the ECM of the septating OFT and associated vessels results in altered gene expression and function of CNCCs and decreased Tgf-beta activity in the OFT. This phenotype reveals a crucial role for Ltbp1L and matrix as extracellular regulators of Tgf-beta activity in heart organogenesis.
7. Expression of syndecan, a putative low affinity fibroblast growth factor receptor, in the early mouse embryo. Syndecan is an integral membrane proteoglycan that binds cells to several interstitial **extracellular matrix** components and binds to basic fibroblast-growth factor (bFGF) thus promoting bFGF association with its high-affinity receptor. We find that syndecan expression undergoes striking spatial and temporal changes during the period from the early cleavage through the late gastrula stages in the mouse embryo. Syndecan is detected initially at the 4-cell stage. Between the 4-cell and late morula stages, syndecan is present intracellularly and on the external surfaces of the blastomeres but is absent from regions of cell-cell contact. At the blastocyst stage, syndecan is first detected at cell-cell boundaries throughout the embryo and then, at the time of endoderm segregation, becomes restricted to the first site of matrix accumulation within the embryo, the interface between the primitive ectoderm and primitive endoderm. During gastrulation, syndecan is distributed uniformly on the basolateral cell surfaces of the embryonic ectoderm and definitive embryonic endoderm, but is expressed with an anteroposterior asymmetry on the surface of embryonic mesoderm cells, suggesting that it contributes to the process of mesoderm specification. In the extraembryonic region, syndecan is not detectable on most cells of the central core of the ectoplacental cone, but is strongly expressed by cells undergoing trophoblast giant cell differentiation and remains prominent on differentiated giant cells, suggesting a role in placental development. Immunoprecipitation studies indicate that the size of the syndecan core protein, although larger than that found in adult tissues (75 versus 69 x 10(3) Mr), does not change during peri-implantation development. The size distribution of the intact proteoglycan does change, however, indicating developmental alterations in its glycosaminoglycan composition. These results indicate potential roles for syndecan in epithelial organization of the embryonic ectoderm, in differential axial patterning of the embryonic mesoderm and in trophoblast giant cell function.

### [Ii]nvasive

#### PubMed

1. LIN28 cooperates with WNT signaling to drive **invasive** intestinal and colorectal adenocarcinoma in mice and humans. Colorectal cancer (CRC) remains a major contributor to cancer-related mortality. LIN28A and LIN28B are highly related RNA-binding protein paralogs that regulate biogenesis of let-7 microRNAs and influence development, metabolism, tissue regeneration, and oncogenesis. Here we demonstrate that overexpression of either LIN28 paralog cooperates with the Wnt pathway to promote **invasive** intestinal adenocarcinoma in murine models. When LIN28 alone is induced genetically, half of the resulting tumors harbor Ctnnb1 (β-catenin) mutation. When overexpressed in Apc(Min/+) mice, LIN28 accelerates tumor formation and enhances proliferation and **invasiv**eness. In conditional genetic models, enforced expression of a LIN28-resistant form of the let-7 microRNA reduces LIN28-induced tumor burden, while silencing of LIN28 expression reduces tumor volume and increases tumor differentiation, indicating that LIN28 contributes to tumor maintenance. We detected aberrant expression of LIN28A and/or LIN28B in 38% of a large series of human CRC samples (n = 595), where LIN28 expression levels were associated with **invasiv**e tumor growth. Our late-stage CRC murine models and analysis of primary human tumors demonstrate prominent roles for both LIN28 paralogs in promoting CRC growth and progression and implicate the LIN28/let-7 pathway as a therapeutic target.
2. Integrin signalling regulates YAP and TAZ to control skin homeostasis. The skin is a squamous epithelium that is continuously renewed by a population of basal layer stem/progenitor cells and can heal wounds. Here, we show that the transcription regulators YAP and TAZ localise to the nucleus in the basal layer of skin and are elevated upon wound healing. Skin-specific deletion of both YAP and TAZ in adult mice slows proliferation of basal layer cells, leads to hair loss and impairs regeneration after wounding. Contact with the basal extracellular matrix and consequent integrin-Src signalling is a key determinant of the nuclear localisation of YAP/TAZ in basal layer cells and in skin tumours. Contact with the basement membrane is lost in differentiating daughter cells, where YAP and TAZ become mostly cytoplasmic. In other types of squamous epithelia and squamous cell carcinomas, a similar control mechanism is present. By contrast, columnar epithelia differentiate an apical domain that recruits CRB3, Merlin (also known as NF2), KIBRA (also known as WWC1) and SAV1 to induce Hippo signalling and retain YAP/TAZ in the cytoplasm despite contact with the basal layer extracellular matrix. When columnar epithelial tumours lose their apical domain and become **invasive**, YAP/TAZ becomes nuclear and tumour growth becomes sensitive to the Src inhibitor Dasatinib.
3. DYRK2 priming phosphorylation of c-Jun and c-Myc modulates cell cycle progression in human cancer cells. Dysregulation of the G(1)/S transition in the cell cycle contributes to tumor development. The oncogenic transcription factors c-Jun and c-Myc are indispensable regulators at this transition, and their aberrant expression is associated with many malignancies. Degradation of c-Jun/c-Myc is a critical process for the G(1)/S transition, which is initiated upon phosphorylation by glycogen synthase kinase 3 β (GSK3β). However, a specific kinase or kinases responsible for priming phosphorylation events that precede this GSK3β modification has not been definitively identified. Here, we found that the dual-specificity tyrosine phosphorylation-regulated kinase DYRK2 functions as a priming kinase of c-Jun and c-Myc. Knockdown of DYRK2 in human cancer cells shortened the G(1) phase and accelerated cell proliferation due to escape of c-Jun and c-Myc from ubiquitination-mediated degradation. In concert with these results, silencing DYRK2 increased cell proliferation in human cancer cells, and this promotion was completely impeded by codeprivation of c-Jun or c-Myc in vivo. We also found marked attenuation of DYRK2 expression in multiple human tumor samples. Downregulation of DYRK2 correlated with high levels of unphosphorylated c-Jun and c-Myc and, importantly, wi**th invas**iveness of human breast cancers. These results reveal that DYRK2 regulates tumor progression through modulation of c-Jun and c-Myc.
4. Defining the role of APC in the mitotic spindle checkpoint in vivo: APC-deficient cells are resistant to Taxol. Mutations in the adenomatous polyposis coli (APC) tumour suppressor are the key initiating event of colorectal cancer. Although the control of WNT signalling is well established as a central tumour-suppressive function, the significance of APC in regulating chromosome instability is less well established. In this study, we test whether APC-deficient cells have a functional spindle assembly checkpoint (SAC) in vivo by examining the response of these cells to Taxol and Vinorelbine. We also show for the first time that APC deficiency compromises the arrest response to Taxol in vivo. This effect is independent of the role that APC has in WNT signalling. At higher levels of Taxol, APC-deficient cells arrest as efficiently as wild-type cells. Importantly, this dose of Taxol strongly suppresses intestinal tumourigenesis in models of benign (APC(Min/+) mouse) and **invasive** (AhCreER(+)APC(fl/+)PTEN(fl/fl)) cancer. In contrast to intestinal enterocytes with a general SAC defect because of Bub1 (budding uninhibited by benzimidazole 1) deletion, APC-deficient enterocytes arrest equivalently to wild type when treated with Vinorelbine. This suggests that the failed arrest in response to Taxol is because of a specific defect in microtubule stabilization following Taxol treatment rather than a general role of the APC protein in the mitotic spindle checkpoint. In summary, this study clarifies the role of APC as a mitotic spindle checkpoint protein in vivo and shows that APC-deficient cells have a compromised response to Taxol.
5. Colorectal cancer in mice genetically deficient in the mucin Muc2. The gastrointestinal tract is lined by a layer of mucus comprised of highly glycosylated proteins called mucins. To evaluate the importance of mucin in intestinal carcinogenesis, we constructed mice genetically deficient in Muc2, the most abundant secreted gastrointestinal mucin. Muc2-/- mice displayed aberrant intestinal crypt morphology and altered cell maturation and migration. Most notably, the mice frequently developed adenomas in the small intestine that progressed to **invasive** adenocarcinoma, as well as rectal tumors. Thus, Muc2 is involved in the suppression of colorectal cancer.
6. DYRK2 priming phosphorylation of c-Jun and c-Myc modulates cell cycle progression in human cancer cells. Dysregulation of the G(1)/S transition in the cell cycle contributes to tumor development. The oncogenic transcription factors c-Jun and c-Myc are indispensable regulators at this transition, and their aberrant expression is associated with many malignancies. Degradation of c-Jun/c-Myc is a critical process for the G(1)/S transition, which is initiated upon phosphorylation by glycogen synthase kinase 3 β (GSK3β). However, a specific kinase or kinases responsible for priming phosphorylation events that precede this GSK3β modification has not been definitively identified. Here, we found that the dual-specificity tyrosine phosphorylation-regulated kinase DYRK2 functions as a priming kinase of c-Jun and c-Myc. Knockdown of DYRK2 in human cancer cells shortened the G(1) phase and accelerated cell proliferation due to escape of c-Jun and c-Myc from ubiquitination-mediated degradation. In concert with these results, silencing DYRK2 increased cell proliferation in human cancer cells, and this promotion was completely impeded by codeprivation of c-Jun or c-Myc in vivo. We also found marked attenuation of DYRK2 expression in multiple human tumor samples. Downregulation of DYRK2 correlated with high levels of unphosphorylated c-Jun and c-Myc and, importantly, wi**th invas**iveness of human breast cancers. These results reveal that DYRK2 regulates tumor progression through modulation of c-Jun and c-Myc.
7. A mouse model of heterogeneous, c-MYC-initiated prostate cancer with loss of Pten and p53. Human tumors are heterogeneous and evolve through a dynamic process of genetic mutation and selection. During this process, the effects of a specific mutation on the incipient cancer cell may dictate the nature of subsequent mutations that can be tolerated or selected for, affecting the rate at which subsequent mutations occur. Here we have used a new mouse model of prostate cancer that recapitulates several salient features of the human disease to examine the relative rates in which the remaining wild-type alleles of Pten and p53 tumor suppressor genes are lost. In this model, focal overexpression of c-MYC in a few prostate luminal epithelial cells provokes a mild proliferative response. In the context of compound Pten/p53 heterozygosity, c-MYC-initiated cells progress to prostatic intraepithelial neoplasia (mPIN) and adenocarcinoma lesions with marked heterogeneity within the same prostate glands. Using laser capture microdissection and gene copy number analyses, we found that the frequency of Pten loss was significantly higher than that of p53 loss in mPIN but not **invasive** carcinoma lesions. c-MYC overexpression, unlike Pten loss, did not activate the p53 pathway in transgenic mouse prostate cells, explaining the lack of selective pressure to lose p53 in the c-MYC-overexpressing cells. This model of heterogeneous prostate cancer based on alterations in genes relevant to the human disease may be useful for understanding pathogenesis of the disease and testing new therapeutic agents.
8. Hepsin cooperates with MYC in the progression of adenocarcinoma in a prostate cancer mouse model. Hepsin is a cell surface protease that is over-expressed in more than 90% of human prostate cancer cases. The previously developed Probasin-hepsin/Large Probasin-T antigen (PB-hepsin/LPB-Tag) bigenic mouse model of prostate cancer demonstrates that hepsin promotes primary tumors that are a mixture of adenocarcinoma and neuroendocrine (NE) lesions, and metastases that are NE in nature. However, since the majority of human prostate tumors are adenocarcinomas, the contribution of hepsin in the progression of adenocarcinoma requires further investigation.We crossed the PB-hepsin mice with PB-Hi-myc transgenic mouse model of prostate adenocarcinoma and characterized the tumor progression in the resulting PB-hepsin/PB-Hi-myc bigenic mice.We report that PB-hepsin/PB-Hi-myc bigenic mice develop **invasive** adenocarcinoma at 4.5 months. Further, histological analysis of the 12- to 17-month-old mice revealed that the PB-hepsin/PB-Hi-myc model develops a higher grade adenocarcinoma compared with age-matched tumors expressing only PB-Hi-myc. Consistent with targeting hepsin to the prostate, the PB-hepsin/PB-Hi-myc tumors showed higher hepsin expression as compared to the age-matched myc tumors. Furthermore, endogenous expression of hepsin increased in the PB-Hi-myc mice as the tumors progressed.Although we did not detect any metastases from the prostates in either the PB-hepsin/PB-Hi-myc or the PB-Hi-myc mice, our data suggests that hepsin and myc cooperate during the progression to high-grade prostatic adenocarcinoma.
9. Alterations in nucleolar structure and gene expression programs in prostatic neoplasia are driven by the MYC oncogene. Increased nucleolar size and number are hallmark features of many cancers. In prostate cancer, nucleolar enlargement and increased numbers are some of the earliest morphological changes associated with development of premalignant prostate intraepithelial neoplasia (PIN) lesions and **invasive** adenocarcinomas. However, the molecular mechanisms that induce nucleolar alterations in PIN and prostate cancer remain largely unknown. We verify that activation of the MYC oncogene, which is overexpressed in most human PIN and prostatic adenocarcinomas, leads to formation of enlarged nucleoli and increased nucleolar number in prostate luminal epithelial cells in vivo. In prostate cancer cells in vitro, MYC expression is needed for maintenance of nucleolar number, and a nucleolar program of gene expression. To begin to decipher the functional relevance of this transcriptional program in prostate cancer, we examined FBL (encoding fibrillarin), a MYC target gene, and report that fibrillarin is required for proliferation, clonogenic survival, and proper ribosomal RNA accumulation/processing in human prostate cancer cells. Further, fibrillarin is overexpressed in PIN lesions induced by MYC overexpression in the mouse prostate, and in human clinical prostate adenocarcinoma and PIN lesions, where its expression correlates with MYC levels. These studies demonstrate that overexpression of the MYC oncogene increases nucleolar number and size and a nucleolar program of gene expression in prostate epithelial cells, thus providing a molecular mechanism responsible for hallmark nucleolar alterations in prostatic neoplasia.
10. Myc inhibition is effective against glioma and reveals a role for Myc in proficient mitosis. Gliomas are the most common primary tumours affecting the adult central nervous system and respond poorly to standard therapy. Myc is causally implicated in most human tumours and the majority of glioblastomas have elevated Myc levels. Using the Myc dominant negative Omomyc, we previously showed that Myc inhibition is a promising strategy for cancer therapy. Here, we preclinically validate Myc inhibition as a therapeutic strategy in mouse and human glioma, using a mouse model of spontaneous multifocal **invasive** astrocytoma and its derived neuroprogenitors, human glioblastoma cell lines, and patient-derived tumours both in vitro and in orthotopic xenografts. Across all these experimental models we find that Myc inhibition reduces proliferation, increases apoptosis and remarkably, elicits the formation of multinucleated cells that then arrest or die by mitotic catastrophe, revealing a new role for Myc in the proficient division of glioma cells.
11. Myc is a Notch1 transcriptional target and a requisite for Notch1-induced mammary tumorigenesis in mice. To explore the potential involvement of aberrant Notch1 signaling in breast cancer pathogenesis, we have used a transgenic mouse model. In these animals, mouse mammary tumor virus LTR-driven expression of the constitutively active intracellular domain of the Notch1 receptor (N1(IC)) causes development of lactation-dependent mammary tumors that regress upon gland involution but progress to nonregressing, **invasive** adenocarcinomas in subsequent pregnancies. Up-regulation of Myc in these tumors prompted a genetic investigation of a potential Notch1/Myc functional relationship in breast carcinogenesis. Conditional ablation of Myc in the mammary epithelium prevented the induction of regressing N1(IC) neoplasms and also reduced the incidence of nonregressing carcinomas, which developed with significantly increased latency. Molecular analyses revealed that both the mouse and human Myc genes are direct transcriptional targets of N1(IC) acting through its downstream Cbf1 transcriptional effector. Consistent with this mechanistic link, Notch1 and Myc expression is positively correlated by immunostaining in 38% of examined human breast carcinomas.
12. β-catenin is required for prostate development and cooperates with Pten loss to drive **invasiv**e carcinoma. Prostate cancer is a major cause of male death in the Western world, but few frequent genetic alterations that drive prostate cancer initiation and progression have been identified. β-Catenin is essential for many developmental processes and has been implicated in tumorigenesis in many tissues, including prostate cancer. However, expression studies on human prostate cancer samples are unclear on the role this protein plays in this disease. We have used in vivo genetic studies in the embryo and adult to extend our understanding of the role of β-Catenin in the normal and neoplastic prostate. Our gene deletion analysis revealed that prostate epithelial β-Catenin is required for embryonic prostate growth and branching but is dispensable in the normal adult organ. During development, β-Catenin controls the number of progenitors in the epithelial buds and regulates a discrete network of genes, including c-Myc and Nkx3.1. Deletion of β-Catenin in a Pten deleted model of castration-resistant prostate cancer demonstrated it is dispensable for disease progression in this setting. Complementary overexpression experiments, through in vivo protein stabilization, showed that β-Catenin promotes the formation of squamous epithelia during prostate development, even in the absence of androgens. β-Catenin overexpression in combination with Pten loss was able to drive progres**sion to** invasive carcinoma together with squamous metaplasia. These studies demonstrate that β-Catenin is essential for prostate development and that an inherent property of high levels of this protein in prostate epithelia is to drive squamous fate differentiation. In addition, they show that β-Catenin overexpression ca**n promot**e invasive prostate cancer in a clinically relevant model of this disease. These data provide novel information on cancer progression pathways that give rise to lethal prostate disease in humans.
13. Integration of a Notch-dependent mesenchymal gene program and Bmp2-driven cell **invasive**ness regulates murine cardiac valve formation. Cardiac valve formation is crucial for embryonic and adult heart function. Valve malformations constitute the most common congenital cardiac defect, but little is known about the molecular mechanisms regulating valve formation and homeostasis. Here, we show that endocardial Notch1 and myocardial Bmp2 signal integration establish a valve-forming field between 2 chamber developmental domains. Patterning occurs through the activation of endocardial epithelial-to-mesenchymal transition (EMT) exclusively in prospective valve territories. Mice with constitutive endocardial Notch1 activity ectopically express Hey1 and Heyl. They also display an activated mesenchymal gene program in ventricles and a partial (non**invasive**) EMT in vitro that becomes **invasive** upon BMP2 treatment. Snail1, TGF-β2, or Notch1 inhibition reduces BMP2-induced ventricular transformation and invasion, whereas BMP2 treatment inhibits endothelial Gsk3β, stabilizing Snail1 and promotin**g invasi**veness. Integration of Notch and Bmp2 signals is consistent with Notch1 signaling being attenuated after myocardial Bmp2 deletion. Notch1 activation in myocardium extends Hey1 expression to nonchamber myocardium, represses Bmp2, and impairs EMT. In contrast, Notch deletion abrogates endocardial Hey gene transcription and extends Bmp2 expression to the ventricular endocardium. This embryonic Notch1-Bmp2-Snail1 relationship may be relevant in adult valve disease, in which decreased NOTCH signaling causes valve mesenchyme cell formation, fibrosis, and calcification.
14. Hepatic stem-like phenotype and interplay of Wnt/beta-catenin and Myc signaling in aggressive childhood liver cancer. Hepatoblastoma, the most common pediatric liver cancer, is tightly linked to excessive Wnt/beta-catenin signaling. Here, we used microarray analysis to identify two tumor subclasses resembling distinct phases of liver development and a discriminating 16-gene signature. beta-catenin activated different transcriptional programs in the two tumor types, with distinctive expression of hepatic stem/progenitor markers in immature tumors. This highly proliferating subclass was typified by gains of chromosomes 8q and 2p and upregulated Myc signaling. Myc-induced hepatoblastoma-like tumors in mice strikingly resembled the human immature subtype, and Myc downregulation in hepatoblastoma cells impaired tumorigenesis in vivo. Remarkably, the 16-gene signature discriminated **invasive** and metastatic hepatoblastomas and predicted prognosis with high accuracy.
15. Non**invasive** molecular imaging of c-Myc activation in living mice. The cytoplasmic Myc protein (c-Myc) regulates various human genes and is dysregulated in many human cancers. Phosphorylation mediates the protein activation of c-Myc and is essential for the function of this transcription factor in normal cell behavior and tumor growth. To date, however, the targeting of Myc as a therapeutic approach for cancer treatment has been achieved primarily at the nonprotein level. We have developed a molecular imaging sensor for non**invasive** imaging of c-Myc activity in living subjects using a split Firefly luciferase (FL) complementation strategy to detect and quantify the phosphorylation-mediated interaction between glycogen synthase kinase 3beta (GSK3beta) and c-Myc. This sensor system consists of two fusion proteins, GSK 35-433-CFL and NFL-c-Myc, in which specific fragments of GSK3beta and c-Myc are fused with C-terminal and N-terminal fragments of the split FL, respectively. The sensor detects phosphorylation-specific GSK3beta-c-Myc interaction, the imaging signal of which correlates with the steady-state and temporal regulation of c-Myc phosphorylation in cell culture. The sensor also detects inhibition of c-Myc activity via differential pathways, allowing non**invasive** monitoring of c-Myc-targeted drug efficacy in intact cells and living mice. Notably, this drug inhibition is detected before changes in tumor size are apparent in mouse xenograft and liver tumor models. This reporter system not only provides an innovative way to investigate the role of functional c-Myc in normal and cancer-related biological processes, but also facilitates c-Myc-targeted drug development by providing a rapid quantitative approach to assessing cancer response to therapy in living subjects.
16. Myc is required for β-catenin-mediated mammary stem cell amplification and tumorigenesis. Basal-like breast cancer is a heterogeneous disease characterized by the expression of basal cell markers, no estrogen or progesterone receptor expression and a lack of HER2 overexpression. Recent studies have linked activation of the Wnt/β-catenin pathway, and its downstream target, Myc, to basal-like breast cancer. Transgenic mice K5ΔNβcat previously generated by our team present a constitutive activation of Wnt/β-catenin signaling in the basal myoepithelial cell layer, resulting in focal mammary hyperplasias that progres**s to inv**asive carcinomas. Mammary lesions developed by K5ΔNβcat mice consist essentially of basal epithelial cells that, in contrast to mammary myoepithelium, do not express smooth muscle markers.Microarray analysis was used to compare K5ΔNβcat mouse tumors to human breast tumors, mammary cancer cell lines and the tumors developed in other mouse models. Cre-Lox approach was employed to delete Myc from the mammary basal cell layer of K5ΔNβcat mice. Stem cell amplification in K5ΔNβcat mouse mammary epithelium was assessed with 3D-culture and transplantation assays.Histological and microarray analyses of the mammary lesions of K5ΔNβcat females revealed their high similarity to a subset of basal-like human breast tumors with squamous differentiation. As in human basal-like carcinomas, the Myc pathway appeared to be activated in the mammary lesions of K5ΔNβcat mice. We found that a basal cell population with stem/progenitor characteristics was amplified in K5ΔNβcat mouse preneoplastic glands. Finally, the deletion of Myc from the mammary basal layer of K5ΔNβcat mice not only abolished the regenerative capacity of basal epithelial cells, but, in addition, completely prevented the tumorigenesis.These results strongly indicate that β-catenin-induced stem cell amplification and tumorigenesis rely ultimately on the Myc pathway activation and reinforce the hypothesis that basal stem/progenitor cells may be at the origin of a subset of basal-like breast tumors.
17. Multiple mechanisms of tumorigenesis in E mu-myc transgenic mice. Transgenic mice bearing a c-myc oncogene under control of the immunoglobulin heavy chain enhancer (E mu-myc mice) reproducibly develop and die from tumors of the B lymphocyte lineage (J.M. Adams, A.W. Harris, C.A. Pinkert, L.M. Corcoran, W.S. Alexander, S. Cory, R.D. Palmiter, and R.L. Brinster, Nature (Lond.), 318: 533-538, 1985; W.Y. Langdon, A. W. Harris, S. Cory, and J.M. Adams, Cell 47: 11-18, 1986; A.W. Harris, C.A. Pinkert, M. Crawford, W.Y. Langdon, R.L. Brinster, and J.M. Adams, J. Exp. Med., 167: 353-371, 1988; reviewed in S. Cory and J.M. Adams, Annu. Rev. Immunol., 6: 25-48, 1988). Analysis of lymphocytes obtained by serial sampling of peripheral blood from individual hemizygous (E mu-myc/0) and homozygous (E mu-myc/E mu-myc) transgenic mice indicates that proliferation in the original host and transplantability into histocompatible recipients are distinct properties that can be acquired independently and in either order. These two types of transgenic mice differ in that homozygous mice have about one-fourth the life span of hemizygous mice and develop polyclonal, non-transplantable tumors in comparison to the oligoclonal, highly transplantable malignancies seen in hemizygous animals. In conclusion, the overall concept of malignancy is best viewed as an aggregate of the separable parameters of cellular proliferation, clonality, tissue **invasive**ness, metastasis, and (experimental) transplantability. The E mu-myc transgenic mouse represents an attractive model in which to investigate the multistep nature and alternative pathways of tumorigenesis.
18. Therapeutic effects of an anti-Myc drug on mouse pancreatic cancer. Pancreatic ductal adenocarcinoma (PDA) is frequently driven by oncogenic KRAS(KRAS\*) mutations. We developed a mouse model of KRAS\*-induced PDA and, based on genetic results demonstrating that KRAS\* tumorigenicity depends on Myc activity, we evaluated the therapeutic potential of an orally administered anti-Myc drug.We tested the efficacy of Mycro3, a small-molecule inhibitor of Myc-Max dimerization, in the treatment of mouse PDA (n = 9) and also of xenografts of human pancreatic cancer cell lines (NOD/SCID mice, n = 3-12). Tumor responses to the drug were evaluated by PET/CT imaging, and histological, immunohistochemical, molecular and microarray analyses. The Student's t test was used for differences between groups. All statistical tests were two-sided.Transgenic overexpression of KRAS\* in the pancreas resulted in pancreatic intraepithelial neoplasia in two-week old mice, which developed **invasive** PDA a week later and became moribund at one month. However, this aggressive form of pancreatic tumorigenesis was effectively prevented by genetic ablation of Myc specifically in the pancreas. We then treated moribund, PDA-bearing mice daily with the Mycro3 Myc-inhibitor. The mice survived until killed at two months. PET/CT image analysis (n = 5) demonstrated marked shrinkage of PDA, while immunohistochemical analyses showed an increase in cancer cell apoptosis and reduction in cell proliferation (treated/untreated proliferation index ratio: 0.29, P < .001, n = 3, each group). Tumor growth was also drastically attenuated in Mycro3-treated NOD/SCID mice (n = 12) carrying orthotopic or heterotopic xenografts of human pancreatic cancer cells (eg, mean tumor weight ± SD of treated heterotopic xenografts vs vehicle-treated controls: 15.2±5.8 mg vs 230.2±43.9 mg, P < .001).These results provide strong justification for eventual clinical evaluation of anti-Myc drugs as potential chemotherapeutic agents for the treatment of PDA.
19. Contrasting patterns of myc and N-myc expression during gastrulation of the mouse embryo. myc and N-myc are related genes whose similar protein products may be used for different purposes in vertebrate organisms. We have explored this possibility by using hybridization in situ to examine the expression of myc and N-myc during gastrulation of mouse embryos. Throughout gastrulation, myc RNA was most abundant in extraembryonic cells; by contrast, N-myc RNA was found at highest levels in the expanding primitive streak and other portions of the embryonic mesoderm. Differentiation of mesoderm to epithelioid cells was accompanied by diminished expression of N-myc. Expression of myc was not an inevitable correlate of cellular proliferation. Instead, the gene appeared to be regulated in concert with changes that affect a diversity of cellular properties, including proliferation, **invasive**ness, and differentiation.
20. Specific requirement for Bax, not Bak, in Myc-induced apoptosis and tumor suppression in vivo. Bax and Bak comprise the mitochondrial gateway for apoptosis induced by diverse stimuli. Loss of both bax and bak is necessary to block cell death induced by such stimuli, indicating a great degree of functional overlap between Bax and Bak. Apoptosis is the major intrinsic pathway that limits the oncogenic potential of Myc. Using a switchable mouse model of Myc-induced apoptosis in pancreatic beta cells, we have shown that Myc induces apoptosis in vivo exclusively through Bax but not Bak. Furthermore, blockade of Myc-induced apoptosis by the inactivation of Bax, but not Bak, eliminates all restraints to the oncogenic potential of Myc, allowing the rapid and synchronous progression of **invasive**, angiogenic tumors.
21. Improving Drug Penetrability with iRGD Leverages the Therapeutic Response to Sorafenib and Doxorubicin in Hepatocellular Carcinoma. iRGD is a derivative of the integrin-binding peptide RGD, which selectively increases the penetrability of tumor tissue to various coadministered substances in several preclinical models. In this study, we investigated the ability of iRGD to improve the delivery of sorafenib and doxorubicin therapy in hepatocellular carcinoma (HCC) using established mouse models of the disease. A contrast-enhanced MRI method was developed in parallel to assess the in vivo effects of iRGD in this setting. We found that iRGD improved the delivery of marker substances to the tumors of HCC-bearing mice about three-fold without a parallel increase in normal tissues. Control peptides lacking the critical CendR motif had no effect. Similarly, iRGD also selectively increased the signal intensity from tumors in Gd-DTPA-enhanced MRI. In terms of antitumor efficacy, iRGD coadministration significantly augmented the individual inhibitory effects of sorafenib and doxorubicin without increasing systemic toxicity. Overall, our results offered a preclinical proof of concept for the use of iRGD coadministration as a strategy to widen the therapeutic window for HCC chemotherapy, as monitored by Gd-DTPA-enhanced MRI as a non**invasive**, clinically applicable method to identify iRGD-reactive tumors.
22. MYC overexpression induces prostatic intraepithelial neoplasia and loss of Nkx3.1 in mouse luminal epithelial cells. Lo-MYC and Hi-MYC mice develop prostatic intraepithelial neoplasia (PIN) and prostatic adenocarcinoma as a result of MYC overexpression in the mouse prostate. However, prior studies have not determined precisely when, and in which cell types, MYC is induced. Using immunohistochemistry (IHC) to localize MYC expression in Lo-MYC transgenic mice, we show that morphological and molecular alterations characteristic of high grade PIN arise in luminal epithelial cells as soon as MYC overexpression is detected. These changes include increased nuclear and nucleolar size and large scale chromatin remodeling. Mouse PIN cells retained a columnar architecture and abundant cytoplasm and appeared as either a single layer of neoplastic cells or as pseudo-stratified/multilayered structures with open glandular lumina-features highly analogous to human high grade PIN. Also using IHC, we show that the onset of MYC overexpression and PIN development coincided precisely with decreased expression of the homeodomain transcription factor and tumor suppressor, Nkx3.1. Virtually all normal appearing prostate luminal cells expressed high levels of Nkx3.1, but all cells expressing MYC in PIN lesions showed marked reductions in Nkx3.1, implicating MYC as a key factor that represses Nkx3.1 in PIN lesions. To determine the effects of less pronounced overexpression of MYC we generated a new line of mice expressing MYC in the prostate under the transcriptional control of the mouse Nkx3.1 control region. These "Super-Lo-MYC" mice also developed PIN, albeit a less aggressive form. We also identified a histologically defined intermediate step in the progression of mouse PIN into **invasive** adenocarcinoma. These lesions are characterized by a loss of cell polarity, multi-layering, and cribriform formation, and by a "paradoxical" increase in Nkx3.1 protein. Similar histopathological changes occurred in Hi-MYC mice, albeit with accelerated kinetics. Our results using IHC provide novel insights that support the contention that MYC overexpression is sufficient to transform prostate luminal epithelial cells into PIN cells in vivo. We also identified a novel histopathologically identifiable intermediate step prior to invasion that should facilitate studies of molecular pathway alterations occurring during early progression of prostatic adenocarcinomas.
23. Global levels of H3K27me3 track with differentiation in vivo and are deregulated by MYC in prostate cancer. Cancer cells and stem cells share a number of biological characteristics including abundant amounts of decondensed chromatin. However, the molecular correlates and the factors involved in altering chromatin structure in cancer cells are not well known. Here, we report that less differentiated stem-like cells in the basal compartment of human and mouse prostate contain lower levels of the polycomb heterochromatin marker H3K27me3 than more differentiated luminal cells. This link to differentiated normal cells is also found in a number of other human and rodent tissues characterized by hierarchical differentiation. In addition to MYC's traditional role as a gene-specific transcription factor, recent studies indicate that MYC also affects global chromatin structure where it is required to maintain "open" or active chromatin. We now demonstrate that in both MYC-driven prostate cancers in mice and human prostate cancers, global levels of H3K27me3 are reduced in prostatic intraepithelial neoplasia and **invasive** adenocarcinoma lesions. Moreover, decreased levels of H3K27me3 correlate with increased markers of disease aggressiveness (eg, Gleason score and pathological stage). In vitro, experimentally forced reductions in MYC levels result in increased global levels of H3K27me3. These findings suggest that increased levels of decondensed chromatin in both normal progenitor cells and cancer cells are associated with global loss of H3K27me3, which is linked to MYC overexpression.
24. Modulation of cellular migration and survival by c-Myc through the downregulation of urokinase (uPA) and uPA receptor. It has been proposed that c-Myc proapoptotic activity accounts for most of its restraint of tumor formation. We established a telomerase-immortalized human epithelial cell line expressing an activatable c-Myc protein. We found that c-Myc activation induces, in addition to increased sensitivity to apoptosis, reductions in cell motility and **invasive**ness. Transcriptome analysis revealed that urokinase (uPA) and uPA receptor (uPAR) were strongly downregulated by c-Myc. Evidence is provided that the repression of uPA and uPAR may account for most of the antimigratory and proapoptotic activities of c-Myc. c-Myc is known to cooperate with Ras in cellular transformation. We therefore investigated if this cooperation could converge in the control of uPA/uPAR expression. We found that Ras is able to block the effects of c-Myc activation on apoptosis and cellular motility but not on cell **invasive**ness. Accordingly, the activation of c-Myc in the context of Ras expression had only minor influence on uPAR expression but still had a profound repressive effect on uPA expression. Thus, the differential regulation of uPA and uPAR by c-Myc and Ras correlates with the effects of these two oncoproteins on cell motility, **invasive**ness, and survival. In conclusion, we have discovered a novel link between c-Myc and uPA/uPAR. We propose that reductions of cell motility and **invasive**ness could contribute to the inhibition of tumorigenesis by c-Myc and that the regulation of uPA and uPAR expression may be a component of the ability of c-Myc to reduce motility and **invasive**ness.
25. c-Myc phosphorylation by PKCζ represses prostate tumorigenesis. Studies showing reduced PKCζ expression or enzymatic activity in different types of human cancers support the clinical relevance of PKCζ as a tumor suppressor. However, the in vivo role of PKCζ and its mechanisms of action in prostate cancer remain unclear. Here we demonstrate that the genetic inactivation of PKCζ in mice result**s in inv**asive prostate carcinoma in vivo in the context of phosphatase and tensin homolog deficiency. Bioinformatic analysis of human prostate cancer gene-expression sets revealed increased c-Myc transcriptional activity in PKCζ-inactive cells, which correlated with increased cell growth, invasion, and metastasis. Interestingly, PKCζ knockdown or the overexpression of a kinase-inactive mutant resulted in enhanced cell proliferation and invasion in vitro through increased c-Myc mRNA and protein levels and decreased Ser-373 phosphorylation of c-Myc. Analysis of prostate cancer samples demonstrated increased expression and decreased phosphorylation of c-Myc at Ser-373 in PKCζ knockout tumors. In vivo xenograft studies revealed that c-Myc phosphorylation by PKCζ is a critical event in the control of metastasis. Collectively, these results establish PKCζ as an important tumor suppressor and regulator of c-Myc function in prostate cancer.
26. Suppression of Myc-induced apoptosis in beta cells exposes multiple oncogenic properties of Myc and triggers carcinogenic progression. To explore the role of c-Myc in carcinogenesis, we have developed a reversible transgenic model of pancreatic beta cell oncogenesis using a switchable form of the c-Myc protein. Activation of c-Myc in adult, mature beta cells induces uniform beta cell proliferation but is accompanied by overwhelming apoptosis that rapidly erodes beta cell mass. Thus, the oncogenic potential of c-Myc in beta cells is masked by apoptosis. Upon suppression of c-Myc-induced beta cell apoptosis by coexpression of Bcl-x(L), c-Myc triggers rapid and uniform progression into angiogenic, **invasive** tumors. Subsequent c-Myc deactivation induces rapid regression associated with vascular degeneration and beta cell apoptosis. Our data indicate that highly complex neoplastic lesions can be both induced and maintained in vivo by a simple combination of two interlocking molecular lesions.

#### GeneRIFs

1. Data suggest that reductions of cell motility and **invasive**ness could contribute to the inhibition of tumorigenesis by c-Myc and that the regulation of uPA and uPAR expression may be a component of the ability of c-Myc to reduce motility and **invasive**ness.

### [Mm]etastasize

#### PubMed

1. Analysis of the tumor-initiating and metastatic capacity of PDX1-positive cells from the adult pancreas. Pancreatic cancer is one of the deadliest human malignancies. A striking feature of pancreatic cancer is that activating Kras mutations are found in ∼90% of cases. However, apart from a restricted population of cells expressing pancreatic and duodenal homeobox 1 (PDX1), most pancreatic cells are refractory to Kras-driven transformation. In the present study, we sought to determine which subsets of PDX1+ cells may be responsible for tumor growth. Using the Lox-Stop-Lox-KrasG12D genetic mouse model of pancreatic carcinogenesis, we isolated a population of KrasG12D-expressing PDX1+ cells with an inherent capacity t**o metastasi**ze. This population of cells bears the surface phenotype of EpCAM+CD24+CD44+CD133-SCA1- and is closer in its properties to stem-like cells than to more mature cell types. We further demonstrate that the tumorigenic capacity of PDX1+ cells is limited, becoming progressively lost as the cells acquire a mature phenotype. These data are consistent with the hypothesis that the adult pancreas harbors a dormant progenitor cell population that is capable of initiating tumor growth under conditions of oncogenic stimulation. We present evidence that constitutive activation of the mitogen-activated protein kinase (MAPK/ERK) signaling and stabilization of the MYC protein are the two main driving forces behind the development of pancreatic cancer cells with stem-cell-like properties and high metastatic potential. Our results suggest that pancreatic cells bearing Kras mutation can be induced to differentiate into quasi-normal cells with suppressed tumorigenicity by selective inhibition of the MAPK/ERK/MYC signaling cascade.
2. Molecular characterizations of Nop16 in murine mammary tumors with varying levels of c-Myc. NOP16, also known as HSPC111, has been identified as a MYC and estrogen regulated gene in in vitro studies, hence coexpression levels were strongly correlated. Importantly, high expression of NOP16 was associated with poor clinical outcome in breast cancer patients. However, coexpression of NOP16, MYC and estrogen receptor (ESR1) varied widely in tumors and cell lines suggesting that transcriptional regulation differed according to pathological environments. The goal of this study was to determine the expression patterns of Nop16, Myc and Esr1 in murine mammary tumors with disparate histopathological and molecular features. We hypothesized that tumor environments with relatively high Myc levels would have different coexpression patterns than tumor environments with relatively low Myc levels. We measured levels of Myc and Nop16 mRNA and protein in tumors from WAP-c-myc mice that were of high grade and **metastasize**d frequently. In contrast, Myc and Nop16 mRNA and proteins levels were significantly lower in the less aggressive tumors that developed in NRL-TGFα mice. Tumors from both mouse lines express ESR1 protein and we found that Esr1 mRNA levels correlated positively with Myc levels in both models. However, Myc and Nop16 transcript levels correlated positively only in tumors from NRL-TGFα mice. We identified prominent NOP16 protein in nuclei and less prominent staining in the cytoplasm of luminal cells of ducts and lobules from normal mammary glands as well as in hyperplasias and tumors obtained from NRL-TGFα mice. This staining pattern was reversed in tumors from WAP-c-Myc mice as nuclear staining was faint or absent and cytoplasmic staining more pronounced. In summary, the regulation of expression and localization of NOP16 varies in tumor environments with high versus low MYC levels and demonstrate the importance of stratifying clinical breast cancers based on MYC levels.

### [Mm]etastasis

#### PubMed

1. c-Myc is essential for vasculogenesis and angiogenesis during development and tumor progression. c-Myc promotes cell growth and transformation by ill-defined mechanisms. c-myc(-/-) mice die by embryonic day 10.5 (E10.5) with defects in growth and in cardiac and neural development. Here we report that the lethality of c-myc(-/-) embryos is also associated with profound defects in vasculogenesis and primitive erythropoiesis. Furthermore, c-myc(-/-) embryonic stem (ES) and yolk sac cells are compromised in their differentiative and growth potential. These defects are intrinsic to c-Myc, and are in part associated with a requirement for c-Myc for the expression of vascular endothelial growth factor (VEGF), as VEGF can partially rescue these defects. However, c-Myc is also required for the proper expression of other angiogenic factors in ES and yolk sac cells, including angiopoietin-2, and the angiogenic inhibitors thrombospondin-1 and angiopoietin-1. Finally, c-myc(-/-) ES cells are dramatically impaired in their ability to form tumors in immune-compromised mice, and the small tumors that sometimes develop are poorly vascularized. Therefore, c-Myc function is also necessary for the angiogenic switch that is indispensable for the progression and **metastasis** of tumors. These findings support the model wherein c-Myc promotes cell growth and transformation, as well as vascular and hematopoietic development, by functioning as a master regulator of angiogenic factors.
2. c-Myc is essential for vasculogenesis and angiogenesis during development and tumor progression. c-Myc promotes cell growth and transformation by ill-defined mechanisms. c-myc(-/-) mice die by embryonic day 10.5 (E10.5) with defects in growth and in cardiac and neural development. Here we report that the lethality of c-myc(-/-) embryos is also associated with profound defects in vasculogenesis and primitive erythropoiesis. Furthermore, c-myc(-/-) embryonic stem (ES) and yolk sac cells are compromised in their differentiative and growth potential. These defects are intrinsic to c-Myc, and are in part associated with a requirement for c-Myc for the expression of vascular endothelial growth factor (VEGF), as VEGF can partially rescue these defects. However, c-Myc is also required for the proper expression of other angiogenic factors in ES and yolk sac cells, including angiopoietin-2, and the angiogenic inhibitors thrombospondin-1 and angiopoietin-1. Finally, c-myc(-/-) ES cells are dramatically impaired in their ability to form tumors in immune-compromised mice, and the small tumors that sometimes develop are poorly vascularized. Therefore, c-Myc function is also necessary for the angiogenic switch that is indispensable for the progression and **metastasis** of tumors. These findings support the model wherein c-Myc promotes cell growth and transformation, as well as vascular and hematopoietic development, by functioning as a master regulator of angiogenic factors.
3. MYC synergizes with activated BRAFV600E in mouse lung tumor development by suppressing senescence. The activated RAS/RAF cascade plays a crucial role in lung cancer, but is also known to induce cellular senescence, a major barrier imposed on tumor cells early in tumorigenesis. MYC is a key factor in suppression of RAS/BRAF(V600E)-induced senescence in vitro. However, it is still unclear whether MYC has the same role during tumor development in vivo. Using a conditional, compound knock-in model of Cre-activated BRAF(V600E) and tamoxifen-regulatable MycER, we show that tamoxifen-induced activation of MYC accelerated the onset and increased the number and size of BRAF(V600E)-driven adenomas in a dose-dependent manner, resulting in reduced survival. Furthermore, MYC activation leads to reduced expression of the senescence markers p16(INK4A), p21(CIP1), and H3K9me3-containing heterochromatin foci, and an increased percentage of Ki67(+) tumor cells. This suggests that MYC already early during tumor formation suppresses a BRAF(V600E)-induced senescence-like state. Initial activation of MYC followed by tamoxifen withdrawal still resulted in an increased number of tumors and reduced survival. However, these tumors were of smaller size, showed increased expression of p16(INK4A) and p21(CIP1), and reduced number of Ki67(+) cells, indicating that MYC inactivation restores BRAF(V600E)-induced senescence. Surprisingly, MYC activation did not promote adenoma to carcinoma progression. This suggests that senescence suppression by MYC is a discrete step in tumor development important for sustained tumor growth but preceding malignant transformation and that additional oncogenic events are required for carcinoma development and **metastasis**. These findings contribute to our understanding of the neoplastic transformation process, with implications for future treatment strategies.
4. B lymphocyte-specific c-Myc expression stimulates early and functional expansion of the vasculature and lymphatics during lymphomagenesis. Expression of the c-myc proto-oncogene is deregulated in many human cancers. We examined the role of c-Myc in stimulating angiogenesis and lymphangiogenesis in a highly metastatic murine model of Burkitt's lymphoma (E micro -c-myc), where c-Myc is expressed exclusively in B lymphocytes. Immunohistochemical analysis of bone marrow and lymph nodes from young (preneoplastic) E micro -c-myc transgenic mice revealed increased growth of blood vessels, which are functional by dye flow assay. Lymphatic sinuses also increased in size and number within the lymph nodes, as demonstrated by immunostaining for with a lymphatic endothelial marker 10.1.1. The 10.1.1 antibody recognizes VEGFR-2- and VEGFR-3-positive lymphatic sinuses and vessels within lymph nodes, and also recognizes lymphatic vessels in other tissues. Subcutaneously injected dye traveled more efficiently through draining lymph nodes in E micro -c-myc mice, indicating that these hypertrophic lymphatic sinuses increase lymph flow. Purified B lymphocytes and lymphoid tissues from E micro -c-myc mice expressed increased levels of vascular endothelial growth factor (VEGF) by immunohistochemical or immunoblot assays, which could promote blood and lymphatic vessel growth through interaction with VEGFR-2, which is expressed on the endothelium of both vessel types. These results indicate that constitutive c-Myc expression stimulates angiogenesis and lymphangiogenesis, which may promote the rapid growth and **metastasis** of c-Myc-expressing cancer cells, respectively.
5. MET and MYC cooperate in mammary tumorigenesis. In human breast cancer, overexpression of the protooncogene MET is strongly associated with poor prognosis and high risk of **metastasis**. It stands out as a reliable prognostic indicator of survival and defines a set of tumors exclusive of those that express HER2 or hormone receptors. Studies have shown that overexpression of mutant forms of MET cause cancer in mice. However, MET mutations have not been found in human breast cancer, and the consequences of overexpression of normal MET are unknown. To investigate the role of MET and other putative oncogenes in breast cancer, we developed an experimental system that involves retroviral delivery of genes into primary mammary epithelial cells, followed by transplantation of the transduced cells into mammary fat pads. Using this approach, we found that overexpression of wild-type MET leads to the development of nonprogressive neoplasms. The lesions progressed to mammary adenocarcinoma when a second protooncogene, MYC, was overexpressed, indicating that MET and MYC cooperate in mammary tumorigenesis. Both the nonprogressive neoplasms and adenocarcinomas display characteristics consistent with transformation and expansion of mammary progenitor cells. The approach described here should provide a useful model with which to efficiently test effects of various genes on tumor development in the breast.
6. Direct reprogramming by oncogenic Ras and Myc. Genetically or epigenetically defined reprogramming is a hallmark of cancer cells. However, a causal association between genome reprogramming and cancer has not yet been conclusively established. In particular, little is known about the mechanisms that underlie **metastasis** of cancer, and even less is known about the identity of metastasizing cancer cells. In this study, we used a model of conditional expression of oncogenic KrasG12D allele in primary mouse cells to show that reprogramming and dedifferentiation is a fundamental early step in malignant transformation and cancer initiation. Our data indicate that stable expression of activated KrasG12D confers on cells a large degree of phenotypic plasticity that predisposes them to neoplastic transformation and acquisition of stem cell characteristics. We have developed a genetically tractable model system to investigate the origins and evolution of metastatic pancreatic cancer cells. We show that metastatic conversion of KrasG12D-expressing cells that exhibit different degrees of differentiation and malignancy can be reconstructed in cell culture, and that the proto-oncogene c-Myc controls the generation of self-renewing metastatic cancer cells. Collectively, our results support a model wherein non-stem cancer cells have the potential to dedifferentiate and acquire stem cell properties as a direct consequence of oncogene-induced plasticity. Moreover, the disturbance in the normally existing dynamic equilibrium between cancer stem cells and non-stem cancer cells allows the formation of cancer stem cells with high metastatic capacity at any time during cancer progression.
7. Effect of recombinant human endostatin on the expression of c-Myc and bFGF in mouse gastric cancer cells. The aim of this study was to observe the effects of re-combinant human endostatin on the proliferation and apoptosis of mouse gastric cancer cells, and explore some possible mechanisms of recom-binant human endostatin inhibition of cancer. A murine gastric cancer xenograft model was established. A total of 20 mice were divided into two groups (control and experimental groups). The expression of c-Myc and basic fibroblast growth factor (bFGF) was determined by reverse transcription-polymerase chain reaction, Western blotting, and immu-nohistochemical staining methods. Tumor volume was measured and a growth curve was calculated. The tumor diameter in the experimental group was significantly smaller than that in the control group after treat-ment with endostatin for 21 days. The expression levels of c-Myc and bFGF in the experimental group were significantly lower than those of the control group (P < 0.05). There was a positive correlation between the expression of c-Myc and bFGF in the experimental group. Microvessel density was significantly inhibited in the experimental group (P < 0.05). These results demonstrated that recombinant human endostatin could in-hibit tumor **metastasis** by inhibition of the expression of c-Myc and bFGF in gastric cancer tissue as well as by inhibition of angiogenesis.
8. Loss of p53 function leads to **metastasis** in ras+myc-initiated mouse prostate cancer. To study the interactions between dominantly acting oncogenes and tumor suppressor genes we used p53 'knockout' mouse urogenital sinus tissue for retroviral transduction of ras and myc in the mouse prostate reconstitution (MPR) model system. Epithelial hyperplasia was observed in all wild-type p53 MPRs with one small focal cancer and no evidence of **metastasis**. Prostatic cancer was found in 100% of the heterozygous and homozygous p53 mutant MPRs with metastatic deposits in 95% of the mice. The pattern of **metastasis** was remarkably similar to that in human prostate cancer with gross metastatic deposits in the lung, lymph nodes, bone and liver of many animals. Progression of carcinomas in the ras+myc-initiated heterozygous p53 mutant MPRs was invariably associated with either complete loss, partial deletion or loss of expression of the wild-type p53 allele. Southern blotting analysis of proviral-cellular DNA junction fragments in primary carcinomas and cell lines derived from metastatic deposits revealed that metastases do not necessarily seed out from the most abundant clone in the primary carcinoma.
9. Synergy between a human c-myc transgene and p53 null genotype in murine thymic lymphomas: contrasting effects of homozygous and heterozygous p53 loss. Activation of the c-myc oncogene and functional loss of the p53 tumour suppressor gene are among the most frequently recorded genetic lesions in neoplasia but their combined effect has not previously been investigated. By breeding together mice transgenic for human c-myc (CD2-myc) and mice carrying an inactive p53 allele (p53-/-) we found that these genetic lesions act synergistically in vivo. Offspring carrying the CD2-myc transgene and the homozygous p53 null mutation (p53-/-/CD2-myc) were viable but developed thymic lymphomas with dramatically increased frequency and reduced latency compared to both parental groups. The tumour phenotype was similar to that previously recorded for CD2-myc mice (predominantly CD3+, CD4+8+) but tumour clonal complexity and **metastasis** was significantly greater in the p53-/-/CD2-myc mice. In contrast, no significant increase in tumour incidence was seen in p53+/-/CD2-myc vs p53+/+/CD2-myc mice over a 6 month observation period. However, the loss of wild type p53 in a proportion of tumour cells in p53+/-/CD2-myc lymphomas suggests that wild type allele loss can occur as a late progression step rather than an initiating step in these tumours. We suggest that p53 loss of function may collaborate with the CD2-myc transgene at more than one stage in thymic lymphoma development.
10. Stress-induced cleavage of Myc promotes cancer cell survival. Evasion of apoptosis is critical in Myc-induced tumor progression. Here we report that cancer cells evade death under stress by activating calpain-mediated proteolysis of Myc. This generates Myc-nick, a cytoplasmic, transcriptionally inactive cleavage product of Myc. We found conversion of Myc into Myc-nick in cell lines and tissues derived from multiple cancers. In colon cancer, the production of Myc-nick is enhanced under stress conditions such as hypoxia and nutrient deprivation. Under these conditions, ectopic expression of Myc-nick promotes anchorage-independent growth and cell survival at least in part by promoting autophagy. Myc-nick also delays colon cancer cell death after treatment with chemotherapeutic drugs such as etoposide, cisplatin, and imatinib. Furthermore, colon cancer cells expressing a cleavage-resistant form of Myc undergo extensive apoptosis but are rescued by overexpression of Myc-nick. We also found that ectopic expression of Myc-nick results in the induction of the actin-bundling protein fascin, formation of filopodia, and increased cell motility-all mediators of tumor **metastasis**. Myc-nick-induced survival, autophagy, and motility require Myc box II (MBII), a region of Myc-nick that recruits acetyltransferases that in turn modify cytoplasmic proteins, including α-tubulin and ATG3. Our results suggest that Myc-nick-induced survival and motility contribute to colon cancer progression and **metastasi**s.
11. Absence of caspase-3 protects pancreatic {beta}-cells from c-Myc-induced apoptosis without leading to tumor formation. c-Myc is a powerful trigger of beta-cell apoptosis, proliferation, and dedifferentiation in rodent islets in vivo. In a transgenic mouse model, c-Myc induction causes rapid beta-cell apoptosis and overt diabetes. When suppression of apoptosis is achieved by overexpression of Bcl-x(L) in an inducible model of c-Myc activation, a full spectrum of tumor development, including distant **metastasis**, occurs. Caspase-3 is a key pro-apoptotic protein involved in the execution phase of multiple apoptotic pathways. To test whether caspase-3 is an essential mediator of apoptosis in this model of tumorigenesis, we generated caspase-3 knock-out mice containing the inducible c-myc transgene (c-Myc(+)Casp3(-/-)). In contrast to Bcl-x(L)-overexpressing c-Myc(+) mice, c-Myc(+)Casp3(-/-) mice remained euglycemic for up to 30 days of c-Myc activation, and there was no evidence of tumor formation. Interestingly, caspase-3 deletion also led to the suppression of proliferation, perhaps through regulation of the cell cycle inhibitory protein p27, suggesting a possible mechanism for maintaining a balance between suppression of apoptosis and excessive proliferation in the context of c-Myc activation. Additionally, c-Myc-activated Casp3(-/-) mice were protected from streptozotocin-induced diabetes. Our studies demonstrate that caspase-3 deletion confers protection from c-Myc-induced apoptosis and diabetes development without unwanted tumorigenic effects. These results may lead to further elucidation of the mechanisms of c-Myc biology relevant to beta-cells, which may result in novel therapeutic strategies for diabetes.
12. In vivo inhibition of c-MYC in myeloid cells impairs tumor-associated macrophage maturation and pro-tumoral activities. Although tumor-associated macrophages (TAMs) are involved in tumor growth and **metastasis**, the mechanisms controlling their pro-tumoral activities remain largely unknown. The transcription factor c-MYC has been recently shown to regulate in vitro human macrophage polarization and be expressed in macrophages infiltrating human tumors. In this study, we exploited the predominant expression of LysM in myeloid cells to generate c-Myc(fl/fl) LysM(cre/+) mice, which lack c-Myc in macrophages, to investigate the role of macrophage c-MYC expression in cancer. Under steady-state conditions, immune system parameters in c-Myc(fl/fl) LysM(cre/+) mice appeared normal, including the abundance of different subsets of bone marrow hematopoietic stem cells, precursors and circulating cells, macrophage density, and immune organ structure. In a model of melanoma, however, TAMs lacking c-Myc displayed a delay in maturation and showed an attenuation of pro-tumoral functions (e.g., reduced expression of VEGF, MMP9, and HIF1α) that was associated with impaired tissue remodeling and angiogenesis and limited tumor growth in c-Myc(fl/fl) LysM(cre/+) mice. Macrophage c-Myc deletion also diminished fibrosarcoma growth. These data identify c-Myc as a positive regulator of the pro-tumoral program of TAMs and suggest c-Myc inactivation as an attractive target for anti-cancer therapy.
13. Multiple mechanisms of tumorigenesis in E mu-myc transgenic mice. Transgenic mice bearing a c-myc oncogene under control of the immunoglobulin heavy chain enhancer (E mu-myc mice) reproducibly develop and die from tumors of the B lymphocyte lineage (J.M. Adams, A.W. Harris, C.A. Pinkert, L.M. Corcoran, W.S. Alexander, S. Cory, R.D. Palmiter, and R.L. Brinster, Nature (Lond.), 318: 533-538, 1985; W.Y. Langdon, A. W. Harris, S. Cory, and J.M. Adams, Cell 47: 11-18, 1986; A.W. Harris, C.A. Pinkert, M. Crawford, W.Y. Langdon, R.L. Brinster, and J.M. Adams, J. Exp. Med., 167: 353-371, 1988; reviewed in S. Cory and J.M. Adams, Annu. Rev. Immunol., 6: 25-48, 1988). Analysis of lymphocytes obtained by serial sampling of peripheral blood from individual hemizygous (E mu-myc/0) and homozygous (E mu-myc/E mu-myc) transgenic mice indicates that proliferation in the original host and transplantability into histocompatible recipients are distinct properties that can be acquired independently and in either order. These two types of transgenic mice differ in that homozygous mice have about one-fourth the life span of hemizygous mice and develop polyclonal, non-transplantable tumors in comparison to the oligoclonal, highly transplantable malignancies seen in hemizygous animals. In conclusion, the overall concept of malignancy is best viewed as an aggregate of the separable parameters of cellular proliferation, clonality, tissue invasiveness, **metastasis**, and (experimental) transplantability. The E mu-myc transgenic mouse represents an attractive model in which to investigate the multistep nature and alternative pathways of tumorigenesis.
14. F-box protein FBXW7 inhibits cancer **metastasis** in a non-cell-autonomous manner. The gene encoding F-box protein FBXW7 is frequently mutated in many human cancers. Although most previous studies have focused on the tumor-suppressive capacity of FBXW7 in tumor cells themselves, we determined that FBXW7 in the host microenvironment also suppresses cancer **metastasis**. Deletion of Fbxw7 in murine BM-derived stromal cells induced accumulation of NOTCH and consequent transcriptional activation of Ccl2. FBXW7-deficient mice exhibited increased serum levels of the chemokine CCL2, which resulted in the recruitment of both monocytic myeloid-derived suppressor cells and macrophages, thereby promoting metastatic tumor growth. Administration of a CCL2 receptor antagonist blocked the enhancement of **metastasis** in FBXW7-deficient mice. Furthermore, in human breast cancer patients, FBXW7 expression in peripheral blood was associated with serum CCL2 concentration and disease prognosis. Together, these results suggest that FBXW7 antagonizes cancer development in not only a cell-autonomous manner, but also a non-cell-autonomous manner, and that modulation of the FBXW7/NOTCH/CCL2 axis may provide a potential approach to suppression of cancer **metastasis**.
15. MYC is a **metastasis** gene for non-small-cell lung cancer. **Metastasis** is a process by which cancer cells learn to form satellite tumors in distant organs and represents the principle cause of death of patients with solid tumors. NSCLC is the most lethal human cancer due to its high rate of **metastasis**.Lack of a suitable animal model has so far hampered analysis of metastatic progression. We have examined c-MYC for its ability to induce **metastasis** in a C-RAF-driven mouse model for non-small-cell lung cancer. c-MYC alone induced frank tumor growth only after long latency at which time secondary mutations in K-Ras or LKB1 were detected reminiscent of human NSCLC. Combination with C-RAF led to immediate acceleration of tumor growth, conversion to papillary epithelial cells and angiogenic switch induction. Moreover, addition of c-MYC was sufficient to induce macro**metastasis** in liver and lymph nodes with short latency associated with lineage switch events. Thus we have generated the first conditional model for **metastasis** of NSCLC and identified a gene, c-MYC that is able to orchestrate all steps of this process.Potential markers for detection of **metastasis** were identified and validated for diagnosis of human biopsies. These markers may represent targets for future therapeutic intervention as they include genes such as Gata4 that are exclusively expressed during lung development.
16. Astrocyte elevated gene-1 and c-Myc cooperate to promote hepatocarcinogenesis in mice. Astrocyte elevated gene-1 (AEG-1) and c-Myc are overexpressed in human hepatocellular carcinoma (HCC) functioning as oncogenes. AEG-1 is transcriptionally regulated by c-Myc, and AEG-1 itself induces c-Myc by activating the Wnt/β-catenin-signaling pathway. We now document the cooperation of AEG-1 and c-Myc in promoting hepatocarcinogenesis by analyzing hepatocyte-specific transgenic mice expressing either AEG-1 (albumin [Alb]/AEG-1), c-Myc (Alb/c-Myc), or both (Alb/AEG-1/c-Myc). Wild-type and Alb/AEG-1 mice did not develop spontaneous HCC. Alb/c-Myc mice developed spontaneous HCC without distant **metastasi**s, whereas Alb/AEG-1/c-Myc mice developed highly aggressive HCC with frank **metastasi**s to the lungs. Induction of carcinogenesis by N-nitrosodiethylamine significantly accelerated the kinetics of tumor formation in all groups. However, in Alb/AEG-1/c-Myc, the effect was markedly pronounced with lung **metastasi**s. In vitro analysis showed that Alb/AEG-1/c-Myc hepatocytes acquired increased proliferation and transformative potential with sustained activation of prosurvival and epithelial-mesenchymal transition-signaling pathways. RNA-sequencing analysis identified a unique gene signature in livers of Alb/AEG-1/c-Myc mice that was not observed when either AEG-1 or c-Myc was overexpressed. Specifically, Alb/AEG-1/c-Myc mice overexpressed maternally imprinted noncoding RNAs (ncRNAs), such as Rian, Meg-3, and Mirg, which are implicated in hepatocarcinogenesis. Knocking down these ncRNAs significantly inhibited proliferation and invasion by Alb/AEG-1/c-Myc hepatocytes.Our studies reveal a novel cooperative oncogenic effect of AEG-1 and c-Myc that might explain the mechanism of aggressive HCC. Alb/AEG-1/c-Myc mice provide a useful model to understand the molecular mechanism of cooperation between these two oncogenes and other molecules involved in hepatocarcinogenesis. This model might also be of use for evaluating novel therapeutic strategies targeting HCC.
17. Somatic cell transfer of c-Myc and Bcl-2 induces large-cell anaplastic medulloblastomas in mice. A highly aggressive subgroup of the pediatric brain tumor medulloblastoma is characterized by overexpression of the proto-oncogene c-Myc, which encodes a transcription factor that normally maintains neural progenitor cells in an undifferentiated, proliferating state during embryonic development. Myc-driven medulloblastomas typically show a large-cell anaplastic (LCA) histological pattern, in which tumor cells display large, round nuclei with prominent nucleoli. This subgroup of medulloblastoma is therapeutically challenging because it is associated with a high rate of metastatic dissemination, which is a powerful predictor of short patient survival times. Genetically engineered mouse models have revealed important insights into the pathogenesis of medulloblastoma and served as preclinical testing platforms for new therapies. Here we report a new mouse model of Myc-driven medulloblastoma, in which tumors arise in situ after retroviral transfer and expression of Myc in Nestin-expressing neural progenitor cells in the cerebella of newborn mice. Tumor induction required concomitant loss of Tp53 or overexpression of the antiapoptotic protein Bcl-2. Like Myc-driven medulloblastomas in humans, the tumors induced in mice by Myc + Bcl-2 and Myc - Tp53 showed LCA cytoarchitecture and a high rate of metastatic dissemination to the spine. The fact that Myc - Tp53 tumors arose only in Tp53(-/-) mice, coupled with the inefficient germline transmission of the Tp53-null allele, made retroviral transfer of Myc + Bcl-2 a more practical method for generating LCA medulloblastomas. The high rate of spinal **metastasis** (87% of brain tumor-bearing mice) will be an asset for testing new therapies that target the most lethal aspect of medulloblastoma.
18. RapidCaP, a novel GEM model for metastatic prostate cancer analysis and therapy, reveals myc as a driver of Pten-mutant **metastasis**. Genetically engineered mouse (GEM) models are a pillar of functional cancer research. Here, we developed RapidCaP, a GEM modeling system that uses surgical injection for viral gene delivery to the prostate. We show that in Pten deficiency, loss of p53 suffices to trigger **metastasis** to distant sites at greater than 50% penetrance by four months, consistent with results from human prostate cancer genome analysis. Live bioluminescence tracking showed that endogenous primary and metastatic disease responds to castration before developing lethal castration resistance. To our surprise, the resulting lesions showed no activation of Akt but activation of the Myc oncogene. Using RapidCaP, we find that Myc drives local prostate **metastasis** and is critical for maintenance of **metastasis**, as shown by using the Brd4 inhibitor JQ1. Taken together, our data suggest that a "MYC-switch" away from AKT forms a critical and druggable event in PTEN-mutant prostate cancer **metastasis** and castration resistance.
19. c-Myc phosphorylation by PKCζ represses prostate tumorigenesis. Studies showing reduced PKCζ expression or enzymatic activity in different types of human cancers support the clinical relevance of PKCζ as a tumor suppressor. However, the in vivo role of PKCζ and its mechanisms of action in prostate cancer remain unclear. Here we demonstrate that the genetic inactivation of PKCζ in mice results in invasive prostate carcinoma in vivo in the context of phosphatase and tensin homolog deficiency. Bioinformatic analysis of human prostate cancer gene-expression sets revealed increased c-Myc transcriptional activity in PKCζ-inactive cells, which correlated with increased cell growth, invasion**, and meta**stasis. Interestingly, PKCζ knockdown or the overexpression of a kinase-inactive mutant resulted in enhanced cell proliferation and invasion in vitro through increased c-Myc mRNA and protein levels and decreased Ser-373 phosphorylation of c-Myc. Analysis of prostate cancer samples demonstrated increased expression and decreased phosphorylation of c-Myc at Ser-373 in PKCζ knockout tumors. In vivo xenograft studies revealed that c-Myc phosphorylation by PKCζ is a critical event in the co**ntrol of m**etastasis. Collectively, these results establish PKCζ as an important tumor suppressor and regulator of c-Myc function in prostate cancer.

#### GeneRIFs

1. These results demonstrated that recombinant human endostatin could in-hibit tumor **metastasis** by inhibition of the expression of c-Myc and bFGF in gastric cancer tissue as well as by inhibition of angiogenesis
2. Results show that Myc drives local prostate **metastasis** and is critical for maintenance of **metastasis** prostate cancer.
3. MYC is a **metastasis** gene for non-small-cell lung cancer
4. Constitutive c-Myc expression stimulates angiogenesis and lymphangiogenesis, which may promote the rapid growth and **metastasis** of c-Myc-expressing cancer cells.

### [Cc]ell [Ii]nvasion

#### PubMed

1. MicroRNA-34a modulates c-Myc transcriptional complexes to suppress malignancy in human prostate cancer cells. MicroRNA-34a (miR-34a), a potent mediator of tumor suppressor p53, has been reported to function as a tumor suppressor and miR-34a was found to be downregulated in prostate cancer tissues. We studied the functional effects of miR-34a on c-Myc transcriptional complexes in PC-3 prostate cancer cells. Transfection of miR-34a into PC-3 cells strongly inhibited in vitro cell proliferation, **cell invasion** and promoted apoptosis. Transfection of miR-34a into PC-3 cells also significantly inhibited in vivo xenograft tumor growth in nude mice. miR-34a downregulated expression of c-Myc oncogene by targeting its 3' UTR as shown by luciferase reporter assays. miR-34a was found to repress RhoA, a regulator of cell migration and invasion, by suppressing c-Myc-Skp2-Miz1 transcriptional complex that activates RhoA. Overexpression of c-Myc reversed miR-34a suppression of RhoA expression, suggesting that miR-34a inhibits invasion by suppressing RhoA through c-Myc. miR-34a was also found to repress c-Myc-pTEFB transcription elongation complex, indicating one of the mechanisms by which miR-34a has profound effects on cellular function. This is the first report to document that miR-34a suppresses assembly and function of the c-Myc-Skp2-Miz1 complex that activates RhoA and the c-Myc-pTEFB complex that elongates transcription of various genes, suggesting a novel role of miR-34a in the regulation of transcription by c-Myc complex.

---

## Fos

- NCBI Gene
- GeneRIF
- Pubmed

| Search Term | Rank | Fields |
| --- | --- | --- |
| [Ff]ilopodia | 1 | PubMed(1) |
| [Mm]igration | 6 | PubMed(11); GeneRIFs(1) |
| [Ee]xtracellular [Mm]atrix | 8 | PubMed(12); GeneRIFs(1) |
| [Ii]nvasive | 10 | PubMed(6) |
| [Mm]etastasis | 12 | PubMed(3) |
| [Cc]ell [Ii]nvasion | 13 | PubMed(1) |

  
hide/show details for Fos

### [Ff]ilopodia

#### PubMed

1. Serum response factor is required for sprouting angiogenesis and vascular integrity. Serum response factor (SRF) is a transcription factor that controls the expression of cytoskeletal proteins and immediate early genes in different cell types. Here, we found that SRF expression is restricted to endothelial cells (ECs) of small vessels such as capillaries in the mouse embryo. EC-specific Srf deletion led to aneurysms and hemorrhages from 11.5 days of mouse development (E11.5) and lethality at E14.5. Mutant embryos presented a reduced capillary density and defects in EC migration, with fewer numbers of **filopodia** in tip cells and ECs showing defects in actin polymerization and intercellular junctions. We show that SRF is essential for the expression of VE-cadherin and beta-actin in ECs both in vivo and in vitro. Moreover, knockdown of SRF in ECs impaired VEGF- and FGF-induced in vitro angiogenesis. Taken together, our results demonstrate that SRF plays an important role in sprouting angiogenesis and small vessel integrity in the mouse embryo.

### [Mm]igration

#### PubMed

1. Serum response factor is required for sprouting angiogenesis and vascular integrity. Serum response factor (SRF) is a transcription factor that controls the expression of cytoskeletal proteins and immediate early genes in different cell types. Here, we found that SRF expression is restricted to endothelial cells (ECs) of small vessels such as capillaries in the mouse embryo. EC-specific Srf deletion led to aneurysms and hemorrhages from 11.5 days of mouse development (E11.5) and lethality at E14.5. Mutant embryos presented a reduced capillary density and defects in EC **migration**, with fewer numbers of filopodia in tip cells and ECs showing defects in actin polymerization and intercellular junctions. We show that SRF is essential for the expression of VE-cadherin and beta-actin in ECs both in vivo and in vitro. Moreover, knockdown of SRF in ECs impaired VEGF- and FGF-induced in vitro angiogenesis. Taken together, our results demonstrate that SRF plays an important role in sprouting angiogenesis and small vessel integrity in the mouse embryo.
2. The goya mouse mutant reveals distinct newly identified roles for MAP3K1 in the development and survival of cochlear sensory hair cells. Mitogen-activated protein kinase, MAP3K1, plays an important role in a number of cellular processes, including epithelial **migration** during eye organogenesis. In addition, studies in keratinocytes indicate that MAP3K1 signalling through JNK is important for actin stress fibre formation and cell **migration**. However, MAP3K1 can also act independently of JNK in the regulation of cell proliferation and apoptosis. We have identified a mouse mutant, goya, which exhibits the eyes-open-at-birth and microphthalmia phenotypes. In addition, these mice also have hearing loss. The goya mice carry a splice site mutation in the Map3k1 gene. We show that goya and kinase-deficient Map3k1 homozygotes initially develop supernumerary cochlear outer hair cells (OHCs) that subsequently degenerate, and a progressive profound hearing loss is observed by 9 weeks of age. Heterozygote mice also develop supernumerary OHCs, but no cellular degeneration or hearing loss is observed. MAP3K1 is expressed in a number of inner-ear cell types, including outer and inner hair cells, stria vascularis and spiral ganglion. Investigation of targets downstream of MAP3K1 identified an increase in p38 phosphorylation (Thr180/Tyr182) in multiple cochlear tissues. We also show that the extra OHCs do not arise from aberrant control of proliferation via p27KIP1. The identification of the goya mutant reveals a signalling molecule involved with hair-cell development and survival. Mammalian hair cells do not have the ability to regenerate after damage, which can lead to irreversible sensorineural hearing loss. Given the observed goya phenotype, and the many diverse cellular processes that MAP3K1 is known to act upon, further investigation of this model might help to elaborate upon the mechanisms underlying sensory hair cell specification, and pathways important for their survival. In addition, MAP3K1 is revealed as a new candidate gene for human sensorineural hearing loss.
3. Changes in protooncogene expression correlated with general and sex-specific differentiation in murine primordial germ cells. Primordial germ cells (PGCs) of the mouse undergo key developmental transitions during embryonic days 12-15. On day 12 they complete **migration** into the gonads. They cease mitotic proliferation on day 13 and subsequently enter sex-specific pathways of development. The molecular mechanisms controlling these transitions are poorly understood, yet they are crucial to production of normal gametes later in life. We have used the polymerase chain reaction (PCR) to directly compare levels of expression of several protooncogenes proposed to be involved in control of cell proliferation and differentiation in proliferating and differentiating PCGs of both sexes over a 4 day time course. We report here that mRNA levels for nuclear protooncogenes c-myc, c-fos, and c-jun increase dramatically in both sexes from little or no detectable expression on day 12 to high expression on days 13-15. We observe c-kit message on day 12 in combined PGCs of both sexes, in female but not male PGCs on day 13, and in both sexes on day 14, c-kit mRNA is undetectable on day 15 in either sex, c-mos is not expressed at detectable levels on day 12 in either sex, but increases gradually in female PGCs to very high levels on day 15. In male PGCs, c-mos is expressed at high levels on days 13-15. Our results are consistent with a role for protooncogenes c-myc, c-fos and c-jun in mediating the initial differentiation of PGCs of both sexes that occurs upon colonization of the gonad. Because c-kit and c-mos are expressed differentially in male and female day 13-15 germ cells, they may play roles in initiating or mediating progress along the sex-specific pathways of development that PGCs embark upon at this time.
4. Podoplanin is regulated by AP-1 and promotes platelet aggregation and cell **migration** in osteosarcoma. Podoplanin is a type-I transmembrane sialomucin-like protein, which is expressed in a wide range of cell types and is involved in platelet aggregation and tumor metastasis. Here, we investigated the function, regulation, and expression of podoplanin in osteosarcoma. Podoplanin expression was observed in three osteosarcoma cell lines (MG-63, HOS, and U-2 OS) with platelet aggregation-inducing ability, which was blocked by podoplanin small-interfering RNA or a neutralizing antibody. Overexpression of podoplanin in nonmetastatic Dunn osteosarcoma cells promoted cell **migration** without attenuating cell proliferation. Both podoplanin and TGF-β1 were up-regulated by c-Fos induction in MC3T3-E1 osteoblastic cells, and were highly expressed in c-Fos transgenic mouse osteosarcomas and c-Fos-transformed osteosarcoma cell lines. Immunohistochemistry of human osteosarcoma tissue microarrays (n = 133) showed staining of tumor cells embedded in an excess of irregular neoplastic bone matrix in 100% of tumors undergoing so-called "normalization/maturation." Podoplanin was also expressed in osteosarcoma subtypes, with 65% of osteoblastic, 100% of chondroblastic, and 79% of fibroblastic tumors. CD44 and pERM immunohistochemistry showed coexpression with podoplanin in both mouse and human osteosarcoma. Podoplanin expression was significantly higher in metastatic osteosarcomas (n = 6) than in primary osteosarcomas (n = 10). Our data suggest that podoplanin, which is not expressed in normal osteoblasts but in osteocytes, is aberrantly expressed in transformed osteoblasts and in osteosarcoma, and is under AP-1 transcriptional control. Thus podoplanin is a candidate molecule for therapeutic targeting.
5. Differential expression of fos and jun family members during murine postnatal intestinal development. We have examined the patterns of expression of members of the fos and jun families of transcription factors during murine postnatal intestinal development. Northern analysis showed increases in fosB, c-fos, and junB mRNA levels in both proximal jejunum and colon. These increases coincide with weaning a period of major changes in intestinal cell proliferation and **migration**. Administration of dexamethasone to suckling mice resulted in transient induction of c-fos mRNA levels in all gut segments. Thus, modifications in expression of certain members of the fos and jun families in vivo correlate with major maturational changes during murine intestinal development and support a role for these transcription factors in the regulation of intestinal functions.
6. Inactivation of PI3Kδ induces vascular injury and promotes aneurysm development by upregulating the AP-1/MMP-12 pathway in macrophages. An aneurysm is an inflammatory vascular condition. Phosphatidylinositol 3-kinases δ is highly expressed in leukocytes, and play a key role in innate immunity. However, the link between phosphatidylinositol 3-kinases δ and aneurysm development has not yet been elucidated.Carotid ligation unexpectedly induced characteristic aneurysm formation beneath the ligation point in p110δ(D910A/D910A) mice (n=25; P<0.001 versus wild-type). Besides, p110δ inactivation exacerbated CaCl2-induced abdominal aortic aneurysms development. A reverse transcription polymerase chain reaction microarray revealed significant extracellular matrix components degradation and matrix metalloproteinases (MMPs) upregulation in the abdominal aorta of p110δ(D910A/D910A) mice. Similarly, the expression of both collagen I and IV was significantly decreased (n=10; P<0.05 versus wild-type) in carotid artery. Western blot assay confirmed that MMP-12 was significantly upregulated in arteries of p110δ(D910A/D910A) mice (n=10; P<0.01 versus wild-type). In vitro, p110δ inactivation marked increase peritoneal macrophages recruitment and synergistically enhance tumor necrosis factor-α-induced recruitment. A specific phosphatidylinositol 3-kinases δ inhibitor (IC87114) or genetic p110δ inactivation upregulated MMP-12 expression and c-Jun phosphorylation (n=6; P<0.05 versus wild-type macrophages). IC87114 also increased activator protein-1 DNA-binding activity (n=6; P<0.001 versus control) and enhanced the effect of tumor necrosis factor-α on activator protein-1-binding activity (n=5; P<0.01 versus tumor necrosis factor-α treatment groups). Knockdown of c-Jun suppressed the effect of the IC87114 and tumor necrosis factor-α on MMP-12 mRNA expression (n=5 in each group; P<0.01 versus scrRNA treatment groups).Our findings demonstrate that p110δ inactivation leads to extracellular matrix degradation in vessels and promotes aneurysm development by induci**ng macrop**hages migration and upregulating the activator protein-1/MMP-12 pathway in macrophages.
7. Arsenic-induced alterations in embryonic transcription factor gene expression: implications for abnormal neural development. We examined the morphological and molecular consequences of acute in utero exposure to teratogenic concentrations of arsenate. The treatment produced a dose-related increase in neural tube defects, along with a significant alteration in the pattern of gene expression for several transcription factors (creb, Hox 3.1, Pax3, and Emx-1) that were examined using in situ transcription and antisense RNA amplification procedures. On gestational day 9:0, there was a significant delay in the embryos progression through neural tube closure, accompanied by a significant downregulation of Hox 3.1 expression and a significant upregulation of Pax3, Emx-1, and creb. As both Hox 3.1 and Pax3 serve to regulate N-CAM expression, it is possible that abnormalities associated with N-CAM may compromise neural crest cell **migration** and normal neural tube closure.
8. Inactivation of PI3Kδ induces vascular injury and promotes aneurysm development by upregulating the AP-1/MMP-12 pathway in macrophages. An aneurysm is an inflammatory vascular condition. Phosphatidylinositol 3-kinases δ is highly expressed in leukocytes, and play a key role in innate immunity. However, the link between phosphatidylinositol 3-kinases δ and aneurysm development has not yet been elucidated.Carotid ligation unexpectedly induced characteristic aneurysm formation beneath the ligation point in p110δ(D910A/D910A) mice (n=25; P<0.001 versus wild-type). Besides, p110δ inactivation exacerbated CaCl2-induced abdominal aortic aneurysms development. A reverse transcription polymerase chain reaction microarray revealed significant extracellular matrix components degradation and matrix metalloproteinases (MMPs) upregulation in the abdominal aorta of p110δ(D910A/D910A) mice. Similarly, the expression of both collagen I and IV was significantly decreased (n=10; P<0.05 versus wild-type) in carotid artery. Western blot assay confirmed that MMP-12 was significantly upregulated in arteries of p110δ(D910A/D910A) mice (n=10; P<0.01 versus wild-type). In vitro, p110δ inactivation marked increase peritoneal macrophages recruitment and synergistically enhance tumor necrosis factor-α-induced recruitment. A specific phosphatidylinositol 3-kinases δ inhibitor (IC87114) or genetic p110δ inactivation upregulated MMP-12 expression and c-Jun phosphorylation (n=6; P<0.05 versus wild-type macrophages). IC87114 also increased activator protein-1 DNA-binding activity (n=6; P<0.001 versus control) and enhanced the effect of tumor necrosis factor-α on activator protein-1-binding activity (n=5; P<0.01 versus tumor necrosis factor-α treatment groups). Knockdown of c-Jun suppressed the effect of the IC87114 and tumor necrosis factor-α on MMP-12 mRNA expression (n=5 in each group; P<0.01 versus scrRNA treatment groups).Our findings demonstrate that p110δ inactivation leads to extracellular matrix degradation in vessels and promotes aneurysm development by induci**ng macrop**hages migration and upregulating the activator protein-1/MMP-12 pathway in macrophages.
9. PRIP promotes tumor formation through enhancing serum-responsive factor-mediated FOS expression. PRIP (peroxisome proliferator-activator receptor interacting protein) is a nuclear receptor coactivator required for mammary gland development. To understand the function of PRIP in breast tumorigenesis, we established a mammary tumor cell line with the PRIP(Loxp/Loxp) genotype. By knocking out the PRIP gene in the tumor cell line, we demonstrated that PRIP deficiency led to inhibited tumor formation without affecting tumor cell proliferation. The PRIP deficiency was associated with decreased cell invasion and **migration** capabilities. We found that PRIP deficiency substantially reduced FOS gene expression. A chromatin immunoprecipitation assay revealed that PRIP was recruited to the FOS promoter. In addition, we demonstrated that PRIP also directly up-regulated the FOS gene expression in human breast cancer cells. Promoter analysis showed that PRIP acted through serum-responsive factor to regulate FOS gene expression. Finally, by re-expressing the FOS gene, we confirmed that the inhibited tumor formation of PRIP-deficient tumor cells was due to reduced expression of the FOS gene.
10. Subcortical visual shell nuclei targeted by ipRGCs develop from a Sox14+-GABAergic progenitor and require Sox14 to regulate daily activity rhythms. Intrinsically photosensitive retinal ganglion cells (ipRGCs) and their nuclear targets in the subcortical visual shell (SVS) are components of the non-image-forming visual system, which regulates important physiological processes, including photoentrainment of the circadian rhythm. While ipRGCs have been the subject of much recent research, less is known about their central targets and how they develop to support specific behavioral functions. We describe Sox14 as a marker to follow the ontogeny of the SVS and find that the complex forms from two narrow stripes of Dlx2-negative GABAergic progenitors in the early diencephalon through sequential waves of tangential **migration**. We characterize the requirement for Sox14 to orchestrate the correct distribution of neurons among the different nuclei of the network and describe how Sox14 expression is required both to ensure robustness in circadian entrainment and for masking of motor activity.
11. Gonadotropin and kisspeptin gene expression, but not GnRH, are impaired in cFOS deficient mice. cFOS is a pleiotropic transcription factor, which binds to the AP1 site in the promoter of target genes. In the pituitary gonadotropes, cFOS mediates induction of FSHβ and GnRH receptor genes. Herein, we analyzed reproductive function in the cFOS-deficient mice to determine its role in vivo. In the pituitary cFOS is necessary for gonadotropin subunit expression, while TSHβ is unaffected. Additionally, cFOS null animals have the same sex-steroid levels, although gametogenesis is impeded. In the brain, cFOS is not necessary for GnRH neurona**l migrati**on, axon targeting, cell number, or mRNA levels. Conversely, cFOS nulls, particularly females, have decreased Kiss1 neuron numbers and lower Kiss1 mRNA levels. Collectively, our novel findings suggest that cFOS plays a cell-specific role at multiple levels of the hypothalamic-pituitary-gonadal axis, affecting gonadotropes but not thyrotropes in the pituitary, and kisspeptin neurons but not GnRH neurons in the hypothalamus, thereby contributing to the overall control of reproduction.

#### GeneRIFs

1. Pik3cd inactivation leads to extracellular matrix degradation in vessels and promotes aneurysm development by inducing macrophage **migration** and upregulating the activator protein-1/MMP-12 pathway in macrophages.

### [Ee]xtracellular [Mm]atrix

#### PubMed

1. Transcriptional regulation of dentin matrix protein 1 (DMP1) by AP-1 (c-fos/c-jun) factors. Dentin matrix protein 1 (DMP1) is an **extracellular matrix** phosphoprotein whose expression is precisely controlled temporally and spatially during the differentiation of the odontoblasts. Odontoblasts are postmitotic cells that differentiate to mature polarized cells and are responsible for the synthesis of a calcified dentin matrix. Although the DMP1 promoter has been isolated from the mouse and the rat, little is actually known about their control by specific transcription factors. Analysis of the rat DMP1 promoter has identified several regulatory transcription factors. These factors include AP-1 family members, SP1 and ETS. Therefore the transcription of DMP1 may be controlled positively or negatively by a variety of stimuli resulting in a developmentally regulated gene expression. This study demonstrates the role of c-fos and c-jun on the transcriptional regulation of DMP1 gene.
2. Inactivation of PI3Kδ induces vascular injury and promotes aneurysm development by upregulating the AP-1/MMP-12 pathway in macrophages. An aneurysm is an inflammatory vascular condition. Phosphatidylinositol 3-kinases δ is highly expressed in leukocytes, and play a key role in innate immunity. However, the link between phosphatidylinositol 3-kinases δ and aneurysm development has not yet been elucidated.Carotid ligation unexpectedly induced characteristic aneurysm formation beneath the ligation point in p110δ(D910A/D910A) mice (n=25; P<0.001 versus wild-type). Besides, p110δ inactivation exacerbated CaCl2-induced abdominal aortic aneurysms development. A reverse transcription polymerase chain reaction microarray revealed signifi**cant extracellular m**atrix components degradation and matrix metalloproteinases (MMPs) upregulation in the abdominal aorta of p110δ(D910A/D910A) mice. Similarly, the expression of both collagen I and IV was significantly decreased (n=10; P<0.05 versus wild-type) in carotid artery. Western blot assay confirmed that MMP-12 was significantly upregulated in arteries of p110δ(D910A/D910A) mice (n=10; P<0.01 versus wild-type). In vitro, p110δ inactivation marked increase peritoneal macrophages recruitment and synergistically enhance tumor necrosis factor-α-induced recruitment. A specific phosphatidylinositol 3-kinases δ inhibitor (IC87114) or genetic p110δ inactivation upregulated MMP-12 expression and c-Jun phosphorylation (n=6; P<0.05 versus wild-type macrophages). IC87114 also increased activator protein-1 DNA-binding activity (n=6; P<0.001 versus control) and enhanced the effect of tumor necrosis factor-α on activator protein-1-binding activity (n=5; P<0.01 versus tumor necrosis factor-α treatment groups). Knockdown of c-Jun suppressed the effect of the IC87114 and tumor necrosis factor-α on MMP-12 mRNA expression (n=5 in each group; P<0.01 versus scrRNA treatment groups).Our findings demonstrate that p110δ inactiv**ation leads to extra**cellular matrix degradation in vessels and promotes aneurysm development by inducing macrophages migration and upregulating the activator protein-1/MMP-12 pathway in macrophages.
3. Phosphorylation of c-Fos by members of the p38 MAPK family. Role in the AP-1 response to UV light. Exposure to sources of UV radiation, such as sunlight, induces a number of cellular alterations that are highly dependent on its ability to affect gene expression. Among them, the rapid activation of genes coding for two subfamilies of proto-oncoproteins, Fos and Jun, which constitute the AP-1 transcription factor, plays a key role in the subsequent regulation of expression of genes involved in DNA repair, cell proliferation, cell cycle arrest, death by apoptosis, and tissue and **extracellular matrix** remodeling proteases. Besides being regulated at the transcriptional level, Jun and Fos transcriptional activities are also regulated by phosphorylation as a result of the activation of intracellular signaling cascades. In this regard, the phosphorylation of c-Jun by UV-induced JNK has been readily documented, whereas a role for Fos proteins in UV-mediated responses and the identification of Fos-activating kinases has remained elusive. Here we identify p38 MAPKs as proteins that can associate with c-Fos and phosphorylate its transactivation domain both in vitro and in vivo. This phosphorylation is transduced into changes in its transcriptional ability as p38-activated c-Fos enhances AP1-driven gene expression. Our findings indicate that as a consequence of the activation of stress pathways induced by UV light, endogenous c-Fos becomes a substrate of p38 MAPKs and, for the first time, provide evidence that support a critical role for p38 MAPKs in mediating stress-induced c-Fos phosphorylation and gene transcription activation. Using a specific pharmacological inhibitor for p38alpha and -beta, we found that most likely these two isoforms mediate UV-induced c-Fos phosphorylation in vivo. Thus, these newly described pathways act concomitantly with the activation of c-Jun by JNK/MAPKs, thereby contributing to the complexity of AP1-driven gene transcription regulation.
4. The AP-1 transcription factor c-Jun prevents stress-imposed maladaptive remodeling of the heart. Systemic hypertension increases cardiac workload and subsequently induces signaling networks in heart that underlie myocyte growth (hypertrophic response) through expansion of sarcomeres with the aim to increase contractility. However, conditions of increased workload can induce both adaptive and maladaptive growth of heart muscle. Previous studies implicate two members of the AP-1 transcription factor family, junD and fra-1, in regulation of heart growth during hypertrophic response. In this study, we investigate the function of the AP-1 transcription factors, c-jun and c-fos, in heart growth. Using pressure overload-induced cardiac hypertrophy in mice and targeted deletion of Jun or Fos in cardiomyocytes, we show that c-jun is required for adaptive cardiac hypertrophy, while c-fos is dispensable in this context. c-jun promotes expression of sarcomere proteins and suppresses expression of **extracellular matrix** proteins. Capacity of cardiac muscle to contract depends on organization of principal thick and thin filaments, myosin and actin, within the sarcomere. In line with decreased expression of sarcomere-associated proteins, Jun-deficient cardiomyocytes present disarrangement of filaments in sarcomeres and actin cytoskeleton disorganization. Moreover, Jun-deficient hearts subjected to pressure overload display pronounced fibrosis and increased myocyte apoptosis finally resulting in dilated cardiomyopathy. In conclusion, c-jun but not c-fos is required to induce a transcriptional program aimed at adapting heart growth upon increased workload.
5. Inactivation of PI3Kδ induces vascular injury and promotes aneurysm development by upregulating the AP-1/MMP-12 pathway in macrophages. An aneurysm is an inflammatory vascular condition. Phosphatidylinositol 3-kinases δ is highly expressed in leukocytes, and play a key role in innate immunity. However, the link between phosphatidylinositol 3-kinases δ and aneurysm development has not yet been elucidated.Carotid ligation unexpectedly induced characteristic aneurysm formation beneath the ligation point in p110δ(D910A/D910A) mice (n=25; P<0.001 versus wild-type). Besides, p110δ inactivation exacerbated CaCl2-induced abdominal aortic aneurysms development. A reverse transcription polymerase chain reaction microarray revealed signifi**cant extracellular m**atrix components degradation and matrix metalloproteinases (MMPs) upregulation in the abdominal aorta of p110δ(D910A/D910A) mice. Similarly, the expression of both collagen I and IV was significantly decreased (n=10; P<0.05 versus wild-type) in carotid artery. Western blot assay confirmed that MMP-12 was significantly upregulated in arteries of p110δ(D910A/D910A) mice (n=10; P<0.01 versus wild-type). In vitro, p110δ inactivation marked increase peritoneal macrophages recruitment and synergistically enhance tumor necrosis factor-α-induced recruitment. A specific phosphatidylinositol 3-kinases δ inhibitor (IC87114) or genetic p110δ inactivation upregulated MMP-12 expression and c-Jun phosphorylation (n=6; P<0.05 versus wild-type macrophages). IC87114 also increased activator protein-1 DNA-binding activity (n=6; P<0.001 versus control) and enhanced the effect of tumor necrosis factor-α on activator protein-1-binding activity (n=5; P<0.01 versus tumor necrosis factor-α treatment groups). Knockdown of c-Jun suppressed the effect of the IC87114 and tumor necrosis factor-α on MMP-12 mRNA expression (n=5 in each group; P<0.01 versus scrRNA treatment groups).Our findings demonstrate that p110δ inactiv**ation leads to extra**cellular matrix degradation in vessels and promotes aneurysm development by inducing macrophages migration and upregulating the activator protein-1/MMP-12 pathway in macrophages.
6. Phosphorylation of c-Fos by members of the p38 MAPK family. Role in the AP-1 response to UV light. Exposure to sources of UV radiation, such as sunlight, induces a number of cellular alterations that are highly dependent on its ability to affect gene expression. Among them, the rapid activation of genes coding for two subfamilies of proto-oncoproteins, Fos and Jun, which constitute the AP-1 transcription factor, plays a key role in the subsequent regulation of expression of genes involved in DNA repair, cell proliferation, cell cycle arrest, death by apoptosis, and tissue and **extracellular matrix** remodeling proteases. Besides being regulated at the transcriptional level, Jun and Fos transcriptional activities are also regulated by phosphorylation as a result of the activation of intracellular signaling cascades. In this regard, the phosphorylation of c-Jun by UV-induced JNK has been readily documented, whereas a role for Fos proteins in UV-mediated responses and the identification of Fos-activating kinases has remained elusive. Here we identify p38 MAPKs as proteins that can associate with c-Fos and phosphorylate its transactivation domain both in vitro and in vivo. This phosphorylation is transduced into changes in its transcriptional ability as p38-activated c-Fos enhances AP1-driven gene expression. Our findings indicate that as a consequence of the activation of stress pathways induced by UV light, endogenous c-Fos becomes a substrate of p38 MAPKs and, for the first time, provide evidence that support a critical role for p38 MAPKs in mediating stress-induced c-Fos phosphorylation and gene transcription activation. Using a specific pharmacological inhibitor for p38alpha and -beta, we found that most likely these two isoforms mediate UV-induced c-Fos phosphorylation in vivo. Thus, these newly described pathways act concomitantly with the activation of c-Jun by JNK/MAPKs, thereby contributing to the complexity of AP1-driven gene transcription regulation.
7. Transcriptional regulation of dentin matrix protein 1 (DMP1) by AP-1 (c-fos/c-jun) factors. Dentin matrix protein 1 (DMP1) is an **extracellular matrix** phosphoprotein whose expression is precisely controlled temporally and spatially during the differentiation of the odontoblasts. Odontoblasts are postmitotic cells that differentiate to mature polarized cells and are responsible for the synthesis of a calcified dentin matrix. Although the DMP1 promoter has been isolated from the mouse and the rat, little is actually known about their control by specific transcription factors. Analysis of the rat DMP1 promoter has identified several regulatory transcription factors. These factors include AP-1 family members, SP1 and ETS. Therefore the transcription of DMP1 may be controlled positively or negatively by a variety of stimuli resulting in a developmentally regulated gene expression. This study demonstrates the role of c-fos and c-jun on the transcriptional regulation of DMP1 gene.
8. Anabolic actions of parathyroid hormone during bone growth are dependent on c-fos. PTH has anabolic and catabolic actions in bone that are not clearly understood. The protooncogene c-fos and other activating protein 1 family members are critical transcriptional mediators in bone, and c-fos is up-regulated by PTH. The purpose of this study was to examine the mechanisms of PTH and the role of c-fos in PTH-mediated anabolic actions in bone. Mice with ablation of c-fos (-/-) and their wild-type (+/+) and heterozygous (+/-) littermates were administered PTH for 17 d. The +/+ mice had increased femoral bone mineral density (BMD), whereas -/- mice had reduced BMD after PTH treatment. PTH increased the ash weight of +/+ and +/-, but not -/-, femurs and decreased the calcium content of -/-, but not +/+ or +/-, femurs. Histomorphometric analysis showed that PTH increased trabecular bone volume in c-fos +/+, +/- vertebrae, but, in contrast, decreased trabecular bone in -/- vertebrae. Serum calcium levels in +/+ mice were greater than those in -/- mice, and PTH increased calcium in -/- mice. Histologically, PTH resulted in an exacerbation of the already widened growth plate and zone of hypertrophic chondrocytes but not the proliferating zone in -/- mice. PTH also increased calvarial thickness in +/+ mice, but not -/- mice. The c-fos -/- mice had lower bone sialoprotein and osteocalcin (OCN), but unaltered PTH-1 receptor mRNA expression in calvaria, suggesting an alteration in **extracellular matrix**. Acute PTH injection (8 h) resulted in a decrease in osteocalcin mRNA expression in wild-type, but unaltered expression in -/-, calvaria. These data indicate that c-fos plays a critical role in the anabolic actions of PTH during endochondral bone growth.
9. SOX9 modulates the expression of key transcription factors required for heart valve development. Heart valve formation initiates when endothelial cells of the heart transform into mesenchyme and populate the cardiac cushions. The transcription factor SOX9 is highly expressed in the cardiac cushion mesenchyme, and is essential for heart valve development. Loss of Sox9 in mouse cardiac cushion mesenchyme alters cell proliferation, embryonic survival, and valve formation. Despite this important role, little is known about how SOX9 regulates heart valve formation or its transcriptional targets. Therefore, we mapped putative SOX9 binding sites by ChIP-Seq in E12.5 heart valves, a stage at which the valve mesenchyme is actively proliferating and initiating differentiation. Embryonic heart valves have been shown to express a high number of genes that are associated with chondrogenesis, including several **extracellular matrix** proteins and transcription factors that regulate chondrogenesis. Therefore, we compared regions of putative SOX9 DNA binding between E12.5 heart valves and E12.5 limb buds. We identified context-dependent and context-independent SOX9-interacting regions throughout the genome. Analysis of context-independent SOX9 binding suggests an extensive role for SOX9 across tissues in regulating proliferation-associated genes including key components of the AP-1 complex. Integrative analysis of tissue-specific SOX9-interacting regions and gene expression profiles on Sox9-deficient heart valves demonstrated that SOX9 controls the expression of several transcription factors with previously identified roles in heart valve development, including Twist1, Sox4, Mecom and Pitx2. Together, our data identify SOX9-coordinated transcriptional hierarchies that control cell proliferation and differentiation during valve formation.
10. Disruption of laminin in the peripheral nervous system impedes nonmyelinating Schwann cell development and impairs nociceptive sensory function. The mechanisms controlling the differentiation of immature Schwann cells (SCs) into nonmyelinating SCs is not known. Laminins are **extracellular matrix** proteins critical for myelinating SC differentiation, but their roles in nonmyelinating SC development have not been established. Here, we show that the peripheral nerves of mutant mice with laminin-deficient SCs do not form Remak bundles, which consist of a single nonmyelinating SC interacting with multiple unmyelinated axons. These mutant nerves show aberrant L1 and neural cell adhesion molecule (N-CAM) expression pattern during development. The homophilic and heterophilic interactions of N-CAM are also impaired in the mutant nerves. Other molecular markers for nonmyelinating SCs, including Egr-1, glial fibrillary acidic protein, and AN2/NG2, are all absent in adult mutant nerves. Analysis of expression of SC lineage markers demonstrates that nonmyelinating SCs do not develop in mutant nerves. Additionally, mutant mice are insensitive to heat stimuli and show a decreased number of C-fiber sensory neurons, indicating reduced nociceptive sensory function. These results show that laminin participates in nonmyelinating SC development and Remak bundle formation and suggest a possible role for laminin deficiency in peripheral sensory neuropathies.
11. Complementary interplay between matrix metalloproteinase-9, vascular endothelial growth factor and osteoclast function drives endochondral bone formation. Long bone development depends on endochondral bone formation, a complex process requiring exquisite balance between hypertrophic cartilage (HC) formation and its ossification. Dysregulation of this process may result in skeletal dysplasias and heterotopic ossification. Endochondral ossification requires the precise orchestration of HC vascularization, **extracellular matrix** remodeling, and the recruitment of osteoclasts and osteoblasts. Matrix metalloproteinase-9 (MMP-9), vascular endothelial growth factor (VEGF) and osteoclasts have all been shown to regulate endochondral ossification, but how their function interrelates is not known. We have investigated the functional relationship among these regulators of endochondral ossification, demonstrating that they have complementary but non-overlapping functions. MMP-9, VEGF and osteoclast deficiency all cause impaired growth plate ossification resulting in the accumulation of HC. VEGF mRNA and protein expression are increased at the MMP-9-/- growth plate, and VEGF activity contributes to endochondral ossification since sequestration of VEGF by soluble receptors results in further inhibition of growth plate vascularization and ossification. However, VEGF bioavailability is still limited in MMP-9 deficiency, as exogenous VEGF is able to rescue the MMP-9-/- phenotype, demonstrating that MMP-9 may partially, but not fully, regulate VEGF bioavailability. The organization of the HC **extracellular matrix** at the MMP-9-/- growth plate is altered, supporting a role for MMP-9 in HC remodeling. Inhibition of VEGF impairs osteoclast recruitment, whereas MMP-9 deficiency leads to an accumulation of osteoclasts at the chondro-osseous junction. Growth plate ossification in osteoclast-deficient mice is impaired in the presence of normal MMP-9 expression, indicating that other osteoclastic functions are also necessary. Our data delineate the complementary interplay between MMP-9, VEGF and osteoclast function that is necessary for normal endochondral bone formation and provide a molecular framework for investigating the molecular defects contributing to disorders of endochondral bone formation.
12. Characterization of E-cadherin-dependent and -independent events in a new model of c-Fos-mediated epithelial-mesenchymal transition. Fos proteins have been implicated in control of tumorigenesis-related genetic programs including invasion, angiogenesis, cell proliferation and apoptosis. In this study, we demonstrate that c-Fos is able to induce mesenchymal transition in murine tumorigenic epithelial cell lines. Expression of c-Fos in MT1TC1 cells led to prominent alterations in cell morphology, increased expression of mesenchymal markers, vimentin and S100A4, DNA methylation-dependent down-regulation of E-cadherin and abrogation of cell-cell adhesion. In addition, c-Fos induced a strong beta-catenin-independent proliferative response in MT1TC1 cells and stimulated cell motility, invasion and adhesion to different **extracellular matrix** proteins. To explore whether loss of E-cadherin plays a role in c-Fos-mediated mesenchymal transition, we expressed wild-type E-cadherin and two different E-cadherin mutants in MT1TC1/c-fos cells. Expression of wild-type E-cadherin restored epithelioid morphology and enhanced cellular levels of catenins. However, exogenous E-cadherin did not influence expression of c-Fos-dependent genes, only partly suppressed growth of MT1TC1/c-fos cells and produced no effect on c-Fos-stimulated cell motility and invasion in matrigel. On the other hand, re-expression of E-cadherin specifically negated c-Fos-induced adhesion to collagen type I, but not to laminin or fibronectin. Of interest, mutant E-cadherin which lacks the ability to form functional adhesive complexes had an opposite, potentiating effect on cell adhesion to collagen I. These data suggest that cell adhesion to collagen I is regulated by the functional state of E-cadherin. Overall, our data demonstrate that, with the exception of adhesion to collagen I, c-Fos is dominant over E-cadherin in relation to the aspects of mesenchymal transition assayed in this study.

#### GeneRIFs

1. Pik3cd inactivation leads to **extracellular matrix** degradation in vessels and promotes aneurysm development by inducing macrophage migration and upregulating the activator protein-1/MMP-12 pathway in macrophages.

### [Ii]nvasive

#### PubMed

1. Rhabdomyosarcoma development in mice lacking Trp53 and Fos: tumor suppression by the Fos protooncogene. The Fos protein, a major component of the AP-1 transcription factor, is essential for osteoclast differentiation, acts as an oncogene, potentiates transforming signals, and controls **invasive** growth and angiogenesis during tumor progression. To investigate a potential genetic interaction between the Trp53 and Fos pathways, Trp53/Fos double knockout mice were generated. These mice develop highly proliferative and **invasive** rhabdomyosarcomas of the facial and orbital regions, with more than 90% penetrance at 6 months of age. Rhabdomyosarcoma cell lines established from the primary tumors express characteristic muscle-specific markers, and reexpression of Fos is associated with enhanced apoptosis in vitro. Moreover, Fos is able to repress Pax7 expression in rhabdomyosarcoma cell lines and primary myoblasts, suggesting a molecular link to genetic alterations involved in human rhabdomyosarcomas.
2. Ecotopic viral integration site 1 (EVI1) regulates multiple cellular processes important for cancer and is a synergistic partner for FOS protein in **invasive** tumors. Ecotropic viral integration site 1 (EVI1) is an oncogenic dual domain zinc finger transcription factor that plays an essential role in the regulation of hematopoietic stem cell renewal, and its overexpression in myeloid leukemia and epithelial cancers is associated with poor patient survival. Despite the discovery of EVI1 in 1988 and its emerging role as a dominant oncogene in various types of cancer, few EVI1 target genes are known. This lack of knowledge has precluded a clear understanding of exactly how EVI1 contributes to cancer. Using a combination of ChIP-Seq and microarray studies in human ovarian carcinoma cells, we show that the two zinc finger domains of EVI1 bind to DNA independently and regulate different sets of target genes. Strikingly, an enriched fraction of EVI1 target genes are cancer genes or genes associated with cancer. We also show that more than 25% of EVI1-occupied genes contain linked EVI1 and activator protein (AP)1 DNA binding sites, and this finding provides evidence for a synergistic cooperative interaction between EVI1 and the AP1 family member FOS in the regulation of cell adhesion, proliferation, and colony formation. An increased number of dual EVI1/AP1 target genes are also differentially regulated in late-stage ovarian carcinomas, further confirming the importance of the functional cooperation between EVI1 and FOS. Collectively, our data indicate that EVI1 is a multipurpose transcription factor that synergizes with FOS in **invasive** tumors.
3. [Rhabdomyosarcoma development in Trp53/fos mutant mice: tumor suppressor functions of the Fos protooncogene]. Rhabdomyosarcoma is the most common soft tissue sarcoma in children, yet the genetic changes causing this disease are poorly understood. The Fos protein, a major component of the AP-1 transcription factor, is essential for osteoclast differentiation, acts as an oncogene, potentiates transforming signals and controls **invasive** growth and angiogenesis during tumor progression. To genetically investigate a potential interaction between the p53 and Fos pathways, Trp53/Fos double knock-out mice were generated. These mice develop highly proliferative and **invasive** rhabdomyosarcomas of the facial and orbital regions with more than 90% penetrance at 6 months of age. Expression of Fos in Trp53/Fos mutant rhabdomyosarcoma cell lines established from primary tumors is associated with enhanced apoptosis and downregulation of Pax7 expression. Our results show that Trp53/Fos double knock-out mice recapitulate major aspects of human rhabdomyosarcoma development and therefore provide a mouse model for the human disease. Furthermore, this study identifies a novel and unexpected tumor suppressive function of the Fos proto-oncogene.
4. The proto-oncoprotein c-Fos negatively regulates hepatocellular tumorigenesis. Hepatocytes adopt an **invasive** and metastatic phenotype caused by the cooperation of transforming growth factor (TGF)-beta and oncogenic Ha-Ras. In the initial phase of this process, c-Fos is rapidly induced by TGF-beta, but then decreases to undetectable levels. Here, we investigated the functional implications of c-Fos activation and its contribution to hepatocellular tumorigenesis. By employing conditional c-Fos expression, we observed that continuous activation of c-Fos and consequently AP-1 activity leads to depolarization of differentiated murine epithelial hepatocytes. Most remarkably, this change in morphology was associated with inhibition of proliferation and induction of cell death. Coexpression of antiapoptotic Bcl-XL or scavenging of reactive oxygen species was sufficient to prevent the c-Fos-mediated phenotype. In contrast, the cooperation of c-Fos with oncogenic Ha-Ras or a Ras mutant selectively activating the MAPK pathway even enhanced c-Fos-induced effects. Showing the negative role in hepatocellular tumorigenesis, c-Fos repressed oncogenic Ras-driven anchorage-independent growth in vitro and strongly suppressed tumour formation in vivo. Taken together, we demonstrate that c-Fos modulates plasticity of epithelial hepatocytes and acts tumour suppressive in neoplastic hepatocytes by stimulating cell cycle inhibition and cell death.
5. Inflammation-mediated skin tumorigenesis induced by epidermal c-Fos. Skin squamous cell carcinomas (SCCs) are the second most prevalent skin cancers. Chronic skin inflammation has been associated with the development of SCCs, but the contribution of skin inflammation to SCC development remains largely unknown. In this study, we demonstrate that inducible expression of c-fos in the epidermis of adult mice is sufficient to promote inflammation-mediated epidermal hyperplasia, leading to the development of preneoplastic lesions. Interestingly, c-Fos transcriptionally controls mmp10 and s100a7a15 expression in keratinocytes, subsequently leading to CD4 T-cell recruitment to the skin, thereby promoting epidermal hyperplasia that is likely induced by CD4 T-cell-derived IL-22. Combining inducible c-fos expression in the epidermis with a single dose of the carcinogen 7,12-dimethylbenz(a)anthracene (DMBA) leads to the development of highly **invasive** SCCs, which are prevented by using the anti-inflammatory drug sulindac. Moreover, human SCCs display a correlation between c-FOS expression and elevated levels of MMP10 and S100A15 proteins as well as CD4 T-cell infiltration. Our studies demonstrate a bidirectional cross-talk between premalignant keratinocytes and infiltrating CD4 T cells in SCC development. Therefore, targeting inflammation along with the newly identified targets, such as MMP10 and S100A15, represents promising therapeutic strategies to treat SCCs.
6. Targeted disruption of the c-fos gene demonstrates c-fos-dependent and -independent pathways for gene expression stimulated by growth factors or oncogenes. The c-fos proto-oncogene is believed to play a pivotal role in transducing growth factor-mediated signals from the extracellular milieu into the nucleus. c-fos protein dimerizes with c-jun and related proteins and mediates transcription via AP-1 sites. Using c-fos-deficient mice generated through gene knockout techniques, we derived 3T3-type cell lines from primary embryonic fibroblasts. The c-fos-deficient cells grow normally under optimal culture conditions and show only a slight reduction in growth rate in low serum culture compared with control cells. They also express mRNA for most of the Fos and Jun family members at normal levels. The overall levels of AP-1 DNA binding activity are normal and several genes (c-jun, MCP1, metallothionein) known to contain functional AP-1 sites are expressed normally in the c-fos-deficient and control cells. In contrast, mRNA for the metalloproteases stromelysin (MMP-3) and type I collagenase (MMP-1), which are often induced by oncogenes and growth factors and have been implicated in tumor **invasive**ness, cannot be induced by epidermal growth factor or platelet-derived growth factor in c-fos-deficient cells. Transformation of mutant cells with polyoma middle T oncogene essentially restores wild-type levels of stromelysin expression, while transformation with v-src leads to only a weak induction of the metalloprotease. These results clearly demonstrate that some AP-1-dependent genes require c-fos for full expression while others do not; oncogenes may activate expression of metalloproteases via either fos-dependent or fos-independent mechanisms. These results also imply that c-fos may play an important regulatory role in the **invasive** behavior of malignant tumors, independent of any role this proto-oncogene might play in cell growth per se.

### [Mm]etastasis

#### PubMed

1. Piperine alleviates osteoclast formation through the p38/c-Fos/NFATc1 signaling axis. Increased bone fracture is one of the health risk factors in patients with bone loss related disorders such as osteoporosis and breast cancer **metastasis** to bone. Over activity of osteoclasts leads to uncoupling of bone remodeling favoring bone loss over bone formation. Receptor activator of nuclear factor-κβ ligand (RANKL) triggers the differentiation pathway leading to multinucleated osteoclast formation. Modulation of RANKL or its downstream signaling pathways involved in osteoclast formation is of significant interest in the development of anti-resorptive agents. In this study, the effects of piperine, an alkaloid present in Piper nigrum L. on osteoclast formation was investigated. Piperine inhibited tartrate-resistant acid phosphatase-positive multinucleated osteoclast formation in murine RAW264.7 macrophages and human CD14+ monocytes induced by RANKL and breast cancer cells. Piperine attenuated the p38-mitogen activated protein kinase pathway activation, while the extracellular-signal-regulated kinase, c-Jun N-terminal kinase, or NF-κβ pathways downstream of RANKL remained unaffected. Concomitantly, expression of c-Fos and nuclear factor of activated T-cells, cytoplasmic 1 (NFATc1), the key transcription factors involved in osteoclastogenesis were remarkably inhibited by piperine. Furthermore, piperine disrupted the actin ring structure and bone resorption, a characteristic hallmark of osteoclasts. Collectively, these results suggested that piperine inhibited osteoclast differentiation by suppressing the p38/NFATc1/c-Fos signaling axis..
2. Podoplanin is regulated by AP-1 and promotes platelet aggregation and cell migration in osteosarcoma. Podoplanin is a type-I transmembrane sialomucin-like protein, which is expressed in a wide range of cell types and is involved in platelet aggregation and tumor **metastasis**. Here, we investigated the function, regulation, and expression of podoplanin in osteosarcoma. Podoplanin expression was observed in three osteosarcoma cell lines (MG-63, HOS, and U-2 OS) with platelet aggregation-inducing ability, which was blocked by podoplanin small-interfering RNA or a neutralizing antibody. Overexpression of podoplanin in nonmetastatic Dunn osteosarcoma cells promoted cell migration without attenuating cell proliferation. Both podoplanin and TGF-β1 were up-regulated by c-Fos induction in MC3T3-E1 osteoblastic cells, and were highly expressed in c-Fos transgenic mouse osteosarcomas and c-Fos-transformed osteosarcoma cell lines. Immunohistochemistry of human osteosarcoma tissue microarrays (n = 133) showed staining of tumor cells embedded in an excess of irregular neoplastic bone matrix in 100% of tumors undergoing so-called "normalization/maturation." Podoplanin was also expressed in osteosarcoma subtypes, with 65% of osteoblastic, 100% of chondroblastic, and 79% of fibroblastic tumors. CD44 and pERM immunohistochemistry showed coexpression with podoplanin in both mouse and human osteosarcoma. Podoplanin expression was significantly higher in metastatic osteosarcomas (n = 6) than in primary osteosarcomas (n = 10). Our data suggest that podoplanin, which is not expressed in normal osteoblasts but in osteocytes, is aberrantly expressed in transformed osteoblasts and in osteosarcoma, and is under AP-1 transcriptional control. Thus podoplanin is a candidate molecule for therapeutic targeting.
3. Piperine alleviates osteoclast formation through the p38/c-Fos/NFATc1 signaling axis. Increased bone fracture is one of the health risk factors in patients with bone loss related disorders such as osteoporosis and breast cancer **metastasis** to bone. Over activity of osteoclasts leads to uncoupling of bone remodeling favoring bone loss over bone formation. Receptor activator of nuclear factor-κβ ligand (RANKL) triggers the differentiation pathway leading to multinucleated osteoclast formation. Modulation of RANKL or its downstream signaling pathways involved in osteoclast formation is of significant interest in the development of anti-resorptive agents. In this study, the effects of piperine, an alkaloid present in Piper nigrum L. on osteoclast formation was investigated. Piperine inhibited tartrate-resistant acid phosphatase-positive multinucleated osteoclast formation in murine RAW264.7 macrophages and human CD14+ monocytes induced by RANKL and breast cancer cells. Piperine attenuated the p38-mitogen activated protein kinase pathway activation, while the extracellular-signal-regulated kinase, c-Jun N-terminal kinase, or NF-κβ pathways downstream of RANKL remained unaffected. Concomitantly, expression of c-Fos and nuclear factor of activated T-cells, cytoplasmic 1 (NFATc1), the key transcription factors involved in osteoclastogenesis were remarkably inhibited by piperine. Furthermore, piperine disrupted the actin ring structure and bone resorption, a characteristic hallmark of osteoclasts. Collectively, these results suggested that piperine inhibited osteoclast differentiation by suppressing the p38/NFATc1/c-Fos signaling axis..

### [Cc]ell [Ii]nvasion

#### PubMed

1. PRIP promotes tumor formation through enhancing serum-responsive factor-mediated FOS expression. PRIP (peroxisome proliferator-activator receptor interacting protein) is a nuclear receptor coactivator required for mammary gland development. To understand the function of PRIP in breast tumorigenesis, we established a mammary tumor cell line with the PRIP(Loxp/Loxp) genotype. By knocking out the PRIP gene in the tumor cell line, we demonstrated that PRIP deficiency led to inhibited tumor formation without affecting tumor cell proliferation. The PRIP deficiency was associated with decreased **cell invasion** and migration capabilities. We found that PRIP deficiency substantially reduced FOS gene expression. A chromatin immunoprecipitation assay revealed that PRIP was recruited to the FOS promoter. In addition, we demonstrated that PRIP also directly up-regulated the FOS gene expression in human breast cancer cells. Promoter analysis showed that PRIP acted through serum-responsive factor to regulate FOS gene expression. Finally, by re-expressing the FOS gene, we confirmed that the inhibited tumor formation of PRIP-deficient tumor cells was due to reduced expression of the FOS gene.

---

## Fosl1

- NCBI Gene
- GeneRIF
- Pubmed

| Search Term | Rank | Fields |
| --- | --- | --- |
| [Mm]igration | 6 | PubMed(4) |
| [Ee]xtracellular [Mm]atrix | 8 | PubMed(4) |
| [Ii]nvasive | 10 | PubMed(1) |
| [Mm]etastasis | 12 | PubMed(4) |
| [Cc]ell [Ii]nvasion | 13 | PubMed(2) |

  
hide/show details for Fosl1

### [Mm]igration

#### PubMed

1. AP-1 (Fra-1/c-Jun)-mediated induction of expression of matrix metalloproteinase-2 is required for 15S-hydroxyeicosatetraenoic acid-induced angiogenesis. To understand the involvement of matrix metalloproteinases (MMPs) in 15(S)-hydroxyeicosatetraenoic acid (15(S)-HETE)-induced angiogenesis, we have studied the role of MMP-2. 15(S)-HETE induced MMP-2 expression and activity in a time-dependent manner in human dermal microvascular endothelial cells (HDMVECs). Inhibition of MMP-2 activity or depletion of its levels attenuated 15(S)-HETE-induced HDMVEC **migration**, tube formation, and Matrigel plug angiogenesis. 15(S)-HETE also induced Fra-1 and c-Jun expression in a Rac1-MEK1-JNK1-dependent manner. In addition, 15(S)-HETE-induced MMP-2 expression and activity were mediated by Rac1-MEK1-JNK1-dependent activation of AP-1 (Fra-1/c-Jun). Cloning and site-directed mutagenesis of MMP-2 promoter revealed that AP-1 site proximal to the transcriptional start site is required for 15(S)-HETE-induced MMP-2 expression, and Fra-1 and c-Jun are the essential components of AP-1 that bind to MMP-2 promoter in response to 15(S)-HETE. Hind limb ischemia led to an increase in MEK1 and JNK1 activation and Fra-1, c-Jun, and MMP-2 expression resulting in enhanced neovascularization and recovery of blood perfusion in wild-type mice as compared with 12/15-Lox(-/-) mice. Together, these results provide the first direct evidence for a role of 12/15-Lox-12/15(S)-HETE axis in the regulation of ischemia-induced angiogenesis.
2. AP-1 (Fra-1/c-Jun)-mediated induction of expression of matrix metalloproteinase-2 is required for 15S-hydroxyeicosatetraenoic acid-induced angiogenesis. To understand the involvement of matrix metalloproteinases (MMPs) in 15(S)-hydroxyeicosatetraenoic acid (15(S)-HETE)-induced angiogenesis, we have studied the role of MMP-2. 15(S)-HETE induced MMP-2 expression and activity in a time-dependent manner in human dermal microvascular endothelial cells (HDMVECs). Inhibition of MMP-2 activity or depletion of its levels attenuated 15(S)-HETE-induced HDMVEC **migration**, tube formation, and Matrigel plug angiogenesis. 15(S)-HETE also induced Fra-1 and c-Jun expression in a Rac1-MEK1-JNK1-dependent manner. In addition, 15(S)-HETE-induced MMP-2 expression and activity were mediated by Rac1-MEK1-JNK1-dependent activation of AP-1 (Fra-1/c-Jun). Cloning and site-directed mutagenesis of MMP-2 promoter revealed that AP-1 site proximal to the transcriptional start site is required for 15(S)-HETE-induced MMP-2 expression, and Fra-1 and c-Jun are the essential components of AP-1 that bind to MMP-2 promoter in response to 15(S)-HETE. Hind limb ischemia led to an increase in MEK1 and JNK1 activation and Fra-1, c-Jun, and MMP-2 expression resulting in enhanced neovascularization and recovery of blood perfusion in wild-type mice as compared with 12/15-Lox(-/-) mice. Together, these results provide the first direct evidence for a role of 12/15-Lox-12/15(S)-HETE axis in the regulation of ischemia-induced angiogenesis.
3. Estrogen-induced expression of Fos-related antigen 1 (FRA-1) regulates uterine stromal differentiation and remodeling. Concerted actions of estrogen and progesterone via their cognate receptors orchestrate changes in the uterine tissue, regulating implantation during early pregnancy. The uterine stromal cells undergo steroid-dependent differentiation into morphologically and functionally distinct decidual cells, which support embryonic growth and survival. The hormone-regulated pathways underlying this unique cellular transformation are not fully understood. Previous studies in the mouse revealed that, following embryo attachment, de novo synthesis of estrogen by the decidual cells is critical for stromal differentiation. In this study we report that Fos-related antigen 1 (FRA-1), a member of the Fos family of transcription factors, is a downstream target of regulation by intrauterine estrogen. FRA-1 expression was localized in the differentiating uterine stromal cells surrounding the implanted embryo. Attenuation of estrogen receptor α (Esr1) expression by siRNA mediated silencing in primary uterine stromal cells suppressed FRA-1 expression. Furthermore, chromatin immunoprecipitation demonstrated direct recruitment of ESR1 to an estrogen response element in the Fra-1 promoter. Down-regulation of Fra-1 expression during in vitro decidualization blocked stromal differentiation and resulted in a marked decrease in stromal cell **migratio**n. Interestingly, FRA-1 controls the expression of matrix metalloproteinases MMP9 and MMP13, which are critical modulators of stromal extracellular matrix remodeling. Collectively, these results suggest that FRA-1, induced in response to estrogen signaling via ESR1, is a key regulator of stromal differentiation and remodeling during early pregnancy.
4. Expression profiling of genes regulated by Fra-1/AP-1 transcription factor during bleomycin-induced pulmonary fibrosis. The Fra-1/AP-1 transcription factor regulates the expression of genes controlling various processes including **migration**, invasion, and survival as well as extracellular remodeling. We recently demonstrated that loss of Fra-1 leads to exacerbated bleomycin-induced pulmonary fibrosis, accompanied by enhanced expression of various inflammatory and fibrotic genes. To better understand the molecular mechanisms by which Fra-1 confers protection during bleomycin-induced lung injury, genome-wide mRNA expression profiling was performed.We found that Fra-1 regulates gene expression programs that include: 1) several cytokines and chemokines involved in inflammation, 2) several genes involved in the extracellular remodeling and cell adhesion, and 3) several genes involved in programmed cell death.Loss of Fra-1 leads to the enhanced expression of genes regulating inflammation and immune responses and decreased the expression of genes involved in apoptosis, suggesting that this transcription factor distinctly modulates early pro-fibrotic cellular responses.

### [Ee]xtracellular [Mm]atrix

#### PubMed

1. Trophoblast stem cell maintenance by fibroblast growth factor 4 requires MEKK4 activation of Jun N-terminal kinase. Trophoblast differentiation during placentation involves an epithelial-mesenchymal transition (EMT) with loss of E-cadherin and gain of trophoblast invasiveness. Mice harboring a point mutation that renders inactive the mitogen-activated protein kinase kinase kinase MEKK4 exhibit dysregulated placental development with increased trophoblast invasion. Isolated MEKK4 kinase-inactive trophoblast stem (TS) cells cultured under undifferentiating, self-renewing conditions in the presence of fibroblast growth factor 4 (FGF4) display increased expression of Slug, Twist, and matrix metalloproteinase 2 (MMP2), loss of E-cadherin, and hyperinvasion of **extracellular matrix**, each a hallmark of EMT. MEKK4 kinase-inactive TS cells show a preferential differentiation to Tpbp alpha- and Gcm1-positive trophoblasts, which are indicative of spongiotrophoblast and syncytiotrophoblast differentiation, respectively. FGF4-stimulated Jun N-terminal kinase (JNK) and p38 activity is markedly reduced in MEKK4 kinase-inactive TS cells. Chemical inhibition of JNK in wild-type TS cells induced a similar EMT response as loss of MEKK4 kinase activity, including inhibition of E-cadherin expression and increased expression of Slug, MMP2, Tpbp alpha, and Gcm1. Chromatin immunoprecipitation analyses revealed changes in AP-1 composition with increased Fra-2 and decreased Fra-1 and JunB binding to the regulatory regions of Gcm1 and MMP2 genes in MEKK4 kinase-inactive TS cells. Our results define MEKK4 as a signaling hub for FGF4 activation of JNK that is required for maintenance of TS cells in an undifferentiated state.
2. Ezh2-mediated repression of a transcriptional pathway upstream of Mmp9 maintains integrity of the developing vasculature. Maintenance of vascular integrity is required for embryogenesis and organ homeostasis. However, the gene expression programs that stabilize blood vessels are poorly understood. Here, we show that the histone methyltransferase Ezh2 maintains integrity of the developing vasculature by repressing a transcriptional program that activates expression of Mmp9. Inactivation of Ezh2 in developing mouse endothelium caused embryonic lethality with compromised vascular integrity and increased **extracellular matrix** degradation. Genome-wide approaches showed that Ezh2 targets Mmp9 and its activators Fosl1 and Klf5. In addition, we uncovered Creb3l1 as an Ezh2 target that directly activates Mmp9 gene expression in the endothelium. Furthermore, genetic inactivation of Mmp9 rescued vascular integrity defects in Ezh2-deficient embryos. Thus, epigenetic repression of Creb3l1, Fosl1, Klf5 and Mmp9 by Ezh2 in endothelial cells maintains the integrity of the developing vasculature, potentially linking this transcriptional network to diseases with compromised vascular integrity.
3. Estrogen-induced expression of Fos-related antigen 1 (FRA-1) regulates uterine stromal differentiation and remodeling. Concerted actions of estrogen and progesterone via their cognate receptors orchestrate changes in the uterine tissue, regulating implantation during early pregnancy. The uterine stromal cells undergo steroid-dependent differentiation into morphologically and functionally distinct decidual cells, which support embryonic growth and survival. The hormone-regulated pathways underlying this unique cellular transformation are not fully understood. Previous studies in the mouse revealed that, following embryo attachment, de novo synthesis of estrogen by the decidual cells is critical for stromal differentiation. In this study we report that Fos-related antigen 1 (FRA-1), a member of the Fos family of transcription factors, is a downstream target of regulation by intrauterine estrogen. FRA-1 expression was localized in the differentiating uterine stromal cells surrounding the implanted embryo. Attenuation of estrogen receptor α (Esr1) expression by siRNA mediated silencing in primary uterine stromal cells suppressed FRA-1 expression. Furthermore, chromatin immunoprecipitation demonstrated direct recruitment of ESR1 to an estrogen response element in the Fra-1 promoter. Down-regulation of Fra-1 expression during in vitro decidualization blocked stromal differentiation and resulted in a marked decrease in stromal cell migration. Interestingly, FRA-1 controls the expression of matrix metalloproteinases MMP9 and MMP13, which are critical modulators of stromal **extracellular matri**x remodeling. Collectively, these results suggest that FRA-1, induced in response to estrogen signaling via ESR1, is a key regulator of stromal differentiation and remodeling during early pregnancy.
4. Inhibition of Osteoarthritis by Adipose-Derived Stromal Cells Overexpressing Fra-1 in Mice. To determine whether overexpression of the activator protein 1 (AP-1) transcription factor Fra-1 in adipose-derived stromal cells (ADSCs) is an effective treatment of collagenase-induced osteoarthritis (OA).OA was induced by injection of collagenase into the knee joints of male C57BL/6 mice. ADSCs were isolated from the inguinal fat pads of 8-week-old wild-type or Fra-1-transgenic mice and injected into the knee joints of mice with collagenase-induced OA 7 days after OA induction. Histologic analyses of cartilage destruction and chondrocyte cell death were performed. Adipogenic differentiation capacity was evaluated, gene expression was analyzed, and cytokine profiling was performed in stromal vascular fractions (SVFs) and ADSCs.OA-related cartilage destruction and chondrocyte cell death were significantly reduced in mouse knee joints treated with ADSCs from Fra-1-transgenic mice compared to mouse knee joints treated with ADSCs from wild-type mice. This effect did not result from the higher number of adipogenic progenitors observed in SVFs from Fra-1-transgenic compared to wild-type mouse fat pads, since injection of wild-type mouse ADSCs enriched for adipogenic progenitors did not show any additional chondroprotective effects compared to nonsorted ADSCs. However, Fra-1-transgenic mouse ADSCs showed decreased adipogenic differentiation capacity. Moreover, Fra-1 significantly inhibited proinflammatory interleukin-6 and pentraxin 3 expression, while increasing the expression of **extracellular matrix** proteins, such as periostin and spondin 1. These findings suggest that Fra-1 overexpression leads to an increased chondroprotective effect of ADSCs in OA.ADSCs overexpressing Fra-1 effectively protect against OA. Our data indicate that genetic modifications of ADSCs, such as Fra-1 overexpression, may improve their potential to protect articular cartilage against OA-mediated damage.

### [Ii]nvasive

#### PubMed

1. Trophoblast stem cell maintenance by fibroblast growth factor 4 requires MEKK4 activation of Jun N-terminal kinase. Trophoblast differentiation during placentation involves an epithelial-mesenchymal transition (EMT) with loss of E-cadherin and gain of trophoblast **invasive**ness. Mice harboring a point mutation that renders inactive the mitogen-activated protein kinase kinase kinase MEKK4 exhibit dysregulated placental development with increased trophoblast invasion. Isolated MEKK4 kinase-inactive trophoblast stem (TS) cells cultured under undifferentiating, self-renewing conditions in the presence of fibroblast growth factor 4 (FGF4) display increased expression of Slug, Twist, and matrix metalloproteinase 2 (MMP2), loss of E-cadherin, and hyperinvasion of extracellular matrix, each a hallmark of EMT. MEKK4 kinase-inactive TS cells show a preferential differentiation to Tpbp alpha- and Gcm1-positive trophoblasts, which are indicative of spongiotrophoblast and syncytiotrophoblast differentiation, respectively. FGF4-stimulated Jun N-terminal kinase (JNK) and p38 activity is markedly reduced in MEKK4 kinase-inactive TS cells. Chemical inhibition of JNK in wild-type TS cells induced a similar EMT response as loss of MEKK4 kinase activity, including inhibition of E-cadherin expression and increased expression of Slug, MMP2, Tpbp alpha, and Gcm1. Chromatin immunoprecipitation analyses revealed changes in AP-1 composition with increased Fra-2 and decreased Fra-1 and JunB binding to the regulatory regions of Gcm1 and MMP2 genes in MEKK4 kinase-inactive TS cells. Our results define MEKK4 as a signaling hub for FGF4 activation of JNK that is required for maintenance of TS cells in an undifferentiated state.

### [Mm]etastasis

#### PubMed

1. MicroRNA-19a-3p inhibits breast cancer progression and **metastasis** by inducing macrophage polarization through downregulated expression of Fra-1 proto-oncogene. One of the hallmarks of malignancy is the polarization of tumor-associated macrophages (TAMs) from a pro-immune (M1-like) phenotype to an immune-suppressive (M2-like) phenotype. However, the molecular basis of the process is still unclear. MicroRNA (miRNA) comprises a group of small, non-coding RNAs that are broadly expressed by a variety of organisms and are involved in cell behaviors such as suppression or promotion of tumorigenesis. Here, we demonstrate that miR-19a-3p, broadly conserved among vertebrates, was downregulated in RAW264.7 macrophage cells of the M2 phenotype in conditoned medium of 4T1 mouse breast tumor cells. This downregulation correlated with an increased expression of the Fra-1 gene, which was reported to act as a pro-oncogene by supporting the invasion and progression of breast tumors. We found significant upregulation of miR-19a-3p in RAW264.7 macrophages after transfection with a miR-19a-3p mimic that resulted in a significant suppression of the expression of this gene. In addition, we could measure the activity of binding between miR-19a-3p and Fra-1 with a psiCHECK luciferase reporter system. Further, transfection of RAW264.7 macrophage cells with the miR-19a-3p mimic decreased the expression of the Fra-1 downstream genes VEGF, STAT3 and pSTAT3. Most importantly, the capacity of 4T1 breast tumor cells to migrate and invade was impaired in vivo by the intratumoral injection of miR-19a-3p. Taken together, these findings indicate that miR-19a-3p is capable of downregulating the M2 phenotype in M2 macrophages and that the low expression of this miRNA has an important role in the upregulation of Fra-1 expression and induction of M2 macrophage polarization.
2. The role of proto-oncogene Fra-1 in remodeling the tumor microenvironment in support of breast tumor cell invasion and progression. A growing body of evidence indicates that interactions between neoplastic cells and tumor-associated macrophages (TAMs) in the tumor microenvironment (TME) are crucial in promoting tumor cell invasion and progression. Macrophages have an ambiguous role in these processes as this M1 phenotype correlates with tumoricidal capacity, whereas TAMs of M2 phenotype exert tumor-promoting effects. In this study, we provide evidence that interactions between mouse breast tumor cells and TAMs remodel the TME, leading to the upregulation of Fra-1, a member of the FOS family of transcription factor. In turn, this proto-oncogene initiates activation of the IL-6/JAK/Stat3 signaling pathway. This creates a malignant switch in breast tumor cells, leading to increased release of proangiogenic factors MMP-9, vascular endothelial growth factor and transforming growth factor-beta from tumor cells and intensified invasion and progression of breast cancer. Proof of the concept for the crucial role played by transcription factor Fra-1 in regulating these processes was established by specific knockdown of Fra-1 with small interfering RNA, which resulted in a marked suppression of tumor cell invasion, angiogenesis and **metastasis** in a mouse breast cancer model. Such a strategy could eventually lead to future efficacious treatments of metastatic breast cancer.
3. Fra-1 protooncogene regulates IL-6 expression in macrophages and promotes the generation of M2d macrophages. The tumor microenvironment (TME) plays a prominent role in the growth of tumor cells. As the major inflammatory component of the TME, M2d macrophages are educated by the TME such that they adopt an immunosuppressive role that promotes tumor **metastasis** and progression. Fra-1 forms activator protein-1 heterodimers with Jun partners and drives gene transcription. Fra-1 is thought to drastically induce tumorigenesis and progression. However, the functional role of Fra-1 in the generation of M2d macrophages is poorly understood to date. Here, we demonstrate that 4T1 mammary carcinoma cells, when co-cultured with RAW264.7 macrophage cells, skew the RAW264.7 macrophage cell differentiation into M2d macrophages. The 4T1 cells stimulate de novo overexpression of Fra-1 in RAW264.7 cells, and then Fra-1 binds to the interleukin 6 (IL-6) promoter to increase the production of the cytokine IL-6 in RAW264.7 cells. IL-6 acts in an autocrine fashion to skew RAW264.7 macrophage cell differentiation into M2d macrophages. These findings open new insights into how to reverse M2d macrophage-induced immune tolerance to improve the efficacy of immunotherapeutic approaches.
4. FRA-1 proto-oncogene induces lung epithelial cell invasion and anchorage-independent growth in vitro, but is insufficient to promote tumor growth in vivo. FRA-1 forms activator protein-1 complexes in association with members of the JUN family and drives gene transcription. FRA-1 has been implicated in the development of airway squamous metaplasia and is frequently overexpressed in squamous cell carcinomas of the esophagus and stomach. We and others have shown a high level of persistent induction of FRA-1 by lung carcinogens, such as cigarette smoke and asbestos, in pulmonary epithelial cells. However, the exact roles of FRA-1 in regulating lung epithelial cell growth and invasion are poorly understood. To examine this aspect, we have stably overexpressed FRA-1 in human type-II-like alveolar malignant cell line (A549) and a nonmalignant bronchial epithelial cell line (BEAS-2B). FRA-1 greatly enhanced the rate of proliferation, motility, and invasion of A549 and BEAS-2B cells. In athymic nude mice, FRA-1, but not the control vector, rapidly enhanced tumor formation and **metastasis** by A549 cells. In contrast, FRA-1 failed to promote tumor formation by BEAS-2B. We suggest that FRA-1 can promote motility, invasion, and anchorage-independent growth of lung epithelial cells in vitro, but is insufficient for tumor formation.

### [Cc]ell [Ii]nvasion

#### PubMed

1. The role of proto-oncogene Fra-1 in remodeling the tumor microenvironment in support of breast tumor **cell invasion** and progression. A growing body of evidence indicates that interactions between neoplastic cells and tumor-associated macrophages (TAMs) in the tumor microenvironment (TME) are crucial in promoting tumor **cell invasion** and progression. Macrophages have an ambiguous role in these processes as this M1 phenotype correlates with tumoricidal capacity, whereas TAMs of M2 phenotype exert tumor-promoting effects. In this study, we provide evidence that interactions between mouse breast tumor cells and TAMs remodel the TME, leading to the upregulation of Fra-1, a member of the FOS family of transcription factor. In turn, this proto-oncogene initiates activation of the IL-6/JAK/Stat3 signaling pathway. This creates a malignant switch in breast tumor cells, leading to increased release of proangiogenic factors MMP-9, vascular endothelial growth factor and transforming growth factor-beta from tumor cells and intensified invasion and progression of breast cancer. Proof of the concept for the crucial role played by transcription factor Fra-1 in regulating these processes was established by specific knockdown of Fra-1 with small interfering RNA, which resulted in a marked suppression of tumor **cell invasion**, angiogenesis and metastasis in a mouse breast cancer model. Such a strategy could eventually lead to future efficacious treatments of metastatic breast cancer.
2. FRA-1 proto-oncogene induces lung epithelial **cell invasion** and anchorage-independent growth in vitro, but is insufficient to promote tumor growth in vivo. FRA-1 forms activator protein-1 complexes in association with members of the JUN family and drives gene transcription. FRA-1 has been implicated in the development of airway squamous metaplasia and is frequently overexpressed in squamous cell carcinomas of the esophagus and stomach. We and others have shown a high level of persistent induction of FRA-1 by lung carcinogens, such as cigarette smoke and asbestos, in pulmonary epithelial cells. However, the exact roles of FRA-1 in regulating lung epithelial cell growth and invasion are poorly understood. To examine this aspect, we have stably overexpressed FRA-1 in human type-II-like alveolar malignant cell line (A549) and a nonmalignant bronchial epithelial cell line (BEAS-2B). FRA-1 greatly enhanced the rate of proliferation, motility, and invasion of A549 and BEAS-2B cells. In athymic nude mice, FRA-1, but not the control vector, rapidly enhanced tumor formation and metastasis by A549 cells. In contrast, FRA-1 failed to promote tumor formation by BEAS-2B. We suggest that FRA-1 can promote motility, invasion, and anchorage-independent growth of lung epithelial cells in vitro, but is insufficient for tumor formation.

---

## Fosl2

- NCBI Gene
- GeneRIF
- Pubmed

| Search Term | Rank | Fields |
| --- | --- | --- |
| [Mm]igration | 6 | PubMed(1) |
| [Ee]xtracellular [Mm]atrix | 8 | PubMed(2) |
| [Ii]nvasive | 10 | PubMed(1) |

  
hide/show details for Fosl2

### [Mm]igration

#### PubMed

1. Transcription factor fos-related antigen-2 induces progressive peripheral vasculopathy in mice closely resembling human systemic sclerosis. Microvascular damage is one of the first pathological changes in systemic sclerosis. In this study, we investigated the role of Fos-related antigen-2 (Fra-2), a transcription factor of the activator protein-1 family, in the peripheral vasculopathy of systemic sclerosis and examined the underlying mechanisms.Expression of Fra-2 protein was significantly increased in skin biopsies of systemic sclerosis patients compared with healthy controls, especially in endothelial and vascular smooth muscle cells. Fra-2 transgenic mice developed a severe loss of small blood vessels in the skin that was paralleled by progressive skin fibrosis at 12 weeks of age. The reduction in capillary density was preceded by a significant increase in apoptosis in endothelial cells at week 9 as detected by immunohistochemistry. Similarly, suppression of Fra-2 by small interfering RNA prevented human microvascular endothelial cells from staurosporine-induced apoptosis and improved both the number of tubes and the cumulative tube lengths in the tube formation assay. In addition, cell **migration** in the scratch assay and vascular endothelial growth factor-dependent chemotaxis in a modified Boyden chamber assay were increased after transfection of human microvascular endothelial cells with Fra-2 small interfering RNA, whereas proliferation was not affected.Fra-2 is present in human systemic sclerosis and may contribute to the development of microvasculopathy by inducing endothelial cell apoptosis and by reducing endothelial cell **migration** and chemotaxis. Fra-2 transgenic mice are a promising preclinical model to study the mechanisms and therapeutic approaches of the peripheral vasculopathy in systemic sclerosis.

### [Ee]xtracellular [Mm]atrix

#### PubMed

1. Trophoblast stem cell maintenance by fibroblast growth factor 4 requires MEKK4 activation of Jun N-terminal kinase. Trophoblast differentiation during placentation involves an epithelial-mesenchymal transition (EMT) with loss of E-cadherin and gain of trophoblast invasiveness. Mice harboring a point mutation that renders inactive the mitogen-activated protein kinase kinase kinase MEKK4 exhibit dysregulated placental development with increased trophoblast invasion. Isolated MEKK4 kinase-inactive trophoblast stem (TS) cells cultured under undifferentiating, self-renewing conditions in the presence of fibroblast growth factor 4 (FGF4) display increased expression of Slug, Twist, and matrix metalloproteinase 2 (MMP2), loss of E-cadherin, and hyperinvasion of **extracellular matrix**, each a hallmark of EMT. MEKK4 kinase-inactive TS cells show a preferential differentiation to Tpbp alpha- and Gcm1-positive trophoblasts, which are indicative of spongiotrophoblast and syncytiotrophoblast differentiation, respectively. FGF4-stimulated Jun N-terminal kinase (JNK) and p38 activity is markedly reduced in MEKK4 kinase-inactive TS cells. Chemical inhibition of JNK in wild-type TS cells induced a similar EMT response as loss of MEKK4 kinase activity, including inhibition of E-cadherin expression and increased expression of Slug, MMP2, Tpbp alpha, and Gcm1. Chromatin immunoprecipitation analyses revealed changes in AP-1 composition with increased Fra-2 and decreased Fra-1 and JunB binding to the regulatory regions of Gcm1 and MMP2 genes in MEKK4 kinase-inactive TS cells. Our results define MEKK4 as a signaling hub for FGF4 activation of JNK that is required for maintenance of TS cells in an undifferentiated state.
2. The transcription factor Fra-2 regulates the production of **extracellular matrix** in systemic sclerosis. Fra-2 belongs to the activator protein 1 family of transcription factors. Mice transgenic for Fra-2 develop a systemic fibrotic disease with vascular manifestations similar to those of systemic sclerosis (SSc). The aim of the present study was to investigate whether Fra-2 plays a role in the pathogenesis of SSc and to identify the molecular mechanisms by which Fra-2 induces fibrosis.Dermal thickness and the number of myofibroblasts were determined in skin sections from Fra-2-transgenic and wild-type mice. The expression of Fra-2 in SSc patients and in animal models of SSc was analyzed by real-time polymerase chain reaction and immunohistochemistry. Fra-2, transforming growth factor beta (TGFbeta), and ERK signaling in SSc fibroblasts were inhibited using small interfering RNA, neutralizing antibodies, and small-molecule inhibitors.Fra-2-transgenic mice developed a skin fibrosis with increases in dermal thickness and increased myofibroblast differentiation starting at age 12 weeks. The expression of Fra-2 was up-regulated in SSc patients and in different mouse models of SSc. Stimulation with TGFbeta and platelet-derived growth factor (PDGF) significantly increased the expression of Fra-2 in SSc fibroblasts and induced DNA binding of Fra-2 in an ERK-dependent manner. Knockdown of Fra-2 potently reduced the stimulatory effects of TGFbeta and PDGF and decreased the release of collagen from SSc fibroblasts.We demonstrate that Fra-2 is overexpressed in SSc and acts as a novel downstream mediator of the profibrotic effects of TGFbeta and PDGF. Since transgenic overexpression of Fra-2 causes not only fibrosis but also vascular disease, Fra-2 might be an interesting novel candidate for molecular-targeted therapies for SSc.

### [Ii]nvasive

#### PubMed

1. Trophoblast stem cell maintenance by fibroblast growth factor 4 requires MEKK4 activation of Jun N-terminal kinase. Trophoblast differentiation during placentation involves an epithelial-mesenchymal transition (EMT) with loss of E-cadherin and gain of trophoblast **invasive**ness. Mice harboring a point mutation that renders inactive the mitogen-activated protein kinase kinase kinase MEKK4 exhibit dysregulated placental development with increased trophoblast invasion. Isolated MEKK4 kinase-inactive trophoblast stem (TS) cells cultured under undifferentiating, self-renewing conditions in the presence of fibroblast growth factor 4 (FGF4) display increased expression of Slug, Twist, and matrix metalloproteinase 2 (MMP2), loss of E-cadherin, and hyperinvasion of extracellular matrix, each a hallmark of EMT. MEKK4 kinase-inactive TS cells show a preferential differentiation to Tpbp alpha- and Gcm1-positive trophoblasts, which are indicative of spongiotrophoblast and syncytiotrophoblast differentiation, respectively. FGF4-stimulated Jun N-terminal kinase (JNK) and p38 activity is markedly reduced in MEKK4 kinase-inactive TS cells. Chemical inhibition of JNK in wild-type TS cells induced a similar EMT response as loss of MEKK4 kinase activity, including inhibition of E-cadherin expression and increased expression of Slug, MMP2, Tpbp alpha, and Gcm1. Chromatin immunoprecipitation analyses revealed changes in AP-1 composition with increased Fra-2 and decreased Fra-1 and JunB binding to the regulatory regions of Gcm1 and MMP2 genes in MEKK4 kinase-inactive TS cells. Our results define MEKK4 as a signaling hub for FGF4 activation of JNK that is required for maintenance of TS cells in an undifferentiated state.

---

## Gpr84

- NCBI Gene
- GeneRIF
- Pubmed

| Search Term | Rank | Fields |
| --- | --- | --- |
| [Mm]igration | 6 | PubMed(1) |

  
hide/show details for Gpr84

### [Mm]igration

#### PubMed

1. Cloning and expression analysis of a novel G-protein-coupled receptor selectively expressed on granulocytes. The **migration** of neutrophils into sites of acute and chronic inflammation is mediated by chemokines. We used degenerate-primer reverse transcriptase-polymerase chain reaction (RT-PCR) to analyze chemokine receptor expression in neutrophils and identify novel receptors. RNA was isolated from human peripheral blood neutrophils and from neutrophils that had been stimulated for 5 h with granulocyte-macrophage colony-stimulating factor or by coculturing with primary human bronchial epithelial cells. Amplification products were cloned, and clone redundancy was determined. Seven known G-protein-coupled receptors were identified among 38 clones-CCR1, CCR4, CXCR1, CXCR2, CXCR4, HM63, and FPR1-as well as a novel gene, EX33. The full-length EX33 clone was obtained, and an in silico approach was used to identify the putative murine homologue. The EX33 gene encodes a 396-amino-acid protein with limited sequence identity to known receptors. Expression studies of several known chemokine receptors and EX33 revealed that resting neutrophils expressed higher levels of CXCRs and EX33 compared with activated neutrophils. Northern blot experiments revealed that EX33 is expressed mainly in bone marrow, lung, and peripheral blood leukocytes. Using RT-PCR analysis, we showed more abundant expression of EX33 in neutrophils and eosinophils, in comparison with that in T- or B-lymphocytes, indicating cell-specific expression among leukocytes.

---

## Arhgef16

- NCBI Gene
- GeneRIF
- Pubmed

| Search Term | Rank | Fields |
| --- | --- | --- |
| [Mm]igration | 6 | PubMed(1) |

  
hide/show details for Arhgef16

### [Mm]igration

#### PubMed

1. Arhgef16, a novel Elmo1 binding partner, promotes clearance of apoptotic cells via RhoG-dependent Rac1 activation. Elmo is an evolutionarily conserved mammalian ortholog of Caenorhabditis elegans CED-12 with proposed roles during the removal of apoptotic cells, cell **migration**, neurite outgrowth, and myoblast fusion (Katoh and Negishi (2003) [1], Park and Tosello (2007) [2], Grimsley et al. (2004) [3], Hamoud et al. (2014) [4]). Elmo mediates these cellular processes by interacting with various proteins located in the plasma membrane, cytoplasm and nucleus, and by modulating their activities although it has no intrinsic catalytic activity (Park and Tosello (2007) [2], Hamoud et al. (2014) [4], Li et al. (2013) [5], Margaron, Fradet and Cote (2013) [6], and Mauldin et al. (2013)[7]). Because there are a limited number of proteins known to interact with Elmo, we performed a yeast two-hybrid screen using Elmo1 as bait to identify Elmo1-interacting proteins and to evaluate their mode of regulation. Arhgef16 was one of the proteins identified through the screen and subsequent analyses revealed that Arhgef16 interacted with Elmo1 in mammalian cells as well. Expression of Arhgef16 in phagocytes promoted engulfment of apoptotic cells, and engulfment mediated by Arhgef16 increased synergistically in the presence of Elmo1 but was abrogated in the absence of Elmo1. In addition, Arhgef16-mediated removal of apoptotic cells was dependent on RhoG, but independent of Dock1. Taken together, this study suggests that the newly identified Elmo1-interacting protein, Arhgef16, functions synergistically with Elmo1 to promote clearance of apoptotic cells in a RhoG-dependent and Dock1-independent manner.

---

## Abcc4

- NCBI Gene
- GeneRIF
- Pubmed

| Search Term | Rank | Fields |
| --- | --- | --- |
| [Mm]igration | 6 | PubMed(3); GeneRIFs(3) |
| [Mm]etastasize | 11 | PubMed(1) |

  
hide/show details for Abcc4

### [Mm]igration

#### PubMed

1. Multi-drug resistance protein 4 (MRP4)-mediated regulation of fibroblast cell **migration** reflects a dichotomous role of intracellular cyclic nucleotides. It has long been known that cyclic nucleotides and cyclic nucleotide-dependent signaling molecules control cell **migration**. However, the concept that it is not just the absence or presence of cyclic nucleotides, but a highly coordinated balance between these molecules that regulates cell **migration**, is new and revolutionary. In this study, we used multidrug resistance protein 4 (MRP4)-expressing cell lines and MRP4 knock-out mice as model systems and wound healing assays as the experimental system to explore this unique and emerging concept. MRP4, a member of a large family of ATP binding cassette transporter proteins, localizes to the plasma membrane and functions as a nucleotide efflux transporter and thus plays a role in the regulation of intracellular cyclic nucleotide levels. Here, we demonstrate that mouse embryonic fibroblasts (MEFs) isolated from Mrp4(-/-) mice have higher intracellular cyclic nucleotide levels and migrate faster compared with MEFs from Mrp4(+/+) mice. Using FRET-based cAMP and cGMP sensors, we show that inhibition of MRP4 with MK571 increases both cAMP and cGMP levels, which results in increased **migration**. In contrast to these moderate increases in cAMP and cGMP levels seen in the absence of MRP4, a robust increase in cAMP levels was observed following treatment with forskolin and isobutylmethylxanthine, which decreases fibroblast **migration**. In response to externally added cell-permeant cyclic nucleotides (cpt-cAMP and cpt-cGMP), MEF **migration** appears to be biphasic. Altogether, our studies provide the first experimental evidence supporting the novel concept that balance between cyclic nucleotides is critical for cell **migration**.
2. PKA and actin play critical roles as downstream effectors in MRP4-mediated regulation of fibroblast **migration**. Multidrug resistance protein 4 (MRP4), a member of the ATP binding cassette transporter family, functions as a plasma membrane exporter of cyclic nucleotides. Recently, we demonstrated that fibroblasts lacking the Mrp4 gene migrate faster and contain higher cyclic-nucleotide levels. Here, we show that cAMP accumulation and protein kinase A (PKA) activity are higher and polarized in Mrp4(-/-) fibroblasts, versus Mrp4(+/+) cells. MRP4-containing macromolecular complexes isolated from these fibroblasts contained several proteins, including actin, which play important roles in cell **migration**. We found that actin interacts with MRP4, predominantly at the plasma membrane, and an intact actin cytoskeleton is required to restrict MRP4 to specific microdomains of the plasma membrane. Our data further indicated that the enhanced accumulation of cAMP in Mrp4(-/-) fibroblasts facilitates cortical actin polymerization in a PKA-dependent manner at the leading edge, which in turn increases the overall rate of cell **migration** to accelerate the process of wound healing. Disruption of actin polymerization or inhibition of PKA activity abolished the effect of MRP4 on cell **migration**. Together, our findings suggest a novel cAMP-dependent mechanism for MRP4-mediated regulation of fibroblast **migration** whereby PKA and actin play critical roles as downstream effectors.
3. Unimpaired immune functions in the absence of Mrp4 (Abcc4). Dendritic cell (DC) **migration** to draining lymph nodes is important for the initiation of an effective immune response. Recently we reported that the human ATP-binding cassette (ABC) transporter multidrug resistance protein 4 (MRP4 and ABCC4) is required for the **migration** of human DC. Since the ABC transporter MRP1 (ABCC1) was previously shown to play a role in both human and mouse DC **migration**, we here studied whether Mrp4 is similarly required for DC **migration** in mice and whether the absence of Mrp4 interferes with the generation of an immune response. Immunological responses were compared in wild-type FVB (FVBwt), FVB Mrp4 knockout (KO) or FVB Mrp4/5 double knockout (dKO) mice. Skin, a preferred immunization site, was analyzed for DC markers, as well as for Mrp1 and Mrp4 expression. Whereas Mrp1 was abundantly present within FVBwt skin, only few Mrp4 expressing cells were detected. In addition, no Mrp4 protein expression was detected on in vitro cultured FVBwt bone marrow-derived DC (BM-DC). DC **migration** from murine ear skin was unaltered between FVBwt and MRP4/5 dKO animals. The absence of Mrp4 also had no effect on immune responses upon allergen sensitization, immunization or oral tolerance induction. We thus conclude that in contrast to its human counterpart, murine Mrp4 is not involved in DC **migration**, nor indeed, in the generation of an effective immune response. These data reveal disparities in the physiological role of ABC transporters between species, which may derive from differences in substrate specificity.

#### GeneRIFs

1. A novel cAMP-dependent mechanism for MRP4-mediated regulation of fibroblast **migration** and indicated that PKA and actin play important roles as downstream effectors.
2. Multi-drug resistance protein 4 (MRP4)-mediated regulation of fibroblast cell **migration** reflects a dichotomous role of intracellular cyclic nucleotides.
3. in contrast to its human counterpart, murine Mrp4 is not involved in dendritic cell **migration**, nor indeed, in the generation of an effective immune response.

### [Mm]etastasize

#### PubMed

1. Upregulation of COX-2 in the lung cancer promotes overexpression of multidrug resistance protein 4 (MRP4) via PGE2-dependent pathway. It is apparent that lung cancer is associated with inflammation, with accompanying hallmark elevations of cyclooxygenase 2 (COX-2) and prostaglandin E2 (PGE2) levels. However, the effects of these changes on MRP efflux transporters have not been thoroughly investigated before. Here, we report that upregulation of COX-2 can induce overexpression of MRP4 in both A549 non-small-cell lung cancer cell lines and mouse lung cancer models. In A549 cells, phorbol 12-myristate 13-acetate (PMA) treatment induced upregulation of COX-2 and MRP4 together, but not other MRP transporters. Transient overexpression of human COX-2 cDNA also specifically increased COX-2 and MRP4. Moreover, COX inhibitor treatment and COX-2-specific siRNA significantly inhibited the upregulation of MRP4. Additionally, PMA-treatment increased extracellular PGE2 levels, likely due to increased MRP4 function. Likewise, COX-2-specific siRNA reduced extracellular PGE2 levels. Furthermore, COX-2 upregulation resulted in an increase in mPGES-1, an enzyme responsible for PGE2 production. Finally, **metastasize**d lung cancer model mice exhibited increased expression levels of COX-2 and MRP4, as well as mPGES-1. In conclusion, the present study suggests that overexpression of MRP4 in lung cancer may be attributable to COX-2 upregulation via a PGE2-dependent pathway.

---

## Hes6

- NCBI Gene
- GeneRIF
- Pubmed

| Search Term | Rank | Fields |
| --- | --- | --- |
| [Mm]etastasis | 12 | PubMed(1) |

  
hide/show details for Hes6

### [Mm]etastasis

#### PubMed

1. Enhancer-bound LDB1 regulates a corticotrope promoter-pausing repression program. Substantial evidence supports the hypothesis that enhancers are critical regulators of cell-type determination, orchestrating both positive and negative transcriptional programs; however, the basic mechanisms by which enhancers orchestrate interactions with cognate promoters during activation and repression events remain incompletely understood. Here we report the required actions of LIM domain-binding protein 1 (LDB1)/cofactor of LIM homeodomain protein 2/nuclear LIM interactor, interacting with the enhancer-binding protein achaete-scute complex homolog 1, to mediate looping to target gene promoters and target gene regulation in corticotrope cells. LDB1-mediated enhancer:promoter looping appears to be required for both activation and repression of these target genes. Although LDB1-dependent activated genes are regulated at the level of transcriptional initiation, the LDB1-dependent repressed transcription units appear to be regulated primarily at the level of promoter pausing, with LDB1 regulating recruitment of **metastasis**-associated 1 family, member 2, a component of the nucleosome remodeling deacetylase complex, on these negative enhancers, required for the repressive enhancer function. These results indicate that LDB1-dependent looping events can deliver repressive cargo to cognate promoters to mediate promoter pausing events in a pituitary cell type.

---

## Uncx

- NCBI Gene
- GeneRIF
- Pubmed

| Search Term | Rank | Fields |
| --- | --- | --- |
| [Mm]igration | 6 | PubMed(9) |
| ECM | 7 | PubMed(1) |
| [Ee]xtracellular [Mm]atrix | 8 | PubMed(1) |
| [Mm]etastasis | 12 | PubMed(1) |

  
hide/show details for Uncx

### [Mm]igration

#### PubMed

1. Neuropilin receptors guide distinct phases of sensory and motor neuronal segmentation. The segmented trunk peripheral nervous system is generated by ventrally migrating neural crest cells that exclusively invade the anterior sclerotome and differentiate into metameric dorsal root and sympathetic ganglia. Meanwhile, ventral spinal motor axons also project through the somites in a segmental fashion. How peripheral nervous system segmentation is generated is unknown. We previously showed that neuropilin 2 (Nrp2)/semaphorin 3F (Sema3F) signaling is required for segmental neural crest **migration**, but not for metameric dorsal root gangliogenesis. We now expand these results to show that Nrp2 patterns initial motor axon outgrowth as well. Later, Nrp1/Sema3A signaling is essential for segmental dorsal root gangliogenesis and motor axonal fasciculation into ventral roots. Strikingly, Nrp/Sema signaling is not required for sympathetic ganglia segmentation. These data show that Nrp2 and Nrp1 work together to produce segmentation of sensory and motor nerves, and that dorsal peripheral nervous system metamerism is generated in a stepwise, Nrp-dependent process.
2. Wnt5a and Wnt11 regulate mammalian anterior-posterior axis elongation. Mesoderm formation and subsequent anterior-posterior (A-P) axis elongation are fundamental aspects of gastrulation, which is initiated by formation of the primitive streak (PS). Convergent extension (CE) movements and epithelial-mesenchymal transition (EMT) are important for A-P axis elongation in vertebrate embryos. The evolutionarily conserved planar cell polarity (PCP) pathway regulates CE, and Wnts regulate many aspects of gastrulation including CE and EMT. However, the Wnt ligands that regulate A-P axis elongation in mammalian development remain unknown. Wnt11 and Wnt5a regulate axis elongation in lower vertebrates, but only Wnt5a, not Wnt11, regulates mammalian PCP signaling and A-P axis elongation in development. Here, by generating Wnt5a; Wnt11 compound mutants, we show that Wnt11 and Wnt5a play redundant roles during mouse A-P axis elongation. Both genes regulate trunk notochord extension through PCP-controlled CE of notochord cells, establishing a role for Wnt11 in mammalian PCP. We show that Wnt5a and Wnt11 are required for proper patterning of the neural tube and somites by regulating notochord formation, and provide evidence that both genes are required for the generation and **migration** of axial and paraxial mesodermal precursor cells by regulating EMT. Axial and paraxial mesodermal precursors ectopically accumulate in the PS at late gastrula stages in Wnt5a(-/-); Wnt11(-/-) embryos and these cells ectopically express epithelial cell adhesion molecules. Our data suggest that Wnt5a and Wnt11 regulate EMT by inducing p38 (Mapk14) phosphorylation. Our findings provide new insights into the role of Wnt5a and Wnt11 in mouse early development and also in cancer metastasis, during which EMT plays a crucial role.
3. Twist function is required for the morphogenesis of the cephalic neural tube and the differentiation of the cranial neural crest cells in the mouse embryo. Loss of Twist function in the cranial mesenchyme of the mouse embryo causes failure of closure of the cephalic neural tube and malformation of the branchial arches. In the Twist(-/-) embryo, the expression of molecular markers that signify dorsal forebrain tissues is either absent or reduced, but those associated with ventral tissues display expanded domains of expression. Dorsoventral organization of the mid- and hindbrain and the anterior-posterior pattern of the neural tube are not affected. In the Twist(-/-) embryo, neural crest cells stray from the subectodermal migratory path and the late-migrating subpopulation invades the cell-free zone separating streams of cells going to the first and second branchial arches. Cell transplantation studies reveal that Twist activity is required in the cranial mesenchyme for directing the **migration** of the neural crest cells, as well as in the neural crest cells within the first branchial arch to achieve correct localization. Twist is also required for the proper differentiation of the first arch tissues into bone, muscle, and teeth.
4. Guidance of trunk neural crest **migration** requires neuropilin 2/semaphorin 3F signaling. In vertebrate embryos, neural crest cells migrate only through the anterior half of each somite while avoiding the posterior half. We demonstrate that neural crest cells express the receptor neuropilin 2 (Npn2), while its repulsive ligand semaphorin 3F (Sema3f) is restricted to the posterior-half somite. In Npn2 and Sema3f mutant mice, neural crest cells lose their segmental **migration** pattern and instead migrate as a uniform sheet, although somite polarity itself remains unchanged. Furthermore, Npn2 is cell autonomously required for neural crest cells to avoid Sema3f in vitro. These data show that Npn2/Sema3f signaling guides neural crest **migration** through the somite. Interestingly, neural crest cells still condense into segmentally arranged dorsal root ganglia in Npn2 nulls, suggesting that segmental neural crest **migration** and segmentation of the peripheral nervous system are separable processes.
5. Identification of Arx transcriptional targets in the developing basal forebrain. Mutations in the aristaless-related homeobox (ARX) gene are associated with multiple neurologic disorders in humans. Studies in mice indicate Arx plays a role in neuronal progenitor proliferation and development of the cerebral cortex, thalamus, hippocampus, striatum, and olfactory bulbs. Specific defects associated with Arx loss of function include abnormal interneuron **migration** and subtype differentiation. How disruptions in ARX result in human disease and how loss of Arx in mice results in these phenotypes remains poorly understood. To gain insight into the biological functions of Arx, we performed a genome-wide expression screen to identify transcriptional changes within the subpallium in the absence of Arx. We have identified 84 genes whose expression was dysregulated in the absence of Arx. This population was enriched in genes involved in cell **migration**, axonal guidance, neurogenesis, and regulation of transcription and includes genes implicated in autism, epilepsy, and mental retardation; all features recognized in patients with ARX mutations. Additionally, we found Arx directly repressed three of the identified transcription factors: Lmo1, Ebf3 and Shox2. To further understand how the identified genes are involved in neural development, we used gene set enrichment algorithms to compare the Arx gene regulatory network (GRN) to the Dlx1/2 GRN and interneuron transcriptome. These analyses identified a subset of genes in the Arx GRN that are shared with that of the Dlx1/2 GRN and that are enriched in the interneuron transcriptome. These data indicate Arx plays multiple roles in forebrain development, both dependent and independent of Dlx1/2, and thus provides further insights into the understanding of the mechanisms underlying the pathology of mental retardation and epilepsy phenotypes resulting from ARX mutations.
6. The T-box transcription factor Tbx18 maintains the separation of anterior and posterior somite compartments. The compartmentalization of somites along their anterior-posterior (AP) axis is pivotal to the segmental organization of the vertebrate axial skeleton and the peripheral nervous system. Anterior and posterior somite halves contribute to different vertebral elements. They are also characterized by different proliferation rates and properties with respect to neural crest cell **migration** and spinal nerve passage. AP-somite polarity is generated in the anterior presomitic mesoderm by Mesp2 and Delta/Notch signaling. Here, we demonstrate that maintenance of AP-somite polarity is mediated by the T-box transcription factor Tbx18. Mice deficient for Tbx18 show expansion of pedicles with transverse processes and proximal ribs, elements derived from the posterior lateral sclerotome. AP-somite polarity is established in Tbx18 mutant embryos but is not maintained. During somite maturation, posterior somite compartments expand most likely because of posterior cells invading the anterior somite half. In the anterior lateral sclerotome, Tbx18 acts as an antiapoptotic factor. Ectopic expression experiments suggest that Tbx18 can promote anterior at the expense of posterior somite compartments. In summary, Tbx18 appears to act downstream of Mesp2 and Delta/Notch signaling to maintain the separation of anterior and posterior somite compartments.
7. Sfrp1 and Sfrp2 regulate anteroposterior axis elongation and somite segmentation during mouse embryogenesis. Regulation of Wnt signaling is essential for embryonic patterning. Sfrps are secreted Wnt antagonists that directly interact with the Wnt ligand to inhibit signaling. Here, we show that Sfrp1 and Sfrp2 are required for anteroposterior (AP) axis elongation and somitogenesis in the thoracic region during mouse embryogenesis. Double homozygous mutations in Sfrp1 and Sfrp2 lead to severe shortening of the thoracic region. By contrast, a homozygous mutation in one or the other exerts no effect on embryogenesis, indicating that Sfrp1 and Sfrp2 are functionally redundant. The defect of a shortened thoracic region appears to be the consequence of AP axis reduction and incomplete somite segmentation. The reduction in the AP axis is partially due to abnormalities in cell **migration** of pre-somitic mesoderm from the end of gastrulation. Aberrant somite segmentation is associated with altered oscillations of Notch signaling, as evidenced by abnormal Lfng and Hes7 expression during somitogenesis in the thoracic region. This study suggests that Wnt regulation by Sfrp1 and Sfrp2 is required for embryonic patterning.
8. Signaling by FGF4 and FGF8 is required for axial elongation of the mouse embryo. Fibroblast growth factor (FGF) signaling has been shown to play critical roles in vertebrate segmentation and elongation of the embryonic axis. Neither the exact roles of FGF signaling, nor the identity of the FGF ligands involved in these processes, has been conclusively determined. Fgf8 is required for cell **migration** away from the primitive streak when gastrulation initiates, but previous studies have shown that drastically reducing the level of FGF8 later in gastrulation has no apparent effect on somitogenesis or elongation of the embryo. In this study, we demonstrate that loss of both Fgf8 and Fgf4 expression during late gastrulation resulted in a dramatic skeletal phenotype. Thoracic vertebrae and ribs had abnormal morphology, lumbar and sacral vertebrae were malformed or completely absent, and no tail vertebrae were present. The expression of Wnt3a in the tail and the amount of nascent mesoderm expressing Brachyury were both severely reduced. Expression of genes in the NOTCH signaling pathway involved in segmentation was significantly affected, and somite formation ceased after the production of about 15-20 somites. Defects seen in the mutants appear to result from a failure to produce sufficient paraxial mesoderm, rather than a failure of mesoderm precursors to migrate away from the primitive streak. Although the epiblast prematurely decreases in size, we did not detect evidence of a change in the proliferation rate of cells in the tail region or excessive apoptosis of epiblast or mesoderm cells. We propose that FGF4 and FGF8 are required to maintain a population of progenitor cells in the epiblast that generates mesoderm and contributes to the stem cell population that is incorporated in the tailbud and required for axial elongation of the mouse embryo after gastrulation.
9. Neurohypophysial dysmorphogenesis in mice lacking the homeobox gene Uncx4.1. A number of transcription factors have been implicated in the development of the hypothalamo-neurohypophysial system (HNS). Null mutations for these factors caused severe defects in proliferation, **migration** and survival during early embryogenesis. While they have informed about early events of HNS developments no insights in mechanisms of late development and maturation of this major peptidergic system have been obtained as yet. In a screen for adult-expressed homeobox genes we identified Uncx4.1 as a gene expressed in adult and embryonic magnocellular neurons of the (HNS). Null mutation of Uncx4.1 left these neurons viable and able to express neuropeptides. However, the connectivity of magnocellular neurons with posterior pituitary elements was compromised. As a consequence neuronal fibres traversed to the adenohypophysis. The penetrance of this phenotype was about 50%. The data show a selective role of Uncx4.1 in controlling the development of connections of hypothalamic neurons to pituitary elements, allowing central neurons to reach the peripheral blood circulation and to deliver hormones for control of peripheral functions.

### ECM

#### PubMed

1. MBTPS1/SKI-1/S1P proprotein convertase is required for **ECM** signaling and axial elongation during somitogenesis and vertebral development†. Caudal regression syndrome (sacral agenesis), which impairs development of the caudal region of the body, occurs with a frequency of about 2 live births per 100 000 newborns although this incidence rises to 1 in 350 infants born to mothers with gestational diabetes. The lower back and limbs can be affected as well as the genitourinary and gastrointestinal tracts. The axial skeleton is formed during embryogenesis through the process of somitogenesis in which the paraxial mesoderm periodically segments into bilateral tissue blocks, called somites. Somites are the precursors of vertebrae and associated muscle, tendons and dorsal dermis. Vertebral anomalies in caudal regression syndrome may arise through perturbation of somitogenesis or, alternatively, could result from defective bone formation and patterning. We discovered that MBTPS1/SKI-1/S1P, which proteolytically activates a class of transmembrane transcription factors, plays a critical role in somitogenesis and the pathogenesis of lumbar/sacral vertebral anomalies. Conditional deletion of Mbtps1 yields a viable mouse with misshapen, fused and reduced number of lumbar and sacral vertebrae, under-developed hind limb bones and a kinky, shortened tail. We show that Mbtps1 is required to (i) maintain the Fgf8 'wavefront' in the presomitic mesoderm that underpins axial elongation, (ii) sustain the Lfng oscillatory 'clock' activity that governs the periodicity of somite formation and (iii) preserve the composition and character of the somitic extracellular matrix containing fibronectin, fibrillin2 and laminin. Based on this spinal phenotype and known functions of MBTPS1, we reason that loss-of-function mutations in Mbtps1 may cause the etiology of caudal regression syndrome.

### [Ee]xtracellular [Mm]atrix

#### PubMed

1. MBTPS1/SKI-1/S1P proprotein convertase is required for ECM signaling and axial elongation during somitogenesis and vertebral development†. Caudal regression syndrome (sacral agenesis), which impairs development of the caudal region of the body, occurs with a frequency of about 2 live births per 100 000 newborns although this incidence rises to 1 in 350 infants born to mothers with gestational diabetes. The lower back and limbs can be affected as well as the genitourinary and gastrointestinal tracts. The axial skeleton is formed during embryogenesis through the process of somitogenesis in which the paraxial mesoderm periodically segments into bilateral tissue blocks, called somites. Somites are the precursors of vertebrae and associated muscle, tendons and dorsal dermis. Vertebral anomalies in caudal regression syndrome may arise through perturbation of somitogenesis or, alternatively, could result from defective bone formation and patterning. We discovered that MBTPS1/SKI-1/S1P, which proteolytically activates a class of transmembrane transcription factors, plays a critical role in somitogenesis and the pathogenesis of lumbar/sacral vertebral anomalies. Conditional deletion of Mbtps1 yields a viable mouse with misshapen, fused and reduced number of lumbar and sacral vertebrae, under-developed hind limb bones and a kinky, shortened tail. We show that Mbtps1 is required to (i) maintain the Fgf8 'wavefront' in the presomitic mesoderm that underpins axial elongation, (ii) sustain the Lfng oscillatory 'clock' activity that governs the periodicity of somite formation and (iii) preserve the composition and character of the somiti**c extracellular matr**ix containing fibronectin, fibrillin2 and laminin. Based on this spinal phenotype and known functions of MBTPS1, we reason that loss-of-function mutations in Mbtps1 may cause the etiology of caudal regression syndrome.

### [Mm]etastasis

#### PubMed

1. Wnt5a and Wnt11 regulate mammalian anterior-posterior axis elongation. Mesoderm formation and subsequent anterior-posterior (A-P) axis elongation are fundamental aspects of gastrulation, which is initiated by formation of the primitive streak (PS). Convergent extension (CE) movements and epithelial-mesenchymal transition (EMT) are important for A-P axis elongation in vertebrate embryos. The evolutionarily conserved planar cell polarity (PCP) pathway regulates CE, and Wnts regulate many aspects of gastrulation including CE and EMT. However, the Wnt ligands that regulate A-P axis elongation in mammalian development remain unknown. Wnt11 and Wnt5a regulate axis elongation in lower vertebrates, but only Wnt5a, not Wnt11, regulates mammalian PCP signaling and A-P axis elongation in development. Here, by generating Wnt5a; Wnt11 compound mutants, we show that Wnt11 and Wnt5a play redundant roles during mouse A-P axis elongation. Both genes regulate trunk notochord extension through PCP-controlled CE of notochord cells, establishing a role for Wnt11 in mammalian PCP. We show that Wnt5a and Wnt11 are required for proper patterning of the neural tube and somites by regulating notochord formation, and provide evidence that both genes are required for the generation and migration of axial and paraxial mesodermal precursor cells by regulating EMT. Axial and paraxial mesodermal precursors ectopically accumulate in the PS at late gastrula stages in Wnt5a(-/-); Wnt11(-/-) embryos and these cells ectopically express epithelial cell adhesion molecules. Our data suggest that Wnt5a and Wnt11 regulate EMT by inducing p38 (Mapk14) phosphorylation. Our findings provide new insights into the role of Wnt5a and Wnt11 in mouse early development and also in cancer **metastasis**, during which EMT plays a crucial role.

---

## Ptprg

- NCBI Gene
- GeneRIF
- Pubmed

| Search Term | Rank | Fields |
| --- | --- | --- |
| [Mm]igration | 6 | Process(1); PubMed(1) |

  
hide/show details for Ptprg

### [Mm]igration

#### Process

1. negative regulation of epithelial cell **migration**

#### PubMed

1. Receptor protein tyrosine phosphatases are expressed by cycling retinal progenitor cells and involved in neuronal development of mouse retina. Receptor protein tyrosine phosphatases (RPTPs) appear to coordinate many aspects of neural development, including cell proliferation, **migration** and differentiation. Here we investigated potential roles of RPTPs in the developing mouse retina. Using a degenerate oligonucleotide-based reverse transcription polymerase chain reaction approach, we identified 11 different RPTPs in the retina at embryonic stage 13 (E13). Subsequently, the expression patterns of RPTPkappa, RPTPJ, RPTPRR, RPTPsigma, RPTPepsilon and RPTPgamma in the retina from embryonic stages to adult were analyzed in detail using quantitative real-time-PCR, in situ hybridization, immunohistochemistry and Western blotting. At E13, all six RPTPs are expressed in actively cycling retinal progenitor cells and postmitotic newborn retinal neurons. With ongoing maturation, RPTPkappa, RPTPJ, RPTPRR, RPTPsigma, RPTPepsilon and RPTPgamma display a different spatiotemporal regulation of mRNAs and proteins in the pre- and postnatal retina. Finally, in adulthood these six RPTPs localize to distinct cellular compartments of multiple retinal neurons. Additional studies in RPTPgamma(-/-) and RPTPbeta/zeta(-/-) (also known as PTPRZ1, RPTPbeta or RPTPzeta) mice at postnatal stage P1 reveal no apparent differences in retinal laminar organization or in the expression pattern of specific retinal cell-type markers when compared with wild type. However, in RPTPbeta/zeta(-/-) retinas, immunoreactivity of vimentin, a marker of Müller glial cells, is selectively reduced and the morphology of vimentin-immunoreactive radial processes of Müller cells is considerably disturbed. Our results suggest distinct roles of RPTPs in cell proliferation and establishing phenotypes of different retinal cells during retinogenesis as well as later in the maintenance of mature retina.

---

## Plekhg5

- NCBI Gene
- GeneRIF
- Pubmed

| Search Term | Rank | Fields |
| --- | --- | --- |
| [Mm]igration | 6 | Process(1); PubMed(4); Summary(1) |

  
hide/show details for Plekhg5

### [Mm]igration

#### Process

1. endothelial cell **migration**

#### PubMed

1. The Amot/Patj/Syx signaling complex spatially controls RhoA GTPase activity in migrating endothelial cells. Controlled regulation of Rho GTPase activity is an essential component mediating growth factor-stimulated **migration**. We have previously shown that angiomotin (Amot), a membrane-associated scaffold protein, plays a critical role during vascular patterning and endothelial **migration** during embryogenesis. However, the signaling pathways by which Amot controls directional **migration** are not known. Here we have used peptide pull-down and yeast 2-hybrid (Y2H) screening to identify proteins that interact with the C-terminal PDZ-binding motifs of Amot and its related proteins AmotL1 and 2. We report that Amot and its related proteins bind to the RhoA GTPase exchange factor (RhoGEF) protein Syx. We show that Amot forms a ternary complex together with Patj (or its paralogue Mupp1) and Syx. Using FRET analysis, we provide evidence that Amot controls targeting of RhoA activity to lamellipodia in vitro. We also report that, similar to Amot, morpholino knockdown of Syx in zebrafish results in inhibition of **migration** of intersegmental arteries. Taken together, our results indicate that the directional **migration** of capillaries in the embryo is governed by the Amot:Patj/Mupp1:Syx signaling that controls local GTPase activity.
2. A PDZ-binding motif as a critical determinant of Rho guanine exchange factor function and cell phenotype. We identified a Rho guanine exchange factor (GEF) expressed as two splice variants, which differ only in either having or lacking a Postsynaptic density 95, Disk large, Zona occludens-1 (PDZ) motif. The PDZ adaptor protein synectin bound the longer splice variant, Syx1, which was targeted to the plasma membrane in a synectin-dependent manner. The shorter variant, Syx2, was diffusely distributed in the cytoplasm. Fluorescence resonance energy transfer (FRET) imaging revealed similar differences between the spatial patterns of active RhoA in Syx1 versus Syx2-expressing cells. Expression of Syx1 augmented endothelial cell (EC) **migration** and tube formation, whereas Syx2 expression did not. It appears, therefore, that synectin-dependent targeting of Syx is critical to its contribution to these EC functions. Although agonist-stimulated global RhoA activity was similar in Syx1- and Syx2-expressing cells, basal RhoA activity was surprisingly higher in the latter. Out of 23 cell types, we found a significant level of endogenous Syx2 expression only in brain tumor cells, which also exhibited high basal RhoA activity. We found that the activity level of JNK, which mediates transcriptional regulation downstream of RhoA, is elevated in a Syx2-dependent manner in these cells, possibly contributing to their tumorigenicity.
3. Rab13-dependent trafficking of RhoA is required for directional **migration** and angiogenesis. Angiogenesis requires concomitant remodeling of cell junctions and **migration**, as exemplified by recent observations of extensive endothelial cell movement along growing blood vessels. We report that a protein complex that regulates cell junctions is required for VEGF-driven directional **migration** and for angiogenesis in vivo. The complex consists of RhoA and Syx, a RhoA guanine exchange factor cross-linked by the Crumbs polarity protein Mupp1 to angiomotin, a phosphatidylinositol-binding protein. The Syx-associated complex translocates to the leading edge of migrating cells by membrane trafficking that requires the tight junction recycling GTPase Rab13. In turn, Rab13 associates with Grb2, targeting Syx and RhoA to Tyr(1175)-phosphorylated VEGFR2 at the leading edge. Rab13 knockdown in zebrafish impeded sprouting of intersegmental vessels and diminished the directionality of their tip cells. These results indicate that endothelial cell mobility in sprouting vessels is facilitated by shuttling the same protein complex from disassembling junctions to the leading edges of cells.
4. Syx, a RhoA guanine exchange factor, is essential for angiogenesis in Vivo. Rho GTPases play an important and versatile role in several biological processes. In this study, we identified the zebrafish ortholog of the mammalian Rho A guanine exchange factor, synectin-binding guanine exchange factor (Syx), and determined its in vivo function in the zebrafish and the mouse. We found that Syx is expressed specifically in the vasculature of these organisms. Loss-of-function studies in the zebrafish and mouse point to a specific role for Syx in angiogenic sprouting in the developing vascular bed. Importantly, vasculogenesis and angioblast differentiation steps were unaffected in syx knockdown zebrafish embryos, and the vascular sprouting defects were partially rescued by the mouse ortholog. Syx knockdown in vitro impairs vascular endothelial growth factor-A-induced endothelial cell **migration** and angiogenesis. We have also uncovered a potential mechanism of endothelial sprout guidance in which angiomotin, a component of endothelial cell junctions, plays an additive role with Syx in directing endothelial sprouts. These results identify Syx as an essential contributor to angiogenesis in vivo.

#### Summary

1. This gene encodes a protein belonging to the Rho guanine exchange factor (GEF) family of proteins, which activate GTPases by replacing GDP with GTP. This family member is a RhoA GEF that plays a role in endothelial cell **migration** and tube formation. It is required for angiogenesis and may function in neuronal cell differentiation. Alternative splicing of this gene results in multiple transcript variants. [provided by RefSeq, Oct 2013]

---

## Pak3

- NCBI Gene
- GeneRIF
- Pubmed

| Search Term | Rank | Fields |
| --- | --- | --- |
| [Ff]ilopodia | 1 | PubMed(2) |
| [Pp]odosome(s)? | 5 | PubMed(1) |
| [Mm]igration | 6 | Process(1); PubMed(7) |
| [Ee]xtracellular [Mm]atrix | 8 | PubMed(1) |
| [Mm]etastasis | 12 | PubMed(1) |

  
hide/show details for Pak3

### [Ff]ilopodia

#### PubMed

1. Cellular functions of TC10, a Rho family GTPase: regulation of morphology, signal transduction and cell growth. The small Ras-related GTPase, TC10, has been classified on the basis of sequence homology to be a member of the Rho family. This family, which includes the Rho, Rac and CDC42 subfamilies, has been shown to regulate a variety of apparently diverse cellular processes such as actin cytoskeletal organization, mitogen-activated protein kinase (MAPK) cascades, cell cycle progression and transformation. In order to begin a study of TC10 biological function, we expressed wild type and various mutant forms of this protein in mammalian cells and investigated both the intracellular localization of the expressed proteins and their abilities to stimulate known Rho family-associated processes. Wild type TC10 was located predominantly in the cell membrane (apparently in the same regions as actin filaments), GTPase defective (75L) and GTP-binding defective (31N) mutants were located predominantly in cytoplasmic perinuclear regions, and a deletion mutant lacking the carboxyl terminal residues required for post-translational prenylation was located predominantly in the nucleus. The GTPase defective (constitutively active) TC10 mutant: (1) stimulated the formation of long **filopodia**; (2) activated c-Jun amino terminal kinase (JNK); (3) activated serum response factor (SRF)-dependent transcription; (4) activated NF-kappaB-dependent transcription; and (5) synergized with an activated Raf-kinase (Raf-CAAX) to transform NIH3T3 cells. In addition, wild type TC10 function is required for full H-Ras transforming potential. We demonstrate that an intact effector domain and carboxyl terminal prenylation signal are required for proper TC10 function and that TC10 signals to at least two separable downstream target pathways. In addition, TC10 interacted with the actin-binding and filament-forming protein, profilin, in both a two-hybrid cDNA library screen, and an in vitro binding assay. Taken together, these data support a classification of TC10 as a member of the Rho family, and in particular, suggest that TC10 functions to regulate cellular signaling to the actin cytoskeleton and processes associated with cell growth.
2. The mental retardation protein PAK3 contributes to synapse formation and plasticity in hippocampus. Mutations of the gene coding for PAK3 (p21-activated kinase 3) are associated with X-linked, nonsyndromic forms of mental retardation (MRX) in which the only distinctive clinical feature is the cognitive deficit. The mechanisms through which PAK3 mutation produces the mental handicap remain unclear, although an involvement in the mechanisms that regulate the formation or plasticity of synaptic networks has been proposed. Here we show, using a transient transfection approach, that antisense and small interfering RNA-mediated suppression of PAK3 or expression of a dominant-negative PAK3 carrying the human MRX30 mutation in rat hippocampal organotypic slice cultures results in the formation of abnormally elongated dendritic spines and **filopodia**-like protrusions and a decrease in mature spine synapses. Ultrastructural analysis of the changes induced by expression of PAK3 carrying the MRX30 mutation reveals that many elongated spines fail to express postsynaptic densities or contact presynaptic terminals. These defects are associated with a reduced spontaneous activity, altered expression of AMPA-type glutamate receptors, and defective long-term potentiation. Together, these data identify PAK3 as a key regulator of synapse formation and plasticity in the hippocampus and support interpretations that these defects might contribute to the cognitive deficits underlying this form of mental retardation.

### [Pp]odosome(s)?

#### PubMed

1. Phosphorylation of cortactin by p21-activated kinase. Cortactin is an F-actin binding protein that is enriched in dynamic cytoskeletal organelles such as **podosomes**, membrane ruffles, and lamellipodia. We have shown previously that Src-phosphorylation of cortactin is not required for its translocation to phorbol-ester induced **podosomes** in A7r5 aortic smooth muscle cells, but may be important for stability and turnover of **podosomes**. However, little is known of the role of Ser/Thr kinases in the regulation of cortactin. Here, we report that p21-associated kinase (PAK), which plays a crucial role in the formation of **podosome** and membrane ruffles, is able to phosphorylate cortactin in vitro. The predominant phosphorylation site is located at Ser113 in the first actin-binding repeat. Phosphorylation by PAK is not required for the translocation of cortactin to **podosomes**, lamellipodia, or membrane ruffles in A7r5 smooth muscle cells. However, binding of cortactin to F-actin is significantly reduced by PAK-phosphorylation. Taken together, these results suggest a role for PAK-phosphorylation of cortactin in the regulation of the dynamics of branched actin filaments in dynamic cytoskeletal organelles.

### [Mm]igration

#### Process

1. positive regulation of fibroblast **migration**

#### PubMed

1. Genes, plasticity and mental retardation. Functional and structural plasticity is a fundamental property of the brain involved in diverse processes ranging from brain construction and repair to storage of experiences during lifetime. Our current understanding of different forms of brain plasticity mechanisms has advanced tremendously in the last decades, benefiting from studies of development and memory storage in adulthood and from investigations of diverse diseased conditions. In this review, we focus on the role of mental retardation (MR) genes and show how this developing area of research can enrich our knowledge of the cellular and molecular mechanisms of brain plasticity and cognitive functions, and of the dysfunctional mechanisms underlying MR. We describe two main groups of MR genes; those leading to dysfunctional neurodevelopmental programs and brain malformations, and those which rely on alterations in molecular mechanisms underlying synaptic organization and plasticity. We first explore the role of MR genes in key mechanisms of neurogenesis and neuronal **migration** during development and in the adult, such as actin and microtubule-cytoskeletal dynamics and signal transduction. We then define the contribution of MR genes to forms of activity-dependent synaptic modifications, such as those involved in molecular organization of the synapse, intracellular signaling regulating gene programs and neuronal cytoskeleton to control network remodeling. We trace the characteristics of MR genes playing key roles in many forms of brain plasticity mechanisms, and highlight specific MR genes that endorse distinct roles in different cell types or brain regions, and at various times of a brain lifetime.
2. CHL1 cooperates with PAK1-3 to regulate morphological differentiation of embryonic cortical neurons. The cell adhesion molecule close homologue of L1 (CHL1) is important for apical dendritic projection and laminar positioning of pyramidal neurons in caudal regions of the cerebral cortex. The p21-activated kinase (PAK1-3) subfamily of serine/threonine kinases has also been implicated in regulating cell adhesion, **migration**, and morphology. Immunofluorescence staining in mouse embryonic brain showed that PAK1-3 was expressed in embryonic cortex and colocalized with CHL1 during neuronal **migration** and differentiation. To investigate a cooperative function for CHL1 and PAK in pyramidal cell differentiation or **migration**, a dominant-negative PAK mutant (PAK1 AID) that inhibits PAK1-3 kinase activity while coexpressing a green fluorescent protein (GFP) reporter was electroporated into the lateral ventricles of wild type (WT) and CHL1 null mutant mouse embryos (E14.5), then brain slices were cultured and neurons analyzed for laminar positioning and morphology by confocal microscopy after 3 days in vitro. Expression of PAK1 AID in CHL1 mutant cortex inactivated PAK and caused embryonic cortical neurons to branch profusely in the intermediate zone (IZ) and cortical plate (CP). The number of nodes, terminals and length of leading processes/apical dendrites of CHL1 mutant embryos expressing PAK1 AID increased dramatically, compared to CHL1 mutants without PAK1 AID, or WT embryos with or without PAK1 AID. These findings suggest that CHL1 and PAK1-3 kinase cooperate, most likely in independent pathways, in regulating morphological development of the leading process/apical dendrite of embryonic cortical neurons.
3. The Rac GTPase effector p21-activated kinase is essential for hematopoietic stem/progenitor cell **migration** and engraftment. The p21-activated kinases (Paks) are serine/threonine kinases that are major effectors of the Rho guanosine 5'\x{2011}triphosphatase, Rac, and Cdc42. Rac and Cdc42 are known regulators of hematopoietic stem and progenitor cell (HSPC) function, however, a direct role for Paks in HSPCs has yet to be elucidated. Lin(-)Sca1(+)c-kit(+) (LSK) cells from wild-type mice were transduced with retrovirus expressing Pak inhibitory domain (PID), a well-characterized inhibitor of Pak activation. Defects in marrow homing and in vitro cell **migration**, assembly of the actin cytoskeleton, proliferation, and survival were associated with engraftment failure of PID-LSK. The PID-LSK demonstrated decreased phosphorylation of extracellular signal-regulated kinase (ERK), whereas constitutive activation of ERK in these cells led to rescue of hematopoietic progenitor cell proliferation in vitro and partial rescue of Pak-deficient HSPC homing and engraftment in vivo. Using conditional knock-out mice, we demonstrate that among group A Paks, Pak2(-/-) HSPC show reduced homing to the bone marrow and altered cell shape similar to PID-LSK cells in vitro and are completely defective in HSPC engraftment. These data demonstrate that Pak proteins are key components of multiple engraftment-associated HSPC functions and play a direct role in activation of ERK in HSPCs, and that Pak2 is specifically essential for HSPC engraftment.
4. Insm1 promotes endocrine cell differentiation by modulating the expression of a network of genes that includes Neurog3 and Ripply3. Insulinoma associated 1 (Insm1) plays an important role in regulating the development of cells in the central and peripheral nervous systems, olfactory epithelium and endocrine pancreas. To better define the role of Insm1 in pancreatic endocrine cell development we generated mice with an Insm1(GFPCre) reporter allele and used them to study Insm1-expressing and null populations. Endocrine progenitor cells lacking Insm1 were less differentiated and exhibited broad defects in hormone production, cell proliferation and cell **migration**. Embryos lacking Insm1 contained greater amounts of a non-coding Neurog3 mRNA splice variant and had fewer Neurog3/Insm1 co-expressing progenitor cells, suggesting that Insm1 positively regulates Neurog3. Moreover, endocrine progenitor cells that express either high or low levels of Pdx1, and thus may be biased towards the formation of specific cell lineages, exhibited cell type-specific differences in the genes regulated by Insm1. Analysis of the function of Ripply3, an Insm1-regulated gene enriched in the Pdx1-high cell population, revealed that it negatively regulates the proliferation of early endocrine cells. Taken together, these findings indicate that in developing pancreatic endocrine cells Insm1 promotes the transition from a ductal progenitor to a committed endocrine cell by repressing a progenitor cell program and activating genes essential for RNA splicing, cell **migration**, controlled cellular proliferation, vasculogenesis, extracellular matrix and hormone secretion.
5. The four mammalian splice variants encoded by the p21-activated kinase 3 gene have different biological properties. The p21-activated kinases (PAK1), PAK2, and PAK3 are members of the PAK group I and share high sequence identity and common biochemical properties. PAK3 is specifically implicated in neuronal plasticity and also regulates cell cycle progression, neuronal **migration**, and apoptosis. Loss of function of PAK3 is responsible for X-linked non-syndromic mental retardation whereas gain of PAK3 function is associated with cancer. To understand the functional specificities of PAK3, we analyzed the structure of PAK3 gene products. We report here the characterization of a new alternatively spliced exon called c located upstream of the previously identified exon b. Exon b is detected in all tetrapods and not in fish, exon c is only present in mammals. Mammalian PAK3 genes encode four splice variants and the corresponding proteins were detected with specific antibodies in brain extracts. All PAK3 transcripts are specifically expressed in brain and in particular in neurons. The presence of the exons b and c renders the kinase constitutively active and decreases interaction with GTPases. The expression of the new splice variants in COS7 cells alters cell morphology and modifies the structure of focal adhesions. We propose that the appearance of new alternatively spliced exons during evolution and the resulting increase of complexity of PAK3 gene products may confer new functions to this kinase and contribute to its specific roles in neuronal signaling.
6. p21-activated kinase 3 is overexpressed in thymic neuroendocrine tumors (carcinoids) with ectopic ACTH syndrome and participates in cell **migration**. Thymic carcinoid is an important component of the tumor spectrum causing Ectopic ACTH Syndrome (EAS) and usually carries a poor prognosis. Efforts have been focused on exploring the mechanism of the excessive ACTH production in non-pituitary tumors, whereas few studies have reported the molecular events underlying the tumor progression. In this study, seven patients with ACTH producing thymic carcinoids were enrolled. Of note is that five of them showed either lymph node metastasis, local invasion or distant metastasis. By using cDNA profiling approach, we evaluated the expression of cell adhesion pathway genes and found a remarkable overexpression of p21-activated kinase 3 (PAK3) in all thymic carcinoids which was further confirmed at both transcriptional and translational level. RAC1, an upstream activator of PAK3, was also overexpressed in thymic carcinoids. Overexpression of PAK3 in NIH3T3 cell enhanced cell **migration** and invasion. Importantly, we observed c-Jun NH(2)-terminal kinase (JNK) was activated in PAK3 transfected cells, and inhibition of JNK activity by SP600125, a JNK pathway inhibitor, abolished PAK3 mediated cell **migration**. Activation of JNK pathway was also detected in thymic carcinoid with high level of PAK3 expression. Our findings suggested a potential role of PAK3 in the progression of ACTH-producing thymic carcinoid.
7. Dlx transcription factors promote **migration** through repression of axon and dendrite growth. In the mouse telencephalon, Dlx homeobox transcription factors are essential for the tangential **migration** of subpallial-derived GABAergic interneurons to neocortex. However, the mechanisms underlying this process are poorly understood. Here, we demonstrate that Dlx1/2 has a central role in restraining neurite growth of subpallial-derived immature interneurons at a stage when they migrate tangentially to cortex. In Dlx1-/-;Dlx2-/- mutants, neurite length is increased and cells fail to migrate. In Dlx1-/-;Dlx2+/- mutants, while the tangential **migration** of immature interneurons appears normal, they develop dendritic and axonal processes with increased length and decreased branching, and have deficits in their neocortical laminar positions. Thus, Dlx1/2 is required for coordinating programs of neurite maturation and **migration**. In this regard, we provide genetic evidence that in immature interneurons Dlx1/2 repression of the p21-activated serine/threonine kinase PAK3, a downstream effector of the Rho family of GTPases, is critical in restraining neurite growth and promoting tangential **migration**.

### [Ee]xtracellular [Mm]atrix

#### PubMed

1. Insm1 promotes endocrine cell differentiation by modulating the expression of a network of genes that includes Neurog3 and Ripply3. Insulinoma associated 1 (Insm1) plays an important role in regulating the development of cells in the central and peripheral nervous systems, olfactory epithelium and endocrine pancreas. To better define the role of Insm1 in pancreatic endocrine cell development we generated mice with an Insm1(GFPCre) reporter allele and used them to study Insm1-expressing and null populations. Endocrine progenitor cells lacking Insm1 were less differentiated and exhibited broad defects in hormone production, cell proliferation and cell migration. Embryos lacking Insm1 contained greater amounts of a non-coding Neurog3 mRNA splice variant and had fewer Neurog3/Insm1 co-expressing progenitor cells, suggesting that Insm1 positively regulates Neurog3. Moreover, endocrine progenitor cells that express either high or low levels of Pdx1, and thus may be biased towards the formation of specific cell lineages, exhibited cell type-specific differences in the genes regulated by Insm1. Analysis of the function of Ripply3, an Insm1-regulated gene enriched in the Pdx1-high cell population, revealed that it negatively regulates the proliferation of early endocrine cells. Taken together, these findings indicate that in developing pancreatic endocrine cells Insm1 promotes the transition from a ductal progenitor to a committed endocrine cell by repressing a progenitor cell program and activating genes essential for RNA splicing, cell migration, controlled cellular proliferation, vasculogenesis, **extracellular matrix** and hormone secretion.

### [Mm]etastasis

#### PubMed

1. p21-activated kinase 3 is overexpressed in thymic neuroendocrine tumors (carcinoids) with ectopic ACTH syndrome and participates in cell migration. Thymic carcinoid is an important component of the tumor spectrum causing Ectopic ACTH Syndrome (EAS) and usually carries a poor prognosis. Efforts have been focused on exploring the mechanism of the excessive ACTH production in non-pituitary tumors, whereas few studies have reported the molecular events underlying the tumor progression. In this study, seven patients with ACTH producing thymic carcinoids were enrolled. Of note is that five of them showed either lymph node **metastasis**, local invasion or distant **metastasis**. By using cDNA profiling approach, we evaluated the expression of cell adhesion pathway genes and found a remarkable overexpression of p21-activated kinase 3 (PAK3) in all thymic carcinoids which was further confirmed at both transcriptional and translational level. RAC1, an upstream activator of PAK3, was also overexpressed in thymic carcinoids. Overexpression of PAK3 in NIH3T3 cell enhanced cell migration and invasion. Importantly, we observed c-Jun NH(2)-terminal kinase (JNK) was activated in PAK3 transfected cells, and inhibition of JNK activity by SP600125, a JNK pathway inhibitor, abolished PAK3 mediated cell migration. Activation of JNK pathway was also detected in thymic carcinoid with high level of PAK3 expression. Our findings suggested a potential role of PAK3 in the progression of ACTH-producing thymic carcinoid.

---

## Pak1

- NCBI Gene
- GeneRIF
- Pubmed

| Search Term | Rank | Fields |
| --- | --- | --- |
| [Ff]ilopodia | 1 | PubMed(2) |
| [Ff]ilopodium | 2 | PubMed(1) |
| [Ii]nvadopodium | 4 | Component(1) |
| [Mm]igration | 6 | Process(3); PubMed(29); GeneRIFs(4) |
| ECM | 7 | PubMed(2) |
| [Ee]xtracellular [Mm]atrix | 8 | PubMed(5) |
| [Ii]nvasive | 10 | PubMed(1) |
| [Mm]etastasis | 12 | PubMed(3) |

  
hide/show details for Pak1

### [Ff]ilopodia

#### PubMed

1. Trihydrophobin 1 Interacts with PAK1 and Regulates ERK/MAPK Activation and Cell Migration. The Rac1/Cdc42 effector, p21-activated kinase (PAK), is activated by various signaling cascades, including receptor-tyrosine kinases and integrins, and regulates a number of processes such as cell proliferation and motility. PAK activity has been shown to be required for maximal activation of the canonical RAF-MEK-MAPK signaling cascade, possibly because of PAK co-activation of RAF and MEK. Here we have shown that trihydrophobin 1 (TH1), originally identified as a negative regulator of A-RAF kinase, also interacted with PAK1 in cultured cells. Confocal microscopy assay indicated that TH1 colocalized with PAK1 in both the cytoplasm and nucleus, which is consistent with our previous results. GST pulldown and coimmunoprecipitation experiments demonstrated that TH1 interacted directly with PAK1 and bound selectively to the carboxyl-terminal kinase domain of PAK1, and the ability of the binding was enhanced along with activation of PAK1. The binding pattern of PAK1 implies that this interaction was mediated in part by PAK1 kinase activity. As indicated by in vitro kinase activity assays and Western blot detections, TH1 inhibited PAK1 kinase activity and negatively regulated MAPK signal transduction. Interestingly, TH1 bound with MEK1/ERK in cells and in vitro without directly suppressing their kinase activity. Furthermore, we observed that TH1 localized to focal adhesions and **filopodia** in the leading edge of cells, where TH1 reduced cell migration through affecting actin and adhesion dynamics. Based on these observations, we propose a model in which TH1 interacts with PAK1 and specifically restricts the activation of MAPK modules through the upstream region of the MAPK pathway, thereby influencing cell migration.
2. Targeted disruption of the gene for the PAK5 kinase in mice. PAK5 is a member of the group B family of PAK serine/threonine kinases and is an effector for the Rho GTPase Cdc42. PAK5 is highly expressed in the brain and is expressed at lower levels in several other tissues. In cell lines, PAK5 has been shown to play a role in **filopodia** formation and neurite outgrowth. To examine the biological function of PAK5, we deleted the PAK5 gene in mice. The phenotypes of the PAK5-null mice are completely different from those of mice null for PAK4, another member of the group B PAK family. Unlike PAK4-null mice, which are embryonic lethal, PAK5-null mice develop normally and are fertile. The nervous system appears normal in the absence of PAK5, as do other tissues in which PAK5 is normally expressed. Our results suggest functional redundancy between PAK5 and other Rho GTPase targets.

### [Ff]ilopodium

#### PubMed

1. The neural cell adhesion molecule (NCAM) associates with and signals through p21-activated kinase 1 (Pak1). The Neural cell adhesion molecule (NCAM) plays an important role in regulation of nervous system development. To expand our understanding of the molecular mechanisms via which NCAM influences differentiation of neurons, we used a yeast two-hybrid screening to search for new binding partners of NCAM and identified p21-activated kinase 1 (Pak1). We show that NCAM interacts with Pak1 in growth cones of neurons. The autophosphorylation and activity of Pak1 were enhanced when isolated growth cones were incubated with NCAM function triggering antibodies, which mimic the interaction between NCAM and its extracellular ligands. The association of Pak1 with cell membranes, the efficiency of Pak1 binding to its activators, and Pak1 activity were inhibited in brains of NCAM-deficient mice. NCAM-dependent Pak1 activation was abolished after lipid raft disruption, suggesting that NCAM promotes Pak1 activation in the lipid raft environment. Phosphorylation of the downstream Pak1 effectors LIMK1 and cofilin was reduced in growth cones from NCAM-deficient neurons, which was accompanied by decreased levels of filamentous actin and inhibited **filopodium** mobility in the growth cones. Dominant-negative Pak1 inhibited and constitutively active Pak1 enhanced the ability of neurons to increase neurite outgrowth in response to the extracellular ligands of NCAM. Our combined observations thus indicate that NCAM activates Pak1 to drive actin polymerization to promote neuronal differentiation.

### [Ii]nvadopodium

#### Component

1. **invadopodium**

### [Mm]igration

#### Process

1. positive regulation of cell **migration**
2. positive regulation of fibroblast **migration**
3. positive regulation of vascular associated smooth muscle cell **migration**

#### PubMed

1. Opposing roles for Akt1 and Akt2 in Rac/Pak signaling and cell **migration**. The Akt/PKB isoforms have different roles in animals, with Akt2 primarily regulating metabolic signaling and Akt1 regulating growth and survival. Here we show distinct roles for Akt1 and Akt2 in mouse embryo fibroblast cell **migration** and regulation of the cytoskeleton. Akt1-deficient cells responded poorly to platelet-derived growth factor while Akt2-deficient cells had a dramatically enhanced response, resulting in a substantial increase in dorsal ruffling. Swapping domains between Akt1 and Akt2 demonstrated that the N-terminal region containing the pleckstrin homology domain and a linker region distinguishes the two isoforms, while the catalytic domains are interchangeable. Akt2 knock-out cells also migrated faster than wild-type cells, especially through extracellular matrix (ECM), while Akt1 knock-out cells migrated more slowly than wild-type cells. Consistently, Akt2 knock-out cells had elevated Pak1 and Rac activities, suggesting that Akt2 inhibits Rac and Pak1. Both Akt2 and Akt1 associated in complexes with Pak1, but only Akt2 inhibited Pak1 in kinase assays, suggesting an underlying molecular basis for the different cellular phenotypes. Together these data provide evidence for an unexpected functional link between Akt2 and Pak1 that opposes the actions of Akt1 on cell **migration**.
2. Myocardin-related transcription factors regulate the Cdk5/Pctaire1 kinase cascade to control neurite outgrowth, neuronal **migration** and brain development. Numerous motile cell functions depend on signaling from the cytoskeleton to the nucleus. Myocardin-related transcription factors (MRTFs) translocate to the nucleus in response to actin polymerization and cooperate with serum response factor (Srf) to regulate the expression of genes encoding actin and other components of the cytoskeleton. Here, we show that MRTF-A (Mkl1) and MRTF-B (Mkl2) redundantly control neuronal **migration** and neurite outgrowth during mouse brain development. Conditional deletion of the genes encoding these Srf coactivators disrupts the formation of multiple brain structures, reflecting a failure in neuronal actin polymerization and cytoskeletal assembly. These abnormalities were accompanied by dysregulation of the actin-severing protein gelsolin and Pctaire1 (Cdk16) kinase, which cooperates with Cdk5 to initiate a kinase cascade that governs cytoskeletal rearrangements essential for neuron **migration** and neurite outgrowth. Thus, the MRTF/Srf partnership interlinks two key signaling pathways that control actin treadmilling and neuronal maturation, thereby fulfilling a regulatory loop that couples cytoskeletal dynamics to nuclear gene transcription during brain development.
3. 14-3-3beta-Rac1-p21 activated kinase signaling regulates Akt1-mediated cytoskeletal organization, lamellipodia formation and fibronectin matrix assembly. Akt1 belongs to the three-gene Akt family and functions as a serine-threonine kinase regulating phosphorylation of an array of substrates and mediating cellular processes such as cell **migration**, proliferation, survival, and cell cycle. Our previous studies have established the importance of Akt1 in angiogenesis and absence of Akt1 resulted in impaired integrin activation, adhesion, **migration**, and extracellular matrix assembly by endothelial cells and fibroblasts. In this study, we identify the downstream signaling pathways activated by Akt1 in the regulation of these cellular events. We demonstrate here that Akt1 is necessary for the growth factor stimulated activation of 14-3-3beta-Rac1-p21 activated kinase (Pak) pathway in endothelial cells and fibroblasts. While activation of Akt1 resulted in translocation of Rac1 to membrane ruffles, enhanced Rac1 activity, Pak1 phosphorylation, and lamellipodia formation, resulting in enhanced adhesion and assembly of fibronectin, inhibition of Akt1 resulted in inhibition of these processes due to impaired Rac1-Pak signaling. Formation of lamellipodia, adhesion, and fibronectin assembly by myristoylated Akt1 expression in NIH 3T3 fibroblasts was inhibited by co-expression with either dominant negative Rac1 or dominant negative Pak1. In contrast, impaired lamellipodia formation, adhesion, and fibronectin assembly by dominant negative-Akt1 expression was rescued by co-expression with either constitutively active-Rac1 or -Pak1. Moreover, previously reported defects in adhesion and extracellular matrix assembly by Akt1(-/-) fibroblasts could be rescued by expression with either active-Rac1 or -Pak1, implying the importance of Rac1-Pak signaling in growth factor stimulated cytoskeletal assembly, lamellipodia formation and cell **migration** in endothelial cells and fibroblasts downstream of Akt1 activation.
4. CHL1 cooperates with PAK1-3 to regulate morphological differentiation of embryonic cortical neurons. The cell adhesion molecule close homologue of L1 (CHL1) is important for apical dendritic projection and laminar positioning of pyramidal neurons in caudal regions of the cerebral cortex. The p21-activated kinase (PAK1-3) subfamily of serine/threonine kinases has also been implicated in regulating cell adhesion, **migration**, and morphology. Immunofluorescence staining in mouse embryonic brain showed that PAK1-3 was expressed in embryonic cortex and colocalized with CHL1 during neuronal **migration** and differentiation. To investigate a cooperative function for CHL1 and PAK in pyramidal cell differentiation or **migration**, a dominant-negative PAK mutant (PAK1 AID) that inhibits PAK1-3 kinase activity while coexpressing a green fluorescent protein (GFP) reporter was electroporated into the lateral ventricles of wild type (WT) and CHL1 null mutant mouse embryos (E14.5), then brain slices were cultured and neurons analyzed for laminar positioning and morphology by confocal microscopy after 3 days in vitro. Expression of PAK1 AID in CHL1 mutant cortex inactivated PAK and caused embryonic cortical neurons to branch profusely in the intermediate zone (IZ) and cortical plate (CP). The number of nodes, terminals and length of leading processes/apical dendrites of CHL1 mutant embryos expressing PAK1 AID increased dramatically, compared to CHL1 mutants without PAK1 AID, or WT embryos with or without PAK1 AID. These findings suggest that CHL1 and PAK1-3 kinase cooperate, most likely in independent pathways, in regulating morphological development of the leading process/apical dendrite of embryonic cortical neurons.
5. The four mammalian splice variants encoded by the p21-activated kinase 3 gene have different biological properties. The p21-activated kinases (PAK1), PAK2, and PAK3 are members of the PAK group I and share high sequence identity and common biochemical properties. PAK3 is specifically implicated in neuronal plasticity and also regulates cell cycle progression, neuronal **migration**, and apoptosis. Loss of function of PAK3 is responsible for X-linked non-syndromic mental retardation whereas gain of PAK3 function is associated with cancer. To understand the functional specificities of PAK3, we analyzed the structure of PAK3 gene products. We report here the characterization of a new alternatively spliced exon called c located upstream of the previously identified exon b. Exon b is detected in all tetrapods and not in fish, exon c is only present in mammals. Mammalian PAK3 genes encode four splice variants and the corresponding proteins were detected with specific antibodies in brain extracts. All PAK3 transcripts are specifically expressed in brain and in particular in neurons. The presence of the exons b and c renders the kinase constitutively active and decreases interaction with GTPases. The expression of the new splice variants in COS7 cells alters cell morphology and modifies the structure of focal adhesions. We propose that the appearance of new alternatively spliced exons during evolution and the resulting increase of complexity of PAK3 gene products may confer new functions to this kinase and contribute to its specific roles in neuronal signaling.
6. Targeted deletion of the mitogen-activated protein kinase kinase 4 gene in the nervous system causes severe brain developmental defects and premature death. The c-Jun NH2-terminal protein kinase (JNK) is a mitogen-activated protein kinase (MAPK) involved in the regulation of various physiological processes. Its activity is increased upon phosphorylation by the MAPK kinases MKK4 and MKK7. The early embryonic death of mice lacking an mkk4 or mkk7 gene has provided genetic evidence that MKK4 and MKK7 have nonredundant functions in vivo. To elucidate the physiological role of MKK4, we generated a novel mouse model in which the mkk4 gene could be specifically deleted in the brain. At birth, the mutant mice were indistinguishable from their control littermates, but they stopped growing a few days later and died prematurely, displaying severe neurological defects. Decreased JNK activity in the absence of MKK4 correlated with impaired phosphorylation of a subset of physiologically relevant JNK substrates and with altered gene expression. These defects resulted in the misalignment of the Purkinje cells in the cerebellum and delayed radial **migration** in the cerebral cortex. Together, our data demonstrate for the first time that MKK4 is an essential activator of JNK required for the normal development of the brain.
7. The Rac GTPase effector p21-activated kinase is essential for hematopoietic stem/progenitor cell **migration** and engraftment. The p21-activated kinases (Paks) are serine/threonine kinases that are major effectors of the Rho guanosine 5'\x{2011}triphosphatase, Rac, and Cdc42. Rac and Cdc42 are known regulators of hematopoietic stem and progenitor cell (HSPC) function, however, a direct role for Paks in HSPCs has yet to be elucidated. Lin(-)Sca1(+)c-kit(+) (LSK) cells from wild-type mice were transduced with retrovirus expressing Pak inhibitory domain (PID), a well-characterized inhibitor of Pak activation. Defects in marrow homing and in vitro cell **migration**, assembly of the actin cytoskeleton, proliferation, and survival were associated with engraftment failure of PID-LSK. The PID-LSK demonstrated decreased phosphorylation of extracellular signal-regulated kinase (ERK), whereas constitutive activation of ERK in these cells led to rescue of hematopoietic progenitor cell proliferation in vitro and partial rescue of Pak-deficient HSPC homing and engraftment in vivo. Using conditional knock-out mice, we demonstrate that among group A Paks, Pak2(-/-) HSPC show reduced homing to the bone marrow and altered cell shape similar to PID-LSK cells in vitro and are completely defective in HSPC engraftment. These data demonstrate that Pak proteins are key components of multiple engraftment-associated HSPC functions and play a direct role in activation of ERK in HSPCs, and that Pak2 is specifically essential for HSPC engraftment.
8. Rac1/PAK1 signaling promotes epithelial-mesenchymal transition of podocytes in vitro via triggering β-catenin transcriptional activity under high glucose conditions. Ras-related C3 botulinum toxin substrate 1 (Rac1), together with its major downstream effector p21-activated kinase 1 (PAK1), has been identified a central role in cellular events such as cell cytoskeletal remodeling that contributed to cell **migratio**n and epithelial-mesenchymal transition (EMT). And there are data implicating that podocytes underwent EMT under pathological conditions. However, little is known about mechanisms of podocytes undergoing EMT. To address this, we assessed the cellular changes of podocytes after high glucose stimulation in vitro, detected the effects of Rac1/PAK1 signaling on podocytes in response to the stimuli, and investigated interactions of Rac1/PAK1 axis with β-catenin and Snail under high glucose conditions. We found that in vitro high glucose treatment led to remarkable down-regulation of nephrin and P-cadherin, as well as significant up-regulation of α-SMA and FSP-1, suggesting that in the presence of high glucose, podocytes underwent EMT, during which Rac1/PAK1 signaling was activated. And these were notably ameliorated by Rac1 gene knockdown. Furthermore, β-catenin and Snail nuclear translocation were triggered by Rac1/PAK1 axis, which were both markedly reversed via Rac1 gene knockdown or pretreatment of IPA-3, a PAK1 inhibitor. These findings elaborated that Rac1/PAK1 signaling contributed to high glucose-induced podocyte EMT via promoting β-catenin and Snail transcriptional activities, which could be a potential mechanism involved in podocytes injury in response to stimuli under diabetic conditions.
9. Rac1/PAK1 signaling promotes epithelial-mesenchymal transition of podocytes in vitro via triggering β-catenin transcriptional activity under high glucose conditions. Ras-related C3 botulinum toxin substrate 1 (Rac1), together with its major downstream effector p21-activated kinase 1 (PAK1), has been identified a central role in cellular events such as cell cytoskeletal remodeling that contributed to cell **migratio**n and epithelial-mesenchymal transition (EMT). And there are data implicating that podocytes underwent EMT under pathological conditions. However, little is known about mechanisms of podocytes undergoing EMT. To address this, we assessed the cellular changes of podocytes after high glucose stimulation in vitro, detected the effects of Rac1/PAK1 signaling on podocytes in response to the stimuli, and investigated interactions of Rac1/PAK1 axis with β-catenin and Snail under high glucose conditions. We found that in vitro high glucose treatment led to remarkable down-regulation of nephrin and P-cadherin, as well as significant up-regulation of α-SMA and FSP-1, suggesting that in the presence of high glucose, podocytes underwent EMT, during which Rac1/PAK1 signaling was activated. And these were notably ameliorated by Rac1 gene knockdown. Furthermore, β-catenin and Snail nuclear translocation were triggered by Rac1/PAK1 axis, which were both markedly reversed via Rac1 gene knockdown or pretreatment of IPA-3, a PAK1 inhibitor. These findings elaborated that Rac1/PAK1 signaling contributed to high glucose-induced podocyte EMT via promoting β-catenin and Snail transcriptional activities, which could be a potential mechanism involved in podocytes injury in response to stimuli under diabetic conditions.
10. The Rac GTPase effector p21-activated kinase is essential for hematopoietic stem/progenitor cell **migration** and engraftment. The p21-activated kinases (Paks) are serine/threonine kinases that are major effectors of the Rho guanosine 5'\x{2011}triphosphatase, Rac, and Cdc42. Rac and Cdc42 are known regulators of hematopoietic stem and progenitor cell (HSPC) function, however, a direct role for Paks in HSPCs has yet to be elucidated. Lin(-)Sca1(+)c-kit(+) (LSK) cells from wild-type mice were transduced with retrovirus expressing Pak inhibitory domain (PID), a well-characterized inhibitor of Pak activation. Defects in marrow homing and in vitro cell **migration**, assembly of the actin cytoskeleton, proliferation, and survival were associated with engraftment failure of PID-LSK. The PID-LSK demonstrated decreased phosphorylation of extracellular signal-regulated kinase (ERK), whereas constitutive activation of ERK in these cells led to rescue of hematopoietic progenitor cell proliferation in vitro and partial rescue of Pak-deficient HSPC homing and engraftment in vivo. Using conditional knock-out mice, we demonstrate that among group A Paks, Pak2(-/-) HSPC show reduced homing to the bone marrow and altered cell shape similar to PID-LSK cells in vitro and are completely defective in HSPC engraftment. These data demonstrate that Pak proteins are key components of multiple engraftment-associated HSPC functions and play a direct role in activation of ERK in HSPCs, and that Pak2 is specifically essential for HSPC engraftment.
11. 14-3-3beta-Rac1-p21 activated kinase signaling regulates Akt1-mediated cytoskeletal organization, lamellipodia formation and fibronectin matrix assembly. Akt1 belongs to the three-gene Akt family and functions as a serine-threonine kinase regulating phosphorylation of an array of substrates and mediating cellular processes such as cell **migration**, proliferation, survival, and cell cycle. Our previous studies have established the importance of Akt1 in angiogenesis and absence of Akt1 resulted in impaired integrin activation, adhesion, **migration**, and extracellular matrix assembly by endothelial cells and fibroblasts. In this study, we identify the downstream signaling pathways activated by Akt1 in the regulation of these cellular events. We demonstrate here that Akt1 is necessary for the growth factor stimulated activation of 14-3-3beta-Rac1-p21 activated kinase (Pak) pathway in endothelial cells and fibroblasts. While activation of Akt1 resulted in translocation of Rac1 to membrane ruffles, enhanced Rac1 activity, Pak1 phosphorylation, and lamellipodia formation, resulting in enhanced adhesion and assembly of fibronectin, inhibition of Akt1 resulted in inhibition of these processes due to impaired Rac1-Pak signaling. Formation of lamellipodia, adhesion, and fibronectin assembly by myristoylated Akt1 expression in NIH 3T3 fibroblasts was inhibited by co-expression with either dominant negative Rac1 or dominant negative Pak1. In contrast, impaired lamellipodia formation, adhesion, and fibronectin assembly by dominant negative-Akt1 expression was rescued by co-expression with either constitutively active-Rac1 or -Pak1. Moreover, previously reported defects in adhesion and extracellular matrix assembly by Akt1(-/-) fibroblasts could be rescued by expression with either active-Rac1 or -Pak1, implying the importance of Rac1-Pak signaling in growth factor stimulated cytoskeletal assembly, lamellipodia formation and cell **migration** in endothelial cells and fibroblasts downstream of Akt1 activation.
12. Opposing roles for Akt1 and Akt2 in Rac/Pak signaling and cell **migration**. The Akt/PKB isoforms have different roles in animals, with Akt2 primarily regulating metabolic signaling and Akt1 regulating growth and survival. Here we show distinct roles for Akt1 and Akt2 in mouse embryo fibroblast cell **migration** and regulation of the cytoskeleton. Akt1-deficient cells responded poorly to platelet-derived growth factor while Akt2-deficient cells had a dramatically enhanced response, resulting in a substantial increase in dorsal ruffling. Swapping domains between Akt1 and Akt2 demonstrated that the N-terminal region containing the pleckstrin homology domain and a linker region distinguishes the two isoforms, while the catalytic domains are interchangeable. Akt2 knock-out cells also migrated faster than wild-type cells, especially through extracellular matrix (ECM), while Akt1 knock-out cells migrated more slowly than wild-type cells. Consistently, Akt2 knock-out cells had elevated Pak1 and Rac activities, suggesting that Akt2 inhibits Rac and Pak1. Both Akt2 and Akt1 associated in complexes with Pak1, but only Akt2 inhibited Pak1 in kinase assays, suggesting an underlying molecular basis for the different cellular phenotypes. Together these data provide evidence for an unexpected functional link between Akt2 and Pak1 that opposes the actions of Akt1 on cell **migration**.
13. Trihydrophobin 1 Interacts with PAK1 and Regulates ERK/MAPK Activation and Cell **Migration**. The Rac1/Cdc42 effector, p21-activated kinase (PAK), is activated by various signaling cascades, including receptor-tyrosine kinases and integrins, and regulates a number of processes such as cell proliferation and motility. PAK activity has been shown to be required for maximal activation of the canonical RAF-MEK-MAPK signaling cascade, possibly because of PAK co-activation of RAF and MEK. Here we have shown that trihydrophobin 1 (TH1), originally identified as a negative regulator of A-RAF kinase, also interacted with PAK1 in cultured cells. Confocal microscopy assay indicated that TH1 colocalized with PAK1 in both the cytoplasm and nucleus, which is consistent with our previous results. GST pulldown and coimmunoprecipitation experiments demonstrated that TH1 interacted directly with PAK1 and bound selectively to the carboxyl-terminal kinase domain of PAK1, and the ability of the binding was enhanced along with activation of PAK1. The binding pattern of PAK1 implies that this interaction was mediated in part by PAK1 kinase activity. As indicated by in vitro kinase activity assays and Western blot detections, TH1 inhibited PAK1 kinase activity and negatively regulated MAPK signal transduction. Interestingly, TH1 bound with MEK1/ERK in cells and in vitro without directly suppressing their kinase activity. Furthermore, we observed that TH1 localized to focal adhesions and filopodia in the leading edge of cells, where TH1 reduced cell **migration** through affecting actin and adhesion dynamics. Based on these observations, we propose a model in which TH1 interacts with PAK1 and specifically restricts the activation of MAPK modules through the upstream region of the MAPK pathway, thereby influencing cell **migration**.
14. Essential role of CIB1 in regulating PAK1 activation and cell **migration**. p21-activated kinases (PAKs) regulate many cellular processes, including cytoskeletal rearrangement and cell **migration**. In this study, we report a direct and specific interaction of PAK1 with a 22-kD Ca2+-binding protein, CIB1, which results in PAK1 activation both in vitro and in vivo. CIB1 binds to PAK1 within discrete regions surrounding the inhibitory switch domain in a calcium-dependent manner, providing a potential mechanism of CIB1-induced PAK1 activation. CIB1 overexpression significantly decreases cell **migration** on fibronectin as a result of a PAK1-and LIM kinase-dependent increase in cofilin phosphorylation. Conversely, the RNA interference-mediated depletion of CIB1 increases cell **migration** and reduces normal adhesion-induced PAK1 activation and cofilin phosphorylation. Together, these results demonstrate that endogenous CIB1 is required for regulated adhesion-induced PAK1 activation and preferentially induces a PAK1-dependent pathway that can negatively regulate cell **migration**. These results point to CIB1 as a key regulator of PAK1 activation and signaling.
15. Localized activation of p21-activated kinase controls neuronal polarity and morphology. In the developing forebrain, neuronal polarization is a stepwise and initially reversible process that underlies correct **migration** and axon specification. Many aspects of cytoskeletal changes that accompany polarization are currently molecularly undefined and thus poorly understood. Here we reveal that the p21-activated kinase (Pak1) is essential for the specification of an axon and dendrites. In hippocampal neurons, activation of Pak1 is spatially restricted to the immature axon despite its uniform presence in all neurites. Hyperactivation of Pak1 at the membrane of all neurites or loss of Pak1 expression disrupts both neuronal morphology and the distinction between an axon and dendrites. We reveal that Pak1 acts on polarity in a kinase-dependent manner, by affecting the F-actin and microtubule cytoskeleton at least in part through Rac1 and cofilin. Our data are the first to demonstrate the importance of localized Pak1 kinase activation for neuronal polarization and differentiation.
16. PAK1 mediates pancreatic cancer cell **migration** and resistance to MET inhibition. Pancreatic adenocarcinoma (PDAC) is a major unmet medical need and a deeper understanding of molecular drivers is needed to advance therapeutic options for patients. We report here that p21-activated kinase 1 (PAK1) is a central node in PDAC cells downstream of multiple growth factor signalling pathways, including hepatocyte growth factor (HGF) and MET receptor tyrosine kinase. PAK1 inhibition blocks signalling to cytoskeletal effectors and tumour cell motility driven by HGF/MET. MET antagonists, such as onartuzumab and crizotinib, are currently in clinical development. Given that even highly effective therapies have resistance mechanisms, we show that combination with PAK1 inhibition overcomes potential resistance mechanisms mediated either by activation of parallel growth factor pathways or by direct amplification of PAK1. Inhibition of PAK1 attenuated in vivo tumour growth and metastasis in a model of pancreatic adenocarcinoma. In human tissues, PAK1 is highly expressed in a proportion of PDACs (33% IHC score 2 or 3; n = 304) and its expression is significantly associated with MET positivity (p < 0.0001) and linked to a widespread metastatic pattern in patients (p = 0.067). Taken together, our results provide evidence for a functional role of MET/PAK1 signalling in pancreatic adenocarcinoma and support further characterization of therapeutic inhibitors in this indication.
17. Scrib regulates PAK activity during the cell **migration** process. Genetic studies have highlighted the key role of Scrib in the development of Metazoans. Deficiency in Scrib impairs many aspects of cell polarity and cell movement although the mechanisms involved remain unclear. In mammals, Scrib belongs to a protein complex containing betaPIX, an exchange factor for Rac/Cdc42, and GIT1, a GTPase activating protein for ARF6 implicated in receptor recycling and exocytosis. Here we show that the Scrib complex associates with PAK, a serine-threonine kinase family crucial for cell **migration**. PAK colocalizes with members of the Scrib complex at the leading edge of heregulin-treated T47D breast cancer cells. We demonstrate that the Scrib complex is required for epithelial cells and primary mouse embryonic fibroblasts to efficiently respond to chemoattractant cues. In Scrib-deficient cells, the pool of cortical PAK is decreased, thereby precluding its proper activation by Rac. Loss of Scrib also impairs the polarized distribution of active Rac at the leading edge and compromises the regulated activation of the GTPase in T47D cells and mouse embryonic fibroblasts. These data underscore the role of Scrib in cell **migration** and show the strong impact of Scrib in the function of PAK and Rac, two key molecules implicated in this process.
18. LKB1 suppresses p21-activated kinase-1 (PAK1) by phosphorylation of Thr109 in the p21-binding domain. The serine/threonine protein kinase LKB1 is a tumor suppressor gene mutated in Peutz-Jeghers syndrome patients. The mutations are found also in several types of sporadic cancer. Although LKB1 is implicated in suppression of cell growth and metastasis, the detailed mechanisms have not yet been elucidated. In this study, we investigated the effect of LKB1 on cell motility, whose acquisition occurs in early metastasis. The knockdown of LKB1 enhanced cell **migration** and PAK1 activity in human colon cancer HCT116 cells, whereas forced expression of LKB1 in Lkb1-null mouse embryonic fibroblasts suppressed PAK1 activity and PAK1-mediated cell **migration** simultaneously. Notably, LKB1 directly phosphorylated PAK1 at Thr(109) in the p21-binding domain in vitro. The phosphomimetic T109E mutant showed significantly lower protein kinase activity than wild-type PAK1, suggesting that the phosphorylation at Thr(109) by LKB1 was responsible for suppression of PAK1. Consistently, the nonphosphorylatable T109A mutant was resistant to suppression by LKB1. Furthermore, we found that PAK1 was activated in the hepatocellular carcinomas and the precancerous liver lesions of Lkb1(+/-) mice. Taken together, these results suggest that PAK1 is a direct downstream target of LKB1 and plays an essential role in LKB1-induced suppression of cell **migration**.
19. Potential compensation among group I PAK members in hindlimb ischemia and wound healing. PAKs are serine/threonine kinases that regulate cytoskeletal dynamics and cell **migration**. PAK1 is activated by binding to the small EF hand protein, CIB1, or to the Rho GTPases Rac1 or Cdc42. The role of PAK1 in angiogenesis was established based only on in vitro studies and its role in angiogenesis in vivo has never been examined. Here we tested the hypothesis that PAK1 is an essential regulator of ischemic neovascularization (arteriogenesis and angiogenesis) and wound healing using a global PAK1 knockout mouse. Neovascularization was assessed using unilateral hindlimb ischemia. We found that plantar perfusion, limb use and appearance were not significantly different between 6-8 week old PAK1-/- and PAK1+/+ mice throughout the 21-day period following hindlimb ischemia; however a slightly delayed healing was observed in 16 week old PAK1-/- mice. In addition, the wound healing rate, as assessed with an ear punch assay, was unchanged in PAK1-/- mice. Surprisingly, however, we observed a notable increase in PAK2 expression and phosphorylation in ischemic gastrocnemius tissue from PAK1-/- but not PAK1+/+ mice. Furthermore, we observed higher levels of activated ERK2, but not AKT, in ischemic and non-ischemic muscle of PAK1-/- mice upon hindlimb ischemic injury. A group I PAK inhibitor, IPA3, significantly inhibited endothelial cell sprouting from aortic rings in both PAK1-/- and PAK1+/+ mice, implying that PAK2 is a potential contributor to this process. Taken together, our data indicate that while PAK1 has the potential to contribute to neovascularization and wound healing, PAK2 may functionally compensate when PAK1 is deficient.
20. Pak1 regulates multiple c-Kit mediated Ras-MAPK gain-in-function phenotypes in Nf1+/- mast cells. Neurofibromatosis type 1 (NF1) is a common genetic disorder caused by mutations in the NF1 locus, which encodes neurofibromin, a negative regulator of Ras. Patients with NF1 develop numerous neurofibromas, which contain many inflammatory mast cells that contribute to tumor formation. Subsequent to c-Kit stimulation, signaling from Ras to Rac1/2 to the MAPK pathway appears to be responsible for multiple hyperactive mast cell phenotypes; however, the specific effectors that mediate these functions remain uncertain. p21-activated kinase 1 (Pak1) is a downstream mediator of Rac1/2 that has been implicated as a positive regulator of MAPK pathway members and is a modulator of cell growth and cytoskeletal dynamics. Using an intercross of Pak 1(-/-) mice with Nf1(+/-) mice, we determined that Pak1 regulates hyperactive Ras-dependent proliferation via a Pak1/Erk pathway, whereas a Pak1/p38 pathway is required for the increased **migration** in Nf1(+/-) mast cells. Furthermore, we confirmed that loss of Pak1 corrects the dermal accumulation of Nf1(+/-) mast cells in vivo to levels found in wild-type mice. Thus, Pak1 is a novel mast cell mediator that functions as a key node in the MAPK signaling network and potential therapeutic target in NF1 patients.
21. p-21-Activated kinase 1 mediates gastrin-stimulated proliferation in the colorectal mucosa via multiple signaling pathways. Gastrins, including amidated (Gamide) and glycine-extended (Ggly) forms, function as growth factors for the gastrointestinal mucosa. The p-21-activated kinase 1 (PAK1) plays important roles in growth factor signaling networks that control cell motility, proliferation, differentiation, and transformation. PAK1, activated by both Gamide and Ggly, mediates gastrin-stimulated proliferation and **migration**, and activation of β-catenin, in gastric epithelial cells. The aim of this study was to investigate the role of PAK1 in the regulation by gastrin of proliferation in the normal colorectal mucosa in vivo. Mucosal proliferation was measured in PAK1 knockout (PAK1 KO) mice by immunohistochemistry. The expression of phosphorylated and unphosphorylated forms of the signaling molecules PAK1, extracellular signal-regulated kinase (ERK), and protein kinase B (AKT), and the expression of β-catenin and its downstream targets c-Myc and cyclin D1, were measured in gastrin knockout (Gas KO) and PAK1 KO mice by Western blotting. The expression and activation of PAK1 are decreased in Gas KO mice, and these decreases are associated with reduced activation of ERK, AKT, and β-catenin. Proliferation in the colorectal mucosa of PAK1 KO mice is reduced, and the reduction is associated with reduced activation of ERK, AKT, and β-catenin. In compensation, antral gastrin mRNA and serum gastrin concentrations are increased in PAK1 KO mice. These results indicate that PAK1 mediates the stimulation of colorectal proliferation by gastrins via multiple signaling pathways involving activation of ERK, AKT, and β-catenin.
22. PAK1 interacts with beta-catenin and is required for the regulation of the beta-catenin signalling pathway by gastrins. Beta-catenin regulates cell-cell adhesion by binding to E-cadherin at the cell membrane and, when translocated into the nucleus, mediates signalling by activation of transcription factors such as TCF4. Mutations of the components of the Wnt/beta-catenin pathway are found in many gastrointestinal cancers. Gastrins, including amidated (Gamide) and glycine-extended (Ggly) gastrin(17), stimulate the proliferation of gastrointestinal cancer cells. Gastrins also regulate beta-catenin signalling through multiple pathways which seem to converge on p21-activated kinase 1 (PAK1). In this study, we have investigated the role of PAK1 in the regulation of beta-catenin signalling by gastrins. Here we report for the first time that PAK1 associated with beta-catenin. Both Gamide and Ggly stimulated the phosphorylation and activation of beta-catenin in a PAK1-dependent manner. A kinase-inactive mutant PAK1(K299A) blocked the gastrin-stimulated dissociation of beta-catenin from E-cadherin, translocation of beta-catenin from the cell membrane to the nucleus, and association of beta-catenin with the transcription factor TCF4. The PAK1(K299A) mutant also inhibited the stimulation of the expression of c-myc and cyclin D1, and of cell proliferation and **migration**, by gastrins. The results indicate that gastrins regulate beta-catenin signalling through a PAK1-dependent pathway. PAK1 seems to be the point of convergence of multiple signalling pathways activated by gastrins.
23. Rac2 regulates neutrophil chemotaxis, superoxide production, and myeloid colony formation through multiple distinct effector pathways. Polymorphonuclear neutrophils (PMN) are an important component of the innate immune system. We have shown previously that **migration** and superoxide (O2\*-) production, as well as some kinase signaling pathways are compromised in mice deficient in the Ras-related Rho GTPase Rac2. In this study, we demonstrate that Rac2 controls chemotaxis and superoxide production via distinct pathways and is critical for development of myeloid colonies in vitro. The Rac2 mutants V36A, F37A, and N39A all bind to both Pak1 and p67(phox), yet are unable to rescue superoxide production and chemotaxis when expressed in Rac2-/- PMN. In contrast, the N43A mutant, which binds to Por1 (Arfaptin 2), p67phox, and Pak1, is able to rescue superoxide production but not chemotaxis. The F37A mutant, demonstrated to have reduced binding to Por1, shows reduced rescue of fMLP-induced chemotaxis. Finally, the Rac2Y40C mutant that is defective in binding to all three potential downstream effectors (Pak1, p67phox, and Por1) is unable to rescue chemotaxis, motility, or superoxide production, but is able to rescue defective growth of myeloid colonies in vitro. These findings suggest that binding to any single effector is not sufficient to rescue the distinct cellular phenotypes of Rac2-/- PMN, implicating multiple, distinct, and potentially parallel effector pathways.
24. PAK1 regulates cortical development via promoting neuronal **migration** and progenitor cell proliferation. p21-activated kinase 1 (PAK1) is a serine/threonine kinase known to be activated by the Rho family small GTPases and to play a key role in cytoskeletal reorganization, spine morphology and synaptic plasticity. PAK1 is also implicated in a number of neurodevelopmental and neurodegenerative diseases, including autism, intellectual disability and Alzheimer's disease. However, the role of PAK1 in early brain development remains unknown.In this study, we employed genetic manipulations to investigate the role of PAK1 in the cerebral cortical development in mice. We showed that compared to the wild type littermates, PAK1 knockout mice have a reduction in the number of pyramidal neurons in several layers of the cerebral cortex, which is associated with a smaller pool of neural progenitor cells and impaired neuronal **migration**.These results suggest that PAK1 regulates cortical development by promoting the proliferation of neural progenitor cells and facilitating the **migration** of these neurons to specific regions of the cortex.
25. Scaffolding function of PAK in the PDK1-Akt pathway. Many extracellular signals stimulate phosphatidylinositol-3-kinase, which in turn activates the Rac1 GTPase, the protein kinase Akt and the Akt Thr 308 upstream kinase PDK1. Active Rac1 stimulates a number of events, including substrate phosphorylation by a subgroup of the PAK family of kinases. The combined effects of Rac1, PDK1 and Akt are crucial for cell **migration**, growth, survival, metabolism and tumorigenesis. Here we show that Rac1 stimulates a second, kinase-independent function of PAK1. The PAK1 kinase domain serves as a scaffold to facilitate Akt stimulation by PDK1 and to aid recruitment of Akt to the membrane. PAK differentially activates subpopulations of Akt. These findings reveal scaffolding functions of PAK that regulate the efficiency, localization and specificity of the PDK1-Akt pathway.
26. The p21-activated kinase is required for neuronal **migration** in the cerebral cortex. The normal formation and function of the mammalian cerebral cortex depend on the positioning of its neurones, which occurs in a highly organized, layer-specific manner. The correct morphology and movement of neurones rely on synchronized regulation of their actin filaments and microtubules. The p21-activated kinase (Pak1), a key cytoskeletal regulator, controls neuronal polarization, elaboration of axons and dendrites, and the formation of dendritic spines. However, its in vivo role in the developing nervous system is unclear. We have utilized in utero electroporation into mouse embryo cortices to reveal that both loss and gain of Pak1 function affect radial **migration** of projection neurones. Overexpression of hyperactivated Pak1 predominantly caused neurones to arrest in the intermediate zone (IZ) with apparently misoriented and disorganized leading projections. Loss of Pak1 disrupted the morphology of migrating neurones, which accumulated in the IZ and deep cortical layers. Unexpectedly, a significant number of neurones with reduced Pak1 expression aberrantly entered into the normally cell-sparse marginal zone, suggesting their inability to cease migrating that may be due to their impaired dissociation from radial glia. Our findings reveal the in vivo importance of temporal and spatial regulation of the Pak1 kinase during key stages of cortical development.
27. Inhibition of Nischarin Expression Promotes Neurite Outgrowth through Regulation of PAK Activity. Nischarin is a cytoplasmic protein expressed in various organs that plays an inhibitory role in cell **migration** and invasion and the carcinogenesis of breast cancer cells. We previously reported that Nischarin is highly expressed in neuronal cell lines and is differentially expressed in the brain tissue of adult rats. However, the physiological function of Nischarin in neural cells remains unknown. Here, we show that Nischarin is expressed in rat primary cortical neurons but not in astrocytes. Nischarin is localized around the nucleus and dendrites. Using shRNA to knockdown the expression of endogenous Nischarin significantly increases the percentage of neurite-bearing cells, remarkably increases neurite length, and accelerates neurite extension in neuronal cells. Silencing Nischarin expression also promotes dendrite elongation in rat cortical neurons where Nischarin interacts with p21-activated kinase 1/2 (PAK1/2) and negatively regulates phosphorylation of both PAK1 and PAK2. The stimulation of neurite growth observed in cells with decreased levels of Nischarin is partially abolished by IPA3-mediated inhibition of PAK1 activity. Our findings indicate that endogenous Nischarin inhibits neurite outgrowth by blocking PAK1 activation in neurons.
28. PAK1-mediated activation of ERK1/2 regulates lamellipodial dynamics. PAK1 is a member of the p21-activated kinase (PAK) family of serine/threonine kinases that are activated by the Rho GTPases Rac and Cdc42, and are implicated in regulating morphological polarity, cell **migration** and adhesion. Here we investigate the function of PAK1 in cell motility using macrophages derived from PAK1-null mice. We show that CSF1, a macrophage chemoattractant, transiently stimulates PAK1 and MAPK activation, and that MAPK activation is reduced in PAK1-/- macrophages. PAK1 regulates the dynamics of lamellipodium extension as cells spread in response to adhesion but is not essential for macrophage **migration** or chemotaxis towards CSF1. Following adhesion, PAK1-/- macrophages spread more rapidly and have more lamellipodia than wild-type cells; however, these lamellipodia were less stable than those in wild-type macrophages. ERK1/2 activity was reduced in PAK1-/- macrophages during adhesion, and inhibition of ERK1/2 activation in wild-type macrophages was sufficient to increase the spread area and mimic the lamellipodial dynamics of PAK1-/- macrophages. Together, these data indicate that PAK1 signals via ERK1/2 to regulate lamellipodial stability.
29. Pak1 and its T212 phosphorylated form accumulate in neurones and epithelial cells of the developing rodent. The serine/threonine kinase Pak1 is a target of the RhoGTPases Rac and Cdc42 and an important regulator of cell morphology and **migration**. Recent work from several laboratories has indicated that Pak1 controls microtubule dynamics as well as the organisation of F-actin microfilaments. Pak1 is phosphorylated on T212 by the p35/Cdk5 or cyclin B1/Cdc2 kinase in postmitotic neurones and mitotic cells, respectively. To understand its function during development, we have carried out a detailed temporal and spatial analysis of Pak1 expression and phosphorylation on T212. In the embryonic forebrain, Pak1 and Pak1T212(PO4) were seen to accumulate in the corpus callosum, intermediate zone, lateral olfactory tracts, and anterior commissures. Epithelial cells of the mouse embryo lung, kidney, intestine, and skin also exhibited high levels of Pak1 and Pak1T212(PO4), suggesting a previously unsuspected role in epithelial differentiation. Pak1T212(PO4) was undetectable in all adult tissues. Together, these data indicate a specific, developmentally regulated role of the Pak1 kinase.

#### GeneRIFs

1. PAK1 is a direct downstream target of LKB1 and plays an essential role in LKB1-induced suppression of cell **migration**
2. Temporal and spatial regulation of Pak1 kinase is highly important during key stages of cortical development; both loss and gain of Pak1 function affect radial **migration** of projection neurons.
3. TH1 interacts with PAK1 and specifically restricts the activation of MAPK modules through the upstream region of the MAPK pathway, thereby influencing cell **migration**.
4. Akt1 and Akt2 have opposing roles in Rac/Pak signaling and cell **migration**

### ECM

#### PubMed

1. Opposing roles for Akt1 and Akt2 in Rac/Pak signaling and cell migration. The Akt/PKB isoforms have different roles in animals, with Akt2 primarily regulating metabolic signaling and Akt1 regulating growth and survival. Here we show distinct roles for Akt1 and Akt2 in mouse embryo fibroblast cell migration and regulation of the cytoskeleton. Akt1-deficient cells responded poorly to platelet-derived growth factor while Akt2-deficient cells had a dramatically enhanced response, resulting in a substantial increase in dorsal ruffling. Swapping domains between Akt1 and Akt2 demonstrated that the N-terminal region containing the pleckstrin homology domain and a linker region distinguishes the two isoforms, while the catalytic domains are interchangeable. Akt2 knock-out cells also migrated faster than wild-type cells, especially through extracellular matrix (**ECM**), while Akt1 knock-out cells migrated more slowly than wild-type cells. Consistently, Akt2 knock-out cells had elevated Pak1 and Rac activities, suggesting that Akt2 inhibits Rac and Pak1. Both Akt2 and Akt1 associated in complexes with Pak1, but only Akt2 inhibited Pak1 in kinase assays, suggesting an underlying molecular basis for the different cellular phenotypes. Together these data provide evidence for an unexpected functional link between Akt2 and Pak1 that opposes the actions of Akt1 on cell migration.
2. Opposing roles for Akt1 and Akt2 in Rac/Pak signaling and cell migration. The Akt/PKB isoforms have different roles in animals, with Akt2 primarily regulating metabolic signaling and Akt1 regulating growth and survival. Here we show distinct roles for Akt1 and Akt2 in mouse embryo fibroblast cell migration and regulation of the cytoskeleton. Akt1-deficient cells responded poorly to platelet-derived growth factor while Akt2-deficient cells had a dramatically enhanced response, resulting in a substantial increase in dorsal ruffling. Swapping domains between Akt1 and Akt2 demonstrated that the N-terminal region containing the pleckstrin homology domain and a linker region distinguishes the two isoforms, while the catalytic domains are interchangeable. Akt2 knock-out cells also migrated faster than wild-type cells, especially through extracellular matrix (**ECM**), while Akt1 knock-out cells migrated more slowly than wild-type cells. Consistently, Akt2 knock-out cells had elevated Pak1 and Rac activities, suggesting that Akt2 inhibits Rac and Pak1. Both Akt2 and Akt1 associated in complexes with Pak1, but only Akt2 inhibited Pak1 in kinase assays, suggesting an underlying molecular basis for the different cellular phenotypes. Together these data provide evidence for an unexpected functional link between Akt2 and Pak1 that opposes the actions of Akt1 on cell migration.

### [Ee]xtracellular [Mm]atrix

#### PubMed

1. Opposing roles for Akt1 and Akt2 in Rac/Pak signaling and cell migration. The Akt/PKB isoforms have different roles in animals, with Akt2 primarily regulating metabolic signaling and Akt1 regulating growth and survival. Here we show distinct roles for Akt1 and Akt2 in mouse embryo fibroblast cell migration and regulation of the cytoskeleton. Akt1-deficient cells responded poorly to platelet-derived growth factor while Akt2-deficient cells had a dramatically enhanced response, resulting in a substantial increase in dorsal ruffling. Swapping domains between Akt1 and Akt2 demonstrated that the N-terminal region containing the pleckstrin homology domain and a linker region distinguishes the two isoforms, while the catalytic domains are interchangeable. Akt2 knock-out cells also migrated faster than wild-type cells, especially through **extracellular matrix** (ECM), while Akt1 knock-out cells migrated more slowly than wild-type cells. Consistently, Akt2 knock-out cells had elevated Pak1 and Rac activities, suggesting that Akt2 inhibits Rac and Pak1. Both Akt2 and Akt1 associated in complexes with Pak1, but only Akt2 inhibited Pak1 in kinase assays, suggesting an underlying molecular basis for the different cellular phenotypes. Together these data provide evidence for an unexpected functional link between Akt2 and Pak1 that opposes the actions of Akt1 on cell migration.
2. 14-3-3beta-Rac1-p21 activated kinase signaling regulates Akt1-mediated cytoskeletal organization, lamellipodia formation and fibronectin matrix assembly. Akt1 belongs to the three-gene Akt family and functions as a serine-threonine kinase regulating phosphorylation of an array of substrates and mediating cellular processes such as cell migration, proliferation, survival, and cell cycle. Our previous studies have established the importance of Akt1 in angiogenesis and absence of Akt1 resulted in impaired integrin activation, adhesion, migration, and **extracellular matrix** assembly by endothelial cells and fibroblasts. In this study, we identify the downstream signaling pathways activated by Akt1 in the regulation of these cellular events. We demonstrate here that Akt1 is necessary for the growth factor stimulated activation of 14-3-3beta-Rac1-p21 activated kinase (Pak) pathway in endothelial cells and fibroblasts. While activation of Akt1 resulted in translocation of Rac1 to membrane ruffles, enhanced Rac1 activity, Pak1 phosphorylation, and lamellipodia formation, resulting in enhanced adhesion and assembly of fibronectin, inhibition of Akt1 resulted in inhibition of these processes due to impaired Rac1-Pak signaling. Formation of lamellipodia, adhesion, and fibronectin assembly by myristoylated Akt1 expression in NIH 3T3 fibroblasts was inhibited by co-expression with either dominant negative Rac1 or dominant negative Pak1. In contrast, impaired lamellipodia formation, adhesion, and fibronectin assembly by dominant negative-Akt1 expression was rescued by co-expression with either constitutively active-Rac1 or -Pak1. Moreover, previously reported defects in adhesion and **extracellular matrix** assembly by Akt1(-/-) fibroblasts could be rescued by expression with either active-Rac1 or -Pak1, implying the importance of Rac1-Pak signaling in growth factor stimulated cytoskeletal assembly, lamellipodia formation and cell migration in endothelial cells and fibroblasts downstream of Akt1 activation.
3. Integrin linked kinase (ILK) is required for lens epithelial cell survival, proliferation and differentiation. While the role of growth factors in lens development has been investigated extensively, the role of **extracellular matrix** signalling is less well understood. The developing lens expresses predominantly laminin-binding integrins (such as α3β1, α6β1), which are cooperatively required in the lens epithelium during development. We investigated the role of ILK, a downstream mediator of integrin signalling in mice conditionally null for Ilk. Mutant lenses showed epithelial thinning at E17.5 with reduced proliferation and epithelial cell number and aberrant fibre differentiation. There was complete loss of the central epithelium from postnatal day (P) 2 due to cell death followed by fibre cell degeneration and death by P10 as well as rupture of the lens capsule between P10 and P21. At E17.5 there was significant inhibition (∼50%) of epithelial cell cycle progression, as shown by BrdU incorporation, cyclin D1/D2 and phospho-histone H3 immunostaining. The epithelial marker, E-cadherin, was decreased progressively from E17.5 to P2, in the central epithelium, but there was no significant change in Pax6 expression. Analyses of ERK and Akt phosphorylation indicated marked depression of MAPK and PI3K-Akt signalling, which correlated with decreased phosphorylation of FRS2α and Shp2, indicating altered activation of FGF receptors. At later postnatal stages there was reduced or delayed expression of fibre cell markers (β-crystallin and p57(kip2)). Loss of Ilk also affected deposi**tion of extracellula**r matrix, with marked retention of collagen IV within differentiating fibre cells. By quantitative RT-PCR array there was significantly decreased expression of 19 genes associated with focal adhesions, actin filament stability and MAPK and PI3K/Akt signalling. Overall, these data indicate that ILK is required for complete activation of signalling cascades downstream of the FGF receptor in lens epithelium and fibre cells during development and thus is involved in epithelial proliferation, survival and subsequent fibre differentiation.
4. 14-3-3beta-Rac1-p21 activated kinase signaling regulates Akt1-mediated cytoskeletal organization, lamellipodia formation and fibronectin matrix assembly. Akt1 belongs to the three-gene Akt family and functions as a serine-threonine kinase regulating phosphorylation of an array of substrates and mediating cellular processes such as cell migration, proliferation, survival, and cell cycle. Our previous studies have established the importance of Akt1 in angiogenesis and absence of Akt1 resulted in impaired integrin activation, adhesion, migration, and **extracellular matrix** assembly by endothelial cells and fibroblasts. In this study, we identify the downstream signaling pathways activated by Akt1 in the regulation of these cellular events. We demonstrate here that Akt1 is necessary for the growth factor stimulated activation of 14-3-3beta-Rac1-p21 activated kinase (Pak) pathway in endothelial cells and fibroblasts. While activation of Akt1 resulted in translocation of Rac1 to membrane ruffles, enhanced Rac1 activity, Pak1 phosphorylation, and lamellipodia formation, resulting in enhanced adhesion and assembly of fibronectin, inhibition of Akt1 resulted in inhibition of these processes due to impaired Rac1-Pak signaling. Formation of lamellipodia, adhesion, and fibronectin assembly by myristoylated Akt1 expression in NIH 3T3 fibroblasts was inhibited by co-expression with either dominant negative Rac1 or dominant negative Pak1. In contrast, impaired lamellipodia formation, adhesion, and fibronectin assembly by dominant negative-Akt1 expression was rescued by co-expression with either constitutively active-Rac1 or -Pak1. Moreover, previously reported defects in adhesion and **extracellular matrix** assembly by Akt1(-/-) fibroblasts could be rescued by expression with either active-Rac1 or -Pak1, implying the importance of Rac1-Pak signaling in growth factor stimulated cytoskeletal assembly, lamellipodia formation and cell migration in endothelial cells and fibroblasts downstream of Akt1 activation.
5. Opposing roles for Akt1 and Akt2 in Rac/Pak signaling and cell migration. The Akt/PKB isoforms have different roles in animals, with Akt2 primarily regulating metabolic signaling and Akt1 regulating growth and survival. Here we show distinct roles for Akt1 and Akt2 in mouse embryo fibroblast cell migration and regulation of the cytoskeleton. Akt1-deficient cells responded poorly to platelet-derived growth factor while Akt2-deficient cells had a dramatically enhanced response, resulting in a substantial increase in dorsal ruffling. Swapping domains between Akt1 and Akt2 demonstrated that the N-terminal region containing the pleckstrin homology domain and a linker region distinguishes the two isoforms, while the catalytic domains are interchangeable. Akt2 knock-out cells also migrated faster than wild-type cells, especially through **extracellular matrix** (ECM), while Akt1 knock-out cells migrated more slowly than wild-type cells. Consistently, Akt2 knock-out cells had elevated Pak1 and Rac activities, suggesting that Akt2 inhibits Rac and Pak1. Both Akt2 and Akt1 associated in complexes with Pak1, but only Akt2 inhibited Pak1 in kinase assays, suggesting an underlying molecular basis for the different cellular phenotypes. Together these data provide evidence for an unexpected functional link between Akt2 and Pak1 that opposes the actions of Akt1 on cell migration.

### [Ii]nvasive

#### PubMed

1. PAK1 hyperactivation is sufficient for mammary gland tumor formation. Emerging data suggest that p21-activated kinase 1 (Pak1), a downstream signaling molecule of the small GTPases, growth factors, and lipid signaling, is upregulated or hyperactivated in human breast cancer. Until now, however, no direct causative role had been found for Pak1 in mammary tumor formation. We therefore sought to identify the role that Pak1 plays in mammary gland tumorigenesis. Our results showed that in a transgenic mouse model, overexpression of catalytically active Pak1 leads to the development of malignant mammary tumors and to a variety of other breast lesions, including focal solid nodules, ductal hyperplasia, and mini-intraductal neoplasm and adenoma. We also found that Pak1 hyperactivation increases the stimulation of downstream proliferative signaling effectors MEK1/2 and p38-MAPK in mammary tumor epithelial cells. Moreover, in our study, we detected expression of estrogen receptor-alpha expression and progesterone receptor expression during early stages of the lesions, but their expression was lost during the cells' transition to malignant **invasive** tumors. Finally, we found that consistent with a role in breast tumor progression, Pak1 expression and its nuclear accumulation was increased progressively during the transition from ductal hyperplasia to ductal carcinoma in situ to adenocarcinoma in widely used multistep polyoma-middle T-antigen transgenic mice. Together, these findings provide the first direct evidence that Pak1 deregulation may be sufficient for the formation of mammary gland tumors.

### [Mm]etastasis

#### PubMed

1. The Retinoblastoma Tumor Suppressor Transcriptionally Represses Pak1 in Osteoblasts. We previously characterized the retinoblastoma tumor suppressor protein (Rb) as a regulator of adherens junction assembly and cell-to-cell adhesion in osteoblasts. This is a novel function since Rb is predominantly known as a cell cycle repressor. Herein, we characterized the molecular mechanisms by which Rb performs this function, hypothesizing that Rb controls the activity of known regulators of adherens junction assembly. We found that Rb represses the expression of the p21-activated protein kinase (Pak1), an effector of the small Rho GTPase Rac1. Rac1 is a well-known regulator of adherens junction assembly whose increased activity in cancer is linked to perturbations of intercellular adhesion. Using nuclear run-on and luciferase reporter transcription assays, we found that Pak1 repression by Rb is transcriptional, without affecting Pak1 mRNA and protein stability. Pak1 promoter bioinformatics showed multiple E2F1 binding sites within 155 base pairs of the transcriptional start site, and a Pak1-promoter region containing these E2F sites is susceptible to transcriptional inhibition by Rb. Chromatin immunoprecipitations showed that an Rb-E2F complex binds to the region of the Pak1 promoter containing the E2F1 binding sites, suggesting that Pak1 is an E2F target and that the repressive effect of Rb on Pak1 involves blocking the trans-activating capacity of E2F. A bioinformatics analysis showed elevated Pak1 expression in several solid tumors relative to adjacent normal tissue, with both Pak1 and E2F increased relative to normal tissue in breast cancer, supporting a cancer etiology for Pak1 up-regulation. Therefore, we propose that by repressing Pak1 expression, Rb prevents Rac1 hyperactivity usually associated with cancer and related to cytoskeletal derangements that disrupt cell adhesion, consequently enhancing cancer cell migratory capacity. This de-regulation of cell adhesion due to Rb loss could be part of the molecular events associated with cancer progression and **metastasis**.
2. PAK1 mediates pancreatic cancer cell migration and resistance to MET inhibition. Pancreatic adenocarcinoma (PDAC) is a major unmet medical need and a deeper understanding of molecular drivers is needed to advance therapeutic options for patients. We report here that p21-activated kinase 1 (PAK1) is a central node in PDAC cells downstream of multiple growth factor signalling pathways, including hepatocyte growth factor (HGF) and MET receptor tyrosine kinase. PAK1 inhibition blocks signalling to cytoskeletal effectors and tumour cell motility driven by HGF/MET. MET antagonists, such as onartuzumab and crizotinib, are currently in clinical development. Given that even highly effective therapies have resistance mechanisms, we show that combination with PAK1 inhibition overcomes potential resistance mechanisms mediated either by activation of parallel growth factor pathways or by direct amplification of PAK1. Inhibition of PAK1 attenuated in vivo tumour growth and **metastasis** in a model of pancreatic adenocarcinoma. In human tissues, PAK1 is highly expressed in a proportion of PDACs (33% IHC score 2 or 3; n = 304) and its expression is significantly associated with MET positivity (p < 0.0001) and linked to a widespread metastatic pattern in patients (p = 0.067). Taken together, our results provide evidence for a functional role of MET/PAK1 signalling in pancreatic adenocarcinoma and support further characterization of therapeutic inhibitors in this indication.
3. LKB1 suppresses p21-activated kinase-1 (PAK1) by phosphorylation of Thr109 in the p21-binding domain. The serine/threonine protein kinase LKB1 is a tumor suppressor gene mutated in Peutz-Jeghers syndrome patients. The mutations are found also in several types of sporadic cancer. Although LKB1 is implicated in suppression of cell growth and **metastasis**, the detailed mechanisms have not yet been elucidated. In this study, we investigated the effect of LKB1 on cell motility, whose acquisition occurs in early **metastasis**. The knockdown of LKB1 enhanced cell migration and PAK1 activity in human colon cancer HCT116 cells, whereas forced expression of LKB1 in Lkb1-null mouse embryonic fibroblasts suppressed PAK1 activity and PAK1-mediated cell migration simultaneously. Notably, LKB1 directly phosphorylated PAK1 at Thr(109) in the p21-binding domain in vitro. The phosphomimetic T109E mutant showed significantly lower protein kinase activity than wild-type PAK1, suggesting that the phosphorylation at Thr(109) by LKB1 was responsible for suppression of PAK1. Consistently, the nonphosphorylatable T109A mutant was resistant to suppression by LKB1. Furthermore, we found that PAK1 was activated in the hepatocellular carcinomas and the precancerous liver lesions of Lkb1(+/-) mice. Taken together, these results suggest that PAK1 is a direct downstream target of LKB1 and plays an essential role in LKB1-induced suppression of cell migration.

---

## Slc17a5

- NCBI Gene
- GeneRIF
- Pubmed

| Search Term | Rank | Fields |
| --- | --- | --- |
| [Mm]igration | 6 | PubMed(1) |

  
hide/show details for Slc17a5

### [Mm]igration

#### PubMed

1. The lysosomal sialic acid transporter sialin is required for normal CNS myelination. Salla disease and infantile sialic acid storage disease are autosomal recessive lysosomal storage disorders caused by mutations in the gene encoding sialin, a membrane protein that transports free sialic acid out of the lysosome after it is cleaved from sialoglycoconjugates undergoing degradation. Accumulation of sialic acid in lysosomes defines these disorders, and the clinical phenotype is characterized by neurodevelopmental defects, including severe CNS hypomyelination. In this study, we used a sialin-deficient mouse to address how loss of sialin leads to the defect in myelination. Behavioral analysis of the sialin(-/-) mouse demonstrates poor coordination, seizures, and premature death. Analysis by histology, electron microscopy, and Western blotting reveals a decrease in myelination of the CNS but normal neuronal cytoarchitecture and normal myelination of the PNS. To investigate potential mechanisms underlying CNS hypomyelination, we studied myelination and oligodendrocyte development in optic nerves. We found reduced numbers of myelinated axons in optic nerves from sialin(-/-) mice, but the myelin that was present appeared grossly normal. **Migration** and density of oligodendrocyte precursor cells were normal; however, a marked decrease in the number of postmitotic oligodendrocytes and an associated increase in the number of apoptotic cells during the later stages of myelinogenesis were observed. These findings suggest that a defect in maturation of cells in the oligodendrocyte lineage leads to increased apoptosis and underlies the myelination defect associated with sialin loss.

---

## Acpp

- NCBI Gene
- GeneRIF
- Pubmed

| Search Term | Rank | Fields |
| --- | --- | --- |
| [Ff]ilopodium | 2 | Component(1) |

  
hide/show details for Acpp

### [Ff]ilopodium

#### Component

1. **filopodium**

---

## Serpini2

- NCBI Gene
- GeneRIF
- Pubmed

| Search Term | Rank | Fields |
| --- | --- | --- |
| [Mm]etastasis | 12 | PubMed(1) |

  
hide/show details for Serpini2

### [Mm]etastasis

#### PubMed

1. Update of the human and mouse SERPIN gene superfamily. The serpin family comprises a structurally similar, yet functionally diverse, set of proteins. Named originally for their function as serine proteinase inhibitors, many of its members are not inhibitors but rather chaperones, involved in storage, transport, and other roles. Serpins are found in genomes of all kingdoms, with 36 human protein-coding genes and five pseudogenes. The mouse has 60 Serpin functional genes, many of which are orthologous to human SERPIN genes and some of which have expanded into multiple paralogous genes. Serpins are found in tissues throughout the body; whereas most are extracellular, there is a class of intracellular serpins. Serpins appear to have roles in inflammation, immune function, tumorigenesis, blood clotting, dementia, and cancer **metastasis**. Further characterization of these proteins will likely reveal potential biomarkers and therapeutic targets for disease.

---

## Hspb2

- NCBI Gene
- GeneRIF
- Pubmed

| Search Term | Rank | Fields |
| --- | --- | --- |
| [Mm]igration | 6 | PubMed(2) |
| [Mm]etastasis | 12 | PubMed(1) |

  
hide/show details for Hspb2

### [Mm]igration

#### PubMed

1. Primary cilia regulates the directional **migration** and barrier integrity of endothelial cells through the modulation of hsp27 dependent actin cytoskeletal organization. Cilia are mechanosensing organelles that communicate extracellular signals into intracellular responses. Altered functions of primary cilia play a key role in the development of various diseases including polycystic kidney disease. Here, we show that endothelial cells from the oak ridge polycystic kidney (Tg737(orpk/orpk) ) mouse, with impaired cilia assembly, exhibit a reduction in the actin stress fibers and focal adhesions compared to wild-type (WT). In contrast, endothelial cells from polycystin-1 deficient mice (pkd1(null/null) ), with impaired cilia function, display robust stress fibers, and focal adhesion assembly. We found that the Tg737(orpk/orpk) cells exhibit impaired directional **migration** and endothelial cell monolayer permeability compared to the WT and pkd1(null/null) cells. Finally, we found that the expression of heat shock protein 27 (hsp27) and the phosphorylation of focal adhesion kinase (FAK) are downregulated in the Tg737(orpk/orpk) cells and overexpression of hsp27 restored both FAK phosphorylation and cell **migration**. Taken together, these results demonstrate that disruption of the primary cilia structure or function compromises the endothelium through the suppression of hsp27 dependent actin organization and focal adhesion formation, which may contribute to the vascular dysfunction in ciliopathies.
2. Hsp27 participates in the maintenance of breast cancer stem cells through regulation of epithelial-mesenchymal transition and nuclear factor-κB. Heat shock proteins (HSPs) are normally induced under environmental stress to serve as chaperones for maintenance of correct protein folding but they are often overexpressed in many cancers, including breast cancer. The expression of Hsp27, an ATP-independent small HSP, is associated with cell **migratio**n and drug resistance of breast cancer cells. Breast cancer stem cells (BCSCs) have been identified as a subpopulation of breast cancer cells with markers of CD24-CD44+ or high intracellular aldehyde dehydrogenase activity (ALDH+) and proved to be associated with radiation resistance and metastasis. However, the involvement of Hsp27 in the maintenance of BCSC is largely unknown.Mitogen-activated protein kinase antibody array and Western blot were used to discover the expression of Hsp27 and its phosphorylation in ALDH + BCSCs. To study the involvement of Hsp27 in BCSC biology, siRNA mediated gene silencing and quercetin treatment were used to inhibit Hsp27 expression and the characters of BCSCs, which include ALDH+ population, mammosphere formation and cell **migratio**n, were analyzed simultaneously. The tumorigenicity of breast cancer cells after knockdown of Hsp27 was analyzed by xenograftment assay in NOD/SCID mice. The epithelial-mesenchymal transition (EMT) of breast cancer cells was analyzed by wound-healing assay and Western blot of snail, vimentin and E-cadherin expression. The activation of nuclear factor kappa B (NF-κB) was analyzed by luciferase-based reporter assay and nuclear translocation.Hsp27 and its phosphorylation were increased in ALDH+ BCSCs in comparison with ALDH- non-BCSCs. Knockdown of Hsp27 in breast cancer cells decreased characters of BCSCs, such as ALDH+ population, mammosphere formation and cel**l migrati**on. In addition, the in vivo CSC frequency could be diminished in Hsp27 knockdown breast cancer cells. The inhibitory effects could also be observed in cells treated with quercetin, a plant flavonoid inhibitor of Hsp27, and it could be reversed by overexpression of Hsp27. Knockdown of Hsp27 also suppressed EMT signatures, such as decreasing the expression of snail and vimentin and increasing the expression of E-cadherin. Furthermore, knockdown of Hsp27 decreased the nuclear translocation as well as the activity of NF-κB in ALDH + BCSCs, which resulted from increasing expression of IκBα. Restored activation of NF-κB by knockdown of IκBα could reverse the inhibitory effect of Hsp27 siRNA in suppression of ALDH+ cells.Our data suggest that Hsp27 regulates the EMT process and NF-κB activity to contribute the maintenance of BCSCs. Targeting Hsp27 may be considered as a novel strategy in breast cancer therapy.

### [Mm]etastasis

#### PubMed

1. Hsp27 participates in the maintenance of breast cancer stem cells through regulation of epithelial-mesenchymal transition and nuclear factor-κB. Heat shock proteins (HSPs) are normally induced under environmental stress to serve as chaperones for maintenance of correct protein folding but they are often overexpressed in many cancers, including breast cancer. The expression of Hsp27, an ATP-independent small HSP, is associated with cell migration and drug resistance of breast cancer cells. Breast cancer stem cells (BCSCs) have been identified as a subpopulation of breast cancer cells with markers of CD24-CD44+ or high intracellular aldehyde dehydrogenase activity (ALDH+) and proved to be associated with radiation resistance and **metastasi**s. However, the involvement of Hsp27 in the maintenance of BCSC is largely unknown.Mitogen-activated protein kinase antibody array and Western blot were used to discover the expression of Hsp27 and its phosphorylation in ALDH + BCSCs. To study the involvement of Hsp27 in BCSC biology, siRNA mediated gene silencing and quercetin treatment were used to inhibit Hsp27 expression and the characters of BCSCs, which include ALDH+ population, mammosphere formation and cell migration, were analyzed simultaneously. The tumorigenicity of breast cancer cells after knockdown of Hsp27 was analyzed by xenograftment assay in NOD/SCID mice. The epithelial-mesenchymal transition (EMT) of breast cancer cells was analyzed by wound-healing assay and Western blot of snail, vimentin and E-cadherin expression. The activation of nuclear factor kappa B (NF-κB) was analyzed by luciferase-based reporter assay and nuclear translocation.Hsp27 and its phosphorylation were increased in ALDH+ BCSCs in comparison with ALDH- non-BCSCs. Knockdown of Hsp27 in breast cancer cells decreased characters of BCSCs, such as ALDH+ population, mammosphere formation and cell migration. In addition, the in vivo CSC frequency could be diminished in Hsp27 knockdown breast cancer cells. The inhibitory effects could also be observed in cells treated with quercetin, a plant flavonoid inhibitor of Hsp27, and it could be reversed by overexpression of Hsp27. Knockdown of Hsp27 also suppressed EMT signatures, such as decreasing the expression of snail and vimentin and increasing the expression of E-cadherin. Furthermore, knockdown of Hsp27 decreased the nuclear translocation as well as the activity of NF-κB in ALDH + BCSCs, which resulted from increasing expression of IκBα. Restored activation of NF-κB by knockdown of IκBα could reverse the inhibitory effect of Hsp27 siRNA in suppression of ALDH+ cells.Our data suggest that Hsp27 regulates the EMT process and NF-κB activity to contribute the maintenance of BCSCs. Targeting Hsp27 may be considered as a novel strategy in breast cancer therapy.

---

## Cryab

- NCBI Gene
- GeneRIF
- Pubmed

| Search Term | Rank | Fields |
| --- | --- | --- |
| [Mm]igration | 6 | PubMed(4) |
| ECM | 7 | PubMed(1) |
| [Ee]xtracellular [Mm]atrix | 8 | PubMed(2) |

  
hide/show details for Cryab

### [Mm]igration

#### PubMed

1. The transcriptional repressor RP58 is crucial for cell-division patterning and neuronal survival in the developing cortex. The neocortex and the hippocampus comprise several specific layers containing distinct neurons that originate from progenitors at specific development times, under the control of an adequate cell-division patterning mechanism. Although many molecules are known to regulate this cell-division patterning process, its details are not well understood. Here, we show that, in the developing cerebral cortex, the RP58 transcription repressor protein was expressed both in postmitotic glutamatergic projection neurons and in their progenitor cells, but not in GABAergic interneurons. Targeted deletion of the RP58 gene led to dysplasia of the neocortex and of the hippocampus, reduction of the number of mature cortical neurons, and defects of laminar organization, which reflect abnormal neuronal **migration** within the cortical plate. We demonstrate an impairment of the cell-division patterning during the late embryonic stage and an enhancement of apoptosis of the postmitotic neurons in the RP58-deficient cortex. These results suggest that RP58 controls cell division of progenitor cells and regulates the survival of postmitotic cortical neurons.
2. Pivotal role of augmented αB-crystallin in tumor development induced by deficient TSC1/2 complex. Tuberous sclerosis complex 1 (TSC1) and TSC2 are suppressors of mechanistic target of rapamycin (mTOR). mTOR is the major component of two protein complexes: mTOR complex 1 (mTORC1) and mTORC2. Inactive mutation of either TSC1 or TSC2 unleashes mTOR signaling and consequently causes TSC, a benign tumor syndrome affecting multiple organs. We report here that expression of αB-crystallin was upregulated in Tsc1-/- or Tsc2-/- mouse embryonic fibroblasts, Eker rat uterine leiomyoma-derived Tsc2-deficient ELT3 cells, mutant Tsc2-associated mouse kidney tumors, and human lung lymphangioleiomyomatosis nodules. αB-crystallin was transcriptionally activated by mTOR complex 2 (mTORC2): nuclear factor-kappa B (NFκB) signaling cascade. The augmented αB-crystallin was critical for **the migr**ation, invasion and apoptotic resistance of Tsc2-defective cells. Disruption of αB-crystallin suppressed Tsc2-null cell proliferation and tumorigenesis. Therefore, enhanced αB-crystallin has an essential role in TSC1/2 complex deficiency-mediated tumorigenesis, and inhibition of αB-crystallin may complement the current therapy for TSC.
3. AlphaB-crystallin is involved in oxidative stress protection determined by VEGF in skeletal myoblasts. Recent studies suggest that the effects of VEGF-A, the prototype VEGF ligand, may extend to a variety of cell types other than endothelial cells. The expression of VEGF-A and its main receptors, Flt-1/VEGFR-1 and KDR/Flk-1/VEGFR-2, was indeed detected in several cell types, including cardiac myocytes and regenerating myotubes. In addition to its proangiogenic activity, evidence indicates that VEGF-A can sustain skeletal muscle regeneration by enhancing the survival and **migration** of myogenic cells and by promoting the growth of myogenic fibers. In this study, our aim was to investigate whether VEGF could protect skeletal muscle satellite cells from apoptotic cell death triggered by reactive oxygen species and to identify the main molecular mechanisms. C2C12 mouse myoblasts, cultured in vitro in the presence of exogenous VEGF or stably transfected with a plasmid vector expressing VEGF-A, were subjected to oxidative stress and analyzed for cell growth and survival, induction of apoptosis, and molecular signaling. The results of our study demonstrated that VEGF protects C2C12 myoblasts from apoptosis induced by oxidative or hypoxic-like stress. This protection did not correlate with the modulation of the expression of VEGF receptors, but is clearly linked to the phosphorylation of the KDR/Flk-1 receptor, the activation of NF-kappaB, and/or the overexpression of the antiapoptotic protein alphaB-crystallin.
4. αB-Crystallin Regulates Subretinal Fibrosis by Modulation of Epithelial-Mesenchymal Transition. Subretinal fibrosis is an end stage of neovascular age-related macular degeneration, characterized by fibrous membrane formation after choroidal neovascularization. An initial step of the pathogenesis is an epithelial-mesenchymal transition (EMT) of retinal pigment epithelium cells. αB-crystallin plays multiple roles in age-related macular degeneration, including cytoprotection and angiogenesis. However, the role of αB-crystallin in subretinal EMT and fibrosis is unknown. Herein, we showed attenuation of subretinal fibrosis after regression of laser-induced choroidal neovascularization and a decrease in mesenchymal retinal pigment epithelium cells in αB-crystallin knockout mice compared with wild-type mice. αB-crystallin was prominently expressed in subretinal fibrotic lesions in mice. In vitro, overexpression of αB-crystallin induced EMT, whereas suppression of αB-crystallin induced a mesenchymal-epithelial transition. Transforming growth factor-β2-induced EMT was further enhanced by overexpression of αB-crystallin but was inhibited by suppression of αB-crystallin. Silencing of αB-crystallin inhibited multiple fibrotic processes, including cell prol**iferation**, migration, and fibronectin production. Bone morphogenetic protein 4 up-regulated αB-crystallin, and its EMT induction was inhibited by knockdown of αB-crystallin. Furthermore, inhibition of αB-crystallin enhanced monotetraubiquitination of SMAD4, which can impair its nuclear localization. Overexpression of αB-crystallin enhanced nuclear translocation and accumulation of SMAD4 and SMAD5. Thus, αB-crystallin is an important regulator of EMT, acting as a molecular chaperone for SMAD4 and as its potential therapeutic target for preventing subretinal fibrosis development in neovascular age-related macular degeneration.

### ECM

#### PubMed

1. The small heat-shock protein αB-crystallin is essential for the nuclear localization of Smad4: impact on pulmonary fibrosis. Idiopathic pulmonary fibrosis (IPF) is a devastating disease characterized by the proliferation of myofibroblasts and the accumulation of extracellular matrix **(EC**M) in the lungs. TGF-β1 is the major profibrotic cytokine involved in IPF and is responsible for myofibroblast proliferation and differentiation an**d E**CM synthesis. αB-crystallin is constitutively expressed in the lungs and is inducible by stress, acts as a chaperone and is known to play a role in cell cytoskeleton architecture homeostasis. The role of αB-crystallin in fibrogenesis remains unknown. The principal signalling pathway involved in this process is the Smad-dependent pathway. We demonstrate here that αB-crystallin is strongly expressed in fibrotic lung tissue from IPF patients and in vivo rodent models of pulmonary fibrosis. We also show that αB-crystallin-deficient mice are protected from bleomycin-induced fibrosis. Similar protection from fibrosis was observed in αB-crystallin KO mice after transient adenoviral-mediated over-expression of IL-1β or TGF-β1. We show in vitro in primary epithelial cells and fibroblasts that αB-crystallin increases the nuclear localization of Smad4, thereby enhancing the TGF-β1-Smad pathway and the consequent activation of TGF-β1 downstream genes. αB-crystallin over-expression disrupts Smad4 mono-ubiquitination by interacting with its E3-ubiquitin ligase, TIF1γ, thus limiting its nuclear export. Conversely, in the absence of αB-crystallin, TIF1γ can freely interact with Smad4. Consequently, Smad4 mono-ubiquitination and nuclear export are favoured and thus TGF-β1-Smad4 pro-fibrotic activity is inhibited. This study demonstrates that αB-crystallin may be a key target for the development of specific drugs in the treatment of IPF or other fibrotic diseases.

### [Ee]xtracellular [Mm]atrix

#### PubMed

1. Differential requirement for beta-catenin in epithelial and fiber cells during lens development. Recent studies implicate Wnt/beta-catenin signaling in lens differentiation (Stump, R. J., et al., 2003. A role for Wnt/beta-catenin signaling in lens epithelial differentiation. Dev Biol;259:48-61). Beta-catenin is a component of adherens junctions and functions as a transcriptional activator in canonical Wnt signaling. We investigated the effects of Cre/LoxP-mediated deletion of beta-catenin during lens development using two Cre lines that specifically deleted beta-catenin in whole lens or only in differentiated fibers, from E13.5. We found that beta-catenin was required in lens epithelium and during early fiber differentiation but appeared to be redundant in differentiated fiber cells. Complete loss of beta-catenin resulted in an abnormal and deficient epithelial layer with loss of E-cadherin and Pax6 expression as well as abnormal expression of c-Maf and p57(kip2) but not Prox1. There was also disrupted fiber cell differentiation, characterized by poor cell elongation, decreased beta-crystallin expression, epithelial cell cycle arrest at G(1)-S transition and premature cell cycle exit. Despite cell cycle arrest there was no induction of apoptosis. Mutant fiber cells displayed altered apical-basal polarity as evidenced by altered distribution of the tight junction protein, ZO1, disruption of apical actin filaments and abnormal deposition of **extracellular matrix**, resulting in a deficient lens capsule. Loss of beta-catenin also affected the formation of adhesion junctions as evidenced by dissociation of N-cadherin and F-actin localization in differentiating fiber cells. However, loss of beta-catenin from terminally differentiating fibers had no apparent effects on adhesion junctions between adjacent embryonic fibers. These data indicate that beta-catenin plays distinct functions during lens fiber differentiation and is involved in both Wnt signaling and adhesion-related mechanisms that regulate lens epithelium and early fiber differentiation.
2. The small heat-shock protein αB-crystallin is essential for the nuclear localization of Smad4: impact on pulmonary fibrosis. Idiopathic pulmonary fibrosis (IPF) is a devastating disease characterized by the proliferation of myofibroblasts and the accumulation of **extracellular matri**x (ECM) in the lungs. TGF-β1 is the major profibrotic cytokine involved in IPF and is responsible for myofibroblast proliferation and differentiation and ECM synthesis. αB-crystallin is constitutively expressed in the lungs and is inducible by stress, acts as a chaperone and is known to play a role in cell cytoskeleton architecture homeostasis. The role of αB-crystallin in fibrogenesis remains unknown. The principal signalling pathway involved in this process is the Smad-dependent pathway. We demonstrate here that αB-crystallin is strongly expressed in fibrotic lung tissue from IPF patients and in vivo rodent models of pulmonary fibrosis. We also show that αB-crystallin-deficient mice are protected from bleomycin-induced fibrosis. Similar protection from fibrosis was observed in αB-crystallin KO mice after transient adenoviral-mediated over-expression of IL-1β or TGF-β1. We show in vitro in primary epithelial cells and fibroblasts that αB-crystallin increases the nuclear localization of Smad4, thereby enhancing the TGF-β1-Smad pathway and the consequent activation of TGF-β1 downstream genes. αB-crystallin over-expression disrupts Smad4 mono-ubiquitination by interacting with its E3-ubiquitin ligase, TIF1γ, thus limiting its nuclear export. Conversely, in the absence of αB-crystallin, TIF1γ can freely interact with Smad4. Consequently, Smad4 mono-ubiquitination and nuclear export are favoured and thus TGF-β1-Smad4 pro-fibrotic activity is inhibited. This study demonstrates that αB-crystallin may be a key target for the development of specific drugs in the treatment of IPF or other fibrotic diseases.

---

## Mid1ip1

- NCBI Gene
- GeneRIF
- Pubmed

| Search Term | Rank | Fields |
| --- | --- | --- |
| [Mm]igration | 6 | PubMed(1) |

  
hide/show details for Mid1ip1

### [Mm]igration

#### PubMed

1. Mig12, a novel Opitz syndrome gene product partner, is expressed in the embryonic ventral midline and co-operates with Mid1 to bundle and stabilize microtubules. Opitz G/BBB syndrome is a genetic disorder characterized by developmental midline abnormalities, such as hypertelorism, cleft palate, and hypospadias. The gene responsible for the X-linked form of this disease, MID1, encodes a TRIM/RBCC protein that is anchored to the microtubules. The association of Mid1 with the cytoskeleton is regulated by dynamic phosphorylation, through the interaction with the alpha4 subunit of phosphatase 2A (PP2A). Mid1 acts as an E3 ubiquitin ligase, regulating PP2A degradation on microtubules.In spite of these findings, the biological role exerted by the Opitz syndrome gene product is still unclear and the presence of other potential interacting moieties in the Mid1 structure prompted us to search for additional cellular partners. Through a yeast two-hybrid screening approach, we identified a novel gene, MIG12, whose protein product interacts with Mid1. We confirmed by immunoprecipitation that this interaction occurs in vivo and that it is mediated by the Mid1 coiled-coil domain. We found that Mig12 is mainly expressed in the neuroepithelial midline, urogenital apparatus, and digits during embryonic development. Transiently expressed Mig12 is found diffusely in both nucleus and cytoplasm, although it is enriched in the microtubule-organizing center region. Consistently with this, endogenous Mig12 protein is partially detected in the polymerized tubulin fraction after microtubule stabilization. When co-transfected with Mid1, Mig12 is massively recruited to thick filamentous structures composed of tubulin. These microtubule bundles are resistant to high doses of depolymerizing agents and are composed of acetylated tubulin, thus representing stabilized microtubule arrays.Our findings suggest that Mig12 co-operates with Mid1 to stabilize microtubules. Mid1-Mig12 complexes might be implicated in cellular processes that require microtubule stabilization, such as cell division and **migration**. Impairment in Mig12/Mid1-mediated microtubule dynamic regulation, during the development of embryonic midline, may cause the pathological signs observed in Opitz syndrome patients.

---

## Tmsb4x

- NCBI Gene
- GeneRIF
- Pubmed

| Search Term | Rank | Fields |
| --- | --- | --- |
| [Mm]igration | 6 | Process(2); PubMed(14); GeneRIFs(2) |
| [Ee]xtracellular [Mm]atrix | 8 | PubMed(3) |
| [Ii]nvasive | 10 | PubMed(3) |
| [Mm]etastasis | 12 | PubMed(3) |

  
hide/show details for Tmsb4x

### [Mm]igration

#### Process

1. positive regulation of smooth muscle cell **migration**
2. regulation of cell **migration**

#### PubMed

1. Thymosin beta4 activates integrin-linked kinase and promotes cardiac cell **migration**, survival and cardiac repair. Heart disease is a leading cause of death in newborn children and in adults. Efforts to promote cardiac repair through the use of stem cells hold promise but typically involve isolation and introduction of progenitor cells. Here, we show that the G-actin sequestering peptide thymosin beta4 promotes myocardial and endothelial cell **migration** in the embryonic heart and retains this property in postnatal cardiomyocytes. Survival of embryonic and postnatal cardiomyocytes in culture was also enhanced by thymosin beta4. We found that thymosin beta4 formed a functional complex with PINCH and integrin-linked kinase (ILK), resulting in activation of the survival kinase Akt (also known as protein kinase B). After coronary artery ligation in mice, thymosin beta4 treatment resulted in upregulation of ILK and Akt activity in the heart, enhanced early myocyte survival and improved cardiac function. These findings suggest that thymosin beta4 promotes cardiomyocyte **migration**, survival and repair and the pathway it regulates may be a new therapeutic target in the setting of acute myocardial damage.
2. Potential target genes of EMX2 include Odz/Ten-M and other gene families with implications for cortical patterning. EMX2 and PAX6 are expressed by cortical progenitors and specify area patterning. We used representational difference analysis (RDA) to compare expressed RNAs from wild type and Emx2-/- cortex and identified 41 unique clones. Using secondary screening by in situ hybridization, we selected five genes for further analysis, Cdk4, Cofilin1, Crmp1, ME2, and Odz4, involved in neuronal proliferation, differentiation, **migration**, and axon guidance. Each exhibits differential expression in wild type cortex. Odz4 is one of four members of a vertebrate gene family homologous to the Drosophila pair-rule patterning gene, Odd Oz (Odz), a transmembrane receptor. We show that Odz genes are expressed in complementary patterns in cortex, as well as in nuclei-specific patterns in thalamus that relates to their area-unique cortical expression. In addition, each of the genes analyzed shows different expression patterns in wild type cortex, Emx2, and Pax6 mutant cortex, consistent with potential roles in area patterning. These findings identify potential targets of EMX2 that might account for its function and the defects in Emx2-/- cortex, and suggest that the Odz family of transmembrane proteins influences cortical area patterning downstream to EMX2 and PAX6.
3. Identification of Thymosin β4 as an effector of Hand1-mediated vascular development. The bHLH transcription factor Hand1 (Heart and neural crest-derived transcript-1) has a fundamental role in cardiovascular development; however, the molecular mechanisms have not been elucidated. In this paper we identify Thymosin β4 (Tβ4/Tmsb4x), which encodes an actin monomer-binding protein implicated in ce**ll migrat**ion and angiogenesis, as a direct target of Hand1. We demonstrate that Hand1 binds an upstream regulatory region proximal to the promoter of Tβ4 at consensus Thing1 and E-Box sites and identify both activation and repression of Tβ4 by Hand1, through direct binding within either non-canonical or canonical E-boxes, providing new insight into gene regulation by bHLH transcription factors. Hand1-mediated activation of Tβ4 is essential for yolk sac vasculogenesis and embryonic survival, and administration of synthetic TB4 partially rescues yolk sac capillary plexus formation in Hand1-null embryos. Thus, we identify an in vivo downstream target of Hand1 and reveal impaired yolk sac vasculogenesis as a primary cause of early embryonic lethality following loss of this critical bHLH factor.
4. Identification of Thymosin β4 as an effector of Hand1-mediated vascular development. The bHLH transcription factor Hand1 (Heart and neural crest-derived transcript-1) has a fundamental role in cardiovascular development; however, the molecular mechanisms have not been elucidated. In this paper we identify Thymosin β4 (Tβ4/Tmsb4x), which encodes an actin monomer-binding protein implicated in ce**ll migrat**ion and angiogenesis, as a direct target of Hand1. We demonstrate that Hand1 binds an upstream regulatory region proximal to the promoter of Tβ4 at consensus Thing1 and E-Box sites and identify both activation and repression of Tβ4 by Hand1, through direct binding within either non-canonical or canonical E-boxes, providing new insight into gene regulation by bHLH transcription factors. Hand1-mediated activation of Tβ4 is essential for yolk sac vasculogenesis and embryonic survival, and administration of synthetic TB4 partially rescues yolk sac capillary plexus formation in Hand1-null embryos. Thus, we identify an in vivo downstream target of Hand1 and reveal impaired yolk sac vasculogenesis as a primary cause of early embryonic lethality following loss of this critical bHLH factor.
5. Thymosin beta4 activates integrin-linked kinase and promotes cardiac cell **migration**, survival and cardiac repair. Heart disease is a leading cause of death in newborn children and in adults. Efforts to promote cardiac repair through the use of stem cells hold promise but typically involve isolation and introduction of progenitor cells. Here, we show that the G-actin sequestering peptide thymosin beta4 promotes myocardial and endothelial cell **migration** in the embryonic heart and retains this property in postnatal cardiomyocytes. Survival of embryonic and postnatal cardiomyocytes in culture was also enhanced by thymosin beta4. We found that thymosin beta4 formed a functional complex with PINCH and integrin-linked kinase (ILK), resulting in activation of the survival kinase Akt (also known as protein kinase B). After coronary artery ligation in mice, thymosin beta4 treatment resulted in upregulation of ILK and Akt activity in the heart, enhanced early myocyte survival and improved cardiac function. These findings suggest that thymosin beta4 promotes cardiomyocyte **migration**, survival and repair and the pathway it regulates may be a new therapeutic target in the setting of acute myocardial damage.
6. Role of thymosin beta 4 in hair growth. Although thymosin beta 4 (Tβ4) is known to play a role in hair growth, its mechanism of action is unclear. We examined the levels of key genes in a Tβ4 epidermal-specific over-expressing mouse model and Tβ4 global knockout mouse model to explore how Tβ4 affects hair growth. By depilation and histological examination of the skin, we confirmed the effect of Tβ4 on hair growth, the number of hair shafts and hair follicle (HF) structure. The mRNA and protein expression of several genes involved in hair growth were detected by real-time PCR and western blotting, respectively. Changes in the expression of β-catenin and Lef-1, the two key molecules in the Wnt signaling pathway, were similar to the changes observed in Tβ4 expression. We also found that compared to the control mice, the mRNA and protein expression of MMP-2 and VEGF were increased in the Tβ4 over-expressing mice, while the level of E-cadherin (E-cad) remained the same. Further, in the Tβ4 global knockout mice, the mRNA and protein levels of MMP-2 and VEGF decreased dramatically and the level of E-cad was stable. Based on the above results, we believe that Tβ4 may regulate the levels of VEGF and MMP-2 via the Wnt/β-catenin/Lef-1 signaling pathway to influence the growth of blood vessels around HFs and to act**ivate cel**l migration. Tβ4 may have potential for the treatment of hair growth problems in adults, and its effects should be further confirmed in future studies.
7. C-terminal variable AGES domain of Thymosin β4: the molecule's primary contribution in support of post-ischemic cardiac function and repair. Repairing defective cardiac cells is important towards improving heart function. Due to the frequency and severity of ischemic heart disease, management of patients featuring this type of cardiac failure receives significant interest. Previously we discovered that Thymosin β4 (TB4), a 43 amino-acid secreted actin sequestering peptide, is beneficial for myocardial cell survival and coronary re-growth after infarction in adult mammals. Considering the regenerative potential of full-length TB4 in the heart, and that minimal structural variations alter TB4's influence on actin assembly and cell movement, we investigated how various TB4 domains affect cardiac cell behavior and post-ischemic mammalian heart function. We synthesized 17 domain combinations of full-length TB4 and analyzed their impact on embryonic cardiac cells in vitro, and after cardiac infarction in vivo. We discovered the domains of TB4 affect cardiac cell behavior distinctly. We revealed TB4 specific C-terminal tetrapeptide, AGES, increases embryonic cardiac cel**l migrati**on and myocyte beating in culture, and improves adult mammalian heart function following ischemia. Investigating the molecular background and mechanism we discovered systemic injection of AGES enhances early myocyte survival by activating Akt-mediated signaling mechanisms, increases coronary vessel growth and inhibits inflammation in mice and pigs. Biodistribution analyses revealed cardiomyocytes uptake AGES efficiently in vitro and in vivo projecting a potential independent clinical utilization for the tetrapeptide. Our comprehensive domain investigations also suggest, preservation and/or restoration of cardiomyocyte communication is a target of TB4 and AGES, and critical to improve post-ischemic heart function in pigs. In summary, we identified the C-terminal four amino-acid variable end of TB4 as the essential and responsible domain for the molecule's full benefits in the hypoxic heart. Additionally, we introduced AGES as a novel, systemically applicable drug candidate to aid cardiac infarction in adult mammals.
8. Muscle injury-induced thymosin β4 acts as a chemoattractant for myoblasts. Thymosin β4 (Tβ4) is a major intracellular G-actin-sequestering peptide. There is increasing evidence to support important extracellular functions of Tβ4 related to angiogenesis, wound healing and cardiovascular regeneration. We investigated the expression of 'Tβ4' and 'thymosin β10', a closely related peptide, during skeletal muscle regeneration in mice and chemotactic responses of myoblasts to these peptides. The mRNA levels of 'Tβ4' and 'thymosin β10' were up-regulated in the early stage of regenerating muscle fibres and inflammatory haematopoietic cells in the injured skeletal muscles of mice. We found that both Tβ4 and its sulphoxized form significantly accelerated wound closure and increased the chemotaxis of C2C12 myoblastic cells. Furthermore, we showed that primary myoblasts and myocytes derived from muscle satellite cells of adult mice were chemoattracted to sulphoxized form of Tβ4. These data indicate that muscle injury enhances the local production of Tβ4, thereby pro**moting th**e migration of myoblasts to facilitate skeletal muscle regeneration.
9. Thymosin β4 has tumor suppressive effects and its decreased expression results in poor prognosis and decreased survival in multiple myeloma. Thymosin beta4 (Tbeta4) is a polypeptide involved in cellular proliferation, differentiation, and **migratio**n, over-expressed in several tumor entities. We evaluated its expression and function in 298 newly diagnosed multiple myeloma patients and the murine 5TMM model. Mean Tbeta4 expression was significantly lower in myeloma cells compared to normal plasma cells (P<0.001). The same observation can be made in the 5TMM-mouse model by qRT-PCR and ELISA. Here, Tbeta4 overexpression by lentiviral transduction of 5T33MMvt-cells led to significantly decreased proliferative and migratory capacities and increased sensitivity to apoptosis-induction. Mice injected with Tbeta4 over-expressing myeloma cells showed a longer survival compared to mice injected with controls (88,9 vs. 65,9 days, P<0.05). In 209 MM patients treated with high-dose therapy and autologous stem cell transplantation, expression of Tbeta4 below the median was associated with a significantly shorter event free survival (37.6 vs. 26.2 months, P<0.05). In conclusion, our results indicate a possible tumor suppressive function of Tbeta4.
10. Expression of thymosin β4 in odontoblasts during mouse tooth development. Thymosin β4 (Tβ4) is expressed in developing tissue, where it stimulates cell differentiation a**nd migrat**ion. Further, Tβ4 is expressed during molar development in mice, but the expression and function of Tβ4 in odontoblasts during mammalian tooth development have not yet been reported. Therefore, this study examined the expression and function of Tβ4 in differentiating odontoblasts during tooth development. As observed by immunohistochemistry, Tβ4 was expressed in the oral epithelium and inside cells of the tooth bud on embryonic day 15 (E15). Further, on E17, Tβ4 was expressed strongly in the dental lamina and oral epithelium, but only expressed in part of the cells in the outer and inner dental epithelium. Tβ4 was strongly expressed in the entire cytoplasm of odontoblasts on postnatal day 1 (PN1) and expressed intensively in the apical area of odontoblasts on PN4. Further, expression of Tβ4 was increased gradually in odontoblasts from PN1 to PN21. In an odontoblast cell line, MDPC-23, expression of Tβ4 mRNA and protein was increased strongly on day 4 and gradually decreased from day 14. The gene expression of dentin sialophosphoprotein (DSPP), bone sialoprotein (BSP), osteocalcin (OCN), osteonectin (ON), and collagen type I, related with mineralization, was significantly decreased in si-Tβ4/MDPC-23 during differentiation compared to that in MDPC-23 cells. Taken together, our results suggest that Tβ4 may be involved in oral epithelial cell proliferation at the initial stage of tooth development and regulates the expression and secretion of proteins during odontoblast differentiation.
11. Over-expression of thymosin beta 4 promotes abnormal tooth development and stimulation of hair growth. Thymosin beta 4 has multi-functional roles in cell physiology. It accelerates wound healing, hair growth and angiogenesis, and increases laminin-5 expression in corneal epithelium. Furthermore, thymosin beta 4 stimulates tumor growth and metastasis by induction of cell **migration** and vascular endothelial growth factor-mediated angiogenesis. Using a construct on the skin-specific keratin-5 promoter, we have developed thymosin beta 4 over-expressing transgenic mice to further study its functional roles. Thymosin beta 4 in adult skin and in embryonic stages of the transgenic mouse was analyzed by both Western blot and immunohistochemistry. The over-expression of thymosin beta 4 was observed especially around hair follicles and in the teeth in the transgenic mice. We examined the phenotype of the thymosin beta 4 over-expressing mice. Hair growth was accelerated. In addition, the transgenic mice had abnormally-shaped white teeth and dull incisors. We found that the expression of laminin-5 was up-regulated in the skin of the transgenic mice. We conclude that thymosin beta 4 has an important physiological role in hair growth and in tooth development.
12. Downregulation of thymosin beta4 in neural progenitor grafts promotes spinal cord regeneration. Thymosin beta4 (Tbeta4) is an actin-binding peptide whose expression in developing brain correlates with **migration** and neurite extension of neurons. Here, we studied the effects of the downregulation of Tbeta4 expression on growth and differentiation of murine neural progenitor cells (NPCs), using an antisense lentiviral vector. In differentiation-promoting medium, we found twice the number of neurons derived from the Tbeta4-antisense-transduced NPCs, which showed enhanced neurite outgrowth accompanied by increased expression of the adhesion complex N-cadherin-beta-catenin and increased ERK activation. Importantly, when the Tbeta4-antisense-transduced NPCs were transplanted in vivo into a mouse model of spinal cord injury, they promoted a significantly greater functional recovery. Locomotory recovery correlated with increased expression of the regeneration-promoting cell adhesion molecule L1 by the grafted Tbeta4-antisense-transduced NPCs. This resulted in an increased number of regenerating axons and in sprouting of serotonergic fibers surrounding and contacting the Tbeta4-antisense-transduced NPCs grafted into the lesion site. In conclusion, our data identify a new role for Tbeta4 in neuronal differentiation of NPCs by regulating fate determination and process outgrowth. Moreover, NPCs with reduced Tbeta4 levels generate an L1-enriched environment in the lesioned spinal cord that favors growth and sprouting of spared host axons and enhances the endogenous tissue-repair processes.
13. Thymosin β4 is not required for embryonic viability or vascular development. Rossdeutsch et al describe a requirement for thymosin β4 (Tβ4) in vascular development. Impaired mural ce**ll migrat**ion, differentiation, partial embryonic lethality, and hemorrhaging were observed after analysis of 2 lines of mice, one of which was germline null for Tβ4 and another in which Tβ4 was knocked down by endothelial-specific expression of Tβ4 short hairpin RNA. These data are in direct contrast to our published global and cardiac-specific Tβ4-knockout lines. Thus, the role of Tβ4 needs to be clarified to understand its importance in cardiovascular development.To investigate and clarify the role of Tβ4 in vascular smooth muscle cell development and vessel stability.Examination of Tβ4 global knockouts did not demonstrate embryonic hemorrhaging, altered mural cell development, or lethality. Endothelial-specific knockouts also did not exhibit any embryonic lethality and were viable to adulthood.Analysis of our Tβ4 global and cardiac- and endothelial-specific knockout models demonstrated that Tβ4 is dispensable for embryonic viability and vascular development.
14. Thymosin beta4 mediated PKC activation is essential to initiate the embryonic coronary developmental program and epicardial progenitor cell activation in adult mice in vivo. Hypoxic heart disease is a predominant cause of disability and death worldwide. Since adult mammalian hearts are incapable of regeneration after hypoxia, attempts to modify this deficiency are critical. As demonstrated in zebrafish, recall of the embryonic developmental program may be the key to success. Because thymosin beta4 (TB4) is beneficial for myocardial cell survival and essential for coronary development in embryos, we hypothesized that it reactivates the embryonic developmental program and initiates epicardial progenitor mobilization in adult mammals. We found that TB4 stimulates capillary-like tube formation of adult coronary endothelial cells and increases embryonic endothelial cell **migration** and proliferation in vitro. The increase of blood vessel/epicardial substance (Bves) expressing cells accompanied by elevated VEGF, Flk-1, TGF-beta, Fgfr-2, Fgfr-4, Fgf-17 and beta-Catenin expression and increase of Tbx-18 and Wt-1 positive myocardial progenitors suggested organ-wide recall of the embryonic program in the adult epicardium. TB4 also positively regulated the expression and phosphorylation of myristoylated alanine-rich C-kinase substrate (Marcks), a direct substrate and indicator of protein kinase C (PKC) activity in vitro and in vivo. PKC inhibition significantly reduced TB4 initiated epicardial thickening, capillary growth and the number of myocardial progenitors. Our results demonstrate that TB4 is the first known molecule capable of organ-wide activation of the embryonic coronary developmental program in the adult mammalian heart after systemic administration and that PKC plays a significant role in the process.

#### GeneRIFs

1. Results provide evidence that Tbeta4 regulate the levels of VEGF and MMP-2 via the Wnt/beta-catenin/Lef-1 signaling pathway to influence the growth of blood vessels around hair follicles and to activate cell **migration**.
2. thymosin beta4 promotes cardiomyocyte **migration**, survival and repair

### [Ee]xtracellular [Mm]atrix

#### PubMed

1. Thymosin beta 4 is dispensable for murine cardiac development and function. Thymosin beta 4 (Tβ4) is a 43-amino acid factor encoded by an X-linked gene. Recent studies have suggested that Tβ4 is a key factor in cardiac development, growth, disease, epicardial integrity, and blood vessel formation. Cardiac-specific short hairpin (sh)RNA knockdown of tβ4 has been reported to result in embryonic lethality at E14.5-16.5, with severe cardiac and angiogenic defects. However, this shRNA tβ4-knockdown model did not completely abrogate Tβ4 expression. To completely ablate Tβ4 and to rule out the possibility of off-target effects associated with shRNA gene silencing, further studies of global or cardiac-specific knockouts are critical.We examined the role of Tβ4 in developing and adult heart through global and cardiac specific tβ4-knockout mouse models.Global tβ4-knockout mice were born at mendelian ratios and exhibited normal heart and blood vessel formation. Furthermore, in adult global tβ4-knockout mice, cardiac function, capillary density, expression of key cardiac fetal and angiogenic genes, epicardial marker expres**sion, and extracellu**lar matrix deposition were indistinguishable from that of controls. Tissue-specific tβ4-deficient mice, generated by crossing tβ4-floxed mice to Nkx2.5-Cre and αMHC-Cre, were also found to have no phenotype.We conclude that Tβ4 is dispensable for embryonic viability, heart development, coronary vessel development, and adult myocardial function.
2. **Extracellular matrix** secretion by cardiac fibroblasts: role of microRNA-29b and microRNA-30c. MicroRNAs (miRNAs), in particular miR-29b and miR-30c, have been implicated as important regulators of cardiac fibrosis.To perform a proteomics comparison of miRNA effects on **extracellular matrix** secretion by cardiac fibroblasts.Mouse cardiac fibroblasts were transfected with pre-/anti-miR of miR-29b and miR-30c, and their conditioned medium was analyzed by mass spectrometry. miR-29b targeted a cadre of proteins involved in fibrosis, including multiple collagens, matrix metalloproteinases, and leukemia inhibitory factor, insulin-like growth factor 1, and pentraxin 3, 3 predicted targets of miR-29b. miR-29b also attenuated the cardiac fibroblast response to transforming growth factor-β. In contrast, miR-30c had little effect on **extracellular matri**x production but opposite effects regarding leukemia inhibitory factor and insulin-like growth factor 1. Both miRNAs indirectly affected cardiac myocytes. On transfection with pre-miR-29b, the conditioned medium of cardiac fibroblasts lost its ability to support adhesion of rat ventricular myocytes and led to a significant reduction of cardiac myocyte proteins (α-actinin, cardiac myosin-binding protein C, and cardiac troponin I). Similarly, cardiomyocytes derived from mouse embryonic stem cells atrophied under pre-miR-29 conditioned medium, whereas pre-miR-30c conditioned medium had a prohypertrophic effect. Levels of miR-29a, miR-29c, and miR-30c, but not miR-29b, were significantly reduced in a mouse model of pathological but not physiological hypertrophy. Treatment with antagomiRs to miR-29b induced excess fibrosis after aortic constriction without overt deterioration in cardiac function.Our proteomic analysis revealed novel molecular targets of miRNAs that are linked to a fibrogenic cardiac phenotype. Such comprehensive screening methods are essential to define the concerted actions of miRNAs in cardiovascular disease.
3. Thymosin beta 4 is dispensable for murine cardiac development and function. Thymosin beta 4 (Tβ4) is a 43-amino acid factor encoded by an X-linked gene. Recent studies have suggested that Tβ4 is a key factor in cardiac development, growth, disease, epicardial integrity, and blood vessel formation. Cardiac-specific short hairpin (sh)RNA knockdown of tβ4 has been reported to result in embryonic lethality at E14.5-16.5, with severe cardiac and angiogenic defects. However, this shRNA tβ4-knockdown model did not completely abrogate Tβ4 expression. To completely ablate Tβ4 and to rule out the possibility of off-target effects associated with shRNA gene silencing, further studies of global or cardiac-specific knockouts are critical.We examined the role of Tβ4 in developing and adult heart through global and cardiac specific tβ4-knockout mouse models.Global tβ4-knockout mice were born at mendelian ratios and exhibited normal heart and blood vessel formation. Furthermore, in adult global tβ4-knockout mice, cardiac function, capillary density, expression of key cardiac fetal and angiogenic genes, epicardial marker expres**sion, and extracellu**lar matrix deposition were indistinguishable from that of controls. Tissue-specific tβ4-deficient mice, generated by crossing tβ4-floxed mice to Nkx2.5-Cre and αMHC-Cre, were also found to have no phenotype.We conclude that Tβ4 is dispensable for embryonic viability, heart development, coronary vessel development, and adult myocardial function.

### [Ii]nvasive

#### PubMed

1. Elevation of intracellular cyclic AMP inhibits NF-kappaB-mediated thymosin beta4 expression in melanoma cells. Thymosin beta4 (Tbeta4) is a major actin-sequestering protein that has been implicated in the growth, survival, motility, and metastasis of certain tumors and is considered an indicator for malignant progression. Therefore, identifying compounds that can downregulate Tbeta4 expression is very important for the development of anti-cancer chemotherapies. In this study, we investigated the effects of elevated cAMP on Tbeta4 expression and the metastatic potential of murine B16 melanoma cells. In addition, we also dissected the mechanism underlying cAMP-mediated Tbeta4 suppression. We found that treatment with the cAMP-inducing compounds alpha-MSH (alpha-melanocyte stimulating hormone) and IBMX (3-isobutyl-1-methylxanthine) significantly suppressed Tbeta4 expression and regulated EMT-associated genes through the suppression of NF-kappaB activation in B16F10 cells. Along with decreased Tbeta4 expression, the in vitro **invasive**ness and anchorage-independent growth in a semi-solid agar of these cells were also inhibited. In animal experiments, the metastatic potential of the alpha-MSH- or IBMX-treated B16F10 melanoma cells was decreased compared to untreated control cells. Collectively, our data demonstrate that elevated intracellular cAMP significantly suppresses Tbeta4 expression and reduces MMP-9 activity, which leads to decreased metastatic potential. Moreover, suppression of NF-kappaB activation by alpha-MSH or IBMX is critical for inhibiting Tbeta4 expression.
2. Elevation of intracellular cyclic AMP inhibits NF-kappaB-mediated thymosin beta4 expression in melanoma cells. Thymosin beta4 (Tbeta4) is a major actin-sequestering protein that has been implicated in the growth, survival, motility, and metastasis of certain tumors and is considered an indicator for malignant progression. Therefore, identifying compounds that can downregulate Tbeta4 expression is very important for the development of anti-cancer chemotherapies. In this study, we investigated the effects of elevated cAMP on Tbeta4 expression and the metastatic potential of murine B16 melanoma cells. In addition, we also dissected the mechanism underlying cAMP-mediated Tbeta4 suppression. We found that treatment with the cAMP-inducing compounds alpha-MSH (alpha-melanocyte stimulating hormone) and IBMX (3-isobutyl-1-methylxanthine) significantly suppressed Tbeta4 expression and regulated EMT-associated genes through the suppression of NF-kappaB activation in B16F10 cells. Along with decreased Tbeta4 expression, the in vitro **invasive**ness and anchorage-independent growth in a semi-solid agar of these cells were also inhibited. In animal experiments, the metastatic potential of the alpha-MSH- or IBMX-treated B16F10 melanoma cells was decreased compared to untreated control cells. Collectively, our data demonstrate that elevated intracellular cAMP significantly suppresses Tbeta4 expression and reduces MMP-9 activity, which leads to decreased metastatic potential. Moreover, suppression of NF-kappaB activation by alpha-MSH or IBMX is critical for inhibiting Tbeta4 expression.
3. Up-regulation of thymosin beta 4 gene expression in experimentally-induced uterine adenomyosis in mice. Degradation of the myometrium contributes to the development of adenomyosis and disruption of the actin cytoskeleton occurs in the myometrium adjacent to the **invasive** endometrium. A subtractive cDNA library that contained genes up-regulated in the uterus with adenomyosis induced by pituitary grafting was constructed in mice. Thymosin beta 4 (Tb4), an actin sequestering protein, was contained in the library and was confirmed to be abundant in the adenomyotic uteri. Tb4-immunoreactivity was shown in the myometrium, with a stronger signal being observed in the area closer to the tip of endometrial tissue that is invading the myometrium. Up-regulation of Tb4 gene expression may be responsible for the disintegration of the musculature during the development of adenomyosis.

### [Mm]etastasis

#### PubMed

1. Elevation of intracellular cyclic AMP inhibits NF-kappaB-mediated thymosin beta4 expression in melanoma cells. Thymosin beta4 (Tbeta4) is a major actin-sequestering protein that has been implicated in the growth, survival, motility, and **metastasis** of certain tumors and is considered an indicator for malignant progression. Therefore, identifying compounds that can downregulate Tbeta4 expression is very important for the development of anti-cancer chemotherapies. In this study, we investigated the effects of elevated cAMP on Tbeta4 expression and the metastatic potential of murine B16 melanoma cells. In addition, we also dissected the mechanism underlying cAMP-mediated Tbeta4 suppression. We found that treatment with the cAMP-inducing compounds alpha-MSH (alpha-melanocyte stimulating hormone) and IBMX (3-isobutyl-1-methylxanthine) significantly suppressed Tbeta4 expression and regulated EMT-associated genes through the suppression of NF-kappaB activation in B16F10 cells. Along with decreased Tbeta4 expression, the in vitro invasiveness and anchorage-independent growth in a semi-solid agar of these cells were also inhibited. In animal experiments, the metastatic potential of the alpha-MSH- or IBMX-treated B16F10 melanoma cells was decreased compared to untreated control cells. Collectively, our data demonstrate that elevated intracellular cAMP significantly suppresses Tbeta4 expression and reduces MMP-9 activity, which leads to decreased metastatic potential. Moreover, suppression of NF-kappaB activation by alpha-MSH or IBMX is critical for inhibiting Tbeta4 expression.
2. Elevation of intracellular cyclic AMP inhibits NF-kappaB-mediated thymosin beta4 expression in melanoma cells. Thymosin beta4 (Tbeta4) is a major actin-sequestering protein that has been implicated in the growth, survival, motility, and **metastasis** of certain tumors and is considered an indicator for malignant progression. Therefore, identifying compounds that can downregulate Tbeta4 expression is very important for the development of anti-cancer chemotherapies. In this study, we investigated the effects of elevated cAMP on Tbeta4 expression and the metastatic potential of murine B16 melanoma cells. In addition, we also dissected the mechanism underlying cAMP-mediated Tbeta4 suppression. We found that treatment with the cAMP-inducing compounds alpha-MSH (alpha-melanocyte stimulating hormone) and IBMX (3-isobutyl-1-methylxanthine) significantly suppressed Tbeta4 expression and regulated EMT-associated genes through the suppression of NF-kappaB activation in B16F10 cells. Along with decreased Tbeta4 expression, the in vitro invasiveness and anchorage-independent growth in a semi-solid agar of these cells were also inhibited. In animal experiments, the metastatic potential of the alpha-MSH- or IBMX-treated B16F10 melanoma cells was decreased compared to untreated control cells. Collectively, our data demonstrate that elevated intracellular cAMP significantly suppresses Tbeta4 expression and reduces MMP-9 activity, which leads to decreased metastatic potential. Moreover, suppression of NF-kappaB activation by alpha-MSH or IBMX is critical for inhibiting Tbeta4 expression.
3. Over-expression of thymosin beta 4 promotes abnormal tooth development and stimulation of hair growth. Thymosin beta 4 has multi-functional roles in cell physiology. It accelerates wound healing, hair growth and angiogenesis, and increases laminin-5 expression in corneal epithelium. Furthermore, thymosin beta 4 stimulates tumor growth and **metastasis** by induction of cell migration and vascular endothelial growth factor-mediated angiogenesis. Using a construct on the skin-specific keratin-5 promoter, we have developed thymosin beta 4 over-expressing transgenic mice to further study its functional roles. Thymosin beta 4 in adult skin and in embryonic stages of the transgenic mouse was analyzed by both Western blot and immunohistochemistry. The over-expression of thymosin beta 4 was observed especially around hair follicles and in the teeth in the transgenic mice. We examined the phenotype of the thymosin beta 4 over-expressing mice. Hair growth was accelerated. In addition, the transgenic mice had abnormally-shaped white teeth and dull incisors. We found that the expression of laminin-5 was up-regulated in the skin of the transgenic mice. We conclude that thymosin beta 4 has an important physiological role in hair growth and in tooth development.

---

## Faah

- NCBI Gene
- GeneRIF
- Pubmed

| Search Term | Rank | Fields |
| --- | --- | --- |
| [Mm]igration | 6 | PubMed(4) |

  
hide/show details for Faah

### [Mm]igration

#### PubMed

1. Endocannabinoid signaling controls pyramidal cell specification and long-range axon patterning. Endocannabinoids (eCBs) have recently been identified as axon guidance cues shaping the connectivity of local GABAergic interneurons in the developing cerebrum. However, eCB functions during pyramidal cell specification and establishment of long-range axonal connections are unknown. Here, we show that eCB signaling is operational in subcortical proliferative zones from embryonic day 12 in the mouse telencephalon and controls the proliferation of pyramidal cell progenitors and radial **migration** of immature pyramidal cells. When layer patterning is accomplished, developing pyramidal cells rely on eCB signaling to initiate the elongation and fasciculation of their long-range axons. Accordingly, CB(1) cannabinoid receptor (CB(1)R) null and pyramidal cell-specific conditional mutant (CB(1)R(f/f,NEX-Cre)) mice develop deficits in neuronal progenitor proliferation and axon fasciculation. Likewise, axonal pathfinding becomes impaired after in utero pharmacological blockade of CB(1)Rs. Overall, eCBs are fundamental developmental cues controlling pyramidal cell development during corticogenesis.
2. Endogenous cannabinoid receptor CB1 activation promotes vascular smooth-muscle cell proliferation and neointima formation. Percutaneous transluminal angioplasty is frequently used in patients with severe arterial narrowing due to atherosclerosis. However, it induces severe arterial injury and an inflammatory response leading to restenosis. Here, we studied a potential activation of the endocannabinoid system and the effect of FA amide hydrolase (FAAH) deficiency, the major enzyme responsible for endocannabinoid anandamide degradation, in arterial injury. We performed carotid balloon injury in atherosclerosis-prone apoE knockout (apoE(-/-)) and apoE(-/-)FAAH(-/-) mice. Anandamide levels were systemically elevated in apoE(-/-) mice after balloon injury. ApoE(-/-)FAAH(-/-) mice had significantly higher baseline anandamide levels and enhanced neointima formation compared with apoE(-/-) controls. The latter effect was inhibited by treatment with CB1 antagonist AM281. Similarly, apoE(-/-) mice treated with AM281 had reduced neointimal areas, reduced lesional vascular smooth-muscle cell (SMC) content, and proliferating cell counts. The lesional macrophage content was unchanged. In vitro proliferation rates were significantly reduced in CB1(-/-) SMCs or when treating apoE(-/-) or apoE(-/-)FAAH(-/-) SMCs with AM281. Macrophage in vitro adhesion and **migration** were marginally affected by CB1 deficiency. Reendothelialization was not inhibited by treatment with AM281. In conclusion, endogenous CB1 activation contributes to vascular SMC proliferation and neointima formation in response to arterial injury.
3. The serine hydrolase ABHD6 controls the accumulation and efficacy of 2-AG at cannabinoid receptors. The endocannabinoid 2-arachidonoylglycerol (2-AG) regulates neurotransmission and neuroinflammation by activating CB1 cannabinoid receptors on neurons and CB2 cannabinoid receptors on microglia. Enzymes that hydrolyze 2-AG, such as monoacylglycerol lipase, regulate the accumulation and efficacy of 2-AG at cannabinoid receptors. We found that the recently described serine hydrolase alpha-beta-hydrolase domain 6 (ABHD6) also controls the accumulation and efficacy of 2-AG at cannabinoid receptors. In cells from the BV-2 microglia cell line, ABHD6 knockdown reduced hydrolysis of 2-AG and increased the efficacy with which 2-AG can stimulate CB2-mediated cell **migration**. ABHD6 was expressed by neurons in primary culture and its inhibition led to activity-dependent accumulation of 2-AG. In adult mouse cortex, ABHD6 was located postsynaptically and its selective inhibition allowed the induction of CB1-dependent long-term depression by otherwise subthreshold stimulation. Our results indicate that ABHD6 is a rate-limiting step of 2-AG signaling and is therefore a bona fide member of the endocannabinoid signaling system.
4. Silencing or amplification of endocannabinoid signaling in blastocysts via CB1 compromises trophoblast cell **migration**. Endocannabinoid signaling plays key roles in multiple female reproductive events. Previous studies have shown an interesting phenomenon, that mice with either silenced or elevated endocannabinoid signaling via Cnr1 encoding CB(1) show similar defects in several pregnancy events, including preimplantation embryo development. To unravel the downstream signaling of this phenomenon, microarray studies were performed using RNAs collected from WT, Cnr1(-/-), and Faah(-/-) mouse blastocysts on day 4 of pregnancy. The results indicate that about 100 genes show unidirectional changes under either silenced or elevated anandamide signaling via CB(1). Functional enrichment analysis of the microarray data predicted that multiple biological functions and pathways are affected under aberrant endocannabinoid signaling. Among them, genes enriched in cell **migration** are suppressed in Cnr1(-/-) or Faah(-/-) blastocysts. Cell **migration** assays validated the prediction of functional enrichment analysis that cell mobility and spreading of either Cnr1(-/-) or Faah(-/-) trophoblast stem cells are compromised. Either silenced or elevated endocannabinoid signaling via CB(1) causes similar changes in downstream targets in preimplantation embryos and trophoblast stem cells. This study provides evidence that a tightly regulated endocannabinoid signaling is critical to normal preimplantation embryo development and **migration** of trophoblast stem cells.

---

## B3gat3

- NCBI Gene
- GeneRIF
- Pubmed

| Search Term | Rank | Fields |
| --- | --- | --- |
| [Ee]xtracellular [Mm]atrix | 8 | PubMed(1) |

  
hide/show details for B3gat3

### [Ee]xtracellular [Mm]atrix

#### PubMed

1. Faulty initiation of proteoglycan synthesis causes cardiac and joint defects. Proteoglycans are a major component of **extracellular matrix** and contribute to normal embryonic and postnatal development by ensuring tissue stability and signaling functions. We studied five patients with recessive joint dislocations and congenital heart defects, including bicuspid aortic valve (BAV) and aortic root dilatation. We identified linkage to chromosome 11 and detected a mutation (c.830G>A, p.Arg277Gln) in B3GAT3, the gene coding for glucuronosyltransferase-I (GlcAT-I). The enzyme catalyzes an initial step in the synthesis of glycosaminoglycan side chains of proteoglycans. Patients' cells as well as recombinant mutant protein showed reduced glucuronyltransferase activity. Patient fibroblasts demonstrated decreased levels of dermatan sulfate, chondroitin sulfate, and heparan sulfate proteoglycans, indicating that the defect in linker synthesis affected all three lines of O-glycanated proteoglycans. Further studies demonstrated that GlcAT-I resides in the cis and cis-medial Golgi apparatus and is expressed in the affected tissues, i.e., heart, aorta, and bone. The study shows that reduced GlcAT-I activity impairs skeletal as well as heart development and results in variable combinations of heart malformations, including mitral valve prolapse, ventricular septal defect, and bicuspid aortic valve. The described family constitutes a syndrome characterized by heart defects and joint dislocations resulting from altered initiation of proteoglycan synthesis (Larsen-like syndrome, B3GAT3 type).

---

## B3gat1

- NCBI Gene
- GeneRIF
- Pubmed

| Search Term | Rank | Fields |
| --- | --- | --- |
| [Ff]ilopodium | 2 | PubMed(1) |
| [Mm]igration | 6 | PubMed(2) |
| [Ee]xtracellular [Mm]atrix | 8 | PubMed(1) |

  
hide/show details for B3gat1

### [Ff]ilopodium

#### PubMed

1. HNK-1 (human natural killer-1) glyco-epitope is essential for normal spine morphogenesis in developing hippocampal neurons. The human natural killer-1 (HNK-1) glyco-epitope possesses a unique structural feature, a sulfated glucuronic acid attached to lactosamine on the non-reducing termini of glycans. The expression of HNK-1 is temporally and spatially regulated by glucuronyltransferase (GlcAT-P) in the brain. Our previous report showed that mice lacking GlcAT-P almost completely lost HNK-1 expression in the brain and exhibited reduced long-term potentiation (LTP) at hippocampal CA1 synapses. GlcAT-P-deficient mice also showed impaired hippocampus-dependent spatial learning. Although HNK-1 plays an essential role in synaptic plasticity and memory formation, it remains unclear how HNK-1 regulates these functions. In this study, we showed that loss of the HNK-1 epitope resulted in an increase of **filopodium**-like immature spines and a decrease of mushroom-like mature spines in both the early postnatal mouse hippocampus and cultured hippocampal neurons. However, HNK-1 had no influence on spine density or **filopodium** formation. Immunofluorescence staining revealed that loss of HNK-1 altered the distribution of postsynaptic proteins such as alpha-amino-3-hydroxy-5-methylisoxazolepropionate (AMPA)-type glutamate receptor subunit GluR2 and PSD-95 from spine heads onto dendritic shafts without affecting synapse formation, resulting in an increase of shaft synapses in cultured GlcAT-P-deficient neurons. GluR2, a major HNK-1 carrier glycoprotein in postsynaptic density, has the ability to promote spine morphogenesis. Overexpression of GluR2 promoted spine growth in both wild-type and GlcAT-P-deficient neurons, but the increase in GlcAT-P-deficient neurons was lower than that in wild-type neurons. This is the first evidence that HNK-1 is a key factor for normal dendritic spine maturation and is involved in the distribution of postsynaptic proteins.

### [Mm]igration

#### PubMed

1. HNK-1 epitope-carrying tenascin-C spliced variant regulates the proliferation of mouse embryonic neural stem cells. Neural stem cells (NSCs) possess high proliferative potential and the capacity for self-renewal with retention of multipotency to differentiate into neuronal and glial cells. NSCs are the source for neurogenesis during central nervous system development from fetal and adult stages. Although the human natural killer-1 (HNK-1) carbohydrate epitope is expressed predominantly in the nervous system and involved in intercellular adhesion, cell **migration**, and synaptic plasticity, the expression patterns and functional roles of HNK-1-containing glycoconjugates in NSCs have not been fully recognized. We found that HNK-1 was expressed in embryonic mouse NSCs and that this expression was lost during the process of differentiation. Based on proteomics analysis, it was revealed that the HNK-1 epitopes were almost exclusively displayed on an extracellular matrix protein, tenascin-C (TNC), in the mouse embryonic NSCs. Furthermore, the HNK-1 epitope was found to be present only on the largest isoform of the TNC molecules. In addition, the expression of HNK-1 was dependent on expression of the largest TNC variant but not by enzymes involved in the biosynthesis of HNK-1. By knocking down HNK-1 sulfotransferase or TNC by small interfering RNA, we further demonstrated that HNK-1 on TNC was involved in the proliferation of NSCs via modulation of the expression level of the epidermal growth factor receptor. Our finding provides insights into the function of HNK-1 carbohydrate epitopes in NSCs to maintain stemness during neural development.
2. Tbx1 controls cardiac neural crest cell **migration** during arch artery development by regulating Gbx2 expression in the pharyngeal ectoderm. Elucidating the gene regulatory networks that govern pharyngeal arch artery (PAA) development is an important goal, as such knowledge can help to identify new genes involved in cardiovascular disease. The transcription factor Tbx1 plays a vital role in PAA development and is a major contributor to cardiovascular disease associated with DiGeorge syndrome. In this report, we used various genetic approaches to reveal part of a signalling network by which Tbx1 controls PAA development in mice. We investigated the crucial role played by the homeobox-containing transcription factor Gbx2 downstream of Tbx1. We found that PAA formation requires the pharyngeal surface ectoderm as a key signalling centre from which Gbx2, in response to Tbx1, triggers essential directional cues to the adjacent cardiac neural crest cells (cNCCs) en route to the caudal PAAs. Abrogation of this signal generates cNCC patterning defects leading to PAA abnormalities. Finally, we showed that the Slit/Robo signalling pathway is activated during cNCC **migration** and that components of this pathway are affected in Gbx2 and Tbx1 mutant embryos at the time of PAA development. We propose that the spatiotemporal control of this tightly orchestrated network of genes participates in crucial aspects of PAA development.

### [Ee]xtracellular [Mm]atrix

#### PubMed

1. HNK-1 epitope-carrying tenascin-C spliced variant regulates the proliferation of mouse embryonic neural stem cells. Neural stem cells (NSCs) possess high proliferative potential and the capacity for self-renewal with retention of multipotency to differentiate into neuronal and glial cells. NSCs are the source for neurogenesis during central nervous system development from fetal and adult stages. Although the human natural killer-1 (HNK-1) carbohydrate epitope is expressed predominantly in the nervous system and involved in intercellular adhesion, cell migration, and synaptic plasticity, the expression patterns and functional roles of HNK-1-containing glycoconjugates in NSCs have not been fully recognized. We found that HNK-1 was expressed in embryonic mouse NSCs and that this expression was lost during the process of differentiation. Based on proteomics analysis, it was revealed that the HNK-1 epitopes were almost exclusively displayed on an **extracellular matrix** protein, tenascin-C (TNC), in the mouse embryonic NSCs. Furthermore, the HNK-1 epitope was found to be present only on the largest isoform of the TNC molecules. In addition, the expression of HNK-1 was dependent on expression of the largest TNC variant but not by enzymes involved in the biosynthesis of HNK-1. By knocking down HNK-1 sulfotransferase or TNC by small interfering RNA, we further demonstrated that HNK-1 on TNC was involved in the proliferation of NSCs via modulation of the expression level of the epidermal growth factor receptor. Our finding provides insights into the function of HNK-1 carbohydrate epitopes in NSCs to maintain stemness during neural development.

---

## Lgals9

- NCBI Gene
- GeneRIF
- Pubmed

| Search Term | Rank | Fields |
| --- | --- | --- |
| [Mm]igration | 6 | Process(1); PubMed(2) |
| ECM | 7 | PubMed(1); GeneRIFs(1) |
| [Ee]xtracellular [Mm]atrix | 8 | PubMed(3) |
| [Mm]etastasis | 12 | PubMed(2) |

  
hide/show details for Lgals9

### [Mm]igration

#### Process

1. positive regulation of T cell **migration**

#### PubMed

1. Galectin-9 suppresses tumor metastasis by blocking adhesion to endothelium and extracellular matrices. We previously described an inverse correlation between galectin-9 (Gal-9) expression and metastasis in patients with malignant melanoma and breast cancer. This study verified the ability of Gal-9 to inhibit lung metastasis in experimental mouse models using highly metastatic B16F10 melanoma and Colon26 colon cancer cells. B16F10 cells transfected with a secreted form of Gal-9 lost their metastatic potential. Intravenous Gal-9 administration reduced the number of metastases of both B16F10 and Colon26 cells in the lung, indicating that secreted Gal-9 suppresses metastasis. Analysis of adhesive molecule expression revealed that B16F10 cells highly express CD44, integrin alpha1, alpha 4, alpha V, and beta1, and that Colon26 cells express CD44, integrin alpha2, alpha 5, alpha V, and beta1, suggesting that Gal-9 may inhibit the adhesion of tumor cells to vascular endothelium and the extracellular matrix (ECM) by binding to such adhesive molecules. Indeed, Gal-9 suppressed the binding of hyaluronic acid to CD44 on both B16F10 and Colon26 cells, and also suppressed the binding of vascular cell adhesion molecule-1 to very late antigen-4 on B16F10 cells. Furthermore, Gal-9 inhibited the binding of tumor cells to ECM components, resulting in the suppression of tumor cell **migration**. The present results suggest that Gal-9 suppresses both attachment and invasion of tumor cells by inhibiting the binding of adhesive molecules on tumor cells to ligands on vascular endothelium and ECM.
2. Galectin-9 binding to cell surface protein disulfide isomerase regulates the redox environment to enhance T-cell **migration** and HIV entry. Interaction of cell surface glycoproteins with endogenous lectins on the cell surface regulates formation and maintenance of plasma membrane domains, clusters signaling complexes, and controls the residency time of glycoproteins on the plasma membrane. Galectin-9 is a soluble, secreted lectin that binds to glycoprotein receptors to form galectin-glycoprotein lattices on the cell surface. Whereas galectin-9 binding to specific glycoprotein receptors induces death of CD4 Th1 cells, CD4 Th2 cells are resistant to galectin-9 death due to alternative glycosylation. On Th2 cells, galectin-9 binds cell surface protein disulfide isomerase (PDI), increasing retention of PDI on the cell surface and altering the redox status at the plasma membrane. Cell surface PDI regulates integrin function on platelets and also enhances susceptibility of T cells to infection with HIV. We find that galectin-9 binding to PDI on Th2 cells results in increased cell **migration** through extracellular matrix via β3 integrins, identifying a unique mechanism to regulate T-cell **migratio**n. In addition, galectin-9 binding to PDI on T cells potentiates infection with HIV. We identify a mechanism for regulating cell surface redox status via a galectin-glycoprotein lattice, to regulate distinct T-cell functions.

### ECM

#### PubMed

1. Galectin-9 suppresses tumor metastasis by blocking adhesion to endothelium and extracellular matrices. We previously described an inverse correlation between galectin-9 (Gal-9) expression and metastasis in patients with malignant melanoma and breast cancer. This study verified the ability of Gal-9 to inhibit lung metastasis in experimental mouse models using highly metastatic B16F10 melanoma and Colon26 colon cancer cells. B16F10 cells transfected with a secreted form of Gal-9 lost their metastatic potential. Intravenous Gal-9 administration reduced the number of metastases of both B16F10 and Colon26 cells in the lung, indicating that secreted Gal-9 suppresses metastasis. Analysis of adhesive molecule expression revealed that B16F10 cells highly express CD44, integrin alpha1, alpha 4, alpha V, and beta1, and that Colon26 cells express CD44, integrin alpha2, alpha 5, alpha V, and beta1, suggesting that Gal-9 may inhibit the adhesion of tumor cells to vascular endothelium and the extracellular matrix (**ECM**) by binding to such adhesive molecules. Indeed, Gal-9 suppressed the binding of hyaluronic acid to CD44 on both B16F10 and Colon26 cells, and also suppressed the binding of vascular cell adhesion molecule-1 to very late antigen-4 on B16F10 cells. Furthermore, Gal-9 inhibited the binding of tumor cells to **ECM** components, resulting in the suppression of tumor cell migration. The present results suggest that Gal-9 suppresses both attachment and invasion of tumor cells by inhibiting the binding of adhesive molecules on tumor cells to ligands on vascular endothelium and **ECM**.

#### GeneRIFs

1. Gal-9 suppresses both attachment and invasion of tumor cells by inhibiting the binding of adhesive molecules on tumor cells to ligands on vascular endothelium and **ECM**.

### [Ee]xtracellular [Mm]atrix

#### PubMed

1. Galectin-9 expands unique macrophages exhibiting plasmacytoid dendritic cell-like phenotypes that activate NK cells in tumor-bearing mice. Galectin-9 (Gal-9) inhibits the metastasis of tumor cells by blocking their adhesion to endothelium and the **extracellular matrix**. In this study, we addressed the involvement of Gal-9 in anti-tumor activity. Gal-9 significantly prolonged the survival of B16F10 melanoma-bearing mice. Gal-9 increased the numbers of NK cells, CD8 T cells and macrophages in tumor-bearing mice. Gal-9-mediated anti-tumor activity was not induced in NK cell-, macrophage- and CD8 T cell-depleted mice. NK cells from Gal-9-treated mice, compared to PBS-treated mice, exhibited significantly higher cytolytic activity. Co-culture of naïve NK cells with macrophages from Gal-9-treated mice resulted in enhanced NK activity, although Gal-9 itself did not enhance the NK activity. We also found that Ly-6C(+)CD11b(+)F4/80(+) macrophages with plasmacytoid cell (pDC)-like phenotypes (PDCA-1 and B220) were responsible for the enhanced NK activity. These results provide evidence that Gal-9 promotes NK cell-mediated anti-tumor activity by expanding unique macrophages with a pDC-like phenotype.
2. Galectin-9 suppresses tumor metastasis by blocking adhesion to endothelium and extracellular matrices. We previously described an inverse correlation between galectin-9 (Gal-9) expression and metastasis in patients with malignant melanoma and breast cancer. This study verified the ability of Gal-9 to inhibit lung metastasis in experimental mouse models using highly metastatic B16F10 melanoma and Colon26 colon cancer cells. B16F10 cells transfected with a secreted form of Gal-9 lost their metastatic potential. Intravenous Gal-9 administration reduced the number of metastases of both B16F10 and Colon26 cells in the lung, indicating that secreted Gal-9 suppresses metastasis. Analysis of adhesive molecule expression revealed that B16F10 cells highly express CD44, integrin alpha1, alpha 4, alpha V, and beta1, and that Colon26 cells express CD44, integrin alpha2, alpha 5, alpha V, and beta1, suggesting that Gal-9 may inhibit the adhesion of tumor cells to vascular endothelium and the **extracellular matrix** (ECM) by binding to such adhesive molecules. Indeed, Gal-9 suppressed the binding of hyaluronic acid to CD44 on both B16F10 and Colon26 cells, and also suppressed the binding of vascular cell adhesion molecule-1 to very late antigen-4 on B16F10 cells. Furthermore, Gal-9 inhibited the binding of tumor cells to ECM components, resulting in the suppression of tumor cell migration. The present results suggest that Gal-9 suppresses both attachment and invasion of tumor cells by inhibiting the binding of adhesive molecules on tumor cells to ligands on vascular endothelium and ECM.
3. Galectin-9 binding to cell surface protein disulfide isomerase regulates the redox environment to enhance T-cell migration and HIV entry. Interaction of cell surface glycoproteins with endogenous lectins on the cell surface regulates formation and maintenance of plasma membrane domains, clusters signaling complexes, and controls the residency time of glycoproteins on the plasma membrane. Galectin-9 is a soluble, secreted lectin that binds to glycoprotein receptors to form galectin-glycoprotein lattices on the cell surface. Whereas galectin-9 binding to specific glycoprotein receptors induces death of CD4 Th1 cells, CD4 Th2 cells are resistant to galectin-9 death due to alternative glycosylation. On Th2 cells, galectin-9 binds cell surface protein disulfide isomerase (PDI), increasing retention of PDI on the cell surface and altering the redox status at the plasma membrane. Cell surface PDI regulates integrin function on platelets and also enhances susceptibility of T cells to infection with HIV. We find that galectin-9 binding to PDI on Th2 cells results in increased cell migration through **extracellular matrix** via β3 integrins, identifying a unique mechanism to regulate T-cell migration. In addition, galectin-9 binding to PDI on T cells potentiates infection with HIV. We identify a mechanism for regulating cell surface redox status via a galectin-glycoprotein lattice, to regulate distinct T-cell functions.

### [Mm]etastasis

#### PubMed

1. Galectin-9 expands unique macrophages exhibiting plasmacytoid dendritic cell-like phenotypes that activate NK cells in tumor-bearing mice. Galectin-9 (Gal-9) inhibits the **metastasis** of tumor cells by blocking their adhesion to endothelium and the extracellular matrix. In this study, we addressed the involvement of Gal-9 in anti-tumor activity. Gal-9 significantly prolonged the survival of B16F10 melanoma-bearing mice. Gal-9 increased the numbers of NK cells, CD8 T cells and macrophages in tumor-bearing mice. Gal-9-mediated anti-tumor activity was not induced in NK cell-, macrophage- and CD8 T cell-depleted mice. NK cells from Gal-9-treated mice, compared to PBS-treated mice, exhibited significantly higher cytolytic activity. Co-culture of naïve NK cells with macrophages from Gal-9-treated mice resulted in enhanced NK activity, although Gal-9 itself did not enhance the NK activity. We also found that Ly-6C(+)CD11b(+)F4/80(+) macrophages with plasmacytoid cell (pDC)-like phenotypes (PDCA-1 and B220) were responsible for the enhanced NK activity. These results provide evidence that Gal-9 promotes NK cell-mediated anti-tumor activity by expanding unique macrophages with a pDC-like phenotype.
2. Galectin-9 suppresses tumor **metastasis** by blocking adhesion to endothelium and extracellular matrices. We previously described an inverse correlation between galectin-9 (Gal-9) expression and **metastasis** in patients with malignant melanoma and breast cancer. This study verified the ability of Gal-9 to inhibit lung **metastasis** in experimental mouse models using highly metastatic B16F10 melanoma and Colon26 colon cancer cells. B16F10 cells transfected with a secreted form of Gal-9 lost their metastatic potential. Intravenous Gal-9 administration reduced the number of metastases of both B16F10 and Colon26 cells in the lung, indicating that secreted Gal-9 suppresses **metastasis**. Analysis of adhesive molecule expression revealed that B16F10 cells highly express CD44, integrin alpha1, alpha 4, alpha V, and beta1, and that Colon26 cells express CD44, integrin alpha2, alpha 5, alpha V, and beta1, suggesting that Gal-9 may inhibit the adhesion of tumor cells to vascular endothelium and the extracellular matrix (ECM) by binding to such adhesive molecules. Indeed, Gal-9 suppressed the binding of hyaluronic acid to CD44 on both B16F10 and Colon26 cells, and also suppressed the binding of vascular cell adhesion molecule-1 to very late antigen-4 on B16F10 cells. Furthermore, Gal-9 inhibited the binding of tumor cells to ECM components, resulting in the suppression of tumor cell migration. The present results suggest that Gal-9 suppresses both attachment and invasion of tumor cells by inhibiting the binding of adhesive molecules on tumor cells to ligands on vascular endothelium and ECM.

---

## Unc13d

- NCBI Gene
- GeneRIF
- Pubmed

| Search Term | Rank | Fields |
| --- | --- | --- |
| [Mm]igration | 6 | PubMed(2) |
| [Mm]etastasis | 12 | PubMed(1) |

  
hide/show details for Unc13d

### [Mm]igration

#### PubMed

1. MST1-dependent vesicle trafficking regulates neutrophil trans**migration** through the vascular basement membrane. Neutrophils need to penetrate the perivascular basement membrane for successful extravasation into inflamed tissue, but this process is incompletely understood. Recent findings have associated mammalian sterile 20-like kinase 1 (MST1) loss of function with a human primary immunodeficiency disorder, suggesting that MST1 may be involved in immune cell **migration**. Here, we have shown that MST1 is a critical regulator of neutrophil extravasation during inflammation. Mst1-deficient (Mst1-/-) neutrophils were unable to migrate into inflamed murine cremaster muscle venules, instead persisting between the endothelium and the basement membrane. Mst1-/- neutrophils also failed to extravasate from gastric submucosal vessels in a murine model of Helicobacter pylori infection. Mechanistically, we observed defective translocation of VLA-3, VLA-6, and neutrophil elastase from intracellular vesicles to the surface of Mst1-/- neutrophils, indicating that MST1 is required for this crucial step in neutrophil trans**migration**. Furthermore, we found that MST1 associates with the Rab27 effector protein synaptotagmin-like protein 1 (JFC1, encoded by Sytl1 in mice), but not Munc13-4, thereby regulating the trafficking of Rab27-positive vesicles to the cellular membrane. Together, these findings highlight a role for MST1 in vesicle trafficking and extravasation in neutrophils, providing an additional mechanistic explanation for the severe immune defect observed in patients with MST1 deficiency.
2. Platelet-derived nucleotides promote tumor-cell transendothelial **migration** and metastasis via P2Y2 receptor. Tumor cells can activate platelets, which in turn facilitate tumor cell survival and dissemination. The exact mechanisms by which platelets promote metastasis have remained unclear. Here, we show that adenine nucleotides released from tumor cell-activated platelets induce opening of the endothelial barrier to allow transendothelial **migration** of tumor cells and thereby promote cancer cell extravasation. We identified the endothelial P2Y2 receptor, which is activated by ATP, as the primary mediator of this effect. Mice deficient in P2Y2 or lacking ATP secretion from platelets show strongly reduced tumor cell metastasis. These findings demonstrate a mechanism by which platelets promote cancer cell metastasis and suggest the P2Y2 receptor and its endothelial downstream signaling mechanisms as a target for antimetastatic therapies.

### [Mm]etastasis

#### PubMed

1. Platelet-derived nucleotides promote tumor-cell transendothelial migration and **metastasis** via P2Y2 receptor. Tumor cells can activate platelets, which in turn facilitate tumor cell survival and dissemination. The exact mechanisms by which platelets promote **metastasis** have remained unclear. Here, we show that adenine nucleotides released from tumor cell-activated platelets induce opening of the endothelial barrier to allow transendothelial migration of tumor cells and thereby promote cancer cell extravasation. We identified the endothelial P2Y2 receptor, which is activated by ATP, as the primary mediator of this effect. Mice deficient in P2Y2 or lacking ATP secretion from platelets show strongly reduced tumor cell **metastasis**. These findings demonstrate a mechanism by which platelets promote cancer cell **metastasis** and suggest the P2Y2 receptor and its endothelial downstream signaling mechanisms as a target for antimetastatic therapies.

---

## Fhl2

- NCBI Gene
- GeneRIF
- Pubmed

| Search Term | Rank | Fields |
| --- | --- | --- |
| [Mm]igration | 6 | PubMed(18); GeneRIFs(3) |
| [Ee]xtracellular [Mm]atrix | 8 | PubMed(5) |
| [Ii]nvasive | 10 | PubMed(1) |
| [Mm]etastasis | 12 | PubMed(1) |
| [Cc]ell [Ii]nvasion | 13 | PubMed(1) |

  
hide/show details for Fhl2

### [Mm]igration

#### PubMed

1. FHL2 prevents cardiac hypertrophy in mice with cardiac-specific deletion of ROCK2. The Rho-associated coiled-coil containing kinases, ROCK1 and ROCK2, are important regulators of cell shape, **migration**, and proliferation through effects on the actin cytoskeleton. However, it is not known whether ROCK2 plays an important role in the development of cardiac hypertrophy. To determine whether the loss of ROCK2 could prevent cardiac hypertrophy, cardiomyocyte-specific ROCK2-null (c-ROCK2(-/-)) were generated using conditional ROCK2(flox/flox) mice and α-myosin heavy-chain promoter-driven Cre recombinase transgenic mice. Cardiac hypertrophy was induced by Ang II infusion (400 ng/kg/min, 28 d) or transverse aortic constriction (TAC). Under basal conditions, hemodynamic parameters, cardiac anatomy, and function of c-ROCK2(-/-) mice were comparable to wild-type (WT) mice. However, following Ang II infusion or TAC, c-ROCK2(-/-) mice exhibited a substantially smaller increase in heart-to-body weight ratio, left ventricular mass, myocyte cross-sectional area, hypertrophy-related fetal gene expression, intraventricular fibrosis, cardiac apoptosis, and oxidative stress compared to control mice. Deletion of ROCK2 in cardiomyocytes leads to increased expression of four-and-a-half LIM-only protein-2 (FHL2) and FHL2-mediated inhibition of serum response factor (SRF) and extracellular signal-regulated mitogen-activated protein kinase (ERK). Knockdown of FHL2 expression in ROCK2-deficient cardiomyocytes or placing ROCK2-haploinsufficient (ROCK2(+/-)) mice on FHL2(+/-)-haploinsufficient background restored the hypertrophic response to Ang II. These results indicate that cardiomyocyte ROCK2 is essential for the development of cardiac hypertrophy and that up-regulation of FHL2 may contribute to the antihypertrophic phenotype that is observed in cardiac-specific ROCK2-deficient mice.
2. The LIM-only proteins FHL2 and FHL3 interact with alpha- and beta-subunits of the muscle alpha7beta1 integrin receptor. FHL1, FHL2, and FHL3 are members of the four and one-half LIM domain protein subclass that are expressed in striated muscles. Here we show that FHL2 and FHL3 are novel alpha(7)beta(1) integrin-interacting proteins. They bind both the alpha- and the beta-subunit as well as different splice isoforms. The minimal binding sites for FHL2 and FHL3 on beta(1A)-chain overlap, whereas on alpha(7A) and alpha(7B) subunits they are situated adjacent. Determining the binding sites for integrins on FHL2 or FHL3 revealed that the suprastructure of the whole molecule is important for these associations, rather than any single LIM domain. Immunofluorescence studies with cells expressing full-length FHL proteins or their deletion mutants showed that FHL2 and FHL3 but not FHL1 colocalize with integrins at cell adhesion sites. Further, their recruitment to the membrane results from binding to either the alpha- or the beta-chain of the integrin receptor. The association of FHL2 or FHL3 with integrin receptors neither influences attachment of cells to different substrates nor changes their **migration** capacity. However, in cardiac and skeletal muscles, FHL2 and FHL3, respectively, are colocalized with alpha(7)beta(1) integrin receptor at the periphery of Z-discs, suggesting a role in mechanical stabilization of muscle cells.
3. Deficiency of the LIM-only protein FHL2 reduces intestinal tumorigenesis in Apc mutant mice. The four and a half LIM-only protein 2 (FHL2) is capable of shuttling between focal adhesion and nucleus where it signals through direct interaction with a number of proteins including beta-catenin. Although FHL2 activation has been found in various human cancers, evidence of its functional contribution to carcinogenesis has been lacking.Here we have investigated the role of FHL2 in intestinal tumorigenesis in which activation of the Wnt pathway by mutations in the adenomatous polyposis coli gene (Apc) or in beta-catenin constitutes the primary transforming event. In this murine model, introduction of a biallelic deletion of FHL2 into mutant Apc(Delta14/+) mice substantially reduces the number of intestinal adenomas but not tumor growth, suggesting a role of FHL2 in the initial steps of tumorigenesis. In the lesions, Wnt signalling is not affected by FHL2 deficiency, remaining constitutively active. Nevertheless, loss of FHL2 activity is associated with increased epithelial cell **migration** in intestinal epithelium, which might allow to eliminate more efficiently deleterious cells and reduce the risk of tumorigenesis. This finding may provide a mechanistic basis for tumor suppression by FHL2 deficiency. In human colorectal carcinoma but not in low-grade dysplasia, we detected up-regulation and enhanced nuclear localization of FHL2, indicating the activation of FHL2 during the development of malignancy.Our data demonstrate that FHL2 represents a critical factor in intestinal tumorigenesis.
4. FHL2 prevents cardiac hypertrophy in mice with cardiac-specific deletion of ROCK2. The Rho-associated coiled-coil containing kinases, ROCK1 and ROCK2, are important regulators of cell shape, **migration**, and proliferation through effects on the actin cytoskeleton. However, it is not known whether ROCK2 plays an important role in the development of cardiac hypertrophy. To determine whether the loss of ROCK2 could prevent cardiac hypertrophy, cardiomyocyte-specific ROCK2-null (c-ROCK2(-/-)) were generated using conditional ROCK2(flox/flox) mice and α-myosin heavy-chain promoter-driven Cre recombinase transgenic mice. Cardiac hypertrophy was induced by Ang II infusion (400 ng/kg/min, 28 d) or transverse aortic constriction (TAC). Under basal conditions, hemodynamic parameters, cardiac anatomy, and function of c-ROCK2(-/-) mice were comparable to wild-type (WT) mice. However, following Ang II infusion or TAC, c-ROCK2(-/-) mice exhibited a substantially smaller increase in heart-to-body weight ratio, left ventricular mass, myocyte cross-sectional area, hypertrophy-related fetal gene expression, intraventricular fibrosis, cardiac apoptosis, and oxidative stress compared to control mice. Deletion of ROCK2 in cardiomyocytes leads to increased expression of four-and-a-half LIM-only protein-2 (FHL2) and FHL2-mediated inhibition of serum response factor (SRF) and extracellular signal-regulated mitogen-activated protein kinase (ERK). Knockdown of FHL2 expression in ROCK2-deficient cardiomyocytes or placing ROCK2-haploinsufficient (ROCK2(+/-)) mice on FHL2(+/-)-haploinsufficient background restored the hypertrophic response to Ang II. These results indicate that cardiomyocyte ROCK2 is essential for the development of cardiac hypertrophy and that up-regulation of FHL2 may contribute to the antihypertrophic phenotype that is observed in cardiac-specific ROCK2-deficient mice.
5. Deficiency of the LIM-only protein FHL2 reduces intestinal tumorigenesis in Apc mutant mice. The four and a half LIM-only protein 2 (FHL2) is capable of shuttling between focal adhesion and nucleus where it signals through direct interaction with a number of proteins including beta-catenin. Although FHL2 activation has been found in various human cancers, evidence of its functional contribution to carcinogenesis has been lacking.Here we have investigated the role of FHL2 in intestinal tumorigenesis in which activation of the Wnt pathway by mutations in the adenomatous polyposis coli gene (Apc) or in beta-catenin constitutes the primary transforming event. In this murine model, introduction of a biallelic deletion of FHL2 into mutant Apc(Delta14/+) mice substantially reduces the number of intestinal adenomas but not tumor growth, suggesting a role of FHL2 in the initial steps of tumorigenesis. In the lesions, Wnt signalling is not affected by FHL2 deficiency, remaining constitutively active. Nevertheless, loss of FHL2 activity is associated with increased epithelial cell **migration** in intestinal epithelium, which might allow to eliminate more efficiently deleterious cells and reduce the risk of tumorigenesis. This finding may provide a mechanistic basis for tumor suppression by FHL2 deficiency. In human colorectal carcinoma but not in low-grade dysplasia, we detected up-regulation and enhanced nuclear localization of FHL2, indicating the activation of FHL2 during the development of malignancy.Our data demonstrate that FHL2 represents a critical factor in intestinal tumorigenesis.
6. The LIM-only proteins FHL2 and FHL3 interact with alpha- and beta-subunits of the muscle alpha7beta1 integrin receptor. FHL1, FHL2, and FHL3 are members of the four and one-half LIM domain protein subclass that are expressed in striated muscles. Here we show that FHL2 and FHL3 are novel alpha(7)beta(1) integrin-interacting proteins. They bind both the alpha- and the beta-subunit as well as different splice isoforms. The minimal binding sites for FHL2 and FHL3 on beta(1A)-chain overlap, whereas on alpha(7A) and alpha(7B) subunits they are situated adjacent. Determining the binding sites for integrins on FHL2 or FHL3 revealed that the suprastructure of the whole molecule is important for these associations, rather than any single LIM domain. Immunofluorescence studies with cells expressing full-length FHL proteins or their deletion mutants showed that FHL2 and FHL3 but not FHL1 colocalize with integrins at cell adhesion sites. Further, their recruitment to the membrane results from binding to either the alpha- or the beta-chain of the integrin receptor. The association of FHL2 or FHL3 with integrin receptors neither influences attachment of cells to different substrates nor changes their **migration** capacity. However, in cardiac and skeletal muscles, FHL2 and FHL3, respectively, are colocalized with alpha(7)beta(1) integrin receptor at the periphery of Z-discs, suggesting a role in mechanical stabilization of muscle cells.
7. Deficiency in four and one half LIM domain protein 2 (FHL2) aggravates liver fibrosis in mice. Four and one half LIM domain protein 2 (FHL2) has been reported to be a key regulator in many cellular processes being associated with fibrogenesis such as cell **migration** and contraction. Moreover, hepatic FHL2 is involved in regulation pathways mediating proliferation and cell death machineries. We here investigated the role of FHL2 in the setting of experimental and clinical liver fibrosis.FHL2(-/-) and wild type (wt) mice were challenged with CCl(4). Fibrotic response was assessed by quantitative real time PCR (qRT-PCR) of fibrotic marker genes, measurement of hydroxyproline content and histological methods. Murine FHL2(-/-) and hepatic stellate cells (HSC) were isolated and investigated via immunofluorescence. Human fibrotic and normal liver samples were analysed immunohistochemically using antibodies directed against FHL2.FHL2(-/-) mice displayed aggravated liver fibrosis compared to wt mice. However, immunofluorescence revealed no significant morphological changes in cultured FHL2(-/-) and wt myofibroblasts (MFB). In human liver samples, FHL2 was strongly expressed both in the nucleus and cytoplasm in MFB of fibrotic livers. In contrast, FHL2 expression was absent in normal liver tissue.Deficiency of FHL2 results in aggravation of murine liver fibrosis. In human liver samples, FHL2 is expressed in activated HSCs and portal fibroblasts in human fibrotic livers, pointing to a central role of FHL2 for human hepatic fibrogenesis as well.
8. Deletion of FHL2 gene impaired ischemia-induced blood flow recovery by modulating circulating proangiogenic cells. The four and a half Lin11, Isl-1 and Mec-3 (LIM) domain protein 2 (FHL2) is a member of the four and a half LIM domain-only (FHL) gene family, and has been shown to play an important role in inhibiting inflammatory angiogenesis. Here, we tested the hypothesis that impaired ischemia-induced neovascularization in mice lacking FHL2 is related to a defect in proangiogenic cell mobilization and functions in vasculogenesis.Unilateral hindlimb ischemia surgery was conducted in FHL2(-/-) mice and wild-type (FHL2(+/+)) mice. After hindlimb ischemia surgery, expression of FHL2 protein was noted in ischemic tissues of wild-type mice. All FHL2-null mice (100%) suffered from spontaneous foot amputation, but only 20% of wild-type mice had ischemia-induced foot amputation after ischemic surgery. Blood flow recovery was significantly impaired in FHL2(-/-) mice when compared with that in wild-type mice as determined by laser Doppler imaging. Histological analysis revealed that the capillary density in the ischemic limb was increased in wild-type mice, whereas no such increase was noted in FHL2(-/-) mice. Flow cytometry demonstrated that the number of CD34(+) or CD34(+)/Sca-1(+)/Flk-1(+) in peripheral blood after ischemic surgery significantly decreased in FHL2-null mice than those in wild-type mice after hindlimb ischemia surgery. FHL2 deficiency impaired ex vivo angiogenesis in mouse aortic-ring culture assay, which revealed that the mean density of the microvessels was significantly higher in the wild-type aorta than in the FHL2(-/-) aorta. Western blot analysis showed that vascular endothelial growth factor (VEGF), interleukin-6, matrix metalloproteinase-2, matrix metalloproteinase-9, vascular cell adhesion molecule-1, and intercellular adhesion molecule-1 levels were significantly downregulated in ischemic muscles in FHL2-null mice compared with wild-type mice. Deletion of FHL2 protein by FHL2 small interfering RNA impaired VEGF production under hypoxia conditions, and also suppressed endothelial progenitor cell angiogenic functions, but these effects could be recovered by administration of VEGF.Deficiency of FHL2 impairs ischemia-induced neovascularization, and these suppressive effects may occur through a reduction in proangiogenic cell mobilization, **migration**, and vasculogenesis functions.
9. The LIM-only protein FHL2 reduces vascular lesion formation involving inhibition of proliferation and **migration** of smooth muscle cells. The LIM-only protein FHL2, also known as DRAL or SLIM3, has a function in fine-tuning multiple physiological processes. FHL2 is expressed in the vessel wall in smooth muscle cells (SMCs) and endothelial cells and conflicting data have been reported on the regulatory function of FHL2 in SMC phenotype transition. At present the function of FHL2 in SMCs in vascular injury is unknown. Therefore, we studied the role of FHL2 in SMC-rich lesion formation. In response to carotid artery ligation FHL2-deficient (FHL2-KO) mice showed accelerated lesion formation with enhanced Ki67 expression compared with wild-type (WT)-mice. Consistent with these findings, cultured SMCs from FHL2-KO mice showed increased proliferation through enhanced phosphorylation of extracellular-regulated kinase-1/2 (ERK1/2) and induction of CyclinD1 expression. Overexpression of FHL2 in SMCs inhibited CyclinD1 expression and CyclinD1-knockdown blocked the enhanced proliferation of FHL2-KO SMCs. We also observed increased CyclinD1 promoter activity in FHL2-KO SMCs, which was reduced upon ERK1/2 inhibition. Furthermore, FHL2-KO SMCs showed enhanced **migration** compared with WT SMCs. In conclusion, FHL2 deficiency in mice results in exacerbated SMC-rich lesion formation involving increased proliferation and **migration** of SMCs via enhanced activation of the ERK1/2-CyclinD1 signaling pathway.
10. Impaired intestinal wound healing in Fhl2-deficient mice is due to disturbed collagen metabolism. Four and one half LIM domain protein FHL2 participates in many cellular processes involved in tissue repair such as regulation of gene expression, cytoarchitecture, cell adhesion, **migration** and signal transduction. The repair process after wounding is initiated by the release of peptides and bioactive lipids. These molecules induce synthesis and deposition of a provisional extracellular matrix. We showed previously that sphingosine-1-phosphate (S1P) triggers a signal transduction cascade mediating nuclear translocation of FHL2 in response to activation of the RhoA GTPase. Our present study shows that FHL2 is an important signal transducer influencing the outcome of intestinal anastomotic healing. Early wound healing is accompanied by reconstitution and remodelling of the extracellular matrix and collagen is primarily responsible for wound strength. Our results show that impaired intestinal wound healing in Fhl2-deficient mice is due to disturbed collagen III metabolism. Impaired collagen III synthesis reduced the mechanical stability of the anastomoses and led to lower bursting pressure in Fhl2-deficient mice after surgery. Our data confirm that FHL2 is an important factor regulating collagen expression in the early phase of wound healing, and thereby is critically involved in the physiologic process of anastomosis healing after bowel surgery and thus may represent a new therapeutic target.
11. The homeobox gene Lbx1 specifies a subpopulation of cardiac neural crest necessary for normal heart development. Cardiac neural crest cells are known to play multiple roles during development of the inflow and outflow tract of the heart and the aortic arch. In addition, cardiac neural crest is required for normal heart tube looping and regulation of myocardial cell proliferation, as well as differentiation and function of the myocardium. We show that the homeobox gene Lbx1 is expressed in a subpopulation of the cardiac neural crest during tubular heart formation. Inactivation of the Lbx1 gene in mice resulted in defects in heart looping, changes in gene expression pattern, and increased cell proliferation ensuing in myocardial hyperplasia. We found that the activity of the Lbx1 promoter, as indicated by a LacZ reporter gene, is upregulated in the hearts of Lbx1(+/-):splotch(1H)/splotch(1H) and Lbx1(-/-) mice, indicating that Pax3 and Lbx1 participate in a negative regulatory feedback that might be necessary for normal differentiation and function of the myocardium during early heart development. Because **migration** of Lbx1-expressing neural crest cells was not altered in Lbx1(-/-) embryos, we postulate that Lbx1 gene function is critical for specification of a subpopulation of cardiac neural crest subsequent to **migration**.
12. Four-and-a-half LIM domain protein 2 is a novel regulator of sphingosine 1-phosphate receptor 1 in CCL19-induced dendritic cell **migration**. We identified the four-and-a-half LIM domain protein 2 (FHL2) as a novel regulator of CCL19-induced dendritic cell (DC) **migration**. Initiation of **migration** is a hallmark of DC function and plays a central role in the induction and regulation of immune responses. In vivo, DCs continuously acquire Ag in the periphery and migrate to draining lymph nodes, under the influence of local environmental chemotactic factors like CCL19/21 or sphingosine 1-phosphate (S1P). We investigated the role of S1P- and RhoA-regulated FHL2 in this process. We found reduced nuclear localization of FHL2 in mature bone marrow-derived DCs (BMDCs), compared with immature BMDCs, following stimulation with CCL19. Furthermore, in vitro-generated murine FHL2(-/-) BMDCs displayed a significantly increased migratory speed, directionality, and migratory persistence toward the chemokine CCL19 compared with wild-type BMDCs. Moreover, in vivo, FHL2(-/-) BMDCs showed increased **migration** toward lymphoid organs. FHL2(-/-) BMDCs increased the expression of S1PR1, which was associated with greater Rac activation. An S1PR1 antagonist and knock-down of S1PR1 abrogated the increased migratory speed of FHL2(-/-) BMDCs. Our results identify FHL2 as an important novel regulator of DC **migration** via regulation of their sensitivity toward environmental migratory cues like S1P and CCL19.
13. FHL2 expression in peritumoural fibroblasts correlates with lymphatic metastasis in sporadic but not in HNPCC-associated colon cancer. Four and a half LIM domain protein-2 (FHL2) is a component of the focal adhesion structures and has been suggested to have an important role in cancer progression. This study analyses the role of FHL2 in peritumoural fibroblasts of sporadic and hereditary non-polyposis colorectal cancer (HNPCC). Tissue specimens of 48 sporadic and 49 hereditary colon cancers, respectively, were stained immunohistochemically for FHL2, transforming growth factor (TGF)-β1 ligand and α-SMA. Myofibroblasts at the tumour invasion front co-expressed α-SMA and FHL2. Sporadic colon cancer but not HNPCC cases showed a correlation between TGF-β1 expression of the invading tumour cells and FHL2 staining of peritumoural myofibroblasts. Overexpression of FHL2 in peritumoural myofibroblasts correlated to lymphatic metastasis in sporadic colon cancer but not in HNPCC. In cultured mouse fibroblasts, TGF-β1 treatment induced myofibroblast differentiation, stimulated FHL2 protein expression and elevated number of migratory cells in transwell motility assays, suggesting that FHL2 is regulated downstream of TGF-β. Physical contact of colon cancer cells and myofibroblasts via FHL2-positive focal adhesions was detected in human colon carcinoma tissue and in co-culture assays using sporadic as well as HNPCC-derived tumour cell lines. Our data provide strong evidence for an important role of FHL2 in the progression of colon cancers. Tumour-secreted TGF-β1 stimulates FHL2 protein expression in peritumoural fibroblasts, probably facilitating the invasion of tumour glands into the surrounding tissue by enhanced myofibr**oblast mi**gration and tight connection of fibroblasts to tumour cells via focal adhesions. These findings are absent in HNPCC-associated colon cancers in vivo and may contribute to a less invasive and more protruding tumour margin of microsatellite instable carcinomas.
14. Deficiency in the LIM-only protein Fhl2 impairs skin wound healing. After skin wounding, the repair process is initiated by the release of growth factors, cytokines, and bioactive lipids from injured vessels and coagulated platelets. These signal molecules induce synthesis and deposition of a provisional extracellular matrix, as well as fibroblast invasion into and contraction of the wounded area. We previously showed that sphingosine-1-phosphate (S1P) triggers a signal transduction cascade mediating nuclear translocation of the LIM-only protein Fhl2 in response to activation of the RhoA GTPase (Muller, J.M., U. Isele, E. Metzger, A. Rempel, M. Moser, A. Pscherer, T. Breyer, C. Holubarsch, R. Buettner, and R. Schule. 2000. EMBO J. 19:359-369; Muller, J.M., E. Metzger, H. Greschik, A.K. Bosserhoff, L. Mercep, R. Buettner, and R. Schule. 2002. EMBO J. 21:736-748.). We demonstrate impaired cutaneous wound healing in Fhl2-deficient mice rescued by transgenic expression of Fhl2. Furthermore, collagen contraction and cell **migration** are severely impaired in Fhl2-deficient cells. Consequently, we show that the expression of alpha-smooth muscle actin, which is regulated by Fhl2, is reduced and delayed in wounds of Fhl2-deficient mice and that the expression of p130Cas, which is essential for cell **migration**, is reduced in Fhl2-deficient cells. In summary, our data demonstrate a function of Fhl2 as a lipid-triggered signaling molecule in mesenchymal cells regulating their **migration** and contraction during cutaneous wound healing.
15. Four and half lim protein 2 (FHL2) stimulates osteoblast differentiation. FHL2, a molecule that interacts with many integrins and transcription factors, was found to play an important role in osteoblast differentiation. Overexpression of FHL2 increases the accumulation of osteoblast differentiation markers and matrix mineralization, whereas FHL2 deficiency results in inhibition of osteoblast differentiation and decreased bone formation.Integrin-matrix interaction plays a critical role in osteoblast function. It has been shown that the cytoplasmic domains of integrin beta subunits mediate signal transduction induced by integrin-matrix interaction. We reasoned that the identification of proteins interacting with beta-cytoplasmic tails followed by analysis of the function of these proteins would enhance our understanding on integrin signaling and the roles of these proteins in osteoblast activities.Yeast two hybrid assay was used to identify proteins interacting with the cytoplasmic domain of integrin beta5 subunit. The association of these proteins with integrin alphavbeta5 was confirmed by confocal analysis and co-immunoprecipitation. A stable MC3T3-E1 cells line overexpressing Four and Half Lim Protein 2 (FHL2) and mouse osteoblasts deficient in FHL2 were used to study the roles of FHL2 in osteoblast differentiation and bone formation. Matrix protein expression was determined by mRNA analysis and Western blotting. Matrix mineralization was detected by Alizarin red staining. Alkaline phosphatase activity was also measured. muCT was used to determine bone histomorphometry.FHL2 and actin-binding proteins, palladin and filamin A, were identified as proteins interacting with beta5 cytoplasmic domain. FHL2 co-localized with alphavbeta5 at the focal adhesion sites in association with palladin and filamin A. FHL2 was also present in nuclei. Osteoblasts overexpressing FHL2 exhibited increased adhesion to and **migration** on matrix proteins. Conversely, FHL2 stimulation of CREB activity was dependent on integrin function because it was inhibited by Gly-Arg-Gly-Asp-Ser (GRGDS) peptide. The expression of osteoblast differentiation markers and Msx2 was upregulated, and bone matrix mineralization was increased in FHL2 overexpressing cells. In contrast, FHL2-deficient bone marrow cells and osteoblasts displayed decreased osteoblast colony formation and differentiation, respectively, compared with wildtype cells. Moreover, FHL2-deficient female mice exhibited greater bone loss than the wildtype littermates after ovariectomy. Thus, FHL2 plays an important role in osteoblast differentiation and bone formation.
16. LIM-only protein FHL2 regulates experimental pulmonary Schistosoma mansoni egg granuloma formation. LIM-only protein FHL2 is associated with several immune and inflammatory diseases such as arthritis, influenza A virus infection, and lung inflammation. However, the role of FHL2 in macrophage differentiation and in the development of granuloma formation is unknown. Here, we show that expression of FHL2 is induced in mouse bone marrow derived macrophages (BMMs) following stimulation with M2 cytokines such as IL-4 and IL-10. FHL2-knockout (FHL2-KO) BMMs exhibit a proinflammatory M1 phenotype after LPS treatment and display a reduced anti-inflammatory M2 phenotype following IL-4 treatment. Furthermore, thioglycollate-induced **migration** of macrophages and B cells is enhanced in FHL2-KO mice. To evaluate the importance of FHL2 in the development of pulmonary granuloma formation, FHL2-KO mice were challenged with Schistosoma mansoni eggs. FHL2-KO mice show an enhanced number of granulomas and display decreased expression of Th2 markers and an exacerbated Th1 type of inflammation, characterized by enhanced expression of neutrophil markers and Th1 cytokines. Furthermore, the expression of barrier proteins is reduced in FHL2-KO lung compared to WT. Collectively, these data identify a previously unrecognized role for FHL2 in the pathogenesis of pulmonary granulomatous inflammation, partly through its effect on macrophage polarization, modulation of the Th1/Th2 balance and regulation of permeability in lung.
17. Loss of the LIM-only protein Fhl2 impairs inflammatory reaction and scar formation after cardiac ischemia leading to better hemodynamic performance. The pathogenesis of myocardial ischemia-reperfusion injury (MI/R) involves an inflammatory response. Since the four-and-a-half LIM domain-containing protein 2 (Fhl2) has been observed to modulate immune cell **migration**, we aimed to study the consequences of Fhl2(-/-) under MI/R with respect to immune reaction, scar formation, and hemodynamic performance.In a closed chest model of 1h MI/R, immune cell invasion of phagocytic monocytes was characterized by flow cytometry and immunohistochemistry. In addition, infarct size was assessed by triphenyltetrazolium chloride/Masson trichrome staining 24h/21days after reperfusion and a set of hemodynamic parameters was recorded by catheterisation in Fhl2(-/-) mice and controls.While flow cytometry did not reveal differences in myocardial CD45(high) immune cell infiltrate, histological analysis showed that infiltrating immune cells in Fhl2(-/-) animals were preferentially located in the perivascular area, whereas in wild type, immune cells were well dispersed within the area at risk. After 24h and 21days of reperfusion, infarct size was significantly reduced in Fhl2(-/-) compared to WT animals. In addition, hemodynamic performance was better in Fhl2(-/-) mice, compared to WT mice up to day 21 of reperfusion. The loss of Fhl2 leads to an altered immune response to myocardial ischemia, which results in smaller infarcts and better hemodynamic performance up to 21days after myocardial ischemia reperfusion.Immune cell invasion plays a pivotal role in the context of MI/R. Fhl2 significantly influences immune cell function and immune cell interaction with injured cardiac tissue leading to altered scar composition.
18. Inhibition of four-and-a-half LIM domain protein-2 increases survival, migratory capacity, and paracrine function of human early outgrowth cells through activation of the sphingosine kinase-1 pathway: implications for endothelial regeneration. Inhibition of four-and-a-half LIM domain protein-2 (FHL2) attenuates atherosclerotic lesion formation and increases endothelial cell **migration**. Early outgrowth cells (EOCs) contribute substantially to endothelial repair.We investigated the role of FHL2 in the regulation of EOCs.Human EOCs were cultured from peripheral blood. FHL2 knockdown in EOCs by siRNA resulted in increased EOC numbers and reduced apoptosis, as indicated by decreased cleaved caspase-III and reduced Bax/Bcl-2 expression ratio. This was mediated through increased phosphorylation and membrane translocation of sphingosine kinase-1, increased sphingosine-1-phosphate levels, and Akt phosphorylation. FHL2 knockdown increased stromal cell-derived factor-1-induced EOC **migration** through upregulation of αv/β3, αv/β5, and β2 integrins, associated with increased cortactin expression. Reduced apoptosis, increased **EOC migr**ation, and cortactin upregulation by FHL2 siRNA were prevented by CAY10621, the sphingosine kinase-1 inhibitor, and the sphingosine-1-phosphate receptor-1/-3 antagonist VPC23019. These findings were confirmed using spleen-derived EOCs from FHL2(-/-) mice. Apoptosis was decreased **and migr**ation increased in endothelial cells exposed to the conditioned medium of FHL2(-/-) versus wild-type (WT) EOCs. These paracrine effects were abolished by VPC23019. Importantly, reendothelialization after focal carotid endothelial injury in WT mice was significantly increased after intravenous injection of FHL2(-/-) versus WT EOCs.Our findings suggest that FHL2 negatively regulates EOC survi**val, migr**ation, and paracrine function. FHL2 inhibition in EOCs reduces apoptosis and enhances survival and migratory capacity of both EOCs and surrounding endothelial cells by activation of the sphingosine kinase-1/sphingosine-1-phosphate pathway, resulting in improvement of endothelial regeneration.

#### GeneRIFs

1. The LIM-only protein FHL2 reduces vascular lesion formation involving inhibition of proliferation and **migration** of smooth muscle cells.
2. Deficiency of FHL2 impairs ischemia-induced neovascularization, and these suppressive effects may occur through a reduction in proangiogenic cell mobilization, **migration**, and vasculogenesis functions.
3. Fhl2 is a lipid-triggered signaling molecule in mesenchymal cells regulating **migration** and contraction during cutaneous wound healing.

### [Ee]xtracellular [Mm]atrix

#### PubMed

1. Impaired intestinal wound healing in Fhl2-deficient mice is due to disturbed collagen metabolism. Four and one half LIM domain protein FHL2 participates in many cellular processes involved in tissue repair such as regulation of gene expression, cytoarchitecture, cell adhesion, migration and signal transduction. The repair process after wounding is initiated by the release of peptides and bioactive lipids. These molecules induce synthesis and deposition of a provisional **extracellular matrix**. We showed previously that sphingosine-1-phosphate (S1P) triggers a signal transduction cascade mediating nuclear translocation of FHL2 in response to activation of the RhoA GTPase. Our present study shows that FHL2 is an important signal transducer influencing the outcome of intestinal anastomotic healing. Early wound healing is accompanied by reconstitution and remodelling of the **extracellular matrix** and collagen is primarily responsible for wound strength. Our results show that impaired intestinal wound healing in Fhl2-deficient mice is due to disturbed collagen III metabolism. Impaired collagen III synthesis reduced the mechanical stability of the anastomoses and led to lower bursting pressure in Fhl2-deficient mice after surgery. Our data confirm that FHL2 is an important factor regulating collagen expression in the early phase of wound healing, and thereby is critically involved in the physiologic process of anastomosis healing after bowel surgery and thus may represent a new therapeutic target.
2. Deficiency in the LIM-only protein FHL2 impairs assembly of **extracellular matrix** proteins. We have described the scaffolding protein FHL2 as a component of focal adhesion structures, to which it is recruited via binding to both alpha- or beta-integrin subunits. Using mesenchymal stem cells from wild-type and FHL2-knockout mice, we show here that inactivation of FHL2 leads to impaired assembly of **extracellular matrix** proteins on the cell surface and to impaired bundling of focal adhesions. Both altered properties can be restored by reexpression of recombinant FHL2 protein in FHL2-null cells. Molecular analysis of integrin-mediated signaling revealed a higher phosphorylation of FAK at tyrosine 925 in FHL2-knockout cells compared to their wild-type counterpart. Consequently, the activation of the mitogenic kinase ERK was more pronounced in knockout cells on cell adhesion. The growth factor-induced activation of ERK, however, was not altered. The perturbed organization of **extracellular matrix** on FHL2-null cells was improved when the increased activation of MAPK was inhibited. Our findings point to a role of FHL2 in bundling of focal adhesion structures, in integrin-mediated ERK activation, and subsequently in proper allocation of matrix proteins on the cell surface.
3. FHL2 mediates dexamethasone-induced mesenchymal cell differentiation into osteoblasts by activating Wnt/beta-catenin signaling-dependent Runx2 expression. The differentiation of bone marrow mesenchymal stem cells (MSCs) into osteoblasts is a crucial step in bone formation. However, the mechanisms involved in the early stages of osteogenic differentiation are not well understood. In this study, we identified FHL2, a member of the LIM-only subclass of the LIM protein superfamily, that is up-regulated during early osteoblast differentiation induced by dexamethasone in murine and human MSCs. Gain-of-function studies showed that FHL2 promotes the expression of the osteoblast transcription factor Runx2, alkaline phosphatase, type I collagen, as well as in vitro **extracellular matrix** mineralization in murine and human mesenchymal cells. Knocking down FHL2 using sh-RNA reduces basal and dexamethasone-induced osteoblast marker gene expression in MSCs. We demonstrate that FHL2 interacts with beta-catenin, a key player involved in bone formation induced by Wnt signaling. FHL2-beta-catenin interaction potentiates beta-catenin nuclear translocation and TCF/LEF transcription, resulting in increased Runx2 and alkaline phosphatase expression, which was inhibited by the Wnt inhibitor DKK1. Reduction of Runx2 transcriptional activity using a mutant Runx2 results in inhibition of FHL2-induced alkaline phosphatase expression in MSCs. These findings reveal that FHL2 acts as an endogenous activator of mesenchymal cell differentiation into osteoblasts and mediates osteogenic differentiation induced by dexamethasone in MSCs through activation of Wnt/beta-catenin signaling- dependent Runx2 expression.
4. The LIM-only protein FHL2 attenuates lung inflammation during bleomycin-induced fibrosis. Fibrogenesis is usually initiated when regenerative processes have failed and/or chronic inflammation occurs. It is characterised by the activation of tissue fibroblasts and dysregulated synthesis of **extracellular matrix** proteins. FHL2 (four-and-a-half LIM domain protein 2) is a scaffolding protein that interacts with numerous cellular proteins, regulating signalling cascades and gene transcription. It is involved in tissue remodelling and tumour progression. Recent data suggest that FHL2 might support fibrogenesis by maintaining the transcriptional expression of alpha smooth muscle actin and the excessive synthesis and assembly of matrix proteins in activated fibroblasts. Here, we present evidence that FHL2 does not promote bleomycin-induced lung fibrosis, but rather suppresses this process by attenuating lung inflammation. Loss of FHL2 results in increased expression of the pro-inflammatory matrix protein tenascin C and downregulation of the macrophage activating C-type lectin receptor DC-SIGN. Consequently, FHL2 knockout mice developed a severe and long-lasting lung pathology following bleomycin administration due to enhanced expression of tenascin C and impaired activation of inflammation-resolving macrophages.
5. Deficiency in the LIM-only protein Fhl2 impairs skin wound healing. After skin wounding, the repair process is initiated by the release of growth factors, cytokines, and bioactive lipids from injured vessels and coagulated platelets. These signal molecules induce synthesis and deposition of a provisional **extracellular matrix**, as well as fibroblast invasion into and contraction of the wounded area. We previously showed that sphingosine-1-phosphate (S1P) triggers a signal transduction cascade mediating nuclear translocation of the LIM-only protein Fhl2 in response to activation of the RhoA GTPase (Muller, J.M., U. Isele, E. Metzger, A. Rempel, M. Moser, A. Pscherer, T. Breyer, C. Holubarsch, R. Buettner, and R. Schule. 2000. EMBO J. 19:359-369; Muller, J.M., E. Metzger, H. Greschik, A.K. Bosserhoff, L. Mercep, R. Buettner, and R. Schule. 2002. EMBO J. 21:736-748.). We demonstrate impaired cutaneous wound healing in Fhl2-deficient mice rescued by transgenic expression of Fhl2. Furthermore, collagen contraction and cell migration are severely impaired in Fhl2-deficient cells. Consequently, we show that the expression of alpha-smooth muscle actin, which is regulated by Fhl2, is reduced and delayed in wounds of Fhl2-deficient mice and that the expression of p130Cas, which is essential for cell migration, is reduced in Fhl2-deficient cells. In summary, our data demonstrate a function of Fhl2 as a lipid-triggered signaling molecule in mesenchymal cells regulating their migration and contraction during cutaneous wound healing.

### [Ii]nvasive

#### PubMed

1. FHL2 expression in peritumoural fibroblasts correlates with lymphatic metastasis in sporadic but not in HNPCC-associated colon cancer. Four and a half LIM domain protein-2 (FHL2) is a component of the focal adhesion structures and has been suggested to have an important role in cancer progression. This study analyses the role of FHL2 in peritumoural fibroblasts of sporadic and hereditary non-polyposis colorectal cancer (HNPCC). Tissue specimens of 48 sporadic and 49 hereditary colon cancers, respectively, were stained immunohistochemically for FHL2, transforming growth factor (TGF)-β1 ligand and α-SMA. Myofibroblasts at the tumour invasion front co-expressed α-SMA and FHL2. Sporadic colon cancer but not HNPCC cases showed a correlation between TGF-β1 expression of the invading tumour cells and FHL2 staining of peritumoural myofibroblasts. Overexpression of FHL2 in peritumoural myofibroblasts correlated to lymphatic metastasis in sporadic colon cancer but not in HNPCC. In cultured mouse fibroblasts, TGF-β1 treatment induced myofibroblast differentiation, stimulated FHL2 protein expression and elevated number of migratory cells in transwell motility assays, suggesting that FHL2 is regulated downstream of TGF-β. Physical contact of colon cancer cells and myofibroblasts via FHL2-positive focal adhesions was detected in human colon carcinoma tissue and in co-culture assays using sporadic as well as HNPCC-derived tumour cell lines. Our data provide strong evidence for an important role of FHL2 in the progression of colon cancers. Tumour-secreted TGF-β1 stimulates FHL2 protein expression in peritumoural fibroblasts, probably facilitating the invasion of tumour glands into the surrounding tissue by enhanced myofibroblast migration and tight connection of fibroblasts to tumour cells via focal adhesions. These findings are absent in HNPCC-associated colon cancers in vivo and may contribute to **a less i**nvasive and more protruding tumour margin of microsatellite instable carcinomas.

### [Mm]etastasis

#### PubMed

1. FHL2 expression in peritumoural fibroblasts correlates with lymphatic **metastasis** in sporadic but not in HNPCC-associated colon cancer. Four and a half LIM domain protein-2 (FHL2) is a component of the focal adhesion structures and has been suggested to have an important role in cancer progression. This study analyses the role of FHL2 in peritumoural fibroblasts of sporadic and hereditary non-polyposis colorectal cancer (HNPCC). Tissue specimens of 48 sporadic and 49 hereditary colon cancers, respectively, were stained immunohistochemically for FHL2, transforming growth factor (TGF)-β1 ligand and α-SMA. Myofibroblasts at the tumour invasion front co-expressed α-SMA and FHL2. Sporadic colon cancer but not HNPCC cases showed a correlation between TGF-β1 expression of the invading tumour cells and FHL2 staining of peritumoural myofibroblasts. Overexpression of FHL2 in peritumoural myofibroblasts correlated to lympha**tic metast**asis in sporadic colon cancer but not in HNPCC. In cultured mouse fibroblasts, TGF-β1 treatment induced myofibroblast differentiation, stimulated FHL2 protein expression and elevated number of migratory cells in transwell motility assays, suggesting that FHL2 is regulated downstream of TGF-β. Physical contact of colon cancer cells and myofibroblasts via FHL2-positive focal adhesions was detected in human colon carcinoma tissue and in co-culture assays using sporadic as well as HNPCC-derived tumour cell lines. Our data provide strong evidence for an important role of FHL2 in the progression of colon cancers. Tumour-secreted TGF-β1 stimulates FHL2 protein expression in peritumoural fibroblasts, probably facilitating the invasion of tumour glands into the surrounding tissue by enhanced myofibroblast migration and tight connection of fibroblasts to tumour cells via focal adhesions. These findings are absent in HNPCC-associated colon cancers in vivo and may contribute to a less invasive and more protruding tumour margin of microsatellite instable carcinomas.

### [Cc]ell [Ii]nvasion

#### PubMed

1. Loss of the LIM-only protein Fhl2 impairs inflammatory reaction and scar formation after cardiac ischemia leading to better hemodynamic performance. The pathogenesis of myocardial ischemia-reperfusion injury (MI/R) involves an inflammatory response. Since the four-and-a-half LIM domain-containing protein 2 (Fhl2) has been observed to modulate immune cell migration, we aimed to study the consequences of Fhl2(-/-) under MI/R with respect to immune reaction, scar formation, and hemodynamic performance.In a closed chest model of 1h MI/R, immune **cell invasion** of phagocytic monocytes was characterized by flow cytometry and immunohistochemistry. In addition, infarct size was assessed by triphenyltetrazolium chloride/Masson trichrome staining 24h/21days after reperfusion and a set of hemodynamic parameters was recorded by catheterisation in Fhl2(-/-) mice and controls.While flow cytometry did not reveal differences in myocardial CD45(high) immune cell infiltrate, histological analysis showed that infiltrating immune cells in Fhl2(-/-) animals were preferentially located in the perivascular area, whereas in wild type, immune cells were well dispersed within the area at risk. After 24h and 21days of reperfusion, infarct size was significantly reduced in Fhl2(-/-) compared to WT animals. In addition, hemodynamic performance was better in Fhl2(-/-) mice, compared to WT mice up to day 21 of reperfusion. The loss of Fhl2 leads to an altered immune response to myocardial ischemia, which results in smaller infarcts and better hemodynamic performance up to 21days after myocardial ischemia reperfusion.Immune **cell invasion** plays a pivotal role in the context of MI/R. Fhl2 significantly influences immune cell function and immune cell interaction with injured cardiac tissue leading to altered scar composition.

---

## Il16

- NCBI Gene
- GeneRIF
- Pubmed

| Search Term | Rank | Fields |
| --- | --- | --- |
| [Mm]igration | 6 | PubMed(3) |

  
hide/show details for Il16

### [Mm]igration

#### PubMed

1. Cutting edge: IL-16/CD4 preferentially induces Th1 cell **migration**: requirement of CCR5. IL-16 binds to CD4 and induces a migratory response in CD4(+) T cells. Although it has been assumed that CD4 is the sole receptor and that IL-16 induces a comparable migratory response in all CD4(+) T cells, this has not been investigated. In this study, we determined that IL-16 preferentially induces a migratory response in Th1 cells. Because chemokine receptor CCR5 is expressed predominantly in Th1 cells and is physically associated with CD4, we investigated whether IL-16/CD4 stimulation was enhanced in the presence of CCR5. Using T cells from CCR5(null) mice, we determined that IL-16-induced **migration** was significantly greater in the presence of CCR5. The presence of CCR5 significantly increased IL-16 binding vs CD4 alone; however, IL-16 could not bind to CCR5 alone. Because CD4(+)CCR5(+) cells are prevalent at sites of inflammation, this intimate functional relationship likely plays a pivotal role for the recruitment and activation of Th1 cells.
2. Molecular cloning and sequence analysis of interleukin 16 from nonhuman primates and from the mouse. Interleukin 16 (IL-16) is synthesized as a 67 000 Mr precursor (pro-IL-16), but only a carboxy terminal part of 12 000-14 000 Mr is secreted by CD8(+) lymphocytes. This lymphokine binds to CD4 and has been shown to induce **migration**, affect the activation state of T cells, and inhibit immunodeficiency virus replication. It has been suggested that CD8(+) cell-derived soluble factors play a pivotal role in protecting natural-host nonhuman primates from developing immunodeficiency following SIV infection. In a first attempt to address this question, we cloned and sequenced the IL-16 cDNA from different primates. Here we report the pro-IL-16 sequence from chimpanzees, African green monkeys (AGM), rhesus macaques, and cynomolgus macaques. In order to compare and analyze structural motifs possibly involved in processing, intracellular targeting, or secretion, we extended our study to the New World monkeys saimiri and aotus and to the mouse. Alignments of deduced amino acids reveal that the human protein shares 99% similarity to that of chimpanzees, approximately 95% to rhesus, cynomolgus and AGM, about 90% to aotus and saimiri, and 77.5% to the mouse. Phylogenetic analyses revealed the expected evolutionary groupings.
3. Decreased renal ischemia-reperfusion injury by IL-16 inactivation. T-cell-mediated renal injury is a major cause of kidney transplant rejection and renal failure; hence, understanding T-cell **migration** within the kidney is important for preventing renal injury. Interleukin (IL)-16 is a T-cell chemoattractant produced by leukocytes. Here we measured IL-16 expression in the kidney and its role in renal ischemia-reperfusion injury induced by different conditions in several strains of mice. IL-16 was strongly expressed in distal and proximal straight tubules of the kidney. The IL-16 precursor protein was cleaved to a chemotactic form in cultured tubular epithelial cells. Inactivation of IL-16 by antibody therapy or IL-16 deficiency prevented ischemia-reperfusion injury as shown by reduced levels of serum creatinine or blood urea nitrogen compared to control mice. Further studies indicated that fewer CD4-cells infiltrated the post-ischemic kidneys of IL-16-deficient mice and that the protective effect of IL-16 antibody treatment was lymphocyte-dependent. Our results suggest that IL-16 is a critical factor in the development of inflammation-mediated renal injury and may be a therapeutic target for prevention of ischemia-reperfusion injury of the kidney.

---

## Cd63

- NCBI Gene
- GeneRIF
- Pubmed

| Search Term | Rank | Fields |
| --- | --- | --- |
| [Mm]igration | 6 | Process(3); PubMed(2) |
| [Mm]etastasis | 12 | PubMed(1) |

  
hide/show details for Cd63

### [Mm]igration

#### Process

1. cell **migration**
2. cell **migration**
3. negative regulation of epithelial cell **migration**

#### PubMed

1. Tetraspanin CD63 promotes vascular endothelial growth factor receptor 2-β1 integrin complex formation, thereby regulating activation and downstream signaling in endothelial cells in vitro and in vivo. CD63 is a member of the transmembrane-4 glycoprotein superfamily (tetraspanins) implicated in the regulation of membrane protein trafficking, leukocyte recruitment, and adhesion processes. We have investigated the involvement of CD63 in endothelial cell (EC) signaling downstream of β1 integrin and VEGF. We report that silencing of CD63 in primary ECs arrested capillary sprouting and tube formation in vitro because of impaired adhesion an**d migrati**on of ECs. Mechanistically, CD63 associated with both β1 integrin and the main VEGF receptor on ECs, VEGFR2. Our data suggest that CD63 serves to bridge between β1 integrin and VEGFR2 because CD63 silencing disrupted VEGFR2-β1 integrin complex formation identified using proximity ligation assays. Signaling downstream of β1 integrin and VEGFR2 was attenuated in CD63-silenced cells, although their cell surface expression levels remained unaffected. CD63 was furthermore required for efficient internalization of VEGFR2 in response to VEGF. Importantly, systemic delivery of VEGF failed to potently induce VEGFR2 phosphorylation and downstream signaling in CD63-deficient mouse lungs. Taken together, our findings demonstrate a previously unrecognized role for CD63 in coordinated integrin and receptor tyrosine kinase signaling in vitro and in vivo.
2. Tetraspanin CD63 promotes vascular endothelial growth factor receptor 2-β1 integrin complex formation, thereby regulating activation and downstream signaling in endothelial cells in vitro and in vivo. CD63 is a member of the transmembrane-4 glycoprotein superfamily (tetraspanins) implicated in the regulation of membrane protein trafficking, leukocyte recruitment, and adhesion processes. We have investigated the involvement of CD63 in endothelial cell (EC) signaling downstream of β1 integrin and VEGF. We report that silencing of CD63 in primary ECs arrested capillary sprouting and tube formation in vitro because of impaired adhesion an**d migrati**on of ECs. Mechanistically, CD63 associated with both β1 integrin and the main VEGF receptor on ECs, VEGFR2. Our data suggest that CD63 serves to bridge between β1 integrin and VEGFR2 because CD63 silencing disrupted VEGFR2-β1 integrin complex formation identified using proximity ligation assays. Signaling downstream of β1 integrin and VEGFR2 was attenuated in CD63-silenced cells, although their cell surface expression levels remained unaffected. CD63 was furthermore required for efficient internalization of VEGFR2 in response to VEGF. Importantly, systemic delivery of VEGF failed to potently induce VEGFR2 phosphorylation and downstream signaling in CD63-deficient mouse lungs. Taken together, our findings demonstrate a previously unrecognized role for CD63 in coordinated integrin and receptor tyrosine kinase signaling in vitro and in vivo.

### [Mm]etastasis

#### PubMed

1. Tetraspanin CD63 acts as a pro-metastatic factor via β-catenin stabilization. The tetraspanin CD63 is implicated in pro-metastatic signaling pathways but, so far, it is unclear, how CD63 levels affect the tumor cell phenotype. Here, we investigated the effect of CD63 modulation in different metastatic tumor cell lines. In vitro, knock down of CD63 induced a more epithelial-like phenotype concomitant with increased E-cadherin expression, downregulation of its repressors Slug and Zeb1, and decreased N-cadherin. In addition, β-catenin protein was markedly reduced, negatively affecting expression of the target genes MMP-2 and PAI-1. β-catenin inhibitors mimicked the epithelial phenotype induced by CD63 knock down. Inhibition of β-catenin upstream regulators PI3K/AKT or GSK3β could rescue the mesenchymal phenotype underlining the importance of the β-catenin pathway in CD63-regulated cell plasticity. CD63 knock down-induced phenotypical changes correlated with a decrease of experim**ental meta**stasis whereas CD63 overexpression enhanced the tumor cell-intrinsic metastatic potential. Taken together, our data show that CD63 is a crucial player in the regulation of the tumor cell-intrinsic metastatic potential by affecting cell plasticity.

---

## Arhgef25

- NCBI Gene
- GeneRIF
- Pubmed

| Search Term | Rank | Fields |
| --- | --- | --- |
| [Ff]ilopodia | 1 | PubMed(1) |
| [Mm]igration | 6 | PubMed(3); GeneRIFs(1) |

  
hide/show details for Arhgef25

### [Ff]ilopodia

#### PubMed

1. A Rac/Cdc42-specific exchange factor, GEFT, induces cell proliferation, transformation, and migration. The Rho family of small GTPases, including Rho, Rac, and Cdc42, play essential roles in diverse cellular functions. The ability of Rho family GTPases to participate in signaling events is determined by the ratio of inactive (GDP-bound) and active (GTP-bound) forms in the cell. The activation of Rho family proteins requires the exchange of bound GDP for GTP, a process catalyzed by the Dbl family of guanine nucleotide exchange factors (GEFs). The GEFs have high affinity for the guanine nucleotide-free state of the GTPases and are thought to promote GDP release by stabilizing an intermediate transition state. In this study, we have identified and characterized a new Rac/Cdc42-specific Dbl family guanine nucleotide exchange factor, named GEFT. GEFT is highly expressed in the excitable tissues, including brain, heart, and muscle. Low or very little expression was detected in other nonexcitable tissues. GEFT has specific exchange activity for Rac and Cdc42 in our in vitro GTPase exchange assays and glutathione S-transferase-PAK pull-down assays with GTP-bound Rac1 and Cdc42. Overexpression of GEFT leads to changes in cell morphology and actin cytoskeleton re-organization, including the formation of membrane microspikes, **filopodia**, and lamilliopodia. Furthermore, expression of GEFT in NIH3T3 cells promotes foci formation, cell proliferation, and cell migration, possibly through the activation of transcriptional factors involved in cell growth and proliferation. Together, our data suggest that GEFT is a Rac/Cdc42-specific GEF protein that regulates cell morphology, cell proliferation, and transformation.

### [Mm]igration

#### PubMed

1. Transcriptional programs in transient embryonic zones of the cerebral cortex defined by high-resolution mRNA sequencing. Characterizing the genetic programs that specify development and evolution of the cerebral cortex is a central challenge in neuroscience. Stem cells in the transient embryonic ventricular and subventricular zones generate neurons that migrate across the intermediate zone to the overlying cortical plate, where they differentiate and form the neocortex. It is clear that not one but a multitude of molecular pathways are necessary to progress through each cellular milestone, yet the underlying transcriptional programs remain unknown. Here, we apply differential transcriptome analysis on microscopically isolated cell populations, to define five transcriptional programs that represent each transient embryonic zone and the progression between these zones. The five transcriptional programs contain largely uncharacterized genes in addition to transcripts necessary for stem cell maintenance, neurogenesis, **migration**, and differentiation. Additionally, we found intergenic transcriptionally active regions that possibly encode unique zone-specific transcripts. Finally, we present a high-resolution transcriptome map of transient zones in the embryonic mouse forebrain.
2. A Rac/Cdc42-specific exchange factor, GEFT, induces cell proliferation, transformation, and **migration**. The Rho family of small GTPases, including Rho, Rac, and Cdc42, play essential roles in diverse cellular functions. The ability of Rho family GTPases to participate in signaling events is determined by the ratio of inactive (GDP-bound) and active (GTP-bound) forms in the cell. The activation of Rho family proteins requires the exchange of bound GDP for GTP, a process catalyzed by the Dbl family of guanine nucleotide exchange factors (GEFs). The GEFs have high affinity for the guanine nucleotide-free state of the GTPases and are thought to promote GDP release by stabilizing an intermediate transition state. In this study, we have identified and characterized a new Rac/Cdc42-specific Dbl family guanine nucleotide exchange factor, named GEFT. GEFT is highly expressed in the excitable tissues, including brain, heart, and muscle. Low or very little expression was detected in other nonexcitable tissues. GEFT has specific exchange activity for Rac and Cdc42 in our in vitro GTPase exchange assays and glutathione S-transferase-PAK pull-down assays with GTP-bound Rac1 and Cdc42. Overexpression of GEFT leads to changes in cell morphology and actin cytoskeleton re-organization, including the formation of membrane microspikes, filopodia, and lamilliopodia. Furthermore, expression of GEFT in NIH3T3 cells promotes foci formation, cell proliferation, and cell **migration**, possibly through the activation of transcriptional factors involved in cell growth and proliferation. Together, our data suggest that GEFT is a Rac/Cdc42-specific GEF protein that regulates cell morphology, cell proliferation, and transformation.
3. Geft is dispensable for the development of the second heart field. Geft is a guanine nucleotide exchange factor, which can specifically activate Rho family of small GTPase by catalyzing the exchange of bound GDP for GTP. Geft is highly expressed in the excitable tissue as heart and skeletal muscle and plays important roles in many cellular processes, such as cell proliferation, **migration**, and cell fate decision. However, the in vivo role of Geft remains unknown. Here, we generated a Geft conditional knockout mouse by flanking exons 5-17 of Geft with loxP sites. Cre-mediated deletion of the Geft gene in heart using Mef2c-Cre transgenic mice resulted in a dramatic decrease of Geft expression. Geft knockout mice develop normally and exhibit no discernable phenotype, suggesting Geft is dispensable for the development of the second heart field in mouse. The Geft conditional knockout mouse will be a valuable genetic tool for uncovering the in vivo roles of Geft during development and in adult homeostasis.

#### GeneRIFs

1. This protein induces cell proliferation, transformation, and **migration**.

---

## Wipf1

- NCBI Gene
- GeneRIF
- Pubmed

| Search Term | Rank | Fields |
| --- | --- | --- |
| [Ff]ilopodia | 1 | PubMed(3) |
| [Ff]ilopodium | 2 | PubMed(2) |
| [Ii]nvadopodia | 3 | PubMed(1) |
| [Ii]nvadopodium | 4 | PubMed(1) |
| [Pp]odosome(s)? | 5 | PubMed(2) |
| [Mm]igration | 6 | PubMed(5); GeneRIFs(2) |
| [Ii]nvasive | 10 | PubMed(1) |

  
hide/show details for Wipf1

### [Ff]ilopodia

#### PubMed

1. Cortactin interacts with WIP in regulating Arp2/3 activation and membrane protrusion. Modulation of actin cytoskeleton assembly is an integral step in many cellular events. A key regulator of actin polymerization is Arp2/3 complex. Cortactin, an F-actin binding protein that localizes to membrane ruffles, is an activator of Arp2/3 complex.A yeast two-hybrid screen revealed the interaction of the cortactin Src homology 3 (SH3) domain with a peptide fragment derived from a cDNA encoding a region of WASp-Interacting Protein (WIP). GST-cortactin interacted with WIP in an SH3-dependent manner. The subcellular localization of cortactin and WIP coincided at the cell periphery. WIP increased the efficiency of cortactin-mediated Arp2/3 complex activation of actin polymerization in a concentration-dependent manner. Lastly, coexpression of cortactin and WIP stimulated membrane protrusions.WIP, a protein involved in **filopodia** formation, binds to both actin monomers and cortactin. Thus, recruitment of actin monomers to a cortactin-activated Arp2/3 complex likely leads to the observed increase in cortactin activation of Arp2/3 complex by WIP. These data suggest that a cortactin-WIP complex functions in regulating actin-based structures at the cell periphery.
2. A role for WASP Interacting Protein, WIP, in fibroblast adhesion, spreading and migration. The WASP (Wiskott Aldrich Syndrome Protein) Interacting Protein, WIP, regulates actin polymerization and the formation of actin-rich structures such as **filopodia** and lamellipodia, each of which is involved in cellular adhesion, spreading and migration. To define the role for WIP in these activities, we analysed cell adhesion and spreading as well as the redistribution of polymerised actin and paxillin that occurred when fibroblasts were plated onto different substrata. We compared the effect of WIP overexpression (gain of function) with that of WIP deficiency (loss of function) on these parameters. WIP-overexpression delayed cellular adhesion and spreading, an effect that could be compensated for by exposure to Y-27632, a well characterized ROCK (Rho kinase) inhibitor. WIP overexpression augmented the phosphorylation of Erk and JNK induced by binding to fibronectin, suggesting that WIP participates in signal transduction pathways initiated by integrin engagement. Conversely, WIP deficiency accelerated fibroblast adhesion to plastic and led to the formation of enlarged focal adhesions. The influence of WIP on fibroblast migration was measured by scratch assay. WIP-overexpression reduced migration while WIP-deficiency increased it, suggesting that WIP acts as a negative regulator of fibroblast migration. Together, these findings suggest a novel role for WIP in fibroblast adhesion, spreading and migration.
3. Neuritic complexity of hippocampal neurons depends on WIP-mediated mTORC1 and Abl family kinases activities. Neuronal morphogenesis is governed mainly by two interconnected processes, cytoskeletal reorganization, and signal transduction. The actin-binding molecule WIP (Wiskott-Aldrich syndrome protein [WASP]-interacting protein) was identified as a negative regulator of neuritogenesis. Although WIP controls activity of the actin-nucleation-promoting factor neural WASP (N-WASP) during neuritic differentiation, its implication in signal transduction remains unknown.Using primary neurons from WIP-deficient and wild-type mice we did an immunofluorescence, morphometric, and biochemical analysis of the signaling modified by WIP deficiency.Here, we describe the WIP contribution to the regulation of neuritic elaboration and ramification through modification in phosphorylation levels of several kinases that participate in the mammalian target of rapamycin complex 1 (mTORC1)-p70S6K (phosphoprotein 70 ribosomal protein S6 kinase, S6K) intracellular signaling pathway. WIP deficiency induces an increase in the number of neuritic bifurcations and **filopodia**l protrusions in primary embryonic neurons. This phenotype is not due to modifications in the activity of the phosphoinositide 3 kinase (PI3K)-Akt pathway, but to reduced phosphorylation of the S6K residues Ser(411) and Thr(389). The resulting decrease in kinase activity leads to reduced S6 phosphorylation in the absence of WIP. Incubation of control neurons with pharmacological inhibitors of mTORC1 or Abl, two S6K regulators, conferred a morphology resembling that of WIP-deficient neurons. Moreover, the preferential co-distribution of phospho-S6K with polymerized actin is altered in WIP-deficient neurons.These experiments identify WIP as a member of a signaling cascade comprised of Abl family kinases, mTORC1 and S6K, which regulates neuron development and specifically, neuritic branching and complexity. Thus, we postulated a new role for WIP protein.

### [Ff]ilopodium

#### PubMed

1. WIP deficiency reveals a differential role for WIP and the actin cytoskeleton in T and B cell activation. WIP stabilizes actin filaments and is important for **filopodium** formation. To define the role of WIP in immunity, we generated WIP-deficient mice. WIP(minus sign/minus sign) mice have normal lymphocyte development, but their T cells fail to proliferate, secrete IL-2, increase their F-actin content, polarize and extend protrusions following T cell receptor ligation, and are deficient in conjugate formation with superantigen-presenting B cells and anti-CD3 bilayers. In contrast, WIP-deficient B lymphocytes have enhanced proliferation and CD69 expression following B cell receptor ligation and mount normal antibody responses to T-independent antigens. Both WIP-deficient T and B cells show a profound defect in their subcortical actin filament networks. These results suggest that WIP is important for immunologic synapse formation and T cell activation.
2. WIP participates in actin reorganization and ruffle formation induced by PDGF. Platelet-derived growth factor (PDGF) is a chemotactic factor for fibroblasts that triggers actin cytoskeleton reorganization by increasing the level of GTP-Rac, the activated form of a small Rho family GTPase. GTP-Rac induces membrane ruffling and lamellipodium formation that are required for adhesion, migration and macropinocytosis, among other functions. We have shown that WIP interacts with members of the Wiskott-Aldrich syndrome protein family and is essential for **filopodium** formation regulated by Cdc42 GTPase. In this report, we show that WIP participates in the actin reorganization that leads to ruffle formation. WIP overexpression in murine fibroblasts (3T3 cells) enhances ruffle formation in response to PDGF stimulation, as shown by immunofluorescence and electron and video microscopy. More importantly, microinjection of anti-WIP antibody or absence of WIP in murine fibroblasts results in decreased ruffle formation in response to PDGF treatment. Finally, overexpression of a modified form of WIP lacking the actin-binding site blocks PDGF-induced membrane ruffling. These data suggest a role for WIP in actin reorganization to form PDGF-induced ruffles. This is the first in vivo evidence in mammalian cells for a function of WIP dependent on its ability to bind actin.

### [Ii]nvadopodia

#### PubMed

1. Molecular mechanisms of invadopodium formation: the role of the N-WASP-Arp2/3 complex pathway and cofilin. **Invadopodia** are actin-rich membrane protrusions with a matrix degradation activity formed by invasive cancer cells. We have studied the molecular mechanisms of invadopodium formation in metastatic carcinoma cells. Epidermal growth factor (EGF) receptor kinase inhibitors blocked invadopodium formation in the presence of serum, and EGF stimulation of serum-starved cells induced invadopodium formation. RNA interference and dominant-negative mutant expression analyses revealed that neural WASP (N-WASP), Arp2/3 complex, and their upstream regulators, Nck1, Cdc42, and WIP, are necessary for invadopodium formation. Time-lapse analysis revealed that **invadopodia** are formed de novo at the cell periphery and their lifetime varies from minutes to several hours. **Invadopodia** with short lifetimes are motile, whereas long-lived **invadopodia** tend to be stationary. Interestingly, suppression of cofilin expression by RNA interference inhibited the formation of long-lived **invadopodia**, resulting in formation of only short-lived **invadopodia** with less matrix degradation activity. These results indicate that EGF receptor signaling regulates invadopodium formation through the N-WASP-Arp2/3 pathway and cofilin is necessary for the stabilization and maturation of **invadopodia**.

### [Ii]nvadopodium

#### PubMed

1. Molecular mechanisms of **invadopodium** formation: the role of the N-WASP-Arp2/3 complex pathway and cofilin. Invadopodia are actin-rich membrane protrusions with a matrix degradation activity formed by invasive cancer cells. We have studied the molecular mechanisms of **invadopodium** formation in metastatic carcinoma cells. Epidermal growth factor (EGF) receptor kinase inhibitors blocked **invadopodium** formation in the presence of serum, and EGF stimulation of serum-starved cells induced **invadopodium** formation. RNA interference and dominant-negative mutant expression analyses revealed that neural WASP (N-WASP), Arp2/3 complex, and their upstream regulators, Nck1, Cdc42, and WIP, are necessary for **invadopodium** formation. Time-lapse analysis revealed that invadopodia are formed de novo at the cell periphery and their lifetime varies from minutes to several hours. Invadopodia with short lifetimes are motile, whereas long-lived invadopodia tend to be stationary. Interestingly, suppression of cofilin expression by RNA interference inhibited the formation of long-lived invadopodia, resulting in formation of only short-lived invadopodia with less matrix degradation activity. These results indicate that EGF receptor signaling regulates **invadopodium** formation through the N-WASP-Arp2/3 pathway and cofilin is necessary for the stabilization and maturation of invadopodia.

### [Pp]odosome(s)?

#### PubMed

1. WIP regulates the stability and localization of WASP to **podosomes** in migrating dendritic cells. The Wiskott-Aldrich Syndrome protein (WASP) is an adaptor protein that is essential for **podosome** formation in hematopoietic cells. Given that 80% of identified Wiskott-Aldrich Syndrome patients result from mutations in the binding site for WASP-interacting-protein (WIP), we examined the possible role of WIP in the regulation of **podosome** architecture and cell motility in dendritic cells (DCs). Our results show that WIP is essential both for the formation of actin cores containing WASP and cortactin and for the organization of integrin and integrin-associated proteins in circular arrays, specific characteristics of **podosome** structure. We also found that WIP is essential for the maintenance of the high turnover of adhesions and polarity in DCs. WIP exerts these functions by regulating calpain-mediated cleavage of WASP and by facilitating the localization of WASP to sites of actin polymerization at **podosomes**. Taken together, our results indicate that WIP is critical for the regulation of both the stability and localization of WASP in migrating DCs and suggest that WASP and WIP operate as a functional unit to control DC motility in response to changes in the extracellular environment.
2. Phosphatidylinositol 4,5-biphosphate (PIP2)-induced vesicle movement depends on N-WASP and involves Nck, WIP, and Grb2. Wiskott-Aldrich syndrome protein (WASP)/Scar family proteins promote actin polymerization by stimulating the actin-nucleating activity of the Arp2/3 complex. While Scar/WAVE proteins are thought to be involved in lamellipodia protrusion, the hematopoietic WASP has been implicated in various actin-based processes such as chemotaxis, **podosome** formation, and phagocytosis. Here we show that the ubiquitously expressed N-WASP is essential for actin assembly at the surface of endomembranes induced as a consequence of increased phosphatidylinositol 4,5-biphosphate (PIP2) levels. This process resulting in the motility of intracellular vesicles at the tips of actin comets involved the recruitment of the Src homology 3 (SH3)-SH2 adaptor proteins Nck and Grb2 as well as of WASP interacting protein (WIP). Reconstitution of vesicle movement in N-WASP-defective cells by expression of various N-WASP mutant proteins revealed three independent domains capable of interaction with the vesicle surface, of which both the WH1 and the polyproline domains contributed significantly to N-WASP recruitment and/or activation. In contrast, the direct interaction of N-WASP with the Rho-GTPase Cdc42 was not required for reconstitution of vesicle motility. Our data reveal a distinct cellular phenotype for N-WASP loss of function, which adds to accumulating evidence that the proposed link between actin and membrane dynamics may, at least partially, be reflected by the actin-based movement of vesicles through the cytoplasm.

### [Mm]igration

#### PubMed

1. WIP participates in actin reorganization and ruffle formation induced by PDGF. Platelet-derived growth factor (PDGF) is a chemotactic factor for fibroblasts that triggers actin cytoskeleton reorganization by increasing the level of GTP-Rac, the activated form of a small Rho family GTPase. GTP-Rac induces membrane ruffling and lamellipodium formation that are required for adhesion, **migration** and macropinocytosis, among other functions. We have shown that WIP interacts with members of the Wiskott-Aldrich syndrome protein family and is essential for filopodium formation regulated by Cdc42 GTPase. In this report, we show that WIP participates in the actin reorganization that leads to ruffle formation. WIP overexpression in murine fibroblasts (3T3 cells) enhances ruffle formation in response to PDGF stimulation, as shown by immunofluorescence and electron and video microscopy. More importantly, microinjection of anti-WIP antibody or absence of WIP in murine fibroblasts results in decreased ruffle formation in response to PDGF treatment. Finally, overexpression of a modified form of WIP lacking the actin-binding site blocks PDGF-induced membrane ruffling. These data suggest a role for WIP in actin reorganization to form PDGF-induced ruffles. This is the first in vivo evidence in mammalian cells for a function of WIP dependent on its ability to bind actin.
2. WIP regulates persistence of cell **migration** and ruffle formation in both mesenchymal and amoeboid modes of motility. The spatial distribution of signals downstream from receptor tyrosine kinases (RTKs) or G-protein coupled receptors (GPCR) regulates fundamental cellular processes that control cell **migration** and growth. Both pathways rely significantly on actin cytoskeleton reorganization mediated by nucleation-promoting factors such as the WASP-(Wiskott-Aldrich Syndrome Protein) family. WIP (WASP Interacting Protein) is essential for the formation of a class of polarised actin microdomain, namely dorsal ruffles, downstream of the RTK for PDGF (platelet-derived growth factor) but the underlying mechanism is poorly understood. Using lentivirally-reconstituted WIP-deficient murine fibroblasts we define the requirement for WIP interaction with N-WASP (neural WASP) and Nck for efficient dorsal ruffle formation and of WIP-Nck binding for fibroblast chemotaxis towards PDGF-AA. The formation of both circular dorsal ruffles in PDGF-AA-stimulated primary fibroblasts and lamellipodia in CXCL13-treated B lymphocytes are also compromised by WIP-deficiency. We provide data to show that a WIP-Nck signalling complex interacts with RTK to promote polarised actin remodelling in fibroblasts and provide the first evidence for WIP involvement in the control of migratory persistence in both mesenchymal (fibroblast) and amoeboid (B lymphocytes) motility.
3. A role for WASP Interacting Protein, WIP, in fibroblast adhesion, spreading and **migration**. The WASP (Wiskott Aldrich Syndrome Protein) Interacting Protein, WIP, regulates actin polymerization and the formation of actin-rich structures such as filopodia and lamellipodia, each of which is involved in cellular adhesion, spreading and **migration**. To define the role for WIP in these activities, we analysed cell adhesion and spreading as well as the redistribution of polymerised actin and paxillin that occurred when fibroblasts were plated onto different substrata. We compared the effect of WIP overexpression (gain of function) with that of WIP deficiency (loss of function) on these parameters. WIP-overexpression delayed cellular adhesion and spreading, an effect that could be compensated for by exposure to Y-27632, a well characterized ROCK (Rho kinase) inhibitor. WIP overexpression augmented the phosphorylation of Erk and JNK induced by binding to fibronectin, suggesting that WIP participates in signal transduction pathways initiated by integrin engagement. Conversely, WIP deficiency accelerated fibroblast adhesion to plastic and led to the formation of enlarged focal adhesions. The influence of WIP on fibroblast **migration** was measured by scratch assay. WIP-overexpression reduced **migration** while WIP-deficiency increased it, suggesting that WIP acts as a negative regulator of fibroblast **migration**. Together, these findings suggest a novel role for WIP in fibroblast adhesion, spreading and **migration**.
4. Binding of the WASP/N-WASP-interacting protein WIP to actin regulates focal adhesion assembly and adhesion. The actin cytoskeleton is essential for cell adhesion and **migration**, functions important for tumor invasion. In addition to binding N-WASP/WASP, WIP binds and stabilizes F-actin. WIP(-/-) fibroblasts were used to test the role of WIP in F-actin function. WIP(-/-) cells had defective focal adhesion (FA), stress fiber assembly, and adherence to substrates, functions that were restored by transduction of wild-type WIP. Protein and mRNA levels of several FA constituents regulated by the myocardin-related transcription factor (MRTF)–serum response factor (SRF) transcription factor complex were reduced in WIP(-/-) fibroblasts. The level of G-actin, which sequesters MRTF in the cytoplasm, was increased, and nuclear localization of MRTF-A and SRF was reduced, in WIP(-/-) fibroblasts. Transfection of an MRTF-A mutant that constitutively translocates to the nucleus or transfection of constitutively active SRF restored FA and stress fiber assembly. Fibroblasts from knock-in mice expressing a WIP mutant that fails to bind actin phenocopied WIP(-/-) fibroblasts. Thus, WIP is a novel regulator of FA assembly and cell adhesion.
5. Binding of WIP to actin is essential for T cell actin cytoskeleton integrity and tissue homing. The Wiskott-Aldrich syndrome protein (WASp) is important for actin polymerization in T cells and for their **migration**. WASp-interacting protein (WIP) binds to and stabilizes WASp and also interacts with actin. Cytoskeletal and functional defects are more severe in WIP(-/-) T cells, which lack WASp, than in WASp(-/-) T cells, suggesting that WIP interaction with actin may be important for T cell cytoskeletal integrity and function. We constructed mice that lack the actin-binding domain of WIP (WIPΔABD mice). WIPΔABD associated normally with WASp but not F-actin. T cells from WIPΔABD mice had normal WASp levels but decreased cellular F-actin content, a disorganized actin cytoskeleton, impaired chemotaxis, and defective homing to lymph nodes. WIPΔABD mice exhibited a T cell intrinsic defect in contact hypersensitivity and impaired responses to cutaneous challenge with protein antigen. Adoptively transferred antigen-specific CD4(+) T cells from WIPΔABD mice had decreased homing to antigen-challenged skin of wild-type recipients. These findings show that WIP binding to actin, independently of its binding to WASp, is critical for the integrity of the actin cytoskeleton in T cells and for t**heir migr**ation into tissues. Disruption of WIP binding to actin could be of therapeutic value in T cell-driven inflammatory diseases.

#### GeneRIFs

1. WIP binding to actin, independently of its binding to Wiskott-Aldrich syndrome protein, is critical for the integrity of the actin cytoskeleton in T cells and for their **migration** into tissues.
2. WIP acts as a negative regulator of fibroblast **migration**.

### [Ii]nvasive

#### PubMed

1. Molecular mechanisms of invadopodium formation: the role of the N-WASP-Arp2/3 complex pathway and cofilin. Invadopodia are actin-rich membrane protrusions with a matrix degradation activity formed by **invasive** cancer cells. We have studied the molecular mechanisms of invadopodium formation in metastatic carcinoma cells. Epidermal growth factor (EGF) receptor kinase inhibitors blocked invadopodium formation in the presence of serum, and EGF stimulation of serum-starved cells induced invadopodium formation. RNA interference and dominant-negative mutant expression analyses revealed that neural WASP (N-WASP), Arp2/3 complex, and their upstream regulators, Nck1, Cdc42, and WIP, are necessary for invadopodium formation. Time-lapse analysis revealed that invadopodia are formed de novo at the cell periphery and their lifetime varies from minutes to several hours. Invadopodia with short lifetimes are motile, whereas long-lived invadopodia tend to be stationary. Interestingly, suppression of cofilin expression by RNA interference inhibited the formation of long-lived invadopodia, resulting in formation of only short-lived invadopodia with less matrix degradation activity. These results indicate that EGF receptor signaling regulates invadopodium formation through the N-WASP-Arp2/3 pathway and cofilin is necessary for the stabilization and maturation of invadopodia.

---

## Tlr3

- NCBI Gene
- GeneRIF
- Pubmed

| Search Term | Rank | Fields |
| --- | --- | --- |
| [Mm]igration | 6 | PubMed(10) |
| [Ee]xtracellular [Mm]atrix | 8 | PubMed(5); GeneRIFs(1) |
| [Ii]nvasive | 10 | PubMed(3) |
| [Mm]etastasis | 12 | PubMed(3) |
| [Cc]ell [Ii]nvasion | 13 | PubMed(1) |

  
hide/show details for Tlr3

### [Mm]igration

#### PubMed

1. Regulation of lung injury and repair by Toll-like receptors and hyaluronan. Mechanisms that regulate inflammation and repair after acute lung injury are incompletely understood. The extracellular matrix glycosaminoglycan hyaluronan is produced after tissue injury and impaired clearance results in unremitting inflammation. Here we report that hyaluronan degradation products require MyD88 and both Toll-like receptor (TLR)4 and TLR2 in vitro and in vivo to initiate inflammatory responses in acute lung injury. Hyaluronan fragments isolated from serum of individuals with acute lung injury stimulated macrophage chemokine production in a TLR4- and TLR2-dependent manner. Myd88(-/-) and Tlr4(-/-)Tlr2(-/-) mice showed impaired transepithelial **migration** of inflammatory cells but decreased survival and enhanced epithelial cell apoptosis after lung injury. Lung epithelial cell-specific overexpression of high-molecular-mass hyaluronan was protective against acute lung injury. Furthermore, epithelial cell-surface hyaluronan was protective against apoptosis, in part, through TLR-dependent basal activation of NF-kappaB. Hyaluronan-TLR2 and hyaluronan-TLR4 interactions provide signals that initiate inflammatory responses, maintain epithelial cell integrity and promote recovery from acute lung injury.
2. Activation of endothelial toll-like receptor 3 impairs endothelial function. Endothelial dysfunction and atherosclerosis are chronic inflammatory diseases characterized by activation of the innate and acquired immune system. Specialized protein receptors of the innate immune system recognize products of microorganisms and endogenous ligands such as nucleic acids. Toll-like receptor 3 (TLR3), for example, detects long double-stranded RNA and is abundantly expressed in endothelial cells. Whether innate immunity contributes to atherogenic mechanisms in endothelial cells is poorly understood.We sought to determine the effects of TLR3 activation in endothelial cells.We first investigated whether stimulation of TLR3 influences endothelial biology in mice. Intravenous injection of polyinosine polycytidylic acid, a synthetic double-stranded RNA analog and TLR3 ligand, impaired endothelium-dependent vasodilation, increased vascular production of reactive oxygen species, and reduced reendothelialization after carotid artery injury in wild-type mice compared with controls but had no effect in TLR3(-/-) animals. TLR3 stimulation not only induced endothelial dysfunction but also enhanced the formation of atherosclerotic plaques in apolipoprotein E-deficient mice. In vitro incubation of endothelial cells with polyinosine polycytidylic acid induced production of the proinflammatory cytokines interleukin-8 and interferon-γ-induced protein 10, increased formation of reactive oxygen species, diminished proliferation, and increased apoptosis, which suggests that endothelial cells are able to directly detect and respond to TLR3 ligands. Neutralization of interleukin-8 and interferon-γ-induced protein 10 antagonizes the observed negative effects of polyinosine polycytidylic acid. We found elevated levels of circulating endothelial progenitor cells in polyinosine polycytidylic acid-treated mice, although they displayed increased endothelial dysfunction. Stimulation of TLR3 in cultured endothelial progenitor cells, however, led to increased formation of reactive oxygen species, increased apoptosis, and reduce**d migrati**on. Injection of endothelial progenitor cells that had been incubated with polyinosine polycytidylic acid ex vivo hindered reendothelialization after carotid artery injury. Therefore, endothelial progenitor cell function was affected by TLR3 stimulation. Finally, apolipoprotein E-deficient/TLR3-deficient mice exhibited improved endothelial function compared with apolipoprotein E-deficient/TLR3(+/+) littermates.Immunorecognition of long double-stranded RNA by endothelial cells may be an important mechanism involved in endothelial cell activation and development of endothelial dysfunction.
3. Lung dendritic cells induce **migration** of protective T cells to the gastrointestinal tract. Developing efficacious vaccines against enteric diseases is a global challenge that requires a better understanding of cellular recruitment dynamics at the mucosal surfaces. The current paradigm of T cell homing to the gastrointestinal (GI) tract involves the induction of α4β7 and CCR9 by Peyer's patch and mesenteric lymph node (MLN) dendritic cells (DCs) in a retinoic acid-dependent manner. This paradigm, however, cannot be reconciled with reports of GI T cell responses after intranasal (i.n.) delivery of antigens that do not directly target the GI lymphoid tissue. To explore alternative pathways of cellula**r migrati**on, we have investigated the ability of DCs from mucosal and nonmucosal tissues to recruit lymphocytes to the GI tract. Unexpectedly, we found that lung DCs, like CD103(+) MLN DCs, up-regulate the gut-homing integrin α4β7 in vitro and in vivo, and induce T c**ell migra**tion to the GI tract in vivo. Consistent with a role for this pathway in generating mucosal immune responses, lung DC targeting by i.n. immunization induced protective immunity against enteric challenge with a highly pathogenic strain of Salmonella. The present report demonstrates novel functional evidence of mucosal cross talk mediated by DCs, which has the potential to inform the design of novel vaccines against mucosal pathogens.
4. The TLR3, PI3K, survivin, FasL, and Fas genes as major risk factors of occurrence and development of cervical cancer disease. To investigate the role of TLR3/PI3K signals in the occurrence and development of cervical cancer disease, TLR3-siRNA was used to block key signaling pathways involved in cervical cancer metastasis that are pivotal to metastatic tumor cells but not to normal cells under ordinary physiologic conditions. Results show that tumor U14 cell growth, **migration** and invasion in TLR3-siRNA treatment group were significantly decreased. Through LY294002 suppressing targeted gene, the LY294002 treatment specifically and significantly knocked down the expressions of tumor TLR3 and PI3K proteins in cervical cancer mice. Furthermore, expressions of tumor Survivin and FasL proteins were markedly suppressed, whereas expressions of Fas protein were upregulated in LY294002 treatment group mice. LY294002 treatment suppressed tumor growth and increased the thymus and spleen indeces and survival days of cervical cancer mice. This study demonstrates that TLR3-siRNA and LY294002 treatments can markedly suppress cervical cancer cell invasion and tumor growth and increase survival life by silencing targeted genes.
5. Damaged spermatogenic cells induce inflammatory gene expression in mouse Sertoli cells through the activation of Toll-like receptors 2 and 4. Testicular inflammation, including noninfectious inflammatory responses in the testis, may impair male fertility. Mechanisms underlying the initiation of noninfectious testicular inflammation are poorly understood. In the current study, we demonstrate that damaged spermatogenic cell products (DSCPs) induce expression of various inflammatory mediators, including TNF-α, IL-1β, IL-6, and macrophage chemotactic protein 1 (MCP-1), in Sertoli cells. Notably, the DSCP-induced inflammatory gene expression was significantly reduced by knockout Toll-like receptor (TLR)2 or TLR4, and abolished by double knockout TLR2 and TLR4 (TLR2(-/-)TLR4(-/-)). MCP-1 secreted by Sertoli cells after stimulation with DSCPs promotes macrophag**e migrati**on. We also provide evidence that busulfan-induced spermatogenic cell damages in vivo upregulate TNF-α and MCP-1 expression in Sertoli cells, and facilitate macrophage infiltration into the testis in wild-type mice. These phenomena were not observed in TLR2(-/-)TLR4(-/-) mice. Data indicate that DSCPs induce inflammatory gene expression in Sertoli cells via the activation of TLR2 and TLR4, which may initiate noninfectious inflammatory responses in the testis. The results provide novel insights into the mechanisms underlying damaged spermatogenic cell-induced testicular inflammation.
6. Toll-like receptor 3 increases allergic and irritant contact dermatitis. There is increasing recognition of the role of Toll-like receptor 3 (TLR3) in noninfectious inflammatory diseases, but the function of TLR3 in inflammatory skin diseases is unclear. We investigated the functions of TLR3 in allergic and irritant contact dermatitis (ICD). The contact hypersensitivity (CHS) response was lower in Toll-like receptor 3 knockout (Tlr3 KO) mice, and was greater in TLR3 transgenic (Tg) mice than in wild-type (WT) mice after challenge with 2,4,6-trinitro-1-chlorobenzene. Adoptive transfer of immunized lymph node cells from Tlr3 KO mice induced CHS in WT recipients. In contrast, adoptive transfer of those from WT mice did not fully induce CHS in Tlr3 KO recipients. The ICD reaction following croton oil application was lower in Tlr3 KO mice, and was greater in TLR3 Tg mice than in WT mice. Maturation, **migration**, and antigen presentation of dendritic cells and proliferation of lymphocytes between WT mice and Tlr3 KO mice were comparable. These results show that TLR3 enhances antigen-independent skin inflammation in the elicitation phase of allergic contact dermatitis and in ICD.
7. TLR3 but not TLR7/8 ligand induces allergic sensitization to inhaled allergen. Epidemiological studies suggest that viral infections during childhood are a risk factor for the development of asthma. However, the role of virus-specific pattern recognition receptors in this process is not well defined. In the current study, we compare the effects of the inhaled viral TLR ligands polyinosinic-polycytidylic acid (TLR3) and resiquimod (TLR7/8) on sensitization to a model allergen (OVA) in a murine model. Both compounds enhance the **migration**, activation, and Ag-processing of myeloid dendritic cells from the lung to the draining lymph nodes comparable to the effects of LPS. Application of polyinosinic-polycytidylic acid [poly(I:C)] or LPS induces production of allergen-specific IgE and IgG1, whereas resiquimod (R848) had no effect. In addition, rechallenge of mice with OVA resulted in airway inflammation and mucus production in animals that received either poly(I:C) or LPS but not after application of R848. In summary, these results show that activation of TLR3 in combination with inhaled allergen results in induction of dendritic cell activation and **migration** similar to the effects of LPS. This leads to the development of allergic airway disease after allergen rechallenge, whereas mice treated with R848 did not develop allergic airway disease. These findings give further insight into the effects of stimulation of different TLRs on the development of asthma.
8. A TRIF-independent branch of TLR3 signaling. dsRNA is a common pathogen-associated molecular pattern that is recognized by cellular TLR3 and used by virus-infected cells to activate specific transcription factors and trigger induction of antiviral genes. In this article, we report a new branch of TLR3 signaling that does not lead to gene induction but affects many cellular properties, such as cell **migration**, adhesion, and proliferation. We demonstrated that the **migration** of multiple cell lineages was affected by dsRNA treatment or influenza virus infection in a TLR3-dependent fashion. Surprisingly, for this effect of TLR3 signaling, the adaptor proteins, TRIF and MyD88, were not required. The effects of the new pathway were mediated by the proto-oncoprotein c-Src, which bound to TLR3 after dsRNA stimulation of cells. The response was biphasic: upon dsRNA treatment, we observed an immediate increase in cell motility followed by its strong inhibition. Our results indicate that the first phase was mediated by dsRNA-induced phosphorylation and activation of Src, whereas the second phase resulted from the sequestration of activated Src in lipid rafts, thus decreasing its active cytoplasmic pool. As expected, two other functions of Src, its effect on cell adhesion and cell proliferation, were also inhibited by dsRNA treatment. These results demonstrate that activated TLR3 can engage Src to trigger multiple cellular effects and reveal a possible link between innate immune response and cell growth regulation. This study also provides a rare example of TLR-mediated cellular effects that do not require gene induction and the first example, to our knowledge, of an adaptor-independent effect of any TLR.
9. Matrix metalloprotease 9 mediates neutrophil **migration** into the airways in response to influenza virus-induced toll-like receptor signaling. The early inflammatory response to influenza virus infection contributes to severe lung disease and continues to pose a serious threat to human health. The mechanisms by which neutrophils gain entry to the respiratory tract and their role during pathogenesis remain unclear. Here, we report that neutrophils significantly contributed to morbidity in a pathological mouse model of influenza virus infection. Using extensive immunohistochemistry, bone marrow transfers, and depletion studies, we identified neutrophils as the predominant pulmonary cellular source of the gelatinase matrix metalloprotease (MMP) 9, which is capable of digesting the extracellular matrix. Furthermore, infection of MMP9-deficient mice showed that MMP9 was functionally required for neutrophil **migration** and control of viral replication in the respiratory tract. Although MMP9 release was toll-like receptor (TLR) signaling-dependent, MyD88-mediated signals in non-hematopoietic cells, rather than neutrophil TLRs themselves, were important for neutrophil **migration**. These results were extended using multiplex analyses of inflammatory mediators to show that neutrophil chemotactic factor, CCL3, and TNFα were reduced in the Myd88⁻/⁻ airways. Furthermore, TNFα induced MMP9 secretion by neutrophils and blocking TNFα in vivo reduced neutrophil recruitment after infection. Innate recognition of influenza virus therefore provides the mechanisms to induce recruitment of neutrophils through chemokines and to enable their motility within the tissue via MMP9-mediated cleavage of the basement membrane. Our results demonstrate a previously unknown contribution of MMP9 to influenza virus pathogenesis by mediating excessive neut**rophil mi**gration into the respiratory tract in response to viral replication that could be exploited for therapeutic purposes.
10. Cardiac RNA induces inflammatory responses in cardiomyocytes and immune cells via Toll-like receptor 7 signaling. We have recently reported that extracellular RNA (exRNA) released from necrotic cells induces cytokine production in cardiomyocytes and immune cells and contributes to myocardial ischemia/reperfusion injury. However, the signaling mechanism by which exRNA exhibits its pro-inflammatory effect is unknown. Here we hypothesize that exRNA directly induces inflammation through specific Toll-like receptors (TLRs). To test the hypothesis, we treated rat neonatal cardiomyocytes, mouse bone marrow-derived macrophages (BMDM), or mouse neutrophils with RNA (2.5-10 μg/ml) isolated from rat cardiomyocytes or the hearts from mouse, rat, and human. We found that cellular RNA induced production of several cytokines such as macrophage inflammatory protein-2 (MIP-2), ILs, TNFα, and the effect was completely diminished by RNase, but not DNase. The RNA-induced cytokine production was partially inhibited in cells treated with TLR7 antagonist or genetically deficient in TLR7. Deletion of myeloid differentiation primary response protein 88 (MyD88), a downstream adapter of TLRs including TLR7, abolished the RNA-induced MIP-2 production. Surprisingly, genetic deletion of TLR3 had no impact on the RNA-induced MIP-2 response. Importantly, extracellular RNA released from damaged cardiomyocytes also induced cytokine production. Finally, mice treated with 50 μg of RNA intraperitoneal injection exhibited acute peritonitis as evidenced by marked neutrophil and monocy**te migrat**ion into the peritoneal space. Together, these data demonstrate that exRNA of cardiac origin exhibits a potent pro-inflammatory property in vitro and in vivo and that exRNA induces cytokine production through TLR7-MyD88 signaling.

### [Ee]xtracellular [Mm]atrix

#### PubMed

1. The role of hyaluronan degradation products as innate alloimmune agonists. Dendritic cells (DCs) play a key role in initiating alloimmunity yet the substances that activate them during the host response to transplantation remain elusive. In this study we examined the potential roles of endogenous innate immune agonists in activating dendritic cell-dependent alloimmunity. Using a murine in vitro culture system, we show that 135 KDa fragments of the **extracellular matrix** glycosaminoglycan hyaluronan induce dendritic cell maturation and initiate alloimmunity. Priming of alloimmunity by hyaluronan-activated DCs was dependent on signaling via TIR-associated protein, a Toll-like receptor (TLR) adaptor downstream of TLRs 2 and 4. However, this effect was independent of alternate TLR adaptors, MyD88 or Trif. Using an in vivo murine transplant model, we show that hyaluronan accumulated during skin transplant rejection. Examination of human lung transplant recipients demonstrated that increased levels of intragraft hyaluronan were associated with bronchiolitis obliterans syndrome. In conclusion, our study suggests that fragments of hyaluronan can act as innate immune agonists that activate alloimmunity.
2. Regulation of lung injury and repair by Toll-like receptors and hyaluronan. Mechanisms that regulate inflammation and repair after acute lung injury are incompletely understood. The **extracellular matrix** glycosaminoglycan hyaluronan is produced after tissue injury and impaired clearance results in unremitting inflammation. Here we report that hyaluronan degradation products require MyD88 and both Toll-like receptor (TLR)4 and TLR2 in vitro and in vivo to initiate inflammatory responses in acute lung injury. Hyaluronan fragments isolated from serum of individuals with acute lung injury stimulated macrophage chemokine production in a TLR4- and TLR2-dependent manner. Myd88(-/-) and Tlr4(-/-)Tlr2(-/-) mice showed impaired transepithelial migration of inflammatory cells but decreased survival and enhanced epithelial cell apoptosis after lung injury. Lung epithelial cell-specific overexpression of high-molecular-mass hyaluronan was protective against acute lung injury. Furthermore, epithelial cell-surface hyaluronan was protective against apoptosis, in part, through TLR-dependent basal activation of NF-kappaB. Hyaluronan-TLR2 and hyaluronan-TLR4 interactions provide signals that initiate inflammatory responses, maintain epithelial cell integrity and promote recovery from acute lung injury.
3. Deleterious role of TLR3 during hyperoxia-induced acute lung injury. Acute respiratory distress syndrome (ARDS) manifests clinically as a consequence of septic and/or traumatic injury in the lung. Oxygen therapy remains a major therapeutic intervention in ARDS, but this can contribute further to lung damage. Patients with ARDS are highly susceptible to viral infection and it may be due to altered Toll-like receptor (TLR) expression.To evaluate the role of TLR3 in ARDS.TLR3 expression and signaling was determined in airway epithelial cells after in vitro hyperoxia challenge. Using a murine model of hyperoxia-induced lung injury, the role of TLR3 was determined using either TLR3-gene deficient mice or a specific neutralizing antibody directed to TLR3.Increased TLR3 expression was observed in airway epithelial cells from patients with ARDS. Further, hyperoxic conditions alone were a major stimulus for increased TLR3 expression and activation in cultured human epithelial cells. Interestingly, TLR3(-/-) mice exhibited less acute lung injury, activation of apoptotic cascades, and **extracellular matrix** deposition after 5 days of 80% oxygen compared with wild-type (TLR3(+/+)) mice under the same conditions. Administration of a monoclonal anti-TLR3 antibody to TLR3(+/+) mice exposed to hyperoxic conditions likewise protected these mice from lung injury and inflammation.The potential for redundancy in function as well as cross-talk between distinct TLRs may indeed contribute to whether the inflammatory cascade can be effectively disrupted once signaling has been initiated. Together, these data show that TLR3 has a major role in the development of ARDS-like pathology in the absence of a viral pathogen.
4. TLR3 deficiency protects against collagen degradation and medial destruction in murine atherosclerotic plaques. Inflammatory cell activation plays a key role in atherosclerotic plaque growth and acute complications. While secretion of proteases and inflammatory cytokines are likely involved in the development of plaque instability, the precise mechanistic pathways are not well understood.Based on our previous study, we crossed Toll-like receptor 3 (Tlr3)(-/-) mice with a unique BALB-Apoe(-/-)Npc1(-/-) plaque complication-susceptible mouse model, as well as the widely-used B6-Ldlr(-/-) atherosclerosis model, to test the role of TLR3 signaling in the development of plaque instability. TLR3-deficient mice showed no change in aortic root lesion area, but displayed a marked increase in collagen and smooth muscle cell (SMC) content of lesions. Notably, Apoe(-/-)Npc1(-/-)Tlr3(-/-) mice exhibited a 50% reduction in the incidence of medial destruction, a precursor to aortic aneurysm formation. MMP-2 activity was markedly reduced in aortic extracts from Apoe(-/-)Npc1(-/-)Tlr3(-/-) compared to controls, while both MMP-2 and -9 activities were reduced in Ldlr(-/-)Tlr3(-/-) extracts. Consistent with the in vivo data, TLR3 deficiency suppressed MMP-2 activity induced by TNF-α or polyinosine-polycytidylic acid in macrophages from Apoe(-/-)Npc1(-/-) mice.TLR3 plays a critical role in regulating the degradation o**f extracellular matr**ix in lesions, in part by modulation of macrophage MMP-2 and -9 activities.
5. Matrix metalloprotease 9 mediates neutrophil migration into the airways in response to influenza virus-induced toll-like receptor signaling. The early inflammatory response to influenza virus infection contributes to severe lung disease and continues to pose a serious threat to human health. The mechanisms by which neutrophils gain entry to the respiratory tract and their role during pathogenesis remain unclear. Here, we report that neutrophils significantly contributed to morbidity in a pathological mouse model of influenza virus infection. Using extensive immunohistochemistry, bone marrow transfers, and depletion studies, we identified neutrophils as the predominant pulmonary cellular source of the gelatinase matrix metalloprotease (MMP) 9, which is capable of digesting the **extracellular matrix**. Furthermore, infection of MMP9-deficient mice showed that MMP9 was functionally required for neutrophil migration and control of viral replication in the respiratory tract. Although MMP9 release was toll-like receptor (TLR) signaling-dependent, MyD88-mediated signals in non-hematopoietic cells, rather than neutrophil TLRs themselves, were important for neutrophil migration. These results were extended using multiplex analyses of inflammatory mediators to show that neutrophil chemotactic factor, CCL3, and TNFα were reduced in the Myd88⁻/⁻ airways. Furthermore, TNFα induced MMP9 secretion by neutrophils and blocking TNFα in vivo reduced neutrophil recruitment after infection. Innate recognition of influenza virus therefore provides the mechanisms to induce recruitment of neutrophils through chemokines and to enable their motility within the tissue via MMP9-mediated cleavage of the basement membrane. Our results demonstrate a previously unknown contribution of MMP9 to influenza virus pathogenesis by mediating excessive neutrophil migration into the respiratory tract in response to viral replication that could be exploited for therapeutic purposes.

#### GeneRIFs

1. TLR3 plays a critical role in regulating the degradation of **extracellular matrix** in lesions, in part by modulation of macrophage MMP-2 and -9 activities.

### [Ii]nvasive

#### PubMed

1. In vivo IFN-γ secretion by NK cells in response to Salmonella typhimurium requires NLRC4 inflammasomes. Natural killer (NK) cells are a critical part of the innate immune defense against viral infections and for the control of tumors. Much less is known about how NK cells contribute to anti-bacterial immunity. NK cell-produced interferon gamma (IFN-γ) contributes to the control of early exponential replication of bacterial pathogens, however the regulation of these events remains poorly resolved. Using a mouse model o**f invasi**ve Salmonellosis, here we report that the activation of the intracellular danger sensor NLRC4 by Salmonella-derived flagellin within CD11c+ cells regulates early IFN-γ secretion by NK cells through the provision of interleukin 18 (IL-18), independently of Toll-like receptor (TLR)-signaling. Although IL18-signalling deficient NK cells improved host protection during S. Typhimurium infection, this increased resistance was inferior to that provided by wild-type NK cells. These findings suggest that although NLRC4 inflammasome-driven secretion of IL18 serves as a potent activator of NK cell mediated IFN-γ secretion, IL18-independent NK cell-mediated mechanisms of IFN-γ secretion contribute to in vivo control of Salmonella replication.
2. Toll-like receptor 3 is required for development of retinopathy caused by impaired all-trans-retinal clearance in mice. Chronic inflammation is an important component that contributes to many age-related neurodegenerative diseases, including macular degeneration. Here, we report a role for toll-like receptor 3 (TLR3) in cone-rod dystrophy (CORD) of mice lacking ATP-binding cassette transporter 4 (ABCA4) and retinol dehydrogenase 8 (RDH8), proteins critical for all-trans-retinal clearance in the retina. Increased expression of toll-like receptor-signaling elements and inflammatory changes were observed in Rdh8(-/-)Abca4(-/-) eyes by RNA expression analysis. Unlike 3-month-old Rdh8(-/-)Abca4(-/-) mice that developed CORD, 6-month-old Tlr3(-/-)Rdh8(-/-)Abca4(-/-) mice did not evidence an abnormal retinal phenotype. Light-induced retinal degeneration in Tlr3(-/-)Rdh8(-/-)Abca4(-/-) mice was milder than that in Rdh8(-/-)Abca4(-/-) mice, and a 2-fold increased TLR3 expression was detected in light-illuminated retinas of Rdh8(-/-)Abca4(-/-) mice compared with nonilluminated retinas. Poly(I-C), a TLR3 ligand, caused caspase-8-independent cellular apoptosis. Whereas poly(I-C) induced retinal cell death in Rdh8(-/-)Abca4(-/-) and WT mice both in vivo and ex vivo, this was not seen in mice lacking Tlr3. Far fewer **invasive** macrophage/microglial cells in the subretinal space and weaker activation of Muller glial cells were exhibited by Tlr3(-/-)Rdh8(-/-) Abca4(-/-) mice compared with Rdh8(-/-)Abca4(-/-) mice at 3 and 6 months of age, indicating that loss of TLR3 inhibits local inflammation in the retina. Both poly(I-C) and endogenous products emanating from dying/dead retinal cells induced NF-κB and IRF3 activation. These findings demonstrate that endogenous products from degenerating retina stimulate TLR3 that causes cellular apoptosis and retinal inflammation and that loss of TLR3 protects mice from CORD.
3. Long-term activation of TLR3 by poly(I:C) induces inflammation and impairs lung function in mice. The immune mechanisms associated with infection-induced disease exacerbations in asthma and COPD are not fully understood. Toll-like receptor (TLR) 3 has an important role in recognition of double-stranded viral RNA, which leads to the production of various inflammatory mediators. Thus, an understanding of TLR3 activation should provide insight into the mechanisms underlying virus-induced exacerbations of pulmonary diseases.TLR3 knock-out (KO) mice and C57B6 (WT) mice were intranasally administered repeated doses of the synthetic double stranded RNA analog poly(I:C).There was a significant increase in total cells, especially neutrophils, in BALF samples from poly(I:C)-treated mice. In addition, IL-6, CXCL10, JE, KC, mGCSF, CCL3, CCL5, and TNFalpha were up regulated. Histological analyses of the lungs revealed a cellular infiltrate in the interstitium and epithelial cell hypertrophy in small bronchioles. Associated with the pro-inflammatory effects of poly(I:C), the mice exhibited significant impairment of lung function both at baseline and in response to methacholine challenge as measured by whole body plethysmography and an **invasive** measure of airway resistance. Importantly, TLR3 KO mice were protected from poly(I:C)-induced changes in lung function at baseline, which correlated with milder inflammation in the lung, and significantly reduced epithelial cell hypertrophy.These findings demonstrate that TLR3 activation by poly(I:C) modulates the local inflammatory response in the lung and suggest a critical role of TLR3 activation in driving lung function impairment. Thus, TLR3 activation may be one mechanism through which viral infections contribute toward exacerbation of respiratory disease.

### [Mm]etastasis

#### PubMed

1. Tumor Exosomal RNAs Promote Lung Pre-metastatic Niche Formation by Activating Alveolar Epithelial TLR3 to Recruit Neutrophils. The pre-metastatic niche educated by primary tumor-derived elements contributes to cancer **metastasis**. However, the role of host stromal cells in metastatic niche formation and organ-specific metastatic tropism is not clearly defined. Here, we demonstrate that lung epithelial cells are critical for initiating neutrophil recruitment and lung metastatic niche formation by sensing tumor exosomal RNAs via Toll-like receptor 3 (TLR3). TLR3-deficient mice show reduced lung **metastasi**s in the spontaneous metastatic models. Mechanistically, primary tumor-derived exosomal RNAs, which are enriched in small nuclear RNAs, activate TLR3 in lung epithelial cells, consequently inducing chemokine secretion in the lung and promoting neutrophil recruitment. Identification of metastatic axis of tumor exosomal RNAs and host lung epithelial cell TLR3 activation provides potential targets to control cancer **metastasi**s to the lung.
2. The TLR3, PI3K, survivin, FasL, and Fas genes as major risk factors of occurrence and development of cervical cancer disease. To investigate the role of TLR3/PI3K signals in the occurrence and development of cervical cancer disease, TLR3-siRNA was used to block key signaling pathways involved in cervical cancer **metastasis** that are pivotal to metastatic tumor cells but not to normal cells under ordinary physiologic conditions. Results show that tumor U14 cell growth, migration and invasion in TLR3-siRNA treatment group were significantly decreased. Through LY294002 suppressing targeted gene, the LY294002 treatment specifically and significantly knocked down the expressions of tumor TLR3 and PI3K proteins in cervical cancer mice. Furthermore, expressions of tumor Survivin and FasL proteins were markedly suppressed, whereas expressions of Fas protein were upregulated in LY294002 treatment group mice. LY294002 treatment suppressed tumor growth and increased the thymus and spleen indeces and survival days of cervical cancer mice. This study demonstrates that TLR3-siRNA and LY294002 treatments can markedly suppress cervical cancer cell invasion and tumor growth and increase survival life by silencing targeted genes.
3. Ultraviolet-radiation-induced inflammation promotes angiotropism and **metastasis** in melanoma. Intermittent intense ultraviolet (UV) exposure represents an important aetiological factor in the development of malignant melanoma. The ability of UV radiation to cause tumour-initiating DNA mutations in melanocytes is now firmly established, but how the microenvironmental effects of UV radiation influence melanoma pathogenesis is not fully understood. Here we report that repetitive UV exposure of primary cutaneous melanomas in a genetically engineered mouse model promotes metastatic progression, independent of its tumour-initiating effects. UV irradiation enhanced the expansion of tumour cells along abluminal blood vessel surfaces and increased the number of lung metastases. This effect depended on the recruitment and activation of neutrophils, initiated by the release of high mobility group box 1 (HMGB1) from UV-damaged epidermal keratinocytes and driven by Toll-like receptor 4 (TLR4). The UV-induced neutrophilic inflammatory response stimulated angiogenesis and promoted the ability of melanoma cells to migrate towards endothelial cells and use selective motility cues on their surfaces. Our results not only reveal how UV irradiation of epidermal keratinocytes is sensed by the innate immune system, but also show that the resulting inflammatory response catalyses reciprocal melanoma-endothelial cell interactions leading to perivascular invasion, a phenomenon originally described as angiotropism in human melanomas by histopathologists. Angiotropism represents a hitherto underappreciated mechanism of **metastasis** that also increases the likelihood of intravasation and haematogenous dissemination. Consistent with our findings, ulcerated primary human melanomas with abundant neutrophils and reactive angiogenesis frequently show angiotropism and a high risk for metastases. Our work indicates that targeting the inflammation-induced phenotypic plasticity of melanoma cells and their association with endothelial cells represent rational strategies to specifically interfere with metastatic progression.

### [Cc]ell [Ii]nvasion

#### PubMed

1. The TLR3, PI3K, survivin, FasL, and Fas genes as major risk factors of occurrence and development of cervical cancer disease. To investigate the role of TLR3/PI3K signals in the occurrence and development of cervical cancer disease, TLR3-siRNA was used to block key signaling pathways involved in cervical cancer metastasis that are pivotal to metastatic tumor cells but not to normal cells under ordinary physiologic conditions. Results show that tumor U14 cell growth, migration and invasion in TLR3-siRNA treatment group were significantly decreased. Through LY294002 suppressing targeted gene, the LY294002 treatment specifically and significantly knocked down the expressions of tumor TLR3 and PI3K proteins in cervical cancer mice. Furthermore, expressions of tumor Survivin and FasL proteins were markedly suppressed, whereas expressions of Fas protein were upregulated in LY294002 treatment group mice. LY294002 treatment suppressed tumor growth and increased the thymus and spleen indeces and survival days of cervical cancer mice. This study demonstrates that TLR3-siRNA and LY294002 treatments can markedly suppress cervical cancer **cell invasion** and tumor growth and increase survival life by silencing targeted genes.

---

## Casp9

- NCBI Gene
- GeneRIF
- Pubmed

| Search Term | Rank | Fields |
| --- | --- | --- |
| [Mm]igration | 6 | PubMed(2) |
| ECM | 7 | PubMed(2) |
| [Ee]xtracellular [Mm]atrix | 8 | PubMed(1) |

  
hide/show details for Casp9

### [Mm]igration

#### PubMed

1. Functions of a jumonji-cyclin D1 pathway in the coordination of cell cycle exit and **migration** during neurogenesis in the mouse hindbrain. During development of the mouse central nervous system (CNS), most neural progenitor cells proliferate in the ventricular zone (VZ). In many regions of the CNS, neural progenitor cells give rise to postmitotic neurons that initiate neuronal differentiation and migrate out of the VZ to the mantle zone (MZ). Thereafter, they remain in a quiescent state. Here, we found many ectopic mitotic cells and cell clusters expressing neural progenitor or proneural marker genes in the MZ of the hindbrain of jumonji (jmj) mutant embryos. When we examined the expression of cyclin D1, which is repressed by jmj in the repression of cardiac myocyte proliferation, we found many ectopic clusters expressing both cyclin D1 and Musashi 1 in the MZ of mutant embryos. jmj is mainly expressed in the cyclin D1 negative region in the hindbrain, and cyclin D1 expression in the VZ was upregulated in jmj mutant mice. In jmj and cyclin D1 double mutant mice, the ectopic mitosis and formation of the abnormal clusters in the MZ were rescued. These results suggest that a jmj-cyclin D1 pathway is required for the precise coordination of cell cycle exit and **migration** during neurogenesis in the mouse hindbrain.
2. Incudomalleal joint formation: the roles of apoptosis, **migration** and downregulation. The middle ear of mammals is composed of three endochondrial ossicles, the stapes, incus and malleus. Joints link the malleus to the incus and the incus to the stapes. In the mouse the first arch derived malleus and incus are formed from a single Sox9 and Type II collagen expressing condensation that later subdivides to give rise to two separate ossicles. In contrast the stapes forms from a separate condensation derived from the second branchial arch. Fusion of the malleus and incus is observed in a number of human syndromes and results in conductive hearing loss. Understanding how this joint forms during normal development is thus an important step in furthering our understanding of such defects.We show that the developing incudomalleal joint is characterised by a lack of proliferation and discrete areas of apoptosis. Apoptosis has been suggested to aid in the removal of pre-cartilaginous cells from the joint region, allowing for the physical separation of the cartilaginous elements, however, we show that joint initiation is unaffected by blocking apoptosis. There is also no evidence of cell **migration** out of the presumptive joint region, as observed by labelling of joint and ossicle cells in culture. Using Type II collagen lacZ reporter mice, however, it is evident that cells in the presumptive joint region remain in place and downregulate cartilage markers.The malleus and incus first appear as a single united condensation expressing early cartilage markers. The incudomalleal joint region forms by cells in the presumptive joint region switching off cartilage markers and turning on joint markers. Failure in this process may result in fusion of this joint, as observed in human syndromes such as Branchio-Oto-Renal Syndrome or Treacher Collins Syndrome.

### ECM

#### PubMed

1. Caspase-9, caspase-3 and caspase-7 have distinct roles during intrinsic apoptosis. Apoptosis is a form of programmed cell death that is regulated by the Bcl-2 family and caspase family of proteins. The caspase cascade responsible for executing cell death following cytochrome c release is well described; however the distinct roles of caspases-9, -3 and -7 during this process are not completely defined.Here we demonstrate several unique functions for each of these caspases during cell death. Specific inhibition of caspase-9 allows for efficient release of cytochrome c, but blocks changes in mitochondrial morphology and ROS production. We show that caspase-9 can cleave Bid into tBid at amino acid 59 and that this cleavage of Bid is required for ROS production following serum withdrawal. We also demonstrate that caspase-3-deficient MEFs are less sensitive to intrinsic cell death stimulation, yet have higher ROS production. In contrast, caspase-7-deficient MEFs are not resistance to intrinsic cell death, but remain attached to the **ECM**.Taken together, these data suggest that caspase-9 is required for mitochondrial morphological changes and ROS production by cleaving and activating Bid into tBid. After activation by caspase-9, caspase-3 inhibits ROS production and is required for efficient execution of apoptosis, while effector caspase-7 is required for apoptotic cell detachment.
2. Caspase-9, caspase-3 and caspase-7 have distinct roles during intrinsic apoptosis. Apoptosis is a form of programmed cell death that is regulated by the Bcl-2 family and caspase family of proteins. The caspase cascade responsible for executing cell death following cytochrome c release is well described; however the distinct roles of caspases-9, -3 and -7 during this process are not completely defined.Here we demonstrate several unique functions for each of these caspases during cell death. Specific inhibition of caspase-9 allows for efficient release of cytochrome c, but blocks changes in mitochondrial morphology and ROS production. We show that caspase-9 can cleave Bid into tBid at amino acid 59 and that this cleavage of Bid is required for ROS production following serum withdrawal. We also demonstrate that caspase-3-deficient MEFs are less sensitive to intrinsic cell death stimulation, yet have higher ROS production. In contrast, caspase-7-deficient MEFs are not resistance to intrinsic cell death, but remain attached to the **ECM**.Taken together, these data suggest that caspase-9 is required for mitochondrial morphological changes and ROS production by cleaving and activating Bid into tBid. After activation by caspase-9, caspase-3 inhibits ROS production and is required for efficient execution of apoptosis, while effector caspase-7 is required for apoptotic cell detachment.

### [Ee]xtracellular [Mm]atrix

#### PubMed

1. Altered apoptosis of inflammatory neutrophils in MMP-9-deficient mice is due to lower expression and activity of caspase-3. Matrix metalloproteinase 9 (MMP-9) is a Zn(2+)-dependent endopeptidase that degrades some of the components of basement membranes and **extracellular matrix** and thus participates in leukocyte infiltration during inflammation. In a model of zymosan peritonitis, neutrophil infiltration in MMP-deficient (MMP-9(-/-)) mice was significantly weaker at the time of their maximal influx in wild-type mice (6h). However, during the late stages of peritonitis (24h) an extended accumulation of neutrophils was observed in MMP-9(-/-)versus the wild-type mice. Recently, we reported that the ratio of apoptosis of inflammatory leukocytes is impaired in MMP-9(-/-) mice during late peritonitis and the process depends on COX-1-driven PGE(2). Here we scrutinized the alterations in apoptotic mechanisms by comparisons between MMP-9(-/-) and the wild-type mice. Altered apoptosis occurred only during late (24h) peritonitis and concerned only neutrophils, and not macrophages, mast cells or lymphocytes. Furthermore, expression and activity of caspases was altered in MMP-9(-/-) animals, delayed for caspase-8 and -9, and decreased in the case of caspase-3. Also the expression of Bax/Bcl-2 proteins was changed in MMP-9(-/-) mice. These changes, and in particular the impaired neutrophil apoptosis and weaker caspase-3 activity, were restored by the selective COX-1 inhibition. We conclude that in mice lacking MMP-9 the enhanced COX-1-PGE(2) decreases caspase-3 expression and activity leading to impaired apoptosis of inflammatory neutrophils resulting in abnormal accumulation of the cells at the inflammatory focus. The data also reinforce the notion that MMP-9 is a key enzyme in neutrophil biology.

---

## Casp2

- NCBI Gene
- GeneRIF
- Pubmed

| Search Term | Rank | Fields |
| --- | --- | --- |
| [Mm]igration | 6 | PubMed(1) |
| [Ee]xtracellular [Mm]atrix | 8 | PubMed(1) |
| [Mm]etastasize | 11 | PubMed(1) |

  
hide/show details for Casp2

### [Mm]igration

#### PubMed

1. Robo1 modulates proliferation and neurogenesis in the developing neocortex. The elaborate cytoarchitecture of the mammalian neocortex requires the timely production of its constituent pyramidal neurons and interneurons and their disposition in appropriate layers. Numerous chemotropic factors present in the forebrain throughout cortical development play important roles in the orchestration of these events. The Roundabout (Robo) family of receptors and their ligands, the Slit proteins, are expressed in the developing forebrain, and are known to play important roles in the generation and **migration** of cortical interneurons. However, few studies have investigated their function(s) in the development of pyramidal cells. Here, we observed expression of Robo1 and Slit genes (Slit1, Slit2) in cells lining the telencephalic ventricles, and found significant increases in progenitor cells (basal and apical) at embryonic day (E)12.5 and E14.5 in the developing cortex of Robo1(-/-), Slit1(-/-), and Slit1(-/-)/Slit2(-/-), but not in mice lacking the other Robo or Slit genes. Using layer-specific markers, we found that both early- and late-born pyramidal neuron populations were significantly increased in the cortices of Robo1(-/-) mice at the end of corticogenesis (E18.5). The excess number of cortical pyramidal neurons generated prenatally appears to die in early postnatal life. The observed increase in pyramidal neurons was due to prolonged proliferative activity of their progenitors and not due to changes in cell cycle events. This finding, confirmed by in utero electroporation with Robo1 short hairpin RNA (shRNA) or control constructs into progenitors along the ventricular zone as well as in dissociated cortical cell cultures, points to a novel role for Robo1 in regulating the proliferation and generation of pyramidal neurons.

### [Ee]xtracellular [Mm]atrix

#### PubMed

1. Oncogenic ras-induced down-regulation of pro-apoptotic protease caspase-2 is required for malignant transformation of intestinal epithelial cells. Resistance of carcinoma cells to anoikis, apoptosis that is normally induced by loss of cell-to-**extracellular matrix** adhesion, is thought to be essential for the ability of these cells to form primary tumors, invade adjacent tissues, and metastasize to distant organs. Current knowledge about the mechanisms by which cancer cells evade anoikis is far from complete. In an effort to understand these mechanisms, we found that ras, a major oncogene, down-regulates protease caspase-2 (which initiates certain steps of the cellular apoptotic program) in malignant human and rat intestinal epithelial cells. This down-regulation could be reversed by inhibition of a protein kinase Mek, a mediator of Ras signaling. We also found that enforced down-regulation of caspase-2 in nonmalignant intestinal epithelial cells by RNA interference protected them from anoikis. Furthermore, the reversal of the effect of Ras on caspase-2 achieved by the expression of exogenous caspase-2 in detached ras-transformed intestinal epithelial cells promoted well established apoptotic events, such as the release of the pro-apoptotic mitochondrial factors cytochrome c and HtrA2/Omi into the cytoplasm of these cells, significantly enhanced their anoikis susceptibility, and blocked their long term growth in the absence of adhesion to the **extracellular matrix**. Finally, the blockade of the effect of Ras on caspase-2 substantially suppressed growth of tumors formed by the ras-transformed cells in mice. We conclude that ras-induced down-regulation of caspase-2 represents a novel mechanism by which oncogenic Ras protects malignant intestinal epithelial cells from anoikis, promotes their anchorage-independent growth, and allows them to form tumors in vivo.

### [Mm]etastasize

#### PubMed

1. Oncogenic ras-induced down-regulation of pro-apoptotic protease caspase-2 is required for malignant transformation of intestinal epithelial cells. Resistance of carcinoma cells to anoikis, apoptosis that is normally induced by loss of cell-to-extracellular matrix adhesion, is thought to be essential for the ability of these cells to form primary tumors, invade adjacent tissues, and **metastasize** to distant organs. Current knowledge about the mechanisms by which cancer cells evade anoikis is far from complete. In an effort to understand these mechanisms, we found that ras, a major oncogene, down-regulates protease caspase-2 (which initiates certain steps of the cellular apoptotic program) in malignant human and rat intestinal epithelial cells. This down-regulation could be reversed by inhibition of a protein kinase Mek, a mediator of Ras signaling. We also found that enforced down-regulation of caspase-2 in nonmalignant intestinal epithelial cells by RNA interference protected them from anoikis. Furthermore, the reversal of the effect of Ras on caspase-2 achieved by the expression of exogenous caspase-2 in detached ras-transformed intestinal epithelial cells promoted well established apoptotic events, such as the release of the pro-apoptotic mitochondrial factors cytochrome c and HtrA2/Omi into the cytoplasm of these cells, significantly enhanced their anoikis susceptibility, and blocked their long term growth in the absence of adhesion to the extracellular matrix. Finally, the blockade of the effect of Ras on caspase-2 substantially suppressed growth of tumors formed by the ras-transformed cells in mice. We conclude that ras-induced down-regulation of caspase-2 represents a novel mechanism by which oncogenic Ras protects malignant intestinal epithelial cells from anoikis, promotes their anchorage-independent growth, and allows them to form tumors in vivo.

---

## Casp8

- NCBI Gene
- GeneRIF
- Pubmed

| Search Term | Rank | Fields |
| --- | --- | --- |
| [Mm]igration | 6 | PubMed(5); GeneRIFs(1) |
| [Ee]xtracellular [Mm]atrix | 8 | PubMed(4) |
| [Ii]nvasive | 10 | PubMed(2); GeneRIFs(1) |
| [Mm]etastasis | 12 | PubMed(3); GeneRIFs(1) |

  
hide/show details for Casp8

### [Mm]igration

#### PubMed

1. Robo1 modulates proliferation and neurogenesis in the developing neocortex. The elaborate cytoarchitecture of the mammalian neocortex requires the timely production of its constituent pyramidal neurons and interneurons and their disposition in appropriate layers. Numerous chemotropic factors present in the forebrain throughout cortical development play important roles in the orchestration of these events. The Roundabout (Robo) family of receptors and their ligands, the Slit proteins, are expressed in the developing forebrain, and are known to play important roles in the generation and **migration** of cortical interneurons. However, few studies have investigated their function(s) in the development of pyramidal cells. Here, we observed expression of Robo1 and Slit genes (Slit1, Slit2) in cells lining the telencephalic ventricles, and found significant increases in progenitor cells (basal and apical) at embryonic day (E)12.5 and E14.5 in the developing cortex of Robo1(-/-), Slit1(-/-), and Slit1(-/-)/Slit2(-/-), but not in mice lacking the other Robo or Slit genes. Using layer-specific markers, we found that both early- and late-born pyramidal neuron populations were significantly increased in the cortices of Robo1(-/-) mice at the end of corticogenesis (E18.5). The excess number of cortical pyramidal neurons generated prenatally appears to die in early postnatal life. The observed increase in pyramidal neurons was due to prolonged proliferative activity of their progenitors and not due to changes in cell cycle events. This finding, confirmed by in utero electroporation with Robo1 short hairpin RNA (shRNA) or control constructs into progenitors along the ventricular zone as well as in dissociated cortical cell cultures, points to a novel role for Robo1 in regulating the proliferation and generation of pyramidal neurons.
2. Caspase-8 mutations in head and neck cancer confer resistance to death receptor-mediated apoptosis and enhance **migration**, invasion, and tumor growth. Little is known regarding molecular markers in head and neck squamous cell carcinoma (HNSCC) that predict responsiveness to different therapeutic regimens or predict HNSCC progression. Mutations in procaspase-8 occur in 9% of HNSCC primary tumors, but the functional consequences of these mutations are poorly understood. In this study, we examined the impact of four, representative, HNSCC-associated procaspase-8 mutations on activation of the extrinsic apoptosis pathway, as well as cellular **migration** and invasion, and in vivo tumor growth. All four mutant proteins acted to potently inhibit activation of apoptosis following treatment with TRAIL or agonistic anti-Fas. In contrast to wild-type procaspase-8, the mutant proteins were not recruited to FADD following treatment with TRAIL or anti-Fas, but may be constitutively bound by FADD. Three of the four procaspase-8 mutants promoted enhanced cellular **migration** and invasion through matrigel, relative to that seen with the wild-type procaspase-8 protein. Procaspase-8 mutation also stimulated the growth of HNSCC xenograft tumors. These findings indicate that HNSCC-associated procaspase-8 mutations inhibit activation of the extrinsic apoptosis pathway and are likely to represent markers for resistance to therapeutic regimens incorporating death receptor activators. Moreover, procaspase-8 mutations may serve as markers of HNSCC tumor progression, as exemplified by enhanced **migration**, invasion, and tumor growth.
3. Caspase-8 is essential for maintaining chromosomal stability and suppressing B-cell lymphomagenesis. In addition to its proapoptotic function, caspase-8 is also important for several other processes, including suppressing necroptosis, cell **migration**, and immune cell survival. In the present study, we report that the loss of caspase-8 in B lymphocytes leads to B-cell malignancies and that the risk for these tumors is further enhanced in the absence of p53. We also report that deficiency of caspase-8 results in impaired cytokinesis and that casp8(-/-) lymphomas display remarkably elevated levels of chromosomal aberrations. Our data support an important role for caspase-8 in the maintenance of genomic integrity and highlight its tumor-suppressive function.
4. Intestinal genetic inactivation of caspase-8 diminishes **migration** of enterocytes. To verify the hypothesis that caspase-8 (Casp8), which regulates cellular apoptosis and necroptosis, is critically involved in enterocyte **migration**.Casp8-silenced Caco2 cells were used in **migration** assays. In addition, enterocyte-specific Casp8 heterozygous (Casp8(+/∆int)) or homozygous knockout mice (Casp8(∆int)) were generated by crossing genetically modified mice carrying loxP recombination sites in intron 2 and 4 of the murine Casp8 gene with transgenic animals expressing a cre-transgene under control of the villin promoter in a pure C57/BL6 genetic background. The nucleoside analog BrdU was injected i.p. in male Casp8(+/∆int) and Casp8(∆int) animals 4 h, 20 h, or 40 h before performing morphometric studies. Locations of anti-BrdU-immunostained cells (cell(max)) in at least 50 hemi-crypts of 6 histoanatomically distinct intestinal mucosal regions were numbered and extracted for statistical procedures. For the mice cohort (n = 28), the walking distance of enterocytes was evaluated from cell(max) within crypt (n = 57), plateau (n = 19), and villus (n = 172) positions, resulting in a total of 6838 observations. Data analysis was performed by fitting a three-level mixed effects model to the data.In cell culture experiments with Caco2 cells, Casp8 knockdown efficiency mediated by RNA interference on Casp8 transcripts was 80% controlled as determined by Western blotting. In the scratch **assay, m**igration of Casp8-deleted Caco2 cells was significantly diminished when compared with controls (Casp8(∆scramble) and Caco2). In BrdU-labeled Casp8(∆int) mice, cell(max) locations were found along the hemi-crypts in a lower position than it was for Casp8(+/∆int) or control (cre-negative) animals. Statistical data analysis with a three-level mixed effects model revealed that in the six different intestinal locations (distinct segments of the small and large intestine), cell movement between the three mice groups differed widely. Especially in duodenal hemi-crypts, enterocyte movement was different between the groups. At 20 h, duodenal cell(max) location was significantly lower in Casp8(∆int) (25.67 ± 2.49) than in Casp8(+/∆int) (35.67 ± 4.78; P < 0.05) or control littermates (44.33 ± 0.94; P < 0**.01).Casp**8-dependent migration of enterocytes is likely involved in intestinal physiology and inflammation-related pathophysiology.
5. Caspase-8 is involved in neovascularization-promoting progenitor cell functions. Endothelial progenitor cells (EPCs) comprise a heterogeneous population of cells, which improve therapeutic neovascularization after ischemia. The neovascularization-promoting potential of progenitor cells depends on survival and retention of the infused cells to the tissue. Caspases mediate apoptosis but are also involved in other critical biological processes. Therefore, we aimed to address the role of caspases in proangiogenic cells.The caspase-8 inhibitor zIETD abrogated the ex vivo formation of EPCs, inhibited EPC adhesion and **migration**, and reduced their capacity to improve neovascularization in vivo. Consistently, cells isolated from caspase-8-deficient mice exhibited a reduced capacity for enhancing neovascularization when transplanted into mice after hindlimb ischemia. Because inhibition of Caspase-8 reduced the adhesion and homing functions of EPCs, we further determined the surface expression of integrins and receptors involved in cell recruitment to ischemic tissues. Pharmacological inhibition of caspase-8 and genetic depletion of caspase-8 reduced the expression of the fibronectin receptor subunits alpha5 and beta1 and the SDF-1 receptor CXCR4. Moreover, we identified the E3 ubiquitin ligase Cbl-b, which negatively regulates integrin and receptor-mediated signaling, as a potential Caspase-8 substrate.In summary, our data demonstrate a novel apoptosis-unrelated role of caspase-8 in proangiogenic cells.

#### GeneRIFs

1. Casp8-dependent **migration** of enterocytes is likely involved in intestinal physiology and inflammation-related pathophysiology.

### [Ee]xtracellular [Mm]atrix

#### PubMed

1. Altered apoptosis of inflammatory neutrophils in MMP-9-deficient mice is due to lower expression and activity of caspase-3. Matrix metalloproteinase 9 (MMP-9) is a Zn(2+)-dependent endopeptidase that degrades some of the components of basement membranes and **extracellular matrix** and thus participates in leukocyte infiltration during inflammation. In a model of zymosan peritonitis, neutrophil infiltration in MMP-deficient (MMP-9(-/-)) mice was significantly weaker at the time of their maximal influx in wild-type mice (6h). However, during the late stages of peritonitis (24h) an extended accumulation of neutrophils was observed in MMP-9(-/-)versus the wild-type mice. Recently, we reported that the ratio of apoptosis of inflammatory leukocytes is impaired in MMP-9(-/-) mice during late peritonitis and the process depends on COX-1-driven PGE(2). Here we scrutinized the alterations in apoptotic mechanisms by comparisons between MMP-9(-/-) and the wild-type mice. Altered apoptosis occurred only during late (24h) peritonitis and concerned only neutrophils, and not macrophages, mast cells or lymphocytes. Furthermore, expression and activity of caspases was altered in MMP-9(-/-) animals, delayed for caspase-8 and -9, and decreased in the case of caspase-3. Also the expression of Bax/Bcl-2 proteins was changed in MMP-9(-/-) mice. These changes, and in particular the impaired neutrophil apoptosis and weaker caspase-3 activity, were restored by the selective COX-1 inhibition. We conclude that in mice lacking MMP-9 the enhanced COX-1-PGE(2) decreases caspase-3 expression and activity leading to impaired apoptosis of inflammatory neutrophils resulting in abnormal accumulation of the cells at the inflammatory focus. The data also reinforce the notion that MMP-9 is a key enzyme in neutrophil biology.
2. Altered apoptosis of inflammatory neutrophils in MMP-9-deficient mice is due to lower expression and activity of caspase-3. Matrix metalloproteinase 9 (MMP-9) is a Zn(2+)-dependent endopeptidase that degrades some of the components of basement membranes and **extracellular matrix** and thus participates in leukocyte infiltration during inflammation. In a model of zymosan peritonitis, neutrophil infiltration in MMP-deficient (MMP-9(-/-)) mice was significantly weaker at the time of their maximal influx in wild-type mice (6h). However, during the late stages of peritonitis (24h) an extended accumulation of neutrophils was observed in MMP-9(-/-)versus the wild-type mice. Recently, we reported that the ratio of apoptosis of inflammatory leukocytes is impaired in MMP-9(-/-) mice during late peritonitis and the process depends on COX-1-driven PGE(2). Here we scrutinized the alterations in apoptotic mechanisms by comparisons between MMP-9(-/-) and the wild-type mice. Altered apoptosis occurred only during late (24h) peritonitis and concerned only neutrophils, and not macrophages, mast cells or lymphocytes. Furthermore, expression and activity of caspases was altered in MMP-9(-/-) animals, delayed for caspase-8 and -9, and decreased in the case of caspase-3. Also the expression of Bax/Bcl-2 proteins was changed in MMP-9(-/-) mice. These changes, and in particular the impaired neutrophil apoptosis and weaker caspase-3 activity, were restored by the selective COX-1 inhibition. We conclude that in mice lacking MMP-9 the enhanced COX-1-PGE(2) decreases caspase-3 expression and activity leading to impaired apoptosis of inflammatory neutrophils resulting in abnormal accumulation of the cells at the inflammatory focus. The data also reinforce the notion that MMP-9 is a key enzyme in neutrophil biology.
3. Th-MYCN mice with caspase-8 deficiency develop advanced neuroblastoma with bone marrow metastasis. Neuroblastoma, the most common extracranial pediatric solid tumor, is responsible for 15% of all childhood cancer deaths. Patients frequently present at diagnosis with metastatic disease, particularly to the bone marrow. Advances in therapy and understanding of the metastatic process have been limited due, in part, to the lack of animal models harboring bone marrow disease. The widely used transgenic model, the Th-MYCN mouse, exhibits limited metastasis to this site. Here, we establish the first genetic immunocompetent mouse model for metastatic neuroblastoma with enhanced secondary tumors in the bone marrow. This model recapitulates 2 frequent alterations in metastatic neuroblastoma, overexpression of MYCN and loss of caspase-8 expression. Mouse caspase-8 gene was deleted in neural crest lineage cells by crossing a Th-Cre transgenic mouse with a caspase-8 conditional knockout mouse. This mouse was then crossed with the neuroblastoma prone Th-MYCN mouse. Although overexpression of MYCN by itself rarely caused bone marrow metastasis, combining MYCN overexpression and caspase-8 deletion significantly enhanced bone marrow metastasis (37% incidence). Microarray expression studies of the primary tumors mRNAs and microRNAs revealed **extracellular matrix** structural changes, increased expression of genes involved in epithelial to mesenchymal transition, inflammation, and downregulation of miR-7a and miR-29b. These molecular changes have been shown to be associated with tumor progression and activation of the cytokine TGF-β pathway in various tumor models. Cytokine TGF-β can preferentially promote single cell motility and blood-borne metastasis and therefore activation of this pathway may explain the enhanced bone marrow metastasis observed in this animal model.
4. IPS-1 is crucial for DAP3-mediated anoikis induction by caspase-8 activation. Detachment of adherent epithelial cells from the **extracellular matrix** induces apoptosis, a process known as anoikis. We have shown that DAP3 is critical for anoikis induction. However, the mechanism for anoikis induction mediated by DAP3 is still unclear. Here, we show that interferon-beta promoter stimulator 1 (IPS-1) binds DAP3 and induces anoikis by caspase activation. Recently, IPS-1 has been shown to be critical for antiviral immune responses, although there has been no report of its function in apoptosis induction. We show that overexpression of IPS-1 induces apoptosis by activation of caspase-3, -8, and -9. In addition, IPS-1 knockout mouse embryonic fibroblasts were shown to be resistant to anoikis. Interestingly, IPS-1 expression, recruitment of caspase-8 to IPS-1, and caspase-8 activation were induced after cell detachment. Furthermore, DAP3-mediated anoikis induction was inhibited by knockdown of IPS-1 expression. Therefore, we elucidated a novel function of IPS-1 for anoikis induction by caspase-8 activation.

### [Ii]nvasive

#### PubMed

1. The non-receptor tyrosine kinase Tec controls assembly and activity of the noncanonical caspase-8 inflammasome. Tec family kinases are intracellular non-receptor tyrosine kinases implicated in numerous functions, including T cell and B cell regulation. However, a role in microbial pathogenesis has not been described. Here, we identified Tec kinase as a novel key mediator of the inflammatory immune response in macrophages invaded by the human fungal pathogen C. albicans. Tec is required for both activation and assembly of the noncanonical caspase-8, but not of the caspase-1 inflammasome, during infections with fungal but not bacterial pathogens, triggering the antifungal response through IL-1β. Furthermore, we identify dectin-1 as the pathogen recognition receptor being required for Syk-dependent Tec activation. Hence, Tec is a novel innate-specific inflammatory kinase, whose genetic ablation or inhibition by small molecule drugs strongly protects mice from fungal sepsis. These data demonstrate a therapeutic potential for Tec kinase inhibition to combat **invasiv**e microbial infections by attenuating the host inflammatory response.
2. Potentiation of neuroblastoma metastasis by loss of caspase-8. Neuroblastoma, the most common paediatric solid tumour, arises from defective neural crest cells. Genetic alterations occur frequently in the most aggressive neuroblastomas. In particular, deletion or suppression of the proapoptotic enzyme caspase-8 is common in malignant, disseminated disease, although the effect of this loss on disease progression is unclear. Here we show that suppression of caspase-8 expression occurs during the establishment of neuroblastoma metastases in vivo, and that reconstitution of caspase-8 expression in deficient neuroblastoma cells suppressed their metastases. Caspase-8 status was not a predictor of primary tumour growth; rather, caspase-8 selectively potentiated apoptosis in neuroblastoma cells invading the collagenous stroma at the tumour margin. Apoptosis was initiated by unligated integrins by means of a process known as integrin-mediated death. Loss of caspase-8 or integrin rendered these cells refractory to integrin-mediated death, allowed cellular survival in the stromal microenvironment, and promoted metastases. These findings define caspase-8 as a metastasis suppressor gene that, together with integrins, regulates the survival and **invasive** capacity of neuroblastoma cells.

#### GeneRIFs

1. findings define caspase-8 as a metastasis suppressor gene that, together with integrins, regulates the survival and **invasive** capacity of neuroblastoma cells

### [Mm]etastasis

#### PubMed

1. TRAIL negatively regulates VEGF-induced angiogenesis via caspase-8-mediated enzymatic and non-enzymatic functions. Solid tumors supply oxygen and nutrients required for angiogenesis by producing vascular endothelial growth factor (VEGF). Thus, inhibitors of VEGF signaling abrogate tumor angiogenesis, resulting in the suppression of tumor growth and **metastasis**. We here investigated the effects of tumor necrosis factor-related apoptosis-inducing ligand (TRAIL) on VEGF-induced angiogenesis. TRAIL inhibited VEGF-induced in vitro angiogenesis of human umbilical vein endothelial cells (HUVECs) and in vivo neovascularization in chicken embryos and mice. TRAIL blocked VEGF-induced angiogenic signaling by inhibiting ERK, Src, FAK, paxillin, Akt, and eNOS. Further, TRAIL blocked intracellular Ca(2+) elevation and actin reorganization in HUVECs stimulated with VEGF, without inhibiting VEGF receptor-2 tyrosine phosphorylation. TRAIL increased caspase-8 activity, without inducing caspase-9/-3 activation and apoptosis. Moreover, TRAIL resulted in cleavage of FAK into FAK-related non-kinase-like fragments in VEGF-stimulated HUVECs, which was blocked by a caspase-8 inhibitor and cellular caspase-8-like inhibitory protein. Biochemical and pharmacological inhibition of caspase-8 and FAK blocked the inhibitory effects of TRAIL on VEGF-stimulated anti-angiogenic signaling and events. In addition, caspase-8 knockdown also suppressed VEGF-mediated signaling and angiogenesis, suggesting that procaspase-8 plays a role of a non-apoptotic modulator in VEGF-induced angiogenic signaling. These results suggest that TRAIL inhibits VEGF-induced angiogenesis by increasing caspase-8 activity and subsequently decreasing non-apoptotic signaling functions of procaspase-8, without inducing caspase-3 activation and endothelial cell cytotoxicity. These data indicate that caspase-8 may be used as an anti-angiogenic drug for solid tumors resistant to TRAIL and anti-tumor drugs.
2. Th-MYCN mice with caspase-8 deficiency develop advanced neuroblastoma with bone marrow **metastasis**. Neuroblastoma, the most common extracranial pediatric solid tumor, is responsible for 15% of all childhood cancer deaths. Patients frequently present at diagnosis with metastatic disease, particularly to the bone marrow. Advances in therapy and understanding of the metastatic process have been limited due, in part, to the lack of animal models harboring bone marrow disease. The widely used transgenic model, the Th-MYCN mouse, exhibits limited **metastasis** to this site. Here, we establish the first genetic immunocompetent mouse model for metastatic neuroblastoma with enhanced secondary tumors in the bone marrow. This model recapitulates 2 frequent alterations in metastatic neuroblastoma, overexpression of MYCN and loss of caspase-8 expression. Mouse caspase-8 gene was deleted in neural crest lineage cells by crossing a Th-Cre transgenic mouse with a caspase-8 conditional knockout mouse. This mouse was then crossed with the neuroblastoma prone Th-MYCN mouse. Although overexpression of MYCN by itself rarely caused bone marrow **metastasis**, combining MYCN overexpression and caspase-8 deletion significantly enhanced bone marrow **metastasis** (37% incidence). Microarray expression studies of the primary tumors mRNAs and microRNAs revealed extracellular matrix structural changes, increased expression of genes involved in epithelial to mesenchymal transition, inflammation, and downregulation of miR-7a and miR-29b. These molecular changes have been shown to be associated with tumor progression and activation of the cytokine TGF-β pathway in various tumor models. Cytokine TGF-β can preferentially promote single cell motility and blood-born**e metastas**is and therefore activation of this pathway may explain the enhanced bone marro**w metastas**is observed in this animal model.
3. Potentiation of neuroblastoma **metastasis** by loss of caspase-8. Neuroblastoma, the most common paediatric solid tumour, arises from defective neural crest cells. Genetic alterations occur frequently in the most aggressive neuroblastomas. In particular, deletion or suppression of the proapoptotic enzyme caspase-8 is common in malignant, disseminated disease, although the effect of this loss on disease progression is unclear. Here we show that suppression of caspase-8 expression occurs during the establishment of neuroblastoma metastases in vivo, and that reconstitution of caspase-8 expression in deficient neuroblastoma cells suppressed their metastases. Caspase-8 status was not a predictor of primary tumour growth; rather, caspase-8 selectively potentiated apoptosis in neuroblastoma cells invading the collagenous stroma at the tumour margin. Apoptosis was initiated by unligated integrins by means of a process known as integrin-mediated death. Loss of caspase-8 or integrin rendered these cells refractory to integrin-mediated death, allowed cellular survival in the stromal microenvironment, and promoted metastases. These findings define caspase-8 as a **metastasis** suppressor gene that, together with integrins, regulates the survival and invasive capacity of neuroblastoma cells.

#### GeneRIFs

1. findings define caspase-8 as a **metastasis** suppressor gene that, together with integrins, regulates the survival and invasive capacity of neuroblastoma cells

---

## Arrdc3

- NCBI Gene
- GeneRIF
- Pubmed

| Search Term | Rank | Fields |
| --- | --- | --- |
| [Ii]nvasive | 10 | PubMed(1) |

  
hide/show details for Arrdc3

### [Ii]nvasive

#### PubMed

1. In vivo and in vitro investigations of heterozygous nebulin knock-out mice disclose a mild skeletal muscle phenotype. Nemaline myopathy is the most common congenital skeletal muscle disease, and mutations in the nebulin gene account for 50% of all cases. Recent studies suggest that the disease severity might be related to the nebulin expression levels. Considering that mutations in the nebulin gene are typically recessive, one would expect that a single functional nebulin allele would maintain nebulin protein expression which would result in preserved skeletal muscle function. We investigated skeletal muscle function of heterozygous nebulin knock-out (i.e., nebulin(+/-)) mice using a multidisciplinary approach including protein and gene expression analysis and combined in vivo and in vitro force measurements. Skeletal muscle anatomy and energy metabolism were studied strictly non-**invasive**ly using magnetic resonance imaging and 31P-magnetic resonance spectroscopy. Maximal force production was reduced by around 16% in isolated muscle of nebulin(+/-) mice while in vivo force generating capacity was preserved. Muscle weakness was associated with a shift toward a slower proteomic phenotype, but was not related to nebulin protein deficiency or to an impaired energy metabolism. Further studies would be warranted in order to determine the mechanisms leading to a mild skeletal muscle phenotype resulting from the expression of a single nebulin allele.

---

## Arrdc2

- NCBI Gene
- GeneRIF
- Pubmed

| Search Term | Rank | Fields |
| --- | --- | --- |
| [Ii]nvasive | 10 | PubMed(1) |

  
hide/show details for Arrdc2

### [Ii]nvasive

#### PubMed

1. In vivo and in vitro investigations of heterozygous nebulin knock-out mice disclose a mild skeletal muscle phenotype. Nemaline myopathy is the most common congenital skeletal muscle disease, and mutations in the nebulin gene account for 50% of all cases. Recent studies suggest that the disease severity might be related to the nebulin expression levels. Considering that mutations in the nebulin gene are typically recessive, one would expect that a single functional nebulin allele would maintain nebulin protein expression which would result in preserved skeletal muscle function. We investigated skeletal muscle function of heterozygous nebulin knock-out (i.e., nebulin(+/-)) mice using a multidisciplinary approach including protein and gene expression analysis and combined in vivo and in vitro force measurements. Skeletal muscle anatomy and energy metabolism were studied strictly non-**invasive**ly using magnetic resonance imaging and 31P-magnetic resonance spectroscopy. Maximal force production was reduced by around 16% in isolated muscle of nebulin(+/-) mice while in vivo force generating capacity was preserved. Muscle weakness was associated with a shift toward a slower proteomic phenotype, but was not related to nebulin protein deficiency or to an impaired energy metabolism. Further studies would be warranted in order to determine the mechanisms leading to a mild skeletal muscle phenotype resulting from the expression of a single nebulin allele.

---

## Txnip

- NCBI Gene
- GeneRIF
- Pubmed

| Search Term | Rank | Fields |
| --- | --- | --- |
| [Mm]igration | 6 | PubMed(1); GeneRIFs(1) |
| [Mm]etastasis | 12 | PubMed(1) |

  
hide/show details for Txnip

### [Mm]igration

#### PubMed

1. Thioredoxin-interacting protein expression is required for VEGF-mediated angiogenic signal in endothelial cells. Thioredoxin-interacting protein (TXNIP) contributes to cellular redox-state homeostasis via binding and inhibiting thioredoxin (TRX). Increasing evidence suggests that cellular redox homeostasis regulates vascular endothelial growth factor (VEGF)-mediated signaling. This study aims to examine the redox-dependant role of TXNIP in regulating VEGF-mediated S-glutathionylation and angiogenic signaling. TXNIP-knockout mice (TKO) or wild-type (WT) treated with the reduced glutathione (GSH)-precursor, N-acetyl cysteine (WT-NAC, 500 mg/kg) were compared to WT using hypoxia-induced neovascularization model.In response to hypoxia, retinas from TKO and WT-NAC mice showed significant decreases in reparative revascularization and pathological neovascularization with similar VEGF expression compared with WT. VEGF failed to stimulate vascular sprouting from aortic rings of TKO compared to WT mice. TKO mice or WT+NAC experienced reductive stress as indicated by twofold increase in TRX reductase activity and fourfold increase in reduced-GSH levels compared with WT. In human microvascular endothelial (HME) cells, VEGF stimulated co-precipitation between vascular endothelial growth factor receptor 2 (VEGFR2) with low molecular weight protein tyrosine phosphatase (LMW-PTP). Silencing TXNIP expression blunted VEGF-induced oxidation of GSH and S-glutathionylation of the LMW-PTP in HME cells. These effects were associated with impaired VEGFR2 phosphorylation that culminated in inhibiting cel**l migrati**on and tube formation. Overexpression of TXNIP restored VEGFR2 phosphorylation and cel**l migrati**on in TKO-endothelial cells.TXNIP expression is required for VEGF-mediated VEGFR2 activation and angiogenic response in vivo and in vitro. TXNIP expression regulates VEGFR-2 phosphorylation via S-glutathionylation of LMW-PTP in endothelial cells.Our results provide novel mechanistic insight into modulating TXNIP expression as a potential therapeutic target in diseases characterized by aberrant angiogenesis.

#### GeneRIFs

1. TXNIP plays a dual role in maintaining long-term quiescence and **migration** of hematopoietic stem cells (HSC) by decreasing Wnt signaling and enhancing HSC interaction with the bone marrow.

### [Mm]etastasis

#### PubMed

1. Deficiency of thioredoxin binding protein-2 (TBP-2) enhances TGF-β signaling and promotes epithelial to mesenchymal transition. Transforming growth factor beta (TGF-β) has critical roles in regulating cell growth, differentiation, apoptosis, invasion and epithelial-mesenchymal transition (EMT) of various cancer cells. TGF-β-induced EMT is an important step during carcinoma progression to invasion state. Thioredoxin binding protein-2 (TBP-2, also called Txnip or VDUP1) is downregulated in various types of human cancer, and its deficiency results in the earlier onset of cancer. However, it remains unclear how TBP-2 suppresses the invasion a**nd metasta**sis of cancer.In this study, we demonstrated that TBP-2 deficiency increases the transcriptional activity in response to TGF-β and also enhances TGF-β-induced Smad2 phosphorylation levels. Knockdown of TBP-2 augmented the TGF-β-responsive expression of Snail and Slug, transcriptional factors related to TGF-β-mediated induction of EMT, and promoted TGF-β-induced spindle-like morphology consistent with the depletion of E-Cadherin in A549 cells.Our results indicate that TBP-2 deficiency enhances TGF-β signaling and promotes TGF-β-induced EMT. The control of TGF-β-induced EMT is critical for the inhibition of the in**vasion and** metastasis. Thus TBP-2, as a novel regulatory molecule of TGF-β signaling, is likely to be a prognostic indicator or a potential therapeutic target for preventing tumor progression.

---

## Serpinh1

- NCBI Gene
- GeneRIF
- Pubmed

| Search Term | Rank | Fields |
| --- | --- | --- |
| [Mm]igration | 6 | PubMed(2); GeneRIFs(1) |
| ECM | 7 | PubMed(1) |
| [Ee]xtracellular [Mm]atrix | 8 | PubMed(6) |

  
hide/show details for Serpinh1

### [Mm]igration

#### PubMed

1. Role for phospholipid flippase complex of ATP8A1 and CDC50A proteins in cell **migration**. Type IV P-type ATPases (P4-ATPases) and CDC50 family proteins form a putative phospholipid flippase complex that mediates the translocation of aminophospholipids such as phosphatidylserine (PS) and phosphatidylethanolamine (PE) from the outer to inner leaflets of the plasma membrane. In Chinese hamster ovary (CHO) cells, at least eight members of P4-ATPases were identified, but only a single CDC50 family protein, CDC50A, was expressed. We demonstrated that CDC50A associated with and recruited P4-ATPase ATP8A1 to the plasma membrane. Overexpression of CDC50A induced extensive cell spreading and greatly enhanced cell **migration**. Depletion of either CDC50A or ATP8A1 caused a severe defect in the formation of membrane ruffles, thereby inhibiting cell **migration**. Analyses of phospholipid translocation at the plasma membrane revealed that the depletion of CDC50A inhibited the inward translocation of both PS and PE, whereas the depletion of ATP8A1 inhibited the translocation of PE but not that of PS, suggesting that the inward translocation of cell-surface PE is involved in cell **migration**. This hypothesis was further examined by using a PE-binding peptide and a mutant cell line with defective PE synthesis; either cell-surface immobilization of PE by the PE-binding peptide or reduction in the cell-surface content of PE inhibited the formation of membrane ruffles, causing a severe defect in cell **migration**. These results indicate that the phospholipid flippase complex of ATP8A1 and CDC50A plays a major role in cell **migration** and suggest that the flippase-mediated translocation of PE at the plasma membrane is involved in the formation of membrane ruffles to promote cell **migration**.
2. Over-expression of HSP47 augments mouse embryonic stem cell smooth muscle differentiation and chemotaxis. In the recent decade, embryonic stem cells (ESC) have emerged as an attractive cell source of smooth muscle cells (SMC) for vascular tissue engineering owing to their unlimited self-renewal and differentiation capacities. Despite their promise in therapy, their efficacy is still hampered by the lack of definitive SMC differentiation mechanisms and difficulties in successful trafficking of the ESC towards a site of injury or target tissue. Heat shock protein 47 (HSP47) is a 47-kDa molecular chaperone that is required for the maturation of various types of collagen and has been shown to be a critical modulator of different pathological and physiological processes. To date, the role of HSP47 on ESC to SMC differentiation or ESC chemotaxis is not known and may represent a potential molecular approach by which ESC can be manipulated to increase their efficacy in clinic. We provide evidence that HSP47 is highly expressed during ESC differentiation into the SMC lineage and that HSP47 reduction results in an attenuation of the differentiation. Our experiments using a HSP47 plasmid transfection system show that gene over-expression is sufficient to induce ESC-SMC differentiation, even in the absence of exogenous stimuli. Furthermore, HSP47 over-expression in ESC also increases their chemotaxis and migratory responses towards a panel of chemokines, likely via the upregulation of chemokine receptors. Our findings provide direct evidence of induced ESC **migration** and differentiation into SMC via the over-expression of HSP47, thus identifying a novel approach of molecular manipulation that can potentially be exploited to improve stem cell therapy for vascular repair and regeneration.

#### GeneRIFs

1. Mouse embryonic stem cell **migration** and differentiation into smooth muscle cells is induced by the over-expression of HSP47.

### ECM

#### PubMed

1. MiR-29b inhibits collagen maturation in hepatic stellate cells through down-regulating the expression of HSP47 and lysyl oxidase. Altered expression of miR-29b is implicated in the pathogenesis and progression of liver fibrosis. We and others previously demonstrated that miR-29b down-regulates the expression of several extracellular-matrix (**ECM**) genes including Col 1A1, Col 3A1 and Elastin via directly targeting their 3'-UTRs. However, whether or not miR-29b plays a role in the post-translational regulation of **ECM** biosynthesis has not been reported. Heat shock protein 47 (HSP47) and lysyl oxidase (LOX) are known to be essential for **ECM** maturation. In this study we have demonstrated that expression of HSP47 and LOX was significantly up-regulated in culture-activated primary rat hepatic stellate cells (HSCs), TGF-β stimulated LX-2 cells and liver tissue of CCl4-treated mice, which was accompanied by a decrease of miR-29b level. In addition, over-expression of miR-29b in LX-2 cells resulted in significant inhibition on HSP47 and LOX expression. Mechanistically, miR-29b inhibited the expression of a reporter gene that contains the respective full-length 3'-UTR from HSP47 and LOX gene, and this inhibitory effect was abolished by the deletion of a putative miR-29b targeting sequence from the 3'-UTRs. Transfection of LX-2 cells with miR-29b led to abnormal collagen structure as shown by electron-microscopy, presumably through down-regulation of the expression of molecules involved in **EC**M maturation including HSP47 and LOX. These results demonstrated that miR-29b is involved in regulating the post-translational processing of **EC**M and fibril formation.

### [Ee]xtracellular [Mm]atrix

#### PubMed

1. Global defects in collagen secretion in a Mia3/TANGO1 knockout mouse. Melanoma inhibitory activity member 3 (MIA3/TANGO1) [corrected] is an evolutionarily conserved endoplasmic reticulum resident transmembrane protein. Recent in vitro studies have shown that it is required for the loading of collagen VII, but not collagen I, into COPII-coated transport vesicles. In this paper, we show that mice lacking Mia3 are defective for the secretion of numerous collagens, including collagens I, II, III, IV, VII, and IX, from chondrocytes, fibroblasts, endothelial cells, and mural cells. Collagen deposition by these cell types is abnormal, and **extracellular matrix** composition is compromised. These changes are associated with intracellular accumulation of collagen and the induction of a strong unfolded protein response, primarily within the developing skeleton. Chondrocyte maturation and bone mineralization are severely compromised in Mia3-null embryos, leading to dwarfism and neonatal lethality. Thus, Mia3's role in protein secretion is much broader than previously realized, and it may, in fact, be required for the efficient secretion of all collagen molecules in higher organisms.
2. Dwarfism and impaired gut development in insulin-like growth factor II mRNA-binding protein 1-deficient mice. Insulin-like growth factor II mRNA-binding protein 1 (IMP1) belongs to a family of RNA-binding proteins implicated in mRNA localization, turnover, and translational control. Mouse IMP1 is expressed during early development, and an increase in expression occurs around embryonic day 12.5 (E12.5). To characterize the physiological role of IMP1, we generated IMP1-deficient mice carrying a gene trap insertion in the Imp1 gene. Imp1(-/-) mice were on average 40% smaller than wild-type and heterozygous sex-matched littermates. Growth retardation was apparent from E17.5 and remained permanent into adult life. Moreover, Imp1(-/-) mice exhibited high perinatal mortality, and only 50% were alive 3 days after birth. In contrast to most other organs, intestinal epithelial cells continue to express IMP1 postnatally, and Imp1(-/-) mice exhibited impaired development of the intestine, with small and misshapen villi and twisted colon crypts. Analysis of target mRNAs and global expression profiling at E12.5 indicated that Igf2 translation was downregulated, whereas the postnatal intestine showed reduced expression of transcripts encoding **extracellular matrix** components, such as galectin- 1, lumican, tenascin-C, procollagen transcripts, and the Hsp47 procollagen chaperone. Taken together, the results demonstrate that IMP1 is essential for normal growth and development. Moreover, IMP1 may facilitate intestinal morphogenesis via regulation of **extracellular matrix** formation.
3. Involvement of collagen-binding heat shock protein 47 in scleroderma-associated fibrosis. Uncontrolled fibrosis of skin and internal organs is the main characteristic of scleroderma, and collagen is a major **extracellular matrix** protein that deposits in the fibrotic organs. As the chaperone of collagen, heat shock protein 47 (HSP47) is closely related with the development of fibrosis. To explore the potential function of HSP47 in the pathogenesis of scleroderma, the clinical, in vivo and in vitro studies were performed. In clinical, the increased mRNA level of HSP47 was observed in the skin fibroblasts and PBMC from scleroderma patients, and the enhanced protein level of HSP47 was also detected in the skin biopsy and plasma of the above patients. Unexpectedly, the enhanced levels of HSP47 were positively correlated with the presence of anti-centromere antibody in scleroderma patients. Moreover, a high expression of HSP47 was found in the skin lesion of BLM-induced scleroderma mouse model. Further in vitro studies demonstrated that HSP47 knockdown could block the intracellular and extracellular collagen over-productions induced by exogenous TGF-β. Therefore, the results in this study provide direct evidence that HSP47 is involved in the pathogenesis of scleroderma. The high expression of HSP47 can be detected in the circulatory system of scleroderma patients, indicating that HSP47 may become a pathological marker to assess the progression of scleroderma, and also explain the systemic fibrosis of scleroderma. Meanwhile, collagen over-expression is blocked by HSP47 knockdown, suggesting the possibility that HSP47 can be a potential therapeutic target for scleroderma.
4. Deletion of the collagen-specific molecular chaperone Hsp47 causes endoplasmic reticulum stress-mediated apoptosis of hepatic stellate cells. Chronic liver injury, often caused by alcoholism and viral hepatitis, causes liver fibrosis via the induction of collagen production. In liver fibrosis, hepatic stellate cells (HSCs) are activated and transform into myofibroblasts, which actively produce and secrete collagen into the **extracellular matrix**. Hsp47 (heat shock protein 47) is a collagen-specific molecular chaperone that is essential for the maturation and secretion of collagen. Here, we used the Cre-LoxP system to disrupt the Hsp47 gene in isolated HSCs from Hsp47 floxed mice. Immature type I procollagen accumulated and partially aggregated in Hsp47-KO HSCs. This accumulation was augmented when autophagy was inhibited, which induced expression of the endoplasmic reticulum (ER) stress-inducible proteins BiP (immunoglobulin heavy chain-binding protein) and Grp94 (94-kDa glucose-regulated protein). The inhibition of autophagy in Hsp47-KO HSCs also induced CHOP (CCAAT/enhancer-binding protein homologous protein), which is an ER stress-induced transcription factor responsible for apoptosis. These data suggest that apoptosis is induced through ER stress by procollagen accumulation in Hsp47-KO HSCs when autophagy is inhibited. Thus, Hsp47 could be a promising therapeutic target in liver fibrosis.
5. Effect of heat shock protein 47 on collagen synthesis of keloid in vivo. Keloid is regarded as a fibroproliferative disorder with excessive accumulation of **extracellular matrix**. However, the molecular mechanism of keloid formation is not well understood and no treatment modality is consistently effective. Heat shock protein 47 (HSP47) is known as a collagen-specific molecular chaperone which plays a critical role in collagen biosynthesis. Results of our previous in vitro experiments demonstrated that HSP47 might be an important reason for excessive collagen accumulation in regard to keloid formation. Our objective is to investigate whether HSP47 has an influence on collagen metabolism in animal keloid models. The constructed plasmids, carrying HSP47-small hairpin RNA (shRNA), were transfected into animal keloid models, in comparison with the control groups. After transfection, the mRNA and protein expression of HSP47 and collage type I were detected by quantitative real-time PCR and Western blot. Both the mRNA and protein levels of HSP47 in animal keloid models were decreased dramatically after transfection of the HSP47- shRNA plasmid, in comparison with the control group. Following the down-regulation of HSP47, we found that the volume of animal keloid models and the major collagen expression were reduced correspondingly. Combining the results of our previous in vitro experiment results, we suggest that overexpression of HSP47 in keloid fibroblast cells could induce excessive collagen accumulation by enhancing collagen synthesis, which not only presents a possible mechanism of keloid formation, but also offers a therapeutic potential of RNA interference to HSP47 for the treatment of keloids and other fibroproliferative disorders.
6. Type I collagen in Hsp47-null cells is aggregated in endoplasmic reticulum and deficient in N-propeptide processing and fibrillogenesis. Heat-shock protein of 47 kDa (Hsp47) is a molecular chaperone that recognizes collagen triple helices in the endoplasmic reticulum (ER). Hsp47-knockout mouse embryos are deficient in the maturation of collagen types I and IV, and collagen triple helices formed in the absence of Hsp47 show increased susceptibility to protease digestion. We show here that the fibrils of type I collagen produced by Hsp47-/- cells are abnormally thin and frequently branched. Type I collagen was highly accumulated in the ER of Hsp47-/- cells, and its secretion rate was much slower than that of Hsp47+/+ cells, leading to accumulation of the insoluble aggregate of type I collagen within the cells. Transient expression of Hsp47 in the Hsp47-/- cells restored normal extracellular fibril formation and intracellular localization of type I collagen. Intriguingly, type I collagen with unprocessed N-terminal propeptide (N-propeptide) was secreted from Hsp47-/- cells and accumulated in the **extracellular matrix**. These results indicate that Hsp47 is required for correct folding and prevention of aggregation of type I collagen in the ER and that this function is indispensable for efficient secretion, processing, and fibril formation of collagen.

---

## Nlgn1

- NCBI Gene
- GeneRIF
- Pubmed

| Search Term | Rank | Fields |
| --- | --- | --- |
| [Ff]ilopodia | 1 | PubMed(1) |
| [Ff]ilopodium | 2 | Process(1); PubMed(1); Component(1) |
| [Mm]igration | 6 | PubMed(2) |

  
hide/show details for Nlgn1

### [Ff]ilopodia

#### PubMed

1. Neurexin-neuroligin cell adhesion complexes contribute to synaptotropic dendritogenesis via growth stabilization mechanisms in vivo. Cell adhesion molecules are well characterized for mediating synapse initiation, specification, differentiation, and maturation, yet their contribution to directing dendritic arborization during early brain circuit formation remains unclear. Using two-photon time-lapse imaging of growing neurons within intact and awake embryonic Xenopus brain, we examine roles of β-neurexin (NRX) and neuroligin-1 (NLG1) in dendritic arbor development. Using methods of dynamic morphometrics for comprehensive 3D quantification of rapid dendritogenesis, we find initial trans-synaptic NRX-NLG1 adhesions confer transient morphologic stabilization independent of NMDA receptor activity, whereas persistent stabilization requires NMDA receptor-dependent synapse maturation. Disrupting NRX-NLG1 function destabilizes **filopodi**a while reducing synaptic density and AMPA receptor mEPSC frequency. Altered dynamic growth culminates in reduced dendritic arbor complexity as neurons mature over days. These results expand the synaptotropic model of dendritogenesis to incorporate cell adhesion molecule-mediated morphological stabilization necessary for directing normal dendritic arborization, providing a potential morphological substrate for developmental cognitive impairment associated with cell adhesion molecule mutations.

### [Ff]ilopodium

#### Process

1. positive regulation of **filopodium** assembly

#### PubMed

1. Neuroligin-1 overexpression in newborn granule cells in vivo. Adult-born dentate granule cells integrate into the hippocampal network, extend neurites and form synapses in otherwise mature tissue. Excitatory and inhibitory inputs innervate these new granule cells in a stereotyped, temporally segregated manner, which presents a unique opportunity to study synapse development in the adult brain. To examine the role of neuroligins as synapse-inducing molecules in vivo, we infected dividing neural precursors in adult mice with a retroviral construct that increased neuroligin-1 levels during granule cell differentiation. By 21 days post-mitosis, exogenous neuroligin-1 was expressed at the tips of dendritic spines and increased the number of dendritic spines. Neuroligin-1-overexpressing cells showed a selective increase in functional excitatory synapses and connection multiplicity by single afferent fibers, as well as an increase in the synaptic AMPA/NMDA receptor ratio. In contrast to its synapse-inducing ability in vitro, neuroligin-1 overexpression did not induce precocious synapse formation in adult-born neurons. However, the dendrites of neuroligin-1-overexpressing cells did have more thin protrusions during an early period of dendritic outgrowth, suggesting enhanced **filopodium** formation or stabilization. Our results indicate that neuroligin-1 expression selectively increases the degree, but not the onset, of excitatory synapse formation in adult-born neurons.

#### Component

1. **filopodium** tip

### [Mm]igration

#### PubMed

1. Neuroligin 1 induces blood vessel maturation by cooperating with the α6 integrin. The synaptic protein Neuroligin 1 (NLGN1), a cell adhesion molecule, is critical for the formation and consolidation of synaptic connectivity and is involved in vascular development. The mechanism through which NLGN1 acts, especially in vascular cells, is unknown. Here, we aimed at deepening our knowledge on the cellular activities and molecular pathways exploited by endothelial NLGN1 both in vitro and in vivo. We analyzed the phenotypic consequences of NLGN1 expression modulation in endothelial cells through in vitro angiogenesis assays and the mouse postnatal retinal angiogenesis model. We demonstrate that NLGN1, whereas not affecting endothelial cell proliferation or **migratio**n, modulates cell adhesion to the vessel stabilizing protein laminin through cooperation with the α6 integrin, a specific laminin receptor. Finally, we show that in vivo, NLGN1 and α6 integrin preferentially colocalize in the mature retinal vessels, whereas NLGN1 deletion causes an aberrant VE-cadherin, laminin and α6 integrin distribution in vessels, along with significant structural defects in the vascular tree.
2. Astn2, a novel member of the astrotactin gene family, regulates the trafficking of ASTN1 during glial-guided neuronal **migration**. Glial-guided neuronal **migration** is a key step in the development of laminar architecture of cortical regions of the mammalian brain. We previously reported that neuronal protein astrotactin (ASTN1) functions as a neuron-glial ligand during CNS glial-guided **migration**. Here, we identify a new Astn family member, Astn2, that is expressed at high levels in migrating, cerebellar granule neurons, along with Astn1, at developmental stages when glial-guided **migration** is ongoing. Biochemical and flow cytometry experiments show that ASTN2 forms a complex with ASTN1 and regulates surface expression of ASTN1. Live imaging of Venus-tagged ASTN1 in migrating cerebellar granule cells reveals the intracellular trafficking of ASTN1-Venus, with ASTN1-Venus accumulating in the forward aspect of the leading process where new sites of adhesion will form. Treatment of migrating neurons with Dynasore, a soluble noncompetitive inhibitor of Dynamin, rapidly arrests the **migration** of immature granule cells in a reversible manner, suggesting the critical importance of receptor trafficking to neuronal locomotion along Bergmann glial fibers in the developing cerebellum. Together, these findings suggest that ASTN2 regulates the levels of ASTN1 in the plasma membrane and that the release of neuronal adhesions to the glial fiber during neuronal locomotion involves the intracellular trafficking of ASTN1.

---

## Hp

- NCBI Gene
- GeneRIF
- Pubmed

| Search Term | Rank | Fields |
| --- | --- | --- |
| [Mm]igration | 6 | PubMed(1) |
| [Ee]xtracellular [Mm]atrix | 8 | PubMed(2) |
| [Cc]ell [Ii]nvasion | 13 | PubMed(1) |

  
hide/show details for Hp

### [Mm]igration

#### PubMed

1. Acute-phase protein haptoglobin is a cell **migration** factor involved in arterial restructuring. Collagen turnover and cell **migration** are fundamental aspects of arterial restructuring. To identify mRNAs involved in blood flow-induced arterial restructuring, we performed subtraction polymerase chain reaction and found expression of haptoglobin mRNA in adventitial fibroblasts of rabbit arteries. Haptoglobin is highly expressed in liver, but its arterial expression and function are unknown. In vitro studies revealed that stimulation of haptoglobin expression by lipopolysaccharides in mice fibroblasts stimulated **migration** of wild-type fibroblasts but had no effect on **migration** of haptoglobin knockout fibroblasts. In vivo studies showed that flow-induced arterial restructuring was delayed in haptoglobin knockout mice. This new function of haptoglobin might be explained by facilitating cell **migration** through accumulation of a temporary gelatin matrix because cell culture showed that haptoglobin is involved in the breakdown of gelatin. We conclude that haptoglobin is highly expressed in arterial tissue and is involved in arterial restructuring. This new haptoglobin function may also apply to other functional and pathological restructuring processes such as angiogenesis, tissue repair, and tumor cell invasion.

### [Ee]xtracellular [Mm]atrix

#### PubMed

1. **Extracellular matrix** secretion by cardiac fibroblasts: role of microRNA-29b and microRNA-30c. MicroRNAs (miRNAs), in particular miR-29b and miR-30c, have been implicated as important regulators of cardiac fibrosis.To perform a proteomics comparison of miRNA effects on **extracellular matrix** secretion by cardiac fibroblasts.Mouse cardiac fibroblasts were transfected with pre-/anti-miR of miR-29b and miR-30c, and their conditioned medium was analyzed by mass spectrometry. miR-29b targeted a cadre of proteins involved in fibrosis, including multiple collagens, matrix metalloproteinases, and leukemia inhibitory factor, insulin-like growth factor 1, and pentraxin 3, 3 predicted targets of miR-29b. miR-29b also attenuated the cardiac fibroblast response to transforming growth factor-β. In contrast, miR-30c had little effect on **extracellular matri**x production but opposite effects regarding leukemia inhibitory factor and insulin-like growth factor 1. Both miRNAs indirectly affected cardiac myocytes. On transfection with pre-miR-29b, the conditioned medium of cardiac fibroblasts lost its ability to support adhesion of rat ventricular myocytes and led to a significant reduction of cardiac myocyte proteins (α-actinin, cardiac myosin-binding protein C, and cardiac troponin I). Similarly, cardiomyocytes derived from mouse embryonic stem cells atrophied under pre-miR-29 conditioned medium, whereas pre-miR-30c conditioned medium had a prohypertrophic effect. Levels of miR-29a, miR-29c, and miR-30c, but not miR-29b, were significantly reduced in a mouse model of pathological but not physiological hypertrophy. Treatment with antagomiRs to miR-29b induced excess fibrosis after aortic constriction without overt deterioration in cardiac function.Our proteomic analysis revealed novel molecular targets of miRNAs that are linked to a fibrogenic cardiac phenotype. Such comprehensive screening methods are essential to define the concerted actions of miRNAs in cardiovascular disease.
2. Identification of cathepsin K as a novel marker of adiposity in white adipose tissue. In obesity, adipocytes undergo dramatic morphological and molecular changes associated with alterations in their gene expression profile. To identify genes differentially modulated in white adipose tissue (WAT) of obese db/db mice compared to wild type (wt) mice, we utilized RNA fingerprinting. Among the 52 candidates that we identified, we focused here on cathepsin K (ctsk), a cysteine protease, prevalently localized in lysosomes and involved in bone **extracellular matrix** degradation. In db/db mice, WAT ctsk mRNA was elevated 5.9-fold, as were Mitf and TFE3 (2- and 3.3-fold respectively), two transcription factors involved in ctsk induction in osteoclasts. Moreover, the level of WAT ctsk mRNA was increased in other obese models including A(y), fat, and tubby (2.8-, 3.2-, and 4.9-fold respectively) and decreased in mice undergoing weight loss. Despite the ubiquitous distribution of the ctsk transcript, we demonstrated that the obesity related increase is specific to the adipocytes. Further, in vitro experiments proved that the abundance of ctsk transcript increases upon adipose conversion of the established cell line of preadipocytes 3T3-F442A. In addition, ctsk gene expression was examined in adipose tissue of 21 lean and obese male subjects and significant correlations with BMI (r = 0.54, P = 0.012) and plasma leptin levels (r = 0.54, P = 0.015) were found. In conclusion, the WAT of obese db/db mice exhibits a different expression profile from that of the wt mice, and cathepsin K can be considered a novel marker of obesity and a target for the inhibition of adipose mass growth.

### [Cc]ell [Ii]nvasion

#### PubMed

1. Acute-phase protein haptoglobin is a cell migration factor involved in arterial restructuring. Collagen turnover and cell migration are fundamental aspects of arterial restructuring. To identify mRNAs involved in blood flow-induced arterial restructuring, we performed subtraction polymerase chain reaction and found expression of haptoglobin mRNA in adventitial fibroblasts of rabbit arteries. Haptoglobin is highly expressed in liver, but its arterial expression and function are unknown. In vitro studies revealed that stimulation of haptoglobin expression by lipopolysaccharides in mice fibroblasts stimulated migration of wild-type fibroblasts but had no effect on migration of haptoglobin knockout fibroblasts. In vivo studies showed that flow-induced arterial restructuring was delayed in haptoglobin knockout mice. This new function of haptoglobin might be explained by facilitating cell migration through accumulation of a temporary gelatin matrix because cell culture showed that haptoglobin is involved in the breakdown of gelatin. We conclude that haptoglobin is highly expressed in arterial tissue and is involved in arterial restructuring. This new haptoglobin function may also apply to other functional and pathological restructuring processes such as angiogenesis, tissue repair, and tumor **cell invasion**.

---

## Ptgs2

- NCBI Gene
- GeneRIF
- Pubmed

| Search Term | Rank | Fields |
| --- | --- | --- |
| [Mm]igration | 6 | Process(1); PubMed(13); GeneRIFs(2) |
| ECM | 7 | PubMed(2) |
| [Ee]xtracellular [Mm]atrix | 8 | PubMed(11); GeneRIFs(4) |
| [Ii]nvasive | 10 | PubMed(16); GeneRIFs(1) |
| [Mm]etastasize | 11 | PubMed(2) |
| [Mm]etastasis | 12 | PubMed(9); GeneRIFs(1) |

  
hide/show details for Ptgs2

### [Mm]igration

#### Process

1. positive regulation of cell **migration** involved in sprouting angiogenesis

#### PubMed

1. Hyaluronic acid is radioprotective in the intestine through a TLR4 and COX-2-mediated mechanism. The intestinal epithelium is sensitive to radiation injury. Damage to the intestinal epithelium is dose limiting in radiation therapy of abdominal cancers. There is a need for agents that can be given before radiation therapy to protect the intestinal epithelium. C57BL6 mice were subjected to 12 Gy of total body radiation. Some mice received intraperitoneal hyaluronic acid (HA) before radiation. Mice were killed 6 h after radiation to assess radiation-induced apoptosis in the intestine; other mice were killed at 84 h to assess crypt survival. Total body radiation (12 Gy) resulted in increased expression of HA synthases and HA in the intestine and increased plasma HA (5-fold). Intraperitoneal injection of HA (30 mg/kg) before radiation resulted in a 1.8-fold increase in intestinal crypt survival and a decrease in radiation-induced apoptosis. The radioprotective effects of HA were not seen in Toll-like receptor 4 (TLR4)- or cyclooxygenase-2 (COX-2)-deficient mice. Intraperitoneal injection of HA induced a 1.5-fold increase in intestinal COX-2 expression, a 1.5-fold increase in intestinal PGE₂, and th**e migrati**on of COX-2-expressing mesenchymal stem cells from the lamina propria in the villi to the lamina propria near the crypt. We conclude that 1) radiation induces increased HA expression through inducing HA synthases, 2) intraperitoneal HA given before radiation reduces radiation-induced apoptosis and increases crypt survival, and 3) these radioprotective effects are mediated through TLR4, COX-2, and th**e migrati**on of COX-2-expressing mesenchymal stem cells.
2. Cyclooxygenase-2 or tumor necrosis factor-α inhibitors attenuate the mechanotransductive effects of pulsed focused ultrasound to suppress mesenchymal stromal cell homing to healthy and dystrophic muscle. Maximal homing of infused stem cells to diseased tissue is critical for regenerative medicine. Pulsed focused ultrasound (pFUS) is a clinically relevant platform to direct stem cell **migratio**n. Through mechanotransduction, pFUS establishes local gradients of cytokines, chemokines, trophic factors (CCTF) and cell adhesion molecules (CAM) in treated skeletal muscle that subsequently infused mesenchymal stromal cells (MSC) can capitalize to migrate into the parenchyma. Characterizing molecular responses to mechanical pFUS effects revealed tumor necrosis factor-alpha (TNFα) drives cyclooxygenase-2 (COX2) signaling to locally increase CCTF/CAM that are necessary for MSC homing. pFUS failed to increase chemoattractants and induce MSC homing to treated muscle in mice pretreated with ibuprofen (nonspecific COX inhibitor) or etanercept (TNFα inhibitor). pFUS-induced MSC homing was also suppressed in COX2-knockout mice, demonstrating ibuprofen blocked the mechanically induced CCTF/CAM by acting on COX2. Anti-inflammatory drugs, including ibuprofen, are administered to muscular dystrophy (MD) patients, and ibuprofen also suppressed pFUS-induced homing to muscle in a mouse model of MD. Drug interactions with cell therapies remain unexplored and are not controlled for during clinical cell therapy trials. This study highlights potentially negative drug-host interactions that suppress stem cell homing and could undermine cell-based approaches for regenerative medicine.
3. Nitric oxide synthase-cyclooxygenase interactions are involved in tumor cell angiogenesis and **migration**. Nitric oxide (NO), produced by distinct nitric oxide synthase (NOS) isoforms, and prostaglandins generated by expression of cyclooxygenases are important mediators in tumor progression. Previous studies have shown that NO can influence the formation of prostaglandin E2 (PGE2). We provide evidence that NO, derived from iNOS and eNOS activity in LMM3 murine mammary adenocarcinoma cell line, is involved in tumor angiogenesis and in tumor cell **migration**. LMM3 cells that also stimulate their neovascularization activity and **migration** liberate high basal amounts of PGE2. There is large amount of evidence that postulates positive regulatory interactions between NOS and cyclooxygenase (COX) isoforms. We here show that, in the LMM3 cell line, while PGE2 exerts a positive modulation on NOS activity, NO closes the loop with a negative feed back on COX activity. We also provide evidence of a positive regulatory effect of protein tyrosine kinases on NOS as well as on COX enzymatic functions affecting tumor induced angiogenesis and cell **migration**.
4. 5-lipoxygenase and cyclooxygenase regulate wound closure in NIH/3T3 fibroblast monolayers. Wound healing involves multiple cell signaling pathways, including those regulating cell-extracellular matrix adhesion. Previous work demonstrated that arachidonate oxidation to leukotriene B(4) (LTB(4)) by 5-lipoxygenase (5-LOX) signals fibroblast spreading on fibronectin, whereas cyclooxygenase-2 (COX-2)-catalyzed prostaglandin E(2) (PGE(2)) formation facilitates subsequent cell **migration**. We investigated arachidonate metabolite signaling in wound closure of perturbed NIH/3T3 fibroblast monolayers. We found that during initial stages of wound closure (0-120 min), all wound margin cells spread into the wound gap perpendicularly to the wound long axis. At regular intervals, between 120 and 300 min, some cells elongated to project across the wound and meet cells from the opposite margin, forming distinct cell bridges spanning the wound that act as foci for later wound-directed cell **migration** and resulting closure. 5-LOX inhibition by AA861 demonstrated a required LTB(4) signal for initial marginal cell spreading and bridge formation, both of which must precede wound-directed cell **migration**. 5-LOX inhibition effects were reversible by exogenous LTB(4). Conversely, COX inhibition by indomethacin reduced directed **migration** into the wound but enhanced early cell spreading and bridge formation. Exogenous PGE(2) reversed this effect and increased cell **migration** into the wound. The differential effects of arachidonic acid metabolites produced by LOX and COX were further confirmed with NIH/3T3 fibroblast cell lines constitutively over- and underexpressing the 5-LOX and COX-2 enzymes. These data suggest that two competing oxidative enzymes in arachidonate metabolism, LOX and COX, differentially regulate sequential aspects of fibroblast wound closure in vitro.
5. Activation of the RON receptor tyrosine kinase by macrophage-stimulating protein inhibits inducible cyclooxygenase-2 expression in murine macrophages. The RON receptor tyrosine kinase is activated by macrophage-stimulating protein, which regulates macrophage **migration**, phagocytosis, and nitric oxide production. We report here the inhibitory effect of RON on lipopolysaccharide (LPS)-induced cyclooxygenase (Cox)-2 expression in mouse macrophages. In RON-expressing macrophages treated with macrophage stimulating protein, LPS-induced prostaglandin E(2) (PGE(2)) production was significantly reduced. The inhibition was accompanied by reduction of Cox-2 protein and mRNA expression. Transcriptional studies indicated that RON activation inhibits LPS-induced luciferase activity driven by the Cox-2 gene promoter. To determine whether RON activation affects LPS-induced NF-kappa B pathway, which is important for Cox-2 expression. Western blot analyses were performed showing that RON activation inhibits LPS-induced I kappa B alpha degradation. The decreased I kappa B alpha degradation was due to reduced I kappa B alpha phosphorylation at Ser-32 as determined by I kappa B alpha (Ser-32) phosphor-antibody. Moreover, we found that LPS-induced IKK beta activity, an enzyme responsible for phosphorylation of I kappa B alpha, was inhibited upon RON activation. Interestingly, these inhibitory effects were not regulated by RON-mediated phosphatidylinositol-3 kinase. These results suggest that RON activation inhibits LPS-induced macrophage Cox-2 expression. The inhibitory effect is mediated by impairing LPS-activated cascade enzymes that activate NF-kappa B. The inhibition of Cox-2 expression might represent a novel mechanism for the inhibitory functions of RON in vivo against LPS-induced inflammation and septic shock.
6. Selective impairment of P2Y signaling by prostaglandin E2 in macrophages: implications for Ca2+-dependent responses. Extracellular nucleotides have been recognized as important modulators of inflammation via their action on specific pyrimidine receptors (P2). This regulation coexists with the temporal framework of proinflammatory and proresolution mediators released by the cells involved in the inflammatory response, including macrophages. Under proinflammatory conditions, the expression of cyclooxygenase-2 leads to the release of large amounts of PGs, such as PGE2, that exert their effects through EP receptors and other intracellular targets. The effect of these PGs on P2 receptors expressed in murine and human macrophages was investigated. In thioglycollate-elicited and alternatively activated macrophages, PGE2 selectively impairs P2Y but not P2X7 Ca(2+) mobilization. This effect is absent in LPS-activated cells and is specific for PGE2 because it cannot be reproduced by other PGs with cyclopentenone structure. The inhibition of P2Y responses by PGE2 involves the activation of nPKCs (PKCε) and PKD that can be abrogated by selective inhibitors or by expression of dominant-negative forms of PKD. The inhibition of P2Y signaling by PGE2 has an impact on the cell **migratio**n elicited by P2Y agonists in thioglycollate-elicited and alternatively activated macrophages, which provide new clues to understand the resolution phase of inflammation, when accumulation of PGE2, anti-inflammatory and proresolving mediators occurs.
7. High glucose-induced hyperosmolarity contributes to COX-2 expression and angiogenesis: implications for diabetic retinopathy. We tested the hypothesis that glucose-induced hyperosmolarity, occurring in diabetic hyperglycemia, promotes retinal angiogenesis, and that interference with osmolarity signaling ameliorates excessive angiogenesis and retinopathy in vitro and in vivo.We incubated human aortic (HAECs) and dermal microvascular endothelial cells (HMVECs) with glucose or mannitol for 24 h and tested them for protein levels and in vitro angiogenesis. We used the Ins2 Akita mice as a model of type 1 diabetes to test the in vivo relevance of in vitro observations. Compared to incubations with normal (5 mmol/L) glucose concentrations, cells exposed to both high glucose and high mannitol (at 30.5 or 50.5 mmol/L) increased expression of the water channel aquaporin-1 (AQP1) and cyclooxygenase (COX)-2. This was preceded by increased activity of the osmolarity-sensitive transcription factor Tonicity enhancer binding protein (TonEBP), and enhanced endothelial **migration** and tubulization in Matrigel, reverted by treatment with AQP1 and TonEBP siRNA. Retinas of Ins2 Akita mice showed increased levels of AQP1 and COX-2, as well as angiogenesis, all reverted by AQP1 siRNA intravitreal injections.Glucose-related hyperosmolarity seems to be able to promote angiogenesis and retinopathy through activation of TonEBP and possibly increasing expression of AQP1 and COX-2. Osmolarity signaling may be a target for therapy.
8. Adiponectin promotes revascularization of ischemic muscle through a cyclooxygenase 2-dependent mechanism. Adiponectin is a fat-derived plasma protein that has cardioprotective roles in obesity-linked diseases. Because cyclooxygenase 2 (COX-2) is an important modulator of endothelial function, we investigated the possible contribution of COX-2 to adiponectin-mediated vascular responses in a mouse hind limb model of vascular insufficiency. Ischemic insult increased COX-2 expression in endothelial cells of wild-type mice, but this induction was attenuated in adiponectin knockout mice. Ischemia-induced revascularization was impaired in mice in which the Cox-2 gene is deleted in Tie2-Cre-expressing cells. Adenovirus-mediated overexpression of adiponectin enhanced COX-2 expression and revascularization of ischemic limbs in control mice, but not in targeted Cox-2-deficient mice. In cultured endothelial cells, adiponectin protein increased COX-2 expression, and ablation of COX-2 abrogated the adiponectin-stimulated increases in endothelial cell **migration**, differentiation, and survival. Ablation of calreticulin (CRT) or its adaptor protein CD91 diminished adiponectin-stimulated COX-2 expression and endothelial cell responses. These observations provide evidence that adiponectin promotes endothelial cell function through CRT/CD91-mediated increases in COX-2 signaling. Thus, disruption of the adiponectin-COX-2 regulatory axis in endothelial cells could participate in the pathogenesis of obesity-related vascular diseases.
9. Cyclooxygenase-2 deficiency in macrophages leads to defective p110γ PI3K signaling and impairs cell adhesion and **migratio**n. Cyclooxygenase (Cox)-2 dependent PGs modulate several functions in many pathophysiological processes, including **migratio**n of immune cells. In this study, we addressed the role of Cox-2 in macrophage **migratio**n by using in vivo and in vitro models. Upon thioglycolate challenge, CD11b(+) F4/80(+) macrophages showed a diminished ability to migrate to the peritoneal cavity in cox-2(-/-) mice. In vivo **migratio**n of cox-2(-/-) macrophages from the peritoneal cavity to lymph nodes, as well as cell adhesion to the mesothelium, was reduced in response to LPS. In vitro **migratio**n of cox-2(-/-) macrophages toward MCP-1, RANTES, MIP-1α, or MIP-1β, as well as cell adhesion to ICAM-1 or fibronectin, was impaired. Defects in ce**ll migrat**ion were not due to changes in chemokine receptor expression. Remarkably, cox-2(-/-) macrophages showed a deficiency in focal adhesion formation, with reduced phosphorylation of paxillin (Tyr(188)). Interestingly, expression of the p110γ catalytic subunit of PI3K was severely reduced in the absence of Cox-2, leading to defective Akt phosphorylation, as well as cdc42 and Rac-1 activation. Our results indicate that the paxillin/p110γ-PI3K/Cdc42/Rac1 axis is defective in cox-2(-/-) macrophages, which results in impaired cell adhesion **and migr**ation.
10. Osteopontin signaling upregulates cyclooxygenase-2 expression in tumor-associated macrophages leading to enhanced angiogenesis and melanoma growth via α9β1 integrin. Tumor-associated macrophages (TAMs) have multifaceted roles in tumor development, particularly linked with tumor angiogenesis and invasion, but the molecular mechanism underlying this association remains unclear. In this study, we report that lack of osteopontin (OPN) suppresses melanoma growth in opn(-/-) mice and macrophages are the crucial component responsible for OPN-regulated melanoma growth. In tumor microenvironment, OPN activates macrophages and influences angiogenesis by enhancing cyclooxygenase-2 (COX-2)-dependent prostaglandin E2 (PGE2) production in an autocrine manner. Furthermore, we identify α9β1 integrin as a functional receptor for OPN that mediates its effect and activates ERK and p38 signaling, which ultimately leads to COX-2 expression in macrophages. The major role played by OPN and PGE2 in angiogenesis are further amplified by upregulation of MMP-9. OPN-activated macrophages promote **the migra**tion of endothelial and cancer cells via PGE2. These findings provide evidence that TAMs serve as source of key components such as OPN and COX-2-derived PGE2 and MMP-9 in melanoma microenvironment. Clinical specimens analyses revealed that increased infiltration of OPN-positive TAMs correlate with melanoma growth and angiogenesis. These data provide compelling evidence that OPN and COX-2 expressing macrophages are obligatory factors in melanoma growth. We conclude that OPN signaling is involved in macrophage recruitment into tumor, and our results emphasize the potential role of macrophage in modulation of tumor microenvironment via secretion of OPN, PGE2 and MMP-9, which trigger angiogenesis and melanoma growth. Thus, blockade of OPN and its regulated signaling network provides unique strategy to eradicate melanoma by manipulating TAMs.
11. Cyclooxygenase-2-derived prostaglandin E₂ promotes injury-induced vascular neointimal hyperplasia through the E-prostanoid 3 receptor. Vascular smooth muscle cell (VSMC**) migrati**on and proliferation are the hallmarks of restenosis pathogenesis after angioplasty. Cyclooxygenase (COX)-derived prostaglandin (PG) E₂ is implicated in the vascular remodeling response to injury. However, its precise molecular role remains unknown.This study investigates the impact of COX-2-derived PGE₂ on neointima formation after injury.Vascular remodeling was induced by wire injury in femoral arteries of mice. Both neointima formation and the restenosis ratio were diminished in COX-2 knockout mice as compared with controls, whereas these parameters were enhanced in COX-1>COX-2 mice, in which COX-1 is governed by COX-2 regulatory elements. PG profile analysis revealed that the reduced PGE₂ by COX-2 deficiency, but not PGI2, could be rescued by COX-1 replacement, indicating COX-2-derived PGE₂ enhanced neointima formation. Through multiple approaches, the EP3 receptor was identified to mediate **the VSMC** migration response to various stimuli. Disruption of EP3 impaired VSMC polarity for di**rectional** migration by decreasing small GTPase activity and restricted vascular neointimal hyperplasia, whereas overexpression of EP3α and EP3β aggravated neointima formation. Inhibition or deletion of EP3α/β, a Gαi protein-coupled receptor, activated the cAMP/protein kinase A pathway and decreased activation of RhoA in VSMCs. PGE₂ could stimulate phosphatidylinositol 3-kinase/Akt/glycogen synthase kinase3β signaling in VSMCs through Gβγ subunits on EP3α/β activation. Ablation of EP3 suppressed phosphatidylinositol 3-kinase signaling and reduced GTPase activity in VSMCs and altered cell pol**arity and** directional migration.COX-2-derived PGE₂ facilitated the neointimal hyperplasia response to injury through EP3α/β-mediated cAMP/protein kinase A and phosphatidylinositol 3-kinase pathways, indicating EP3 inhibition may be a promising therapeutic strategy for percutaneous transluminal coronary angioplasty.
12. Cyclosporin A impairs dendritic cell **migration** by regulating chemokine receptor expression and inhibiting cyclooxygenase-2 expression. **Migration** of dendritic cells (DCs) into tissues and secondary lymphoid organs plays a crucial role in the initiation of innate and adaptive immunity. In this article, we show that cyclosporin A (CsA) impairs the **migration** of DCs both in vitro and in vivo. Exposure of DCs to clinical concentrations of CsA neither induces apoptosis nor alters development but does impair cytokine secretion, chemokine receptor expression, and **migration**. In vitro, CsA impairs the **migration** of mouse bone marrow-derived DCs toward macrophage inflammatory protein-3beta (MIP-3beta) and induces them to retain responsiveness to MIP-1alpha after lipopolysaccharide (LPS)-stimulated DC maturation, while in vivo administration of CsA inhibits the **migration** of DCs out of skin and into the secondary lymphoid organs. CsA impairs chemokine receptor and cyclooxygenase-2 (COX-2) expression normally triggered in LPS-stimulated DCs; administration of exogenous prostaglandin E2 (PGE2) reverses the effects of CsA on chemokine receptor expression and DC **migration**. Inhibition of nuclear factor-kappaB (NF-kappaB) and mitogen-activated protein kinase (MAPK) pathway signaling by CsA may be responsible for the CsA-mediated effects on the regulation of chemokine receptor and cyclooxygenase-2 (COX-2) expression. Impairment of DC **migration** due to inhibition of PGE2 production and regulation of chemokine receptor expression may contribute, in part, to CsA-mediated immunosuppression.
13. Prostaglandin E2 stimulates granulocyte colony-stimulating factor production via the prostanoid EP2 receptor in mouse peritoneal neutrophils. G-CSF is a hemopoietic growth factor involved in granulocytic differentiation of progenitor cells. In this study, we investigated the effects of PGE2 on G-CSF production in murine peritoneal neutrophils in vitro and in vivo. PGE2 augmented LPS-primed G-CSF release from peritoneal neutrophils. This augmentation was mimicked by a type E prostanoid receptor (EP)2-selective agonist but not by other EP-specific agonists. Indeed, the effect of PGE2 on G-CSF release was abolished in neutrophils isolated from EP2-deficient mice. PGE2 and an EP2 agonist have the ability to stimulate G-CSF gene expression even in the absence of LPS. In the casein-induced peritonitis model, the appearance of G-CSF in the casein-injected peritoneal cavity associated well with the timing of neutrophil infiltration as well as PGE2 levels in exudates, with a peak value at 6 h postinjection. Inhibition of endogenous PG synthesis by indomethacin resulted in a marked decrease in G-CSF content and neutrophil number in the peritoneal cavity. Moreover, EP2-deficient mice exhibited a strikingly reduced G-CSF content in peritoneal exudates with comparable responses in neutrophil **migration** and local PGE2 production at 6 h postinjection. These results suggest that the PGE2-EP2 system contributes to the local production of G-CSF during acute inflammation.

#### GeneRIFs

1. The phosphatidylinositol (PI)3-kinase/Cdc42/Rac1 axis can be defective in cox2-deficient macrophages, resulting in impaired cell adhesion and **migration**.
2. inducible nitric oxide synthase-cyclooxygenase 2 interactions are involved in tumor cell angiogenesis and **migration**

### ECM

#### PubMed

1. Extracellular matrix-induced cyclooxygenase-2 regulates macrophage proteinase expression. Chronic inflammatory diseases are characterized by the persistent presence of macrophages and other mononuclear cells, tissue destruction, cell proliferation, and the deposition of extracellular matrix (**ECM**). The tissue degradation is mediated, in part, by enhanced proteinase expression by macrophages. It has been demonstrated recently that macrophage proteinase expression can be stimulated or inhibited by purified **ECM** components. However, in an intact **ECM** the biologically active domains of matrix components may be masked either by tertiary conformation or by complex association with other matrix molecules. In an effort to determine whether a complex **ECM** produced by vascular smooth muscle cells (SMC) regulates macrophage degradative phenotype, we prepared insoluble SMC matrices and examined their ability to regulate proteinase expression by RAW264.7 and thioglycollate-elicited peritoneal macrophages. Here we demonstrate that macrophage engagement of SMC-**ECM** triggers PKC-dependent activation of MAPK(erk1/2) leading to increased expression of cyclooxygenase (COX)-2 and prostaglandin (PG) E(2) synthesis. The addition of PGE(2) to macrophage cultures stimulates their expression of both urokinase-type plasminogen activator and MMP-9, and the selective COX-2 inhibitor NS-398 blocks **ECM**-induced proteinase expression. Moreover, **ECM**-induced PGE(2) and MMP-9 expression by elicited COX-2(-/-) macrophages is markedly reduced when compared with the response of either COX-2(+/-) or COX-2(+/+) macrophages. These data clearly demonstrate that SMC-**ECM** exerts a regulatory role on the degradative phenotype of macrophages via enhanced urokinase-type plasminogen activator and MMP-9 expression, and identify COX-2 as a targetable component of the signaling pathway leading to increased proteinase expression.
2. Targeting prostaglandin E2 receptors as an alternative strategy to block cyclooxygenase-2-dependent extracellular matrix-induced matrix metalloproteinase-9 expression by macrophages. COX-2-dependent prostaglandin (PG) E2 synthesis regulates macrophage MMP expression, which is thought to destabilize atherosclerotic plaques. However, the administration of selective COX-2 inhibitors paradoxically increases the frequency of adverse cardiovascular events potentially through the loss of anti-inflammatory prostanoids and/or disturbance in the balance of pro- and anti-thrombotic prostanoids. To avoid these collateral effects of COX-2 inhibition, a strategy to identify and block specific prostanoid-receptor interactions may be required. We previously reported that macrophage engagement of vascular extracellular matrix (**ECM**) triggers proteinase expression through a MAPKerk1/2-dependent increase in COX-2 expression and PGE2 synthesis. Here we demonstrate that elicited macrophages express the PGE2 receptors EP1-4. When plated on **ECM**, their expression of EP2 and EP4, receptors linked to PGE2-induced activation of adenylyl cyclase, is strongly stimulated. Forskolin and dibutryl cyclic-AMP stimulate macrophage matrix metalloproteinase (MMP)-9 expression in a dose-dependent manner. However, an EP2 agonist (butaprost) has no effect on MMP-9 expression, and macrophages from EP2 null mice exhibited enhanced COX-2 and MMP-9 expression when plated on **ECM**. In contrast, the EP4 agonist (PGE1-OH) stimulated macrophage MMP-9 expression, which was inhibited by the EP4 antagonist ONO-AE3-208. When compared with COX-2 silencing by small interfering RNA or inhibition by celecoxib, the EP4 antagonist was as effective in inhibiting **ECM**-induced proteinase expression. In addition, **ECM**-induced MMP-9 expression was blocked in macrophages in which EP4 was silenced by small interfering RNA. Thus, COX-2-dependent **ECM**-induced proteinase expression is effectively blocked by selective inhibition of EP4, a member of the PGE2 family of receptors.

### [Ee]xtracellular [Mm]atrix

#### PubMed

1. Absence of cyclooxygenase-2 exacerbates hypoxia-induced pulmonary hypertension and enhances contractility of vascular smooth muscle cells. Cyclooxygenase-2 (COX-2) is upregulated in pulmonary artery smooth muscle cells (PASMCs) during hypoxia and may play a protective role in the response of the lung to hypoxia. Selective COX-2 inhibition may have detrimental pulmonary vascular consequences during hypoxia.To investigate the role of COX-2 in the pulmonary vascular response to hypoxia, we subjected wild-type and COX-2-deficient mice to a model of chronic normobaric hypoxia. COX-2-null mice developed severe pulmonary hypertension with exaggerated elevation of right ventricular systolic pressure, significant right ventricular hypertrophy, and striking vascular remodeling after hypoxia. Pulmonary vascular remodeling in COX-2-deficient mice was characterized by PASMC hypertrophy but not increased proliferation. Furthermore, COX-2-deficient mice had significant upregulation of the endothelin-1 receptor (ET(A)) in the lung after hypoxia. Similarly, selective pharmacological inhibition of COX-2 in wild-type mice exacerbated hypoxia-induced pulmonary hypertension and resulted in PASMC hypertrophy and increased ET(A) receptor expression in pulmonary arterioles. The absence of COX-2 in vascular smooth muscle cells during hypoxia in vitro augmented traction forces and enhanced contractility of an **extracellular matrix**. Treatment of COX-2-deficient PASMCs with iloprost, a prostaglandin I(2) analog, and prostaglandin E(2) abrogated the potent contractile response to hypoxia and restored the wild-type phenotype.Our findings reveal that hypoxia-induced pulmonary hypertension and vascular remodeling are exacerbated in the absence of COX-2 with enhanced ET(A) receptor expression and increased PASMC hypertrophy. COX-2-deficient PASMCs have a maladaptive response to hypoxia manifested by exaggerated contractility, which may be rescued by either COX-2-derived prostaglandin I(2) or prostaglandin E(2).
2. Decreased expression of tumor necrosis factor-alpha-stimulated gene 6 in cumulus cells of the cyclooxygenase-2 and EP2 null mice. Ovulation, the release of fertilizable oocytes from mature follicles, involves tissue remodeling and increased prostaglandin (PG) signaling. Cyclooxygenase (COX)-2 is the rate-limiting enzyme during PG synthesis. Female mice null for either COX-2 or the PGE(2) receptor EP2 are infertile, show decreased ovulation, and exhibit abnormal cumulus expansion. Cumulus expansion is the production of a complex **extracellular matrix** surrounding the cumulus-oocyte complex (COC). Matrix components consist of hyaluronan, proteoglycans, and proteins with hyaluronan binding domains. One such hyaluronan binding protein is TNFalpha-stimulated gene 6 (TSG-6). By various methods, we show induction of TSG-6 and hyaluronan synthase-2 mRNA in ovaries of mice treated with pregnant mare serum gonadotropin and human chorionic gonadotropin. By in situ hybridization, we show that both genes are expressed in periantral mural granulosa cells and cumulus cells of the mouse ovary. Notably, RT-PCR and in situ hybridization show that TSG-6 mRNA but not hyaluronan synthase-2 mRNA expression is selectively reduced in cumulus cells of COX-2 and EP2 null mice. Western analysis further confirms that TSG-6 protein is reduced in isolated COCs but remains covalently associated with inter alpha-trypsin inhibitor in COX-2 null mice. These observations identify TSG-6 as a target of PG action and show that its production in ovulatory follicles is associated with proper formation of the cumulus-derived **extracellular matrix**.
3. Heterogeneous nuclear ribonucleoprotein K (hnRNP-K) promotes tumor metastasis by induction of genes involved in **extracellular matrix**, cell movement, and angiogenesis. Cancer is a leading cause of death and still awaits effective therapies. Rapid industrialization has contributed to increase in incidence of cancer. One of the reasons why most of the cancers fail therapy is due to their metastatic property. Hence identification of factors leading to metastasis is highly important to design effective and novel anti-cancer therapeutics. In our earlier study (Inoue, A., Sawata, S. Y., Taira, K., and Wadhwa, R. (2007) Loss-of-function screening by randomized intracellular antibodies: identification of hnRNP-K as a potential target for metastasis. Proc. Natl. Acad. Sci. U.S.A. 104, 8983-8988), we had reported that the involvement of heterogeneous nuclear ribonucleoprotein K (hnRNP-K) in metastasis. Here, we established hnRNP-K-overexpressing and -underexpressing derivative cell lines and examined their proliferation and metastatic properties in vitro and in vivo. Whereas hnRNP-K compromised cells showed delayed tumor growth, its overexpression resulted in enhanced malignancy and metastasis. Molecular basis of the hnRNP-K induced malignant and metastatic phenotypes was dissected by cDNA microarray and pathway analyses. We found that the hnRNP-K regulates **extracellular matrix**, cell motility, and angiogenesis pathways. Involvement of the selected genes (Cck, Mmp-3, Ptgs2, and Ctgf) and pathways was validated by gene-specific expression analysis. Our results demonstrated that the hnRNP-K is a potential target for metastasis therapy.
4. 5-lipoxygenase and cyclooxygenase regulate wound closure in NIH/3T3 fibroblast monolayers. Wound healing involves multiple cell signaling pathways, including those regulating cell-**extracellular matrix** adhesion. Previous work demonstrated that arachidonate oxidation to leukotriene B(4) (LTB(4)) by 5-lipoxygenase (5-LOX) signals fibroblast spreading on fibronectin, whereas cyclooxygenase-2 (COX-2)-catalyzed prostaglandin E(2) (PGE(2)) formation facilitates subsequent cell migration. We investigated arachidonate metabolite signaling in wound closure of perturbed NIH/3T3 fibroblast monolayers. We found that during initial stages of wound closure (0-120 min), all wound margin cells spread into the wound gap perpendicularly to the wound long axis. At regular intervals, between 120 and 300 min, some cells elongated to project across the wound and meet cells from the opposite margin, forming distinct cell bridges spanning the wound that act as foci for later wound-directed cell migration and resulting closure. 5-LOX inhibition by AA861 demonstrated a required LTB(4) signal for initial marginal cell spreading and bridge formation, both of which must precede wound-directed cell migration. 5-LOX inhibition effects were reversible by exogenous LTB(4). Conversely, COX inhibition by indomethacin reduced directed migration into the wound but enhanced early cell spreading and bridge formation. Exogenous PGE(2) reversed this effect and increased cell migration into the wound. The differential effects of arachidonic acid metabolites produced by LOX and COX were further confirmed with NIH/3T3 fibroblast cell lines constitutively over- and underexpressing the 5-LOX and COX-2 enzymes. These data suggest that two competing oxidative enzymes in arachidonate metabolism, LOX and COX, differentially regulate sequential aspects of fibroblast wound closure in vitro.
5. **Extracellular matrix**-induced cyclooxygenase-2 regulates macrophage proteinase expression. Chronic inflammatory diseases are characterized by the persistent presence of macrophages and other mononuclear cells, tissue destruction, cell proliferation, and the deposition of **extracellular matrix** (ECM). The tissue degradation is mediated, in part, by enhanced proteinase expression by macrophages. It has been demonstrated recently that macrophage proteinase expression can be stimulated or inhibited by purified ECM components. However, in an intact ECM the biologically active domains of matrix components may be masked either by tertiary conformation or by complex association with other matrix molecules. In an effort to determine whether a complex ECM produced by vascular smooth muscle cells (SMC) regulates macrophage degradative phenotype, we prepared insoluble SMC matrices and examined their ability to regulate proteinase expression by RAW264.7 and thioglycollate-elicited peritoneal macrophages. Here we demonstrate that macrophage engagement of SMC-ECM triggers PKC-dependent activation of MAPK(erk1/2) leading to increased expression of cyclooxygenase (COX)-2 and prostaglandin (PG) E(2) synthesis. The addition of PGE(2) to macrophage cultures stimulates their expression of both urokinase-type plasminogen activator and MMP-9, and the selective COX-2 inhibitor NS-398 blocks ECM-induced proteinase expression. Moreover, ECM-induced PGE(2) and MMP-9 expression by elicited COX-2(-/-) macrophages is markedly reduced when compared with the response of either COX-2(+/-) or COX-2(+/+) macrophages. These data clearly demonstrate that SMC-ECM exerts a regulatory role on the degradative phenotype of macrophages via enhanced urokinase-type plasminogen activator and MMP-9 expression, and identify COX-2 as a targetable component of the signaling pathway leading to increased proteinase expression.
6. Role of COX-2-derived PGE2 on vascular stiffness and function in hypertension. Prostanoids derived from COX-2 and EP receptors are involved in vascular remodelling in different cardiovascular pathologies. This study evaluates the contribution of COX-2 and EP1 receptors to vascular remodelling and function in hypertension.Spontaneously hypertensive rats (SHR) and angiotensin II (AngII)-infused (1.44 mg · kg(-1) · day(-1), 2 weeks) mice were treated with the COX-2 inhibitor celecoxib (25 mg · kg(-1) · day(-1) i.p) or with the EP1 receptor antagonist SC19220 (10 mg · kg(-1) · day(-1) i.p.). COX-2(-/-) mice with or without AngII infusion were also used.Celecoxib and SC19220 treatment did not modify the altered lumen diameter and wall : lumen ratio in mesenteric resistance arteries from SHR-infused and/or AngII-infused animals. However, both treatments and COX-2 deficiency decreased the augmented vascular stiffness in vessels from hypertensive animals. This was accompanied by diminished vascular collagen deposition, normalization of altered elastin structure and decreased connective tissue growth factor and plasminogen activator inhibitor-1 gene expression. COX-2 deficiency and SC19220 treatment diminished the increased vasoconstrictor responses and endothelial dysfunction induced by AngII infusion. Hypertensive animals showed increased mPGES-1 expression and PGE2 production in vascular tissue, normalized by celecoxib. Celecoxib treatment also decreased AngII-induced macrophage infiltration and TNF-α expression. Macrophage conditioned media (MCM) increased COX-2 and collagen type I expression in vascular smooth muscle cells; the latter was reduced by celecoxib treatment.COX-2 and EP1 receptors participate in the inc**reased extracellular** matrix deposition and vascular stiffness, the impaired vascular function and inflammation in hypertension. Targeting PGE2 receptors might have benefits in hypertension-associated vascular damage.
7. Increased prostaglandin E₂-EP2 signalling in cumulus cells of female mice sired by males with the Y-chromosome long-arm deletion. Cumuli oophori surrounding ovulated oocytes of B10.BR(Y(del)) females (sired by males with the Y-chromosome long-arm deletion) are more resistant to hyaluronidase digestion than cumuli oophori around eggs of genetically identical females but sired by males with the intact Y chromosome (B10.BR). This has been interpreted as a result of differences in paternal genome imprinting, which females of both groups inherit from their fathers. The following study shows that it is not hyaluronan, but rather excessive protein concentration, that makes the cumulu**s extracellular matr**ix of B10.BR(Y(del)) oocytes more resistant to enzymatic treatment. It was revealed, additionally, that cumulus cells around ovulating oocytes of B10.BR(Y(del)) females display higher surface accumulation of prostaglandin EP2 subtype receptors and higher expression of the Ptgs2 gene (encoding a rate-limiting enzyme of prostaglandin E₂ synthesis) in relation to the cells of control B10.BR females. The expression levels of the prostaglandin-dependent Tnfaip6 and Ccl2 genes were also altered in B10.BR(Y(del)) cumulus cells in a manner indicating increased prostaglandin signalling. The study provides further evidence for the divergence in reproductive phenotypes between B10.BR and B10.BR(Y(del)) female mice. It supports the hypothesis that genes of the Y-chromosome long arm may be involved in establishment of epigenetic marks in X-bearing spermatozoa.
8. MAPK-activated protein kinase-2 in cardiac hypertrophy and cyclooxygenase-2 regulation in heart. Activation of p38 mitogen-activated protein kinase (MAPK) has a significant impact on cardiac gene expression, contractility, **extracellular matrix** remodeling, and inflammatory response in heart. The p38 kinase pathway also has a controversial role in cardiac hypertrophy. MAPK-activated protein kinase-2 (MK2) is a well-established p38 downstream kinase, yet its contribution to p38-mediated pathological response in heart has not been investigated.We examined the specific contribution of MK2 to the pathological remodeling induced by p38.We used a cardiomyocyte specific and inducible transgenic approach to determine the functional and molecular impact of acute activation of the p38 pathway in heart in either a MK2 wild-type or a MK2-null background. p38 activation in wild-type mice led to a rapid onset of lethal cardiomyopathy associated with cardiomyocyte hypertrophy, interstitial fibrosis, and contractile dysfunction. Inactivation of MK2 partially but significantly reduced cardiomyocyte hypertrophy, improved contractile performance, and prevented early lethality. MK2 inactivation had no effect on the mRNA levels of hypertrophic marker genes or the proinflammatory gene cyclooxygenase (COX)-2. However, MK2 had a major role in COX-2 protein synthesis without affecting the mRNA level or protein stability.p38 activity in adult myocytes can contribute to pathological hypertrophy and remodeling in adult heart and that MK2 is an important downstream molecule responsible for specific features of p38-induced cardiac pathology.
9. Targeting prostaglandin E2 receptors as an alternative strategy to block cyclooxygenase-2-dependent **extracellular matrix**-induced matrix metalloproteinase-9 expression by macrophages. COX-2-dependent prostaglandin (PG) E2 synthesis regulates macrophage MMP expression, which is thought to destabilize atherosclerotic plaques. However, the administration of selective COX-2 inhibitors paradoxically increases the frequency of adverse cardiovascular events potentially through the loss of anti-inflammatory prostanoids and/or disturbance in the balance of pro- and anti-thrombotic prostanoids. To avoid these collateral effects of COX-2 inhibition, a strategy to identify and block specific prostanoid-receptor interactions may be required. We previously reported that macrophage engagement of vascular **extracellular matrix** (ECM) triggers proteinase expression through a MAPKerk1/2-dependent increase in COX-2 expression and PGE2 synthesis. Here we demonstrate that elicited macrophages express the PGE2 receptors EP1-4. When plated on ECM, their expression of EP2 and EP4, receptors linked to PGE2-induced activation of adenylyl cyclase, is strongly stimulated. Forskolin and dibutryl cyclic-AMP stimulate macrophage matrix metalloproteinase (MMP)-9 expression in a dose-dependent manner. However, an EP2 agonist (butaprost) has no effect on MMP-9 expression, and macrophages from EP2 null mice exhibited enhanced COX-2 and MMP-9 expression when plated on ECM. In contrast, the EP4 agonist (PGE1-OH) stimulated macrophage MMP-9 expression, which was inhibited by the EP4 antagonist ONO-AE3-208. When compared with COX-2 silencing by small interfering RNA or inhibition by celecoxib, the EP4 antagonist was as effective in inhibiting ECM-induced proteinase expression. In addition, ECM-induced MMP-9 expression was blocked in macrophages in which EP4 was silenced by small interfering RNA. Thus, COX-2-dependent ECM-induced proteinase expression is effectively blocked by selective inhibition of EP4, a member of the PGE2 family of receptors.
10. EC-SOD induces apoptosis through COX-2 and galectin-7 in the epidermis. Extracellular superoxide dismutase (EC-SOD) is an anti-oxidant enzyme found in the **extracellular matrix** of tissues, and plays an important role in the prevention of many diseases caused by oxidative stress. However, other functions of EC-SOD in epidermis are not well known.We investigated the functions of EC-SOD in epidermis using keratinocyte cell line and EC-SOD transgenic mice.Expression of galectin-7 in pEC-SOD transfected cells or skin of EC-SOD transgenic mice was detected by western blot analysis. The percentage of apoptotic cells was determined by propidium iodide staining and subsequent FACS analysis. COX-2 siRNA or scrambled siRNA was transfected into HaCaT cells and western blot analysis was performed to detect pro-apoptotic protein levels.The epidermis of EC-SOD transgenic mice was thinner than wild type mice. In addition, we showed that the thin epidermis of EC-SOD transgenic mice results from the apoptosis of epidermal cells. To elucidate which molecules are involved in EC-SOD-induced apoptosis, we utilized two-dimensional electrophoresis; the results showed that the epidermis of EC-SOD transgenic mice produces more galectin-7, a pro-apoptotic factor, than the wild type. Furthermore, we showed that the transfection of EC-SOD-expressing plasmids induces the production of galectin-7, and pro-apoptotic proteins in keratinocytes. This suggests that EC-SOD induces apoptosis through increased galectin-7 expression. Finally, we demonstrated that EC-SOD-induced galectin-7 results from the production of COX-2.Our results imply that EC-SOD plays a role not only as a reactive oxygen species scavenger, but also as a pro-apoptotic factor via COX-2/galectin-7 pathways in the epidermis.
11. Cyclooxygenase inhibitors repress vascular hyaluronan-synthesis in murine atherosclerosis and neointimal thickening. Hyaluronan (HA) is a key molecule of the **extracellular matrix** that is thought to be critically involved in both atherosclerosis and restenosis. Recently, it has been demonstrated that the cyclooxygenase (COX) products, prostacyclin and prostaglandin E(2), induce HA synthesis in vitro by transcriptional up-regulation of HA-synthase 2 (HAS2) and HAS1. The relative roles in atherosclerotic and restenotic artery disease of tissue specifically expressed COX-1 and COX-2 are still under debate. Thus, the present study aimed to investigate the effect of COX isoform inhibition on HA-accumulation and regulation of HAS isoform expression in two models of pathologic artery remodelling in vivo. Firstly, ApoE-deficient mice were treated with a prototypic isoform non-selective inhibitor, indomethacin or with a prototypic COX-2 selective inhibitor, rofecoxib, for 8 weeks. Aortic HAS mRNA expression and HA-accumulation in atherosclerotic aortic root lesions were analyzed. Secondly, neointimal hyperplasia was induced by carotid artery ligation in ApoE-deficient mice on a high fat diet and the effects of the COX inhibitors were determined after 4 weeks of treatment. Intimal HA-accumulation was markedly reduced in both models by indomethacin and rofecoxib. This coincided with a strong inhibition of HAS1 mRNA expression in both models and with decreased HAS2 mRNA in the aorta of ApoE-deficient mice. HAS3 was not affected. The repression of HA-accumulation by both COX-2 selective and non-selective COX inhibition implicates COX-2 in the regulation of HA synthesis via stimulation of HAS1 and HAS2 expression in vivo. Modulation of vascular HA-accumulation might play a role in chronic effects of COX inhibitors on the progression of atherosclerosis.

#### GeneRIFs

1. COX-2 and EP1 receptors participate in the increased **extracellular matrix** deposition and vascular stiffness, the impaired vascular function and inflammation in hypertension. Targeting PGE2 receptors might have benefits in hypertension-associated vascular damage.
2. hnRNP-K regulates **extracellular matrix**, cell motility, and angiogenesis pathways. Involvement of the selected genes (Cck, Mmp-3, Ptgs2, and Ctgf) and pathways was validated by gene-specific expression analysis
3. cyclooxygenase-2-dependent **extracellular matrix**-induced matrix metalloproteinase-9 expression by macrophages is blocked through prostaglandin E2 receptors
4. **Extracellular matrix**-induced cyclooxygenase-2 regulates macrophage proteinase expression

### [Ii]nvasive

#### PubMed

1. The cyclooxygenase-2-mediated prostaglandin signaling is causally related to epithelial carcinogenesis. Epidemiologic, pharmacologic, clinical, and experimental studies document the importance of prostaglandin (PG) signaling in cancer development, including non-melanoma skin cancer lesions in humans and mice. First of all, enzymes involved in PG biosynthesis, such as cyclooxygenase (COX)-2 and/or membrane prostaglandin E synthase (mPGES)-1, were found to be overexpressed in a wide range of premalignant and malignant epithelial tumors, including those of the skin, breast, esophagus, stomach, colorectum, pancreas, and bladder. On the other hand, 15-hydroxy-prostaglandin dehydrogenase (15-PGDH), which is involved in the degradation pathway of PG including PGE(2,) thus counteracting the activities of COX-2 and PGES, was found to be downregulated in human epithelial tumors, indicating a tumor suppressor activity of this enzyme. Most remarkably, genetic studies showed that mice, which are deficient in COX-2 and/or PGES are resistant to the development of cancer of skin, colon, and stomach. In contrast, the forced overexpression of COX-2 in proliferative compartments of simple or stratified epithelia such as skin epidermis, urinary bladder, mammary gland, and pancreas results in spontaneous hyperplasia and dysplasia in transgenic mice. In skin, the pathological changes are found to be due to an abnormal process of terminal differentiation, while in other tissues, hyperproliferation seems to be the main contributor to the pre-**invasive** neoplasms. Moreover, the COX-2 transgenic mouse lines are sensitized for cancer development.
2. Cyclooxygenase-two (COX-2) modulates proliferation in aggressive fibromatosis (desmoid tumor). Aggressive fibromatosis is a locally **invasive** soft tissue lesion. Seventy-five per cent of cases harbor a somatic mutation in either the APC or beta-catenin genes, resulting in beta-catenin protein stabilization. Cyclooxygenase-2 (COX-2) is an enzyme involved in prostaglandin synthesis that modulates the formation of colonic neoplasia, especially in cases due to mutations resulting in beta-catenin stabilization. Human aggressive fibromatoses and lesions from the Apc+/Apc1638N mouse (a murine model for Apc-driven fibromatosis) demonstrated elevated COX-2 levels. COX-2 blockade either by the selective agent DFU or by non-selective COX blocking agents results in reduced proliferation in human tumor cell cultures. Breeding mice with Cox-2-/- mice resulted in no difference in number of aggressive fibromatoses formed, but in a smaller tumor size, while there was a decrease in number of GI lesions by 50%. Mice fed various COX blocking agents also showed a decline in tumor size. COX-2 expression was regulated by tcf-dependent transcription in this lesion. COX-2 partially regulates proliferation due to beta-catenin stabilization in aggressive fibromatosis. Although COX blockade alone does not cause tumor regression, this data suggests that it may have a role as an adjuvant therapy to slow tumor growth in this lesion.
3. Differential expression of matrilysin and cyclooxygenase-2 in intestinal and colorectal neoplasms. Both the matrix metalloproteinase matrilysin and the prostaglandin H synthase cyclooxygenase-2 (Cox-2), are thought to play key roles in colorectal carcinogenesis. These enzymes are overexpressed in 85-90% of human colorectal cancers. Furthermore, mice carrying an adenomatous polyposis coli germline mutation that are also nullizygous for either matrilysin or Cox-2 display a significant reduction in tumor multiplicity. To determine if there is a direct link between matrilysin and Cox-2, their expression was characterized in two mouse models of intestinal carcinogenesis and in human colorectal tumor samples. Both matrilysin and Cox-2 expression was increased in the mouse models and in the human colorectal cancers; however, immunohistochemistry and in situ hybridization indicated that their localization within the tumors was different. In the mouse models, Cox-2 was expressed in the superficial stroma, whereas matrilysin expression was localized exclusively to the neoplastic epithelium. In contrast, in human colorectal cancers, both Cox-2 and matrilysin were expressed in the neoplastic epithelium. Although over 80% of the specimens expressed both matrilysin and Cox-2, the levels and localization of matrilysin and Cox-2 expression were distinct. Cox-2 expression was strongest in well-differentiated areas, and matrilysin immunostaining was strongest in the more dysplastic and **invasive** regions of the tumor. These results indicate that these two important modulators of colorectal tumorigenesis are differentially expressed and imply that the therapeutic benefit may be improved by combination therapy utilizing selective Cox-2 and matrilysin inhibitors.
4. Cyclooxygenase 2- and prostaglandin E(2) receptor EP(2)-dependent angiogenesis in Apc(Delta716) mouse intestinal polyps. To investigate angiogenesis during intestinal polyp development, we determined the microvessel density (MVD) in polyps of Apc knockout (Apc(Delta716)) mice, a model for human familial adenomatous polyposis. We scored MVD also in several compound mutants carrying Apc(Delta716), namely, mice with an additional mutation in Smad4, in which the polyps progress into **invasive** adenocarcinomas; mice with a cyclooxygenase (COX)-2 gene (Ptgs2) mutation, in which adenoma growth is suppressed; and mice with prostaglandin E(2) EP receptor gene mutations. In both simple Apc(Delta716) and compound Apc(Delta716) Smad4 mutants, MVD increased in a polyp size-dependent manner only in the polyps expanded beyond a threshold of about 1 mm in diameter. These results indicate that tumor angiogenesis is stimulated only after tumors grow to a certain size, and this angiogenic switch is common to both benign adenomas and malignant adenocarcinomas. In Apc(Delta716) polyposis attenuated by the COX-2 gene mutation, in contrast, MVD did not increase even in polyps larger than 1 mm. The same phenomenon was observed in the compound mutant mice with Apc(Delta716) and the EP(2) receptor gene mutations, but not in other EP compound mutants. We also immunohistochemically studied COX-2 and angiogenic factors, vascular endothelial growth factor and basic fibroblast growth factor. Interestingly, expression of these proteins was also increased in polyps larger than 1 mm. These results suggest that, in both benign and malignant mouse intestinal tumors, stromal expression of COX-2 results in elevated prostaglandin E(2) levels that stimulate cell surface receptor EP(2), followed by induction of vascular endothelial growth factor that causes tumor angiogenesis.
5. PGE2 receptor EP2 mediates the antagonistic effect of COX-2 on TGF-beta signaling during mammary tumorigenesis. The molecular mechanisms that enable cyclooxygenase-2 (COX-2) and its mediator prostaglandin E2 (PGE2) to inhibit transforming growth factor-beta (TGF-beta) signaling during mammary tumorigenesis remain unknown. We show here that TGF-beta selectively stimulated the expression of the PGE2 receptor EP2, which increased normal and malignant mammary epithelial cell (MEC) invasion, anchorage-independent growth, and resistance to TGF-beta-induced cytostasis. Mechanistically, elevated EP2 expression in normal MECs inhibited the coupling of TGF-beta to Smad2/3 activation and plasminogen activator inhibitor-1 (PAI1) expression, while EP2 deficiency in these same MECs augmented Smad2/3 activation and PAI expression stimulated by TGF-beta. Along these lines, engineering malignant MECs to lack EP2 expression prevented their growth in soft agar, restored their cytostatic response to TGF-beta, decreased their **invasive**ness in response to TGF-beta, and potentiated their activation of Smad2/3 and expression of PAI stimulated by TGF-beta. More important, we show that COX-2 or EP2 deficiency both significantly decreased the growth, angiogenesis, and pulmonary metastasis of mammary tumors produced in mice. Collectively, this investigation establishes EP2 as a potent mediator of the anti-TGF-beta activities elicited by COX-2/PGE2 in normal and malignant MECs. Our findings also suggest that pharmacological targeting of EP2 receptors may provide new inroads to antagonize the oncogenic activities of TGF-beta during mammary tumorigenesis.-Tian, M., Schiemann, W. P. PGE2 receptor EP2 mediates the antagonistic effect of COX-2 on TGF-beta signaling during mammary tumorigenesis.
6. Significance of COX-2 expression in human esophageal squamous cell carcinoma. Cyclooxygenase-2 (COX-2) is well established to play an important role in the tumorigenesis of a variety of human cancers; however, the function of COX-2 in the development of esophageal squamous cell carcinoma (ESCC) remains less clear. Here, we determined, first, the pattern of COX-2 expression in normal esophageal mucosa, dysplasia, carcinoma in situ (CIS) and **invasive** SCC. Immunohistochemical analysis showed that, while COX-2 was weakly expressed, if at all, in normal squamous epithelium, strong COX-2 expression was detected as early as the stage of dysplasia and frequently in 20 of 26 (77%) CIS and 86 of 111 (77%) **invasive** SCC. Upregulation of COX-2 in ESCC was found to be significantly associated with tumor progression (R = 0.493, P < 0.01). Further, treatment of human ESCC cell lines (KYSE450 and KYSE510) with NS-398, a COX-2 specific chemical inhibitor, suppressed the production of prostaglandin E2 (PGE2) and induced cell growth inhibition, cell cycle arrest at the G1-S checkpoint, and the expression of cyclin-dependent kinase inhibitors p21waf1/cip1 and p27kip1. Finally, knockdown expression of COX-2 in KYSE450 cells by a specific COX-2 siRNA dramatically inhibited PGE2 production, cell growth and, more importantly, colony formation and tumorigenesis in nude mice. Together, this study suggested that COX-2 may be involved in an early stage of squamous cell carcinogenesis of the esophagus and has a non-redundant role in the regulation of cellular proliferation and tumorigenesis of esophageal epithelial cells.
7. Role of COX-2 in epithelial-stromal cell interactions and progression of ductal carcinoma in situ of the breast. Epithelial-stromal cell interactions have an important role in breast tumor progression, but the molecular mechanisms underlying these effects are just beginning to be understood. We previously described that fibroblasts promote, whereas normal myoepithelial cells inhibit, the progression of ductal carcinoma in situ (DCIS) to **invasive** breast carcinomas by using a xenograft model of human DCIS. Here, we report that the tumor growth and progression-promoting effects of fibroblasts are at least in part due to increased COX-2 expression in tumor epithelial cells provoked by their interaction with fibroblasts. Up-regulation of COX-2 in DCIS xenografts resulted in increased VEGF and MMP14 expression, which may contribute to the larger weight and **invasive** histology of COX-2-expressing tumors. Administration of celecoxib, a selective COX-2 inhibitor, to tumor-bearing mice decreased xenograft tumor weight and inhibited progression to invasion. Coculture of fibroblasts with DCIS epithelial cells enhanced their motility and invasion, and this change was associated with increased MMP14 expression and MMP9 protease activity. We identified the NF-kappaB pathway as one of the mediators of stromal fibroblast-derived signals regulating COX-2 expression in tumor epithelial cells. Inhibition of NF-kappaB and COX-2 activity and down-regulation of MMP9 expression attenuated the invasion-promoting effects of fibroblasts. These findings support a role for COX-2 in promoting the progression of DCIS to **invasive** breast carcinomas, and suggest that therapeutic targeting of the NF-kappaB and prostaglandin signaling pathways might be used for the treatment and prevention of breast cancer.
8. Inducible cyclooxygenase released prostaglandin E2 modulates the severity of infection caused by Streptococcus pyogenes. Streptococcus pyogenes is a significant human pathogen that can cause life-threatening **invasive** infections. Understanding the mechanism of disease is crucial to the development of more effective therapies. In this report, we explored the role of PGE(2), an arachidonic acid metabolite, and its rate-limiting enzyme cyclooxygenase 2 (COX-2) in the pathogenesis of severe S. pyogenes infections. We found that the COX-2 expression levels in tissue biopsies from S. pyogenes-infected patients, as well as in tissue of experimentally infected mice, strongly correlated with the severity of infection. This harmful effect was attributed to PGE(2)-mediated suppression of the bactericidial activity of macrophages through interaction with the G2-coupled E prostanoid receptor. The suppressive effect of PGE(2) was associated with enhanced intracellular cAMP production and was mimicked by the cAMP-elevating agent, forskolin. Activation of protein kinase A (PKA) was the downstream effector mechanisms of cAMP because treatment with PKI(14-22), a highly specific inhibitor of PKA, prevented the PGE(2)-mediated inhibition of S. pyogenes killing in macrophages. The inhibitory effect exerted by PKA in the generation of antimicrobial oxygen radical species seems to be the ultimate effector mechanism responsible for the PGE(2)-mediated downregulation of the macrophage bactericidal activity. Importantly, either genetic ablation of COX-2, pharmacological inhibition of COX-2 or treatment with the G2-coupled E prostanoid antagonist, AH6809, significantly improved the disease outcome in S. pyogenes infected mice. Therefore, the results of this study open up new perspectives on potential molecular pathways that are prone to pharmacological manipulation during severe streptococcal infections.
9. Nucleotide-binding domain of phosphoglycerate kinase 1 reduces tumor growth by suppressing COX-2 expression. Phosphoglycerate kinase 1 (PGK-1) is a multifunctional protein that is involved in the glycolytic pathway and the generation of the angiogenesis inhibitor angiostatin. In a previous study, we showed that the overexpression of full-length PGK-1 in Lewis lung carcinoma (LLC-1) can reduce tumor growth in vivo by downregulation of COX-2 expression. Phosphoglycerate kinase 1 has two functional domains: a catalytic domain (CD); and a nucleotide-binding domain (NBD). To identify the functional domain of PGK-1 responsible for its antitumor effects, we evaluated the tumorigenicity of LLC-1 cells overexpressing full-length PGK-1 (LLC-1/PGK), CD (LLC-1/CD), and NBD (LLC-1/NBD). Although no difference in tumor cell growth was observed in vitro, the tumor **invasive**ness was reduced in the LLC-1/PGK, LLC-1/CD, and LLC-1/NBD cells compared to parental LLC-1 cells in vivo. In addition, in vivo tumor growth retardation by LLC-1/CD and LLC-1/NBD cells was observed, similar to that by LLC-1/PGK cells. However, the reduced stability of COX-2 mRNA and downregulation of the COX-2 protein and its metabolite, prostaglandin E2, was only found in LLC-1/PGK and LLC-1/NBD cells. Low levels of COX-2 were also observed in the tumor mass formed by the modified cells when injected into mice. The results indicate that COX-2 suppression by PGK-1 is independent of its catalytic activity. COX-2 targeting by PGK-1 can be attributed to its NBD and is probably a result of the destabilization of COX-2 gene transcripts brought about by the mRNA-binding property of PGK-1.
10. HER2/neu-induced mammary tumorigenesis and angiogenesis are reduced in cyclooxygenase-2 knockout mice. The inducible prostaglandin synthase cyclooxygenase-2 (Cox-2) is overexpressed in approximately 40% of human breast cancers and at higher frequencies in pre**invasive** ductal carcinoma in situ (DCIS). Cox-2 expression is particularly associated with overexpression of human epidermal growth factor receptor 2 (HER2/neu). To definitively interrogate the role of Cox-2 in mammary neoplasia, we have used a genetic approach, crossing Cox-2-deficient mice with a HER2/neu transgenic strain, MMTV/NDL. At 20 weeks of age, mammary glands from virgin MMTV/NDL females contained multiple focal tumors, or mammary intraepithelial neoplasias, which histologically resembled human DCIS. Mammary tumor multiplicity and prostaglandin E2 (PGE2) levels were significantly decreased in Cox-2 heterozygous and knockout animals relative to Cox-2 wild-type controls. Notably, the proportion of larger tumors was decreased in Cox-2-deficient mice. HER2/neu-induced mammary hyperplasia was also substantially reduced in Cox-2 null mice. Additionally, mammary glands from Cox-2 knockout mice exhibited a striking reduction in vascularization, and expression of proangiogenic genes was correspondingly reduced. Decreased vascularization was observed both in dysplastic and normal-appearing regions of Cox-2-null mammary glands. Our data provide the first genetic evidence that Cox-2 contributes to HER2/neu-induced mammary tumorigenesis. This finding may help to explain the reduced risk of breast cancer associated with regular use of nonsteroidal anti-inflammatory drugs.
11. Imaging cyclooxygenase-2 (Cox-2) gene expression in living animals with a luciferase knock-in reporter gene. The cyclooxygenase-2 (Cox-2) gene plays a role in a variety of normal and pathophysiological conditions. Expression of the Cox-2 gene is induced in a broad range of cells, in response to many distinct stimuli. The ability to monitor and quantify Cox-2 expression non**invasive**ly in vivo may facilitate a better understanding of the role of Cox-2, both in normal physiology and in different diseases. We generated a "knock-in" mouse in which the firefly luciferase reporter enzyme is expressed at the start site of translation of the endogenous Cox-2 gene. Correlation of luciferase and Cox-2 expression was confirmed in heterozygous Cox-2luc/+ mouse embryonic fibroblasts isolated from the knock-in mouse. In an acute sepsis model, following injection of interferon gamma and endotoxin, ex vivo imaging and Western blotting demonstrated coordinate Cox-2 and luciferase induction in multiple organs. Using both paw and air pouch inflammation models, we can monitor repeatedly localized luciferase expression in the same living mouse. Cox-2luc/+ knock-in mice should provide a valuable tool to analyze Cox-2 expression in many disease models.
12. Feedback regulation of cyclooxygenase-2 transcription ex vivo and in vivo. Cyclooxygenase-2 (COX-2) is a rate-limiting enzyme for prostaglandin biosynthesis. Its inducible expression is regulated by complex pathways. To monitor Cox-2 transcriptional activity in vivo, we generated a knock-in mouse expressing a firefly luciferase reporter. In this study we examined, by comparing luciferase activity of Cox-2(luc/+) and Cox-2(luc/-) cells and mice, effects of prostanoid products on Cox-2 promoter transcriptional activation. In peritoneal macrophages, luciferase induction by LPS in Cox-2(luc/-) cells was less than that of Cox-2(luc/+) cells. However, in the presence of PGE(2), induction was comparable, suggesting positive Cox-2 feedback regulation by PGE(2) occurs for macrophages. In contrast, feedback modulation was not observed in TPA-induced Cox-2(luc/+) and Cox-2(luc/-) mouse embryonic fibroblasts (MEFs). Using non-**invasive** in vivo imaging, we observed negative feedback regulation of Cox-2 expression during paw inflammation in living mice. Our results suggest Cox-2 expression is regulated by cell type specific feedback mechanisms, both in cultured cells and in living animals.
13. Delayed progression of pancreatic intraepithelial neoplasia in a conditional Kras(G12D) mouse model by a selective cyclooxygenase-2 inhibitor. Pancreatic ductal adenocarcinomas are thought to arise from non**invasive**, intraductal precursor lesions called pancreatic intraepithelial neoplasias (PanIN). The study of PanINs holds great promise for the identification of early detection markers and effective cancer-preventing strategies. Cyclooxygenase-2 (COX-2) represents an intriguing target for therapeutic and preventive approaches in various human malignancies. The aim of the present study was to evaluate the efficacy of a selective COX-2 inhibitor to prevent the progression of PanINs in a conditional Kras(G12D) mouse model. Offspring of LSL-KRAS(G12D) x PDX-1-Cre intercrosses were randomly allocated to a diet supplemented with the selective COX-2 inhibitor nimesulide (400 ppm) or a control diet. After 10 months, animals were sacrificed. Successful recombination in the pancreas was evaluated by PCR. The pancreas of KRAS(G12D);PDX-1-Cre mice was analyzed for the presence of murine PanINs. Animals fed the COX-2 inhibitor had significantly fewer PanIN-2 and PanIN-3 lesions than control animals (P < 0.05). Ten percent of all pancreatic ducts in the nimesulide-fed animals showed PanIN-2 or PanIN-3 lesions, whereas 40% of the pancreatic ducts in the control animals had PanIN-2 or PanIN-3 lesions. Intrapancreatic prostaglandin E(2) levels were reduced in nimesulide-fed animals. Immunohistochemistry confirmed COX-2 expression in early and late PanINs. In summary, we found that the selective COX-2 inhibitor nimesulide delays the progression of pancreatic cancer precursor lesions in a preclinical animal model. These data highlight the importance of COX-2 in the development of pancreatic cancer. Inhibition of COX-2 may represent an intriguing strategy to prevent pancreatic cancer in high-risk patients.
14. Overexpression of cyclooxygenase-2 (COX-2) in the mouse urinary bladder induces the expression of immune- and cell proliferation-related genes. The mechanisms whereby cyclooxygenase-2 (COX-2) overexpression may contribute to bladder carcinogenesis remain unknown. We recently developed a transgenic mouse model overexpressing COX-2 under the control of a bovine keratin 5 (BK5) promoter causing a high incidence of transitional cell hyperplasia (TCH) in the bladder with a proportion of lesions progressing to **invasive** carcinoma. Microarray gene analysis was employed to determine the effects of COX-2 overexpression on gene expression profiles in the urinary bladder. Statistical analysis revealed that 70 genes were upregulated and 60 were downregulated by twofold or more in bladders from transgenic compared to wild-type mice. Expression Analysis Systematic Explorer (EASE) analysis revealed that genes associated with Immune/Stress Response and Cell Cycle/Proliferation biological processes were overexpressed in the transgenic mice. Relevant downregulated genes included three transforming growth factor (TGF)-beta related genes, Tgfb2, Tgfb3, and Tgfbi. The growth factor epiregulin was the most highly induced gene among those validated by qRT-PCR in TCH of BK5.COX-2 mouse bladders in parallel with increased staining for Ki67. Prostaglandin E(2) (PGE(2)) directly induced the expression of epiregulin mRNA in bladders from wild-type FVB mice ex vivo. We further determined that recombinant epiregulin increased both cell proliferation and Erk phosphorylation in UMUC-3 bladder cancer cells. These results indicate that the response of the mouse urinary bladder to elevated COX-2 expression includes enhanced inflammatory response and induction of cell proliferation. The growth factor epiregulin may play a role in bladder carcinogenesis and may serve as a novel target for the prevention and treatment of bladder cancer.
15. Oncostatin M stimulates the detachment of a reservoir of **invasive** mammary carcinoma cells: role of cyclooxygenase-2. Previously, oncostatin M (OSM) has been shown to inhibit the proliferation of breast cancer cells in vitro. Circumstantial evidence, however, suggests that OSM could be involved in the development of a metastatic phenotype in vivo. We examined the effects of OSM on the proliferation and metastatic potential of the murine mammary carcinoma cell lines M6 (adenocarcinoma) and M6c (metastatic adenocarcinoma). OSM inhibits the proliferation of both cell lines by 43%, but also causes a loss of cell-cell and cell-substratum adhesion that culminates in cell detachment from monolayer culture. OSM treatment results in a 258% and 550% increase in the detachment of M6 and M6c, respectively, in 32 hours. This effect was abrogated by the selective Cox-2 inhibitor NS-398, and by the anti-inflammatory glucocorticoid dexamethasone. Exogenous prostaglandin E2 (PGE2) partially reverses NS-398's inhibition of OSM-induced cell detachment, indicating Cox-2 involvement. In addition, OSM induces the expression of Cox-2 mRNA, and of the 74 kDa form of Cox-2 protein. M6 and M6c cells detached by OSM are viable and will re-adhere and proliferate in the absence of OSM. OSM-detached cells (M6DET and M6cDET) were collected and maintained in culture and their **invasive**ness was assessed in vitro. Importantly, M6DET and M6cDET are both significantly more **invasive** that their respective parental cells. These data suggest that OSM could contribute to the development of a metastatic phenotype in vivo, which would render OSM unsuitable as a cancer therapy and suggest that OSM itself is a potential therapeutic target.
16. Transgenic mouse for conditional, tissue-specific Cox-2 overexpression. We constructed a cyclooxygenase-2 (Cox-2) conditional overexpression transgenic mouse (Cox-2 COE). The transgene contains a CAG promoter driving the Cox-2 and humanized Renilla luciferase (hRL) coding regions, linked by an internal ribosomal entry site. The promoter is followed by a loxP-flanked sequence containing enhanced green fluorescent protein (EGFP), a neomycin selection cassette, and a transcriptional/translational STOP sequence. In the presence of Cre recombinase the loxP-flanked sequence is excised. Cox-2/hRL expression can be monitored repeatedly and non**invasive**ly in vivo by imaging hRL activity. To demonstrate conditional Cox-2 and hRL expression, a nonreplicating adenovirus carrying Cre recombinase (Ad.CMV.Cre) was injected intravenously; hepatic Cox-2 expression and hRL signal were elevated. Cox-2 COE embryonic fibroblasts express both Cox-2 and hRL following Ad.CMV.Cre infection. PGE(2) production is also increased following Ad.CMV.Cre infection of Cox-2 COE embryo fibroblasts. Cox-2 COE mice should be valuable for the study of Cox-2 overexpression in cardiovascular disease, acute and chronic inflammatory responses, neurodegenerative diseases, and cancer.

#### GeneRIFs

1. support a role for COX-2 in promoting the progression of ductal carcinoma in situ to **invasive** breast carcinomas

### [Mm]etastasize

#### PubMed

1. Upregulation of COX-2 in the lung cancer promotes overexpression of multidrug resistance protein 4 (MRP4) via PGE2-dependent pathway. It is apparent that lung cancer is associated with inflammation, with accompanying hallmark elevations of cyclooxygenase 2 (COX-2) and prostaglandin E2 (PGE2) levels. However, the effects of these changes on MRP efflux transporters have not been thoroughly investigated before. Here, we report that upregulation of COX-2 can induce overexpression of MRP4 in both A549 non-small-cell lung cancer cell lines and mouse lung cancer models. In A549 cells, phorbol 12-myristate 13-acetate (PMA) treatment induced upregulation of COX-2 and MRP4 together, but not other MRP transporters. Transient overexpression of human COX-2 cDNA also specifically increased COX-2 and MRP4. Moreover, COX inhibitor treatment and COX-2-specific siRNA significantly inhibited the upregulation of MRP4. Additionally, PMA-treatment increased extracellular PGE2 levels, likely due to increased MRP4 function. Likewise, COX-2-specific siRNA reduced extracellular PGE2 levels. Furthermore, COX-2 upregulation resulted in an increase in mPGES-1, an enzyme responsible for PGE2 production. Finally, **metastasize**d lung cancer model mice exhibited increased expression levels of COX-2 and MRP4, as well as mPGES-1. In conclusion, the present study suggests that overexpression of MRP4 in lung cancer may be attributable to COX-2 upregulation via a PGE2-dependent pathway.
2. Upregulation of COX-2 in the lung cancer promotes overexpression of multidrug resistance protein 4 (MRP4) via PGE2-dependent pathway. It is apparent that lung cancer is associated with inflammation, with accompanying hallmark elevations of cyclooxygenase 2 (COX-2) and prostaglandin E2 (PGE2) levels. However, the effects of these changes on MRP efflux transporters have not been thoroughly investigated before. Here, we report that upregulation of COX-2 can induce overexpression of MRP4 in both A549 non-small-cell lung cancer cell lines and mouse lung cancer models. In A549 cells, phorbol 12-myristate 13-acetate (PMA) treatment induced upregulation of COX-2 and MRP4 together, but not other MRP transporters. Transient overexpression of human COX-2 cDNA also specifically increased COX-2 and MRP4. Moreover, COX inhibitor treatment and COX-2-specific siRNA significantly inhibited the upregulation of MRP4. Additionally, PMA-treatment increased extracellular PGE2 levels, likely due to increased MRP4 function. Likewise, COX-2-specific siRNA reduced extracellular PGE2 levels. Furthermore, COX-2 upregulation resulted in an increase in mPGES-1, an enzyme responsible for PGE2 production. Finally, **metastasize**d lung cancer model mice exhibited increased expression levels of COX-2 and MRP4, as well as mPGES-1. In conclusion, the present study suggests that overexpression of MRP4 in lung cancer may be attributable to COX-2 upregulation via a PGE2-dependent pathway.

### [Mm]etastasis

#### PubMed

1. PGE2 receptor EP2 mediates the antagonistic effect of COX-2 on TGF-beta signaling during mammary tumorigenesis. The molecular mechanisms that enable cyclooxygenase-2 (COX-2) and its mediator prostaglandin E2 (PGE2) to inhibit transforming growth factor-beta (TGF-beta) signaling during mammary tumorigenesis remain unknown. We show here that TGF-beta selectively stimulated the expression of the PGE2 receptor EP2, which increased normal and malignant mammary epithelial cell (MEC) invasion, anchorage-independent growth, and resistance to TGF-beta-induced cytostasis. Mechanistically, elevated EP2 expression in normal MECs inhibited the coupling of TGF-beta to Smad2/3 activation and plasminogen activator inhibitor-1 (PAI1) expression, while EP2 deficiency in these same MECs augmented Smad2/3 activation and PAI expression stimulated by TGF-beta. Along these lines, engineering malignant MECs to lack EP2 expression prevented their growth in soft agar, restored their cytostatic response to TGF-beta, decreased their invasiveness in response to TGF-beta, and potentiated their activation of Smad2/3 and expression of PAI stimulated by TGF-beta. More important, we show that COX-2 or EP2 deficiency both significantly decreased the growth, angiogenesis, and pulmonary **metastasis** of mammary tumors produced in mice. Collectively, this investigation establishes EP2 as a potent mediator of the anti-TGF-beta activities elicited by COX-2/PGE2 in normal and malignant MECs. Our findings also suggest that pharmacological targeting of EP2 receptors may provide new inroads to antagonize the oncogenic activities of TGF-beta during mammary tumorigenesis.-Tian, M., Schiemann, W. P. PGE2 receptor EP2 mediates the antagonistic effect of COX-2 on TGF-beta signaling during mammary tumorigenesis.
2. Heterogeneous nuclear ribonucleoprotein K (hnRNP-K) promotes tumor **metastasis** by induction of genes involved in extracellular matrix, cell movement, and angiogenesis. Cancer is a leading cause of death and still awaits effective therapies. Rapid industrialization has contributed to increase in incidence of cancer. One of the reasons why most of the cancers fail therapy is due to their metastatic property. Hence identification of factors leading to **metastasis** is highly important to design effective and novel anti-cancer therapeutics. In our earlier study (Inoue, A., Sawata, S. Y., Taira, K., and Wadhwa, R. (2007) Loss-of-function screening by randomized intracellular antibodies: identification of hnRNP-K as a potential target for **metastasis**. Proc. Natl. Acad. Sci. U.S.A. 104, 8983-8988), we had reported that the involvement of heterogeneous nuclear ribonucleoprotein K (hnRNP-K) in **metastasis**. Here, we established hnRNP-K-overexpressing and -underexpressing derivative cell lines and examined their proliferation and metastatic properties in vitro and in vivo. Whereas hnRNP-K compromised cells showed delayed tumor growth, its overexpression resulted in enhanced malignancy and **metastasis**. Molecular basis of the hnRNP-K induced malignant and metastatic phenotypes was dissected by cDNA microarray and pathway analyses. We found that the hnRNP-K regulates extracellular matrix, cell motility, and angiogenesis pathways. Involvement of the selected genes (Cck, Mmp-3, Ptgs2, and Ctgf) and pathways was validated by gene-specific expression analysis. Our results demonstrated that the hnRNP-K is a potential target for **metastasis** therapy.
3. Cyclooxygenase-2 is a target of KRASD12, which facilitates the outgrowth of murine C26 colorectal liver metastases. Mutational activation of the KRAS oncogene and overexpression of cyclooxygenase-2 (COX-2) contribute to colorectal carcinoma (CRC) development, but the relationship between these two events is unclear. This study was designed to clarify that relationship and to assess the contribution of KRAS-dependent COX-2 to the seeding of CRC cells in the liver and to their outgrowth as liver metastases in an experimental mouse model.The effect of RNA interference-mediated KRAS knockdown on COX-2 expression and activity was tested in murine C26 CRC cells. The contribution of KRAS-dependent COX-2 to early metastatic tumor cell seeding (by intravital microscopy) and outgrowth of metastases in the liver (by bioluminescence imaging) was studied by using parecoxib, a novel and highly selective liver-activated COX-2 inhibitor. Intratumoral cell proliferation, apoptosis, and tumor-associated angiogenesis were assessed by immunohistochemistry on liver tissue sections.Stable knockdown of mutant KRAS(D12) in murine C26 CRC cells by RNA interference lead to a dramatic reduction of COX-2 synthesis and prostaglandin E2 production. Inhibition of host or tumor cell COX-2 activity had no effect on early metastatic cell seeding in the liver but greatly reduced intrahepatic tumor cell proliferation and the rate of liver **metastasis** outgrowth. COX-2 inhibition had no effect on early tumor vascularization or on tumor cell apoptosis.The high levels of COX-2 enzyme and prostaglandin production in C26 CRC cells are primarily caused by the presence of endogenous mutant KRAS(D12). Furthermore, COX-2 inhibition affects the tumoral rather than the vascular compartment during the early stages of C26 liver **metastasis** outgrowth.
4. COX-2 modulates mammary tumor progression in response to collagen density. High breast density is linked to an increased risk of breast cancer, and correlates with changes in collagen. In a mouse model of mammary carcinoma in the context of increased collagen deposition, the MMTV-PyMT/Col1a1 (tm1jae) , there is accelerated mammary tumor formation and progression. Previous gene expression analysis suggests that increased collagen density elevates expression of PTGS2 (prostaglandin-endoperoxide synthase 2), the gene for cyclooxygenase-2 (COX-2).To understand the role of COX-2 in tumor progression within a collagen-dense microenvironment, we treated MMTV-PyMT or MMTV-PyMT/Col1a1 (tm1jae) tumors prior to and after tumor formation. Animals received treatment with celecoxib, a specific COX-2 inhibitor, or placebo. Mammary tumors were examined for COX-2, inflammatory and stromal cell components, and collagen deposition through immunohistochemical analysis, immunofluorescence, multiplex cytokine ELISA and tissue imaging techniques.PyMT/Col1a1 (tm1jae) tumors were larger, more proliferative, and expressed higher levels of COX-2 and PGE2 than PyMT tumors in wild type (WT) mice. Treatment with celecoxib significantly decreased the induced tumor size and **metastasis** of the PyMT/Col1a1 tumors, such that their size was not different from the smaller PyMT tumors. Celecoxib had minimal effect on the PyMT tumors. Celecoxib decreased expression levels of COX-2, PGE2, and Ki-67. Several cytokines were over-expressed in PyMT/Col1a1 compared to PyMT, and celecoxib treatment prevented their over-expression. Furthermore, macrophage and neutrophil recruitment were enhanced in PyMT/Col1a1 tumors, and this effect was inhibited by celecoxib. Notably, COX-2 inhibition reduced overall collagen deposition. Finally, when celecoxib was used prior to tumor formation, PyMT/Col1a1 tumors were fewer and smaller than in untreated animals.These findings suggest that COX-2 has a direct role in modulating tumor progression in tumors arising within collagen-dense microenvironments, and suggest that COX-2 may be an effective therapeutic target for women with dense breast tissue and early-stage breast cancer.
5. Vascular endothelial growth factor regulates melanoma cell adhesion and growth in the bone marrow microenvironment via tumor cyclooxygenase-2. Human melanoma frequently colonizes bone marrow (BM) since its earliest stage of systemic dissemination, prior to clinical **metastasis** occurrence. However, how melanoma cell adhesion and proliferation mechanisms are regulated within bone marrow stromal cell (BMSC) microenvironment remain unclear. Consistent with the prometastatic role of inflammatory and angiogenic factors, several studies have reported elevated levels of cyclooxygenase-2 (COX-2) in melanoma although its pathogenic role in bone marrow melanoma **metastasis** is unknown.Herein we analyzed the effect of cyclooxygenase-2 (COX-2) inhibitor celecoxib in a model of generalized BM dissemination of left cardiac ventricle-injected B16 melanoma (B16M) cells into healthy and bacterial endotoxin lipopolysaccharide (LPS)-pretreated mice to induce inflammation. In addition, B16M and human A375 melanoma (A375M) cells were exposed to conditioned media from basal and LPS-treated primary cultured murine and human BMSCs, and the contribution of COX-2 to the adhesion and proliferation of melanoma cells was also studied.Mice given one single intravenous injection of LPS 6 hour prior to cancer cells significantly increased B16M **metastasis** in BM compared to untreated mice; however, administration of oral celecoxib reduced BM **metastasis** incidence and volume in healthy mice, and almost completely abrogated LPS-dependent melanoma metastases. In vitro, untreated and LPS-treated murine and human BMSC-conditioned medium (CM) increased VCAM-1-dependent BMSC adherence and proliferation of B16M and A375M cells, respectively, as compared to basal medium-treated melanoma cells. Addition of celecoxib to both B16M and A375M cells abolished adhesion and proliferation increments induced by BMSC-CM. TNFα and VEGF secretion increased in the supernatant of LPS-treated BMSCs; however, anti-VEGF neutralizing antibodies added to B16M and A375M cells prior to LPS-treated BMSC-CM resulted in a complete abrogation of both adhesion- and proliferation-stimulating effect of BMSC on melanoma cells. Conversely, recombinant VEGF increased adherence to BMSC and proliferation of both B16M and A375M cells, compared to basal medium-treated cells, while addition of celecoxib neutralized VEGF effects on melanoma. Recombinant TNFα induced B16M production of VEGF via COX-2-dependent mechanism. Moreover, exogenous PGE2 also increased B16M cell adhesion to immobilized recombinant VCAM-1.We demonstrate the contribution of VEGF-induced tumor COX-2 to the regulation of adhesion- and proliferation-stimulating effects of TNFα, from endotoxin-activated bone marrow stromal cells, on VLA-4-expressing melanoma cells. These data suggest COX-2 neutralization as a potential anti-metastatic therapy in melanoma patients at high risk of systemic and bone dissemination due to intercurrent infectious and inflammatory diseases.
6. Cox-2 inactivates Smad signaling and enhances EMT stimulated by TGF-beta through a PGE2-dependent mechanisms. Although it is well established that mammary tumorigenesis converts transforming growth factor-beta (TGF-beta) from a tumor suppressor to a tumor promoter, the molecular, cellular and microenvironmental mechanisms underlying the dichotomous nature of TGF-beta in mammary epithelial cells (MECs) remains to be determined definitively. Aberrant upregulation of the inducible cyclooxygenase, Cox-2, occurs frequently in breast cancers and is associated with increasing disease severity and the acquisition of **metastasis**; however, the impact of Cox-2 expression on normal and malignant MEC response to TGF-beta remains unknown. We show here that TGF-beta induced Cox-2 expression in normal MECs during their acquisition of an epithelial-mesenchymal transition (EMT) phenotype. Moreover, stable Cox-2 expression in normal MECs stimulated their invasion, EMT and anchorage-independent growth and inhibited their activation of Smad2/3 by TGF-beta. Conversely, antagonizing TGF-beta signaling in malignant, metastatic MECs significantly reduced their expression of Cox-2 as well as enhanced their activation of Smad2/3 by TGF-beta. Along these lines, elevated Cox-2 expression elicited prostaglandin E(2) (PGE(2)) production and the autocrine activation of EP receptors, which antagonized Smad2/3 signaling in normal and malignant MECs. Importantly, rendering normal and malignant MECs Cox-2 deficient inhibited their production of PGE(2) and acquisition of an EMT morphology as well as potentiated their nuclear accumulation of Smad2/3 and transcription of plasminogen activator inhibitor-1 and p15 messenger RNA. Collectively, our findings establish Cox-2 as a novel antagonist of Smad2/3 signaling in normal and malignant MECs; they also suggest that chemotherapeutic targeting of Cox-2 may offer new inroads in restoring the tumor-suppressing activities of TGF-beta in malignant, metastatic breast cancers.
7. Dual inhibition of cyclooxygenase-2 and soluble epoxide hydrolase synergistically suppresses primary tumor growth and **metastasis**. Prostaglandins derived from the cyclooxygenase (COX) pathway and epoxyeicosatrienoic acids (EETs) from the cytochrome P450/soluble epoxide hydrolase (sEH) pathway are important eicosanoids that regulate angiogenesis and tumorigenesis. COX-2 inhibitors, which block the formation of prostaglandins, suppress tumor growth, whereas sEH inhibitors, which increase endogenous EETs, stimulate primary tumor growth and **metastasis**. However, the functional interactions of these two pathways in cancer are unknown. Using pharmacological inhibitors as probes, we show here that dual inhibition of COX-2 and sEH synergistically inhibits primary tumor growth and **metastasis** by suppressing tumor angiogenesis. COX-2/sEH dual pharmacological inhibitors also potently suppress primary tumor growth and **metastasis** by inhibiting tumor angiogenesis via selective inhibition of endothelial cell proliferation. These results demonstrate a critical interaction of these two lipid metabolism pathways on tumorigenesis and suggest dual inhibition of COX-2 and sEH as a potential therapeutic strategy for cancer therapy.
8. Increased COX2 expression enhances tumor-induced osteoclastic lesions in breast cancer bone **metastasis**. **Metastasis** is a complicated process that depends largely on the interaction of primary tumors and the local host environment. In this study, we have established a new mouse model to mimic the process of tumor **metastasis** in breast cancer patients. TM40D-MB mouse mammary tumor cells were transplanted to the mammary glands of syngeneic mice. Mammary tumors developed in the glands in situ and were metastatic to bone with over 53% efficiency. We compared the gene expression profile of high (TM40D-MB) vs. low metastatic (TM40D) tumor cells using Affymetrix microarray chips. Microarray analysis revealed a list of genes that were consistently up-/down-regulated in TM40D-MB tumor cells. One of the genes, COX2 was selected for functional study. Injection of TM40D-COX2 cells to mouse femur caused a significant increase in bone destruction compared to TM40D-C control, as measured by the micro-computed tomography (microCT) scanning for the trabecular bone volume. In vitro assays showed that COX2 significantly increased the rate of osteoclast formation, and this effect was reversible by the specific COX2 inhibitor NS-398. These data indicate that COX2 is one gene important for breast cancer bone **metastasis**. The availability of this mouse model and the comparative study of gene expression signatures between TM40D and other bone **metastasis** models will likely generate important information about common pathways for breast cancer bone **metastasis**. Further functional studies of **metastasis** signature genes should help to develop means to curb the **metastasis** process.
9. Regulation of transforming growth factor-beta-dependent cyclooxygenase-2 expression in fibroblasts. Abnormal transforming growth factor-beta (TGF-beta) signaling is a critical contributor to the pathogenesis of various human diseases ranging from tissue fibrosis to tumor formation. Excessive TGF-beta signaling stimulates fibrotic responses. Recent research has focused in the main on the antiproliferative effects of TGF-beta in fibroblasts, and it is presently understood that TGF-beta-stimulated cyclooxygenase-2 (COX-2) induction in fibroblasts is essential for antifibroproliferative effects of TGF-beta. Both TGF-beta and COX-2 have been implicated in tumor growth, invasion, and **metastasis**, and therefore tumor-associated fibroblasts are a recent topic of interest. Here we report the identification of positive and negative regulatory factors of COX-2 expression induced by TGF-beta as determined using proteomic approaches. We show that TGF-beta coordinately up-regulates three factors, heterogeneous nuclear ribonucleoprotein A/B (HNRPAB), nucleotide diphosphate kinase A (NDPK A), and nucleotide diphosphate kinase A (NDPK B). Functional pathway analysis showed that HNRPAB augments mRNA and protein levels of COX-2 and subsequent prostaglandin E(2) (PGE(2)) production by suppressing degradation of COX-2 mRNA. In contrast, NDPK A and NDPK B attenuated mRNA and protein levels of COX-2 by affecting TGF-beta-Smad2/3/4 signaling at the receptor level. Collectively, we report on a new regulatory pathway of TGF-beta in controlling expression of COX-2 in fibroblasts, which advances our understanding of pathophysiological mechanisms of TGF-beta.

#### GeneRIFs

1. Increased COX2 expression enhances tumor-induced osteoclastic lesions in breast cancer bone **metastasis**.

---

## Trpa1

- NCBI Gene
- GeneRIF
- Pubmed

| Search Term | Rank | Fields |
| --- | --- | --- |
| [Mm]igration | 6 | PubMed(1) |

  
hide/show details for Trpa1

### [Mm]igration

#### PubMed

1. Superoxide generation and leukocyte accumulation: key elements in the mediation of leukotriene B₄-induced itch by transient receptor potential ankyrin 1 and transient receptor potential vanilloid 1. The underlying mechanisms of itch are poorly understood. We have investigated a model involving the chemoattractant leukotriene B₄ (LTB₄) that is up-regulated in common skin diseases. Intradermal injection of LTB4 (0.1 nmol/site) into female CD1 mice induced significant scratching movements (used as an itch index) compared with vehicle-injected (0.1% bovine serum albumin-saline) mice. Intraperitoneal transient receptor potential (TRP) channel antagonist treatment significantly inhibited itch as follows: TRP vanilloid 1 (TRPV1) antagonist SB366791 (0.5 mg/kg, by 97%) and the TRP ankyrin 1 (TRPA1) antagonists TCS 5861528 (10 mg/kg; 82%) and HC-030031 (100 mg/kg; 76%). Leukotriene B₄ receptor 2 antagonism by LY255283 (5 mg/kg i.p.; 62%) reduced itch. Neither TRPV1-knockout (TRPV1-KO) nor TRPA1-knockout (TRPA1-KO mice exhibited LTB₄-induced itch compared with their wild-type counterparts. The reactive oxygen species scavengers N-acetylcysteine (NAC; 204 mg/kg i.p.; 86%) or superoxide dismutase (SOD; 10 mg/kg i.p.; 83%) also inhibited itch. LTB4-induced superoxide release was attenuated by TCS 5861528 (56%) and HC-030031 (66%), NAC (58%), SOD (50%), and LY255283 (59%) but not by the leukotriene B4 receptor 1 antagonist U-75302 (9 nmol/site) or SB366791. Itch, superoxide, and myeloperoxidase generation were inhibited by the **leukocyte** migration inhibitor fucoidan (10 mg/kg i.v.) by 80, 61, and 34%, respectively. Myeloperoxidase activity was also reduced by SB366791 (35%) and SOD (28%). TRPV1-KO mice showed impaired myeloperoxidase release, whereas TRPA1-KO mice exhibited diminished production of superoxide. This result provides novel evidence that TRPA1 and TRPV1 contribute to itch via distinct mechanisms.

---

## Ctsh

- NCBI Gene
- GeneRIF
- Pubmed

| Search Term | Rank | Fields |
| --- | --- | --- |
| [Mm]igration | 6 | Process(2); PubMed(1) |
| [Ee]xtracellular [Mm]atrix | 8 | PubMed(1) |
| [Mm]etastasis | 12 | PubMed(2) |
| [Cc]ell [Ii]nvasion | 13 | PubMed(1) |

  
hide/show details for Ctsh

### [Mm]igration

#### Process

1. positive regulation of cell **migration**
2. positive regulation of epithelial cell **migration**

#### PubMed

1. Cathepsin H regulated by the thyroid hormone receptors associate with tumor invasion in human hepatoma cells. Thyroid hormone, 3, 3', 5-triiodo-L-thyronine (T(3)), mediates cell growth, development and differentiation by binding to its nuclear receptors (TRs). The role of TRs in cancer is still undefined. Notably, hyperthyroxinemia has been reported to influence the rate of colon cancer in an experimental model of carcinogenesis in rats. Previous microarray analysis revealed that cathepsin H (CTSH) is upregulated by T(3) in HepG2-TR cells. We verified that mRNA and protein expression of CTSH are induced by T(3) in HepG2-TR cells and in thyroidectomized rats following administration of T(3). The possible thyroid hormone-responsive elements of the CTSH promoter localized to the nucleotides -2038 to -1966 and -1565 to -1501 regions. An in vitro functional assay showed that CTSH can increase metastasis. J7 cells overexpressing CTSH were inoculated into severe combined immune-deficient mice and these J7-CTSH mice displayed a greater metastatic potential than did J7-control mice. The clinicopathologic significance of CTSH expression in hepatocellular carcinoma (HCC) was also investigated. The CTSH overexpressing in HCC was associated with the presence of microvascular invasion (P=0.037). The microvascular invasion characteristic is closely related to our in vitro characterization of CTSH function. Our results show that T(3)-mediated upregulation of CTSH led to matrix metallopeptidase or extracellular signal-regulated kinase activation and increased cell **migration**. This study demonstrated that CTSH overexpression in a subset hepatoma may be TR dependent and suggests that this overexpression has an important role in hepatoma progression.

### [Ee]xtracellular [Mm]atrix

#### PubMed

1. Deletion of cathepsin H perturbs angiogenic switching, vascularization and growth of tumors in a mouse model of pancreatic islet cell cancer. Proteases can regulate many aspects of tumor development as their actions, which include degradation of the **extracellular matrix**, proteolytic processing of chemokines and activation of other enzymes, influence several key tumorigenic processes. Members of one protease class, the cysteine cathepsins, have received increasing recognition for their involvement in cancer development, and numerous clinical studies have reported correlations between elevated cathepsin levels and malignant progression. This is also the case for cathepsin H, a member of the cysteine cathepsin family, and its utility as a prognostic marker has been analyzed extensively. However, there is limited information available on its specific functions in tumor development and progression. To gain further insight into the role of this protease in cancer, we crossed cathepsin H-deficient mice with the RIP1-Tag2 model of pancreatic islet carcinogenesis. Deletion of cathepsin H significantly impaired angiogenic switching of the pre-malignant hyperplastic islets and resulted in a reduction in the subsequent number of tumors that formed. Moreover, the tumor burden in cathepsin H null RT2 mice was significantly reduced, in association with defects in the blood vasculature and increased apoptosis. Thus, we demonstrate here for the first time important tumor-promoting roles for cathepsin H in vivo using a mouse model of human cancer.

### [Mm]etastasis

#### PubMed

1. STAT3 and STAT6 Signaling Pathways Synergize to Promote Cathepsin Secretion from Macrophages via IRE1α Activation. Tumor-associated macrophages play critical roles during tumor progression by promoting angiogenesis, cancer cell proliferation, invasion, and **metastasi**s. Cysteine cathepsin proteases, produced by macrophages and cancer cells, modulate these processes, but it remains unclear how these typically lysosomal enzymes are regulated and secreted within the tumor microenvironment. Here, we identify a STAT3 and STAT6 synergy that potently upregulates cathepsin secretion by macrophages via engagement of an unfolded protein response (UPR) pathway. Whole-genome expression analyses revealed that the TH2 cytokine interleukin (IL)-4 synergizes with IL-6 or IL-10 to activate UPR via STAT6 and STAT3. Pharmacological inhibition of the UPR sensor IRE1α blocks cathepsin secretion and blunts macrophage-mediated cancer cell invasion. Similarly, genetic deletion of STAT3 and STAT6 signaling components impairs tumor development and invasion in vivo. Together, these findings demonstrate that cytokine-activated STAT3 and STAT6 cooperate in macrophages to promote a secretory phenotype that enhances tumor progression in a cathepsin-dependent manner.
2. Cathepsin H regulated by the thyroid hormone receptors associate with tumor invasion in human hepatoma cells. Thyroid hormone, 3, 3', 5-triiodo-L-thyronine (T(3)), mediates cell growth, development and differentiation by binding to its nuclear receptors (TRs). The role of TRs in cancer is still undefined. Notably, hyperthyroxinemia has been reported to influence the rate of colon cancer in an experimental model of carcinogenesis in rats. Previous microarray analysis revealed that cathepsin H (CTSH) is upregulated by T(3) in HepG2-TR cells. We verified that mRNA and protein expression of CTSH are induced by T(3) in HepG2-TR cells and in thyroidectomized rats following administration of T(3). The possible thyroid hormone-responsive elements of the CTSH promoter localized to the nucleotides -2038 to -1966 and -1565 to -1501 regions. An in vitro functional assay showed that CTSH can increase **metastasis**. J7 cells overexpressing CTSH were inoculated into severe combined immune-deficient mice and these J7-CTSH mice displayed a greater metastatic potential than did J7-control mice. The clinicopathologic significance of CTSH expression in hepatocellular carcinoma (HCC) was also investigated. The CTSH overexpressing in HCC was associated with the presence of microvascular invasion (P=0.037). The microvascular invasion characteristic is closely related to our in vitro characterization of CTSH function. Our results show that T(3)-mediated upregulation of CTSH led to matrix metallopeptidase or extracellular signal-regulated kinase activation and increased cell migration. This study demonstrated that CTSH overexpression in a subset hepatoma may be TR dependent and suggests that this overexpression has an important role in hepatoma progression.

### [Cc]ell [Ii]nvasion

#### PubMed

1. STAT3 and STAT6 Signaling Pathways Synergize to Promote Cathepsin Secretion from Macrophages via IRE1α Activation. Tumor-associated macrophages play critical roles during tumor progression by promoting angiogenesis, cancer cell proliferation, invasion, and metastasis. Cysteine cathepsin proteases, produced by macrophages and cancer cells, modulate these processes, but it remains unclear how these typically lysosomal enzymes are regulated and secreted within the tumor microenvironment. Here, we identify a STAT3 and STAT6 synergy that potently upregulates cathepsin secretion by macrophages via engagement of an unfolded protein response (UPR) pathway. Whole-genome expression analyses revealed that the TH2 cytokine interleukin (IL)-4 synergizes with IL-6 or IL-10 to activate UPR via STAT6 and STAT3. Pharmacological inhibition of the UPR sensor IRE1α blocks cathepsin secretion and blunts macrophage-mediated cance**r cell invasi**on. Similarly, genetic deletion of STAT3 and STAT6 signaling components impairs tumor development and invasion in vivo. Together, these findings demonstrate that cytokine-activated STAT3 and STAT6 cooperate in macrophages to promote a secretory phenotype that enhances tumor progression in a cathepsin-dependent manner.

---

## Prss12

- NCBI Gene
- GeneRIF
- Pubmed

| Search Term | Rank | Fields |
| --- | --- | --- |
| [Ff]ilopodia | 1 | PubMed(2) |

  
hide/show details for Prss12

### [Ff]ilopodia

#### PubMed

1. Zymogen activation of neurotrypsin and neurotrypsin-dependent agrin cleavage on the cell surface are enhanced by glycosaminoglycans. The serine peptidase neurotrypsin is stored in presynaptic nerve endings and secreted in an inactive zymogenic form by synaptic activity. After activation, which requires activity of postsynaptic NMDA (N-methyl-D-aspartate) receptors, neurotrypsin cleaves the heparan sulfate proteoglycan agrin at active synapses. The resulting C-terminal 22-kDa fragment of agrin induces dendritic **filopodia**, which are considered to be precursors of new synapses. In the present study, we investigated the role of GAGs (glycosaminoglycans) in the activation of neurotrypsin and neurotrypsin-dependent agrin cleavage. We found binding of neurotrypsin to the GAG side chains of agrin, which in turn enhanced the activation of neurotrypsin by proprotein convertases and resulted in enhanced agrin cleavage. A similar enhancement of neurotrypsin binding to agrin, neurotrypsin activation and agrin cleavage was induced by the four-amino-acid insert at the y splice site of agrin, which is crucial for the formation of a heparin-binding site. Non-agrin GAGs also contributed to binding and activation of neurotrypsin and, thereby, to agrin cleavage, albeit to a lesser extent. Binding of neurotrypsin to cell-surface glycans locally restricts its conversion from zymogen into active peptidase. This provides the molecular foundation for the local action of neurotrypsin at or in the vicinity of its site of synaptic secretion. By its local action at synapses with correlated pre- and post-synaptic activity, the neurotrypsin-agrin system fulfils the requirements for a mechanism serving experience-dependent modification of activated synapses, which is essential for adaptive structural reorganizations of neuronal circuits in the developing and/or adult brain.
2. Coincident pre- and postsynaptic activation induces dendritic **filopodia** via neurotrypsin-dependent agrin cleavage. The synaptic serine protease neurotrypsin is essential for cognitive function, as its deficiency in humans results in severe mental retardation. Recently, we demonstrated the activity-dependent release of neurotrypsin from presynaptic terminals and proteolytical cleavage of agrin at the synapse. Here we show that the activity-dependent formation of dendritic **filopodia** is abolished in hippocampal neurons from neurotrypsin-deficient mice. Administration of the neurotrypsin-dependent 22 kDa fragment of agrin rescues the **filopodia**l response. Detailed analyses indicated that presynaptic action potential firing is necessary for the release of neurotrypsin, whereas postsynaptic NMDA receptor activation is necessary for the neurotrypsin-dependent cleavage of agrin. This contingency characterizes the neurotrypsin-agrin system as a coincidence detector of pre- and postsynaptic activation. As the resulting dendritic **filopodia** are thought to represent precursors of synapses, the neurotrypsin-dependent cleavage of agrin at the synapse may be instrumental for a Hebbian organization and remodeling of synaptic circuits in the CNS.

---

## Hspb1

- NCBI Gene
- GeneRIF
- Pubmed

| Search Term | Rank | Fields |
| --- | --- | --- |
| [Mm]igration | 6 | Process(1); PubMed(1) |
| [Ee]xtracellular [Mm]atrix | 8 | Component(1) |

  
hide/show details for Hspb1

### [Mm]igration

#### Process

1. positive regulation of blood vessel endothelial cell **migration**

#### PubMed

1. A key role for the HLH transcription factor EBF2COE2,O/E-3 in Purkinje neuron **migration** and cerebellar cortical topography. Early B-cell factor 2 (EBF2) is one of four mammalian members of an atypical helix-loop-helix transcription factor family (COE). COE proteins have been implicated in various aspects of nervous and immune system development. We and others have generated and described mice carrying a null mutation of Ebf2, a gene previously characterized in the context of Xenopus laevis primary neurogenesis and neuronal differentiation. In addition to deficits in neuroendocrine and olfactory development, and peripheral nerve maturation, Ebf2 null mice feature an ataxic gait and obvious motor deficits associated with clear-cut abnormalities of cerebellar development. The number of Purkinje cells (PCs) in the Ebf2 null is markedly decreased, resulting in a small cerebellum with notable foliation defects, particularly in the anterior vermis. We show that this stems from the defective **migration** of a molecularly defined PC subset that subsequently dies by apoptosis. Part of the striped cerebellar topography is disrupted due to cell death and, in addition, many of the surviving PCs, that would normally adopt a zebrin II-negative phenotype, transdifferentiate to Zebrin II-positive, an unprecedented finding suggesting that Ebf2 is required for the establishment of a proper cerebellar cortical map.

### [Ee]xtracellular [Mm]atrix

#### Component

1. **extracellular matrix**

---

## Cryaa

- NCBI Gene
- GeneRIF
- Pubmed

| Search Term | Rank | Fields |
| --- | --- | --- |
| [Mm]igration | 6 | PubMed(5); GeneRIFs(1) |
| [Ee]xtracellular [Mm]atrix | 8 | PubMed(2) |

  
hide/show details for Cryaa

### [Mm]igration

#### PubMed

1. Dosage effects of cohesin regulatory factor PDS5 on mammalian development: implications for cohesinopathies. Cornelia de Lange syndrome (CdLS), a disorder caused by mutations in cohesion proteins, is characterized by multisystem developmental abnormalities. PDS5, a cohesion protein, is important for proper chromosome segregation in lower organisms and has two homologues in vertebrates (PDS5A and PDS5B). Pds5B mutant mice have developmental abnormalities resembling CdLS; however the role of Pds5A in mammals and the association of PDS5 proteins with CdLS are unknown. To delineate genetic interactions between Pds5A and Pds5B and explore mechanisms underlying phenotypic variability, we generated Pds5A-deficient mice. Curiously, these mice exhibit multiple abnormalities that were previously observed in Pds5B-deficient mice, including cleft palate, skeletal patterning defects, growth retardation, congenital heart defects and delayed **migration** of enteric neuron precursors. They also frequently display renal agenesis, an abnormality not observed in Pds5B(-/-) mice. While Pds5A(-/-) and Pds5B(-/-) mice die at birth, embryos harboring 3 mutant Pds5 alleles die between E11.5 and E12.5 most likely of heart failure, indicating that total Pds5 gene dosage is critical for normal development. In addition, characterization of these compound homozygous-heterozygous mice revealed a severe abnormality in lens formation that does not occur in either Pds5A(-/-) or Pds5B(-/-) mice. We further identified a functional missense mutation (R1292Q) in the PDS5B DNA-binding domain in a familial case of CdLS, in which affected individuals also develop megacolon. This study shows that PDS5A and PDS5B functions other than those involving chromosomal dynamics are important for normal development, highlights the sensitivity of key developmental processes on PDS5 signaling, and provides mechanistic insights into how PDS5 mutations may lead to CdLS.
2. The transcriptional repressor RP58 is crucial for cell-division patterning and neuronal survival in the developing cortex. The neocortex and the hippocampus comprise several specific layers containing distinct neurons that originate from progenitors at specific development times, under the control of an adequate cell-division patterning mechanism. Although many molecules are known to regulate this cell-division patterning process, its details are not well understood. Here, we show that, in the developing cerebral cortex, the RP58 transcription repressor protein was expressed both in postmitotic glutamatergic projection neurons and in their progenitor cells, but not in GABAergic interneurons. Targeted deletion of the RP58 gene led to dysplasia of the neocortex and of the hippocampus, reduction of the number of mature cortical neurons, and defects of laminar organization, which reflect abnormal neuronal **migration** within the cortical plate. We demonstrate an impairment of the cell-division patterning during the late embryonic stage and an enhancement of apoptosis of the postmitotic neurons in the RP58-deficient cortex. These results suggest that RP58 controls cell division of progenitor cells and regulates the survival of postmitotic cortical neurons.
3. Regulation of c-Maf and αA-Crystallin in Ocular Lens by Fibroblast Growth Factor Signaling. Fibroblast growth factor (FGF) signaling regulates a multitude of cellular processes, including cell proliferation, survival, **migratio**n, and differentiation. In the vertebrate lens, FGF signaling regulates fiber cell differentiation characterized by high expression of crystallin proteins. However, a direct link between FGF signaling and crystallin gene transcriptional machinery remains to be established. Previously, we have shown that the bZIP proto-oncogene c-Maf regulates expression of αA-crystallin (Cryaa) through binding to its promoter and distal enhancer, DCR1, both activated by FGF2 in cell culture. Herein, we identified and characterized a novel FGF2-responsive region in the c-Maf promoter (-272/-70, FRE). Both c-Maf and Cryaa regulatory regions contain arrays of AP-1 and Ets-binding sites. Chromatin immunoprecipitation (ChIP) assays established binding of c-Jun (an AP-1 factor) and Etv5/ERM (an Ets factor) to these regions in lens chromatin. Analysis of temporal and spatial expression of c-Jun, phospho-c-Jun, and Etv5/ERM in wild type and ERK1/2 deficient lenses supports their roles as nuclear effectors of FGF signaling in mouse embryonic lens. Collectively, these studies show that FGF signaling up-regulates expression of αA-crystallin both directly and indirectly via up-regulation of c-Maf. These molecular mechanisms are applicable for other crystallins and genes highly expressed in terminally differentiated lens fibers.
4. Knockout of αA-crystallin inhibits ocular neovascularization. The present study aimed to investigate the effects of αA-crystallin on pathologic ocular neovascularization.Human umbilical vein endothelial cells (HUVECs) were used for the in vitro study, and αA-crystallin-knockout (CRYAA[-/-]) mice were used for the in vivo study. αA-crystallin was knocked down in HUVECs by using a specific small interfering RNA (siRNA), and the effects of αA-crystallin knockdown on proliferat**ion, migr**ation, tube formation, and apoptosis were evaluated. Enzyme-linked immunosorbent assays were performed to investigate extracellular concentrations of vascular endothelial growth factor (VEGF). In vivo mouse models of oxygen-induced retinopathy (OIR) and laser-induced choroidal neovascularization (CNV) were generated by using CRYAA(-/-) mice. The nonperfused area in the OIR model was measured in flat-mounted retinas, and angiogenesis resulting in CNV was evaluated in retinal sections. Western blot analysis was performed to investigate the phosphorylation status of vascular endothelial growth factor receptor 2 (VEGFR2, high affinity receptor for VEGF), AKT, PLCγ1, FAK, Src, p42/p44MAPK, p38MAPK, caspase-3, and caspase-9 in cultured HUVECs, as well as in the OIR and CNV models.The CRYAA siRNA not only induced HUVEC apoptosis but also inhibited exogenous and endogenous VEGF-induced cell activities, including prolifera**tion, mig**ration, and tube formation. Pathologic neovascularization was attenuated in the CRYAA(-/-) murine OIR and CNV models. Both in vitro and in vivo, the inhibition of angiogenesis was mediated by the suppression of VEGF secretion and the inhibition of the VEGFR2 signaling pathway; VEGFR2, AKT, PLCγ1, FAK, Src, p42/p44 MAPK, and p38 MAPK all showed reduced phosphorylation levels. In addition, CRYAA knockout led to increased levels of cleaved caspase-9 and caspase-3.Knockout of αA-crystallin inhibited pathologic neovascularization through the VEGF and VEGFR2 signaling pathways both in vitro and in vivo. These results suggest that αA-crystallin could be a novel pharmaceutical target for the prevention of ocular neovascularization.
5. AlphaA-crystallin R49Cneo mutation influences the architecture of lens fiber cell membranes and causes posterior and nuclear cataracts in mice. AlphaA-crystallin (CRYAA/HSPB4), a major component of all vertebrate eye lenses, is a small heat shock protein responsible for maintaining lens transparency. The R49C mutation in the alphaA-crystallin protein is linked with non-syndromic, hereditary human cataracts in a four-generation Caucasian family.This study describes a mouse cataract model generated by insertion of a neomycin-resistant (neor) gene into an intron of the gene encoding mutant R49C alphaA-crystallin. Mice carrying the neor gene and wild-type Cryaa were also generated as controls. Heterozygous knock-in mice containing one wild type gene and one mutated gene for alphaA-crystallin (WT/R49Cneo) and homozygous knock-in mice containing two mutated genes (R49Cneo/R49Cneo) were compared.By 3 weeks, WT/R49Cneo mice exhibited large vacuoles in the cortical region 100 mum from the lens surface, and by 3 months posterior and nuclear cataracts had developed. WT/R49Cneo mice demonstrated severe posterior cataracts at 9 months of age, with considerable posterior nuclear **migration** evident in histological sections. R49Cneo/R49Cneo mice demonstrated nearly complete lens opacities by 5 months of age. In contrast, R49C mice in which the neor gene was deleted by breeding with CreEIIa mice developed lens abnormalities at birth, suggesting that the neor gene may suppress expression of mutant R49C alphaA-crystallin protein.It is apparent that modification of membrane and cell-cell interactions occurs in the presence of the alphaA-crystallin mutation and rapidly leads to lens cell pathology in vivo.

#### GeneRIFs

1. expression of R49C CRYAA mutant protein in the presence of the neor gene causes posterior cataracts with posterior **migration** of epithelial cell nuclei, and demonstrates that the mutant protein affects the integrity of lens fiber cells in the deep cortex.

### [Ee]xtracellular [Mm]atrix

#### PubMed

1. Differential requirement for beta-catenin in epithelial and fiber cells during lens development. Recent studies implicate Wnt/beta-catenin signaling in lens differentiation (Stump, R. J., et al., 2003. A role for Wnt/beta-catenin signaling in lens epithelial differentiation. Dev Biol;259:48-61). Beta-catenin is a component of adherens junctions and functions as a transcriptional activator in canonical Wnt signaling. We investigated the effects of Cre/LoxP-mediated deletion of beta-catenin during lens development using two Cre lines that specifically deleted beta-catenin in whole lens or only in differentiated fibers, from E13.5. We found that beta-catenin was required in lens epithelium and during early fiber differentiation but appeared to be redundant in differentiated fiber cells. Complete loss of beta-catenin resulted in an abnormal and deficient epithelial layer with loss of E-cadherin and Pax6 expression as well as abnormal expression of c-Maf and p57(kip2) but not Prox1. There was also disrupted fiber cell differentiation, characterized by poor cell elongation, decreased beta-crystallin expression, epithelial cell cycle arrest at G(1)-S transition and premature cell cycle exit. Despite cell cycle arrest there was no induction of apoptosis. Mutant fiber cells displayed altered apical-basal polarity as evidenced by altered distribution of the tight junction protein, ZO1, disruption of apical actin filaments and abnormal deposition of **extracellular matrix**, resulting in a deficient lens capsule. Loss of beta-catenin also affected the formation of adhesion junctions as evidenced by dissociation of N-cadherin and F-actin localization in differentiating fiber cells. However, loss of beta-catenin from terminally differentiating fibers had no apparent effects on adhesion junctions between adjacent embryonic fibers. These data indicate that beta-catenin plays distinct functions during lens fiber differentiation and is involved in both Wnt signaling and adhesion-related mechanisms that regulate lens epithelium and early fiber differentiation.
2. Deletion of G protein-coupled receptor 48 leads to ocular anterior segment dysgenesis (ASD) through down-regulation of Pitx2. The development of the anterior segment of the mammalian eye is critical for normal ocular function, whereas abnormal development can cause glaucoma, a leading cause of blindness in the world. We report that orphan G protein-coupled receptor 48 (Gpr48/LGR4) plays an important role in the development of the anterior segment structure. Disruption of Gpr48 causes a wide spectrum of anterior segment dysgenesis (ASD), including microphthalmia, iris hypoplasia, irdiocorneal angle malformation, cornea dysgenesis, and cataract. Detailed analyses reveal that defective iris myogenesis and ocular **extracellular matrix** homeostasis are detected at early postnatal stages of eye development, whereas ganglion cell loss, inner nuclear layer thinness, and early onset of glaucoma were detected in 6-month-old Gpr48(-/-) mice. To determine the molecular mechanism of ASD caused by the deletion of Gpr48, we performed gene expression analyses and revealed dramatic down-regulation of Pitx2 in homozygous knockout mice. In vitro studies with the constitutively active Gpr48 mutant receptor demonstrate that Pitx2 is a direct target of the Gpr48-mediated cAMP-CREB signaling pathway in regulating anterior segment development, suggesting a role of Gpr48 as a potential therapeutic target of ASD.

---

## Opcml

- NCBI Gene
- GeneRIF
- Pubmed

| Search Term | Rank | Fields |
| --- | --- | --- |
| [Mm]igration | 6 | PubMed(1) |
| [Ee]xtracellular [Mm]atrix | 8 | PubMed(1) |

  
hide/show details for Opcml

### [Mm]igration

#### PubMed

1. Genomic cloning of mouse MIF (macrophage inhibitory factor) and genetic mapping of the human and mouse expressed gene and nine mouse pseudogenes. The single functional mouse gene for MIF (macrophage **migration** inhibitory factor) has been cloned from a P1 library, and its exon/intron structure determined and shown to resemble that of the human gene. The gene was mapped to chromosome 10 using two multilocus crosses between laboratory strains and either Mus musculus musculus or Mus spretus. Nine additional loci containing related sequences, apparently all processed pseudogenes, were also mapped to chromosomes 1, 2, 3, 7, 8, 9, 12, 17, and 19. While most of these pseudogenes were found in inbred mice and M. spretus, some are species specific. This suggests that there have been active phases of pseudogene formation in Mus both before and after the separation of musculus and spretus. The human genome contains no pseudogenes; we assigned the human gene to chromosome 19, consistent with the location of mouse and human functional genes for MIF in a region of conserved linkage.

### [Ee]xtracellular [Mm]atrix

#### PubMed

1. Isolation, characterization, and chromosomal location of the mouse enamelysin gene. Mouse enamelysin (Mmp20), a member of the matrix metalloproteinase (MMP) family of **extracellular matrix** degrading enzymes, shows a high degree of homology with other MMPs, particularly those of the stromelysin/collagenase subfamilies. It is expressed exclusively in ameloblasts and odontoblasts. The mouse enamelysin gene (Mmp20) is made up of 10 exons spanning approximately 65 kb within the MMP gene cluster at the centromeric end of chromosome 9.

---

## Hes7

- NCBI Gene
- GeneRIF
- Pubmed

| Search Term | Rank | Fields |
| --- | --- | --- |
| [Mm]igration | 6 | PubMed(1) |
| [Mm]etastasis | 12 | PubMed(1) |

  
hide/show details for Hes7

### [Mm]igration

#### PubMed

1. Wnt5a and Wnt11 regulate mammalian anterior-posterior axis elongation. Mesoderm formation and subsequent anterior-posterior (A-P) axis elongation are fundamental aspects of gastrulation, which is initiated by formation of the primitive streak (PS). Convergent extension (CE) movements and epithelial-mesenchymal transition (EMT) are important for A-P axis elongation in vertebrate embryos. The evolutionarily conserved planar cell polarity (PCP) pathway regulates CE, and Wnts regulate many aspects of gastrulation including CE and EMT. However, the Wnt ligands that regulate A-P axis elongation in mammalian development remain unknown. Wnt11 and Wnt5a regulate axis elongation in lower vertebrates, but only Wnt5a, not Wnt11, regulates mammalian PCP signaling and A-P axis elongation in development. Here, by generating Wnt5a; Wnt11 compound mutants, we show that Wnt11 and Wnt5a play redundant roles during mouse A-P axis elongation. Both genes regulate trunk notochord extension through PCP-controlled CE of notochord cells, establishing a role for Wnt11 in mammalian PCP. We show that Wnt5a and Wnt11 are required for proper patterning of the neural tube and somites by regulating notochord formation, and provide evidence that both genes are required for the generation and **migration** of axial and paraxial mesodermal precursor cells by regulating EMT. Axial and paraxial mesodermal precursors ectopically accumulate in the PS at late gastrula stages in Wnt5a(-/-); Wnt11(-/-) embryos and these cells ectopically express epithelial cell adhesion molecules. Our data suggest that Wnt5a and Wnt11 regulate EMT by inducing p38 (Mapk14) phosphorylation. Our findings provide new insights into the role of Wnt5a and Wnt11 in mouse early development and also in cancer metastasis, during which EMT plays a crucial role.

### [Mm]etastasis

#### PubMed

1. Wnt5a and Wnt11 regulate mammalian anterior-posterior axis elongation. Mesoderm formation and subsequent anterior-posterior (A-P) axis elongation are fundamental aspects of gastrulation, which is initiated by formation of the primitive streak (PS). Convergent extension (CE) movements and epithelial-mesenchymal transition (EMT) are important for A-P axis elongation in vertebrate embryos. The evolutionarily conserved planar cell polarity (PCP) pathway regulates CE, and Wnts regulate many aspects of gastrulation including CE and EMT. However, the Wnt ligands that regulate A-P axis elongation in mammalian development remain unknown. Wnt11 and Wnt5a regulate axis elongation in lower vertebrates, but only Wnt5a, not Wnt11, regulates mammalian PCP signaling and A-P axis elongation in development. Here, by generating Wnt5a; Wnt11 compound mutants, we show that Wnt11 and Wnt5a play redundant roles during mouse A-P axis elongation. Both genes regulate trunk notochord extension through PCP-controlled CE of notochord cells, establishing a role for Wnt11 in mammalian PCP. We show that Wnt5a and Wnt11 are required for proper patterning of the neural tube and somites by regulating notochord formation, and provide evidence that both genes are required for the generation and migration of axial and paraxial mesodermal precursor cells by regulating EMT. Axial and paraxial mesodermal precursors ectopically accumulate in the PS at late gastrula stages in Wnt5a(-/-); Wnt11(-/-) embryos and these cells ectopically express epithelial cell adhesion molecules. Our data suggest that Wnt5a and Wnt11 regulate EMT by inducing p38 (Mapk14) phosphorylation. Our findings provide new insights into the role of Wnt5a and Wnt11 in mouse early development and also in cancer **metastasis**, during which EMT plays a crucial role.

---

## Bhlhe40

- NCBI Gene
- GeneRIF
- Pubmed

| Search Term | Rank | Fields |
| --- | --- | --- |
| [Mm]igration | 6 | PubMed(2) |
| [Ee]xtracellular [Mm]atrix | 8 | PubMed(1) |

  
hide/show details for Bhlhe40

### [Mm]igration

#### PubMed

1. Arid3b is essential for second heart field cell deployment and heart patterning. Arid3b, a member of the conserved ARID family of transcription factors, is essential for mouse embryonic development but its precise roles are poorly understood. Here, we show that Arid3b is expressed in the myocardium of the tubular heart and in second heart field progenitors. Arid3b-deficient embryos show cardiac abnormalities, including a notable shortening of the poles, absence of myocardial differentiation and altered patterning of the atrioventricular canal, which also lacks epithelial-to-mesenchymal transition. Proliferation and death of progenitors as well as early patterning of the heart appear normal. However, DiI labelling of second heart field progenitors revealed a defect in the addition of cells to the heart. RNA microarray analysis uncovered a set of differentially expressed genes in Arid3b-deficient tissues, including Bhlhb2, a regulator of cardiomyocyte differentiation, and Lims2, a gene involved in cell **migration**. Arid3b is thus required for heart development by regulating the motility and differentiation of heart progenitors. These findings identify Arid3b as a candidate gene involved in the aetiology of human congenital malformations.
2. Basic helix-loop-helix transcription factor DEC1 negatively regulates cyclin D1. DEC1 (also known as Stra13/Bhlhb2/Sharp2) and DEC2 (also known as Bhlhb3/Sharp1) are two paralogous basic helix-loop-helix (bHLH) transcriptional regulators which exhibit a robust circadian gene expression pattern in the suprachiasmatic nucleus (SCN) and in peripheral organs. DEC1 has been suggested to play key roles in mammalian cell differentiation, the cell cycle and circadian regulation, hypoxia response, and carcinogenesis. Here we show that DEC1 overexpression exhibits delayed wound healing and reduces cell proliferation, **migration**, and invasion. DEC1 strongly repressed the promoter activity of cyclin D1. We further identify a possible DEC-response element in the cyclin D1 promoter region, and confirmed the direct binding of DEC1 to that element. Forced expression of DEC1 efficiently repressed the cyclin D1 promoter and expression. Our clinical data provide the first evidence that there is a strong inverse correlation between DEC1 and cyclin D1 expression in oral cancer, and DEC1 expression significantly correlated with clinicopathological parameters. We suggest that radiation-induced DEC1 overexpression and Akt phosphorylation in cancer cells are mediated via PI-3K signalling. Overexpression of DEC1 activates the PI-3K/Akt signalling pathway through reactive oxygen species (ROS).

### [Ee]xtracellular [Mm]atrix

#### PubMed

1. SOX9 modulates the expression of key transcription factors required for heart valve development. Heart valve formation initiates when endothelial cells of the heart transform into mesenchyme and populate the cardiac cushions. The transcription factor SOX9 is highly expressed in the cardiac cushion mesenchyme, and is essential for heart valve development. Loss of Sox9 in mouse cardiac cushion mesenchyme alters cell proliferation, embryonic survival, and valve formation. Despite this important role, little is known about how SOX9 regulates heart valve formation or its transcriptional targets. Therefore, we mapped putative SOX9 binding sites by ChIP-Seq in E12.5 heart valves, a stage at which the valve mesenchyme is actively proliferating and initiating differentiation. Embryonic heart valves have been shown to express a high number of genes that are associated with chondrogenesis, including several **extracellular matrix** proteins and transcription factors that regulate chondrogenesis. Therefore, we compared regions of putative SOX9 DNA binding between E12.5 heart valves and E12.5 limb buds. We identified context-dependent and context-independent SOX9-interacting regions throughout the genome. Analysis of context-independent SOX9 binding suggests an extensive role for SOX9 across tissues in regulating proliferation-associated genes including key components of the AP-1 complex. Integrative analysis of tissue-specific SOX9-interacting regions and gene expression profiles on Sox9-deficient heart valves demonstrated that SOX9 controls the expression of several transcription factors with previously identified roles in heart valve development, including Twist1, Sox4, Mecom and Pitx2. Together, our data identify SOX9-coordinated transcriptional hierarchies that control cell proliferation and differentiation during valve formation.

---

## Bhlhe41

- NCBI Gene
- GeneRIF
- Pubmed

| Search Term | Rank | Fields |
| --- | --- | --- |
| [Ee]xtracellular [Mm]atrix | 8 | PubMed(1) |

  
hide/show details for Bhlhe41

### [Ee]xtracellular [Mm]atrix

#### PubMed

1. Sharp-1 regulates TGF-β signaling and skeletal muscle regeneration. Sharp-1 is a basic helix-loop-helix (bHLH) transcriptional repressor that is involved in a number of cellular processes. Our previous studies have demonstrated that Sharp-1 is a negative regulator of skeletal myogenesis and it blocks differentiation of muscle precursor cells by modulating the activity of MyoD. In order to understand its role in pre- and post-natal myogenesis, we assessed skeletal muscle development and freeze-injury-induced regeneration in Sharp-1-deficient mice. We show that embryonic skeletal muscle development is not impaired in the absence of Sharp-1; however, post-natally, the regenerative capacity is compromised. Although the initial phases of injury-induced regeneration proceed normally in Sharp-1(-/-) mice, during late stages, the mutant muscle exhibits necrotic fibers, calcium deposits and fibrosis. TGF-β expression, as well as levels of phosphorylated Smad2 and Smad3, are sustained in the mutant tissue and treatment with decorin, which blocks TGF-β signaling, improves the histopathology of Sharp-1(-/-) injured muscles. In vitro, Sharp-1 associates with Smad3, and its overexpression inhibits TGF-β- and Smad3-mediated expression **of extracellular ma**trix genes in myofibroblasts. These results demonstrate that Sharp-1 regulates muscle regenerative capacity, at least in part, by modulation of TGF-β signaling.

---

## Helt

- NCBI Gene
- GeneRIF
- Pubmed

| Search Term | Rank | Fields |
| --- | --- | --- |
| [Mm]igration | 6 | PubMed(3) |

  
hide/show details for Helt

### [Mm]igration

#### PubMed

1. Subcortical visual shell nuclei targeted by ipRGCs develop from a Sox14+-GABAergic progenitor and require Sox14 to regulate daily activity rhythms. Intrinsically photosensitive retinal ganglion cells (ipRGCs) and their nuclear targets in the subcortical visual shell (SVS) are components of the non-image-forming visual system, which regulates important physiological processes, including photoentrainment of the circadian rhythm. While ipRGCs have been the subject of much recent research, less is known about their central targets and how they develop to support specific behavioral functions. We describe Sox14 as a marker to follow the ontogeny of the SVS and find that the complex forms from two narrow stripes of Dlx2-negative GABAergic progenitors in the early diencephalon through sequential waves of tangential **migration**. We characterize the requirement for Sox14 to orchestrate the correct distribution of neurons among the different nuclei of the network and describe how Sox14 expression is required both to ensure robustness in circadian entrainment and for masking of motor activity.
2. Nkx6-1 controls the identity and fate of red nucleus and oculomotor neurons in the mouse midbrain. Little is known about the cues controlling the generation of motoneuron populations in the mammalian ventral midbrain. We show that Otx2 provides the crucial anterior-posterior positional information for the generation of red nucleus neurons in the murine midbrain. Moreover, the homeodomain transcription factor Nkx6-1 controls the proper development of the red nucleus and of the oculomotor and trochlear nucleus neurons. Nkx6-1 is expressed in ventral midbrain progenitors and acts as a fate determinant of the Brn3a(+) (also known as Pou4f1) red nucleus neurons. These progenitors are partially dorsalized in the absence of Nkx6-1, and a fraction of their postmitotic offspring adopts an alternative cell fate, as revealed by the activation of Dbx1 and Otx2 in these cells. Nkx6-1 is also expressed in postmitotic Isl1(+) oculomotor and trochlear neurons. Similar to hindbrain visceral (branchio-) motoneurons, Nkx6-1 controls the proper **migration** and axon outgrowth of these neurons by regulating the expression of at least three axon guidance/neuronal **migration** molecules. Based on these findings, we provide additional evidence that the developmental mechanism of the oculomotor and trochlear neurons exhibits more similarity with that of special visceral motoneurons than with that controlling the generation of somatic motoneurons located in the murine caudal hindbrain and spinal cord.
3. Loss of Gsx1 and Gsx2 function rescues distinct phenotypes in Dlx1/2 mutants. Mice lacking the Dlx1 and Dlx2 homeobox genes (Dlx1/2 mutants) have severe deficits in subpallial differentiation, including overexpression of the Gsx1 and Gsx2 homeobox genes. To investigate whether Gsx overexpression contributes to the Dlx1/2 mutant phenotypes, we made compound loss-of-function mutants. Eliminating Gsx2 function from the Dlx1/2 mutants rescued the increased expression of Ascl1 and Hes5 (Notch signaling mediators) and Olig2 (oligodendrogenesis mediator). In addition, Dlx1/2;Gsx2 mutants, like Dlx1/2;Ascl1 mutants, exacerbated the Gsx2 and Dlx1/2 patterning and differentiation phenotypes, particularly in the lateral ganglionic eminence (LGE) caudal ganglionic eminence (CGE), and septum, including loss of GAD1 expression. On the other hand, eliminating Gsx1 function from the Dlx1/2 mutants (Dlx1/2;Gsx1 mutants) did not severely exacerbate their phenotype; on the contrary, it resulted in a partial rescue of medial ganglionic eminence (MGE) properties, including interneuron **migration** to the cortex. Thus, despite their redundant properties, Gsx1 and -2 have distinct interactions with Dlx1 and -2. Gsx2 interaction is strongest in the LGE, CGE, and septum, whereas the Gsx1 interaction is strongest in the MGE. From these studies, and earlier studies, we present a model of the transcriptional network that regulates early steps of subcortical development.

---

## Traf6

- NCBI Gene
- GeneRIF
- Pubmed

| Search Term | Rank | Fields |
| --- | --- | --- |
| [Ff]ilopodia | 1 | PubMed(1) |
| [Mm]igration | 6 | PubMed(6) |
| [Mm]etastasis | 12 | PubMed(1) |

  
hide/show details for Traf6

### [Ff]ilopodia

#### PubMed

1. Unc-51-like kinase 1/2-mediated endocytic processes regulate **filopodia** extension and branching of sensory axons. The molecular mechanism and significance of endocytic processes involved in directional axon elongation are not well understood. The Unc-51 family of serine/threonine kinases was shown to be important for axon growth and was also linked to endocytosis, providing an entry point to study this problem. We found that mouse Unc-51-like kinase 1/2 (Ulk1/2) proteins are localized to vesicular structures in growth cones of mouse spinal sensory neurons. RNAi-mediated knockdown of Ulk1 and/or Ulk2 resulted in impaired endocytosis of nerve growth factor (NGF), excessive axon arborization, and severely stunted axon elongation. The evidence also indicates that Ulk1/2 mediates a non-clathrin-coated endocytosis in sensory growth cones. Interestingly, NGF can induce the interaction of Ulk1 with TrkA receptor complexes through promoting K63-polyubiquitination of Ulk1 and binding of Ulk1 to the scaffolding protein p62. These results and additional studies suggest that Ulk1/2 proteins regulate **filopodia** extension and neurite branching during sensory axon outgrowth, probably through regulating TrkA receptor trafficking and signaling.

### [Mm]igration

#### PubMed

1. SASH1 is a scaffold molecule in endothelial TLR4 signaling. Recognition of microbial products by TLRs is critical for mediating innate immune responses to invading pathogens. In this study, we identify a novel scaffold protein in TLR4 signaling called SAM and SH3 domain containing protein 1 (SASH1). Sash1 is expressed across all microvascular beds and functions as a scaffold molecule to independently bind TRAF6, TAK1, IκB kinase α, and IκB kinase β. This interaction fosters ubiquitination of TRAF6 and TAK1 and promotes LPS-induced NF-κB, JNK, and p38 activation, culminating in increased production of proinflammatory cytokines and increased LPS-induced endothe**lial migr**ation. Our findings suggest that SASH1 acts to assemble a signaling complex downstream of TLR4 to activate early endothelial responses to receptor activation.
2. The LIM protein, Limd1, regulates AP-1 activation through an interaction with Traf6 to influence osteoclast development. Increasingly a number of proteins important in the regulation of bone osteoclast development have been shown primarily influence osteoclastogenesis under conditions of physiologic or pathologic stress. Why basal osteoclastogenesis is normal and how these proteins regulate stress osteoclastogenic responses, as opposed to basal osteoclastogenesis, is unclear. LIM proteins of the Ajuba/Zyxin family localize to cellular sites of cell adhesion where they contribute to the regulation of cell adhesion and **migration**, translocate into the nucleus where they can affect cell fate, but are also found in the cytoplasm where their function is largely unknown. We show that one member of this LIM protein family, Limd1, is uniquely up-regulated during osteoclast differentiation and interacts with Traf6, a critical cytosolic regulator of RANK-L-regulated osteoclast development. Limd1 positively affects the capacity of Traf6 to activate AP-1, and Limd1(-/-) osteoclast precursor cells are defective in the activation of AP-1 and thus induction of NFAT2. Limd1(-/-) mice, although having normal basal bone osteoclast numbers and bone density, are resistant to physiological and pathologic osteoclastogenic stimuli. These results implicate Limd1 as a potentially important regulator of osteoclast development under conditions of stress.
3. The LIM protein, Limd1, regulates AP-1 activation through an interaction with Traf6 to influence osteoclast development. Increasingly a number of proteins important in the regulation of bone osteoclast development have been shown primarily influence osteoclastogenesis under conditions of physiologic or pathologic stress. Why basal osteoclastogenesis is normal and how these proteins regulate stress osteoclastogenic responses, as opposed to basal osteoclastogenesis, is unclear. LIM proteins of the Ajuba/Zyxin family localize to cellular sites of cell adhesion where they contribute to the regulation of cell adhesion and **migration**, translocate into the nucleus where they can affect cell fate, but are also found in the cytoplasm where their function is largely unknown. We show that one member of this LIM protein family, Limd1, is uniquely up-regulated during osteoclast differentiation and interacts with Traf6, a critical cytosolic regulator of RANK-L-regulated osteoclast development. Limd1 positively affects the capacity of Traf6 to activate AP-1, and Limd1(-/-) osteoclast precursor cells are defective in the activation of AP-1 and thus induction of NFAT2. Limd1(-/-) mice, although having normal basal bone osteoclast numbers and bone density, are resistant to physiological and pathologic osteoclastogenic stimuli. These results implicate Limd1 as a potentially important regulator of osteoclast development under conditions of stress.
4. Interleukin-33 induces angiogenesis and vascular permeability through ST2/TRAF6-mediated endothelial nitric oxide production. Interleukin-33 (IL-33), a member of the IL-1 cytokine family, is emerging as a new regulator of immune responses and inflammatory vascular diseases. Although IL-33 and its cognate receptor ST2 appear to be expressed in vascular cells, the precise role of IL-33 in the vasculature has not been determined. In this study, we report a novel role of IL-33 as a potent endothelial activator, promoting both angiogenesis and vascular permeability. IL-33 increased proliferation, **migration**, and morphologic differentiation of human endothelial cells, consistently with increased angiogenesis in vivo. IL-33 also increased endothelial permeability with reduced vascular endothelial-cadherin-facilitated cell-cell junctions in vitro and induced vascular leakage in mouse skin. These effects of IL-33 were blocked by knockdown of ST2. Ligation of IL-33 with ST2 rapidly increased endothelial nitric oxide (NO) production through TRAF6-mediated activation of phosphoinoside-3-kinase, Akt, and endothelial NO synthase. Moreover, pharmacologic or genetic blockage of endothelial NO generation resulted in the inhibition of angiogenesis and vascular hyperpermeability induced by IL-33. These data demonstrate that IL-33 promotes angiogenesis and vascular leakage by stimulating endothelial NO production via the ST2/TRAF6-Akt-eNOS signaling pathway. These findings open new perspectives for the role of IL-33 in the pathogenesis of angiogenesis-dependent and inflammatory vascular diseases.
5. Tumor necrosis factor receptor-associated factor 6 (TRAF6) deficiency results in exencephaly and is required for apoptosis within the developing CNS. Tumor necrosis factor receptor-associated factors (TRAFs) are adaptor proteins important in mediating intracellular signaling. We report here that targeted deletion of traf6 greatly increases the frequency of failure of neural tube closure and exencephaly in traf6 (-/-) mice. The penetrance of this defect is influenced by genetic background. Neural tube fusion requires the coordination of several biological processes, including cell **migration** invoked by contact-dependent signaling, cell proliferation, and programmed cell death (PCD). To gain greater insight into the role of TRAF6 in these processes, neural development and **migration** within the CNS of traf6 (-/-) mice and controls were assessed through temporal examination of a number of immunohistochemical markers. In addition, relative levels of cellular proliferation and PCD were examined throughout embryonic development using bromodeoxyuridine (BrdU) and in situ terminal deoxynucleotidyl transferase-mediated dUTP biotinylated nick end labeling (TUNEL), respectively. The data suggest that loss of TRAF6 does not significantly alter the level of cellular proliferation or the pattern of neural differentiation per se, but rather regulates the level of PCD within specific regions of the developing CNS. Substantial reductions in TUNEL were observed within the ventral diencephalon and mesencephalon in exencephalic traf6 (-/-) embryos. Our results demonstrate a novel and prominent role for TRAF6 in the regional control of PCD within the developing CNS.
6. Interleukin-1 assembles a proangiogenic signaling module consisting of caveolin-1, tumor necrosis factor receptor-associated factor 6, p38-mitogen-activated protein kinase (MAPK), and MAPK-activated protein kinase 2 in endothelial cells. Interleukin-1β (IL-1β) is a major cytokine linking inflammation and angiogenesis in pathological vascular processes, such as atherosclerosis and tumor neoangiogenesis. However, signaling pathways mediating IL-1β-induced proangiogenic processes in endothelial cells (ECs) have barely been elucidated yet. Therefore, the present study investigated IL-1β-induced proangiogenic signaling in ECs.IL-1β potently induced tube formation **and migr**ation of ECs. This was associated with and dependent on activation of p38-mitogen-activated protein kinase (MAPK) and MAPK-activated protein kinase 2 (MK2) as determined by pharmacological inhibition and gene silencing. Furthermore, silencing of the adaptor protein tumor necrosis factor receptor-associated factor 6 (TRAF6) (lentiviral short hairpin RNA) inhibited these IL-1β-induced processes. Moreover, IL-1β promoted translocation of TRAF6 to insoluble cellular fractions (containing membrane rafts/caveolae) and interaction of TRAF6 with caveolin-1. Accordingly, cellular cholesterol depletion (cyclodextrin) and silencing of caveolin-1 (small interfering RNA) inhibited IL-1β-induced activation of p38-MAPK and MK2, as well as IL-1β-induced tube forma**tion and** migration. Finally, silencing of TRAF6 and MK2 deficiency inhibited IL-1β-induced microvessel outgrowth in murine aortic rings ex vivo, and deficiency of MK2 or caveolin-1 significantly reduced IL-1β-induced angiogenesis in mice in vivo (Matrigel plug assay).IL-1β assembles a proangiogenic signaling module consisting of caveolin-1, TRAF6, p38-MAPK, and MK2 in ECs, representing a potential target to intervene into angiogenesis-dependent processes and diseases.

### [Mm]etastasis

#### PubMed

1. Carboxyl terminus of Hsp70-interacting protein regulation of osteoclast formation in mice through promotion of tumor necrosis factor receptor-associated factor 6 protein degradation. Carboxyl terminus of Hsp70-interacting protein (CHIP or STUB1) is an E3 ligase that regulates the stability of several proteins involved in tumor growth and **metastasis**. However, the role of CHIP in bone growth and bone remodeling in vivo has not been reported. This study was undertaken to investigate the role and mechanism of CHIP in regulation of bone mass and bone remodeling.The bone phenotype of Chip(-/-) mice was assessed by histologic, histomorphometric, and micro-computed tomographic analyses. The mechanism by which CHIP regulates the degradation of tumor necrosis factor receptor-associated factor 6 (TRAF6) and the inhibition of NF-κB signaling was examined by immunoprecipitation, Western blot, and luciferase reporter assays.Deletion of the Chip gene led to an osteopenic phenotype and increased osteoclast formation. TRAF6, an adaptor protein that is a key regulator of NF-κB signaling and is critical for RANKL-induced osteoclastogenesis, was up-regulated in osteoclasts from Chip(-/-) mice. CHIP interacted with TRAF6 to promote TRAF6 ubiquitination and proteasome degradation. Further, CHIP inhibited p65 nuclear translocation, leading to the repression of TRAF6-mediated NF-κB transcription.CHIP inhibits NF-κB signaling by promoting TRAF6 degradation and plays an important role in osteoclastogenesis and bone remodeling. These findings suggest that CHIP may be a novel therapeutic target in bone loss-associated disorders.

---

## Rab5a

- NCBI Gene
- GeneRIF
- Pubmed

| Search Term | Rank | Fields |
| --- | --- | --- |
| [Ff]ilopodia | 1 | PubMed(1) |
| [Ff]ilopodium | 2 | Process(2) |
| [Mm]igration | 6 | Process(1); PubMed(1) |

  
hide/show details for Rab5a

### [Ff]ilopodia

#### PubMed

1. Optogenetic control of PIP3: PIP3 is sufficient to induce the actin-based active part of growth cones and is regulated via endocytosis. Phosphatidylinositol-3,4,5-trisphosphate (PIP3) is highly regulated in a spatiotemporal manner and plays multiple roles in individual cells. However, the local dynamics and primary functions of PIP3 in developing neurons remain unclear because of a lack of techniques for manipulating PIP3 spatiotemporally. We addressed this issue by combining optogenetic control and observation of endogenous PIP3 signaling. Endogenous PIP3 was abundant in actin-rich structures such as growth cones and "waves", and PIP3-rich plasma membranes moved actively within growth cones. To study the role of PIP3 in developing neurons, we developed a PI3K photoswitch that can induce production of PIP3 at specific locations upon blue light exposure. We succeeded in producing PIP3 locally in mouse hippocampal neurons. Local PIP3 elevation at neurite tips did not induce neurite elongation, but it was sufficient to induce the formation of **filopodia** and lamellipodia. Interestingly, ectopic PIP3 elevation alone activated membranes to form actin-based structures whose behavior was similar to that of growth-cone-like "waves". We also found that endocytosis regulates effective PIP3 concentration at plasma membranes. These results revealed the local dynamics and primary functions of PIP3, providing fundamental information about PIP3 signaling in neurons.

### [Ff]ilopodium

#### Process

1. regulation of **filopodium** assembly
2. regulation of **filopodium** assembly

### [Mm]igration

#### Process

1. positive regulation of smooth muscle cell **migration**

#### PubMed

1. SARA regulates neuronal **migration** during neocortical development through L1 trafficking. Emerging evidence suggests that endocytic trafficking of adhesion proteins plays a crucial role in neuronal **migration** during neocortical development. However, molecular insights into these processes remain elusive. Here, we study the early endosomal protein Smad anchor for receptor activation (SARA) in the developing mouse brain. SARA is enriched at the apical endfeet of radial glia of the neocortex. Although SARA knockdown did not lead to detectable neurogenic phenotypes, SARA-suppressed neurons exhibited impaired orientation and **migration** across the intermediate zone. Mechanistically, we show that SARA knockdown neurons exhibit increased surface expression of the L1 cell adhesion molecule. Neurons ectopically expressing L1 phenocopy the **migration** and orientation defects caused by SARA knockdown and display increased contact with neighboring neurites. L1 knockdown effectively rescues SARA suppression-induced phenotypes. SARA knockdown neurons eventually overcome their **migration** defect and enter later into the cortical plate. Nevertheless, these neurons localize at more superficial cortical layers than their control counterparts. These results suggest that SARA regulates the orientation, multipolar-to-bipolar transition and the positioning of cortical neurons via modulating surface L1 expression.

---

## Xrcc5

- NCBI Gene
- GeneRIF
- Pubmed

| Search Term | Rank | Fields |
| --- | --- | --- |
| [Ii]nvasive | 10 | PubMed(1) |

  
hide/show details for Xrcc5

### [Ii]nvasive

#### PubMed

1. In vivo and in vitro investigations of heterozygous nebulin knock-out mice disclose a mild skeletal muscle phenotype. Nemaline myopathy is the most common congenital skeletal muscle disease, and mutations in the nebulin gene account for 50% of all cases. Recent studies suggest that the disease severity might be related to the nebulin expression levels. Considering that mutations in the nebulin gene are typically recessive, one would expect that a single functional nebulin allele would maintain nebulin protein expression which would result in preserved skeletal muscle function. We investigated skeletal muscle function of heterozygous nebulin knock-out (i.e., nebulin(+/-)) mice using a multidisciplinary approach including protein and gene expression analysis and combined in vivo and in vitro force measurements. Skeletal muscle anatomy and energy metabolism were studied strictly non-**invasive**ly using magnetic resonance imaging and 31P-magnetic resonance spectroscopy. Maximal force production was reduced by around 16% in isolated muscle of nebulin(+/-) mice while in vivo force generating capacity was preserved. Muscle weakness was associated with a shift toward a slower proteomic phenotype, but was not related to nebulin protein deficiency or to an impaired energy metabolism. Further studies would be warranted in order to determine the mechanisms leading to a mild skeletal muscle phenotype resulting from the expression of a single nebulin allele.

---

## Cyp3a11

- NCBI Gene
- GeneRIF
- Pubmed

| Search Term | Rank | Fields |
| --- | --- | --- |
| [Mm]igration | 6 | PubMed(1) |

  
hide/show details for Cyp3a11

### [Mm]igration

#### PubMed

1. Prox1 ablation in hepatic progenitors causes defective hepatocyte specification and increases biliary cell commitment. The liver has multiple functions that preserve homeostasis. Liver diseases are debilitating, costly and often result in death. Elucidating the developmental mechanisms that establish the liver's architecture or generate the cellular diversity of this organ should help advance the prevention, diagnosis and treatment of hepatic diseases. We previously reported that **migration** of early hepatic precursors away from the gut epithelium requires the activity of the homeobox gene Prox1. Here, we show that Prox1 is a novel regulator of cell differentiation and morphogenesis during hepatogenesis. Prox1 ablation in bipotent hepatoblasts dramatically reduced the expression of multiple hepatocyte genes and led to very defective hepatocyte morphogenesis. As a result, abnormal epithelial structures expressing hepatocyte and cholangiocyte markers or resembling ectopic bile ducts developed in the Prox1-deficient liver parenchyma. By contrast, excessive commitment of hepatoblasts into cholangiocytes, premature intrahepatic bile duct morphogenesis, and biliary hyperplasia occurred in periportal areas of Prox1-deficient livers. Together, these abnormalities indicate that Prox1 activity is necessary to correctly allocate cell fates in liver precursors. These results increase our understanding of differentiation anomalies in pathological conditions and will contribute to improving stem cell protocols in which differentiation is directed towards hepatocytes and cholangiocytes.

---

## Tbxas1

- NCBI Gene
- GeneRIF
- Pubmed

| Search Term | Rank | Fields |
| --- | --- | --- |
| [Mm]igration | 6 | PubMed(1) |

  
hide/show details for Tbxas1

### [Mm]igration

#### PubMed

1. 12-Hydroxyheptadecatrienoic acid promotes epidermal wound healing by accelerating keratinocyte **migration** via the BLT2 receptor. Leukotriene B4 (LTB4) receptor type 2 (BLT2) is a G protein-coupled receptor (GPCR) for 12(S)-hydroxyheptadeca-5Z,8E,10E-trienoic acid (12-HHT) and LTB4. Despite the well-defined proinflammatory roles of BLT1, the in vivo functions of BLT2 remain elusive. As mouse BLT2 is highly expressed in epidermal keratinocytes, we investigated the role of the 12-HHT/BLT2 axis in skin wound healing processes. 12-HHT accumulated in the wound fluid in mice, and BLT2-deficient mice exhibited impaired re-epithelialization and delayed wound closure after skin punching. Aspirin administration reduced 12-HHT production and resulted in delayed wound closure in wild-type mice, which was abrogated in BLT2-deficient mice. In vitro scratch assay using primary keratinocytes and a keratinocyte cell line also showed that the 12-HHT/BLT2 axis accelerated wound closure through the production of tumor necrosis factor α (TNF) and matrix metalloproteinases (MMPs). A synthetic BLT2 agonist accelerated wound closure in cultured cells as well as in C57BL/6J and diabetic mice. These results identify a novel mechanism underlying the action of the 12-HHT/BLT2 axis in epidermal keratinocytes and accordingly suggest the use of BLT2 agonists as therapeutic agents to accelerate wound healing, particularly for intractable wounds, such as diabetic ulcers.

---

## Zfand5

- NCBI Gene
- GeneRIF
- Pubmed

| Search Term | Rank | Fields |
| --- | --- | --- |
| [Mm]igration | 6 | Process(1) |
| [Ii]nvasive | 10 | PubMed(1) |
| [Mm]etastasis | 12 | PubMed(1) |

  
hide/show details for Zfand5

### [Mm]igration

#### Process

1. fibroblast **migration**

### [Ii]nvasive

#### PubMed

1. In vivo and in vitro investigations of heterozygous nebulin knock-out mice disclose a mild skeletal muscle phenotype. Nemaline myopathy is the most common congenital skeletal muscle disease, and mutations in the nebulin gene account for 50% of all cases. Recent studies suggest that the disease severity might be related to the nebulin expression levels. Considering that mutations in the nebulin gene are typically recessive, one would expect that a single functional nebulin allele would maintain nebulin protein expression which would result in preserved skeletal muscle function. We investigated skeletal muscle function of heterozygous nebulin knock-out (i.e., nebulin(+/-)) mice using a multidisciplinary approach including protein and gene expression analysis and combined in vivo and in vitro force measurements. Skeletal muscle anatomy and energy metabolism were studied strictly non-**invasive**ly using magnetic resonance imaging and 31P-magnetic resonance spectroscopy. Maximal force production was reduced by around 16% in isolated muscle of nebulin(+/-) mice while in vivo force generating capacity was preserved. Muscle weakness was associated with a shift toward a slower proteomic phenotype, but was not related to nebulin protein deficiency or to an impaired energy metabolism. Further studies would be warranted in order to determine the mechanisms leading to a mild skeletal muscle phenotype resulting from the expression of a single nebulin allele.

### [Mm]etastasis

#### PubMed

1. Lack of periostin leads to suppression of Notch1 signaling and calcific aortic valve disease. The Postn gene encodes protein periostin. During embryonic development, it is highly expressed in the outflow tract (OFT) endocardial cushions of the developing heart, which give rise to several structures of the mature heart including the aortic valve. Periostin was previously implicated in osteoblast differentiation, cancer **metastasis**, and tooth and bone development, but its role in cardiac OFT development is unclear. To elucidate the role that periostin plays in the developing heart we analyzed cardiac OFT phenotype in mice after deletion of the Postn gene. We found that lack of periostin in the embryonic OFT leads to ectopic expression of the proosteogenic growth factor pleiotrophin (Ptn) and overexpression of delta-like 1 homolog (Dlk1), a negative regulator of Notch1, in the distal (prevalvular) cushions of the OFT. This resulted in suppression of Notch1 signaling, strong induction of the central transcriptional regulator of osteoblast cell fate Runx2, upregulation of osteopontin and osteocalcin expression, and subsequent calcification of the aortic valve. Our data suggest that periostin represses a default osteogenic program in the OFT cushion mesenchyme and promotes differentiation along a fibrogenic lineage. Lack of periostin causes derepression of the osteogenic potential of OFT mesenchymal cells, calcium deposition, and calcific aortic valve disease. These results establish periostin as a key regulator of OFT endocardial cushion mesenchymal cell fate during embryonic development.

---

## Klra8

- NCBI Gene
- GeneRIF
- Pubmed

| Search Term | Rank | Fields |
| --- | --- | --- |
| ECM | 7 | PubMed(1) |

  
hide/show details for Klra8

### ECM

#### PubMed

1. The contribution of natural killer complex loci to the development of experimental cerebral malaria. The Natural Killer Complex (NKC) is a genetic region of highly linked genes encoding several receptors involved in the control of NK cell function. The NKC is highly polymorphic and allelic variability of various NKC loci has been demonstrated in inbred mice, providing evidence for NKC haplotypes. Using BALB.B6-Cmv1r congenic mice, in which NKC genes from C57BL/6 mice were introduced into the BALB/c background, we have previously shown that the NKC is a genetic determinant of malarial pathogenesis. C57BL/6 alleles are associated with increased disease-susceptibility as BALB.B6-Cmv1r congenic mice had increased cerebral pathology and death rates during P. berghei ANKA infection than cerebral malaria-resistant BALB/c controls.To investigate which regions of the NKC are involved in susceptibility to experimental cerebral malaria (**ECM**), intra-NKC congenic mice generated by backcrossing recombinant F2 progeny from a (BALB/c x BALB.B6-Cmv1r) F1 intercross to BALB/c mice were infected with P. berghei ANKA.Our results revealed that C57BL/6 alleles at two locations in the NKC contribute to the development of **ECM**. The increased severity to severe disease in intra-NKC congenic mice was not associated with higher parasite burdens but correlated with a significantly enhanced systemic IFN-γ response to infection and an increased recruitment of CD8+ T cells to the brain of infected animals.Polymorphisms within the NKC modulate malarial pathogenesis and acquired immune responses to infection.

---

## Reg2

- NCBI Gene
- GeneRIF
- Pubmed

| Search Term | Rank | Fields |
| --- | --- | --- |
| [Mm]igration | 6 | PubMed(1) |
| ECM | 7 | PubMed(1) |

  
hide/show details for Reg2

### [Mm]igration

#### PubMed

1. Gene expression profiling of a mouse model of pancreatic islet dysmorphogenesis. In the past decade, several transcription factors critical for pancreas organogenesis have been identified. Despite this success, many of the factors necessary for proper islet morphogenesis and function remain uncharacterized. Previous studies have shown that transgenic over-expression of the transcription factor Hnf6 specifically in the pancreatic endocrine cell lineage resulted in disruptions in islet morphogenesis, including dysfunctional endocrine cell sorting, increased individual islet size, increased number of peripheral endocrine cell types, and failure of islets to migrate away from the ductal epithelium. The mechanisms whereby maintained Hnf6 causes defects in islet morphogenesis have yet to be elucidated.We exploited the dysmorphic islets in Hnf6 transgenic animals as a tool to identify factors important for islet morphogenesis. Genome-wide microarray analysis was used to identify differences in the gene expression profiles of late gestation and early postnatal total pancreas tissue from wild type and Hnf6 transgenic animals. Here we report the identification of genes with an altered expression in Hnf6 transgenic animals and highlight factors with potential importance in islet morphogenesis. Importantly, gene products involved in cell adhesion, cell **migration**, ECM remodeling and proliferation were found to be altered in Hnf6 transgenic pancreata, revealing specific candidates that can now be analyzed directly for their role in these processes during islet development.This study provides a unique dataset that can act as a starting point for other investigators to explore the role of the identified genes in pancreatogenesis, islet morphogenesis and mature beta cell function.

### ECM

#### PubMed

1. Gene expression profiling of a mouse model of pancreatic islet dysmorphogenesis. In the past decade, several transcription factors critical for pancreas organogenesis have been identified. Despite this success, many of the factors necessary for proper islet morphogenesis and function remain uncharacterized. Previous studies have shown that transgenic over-expression of the transcription factor Hnf6 specifically in the pancreatic endocrine cell lineage resulted in disruptions in islet morphogenesis, including dysfunctional endocrine cell sorting, increased individual islet size, increased number of peripheral endocrine cell types, and failure of islets to migrate away from the ductal epithelium. The mechanisms whereby maintained Hnf6 causes defects in islet morphogenesis have yet to be elucidated.We exploited the dysmorphic islets in Hnf6 transgenic animals as a tool to identify factors important for islet morphogenesis. Genome-wide microarray analysis was used to identify differences in the gene expression profiles of late gestation and early postnatal total pancreas tissue from wild type and Hnf6 transgenic animals. Here we report the identification of genes with an altered expression in Hnf6 transgenic animals and highlight factors with potential importance in islet morphogenesis. Importantly, gene products involved in cell adhesion, cell migration, **ECM** remodeling and proliferation were found to be altered in Hnf6 transgenic pancreata, revealing specific candidates that can now be analyzed directly for their role in these processes during islet development.This study provides a unique dataset that can act as a starting point for other investigators to explore the role of the identified genes in pancreatogenesis, islet morphogenesis and mature beta cell function.

---

## Reg1

- NCBI Gene
- GeneRIF
- Pubmed

| Search Term | Rank | Fields |
| --- | --- | --- |
| [Mm]igration | 6 | PubMed(1) |

  
hide/show details for Reg1

### [Mm]igration

#### PubMed

1. Reg I-knockout mice reveal its role in regulation of cell growth that is required in generation and maintenance of the villous structure of small intestine. Reg I (regenerating gene product I) is a growth factor that plays a central role in the generation and regeneration of the gastric mucosal architecture. On the other hand, mouse Reg I mRNA is expressed at the highest levels in the small intestine among the gastrointestinal tissues. In the current study, with the aim to clarify the role of Reg I protein in the small intestine, the temporal and spatial pattern of Reg I expression and the phenotype of Reg I-knockout mice in the tissue were examined. In the wild-type mice, immunohistochemistry localized Reg I protein expression in absorptive cells located in the lower half of the intestinal villi. Reg I expression was undetectable until embryonic day 13 (E13), when the fetal intestine still lacks villous structure; however, it dramatically increased at E17 along with the formation and maturation of the fetal intestinal villi. In the small intestine of the adult Reg I-knockout mice, less densely packed, round-shaped aberrant morphology of the absorptive cells was observed light microscopically, and electron microscopical examination revealed a strikingly loose connection of these cells to the basement membrane. Antiproliferating cell nuclear antigen staining and anti-Ki67 staining demonstrated the marked decrease in the number of proliferating cells in the small intestinal mucosa of the knockout mice. The cell **migration** speed visualized by one shot labeling of 5-bromodeoxyuridine was significantly slower in the knockout mice. These phenotypes of Reg I-knockout mice emerged, in accordance with the temporal pattern of Reg I expression described above, from E17. Reg I was considered to be a regulator of cell growth that is required to generate and maintain the villous structure of the small intestine.

---

## Omg

- NCBI Gene
- GeneRIF
- Pubmed

| Search Term | Rank | Fields |
| --- | --- | --- |
| ECM | 7 | PubMed(1) |
| [Ee]xtracellular [Mm]atrix | 8 | PubMed(1); Component(1) |

  
hide/show details for Omg

### ECM

#### PubMed

1. Oligodendrocyte myelin glycoprotein does not influence node of ranvier structure or assembly. Oligodendrocyte myelin glycoprotein (OMgp) is expressed by both neurons and oligodendrocytes in the CNS. It has been implicated in growth cone collapse and neurite outgrowth inhibition by signaling through the Nogo receptor and paired Ig-like receptor B (PirB). OMgp was also reported to be an extracellular matrix (**ECM**) protein surrounding CNS nodes of Ranvier and proposed to function as (1) an inhibitor of nodal collateral sprouting and (2) an important contributor to proper nodal and paranodal architecture. However, we show here that the anti-OMgp antiserum used in previous studies to define the functions of OMgp at nodes is not specific. Among all reported nodal **ECM** components, the antiserum exhibited strong cross-reactivity against versican V2 isoform, a chondroitin sulfate proteoglycan. Furthermore, the OMgp antiserum labeled OMgp-null nodes, but not nodes from versican V2-deficient mice, and preadsorption of the OMgp antiserum with recombinant versican V2 blocked nodal labeling. Analysis of CNS nodes in OMgp-null mice failed to reveal any nodal or paranodal defects, or increased nodal collateral sprouting, indicating that OMgp does not participate in CNS node of Ranvier assembly or maintenance. We successfully identified a highly specific anti-OMgp antibody and observed OMgp staining in white matter only after initiation of myelination. OMgp immunoreactivity decorated the surface of mature myelinated axons, but was excluded from compact myelin and nodes. Together, our results strongly argue against the nodal localization of OMgp and its proposed functions at nodes, and reveal OMgp's authentic localization relative to nodes and myelin.

### [Ee]xtracellular [Mm]atrix

#### PubMed

1. Oligodendrocyte myelin glycoprotein does not influence node of ranvier structure or assembly. Oligodendrocyte myelin glycoprotein (OMgp) is expressed by both neurons and oligodendrocytes in the CNS. It has been implicated in growth cone collapse and neurite outgrowth inhibition by signaling through the Nogo receptor and paired Ig-like receptor B (PirB). OMgp was also reported to be an **extracellular matrix** (ECM) protein surrounding CNS nodes of Ranvier and proposed to function as (1) an inhibitor of nodal collateral sprouting and (2) an important contributor to proper nodal and paranodal architecture. However, we show here that the anti-OMgp antiserum used in previous studies to define the functions of OMgp at nodes is not specific. Among all reported nodal ECM components, the antiserum exhibited strong cross-reactivity against versican V2 isoform, a chondroitin sulfate proteoglycan. Furthermore, the OMgp antiserum labeled OMgp-null nodes, but not nodes from versican V2-deficient mice, and preadsorption of the OMgp antiserum with recombinant versican V2 blocked nodal labeling. Analysis of CNS nodes in OMgp-null mice failed to reveal any nodal or paranodal defects, or increased nodal collateral sprouting, indicating that OMgp does not participate in CNS node of Ranvier assembly or maintenance. We successfully identified a highly specific anti-OMgp antibody and observed OMgp staining in white matter only after initiation of myelination. OMgp immunoreactivity decorated the surface of mature myelinated axons, but was excluded from compact myelin and nodes. Together, our results strongly argue against the nodal localization of OMgp and its proposed functions at nodes, and reveal OMgp's authentic localization relative to nodes and myelin.

#### Component

1. proteinaceous **extracellular matrix**

---

## Lrrc55

- NCBI Gene
- GeneRIF
- Pubmed

| Search Term | Rank | Fields |
| --- | --- | --- |
| [Ee]xtracellular [Mm]atrix | 8 | Component(1) |

  
hide/show details for Lrrc55

### [Ee]xtracellular [Mm]atrix

#### Component

1. proteinaceous **extracellular matrix**

---

## Pla1a

- NCBI Gene
- GeneRIF
- Pubmed

| Search Term | Rank | Fields |
| --- | --- | --- |
| [Ee]xtracellular [Mm]atrix | 8 | PubMed(1) |

  
hide/show details for Pla1a

### [Ee]xtracellular [Mm]atrix

#### PubMed

1. **Extracellular matrix** secretion by cardiac fibroblasts: role of microRNA-29b and microRNA-30c. MicroRNAs (miRNAs), in particular miR-29b and miR-30c, have been implicated as important regulators of cardiac fibrosis.To perform a proteomics comparison of miRNA effects on **extracellular matrix** secretion by cardiac fibroblasts.Mouse cardiac fibroblasts were transfected with pre-/anti-miR of miR-29b and miR-30c, and their conditioned medium was analyzed by mass spectrometry. miR-29b targeted a cadre of proteins involved in fibrosis, including multiple collagens, matrix metalloproteinases, and leukemia inhibitory factor, insulin-like growth factor 1, and pentraxin 3, 3 predicted targets of miR-29b. miR-29b also attenuated the cardiac fibroblast response to transforming growth factor-β. In contrast, miR-30c had little effect on **extracellular matri**x production but opposite effects regarding leukemia inhibitory factor and insulin-like growth factor 1. Both miRNAs indirectly affected cardiac myocytes. On transfection with pre-miR-29b, the conditioned medium of cardiac fibroblasts lost its ability to support adhesion of rat ventricular myocytes and led to a significant reduction of cardiac myocyte proteins (α-actinin, cardiac myosin-binding protein C, and cardiac troponin I). Similarly, cardiomyocytes derived from mouse embryonic stem cells atrophied under pre-miR-29 conditioned medium, whereas pre-miR-30c conditioned medium had a prohypertrophic effect. Levels of miR-29a, miR-29c, and miR-30c, but not miR-29b, were significantly reduced in a mouse model of pathological but not physiological hypertrophy. Treatment with antagomiRs to miR-29b induced excess fibrosis after aortic constriction without overt deterioration in cardiac function.Our proteomic analysis revealed novel molecular targets of miRNAs that are linked to a fibrogenic cardiac phenotype. Such comprehensive screening methods are essential to define the concerted actions of miRNAs in cardiovascular disease.

---

## Lipg

- NCBI Gene
- GeneRIF
- Pubmed

| Search Term | Rank | Fields |
| --- | --- | --- |
| [Mm]igration | 6 | PubMed(2) |

  
hide/show details for Lipg

### [Mm]igration

#### PubMed

1. Endothelial lipase is a critical determinant of high-density lipoprotein-stimulated sphingosine 1-phosphate-dependent signaling in vascular endothelium. In addition to an extensively characterized role of high-density lipoprotein (HDL) in reverse cholesterol transport, bioactive lipids bound to HDL can also exert diverse vascular effects. Despite this, integration of HDL action in the vasculature with pathways that metabolize HDL and release bioactive lipids has been much less explored. The effects of HDL on endothelial cells are mediated in part by HDL-associated sphingosine 1-phosphate (S1P), which binds to S1P1 receptors and promotes activation of endothelial NO synthase (eNOS) and the kinase Akt. In these studies, we characterized the role of endothelial lipase (EL) in the control of endothelial signaling and biology, including those mediated by HDL-associated S1P.HDL-induced angiogenesis in aortic rings from EL-deficient (EL(-/-)) mice was markedly decreased compared with wild-type controls. In cultured endothelial cells, small interfering RNA-mediated knockdown of EL abrogated HDL-promoted endothelial cell **migration** and tube formation. Small interfering RNA-mediated EL knockdown also attenuated HDL-induced phosphorylation of eNOS(1179) and Akt(473). S1P stimulation restored HDL-induced endothelial **migration** and Akt/eNOS phosphorylation that had been blocked by small interfering RNA-mediated EL knockdown. HDL-induced endothelial cell **migration** and Akt/eNOS phosphorylation were completely inhibited by the S1P1 antagonist W146 but not by the S1P3 antagonist CAY10444.EL is a critical determinant of the effects of HDL on S1P-mediated vascular responses and acts on HDL to promote activation of S1P1, leading to Akt/eNOS phosphorylation and subsequent endothelial **migration** and angiogenesis. The role of EL in HDL-associated S1P effects provides new insights into EL action, the responses seen through EL and HDL interaction, and S1P signaling.
2. Expression of endothelial lipase correlates with the size of neointima in a murine model of vascular remodeling. Endothelial lipase (EL) regulates plasma high-density lipoprotein-cholesterol (HDL-C) levels by promoting HDL catabolism. However, it remains unknown whether the inhibition of EL has beneficial effects on the genesis of vascular diseases. Here, we investigated the role of EL on vascular remodeling in mice.Vascular remodeling was developed by ligation of the left common carotid artery and neointimal lesions were histologically compared between EL-knockout (ELKO), EL-transgenic (ELTg), and wild-type (WT) mice. HDL was isolated from these mice, and effects of the HDL on cell growth and Erk activation were evaluated in vitro using cultured vascular smooth muscle cells.Plasma HDL-C levels were 62% higher in ELKO and 13% lower in ELTg than in WT mice, after the carotid ligation. The size of neointimal lesion was significantly larger in ELTg and smaller in ELKO than in WT mice. Vascular expression of adhesion molecules was lower in ELKO and higher in ELTg compared with WT mice. Moreover, oxidative stress was attenuated in ELKO mice. HDL isolated from ELKO, ELTg, and WT mice inhibited expression of intercellular adhesion molecule-1, angiotensin II-induced activation of Erk, and growth of cultured vascular smooth muscle cells, whereas EL expression itself did not affect cell **migration** or growth.EL expression modulates vascular remodeling as well as plasma HDL-C levels. EL inactivation may increase HDL particles that can inhibit smooth muscle cell growth and **migration**.

---

## Lpl

- NCBI Gene
- GeneRIF
- Pubmed

| Search Term | Rank | Fields |
| --- | --- | --- |
| [Mm]igration | 6 | PubMed(1) |
| [Ee]xtracellular [Mm]atrix | 8 | Component(1) |

  
hide/show details for Lpl

### [Mm]igration

#### PubMed

1. Induction of tumor necrosis factor alpha gene expression by lipoprotein lipase. In view of the suppressive effect of tumor necrosis factor alpha (TNF alpha) on lipoprotein lipase (LPL) and of the potential proatherogenic effects of these two macrophage secretory products, we have tested the possibility that LPL could modulate the production of TNF alpha. Treatment of macrophages with lipoprotein lipase induced tumor necrosis factor alpha gene expression and protein secretion. Maximal increase of TNF alpha mRNA levels occurred after a 3-h treatment with 200 ng/ml LPL. An additive effect of interferon gamma (IFN gamma) was observed on LPL-induced TNF alpha mRNA expression. De novo mRNA synthesis was required for induction of TNF alpha mRNA by LPL as no induction was observed when macrophages were pretreated with actinomycin D before adding LPL. We further established that LPL induced the transcription of the TNF alpha gene in macrophages. We also found that LPL caused the nuclear **migration** of one member of the NFkB family that appears to bind to a site in the murine TNF alpha gene promoter. Furthermore, we demonstrated that the treatment of macrophages with LPL increased the stability of TNF alpha mRNA. These results show that the TNF alpha gene induction in response to LPL involves both transcriptional activation and the enhancement of TNF alpha mRNA stability. Overall, our data demonstrate a new role for LPL, that of modulating macrophage TNF alpha gene expression. This effect may represent one of the mechanisms by which LPL may favor the development of atherosclerosis.

### [Ee]xtracellular [Mm]atrix

#### Component

1. **extracellular matrix**

---

## Cela3b

- NCBI Gene
- GeneRIF
- Pubmed

| Search Term | Rank | Fields |
| --- | --- | --- |
| [Mm]igration | 6 | PubMed(1) |

  
hide/show details for Cela3b

### [Mm]igration

#### PubMed

1. Osteopontin is a novel marker of pancreatic ductal tissues and of undifferentiated pancreatic precursors in mice. Matricellular proteins mediate both tissue morphogenesis and tissue homeostasis in important ways because they modulate cell-matrix and cell-cell interactions. In this study, we found that the matricellular protein osteopontin (Opn) is a novel marker of undifferentiated pancreatic precursors and pancreatic ductal tissues in mice. Our analysis also underscored a specific, dynamic profile of Opn expression in embryonic pancreatic tissues that suggests the participation of this protein's function in processes involving cell **migration**, cell-cell interactions, or both. Surprisingly, our analysis of Opn-deficient pancreata did not reveal obvious alterations in the morphology or differentiation of these tissues. Therefore, in embryonic pancreatic tissues, it is possible that other proteins act redundantly to Opn or that this protein's function is dispensable for pancreas development. Finally, the maintenance of Opn expression in pancreatic tissues of adults argues for a possible function of this protein in injury and pathologic responses.

---

## Ctrb1

- NCBI Gene
- GeneRIF
- Pubmed

| Search Term | Rank | Fields |
| --- | --- | --- |
| [Mm]igration | 6 | PubMed(1) |

  
hide/show details for Ctrb1

### [Mm]igration

#### PubMed

1. A microarray analysis of the XX Wnt4 mutant gonad targeted at the identification of genes involved in testis vascular differentiation. One of the earliest morphological changes during testicular differentiation is the establishment of an XY specific vasculature. The testis vascular system is derived from mesonephric endothelial cells that migrate into the gonad. In the XX gonad, mesonephric cell **migration** and testis vascular development are inhibited by WNT4 signaling. In Wnt4 mutant XX gonads, endothelial cells migrate from the mesonephros and form a male-like coelomic vessel. Interestingly, this process occurs in the absence of other obvious features of testis differentiation, suggesting that Wnt4 specifically inhibits XY vascular development. Consequently, the XX Wnt4 mutant mice presented an opportunity to focus a gene expression screen on the processes of mesonephric cell **migration** and testicular vascular development. We compared differences in gene expression between XY Wnt4+/+ and XX Wnt4+/+ gonads and between XX Wnt4+/+ and XX Wnt4+/+ gonads to identify sets of genes similarly upregulated in wildtype XY gonads and XX mutant gonads or upregulated in XX gonads as compared to XY gonads and XX mutant gonads. We show that several genes identified in the first set are expressed in vascular domains, and have predicted functions related to cell **migration** or vascular development. However, the expression patterns and known functions of other genes are not consistent with roles in these processes. This screen has identified candidates for regulation of sex specific vascular development, and has implicated a role for WNT4 signaling in the development of Sertoli and germ cell lineages not immediately obvious from previous phenotypic analyses.

---

## Cela1

- NCBI Gene
- GeneRIF
- Pubmed

| Search Term | Rank | Fields |
| --- | --- | --- |
| [Mm]igration | 6 | PubMed(1) |
| [Ee]xtracellular [Mm]atrix | 8 | PubMed(2); GeneRIFs(1) |

  
hide/show details for Cela1

### [Mm]igration

#### PubMed

1. Osteopontin is a novel marker of pancreatic ductal tissues and of undifferentiated pancreatic precursors in mice. Matricellular proteins mediate both tissue morphogenesis and tissue homeostasis in important ways because they modulate cell-matrix and cell-cell interactions. In this study, we found that the matricellular protein osteopontin (Opn) is a novel marker of undifferentiated pancreatic precursors and pancreatic ductal tissues in mice. Our analysis also underscored a specific, dynamic profile of Opn expression in embryonic pancreatic tissues that suggests the participation of this protein's function in processes involving cell **migration**, cell-cell interactions, or both. Surprisingly, our analysis of Opn-deficient pancreata did not reveal obvious alterations in the morphology or differentiation of these tissues. Therefore, in embryonic pancreatic tissues, it is possible that other proteins act redundantly to Opn or that this protein's function is dispensable for pancreas development. Finally, the maintenance of Opn expression in pancreatic tissues of adults argues for a possible function of this protein in injury and pathologic responses.

### [Ee]xtracellular [Mm]atrix

#### PubMed

1. Identification of glucocorticoid-regulated genes that control cell proliferation during murine respiratory development. Glucocorticoids play a vital role in fetal respiratory development and act via the intracellular glucocorticoid receptor (GR) to regulate transcription of key target genes. GR-null mice die at birth due to respiratory dysfunction associated with hypercellularity and atelectasis. To identify events associated with this lung phenotype we examined perinatal cellular proliferation rates and apoptotic indices. We demonstrate that compared to wild-type controls, day 18.5 postcoitum (p.c.) GR-null mouse lungs display significantly increased cell proliferation rates (1.8-fold P < 0.05) and no change in apoptosis. To examine underlying molecular mechanisms, we compared whole genome expression profiles by microarray analysis at 18.5 days p.c. Pathways relating to cell proliferation, division and cell cycle were significantly down-regulated while pathways relating to carbohydrate metabolism, kinase activities and immune responses were significantly up-regulated. Differential levels of gene expression were verified by quantitative-RT-PCR and/or Northern analysis. Key regulators of proliferation differentially expressed in the lung of 18.5 p.c. GR-null lungs included p21 CIP1 (decreased 2.9-fold, P < 0.05), a negative regulator of the cell cycle, and Mdk (increased 6.0-fold, P < 0.05), a lung growth factor. The more under-expressed genes in 18.5 p.c. GR-null lungs included Chi3l3 (11-fold, P < 0.05), a macrophage inflammatory response gene and Ela1 (9.4-fold, P < 0.05), an **extracellular matrix** remodeling enzyme. Our results demonstrate that GR affects the transcriptional status of a number of regulatory processes during late fetal lung development. Amongst these processes is cell proliferation whereby GR induces expression of cell cycle repressors while suppressing induction of a well characterized cell cycle stimulator.
2. Identification of glucocorticoid-regulated genes that control cell proliferation during murine respiratory development. Glucocorticoids play a vital role in fetal respiratory development and act via the intracellular glucocorticoid receptor (GR) to regulate transcription of key target genes. GR-null mice die at birth due to respiratory dysfunction associated with hypercellularity and atelectasis. To identify events associated with this lung phenotype we examined perinatal cellular proliferation rates and apoptotic indices. We demonstrate that compared to wild-type controls, day 18.5 postcoitum (p.c.) GR-null mouse lungs display significantly increased cell proliferation rates (1.8-fold P < 0.05) and no change in apoptosis. To examine underlying molecular mechanisms, we compared whole genome expression profiles by microarray analysis at 18.5 days p.c. Pathways relating to cell proliferation, division and cell cycle were significantly down-regulated while pathways relating to carbohydrate metabolism, kinase activities and immune responses were significantly up-regulated. Differential levels of gene expression were verified by quantitative-RT-PCR and/or Northern analysis. Key regulators of proliferation differentially expressed in the lung of 18.5 p.c. GR-null lungs included p21 CIP1 (decreased 2.9-fold, P < 0.05), a negative regulator of the cell cycle, and Mdk (increased 6.0-fold, P < 0.05), a lung growth factor. The more under-expressed genes in 18.5 p.c. GR-null lungs included Chi3l3 (11-fold, P < 0.05), a macrophage inflammatory response gene and Ela1 (9.4-fold, P < 0.05), an **extracellular matrix** remodeling enzyme. Our results demonstrate that GR affects the transcriptional status of a number of regulatory processes during late fetal lung development. Amongst these processes is cell proliferation whereby GR induces expression of cell cycle repressors while suppressing induction of a well characterized cell cycle stimulator.

#### GeneRIFs

1. Glucocorticoid receptor-null lungs included Chi3l3 (11-fold, P < 0.05), a macrophage inflammatory response gene and Ela1 (9.4-fold, P < 0.05), an **extracellular matrix** remodeling enzyme.

---

## Ctrl

- NCBI Gene
- GeneRIF
- Pubmed

| Search Term | Rank | Fields |
| --- | --- | --- |
| ECM | 7 | PubMed(1) |
| [Ee]xtracellular [Mm]atrix | 8 | PubMed(2) |

  
hide/show details for Ctrl

### ECM

#### PubMed

1. Transforming growth factor Beta2 is required for valve remodeling during heart development. Although the function of transforming growth factor beta2 (TGFβ2) in epithelial mesenchymal transition (EMT) is well studied, its role in valve remodeling remains to be fully explored. Here, we used histological, morphometric, immunohistochemical and molecular approaches and showed that significant dysregulation of major extracellular matrix **(EC**M) components contributed to valve remodeling defects in Tgfb2(-/-) embryos. The data indicated that cushion mesenchymal cell differentiation was impaired in Tgfb2(-/-) embryos. Hyaluronan and cartilage link protein-1 (CRTL1) were increased in hyperplastic valves of Tgfb2(-/-) embryos, indicating increased expansion and diversification of cushion mesenchyme into the cartilage cell lineage during heart development. Finally, Western blot and immunohistochemistry analyses indicate that the activation of SMAD2/3 was decreased in Tgfb2(-/-) embryos during valve remodeling. Collectively, the data indicate that TGFβ2 promotes valve remodeling and differentiation by inducing matrix organization and suppressing cushion mesenchyme differentiation into cartilage cell lineage during heart development.

### [Ee]xtracellular [Mm]atrix

#### PubMed

1. Transforming growth factor Beta2 is required for valve remodeling during heart development. Although the function of transforming growth factor beta2 (TGFβ2) in epithelial mesenchymal transition (EMT) is well studied, its role in valve remodeling remains to be fully explored. Here, we used histological, morphometric, immunohistochemical and molecular approaches and showed that significant dysregulation of major **extracellular matri**x (ECM) components contributed to valve remodeling defects in Tgfb2(-/-) embryos. The data indicated that cushion mesenchymal cell differentiation was impaired in Tgfb2(-/-) embryos. Hyaluronan and cartilage link protein-1 (CRTL1) were increased in hyperplastic valves of Tgfb2(-/-) embryos, indicating increased expansion and diversification of cushion mesenchyme into the cartilage cell lineage during heart development. Finally, Western blot and immunohistochemistry analyses indicate that the activation of SMAD2/3 was decreased in Tgfb2(-/-) embryos during valve remodeling. Collectively, the data indicate that TGFβ2 promotes valve remodeling and differentiation by inducing matrix organization and suppressing cushion mesenchyme differentiation into cartilage cell lineage during heart development.
2. Genetic rescue of chondrodysplasia and the perinatal lethal effect of cartilage link protein deficiency. The targeted disruption of cartilage link protein gene (Crtl1) in homozygous mice resulted in a severe chondrodysplasia and perinatal lethality. This raised the question of whether the abnormalities seen in Crtl1 null mice are all caused by the absence of link protein in cartilage or whether the deficiency of the protein in other tissues and organs contributed to the phenotype. To address this question we have generated transgenic mice overexpressing cartilage link protein under the control of a cartilage-specific promoter, and then these transgenic mice were used for a genetic rescue of abnormalities in Crtl1 null mice. While the overexpression of cartilage link protein resulted in no abnormal phenotype, the cartilage-specific transgene expression of link protein could completely prevent the perinatal mortality of link protein-deficient mice and, depending on the level of the link protein expression, rescue skeletal abnormalities. Although link protein was originally isolated from cartilage, we found and determined Crtl1 transcripts and corresponding proteins in every organ tested from mouse embryos to aging animals. We also identified three additional members of the link protein family, all co-localized with hyaluronic acid-binding proteoglycans in the mouse genome. The ubiquitous presence of link protein suggests a general and systemic function of link protein in the organization of **extracellular matrix** in a number of tissues, possibly interacting with other proteoglycans, such as versican, brevican, and neurocan.

---

## Gstt3

- NCBI Gene
- GeneRIF
- Pubmed

| Search Term | Rank | Fields |
| --- | --- | --- |
| [Mm]igration | 6 | PubMed(1) |

  
hide/show details for Gstt3

### [Mm]igration

#### PubMed

1. A mutation in the pericentrin gene causes abnormal interneuron **migration** to the olfactory bulb in mice. Precise control of neuronal **migration** is essential for proper function of the brain. Taking a forward genetic screen, we isolated a mutant mouse with defects in interneuron **migration**. By genetic mapping, we identified a frame shift mutation in the pericentrin (Pcnt) gene. The Pcnt gene encodes a large centrosomal coiled-coil protein that has been implicated in schizophrenia. Recently, frame shift and premature termination mutations in the pericentrin (PCNT) gene were identified in individuals with Seckel syndrome and microcephalic osteodysplastic primordial dwarfism (MOPD II), both of which are characterized by greatly reduced body and brain sizes. The mouse Pcnt mutant shares features with the human syndromes in its overall growth retardation and reduced brain size. We found that dorsal lateral ganglionic eminence (dLGE)-derived olfactory bulb interneurons are severely affected and distributed abnormally in the rostral forebrain in the mutant. Furthermore, mutant interneurons exhibit abnormal **migration** behavior and RNA interference knockdown of Pcnt impairs cell **migration** along the rostal migratory stream (RMS) into the olfactory bulb. These findings indicate that pericentrin is required for proper **migration** of olfactory bulb interneurons and provide a developmental basis for association of pericentrin function with interneuron defects in human schizophrenia.

---

## Gstt2

- NCBI Gene
- GeneRIF
- Pubmed

| Search Term | Rank | Fields |
| --- | --- | --- |
| [Mm]igration | 6 | PubMed(1) |

  
hide/show details for Gstt2

### [Mm]igration

#### PubMed

1. A mutation in the pericentrin gene causes abnormal interneuron **migration** to the olfactory bulb in mice. Precise control of neuronal **migration** is essential for proper function of the brain. Taking a forward genetic screen, we isolated a mutant mouse with defects in interneuron **migration**. By genetic mapping, we identified a frame shift mutation in the pericentrin (Pcnt) gene. The Pcnt gene encodes a large centrosomal coiled-coil protein that has been implicated in schizophrenia. Recently, frame shift and premature termination mutations in the pericentrin (PCNT) gene were identified in individuals with Seckel syndrome and microcephalic osteodysplastic primordial dwarfism (MOPD II), both of which are characterized by greatly reduced body and brain sizes. The mouse Pcnt mutant shares features with the human syndromes in its overall growth retardation and reduced brain size. We found that dorsal lateral ganglionic eminence (dLGE)-derived olfactory bulb interneurons are severely affected and distributed abnormally in the rostral forebrain in the mutant. Furthermore, mutant interneurons exhibit abnormal **migration** behavior and RNA interference knockdown of Pcnt impairs cell **migration** along the rostal migratory stream (RMS) into the olfactory bulb. These findings indicate that pericentrin is required for proper **migration** of olfactory bulb interneurons and provide a developmental basis for association of pericentrin function with interneuron defects in human schizophrenia.

---

## Eef1e1

- NCBI Gene
- GeneRIF
- Pubmed

| Search Term | Rank | Fields |
| --- | --- | --- |
| [Mm]etastasis | 12 | PubMed(1) |

  
hide/show details for Eef1e1

### [Mm]etastasis

#### PubMed

1. Lack of periostin leads to suppression of Notch1 signaling and calcific aortic valve disease. The Postn gene encodes protein periostin. During embryonic development, it is highly expressed in the outflow tract (OFT) endocardial cushions of the developing heart, which give rise to several structures of the mature heart including the aortic valve. Periostin was previously implicated in osteoblast differentiation, cancer **metastasis**, and tooth and bone development, but its role in cardiac OFT development is unclear. To elucidate the role that periostin plays in the developing heart we analyzed cardiac OFT phenotype in mice after deletion of the Postn gene. We found that lack of periostin in the embryonic OFT leads to ectopic expression of the proosteogenic growth factor pleiotrophin (Ptn) and overexpression of delta-like 1 homolog (Dlk1), a negative regulator of Notch1, in the distal (prevalvular) cushions of the OFT. This resulted in suppression of Notch1 signaling, strong induction of the central transcriptional regulator of osteoblast cell fate Runx2, upregulation of osteopontin and osteocalcin expression, and subsequent calcification of the aortic valve. Our data suggest that periostin represses a default osteogenic program in the OFT cushion mesenchyme and promotes differentiation along a fibrogenic lineage. Lack of periostin causes derepression of the osteogenic potential of OFT mesenchymal cells, calcium deposition, and calcific aortic valve disease. These results establish periostin as a key regulator of OFT endocardial cushion mesenchymal cell fate during embryonic development.

---

## Gstt4

- NCBI Gene
- GeneRIF
- Pubmed

| Search Term | Rank | Fields |
| --- | --- | --- |
| [Mm]igration | 6 | PubMed(1) |

  
hide/show details for Gstt4

### [Mm]igration

#### PubMed

1. A mutation in the pericentrin gene causes abnormal interneuron **migration** to the olfactory bulb in mice. Precise control of neuronal **migration** is essential for proper function of the brain. Taking a forward genetic screen, we isolated a mutant mouse with defects in interneuron **migration**. By genetic mapping, we identified a frame shift mutation in the pericentrin (Pcnt) gene. The Pcnt gene encodes a large centrosomal coiled-coil protein that has been implicated in schizophrenia. Recently, frame shift and premature termination mutations in the pericentrin (PCNT) gene were identified in individuals with Seckel syndrome and microcephalic osteodysplastic primordial dwarfism (MOPD II), both of which are characterized by greatly reduced body and brain sizes. The mouse Pcnt mutant shares features with the human syndromes in its overall growth retardation and reduced brain size. We found that dorsal lateral ganglionic eminence (dLGE)-derived olfactory bulb interneurons are severely affected and distributed abnormally in the rostral forebrain in the mutant. Furthermore, mutant interneurons exhibit abnormal **migration** behavior and RNA interference knockdown of Pcnt impairs cell **migration** along the rostal migratory stream (RMS) into the olfactory bulb. These findings indicate that pericentrin is required for proper **migration** of olfactory bulb interneurons and provide a developmental basis for association of pericentrin function with interneuron defects in human schizophrenia.

---

## Gstt1

- NCBI Gene
- GeneRIF
- Pubmed

| Search Term | Rank | Fields |
| --- | --- | --- |
| [Mm]igration | 6 | PubMed(1) |

  
hide/show details for Gstt1

### [Mm]igration

#### PubMed

1. A mutation in the pericentrin gene causes abnormal interneuron **migration** to the olfactory bulb in mice. Precise control of neuronal **migration** is essential for proper function of the brain. Taking a forward genetic screen, we isolated a mutant mouse with defects in interneuron **migration**. By genetic mapping, we identified a frame shift mutation in the pericentrin (Pcnt) gene. The Pcnt gene encodes a large centrosomal coiled-coil protein that has been implicated in schizophrenia. Recently, frame shift and premature termination mutations in the pericentrin (PCNT) gene were identified in individuals with Seckel syndrome and microcephalic osteodysplastic primordial dwarfism (MOPD II), both of which are characterized by greatly reduced body and brain sizes. The mouse Pcnt mutant shares features with the human syndromes in its overall growth retardation and reduced brain size. We found that dorsal lateral ganglionic eminence (dLGE)-derived olfactory bulb interneurons are severely affected and distributed abnormally in the rostral forebrain in the mutant. Furthermore, mutant interneurons exhibit abnormal **migration** behavior and RNA interference knockdown of Pcnt impairs cell **migration** along the rostal migratory stream (RMS) into the olfactory bulb. These findings indicate that pericentrin is required for proper **migration** of olfactory bulb interneurons and provide a developmental basis for association of pericentrin function with interneuron defects in human schizophrenia.

---

## Rac3

- NCBI Gene
- GeneRIF
- Pubmed

| Search Term | Rank | Fields |
| --- | --- | --- |
| [Mm]igration | 6 | PubMed(11); GeneRIFs(1) |
| ECM | 7 | PubMed(1) |
| [Ee]xtracellular [Mm]atrix | 8 | PubMed(1) |
| [Mm]etastasis | 12 | PubMed(2) |

  
hide/show details for Rac3

### [Mm]igration

#### PubMed

1. Loss of Either Rac1 or Rac3 GTPase Differentially Affects the Behavior of Mutant Mice and the Development of Functional GABAergic Networks. Rac GTPases regulate the development of cortical/hippocampal GABAergic interneurons by affecting the early development and **migration** of GABAergic precursors. We have addressed the function of Rac1 and Rac3 proteins during the late maturation of hippocampal interneurons. We observed specific phenotypic differences between conditional Rac1 and full Rac3 knockout mice. Rac1 deletion caused greater generalized hyperactivity and cognitive impairment compared with Rac3 deletion. This phenotype matched with a more evident functional impairment of the inhibitory circuits in Rac1 mutants, showing higher excitability and reduced spontaneous inhibitory currents in the CA hippocampal pyramidal neurons. Morphological analysis confirmed a differential modification of the inhibitory circuits: deletion of either Rac caused a similar reduction of parvalbumin-positive inhibitory terminals in the pyramidal layer. Intriguingly, cannabinoid receptor-1-positive terminals were strongly increased only in the CA1 of Rac1-depleted mice. This increase may underlie the stronger electrophysiological defects in this mutant. Accordingly, incubation with an antagonist for cannabinoid receptors partially rescued the reduction of spontaneous inhibitory currents in the pyramidal cells of Rac1 mutants. Our results show that Rac1 and Rac3 have independent roles in the formation of GABAergic circuits, as highlighted by the differential effects of their deletion on the late maturation of specific populations of interneurons.
2. Regulation of lymphatic-blood vessel separation by endothelial Rac1. Sprouting angiogenesis and lymphatic-blood vessel segregation both involve the **migration** of endothelial cells, but the precise migratory molecules that govern the decision of blood vascular endothelial cells to segregate into lymphatic vasculature are unknown. Here, we deleted endothelial Rac1 in mice (Tie1-Cre(+);Rac1(fl/fl)) and revealed, unexpectedly, that whereas blood vessel morphology appeared normal, lymphatic-blood vessel separation was impaired, with corresponding edema, haemorrhage and embryonic lethality. Importantly, normal levels of Rac1 were essential for directed endothelial cell migratory responses to lymphatic-inductive signals. Our studies identify Rac1 as a crucial part of the migratory machinery required for endothelial cells to separate and form lymphatic vasculature.
3. Rac-GTPases Regulate Microtubule Stability and Axon Growth of Cortical GABAergic Interneurons. Cortical interneurons are characterized by extraordinary functional and morphological diversity. Although tremendous progress has been made in uncovering molecular and cellular mechanisms implicated in interneuron generation and function, several questions still remain open. Rho-GTPases have been implicated as intracellular mediators of numerous developmental processes such as cytoskeleton organization, vesicle trafficking, transcription, cell cycle progression, and apoptosis. Specifically in cortical interneurons, we have recently shown a cell-autonomous and stage-specific requirement for Rac1 activity within proliferating interneuron precursors. Conditional ablation of Rac1 in the medial ganglionic eminence leads to a 50% reduction of GABAergic interneurons in the postnatal cortex. Here we examine the additional role of Rac3 by analyzing Rac1/Rac3 double-mutant mice. We show that in the absence of both Rac proteins, the embryonic **migration** of medial ganglionic eminence-derived interneurons is further impaired. Postnatally, double-mutant mice display a dramatic loss of cortical interneurons. In addition, Rac1/Rac3-deficient interneurons show gross cytoskeletal defects in vitro, with the length of their leading processes significantly reduced and a clear multipolar morphology. We propose that in the absence of Rac1/Rac3, cortical interneurons fail to migrate tangentially towards the pallium due to defects in actin and microtubule cytoskeletal dynamics.
4. Rac1 and Rac3 have opposing functions in cell adhesion and differentiation of neuronal cells. Rac1 and Rac3 are highly homologous members of the Rho small GTPase family. Rac1 is ubiquitously expressed and regulates cell adhesion, **migration** and differentiation in various cell types. Rac3 is primarily expressed in brain and may therefore have a specific function in neuronal cells. We found that depletion of Rac1 by short interference RNA leads to decreased cell-matrix adhesions and cell rounding in neuronal N1E-115 cells. By contrast, depletion of Rac3 induces stronger cell adhesions and dramatically increases the outgrowth of neurite-like protrusions, suggesting opposite functions for Rac1 and Rac3 in neuronal cells. Consistent with this, overexpression of Rac1 induces cell spreading, whereas overexpression of Rac3 results in a contractile round morphology. Rac1 is mainly found at the plasma membrane, whereas Rac3 is predominantly localized in the perinuclear region. Residues 185-187, present in the variable polybasic rich region at the carboxyl terminus are responsible for the difference in phenotype induced by Rac1 and Rac3 as well as for their different intracellular localization. The Rac1-opposing function of Rac3 is not mediated by or dependent on components of the RhoA signaling pathway. It rather seems that Rac3 exerts its function through negatively affecting integrin-mediated cell-matrix adhesions. Together, our data reveal that Rac3 opposes Rac1 in the regulation of cell adhesion and differentiation of neuronal cells.
5. Rac1 and rac3 GTPases control synergistically the development of cortical and hippocampal GABAergic interneurons. The intracellular mechanisms driving postmitotic development of cortical γ-aminobutyric acid (GABA)ergic interneurons are poorly understood. We have addressed the function of Rac GTPases in cortical and hippocampal interneuron development. Developing neurons express both Rac1 and Rac3. Previous work has shown that Rac1 ablation does not affect the development of migrating cortical interneurons. Analysis of mice with double deletion of Rac1 and Rac3 shows that these GTPases are required during postmitotic interneuron development. The number of parvalbumin-positive cells was affected in the hippocampus and cortex of double knockout mice. Rac depletion also influences the maturation of interneurons that reach their destination, with reduction of inhibitory synapses in both hippocampal CA1 and cortical pyramidal cells. The decreased number of cortical migrating interneurons and their altered morphology indicate a role of Rac1 and Rac3 in regulating the motility of cortical interneurons, thus interfering with their final localization. While electrophysiological passive and active properties of pyramidal neurons including membrane capacity, resting potential, and spike amplitude and duration were normal, these cells showed reduced spontaneous inhibitory currents and increased excitability. Our results show that Rac1 and Rac3 contribute synergistically to postmitotic development of specific populations of GABAergic cells, suggesting that these proteins regulate their **migratio**n and differentiation.
6. Rac1 controls the formation of midline commissures and the competency of tangential **migration** in ventral telencephalic neurons. Previous studies using dominant-mutant constructs have implicated Rac1 GTPase in neuritogenesis and neuronal **migration**. However, overexpression of dominant mutants generally blocks Rho-GTPase activity; thus, it may not reveal the specific or physiological functions of Rac1. To address this issue, we have applied a conditional gene-targeting strategy, using Foxg1-Cre mice to delete Rac1 in the ventricular zone (VZ) of telencephalon and Dlx5/6-Cre-IRES (internal ribosomal entry site)-EGFP (enhanced green fluorescent protein) (Dlx5/6-CIE) in the subventricular zone (SVZ) of ventral telencephalon, respectively. Surprisingly, the deletion of Rac1 in VZ progenitors did not prevent axonal outgrowth of telencephalic neurons. However, the anterior commissure was absent, and the corpus callosal as well as hippocampal commissural axons failed to cross the midline in Rac1/Foxg1-Cre knock-out embryos. The thalamocortical and corticothalamic axons also showed defasciculation or projection defects. These results suggest that Rac1 controls axon guidance rather than neuritogenesis. In addition, although Rac1/Foxg1-Cre knock-out embryos showed delayed radial **migration** of cortical projection neurons and severe impairment of tangential **migration** by the ventral telencephalon-derived interneurons, deletion of Rac1 in the SVZ by Dlx5/6-CIE mice produced no discernible defects in tangential **migration**. These contrasting effects of Rac1 deletion on tangential **migration** suggest that Rac1 is dispensable for cellular motility per se during neuronal **migration**. Together, these results underscore the challenge of deciphering the biological functions of Rac1, and Rho-GTPases in general, during mammalian brain development. Moreover, they indicate that Rac1 has a critical role in axon guidance and in acquisition of migratory competency during differentiation of the progenitors for the ventral telencephalon-derived interneurons.
7. Rac1 and Rac3 GTPases regulate the development of hilar mossy cells by affecting the **migration** of their precursors to the hilus. We have previously shown that double deletion of the genes for Rac1 and Rac3 GTPases during neuronal development affects late developmental events that perturb the circuitry of the hippocampus, with ensuing epileptic phenotype. These effects include a defect in mossy cells, the major class of excitatory neurons of the hilus. Here, we have addressed the mechanisms that affect the loss of hilar mossy cells in the dorsal hippocampus of mice depleted of the two Rac GTPases. Quantification showed that the loss of mossy cells was evident already at postnatal day 8, soon after these cells become identifiable by a specific marker in the dorsal hilus. Comparative analysis of the hilar region from control and double mutant mice revealed that synaptogenesis was affected in the double mutants, with strongly reduced presynaptic input from dentate granule cells. We found that apoptosis was equally low in the hippocampus of both control and double knockout mice. Labelling with bromodeoxyuridine at embryonic day 12.5 showed no evident difference in the proliferation of neuronal precursors in the hippocampal primordium, while differences in the number of bromodeoxyuridine-labelled cells in the developing hilus revealed a defect in the **migration** of immature, developing mossy cells in the brain of double knockout mice. Overall, our data show that Rac1 and Rac3 GTPases participate in the normal development of hilar mossy cells, and indicate that they are involved in the regulation of the **migration** of the mossy cell precursor by preventing their arrival to the dorsal hilus.
8. Loss of Either Rac1 or Rac3 GTPase Differentially Affects the Behavior of Mutant Mice and the Development of Functional GABAergic Networks. Rac GTPases regulate the development of cortical/hippocampal GABAergic interneurons by affecting the early development and **migration** of GABAergic precursors. We have addressed the function of Rac1 and Rac3 proteins during the late maturation of hippocampal interneurons. We observed specific phenotypic differences between conditional Rac1 and full Rac3 knockout mice. Rac1 deletion caused greater generalized hyperactivity and cognitive impairment compared with Rac3 deletion. This phenotype matched with a more evident functional impairment of the inhibitory circuits in Rac1 mutants, showing higher excitability and reduced spontaneous inhibitory currents in the CA hippocampal pyramidal neurons. Morphological analysis confirmed a differential modification of the inhibitory circuits: deletion of either Rac caused a similar reduction of parvalbumin-positive inhibitory terminals in the pyramidal layer. Intriguingly, cannabinoid receptor-1-positive terminals were strongly increased only in the CA1 of Rac1-depleted mice. This increase may underlie the stronger electrophysiological defects in this mutant. Accordingly, incubation with an antagonist for cannabinoid receptors partially rescued the reduction of spontaneous inhibitory currents in the pyramidal cells of Rac1 mutants. Our results show that Rac1 and Rac3 have independent roles in the formation of GABAergic circuits, as highlighted by the differential effects of their deletion on the late maturation of specific populations of interneurons.
9. Rac-GTPases Regulate Microtubule Stability and Axon Growth of Cortical GABAergic Interneurons. Cortical interneurons are characterized by extraordinary functional and morphological diversity. Although tremendous progress has been made in uncovering molecular and cellular mechanisms implicated in interneuron generation and function, several questions still remain open. Rho-GTPases have been implicated as intracellular mediators of numerous developmental processes such as cytoskeleton organization, vesicle trafficking, transcription, cell cycle progression, and apoptosis. Specifically in cortical interneurons, we have recently shown a cell-autonomous and stage-specific requirement for Rac1 activity within proliferating interneuron precursors. Conditional ablation of Rac1 in the medial ganglionic eminence leads to a 50% reduction of GABAergic interneurons in the postnatal cortex. Here we examine the additional role of Rac3 by analyzing Rac1/Rac3 double-mutant mice. We show that in the absence of both Rac proteins, the embryonic **migration** of medial ganglionic eminence-derived interneurons is further impaired. Postnatally, double-mutant mice display a dramatic loss of cortical interneurons. In addition, Rac1/Rac3-deficient interneurons show gross cytoskeletal defects in vitro, with the length of their leading processes significantly reduced and a clear multipolar morphology. We propose that in the absence of Rac1/Rac3, cortical interneurons fail to migrate tangentially towards the pallium due to defects in actin and microtubule cytoskeletal dynamics.
10. Rac1 and rac3 GTPases control synergistically the development of cortical and hippocampal GABAergic interneurons. The intracellular mechanisms driving postmitotic development of cortical γ-aminobutyric acid (GABA)ergic interneurons are poorly understood. We have addressed the function of Rac GTPases in cortical and hippocampal interneuron development. Developing neurons express both Rac1 and Rac3. Previous work has shown that Rac1 ablation does not affect the development of migrating cortical interneurons. Analysis of mice with double deletion of Rac1 and Rac3 shows that these GTPases are required during postmitotic interneuron development. The number of parvalbumin-positive cells was affected in the hippocampus and cortex of double knockout mice. Rac depletion also influences the maturation of interneurons that reach their destination, with reduction of inhibitory synapses in both hippocampal CA1 and cortical pyramidal cells. The decreased number of cortical migrating interneurons and their altered morphology indicate a role of Rac1 and Rac3 in regulating the motility of cortical interneurons, thus interfering with their final localization. While electrophysiological passive and active properties of pyramidal neurons including membrane capacity, resting potential, and spike amplitude and duration were normal, these cells showed reduced spontaneous inhibitory currents and increased excitability. Our results show that Rac1 and Rac3 contribute synergistically to postmitotic development of specific populations of GABAergic cells, suggesting that these proteins regulate their **migratio**n and differentiation.
11. Rac1 and Rac3 GTPases regulate the development of hilar mossy cells by affecting the **migration** of their precursors to the hilus. We have previously shown that double deletion of the genes for Rac1 and Rac3 GTPases during neuronal development affects late developmental events that perturb the circuitry of the hippocampus, with ensuing epileptic phenotype. These effects include a defect in mossy cells, the major class of excitatory neurons of the hilus. Here, we have addressed the mechanisms that affect the loss of hilar mossy cells in the dorsal hippocampus of mice depleted of the two Rac GTPases. Quantification showed that the loss of mossy cells was evident already at postnatal day 8, soon after these cells become identifiable by a specific marker in the dorsal hilus. Comparative analysis of the hilar region from control and double mutant mice revealed that synaptogenesis was affected in the double mutants, with strongly reduced presynaptic input from dentate granule cells. We found that apoptosis was equally low in the hippocampus of both control and double knockout mice. Labelling with bromodeoxyuridine at embryonic day 12.5 showed no evident difference in the proliferation of neuronal precursors in the hippocampal primordium, while differences in the number of bromodeoxyuridine-labelled cells in the developing hilus revealed a defect in the **migration** of immature, developing mossy cells in the brain of double knockout mice. Overall, our data show that Rac1 and Rac3 GTPases participate in the normal development of hilar mossy cells, and indicate that they are involved in the regulation of the **migration** of the mossy cell precursor by preventing their arrival to the dorsal hilus.

#### GeneRIFs

1. This study showed that in the absence of both Rac1 and rac3, the embryonic **migration** of medial ganglionic eminence-derived interneurons is further impaired.

### ECM

#### PubMed

1. Blood vessel tubulogenesis requires Rasip1 regulation of GTPase signaling. Cardiovascular function depends on patent blood vessel formation by endothelial cells (ECs). However, the mechanisms underlying vascular "tubulogenesis" are only beginning to be unraveled. We show that endothelial tubulogenesis requires the Ras interacting protein 1, Rasip1, and its binding partner, the RhoGAP Arhgap29. Mice lacking Rasip1 fail to form patent lumens in all blood vessels, including the early endocardial tube. Rasipl null angioblasts fail to properly localize the polarity determinant Par3 and display defective cell polarity, resulting in mislocalized junctional complexes and loss of adhesion to extracellular matrix (**ECM**). Similarly, depletion of either Rasip1 or Arhgap29 in cultured ECs blocks in vitro lumen formation, fundamentally alters the cytoskeleton, and reduces integrin-dependent adhesion to **ECM**. These defects result from increased RhoA/ROCK/myosin II activity and blockade of Cdc42 and Rac1 signaling. This study identifies Rasip1 as a unique, endothelial-specific regulator of Rho GTPase signaling, which is essential for blood vessel morphogenesis.

### [Ee]xtracellular [Mm]atrix

#### PubMed

1. Blood vessel tubulogenesis requires Rasip1 regulation of GTPase signaling. Cardiovascular function depends on patent blood vessel formation by endothelial cells (ECs). However, the mechanisms underlying vascular "tubulogenesis" are only beginning to be unraveled. We show that endothelial tubulogenesis requires the Ras interacting protein 1, Rasip1, and its binding partner, the RhoGAP Arhgap29. Mice lacking Rasip1 fail to form patent lumens in all blood vessels, including the early endocardial tube. Rasipl null angioblasts fail to properly localize the polarity determinant Par3 and display defective cell polarity, resulting in mislocalized junctional complexes and loss of adhesion to **extracellular matrix** (ECM). Similarly, depletion of either Rasip1 or Arhgap29 in cultured ECs blocks in vitro lumen formation, fundamentally alters the cytoskeleton, and reduces integrin-dependent adhesion to ECM. These defects result from increased RhoA/ROCK/myosin II activity and blockade of Cdc42 and Rac1 signaling. This study identifies Rasip1 as a unique, endothelial-specific regulator of Rho GTPase signaling, which is essential for blood vessel morphogenesis.

### [Mm]etastasis

#### PubMed

1. Matrix compliance regulates Rac1b localization, NADPH oxidase assembly, and epithelial-mesenchymal transition. Epithelial-mesenchymal transition (EMT) is a form of epithelial plasticity implicated in fibrosis and tumor **metastasis**. Here we show that the mechanical rigidity of the microenvironment plays a pivotal role in the promotion of EMT by controlling the subcellular localization and downstream signaling of Rac GTPases. Soft substrata, with compliances comparable to that of normal mammary tissue, are protective against EMT, whereas stiffer substrata, with compliances characteristic of breast tumors, promote EMT. Rac1b, a highly activated splice variant of Rac1 found in tumors, localizes to the plasma membrane in cells cultured on stiff substrata or in collagen-rich regions of human breast tumors. At the membrane, Rac1b forms a complex with NADPH oxidase and promotes the production of reactive oxygen species, expression of Snail, and activation of the EMT program. In contrast, soft microenvironments inhibit the membrane localization of Rac1b and subsequent redox changes. These results reveal a novel mechanotransduction pathway in the regulation of epithelial plasticity via EMT.
2. Matrix compliance regulates Rac1b localization, NADPH oxidase assembly, and epithelial-mesenchymal transition. Epithelial-mesenchymal transition (EMT) is a form of epithelial plasticity implicated in fibrosis and tumor **metastasis**. Here we show that the mechanical rigidity of the microenvironment plays a pivotal role in the promotion of EMT by controlling the subcellular localization and downstream signaling of Rac GTPases. Soft substrata, with compliances comparable to that of normal mammary tissue, are protective against EMT, whereas stiffer substrata, with compliances characteristic of breast tumors, promote EMT. Rac1b, a highly activated splice variant of Rac1 found in tumors, localizes to the plasma membrane in cells cultured on stiff substrata or in collagen-rich regions of human breast tumors. At the membrane, Rac1b forms a complex with NADPH oxidase and promotes the production of reactive oxygen species, expression of Snail, and activation of the EMT program. In contrast, soft microenvironments inhibit the membrane localization of Rac1b and subsequent redox changes. These results reveal a novel mechanotransduction pathway in the regulation of epithelial plasticity via EMT.

---

## Cdc42

- NCBI Gene
- GeneRIF
- Pubmed

| Search Term | Rank | Fields |
| --- | --- | --- |
| [Ff]ilopodia | 1 | PubMed(41); GeneRIFs(6) |
| [Ff]ilopodium | 2 | Process(2); PubMed(3); GeneRIFs(1) |
| [Ii]nvadopodia | 3 | PubMed(2) |
| [Ii]nvadopodium | 4 | PubMed(2) |
| [Pp]odosome(s)? | 5 | PubMed(1) |
| [Mm]igration | 6 | Process(2); PubMed(98); GeneRIFs(16) |
| ECM | 7 | PubMed(7); GeneRIFs(1) |
| [Ee]xtracellular [Mm]atrix | 8 | PubMed(16); GeneRIFs(2) |
| [Ii]nvasive | 10 | PubMed(6) |
| [Mm]etastasis | 12 | PubMed(6); GeneRIFs(1) |
| [Cc]ell [Ii]nvasion | 13 | PubMed(1) |

  
hide/show details for Cdc42

### [Ff]ilopodia

#### PubMed

1. Imaging **filopodia** dynamics in the mouse blastocyst. During mammalian development, the first cell lineage diversification event occurs in the blastocyst, when the trophectoderm (TE) and the inner cell mass (ICM) become established. Part of the TE (polar) remains in contact with the ICM and differs from the mural TE (mTE) which is separated from the ICM by a cavity known as the blastocoele. The presence of **filopodia** connecting ICM cells with the distant mural TE cells through the blastocoelic fluid was investigated in this work. We describe two types of actin-based cell projections found in freshly dissected and in vitro cultured expanding blastocysts: abundant short **filopodia** projecting into the blastocoelic cavity that present a continuous undulating behavior; and long, thin traversing **filopodia** connecting the mural TE with the ICM. Videomicroscopy analyses revealed the presence of vesicle-like structures moving along traversing **filopodia** and dynamic cytoskeletal rearrangements. These observations, together with immunolocalization of the FGFR2 and the ErbB3 receptors to these cell extensions, suggest that they display signal transduction activity. We propose that traversing **filopodia** are employed by mitotic mTE cells to receive the required signals for cell division after they become distant to the ICM.
2. The cytotoxic necrotizing factor of Yersinia pseudotuberculosis (CNFY) enhances inflammation and Yop delivery during infection by activation of Rho GTPases. Some isolates of Yersinia pseudotuberculosis produce the cytotoxic necrotizing factor (CNFY), but the functional consequences of this toxin for host-pathogen interactions during the infection are unknown. In the present study we show that CNFY has a strong influence on virulence. We demonstrate that the CNFY toxin is thermo-regulated and highly expressed in all colonized lymphatic tissues and organs of orally infected mice. Most strikingly, we found that a cnfY knock-out variant of a naturally toxin-expressing Y. pseudotuberculosis isolate is strongly impaired in its ability to disseminate into the mesenteric lymph nodes, liver and spleen, and has fully lost its lethality. The CNFY toxin contributes significantly to the induction of acute inflammatory responses and to the formation of necrotic areas in infected tissues. The analysis of the host immune response demonstrated that presence of CNFY leads to a strong reduction of professional phagocytes and natural killer cells in particular in the spleen, whereas loss of the toxin allows efficient tissue infiltration of these immune cells and rapid killing of the pathogen. Addition of purified CNFY triggers formation of actin-rich membrane ruffles and **filopodia**, which correlates with the activation of the Rho GTPases, RhoA, Rac1 and Cdc42. The analysis of type III effector delivery into epithelial and immune cells in vitro and during the course of the infection further demonstrated that CNFY enhances the Yop translocation process and supports a role for the toxin in the suppression of the antibacterial host response. In summary, we highlight the importance of CNFY for pathogenicity by showing that this toxin modulates inflammatory responses, protects the bacteria from attacks of innate immune effectors and enhances the severity of a Yersinia infection.
3. cIAP1 regulates TNF-mediated cdc42 activation and **filopodia** formation. Tumour necrosis factor-α (TNF) is a cytokine endowed with multiple functions, depending on the cellular and environmental context. TNF receptor engagement induces the formation of a multimolecular complex including the TNFR-associated factor TRAF2, the receptor-interaction protein kinase RIP1 and the cellular inhibitor of apoptosis cIAP1, the latter being essential for NF-κB activation. Here, we show that cIAP1 also regulates TNF-induced actin cytoskeleton reorganization through a cdc42-dependent, NF-κB-independent pathway. Deletion of cIAP1 prevents TNF-induc**ed filopo**dia and cdc42 activation. The expression of cIAP1 or its E3-ubiquitin ligase-defective mutant restores the ability of cIAP1(-/-) MEFs to produ**ce filopo**dia, whereas a cIAP1 mutant unable to bind TRAF2 does not. Accordingly, the silencing of TRAF2 inhibits TNF-mediat**ed filopo**dia formation, whereas silencing of RIP1 does not. cIAP1 directly binds cdc42 and promotes its RhoGDIα-mediated stabilization. TNF decreases cIAP1-cdc42 interaction, suggesting that TNF-induced recruitment of cIAP1/TRAF2 to the receptor releases cdc42, which in turn triggers actin remodeling. cIAP1 also regulates cdc42 activation in response to EGF and HRas-V12 expression. A downregulation of cIAP1 altered the cell polarization, the cell adhesion to endothelial cells and cell intercalation, which are cdc42-dependent processes. Finally, we demonstrated that the deletion of cIAP1 regulated the HRas-V12-mediated transformation process, including anchorage-dependent cell growth, tumour growth in a xenograft model and the development of experimental metastasis in the lung.
4. NRP1 Regulates CDC42 Activation to Promote **Filopodia** Formation in Endothelial Tip Cells. Sprouting blood vessels are led by **filopodia**-studded endothelial tip cells that respond to angiogenic signals. Mosaic lineage tracing previously revealed that NRP1 is essential for tip cell function, although its mechanistic role in tip cells remains poorly defined. Here, we show that NRP1 is dispensable for genetic tip cell identity. Instead, we find that NRP1 is essential to form the **filopodia**l bursts that distinguish tip cells morphologically from neighboring stalk cells, because it enables the extracellular matrix (ECM)-induced activation of CDC42, a key regulator of **filopodia** formation. Accordingly, NRP1 knockdown and pharmacological CDC42 inhibition similarly impaired **filopodia** formation in vitro and in developing zebrafish in vivo. During mouse retinal angiogenesis, CDC42 inhibition impaired tip cell and vascular network formation, causing defects that resembled those due to loss of ECM-induced, but not VEGF-induced, NRP1 signaling. We conclude that NRP1 enables ECM-induced **filopodia** formation for tip cell function during sprouting angiogenesis.
5. A Rac/Cdc42-specific exchange factor, GEFT, induces cell proliferation, transformation, and migration. The Rho family of small GTPases, including Rho, Rac, and Cdc42, play essential roles in diverse cellular functions. The ability of Rho family GTPases to participate in signaling events is determined by the ratio of inactive (GDP-bound) and active (GTP-bound) forms in the cell. The activation of Rho family proteins requires the exchange of bound GDP for GTP, a process catalyzed by the Dbl family of guanine nucleotide exchange factors (GEFs). The GEFs have high affinity for the guanine nucleotide-free state of the GTPases and are thought to promote GDP release by stabilizing an intermediate transition state. In this study, we have identified and characterized a new Rac/Cdc42-specific Dbl family guanine nucleotide exchange factor, named GEFT. GEFT is highly expressed in the excitable tissues, including brain, heart, and muscle. Low or very little expression was detected in other nonexcitable tissues. GEFT has specific exchange activity for Rac and Cdc42 in our in vitro GTPase exchange assays and glutathione S-transferase-PAK pull-down assays with GTP-bound Rac1 and Cdc42. Overexpression of GEFT leads to changes in cell morphology and actin cytoskeleton re-organization, including the formation of membrane microspikes, **filopodia**, and lamilliopodia. Furthermore, expression of GEFT in NIH3T3 cells promotes foci formation, cell proliferation, and cell migration, possibly through the activation of transcriptional factors involved in cell growth and proliferation. Together, our data suggest that GEFT is a Rac/Cdc42-specific GEF protein that regulates cell morphology, cell proliferation, and transformation.
6. Laminin alpha5 is required for dental epithelium growth and polarity and the development of tooth bud and shape. In tooth development, the oral ectoderm and mesenchyme coordinately and reciprocally interact through the basement membrane for their growth and differentiation to form the proper shape and size of the tooth. Laminin alpha5 subunit-containing laminin-10/11 (LM-511/521) is the major laminin in the tooth germ basement membrane. Here, we have examined the role of laminin alpha5 (Lama5) in tooth development using laminin alpha5-null mouse primary dental epithelium and tooth germ organ cultures. Lama5-null mice develop a small tooth germ with defective cusp formation and have reduced proliferation of dental epithelium. Also, cell polarity and formation of the monolayer of the inner dental epithelium are disturbed. The enamel knot, a signaling center for tooth germ development, is defective, and there is a significant reduction of Shh and Fgf4 expression in the dental epithelium. In the absence of laminin alpha5, the basement membrane in the inner dental epithelium becomes discontinuous. In normal mice, integrin alpha6beta4, a receptor for laminin alpha5, is strongly localized at the basal layer of the epithelium, whereas in mutant mice, integrin alpha6beta4 is expressed around the cell surface. In primary dental epithelium culture, laminin-10/11 promotes cell growth, spreading, and **filopodia**-like microspike formation. This promotion is inhibited by anti-integrin alpha6 and beta4 antibodies and by phosphatidylinositol 3-kinase inhibitors and dominant negative Rho-GTPase family proteins Cdc42 and Rac. In organ culture, anti-integrin alpha6 antibody and wortmannin reduce tooth germ size and shape. Our studies demonstrate that laminin alpha5 is required for the proliferation and polarity of basal epithelial cells and suggest that the interaction between laminin-10/11-integrin alpha6beta4 and the phosphatidylinositol 3-kinase-Cdc42/Rac pathways play an important role in determining the size and shape of tooth germ.
7. Functional analysis of ARHGAP6, a novel GTPase-activating protein for RhoA. Microphthalmia with linear skin defects (MLS) is an X-linked dominant, male-lethal syndrome characterized by microphthalmia, aplastic skin and agenesis of the corpus callosum, and is caused by the deletion of a 500 kb critical region in Xp22.3. Our laboratory isolated a novel rho GTPase-activating protein (rhoGAP) gene named ARHGAP6 from the MLS region. ARHGAP6 contains 14 exons encoding a 974 amino acid protein with three putative SH3-binding domains. Because exons 2-14 are deleted in all MLS patients, we hypothesized that ARHGAP6 may be responsible for some of the phenotypic features of MLS. We pursued two approaches to study the function of ARHGAP6 and its role in the pathogenesis of MLS: gene targeting of the rhoGAP domain in mouse embryonic stem cells and in vitro expression studies. Surprisingly, loss of the rhoGAP function of Arhgap6 does not cause any detectable phenotypic or behavioral abnormalities in the mutant mice. Transfected mammalian cells expressing ARHGAP6 lose their actin stress fibers, retract from the growth surface and extend thin, branching processes resembling **filopodia**. The ARHGAP6 protein co-localizes with actin filaments through an N-terminal domain and recruits F-actin into the growing processes. Mutation of a conserved arginine residue in the rhoGAP domain prevents the loss of stress fibers but has little effect on process outgrowth. These results suggest that ARHGAP6 has two independent functions: one as a GAP with specificity for RhoA and the other as a cytoskeletal protein that promotes actin remodeling.
8. Tubulin tyrosination is required for the proper organization and pathfinding of the growth cone. During development, neuronal growth cones integrate diffusible and contact guidance cues that are conveyed to both actin and microtubule (MT) cytoskeletons and ensure axon outgrowth and pathfinding. Although several post-translational modifications of tubulin have been identified and despite their strong conservation among species, their physiological roles during development, especially in the nervous sytem, are still poorly understood.Here, we have dissected the role of a post-translational modification of the last amino acid of the alpha-tubulin on axonal growth by analyzing the phenotype of precerebellar neurons in Tubulin tyrosin ligase knock-out mice (TTL(-/-)) through in vivo, ex vivo and in vitro analyses. TTL(-/-) neurons are devoid of tyrosinated tubulin. Their pathway shows defects in vivo, ex vivo, in hindbrains open-book preparations or in vitro, in a collagen matrix. Their axons still orient toward tropic cues, but they emit supernumerary branches and their growth cones are enlarged and exhibit an emission of mis-oriented **filopodia**. Further analysis of the TTL(-/-) growth cone intracellular organization also reveals that the respective localization of actin and MT filaments is disturbed, with a decrease in the distal accumulation of Myosin IIB, as well as a concomitant Rac1 over-activation in the hindbrain. Pharmacological inhibition of Rac1 over-activation in TTL(-/-) neurons can rescue Myosin IIB localization.In the growth cone, we propose that tubulin tyrosination takes part in the relative arrangement of actin and MT cytoskeletons, in the regulation of small GTPases activity, and consequently, in the proper morphogenesis, organization and pathfinding of the growth cone during development.
9. Cdc42- and IRSp53-dependent contractile **filopodia** tether presumptive lens and retina to coordinate epithelial invagination. The vertebrate lens provides an excellent model with which to study the mechanisms required for epithelial invagination. In the mouse, the lens forms from the head surface ectoderm. A domain of ectoderm first thickens to form the lens placode and then invaginates to form the lens pit. The epithelium of the lens placode remains in close apposition to the epithelium of the presumptive retina as these structures undergo a coordinated invagination. Here, we show that F-actin-rich basal **filopodia** that link adjacent presumptive lens and retinal epithelia function as physical tethers that coordinate invagination. The **filopodia**, most of which originate in the presumptive lens, form at E9.5 when presumptive lens and retinal epithelia first come into close contact, and have retracted by E11.5 when invagination is complete. At E10.5--the lens pit stage--there is approximately one filopodium per epithelial cell. Formation of **filopodia** is dependent on the Rho family GTPase Cdc42 and the Cdc42 effector IRSp53 (Baiap2). Loss of **filopodia** results in reduced lens pit invagination. Pharmacological manipulation of the actin-myosin contraction pathway showed that the **filopodia** can respond rapidly in length to change inter-epithelial distance. These data suggest that the lens-retina inter-epithelial **filopodia** are a fine-tuning mechanism to assist in lens pit invagination by transmitting the forces between presumptive lens and retina. Although invagination of the archenteron in sea urchins and dorsal closure in Drosophila are known to be partly dependent on **filopodia**, this mechanism of morphogenesis has not previously been identified in vertebrates.
10. Regulation of cell protrusions by small GTPases during fusion of the neural folds. Epithelial fusion is a crucial process in embryonic development, and its failure underlies several clinically important birth defects. For example, failure of neural fold fusion during neurulation leads to open neural tube defects including spina bifida. Using mouse embryos, we show that cell protrusions emanating from the apposed neural fold tips, at the interface between the neuroepithelium and the surface ectoderm, are required for completion of neural tube closure. By genetically ablating the cytoskeletal regulators Rac1 or Cdc42 in the dorsal neuroepithelium, or in the surface ectoderm, we show that these protrusions originate from surface ectodermal cells and that Rac1 is necessary for the formation of membrane ruffles which typify late closure stages, whereas Cdc42 is required for the predominance of **filopodia** in early neurulation. This study provides evidence for the essential role and molecular regulation of membrane protrusions prior to fusion of a key organ primordium in mammalian development.
11. Regulation of cell protrusions by small GTPases during fusion of the neural folds. Epithelial fusion is a crucial process in embryonic development, and its failure underlies several clinically important birth defects. For example, failure of neural fold fusion during neurulation leads to open neural tube defects including spina bifida. Using mouse embryos, we show that cell protrusions emanating from the apposed neural fold tips, at the interface between the neuroepithelium and the surface ectoderm, are required for completion of neural tube closure. By genetically ablating the cytoskeletal regulators Rac1 or Cdc42 in the dorsal neuroepithelium, or in the surface ectoderm, we show that these protrusions originate from surface ectodermal cells and that Rac1 is necessary for the formation of membrane ruffles which typify late closure stages, whereas Cdc42 is required for the predominance of **filopodia** in early neurulation. This study provides evidence for the essential role and molecular regulation of membrane protrusions prior to fusion of a key organ primordium in mammalian development.
12. NRP1 Regulates CDC42 Activation to Promote **Filopodia** Formation in Endothelial Tip Cells. Sprouting blood vessels are led by **filopodia**-studded endothelial tip cells that respond to angiogenic signals. Mosaic lineage tracing previously revealed that NRP1 is essential for tip cell function, although its mechanistic role in tip cells remains poorly defined. Here, we show that NRP1 is dispensable for genetic tip cell identity. Instead, we find that NRP1 is essential to form the **filopodia**l bursts that distinguish tip cells morphologically from neighboring stalk cells, because it enables the extracellular matrix (ECM)-induced activation of CDC42, a key regulator of **filopodia** formation. Accordingly, NRP1 knockdown and pharmacological CDC42 inhibition similarly impaired **filopodia** formation in vitro and in developing zebrafish in vivo. During mouse retinal angiogenesis, CDC42 inhibition impaired tip cell and vascular network formation, causing defects that resembled those due to loss of ECM-induced, but not VEGF-induced, NRP1 signaling. We conclude that NRP1 enables ECM-induced **filopodia** formation for tip cell function during sprouting angiogenesis.
13. cIAP1 regulates TNF-mediated cdc42 activation and **filopodia** formation. Tumour necrosis factor-α (TNF) is a cytokine endowed with multiple functions, depending on the cellular and environmental context. TNF receptor engagement induces the formation of a multimolecular complex including the TNFR-associated factor TRAF2, the receptor-interaction protein kinase RIP1 and the cellular inhibitor of apoptosis cIAP1, the latter being essential for NF-κB activation. Here, we show that cIAP1 also regulates TNF-induced actin cytoskeleton reorganization through a cdc42-dependent, NF-κB-independent pathway. Deletion of cIAP1 prevents TNF-induc**ed filopo**dia and cdc42 activation. The expression of cIAP1 or its E3-ubiquitin ligase-defective mutant restores the ability of cIAP1(-/-) MEFs to produ**ce filopo**dia, whereas a cIAP1 mutant unable to bind TRAF2 does not. Accordingly, the silencing of TRAF2 inhibits TNF-mediat**ed filopo**dia formation, whereas silencing of RIP1 does not. cIAP1 directly binds cdc42 and promotes its RhoGDIα-mediated stabilization. TNF decreases cIAP1-cdc42 interaction, suggesting that TNF-induced recruitment of cIAP1/TRAF2 to the receptor releases cdc42, which in turn triggers actin remodeling. cIAP1 also regulates cdc42 activation in response to EGF and HRas-V12 expression. A downregulation of cIAP1 altered the cell polarization, the cell adhesion to endothelial cells and cell intercalation, which are cdc42-dependent processes. Finally, we demonstrated that the deletion of cIAP1 regulated the HRas-V12-mediated transformation process, including anchorage-dependent cell growth, tumour growth in a xenograft model and the development of experimental metastasis in the lung.
14. The cytotoxic necrotizing factor of Yersinia pseudotuberculosis (CNFY) enhances inflammation and Yop delivery during infection by activation of Rho GTPases. Some isolates of Yersinia pseudotuberculosis produce the cytotoxic necrotizing factor (CNFY), but the functional consequences of this toxin for host-pathogen interactions during the infection are unknown. In the present study we show that CNFY has a strong influence on virulence. We demonstrate that the CNFY toxin is thermo-regulated and highly expressed in all colonized lymphatic tissues and organs of orally infected mice. Most strikingly, we found that a cnfY knock-out variant of a naturally toxin-expressing Y. pseudotuberculosis isolate is strongly impaired in its ability to disseminate into the mesenteric lymph nodes, liver and spleen, and has fully lost its lethality. The CNFY toxin contributes significantly to the induction of acute inflammatory responses and to the formation of necrotic areas in infected tissues. The analysis of the host immune response demonstrated that presence of CNFY leads to a strong reduction of professional phagocytes and natural killer cells in particular in the spleen, whereas loss of the toxin allows efficient tissue infiltration of these immune cells and rapid killing of the pathogen. Addition of purified CNFY triggers formation of actin-rich membrane ruffles and **filopodia**, which correlates with the activation of the Rho GTPases, RhoA, Rac1 and Cdc42. The analysis of type III effector delivery into epithelial and immune cells in vitro and during the course of the infection further demonstrated that CNFY enhances the Yop translocation process and supports a role for the toxin in the suppression of the antibacterial host response. In summary, we highlight the importance of CNFY for pathogenicity by showing that this toxin modulates inflammatory responses, protects the bacteria from attacks of innate immune effectors and enhances the severity of a Yersinia infection.
15. Cdc42- and IRSp53-dependent contractile **filopodia** tether presumptive lens and retina to coordinate epithelial invagination. The vertebrate lens provides an excellent model with which to study the mechanisms required for epithelial invagination. In the mouse, the lens forms from the head surface ectoderm. A domain of ectoderm first thickens to form the lens placode and then invaginates to form the lens pit. The epithelium of the lens placode remains in close apposition to the epithelium of the presumptive retina as these structures undergo a coordinated invagination. Here, we show that F-actin-rich basal **filopodia** that link adjacent presumptive lens and retinal epithelia function as physical tethers that coordinate invagination. The **filopodia**, most of which originate in the presumptive lens, form at E9.5 when presumptive lens and retinal epithelia first come into close contact, and have retracted by E11.5 when invagination is complete. At E10.5--the lens pit stage--there is approximately one filopodium per epithelial cell. Formation of **filopodia** is dependent on the Rho family GTPase Cdc42 and the Cdc42 effector IRSp53 (Baiap2). Loss of **filopodia** results in reduced lens pit invagination. Pharmacological manipulation of the actin-myosin contraction pathway showed that the **filopodia** can respond rapidly in length to change inter-epithelial distance. These data suggest that the lens-retina inter-epithelial **filopodia** are a fine-tuning mechanism to assist in lens pit invagination by transmitting the forces between presumptive lens and retina. Although invagination of the archenteron in sea urchins and dorsal closure in Drosophila are known to be partly dependent on **filopodia**, this mechanism of morphogenesis has not previously been identified in vertebrates.
16. Laminin alpha5 is required for dental epithelium growth and polarity and the development of tooth bud and shape. In tooth development, the oral ectoderm and mesenchyme coordinately and reciprocally interact through the basement membrane for their growth and differentiation to form the proper shape and size of the tooth. Laminin alpha5 subunit-containing laminin-10/11 (LM-511/521) is the major laminin in the tooth germ basement membrane. Here, we have examined the role of laminin alpha5 (Lama5) in tooth development using laminin alpha5-null mouse primary dental epithelium and tooth germ organ cultures. Lama5-null mice develop a small tooth germ with defective cusp formation and have reduced proliferation of dental epithelium. Also, cell polarity and formation of the monolayer of the inner dental epithelium are disturbed. The enamel knot, a signaling center for tooth germ development, is defective, and there is a significant reduction of Shh and Fgf4 expression in the dental epithelium. In the absence of laminin alpha5, the basement membrane in the inner dental epithelium becomes discontinuous. In normal mice, integrin alpha6beta4, a receptor for laminin alpha5, is strongly localized at the basal layer of the epithelium, whereas in mutant mice, integrin alpha6beta4 is expressed around the cell surface. In primary dental epithelium culture, laminin-10/11 promotes cell growth, spreading, and **filopodia**-like microspike formation. This promotion is inhibited by anti-integrin alpha6 and beta4 antibodies and by phosphatidylinositol 3-kinase inhibitors and dominant negative Rho-GTPase family proteins Cdc42 and Rac. In organ culture, anti-integrin alpha6 antibody and wortmannin reduce tooth germ size and shape. Our studies demonstrate that laminin alpha5 is required for the proliferation and polarity of basal epithelial cells and suggest that the interaction between laminin-10/11-integrin alpha6beta4 and the phosphatidylinositol 3-kinase-Cdc42/Rac pathways play an important role in determining the size and shape of tooth germ.
17. A Rac/Cdc42-specific exchange factor, GEFT, induces cell proliferation, transformation, and migration. The Rho family of small GTPases, including Rho, Rac, and Cdc42, play essential roles in diverse cellular functions. The ability of Rho family GTPases to participate in signaling events is determined by the ratio of inactive (GDP-bound) and active (GTP-bound) forms in the cell. The activation of Rho family proteins requires the exchange of bound GDP for GTP, a process catalyzed by the Dbl family of guanine nucleotide exchange factors (GEFs). The GEFs have high affinity for the guanine nucleotide-free state of the GTPases and are thought to promote GDP release by stabilizing an intermediate transition state. In this study, we have identified and characterized a new Rac/Cdc42-specific Dbl family guanine nucleotide exchange factor, named GEFT. GEFT is highly expressed in the excitable tissues, including brain, heart, and muscle. Low or very little expression was detected in other nonexcitable tissues. GEFT has specific exchange activity for Rac and Cdc42 in our in vitro GTPase exchange assays and glutathione S-transferase-PAK pull-down assays with GTP-bound Rac1 and Cdc42. Overexpression of GEFT leads to changes in cell morphology and actin cytoskeleton re-organization, including the formation of membrane microspikes, **filopodia**, and lamilliopodia. Furthermore, expression of GEFT in NIH3T3 cells promotes foci formation, cell proliferation, and cell migration, possibly through the activation of transcriptional factors involved in cell growth and proliferation. Together, our data suggest that GEFT is a Rac/Cdc42-specific GEF protein that regulates cell morphology, cell proliferation, and transformation.
18. Hydrogen peroxide formation and actin filament reorganization by Cdc42 are essential for ethanol-induced in vitro angiogenesis. This report focuses on the identification of the molecular mechanisms of ethanol-induced in vitro angiogenesis. The manipulation of angiogenesis is an important therapeutic approach for the treatment of cancer, cardiovascular diseases, and chronic inflammation. Our results showed that ethanol stimulation altered the integrity of actin filaments and increased the formation of lamellipodia and **filopodia** in SVEC4-10 cells. Further experiments demonstrated that ethanol stimulation increased cell migration and invasion and induced in vitro angiogenesis in SVEC4-10 cells. Mechanistically, ethanol stimulation activated Cdc42 and produced H(2)O(2) a reactive oxygen species intermediate in SVEC4-10 cells. Measuring the time course of Cdc42 activation and H(2)O(2) production upon ethanol stimulation revealed that the Cdc42 activation and the increase of H(2)O(2) lasted more than 3 h, which indicates the mechanisms of the long duration effects of ethanol on the cells. Furthermore, either overexpression of a constitutive dominant negative Cdc42 or inhibition of H(2)O(2) production abrogated the effects of ethanol on SVEC4-10 cells, indicating that both the activation of Cdc42 and the production of H(2)O(2) are essential for the actions of ethanol. Interestingly, we also found that overexpression of a constitutive dominant positive Cdc42 itself was sufficient to produce H(2)O(2) and to induce in vitro angiogenesis. Taken together, our results suggest that ethanol stimulation can induce H(2)O(2) production through the activation of Cdc42, which results in reorganizing actin filaments and increasing cell motility and in vitro angiogenesis.
19. p21-activated kinase 2 regulates HSPC cytoskeleton, migration, and homing via CDC42 activation and interaction with β-Pix. Cytoskeletal remodeling of hematopoietic stem and progenitor cells (HSPCs) is essential for homing to the bone marrow (BM). The Ras-related C3 botulinum toxin substrate (Rac)/cell division control protein 42 homolog (CDC42) effector p21-activated kinase (Pak2) has been implicated in HSPC homing and engraftment. However, the molecular pathways mediating Pak2 functions in HSPCs are unknown. Here, we demonstrate that both Pak2 kinase activity and its interaction with the PAK-interacting exchange factor-β (β-Pix) are required to reconstitute defective ITALIC! Pak2 (ITALIC! Δ/Δ)HSPC homing to the BM. Pak2 serine/threonine kinase activity is required for stromal-derived factor-1 (SDF1α) chemokine-induced HSPC directional migration, whereas Pak2 interaction with β-Pix is required to regulate the velocity of HSPC migration and precise F-actin assembly. Lack of SDF1α-**induced f**ilopodia and associated abnormal cell protrusions seen in ITALIC! Pak2 (ITALIC! Δ/Δ)HSPCs were rescued by wild-type (WT) Pak2 but not by a Pak2-kinase dead mutant (KD). Expression of a β-Pix interaction-defective mutant of Pa**k2 rescue**d filopodia formation but led to abnormal F-actin bundles. Although CDC42 has previously been considered an upstream regulator of Pak2, we found a paradoxical decrease in baseline activation of CDC42 in ITALIC! Pak2 (ITALIC! Δ/Δ)HSPCs, which was rescued by expression of Pak2-WT but not by Pak2-KD; defective homing of ITALIC! Pak2-deleted HSPCs was rescued by constitutive active CDC42. These data demonstrate that both Pak2 kinase activity and its interaction with β-Pix are essen**tial for** HSPC filopodia formation, cytoskeletal integrity, and homing via activation of CDC42. Taken together, we provide mechanistic insights into the role of Pak2 in HSPC migration and homing.
20. Regulation of Cdc42-mediated morphological effects: a novel function for p53. The tumour suppressor functions of p53 that are important for its activity depend on its role as a cell cycle arrest mediator and apoptosis inducer. Here we identify a novel function for p53 in regulating cell morphology and movement. We investigated the overall effect of p53 on morphological changes induced by RhoA, Rac1 and Cdc42 GTPases in mouse embryonic fibroblasts (MEFs). Interestingly, p53 exerted a selective effect on Cdc42-mediated cell functions. (i) Both overexpression of wild-type p53 and activation of endogenous p53 counteracted Cdc42-induced **filopodia** formation. Conversely, p53-deficient MEFs exhibited constitutive membrane **filopodia**. Mechanistic studies indicate that p53 prevents the initiating steps of **filopodia** formation downstream of Cdc42. (ii) Over expression of p53 modulates cell spreading of MEFs on fibronectin. (iii) During cell migration, the reorientation of the Golgi apparatus in the direction of movement is abolished by wild-type p53 expression, thus preventing cell polarity. Our data demonstrate a previously uncharacterized role for p53 in regulating Cdc42-dependent cell effects that control actin cytoskeletal dynamics and cell movement. This novel function may contribute to p53 anti-tumour activity.
21. Cdc42 and Gsk3 modulate the dynamics of radial glial growth, inter-radial glial interactions and polarity in the developing cerebral cortex. Polarized radial glia are crucial to the formation of the cerebral cortex. They serve as neural progenitors and as guides for neuronal placement in the developing cerebral cortex. The maintenance of polarized morphology is essential for radial glial functions, but the extent to which the polarized radial glial scaffold is static or dynamic during corticogenesis remains an open question. The developmental dynamics of radial glial morphology, inter-radial glial interactions during corticogenesis, and the role of the cell polarity complexes in these activities remain undefined. Here, using real-time imaging of cohorts of mouse radial glia cells, we show that the radial glial scaffold, upon which the cortex is constructed, is highly dynamic. Radial glial cells within the scaffold constantly interact with one another. These interactions are mediated by growth cone-like endfeet and **filopodia**-like protrusions. Polarized expression of the cell polarity regulator Cdc42 in radial glia regulates glial endfeet activities and inter-radial glial interactions. Furthermore, appropriate regulation of Gsk3 activity is required to maintain the overall polarity of the radial glia scaffold. These findings reveal dynamism and interactions among radial glia that appear to be crucial contributors to the formation of the cerebral cortex. Related cell polarity determinants (Cdc42, Gsk3) differentially influence radial glial activities within the evolving radial glia scaffold to coordinate the formation of cerebral cortex.
22. A role for Cdc42 in macrophage chemotaxis. Three members of the Rho family, Cdc42, Rac, and Rho are known to regulate the organization of actin-based cytoskeletal structures. In Bac1.2F5 macrophages, we have shown that Rho regulates cell contraction, whereas Rac and Cdc42 regulate the formation of lamellipodia and **filopodia**, respectively. We have now tested the roles of Cdc42, Rac, and Rho in colony stimulating factor-1 (CSF-1)-induced macrophage migration and chemotaxis using the Dunn chemotaxis chamber. Microinjection of constitutively activated RhoA, Rac1, or Cdc42 inhibited cell migration, presumably because the cells were unable to polarize significantly in response to CSF-1. Both Rho and Rac were required for CSF-1-induced migration, since migration speed was reduced to background levels in cells injected with C3 transferase, an inhibitor of Rho, or with the dominant-negative Rac mutant, N17Rac1. In contrast, cells injected with the dominant-negative Cdc42 mutant, N17Cdc42, were able to migrate but did not polarize in the direction of the gradient, and chemotaxis towards CSF-1 was abolished. We conclude that Rho and Rac are required for the process of cell migration, whereas Cdc42 is required for cells to respond to a gradient of CSF-1 but is not essential for cell locomotion.
23. Gene targeting of Cdc42 and Cdc42GAP affirms the critical involvement of Cdc42 in **filopodia** induction, directed migration, and proliferation in primary mouse embryonic fibroblasts. Recent studies in Cdc42 knockout mouse embryonic stem (ES) cells and ES-derived fibroblastoid cell lines raise concern on a body of literature derived by dominant mutant expression approach in a variety of cell lines implicating mammalian Cdc42 as a key regulator of **filopodia** induction, directional migration and cell cycle progression. To resolve the physiological function of mammalian Cdc42, we have characterized the Cdc42(-/-) and Cdc42GAP(-/-) primary mouse embryonic fibroblasts (MEFs) produced by gene targeting as the Cdc42 loss- or gain-of-activity cell model. The Cdc42(-/-) cells were defective in **filopodia** formation stimulated by bradykinin and in dorsal membrane ruffling stimulated by PDGF, whereas the Cdc42GAP(-/-) cells displayed spontaneous **filopodia**. The Cdc42 loss- or gain-of-activity cells were defective in adhesion to fibronectin, wound-healing, polarity establishment, and migration toward a serum gradient. These defects were associated with deficiencies of PAK1, GSK3beta, myosin light chain, and FAK phosphorylation. Furthermore, Cdc42(-/-) cells were defective in G1/S-phase transition and survival, correlating with deficient NF-kappaB transcription and defective JNK, p70 S6K, and ERK1/2 activation. These results demonstrate a different requirement of Cdc42 activity in primary MEFs from ES or ES-derived clonal fibroblastoid cells and suggest that Cdc42 plays cell-type-specific signaling roles.
24. Influencing cellular transformation by modulating the rates of GTP hydrolysis by Cdc42. The small GTPase Cdc42 has been implicated in a number of cellular responses ranging from the regulation of the actin cytoskeletal architecture to intracellular trafficking and cell cycle progression. Cdc42 mutants that constitutively exchange GDP for GTP but still hydrolyze GTP (called 'fast-cycling' mutants) promote cellular transformation, whereas Cdc42 mutants that are unable to hydrolyze GTP and are irreversibly trapped in the GTP-bound state often inhibit cell growth. In this work, we have set out to further establish that Cdc42 needs to cycle between its 'on' and 'off' states to stimulate cell growth, by examining the consequences of manipulating its GTP-binding/GTP hydrolytic cycle in two different ways. One approach was to examine whether substitutions that act in a manner opposite to the 'fast cyclers', and extend the lifetime of the activated GTP-bound state by slowing the GTP hydrolytic reaction (i.e., 'slow-cycling' mutations), positively influence cell growth. Indeed we show that one such slow-cycling mutant, Cdc42[Y32A], which is insensitive to Cdc42GAP but still exhibits a measurable intrinsic GTP hydrolytic activity, gives rise to increased levels of activated Cdc42 in NIH 3T3 cells. We go on to show that the Y32A mutant stimulates the actin cytoskeletal changes that lead to **filopodia** formation, confer growth advantages to fibroblasts under low serum conditions, and enable cells to grow to high densities when exposed to normal levels of serum. The second approach was to determine whether the transforming activity of the fast-cycling Cdc42[F28L] mutant can be reversed by compensating for its accelerated nucleotide exchange reaction through the expression of the GTPase-activating protein (Cdc42GAP) and the ensuing stimulation of GTP hydrolytic activity. We showed that expression of the limit functional domain of Cdc42GAP inhibited Cdc42[F28L]-induced transformation, as well as selectively reversed the transformed phenotypes caused by the hyperactivation of wild-type Cdc42 in cells expressing the oncogenic version of Dbl (for Diffuse B cell lymphoma), a guanine nucleotide exchange factor for Cdc42 and the related Rac and Rho GTPases. Overall, the results reported here establish the requirement for Cdc42 to cycle between its signaling-on and -off states in order to positively influence cell growth and highlight how the Cdc42GAP can play an important role in regulating cell proliferation.
25. Multiple alterations of platelet functions dominated by increased secretion in mice lacking Cdc42 in platelets. Platelet activation at sites of vascular injury is crucial for hemostasis, but it may also cause myocardial infarction or stroke. Cytoskeletal reorganization is essential for platelet activation and secretion. The small GTPase Cdc42 has been implicated as an important mediator of **filopodia** formation and exocytosis in various cell types, but its exact function in platelets is not established. Here, we show that the megakaryocyte/platelet-specific loss of Cdc42 leads to mild thrombocytopenia and a small increase in platelet size in mice. Unexpectedly, Cdc42-deficient platelets were able to form normally shaped **filopodia** and spread fully on fibrinogen upon activation, whereas **filopodia** formation upon selective induction of GPIb signaling was reduced compared with wild-type platelets. Furthermore, Cdc42-deficient platelets showed enhanced secretion of alpha granules, a higher adenosine diphosphate (ADP)/adenosine triphosphate (ATP) content, increased aggregation at low agonist concentrations, and enhanced aggregate formation on collagen under flow. In vivo, lack of Cdc42 resulted in faster occlusion of ferric chloride-injured arterioles. The life span of Cdc42-deficient platelets was markedly reduced, suggesting increased clearing of the cells under physiologic conditions. These data point to novel multiple functions of Cdc42 in the regulation of platelet activation, granule organization, degranulation, and a specific role in GPIb signaling.
26. SNX26, a GTPase-activating protein for Cdc42, interacts with PSD-95 protein and is involved in activity-dependent dendritic spine formation in mature neurons. SNX26, a brain-enriched RhoGAP, plays a key role in dendritic arborization during early neuronal development in the neocortex. In mature neurons, it is localized to dendritic spines, but little is known about its role in later stages of development. Our results show that SNX26 interacts with PSD-95 in dendritic spines of cultured hippocampal neurons, and as a GTPase-activating protein for Cdc42, it decreased the F-actin content in COS-7 cells and in dendritic spines of neurons. Overexpression of SNX26 resulted in a GTPase-activating protein activity-dependent decrease in total protrusions and spine density together with dramatic inhibition of **filopodia**-to-spine transformations. Such effects of SNX26 were largely rescued by a constitutively active mutant of Cdc42. Consistently, an shRNA-mediated knockdown of SNX26 significantly increased total protrusions and spine density, resulting in an increase in thin or stubby type spines at the expense of the mushroom spine type. Moreover, endogenous expression of SNX26 was shown to be bi-directionally modulated by neuronal activity. Therefore, we propose that in addition to its key role in neuronal development, SNX26 also has a role in the activity-dependent structural change of dendritic spines in mature neurons.
27. Dual lipidation of the brain-specific Cdc42 isoform regulates its functional properties. Cdc42 (cell division cycle 42) is a member of the Rho GTPase family which regulates a variety of cellular activities by controlling actin cytoskeleton and gene expression. Cdc42 is expressed in the form of two splice variants. The canonical Cdc42 isoform is prenylated (Cdc42-prenyl), whereas the brainspecific isoform can be palmitoylated (Cdc42-palm). In the present study we have demonstrated palmitoylation of endogenous Cdc42 in rodent and human brains and identified Cys(188) and Cys(189) as acylation sites of Cdc42-palm. Moreover, we have shown that Cys(188) can also be prenylated. Analysis of acylation-deficient mutants revealed that lipidation of Cys(188) is essential for proper membrane binding of Cdc42-palm as well as for Cdc42-mediated regulation of gene transcription and induction of densely packed **filopodia** in neuroblastoma cells. We also found that Cdc42-prenyl is a dominant splice variant in a wide range of commonly used cell lines as well as in the cerebellum, whereas Cdc42-palm is the main Cdc42 isoform in hippocampus, where it is critically involved in the formation of dendritic **filopodia** and spines. Replacement of endogenous Cdc42 by its acylation-deficient mutants revealed the importance of Cdc42-palm lipidation for its morphogenic and synaptogenic effects in neurons. These findings demonstrate that dual lipidation of Cdc42-palm represents an important regulator of morphogenic signalling in hippocampal neurons.
28. Cdc42 is required for cytoskeletal support of endothelial cell adhesion during blood vessel formation in mice. The Rho family of small GTPases has been shown to be required in endothelial cells (ECs) during blood vessel formation. However, the underlying cellular events controlled by different GTPases remain unclear. Here, we assess the cellular mechanisms by which Cdc42 regulates mammalian vascular morphogenesis and maintenance. In vivo deletion of Cdc42 in embryonic ECs (Cdc42(Tie2KO)) results in blocked lumen formation and endothelial tearing, leading to lethality of mutant embryos by E9-10 due to failed blood circulation. Similarly, inducible deletion of Cdc42 (Cdc42(Cad5KO)) at mid-gestation blocks angiogenic tubulogenesis. By contrast, deletion of Cdc42 in postnatal retinal vessels leads to aberrant vascular remodeling and sprouting, as well as markedly reduced **filopodia** formation. We find that Cdc42 is essential for organization of EC adhesion, as its loss results in disorganized cell-cell junctions and reduced focal adhesions. Endothelial polarity is also rapidly lost upon Cdc42 deletion, as seen by failed localization of apical podocalyxin (PODXL) and basal actin. We link observed failures to a defect in F-actin organization, both in vitro and in vivo, which secondarily impairs EC adhesion and polarity. We also identify Cdc42 effectors Pak2/4 and N-WASP, as well as the actomyosin machinery, to be crucial for EC actin organization. This work supports the notion of Cdc42 as a central regulator of the cellular machinery in ECs that drives blood vessel formation.
29. Vav2 as a Rac-GDP/GTP exchange factor responsible for the nectin-induced, c-Src- and Cdc42-mediated activation of Rac. Nectins are Ca2+-independent immunoglobulin-like cell-cell adhesion molecules that form homo- and hetero-trans-dimers (trans-interactions). Nectins first form cell-cell contact and then recruit cadherins to the nectin-based cell-cell contact sites to form adherens junctions cooperatively with cadherins. In addition, the trans-interactions of nectins induce the activation of Cdc42 and Rac small G proteins, which enhances the formation of adherens junctions by forming **filopodia** and lamellipodia, respectively. The trans-interactions of nectins first recruit and activate c-Src at the nectin-based cell-cell contact sites. c-Src then phosphorylates and activates FRG, a Cdc42-GDP/GTP exchange factor (GEF) for Cdc42. The activation of both c-Src and Cdc42 by FRG is necessary for the activation of Rac, but the Rac-GEF responsible for this activation of Rac remains unknown. We showed here that the nectin-induced activation of Rac was inhibited by a dominant negative mutant of Vav2, a Rac-GEF. Nectins recruited and tyrosine-phosphorylated Vav2 through c-Src at the nectin-based cell-cell contact sites, whereas Cdc42 was not necessary for the nectin-induced recruitment of Vav2 or the nectin-induced, c-Src-mediated tyrosine phosphorylation of Vav2. Cdc42 activated through c-Src then enhanced the GEF activity of tyrosine-phosphorylated Vav2 on Rac1. These results indicate that Vav2 is a GEF responsible for the nectin-induced, c-Src-, and Cdc42-mediated activation of Rac.
30. Daphnetin inhibits invasion and migration of LM8 murine osteosarcoma cells by decreasing RhoA and Cdc42 expression. Daphnetin, 7,8-dihydroxycoumarin, present in main constituents of Daphne odora var. marginatai, has multiple pharmacological activities including anti-proliferative effects in cancer cells. In this study, using a Transwell system, we showed that daphnetin inhibited invasion and migration of highly metastatic murine osteosarcoma LM8 cells in a dose-dependent manner. Following treatment by daphnetin, cells that penetrated the Transwell membrane were rounder than non-treated cells. Immunofluorescence analysis revealed that daphnetin decreased the numbers of intracellular stress fibers and **filopodia**. Moreover, daphnetin treatment dramatically decreased the expression levels of RhoA and Cdc42. In summary, the dihydroxycoumarin derivative daphnetin inhibits the invasion and migration of LM8 cells, and therefore represents a promising agent for use against metastatic cancer.
31. Cdc42 regulates arsenic-induced NADPH oxidase activation and cell migration through actin filament reorganization. Although arsenic is a human carcinogen, the molecular mechanisms of its action remain to be understood. The present study reports that exposure to arsenic induced actin filament reorganization, resulting in lamellipodia and **filopodia** structures through the activation of Cdc42 in SVEC4-10 endothelial cells. It was also found that arsenic induced the formation of the superoxide anion (O2\*) in SVEC4-10 cells. Immunoprecipitation and Western blotting analysis demonstrated that arsenic stimulation induced serine phosphorylation of p47phox, a key component of NADPH oxidase, indicating that arsenic induces O2\* formation through NADPH oxidase activation. Inhibition of arsenic-induced actin filament reorganization by either overexpression of a dominant negative Cdc42 or pretreatment of an actin filament stabilizing regent, jasplakinolide, abrogated arsenic-induced NADPH oxidase activation, showing that the activation of NADPH oxidase was regulated by Cdc42-mediated actin filament reorganization. This study also showed that overexpression of a dominant negative Rac1 was sufficient to abolish arsenic-induced O2\*- production, implying that Rac1 activities are required for Cdc42-mediated NADPH oxidase activation in response to arsenic stimulation. Furthermore, arsenic stimulation induced cell migration, which can be inhibited by the inactivation of either Cdc42 or NADPH oxidase. Taken together, the results indicate that arsenic is able to activate NADPH oxidase through Cdc42-mediated actin filament reorganization, leading to the induction of an increase in cell migration in SVEC4-10 endothelial cells.
32. Reelin signals through apolipoprotein E receptor 2 and Cdc42 to increase growth cone motility and **filopodia** formation. Lipoprotein receptor signaling regulates the positioning and differentiation of postmitotic neurons during development and modulates neuronal plasticity in the mature brain. Depending on the contextual situation, the lipoprotein receptor ligand Reelin can have opposing effects on cortical neurons. We show that Reelin increases growth cone motility and **filopodia** formation, and identify the underlying signaling cascade. Reelin activates the Rho GTPase Cdc42, known for its role in neuronal morphogenesis and directed migration, in an apolipoprotein E receptor 2-, Disabled-1-, and phosphatidylinositol 3-kinase-dependent manner. We demonstrate that neuronal vesicle trafficking, a Cdc42-controlled process, is increased after Reelin treatment and further provide evidence that the peptidergic VIP/PACAP38 system and Reelin can functionally interact to promote axonal branching. In conclusion, Reelin-induced activation of Cdc42 contributes to the regulation of the cytoskeleton of individual responsive neurons and converges with other signaling cascades to orchestrate Rho GTPase activity and promote neuronal development. Our data link the observation that defects in Rho GTPases and Reelin signaling are responsible for developmental defects leading to neurological and psychiatric disorders.
33. Cdc42 regulates cofilin during the establishment of neuronal polarity. The establishment of polarity is an essential process in early neuronal development. Although a number of molecules controlling neuronal polarity have been identified, genetic evidence about their physiological roles in this process is mostly lacking. We analyzed the consequences of loss of Cdc42, a central regulator of polarity in multiple systems, on the polarization of mammalian neurons. Genetic ablation of Cdc42 in the brain led to multiple abnormalities, including striking defects in the formation of axonal tracts. Neurons from the Cdc42 null animals sprouted neurites but had a strongly suppressed ability to form axons both in vivo and in culture. This was accompanied by disrupted cytoskeletal organization, enlargement of the growth cones, and inhibition of **filopodia**l dynamics. Axon formation in the knock-out neurons was rescued by manipulation of the actin cytoskeleton, indicating that the effects of Cdc42 ablation are exerted through modulation of actin dynamics. In addition, the knock-outs showed a specific increase in the phosphorylation (inactivation) of the Cdc42 effector cofilin. Furthermore, the active, nonphosphorylated form of cofilin was enriched in the axonal growth cones of wild-type, but not of mutant, neurons. Importantly, cofilin knockdown resulted in polarity defects quantitatively analogous to the ones seen after Cdc42 ablation. We conclude that Cdc42 is a key regulator of axon specification, and that cofilin is a physiological downstream effector of Cdc42 in this process.
34. Two novel human NUMB isoforms provide a potential link between development and cancer. We previously identified four functionally distinct human NUMB isoforms. Here, we report the identification of two additional isoforms and propose a link between the expression of these isoforms and cancer. These novel isoforms, NUMB5 and NUMB6, lack exon 10 and are expressed in cells known for polarity and migratory behavior, such as human amniotic fluid cells, glioblastoma and metastatic tumor cells. RT-PCR and luciferase assays demonstrate that NUMB5 and NUMB6 are less antagonistic to NOTCH signaling than other NUMB isoforms. Immunocytochemistry analyses show that NUMB5 and NUMB6 interact and complex with CDC42, vimentin and the CDC42 regulator IQGAP1 (IQ (motif) GTPase activating protein 1). Furthermore, the ectopic expression of NUMB5 and NUMB6 induces the formation of lamellipodia (NUMB5) and **filopodia** (NUMB6) in a CDC42- and RAC1-dependent manner. These results are complemented by in vitro and in vivo studies, demonstrating that NUMB5 and NUMB6 alter the migratory behavior of cells. Together, these novel isoforms may play a role in further understanding the NUMB function in development and cancer.
35. ROCK/Cdc42-mediated microglial motility and gliapse formation lead to phagocytosis of degenerating dopaminergic neurons in vivo. The role of microglial motility in the context of adult neurodegeneration is poorly understood. In the present work, we investigated the microanatomical details of microglia-neuron interactions in an experimental mouse model of Parkinson's disease following the intraperitoneal injection of MPTP. The specific intoxication of dopaminergic neurons induces the cellular polarization of microglia, leading to the formation of body-to-body neuron-glia contacts, called gliapses, which precede neuron elimination. Inhibiting ROCK/Cdc42-mediated microglial motility in vivo blocks the activating features of microglia, such as increased cell size and number of **filopodia** and diminishes their phagocyting/secreting domains, as the reduction of the Golgi apparatus and the number of microglia-neuron contacts has shown. High-resolution confocal images and three-dimensional rendering demonstrate that microglia engulf entire neurons at one-to-one ratio, and the microglial cell body participates in the formation of the phagocytic cup, engulfing and eliminating neurons in areas of dopaminergic degeneration in adult mammals.
36. Age-related guanine nucleotide exchange factor, mouse Zizimin2, induces **filopodia** in bone marrow-derived dendritic cells. We recently isolated and identified Zizimin2 as a functional factor that is highly expressed in murine splenic germinal center B cells after immunization with T-cell-dependent antigen. Zizimin2 was revealed to be a new family member of Dock (dedicator of cytokinesis), Dock11, which is the guanine nucleotide exchange factor for Cdc42, a low-molecular-weight GTPase. However, the molecular function of Zizimin2 in acquired immunity has not been elucidated.In this study, we show that the protein expression of Zizimin2, which is also restricted to lymphoid tissues and lymphocytes, is reduced in aged mice. Over-expression of full-length Zizimin2 induced **filopodia**l formation in 293T cells, whereas expression of CZH2 domain inhibited it. Stimulation of Fcγ receptor and Toll-like receptor 4 triggered Zizimin2 up-regulation and Cdc42 activation in bone marrow-derived dendritic cells.These data suggest that Zizimin2 is an immune-related and age-regulated guanine nucleotide exchange factor, which facilitates **filopodi**al formation through activation of Cdc42, which results in activation of cell migration.
37. Rif-mDia1 interaction is involved in filopodium formation independent of Cdc42 and Rac effectors. **Filopodia** are cellular protrusions important for axon guidance, embryonic development, and wound healing. The Rho GTPase Cdc42 is the best studied inducer of filopodium formation, and several of its effectors and their interacting partners have been linked to the process. These include IRSp53, N-WASP, Mena, and Eps8. The Rho GTPase, Rif, also drives filopodium formation. The signaling pathway by which Rif induces **filopodia** is poorly understood, with mDia2 being the only protein implicated to date. It is thus not clear how distinct the Rif-driven pathway for filopodium formation is from the one mediated by Cdc42. In this study, we characterize the dynamics of Rif-induced **filopodia** by time lapse imaging of live neuronal cells and show that Rif drives filopodium formation via an independent pathway that does not involve the Cdc42 effectors N-WASP and IRSp53, the IRSp53 binding partner Mena, or the Rac effectors WAVE1 and WAVE2. Rif formed **filopodia** in the absence of N-WASP or Mena and when IRSp53, WAVE1, or WAVE2 was knocked down by RNAi. Rif-mediated **filopodia**l protrusion was instead reduced by silencing mDia1 expression or overexpressing a dominant negative mutant of mDia1. mDia1 on its own was able to form **filopodia**. Data from acceptor photobleaching FRET studies of protein-protein interaction demonstrate that Rif interacts directly with mDia1 in **filopodia** but not with mDia2. Taken together, these results suggest a novel pathway for **filopodia** formation via Rif and mDia1.
38. CTL-associated antigen-4 ligation induces rapid T cell polarization that depends on phosphatidylinositol 3-kinase, Vav-1, Cdc42, and myosin light chain kinase. CTLA-4 can negatively regulate cytokine production and proliferation, increase motility, and override the TCR-induced stop-signal needed for stable T cell-APC conjugation. Despite this, little is known regarding whether CTLA-4 can alter T cell morphology and the nature of the signaling events that could account for this event. In this study, we demonstrate that anti-CTLA-4 and CD3/CTLA-4 induce rapid T cell polarization (i.e., within 15-30 min) with increases in lamellipodia, **filopodia**, and uropod formation. This was observed with anti-CTLA-4 and CD80-Ig ligation of CTLA-4, but not with anti-CD3 alone, or anti-CD3/CD28 coligation. Polarization required PI3K, the guanine nucleotide exchange factor Vav1, the GTP-binding protein Cdc42, as well as myosin L chain kinase. By contrast, a key downstream target of PI3K, protein kinase B, as well as Rho kinase and RhoA, were not needed. Our results demonstrate that CTLA-4 is a potent activator T cell polarization needed for motility, and this process involves specific set of signaling proteins that might contribute to coreceptor regulation of T cell function.
39. Disruption of the Diaphanous-related formin Drf1 gene encoding mDia1 reveals a role for Drf3 as an effector for Cdc42. Mammalian Diaphanous-related formins (Drfs) act as Rho small GTPase effectors during growth factor-induced cytoskeletal remodeling and cell division. While both p140 mDia1 (herein called Drf1) and p134 mDia2 (Drf3) have been shown to bind in vitro to activated RhoA-C, and Drf3 has also been shown to bind to Cdc42, little is known about the cellular function of these GTPase effector pairs. Thus, we have begun targeting the murine Drf genes to address their various contributions to small GTPase signaling in cytoskeletal remodeling and development.Drf1 +/+, +/-, and -/- cell lines were derived from embryonic stem cells. While some Drf1 +/- lines had fewer actin stress fibers, several Drf1 +/- and -/- cells were more motile and had more abundant lamella and **filopodia**. Because the apparent "gain-of-function" corresponded with elevated levels of Drf3 protein expression, we hypothesized that the effects on the actin cytoskeleton were due to Cdc42 utilization of Drf3 as an effector. In this study, we found that inactive Drf3 variants and microinjected Drf3 antibodies interfered with Cdc42-induced **filopodia**. In addition, we observed that Drf3 contains a previously unidentified CRIB-like motif within its GTPase binding domain (GBD). By fluorescent resonance energy transfer (FRET) analysis, we demonstrate that this motif is required for Cdc42 binding and Drf3 recruitment to the leading edge and, surprisingly, to the microtubule organizing center (MTOC) of migrating fibroblasts.Our observations extend the role of the mammalian Drfs in cell signaling and demonstrate that Cdc42 not only activates Drf3, but guides the effector to sites at the cell cortex where it remodels the actin cytoskeleton.
40. CDC42 switches IRSp53 from inhibition of actin growth to elongation by clustering of VASP. **Filopodia** explore the environment, sensing soluble and mechanical cues during directional motility and tissue morphogenesis. How **filopodia** are initiated and spatially restricted to specific sites on the plasma membrane is still unclear. Here, we show that the membrane deforming and curvature sensing IRSp53 (Insulin Receptor Substrate of 53 kDa) protein slows down actin filament barbed end growth. This inhibition is relieved by CDC42 and counteracted by VASP, which also binds to IRSp53. The VASP:IRSp53 interaction is regulated by activated CDC42 and promotes high-density clustering of VASP, which is required for processive actin filament elongation. The interaction also mediates VASP recruitment to liposomes. In cells, IRSp53 and VASP accumulate at discrete foci at the leading edge, wher**e filopod**ia are initiated. Genetic removal of IRSp53 impairs the formation of VASP foci**, filopod**ia and chemotactic motility, while IRSp53 null mice display defective wound healing. Thus, IRSp53 dampens barbed end growth. CDC42 activation inhibits this activity and promotes IRSp53-dependent recruitment and clustering of VASP to drive actin assembly. These events result in spatial restriction of VASP filament elongation for initiation o**f filopod**ia during cell migration, invasion, and tissue repair.
41. Gene targeting implicates Cdc42 GTPase in GPVI and non-GPVI mediated platelet **filopodia** formation, secretion and aggregation. Cdc42 and Rac1, members of the Rho family of small GTPases, play critical roles in actin cytoskeleton regulation. We have shown previously that Rac1 is involved in regulation of platelet secretion and aggregation. However, the role of Cdc42 in platelet activation remains controversial. This study was undertaken to better understand the role of Cdc42 in platelet activation.We utilized the Mx-cre;Cdc42(lox/lox) inducible mice with transient Cdc42 deletion to investigate the involvement of Cdc42 in platelet function. The Cdc42-deficient mice exhibited a significantly reduced platelet count than the matching Cdc42(+/+) mice. Platelets isolated from Cdc42(-/-), as compared to Cdc42(+/+), mice exhibited (a) diminished phosphorylation of PAK1/2, an effector molecule of Cdc42, (b) inhibition of **filopodia** formation on immobilized CRP or fibrinogen, (c) inhibition of CRP- or thrombin-induced secretion of ATP and release of P-selectin, (d) inhibition of CRP, collagen or thrombin induced platelet aggregation, and (e) minimal phosphorylation of Akt upon stimulation with CRP or thrombin. The bleeding times were significantly prolonged in Cdc42(-/-) mice compared with Cdc42(+/+) mice.Our data demonstrate that Cdc42 is required for platelet **filopodia** formation, secretion and aggregation and therefore plays a critical role in platelet mediated hemostasis and thrombosis.

#### GeneRIFs

1. These data show that membrane protrusions originate from surface ectodermal cells and that Rac1 is necessary for the formation of membrane ruffles which typify late neural fold closure stages, whereas Cdc42 is required for the predominance of **filopodia** in early neurulation.
2. NRP1 enables the extracellular matrix (ECM)-induced activation of CDC42, a key regulator of **filopodia** formation.
3. Addition of purified CNFY triggers formation of actin-rich membrane ruffles and **filopodia**, which correlates with the activation of the Rho GTPases, RhoA, Rac1 and Cdc42.
4. Cdc42 is required for platelet **filopodia** formation, secretion and aggregation and therefore plays a critical role in platelet mediated hemostasis and thrombosis.
5. Formation of **filopodia** is dependent on the Rho family GTPase Cdc42 and the Cdc42 effector IRSp53 (Baiap2).
6. Activated Cdc42 binds to Drf3/mDia2 in cells in regions associated with the generation of **filopodia**.

### [Ff]ilopodium

#### Process

1. **filopodium** assembly
2. positive regulation of **filopodium** assembly

#### PubMed

1. Cdc42- and IRSp53-dependent contractile filopodia tether presumptive lens and retina to coordinate epithelial invagination. The vertebrate lens provides an excellent model with which to study the mechanisms required for epithelial invagination. In the mouse, the lens forms from the head surface ectoderm. A domain of ectoderm first thickens to form the lens placode and then invaginates to form the lens pit. The epithelium of the lens placode remains in close apposition to the epithelium of the presumptive retina as these structures undergo a coordinated invagination. Here, we show that F-actin-rich basal filopodia that link adjacent presumptive lens and retinal epithelia function as physical tethers that coordinate invagination. The filopodia, most of which originate in the presumptive lens, form at E9.5 when presumptive lens and retinal epithelia first come into close contact, and have retracted by E11.5 when invagination is complete. At E10.5--the lens pit stage--there is approximately one **filopodium** per epithelial cell. Formation of filopodia is dependent on the Rho family GTPase Cdc42 and the Cdc42 effector IRSp53 (Baiap2). Loss of filopodia results in reduced lens pit invagination. Pharmacological manipulation of the actin-myosin contraction pathway showed that the filopodia can respond rapidly in length to change inter-epithelial distance. These data suggest that the lens-retina inter-epithelial filopodia are a fine-tuning mechanism to assist in lens pit invagination by transmitting the forces between presumptive lens and retina. Although invagination of the archenteron in sea urchins and dorsal closure in Drosophila are known to be partly dependent on filopodia, this mechanism of morphogenesis has not previously been identified in vertebrates.
2. Cdc42- and IRSp53-dependent contractile filopodia tether presumptive lens and retina to coordinate epithelial invagination. The vertebrate lens provides an excellent model with which to study the mechanisms required for epithelial invagination. In the mouse, the lens forms from the head surface ectoderm. A domain of ectoderm first thickens to form the lens placode and then invaginates to form the lens pit. The epithelium of the lens placode remains in close apposition to the epithelium of the presumptive retina as these structures undergo a coordinated invagination. Here, we show that F-actin-rich basal filopodia that link adjacent presumptive lens and retinal epithelia function as physical tethers that coordinate invagination. The filopodia, most of which originate in the presumptive lens, form at E9.5 when presumptive lens and retinal epithelia first come into close contact, and have retracted by E11.5 when invagination is complete. At E10.5--the lens pit stage--there is approximately one **filopodium** per epithelial cell. Formation of filopodia is dependent on the Rho family GTPase Cdc42 and the Cdc42 effector IRSp53 (Baiap2). Loss of filopodia results in reduced lens pit invagination. Pharmacological manipulation of the actin-myosin contraction pathway showed that the filopodia can respond rapidly in length to change inter-epithelial distance. These data suggest that the lens-retina inter-epithelial filopodia are a fine-tuning mechanism to assist in lens pit invagination by transmitting the forces between presumptive lens and retina. Although invagination of the archenteron in sea urchins and dorsal closure in Drosophila are known to be partly dependent on filopodia, this mechanism of morphogenesis has not previously been identified in vertebrates.
3. Rif-mDia1 interaction is involved in **filopodium** formation independent of Cdc42 and Rac effectors. Filopodia are cellular protrusions important for axon guidance, embryonic development, and wound healing. The Rho GTPase Cdc42 is the best studied inducer of **filopodium** formation, and several of its effectors and their interacting partners have been linked to the process. These include IRSp53, N-WASP, Mena, and Eps8. The Rho GTPase, Rif, also drives **filopodium** formation. The signaling pathway by which Rif induces filopodia is poorly understood, with mDia2 being the only protein implicated to date. It is thus not clear how distinct the Rif-driven pathway for **filopodium** formation is from the one mediated by Cdc42. In this study, we characterize the dynamics of Rif-induced filopodia by time lapse imaging of live neuronal cells and show that Rif drives **filopodium** formation via an independent pathway that does not involve the Cdc42 effectors N-WASP and IRSp53, the IRSp53 binding partner Mena, or the Rac effectors WAVE1 and WAVE2. Rif formed filopodia in the absence of N-WASP or Mena and when IRSp53, WAVE1, or WAVE2 was knocked down by RNAi. Rif-mediated filopodial protrusion was instead reduced by silencing mDia1 expression or overexpressing a dominant negative mutant of mDia1. mDia1 on its own was able to form filopodia. Data from acceptor photobleaching FRET studies of protein-protein interaction demonstrate that Rif interacts directly with mDia1 in filopodia but not with mDia2. Taken together, these results suggest a novel pathway for filopodia formation via Rif and mDia1.

#### GeneRIFs

1. Rif-mDia1 interaction is involved in **filopodium** formation independent of Cdc42 and Rac effectors.

### [Ii]nvadopodia

#### PubMed

1. Phosphoinositide 3-kinase p85beta regulates invadopodium formation. The acquisition of invasiveness is characteristic of tumor progression. Numerous genetic changes are associated with metastasis, but the mechanism by which a cell becomes invasive remains unclear. Expression of p85β, a regulatory subunit of phosphoinositide-3-kinase, markedly increases in advanced carcinoma, but its mode of action is unknown. We postulated that p85β might facilitate cell invasion. We show that p85β localized at cell adhesions in complex with focal adhesion kinase and enhanced stability and maturation of cell adhesions. In addition, p85β induced development at cell adhesions of an F-actin core that extended several microns into the cell z-axis resembling the skeleton **of invadop**odia. p85β lead to F-actin polymerization at cell adhesions by recruiting active Cdc42/Rac at these structures. In accordance with p85β function in invadopodium-like formation, p85β levels increased in metastatic melanoma and p85β depletion reduced invadopodium formation and invasion. These results show that p85β enhances invasion by inducing cell adhesion developm**ent into in**vadopodia-like structures explaining the metastatic potential of tumors with increased p85β levels.
2. Molecular mechanisms of invadopodium formation: the role of the N-WASP-Arp2/3 complex pathway and cofilin. **Invadopodia** are actin-rich membrane protrusions with a matrix degradation activity formed by invasive cancer cells. We have studied the molecular mechanisms of invadopodium formation in metastatic carcinoma cells. Epidermal growth factor (EGF) receptor kinase inhibitors blocked invadopodium formation in the presence of serum, and EGF stimulation of serum-starved cells induced invadopodium formation. RNA interference and dominant-negative mutant expression analyses revealed that neural WASP (N-WASP), Arp2/3 complex, and their upstream regulators, Nck1, Cdc42, and WIP, are necessary for invadopodium formation. Time-lapse analysis revealed that **invadopodia** are formed de novo at the cell periphery and their lifetime varies from minutes to several hours. **Invadopodia** with short lifetimes are motile, whereas long-lived **invadopodia** tend to be stationary. Interestingly, suppression of cofilin expression by RNA interference inhibited the formation of long-lived **invadopodia**, resulting in formation of only short-lived **invadopodia** with less matrix degradation activity. These results indicate that EGF receptor signaling regulates invadopodium formation through the N-WASP-Arp2/3 pathway and cofilin is necessary for the stabilization and maturation of **invadopodia**.

### [Ii]nvadopodium

#### PubMed

1. Phosphoinositide 3-kinase p85beta regulates **invadopodium** formation. The acquisition of invasiveness is characteristic of tumor progression. Numerous genetic changes are associated with metastasis, but the mechanism by which a cell becomes invasive remains unclear. Expression of p85β, a regulatory subunit of phosphoinositide-3-kinase, markedly increases in advanced carcinoma, but its mode of action is unknown. We postulated that p85β might facilitate cell invasion. We show that p85β localized at cell adhesions in complex with focal adhesion kinase and enhanced stability and maturation of cell adhesions. In addition, p85β induced development at cell adhesions of an F-actin core that extended several microns into the cell z-axis resembling the skeleton of invadopodia. p85β lead to F-actin polymerization at cell adhesions by recruiting active Cdc42/Rac at these structures. In accordance with p85β functi**on in invado**podium-like formation, p85β levels increased in metastatic melanoma and p85β depletion **reduced inva**dopodium formation and invasion. These results show that p85β enhances invasion by inducing cell adhesion development into invadopodia-like structures explaining the metastatic potential of tumors with increased p85β levels.
2. Molecular mechanisms of **invadopodium** formation: the role of the N-WASP-Arp2/3 complex pathway and cofilin. Invadopodia are actin-rich membrane protrusions with a matrix degradation activity formed by invasive cancer cells. We have studied the molecular mechanisms of **invadopodium** formation in metastatic carcinoma cells. Epidermal growth factor (EGF) receptor kinase inhibitors blocked **invadopodium** formation in the presence of serum, and EGF stimulation of serum-starved cells induced **invadopodium** formation. RNA interference and dominant-negative mutant expression analyses revealed that neural WASP (N-WASP), Arp2/3 complex, and their upstream regulators, Nck1, Cdc42, and WIP, are necessary for **invadopodium** formation. Time-lapse analysis revealed that invadopodia are formed de novo at the cell periphery and their lifetime varies from minutes to several hours. Invadopodia with short lifetimes are motile, whereas long-lived invadopodia tend to be stationary. Interestingly, suppression of cofilin expression by RNA interference inhibited the formation of long-lived invadopodia, resulting in formation of only short-lived invadopodia with less matrix degradation activity. These results indicate that EGF receptor signaling regulates **invadopodium** formation through the N-WASP-Arp2/3 pathway and cofilin is necessary for the stabilization and maturation of invadopodia.

### [Pp]odosome(s)?

#### PubMed

1. Regulation of **podosome** dynamics by WASp phosphorylation: implication in matrix degradation and chemotaxis in macrophages. **Podosomes**, adhesion structures capable of matrix degradation, have been linked with the ability of cells to perform chemotaxis and invade tissues. Wiskott-Aldrich Syndrome protein (WASp), an effector of the RhoGTPase Cdc42 and a Src family kinase substrate, regulates macrophage **podosome** formation. In this study, we demonstrate that WASp is active in **podosomes** by using TIRF-FRET microscopy. Pharmacological and RNA interference approaches suggested that continuous WASp activity is required for **podosome** formation and function. Rescue experiments using point mutations demonstrate an absolute requirement for Cdc42 binding to WASp in **podosome** formation. Although tyrosine phosphorylation was not absolutely required for **podosome** formation, phosphorylation did regulate the rate of **podosome** nucleation and actin filament stability. Importantly, WASp tyrosine phosphorylation does not alter WASp activation, instead phosphorylation appears to be important for the restriction of WASp activity to **podosomes**. In addition, the matrix-degrading ability of cells requires WASp phosphorylation. Chemotactic responses to CSF-1 were also attenuated in the absence of endogenous WASp, which could not be rescued with either tyrosine mutation. These results suggest a more complex role for tyrosine phosphorylation than simply in the regulation of WASp activity, and suggest a link between **podosome** dynamics and macrophage migration.

### [Mm]igration

#### Process

1. dendritic cell **migration**
2. nuclear **migration**

#### PubMed

1. ACF7 regulates cytoskeletal-focal adhesion dynamics and **migration** and has ATPase activity. Coordinated interactions between microtubule (MT) and actin cytoskeletons are involved in many polarized cellular processes. Spectraplakins are enormous (>500 kDa) proteins able to bind both MTs and actin filaments (F-actin) directly. To elucidate the physiological significance and functions of mammalian spectraplakin ACF7, we've conditionally targeted it in skin epidermis. Intriguingly, ACF7 deficiency compromises the targeting of microtubules along F-actin to focal adhesions (FAs), stabilizes FA-actin networks, and impairs epidermal **migration**. Exploring underlying mechanisms, we show that ACF7's binding domains for F-actin, MTs, and MT plus-end proteins are not sufficient to rescue the defects in FA-cytoskeletal dynamics and **migration** functions of ACF7 null keratinocytes. We've uncovered an intrinsic actin-regulated ATPase domain in ACF7 and demonstrate that it is both functional and essential for these roles. Our findings provide insight into the functions of this important cytoskeletal crosslinking protein in regulating dynamic interactions between MTs and F-actin to sustain directional cell movement.
2. Membrane localization of adenomatous polyposis coli protein at cellular protrusions: targeting sequences and regulation by beta-catenin. Adenomatous polyposis coli protein (APC) translocates to, and stabilizes, the plus-ends of microtubules. In microtubule-dependent cellular protrusions, APC frequently accumulates in peripheral clusters at the basal membrane. APC targeting to membrane clusters is important for cell **migration**, but the localization mechanism is poorly understood. In this study, we performed deletion mapping and defined a minimal sequence (amino acids 1-2226) that efficiently targets APC to membrane clusters. This sequence lacks DLG-1 and EB1 binding sites, suggesting that these partners are not absolutely required for APC membrane targeting. A series of APC sequences were transiently expressed in cells and compared for their ability to compete endogenous APC at the membrane; potent inhibition of endogenous APC targeting was elicited by the Armadillo- (binds KAP3A, B56alpha, and ASEF) and beta-catenin-binding domains. The Armadillo domain was predicted to inhibit APC membrane localization through sequestration of the kinesin-KAP3A complex. The role of beta-catenin in APC membrane localization was unexpected but affirmed by overexpressing the APC binding sequence of beta-catenin, which similarly reduced APC membrane staining. Furthermore, we used RNA interference to show that loss of beta-catenin reduced APC at membrane clusters in migrating cells. In addition, we report that transiently expressed APC-yellow fluorescent protein co-localized with beta-catenin, KAP3A, EB1, and DLG-1 at membrane clusters, but only beta-catenin stimulated APC anchorage at the membrane. Our findings identify beta-catenin as a regulator of APC targeting to membrane clusters and link these two proteins to cell **migration**.
3. PtdIns(3,4,5)P3 is constitutively synthesized and required for spindle translocation during meiosis in mouse oocytes. Prior to ovulation, mammalian oocytes complete their first meiotic division and arrest at metaphase II. During this marked asymmetric cell division, the meiotic spindle moves dramatically from the center of the oocyte to the cortex to facilitate segregation of half of its chromosomal content into the diminutive first polar body. Recent investigations have documented crucial roles for filamentous actin (F-actin) in meiotic spindle translocation. However, the identity of the upstream regulators responsible for these carefully orchestrated movements has remained elusive. Utilizing fluorescently tagged probes and time-lapse confocal microscopy, we document that phosphatidylinositol 3,4,5-trisphosphate [PtdIns(3,4,5)P3] is constitutively synthesized with spatial and temporal dynamics similar to that of F-actin and Formin 2 (Fmn2). Blockage of PtdIns(3,4,5)P3 synthesis by LY294002, a specific inhibitor of phosphoinositide 3-kinase (PI3K), disrupts cytoplasmic F-actin organization and meiotic spindle **migration** to the cortex. F-actin nucleator Fmn2 and Rho GTPase Cdc42 play roles in mediating the effect of PtdIns(3,4,5)P3 on F-actin assembly. Moreover, the spatial and temporal dynamics of PtdIns(3,4,5)P3 is impaired by depletion of MATER or Filia, two oocyte proteins encoded by maternal effect genes. Thus, PtdIns(3,4,5)P3 is synthesized during meiotic maturation and acts upstream of Cdc42 and Fmn2, but downstream of MATER/Filia proteins to regulate the F-actin organization and spindle translocation to the cortex during mouse oocyte meiosis.
4. Cdc42 and Rac family GTPases regulate mode and speed but not direction of primary fibroblast **migration** during platelet-derived growth factor-dependent chemotaxis. Cdc42 and Rac family GTPases are important regulators of morphology, motility, and polarity in a variety of mammalian cell types. However, comprehensive analysis of their roles in the morphological and behavioral aspects of chemotaxis within a single experimental system is still lacking. Here we demonstrate using a direct viewing chemotaxis assay that of all of the Cdc42/Rac1-related GTPases expressed in primary fibroblasts, Cdc42, Rac1, and RhoG are required for efficient **migration** towards platelet-derived growth factor (PDGF). During **migration**, Cdc42-, Rac1-, and RhoG-deficient cells show aberrant morphology characterized as cell elongation and cell body rounding, loss of lamellipodia, and formation of thick membrane extensions, respectively. Analysis of individual cell trajectories reveals that cell speed is significantly reduced, as well as persistence, but to a smaller degree, while the directional response to the gradient of PDGF is not affected. Combined knockdown of Cdc42, Rac1, and RhoG results in greater inhibition of cell speed than when each protein is knocked down alone, but the cells are still capable of migrating toward PDGF. We conclude that, Cdc42, Rac1, and RhoG function cooperatively during cell **migration** and that, while each GTPase is implicated in the control of morphology and cell speed, these and other Cdc42/Rac-related GTPases are not essential for the directional response toward PDGF.
5. MYC-nick promotes cell **migration** by inducing fascin expression and Cdc42 activation. MYC-nick is a cytoplasmic, transcriptionally inactive member of the MYC oncoprotein family, generated by a proteolytic cleavage of full-length MYC. MYC-nick promotes **migration** and survival of cells in response to chemotherapeutic agents or withdrawal of glucose. Here we report that MYC-nick is abundant in colonic and intestinal tumors derived from mouse models with mutations in the Wnt, TGF-β, and PI3K pathways. Moreover, MYC-nick is elevated in colon cancer cells deleted for FBWX7, which encodes the major E3 ligase of full-length MYC frequently mutated in colorectal cancers. MYC-nick promotes the **migratio**n of colon cancer cells assayed in 3D cultures or grown as xenografts in a zebrafish metastasis model. MYC-nick accelerates **migratio**n by activating the Rho GTPase Cdc42 and inducing fascin expression. MYC-nick, fascin, and Cdc42 are frequently up-regulated in cells present at the invasive front of human colorectal tumors, suggesting a coordinated role for these proteins in tumor **migratio**n.
6. Genetic deletion of Cdc42GAP reveals a role of Cdc42 in erythropoiesis and hematopoietic stem/progenitor cell survival, adhesion, and engraftment. Rho family GTPases are key signal transducers in cell regulation. Although a body of literature has implicated the Rho family members Rac1 and Rac2 in multiple hematopoietic-cell functions, the role of Cdc42 in hematopoiesis remains unclear. Here we have examined the hematopoietic properties and the hematopoietic stem/progenitor cell (HSP) functions of gene-targeted mice carrying null alleles of cdc42gap, a negative regulator of Cdc42. The Cdc42GAP-/- fetal liver and bone marrow cells showed a 3-fold increase in Cdc42 activity but normal Rac and RhoA activities, indicating that Cdc42GAP knockout resulted in a gain of Cdc42 activity in the hematopoietic tissues. Cdc42GAP-/- mice were anemic. The cellularity of fetal liver and bone marrow, the number and composition percentage of HSPs, and the erythroid blast-forming unit and colony-forming unit (BFU-E/CFU-E) activities were significantly reduced in the homozygous mice. The decrease in HSP number was associated with increased apoptosis of the Cdc42GAP-/- HSPs and the activation of JNK-mediated apoptotic machinery. Moreover, homozygous HSPs showed impaired cortical F-actin assembly, deficiency in adhesion and **migration**, and defective engraftment. These results provide evidence that Cdc42 activity is important for erythropoiesis and for multiple HSP functions, including survival, adhesion, and engraftment.
7. Integrin-linked kinase is required for radial sorting of axons and Schwann cell remyelination in the peripheral nervous system. During development, Schwann cells (SCs) interpret different extracellular cues to regulate their **migration**, proliferation, and the remarkable morphological changes associated with the sorting, ensheathment, and myelination of axons. Although interactions between extracellular matrix proteins and integrins are critical to some of these processes, the downstream signaling pathways they control are still poorly understood. Integrin-linked kinase (ILK) is a focal adhesion protein that associates with multiple binding partners to link integrins to the actin cytoskeleton and is thought to participate in integrin and growth factor-mediated signaling. Using SC-specific gene ablation, we report essential functions for ILK in radial sorting of axon bundles and in remyelination in the peripheral nervous system. Our in vivo and in vitro experiments show that ILK negatively regulates Rho/Rho kinase signaling to promote SC process extension and to initiate radial sorting. ILK also facilitates axon remyelination, likely by promoting the activation of downstream molecules such as AKT/protein kinase B.
8. Novel Activities of Select NSAID R-Enantiomers against Rac1 and Cdc42 GTPases. Rho family GTPases (including Rac, Rho and Cdc42) collectively control cell proliferation, adhesion and **migration** and are of interest as functional therapeutic targets in numerous epithelial cancers. Based on high throughput screening of the Prestwick Chemical Library® and cheminformatics we identified the R-enantiomers of two approved drugs (naproxen and ketorolac) as inhibitors of Rac1 and Cdc42. The corresponding S-enantiomers are considered the active component in racemic drug formulations, acting as non-steroidal anti-inflammatory drugs (NSAIDs) with selective activity against cyclooxygenases. Here, we show that the S-enantiomers of naproxen and ketorolac are inactive against the GTPases. Additionally, more than twenty other NSAIDs lacked inhibitory action against the GTPases, establishing the selectivity of the two identified NSAIDs. R-naproxen was first identified as a lead compound and tested in parallel with its S-enantiomer and the non-chiral 6-methoxy-naphthalene acetic acid (active metabolite of nabumetone, another NSAID) as a structural series. Cheminformatics-based substructure analyses-using the rotationally constrained carboxylate in R-naproxen-led to identification of racemic [R/S] ketorolac as a suitable FDA-approved candidate. Cell based measurement of GTPase activity (in animal and human cell lines) demonstrated that the R-enantiomers specifically inhibit epidermal growth factor stimulated Rac1 and Cdc42 activation. The GTPase inhibitory effects of the R-enantiomers in cells largely mimic those of established Rac1 (NSC23766) and Cdc42 (CID2950007/ML141) specific inhibitors. Docking predicts that rotational constraints position the carboxylate moieties of the R-enantiomers to preferentially coordinate the magnesium ion, thereby destabilizing nucleotide binding to Rac1 and Cdc42. The S-enantiomers can be docked but are less favorably positioned in proximity to the magnesium. R-naproxen and R-ketorolac have potential for rapid translation and efficacy in the treatment of several epithelial cancer types on account of established human toxicity profiles and novel activities against Rho-family GTPases.
9. CBAP functions as a novel component in chemokine-induced ZAP70-mediated T-cell adhesion and **migration**. Activated chemokine receptor initiates inside-out signaling to transiently trigger activation of integrins, a process involving multiple components that have not been fully characterized. Here we report that GM-CSF/IL-3/IL-5 receptor common beta-chain-associated protein (CBAP) is required to optimize this inside-out signaling and activation of integrins. First, knockdown of CBAP expression in human Jurkat T cells caused attenuated CXC chemokine ligand-12 (CXCL12)-induced cell **migration** and integrin α4β1- and αLβ2-mediated cell adhesion in vitro, which could be rescued sufficiently upon expression of murine CBAP proteins. Freshly isolated CBAP-deficient primary T cells also exhibited diminution of chemotaxis toward CC chemokine ligand-21 (CCL21) and CXCL12, and these chemokines-induced T-cell adhesions in vitro. Adoptive transfer of isolated naive T cells demonstrated that CBAP deficiency significantly reduced lymph node homing ability in vivo. Final**ly, migra**tion of T cell-receptor-activated T cells induced by inflammatory chemokines was also attenuated in CBAP-deficient cells. Further analyses revealed that CBAP constitutively associated with both integrin β1 and ZAP70 and that CBAP is required for chemokine-induced initial binding of the talin-Vav1 complex to integrin β1 and to facilitate subsequent ZAP70-mediated dissociation of the talin-Vav1 complex and Vav1 phosphorylation. Within such an integrin signaling complex, CBAP likely functions as an adaptor and ultimately leads to activation of both integrin α4β1 and Rac1. Taken together, our data suggest that CBAP indeed can function as a novel signaling component within the ZAP70/Vav1/talin complex and plays an important role in regulating chemokine-promoted T-cell trafficking.
10. Rho GTPases in hematopoietic stem/progenitor cell **migration**. Rho GTPases including RhoA, Rac1, and Cdc42 are a class of intracellular signaling proteins critical for the regulation of cytoskeleton organization, adhesion, and **migration**. Molecular mechanisms of mammalian cell **migration** were first revealed in fibroblasts where RhoA, Rac1, and Cdc42 facilitate in the multistep process including establishment and maintenance of polarity, formation of actin-rich protrusions, remodeling of adhesive contacts, and generation of force. In hematopoietic stem/progenitor cells, Rho GTPases relay signals from chemokines and cytokines such as SDF-1α and SCF to the actin and microtubule cytoskeleton through effector kinases and/or adaptor molecules that affect adhesion or transcription. Comprehensive use of murine conditional gene knockout technology combined with biochemical approaches in recent studies allows for physiologically relevant investigations of the involvement of Rho GTPases in hematopoietic stem/progenitor cell **migratio**n, providing important mechanisms for the stem/progenitor maintenance.
11. RhoA and Cdc42 are required in pre-migratory progenitors of the medial ganglionic eminence ventricular zone for proper cortical interneuron **migration**. Cortical interneurons arise from the ganglionic eminences in the ventral telencephalon and migrate tangentially to the cortex. Although RhoA and Cdc42, members of the Rho family of small GTPases, have been implicated in regulating neuronal **migration**, their respective roles in the tangential **migration** of cortical interneurons remain unknown. Here we show that loss of RhoA and Cdc42 in the ventricular zone (VZ) of the medial ganglionic eminence (MGE) using Olig2-Cre mice causes moderate or severe defects in the **migration** of cortical interneurons, respectively. Furthermore, RhoA- or Cdc42-deleted MGE cells exhibit impaired **migration** in vitro. To determine whether RhoA and Cdc42 directly regulate the motility of cortical interneurons during **migration**, we deleted RhoA and Cdc42 in the subventricular zone (SVZ), where more fate-restricted progenitors are located within the ganglionic eminences, using Dlx5/6-Cre-ires-EGFP (Dlx5/6-CIE) mice. Deletion of either gene within the SVZ does not cause any obvious defects in cortical interneuron **migration**, indicating that cell motility is not dependent upon RhoA or Cdc42. These findings provide genetic evidence that RhoA and Cdc42 are required in progenitors of the MGE in the VZ, but not the SVZ, for proper cortical interneuron **migration**.
12. Inactivation of Cdc42 in embryonic brain results in hydrocephalus with ependymal cell defects in mice. The establishment of a polarized cellular morphology is essential for a variety of processes including neural tube morphogenesis and the development of the brain. Cdc42 is a Ras-related GTPase that plays an essential role in controlling cell polarity through the regulation of the actin and microtubule cytoskeleton architecture. Previous studies have shown that Cdc42 plays an indispensable role in telencephalon development in earlier embryo developmental stage (before E12.5). However, the functions of Cdc42 in other parts of brain in later embryo developmental stage or in adult brain remain unclear. Thus, in order to address the role of Cdc42 in the whole brain in later embryo developmental stage or in adulthood, we used Cre/loxP technology to generate two lines of tissue-specific Cdc42-knock-out mice. Inactivation of Cdc42 was achieved in neuroepithelial cells by crossing Cdc42/ flox mice with Nestin-Cre mice and resulted in hydrocephalus, causing death to occur within the postnatal stage. Histological analyses of the brains from these mice showed that ependymal cell differentiation was disrupted, resulting in aqueductal stenosis. Deletion of Cdc42 in the cerebral cortex also induced obvious defects in interkinetic nuclear **migration** and hypoplasia. To further explore the role of Cdc42 in adult mice brain, we examined the effects of knocking-out Cdc42 in radial glial cells by crossing Cdc42/flox mice with human glial fibrillary acidic protein (GFAP)-Cre mice. Inactivation of Cdc42 in radial glial cells resulted in hydrocephalus and ependymal cell denudation. Taken together, these results highlight the importance of Cdc42 for ependymal cell differentiation and maintaining, and suggest that these functions likely contribute to the essential roles played by Cdc42 in the development of the brain.
13. GPR124, an orphan G protein-coupled receptor, is required for CNS-specific vascularization and establishment of the blood-brain barrier. Every organ in the body requires blood vessels for efficient delivery of oxygen and nutrients, but independent vascular beds are highly specialized to meet the individual needs of specific organs. The vasculature of the brain is tightly sealed, with blood-brain barrier (BBB) properties developing coincident with neural vascularization. G protein-coupled receptor 124 (GPR124) (tumor endothelial marker 5, TEM5), an orphan member of the adhesion family of G protein-coupled receptors, was previously identified on the basis of its overexpression in tumor vasculature. Here, we show that global deletion or endothelial-specific deletion of GPR124 in mice results in embryonic lethality associated with abnormal angiogenesis of the forebrain and spinal cord. Expression of GPR124 was found to be required for invasion and **migration** of blood vessels into neuroepithelium, establishment of BBB properties, and expansion of the cerebral cortex. Thus, GPR124 is an important regulator of neurovasculature development and a potential drug target for cerebrovascular diseases.
14. The Rho-GTPase cdc42 regulates neural progenitor fate at the apical surface. Stem cell persistence into adulthood requires self-renewal from early developmental stages. In the developing mouse brain, only apical progenitors located at the ventricle are self-renewing, whereas basal progenitors gradually deplete. However, nothing is known about the mechanisms regulating the fundamental difference between these progenitors. Here we show that the conditional deletion of the small Rho-GTPase cdc42 at different stages of neurogenesis in mouse telencephalon results in an immediate increase in basal mitoses. Whereas cdc42-deficient progenitors have normal cell cycle length, orientation of cell division and basement membrane contact, the apical location of the Par complex and adherens junctions are gradually lost, leading to an increasing failure of apically directed interkinetic nuclear **migration**. These cells then undergo mitoses at basal positions and acquire the fate of basal progenitors. Thus, cdc42 has a crucial role at the apical pole of progenitors, thereby regulating the position of mitoses and cell fate.
15. Essential role of Cdc42 in cardiomyocyte proliferation and cell-cell adhesion during heart development. Cdc42 is a member of the Rho GTPase family and functions as a molecular switch in regulating cell **migration**, proliferation, differentiation and survival. However, the role of Cdc42 in heart development remains largely unknown. To determine the function of Cdc42 in heart formation, we have generated a Cdc42 cardiomyocyte knockout (CCKO) mouse line by crossing Cdc42 flox mice with myosin light chain (MLC) 2a-Cre mice. The inactivation of Cdc42 in embryonic cardiomyocytes induced lethality after embryonic day 12.5. Histological analysis of CCKO embryos showed cardiac developmental defects that included thin ventricular walls and ventricular septum defects. Microarray and real-time PCR data also revealed that the expression level of p21 was significantly increased and cyclin B1 was dramatically decreased, suggesting that Cdc42 is required for cardiomyocyte proliferation. Phosphorylated Histone H3 staining confirmed that the inactivation of Cdc42 inhibited cardiomyocytes proliferation. In addition, transmission electron microscope studies showed disorganized sarcomere structure and disruption of cell-cell contact among cardiomyocytes in CCKO hearts. Accordingly, we found that the distribution of N-cadherin/β-Catenin in CCKO cardiomyocytes was impaired. Taken together, our data indicate that Cdc42 is essential for cardiomyocyte proliferation, sarcomere organization and cell-cell adhesion during heart development.
16. Plexin-B2 negatively regulates macrophage motility, Rac, and Cdc42 activation. Plexins are cell surface receptors widely studied in the nervous system, where they mediate **migration** and morphogenesis though the Rho family of small GTPases. More recently, plexins have been implicated in immune processes including cell-cell interaction, immune activation, **migration**, and cytokine production. Plexin-B2 facilitates ligand induced cell guidance and **migration** in the nervous system, and induces cytoskeletal changes in overexpression assays through RhoGTPase. The function of Plexin-B2 in the immune system is unknown. This report shows that Plexin-B2 is highly expressed on cells of the innate immune system in the mouse, including macrophages, conventional dendritic cells, and plasmacytoid dendritic cells. However, Plexin-B2 does not appear to regulate the production of proinflammatory cytokines, phagocytosis of a variety of targets, or directional **migration** towards chemoattractants or extracellular matrix in mouse macrophages. Instead, Plxnb2(-/-) macrophages have greater cellular motility than wild type in the unstimulated state that is accompanied by more active, GTP-bound Rac and Cdc42. Additionally, Plxnb2(-/-) macrophages demonstrate faster in vitro wound closure activity. Studies have shown that a closely related family member, Plexin-B1, binds to active Rac and sequesters it from downstream signaling. The interaction of Plexin-B2 with Rac has only been previously confirmed in yeast and bacterial overexpression assays. The data presented here show that Plexin-B2 functions in mouse macrophages as a negative regulator of the GTPases Rac and Cdc42 and as a negative regulator of basal cell motility and wound healing.
17. The N-BAR domain protein, Bin3, regulates Rac1- and Cdc42-dependent processes in myogenesis. Actin dynamics are necessary at multiple steps in the formation of multinucleated muscle cells. BAR domain proteins can regulate actin dynamics in several cell types, but have been little studied in skeletal muscle. Here, we identify novel functions for the N-BAR domain protein, Bridging integrator 3 (Bin3), during myogenesis in mice. Bin3 plays an important role in regulating myofiber size in vitro and in vivo. During early myogenesis, Bin3 promotes **migration** of differentiated muscle cells, where it colocalizes with F-actin in lamellipodia. In addition, Bin3 forms a complex with Rac1 and Cdc42, Rho GTPases involved in actin polymerization, which are known to be essential for myotube formation. Importantly, a Bin3-dependent pathway is a major regulator of Rac1 and Cdc42 activity in differentiated muscle cells. Overall, these data classify N-BAR domain proteins as novel regulators of actin-dependent processes in myogenesis, and further implicate BAR domain proteins in muscle growth and repair.
18. A Rac/Cdc42-specific exchange factor, GEFT, induces cell proliferation, transformation, and **migration**. The Rho family of small GTPases, including Rho, Rac, and Cdc42, play essential roles in diverse cellular functions. The ability of Rho family GTPases to participate in signaling events is determined by the ratio of inactive (GDP-bound) and active (GTP-bound) forms in the cell. The activation of Rho family proteins requires the exchange of bound GDP for GTP, a process catalyzed by the Dbl family of guanine nucleotide exchange factors (GEFs). The GEFs have high affinity for the guanine nucleotide-free state of the GTPases and are thought to promote GDP release by stabilizing an intermediate transition state. In this study, we have identified and characterized a new Rac/Cdc42-specific Dbl family guanine nucleotide exchange factor, named GEFT. GEFT is highly expressed in the excitable tissues, including brain, heart, and muscle. Low or very little expression was detected in other nonexcitable tissues. GEFT has specific exchange activity for Rac and Cdc42 in our in vitro GTPase exchange assays and glutathione S-transferase-PAK pull-down assays with GTP-bound Rac1 and Cdc42. Overexpression of GEFT leads to changes in cell morphology and actin cytoskeleton re-organization, including the formation of membrane microspikes, filopodia, and lamilliopodia. Furthermore, expression of GEFT in NIH3T3 cells promotes foci formation, cell proliferation, and cell **migration**, possibly through the activation of transcriptional factors involved in cell growth and proliferation. Together, our data suggest that GEFT is a Rac/Cdc42-specific GEF protein that regulates cell morphology, cell proliferation, and transformation.
19. P-cadherin promotes collective cell **migration** via a Cdc42-mediated increase in mechanical forces. Collective cell **migration** (CCM) is essential for organism development, wound healing, and metastatic transition, the primary cause of cancer-related death, and it involves cell-cell adhesion molecules of the cadherin family. Increased P-cadherin expression levels are correlated with tumor aggressiveness in carcinoma and aggressive sarcoma; however, how P-cadherin promotes tumor malignancy remains unknown. Here, using integrated cell biology and biophysical approaches, we determined that P-cadherin specifically induces polarization and CCM through an increase in the strength and anisotropy of mechanical forces. We show that this mechanical regulation is mediated by the P-cadherin/β-PIX/Cdc42 axis; P-cadherin specifically activates Cdc42 through β-PIX, which is specifically recruited at cell-cell contacts upon CCM. This mechanism of cell polarization an**d migrati**on is absent in cells expressing E- or R-cadherin. Thus, we identify a specific role of P-cadherin through β-PIX-mediated Cdc42 activation in the regulation of cell polarity and force anisotropy that drives CCM.
20. FAT1 mutations cause a glomerulotubular nephropathy. Steroid-resistant nephrotic syndrome (SRNS) causes 15% of chronic kidney disease (CKD). Here we show that recessive mutations in FAT1 cause a distinct renal disease entity in four families with a combination of SRNS, tubular ectasia, haematuria and facultative neurological involvement. Loss of FAT1 results in decreased cell adhesion and **migration** in fibroblasts and podocytes and the decreased **migration** is partially reversed by a RAC1/CDC42 activator. Podocyte-specific deletion of Fat1 in mice induces abnormal glomerular filtration barrier development, leading to podocyte foot process effacement. Knockdown of Fat1 in renal tubular cells reduces **migration**, decreases active RAC1 and CDC42, and induces defects in lumen formation. Knockdown of fat1 in zebrafish causes pronephric cysts, which is partially rescued by RAC1/CDC42 activators, confirming a role of the two small GTPases in the pathogenesis. These findings provide new insights into the pathogenesis of SRNS and tubulopathy, linking FAT1 and RAC1/CDC42 to podocyte and tubular cell function.
21. Regulation of ephexin1, a guanine nucleotide exchange factor of Rho family GTPases, by fibroblast growth factor receptor-mediated tyrosine phosphorylation. Fibroblast growth factor (FGF) signal is implicated in not only cell proliferation, but cell **migration** and morphological changes. Several different Rho family GTPases downstream of the Ras/ERK pathway are postulated to mediate the latter functions. However, none have been recognized to be directly coupled to FGF receptors (FGFRs). We have previously reported that EphA4 and FGFRs hetero-oligomerize through their cytoplasmic domains, trans-activate each other, and transduce a signal for cell proliferation through a docking protein, FRS2alpha (Yokote, H., Fujita, K., Jing, X., Sawada, T., Liang, S., Yao, L., Yan, X., Zhang, Y., Schlessinger, J., and Sakaguchi, K. (2005) Proc. Natl. Acad. Sci. U. S. A. 102, 18866-18871). Here, we have found that ephexin1, a guanine nucleotide exchange factor for Rho family GTPases, constitutes another downstream component of the receptor complex. Ephexin1 directly binds to the kinase domain of FGFR mainly through its DH and PH domains. The binding appears to become weaker and limited to the DH domain when FGFRs become activated. FGFR-mediated phosphorylation of ephexin1 enhances the guanine nucleotide exchange activity toward RhoA without affecting the activity to Rac1 or Cdc42. The FGFR-mediated tyrosine phosphorylation includes, but is not limited to, the residue (Tyr-87) phosphorylated by Src family kinase, which is known to be activated following EphA4 activation. The Tyr-to-Asp mutations that mimic the tyrosine phosphorylation in some of the putative FGFR-mediated phosphorylation sites increase the nucleotide exchange activity for RhoA without changing the activity for Rac1 or Cdc42. From these results, we conclude that ephexin1 is located immediately downstream of the EphA4-FGFR complex and the function is altered by the FGFR-mediated tyrosine phosphorylation at multiple sites.
22. Involvement of the phosphatidylinositol 3-kinase/rac1 and cdc42 pathways in radial **migration** of cortical neurons. During cortical development, newly generated neurons migrate radially toward their final positions. Although several candidate genes essential for this radial **migration** have been reported, the signaling pathways regulating it are largely unclear. Here we studied the role of phosphatidylinositol (PI) 3-kinase and its downstream signaling molecules in the radial **migration** of cortical neurons in vivo and in vitro. The expression of constitutively active and dominant-negative PI 3-kinases markedly inhibited radial **migration**. In the neocortical slice culture, a PI 3-kinase inhibitor suppressed the formation of GTP-bound Rac1 and Cdc42 and radial **migration**. Constitutively active and dominant-negative forms of Rac1 and Cdc42 but not Akt also significantly inhibited radial **migration**. In migrating neurons, wild-type Rac1 and Cdc42 showed different localizations; Rac1 localized to the plasma membrane and Cdc42 to the perinuclear region on the side of the leading processes. These results suggest that both the PI 3-kinase/Rac1 and Cdc42 pathways are involved in the radial **migration** of cortical neurons and that they have different roles.
23. Effects of pharmacological inhibition of small GTPases on axon extension and **migration** of enteric neural crest-derived cells. In the developing enteric nervous system, there is a close association between migrating neural crest-derived cells and the axons of early differentiating neurons. We used pharmacological inhibitors of small GTPases to determine if crest cell **migration** and axon growth could be uncoupled in cultured intact explants of embryonic mouse gut and slices of embryonic gut grown on collagen gels containing GDNF. Inhibition of the Rho effectors, ROCKI/II, or Rac/Cdc42 inhibited both cell **migration** and neurite growth in intact explants of embryonic gut. The effects of both ROCKI/II and Rac/Cdc42 inhibitors were more severe on cell **migration** and axon extension in gut explants from Ret(+/-) mice than in explants from wildtype mice, indicating that Rho GTPases probably act downstream of the receptor tyrosine kinase, Ret. Inhibition of ROCKI/II had different effects on **migration** and axon extension in gut slices grown on collagen gels containing GDNF from that seen in intact explants of gut. We conclude that ROCKI/II and Rac/Cdc42 are required for both neural crest-derived cell **migration** and axon growth in the developing gut.
24. HER2/ErbB2-induced breast cancer cell **migration** and invasion require p120 catenin activation of Rac1 and Cdc42. Breast cancers that overexpress the receptor tyrosine kinase ErbB2/HER2/Neu result in poor patient outcome because of extensive metastatic progression. Herein, we delineate a molecular mechanism that may govern this malignant phenotype. ErbB2 induction of **migration** requires activation of the small GTPases Rac1 and Cdc42. The ability of ErbB2 to activate these small GTPases necessitated expression of p120 catenin, which is itself up-regulated by signaling through ErbB2 and the tyrosine kinase Src. Silencing p120 in ErbB2-dependent breast cancer cell lines dramatically inhibited **migration** and invasion as well as activation of Rac1 and Cdc42. In contrast, overexpression of constitutively active mutants of these GTPases reversed the effects of p120 silencing. Lastly, ectopic expression of p120 promoted **migration** and invasion and potentiated metastatic progression of a weakly metastatic, ErbB2-dependent breast cancer cell line. These results suggest that p120 acts as an obligate intermediate between ErbB2 and Rac1/Cdc42 to modulate the metastatic potential of breast cancer cells.
25. Vav GEFs are required for beta2 integrin-dependent functions of neutrophils. Integrin regulation of neutrophils is essential for appropriate adhesion and trans**migration** into tissues. Vav proteins are Rho family guanine nucleotide exchange factors that become tyrosine phosphorylated in response to adhesion. Using Vav1/Vav3-deficient neutrophils (Vav1/3ko), we show that Vav proteins are required for multiple beta2 integrin-dependent functions, including sustained adhesion, spreading, and complement-mediated phagocytosis. These defects are not attributable to a lack of initial beta2 activation as Vav1/3ko neutrophils undergo chemoattractant-induced arrest on intercellular adhesion molecule-1 under flow. Accordingly, in vivo, Vav1/3ko leukocytes arrest on venular endothelium yet are unable to sustain adherence. Thus, Vav proteins are specifically required for stable adhesion. beta2-induced activation of Cdc42, Rac1, and RhoA is defective in Vav1/3ko neutrophils, and phosphorylation of Pyk2, paxillin, and Akt is also significantly reduced. In contrast, Vav proteins are largely dispensable for G protein-coupled receptor-induced signaling events and chemotaxis. Thus, Vav proteins play an essential role coupling beta2 to Rho GTPases and regulating multiple integrin-induced events important in leukocyte adhesion and phagocytosis.
26. Cdc42 and Rac1 signaling are both required for and act synergistically in the correct formation of myelin sheaths in the CNS. The formation of myelin sheaths in the CNS is the result of a complex series of events involving oligodendrocyte progenitor cell (OPC) proliferation, directed **migration**, and the morphological changes associated with axon ensheathment and myelination. To examine the role of Rho GTPases in oligodendrocyte biology, we have used a conditional tissue-specific gene-targeting approach. Ablation of Cdc42 in cells of the oligodendrocyte lineage did not affect OPC proliferation, directed **migration**, or in vitro differentiation, but it led to the formation of a unique and stage-specific myelination phenotype. This was characterized by the extraordinary enlargement of the inner tongue of the oligodendrocyte process and concomitant formation of a myelin outfolding as a result of abnormal accumulation of cytoplasm in this region. Ablation of Rac1 also resulted in the abnormal accumulation of cytoplasm in the inner tongue of the oligodendrocyte process, and we provide genetic evidence that rac1 synergizes with cdc42 in a gene dosage-dependent way to regulate myelination.
27. Distinct roles of Rac1/Cdc42 and Rho/Rock for axon outgrowth and nucleokinesis of precerebellar neurons toward netrin 1. During embryonic development, tangentially migrating precerebellar neurons emit a leading process and then translocate their nuclei inside it (nucleokinesis). Netrin 1 (also known as netrin-1) acts as a chemoattractant factor for neurophilic **migration** of precerebellar neurons (PCN) both in vivo and in vitro. In the present work, we analyzed Rho GTPases that could direct axon outgrowth and/or nuclear **migration**. We show that the expression pattern of Rho GTPases in developing PCN is consistent with their involvement in the **migration** of PCN from the rhombic lips. We report that pharmacological inhibition of Rho enhances axon outgrowth of PCN and prevents nuclei **migration** toward a netrin 1 source, whereas inhibition of Rac and Cdc42 sub-families impair neurite outgrowth of PCN without affecting **migration**. We show, through pharmacological inhibition, that Rho signaling directs neurophilic **migration** through Rock activation. Altogether, our results indicate that Rho/Rock acts on signaling pathways favoring nuclear translocation during tangential **migration** of PCN. Thus, axon extension and nuclear **migration** of PCN in response to netrin 1 are not strictly dependent processes because: (1) distinct small GTPases are involved; (2) axon extension can occur when **migration** is blocked; and (3) **migration** can occur when axon outgrowth is impaired.
28. Redundant and nonredundant roles for Cdc42 and Rac1 in lymphomas developed in NPM-ALK transgenic mice. Increasing evidence suggests that Rho family GTPases could have a critical role in the biology of T-cell lymphoma. In ALK-rearranged anaplastic large cell lymphoma (ALCL), a specific subtype of T-cell lymphoma, the Rho family GTPases Cdc42 and Rac1 are activated by the ALK oncogenic activity. In vitro studies have shown that Cdc42 and Rac1 control rather similar phenotypes of ALCL biology such as the proliferation, survival, and **migration** of lymphoma cells. However, their role and possible redundancy in ALK-driven lymphoma development in vivo are still undetermined. We genetically deleted Cdc42 or Rac1 in a mouse model of ALK-rearranged ALCL to show that either Cdc42 or Rac1 deletion impaired lymphoma development, modified lymphoma morphology, actin filament distribution, and **migration** properties of lymphoma cells. Cdc42 or Rac1 deletion primarily affected survival rather than proliferation of lymphoma cells. Apoptosis of lymphoma cells was equally induced following Cdc42 or Rac1 deletion, was associated with upregulation of the proapoptotic molecule Bid, and was blocked by Bcl2 overexpression. Remarkably, Cdc42/Rac1 double deletion, but not Cdc42 or Rac1 single deletions, completely prevented NPM-ALK lymphoma dissemination in vivo. Thus, Cdc42 and Rac1 have nonredundant roles in controlling ALK-rearranged lymphoma survival and morphology but are redundant for lymphoma dissemination, suggesting that targeting both GTPases could represent a preferable therapeutic option for ALCL treatment.
29. Impaired Rho GTPase activation abrogates cell polarization and **migration** in macrophages with defective lipolysis. Infiltration of monocytes and macrophages into the site of inflammation is critical in the progression of inflammatory diseases such as atherosclerosis. Cell **migration** is dependent on the continuous organization of the actin cytoskeleton, which is regulated by members of the small Rho GTPase family (RhoA, Cdc42, Rac) that are also important for the regulation of signal transduction pathways. We have recently reported on reduced plaque formation in an atherosclerotic mouse model transplanted with bone marrow from adipose triglyceride lipase-deficient (Atgl-/-) mice. Here we provide evidence that defective lipolysis in macrophages lacking ATGL, the major enzyme responsible for triacylglycerol hydrolysis, favors an anti-inflammatory M2-like macrophage phenotype. Our data implicate an as yet unrecognized principle that insufficient lipolysis influences macrophage polarization and actin polymerization, resulting in impaired macrophage **migration**. Sustained phosphorylation of focal adhesion kinase [due to inactivation of its phosphatase by elevated levels of reactive oxygen species (ROS)] results in defective Cdc42, Rac1 and RhoA activation and in increased and sustained activation of Rac2. Inhibition of ROS production restores the migratory capacity of Atgl-/- macrophages. Since monocyte and macrophage **migration** are a prerequisite for infiltrating the arterial wall, our results provide a molecular link between lipolysis and the development of atherosclerosis.
30. The atypical Guanine-nucleotide exchange factor, dock7, negatively regulates schwann cell differentiation and myelination. In development of the peripheral nervous system, Schwann cells proliferate, migrate, and ultimately differentiate to form myelin sheath. In all of the myelination stages, Schwann cells continuously undergo morphological changes; however, little is known about their underlying molecular mechanisms. We previously cloned the dock7 gene encoding the atypical Rho family guanine-nucleotide exchange factor (GEF) and reported the positive role of Dock7, the target Rho GTPases Rac/Cdc42, and the downstream c-Jun N-terminal kinase in Schwann cell **migration** (Yamauchi et al., 2008). We investigated the role of Dock7 in Schwann cell differentiation and myelination. Knockdown of Dock7 by the specific small interfering (si)RNA in primary Schwann cells promotes dibutyryl cAMP-induced morphological differentiation, indicating the negative role of Dock7 in Schwann cell differentiation. It also results in a shorter duration of activation of Rac/Cdc42 and JNK, which is the negative regulator of myelination, and the earlier activation of Rho and Rho-kinase, which is the positive regulator of myelination. To obtain the in vivo evidence, we generated Dock7 short hairpin (sh)RNA transgenic mice. They exhibited a decreased expression of Dock7 in the sciatic nerves and enhanced myelin thickness, consistent with in vitro observation. The effects of the in vivo knockdown on the signals to Rho GTPases are similar to those of the in vitro knockdown. Collectively, the signaling through Dock7 negatively regulates Schwann cell differentiation and the onset of myelination, demonstrating the unexpected role of Dock7 in the interplay between Schwann cell **migration** and myelination.
31. Rac1 controls the formation of midline commissures and the competency of tangential **migration** in ventral telencephalic neurons. Previous studies using dominant-mutant constructs have implicated Rac1 GTPase in neuritogenesis and neuronal **migration**. However, overexpression of dominant mutants generally blocks Rho-GTPase activity; thus, it may not reveal the specific or physiological functions of Rac1. To address this issue, we have applied a conditional gene-targeting strategy, using Foxg1-Cre mice to delete Rac1 in the ventricular zone (VZ) of telencephalon and Dlx5/6-Cre-IRES (internal ribosomal entry site)-EGFP (enhanced green fluorescent protein) (Dlx5/6-CIE) in the subventricular zone (SVZ) of ventral telencephalon, respectively. Surprisingly, the deletion of Rac1 in VZ progenitors did not prevent axonal outgrowth of telencephalic neurons. However, the anterior commissure was absent, and the corpus callosal as well as hippocampal commissural axons failed to cross the midline in Rac1/Foxg1-Cre knock-out embryos. The thalamocortical and corticothalamic axons also showed defasciculation or projection defects. These results suggest that Rac1 controls axon guidance rather than neuritogenesis. In addition, although Rac1/Foxg1-Cre knock-out embryos showed delayed radial **migration** of cortical projection neurons and severe impairment of tangential **migration** by the ventral telencephalon-derived interneurons, deletion of Rac1 in the SVZ by Dlx5/6-CIE mice produced no discernible defects in tangential **migration**. These contrasting effects of Rac1 deletion on tangential **migration** suggest that Rac1 is dispensable for cellular motility per se during neuronal **migration**. Together, these results underscore the challenge of deciphering the biological functions of Rac1, and Rho-GTPases in general, during mammalian brain development. Moreover, they indicate that Rac1 has a critical role in axon guidance and in acquisition of migratory competency during differentiation of the progenitors for the ventral telencephalon-derived interneurons.
32. Inactivation of Cdc42 in neural crest cells causes craniofacial and cardiovascular morphogenesis defects. Neural crest cells (NCCs) are physically responsible for craniofacial skeleton formation, pharyngeal arch artery remodeling and cardiac outflow tract septation during vertebrate development. Cdc42 (cell division cycle 42) is a Rho family small GTP-binding protein that works as a molecular switch to regulate cytoskeleton remodeling and the establishment of cell polarity. To investigate the role of Cdc42 in NCCs during embryonic development, we deleted Cdc42 in NCCs by crossing Cdc42 flox mice with Wnt1-cre mice. We found that the inactivation of Cdc42 in NCCs caused embryonic lethality with craniofacial deformities and cardiovascular developmental defects. Specifically, Cdc42 NCC knockout embryos showed fully penetrant cleft lips and short snouts. Alcian Blue and Alizarin Red staining of the cranium exhibited an unfused nasal capsule and palatine in the mutant embryos. India ink intracardiac injection analysis displayed a spectrum of cardiovascular developmental defects, including persistent truncus arteriosus, hypomorphic pulmonary arteries, interrupted aortic arches, and right-sided aortic arches. To explore the underlying mechanisms of Cdc42 in the formation of the great blood vessels, we generated Wnt1Cre-Cdc42-Rosa26 reporter mice. By beta-galactosidase staining, a subpopulation of Cdc42-null NCCs was observed halting in their **migration** midway from the pharyngeal arches to the conotruncal cushions. Phalloidin staining revealed dispersed, shorter and disoriented stress fibers in Cdc42-null NCCs. Finally, we demonstrated that the inactivation of Cdc42 in NCCs impaired bone morphogenetic protein 2 (BMP2)-induced NCC cytoskeleton remodeling and **migration**. In summary, our results demonstrate that Cdc42 plays an essential role in NCC **migration**, and inactivation of Cdc42 in NCCs impairs craniofacial and cardiovascular development in mice.
33. P-cadherin promotes collective cell **migration** via a Cdc42-mediated increase in mechanical forces. Collective cell **migration** (CCM) is essential for organism development, wound healing, and metastatic transition, the primary cause of cancer-related death, and it involves cell-cell adhesion molecules of the cadherin family. Increased P-cadherin expression levels are correlated with tumor aggressiveness in carcinoma and aggressive sarcoma; however, how P-cadherin promotes tumor malignancy remains unknown. Here, using integrated cell biology and biophysical approaches, we determined that P-cadherin specifically induces polarization and CCM through an increase in the strength and anisotropy of mechanical forces. We show that this mechanical regulation is mediated by the P-cadherin/β-PIX/Cdc42 axis; P-cadherin specifically activates Cdc42 through β-PIX, which is specifically recruited at cell-cell contacts upon CCM. This mechanism of cell polarization an**d migrati**on is absent in cells expressing E- or R-cadherin. Thus, we identify a specific role of P-cadherin through β-PIX-mediated Cdc42 activation in the regulation of cell polarity and force anisotropy that drives CCM.
34. Redundant and nonredundant roles for Cdc42 and Rac1 in lymphomas developed in NPM-ALK transgenic mice. Increasing evidence suggests that Rho family GTPases could have a critical role in the biology of T-cell lymphoma. In ALK-rearranged anaplastic large cell lymphoma (ALCL), a specific subtype of T-cell lymphoma, the Rho family GTPases Cdc42 and Rac1 are activated by the ALK oncogenic activity. In vitro studies have shown that Cdc42 and Rac1 control rather similar phenotypes of ALCL biology such as the proliferation, survival, and **migration** of lymphoma cells. However, their role and possible redundancy in ALK-driven lymphoma development in vivo are still undetermined. We genetically deleted Cdc42 or Rac1 in a mouse model of ALK-rearranged ALCL to show that either Cdc42 or Rac1 deletion impaired lymphoma development, modified lymphoma morphology, actin filament distribution, and **migration** properties of lymphoma cells. Cdc42 or Rac1 deletion primarily affected survival rather than proliferation of lymphoma cells. Apoptosis of lymphoma cells was equally induced following Cdc42 or Rac1 deletion, was associated with upregulation of the proapoptotic molecule Bid, and was blocked by Bcl2 overexpression. Remarkably, Cdc42/Rac1 double deletion, but not Cdc42 or Rac1 single deletions, completely prevented NPM-ALK lymphoma dissemination in vivo. Thus, Cdc42 and Rac1 have nonredundant roles in controlling ALK-rearranged lymphoma survival and morphology but are redundant for lymphoma dissemination, suggesting that targeting both GTPases could represent a preferable therapeutic option for ALCL treatment.
35. FAT1 mutations cause a glomerulotubular nephropathy. Steroid-resistant nephrotic syndrome (SRNS) causes 15% of chronic kidney disease (CKD). Here we show that recessive mutations in FAT1 cause a distinct renal disease entity in four families with a combination of SRNS, tubular ectasia, haematuria and facultative neurological involvement. Loss of FAT1 results in decreased cell adhesion and **migration** in fibroblasts and podocytes and the decreased **migration** is partially reversed by a RAC1/CDC42 activator. Podocyte-specific deletion of Fat1 in mice induces abnormal glomerular filtration barrier development, leading to podocyte foot process effacement. Knockdown of Fat1 in renal tubular cells reduces **migration**, decreases active RAC1 and CDC42, and induces defects in lumen formation. Knockdown of fat1 in zebrafish causes pronephric cysts, which is partially rescued by RAC1/CDC42 activators, confirming a role of the two small GTPases in the pathogenesis. These findings provide new insights into the pathogenesis of SRNS and tubulopathy, linking FAT1 and RAC1/CDC42 to podocyte and tubular cell function.
36. Novel Activities of Select NSAID R-Enantiomers against Rac1 and Cdc42 GTPases. Rho family GTPases (including Rac, Rho and Cdc42) collectively control cell proliferation, adhesion and **migration** and are of interest as functional therapeutic targets in numerous epithelial cancers. Based on high throughput screening of the Prestwick Chemical Library® and cheminformatics we identified the R-enantiomers of two approved drugs (naproxen and ketorolac) as inhibitors of Rac1 and Cdc42. The corresponding S-enantiomers are considered the active component in racemic drug formulations, acting as non-steroidal anti-inflammatory drugs (NSAIDs) with selective activity against cyclooxygenases. Here, we show that the S-enantiomers of naproxen and ketorolac are inactive against the GTPases. Additionally, more than twenty other NSAIDs lacked inhibitory action against the GTPases, establishing the selectivity of the two identified NSAIDs. R-naproxen was first identified as a lead compound and tested in parallel with its S-enantiomer and the non-chiral 6-methoxy-naphthalene acetic acid (active metabolite of nabumetone, another NSAID) as a structural series. Cheminformatics-based substructure analyses-using the rotationally constrained carboxylate in R-naproxen-led to identification of racemic [R/S] ketorolac as a suitable FDA-approved candidate. Cell based measurement of GTPase activity (in animal and human cell lines) demonstrated that the R-enantiomers specifically inhibit epidermal growth factor stimulated Rac1 and Cdc42 activation. The GTPase inhibitory effects of the R-enantiomers in cells largely mimic those of established Rac1 (NSC23766) and Cdc42 (CID2950007/ML141) specific inhibitors. Docking predicts that rotational constraints position the carboxylate moieties of the R-enantiomers to preferentially coordinate the magnesium ion, thereby destabilizing nucleotide binding to Rac1 and Cdc42. The S-enantiomers can be docked but are less favorably positioned in proximity to the magnesium. R-naproxen and R-ketorolac have potential for rapid translation and efficacy in the treatment of several epithelial cancer types on account of established human toxicity profiles and novel activities against Rho-family GTPases.
37. Inactivation of Cdc42 in neural crest cells causes craniofacial and cardiovascular morphogenesis defects. Neural crest cells (NCCs) are physically responsible for craniofacial skeleton formation, pharyngeal arch artery remodeling and cardiac outflow tract septation during vertebrate development. Cdc42 (cell division cycle 42) is a Rho family small GTP-binding protein that works as a molecular switch to regulate cytoskeleton remodeling and the establishment of cell polarity. To investigate the role of Cdc42 in NCCs during embryonic development, we deleted Cdc42 in NCCs by crossing Cdc42 flox mice with Wnt1-cre mice. We found that the inactivation of Cdc42 in NCCs caused embryonic lethality with craniofacial deformities and cardiovascular developmental defects. Specifically, Cdc42 NCC knockout embryos showed fully penetrant cleft lips and short snouts. Alcian Blue and Alizarin Red staining of the cranium exhibited an unfused nasal capsule and palatine in the mutant embryos. India ink intracardiac injection analysis displayed a spectrum of cardiovascular developmental defects, including persistent truncus arteriosus, hypomorphic pulmonary arteries, interrupted aortic arches, and right-sided aortic arches. To explore the underlying mechanisms of Cdc42 in the formation of the great blood vessels, we generated Wnt1Cre-Cdc42-Rosa26 reporter mice. By beta-galactosidase staining, a subpopulation of Cdc42-null NCCs was observed halting in their **migration** midway from the pharyngeal arches to the conotruncal cushions. Phalloidin staining revealed dispersed, shorter and disoriented stress fibers in Cdc42-null NCCs. Finally, we demonstrated that the inactivation of Cdc42 in NCCs impaired bone morphogenetic protein 2 (BMP2)-induced NCC cytoskeleton remodeling and **migration**. In summary, our results demonstrate that Cdc42 plays an essential role in NCC **migration**, and inactivation of Cdc42 in NCCs impairs craniofacial and cardiovascular development in mice.
38. The N-BAR domain protein, Bin3, regulates Rac1- and Cdc42-dependent processes in myogenesis. Actin dynamics are necessary at multiple steps in the formation of multinucleated muscle cells. BAR domain proteins can regulate actin dynamics in several cell types, but have been little studied in skeletal muscle. Here, we identify novel functions for the N-BAR domain protein, Bridging integrator 3 (Bin3), during myogenesis in mice. Bin3 plays an important role in regulating myofiber size in vitro and in vivo. During early myogenesis, Bin3 promotes **migration** of differentiated muscle cells, where it colocalizes with F-actin in lamellipodia. In addition, Bin3 forms a complex with Rac1 and Cdc42, Rho GTPases involved in actin polymerization, which are known to be essential for myotube formation. Importantly, a Bin3-dependent pathway is a major regulator of Rac1 and Cdc42 activity in differentiated muscle cells. Overall, these data classify N-BAR domain proteins as novel regulators of actin-dependent processes in myogenesis, and further implicate BAR domain proteins in muscle growth and repair.
39. RhoA and Cdc42 are required in pre-migratory progenitors of the medial ganglionic eminence ventricular zone for proper cortical interneuron **migration**. Cortical interneurons arise from the ganglionic eminences in the ventral telencephalon and migrate tangentially to the cortex. Although RhoA and Cdc42, members of the Rho family of small GTPases, have been implicated in regulating neuronal **migration**, their respective roles in the tangential **migration** of cortical interneurons remain unknown. Here we show that loss of RhoA and Cdc42 in the ventricular zone (VZ) of the medial ganglionic eminence (MGE) using Olig2-Cre mice causes moderate or severe defects in the **migration** of cortical interneurons, respectively. Furthermore, RhoA- or Cdc42-deleted MGE cells exhibit impaired **migration** in vitro. To determine whether RhoA and Cdc42 directly regulate the motility of cortical interneurons during **migration**, we deleted RhoA and Cdc42 in the subventricular zone (SVZ), where more fate-restricted progenitors are located within the ganglionic eminences, using Dlx5/6-Cre-ires-EGFP (Dlx5/6-CIE) mice. Deletion of either gene within the SVZ does not cause any obvious defects in cortical interneuron **migration**, indicating that cell motility is not dependent upon RhoA or Cdc42. These findings provide genetic evidence that RhoA and Cdc42 are required in progenitors of the MGE in the VZ, but not the SVZ, for proper cortical interneuron **migration**.
40. Inactivation of Cdc42 in embryonic brain results in hydrocephalus with ependymal cell defects in mice. The establishment of a polarized cellular morphology is essential for a variety of processes including neural tube morphogenesis and the development of the brain. Cdc42 is a Ras-related GTPase that plays an essential role in controlling cell polarity through the regulation of the actin and microtubule cytoskeleton architecture. Previous studies have shown that Cdc42 plays an indispensable role in telencephalon development in earlier embryo developmental stage (before E12.5). However, the functions of Cdc42 in other parts of brain in later embryo developmental stage or in adult brain remain unclear. Thus, in order to address the role of Cdc42 in the whole brain in later embryo developmental stage or in adulthood, we used Cre/loxP technology to generate two lines of tissue-specific Cdc42-knock-out mice. Inactivation of Cdc42 was achieved in neuroepithelial cells by crossing Cdc42/ flox mice with Nestin-Cre mice and resulted in hydrocephalus, causing death to occur within the postnatal stage. Histological analyses of the brains from these mice showed that ependymal cell differentiation was disrupted, resulting in aqueductal stenosis. Deletion of Cdc42 in the cerebral cortex also induced obvious defects in interkinetic nuclear **migration** and hypoplasia. To further explore the role of Cdc42 in adult mice brain, we examined the effects of knocking-out Cdc42 in radial glial cells by crossing Cdc42/flox mice with human glial fibrillary acidic protein (GFAP)-Cre mice. Inactivation of Cdc42 in radial glial cells resulted in hydrocephalus and ependymal cell denudation. Taken together, these results highlight the importance of Cdc42 for ependymal cell differentiation and maintaining, and suggest that these functions likely contribute to the essential roles played by Cdc42 in the development of the brain.
41. Plexin-B2 negatively regulates macrophage motility, Rac, and Cdc42 activation. Plexins are cell surface receptors widely studied in the nervous system, where they mediate **migration** and morphogenesis though the Rho family of small GTPases. More recently, plexins have been implicated in immune processes including cell-cell interaction, immune activation, **migration**, and cytokine production. Plexin-B2 facilitates ligand induced cell guidance and **migration** in the nervous system, and induces cytoskeletal changes in overexpression assays through RhoGTPase. The function of Plexin-B2 in the immune system is unknown. This report shows that Plexin-B2 is highly expressed on cells of the innate immune system in the mouse, including macrophages, conventional dendritic cells, and plasmacytoid dendritic cells. However, Plexin-B2 does not appear to regulate the production of proinflammatory cytokines, phagocytosis of a variety of targets, or directional **migration** towards chemoattractants or extracellular matrix in mouse macrophages. Instead, Plxnb2(-/-) macrophages have greater cellular motility than wild type in the unstimulated state that is accompanied by more active, GTP-bound Rac and Cdc42. Additionally, Plxnb2(-/-) macrophages demonstrate faster in vitro wound closure activity. Studies have shown that a closely related family member, Plexin-B1, binds to active Rac and sequesters it from downstream signaling. The interaction of Plexin-B2 with Rac has only been previously confirmed in yeast and bacterial overexpression assays. The data presented here show that Plexin-B2 functions in mouse macrophages as a negative regulator of the GTPases Rac and Cdc42 and as a negative regulator of basal cell motility and wound healing.
42. Impaired Rho GTPase activation abrogates cell polarization and **migration** in macrophages with defective lipolysis. Infiltration of monocytes and macrophages into the site of inflammation is critical in the progression of inflammatory diseases such as atherosclerosis. Cell **migration** is dependent on the continuous organization of the actin cytoskeleton, which is regulated by members of the small Rho GTPase family (RhoA, Cdc42, Rac) that are also important for the regulation of signal transduction pathways. We have recently reported on reduced plaque formation in an atherosclerotic mouse model transplanted with bone marrow from adipose triglyceride lipase-deficient (Atgl-/-) mice. Here we provide evidence that defective lipolysis in macrophages lacking ATGL, the major enzyme responsible for triacylglycerol hydrolysis, favors an anti-inflammatory M2-like macrophage phenotype. Our data implicate an as yet unrecognized principle that insufficient lipolysis influences macrophage polarization and actin polymerization, resulting in impaired macrophage **migration**. Sustained phosphorylation of focal adhesion kinase [due to inactivation of its phosphatase by elevated levels of reactive oxygen species (ROS)] results in defective Cdc42, Rac1 and RhoA activation and in increased and sustained activation of Rac2. Inhibition of ROS production restores the migratory capacity of Atgl-/- macrophages. Since monocyte and macrophage **migration** are a prerequisite for infiltrating the arterial wall, our results provide a molecular link between lipolysis and the development of atherosclerosis.
43. Rho GTPases in hematopoietic stem/progenitor cell **migration**. Rho GTPases including RhoA, Rac1, and Cdc42 are a class of intracellular signaling proteins critical for the regulation of cytoskeleton organization, adhesion, and **migration**. Molecular mechanisms of mammalian cell **migration** were first revealed in fibroblasts where RhoA, Rac1, and Cdc42 facilitate in the multistep process including establishment and maintenance of polarity, formation of actin-rich protrusions, remodeling of adhesive contacts, and generation of force. In hematopoietic stem/progenitor cells, Rho GTPases relay signals from chemokines and cytokines such as SDF-1α and SCF to the actin and microtubule cytoskeleton through effector kinases and/or adaptor molecules that affect adhesion or transcription. Comprehensive use of murine conditional gene knockout technology combined with biochemical approaches in recent studies allows for physiologically relevant investigations of the involvement of Rho GTPases in hematopoietic stem/progenitor cell **migratio**n, providing important mechanisms for the stem/progenitor maintenance.
44. HER2/ErbB2-induced breast cancer cell **migration** and invasion require p120 catenin activation of Rac1 and Cdc42. Breast cancers that overexpress the receptor tyrosine kinase ErbB2/HER2/Neu result in poor patient outcome because of extensive metastatic progression. Herein, we delineate a molecular mechanism that may govern this malignant phenotype. ErbB2 induction of **migration** requires activation of the small GTPases Rac1 and Cdc42. The ability of ErbB2 to activate these small GTPases necessitated expression of p120 catenin, which is itself up-regulated by signaling through ErbB2 and the tyrosine kinase Src. Silencing p120 in ErbB2-dependent breast cancer cell lines dramatically inhibited **migration** and invasion as well as activation of Rac1 and Cdc42. In contrast, overexpression of constitutively active mutants of these GTPases reversed the effects of p120 silencing. Lastly, ectopic expression of p120 promoted **migration** and invasion and potentiated metastatic progression of a weakly metastatic, ErbB2-dependent breast cancer cell line. These results suggest that p120 acts as an obligate intermediate between ErbB2 and Rac1/Cdc42 to modulate the metastatic potential of breast cancer cells.
45. Cdc42 and Rac family GTPases regulate mode and speed but not direction of primary fibroblast **migration** during platelet-derived growth factor-dependent chemotaxis. Cdc42 and Rac family GTPases are important regulators of morphology, motility, and polarity in a variety of mammalian cell types. However, comprehensive analysis of their roles in the morphological and behavioral aspects of chemotaxis within a single experimental system is still lacking. Here we demonstrate using a direct viewing chemotaxis assay that of all of the Cdc42/Rac1-related GTPases expressed in primary fibroblasts, Cdc42, Rac1, and RhoG are required for efficient **migration** towards platelet-derived growth factor (PDGF). During **migration**, Cdc42-, Rac1-, and RhoG-deficient cells show aberrant morphology characterized as cell elongation and cell body rounding, loss of lamellipodia, and formation of thick membrane extensions, respectively. Analysis of individual cell trajectories reveals that cell speed is significantly reduced, as well as persistence, but to a smaller degree, while the directional response to the gradient of PDGF is not affected. Combined knockdown of Cdc42, Rac1, and RhoG results in greater inhibition of cell speed than when each protein is knocked down alone, but the cells are still capable of migrating toward PDGF. We conclude that, Cdc42, Rac1, and RhoG function cooperatively during cell **migration** and that, while each GTPase is implicated in the control of morphology and cell speed, these and other Cdc42/Rac-related GTPases are not essential for the directional response toward PDGF.
46. Regulation of ephexin1, a guanine nucleotide exchange factor of Rho family GTPases, by fibroblast growth factor receptor-mediated tyrosine phosphorylation. Fibroblast growth factor (FGF) signal is implicated in not only cell proliferation, but cell **migration** and morphological changes. Several different Rho family GTPases downstream of the Ras/ERK pathway are postulated to mediate the latter functions. However, none have been recognized to be directly coupled to FGF receptors (FGFRs). We have previously reported that EphA4 and FGFRs hetero-oligomerize through their cytoplasmic domains, trans-activate each other, and transduce a signal for cell proliferation through a docking protein, FRS2alpha (Yokote, H., Fujita, K., Jing, X., Sawada, T., Liang, S., Yao, L., Yan, X., Zhang, Y., Schlessinger, J., and Sakaguchi, K. (2005) Proc. Natl. Acad. Sci. U. S. A. 102, 18866-18871). Here, we have found that ephexin1, a guanine nucleotide exchange factor for Rho family GTPases, constitutes another downstream component of the receptor complex. Ephexin1 directly binds to the kinase domain of FGFR mainly through its DH and PH domains. The binding appears to become weaker and limited to the DH domain when FGFRs become activated. FGFR-mediated phosphorylation of ephexin1 enhances the guanine nucleotide exchange activity toward RhoA without affecting the activity to Rac1 or Cdc42. The FGFR-mediated tyrosine phosphorylation includes, but is not limited to, the residue (Tyr-87) phosphorylated by Src family kinase, which is known to be activated following EphA4 activation. The Tyr-to-Asp mutations that mimic the tyrosine phosphorylation in some of the putative FGFR-mediated phosphorylation sites increase the nucleotide exchange activity for RhoA without changing the activity for Rac1 or Cdc42. From these results, we conclude that ephexin1 is located immediately downstream of the EphA4-FGFR complex and the function is altered by the FGFR-mediated tyrosine phosphorylation at multiple sites.
47. Effects of pharmacological inhibition of small GTPases on axon extension and **migration** of enteric neural crest-derived cells. In the developing enteric nervous system, there is a close association between migrating neural crest-derived cells and the axons of early differentiating neurons. We used pharmacological inhibitors of small GTPases to determine if crest cell **migration** and axon growth could be uncoupled in cultured intact explants of embryonic mouse gut and slices of embryonic gut grown on collagen gels containing GDNF. Inhibition of the Rho effectors, ROCKI/II, or Rac/Cdc42 inhibited both cell **migration** and neurite growth in intact explants of embryonic gut. The effects of both ROCKI/II and Rac/Cdc42 inhibitors were more severe on cell **migration** and axon extension in gut explants from Ret(+/-) mice than in explants from wildtype mice, indicating that Rho GTPases probably act downstream of the receptor tyrosine kinase, Ret. Inhibition of ROCKI/II had different effects on **migration** and axon extension in gut slices grown on collagen gels containing GDNF from that seen in intact explants of gut. We conclude that ROCKI/II and Rac/Cdc42 are required for both neural crest-derived cell **migration** and axon growth in the developing gut.
48. Genetic deletion of Cdc42GAP reveals a role of Cdc42 in erythropoiesis and hematopoietic stem/progenitor cell survival, adhesion, and engraftment. Rho family GTPases are key signal transducers in cell regulation. Although a body of literature has implicated the Rho family members Rac1 and Rac2 in multiple hematopoietic-cell functions, the role of Cdc42 in hematopoiesis remains unclear. Here we have examined the hematopoietic properties and the hematopoietic stem/progenitor cell (HSP) functions of gene-targeted mice carrying null alleles of cdc42gap, a negative regulator of Cdc42. The Cdc42GAP-/- fetal liver and bone marrow cells showed a 3-fold increase in Cdc42 activity but normal Rac and RhoA activities, indicating that Cdc42GAP knockout resulted in a gain of Cdc42 activity in the hematopoietic tissues. Cdc42GAP-/- mice were anemic. The cellularity of fetal liver and bone marrow, the number and composition percentage of HSPs, and the erythroid blast-forming unit and colony-forming unit (BFU-E/CFU-E) activities were significantly reduced in the homozygous mice. The decrease in HSP number was associated with increased apoptosis of the Cdc42GAP-/- HSPs and the activation of JNK-mediated apoptotic machinery. Moreover, homozygous HSPs showed impaired cortical F-actin assembly, deficiency in adhesion and **migration**, and defective engraftment. These results provide evidence that Cdc42 activity is important for erythropoiesis and for multiple HSP functions, including survival, adhesion, and engraftment.
49. Involvement of the phosphatidylinositol 3-kinase/rac1 and cdc42 pathways in radial **migration** of cortical neurons. During cortical development, newly generated neurons migrate radially toward their final positions. Although several candidate genes essential for this radial **migration** have been reported, the signaling pathways regulating it are largely unclear. Here we studied the role of phosphatidylinositol (PI) 3-kinase and its downstream signaling molecules in the radial **migration** of cortical neurons in vivo and in vitro. The expression of constitutively active and dominant-negative PI 3-kinases markedly inhibited radial **migration**. In the neocortical slice culture, a PI 3-kinase inhibitor suppressed the formation of GTP-bound Rac1 and Cdc42 and radial **migration**. Constitutively active and dominant-negative forms of Rac1 and Cdc42 but not Akt also significantly inhibited radial **migration**. In migrating neurons, wild-type Rac1 and Cdc42 showed different localizations; Rac1 localized to the plasma membrane and Cdc42 to the perinuclear region on the side of the leading processes. These results suggest that both the PI 3-kinase/Rac1 and Cdc42 pathways are involved in the radial **migration** of cortical neurons and that they have different roles.
50. Cdc42 and Rac1 signaling are both required for and act synergistically in the correct formation of myelin sheaths in the CNS. The formation of myelin sheaths in the CNS is the result of a complex series of events involving oligodendrocyte progenitor cell (OPC) proliferation, directed **migration**, and the morphological changes associated with axon ensheathment and myelination. To examine the role of Rho GTPases in oligodendrocyte biology, we have used a conditional tissue-specific gene-targeting approach. Ablation of Cdc42 in cells of the oligodendrocyte lineage did not affect OPC proliferation, directed **migration**, or in vitro differentiation, but it led to the formation of a unique and stage-specific myelination phenotype. This was characterized by the extraordinary enlargement of the inner tongue of the oligodendrocyte process and concomitant formation of a myelin outfolding as a result of abnormal accumulation of cytoplasm in this region. Ablation of Rac1 also resulted in the abnormal accumulation of cytoplasm in the inner tongue of the oligodendrocyte process, and we provide genetic evidence that rac1 synergizes with cdc42 in a gene dosage-dependent way to regulate myelination.
51. A Rac/Cdc42-specific exchange factor, GEFT, induces cell proliferation, transformation, and **migration**. The Rho family of small GTPases, including Rho, Rac, and Cdc42, play essential roles in diverse cellular functions. The ability of Rho family GTPases to participate in signaling events is determined by the ratio of inactive (GDP-bound) and active (GTP-bound) forms in the cell. The activation of Rho family proteins requires the exchange of bound GDP for GTP, a process catalyzed by the Dbl family of guanine nucleotide exchange factors (GEFs). The GEFs have high affinity for the guanine nucleotide-free state of the GTPases and are thought to promote GDP release by stabilizing an intermediate transition state. In this study, we have identified and characterized a new Rac/Cdc42-specific Dbl family guanine nucleotide exchange factor, named GEFT. GEFT is highly expressed in the excitable tissues, including brain, heart, and muscle. Low or very little expression was detected in other nonexcitable tissues. GEFT has specific exchange activity for Rac and Cdc42 in our in vitro GTPase exchange assays and glutathione S-transferase-PAK pull-down assays with GTP-bound Rac1 and Cdc42. Overexpression of GEFT leads to changes in cell morphology and actin cytoskeleton re-organization, including the formation of membrane microspikes, filopodia, and lamilliopodia. Furthermore, expression of GEFT in NIH3T3 cells promotes foci formation, cell proliferation, and cell **migration**, possibly through the activation of transcriptional factors involved in cell growth and proliferation. Together, our data suggest that GEFT is a Rac/Cdc42-specific GEF protein that regulates cell morphology, cell proliferation, and transformation.
52. Hydrogen peroxide formation and actin filament reorganization by Cdc42 are essential for ethanol-induced in vitro angiogenesis. This report focuses on the identification of the molecular mechanisms of ethanol-induced in vitro angiogenesis. The manipulation of angiogenesis is an important therapeutic approach for the treatment of cancer, cardiovascular diseases, and chronic inflammation. Our results showed that ethanol stimulation altered the integrity of actin filaments and increased the formation of lamellipodia and filopodia in SVEC4-10 cells. Further experiments demonstrated that ethanol stimulation increased cell **migration** and invasion and induced in vitro angiogenesis in SVEC4-10 cells. Mechanistically, ethanol stimulation activated Cdc42 and produced H(2)O(2) a reactive oxygen species intermediate in SVEC4-10 cells. Measuring the time course of Cdc42 activation and H(2)O(2) production upon ethanol stimulation revealed that the Cdc42 activation and the increase of H(2)O(2) lasted more than 3 h, which indicates the mechanisms of the long duration effects of ethanol on the cells. Furthermore, either overexpression of a constitutive dominant negative Cdc42 or inhibition of H(2)O(2) production abrogated the effects of ethanol on SVEC4-10 cells, indicating that both the activation of Cdc42 and the production of H(2)O(2) are essential for the actions of ethanol. Interestingly, we also found that overexpression of a constitutive dominant positive Cdc42 itself was sufficient to produce H(2)O(2) and to induce in vitro angiogenesis. Taken together, our results suggest that ethanol stimulation can induce H(2)O(2) production through the activation of Cdc42, which results in reorganizing actin filaments and increasing cell motility and in vitro angiogenesis.
53. p21-activated kinase 2 regulates HSPC cytoskeleton, **migration**, and homing via CDC42 activation and interaction with β-Pix. Cytoskeletal remodeling of hematopoietic stem and progenitor cells (HSPCs) is essential for homing to the bone marrow (BM). The Ras-related C3 botulinum toxin substrate (Rac)/cell division control protein 42 homolog (CDC42) effector p21-activated kinase (Pak2) has been implicated in HSPC homing and engraftment. However, the molecular pathways mediating Pak2 functions in HSPCs are unknown. Here, we demonstrate that both Pak2 kinase activity and its interaction with the PAK-interacting exchange factor-β (β-Pix) are required to reconstitute defective ITALIC! Pak2 (ITALIC! Δ/Δ)HSPC homing to the BM. Pak2 serine/threonine kinase activity is required for stromal-derived factor-1 (SDF1α) chemokine-induced HSPC direct**ional mig**ration, whereas Pak2 interaction with β-Pix is required to regulate the velocity o**f HSPC mi**gration and precise F-actin assembly. Lack of SDF1α-induced filopodia and associated abnormal cell protrusions seen in ITALIC! Pak2 (ITALIC! Δ/Δ)HSPCs were rescued by wild-type (WT) Pak2 but not by a Pak2-kinase dead mutant (KD). Expression of a β-Pix interaction-defective mutant of Pak2 rescued filopodia formation but led to abnormal F-actin bundles. Although CDC42 has previously been considered an upstream regulator of Pak2, we found a paradoxical decrease in baseline activation of CDC42 in ITALIC! Pak2 (ITALIC! Δ/Δ)HSPCs, which was rescued by expression of Pak2-WT but not by Pak2-KD; defective homing of ITALIC! Pak2-deleted HSPCs was rescued by constitutive active CDC42. These data demonstrate that both Pak2 kinase activity and its interaction with β-Pix are essential for HSPC filopodia formation, cytoskeletal integrity, and homing via activation of CDC42. Taken together, we provide mechanistic insights into the role of **Pak2 in** HSPC migration and homing.
54. Cdc42 protein acts upstream of IQGAP1 and regulates cytokinesis in mouse oocytes and embryos. Cdc42 and Rac1 Rho family GTPases, and their interacting protein IQGAP1 are the key regulators of cell polarity. We examined the role of Cdc42 and IQGAP1 in establishing the polarity of mouse oocyte and regulation of meiotic and mitotic divisions. We showed that Cdc42 was localized on the microtubules of meiotic and mitotic spindle and in the cortex of mouse oocytes and cleaving embryos. IQGAP1 was present in the cytoplasm and cortex of growing and fully-grown oocytes. During maturation it disappeared from the cortex and during meiotic and mitotic cytokinesis it concentrated in the contractile ring. Toxin B inhibition of the binding activity of Cdc42 changed the localization of IQGAP1, inhibited emission of the first polar body, and caused disappearance of the cortical actin without affecting the **migration** of meiotic spindle. This indicates, that in maturing oocytes accumulation of cortical actin is not indispensable for spindle **migration**. In zygotes treated with toxin B actin cytoskeleton was rearranged and the first and/or subsequent cytokinesis were inhibited. Our results indicate that Cdc42 acts upstream of IQGAP1 and is involved in regulation of cytokinesis in mouse oocytes and cleaving embryos, rather than in establishing the polarity of the oocyte.
55. Regulation of Cdc42-mediated morphological effects: a novel function for p53. The tumour suppressor functions of p53 that are important for its activity depend on its role as a cell cycle arrest mediator and apoptosis inducer. Here we identify a novel function for p53 in regulating cell morphology and movement. We investigated the overall effect of p53 on morphological changes induced by RhoA, Rac1 and Cdc42 GTPases in mouse embryonic fibroblasts (MEFs). Interestingly, p53 exerted a selective effect on Cdc42-mediated cell functions. (i) Both overexpression of wild-type p53 and activation of endogenous p53 counteracted Cdc42-induced filopodia formation. Conversely, p53-deficient MEFs exhibited constitutive membrane filopodia. Mechanistic studies indicate that p53 prevents the initiating steps of filopodia formation downstream of Cdc42. (ii) Over expression of p53 modulates cell spreading of MEFs on fibronectin. (iii) During cell **migration**, the reorientation of the Golgi apparatus in the direction of movement is abolished by wild-type p53 expression, thus preventing cell polarity. Our data demonstrate a previously uncharacterized role for p53 in regulating Cdc42-dependent cell effects that control actin cytoskeletal dynamics and cell movement. This novel function may contribute to p53 anti-tumour activity.
56. RhoB links PDGF signaling to cell **migration** by coordinating activation and localization of Cdc42 and Rac. The small GTPase RhoB regulates endocytic trafficking of receptor tyrosine kinases (RTKs) and the non-receptor kinases Src and Akt. While receptor-mediated endocytosis is critical for signaling processes driving cell **migration**, mechanisms that coordinate endocytosis with the propagation of migratory signals remain relatively poorly understood. In this study, we show that RhoB is essential for activation and trafficking of the key migratory effectors Cdc42 and Rac in mediating the ability of platelet-derived growth factor (PDGF) to stimulate cell movement. Stimulation of the PDGF receptor-β on primary vascular smooth muscle cells (VSMCs) results in RhoB-dependent trafficking of endosome-bound Cdc42 from the perinuclear region to the cell periphery, where the RhoGEF Vav2 and Rac are also recruited to drive formation of circular dorsal and peripheral ruffles necessary for cell **migratio**n. Our findings identify a novel RhoB-dependent endosomal trafficking pathway that integrates RTK endocytosis with Cdc42/Rac localization and cell movement.
57. Calcium-dependent interaction of Lis1 with IQGAP1 and Cdc42 promotes neuronal motility. Lis1 gene defects impair neuronal **migration**, causing the severe human brain malformation lissencephaly. Although much is known about its interactions with microtubules, microtubule-binding proteins such as CLIP-170, and with the dynein motor complex, the response of Lis1 to neuronal motility signals has not been elucidated. Lis1 deficiency is associated with deregulation of the Rho-family GTPases Cdc42, Rac1 and RhoA, and ensuing actin cytoskeletal defects, but the link between Lis1 and Rho GTPases remains unclear. We report here that calcium influx enhances neuronal motility through Lis1-dependent regulation of Rho GTPases. Lis1 promotes Cdc42 activation through interaction with the calcium sensitive GTPase scaffolding protein IQGAP1, maintaining the perimembrane localization of IQGAP1 and CLIP170 and thereby tethering microtubule ends to the cortical actin cytoskeleton. Lis1 thus is a key component of neuronal motility signal transduction that regulates the cytoskeleton by complexing with IQGAP1, active Cdc42 and CLIP-170 upon calcium influx.
58. DOCK8 is a Cdc42 activator critical for interstitial dendritic cell **migration** during immune responses. To migrate efficiently through the interstitium, dendritic cells (DCs) constantly adapt their shape to the given structure of the extracellular matrix and follow the path of least resistance. It is known that this amoeboid **migration** of DCs requires Cdc42, yet the upstream regulators critical for localization and activation of Cdc42 remain to be determined. Mutations of DOCK8, a member of the atypical guanine nucleotide exchange factor family, causes combined immunodeficiency in humans. In the present study, we show that DOCK8 is a Cdc42-specific guanine nucleotide exchange factor that is critical for interstitial DC **migration**. By generating the knockout mice, we found that in the absence of DOCK8, DCs failed to accumulate in the lymph node parenchyma for T-cell priming. Although DOCK8-deficient DCs migrated normally on 2-dimensional surfaces, DOCK8 was required for DCs to crawl within 3-dimensional fibrillar networks and to transmigrate through the subcapsular sinus floor. This function of DOCK8 depended on the DHR-2 domain mediating Cdc42 activation. DOCK8 deficiency did not affect global Cdc42 activity. However, Cdc42 activation at the leading edge membrane was impaired in DOCK8-deficient DCs, resulting in a severe defect in amoeboid polarization and **migration**. Therefore, DOCK8 regulates interstitial DC **migration** by controlling Cdc42 activity spatially.
59. A role for Cdc42 in macrophage chemotaxis. Three members of the Rho family, Cdc42, Rac, and Rho are known to regulate the organization of actin-based cytoskeletal structures. In Bac1.2F5 macrophages, we have shown that Rho regulates cell contraction, whereas Rac and Cdc42 regulate the formation of lamellipodia and filopodia, respectively. We have now tested the roles of Cdc42, Rac, and Rho in colony stimulating factor-1 (CSF-1)-induced macrophage **migration** and chemotaxis using the Dunn chemotaxis chamber. Microinjection of constitutively activated RhoA, Rac1, or Cdc42 inhibited cell **migration**, presumably because the cells were unable to polarize significantly in response to CSF-1. Both Rho and Rac were required for CSF-1-induced **migration**, since **migration** speed was reduced to background levels in cells injected with C3 transferase, an inhibitor of Rho, or with the dominant-negative Rac mutant, N17Rac1. In contrast, cells injected with the dominant-negative Cdc42 mutant, N17Cdc42, were able to migrate but did not polarize in the direction of the gradient, and chemotaxis towards CSF-1 was abolished. We conclude that Rho and Rac are required for the process of cell **migration**, whereas Cdc42 is required for cells to respond to a gradient of CSF-1 but is not essential for cell locomotion.
60. Deletion of Cdc42 enhances ADAM17-mediated vascular endothelial growth factor receptor 2 shedding and impairs vascular endothelial cell survival and vasculogenesis. Cdc42 is a Ras-related GTPase that plays an important role in the regulation of a range of cellular functions, including cell **migration**, proliferation, and survival. Consistent with its critical functions in vitro, the inactivation of Cdc42 in mice has been shown to result in embryonic lethality at embryonic day 6.5 (E6.5) before blood vessel formation. To determine the role of Cdc42 in new blood vessel formation, we have generated vascular endothelial cell (EC)-specific Cdc42 knockout mice by crossing Cdc42(flox/flox) mice with Tie2-Cre mice. The deletion of Cdc42 in ECs caused embryonic lethality with vasculogenesis and angiogenesis defects. We observed that Cdc42 is critical for EC **migration** and survival but not for cell cycle progression. Moreover, we found that the inactivation of Cdc42 in ECs decreased the level of vascular endothelial growth factor receptor 2 (VEGFR2) protein on the EC surface and promoted the production of a 75-kDa membrane-associated C-terminal VEGFR2 fragment. Using cultured primary mouse ECs and human umbilical vein ECs, we have demonstrated that the deletion of Cdc42 increased ADAM17-mediated VEGFR2 shedding. Notably, inhibition of ADAM17 or overexpression of VEGFR2 can partially reverse Cdc42 deletion-induced EC apoptosis. These data indicate that Cdc42 is essential for VEGFR2-mediated signal transduction in blood vessel formation.
61. Gene targeting of Cdc42 and Cdc42GAP affirms the critical involvement of Cdc42 in filopodia induction, directed **migration**, and proliferation in primary mouse embryonic fibroblasts. Recent studies in Cdc42 knockout mouse embryonic stem (ES) cells and ES-derived fibroblastoid cell lines raise concern on a body of literature derived by dominant mutant expression approach in a variety of cell lines implicating mammalian Cdc42 as a key regulator of filopodia induction, directional **migration** and cell cycle progression. To resolve the physiological function of mammalian Cdc42, we have characterized the Cdc42(-/-) and Cdc42GAP(-/-) primary mouse embryonic fibroblasts (MEFs) produced by gene targeting as the Cdc42 loss- or gain-of-activity cell model. The Cdc42(-/-) cells were defective in filopodia formation stimulated by bradykinin and in dorsal membrane ruffling stimulated by PDGF, whereas the Cdc42GAP(-/-) cells displayed spontaneous filopodia. The Cdc42 loss- or gain-of-activity cells were defective in adhesion to fibronectin, wound-healing, polarity establishment, and **migration** toward a serum gradient. These defects were associated with deficiencies of PAK1, GSK3beta, myosin light chain, and FAK phosphorylation. Furthermore, Cdc42(-/-) cells were defective in G1/S-phase transition and survival, correlating with deficient NF-kappaB transcription and defective JNK, p70 S6K, and ERK1/2 activation. These results demonstrate a different requirement of Cdc42 activity in primary MEFs from ES or ES-derived clonal fibroblastoid cells and suggest that Cdc42 plays cell-type-specific signaling roles.
62. CLN3 deficient cells display defects in the ARF1-Cdc42 pathway and actin-dependent events. Juvenile Batten disease (juvenile neuronal ceroid lipofuscinosis, JNCL) is a devastating neurodegenerative disease caused by mutations in CLN3, a protein of undefined function. Cell lines derived from patients or mice with CLN3 deficiency have impairments in actin-regulated processes such as endocytosis, autophagy, vesicular trafficking, and cell **migration**. Here we demonstrate the small GTPase Cdc42 is misregulated in the absence of CLN3, and thus may be a common link to multiple cellular defects. We discover that active Cdc42 (Cdc42-GTP) is elevated in endothelial cells from CLN3 deficient mouse brain, and correlates with enhanced PAK-1 phosphorylation, LIMK membrane recruitment, and altered actin-driven events. We also demonstrate dramatically reduced plasma membrane recruitment of the Cdc42 GTPase activating protein, ARHGAP21. In line with this, GTP-loaded ARF1, an effector of ARHGAP21 recruitment, is depressed. Together these data implicate misregulated ARF1-Cdc42 signaling as a central defect in JNCL cells, which in-turn impairs various cell functions. Furthermore our findings support concerted action of ARF1, ARHGAP21, and Cdc42 to regulate fluid phase endocytosis in mammalian cells. The ARF1-Cdc42 pathway presents a promising new avenue for JNCL therapeutic development.
63. Nuclear movement regulated by Cdc42, MRCK, myosin, and actin flow establishes MTOC polarization in migrating cells. The microtubule-organizing center (MTOC) is reoriented between the nucleus and the leading edge in many migrating cells and contributes to directional **migration**. Models suggest that the MTOC is moved to its position during reorientation. By direct imaging of wound-edge fibroblasts after triggering MTOC reorientation with soluble factors, we found instead that the nucleus moved away from the leading edge to reorient the MTOC, while the MTOC remained stationary. Rearward nuclear movement was coupled with actin retrograde flow and was regulated by a pathway involving Cdc42, MRCK, myosin, and actin. Nuclear movement was unaffected by the inhibition of dynein, Par6, or PKCzeta, yet these components were essential for MTOC reorientation, as they maintained the MTOC at the cell centroid. These results show that nuclear repositioning is an initial polarizing event in migrating cells and that the positions of the nucleus and the MTOC are established by separate regulatory pathways.
64. Small molecule targeting Cdc42-intersectin interaction disrupts Golgi organization and suppresses cell motility. Signaling through the Rho family of small GTPases has been intensely investigated for its crucial roles in a wide variety of human diseases. Although RhoA and Rac1 signaling pathways are frequently exploited with the aid of effective small molecule modulators, studies of the Cdc42 subclass have lagged because of a lack of such means. We have applied high-throughput in silico screening and identified compounds that are able to fit into the surface groove of Cdc42, which is critical for guanine nucleotide exchange factor binding. Based on the interaction between Cdc42 and intersectin (ITSN), a specific Cdc42 guanine nucleotide exchange factor, we discovered compounds that rendered ITSN-like interactions in the binding pocket. By using in vitro binding and imaging as well as biochemical and cell-based assays, we demonstrated that ZCL278 has emerged as a selective Cdc42 small molecule modulator that directly binds to Cdc42 and inhibits its functions. In Swiss 3T3 fibroblast cultures, ZCL278 abolished microspike formation and disrupted GM130-docked Golgi structures, two of the most prominent Cdc42-mediated subcellular events. ZCL278 reduces the perinuclear accumulation of active Cdc42 in contrast to NSC23766, a selective Rac inhibitor. ZCL278 suppresses Cdc42-mediated neuronal branching and growth cone dynamics as well as actin-based motility and **migration** in a metastatic prostate cancer cell line (i.e., PC-3) without disrupting cell viability. Thus, ZCL278 is a small molecule that specifically targets Cdc42-ITSN interaction and inhibits Cdc42-mediated cellular processes, thus providing a powerful tool for research of Cdc42 subclass of Rho GTPases in human pathogenesis, such as those of cancer and neurological disorders.
65. Cas utilizes Nck2 to activate Cdc42 and regulate cell polarization during cell **migration** in response to wound healing. Integrin-mediated activation of Cdc42 is essential for cell polarization, whereas the integrin adaptor protein Cas is required for cell **migration** during wound healing. After phosphorylation on tyrosine residues, Cas recruits the adaptor proteins Crk and Nck to execute integrin-mediated signals. However, the mechanisms leading to Cdc42 activation and its relationship with Cas, Crk and Nck have not been elucidated clearly. In the present study, we demonstrate that Cas utilizes Nck2 to activate Cdc42 and induce cell polarization in response to wounding. By contrast, Cas recruits CrkII to activate Rac1 and promote the extension of cell protrusions needed for cell motility. These results indicate that Cas utilizes Nck2 and CrkII in a coordinated set of distinct pathways leading to cell **migration**.
66. Cdc42 critically regulates the balance between myelopoiesis and erythropoiesis. The Rho GTPase Cdc42 regulates adhesion, **migration**, and homing, as well as cell cycle progression, of hematopoietic stem cells, but its role in multilineage blood development remains unclear. We report here that inducible deletion of cdc42 in cdc42-floxed mouse bone marrow by the interferon-responsive, Mx1-Cre-mediated excision led to myeloid and erythroid developmental defects. Cdc42 deletion affected the number of early myeloid progenitors while suppressing erythroid differentiation. Cdc42-deficient mice developed a fatal myeloproliferative disorder manifested by significant leukocytosis with neutrophilia, myeloid hyperproliferation, and myeloid cell infiltration into distal organs. Concurrently, Cdc42 deficiency caused anemia and splenomegaly accompanied with decreased bone marrow erythroid burst-forming units (BFU-Es) and colony-forming units-erythroid (CFU-Es) activities and reduced immature erythroid progenitors, suggesting that Cdc42 deficiency causes a block in the early stage of erythropoiesis. Cdc42 activity is responsive to stimulation by SCF, IL3, SDF-1alpha, and fibronectin. The increased myelopoiesis and decreased erythropoiesis of the knockout mice are associated with an altered gene transcription program in hematopoietic progenitors, including up-regulation of promyeloid genes such as PU.1, C/EBP1alpha, and Gfi-1 in the common myeloid progenitors and granulocyte-macrophage progenitors and down-regulation of proerythroid gene such as GATA-2 in the megakaryocyte-erythroid progenitors. Thus, Cdc42 is an essential regulator of the balance between myelopoiesis and erythropoiesis.
67. Rho GTPase protein Cdc42 is critical for postnatal cartilage development. Cdc42, a small Rho GTPase family member, has been shown to regulate multiple cellular functions in vitro, including actin cytoskeletal reorganization, cell **migration**, proliferation, and gene expression. However, its tissue-specific roles in vivo remain largely unknown, especially in postnatal cartilage development, as cartilage-specific Cdc42 inactivated mice die within a few days after birth. In this study, we investigated the physiological functions of Cdc42 during cartilage development after birth using tamoxifen-induced cartilage-specific inactivated Cdc42 conditional knockout (Cdc42 (fl/fl); Col2-CreERT) mice, which were generated by crossing Cdc42 flox mice (Cdc42 (fl/fl)) with tamoxifen-induced type II collagen (Col2) Cre transgenic mice using a Cre/loxP system. The gross morphology of the Cdc42 cKO mice was shorter limbs and body, as well as reduced body weight as compared with the controls. In addition, severe defects were found in growth plate chondrocytes of the long bones, characterized by a shorter proliferating zone (PZ), wider hypertrophic zone (HZ), and loss of columnar organization of proliferating chondrocytes, resulting in delayed endochondral bone formation associated with abnormal bone growth. Our findings demonstrate the importance of Cdc42 for cartilage development during both embryonic and postnatal stages.
68. CDC42 is required for structural patterning of the lung during development. The formation of highly branched epithelial structures is critical for the development of many essential organs, including lung, liver, pancreas, kidney and mammary glands. Elongation and branching of these structures require precise control of complex morphogenetic processes that are dependent upon coordinate regulation of cell shape, apical-basal polarity, proliferation, **migration**, and interactions among multiple cell types. Herein, we demonstrate that temporal-spatial regulation of epithelial cell polarity by the small GTPase, CDC42, is essential for branching morphogenesis of the developing lung. Epithelial cell-specific deletion of CDC42 in fetal mice disrupted epithelial cell polarity, the actin cytoskeleton, intercellular contacts, directional trafficking of proteins, proliferation and mitotic spindle orientation, impairing the organization and patterning of the developing respiratory epithelium and adjacent mesenchyme. Transition from a pseudostratified to a simple columnar epithelium was impaired, consistent with coordinate dysregulation of epithelial cell polarity, mitotic spindle orientation, and repositioning of mitotic cells within the epithelium during cell cycle progression. Expression of sonic hedgehog and its receptor, patched-1, was decreased, while fibroblast growth factor 10 expression in the mesenchyme was expanded, resulting in disruption of branching morphogenesis and bronchiolar smooth muscle formation in this model. CDC42 is required for spatial positioning of proliferating epithelial cells, as well as signaling interactions between the epithelium and mesenchyme and is, therefore, essential for formation and maintenance of the respiratory tract during morphogenesis of the fetal lung.
69. Cdc42 regulates neutrophil **migration** via crosstalk between WASp, CD11b, and microtubules. Chemotaxis promotes neutrophil participation in cellular defense by enabling neutrophil **migration** to infected tissue and is controlled by persistent cell polarization. One long-standing question of neutrophil polarity has been how the pseudopod and the uropod are coordinated. In our previous report, we suggested that Rho GTPase Cdc42 controls neutrophil polarity through CD11b signaling at the uropod, albeit through an unknown mechanism. Here, we show that Cdc42 controls polarity, unexpectedly, via its effector WASp. Cdc42 controls WASp activation and its distant localization to the uropod. At the uropod, WASp regulates the reorganization of CD11b integrin into detergent resistant membrane domains; in turn, CD11b recruits the microtubule end binding protein EB1 to capture and stabilize microtubules at the uropod. This organization is necessary to maintain neutrophil polarity during **migration** and is critical for neutrophil e**migration** into inflamed lungs. These results suggest unrecognized mechanism of neutrophil polarity in which WASp mediates long-distance control of the uropod by Cdc42 to maintain a proper balance between the pseudopod and the uropod. Our study reveals a new function for WASp in the control of neutrophil polarity via crosstalk between CD11b and microtubules.
70. Cdc42-dependent leading edge coordination is essential for interstitial dendritic cell **migration**. Mature dendritic cells (DCs) moving from the skin to the lymph node are a prototypic example of rapidly migrating amoeboid leukocytes. Interstitial DC **migration** is directionally guided by chemokines, but independent of specific adhesive interactions with the tissue as well as pericellular proteolysis. Instead, the protrusive flow of the actin cytoskeleton directly drives a basal mode of locomotion that is occasionally supported by actomyosin contractions at the trailing edge to propel the cell's rigid nucleus. We here delete the small GTPase Cdc42 in DCs and find that actin flow and actomyosin contraction are still initiated in response to chemotactic cues. Accordingly, the cells are able to polarize and form protrusions. However, in the absence of Cdc42 the protrusions are temporally and spatially dysregulated, which leads to impaired leading edge coordination. Although this defect still allows the cells to move on 2-dimensional surfaces, their in vivo motility is completely abrogated. We show that this difference is entirely caused by the geometric complexity of the environment, as multiple competing protrusions lead to instantaneous entanglement within 3-dimensional extracellular matrix scaffolds. This demonstrates that the decisive factor for migrating DCs is not specific interaction with the extracellular environment, but adequate coordination of cytoskeletal flow.
71. Cdc42 regulates epithelial cell polarity and cytoskeletal function during kidney tubule development. The Rho GTPase Cdc42 regulates key signaling pathways required for multiple cell functions, including maintenance of shape, polarity, proliferation, **migration**, differentiation and morphogenesis. Although previous studies have shown that Cdc42 is required for proper epithelial development and maintenance, its exact molecular function in kidney development is not well understood. In this study, we define the specific role of Cdc42 during murine kidney epithelial tubulogenesis by deleting it selectively at the initiation of ureteric bud or metanephric mesenchyme development. Deletion in either lineage results in abnormal tubulogenesis, with profound defects in polarity, lumen formation and the actin cytoskeleton. Ultimately, these defects lead to renal failure. Additionally, in vitro analysis of Cdc42-null collecting duct cells shows that Cdc42 controls these processes by regulating the polarity Par complex (Par3-Par6-aPKC-Cdc42) and the cytoskeletal proteins N-Wasp and ezrin. Thus, we conclude that the principal role of Cdc42 in ureteric bud and metanephric mesenchyme development is to regulate epithelial cell polarity and the actin cytoskeleton.
72. Promyogenic function of Integrin/FAK signaling is mediated by Cdo, Cdc42 and MyoD. The Integrin-mediated cell adhesion to the extracellular matrix is implicated in the control of proliferation, survival, **migration** and differentiation of myoblasts. Focal adhesion kinase (FAK) mediates signals from Integrins and plays an essential role in myotube formation. Cdo forms a multiprotein complex that includes other cell adhesion molecules like Cadherins and Boc. Multiple signals emanate from such complexes, including Cdc42 and p38MAPK pathways to activate MyoD. Here we show that C2C12 myoblasts cultured in suspension or on Poly-L-Lysine (PLL), a well known Integrin-independent substratum, failed to express Cdo and MyoD, while the expression of Cadherins and Boc was unchanged. In addition, the activation of Akt and p38MAPK as well as the expression of Cdc42 was affected in these cells. Overexpression of FAK rescued MyoD and Cdo expression as well as myotube formation of C2C12 cells on PLL. Furthermore, reintroduction of Cdo induced enhanced myotube formation on PLL and increased the expression of myogenic markers. Inhibition of ROCK or overexpression of Cdc42-V12 in C2C12 cells upregulated Cdc42 and MyoD expression and rescued defective myoblast differentiation. Taken together, these data indicate that the Integrin/FAK signaling pathway is required for myoblast differentiation by regulating the expression of the promyogenic factors, Cdo, MyoD and Cdc42.
73. Cdc42 is required for chondrogenesis and interdigital programmed cell death during limb development. Cdc42, a member of the Rho subfamily of small GTPases, is known to be a regulator of multiple cellular functions, including cytoskeletal organization, cell **migration**, proliferation, and apoptosis. However, its tissue-specific roles, especially in mammalian limb development, remain unclear. To investigate the physiological function of Cdc42 during limb development, we generated limb bud mesenchyme-specific inactivated Cdc42 (Cdc42(fl/fl); Prx1-Cre) mice. Cdc42(fl/fl); Prx1-Cre mice demonstrated short limbs and body, abnormal calcification of the cranium, cleft palate, disruption of the xiphoid process, and syndactyly. Severe defects were also found in long bone growth plate cartilage, characterized by loss of columnar organization of chondrocytes, and thickening and massive accumulation of hypertrophic chondrocytes, resulting in delayed endochondral bone formation associated with reduced bone growth. In situ hybridization analysis revealed that expressions of Col10 and Mmp13 were reduced in non-resorbed hypertrophic cartilage, indicating that deletion of Cdc42 inhibited their terminal differentiation. Syndactyly in Cdc42(fl/fl); Prx1-Cre mice was caused by fusion of metacarpals and a failure of interdigital programmed cell death (ID-PCD). Whole mount in situ hybridization analysis of limb buds showed that the expression patterns of Sox9 were ectopic, while those of Bmp2, Msx1, and Msx2, known to promote apoptosis in the interdigital mesenchyme, were down-regulated. These results demonstrate that Cdc42 is essential for chondrogenesis and ID-PCD during limb development.
74. Cdc42 expression in keratinocytes is required for the maintenance of the basement membrane in skin. Cdc42 is a small GTPase, which acts as a molecular switch to regulate a wide variety of cellular functions, such as actin cytoskeleton organization, cell proliferation, apoptosis, cell **migration** and in particular, cell polarity. Formation and maintenance of the basement membrane is a polarized process, which requires directed secretion, deposition and organization of basement membrane components at the basal side of epithelial cells. In the current study, we analyzed the maintenance of skin basement membrane in mice with a keratinocyte-restricted deletion of the Cdc42 gene. In the absence of Cdc42, basement membrane components became aberrantly deposited and the processing of laminin 5 was impaired in parts of the dermal-epidermal junction. These impairments became more severe with age and corresponded to local defects of the basement membrane in 4.5-month-old mutant mice. However, both, structure and number of hemidesomosomes were not significantly changed in the Cdc42 mutant skin compared with the control mice and no blister formation was observed in mutant skin. These data indicate that Cdc42 in keratinocytes is important for maintenance of the basement membrane of skin.
75. Rho GTPase Cdc42 is essential for B-lymphocyte development and activation. Cdc42 is a member of the Rho GTPase family that has been implicated in several cell functions including proliferation and **migration**, but its physiologic role needs to be dissected in each cell type. We achieved B-cell and hematopoietic stem cell deletion of Cdc42 by conditional gene targeting in mice. Deletion of Cdc42 from proB/preB-cell stage significantly blocked B-cell development at T1 and later stages, resulting in reduced mature B-cell populations and reduced antigen-specific immunoglobulin M (IgM), IgG1, and IgG3 production. The Cdc42(-/-) B cells, themselves, were abnormal with impaired proliferation and survival. The mutant B cells were further characterized by a B-cell receptor (BCR) signaling defect with increased Erk and decreased Akt activation, as well as a defect in BCR-mediated B-cell-activating factor (BAFF) receptor up-regulation and subsequent BAFF receptor signaling in mature resting B cells. Surprisingly, Cdc42 was dispensable for stromal cell-derived factor-1alpha (SDF-1alpha)- or B-lymphocyte chemoattractant (BLC)-induced B-cell **migration**. Finally, loss of Cdc42 from hematopoietic stem cells did not alter common lymphoid progenitor production but severely reduced proB/preB- and immature B-cell populations, indicating that Cdc42 is also involved in B-cell precursor differentiation. These results reveal multifaceted roles of Cdc42 in B-cell development and activation.
76. Daphnetin inhibits invasion and **migration** of LM8 murine osteosarcoma cells by decreasing RhoA and Cdc42 expression. Daphnetin, 7,8-dihydroxycoumarin, present in main constituents of Daphne odora var. marginatai, has multiple pharmacological activities including anti-proliferative effects in cancer cells. In this study, using a Transwell system, we showed that daphnetin inhibited invasion and **migration** of highly metastatic murine osteosarcoma LM8 cells in a dose-dependent manner. Following treatment by daphnetin, cells that penetrated the Transwell membrane were rounder than non-treated cells. Immunofluorescence analysis revealed that daphnetin decreased the numbers of intracellular stress fibers and filopodia. Moreover, daphnetin treatment dramatically decreased the expression levels of RhoA and Cdc42. In summary, the dihydroxycoumarin derivative daphnetin inhibits the invasion and **migration** of LM8 cells, and therefore represents a promising agent for use against metastatic cancer.
77. Cdc42 regulates arsenic-induced NADPH oxidase activation and cell **migration** through actin filament reorganization. Although arsenic is a human carcinogen, the molecular mechanisms of its action remain to be understood. The present study reports that exposure to arsenic induced actin filament reorganization, resulting in lamellipodia and filopodia structures through the activation of Cdc42 in SVEC4-10 endothelial cells. It was also found that arsenic induced the formation of the superoxide anion (O2\*) in SVEC4-10 cells. Immunoprecipitation and Western blotting analysis demonstrated that arsenic stimulation induced serine phosphorylation of p47phox, a key component of NADPH oxidase, indicating that arsenic induces O2\* formation through NADPH oxidase activation. Inhibition of arsenic-induced actin filament reorganization by either overexpression of a dominant negative Cdc42 or pretreatment of an actin filament stabilizing regent, jasplakinolide, abrogated arsenic-induced NADPH oxidase activation, showing that the activation of NADPH oxidase was regulated by Cdc42-mediated actin filament reorganization. This study also showed that overexpression of a dominant negative Rac1 was sufficient to abolish arsenic-induced O2\*- production, implying that Rac1 activities are required for Cdc42-mediated NADPH oxidase activation in response to arsenic stimulation. Furthermore, arsenic stimulation induced cell **migration**, which can be inhibited by the inactivation of either Cdc42 or NADPH oxidase. Taken together, the results indicate that arsenic is able to activate NADPH oxidase through Cdc42-mediated actin filament reorganization, leading to the induction of an increase in cell **migration** in SVEC4-10 endothelial cells.
78. Reelin signals through apolipoprotein E receptor 2 and Cdc42 to increase growth cone motility and filopodia formation. Lipoprotein receptor signaling regulates the positioning and differentiation of postmitotic neurons during development and modulates neuronal plasticity in the mature brain. Depending on the contextual situation, the lipoprotein receptor ligand Reelin can have opposing effects on cortical neurons. We show that Reelin increases growth cone motility and filopodia formation, and identify the underlying signaling cascade. Reelin activates the Rho GTPase Cdc42, known for its role in neuronal morphogenesis and directed **migration**, in an apolipoprotein E receptor 2-, Disabled-1-, and phosphatidylinositol 3-kinase-dependent manner. We demonstrate that neuronal vesicle trafficking, a Cdc42-controlled process, is increased after Reelin treatment and further provide evidence that the peptidergic VIP/PACAP38 system and Reelin can functionally interact to promote axonal branching. In conclusion, Reelin-induced activation of Cdc42 contributes to the regulation of the cytoskeleton of individual responsive neurons and converges with other signaling cascades to orchestrate Rho GTPase activity and promote neuronal development. Our data link the observation that defects in Rho GTPases and Reelin signaling are responsible for developmental defects leading to neurological and psychiatric disorders.
79. Asymmetric positioning and organization of the meiotic spindle of mouse oocytes requires CDC42 function. The mature mammalian oocyte is highly polarized because asymmetrical spindle **migration** to the oocyte cortex ensures extrusion of small polar bodies in the two meiotic divisions, essential for generation of the large egg. Actin filaments, myosin motors, and formin-2, but not microtubules, are required for spindle **migration**. Here, we show that Cdc42, a key regulator of cytoskeleton and cell polarity in other systems , is essential for meiotic maturation and oocyte asymmetry. Disrupting CDC42 function by ectopic expression of its GTPase-defective mutants causes both halves of the first meiotic spindle to extend symmetrically toward opposing cortical regions and prevents an asymmetrical division. The elongated spindle has numerous astral-like microtubules, and aPKCzeta, normally associated with the spindle poles, is distributed along its length. Dynactin is displaced from kinetochores, consistently homologous chromosomes do not segregate, and polar body extrusion is prevented. Perturbing the function of aPKCzeta also causes elongation of the meiotic spindle but still permits spindle **migration** and polar body extrusion. Thus, at least two pathways appear to be downstream of CDC42: one affecting the actin cytoskeleton and required for **migration** of the meiotic spindle, and a second affecting the spindle microtubules in which aPKCzeta plays a role.
80. The orphan adhesion G protein-coupled receptor GPR97 regulates **migration** of lymphatic endothelial cells via the small GTPases RhoA and Cdc42. The important role of the lymphatic vascular system in pathological conditions such as inflammation and cancer has been increasingly recognized, but its potential as a pharmacological target is poorly exploited. Our study aimed at the identification and molecular characterization of lymphatic-specific G protein-coupled receptors (GPCRs) to assess new targets for pharmacological manipulation of the lymphatic vascular system. We used a TaqMan quantitative RT-PCR-based low density array to determine the GPCR expression profiles of ex vivo isolated intestinal mouse lymphatic (LECs) and blood vascular endothelial cells (BECs). GPR97, an orphan adhesion GPCR of unknown function, was the most highly and specifically expressed GPCR in mouse lymphatic endothelium. Using siRNA silencing, we found that GPR97-deficient primary human LECs displayed increased adhesion and collective cell **migration**, whereas single cell **migration** was decreased as compared with nontargeting siRNA-transfected control LECs. Loss of GPR97 shifted the ratio of active Cdc42 and RhoA and initiated cytoskeletal rearrangements, including F-actin redistribution, paxillin and PAK4 phosphorylation, and β1-integrin activation. Our data suggest a possible role of GPR97 in lymphatic remodeling and furthermore provide the first insights into the biological functions of GPR97.
81. Distinct roles of Cdc42 in thymopoiesis and effector and memory T cell differentiation. Cdc42 of the Rho GTPase family has been implicated in cell actin organization, proliferation, survival, and **migration** but its physiological role is likely cell-type specific. By a T cell-specific deletion of Cdc42 in mouse, we have recently shown that Cdc42 maintains naïve T cell homeostasis through promoting cell survival and suppressing T cell activation. Here we have further investigated the involvement of Cdc42 in multiple stages of T cell differentiation. We found that in Cdc42(-/-) thymus, positive selection of CD4(+)CD8(+) double-positive thymocytes was defective, CD4(+) and CD8(+) single-positive thymocytes were impaired in **migratio**n and showed an increase in cell apoptosis triggered by anti-CD3/-CD28 antibodies, and thymocytes were hyporesponsive to anti-CD3/-CD28-induced cell proliferation and hyperresponsive to anti-CD3/-CD28-stimulated MAP kinase activation. At the periphery, Cdc42-deficient naive T cells displayed an impaired actin polymerization and TCR clustering during the formation of mature immunological synapse, and showed an enhanced differentiation to Th1 and CD8(+) effector and memory cells in vitro and in vivo. Finally, Cdc42(-/-) mice exhibited exacerbated liver damage in an induced autoimmune disease model. Collectively, these data establish that Cdc42 is critically involved in thymopoiesis and plays a restrictive role in effector and memory T cell differentiation and autoimmunity.
82. Cdc42 promotes host defenses against fatal infection. The small Rho GTPase Cdc42 regulates key signaling pathways required for multiple cell functions, including maintenance of shape, polarity, proliferation, invasion, **migration**, differentiation, and morphogenesis. As the role of Cdc42-dependent signaling in fibroblasts in vivo is unknown, we attempted to specifically delete it in these cells by crossing the Cdc42(fl/fl) mouse with an fibroblast-specific protein 1 (FSP1)-Cre mouse, which is thought to mediate recombination exclusively in fibroblasts. Surprisingly, the FSP1-Cre;Cdc42(fl/fl) mice died at 3 weeks of age due to overwhelming suppurative upper airway infections that were associated with neutrophilia and lymphopenia. Even though major aberrations in lymphoid tissue development were present in the mice, the principal cause of death was severe **migration** and killing abnormalities of the neutrophil population resulting in an inability to control infection. We also show that in addition to fibroblasts, FSP1-Cre deleted Cdc42 very efficiently in all leukocytes. Thus, by using this nonspecific Cre mouse, we inadvertently demonstrated the importance of Cdc42 in host protection from lethal infections and suggest a critical role for this small GTPase in innate immunity.
83. Cdc42-dependent actin dynamics controls maturation and secretory activity of dendritic cells. Cell division cycle 42 (Cdc42) is a member of the Rho guanosine triphosphatase family and has pivotal functions in actin organization, cell **migration**, and proliferation. To further study the molecular mechanisms of dendritic cell (DC) regulation by Cdc42, we used Cdc42-deficient DCs. Cdc42 deficiency renders DCs phenotypically mature as they up-regulate the co-stimulatory molecule CD86 from intracellular storages to the cell surface. Cdc42 knockout DCs also accumulate high amounts of invariant chain-major histocompatibility complex (MHC) class II complexes at the cell surface, which cannot efficiently present peptide antigens (Ag's) for priming of Ag-specific CD4 T cells. Proteome analyses showed a significant reduction in lysosomal MHC class II-processing proteins, such as cathepsins, which are lost from DCs by enhanced secretion. As these effects on DCs can be mimicked by chemical actin disruption, our results propose that Cdc42 control of actin dynamics keeps DCs in an immature state, and cessation of Cdc42 activity during DC maturation facilitates secretion as well as rapid up-regulation of intracellular molecules to the cell surface.
84. Cdc42 controls vascular network assembly through protein kinase Cι during embryonic vasculogenesis. The goal of this study was to determine the role of Cdc42 in embryonic vasculogenesis and the underlying mechanisms.By using genetically modified mouse embryonic stem (ES) cells, we demonstrate that ablation of the Rho GTPase Cdc42 blocks vascular network assembly during embryoid body (EB) vasculogenesis without affecting endothelial lineage differentiation. Reexpression of Cdc42 in mutant EBs rescues the mutant phenotype, establishing an essential role for Cdc42 in vasculogenesis. Chimeric analysis revealed that the vascular phenotype is caused by inactivation of Cdc42 in endothelial cells rather than surrounding cells. Endothelial cells isolated from Cdc42-null EBs are defective in directional **migratio**n and network assembly. In addition, activation of atypical protein kinase Cι (PKCι) is abolished in Cdc42-null endothelial cells, and PKCι ablation phenocopies the vascular abnormalities of the Cdc42-null EBs. Moreover, the inhibitory phosphorylation of glycogen synthase kinase-3β (GSK-3β) at Ser9 depends on Cdc42 and PKCι, and expression of kinase-dead GSK-3β in Cdc42-null EBs promotes the formation of linear endothelial segments without branches. These results suggest that PKCι and GSK-3β are downstream effectors of Cdc42 during vascular morphogenesis.Cdc42 controls vascular network assembly but not endothelial lineage differentiation by activating PKCι during embryonic vasculogenesis.
85. CCR7 ligands induce rapid endocytosis in mature dendritic cells with concomitant up-regulation of Cdc42 and Rac activities. Although chemokines are well known to function in chemotaxis, additional roles for these molecules in the immune system are not well understood. Dendritic cells (DCs) developmentally regulate the expression of chemokine receptors to facilitate their **migration** from the peripheral tissues to regional lymph nodes. Expressions of CCR1 and CCR5 on immature DCs are down-regulated on maturation, whereas CCR7 is selectively expressed on mature DCs. In the present study, we examined the effects of CCL19 and CCL21, 2 CCR7 ligands, on endocytosis of fluorescein isothiocyanate (FITC)-dextran by murine DCs. Both CCL19 and CCL21 markedly induced rapid uptake of FITC-dextran by mature DCs but not immature DCs. In contrast, CCL3, a ligand of CCR1 and CCR5, induced rapid uptake of FITC-dextran by immature DCs but not mature DCs. CCL19-induced endocytosis could be completely blocked by Clostridium difficile toxin B, which inhibits the Rho guanosine triphosphatase proteins, Rho, Rac, and Cdc42. This process was not abrogated by Y-27632, a specific inhibitor of Rho-associated kinase. In addition, CCL19 rapidly enhanced Cdc42 and Rac activity in mature DCs. These findings demonstrate that certain chemokines induce rapid endocytosis in each relevant DC population. It is suggested that CCR7 ligands activate Cdc42 and Rac, thereby inducing the endocytosis in mature DCs.
86. Rho-family GTPase Cdc42 controls **migration** of Langerhans cells in vivo. Epidermal Langerhans cells (LCs) of the skin represent the prototype migratory dendritic cell (DC) subtype. In the skin, they take up Ag, migrate to the draining lymph nodes, and contribute to Ag transport and immunity. Different depletion models for LCs have revealed contrasting roles and contributions of this cell type. To target the migratory properties of DCs, we generated mice lacking the Rho-family GTPase Cdc42 specifically in DCs. In these animals, the initial seeding of the epidermis with LCs is functional, resulting in slightly reduced Langerhans cell numbers. However, Cdc42-deficient LCs fail to leave the skin in steady state as well as upon stimulation, as they do not enter the skin-draining afferent lymph vessels. Similarly, also other Cdc42-deficient migratory DC subsets fail to home properly to the corresponding draining lymph nodes. We used this novel mouse model, in which LCs are locked out, to demonstrate that these cells contribute substantially to priming of Ag-specific CD4 and CD8 T cell responses upon epicutaneous immunization, but could not detect a role in the induction of contact hypersensitivity to various doses of hapten.
87. The serotonin receptor 5-HT₇R regulates the morphology and migratory properties of dendritic cells. Dendritic cells are potent antigen-presenting cells endowed with the unique ability to initiate adaptive immune responses upon inflammation. Inflammatory processes are often associated with an increased production of serotonin, which operates by activating specific receptors. However, the functional role of serotonin receptors in regulation of dendritic cell functions is poorly understood. Here, we demonstrate that expression of serotonin receptor 5-HT7 (5-HT7R) as well as its downstream effector Cdc42 is upregulated in dendritic cells upon maturation. Although dendritic cell maturation was independent of 5-HT7R, receptor stimulation affected dendritic cell morphology through Cdc42-mediated signaling. In addition, basal activity of 5-HT7R was required for the proper expression of the chemokine receptor CCR7, which is a key factor that controls dendritic cel**l migrati**on. Consistent with this, we observed that 5-HT7R enhances chemotactic motility of dendritic cells in vitro by modulating their directionality an**d migrati**on velocity. Accordingly**, migrati**on of dendritic cells in murine colon explants was abolished after pharmacological receptor inhibition. Our results indicate that there is a crucial role for 5-HT7R-Cdc42-mediated signaling in the regulation of dendritic cell morphology and motility, suggesting that 5-HT7R could be a new target for treatment of a variety of inflammatory and immune disorders.
88. Regulation of podosome dynamics by WASp phosphorylation: implication in matrix degradation and chemotaxis in macrophages. Podosomes, adhesion structures capable of matrix degradation, have been linked with the ability of cells to perform chemotaxis and invade tissues. Wiskott-Aldrich Syndrome protein (WASp), an effector of the RhoGTPase Cdc42 and a Src family kinase substrate, regulates macrophage podosome formation. In this study, we demonstrate that WASp is active in podosomes by using TIRF-FRET microscopy. Pharmacological and RNA interference approaches suggested that continuous WASp activity is required for podosome formation and function. Rescue experiments using point mutations demonstrate an absolute requirement for Cdc42 binding to WASp in podosome formation. Although tyrosine phosphorylation was not absolutely required for podosome formation, phosphorylation did regulate the rate of podosome nucleation and actin filament stability. Importantly, WASp tyrosine phosphorylation does not alter WASp activation, instead phosphorylation appears to be important for the restriction of WASp activity to podosomes. In addition, the matrix-degrading ability of cells requires WASp phosphorylation. Chemotactic responses to CSF-1 were also attenuated in the absence of endogenous WASp, which could not be rescued with either tyrosine mutation. These results suggest a more complex role for tyrosine phosphorylation than simply in the regulation of WASp activity, and suggest a link between podosome dynamics and macrophage **migration**.
89. Age-related guanine nucleotide exchange factor, mouse Zizimin2, induces filopodia in bone marrow-derived dendritic cells. We recently isolated and identified Zizimin2 as a functional factor that is highly expressed in murine splenic germinal center B cells after immunization with T-cell-dependent antigen. Zizimin2 was revealed to be a new family member of Dock (dedicator of cytokinesis), Dock11, which is the guanine nucleotide exchange factor for Cdc42, a low-molecular-weight GTPase. However, the molecular function of Zizimin2 in acquired immunity has not been elucidated.In this study, we show that the protein expression of Zizimin2, which is also restricted to lymphoid tissues and lymphocytes, is reduced in aged mice. Over-expression of full-length Zizimin2 induced filopodial formation in 293T cells, whereas expression of CZH2 domain inhibited it. Stimulation of Fcγ receptor and Toll-like receptor 4 triggered Zizimin2 up-regulation and Cdc42 activation in bone marrow-derived dendritic cells.These data suggest that Zizimin2 is an immune-related and age-regulated guanine nucleotide exchange factor, which facilitates filopodial formation through activation of Cdc42, which results in activation of cell **migratio**n.
90. Deregulation of SLIT2-mediated Cdc42 activity is associated with esophageal cancer metastasis and poor prognosis. SLIT2, a secreted protein, has been found to inactivate Cdc42 GTPase to modulate neural cell **migration**. However, alteration of SLIT2-mediated Cdc42 in terms of **migration** regulation remains undefined in esophageal squamous cell carcinoma (ESCC).We report here in ESCC cell, animal, and clinical models that SLIT2 acts as a **migration** suppressor and serves as a prognostic biomarker.The immunohistochemistry data indicated that 31.8% (49 of 154) of tumors from ESCC patients showed low expression of SLIT2 protein which correlated with poor overall survival and disease-free survival. DNA methylation analysis suggested that promoter hypermethylation is responsible for low expression of SLIT2 in ESCC. Knockdown of SLIT2 increased ESCC cell **migration**, while SLIT2 stable overexpression reduced cell **migration**. ESCC cells treated with conditioned media from cells overexpressing SLIT2 also suppressed cell **migration**. Importantly, silencing of SLIT2 decreased the complex formation, and thus induced Cdc42 activity and promoted membrane localization of focal adhesion kinase and Paxillin. Anti-metastatic effect of SLIT2 was confirmed in an experimental metastasis model of SLIT2 knockdown ESCC cells.Our results provide novel evidence that low expression of SLIT2 correlates with poor prognosis and promotes metastasis in ESCC, which may be regulated by the Cdc42-mediated pathways.
91. The small Rho GTPase Cdc42 regulates neutrophil polarity via CD11b integrin signaling. Neutrophil **migration** to sites of infection is the first line of cellular defense. A key event of **migration** is the maintenance of a polarized morphology, which is characterized by a single leading edge of filamentous actin and a contractile uropod devoid of filamentous actin protrusions. Using a mouse model of high Cdc42 activity, we previously demonstrated the importance of Cdc42 activity in neutrophil **migration**. However, the specific functions of Cdc42 in this process remain to be understood. Using neutrophils genetically deficient in Cdc42, we show that Cdc42 regulates directed **migration** by maintaining neutrophil polarity. Although it is known to be activated at the front, Cdc42 suppresses protrusions at the uropod. Interestingly, Cdc42 makes use of the integrin CD11b during this process. Cdc42 determines the redistribution of CD11b at the uropod. In turn, using CD11b-null cells and CD11b crosslinking experiments, we show that CD11b modulates myosin light chain phosphorylation to suppress lateral protrusions. Our results uncover a new mechanism in which Cdc42 regulates the uropod through CD11b signaling to maintain polarity in migrating neutrophils. It also reveals new functions for CD11b in neutrophil polarity.
92. Nudel binds Cdc42GAP to modulate Cdc42 activity at the leading edge of migrating cells. Cdc42GAP promotes inactivation of Cdc42, a small GTPase whose activation at the leading edge by guanine nucleotide exchange factors is critical for cell **migration**. How Cdc42GAP is regulated to ensure proper levels of active Cdc42 is poorly understood. Here we show that Nudel, a cytoplasmic dynein regulator, competes with Cdc42 for binding Cdc42GAP. Consequently, Nudel can inhibit Cdc42GAP-mediated inactivation of Cdc42 in a dose-dependent manner. Both Nudel and Cdc42GAP exhibit leading-edge localization in migrating cells. The localization of Nudel requires its phosphorylation by Erk1/2. Depleting Nudel by RNAi or overexpression of a nonphosphorylatable mutant abolishes Cdc42 activation and cell **migration**. Our data thus uncover Nudel as a regulator of Cdc42 during cell **migration**. Nudel facilitates cell **migration** by sequestering Cdc42GAP at the leading edge to stabilize active Cdc42 in response to extracellular stimuli. Excess active Cdc42 may in turn control its own activity by recruiting Cdc42GAP from Nudel.
93. C(16)-Ceramide-induced F-actin regulation stimulates mouse embryonic stem cell **migration**: involvement of N-WASP/Cdc42/Arp2/3 complex and cofilin-1/α-actinin. Ceramide, a major structural element in the cellular membrane, is a key regulatory factor in various cellular behaviors that are dependent on ceramide-induced association of specific proteins. However, molecular mechanisms that regulate ceramide-induced embryonic stem cell (ESC) **migratio**n are still not well understood. Thus, we investigated the effect of ceramide on **migratio**n and its related signal pathways in mouse ESCs. Among ceramide species with different fatty acid chain lengths, C(16)-Cer increased **migratio**n of mouse ESCs in a dose- (≥1μM) and time-dependent (≥8h) manners, as determined by the **cell mig**ration assay. C(16)-Cer (10μM) increased protein-kinase C (PKC) phosphorylation. Subsequently, C(16)-Cer increased focal adhesion kinase (FAK) and Paxillin phosphorylation, which were inhibited by PKC inhibitor Bisindolylmaleimide I (1μM). When we examined for the downstream signaling molecules, C(16)-Cer activated small G protein (Cdc42) and increased the formation of complex with Neural Wiskott-Aldrich Syndrome Protein (N-WASP)/Cdc42/Actin-Related Protein 2/3 (Arp2/3). This complex formation was disrupted by FAK- and Paxillin-specific siRNAs. Furthermore, C(16)-Cer-induced increase of filamentous actin (F-actin) expression was inhibited by Cdc42-, N-WASP-, and Arp2/3-specific siRNAs, respectively. Indeed, C(16)-Cer increased cofilin-1/F-actin interaction or F-actin/α-actinin-1 and α-actinin-4 interactions in the cytoskeleton compartment, which was reversed by Cdc42-specific siRNA. Finally, C(16)-Cer-induced increas**e of cell** migration was inhibited by knocking down each signal pathway-related molecules with siRNA or inhibitors. In conclusion, C(16)-Cer enhances **mouse ESC** migration through the regulation of PKC and FAK/Paxillin-dependent N-WASP/Cdc42/Arp2/3 complex formation as well as through promoting the interaction between cofilin-1 or α-actinin-1/-4 and F-actin.
94. BMP2 induction of actin cytoskeleton reorganization and cell **migration** requires PI3-kinase and Cdc42 activity. Bone morphogenetic proteins (BMPs) are potent regulators of several cellular events. We report that exposure of C2C12 cells to BMP2 leads to an increase in cell **migration** and a rapid rearrangement of the actin filaments into cortical protrusions. These effects required independent and parallel activation of the Cdc42 small GTPase and the alpha-isoform of the phosphoinositide 3-kinase (PI3Kalpha), because ectopic expression of a dominant-negative form of Cdc42 or distinct pharmacological PI3K inhibitors abrogated these responses. Furthermore, we demonstrate that BMP2 activates different group I and group II PAK isoforms as well as LIMK1 with similar kinetics to Cdc42 or PI3K activation. BMP2 activation of PAK and LIMK1, measured by either kinase activity or with antibodies raised against phosphorylated residues at their activation loops, were abolished by blocking PI3K-signaling pathways. Together, these findings suggest that Cdc42 and PI3K signals emanating from BMP receptors are involved in specific regulation of actin assembly and cell **migration**.
95. Specific deletion of Cdc42 does not affect meiotic spindle organization/**migration** and homologous chromosome segregation but disrupts polarity establishment and cytokinesis in mouse oocytes. Mammalian oocyte maturation is distinguished by highly asymmetric meiotic divisions during which a haploid female gamete is produced and almost all the cytoplasm is maintained in the egg for embryo development. Actin-dependent meiosis I spindle positioning to the cortex induces the formation of a polarized actin cap and oocyte polarity, and it determines asymmetric divisions resulting in two polar bodies. Here we investigate the functions of Cdc42 in oocyte meiotic maturation by oocyte-specific deletion of Cdc42 through Cre-loxP conditional knockout technology. We find that Cdc42 deletion causes female infertility in mice. Cdc42 deletion has little effect on meiotic spindle organization and **migration** to the cortex but inhibits polar body emission, although homologous chromosome segregation occurs. The failure of cytokinesis is due to the loss of polarized Arp2/3 accumulation and actin cap formation; thus the defective contract ring. In addition, we correlate active Cdc42 dynamics with its function during polar body emission and find a relationship between Cdc42 and polarity, as well as polar body emission, in mouse oocytes.
96. Krüppel-like factor 5 controls keratinocyte **migratio**n via the integrin-linked kinase. **Migratio**n of epithelial cells is critical for normal homeostasis in gut and skin, but the factors regulating this process are not completely understood. The zinc finger transcription factor Klf5 (IKLF; BTEB2) is highly expressed in proliferating cells of esophagus, skin, and other organs. We hypothesized that Klf5 regulates keratinocyte **migratio**n via the integrin-linked kinase (ILK), which, like Klf5, is localized to basal keratinocytes. We stably transduced mouse primary esophageal keratinocytes to overexpress Klf5 or small interfering RNA against Klf5. Klf5 overexpression in keratinocytes increased **migratio**n and correlated directly with ILK expression and activation. ILK expression restored migratory capacity in keratinocytes with suppression of Klf5, whereas ILK small interfering RNA blocked the increased **migratio**n resulting from Klf5 overexpression. By chromatin immunoprecipitation, electromobility shift assay, and luciferase reporter assays, we confirmed that ILK was a direct target for Klf5. In addition, Klf5 induced the activation of the ILK targets Cdc42 and myosin light chain, which are critical for cell **migratio**n and motility but not Rac1, AKT, or GSK3beta. Overall, these results demonstrate that Klf5 is a key regulator of cell **migratio**n via ILK and provide new insight into the regulation of epithelial cell **migratio**n.
97. CDC42 switches IRSp53 from inhibition of actin growth to elongation by clustering of VASP. Filopodia explore the environment, sensing soluble and mechanical cues during directional motility and tissue morphogenesis. How filopodia are initiated and spatially restricted to specific sites on the plasma membrane is still unclear. Here, we show that the membrane deforming and curvature sensing IRSp53 (Insulin Receptor Substrate of 53 kDa) protein slows down actin filament barbed end growth. This inhibition is relieved by CDC42 and counteracted by VASP, which also binds to IRSp53. The VASP:IRSp53 interaction is regulated by activated CDC42 and promotes high-density clustering of VASP, which is required for processive actin filament elongation. The interaction also mediates VASP recruitment to liposomes. In cells, IRSp53 and VASP accumulate at discrete foci at the leading edge, where filopodia are initiated. Genetic removal of IRSp53 impairs the formation of VASP foci, filopodia and chemotactic motility, while IRSp53 null mice display defective wound healing. Thus, IRSp53 dampens barbed end growth. CDC42 activation inhibits this activity and promotes IRSp53-dependent recruitment and clustering of VASP to drive actin assembly. These events result in spatial restriction of VASP filament elongation for initiation of filopodia during cel**l migrati**on, invasion, and tissue repair.
98. Identification of a DOCK180-related guanine nucleotide exchange factor that is capable of mediating a positive feedback activation of Cdc42. Cdc42, a member of the Rho subfamily of small GTPases, influences a wide range of activities including the establishment of cell polarity, **migration**, and the regulation of cell growth and differentiation. Here we describe the identification of an approximately 220-kDa protein that formed a stable complex with activated forms of Cdc42 and thereby showed characteristics of a downstream target/effector for this GTPase. However, molecular cloning of the cDNA encoding this protein (p220) revealed that it was highly related to Zizimin-1 and identical in sequence to a gene product in the data base designated DOCK11, which are members of the DOCK180 family of guanine nucleotide exchange factors (GEFs) for Cdc42 and Rac. Biochemical characterization shows that p220 is a specific GEF for Cdc42, with the GEF activity originating from its DHR2 (for DOCK homology region 2) domain. Nucleotide-depleted Cdc42 forms a stable complex with the DHR2 domain, whereas the binding of activated Cdc42 requires both the DHR2 domain and residues 66-126 within the amino-terminal portion of p220. Moreover, the full-length protein shows markedly higher GEF activity than the isolated DHR2 domain, whereas removal of the amino-terminal 126 amino acids necessary for binding-activated Cdc42 dramatically diminishes the activity. These and other results point to activated Cdc42 providing a positive feedback regulation of the GEF activity of p220. Thus, we refer to p220/DOCK11 as activated Cdc42-associated GEF, befitting its functional activity.

#### GeneRIFs

1. In summary, the dihydroxycoumarin derivative daphnetin inhibits the invasion and **migration** of LM8 cells, and therefore represents a promising agent for use against metastatic cancer.
2. Cdc42 plays an essential role in neural crest cells (NCC) **migration**, and inactivation of Cdc42 in NCCs impairs craniofacial and cardiovascular development in mice.
3. To determine whether RhoA and Cdc42 directly regulate the motility of cortical interneurons during **migration**, we deleted RhoA and Cdc42 in the subventricular zone, where more fate-restricted progenitors are located within the ganglionic eminences.
4. Cdc42 regulates neutrophil **migration** via crosstalk between WASp, CD11b, and microtubules in the process of chemotaxis.
5. DOCK8 regulates interstitial DC **migration** by controlling Cdc42 activity spatially.
6. The cell **migration** is dependent on the continuous organization of the actin cytoskeleton, which is regulated by Cdc42.
7. Hematopoietic stem cell **migration** is essentially regulated by Rho GTPase, including RhoA, Rac1, and Cdc42, and necessitates the rearrangement of cytoskeleton structures, i.e., F-actin and microtubules.
8. RhoB links PDGF signaling to cell **migration** by coordinating activation and localization of Cdc42 and Rac.
9. Study demonstrate that Cdc42 is required for thymocyte development by impact on positive selection, cell **migration**, and proliferation.
10. Data suggest that Cdc42, Rac1, and RhoG function cooperatively during cell **migration** and but are not essential for the directional response toward PDGF.
11. Klf5 induced the activation of the ILK targets Cdc42 and myosin light chain, which are critical for cell **migration** and motility
12. Nudel facilitates cell **migration** by sequestering Cdc42GAP at the leading edge to stabilize active Cdc42 in response to extracellular stimuli.
13. ROCKI/II and Rac/Cdc42 are required for both neural crest-derived cell **migration** and axon growth in the developing gut.
14. homozygous Cdc42GAP-/- hematopoietic stem cells showed impaired cortical F-actin assembly, deficiency in adhesion and **migration**, and defective engraftment.
15. the PI 3-kinase/Rac1 and Cdc42 pathways are involved in the radial **migration** of cortical neurons but have different roles
16. This protein is specific for GEFT, which induces cell proliferation, transformation, and **migration**.

### ECM

#### PubMed

1. NRP1 Regulates CDC42 Activation to Promote Filopodia Formation in Endothelial Tip Cells. Sprouting blood vessels are led by filopodia-studded endothelial tip cells that respond to angiogenic signals. Mosaic lineage tracing previously revealed that NRP1 is essential for tip cell function, although its mechanistic role in tip cells remains poorly defined. Here, we show that NRP1 is dispensable for genetic tip cell identity. Instead, we find that NRP1 is essential to form the filopodial bursts that distinguish tip cells morphologically from neighboring stalk cells, because it enables the extracellular matrix (**ECM**)-induced activation of CDC42, a key regulator of filopodia formation. Accordingly, NRP1 knockdown and pharmacological CDC42 inhibition similarly impaired filopodia formation in vitro and in developing zebrafish in vivo. During mouse retinal angiogenesis, CDC42 inhibition impaired tip cell and vascular network formation, causing defects that resembled those due to loss of **ECM**-induced, but not VEGF-induced, NRP1 signaling. We conclude that NRP1 enables **ECM**-induced filopodia formation for tip cell function during sprouting angiogenesis.
2. Arhgap28 is a RhoGAP that inactivates RhoA and downregulates stress fibers. The small GTPase RhoA is a major regulator of actin reorganization during the formation of stress fibers; thus identifying molecules that regulate Rho activity is necessary for a complete understanding of the mechanisms that determine cell contractility. Here, we have identified Arhgap28 as a Rho GTPase activating protein (RhoGAP) that switches RhoA to its inactive form. We generated an Arhgap28-LacZ reporter mouse that revealed gene expression in soft tissues at E12.5, pre-bone structures of the limb at E15.5, and prominent expression restricted mostly to ribs and limb long bones at E18.5 days of development. Expression of recombinant Arhgap28-V5 in human osteosarcoma SaOS-2 cells caused a reduction in the basal level of RhoA activation and disruption of actin stress fibers. Extracellular matrix assembly studies using a 3-dimensional cell culture system showed that Arhgap28 was upregulated during Rho-dependent assembly of the **ECM**. Taken together, these observations led to the hypothesis that an Arhgap28 knockout mouse model would show a connective tissue phenotype, perhaps affecting bone. Arhgap28-null mice were viable and appeared normal, suggesting that there could be compensation from other RhoGAPs. Indeed, we showed that expression of Arhgap6 (a closely related RhoGAP) was upregulated in Arhgap28-null bone tissue. An upregulation in RhoA expression was also detected suggesting that Arhgap28 may be able to additionally regulate Rho signaling at a transcriptional level. Microarray analyses revealed that Col2a1, Col9a1, Matn3, and Comp that encode extracellular matrix proteins were downregulated in Arhgap28-null bone. Although mutations in these genes cause bone dysplasias no bone phenotype was detected in the Arhgap-28 null mice. Together, these data suggest that the regulation of Rho by RhoGAPs, including Arhgap28, during the assembly and development of mechanically strong tissues is complex and may involve multiple RhoGAPs.
3. Rac1 links integrin-mediated adhesion to the control of lactational differentiation in mammary epithelia. The expression of tissue-specific genes during mammary gland differentiation relies on the coincidence of two distinct signaling events: the continued engagement of beta1 integrins with the extracellular matrix (**ECM**) and a hormonal stimulus from prolactin (Prl). How the integrin and Prl receptor (PrlR) systems integrate to regulate milk protein gene synthesis is unknown. In this study, we identify Rac1 as a key link. Dominant-negative Rac1 prevents Prl-induced synthesis of the milk protein beta-casein in primary mammary epithelial cells cultured as three-dimensional acini on basement membrane. Conversely, activated Rac1 rescues the defective beta-casein synthesis that occurs under conditions not normally permissive for mammary differentiation, either in beta1 integrin-null cells or in wild-type cells cultured on collagen. Rac1 is required downstream of integrins for activation of the PrlR/Stat5 signaling cascade. Cdc42 is also necessary for milk protein synthesis but functions via a distinct mechanism to Rac1. This study identifies the integration of signals provided by **ECM** and hormones as a novel role for Rho family guanosine triphosphatases.
4. Loss of alpha3beta1 integrin function results in an altered differentiation program in the mouse submandibular gland. Mammalian submandibular gland (SMG) development leads to the establishment of highly organized secretory acinar and nonsecretory ductal epithelial cells. The ability of maturing salivary epithelial cells to attain their differentiated state has been shown to depend, in part, on interactions between extracellular matrix (**ECM**) proteins and their integrin receptors. In a search for key regulators of salivary cell lineage, we have studied alpha3beta1 integrin, a receptor for the basement membrane protein laminin, by characterizing embryonic day 18 (E18) SMGs isolated from mice carrying a targeted mutation in the alpha3 integrin gene. Transmission electron microscopy studies showed that the mutant SMGs exhibited an aberrant differentiation phenotype with defects in the apical-basal polarity axis and in the basement membrane. Based on immunohistochemistry and Western blot analyses, the alpha3beta1-deficient SMGs had altered expression and/or localization of several **ECM** and adhesive molecules, including laminin beta1, fibronectin, alpha5 integrin, and E-cadherin. These changes correlated with alterations in the activation state of Ras-extracellular signal-regulated kinase (ERK), as well as the expression and/or localization of Cdc42 and RhoA, two Rho GTPases that regulate the organization of the actin cytoskeleton. We conclude that alpha3beta1 is required for normal salivary cell differentiation and that its absence affects multiple components of adhesive complexes and their associated signalling pathways.
5. NRP1 Regulates CDC42 Activation to Promote Filopodia Formation in Endothelial Tip Cells. Sprouting blood vessels are led by filopodia-studded endothelial tip cells that respond to angiogenic signals. Mosaic lineage tracing previously revealed that NRP1 is essential for tip cell function, although its mechanistic role in tip cells remains poorly defined. Here, we show that NRP1 is dispensable for genetic tip cell identity. Instead, we find that NRP1 is essential to form the filopodial bursts that distinguish tip cells morphologically from neighboring stalk cells, because it enables the extracellular matrix (**ECM**)-induced activation of CDC42, a key regulator of filopodia formation. Accordingly, NRP1 knockdown and pharmacological CDC42 inhibition similarly impaired filopodia formation in vitro and in developing zebrafish in vivo. During mouse retinal angiogenesis, CDC42 inhibition impaired tip cell and vascular network formation, causing defects that resembled those due to loss of **ECM**-induced, but not VEGF-induced, NRP1 signaling. We conclude that NRP1 enables **ECM**-induced filopodia formation for tip cell function during sprouting angiogenesis.
6. Rac1 links integrin-mediated adhesion to the control of lactational differentiation in mammary epithelia. The expression of tissue-specific genes during mammary gland differentiation relies on the coincidence of two distinct signaling events: the continued engagement of beta1 integrins with the extracellular matrix (**ECM**) and a hormonal stimulus from prolactin (Prl). How the integrin and Prl receptor (PrlR) systems integrate to regulate milk protein gene synthesis is unknown. In this study, we identify Rac1 as a key link. Dominant-negative Rac1 prevents Prl-induced synthesis of the milk protein beta-casein in primary mammary epithelial cells cultured as three-dimensional acini on basement membrane. Conversely, activated Rac1 rescues the defective beta-casein synthesis that occurs under conditions not normally permissive for mammary differentiation, either in beta1 integrin-null cells or in wild-type cells cultured on collagen. Rac1 is required downstream of integrins for activation of the PrlR/Stat5 signaling cascade. Cdc42 is also necessary for milk protein synthesis but functions via a distinct mechanism to Rac1. This study identifies the integration of signals provided by **ECM** and hormones as a novel role for Rho family guanosine triphosphatases.
7. Cdc42 regulates extracellular matrix remodeling in three dimensions. Extracellular matrix (**ECM**) actively participates in normal cell regulation and in the process of tumor progression. The Rho GTPase Cdc42 has been shown to regulate cell-**ECM** interaction in conventional two-dimensional culture conditions by using dominant mutants of Cdc42 in immortalized cell lines that may introduce nonspecific effects. Here, we employ three-dimensional culture systems for conditional gene targeted primary mouse embryonic fibroblasts that better simulate the reciprocal and adaptive interactions between cells and surrounding matrix to define the role of Cdc42 signaling pathways in **ECM** organization. Cdc42 deficiency leads to a defect in global cell-matrix interactions reflected by a decrease in collagen gel contraction. The defect is associated with an altered cell-matrix interaction that is evident by morphologic changes and reduced focal adhesion complex formation. The matrix defect is also associated with a reduction in synthesis and activation of matrix metalloproteinase 9 (MMP9) and altered fibronectin deposition patterning. A Cdc42 mutant rescue experiment found that downstream of Cdc42, p21-activated kinase (PAK), but not Par6 or WASP, may be involved in regulating collagen gel contraction and fibronectin organization. Thus, in addition to the previously implicated roles in intracellular regulation of actin organization, proliferation, and vesicle trafficking, Cdc42 is essential in **ECM** remodeling in three dimensions.

#### GeneRIFs

1. NRP1 enables the extracellular matrix (**ECM**)-induced activation of CDC42, a key regulator of filopodia formation.

### [Ee]xtracellular [Mm]atrix

#### PubMed

1. Integrin-linked kinase is required for radial sorting of axons and Schwann cell remyelination in the peripheral nervous system. During development, Schwann cells (SCs) interpret different extracellular cues to regulate their migration, proliferation, and the remarkable morphological changes associated with the sorting, ensheathment, and myelination of axons. Although interactions between **extracellular matrix** proteins and integrins are critical to some of these processes, the downstream signaling pathways they control are still poorly understood. Integrin-linked kinase (ILK) is a focal adhesion protein that associates with multiple binding partners to link integrins to the actin cytoskeleton and is thought to participate in integrin and growth factor-mediated signaling. Using SC-specific gene ablation, we report essential functions for ILK in radial sorting of axon bundles and in remyelination in the peripheral nervous system. Our in vivo and in vitro experiments show that ILK negatively regulates Rho/Rho kinase signaling to promote SC process extension and to initiate radial sorting. ILK also facilitates axon remyelination, likely by promoting the activation of downstream molecules such as AKT/protein kinase B.
2. NRP1 Regulates CDC42 Activation to Promote Filopodia Formation in Endothelial Tip Cells. Sprouting blood vessels are led by filopodia-studded endothelial tip cells that respond to angiogenic signals. Mosaic lineage tracing previously revealed that NRP1 is essential for tip cell function, although its mechanistic role in tip cells remains poorly defined. Here, we show that NRP1 is dispensable for genetic tip cell identity. Instead, we find that NRP1 is essential to form the filopodial bursts that distinguish tip cells morphologically from neighboring stalk cells, because it enables the **extracellular matrix** (ECM)-induced activation of CDC42, a key regulator of filopodia formation. Accordingly, NRP1 knockdown and pharmacological CDC42 inhibition similarly impaired filopodia formation in vitro and in developing zebrafish in vivo. During mouse retinal angiogenesis, CDC42 inhibition impaired tip cell and vascular network formation, causing defects that resembled those due to loss of ECM-induced, but not VEGF-induced, NRP1 signaling. We conclude that NRP1 enables ECM-induced filopodia formation for tip cell function during sprouting angiogenesis.
3. Arhgap28 is a RhoGAP that inactivates RhoA and downregulates stress fibers. The small GTPase RhoA is a major regulator of actin reorganization during the formation of stress fibers; thus identifying molecules that regulate Rho activity is necessary for a complete understanding of the mechanisms that determine cell contractility. Here, we have identified Arhgap28 as a Rho GTPase activating protein (RhoGAP) that switches RhoA to its inactive form. We generated an Arhgap28-LacZ reporter mouse that revealed gene expression in soft tissues at E12.5, pre-bone structures of the limb at E15.5, and prominent expression restricted mostly to ribs and limb long bones at E18.5 days of development. Expression of recombinant Arhgap28-V5 in human osteosarcoma SaOS-2 cells caused a reduction in the basal level of RhoA activation and disruption of actin stress fibers. **Extracellular matrix** assembly studies using a 3-dimensional cell culture system showed that Arhgap28 was upregulated during Rho-dependent assembly of the ECM. Taken together, these observations led to the hypothesis that an Arhgap28 knockout mouse model would show a connective tissue phenotype, perhaps affecting bone. Arhgap28-null mice were viable and appeared normal, suggesting that there could be compensation from other RhoGAPs. Indeed, we showed that expression of Arhgap6 (a closely related RhoGAP) was upregulated in Arhgap28-null bone tissue. An upregulation in RhoA expression was also detected suggesting that Arhgap28 may be able to additionally regulate Rho signaling at a transcriptional level. Microarray analyses revealed that Col2a1, Col9a1, Matn3, and Comp that encode **extracellular matrix** proteins were downregulated in Arhgap28-null bone. Although mutations in these genes cause bone dysplasias no bone phenotype was detected in the Arhgap-28 null mice. Together, these data suggest that the regulation of Rho by RhoGAPs, including Arhgap28, during the assembly and development of mechanically strong tissues is complex and may involve multiple RhoGAPs.
4. Plexin-B2 negatively regulates macrophage motility, Rac, and Cdc42 activation. Plexins are cell surface receptors widely studied in the nervous system, where they mediate migration and morphogenesis though the Rho family of small GTPases. More recently, plexins have been implicated in immune processes including cell-cell interaction, immune activation, migration, and cytokine production. Plexin-B2 facilitates ligand induced cell guidance and migration in the nervous system, and induces cytoskeletal changes in overexpression assays through RhoGTPase. The function of Plexin-B2 in the immune system is unknown. This report shows that Plexin-B2 is highly expressed on cells of the innate immune system in the mouse, including macrophages, conventional dendritic cells, and plasmacytoid dendritic cells. However, Plexin-B2 does not appear to regulate the production of proinflammatory cytokines, phagocytosis of a variety of targets, or directional migration towards chemoattractants or **extracellular matrix** in mouse macrophages. Instead, Plxnb2(-/-) macrophages have greater cellular motility than wild type in the unstimulated state that is accompanied by more active, GTP-bound Rac and Cdc42. Additionally, Plxnb2(-/-) macrophages demonstrate faster in vitro wound closure activity. Studies have shown that a closely related family member, Plexin-B1, binds to active Rac and sequesters it from downstream signaling. The interaction of Plexin-B2 with Rac has only been previously confirmed in yeast and bacterial overexpression assays. The data presented here show that Plexin-B2 functions in mouse macrophages as a negative regulator of the GTPases Rac and Cdc42 and as a negative regulator of basal cell motility and wound healing.
5. Atypical protein kinase C and Par3 are required for proteoglycan-induced axon growth inhibition. When the CNS is injured, damaged axons do not regenerate. This failure is due in part to the growth-inhibitory environment that forms at the injury site. Myelin-associated molecules, repulsive axon guidance molecules, and **extracellular matrix** molecules including chondroitin sulfate proteoglycans (CSPGs) found within the glial scar inhibit axon regeneration but the intracellular signaling mechanisms triggered by these diverse molecules remain largely unknown. Here we provide biochemical and functional evidence that atypical protein kinase C (PKCζ) and polarity (Par) complex proteins mediate axon growth inhibition. Treatment of postnatal rat neurons in vitro with the NG2 CSPG, a major component of the glial scar, activates PKCζ, and this activation is both necessary and sufficient to inhibit axonal growth. NG2 treatment also activates Cdc42, increases the association of Par6 with PKCζ, and leads to a Par3-dependent activation of Rac1. Transfection of neurons with kinase-dead forms of PKCζ, dominant-negative forms of Cdc42, or mutant forms of Par6 that do not bind to Cdc42 prevent NG2-induced growth inhibition. Similarly, transfection with either a phosphomutant Par3 (S824A) or dominant-negative Rac1 prevent inhibition, whereas expression of constitutively active Rac1 inhibits axon growth on control surfaces. These results suggest a model in which NG2 binding to neurons activates PKCζ and modifies Par complex function. They also identify the Par complex as a novel therapeutic target for promoting axon regeneration after CNS injury.
6. Rac1 links integrin-mediated adhesion to the control of lactational differentiation in mammary epithelia. The expression of tissue-specific genes during mammary gland differentiation relies on the coincidence of two distinct signaling events: the continued engagement of beta1 integrins with the **extracellular matrix** (ECM) and a hormonal stimulus from prolactin (Prl). How the integrin and Prl receptor (PrlR) systems integrate to regulate milk protein gene synthesis is unknown. In this study, we identify Rac1 as a key link. Dominant-negative Rac1 prevents Prl-induced synthesis of the milk protein beta-casein in primary mammary epithelial cells cultured as three-dimensional acini on basement membrane. Conversely, activated Rac1 rescues the defective beta-casein synthesis that occurs under conditions not normally permissive for mammary differentiation, either in beta1 integrin-null cells or in wild-type cells cultured on collagen. Rac1 is required downstream of integrins for activation of the PrlR/Stat5 signaling cascade. Cdc42 is also necessary for milk protein synthesis but functions via a distinct mechanism to Rac1. This study identifies the integration of signals provided by ECM and hormones as a novel role for Rho family guanosine triphosphatases.
7. Loss of alpha3beta1 integrin function results in an altered differentiation program in the mouse submandibular gland. Mammalian submandibular gland (SMG) development leads to the establishment of highly organized secretory acinar and nonsecretory ductal epithelial cells. The ability of maturing salivary epithelial cells to attain their differentiated state has been shown to depend, in part, on interactions between **extracellular matrix** (ECM) proteins and their integrin receptors. In a search for key regulators of salivary cell lineage, we have studied alpha3beta1 integrin, a receptor for the basement membrane protein laminin, by characterizing embryonic day 18 (E18) SMGs isolated from mice carrying a targeted mutation in the alpha3 integrin gene. Transmission electron microscopy studies showed that the mutant SMGs exhibited an aberrant differentiation phenotype with defects in the apical-basal polarity axis and in the basement membrane. Based on immunohistochemistry and Western blot analyses, the alpha3beta1-deficient SMGs had altered expression and/or localization of several ECM and adhesive molecules, including laminin beta1, fibronectin, alpha5 integrin, and E-cadherin. These changes correlated with alterations in the activation state of Ras-extracellular signal-regulated kinase (ERK), as well as the expression and/or localization of Cdc42 and RhoA, two Rho GTPases that regulate the organization of the actin cytoskeleton. We conclude that alpha3beta1 is required for normal salivary cell differentiation and that its absence affects multiple components of adhesive complexes and their associated signalling pathways.
8. NRP1 Regulates CDC42 Activation to Promote Filopodia Formation in Endothelial Tip Cells. Sprouting blood vessels are led by filopodia-studded endothelial tip cells that respond to angiogenic signals. Mosaic lineage tracing previously revealed that NRP1 is essential for tip cell function, although its mechanistic role in tip cells remains poorly defined. Here, we show that NRP1 is dispensable for genetic tip cell identity. Instead, we find that NRP1 is essential to form the filopodial bursts that distinguish tip cells morphologically from neighboring stalk cells, because it enables the **extracellular matrix** (ECM)-induced activation of CDC42, a key regulator of filopodia formation. Accordingly, NRP1 knockdown and pharmacological CDC42 inhibition similarly impaired filopodia formation in vitro and in developing zebrafish in vivo. During mouse retinal angiogenesis, CDC42 inhibition impaired tip cell and vascular network formation, causing defects that resembled those due to loss of ECM-induced, but not VEGF-induced, NRP1 signaling. We conclude that NRP1 enables ECM-induced filopodia formation for tip cell function during sprouting angiogenesis.
9. Plexin-B2 negatively regulates macrophage motility, Rac, and Cdc42 activation. Plexins are cell surface receptors widely studied in the nervous system, where they mediate migration and morphogenesis though the Rho family of small GTPases. More recently, plexins have been implicated in immune processes including cell-cell interaction, immune activation, migration, and cytokine production. Plexin-B2 facilitates ligand induced cell guidance and migration in the nervous system, and induces cytoskeletal changes in overexpression assays through RhoGTPase. The function of Plexin-B2 in the immune system is unknown. This report shows that Plexin-B2 is highly expressed on cells of the innate immune system in the mouse, including macrophages, conventional dendritic cells, and plasmacytoid dendritic cells. However, Plexin-B2 does not appear to regulate the production of proinflammatory cytokines, phagocytosis of a variety of targets, or directional migration towards chemoattractants or **extracellular matrix** in mouse macrophages. Instead, Plxnb2(-/-) macrophages have greater cellular motility than wild type in the unstimulated state that is accompanied by more active, GTP-bound Rac and Cdc42. Additionally, Plxnb2(-/-) macrophages demonstrate faster in vitro wound closure activity. Studies have shown that a closely related family member, Plexin-B1, binds to active Rac and sequesters it from downstream signaling. The interaction of Plexin-B2 with Rac has only been previously confirmed in yeast and bacterial overexpression assays. The data presented here show that Plexin-B2 functions in mouse macrophages as a negative regulator of the GTPases Rac and Cdc42 and as a negative regulator of basal cell motility and wound healing.
10. Rac1 links integrin-mediated adhesion to the control of lactational differentiation in mammary epithelia. The expression of tissue-specific genes during mammary gland differentiation relies on the coincidence of two distinct signaling events: the continued engagement of beta1 integrins with the **extracellular matrix** (ECM) and a hormonal stimulus from prolactin (Prl). How the integrin and Prl receptor (PrlR) systems integrate to regulate milk protein gene synthesis is unknown. In this study, we identify Rac1 as a key link. Dominant-negative Rac1 prevents Prl-induced synthesis of the milk protein beta-casein in primary mammary epithelial cells cultured as three-dimensional acini on basement membrane. Conversely, activated Rac1 rescues the defective beta-casein synthesis that occurs under conditions not normally permissive for mammary differentiation, either in beta1 integrin-null cells or in wild-type cells cultured on collagen. Rac1 is required downstream of integrins for activation of the PrlR/Stat5 signaling cascade. Cdc42 is also necessary for milk protein synthesis but functions via a distinct mechanism to Rac1. This study identifies the integration of signals provided by ECM and hormones as a novel role for Rho family guanosine triphosphatases.
11. DOCK8 is a Cdc42 activator critical for interstitial dendritic cell migration during immune responses. To migrate efficiently through the interstitium, dendritic cells (DCs) constantly adapt their shape to the given structure of the **extracellular matrix** and follow the path of least resistance. It is known that this amoeboid migration of DCs requires Cdc42, yet the upstream regulators critical for localization and activation of Cdc42 remain to be determined. Mutations of DOCK8, a member of the atypical guanine nucleotide exchange factor family, causes combined immunodeficiency in humans. In the present study, we show that DOCK8 is a Cdc42-specific guanine nucleotide exchange factor that is critical for interstitial DC migration. By generating the knockout mice, we found that in the absence of DOCK8, DCs failed to accumulate in the lymph node parenchyma for T-cell priming. Although DOCK8-deficient DCs migrated normally on 2-dimensional surfaces, DOCK8 was required for DCs to crawl within 3-dimensional fibrillar networks and to transmigrate through the subcapsular sinus floor. This function of DOCK8 depended on the DHR-2 domain mediating Cdc42 activation. DOCK8 deficiency did not affect global Cdc42 activity. However, Cdc42 activation at the leading edge membrane was impaired in DOCK8-deficient DCs, resulting in a severe defect in amoeboid polarization and migration. Therefore, DOCK8 regulates interstitial DC migration by controlling Cdc42 activity spatially.
12. Cdc42-dependent leading edge coordination is essential for interstitial dendritic cell migration. Mature dendritic cells (DCs) moving from the skin to the lymph node are a prototypic example of rapidly migrating amoeboid leukocytes. Interstitial DC migration is directionally guided by chemokines, but independent of specific adhesive interactions with the tissue as well as pericellular proteolysis. Instead, the protrusive flow of the actin cytoskeleton directly drives a basal mode of locomotion that is occasionally supported by actomyosin contractions at the trailing edge to propel the cell's rigid nucleus. We here delete the small GTPase Cdc42 in DCs and find that actin flow and actomyosin contraction are still initiated in response to chemotactic cues. Accordingly, the cells are able to polarize and form protrusions. However, in the absence of Cdc42 the protrusions are temporally and spatially dysregulated, which leads to impaired leading edge coordination. Although this defect still allows the cells to move on 2-dimensional surfaces, their in vivo motility is completely abrogated. We show that this difference is entirely caused by the geometric complexity of the environment, as multiple competing protrusions lead to instantaneous entanglement within 3-dimensional **extracellular matrix** scaffolds. This demonstrates that the decisive factor for migrating DCs is not specific interaction with the extracellular environment, but adequate coordination of cytoskeletal flow.
13. Promyogenic function of Integrin/FAK signaling is mediated by Cdo, Cdc42 and MyoD. The Integrin-mediated cell adhesion to the **extracellular matrix** is implicated in the control of proliferation, survival, migration and differentiation of myoblasts. Focal adhesion kinase (FAK) mediates signals from Integrins and plays an essential role in myotube formation. Cdo forms a multiprotein complex that includes other cell adhesion molecules like Cadherins and Boc. Multiple signals emanate from such complexes, including Cdc42 and p38MAPK pathways to activate MyoD. Here we show that C2C12 myoblasts cultured in suspension or on Poly-L-Lysine (PLL), a well known Integrin-independent substratum, failed to express Cdo and MyoD, while the expression of Cadherins and Boc was unchanged. In addition, the activation of Akt and p38MAPK as well as the expression of Cdc42 was affected in these cells. Overexpression of FAK rescued MyoD and Cdo expression as well as myotube formation of C2C12 cells on PLL. Furthermore, reintroduction of Cdo induced enhanced myotube formation on PLL and increased the expression of myogenic markers. Inhibition of ROCK or overexpression of Cdc42-V12 in C2C12 cells upregulated Cdc42 and MyoD expression and rescued defective myoblast differentiation. Taken together, these data indicate that the Integrin/FAK signaling pathway is required for myoblast differentiation by regulating the expression of the promyogenic factors, Cdo, MyoD and Cdc42.
14. Class A scavenger receptor-mediated macrophage adhesion requires coupling of calcium-independent phospholipase A(2) and 12/15-lipoxygenase to Rac and Cdc42 activation. Class A scavenger receptors (SR-A) participate in multiple macrophage functions including adhesion to modified **extracellular matrix** proteins present in various inflammatory disorders such as atherosclerosis and diabetes. By mediating macrophage adhesion to modified proteins and increasing macrophage retention, SR-A may contribute to the inflammatory process. Eicosanoids produced after phospholipase A(2) (PLA(2))-catalyzed release of arachidonic acid (AA) are important regulators of macrophage function and inflammatory responses. The potential roles of AA release and metabolism in SR-A-mediated macrophage adhesion were determined using macrophages adherent to modified protein. SR-A-dependent macrophage adhesion was abolished by selectively inhibiting calcium-independent PLA(2) (iPLA(2)) activity and absent in macrophages isolated from iPLA(2) beta(-/-) mice. Our results further demonstrate that 12/15-lipoxygenase (12/15-LOX)-derived, but not cyclooxygenase- or cytochrome P450-dependent epoxygenase-derived AA metabolites, are specifically required for SR-A-dependent adhesion. Because of their role in regulating actin polymerization and cell adhesion, Rac and Cdc42 activation were also examined and shown to be increased via an iPLA(2)- and LOX-dependent pathway. Together, our results identify a novel role for iPLA(2)-catalyzed AA release and its metabolism by 12/15-LOX in coupling SR-A-mediated macrophage adhesion to Rac and Cdc42 activation.
15. Cdc42 regulates **extracellular matrix** remodeling in three dimensions. **Extracellular matrix** (ECM) actively participates in normal cell regulation and in the process of tumor progression. The Rho GTPase Cdc42 has been shown to regulate cell-ECM interaction in conventional two-dimensional culture conditions by using dominant mutants of Cdc42 in immortalized cell lines that may introduce nonspecific effects. Here, we employ three-dimensional culture systems for conditional gene targeted primary mouse embryonic fibroblasts that better simulate the reciprocal and adaptive interactions between cells and surrounding matrix to define the role of Cdc42 signaling pathways in ECM organization. Cdc42 deficiency leads to a defect in global cell-matrix interactions reflected by a decrease in collagen gel contraction. The defect is associated with an altered cell-matrix interaction that is evident by morphologic changes and reduced focal adhesion complex formation. The matrix defect is also associated with a reduction in synthesis and activation of matrix metalloproteinase 9 (MMP9) and altered fibronectin deposition patterning. A Cdc42 mutant rescue experiment found that downstream of Cdc42, p21-activated kinase (PAK), but not Par6 or WASP, may be involved in regulating collagen gel contraction and fibronectin organization. Thus, in addition to the previously implicated roles in intracellular regulation of actin organization, proliferation, and vesicle trafficking, Cdc42 is essential in ECM remodeling in three dimensions.
16. Protein kinase C δ regulates the release of collagen type I from vascular smooth muscle cells via regulation of Cdc42. Collagen type I is the most abundant component of **extracellular matri**x in the arterial wall. Mice knocked out for the protein kinase C δ gene (PKCδ KO) show a marked reduction of collagen I in the arterial wall. The lack of PKCδ diminished the ability of arterial smooth muscle cells (SMCs) to secrete collagen I without significantly altering the intracellular collagen content. Moreover, the unsecreted collagen I molecules accumulate in large perinuclear puncta. These perinuclear structures colocalize with the trans-Golgi network (TGN) marker TGN38 and to a lesser degree with cis-Golgi marker (GM130) but not with early endosomal marker (EEA1). Associated with diminished collagen I secretion, PKCδ KO SMCs exhibit a significant reduction in levels of cell division cycle 42 (Cdc42) protein and mRNA. Restoring PKCδ expression partially rescues Cdc42 expression and collagen I secretion in PKCδ KO SMCs. Inhibition of Cdc42 expression or activity with small interfering RNA or secramine A in PKCδ WT SMCs eliminates collagen I secretion. Conversely, restoring Cdc42 expression in PKCδ KO SMCs enables collagen I secretion. Taken together, our data demonstrate that PKCδ mediates collagen I secretion from SMCs, likely through a Cdc42-dependent mechanism.

#### GeneRIFs

1. NRP1 enables the **extracellular matrix** (ECM)-induced activation of CDC42, a key regulator of filopodia formation.
2. Cdc42 regulates **extracellular matrix** remodeling in three dimensions.

### [Ii]nvasive

#### PubMed

1. MYC-nick promotes cell migration by inducing fascin expression and Cdc42 activation. MYC-nick is a cytoplasmic, transcriptionally inactive member of the MYC oncoprotein family, generated by a proteolytic cleavage of full-length MYC. MYC-nick promotes migration and survival of cells in response to chemotherapeutic agents or withdrawal of glucose. Here we report that MYC-nick is abundant in colonic and intestinal tumors derived from mouse models with mutations in the Wnt, TGF-β, and PI3K pathways. Moreover, MYC-nick is elevated in colon cancer cells deleted for FBWX7, which encodes the major E3 ligase of full-length MYC frequently mutated in colorectal cancers. MYC-nick promotes the migration of colon cancer cells assayed in 3D cultures or grown as xenografts in a zebrafish metastasis model. MYC-nick accelerates migration by activating the Rho GTPase Cdc42 and inducing fascin expression. MYC-nick, fascin, and Cdc42 are frequently up-regulated in cells present at the **invasiv**e front of human colorectal tumors, suggesting a coordinated role for these proteins in tumor migration.
2. The adenomatous polyposis coli-associated exchange factors Asef and Asef2 are required for adenoma formation in Apc(Min/+)mice. Sporadic and familial colorectal tumours usually harbour biallelic adenomatous polyposis coli (APC)-associated mutations that result in constitutive activation of Wnt signalling. Furthermore, APC activates Asef and Asef2, which are guanine-nucleotide exchange factors specific for Rac1 and Cdc42. Here, we show that Asef and Asef2 expression is aberrantly enhanced in intestinal adenomas and tumours. We also show that deficiency of either Asef or Asef2 significantly reduces the number and size of adenomas in Apc(Min/+) mice, which are heterozygous for an APC mutation and spontaneously develop adenomas in the intestine. We observed that the APC-Asef/Asef2 complex induces c-Jun amino-terminal kinase-mediated transactivation of matrix metalloproteinase 9, and is required for the **invasive** activity of colorectal tumour cells. Furthermore, we show that Asef and Asef2 are required for tumour angiogenesis. These results suggest that Asef and Asef2 have a crucial role in intestinal adenoma formation and tumour progression, and might be promising molecular targets for the treatment of colorectal tumours.
3. Phosphoinositide 3-kinase p85beta regulates invadopodium formation. The acquisition of **invasive**ness is characteristic of tumor progression. Numerous genetic changes are associated with metastasis, but the mechanism by which a cell becomes **invasive** remains unclear. Expression of p85β, a regulatory subunit of phosphoinositide-3-kinase, markedly increases in advanced carcinoma, but its mode of action is unknown. We postulated that p85β might facilitate cell invasion. We show that p85β localized at cell adhesions in complex with focal adhesion kinase and enhanced stability and maturation of cell adhesions. In addition, p85β induced development at cell adhesions of an F-actin core that extended several microns into the cell z-axis resembling the skeleton of invadopodia. p85β lead to F-actin polymerization at cell adhesions by recruiting active Cdc42/Rac at these structures. In accordance with p85β function in invadopodium-like formation, p85β levels increased in metastatic melanoma and p85β depletion reduced invadopodium formation and invasion. These results show that p85β enhances invasion by inducing cell adhesion development into invadopodia-like structures explaining the metastatic potential of tumors with increased p85β levels.
4. Cdc42 and Rac1 are necessary for autotaxin-induced tumor cell motility in A2058 melanoma cells. Autotaxin (ATX) is a strong motogen that can increase **invasive**ness and angiogenesis. In the present study, we investigated the signal transduction mechanism of ATX-induced tumor cell motility. Unlike N19RhoA expressing cells, the cells expressing N17Cdc42 or N17Rac1 showed reduced motility against ATX. ATX activated Cdc42 and Rac1 and increased complex formation between these small G proteins and p21-activated kinase (PAK). Furthermore, ATX phosphorylated focal adhesion kinase (FAK) that was not shown in cells expressing dominant negative mutants of Cdc42 or Rac1. Collectively, these data strongly indicate that Cdc42 and Rac1 are essential for ATX-induced tumor cell motility in A2058 melanoma cells, and that PAK and FAK might be also involved in the process.
5. Molecular mechanisms of invadopodium formation: the role of the N-WASP-Arp2/3 complex pathway and cofilin. Invadopodia are actin-rich membrane protrusions with a matrix degradation activity formed by **invasive** cancer cells. We have studied the molecular mechanisms of invadopodium formation in metastatic carcinoma cells. Epidermal growth factor (EGF) receptor kinase inhibitors blocked invadopodium formation in the presence of serum, and EGF stimulation of serum-starved cells induced invadopodium formation. RNA interference and dominant-negative mutant expression analyses revealed that neural WASP (N-WASP), Arp2/3 complex, and their upstream regulators, Nck1, Cdc42, and WIP, are necessary for invadopodium formation. Time-lapse analysis revealed that invadopodia are formed de novo at the cell periphery and their lifetime varies from minutes to several hours. Invadopodia with short lifetimes are motile, whereas long-lived invadopodia tend to be stationary. Interestingly, suppression of cofilin expression by RNA interference inhibited the formation of long-lived invadopodia, resulting in formation of only short-lived invadopodia with less matrix degradation activity. These results indicate that EGF receptor signaling regulates invadopodium formation through the N-WASP-Arp2/3 pathway and cofilin is necessary for the stabilization and maturation of invadopodia.
6. Cdc42 and Rac1 are necessary for autotaxin-induced tumor cell motility in A2058 melanoma cells. Autotaxin (ATX) is a strong motogen that can increase **invasive**ness and angiogenesis. In the present study, we investigated the signal transduction mechanism of ATX-induced tumor cell motility. Unlike N19RhoA expressing cells, the cells expressing N17Cdc42 or N17Rac1 showed reduced motility against ATX. ATX activated Cdc42 and Rac1 and increased complex formation between these small G proteins and p21-activated kinase (PAK). Furthermore, ATX phosphorylated focal adhesion kinase (FAK) that was not shown in cells expressing dominant negative mutants of Cdc42 or Rac1. Collectively, these data strongly indicate that Cdc42 and Rac1 are essential for ATX-induced tumor cell motility in A2058 melanoma cells, and that PAK and FAK might be also involved in the process.

### [Mm]etastasis

#### PubMed

1. MYC-nick promotes cell migration by inducing fascin expression and Cdc42 activation. MYC-nick is a cytoplasmic, transcriptionally inactive member of the MYC oncoprotein family, generated by a proteolytic cleavage of full-length MYC. MYC-nick promotes migration and survival of cells in response to chemotherapeutic agents or withdrawal of glucose. Here we report that MYC-nick is abundant in colonic and intestinal tumors derived from mouse models with mutations in the Wnt, TGF-β, and PI3K pathways. Moreover, MYC-nick is elevated in colon cancer cells deleted for FBWX7, which encodes the major E3 ligase of full-length MYC frequently mutated in colorectal cancers. MYC-nick promotes the migration of colon cancer cells assayed in 3D cultures or grown as xenografts in a zebrafish **metastasi**s model. MYC-nick accelerates migration by activating the Rho GTPase Cdc42 and inducing fascin expression. MYC-nick, fascin, and Cdc42 are frequently up-regulated in cells present at the invasive front of human colorectal tumors, suggesting a coordinated role for these proteins in tumor migration.
2. cIAP1 regulates TNF-mediated cdc42 activation and filopodia formation. Tumour necrosis factor-α (TNF) is a cytokine endowed with multiple functions, depending on the cellular and environmental context. TNF receptor engagement induces the formation of a multimolecular complex including the TNFR-associated factor TRAF2, the receptor-interaction protein kinase RIP1 and the cellular inhibitor of apoptosis cIAP1, the latter being essential for NF-κB activation. Here, we show that cIAP1 also regulates TNF-induced actin cytoskeleton reorganization through a cdc42-dependent, NF-κB-independent pathway. Deletion of cIAP1 prevents TNF-induced filopodia and cdc42 activation. The expression of cIAP1 or its E3-ubiquitin ligase-defective mutant restores the ability of cIAP1(-/-) MEFs to produce filopodia, whereas a cIAP1 mutant unable to bind TRAF2 does not. Accordingly, the silencing of TRAF2 inhibits TNF-mediated filopodia formation, whereas silencing of RIP1 does not. cIAP1 directly binds cdc42 and promotes its RhoGDIα-mediated stabilization. TNF decreases cIAP1-cdc42 interaction, suggesting that TNF-induced recruitment of cIAP1/TRAF2 to the receptor releases cdc42, which in turn triggers actin remodeling. cIAP1 also regulates cdc42 activation in response to EGF and HRas-V12 expression. A downregulation of cIAP1 altered the cell polarization, the cell adhesion to endothelial cells and cell intercalation, which are cdc42-dependent processes. Finally, we demonstrated that the deletion of cIAP1 regulated the HRas-V12-mediated transformation process, including anchorage-dependent cell growth, tumour growth in a xenograft model and the development of experimen**tal metast**asis in the lung.
3. Phosphoinositide 3-kinase p85beta regulates invadopodium formation. The acquisition of invasiveness is characteristic of tumor progression. Numerous genetic changes are associated with **metastasis**, but the mechanism by which a cell becomes invasive remains unclear. Expression of p85β, a regulatory subunit of phosphoinositide-3-kinase, markedly increases in advanced carcinoma, but its mode of action is unknown. We postulated that p85β might facilitate cell invasion. We show that p85β localized at cell adhesions in complex with focal adhesion kinase and enhanced stability and maturation of cell adhesions. In addition, p85β induced development at cell adhesions of an F-actin core that extended several microns into the cell z-axis resembling the skeleton of invadopodia. p85β lead to F-actin polymerization at cell adhesions by recruiting active Cdc42/Rac at these structures. In accordance with p85β function in invadopodium-like formation, p85β levels increased in metastatic melanoma and p85β depletion reduced invadopodium formation and invasion. These results show that p85β enhances invasion by inducing cell adhesion development into invadopodia-like structures explaining the metastatic potential of tumors with increased p85β levels.
4. cIAP1 regulates TNF-mediated cdc42 activation and filopodia formation. Tumour necrosis factor-α (TNF) is a cytokine endowed with multiple functions, depending on the cellular and environmental context. TNF receptor engagement induces the formation of a multimolecular complex including the TNFR-associated factor TRAF2, the receptor-interaction protein kinase RIP1 and the cellular inhibitor of apoptosis cIAP1, the latter being essential for NF-κB activation. Here, we show that cIAP1 also regulates TNF-induced actin cytoskeleton reorganization through a cdc42-dependent, NF-κB-independent pathway. Deletion of cIAP1 prevents TNF-induced filopodia and cdc42 activation. The expression of cIAP1 or its E3-ubiquitin ligase-defective mutant restores the ability of cIAP1(-/-) MEFs to produce filopodia, whereas a cIAP1 mutant unable to bind TRAF2 does not. Accordingly, the silencing of TRAF2 inhibits TNF-mediated filopodia formation, whereas silencing of RIP1 does not. cIAP1 directly binds cdc42 and promotes its RhoGDIα-mediated stabilization. TNF decreases cIAP1-cdc42 interaction, suggesting that TNF-induced recruitment of cIAP1/TRAF2 to the receptor releases cdc42, which in turn triggers actin remodeling. cIAP1 also regulates cdc42 activation in response to EGF and HRas-V12 expression. A downregulation of cIAP1 altered the cell polarization, the cell adhesion to endothelial cells and cell intercalation, which are cdc42-dependent processes. Finally, we demonstrated that the deletion of cIAP1 regulated the HRas-V12-mediated transformation process, including anchorage-dependent cell growth, tumour growth in a xenograft model and the development of experimen**tal metast**asis in the lung.
5. GLI activation by atypical protein kinase C ι/λ regulates the growth of basal cell carcinomas. Growth of basal cell carcinomas (BCCs) requires high levels of hedgehog (HH) signalling through the transcription factor GLI. Although inhibitors of membrane protein smoothened (SMO) effectively suppress HH signalling, early tumour resistance illustrates the need for additional downstream targets for therapy. Here we identify atypical protein kinase C ι/λ (aPKC-ι/λ) as a novel GLI regulator in mammals. aPKC-ι/λ and its polarity signalling partners co-localize at the centrosome and form a complex with mis**sing-in-me**tastasis (MIM), a scaffolding protein that potentiates HH signalling. Genetic or pharmacological loss of aPKC-ι/λ function blocks HH signalling and proliferation of BCC cells. Prkci is a HH target gene that forms a positive feedback loop with GLI and exists at increased levels in BCCs. Genome-wide transcriptional profiling shows that aPKC-ι/λ and SMO control the expression of similar genes in tumour cells. aPKC-ι/λ functions downstream of SMO to phosphorylate and activate GLI1, resulting in maximal DNA binding and transcriptional activation. Activated aPKC-ι/λ is upregulated in SMO-inhibitor-resistant tumours and targeting aPKC-ι/λ suppresses signalling and growth of resistant BCC cell lines. These results demonstrate that aPKC-ι/λ is critical for HH-dependent processes and implicates aPKC-ι/λ as a new, tumour-selective therapeutic target for the treatment of SMO-inhibitor-resistant cancers.
6. Deregulation of SLIT2-mediated Cdc42 activity is associated with esophageal cancer **metastasis** and poor prognosis. SLIT2, a secreted protein, has been found to inactivate Cdc42 GTPase to modulate neural cell migration. However, alteration of SLIT2-mediated Cdc42 in terms of migration regulation remains undefined in esophageal squamous cell carcinoma (ESCC).We report here in ESCC cell, animal, and clinical models that SLIT2 acts as a migration suppressor and serves as a prognostic biomarker.The immunohistochemistry data indicated that 31.8% (49 of 154) of tumors from ESCC patients showed low expression of SLIT2 protein which correlated with poor overall survival and disease-free survival. DNA methylation analysis suggested that promoter hypermethylation is responsible for low expression of SLIT2 in ESCC. Knockdown of SLIT2 increased ESCC cell migration, while SLIT2 stable overexpression reduced cell migration. ESCC cells treated with conditioned media from cells overexpressing SLIT2 also suppressed cell migration. Importantly, silencing of SLIT2 decreased the complex formation, and thus induced Cdc42 activity and promoted membrane localization of focal adhesion kinase and Paxillin. Anti-metastatic effect of SLIT2 was confirmed in an experimental **metastasis** model of SLIT2 knockdown ESCC cells.Our results provide novel evidence that low expression of SLIT2 correlates with poor prognosis and promotes **metastasis** in ESCC, which may be regulated by the Cdc42-mediated pathways.

#### GeneRIFs

1. Results provide novel evidence that low expression of SLIT2 correlates with poor prognosis and promotes **metastasis** in ESCC, which may be regulated by the Cdc42-mediated pathways.

### [Cc]ell [Ii]nvasion

#### PubMed

1. Phosphoinositide 3-kinase p85beta regulates invadopodium formation. The acquisition of invasiveness is characteristic of tumor progression. Numerous genetic changes are associated with metastasis, but the mechanism by which a cell becomes invasive remains unclear. Expression of p85β, a regulatory subunit of phosphoinositide-3-kinase, markedly increases in advanced carcinoma, but its mode of action is unknown. We postulated that p85β might facilitat**e cell invasi**on. We show that p85β localized at cell adhesions in complex with focal adhesion kinase and enhanced stability and maturation of cell adhesions. In addition, p85β induced development at cell adhesions of an F-actin core that extended several microns into the cell z-axis resembling the skeleton of invadopodia. p85β lead to F-actin polymerization at cell adhesions by recruiting active Cdc42/Rac at these structures. In accordance with p85β function in invadopodium-like formation, p85β levels increased in metastatic melanoma and p85β depletion reduced invadopodium formation and invasion. These results show that p85β enhances invasion by inducing cell adhesion development into invadopodia-like structures explaining the metastatic potential of tumors with increased p85β levels.

---

## Rnd2

- NCBI Gene
- GeneRIF
- Pubmed

| Search Term | Rank | Fields |
| --- | --- | --- |
| [Mm]igration | 6 | PubMed(12); GeneRIFs(2) |

  
hide/show details for Rnd2

### [Mm]igration

#### PubMed

1. LMO4 functions as a co-activator of neurogenin 2 in the developing cortex. The proneural protein neurogenin 2 (NGN2) is a key transcription factor in regulating both neurogenesis and neuronal radial **migration** in the embryonic cerebral cortex. However, the co-factors that support the action of NGN2 in the cortex remain unclear. Here, we show that the LIM-only protein LMO4 functions as a novel co-factor of NGN2 in the developing cortex. LMO4 and its binding partner nuclear LIM interactor (NLI/LDB1/CLIM2) interact with NGN2 simultaneously, forming a multi-protein transcription complex. This complex is recruited to the E-box containing enhancers of NGN2-target genes, which regulate various aspects of cortical development, and activates NGN2-mediated transcription. Correspondingly, analysis of Lmo4-null embryos shows that the loss of LMO4 leads to impairments of neuronal differentiation in the cortex. In addition, expression of LMO4 facilitates NGN2-mediated radial **migration** of cortical neurons in the embryonic cortex. Our results indicate that LMO4 promotes the acquisition of cortical neuronal identities by forming a complex with NGN2 and subsequently activating NGN2-dependent gene expression.
2. The zinc finger transcription factor RP58 negatively regulates Rnd2 for the control of neuronal **migration** during cerebral cortical development. The zinc finger transcription factor RP58 (also known as ZNF238) regulates neurogenesis of the mouse neocortex and cerebellum (Okado et al. 2009; Xiang et al. 2011; Baubet et al. 2012; Ohtaka-Maruyama et al. 2013), but its mechanism of action remains unclear. In this study, we report a cell-autonomous function for RP58 during the differentiation of embryonic cortical projection neurons via its activities as a transcriptional repressor. Disruption of RP58 expression alters the differentiation of immature neurons and impairs their **migration** and positioning within the mouse cerebral cortex. Loss of RP58 within the embryonic cortex also leads to elevated mRNA for Rnd2, a member of the Rnd family of atypical RhoA-like GTPase proteins important for cortical neuron **migration** (Heng et al. 2008). Mechanistically, RP58 represses transcription of Rnd2 via binding to a 3'-regulatory enhancer in a sequence-specific fashion. Using reporter assays, we found that RP58 repression of Rnd2 is competed by proneural basic helix-loop-helix transcriptional activators. Finally, our rescue experiments revealed that negative regulation of Rnd2 by RP58 was important for cortical cell **migration** in vivo. Taken together, these studies demonstrate that RP58 is a key player in the transcriptional control of cell **migration** in the developing cerebral cortex.
3. COUP-TFI is required for the formation of commissural projections in the forebrain by regulating axonal growth. The transcription factor COUP-TFI (NR2F1), an orphan member of the nuclear receptor superfamily, is an important regulator of neurogenesis, cellular differentiation and cell **migration**. In the forebrain, COUP-TFI controls the connectivity between thalamus and cortex and neuronal tangential **migration** in the basal telencephalon. Here, we show that COUP-TFI is required for proper axonal growth and guidance of all major forebrain commissures. Fibres of the corpus callosum, the hippocampal commissure and the anterior commissure project aberrantly and fail to cross the midline in COUP-TFI null mutants. Moreover, hippocampal neurons lacking COUP-TFI have a defect in neurite outgrowth and show an abnormal axonal morphology. To search for downstream effectors, we used microarray analysis and showed that, in the absence of COUP-TFI, expression of various cytoskeleton molecules involved in neuronal morphogenesis is affected. Diminished protein levels of the microtubule-associated protein MAP1B and increased levels of the GTP-binding protein RND2 were confirmed in the developing cortex in vivo and in primary hippocampal neurons in vitro. Therefore, based on morphological studies, gene expression profiling and primary cultured neurons, the present data uncover a previously unappreciated intrinsic role for COUP-TFI in axonal growth in vivo and supply one of the premises for COUP-TFI coordination of neuronal morphogenesis in the developing forebrain.
4. Neurogenin 2 controls cortical neuron **migration** through regulation of Rnd2. Motility is a universal property of newly generated neurons. How cell **migration** is coordinately regulated with other aspects of neuron production is not well understood. Here we show that the proneural protein neurogenin 2 (Neurog2), which controls neurogenesis in the embryonic cerebral cortex, directly induces the expression of the small GTP-binding protein Rnd2 (ref. 3) in newly generated mouse cortical neurons before they initiate **migration**. Rnd2 silencing leads to a defect in radial **migration** of cortical neurons similar to that observed when the Neurog2 gene is deleted. Remarkably, restoring Rnd2 expression in Neurog2-mutant neurons is sufficient to rescue their ability to migrate. Our results identify Rnd2 as a novel essential regulator of neuronal **migration** in the cerebral cortex and demonstrate that Rnd2 is a major effector of Neurog2 function in the promotion of **migration**. Thus, a proneural protein controls the complex cellular behaviour of cell **migration** through a remarkably direct pathway involving the transcriptional activation of a small GTP-binding protein.
5. The zinc finger transcription factor RP58 negatively regulates Rnd2 for the control of neuronal **migration** during cerebral cortical development. The zinc finger transcription factor RP58 (also known as ZNF238) regulates neurogenesis of the mouse neocortex and cerebellum (Okado et al. 2009; Xiang et al. 2011; Baubet et al. 2012; Ohtaka-Maruyama et al. 2013), but its mechanism of action remains unclear. In this study, we report a cell-autonomous function for RP58 during the differentiation of embryonic cortical projection neurons via its activities as a transcriptional repressor. Disruption of RP58 expression alters the differentiation of immature neurons and impairs their **migration** and positioning within the mouse cerebral cortex. Loss of RP58 within the embryonic cortex also leads to elevated mRNA for Rnd2, a member of the Rnd family of atypical RhoA-like GTPase proteins important for cortical neuron **migration** (Heng et al. 2008). Mechanistically, RP58 represses transcription of Rnd2 via binding to a 3'-regulatory enhancer in a sequence-specific fashion. Using reporter assays, we found that RP58 repression of Rnd2 is competed by proneural basic helix-loop-helix transcriptional activators. Finally, our rescue experiments revealed that negative regulation of Rnd2 by RP58 was important for cortical cell **migration** in vivo. Taken together, these studies demonstrate that RP58 is a key player in the transcriptional control of cell **migration** in the developing cerebral cortex.
6. Neurogenin 2 controls cortical neuron **migration** through regulation of Rnd2. Motility is a universal property of newly generated neurons. How cell **migration** is coordinately regulated with other aspects of neuron production is not well understood. Here we show that the proneural protein neurogenin 2 (Neurog2), which controls neurogenesis in the embryonic cerebral cortex, directly induces the expression of the small GTP-binding protein Rnd2 (ref. 3) in newly generated mouse cortical neurons before they initiate **migration**. Rnd2 silencing leads to a defect in radial **migration** of cortical neurons similar to that observed when the Neurog2 gene is deleted. Remarkably, restoring Rnd2 expression in Neurog2-mutant neurons is sufficient to rescue their ability to migrate. Our results identify Rnd2 as a novel essential regulator of neuronal **migration** in the cerebral cortex and demonstrate that Rnd2 is a major effector of Neurog2 function in the promotion of **migration**. Thus, a proneural protein controls the complex cellular behaviour of cell **migration** through a remarkably direct pathway involving the transcriptional activation of a small GTP-binding protein.
7. Bacurd2 is a novel interacting partner to Rnd2 which controls radial **migration** within the developing mammalian cerebral cortex. During fetal brain development in mammals, newborn neurons undergo cell **migration** to reach their appropriate positions and form functional circuits. We previously reported that the atypical RhoA GTPase Rnd2 promotes the radial **migration** of mouse cerebral cortical neurons (Nature 455(7209):114-8, 2008; Neuron 69(6):1069-84, 2011), but its downstream signalling pathway is not well understood.We have identified BTB-domain containing adaptor for Cul3-mediated RhoA degradation 2 (Bacurd2) as a novel interacting partner to Rnd2, which promotes radial **migration** within the developing cerebral cortex. We find that Bacurd2 binds Rnd2 at its C-terminus, and this interaction is critical to its cell **migration** function. We show that forced expression or knockdown of Bacurd2 impairs neuronal **migration** within the embryonic cortex and alters the morphology of immature neurons. Our in vivo cellular analysis reveals that Bacurd2 influences the multipolar-to-bipolar transition of radially migrating neurons in a cell autonomous fashion. When we addressed the potential signalling relationship between Bacurd2 and Rnd2 using a Bacurd2-Rnd2 chimeric construct, our results suggest that Bacurd2 and Rnd2 could interact to promote radial **migration** within the embryonic cortex.Our studies demonstrate that Bacurd2 is a novel player in neuronal development and influences radial **migration** within the embryonic cerebral cortex.
8. Different requirement for Rnd GTPases of R-Ras GAP activity of Plexin-C1 and Plexin-D1. Plexins, comprising Plexin-A, -B, -C, and -D subfamilies, are receptors for semaphorins governing cell adhesion, **migration**, and axon guidance. Among plexin subfamilies, Plexin-A1 and Plexin-B1 have been shown to function as an R-Ras GAP, inducing repulsive responses, and the expression of R-Ras GAP activity requires the binding of Rnd1, a member of Rnd subfamily of Rho GTPases. However, signaling pathways of Plexin-D1 and Plexin-C1 still remain obscure. Here, we found that Plexin-D1 displayed R-Ras GAP activity and inhibited **migration** of COS-7 cells, and these actions required Rnd2, another Rnd subfamily GTPase. Rnd2 bound to Plexin-D1 in cortical neurons, and Sema3E/Plexin-D1-induced inhibition of axon outgrowth of cortical neurons required Rnd2 and down-regulation of R-Ras activity. On the other hand, Plexin-C1 displayed R-Ras GAP activity and inhibited cell **migration** of COS-7 cells without Rnd proteins. Therefore, R-Ras GAP activity is a common function of plexin subfamilies but the regulation of R-Ras GAP activity of plexins by Rnd proteins is different among plexin subfamilies.
9. Proneural transcription factors regulate different steps of cortical neuron **migration** through Rnd-mediated inhibition of RhoA signaling. Little is known of the intracellular machinery that controls the motility of newborn neurons. We have previously shown that the proneural protein Neurog2 promotes the **migration** of nascent cortical neurons by inducing the expression of the atypical Rho GTPase Rnd2. Here, we show that another proneural factor, Ascl1, promotes neuronal **migration** in the cortex through direct regulation of a second Rnd family member, Rnd3. Both Rnd2 and Rnd3 promote neuronal **migration** by inhibiting RhoA signaling, but they control distinct steps of the migratory process, multipolar to bipolar transition in the intermediate zone and locomotion in the cortical plate, respectively. Interestingly, these divergent functions directly result from the distinct subcellular distributions of the two Rnd proteins. Because Rnd proteins also regulate progenitor divisions and neurite outgrowth, we propose that proneural factors, through spatiotemporal regulation of Rnd proteins, integrate the process of neuronal **migration** with other events in the neurogenic program.
10. Dicer is required for proliferation, viability, **migration** and differentiation in corticoneurogenesis. In mice, microRNAs (miRNAs) are required for embryonic viability, and previous reports implicate miRNA participation in brain cortical neurogenesis. Here, we provide a more comprehensive analysis of miRNA involvement in cortical brain development. To accomplish this we used mice in which Dicer, the RNase III enzyme necessary for canonical miRNA biogenesis, is depleted from Nestin-expressing progenitors and progeny cells. We systematically assessed how Dicer depletion impacts proliferation, cell death, **migration** and differentiation in the developing brain. Using markers for proliferation and in vivo labeling with thymidine analogs, we found reduced numbers of proliferating cells, and altered cell cycle kinetics from embryonic day 15.5 (E15.5). Progenitor cells were distributed aberrantly throughout the cortex rather than restricted to the ventricular and subventricular zones. Activated Caspase3 was elevated, reflecting increased cortical cell death as early as E15.5. Cajal-Retzius-positive cells were more numerous at E15.5 and were dysmorphic relative to control cortices. Consistent with this, Reelin levels were enhanced. Doublecortin and Rnd2 were also increased and showed altered distribution, supporting a strong regulatory role for miRNAs in both early and late neuronal **migration**. In addition, GFAP staining at E15.5 was more intense and disorganized throughout the cortex with Dicer depletion. These results significantly extend earlier works, and emphasize the impact of miRNAs on neural progenitor cell proliferation, apoptosis, **migration**, and differentiation in the developing mammalian brain.
11. An antagonistic interaction between PlexinB2 and Rnd3 controls RhoA activity and cortical neuron **migration**. A transcriptional programme initiated by the proneural factors Neurog2 and Ascl1 controls successive steps of neurogenesis in the embryonic cerebral cortex. Previous work has shown that proneural factors also confer a migratory behaviour to cortical neurons by inducing the expression of the small GTP-binding proteins such as Rnd2 and Rnd3. However, the directionality of radial **migration** suggests that migrating neurons also respond to extracellular signal-regulated pathways. Here we show that the Plexin B2 receptor interacts physically and functionally with Rnd3 and stimulates RhoA activity in migrating cortical neurons. Plexin B2 competes with p190RhoGAP for binding to Rnd3, thus blocking the Rnd3-mediated inhibition of RhoA and also recruits RhoGEFs to directly stimulate RhoA activity. Thus, an interaction between the cell-extrinsic Plexin signalling pathway and the cell-intrinsic Ascl1-Rnd3 pathway determines the level of RhoA activity appropriate for cortical neuron **migration**.
12. COUP-TFI promotes radial **migration** and proper morphology of callosal projection neurons by repressing Rnd2 expression. During corticogenesis, late-born callosal projection neurons (CPNs) acquire their laminar position through glia-guided radial **migration** and then undergo final differentiation. However, the mechanisms controlling radial **migration** and final morphology of CPNs are poorly defined. Here, we show that in COUP-TFI mutant mice CPNs are correctly specified, but are delayed in reaching the cortical plate and have morphological defects during **migration**. Interestingly, we observed that the rate of neuronal **migration** to the cortical plate normally follows a low-rostral to high-caudal gradient, similar to that described for COUP-TFI. This gradient is strongly impaired in COUP-TFI(-/-) brains. Moreover, the expression of the Rho-GTPase Rnd2, a modulator of radial **migration**, is complementary to both these gradients and strongly increases in the absence of COUP-TFI function. We show that COUP-TFI directly represses Rnd2 expression at the post-mitotic level along the rostrocaudal axis of the neocortex. Restoring correct Rnd2 levels in COUP-TFI(-/-) brains cell-autonomously rescues neuron radial **migration** and morphological transitions. We also observed impairments in axonal elongation and dendritic arborization of COUP-TFI-deficient CPNs, which were rescued by lowering Rnd2 expression levels. Thus, our data demonstrate that COUP-TFI modulates late-born neuron **migration** and favours proper differentiation of CPNs by finely regulating Rnd2 expression levels.

#### GeneRIFs

1. Data demonstrate that COUP-TFI modulates late-born neuron **migration** and favours proper differentiation of callosal projection neurons by finely regulating Rnd2 expression levels.
2. results identify Rnd2 as a novel essential regulator of neuronal **migration** in the cerebral cortex and demonstrate that Rnd2 is a major effector of Neurog2 function in the promotion of **migration**

---

## Rnd3

- NCBI Gene
- GeneRIF
- Pubmed

| Search Term | Rank | Fields |
| --- | --- | --- |
| [Pp]odosome(s)? | 5 | PubMed(1); GeneRIFs(1) |
| [Mm]igration | 6 | PubMed(12); GeneRIFs(1) |
| [Ee]xtracellular [Mm]atrix | 8 | PubMed(1) |
| [Mm]etastasis | 12 | PubMed(1) |

  
hide/show details for Rnd3

### [Pp]odosome(s)?

#### PubMed

1. Comparative transcriptomics reveals RhoE as a novel regulator of actin dynamics in bone-resorbing osteoclasts. The function of osteoclasts (OCs), multinucleated giant cells (MGCs) of the monocytic lineage, is bone resorption. To resorb bone, OCs form **podosomes**. These are actin-rich adhesive structures that pattern into rings that drive OC migration and into "sealing-zones" (SZs) that confine the resorption lacuna. Although changes in actin dynamics during **podosome** patterning have been documented, the mechanisms that regulate these changes are largely unknown. From human monocytic precursors, we differentiated MGCs that express OC degradation enzymes but are unable to resorb the mineral matrix. We demonstrated that, despite exhibiting bona fide **podosomes**, these cells presented dysfunctional SZs. We then performed two-step differential transcriptomic profiling of bone-resorbing OCs versus nonresorbing MGCs to generate a list of genes implicated in bone resorption. From this list of candidate genes, we investigated the role of Rho/Rnd3. Using primary RhoE-deficient OCs, we demonstrated that RhoE is indispensable for OC migration and bone resorption by maintaining fast actin turnover in **podosomes**. We further showed that RhoE activates **podosome** component cofilin by inhibiting its Rock-mediated phosphorylation. We conclude that the RhoE-Rock-cofilin pathway, by promoting **podosome** dynamics and patterning, is central for OC migration, SZ formation, and, ultimately, bone resorption.

#### GeneRIFs

1. RhoE is indispensable for osteoclast migration and bone resorption by maintaining fast actin turnover in **podosomes**. RhoE activates **podosome** component cofilin by inhibiting its Rock-mediated phosphorylation.

### [Mm]igration

#### PubMed

1. Integrin-linked kinase is required for radial sorting of axons and Schwann cell remyelination in the peripheral nervous system. During development, Schwann cells (SCs) interpret different extracellular cues to regulate their **migration**, proliferation, and the remarkable morphological changes associated with the sorting, ensheathment, and myelination of axons. Although interactions between extracellular matrix proteins and integrins are critical to some of these processes, the downstream signaling pathways they control are still poorly understood. Integrin-linked kinase (ILK) is a focal adhesion protein that associates with multiple binding partners to link integrins to the actin cytoskeleton and is thought to participate in integrin and growth factor-mediated signaling. Using SC-specific gene ablation, we report essential functions for ILK in radial sorting of axon bundles and in remyelination in the peripheral nervous system. Our in vivo and in vitro experiments show that ILK negatively regulates Rho/Rho kinase signaling to promote SC process extension and to initiate radial sorting. ILK also facilitates axon remyelination, likely by promoting the activation of downstream molecules such as AKT/protein kinase B.
2. Proneural transcription factors regulate different steps of cortical neuron **migration** through Rnd-mediated inhibition of RhoA signaling. Little is known of the intracellular machinery that controls the motility of newborn neurons. We have previously shown that the proneural protein Neurog2 promotes the **migration** of nascent cortical neurons by inducing the expression of the atypical Rho GTPase Rnd2. Here, we show that another proneural factor, Ascl1, promotes neuronal **migration** in the cortex through direct regulation of a second Rnd family member, Rnd3. Both Rnd2 and Rnd3 promote neuronal **migration** by inhibiting RhoA signaling, but they control distinct steps of the migratory process, multipolar to bipolar transition in the intermediate zone and locomotion in the cortical plate, respectively. Interestingly, these divergent functions directly result from the distinct subcellular distributions of the two Rnd proteins. Because Rnd proteins also regulate progenitor divisions and neurite outgrowth, we propose that proneural factors, through spatiotemporal regulation of Rnd proteins, integrate the process of neuronal **migration** with other events in the neurogenic program.
3. An antagonistic interaction between PlexinB2 and Rnd3 controls RhoA activity and cortical neuron **migration**. A transcriptional programme initiated by the proneural factors Neurog2 and Ascl1 controls successive steps of neurogenesis in the embryonic cerebral cortex. Previous work has shown that proneural factors also confer a migratory behaviour to cortical neurons by inducing the expression of the small GTP-binding proteins such as Rnd2 and Rnd3. However, the directionality of radial **migration** suggests that migrating neurons also respond to extracellular signal-regulated pathways. Here we show that the Plexin B2 receptor interacts physically and functionally with Rnd3 and stimulates RhoA activity in migrating cortical neurons. Plexin B2 competes with p190RhoGAP for binding to Rnd3, thus blocking the Rnd3-mediated inhibition of RhoA and also recruits RhoGEFs to directly stimulate RhoA activity. Thus, an interaction between the cell-extrinsic Plexin signalling pathway and the cell-intrinsic Ascl1-Rnd3 pathway determines the level of RhoA activity appropriate for cortical neuron **migration**.
4. Genetic deletion of Rnd3 results in aqueductal stenosis leading to hydrocephalus through up-regulation of Notch signaling. Rho family guanosine triphosphatase (GTPase) 3 (Rnd3), a member of the small Rho GTPase family, is involved in the regulation of cell actin cytoskeleton dynamics, cell **migration**, and proliferation through the Rho kinase-dependent signaling pathway. We report a role of Rnd3 in the pathogenesis of hydrocephalus disorder. Mice with Rnd3 genetic deletion developed severe obstructive hydrocephalus with enlargement of the lateral and third ventricles, but not of the fourth ventricles. The cerebral aqueducts in Rnd3-null mice were partially or completely blocked by the overgrowth of ependymal epithelia. We examined the molecular mechanism contributing to this Rnd3-deficiency-mediated hydrocephalus and found that Rnd3 is a regulator of Notch signaling. Rnd3 deficiency, through either gene deletion or siRNA knockdown, resulted in a decrease in Notch intracellular domain (NICD) protein degradation. However, there was no correlated change in mRNA change, which in turn led to an increase in NICD protein levels. Immunoprecipitation analysis demonstrated that Rnd3 and NICD physically interacted, and that down-regulation of Rnd3 attenuated NICD protein ubiquitination. This eventually enhanced Notch signaling activity and promoted aberrant growth of aqueduct ependymal cells, resulting in aqueduct stenosis and the development of congenital hydrocephalus. Inhibition of Notch activity rescued the hydrocephalus disorder in the mutant animals.
5. An antagonistic interaction between PlexinB2 and Rnd3 controls RhoA activity and cortical neuron **migration**. A transcriptional programme initiated by the proneural factors Neurog2 and Ascl1 controls successive steps of neurogenesis in the embryonic cerebral cortex. Previous work has shown that proneural factors also confer a migratory behaviour to cortical neurons by inducing the expression of the small GTP-binding proteins such as Rnd2 and Rnd3. However, the directionality of radial **migration** suggests that migrating neurons also respond to extracellular signal-regulated pathways. Here we show that the Plexin B2 receptor interacts physically and functionally with Rnd3 and stimulates RhoA activity in migrating cortical neurons. Plexin B2 competes with p190RhoGAP for binding to Rnd3, thus blocking the Rnd3-mediated inhibition of RhoA and also recruits RhoGEFs to directly stimulate RhoA activity. Thus, an interaction between the cell-extrinsic Plexin signalling pathway and the cell-intrinsic Ascl1-Rnd3 pathway determines the level of RhoA activity appropriate for cortical neuron **migration**.
6. Genetic deletion of Rnd3 results in aqueductal stenosis leading to hydrocephalus through up-regulation of Notch signaling. Rho family guanosine triphosphatase (GTPase) 3 (Rnd3), a member of the small Rho GTPase family, is involved in the regulation of cell actin cytoskeleton dynamics, cell **migration**, and proliferation through the Rho kinase-dependent signaling pathway. We report a role of Rnd3 in the pathogenesis of hydrocephalus disorder. Mice with Rnd3 genetic deletion developed severe obstructive hydrocephalus with enlargement of the lateral and third ventricles, but not of the fourth ventricles. The cerebral aqueducts in Rnd3-null mice were partially or completely blocked by the overgrowth of ependymal epithelia. We examined the molecular mechanism contributing to this Rnd3-deficiency-mediated hydrocephalus and found that Rnd3 is a regulator of Notch signaling. Rnd3 deficiency, through either gene deletion or siRNA knockdown, resulted in a decrease in Notch intracellular domain (NICD) protein degradation. However, there was no correlated change in mRNA change, which in turn led to an increase in NICD protein levels. Immunoprecipitation analysis demonstrated that Rnd3 and NICD physically interacted, and that down-regulation of Rnd3 attenuated NICD protein ubiquitination. This eventually enhanced Notch signaling activity and promoted aberrant growth of aqueduct ependymal cells, resulting in aqueduct stenosis and the development of congenital hydrocephalus. Inhibition of Notch activity rescued the hydrocephalus disorder in the mutant animals.
7. Neuronal polarization is impaired in mice lacking RhoE expression. Proper development of neuronal networks relies on the polarization of the neurons, thus the establishment of two compartments, axons and dendrites, whose formation depends on cytoskeletal rearrangements. Rnd proteins are regulators of actin organization and they are important players in several aspects of brain development as neurite formation, axon guidance and neuron **migration**. We have recently demonstrated that mice lacking RhoE/Rnd3 expression die shortly after birth and have neuromotor impairment and neuromuscular alterations, indicating an abnormal development of the nervous system. In this study, we have further investigated the specific role played by RhoE in several aspects of neuronal development by using hippocampal neuron cultures. Our findings show that neurons from a mice lacking RhoE expression exhibit a decrease in the number and the total length of the neurites. We also show that RhoE-deficient neurons display a reduction in axon outgrowth and a delay in the process of neuronal polarization. In addition, our results suggest an involvement of the RHOA/ROCK/LIMK/COFILIN signaling pathway in the neuronal alterations induced by the lack of RhoE. These findings support our previous report revealing the important role of RhoE in the normal development of the nervous system and may provide novel therapeutic targets in neurodegenerative disorders.
8. Comparative transcriptomics reveals RhoE as a novel regulator of actin dynamics in bone-resorbing osteoclasts. The function of osteoclasts (OCs), multinucleated giant cells (MGCs) of the monocytic lineage, is bone resorption. To resorb bone, OCs form podosomes. These are actin-rich adhesive structures that pattern into rings that drive OC **migration** and into "sealing-zones" (SZs) that confine the resorption lacuna. Although changes in actin dynamics during podosome patterning have been documented, the mechanisms that regulate these changes are largely unknown. From human monocytic precursors, we differentiated MGCs that express OC degradation enzymes but are unable to resorb the mineral matrix. We demonstrated that, despite exhibiting bona fide podosomes, these cells presented dysfunctional SZs. We then performed two-step differential transcriptomic profiling of bone-resorbing OCs versus nonresorbing MGCs to generate a list of genes implicated in bone resorption. From this list of candidate genes, we investigated the role of Rho/Rnd3. Using primary RhoE-deficient OCs, we demonstrated that RhoE is indispensable for OC **migration** and bone resorption by maintaining fast actin turnover in podosomes. We further showed that RhoE activates podosome component cofilin by inhibiting its Rock-mediated phosphorylation. We conclude that the RhoE-Rock-cofilin pathway, by promoting podosome dynamics and patterning, is central for OC **migration**, SZ formation, and, ultimately, bone resorption.
9. Rnd3 coordinates early steps of cortical neurogenesis through actin-dependent and -independent mechanisms. The generation of neurons by neural stem cells is a highly choreographed process that requires extensive and dynamic remodelling of the cytoskeleton at each step of the process. The atypical RhoGTPase Rnd3 is expressed by progenitors in the embryonic brain but its role in early steps of neurogenesis has not been addressed. Here we show that silencing Rnd3 in the embryonic cerebral cortex interferes with the interkinetic nuclear **migration** of radial glial stem cells, disrupts their apical attachment and modifies the orientation of their cleavage plane. These defects are rescued by co-expression of a constitutively active form of cofilin, demonstrating that Rnd3-mediated disassembly of actin filaments coordinates the cellular behaviour of radial glial. Rnd3 also limits the divisions of basal progenitors via a distinct mechanism involving the suppression of cyclin D1 translation. Interestingly, although Rnd3 expression is controlled transcriptionally by Ascl1, this proneural factor is itself required in radial glial progenitors only for proper orientation of cell divisions.
10. Genetic deletion of Rnd3/RhoE results in mouse heart calcium leakage through upregulation of protein kinase A signaling. Rnd3, a small Rho GTPase, is involved in the regulation of cell actin cytoskeleton dynamics, cell **migration**, and proliferation. The biological function of Rnd3 in the heart remains unexplored.To define the functional role of the Rnd3 gene in the animal heart and investigate the associated molecular mechanism.By loss-of-function approaches, we discovered that Rnd3 is involved in calcium regulation in cardiomyocytes. Rnd3-null mice died at the embryonic stage with fetal arrhythmias. The deletion of Rnd3 resulted in severe Ca(2+) leakage through destabilized ryanodine receptor type 2 Ca(2+) release channels. We further found that downregulation of Rnd3 attenuated β2-adrenergic receptor lysosomal targeting and ubiquitination, which in turn resulted in the elevation of β2-adrenergic receptor protein levels leading to the hyperactivation of protein kinase A (PKA) signaling. The PKA activation destabilized ryanodine receptor type 2 channels. This irregular spontaneous Ca(2+) release can be curtailed by PKA inhibitor treatment. Increases in the PKA activity along with elevated cAMP levels were detected in Rnd3-null embryos, in neonatal rat cardiomyocytes, and noncardiac cell lines with Rnd3 knockdown, suggesting a general mechanism for Rnd3-mediated PKA signaling activation. β2-Adrenergic receptor blocker treatment reduced arrhythmia and improved cardiac function.Rnd3 is a novel factor involved in intracellular Ca(2+) homeostasis regulation in the heart. Deficiency of the protein induces ryanodine receptor type 2 dysfunction by a mechanism that attenuates Rnd3-mediated β2-adrenergic receptor ubiquitination, which leads to the activation of PKA signaling. Increased PKA signaling in turn promotes ryanodine receptor type 2 hyperphosphorylation, which contributes to arrhythmogenesis and heart failure.
11. Rnd3 haploinsufficient mice are predisposed to hemodynamic stress and develop apoptotic cardiomyopathy with heart failure. Rho family guanosine triphosphatase (GTPase) 3 (Rnd3), a member of the small Rho GTPase family, has been suggested to regulate cell actin cytoskeleton dynamics, cell **migration**, and apoptosis through the Rho kinase-dependent signaling pathway. The biological function of Rnd3 in the heart is unknown. The downregulation of small GTPase Rnd3 transcripts was found in patients with end-stage heart failure. The pathological significance of Rnd3 loss in the transition to heart failure remains unexplored. To investigate the functional consequence of Rnd3 downregulation and the associated molecular mechanism, we generated Rnd3(+/-) haploinsufficient mice to mimic the downregulation of Rnd3 observed in the failing human heart. Rnd3(+/-) mice were viable; however, the mice developed heart failure after pressure overload by transverse aortic constriction (TAC). Remarkable apoptosis, increased caspase-3 activity, and elevated Rho kinase activity were detected in the Rnd3(+/-) haploinsufficient animal hearts. Pharmacological inhibition of Rho kinase by fasudil treatment partially improved Rnd3(+/-) mouse cardiac functions and attenuated myocardial apoptosis. To determine if Rho-associated coiled-coil kinase 1 (ROCK1) was responsible for Rnd3 deficiency-mediated apoptotic cardiomyopathy, we established a double-knockout mouse line, the Rnd3 haploinsufficient mice with ROCK1-null background (Rnd3(+/-/ROCK1-/-)). Again, genetic deletion of ROCK1 partially but not completely rescued Rnd3 deficiency-mediated heart failure phenotype. These data suggest that downregulation of Rnd3 correlates with cardiac loss of function as in heart failure patients. Animals with Rnd3 haploinsufficiency are predisposed to hemodynamic stress. Hyperactivation of Rho kinase activity is responsible in part for the apoptotic cardiomyopathy development. Further investigation of ROCK1-independent mechanisms in Rnd3-mediated cardiac remodeling should be the focus for future study.
12. Rnd3 in Cancer: A Review of the Evidence for Tumor Promoter or Suppressor. Rho-GTPases are members of the Ras superfamily of small GTPases and are general modulators of important cellular processes in tumor biology such as **migration** and proliferation. Among these proteins, Rnd3/RhoE, an atypical Rho-GTPase devoid of GTP hydrolytic activity, has recently been studied for its putative role in tumorigenesis. Indeed, Rnd3 is implicated in processes, such as proliferation and **migration**, whose deregulation is linked to cancer development and metastasis. The aim of this review is to provide an overview of the data surrounding Rnd3 deregulation in cancers, its origin, and consequences. Presented here is a comprehensive account of the expression status and biological output obtained in prostate, liver, stomach, colon, lung, and brain cancers as well as in melanoma and squamous cell carcinoma. Although there appears to be no general consensus about Rnd3 expression in cancers as this protein is differently altered according to the tumor context, these alterations overwhelmingly favor a protumorigenic role. Thus, depending on the tumor type, it may behave either as a tumor suppressor or as a tumor promoter. Importantly, the deregulation of Rnd3, in most cases, is linked to patient poor outcome.Rnd3 has prognostic marker potential as exemplified in lung cancers and Rnd3 or Rnd3-associated signaling pathways may represent a new putative therapeutic target. Mol Cancer Res; 14(11); 1033-44. ©2016 AACR.

#### GeneRIFs

1. RhoE is indispensable for osteoclast **migration** and bone resorption by maintaining fast actin turnover in podosomes. RhoE activates podosome component cofilin by inhibiting its Rock-mediated phosphorylation.

### [Ee]xtracellular [Mm]atrix

#### PubMed

1. Integrin-linked kinase is required for radial sorting of axons and Schwann cell remyelination in the peripheral nervous system. During development, Schwann cells (SCs) interpret different extracellular cues to regulate their migration, proliferation, and the remarkable morphological changes associated with the sorting, ensheathment, and myelination of axons. Although interactions between **extracellular matrix** proteins and integrins are critical to some of these processes, the downstream signaling pathways they control are still poorly understood. Integrin-linked kinase (ILK) is a focal adhesion protein that associates with multiple binding partners to link integrins to the actin cytoskeleton and is thought to participate in integrin and growth factor-mediated signaling. Using SC-specific gene ablation, we report essential functions for ILK in radial sorting of axon bundles and in remyelination in the peripheral nervous system. Our in vivo and in vitro experiments show that ILK negatively regulates Rho/Rho kinase signaling to promote SC process extension and to initiate radial sorting. ILK also facilitates axon remyelination, likely by promoting the activation of downstream molecules such as AKT/protein kinase B.

### [Mm]etastasis

#### PubMed

1. Rnd3 in Cancer: A Review of the Evidence for Tumor Promoter or Suppressor. Rho-GTPases are members of the Ras superfamily of small GTPases and are general modulators of important cellular processes in tumor biology such as migration and proliferation. Among these proteins, Rnd3/RhoE, an atypical Rho-GTPase devoid of GTP hydrolytic activity, has recently been studied for its putative role in tumorigenesis. Indeed, Rnd3 is implicated in processes, such as proliferation and migration, whose deregulation is linked to cancer development and **metastasis**. The aim of this review is to provide an overview of the data surrounding Rnd3 deregulation in cancers, its origin, and consequences. Presented here is a comprehensive account of the expression status and biological output obtained in prostate, liver, stomach, colon, lung, and brain cancers as well as in melanoma and squamous cell carcinoma. Although there appears to be no general consensus about Rnd3 expression in cancers as this protein is differently altered according to the tumor context, these alterations overwhelmingly favor a protumorigenic role. Thus, depending on the tumor type, it may behave either as a tumor suppressor or as a tumor promoter. Importantly, the deregulation of Rnd3, in most cases, is linked to patient poor outcome.Rnd3 has prognostic marker potential as exemplified in lung cancers and Rnd3 or Rnd3-associated signaling pathways may represent a new putative therapeutic target. Mol Cancer Res; 14(11); 1033-44. ©2016 AACR.

---

## Rhoq

- NCBI Gene
- GeneRIF
- Pubmed

| Search Term | Rank | Fields |
| --- | --- | --- |
| [Ff]ilopodia | 1 | PubMed(1) |
| [Ff]ilopodium | 2 | Process(1) |
| ECM | 7 | PubMed(1) |
| [Ee]xtracellular [Mm]atrix | 8 | PubMed(1) |

  
hide/show details for Rhoq

### [Ff]ilopodia

#### PubMed

1. Small GTPase Tc10 and its homologue RhoT induce N-WASP-mediated long process formation and neurite outgrowth. Rho family small GTPases regulate multiple cellular functions through reorganization of the actin cytoskeleton. Among them, Cdc42 and Tc10 induce **filopodia** or peripheral processes in cultured cells. We have identified a member of the family, designated as RhoT, which is closely related to Tc10. Tc10 was highly expressed in muscular tissues and brain and remarkably induced during differentiation of C2 skeletal muscle cells and neuronal differentiation of PC12 and N1E-115 cells. On the other hand, RhoT was predominantly expressed in heart and uterus and induced during neuronal differentiation of N1E-115 cells. Tc10 exogenously expressed in fibroblasts generated actin-filament-containing peripheral processes longer than the Cdc42-formed **filopodia**, whereas RhoT produced much longer and thicker processes containing actin filaments. Furthermore, both Tc10 and RhoT induced neurite outgrowth in PC12 and N1E-115 cells, but Cdc42 did not do this by itself. Tc10 and RhoT as well as Cdc42 bound to the N-terminal CRIB-motif-containing portion of N-WASP and activated N-WASP to induce Arp2/3-complex-mediated actin polymerization. The formation of peripheral processes and neurites by Tc10 and RhoT was prevented by the coexpression of dominant-negative mutants of N-WASP. Thus, N-WASP is essential for the process formation and neurite outgrowth induced by Tc10 and RhoT. Neuronal differentiation of PC12 and N1E-115 cells induced by dibutyryl cyclic AMP and by serum starvation, respectively, was prevented by dominant-negative Cdc42, Tc10 and RhoT. Taken together, all these Rho family proteins are required for neuronal differentiation, but they exert their functions differentially in process formation and neurite extension. Consequently, N-WASP activated by these small GTPases mediates neuronal differentiation in addition to its recently identified role in glucose uptake.

### [Ff]ilopodium

#### Process

1. positive regulation of **filopodium** assembly

### ECM

#### PubMed

1. Arhgap28 is a RhoGAP that inactivates RhoA and downregulates stress fibers. The small GTPase RhoA is a major regulator of actin reorganization during the formation of stress fibers; thus identifying molecules that regulate Rho activity is necessary for a complete understanding of the mechanisms that determine cell contractility. Here, we have identified Arhgap28 as a Rho GTPase activating protein (RhoGAP) that switches RhoA to its inactive form. We generated an Arhgap28-LacZ reporter mouse that revealed gene expression in soft tissues at E12.5, pre-bone structures of the limb at E15.5, and prominent expression restricted mostly to ribs and limb long bones at E18.5 days of development. Expression of recombinant Arhgap28-V5 in human osteosarcoma SaOS-2 cells caused a reduction in the basal level of RhoA activation and disruption of actin stress fibers. Extracellular matrix assembly studies using a 3-dimensional cell culture system showed that Arhgap28 was upregulated during Rho-dependent assembly of the **ECM**. Taken together, these observations led to the hypothesis that an Arhgap28 knockout mouse model would show a connective tissue phenotype, perhaps affecting bone. Arhgap28-null mice were viable and appeared normal, suggesting that there could be compensation from other RhoGAPs. Indeed, we showed that expression of Arhgap6 (a closely related RhoGAP) was upregulated in Arhgap28-null bone tissue. An upregulation in RhoA expression was also detected suggesting that Arhgap28 may be able to additionally regulate Rho signaling at a transcriptional level. Microarray analyses revealed that Col2a1, Col9a1, Matn3, and Comp that encode extracellular matrix proteins were downregulated in Arhgap28-null bone. Although mutations in these genes cause bone dysplasias no bone phenotype was detected in the Arhgap-28 null mice. Together, these data suggest that the regulation of Rho by RhoGAPs, including Arhgap28, during the assembly and development of mechanically strong tissues is complex and may involve multiple RhoGAPs.

### [Ee]xtracellular [Mm]atrix

#### PubMed

1. Arhgap28 is a RhoGAP that inactivates RhoA and downregulates stress fibers. The small GTPase RhoA is a major regulator of actin reorganization during the formation of stress fibers; thus identifying molecules that regulate Rho activity is necessary for a complete understanding of the mechanisms that determine cell contractility. Here, we have identified Arhgap28 as a Rho GTPase activating protein (RhoGAP) that switches RhoA to its inactive form. We generated an Arhgap28-LacZ reporter mouse that revealed gene expression in soft tissues at E12.5, pre-bone structures of the limb at E15.5, and prominent expression restricted mostly to ribs and limb long bones at E18.5 days of development. Expression of recombinant Arhgap28-V5 in human osteosarcoma SaOS-2 cells caused a reduction in the basal level of RhoA activation and disruption of actin stress fibers. **Extracellular matrix** assembly studies using a 3-dimensional cell culture system showed that Arhgap28 was upregulated during Rho-dependent assembly of the ECM. Taken together, these observations led to the hypothesis that an Arhgap28 knockout mouse model would show a connective tissue phenotype, perhaps affecting bone. Arhgap28-null mice were viable and appeared normal, suggesting that there could be compensation from other RhoGAPs. Indeed, we showed that expression of Arhgap6 (a closely related RhoGAP) was upregulated in Arhgap28-null bone tissue. An upregulation in RhoA expression was also detected suggesting that Arhgap28 may be able to additionally regulate Rho signaling at a transcriptional level. Microarray analyses revealed that Col2a1, Col9a1, Matn3, and Comp that encode **extracellular matrix** proteins were downregulated in Arhgap28-null bone. Although mutations in these genes cause bone dysplasias no bone phenotype was detected in the Arhgap-28 null mice. Together, these data suggest that the regulation of Rho by RhoGAPs, including Arhgap28, during the assembly and development of mechanically strong tissues is complex and may involve multiple RhoGAPs.

---

## Rhou

- NCBI Gene
- GeneRIF
- Pubmed

| Search Term | Rank | Fields |
| --- | --- | --- |
| [Ff]ilopodia | 1 | PubMed(1) |
| [Ff]ilopodium | 2 | PubMed(1) |
| [Mm]igration | 6 | PubMed(2) |
| [Mm]etastasis | 12 | PubMed(1) |

  
hide/show details for Rhou

### [Ff]ilopodia

#### PubMed

1. The atypical Rho GTPase Wrch1 collaborates with the nonreceptor tyrosine kinases Pyk2 and Src in regulating cytoskeletal dynamics. The Cdc42-like GTPase Wnt responsive Cdc42 homolog 1 (Wrch1) has several atypical features; it has an N-terminal proline-rich extension that confers binding to SH3 domains, and it harbors an extremely high intrinsic nucleotide exchange activity, which overrides the normal GTPase activity. As a result, Wrch1 resides mainly in the active, GTP-loaded conformation under normal cellular conditions. We have previously shown that ectopic expression of Wrch1 in fibroblasts resulted in an altered cell morphology visible as a formation of **filopodia**, a loss of stress fibers, and a reduction in focal adhesions. Here, we show that Wrch1 binds to the nonreceptor tyrosine kinase Pyk2. The interaction required Wrch1 to be in a GTP conformation and also required an intact N-terminal proline-rich extension as well as an intact effector loop. Wrch1 requires Pyk2 in imposing the cytoskeletal effects, seen as the formation of **filopodia**, since treatment of cells with a Pyk2-specific small interfering RNA abrogated this response. Interestingly, we found that the presence and activity of Src were needed for the formation of a Wrch1-Pyk2 complex as well as for the Wrch1-induced formation of **filopodia**. We propose a model in which Pyk2 and Src function to coordinate the Wrch1-dependent effects on cytoskeletal dynamics.

### [Ff]ilopodium

#### PubMed

1. Wrch-1, a novel member of the Rho gene family that is regulated by Wnt-1. We report the isolation and cloning of the Wrch-1 (Wnt-1 responsive Cdc42 homolog) cDNA. Wrch-1 is a novel gene whose mRNA level increases in response to Wnt-1 signaling in Wnt-1 transformed cells, Wnt-1 transgene induced mouse mammary tumors, and Wnt-1 retrovirus infected cells. Wrch-1 encodes a homolog of the Rho family of GTPases. It shares 57% amino acid sequence identity with Cdc42, but possesses a unique N-terminal domain that contains several putative PXXP SH3-binding motifs. Like Cdc42, Wrch-1 can activate PAK-1 and JNK-1, and induce **filopodium** formation and stress fiber dissolution. Active Wrch-1 stimulates quiescent cells to reenter the cell cycle. Moreover, overexpression of Wrch-1 phenocopies Wnt-1 in morphological transformation of mouse mammary epithelial cells. Taken together, Wrch-1 could mediate the effects of Wnt-1 signaling in the regulation of cell morphology, cytoskeletal organization, and cell proliferation.

### [Mm]igration

#### PubMed

1. Identification of molecules preferentially expressed beneath the marginal zone in the developing cerebral cortex. During cerebral cortical development, the majority of excitatory neurons are born near the ventricle and migrate radially toward the marginal zone (MZ). Since the cells invariably stop migrating beneath the MZ, neurons are aligned in an "inside-out" manner in the cortical plate (CP); that is, the early-born and late-born neurons are ultimately positioned in the deep and superficial layers, respectively. Since dramatic morphological changes occur in cells beneath the MZ, several events critical for proper neuronal maturation and layer formation must take place. In this study, we screened for molecules strongly expressed beneath the MZ, and identified 28 genes that are preferentially expressed in the upper half of the mouse CP on both embryonic day (E) 16.5 and E18.5. Expression analyses in reeler and yotari mice, in which neurons terminate **migration** throughout the CP, suggested that these genes were indeed related to the events beneath the MZ rather than unrelatedly induced by the structures near the brain surface. Pathway analyses suggested calcium signaling to have an important role in cells beneath the MZ. The gene list presented here will be useful for clarifying the molecular mechanisms that control cortical development.
2. ARHGAP30 is a Wrch-1-interacting protein involved in actin dynamics and cell adhesion. The atypical Rho GTPase Wrch-1 has been proposed roles in cell **migration**, focal adhesion dissolution, stress fibre break down and tight junction heterogeneity. A screen for Wrch-1 binding-partners identified the novel RhoGAP protein, ARHGAP30, as a Wrch-1 interactor. ARHGAP30 is related to the Cdc42- and Rac1-specific RhoGAP CdGAP, which was likewise found to bind Wrch-1. In contrast to CdGAP, ARHGAP30 serves as a Rac1- and RhoA-specific RhoGAP. Ectopic expression of ARHGAP30 results in membrane blebbing and dissolution of stress-fibres and focal adhesions. Our data suggest roles for ARHGAP30 and CdGAP in regulation of cell adhesion downstream of Wrch-1.

### [Mm]etastasis

#### PubMed

1. Lack of periostin leads to suppression of Notch1 signaling and calcific aortic valve disease. The Postn gene encodes protein periostin. During embryonic development, it is highly expressed in the outflow tract (OFT) endocardial cushions of the developing heart, which give rise to several structures of the mature heart including the aortic valve. Periostin was previously implicated in osteoblast differentiation, cancer **metastasis**, and tooth and bone development, but its role in cardiac OFT development is unclear. To elucidate the role that periostin plays in the developing heart we analyzed cardiac OFT phenotype in mice after deletion of the Postn gene. We found that lack of periostin in the embryonic OFT leads to ectopic expression of the proosteogenic growth factor pleiotrophin (Ptn) and overexpression of delta-like 1 homolog (Dlk1), a negative regulator of Notch1, in the distal (prevalvular) cushions of the OFT. This resulted in suppression of Notch1 signaling, strong induction of the central transcriptional regulator of osteoblast cell fate Runx2, upregulation of osteopontin and osteocalcin expression, and subsequent calcification of the aortic valve. Our data suggest that periostin represses a default osteogenic program in the OFT cushion mesenchyme and promotes differentiation along a fibrogenic lineage. Lack of periostin causes derepression of the osteogenic potential of OFT mesenchymal cells, calcium deposition, and calcific aortic valve disease. These results establish periostin as a key regulator of OFT endocardial cushion mesenchymal cell fate during embryonic development.

---

## Rhoh

- NCBI Gene
- GeneRIF
- Pubmed

| Search Term | Rank | Fields |
| --- | --- | --- |
| [Mm]igration | 6 | PubMed(5) |
| [Ee]xtracellular [Mm]atrix | 8 | PubMed(1) |

  
hide/show details for Rhoh

### [Mm]igration

#### PubMed

1. Cross-talk between RhoH and Rac1 in regulation of actin cytoskeleton and chemotaxis of hematopoietic progenitor cells. RhoH, a hematopoietic-specific and constitutively active member of the Rho guanosine triphosphatase (GTPase) family, has been implicated in the negative regulation of Rac GTPase-mediated signaling in hematopoietic cells. However, the molecular mechanisms underlying the functional interaction between RhoH and Rac in primary cells are poorly understood. Here we show that deletion of Rhoh in hematopoietic progenitor cells (HPCs) leads to increased stromal-derived factor-1alpha (SDF-1alpha)-induced chemotaxis and chemokinesis (random **migration**). The abnormally enhanced **migration** of Rhoh(-/-) HPCs is associated with increased Rac1 activity and translocation of Rac1 protein to the cell membrane, where it colocalizes with cortical filamentous-actin (F-actin) and lipid rafts. Expression of the dominant-negative mutant Rac1N17 inhibits the cortical F-actin assembly and chemotaxis of wild-type and Rhoh(-/-) HPCs to the same extent. Conversely, overexpression of RhoH in HPCs blocks the membrane translocation of Rac1-enhanced green fluorescence protein (EGFP) and active Rac1V12-EGFP proteins and impairs cortical F-actin assembly and chemotaxis in response to SDF-1alpha stimulation. Furthermore, we demonstrate that the subcellular localization and inhibitory function of RhoH in HPCs are regulated by C-terminal motifs, including a CKIF prenylation site. Together, we have identified an antagonistic role of RhoH in regulation of cortical F-actin assembly and chemotaxis via suppressing Rac1 membrane targeting and activation in primary HPCs.
2. Cross-talk between RhoH and Rac1 in regulation of actin cytoskeleton and chemotaxis of hematopoietic progenitor cells. RhoH, a hematopoietic-specific and constitutively active member of the Rho guanosine triphosphatase (GTPase) family, has been implicated in the negative regulation of Rac GTPase-mediated signaling in hematopoietic cells. However, the molecular mechanisms underlying the functional interaction between RhoH and Rac in primary cells are poorly understood. Here we show that deletion of Rhoh in hematopoietic progenitor cells (HPCs) leads to increased stromal-derived factor-1alpha (SDF-1alpha)-induced chemotaxis and chemokinesis (random **migration**). The abnormally enhanced **migration** of Rhoh(-/-) HPCs is associated with increased Rac1 activity and translocation of Rac1 protein to the cell membrane, where it colocalizes with cortical filamentous-actin (F-actin) and lipid rafts. Expression of the dominant-negative mutant Rac1N17 inhibits the cortical F-actin assembly and chemotaxis of wild-type and Rhoh(-/-) HPCs to the same extent. Conversely, overexpression of RhoH in HPCs blocks the membrane translocation of Rac1-enhanced green fluorescence protein (EGFP) and active Rac1V12-EGFP proteins and impairs cortical F-actin assembly and chemotaxis in response to SDF-1alpha stimulation. Furthermore, we demonstrate that the subcellular localization and inhibitory function of RhoH in HPCs are regulated by C-terminal motifs, including a CKIF prenylation site. Together, we have identified an antagonistic role of RhoH in regulation of cortical F-actin assembly and chemotaxis via suppressing Rac1 membrane targeting and activation in primary HPCs.
3. RhoH, a hematopoietic-specific Rho GTPase, regulates proliferation, survival, **migration**, and engraftment of hematopoietic progenitor cells. Rho guanosine triphosphatases (GT-Pases) are recognized as critical mediators of signaling pathways regulating actin assembly, **migration**, proliferation, and survival in hematopoietic cells. Here, we have studied a recently identified hematopoietic-specific Rho GTPase, RhoH. Unlike most members of the Rho GTPase family, RhoH is GTPase deficient and does not cycle between GTP- and guanosine diphosphate (GDP)-bound forms, suggesting that regulation of RhoH expression may be critical in its activity. We found that RhoH is expressed in murine hematopoietic progenitor cells (HPCs) and fully differentiated myeloid and lymphoid lineages. In cytokine-stimulated HPCs, knockdown of RhoH expression via RNA interference stimulates proliferation, survival, and stromal cell-derived factor-1 alpha (SDF-1 alpha)-induced **migration** in vitro. Conversely, RhoH overexpression in these cells via retrovirus-mediated gene transfer is associated with impaired activation of Rac GTPases, reduced proliferation, increased apoptosis, and defective actin polymerization and chemotaxis. In vivo, HPCs with RhoH overexpression demonstrate defective hematopoietic reconstitution capability compared with control vector-transduced cells. Our results suggest that RhoH serves as a negative regulator of both growth and actin-based function of HPCs possibly via suppression of Rac-mediated signaling.
4. Opposing roles for RhoH GTPase during T-cell **migration** and activation. T cells spend the majority of their time perusing lymphoid organs in search of cognate antigen presented by antigen presenting cells (APCs) and then quickly recirculate through the bloodstream to another lymph node. Therefore, regulation of a T-cell response is dependent upon the ability of cells to arrive in the correct location following chemokine gradients ("go" signal) as well as to receive appropriate T-cell receptor (TCR) activation signals upon cognate antigen recognition ("stop" signal). However, the mechanisms by which T cells regulate these go and stop signals remain unclear. We found that overexpression of the hematopoietic-specific RhoH protein in the presence of chemokine signals resulted in decreased Rap1-GTP and LFA-1 adhesiveness to ICAM-1, thus impairing T-cell chemotaxis; while in the presence of TCR signals, there were enhanced and sustained Rap1-GTP and LFA-1 activation as well as prolonged T:APC conjugates. RT-PCR analyses of activated CD4(+) T cells and live images of T-cell **migration** and immunological synapse (IS) formation revealed that functions of RhoH took place primarily at the levels of transcription and intracellular distribution. Thus, we conclude that RhoH expression provides a key molecular determinant that allows T cells to switch between sensing chemokine-mediated go signals and TCR-dependent stop signals.
5. RhoH is critical for cell-microenvironment interactions in chronic lymphocytic leukemia in mice and humans. Trafficking of B-cell chronic lymphocytic leukemia (CLL) cells to the bone marrow and interaction with supporting stromal cells mediates important survival and proliferation signals. Previous studies have demonstrated that deletion of Rhoh led to a delayed disease onset in a murine model of CLL. Here we assessed the impact of RhoH on homing, **migration**, and cell-contact dependent interactions of CLL cells. Rhoh(-/-) CLL cells exhibited reduced marrow homing and subsequent engraftment. In vitro **migration** toward the chemokines CXCL12 and CXCL13 and cell-cell interactions between Rhoh(-/-) CLL cells and the supporting microenvironment was reduced. In the absence of RhoH the distribution of phosphorylated focal adhesion kinase, a protein known to coordinate activation of the Rho GTPases RhoA and Rac, appeared less polarized in chemokine-stimulated Rhoh(-/-) CLL cells, and activation and localization of RhoA and Rac was dysregulated leading to defective integrin function. These findings in the Rhoh(-/-) CLL cells were subsequently demonstrated to closely resemble changes in GTPase activation observed in human CLL samples after in vitro and in vivo treatment with lenalidomide, an agent with known influence on microenvironment protection, and suggest that RhoH plays a critical role in prosurvival CLL cell-cell and cell-microenvironment interactions with this agent.

### [Ee]xtracellular [Mm]atrix

#### PubMed

1. Absence of cyclooxygenase-2 exacerbates hypoxia-induced pulmonary hypertension and enhances contractility of vascular smooth muscle cells. Cyclooxygenase-2 (COX-2) is upregulated in pulmonary artery smooth muscle cells (PASMCs) during hypoxia and may play a protective role in the response of the lung to hypoxia. Selective COX-2 inhibition may have detrimental pulmonary vascular consequences during hypoxia.To investigate the role of COX-2 in the pulmonary vascular response to hypoxia, we subjected wild-type and COX-2-deficient mice to a model of chronic normobaric hypoxia. COX-2-null mice developed severe pulmonary hypertension with exaggerated elevation of right ventricular systolic pressure, significant right ventricular hypertrophy, and striking vascular remodeling after hypoxia. Pulmonary vascular remodeling in COX-2-deficient mice was characterized by PASMC hypertrophy but not increased proliferation. Furthermore, COX-2-deficient mice had significant upregulation of the endothelin-1 receptor (ET(A)) in the lung after hypoxia. Similarly, selective pharmacological inhibition of COX-2 in wild-type mice exacerbated hypoxia-induced pulmonary hypertension and resulted in PASMC hypertrophy and increased ET(A) receptor expression in pulmonary arterioles. The absence of COX-2 in vascular smooth muscle cells during hypoxia in vitro augmented traction forces and enhanced contractility of an **extracellular matrix**. Treatment of COX-2-deficient PASMCs with iloprost, a prostaglandin I(2) analog, and prostaglandin E(2) abrogated the potent contractile response to hypoxia and restored the wild-type phenotype.Our findings reveal that hypoxia-induced pulmonary hypertension and vascular remodeling are exacerbated in the absence of COX-2 with enhanced ET(A) receptor expression and increased PASMC hypertrophy. COX-2-deficient PASMCs have a maladaptive response to hypoxia manifested by exaggerated contractility, which may be rescued by either COX-2-derived prostaglandin I(2) or prostaglandin E(2).

---

## Rhob

- NCBI Gene
- GeneRIF
- Pubmed

| Search Term | Rank | Fields |
| --- | --- | --- |
| [Pp]odosome(s)? | 5 | PubMed(1) |
| [Mm]igration | 6 | Process(3); PubMed(14); GeneRIFs(3) |
| [Ee]xtracellular [Mm]atrix | 8 | PubMed(1) |
| [Ii]nvasive | 10 | PubMed(3) |
| [Mm]etastasis | 12 | PubMed(2); GeneRIFs(1) |

  
hide/show details for Rhob

### [Pp]odosome(s)?

#### PubMed

1. RhoB affects macrophage adhesion, integrin expression and migration. Rho GTPases regulate multiple cellular responses, including cell motility and cell cycle progression. The Rho isoform RhoB represses transformation and affects endosomal trafficking, but its effects on cell adhesion and migration have not been investigated in detail. Here we show that RhoB-null macrophages are more rounded than wild-type macrophages on fibronectin and uncoated glass, and have reduced adhesion to ICAM-1 and glass but not fibronectin. This correlated with lower cell surface expression of beta2 and beta3 integrins but not beta1 integrin. RhoB-null cells migrated faster than Wt cells on fibronectin, consistent with their smaller spread area, but slower than Wt cells on glass, reflecting their reduced adhesion. C3 transferase, which inhibits RhoA, RhoB and RhoC, induced cell spreading but this effect was reduced in RhoB-null cells. However, RhoB is not required for assembly of **podosomes**, which are integrin-based adhesion sites, whereas C3 transferase induced a decrease in **podosomes** and defects in tail retraction. Since macrophages do not express RhoC, these effects of C3 transferase are due to inhibition of RhoA rather than RhoB. Our results suggest that RhoB affects cell shape and migration by regulating surface integrin levels.

### [Mm]igration

#### Process

1. negative regulation of cell **migration**
2. positive regulation of endothelial cell **migration**
3. regulation of cell **migration**

#### PubMed

1. RhoB links PDGF signaling to cell **migration** by coordinating activation and localization of Cdc42 and Rac. The small GTPase RhoB regulates endocytic trafficking of receptor tyrosine kinases (RTKs) and the non-receptor kinases Src and Akt. While receptor-mediated endocytosis is critical for signaling processes driving cell **migration**, mechanisms that coordinate endocytosis with the propagation of migratory signals remain relatively poorly understood. In this study, we show that RhoB is essential for activation and trafficking of the key migratory effectors Cdc42 and Rac in mediating the ability of platelet-derived growth factor (PDGF) to stimulate cell movement. Stimulation of the PDGF receptor-β on primary vascular smooth muscle cells (VSMCs) results in RhoB-dependent trafficking of endosome-bound Cdc42 from the perinuclear region to the cell periphery, where the RhoGEF Vav2 and Rac are also recruited to drive formation of circular dorsal and peripheral ruffles necessary for cell **migratio**n. Our findings identify a novel RhoB-dependent endosomal trafficking pathway that integrates RTK endocytosis with Cdc42/Rac localization and cell movement.
2. Autotaxin/lysophospholipase D-mediated lysophosphatidic acid signaling is required to form distinctive large lysosomes in the visceral endoderm cells of the mouse yolk sac. Autotaxin, a lysophospholipase D encoded by the Enpp2 gene, is an exoenzyme that produces lysophosphatidic acid in the extracellular space. Lysophosphatidic acid acts on specific G protein-coupled receptors, thereby regulating cell growth, **migration**, and survival. Previous studies have revealed that Enpp2(-/-) mouse embryos die at about embryonic day (E) 9.5 because of angiogenic defects in the yolk sac. However, what cellular defects occur in Enpp2(-/-) embryos and what intracellular signaling pathways are involved in the phenotype manifestation remain unknown. Here, we show that Enpp2 is required to form distinctive large lysosomes in the yolk sac visceral endoderm cells. From E7.5 to E9.5, Enpp2 mRNA is abundantly expressed in the visceral endoderm cells. In Enpp2(-/-) mouse embryos, lysosomes in the visceral endoderm cells are fragmented. By using a whole embryo culture system combined with specific pharmacological inhibitors for intracellular signaling molecules, we show that lysophosphatidic acid receptors and the Rho-Rho-associated coiled-coil containing protein kinase (ROCK)-LIM kinase pathway are required to form large lysosomes. In addition, electroporation of dominant negative forms of Rho, ROCK, or LIM kinase also leads to the size reduction of lysosomes in wild-type visceral endoderm cells. In Enpp2(-/-) visceral endoderm cells, the steady-state levels of cofilin phosphorylation and actin polymerization are reduced. In addition, perturbations of actin turnover dynamics by actin inhibitors cytochalasin B and jasplakinolide result in the defect in lysosome formation. These results suggest that constitutive activation of the Rho-ROCK-LIM kinase pathway by extracellular production of lysophosphatidic acid by the action of autotaxin is required to maintain the large size of lysosomes in visceral endoderm cells.
3. Potential target genes of EMX2 include Odz/Ten-M and other gene families with implications for cortical patterning. EMX2 and PAX6 are expressed by cortical progenitors and specify area patterning. We used representational difference analysis (RDA) to compare expressed RNAs from wild type and Emx2-/- cortex and identified 41 unique clones. Using secondary screening by in situ hybridization, we selected five genes for further analysis, Cdk4, Cofilin1, Crmp1, ME2, and Odz4, involved in neuronal proliferation, differentiation, **migration**, and axon guidance. Each exhibits differential expression in wild type cortex. Odz4 is one of four members of a vertebrate gene family homologous to the Drosophila pair-rule patterning gene, Odd Oz (Odz), a transmembrane receptor. We show that Odz genes are expressed in complementary patterns in cortex, as well as in nuclei-specific patterns in thalamus that relates to their area-unique cortical expression. In addition, each of the genes analyzed shows different expression patterns in wild type cortex, Emx2, and Pax6 mutant cortex, consistent with potential roles in area patterning. These findings identify potential targets of EMX2 that might account for its function and the defects in Emx2-/- cortex, and suggest that the Odz family of transmembrane proteins influences cortical area patterning downstream to EMX2 and PAX6.
4. The transcriptional control of trunk neural crest induction, survival, and delamination. Trunk neural crest cells are generated at the border between the neural plate and nonneural ectoderm, where they initiate a distinct program of gene expression, undergo an epithelial-mesenchymal transition (EMT), and delaminate from the neuroepithelium. Here, we provide evidence that members of three families of transcription induce these properties in premigratory neural crest cells. Sox9 acts to provide the competence for neural crest cells to undergo an EMT and is required for trunk neural crest survival. In the absence of Sox9, cells apoptose prior to or shortly after delamination. Slug/Snail, in the presence of Sox9, is sufficient to induce an EMT in neural epithelial cells, while FoxD3 regulates the expression of cell-cell adhesion molecules required for neural crest **migration**. Together, the data suggest a model in which a combination of transcription factors regulates the acquisition of the diverse properties of neural crest cells.
5. Distinct roles of Rac1/Cdc42 and Rho/Rock for axon outgrowth and nucleokinesis of precerebellar neurons toward netrin 1. During embryonic development, tangentially migrating precerebellar neurons emit a leading process and then translocate their nuclei inside it (nucleokinesis). Netrin 1 (also known as netrin-1) acts as a chemoattractant factor for neurophilic **migration** of precerebellar neurons (PCN) both in vivo and in vitro. In the present work, we analyzed Rho GTPases that could direct axon outgrowth and/or nuclear **migration**. We show that the expression pattern of Rho GTPases in developing PCN is consistent with their involvement in the **migration** of PCN from the rhombic lips. We report that pharmacological inhibition of Rho enhances axon outgrowth of PCN and prevents nuclei **migration** toward a netrin 1 source, whereas inhibition of Rac and Cdc42 sub-families impair neurite outgrowth of PCN without affecting **migration**. We show, through pharmacological inhibition, that Rho signaling directs neurophilic **migration** through Rock activation. Altogether, our results indicate that Rho/Rock acts on signaling pathways favoring nuclear translocation during tangential **migration** of PCN. Thus, axon extension and nuclear **migration** of PCN in response to netrin 1 are not strictly dependent processes because: (1) distinct small GTPases are involved; (2) axon extension can occur when **migration** is blocked; and (3) **migration** can occur when axon outgrowth is impaired.
6. RhoB links PDGF signaling to cell **migration** by coordinating activation and localization of Cdc42 and Rac. The small GTPase RhoB regulates endocytic trafficking of receptor tyrosine kinases (RTKs) and the non-receptor kinases Src and Akt. While receptor-mediated endocytosis is critical for signaling processes driving cell **migration**, mechanisms that coordinate endocytosis with the propagation of migratory signals remain relatively poorly understood. In this study, we show that RhoB is essential for activation and trafficking of the key migratory effectors Cdc42 and Rac in mediating the ability of platelet-derived growth factor (PDGF) to stimulate cell movement. Stimulation of the PDGF receptor-β on primary vascular smooth muscle cells (VSMCs) results in RhoB-dependent trafficking of endosome-bound Cdc42 from the perinuclear region to the cell periphery, where the RhoGEF Vav2 and Rac are also recruited to drive formation of circular dorsal and peripheral ruffles necessary for cell **migratio**n. Our findings identify a novel RhoB-dependent endosomal trafficking pathway that integrates RTK endocytosis with Cdc42/Rac localization and cell movement.
7. Autotaxin/lysophospholipase D-mediated lysophosphatidic acid signaling is required to form distinctive large lysosomes in the visceral endoderm cells of the mouse yolk sac. Autotaxin, a lysophospholipase D encoded by the Enpp2 gene, is an exoenzyme that produces lysophosphatidic acid in the extracellular space. Lysophosphatidic acid acts on specific G protein-coupled receptors, thereby regulating cell growth, **migration**, and survival. Previous studies have revealed that Enpp2(-/-) mouse embryos die at about embryonic day (E) 9.5 because of angiogenic defects in the yolk sac. However, what cellular defects occur in Enpp2(-/-) embryos and what intracellular signaling pathways are involved in the phenotype manifestation remain unknown. Here, we show that Enpp2 is required to form distinctive large lysosomes in the yolk sac visceral endoderm cells. From E7.5 to E9.5, Enpp2 mRNA is abundantly expressed in the visceral endoderm cells. In Enpp2(-/-) mouse embryos, lysosomes in the visceral endoderm cells are fragmented. By using a whole embryo culture system combined with specific pharmacological inhibitors for intracellular signaling molecules, we show that lysophosphatidic acid receptors and the Rho-Rho-associated coiled-coil containing protein kinase (ROCK)-LIM kinase pathway are required to form large lysosomes. In addition, electroporation of dominant negative forms of Rho, ROCK, or LIM kinase also leads to the size reduction of lysosomes in wild-type visceral endoderm cells. In Enpp2(-/-) visceral endoderm cells, the steady-state levels of cofilin phosphorylation and actin polymerization are reduced. In addition, perturbations of actin turnover dynamics by actin inhibitors cytochalasin B and jasplakinolide result in the defect in lysosome formation. These results suggest that constitutive activation of the Rho-ROCK-LIM kinase pathway by extracellular production of lysophosphatidic acid by the action of autotaxin is required to maintain the large size of lysosomes in visceral endoderm cells.
8. RhoA determines disease progression by controlling neutrophil motility and restricting hyperresponsiveness. Neutrophil responses are central to host protection and inflammation. Neutrophil activation follows a 2-step process in which priming amplifies responses to activating stimuli. Priming is essential for life span extension, chemotaxis, and respiratory burst activity. Here we show that the cytoskeletal organizer RhoA suppresses neutrophil priming via formins. Premature granule exocytosis in Rho-deficient neutrophils activated numerous signaling pathways and amplified superoxide generation. Deletion of Rho altered front-to-back coordination by simultaneously increasing uropod elongation, leading edge formation, and random **migration**. Concomitant negative and positive regulation of β2 integrin-independent and β2 integrin-dependen**t migrati**on, respectively, reveal Rho as a key decision point in the neutrophil response to discrete chemotactic agents. Although even restricted influx of Rho-deficient hyperactive neutrophils exacerbated lipopolysaccharide-mediated lung injury, deleting Rho in innate immune cells was highly protective in influenza A virus infection. Hence, Rho is a key regulator of disease progression by maintaining neutrophil quiescence and suppressing hyperresponsiveness.
9. Role of RhoB in the regulation of pulmonary endothelial and smooth muscle cell responses to hypoxia. RhoA and Rho kinase contribute to pulmonary vasoconstriction and vascular remodeling in pulmonary hypertension. RhoB, a protein homologous to RhoA and activated by hypoxia, regulates neoplastic growth and vasoconstriction but its role in the regulation of pulmonary vascular function is not known.To determine the role of RhoB in pulmonary endothelial and smooth muscle cell responses to hypoxia and in pulmonary vascular remodeling in chronic hypoxia-induced pulmonary hypertension.Hypoxia increased expression and activity of RhoB in human pulmonary artery endothelial and smooth muscle cells, coincidental with activation of RhoA. Hypoxia or adenoviral overexpression of constitutively activated RhoB increased actomyosin contractility, induced endothelial permeability, and promoted cell growth; dominant negative RhoB or manumycin, a farnesyltransferase inhibitor that targets the vascular function of RhoB, inhibited the effects of hypoxia. Coordinated activation of RhoA and RhoB maximized the hypoxia-induced stress fiber formation caused by RhoB/mammalian homolog of Drosophila diaphanous-induced actin polymerization and RhoA/Rho kinase-induced phosphorylation of myosin light chain on Ser19. Notably, RhoB was specifically required for hypoxia-induced factor-1α stabilization and for hypoxia- and platelet-derived growth factor-induced cell proliferation and **migratio**n. RhoB deficiency in mice markedly attenuated development of chronic hypoxia-induced pulmonary hypertension, despite compensatory expression of RhoA in the lung.RhoB mediates adaptational changes to acute hypoxia in the vasculature, but its continual activation by chronic hypoxia can accentuate vascular remodeling to promote development of pulmonary hypertension. RhoB is a potential target for novel approaches (eg, farnesyltransferase inhibitors) aimed at regulating pulmonary vascular tone and structure.
10. MicroRNA-21 exhibits antiangiogenic function by targeting RhoB expression in endothelial cells. MicroRNAs (miRNAs) are endogenously expressed small non-coding RNAs that regulate gene expression at post-transcriptional level. The recent discovery of the involvement of these RNAs in the control of angiogenesis renders them very attractive in the development of new approaches for restoring the angiogenic balance. Whereas miRNA-21 has been demonstrated to be highly expressed in endothelial cells, the potential function of this miRNA in angiogenesis has never been investigated.We first observed in endothelial cells a negative regulation of miR-21 expression by serum and bFGF, two pro-angiogenic factors. Then using in vitro angiogenic assays, we observed that miR-21 acts as a negative modulator of angiogenesis. miR-21 overexpression reduced endothelial cell proliferation, **migration** and the ability of these cells to form tubes whereas miR-21 inhibition using a LNA-anti-miR led to opposite effects. Expression of miR-21 in endothelial cells also led to a reduction in the organization of actin into stress fibers, which may explain the decrease in cell **migration**. Further mechanistic studies showed that miR-21 targets RhoB, as revealed by a decrease in RhoB expression and activity in miR-21 overexpressing cells. RhoB silencing impairs endothelial cell **migration** and tubulogenesis, thus providing a possible mechanism for miR-21 to inhibit angiogenesis. Finally, the therapeutic potential of miR-21 as an angiogenesis inhibitor was demonstrated in vivo in a mouse model of choroidal neovascularization.Our results identify miR-21 as a new angiogenesis inhibitor and suggest that inhibition of cell **migration** and tubulogenesis is mediated through repression of RhoB.
11. RhoB is expressed in migrating neural crest and endocardial cushions of the developing mouse embryo. RhoB mRNA expression was examined in the developing mouse embryo between E8.5 and E11.5. Specific expression was found in migrating neural crest (NC) cells, from the first stages of their **migration** at E9.5, throughout the **migration** period. Expression is maintained in NC derivatives for at least one embryonic day after they reach their final destinations, but is then down-regulated. RhoB is also expressed in non NC-derived neural tissues, including motor neurones and the floor plate of the neural tube. RhoB mRNA expression is also found in the developing endocardial cushions of the atrioventricular and outflow regions of the developing heart.
12. Mouse macrophages completely lacking Rho subfamily GTPases (RhoA, RhoB, and RhoC) have severe lamellipodial retraction defects, but robust chemotactic navigation and altered motility. RhoA is thought to be essential for coordination of the membrane protrusions and retractions required for immune cell motility and directed **migration**. Whether the subfamily of Rho (Ras homolog) GTPases (RhoA, RhoB, and RhoC) is actually required for the directed **migration** of primary cells is difficult to predict. Macrophages isolated from myeloid-restricted RhoA/RhoB (conditional) double knock-out (dKO) mice did not express RhoC and were essentially "pan-Rho"-deficient. Using real-time chemotaxis assays, we found that retraction of the trailing edge was dissociated from the advance of the cell body in dKO cells, which developed extremely elongated tails. Surprisingly, velocity (of the cell body) was increased, whereas chemotactic efficiency was preserved, when compared with WT macrophages. Randomly migrating RhoA/RhoB dKO macrophages exhibited multiple small protrusions and developed large "branches" due to impaired lamellipodial retraction. A mouse model of peritonitis indicated that monocyte/macrophage recruitment was, surprisingly, more rapid in RhoA/RhoB dKO mice than in WT mice. In comparison with dKO cells, the phenotypes of single RhoA- or RhoB-deficient macrophages were mild due to mutual compensation. Furthermore, genetic deletion of RhoB partially reversed the motility defect of macrophages lacking the RhoGAP (Rho GTPase-activating protein) myosin IXb (Myo9b). In conclusion, the Rho subfamily is not required for "front end" functions (motility and chemotaxis), although both RhoA and RhoB are involved in pulling up the "back end" and resorbing lamellipodial membrane protrusions. Macrophages lacking Rho proteins migrate faster in vitro, which, in the case of the peritoneum, translates to more rapid in vivo monocyte/macrophage recruitment.
13. RhoB affects macrophage adhesion, integrin expression and **migration**. Rho GTPases regulate multiple cellular responses, including cell motility and cell cycle progression. The Rho isoform RhoB represses transformation and affects endosomal trafficking, but its effects on cell adhesion and **migration** have not been investigated in detail. Here we show that RhoB-null macrophages are more rounded than wild-type macrophages on fibronectin and uncoated glass, and have reduced adhesion to ICAM-1 and glass but not fibronectin. This correlated with lower cell surface expression of beta2 and beta3 integrins but not beta1 integrin. RhoB-null cells migrated faster than Wt cells on fibronectin, consistent with their smaller spread area, but slower than Wt cells on glass, reflecting their reduced adhesion. C3 transferase, which inhibits RhoA, RhoB and RhoC, induced cell spreading but this effect was reduced in RhoB-null cells. However, RhoB is not required for assembly of podosomes, which are integrin-based adhesion sites, whereas C3 transferase induced a decrease in podosomes and defects in tail retraction. Since macrophages do not express RhoC, these effects of C3 transferase are due to inhibition of RhoA rather than RhoB. Our results suggest that RhoB affects cell shape and **migration** by regulating surface integrin levels.
14. Akt mediates Ras downregulation of RhoB, a suppressor of transformation, invasion, and metastasis. Although recent evidence supports a tumor-suppressive role for the GTPase RhoB, little is known about its regulation by signal transduction pathways. Here we demonstrate that Ras downregulates RhoB expression by a phosphatidylinositol 3-kinase (PI3K)- and Akt- but not Mek-dependent mechanism. Furthermore, genetic and pharmacological blockade of PI3K/Akt results in upregulation of RhoB expression. We also provide evidence for the importance of the downregulation of RhoB in oncogenesis by demonstrating that RhoB antagonizes Ras/PI3K/Akt malignancy. Ectopic expression of RhoB, but not the close relative RhoA, inhibits Ras, PI3K, and Akt induction of transformation, **migration**, and invasion and induces apoptosis and anoikis. Finally, RhoB inhibits melanoma metastasis to the lung in a mouse model. These studies identify suppression of RhoB as a mechanism by which the Ras/PI3K/Akt pathway induces tumor survival, transformation, invasion, and metastasis.

#### GeneRIFs

1. Active RhoA and RhoB are involved in back end functions, such as retracting the tail and lamellipodial membrane extensions during macrophage **migration**.
2. RhoB links PDGF signaling to cell **migration** by coordinating activation and localization of Cdc42 and Rac.
3. RhoB affects cell shape and **migration** by regulating surface integrin levels.

### [Ee]xtracellular [Mm]atrix

#### PubMed

1. Laminin and integrin expression in the ventral ectodermal ridge of the mouse embryo: implications for regulation of BMP signalling. The ventral ectodermal ridge (VER) is an important signalling centre in the mouse tail-bud following completion of gastrulation. BMP regulation is essential for VER function, but how these signals are transmitted between adjacent tissues is unclear.We investigated the idea that **extracellular matrix** components might be involved, using immunohistochemistry and in situ hybridisation to detect all known α, β, and γ laminin chains and their mRNAs in the early tail bud. We identified an apparently novel laminin variant, comprising α5, β3 and γ2 chains, as a major component of the VER basement membrane at E9.5. Strikingly, only the mRNAs for these chains were co-expressed in VER cells, suggesting that lamin532 may be the sole basement membrane laminin at this stage. Since α6 integrin was also expressed in VER cells, this raises the possibility of cell-matrix interactions regulating BMP signalling at this site of caudal morphogenesis.Laminin532 could interact with α6-containing integrin to direct differentiation of the specialised VER cells from surface ectoderm.

### [Ii]nvasive

#### PubMed

1. In vivo and in vitro investigations of heterozygous nebulin knock-out mice disclose a mild skeletal muscle phenotype. Nemaline myopathy is the most common congenital skeletal muscle disease, and mutations in the nebulin gene account for 50% of all cases. Recent studies suggest that the disease severity might be related to the nebulin expression levels. Considering that mutations in the nebulin gene are typically recessive, one would expect that a single functional nebulin allele would maintain nebulin protein expression which would result in preserved skeletal muscle function. We investigated skeletal muscle function of heterozygous nebulin knock-out (i.e., nebulin(+/-)) mice using a multidisciplinary approach including protein and gene expression analysis and combined in vivo and in vitro force measurements. Skeletal muscle anatomy and energy metabolism were studied strictly non-**invasive**ly using magnetic resonance imaging and 31P-magnetic resonance spectroscopy. Maximal force production was reduced by around 16% in isolated muscle of nebulin(+/-) mice while in vivo force generating capacity was preserved. Muscle weakness was associated with a shift toward a slower proteomic phenotype, but was not related to nebulin protein deficiency or to an impaired energy metabolism. Further studies would be warranted in order to determine the mechanisms leading to a mild skeletal muscle phenotype resulting from the expression of a single nebulin allele.
2. RASSF1A Suppresses the Invasion and Metastatic Potential of Human Non-Small Cell Lung Cancer Cells by Inhibiting YAP Activation through the GEF-H1/RhoB Pathway. Inactivation of the tumor suppressor gene RASSF1A by promoter hypermethylation represents a key event underlying the initiation and progression of lung cancer. RASSF1A inactivation is also associated with poor prognosis and may promote metastatic spread. In this study, we investigated how RASSF1A inactivation conferred **invasive** phenotypes to human bronchial cells. RNAi-mediated silencing of RASSF1A induced epithelial-to-mesenchymal transition (EMT), fomenting a motile and **invasive** cellular phenotype in vitro and increased metastatic prowess in vivo. Mechanistic investigations revealed that RASSF1A blocked tumor growth by stimulating cofilin/PP2A-mediated dephosphorylation of the guanine nucleotide exchange factor GEF-H1, thereby stimulating its ability to activate the antimetastatic small GTPase RhoB. Furthermore, RASSF1A reduced nuclear accumulation of the Hippo pathway transcriptional cofactor Yes-associated protein (YAP), which was reinforced by RhoB activation. Collectively, our results indicated that RASSF1 acts to restrict EMT and invasion by indirectly controlling YAP nuclear shuttling and activation through a RhoB-regulated cytoskeletal remodeling process, with potential implications to delay the progression of RASSF1-hypermethylated lung tumors.
3. Loss of RhoB expression in human lung cancer progression. RhoB is a low molecular weight GTPase belonging to the Ras protein superfamily. Whereas most Rho proteins have been shown to have a positive role in proliferation and malignant transformation, the specific role of RhoB appears more divergent. We reported previously that RhoB inhibits cell proliferation in various human cancer cells. Here, we studied the specific role played by RhoB in human lung cancer.We analyzed the expression of RhoB protein by immunostaining in human lung tissues ranging from normal to **invasive** carcinoma from different histological types in two large independent studies of, respectively, 94 and 45 samples. We then studied the cellular effect of RhoB overexpression in a model of lung cancer (A549, adenocarcinoma) and tumorigenicity in nude mice.We showed in both studies that RhoB protein was expressed in normal lung and decreased dramatically through lung cancer progression (P < 0.01). Interestingly, RhoB expression was lost in 96% of **invasive** tumors and reduced by 86% in poorly differentiated tumors compared with the nonneoplastic epithelium. Moreover, the loss of expression of RhoB correlated significantly with tumor stage and proliferative index, whereas no correlation was found between RhoB and p53 or Bcl-2 expression. We then showed that ectopic expression of RhoB in lung cancer cell line A549 suppressed cell proliferation, anchorage-independent growth, and xenograft tumor growth in nude mice.RhoB loss of expression occurs very frequently in lung carcinogenesis, reinforcing its putative tumor suppressive activity, and raising the value of its potential use in cancer therapy.

### [Mm]etastasis

#### PubMed

1. In vivo restoration of RhoB expression leads to ovarian tumor regression. Ovarian cancers are very aggressive cancers most often diagnosed when **metastasis** has already occurred in the entire peritoneal cavity. Ovarian adenocarcinoma cells present an undetectable level of RhoB GTPase. Using preclinical ovarian cancer models, we aimed to evaluate the potential use of RhoB cDNA as a tumor suppressor gene in gene therapy. RhoB restoration in vitro, through recombinant adenovirus transduction, resulted in the apoptosis of endogenous RhoB protein low-expressing cell lines (OVCAR-3 and IGROV-1) through the activation of the intrinsic apoptotic caspase cascade. We showed that a single injection of 10(8) p.f.u. of adenoviral vector encoding a reporter gene into the peritoneal cavity of ovarian tumor bearing mice can induce the gene modification of a large quantity of cells throughout the cavity. We thereby tested the effect of AdRhoB injections to treat ovarian cancer-bearing mice. The ectopic expression of RhoB, following its introduction via viral transduction into nude mice in vivo, was highly effective in suppressing tumor growth of ovarian cancer xenografts. Therapeutic agents designed to correct defects of RhoB at the molecular level may thereby provide innovative treatment options for patients not responding to standard therapies.
2. Akt mediates Ras downregulation of RhoB, a suppressor of transformation, invasion, and **metastasis**. Although recent evidence supports a tumor-suppressive role for the GTPase RhoB, little is known about its regulation by signal transduction pathways. Here we demonstrate that Ras downregulates RhoB expression by a phosphatidylinositol 3-kinase (PI3K)- and Akt- but not Mek-dependent mechanism. Furthermore, genetic and pharmacological blockade of PI3K/Akt results in upregulation of RhoB expression. We also provide evidence for the importance of the downregulation of RhoB in oncogenesis by demonstrating that RhoB antagonizes Ras/PI3K/Akt malignancy. Ectopic expression of RhoB, but not the close relative RhoA, inhibits Ras, PI3K, and Akt induction of transformation, migration, and invasion and induces apoptosis and anoikis. Finally, RhoB inhibits melanoma **metastasis** to the lung in a mouse model. These studies identify suppression of RhoB as a mechanism by which the Ras/PI3K/Akt pathway induces tumor survival, transformation, invasion, and **metastasis**.

#### GeneRIFs

1. suppression of RhoB as a mechanism by which the Ras/PI3K/Akt pathway induces tumor survival, transformation, invasion, and **metastasis**

---

## Rhof

- NCBI Gene
- GeneRIF
- Pubmed

| Search Term | Rank | Fields |
| --- | --- | --- |
| [Ff]ilopodia | 1 | PubMed(3); Description(1); Alternative Name(1) |
| [Ff]ilopodium | 2 | PubMed(2); GeneRIFs(1) |

  
hide/show details for Rhof

### [Ff]ilopodia

#### PubMed

1. Rif-mDia1 interaction is involved in filopodium formation independent of Cdc42 and Rac effectors. **Filopodia** are cellular protrusions important for axon guidance, embryonic development, and wound healing. The Rho GTPase Cdc42 is the best studied inducer of filopodium formation, and several of its effectors and their interacting partners have been linked to the process. These include IRSp53, N-WASP, Mena, and Eps8. The Rho GTPase, Rif, also drives filopodium formation. The signaling pathway by which Rif induces **filopodia** is poorly understood, with mDia2 being the only protein implicated to date. It is thus not clear how distinct the Rif-driven pathway for filopodium formation is from the one mediated by Cdc42. In this study, we characterize the dynamics of Rif-induced **filopodia** by time lapse imaging of live neuronal cells and show that Rif drives filopodium formation via an independent pathway that does not involve the Cdc42 effectors N-WASP and IRSp53, the IRSp53 binding partner Mena, or the Rac effectors WAVE1 and WAVE2. Rif formed **filopodia** in the absence of N-WASP or Mena and when IRSp53, WAVE1, or WAVE2 was knocked down by RNAi. Rif-mediated **filopodia**l protrusion was instead reduced by silencing mDia1 expression or overexpressing a dominant negative mutant of mDia1. mDia1 on its own was able to form **filopodia**. Data from acceptor photobleaching FRET studies of protein-protein interaction demonstrate that Rif interacts directly with mDia1 in **filopodia** but not with mDia2. Taken together, these results suggest a novel pathway for **filopodia** formation via Rif and mDia1.
2. Rif-mDia1 interaction is involved in filopodium formation independent of Cdc42 and Rac effectors. **Filopodia** are cellular protrusions important for axon guidance, embryonic development, and wound healing. The Rho GTPase Cdc42 is the best studied inducer of filopodium formation, and several of its effectors and their interacting partners have been linked to the process. These include IRSp53, N-WASP, Mena, and Eps8. The Rho GTPase, Rif, also drives filopodium formation. The signaling pathway by which Rif induces **filopodia** is poorly understood, with mDia2 being the only protein implicated to date. It is thus not clear how distinct the Rif-driven pathway for filopodium formation is from the one mediated by Cdc42. In this study, we characterize the dynamics of Rif-induced **filopodia** by time lapse imaging of live neuronal cells and show that Rif drives filopodium formation via an independent pathway that does not involve the Cdc42 effectors N-WASP and IRSp53, the IRSp53 binding partner Mena, or the Rac effectors WAVE1 and WAVE2. Rif formed **filopodia** in the absence of N-WASP or Mena and when IRSp53, WAVE1, or WAVE2 was knocked down by RNAi. Rif-mediated **filopodia**l protrusion was instead reduced by silencing mDia1 expression or overexpressing a dominant negative mutant of mDia1. mDia1 on its own was able to form **filopodia**. Data from acceptor photobleaching FRET studies of protein-protein interaction demonstrate that Rif interacts directly with mDia1 in **filopodia** but not with mDia2. Taken together, these results suggest a novel pathway for **filopodia** formation via Rif and mDia1.
3. The small GTPase Rif is dispensable for platelet **filopodia** generation in mice. Formation of **filopodia** and other shape change events are vital for platelet hemostatic function. The mechanisms regulating **filopodia** formation by platelets are incompletely understood however. In particular the small GTPase responsible for initiating **filopodia** formation by platelets remains elusive. The canonical pathway involving Cdc42 is not essential for **filopodia** formation in mouse platelets. The small GTPase Rif (RhoF) provides an alternative route to **filopodia** generation in other cell types and is expressed in both human and mouse platelets.We hypothesized that Rif might be responsible for generating **filopodia** by platelets and generated a novel knockout mouse model to investigate the functional role of Rif in platelets.Constitutive RhoF(-/-) mice are viable and have normal platelet, leukocyte and erythrocyte counts and indices. RhoF(-/-) platelets form **filopodia** and spread normally on various agonist surfaces in static conditions and under arterial shear. In addition, RhoF(-/-) platelets have normal actin dynamics, are able to activate and aggregate normally and secrete from alpha and dense granules in response to collagen related peptide and thrombin stimulation.The small GTPase Rif does not appear to be critical for platelet function in mice. Functional overlap between Rif and other small GTPases may be responsible for the non-essential role of Rif in platelets.

#### Description

1. ras homolog family member F (in **filopodia**)

#### Alternative Name

1. ras homolog gene family, member f (in **filopodia**)

### [Ff]ilopodium

#### PubMed

1. Rif-mDia1 interaction is involved in **filopodium** formation independent of Cdc42 and Rac effectors. Filopodia are cellular protrusions important for axon guidance, embryonic development, and wound healing. The Rho GTPase Cdc42 is the best studied inducer of **filopodium** formation, and several of its effectors and their interacting partners have been linked to the process. These include IRSp53, N-WASP, Mena, and Eps8. The Rho GTPase, Rif, also drives **filopodium** formation. The signaling pathway by which Rif induces filopodia is poorly understood, with mDia2 being the only protein implicated to date. It is thus not clear how distinct the Rif-driven pathway for **filopodium** formation is from the one mediated by Cdc42. In this study, we characterize the dynamics of Rif-induced filopodia by time lapse imaging of live neuronal cells and show that Rif drives **filopodium** formation via an independent pathway that does not involve the Cdc42 effectors N-WASP and IRSp53, the IRSp53 binding partner Mena, or the Rac effectors WAVE1 and WAVE2. Rif formed filopodia in the absence of N-WASP or Mena and when IRSp53, WAVE1, or WAVE2 was knocked down by RNAi. Rif-mediated filopodial protrusion was instead reduced by silencing mDia1 expression or overexpressing a dominant negative mutant of mDia1. mDia1 on its own was able to form filopodia. Data from acceptor photobleaching FRET studies of protein-protein interaction demonstrate that Rif interacts directly with mDia1 in filopodia but not with mDia2. Taken together, these results suggest a novel pathway for filopodia formation via Rif and mDia1.
2. Rif-mDia1 interaction is involved in **filopodium** formation independent of Cdc42 and Rac effectors. Filopodia are cellular protrusions important for axon guidance, embryonic development, and wound healing. The Rho GTPase Cdc42 is the best studied inducer of **filopodium** formation, and several of its effectors and their interacting partners have been linked to the process. These include IRSp53, N-WASP, Mena, and Eps8. The Rho GTPase, Rif, also drives **filopodium** formation. The signaling pathway by which Rif induces filopodia is poorly understood, with mDia2 being the only protein implicated to date. It is thus not clear how distinct the Rif-driven pathway for **filopodium** formation is from the one mediated by Cdc42. In this study, we characterize the dynamics of Rif-induced filopodia by time lapse imaging of live neuronal cells and show that Rif drives **filopodium** formation via an independent pathway that does not involve the Cdc42 effectors N-WASP and IRSp53, the IRSp53 binding partner Mena, or the Rac effectors WAVE1 and WAVE2. Rif formed filopodia in the absence of N-WASP or Mena and when IRSp53, WAVE1, or WAVE2 was knocked down by RNAi. Rif-mediated filopodial protrusion was instead reduced by silencing mDia1 expression or overexpressing a dominant negative mutant of mDia1. mDia1 on its own was able to form filopodia. Data from acceptor photobleaching FRET studies of protein-protein interaction demonstrate that Rif interacts directly with mDia1 in filopodia but not with mDia2. Taken together, these results suggest a novel pathway for filopodia formation via Rif and mDia1.

#### GeneRIFs

1. Rif-mDia1 interaction is involved in **filopodium** formation independent of Cdc42 and Rac effectors.

---

## Rhoj

- NCBI Gene
- GeneRIF
- Pubmed

| Search Term | Rank | Fields |
| --- | --- | --- |
| [Ff]ilopodia | 1 | PubMed(2) |
| [Mm]igration | 6 | PubMed(1); GeneRIFs(1) |

  
hide/show details for Rhoj

### [Ff]ilopodia

#### PubMed

1. Small GTPase Tc10 and its homologue RhoT induce N-WASP-mediated long process formation and neurite outgrowth. Rho family small GTPases regulate multiple cellular functions through reorganization of the actin cytoskeleton. Among them, Cdc42 and Tc10 induce **filopodia** or peripheral processes in cultured cells. We have identified a member of the family, designated as RhoT, which is closely related to Tc10. Tc10 was highly expressed in muscular tissues and brain and remarkably induced during differentiation of C2 skeletal muscle cells and neuronal differentiation of PC12 and N1E-115 cells. On the other hand, RhoT was predominantly expressed in heart and uterus and induced during neuronal differentiation of N1E-115 cells. Tc10 exogenously expressed in fibroblasts generated actin-filament-containing peripheral processes longer than the Cdc42-formed **filopodia**, whereas RhoT produced much longer and thicker processes containing actin filaments. Furthermore, both Tc10 and RhoT induced neurite outgrowth in PC12 and N1E-115 cells, but Cdc42 did not do this by itself. Tc10 and RhoT as well as Cdc42 bound to the N-terminal CRIB-motif-containing portion of N-WASP and activated N-WASP to induce Arp2/3-complex-mediated actin polymerization. The formation of peripheral processes and neurites by Tc10 and RhoT was prevented by the coexpression of dominant-negative mutants of N-WASP. Thus, N-WASP is essential for the process formation and neurite outgrowth induced by Tc10 and RhoT. Neuronal differentiation of PC12 and N1E-115 cells induced by dibutyryl cyclic AMP and by serum starvation, respectively, was prevented by dominant-negative Cdc42, Tc10 and RhoT. Taken together, all these Rho family proteins are required for neuronal differentiation, but they exert their functions differentially in process formation and neurite extension. Consequently, N-WASP activated by these small GTPases mediates neuronal differentiation in addition to its recently identified role in glucose uptake.
2. Sema3E-PlexinD1 signaling selectively suppresses disoriented angiogenesis in ischemic retinopathy in mice. During development, the retinal vasculature grows toward hypoxic areas in an organized fashion. By contrast, in ischemic retinopathies, new blood vessels grow out of the retinal surfaces without ameliorating retinal hypoxia. Restoration of proper angiogenic directionality would be of great benefit to reoxygenize the ischemic retina and resolve disease pathogenesis. Here, we show that binding of the semaphorin 3E (Sema3E) ligand to the transmembrane PlexinD1 receptor initiates a signaling pathway that normalizes angiogenic directionality in both developing retinas and ischemic retinopathy. In developing mouse retinas, inhibition of VEGF signaling resulted in downregulation of endothelial PlexinD1 expression, suggesting that astrocyte-derived VEGF normally promotes PlexinD1 expression in growing blood vessels. Neuron-derived Sema3E signaled to PlexinD1 and activated the small GTPase RhoJ in ECs, thereby counteracting VEGF-induced **filopodia** projections and defining the retinal vascular pathfinding. In a mouse model of ischemic retinopathy, enhanced expression of PlexinD1 and RhoJ in extraretinal vessels prevented VEGF-induced disoriented projections of the endothelial **filopodia**. Remarkably, intravitreal administration of Sema3E protein selectively suppressed extraretinal vascular outgrowth without affecting the desired regeneration of the retinal vasculature. Our study suggests a new paradigm for vascular regeneration therapy that guides angiogenesis precisely toward the ischemic retina.

### [Mm]igration

#### PubMed

1. RhoJ/TCL regulates endothelial motility and tube formation and modulates actomyosin contractility and focal adhesion numbers. RhoJ/TCL was identified by our group as an endothelial-expressed Rho GTPase. The aim of this study was to determine its tissue distribution, subcellular localization, and function in endothelial **migration** and tube formation.Using in situ hybridization, RhoJ was localized to endothelial cells in a set of normal and cancerous tissues and in the vasculature of mouse embryos; endogenous RhoJ was localized to focal adhesions by immunofluorescence. The proangiogenic factor vascular endothelial growth factor activated RhoJ in endothelial cells. Using either small interfering (si)RNA-mediated knockdown of RhoJ expression or overexpression of constitutively active RhoJ (daRhoJ), RhoJ was found to positively regulate endothelial motility and tubule formation. Downregulating RhoJ expression increased focal adhesions and stress fibers in migrating cells, whereas daRhoJ overexpression resulted in the converse. RhoJ downregulation resulted in increased contraction of a collagen gel and increased phospho-myosin light chain, indicative of increased actomyosin contractility. Pharmacological inhibition of Rho-kinase (which phosphorylates myosin light chain) or nonmuscle myosin II reversed the defective tube formation and **migration** of RhoJ knockdown cells.RhoJ is endothelial-expressed in vivo, activated by vascular endothelial growth factor, localizes to focal adhesions, regulates endothelial cell **migration** and tube formation, and modulates actomyosin contractility and focal adhesion numbers.

#### GeneRIFs

1. RhoJ is endothelial-expressed in vivo, activated by vascular endothelial growth factor, localizes to focal adhesions, regulates endothelial cell **migration** and tube formation, and modulates actomyosin contractility and focal adhesion numbers.

---

## Rac2

- NCBI Gene
- GeneRIF
- Pubmed

| Search Term | Rank | Fields |
| --- | --- | --- |
| [Ff]ilopodia | 1 | PubMed(1) |
| [Pp]odosome(s)? | 5 | PubMed(1) |
| [Mm]igration | 6 | Process(1); PubMed(42); GeneRIFs(6) |
| [Ee]xtracellular [Mm]atrix | 8 | PubMed(2) |
| [Ii]nvasive | 10 | PubMed(2); GeneRIFs(1) |
| [Mm]etastasis | 12 | PubMed(2); GeneRIFs(1) |

  
hide/show details for Rac2

### [Ff]ilopodia

#### PubMed

1. Rac1 is essential for platelet lamellipodia formation and aggregate stability under flow. The role of Rac family proteins in platelet spreading on matrix proteins under static and flow conditions has been investigated by using Rac-deficient platelets. Murine platelets form **filopodia** and undergo limited spreading on fibrinogen independent of Rac1 and Rac2. In the presence of thrombin, marked lamellipodia formation is observed on fibrinogen, which is abrogated in the absence of Rac1. However, Rac1 is not required for thrombin-induced aggregation or elevation of F-actin levels. Formation of lamellipodia on collagen and laminin is also Rac1-dependent. Analysis of platelet adhesion dynamics on collagen under flow conditions in vitro revealed that Rac1 is required for platelet aggregate stability at arterial rates of shear, as evidenced by a dramatic increase in platelet embolization. Furthermore, studies employing intravital microscopy demonstrated that Rac1 plays a critical role in the development of stable thrombi at sites of vascular injury in vivo. Thus, our data demonstrated that Rac1 is essential for lamellipodia formation in platelets and indicated that Rac1 is required for aggregate integrity leading to thrombus formation under physiologically relevant levels of shear both in vitro and in vivo.

### [Pp]odosome(s)?

#### PubMed

1. Rac1 and Rac2 regulate macrophage morphology but are not essential for migration. Rac GTPases are believed to contribute to migration in leukocytes by transducing signals from cell surface receptors to the actin and microtubule cytoskeletons. Mammals have three closely related Rac isoforms, Rac1, Rac2 and Rac3, and it is widely assumed that cell migration requires the activity of these Rac GTPases. We have previously shown that Rac1-null mouse macrophages have altered cell shape and reduced membrane ruffling but normal migration speed. Here we investigate the behaviour of macrophages lacking Rac2 (Rac2(-/-)) or Rac1 and Rac2 (Rac1/2(-/-)). Rac2(-/-) macrophages have reduced F-actin levels and lack **podosomes**, which are integrin-based adhesion sites, and their migration speed is similar to or slightly slower than wild-type macrophages, depending on the substrate. Unexpectedly, Rac1/2(-/-) macrophages, which do not express Rac1, Rac2 or Rac3, migrate at a similar speed to wild-type macrophages on a variety of substrates and perform chemotaxis normally, although their morphology and mode of migration is altered. However, Rac1(-/-) and Rac1/2(-/-) but not Rac2(-/-) macrophages are impaired in their ability to invade through Matrigel. Together, these data show that Rac1 and Rac2 have distinct roles in regulating cell morphology, migration and invasion, but are not essential for macrophage migration or chemotaxis.

### [Mm]igration

#### Process

1. regulation of neutrophil **migration**

#### PubMed

1. Rac GTPase isoforms Rac1 and Rac2 play a redundant and crucial role in T-cell development. Rac GTPases have been implicated in the regulation of diverse functions in various blood cell lineages, but their role in T-cell development is not well understood. We have carried out conditional gene targeting to achieve hematopoietic stem cell (HSC)- or T-cell lineage-specific deletion of Rac1 or Rac1/Rac2 by crossbreeding the Mx-Cre or Lck-Cre transgenic mice with Rac1(loxp/loxp) or Rac1(loxp/loxp);Rac2(-/-) mice. We found that (1) HSC deletion of both Rac1 and Rac2 inhibited production of common lymphoid progenitors (CLPs) in bone marrow and suppressed T-cell development in thymus and peripheral organs, whereas deletion of Rac1 moderately affected CLP production and T-cell development. (2) T cell-specific deletion of Rac1 did not affect T-cell development, whereas deletion of both Rac1 and Rac2 reduced immature CD4(+)CD8(+) and mature CD4(+) populations in thymus as well as CD4(+) and CD8(+) populations in spleen. (3) The developmental defects of Rac1/Rac2 knockout T cells were associated with proliferation, survival, adhesion, and **migration** defects. (4) Rac1/Rac2 deletion suppressed T-cell receptor-mediated proliferation, IL-2 production, and Akt activation in thymocytes. Thus, Rac1 and Rac2 have unique roles in CLP production and share a redundant but essential role in later stages of T-cell development by regulating survival and proliferation signals.
2. Regulation of lymphatic-blood vessel separation by endothelial Rac1. Sprouting angiogenesis and lymphatic-blood vessel segregation both involve the **migration** of endothelial cells, but the precise migratory molecules that govern the decision of blood vascular endothelial cells to segregate into lymphatic vasculature are unknown. Here, we deleted endothelial Rac1 in mice (Tie1-Cre(+);Rac1(fl/fl)) and revealed, unexpectedly, that whereas blood vessel morphology appeared normal, lymphatic-blood vessel separation was impaired, with corresponding edema, haemorrhage and embryonic lethality. Importantly, normal levels of Rac1 were essential for directed endothelial cell migratory responses to lymphatic-inductive signals. Our studies identify Rac1 as a crucial part of the migratory machinery required for endothelial cells to separate and form lymphatic vasculature.
3. Critical roles for Rac1 and Rac2 GTPases in B cell development and signaling. The Rac1 guanosine triphosphatase (GTPase) has been implicated in multiple cellular functions, including actin dynamics, proliferation, apoptosis, adhesion, and **migration** resulting from signaling by multiple receptors, including the B cell antigen receptor (BCR). We used conditional gene targeting to generate mice with specific Rac1 deficiency in the B cell lineage. In the absence of both Rac1 and the highly related Rac2, B cell development was almost completely blocked. Both GTPases were required to transduce BCR signals leading to proliferation, survival and up-regulation of BAFF-R, a receptor for BAFF, a key survival molecule required for B cell development and maintenance.
4. Genetic evidence for convergence of c-Kit- and alpha4 integrin-mediated signals on class IA PI-3kinase and the Rac pathway in regulating integrin-directed **migration** in mast cells. Mast cells play a critical role in host defense against a number of pathogens. Increased mast cell infiltration has been described in allergic asthma, in rheumatoid arthritis, and during helminthes infection. Despite the importance of mast cells in allergic disease and defense against infection, little is known about the mechanisms by which mast cells migrate to various tissues under steady state conditions or during infection or inflammation. Here, we show that activation of c-Kit by its ligand, stem cell factor (SCF), cooperates with alpha4 integrin in inducing directed **migration** of mast cells on fibronectin. A reduction in **migration** and activation of a small G protein, Rac, was observed in mast cells derived from class IA phosphoinositide-3 kinase (PI-3kinase)-deficient mice in response to SCF stimulation and in mast cells expressing the dominant-negative Rac (RacN17), as well as in mast cells deficient in the hematopoietic-specific small G protein, Rac2. In addition, a PI-3kinase inhibitor inhibited alpha4- as well as SCF-induced **migration** in a dose-dependent fashion. In contrast, a mitogen-activated protein kinase (MAPK) inhibitor had little effect. Consistent with the pharmacologic results, abrogating the binding of the p85alpha subunit of class IA PI-3kinase to c-Kit also resulted in inhibition of SCF-induced **migration** on fibronectin. These genetic and biochemical data demonstrate that both c-Kit and alpha4 integrin signaling are linked to class IA PI-3kinase and Rac pathways and regulate integrin-directed (haptotactic) **migration** in mast cells.
5. Requirement of Rac1 and Rac2 expression by mature dendritic cells for T cell priming. Upon maturation, dendritic cells (DCs) acquire the unique ability to activate naïve T cells. We used time-lapse video microscopy and two-photon imaging of intact lymph nodes to show that after establishing initial contact between their dendrites and naïve T lymphocytes, mature DCs migrate toward the contacted lymphocytes. Subsequently, the DCs tightly entrap the T cells within a complex net of membrane extensions. The Rho family guanosine triphosphatases Rac1 and Rac2 but not Rho itself control the formation of dendrites in mature DCs, their polarized short-rang**e migrati**on toward T cells, and T cell priming.
6. The role of chemokine activation of Rac GTPases in hematopoietic stem cell marrow homing, retention, and peripheral mobilization. Signaling downstream from the chemokine receptor CXCR4, the tyrosine kinase receptor c-kit and beta1-integrins has been shown to be crucial in the regulation of **migration**, homing, and engraftment of hematopoietic stem cells and progenitors. Each of these receptors signal through Rac-type Rho guanosine triphosphatases (GTPases). Rac GTPases play a major role in the organization of the actin cytoskeleton and also in the control of gene expression and the activation of proliferation and survival pathways. Here we review the specific roles of the members of the Rac subfamily of the Rho GTPase family in regulating the intracellular signaling of hematopoietic cells responsible for regulation of homing, marrow retention, and peripheral mobilization.
7. Inhibition of Rac GTPase signaling and downstream prosurvival Bcl-2 proteins as combination targeted therapy in MLL-AF9 leukemia. The Rac family of small Rho GTPases coordinates diverse cellular functions in hematopoietic cells including adhesion, **migration**, cytoskeleton rearrangements, gene transcription, proliferation, and survival. The integrity of Rac signaling has also been found to critically regulate cellular functions in the initiation and maintenance of hematopoietic malignancies. Using an in vivo gene targeting approach, we demonstrate that Rac2, but not Rac1, is critical to the initiation of acute myeloid leukemia in a retroviral expression model of MLL-AF9 leukemogenesis. However, loss of either Rac1 or Rac2 is sufficient to impair survival and growth of the transformed MLL-AF9 leukemia. Rac2 is known to positively regulate expression of Bcl-2 family proteins toward a prosurvival balance. We demonstrate that disruption of downstream survival signaling through antiapoptotic Bcl-2 proteins is implicated in mediating the effects of Rac2 deficiency in MLL-AF9 leukemia. Indeed, overexpression of Bcl-xL is able to rescue the effects of Rac2 deficiency and MLL-AF9 cells are exquisitely sensitive to direct inhibition of Bcl-2 family proteins by the BH3-mimetic, ABT-737. Furthermore, concurrent exposure to NSC23766, a small-molecule inhibitor of Rac activation, increases the apoptotic effect of ABT-737, indicating the Rac/Bcl-2 survival pathway may be targeted synergistically.
8. Critical roles for Rac GTPases in T-cell **migration** to and within lymph nodes. Naive T cells continuously recirculate between secondary lymphoid tissue via the blood and lymphatic systems, a process that maximizes the chances of an encounter between a T cell and its cognate antigen. This recirculation depends on signals from chemokine receptors, integrins, and the sphingosine-1-phosphate receptor. The authors of previous studies in other cell types have shown that Rac GTPases transduce signals leading to cell **migration** and adhesion; however, their roles in T cells are unknown. By using both 3-dimensional intravital and in vitro approaches, we show that Rac1- and Rac2-deficient T cells have multiple defects in this recirculation process. Rac-deficient T cells home very inefficiently to lymph nodes and the white pulp of the spleen, show reduced interstitial **migration** within lymph node parenchyma, and are defective in egress from lymph nodes. These mutant T cells show defective chemokine-induced chemotaxis, chemokinesis, and adhesion to integrin ligands. They have reduced lateral motility on endothelial cells and transmigrate in-efficiently. These multiple defects stem from critical roles for Rac1 and Rac2 in transducing chemokine and sphingosine-1-phosphate receptor 1 signals leading to motility and adhesion.
9. Human neutrophils coordinate chemotaxis by differential activation of Rac1 and Rac2. Rac1 and Rac2, members of the small Rho GTPase family, play essential roles in coordinating directional **migration** and superoxide production during neutrophil responses to chemoattractants. Although earlier studies in Rac1 and Rac2 knockout mice have demonstrated unique roles for each Rac isoform in chemotaxis and NADPH oxidase activation, it is still unclear how human neutrophils use Rac1 and Rac2 to achieve their immunological responses to foreign agent stimulation. In the current study, we used TAT dominant-negative Rac1-T17N and Rac2-T17N fusion proteins to acutely alter the activity of Rac1 and Rac2 individually in human neutrophils. We demonstrate distinct activation kinetics and different roles for Rac1 and Rac2 in response to low vs high concentrations of fMLP. These observations were verified using neutrophils from mice in which Rac1 or Rac2 was genetically absent. Based on these results, we propose a model to explain how human neutrophils kill invading microbes while limiting oxidative damage to the adjacent surrounding healthy tissue through the differential activation of Rac1 and Rac2 in response to different concentrations of chemoattractant.
10. A novel Rac-dependent checkpoint in B cell development controls entry into the splenic white pulp and cell survival. Rac1 and Rac2 GTPases transduce signals from multiple receptors leading to cell **migration**, adhesion, proliferation, and survival. In the absence of Rac1 and Rac2, B cell development is arrested at an IgD- transitional B cell stage that we term transitional type 0 (T0). We show that T0 cells cannot enter the white pulp of the spleen until they mature into the T1 and T2 stages, and that this entry into the white pulp requires integrin and chemokine receptor signaling and is required for cell survival. In the absence of Rac1 and Rac2, transitional B cells are unable to migrate in response to chemokines and cannot enter the splenic white pulp. We propose that loss of Rac1 and Rac2 causes arrest at the T0 stage at least in part because transitional B cells need to migrate into the white pulp to receive survival signals. Finally, we show that in the absence of Syk, a kinase that transduces B cell antigen receptor signals required for positive selection, development is arrested at the same T0 stage, with transitional B cells excluded from the white pulp. Thus, these studies identify a novel developmental checkpoint that coincides with B cell positive selection.
11. Rac2 regulates neutrophil chemotaxis, superoxide production, and myeloid colony formation through multiple distinct effector pathways. Polymorphonuclear neutrophils (PMN) are an important component of the innate immune system. We have shown previously that **migration** and superoxide (O2\*-) production, as well as some kinase signaling pathways are compromised in mice deficient in the Ras-related Rho GTPase Rac2. In this study, we demonstrate that Rac2 controls chemotaxis and superoxide production via distinct pathways and is critical for development of myeloid colonies in vitro. The Rac2 mutants V36A, F37A, and N39A all bind to both Pak1 and p67(phox), yet are unable to rescue superoxide production and chemotaxis when expressed in Rac2-/- PMN. In contrast, the N43A mutant, which binds to Por1 (Arfaptin 2), p67phox, and Pak1, is able to rescue superoxide production but not chemotaxis. The F37A mutant, demonstrated to have reduced binding to Por1, shows reduced rescue of fMLP-induced chemotaxis. Finally, the Rac2Y40C mutant that is defective in binding to all three potential downstream effectors (Pak1, p67phox, and Por1) is unable to rescue chemotaxis, motility, or superoxide production, but is able to rescue defective growth of myeloid colonies in vitro. These findings suggest that binding to any single effector is not sufficient to rescue the distinct cellular phenotypes of Rac2-/- PMN, implicating multiple, distinct, and potentially parallel effector pathways.
12. Rac1 deletion in mouse neutrophils has selective effects on neutrophil functions. Defects in myeloid cell function in Rac2 knockout mice underline the importance of this isoform in activation of NADPH oxidase and cell motility. However, the specific role of Rac1 in neutrophil function has been difficult to assess since deletion of Rac1 results in embryonic lethality in mice. To elucidate the specific role of Rac1 in neutrophils, we generated mice with a conditional Rac1 deficiency restricted to cells of the granulocyte/monocyte lineage. As observed in Rac2-deficient neutrophils, Rac1-deficient neutrophils demonstrated profound defects in inflammatory recruitment in vivo, **migration** to chemotactic stimuli, and chemoattractant-mediated actin assembly. In contrast, superoxide production is normal in Rac1-deficient neutrophils but markedly diminished in Rac2 null cells. These data demonstrate that although Rac1 and Rac2 are both required for actin-mediated functions, Rac2 is specifically required for activation of the neutrophil NADPH oxidase.
13. Rac1 links leading edge and uropod events through Rho and myosin activation during chemotaxis. Chemotactic responsiveness is crucial to neutrophil recruitment to sites of infection. During chemotaxis, highly divergent cytoskeletal programs are executed at the leading and trailing edge of motile neutrophils. The Rho family of small GTPases plays a critical role in cell **migration**, and recent work has focused on elucidating the specific roles played by Rac1, Rac2, Cdc42, and Rho during cellular chemotaxis. Rac GTPases regulate actin polymerization and extension of the leading edge, whereas Rho GTPases control myosin-based contraction of the trailing edge. Rac and Rho signaling are thought to crosstalk with one another, and previous research has focused on mutual inhibition of Rac and Rho signaling during chemotaxis. Indeed, polarization of neutrophils has been proposed to involve the activity of a negative feedback system where Rac activation at the front of the cell inhibits local Rho activation, and vice versa. Using primary human neutrophils and neutrophils derived from a Rac1/Rac2-null transgenic mouse model, we demonstrate here that Rac1 (and not Rac2) is essential for Rho and myosin activation at the trailing edge to regulate uropod function. We conclude that Rac plays both positive and negative roles in the organization of the Rhomyosin "backness" program, thereby promoting stable polarity in chemotaxing neutrophils.
14. Genetic deletion of Cdc42GAP reveals a role of Cdc42 in erythropoiesis and hematopoietic stem/progenitor cell survival, adhesion, and engraftment. Rho family GTPases are key signal transducers in cell regulation. Although a body of literature has implicated the Rho family members Rac1 and Rac2 in multiple hematopoietic-cell functions, the role of Cdc42 in hematopoiesis remains unclear. Here we have examined the hematopoietic properties and the hematopoietic stem/progenitor cell (HSP) functions of gene-targeted mice carrying null alleles of cdc42gap, a negative regulator of Cdc42. The Cdc42GAP-/- fetal liver and bone marrow cells showed a 3-fold increase in Cdc42 activity but normal Rac and RhoA activities, indicating that Cdc42GAP knockout resulted in a gain of Cdc42 activity in the hematopoietic tissues. Cdc42GAP-/- mice were anemic. The cellularity of fetal liver and bone marrow, the number and composition percentage of HSPs, and the erythroid blast-forming unit and colony-forming unit (BFU-E/CFU-E) activities were significantly reduced in the homozygous mice. The decrease in HSP number was associated with increased apoptosis of the Cdc42GAP-/- HSPs and the activation of JNK-mediated apoptotic machinery. Moreover, homozygous HSPs showed impaired cortical F-actin assembly, deficiency in adhesion and **migration**, and defective engraftment. These results provide evidence that Cdc42 activity is important for erythropoiesis and for multiple HSP functions, including survival, adhesion, and engraftment.
15. Vav GEFs are required for beta2 integrin-dependent functions of neutrophils. Integrin regulation of neutrophils is essential for appropriate adhesion and trans**migration** into tissues. Vav proteins are Rho family guanine nucleotide exchange factors that become tyrosine phosphorylated in response to adhesion. Using Vav1/Vav3-deficient neutrophils (Vav1/3ko), we show that Vav proteins are required for multiple beta2 integrin-dependent functions, including sustained adhesion, spreading, and complement-mediated phagocytosis. These defects are not attributable to a lack of initial beta2 activation as Vav1/3ko neutrophils undergo chemoattractant-induced arrest on intercellular adhesion molecule-1 under flow. Accordingly, in vivo, Vav1/3ko leukocytes arrest on venular endothelium yet are unable to sustain adherence. Thus, Vav proteins are specifically required for stable adhesion. beta2-induced activation of Cdc42, Rac1, and RhoA is defective in Vav1/3ko neutrophils, and phosphorylation of Pyk2, paxillin, and Akt is also significantly reduced. In contrast, Vav proteins are largely dispensable for G protein-coupled receptor-induced signaling events and chemotaxis. Thus, Vav proteins play an essential role coupling beta2 to Rho GTPases and regulating multiple integrin-induced events important in leukocyte adhesion and phagocytosis.
16. Inhibition of Rac activity alleviates lipopolysaccharide-induced acute pulmonary injury in mice. Rac small GTPases play important roles in cytoskeleton and many cell functions including cell cycle, cell growth, cell adhesion and gene transcription. Here, we investigated the roles of Rac including Rac1 and Rac2 in lipopolysaccharide (LPS)-induced pulmonary injury.After LPS was intratracheally instilled to lungs in mice, Rac, CDC42 and RhoA activation assay by pull-down and West blot, inflammatory cell infiltration assay by counting cell numbers and lung histological examination, pro-inflammatory mediator mRNA expression assay by quantitative RT-PCR, measurement of myeloperoxidase (MPO) activity, Evans Blue and albumin accumulation by spectrophotometry were performed to evaluate the roles of Rac in pulmonary injury by using its specific inhibitor, NSC23766.LPS challenge led to increases of both Rac1 and Rac2, but not CDC42 or RhoA activities in lungs, and intraperitoneal administration with NSC23766 inhibited both Rac1 and Rac2, but not CDC42 or RhoA activities. Treatment with NSC23766 at 1 or 3mg/kg not only reduced the inflammatory cells infiltration and MPO activities, but also inhibited pro-inflammatory mediators, tumor necrosis factor-α and interleukin-1β, mRNA expression. Moreover, in vitro neutrophi**l migrati**on assay and in vivo microvascular permeability assay indicated that NSC23766 not only inhibited neutrophil transwel**l migrati**on toward a chemoattractant, fMLP, but also reduced Evans Blue and albumin accumulation in LPS-challenged lungs. LPS activated both Rac1 and Rac2, but not CDC42 or RhoA activities in lungs, and specific inhibition of Rac activities by NSC23766 effectively alleviated LPS-induced injury.Rac could be a potential target for therapeutic intervention of pulmonary inflammation.
17. Hematopoietic cell regulation by Rac1 and Rac2 guanosine triphosphatases. The Rho guanosine triphosphatases (GTPases) Rac1 and Rac2 are critical signaling regulators in mammalian cells. The deletion of both Rac1 and Rac2 murine alleles leads to a massive egress of hematopoietic stem/progenitor cells (HSC/Ps) into the blood from the marrow, whereas Rac1-/- but not Rac2-/- HSC/Ps fail to engraft in the bone marrow of irradiated recipient mice. In contrast, Rac2, but not Rac1, regulates superoxide production and directed **migration** in neutrophils, and in each cell type, the two GTPases play distinct roles in actin organization, cell survival, and proliferation. Thus, Rac1 and Rac2 regulate unique aspects of hematopoietic development and function.
18. Rac regulates PtdInsP₃ signaling and the chemotactic compass through a redox-mediated feedback loop. Directional cel**l migrati**on is an essential requirement for efficient neutrophil translocation to sites of infection and requires the establishment of a polarized cell characterized by an actin-rich leading edge facing the chemoattractant gradient. The asymmetrical accumulation of phosphatidylinositol(3,4,5)-trisphosphate [PtdIns(3,4,5)P(3)] in the up-gradient leading edge is a hallmark of polarization and regulates the recruitment and localization of various effector proteins at the leading-edge plasma membrane. How shallow gradients of chemoattractants trigger and maintain a much steeper intracellular gradient of PtdIns(3,4,5)P(3) is a critical question in the study of leukocyte chemotaxis. Our data demonstrate that th**e migrati**on of neutrophils toward the chemoattractant N-formyl-L-methionyl-L-leucyl-L-phenylalanine depends on the generation of reactive oxygen species by the phagocytic NADPH oxidase (NOX2) and subsequent oxidation and inhibition of phosphatase and tensin homolog. Moreover, we show that events downstream of PtdIns(3,4,5)P(3), including phosphorylation of AKT, Rac activation, uncapping of actin filaments, and directiona**l migrati**on, can be attenuated by ROS scavengers or genetic ablation of NOX2. Using Rac mutants that are defective in their ability to activate NOX2, we show that Rac regulates a redox-mediated feedback loop that mediates directiona**l migrati**on of neutrophils.
19. Aquaporin 9 phosphorylation mediates membrane localization and neutrophil polarization. Neutrophils are of prime importance in the host innate defense against invading microorganisms by using two primary mechanisms-locomotion toward and phagocytosis of the prey. Recent research points to pivotal roles for water channels known as AQPs in cell motility. Here, we focused on the role of AQP9 in chemoattractant-induced polarization and **migration** of primary mouse neutrophils and neutrophil-like HL60 cells. We found that AQP9 is phosphorylated downstream of fMLFR or PMA stimulation in primary human neutrophils. The dynamics of AQP9 were assessed using GFP-tagged AQP9 constructs and other fluorescent markers through various live-cell imaging techniques. Expression of WT or the phosphomimic S11D AQP9 changed cell volume regulation as a response to hyperosmotic changes and enhanced neutrophil polarization and chemotaxis. WT AQP9 and S11D AQP9 displayed a very dynamic distribution at the cell membrane, whereas the phosphorylation-deficient S11A AQP9 failed to localize to the plasma membrane. Furthermore, we found that Rac1 regulated the translocation of AQP9 to the plasma membrane. Our results show that AQP9 plays an active role in neutrophil volume regulation and **migration**. The display of AQP9 at the plasma membrane depends on AQP9 phosphorylation, which appeared to be regulated through a Rac1-dependent pathway.
20. Rac regulates PtdInsP₃ signaling and the chemotactic compass through a redox-mediated feedback loop. Directional cel**l migrati**on is an essential requirement for efficient neutrophil translocation to sites of infection and requires the establishment of a polarized cell characterized by an actin-rich leading edge facing the chemoattractant gradient. The asymmetrical accumulation of phosphatidylinositol(3,4,5)-trisphosphate [PtdIns(3,4,5)P(3)] in the up-gradient leading edge is a hallmark of polarization and regulates the recruitment and localization of various effector proteins at the leading-edge plasma membrane. How shallow gradients of chemoattractants trigger and maintain a much steeper intracellular gradient of PtdIns(3,4,5)P(3) is a critical question in the study of leukocyte chemotaxis. Our data demonstrate that th**e migrati**on of neutrophils toward the chemoattractant N-formyl-L-methionyl-L-leucyl-L-phenylalanine depends on the generation of reactive oxygen species by the phagocytic NADPH oxidase (NOX2) and subsequent oxidation and inhibition of phosphatase and tensin homolog. Moreover, we show that events downstream of PtdIns(3,4,5)P(3), including phosphorylation of AKT, Rac activation, uncapping of actin filaments, and directiona**l migrati**on, can be attenuated by ROS scavengers or genetic ablation of NOX2. Using Rac mutants that are defective in their ability to activate NOX2, we show that Rac regulates a redox-mediated feedback loop that mediates directiona**l migrati**on of neutrophils.
21. Inhibition of Rac GTPase signaling and downstream prosurvival Bcl-2 proteins as combination targeted therapy in MLL-AF9 leukemia. The Rac family of small Rho GTPases coordinates diverse cellular functions in hematopoietic cells including adhesion, **migration**, cytoskeleton rearrangements, gene transcription, proliferation, and survival. The integrity of Rac signaling has also been found to critically regulate cellular functions in the initiation and maintenance of hematopoietic malignancies. Using an in vivo gene targeting approach, we demonstrate that Rac2, but not Rac1, is critical to the initiation of acute myeloid leukemia in a retroviral expression model of MLL-AF9 leukemogenesis. However, loss of either Rac1 or Rac2 is sufficient to impair survival and growth of the transformed MLL-AF9 leukemia. Rac2 is known to positively regulate expression of Bcl-2 family proteins toward a prosurvival balance. We demonstrate that disruption of downstream survival signaling through antiapoptotic Bcl-2 proteins is implicated in mediating the effects of Rac2 deficiency in MLL-AF9 leukemia. Indeed, overexpression of Bcl-xL is able to rescue the effects of Rac2 deficiency and MLL-AF9 cells are exquisitely sensitive to direct inhibition of Bcl-2 family proteins by the BH3-mimetic, ABT-737. Furthermore, concurrent exposure to NSC23766, a small-molecule inhibitor of Rac activation, increases the apoptotic effect of ABT-737, indicating the Rac/Bcl-2 survival pathway may be targeted synergistically.
22. Inhibition of Rac activity alleviates lipopolysaccharide-induced acute pulmonary injury in mice. Rac small GTPases play important roles in cytoskeleton and many cell functions including cell cycle, cell growth, cell adhesion and gene transcription. Here, we investigated the roles of Rac including Rac1 and Rac2 in lipopolysaccharide (LPS)-induced pulmonary injury.After LPS was intratracheally instilled to lungs in mice, Rac, CDC42 and RhoA activation assay by pull-down and West blot, inflammatory cell infiltration assay by counting cell numbers and lung histological examination, pro-inflammatory mediator mRNA expression assay by quantitative RT-PCR, measurement of myeloperoxidase (MPO) activity, Evans Blue and albumin accumulation by spectrophotometry were performed to evaluate the roles of Rac in pulmonary injury by using its specific inhibitor, NSC23766.LPS challenge led to increases of both Rac1 and Rac2, but not CDC42 or RhoA activities in lungs, and intraperitoneal administration with NSC23766 inhibited both Rac1 and Rac2, but not CDC42 or RhoA activities. Treatment with NSC23766 at 1 or 3mg/kg not only reduced the inflammatory cells infiltration and MPO activities, but also inhibited pro-inflammatory mediators, tumor necrosis factor-α and interleukin-1β, mRNA expression. Moreover, in vitro neutrophi**l migrati**on assay and in vivo microvascular permeability assay indicated that NSC23766 not only inhibited neutrophil transwel**l migrati**on toward a chemoattractant, fMLP, but also reduced Evans Blue and albumin accumulation in LPS-challenged lungs. LPS activated both Rac1 and Rac2, but not CDC42 or RhoA activities in lungs, and specific inhibition of Rac activities by NSC23766 effectively alleviated LPS-induced injury.Rac could be a potential target for therapeutic intervention of pulmonary inflammation.
23. Critical roles for Rac GTPases in T-cell **migration** to and within lymph nodes. Naive T cells continuously recirculate between secondary lymphoid tissue via the blood and lymphatic systems, a process that maximizes the chances of an encounter between a T cell and its cognate antigen. This recirculation depends on signals from chemokine receptors, integrins, and the sphingosine-1-phosphate receptor. The authors of previous studies in other cell types have shown that Rac GTPases transduce signals leading to cell **migration** and adhesion; however, their roles in T cells are unknown. By using both 3-dimensional intravital and in vitro approaches, we show that Rac1- and Rac2-deficient T cells have multiple defects in this recirculation process. Rac-deficient T cells home very inefficiently to lymph nodes and the white pulp of the spleen, show reduced interstitial **migration** within lymph node parenchyma, and are defective in egress from lymph nodes. These mutant T cells show defective chemokine-induced chemotaxis, chemokinesis, and adhesion to integrin ligands. They have reduced lateral motility on endothelial cells and transmigrate in-efficiently. These multiple defects stem from critical roles for Rac1 and Rac2 in transducing chemokine and sphingosine-1-phosphate receptor 1 signals leading to motility and adhesion.
24. A novel Rac-dependent checkpoint in B cell development controls entry into the splenic white pulp and cell survival. Rac1 and Rac2 GTPases transduce signals from multiple receptors leading to cell **migration**, adhesion, proliferation, and survival. In the absence of Rac1 and Rac2, B cell development is arrested at an IgD- transitional B cell stage that we term transitional type 0 (T0). We show that T0 cells cannot enter the white pulp of the spleen until they mature into the T1 and T2 stages, and that this entry into the white pulp requires integrin and chemokine receptor signaling and is required for cell survival. In the absence of Rac1 and Rac2, transitional B cells are unable to migrate in response to chemokines and cannot enter the splenic white pulp. We propose that loss of Rac1 and Rac2 causes arrest at the T0 stage at least in part because transitional B cells need to migrate into the white pulp to receive survival signals. Finally, we show that in the absence of Syk, a kinase that transduces B cell antigen receptor signals required for positive selection, development is arrested at the same T0 stage, with transitional B cells excluded from the white pulp. Thus, these studies identify a novel developmental checkpoint that coincides with B cell positive selection.
25. Human neutrophils coordinate chemotaxis by differential activation of Rac1 and Rac2. Rac1 and Rac2, members of the small Rho GTPase family, play essential roles in coordinating directional **migration** and superoxide production during neutrophil responses to chemoattractants. Although earlier studies in Rac1 and Rac2 knockout mice have demonstrated unique roles for each Rac isoform in chemotaxis and NADPH oxidase activation, it is still unclear how human neutrophils use Rac1 and Rac2 to achieve their immunological responses to foreign agent stimulation. In the current study, we used TAT dominant-negative Rac1-T17N and Rac2-T17N fusion proteins to acutely alter the activity of Rac1 and Rac2 individually in human neutrophils. We demonstrate distinct activation kinetics and different roles for Rac1 and Rac2 in response to low vs high concentrations of fMLP. These observations were verified using neutrophils from mice in which Rac1 or Rac2 was genetically absent. Based on these results, we propose a model to explain how human neutrophils kill invading microbes while limiting oxidative damage to the adjacent surrounding healthy tissue through the differential activation of Rac1 and Rac2 in response to different concentrations of chemoattractant.
26. Rac GTPase isoforms Rac1 and Rac2 play a redundant and crucial role in T-cell development. Rac GTPases have been implicated in the regulation of diverse functions in various blood cell lineages, but their role in T-cell development is not well understood. We have carried out conditional gene targeting to achieve hematopoietic stem cell (HSC)- or T-cell lineage-specific deletion of Rac1 or Rac1/Rac2 by crossbreeding the Mx-Cre or Lck-Cre transgenic mice with Rac1(loxp/loxp) or Rac1(loxp/loxp);Rac2(-/-) mice. We found that (1) HSC deletion of both Rac1 and Rac2 inhibited production of common lymphoid progenitors (CLPs) in bone marrow and suppressed T-cell development in thymus and peripheral organs, whereas deletion of Rac1 moderately affected CLP production and T-cell development. (2) T cell-specific deletion of Rac1 did not affect T-cell development, whereas deletion of both Rac1 and Rac2 reduced immature CD4(+)CD8(+) and mature CD4(+) populations in thymus as well as CD4(+) and CD8(+) populations in spleen. (3) The developmental defects of Rac1/Rac2 knockout T cells were associated with proliferation, survival, adhesion, and **migration** defects. (4) Rac1/Rac2 deletion suppressed T-cell receptor-mediated proliferation, IL-2 production, and Akt activation in thymocytes. Thus, Rac1 and Rac2 have unique roles in CLP production and share a redundant but essential role in later stages of T-cell development by regulating survival and proliferation signals.
27. Requirement of Rac1 and Rac2 expression by mature dendritic cells for T cell priming. Upon maturation, dendritic cells (DCs) acquire the unique ability to activate naïve T cells. We used time-lapse video microscopy and two-photon imaging of intact lymph nodes to show that after establishing initial contact between their dendrites and naïve T lymphocytes, mature DCs migrate toward the contacted lymphocytes. Subsequently, the DCs tightly entrap the T cells within a complex net of membrane extensions. The Rho family guanosine triphosphatases Rac1 and Rac2 but not Rho itself control the formation of dendrites in mature DCs, their polarized short-rang**e migrati**on toward T cells, and T cell priming.
28. Rac2 regulates neutrophil chemotaxis, superoxide production, and myeloid colony formation through multiple distinct effector pathways. Polymorphonuclear neutrophils (PMN) are an important component of the innate immune system. We have shown previously that **migration** and superoxide (O2\*-) production, as well as some kinase signaling pathways are compromised in mice deficient in the Ras-related Rho GTPase Rac2. In this study, we demonstrate that Rac2 controls chemotaxis and superoxide production via distinct pathways and is critical for development of myeloid colonies in vitro. The Rac2 mutants V36A, F37A, and N39A all bind to both Pak1 and p67(phox), yet are unable to rescue superoxide production and chemotaxis when expressed in Rac2-/- PMN. In contrast, the N43A mutant, which binds to Por1 (Arfaptin 2), p67phox, and Pak1, is able to rescue superoxide production but not chemotaxis. The F37A mutant, demonstrated to have reduced binding to Por1, shows reduced rescue of fMLP-induced chemotaxis. Finally, the Rac2Y40C mutant that is defective in binding to all three potential downstream effectors (Pak1, p67phox, and Por1) is unable to rescue chemotaxis, motility, or superoxide production, but is able to rescue defective growth of myeloid colonies in vitro. These findings suggest that binding to any single effector is not sufficient to rescue the distinct cellular phenotypes of Rac2-/- PMN, implicating multiple, distinct, and potentially parallel effector pathways.
29. Critical roles for Rac1 and Rac2 GTPases in B cell development and signaling. The Rac1 guanosine triphosphatase (GTPase) has been implicated in multiple cellular functions, including actin dynamics, proliferation, apoptosis, adhesion, and **migration** resulting from signaling by multiple receptors, including the B cell antigen receptor (BCR). We used conditional gene targeting to generate mice with specific Rac1 deficiency in the B cell lineage. In the absence of both Rac1 and the highly related Rac2, B cell development was almost completely blocked. Both GTPases were required to transduce BCR signals leading to proliferation, survival and up-regulation of BAFF-R, a receptor for BAFF, a key survival molecule required for B cell development and maintenance.
30. Hematopoietic cell regulation by Rac1 and Rac2 guanosine triphosphatases. The Rho guanosine triphosphatases (GTPases) Rac1 and Rac2 are critical signaling regulators in mammalian cells. The deletion of both Rac1 and Rac2 murine alleles leads to a massive egress of hematopoietic stem/progenitor cells (HSC/Ps) into the blood from the marrow, whereas Rac1-/- but not Rac2-/- HSC/Ps fail to engraft in the bone marrow of irradiated recipient mice. In contrast, Rac2, but not Rac1, regulates superoxide production and directed **migration** in neutrophils, and in each cell type, the two GTPases play distinct roles in actin organization, cell survival, and proliferation. Thus, Rac1 and Rac2 regulate unique aspects of hematopoietic development and function.
31. Rac2 stimulates Akt activation affecting BAD/Bcl-XL expression while mediating survival and actin function in primary mast cells. Mast cells generated from Rac2-deficient (-/-) mice demonstrated defective actin-based functions, including adhesion, **migration**, and degranulation. Rac2(-/-) mast cells generated lower numbers and less mast cell colonies in response to growth factors and were deficient in vivo. Rac2(-/-) mast cells demonstrated a significant reduction in growth factor-induced survival, which correlated with the lack of activation of Akt and significant changes in the expression of the Bcl-2 family members BAD and Bcl-XL, in spite of a 3-fold induction of Rac1 protein. These results suggest that Rac2 plays a unique role in multiple cellular functions and describe an essential role for Rac2 in growth factor-dependent survival and expression of BAD/Bcl-XL.
32. Rac2 specificity in macrophage integrin signaling: potential role for Syk kinase. Herein we report that, despite the similarity of Rac2 to Rac1 (92% amino acid identity), macrophages derived from Rac2-/- mice, which continue to express Rac1, display a marked defect in alphavbeta3/alphavbeta5 and alpha4beta1 integrin-directed **migration** measured on vitronectin and fibronectin fragments (FN-H296), respectively. In contrast, mouse embryo fibroblasts derived from the Rac2 knockout mice utilize Rac1 for **migration** via alphavbeta3/alphavbeta5 and alpha4beta1. The genetic reconstitution of bone marrow-derived macrophages (BMM) with Rac2 restores the integrin-dependent **migration** of Rac2-deficient macrophages on vitronectin (VN) and FN-H296. The levels of GTP-Rac2 generated upon specific integrin engagement in wild type macrophages parallels the phenotypic defect observed in Rac2-deficient macrophages; i.e. FN-H296, alpha4beta1 > VN, alphavbeta3/alphavbeta5 > FN-CH271, alpha5beta1 > intact FN. In a COS7 cell system, the expression of Syk kinase alone is sufficient to convert the alpha4beta1 **migration** response to Rac2 dependence. Therefore, we present the first evidence that the alpha4beta1 receptor in blood cells has evolved a Syk-Rac2 signaling axis to transmit signals required for integrin-directed **migration** suggesting that Syk kinase in part encodes myeloid Rac2 specificity in vivo.
33. Rac1 and Rac2 regulate macrophage morphology but are not essential for **migration**. Rac GTPases are believed to contribute to **migration** in leukocytes by transducing signals from cell surface receptors to the actin and microtubule cytoskeletons. Mammals have three closely related Rac isoforms, Rac1, Rac2 and Rac3, and it is widely assumed that cell **migration** requires the activity of these Rac GTPases. We have previously shown that Rac1-null mouse macrophages have altered cell shape and reduced membrane ruffling but normal **migration** speed. Here we investigate the behaviour of macrophages lacking Rac2 (Rac2(-/-)) or Rac1 and Rac2 (Rac1/2(-/-)). Rac2(-/-) macrophages have reduced F-actin levels and lack podosomes, which are integrin-based adhesion sites, and their **migration** speed is similar to or slightly slower than wild-type macrophages, depending on the substrate. Unexpectedly, Rac1/2(-/-) macrophages, which do not express Rac1, Rac2 or Rac3, migrate at a similar speed to wild-type macrophages on a variety of substrates and perform chemotaxis normally, although their morphology and mode of **migration** is altered. However, Rac1(-/-) and Rac1/2(-/-) but not Rac2(-/-) macrophages are impaired in their ability to invade through Matrigel. Together, these data show that Rac1 and Rac2 have distinct roles in regulating cell morphology, **migration** and invasion, but are not essential for macrophage **migration** or chemotaxis.
34. Rac2 GTPase deficiency depletes BCR-ABL+ leukemic stem cells and progenitors in vivo. Chronic myelogenous leukemia (CML) is a clonal myeloproliferative disease (MPD) initiated by p210-BCR-ABL-mediated transformation of hematopoietic stem cells (HSCs). Inhibition of the ABL kinase alone is not sufficient to eradicate leukemic stem cells (LSCs). We have previously shown that the deficiency of Rac2 GTPase signaling, but not Rac1, in p210-BCR-ABL-transduced hematopoietic cells prolonged survival of mice with MPD. Here we demonstrate that absence of Rac2 GTPase prolongs survival of HSC-initiated, inducible Scl/p210-BCR-ABL (Scl/p210) binary transgenic mice, it induces apoptosis, and, unlike in normal HSC and progenitor (HSC/P), impairs LSC and progenitor (LSC/P) proliferation in vivo. As a result, Rac2 deficiency causes functional exhaustion of the LSC pool in vivo. This defect is not due to impaired interaction with the hematopoietic microenvironment as reflected by its unaltered adhesion, **migration**, and homing to recipient organs. In summary, Rac2 deficiency exhausts the LSC pool in vivo through impairment of oncogene-induced proliferation and survival signals.
35. The TRQQKRP motif located near the C-terminus of Rac2 is essential for Rac2 biologic functions and intracellular localization. Rac GTPases regulate a wide variety of cellular processes including actin cytoskeleton organization, gene expression, cell-cycle progression, and apoptosis. Here we report that the TRQQKRP motif of Rac2 located near the C-terminus, a region of sequence disparity among Rac proteins, is essential for complementation of Rac2 function in Rac2-deficient cells. Deletion of this sequence can also intragenically suppress the dominant-negative Rac2(D57N) mutation in a variety of functional assays. In Rac2-deficient cells, expression of TRQQKRP-deleted Rac2 protein is unable to completely rescue **migration** and nicotinamide adenine dinucleotide phosphate oxidase deficiencies previously described in these cells. In fibroblasts, the Rac2(D57N) mutant phenotypes of abnormal proliferation, cell morphology, and membrane ruffling are suppressed by the TRQQKRP motif deletion. In myeloid hematopoietic cells, the deletion of the TRQQKRP motif eliminates a Rac2(D57N)-induced block in in vitro differentiation of neutrophils not previously described with this mutant. Mechanistically, deletion of the TRQQKRP motif results in diminished geranylgeranylation and delocalization of intracellular Rac2 protein. Taken together, these results indicate that the TRQQKRP motif in Rac2 protein is required for efficient prenylation and correct intracellular localization of Rac2 protein and is essential for Rac2 to mediate a variety of its biologic functions. These data suggest that precise localization of Rac2 protein in intracellular compartments and/or with other proteins/lipids is a prerequisite for its diverse functions.
36. CXCL9 inhibits eosinophil responses by a CCR3- and Rac2-dependent mechanism. Recently, inhibitory cytokine pathways for leukocyte chemoattraction and activation have been identified, but there is little insight into the operational mechanisms except for models that rely on simple receptor antagonism. We have previously identified the existence of a murine eosinophil inhibitory pathway mediated by the CXC chemokine ligand 9 (CXCL9, Mig [monokine induced by interferon-gamma]) that impressively blocks eosinophil chemoattraction and function, but the mechanism has remained elusive. We now demonstrate that Mig's inhibitory action extends beyond receptor antagonism alone. Notably, in addition to inhibiting eotaxin-induced filamentous actin (F-actin) formation and chemoattraction, Mig potently blocks platelet activating factor (PAF)- and leukotriene B4 (LTB4)-induced responses. Remarkably, Mig-treated eosinophils display an abnormal F-actin assembly in the absence of agonist stimulation. Additionally, Mig pretreatment inhibits eotaxin-induced activation of the Rho-guanosine triphosphatase (GTPase) Rac, and Rac2-deficient eosinophils demonstrate an impaired trans**migration** and actin polymerization response to eotaxin stimulation. Furthermore, Mig was unable to inhibit eotaxin-induced responses in Rac2-deficient eosinophils. Finally, using CCR3 gene-targeted cells, Mig's inhibitory activity is demonstrated to be mediated by CC chemokine receptor 3 (CCR3). Thus, by altering agonist-induced signaling and abrogating cytoskeletal reorganization by a Rac2-dependent mechanism, Mig markedly inhibits eosinophil responses to diverse stimuli. These results establish evidence that distinct chemokines can use CCR3 to induce opposing signals in eosinophils.
37. Macrophage **migration** arrest due to a winning balance of Rac2/Sp1 repression over β-catenin-induced PLD expression. Monocytes and neutrophils infiltrate into tissues during inflammation and stay for extended periods of time until the initial insult is resolved or sometimes remain even longer in the case of chronic inflammation. The mechanism as to why phagocytes become immobilized after the initial cell **migratio**n event is not understood completely. Here, we show that overexpression or hyperactivation of Rac2 decreases sustained chemotactic responses of macrophages to MCP-1/CCL2. The resulting leukocyte arrest is not caused by a diminished availability of the cytokine receptor CCR2 that remains intact during MCP-1 stimulation. We show a novel mechanism that links the Rac2-dependent arrest of chemotaxis to decreased expression of PLD2 through the transcription regulator Sp1. Prolonged Rac2 activity leads to nuclear overactivation of Sp1, which acts as a repressor for PLD2. Also, another signaling component plays a regulatory role: β-catenin. Although early times of stimulation (≈ 20 min) with MCP-1/CCL2 resulted in activation of β-catenin with a positive effect on PLD2, after ≈ 3 h of stimulation, the levels of β-catenin were reduced and not able to prevent the negative effect of Rac2 on PLD2 activity. This is a novel molecular mechanism underlying immobilization of monocyte/mac**rophage m**igration that is important for the physiological maintenance of leukocytes at the site of inflammation. If this immobilization is prolonged enough, it could lead to chronic inflammation.
38. Expression of RAC2 in endothelial cells is required for the postnatal neovascular response. Herein, we describe an obligate role for the hematopoietic specific GTPase, RAC2 in endothelial integrin signaling and the postnatal neovascularization response in vivo. Using a Rac2 knockout mouse model, we discovered that despite the presence of both RAC1 and RAC2 protein in endothelial cells, RAC2 is obligately required for the postnatal neovascular response and alphavbeta3/ alpha4beta1/alpha5beta1 integrin-directed **migration** on vitronectin, H296 and CH271, fibronectin fragments, respectively. The molecular basis for RAC2 specificity was explored. A genetic analysis of Syk -/+ or Syk-/+;Rac2 -/+ mice revealed that SYK kinase is required for the integrin induced activation of RAC2. The analysis of endothelial cells from Rac2-/+ versus Syk-/+;Rac2-/+ mice provided genetic evidence that SYK-RAC2 signaling axis regulates integrin (alphavbeta3, alpha4beta1 and alpha5beta1) dependent **migration**. Our results provide evidence that a specific region of the nonreceptor protein tyrosine kinase, SYK, the B linker region containing Y342 and Y346 is required for SYK's regulation of RAC2 and integrin dependent **migration**. Moreover, the capacity of mice to vascularize the ischemic hindlimb following femoral artery ligation or matrigel plugs was markedly reduced in mice homozygous deficient for the Rac2 gene. These findings identify a novel signaling axis for the induction and potential modulation of postnatal angiogenesis.
39. The dual effect of Rac2 on phospholipase D2 regulation that explains both the onset and termination of chemotaxis. We document a biphasic effect of Rac2 on the activation and inhibition of PLD2. Cells overexpressing Rac2 and PLD2 simultaneously show a robust initial (<10 min) response toward a chemoattractant that is later (>30 min) greatly diminished over PLD2-only controls. The first phase is due to the presence of a Rac2-PLD2 positive-feedback loop. To explain the mechanism for the Rac2-led PLD2 inhibition (the second phase), we used leukocytes from wild-type (WT) and Rac2(-/-) knockout mice. Rac2(-/-) cells displayed an enhanced PLD2 (but not PLD1) enzymatic activity, confirming the inhibitory role of Rac2. Late inhibitory responses on PLD2 due to Rac2 were reversed in the presence of phosphatidylinositol 4,5-bisphosphate (PIP(2)) both in vitro (purified GST-PH-PLD2, where GST is glutathione S-transferase and PH is pleckstrin homology) and in vivo. Coimmunoprecipitation and immunofluorescence microscopy indicated that PLD2 and Rac2 remain together. The presence of an "arc" of Rac2 at the leading edge of leukocyte pseudopodia and PLD2 physically posterior to this wave of Rac2 was observed in late chemotaxis. We propose Rac-led inhibition of PLD2 function is due to sterical interference of Rac with PLD2's PH binding site to the membrane and deprivation of the PIP(2). This work supports the importance of functional interactions between PLD and Rac in the biological response of cell **migration**.
40. Sbds is required for Rac2-mediated monocyte **migration** and signaling downstream of RANK during osteoclastogenesis. Shwachman-Diamond syndrome (SDS) results from mutations in the SBDS gene, characterized by exocrine pancreatic insufficiency and hematologic and skeletal abnormalities. Neutropenia and neutrophil dysfunction are hallmark features of SDS; however, causes for the bone defects are unknown. Dysfunction of bone-resorbing osteoclasts, formed by the fusion of monocytic progenitors derived from the same granulocytic precursors as neutrophils, could be responsible. We report that Sbds is required for in vitro and in vivo osteoclastogenesis (OCG). Sbds-null murine monocytes formed osteoclasts of reduced number and size because of impaired **migration** and fusion required for OCG. Phenotypically, Sbds-null mice exhibited low-turnover osteoporosis consistent with findings in SDS patients. Western blotting of Rho GTPases that control actin dynamics and **migration** showed a 5-fold decrease in Rac2, whereas Rac1, Cdc42, and RhoA were unchanged or only mildly reduced. Although **migration** was rescued on Rac2 supplementation, OCG was not. This was attributed to impaired signaling downstream of receptor activator of nuclear factor-κB (RANK) and reduced expression of the RANK-ligand-dependent fusion receptor DC-STAMP. We conclude that Sbds is required for OCG by regulating monocyte **migratio**n via Rac2 and osteoclast differentiation signaling downstream of RANK. Impaired osteoclast formation could disrupt bone homeostasis, resulting in skeletal abnormalities seen in SDS patients.
41. Rac2-deficient mice display perturbed T-cell distribution and chemotaxis, but only minor abnormalities in T(H)1 responses. The haematopoietic-specific RhoGTPase, Rac2, has been indirectly implicated in T-lymphocyte development and function, and as a pivotal regulator of T Helper 1 (T(H)1) responses. In other haematopoietic cells it regulates cytoskeletal rearrangement downstream of extracellular signals. Here we demonstrate that Rac2 deficiency results in an abnormal distribution of T lymphocytes in vivo and defects in T-lymphocyte **migration** and filamentous actin generation in response to chemoattractants in vitro. To investigate the requirement for Rac2 in IFN-gamma production and TH1 responses in vivo, Rac2-deficient mice were challenged with Leishmania major and immunized with ovalbumin-expressing cytomegalovirus. Despite a minor skewing towards a T(H)2 phenotype, Rac2-deficient mice displayed no increased susceptibility to L. major infection. Cytotoxic T-lymphocyte responses to cytomegalovirus and ovalbumin were also normal. Although Rac2 is required for normal T-lymphocyte **migration**, its role in the generation of T(H)1 responses to infection in vivo is largely redundant.
42. P-Rex and Vav Rac-GEFs in platelets control leukocyte recruitment to sites of inflammation. The small GTPase Rac is required for neutrophil recruitment during inflammation, but its guanine-nucleotide exchange factor (GEF) activators seem dispensable for this process, which led us to investigate the possibility of cooperation between Rac-GEF families. Thioglycollate-induced neutrophil recruitment into the peritoneum was more severely impaired in P-Rex1(-/-) Vav1(-/-) (P1V1) or P-Rex1(-/-) Vav3(-/-) (P1V3) mice than in P-Rex null or Vav null mice, suggesting cooperation between P-Rex and Vav Rac-GEFs in this process. Neutrophil trans**migration** and airway infiltration were all but lost in P1V1 and P1V3 mice during lipopolysaccharide (LPS)-induced pulmonary inflammation, with altered intercellular adhesion molecule 1-dependent slow neutrophil rolling and strongly reduced L- and E-selectin-dependent adhesion in airway postcapillary venules. Analysis of adhesion molecule expression, neutrophil adhesion, spreading, and **migration** suggested that these defects were only partially neutrophil-intrinsic and were not obviously involving vascular endothelial cells. Instead, P1V1 and P1V3 platelets recapitulated the impairment of LPS-induced intravascular neutrophil adhesion and recruitment, showing P-Rex and Vav expression in platelets to be crucial. Similarly, during ovalbumin-induced allergic inflammation, pulmonary recruitment of P1V1 and P1V3 eosinophils, monocytes, and lymphocytes was compromised in a platelet-dependent manner, and airway inflammation was essentially abolished, resulting in improved airway responsiveness. Therefore, platelet P-Rex and Vav family Rac-GEFs play important proinflammatory roles in leukocyte recruitment.

#### GeneRIFs

1. Using Rac mutants that are defective in their ability to activate NOX2, we show that Rac regulates a redox-mediated feedback loop that mediates directional **migration** of neutrophils.
2. Sbds is required for osteoclastogenesis by regulating monocyte **migration** via Rac2 and osteoclast differentiation signaling downstream of RANK.
3. Rac1-deficient T cells home very inefficiently to lymph nodes and show reduced interstitial **migration** within lymph node parenchyma, and are defective in egress from lymph nodes.
4. RAC2 in vascular endothelial cells is obligately required for the postnatal neovascular response and alphavbeta3/ alpha4beta1/alpha5beta1 integrin-directed **migration** on vitronectin, H296 and CH271, fibronectin fragments, respectively.
5. evidence that the alpha4beta1 receptor in blood cells has evolved a Syk-Rac2 signaling axis to transmit signals required for integrin-directed **migration** suggesting that Syk kinase in part encodes myeloid Rac2 specificity in vivo
6. Rac1 and Rac2 control the formation of dendrites in mature dendritic cells, their polarized short-range **migration** toward T cells, and T cell priming

### [Ee]xtracellular [Mm]atrix

#### PubMed

1. Rac2 controls tumor growth, metastasis and M1-M2 macrophage differentiation in vivo. Although it is well-established that the macrophage M1 to M2 transition plays a role in tumor progression, the molecular basis for this process remains incompletely understood. Herein, we demonstrate that the small GTPase, Rac2 controls macrophage M1 to M2 differentiation and the metastatic phenotype in vivo. Using a genetic approach, combined with syngeneic and orthotopic tumor models we demonstrate that Rac2-/- mice display a marked defect in tumor growth, angiogenesis and metastasis. Microarray, RT-PCR and metabolomic analysis on bone marrow derived macrophages isolated from the Rac2-/- mice identify an important role for Rac2 in M2 macrophage differentiation. Furthermore, we define a novel molecular mechanism by which signals transmitted from the **extracellular matrix** via the α4β1 integrin and MCSF receptor lead to the activation of Rac2 and potentially regulate macrophage M2 differentiation. Collectively, our findings demonstrate a macrophage autonomous process by which the Rac2 GTPase is activated downstream of the α4β1 integrin and the MCSF receptor to control tumor growth, metastasis and macrophage differentiation into the M2 phenotype. Finally, using gene expression and metabolomic data from our Rac2-/- model, and information related to M1-M2 macrophage differentiation curated from the literature we executed a systems biologic analysis of hierarchical protein-protein interaction networks in an effort to develop an iterative interactome map which will predict additional mechanisms by which Rac2 may coordinately control macrophage M1 to M2 differentiation and metastasis.
2. Rac2 controls tumor growth, metastasis and M1-M2 macrophage differentiation in vivo. Although it is well-established that the macrophage M1 to M2 transition plays a role in tumor progression, the molecular basis for this process remains incompletely understood. Herein, we demonstrate that the small GTPase, Rac2 controls macrophage M1 to M2 differentiation and the metastatic phenotype in vivo. Using a genetic approach, combined with syngeneic and orthotopic tumor models we demonstrate that Rac2-/- mice display a marked defect in tumor growth, angiogenesis and metastasis. Microarray, RT-PCR and metabolomic analysis on bone marrow derived macrophages isolated from the Rac2-/- mice identify an important role for Rac2 in M2 macrophage differentiation. Furthermore, we define a novel molecular mechanism by which signals transmitted from the **extracellular matrix** via the α4β1 integrin and MCSF receptor lead to the activation of Rac2 and potentially regulate macrophage M2 differentiation. Collectively, our findings demonstrate a macrophage autonomous process by which the Rac2 GTPase is activated downstream of the α4β1 integrin and the MCSF receptor to control tumor growth, metastasis and macrophage differentiation into the M2 phenotype. Finally, using gene expression and metabolomic data from our Rac2-/- model, and information related to M1-M2 macrophage differentiation curated from the literature we executed a systems biologic analysis of hierarchical protein-protein interaction networks in an effort to develop an iterative interactome map which will predict additional mechanisms by which Rac2 may coordinately control macrophage M1 to M2 differentiation and metastasis.

### [Ii]nvasive

#### PubMed

1. Assessment of wound-site redox environment and the significance of Rac2 in cutaneous healing. We have previously reported that H(2)O(2) is actively generated by cells at the wound site and that H(2)O(2)-driven redox signaling supports wound angiogenesis and healing. In this study, we have standardized a novel and effective electron paramagnetic resonance spectroscopy-based approach to assess the redox environment of the dermal wound site in vivo. Rac2 regulates inducible NADPH oxidase activation and other functional responses in neutrophils. Using Rac2-deficient mice we sought to investigate the significance of Rac2 in the wound-site redox environment and healing responses. Non**invasive** measurements of metabolism of topically applied nitroxide (15)N-perdeuterated tempone in murine excisional dermal wounds demonstrated that the wound site is rich in oxidants, the levels of which peak 2 days postwounding in the inflammatory phase. Rac2-deficient mice had threefold lower production of superoxide compared to controls with similar wounds. In these mice, a lower wound-site superoxide level was associated with compromised wound closure. Immunostaining of wound edges harvested during the inflammatory phase showed that the numbers of phagocytic cells recruited to the wound site in Rac2-deficient and control mice were similar, but the amount of lipid peroxidation was significantly lower in Rac2-deficient mice, indicating compromised NADPH oxidase activity. Taken together, the findings of this study support that the wound site is rich in oxidants. Rac2 significantly contributes to oxidant production at the wound site and supports the healing process.
2. RAC2, AEP, and ICAM1 expression are associated with CNS disease in a mouse model of pre-B childhood acute lymphoblastic leukemia. We developed a murine model of CNS disease to obtain a better understanding of the pathogenesis of CNS involvement in pre-B-cell acute lymphoblastic leukemia (ALL). Semiquantitative proteomic discovery-based approaches identified unique expression of asparaginyl endopeptidase (AEP), intercellular adhesion molecule 1 (ICAM1), and ras-related C3 botulinum toxin substrate 2 (RAC2), among others, in an **invasive** pre-B-cell line that produced CNS leukemia in NOD-SCID mice. Targeting RAC2 significantly inhibited in vitro invasion and delayed disease onset in mice. Induced expression of RAC2 in cell lines with low/absent expression of AEP and ICAM1 did not result in an **invasive** phenotype or murine CNS disease. Flow cytometric analysis identified an enriched population of blast cells expressing ICAM1/lymphocyte function associated antigen-1 (LFA-1)/CD70 in the CD10(+)/CD19(+) fraction of bone marrow aspirates obtained from relapsed compared with normal controls and those with primary disease. CD10(+)/CD19(+) fractions obtained from relapsed patients also express RAC2 and give rise to CNS disease in mice. Our data suggest that combinations of processes are involved in the pathogenesis of CNS disease in pre-B-cell ALL, support a model in which CNS disease occurs as a result of external invasion, and suggest that targeting the processes of adhesion and invasion unique to pre-B cells may prevent recurrences within the CNS.

#### GeneRIFs

1. We identified unique expression of asparaginyl endopeptidase (AEP), intercellular adhesion molecule 1 (ICAM1), and ras-related C3 botulinum toxin substrate 2 (RAC2), among others, in an **invasive** pre-B-cell line that produced leukemia in NOD-SCID mice

### [Mm]etastasis

#### PubMed

1. Rac2 controls tumor growth, **metastasis** and M1-M2 macrophage differentiation in vivo. Although it is well-established that the macrophage M1 to M2 transition plays a role in tumor progression, the molecular basis for this process remains incompletely understood. Herein, we demonstrate that the small GTPase, Rac2 controls macrophage M1 to M2 differentiation and the metastatic phenotype in vivo. Using a genetic approach, combined with syngeneic and orthotopic tumor models we demonstrate that Rac2-/- mice display a marked defect in tumor growth, angiogenesis and **metastasis**. Microarray, RT-PCR and metabolomic analysis on bone marrow derived macrophages isolated from the Rac2-/- mice identify an important role for Rac2 in M2 macrophage differentiation. Furthermore, we define a novel molecular mechanism by which signals transmitted from the extracellular matrix via the α4β1 integrin and MCSF receptor lead to the activation of Rac2 and potentially regulate macrophage M2 differentiation. Collectively, our findings demonstrate a macrophage autonomous process by which the Rac2 GTPase is activated downstream of the α4β1 integrin and the MCSF receptor to control tumor grow**th, metast**asis and macrophage differentiation into the M2 phenotype. Finally, using gene expression and metabolomic data from our Rac2-/- model, and information related to M1-M2 macrophage differentiation curated from the literature we executed a systems biologic analysis of hierarchical protein-protein interaction networks in an effort to develop an iterative interactome map which will predict additional mechanisms by which Rac2 may coordinately control macrophage M1 to M2 differentiation **and metast**asis.
2. Rac2 controls tumor growth, **metastasis** and M1-M2 macrophage differentiation in vivo. Although it is well-established that the macrophage M1 to M2 transition plays a role in tumor progression, the molecular basis for this process remains incompletely understood. Herein, we demonstrate that the small GTPase, Rac2 controls macrophage M1 to M2 differentiation and the metastatic phenotype in vivo. Using a genetic approach, combined with syngeneic and orthotopic tumor models we demonstrate that Rac2-/- mice display a marked defect in tumor growth, angiogenesis and **metastasis**. Microarray, RT-PCR and metabolomic analysis on bone marrow derived macrophages isolated from the Rac2-/- mice identify an important role for Rac2 in M2 macrophage differentiation. Furthermore, we define a novel molecular mechanism by which signals transmitted from the extracellular matrix via the α4β1 integrin and MCSF receptor lead to the activation of Rac2 and potentially regulate macrophage M2 differentiation. Collectively, our findings demonstrate a macrophage autonomous process by which the Rac2 GTPase is activated downstream of the α4β1 integrin and the MCSF receptor to control tumor grow**th, metast**asis and macrophage differentiation into the M2 phenotype. Finally, using gene expression and metabolomic data from our Rac2-/- model, and information related to M1-M2 macrophage differentiation curated from the literature we executed a systems biologic analysis of hierarchical protein-protein interaction networks in an effort to develop an iterative interactome map which will predict additional mechanisms by which Rac2 may coordinately control macrophage M1 to M2 differentiation **and metast**asis.

#### GeneRIFs

1. Rac2 GTPase is activated downstream of alpha4beta1 integrin and the MCSF receptor to control tumor growth, **metastasis** and macrophage differentiation.

---

## Rhoc

- NCBI Gene
- GeneRIF
- Pubmed

| Search Term | Rank | Fields |
| --- | --- | --- |
| [Mm]igration | 6 | PubMed(4) |
| [Mm]etastasis | 12 | PubMed(5); GeneRIFs(3) |
| [Cc]ell [Ii]nvasion | 13 | PubMed(1) |

  
hide/show details for Rhoc

### [Mm]igration

#### PubMed

1. Distinct roles of Rac1/Cdc42 and Rho/Rock for axon outgrowth and nucleokinesis of precerebellar neurons toward netrin 1. During embryonic development, tangentially migrating precerebellar neurons emit a leading process and then translocate their nuclei inside it (nucleokinesis). Netrin 1 (also known as netrin-1) acts as a chemoattractant factor for neurophilic **migration** of precerebellar neurons (PCN) both in vivo and in vitro. In the present work, we analyzed Rho GTPases that could direct axon outgrowth and/or nuclear **migration**. We show that the expression pattern of Rho GTPases in developing PCN is consistent with their involvement in the **migration** of PCN from the rhombic lips. We report that pharmacological inhibition of Rho enhances axon outgrowth of PCN and prevents nuclei **migration** toward a netrin 1 source, whereas inhibition of Rac and Cdc42 sub-families impair neurite outgrowth of PCN without affecting **migration**. We show, through pharmacological inhibition, that Rho signaling directs neurophilic **migration** through Rock activation. Altogether, our results indicate that Rho/Rock acts on signaling pathways favoring nuclear translocation during tangential **migration** of PCN. Thus, axon extension and nuclear **migration** of PCN in response to netrin 1 are not strictly dependent processes because: (1) distinct small GTPases are involved; (2) axon extension can occur when **migration** is blocked; and (3) **migration** can occur when axon outgrowth is impaired.
2. Inhibition of Ovarian Epithelial Carcinoma Tumorigenesis and Progression by microRNA 106b Mediated through the RhoC Pathway. Epithelial ovarian cancer (EOC) is the most lethal of the gynecological malignancies. Exploring the molecular mechanisms and major factors of invasion and metastasis could have great significance for the treatment and prognosis of EOC. Studies have demonstrated that microRNA 106b (miR-106b) may be a promising therapeutic target for inhibiting breast cancer bone metastasis, but the role of miR-106b in EOC is largely unknown. In this work, miRNA-106b expression was quantified in various ovarian tissues and tumors. Ovarian carcinoma cell lines were transfected with miR-106b, after which, cell phenotype and expression of relevant molecules was assayed. Dual-luciferase reporter assays and xenograft mouse models were also used to investigate miR-106b and its target gene. MiR-106b mRNA expression was found to be significantly higher in normal ovarian tissues and benign tumors than in ovarian carcinomas and borderline tumors (p < 0.01), and was negatively associated with differentiation (Well vs. Por & Mod) and the International Federation of Gynecology and Obstetrics (FIGO) staging (stage I/II vs. stage III/IV) in ovarian carcinoma (p < 0.05). MiR-106b transfection reduced cell proliferation; promoted G1 or S arrest and apoptosis (p < 0.05); suppressed cell **migration** and invasion (p < 0.05); reduced Ras homolog gene family member C (RhoC), P70 ribosomal S6 kinase (P70S6K), Bcl-xL, Matrix metallopeptidase 2 (MMP2), MMP9 mRNA and protein expression; and induced p53 expression (p < 0.05). Dual-luciferase reporter assays indicated that miR-106b directly targets RhoC by binding its 3'UTR. MiR-106b transfection also suppressed tumor development and RhoC expression in vivo in xenograft mouse models. This is the first demonstration that miR-106b may inhibit tumorigenesis and progression of EOC by targeting RhoC. The involvement of miR-106b-mediated RhoC downregulation in EOC aggression may give extended insights into molecular mechanisms underlying cancer aggression. Approaches aimed at overexpressing miR-106b may serve as promising therapeutic strategies for treating EOC patients.
3. A definitive role of RhoC in metastasis of orthotopic lung cancer in mice. Lung cancer is a major cause of cancer death, and its incidence is increasing in the world. Conventional therapies remain less effective for metastases of lung cancer, leading to poor prognosis of this disorder. The present study investigates pathological roles of RhoC in metastasis of lung cancer using a clinically relevant mouse model of lung cancer.RhoA, RhoC, dominant-negative Rho (dnRho) or green fluorescent protein gene was retrovirally transduced to murine lung cancer cells. For in vivo study, these transduced cells were intrapulmonary inoculated in syngeneic mice, and subsequently, growth and metastasis were analyzed. **Migration** and invasion activities were further investigated by in vitro chemotaxic chamber assays. Expression levels and activities of certain matrix metalloproteinases (MMPs) were explored by reverse transcription-PCR and gelatin zymography.Metastasis of lung cancer in the animal model, as well as in vitro **migration** and invasion, were significantly enhanced or inhibited by overexpression of RhoC or dnRho, respectively, without affecting the growth of primary tumors. Expression levels of certain MMPs and the activity of MMP-2 were significantly enhanced or suppressed by overexpression of RhoC or dnRho, respectively.RhoC plays a crucial role in metastasis of lung cancer. RhoC does not affect tumor growth but enhances the metastatic nature of lung cancer by not only stimulating cell motility but also up-regulating certain MMPs. Attenuation of RhoC activity may be a potential target in the development of a novel strategy for treating metastasis of lung cancer.
4. RhoC is dispensable for embryogenesis and tumor initiation but essential for metastasis. The Rho proteins are Ras-related guanosine triphosphatases (GTPases) that function in cytoskeletal reorganization, cell **migration**, and stress fiber and focal adhesion formation. Overexpression of RhoC enhances the ability of melanoma cells to exit the blood and colonize the lungs. However, in vivo confirmation of RhoC's role in metastasis has awaited a RhoC-deficient mouse model. Here we report the generation of RhoC-deficient mice and show that RhoC is dispensable for embryonic and post-natal development. We demonstrate that loss of RhoC does not affect tumor development but decreases tumor cell motility and metastatic cell survival leading to a drastic inhibition of metastasis.

### [Mm]etastasis

#### PubMed

1. A p27(kip1)-binding protein, p27RF-Rho, promotes cancer **metastasis** via activation of RhoA and RhoC. Rho family proteins regulate multiple cellular functions including motility and invasion through regulation of the actin cytoskeleton and gene expression. Activation of Rho proteins is controlled precisely by multiple regulators in a spatiotemporal manner. RhoA and/or RhoC are key players that regulate the metastatic activity of malignant tumor cells, and it is therefore of particular interest to understand how activation of these Rho proteins is controlled. We recently identified an upstream regulator of RhoA activation, p27RF-Rho (p27(kip1) releasing factor from RhoA) that acts by freeing RhoA from inhibition by p27(kip1). p27(kip1) is a cell cycle regulator when it is localized to the nucleus, but it binds RhoA and inhibits activation of the latter when it is localized to the cytoplasm. Here, we show that a metastatic variant of mouse melanoma B16 cells (F10) exhibits greater expression of p27RF-Rho, RhoA, and RhoC than the nonmetastatic parental cells (F0). Injection of F10 cells into mouse tail vein resulted in the formation of metastatic lung colonies, whereas prior knockdown of expression of either one of the three proteins using specific shRNA sequences decreased **metastasis** markedly. p27RF-Rho regulated the activation of RhoA and RhoC and thereby modulated cellular adhesion and motility, in addition to pericellular proteolysis. The Rho activities enhanced by p27RF-Rho had a marked effect upon efficiency of lodging of F10 cells in the lung, which represents an early step of **metastasis**. p27RF-Rho also regulated **metastasis** of human melanoma and fibrosarcoma cells. Thus, p27RF-Rho is a key upstream regulator of RhoA and RhoC that controls spreading of tumor cells.
2. Inhibition of Ovarian Epithelial Carcinoma Tumorigenesis and Progression by microRNA 106b Mediated through the RhoC Pathway. Epithelial ovarian cancer (EOC) is the most lethal of the gynecological malignancies. Exploring the molecular mechanisms and major factors of invasion and **metastasis** could have great significance for the treatment and prognosis of EOC. Studies have demonstrated that microRNA 106b (miR-106b) may be a promising therapeutic target for inhibiting breast cancer bone **metastasis**, but the role of miR-106b in EOC is largely unknown. In this work, miRNA-106b expression was quantified in various ovarian tissues and tumors. Ovarian carcinoma cell lines were transfected with miR-106b, after which, cell phenotype and expression of relevant molecules was assayed. Dual-luciferase reporter assays and xenograft mouse models were also used to investigate miR-106b and its target gene. MiR-106b mRNA expression was found to be significantly higher in normal ovarian tissues and benign tumors than in ovarian carcinomas and borderline tumors (p < 0.01), and was negatively associated with differentiation (Well vs. Por & Mod) and the International Federation of Gynecology and Obstetrics (FIGO) staging (stage I/II vs. stage III/IV) in ovarian carcinoma (p < 0.05). MiR-106b transfection reduced cell proliferation; promoted G1 or S arrest and apoptosis (p < 0.05); suppressed cell migration and invasion (p < 0.05); reduced Ras homolog gene family member C (RhoC), P70 ribosomal S6 kinase (P70S6K), Bcl-xL, Matrix metallopeptidase 2 (MMP2), MMP9 mRNA and protein expression; and induced p53 expression (p < 0.05). Dual-luciferase reporter assays indicated that miR-106b directly targets RhoC by binding its 3'UTR. MiR-106b transfection also suppressed tumor development and RhoC expression in vivo in xenograft mouse models. This is the first demonstration that miR-106b may inhibit tumorigenesis and progression of EOC by targeting RhoC. The involvement of miR-106b-mediated RhoC downregulation in EOC aggression may give extended insights into molecular mechanisms underlying cancer aggression. Approaches aimed at overexpressing miR-106b may serve as promising therapeutic strategies for treating EOC patients.
3. A definitive role of RhoC in **metastasis** of orthotopic lung cancer in mice. Lung cancer is a major cause of cancer death, and its incidence is increasing in the world. Conventional therapies remain less effective for metastases of lung cancer, leading to poor prognosis of this disorder. The present study investigates pathological roles of RhoC in **metastasis** of lung cancer using a clinically relevant mouse model of lung cancer.RhoA, RhoC, dominant-negative Rho (dnRho) or green fluorescent protein gene was retrovirally transduced to murine lung cancer cells. For in vivo study, these transduced cells were intrapulmonary inoculated in syngeneic mice, and subsequently, growth and **metastasis** were analyzed. Migration and invasion activities were further investigated by in vitro chemotaxic chamber assays. Expression levels and activities of certain matrix metalloproteinases (MMPs) were explored by reverse transcription-PCR and gelatin zymography.**Metastasis** of lung cancer in the animal model, as well as in vitro migration and invasion, were significantly enhanced or inhibited by overexpression of RhoC or dnRho, respectively, without affecting the growth of primary tumors. Expression levels of certain MMPs and the activity of MMP-2 were significantly enhanced or suppressed by overexpression of RhoC or dnRho, respectively.RhoC plays a crucial role in **metastasis** of lung cancer. RhoC does not affect tumor growth but enhances the metastatic nature of lung cancer by not only stimulating cell motility but also up-regulating certain MMPs. Attenuation of RhoC activity may be a potential target in the development of a novel strategy for treating **metastasis** of lung cancer.
4. The collection of NFATc1-dependent transcripts in the osteoclast includes numerous genes non-essential to physiologic bone resorption. Osteoclasts are specialized secretory cells of the myeloid lineage important for normal skeletal homeostasis as well as pathologic conditions of bone including osteoporosis, inflammatory arthritis and cancer **metastasis**. Differentiation of these multinucleated giant cells from precursors is controlled by the cytokine RANKL, which through its receptor RANK initiates a signaling cascade culminating in the activation of transcriptional regulators which induce the expression of the bone degradation machinery. The transcription factor nuclear factor of activated T-cells c1 (NFATc1) is the master regulator of this process and in its absence osteoclast differentiation is aborted both in vitro and in vivo. Differential mRNA expression analysis by microarray is used to identify genes of potential physiologic relevance across nearly all biologic systems. We compared the gene expression profile of murine wild-type and NFATc1-deficient osteoclast precursors stimulated with RANKL and identified that the majority of the known genes important for osteoclastic bone resorption require NFATc1 for induction. Here, five novel RANKL-induced, NFATc1-dependent transcripts in the osteoclast are described: Nhedc2, Rhoc, Serpind1, Adcy3 and Rab38. Despite reasonable hypotheses for the importance of these molecules in the bone resorption pathway and their dramatic induction during differentiation, the analysis of mice with mutations in these genes failed to reveal a function in osteoclast biology. Compared to littermate controls, none of these mutants demonstrated a skeletal phenotype in vivo or alterations in osteoclast differentiation or function in vitro. These data highlight the need for rigorous validation studies to complement expression profiling results before functional importance can be assigned to highly regulated genes in any biologic process.
5. RhoC is dispensable for embryogenesis and tumor initiation but essential for **metastasis**. The Rho proteins are Ras-related guanosine triphosphatases (GTPases) that function in cytoskeletal reorganization, cell migration, and stress fiber and focal adhesion formation. Overexpression of RhoC enhances the ability of melanoma cells to exit the blood and colonize the lungs. However, in vivo confirmation of RhoC's role in **metastasis** has awaited a RhoC-deficient mouse model. Here we report the generation of RhoC-deficient mice and show that RhoC is dispensable for embryonic and post-natal development. We demonstrate that loss of RhoC does not affect tumor development but decreases tumor cell motility and metastatic cell survival leading to a drastic inhibition of **metastasis**.

#### GeneRIFs

1. A p27(kip1)-binding protein, p27RF-Rho, promotes cancer **metastasis** via activation of RhoA and RhoC.
2. RhoC has a role in **metastasis** of lung cancer in mice
3. loss of RhoC does not affect tumor development but decreases tumor cell motility and metastatic cell survival leading to a drastic inhibition of **metastasis**

### [Cc]ell [Ii]nvasion

#### PubMed

1. RhoC promotes human melanoma invasion in a PI3K/Akt-dependent pathway. Overexpression of the small GTPase, RhoC, in various human cancers has been correlated with high metastatic ability and poor prognosis. Rho-kinase (ROCK) is an important effector of Rho GTPases. The oncogenic serine/threonine kinase Akt (also known as PKB) is a downstream effector of phosphatidylinositol-3 kinase (PI3K). Akt activation contributes to the neoplastic phenotype by promoting cell cycle progression, increasing antiapoptotic functions, and enhancing tumor **cell invasion**. Rho signaling via ROCK has been previously shown either to activate or to downregulate PI3K/Akt. Using a human radial growth phase melanoma cell line, WM35, we have established stable transfectants that overexpress RhoC (called WM35RhoC). We found that overexpression of RhoC increased phosphorylated-Akt (Ser473/474/472, pAkt) expression and promoted **cell invasion**. Inhibition of RhoC with C3 transferase downregulated pAkt expression and decreased **cell invasion** in these cells. In addition, inhibition of PI3K, Akt, or ROCK partially decreased invasion. Further, inhibition of PI3K but not ROCK decreased the pAkt level. These results suggest that RhoC promotes invasion in part via activation of a PI3K/Akt pathway, in a manner independent of ROCK signaling. We propose that RhoC promotes melanoma progression via separate mechanisms that regulate the PI3K/Akt pathway and the ROCK signaling pathway.

---

## Rhoa

- NCBI Gene
- GeneRIF
- Pubmed

| Search Term | Rank | Fields |
| --- | --- | --- |
| [Ff]ilopodia | 1 | PubMed(13); GeneRIFs(1) |
| [Ii]nvadopodia | 3 | PubMed(1) |
| [Ii]nvadopodium | 4 | PubMed(1) |
| [Pp]odosome(s)? | 5 | Process(1); PubMed(2) |
| [Mm]igration | 6 | Process(6); PubMed(116); GeneRIFs(25) |
| ECM | 7 | PubMed(13) |
| [Ee]xtracellular [Mm]atrix | 8 | PubMed(29); GeneRIFs(3) |
| [Ii]nvasive | 10 | PubMed(10); GeneRIFs(1) |
| [Mm]etastasis | 12 | PubMed(12); GeneRIFs(1); Summary(1) |
| [Cc]ell [Ii]nvasion | 13 | PubMed(6); GeneRIFs(1) |

  
hide/show details for Rhoa

### [Ff]ilopodia

#### PubMed

1. Daphnetin inhibits invasion and migration of LM8 murine osteosarcoma cells by decreasing RhoA and Cdc42 expression. Daphnetin, 7,8-dihydroxycoumarin, present in main constituents of Daphne odora var. marginatai, has multiple pharmacological activities including anti-proliferative effects in cancer cells. In this study, using a Transwell system, we showed that daphnetin inhibited invasion and migration of highly metastatic murine osteosarcoma LM8 cells in a dose-dependent manner. Following treatment by daphnetin, cells that penetrated the Transwell membrane were rounder than non-treated cells. Immunofluorescence analysis revealed that daphnetin decreased the numbers of intracellular stress fibers and **filopodia**. Moreover, daphnetin treatment dramatically decreased the expression levels of RhoA and Cdc42. In summary, the dihydroxycoumarin derivative daphnetin inhibits the invasion and migration of LM8 cells, and therefore represents a promising agent for use against metastatic cancer.
2. Cadherin-11 coordinates cellular migration and extracellular matrix remodeling during aortic valve maturation. Proper remodeling of the endocardial cushions into thin fibrous valves is essential for gestational progression and long-term function. This process involves dynamic interactions between resident cells and their local environment, much of which is not understood. In this study, we show that deficiency of the cell-cell adhesion protein cadherin-11 (Cad-11) results in significant embryonic and perinatal lethality primarily due to valve related cardiac dysfunction. While endocardial to mesenchymal transformation is not abrogated, mesenchymal cells do not homogeneously cellularize the cushions. These cushions remain thickened with disorganized ECM, resulting in pronounced aortic valve insufficiency. Mice that survive to adulthood maintain thickened and stenotic semilunar valves, but interestingly do not develop calcification. Cad-11 (-/-) aortic valve leaflets contained reduced Sox9 activity, β1 integrin expression, and RhoA-GTP activity, suggesting that remodeling defects are due to improper migration and/or cellular contraction. Cad-11 deletion or siRNA knockdown reduced migration, eliminated collective migration, and impaired 3D matrix compaction by aortic valve interstitial cells (VIC). Cad-11 depleted cells in culture contained few **filopodi**a, stress fibers, or contact inhibited locomotion. Transfection of Cad-11 depleted cells with constitutively active RhoA restored cell phenotypes. Together, these results identify cadherin-11 mediated adhesive signaling for proper remodeling of the embryonic semilunar valves.
3. The cytotoxic necrotizing factor of Yersinia pseudotuberculosis (CNFY) enhances inflammation and Yop delivery during infection by activation of Rho GTPases. Some isolates of Yersinia pseudotuberculosis produce the cytotoxic necrotizing factor (CNFY), but the functional consequences of this toxin for host-pathogen interactions during the infection are unknown. In the present study we show that CNFY has a strong influence on virulence. We demonstrate that the CNFY toxin is thermo-regulated and highly expressed in all colonized lymphatic tissues and organs of orally infected mice. Most strikingly, we found that a cnfY knock-out variant of a naturally toxin-expressing Y. pseudotuberculosis isolate is strongly impaired in its ability to disseminate into the mesenteric lymph nodes, liver and spleen, and has fully lost its lethality. The CNFY toxin contributes significantly to the induction of acute inflammatory responses and to the formation of necrotic areas in infected tissues. The analysis of the host immune response demonstrated that presence of CNFY leads to a strong reduction of professional phagocytes and natural killer cells in particular in the spleen, whereas loss of the toxin allows efficient tissue infiltration of these immune cells and rapid killing of the pathogen. Addition of purified CNFY triggers formation of actin-rich membrane ruffles and **filopodia**, which correlates with the activation of the Rho GTPases, RhoA, Rac1 and Cdc42. The analysis of type III effector delivery into epithelial and immune cells in vitro and during the course of the infection further demonstrated that CNFY enhances the Yop translocation process and supports a role for the toxin in the suppression of the antibacterial host response. In summary, we highlight the importance of CNFY for pathogenicity by showing that this toxin modulates inflammatory responses, protects the bacteria from attacks of innate immune effectors and enhances the severity of a Yersinia infection.
4. Imaging **filopodia** dynamics in the mouse blastocyst. During mammalian development, the first cell lineage diversification event occurs in the blastocyst, when the trophectoderm (TE) and the inner cell mass (ICM) become established. Part of the TE (polar) remains in contact with the ICM and differs from the mural TE (mTE) which is separated from the ICM by a cavity known as the blastocoele. The presence of **filopodia** connecting ICM cells with the distant mural TE cells through the blastocoelic fluid was investigated in this work. We describe two types of actin-based cell projections found in freshly dissected and in vitro cultured expanding blastocysts: abundant short **filopodia** projecting into the blastocoelic cavity that present a continuous undulating behavior; and long, thin traversing **filopodia** connecting the mural TE with the ICM. Videomicroscopy analyses revealed the presence of vesicle-like structures moving along traversing **filopodia** and dynamic cytoskeletal rearrangements. These observations, together with immunolocalization of the FGFR2 and the ErbB3 receptors to these cell extensions, suggest that they display signal transduction activity. We propose that traversing **filopodia** are employed by mitotic mTE cells to receive the required signals for cell division after they become distant to the ICM.
5. Vangl2 acts via RhoA signaling to regulate polarized cell movements during development of the proximal outflow tract. Loop-tail (Lp) mice develop double outlet right ventricle and aortic arch defects, and the defects are caused by mutations in the Vangl2 gene. Vangl2 mRNA is found in the outflow tract myocardium, including the myocardializing cells that migrate into the outflow tract cushions. Analysis of muscularization of the outflow tract septum showed that this process is compromised in Lp/Lp fetuses. Vangl2 is a component of the noncanonical Wnt, planar cell polarity (PCP) pathway that signals via RhoA. We therefore looked for evidence of polarization in myocardializing cells. In wild-type fetuses, myocardializing cells extend lamellipodia and **filopodia** into the cushion tissue and reorganize their actin cytoskeleton from a cortical form to stress fibers; behaviors that are characteristic of polarized cells. In contrast, Lp/Lp littermates do not extend lamellipodia or **filopodia** into the cushion tissue, and their actin remains in a cortical form, suggesting that polarized cell migration of myocardializing cells is inhibited in Lp/Lp. Several other components of the PCP pathway are also localized in the outflow tract myocardium. In wild-type fetuses, the myocardializing cells coexpress RhoA and one of its downstream mediators, ROCK1. RhoA expression is disrupted in Lp/Lp, and is lost from the myocardial-cushion tissue interface, including the presumptive myocardializing cells. These data suggest that Vangl2 is required for the polarization and movement of myocardializing cells into the outflow tract cushions, and that RhoA and ROCK1 are downstream mediators of the PCP signaling pathway in the developing outflow tract.
6. Functional analysis of ARHGAP6, a novel GTPase-activating protein for RhoA. Microphthalmia with linear skin defects (MLS) is an X-linked dominant, male-lethal syndrome characterized by microphthalmia, aplastic skin and agenesis of the corpus callosum, and is caused by the deletion of a 500 kb critical region in Xp22.3. Our laboratory isolated a novel rho GTPase-activating protein (rhoGAP) gene named ARHGAP6 from the MLS region. ARHGAP6 contains 14 exons encoding a 974 amino acid protein with three putative SH3-binding domains. Because exons 2-14 are deleted in all MLS patients, we hypothesized that ARHGAP6 may be responsible for some of the phenotypic features of MLS. We pursued two approaches to study the function of ARHGAP6 and its role in the pathogenesis of MLS: gene targeting of the rhoGAP domain in mouse embryonic stem cells and in vitro expression studies. Surprisingly, loss of the rhoGAP function of Arhgap6 does not cause any detectable phenotypic or behavioral abnormalities in the mutant mice. Transfected mammalian cells expressing ARHGAP6 lose their actin stress fibers, retract from the growth surface and extend thin, branching processes resembling **filopodia**. The ARHGAP6 protein co-localizes with actin filaments through an N-terminal domain and recruits F-actin into the growing processes. Mutation of a conserved arginine residue in the rhoGAP domain prevents the loss of stress fibers but has little effect on process outgrowth. These results suggest that ARHGAP6 has two independent functions: one as a GAP with specificity for RhoA and the other as a cytoskeletal protein that promotes actin remodeling.
7. Daphnetin inhibits invasion and migration of LM8 murine osteosarcoma cells by decreasing RhoA and Cdc42 expression. Daphnetin, 7,8-dihydroxycoumarin, present in main constituents of Daphne odora var. marginatai, has multiple pharmacological activities including anti-proliferative effects in cancer cells. In this study, using a Transwell system, we showed that daphnetin inhibited invasion and migration of highly metastatic murine osteosarcoma LM8 cells in a dose-dependent manner. Following treatment by daphnetin, cells that penetrated the Transwell membrane were rounder than non-treated cells. Immunofluorescence analysis revealed that daphnetin decreased the numbers of intracellular stress fibers and **filopodia**. Moreover, daphnetin treatment dramatically decreased the expression levels of RhoA and Cdc42. In summary, the dihydroxycoumarin derivative daphnetin inhibits the invasion and migration of LM8 cells, and therefore represents a promising agent for use against metastatic cancer.
8. The cytotoxic necrotizing factor of Yersinia pseudotuberculosis (CNFY) enhances inflammation and Yop delivery during infection by activation of Rho GTPases. Some isolates of Yersinia pseudotuberculosis produce the cytotoxic necrotizing factor (CNFY), but the functional consequences of this toxin for host-pathogen interactions during the infection are unknown. In the present study we show that CNFY has a strong influence on virulence. We demonstrate that the CNFY toxin is thermo-regulated and highly expressed in all colonized lymphatic tissues and organs of orally infected mice. Most strikingly, we found that a cnfY knock-out variant of a naturally toxin-expressing Y. pseudotuberculosis isolate is strongly impaired in its ability to disseminate into the mesenteric lymph nodes, liver and spleen, and has fully lost its lethality. The CNFY toxin contributes significantly to the induction of acute inflammatory responses and to the formation of necrotic areas in infected tissues. The analysis of the host immune response demonstrated that presence of CNFY leads to a strong reduction of professional phagocytes and natural killer cells in particular in the spleen, whereas loss of the toxin allows efficient tissue infiltration of these immune cells and rapid killing of the pathogen. Addition of purified CNFY triggers formation of actin-rich membrane ruffles and **filopodia**, which correlates with the activation of the Rho GTPases, RhoA, Rac1 and Cdc42. The analysis of type III effector delivery into epithelial and immune cells in vitro and during the course of the infection further demonstrated that CNFY enhances the Yop translocation process and supports a role for the toxin in the suppression of the antibacterial host response. In summary, we highlight the importance of CNFY for pathogenicity by showing that this toxin modulates inflammatory responses, protects the bacteria from attacks of innate immune effectors and enhances the severity of a Yersinia infection.
9. CellGeo: a computational platform for the analysis of shape changes in cells with complex geometries. Cell biologists increasingly rely on computer-aided image analysis, allowing them to collect precise, unbiased quantitative results. However, despite great progress in image processing and computer vision, current computational approaches fail to address many key aspects of cell behavior, including the cell protrusions that guide cell migration and drive morphogenesis. We developed the open source MATLAB application CellGeo, a user-friendly computational platform to allow simultaneous, automated tracking and analysis of dynamic changes in cell shape, including protrusions ranging from **filopodia** to lamellipodia. Our method maps an arbitrary cell shape onto a tree graph that, unlike traditional skeletonization algorithms, preserves complex boundary features. CellGeo allows rigorous but flexible definition and accurate automated detection and tracking of geometric features of interest. We demonstrate CellGeo's utility by deriving new insights into (a) the roles of Diaphanous, Enabled, and Capping protein in regulating **filopodia** and lamellipodia dynamics in Drosophila melanogaster cells and (b) the dynamic properties of growth cones in catecholaminergic a-differentiated neuroblastoma cells.
10. A novel Rho-dependent pathway that drives interaction of fascin-1 with p-Lin-11/Isl-1/Mec-3 kinase (LIMK) 1/2 to promote fascin-1/actin binding and **filopodia** stability. Fascin-1 is an actin crosslinking protein that is important for the assembly of cell protrusions in neurons, skeletal and smooth muscle, fibroblasts, and dendritic cells. Although absent from most normal adult epithelia, fascin-1 is upregulated in many human carcinomas, and is associated with poor prognosis because of its promotion of carcinoma cell migration, invasion, and metastasis. Rac and Cdc42 small guanine triphosphatases have been identified as upstream regulators of the association of fascin-1 with actin, but the possible role of Rho has remained obscure. Additionally, experiments have been hampered by the inability to measure the fascin-1/actin interaction directly in intact cells. We investigated the hypothesis that fascin-1 is a functional target of Rho in normal and carcinoma cells, using experimental approaches that included a novel fluorescence resonance energy transfer (FRET)/fluorescence lifetime imaging (FLIM) method to measure the interaction of fascin-1 with actin.Rho activity modulates the interaction of fascin-1 with actin, as detected by a novel FRET method, in skeletal myoblasts and human colon carcinoma cells. Mechanistically, Rho regulation depends on Rho kinase activity, is independent of the status of myosin II activity, and is not mediated by promotion of the fascin/PKC complex. The p-Lin-11/Isl-1/Mec-3 kinases (LIMK), LIMK1 and LIMK2, act downstream of Rho kinases as novel binding partners of fascin-1, and this complex regulates the stability of **filopodia**.We have identified a novel activity of Rho in promoting a complex between fascin-1 and LIMK1/2 that modulates the interaction of fascin-1 with actin. These data provide new mechanistic insight into the intracellular coordination of contractile and protrusive actin-based structures. During the course of the study, we developed a novel FRET method for analysis of the fascin-1/actin interaction, with potential general applicability for analyzing the activities of actin-binding proteins in intact cells.
11. EphB receptors regulate dendritic spine morphogenesis through the recruitment/phosphorylation of focal adhesion kinase and RhoA activation. Dendritic **filopodia** are small protrusions on the surface of neuronal dendrites that transform into dendritic spines upon synaptic contact with axon terminals. The formation of dendritic spines is a critical aspect of synaptic development. Dendritic spine morphogenesis is characterized by **filopodia** shortening followed by the formation of mature mushroom-shaped spines. Here we show that activation of the EphB receptor tyrosine kinases in cultured hippocampal neurons by their ephrinB ligands induces morphogenesis of dendritic **filopodia** into dendritic spines. This appears to occur through assembly of an EphB-associated protein complex that includes focal adhesion kinase (FAK), Src, Grb2, and paxillin and the subsequent activations of FAK, Src, paxillin, and RhoA. Furthermore, Cre-mediated knock-out of loxP-flanked fak or RhoA inhibition blocks EphB-mediated morphogenesis of dendritic **filopodia**. Finally, EphB-mediated RhoA activation is disrupted by FAK knock-down. These data suggest that EphB receptors are upstream regulators of FAK in dendritic **filopodia** and that FAK-mediated RhoA activation contributes to assembly of actin filaments in dendritic spines.
12. ProBDNF collapses neurite outgrowth of primary neurons by activating RhoA. Neurons extend their dendrites and axons to build functional neural circuits, which are regulated by both positive and negative signals during development. Brain-derived neurotrophic factor (BDNF) is a positive regulator for neurite outgrowth and neuronal survival but the functions of its precursor (proBDNF) are less characterized.Here we show that proBDNF collapses neurite outgrowth in murine dorsal root ganglion (DRG) neurons and cortical neurons by activating RhoA via the p75 neurotrophin receptor (p75NTR). We demonstrated that the receptor proteins for proBDNF, p75NTR and sortilin, were highly expressed in cultured DRG or cortical neurons. ProBDNF caused a dramatic neurite collapse in a dose-dependent manner and this effect was about 500 fold more potent than myelin-associated glycoprotein. Neutralization of endogenous proBDNF by using antibodies enhanced neurite outgrowth in vitro and in vivo, but this effect was lost in p75NTR(-/-) mice. The neurite outgrowth of cortical neurons from p75NTR deficient (p75NTR(-/-)) mice was insensitive to proBDNF. There was a time-dependent reduction of length and number of **filopodia** in response to proBDNF which was accompanied with a polarized RhoA activation in growth cones. Moreover, proBDNF treatment of cortical neurons resulted in a time-dependent activation of RhoA but not Cdc42 and the effect was absent in p75NTR(-/-) neurons. Rho kinase (ROCK) and the collapsin response mediator protein-2 (CRMP-2) were also involved in the proBDNF action.proBDNF has an opposing role in neurite outgrowth to that of mature BDNF. Our observations suggest that proBDNF collapses neurites outgrowth and **filopodia**l growth cones by activating RhoA through the p75NTR signaling pathway.
13. Control of the direction of lamellipodia extension through changes in the balance between Rac and Rho activities. The direction in which cells extend new motile processes, such as lamellipodia and **filopodia**, can be controlled by altering the geometry of extracellular matrix adhesive islands on which individual cells are cultured, thereby altering mechanical interactions between cells and the adhesive substrate [Parker (2002)]. Here we specifically investigate the intracellular molecular signals that mediate the mechanism by which cells selectively extend these processes from the corners of polygonal-shaped adhesive islands. Constitutive activation of the small GTPase Rac within cells cultured on square-shaped islands of fibronectin resulted in the elimination of preferential extension from corners. This loss of directionality was accompanied by a re-distribution of focal adhesions: the large focal adhesions normally found within the corner regions of square cells were lost and replaced by many smaller focal contacts that were distributed along the entire cell perimeter. Inhibition of the small GTPase, Rho, using C3 exoenzyme blocked lamellipodia extension entirely. However, inhibition of Rho signaling in combination with ectopic Rac activation rescued the corner localization of motile processes and focal adhesions. These results suggest that the ability of cells to sense their physical surroundings and respond by moving in a spatially oriented manner is mediated by a balance between Rho and Rac activities.

#### GeneRIFs

1. Addition of purified CNFY triggers formation of actin-rich membrane ruffles and **filopodia**, which correlates with the activation of the Rho GTPases, RhoA, Rac1 and Cdc42.

### [Ii]nvadopodia

#### PubMed

1. The guanine nucleotide exchange factor Arhgef5 plays crucial roles in Src-induced podosome formation. Podosomes and **invadopodia** are actin-rich membrane protrusions that play a crucial role in cell adhesion and migration, and extracellular matrix remodeling in normal and cancer cells. The formation of podosomes and **invadopodia** is promoted by upregulation of some oncogenic molecules and is closely related to the invasive potential of cancer cells. However, the molecular mechanisms underlying the podosome and invadopodium formation still remain unclear. Here, we show that a guanine nucleotide exchange factor (GEF) for Rho family GTPases (Arhgef5) is crucial for Src-induced podosome formation. Using an inducible system for Src activation, we found that Src-induced podosome formation depends upon the Src SH3 domain, and identified Arhgef5 as a Src SH3-binding protein. RNA interference (RNAi)-mediated depletion of Arhgef5 caused robust inhibition of Src-dependent podosome formation. Overexpression of Arhgef5 promoted actin stress fiber remodeling through activating RhoA, and the activation of RhoA or Cdc42 was required for Src-induced podosome formation. Arhgef5 was tyrosine-phosphorylated by Src and bound to Src to positively regulate its activity. Furthermore, the pleckstrin homology (PH) domain of Arhgef5 was required for podosome formation, and Arhgef5 formed a ternary complex with Src and phosphoinositide 3-kinase when Src and/or Arhgef5 were upregulated. These findings provide novel insights into the molecular mechanisms of podosome and invadopodium formation induced by Src upregulation.

### [Ii]nvadopodium

#### PubMed

1. The guanine nucleotide exchange factor Arhgef5 plays crucial roles in Src-induced podosome formation. Podosomes and invadopodia are actin-rich membrane protrusions that play a crucial role in cell adhesion and migration, and extracellular matrix remodeling in normal and cancer cells. The formation of podosomes and invadopodia is promoted by upregulation of some oncogenic molecules and is closely related to the invasive potential of cancer cells. However, the molecular mechanisms underlying the podosome and **invadopodium** formation still remain unclear. Here, we show that a guanine nucleotide exchange factor (GEF) for Rho family GTPases (Arhgef5) is crucial for Src-induced podosome formation. Using an inducible system for Src activation, we found that Src-induced podosome formation depends upon the Src SH3 domain, and identified Arhgef5 as a Src SH3-binding protein. RNA interference (RNAi)-mediated depletion of Arhgef5 caused robust inhibition of Src-dependent podosome formation. Overexpression of Arhgef5 promoted actin stress fiber remodeling through activating RhoA, and the activation of RhoA or Cdc42 was required for Src-induced podosome formation. Arhgef5 was tyrosine-phosphorylated by Src and bound to Src to positively regulate its activity. Furthermore, the pleckstrin homology (PH) domain of Arhgef5 was required for podosome formation, and Arhgef5 formed a ternary complex with Src and phosphoinositide 3-kinase when Src and/or Arhgef5 were upregulated. These findings provide novel insights into the molecular mechanisms of podosome and **invadopodium** formation induced by Src upregulation.

### [Pp]odosome(s)?

#### Process

1. positive regulation of **podosome** assembly

#### PubMed

1. The guanine nucleotide exchange factor Arhgef5 plays crucial roles in Src-induced **podosome** formation. **Podosomes** and invadopodia are actin-rich membrane protrusions that play a crucial role in cell adhesion and migration, and extracellular matrix remodeling in normal and cancer cells. The formation of **podosomes** and invadopodia is promoted by upregulation of some oncogenic molecules and is closely related to the invasive potential of cancer cells. However, the molecular mechanisms underlying the **podosome** and invadopodium formation still remain unclear. Here, we show that a guanine nucleotide exchange factor (GEF) for Rho family GTPases (Arhgef5) is crucial for Src-induced **podosome** formation. Using an inducible system for Src activation, we found that Src-induced **podosome** formation depends upon the Src SH3 domain, and identified Arhgef5 as a Src SH3-binding protein. RNA interference (RNAi)-mediated depletion of Arhgef5 caused robust inhibition of Src-dependent **podosome** formation. Overexpression of Arhgef5 promoted actin stress fiber remodeling through activating RhoA, and the activation of RhoA or Cdc42 was required for Src-induced **podosome** formation. Arhgef5 was tyrosine-phosphorylated by Src and bound to Src to positively regulate its activity. Furthermore, the pleckstrin homology (PH) domain of Arhgef5 was required for **podosome** formation, and Arhgef5 formed a ternary complex with Src and phosphoinositide 3-kinase when Src and/or Arhgef5 were upregulated. These findings provide novel insights into the molecular mechanisms of **podosome** and invadopodium formation induced by Src upregulation.
2. miR-31 controls osteoclast formation and bone resorption by targeting RhoA. Increased activity of osteoclasts is responsible for bone loss and joint destruction in rheumatoid arthritis. For osteoclast development and bone resorption activity, cytoskeletal organization must be properly regulated. MicroRNAs (miRNAs) are endogenous small noncoding RNAs that suppress expression of their target genes. This study was conducted to identify crucial miRNAs to control osteoclasts.miRNA expression in the bone marrow-derived macrophages (BMM) with or without receptor activator of nuclear factor κB ligand (RANKL) stimulation was analyzed by miRNA array. To examine the role of specific miRNAs in osteoclast formation, bone resorption activity and actin ring formation, the BMM were retrovirally transduced with miRNA antagomirs. To confirm whether the suppressive effects on osteoclastogenesis by miR-31 inhibition were mediated by targeting RhoA, osteoclast formation was analyzed in the presence of the RhoA inhibitor, exoenzyme C3.miR-31 was identified as one of the highly upregulated miRNAs during osteoclast development under RANKL stimulation. Inhibition of miR-31 by specific antagomirs suppressed the RANKL-induced formation of osteoclasts and bone resorption. Phalloidin staining of osteoclasts revealed that actin ring formation at the cell periphery was severely impaired by miR-31 inhibition, and clusters of small ringed **podosome**s were observed instead. In these osteoclasts, expression of RhoA, one of the miR-31 target genes, was upregulated by miR-31 inhibition in spite of the impaired osteoclastogenesis. Treatment with the RhoA inhibitor, exoenzyme C3, rescued the osteoclastogenesis impaired by miR-31 inhibition.miR-31 controls cytoskeleton organization in osteoclasts for optimal bone resorption activity by regulating the expression of RhoA.

### [Mm]igration

#### Process

1. cell **migration**
2. cerebral cortex cell **migration**
3. endothelial cell **migration**
4. negative regulation of cell **migration** involved in sprouting angiogenesis
5. positive regulation of T cell **migration**
6. regulation of cell **migration**

#### PubMed

1. The small GTPases RhoA and Rac1 regulate cerebellar development by controlling cell morphogenesis, **migration** and foliation. The small GTPases RhoA and Rac1 are key cytoskeletal regulators that function in a mutually antagonistic manner to control the **migration** and morphogenesis of a broad range of cell types. However, their role in shaping the cerebellum, a unique brain structure composed of an elaborate set of folia separated by fissures of different lengths, remains largely unexplored. Here we show that dysregulation of both RhoA and Rac1 signaling results in abnormal cerebellar ontogenesis. Ablation of RhoA from neuroprogenitor cells drastically alters the timing and placement of fissure formation, the **migration** and positioning of granule and Purkinje cells, the alignment of Bergmann glia, and the integrity of the basement membrane, primarily in the anterior lobules. Furthermore, in the absence of RhoA, granule cell precursors located at the base of fissures fail to undergo cell shape changes required for fissure initiation. Many of these abnormalities can be recapitulated by deleting RhoA specifically from granule cell precursors but not postnatal glia, indicating that RhoA functions in granule cell precursors to control cerebellar morphogenesis. Notably, mice with elevated Rac1 activity due to loss of the Rac1 inhibitors Bcr and Abr show similar anterior cerebellar deficits, including ectopic neurons and defects in fissure formation, Bergmann glia organization and basement membrane integrity. Together, our results suggest that RhoA and Rac1 play indispensable roles in patterning cerebellar morphology.
2. RhoA and Cdc42 are required in pre-migratory progenitors of the medial ganglionic eminence ventricular zone for proper cortical interneuron **migration**. Cortical interneurons arise from the ganglionic eminences in the ventral telencephalon and migrate tangentially to the cortex. Although RhoA and Cdc42, members of the Rho family of small GTPases, have been implicated in regulating neuronal **migration**, their respective roles in the tangential **migration** of cortical interneurons remain unknown. Here we show that loss of RhoA and Cdc42 in the ventricular zone (VZ) of the medial ganglionic eminence (MGE) using Olig2-Cre mice causes moderate or severe defects in the **migration** of cortical interneurons, respectively. Furthermore, RhoA- or Cdc42-deleted MGE cells exhibit impaired **migration** in vitro. To determine whether RhoA and Cdc42 directly regulate the motility of cortical interneurons during **migration**, we deleted RhoA and Cdc42 in the subventricular zone (SVZ), where more fate-restricted progenitors are located within the ganglionic eminences, using Dlx5/6-Cre-ires-EGFP (Dlx5/6-CIE) mice. Deletion of either gene within the SVZ does not cause any obvious defects in cortical interneuron **migration**, indicating that cell motility is not dependent upon RhoA or Cdc42. These findings provide genetic evidence that RhoA and Cdc42 are required in progenitors of the MGE in the VZ, but not the SVZ, for proper cortical interneuron **migration**.
3. N-acetylglucosaminyltransferase V mediates cell **migration** and invasion of mouse mammary tumor cells 4TO7 via RhoA and Rac1 signaling pathway. In tumor cells, alterations in cellular glycosylation may play a key role in their metastatic behavior. Using small interfering RNA against GnT-V, we found that the expression of GnT-V and beta1,6GlcNAc branching were significantly reduced which was particularly accompanied by the arrest in both cell **migration** and invasion as compared to the negative control. Moreover, the suppressed GnT-V expression by siRNA technique inactivated the signaling molecules including Rac1, cofilin, Erk and Akt, and activated RhoA levels in cells lacking GnT-V, but revealed no impact on Cdc42 activity. All these notions disclose for the first time that GnT-V and beta1, 6GlcNAc branching mediate the cell **migration** and invasion in Rac1-positive and RhoA-negative regulatory manners.
4. Daphnetin inhibits invasion and **migration** of LM8 murine osteosarcoma cells by decreasing RhoA and Cdc42 expression. Daphnetin, 7,8-dihydroxycoumarin, present in main constituents of Daphne odora var. marginatai, has multiple pharmacological activities including anti-proliferative effects in cancer cells. In this study, using a Transwell system, we showed that daphnetin inhibited invasion and **migration** of highly metastatic murine osteosarcoma LM8 cells in a dose-dependent manner. Following treatment by daphnetin, cells that penetrated the Transwell membrane were rounder than non-treated cells. Immunofluorescence analysis revealed that daphnetin decreased the numbers of intracellular stress fibers and filopodia. Moreover, daphnetin treatment dramatically decreased the expression levels of RhoA and Cdc42. In summary, the dihydroxycoumarin derivative daphnetin inhibits the invasion and **migration** of LM8 cells, and therefore represents a promising agent for use against metastatic cancer.
5. Rab13-dependent trafficking of RhoA is required for directional **migration** and angiogenesis. Angiogenesis requires concomitant remodeling of cell junctions and **migration**, as exemplified by recent observations of extensive endothelial cell movement along growing blood vessels. We report that a protein complex that regulates cell junctions is required for VEGF-driven directional **migration** and for angiogenesis in vivo. The complex consists of RhoA and Syx, a RhoA guanine exchange factor cross-linked by the Crumbs polarity protein Mupp1 to angiomotin, a phosphatidylinositol-binding protein. The Syx-associated complex translocates to the leading edge of migrating cells by membrane trafficking that requires the tight junction recycling GTPase Rab13. In turn, Rab13 associates with Grb2, targeting Syx and RhoA to Tyr(1175)-phosphorylated VEGFR2 at the leading edge. Rab13 knockdown in zebrafish impeded sprouting of intersegmental vessels and diminished the directionality of their tip cells. These results indicate that endothelial cell mobility in sprouting vessels is facilitated by shuttling the same protein complex from disassembling junctions to the leading edges of cells.
6. Therapeutic ultrasound bypasses canonical syndecan-4 signaling to activate rac1. The application of pulsed, low intensity ultrasound is emerging as a potent therapy for the treatment of complex bone fractures and tissue damage. Ultrasonic stimuli accelerate fracture healing by up to 40% and enhance tendon and ligament healing by promoting cell proliferation, **migration**, and matrix synthesis through an unresolved mechanism. Ultrasound treatment also induces closure of nonunion fractures, at a success rate (85% of cases) similar to that of surgical intervention (68-96%) while avoiding the complications associated with surgery. The regulation of cell adhesion necessary for wound healing depends on cooperative engagement of the extracellular matrix receptors, integrin and syndecan, as exemplified by the wound healing defects observed in syndecan- and integrin-knock-out mice. This report distinguishes the influence of ultrasound on signals downstream of the prototypic fibronectin receptors, alpha(5)beta(1) integrin and syndecan-4, which cooperate to regulate Rac1 and RhoA. Ultrasonic stimulation fails to activate integrins or induce cell spreading on poor, electrostatic ligands. By contrast, ultrasound treatment overcomes the necessity of engagement or expression of syndecan-4 during the process of focal adhesion formation, which normally requires simultaneous engagement of both receptors. Ultrasound exerts an influence downstream of syndecan-4 and PKCalpha to specifically activate Rac1, itself a critical regulator of tissue repair, and to a lesser extent RhoA. The ability of ultrasound to bypass syndecan-4 signaling, which is known to facilitate efficient tissue repair, explains the reduction in healing times observed in ultrasound-treated patients. By substituting for one of the key axes of adhesion-dependent signaling, ultrasound therapy has considerable potential as a clinical technique.
7. RhoA determines disease progression by controlling neutrophil motility and restricting hyperresponsiveness. Neutrophil responses are central to host protection and inflammation. Neutrophil activation follows a 2-step process in which priming amplifies responses to activating stimuli. Priming is essential for life span extension, chemotaxis, and respiratory burst activity. Here we show that the cytoskeletal organizer RhoA suppresses neutrophil priming via formins. Premature granule exocytosis in Rho-deficient neutrophils activated numerous signaling pathways and amplified superoxide generation. Deletion of Rho altered front-to-back coordination by simultaneously increasing uropod elongation, leading edge formation, and random **migration**. Concomitant negative and positive regulation of β2 integrin-independent and β2 integrin-dependen**t migrati**on, respectively, reveal Rho as a key decision point in the neutrophil response to discrete chemotactic agents. Although even restricted influx of Rho-deficient hyperactive neutrophils exacerbated lipopolysaccharide-mediated lung injury, deleting Rho in innate immune cells was highly protective in influenza A virus infection. Hence, Rho is a key regulator of disease progression by maintaining neutrophil quiescence and suppressing hyperresponsiveness.
8. A radial glia-specific role of RhoA in double cortex formation. The positioning of neurons in the cerebral cortex is of crucial importance for its function as highlighted by the severe consequences of **migration**al disorders in patients. Here we show that genetic deletion of the small GTPase RhoA in the developing cerebral cortex results in two **migration**al disorders: subcortical band heterotopia (SBH), a heterotopic cortex underlying the normotopic cortex, and cobblestone lissencephaly, in which neurons protrude beyond layer I at the pial surface of the brain. Surprisingly, RhoA(-/-) neurons migrated normally when transplanted into wild-type cerebral cortex, whereas the converse was not the case. Alterations in the radial glia scaffold are demonstrated to cause these **migration**al defects through destabilization of both the actin and the microtubules cytoskeleton. These data not only demonstrate that RhoA is largely dispensable for **migration** in neurons but also showed that defects in radial glial cells, rather than neurons, can be sufficient to produce SBH.
9. Distinct roles of Rac1/Cdc42 and Rho/Rock for axon outgrowth and nucleokinesis of precerebellar neurons toward netrin 1. During embryonic development, tangentially migrating precerebellar neurons emit a leading process and then translocate their nuclei inside it (nucleokinesis). Netrin 1 (also known as netrin-1) acts as a chemoattractant factor for neurophilic **migration** of precerebellar neurons (PCN) both in vivo and in vitro. In the present work, we analyzed Rho GTPases that could direct axon outgrowth and/or nuclear **migration**. We show that the expression pattern of Rho GTPases in developing PCN is consistent with their involvement in the **migration** of PCN from the rhombic lips. We report that pharmacological inhibition of Rho enhances axon outgrowth of PCN and prevents nuclei **migration** toward a netrin 1 source, whereas inhibition of Rac and Cdc42 sub-families impair neurite outgrowth of PCN without affecting **migration**. We show, through pharmacological inhibition, that Rho signaling directs neurophilic **migration** through Rock activation. Altogether, our results indicate that Rho/Rock acts on signaling pathways favoring nuclear translocation during tangential **migration** of PCN. Thus, axon extension and nuclear **migration** of PCN in response to netrin 1 are not strictly dependent processes because: (1) distinct small GTPases are involved; (2) axon extension can occur when **migration** is blocked; and (3) **migration** can occur when axon outgrowth is impaired.
10. Paxillin and Hic-5 interaction with vinculin is differentially regulated by Rac1 and RhoA. Cell **migration** is of paramount importance to organism development and maintenance as well as multiple pathological processes, including cancer metastasis. The RhoGTPases Rac1 and RhoA are indispensable for cell **migration** as they regulate cell protrusion, cell-extracellular matrix (ECM) interactions and force transduction. However, the consequences of their activity at a molecular level within the cell remain undetermined. Using a combination of FRET, FRAP and biochemical analyses we show that the interactions between the focal adhesion proteins vinculin and paxillin, as well as the closely related family member Hic-5 are spatially and reciprocally regulated by the activity of Rac1 and RhoA. Vinculin in its active conformation interacts with either paxillin or Hic-5 in adhesions in response to Rac1 and RhoA activation respectively, while inactive vinculin interacts with paxillin in the membrane following Rac1 inhibition. Additionally, Rac1 specifically regulates the dynamics of paxillin as well as its binding partner and F-actin interacting protein actopaxin (α-parvin) in adhesions. Furthermore, FRET analysis of protein:protein interactions within cell adhesions formed in 3D matrices revealed that, in contrast to 2D systems vinculin interacts preferentially with Hic-5. This study provides new insight into the complexity of cell-ECM adhesions in both 2D and 3D matrices by providing the first description of RhoGTPase-coordinated protein:protein interactions in a cellular microenvironment. These data identify discrete roles for paxillin and Hic-5 in Rac1 and RhoA-dependent cell adhesion formation and maturation; processes essential for productive cell **migratio**n.
11. Cadherin-11 coordinates cellular **migration** and extracellular matrix remodeling during aortic valve maturation. Proper remodeling of the endocardial cushions into thin fibrous valves is essential for gestational progression and long-term function. This process involves dynamic interactions between resident cells and their local environment, much of which is not understood. In this study, we show that deficiency of the cell-cell adhesion protein cadherin-11 (Cad-11) results in significant embryonic and perinatal lethality primarily due to valve related cardiac dysfunction. While endocardial to mesenchymal transformation is not abrogated, mesenchymal cells do not homogeneously cellularize the cushions. These cushions remain thickened with disorganized ECM, resulting in pronounced aortic valve insufficiency. Mice that survive to adulthood maintain thickened and stenotic semilunar valves, but interestingly do not develop calcification. Cad-11 (-/-) aortic valve leaflets contained reduced Sox9 activity, β1 integrin expression, and RhoA-GTP activity, suggesting that remodeling defects are due to improper **migratio**n and/or cellular contraction. Cad-11 deletion or siRNA knockdown reduced **migratio**n, eliminated collective **migratio**n, and impaired 3D matrix compaction by aortic valve interstitial cells (VIC). Cad-11 depleted cells in culture contained few filopodia, stress fibers, or contact inhibited locomotion. Transfection of Cad-11 depleted cells with constitutively active RhoA restored cell phenotypes. Together, these results identify cadherin-11 mediated adhesive signaling for proper remodeling of the embryonic semilunar valves.
12. Mouse macrophages completely lacking Rho subfamily GTPases (RhoA, RhoB, and RhoC) have severe lamellipodial retraction defects, but robust chemotactic navigation and altered motility. RhoA is thought to be essential for coordination of the membrane protrusions and retractions required for immune cell motility and directed **migration**. Whether the subfamily of Rho (Ras homolog) GTPases (RhoA, RhoB, and RhoC) is actually required for the directed **migration** of primary cells is difficult to predict. Macrophages isolated from myeloid-restricted RhoA/RhoB (conditional) double knock-out (dKO) mice did not express RhoC and were essentially "pan-Rho"-deficient. Using real-time chemotaxis assays, we found that retraction of the trailing edge was dissociated from the advance of the cell body in dKO cells, which developed extremely elongated tails. Surprisingly, velocity (of the cell body) was increased, whereas chemotactic efficiency was preserved, when compared with WT macrophages. Randomly migrating RhoA/RhoB dKO macrophages exhibited multiple small protrusions and developed large "branches" due to impaired lamellipodial retraction. A mouse model of peritonitis indicated that monocyte/macrophage recruitment was, surprisingly, more rapid in RhoA/RhoB dKO mice than in WT mice. In comparison with dKO cells, the phenotypes of single RhoA- or RhoB-deficient macrophages were mild due to mutual compensation. Furthermore, genetic deletion of RhoB partially reversed the motility defect of macrophages lacking the RhoGAP (Rho GTPase-activating protein) myosin IXb (Myo9b). In conclusion, the Rho subfamily is not required for "front end" functions (motility and chemotaxis), although both RhoA and RhoB are involved in pulling up the "back end" and resorbing lamellipodial membrane protrusions. Macrophages lacking Rho proteins migrate faster in vitro, which, in the case of the peritoneum, translates to more rapid in vivo monocyte/macrophage recruitment.
13. Genetic deletion of Cdc42GAP reveals a role of Cdc42 in erythropoiesis and hematopoietic stem/progenitor cell survival, adhesion, and engraftment. Rho family GTPases are key signal transducers in cell regulation. Although a body of literature has implicated the Rho family members Rac1 and Rac2 in multiple hematopoietic-cell functions, the role of Cdc42 in hematopoiesis remains unclear. Here we have examined the hematopoietic properties and the hematopoietic stem/progenitor cell (HSP) functions of gene-targeted mice carrying null alleles of cdc42gap, a negative regulator of Cdc42. The Cdc42GAP-/- fetal liver and bone marrow cells showed a 3-fold increase in Cdc42 activity but normal Rac and RhoA activities, indicating that Cdc42GAP knockout resulted in a gain of Cdc42 activity in the hematopoietic tissues. Cdc42GAP-/- mice were anemic. The cellularity of fetal liver and bone marrow, the number and composition percentage of HSPs, and the erythroid blast-forming unit and colony-forming unit (BFU-E/CFU-E) activities were significantly reduced in the homozygous mice. The decrease in HSP number was associated with increased apoptosis of the Cdc42GAP-/- HSPs and the activation of JNK-mediated apoptotic machinery. Moreover, homozygous HSPs showed impaired cortical F-actin assembly, deficiency in adhesion and **migration**, and defective engraftment. These results provide evidence that Cdc42 activity is important for erythropoiesis and for multiple HSP functions, including survival, adhesion, and engraftment.
14. Vav GEFs regulate macrophage morphology and adhesion-induced Rac and Rho activation. The Vav family of proteins have the potential to act as both signalling adapters and GEFs for Rho GTPases. They have therefore been proposed as regulators of the cytoskeleton in various cell types. We have used macrophages from mice deficient in all three Vav isoforms to determine how their function affects cell morphology and **migration**. Macrophages lacking Vav proteins adopt an elongated morphology and have enhanced migratory persistence in culture. To investigate the pathways through which Vav proteins exert their effects we analysed the responses of macrophages to the chemoattractant CSF-1 and to adhesion. We found that morphological and signalling responses of macrophages to CSF-1 did not require Vav proteins. In contrast, adhesion-induced cell spreading, RhoA and Rac1 activation and cell signalling were all dependent on Vav proteins. We propose that Vav proteins affect macrophage morphology and motile behaviour by coupling adhesion receptors to Rac1 and RhoA activity and regulating adhesion signalling events such as paxillin and ERK1/2 phosphorylation by acting as adapters.
15. Rho GTPases in hematopoietic stem/progenitor cell **migration**. Rho GTPases including RhoA, Rac1, and Cdc42 are a class of intracellular signaling proteins critical for the regulation of cytoskeleton organization, adhesion, and **migration**. Molecular mechanisms of mammalian cell **migration** were first revealed in fibroblasts where RhoA, Rac1, and Cdc42 facilitate in the multistep process including establishment and maintenance of polarity, formation of actin-rich protrusions, remodeling of adhesive contacts, and generation of force. In hematopoietic stem/progenitor cells, Rho GTPases relay signals from chemokines and cytokines such as SDF-1α and SCF to the actin and microtubule cytoskeleton through effector kinases and/or adaptor molecules that affect adhesion or transcription. Comprehensive use of murine conditional gene knockout technology combined with biochemical approaches in recent studies allows for physiologically relevant investigations of the involvement of Rho GTPases in hematopoietic stem/progenitor cell **migratio**n, providing important mechanisms for the stem/progenitor maintenance.
16. ACF7 regulates cytoskeletal-focal adhesion dynamics and **migration** and has ATPase activity. Coordinated interactions between microtubule (MT) and actin cytoskeletons are involved in many polarized cellular processes. Spectraplakins are enormous (>500 kDa) proteins able to bind both MTs and actin filaments (F-actin) directly. To elucidate the physiological significance and functions of mammalian spectraplakin ACF7, we've conditionally targeted it in skin epidermis. Intriguingly, ACF7 deficiency compromises the targeting of microtubules along F-actin to focal adhesions (FAs), stabilizes FA-actin networks, and impairs epidermal **migration**. Exploring underlying mechanisms, we show that ACF7's binding domains for F-actin, MTs, and MT plus-end proteins are not sufficient to rescue the defects in FA-cytoskeletal dynamics and **migration** functions of ACF7 null keratinocytes. We've uncovered an intrinsic actin-regulated ATPase domain in ACF7 and demonstrate that it is both functional and essential for these roles. Our findings provide insight into the functions of this important cytoskeletal crosslinking protein in regulating dynamic interactions between MTs and F-actin to sustain directional cell movement.
17. Type VIII collagen signals via β1 integrin and RhoA to regulate MMP-2 expression and smooth muscle cell **migratio**n. The extracellular matrix signals and regulates the behavior of vascular cells during the pathogenesis of atherosclerosis. Type VIII collagen, a short chain collagen, is scarcely present in normal arteries, but is dramatically upregulated in atherosclerosis and after other types of vascular injury. Cell culture studies have revealed that this protein supports smooth muscle cell (SMC) adhesion and stimulates **migratio**n, however little is known about the signaling or the mechanisms by which this occurs. SMCs isolated from wild-type C57BL/6 and type VIII collagen deficient mice were studied using assays to measure chemotactic and haptotactic **migratio**n, and remodeling and contraction of 3-dimensional type I collagen gels. Col8(-/-) SMCs exhibited impairments in **migratio**n, and a strongly adhesive phenotype with prominent stress fibers, stable microtubules and pronounced central basal focal adhesions. The addition of exogenous type VIII collagen to the Col8(-/-) SMCs rescued the impairments in **migratio**n, and restored cytoskeletal architecture so that it was similar to Col8(+/+) cells. We measured elevated levels of active GTP-RhoA in the Col8(-/-) cells, and this too was reversed by treatment with exogenous type VIII collagen. We showed that type VIII collagen normally suppresses RhoA activation through a beta-1 integrin dependent mechanism. MMP-2 levels were reduced in the Col8(-/-) SMCs, and knockdown of MMP-2 in Col8(+/+) SMCs partially recapitulated the decreases in **migratio**n and 3D gel contraction seen in Col8(-/-) cells, showing that type VIII collagen-stimulated **migratio**n was dependent on MMP-2. Inhibition of Rho restored MMP-2 activity in the Col8(-/-) cells, and partially rescued **migratio**n, demonstrating that the elevations in RhoA activity were responsible for the suppression of **migratio**n of these cells. In conclusion, we have shown that type VIII collagen signals through beta-1 integrin receptors to suppress RhoA, allowing optimal configuration of the cytoskeleton, and the stimulation of MMP-2-dependent cell **migratio**n.
18. Essential role of Cdc42 in cardiomyocyte proliferation and cell-cell adhesion during heart development. Cdc42 is a member of the Rho GTPase family and functions as a molecular switch in regulating cell **migration**, proliferation, differentiation and survival. However, the role of Cdc42 in heart development remains largely unknown. To determine the function of Cdc42 in heart formation, we have generated a Cdc42 cardiomyocyte knockout (CCKO) mouse line by crossing Cdc42 flox mice with myosin light chain (MLC) 2a-Cre mice. The inactivation of Cdc42 in embryonic cardiomyocytes induced lethality after embryonic day 12.5. Histological analysis of CCKO embryos showed cardiac developmental defects that included thin ventricular walls and ventricular septum defects. Microarray and real-time PCR data also revealed that the expression level of p21 was significantly increased and cyclin B1 was dramatically decreased, suggesting that Cdc42 is required for cardiomyocyte proliferation. Phosphorylated Histone H3 staining confirmed that the inactivation of Cdc42 inhibited cardiomyocytes proliferation. In addition, transmission electron microscope studies showed disorganized sarcomere structure and disruption of cell-cell contact among cardiomyocytes in CCKO hearts. Accordingly, we found that the distribution of N-cadherin/β-Catenin in CCKO cardiomyocytes was impaired. Taken together, our data indicate that Cdc42 is essential for cardiomyocyte proliferation, sarcomere organization and cell-cell adhesion during heart development.
19. Blockade of the RhoA-JNK-c-Jun-MMP2 cascade by atorvastatin reduces osteosarcoma cell invasion. Osteosarcoma is characterized by a high malignant and metastatic potential, which points to the need for new therapeutic strategies to prevent cell metastasis. In this study, we show that statin-induced HMG-CoA reductase inhibition reduces cell **migration** and invasion in human and murine osteosarcoma cells, independently of the genotype. The statin-induced reduction of cell **migration** and invasion was independent of induction of apoptosis and was geranylgeranylpyrophosphate-dependent. The statin reduced matrix metalloproteinase (MMP) 2, 9, and 14 and TIMP2 expression or activity in invading cells. Forced expression of MMP2 and MMP14 overcame the inhibitory effect of the statin on cell invasion, suggesting a role for these MMPs in invasive potential. We also investigated the mechanisms involved in the reduced MMP2 activity and cell invasion. Inhibition of JNK, but not ERK1/2 signaling, reduced MMP2 activity. Pharmacological or constitutive activation of JNK overcame the reduced MMP2 activity and cell invasion induced by the statin. The statin decreased JNK phosphorylation and c-Jun nuclear translocation, suggesting that HMG-CoA reductase inhibition targets the JNK-c-Jun signaling pathway. We showed that mevalonate or geranylgeranylpyrophosphate treatment prevented the statin-induced reduction in JNK phosphorylation, MMP2 activity, and cell invasion. Forced expression of a constitutively active form of RhoA increased JNK phosphorylation and overcame the inhibitory effect of atorvastatin on MMP2 activity and cell invasion. The data establish a link between RhoA, JNK, c-Jun, and MMP2 activity that is functionally involved in the reduction in osteosarcoma cell invasion by the statin. This suggests a novel strategy targeting RhoA-JNK-c-Jun signaling to reduce osteosarcoma cell tumorigenesis.
20. Regulation of ephexin1, a guanine nucleotide exchange factor of Rho family GTPases, by fibroblast growth factor receptor-mediated tyrosine phosphorylation. Fibroblast growth factor (FGF) signal is implicated in not only cell proliferation, but cell **migration** and morphological changes. Several different Rho family GTPases downstream of the Ras/ERK pathway are postulated to mediate the latter functions. However, none have been recognized to be directly coupled to FGF receptors (FGFRs). We have previously reported that EphA4 and FGFRs hetero-oligomerize through their cytoplasmic domains, trans-activate each other, and transduce a signal for cell proliferation through a docking protein, FRS2alpha (Yokote, H., Fujita, K., Jing, X., Sawada, T., Liang, S., Yao, L., Yan, X., Zhang, Y., Schlessinger, J., and Sakaguchi, K. (2005) Proc. Natl. Acad. Sci. U. S. A. 102, 18866-18871). Here, we have found that ephexin1, a guanine nucleotide exchange factor for Rho family GTPases, constitutes another downstream component of the receptor complex. Ephexin1 directly binds to the kinase domain of FGFR mainly through its DH and PH domains. The binding appears to become weaker and limited to the DH domain when FGFRs become activated. FGFR-mediated phosphorylation of ephexin1 enhances the guanine nucleotide exchange activity toward RhoA without affecting the activity to Rac1 or Cdc42. The FGFR-mediated tyrosine phosphorylation includes, but is not limited to, the residue (Tyr-87) phosphorylated by Src family kinase, which is known to be activated following EphA4 activation. The Tyr-to-Asp mutations that mimic the tyrosine phosphorylation in some of the putative FGFR-mediated phosphorylation sites increase the nucleotide exchange activity for RhoA without changing the activity for Rac1 or Cdc42. From these results, we conclude that ephexin1 is located immediately downstream of the EphA4-FGFR complex and the function is altered by the FGFR-mediated tyrosine phosphorylation at multiple sites.
21. Vav GEFs are required for beta2 integrin-dependent functions of neutrophils. Integrin regulation of neutrophils is essential for appropriate adhesion and trans**migration** into tissues. Vav proteins are Rho family guanine nucleotide exchange factors that become tyrosine phosphorylated in response to adhesion. Using Vav1/Vav3-deficient neutrophils (Vav1/3ko), we show that Vav proteins are required for multiple beta2 integrin-dependent functions, including sustained adhesion, spreading, and complement-mediated phagocytosis. These defects are not attributable to a lack of initial beta2 activation as Vav1/3ko neutrophils undergo chemoattractant-induced arrest on intercellular adhesion molecule-1 under flow. Accordingly, in vivo, Vav1/3ko leukocytes arrest on venular endothelium yet are unable to sustain adherence. Thus, Vav proteins are specifically required for stable adhesion. beta2-induced activation of Cdc42, Rac1, and RhoA is defective in Vav1/3ko neutrophils, and phosphorylation of Pyk2, paxillin, and Akt is also significantly reduced. In contrast, Vav proteins are largely dispensable for G protein-coupled receptor-induced signaling events and chemotaxis. Thus, Vav proteins play an essential role coupling beta2 to Rho GTPases and regulating multiple integrin-induced events important in leukocyte adhesion and phagocytosis.
22. The Amot/Patj/Syx signaling complex spatially controls RhoA GTPase activity in migrating endothelial cells. Controlled regulation of Rho GTPase activity is an essential component mediating growth factor-stimulated **migration**. We have previously shown that angiomotin (Amot), a membrane-associated scaffold protein, plays a critical role during vascular patterning and endothelial **migration** during embryogenesis. However, the signaling pathways by which Amot controls directional **migration** are not known. Here we have used peptide pull-down and yeast 2-hybrid (Y2H) screening to identify proteins that interact with the C-terminal PDZ-binding motifs of Amot and its related proteins AmotL1 and 2. We report that Amot and its related proteins bind to the RhoA GTPase exchange factor (RhoGEF) protein Syx. We show that Amot forms a ternary complex together with Patj (or its paralogue Mupp1) and Syx. Using FRET analysis, we provide evidence that Amot controls targeting of RhoA activity to lamellipodia in vitro. We also report that, similar to Amot, morpholino knockdown of Syx in zebrafish results in inhibition of **migration** of intersegmental arteries. Taken together, our results indicate that the directional **migration** of capillaries in the embryo is governed by the Amot:Patj/Mupp1:Syx signaling that controls local GTPase activity.
23. p27(Kip1) is a microtubule-associated protein that promotes microtubule polymerization during neuron **migration**. The **migration** of cortical interneurons is characterized by extensive morphological changes that result from successive cycles of nucleokinesis and neurite branching. Their molecular bases remain elusive, and the present work describes how p27(Kip1) controls cell-cycle-unrelated signaling pathways to regulate these morphological remodelings. Live imaging reveals that interneurons lacking p27(Kip1) show delayed tangential **migration** resulting from defects in both nucleokinesis and dynamic branching of the leading process. At the molecular level, p27(Kip1) is a microtubule-associated protein that promotes polymerization of microtubules in extending neurites, thereby contributing to tangential **migration**. Furthermore, we show that p27(Kip1) controls actomyosin contractions that drive both forward translocation of the nucleus and growth cone splitting. Thus, p27(Kip1) cell-autonomously controls nucleokinesis and neurite branching by regulating both actin and microtubule cytoskeletons.
24. GPR124, an orphan G protein-coupled receptor, is required for CNS-specific vascularization and establishment of the blood-brain barrier. Every organ in the body requires blood vessels for efficient delivery of oxygen and nutrients, but independent vascular beds are highly specialized to meet the individual needs of specific organs. The vasculature of the brain is tightly sealed, with blood-brain barrier (BBB) properties developing coincident with neural vascularization. G protein-coupled receptor 124 (GPR124) (tumor endothelial marker 5, TEM5), an orphan member of the adhesion family of G protein-coupled receptors, was previously identified on the basis of its overexpression in tumor vasculature. Here, we show that global deletion or endothelial-specific deletion of GPR124 in mice results in embryonic lethality associated with abnormal angiogenesis of the forebrain and spinal cord. Expression of GPR124 was found to be required for invasion and **migration** of blood vessels into neuroepithelium, establishment of BBB properties, and expansion of the cerebral cortex. Thus, GPR124 is an important regulator of neurovasculature development and a potential drug target for cerebrovascular diseases.
25. The p110delta isoform of PI 3-kinase negatively controls RhoA and PTEN. Inactivation of PI 3-kinase (PI3K) signalling is critical for tumour suppression by PTEN. This is thought to be a unidirectional relationship in which PTEN degrades the lipids produced by PI3K, thus controlling cell proliferation, survival and **migration**. We now show that this relationship is in fact bidirectional, whereby PI3K reciprocally controls PTEN. We report that the p110delta PI3K negatively regulates PTEN, through a pathway involving inhibition of RhoA. Inactivation of p110delta in macrophages led to reduced Akt and Rac1 activation, but paradoxically to increased RhoA and PTEN activity. Partial inactivation of p190RhoGAP and a reduced binding of cytoplasmic RhoA to the cyclin-dependent kinase inhibitor p27 both contributed to the increased RhoA-GTP levels upon p110delta inactivation. Pharmacological inhibition of ROCK, a downstream effector kinase of RhoA, restored all signalling and functional defects of p110delta inactivation, including Akt phosphorylation, chemotaxis and proliferation. This work identifies the RhoA/ROCK pathway as a major target of p110delta-mediated PI3K signalling, and establishes for the first time that PI3K controls itself, via a feedback loop involving PTEN.
26. Neurogenin 2 controls cortical neuron **migration** through regulation of Rnd2. Motility is a universal property of newly generated neurons. How cell **migration** is coordinately regulated with other aspects of neuron production is not well understood. Here we show that the proneural protein neurogenin 2 (Neurog2), which controls neurogenesis in the embryonic cerebral cortex, directly induces the expression of the small GTP-binding protein Rnd2 (ref. 3) in newly generated mouse cortical neurons before they initiate **migration**. Rnd2 silencing leads to a defect in radial **migration** of cortical neurons similar to that observed when the Neurog2 gene is deleted. Remarkably, restoring Rnd2 expression in Neurog2-mutant neurons is sufficient to rescue their ability to migrate. Our results identify Rnd2 as a novel essential regulator of neuronal **migration** in the cerebral cortex and demonstrate that Rnd2 is a major effector of Neurog2 function in the promotion of **migration**. Thus, a proneural protein controls the complex cellular behaviour of cell **migration** through a remarkably direct pathway involving the transcriptional activation of a small GTP-binding protein.
27. The orphan adhesion G protein-coupled receptor GPR97 regulates **migration** of lymphatic endothelial cells via the small GTPases RhoA and Cdc42. The important role of the lymphatic vascular system in pathological conditions such as inflammation and cancer has been increasingly recognized, but its potential as a pharmacological target is poorly exploited. Our study aimed at the identification and molecular characterization of lymphatic-specific G protein-coupled receptors (GPCRs) to assess new targets for pharmacological manipulation of the lymphatic vascular system. We used a TaqMan quantitative RT-PCR-based low density array to determine the GPCR expression profiles of ex vivo isolated intestinal mouse lymphatic (LECs) and blood vascular endothelial cells (BECs). GPR97, an orphan adhesion GPCR of unknown function, was the most highly and specifically expressed GPCR in mouse lymphatic endothelium. Using siRNA silencing, we found that GPR97-deficient primary human LECs displayed increased adhesion and collective cell **migration**, whereas single cell **migration** was decreased as compared with nontargeting siRNA-transfected control LECs. Loss of GPR97 shifted the ratio of active Cdc42 and RhoA and initiated cytoskeletal rearrangements, including F-actin redistribution, paxillin and PAK4 phosphorylation, and β1-integrin activation. Our data suggest a possible role of GPR97 in lymphatic remodeling and furthermore provide the first insights into the biological functions of GPR97.
28. Regulation of mesenchymal-to-epithelial transition by PARAXIS during somitogenesis. Dynamic alterations in cell shape, **migration**, and adhesion play a central role in tissue morphogenesis during embryonic development and congenital disease. The mesenchymal-to-epithelial transition that occurs during vertebrate somitogenesis is required for proper patterning of the axial musculoskeletal system. Somitic MET is initiated in the presomitic mesoderm by PARAXIS-dependent changes in cell adhesion, cell polarity, and the composition of the extracellular matrix. However, the target genes downstream of the transcription factor PARAXIS remain poorly described.A genome-wide comparison of gene expression in the anterior presomitic mesoderm and newly formed somites of Paraxis(-/-) embryos resulted in a set of deregulated genes enriched for factors associated with extracellular matrix and cytoskeletal organization and cell-cell and cell-ECM adhesion. The greatest change in expression was seen in fibroblast activation protein alpha (Fap), encoding a dipeptidyl peptidase capable of increasing fibronectin and collagen fiber organization in extracellular matrix. Further, downstream genes in the Wnt and Notch signaling pathways were downregulated, predicting that PARAXIS participates in positive feedback loops in both pathways.These data demonstrate that PARAXIS initiates and stabilizes somite epithelialization by integrating signals from multiple pathways to control the reorganization of the ECM, cytoskeleton, and adhesion junctions during MET.
29. The atypical Guanine-nucleotide exchange factor, dock7, negatively regulates schwann cell differentiation and myelination. In development of the peripheral nervous system, Schwann cells proliferate, migrate, and ultimately differentiate to form myelin sheath. In all of the myelination stages, Schwann cells continuously undergo morphological changes; however, little is known about their underlying molecular mechanisms. We previously cloned the dock7 gene encoding the atypical Rho family guanine-nucleotide exchange factor (GEF) and reported the positive role of Dock7, the target Rho GTPases Rac/Cdc42, and the downstream c-Jun N-terminal kinase in Schwann cell **migration** (Yamauchi et al., 2008). We investigated the role of Dock7 in Schwann cell differentiation and myelination. Knockdown of Dock7 by the specific small interfering (si)RNA in primary Schwann cells promotes dibutyryl cAMP-induced morphological differentiation, indicating the negative role of Dock7 in Schwann cell differentiation. It also results in a shorter duration of activation of Rac/Cdc42 and JNK, which is the negative regulator of myelination, and the earlier activation of Rho and Rho-kinase, which is the positive regulator of myelination. To obtain the in vivo evidence, we generated Dock7 short hairpin (sh)RNA transgenic mice. They exhibited a decreased expression of Dock7 in the sciatic nerves and enhanced myelin thickness, consistent with in vitro observation. The effects of the in vivo knockdown on the signals to Rho GTPases are similar to those of the in vitro knockdown. Collectively, the signaling through Dock7 negatively regulates Schwann cell differentiation and the onset of myelination, demonstrating the unexpected role of Dock7 in the interplay between Schwann cell **migration** and myelination.
30. Prickle1 mutation causes planar cell polarity and directional cell **migration** defects associated with cardiac outflow tract anomalies and other structural birth defects. Planar cell polarity (PCP) is controlled by a conserved pathway that regulates directional cell behavior. Here, we show that mutant mice harboring a newly described mutation termed Beetlejuice (Bj) in Prickle1 (Pk1), a PCP component, exhibit developmental phenotypes involving cell polarity defects, including skeletal, cochlear and congenital cardiac anomalies. Bj mutants die neonatally with cardiac outflow tract (OFT) malalignment. This is associated with OFT shortening due to loss of polarized cell orientation and failure of second heart field cell intercalation mediating OFT lengthening. OFT myocardialization was disrupted with cardiomyocytes failing to align with the direction of cell invasion into the outflow cushions. The expression of genes mediating Wnt signaling was altered. Also noted were shortened but widened bile ducts and disruption in canonical Wnt signaling. Using an in vitro wound closure assay, we showed Bj mutant fibroblasts cannot establish polarized cell morphology or engage in directional cell **migration**, and their actin cytoskeleton failed to align with the direction of wound closure. Unexpectedly, Pk1 mutants exhibited primary and motile cilia defects. Given Bj mutant phenotypes are reminiscent of ciliopathies, these findings suggest Pk1 may also regulate ciliogenesis. Together these findings show Pk1 plays an essential role in regulating cell polarity and directional cell **migration** during development.
31. MEKK1 transduces activin signals in keratinocytes to induce actin stress fiber formation and **migration**. Activins and other members of the transforming growth factor beta family play a critical role in morphological changes of the epidermis that require epithelial cell movement. We investigated the molecular pathways in the transmission of activin signals that lead to actin reorganization and epithelial cell **migration**. We found that activins cause the activation of RhoA but not of Rac and CDC42, leading to MEKK1-dependent phosphorylation of JNK and transcription factor c-Jun. Through a RhoA-independent mechanism, the activins also induce p38 activity in keratinocytes from wild-type but not from MEKK1-deficient mice. Although neither pathway is dependent on Smad activation, the MEKK1-mediated JNK and p38 activities are both essential for activin-stimulated and transcription-dependent keratinocyte **migration**. Only JNK is involved in transcription-independent actin stress fiber formation, which needs also the activity of ROCK. Because ROCK is required for JNK activation by RhoA and its overexpression leads to MEKK1 activation, we propose a RhoA-ROCK-MEKK1-JNK pathway and a MEKK1-p38 pathway as Smad-independent mechanisms in the transmission of activin signals. Together, these pathways lead to the control of actin cytoskeleton reorganization and epithelial cell **migration**, contributing to the physiologic and pathological effects of activins on epithelial morphogenesis.
32. Cooperation of matrix metalloproteinases with the RhoA/Rho kinase and mitogen-activated protein kinase kinase-1/extracellular signal-regulated kinase signaling pathways is required for the sphingosine-1-phosphate-induced mobilization of marrow-derived stromal cells. The ease of isolation and ex vivo culture of marrow-derived stromal cells (MSCs) from adult bone marrow renders them a very promising source of adult stem cells for gene transfer and cell therapy. However, little is known about the signaling pathways that control their in vivo mobilization and subsequent biodistribution. Platelet-derived sphingosine-1-phosphate (S1P), a bioactive lipid that acts via G-protein-coupled-receptors, exerts strong chemoattraction upon MSCs through yet-uncharacterized signaling pathways. We show that the S1P-induced **migration** and morphological changes of MSCs in vitro require the activities of extracellular signal-regulated kinase (ERK), Rho kinase (ROCK), and matrix metalloproteinase (MMP) signaling molecules. Specifically, S1P-induced remodeling of the MSC cytoskeleton led to the rapid (<1 minute) formation of actin stress fibers via activation of the RhoA/ROCK pathway and required the catalytic activity of MMPs. S1P-induced activation of the mitogen-activated protein kinase kinase-1 (MEK1)/ERK pathway also contributed to the induction of the actin stress fibers and to the redistribution of paxillin at the focal adhesions through tyrosine phosphorylation of focal adhesion kinase in an MMP-dependent manner. Moreover, MMP- and ROCK-dependent molecular events are implicated in the regulation of the S1P-induced activation of ERK. Our results demonstrate that MSC mobilization in response to S1P requires cooperation between MMP-mediated signaling events and the RhoA/ROCK and MEK1/ERK intracellular pathways. Therefore, the characterization of the cellular factors and the intracellular signaling pathways underlying MSC mobilization is crucial to achieve high efficacy in therapeutic use.
33. Impaired Rho GTPase activation abrogates cell polarization and **migration** in macrophages with defective lipolysis. Infiltration of monocytes and macrophages into the site of inflammation is critical in the progression of inflammatory diseases such as atherosclerosis. Cell **migration** is dependent on the continuous organization of the actin cytoskeleton, which is regulated by members of the small Rho GTPase family (RhoA, Cdc42, Rac) that are also important for the regulation of signal transduction pathways. We have recently reported on reduced plaque formation in an atherosclerotic mouse model transplanted with bone marrow from adipose triglyceride lipase-deficient (Atgl-/-) mice. Here we provide evidence that defective lipolysis in macrophages lacking ATGL, the major enzyme responsible for triacylglycerol hydrolysis, favors an anti-inflammatory M2-like macrophage phenotype. Our data implicate an as yet unrecognized principle that insufficient lipolysis influences macrophage polarization and actin polymerization, resulting in impaired macrophage **migration**. Sustained phosphorylation of focal adhesion kinase [due to inactivation of its phosphatase by elevated levels of reactive oxygen species (ROS)] results in defective Cdc42, Rac1 and RhoA activation and in increased and sustained activation of Rac2. Inhibition of ROS production restores the migratory capacity of Atgl-/- macrophages. Since monocyte and macrophage **migration** are a prerequisite for infiltrating the arterial wall, our results provide a molecular link between lipolysis and the development of atherosclerosis.
34. Proneural transcription factors regulate different steps of cortical neuron **migration** through Rnd-mediated inhibition of RhoA signaling. Little is known of the intracellular machinery that controls the motility of newborn neurons. We have previously shown that the proneural protein Neurog2 promotes the **migration** of nascent cortical neurons by inducing the expression of the atypical Rho GTPase Rnd2. Here, we show that another proneural factor, Ascl1, promotes neuronal **migration** in the cortex through direct regulation of a second Rnd family member, Rnd3. Both Rnd2 and Rnd3 promote neuronal **migration** by inhibiting RhoA signaling, but they control distinct steps of the migratory process, multipolar to bipolar transition in the intermediate zone and locomotion in the cortical plate, respectively. Interestingly, these divergent functions directly result from the distinct subcellular distributions of the two Rnd proteins. Because Rnd proteins also regulate progenitor divisions and neurite outgrowth, we propose that proneural factors, through spatiotemporal regulation of Rnd proteins, integrate the process of neuronal **migration** with other events in the neurogenic program.
35. An antagonistic interaction between PlexinB2 and Rnd3 controls RhoA activity and cortical neuron **migration**. A transcriptional programme initiated by the proneural factors Neurog2 and Ascl1 controls successive steps of neurogenesis in the embryonic cerebral cortex. Previous work has shown that proneural factors also confer a migratory behaviour to cortical neurons by inducing the expression of the small GTP-binding proteins such as Rnd2 and Rnd3. However, the directionality of radial **migration** suggests that migrating neurons also respond to extracellular signal-regulated pathways. Here we show that the Plexin B2 receptor interacts physically and functionally with Rnd3 and stimulates RhoA activity in migrating cortical neurons. Plexin B2 competes with p190RhoGAP for binding to Rnd3, thus blocking the Rnd3-mediated inhibition of RhoA and also recruits RhoGEFs to directly stimulate RhoA activity. Thus, an interaction between the cell-extrinsic Plexin signalling pathway and the cell-intrinsic Ascl1-Rnd3 pathway determines the level of RhoA activity appropriate for cortical neuron **migration**.
36. Vangl2 acts via RhoA signaling to regulate polarized cell movements during development of the proximal outflow tract. Loop-tail (Lp) mice develop double outlet right ventricle and aortic arch defects, and the defects are caused by mutations in the Vangl2 gene. Vangl2 mRNA is found in the outflow tract myocardium, including the myocardializing cells that migrate into the outflow tract cushions. Analysis of muscularization of the outflow tract septum showed that this process is compromised in Lp/Lp fetuses. Vangl2 is a component of the noncanonical Wnt, planar cell polarity (PCP) pathway that signals via RhoA. We therefore looked for evidence of polarization in myocardializing cells. In wild-type fetuses, myocardializing cells extend lamellipodia and filopodia into the cushion tissue and reorganize their actin cytoskeleton from a cortical form to stress fibers; behaviors that are characteristic of polarized cells. In contrast, Lp/Lp littermates do not extend lamellipodia or filopodia into the cushion tissue, and their actin remains in a cortical form, suggesting that polarized cell **migration** of myocardializing cells is inhibited in Lp/Lp. Several other components of the PCP pathway are also localized in the outflow tract myocardium. In wild-type fetuses, the myocardializing cells coexpress RhoA and one of its downstream mediators, ROCK1. RhoA expression is disrupted in Lp/Lp, and is lost from the myocardial-cushion tissue interface, including the presumptive myocardializing cells. These data suggest that Vangl2 is required for the polarization and movement of myocardializing cells into the outflow tract cushions, and that RhoA and ROCK1 are downstream mediators of the PCP signaling pathway in the developing outflow tract.
37. Cytohesin-1 controls the activation of RhoA and modulates integrin-dependent adhesion and **migration** of dendritic cells. Adhesion and motility of mammalian leukocytes are essential requirements for innate and adaptive immune defense mechanisms. We show here that the guanine nucleotide exchange factor cytohesin-1, which had previously been demonstrated to be an important component of beta-2 integrin activation in lymphocytes, regulates the activation of the small GTPase RhoA in primary dendritic cells (DCs). Cytohesin-1 and RhoA are both required for the induction of chemokine-dependent conformational changes of the integrin beta-2 subunit of DCs during adhesion under physiological flow conditions. Furthermore, use of RNAi in murine bone marrow DCs (BM-DCs) revealed that interference with cytohesin-1 signaling impairs **migration** of wild-type dendritic cells in complex 3D environments and in vivo. This phenotype was not observed in the complete absence of integrins. We thus demonstrate an essential role of cytohesin-1/RhoA during ameboid **migration** in the presence of integrins and further suggest that DCs without integrins switch to a different **migration** mode.
38. Tensin1 positively regulates RhoA activity through its interaction with DLC1. DLC1 is a RhoGAP-containing tumor suppressor and many of DLC1's functions are absolutely dependent on its RhoGAP activity. Through its RhoGAP domain, DLC1 inhibits the activity of RhoA GTPase, which regulates actin cytoskeleton networks and dis/assembly of focal adhesions. Tensin1 (TNS1) is a focal adhesion molecule that links the actin cytoskeleton to integrins and forms signaling complexes through its multiple binding domains. Here, we report that TNS1 enhances RhoA activity in a DLC1-dependent manner. This is accomplished by binding to DLC1 through TNS1's C2, SH2, and PTB domains. Point mutations at these three sites disrupt TNS1's interaction with DLC1 as well as its effect on RhoA activity. The biological relevance of this TNS1-DLC1-RhoA signaling axis is investigated in TNS1 knockout (KO) cells and mice. Endothelial cells isolated from TNS1 KO mice or those silenced with TNS1 siRNA show significant reduction in proliferation, **migration**, and tube formation activities. Concomitantly, the RhoA activity is down-regulated in TNS1 KO cells and this reduction is restored by further silencing of DLC1. Furthermore, the angiogenic process is compromised in TNS1 KO mice. These studies demonstrate that TNS1 binds to DLC1 and fine-tunes its RhoGAP activity toward RhoA and that the TNS1-DLC1-RhoA signaling axis is critical in regulating cellular functions that lead to angiogenesis.
39. Endothelial cell **migration** during murine yolk sac vascular remodeling occurs by means of a Rac1 and FAK activation pathway in vivo. The molecular mechanism(s) controlling cell **migration** during vascular morphogenesis in vivo remain largely undefined. To address this within a physiological context, we used retinaldehyde dehydrogenase-2 (Raldh2) null mouse embryos and demonstrate that retinoic acid (RA) deficiency results in abnormal yolk sac vascular remodeling due to decreased Rac1 activation, increased RhoA activation, and increased focal adhesions. Vinculin was increased in Raldh2-/- yolk sacs, and molecular events important for focal adhesion turnover, FAK phosphorylation (Tyr397) and FAK-paxillin association, were decreased. RA-rescue of vascular remodeling down-regulated vinculin and restored FAK phosphorylation (Tyr397) and FAK-paxillin association. Furthermore, vascular rescue with vascular endothelial growth factor-A, Indian hedgehog, and basic fibroblast growth factor restored FAK phosphorylation (Tyr397) in the endothelium of Raldh2-/- yolk sacs. Our results provide new insights into the regulation of endothelial cell **migration** during vascular remodeling in vivo by adding the Rac1 and FAK activation pathway as a critical mediator of focal adhesion formation and turnover during vascular remodeling.
40. Daphnetin inhibits invasion and **migration** of LM8 murine osteosarcoma cells by decreasing RhoA and Cdc42 expression. Daphnetin, 7,8-dihydroxycoumarin, present in main constituents of Daphne odora var. marginatai, has multiple pharmacological activities including anti-proliferative effects in cancer cells. In this study, using a Transwell system, we showed that daphnetin inhibited invasion and **migration** of highly metastatic murine osteosarcoma LM8 cells in a dose-dependent manner. Following treatment by daphnetin, cells that penetrated the Transwell membrane were rounder than non-treated cells. Immunofluorescence analysis revealed that daphnetin decreased the numbers of intracellular stress fibers and filopodia. Moreover, daphnetin treatment dramatically decreased the expression levels of RhoA and Cdc42. In summary, the dihydroxycoumarin derivative daphnetin inhibits the invasion and **migration** of LM8 cells, and therefore represents a promising agent for use against metastatic cancer.
41. Tensin1 positively regulates RhoA activity through its interaction with DLC1. DLC1 is a RhoGAP-containing tumor suppressor and many of DLC1's functions are absolutely dependent on its RhoGAP activity. Through its RhoGAP domain, DLC1 inhibits the activity of RhoA GTPase, which regulates actin cytoskeleton networks and dis/assembly of focal adhesions. Tensin1 (TNS1) is a focal adhesion molecule that links the actin cytoskeleton to integrins and forms signaling complexes through its multiple binding domains. Here, we report that TNS1 enhances RhoA activity in a DLC1-dependent manner. This is accomplished by binding to DLC1 through TNS1's C2, SH2, and PTB domains. Point mutations at these three sites disrupt TNS1's interaction with DLC1 as well as its effect on RhoA activity. The biological relevance of this TNS1-DLC1-RhoA signaling axis is investigated in TNS1 knockout (KO) cells and mice. Endothelial cells isolated from TNS1 KO mice or those silenced with TNS1 siRNA show significant reduction in proliferation, **migration**, and tube formation activities. Concomitantly, the RhoA activity is down-regulated in TNS1 KO cells and this reduction is restored by further silencing of DLC1. Furthermore, the angiogenic process is compromised in TNS1 KO mice. These studies demonstrate that TNS1 binds to DLC1 and fine-tunes its RhoGAP activity toward RhoA and that the TNS1-DLC1-RhoA signaling axis is critical in regulating cellular functions that lead to angiogenesis.
42. Mouse macrophages completely lacking Rho subfamily GTPases (RhoA, RhoB, and RhoC) have severe lamellipodial retraction defects, but robust chemotactic navigation and altered motility. RhoA is thought to be essential for coordination of the membrane protrusions and retractions required for immune cell motility and directed **migration**. Whether the subfamily of Rho (Ras homolog) GTPases (RhoA, RhoB, and RhoC) is actually required for the directed **migration** of primary cells is difficult to predict. Macrophages isolated from myeloid-restricted RhoA/RhoB (conditional) double knock-out (dKO) mice did not express RhoC and were essentially "pan-Rho"-deficient. Using real-time chemotaxis assays, we found that retraction of the trailing edge was dissociated from the advance of the cell body in dKO cells, which developed extremely elongated tails. Surprisingly, velocity (of the cell body) was increased, whereas chemotactic efficiency was preserved, when compared with WT macrophages. Randomly migrating RhoA/RhoB dKO macrophages exhibited multiple small protrusions and developed large "branches" due to impaired lamellipodial retraction. A mouse model of peritonitis indicated that monocyte/macrophage recruitment was, surprisingly, more rapid in RhoA/RhoB dKO mice than in WT mice. In comparison with dKO cells, the phenotypes of single RhoA- or RhoB-deficient macrophages were mild due to mutual compensation. Furthermore, genetic deletion of RhoB partially reversed the motility defect of macrophages lacking the RhoGAP (Rho GTPase-activating protein) myosin IXb (Myo9b). In conclusion, the Rho subfamily is not required for "front end" functions (motility and chemotaxis), although both RhoA and RhoB are involved in pulling up the "back end" and resorbing lamellipodial membrane protrusions. Macrophages lacking Rho proteins migrate faster in vitro, which, in the case of the peritoneum, translates to more rapid in vivo monocyte/macrophage recruitment.
43. An antagonistic interaction between PlexinB2 and Rnd3 controls RhoA activity and cortical neuron **migration**. A transcriptional programme initiated by the proneural factors Neurog2 and Ascl1 controls successive steps of neurogenesis in the embryonic cerebral cortex. Previous work has shown that proneural factors also confer a migratory behaviour to cortical neurons by inducing the expression of the small GTP-binding proteins such as Rnd2 and Rnd3. However, the directionality of radial **migration** suggests that migrating neurons also respond to extracellular signal-regulated pathways. Here we show that the Plexin B2 receptor interacts physically and functionally with Rnd3 and stimulates RhoA activity in migrating cortical neurons. Plexin B2 competes with p190RhoGAP for binding to Rnd3, thus blocking the Rnd3-mediated inhibition of RhoA and also recruits RhoGEFs to directly stimulate RhoA activity. Thus, an interaction between the cell-extrinsic Plexin signalling pathway and the cell-intrinsic Ascl1-Rnd3 pathway determines the level of RhoA activity appropriate for cortical neuron **migration**.
44. The small GTPases RhoA and Rac1 regulate cerebellar development by controlling cell morphogenesis, **migration** and foliation. The small GTPases RhoA and Rac1 are key cytoskeletal regulators that function in a mutually antagonistic manner to control the **migration** and morphogenesis of a broad range of cell types. However, their role in shaping the cerebellum, a unique brain structure composed of an elaborate set of folia separated by fissures of different lengths, remains largely unexplored. Here we show that dysregulation of both RhoA and Rac1 signaling results in abnormal cerebellar ontogenesis. Ablation of RhoA from neuroprogenitor cells drastically alters the timing and placement of fissure formation, the **migration** and positioning of granule and Purkinje cells, the alignment of Bergmann glia, and the integrity of the basement membrane, primarily in the anterior lobules. Furthermore, in the absence of RhoA, granule cell precursors located at the base of fissures fail to undergo cell shape changes required for fissure initiation. Many of these abnormalities can be recapitulated by deleting RhoA specifically from granule cell precursors but not postnatal glia, indicating that RhoA functions in granule cell precursors to control cerebellar morphogenesis. Notably, mice with elevated Rac1 activity due to loss of the Rac1 inhibitors Bcr and Abr show similar anterior cerebellar deficits, including ectopic neurons and defects in fissure formation, Bergmann glia organization and basement membrane integrity. Together, our results suggest that RhoA and Rac1 play indispensable roles in patterning cerebellar morphology.
45. RhoA determines disease progression by controlling neutrophil motility and restricting hyperresponsiveness. Neutrophil responses are central to host protection and inflammation. Neutrophil activation follows a 2-step process in which priming amplifies responses to activating stimuli. Priming is essential for life span extension, chemotaxis, and respiratory burst activity. Here we show that the cytoskeletal organizer RhoA suppresses neutrophil priming via formins. Premature granule exocytosis in Rho-deficient neutrophils activated numerous signaling pathways and amplified superoxide generation. Deletion of Rho altered front-to-back coordination by simultaneously increasing uropod elongation, leading edge formation, and random **migration**. Concomitant negative and positive regulation of β2 integrin-independent and β2 integrin-dependen**t migrati**on, respectively, reveal Rho as a key decision point in the neutrophil response to discrete chemotactic agents. Although even restricted influx of Rho-deficient hyperactive neutrophils exacerbated lipopolysaccharide-mediated lung injury, deleting Rho in innate immune cells was highly protective in influenza A virus infection. Hence, Rho is a key regulator of disease progression by maintaining neutrophil quiescence and suppressing hyperresponsiveness.
46. Type VIII collagen signals via β1 integrin and RhoA to regulate MMP-2 expression and smooth muscle cell **migratio**n. The extracellular matrix signals and regulates the behavior of vascular cells during the pathogenesis of atherosclerosis. Type VIII collagen, a short chain collagen, is scarcely present in normal arteries, but is dramatically upregulated in atherosclerosis and after other types of vascular injury. Cell culture studies have revealed that this protein supports smooth muscle cell (SMC) adhesion and stimulates **migratio**n, however little is known about the signaling or the mechanisms by which this occurs. SMCs isolated from wild-type C57BL/6 and type VIII collagen deficient mice were studied using assays to measure chemotactic and haptotactic **migratio**n, and remodeling and contraction of 3-dimensional type I collagen gels. Col8(-/-) SMCs exhibited impairments in **migratio**n, and a strongly adhesive phenotype with prominent stress fibers, stable microtubules and pronounced central basal focal adhesions. The addition of exogenous type VIII collagen to the Col8(-/-) SMCs rescued the impairments in **migratio**n, and restored cytoskeletal architecture so that it was similar to Col8(+/+) cells. We measured elevated levels of active GTP-RhoA in the Col8(-/-) cells, and this too was reversed by treatment with exogenous type VIII collagen. We showed that type VIII collagen normally suppresses RhoA activation through a beta-1 integrin dependent mechanism. MMP-2 levels were reduced in the Col8(-/-) SMCs, and knockdown of MMP-2 in Col8(+/+) SMCs partially recapitulated the decreases in **migratio**n and 3D gel contraction seen in Col8(-/-) cells, showing that type VIII collagen-stimulated **migratio**n was dependent on MMP-2. Inhibition of Rho restored MMP-2 activity in the Col8(-/-) cells, and partially rescued **migratio**n, demonstrating that the elevations in RhoA activity were responsible for the suppression of **migratio**n of these cells. In conclusion, we have shown that type VIII collagen signals through beta-1 integrin receptors to suppress RhoA, allowing optimal configuration of the cytoskeleton, and the stimulation of MMP-2-dependent cell **migratio**n.
47. The orphan adhesion G protein-coupled receptor GPR97 regulates **migration** of lymphatic endothelial cells via the small GTPases RhoA and Cdc42. The important role of the lymphatic vascular system in pathological conditions such as inflammation and cancer has been increasingly recognized, but its potential as a pharmacological target is poorly exploited. Our study aimed at the identification and molecular characterization of lymphatic-specific G protein-coupled receptors (GPCRs) to assess new targets for pharmacological manipulation of the lymphatic vascular system. We used a TaqMan quantitative RT-PCR-based low density array to determine the GPCR expression profiles of ex vivo isolated intestinal mouse lymphatic (LECs) and blood vascular endothelial cells (BECs). GPR97, an orphan adhesion GPCR of unknown function, was the most highly and specifically expressed GPCR in mouse lymphatic endothelium. Using siRNA silencing, we found that GPR97-deficient primary human LECs displayed increased adhesion and collective cell **migration**, whereas single cell **migration** was decreased as compared with nontargeting siRNA-transfected control LECs. Loss of GPR97 shifted the ratio of active Cdc42 and RhoA and initiated cytoskeletal rearrangements, including F-actin redistribution, paxillin and PAK4 phosphorylation, and β1-integrin activation. Our data suggest a possible role of GPR97 in lymphatic remodeling and furthermore provide the first insights into the biological functions of GPR97.
48. RhoA and Cdc42 are required in pre-migratory progenitors of the medial ganglionic eminence ventricular zone for proper cortical interneuron **migration**. Cortical interneurons arise from the ganglionic eminences in the ventral telencephalon and migrate tangentially to the cortex. Although RhoA and Cdc42, members of the Rho family of small GTPases, have been implicated in regulating neuronal **migration**, their respective roles in the tangential **migration** of cortical interneurons remain unknown. Here we show that loss of RhoA and Cdc42 in the ventricular zone (VZ) of the medial ganglionic eminence (MGE) using Olig2-Cre mice causes moderate or severe defects in the **migration** of cortical interneurons, respectively. Furthermore, RhoA- or Cdc42-deleted MGE cells exhibit impaired **migration** in vitro. To determine whether RhoA and Cdc42 directly regulate the motility of cortical interneurons during **migration**, we deleted RhoA and Cdc42 in the subventricular zone (SVZ), where more fate-restricted progenitors are located within the ganglionic eminences, using Dlx5/6-Cre-ires-EGFP (Dlx5/6-CIE) mice. Deletion of either gene within the SVZ does not cause any obvious defects in cortical interneuron **migration**, indicating that cell motility is not dependent upon RhoA or Cdc42. These findings provide genetic evidence that RhoA and Cdc42 are required in progenitors of the MGE in the VZ, but not the SVZ, for proper cortical interneuron **migration**.
49. Paxillin and Hic-5 interaction with vinculin is differentially regulated by Rac1 and RhoA. Cell **migration** is of paramount importance to organism development and maintenance as well as multiple pathological processes, including cancer metastasis. The RhoGTPases Rac1 and RhoA are indispensable for cell **migration** as they regulate cell protrusion, cell-extracellular matrix (ECM) interactions and force transduction. However, the consequences of their activity at a molecular level within the cell remain undetermined. Using a combination of FRET, FRAP and biochemical analyses we show that the interactions between the focal adhesion proteins vinculin and paxillin, as well as the closely related family member Hic-5 are spatially and reciprocally regulated by the activity of Rac1 and RhoA. Vinculin in its active conformation interacts with either paxillin or Hic-5 in adhesions in response to Rac1 and RhoA activation respectively, while inactive vinculin interacts with paxillin in the membrane following Rac1 inhibition. Additionally, Rac1 specifically regulates the dynamics of paxillin as well as its binding partner and F-actin interacting protein actopaxin (α-parvin) in adhesions. Furthermore, FRET analysis of protein:protein interactions within cell adhesions formed in 3D matrices revealed that, in contrast to 2D systems vinculin interacts preferentially with Hic-5. This study provides new insight into the complexity of cell-ECM adhesions in both 2D and 3D matrices by providing the first description of RhoGTPase-coordinated protein:protein interactions in a cellular microenvironment. These data identify discrete roles for paxillin and Hic-5 in Rac1 and RhoA-dependent cell adhesion formation and maturation; processes essential for productive cell **migratio**n.
50. A radial glia-specific role of RhoA in double cortex formation. The positioning of neurons in the cerebral cortex is of crucial importance for its function as highlighted by the severe consequences of **migration**al disorders in patients. Here we show that genetic deletion of the small GTPase RhoA in the developing cerebral cortex results in two **migration**al disorders: subcortical band heterotopia (SBH), a heterotopic cortex underlying the normotopic cortex, and cobblestone lissencephaly, in which neurons protrude beyond layer I at the pial surface of the brain. Surprisingly, RhoA(-/-) neurons migrated normally when transplanted into wild-type cerebral cortex, whereas the converse was not the case. Alterations in the radial glia scaffold are demonstrated to cause these **migration**al defects through destabilization of both the actin and the microtubules cytoskeleton. These data not only demonstrate that RhoA is largely dispensable for **migration** in neurons but also showed that defects in radial glial cells, rather than neurons, can be sufficient to produce SBH.
51. Impaired Rho GTPase activation abrogates cell polarization and **migration** in macrophages with defective lipolysis. Infiltration of monocytes and macrophages into the site of inflammation is critical in the progression of inflammatory diseases such as atherosclerosis. Cell **migration** is dependent on the continuous organization of the actin cytoskeleton, which is regulated by members of the small Rho GTPase family (RhoA, Cdc42, Rac) that are also important for the regulation of signal transduction pathways. We have recently reported on reduced plaque formation in an atherosclerotic mouse model transplanted with bone marrow from adipose triglyceride lipase-deficient (Atgl-/-) mice. Here we provide evidence that defective lipolysis in macrophages lacking ATGL, the major enzyme responsible for triacylglycerol hydrolysis, favors an anti-inflammatory M2-like macrophage phenotype. Our data implicate an as yet unrecognized principle that insufficient lipolysis influences macrophage polarization and actin polymerization, resulting in impaired macrophage **migration**. Sustained phosphorylation of focal adhesion kinase [due to inactivation of its phosphatase by elevated levels of reactive oxygen species (ROS)] results in defective Cdc42, Rac1 and RhoA activation and in increased and sustained activation of Rac2. Inhibition of ROS production restores the migratory capacity of Atgl-/- macrophages. Since monocyte and macrophage **migration** are a prerequisite for infiltrating the arterial wall, our results provide a molecular link between lipolysis and the development of atherosclerosis.
52. Rho GTPases in hematopoietic stem/progenitor cell **migration**. Rho GTPases including RhoA, Rac1, and Cdc42 are a class of intracellular signaling proteins critical for the regulation of cytoskeleton organization, adhesion, and **migration**. Molecular mechanisms of mammalian cell **migration** were first revealed in fibroblasts where RhoA, Rac1, and Cdc42 facilitate in the multistep process including establishment and maintenance of polarity, formation of actin-rich protrusions, remodeling of adhesive contacts, and generation of force. In hematopoietic stem/progenitor cells, Rho GTPases relay signals from chemokines and cytokines such as SDF-1α and SCF to the actin and microtubule cytoskeleton through effector kinases and/or adaptor molecules that affect adhesion or transcription. Comprehensive use of murine conditional gene knockout technology combined with biochemical approaches in recent studies allows for physiologically relevant investigations of the involvement of Rho GTPases in hematopoietic stem/progenitor cell **migratio**n, providing important mechanisms for the stem/progenitor maintenance.
53. Rab13-dependent trafficking of RhoA is required for directional **migration** and angiogenesis. Angiogenesis requires concomitant remodeling of cell junctions and **migration**, as exemplified by recent observations of extensive endothelial cell movement along growing blood vessels. We report that a protein complex that regulates cell junctions is required for VEGF-driven directional **migration** and for angiogenesis in vivo. The complex consists of RhoA and Syx, a RhoA guanine exchange factor cross-linked by the Crumbs polarity protein Mupp1 to angiomotin, a phosphatidylinositol-binding protein. The Syx-associated complex translocates to the leading edge of migrating cells by membrane trafficking that requires the tight junction recycling GTPase Rab13. In turn, Rab13 associates with Grb2, targeting Syx and RhoA to Tyr(1175)-phosphorylated VEGFR2 at the leading edge. Rab13 knockdown in zebrafish impeded sprouting of intersegmental vessels and diminished the directionality of their tip cells. These results indicate that endothelial cell mobility in sprouting vessels is facilitated by shuttling the same protein complex from disassembling junctions to the leading edges of cells.
54. Vav GEFs regulate macrophage morphology and adhesion-induced Rac and Rho activation. The Vav family of proteins have the potential to act as both signalling adapters and GEFs for Rho GTPases. They have therefore been proposed as regulators of the cytoskeleton in various cell types. We have used macrophages from mice deficient in all three Vav isoforms to determine how their function affects cell morphology and **migration**. Macrophages lacking Vav proteins adopt an elongated morphology and have enhanced migratory persistence in culture. To investigate the pathways through which Vav proteins exert their effects we analysed the responses of macrophages to the chemoattractant CSF-1 and to adhesion. We found that morphological and signalling responses of macrophages to CSF-1 did not require Vav proteins. In contrast, adhesion-induced cell spreading, RhoA and Rac1 activation and cell signalling were all dependent on Vav proteins. We propose that Vav proteins affect macrophage morphology and motile behaviour by coupling adhesion receptors to Rac1 and RhoA activity and regulating adhesion signalling events such as paxillin and ERK1/2 phosphorylation by acting as adapters.
55. Cytohesin-1 controls the activation of RhoA and modulates integrin-dependent adhesion and **migration** of dendritic cells. Adhesion and motility of mammalian leukocytes are essential requirements for innate and adaptive immune defense mechanisms. We show here that the guanine nucleotide exchange factor cytohesin-1, which had previously been demonstrated to be an important component of beta-2 integrin activation in lymphocytes, regulates the activation of the small GTPase RhoA in primary dendritic cells (DCs). Cytohesin-1 and RhoA are both required for the induction of chemokine-dependent conformational changes of the integrin beta-2 subunit of DCs during adhesion under physiological flow conditions. Furthermore, use of RNAi in murine bone marrow DCs (BM-DCs) revealed that interference with cytohesin-1 signaling impairs **migration** of wild-type dendritic cells in complex 3D environments and in vivo. This phenotype was not observed in the complete absence of integrins. We thus demonstrate an essential role of cytohesin-1/RhoA during ameboid **migration** in the presence of integrins and further suggest that DCs without integrins switch to a different **migration** mode.
56. Therapeutic ultrasound bypasses canonical syndecan-4 signaling to activate rac1. The application of pulsed, low intensity ultrasound is emerging as a potent therapy for the treatment of complex bone fractures and tissue damage. Ultrasonic stimuli accelerate fracture healing by up to 40% and enhance tendon and ligament healing by promoting cell proliferation, **migration**, and matrix synthesis through an unresolved mechanism. Ultrasound treatment also induces closure of nonunion fractures, at a success rate (85% of cases) similar to that of surgical intervention (68-96%) while avoiding the complications associated with surgery. The regulation of cell adhesion necessary for wound healing depends on cooperative engagement of the extracellular matrix receptors, integrin and syndecan, as exemplified by the wound healing defects observed in syndecan- and integrin-knock-out mice. This report distinguishes the influence of ultrasound on signals downstream of the prototypic fibronectin receptors, alpha(5)beta(1) integrin and syndecan-4, which cooperate to regulate Rac1 and RhoA. Ultrasonic stimulation fails to activate integrins or induce cell spreading on poor, electrostatic ligands. By contrast, ultrasound treatment overcomes the necessity of engagement or expression of syndecan-4 during the process of focal adhesion formation, which normally requires simultaneous engagement of both receptors. Ultrasound exerts an influence downstream of syndecan-4 and PKCalpha to specifically activate Rac1, itself a critical regulator of tissue repair, and to a lesser extent RhoA. The ability of ultrasound to bypass syndecan-4 signaling, which is known to facilitate efficient tissue repair, explains the reduction in healing times observed in ultrasound-treated patients. By substituting for one of the key axes of adhesion-dependent signaling, ultrasound therapy has considerable potential as a clinical technique.
57. Blockade of the RhoA-JNK-c-Jun-MMP2 cascade by atorvastatin reduces osteosarcoma cell invasion. Osteosarcoma is characterized by a high malignant and metastatic potential, which points to the need for new therapeutic strategies to prevent cell metastasis. In this study, we show that statin-induced HMG-CoA reductase inhibition reduces cell **migration** and invasion in human and murine osteosarcoma cells, independently of the genotype. The statin-induced reduction of cell **migration** and invasion was independent of induction of apoptosis and was geranylgeranylpyrophosphate-dependent. The statin reduced matrix metalloproteinase (MMP) 2, 9, and 14 and TIMP2 expression or activity in invading cells. Forced expression of MMP2 and MMP14 overcame the inhibitory effect of the statin on cell invasion, suggesting a role for these MMPs in invasive potential. We also investigated the mechanisms involved in the reduced MMP2 activity and cell invasion. Inhibition of JNK, but not ERK1/2 signaling, reduced MMP2 activity. Pharmacological or constitutive activation of JNK overcame the reduced MMP2 activity and cell invasion induced by the statin. The statin decreased JNK phosphorylation and c-Jun nuclear translocation, suggesting that HMG-CoA reductase inhibition targets the JNK-c-Jun signaling pathway. We showed that mevalonate or geranylgeranylpyrophosphate treatment prevented the statin-induced reduction in JNK phosphorylation, MMP2 activity, and cell invasion. Forced expression of a constitutively active form of RhoA increased JNK phosphorylation and overcame the inhibitory effect of atorvastatin on MMP2 activity and cell invasion. The data establish a link between RhoA, JNK, c-Jun, and MMP2 activity that is functionally involved in the reduction in osteosarcoma cell invasion by the statin. This suggests a novel strategy targeting RhoA-JNK-c-Jun signaling to reduce osteosarcoma cell tumorigenesis.
58. N-acetylglucosaminyltransferase V mediates cell **migration** and invasion of mouse mammary tumor cells 4TO7 via RhoA and Rac1 signaling pathway. In tumor cells, alterations in cellular glycosylation may play a key role in their metastatic behavior. Using small interfering RNA against GnT-V, we found that the expression of GnT-V and beta1,6GlcNAc branching were significantly reduced which was particularly accompanied by the arrest in both cell **migration** and invasion as compared to the negative control. Moreover, the suppressed GnT-V expression by siRNA technique inactivated the signaling molecules including Rac1, cofilin, Erk and Akt, and activated RhoA levels in cells lacking GnT-V, but revealed no impact on Cdc42 activity. All these notions disclose for the first time that GnT-V and beta1, 6GlcNAc branching mediate the cell **migration** and invasion in Rac1-positive and RhoA-negative regulatory manners.
59. Regulation of ephexin1, a guanine nucleotide exchange factor of Rho family GTPases, by fibroblast growth factor receptor-mediated tyrosine phosphorylation. Fibroblast growth factor (FGF) signal is implicated in not only cell proliferation, but cell **migration** and morphological changes. Several different Rho family GTPases downstream of the Ras/ERK pathway are postulated to mediate the latter functions. However, none have been recognized to be directly coupled to FGF receptors (FGFRs). We have previously reported that EphA4 and FGFRs hetero-oligomerize through their cytoplasmic domains, trans-activate each other, and transduce a signal for cell proliferation through a docking protein, FRS2alpha (Yokote, H., Fujita, K., Jing, X., Sawada, T., Liang, S., Yao, L., Yan, X., Zhang, Y., Schlessinger, J., and Sakaguchi, K. (2005) Proc. Natl. Acad. Sci. U. S. A. 102, 18866-18871). Here, we have found that ephexin1, a guanine nucleotide exchange factor for Rho family GTPases, constitutes another downstream component of the receptor complex. Ephexin1 directly binds to the kinase domain of FGFR mainly through its DH and PH domains. The binding appears to become weaker and limited to the DH domain when FGFRs become activated. FGFR-mediated phosphorylation of ephexin1 enhances the guanine nucleotide exchange activity toward RhoA without affecting the activity to Rac1 or Cdc42. The FGFR-mediated tyrosine phosphorylation includes, but is not limited to, the residue (Tyr-87) phosphorylated by Src family kinase, which is known to be activated following EphA4 activation. The Tyr-to-Asp mutations that mimic the tyrosine phosphorylation in some of the putative FGFR-mediated phosphorylation sites increase the nucleotide exchange activity for RhoA without changing the activity for Rac1 or Cdc42. From these results, we conclude that ephexin1 is located immediately downstream of the EphA4-FGFR complex and the function is altered by the FGFR-mediated tyrosine phosphorylation at multiple sites.
60. The p110delta isoform of PI 3-kinase negatively controls RhoA and PTEN. Inactivation of PI 3-kinase (PI3K) signalling is critical for tumour suppression by PTEN. This is thought to be a unidirectional relationship in which PTEN degrades the lipids produced by PI3K, thus controlling cell proliferation, survival and **migration**. We now show that this relationship is in fact bidirectional, whereby PI3K reciprocally controls PTEN. We report that the p110delta PI3K negatively regulates PTEN, through a pathway involving inhibition of RhoA. Inactivation of p110delta in macrophages led to reduced Akt and Rac1 activation, but paradoxically to increased RhoA and PTEN activity. Partial inactivation of p190RhoGAP and a reduced binding of cytoplasmic RhoA to the cyclin-dependent kinase inhibitor p27 both contributed to the increased RhoA-GTP levels upon p110delta inactivation. Pharmacological inhibition of ROCK, a downstream effector kinase of RhoA, restored all signalling and functional defects of p110delta inactivation, including Akt phosphorylation, chemotaxis and proliferation. This work identifies the RhoA/ROCK pathway as a major target of p110delta-mediated PI3K signalling, and establishes for the first time that PI3K controls itself, via a feedback loop involving PTEN.
61. MEKK1 transduces activin signals in keratinocytes to induce actin stress fiber formation and **migration**. Activins and other members of the transforming growth factor beta family play a critical role in morphological changes of the epidermis that require epithelial cell movement. We investigated the molecular pathways in the transmission of activin signals that lead to actin reorganization and epithelial cell **migration**. We found that activins cause the activation of RhoA but not of Rac and CDC42, leading to MEKK1-dependent phosphorylation of JNK and transcription factor c-Jun. Through a RhoA-independent mechanism, the activins also induce p38 activity in keratinocytes from wild-type but not from MEKK1-deficient mice. Although neither pathway is dependent on Smad activation, the MEKK1-mediated JNK and p38 activities are both essential for activin-stimulated and transcription-dependent keratinocyte **migration**. Only JNK is involved in transcription-independent actin stress fiber formation, which needs also the activity of ROCK. Because ROCK is required for JNK activation by RhoA and its overexpression leads to MEKK1 activation, we propose a RhoA-ROCK-MEKK1-JNK pathway and a MEKK1-p38 pathway as Smad-independent mechanisms in the transmission of activin signals. Together, these pathways lead to the control of actin cytoskeleton reorganization and epithelial cell **migration**, contributing to the physiologic and pathological effects of activins on epithelial morphogenesis.
62. Cooperation of matrix metalloproteinases with the RhoA/Rho kinase and mitogen-activated protein kinase kinase-1/extracellular signal-regulated kinase signaling pathways is required for the sphingosine-1-phosphate-induced mobilization of marrow-derived stromal cells. The ease of isolation and ex vivo culture of marrow-derived stromal cells (MSCs) from adult bone marrow renders them a very promising source of adult stem cells for gene transfer and cell therapy. However, little is known about the signaling pathways that control their in vivo mobilization and subsequent biodistribution. Platelet-derived sphingosine-1-phosphate (S1P), a bioactive lipid that acts via G-protein-coupled-receptors, exerts strong chemoattraction upon MSCs through yet-uncharacterized signaling pathways. We show that the S1P-induced **migration** and morphological changes of MSCs in vitro require the activities of extracellular signal-regulated kinase (ERK), Rho kinase (ROCK), and matrix metalloproteinase (MMP) signaling molecules. Specifically, S1P-induced remodeling of the MSC cytoskeleton led to the rapid (<1 minute) formation of actin stress fibers via activation of the RhoA/ROCK pathway and required the catalytic activity of MMPs. S1P-induced activation of the mitogen-activated protein kinase kinase-1 (MEK1)/ERK pathway also contributed to the induction of the actin stress fibers and to the redistribution of paxillin at the focal adhesions through tyrosine phosphorylation of focal adhesion kinase in an MMP-dependent manner. Moreover, MMP- and ROCK-dependent molecular events are implicated in the regulation of the S1P-induced activation of ERK. Our results demonstrate that MSC mobilization in response to S1P requires cooperation between MMP-mediated signaling events and the RhoA/ROCK and MEK1/ERK intracellular pathways. Therefore, the characterization of the cellular factors and the intracellular signaling pathways underlying MSC mobilization is crucial to achieve high efficacy in therapeutic use.
63. Hic-5 contributes to epithelial-mesenchymal transformation through a RhoA/ROCK-dependent pathway. Epithelial-mesenchymal transformation (EMT) in response to TGFbeta1 is a coordinated process of tissue morphogenesis that occurs during embryonic development as well as during certain pathologic events including kidney tubulointerstitial fibrosis. It is characterized by the disassembly of cell-cell junctions and dramatic alterations in the actin cytoskeleton that facilitates cell-matrix adhesion and stimulates **migration**. The focal adhesion adapter protein, Hic-5, has previously been reported to be upregulated during TGFbeta1-induced EMT in mouse mammary epithelial cells and the current study recapitulates this result in both mouse kidney proximal tubule epithelial, MCT, cells and human mammary epithelial, MCF10A, cells. To evaluate a causative role for Hic-5 in EMT, Hic-5 RNA interference (siRNA) was used to prevent Hic-5 expression in response to TGFbeta1 stimulation and was shown to suppress cell **migration** and actin stress fiber formation. It also resulted in the retention of a robust epithelial cell morphology characterized by elevated E-cadherin protein expression and well-organized adherens junctions. In addition, Hic-5 siRNA treatment led to the suppression of TGFbeta1 induction of RhoA activation. In contrast, forced expression of Hic-5 led to the formation of ROCK-dependent actin stress fibers. Furthermore, the induction of Hic-5 expression in response to TGFbeta1 was shown to be a RhoA/ROCK I-dependent process. Together, these data implicate Hic-5 as a key regulator of EMT and suggest that RhoA stimulated Hic-5 expression in response to TGFbeta1 may be functioning in a feed forward mechanism whereby Hic-5 maintains the mesenchymal phenotype through sustained RhoA activation and signaling.
64. The guanine nucleotide exchange factor Arhgef5 plays crucial roles in Src-induced podosome formation. Podosomes and invadopodia are actin-rich membrane protrusions that play a crucial role in cell adhesion and **migration**, and extracellular matrix remodeling in normal and cancer cells. The formation of podosomes and invadopodia is promoted by upregulation of some oncogenic molecules and is closely related to the invasive potential of cancer cells. However, the molecular mechanisms underlying the podosome and invadopodium formation still remain unclear. Here, we show that a guanine nucleotide exchange factor (GEF) for Rho family GTPases (Arhgef5) is crucial for Src-induced podosome formation. Using an inducible system for Src activation, we found that Src-induced podosome formation depends upon the Src SH3 domain, and identified Arhgef5 as a Src SH3-binding protein. RNA interference (RNAi)-mediated depletion of Arhgef5 caused robust inhibition of Src-dependent podosome formation. Overexpression of Arhgef5 promoted actin stress fiber remodeling through activating RhoA, and the activation of RhoA or Cdc42 was required for Src-induced podosome formation. Arhgef5 was tyrosine-phosphorylated by Src and bound to Src to positively regulate its activity. Furthermore, the pleckstrin homology (PH) domain of Arhgef5 was required for podosome formation, and Arhgef5 formed a ternary complex with Src and phosphoinositide 3-kinase when Src and/or Arhgef5 were upregulated. These findings provide novel insights into the molecular mechanisms of podosome and invadopodium formation induced by Src upregulation.
65. HDAC6 and RhoA are novel players in Abeta-driven disruption of neuronal polarity. Maintenance of neuronal polarity and regulation of cytoskeletal dynamics are vital during development and to uphold synaptic activity in neuronal networks. Here we show that soluble β-amyloid (Aβ) disrupts actin and microtubule (MT) dynamics via activation of RhoA and inhibition of histone deacetylase 6 (HDAC6) in cultured hippocampal neurons. The contact of Aβ with the extracellular membrane promotes RhoA activation, leading to growth cone collapse and neurite retraction, which might be responsible for hampered neuronal pathfinding a**nd migrat**ion in Alzheimer's disease (AD). The inhibition of HDAC6 by Aβ increases the level of heterodimeric acetylated tubulin and acetylated tau, both of which have been found altered in AD. We also find that the loss of HDAC6 activity perturbs the integrity of axon initial segment (AIS), resulting in mislocalization of ankyrin G and increased MT instability in the AIS concomitant with loss of polarized localization of tau and impairment of action potential firing.
66. Muscle-specific signaling mechanism that links actin dynamics to serum response factor. Myocardin and the myocardin-related transcription factors (MRTFs) MRTF-A and MRTF-B are coactivators for serum response factor (SRF), which regulates genes involved in cell proliferation, **migration**, cytoskeletal dynamics, and myogenesis. MRTF-A has been shown to translocate to the nucleus and activate SRF in response to Rho signaling and actin polymerization. Previously, we described a muscle-specific actin-binding protein named striated muscle activator of Rho signaling (STARS) that also activates SRF through a Rho-dependent mechanism. Here we show that STARS activates SRF by inducing the nuclear translocation of MRTFs. The STARS-dependent nuclear import of MRTFs requires RhoA and actin polymerization, and the actin-binding domain of STARS is necessary and sufficient for this activity. A knockdown of endogenous STARS expression by using small interfering RNA significantly reduced SRF activity in differentiated C2C12 skeletal muscle cells and cardiac myocytes. The ability of STARS to promote the nuclear localization of MRTFs and SRF-mediated transcription provides a potential muscle-specific mechanism for linking changes in actin dynamics and sarcomere structure with striated muscle gene expression.
67. Geranylgeranylation but not GTP loading determines rho migratory function in T cells. Rho GTPases orchestrate signaling pathways leading to cell **migration**. Their function depends on GTP loading and isoprenylation by geranylgeranyl pyrophosphate (GGpp). In this study, we show that in human T cells, geranylgeranylation-and not GTP loading-is necessary for RhoA-mediated downstream events. As a result of GGpp depletion with the 3-hydroxy-3-methylglutaryl-CoA reductase inhibitor atorvastatin, RhoA was sequestered from the membrane to the cytosol and, notwithstanding increased GTP loading, the constitutive activation of its substrate Rho-associated coiled-coil protein kinase-1 was blocked. In line with this, T cells expressing increased GTP-RhoA failed to form an intact cytoskeleton and to migrate toward a chemokine gradient. In vivo treatment with atorvastatin in the rodent model of multiple sclerosis markedly decreased the capacity of activated T cells to traffic within the brain, as demonstrated by multiphoton analysis. Thus, tethering of RhoA to the membrane by GGpp is determinant for T cell **migration** and provides a mechanism for preventing T cell infiltration into inflamed compartments by 3-hydroxy-3-methylglutaryl-CoA reductase inhibitors.
68. Acute activation of β2-adrenergic receptor regulates focal adhesions through βArrestin2- and p115RhoGEF protein-mediated activation of RhoA. β(2)-Adrenergic receptors (β(2)ARs) regulate cellular functions through G protein-transduced and βArrestin-transduced signals. β(2)ARs have been shown to regulate cancer **cell mig**ration, but the underlying mechanisms are not well understood. Here, we report that β(2)AR regulates formation of focal adhesions, whose dynamic remodeling is critical for directe**d cell mi**gration. β(2)ARs induce activation of RhoA, which is dependent on βArrestin2 but not G(s). βArrestin2 forms a complex with p115RhoGEF, a guanine nucleotide exchange factor for RhoA that is well known to be activated by G(12/13)-coupled receptors. Our results show that βArrestin2 forms a complex with p115RhoGEF in the cytosol in resting cells. Upon β(2)AR activation, both βArrestin2 and p115RhoGEF translocate to the plasma membrane, with concomitant activation of RhoA and formation of focal adhesions and stress fibers. Activation of RhoA and focal adhesion remodeling may explain, at least in part, the role of β(**2)ARs in** cell migration. These results suggest that βArrestin2 may serve as a convergence point for non-G(12/13) and non-G(q) protein-coupled receptors to activate RhoA.
69. The Meckel-Gruber syndrome protein TMEM67 controls basal body positioning and epithelial branching morphogenesis in mice via the non-canonical Wnt pathway. Ciliopathies are a group of developmental disorders that manifest with multi-organ anomalies. Mutations in TMEM67 (MKS3) cause a range of human ciliopathies, including Meckel-Gruber and Joubert syndromes. In this study we describe multi-organ developmental abnormalities in the Tmem67(tm1Dgen/H1) knockout mouse that closely resemble those seen in Wnt5a and Ror2 knockout mice. These include pulmonary hypoplasia, ventricular septal defects, shortening of the body longitudinal axis, limb abnormalities, and cochlear hair cell stereociliary bundle orientation and basal body/kinocilium positioning defects. The basal body/kinocilium complex was often uncoupled from the hair bundle, suggesting aberrant basal body **migration**, although planar cell polarity and apical planar asymmetry in the organ of Corti were normal. TMEM67 (meckelin) is essential for phosphorylation of the non-canonical Wnt receptor ROR2 (receptor-tyrosine-kinase-like orphan receptor 2) upon stimulation with Wnt5a-conditioned medium. ROR2 also colocalises and interacts with TMEM67 at the ciliary transition zone. Additionally, the extracellular N-terminal domain of TMEM67 preferentially binds to Wnt5a in an in vitro binding assay. Cultured lungs of Tmem67 mutant mice failed to respond to stimulation of epithelial branching morphogenesis by Wnt5a. Wnt5a also inhibited both the Shh and canonical Wnt/β-catenin signalling pathways in wild-type embryonic lung. Pulmonary hypoplasia phenotypes, including loss of correct epithelial branching morphogenesis and cell polarity, were rescued by stimulating the non-canonical Wnt pathway downstream of the Wnt5a-TMEM67-ROR2 axis by activating RhoA. We propose that TMEM67 is a receptor that has a main role in non-canonical Wnt signalling, mediated by Wnt5a and ROR2, and normally represses Shh signalling. Downstream therapeutic targeting of the Wnt5a-TMEM67-ROR2 axis might, therefore, reduce or prevent pulmonary hypoplasia in ciliopathies and other congenital conditions.
70. Negative regulation of RhoA translation and signaling by hnRNP-Q1 affects cellular morphogenesis. The small GTPase RhoA has critical functions in regulating actin dynamics affecting cellular morphogenesis through the RhoA/Rho kinase (ROCK) signaling cascade. RhoA signaling controls stress fiber and focal adhesion formation and cell motility in fibroblasts. RhoA signaling is involved in several aspects of neuronal development, including neuronal **migration**, growth cone collapse, dendrite branching, and spine growth. Altered RhoA signaling is implicated in cancer and neurodegenerative disease and is linked to inherited intellectual disabilities. Although much is known about factors regulating RhoA activity and/or degradation, little is known about molecular mechanisms regulating RhoA expression and the subsequent effects on RhoA signaling. We hypothesized that posttranscriptional control of RhoA expression may provide a mechanism to regulate RhoA signaling and downstream effects on cell morphology. Here we uncover a cellular function for the mRNA-binding protein heterogeneous nuclear ribonucleoprotein (hnRNP) Q1 in the control of dendritic development and focal adhesion formation that involves the negative regulation of RhoA synthesis and signaling. We show that hnRNP-Q1 represses RhoA translation and knockdown of hnRNP-Q1 induced phenotypes associated with elevated RhoA protein levels and RhoA/ROCK signaling. These morphological changes were rescued by ROCK inhibition and/or RhoA knockdown. These findings further suggest that negative modulation of RhoA mRNA translation can provide control over downstream signaling and cellular morphogenesis.
71. AMF/PGI transactivates the MMP-3 gene through the activation of Src-RhoA-phosphatidylinositol 3-kinase signaling to induce hepatoma cell **migration**. We have previously shown that AMF/PGI induces hepatoma cell **migration** through the induction of MMP-3. This work investigates how AMF/PGI activates the MMP-3 gene. We demonstrated that AMF/PGI transactivates the MMP-3 gene promoter through AP-1. The transactivation and induction of cell **migration** effect of AMF/PGI directly correlates with its enzymatic activity. Various analyses showed that AMF/PGI stimulated the Src-RhoA-PI3-kinase signaling pathway, and these three signaling molecules could form a complex. Our results demonstrate a new mechanism of AMF/PGI-induced cell **migration** and a link between Src-RhoA-PI3-kinase, AP-1, MMP-3 and hepatoma cell **migration**.
72. Kank regulates RhoA-dependent formation of actin stress fibers and cell **migration** via 14-3-3 in PI3K-Akt signaling. Phosphoinositide-3 kinase (PI3K)/Akt signaling is activated by growth factors such as insulin and epidermal growth factor (EGF) and regulates several functions such as cell cycling, apoptosis, cell growth, and cell **migration**. Here, we find that Kank is an Akt substrate located downstream of PI3K and a 14-3-3-binding protein. The interaction between Kank and 14-3-3 is regulated by insulin and EGF and is mediated through phosphorylation of Kank by Akt. In NIH3T3 cells expressing Kank, the amount of actin stress fibers is reduced, and the coexpression of 14-3-3 disrupted this effect. Kank also inhibits insulin-induced cell **migration** via 14-3-3 binding. Furthermore, Kank inhibits insulin and active Akt-dependent activation of RhoA through binding to 14-3-3. Based on these findings, we hypothesize that Kank negatively regulates the formation of actin stress fibers and cell **migration** through the inhibition of RhoA activity, which is controlled by binding of Kank to 14-3-3 in PI3K-Akt signaling.
73. CellGeo: a computational platform for the analysis of shape changes in cells with complex geometries. Cell biologists increasingly rely on computer-aided image analysis, allowing them to collect precise, unbiased quantitative results. However, despite great progress in image processing and computer vision, current computational approaches fail to address many key aspects of cell behavior, including the cell protrusions that guide cell **migration** and drive morphogenesis. We developed the open source MATLAB application CellGeo, a user-friendly computational platform to allow simultaneous, automated tracking and analysis of dynamic changes in cell shape, including protrusions ranging from filopodia to lamellipodia. Our method maps an arbitrary cell shape onto a tree graph that, unlike traditional skeletonization algorithms, preserves complex boundary features. CellGeo allows rigorous but flexible definition and accurate automated detection and tracking of geometric features of interest. We demonstrate CellGeo's utility by deriving new insights into (a) the roles of Diaphanous, Enabled, and Capping protein in regulating filopodia and lamellipodia dynamics in Drosophila melanogaster cells and (b) the dynamic properties of growth cones in catecholaminergic a-differentiated neuroblastoma cells.
74. p120-catenin differentially regulates cell **migration** by Rho-dependent intracellular and secreted signals. The adherens junction protein p120-catenin is implicated in the regulation of cadherin stability, cell **migration** and inflammatory responses in mammalian epithelial tissues. How these events are coordinated to promote wound repair is not understood. We show that p120 catenin regulates the intrinsic migratory properties of primary mouse keratinocytes, but also influences the migratory behavior of neighboring cells by secreted signals. These events are rooted in the ability of p120-catenin to regulate RhoA GTPase activity, which leads to a two-tiered control of cell **migration**. One restrains cell motility via an increase in actin stress fibers, reduction in integrin turnover and an increase in the robustness of focal adhesions. The other is coupled to the secretion of inflammatory cytokines including interleukin-24, which causally enhances randomized cell movements. Taken together, our results indicate that p120-RhoA-GTPase-mediated signaling can differentially regulate the migratory behavior of epidermal cells, which has potential implications for chronic wound responses and cancer.
75. Thrombospondin induces RhoA inactivation through FAK-dependent signaling to stimulate focal adhesion disassembly. Cells utilize dynamic interactions with the extracellular matrix to adapt to changing environmental conditions. Thrombospondin 1 (TSP1) induces focal adhesion disassembly and cell **migration** through a sequence (hep I) in its heparin-binding domain signaling through the calreticulin-low density lipoprotein receptor-related protein receptor complex. This involves the Galphai-dependent activation of ERK and phosphoinositide (PI) 3-kinase, both of which are required for focal adhesion disassembly. Focal adhesion kinase (FAK) regulates adhesion dynamics, acting in part by modulating RhoA activity, and FAK is implicated in ERK and PI 3-kinase activation. In this work, we sought to determine the role of FAK in TSP1-induced focal adhesion disassembly. TSP1/hep I does not stimulate focal adhesion disassembly in FAK knockout fibroblasts, whereas re-expressing FAK rescues responsiveness. Inhibiting FAK signaling through FRNK or FAK Y397F expression in endothelial cells also abrogates this response. TSP1/hep I stimulates a transient increase in FAK phosphorylation that requires calreticulin and Galphai, but not ERK or PI 3-kinase. Hep I does not activate ERK or PI 3-kinase in FAK knockout fibroblasts, suggesting activation occurs downstream of FAK. TSP1/hep I stimulates RhoA inactivation with kinetics corresponding to focal adhesion disassembly in a FAK, ERK, and PI 3-kinase-dependent manner. Furthermore, hep I does not stimulate focal adhesion disassembly in cells expressing constitutively active RhoA, suggesting that RhoA inactivation is required for this response. This is the first work to illustrate a connection between FAK phosphorylation in response to a soluble factor and RhoA inactivation, as well as the first report of PI 3-kinase and ERK in FAK regulation of RhoA activity.
76. A novel Rho-dependent pathway that drives interaction of fascin-1 with p-Lin-11/Isl-1/Mec-3 kinase (LIMK) 1/2 to promote fascin-1/actin binding and filopodia stability. Fascin-1 is an actin crosslinking protein that is important for the assembly of cell protrusions in neurons, skeletal and smooth muscle, fibroblasts, and dendritic cells. Although absent from most normal adult epithelia, fascin-1 is upregulated in many human carcinomas, and is associated with poor prognosis because of its promotion of carcinoma cell **migration**, invasion, and metastasis. Rac and Cdc42 small guanine triphosphatases have been identified as upstream regulators of the association of fascin-1 with actin, but the possible role of Rho has remained obscure. Additionally, experiments have been hampered by the inability to measure the fascin-1/actin interaction directly in intact cells. We investigated the hypothesis that fascin-1 is a functional target of Rho in normal and carcinoma cells, using experimental approaches that included a novel fluorescence resonance energy transfer (FRET)/fluorescence lifetime imaging (FLIM) method to measure the interaction of fascin-1 with actin.Rho activity modulates the interaction of fascin-1 with actin, as detected by a novel FRET method, in skeletal myoblasts and human colon carcinoma cells. Mechanistically, Rho regulation depends on Rho kinase activity, is independent of the status of myosin II activity, and is not mediated by promotion of the fascin/PKC complex. The p-Lin-11/Isl-1/Mec-3 kinases (LIMK), LIMK1 and LIMK2, act downstream of Rho kinases as novel binding partners of fascin-1, and this complex regulates the stability of filopodia.We have identified a novel activity of Rho in promoting a complex between fascin-1 and LIMK1/2 that modulates the interaction of fascin-1 with actin. These data provide new mechanistic insight into the intracellular coordination of contractile and protrusive actin-based structures. During the course of the study, we developed a novel FRET method for analysis of the fascin-1/actin interaction, with potential general applicability for analyzing the activities of actin-binding proteins in intact cells.
77. TGFBR1\*6A enhances the **migration** and invasion of MCF-7 breast cancer cells through RhoA activation. TGFBR1\*6A is a common hypomorphic variant of the type 1 transforming growth factor beta receptor (TGFBR1), which has been associated with increased cancer risk in some studies. Although TGFBR1\*6A is capable of switching TGF-beta growth-inhibitory signals into growth-stimulatory signals when stably transfected into MCF-7 breast cancer cells, the biological effects of TGFBR1\*6A are largely unknown. To broadly explore the potential oncogenic properties of TGFBR1\*6A, we assessed its effects on NIH-3T3 cells as well as its effect on the **migration** and invasion of MCF-7 cells. We found that TGFBR1\*6A has decreased oncogenic properties compared with TGFBR1. However, TGFBR1\*6A significantly enhances MCF-7 cell **migration** and invasion in a TGF-beta signaling-independent manner. Gene expression profiling studies identified two down-regulated genes involved in cell **migration** and invasion: ARHGAP5, encoding ARHGAP5, and FN1, encoding fibronectin-1 (FN1). ARHGAP5 and FN1 expression was similarly down-regulated in MCF-7 cells stably transfected with a kinase-inactivated TGFBR1\*6A construct. Functional assays show that TGFBR1\*6A-mediated decreased ARHGAP5 expression is associated with higher RhoA activation, a crucial mediator of cell **migration**. Extracellular signal-regulated kinase (ERK) activation is also higher in cells that harbor the TGFBR1\*6A allele. We conclude that TGFBR1\*6A is not an oncogene but enhances MCF-7 cell **migration** and invasion through RhoA and ERK pathway activation and down-regulates two crucial mediators of this phenotype. These results provide the first evidence that TGFBR1\*6A may contribute to cancer progression in a TGF-beta signaling-independent manner.
78. Lens fiber cell elongation and differentiation is associated with a robust increase in myosin light chain phosphorylation in the developing mouse. Myosin II, a molecular motor, plays a critical role in cell **migration**, cell shape changes, cell adhesion, and cytokinesis. To understand the role of myosin II in lens fiber cell elongation and differentiation, we determined the distribution pattern of nonmuscle myosin IIA, IIB, and phosphorylated regulatory myosin light chain-2 (phospho-MLC) in frozen sections of the developing mouse lens by immunofluorescence analysis. While myosin IIA was distributed uniformly throughout the differentiating lens, including the epithelium and fibers, myosin IIB was localized predominantly to the epithelium and the posterior tips of the lens fibers. In contrast, immunostaining with a di-phospho-MLC antibody localized intensely and precisely to the elongating and differentiating primary and secondary lens fibers, co-localizing with actin filaments. An in situ analysis of Rho GTPase activation revealed that Rho-GTP was distributed uniformly throughout the embryonic lens, including epithelium and fibers. Inhibition of myosin light chain kinase (MLCK) activity by ML-7 in organ cultured mouse lenses led to development of nuclear lens opacity in association with abnormal fiber cell organization. Taken together, these data reveal a distinct spatial distribution pattern of myosin II isoforms in the developing lens and a robust activation of MLC phosphorylation in the differentiating lens fibers. Moreover, the regulation of MLC phosphorylation by MLCK appears to be critical for crystallin organization and for maintenance of lens transparency and lens membrane function.
79. Roles of Rho-associated kinase and myosin light chain kinase in morphological and migratory defects of focal adhesion kinase-null cells. Fibroblasts derived from focal adhesion kinase (FAK)-null mouse embryos have a reduced **migration** rate and an increase in the number and size of peripherally localized adhesions (Ilic, D., Furuta, Y., Kanazawa, S., Takeda, N., Sobue, K., Nakatsuji, N., Nomura, S., Fujimoto, J., Okada, M., and Yamamoto, T. (1995) Nature 377, 539-544). In this study, we have found that Y27632, a specific inhibitor for Rho-associated kinase (Rho-kinase), dramatically reversed the round cell morphology of FAK(-/-) cells to a spread fibroblast-like shape in 30 min and significantly enhanced their motility. The effects of Y27632 on the FAK(-/-) cell morphology and motility were concomitant with reorganization of the actin cytoskeleton and redistribution of focal adhesions. Conversely, the expression of the constitutively active Rho-kinase in FAK(+/+) cells led to round cell shape and inhibition of cell motility. Furthermore, coincident with the formation of cortical actin filaments, myosin light chain (MLC), Ser-19-phosphorylated MLC, and MLC kinase mainly accumulated at the FAK(-/-) cell periphery. We found that the disruption of actin filaments by cytochalasin D prevented the peripheral accumulation of MLC kinase and that inhibition of myosin-mediated contractility by 2,3-butanedione monoxime induced FAK(-/-) cells to spread. Taken together, our results suggest that Rho-kinase may mediate the formation of cortical actomyosin filaments at the FAK(-/-) cell periphery, which further recruits MLC kinase to the cell periphery and generates a non-polar contractile force surrounding the cell, leading to cell rounding and decreased motility.
80. Transforming growth factor-beta1 regulates macrophage **migration** via RhoA. Brief treatment with transforming growth factor (TGF)-beta1 stimulated the **migration** of macrophages, whereas long-term exposure decreased their **migration**. Cell **migration** stimulated by TGF-beta1 was markedly inhibited by 10 mug/mL Tat-C3 exoenzyme. TGF-beta1 increased mRNA and protein levels of macrophage inflammatory protein (MIP)-1alpha in the initial period, and these effects also were inhibited by 10 mug/mL Tat-C3 and a dominant-negative (DN)-RhoA (N19RhoA). Cycloheximide, actinomycin D, and antibodies against MIP-1alpha and monocyte chemoattractant protein-1 (MCP-1) abolished the stimulation of cell **migration** by TGF-beta1. These findings suggest that **migration** of these cells is regulated directly and indirectly via the expression of chemokines such as MIP-1alpha and MCP-1 mediated by RhoA in response to TGF-beta1. TGF-beta1 activated RhoA in the initial period, and thereafter inactivated them, suggesting that the inactivation of RhoA may be the cause of the reduced cell **migration** in response to TGF-beta1 at later times. We therefore attempted to elucidate the molecular mechanism of the inactivation of RhoA by TGF-beta1. First, TGF-beta1 phosphorylated RhoA via protein kinase A, leading to inactivation of RhoA. Second, wild-type p190 Rho GTPase activating protein (p190RhoGAP) reduced and DN-p190RhoGAP reversed the reduction of cell **migration** induced by TGF-beta, suggesting that it inactivated RhoA via p190 Rho GAP.
81. Phospholipase Cdelta3 regulates RhoA/Rho kinase signaling and neurite outgrowth. Phospholipase Cδ3 (PLCδ3) is a key enzyme regulating phosphoinositide metabolism; however, its physiological function remains unknown. Because PLCδ3 is highly enriched in the cerebellum and cerebral cortex, we examined the role of PLCδ3 in neuro**nal migra**tion and outgrowth. PLCδ3 knockdown (KD) inhibits neurite formation of cerebellar granule cells, and application of PLCδ3KD using in utero electroporation in the developing brain results in the retardation of the r**adial mig**ration of neurons in the cerebral cortex. In addition, PLCδ3KD inhibits axon and dendrite outgrowth in primary cortical neurons. PLCδ3KD also suppresses neurite formation of Neuro2a neuroblastoma cells induced by serum withdrawal or treatment with retinoic acid. This inhibition is released by the reintroduction of wild-type PLCδ3. Interestingly, the H393A mutant lacking phosphatidylinositol 4,5-bisphosphate hydrolyzing activity generates supernumerary protrusions, and a constitutively active mutant promotes extensive neurite outgrowth, indicating that PLC activity is important for normal neurite outgrowth. The introduction of dominant negative RhoA (RhoA-DN) or treatment with Y-27632, a Rho kinase-specific inhibitor, rescues the neurite extension in PLCδ3KD Neuro2a cells. Similar effects were also detected in primary cortical neurons. Furthermore, the RhoA expression level was significantly decreased by serum withdrawal or retinoic acid in control cells, although this decrease was not observed in PLCδ3KD cells. We also found that exogenous expression of PLCδ3 down-regulated RhoA protein, and constitutively active PLCδ3 promotes the RhoA down-regulation more significantly than PLCδ3 upon differentiation. These results indicate that PLCδ3 negatively regulates RhoA expression, inhibits RhoA/Rho kinase signaling, and thereby promotes neurite extension.
82. CD146, an epithelial-mesenchymal transition inducer, is associated with triple-negative breast cancer. The epithelial-mesenchymal transition (EMT) plays an important role in breast cancer metastasis, especially in the most aggressive and lethal subtype, "triple-negative breast cancer" (TNBC). Here, we report that CD146 is a unique activator of EMTs and significantly correlates with TNBC. In epithelial breast cancer cells, overexpression of CD146 down-regulated epithelial markers and up-regulated mesenchymal markers, significantly promoted cell **migration** and invasion, and induced cancer stem cell-like properties. We further found that RhoA pathways positively regulated CD146-induced EMTs via the key EMT transcriptional factor Slug. An orthotopic breast tumor model demonstrated that CD146-overexpressing breast tumors showed a poorly differentiated phenotype and displayed increased tumor invasion and metastasis. We confirmed these findings by conducting an immunohistochemical analysis of 505 human primary breast tumor tissues and found that CD146 expression was significantly associated with high tumor stage, poor prognosis, and TNBC. CD146 was expressed at abnormally high levels (68.9%), and was strongly associated with E-cadherin down-regulation in TNBC samples. Taken together, these findings provide unique evidence that CD146 promotes breast cancer progression by induction of EMTs via the activation of RhoA and up-regulation of Slug. Thus, CD146 could be a therapeutic target for breast cancer, especially for TNBC.
83. Involvement of p114-RhoGEF and Lfc in Wnt-3a- and dishevelled-induced RhoA activation and neurite retraction in N1E-115 mouse neuroblastoma cells. The Wnt-induced planar cell polarity (PCP) signaling pathway is essential for polarized cell **migration** and morphogenesis. Dishevelled (Dvl) and its binding protein Daam1 mediate RhoA activation in this pathway. WGEF, a member of the Rho-guanine nucleotide exchange factor (Rho-GEF) family, was shown to play a role in Wnt-induced RhoA activation in Xenopus embryos. However, it has remained unknown which member(s) of a Rho-GEF family are involved in Wnt/Dvl-induced RhoA activation in mammalian cells. Here we identified p114-RhoGEF and Lfc (also called GEF-H1) as the Rho-GEFs responsible for Wnt-3a-induced RhoA activation in N1E-115 mouse neuroblastoma cells. We screened for Rho-GEF-silencing short-hairpin RNAs (shRNAs) that are capable of suppressing Dvl-induced neurite retraction in N1E-115 cells and found that p114-RhoGEF and Lfc shRNAs, but not WGEF shRNA, suppressed Dvl- and Wnt-3a-induced neurite retraction. p114-RhoGEF and Lfc shRNAs also inhibited Dvl- and Wnt-3a-induced RhoA activation, and p114-RhoGEF and Lfc proteins were capable of binding to Dvl and Daam1. Additionally, the Dvl-binding domains of p114-RhoGEF and Lfc inhibited Dvl-induced neurite retraction. Our results suggest that p114-RhoGEF and Lfc are critically involved in Wnt-3a- and Dvl-induced RhoA activation and neurite retraction in N1E-115 cells.
84. RhoA is dispensable for skin development, but crucial for contraction and directed **migration** of keratinocytes. RhoA is a small guanosine-5'-triphosphatase (GTPase) suggested to be essential for cytokinesis, stress fiber formation, and epithelial cell-cell contacts. In skin, loss of RhoA was suggested to underlie pemphigus skin blistering. To analyze RhoA function in vivo, we generated mice with a keratinocyte-restricted deletion of the RhoA gene. Despite a severe reduction of cofilin and myosin light chain (MLC) phosphorylation, these mice showed normal skin development. Primary RhoA-null keratinocytes, however, displayed an increased percentage of multinucleated cells, defective maturation of cell-cell contacts. Furthermore we observed increased cell spreading due to impaired RhoA-ROCK (Rho-associated protein kinase)-MLC phosphatase-MLC-mediated cell contraction, independent of Rac1. Rho-inhibiting toxins further increased multinucleation of RhoA-null cells but had no significant effect on spreading, suggesting that RhoB and RhoC have partially overlapping functions with RhoA. Loss of RhoA decreased directed cell **migration** in vitro caused by reduced **migration** speed and directional persistence. These defects were not related to the decreased cell contraction and were independent of ROCK, as ROCK inhibition by Y27632 increased directed **migration** of both control and RhoA-null keratinocytes. Our data indicate a crucial role for RhoA and contraction in regulating cell spreading and a contraction-independent function of RhoA in keratinocyte **migration**. In addition, our data show that RhoA is dispensable for skin development.
85. Endotoxin inhibits intestinal epithelial restitution through activation of Rho-GTPase and increased focal adhesions. Diseases of gut inflammation such as neonatal necrotizing enterocolitis (NEC) result after an injury to the mucosal lining of the intestine, leading to translocation of bacteria and endotoxin (lipopolysaccharide). Intestinal mucosal defects are repaired by the process of intestinal restitution, during which enterocytes migrate from healthy areas to sites of injury. In an animal model of NEC, we determined that intestinal restitution was significantly impaired compared with control animals. We therefore sought to determine the mechanisms governing enterocyte **migration** under basal conditions and after an endotoxin challenge. Here we show that the cytoskeletal reorganization and stress fiber formation required for **migration** in IEC-6 enterocytes requires RhoA. Enterocytes were found to express the endotoxin receptor Toll-like receptor 4, which served to bind and internalize lipopolysaccharide. Strikingly, endotoxin treatment significantly inhibited intestinal restitution, as measured by impaired IEC-6 cell **migration** across a scraped wound. Lipopolysaccharide was found to increase RhoA activity in a phosphatidylinositol 3-kinase-dependent manner, leading to an increase in phosphorylation of focal adhesion kinase and an enhanced number of focal adhesions. Importantly, endotoxin caused a progressive, RhoA-dependent increase in cell matrix tension/contractility, which correlated with the observed impairment in enterocyte **migration**. We therefore conclude that endotoxin inhibits enterocyte **migration** through a RhoA-dependent increase in focal adhesions and enhanced cell adhesiveness, which may participate in the impaired restitution observed in experimental NEC.
86. MIP-2 causes differential activation of RhoA in mouse aortic versus pulmonary artery endothelial cells. Previously, we have shown that endothelial cell chemotaxis to the proangiogenic chemokine MIP-2 (macrophage inflammatory protein-2) is much greater in mouse aortic endothelial cells (EC) than pulmonary arterial endothelial cells (PA EC). This was true despite the observation that both cell types display comparable levels of the ligand receptor, CXCR(2) (8). Since the systemic arterial circulation is proangiogenic in the adult lung and the pulmonary circulation is relatively resistant to neovascularization, we questioned whether the observed functional heterogeneity is related to inherent differences in cell signaling cascades of the two EC subtypes. Specifically, we measured activation of Rac1 and RhoA, both thought to be involved in EC cell **migration**. Rac1 showed inconsistent and minimal changes in both cell types after MIP-2 treatment (p>0.05). However, activated RhoA was increased upon exposure to MIP-2 only in aortic EC (61% increase; p<0.05). Decreased RhoA activation after treatment of aortic EC with specific siRNA for RhoA resulted in a functional decrease in EC chemotaxis to MIP-2 (17% increase; p<0.05). Additionally, increased RhoA activation in PA EC with adenoviral infection of RhoA caused an increase in PA EC chemotaxis to MIP-2 (46% increase; p<0.05). Inhibition of RhoA activity with the Rho kinase inhibitor, Y27632, blocked aortic EC chemotaxis and stress fiber formation. Thus, RhoA activation is increased after MIP-2 treatment in mouse aortic endothelial cells but not in pulmonary artery endothelial cells. We conclude that RhoA is part of a signaling pathway essential for aortic cell **migration** after CXCR(2) ligation. This result provides one explanation for the difference in chemotaxis observed in these two endothelial subtypes that express similar levels of CXCR(2).
87. Galectin 3 regulates HCC cell invasion by RhoA and MLCK activation. Hepatocellular carcinoma (HCC) carries a poor prognosis with no effective treatment available other than liver transplantation for selected patients. Vascular invasion of HCC is one of the most important negative predictor of survival. As the regulation of invasion of HCC cells is not well understood, our aim was to study the mechanisms by which galectin 3, a β-galactosidase-binding lectin mediates HCC cell **migratio**n. HCC was induced by N-diethylnitrosamine in wild-type and galectin 3(-/-) mice, and tumor formation, histology, and tumor cell invasion were assessed. The galectin 3(-/-) mice developed significantly smaller tumor burden with a less invasive phenotype than the wild-type animals. Galectin 3 was upregulated in the wild-type HCC tumor tissue, but not in the surrounding parenchyma. Galectin 3 expression in HCC was induced by NF-κB transactivation as determined by chromatin immunoprecipitation assays. In vitro studies assessed the pro-migratory effects of galectin 3. Th**e migrati**on of hepatoma cells was significantly decreased after transfection by the galectin 3 siRNA and also after using the Rho kinase inhibitor Y-27632. The reorganization of the actin cytoskeleton, RhoA GTPase activity and the phosphorylation of MLC2 (myosin light chain 2) were decreased in the galectin 3 siRNA-transfected cells. In addition, in vitro and in vivo evidence showed that galectin 3 deficiency reduced hepatoma cell proliferation and increased their apoptosis rate. In conclusion, galectin 3 is an important lectin that is induced in HCC cells, and promotes hepatoma cell motility and invasion by an autocrine pathway. Targeting galectin 3 therefore could be an important novel treatment strategy to halt disease progression.
88. Sphingosine 1-phosphate-induced motility and endocytosis of dendritic cells is regulated by SWAP-70 through RhoA. The phospholipid mediator sphingosine 1-phosphate (S1P) enhances motility and endocytosis of mature dendritic cells (DCs). We show that in vitro **migration** of Swap-70(-/-) bone marrow-derived DCs (BMDCs) in response to S1P and S1P-induced upregulation of endocytosis are significantly reduced. S1P-stimulated movement of Swap-70(-/-) BMDCs, specifically retraction of their trailing edge, in a collagen three-dimensional environment is impaired. These in vitro observations correlate with delayed entry into lymphatic vessels and **migration** to lymph nodes of skin DCs in Swap-70(-/-) mice. Expression of S1P receptors (S1P(1-3)) by wild-type and Swap-70(-/-) BMDCs is similar, but Swap-70(-/-) BMDCs fail to activate RhoA and to localize Rac1 and RhoA into areas of actin polymerization after S1P stimulus. The Rho-activating G protein Gα(i) interacts with SWAP-70, which also supports the localization of Gα(13) to membrane rafts in BMDCs. LPS-matured Swap-70(-/-) BMDCs contain significantly more active RhoA than wild-type DCs. Preinhibition of Rho activation restore**d migrati**on to S1P, S1P-induced upregulation of endocytosis in mature Swap-70(-/-) BMDCs, and localization of Gα(13) to membrane rafts. These data demonstrate SWAP-70 as a novel regulator of S1P signaling necessary for DC motility and endocytosis.
89. Glycine-extended gastrin stimulates cell proliferation and **migration** through a Rho- and ROCK-dependent pathway, not a Rac/Cdc42-dependent pathway. Both amidated gastrin (Gamide) and glycine-extended gastrin (Ggly) stimulate gastrointestinal cell proliferation and **migration**. Binding of Gamide to the cholecystokinin-2 receptor activates small GTP-binding proteins of the Rho family (Rho, Rac, and Cdc42), and dominant-negative mutants of Rho or Cdc42 block Gamide-stimulated cell proliferation and survival. In comparison, little is known about the Ggly signaling transduction pathway leading to cell proliferation and **migration**. The present study examined the roles of the small G proteins Rho, Rac, and Cdc42 in Ggly-induced proliferation and **migration** of the mouse gastric epithelial cell line IMGE-5. Ggly stimulated the activation of Rho and its downstream effector protein ROCK. The activation of Rho and ROCK mediated Ggly-induced cell proliferation and **migration** as inhibition of Rho by C3, or ROCK by Y-27632, completely blocked these effects of Ggly. Ggly also stimulated tyrosine phosphorylation of focal adhesion kinase, and stimulation was reversed by addition of C3 and Y-27632. In contrast to the effects of Rho and ROCK, inhibition of the Rac or Cdc42 pathways by expression of dominant-negative mutants of Rac or Cdc42 did not affect Ggly-induced cell proliferation and **migration**. These results demonstrate that Ggly stimulates IMGE-5 cell proliferation and **migration** through a Rho/ROCK-dependent pathway but not via Rac- or Cdc42-dependent pathways.
90. Small GTPase Rap1 regulates cell **migration** through regulation of small GTPase RhoA activity in response to transforming growth factor-β1. Transforming growth factor (TGF)-β1 regulates diverse cellular functions. Particularly, TGF-β1 induces monocy**te migrat**ion to sites of injury or inflammation in early period, whereas TGF-β1 inhibits c**ell migra**tion in late phase. In this study, we attempted to understand how TGF-β1 suppresses **cell migr**ation in late phase. We found that TGF-β1 of short exposure induces the production of chemokines, such as macrophage inflammatory protein (MIP)-1α, by Raw 264.7 cells. However, knock-down of small GTPase RhoA by sh-RhoA inhibited the production of MIP-1α and mac**rophage m**igration, suggesting that RhoA is essential for expression of this chemokine. An activator of Epac (exchange proteins directly activated by cAMP; a guanine nucleotide exchange factor of Rap1), 8CPT-2Me-cAMP which leads to Rap1 activation abrogated MIP-1α expression and ma**crophage** migration. Indeed, GTP-RhoA and GTP-Rap1 levels were reciprocally regulated in a time-dependent manner following TGF-β1 stimulation. 8CPT-2Me-cAMP suppressed GTP-RhoA levels, whereas si-Rap1 augmented GTP-RhoA levels **and cell** migration. TGF-β1 produced cAMP in late period and si-RNAs of Epac1 and Epac2 reduced GTP-Rap1 levels leading to promotion of GTP-RhoA levels. Furthermore, si-RNA of ARAP3 (Rap-dependent RhoGAP) increased GTP-RhoA leve**l and cel**l migration. Therefore, we propose the mechanism that prolonged TGF-β1 treatment produce cAMP, which activates sequentially Epac, Rap1 and ARAP3, resulting in suppression of RhoA, chemokine expression, and **macropha**ge migration. Contrary to the general concept that Rap1 stim**ulates ce**ll migration, we demonstrated in this study that Rap1 in**hibits ce**ll migration by suppression of RhoA activity in response to TGF-β1.
91. Puromycin insensitive leucyl-specific aminopeptidase (PILSAP) affects RhoA activation in endothelial cells. Puromycin insensitive leucyl-specific aminopeptidase (PILSAP) expressed in endothelial cells (ECs) plays an important role in angiogenesis due to its involvement in **migration**, proliferation and network formation. Here we examined the biological function of PILSAP with respect to EC morphogenesis and the related intracellular signaling for this process. When mouse endothelial MSS31 cells were cultured, a dominant negative PILSAP mutant converted cell shape to disk-like morphology, blocked stress fiber formation, and augmented membrane ruffling in random directions. These phenotypic changes led us to test whether PILSAP affected activities of Rho family small G-proteins. Abrogation of PILSAP enzymatic activity or its expression attenuated RhoA but not Rac1 activation during cell adhesion. This attenuation of RhoA activation was also evident when G-protein coupled receptors such as proteinase-activated receptor or lysophosphatidic acid receptor were activated in ECs. These results indicate that PILSAP affects RhoA activation and that influences the proper function of ECs.
92. Force-induced cell polarisation is linked to RhoA-driven microtubule-independent focal-adhesion sliding. Mechanical forces play a crucial role in controlling the integrity and functionality of cells and tissues. External forces are sensed by cells and translated into signals that induce various responses. To increase the detailed understanding of these processes, we investigated cell **migration** and dynamic cellular reorganisation of focal adhesions and cytoskeleton upon application of cyclic stretching forces. Of particular interest was the role of microtubules and GTPase activation in the course of mechanotransduction. We showed that focal adhesions and the actin cytoskeleton undergo dramatic reorganisation perpendicular to the direction of stretching forces even without microtubules. Rather, we found that microtubule orientation is controlled by the actin cytoskeleton. Using biochemical assays and fluorescence resonance energy transfer (FRET) measurements, we revealed that Rac1 and Cdc42 activities did not change upon stretching, whereas overall RhoA activity increased dramatically, but independently of intact microtubules. In conclusion, we demonstrated that key players in force-induced cellular reorganisation are focal-adhesion sliding, RhoA activation and the actomyosin machinery. In contrast to the importance of microtubules in **migration**, the force-induced cellular reorganisation, including focal-adhesion sliding, is independent of a dynamic microtubule network. Consequently, the elementary molecular mechanism of cellular reorganisation during **migration** is different to the one in force-induced cell reorganisation.
93. Spatiotemporal dynamics of RhoA activity in migrating cells. Rho family GTPases regulate the actin and adhesion dynamics that control cell **migration**. Current models postulate that Rac promotes membrane protrusion at the leading edge and that RhoA regulates contractility in the cell body. However, there is evidence that RhoA also regulates membrane protrusion. Here we use a fluorescent biosensor, based on a novel design preserving reversible membrane interactions, to visualize the spatiotemporal dynamics of RhoA activity during cell **migration**. In randomly migrating cells, RhoA activity is concentrated in a sharp band directly at the edge of protrusions. It is observed sporadically in retracting tails, and is low in the cell body. RhoA activity is also associated with peripheral ruffles and pinocytic vesicles, but not with dorsal ruffles induced by platelet-derived growth factor (PDGF). In contrast to randomly migrating cells, PDGF-induced membrane protrusions have low RhoA activity, potentially because PDGF strongly activates Rac, which has previously been shown to antagonize RhoA activity. Our data therefore show that different extracellular cues induce distinct patterns of RhoA signalling during membrane protrusion.
94. Geranylgeranylation but not GTP-loading of Rho GTPases determines T cell function. Rho guanosine triphosphatases (GTPases) orchestrate signaling pathways leading to cell **migration**. They are typically responsible for the organization of actin filaments that support actomyosin contractility and cell-body translocation. The function of Rho GTPases depends on GTP-loading and isoprenylation by geranylgeranyl pyrophosphate (GGpp). The latter posttranslational modification may be manipulated by agents such as 3-hydroxy-3-methylglutaryl-CoA reductase inhibitors (HMGCRIs) that prevent de novo synthesis of isoprenoids such as GGpp. HMGCRIs have anti-inflammatory properties and substantially reduce infiltration of inflammatory immune cells into target tissues, including the central nervous system (CNS) during neuroinflammation. The depletion of the cellular isoprenoid pool is believed to result in the regulation of antigen-specific T cells outside the target organ and also to prevent **migration** of these cells into target organs, such as the CNS. In vivo treatment with HMGCRI in the experimental autoimmune encephalitis (EAE) rodent model of multiple sclerosis reduces the capacity of activated T cells to traffic to and within the brain. This presentation shows that geranylgeranylation is fundamental for RhoA-mediated downstream events such as influencing cytoskeletal organization and the **migration** of T cells. Tethering of RhoA to the membrane by GGpp is necessary for T cell **migration** and provides a mechanism by which HMGCRI may prevent T cell infiltration into inflamed compartments.
95. Staphylococcus aureus protein A mediates invasion across airway epithelial cells through activation of RhoA GTPase signaling and proteolytic activity. Staphyococcus aureus and especially the epidemic methicillin-resistant S. aureus strains cause severe necrotizing pneumonia. The mechanisms whereby these organisms invade across the mucosal epithelial barrier to initiate invasive infection are not well understood. Protein A (SpA), a highly conserved and abundant surface protein of S. aureus, activates TNF receptor 1 and EGF receptor (EGFR) signaling cascades that can perturb the cytoskeleton. We demonstrate that wild-type S. aureus, but not spa mutants, invade across polarized airway epithelial cell monolayers via the paracellular junctions. SpA stimulated a RhoA/ROCK/MLC cascade, resulting in the contraction of the cytoskeleton. SpA(+) but not SpA(-) mutants stimulated activation of EGFR and along with subsequent calpain activity cleaved the membrane-spanning junctional proteins occludin and E-cadherin, facilitating staphylococcal trans**migration** through the cell-cell junctions. Treatment of polarized human airway epithelial monolayers with inhibitors of ROCK, EGFR, MAPKs, or calpain prevented staphylococcal penetration through the monolayers. In vivo, blocking calpain activity impeded bacterial invasion into the lung parenchyma. Thus, S. aureus exploits multiple receptors available on the airway mucosal surface to facilitate invasion across epithelial barriers.
96. DLC2 modulates angiogenic responses in vascular endothelial cells by regulating cell attachment and **migration**. Deleted in liver cancer 1 (DLC1) is a RhoGTPase activation protein-containing tumor suppressor that associates with various types of cancer. Although DLC2 shares a similar domain structure with that of DLC1, the function of DLC2 is not well characterized. Here, we describe the expression and ablation of DLC2 in mice using a reporter-knockout approach. DLC2 is expressed in several tissues and in endothelial cells (ECs) of blood vessels. Although ECs and blood vessels show no histological abnormalities and mice appear overall healthy, DLC2-mutant mice display enhanced angiogenic responses induced by matrigel and by tumor cells. Silencing of DLC2 in human ECs has reduced cell attachment, increased **migration**, and tube formation. These changes are rescued by silencing of RhoA, suggesting that the process is RhoA pathway dependent. These results indicate that DLC2 is not required for mouse development and normal vessel formation, but may protect mouse from unwanted angiogenesis induced by, for example, tumor cells.
97. Fibronectin stimulates **migration** through lipid raft dependent NHE-1 activation in mouse embryonic stem cells: involvement of RhoA, Ca(2+)/CaM, and ERK. Extracellular matrix (ECM) components and intracellular pH (pH(i)) may serve as regulators of cell **migration** in various cell types.The Oris **migration** assay was used to assess the effect of fibronectin (FN) on cell motility. The Na(+)/H(+) exchanger (NHE)-1 activity was evaluated by measuring pH(i) and [(22)Na(+)] uptake. To examine activated signaling molecules, western blot analysis and immunoprecipitation was performed.ECM components (FN, laminin, fibrinogen, and collagen type I) increased [(22)Na(+)] uptake, pH(i), and cell **migration**. In addition, FN-induced increase of cell **migration** was inhibited by NHE-1 inhibitor amiloride or NHE-1-specific siRNA. FN selectively increased the mRNA and protein expression of NHE-1, but not that of NHE-2 or NHE-3. FN binds integrin β1 and subsequently stimulates caveolin-1 phosphorylation and Ca(2+) influx. Then, NHE-1 is phosphorylated by RhoA and Rho kinases, and Ca(2+)/calmodulin (CaM) signaling elicits complex formation with NHE-1, which is enriched in lipid raft/caveolae microdomains of the plasma membrane. Activation of NHE-1 continuously induces an increase of [(22)Na(+)] uptake and pH(i). Finally, NHE-1-dependent extracellular signal-regulated kinase (ERK) 1/2 phosphorylation enhanced matrix metalloproteinase-2 (MMP-2) and filamentous-actin (F-actin) expression, partially contributing to the regulation of embryonic stem cells (ESCs) **migratio**n.FN stimulated mESCs **migratio**n and proliferation through NHE-1 activation, which were mediated by lipid raft-associated caveolin-1, RhoA/ROCK, and Ca(2+)/CaM signaling pathways.The precise role of NHE in the modulation of ECM-related physiological functions such as proliferation and **migratio**n remains poorly understood. Thus, this study analyzed the relationship between FN and NHE in regulating the **migratio**n of mouse ESCs and their related signaling pathways.
98. Loss of p53 promotes RhoA-ROCK-dependent cell **migration** and invasion in 3D matrices. In addition to its role in controlling cell cycle progression, the tumor suppressor protein p53 can also affect other cellular functions such as cell **migration**. In this study, we show that p53 deficiency in mouse embryonic fibroblasts cultured in three-dimensional matrices induces a switch from an elongated spindle morphology to a markedly spherical and flexible one associated with highly dynamic membrane blebs. These rounded, motile cells exhibit amoeboid-like movement and have considerably increased invasive properties. The morphological transition requires the RhoA-ROCK (Rho-associated coil-containing protein kinase) pathway and is prevented by RhoE. A similar p53-mediated transition is observed in melanoma A375P cancer cells. Our data suggest that genetic alterations of p53 in tumors are sufficient to promote motility and invasion, thereby contributing to metastasis.
99. RhoA signaling through platelet P2Y₁ receptor controls leukocyte recruitment in allergic mice. Clinical studies reveal platelet activation in patients with asthma, allergic rhinitis, and eczema. This is distinct from platelet aggregation, which is critical for the maintenance of hemostasis and in which a role for platelet purinergic receptors is well documented. However, purines are also essential for inflammatory cell trafficking in animal models of allergic lung inflammation, which are known to be platelet dependent, yet the role of purines in the platelet activation accompanying inflammation is unknown.We investigated whether the involvement of purine activation of platelets during allergic inflammation is distinct from purine involvement in platelet aggregation.BALB/c mice were sensitized to ovalbumin and subsequent airway ovalbumin challenge. Bronchoalveolar lavage fluid was analyzed for inflammatory cells, and blood samples were assessed for platelet activation. The role of platelet purinergic receptors and associated signaling mechanisms (RhoA) were assessed.P2Y₁, but not P2Y₁₂ or P2X₁, antagonism inhibited pulmonary leukocyte recruitment. The formation of platelet-leukocyte complexes in vivo and platelet/P-selectin-dependent polymorphonuc**lear cell** migration in vitro were exclusively platelet P2Y₁ receptor dependent. Furthermore, platelet P2Y₁ activation resulted in RhoA activity in vivo after allergen challenge, and RhoA signaling in platelets through P2Y₁ stimulation was required for platelet-dependent leukocyte chemotaxis in vitro. Leukocyte recruitment in thrombocytopenic mice remained suppressed after reinfusion of platelets pretreated with a P2Y₁ antagonist or a Rho-associated kinase 1 inhibitor, confirming the crucial role of platelet P2Y₁ receptor and subsequent activation of RhoA.RhoA signaling downstream of platelet P2Y₁, but not P2Y₁₂, represents a clear dichotomy in platelet activation during allergic inflammation versus hemostasis.
100. Small Rho-GTPases and cortical malformations: fine-tuning the cytoskeleton stability. Rho-GTPases have been found to be crucial for cytoskeleton remodelling and cell polarity, as well as key players in directed cell **migration** in various tissues and organs, therefore becoming good candidates for involvement in neuronal **migration** disorders. We recently found that genetic deletion of the small GTPase RhoA in the developing mouse cerebral cortex results in three distinct cortical malformations: a defect in the proliferation of progenitor cells during development that leads to a bigger cerebral cortex in the adult mouse, a change in the morphology of radial glial cells that results in the formation of a subcortical band heterotopia (SBH, also called Double Cortex) and an increase in the speed of migrating newborn neurons. The latter, together with the aberrant radial glial shape, is likely to be the cause of cobblestone lissencephaly, where neurons protrude beyond layer I at the pial surface of the brain.
101. The transmembrane adaptor protein NTAL signals to mast cell cytoskeleton via the small GTPase Rho. The transmembrane adaptor protein NTAL (non-T-cell activation linker) participates in signalosome assembly in hematopoietic cells, but its exact role in cell physiology remains enigmatic. We report here that BM-derived mast cells from NTAL-deficient mice, responding to Ag alone or in combination with SCF, exhibit reduced spreading on fibronectin, enhanced filamentous actin depolymerization and enhanced **migration** towards Ag relative to WT cells. No such differences between WT and NTAL(-/-) BM-derived mast cells were observed when SCF alone was used as activator. We have examined the activities of two small GTPases, Rac and Rho, which are important regulators of actin polymerization. Stimulation with Ag and/or SCF enhanced activity of Rac(1,2,3) in both NTAL(-/-) and WT cells. In contrast, RhoA activity decreased and this trend was much faster and more extensive in NTAL(-/-) cells, indicating a positive regulatory role of NTAL in the recovery of RhoA activity. After restoring NTAL into NTAL(-/-) cells, both spreading and actin responses were rescued. This is the first report of a crucial role of NTAL in signaling, via RhoA, to mast cell cytoskeleton.
102. RhoA-Mediated Functions in C3H10T1/2 Osteoprogenitors Are Substrate Topography Dependent. Surface topography broadly influences cellular responses. Adherent cell activities are regulated, in part, by RhoA, a member of the Rho-family of GTPases. In this study, we evaluated the influence of surface topography on RhoA activity and associated cellular functions. The murine mesenchymal stem cell line C3H10T1/2 cells (osteoprogenitor cells) were cultured on titanium substrates with smooth topography (S), microtopography (M), and nanotopography (N) to evaluate the effect of surface topography on RhoA-mediated functions (cell spreading, adhesion, **migration**, and osteogenic differentiation). The influence of RhoA activity in the context of surface topography was also elucidated using RhoA pharmacologic inhibitor. Following adhesion, M and N adherent cells developed multiple projections, while S adherent cells had flattened and widespread morphology. RhoA inhibitor induced remarkable longer and thinner cytoplasmic projections on all surfaces. Cell adhesion and osteogenic differentiation was topography dependent with S < M and N surfaces. RhoA inhibition increased adhesion on S and M surfaces, but not N surfaces. C**ell migra**tion in a wound healing assay was greater on S versus M versus N surfaces and RhoA inhibitor increased S adherent c**ell migra**tion, but not N adherent c**ell migra**tion. RhoA inhibitor enhanced osteogenic differentiation in S adherent cells, but not M or N adherent cells. RhoA activity was surface topography roughness dependent (S < M, N). RhoA activity and -mediated functions are influenced by surface topography. Smooth surface adherent cells appear highly sensitive to RhoA function, while nano-scale topography adherent cell may utilize alternative cellular signaling pathway(s) to influence adherent cellular functions regardless of RhoA activity.
103. Coupling of cell **migration** with neurogenesis by proneural bHLH factors. After cell birth, almost all neurons in the mammalian central nervous system migrate. It is unclear whether and how cell **migration** is coupled with neurogenesis. Here we report that proneural basic helix-loop-helix (bHLH) transcription factors not only initiate neuronal differentiation but also potentiate cell **migration**. Mechanistically, proneural bHLH factors regulate the expression of genes critically involved in **migration**, including down-regulation of RhoA small GTPase and up-regulation of doublecortin and p35, which, in turn, modulate the actin and microtubule cytoskeleton assembly and enable newly generated neurons to migrate. In addition, we report that several DNA-binding-deficient proneural genes that fail to initiate neuronal differentiation still activate **migration**, whereas a different mutation of a proneural gene that causes a failure in initiating cell **migration** still leads to robust neuronal differentiation. Collectively, these data suggest that transcription programs for neurogenesis and **migration** are regulated by bHLH factors through partially distinct mechanisms.
104. Gene targeting RhoA reveals its essential role in coordinating mitochondrial function and thymocyte development. Thymocyte development is regulated by complex signaling pathways. How these signaling cascades are coordinated remains elusive. RhoA of the Rho family small GTPases plays an important role in actin cytoskeleton organization, cell adhesion, **migration**, proliferation, and survival. Nonetheless, the physiological function of RhoA in thymocyte development is not clear. By characterizing a conditional gene targeting mouse model bearing T cell deletion of RhoA, we show that RhoA critically regulates thymocyte development by coordinating multiple developmental events. RhoA gene disruption caused a strong developmental block at the pre-TCR checkpoint and during positive selection. Ablation of RhoA led to reduced DNA synthesis in CD4(-)CD8(-), CD4(+)CD8(-), and CD4(-)CD8(+) thymocytes but not in CD4(+)CD8(+) thymocytes. Instead, RhoA-deficient CD4(+)CD8(+) thymocytes showed an impaired mitosis. Furthermore, we found that abrogation of RhoA led to an increased apoptosis in all thymocyte subpopulations. Importantly, we show that the increased apoptosis was resulted from reduced pre-TCR expression and increased production of reactive oxygen species (ROS), which may be because of an enhanced mitochondrial function, as manifested by increased oxidative phosphorylation, glycolysis, mitochondrial membrane potential, and mitochondrial biogenesis in RhoA-deficient thymocytes. Restoration of pre-TCR expression or treatment of RhoA-deficient mice with a ROS scavenger N-acetylcysteine partially restored thymocyte development. These results suggest that RhoA is required for thymocyte development and indicate, to our knowledge, for the first time that fine-tuning of ROS production by RhoA, through a delicate control of metabolic circuit, may contribute to thymopoiesis.
105. Autophagy and SQSTM1 on the RHOA(d) again: emerging roles of autophagy in the degradation of signaling proteins. Degradation of signaling proteins is one of the most powerful tumor-suppressive mechanisms by which a cell can control its own growth, its survival, and its motility. Emerging evidence suggests that autophagy limits several signaling pathways by degrading kinases, downstream components, and transcription factors; however, this often occurs under stressful conditions. Our recent studies revealed that constitutive autophagy temporally and spatially controls the RHOA pathway. Specifically, inhibition of autophagosome degradation induces the accumulation of the GTP-bound form of RHOA. The active RHOA is sequestered via SQSTM1/p62 within autolysosomes, and accordingly fails to localize to the spindle midbody or to the cell surface, as we demonstrate herein. As a result, all RHOA-downstream responses are deregulated, thus driving cytokinesis failure, aneuploidy and motility, three processes that directly have an impact upon cancer progression. We therefore propose that autophagy acts as a degradative brake for RHOA signaling and thereby controls cell proliferation, **migration**, and genome stability.
106. Diabetes causes bone marrow endothelial barrier dysfunction by activation of the RhoA-Rho-associated kinase signaling pathway. Diabetes mellitus causes bone marrow (BM) microangiopathy. This study aimed to investigate the mechanisms responsible for BM endothelial dysfunction in diabetes mellitus.The analysis of differentially expressed transcripts in BM endothelial cells (BMECs) from type-1 diabetic and nondiabetic mice showed an effect of diabetes mellitus on signaling pathways controlling cell death, **migration**, and cytoskeletal rearrangement. Type-1 diabetic-BMECs displayed high reactive oxygen species levels, increased expression and activity of RhoA and its associated protein kinases Rho-associated kinase 1/Rho-associated kinase 2, and reduced Akt phosphorylation/activity. Likewise, diabetes mellitus impaired Akt-related BMEC functions, such as **migration**, network formation, and angiocrine factor-releasing activity, and increased vascular permeability. Moreover, high glucose disrupted BMEC contacts through Src tyrosine kinase phosphorylation of vascular endothelial cadherin. These alterations were prevented by constitutively active Akt (myristoylated Akt), Rho-associated kinase inhibitor Y-27632, and Src inhibitors. Insulin replacement restored BMEC abundance, as assessed by flow cytometry analysis of the endothelial marker MECA32, and endothelial barrier function in BM of type-1 diabetic mice.Redox-dependent activation of RhoA/Rho-associated kinase and Src/vascular endothelial cadherin signaling pathways, together with Akt inactivation, contribute to endothelial dysfunction in diabetic BM. Metabolic control is crucial for maintenance of endothelial cell homeostasis and endothelial barrier function in BM of diabetic mice.
107. PRL-1 protein promotes ERK1/2 and RhoA protein activation through a non-canonical interaction with the Src homology 3 domain of p115 Rho GTPase-activating protein. Phosphatases of the regenerating liver (PRL) play oncogenic roles in cancer development and metastasis. Although previous studies indicate that PRL-1 promotes cell growth and **migration** by activating both the ERK1/2 and RhoA pathways, the mechanism by which it activates these signaling events remains unclear. We have identified a PRL-1-binding peptide (Peptide 1) that shares high sequence identity with a conserved motif in the Src homology 3 (SH3) domain of p115 Rho GTPase-activating protein (GAP). p115 RhoGAP directly binds PRL-1 in vitro and in cells via its SH3 domain. Structural analyses of the PRL-1·Peptide 1 complex revealed a novel protein-protein interaction whereby a sequence motif within the PxxP ligand-binding site of the p115 RhoGAP SH3 domain occupies a folded groove within PRL-1. This prevents the canonical interaction between the SH3 domain of p115 RhoGAP and MEKK1 and results in activation of ERK1/2. Furthermore, PRL-1 binding activates RhoA signaling by inhibiting the catalytic activity of p115 RhoGAP. The results demonstrate that PRL-1 binding to p115 RhoGAP provides a coordinated mechanism underlying ERK1/2 and RhoA activation.
108. RhoA-mediated FMNL1 regulates GM130 for actin assembly and phosphorylates MAPK for spindle formation in mouse oocyte meiosis. Formin-like 1 (FMNL1) is a member of Formin family proteins which are the actin nucleators. Although FMNL1 activities have been shown to be essential for cell adhesion, cytokinesis, cell polarization and **migration** in mitosis, the functional roles of mammalian FMNL1 during oocyte meiosis remain uncertain. In this study, we investigated the functions of FMNL1 in mouse oocytes using specific morpholino (MO) microinjection and live cell imaging. Immunofluorescent staining showed that in addition to its cytoplasmic distribution, FMNL1 was primarily localized at the spindle poles after germinal vesicle breakdown (GVBD). FMNL1 knockdown caused the low rate of polar body extrusion and resulted in large polar bodies. Time-lapse microscopic and immunofluorescence intensity analysis indicated that this might be due to the aberrant actin expression levels. Cortical polarity was disrupted as shown by a loss of actin cap and cortical granule free domain (CGFD) formation, which was confirmed by a failure of meiotic spindle positioning. And this might be the reason for the large polar body formation. Spindle formation was also disrupted, which might be due to the abnormal localization of p-MAPK. These results indicated that FMNL1 affected both actin dynamics and spindle formation for the oocyte polar body extrusion. Moreover, FMNL1 depletion resulted in aberrant localization and expression patterns of a cis-Golgi marker protein, GM130. Finally, we found that the small GTPase RhoA might be the upstream regulator of FMNL1. Taken together, our data indicate that FMNL1 is required for spindle organization and actin assembly through a RhoA-FMNL1-GM130 pathway during mouse oocyte meiosis.
109. Deleted in liver cancer 2 (DLC2) suppresses cell transformation by means of inhibition of RhoA activity. The deleted in liver cancer 2 (DLC2) gene, located at chromosome 13q12.3, is a recently identified tumor suppressor gene. The gene is frequently underexpressed in human hepatocellular carcinoma, and its chromosomal region shows frequent deletion. DLC2 encodes a unique RhoGTPase-activating protein (RhoGAP) specific for small RhoGTPases, RhoA, and Cdc42. With bioinformatic analysis, we have identified four different isoforms of DLC2, which we named DLC2alpha, DLC2beta, DLC2gamma, and DLC2delta. Three of the isoforms contain the RhoGAP domain, namely, DLC2alpha, DLC2beta, and DLC2gamma. Ectopic expression of these three isoforms in mouse fibroblasts showed cytoplasmic localization. Of interest, overexpression of these isoforms suppressed the lysophosphatidic acid-induced stress fiber formation in mouse fibroblasts and changed the morphology of the transfected cells from angular and spindle to round. Furthermore, the RhoA pull-down assay demonstrated a remarkable reduction in RhoA activity in the DLC2 transiently transfected cells. In contrast, cells transfected with inactive DLC2 GAP-mutant remained unchanged in cell morphology, actin stress fiber formation, and RhoA activity. HepG2 hepatoma cells stably transfected with the DLC2gamma isoform also changed to a round morphology, as in mouse fibroblasts. Of significance, these DLC2gamma stable transfectants showed marked suppression in cell proliferation, motility, and transformation, and there was a remarkable reduction in in vivo RhoA activity in these cells. These results suggest that DLC2 exhibits its tumor suppressor functions in vivo as a GAP specific for RhoA, exerting its effects in suppression of cytoskeleton reorganization, cell growth, cell **migration**, and transformation.
110. Nitric oxide inhibits enterocyte **migration** through activation of RhoA-GTPase in a SHP-2-dependent manner. Diseases of intestinal inflammation like necrotizing enterocolitis (NEC) are associated with impaired epithelial barrier integrity and the sustained release of intestinal nitric oxide (NO). NO modifies the cytoskeletal regulator RhoA-GTPase, suggesting that NO could affect barrier healing by inhibiting intestinal restitution. We now hypothesize that NO inhibits enterocyte **migration** through RhoA-GTPase and sought to determine the pathways involved. The induction of NEC was associated with increased enterocyte NO release and impaired **migration** of bromodeoxyuridine-labeled enterocytes from terminal ileal crypts to villus tips. In IEC-6 enterocytes, NO significantly inhibited enterocyte **migration** and activated RhoA-GTPase while increasing the formation of stress fibers. In parallel, exposure of IEC-6 cells to NO increased the phosphorylation of focal adhesion kinase (pFAK) and caused a striking increase in cell-matrix adhesiveness, suggesting a mechanism by which NO could impair enterocyte **migration**. NEC was associated with increased expression of pFAK in the terminal ileal mucosa of wild-type mice and a corresponding increase in disease severity compared with inducible NO synthase knockout mice, confirming the dependence of NO for FAK phosphorylation in vivo and its role in the pathogenesis of NEC. Strikingly, inhibition of the protein tyrosine phosphatase SHP-2 in IEC-6 cells prevented the activation of RhoA by NO, restored focal adhesions, and reversed the inhibitory effects of NO on enterocyte **migration**. These data indicate that NO impairs mucosal healing by inhibiting enterocyte **migration** through activation of RhoA in a SHP-2-dependent manner and support a possible role for SHP-2 as a therapeutic target in diseases of intestinal inflammation like NEC.
111. MicroRNA-155 is regulated by the transforming growth factor beta/Smad pathway and contributes to epithelial cell plasticity by targeting RhoA. Transforming growth factor beta (TGF-beta) signaling facilitates metastasis in advanced malignancy. While a number of protein-encoding genes are known to be involved in this process, information on the role of microRNAs (miRNAs) in TGF-beta-induced cell **migration** and invasion is still limited. By hybridizing a 515-miRNA oligonucleotide-based microarray library, a total of 28 miRNAs were found to be significantly deregulated in TGF-beta-treated normal murine mammary gland (NMuMG) epithelial cells but not Smad4 knockdown NMuMG cells. Among upregulated miRNAs, miR-155 was the most significantly elevated miRNA. TGF-beta induces miR-155 expression and promoter activity through Smad4. The knockdown of miR-155 suppressed TGF-beta-induced epithelial-mesenchymal transition (EMT) and tight junction dissolution, as well as cell **migration** and invasion. Further, the ectopic expression of miR-155 reduced RhoA protein and disrupted tight junction formation. Reintroducing RhoA cDNA without the 3' untranslated region largely reversed the phenotype induced by miR-155 and TGF-beta. In addition, elevated levels of miR-155 were frequently detected in invasive breast cancer tissues. These data suggest that miR-155 may play an important role in TGF-beta-induced EMT and cell **migration** and invasion by targeting RhoA and indicate that it is a potential therapeutic target for breast cancer intervention.
112. Cdk5-dependent Mst3 phosphorylation and activity regulate neuronal **migration** through RhoA inhibition. The radial **migration** of newborn neurons is critical for the lamination of the cerebral cortex. Proper neuronal **migration** requires precise and rapid reorganization of the actin and microtubule cytoskeleton. However, the underlying signaling mechanisms controlling cytoskeletal reorganization are not well understood. Here, we show that Mst3, a serine/threonine kinase highly expressed in the developing mouse brain, is essential for radial neuronal **migration** and final neuronal positioning in the developing mouse neocortex. Mst3 silencing by in utero electroporation perturbed the multipolar-to-bipolar transition of migrating neurons and significantly retards radial **migration**. Although the kinase activity of Mst3 is essential for its functions in neuronal morphogenesis and **migration**, it is regulated via its phosphorylation at Ser79 by a serine/threonine kinase, cyclin-dependent kinase 5 (Cdk5). Our results show that Mst3 regulates neuronal **migration** through modulating the activity of RhoA, a Rho-GTPase critical for actin cytoskeletal reorganization. Mst3 phosphorylates RhoA at Ser26, thereby negatively regulating the GTPase activity of RhoA. Importantly, RhoA knockdown successfully rescues neuronal **migration** defect in Mst3-knockdown cortices. Our findings collectively suggest that Cdk5-Mst3 signaling regulates neuronal **migration** via RhoA-dependent actin dynamics.
113. Integrin regulation by RhoA in thymocytes. The guanine nucleotide-binding protein Rho has essential functions in T cell development and is important for the survival and proliferation of T cell progenitors in the thymus. To explore the mechanisms used by RhoA to control thymocyte biology, the role of this GTPase in the regulation of integrin-mediated cell adhesion was examined. The data show that RhoA activation is sufficient to stimulate beta(1) and beta(2) integrin-mediated adhesion in murine thymocytes. RhoA is also needed for integrin activation in vivo as loss of Rho function impaired the ability of thymocytes to adhere to the extracellular matrix protein VCAM-1 and prevented integrin activation induced by the GTPases Rac-1 and Rap1A in vivo. The regulated activity of integrins is needed for cell motility and in the present study it was seen that RhoA activity is critical for integrin-mediated thymocyte **migration** to chemokines in vitro. Thus, RhoA has a critical role in regulating cell adhesion and **migration** during T cell development.
114. RhoA-mediated inhibition of vascular endothelial cell mobility: positive feedback through reduced cytosolic p21 and p27. We previously identified that activation of the aryl hydrocarbon receptor (AhR) by 3-methylcholanthrene (3MC) exerts antiproliferative and antimigratory effects on human umbilical vein endothelial cells (HUVECs) through the upregulation of p21/p27 transcription and RhoA activation. In this study, we investigated the mechanisms of 3MC-mediated downregulation of cytosolic p21/ p27, and the effects of 3MC on RhoA activation and cell **migration**, in mouse cerebral vascular endothelial cells (MCVECs). Our results indicated that 3MC reduced the phosphorylation of p21/p27 through AhR/RhoA/PTEN-mediated PI3K/Akt inactivation, which reduced cytosolic p21/p27 retention, causing RhoA activation through positive feedback. Downregulation of p21/p27 by siRNA, and cytosolic p21/p27 by the nuclear export blocker leptomycin B, further reduced cell **migration** in the 3MC-treated cells. Reduced cytosolic p21/p27 expression led to reduced interaction between RhoA and the RhoA inhibitor p190RhoGAP, causing RhoA activation. Treatment with YS-49 activated PI3K/Akt, a downstream target of RhoA, to reduce RhoA/PTEN activation in the 3MC-treated cells, whereas treatment with wortmannin, a PI3K inhibitor, activated RhoA/PTEN. Gain- and loss-of-function analyses revealed that constitutively active (CA) Akt1, but not CA Akt2, inactivated RhoA and stimulated migratory activity. Considering the essential role of RhoA activation in cell **migration**, we evaluated the potential use of simvastatin, a RhoA inhibitor, as a therapeutic intervention in vivo using matrigel plug formation assays. Our results provide a molecular basis for the therapeutic application of simvastatin to reduce RhoA/PTEN activation, restore cytosolic levels of phosphorylated p21/p27, and induce angiogenic processes.
115. Afamin secreted from nonresorbing osteoclasts acts as a chemokine for preosteoblasts via the Akt-signaling pathway. Although it is well known that osteoclastic bone resorption is followed by osteoblastic bone formation, questions remain as to when coupling factors are produced during bone resorption and which stages of bone formation are affected by these factors. To clarify these mechanisms, we established an in vitro system to investigate the coupling phenomenon. We obtained conditioned media (CM) from osteoclasts in the early and late stages of differentiation and from bone resorption stages. The collected CM was used to treat primary mouse calvarial osteoblasts and preosteoblastic MC3T3-E1 cells and to evaluate its influence on the **migration**, viability, proliferation, and differentiation of osteoblasts. We found that CM from osteoclasts in the early stage of differentiation predominantly stimulated the **migration** of osteoblastic lineages. By further performing fractional analyses of the CM with liquid chromatography-tandem mass spectrometry, we identified afamin, which has binding activity with vitamin E, as a possible coupling factor. The CM collected from afamin siRNA-transfected osteoclasts significantly suppressed preosteoblast **migration**. Afamin activated Akt in preosteoblasts, and pretreatment with Akt inhibitor significantly blocked afamin-stimulated preosteoblast **migration**. In conclusion, these results indicate that osteoclasts themselves play a central role in the coupling of bone resorption and formation by stimulating preosteoblast **migration**. In addition, we identified afamin as one of osteoclast-derived chemokines that affect preosteoblasts through the activation of the Akt-signaling pathway.
116. IκB kinase γ/nuclear factor-κB-essential modulator (IKKγ/NEMO) facilitates RhoA GTPase activation, which, in turn, activates Rho-associated KINASE (ROCK) to phosphorylate IKKβ in response to transforming growth factor (TGF)-β1. Transforming growth factor (TGF)-β1 plays several roles in a variety of cellular functions. TGF-β1 transmits its signal through Smad transcription factor-dependent and -independent pathways. It was reported that TGF-β1 activates NF-κB and RhoA, and RhoA activates NF-κB in several kinds of cells in a Smad-independent pathway. However, the activation molecular mechanism of NF-κB by RhoA upon TGF-β1 has not been clearly elucidated. We observed that RhoA-GTP level was increased by TGF-β1 in RAW264.7 cells. RhoA-GDP and RhoGDI were bound to N- and C-terminal domains of IKKγ, respectively. Purified IKKγ facilitated GTP binding to RhoA complexed with RhoGDI. Furthermore, Dbs, a guanine nucletotide exchange factor of RhoA much more enhanced GTP binding to RhoA complexed with RhoGDI in the presence of IKKγ. Indeed, si-IKKγ abolished RhoA activation in response to TGF-β1 in cells. However, TGF-β1 stimulated the release of RhoA-GTP from IKKγ and Rho-associated kinase (ROCK), an active RhoA effector protein, directly phosphorylated IKKβ in vitro, whereas TGF-β1-activated kinase 1 activated RhoA upon TGF-β1 stimulation. Taken together, our data indicate that IKKγ facilitates RhoA activation via a guanine nucletotide exchange factor, which in turn activates ROCK to phosphorylate IKKβ, leading to NF-κB activation that induced the che**mokine ex**pression and cell migration upon TGF-β1.

#### GeneRIFs

1. In summary, the dihydroxycoumarin derivative daphnetin inhibits the invasion and **migration** of LM8 cells, and therefore represents a promising agent for use against metastatic cancer.
2. Data suggest galectin-3 in hepatocellular carcinoma cells promotes cell **migration** by inducing RhoA GTPase, activating Mlck (myosin light chain kinase 3), up-regulating phosphorylation of MLC2 (myosin light chain 2), and inducing actin rearrangement.
3. Active RhoA and RhoB are involved in back end functions, such as retracting the tail and lamellipodial membrane extensions during macrophage **migration**.
4. Ablation of RhoA drastically alters the timing and placement of fissure formation, the **migration** and positioning of granule and Purkinje cells, the alignment of Bergmann glia, and the integrity of the basement membrane, primarily in the anterior lobules.
5. RhoA knockdown successfully rescues the neuronal **migration** defect in Mst3-knockdown neocortex.
6. IKKgamma facilitates RhoA activation via a guanine nucletotide exchange factor, which in turn activates ROCK to phosphorylate IKKbeta, leading to NF-kappaB activation that induced the chemokine expression and cell **migration** upon TGF-beta1.
7. Type VIII collagen signals via beta1 integrin and RhoA to regulate MMP-2 expression and smooth muscle cell **migration**.
8. Rap1 inhibits cell **migration** by suppression of RhoA activity in response to TGF-beta1.
9. To determine whether RhoA and Cdc42 directly regulate the motility of cortical interneurons during **migration**, we deleted RhoA and Cdc42 in the subventricular zone, where more fate-restricted progenitors are located within the ganglionic eminences.
10. Fibronectin stimulates **migration** through lipid raft dependent NHE-1 activation in mouse embryonic stem cells: involvement of RhoA, calcium/ caM and ERK.
11. These data identify discrete roles for paxillin and Hic-5 in Rac1 and RhoA-dependent cell adhesion formation and maturation; processes essential for productive cell **migration**.
12. The cell **migration** is dependent on the continuous organization of the actin cytoskeleton, which is regulated by RhoA.
13. Hematopoietic stem cell **migration** is essentially regulated by Rho GTPase, including RhoA, Rac1, and Cdc42, and necessitates the rearrangement of cytoskeleton structures, i.e., F-actin and microtubules.
14. These data indicate a crucial role for RhoA and contraction in regulating cell spreading and a contraction-independent function of RhoA in keratinocyte **migration** and that RhoA is dispensable for skin development
15. demonstrate an essential role of cytohesin-1/RhoA during ameboid **migration** in the presence of integrins
16. Autocrine motility factor transactivates the MMP-3 gene through the activation of Src-RhoA-phosphatidylinositol 3-kinase signaling to induce hepatoma cell **migration**.
17. These data suggest that miR-155 may play an important role in Smad4/TGF-beta-induced EMT and cell **migration** and invasion by targeting RhoA and indicate that it is a potential therapeutic target for breast cancer intervention.
18. Tethering of RhoA to the membrane by GGpp is necessary for T cell **migration** and provides a mechanism by which HMGCRI may prevent T cell infiltration into inflamed compartments.
19. Data suggest that Kank negatively regulates the formation of actin stress fibers and cell **migration** through the inhibition of RhoA activity, which is controlled by binding of Kank to 14-3-3 in PI3K-Akt signaling.
20. GnT-V and beta1, 6GlcNAc branching mediate the cell **migration** and invasion in Rac1-positive and RhoA-negative regulatory manners.
21. RhoA activation increased after MIP-2 treatment in aortic endothelial cells but not in pulmonary artery endothelial cells. RhoA is part of a signaling pathway essential for aortic cell **migration** after CXCR(2) ligation.
22. tethering of RhoA to the membrane by GGpp is determinant for T cell **migration**
23. Loss of p53 promotes Rhoa-Rock1-dependent cell **migration** and invasion in 3D matrices.
24. Results suggest that DLC2 exhibits its tumor suppressor functions in vivo as a GAP specific for RhoA, exerting its effects in suppression of cytoskeleton reorganization, cell growth, cell **migration**, and transformation.
25. In IEC-6 enterocytes, NO significantly inhibited enterocyte **migration** and activated RhoA-GTPase while increasing the formation of stress fibers.

### ECM

#### PubMed

1. Arhgap28 is a RhoGAP that inactivates RhoA and downregulates stress fibers. The small GTPase RhoA is a major regulator of actin reorganization during the formation of stress fibers; thus identifying molecules that regulate Rho activity is necessary for a complete understanding of the mechanisms that determine cell contractility. Here, we have identified Arhgap28 as a Rho GTPase activating protein (RhoGAP) that switches RhoA to its inactive form. We generated an Arhgap28-LacZ reporter mouse that revealed gene expression in soft tissues at E12.5, pre-bone structures of the limb at E15.5, and prominent expression restricted mostly to ribs and limb long bones at E18.5 days of development. Expression of recombinant Arhgap28-V5 in human osteosarcoma SaOS-2 cells caused a reduction in the basal level of RhoA activation and disruption of actin stress fibers. Extracellular matrix assembly studies using a 3-dimensional cell culture system showed that Arhgap28 was upregulated during Rho-dependent assembly of the **ECM**. Taken together, these observations led to the hypothesis that an Arhgap28 knockout mouse model would show a connective tissue phenotype, perhaps affecting bone. Arhgap28-null mice were viable and appeared normal, suggesting that there could be compensation from other RhoGAPs. Indeed, we showed that expression of Arhgap6 (a closely related RhoGAP) was upregulated in Arhgap28-null bone tissue. An upregulation in RhoA expression was also detected suggesting that Arhgap28 may be able to additionally regulate Rho signaling at a transcriptional level. Microarray analyses revealed that Col2a1, Col9a1, Matn3, and Comp that encode extracellular matrix proteins were downregulated in Arhgap28-null bone. Although mutations in these genes cause bone dysplasias no bone phenotype was detected in the Arhgap-28 null mice. Together, these data suggest that the regulation of Rho by RhoGAPs, including Arhgap28, during the assembly and development of mechanically strong tissues is complex and may involve multiple RhoGAPs.
2. Paxillin and Hic-5 interaction with vinculin is differentially regulated by Rac1 and RhoA. Cell migration is of paramount importance to organism development and maintenance as well as multiple pathological processes, including cancer metastasis. The RhoGTPases Rac1 and RhoA are indispensable for cell migration as they regulate cell protrusion, cell-extracellular matrix (**ECM**) interactions and force transduction. However, the consequences of their activity at a molecular level within the cell remain undetermined. Using a combination of FRET, FRAP and biochemical analyses we show that the interactions between the focal adhesion proteins vinculin and paxillin, as well as the closely related family member Hic-5 are spatially and reciprocally regulated by the activity of Rac1 and RhoA. Vinculin in its active conformation interacts with either paxillin or Hic-5 in adhesions in response to Rac1 and RhoA activation respectively, while inactive vinculin interacts with paxillin in the membrane following Rac1 inhibition. Additionally, Rac1 specifically regulates the dynamics of paxillin as well as its binding partner and F-actin interacting protein actopaxin (α-parvin) in adhesions. Furthermore, FRET analysis of protein:protein interactions within cell adhesions formed in 3D matrices revealed that, in contrast to 2D systems vinculin interacts preferentially with Hic-5. This study provides new insight into the complexity of cell**-EC**M adhesions in both 2D and 3D matrices by providing the first description of RhoGTPase-coordinated protein:protein interactions in a cellular microenvironment. These data identify discrete roles for paxillin and Hic-5 in Rac1 and RhoA-dependent cell adhesion formation and maturation; processes essential for productive cell migration.
3. Cadherin-11 coordinates cellular migration and extracellular matrix remodeling during aortic valve maturation. Proper remodeling of the endocardial cushions into thin fibrous valves is essential for gestational progression and long-term function. This process involves dynamic interactions between resident cells and their local environment, much of which is not understood. In this study, we show that deficiency of the cell-cell adhesion protein cadherin-11 (Cad-11) results in significant embryonic and perinatal lethality primarily due to valve related cardiac dysfunction. While endocardial to mesenchymal transformation is not abrogated, mesenchymal cells do not homogeneously cellularize the cushions. These cushions remain thickened with disorganized **ECM**, resulting in pronounced aortic valve insufficiency. Mice that survive to adulthood maintain thickened and stenotic semilunar valves, but interestingly do not develop calcification. Cad-11 (-/-) aortic valve leaflets contained reduced Sox9 activity, β1 integrin expression, and RhoA-GTP activity, suggesting that remodeling defects are due to improper migration and/or cellular contraction. Cad-11 deletion or siRNA knockdown reduced migration, eliminated collective migration, and impaired 3D matrix compaction by aortic valve interstitial cells (VIC). Cad-11 depleted cells in culture contained few filopodia, stress fibers, or contact inhibited locomotion. Transfection of Cad-11 depleted cells with constitutively active RhoA restored cell phenotypes. Together, these results identify cadherin-11 mediated adhesive signaling for proper remodeling of the embryonic semilunar valves.
4. Loss of alpha3beta1 integrin function results in an altered differentiation program in the mouse submandibular gland. Mammalian submandibular gland (SMG) development leads to the establishment of highly organized secretory acinar and nonsecretory ductal epithelial cells. The ability of maturing salivary epithelial cells to attain their differentiated state has been shown to depend, in part, on interactions between extracellular matrix (**ECM**) proteins and their integrin receptors. In a search for key regulators of salivary cell lineage, we have studied alpha3beta1 integrin, a receptor for the basement membrane protein laminin, by characterizing embryonic day 18 (E18) SMGs isolated from mice carrying a targeted mutation in the alpha3 integrin gene. Transmission electron microscopy studies showed that the mutant SMGs exhibited an aberrant differentiation phenotype with defects in the apical-basal polarity axis and in the basement membrane. Based on immunohistochemistry and Western blot analyses, the alpha3beta1-deficient SMGs had altered expression and/or localization of several **ECM** and adhesive molecules, including laminin beta1, fibronectin, alpha5 integrin, and E-cadherin. These changes correlated with alterations in the activation state of Ras-extracellular signal-regulated kinase (ERK), as well as the expression and/or localization of Cdc42 and RhoA, two Rho GTPases that regulate the organization of the actin cytoskeleton. We conclude that alpha3beta1 is required for normal salivary cell differentiation and that its absence affects multiple components of adhesive complexes and their associated signalling pathways.
5. Plakoglobin regulates cell motility through Rho- and fibronectin-dependent Src signaling. We previously showed that the cell-cell junction protein plakoglobin (PG) not only suppresses motility of keratinocytes in contact with each other, but also, unexpectedly, of single cells. Here we show that PG deficiency results in extracellular matrix (**ECM**)-dependent disruption of mature focal adhesions and cortical actin organization. Plating PG⁻/⁻ cells o**nto** ECM deposited by PG+/⁻ cells partially restored normal cell morphology and inhibited PG⁻/⁻ cell motility. In over 70 adhesion molecules whose expression we previously showed to be altered in PG⁻/⁻ cells, a substantial decrease in fibronectin (FN) in PG⁻/⁻ cells stood out. Re-introduction of PG into PG⁻/⁻ cells restored FN expression, and keratinocyte motility was reversed by plating PG⁻/⁻ cells onto FN. Somewhat surprisingly, based on previously reported roles for PG in regulating gene transcription, PG-null cells exhibited an increase, not a decrease, in FN promoter activity. Instead, PG was required for maintenance of FN mRNA stability. PG⁻/⁻ cells exhibited an increase in activated Src, one of the kinases controlled by FN, a phenotype rever**sed** by plating PG⁻/⁻ cells on ECM deposited by PG+/⁻ keratinocytes. PG⁻/⁻ cells also exhibited Src-independent activation of the small GTPases Rac1 and RhoA. Both Src and RhoA inhibition attenuated PG⁻/⁻ keratinocyte motility. We propose a novel role for PG i**n r**egulating cell motility through distinct ECM-Src and RhoGTPase-dependent pathways, influenced in part by PG-dependent regulation of FN mRNA stability.
6. Regulation of mesenchymal-to-epithelial transition by PARAXIS during somitogenesis. Dynamic alterations in cell shape, migration, and adhesion play a central role in tissue morphogenesis during embryonic development and congenital disease. The mesenchymal-to-epithelial transition that occurs during vertebrate somitogenesis is required for proper patterning of the axial musculoskeletal system. Somitic MET is initiated in the presomitic mesoderm by PARAXIS-dependent changes in cell adhesion, cell polarity, and the composition of the extracellular matrix. However, the target genes downstream of the transcription factor PARAXIS remain poorly described.A genome-wide comparison of gene expression in the anterior presomitic mesoderm and newly formed somites of Paraxis(-/-) embryos resulted in a set of deregulated genes enriched for factors associated with extracellular matrix and cytoskeletal organization and cell-cell and cell-**ECM** adhesion. The greatest change in expression was seen in fibroblast activation protein alpha (Fap), encoding a dipeptidyl peptidase capable of increasing fibronectin and collagen fiber organization in extracellular matrix. Further, downstream genes in the Wnt and Notch signaling pathways were downregulated, predicting that PARAXIS participates in positive feedback loops in both pathways.These data demonstrate that PARAXIS initiates and stabilizes somite epithelialization by integrating signals from multiple pathways to control the reorganization of the **ECM**, cytoskeleton, and adhesion junctions during MET.
7. Paxillin and Hic-5 interaction with vinculin is differentially regulated by Rac1 and RhoA. Cell migration is of paramount importance to organism development and maintenance as well as multiple pathological processes, including cancer metastasis. The RhoGTPases Rac1 and RhoA are indispensable for cell migration as they regulate cell protrusion, cell-extracellular matrix (**ECM**) interactions and force transduction. However, the consequences of their activity at a molecular level within the cell remain undetermined. Using a combination of FRET, FRAP and biochemical analyses we show that the interactions between the focal adhesion proteins vinculin and paxillin, as well as the closely related family member Hic-5 are spatially and reciprocally regulated by the activity of Rac1 and RhoA. Vinculin in its active conformation interacts with either paxillin or Hic-5 in adhesions in response to Rac1 and RhoA activation respectively, while inactive vinculin interacts with paxillin in the membrane following Rac1 inhibition. Additionally, Rac1 specifically regulates the dynamics of paxillin as well as its binding partner and F-actin interacting protein actopaxin (α-parvin) in adhesions. Furthermore, FRET analysis of protein:protein interactions within cell adhesions formed in 3D matrices revealed that, in contrast to 2D systems vinculin interacts preferentially with Hic-5. This study provides new insight into the complexity of cell**-EC**M adhesions in both 2D and 3D matrices by providing the first description of RhoGTPase-coordinated protein:protein interactions in a cellular microenvironment. These data identify discrete roles for paxillin and Hic-5 in Rac1 and RhoA-dependent cell adhesion formation and maturation; processes essential for productive cell migration.
8. Plakoglobin regulates cell motility through Rho- and fibronectin-dependent Src signaling. We previously showed that the cell-cell junction protein plakoglobin (PG) not only suppresses motility of keratinocytes in contact with each other, but also, unexpectedly, of single cells. Here we show that PG deficiency results in extracellular matrix (**ECM**)-dependent disruption of mature focal adhesions and cortical actin organization. Plating PG⁻/⁻ cells o**nto** ECM deposited by PG+/⁻ cells partially restored normal cell morphology and inhibited PG⁻/⁻ cell motility. In over 70 adhesion molecules whose expression we previously showed to be altered in PG⁻/⁻ cells, a substantial decrease in fibronectin (FN) in PG⁻/⁻ cells stood out. Re-introduction of PG into PG⁻/⁻ cells restored FN expression, and keratinocyte motility was reversed by plating PG⁻/⁻ cells onto FN. Somewhat surprisingly, based on previously reported roles for PG in regulating gene transcription, PG-null cells exhibited an increase, not a decrease, in FN promoter activity. Instead, PG was required for maintenance of FN mRNA stability. PG⁻/⁻ cells exhibited an increase in activated Src, one of the kinases controlled by FN, a phenotype rever**sed** by plating PG⁻/⁻ cells on ECM deposited by PG+/⁻ keratinocytes. PG⁻/⁻ cells also exhibited Src-independent activation of the small GTPases Rac1 and RhoA. Both Src and RhoA inhibition attenuated PG⁻/⁻ keratinocyte motility. We propose a novel role for PG i**n r**egulating cell motility through distinct ECM-Src and RhoGTPase-dependent pathways, influenced in part by PG-dependent regulation of FN mRNA stability.
9. RhoA determines lineage fate of mesenchymal stem cells by modulating CTGF-VEGF complex in extracellular matrix. Mesenchymal stem cells (MSCs) participate in the repair/remodelling of many tissues, where MSCs commit to different lineages dependent on the cues in the local microenvironment. Here we show that TGFβ-activated RhoA/ROCK signalling functions as a molecular switch regarding the fate of MSCs in arterial repair/remodelling after injury. MSCs differentiate into myofibroblasts when RhoA/ROCK is turned on, endothelial cells when turned off. The former is pathophysiologic resulting in intimal hyperplasia, whereas the latter is physiological leading to endothelial repair. Further analysis revealed that MSC RhoA activation promotes formation of an extracellular matrix **(EC**M) complex consisting of connective tissue growth factor (CTGF) and vascular endothelial growth factor (VEGF). Inactivation of RhoA/ROCK in MSCs induces matrix metalloproteinase-3-mediated CTGF cleavage, resulting in VEGF release and MSC endothelial differentiation. Our findings uncover a novel mechanism by which cell**-EC**M interactions determine stem cell lineage specificity and offer additional molecular targets to manipulate MSC-involved tissue repair/regeneration.
10. Laminin-111 stimulates proliferation of mouse embryonic stem cells through a reduction of gap junctional intercellular communication via RhoA-mediated Cx43 phosphorylation and dissociation of Cx43/ZO-1/drebrin complex. Gap junctions within extracellular matrix (**ECM**)-defined boundaries ensure synchronous activity between cells destined to become functional mediators that regulate cell behavior. However, the role of **ECM** in connexin (Cx) function in mouse embryonic stem cells (mESCs) has not been elucidated. Therefore, we examined the role of laminin-111 in the control of Cx43 functions and related signal pathways in mESCs. **ECM** components (laminin-111, fibronectin, and collagen I) increased Cx43 phosphorylation and decreased Lucifer yellow (Ly) diffusion. In addition, laminin-111 increased the proliferation index through reduction of gap junctional intercellular communication (GJIC), which was confirmed by 18α-glycyrrhetinic acid (18α-GA). Laminin-111 increased phosphorylation of focal adhesion kinase (FAK)/Src and protein kinase C (PKC), which were inhibited by integrin β1 antibody (Ab) and laminin receptor-1 (LR-1) Ab, respectively. In addition, inhibition of both FAK/Src and PKC blocked Cx43 phosphorylation. Laminin-111 increased the Ras homolog gene family, member A (RhoA) activation, which was blocked by FAK/Src and PKC inhibitors, suggesting the existence of parallel pathways that merge at RhoA. Inhibition of RhoA reversed the laminin-111-induced increase of Cx43 phosphorylation and reduction of GJIC. Laminin-111 also stimulated the dissociation of Cx43/ZO-1 complex followed by disruption of Cx43/drebrin and Cx43/F-actin complexes, which were reversed by C3 (RhoA inhibitor). ZO-1 small interfering (si) RNA significantly decreased Ly diffusion. Moreover, laminin-111 decreased Cx43 labeling at the intercellular junction, whereas pretreatment with degradation inhibitors (lysosomal protease inhibitor, chloroquine; proteasome inhibitor, lactacystin) increased Cx43 expression, reversely. In conclusion, laminin-111 stimulated mESC proliferation through a reduction of GJIC via RhoA-mediated Cx43 phosphorylation and Cx43/ZO-1/drebrin complex instability-mediated Cx43 degradation.
11. **ECM** compliance regulates osteogenesis by influencing MAPK signaling downstream of RhoA and ROCK. The compliance of the extracellular matrix (**ECM**) regulates osteogenic differentiation by modulating extracellular signal-regulated kinase (ERK) activity. However, the molecular mechanism linking **ECM** compliance to the ERK-mitogen-activated protein kinase (MAPK) pathway remains unclear. Furthermore, RhoA has been widely implicated in integrin-mediated signaling and mechanotransduction. We studied the relationship between RhoA and ERK-MAPK signaling to determine their roles in the regulation of osteogenesis by **ECM** compliance. Inhibition of RhoA and ROCK in MC3T3-E1 pre-osteoblasts cultured on substrates of varying compliance reduced ERK activity, whereas constitutively active RhoA enhanced it. The expression of RUNX2, a potent osteogenic transcription factor, was increased on stiffer matrices and correlated with elevated ERK activity. Inhibition of RhoA, ROCK, or the MAPK pathway diminished RUNX2 activity and delayed the onset of osteogenesis as shown by altered osteocalcin (OCN) and bone sialoprotein (BSP) gene expression, alkaline phosphatase (ALP) activity, and matrix mineralization. These data establish that one possible mechanism by which **ECM** rigidity regulates osteogenic differentiation involves MAPK activation downstream of the RhoA-ROCK signaling pathway.
12. Fibronectin stimulates migration through lipid raft dependent NHE-1 activation in mouse embryonic stem cells: involvement of RhoA, Ca(2+)/CaM, and ERK. Extracellular matrix (**ECM**) components and intracellular pH (pH(i)) may serve as regulators of cell migration in various cell types.The Oris migration assay was used to assess the effect of fibronectin (FN) on cell motility. The Na(+)/H(+) exchanger (NHE)-1 activity was evaluated by measuring pH(i) and [(22)Na(+)] uptake. To examine activated signaling molecules, western blot analysis and immunoprecipitation was performed.**ECM** components (FN, laminin, fibrinogen, and collagen type I) increased [(22)Na(+)] uptake, pH(i), and cell migration. In addition, FN-induced increase of cell migration was inhibited by NHE-1 inhibitor amiloride or NHE-1-specific siRNA. FN selectively increased the mRNA and protein expression of NHE-1, but not that of NHE-2 or NHE-3. FN binds integrin β1 and subsequently stimulates caveolin-1 phosphorylation and Ca(2+) influx. Then, NHE-1 is phosphorylated by RhoA and Rho kinases, and Ca(2+)/calmodulin (CaM) signaling elicits complex formation with NHE-1, which is enriched in lipid raft/caveolae microdomains of the plasma membrane. Activation of NHE-1 continuously induces an increase of [(22)Na(+)] uptake and pH(i). Finally, NHE-1-dependent extracellular signal-regulated kinase (ERK) 1/2 phosphorylation enhanced matrix metalloproteinase-2 (MMP-2) and filamentous-actin (F-actin) expression, partially contributing to the regulation of embryonic stem cells (ESCs) migration.FN stimulated mESCs migration and proliferation through NHE-1 activation, which were mediated by lipid raft-associated caveolin-1, RhoA/ROCK, and Ca(2+)/CaM signaling pathways.The precise role of NHE in the modulation of **EC**M-related physiological functions such as proliferation and migration remains poorly understood. Thus, this study analyzed the relationship between FN and NHE in regulating the migration of mouse ESCs and their related signaling pathways.
13. RhoA/Rho kinase signaling in the cumulus mediates extracellular matrix assembly. Cumulus cells surround the oocyte and regulate the production and assembly of the extracellular matrix (**ECM**) around the cumulus-oocyte complex for its timely interaction with sperm in the oviduct. We recently found that C-C chemokines such as CCL2, CCL7, and CCL9 are produced and stimulate integrin-mediated **ECM** assembly in the postovulatory cumulus to protect eggs and that prostaglandin E(2)-EP2 signaling in the cumulus cells facilitates fertilization by suppressing this chemokine signaling, which otherwise results in fertilization failure by preventing sperm penetration through the cumulus **ECM**. However, it remains unknown as to what mechanisms underlie chemokine-induced cumulus **ECM** assembly. Here we report that inhibition of EP2 signaling or addition of CCL7 augments RhoA activation and induces the surface accumulation of integrin and the contraction of cumulus cells. Enhanced surface accumulation of integrin then stimulates the formation and assembly of fibronectin fibrils as well as induces cumulus **ECM** resistance to hyaluronidase and sperm penetration. These changes in the cumulus **ECM** as well as cell contraction are relieved by the addition of Y27632 or blebbistatin. These results suggest that chemokines induce integrin engagement to the **ECM** and consequent **ECM** remodeling through the RhoA/Rho kinase/actomyosin pathway, making the cumulus **ECM** barrier resistant to sperm penetration. Based on these results, we propose that prostaglandin E(2)-EP2 signaling negatively regulates chemokine-induced Rho/ROCK signaling in cumulus cells for successful fertilization.

### [Ee]xtracellular [Mm]atrix

#### PubMed

1. Arhgap28 is a RhoGAP that inactivates RhoA and downregulates stress fibers. The small GTPase RhoA is a major regulator of actin reorganization during the formation of stress fibers; thus identifying molecules that regulate Rho activity is necessary for a complete understanding of the mechanisms that determine cell contractility. Here, we have identified Arhgap28 as a Rho GTPase activating protein (RhoGAP) that switches RhoA to its inactive form. We generated an Arhgap28-LacZ reporter mouse that revealed gene expression in soft tissues at E12.5, pre-bone structures of the limb at E15.5, and prominent expression restricted mostly to ribs and limb long bones at E18.5 days of development. Expression of recombinant Arhgap28-V5 in human osteosarcoma SaOS-2 cells caused a reduction in the basal level of RhoA activation and disruption of actin stress fibers. **Extracellular matrix** assembly studies using a 3-dimensional cell culture system showed that Arhgap28 was upregulated during Rho-dependent assembly of the ECM. Taken together, these observations led to the hypothesis that an Arhgap28 knockout mouse model would show a connective tissue phenotype, perhaps affecting bone. Arhgap28-null mice were viable and appeared normal, suggesting that there could be compensation from other RhoGAPs. Indeed, we showed that expression of Arhgap6 (a closely related RhoGAP) was upregulated in Arhgap28-null bone tissue. An upregulation in RhoA expression was also detected suggesting that Arhgap28 may be able to additionally regulate Rho signaling at a transcriptional level. Microarray analyses revealed that Col2a1, Col9a1, Matn3, and Comp that encode **extracellular matrix** proteins were downregulated in Arhgap28-null bone. Although mutations in these genes cause bone dysplasias no bone phenotype was detected in the Arhgap-28 null mice. Together, these data suggest that the regulation of Rho by RhoGAPs, including Arhgap28, during the assembly and development of mechanically strong tissues is complex and may involve multiple RhoGAPs.
2. Therapeutic ultrasound bypasses canonical syndecan-4 signaling to activate rac1. The application of pulsed, low intensity ultrasound is emerging as a potent therapy for the treatment of complex bone fractures and tissue damage. Ultrasonic stimuli accelerate fracture healing by up to 40% and enhance tendon and ligament healing by promoting cell proliferation, migration, and matrix synthesis through an unresolved mechanism. Ultrasound treatment also induces closure of nonunion fractures, at a success rate (85% of cases) similar to that of surgical intervention (68-96%) while avoiding the complications associated with surgery. The regulation of cell adhesion necessary for wound healing depends on cooperative engagement of the **extracellular matrix** receptors, integrin and syndecan, as exemplified by the wound healing defects observed in syndecan- and integrin-knock-out mice. This report distinguishes the influence of ultrasound on signals downstream of the prototypic fibronectin receptors, alpha(5)beta(1) integrin and syndecan-4, which cooperate to regulate Rac1 and RhoA. Ultrasonic stimulation fails to activate integrins or induce cell spreading on poor, electrostatic ligands. By contrast, ultrasound treatment overcomes the necessity of engagement or expression of syndecan-4 during the process of focal adhesion formation, which normally requires simultaneous engagement of both receptors. Ultrasound exerts an influence downstream of syndecan-4 and PKCalpha to specifically activate Rac1, itself a critical regulator of tissue repair, and to a lesser extent RhoA. The ability of ultrasound to bypass syndecan-4 signaling, which is known to facilitate efficient tissue repair, explains the reduction in healing times observed in ultrasound-treated patients. By substituting for one of the key axes of adhesion-dependent signaling, ultrasound therapy has considerable potential as a clinical technique.
3. Paxillin and Hic-5 interaction with vinculin is differentially regulated by Rac1 and RhoA. Cell migration is of paramount importance to organism development and maintenance as well as multiple pathological processes, including cancer metastasis. The RhoGTPases Rac1 and RhoA are indispensable for cell migration as they regulate cell protrusion, cell-**extracellular matrix** (ECM) interactions and force transduction. However, the consequences of their activity at a molecular level within the cell remain undetermined. Using a combination of FRET, FRAP and biochemical analyses we show that the interactions between the focal adhesion proteins vinculin and paxillin, as well as the closely related family member Hic-5 are spatially and reciprocally regulated by the activity of Rac1 and RhoA. Vinculin in its active conformation interacts with either paxillin or Hic-5 in adhesions in response to Rac1 and RhoA activation respectively, while inactive vinculin interacts with paxillin in the membrane following Rac1 inhibition. Additionally, Rac1 specifically regulates the dynamics of paxillin as well as its binding partner and F-actin interacting protein actopaxin (α-parvin) in adhesions. Furthermore, FRET analysis of protein:protein interactions within cell adhesions formed in 3D matrices revealed that, in contrast to 2D systems vinculin interacts preferentially with Hic-5. This study provides new insight into the complexity of cell-ECM adhesions in both 2D and 3D matrices by providing the first description of RhoGTPase-coordinated protein:protein interactions in a cellular microenvironment. These data identify discrete roles for paxillin and Hic-5 in Rac1 and RhoA-dependent cell adhesion formation and maturation; processes essential for productive cell migration.
4. Cadherin-11 coordinates cellular migration and **extracellular matrix** remodeling during aortic valve maturation. Proper remodeling of the endocardial cushions into thin fibrous valves is essential for gestational progression and long-term function. This process involves dynamic interactions between resident cells and their local environment, much of which is not understood. In this study, we show that deficiency of the cell-cell adhesion protein cadherin-11 (Cad-11) results in significant embryonic and perinatal lethality primarily due to valve related cardiac dysfunction. While endocardial to mesenchymal transformation is not abrogated, mesenchymal cells do not homogeneously cellularize the cushions. These cushions remain thickened with disorganized ECM, resulting in pronounced aortic valve insufficiency. Mice that survive to adulthood maintain thickened and stenotic semilunar valves, but interestingly do not develop calcification. Cad-11 (-/-) aortic valve leaflets contained reduced Sox9 activity, β1 integrin expression, and RhoA-GTP activity, suggesting that remodeling defects are due to improper migration and/or cellular contraction. Cad-11 deletion or siRNA knockdown reduced migration, eliminated collective migration, and impaired 3D matrix compaction by aortic valve interstitial cells (VIC). Cad-11 depleted cells in culture contained few filopodia, stress fibers, or contact inhibited locomotion. Transfection of Cad-11 depleted cells with constitutively active RhoA restored cell phenotypes. Together, these results identify cadherin-11 mediated adhesive signaling for proper remodeling of the embryonic semilunar valves.
5. Loss of alpha3beta1 integrin function results in an altered differentiation program in the mouse submandibular gland. Mammalian submandibular gland (SMG) development leads to the establishment of highly organized secretory acinar and nonsecretory ductal epithelial cells. The ability of maturing salivary epithelial cells to attain their differentiated state has been shown to depend, in part, on interactions between **extracellular matrix** (ECM) proteins and their integrin receptors. In a search for key regulators of salivary cell lineage, we have studied alpha3beta1 integrin, a receptor for the basement membrane protein laminin, by characterizing embryonic day 18 (E18) SMGs isolated from mice carrying a targeted mutation in the alpha3 integrin gene. Transmission electron microscopy studies showed that the mutant SMGs exhibited an aberrant differentiation phenotype with defects in the apical-basal polarity axis and in the basement membrane. Based on immunohistochemistry and Western blot analyses, the alpha3beta1-deficient SMGs had altered expression and/or localization of several ECM and adhesive molecules, including laminin beta1, fibronectin, alpha5 integrin, and E-cadherin. These changes correlated with alterations in the activation state of Ras-extracellular signal-regulated kinase (ERK), as well as the expression and/or localization of Cdc42 and RhoA, two Rho GTPases that regulate the organization of the actin cytoskeleton. We conclude that alpha3beta1 is required for normal salivary cell differentiation and that its absence affects multiple components of adhesive complexes and their associated signalling pathways.
6. Type VIII collagen signals via β1 integrin and RhoA to regulate MMP-2 expression and smooth muscle cell migration. The **extracellular matri**x signals and regulates the behavior of vascular cells during the pathogenesis of atherosclerosis. Type VIII collagen, a short chain collagen, is scarcely present in normal arteries, but is dramatically upregulated in atherosclerosis and after other types of vascular injury. Cell culture studies have revealed that this protein supports smooth muscle cell (SMC) adhesion and stimulates migration, however little is known about the signaling or the mechanisms by which this occurs. SMCs isolated from wild-type C57BL/6 and type VIII collagen deficient mice were studied using assays to measure chemotactic and haptotactic migration, and remodeling and contraction of 3-dimensional type I collagen gels. Col8(-/-) SMCs exhibited impairments in migration, and a strongly adhesive phenotype with prominent stress fibers, stable microtubules and pronounced central basal focal adhesions. The addition of exogenous type VIII collagen to the Col8(-/-) SMCs rescued the impairments in migration, and restored cytoskeletal architecture so that it was similar to Col8(+/+) cells. We measured elevated levels of active GTP-RhoA in the Col8(-/-) cells, and this too was reversed by treatment with exogenous type VIII collagen. We showed that type VIII collagen normally suppresses RhoA activation through a beta-1 integrin dependent mechanism. MMP-2 levels were reduced in the Col8(-/-) SMCs, and knockdown of MMP-2 in Col8(+/+) SMCs partially recapitulated the decreases in migration and 3D gel contraction seen in Col8(-/-) cells, showing that type VIII collagen-stimulated migration was dependent on MMP-2. Inhibition of Rho restored MMP-2 activity in the Col8(-/-) cells, and partially rescued migration, demonstrating that the elevations in RhoA activity were responsible for the suppression of migration of these cells. In conclusion, we have shown that type VIII collagen signals through beta-1 integrin receptors to suppress RhoA, allowing optimal configuration of the cytoskeleton, and the stimulation of MMP-2-dependent cell migration.
7. Role of the Rho-ROCK (Rho-associated kinase) signaling pathway in the regulation of pancreatic beta-cell function. **Extracellular matrix** has a beneficial impact on beta-cell spreading and function, but the underlying signaling pathways have yet to be fully elucidated. In other cell types, Rho, a well-characterized member of the family of Rho GTPases, and its effector Rho-associated kinase (ROCK), play an important role as downstream mediators of outside in signaling from **extracellular matrix**. Therefore, a possible role of the Rho-ROCK pathway in beta-cell spreading, actin cytoskeleton dynamics, and function was investigated. Rho was inhibited using a new cell-permeable version of C3 transferase, whereas the activity of ROCK was repressed using the specific ROCK inhibitors H-1152 and Y-27632. Inhibition of Rho and of ROCK increased spreading and improved both short-term and prolonged glucose-stimulated insulin secretion but had no impact on basal secretion. Inhibition of this pathway led to a depolymerization of the actin cytoskeleton. Furthermore, the impact of the inhibition of ROCK on stimulated insulin secretion was acute and reversible, suggesting that rapid signaling such as phosphorylation is involved. Finally, quantification of the activity of RhoA indicated that the **extracellular matrix** represses RhoA activity. Overall these results show for the first time that the Rho-ROCK signaling pathway contributes to the stabilization of the actin cytoskeleton and inhibits glucose-stimulated insulin secretion in primary pancreatic beta-cells. Furthermore, they indicate that inhibition of this pathway might be one of the mechanisms by which the **extracellular matrix** exerts its beneficial effects on pancreatic beta-cell function.
8. Plakoglobin regulates cell motility through Rho- and fibronectin-dependent Src signaling. We previously showed that the cell-cell junction protein plakoglobin (PG) not only suppresses motility of keratinocytes in contact with each other, but also, unexpectedly, of single cells. Here we show that PG deficiency results in **extracellular matrix** (ECM)-dependent disruption of mature focal adhesions and cortical actin organization. Plating PG⁻/⁻ cells onto ECM deposited by PG+/⁻ cells partially restored normal cell morphology and inhibited PG⁻/⁻ cell motility. In over 70 adhesion molecules whose expression we previously showed to be altered in PG⁻/⁻ cells, a substantial decrease in fibronectin (FN) in PG⁻/⁻ cells stood out. Re-introduction of PG into PG⁻/⁻ cells restored FN expression, and keratinocyte motility was reversed by plating PG⁻/⁻ cells onto FN. Somewhat surprisingly, based on previously reported roles for PG in regulating gene transcription, PG-null cells exhibited an increase, not a decrease, in FN promoter activity. Instead, PG was required for maintenance of FN mRNA stability. PG⁻/⁻ cells exhibited an increase in activated Src, one of the kinases controlled by FN, a phenotype reversed by plating PG⁻/⁻ cells on ECM deposited by PG+/⁻ keratinocytes. PG⁻/⁻ cells also exhibited Src-independent activation of the small GTPases Rac1 and RhoA. Both Src and RhoA inhibition attenuated PG⁻/⁻ keratinocyte motility. We propose a novel role for PG in regulating cell motility through distinct ECM-Src and RhoGTPase-dependent pathways, influenced in part by PG-dependent regulation of FN mRNA stability.
9. Regulation of mesenchymal-to-epithelial transition by PARAXIS during somitogenesis. Dynamic alterations in cell shape, migration, and adhesion play a central role in tissue morphogenesis during embryonic development and congenital disease. The mesenchymal-to-epithelial transition that occurs during vertebrate somitogenesis is required for proper patterning of the axial musculoskeletal system. Somitic MET is initiated in the presomitic mesoderm by PARAXIS-dependent changes in cell adhesion, cell polarity, and the composition of the **extracellular matrix**. However, the target genes downstream of the transcription factor PARAXIS remain poorly described.A genome-wide comparison of gene expression in the anterior presomitic mesoderm and newly formed somites of Paraxis(-/-) embryos resulted in a set of deregulated genes enriched for factors associated with **extracellular matrix** and cytoskeletal organization and cell-cell and cell-ECM adhesion. The greatest change in expression was seen in fibroblast activation protein alpha (Fap), encoding a dipeptidyl peptidase capable of increasing fibronectin and collagen fiber organization in **extracellular matrix**. Further, downstream genes in the Wnt and Notch signaling pathways were downregulated, predicting that PARAXIS participates in positive feedback loops in both pathways.These data demonstrate that PARAXIS initiates and stabilizes somite epithelialization by integrating signals from multiple pathways to control the reorganization of the ECM, cytoskeleton, and adhesion junctions during MET.
10. Type VIII collagen signals via β1 integrin and RhoA to regulate MMP-2 expression and smooth muscle cell migration. The **extracellular matri**x signals and regulates the behavior of vascular cells during the pathogenesis of atherosclerosis. Type VIII collagen, a short chain collagen, is scarcely present in normal arteries, but is dramatically upregulated in atherosclerosis and after other types of vascular injury. Cell culture studies have revealed that this protein supports smooth muscle cell (SMC) adhesion and stimulates migration, however little is known about the signaling or the mechanisms by which this occurs. SMCs isolated from wild-type C57BL/6 and type VIII collagen deficient mice were studied using assays to measure chemotactic and haptotactic migration, and remodeling and contraction of 3-dimensional type I collagen gels. Col8(-/-) SMCs exhibited impairments in migration, and a strongly adhesive phenotype with prominent stress fibers, stable microtubules and pronounced central basal focal adhesions. The addition of exogenous type VIII collagen to the Col8(-/-) SMCs rescued the impairments in migration, and restored cytoskeletal architecture so that it was similar to Col8(+/+) cells. We measured elevated levels of active GTP-RhoA in the Col8(-/-) cells, and this too was reversed by treatment with exogenous type VIII collagen. We showed that type VIII collagen normally suppresses RhoA activation through a beta-1 integrin dependent mechanism. MMP-2 levels were reduced in the Col8(-/-) SMCs, and knockdown of MMP-2 in Col8(+/+) SMCs partially recapitulated the decreases in migration and 3D gel contraction seen in Col8(-/-) cells, showing that type VIII collagen-stimulated migration was dependent on MMP-2. Inhibition of Rho restored MMP-2 activity in the Col8(-/-) cells, and partially rescued migration, demonstrating that the elevations in RhoA activity were responsible for the suppression of migration of these cells. In conclusion, we have shown that type VIII collagen signals through beta-1 integrin receptors to suppress RhoA, allowing optimal configuration of the cytoskeleton, and the stimulation of MMP-2-dependent cell migration.
11. Paxillin and Hic-5 interaction with vinculin is differentially regulated by Rac1 and RhoA. Cell migration is of paramount importance to organism development and maintenance as well as multiple pathological processes, including cancer metastasis. The RhoGTPases Rac1 and RhoA are indispensable for cell migration as they regulate cell protrusion, cell-**extracellular matrix** (ECM) interactions and force transduction. However, the consequences of their activity at a molecular level within the cell remain undetermined. Using a combination of FRET, FRAP and biochemical analyses we show that the interactions between the focal adhesion proteins vinculin and paxillin, as well as the closely related family member Hic-5 are spatially and reciprocally regulated by the activity of Rac1 and RhoA. Vinculin in its active conformation interacts with either paxillin or Hic-5 in adhesions in response to Rac1 and RhoA activation respectively, while inactive vinculin interacts with paxillin in the membrane following Rac1 inhibition. Additionally, Rac1 specifically regulates the dynamics of paxillin as well as its binding partner and F-actin interacting protein actopaxin (α-parvin) in adhesions. Furthermore, FRET analysis of protein:protein interactions within cell adhesions formed in 3D matrices revealed that, in contrast to 2D systems vinculin interacts preferentially with Hic-5. This study provides new insight into the complexity of cell-ECM adhesions in both 2D and 3D matrices by providing the first description of RhoGTPase-coordinated protein:protein interactions in a cellular microenvironment. These data identify discrete roles for paxillin and Hic-5 in Rac1 and RhoA-dependent cell adhesion formation and maturation; processes essential for productive cell migration.
12. Plakoglobin regulates cell motility through Rho- and fibronectin-dependent Src signaling. We previously showed that the cell-cell junction protein plakoglobin (PG) not only suppresses motility of keratinocytes in contact with each other, but also, unexpectedly, of single cells. Here we show that PG deficiency results in **extracellular matrix** (ECM)-dependent disruption of mature focal adhesions and cortical actin organization. Plating PG⁻/⁻ cells onto ECM deposited by PG+/⁻ cells partially restored normal cell morphology and inhibited PG⁻/⁻ cell motility. In over 70 adhesion molecules whose expression we previously showed to be altered in PG⁻/⁻ cells, a substantial decrease in fibronectin (FN) in PG⁻/⁻ cells stood out. Re-introduction of PG into PG⁻/⁻ cells restored FN expression, and keratinocyte motility was reversed by plating PG⁻/⁻ cells onto FN. Somewhat surprisingly, based on previously reported roles for PG in regulating gene transcription, PG-null cells exhibited an increase, not a decrease, in FN promoter activity. Instead, PG was required for maintenance of FN mRNA stability. PG⁻/⁻ cells exhibited an increase in activated Src, one of the kinases controlled by FN, a phenotype reversed by plating PG⁻/⁻ cells on ECM deposited by PG+/⁻ keratinocytes. PG⁻/⁻ cells also exhibited Src-independent activation of the small GTPases Rac1 and RhoA. Both Src and RhoA inhibition attenuated PG⁻/⁻ keratinocyte motility. We propose a novel role for PG in regulating cell motility through distinct ECM-Src and RhoGTPase-dependent pathways, influenced in part by PG-dependent regulation of FN mRNA stability.
13. Therapeutic ultrasound bypasses canonical syndecan-4 signaling to activate rac1. The application of pulsed, low intensity ultrasound is emerging as a potent therapy for the treatment of complex bone fractures and tissue damage. Ultrasonic stimuli accelerate fracture healing by up to 40% and enhance tendon and ligament healing by promoting cell proliferation, migration, and matrix synthesis through an unresolved mechanism. Ultrasound treatment also induces closure of nonunion fractures, at a success rate (85% of cases) similar to that of surgical intervention (68-96%) while avoiding the complications associated with surgery. The regulation of cell adhesion necessary for wound healing depends on cooperative engagement of the **extracellular matrix** receptors, integrin and syndecan, as exemplified by the wound healing defects observed in syndecan- and integrin-knock-out mice. This report distinguishes the influence of ultrasound on signals downstream of the prototypic fibronectin receptors, alpha(5)beta(1) integrin and syndecan-4, which cooperate to regulate Rac1 and RhoA. Ultrasonic stimulation fails to activate integrins or induce cell spreading on poor, electrostatic ligands. By contrast, ultrasound treatment overcomes the necessity of engagement or expression of syndecan-4 during the process of focal adhesion formation, which normally requires simultaneous engagement of both receptors. Ultrasound exerts an influence downstream of syndecan-4 and PKCalpha to specifically activate Rac1, itself a critical regulator of tissue repair, and to a lesser extent RhoA. The ability of ultrasound to bypass syndecan-4 signaling, which is known to facilitate efficient tissue repair, explains the reduction in healing times observed in ultrasound-treated patients. By substituting for one of the key axes of adhesion-dependent signaling, ultrasound therapy has considerable potential as a clinical technique.
14. RhoA determines lineage fate of mesenchymal stem cells by modulating CTGF-VEGF complex in **extracellular matrix**. Mesenchymal stem cells (MSCs) participate in the repair/remodelling of many tissues, where MSCs commit to different lineages dependent on the cues in the local microenvironment. Here we show that TGFβ-activated RhoA/ROCK signalling functions as a molecular switch regarding the fate of MSCs in arterial repair/remodelling after injury. MSCs differentiate into myofibroblasts when RhoA/ROCK is turned on, endothelial cells when turned off. The former is pathophysiologic resulting in intimal hyperplasia, whereas the latter is physiological leading to endothelial repair. Further analysis revealed that MSC RhoA activation promotes formation of an **extracellular matri**x (ECM) complex consisting of connective tissue growth factor (CTGF) and vascular endothelial growth factor (VEGF). Inactivation of RhoA/ROCK in MSCs induces matrix metalloproteinase-3-mediated CTGF cleavage, resulting in VEGF release and MSC endothelial differentiation. Our findings uncover a novel mechanism by which cell-ECM interactions determine stem cell lineage specificity and offer additional molecular targets to manipulate MSC-involved tissue repair/regeneration.
15. The guanine nucleotide exchange factor Arhgef5 plays crucial roles in Src-induced podosome formation. Podosomes and invadopodia are actin-rich membrane protrusions that play a crucial role in cell adhesion and migration, and **extracellular matrix** remodeling in normal and cancer cells. The formation of podosomes and invadopodia is promoted by upregulation of some oncogenic molecules and is closely related to the invasive potential of cancer cells. However, the molecular mechanisms underlying the podosome and invadopodium formation still remain unclear. Here, we show that a guanine nucleotide exchange factor (GEF) for Rho family GTPases (Arhgef5) is crucial for Src-induced podosome formation. Using an inducible system for Src activation, we found that Src-induced podosome formation depends upon the Src SH3 domain, and identified Arhgef5 as a Src SH3-binding protein. RNA interference (RNAi)-mediated depletion of Arhgef5 caused robust inhibition of Src-dependent podosome formation. Overexpression of Arhgef5 promoted actin stress fiber remodeling through activating RhoA, and the activation of RhoA or Cdc42 was required for Src-induced podosome formation. Arhgef5 was tyrosine-phosphorylated by Src and bound to Src to positively regulate its activity. Furthermore, the pleckstrin homology (PH) domain of Arhgef5 was required for podosome formation, and Arhgef5 formed a ternary complex with Src and phosphoinositide 3-kinase when Src and/or Arhgef5 were upregulated. These findings provide novel insights into the molecular mechanisms of podosome and invadopodium formation induced by Src upregulation.
16. The RhoA-Rok-myosin II pathway is involved in **extracellular matrix**-mediated regulation of prolactin signaling in mammary epithelial cells. In mammary epithelial cells (MECs), prolactin-induced signaling and gene expression requires integrin-mediated cell adhesion to basement membrane (BM). In the absence of proper cell-BM interactions, for example, culturing cells on collagen-coated plastic dishes, signal propagation is substantially impaired. Here we demonstrate that the RhoA-Rok-myosin II pathway accounts for the ineffectiveness of prolactin signaling in MECs cultured on collagen I. Under these culture conditions, the RhoA pathway is activated, leading to downregulation of prolactin receptor expression and reduced prolactin signaling. Enforced activation of RhoA in MECs cultured on BM suppresses prolactin receptor levels, and prevents prolactin-induced Stat5 tyrosine phosphorylation and β-casein expression. Overexpression of dominant negative RhoA in MECs cultured on collagen I, or inhibiting Rok activity, increases prolactin receptor expression, and enhances prolactin signaling. In addition, inhibition of myosin II ATPase activity by blebbistatin also exerts a beneficial effect on prolactin receptor expression and prolactin signaling, suggesting that tension exerted by the collagen substratum, in collaboration with the RhoA-Rok-myosin II pathway, contributes to the failure of prolactin signaling. Furthermore, MECs cultured on laminin-coated plastic have similar morphology and response to prolactin as those cultured on collagen I. They display high levels of RhoA activity and are inefficient in prolactin signaling, stressing the importance of matrix stiffness in signal transduction. Our results reveal that RhoA has a central role in determining the fate decisions of MECs in response to cell-matrix interactions.
17. Cytokine-induced F-actin reorganization in endothelial cells involves RhoA activation. Acute ischemic kidney injury results in marked increases in local and systemic cytokine levels. IL-1alpha, IL-6, and TNF-alpha orchestrate various inflammatory reactions influencing endothelial permeability by altering cell-to-cell and cell-to-**extracellular matrix** attachments. To explore the role of actin and the regulatory proteins RhoA and cofilin in this process, microvascular endothelial cells (MS1) were exposed to individual cytokines or a cytokine cocktail. Within minutes, a marked, time-dependent redistribution of the actin cytoskeleton occurred with the formation of long, dense F-actin basal stress fibers. The concentration of F-actin, normalized to nuclear staining, significantly increased compared with untreated cells (up 20%, P < or = 0.05). Western blot analysis of MS1 lysates incubated with the cytokine cocktail for 4 h showed an increase in phosphorylated/inactive cofilin (up 25 +/- 15%, P < or = 0.05) and RhoA activation (up to 227 +/- 26% increase, P < or = 0.05) compared with untreated cells. Decreasing RhoA levels using small interfering RNA blocked the effect of cytokines on stress fiber organization. Treatment with Y-27632, an inhibitor of the RhoA effector p160-ROCK, decreased levels of phosphorylated cofilin and reduced stress fiber fluorescence by 22%. In cells treated with Y-27632 followed by treatment with the cytokine cocktail, stress fiber levels were similar to control cells and cofilin phosphorylation was 55% of control levels. Taken together, these studies demonstrate cytokine stimulation of RhoA, which in turn leads to cofilin phosphorylation and formation of numerous basal actin stress fibers. These results suggest cytokines signal through the Rho-ROCK pathway, but also through another pathway to affect actin dynamics.
18. Thrombospondin induces RhoA inactivation through FAK-dependent signaling to stimulate focal adhesion disassembly. Cells utilize dynamic interactions with the **extracellular matrix** to adapt to changing environmental conditions. Thrombospondin 1 (TSP1) induces focal adhesion disassembly and cell migration through a sequence (hep I) in its heparin-binding domain signaling through the calreticulin-low density lipoprotein receptor-related protein receptor complex. This involves the Galphai-dependent activation of ERK and phosphoinositide (PI) 3-kinase, both of which are required for focal adhesion disassembly. Focal adhesion kinase (FAK) regulates adhesion dynamics, acting in part by modulating RhoA activity, and FAK is implicated in ERK and PI 3-kinase activation. In this work, we sought to determine the role of FAK in TSP1-induced focal adhesion disassembly. TSP1/hep I does not stimulate focal adhesion disassembly in FAK knockout fibroblasts, whereas re-expressing FAK rescues responsiveness. Inhibiting FAK signaling through FRNK or FAK Y397F expression in endothelial cells also abrogates this response. TSP1/hep I stimulates a transient increase in FAK phosphorylation that requires calreticulin and Galphai, but not ERK or PI 3-kinase. Hep I does not activate ERK or PI 3-kinase in FAK knockout fibroblasts, suggesting activation occurs downstream of FAK. TSP1/hep I stimulates RhoA inactivation with kinetics corresponding to focal adhesion disassembly in a FAK, ERK, and PI 3-kinase-dependent manner. Furthermore, hep I does not stimulate focal adhesion disassembly in cells expressing constitutively active RhoA, suggesting that RhoA inactivation is required for this response. This is the first work to illustrate a connection between FAK phosphorylation in response to a soluble factor and RhoA inactivation, as well as the first report of PI 3-kinase and ERK in FAK regulation of RhoA activity.
19. Role of RhoA/ROCK-dependent actin contractility in the induction of tenascin-C by cyclic tensile strain. In chick embryo fibroblasts, the mRNA for **extracellular matrix** protein tenascin-C is induced 2-fold by cyclic strain (10%, 0.3 Hz, 6 h). This response is attenuated by inhibiting Rho-dependent kinase (ROCK). The RhoA/ROCK signaling pathway is primarily involved in actin dynamics. Here, we demonstrate its crucial importance in regulating tenascin-C expression. Cyclic strain stimulated RhoA activation and induced fibroblast contraction. Chemical activators of RhoA synergistically enhanced the effects of cyclic strain on cell contractility. Interestingly, tenascin-C mRNA levels perfectly matched the extent of RhoA/ROCK-mediated actin contraction. First, RhoA activation by thrombin, lysophosphatidic acid, or colchicine induced tenascin-C mRNA to a similar extent as strain. Second, RhoA activating drugs in combination with cyclic strain caused a super-induction (4- to 5-fold) of tenascin-C mRNA, which was again suppressed by ROCK inhibition. Third, disruption of the actin cytoskeleton with latrunculin A abolished induction of tenascin-C mRNA by chemical RhoA activators in combination with cyclic strain. Lastly, we found that myosin II activity is required for tenascin-C induction by cyclic strain. We conclude that RhoA/ROCK-controlled actin contractility has a mechanosensory function in fibroblasts that correlates directly with tenascin-C gene expression. Previous RhoA/ROCK activation, either by chemical or mechanical signals, might render fibroblasts more sensitive to external tensile stress, e.g., during wound healing.
20. Laminin-111 stimulates proliferation of mouse embryonic stem cells through a reduction of gap junctional intercellular communication via RhoA-mediated Cx43 phosphorylation and dissociation of Cx43/ZO-1/drebrin complex. Gap junctions within **extracellular matrix** (ECM)-defined boundaries ensure synchronous activity between cells destined to become functional mediators that regulate cell behavior. However, the role of ECM in connexin (Cx) function in mouse embryonic stem cells (mESCs) has not been elucidated. Therefore, we examined the role of laminin-111 in the control of Cx43 functions and related signal pathways in mESCs. ECM components (laminin-111, fibronectin, and collagen I) increased Cx43 phosphorylation and decreased Lucifer yellow (Ly) diffusion. In addition, laminin-111 increased the proliferation index through reduction of gap junctional intercellular communication (GJIC), which was confirmed by 18α-glycyrrhetinic acid (18α-GA). Laminin-111 increased phosphorylation of focal adhesion kinase (FAK)/Src and protein kinase C (PKC), which were inhibited by integrin β1 antibody (Ab) and laminin receptor-1 (LR-1) Ab, respectively. In addition, inhibition of both FAK/Src and PKC blocked Cx43 phosphorylation. Laminin-111 increased the Ras homolog gene family, member A (RhoA) activation, which was blocked by FAK/Src and PKC inhibitors, suggesting the existence of parallel pathways that merge at RhoA. Inhibition of RhoA reversed the laminin-111-induced increase of Cx43 phosphorylation and reduction of GJIC. Laminin-111 also stimulated the dissociation of Cx43/ZO-1 complex followed by disruption of Cx43/drebrin and Cx43/F-actin complexes, which were reversed by C3 (RhoA inhibitor). ZO-1 small interfering (si) RNA significantly decreased Ly diffusion. Moreover, laminin-111 decreased Cx43 labeling at the intercellular junction, whereas pretreatment with degradation inhibitors (lysosomal protease inhibitor, chloroquine; proteasome inhibitor, lactacystin) increased Cx43 expression, reversely. In conclusion, laminin-111 stimulated mESC proliferation through a reduction of GJIC via RhoA-mediated Cx43 phosphorylation and Cx43/ZO-1/drebrin complex instability-mediated Cx43 degradation.
21. ECM compliance regulates osteogenesis by influencing MAPK signaling downstream of RhoA and ROCK. The compliance of the **extracellular matrix** (ECM) regulates osteogenic differentiation by modulating extracellular signal-regulated kinase (ERK) activity. However, the molecular mechanism linking ECM compliance to the ERK-mitogen-activated protein kinase (MAPK) pathway remains unclear. Furthermore, RhoA has been widely implicated in integrin-mediated signaling and mechanotransduction. We studied the relationship between RhoA and ERK-MAPK signaling to determine their roles in the regulation of osteogenesis by ECM compliance. Inhibition of RhoA and ROCK in MC3T3-E1 pre-osteoblasts cultured on substrates of varying compliance reduced ERK activity, whereas constitutively active RhoA enhanced it. The expression of RUNX2, a potent osteogenic transcription factor, was increased on stiffer matrices and correlated with elevated ERK activity. Inhibition of RhoA, ROCK, or the MAPK pathway diminished RUNX2 activity and delayed the onset of osteogenesis as shown by altered osteocalcin (OCN) and bone sialoprotein (BSP) gene expression, alkaline phosphatase (ALP) activity, and matrix mineralization. These data establish that one possible mechanism by which ECM rigidity regulates osteogenic differentiation involves MAPK activation downstream of the RhoA-ROCK signaling pathway.
22. Fibronectin stimulates migration through lipid raft dependent NHE-1 activation in mouse embryonic stem cells: involvement of RhoA, Ca(2+)/CaM, and ERK. **Extracellular matrix** (ECM) components and intracellular pH (pH(i)) may serve as regulators of cell migration in various cell types.The Oris migration assay was used to assess the effect of fibronectin (FN) on cell motility. The Na(+)/H(+) exchanger (NHE)-1 activity was evaluated by measuring pH(i) and [(22)Na(+)] uptake. To examine activated signaling molecules, western blot analysis and immunoprecipitation was performed.ECM components (FN, laminin, fibrinogen, and collagen type I) increased [(22)Na(+)] uptake, pH(i), and cell migration. In addition, FN-induced increase of cell migration was inhibited by NHE-1 inhibitor amiloride or NHE-1-specific siRNA. FN selectively increased the mRNA and protein expression of NHE-1, but not that of NHE-2 or NHE-3. FN binds integrin β1 and subsequently stimulates caveolin-1 phosphorylation and Ca(2+) influx. Then, NHE-1 is phosphorylated by RhoA and Rho kinases, and Ca(2+)/calmodulin (CaM) signaling elicits complex formation with NHE-1, which is enriched in lipid raft/caveolae microdomains of the plasma membrane. Activation of NHE-1 continuously induces an increase of [(22)Na(+)] uptake and pH(i). Finally, NHE-1-dependent extracellular signal-regulated kinase (ERK) 1/2 phosphorylation enhanced matrix metalloproteinase-2 (MMP-2) and filamentous-actin (F-actin) expression, partially contributing to the regulation of embryonic stem cells (ESCs) migration.FN stimulated mESCs migration and proliferation through NHE-1 activation, which were mediated by lipid raft-associated caveolin-1, RhoA/ROCK, and Ca(2+)/CaM signaling pathways.The precise role of NHE in the modulation of ECM-related physiological functions such as proliferation and migration remains poorly understood. Thus, this study analyzed the relationship between FN and NHE in regulating the migration of mouse ESCs and their related signaling pathways.
23. Suppression of RhoA activity by focal adhesion kinase-induced activation of p190RhoGAP: role in regulation of endothelial permeability. The interaction of endothelial cells with **extracellular matrix** proteins at focal adhesions sites contributes to the integrity of vascular endothelial barrier. Although focal adhesion kinase (FAK) activation is required for the recovery of the barrier function after increased endothelial junctional permeability, the basis for the recovery remains unclear. We tested the hypothesis that FAK activates p190RhoGAP and, thus, negatively regulates RhoA activity and promotes endothelial barrier restoration in response to the permeability-increasing mediator thrombin. We observed that thrombin caused a transient activation of RhoA but a more prolonged FAK activation temporally coupled to the recovery of barrier function. Thrombin also induced tyrosine phosphorylation of p190RhoGAP, which coincided with decrease in RhoA activity. We further showed that FAK was associated with p190RhoGAP, and importantly, recombinant FAK phosphorylated p190RhoGAP in vitro. Inhibition of FAK by adenoviral expression of FRNK (a dominant negative FAK construct) in monolayers prevented p190RhoGAP phosphorylation, increased RhoA activity, induced actin stress fiber formation, and produced an irreversible increase in endothelial permeability in response to thrombin. We also observed that p190RhoGAP was unable to attenuate RhoA activation in the absence of FAK activation induced by FRNK. The inhibition of RhoA by the C3 toxin (Clostridium botulinum toxin) restored endothelial barrier function in the FRNK-expressing cells. These findings in endothelial cells were recapitulated in the lung microcirculation in which FRNK expression in microvessel endothelia increased vascular permeability. Our studies demonstrate that FAK-induced down-modulation of RhoA activity via p190RhoGAP is a crucial step in signaling endothelial barrier restoration after increased endothelial permeability.
24. Leukocyte common antigen-related phosphatase is a functional receptor for chondroitin sulfate proteoglycan axon growth inhibitors. Chondroitin sulfate proteoglycans (CSPGs) are a family of **extracellular matrix** molecules with various functions in regulating tissue morphogenesis, cell division, and axon guidance. A number of CSPGs are highly upregulated by reactive glial scar tissues after injuries and form a strong barrier for axonal regeneration in the adult vertebrate CNS. Although CSPGs may negatively regulate axonal growth via binding and altering activity of other growth-regulating factors, the molecular mechanisms by which CSPGs restrict axonal elongation are not well understood. Here, we identified a novel receptor mechanism whereby CSPGs inhibit axonal growth via interactions with neuronal transmembrane leukocyte common antigen-related phosphatase (LAR). CSPGs bind LAR with high affinity in transfected COS-7 cells and coimmunoprecipitate with LAR expressed in various tissues including the brain and spinal cord. CSPG stimulation enhances activity of LAR phosphatase in vitro. Deletion of LAR in knock-out mice or blockade of LAR with sequence-selective peptides significantly overcomes neurite growth restrictions of CSPGs in neuronal cultures. Intracellularly, CSPG-LAR interaction mediates axonal growth inhibition of neurons partially via inactivating Akt and activating RhoA signals. Systemic treatments with LAR-targeting peptides in mice with thoracic spinal cord transection injuries induce significant axon growth of descending serotonergic fibers in the vicinity of the lesion and beyond in the caudal spinal cord and promote locomotor functional recovery. Identification of LAR as a novel CSPG functional receptor provides a therapeutic basis for enhancing axonal regeneration and functional recovery after CNS injuries in adult mammals.
25. High RhoA activity maintains the undifferentiated mesenchymal cell phenotype, whereas RhoA down-regulation by laminin-2 induces smooth muscle myogenesis. Round embryonic mesenchymal cells have the potential to differentiate into smooth muscle (SM) cells upon spreading/elongation (Yang, Y., K.C. Palmer, N. Relan, C. Diglio, and L. Schuger. 1998. Development. 125:2621-2629; Yang, Y., N.K. Relan, D.A. Przywara, and L. Schuger. 1999. Development. 126:3027-3033; Yang, Y., S. Beqaj, P. Kemp, I. Ariel, and L. Schuger. 2000. J. Clin. Invest. 106:1321-1330). In the developing lung, this process is stimulated by peribronchial accumulation of laminin (LN)-2 (Relan, N.K., Y. Yang, S. Beqaj, J.H. Miner, and L. Schuger. 1999. J. Cell Biol. 147:1341-1350). Here we show that LN-2 stimulates bronchial myogenesis by down-regulating RhoA activity. Immunohistochemistry, immunoblotting, and reverse transcriptase-PCR indicated that RhoA, a small GTPase signaling protein, is abundant in undifferentiated embryonic mesenchymal cells and that its levels decrease along with SM myogenesis. Functional studies using agonists and antagonists of RhoA activation and dominant positive and negative plasmid constructs demonstrated that high RhoA activity was required to maintain the round undifferentiated mesenchymal cell phenotype. This was in part achieved by restricting the localization of the myogenic transcription factor serum response factor (SRF) mostly to the mesenchymal cell cytoplasm. Upon spreading on LN-2 but not on other main components of the **extracellular matrix**, the activity and level of RhoA decreased rapidly, resulting in translocation of SRF to the nucleus. Both cell elongation and SRF translocation were prevented by overexpression of dominant positive RhoA. Once the cells underwent SM differentiation, up-regulation of RhoA activity induced rather than inhibited SM gene expression. Therefore, our studies suggest a novel mechanism whereby LN-2 and RhoA modulate SM myogenesis.
26. Nuclear factor of activated T cells mediates RhoA-induced fibronectin upregulation in glomerular podocytes. Glomerulosclerosis is featured by accumulation of the **extracellular matrix**es in the glomerulus. We showed previously that activation of the small GTPase RhoA in podocytes induces heavy proteinuria and glomerulosclerosis in the mouse. In the current study, we investigated the mechanism by which RhoA stimulates the production of one of the **extracellular matrix**es, fibronectin, by podocytes, specifically testing the role of nuclear factor of activated T cells (NFAT). Expression of constitutively active RhoA in cultured podocytes activated the fibronectin promoter, upregulated fibronectin protein, and activated NFAT. Expression of constitutively active NFAT in podocytes also activated the fibronectin promoter and upregulated fibronectin protein. RhoA-induced NFAT activation and fibronectin upregulation were both dependent on the calcium/calmodulin pathway and Rho kinase. NFAT activation was also observed in vivo in the rat and mouse models of podocyte injury and proteinuria, and NFAT inhibition ameliorated fibronectin upregulation in the latter. RhoA activation induced a rise of intracellular calcium ion concentration ([Ca(2+)]i), which was at least in part dependent on the transient receptor potential canonical 6 (TRPC6) cation channel. The results indicate that RhoA activates NFAT by inducing a rise of [Ca(2+)]i in podocytes, which in turn contributes to fibronectin upregulation. This pathway may be responsible for the pathogenesis of certain glomerular diseases such as hypertension-mediated glomerulosclerosis.
27. Control of the direction of lamellipodia extension through changes in the balance between Rac and Rho activities. The direction in which cells extend new motile processes, such as lamellipodia and filopodia, can be controlled by altering the geometry of **extracellular matrix** adhesive islands on which individual cells are cultured, thereby altering mechanical interactions between cells and the adhesive substrate [Parker (2002)]. Here we specifically investigate the intracellular molecular signals that mediate the mechanism by which cells selectively extend these processes from the corners of polygonal-shaped adhesive islands. Constitutive activation of the small GTPase Rac within cells cultured on square-shaped islands of fibronectin resulted in the elimination of preferential extension from corners. This loss of directionality was accompanied by a re-distribution of focal adhesions: the large focal adhesions normally found within the corner regions of square cells were lost and replaced by many smaller focal contacts that were distributed along the entire cell perimeter. Inhibition of the small GTPase, Rho, using C3 exoenzyme blocked lamellipodia extension entirely. However, inhibition of Rho signaling in combination with ectopic Rac activation rescued the corner localization of motile processes and focal adhesions. These results suggest that the ability of cells to sense their physical surroundings and respond by moving in a spatially oriented manner is mediated by a balance between Rho and Rac activities.
28. RhoA/Rho kinase signaling in the cumulus mediates **extracellular matrix** assembly. Cumulus cells surround the oocyte and regulate the production and assembly of the **extracellular matrix** (ECM) around the cumulus-oocyte complex for its timely interaction with sperm in the oviduct. We recently found that C-C chemokines such as CCL2, CCL7, and CCL9 are produced and stimulate integrin-mediated ECM assembly in the postovulatory cumulus to protect eggs and that prostaglandin E(2)-EP2 signaling in the cumulus cells facilitates fertilization by suppressing this chemokine signaling, which otherwise results in fertilization failure by preventing sperm penetration through the cumulus ECM. However, it remains unknown as to what mechanisms underlie chemokine-induced cumulus ECM assembly. Here we report that inhibition of EP2 signaling or addition of CCL7 augments RhoA activation and induces the surface accumulation of integrin and the contraction of cumulus cells. Enhanced surface accumulation of integrin then stimulates the formation and assembly of fibronectin fibrils as well as induces cumulus ECM resistance to hyaluronidase and sperm penetration. These changes in the cumulus ECM as well as cell contraction are relieved by the addition of Y27632 or blebbistatin. These results suggest that chemokines induce integrin engagement to the ECM and consequent ECM remodeling through the RhoA/Rho kinase/actomyosin pathway, making the cumulus ECM barrier resistant to sperm penetration. Based on these results, we propose that prostaglandin E(2)-EP2 signaling negatively regulates chemokine-induced Rho/ROCK signaling in cumulus cells for successful fertilization.
29. Integrin regulation by RhoA in thymocytes. The guanine nucleotide-binding protein Rho has essential functions in T cell development and is important for the survival and proliferation of T cell progenitors in the thymus. To explore the mechanisms used by RhoA to control thymocyte biology, the role of this GTPase in the regulation of integrin-mediated cell adhesion was examined. The data show that RhoA activation is sufficient to stimulate beta(1) and beta(2) integrin-mediated adhesion in murine thymocytes. RhoA is also needed for integrin activation in vivo as loss of Rho function impaired the ability of thymocytes to adhere to the **extracellular matrix** protein VCAM-1 and prevented integrin activation induced by the GTPases Rac-1 and Rap1A in vivo. The regulated activity of integrins is needed for cell motility and in the present study it was seen that RhoA activity is critical for integrin-mediated thymocyte migration to chemokines in vitro. Thus, RhoA has a critical role in regulating cell adhesion and migration during T cell development.

#### GeneRIFs

1. RhoA determines lineage fate of mesenchymal stem cells by modulating CTGF-VEGF complex in **extracellular matrix**.
2. These data establish that one possible mechanism by which **extracellular matrix** rigidity regulates osteogenic differentiation involves mitogen-activated protein kinase activation downstream of the RhoA-ROCK signaling pathway.
3. study shows that sustained chemokine stimulation in cumulus cells activates RhoA-ROCK signaling, which facilitates formation of actomyosin bundles, surface accumulation of integrins, and excessive **extracellular matrix** assembly

### [Ii]nvasive

#### PubMed

1. Blockade of the RhoA-JNK-c-Jun-MMP2 cascade by atorvastatin reduces osteosarcoma cell invasion. Osteosarcoma is characterized by a high malignant and metastatic potential, which points to the need for new therapeutic strategies to prevent cell metastasis. In this study, we show that statin-induced HMG-CoA reductase inhibition reduces cell migration and invasion in human and murine osteosarcoma cells, independently of the genotype. The statin-induced reduction of cell migration and invasion was independent of induction of apoptosis and was geranylgeranylpyrophosphate-dependent. The statin reduced matrix metalloproteinase (MMP) 2, 9, and 14 and TIMP2 expression or activity in invading cells. Forced expression of MMP2 and MMP14 overcame the inhibitory effect of the statin on cell invasion, suggesting a role for these MMPs in **invasive** potential. We also investigated the mechanisms involved in the reduced MMP2 activity and cell invasion. Inhibition of JNK, but not ERK1/2 signaling, reduced MMP2 activity. Pharmacological or constitutive activation of JNK overcame the reduced MMP2 activity and cell invasion induced by the statin. The statin decreased JNK phosphorylation and c-Jun nuclear translocation, suggesting that HMG-CoA reductase inhibition targets the JNK-c-Jun signaling pathway. We showed that mevalonate or geranylgeranylpyrophosphate treatment prevented the statin-induced reduction in JNK phosphorylation, MMP2 activity, and cell invasion. Forced expression of a constitutively active form of RhoA increased JNK phosphorylation and overcame the inhibitory effect of atorvastatin on MMP2 activity and cell invasion. The data establish a link between RhoA, JNK, c-Jun, and MMP2 activity that is functionally involved in the reduction in osteosarcoma cell invasion by the statin. This suggests a novel strategy targeting RhoA-JNK-c-Jun signaling to reduce osteosarcoma cell tumorigenesis.
2. Blockade of the RhoA-JNK-c-Jun-MMP2 cascade by atorvastatin reduces osteosarcoma cell invasion. Osteosarcoma is characterized by a high malignant and metastatic potential, which points to the need for new therapeutic strategies to prevent cell metastasis. In this study, we show that statin-induced HMG-CoA reductase inhibition reduces cell migration and invasion in human and murine osteosarcoma cells, independently of the genotype. The statin-induced reduction of cell migration and invasion was independent of induction of apoptosis and was geranylgeranylpyrophosphate-dependent. The statin reduced matrix metalloproteinase (MMP) 2, 9, and 14 and TIMP2 expression or activity in invading cells. Forced expression of MMP2 and MMP14 overcame the inhibitory effect of the statin on cell invasion, suggesting a role for these MMPs in **invasive** potential. We also investigated the mechanisms involved in the reduced MMP2 activity and cell invasion. Inhibition of JNK, but not ERK1/2 signaling, reduced MMP2 activity. Pharmacological or constitutive activation of JNK overcame the reduced MMP2 activity and cell invasion induced by the statin. The statin decreased JNK phosphorylation and c-Jun nuclear translocation, suggesting that HMG-CoA reductase inhibition targets the JNK-c-Jun signaling pathway. We showed that mevalonate or geranylgeranylpyrophosphate treatment prevented the statin-induced reduction in JNK phosphorylation, MMP2 activity, and cell invasion. Forced expression of a constitutively active form of RhoA increased JNK phosphorylation and overcame the inhibitory effect of atorvastatin on MMP2 activity and cell invasion. The data establish a link between RhoA, JNK, c-Jun, and MMP2 activity that is functionally involved in the reduction in osteosarcoma cell invasion by the statin. This suggests a novel strategy targeting RhoA-JNK-c-Jun signaling to reduce osteosarcoma cell tumorigenesis.
3. The guanine nucleotide exchange factor Arhgef5 plays crucial roles in Src-induced podosome formation. Podosomes and invadopodia are actin-rich membrane protrusions that play a crucial role in cell adhesion and migration, and extracellular matrix remodeling in normal and cancer cells. The formation of podosomes and invadopodia is promoted by upregulation of some oncogenic molecules and is closely related to the **invasive** potential of cancer cells. However, the molecular mechanisms underlying the podosome and invadopodium formation still remain unclear. Here, we show that a guanine nucleotide exchange factor (GEF) for Rho family GTPases (Arhgef5) is crucial for Src-induced podosome formation. Using an inducible system for Src activation, we found that Src-induced podosome formation depends upon the Src SH3 domain, and identified Arhgef5 as a Src SH3-binding protein. RNA interference (RNAi)-mediated depletion of Arhgef5 caused robust inhibition of Src-dependent podosome formation. Overexpression of Arhgef5 promoted actin stress fiber remodeling through activating RhoA, and the activation of RhoA or Cdc42 was required for Src-induced podosome formation. Arhgef5 was tyrosine-phosphorylated by Src and bound to Src to positively regulate its activity. Furthermore, the pleckstrin homology (PH) domain of Arhgef5 was required for podosome formation, and Arhgef5 formed a ternary complex with Src and phosphoinositide 3-kinase when Src and/or Arhgef5 were upregulated. These findings provide novel insights into the molecular mechanisms of podosome and invadopodium formation induced by Src upregulation.
4. TGFbeta3-induced activation of RhoA/Rho-kinase pathway is necessary but not sufficient for epithelio-mesenchymal transdifferentiation: implications for palatogenesis. TGFbeta-induced epithelio-mesenchymal transdifferentiation (EMT) has been shown to play a pivotal role in developmental processes such as palatogenesis and heart organogenesis. Interestingly, EMT has also been shown to contribute to the promotion of **invasive**ness during the later stages of tumorigenesis. Here we show that cells induced to undergo EMT by TGFbeta3 demonstrate increased motility on fibronectin and display a rapid activation of RhoA followed by a gradual downregulation of Cdc42 and Rac3 activity. The induced expression of a fast-cycling RhoA mutant (RhoA F30) stimulates the formation of stress fibers and spreading, and therefore the generation of mesenchymal phenotype. Inactivation of the Rho effector Rho-kinase, interferes with TGFbeta3-induced EMT by inhibiting stress fiber formation, whereas disruption of adherens and tight junctions was not significantly affected. Moreover, we show that TGFbeta3-induced RhoA/Rho-kinase activation is biologically significant, since palatal shelves from pre-fusion mouse embryos cultured in the presence of Rho-kinase inhibitors failed to fuse and midline epithelial cells did not undergo EMT. To conclude, our results indicate that TGFbeta3 induces rapid activation of the RhoA/Rho-kinase pathway and subsequently reorganization of the actin cytoskeleton. These changes are likely to be necessary in biological processes in which EMT has been shown to play a critical role, such as palatal fusion.
5. High activity of serum response factor in the mesenchymal transition of epithelial tumor cells is regulated by RhoA signaling. The serum response factor (SRF) regulates the transcription of target genes by binding to serum response elements in dimeric form and by interacting with ternary complex factors. In this study, we have analyzed the role of the serum response factor and mechanisms that regulate its activity in tumor progression utilizing a multistage model of mouse skin carcinogenesis. We demonstrate elevated SRF DNA binding activity only in the cell lines that have undergone an epithelial to mesenchymal transition and have increased actin stress fiber formation. Transient transfection experiments of activated or dominant negative forms of RhoA showed that the high activity of SRF and the induced formation of actin stress fibers in cells with spindle morphology were mediated by RhoA signaling. A dominant negative form of SRF inhibited RhoA-induced actin polymerization and stress fiber formation. The DNA binding activity of SRF in mesenchymal tumor cells was also correlated with elevated expression of SRF target genes, similar to SRF itself, actin, and vinculin. These observations suggest for the first time that SRF may play an important role in tumor progression, specifically at the transition to an **invasive** metastatic stage of carcinogenesis.
6. Netrin-1 promotes glioblastoma cell **invasive**ness and angiogenesis by multiple pathways including activation of RhoA, cathepsin B, and cAMP-response element-binding protein. Glioblastomas are very difficult tumors to treat because they are highly **invasive** and disseminate within the normal brain, resulting in newly growing tumors. We have identified netrin-1 as a molecule that promotes glioblastoma **invasive**ness. As evidence, netrin-1 stimulates glioblastoma cell invasion directly through Matrigel-coated transwells, promotes tumor cell sprouting and enhances metastasis to lymph nodes in vivo. Furthermore, netrin-1 regulates angiogenesis as shown in specific angiogenesis assays such as enhanced capillary endothelial cells (EC) sprouting and by increased EC infiltration into Matrigel plugs in vivo, as does VEGF-A. This netrin-1 signaling pathway in glioblastoma cells includes activation of RhoA and cyclic AMP response element-binding protein (CREB). A novel finding is that netrin-1-induced glioblastoma **invasive**ness and angiogenesis are mediated by activated cathepsin B (CatB), a cysteine protease that translocates to the cell surface as an active enzyme and co-localizes with cell surface annexin A2 (ANXA2). The specific CatB inhibitor CA-074Me inhibits netrin-1-induced cell invasion, sprouting, and Matrigel plug angiogenesis. Silencing of CREB suppresses netrin-1-induced glioblastoma cell invasion, sprouting, and CatB expression. It is concluded that netrin-1 plays an important dual role in glioblastoma progression by promoting both glioblastoma cell **invasive**ness and angiogenesis in a RhoA-, CREB-, and CatB-dependent manner. Targeting netrin-1 pathways may be a promising strategy for brain cancer therapy.
7. Galectin 3 regulates HCC cell invasion by RhoA and MLCK activation. Hepatocellular carcinoma (HCC) carries a poor prognosis with no effective treatment available other than liver transplantation for selected patients. Vascular invasion of HCC is one of the most important negative predictor of survival. As the regulation of invasion of HCC cells is not well understood, our aim was to study the mechanisms by which galectin 3, a β-galactosidase-binding lectin mediates HCC cell migration. HCC was induced by N-diethylnitrosamine in wild-type and galectin 3(-/-) mice, and tumor formation, histology, and tumor cell invasion were assessed. The galectin 3(-/-) mice developed significantly smaller tumor burden with a less **invasiv**e phenotype than the wild-type animals. Galectin 3 was upregulated in the wild-type HCC tumor tissue, but not in the surrounding parenchyma. Galectin 3 expression in HCC was induced by NF-κB transactivation as determined by chromatin immunoprecipitation assays. In vitro studies assessed the pro-migratory effects of galectin 3. The migration of hepatoma cells was significantly decreased after transfection by the galectin 3 siRNA and also after using the Rho kinase inhibitor Y-27632. The reorganization of the actin cytoskeleton, RhoA GTPase activity and the phosphorylation of MLC2 (myosin light chain 2) were decreased in the galectin 3 siRNA-transfected cells. In addition, in vitro and in vivo evidence showed that galectin 3 deficiency reduced hepatoma cell proliferation and increased their apoptosis rate. In conclusion, galectin 3 is an important lectin that is induced in HCC cells, and promotes hepatoma cell motility and invasion by an autocrine pathway. Targeting galectin 3 therefore could be an important novel treatment strategy to halt disease progression.
8. Staphylococcus aureus protein A mediates invasion across airway epithelial cells through activation of RhoA GTPase signaling and proteolytic activity. Staphyococcus aureus and especially the epidemic methicillin-resistant S. aureus strains cause severe necrotizing pneumonia. The mechanisms whereby these organisms invade across the mucosal epithelial barrier to initiate **invasive** infection are not well understood. Protein A (SpA), a highly conserved and abundant surface protein of S. aureus, activates TNF receptor 1 and EGF receptor (EGFR) signaling cascades that can perturb the cytoskeleton. We demonstrate that wild-type S. aureus, but not spa mutants, invade across polarized airway epithelial cell monolayers via the paracellular junctions. SpA stimulated a RhoA/ROCK/MLC cascade, resulting in the contraction of the cytoskeleton. SpA(+) but not SpA(-) mutants stimulated activation of EGFR and along with subsequent calpain activity cleaved the membrane-spanning junctional proteins occludin and E-cadherin, facilitating staphylococcal transmigration through the cell-cell junctions. Treatment of polarized human airway epithelial monolayers with inhibitors of ROCK, EGFR, MAPKs, or calpain prevented staphylococcal penetration through the monolayers. In vivo, blocking calpain activity impeded bacterial invasion into the lung parenchyma. Thus, S. aureus exploits multiple receptors available on the airway mucosal surface to facilitate invasion across epithelial barriers.
9. Loss of p53 promotes RhoA-ROCK-dependent cell migration and invasion in 3D matrices. In addition to its role in controlling cell cycle progression, the tumor suppressor protein p53 can also affect other cellular functions such as cell migration. In this study, we show that p53 deficiency in mouse embryonic fibroblasts cultured in three-dimensional matrices induces a switch from an elongated spindle morphology to a markedly spherical and flexible one associated with highly dynamic membrane blebs. These rounded, motile cells exhibit amoeboid-like movement and have considerably increased **invasive** properties. The morphological transition requires the RhoA-ROCK (Rho-associated coil-containing protein kinase) pathway and is prevented by RhoE. A similar p53-mediated transition is observed in melanoma A375P cancer cells. Our data suggest that genetic alterations of p53 in tumors are sufficient to promote motility and invasion, thereby contributing to metastasis.
10. MicroRNA-155 is regulated by the transforming growth factor beta/Smad pathway and contributes to epithelial cell plasticity by targeting RhoA. Transforming growth factor beta (TGF-beta) signaling facilitates metastasis in advanced malignancy. While a number of protein-encoding genes are known to be involved in this process, information on the role of microRNAs (miRNAs) in TGF-beta-induced cell migration and invasion is still limited. By hybridizing a 515-miRNA oligonucleotide-based microarray library, a total of 28 miRNAs were found to be significantly deregulated in TGF-beta-treated normal murine mammary gland (NMuMG) epithelial cells but not Smad4 knockdown NMuMG cells. Among upregulated miRNAs, miR-155 was the most significantly elevated miRNA. TGF-beta induces miR-155 expression and promoter activity through Smad4. The knockdown of miR-155 suppressed TGF-beta-induced epithelial-mesenchymal transition (EMT) and tight junction dissolution, as well as cell migration and invasion. Further, the ectopic expression of miR-155 reduced RhoA protein and disrupted tight junction formation. Reintroducing RhoA cDNA without the 3' untranslated region largely reversed the phenotype induced by miR-155 and TGF-beta. In addition, elevated levels of miR-155 were frequently detected in **invasive** breast cancer tissues. These data suggest that miR-155 may play an important role in TGF-beta-induced EMT and cell migration and invasion by targeting RhoA and indicate that it is a potential therapeutic target for breast cancer intervention.

#### GeneRIFs

1. Netrin-1 promotes glioblastoma cell **invasive**ness and angiogenesis by multiple pathways including activation of RhoA, cathepsin B, and cAMP-response element-binding protein

### [Mm]etastasis

#### PubMed

1. A p27(kip1)-binding protein, p27RF-Rho, promotes cancer **metastasis** via activation of RhoA and RhoC. Rho family proteins regulate multiple cellular functions including motility and invasion through regulation of the actin cytoskeleton and gene expression. Activation of Rho proteins is controlled precisely by multiple regulators in a spatiotemporal manner. RhoA and/or RhoC are key players that regulate the metastatic activity of malignant tumor cells, and it is therefore of particular interest to understand how activation of these Rho proteins is controlled. We recently identified an upstream regulator of RhoA activation, p27RF-Rho (p27(kip1) releasing factor from RhoA) that acts by freeing RhoA from inhibition by p27(kip1). p27(kip1) is a cell cycle regulator when it is localized to the nucleus, but it binds RhoA and inhibits activation of the latter when it is localized to the cytoplasm. Here, we show that a metastatic variant of mouse melanoma B16 cells (F10) exhibits greater expression of p27RF-Rho, RhoA, and RhoC than the nonmetastatic parental cells (F0). Injection of F10 cells into mouse tail vein resulted in the formation of metastatic lung colonies, whereas prior knockdown of expression of either one of the three proteins using specific shRNA sequences decreased **metastasis** markedly. p27RF-Rho regulated the activation of RhoA and RhoC and thereby modulated cellular adhesion and motility, in addition to pericellular proteolysis. The Rho activities enhanced by p27RF-Rho had a marked effect upon efficiency of lodging of F10 cells in the lung, which represents an early step of **metastasis**. p27RF-Rho also regulated **metastasis** of human melanoma and fibrosarcoma cells. Thus, p27RF-Rho is a key upstream regulator of RhoA and RhoC that controls spreading of tumor cells.
2. Paxillin and Hic-5 interaction with vinculin is differentially regulated by Rac1 and RhoA. Cell migration is of paramount importance to organism development and maintenance as well as multiple pathological processes, including cancer **metastasis**. The RhoGTPases Rac1 and RhoA are indispensable for cell migration as they regulate cell protrusion, cell-extracellular matrix (ECM) interactions and force transduction. However, the consequences of their activity at a molecular level within the cell remain undetermined. Using a combination of FRET, FRAP and biochemical analyses we show that the interactions between the focal adhesion proteins vinculin and paxillin, as well as the closely related family member Hic-5 are spatially and reciprocally regulated by the activity of Rac1 and RhoA. Vinculin in its active conformation interacts with either paxillin or Hic-5 in adhesions in response to Rac1 and RhoA activation respectively, while inactive vinculin interacts with paxillin in the membrane following Rac1 inhibition. Additionally, Rac1 specifically regulates the dynamics of paxillin as well as its binding partner and F-actin interacting protein actopaxin (α-parvin) in adhesions. Furthermore, FRET analysis of protein:protein interactions within cell adhesions formed in 3D matrices revealed that, in contrast to 2D systems vinculin interacts preferentially with Hic-5. This study provides new insight into the complexity of cell-ECM adhesions in both 2D and 3D matrices by providing the first description of RhoGTPase-coordinated protein:protein interactions in a cellular microenvironment. These data identify discrete roles for paxillin and Hic-5 in Rac1 and RhoA-dependent cell adhesion formation and maturation; processes essential for productive cell migration.
3. Blockade of the RhoA-JNK-c-Jun-MMP2 cascade by atorvastatin reduces osteosarcoma cell invasion. Osteosarcoma is characterized by a high malignant and metastatic potential, which points to the need for new therapeutic strategies to prevent cell **metastasis**. In this study, we show that statin-induced HMG-CoA reductase inhibition reduces cell migration and invasion in human and murine osteosarcoma cells, independently of the genotype. The statin-induced reduction of cell migration and invasion was independent of induction of apoptosis and was geranylgeranylpyrophosphate-dependent. The statin reduced matrix metalloproteinase (MMP) 2, 9, and 14 and TIMP2 expression or activity in invading cells. Forced expression of MMP2 and MMP14 overcame the inhibitory effect of the statin on cell invasion, suggesting a role for these MMPs in invasive potential. We also investigated the mechanisms involved in the reduced MMP2 activity and cell invasion. Inhibition of JNK, but not ERK1/2 signaling, reduced MMP2 activity. Pharmacological or constitutive activation of JNK overcame the reduced MMP2 activity and cell invasion induced by the statin. The statin decreased JNK phosphorylation and c-Jun nuclear translocation, suggesting that HMG-CoA reductase inhibition targets the JNK-c-Jun signaling pathway. We showed that mevalonate or geranylgeranylpyrophosphate treatment prevented the statin-induced reduction in JNK phosphorylation, MMP2 activity, and cell invasion. Forced expression of a constitutively active form of RhoA increased JNK phosphorylation and overcame the inhibitory effect of atorvastatin on MMP2 activity and cell invasion. The data establish a link between RhoA, JNK, c-Jun, and MMP2 activity that is functionally involved in the reduction in osteosarcoma cell invasion by the statin. This suggests a novel strategy targeting RhoA-JNK-c-Jun signaling to reduce osteosarcoma cell tumorigenesis.
4. Paxillin and Hic-5 interaction with vinculin is differentially regulated by Rac1 and RhoA. Cell migration is of paramount importance to organism development and maintenance as well as multiple pathological processes, including cancer **metastasis**. The RhoGTPases Rac1 and RhoA are indispensable for cell migration as they regulate cell protrusion, cell-extracellular matrix (ECM) interactions and force transduction. However, the consequences of their activity at a molecular level within the cell remain undetermined. Using a combination of FRET, FRAP and biochemical analyses we show that the interactions between the focal adhesion proteins vinculin and paxillin, as well as the closely related family member Hic-5 are spatially and reciprocally regulated by the activity of Rac1 and RhoA. Vinculin in its active conformation interacts with either paxillin or Hic-5 in adhesions in response to Rac1 and RhoA activation respectively, while inactive vinculin interacts with paxillin in the membrane following Rac1 inhibition. Additionally, Rac1 specifically regulates the dynamics of paxillin as well as its binding partner and F-actin interacting protein actopaxin (α-parvin) in adhesions. Furthermore, FRET analysis of protein:protein interactions within cell adhesions formed in 3D matrices revealed that, in contrast to 2D systems vinculin interacts preferentially with Hic-5. This study provides new insight into the complexity of cell-ECM adhesions in both 2D and 3D matrices by providing the first description of RhoGTPase-coordinated protein:protein interactions in a cellular microenvironment. These data identify discrete roles for paxillin and Hic-5 in Rac1 and RhoA-dependent cell adhesion formation and maturation; processes essential for productive cell migration.
5. A p27(kip1)-binding protein, p27RF-Rho, promotes cancer **metastasis** via activation of RhoA and RhoC. Rho family proteins regulate multiple cellular functions including motility and invasion through regulation of the actin cytoskeleton and gene expression. Activation of Rho proteins is controlled precisely by multiple regulators in a spatiotemporal manner. RhoA and/or RhoC are key players that regulate the metastatic activity of malignant tumor cells, and it is therefore of particular interest to understand how activation of these Rho proteins is controlled. We recently identified an upstream regulator of RhoA activation, p27RF-Rho (p27(kip1) releasing factor from RhoA) that acts by freeing RhoA from inhibition by p27(kip1). p27(kip1) is a cell cycle regulator when it is localized to the nucleus, but it binds RhoA and inhibits activation of the latter when it is localized to the cytoplasm. Here, we show that a metastatic variant of mouse melanoma B16 cells (F10) exhibits greater expression of p27RF-Rho, RhoA, and RhoC than the nonmetastatic parental cells (F0). Injection of F10 cells into mouse tail vein resulted in the formation of metastatic lung colonies, whereas prior knockdown of expression of either one of the three proteins using specific shRNA sequences decreased **metastasis** markedly. p27RF-Rho regulated the activation of RhoA and RhoC and thereby modulated cellular adhesion and motility, in addition to pericellular proteolysis. The Rho activities enhanced by p27RF-Rho had a marked effect upon efficiency of lodging of F10 cells in the lung, which represents an early step of **metastasis**. p27RF-Rho also regulated **metastasis** of human melanoma and fibrosarcoma cells. Thus, p27RF-Rho is a key upstream regulator of RhoA and RhoC that controls spreading of tumor cells.
6. Blockade of the RhoA-JNK-c-Jun-MMP2 cascade by atorvastatin reduces osteosarcoma cell invasion. Osteosarcoma is characterized by a high malignant and metastatic potential, which points to the need for new therapeutic strategies to prevent cell **metastasis**. In this study, we show that statin-induced HMG-CoA reductase inhibition reduces cell migration and invasion in human and murine osteosarcoma cells, independently of the genotype. The statin-induced reduction of cell migration and invasion was independent of induction of apoptosis and was geranylgeranylpyrophosphate-dependent. The statin reduced matrix metalloproteinase (MMP) 2, 9, and 14 and TIMP2 expression or activity in invading cells. Forced expression of MMP2 and MMP14 overcame the inhibitory effect of the statin on cell invasion, suggesting a role for these MMPs in invasive potential. We also investigated the mechanisms involved in the reduced MMP2 activity and cell invasion. Inhibition of JNK, but not ERK1/2 signaling, reduced MMP2 activity. Pharmacological or constitutive activation of JNK overcame the reduced MMP2 activity and cell invasion induced by the statin. The statin decreased JNK phosphorylation and c-Jun nuclear translocation, suggesting that HMG-CoA reductase inhibition targets the JNK-c-Jun signaling pathway. We showed that mevalonate or geranylgeranylpyrophosphate treatment prevented the statin-induced reduction in JNK phosphorylation, MMP2 activity, and cell invasion. Forced expression of a constitutively active form of RhoA increased JNK phosphorylation and overcame the inhibitory effect of atorvastatin on MMP2 activity and cell invasion. The data establish a link between RhoA, JNK, c-Jun, and MMP2 activity that is functionally involved in the reduction in osteosarcoma cell invasion by the statin. This suggests a novel strategy targeting RhoA-JNK-c-Jun signaling to reduce osteosarcoma cell tumorigenesis.
7. A novel Rho-dependent pathway that drives interaction of fascin-1 with p-Lin-11/Isl-1/Mec-3 kinase (LIMK) 1/2 to promote fascin-1/actin binding and filopodia stability. Fascin-1 is an actin crosslinking protein that is important for the assembly of cell protrusions in neurons, skeletal and smooth muscle, fibroblasts, and dendritic cells. Although absent from most normal adult epithelia, fascin-1 is upregulated in many human carcinomas, and is associated with poor prognosis because of its promotion of carcinoma cell migration, invasion, and **metastasis**. Rac and Cdc42 small guanine triphosphatases have been identified as upstream regulators of the association of fascin-1 with actin, but the possible role of Rho has remained obscure. Additionally, experiments have been hampered by the inability to measure the fascin-1/actin interaction directly in intact cells. We investigated the hypothesis that fascin-1 is a functional target of Rho in normal and carcinoma cells, using experimental approaches that included a novel fluorescence resonance energy transfer (FRET)/fluorescence lifetime imaging (FLIM) method to measure the interaction of fascin-1 with actin.Rho activity modulates the interaction of fascin-1 with actin, as detected by a novel FRET method, in skeletal myoblasts and human colon carcinoma cells. Mechanistically, Rho regulation depends on Rho kinase activity, is independent of the status of myosin II activity, and is not mediated by promotion of the fascin/PKC complex. The p-Lin-11/Isl-1/Mec-3 kinases (LIMK), LIMK1 and LIMK2, act downstream of Rho kinases as novel binding partners of fascin-1, and this complex regulates the stability of filopodia.We have identified a novel activity of Rho in promoting a complex between fascin-1 and LIMK1/2 that modulates the interaction of fascin-1 with actin. These data provide new mechanistic insight into the intracellular coordination of contractile and protrusive actin-based structures. During the course of the study, we developed a novel FRET method for analysis of the fascin-1/actin interaction, with potential general applicability for analyzing the activities of actin-binding proteins in intact cells.
8. CD146, an epithelial-mesenchymal transition inducer, is associated with triple-negative breast cancer. The epithelial-mesenchymal transition (EMT) plays an important role in breast cancer **metastasis**, especially in the most aggressive and lethal subtype, "triple-negative breast cancer" (TNBC). Here, we report that CD146 is a unique activator of EMTs and significantly correlates with TNBC. In epithelial breast cancer cells, overexpression of CD146 down-regulated epithelial markers and up-regulated mesenchymal markers, significantly promoted cell migration and invasion, and induced cancer stem cell-like properties. We further found that RhoA pathways positively regulated CD146-induced EMTs via the key EMT transcriptional factor Slug. An orthotopic breast tumor model demonstrated that CD146-overexpressing breast tumors showed a poorly differentiated phenotype and displayed increased tumor invasion and **metastasis**. We confirmed these findings by conducting an immunohistochemical analysis of 505 human primary breast tumor tissues and found that CD146 expression was significantly associated with high tumor stage, poor prognosis, and TNBC. CD146 was expressed at abnormally high levels (68.9%), and was strongly associated with E-cadherin down-regulation in TNBC samples. Taken together, these findings provide unique evidence that CD146 promotes breast cancer progression by induction of EMTs via the activation of RhoA and up-regulation of Slug. Thus, CD146 could be a therapeutic target for breast cancer, especially for TNBC.
9. Netrin-1 promotes glioblastoma cell invasiveness and angiogenesis by multiple pathways including activation of RhoA, cathepsin B, and cAMP-response element-binding protein. Glioblastomas are very difficult tumors to treat because they are highly invasive and disseminate within the normal brain, resulting in newly growing tumors. We have identified netrin-1 as a molecule that promotes glioblastoma invasiveness. As evidence, netrin-1 stimulates glioblastoma cell invasion directly through Matrigel-coated transwells, promotes tumor cell sprouting and enhances **metastasis** to lymph nodes in vivo. Furthermore, netrin-1 regulates angiogenesis as shown in specific angiogenesis assays such as enhanced capillary endothelial cells (EC) sprouting and by increased EC infiltration into Matrigel plugs in vivo, as does VEGF-A. This netrin-1 signaling pathway in glioblastoma cells includes activation of RhoA and cyclic AMP response element-binding protein (CREB). A novel finding is that netrin-1-induced glioblastoma invasiveness and angiogenesis are mediated by activated cathepsin B (CatB), a cysteine protease that translocates to the cell surface as an active enzyme and co-localizes with cell surface annexin A2 (ANXA2). The specific CatB inhibitor CA-074Me inhibits netrin-1-induced cell invasion, sprouting, and Matrigel plug angiogenesis. Silencing of CREB suppresses netrin-1-induced glioblastoma cell invasion, sprouting, and CatB expression. It is concluded that netrin-1 plays an important dual role in glioblastoma progression by promoting both glioblastoma cell invasiveness and angiogenesis in a RhoA-, CREB-, and CatB-dependent manner. Targeting netrin-1 pathways may be a promising strategy for brain cancer therapy.
10. Loss of p53 promotes RhoA-ROCK-dependent cell migration and invasion in 3D matrices. In addition to its role in controlling cell cycle progression, the tumor suppressor protein p53 can also affect other cellular functions such as cell migration. In this study, we show that p53 deficiency in mouse embryonic fibroblasts cultured in three-dimensional matrices induces a switch from an elongated spindle morphology to a markedly spherical and flexible one associated with highly dynamic membrane blebs. These rounded, motile cells exhibit amoeboid-like movement and have considerably increased invasive properties. The morphological transition requires the RhoA-ROCK (Rho-associated coil-containing protein kinase) pathway and is prevented by RhoE. A similar p53-mediated transition is observed in melanoma A375P cancer cells. Our data suggest that genetic alterations of p53 in tumors are sufficient to promote motility and invasion, thereby contributing to **metastasis**.
11. PRL-1 protein promotes ERK1/2 and RhoA protein activation through a non-canonical interaction with the Src homology 3 domain of p115 Rho GTPase-activating protein. Phosphatases of the regenerating liver (PRL) play oncogenic roles in cancer development and **metastasis**. Although previous studies indicate that PRL-1 promotes cell growth and migration by activating both the ERK1/2 and RhoA pathways, the mechanism by which it activates these signaling events remains unclear. We have identified a PRL-1-binding peptide (Peptide 1) that shares high sequence identity with a conserved motif in the Src homology 3 (SH3) domain of p115 Rho GTPase-activating protein (GAP). p115 RhoGAP directly binds PRL-1 in vitro and in cells via its SH3 domain. Structural analyses of the PRL-1·Peptide 1 complex revealed a novel protein-protein interaction whereby a sequence motif within the PxxP ligand-binding site of the p115 RhoGAP SH3 domain occupies a folded groove within PRL-1. This prevents the canonical interaction between the SH3 domain of p115 RhoGAP and MEKK1 and results in activation of ERK1/2. Furthermore, PRL-1 binding activates RhoA signaling by inhibiting the catalytic activity of p115 RhoGAP. The results demonstrate that PRL-1 binding to p115 RhoGAP provides a coordinated mechanism underlying ERK1/2 and RhoA activation.
12. MicroRNA-155 is regulated by the transforming growth factor beta/Smad pathway and contributes to epithelial cell plasticity by targeting RhoA. Transforming growth factor beta (TGF-beta) signaling facilitates **metastasis** in advanced malignancy. While a number of protein-encoding genes are known to be involved in this process, information on the role of microRNAs (miRNAs) in TGF-beta-induced cell migration and invasion is still limited. By hybridizing a 515-miRNA oligonucleotide-based microarray library, a total of 28 miRNAs were found to be significantly deregulated in TGF-beta-treated normal murine mammary gland (NMuMG) epithelial cells but not Smad4 knockdown NMuMG cells. Among upregulated miRNAs, miR-155 was the most significantly elevated miRNA. TGF-beta induces miR-155 expression and promoter activity through Smad4. The knockdown of miR-155 suppressed TGF-beta-induced epithelial-mesenchymal transition (EMT) and tight junction dissolution, as well as cell migration and invasion. Further, the ectopic expression of miR-155 reduced RhoA protein and disrupted tight junction formation. Reintroducing RhoA cDNA without the 3' untranslated region largely reversed the phenotype induced by miR-155 and TGF-beta. In addition, elevated levels of miR-155 were frequently detected in invasive breast cancer tissues. These data suggest that miR-155 may play an important role in TGF-beta-induced EMT and cell migration and invasion by targeting RhoA and indicate that it is a potential therapeutic target for breast cancer intervention.

#### GeneRIFs

1. A p27(kip1)-binding protein, p27RF-Rho, promotes cancer **metastasis** via activation of RhoA and RhoC.

#### Summary

1. This gene encodes a member of the Rho family of small GTPases, which cycle between inactive GDP-bound and active GTP-bound states and function as molecular switches in signal transduction cascades. Rho proteins promote reorganization of the actin cytoskeleton and regulate cell shape, attachment, and motility. Overexpression of this gene is associated with tumor cell proliferation and **metastasis**. Multiple alternatively spliced variants have been identified. [provided by RefSeq, Sep 2015]

### [Cc]ell [Ii]nvasion

#### PubMed

1. Blockade of the RhoA-JNK-c-Jun-MMP2 cascade by atorvastatin reduces osteosarcoma **cell invasion**. Osteosarcoma is characterized by a high malignant and metastatic potential, which points to the need for new therapeutic strategies to prevent cell metastasis. In this study, we show that statin-induced HMG-CoA reductase inhibition reduces cell migration and invasion in human and murine osteosarcoma cells, independently of the genotype. The statin-induced reduction of cell migration and invasion was independent of induction of apoptosis and was geranylgeranylpyrophosphate-dependent. The statin reduced matrix metalloproteinase (MMP) 2, 9, and 14 and TIMP2 expression or activity in invading cells. Forced expression of MMP2 and MMP14 overcame the inhibitory effect of the statin on **cell invasion**, suggesting a role for these MMPs in invasive potential. We also investigated the mechanisms involved in the reduced MMP2 activity and **cell invasion**. Inhibition of JNK, but not ERK1/2 signaling, reduced MMP2 activity. Pharmacological or constitutive activation of JNK overcame the reduced MMP2 activity and **cell invasion** induced by the statin. The statin decreased JNK phosphorylation and c-Jun nuclear translocation, suggesting that HMG-CoA reductase inhibition targets the JNK-c-Jun signaling pathway. We showed that mevalonate or geranylgeranylpyrophosphate treatment prevented the statin-induced reduction in JNK phosphorylation, MMP2 activity, and **cell invasion**. Forced expression of a constitutively active form of RhoA increased JNK phosphorylation and overcame the inhibitory effect of atorvastatin on MMP2 activity and **cell invasion**. The data establish a link between RhoA, JNK, c-Jun, and MMP2 activity that is functionally involved in the reduction in osteosarcoma **cell invasion** by the statin. This suggests a novel strategy targeting RhoA-JNK-c-Jun signaling to reduce osteosarcoma cell tumorigenesis.
2. Prickle1 mutation causes planar cell polarity and directional cell migration defects associated with cardiac outflow tract anomalies and other structural birth defects. Planar cell polarity (PCP) is controlled by a conserved pathway that regulates directional cell behavior. Here, we show that mutant mice harboring a newly described mutation termed Beetlejuice (Bj) in Prickle1 (Pk1), a PCP component, exhibit developmental phenotypes involving cell polarity defects, including skeletal, cochlear and congenital cardiac anomalies. Bj mutants die neonatally with cardiac outflow tract (OFT) malalignment. This is associated with OFT shortening due to loss of polarized cell orientation and failure of second heart field cell intercalation mediating OFT lengthening. OFT myocardialization was disrupted with cardiomyocytes failing to align with the direction of **cell invasion** into the outflow cushions. The expression of genes mediating Wnt signaling was altered. Also noted were shortened but widened bile ducts and disruption in canonical Wnt signaling. Using an in vitro wound closure assay, we showed Bj mutant fibroblasts cannot establish polarized cell morphology or engage in directional cell migration, and their actin cytoskeleton failed to align with the direction of wound closure. Unexpectedly, Pk1 mutants exhibited primary and motile cilia defects. Given Bj mutant phenotypes are reminiscent of ciliopathies, these findings suggest Pk1 may also regulate ciliogenesis. Together these findings show Pk1 plays an essential role in regulating cell polarity and directional cell migration during development.
3. Blockade of the RhoA-JNK-c-Jun-MMP2 cascade by atorvastatin reduces osteosarcoma **cell invasion**. Osteosarcoma is characterized by a high malignant and metastatic potential, which points to the need for new therapeutic strategies to prevent cell metastasis. In this study, we show that statin-induced HMG-CoA reductase inhibition reduces cell migration and invasion in human and murine osteosarcoma cells, independently of the genotype. The statin-induced reduction of cell migration and invasion was independent of induction of apoptosis and was geranylgeranylpyrophosphate-dependent. The statin reduced matrix metalloproteinase (MMP) 2, 9, and 14 and TIMP2 expression or activity in invading cells. Forced expression of MMP2 and MMP14 overcame the inhibitory effect of the statin on **cell invasion**, suggesting a role for these MMPs in invasive potential. We also investigated the mechanisms involved in the reduced MMP2 activity and **cell invasion**. Inhibition of JNK, but not ERK1/2 signaling, reduced MMP2 activity. Pharmacological or constitutive activation of JNK overcame the reduced MMP2 activity and **cell invasion** induced by the statin. The statin decreased JNK phosphorylation and c-Jun nuclear translocation, suggesting that HMG-CoA reductase inhibition targets the JNK-c-Jun signaling pathway. We showed that mevalonate or geranylgeranylpyrophosphate treatment prevented the statin-induced reduction in JNK phosphorylation, MMP2 activity, and **cell invasion**. Forced expression of a constitutively active form of RhoA increased JNK phosphorylation and overcame the inhibitory effect of atorvastatin on MMP2 activity and **cell invasion**. The data establish a link between RhoA, JNK, c-Jun, and MMP2 activity that is functionally involved in the reduction in osteosarcoma **cell invasion** by the statin. This suggests a novel strategy targeting RhoA-JNK-c-Jun signaling to reduce osteosarcoma cell tumorigenesis.
4. Activation of a RhoA/myosin II-dependent but Arp2/3 complex-independent pathway facilitates Salmonella invasion. Salmonella stimulates host **cell invasion** using virulence effectors translocated by the pathogen's type-three secretion system (T3SS). These factors manipulate host signaling pathways, primarily driven by Rho family GTPases, which culminates in Arp2/3 complex-dependent activation of host actin nucleation to mediate the uptake of Salmonella into host cells. However, recent data argue for the existence of additional mechanisms that cooperate in T3SS-dependent Salmonella invasion. We identify a myosin II-mediated mechanism, operating independent of but complementary to the Arp2/3-dependent pathway, as contributing to Salmonella invasion into nonphagocytic cells. We also establish that the T3SS effector SopB constitutes an important regulator of this Rho/Rho kinase and myosin II-dependent invasion pathway. Thus, Salmonella enters nonphagocytic cells by manipulating the two core machineries of actin-based motility in the host: Arp2/3 complex-driven actin polymerization and actomyosin-mediated contractility.
5. Netrin-1 promotes glioblastoma cell invasiveness and angiogenesis by multiple pathways including activation of RhoA, cathepsin B, and cAMP-response element-binding protein. Glioblastomas are very difficult tumors to treat because they are highly invasive and disseminate within the normal brain, resulting in newly growing tumors. We have identified netrin-1 as a molecule that promotes glioblastoma invasiveness. As evidence, netrin-1 stimulates glioblastoma **cell invasion** directly through Matrigel-coated transwells, promotes tumor cell sprouting and enhances metastasis to lymph nodes in vivo. Furthermore, netrin-1 regulates angiogenesis as shown in specific angiogenesis assays such as enhanced capillary endothelial cells (EC) sprouting and by increased EC infiltration into Matrigel plugs in vivo, as does VEGF-A. This netrin-1 signaling pathway in glioblastoma cells includes activation of RhoA and cyclic AMP response element-binding protein (CREB). A novel finding is that netrin-1-induced glioblastoma invasiveness and angiogenesis are mediated by activated cathepsin B (CatB), a cysteine protease that translocates to the cell surface as an active enzyme and co-localizes with cell surface annexin A2 (ANXA2). The specific CatB inhibitor CA-074Me inhibits netrin-1-induced **cell invasion**, sprouting, and Matrigel plug angiogenesis. Silencing of CREB suppresses netrin-1-induced glioblastoma **cell invasion**, sprouting, and CatB expression. It is concluded that netrin-1 plays an important dual role in glioblastoma progression by promoting both glioblastoma cell invasiveness and angiogenesis in a RhoA-, CREB-, and CatB-dependent manner. Targeting netrin-1 pathways may be a promising strategy for brain cancer therapy.
6. Galectin 3 regulates HCC **cell invasion** by RhoA and MLCK activation. Hepatocellular carcinoma (HCC) carries a poor prognosis with no effective treatment available other than liver transplantation for selected patients. Vascular invasion of HCC is one of the most important negative predictor of survival. As the regulation of invasion of HCC cells is not well understood, our aim was to study the mechanisms by which galectin 3, a β-galactosidase-binding lectin mediates HCC cell migration. HCC was induced by N-diethylnitrosamine in wild-type and galectin 3(-/-) mice, and tumor formation, histology, and tumor **cell invasio**n were assessed. The galectin 3(-/-) mice developed significantly smaller tumor burden with a less invasive phenotype than the wild-type animals. Galectin 3 was upregulated in the wild-type HCC tumor tissue, but not in the surrounding parenchyma. Galectin 3 expression in HCC was induced by NF-κB transactivation as determined by chromatin immunoprecipitation assays. In vitro studies assessed the pro-migratory effects of galectin 3. The migration of hepatoma cells was significantly decreased after transfection by the galectin 3 siRNA and also after using the Rho kinase inhibitor Y-27632. The reorganization of the actin cytoskeleton, RhoA GTPase activity and the phosphorylation of MLC2 (myosin light chain 2) were decreased in the galectin 3 siRNA-transfected cells. In addition, in vitro and in vivo evidence showed that galectin 3 deficiency reduced hepatoma cell proliferation and increased their apoptosis rate. In conclusion, galectin 3 is an important lectin that is induced in HCC cells, and promotes hepatoma cell motility and invasion by an autocrine pathway. Targeting galectin 3 therefore could be an important novel treatment strategy to halt disease progression.

#### GeneRIFs

1. a link between RhoA, JNK, c-Jun, and MMP2 activity that is functionally involved in the reduction in osteosarcoma **cell invasion** by the statin. This suggests a novel strategy targeting RhoA-JNK-c-Jun signaling to reduce osteosarcoma cell tumorigenesis.

---

## Rhod

- NCBI Gene
- GeneRIF
- Pubmed

| Search Term | Rank | Fields |
| --- | --- | --- |
| [Ff]ilopodia | 1 | PubMed(2) |
| [Pp]odosome(s)? | 5 | PubMed(1); GeneRIFs(1) |
| [Mm]igration | 6 | Process(1); PubMed(4) |
| [Ii]nvasive | 10 | PubMed(1) |

  
hide/show details for Rhod

### [Ff]ilopodia

#### PubMed

1. RhoD is a Golgi component with a role in anterograde protein transport from the ER to the plasma membrane. RhoD is a member of the Rho GTPase family and it coordinates actin dynamics and membrane trafficking. Activation of RhoD results in formation of **filopodia**, dissolution of stress fibers, and the subsequent formation of short actin bundles. In addition, RhoD localizes to early endosomes and recycling endosomes, and has a regulatory role in endosome trafficking. In this study, we report on a function of RhoD in the regulation of Golgi homeostasis. We show that manipulation of protein and activation levels of RhoD, as well as of its binding partner WHAMM, result in derailed localization of Golgi stacks. Moreover, vesicle trafficking from the endoplasmic reticulum to the plasma membrane via the Golgi apparatus measured by the VSV-G protein is severely hampered by manipulation of RhoD or WHAMM. In summary, our studies demonstrate a novel role for this member of the Rho GTPases in the regulation of Golgi function.
2. RhoD activated by fibroblast growth factor induces cytoneme-like cellular protrusions through mDia3C. The small GTPase RhoD regulates actin cytoskeleton to collapse actin stress fibers and focal adhesions, resulting in suppression of cell migration and cytokinesis. It also induces alignment of early endosomes along actin filaments and reduces their motility. We show here that a constitutively activated RhoD generated two types of actin-containing thin peripheral cellular protrusions distinct from Cdc42-induced **filopodia**. One was longer, almost straight, immotile, and sensitive to fixation, whereas the other was shorter, undulating, motile, and resistant to fixation. Moreover, cells expressing wild-type RhoD extended protrusions toward fibroblast growth factor (FGF) 2/4/8-coated beads. Stimulation of wild-type RhoD-expressing cells with these FGFs also caused formation of cellular protrusions. Nodules moved through the RhoD-induced longer protrusions, mainly toward the cell body. Exogenously expressed FGF receptor was associated with these moving nodules containing endosome-like vesicles. These results suggest that the protrusions are responsible for intercellular communication mediated by FGF and its receptor. Accordingly, the protrusions are morphologically and functionally equivalent to cytonemes. RhoD was activated by FGF2/4/8. Knockdown of RhoD interfered with FGF-induced protrusion formation. Activated RhoD specifically bound to mDia3C and facilitated actin polymerization together with mDia3C. mDia3C was localized to the tips or stems of the protrusions. In addition, constitutively activated mDia3C formed protrusions without RhoD or FGF stimulation. Knockdown of mDia3 obstructed RhoD-induced protrusion formation. These results imply that RhoD activated by FGF signaling forms cytoneme-like protrusions through activation of mDia3C, which induces actin filament formation.

### [Pp]odosome(s)?

#### PubMed

1. ERK5 promotes Src-induced **podosome** formation by limiting Rho activation. Increased Src activity, often associated with tumorigenesis, leads to the formation of invasive adhesions termed **podosomes**. **Podosome** formation requires the function of Rho family guanosine triphosphatases and reorganization of the actin cytoskeleton. In addition, Src induces changes in gene expression required for transformation, in part by activating mitogen-activated protein kinase (MAPK) signaling pathways. We sought to determine whether MAPK signaling regulates **podosome** formation. Unlike extracellular signal-regulated kinase 1/2 (ERK1/2), ERK5 is constitutively activated in Src-transformed fibroblasts. ERK5-deficient cells expressing v-Src exhibited increased RhoA activation and signaling, which lead to cellular retraction and an inability to form **podosomes** or induce invasion. Addition of the Rho-kinase inhibitor Y27632 to ERK5-deficient cells expressing v-Src led to cellular extension and restored **podosome** formation. In Src-transformed cells, ERK5 induced the expression of a Rho GTPase-activating protein (RhoGAP), RhoGAP7/DLC-1, via activation of the transcription factor myocyte enhancing factor 2C, and RhoGAP7 expression restored **podosome** formation in ERK5-deficient cells. We conclude that ERK5 promotes Src-induced **podosome** formation by inducing RhoGAP7 and thereby limiting Rho activation.

#### GeneRIFs

1. ERK5 promotes Src-induced **podosome** formation by inducing RhoGAP7 and thereby limiting Rho activation.

### [Mm]igration

#### Process

1. positive regulation of cell **migration**

#### PubMed

1. RhoD activated by fibroblast growth factor induces cytoneme-like cellular protrusions through mDia3C. The small GTPase RhoD regulates actin cytoskeleton to collapse actin stress fibers and focal adhesions, resulting in suppression of cell **migration** and cytokinesis. It also induces alignment of early endosomes along actin filaments and reduces their motility. We show here that a constitutively activated RhoD generated two types of actin-containing thin peripheral cellular protrusions distinct from Cdc42-induced filopodia. One was longer, almost straight, immotile, and sensitive to fixation, whereas the other was shorter, undulating, motile, and resistant to fixation. Moreover, cells expressing wild-type RhoD extended protrusions toward fibroblast growth factor (FGF) 2/4/8-coated beads. Stimulation of wild-type RhoD-expressing cells with these FGFs also caused formation of cellular protrusions. Nodules moved through the RhoD-induced longer protrusions, mainly toward the cell body. Exogenously expressed FGF receptor was associated with these moving nodules containing endosome-like vesicles. These results suggest that the protrusions are responsible for intercellular communication mediated by FGF and its receptor. Accordingly, the protrusions are morphologically and functionally equivalent to cytonemes. RhoD was activated by FGF2/4/8. Knockdown of RhoD interfered with FGF-induced protrusion formation. Activated RhoD specifically bound to mDia3C and facilitated actin polymerization together with mDia3C. mDia3C was localized to the tips or stems of the protrusions. In addition, constitutively activated mDia3C formed protrusions without RhoD or FGF stimulation. Knockdown of mDia3 obstructed RhoD-induced protrusion formation. These results imply that RhoD activated by FGF signaling forms cytoneme-like protrusions through activation of mDia3C, which induces actin filament formation.
2. Small GTPase RhoD suppresses cell **migration** and cytokinesis. Rho family small GTPases regulate organization of the actin cytoskeleton. Among them, RhoA plays essential roles in the formation of the actin stress fibers, the associated focal adhesions, and the contractile rings necessary for cytokinesis. Recently, RhoD, a novel member of Rho family has been identified. The amino acid sequences of its effector domain is distinct from those of the other Rho family proteins, suggesting its unique cellular functions. Introduction of the constitutively active form of RhoD(G26V) into fibroblasts by microinjection or transfection resulted in disassembly of the actin stress fibers and the focal adhesions, whereas the dominant negative form of RhoD(T31K) did not affect these structures. The degree of cell **migration** assessed by the phagokinetic tracks on a substrate covered with gold particles was diminished by the expression of RhoD(G26V) but not by RhoD(T31K). Thus, cytoskeletal alterations including the loss of stress fibers and focal adhesions by RhoD seems to lead to the retardation of cell **migration**. Transfection of RhoD(G26V) cDNA into cultured cells also induced multinucleation. Moreover, RhoD(G26V) microinjected into fertilized eggs and embryos of Xenopus laevis caused cleavage arrest only in the injected cells, and the uncleaved cells contained multiple nuclei. These results imply that RhoD does not affect nuclear division but can interfere with cytokinesis presumably by preventing the formation of the actin-based contractile ring. Enhancement of the stress fibers by RhoA or RhoA-activating lysophosphatidic acid was reversed by the transfection of RhoD cDNA. Accordingly, the cellular functions of RhoD are likely to be antagonistic to those of RhoA.
3. Atypical Rho GTPases RhoD and Rif integrate cytoskeletal dynamics and membrane trafficking. The Rho GTPases are essential regulators of basic cellular processes, including cell **migration**, cell contraction and cell division. Most studies still involve just the three canonical members, RhoA, Rac1 and Cdc42, although the Rho GTPases comprise at least 20 members. The aim of this review is to highlight some of the recent advances in our knowledge regarding the less-studied Rho members, with the focus on RhoD and Rif. The phenotypic alterations to cell behaviour that are triggered by RhoD and Rif suggest that they have unique impacts on cytoskeletal dynamics that distinguish them from the well-studied members of the Rho GTPases. In addition, RhoD has a role in the regulation of intracellular transport of vesicles. Taken together, the available data indicate that RhoD and Rif have functions as master regulators in the integration of cytoskeletal reorganisation and membrane trafficking.
4. Potential role of MCP-1 in endothelial cell tight junction 'opening': signaling via Rho and Rho kinase. The expression of the monocyte chemoattractant protein-1 (MCP-1) receptor CCR2 by brain endothelial cells suggests that MCP-1 may have other functions than purely driving leukocyte **migration** into brain parenchyma during inflammation. This study examines one of these potential novel roles of MCP-1 regulation of endothelial permeability using primary cultures of mouse brain endothelial cells. MCP-1 induces reorganization of actin cytoskeleton (stress fiber formation) and redistribution of tight junction proteins, ZO-1, ZO-2 occludin and claudin-5, from the Triton X-100-soluble to the Triton X-100-insoluble fractions. These morphological changes are associated with a decrease in transendothelial electrical membrane resistance and an increase in [14C]inulin permeability. MCP-1 did not induce these events in brain endothelial cells prepared from mice genotype CCR2-/-. The Rho kinase inhibitor Y27632 and inhibition of Rho (C3 exoenzyme, and dominant negative mutant of Rho, RhoT19N) prevented MCP-1-induced stress fiber assembly, reorganization of tight junction proteins and alterations in endothelial permeability. In all, this suggests that a small GTPase Rho and Rho kinase have a pivotal role in MCP-1-induced junction disarrangement. These data are the first to strongly suggest that MCP-1, via CCR2 present on brain endothelial cells, contributes to increased brain endothelial permeability.

### [Ii]nvasive

#### PubMed

1. ERK5 promotes Src-induced podosome formation by limiting Rho activation. Increased Src activity, often associated with tumorigenesis, leads to the formation of **invasive** adhesions termed podosomes. Podosome formation requires the function of Rho family guanosine triphosphatases and reorganization of the actin cytoskeleton. In addition, Src induces changes in gene expression required for transformation, in part by activating mitogen-activated protein kinase (MAPK) signaling pathways. We sought to determine whether MAPK signaling regulates podosome formation. Unlike extracellular signal-regulated kinase 1/2 (ERK1/2), ERK5 is constitutively activated in Src-transformed fibroblasts. ERK5-deficient cells expressing v-Src exhibited increased RhoA activation and signaling, which lead to cellular retraction and an inability to form podosomes or induce invasion. Addition of the Rho-kinase inhibitor Y27632 to ERK5-deficient cells expressing v-Src led to cellular extension and restored podosome formation. In Src-transformed cells, ERK5 induced the expression of a Rho GTPase-activating protein (RhoGAP), RhoGAP7/DLC-1, via activation of the transcription factor myocyte enhancing factor 2C, and RhoGAP7 expression restored podosome formation in ERK5-deficient cells. We conclude that ERK5 promotes Src-induced podosome formation by inducing RhoGAP7 and thereby limiting Rho activation.

---

## Rhog

- NCBI Gene
- GeneRIF
- Pubmed

| Search Term | Rank | Fields |
| --- | --- | --- |
| [Ii]nvadopodia | 3 | PubMed(1); GeneRIFs(1) |
| [Mm]igration | 6 | PubMed(9) |
| [Ee]xtracellular [Mm]atrix | 8 | PubMed(1) |
| [Ii]nvasive | 10 | PubMed(1); GeneRIFs(1) |
| [Mm]etastasis | 12 | PubMed(3) |
| [Cc]ell [Ii]nvasion | 13 | PubMed(1) |

  
hide/show details for Rhog

### [Ii]nvadopodia

#### PubMed

1. The small GTPase RhoG mediates glioblastoma cell invasion. The invasion of glioblastoma cells into regions of the normal brain is a critical factor that limits current therapies for malignant astrocytomas. Previous work has identified roles for the Rho family guanine nucleotide exchange factors Trio and Vav3 in glioblastoma invasion. Both Trio and Vav3 act on the small GTPase RhoG. We therefore examined the role of RhoG in the invasive behavior of glioblastoma cells.We found that siRNA-mediated depletion of RhoG strongly inhibits invasion of glioblastoma cells through brain slices ex vivo. In addition, depletion of RhoG has a marginal effect on glioblastoma cell proliferation, but significantly inhibits glioblastoma cell survival in colony formation assays. We also observed that RhoG is activated by both HGF and EGF, two factors that are thought to be clinically relevant drivers of glioblastoma invasive behavior, and that RhoG is overexpressed in human glioblastoma tumors versus non-neoplastic brain. In search of a mechanism for the contribution of RhoG to the malignant behavior of glioblastoma cells, we found that depletion of RhoG strongly inhibits activation of the Rac1 GTPase by both HGF and EGF. In line with this observation, we also show that RhoG contributes to the formation of lamellipodia and **invadopodia**, two functions that have been shown to be Rac1-dependent.Our functional analysis of RhoG in the context of glioblastoma revealed a critical role for RhoG in tumor cell invasion and survival. These results suggest that targeting RhoG-mediated signaling presents a novel avenue for glioblastoma therapy.

#### GeneRIFs

1. Examined the role of RhoG in the invasive behavior of glioblastoma cells; found that depletion of RhoG strongly inhibits activation of the Rac1 GTPase by both HGF and EGF; also RhoG contributes to the formation of lamellipodia and **invadopodia**.

### [Mm]igration

#### PubMed

1. Suppression of RhoG activity is mediated by a syndecan 4-synectin-RhoGDI1 complex and is reversed by PKCalpha in a Rac1 activation pathway. Fibroblast growth factor 2 (FGF2) is a major regulator of developmental, pathological, and therapeutic angiogenesis. Its activity is partially mediated by binding to syndecan 4 (S4), a proteoglycan receptor. Angiogenesis requires polarized activation of the small guanosine triphosphatase Rac1, which involves localized dissociation from RhoGDI1 and association with the plasma membrane. Previous work has shown that genetic deletion of S4 or its adapter, synectin, leads to depolarized Rac activation, decreased endothelial **migration**, and other physiological defects. In this study, we show that Rac1 activation downstream of S4 is mediated by the RhoG activation pathway. RhoG is maintained in an inactive state by RhoGDI1, which is found in a ternary complex with synectin and S4. Binding of S4 to synectin increases the latter's binding to RhoGDI1, which in turn enhances RhoGDI1's affinity for RhoG. S4 clustering activates PKCalpha, which phosphorylates RhoGDI1 at Ser(96). This phosphorylation triggers release of RhoG, leading to polarized activation of Rac1. Thus, FGF2-induced Rac1 activation depends on the suppression of RhoG by a previously uncharacterized ternary S4-synectin-RhoGDI1 protein complex and activation via PKCalpha.
2. Cdc42 and Rac family GTPases regulate mode and speed but not direction of primary fibroblast **migration** during platelet-derived growth factor-dependent chemotaxis. Cdc42 and Rac family GTPases are important regulators of morphology, motility, and polarity in a variety of mammalian cell types. However, comprehensive analysis of their roles in the morphological and behavioral aspects of chemotaxis within a single experimental system is still lacking. Here we demonstrate using a direct viewing chemotaxis assay that of all of the Cdc42/Rac1-related GTPases expressed in primary fibroblasts, Cdc42, Rac1, and RhoG are required for efficient **migration** towards platelet-derived growth factor (PDGF). During **migration**, Cdc42-, Rac1-, and RhoG-deficient cells show aberrant morphology characterized as cell elongation and cell body rounding, loss of lamellipodia, and formation of thick membrane extensions, respectively. Analysis of individual cell trajectories reveals that cell speed is significantly reduced, as well as persistence, but to a smaller degree, while the directional response to the gradient of PDGF is not affected. Combined knockdown of Cdc42, Rac1, and RhoG results in greater inhibition of cell speed than when each protein is knocked down alone, but the cells are still capable of migrating toward PDGF. We conclude that, Cdc42, Rac1, and RhoG function cooperatively during cell **migration** and that, while each GTPase is implicated in the control of morphology and cell speed, these and other Cdc42/Rac-related GTPases are not essential for the directional response toward PDGF.
3. Endogenous RhoG is dispensable for integrin-mediated cell spreading but contributes to Rac-independent **migration**. Rac activation by integrins is essential for cell spreading, **migration**, growth and survival. Based mainly on overexpression of dominant-negative mutants, RhoG has been proposed to mediate integrin-dependent Rac activation upstream of ELMO and Dock180. RhoG-knockout mice, however, display no significant developmental or functional abnormalities. To clarify the role of RhoG in integrin-mediated signaling, we developed a RhoG-specific antibody, which, together with shRNA-mediated knockdown, allowed analysis of the endogenous protein. Despite dramatic effects of dominant-negative constructs, nearly complete RhoG depletion did not substantially inhibit cell adhesion, spreading, **migration** or Rac activation. Additionally, RhoG was not detectably activated by adhesion to fibronectin. Using Rac1(-/-) cells, we found that constitutively active RhoG induced membrane ruffling via both Rac-dependent and -independent pathways. Additionally, endogenous RhoG was important for Rac-independent cell **migration**. However, RhoG did not significantly contribute to cell spreading even in these cells. These data therefore clarify the role of RhoG in integrin signaling and cell motility.
4. Suppression of RhoG activity is mediated by a syndecan 4-synectin-RhoGDI1 complex and is reversed by PKCalpha in a Rac1 activation pathway. Fibroblast growth factor 2 (FGF2) is a major regulator of developmental, pathological, and therapeutic angiogenesis. Its activity is partially mediated by binding to syndecan 4 (S4), a proteoglycan receptor. Angiogenesis requires polarized activation of the small guanosine triphosphatase Rac1, which involves localized dissociation from RhoGDI1 and association with the plasma membrane. Previous work has shown that genetic deletion of S4 or its adapter, synectin, leads to depolarized Rac activation, decreased endothelial **migration**, and other physiological defects. In this study, we show that Rac1 activation downstream of S4 is mediated by the RhoG activation pathway. RhoG is maintained in an inactive state by RhoGDI1, which is found in a ternary complex with synectin and S4. Binding of S4 to synectin increases the latter's binding to RhoGDI1, which in turn enhances RhoGDI1's affinity for RhoG. S4 clustering activates PKCalpha, which phosphorylates RhoGDI1 at Ser(96). This phosphorylation triggers release of RhoG, leading to polarized activation of Rac1. Thus, FGF2-induced Rac1 activation depends on the suppression of RhoG by a previously uncharacterized ternary S4-synectin-RhoGDI1 protein complex and activation via PKCalpha.
5. An ELMO2-RhoG-ILK network modulates microtubule dynamics. ELMO2 belongs to a family of scaffold proteins involved in phagocytosis and cell motility. ELMO2 can simultaneously bind integrin-linked kinase (ILK) and RhoG, forming tripartite ERI complexes. These complexes are involved in promoting β1 integrin-dependent directional **migratio**n in undifferentiated epidermal keratinocytes. ELMO2 and ILK have also separately been implicated in microtubule regulation at integrin-containing focal adhesions. During differentiation, epidermal keratinocytes cease to express integrins, but ERI complexes persist. Here we show an integrin-independent role of ERI complexes in modulation of microtubule dynamics in differentiated keratinocytes. Depletion of ERI complexes by inactivating the Ilk gene in these cells reduces microtubule growth and increases the frequency of catastrophe. Reciprocally, exogenous expression of ELMO2 or RhoG stabilizes microtubules, but only if ILK is also present. Mechanistically, activation of Rac1 downstream from ERI complexes mediates their effects on microtubule stability. In this pathway, Rac1 serves as a hub to modulate microtubule dynamics through two different routes: 1) phosphorylation and inactivation of the microtubule-destabilizing protein stathmin and 2) phosphorylation and inactivation of GSK-3β, which leads to the activation of CRMP2, promoting microtubule growth. At the cellular level, the absence of ERI species impairs Ca(2+)-mediated formation of adherens junctions, critical to maintaining mechanical integrity in the epidermis. Our findings support a key role for ERI species in integrin-independent stabilization of the microtubule network in differentiated keratinocytes.
6. Integrin-linked kinase interactions with ELMO2 modulate cell polarity. Cell polarization is a key prerequisite for directed **migration** during development, tissue regeneration, and metastasis. Integrin-linked kinase (ILK) is a scaffold protein essential for cell polarization, but very little is known about the precise mechanisms whereby ILK modulates polarization in normal epithelia. Elucidating these mechanisms is essential to understand tissue morphogenesis, transformation, and repair. Here we identify a novel ILK protein complex that includes Engulfment and Cell Motility 2 (ELMO2). We also demonstrate the presence of RhoG in ILK-ELMO2 complexes, and the localization of this multiprotein species specifically to the leading lamellipodia of polarized cells. Significantly, the ability of RhoG to bind ELMO is crucial for ILK induction of cell polarization, and the joint expression of ILK and ELMO2 synergistically promotes the induction of front-rear polarity and haptotactic **migration**. This places RhoG-ELMO2-ILK complexes in a key position for the development of cell polarity and forward movement. Although ILK is a component of many diverse multiprotein species that may contribute to cell polarization, expression of dominant-negative ELMO2 mutants is sufficient to abolish the ability of ILK to promote cell polarization. Thus, its interaction with ELMO2 and RhoG is essential for the ability of ILK to induce front-rear cell polarity.
7. The guanine-nucleotide exchange factor SGEF plays a crucial role in the formation of atherosclerosis. The passage of leukocytes across the endothelium and into arterial walls is a critical step in the development of atherosclerosis. Previously, we showed in vitro that the RhoG guanine nucleotide exchange factor SGEF (Arhgef26) contributes to the formation of ICAM-1-induced endothelial docking structures that facilitate leukocyte transendothelial **migration**. To further explore the in vivo role of this protein during inflammation, we generated SGEF-deficient mice. When crossed with ApoE null mice and fed a Western diet, mice lacking SGEF showed a significant decrease in the formation of atherosclerosis in multiple aortic areas. A fluorescent biosensor revealed local activation of RhoG around bead-clustered ICAM-1 in mouse aortic endothelial cells. Notably, this activation was decreased in cells from SGEF-deficient aortas compared to wild type. In addition, scanning electron microscopy of intimal surfaces of SGEF(-/-) mouse aortas revealed reduced docking structures around beads that were coated with ICAM-1 antibody. Similarly, under conditions of flow, these beads adhered less stably to the luminal surface of carotid arteries from SGEF(-/-) mice. Taken together, these results show for the first time that a Rho-GEF, namely SGEF, contributes to the formation of atherosclerosis by promoting endothelial docking structures and thereby retention of leukocytes at athero-prone sites of inflammation experiencing high shear flow. SGEF may therefore provide a novel therapeutic target for inhibiting the development of atherosclerosis.
8. A syndecan-4 hair trigger initiates wound healing through caveolin- and RhoG-regulated integrin endocytosis. Cell **migration** during wound healing requires adhesion receptor turnover to enable the formation and disassembly of cell-extracellular matrix contacts. Although recent advances have improved our understanding of integrin trafficking pathways, it is not known how extracellular ligand engagement controls receptor dynamics. Using atomic force microscopy, we have measured cell avidity for fibronectin and defined a mechanism for the outside-in regulation of α(5)β(1)-integrin. Surprisingly, adhesive strength was attenuated by the syndecan-4-binding domain of fibronectin due to a rapid triggering of α(5)β(1)-integrin endocytosis. Association of syndecan-4 with PKCα was found to trigger RhoG activation and subsequent dynamin- and caveolin-dependent integrin uptake. Like disruption of syndecan-4 or caveolin, gene disruption of RhoG in mice was found to retard closure of dermal wounds due **to a migr**ation defect of the fibroblasts and keratinocytes of RhoG null mice. Thus, this syndecan-4-regulated integrin endocytic pathway appears to play a key role in tissue repair.
9. miR-124-regulated RhoG reduces neuronal process complexity via ELMO/Dock180/Rac1 and Cdc42 signalling. The small GTPase RhoG plays a central role in actin remodelling during diverse biological processes such as neurite outgrowth, cell **migration**, phagocytosis of apoptotic cells, and the invasion of pathogenic bacteria. Although it is known that RhoG stimulates neurite outgrowth in the rat pheochromocytoma PC12 cell line, neither the physiological function nor the regulation of this GTPase in neuronal differentiation is clear. Here, we identify RhoG as an inhibitor of neuronal process complexity, which is regulated by the microRNA miR-124. We find that RhoG inhibits dendritic branching in hippocampal neurons in vitro and in vivo. RhoG also inhibits axonal branching, acting via an ELMO/Dock180/Rac1 signalling pathway. However, RhoG inhibits dendritic branching dependent on the small GTPase Cdc42. Finally, we show that the expression of RhoG in neurons is suppressed by the CNS-specific microRNA miR-124 and connect the regulation of RhoG expression by miR-124 to the stimulation of neuronal process complexity. Thus, RhoG emerges as a cellular conductor of Rac1 and Cdc42 activity, in turn regulated by miR-124 to control axonal and dendritic branching.

### [Ee]xtracellular [Mm]atrix

#### PubMed

1. A syndecan-4 hair trigger initiates wound healing through caveolin- and RhoG-regulated integrin endocytosis. Cell migration during wound healing requires adhesion receptor turnover to enable the formation and disassembly of cell-**extracellular matrix** contacts. Although recent advances have improved our understanding of integrin trafficking pathways, it is not known how extracellular ligand engagement controls receptor dynamics. Using atomic force microscopy, we have measured cell avidity for fibronectin and defined a mechanism for the outside-in regulation of α(5)β(1)-integrin. Surprisingly, adhesive strength was attenuated by the syndecan-4-binding domain of fibronectin due to a rapid triggering of α(5)β(1)-integrin endocytosis. Association of syndecan-4 with PKCα was found to trigger RhoG activation and subsequent dynamin- and caveolin-dependent integrin uptake. Like disruption of syndecan-4 or caveolin, gene disruption of RhoG in mice was found to retard closure of dermal wounds due to a migration defect of the fibroblasts and keratinocytes of RhoG null mice. Thus, this syndecan-4-regulated integrin endocytic pathway appears to play a key role in tissue repair.

### [Ii]nvasive

#### PubMed

1. The small GTPase RhoG mediates glioblastoma cell invasion. The invasion of glioblastoma cells into regions of the normal brain is a critical factor that limits current therapies for malignant astrocytomas. Previous work has identified roles for the Rho family guanine nucleotide exchange factors Trio and Vav3 in glioblastoma invasion. Both Trio and Vav3 act on the small GTPase RhoG. We therefore examined the role of RhoG in the **invasive** behavior of glioblastoma cells.We found that siRNA-mediated depletion of RhoG strongly inhibits invasion of glioblastoma cells through brain slices ex vivo. In addition, depletion of RhoG has a marginal effect on glioblastoma cell proliferation, but significantly inhibits glioblastoma cell survival in colony formation assays. We also observed that RhoG is activated by both HGF and EGF, two factors that are thought to be clinically relevant drivers of glioblastoma **invasive** behavior, and that RhoG is overexpressed in human glioblastoma tumors versus non-neoplastic brain. In search of a mechanism for the contribution of RhoG to the malignant behavior of glioblastoma cells, we found that depletion of RhoG strongly inhibits activation of the Rac1 GTPase by both HGF and EGF. In line with this observation, we also show that RhoG contributes to the formation of lamellipodia and invadopodia, two functions that have been shown to be Rac1-dependent.Our functional analysis of RhoG in the context of glioblastoma revealed a critical role for RhoG in tumor cell invasion and survival. These results suggest that targeting RhoG-mediated signaling presents a novel avenue for glioblastoma therapy.

#### GeneRIFs

1. Examined the role of RhoG in the **invasive** behavior of glioblastoma cells; found that depletion of RhoG strongly inhibits activation of the Rac1 GTPase by both HGF and EGF; also RhoG contributes to the formation of lamellipodia and invadopodia.

### [Mm]etastasis

#### PubMed

1. Lack of periostin leads to suppression of Notch1 signaling and calcific aortic valve disease. The Postn gene encodes protein periostin. During embryonic development, it is highly expressed in the outflow tract (OFT) endocardial cushions of the developing heart, which give rise to several structures of the mature heart including the aortic valve. Periostin was previously implicated in osteoblast differentiation, cancer **metastasis**, and tooth and bone development, but its role in cardiac OFT development is unclear. To elucidate the role that periostin plays in the developing heart we analyzed cardiac OFT phenotype in mice after deletion of the Postn gene. We found that lack of periostin in the embryonic OFT leads to ectopic expression of the proosteogenic growth factor pleiotrophin (Ptn) and overexpression of delta-like 1 homolog (Dlk1), a negative regulator of Notch1, in the distal (prevalvular) cushions of the OFT. This resulted in suppression of Notch1 signaling, strong induction of the central transcriptional regulator of osteoblast cell fate Runx2, upregulation of osteopontin and osteocalcin expression, and subsequent calcification of the aortic valve. Our data suggest that periostin represses a default osteogenic program in the OFT cushion mesenchyme and promotes differentiation along a fibrogenic lineage. Lack of periostin causes derepression of the osteogenic potential of OFT mesenchymal cells, calcium deposition, and calcific aortic valve disease. These results establish periostin as a key regulator of OFT endocardial cushion mesenchymal cell fate during embryonic development.
2. P-Rex1 directly activates RhoG to regulate GPCR-driven Rac signalling and actin polarity in neutrophils. G-protein-coupled receptors (GPCRs) regulate the organisation of the actin cytoskeleton by activating the Rac subfamily of small GTPases. The guanine-nucleotide-exchange factor (GEF) P-Rex1 is engaged downstream of GPCRs and phosphoinositide 3-kinase (PI3K) in many cell types, and promotes tumorigenic signalling and **metastasis** in breast cancer and melanoma, respectively. Although P-Rex1-dependent functions have been attributed to its GEF activity towards Rac1, we show that P-Rex1 also acts as a GEF for the Rac-related GTPase RhoG, both in vitro and in GPCR-stimulated primary mouse neutrophils. Furthermore, loss of either P-Rex1 or RhoG caused equivalent reductions in GPCR-driven Rac activation and Rac-dependent NADPH oxidase activity, suggesting they both function upstream of Rac in this system. Loss of RhoG also impaired GPCR-driven recruitment of the Rac GEF DOCK2, and F-actin, to the leading edge of migrating neutrophils. Taken together, our results reveal a new signalling hierarchy in which P-Rex1, acting as a GEF for RhoG, regulates Rac-dependent functions indirectly through RhoG-dependent recruitment of DOCK2. These findings thus have broad implications for our understanding of GPCR signalling to Rho GTPases and the actin cytoskeleton.
3. Integrin-linked kinase interactions with ELMO2 modulate cell polarity. Cell polarization is a key prerequisite for directed migration during development, tissue regeneration, and **metastasis**. Integrin-linked kinase (ILK) is a scaffold protein essential for cell polarization, but very little is known about the precise mechanisms whereby ILK modulates polarization in normal epithelia. Elucidating these mechanisms is essential to understand tissue morphogenesis, transformation, and repair. Here we identify a novel ILK protein complex that includes Engulfment and Cell Motility 2 (ELMO2). We also demonstrate the presence of RhoG in ILK-ELMO2 complexes, and the localization of this multiprotein species specifically to the leading lamellipodia of polarized cells. Significantly, the ability of RhoG to bind ELMO is crucial for ILK induction of cell polarization, and the joint expression of ILK and ELMO2 synergistically promotes the induction of front-rear polarity and haptotactic migration. This places RhoG-ELMO2-ILK complexes in a key position for the development of cell polarity and forward movement. Although ILK is a component of many diverse multiprotein species that may contribute to cell polarization, expression of dominant-negative ELMO2 mutants is sufficient to abolish the ability of ILK to promote cell polarization. Thus, its interaction with ELMO2 and RhoG is essential for the ability of ILK to induce front-rear cell polarity.

### [Cc]ell [Ii]nvasion

#### PubMed

1. The small GTPase RhoG mediates glioblastoma **cell invasion**. The invasion of glioblastoma cells into regions of the normal brain is a critical factor that limits current therapies for malignant astrocytomas. Previous work has identified roles for the Rho family guanine nucleotide exchange factors Trio and Vav3 in glioblastoma invasion. Both Trio and Vav3 act on the small GTPase RhoG. We therefore examined the role of RhoG in the invasive behavior of glioblastoma cells.We found that siRNA-mediated depletion of RhoG strongly inhibits invasion of glioblastoma cells through brain slices ex vivo. In addition, depletion of RhoG has a marginal effect on glioblastoma cell proliferation, but significantly inhibits glioblastoma cell survival in colony formation assays. We also observed that RhoG is activated by both HGF and EGF, two factors that are thought to be clinically relevant drivers of glioblastoma invasive behavior, and that RhoG is overexpressed in human glioblastoma tumors versus non-neoplastic brain. In search of a mechanism for the contribution of RhoG to the malignant behavior of glioblastoma cells, we found that depletion of RhoG strongly inhibits activation of the Rac1 GTPase by both HGF and EGF. In line with this observation, we also show that RhoG contributes to the formation of lamellipodia and invadopodia, two functions that have been shown to be Rac1-dependent.Our functional analysis of RhoG in the context of glioblastoma revealed a critical role for RhoG in tumor **cell invasion** and survival. These results suggest that targeting RhoG-mediated signaling presents a novel avenue for glioblastoma therapy.

---

## F10

- NCBI Gene
- GeneRIF
- Pubmed

| Search Term | Rank | Fields |
| --- | --- | --- |
| [Mm]igration | 6 | PubMed(1) |
| ECM | 7 | PubMed(1) |
| [Ee]xtracellular [Mm]atrix | 8 | PubMed(1) |

  
hide/show details for F10

### [Mm]igration

#### PubMed

1. Factor Xa inhibits HMGB1-induced septic responses in human umbilical vein endothelial cells and in mice. Nuclear DNA-binding protein high mobility group box 1 (HMGB1) acts as a late mediator of severe vascular inflammatory conditions, such as sepsis. Activated factor X (FXa) is an important player in the coagulation cascade responsible for thrombin generation, and it influences cell signalling in various cell types by activating protease-activated receptors (PARs). However, the effect of FXa on HMGB1-induced inflammatory response has not been studied. First, we addressed this issue by monitoring the effects of post-treatment with FXa on lipopolysaccharide (LPS)- and cecal ligation and puncture (CLP)-mediated release of HMGB1 and HMGB1-mediated regulation of pro-inflammatory responses in human umbilical vein endothelial cells (HUVECs) and septic mice. Post-treatment with FXa was found to suppress LPS-mediated release of HMGB1 and HMGB1-mediated cytoskeletal rearrangements. FXa also inhibited HMGB1-mediated hyperpermeability and leukocyte **migration** in septic mice. In addition, FXa inhibited the production of tumour necrosis factor-α and interleukin (IL)-1β. FXa also facilitated the downregulation of CLP-induced release of HMGB1, production of IL-6, and mortality. Collectively, these results suggest that FXa may be regarded as a candidate therapeutic agent for treating vascular inflammatory diseases by inhibiting the HMGB1 signalling pathway.

### ECM

#### PubMed

1. Roles of coagulation pathway and factor Xa in the progression of diabetic nephropathy in db/db mice. The active type of coagulation factor X (factor Xa) activates various cell-types through protease-activated receptor 2 (PAR2). We previously reported that a factor Xa inhibitor could suppress Thy-1 nephritis. Considering that fibrin deposition is observed in diabetic nephropathy as well as in glomerulonephritis, this study examined the roles of the coagulation pathway and factor Xa in the development of diabetic nephropathy using type 2 diabetic model mice. Diabetic (db/db) and normoglycemic (m+/m+) mice were immunohistochemically evaluated for their expression/deposition of PAR2, transforming growth factor (TGF)-β, fibrin, extracellular matrix **(EC**M) proteins, and CD31 at week 20. Significantly greater numbers of PAR2-positive cells and larger amounts of fibronectin, and collagen IV depositions were observed in the glomeruli of db/db mice than those in m+/m+ mice. Next, expression of PAR2 versus deposition of collagen IV and fibronectin was compared between week 20 and week 30, and the number of PAR2-positive cells in the glomeruli decreased in contrast with the increased accumulation of **EC**M proteins. In an intervention study, fondaparinux, a factor Xa inhibitor, was subcutaneously administered for ten weeks from week 10 to 20. Fondaparinux treatment significantly suppressed urinary protein, glomerular hypertrophy, fibrin deposition, expression of connective tissue growth factor, and **EC**M proteins deposition together with CD31-positive capillaries. These results suggest that coagulation pathway and glomerular PAR2 expression are upregulated in the early phase of diabetes, together with the increase of profibrotic cytokines expression, **EC**M proteins deposition and CD-31-positive vessels. Factor Xa inhibition may ameliorate glomerular neoangiogenesis and **EC**M accumulation in diabetic nephropathy.

### [Ee]xtracellular [Mm]atrix

#### PubMed

1. Roles of coagulation pathway and factor Xa in the progression of diabetic nephropathy in db/db mice. The active type of coagulation factor X (factor Xa) activates various cell-types through protease-activated receptor 2 (PAR2). We previously reported that a factor Xa inhibitor could suppress Thy-1 nephritis. Considering that fibrin deposition is observed in diabetic nephropathy as well as in glomerulonephritis, this study examined the roles of the coagulation pathway and factor Xa in the development of diabetic nephropathy using type 2 diabetic model mice. Diabetic (db/db) and normoglycemic (m+/m+) mice were immunohistochemically evaluated for their expression/deposition of PAR2, transforming growth factor (TGF)-β, fibrin, **extracellular matri**x (ECM) proteins, and CD31 at week 20. Significantly greater numbers of PAR2-positive cells and larger amounts of fibronectin, and collagen IV depositions were observed in the glomeruli of db/db mice than those in m+/m+ mice. Next, expression of PAR2 versus deposition of collagen IV and fibronectin was compared between week 20 and week 30, and the number of PAR2-positive cells in the glomeruli decreased in contrast with the increased accumulation of ECM proteins. In an intervention study, fondaparinux, a factor Xa inhibitor, was subcutaneously administered for ten weeks from week 10 to 20. Fondaparinux treatment significantly suppressed urinary protein, glomerular hypertrophy, fibrin deposition, expression of connective tissue growth factor, and ECM proteins deposition together with CD31-positive capillaries. These results suggest that coagulation pathway and glomerular PAR2 expression are upregulated in the early phase of diabetes, together with the increase of profibrotic cytokines expression, ECM proteins deposition and CD-31-positive vessels. Factor Xa inhibition may ameliorate glomerular neoangiogenesis and ECM accumulation in diabetic nephropathy.

---

## Plp2

- NCBI Gene
- GeneRIF
- Pubmed

| Search Term | Rank | Fields |
| --- | --- | --- |
| [Mm]etastasis | 12 | PubMed(1); GeneRIFs(1) |

  
hide/show details for Plp2

### [Mm]etastasis

#### PubMed

1. Proteolipid protein 2 is associated with melanoma **metastasis**. The **metastasis** of malignant tumor cells from the primary tumor to distant sites in the body is a complex process. To identify genes that may be essential for **metastasis**, we established poorly metastatic mouse melanoma cells, namely Y925F-mutated FAK-transfected cells (Y925F cells), from the highly metastatic mouse melanoma cell line B16F10, and performed expression analyses. The expression of phospholipid protein 2 (PLP2) was markedly down-regulated in the Y925F cells. To elucidate the function of PLP2, we established melanoma cells overexpressing PLP2. We found that PLP2 enhanced proliferation, adhesion, invasion, and MMP-2 secretion in vitro, and tumor **metastasis** in vivo. These results suggest that PLP2 aids **metastasis**. Furthermore, we showed that PLP2 binds specifically to PI3K, thus activating Akt.

#### GeneRIFs

1. PLP2 enhanced proliferation, adhesion, invasion, and MMP-2 secretion in vitro, and tumor **metastasis** in vivo.

---

## Cmtm2a

- NCBI Gene
- GeneRIF
- Pubmed

| Search Term | Rank | Fields |
| --- | --- | --- |
| [Mm]igration | 6 | PubMed(1); GeneRIFs(1) |

  
hide/show details for Cmtm2a

### [Mm]igration

#### PubMed

1. Developmental expression of chemokine-like factor 1, a novel member of chemokines family, in postnatal rat cerebral cortex. Chemokine-like factor 1 (CKLF1) has been implicated to induce the **migration** of neuroblastoma cells and is abundant in fetal brain but scarce in adult brain. Given the importance of neural cell **migration** in brain development, it is possible that the chemotaxis of CKLF1 is required during brain development. Therefore, it is essential to know the detailed expression profiles of CKLF1 during brain development first. However, the developmental expression patterns of CKLF1 still remain unclear. We aimed to investigate the temporal and spatial expressions of CKLF1 during cerebral cortex postnatal development in rats. By reverse-transcription PCR/immunoblotting at multiple time points, the mRNA/protein expressions of CKLF1 were in abundance at birth, then decreased progressively within the next two weeks and almost disappeared in adulthood. By immunohistochemistry staining, an obvious expression of CKLF1 was observed in the cerebral cortex, hippocampus, olfactory bulb, some specific nuclei and commissural fibers. Concluding, the temporal expression pattern of CKLF1 was coincident with the postnatal developmental stages and the spatial locations of CKLF1 were some destinations of neural cell **migration** or regions where myelination normally occurs during cerebrum postnatal development.

#### GeneRIFs

1. Concluding, the temporal expression pattern of CKLF1 was coincident with the postnatal developmental stages and the spatial locations of CKLF1 were some destinations of neural cell **migration** or regions where myelination normally occurs.

---

## Vat1l

- NCBI Gene
- GeneRIF
- Pubmed

| Search Term | Rank | Fields |
| --- | --- | --- |
| [Mm]igration | 6 | PubMed(2) |
| [Ee]xtracellular [Mm]atrix | 8 | PubMed(1) |

  
hide/show details for Vat1l

### [Mm]igration

#### PubMed

1. Insm1 promotes endocrine cell differentiation by modulating the expression of a network of genes that includes Neurog3 and Ripply3. Insulinoma associated 1 (Insm1) plays an important role in regulating the development of cells in the central and peripheral nervous systems, olfactory epithelium and endocrine pancreas. To better define the role of Insm1 in pancreatic endocrine cell development we generated mice with an Insm1(GFPCre) reporter allele and used them to study Insm1-expressing and null populations. Endocrine progenitor cells lacking Insm1 were less differentiated and exhibited broad defects in hormone production, cell proliferation and cell **migration**. Embryos lacking Insm1 contained greater amounts of a non-coding Neurog3 mRNA splice variant and had fewer Neurog3/Insm1 co-expressing progenitor cells, suggesting that Insm1 positively regulates Neurog3. Moreover, endocrine progenitor cells that express either high or low levels of Pdx1, and thus may be biased towards the formation of specific cell lineages, exhibited cell type-specific differences in the genes regulated by Insm1. Analysis of the function of Ripply3, an Insm1-regulated gene enriched in the Pdx1-high cell population, revealed that it negatively regulates the proliferation of early endocrine cells. Taken together, these findings indicate that in developing pancreatic endocrine cells Insm1 promotes the transition from a ductal progenitor to a committed endocrine cell by repressing a progenitor cell program and activating genes essential for RNA splicing, cell **migration**, controlled cellular proliferation, vasculogenesis, extracellular matrix and hormone secretion.
2. Identification of Arx transcriptional targets in the developing basal forebrain. Mutations in the aristaless-related homeobox (ARX) gene are associated with multiple neurologic disorders in humans. Studies in mice indicate Arx plays a role in neuronal progenitor proliferation and development of the cerebral cortex, thalamus, hippocampus, striatum, and olfactory bulbs. Specific defects associated with Arx loss of function include abnormal interneuron **migration** and subtype differentiation. How disruptions in ARX result in human disease and how loss of Arx in mice results in these phenotypes remains poorly understood. To gain insight into the biological functions of Arx, we performed a genome-wide expression screen to identify transcriptional changes within the subpallium in the absence of Arx. We have identified 84 genes whose expression was dysregulated in the absence of Arx. This population was enriched in genes involved in cell **migration**, axonal guidance, neurogenesis, and regulation of transcription and includes genes implicated in autism, epilepsy, and mental retardation; all features recognized in patients with ARX mutations. Additionally, we found Arx directly repressed three of the identified transcription factors: Lmo1, Ebf3 and Shox2. To further understand how the identified genes are involved in neural development, we used gene set enrichment algorithms to compare the Arx gene regulatory network (GRN) to the Dlx1/2 GRN and interneuron transcriptome. These analyses identified a subset of genes in the Arx GRN that are shared with that of the Dlx1/2 GRN and that are enriched in the interneuron transcriptome. These data indicate Arx plays multiple roles in forebrain development, both dependent and independent of Dlx1/2, and thus provides further insights into the understanding of the mechanisms underlying the pathology of mental retardation and epilepsy phenotypes resulting from ARX mutations.

### [Ee]xtracellular [Mm]atrix

#### PubMed

1. Insm1 promotes endocrine cell differentiation by modulating the expression of a network of genes that includes Neurog3 and Ripply3. Insulinoma associated 1 (Insm1) plays an important role in regulating the development of cells in the central and peripheral nervous systems, olfactory epithelium and endocrine pancreas. To better define the role of Insm1 in pancreatic endocrine cell development we generated mice with an Insm1(GFPCre) reporter allele and used them to study Insm1-expressing and null populations. Endocrine progenitor cells lacking Insm1 were less differentiated and exhibited broad defects in hormone production, cell proliferation and cell migration. Embryos lacking Insm1 contained greater amounts of a non-coding Neurog3 mRNA splice variant and had fewer Neurog3/Insm1 co-expressing progenitor cells, suggesting that Insm1 positively regulates Neurog3. Moreover, endocrine progenitor cells that express either high or low levels of Pdx1, and thus may be biased towards the formation of specific cell lineages, exhibited cell type-specific differences in the genes regulated by Insm1. Analysis of the function of Ripply3, an Insm1-regulated gene enriched in the Pdx1-high cell population, revealed that it negatively regulates the proliferation of early endocrine cells. Taken together, these findings indicate that in developing pancreatic endocrine cells Insm1 promotes the transition from a ductal progenitor to a committed endocrine cell by repressing a progenitor cell program and activating genes essential for RNA splicing, cell migration, controlled cellular proliferation, vasculogenesis, **extracellular matrix** and hormone secretion.

---

## Serpinf2

- NCBI Gene
- GeneRIF
- Pubmed

| Search Term | Rank | Fields |
| --- | --- | --- |
| [Ff]ilopodia | 1 | PubMed(1) |
| [Mm]igration | 6 | PubMed(2) |
| [Ee]xtracellular [Mm]atrix | 8 | PubMed(3) |
| [Mm]etastasis | 12 | PubMed(2) |
| [Cc]ell [Ii]nvasion | 13 | PubMed(1) |

  
hide/show details for Serpinf2

### [Ff]ilopodia

#### PubMed

1. Involvement of α2-antiplasmin in dendritic growth of hippocampal neurons. The α2-Antiplasmin (α2AP) protein is known as a principal physiological inhibitor of plasmin, but we previously demonstrated that it acts as a regulatory factor for cellular functions independent of plasmin. α2AP is highly expressed in the hippocampus, suggesting a potential role for α2AP in hippocampal neuronal functions. However, the role for α2AP was unclear. This study is the first to investigate the involvement of α2AP in the dendritic growth of hippocampal neurons. The expression of microtubule-associated protein 2, which contributes to neurite initiation and neuronal growth, was lower in the neurons from α2AP⁻/⁻ mice than in the neurons from α2AP⁺/⁺ mice. Exogenous treatment with α2AP enhanced the microtubule-associated protein 2 expression, den**dritic gr**owth and filopodia formation in the neurons. This study also elucidated the mechanism underlying the α2AP-induced dendritic growth. Aprotinin, another plasmin inhibitor, had little effect on the dendritic growth of neurons, and α2AP induced its expression in the neurons from plaminogen⁻/⁻ mice. The activation of p38 MAPK was involved in the α2AP-induced dendritic growth. Therefore, our findings suggest that α2AP induces dendritic growth in hippocampal neurons through p38 MAPK activation, independent of plasmin, providing new insights into the role of α2AP in the CNS.

### [Mm]igration

#### PubMed

1. Alpha(2)-antiplasmin gene deficiency in mice does not affect neointima formation after vascular injury. The hypothesis that alpha(2)-antiplasmin (alpha(2)-AP), the main physiological plasmin inhibitor, plays a role in neointima formation was tested with use of a vascular injury model in wild-type (alpha(2)-AP(+/+)) and alpha(2)-AP-deficient (alpha(2)-AP(-/-)) mice. The neointimal and medial areas were similar 1 to 3 weeks after electric injury of the femoral artery in alpha(2)-AP(+/+) and alpha(2)-AP(-/-) mice, resulting in comparable intima/media ratios (eg, 0.43+/-0.12 and 0.42+/-0.11 2 weeks after injury). Nuclear cell counts in cross-sectional areas of the intima of the injured region were also comparable in arteries from alpha(2)-AP(+/+) and alpha(2)-AP(-/-) mice (78+/-19 and 69+/-8). Fibrin deposition was not significantly different in arteries of both genotypes 1 day after injury, and no mural thrombosis was detected 1 week after injury. Fibrinolytic activity in femoral arterial sections, as monitored by fibrin zymography, was higher in alpha(2)-AP(-/-) mice 1 week after injury (P<0.001) but was comparable in both genotypes 2 and 3 weeks after injury. Staining for elastin did not reveal significant degradation of the internal elastica lamina in either genotype. Immunocytochemical analysis revealed a comparable distribution pattern of alpha-actin-positive smooth muscle cells in both genotypes. These findings indicate that the endogenous fibrinolytic system of alpha(2)-AP(+/+) mice is capable of preventing fibrin deposition after vascular injury and suggest that alpha(2)-AP does not play a major role in smooth muscle cell **migration** and neointima formation in vivo.
2. Antiprotease inactivation by Salmonella enterica released from infected macrophages. The mammalian serine protease plasmin, which has an important role in extracellular matrix degradation during cell **migration**, is regulated by the plasma antiprotease alpha(2)-antiplasmin (alpha(2)AP). The surface protease PgtE of Salmonella enterica serovar Typhimurium proteolytically inactivated alpha(2)AP. PgtE also activates the plasma zymogen plasminogen to plasmin, and bacteria expressing PgtE promoted degradation of extracellular matrix laminin in the presence of plasminogen and alpha(2)AP. alpha(2)AP inactivation was detected with the rough derivative of S. enterica 14028, but not with the smooth wild-type strain, suggesting that the O-antigen of lipopolysaccharide prevented contact of PgtE with the substrate molecule. After growth of S. enterica 14028 in murine J774A.1 macrophage-like cells, the infected cell lysate as well as bacteria from isolated Salmonella-containing vacuoles (SCVs) cleaved alpha(2)AP. Bacteria from SCVs produced an elevated level of PgtE and had a reduced O-antigen chain length. The lysate from S. enterica 14028-infected macrophages promoted formation of plasmin in the presence of alpha(2)AP, whereas plasmin formation by lysates from uninfected macrophages, or from macrophages infected with the pgtE-negative derivative of 14028, was inhibited by alpha(2)AP. Salmonella disseminates in the host within macrophages, which utilize plasmin for **migration** through tissue barriers. The results suggest that intracellular enhancement of PgtE activity in Salmonella may promote macrophage-associated proteolysis and cellular **migration** by altering the balance between host plasmin and alpha(2)AP.

### [Ee]xtracellular [Mm]atrix

#### PubMed

1. alpha2-antiplasmin is associated with the progression of fibrosis. Systemic sclerosis results in tissue fibrosis due to the activation of fibroblasts and the ensuing overproduction of the **extracellular matrix**. We previously reported that the absence of alpha2-antiplasmin (alpha2AP) attenuated the process of dermal fibrosis; however, the detailed mechanism of how alpha2AP affects the progression of fibrosis remained unclear. The goal of the present study was to examine the role of alpha2AP in fibrotic change. We observed significantly higher levels of alpha2AP expression in the skin of bleomycin-injected systemic sclerosis model mice in comparison with the levels seen in control mice. We also demonstrated that alpha2AP induced myofibroblast differentiation, and the absence of alpha2AP attenuated the induction of myofibroblast differentiation. Moreover, we found that connective tissue growth factor induced the expression of alpha2AP through both the extracellular signal-regulated kinase 1/2 (ERK1/2) and c-Jun N-terminal kinase (JNK) pathways in fibroblasts. Interestingly, alpha2AP also induced transforming growth factor-beta expression through the same pathways, and the inhibition of ERK1/2 and JNK slowed the progression of bleomycin-induced fibrosis. Our findings suggest that alpha2AP is associated with the progression of fibrosis, and regulation of alpha2AP expression by the ERK1/2 and JNK pathways may be an effective antifibrotic therapy for the treatment of systemic sclerosis.
2. Lack of alpha 2-antiplasmin promotes re-endothelialization via over-release of VEGF after vascular injury in mice. We here report that the arterial blood flow after endothelial injury in mice deficient in alpha 2-antiplasmin (alpha 2-AP-/- mice) was well maintained compared with that of wild-type mice. Moreover, the development of neointima 4 weeks after injury in alpha 2-AP-/- mice was significantly decreased. Histologic observations showed a prompt recovery of endothelial cells with a much higher proliferating index in repaired endothelium in alpha 2-AP-/- mice. The amount of secreted vascular endothelial growth factor (VEGF) by explanted vascular smooth muscle cells (SMCs) from alpha 2-AP-/- mice was significantly increased. In separate experiments using a human endothelial cell (EC) line, we could demonstrate that plasminogen binds to ECs and that this binding can be prevented by alpha 2-AP. Finally, an injection of either an anti-VEGF receptor-1 antibody or alpha 2-AP reduced the prompt endothelial healing. alpha 2-AP is the main inactivator of plasmin, which cleaves **extracellular matrix**-bound VEGF to release a diffusible proteolytic fragment. Lack of alpha 2-AP, therefore, could lead to a local over-release of VEGF by the continuously active plasmin in the injured area, which could result in a prompt re-endothelialization after vascular injury. Our results provide new insight into the role of alpha 2-AP and VEGF in the pathogenesis of re-endothelialization following vascular injury.
3. Antiprotease inactivation by Salmonella enterica released from infected macrophages. The mammalian serine protease plasmin, which has an important role in **extracellular matrix** degradation during cell migration, is regulated by the plasma antiprotease alpha(2)-antiplasmin (alpha(2)AP). The surface protease PgtE of Salmonella enterica serovar Typhimurium proteolytically inactivated alpha(2)AP. PgtE also activates the plasma zymogen plasminogen to plasmin, and bacteria expressing PgtE promoted degradation of **extracellular matrix** laminin in the presence of plasminogen and alpha(2)AP. alpha(2)AP inactivation was detected with the rough derivative of S. enterica 14028, but not with the smooth wild-type strain, suggesting that the O-antigen of lipopolysaccharide prevented contact of PgtE with the substrate molecule. After growth of S. enterica 14028 in murine J774A.1 macrophage-like cells, the infected cell lysate as well as bacteria from isolated Salmonella-containing vacuoles (SCVs) cleaved alpha(2)AP. Bacteria from SCVs produced an elevated level of PgtE and had a reduced O-antigen chain length. The lysate from S. enterica 14028-infected macrophages promoted formation of plasmin in the presence of alpha(2)AP, whereas plasmin formation by lysates from uninfected macrophages, or from macrophages infected with the pgtE-negative derivative of 14028, was inhibited by alpha(2)AP. Salmonella disseminates in the host within macrophages, which utilize plasmin for migration through tissue barriers. The results suggest that intracellular enhancement of PgtE activity in Salmonella may promote macrophage-associated proteolysis and cellular migration by altering the balance between host plasmin and alpha(2)AP.

### [Mm]etastasis

#### PubMed

1. Update of the human and mouse SERPIN gene superfamily. The serpin family comprises a structurally similar, yet functionally diverse, set of proteins. Named originally for their function as serine proteinase inhibitors, many of its members are not inhibitors but rather chaperones, involved in storage, transport, and other roles. Serpins are found in genomes of all kingdoms, with 36 human protein-coding genes and five pseudogenes. The mouse has 60 Serpin functional genes, many of which are orthologous to human SERPIN genes and some of which have expanded into multiple paralogous genes. Serpins are found in tissues throughout the body; whereas most are extracellular, there is a class of intracellular serpins. Serpins appear to have roles in inflammation, immune function, tumorigenesis, blood clotting, dementia, and cancer **metastasis**. Further characterization of these proteins will likely reveal potential biomarkers and therapeutic targets for disease.
2. Induction of alpha2-antiplasmin inhibits E-cadherin processing mediated by the plasminogen activator/plasmin system, leading to suppression of progression of oral squamous cell carcinoma via upregulation of cell-cell adhesion. The plasminogen activator/plasmin system is one of the main protease systems involved in tumor cell invasion and **metastasis**. Our previous study has shown that plasmin degrades E-cadherin and promotes cell dissemination by downregulation of E-cadherin-mediated cell-cell adhesion in oral squamous cell carcinoma (SCC) cells. To examine the effect of downregulation of the plasminogen activator/plasmin system by alpha2-antiplasmin (alpha2-AP) on cell-cell adhesion mediated by E-cadherin in oral SCC cells, the oral SCC cell line SCCKN was stably transfected with alpha2-AP cDNA. Induction of alpha2-AP expression led to the inhibition of the proteolysis of E-cadherin by plasminogen activator/plasmin in SCC cells, resulting in the enhancement of the cell aggregation and the suppression of the cell motility. Moreover, alpha2-AP also reduced the ability of SCC cells to invade type I collagen gel, and suppressed tumorigenicity in vivo. These results suggested that downregulation of the plasminogen activator/ plasmin system by alpha2-AP might be a potent therapeutic approach to prevent the progression of oral SCC.

### [Cc]ell [Ii]nvasion

#### PubMed

1. Induction of alpha2-antiplasmin inhibits E-cadherin processing mediated by the plasminogen activator/plasmin system, leading to suppression of progression of oral squamous cell carcinoma via upregulation of cell-cell adhesion. The plasminogen activator/plasmin system is one of the main protease systems involved in tumor **cell invasion** and metastasis. Our previous study has shown that plasmin degrades E-cadherin and promotes cell dissemination by downregulation of E-cadherin-mediated cell-cell adhesion in oral squamous cell carcinoma (SCC) cells. To examine the effect of downregulation of the plasminogen activator/plasmin system by alpha2-antiplasmin (alpha2-AP) on cell-cell adhesion mediated by E-cadherin in oral SCC cells, the oral SCC cell line SCCKN was stably transfected with alpha2-AP cDNA. Induction of alpha2-AP expression led to the inhibition of the proteolysis of E-cadherin by plasminogen activator/plasmin in SCC cells, resulting in the enhancement of the cell aggregation and the suppression of the cell motility. Moreover, alpha2-AP also reduced the ability of SCC cells to invade type I collagen gel, and suppressed tumorigenicity in vivo. These results suggested that downregulation of the plasminogen activator/ plasmin system by alpha2-AP might be a potent therapeutic approach to prevent the progression of oral SCC.

---

## Serpind1

- NCBI Gene
- GeneRIF
- Pubmed

| Search Term | Rank | Fields |
| --- | --- | --- |
| [Mm]igration | 6 | PubMed(1) |
| [Ii]nvasive | 10 | PubMed(1) |
| [Mm]etastasis | 12 | PubMed(2) |

  
hide/show details for Serpind1

### [Mm]igration

#### PubMed

1. Heparin cofactor II, a serine protease inhibitor, promotes angiogenesis via activation of the AMP-activated protein kinase-endothelial nitric-oxide synthase signaling pathway. We previously clarified that heparin cofactor II (HCII), a serine proteinase inhibitor, exerts various protective actions on cardiovascular diseases in both experimental and clinical studies. In the present study, we aimed to clarify whether HCII participates in the regulation of angiogenesis. Male heterozygous HCII-deficient (HCII(+/-)) mice and male littermate wild-type (HCII(+/+)) mice at the age of 12-16 weeks were subjected to unilateral hindlimb ligation surgery. Laser speckle blood flow analysis showed that blood flow recovery in response to hindlimb ischemia was delayed in HCII(+/-) mice compared with that in HCII(+/+) mice. Capillary number, arteriole number, and endothelial nitric-oxide synthase (eNOS), AMP-activated protein kinase (AMPK), and liver kinase B1 (LKB1) phosphorylation in ischemic muscles were decreased in HCII(+/-) mice. Human purified HCII (h-HCII) administration almost restored blood flow recovery, capillary density, and arteriole number as well as phosphorylation levels of eNOS, AMPK, and LKB1 in ischemic muscles of HCII(+/-) mice. Although treatment with h-HCII increased phosphorylation levels of eNOS, AMPK, and LKB1 in human aortic endothelial cells (HAECs), the h-HCII-induced eNOS phosphorylation was abolished by compound C, an AMPK inhibitor, and by AMPK siRNA. In a similar fashion, tube formation, proliferation, and **migration** of HAECs were also promoted by h-HCII treatment and were abrogated by pretreatment with compound C. HCII potentiates the activation of vascular endothelial cells and the promotion of angiogenesis in response to hindlimb ischemia via an AMPK-eNOS signaling pathway. These findings suggest that HCII is a novel therapeutic target for treatment of patients with peripheral circulation insufficiency.

### [Ii]nvasive

#### PubMed

1. Proteolytic activation transforms heparin cofactor II into a host defense molecule. The abundant serine proteinase inhibitor heparin cofactor II (HCII) has been proposed to inhibit extravascular thrombin. However, the exact physiological role of this plasma protein remains enigmatic. In this study, we demonstrate a previously unknown role for HCII in host defense. Proteolytic cleavage of the molecule induced a conformational change, thereby inducing endotoxin-binding and antimicrobial properties. Analyses employing representative peptide epitopes mapped these effects to helices A and D. Mice deficient in HCII showed increased susceptibility to **invasive** infection by Pseudomonas aeruginosa, along with a significantly increased cytokine response. Correspondingly, decreased levels of HCII were observed in wild-type animals challenged with bacteria or endotoxin. In humans, proteolytically cleaved HCII forms were detected during wounding and in association with bacteria. Thus, the protease-induced uncovering of cryptic epitopes in HCII, which transforms the molecule into a host defense factor, represents a previously unknown regulatory mechanism in HCII biology and innate immunity.

### [Mm]etastasis

#### PubMed

1. The collection of NFATc1-dependent transcripts in the osteoclast includes numerous genes non-essential to physiologic bone resorption. Osteoclasts are specialized secretory cells of the myeloid lineage important for normal skeletal homeostasis as well as pathologic conditions of bone including osteoporosis, inflammatory arthritis and cancer **metastasis**. Differentiation of these multinucleated giant cells from precursors is controlled by the cytokine RANKL, which through its receptor RANK initiates a signaling cascade culminating in the activation of transcriptional regulators which induce the expression of the bone degradation machinery. The transcription factor nuclear factor of activated T-cells c1 (NFATc1) is the master regulator of this process and in its absence osteoclast differentiation is aborted both in vitro and in vivo. Differential mRNA expression analysis by microarray is used to identify genes of potential physiologic relevance across nearly all biologic systems. We compared the gene expression profile of murine wild-type and NFATc1-deficient osteoclast precursors stimulated with RANKL and identified that the majority of the known genes important for osteoclastic bone resorption require NFATc1 for induction. Here, five novel RANKL-induced, NFATc1-dependent transcripts in the osteoclast are described: Nhedc2, Rhoc, Serpind1, Adcy3 and Rab38. Despite reasonable hypotheses for the importance of these molecules in the bone resorption pathway and their dramatic induction during differentiation, the analysis of mice with mutations in these genes failed to reveal a function in osteoclast biology. Compared to littermate controls, none of these mutants demonstrated a skeletal phenotype in vivo or alterations in osteoclast differentiation or function in vitro. These data highlight the need for rigorous validation studies to complement expression profiling results before functional importance can be assigned to highly regulated genes in any biologic process.
2. Update of the human and mouse SERPIN gene superfamily. The serpin family comprises a structurally similar, yet functionally diverse, set of proteins. Named originally for their function as serine proteinase inhibitors, many of its members are not inhibitors but rather chaperones, involved in storage, transport, and other roles. Serpins are found in genomes of all kingdoms, with 36 human protein-coding genes and five pseudogenes. The mouse has 60 Serpin functional genes, many of which are orthologous to human SERPIN genes and some of which have expanded into multiple paralogous genes. Serpins are found in tissues throughout the body; whereas most are extracellular, there is a class of intracellular serpins. Serpins appear to have roles in inflammation, immune function, tumorigenesis, blood clotting, dementia, and cancer **metastasis**. Further characterization of these proteins will likely reveal potential biomarkers and therapeutic targets for disease.

---

## Serpinf1

- NCBI Gene
- GeneRIF
- Pubmed

| Search Term | Rank | Fields |
| --- | --- | --- |
| [Ff]ilopodia | 1 | PubMed(2) |
| [Mm]igration | 6 | Process(1); PubMed(4) |
| ECM | 7 | PubMed(1) |
| [Ee]xtracellular [Mm]atrix | 8 | PubMed(8); Component(1) |
| [Ii]nvasive | 10 | PubMed(1); GeneRIFs(1) |
| [Mm]etastasis | 12 | PubMed(4); GeneRIFs(1) |

  
hide/show details for Serpinf1

### [Ff]ilopodia

#### PubMed

1. Involvement of α2-antiplasmin in dendritic growth of hippocampal neurons. The α2-Antiplasmin (α2AP) protein is known as a principal physiological inhibitor of plasmin, but we previously demonstrated that it acts as a regulatory factor for cellular functions independent of plasmin. α2AP is highly expressed in the hippocampus, suggesting a potential role for α2AP in hippocampal neuronal functions. However, the role for α2AP was unclear. This study is the first to investigate the involvement of α2AP in the dendritic growth of hippocampal neurons. The expression of microtubule-associated protein 2, which contributes to neurite initiation and neuronal growth, was lower in the neurons from α2AP⁻/⁻ mice than in the neurons from α2AP⁺/⁺ mice. Exogenous treatment with α2AP enhanced the microtubule-associated protein 2 expression, den**dritic gr**owth and filopodia formation in the neurons. This study also elucidated the mechanism underlying the α2AP-induced dendritic growth. Aprotinin, another plasmin inhibitor, had little effect on the dendritic growth of neurons, and α2AP induced its expression in the neurons from plaminogen⁻/⁻ mice. The activation of p38 MAPK was involved in the α2AP-induced dendritic growth. Therefore, our findings suggest that α2AP induces dendritic growth in hippocampal neurons through p38 MAPK activation, independent of plasmin, providing new insights into the role of α2AP in the CNS.
2. Involvement of α2-antiplasmin in dendritic growth of hippocampal neurons. The α2-Antiplasmin (α2AP) protein is known as a principal physiological inhibitor of plasmin, but we previously demonstrated that it acts as a regulatory factor for cellular functions independent of plasmin. α2AP is highly expressed in the hippocampus, suggesting a potential role for α2AP in hippocampal neuronal functions. However, the role for α2AP was unclear. This study is the first to investigate the involvement of α2AP in the dendritic growth of hippocampal neurons. The expression of microtubule-associated protein 2, which contributes to neurite initiation and neuronal growth, was lower in the neurons from α2AP⁻/⁻ mice than in the neurons from α2AP⁺/⁺ mice. Exogenous treatment with α2AP enhanced the microtubule-associated protein 2 expression, den**dritic gr**owth and filopodia formation in the neurons. This study also elucidated the mechanism underlying the α2AP-induced dendritic growth. Aprotinin, another plasmin inhibitor, had little effect on the dendritic growth of neurons, and α2AP induced its expression in the neurons from plaminogen⁻/⁻ mice. The activation of p38 MAPK was involved in the α2AP-induced dendritic growth. Therefore, our findings suggest that α2AP induces dendritic growth in hippocampal neurons through p38 MAPK activation, independent of plasmin, providing new insights into the role of α2AP in the CNS.

### [Mm]igration

#### Process

1. negative regulation of endothelial cell **migration**

#### PubMed

1. Pigment epithelium-derived factor mediates impaired lung vascular development in neonatal hyperoxia. Bronchopulmonary dysplasia is a chronic lung disease of preterm infants characterized by arrested microvascularization and alveolarization. Studies show the importance of proangiogenic factors for alveolarization, but the importance of antiangiogenic factors is unknown. We proposed that hyperoxia increases the potent angiostatin, pigment epithelium-derived factor (PEDF), in neonatal lungs, inhibiting alveolarization and microvascularization. Wild-type (WT) and PEDF(-/-) mice were exposed to room air (RA) or 0.9 fraction of inspired oxygen from Postnatal Day 5 to 13. PEDF protein was increased in hyperoxic lungs compared with RA-exposed lungs (P < 0.05). In situ hybridization and immunofluorescence identified PEDF production primarily in alveolar epithelium. Hyperoxia reduced alveolarization in WT mice (P < 0.05) but not in PEDF(-/-) mice. WT hyperoxic mice had fewer platelet endothelial cell adhesion molecule (PECAM)-positive cells per alveolus (1.4 ± 0.4) than RA-exposed mice (4.3 ± 0.3; P < 0.05); this reduction was absent in hyperoxic PEDF(-/-) mice. The interactive regulation of lung microvascularization by vascular endothelial growth factor and PEDF was studied in vitro using MFLM-91U cells, a fetal mouse lung endothelial cell line. Vascular endothelial growth factor stimulation of proliferation**, migrati**on, and capillary tube formation was inhibited by PEDF. MFLM-91U cells exposed to conditioned medium (CM) from E17 fetal mouse lung type II (T2) cells cultured in 0.9 fraction of inspired oxygen formed fewer capillary tubes than CM from T2 cells cultured in RA (hyperoxia CM, 51 ± 10% of RA CM, P < 0.05), an effect abolished by PEDF antibody. We conclude that PEDF mediates reduced vasculogenesis and alveolarization in neonatal hyperoxia. Bronchopulmonary dysplasia likely results from an altered balance between pro- and antiangiogenic factors.
2. Constitutive overexpression of pigment epithelium-derived factor inhibition of ocular melanoma growth and metastasis. Pigment epithelium-derived factor (PEDF) is known to be an angiogenesis suppressor and to have antitumor effects. This study investigates whether constitutive overexpression of PEDF inhibits the growth and hepatic micrometastasis of ocular melanoma.Real-time RT-PCR was used to detect endogenous PEDF expression in human uveal melanoma cell lines and mouse melanoma cells. A lentiviral vector containing a mouse PEDF expression sequence was constructed and transduced into mouse melanoma cells in vitro. Transgene expression was assessed by Western blot analysis. Angiogenesis and transendothelial **migration** assays were performed in constitutively stable PEDF-overexpressing cells and transduced lentiviral vector control cells. The size and microvessel density of the ocular tumor and the number of hepatic micrometastasis were compared between the mice inoculated with PEDF-overexpressing tumor cells and those mice with the control cell line.Four human uveal melanoma and three mouse melanoma cell lines were found to express PEDF mRNA. Endogenous overexpressing PEDF melanoma cells lost the ability to migrate and form tubes in vitro. In the animal experiment, the size of the ocular melanoma and the number of hepatic micrometastasis were decreased and microvessel density was also reduced in mice inoculated with constitutively overexpressing PEDF melanoma cells.Lentivirus-mediated gene transfer of PEDF decreased the growth of ocular melanoma and its hepatic micrometastasis in a mouse ocular melanoma model. Dual antitumor/antiangiogenic activities of PEDF suggest that PEDF gene therapy may be considered an approach for the treatment of ocular melanoma.
3. PEDF from mouse mesenchymal stem cell secretome attracts fibroblasts. Conditioned medium (secretome) derived from an enriched stem cell culture stimulates chemotaxis of human fibroblasts. These cells are classified as multipotent murine mesenchymal stromal cells (mMSC) by immunochemical analysis of marker proteins. Proteomic analysis of mMSC secretome identifies nineteen secreted proteins, including extracellular matrix structural proteins, collagen processing enzymes, pigment epithelium-derived factor (PEDF) and cystatin C. Immunodepletion and reconstitution experiments show that PEDF is the predominant fibroblast chemoattractant in the conditioned medium, and immunofluorescence microscopy shows strong staining for PEDF in the cytoplasm, at the cell surface, and in intercellular space between mMSCs. This stimulatory effect of PEDF on fibroblast chemotaxis is in contrast to the PEDF-mediated inhibition of endothelial cell **migration**, reported previously. These differential functional effects of PEDF toward fibroblasts and endothelial cells may serve to program an ordered temporal sequence of scaffold building followed by angiogenesis during wound healing.
4. Hormonal regulation of pigment epithelium-derived factor (PEDF) in granulosa cells. Angiogenesis is critical for the development of ovarian follicles. Blood vessels are abrogated from the follicle until ovulation, when they invade it to support the developing corpus luteum. Granulosa cells are known to secrete anti-angiogenic factors that shield against premature vascularization; however, their molecular identity is yet to be defined. In this study we address the physiological role of pigment epithelium-derived factor (PEDF), a well-known angiogenic inhibitor, in granulosa cells. We have shown that human and mouse primary granulosa cells express and secrete PEDF, and characterized its hormonal regulation. Stimulation of granulosa cells with increasing doses of estrogen caused a gradual decrease in the PEDF secretion, while stimulation with progesterone caused an abrupt decrease in its secretion. Moreover, We have shown, by time- and dose-response experiments, that the secreted PEDF and vascular endothelial growth factor (VEGF) were inversely regulated by hCG; namely, PEDF level was nearly undetectable under high doses of hCG, while VEGF level was significantly elevated. The anti-angiogenic nature of the PEDF secreted from granulosa cells was examined by **migration**, proliferation and tube formation assays in cultures of human umbilical vein endothelial cells. Depleting PEDF from primary granulosa cells conditioned media accelerated endothelial cells proliferation, **migration** and tube formation. Collectively, the dynamic expression of PEDF that inversely portrays VEGF expression may imply its putative role as a physiological negative regulator of follicular angiogenesis.

### ECM

#### PubMed

1. Dual-site recognition of different extracellular matrix components by anti-angiogenic/neurotrophic serpin, PEDF. Pigment epithelium-derived factor (PEDF), a member of the serine protease inhibitor (serpin) superfamily, possesses anti-angiogenic and neurotrophic activities. PEDF has been reported to bind to extracellular matrix (**ECM**) components such as collagens and glycosaminoglycans (GAGs). In this study, to determine the binding sites for collagens and GAGs, we analyzed the interaction of recombinant mouse PEDF (rPEDF) with collagen I and heparin. By utilizing residue-specific chemical modification and site-directed mutagenesis techniques, we revealed that the acidic amino acid residues on PEDF (Asp(255), Asp(257), and Asp(299)) are critical to collagen binding, and three clustered basic amino acid residues (Arg(145), Lys(146), and Arg(148)) are necessary for heparin binding. Mapping of these residues on the crystal structure of human PEDF (Simonovic, M., Gettins, P. G. W., and Volz, K. (2001) Proc. Natl. Acad. Sci. U.S.A. 98, 11131-11135) demonstrated that the collagen-binding site is oriented toward the opposite side of the highly basic surface where the heparin-binding site is localized. These results indicate that PEDF possesses dual binding sites for different **ECM** components, and this unique localization of **ECM**-binding sites implies that the binding to **ECM** components could regulate PEDF activities.

### [Ee]xtracellular [Mm]atrix

#### PubMed

1. Forkhead box transcription factor FoxC1 preserves corneal transparency by regulating vascular growth. Normal vision requires the precise control of vascular growth to maintain corneal transparency. Here we provide evidence for a unique mechanism by which the Forkhead box transcription factor FoxC1 regulates corneal vascular development. Murine Foxc1 is essential for development of the ocular anterior segment, and in humans, mutations have been identified in Axenfeld-Rieger syndrome, a disorder characterized by anterior segment dysgenesis. We show that FOXC1 mutations also lead to corneal angiogenesis, and that mice homozygous for either a global (Foxc1(-/-)) or neural crest (NC)-specific (NC-Foxc1(-/-)) null mutation display excessive growth of corneal blood and lymphatic vessels. This is associated with disorganization of the **extracellular matrix** and increased expression of multiple matrix metalloproteinases. Heterozygous mutants (Foxc1(+/-) and NC-Foxc1(+/-)) exhibit milder phenotypes, such as disrupted limbal vasculature. Moreover, environmental exposure to corneal injury significantly increases growth of both blood and lymphatic vessels in both Foxc1(+/-) and NC-Foxc1(+/-) mice compared with controls. Notably, this amplification of the angiogenic response is abolished by inhibition of VEGF receptor 2. Collectively, these findings identify a role for FoxC1 in inhibiting corneal angiogenesis, thereby maintaining corneal transparency by regulating VEGF signaling.
2. **Extracellular matrix** secretion by cardiac fibroblasts: role of microRNA-29b and microRNA-30c. MicroRNAs (miRNAs), in particular miR-29b and miR-30c, have been implicated as important regulators of cardiac fibrosis.To perform a proteomics comparison of miRNA effects on **extracellular matrix** secretion by cardiac fibroblasts.Mouse cardiac fibroblasts were transfected with pre-/anti-miR of miR-29b and miR-30c, and their conditioned medium was analyzed by mass spectrometry. miR-29b targeted a cadre of proteins involved in fibrosis, including multiple collagens, matrix metalloproteinases, and leukemia inhibitory factor, insulin-like growth factor 1, and pentraxin 3, 3 predicted targets of miR-29b. miR-29b also attenuated the cardiac fibroblast response to transforming growth factor-β. In contrast, miR-30c had little effect on **extracellular matri**x production but opposite effects regarding leukemia inhibitory factor and insulin-like growth factor 1. Both miRNAs indirectly affected cardiac myocytes. On transfection with pre-miR-29b, the conditioned medium of cardiac fibroblasts lost its ability to support adhesion of rat ventricular myocytes and led to a significant reduction of cardiac myocyte proteins (α-actinin, cardiac myosin-binding protein C, and cardiac troponin I). Similarly, cardiomyocytes derived from mouse embryonic stem cells atrophied under pre-miR-29 conditioned medium, whereas pre-miR-30c conditioned medium had a prohypertrophic effect. Levels of miR-29a, miR-29c, and miR-30c, but not miR-29b, were significantly reduced in a mouse model of pathological but not physiological hypertrophy. Treatment with antagomiRs to miR-29b induced excess fibrosis after aortic constriction without overt deterioration in cardiac function.Our proteomic analysis revealed novel molecular targets of miRNAs that are linked to a fibrogenic cardiac phenotype. Such comprehensive screening methods are essential to define the concerted actions of miRNAs in cardiovascular disease.
3. Dual-site recognition of different **extracellular matrix** components by anti-angiogenic/neurotrophic serpin, PEDF. Pigment epithelium-derived factor (PEDF), a member of the serine protease inhibitor (serpin) superfamily, possesses anti-angiogenic and neurotrophic activities. PEDF has been reported to bind to **extracellular matrix** (ECM) components such as collagens and glycosaminoglycans (GAGs). In this study, to determine the binding sites for collagens and GAGs, we analyzed the interaction of recombinant mouse PEDF (rPEDF) with collagen I and heparin. By utilizing residue-specific chemical modification and site-directed mutagenesis techniques, we revealed that the acidic amino acid residues on PEDF (Asp(255), Asp(257), and Asp(299)) are critical to collagen binding, and three clustered basic amino acid residues (Arg(145), Lys(146), and Arg(148)) are necessary for heparin binding. Mapping of these residues on the crystal structure of human PEDF (Simonovic, M., Gettins, P. G. W., and Volz, K. (2001) Proc. Natl. Acad. Sci. U.S.A. 98, 11131-11135) demonstrated that the collagen-binding site is oriented toward the opposite side of the highly basic surface where the heparin-binding site is localized. These results indicate that PEDF possesses dual binding sites for different ECM components, and this unique localization of ECM-binding sites implies that the binding to ECM components could regulate PEDF activities.
4. Lack of alpha2-antiplasmin promotes pulmonary heart failure via overrelease of VEGF after acute myocardial infarction. Identification of a novel therapy for prevention of sudden death by ischemic cardiac infarction is an area of intensive investigation. We here report that the mortality due to an experimental acute myocardial infarction (AMI) was markedly increased in mice deficient in alpha2-antiplasmin (alpha2-AP(-/-) mice) but not in mice deficient in other components acting in fibrinolysis (tissue-type PA, urokinase type PA, or plasminogen activator inhibitor-1) even if the infarct area in alpha2-AP(-/-) mice was not different from those in the other mice. Echocardiography showed in alpha2-AP(-/-) mice after AMI an overload of the right ventricle and that pulmonary permeability was increased. According to the experiments using explanted myocytes and vascular smooth muscle cells, it was found that the amount of secreted vascular endothelial cell growth factor (VEGF) in alpha2-AP(-/-) mice was markedly increased compared with that in wild-type mice. Finally, an injection of an anti-VEGF antibody decreased the mortality after AMI in alpha2-AP(-/-) mice. Plasmin cleaves **extracellular matrix**-bound VEGF to release a diffusible proteolytic fragment and is inactivated mainly by alpha2-AP. Therefore, lack of alpha2-AP could markedly result in overrelease of VEGF by the continuous activation of plasmin because of AMI and could result in an acute cor pulmonale. Our results provide new aspects on the role of alpha2-AP and VEGF in the pathogenesis of cardiac events.
5. Isolation, purification, and characterization of a collagen-associated serpin, caspin, produced by murine colon adenocarcinoma cells. A 45-kDa serpin secreted by a murine colon adenocarcinoma cell line, colon26, was isolated, purified, and characterized. It was found to bind specifically to type I collagen with high affinity and to type III collagen with lower affinity. Immunohistochemical studies of murine embryonic tissues showed a specific distribution of this collagen-associated serpin, named caspin, in relation to the formation of bone, cartilage, teeth, and basement membrane. The expression of caspin in high and low lung metastatic subclones of colon26 cell lines was inversely correlated with their metastatic capacity: low lung metastatic cells secreted higher amounts of caspin than their high lung metastatic counterparts. Caspin also demonstrated high homology with human pigment epithelium-derived factor/early population doubling level cDNA-1, which reportedly induces neuronal differentiation of human retinoblastoma cells and is expressed in association with G0 growth arrest. These findings suggest that caspin/pigment epithelium-derived factor/early population doubling level cDNA-1 is a novel factor that might play a crucial role in embryogenesis and tumor metastasis through binding to the **extracellular matrix**.
6. PEDF from mouse mesenchymal stem cell secretome attracts fibroblasts. Conditioned medium (secretome) derived from an enriched stem cell culture stimulates chemotaxis of human fibroblasts. These cells are classified as multipotent murine mesenchymal stromal cells (mMSC) by immunochemical analysis of marker proteins. Proteomic analysis of mMSC secretome identifies nineteen secreted proteins, including **extracellular matrix** structural proteins, collagen processing enzymes, pigment epithelium-derived factor (PEDF) and cystatin C. Immunodepletion and reconstitution experiments show that PEDF is the predominant fibroblast chemoattractant in the conditioned medium, and immunofluorescence microscopy shows strong staining for PEDF in the cytoplasm, at the cell surface, and in intercellular space between mMSCs. This stimulatory effect of PEDF on fibroblast chemotaxis is in contrast to the PEDF-mediated inhibition of endothelial cell migration, reported previously. These differential functional effects of PEDF toward fibroblasts and endothelial cells may serve to program an ordered temporal sequence of scaffold building followed by angiogenesis during wound healing.
7. Pigment epithelium-derived factor (PEDF) shares binding sites in collagen with heparin/heparan sulfate proteoglycans. Pigment epithelium-derived factor (PEDF) is a collagen-binding protein that is abundantly distributed in various tissues, including the eye. It exhibits various biological functions, such as anti-angiogenic, neurotrophic, and neuroprotective activities. PEDF also interacts with **extracellular matrix** components such as collagen, heparan sulfate proteoglycans (HSPGs), and hyaluronan. The collagen-binding property has been elucidated to be important for the anti-angiogenic activity in vivo (Hosomichi, J., Yasui, N., Koide, T., Soma, K., and Morita, I. (2005) Biochem. Biophys. Res. Commun. 335, 756-761). Here, we investigated the collagen recognition mechanism by PEDF. We first narrowed down candidate PEDF-binding sequences by taking advantage of previously reported structural requirements in collagen. Subsequent searches for PEDF-binding sequences employing synthetic collagen-like peptides resulted in the identification of one of the critical binding sites for PEDF, human α1(I)(929-938) (IKGHRGFSGL). Further analysis revealed that the collagen recognition by PEDF is sequence- and conformation-specific, and the high affinity binding motif is KGXRGFXGL in the triple helix. The PEDF-binding motif significantly overlapped with the heparin/HSPG-binding motif, KGHRG(F/Y). The interaction of PEDF with collagen I was specifically competed with by heparin but not by chondroitin sulfate-C or hyaluronan. The binding sequences for PEDF and heparin/HSPG also overlapped with the covalent cross-linking sites between collagen molecules. These findings imply a functional relationship between PEDF and HSPGs during angiogenesis, and the interaction of these molecules is regulated by collagen modifications.
8. Pigment epithelium-derived factor regulates early pancreatic fibrotic responses and suppresses the profibrotic cytokine thrombospondin-1. Pigment epithelium-derived factor (PEDF) is important for maintaining the normal **extracellular matrix**. We hypothesized that the initiation of pancreatic fibrosis is dependent on the loss of PEDF. Pancreatic PEDF expression was assessed in wild-type mice fed either a control or ethanol diet using an intragastric feeding model. Pancreatitis responses were elicited with either a single episode or a repetitive cerulein-induced (50 μg/kg, 6 hourly i.p. injections) protocol in wild-type and PEDF-null mice. Quantitative real-time PCR and immunoblotting were performed to assess fibrogenic responses. In wild-type animals, PEDF expression increased with pancreatitis and was more pronounced in mice fed ethanol. Compared with wild-type mice, α-smooth muscle actin staining and expression levels of fibrogenic markers (eg, transforming growth factor-β1, platelet-derived growth factor, collagen I, and thrombospondin-1) were higher in PEDF-null mice at baseline. Sirius red staining revealed more fibrosis in PEDF-null versus wild-type pancreas 1 week after pancreatitis. Differences in tissue fibrosis resolved with longer recovery periods. PEDF overexpression suppressed thrombospondin-1 levels in vitro. Ethanol feeding and experimental pancreatitis increased PEDF expression in wild-type mice. PEDF-null mice, however, demonstrated enhanced early fibrotic responses compared with wild-type mice with pancreatitis. These findings indicate that PEDF acts as a compensatory antifibrotic cytokine in pancreatitis.

#### Component

1. **extracellular matrix**

### [Ii]nvasive

#### PubMed

1. Concurrent PEDF deficiency and Kras mutation induce **invasive** pancreatic cancer and adipose-rich stroma in mice. Pigment epithelium-derived factor (PEDF), a non-inhibitory SERPIN with potent antiangiogenic activity, has been recently implicated in metabolism and adipogenesis, both of which are known to influence pancreatic cancer progression. Increased pancreatic fat in human pancreatic tumour correlates with greater tumour dissemination while PEDF deficiency in mice promotes pancreatic hyperplasia and visceral obesity. Oncogenic Ras, the most common mutation in pancreatic ductal adenocarcinoma (PDAC), has similarly been shown to promote adipogenesis and premalignant lesions.In order to determine whether concurrent loss of PEDF is sufficient to promote adipogenesis and tumorigenesis in the pancreas, the authors ablated PEDF in an EL-Kras(G12D) mouse model of non-**invasive** cystic papillary neoplasms.EL-Kras(G12D)/PEDF deficient mice developed **invasive** PDAC associated with enhanced matrix metalloproteinase (MMP)-2 and MMP-9 expression and increased peripancreatic fat with adipocyte hypertrophy and intrapancreatic adipocyte infiltration (pancreatic steatosis). In support of increased adipogenesis, the stroma of the pancreas of EL-Kras(G12D)/PEDF deficient mice demonstrated higher tissue levels of two lipid droplet associated proteins, tail-interacting protein 47 (TIP47, perilipin 3) and adipose differentiation-related protein (ADRP, Pperilipin 2), while adipose triglyceride lipase, a key factor in lipolysis, was decreased. In patients with PDAC, both tissue and serum levels of PEDF were decreased, stromal TIP47 expression was higher and the tissue VEGF to PEDF ratio was increased (p<0.05).These data highlight the importance of lipid metabolism in the tumour microenvironment and identify PEDF as a critical negative regulator of both adiposity and tumour invasion in the pancreas.

#### GeneRIFs

1. EL-Kras(G12D)/PEDF deficient mice developed **invasive** pancreatic ductal adenocarcinoma with increased peripancreatic fat with adipocyte hypertrophy and intrapancreatic adipocyte infiltration.

### [Mm]etastasis

#### PubMed

1. Update of the human and mouse SERPIN gene superfamily. The serpin family comprises a structurally similar, yet functionally diverse, set of proteins. Named originally for their function as serine proteinase inhibitors, many of its members are not inhibitors but rather chaperones, involved in storage, transport, and other roles. Serpins are found in genomes of all kingdoms, with 36 human protein-coding genes and five pseudogenes. The mouse has 60 Serpin functional genes, many of which are orthologous to human SERPIN genes and some of which have expanded into multiple paralogous genes. Serpins are found in tissues throughout the body; whereas most are extracellular, there is a class of intracellular serpins. Serpins appear to have roles in inflammation, immune function, tumorigenesis, blood clotting, dementia, and cancer **metastasis**. Further characterization of these proteins will likely reveal potential biomarkers and therapeutic targets for disease.
2. Pigment epithelium-derived factor regulates the vasculature and mass of the prostate and pancreas. Angiogenesis sustains tumor growth and **metastasis**, and recent studies indicate that the vascular endothelium regulates tissue mass. In the prostate, androgens drive angiogenic inducers to stimulate growth, whereas androgen withdrawal leads to decreased vascular endothelial growth factor, vascular regression and epithelial cell apoptosis. Here, we identify the angiogenesis inhibitor pigment epithelium-derived factor (PEDF) as a key inhibitor of stromal vasculature and epithelial tissue growth in mouse prostate and pancreas. In PEDF-deficient mice, stromal vessels were increased and associated with epithelial cell hyperplasia. Androgens inhibited prostatic PEDF expression in cultured cells. In vivo, androgen ablation increased PEDF in normal rat prostates and in human cancer biopsies. Exogenous PEDF induced tumor epithelial apoptosis in vitro and limited in vivo tumor xenograft growth, triggering endothelial apoptosis. Thus, PEDF regulates normal pancreas and prostate mass. Its androgen sensitivity makes PEDF a likely contributor to the anticancer effects of androgen ablation.
3. Isolation, purification, and characterization of a collagen-associated serpin, caspin, produced by murine colon adenocarcinoma cells. A 45-kDa serpin secreted by a murine colon adenocarcinoma cell line, colon26, was isolated, purified, and characterized. It was found to bind specifically to type I collagen with high affinity and to type III collagen with lower affinity. Immunohistochemical studies of murine embryonic tissues showed a specific distribution of this collagen-associated serpin, named caspin, in relation to the formation of bone, cartilage, teeth, and basement membrane. The expression of caspin in high and low lung metastatic subclones of colon26 cell lines was inversely correlated with their metastatic capacity: low lung metastatic cells secreted higher amounts of caspin than their high lung metastatic counterparts. Caspin also demonstrated high homology with human pigment epithelium-derived factor/early population doubling level cDNA-1, which reportedly induces neuronal differentiation of human retinoblastoma cells and is expressed in association with G0 growth arrest. These findings suggest that caspin/pigment epithelium-derived factor/early population doubling level cDNA-1 is a novel factor that might play a crucial role in embryogenesis and tumor **metastasis** through binding to the extracellular matrix.
4. Constitutive overexpression of pigment epithelium-derived factor inhibition of ocular melanoma growth and **metastasis**. Pigment epithelium-derived factor (PEDF) is known to be an angiogenesis suppressor and to have antitumor effects. This study investigates whether constitutive overexpression of PEDF inhibits the growth and hepatic micro**metastasis** of ocular melanoma.Real-time RT-PCR was used to detect endogenous PEDF expression in human uveal melanoma cell lines and mouse melanoma cells. A lentiviral vector containing a mouse PEDF expression sequence was constructed and transduced into mouse melanoma cells in vitro. Transgene expression was assessed by Western blot analysis. Angiogenesis and transendothelial migration assays were performed in constitutively stable PEDF-overexpressing cells and transduced lentiviral vector control cells. The size and microvessel density of the ocular tumor and the number of hepatic micro**metastasis** were compared between the mice inoculated with PEDF-overexpressing tumor cells and those mice with the control cell line.Four human uveal melanoma and three mouse melanoma cell lines were found to express PEDF mRNA. Endogenous overexpressing PEDF melanoma cells lost the ability to migrate and form tubes in vitro. In the animal experiment, the size of the ocular melanoma and the number of hepatic micro**metastasis** were decreased and microvessel density was also reduced in mice inoculated with constitutively overexpressing PEDF melanoma cells.Lentivirus-mediated gene transfer of PEDF decreased the growth of ocular melanoma and its hepatic micro**metastasis** in a mouse ocular melanoma model. Dual antitumor/antiangiogenic activities of PEDF suggest that PEDF gene therapy may be considered an approach for the treatment of ocular melanoma.

#### GeneRIFs

1. Lentivirus-mediated gene transfer of PEDF decreased the growth of ocular melanoma and its hepatic micro**metastasis** in a mouse ocular melanoma model.

---

## Agt

- NCBI Gene
- GeneRIF
- Pubmed

| Search Term | Rank | Fields |
| --- | --- | --- |
| [Mm]igration | 6 | Process(2); PubMed(11); GeneRIFs(1) |
| ECM | 7 | PubMed(7) |
| [Ee]xtracellular [Mm]atrix | 8 | Process(3); PubMed(11); GeneRIFs(1) |
| [Ii]nvasive | 10 | PubMed(1) |
| [Mm]etastasis | 12 | PubMed(2); GeneRIFs(1) |

  
hide/show details for Agt

### [Mm]igration

#### Process

1. positive regulation of endothelial cell **migration**
2. positive regulation of vascular associated smooth muscle cell **migration**

#### PubMed

1. Angiotensin-II and MARCKS: a hydrogen peroxide- and RAC1-dependent signaling pathway in vascular endothelium. MARCKS is an actin-binding protein that modulates vascular endothelial cell **migration** and cytoskeleton signaling (Kalwa, H., and Michel, T. (2011) J. Biol. Chem. 286, 2320-2330). Angiotensin-II is a vasoactive peptide implicated in vascular physiology as well as pathophysiology; the pathways connecting angiotensin-II and cytoskeletal remodeling are incompletely understood. Here we show that MARCKS is expressed in intact arterial preparations, with prominent staining of the endothelium. In endothelial cells, angiotensin-II-promoted MARCKS phosphorylation is abrogated by PEG-catalase, implicating endogenous H(2)O(2) in the angiotensin-II response. Studies using the H(2)O(2) biosensor HyPer2 reveal that angiotensin-II promotes increases in intracellular H(2)O(2). We used a Rac1 FRET biosensor to show that angiotensin-II promotes Rac1 activation that is attenuated by PEG-catalase. siRNA-mediated Rac1 knockdown blocks angiotensin-II-stimulated MARCKS phosphorylation. Cell imaging studies using a phosphoinositide 4,5-bisphosphate (PIP(2)) biosensor revealed that angiotensin-II PIP(2) regulation depends on MARCKS and H(2)O(2). siRNA-mediated knockdown of MARCKS or Rac1 attenuates receptor-mediated activation of the tyrosine kinase c-Abl and disrupts actin fiber formation. These studies establish a critical role for H(2)O(2) in angiotensin-II signaling to the endothelial cytoskeleton in a novel pathway that is critically dependent on MARCKS, Rac1, and c-Abl.
2. Role of Rho-kinase and p27 in angiotensin II-induced vascular injury. Angiotensin II enhances the development of atherosclerotic lesion in which cellular proliferation and/or **migration** are critical steps. Although cyclin-dependent kinase inhibitor, p27, and Rho/Rho-kinase pathway have recently been implicated as factors regulating these events cooperatively, their role in vivo has not been fully elucidated. We evaluated the contribution of p27 and Rho-kinase to angiotensin II-induced vascular injury using p27-deficient mice. Two-week angiotensin II (1500 ng/kg per minute SC) infusion elicited similar degrees of elevation in systolic blood pressure in wild-type mice (159+/-5 mm Hg) and p27-deficient mice (157+/-5 mm Hg; P>0.05). Angiotensin II infusion to wild-type mice resulted in increases in the medial thickness of aorta, proliferating cell number, and monocyte/macrophage infiltration within the vasculature. In p27-deficient mice, however, these changes were more prominent than those in wild-type mice. Treatment of wild-type mice with fasudil, a selective Rho-kinase inhibitor, did not alter blood pressure but significantly upregulated p27 expression, decreased medial thickness of aorta, reduced proliferating cell number, and prevented monocyte/macrophage infiltration. These protective effects of fasudil were attenuated in p27-deficient mice. In conclusion, p27 constitutes an important modulator of angiotensin II-induced monocyte/macrophage infiltration and vascular remodeling, which is mediated in part by Rho-kinase stimulation. Inhibition of Rho-kinase activity improves angiotensin II-induced vascular injury through p27-dependent and p27-independent mechanisms.
3. XIAP-associated factor 1 (XAF1) suppresses angiogenesis in mouse endothelial cells. XIAP-associated factor 1 (XAF1) is a tumor suppressor gene, but its role in angiogenesis is unknown. We investigated whether XAF1 has any antiangiogenesis effect.MS1 (a mouse endothelial cell line) was infected with an adenoviral vector ZD55-XAF1. Controls were uninfected or infected with ZD55-EGFP. Wound healing assay and tube formation assay were used to assess angiogenesis. Cell proliferation was detected by WST-1 assay, and apoptosis was detected by TUNEL and APOPercentagetrade mark assays.ZD55-XAF1 significantly upregulated XAF1, which was associated with decreased cell proliferation, **migration** and tube formation of MS1 cells. Ectopic overexpression of XAF1 induced apoptosis in MS1 and also sensitized cells to 5-fluorouracil-induced apoptosis. A significant decrease in the expression of Tie-1, Ang-1, Ang-2 and c-Myc was observed.Our data suggest that XAF1 possesses a potential antiangiogenesis effect. Suppressed expression of Tie-1, Ang-1, Ang-2 and c-Myc may be mechanistically responsible for the observed antiangiogenesis effect.
4. Angiotensin-II and MARCKS: a hydrogen peroxide- and RAC1-dependent signaling pathway in vascular endothelium. MARCKS is an actin-binding protein that modulates vascular endothelial cell **migration** and cytoskeleton signaling (Kalwa, H., and Michel, T. (2011) J. Biol. Chem. 286, 2320-2330). Angiotensin-II is a vasoactive peptide implicated in vascular physiology as well as pathophysiology; the pathways connecting angiotensin-II and cytoskeletal remodeling are incompletely understood. Here we show that MARCKS is expressed in intact arterial preparations, with prominent staining of the endothelium. In endothelial cells, angiotensin-II-promoted MARCKS phosphorylation is abrogated by PEG-catalase, implicating endogenous H(2)O(2) in the angiotensin-II response. Studies using the H(2)O(2) biosensor HyPer2 reveal that angiotensin-II promotes increases in intracellular H(2)O(2). We used a Rac1 FRET biosensor to show that angiotensin-II promotes Rac1 activation that is attenuated by PEG-catalase. siRNA-mediated Rac1 knockdown blocks angiotensin-II-stimulated MARCKS phosphorylation. Cell imaging studies using a phosphoinositide 4,5-bisphosphate (PIP(2)) biosensor revealed that angiotensin-II PIP(2) regulation depends on MARCKS and H(2)O(2). siRNA-mediated knockdown of MARCKS or Rac1 attenuates receptor-mediated activation of the tyrosine kinase c-Abl and disrupts actin fiber formation. These studies establish a critical role for H(2)O(2) in angiotensin-II signaling to the endothelial cytoskeleton in a novel pathway that is critically dependent on MARCKS, Rac1, and c-Abl.
5. XIAP-associated factor 1 (XAF1) suppresses angiogenesis in mouse endothelial cells. XIAP-associated factor 1 (XAF1) is a tumor suppressor gene, but its role in angiogenesis is unknown. We investigated whether XAF1 has any antiangiogenesis effect.MS1 (a mouse endothelial cell line) was infected with an adenoviral vector ZD55-XAF1. Controls were uninfected or infected with ZD55-EGFP. Wound healing assay and tube formation assay were used to assess angiogenesis. Cell proliferation was detected by WST-1 assay, and apoptosis was detected by TUNEL and APOPercentagetrade mark assays.ZD55-XAF1 significantly upregulated XAF1, which was associated with decreased cell proliferation, **migration** and tube formation of MS1 cells. Ectopic overexpression of XAF1 induced apoptosis in MS1 and also sensitized cells to 5-fluorouracil-induced apoptosis. A significant decrease in the expression of Tie-1, Ang-1, Ang-2 and c-Myc was observed.Our data suggest that XAF1 possesses a potential antiangiogenesis effect. Suppressed expression of Tie-1, Ang-1, Ang-2 and c-Myc may be mechanistically responsible for the observed antiangiogenesis effect.
6. Regulation of muscle satellite cell activation and chemotaxis by angiotensin II. The role of angiotensin II (Ang II) in skeletal muscle is poorly understood. We report that pharmacological inhibition of Ang II signaling or ablation of the AT1a receptor significantly impaired skeletal muscle growth following myotrauma, in vivo, likely due to impaired satellite cell activation and chemotaxis. In vitro experiments demonstrated that Ang II treatment activated quiescent myoblasts as evidenced by the upregulation of myogenic regulatory factors, increased number of β-gal+, Myf5-LacZ myoblasts and the acquisition of cellular motility. Furthermore, exogenous treatment with Ang II significantly increased the chemotactic capacity of C2C12 and primary cells while AT1a(-/-) myoblasts demonstrated a severe impairment in basal **migratio**n and were not responsive to Ang II treatment. Additionally, Ang II interacted with myoblasts in a paracrine-mediated fashion as 4 h of cyclic mechanical stimulation resulted in Ang II-induced **migratio**n of cocultured myoblasts. Ang II-induced chemotaxis appeared to be regulated by multiple mechanisms including reorganization of the actin cytoskeleton and augmentation of MMP2 activity. Collectively, these results highlight a novel role for Ang II and ACE inhibitors in the regulation of skeletal muscle growth and satellite cell function.
7. Unique mechanistic insights into the beneficial effects of soluble epoxide hydrolase inhibitors in the prevention of cardiac fibrosis. Tissue fibrosis represents one of the largest groups of diseases for which there are very few effective therapies. In the heart, myocardial infarction (MI) resulting in the loss of cardiac myocytes can culminate in adverse cardiac remodeling leading to eventual heart failure. Adverse cardiac remodeling includes myocyte hypertrophy, fibrosis, and electrical remodeling. We have previously demonstrated the beneficial effects of several potent soluble epoxide hydrolase inhibitors (sEHIs) in different models of cardiac hypertrophy and failure. Here, we directly determine the molecular mechanisms underlying the beneficial effects of sEHIs in cardiac remodeling post-MI. Treatment with a potent sEHI, 1-trifluoromethoxyphenyl-3-(1-propionylpiperidine-4-yl)urea (TPPU), which was started 1 wk post-MI in a murine model, results in a significant improvement in cardiac function. Importantly, treatment with TPPU results in a decrease in cardiac fibrosis as quantified using histological and immunostaining techniques. Moreover, single-cell-based assays demonstrate that treatment with TPPU results in a significant decrease not only in the percentages but also the proliferative capacity of different populations of cardiac fibroblasts as well as a reduction in the **migration** of fibroblasts into the heart from the bone marrow. Our study provides evidence for a possible unique therapeutic strategy to reduce cardiac fibrosis and improve cardiac function post-MI.
8. Skeletal muscle-endothelial cell cross talk through angiotensin II. The role of angiotensin II (ANG II) in postnatal vasculogenesis and angiogenesis during skeletal muscle (SKM) regeneration is unknown. We examined the capacity of ANG II to stimulate capillary formation and growth during cardiotoxin-induced muscle regeneration in ACE inhibitor-treated ANG II type 1a receptor knockout (AT1a(-/-)) and C57Bl/6 control mice. Analysis of tibialis anterior (TA) cross-sections revealed 17% and 23% reductions in capillarization in AT1a(-/-) and captopril treated mice, respectively, when compared with controls, 21 days postinjury. Conversely, no differences in capillarization were detected at early time points (7 and 10 days). These results identify ANG II as a regulator of angiogenesis but not vasculogenesis in vivo. In vitro angiogenesis assays of human umbilical vein endothelial cells (HUVECs) further confirmed ANG II as proangiogeneic as 71% and 124% increases in tube length and branch point number were observed following ANG II treatment. Importantly, treatment of HUVECs with conditioned media from differentiated muscle cells resulted in an 84% and 203% increase in tube length and branch point number compared with controls, which was abolished following pretreatment of the cells with an angiotensin-converting enzyme inhibitor. The pro-angiogenic effect of ANG II can be attributed to an enhanced endothelial cell **migration** because both transwell and under agarose **migration** assays revealed a 37% and 101% increase in cell motility, respectively. Collectively, these data highlight ANG II as a proangiogenic regulator during SKM regeneration in vivo and more importantly demonstrates that ANG II released from SKM can signal endothelial cells and regulate angiogenesis through the induction of endothelial cell **migration**.
9. Hypoxia-induced endothelial progenitor cell function is blunted in angiotensinogen knockout mice. Angiotensinogen (AGT), the precursor of angiotensin I, is known to be involved in tumor angiogenesis and associated with the pathogenesis of coronary atherosclerosis. This study was undertaken to determine the role played by AGT in endothelial progenitor cells (EPCs) in tumor progression and metastasis. It was found that the number of EPC colonies formed by AGT heterozygous knockout (AGT(+/-)) cells was less than that formed by wild-type (WT) cells, and that the **migration** and tube formation abilities of AGT(+/-) EPCs were significantly lower than those of WT EPCs. In addition, the gene expressions of vascular endothelial growth factor (VEGF), Flk1, angiopoietin (Ang)-1, Ang-2, Tie-2, stromal derived factor (SDF)-1, C-X-C chemokine receptor type 4 (CXCR4), and of endothelial nitric oxide synthase (eNOS) were suppressed in AGT(+/-) EPCs. Furthermore, the expressions of hypoxia-inducible factor (HIF)-1α and -2α were downregulated in AGT(+/-) early EPCs under hypoxic conditions, suggesting a blunting of response to hypoxia. Moreover, the activation of Akt/eNOS signaling pathways induced by VEGF, epithelial growth factor (EGF), or SDF-1α were suppressed in AGT(+/-) EPCs. In AGT(+/-) mice, the incorporation of EPCs into the tumor vasculature was significantly reduced, and lung tumor growth and melanoma metastasis were attenuated. In conclusion, AGT is required for hypoxia-induced vasculogenesis.
10. ACE2 and Ang-(1-7) protect endothelial cell function and prevent early atherosclerosis by inhibiting inflammatory response. Angiotensin-converting enzyme 2 (ACE2) is a counter-regulator against ACE by converting angiotensin II (Ang-II) to Ang-(1-7), but the effect of ACE2 and Ang-(1-7) on endothelial cell function and atherosclerotic evolution is unknown. We hypothesized that ACE2 overexpression and Ang-(1-7) may protect endothelial cell function by counterregulation of angiotensin II signaling and inhibition of inflammatory response.We used a recombinant adenovirus vector to locally overexpress ACE2 gene (Ad-ACE2) in human endothelial cells in vitro and in apoE-deficient mice in vivo. The Ang II-induced MCP-1, VCAM-1 and E-selectin expression, endothelial cell **migration** and adhesion of human monocytic cells (U-937) to HUVECs by ACE2 gene transfer were evaluated in vitro. Accelerated atherosclerosis was studied in vivo, and atherosclerosis was induced in apoE-deficient mice which were divided randomly into four groups that received respectively a ACE2 gene transfer, Ad-ACE2, Ad-EGFP, Ad-ACE2 + A779, an Ang-(1-7) receptor antagonist, control group. After a gene transfer for 4 weeks, atherosclerotic pathology was evaluated.ACE2 gene transfer not only promoted HUVECs **migration**, inhibited adhesion of monocyte to HUVECs and decreased Ang II-induced MCP-1, VCAM-1 and E-selectin protein production in vitro, but also decreased the level of MCP-1, VCAM-1 and interleukin 6 and inhibit atherosclerotic plaque evolution in vivo. Further, administration of A779 increased the level of MCP-1, VCAM-1 and interleukin 6 in vivo and led to further advancements in atherosclerotic extent.ACE2 and Ang-(1-7) significantly inhibit early atherosclerotic lesion formation via protection of endothelial function and inhibition of inflammatory response.
11. Angiotensin II-induced Egr-1 expression is suppressed by peroxisome proliferator-activated receptor-γ ligand 15d-PGJ₂ in macrophages. Angiotensin II (Ang II) plays a critical role in regulating vascular inflammatory diseases, such as atherosclerosis and hypertension. Early growth response-1 (Egr-1) is an immediate early gene that acts as a master switch for the inflammatory response. However, the role of Ang II in regulating Egr-1 expression in macrophages, and the effect of peroxisome proliferators-activated receptor-γ (PPAR-γ) ligand 15d-PGJ2 in this process remain to be investigated.We showed that Ang II significantly up-regulated the expression of Egr-1 in RAW264.7 macrophages, and this effect was markedly attenuated by Eprosartan (an AT1R blocker), SP600125 (a JNK-specific inhibitor) and PD98059 (an ERK-specific inhibitor). Moreover, treatment of macrophages with 15d-PGJ2, a natural PPAR-γ ligand, significantly reduced Ang II-induced expression of Egr-1 and its inflammatory gene targets (IL-1β, TNF-α, TGF-β, MCP-1 and ICAM-1) through PPAR-γ activation and ROS formation. In addition, 15d-PGJ2 treatment markedly inhibited Ang II-induced m**acrophage** migration and proliferation.This study for the first time demonstrates that Ang II can induce the expression of Egr-1 via AT1R/JNK and ERK signaling pathways. Activation of PPAR-γ by 15d-PGJ2 suppresses Egr-1-mediated proinflammatory response, and may be a novel therapeutic strategy for treatment of vascular inflammatory diseases.

#### GeneRIFs

1. ANG II is a proangiogenic regulator during SKM regeneration in vivo; ANG II released from SKM can signal endothelial cells and regulate angiogenesis through the induction of endothelial cell **migration**.

### ECM

#### PubMed

1. Loss of Timp3 gene leads to abdominal aortic aneurysm formation in response to angiotensin II. Aortic aneurysm is dilation of the aorta primarily due to degradation of the aortic wall extracellular matrix (**ECM**). Tissue inhibitors of metalloproteinases (TIMPs) inhibit matrix metalloproteinases (MMPs), the proteases that degrade the **ECM**. Timp3 is the only **ECM**-bound Timp, and its levels are altered in the aorta from patients with abdominal aortic aneurysm (AAA). We investigated the causal role of Timp3 in AAA formation. Infusion of angiotensin II (Ang II) using micro-osmotic (Alzet) pumps in Timp3(-/-) male mice, but not in wild type control mice, led to adverse remodeling of the abdominal aorta, reduced collagen and elastin proteins but not mRNA, and elevated proteolytic activities, suggesting excess protein degradation within 2 weeks that led to formation of AAA by 4 weeks. Intriguingly, despite early up-regulation of MMP2 in Timp3(-/-)Ang II aortas, additional deletion of Mmp2 in these mice (Timp3(-/-)/Mmp2(-/-)) resulted in exacerbated AAA, compromised survival due to aortic rupture, and inflammation in the abdominal aorta. Reconstitution of WT bone marrow in Timp3(-/-)/Mmp2(-/-) mice reduced inflammation and prevented AAA in these animals following Ang II infusion. Treatment with a broad spectrum MMP inhibitor (PD166793) prevented the Ang II-induced AAA in Timp3(-/-) and Timp3(-/-)/Mmp2(-/-) mice. Our study demonstrates that the regulatory function of TIMP3 is critical in preventing adverse vascular remodeling and AAA. Hence, replenishing TIMP3, a physiological inhibitor of a number of metalloproteinases, could serve as a therapeutic approach in limiting AAA development or expansion.
2. Angiotensin II-induced MMP-2 activity and MMP-14 and basigin protein expression are mediated via the angiotensin II receptor type 1-mitogen-activated protein kinase 1 pathway in retinal pigment epithelium: implications for age-related macular degeneration. Accumulation of various lipid-rich extracellular matrix (**ECM**) deposits under the retinal pigment epithelium (RPE) has been observed in eyes with age-related macular degeneration (AMD). RPE-derived matrix metalloproteinase (MMP)-2, MMP-14, and basigin (BSG) are major enzymes involved in the maintenance of **ECM** turnover. Hypertension (HTN) is a systemic risk factor for AMD. It has previously been reported that angiotensin II (Ang II), one of the most important hormones associated with HTN, increases MMP-2 activity and its key regulator, MMP-14, in RPE, inducing breakdown of the RPE basement membrane, which may lead to progression of sub-RPE deposits. Ang II exerts most of its actions by activating the mitogen-activated protein kinase (MAPK) signaling pathway. Herein is explored the MAPK signaling pathway as a potential key intracellular modulator of Ang II-induced increase in MMP-2 activity and MMP-14 and BSG protein expression. It was observed that Ang II stimulates phosphorylation of extracellular signal-regulated kinase (ERK) and p38 MAPK in RPE cells and ERK/p38 and Jun N-terminal kinase (JNK) in mice. These effects were mediated by Ang II type 1 receptors. Blockade of ERK or p38 MAPK abrogated the increase in MMP-2 activity and MMP-14 and BSG proteins in ARPE-19 cells. A better understanding of the molecular events by which Ang II induces **ECM** dysregulation is of critical importance to further define its contribution to the progression of sub-RPE deposits in AMD patients with HTN.
3. Loss of Timp3 gene leads to abdominal aortic aneurysm formation in response to angiotensin II. Aortic aneurysm is dilation of the aorta primarily due to degradation of the aortic wall extracellular matrix (**ECM**). Tissue inhibitors of metalloproteinases (TIMPs) inhibit matrix metalloproteinases (MMPs), the proteases that degrade the **ECM**. Timp3 is the only **ECM**-bound Timp, and its levels are altered in the aorta from patients with abdominal aortic aneurysm (AAA). We investigated the causal role of Timp3 in AAA formation. Infusion of angiotensin II (Ang II) using micro-osmotic (Alzet) pumps in Timp3(-/-) male mice, but not in wild type control mice, led to adverse remodeling of the abdominal aorta, reduced collagen and elastin proteins but not mRNA, and elevated proteolytic activities, suggesting excess protein degradation within 2 weeks that led to formation of AAA by 4 weeks. Intriguingly, despite early up-regulation of MMP2 in Timp3(-/-)Ang II aortas, additional deletion of Mmp2 in these mice (Timp3(-/-)/Mmp2(-/-)) resulted in exacerbated AAA, compromised survival due to aortic rupture, and inflammation in the abdominal aorta. Reconstitution of WT bone marrow in Timp3(-/-)/Mmp2(-/-) mice reduced inflammation and prevented AAA in these animals following Ang II infusion. Treatment with a broad spectrum MMP inhibitor (PD166793) prevented the Ang II-induced AAA in Timp3(-/-) and Timp3(-/-)/Mmp2(-/-) mice. Our study demonstrates that the regulatory function of TIMP3 is critical in preventing adverse vascular remodeling and AAA. Hence, replenishing TIMP3, a physiological inhibitor of a number of metalloproteinases, could serve as a therapeutic approach in limiting AAA development or expansion.
4. Effect of angiotensin II on primary cardiac fibroblast matrix metalloproteinase activities. Left ventricular dysfunction is associated with reperfusion injury occurring during open-heart surgery. There is an increased secretion of angiotensin II (Ang II) and increased matrix metalloproteinases (MMPs) activities associated with open-heart surgery that may affect the cardiac extracellular matrix (**ECM**). The goal of this study was to determine the effects of Ang II and selective angiotensin II receptor (AT1-R and AT2-R) blockers on the enzymatic activities of MMPs in primary adult murine cardiac fibroblasts (CF). Our hypothesis is that Aug II, with and without a selective receptor blocker, differentially affects CF MMPs activities. The CF were treated with Ang II (10(-6) M) and doses of AT1-R and AT2-R blockers (losartan and PD123319, respectively) at doses of 10(-7) to 10(-5) M for 48 hours. The Ang II-stimulated CF reduced collagenase activities by only 24% (p = 0.004); however, the MMP-2 and MMP-9 gelatinase activities were reduced by 42% and 39%, respectively (p = 0.022). The losartan dose dependently increased MMP-2 (p = 0.02) and MMP-9 (ns). PD123319 at 10(-5) M significantly reduced MMP-2 and MMP-9 activities compared with the Ang II group (p = 0.014 and p = 0.02, respectively). The doses of PD123319 at 10(-6) and 10(-7) M increased the MMP-2 and MMP-9 enzymatic activities significantly above the Ang II only group. Thus, Ang II and AT1-R and AT2-R differentially affect the collagenase and gelatinase MMPs activities released by cardiac fibroblasts.
5. Interactions between angiotensin ll and atrial natriuretic peptide in renomedullary interstitial cells: the role of neutral endopeptidase. Neutral endopeptidase (NEP) inhibition attenuates renal damage in the diabetic kidney, but little is known about the mechanisms of this renoprotective effect.We examined the interaction between angiotensin II (Ang II) and atrial natriuretic peptide (ANP) under low (5 mM) and high (30 mM) glucose conditions, on cell proliferation and extracellular matrix (**ECM**) synthesis in renomedullary interstitial cells (RMICs) derived from wild-type (WT) and NEP-deficient (NEP-) mice.Under high glucose conditions, Ang II (10(-6)M) increased cell proliferation (control, 174.3 +/- 16.9; Ang II, 846.3 +/- 91.0 cpm/well) and **ECM** synthesis (control, 22.3 +/- 3.1; Ang II, 79.0 +/- 9.6 cpm/cell) in RMICs derived from WT and NEP- mice to a similar extent. ANP (10(-7)M) reduced Ang II-induced cell proliferation and **ECM** synthesis in RMICs derived from both strains, but more efficiently in RMICs derived from NEP- mice. The Ang II-induced cell proliferation and **ECM** synthesis was attenuated with AT1 receptor blockade, but more efficiently in RMICs-derived NEP- mice.This data shows that ANP and AT1 receptor blockade attenuate Ang II-induced RMIC proliferation and **ECM** synthesis more efficiently in the absence of NEP. These results support the concept that NEP inhibition is beneficial in attenuating abnormal cell growth and **ECM** metabolism associated with diabetic nephropathy.
6. Gene expression profile in the early stage of angiotensin II-induced cardiac remodeling: a time series microarray study in a mouse model. Angiotensin II (Ang II) plays a critical role in the cardiac remodeling contributing to heart failure. However, the gene expression profiles induced by Ang II in the early stage of cardiac remodeling remain unknown.Wild-type male mice (C57BL/6 background, 10-weeek-old) were infused with Ang II (1500 ng/kg/min) for 7 days. Blood pressure was measured. Cardiac function and remodeling were examined by echocardiography, H&E and Masson staining. The time series microarrays were then conducted to detected gene expression profiles.Microarray results identified that 1,489 genes were differentially expressed in the hearts at day 1, 3 and 7 of Ang II injection. These genes were further classified into 26 profiles by hierarchical cluster analysis. Of them, 4 profiles were significant (No. 19, 8, 21 and 22) and contained 904 genes. Gene Ontology showed that these genes mainly participate in metabolic process, oxidation-reduction process, extracellular matrix organization, apoptotic process, immune response, and others. Significant pathways included focal adhesion, **ECM**-receptor interaction, cytokine-cytokine receptor interaction, MAPK and insulin signaling pathways, which were known to play important roles in Ang II-induced cardiac remodeling. Moreover, gene co-expression networks analysis suggested that serine/cysteine peptidase inhibitor, member 1 (Serpine1, also known as PAI-1) localized in the core of the network.Our results indicate that many genes are mainly involved in metabolism, inflammation, cardiac fibrosis and hypertrophy. Serpine1 may play a central role in the development of Ang II-induced cardiac remodeling at the early stage.
7. Plasminogen activator inhibitor-1 deficient mice are protected from angiotensin II-induced fibrosis. PAI-1 has been shown to be both profibrotic and antifibrotic in animal models of hepatic fibrosis. Although these models have similarities to human fibrotic liver disease, no rodent model completely recapitulates the clinical situation; indeed, transaminase values in most models of hepatic fibrosis are much higher than in chronic liver diseases in humans. Here, wild-type and PAI-1(-/-) mice were administered AngII (500 ng/kg/min) for 4 weeks. **ECM** accumulation was evaluated by Sirius red staining, hydroxyproline content, and fibrin and collagen immunostaining. Induction of pro-fibrotic genes was assessed by real-time RT-PCR. Despite the absence of any significant liver damage, AngII infusion increased the deposition of hepatic collagen and fibrin **ECM**, with a perisinusoidal pattern. PAI-1(-/-) mice were protected from these **ECM** changes, indicating a causal role of PAI-1 in this fibrosis model. Protection in the knockout strain correlated with a blunted increase in αSMA, and elevated activities of matrix metalloproteinases (MMP2, MMP9). These data suggest that PAI-1 plays a critical role in mediating fibrosis caused by AngII and lends weight-of-evidence to a pro-fibrotic role of this protein in liver. Furthermore, the current study proposes a new model of 'pure' hepatic fibrosis in mice with little inflammation or hepatocyte death.

### [Ee]xtracellular [Mm]atrix

#### Process

1. **extracellular matrix** organization
2. positive regulation of **extracellular matrix** constituent secretion
3. regulation of **extracellular matrix** assembly

#### PubMed

1. Loss of Timp3 gene leads to abdominal aortic aneurysm formation in response to angiotensin II. Aortic aneurysm is dilation of the aorta primarily due to degradation of the aortic wall **extracellular matrix** (ECM). Tissue inhibitors of metalloproteinases (TIMPs) inhibit matrix metalloproteinases (MMPs), the proteases that degrade the ECM. Timp3 is the only ECM-bound Timp, and its levels are altered in the aorta from patients with abdominal aortic aneurysm (AAA). We investigated the causal role of Timp3 in AAA formation. Infusion of angiotensin II (Ang II) using micro-osmotic (Alzet) pumps in Timp3(-/-) male mice, but not in wild type control mice, led to adverse remodeling of the abdominal aorta, reduced collagen and elastin proteins but not mRNA, and elevated proteolytic activities, suggesting excess protein degradation within 2 weeks that led to formation of AAA by 4 weeks. Intriguingly, despite early up-regulation of MMP2 in Timp3(-/-)Ang II aortas, additional deletion of Mmp2 in these mice (Timp3(-/-)/Mmp2(-/-)) resulted in exacerbated AAA, compromised survival due to aortic rupture, and inflammation in the abdominal aorta. Reconstitution of WT bone marrow in Timp3(-/-)/Mmp2(-/-) mice reduced inflammation and prevented AAA in these animals following Ang II infusion. Treatment with a broad spectrum MMP inhibitor (PD166793) prevented the Ang II-induced AAA in Timp3(-/-) and Timp3(-/-)/Mmp2(-/-) mice. Our study demonstrates that the regulatory function of TIMP3 is critical in preventing adverse vascular remodeling and AAA. Hence, replenishing TIMP3, a physiological inhibitor of a number of metalloproteinases, could serve as a therapeutic approach in limiting AAA development or expansion.
2. Angiotensin II-induced MMP-2 activity and MMP-14 and basigin protein expression are mediated via the angiotensin II receptor type 1-mitogen-activated protein kinase 1 pathway in retinal pigment epithelium: implications for age-related macular degeneration. Accumulation of various lipid-rich **extracellular matrix** (ECM) deposits under the retinal pigment epithelium (RPE) has been observed in eyes with age-related macular degeneration (AMD). RPE-derived matrix metalloproteinase (MMP)-2, MMP-14, and basigin (BSG) are major enzymes involved in the maintenance of ECM turnover. Hypertension (HTN) is a systemic risk factor for AMD. It has previously been reported that angiotensin II (Ang II), one of the most important hormones associated with HTN, increases MMP-2 activity and its key regulator, MMP-14, in RPE, inducing breakdown of the RPE basement membrane, which may lead to progression of sub-RPE deposits. Ang II exerts most of its actions by activating the mitogen-activated protein kinase (MAPK) signaling pathway. Herein is explored the MAPK signaling pathway as a potential key intracellular modulator of Ang II-induced increase in MMP-2 activity and MMP-14 and BSG protein expression. It was observed that Ang II stimulates phosphorylation of extracellular signal-regulated kinase (ERK) and p38 MAPK in RPE cells and ERK/p38 and Jun N-terminal kinase (JNK) in mice. These effects were mediated by Ang II type 1 receptors. Blockade of ERK or p38 MAPK abrogated the increase in MMP-2 activity and MMP-14 and BSG proteins in ARPE-19 cells. A better understanding of the molecular events by which Ang II induces ECM dysregulation is of critical importance to further define its contribution to the progression of sub-RPE deposits in AMD patients with HTN.
3. Loss of Timp3 gene leads to abdominal aortic aneurysm formation in response to angiotensin II. Aortic aneurysm is dilation of the aorta primarily due to degradation of the aortic wall **extracellular matrix** (ECM). Tissue inhibitors of metalloproteinases (TIMPs) inhibit matrix metalloproteinases (MMPs), the proteases that degrade the ECM. Timp3 is the only ECM-bound Timp, and its levels are altered in the aorta from patients with abdominal aortic aneurysm (AAA). We investigated the causal role of Timp3 in AAA formation. Infusion of angiotensin II (Ang II) using micro-osmotic (Alzet) pumps in Timp3(-/-) male mice, but not in wild type control mice, led to adverse remodeling of the abdominal aorta, reduced collagen and elastin proteins but not mRNA, and elevated proteolytic activities, suggesting excess protein degradation within 2 weeks that led to formation of AAA by 4 weeks. Intriguingly, despite early up-regulation of MMP2 in Timp3(-/-)Ang II aortas, additional deletion of Mmp2 in these mice (Timp3(-/-)/Mmp2(-/-)) resulted in exacerbated AAA, compromised survival due to aortic rupture, and inflammation in the abdominal aorta. Reconstitution of WT bone marrow in Timp3(-/-)/Mmp2(-/-) mice reduced inflammation and prevented AAA in these animals following Ang II infusion. Treatment with a broad spectrum MMP inhibitor (PD166793) prevented the Ang II-induced AAA in Timp3(-/-) and Timp3(-/-)/Mmp2(-/-) mice. Our study demonstrates that the regulatory function of TIMP3 is critical in preventing adverse vascular remodeling and AAA. Hence, replenishing TIMP3, a physiological inhibitor of a number of metalloproteinases, could serve as a therapeutic approach in limiting AAA development or expansion.
4. Effect of angiotensin II on primary cardiac fibroblast matrix metalloproteinase activities. Left ventricular dysfunction is associated with reperfusion injury occurring during open-heart surgery. There is an increased secretion of angiotensin II (Ang II) and increased matrix metalloproteinases (MMPs) activities associated with open-heart surgery that may affect the cardiac **extracellular matrix** (ECM). The goal of this study was to determine the effects of Ang II and selective angiotensin II receptor (AT1-R and AT2-R) blockers on the enzymatic activities of MMPs in primary adult murine cardiac fibroblasts (CF). Our hypothesis is that Aug II, with and without a selective receptor blocker, differentially affects CF MMPs activities. The CF were treated with Ang II (10(-6) M) and doses of AT1-R and AT2-R blockers (losartan and PD123319, respectively) at doses of 10(-7) to 10(-5) M for 48 hours. The Ang II-stimulated CF reduced collagenase activities by only 24% (p = 0.004); however, the MMP-2 and MMP-9 gelatinase activities were reduced by 42% and 39%, respectively (p = 0.022). The losartan dose dependently increased MMP-2 (p = 0.02) and MMP-9 (ns). PD123319 at 10(-5) M significantly reduced MMP-2 and MMP-9 activities compared with the Ang II group (p = 0.014 and p = 0.02, respectively). The doses of PD123319 at 10(-6) and 10(-7) M increased the MMP-2 and MMP-9 enzymatic activities significantly above the Ang II only group. Thus, Ang II and AT1-R and AT2-R differentially affect the collagenase and gelatinase MMPs activities released by cardiac fibroblasts.
5. Loss of angiotensin-converting enzyme-2 exacerbates diabetic cardiovascular complications and leads to systolic and vascular dysfunction: a critical role of the angiotensin II/AT1 receptor axis. Diabetic cardiovascular complications are reaching epidemic proportions. Angiotensin-converting enzyme-2 (ACE2) is a negative regulator of the renin-angiotensin system. We hypothesize that loss of ACE2 exacerbates cardiovascular complications induced by diabetes.To define the role of ACE2 in diabetic cardiovascular complications.We used the well-validated Akita mice, a model of human diabetes, and generated double-mutant mice using the ACE2 knockout (KO) mice (Akita/ACE2(-/y)). Diabetic state was associated with increased ACE2 in Akita mice, whereas additional loss of ACE2 in these mice leads to increased plasma and tissue angiotensin II levels, resulting in systolic dysfunction on a background of impaired diastolic function. Downregulation of SERCA2 and lipotoxicity were equivalent in Akita and Akita/ACE2KO hearts and are likely mediators of the diastolic dysfunction. However, greater activation of protein kinase C and loss of Akt and endothelial nitric oxide synthase phosphorylation occurred in the Akita/ACE2KO hearts. Systolic dysfunction in Akita/ACE2KO mice was linked to enhanced activation of NADPH oxidase and metalloproteinases, resulting in greater oxidative stress and degradation of the **extracellular matrix**. Impaired flow-mediated dilation in vivo correlated with increased vascular oxidative stress in Akita/ACE2KO mice. Treatment with the AT1 receptor blocker, irbesartan rescued the systolic dysfunction, normalized altered signaling pathways, flow-mediated dilation, and the increased oxidative stress in the cardiovascular system.Loss of ACE2 disrupts the balance of the renin-angiotensin system in a diabetic state and leads to an angiotensin II/AT1 receptor-dependent systolic dysfunction and impaired vascular function. Our study demonstrates that ACE2 serves as a protective mechanism against diabetes-induced cardiovascular complications.
6. Enhanced expression of fibrillin-1, a constituent of the myocardial **extracellular matrix** in fibrosis. Fibrillin-1 localization in the myocardium and the modulation of its expression in cardiac fibrosis were examined. In normal rat hearts, fibrillin-1 was abundant throughout the myocardium as thin fibers that crossed over the perimysium and around arteries. After cardiac fibrosis was induced in rats by either 14-day ANG II infusion or 21-day DOCA-salt treatment [a high endothelin-1 (ET-1) model], fibrillin-1 immunostaining was stronger in the interstitium (2.8-fold and 4.4-fold increases, respectively, in each model), extended between myocytes, and accumulated in microscopic scars and in the perivascular area of both ventricles. mRNA analysis confirmed its enhanced ventricular expression in both groups of rats (2.5-fold and 6.6-fold increments, respectively, in each model). In 1B normotensive and 2C hypertensive transgenic mice, two lines expressing an ANG II fusion protein in cardiac myocytes, strong fibrillin-1 immunoreactivity was observed in the interstitium and around arteries (3.7-fold and 7-fold increases, respectively). ANG II and transforming growth factor-beta1 enhanced fibrillin-1 synthesis by cardiac fibroblasts. Some fibrillin-1 fragments interacted with RGD-dependent integrins, including alpha(8)beta(1)-integrin, of cardiac fibroblasts but not necessarily through the RGD motif. Our findings illustrate that fibrillin-1 is an important constituent of the myocardium. In vitro and in vivo evidence suggests that ANG II can directly induce fibrillin-1 expression in cardiac fibroblasts. This protein can thus contribute to reactive and reparative processes.
7. Interactions between angiotensin ll and atrial natriuretic peptide in renomedullary interstitial cells: the role of neutral endopeptidase. Neutral endopeptidase (NEP) inhibition attenuates renal damage in the diabetic kidney, but little is known about the mechanisms of this renoprotective effect.We examined the interaction between angiotensin II (Ang II) and atrial natriuretic peptide (ANP) under low (5 mM) and high (30 mM) glucose conditions, on cell proliferation and **extracellular matrix** (ECM) synthesis in renomedullary interstitial cells (RMICs) derived from wild-type (WT) and NEP-deficient (NEP-) mice.Under high glucose conditions, Ang II (10(-6)M) increased cell proliferation (control, 174.3 +/- 16.9; Ang II, 846.3 +/- 91.0 cpm/well) and ECM synthesis (control, 22.3 +/- 3.1; Ang II, 79.0 +/- 9.6 cpm/cell) in RMICs derived from WT and NEP- mice to a similar extent. ANP (10(-7)M) reduced Ang II-induced cell proliferation and ECM synthesis in RMICs derived from both strains, but more efficiently in RMICs derived from NEP- mice. The Ang II-induced cell proliferation and ECM synthesis was attenuated with AT1 receptor blockade, but more efficiently in RMICs-derived NEP- mice.This data shows that ANP and AT1 receptor blockade attenuate Ang II-induced RMIC proliferation and ECM synthesis more efficiently in the absence of NEP. These results support the concept that NEP inhibition is beneficial in attenuating abnormal cell growth and ECM metabolism associated with diabetic nephropathy.
8. Apocynin attenuates tubular apoptosis and tubulointerstitial fibrosis in transgenic mice independent of hypertension. Angiotensin II stimulates the formation of reactive oxygen species by increased NADPH oxidase activity, which contributes to proapoptotic and profibrotic mechanisms critical in renal injury. Here we determine if apocynin, an inhibitor of NADPH oxidase, interferes with the action of the intrarenal renin-angiotensin system to minimize the progression of renal disease. Transgenic mice that overexpress rat angiotensinogen in their proximal tubule cells were given either apocynin, perindopril, or hydralazine while untreated or apocynin-treated non-transgenic littermates served as controls. Untreated transgenic mice had significant elevations of their systolic blood pressure, albuminuria, reactive oxygen species production, NADPH oxidase activity, tubular apoptosis, active caspase-3, Bax, transforming growth factor-beta1, plasminogen activator inhibitor-1, **extracellular matrix** proteins, collagen type IV, and phosphorylated p47phox expression compared to untreated non-transgenic mice. Apocynin and perindopril blunted these changes; however, apocynin had no effect on the systolic blood pressure whereas hydralazine prevented hypertension and tubulointerstitial fibrosis but not proximal tubule cell apoptosis. Our study shows that the intrarenal renin-angiotensin system stimulates proximal tubule cell apoptosis and tubulointerstitial fibrosis, in part, by enhanced NADPH oxidase activity and reactive oxygen species generation independent of systemic hypertension.
9. Angiotensin II upregulates toll-like receptor 4 on mesangial cells. Angiotensin II (AngII) mediates proinflammatory properties by activating NF-kappaB transcription factor nuclear translocation and inducing the expression of chemokines. For examination of whether AngII modulates the expression of Toll-like receptor 4 (TLR4), a key element of the innate immune system that senses LPS, mouse mesangial cells (MMC) were treated with AngII. AngII upregulated TLR4 mRNA and protein in MMC, and this effect was mediated through AngII type 1 receptors. Reporter gene experiments indicate that an activating protein-1 (AP-1) as well as an E-26 specific sequence (Ets) binding site in the TLR4 promoter are responsible for the AngII-stimulated transcriptional activity of the TLR4 gene. Preincubation of MMC with AngII enhanced LPS-induced NF-kappaB activation and chemokine expression. Immunohistochemical analyses revealed that double-transgenic rats that overexpressed human renin and angiotensinogen expressed higher levels of glomerular TLR4 compared with normal Sprague-Dawley rats. In vivo, infusion with AngII but not with norepinephrine into rats for 7 d also enhanced glomerular NF-kappaB activation after systemic application of LPS, suggesting that the effects are independent of concomitantly induced hypertension. Together, these observations suggest that AngII leads to an activation of the innate immune system by a novel mechanism involving the upregulation of TLR4. Our data contribute to a better understanding of how exogenous infections may trigger renal autoimmune processes, particularly in pathophysiologic situations with high renal AngII concentrations. Because TLR4 binds endogenous ligands (e.g., **extracellular matrix** components) in addition to microbial products, AngII-mediated upregulation of TLR4 also could be relevant for the development of inflammation in many noninfectious renal diseases.
10. Gene expression profile in the early stage of angiotensin II-induced cardiac remodeling: a time series microarray study in a mouse model. Angiotensin II (Ang II) plays a critical role in the cardiac remodeling contributing to heart failure. However, the gene expression profiles induced by Ang II in the early stage of cardiac remodeling remain unknown.Wild-type male mice (C57BL/6 background, 10-weeek-old) were infused with Ang II (1500 ng/kg/min) for 7 days. Blood pressure was measured. Cardiac function and remodeling were examined by echocardiography, H&E and Masson staining. The time series microarrays were then conducted to detected gene expression profiles.Microarray results identified that 1,489 genes were differentially expressed in the hearts at day 1, 3 and 7 of Ang II injection. These genes were further classified into 26 profiles by hierarchical cluster analysis. Of them, 4 profiles were significant (No. 19, 8, 21 and 22) and contained 904 genes. Gene Ontology showed that these genes mainly participate in metabolic process, oxidation-reduction process, **extracellular matrix** organization, apoptotic process, immune response, and others. Significant pathways included focal adhesion, ECM-receptor interaction, cytokine-cytokine receptor interaction, MAPK and insulin signaling pathways, which were known to play important roles in Ang II-induced cardiac remodeling. Moreover, gene co-expression networks analysis suggested that serine/cysteine peptidase inhibitor, member 1 (Serpine1, also known as PAI-1) localized in the core of the network.Our results indicate that many genes are mainly involved in metabolism, inflammation, cardiac fibrosis and hypertrophy. Serpine1 may play a central role in the development of Ang II-induced cardiac remodeling at the early stage.
11. Impaired blood-brain barrier function in angiotensinogen-deficient mice. Astrocytes in the central nervous system have physiologically important roles in the response to brain injury. Brain damage results in disruption of the blood-brain barrier (BBB), producing detachment of astrocyte endfeet from endothelial cells. The resultant leakage of serum proteins from loosened tight junctions between endothelial cells produces brain edema. At the same time, reactive astrocytes migrate to the injured area, where they proliferate and produce **extracellular matrix**, thereby reconstituting the BBB. As astrocytes are known to express angiotensinogen, which is the precursor of angiotensins (AI to AIV), we have investigated a possible functional contribution of angiotensinogen or one of its metabolites to BBB reconstitution. The astrocytes of angiotensinogen knockout mice had very attenuated expression of glial fibrially acidic protein and decreased laminin production in response to cold injury, and ultimately incomplete reconstitution of impaired BBB function. Although these abnormalities were rescued by administration of AII or AIV, the restoration of BBB function was not inhibited by AII type 1 and 2 receptor antagonists. These findings provide evidence that astrocytes with angiotensins are required for functional maintenance of the BBB.

#### GeneRIFs

1. ANP and AT1 receptor blockade attenuate angiotensin II-induced renomedullary interstitial cells proliferation and **extracellular matrix** synthesis more efficiently in the absence of neutral endopeptidase.

### [Ii]nvasive

#### PubMed

1. Protein microarrays discover angiotensinogen and PRKRIP1 as novel targets for autoantibodies in chronic renal disease. Biomarkers for early detection of chronic kidney disease are needed, as millions of patients suffer from chronic diseases predisposing them to kidney failure. Protein microarrays may also hold utility in the discovery of auto-antibodies in other conditions not commonly considered auto-immune diseases. We hypothesized that proteins are released as a consequence of damage at a cellular level during end-organ damage from renal injury, not otherwise recognized as self-antigens, and an adaptive humoral immune response to these proteins might be detected in the blood, as a non**invasive** tracker of this injury. The resultant antibodies (Ab) detected in the blood would serve as effective biomarkers for occult renal injury, enabling earlier clinical detection of chronic kidney disease than currently possible, because of the redundancy of the serum creatinine as a biomarker for early kidney injury. To screen for novel autoantibodies in chronic kidney disease, 24 protein microarrays were used to compare serum Ab from patients with chronic kidney disease against matched controls. From a panel of 38 antigens with increased Ab binding, four were validated in 71 individuals, with (n=50) and without (n=21) renal insufficiency. Significant elevations in the titer of novel auto-Ab were noted against angiotensinogen and PRKRIP1 in renal insufficiency. Current validation is underway to evaluate if these auto-Ab can provide means to follow the evolution of chronic kidney disease in patients with early stages of renal insufficiency, and if these rising titers of these auto-Ab correlate with the rate of progression of chronic kidney disease.

### [Mm]etastasis

#### PubMed

1. Update of the human and mouse SERPIN gene superfamily. The serpin family comprises a structurally similar, yet functionally diverse, set of proteins. Named originally for their function as serine proteinase inhibitors, many of its members are not inhibitors but rather chaperones, involved in storage, transport, and other roles. Serpins are found in genomes of all kingdoms, with 36 human protein-coding genes and five pseudogenes. The mouse has 60 Serpin functional genes, many of which are orthologous to human SERPIN genes and some of which have expanded into multiple paralogous genes. Serpins are found in tissues throughout the body; whereas most are extracellular, there is a class of intracellular serpins. Serpins appear to have roles in inflammation, immune function, tumorigenesis, blood clotting, dementia, and cancer **metastasis**. Further characterization of these proteins will likely reveal potential biomarkers and therapeutic targets for disease.
2. Hypoxia-induced endothelial progenitor cell function is blunted in angiotensinogen knockout mice. Angiotensinogen (AGT), the precursor of angiotensin I, is known to be involved in tumor angiogenesis and associated with the pathogenesis of coronary atherosclerosis. This study was undertaken to determine the role played by AGT in endothelial progenitor cells (EPCs) in tumor progression and **metastasis**. It was found that the number of EPC colonies formed by AGT heterozygous knockout (AGT(+/-)) cells was less than that formed by wild-type (WT) cells, and that the migration and tube formation abilities of AGT(+/-) EPCs were significantly lower than those of WT EPCs. In addition, the gene expressions of vascular endothelial growth factor (VEGF), Flk1, angiopoietin (Ang)-1, Ang-2, Tie-2, stromal derived factor (SDF)-1, C-X-C chemokine receptor type 4 (CXCR4), and of endothelial nitric oxide synthase (eNOS) were suppressed in AGT(+/-) EPCs. Furthermore, the expressions of hypoxia-inducible factor (HIF)-1α and -2α were downregulated in AGT(+/-) early EPCs under hypoxic conditions, suggesting a blunting of response to hypoxia. Moreover, the activation of Akt/eNOS signaling pathways induced by VEGF, epithelial growth factor (EGF), or SDF-1α were suppressed in AGT(+/-) EPCs. In AGT(+/-) mice, the incorporation of EPCs into the tumor vasculature was significantly reduced, and lung tumor growth and melano**ma metasta**sis were attenuated. In conclusion, AGT is required for hypoxia-induced vasculogenesis.

#### GeneRIFs

1. Results suggest that AGT is importantly required for the participation of endothelial progenitor cells in tumor neovascularization and **metastasis**.

---

## Serpinb7

- NCBI Gene
- GeneRIF
- Pubmed

| Search Term | Rank | Fields |
| --- | --- | --- |
| ECM | 7 | PubMed(1) |
| [Ee]xtracellular [Mm]atrix | 8 | PubMed(1) |
| [Mm]etastasis | 12 | PubMed(1) |

  
hide/show details for Serpinb7

### ECM

#### PubMed

1. Overexpression of the serpin megsin induces progressive mesangial cell proliferation and expansion. Mesangial cells maintain normal glomerular function by mediating **ECM** remodeling and immune complex disposal. We have recently identified megsin, a novel member of the serine protease inhibitor (serpin) superfamily predominantly expressed in the mesangium. While our previous studies suggested a role for megsin in the pathogenesis of human glomerular diseases, its exact biological significance remained unknown. Here we produced two lines of megsin transgenic mice. Overexpression of megsin led to progressive mesangial matrix expansion and an increase in the number of mesangial cells. These glomerular lesions were accompanied by an augmented immune complex deposition, together with Ig's and complement. Binding and functional assays in vitro identified plasmin as one biological substrate of megsin and confirmed its activity as a proteinase inhibitor. Transgenic animals exhibiting nephritis as a result of treatment with anti--glomerular basement membrane antiserum showed significantly more persistent expansion of the mesangial **ECM** than was seen in parental mice. Megsin therefore exerts a biologically relevant influence on mesangial function, and on the mesangial microenvironment, such that simple overexpression of this endogenous serpin engenders elementary mesangial lesions.

### [Ee]xtracellular [Mm]atrix

#### PubMed

1. Cloning of rodent megsin revealed its up-regulation in mesangioproliferative nephritis. We recently cloned a new human mesangium-predominant gene, megsin. Megsin is a novel member of the serine protease inhibitor (serpin) superfamily. To elucidate functional roles of this gene, we cloned megsin in rodents and investigated its role in a rat nephritis model.Megsin homologues were cloned from cultured rat and mouse mesangial cDNAs utilizing polymerase chain reaction (PCR) with degenerative primers. Expression of megsin in three different types of resident glomerular cells was investigated by PCR. Levels of megsin mRNA expression at various time points in the anti-Thy1 rat nephritis model were studied by semiquantitative PCR and Northern blotting analysis. In order to investigate megsin protein expression in anti-Thy1 nephritis rats, we raised antibody against rat megsin-specific synthetic peptide, with which immunohistochemical studies were performed.Rat and mouse megsins were composed of 380 amino acids, which revealed 75.3 and 73.9% identity, respectively, with human megsin at the amino acid level. Characteristic features as an inhibitory serpin were conserved in both rat and megsin megsins. PCR analysis revealed expression of megsin in cultured mesangial cells but not in glomerular epithelial or endothelial cells. In anti-Thy1 nephritis rats, semiquantitative PCR and Northern blotting showed that expression of megsin mRNA was up-regulated in glomeruli at day 8. Immunohistochemical studies demonstrated the prominent accumulation of megsin in glomeruli at the same time point. Megsin was mainly localized in mesangial area. The megsin expression level returned to the basal level at day 28.Sequences of megsin were well conserved among different species. Rat megsin was also predominantly expressed in mesangial cells. Expression of megsin was up-regulated at the peak of hypercellularity and matrix accumulation in the mesangioproliferative glomerulonephritis model, suggesting that megsin may participate in the process of glomerulosclerosis by modulating **extracellular matrix** deposition or cell survival.

### [Mm]etastasis

#### PubMed

1. Update of the human and mouse SERPIN gene superfamily. The serpin family comprises a structurally similar, yet functionally diverse, set of proteins. Named originally for their function as serine proteinase inhibitors, many of its members are not inhibitors but rather chaperones, involved in storage, transport, and other roles. Serpins are found in genomes of all kingdoms, with 36 human protein-coding genes and five pseudogenes. The mouse has 60 Serpin functional genes, many of which are orthologous to human SERPIN genes and some of which have expanded into multiple paralogous genes. Serpins are found in tissues throughout the body; whereas most are extracellular, there is a class of intracellular serpins. Serpins appear to have roles in inflammation, immune function, tumorigenesis, blood clotting, dementia, and cancer **metastasis**. Further characterization of these proteins will likely reveal potential biomarkers and therapeutic targets for disease.

---

## Serpina3a

- NCBI Gene
- GeneRIF
- Pubmed

| Search Term | Rank | Fields |
| --- | --- | --- |
| [Mm]etastasis | 12 | PubMed(1) |

  
hide/show details for Serpina3a

### [Mm]etastasis

#### PubMed

1. Update of the human and mouse SERPIN gene superfamily. The serpin family comprises a structurally similar, yet functionally diverse, set of proteins. Named originally for their function as serine proteinase inhibitors, many of its members are not inhibitors but rather chaperones, involved in storage, transport, and other roles. Serpins are found in genomes of all kingdoms, with 36 human protein-coding genes and five pseudogenes. The mouse has 60 Serpin functional genes, many of which are orthologous to human SERPIN genes and some of which have expanded into multiple paralogous genes. Serpins are found in tissues throughout the body; whereas most are extracellular, there is a class of intracellular serpins. Serpins appear to have roles in inflammation, immune function, tumorigenesis, blood clotting, dementia, and cancer **metastasis**. Further characterization of these proteins will likely reveal potential biomarkers and therapeutic targets for disease.

---

## Cd226

- NCBI Gene
- GeneRIF
- Pubmed

| Search Term | Rank | Fields |
| --- | --- | --- |
| [Mm]igration | 6 | PubMed(2) |

  
hide/show details for Cd226

### [Mm]igration

#### PubMed

1. Expression and possible role of PVR/CD155/Necl-5 in osteoclastogenesis. Osteoclasts, the bone-resorbing cells, are differentiated from hematopoietic precursors via two-step cell-cell interactions. One is the interaction between the osteoclast precursor and the stromal cell to initiate differentiation. The other is the interaction among osteoclast precursors to form multinucleated osteoclasts. Recently, the poliovirus receptor (PVR, CD155, Necl-5) was reported to play important roles in cell adhesion and **migration**. However, there are no reports of PVR in osteoclastogenesis. In this paper, we examined the expression of PVR and its ligand, DNAX accessory molecule-1 (DNAM-1, CD226), in osteoclast precursors, mature osteoclasts, and stromal cells. We found that the PVR was constitutively expressed in both osteoclast cells and stromal cells. The expression of PVR was not changed at various stages of osteoclast formation. In contrast, the expression of DNAM-1 was observed in mononuclear cells and was down-regulated during osteoclastogenesis. Moreover, multinucleated osteoclast formation was inhibited by treatment with the extracellular domain of DNAM-1 (ED-DNAM-1) as a soluble ligand for PVR, but mononuclear preosteoclast formation was not affected. Especially, during the 7-day cultivation, osteoclast formation was suppressed by the treatment with ED-DNAM-1 on days 6 and 7, when the mononuclear preosteoclasts fused into multinucleated osteoclasts. This suppression was abrogated partially by a small interfering RNA specific for PVR. These results suggest that, at least in part, the binding of PVR with DNAM-1 negatively regulates osteoclast formation. Furthermore, our results indicate that the cellular fusion process may be inhibited by the PVR-mediated signaling.
2. DNAM-1 promotes activation of cytotoxic lymphocytes by nonprofessional antigen-presenting cells and tumors. Natural killer (NK) cells and CD8 T cells require adhesion molecules for **migration**, activation, expansion, differentiation, and effector functions. DNAX accessory molecule 1 (DNAM-1), an adhesion molecule belonging to the immunoglobulin superfamily, promotes many of these functions in vitro. However, because NK cells and CD8 T cells express multiple adhesion molecules, it is unclear whether DNAM-1 has a unique function or is effectively redundant in vivo. To address this question, we generated mice lacking DNAM-1 and evaluated DNAM-1-deficient CD8 T cell and NK cell function in vitro and in vivo. Our results demonstrate that CD8 T cells require DNAM-1 for co-stimulation when recognizing antigen presented by nonprofessional antigen-presenting cells; in contrast, DNAM-1 is dispensable when dendritic cells present the antigen. Similarly, NK cells require DNAM-1 for the elimination of tumor cells that are comparatively resistant to NK cell-mediated cytotoxicity caused by the paucity of other NK cell-activating ligands. We conclude that DNAM-1 serves to extend the range of target cells that can activate CD8 T cell and NK cells and, hence, may be essential for immunosurveillance against tumors and/or viruses that evade recognition by other activating or accessory molecules.

---

## Nectin3

- NCBI Gene
- GeneRIF
- Pubmed

| Search Term | Rank | Fields |
| --- | --- | --- |
| [Mm]igration | 6 | PubMed(5); GeneRIFs(2) |
| [Ee]xtracellular [Mm]atrix | 8 | PubMed(1) |
| [Mm]etastasis | 12 | PubMed(1) |

  
hide/show details for Nectin3

### [Mm]igration

#### PubMed

1. Cajal-Retzius cells instruct neuronal **migration** by coincidence signaling between secreted and contact-dependent guidance cues. Cajal-Retzius (CR) cells are a transient cell population of the CNS that is critical for brain development. In the neocortex, CR cells secrete reelin to instruct the radial **migration** of projection neurons. It has remained unexplored, however, whether CR cells provide additional molecular cues important for brain development. Here, we show that CR cells express the immunoglobulin-like adhesion molecule nectin1, whereas neocortical projection neurons express its preferred binding partner, nectin3. We demonstrate that nectin1- and nectin3-mediated interactions between CR cells and migrating neurons are critical for radial **migration**. Furthermore, reelin signaling to Rap1 promotes neuronal Cdh2 function via nectin3 and afadin, thus directing the broadly expressed homophilic cell adhesion molecule Cdh2 toward mediating heterotypic cell-cell interactions between neurons and CR cells. Our findings identify nectins and afadin as components of the reelin signaling pathway and demonstrate that coincidence signaling between CR cell-derived secreted and short-range guidance cues direct neuronal **migration**.
2. Cajal-Retzius cells instruct neuronal **migration** by coincidence signaling between secreted and contact-dependent guidance cues. Cajal-Retzius (CR) cells are a transient cell population of the CNS that is critical for brain development. In the neocortex, CR cells secrete reelin to instruct the radial **migration** of projection neurons. It has remained unexplored, however, whether CR cells provide additional molecular cues important for brain development. Here, we show that CR cells express the immunoglobulin-like adhesion molecule nectin1, whereas neocortical projection neurons express its preferred binding partner, nectin3. We demonstrate that nectin1- and nectin3-mediated interactions between CR cells and migrating neurons are critical for radial **migration**. Furthermore, reelin signaling to Rap1 promotes neuronal Cdh2 function via nectin3 and afadin, thus directing the broadly expressed homophilic cell adhesion molecule Cdh2 toward mediating heterotypic cell-cell interactions between neurons and CR cells. Our findings identify nectins and afadin as components of the reelin signaling pathway and demonstrate that coincidence signaling between CR cell-derived secreted and short-range guidance cues direct neuronal **migration**.
3. Nectin-like molecule-5/Tage4 enhances cell **migration** in an integrin-dependent, Nectin-3-independent manner. Cell **migration** plays roles in invasion of transformed cells and scattering of embryonic mesenchymal cells into surrounding tissues. We have found that Ig-like Necl-5/Tage4 is up-regulated in NIH3T3 cells transformed by an oncogenic Ras (V12Ras-NIH3T3 cells) and heterophilically trans-interacts with a Ca(2+)-independent Ig-like cell adhesion molecule nectin-3, eventually enhancing their intercellular motility. We show here that Necl-5 furthermore enhances cell **migration** in a nectin-3-independent manner. Studies using L fibroblasts expressing various mutants of Necl-5, NIH3T3 cells, and V12Ras-NIH3T3 cells have revealed that Necl-5 enhances serum- and platelet-derived growth factor-induced cell **migration**. The extracellular region of Necl-5 is necessary for directional cell **migration**, but not for random cell motility. The cytoplasmic region of Necl-5 is necessary for both directional and random cell movement. Necl-5 colocalizes with integrin alpha(V)beta(3) at leading edges of migrating cells. Analyses using an inhibitor or an activator of integrin alpha(V)beta(3) or a dominant negative mutant of Necl-5 have shown the functional association of Necl-5 with integrin alpha(V)beta(3) in cell motility. Cdc42 and Rac small G proteins are activated by the action of Necl-5 and required for the serum-induced, Necl-5-enhanced cell motility. These results indicate that Necl-5 regulates serum- and platelet-derived growth factor-induced cell **migration** in an integrin-dependent, nectin-3-independent manner, when cells do not contact other cells. We furthermore show here that enhanced motility and metastasis of V12Ras-NIH3T3 cells are at least partly the result of up-regulated Necl-5.
4. Tage4/Nectin-like molecule-5 heterophilically trans-interacts with cell adhesion molecule Nectin-3 and enhances cell **migration**. Malignant transformation of cells causes disruption of cell-cell adhesion, enhancement of cell motility, and invasion into surrounding tissues. Nectins have both homophilic and heterophilic cell-cell adhesion activities and organize adherens junctions in cooperation with cadherins. We examined here whether Tage4, which was originally identified to be a gene overexpressed in colon carcinoma and has a domain structure similar to those of nectins, is involved in cell adhesion and/or **migration**. Tage4 heterophilically trans-interacted with nectin-3, but not homophilically with Tage4. Expression of Tage4 was markedly elevated in NIH3T3 cells transformed by an oncogenic Ki-Ras (V12Ras-NIH3T3 cells) as compared with that of wild-type NIH3T3 cells. trans-Interaction of Tage4 with nectin-3 enhanced motility of V12Ras-NIH3T3 cells. Tage4 did not bind afadin, a nectin- and actin filament-binding protein that connects nectins to the actin cytoskeleton and cadherins through catenins. Thus, Tage4 heterophilically trans-interacts with nectin-3 and regulates cell **migration**. Tage4 is tentatively re-named here nectin-like molecule-5 (necl-5) on the basis of its function and domain structure similar to those of nectins.
5. Involvement of the nectin-afadin complex in PDGF-induced cell survival. The nectin-afadin complex is involved in the formation of cell-cell junctions, such as adherens junctions (AJs) and tight junctions (TJs). Nectins are Ca(2+)-independent immunoglobulin-like cell-cell adhesion molecules, whereas afadin is an intracellular nectin-binding protein that connects nectins to the cadherin-catenin system at AJs and to the claudin-zona-occludens (ZO) protein system at TJs. Afadin(-/-) mice show embryonic lethality, resulting from impaired **migration** and improper differentiation of cells due to disorganization of cell-cell junctions during gastrulation. However, it remains to be elucidated whether disruption of afadin affects apoptosis. In the present study, we first found that embryoid bodies derived from afadin-knockout embryonic stem (ES) cells contained many more apoptotic cells than those derived from wild-type ES cells. We also revealed that apoptosis induced by serum starvation or Fas-ligand stimulation was increased in cultured NIH3T3 cells when afadin or nectin-3 was knocked down. The nectin-afadin complex was involved in the platelet-derived growth factor (PDGF)-induced activation of phosphatidylinositol 3-kinase (PI3K)-Akt signaling for cell survival. This complex was associated with PDGF receptor on the plasma membrane at cell-cell adhesion sites. Thus, the nectin-afadin complex is involved in PDGF-induced cell survival, at least through the PI3K-Akt signaling pathway.

#### GeneRIFs

1. This study demonstrated that nectin1- and nectin3-mediated interactions between Cajal-Retzius cells cells and migrating neurons are critical for radial **migration**.
2. Nectin-3 does not have a role in Necl-5/Tage4- enhanced cell **migration**

### [Ee]xtracellular [Mm]atrix

#### PubMed

1. Recruitment of nectin-3 to cell-cell junctions through trans-heterophilic interaction with CD155, a vitronectin and poliovirus receptor that localizes to alpha(v)beta3 integrin-containing membrane microdomains. Nectins present a novel class of Ig superfamily adhesion molecules that, cooperatively with cadherins, establish and maintain cell-cell adherens junctions. CD155, the cognate receptor for poliovirus, undergoes cell-matrix contacts by binding to the **extracellular matrix** protein vitronectin. The significant homology of nectins with CD155 prompted us to investigate the possibility of their interaction. We determined that nectin-3 binds CD155 and its putative mouse homologue Tage4 in cell-based ligand binding assays. Coculture of nectin-3- and CD155-expressing HeLa cells led to CD155-dependent recruitment of nectin-3 to cell-cell contacts. In a heterologous coculture system with CD155 expressing mouse neuroblastoma cells, HeLa cell-expressed nectin-3 was recruited to contact sites with CD155 bearing neurites. CD155 and nectin-3 colocalized to epithelial cell-cell junctions in renal proximal tubules and in the amniotic membrane. Efficient interaction depended on CD155 dimerization, which appears to be aided by cell type-specific cofactors. We furthermore found CD155 to codistribute with alpha(v) integrin microdomains on the surface of transfected mouse fibroblasts and at amniotic epithelial cell junctions. Our findings demonstrate the possible trans-interaction between the bona fide cell-cell adherens type adhesion system (cadherin/nectin) and the cell-matrix adhesion system (integrin/CD155) by virtue of their nectin-3 and CD155 components, respectively.

### [Mm]etastasis

#### PubMed

1. Nectin-like molecule-5/Tage4 enhances cell migration in an integrin-dependent, Nectin-3-independent manner. Cell migration plays roles in invasion of transformed cells and scattering of embryonic mesenchymal cells into surrounding tissues. We have found that Ig-like Necl-5/Tage4 is up-regulated in NIH3T3 cells transformed by an oncogenic Ras (V12Ras-NIH3T3 cells) and heterophilically trans-interacts with a Ca(2+)-independent Ig-like cell adhesion molecule nectin-3, eventually enhancing their intercellular motility. We show here that Necl-5 furthermore enhances cell migration in a nectin-3-independent manner. Studies using L fibroblasts expressing various mutants of Necl-5, NIH3T3 cells, and V12Ras-NIH3T3 cells have revealed that Necl-5 enhances serum- and platelet-derived growth factor-induced cell migration. The extracellular region of Necl-5 is necessary for directional cell migration, but not for random cell motility. The cytoplasmic region of Necl-5 is necessary for both directional and random cell movement. Necl-5 colocalizes with integrin alpha(V)beta(3) at leading edges of migrating cells. Analyses using an inhibitor or an activator of integrin alpha(V)beta(3) or a dominant negative mutant of Necl-5 have shown the functional association of Necl-5 with integrin alpha(V)beta(3) in cell motility. Cdc42 and Rac small G proteins are activated by the action of Necl-5 and required for the serum-induced, Necl-5-enhanced cell motility. These results indicate that Necl-5 regulates serum- and platelet-derived growth factor-induced cell migration in an integrin-dependent, nectin-3-independent manner, when cells do not contact other cells. We furthermore show here that enhanced motility and **metastasis** of V12Ras-NIH3T3 cells are at least partly the result of up-regulated Necl-5.

---

## Nectin1

- NCBI Gene
- GeneRIF
- Pubmed

| Search Term | Rank | Fields |
| --- | --- | --- |
| [Ff]ilopodia | 1 | PubMed(2) |
| [Mm]igration | 6 | PubMed(8); GeneRIFs(1) |
| ECM | 7 | PubMed(1) |
| [Ee]xtracellular [Mm]atrix | 8 | PubMed(2) |

  
hide/show details for Nectin1

### [Ff]ilopodia

#### PubMed

1. Vav2 as a Rac-GDP/GTP exchange factor responsible for the nectin-induced, c-Src- and Cdc42-mediated activation of Rac. Nectins are Ca2+-independent immunoglobulin-like cell-cell adhesion molecules that form homo- and hetero-trans-dimers (trans-interactions). Nectins first form cell-cell contact and then recruit cadherins to the nectin-based cell-cell contact sites to form adherens junctions cooperatively with cadherins. In addition, the trans-interactions of nectins induce the activation of Cdc42 and Rac small G proteins, which enhances the formation of adherens junctions by forming **filopodia** and lamellipodia, respectively. The trans-interactions of nectins first recruit and activate c-Src at the nectin-based cell-cell contact sites. c-Src then phosphorylates and activates FRG, a Cdc42-GDP/GTP exchange factor (GEF) for Cdc42. The activation of both c-Src and Cdc42 by FRG is necessary for the activation of Rac, but the Rac-GEF responsible for this activation of Rac remains unknown. We showed here that the nectin-induced activation of Rac was inhibited by a dominant negative mutant of Vav2, a Rac-GEF. Nectins recruited and tyrosine-phosphorylated Vav2 through c-Src at the nectin-based cell-cell contact sites, whereas Cdc42 was not necessary for the nectin-induced recruitment of Vav2 or the nectin-induced, c-Src-mediated tyrosine phosphorylation of Vav2. Cdc42 activated through c-Src then enhanced the GEF activity of tyrosine-phosphorylated Vav2 on Rac1. These results indicate that Vav2 is a GEF responsible for the nectin-induced, c-Src-, and Cdc42-mediated activation of Rac.
2. Vav2 as a Rac-GDP/GTP exchange factor responsible for the nectin-induced, c-Src- and Cdc42-mediated activation of Rac. Nectins are Ca2+-independent immunoglobulin-like cell-cell adhesion molecules that form homo- and hetero-trans-dimers (trans-interactions). Nectins first form cell-cell contact and then recruit cadherins to the nectin-based cell-cell contact sites to form adherens junctions cooperatively with cadherins. In addition, the trans-interactions of nectins induce the activation of Cdc42 and Rac small G proteins, which enhances the formation of adherens junctions by forming **filopodia** and lamellipodia, respectively. The trans-interactions of nectins first recruit and activate c-Src at the nectin-based cell-cell contact sites. c-Src then phosphorylates and activates FRG, a Cdc42-GDP/GTP exchange factor (GEF) for Cdc42. The activation of both c-Src and Cdc42 by FRG is necessary for the activation of Rac, but the Rac-GEF responsible for this activation of Rac remains unknown. We showed here that the nectin-induced activation of Rac was inhibited by a dominant negative mutant of Vav2, a Rac-GEF. Nectins recruited and tyrosine-phosphorylated Vav2 through c-Src at the nectin-based cell-cell contact sites, whereas Cdc42 was not necessary for the nectin-induced recruitment of Vav2 or the nectin-induced, c-Src-mediated tyrosine phosphorylation of Vav2. Cdc42 activated through c-Src then enhanced the GEF activity of tyrosine-phosphorylated Vav2 on Rac1. These results indicate that Vav2 is a GEF responsible for the nectin-induced, c-Src-, and Cdc42-mediated activation of Rac.

### [Mm]igration

#### PubMed

1. Cajal-Retzius cells instruct neuronal **migration** by coincidence signaling between secreted and contact-dependent guidance cues. Cajal-Retzius (CR) cells are a transient cell population of the CNS that is critical for brain development. In the neocortex, CR cells secrete reelin to instruct the radial **migration** of projection neurons. It has remained unexplored, however, whether CR cells provide additional molecular cues important for brain development. Here, we show that CR cells express the immunoglobulin-like adhesion molecule nectin1, whereas neocortical projection neurons express its preferred binding partner, nectin3. We demonstrate that nectin1- and nectin3-mediated interactions between CR cells and migrating neurons are critical for radial **migration**. Furthermore, reelin signaling to Rap1 promotes neuronal Cdh2 function via nectin3 and afadin, thus directing the broadly expressed homophilic cell adhesion molecule Cdh2 toward mediating heterotypic cell-cell interactions between neurons and CR cells. Our findings identify nectins and afadin as components of the reelin signaling pathway and demonstrate that coincidence signaling between CR cell-derived secreted and short-range guidance cues direct neuronal **migration**.
2. Rac1 GTPase-deficient mouse lens exhibits defects in shape, suture formation, fiber cell **migration** and survival. Morphogenesis and shape of the ocular lens depend on epithelial cell elongation and differentiation into fiber cells, followed by the symmetric and compact organization of fiber cells within an enclosed extracellular matrix-enriched elastic capsule. The cellular mechanisms orchestrating these different events however, remain obscure. We investigated the role of the Rac1 GTPase in these processes by targeted deletion of expression using the conditional gene knockout (cKO) approach. Rac1 cKO mice were derived from two different Cre (Le-Cre and MLR-10) transgenic mice in which lens-specific Cre expression starts at embryonic day 8.75 and 10.5, respectively, in both the lens epithelium and fiber cells. The Le-Cre/Rac1 cKO mice exhibited an early-onset (E12.5) and severe lens phenotype compared to the MLR-10/Rac1 cKO (E15.5) mice. While the Le-Cre/Rac1 cKO lenses displayed delayed primary fiber cell elongation, lenses from both Rac1 cKO strains were characterized by abnormal shape, impaired secondary fiber cell **migration**, sutural defects and thinning of the posterior capsule which often led to rupture. Lens fiber cell N-cadherin/β-catenin/Rap1/Nectin-based cell-cell junction formation and WAVE-2/Abi-2/Nap1-regulated actin polymerization were impaired in the Rac1 deficient mice. Additionally, the Rac1 cKO lenses were characterized by a shortened epithelial sheet, reduced levels of extracellular matrix (ECM) proteins and increased apoptosis. Taken together, these data uncover the essential role of Rac1 GTPase activity in establishment and maintenance of lens shape, suture formation and capsule integrity, and in fiber cell **migratio**n, adhesion and survival, via regulation of actin cytoskeletal dynamics, cell adhesive interactions and ECM turnover.
3. Impairment of radial glial scaffold-dependent neuronal **migration** and formation of double cortex by genetic ablation of afadin. Studies of human brain malformations, such as lissencephaly and double cortex, have revealed the importance of neuronal **migration** during cortical development. Afadin, a membrane scaffolding protein, regulates the formation of adherens junctions (AJs) and cell **migration** to form and maintain tissue structures. Here, we report that mice with dorsal telencephalon-specific ablation of afadin gene exhibited defects similar to human double cortex, in which the heterotopic cortex was located underneath the normotopic cortex. The normotopic cortex of the mutant mice was arranged in the pattern similar to the cortex of the control mice, while the heterotopic cortex was disorganized. As seen in human patients, double cortex in the mutant mice was formed by impaired neuronal **migration** during cortical development. Genetic ablation of afadin in the embryonic cerebral cortex disrupted AJs of radial glial cells, likely resulting in the retraction of the apical endfeet from the ventricular surface and the dispersion of radial glial cells from the ventricular zone to the subventricular and intermediate zones. These results indicate that afadin is required for the maintenance of AJs of radial glial cells and that the disruption of AJs might cause an abnormal radial scaffold for neuronal **migration**. In contrast, the proliferation or differentiation of radial glial cells was not significantly affected. Taken together, these findings indicate that afadin is required for the maintenance of the radial glial scaffold for neuronal **migration** and that the genetic ablation of afadin leads to the formation of double cortex.
4. Rap1 GTPase is required for mouse lens epithelial maintenance and morphogenesis. Rap1, a Ras-like small GTPase, plays a crucial role in cell-matrix adhesive interactions, cell-cell junction formation, cell polarity and **migration**. The role of Rap1 in vertebrate organ development and tissue architecture, however, remains elusive. We addressed this question in a mouse lens model system using a conditional gene targeting approach. While individual germline deficiency of either Rap1a or Rap1b did not cause overt defects in mouse lens, conditional double deficiency (Rap1 cKO) prior to lens placode formation led to an ocular phenotype including microphthalmia and lens opacification in embryonic mice. The embryonic Rap1 cKO mouse lens exhibited striking defects including loss of E-cadherin- and ZO-1-based cell-cell junctions, disruption of paxillin and β1-integrin-based cell adhesive interactions along with abnormalities in cell shape and apical-basal polarity of epithelium. These epithelial changes were accompanied by increased levels of α-smooth muscle actin, vimentin and N-cadherin, and expression of transcriptional suppressors of E-cadherin (Snai1, Slug and Zeb2), and a mesenchymal metabolic protein (Dihydropyrimidine dehydrogenase). Additionally, while lens differentiation was not overtly affected, increased apoptosis and dysregulated cell cycle progression were noted in epithelium and fibers in Rap1 cKO mice. Collectively these observations uncover a requirement for Rap1 in maintenance of lens epithelial phenotype and morphogenesis.
5. MPP3 is required for maintenance of the apical junctional complex, neuronal **migration**, and stratification in the developing cortex. During mammalian cortical development, division of progenitor cells occurs at the apical ventricular zone. Apical complex proteins and adherens junctions regulate the different modes of division. Here, we have identified the membrane-associated guanylate kinase protein membrane palmitoylated protein 3 (MPP3) as an essential protein for the maintenance of these complexes. MPP3 localizes at the apical membrane in which it shows partial colocalization with adherens junction proteins and apical proteins. We generated Mpp3 conditional knock-out mice and specifically ablated Mpp3 expression in cortical progenitor cells. Conditional deletion of Mpp3 during cortical development resulted in a gradual loss of the apical complex proteins and disrupted adherens junctions. Although there is cellular disorganization in the ventricular zone, gross morphology of the cortex was unaffected during loss of MPP3. However, in the ventricular zone, removal of MPP3 resulted in randomization of spindle orientation and ectopically localized mitotic cells. Loss of MPP3 in the developing cortex resulted in delayed **migration** of progenitor cells, whereas the rate of cell division and exit from the cell cycle was not affected. This resulted in defects in cortical stratification and ectopically localized layer II-IV pyramidal neurons and interneurons. These data show that MPP3 is required for maintenance of the apical protein complex and adherens junctions and for stratification and proper **migration** of neurons during the development of the cortex.
6. Cajal-Retzius cells instruct neuronal **migration** by coincidence signaling between secreted and contact-dependent guidance cues. Cajal-Retzius (CR) cells are a transient cell population of the CNS that is critical for brain development. In the neocortex, CR cells secrete reelin to instruct the radial **migration** of projection neurons. It has remained unexplored, however, whether CR cells provide additional molecular cues important for brain development. Here, we show that CR cells express the immunoglobulin-like adhesion molecule nectin1, whereas neocortical projection neurons express its preferred binding partner, nectin3. We demonstrate that nectin1- and nectin3-mediated interactions between CR cells and migrating neurons are critical for radial **migration**. Furthermore, reelin signaling to Rap1 promotes neuronal Cdh2 function via nectin3 and afadin, thus directing the broadly expressed homophilic cell adhesion molecule Cdh2 toward mediating heterotypic cell-cell interactions between neurons and CR cells. Our findings identify nectins and afadin as components of the reelin signaling pathway and demonstrate that coincidence signaling between CR cell-derived secreted and short-range guidance cues direct neuronal **migration**.
7. Nectin-like molecule-5 regulates intimal thickening after carotid artery ligation in mice. Intimal thickening is considered to result from the dedifferentiation of medial smooth muscle cells (SMCs) from a contractile to a synthetic phenotype, and their subsequent **migration** and proliferation. It is unknown whether nectin-like molecule (Necl)-5, which is overexpressed in cancer cells, is involved in intimal thickening.Necl-5 was upregulated in mouse carotid artery after ligation. Compared with wild-type mice, intimal thickening after carotid artery ligation was milder in Necl-5 knockout mice. In vitro, the expression levels of SMC differentiation markers were higher, whereas the expression level of an SMC dedifferentiation marker was lower, in Necl-5 knockout mouse aortic SMCs (MASMCs) compared with wild-type MASMCs. The **migration**, proliferation, and extracellular signal-regulated kinase activity in response to serum were decreased in Necl-5 knockout MASMCs compared with wild-type MASMCs. In wild-type MASMCs, inhibition of extracellular signal-regulated kinase activity increased the expression levels of SMC differentiation markers and decreased their **migration** and proliferation in response to serum.The present findings indicate that Necl-5 plays a role in the formation of intimal thickening after carotid artery ligation by regulating dedifferentiation, **migration**, and proliferation of SMCs in an extracellular signal-regulated kinase-dependent manner. Our results suggest that Necl-5 may represent a potential therapeutic target to limit intimal thickening after vascular injury.
8. Spatiotemporal changes of the herpes simplex virus entry receptor nectin-1 in murine brain during postnatal development. Herpes simplex virus (HSV) is known to replicate within the limbic system and to alter behavior in both humans and experimental animals. However, the reason why the virus selectively damages this anatomical, developmental, and functional neural unit remains a mystery. Nor is it known why herpes simplex encephalitis fails to respect these neuroanatomical boundaries in newborns. In the present study, the authors determined the spatiotemporal changes in the distribution of the major neural entry receptor for HSV (nectin-1) in postnatal mouse and rat brains. Discrete nectin-1 immunopositivity was observed in regions susceptible to HSV infection in specific developmental phases of central nervous system. The authors also describe nectin-1-related pathways controlling neuronal cell **migration**/brain morphogenesis, the disruption of which might lead to the emergence of mental disorders with a rapid cognitive decline.

#### GeneRIFs

1. This study demonstrated that nectin1- and nectin3-mediated interactions between Cajal-Retzius cells cells and migrating neurons are critical for radial **migration**

### ECM

#### PubMed

1. Rac1 GTPase-deficient mouse lens exhibits defects in shape, suture formation, fiber cell migration and survival. Morphogenesis and shape of the ocular lens depend on epithelial cell elongation and differentiation into fiber cells, followed by the symmetric and compact organization of fiber cells within an enclosed extracellular matrix-enriched elastic capsule. The cellular mechanisms orchestrating these different events however, remain obscure. We investigated the role of the Rac1 GTPase in these processes by targeted deletion of expression using the conditional gene knockout (cKO) approach. Rac1 cKO mice were derived from two different Cre (Le-Cre and MLR-10) transgenic mice in which lens-specific Cre expression starts at embryonic day 8.75 and 10.5, respectively, in both the lens epithelium and fiber cells. The Le-Cre/Rac1 cKO mice exhibited an early-onset (E12.5) and severe lens phenotype compared to the MLR-10/Rac1 cKO (E15.5) mice. While the Le-Cre/Rac1 cKO lenses displayed delayed primary fiber cell elongation, lenses from both Rac1 cKO strains were characterized by abnormal shape, impaired secondary fiber cell migration, sutural defects and thinning of the posterior capsule which often led to rupture. Lens fiber cell N-cadherin/β-catenin/Rap1/Nectin-based cell-cell junction formation and WAVE-2/Abi-2/Nap1-regulated actin polymerization were impaired in the Rac1 deficient mice. Additionally, the Rac1 cKO lenses were characterized by a shortened epithelial sheet, reduced levels of extracellular matrix **(EC**M) proteins and increased apoptosis. Taken together, these data uncover the essential role of Rac1 GTPase activity in establishment and maintenance of lens shape, suture formation and capsule integrity, and in fiber cell migration, adhesion and survival, via regulation of actin cytoskeletal dynamics, cell adhesive interactions and **EC**M turnover.

### [Ee]xtracellular [Mm]atrix

#### PubMed

1. Rac1 GTPase-deficient mouse lens exhibits defects in shape, suture formation, fiber cell migration and survival. Morphogenesis and shape of the ocular lens depend on epithelial cell elongation and differentiation into fiber cells, followed by the symmetric and compact organization of fiber cells within an enclosed **extracellular matrix**-enriched elastic capsule. The cellular mechanisms orchestrating these different events however, remain obscure. We investigated the role of the Rac1 GTPase in these processes by targeted deletion of expression using the conditional gene knockout (cKO) approach. Rac1 cKO mice were derived from two different Cre (Le-Cre and MLR-10) transgenic mice in which lens-specific Cre expression starts at embryonic day 8.75 and 10.5, respectively, in both the lens epithelium and fiber cells. The Le-Cre/Rac1 cKO mice exhibited an early-onset (E12.5) and severe lens phenotype compared to the MLR-10/Rac1 cKO (E15.5) mice. While the Le-Cre/Rac1 cKO lenses displayed delayed primary fiber cell elongation, lenses from both Rac1 cKO strains were characterized by abnormal shape, impaired secondary fiber cell migration, sutural defects and thinning of the posterior capsule which often led to rupture. Lens fiber cell N-cadherin/β-catenin/Rap1/Nectin-based cell-cell junction formation and WAVE-2/Abi-2/Nap1-regulated actin polymerization were impaired in the Rac1 deficient mice. Additionally, the Rac1 cKO lenses were characterized by a shortened epithelial sheet, reduced levels of **extracellular matri**x (ECM) proteins and increased apoptosis. Taken together, these data uncover the essential role of Rac1 GTPase activity in establishment and maintenance of lens shape, suture formation and capsule integrity, and in fiber cell migration, adhesion and survival, via regulation of actin cytoskeletal dynamics, cell adhesive interactions and ECM turnover.
2. Alteration of medial-edge epithelium cell adhesion in two Tgf-beta3 null mouse strains. Although palatal shelf adhesion is a crucial event during palate development, little work has been carried out to determine which molecules are responsible for this process. Furthermore, whether altered palatal shelf adhesion causes the cleft palate presented by Tgf-beta3 null mutant mice has not yet been clarified. Here, we study the presence/distribution of some **extracellular matrix** and cell adhesion molecules at the time of the contact of palatal shelves in both wild-type and Tgf-beta3 null mutant palates of two strains of mice (C57/BL/6J (C57), and MF1) that develop cleft palates of different severity. We have performed immunohistochemistry with antibodies against collagens IV and IX, laminin, fibronectin, the alpha5- and beta1-integrins, and ICAM-1; in situ hybridization with a Nectin-1 riboprobe; and palatal shelf cultures treated or untreated with TGF-beta3 or neutralizing antibodies against fibronectin or the alpha5-integrin. Our results show the location of these molecules in the wild-type mouse medial edge epithelium (MEE) of both strains at the time of the contact of palatal shelves; the heavier (C57) and milder (MF1) alteration of their presence in the Tgf-beta3 null mutants; the importance of TGF-beta3 to restore their normal pattern of expression; and the crucial role of fibronectin and the alpha5-integrin in palatal shelf adhesion. We thus provide insight into the molecular bases of this important process and the cleft palate presented by Tgf-beta3 null mutant mice.

---

## Cd200

- NCBI Gene
- GeneRIF
- Pubmed

| Search Term | Rank | Fields |
| --- | --- | --- |
| [Mm]igration | 6 | PubMed(2) |
| [Mm]etastasis | 12 | PubMed(2); GeneRIFs(1) |

  
hide/show details for Cd200

### [Mm]igration

#### PubMed

1. Early specification of limb muscle precursor cells by the homeobox gene Lbx1h. During vertebrate embryogenesis, myogenic precursor cells of limb muscles delaminate from the ventro-lateral edge of the somitic dermomyotome and migrate to the limb buds, where they congregate into dorsal and ventral muscle masses. It has been proposed that the surrounding connective tissue controls muscle pattern formation in limbs. Regulatory molecules such as receptor tyrosine kinases like c-Met ( ref. 6) and those encoded by homeobox-containing genes, including c-Met (ref. 6), Tbx1 (ref. 7), Mox2 (ref. 8), Six1 and Six2 (ref. 9), Pitx2, Pax3 (refs 10,11) and Lbx1h (refs 12,13), are expressed in migrating limb precursor cells. The role of these genes in the patterning of limb muscles is unknown, although mutation of Pax3 or Met causes disruption of limb muscle development at an initial step, disturbing the epithelial-to-mesenchymal transition of the somitic epithelium. No limb muscle cells form in these mutants, and the early loss of myogenic precursor cells prevented an analysis of later functions of these genes during limb muscle development. Based on quail-chick chimaera studies, it was assumed that a cell-autonomous contribution of myogenic cells to the formation of individual limb muscles is negligible, and that an instructive role of limb mesenchyme is critical in this process. Here we show that Lbx1h determines migratory routes of muscle precursor cells in a cell-autonomous manner, thereby leading to the formation of distinct limb muscle patterns. Inactivation of Lbx1h, which is specifically expressed in migrating muscle precursor cells, led to a lack of extensor muscles in forelimbs and an absence of muscles in hindlimbs. The defect was caused by the failure of all muscle precursor cells of hindlimbs and of precursor cells of extensor muscles of forelimbs to migrate to their corresponding muscle anlagen. Our results demonstrate that Lbx1h is a key regulator of muscle precursor cell **migration** and is required for the acquisition of dorsal identities of forelimb muscles.
2. Bidirectional effect of CD200 on breast cancer development and metastasis, with ultimate outcome determined by tumor aggressiveness and a cancer-induced inflammatory response. CD200 acts through its receptor (CD200R) to inhibit excessive inflammation. The role of CD200-CD200R1 interaction in tumor immunity is poorly understood. In this study, we examined the role of CD200-CD200R1 interaction in the progression and metastasis of highly aggressive 4THM murine-breast carcinoma using CD200 transgenic (CD200(tg)) and CD200R1 knock-out (CD200R1(-)(/-)) BALB/c mice. 4THM cells induce extensive visceral metastasis and neutrophil infiltration in affected tissues. CD200 overexpression in the host was associated with decreased primary tumor growth and metastasis, whereas lack of CD200R1 expression by host cells was associated with enhanced visceral metastasis. Absence of CD200R1 expression led to decreased tumor-infiltrating-cytotoxic T cells and increased the release of inflammatory cytokines, such as tumor necrosis factor-α (TNF-α) and interleukin (IL)-6. In contrast, CD200 overexpression led to increased tumor-induced interferon-γ and IL-10 response and decreased TNF-α and IL-6 release. Neutrophil infiltration of tissues was markedly decreased in CD200(tg) animals and increased in CD200R1(-/-) mice. These findings are contradictory to what has been reported in the EMT6 mouse breast-cancer model. Other distinguishing features of tumor elicited by EMT6 and 4THM cell injections were also examined. Visceral tissues from mice bearing EMT6 tumors showed a lack of neutrophil infiltration and decreased IL-6 release in CD200R1(-/-) mice. EMT6 and 4THM cells also differed in vimentin expression and in vi**tro migra**tion rate, which was markedly lower in EMT6 tumors. These results support the hypothesis that CD200 expression can alter immune responses, and can inhibit metastatic growth of tumor cells that induce systemic and local inflammatory response. Increasing CD200 activity/signaling might be an important therapeutic strategy for treatment of aggressive breast carcinomas.

### [Mm]etastasis

#### PubMed

1. Bidirectional effect of CD200 on breast cancer development and **metastasis**, with ultimate outcome determined by tumor aggressiveness and a cancer-induced inflammatory response. CD200 acts through its receptor (CD200R) to inhibit excessive inflammation. The role of CD200-CD200R1 interaction in tumor immunity is poorly understood. In this study, we examined the role of CD200-CD200R1 interaction in the progression and **metastasis** of highly aggressive 4THM murine-breast carcinoma using CD200 transgenic (CD200(tg)) and CD200R1 knock-out (CD200R1(-)(/-)) BALB/c mice. 4THM cells induce extensive visceral **metastasis** and neutrophil infiltration in affected tissues. CD200 overexpression in the host was associated with decreased primary tumor growth and **metastasis**, whereas lack of CD200R1 expression by host cells was associated with enhanced visceral **metastasis**. Absence of CD200R1 expression led to decreased tumor-infiltrating-cytotoxic T cells and increased the release of inflammatory cytokines, such as tumor necrosis factor-α (TNF-α) and interleukin (IL)-6. In contrast, CD200 overexpression led to increased tumor-induced interferon-γ and IL-10 response and decreased TNF-α and IL-6 release. Neutrophil infiltration of tissues was markedly decreased in CD200(tg) animals and increased in CD200R1(-/-) mice. These findings are contradictory to what has been reported in the EMT6 mouse breast-cancer model. Other distinguishing features of tumor elicited by EMT6 and 4THM cell injections were also examined. Visceral tissues from mice bearing EMT6 tumors showed a lack of neutrophil infiltration and decreased IL-6 release in CD200R1(-/-) mice. EMT6 and 4THM cells also differed in vimentin expression and in vitro migration rate, which was markedly lower in EMT6 tumors. These results support the hypothesis that CD200 expression can alter immune responses, and can inhibit metastatic growth of tumor cells that induce systemic and local inflammatory response. Increasing CD200 activity/signaling might be an important therapeutic strategy for treatment of aggressive breast carcinomas.
2. Role of CD200 expression in regulation of **metastasis** of EMT6 tumor cells in mice. Previous studies have confirmed that levels of CD200 expression on the cells of the transplantable EMT6 mouse breast cancer line are increased markedly during growth in immunocompetent mice, unlike the persistent low levels of expression observed in NOD-SCID.IL-2(γr-/-) mice or mice with generalized over-expression of a CD200 transgene (CD200(tg) mice). Faster tumor growth occurs in both of these latter mice, with decreased evidence for a host immune reaction in lymph nodes draining the tumor (DLN). We now report evidence for a role for CD200 expression (by the host and/or tumor cells) in increased seeding of tumor cells to DLN in immunocompromised (CD200(tg) or NOD-SCID.IL-2(γr-/-)) vs immunocompetent mice, by limiting dilution cloning of tumor cells from DLN (vs contralateral lymph nodes, CLN), using control and GFP-tagged EMT6 cells. Neutralization of expressed CD200 by anti-CD200mAbs decreased the tumo**r metastas**is at the same time as increasing detection of cytotoxic anti-tumor immune cells in DLN. Infusion of either anti-CD4 to deplete T-effector cells, or anti-TGFβ antibody, increas**ed metasta**sis to DLN, as did indeed the infusion of EMT6 cells selected for the loss of TGFβRII expression. It is concluded that the increased CD200 expression by breast cancer cells (and/or host tissue) may be an important variable involved in determining the risk **of metast**asis.

#### GeneRIFs

1. the increased CD200 expression by breast cancer cells (and/or host tissue) may be an important variable involved in determining the risk of **metastasis**.

---

## Cadm1

- NCBI Gene
- GeneRIF
- Pubmed

| Search Term | Rank | Fields |
| --- | --- | --- |
| [Ff]ilopodia | 1 | PubMed(3) |
| [Mm]igration | 6 | PubMed(3); GeneRIFs(1) |
| [Ee]xtracellular [Mm]atrix | 8 | PubMed(1) |
| [Ii]nvasive | 10 | PubMed(1) |
| [Mm]etastasis | 12 | PubMed(2) |

  
hide/show details for Cadm1

### [Ff]ilopodia

#### PubMed

1. SynCAM 1 participates in axo-dendritic contact assembly and shapes neuronal growth cones. Neuronal growth cones are highly motile structures that tip developing neurites and explore their surroundings before axo-dendritic contact and synaptogenesis. However, the membrane proteins organizing these processes remain insufficiently understood. Here we identify that the synaptic cell adhesion molecule 1 (SynCAM 1), an immunoglobulin superfamily member, is already expressed in developing neurons and localizes to their growth cones. Upon interaction of growth cones with target neurites, SynCAM 1 rapidly assembles at these contacts to form stable adhesive clusters. Synaptic markers can also be detected at these sites. Addressing the functions of SynCAM 1 in growth cones preceding contact, we determine that it is required and sufficient to restrict the number of active **filopodia**. Further, SynCAM 1 negatively regulates the morphological complexity of migrating growth cones. Focal adhesion kinase, a binding partner of SynCAM 1, is implicated in its morphogenetic activities. These results reveal that SynCAM 1 acts in developing neurons to shape migrating growth cones and contributes to the adhesive differentiation of their axo-dendritic contacts.
2. In vivo imaging demonstrates dendritic spine stabilization by SynCAM 1. Formation and stability of synapses are required for proper brain function. While it is well established that synaptic adhesion molecules are important regulators of synapse formation, their specific role during different phases of synapse development remains unclear. To investigate the function of the synaptic cell adhesion molecule SynCAM 1 in the formation, stability, and maintenance of spines we used 2-photon in vivo imaging to follow individual spines over a long period of time. In SynCAM 1 knockout mice the survival rate of existing spines was reduced and fewer **filopodia**-like structures were converted into stable spines. SynCAM 1(flag) overexpression resulted in more stable spines and fewer **filopodia**-like structures. When SynCAM 1(flag) overexpression is turned on the spine density rapidly increases within a few days. Interestingly, the spine density stayed at an elevated level when SynCAM 1(flag) overexpression was turned off. Our data indicate that the SynCAM 1 induced altered spine density is not caused by the formation of newly emerging protrusions, instead SynCAM 1 stabilizes nascent synaptic contacts which promotes their maturation. Concomitant with the synaptic stabilization, SynCAM 1 generally prolongs the lifetime of spines. In summary, we demonstrate that SynCAM 1 is a key regulator of spine stability.
3. The novel synaptogenic protein Farp1 links postsynaptic cytoskeletal dynamics and transsynaptic organization. Synaptic adhesion organizes synapses, yet the signaling pathways that drive and integrate synapse development remain incompletely understood. We screened for regulators of these processes by proteomically analyzing synaptic membranes lacking the synaptogenic adhesion molecule SynCAM 1. This identified FERM, Rho/ArhGEF, and Pleckstrin domain protein 1 (Farp1) as strongly reduced in SynCAM 1 knockout mice. Farp1 regulates dendritic **filopodia**l dynamics in immature neurons, indicating roles in synapse formation. Later in development, Farp1 is postsynaptic and its 4.1 protein/ezrin/radixin/moesin (FERM) domain binds SynCAM 1, assembling a synaptic complex. Farp1 increases synapse number and modulates spine morphology, and SynCAM 1 requires Farp1 for promoting spines. In turn, SynCAM 1 loss reduces the ability of Farp1 to elevate spine density. Mechanistically, Farp1 activates the GTPase Rac1 in spines downstream of SynCAM 1 clustering, and promotes F-actin assembly. Farp1 furthermore triggers a retrograde signal regulating active zone composition via SynCAM 1. These results reveal a postsynaptic signaling pathway that engages transsynaptic interactions to coordinate synapse development.

### [Mm]igration

#### PubMed

1. Dlx transcription factors promote **migration** through repression of axon and dendrite growth. In the mouse telencephalon, Dlx homeobox transcription factors are essential for the tangential **migration** of subpallial-derived GABAergic interneurons to neocortex. However, the mechanisms underlying this process are poorly understood. Here, we demonstrate that Dlx1/2 has a central role in restraining neurite growth of subpallial-derived immature interneurons at a stage when they migrate tangentially to cortex. In Dlx1-/-;Dlx2-/- mutants, neurite length is increased and cells fail to migrate. In Dlx1-/-;Dlx2+/- mutants, while the tangential **migration** of immature interneurons appears normal, they develop dendritic and axonal processes with increased length and decreased branching, and have deficits in their neocortical laminar positions. Thus, Dlx1/2 is required for coordinating programs of neurite maturation and **migration**. In this regard, we provide genetic evidence that in immature interneurons Dlx1/2 repression of the p21-activated serine/threonine kinase PAK3, a downstream effector of the Rho family of GTPases, is critical in restraining neurite growth and promoting tangential **migration**.
2. Distribution of RA175/TSLC1/SynCAM, a member of the immunoglobulin superfamily, in the developing nervous system. RA175 is a new member of the immunoglobulin superfamily with trans interaction activity, and it plays a role as a tumor suppressor in lung carcinoma (TSLC1) and as a cell adhesion molecule promoting the formation of functional synapses (SynCAM). Little is known about the biological function of RA175/TSLC1/SynCAM neural network formation during neurogenesis. We examined the distribution and colocalization of the RA175/TSLC1/SynCAM protein with other members of the immunoglobulin superfamily such as NCAM, L1, and TAG-1 in the mouse developing nervous system. Consistent with the expression of RA175/TSLC1/SynCAM mRNA, the protein was localized in the brain neuroepithelium at embryonic day (E) 9.5, neural crest at E10.5, motor neurons at E10.5, and olfactory epithelium at E16.5. In contrast with its mRNA, the protein was intensely detected on the fasciculated axons in the floor plates, ventral root, and dorsal funiculus in the E10.5-11.5 spinal cord and colocalized with NCAM and L1 on the ventral root and dorsal funiculus and partly colocalized with TAG-1 on the commissural axons and dorsal funiculus. In the E13.5-15.5 brain, RA175/TSLC1/SynCAM colocalized with NCAM and L1 on the developing thalamocortical fibers from the internal capsule (IC) and partly colocalized with TAG-1 on the cortical efferent axons in the intermediate zone (IZ). RA175/TSLC1/SynCAM was localized on the axons of some of the cortical neurons cultured in vitro. Thus, in addition to cell adhesion activity in the neuroepithelium and the synapses, RA175/TSLC1/SynCAM may be involved in neuronal **migration**, axon growth, pathfinding, and fasciculation on the axons of differentiating neurons.
3. Distinct roles for the SgIGSF adhesion molecule and c-kit receptor tyrosine kinase in the interaction between mast cells and the mesentery. Intraperitoneal injection of bone marrow-derived mast cells (BMMCs) has therapeutic efficacy against acute bacterial peritonitis. For this role, BMMCs need to settle down the mesentery from the peritoneal cavity. Interaction between BMMCs and the mesentery was examined by using mast cell deficient WBB6F1(F1)-W/Wv [c-kit receptor tyrosine kinase (KIT) mutant], F1-Sl/Sld [KIT ligand stem cell factor mutant], and F1-tg/tg [a practically microphthalmia transcription factor (MITF)-null mutant] mice. Three parameters were measured: the number of BMMCs: (1) developed in the mesentery 5 weeks after intraperitoneal injection into mast cell deficient mice, (2) adhered to mesenteric mesothelial cells, and (3) transmigrated across the mesenteric mesothelial cell monolayer when coculturing both cells for 3 and 18 h, respectively. After intraperitoneal injection, F1-wild type (+/+) BMMCs developed in the mesentery of F1-W/Wv mice but not in that of F1-Sl/Sld mice, while F1-tg/tg BMMCs did not develop, even in the mesentery of WBB6F1-W/Wv mice. In the coculture, WB-W/W BMMCs normally adhered to but poorly transmigrated across F1-+/+ mesothelial cells, and in accordance, F1-+/+ BMMCs normally adhered to but poorly transmigrated across F1-Sl/Sld mesothelial cells. F1-tg/tg BMMCs showed poor adhesion and trans**migration**, but both parameters were partially but significantly improved by ectopic expression of spermatogenic immunoglobulin superfamily (SgIGSF), a mast-cell adhesion molecule critically regulated by MITF. Since F1-tg/tg BMMCs expressed reduced levels of KIT, these results suggested that SgIGSF and KIT independently played a significant role in the trans**migration**. Among three parameters, development of mast cells in the mesentery well correlated with the trans**migration**. This process seemed important for mast cells to settle down from the peritoneal cavity to the mesentery.

#### GeneRIFs

1. SgIGSF and KIT independently play significant roles in cell **migration**

### [Ee]xtracellular [Mm]atrix

#### PubMed

1. Interaction of nectin-like molecule 2 with integrin alpha6beta4 and inhibition of disassembly of integrin alpha6beta4 from hemidesmosomes. In normal epithelial cells, integrin α(6)β(4) is abundantly expressed and forms hemidesmosomes, which is a cellular structure that mediates cel**l-extracellular matr**ix binding. In many types of cancer cells, integrin α(6)β(4) is up-regulated, laminin is cleaved, and hemidesmosomes are disrupted, eventually causing an enhancement of cancer cell movement and facilitation of their invasion. We previously showed that the immunoglobulin-like cell adhesion molecule Necl-2 (Nectin-like molecule 2), known as a tumor suppressor, inhibits cancer cell movement by suppressing the ErbB3/ErbB2 signaling. We show here that Necl-2 interacts in cis with integrin α(6)β(4). The binding of Necl-2 with integrin β(4) was mediated by its extracellular region. In human colorectal adenocarcinoma Caco-2 cells, integrin α(6)β(4) was localized at hemidesmosomes. Small interfering RNA-mediated suppression of Necl-2 expression enhanced the phorbol ester-induced disruption of the integrin α(6)β(4) complex at hemidesmosomes, whereas expression of Necl-2 suppressed the disruption of this structure. These results indicate that tumor-suppressive functions of Necl-2 are mediated by the stabilization of the hemidesmosome structure in addition to the inhibition of the ErbB3/ErbB2 signaling.

### [Ii]nvasive

#### PubMed

1. Optimising experimental design for high-throughput phenotyping in mice: a case study. To further the functional annotation of the mammalian genome, the Sanger Mouse Genetics Programme aims to generate and characterise knockout mice in a high-throughput manner. Annually, approximately 200 lines of knockout mice will be characterised using a standardised battery of phenotyping tests covering key disease indications ranging from obesity to sensory acuity. From these findings secondary centres will select putative mutants of interest for more in-depth, confirmatory experiments. Optimising experimental design and data analysis is essential to maximise output using the resources with greatest efficiency, thereby attaining our biological objective of understanding the role of genes in normal development and disease. This study uses the example of the non**invasive** blood pressure test to demonstrate how statistical investigation is important for generating meaningful, reliable results and assessing the design for the defined research objectives. The analysis adjusts for the multiple-testing problem by applying the false discovery rate, which controls the number of false calls within those highlighted as significant. A variance analysis finds that the variation between mice dominates this assay. These variance measures were used to examine the interplay between days, readings, and number of mice on power, the ability to detect change. If an experiment is underpowered, we cannot conclude whether failure to detect a biological difference arises from low power or lack of a distinct phenotype, hence the mice are subjected to testing without gain. Consequently, in confirmatory studies, a power analysis along with the 3Rs can provide justification to increase the number of mice used.

### [Mm]etastasis

#### PubMed

1. Silencing of ErbB3/ErbB2 signaling by immunoglobulin-like Necl-2. ErbB2 and ErbB3, members of the EGF receptor/ErbB family, form a heterodimer upon binding of a ligand, inducing the activation of Rac small G protein and Akt protein kinase for cell movement and survival, respectively. The enhanced ErbB3/ErbB2 signaling causes tumorigenesis, invasion, and **metastasis**. We found here that the ErbB3/ErbB2 signaling is regulated by immunoglobulin-like Necl-2, which is down-regulated in various cancer cells and serves as a tumor suppressor. The extracellular region of ErbB3, but not ErbB2, interacted in cis with that of Necl-2. This interaction reduced the ligand-induced, ErbB2-catalyzed tyrosine phosphorylation of ErbB3 and inhibited the consequent ErbB3-mediated activation of Rac and Akt, resulting in the inhibition of cancer cell movement and survival. These inhibitory effects of Necl-2 were mediated by the protein-tyrosine phosphatase PTPN13 which interacted with the cytoplasmic tail of Necl-2. We describe here this novel mechanism for silencing of the ErbB3/ErbB2 signaling by Necl-2.
2. RA175, which is the mouse ortholog of TSLC1, a tumor suppressor gene in human lung cancer, is a cell adhesion molecule. RA175, a new immunoglobulin superfamily member, is preferentially expressed during differentiation of P19 embryonic carcinoma (EC) cells induced by retinoic acid. In the present study, we isolated mouse RA175 cDNA in its entirety and showed that RA175 is the mouse ortholog of TSLC1, a tumor suppressor gene in human lung cancer. RA175/TSLC1 was localized in the adherent region of human lung squamous carcinoma cells and in the differentiated P19 EC cells. RA175/TSLC1 showed homophilic trans-interaction activity in a Ca(2+)-independent manner. RA175/TSLC1 was preferentially expressed in the polarized cells lining the lumen of developing mouse lung epithelium. This suggests that RA175/TSLC1 is a cell adhesion molecule that is acting as a tumor suppressor gene in the **metastasis** of lung tumors. RA175/TSLC1 may be necessary for cells to remain tightly associated in the epithelium, thereby suppressing **metastasis**.

---

## Pvr

- NCBI Gene
- GeneRIF
- Pubmed

| Search Term | Rank | Fields |
| --- | --- | --- |
| [Mm]igration | 6 | Process(1); PubMed(9); GeneRIFs(1) |
| [Ee]xtracellular [Mm]atrix | 8 | PubMed(1) |
| [Ii]nvasive | 10 | PubMed(1) |
| [Mm]etastasize | 11 | PubMed(1) |
| [Mm]etastasis | 12 | PubMed(3); GeneRIFs(1) |

  
hide/show details for Pvr

### [Mm]igration

#### Process

1. cell **migration**

#### PubMed

1. Expression and possible role of PVR/CD155/Necl-5 in osteoclastogenesis. Osteoclasts, the bone-resorbing cells, are differentiated from hematopoietic precursors via two-step cell-cell interactions. One is the interaction between the osteoclast precursor and the stromal cell to initiate differentiation. The other is the interaction among osteoclast precursors to form multinucleated osteoclasts. Recently, the poliovirus receptor (PVR, CD155, Necl-5) was reported to play important roles in cell adhesion and **migration**. However, there are no reports of PVR in osteoclastogenesis. In this paper, we examined the expression of PVR and its ligand, DNAX accessory molecule-1 (DNAM-1, CD226), in osteoclast precursors, mature osteoclasts, and stromal cells. We found that the PVR was constitutively expressed in both osteoclast cells and stromal cells. The expression of PVR was not changed at various stages of osteoclast formation. In contrast, the expression of DNAM-1 was observed in mononuclear cells and was down-regulated during osteoclastogenesis. Moreover, multinucleated osteoclast formation was inhibited by treatment with the extracellular domain of DNAM-1 (ED-DNAM-1) as a soluble ligand for PVR, but mononuclear preosteoclast formation was not affected. Especially, during the 7-day cultivation, osteoclast formation was suppressed by the treatment with ED-DNAM-1 on days 6 and 7, when the mononuclear preosteoclasts fused into multinucleated osteoclasts. This suppression was abrogated partially by a small interfering RNA specific for PVR. These results suggest that, at least in part, the binding of PVR with DNAM-1 negatively regulates osteoclast formation. Furthermore, our results indicate that the cellular fusion process may be inhibited by the PVR-mediated signaling.
2. Nectin-like molecule-5 regulates intimal thickening after carotid artery ligation in mice. Intimal thickening is considered to result from the dedifferentiation of medial smooth muscle cells (SMCs) from a contractile to a synthetic phenotype, and their subsequent **migration** and proliferation. It is unknown whether nectin-like molecule (Necl)-5, which is overexpressed in cancer cells, is involved in intimal thickening.Necl-5 was upregulated in mouse carotid artery after ligation. Compared with wild-type mice, intimal thickening after carotid artery ligation was milder in Necl-5 knockout mice. In vitro, the expression levels of SMC differentiation markers were higher, whereas the expression level of an SMC dedifferentiation marker was lower, in Necl-5 knockout mouse aortic SMCs (MASMCs) compared with wild-type MASMCs. The **migration**, proliferation, and extracellular signal-regulated kinase activity in response to serum were decreased in Necl-5 knockout MASMCs compared with wild-type MASMCs. In wild-type MASMCs, inhibition of extracellular signal-regulated kinase activity increased the expression levels of SMC differentiation markers and decreased their **migration** and proliferation in response to serum.The present findings indicate that Necl-5 plays a role in the formation of intimal thickening after carotid artery ligation by regulating dedifferentiation, **migration**, and proliferation of SMCs in an extracellular signal-regulated kinase-dependent manner. Our results suggest that Necl-5 may represent a potential therapeutic target to limit intimal thickening after vascular injury.
3. Nectin-like molecule-5/Tage4 enhances cell **migration** in an integrin-dependent, Nectin-3-independent manner. Cell **migration** plays roles in invasion of transformed cells and scattering of embryonic mesenchymal cells into surrounding tissues. We have found that Ig-like Necl-5/Tage4 is up-regulated in NIH3T3 cells transformed by an oncogenic Ras (V12Ras-NIH3T3 cells) and heterophilically trans-interacts with a Ca(2+)-independent Ig-like cell adhesion molecule nectin-3, eventually enhancing their intercellular motility. We show here that Necl-5 furthermore enhances cell **migration** in a nectin-3-independent manner. Studies using L fibroblasts expressing various mutants of Necl-5, NIH3T3 cells, and V12Ras-NIH3T3 cells have revealed that Necl-5 enhances serum- and platelet-derived growth factor-induced cell **migration**. The extracellular region of Necl-5 is necessary for directional cell **migration**, but not for random cell motility. The cytoplasmic region of Necl-5 is necessary for both directional and random cell movement. Necl-5 colocalizes with integrin alpha(V)beta(3) at leading edges of migrating cells. Analyses using an inhibitor or an activator of integrin alpha(V)beta(3) or a dominant negative mutant of Necl-5 have shown the functional association of Necl-5 with integrin alpha(V)beta(3) in cell motility. Cdc42 and Rac small G proteins are activated by the action of Necl-5 and required for the serum-induced, Necl-5-enhanced cell motility. These results indicate that Necl-5 regulates serum- and platelet-derived growth factor-induced cell **migration** in an integrin-dependent, nectin-3-independent manner, when cells do not contact other cells. We furthermore show here that enhanced motility and metastasis of V12Ras-NIH3T3 cells are at least partly the result of up-regulated Necl-5.
4. Tage4/Nectin-like molecule-5 heterophilically trans-interacts with cell adhesion molecule Nectin-3 and enhances cell **migration**. Malignant transformation of cells causes disruption of cell-cell adhesion, enhancement of cell motility, and invasion into surrounding tissues. Nectins have both homophilic and heterophilic cell-cell adhesion activities and organize adherens junctions in cooperation with cadherins. We examined here whether Tage4, which was originally identified to be a gene overexpressed in colon carcinoma and has a domain structure similar to those of nectins, is involved in cell adhesion and/or **migration**. Tage4 heterophilically trans-interacted with nectin-3, but not homophilically with Tage4. Expression of Tage4 was markedly elevated in NIH3T3 cells transformed by an oncogenic Ki-Ras (V12Ras-NIH3T3 cells) as compared with that of wild-type NIH3T3 cells. trans-Interaction of Tage4 with nectin-3 enhanced motility of V12Ras-NIH3T3 cells. Tage4 did not bind afadin, a nectin- and actin filament-binding protein that connects nectins to the actin cytoskeleton and cadherins through catenins. Thus, Tage4 heterophilically trans-interacts with nectin-3 and regulates cell **migration**. Tage4 is tentatively re-named here nectin-like molecule-5 (necl-5) on the basis of its function and domain structure similar to those of nectins.
5. Expression and possible role of PVR/CD155/Necl-5 in osteoclastogenesis. Osteoclasts, the bone-resorbing cells, are differentiated from hematopoietic precursors via two-step cell-cell interactions. One is the interaction between the osteoclast precursor and the stromal cell to initiate differentiation. The other is the interaction among osteoclast precursors to form multinucleated osteoclasts. Recently, the poliovirus receptor (PVR, CD155, Necl-5) was reported to play important roles in cell adhesion and **migration**. However, there are no reports of PVR in osteoclastogenesis. In this paper, we examined the expression of PVR and its ligand, DNAX accessory molecule-1 (DNAM-1, CD226), in osteoclast precursors, mature osteoclasts, and stromal cells. We found that the PVR was constitutively expressed in both osteoclast cells and stromal cells. The expression of PVR was not changed at various stages of osteoclast formation. In contrast, the expression of DNAM-1 was observed in mononuclear cells and was down-regulated during osteoclastogenesis. Moreover, multinucleated osteoclast formation was inhibited by treatment with the extracellular domain of DNAM-1 (ED-DNAM-1) as a soluble ligand for PVR, but mononuclear preosteoclast formation was not affected. Especially, during the 7-day cultivation, osteoclast formation was suppressed by the treatment with ED-DNAM-1 on days 6 and 7, when the mononuclear preosteoclasts fused into multinucleated osteoclasts. This suppression was abrogated partially by a small interfering RNA specific for PVR. These results suggest that, at least in part, the binding of PVR with DNAM-1 negatively regulates osteoclast formation. Furthermore, our results indicate that the cellular fusion process may be inhibited by the PVR-mediated signaling.
6. Tage4/Nectin-like molecule-5 heterophilically trans-interacts with cell adhesion molecule Nectin-3 and enhances cell **migration**. Malignant transformation of cells causes disruption of cell-cell adhesion, enhancement of cell motility, and invasion into surrounding tissues. Nectins have both homophilic and heterophilic cell-cell adhesion activities and organize adherens junctions in cooperation with cadherins. We examined here whether Tage4, which was originally identified to be a gene overexpressed in colon carcinoma and has a domain structure similar to those of nectins, is involved in cell adhesion and/or **migration**. Tage4 heterophilically trans-interacted with nectin-3, but not homophilically with Tage4. Expression of Tage4 was markedly elevated in NIH3T3 cells transformed by an oncogenic Ki-Ras (V12Ras-NIH3T3 cells) as compared with that of wild-type NIH3T3 cells. trans-Interaction of Tage4 with nectin-3 enhanced motility of V12Ras-NIH3T3 cells. Tage4 did not bind afadin, a nectin- and actin filament-binding protein that connects nectins to the actin cytoskeleton and cadherins through catenins. Thus, Tage4 heterophilically trans-interacts with nectin-3 and regulates cell **migration**. Tage4 is tentatively re-named here nectin-like molecule-5 (necl-5) on the basis of its function and domain structure similar to those of nectins.
7. Nectin-like molecule-5/Tage4 enhances cell **migration** in an integrin-dependent, Nectin-3-independent manner. Cell **migration** plays roles in invasion of transformed cells and scattering of embryonic mesenchymal cells into surrounding tissues. We have found that Ig-like Necl-5/Tage4 is up-regulated in NIH3T3 cells transformed by an oncogenic Ras (V12Ras-NIH3T3 cells) and heterophilically trans-interacts with a Ca(2+)-independent Ig-like cell adhesion molecule nectin-3, eventually enhancing their intercellular motility. We show here that Necl-5 furthermore enhances cell **migration** in a nectin-3-independent manner. Studies using L fibroblasts expressing various mutants of Necl-5, NIH3T3 cells, and V12Ras-NIH3T3 cells have revealed that Necl-5 enhances serum- and platelet-derived growth factor-induced cell **migration**. The extracellular region of Necl-5 is necessary for directional cell **migration**, but not for random cell motility. The cytoplasmic region of Necl-5 is necessary for both directional and random cell movement. Necl-5 colocalizes with integrin alpha(V)beta(3) at leading edges of migrating cells. Analyses using an inhibitor or an activator of integrin alpha(V)beta(3) or a dominant negative mutant of Necl-5 have shown the functional association of Necl-5 with integrin alpha(V)beta(3) in cell motility. Cdc42 and Rac small G proteins are activated by the action of Necl-5 and required for the serum-induced, Necl-5-enhanced cell motility. These results indicate that Necl-5 regulates serum- and platelet-derived growth factor-induced cell **migration** in an integrin-dependent, nectin-3-independent manner, when cells do not contact other cells. We furthermore show here that enhanced motility and metastasis of V12Ras-NIH3T3 cells are at least partly the result of up-regulated Necl-5.
8. The CD155/poliovirus receptor enhances the proliferation of ras-mutated cells. Stimulation of the CD155/poliovirus receptor, which localizes in the cell-matrix and at cell-cell junctions, inhibits cell adhesion and enhances cell **migration**. Necl-5, a mouse homolog of CD155, is implicated in the formation of adherence junctions. Recently, Necl-5 has also been found to enhance cell proliferation via the stimulation of serum and platelet-derived growth factor through the Ras-Raf-MEK-ERK signaling pathway. In our present study, we find that CD155 significantly enhances the serum-induced cell proliferation of NIH3T3 cells which have been transformed by an oncogenic Ras (V12Ras-NIH3T3), but not the parental cells. CD155 expression in V12Ras-NIH3T3 cells is also found to upregulate cyclin D2, downregulate p27(Kip1) and shorten the G0/G1 phase of the cell cycle. An inhibitor of focal adhesion kinase does not reduce this CD155-mediated enhancement of V12Ras-NIH3T3 cell proliferation. The expression of CD155DeltaCP, which lacks the cytoplasmic region including the immunoreceptor tyrosine-based inhibitory motif (ITIM), has a reduced ability to enhance the serum responsiveness of V12Ras-NIH3T3 cells, suggesting that the ITIM might be required for this effect of CD155. In addition, the overexpression of exogenous CD155 enhances the serum responsiveness of HT1080 cells, which harbor a mutant N-ras gene. On the other hand, siRNA-induced knockdown of endogenous CD155 and/or CD155DeltaCP expression significantly repress the serum responsiveness of DLD-1 cells, which express endogenous CD155 and harbor a mutant K-ras gene, suggesting that this mutant may function in a dominant negative manner. Taken together, our present data suggest that CD155, at least in part, enhances the proliferation of ras-mutated cells.
9. Necl-5/poliovirus receptor interacts with VEGFR2 and regulates VEGF-induced angiogenesis. Vascular endothelial growth factor (VEGF), a major proangiogenic agent, exerts its proangiogenic action by binding to VEGF receptor 2 (VEGFR2), the activity of which is regulated by direct interactions with other cell surface proteins, including integrin α(V)β(3). However, how the interaction between VEGFR2 and integrin α(V)β(3) is regulated is not clear.To investigate whether Necl-5/poliovirus receptor, an immunoglobulin-like molecule that is known to bind integrin α(V)β(3), regulates the interaction between VEGFR2 and integrin α(V)β(3), and to clarify the role of Necl-5 in the VEGF-induced angiogenesis.Necl-5-knockout mice displayed no obvious defect in vascular development; however, recovery of blood flow after hindlimb ischemia and the VEGF-induced neovascularization in implanted Matrigel plugs were impaired in Necl-5-knockout mice. To clarify the mechanism of the regulation of angiogenesis by Necl-5, we investigated the roles of Necl-5 in the VEGF-induced angiogenic responses in vitro. Knockdown of Necl-5 by siRNAs in human umbilical vein endothelial cells (HUVECs) inhibited the VEGF-induced capillary-like network formation on Ma**trigel, m**igration, and proliferation, and conversely, enhanced apoptosis. Coimmunoprecipitation assays showed the interaction of Necl-5 with VEGFR2, and knockdown of Necl-5 prevented the VEGF-induced interaction of integrin α(V)β(3) with VEGFR2. Knockdown of Necl-5 suppressed the VEGFR2-mediated activation of downstream proangiogenic and survival signals, including Rap1, Akt, and endothelial nitric oxide synthase.These results demonstrate the critical role of Necl-5 in angiogenesis and suggest that Necl-5 may regulate the VEGF-induced angiogenesis by controlling the interaction of VEGFR2 with integrin α(v)β(3), and the VEGFR2-mediated Rap1-Akt signaling pathway.

#### GeneRIFs

1. Necl-5/Tage4 regulates serum- and platelet-derived growth factor-induced cell **migration** in an integrin-dependent, nectin-3-independent manner, when cells do not contact other cells

### [Ee]xtracellular [Mm]atrix

#### PubMed

1. Recruitment of nectin-3 to cell-cell junctions through trans-heterophilic interaction with CD155, a vitronectin and poliovirus receptor that localizes to alpha(v)beta3 integrin-containing membrane microdomains. Nectins present a novel class of Ig superfamily adhesion molecules that, cooperatively with cadherins, establish and maintain cell-cell adherens junctions. CD155, the cognate receptor for poliovirus, undergoes cell-matrix contacts by binding to the **extracellular matrix** protein vitronectin. The significant homology of nectins with CD155 prompted us to investigate the possibility of their interaction. We determined that nectin-3 binds CD155 and its putative mouse homologue Tage4 in cell-based ligand binding assays. Coculture of nectin-3- and CD155-expressing HeLa cells led to CD155-dependent recruitment of nectin-3 to cell-cell contacts. In a heterologous coculture system with CD155 expressing mouse neuroblastoma cells, HeLa cell-expressed nectin-3 was recruited to contact sites with CD155 bearing neurites. CD155 and nectin-3 colocalized to epithelial cell-cell junctions in renal proximal tubules and in the amniotic membrane. Efficient interaction depended on CD155 dimerization, which appears to be aided by cell type-specific cofactors. We furthermore found CD155 to codistribute with alpha(v) integrin microdomains on the surface of transfected mouse fibroblasts and at amniotic epithelial cell junctions. Our findings demonstrate the possible trans-interaction between the bona fide cell-cell adherens type adhesion system (cadherin/nectin) and the cell-matrix adhesion system (integrin/CD155) by virtue of their nectin-3 and CD155 components, respectively.

### [Ii]nvasive

#### PubMed

1. Necl-5/poliovirus receptor interacts in cis with integrin alphaVbeta3 and regulates its clustering and focal complex formation. Integrin alphavbeta3, which forms focal complexes at leading edges in moving cells, is up-regulated in cancer cells and so is implicated in their **invasive**ness. Necl-5, originally identified as a poliovirus receptor and also up-regulated in cancer cells, colocalizes with integrin alphavbeta3 at leading edges in moving cells and enhances growth factor-induced cell movement. Here, we show that Necl-5 interacts directly, in cis, with integrin alphavbeta3, and enhances integrin alphavbeta3 clustering and focal complex formation at leading edges in NIH3T3 cells. The extracellular region of Necl-5, but not the cytoplasmic region, is necessary for its interaction with integrin alphavbeta3; however, both regions are necessary for its action. An interaction between integrin alphavbeta3 and vitronectin and PDGF-induced activation of Rac are also necessary for integrin alphavbeta3 clustering. The interaction between Necl-5 and integrin alphavbeta3 enhances PDGF-induced Rac activation, facilitating integrin alphavbeta3 clustering presumably in a feedback amplification manner. Thus, Necl-5 has a critical role in integrin alphavbeta3 clustering and focal complex formation.

### [Mm]etastasize

#### PubMed

1. Interaction of cancer cells with platelets mediated by Necl-5/poliovirus receptor enhances cancer cell metastasis to the lungs. Necl-5 is an immunoglobulin (Ig)-like molecule that was originally identified as a poliovirus receptor and is often upregulated in cancer cells. We recently found that it colocalizes with integrin alpha(v)beta(3) at the leading edges of moving cells and enhances growth factor-induced cell movement and proliferation. Upon cell-cell contact, Necl-5 is removed from the cell surface by its trans-interaction with the cell adhesion molecule nectin-3, resulting in reduced cell movement and proliferation. Here, we investigated the role of Necl-5 in the interaction of cancer cells with platelets. Necl-5 was upregulated in CT26 cells, a colon adenocarcinoma cell line. When CT26 cells were injected into the tail vein of mice, they were arrested in the pulmonary vessels by adhering to platelets and subsequently **metastasize**d to the lungs. Overexpression of Necl-5 in CT26 cells enhanced this metastasis, while inhibition of the trans-interaction of Necl-5 with CD226 by an anti-Necl-5 monoclonal antibody reduced the metastasis. Depletion of platelets by treatment with a rabbit anti-mouse platelet serum reduced the Necl-5-enhanced metastasis in mice. Thus, the trans-interaction of upregulated Necl-5 in cancer cells with its counter-receptor in platelets, probably CD226, is critical for efficient metastasis of cancer cells to the lungs.

### [Mm]etastasis

#### PubMed

1. Nectin-like molecule-5/Tage4 enhances cell migration in an integrin-dependent, Nectin-3-independent manner. Cell migration plays roles in invasion of transformed cells and scattering of embryonic mesenchymal cells into surrounding tissues. We have found that Ig-like Necl-5/Tage4 is up-regulated in NIH3T3 cells transformed by an oncogenic Ras (V12Ras-NIH3T3 cells) and heterophilically trans-interacts with a Ca(2+)-independent Ig-like cell adhesion molecule nectin-3, eventually enhancing their intercellular motility. We show here that Necl-5 furthermore enhances cell migration in a nectin-3-independent manner. Studies using L fibroblasts expressing various mutants of Necl-5, NIH3T3 cells, and V12Ras-NIH3T3 cells have revealed that Necl-5 enhances serum- and platelet-derived growth factor-induced cell migration. The extracellular region of Necl-5 is necessary for directional cell migration, but not for random cell motility. The cytoplasmic region of Necl-5 is necessary for both directional and random cell movement. Necl-5 colocalizes with integrin alpha(V)beta(3) at leading edges of migrating cells. Analyses using an inhibitor or an activator of integrin alpha(V)beta(3) or a dominant negative mutant of Necl-5 have shown the functional association of Necl-5 with integrin alpha(V)beta(3) in cell motility. Cdc42 and Rac small G proteins are activated by the action of Necl-5 and required for the serum-induced, Necl-5-enhanced cell motility. These results indicate that Necl-5 regulates serum- and platelet-derived growth factor-induced cell migration in an integrin-dependent, nectin-3-independent manner, when cells do not contact other cells. We furthermore show here that enhanced motility and **metastasis** of V12Ras-NIH3T3 cells are at least partly the result of up-regulated Necl-5.
2. Nectin-like molecule-5/Tage4 enhances cell migration in an integrin-dependent, Nectin-3-independent manner. Cell migration plays roles in invasion of transformed cells and scattering of embryonic mesenchymal cells into surrounding tissues. We have found that Ig-like Necl-5/Tage4 is up-regulated in NIH3T3 cells transformed by an oncogenic Ras (V12Ras-NIH3T3 cells) and heterophilically trans-interacts with a Ca(2+)-independent Ig-like cell adhesion molecule nectin-3, eventually enhancing their intercellular motility. We show here that Necl-5 furthermore enhances cell migration in a nectin-3-independent manner. Studies using L fibroblasts expressing various mutants of Necl-5, NIH3T3 cells, and V12Ras-NIH3T3 cells have revealed that Necl-5 enhances serum- and platelet-derived growth factor-induced cell migration. The extracellular region of Necl-5 is necessary for directional cell migration, but not for random cell motility. The cytoplasmic region of Necl-5 is necessary for both directional and random cell movement. Necl-5 colocalizes with integrin alpha(V)beta(3) at leading edges of migrating cells. Analyses using an inhibitor or an activator of integrin alpha(V)beta(3) or a dominant negative mutant of Necl-5 have shown the functional association of Necl-5 with integrin alpha(V)beta(3) in cell motility. Cdc42 and Rac small G proteins are activated by the action of Necl-5 and required for the serum-induced, Necl-5-enhanced cell motility. These results indicate that Necl-5 regulates serum- and platelet-derived growth factor-induced cell migration in an integrin-dependent, nectin-3-independent manner, when cells do not contact other cells. We furthermore show here that enhanced motility and **metastasis** of V12Ras-NIH3T3 cells are at least partly the result of up-regulated Necl-5.
3. Interaction of cancer cells with platelets mediated by Necl-5/poliovirus receptor enhances cancer cell **metastasis** to the lungs. Necl-5 is an immunoglobulin (Ig)-like molecule that was originally identified as a poliovirus receptor and is often upregulated in cancer cells. We recently found that it colocalizes with integrin alpha(v)beta(3) at the leading edges of moving cells and enhances growth factor-induced cell movement and proliferation. Upon cell-cell contact, Necl-5 is removed from the cell surface by its trans-interaction with the cell adhesion molecule nectin-3, resulting in reduced cell movement and proliferation. Here, we investigated the role of Necl-5 in the interaction of cancer cells with platelets. Necl-5 was upregulated in CT26 cells, a colon adenocarcinoma cell line. When CT26 cells were injected into the tail vein of mice, they were arrested in the pulmonary vessels by adhering to platelets and subsequently metastasized to the lungs. Overexpression of Necl-5 in CT26 cells enhanced this **metastasis**, while inhibition of the trans-interaction of Necl-5 with CD226 by an anti-Necl-5 monoclonal antibody reduced the **metastasis**. Depletion of platelets by treatment with a rabbit anti-mouse platelet serum reduced the Necl-5-enhanced **metastasis** in mice. Thus, the trans-interaction of upregulated Necl-5 in cancer cells with its counter-receptor in platelets, probably CD226, is critical for efficient **metastasis** of cancer cells to the lungs.

#### GeneRIFs

1. The trans-interaction of upregulated Necl-5 in cancer cells with its counter-receptor in platelets, probably CD226, is critical for efficient **metastasis** of cancer cells to the lungs.

---

## Pvr

- NCBI Gene
- GeneRIF
- Pubmed

| Search Term | Rank | Fields |
| --- | --- | --- |
| [Mm]igration | 6 | PubMed(2) |
| [Ee]xtracellular [Mm]atrix | 8 | PubMed(1) |
| [Mm]etastasis | 12 | PubMed(1) |

  
hide/show details for Pvr

### [Mm]igration

#### PubMed

1. Nectin-like molecule-5/Tage4 enhances cell **migration** in an integrin-dependent, Nectin-3-independent manner. Cell **migration** plays roles in invasion of transformed cells and scattering of embryonic mesenchymal cells into surrounding tissues. We have found that Ig-like Necl-5/Tage4 is up-regulated in NIH3T3 cells transformed by an oncogenic Ras (V12Ras-NIH3T3 cells) and heterophilically trans-interacts with a Ca(2+)-independent Ig-like cell adhesion molecule nectin-3, eventually enhancing their intercellular motility. We show here that Necl-5 furthermore enhances cell **migration** in a nectin-3-independent manner. Studies using L fibroblasts expressing various mutants of Necl-5, NIH3T3 cells, and V12Ras-NIH3T3 cells have revealed that Necl-5 enhances serum- and platelet-derived growth factor-induced cell **migration**. The extracellular region of Necl-5 is necessary for directional cell **migration**, but not for random cell motility. The cytoplasmic region of Necl-5 is necessary for both directional and random cell movement. Necl-5 colocalizes with integrin alpha(V)beta(3) at leading edges of migrating cells. Analyses using an inhibitor or an activator of integrin alpha(V)beta(3) or a dominant negative mutant of Necl-5 have shown the functional association of Necl-5 with integrin alpha(V)beta(3) in cell motility. Cdc42 and Rac small G proteins are activated by the action of Necl-5 and required for the serum-induced, Necl-5-enhanced cell motility. These results indicate that Necl-5 regulates serum- and platelet-derived growth factor-induced cell **migration** in an integrin-dependent, nectin-3-independent manner, when cells do not contact other cells. We furthermore show here that enhanced motility and metastasis of V12Ras-NIH3T3 cells are at least partly the result of up-regulated Necl-5.
2. Tage4/Nectin-like molecule-5 heterophilically trans-interacts with cell adhesion molecule Nectin-3 and enhances cell **migration**. Malignant transformation of cells causes disruption of cell-cell adhesion, enhancement of cell motility, and invasion into surrounding tissues. Nectins have both homophilic and heterophilic cell-cell adhesion activities and organize adherens junctions in cooperation with cadherins. We examined here whether Tage4, which was originally identified to be a gene overexpressed in colon carcinoma and has a domain structure similar to those of nectins, is involved in cell adhesion and/or **migration**. Tage4 heterophilically trans-interacted with nectin-3, but not homophilically with Tage4. Expression of Tage4 was markedly elevated in NIH3T3 cells transformed by an oncogenic Ki-Ras (V12Ras-NIH3T3 cells) as compared with that of wild-type NIH3T3 cells. trans-Interaction of Tage4 with nectin-3 enhanced motility of V12Ras-NIH3T3 cells. Tage4 did not bind afadin, a nectin- and actin filament-binding protein that connects nectins to the actin cytoskeleton and cadherins through catenins. Thus, Tage4 heterophilically trans-interacts with nectin-3 and regulates cell **migration**. Tage4 is tentatively re-named here nectin-like molecule-5 (necl-5) on the basis of its function and domain structure similar to those of nectins.

### [Ee]xtracellular [Mm]atrix

#### PubMed

1. Recruitment of nectin-3 to cell-cell junctions through trans-heterophilic interaction with CD155, a vitronectin and poliovirus receptor that localizes to alpha(v)beta3 integrin-containing membrane microdomains. Nectins present a novel class of Ig superfamily adhesion molecules that, cooperatively with cadherins, establish and maintain cell-cell adherens junctions. CD155, the cognate receptor for poliovirus, undergoes cell-matrix contacts by binding to the **extracellular matrix** protein vitronectin. The significant homology of nectins with CD155 prompted us to investigate the possibility of their interaction. We determined that nectin-3 binds CD155 and its putative mouse homologue Tage4 in cell-based ligand binding assays. Coculture of nectin-3- and CD155-expressing HeLa cells led to CD155-dependent recruitment of nectin-3 to cell-cell contacts. In a heterologous coculture system with CD155 expressing mouse neuroblastoma cells, HeLa cell-expressed nectin-3 was recruited to contact sites with CD155 bearing neurites. CD155 and nectin-3 colocalized to epithelial cell-cell junctions in renal proximal tubules and in the amniotic membrane. Efficient interaction depended on CD155 dimerization, which appears to be aided by cell type-specific cofactors. We furthermore found CD155 to codistribute with alpha(v) integrin microdomains on the surface of transfected mouse fibroblasts and at amniotic epithelial cell junctions. Our findings demonstrate the possible trans-interaction between the bona fide cell-cell adherens type adhesion system (cadherin/nectin) and the cell-matrix adhesion system (integrin/CD155) by virtue of their nectin-3 and CD155 components, respectively.

### [Mm]etastasis

#### PubMed

1. Nectin-like molecule-5/Tage4 enhances cell migration in an integrin-dependent, Nectin-3-independent manner. Cell migration plays roles in invasion of transformed cells and scattering of embryonic mesenchymal cells into surrounding tissues. We have found that Ig-like Necl-5/Tage4 is up-regulated in NIH3T3 cells transformed by an oncogenic Ras (V12Ras-NIH3T3 cells) and heterophilically trans-interacts with a Ca(2+)-independent Ig-like cell adhesion molecule nectin-3, eventually enhancing their intercellular motility. We show here that Necl-5 furthermore enhances cell migration in a nectin-3-independent manner. Studies using L fibroblasts expressing various mutants of Necl-5, NIH3T3 cells, and V12Ras-NIH3T3 cells have revealed that Necl-5 enhances serum- and platelet-derived growth factor-induced cell migration. The extracellular region of Necl-5 is necessary for directional cell migration, but not for random cell motility. The cytoplasmic region of Necl-5 is necessary for both directional and random cell movement. Necl-5 colocalizes with integrin alpha(V)beta(3) at leading edges of migrating cells. Analyses using an inhibitor or an activator of integrin alpha(V)beta(3) or a dominant negative mutant of Necl-5 have shown the functional association of Necl-5 with integrin alpha(V)beta(3) in cell motility. Cdc42 and Rac small G proteins are activated by the action of Necl-5 and required for the serum-induced, Necl-5-enhanced cell motility. These results indicate that Necl-5 regulates serum- and platelet-derived growth factor-induced cell migration in an integrin-dependent, nectin-3-independent manner, when cells do not contact other cells. We furthermore show here that enhanced motility and **metastasis** of V12Ras-NIH3T3 cells are at least partly the result of up-regulated Necl-5.

---

## Nectin2

- NCBI Gene
- GeneRIF
- Pubmed

| Search Term | Rank | Fields |
| --- | --- | --- |
| [Mm]igration | 6 | PubMed(1); GeneRIFs(1) |
| [Ee]xtracellular [Mm]atrix | 8 | PubMed(1) |

  
hide/show details for Nectin2

### [Mm]igration

#### PubMed

1. Loss of nectin-2 at Sertoli-spermatid junctions leads to male infertility and correlates with severe spermatozoan head and midpiece malformation, impaired binding to the zona pellucida, and oocyte penetration. The members of the nectin/CD155 gene family represent a growing class of novel cell adhesion molecules of the immunoglobulin superfamily. In the present study, we describe the generation of a mouse line lacking a functional nectin-2 gene (nectin-2LacZ/LacZ) and analyze the resulting male-specific infertility phenotype. Although nectin-2LacZ/LacZ males produced normal amounts of motile spermatozoa, scanning electron microscopy revealed severe malformations of the spermatozoan head and midpiece. Besides a 4-fold reduction in **migration** of nectin-2LacZ/LacZ spermatozoa to the oviducts, in vitro binding to zona-intact mouse oocytes was reduced 6-fold. On the other hand, nectin-2LacZ/LacZ spermatozoa bound to zona-free hamster oocytes at near-wild type levels but, remarkably, failed to penetrate. In addition to the previously reported expression of nectin-2 and nectin-3 at Sertoli-spermatid junctions and of nectin-2 at inter-Sertoli cell junctions, we also found nectin-2 to localize at apical cell-cell junctions of the epididymal epithelium. Expression analysis of a LacZ knockin gene into the defunct nectin-2 gene in nectin-2LacZ/LacZ mice provided additional support for our earlier conjecture that in normal testis, nectin-2 is produced exclusively by Sertoli cells. Finally, we found Sertoli-spermatid junctions in nectin-2LacZ/LacZ mice to be virtually devoid of the actin-bundling protein espin, suggesting that ectoplasmic specializations fail to form in the absence of nectin-2. Our functional analyses indicate that the infertility phenotype of nectin-2-deficient male mice is caused by a combination of reduced **migration** to the oviduct, spermatozoa-zona binding, and sperm-oocyte fusion. We corroborate our previous description of a heterotypic adhesion complex between Sertoli cells and elongated spermatids that is maintained by nectin-2 and nectin-3, respectively.

#### GeneRIFs

1. Our functional analyses indicate that the infertility phenotype of nectin-2-deficient male mice is caused by a combination of reduced **migration** to the oviduct, spermatozoa-zona binding, and sperm-oocyte fusion

### [Ee]xtracellular [Mm]atrix

#### PubMed

1. Recruitment of nectin-3 to cell-cell junctions through trans-heterophilic interaction with CD155, a vitronectin and poliovirus receptor that localizes to alpha(v)beta3 integrin-containing membrane microdomains. Nectins present a novel class of Ig superfamily adhesion molecules that, cooperatively with cadherins, establish and maintain cell-cell adherens junctions. CD155, the cognate receptor for poliovirus, undergoes cell-matrix contacts by binding to the **extracellular matrix** protein vitronectin. The significant homology of nectins with CD155 prompted us to investigate the possibility of their interaction. We determined that nectin-3 binds CD155 and its putative mouse homologue Tage4 in cell-based ligand binding assays. Coculture of nectin-3- and CD155-expressing HeLa cells led to CD155-dependent recruitment of nectin-3 to cell-cell contacts. In a heterologous coculture system with CD155 expressing mouse neuroblastoma cells, HeLa cell-expressed nectin-3 was recruited to contact sites with CD155 bearing neurites. CD155 and nectin-3 colocalized to epithelial cell-cell junctions in renal proximal tubules and in the amniotic membrane. Efficient interaction depended on CD155 dimerization, which appears to be aided by cell type-specific cofactors. We furthermore found CD155 to codistribute with alpha(v) integrin microdomains on the surface of transfected mouse fibroblasts and at amniotic epithelial cell junctions. Our findings demonstrate the possible trans-interaction between the bona fide cell-cell adherens type adhesion system (cadherin/nectin) and the cell-matrix adhesion system (integrin/CD155) by virtue of their nectin-3 and CD155 components, respectively.

---

## Crtam

- NCBI Gene
- GeneRIF
- Pubmed

| Search Term | Rank | Fields |
| --- | --- | --- |
| [Mm]igration | 6 | PubMed(3) |

  
hide/show details for Crtam

### [Mm]igration

#### PubMed

1. Regulation of a late phase of T cell polarity and effector functions by Crtam. Spatial organization of cellular proteins plays an important role in establishment of cellular polarity to regulate cell division, differentiation, **migration**, and organogenesis. Activation of T cells by antigen-presenting cells (APCs) results in the formation of an immunological synapse (IS), assembly of a signaling scaffold at the T cell receptor (TCR) contact, cytoskeletal reorganization, and generation of second messengers within the first hours following intercellular contact. We demonstrate here that Crtam (class-I MHC-restricted T-cell associated molecule), an immunoglobulin-superfamily transmembrane protein, coordinates a signaling complex anchored by the Scrib polarity protein to establish a later phase of T cell polarity on a subset of CD4+ T cells >6 hours following activation. Maintenance of this late cellular polarity results in the ability of CD4+Crtam+ T cells to selectively produce more IFNgamma and IL22. Crtam engagement thus modulates signals many hours beyond the initial activation event and dynamically influences the adaptive immune response.
2. Heterotypic interaction of CRTAM with Necl2 induces cell adhesion on activated NK cells and CD8+ T cells. NK cells and CD8+ T cells exhibit cytotoxicity and cytokine production upon recognizing target cells through cell-cell interaction. We screened the molecules involved in the recognition and regulation of these cells using cDNA subtraction between naive and activated NK cells. We identified class I-restricted T cell-associated molecule (CRTAM), a two Ig domain-bearing surface receptor, as a molecule rapidly and transiently expressed on NK cells and CD8+ T cells upon activation. CRTAM is expressed as a dimer on the cell surface, and its expression is transcriptionally regulated. Using an expression-cloning system, we then further identified Nectin-like (Necl) molecule 2, a three Ig domain-containing receptor, as a ligand of CRTAM. While Necl2 mediates homotypic interaction, CRTAM interacts with Necl2 but not with CRTAM itself. The heterotypic CRTAM-Necl2 interaction has a higher affinity than the homotypic Necl2 interaction. Although there was no clear alteration in the cytotoxic function of the NK cells and CD8+ T cells against the Necl2-expressing target cells, T cells expressing CRTAM tightly bound to Necl2-expressing cells. CRTAM+ cells did not induce homotypic aggregation but they did exert strong heterotypic binding with Necl2+ cells, which was inhibited by the addition of the CRTAM-Ig fusion protein. These results suggest that the heterotypic interaction between CRTAM and Necl2 plays an important role in the adhesion, interaction or **migration** of NK cells and CD8+ T cells upon stimulation.
3. CRTAM confers late-stage activation of CD8+ T cells to regulate retention within lymph node. In vivo immune response is triggered in the lymph node, where lymphocytes for entry into, retention at, and **migration** to effector sites are dynamically regulated. The molecular mechanism underlying retention regulation is the key to elucidating in vivo regulation of immune response. In this study, we describe the function of the adhesion molecule class I-restricted T cell-associated molecule (CRTAM) in regulating CD8+ T cell retention within the lymph node and eventually effector function. We previously identified CRTAM as a receptor predominantly expressed on activated CD8+ T cells, and nectin-like molecule-2 (Necl2) as its ligand. In vivo function of CRTAM-Necl2 interaction was analyzed by generating CRTAM(-/-) mice. CRTAM(-/-) mice exhibited reduced protective immunity against viral infection and impaired autoimmune diabetes induction in vivo. Although Ag-specific CRTAM(-/-) CD8+ T cells showed normal CTL functions in vitro, their number in the draining lymph node was reduced. Because CRTAM+ T cells bound efficiently to Necl2-expressing CD8+ dendritic cells (DCs) that reside in T cell area of lymph node, CRTAM may induce retention by binding to CD8+ DCs at the late stage of activation before proliferation. The CRTAM-mediated late interaction with DCs induced retention of activated CD8+ T cells in an Ag-independent fashion, and this possibly resulted in effective CTL development in the draining lymph node.

---

## Kirrel

- NCBI Gene
- GeneRIF
- Pubmed

| Search Term | Rank | Fields |
| --- | --- | --- |
| [Mm]igration | 6 | PubMed(1) |
| ECM | 7 | PubMed(1) |
| [Ee]xtracellular [Mm]atrix | 8 | PubMed(2) |

  
hide/show details for Kirrel

### [Mm]igration

#### PubMed

1. Role of Neph2 in pontine nuclei formation in the developing hindbrain. Nuclei are anatomical units of the central nervous system (CNS). Their formation sets the structural basis for the functional organization of the brain, a process known as nucleogenesis. In the present study, we investigated the role of the transmembrane immunoglobulin superfamily molecule Neph2 in the nucleogenesis of the pontine nucleus (PN). Neph2 expression is turned on in migrating PN neurons only after they enter the presumptive nuclear region. Neph2 knockdown disrupted the nuclear organization of PN presumably by changing the migratory behavior of PN neurons inside the nuclear region. Moreover, overexpression of the cytoplasmic region of Neph2, which can sequester intracellular signaling of endogenous Neph2, resulted in similar phenotypes. Overall, these results suggest Neph2 is involved in the nucleogenesis of the PN through the control of neuronal **migration** inside the nucleus.

### ECM

#### PubMed

1. Beta1 integrin expression by podocytes is required to maintain glomerular structural integrity. Integrins are transmembrane heteromeric receptors that mediate interactions between cells and extracellular matrix (**ECM**). beta1, the most abundantly expressed integrin subunit, binds at least 12 alpha subunits. beta1 containing integrins are highly expressed in the glomerulus of the kidney; however their role in glomerular morphogenesis and maintenance of glomerular filtration barrier integrity is poorly understood. To study these questions we selectively deleted beta1 integrin in the podocyte by crossing beta1(flox/flox) mice with podocyte specific podocin-cre mice (pod-Cre), which express cre at the time of glomerular capillary formation. We demonstrate that podocyte abnormalities are visualized during glomerulogenesis of the pod-Cre;beta1(flox/flox) mice and proteinuria is present at birth, despite a grossly normal glomerular basement membrane. Following the advent of glomerular filtration there is progressive podocyte loss and the mice develop capillary loop and mesangium degeneration with little evidence of glomerulosclerosis. By 3 weeks of age the mice develop severe end stage renal failure characterized by both tubulointerstitial and glomerular pathology. Thus, expression of beta1 containing integrins by the podocyte is critical for maintaining the structural integrity of the glomerulus.

### [Ee]xtracellular [Mm]atrix

#### PubMed

1. Beta1 integrin expression by podocytes is required to maintain glomerular structural integrity. Integrins are transmembrane heteromeric receptors that mediate interactions between cells and **extracellular matrix** (ECM). beta1, the most abundantly expressed integrin subunit, binds at least 12 alpha subunits. beta1 containing integrins are highly expressed in the glomerulus of the kidney; however their role in glomerular morphogenesis and maintenance of glomerular filtration barrier integrity is poorly understood. To study these questions we selectively deleted beta1 integrin in the podocyte by crossing beta1(flox/flox) mice with podocyte specific podocin-cre mice (pod-Cre), which express cre at the time of glomerular capillary formation. We demonstrate that podocyte abnormalities are visualized during glomerulogenesis of the pod-Cre;beta1(flox/flox) mice and proteinuria is present at birth, despite a grossly normal glomerular basement membrane. Following the advent of glomerular filtration there is progressive podocyte loss and the mice develop capillary loop and mesangium degeneration with little evidence of glomerulosclerosis. By 3 weeks of age the mice develop severe end stage renal failure characterized by both tubulointerstitial and glomerular pathology. Thus, expression of beta1 containing integrins by the podocyte is critical for maintaining the structural integrity of the glomerulus.
2. Integrin beta1-mediated matrix assembly and signaling are critical for the normal development and function of the kidney glomerulus. The human kidneys filter 180 l of blood every day via about 2.5 million glomeruli. The three layers of the glomerular filtration apparatus consist of fenestrated endothelium, specialized **extracellular matrix** known as the glomerular basement membrane (GBM) and the podocyte foot processes with their modified adherens junctions known as the slit diaphragm (SD). In this study we explored the contribution of podocyte beta1 integrin signaling for normal glomerular function. Mice with podocyte specific deletion of integrin beta1 (podocin-Cre beta1-fl/fl mice) are born normal but cannot complete postnatal renal development. They exhibit detectable proteinuria on day 1 and die within a week. The kidneys of podocin-Cre beta1-fl/fl mice exhibit normal glomerular endothelium but show severe GBM defects with multilaminations and splitting including podocyte foot process effacement. The integrin linked kinase (ILK) is a downstream mediator of integrin beta1 activity in epithelial cells. To further explore whether integrin beta1-mediated signaling facilitates proper glomerular filtration, we generated mice deficient of ILK in the podocytes (podocin-Cre ILK-fl/fl mice). These mice develop normally but exhibit postnatal proteinuria at birth and die within 15 weeks of age due to renal failure. Collectively, our studies demonstrate that podocyte beta1 integrin and ILK signaling is critical for postnatal development and function of the glomerular filtration apparatus.

---

## Kirrel2

- NCBI Gene
- GeneRIF
- Pubmed

| Search Term | Rank | Fields |
| --- | --- | --- |
| [Mm]igration | 6 | PubMed(1) |

  
hide/show details for Kirrel2

### [Mm]igration

#### PubMed

1. Role of Neph2 in pontine nuclei formation in the developing hindbrain. Nuclei are anatomical units of the central nervous system (CNS). Their formation sets the structural basis for the functional organization of the brain, a process known as nucleogenesis. In the present study, we investigated the role of the transmembrane immunoglobulin superfamily molecule Neph2 in the nucleogenesis of the pontine nucleus (PN). Neph2 expression is turned on in migrating PN neurons only after they enter the presumptive nuclear region. Neph2 knockdown disrupted the nuclear organization of PN presumably by changing the migratory behavior of PN neurons inside the nuclear region. Moreover, overexpression of the cytoplasmic region of Neph2, which can sequester intracellular signaling of endogenous Neph2, resulted in similar phenotypes. Overall, these results suggest Neph2 is involved in the nucleogenesis of the PN through the control of neuronal **migration** inside the nucleus.

---

## Ptprq

- NCBI Gene
- GeneRIF
- Pubmed

| Search Term | Rank | Fields |
| --- | --- | --- |
| [Mm]igration | 6 | PubMed(1) |

  
hide/show details for Ptprq

### [Mm]igration

#### PubMed

1. Proliferating and migrating mesangial cells responding to injury express a novel receptor protein-tyrosine phosphatase in experimental mesangial proliferative glomerulonephritis. The mesangial cell provides structural support to the kidney glomerulus. A polymerase chain reaction-based cDNA display approach identified a novel protein-tyrosine phosphatase, rPTP-GMC1, whose transcript expression is transiently and dramatically up-regulated during the period of mesangial cell **migration** and proliferation that follows mesangial cell injury in the anti-Thy 1 model of mesangial proliferative glomerulonephritis in the rat. In situ hybridization analysis confirmed that rPTP-GMC1 mRNA is up-regulated specifically by mesangial cells responding to the injury and is not detectable in other cells in the kidney or in many normal tissues. In cell culture, rPTP-GMC1 is expressed by mesangial cells but not by glomerular endothelial or epithelial cells (podocytes). The longest transcript (7.5 kilobases) encodes a receptor-like protein-tyrosine phosphatase consisting of a single catalytic domain, a transmembrane segment, and 18 fibronectin type III-like repeats in the extracellular segment. A splice variant predicts a truncated molecule missing the catalytic domain. rPTP-GMC1 maps to human chromosome 12q15 and to the distal end of mouse chromosome 10. The predicted structure of rPTP-GMC1 and its pattern of expression in vivo and in culture suggest that it plays a role in regulating the adhesion and **migration** of mesangial cells in response to injury.

---

## Ptprq

- NCBI Gene
- GeneRIF
- Pubmed

| Search Term | Rank | Fields |
| --- | --- | --- |
| [Mm]igration | 6 | PubMed(1) |

  
hide/show details for Ptprq

### [Mm]igration

#### PubMed

1. Proliferating and migrating mesangial cells responding to injury express a novel receptor protein-tyrosine phosphatase in experimental mesangial proliferative glomerulonephritis. The mesangial cell provides structural support to the kidney glomerulus. A polymerase chain reaction-based cDNA display approach identified a novel protein-tyrosine phosphatase, rPTP-GMC1, whose transcript expression is transiently and dramatically up-regulated during the period of mesangial cell **migration** and proliferation that follows mesangial cell injury in the anti-Thy 1 model of mesangial proliferative glomerulonephritis in the rat. In situ hybridization analysis confirmed that rPTP-GMC1 mRNA is up-regulated specifically by mesangial cells responding to the injury and is not detectable in other cells in the kidney or in many normal tissues. In cell culture, rPTP-GMC1 is expressed by mesangial cells but not by glomerular endothelial or epithelial cells (podocytes). The longest transcript (7.5 kilobases) encodes a receptor-like protein-tyrosine phosphatase consisting of a single catalytic domain, a transmembrane segment, and 18 fibronectin type III-like repeats in the extracellular segment. A splice variant predicts a truncated molecule missing the catalytic domain. rPTP-GMC1 maps to human chromosome 12q15 and to the distal end of mouse chromosome 10. The predicted structure of rPTP-GMC1 and its pattern of expression in vivo and in culture suggest that it plays a role in regulating the adhesion and **migration** of mesangial cells in response to injury.

---

## Papln

- NCBI Gene
- GeneRIF
- Pubmed

| Search Term | Rank | Fields |
| --- | --- | --- |
| ECM | 7 | PubMed(3) |
| [Ee]xtracellular [Mm]atrix | 8 | PubMed(4); Component(2) |

  
hide/show details for Papln

### ECM

#### PubMed

1. Nell1-deficient mice have reduced expression of extracellular matrix proteins causing cranial and vertebral defects. The mammalian Nell1 gene encodes a protein kinase C-beta1 (PKC-beta1) binding protein that belongs to a new class of cell-signaling molecules controlling cell growth and differentiation. Over-expression of Nell1 in the developing cranial sutures in both human and mouse induces craniosynostosis, the premature fusion of the growing cranial bone fronts. Here, we report the generation, positional cloning and characterization of Nell1(6R), a recessive, neonatal-lethal point mutation in the mouse Nell1 gene, induced by N-ethyl-N-nitrosourea. Nell1(6R) has a T-->A base change that converts a codon for cysteine into a premature stop codon [Cys(502)Ter], resulting in severe truncation of the predicted protein product and marked reduction in steady-state levels of the transcript. In addition to the expected alteration of cranial morphology, Nell1(6R) mutants manifest skeletal defects in the vertebral column and ribcage, revealing a hitherto undefined role for Nell1 in signal transduction in endochondral ossification. Real-time quantitative reverse transcription-PCR assays of 219 genes showed an association between the loss of Nell1 function and reduced expression of genes for extracellular matrix (**ECM**) proteins critical for chondrogenesis and osteogenesis. Several affected genes are involved in the human cartilage disorder Ehlers-Danlos Syndrome and other disorders associated with spinal curvature anomalies. Nell1(6R) mutant mice are a new tool for elucidating basic mechanisms in osteoblast and chrondrocyte differentiation in the developing skull and vertebral column and understanding how perturbations in the production of **ECM** proteins can lead to anomalies in these structures.
2. O-fucosylation of thrombospondin type 1 repeats restricts epithelial to mesenchymal transition (EMT) and maintains epiblast pluripotency during mouse gastrulation. Thrombospondin type 1 repeat (TSR) superfamily members regulate diverse biological activities ranging from cell motility to inhibition of angiogenesis. In this study, we verified that mouse protein O-fucosyltransferase-2 (POFUT2) specifically adds O-fucose to TSRs. Using two Pofut2 gene-trap lines, we demonstrated that O-fucosylation of TSRs was essential for restricting epithelial to mesenchymal transition in the primitive streak, correct patterning of mesoderm, and localization of the definitive endoderm. Although Pofut2 mutant embryos established anterior/posterior polarity, they underwent extensive mesoderm differentiation at the expense of maintaining epiblast pluripotency. Moreover, mesoderm differentiation was biased towards the vascular endothelial cell lineage. Localization of Foxa2 and Cer1 expressing cells within the interior of Pofut2 mutant embryos suggested that POFUT2 activity was also required for the displacement of the primitive endoderm by definitive endoderm. Notably, Nodal, BMP4, Fgf8, and Wnt3 expression were markedly elevated and expanded in Pofut2 mutants, providing evidence that O-fucose modification of TSRs was essential for modulation of growth factor signaling during gastrulation. The ability of Pofut2 mutant embryos to form teratomas comprised of tissues from all three germ layer origins suggested that defects in Pofut2 mutant embryos resulted from abnormalities in the extracellular environment. This prediction is consistent with the observation that POFUT2 targets are constitutive components of the extracellular matrix (**ECM**) or associate with the **ECM**. For this reason, the Pofut2 mutants represent a valuable tool for studying the role of O-fucosylation in **ECM** synthesis and remodeling, and will be a valuable model to study how post-translational modification of **ECM** components regulates the formation of tissue boundaries, cell movements, and signaling.
3. Transcriptome-based systematic identification of extracellular matrix proteins. Extracellular matrix (**ECM**), which provides critical scaffolds for all adhesive cells, regulates proliferation, differentiation, and apoptosis. Different cell types employ customized **ECM**s, which are thought to play important roles in the generation of so-called niches that contribute to cell-specific functions. The molecular entities of these customized **ECM**s, however, have not been elucidated. Here, we describe a strategy for transcriptome-wide identification of **ECM** proteins based on computational screening of >60,000 full-length mouse cDNAs for secreted proteins, followed by in vitro functional assays. These assays screened the candidate proteins for **ECM**-assembling activities, interactions with other **ECM** molecules, modifications with glycosaminoglycans, and cell-adhesive activities, and were then complemented with immunohistochemical analysis. We identified 16 **ECM** proteins, of which seven were localized in basement membrane (BM) zones. The identification of these previously unknown BM proteins allowed us to construct a body map of BM proteins, which represents the comprehensive immunohistochemistry-based expression profiles of the tissue-specific customization of BMs.

### [Ee]xtracellular [Mm]atrix

#### PubMed

1. Nell1-deficient mice have reduced expression of **extracellular matrix** proteins causing cranial and vertebral defects. The mammalian Nell1 gene encodes a protein kinase C-beta1 (PKC-beta1) binding protein that belongs to a new class of cell-signaling molecules controlling cell growth and differentiation. Over-expression of Nell1 in the developing cranial sutures in both human and mouse induces craniosynostosis, the premature fusion of the growing cranial bone fronts. Here, we report the generation, positional cloning and characterization of Nell1(6R), a recessive, neonatal-lethal point mutation in the mouse Nell1 gene, induced by N-ethyl-N-nitrosourea. Nell1(6R) has a T-->A base change that converts a codon for cysteine into a premature stop codon [Cys(502)Ter], resulting in severe truncation of the predicted protein product and marked reduction in steady-state levels of the transcript. In addition to the expected alteration of cranial morphology, Nell1(6R) mutants manifest skeletal defects in the vertebral column and ribcage, revealing a hitherto undefined role for Nell1 in signal transduction in endochondral ossification. Real-time quantitative reverse transcription-PCR assays of 219 genes showed an association between the loss of Nell1 function and reduced expression of genes for **extracellular matrix** (ECM) proteins critical for chondrogenesis and osteogenesis. Several affected genes are involved in the human cartilage disorder Ehlers-Danlos Syndrome and other disorders associated with spinal curvature anomalies. Nell1(6R) mutant mice are a new tool for elucidating basic mechanisms in osteoblast and chrondrocyte differentiation in the developing skull and vertebral column and understanding how perturbations in the production of ECM proteins can lead to anomalies in these structures.
2. O-fucosylation of thrombospondin type 1 repeats restricts epithelial to mesenchymal transition (EMT) and maintains epiblast pluripotency during mouse gastrulation. Thrombospondin type 1 repeat (TSR) superfamily members regulate diverse biological activities ranging from cell motility to inhibition of angiogenesis. In this study, we verified that mouse protein O-fucosyltransferase-2 (POFUT2) specifically adds O-fucose to TSRs. Using two Pofut2 gene-trap lines, we demonstrated that O-fucosylation of TSRs was essential for restricting epithelial to mesenchymal transition in the primitive streak, correct patterning of mesoderm, and localization of the definitive endoderm. Although Pofut2 mutant embryos established anterior/posterior polarity, they underwent extensive mesoderm differentiation at the expense of maintaining epiblast pluripotency. Moreover, mesoderm differentiation was biased towards the vascular endothelial cell lineage. Localization of Foxa2 and Cer1 expressing cells within the interior of Pofut2 mutant embryos suggested that POFUT2 activity was also required for the displacement of the primitive endoderm by definitive endoderm. Notably, Nodal, BMP4, Fgf8, and Wnt3 expression were markedly elevated and expanded in Pofut2 mutants, providing evidence that O-fucose modification of TSRs was essential for modulation of growth factor signaling during gastrulation. The ability of Pofut2 mutant embryos to form teratomas comprised of tissues from all three germ layer origins suggested that defects in Pofut2 mutant embryos resulted from abnormalities in the extracellular environment. This prediction is consistent with the observation that POFUT2 targets are constitutive components of the **extracellular matrix** (ECM) or associate with the ECM. For this reason, the Pofut2 mutants represent a valuable tool for studying the role of O-fucosylation in ECM synthesis and remodeling, and will be a valuable model to study how post-translational modification of ECM components regulates the formation of tissue boundaries, cell movements, and signaling.
3. Transcriptome-based systematic identification of **extracellular matrix** proteins. **Extracellular matrix** (ECM), which provides critical scaffolds for all adhesive cells, regulates proliferation, differentiation, and apoptosis. Different cell types employ customized ECMs, which are thought to play important roles in the generation of so-called niches that contribute to cell-specific functions. The molecular entities of these customized ECMs, however, have not been elucidated. Here, we describe a strategy for transcriptome-wide identification of ECM proteins based on computational screening of >60,000 full-length mouse cDNAs for secreted proteins, followed by in vitro functional assays. These assays screened the candidate proteins for ECM-assembling activities, interactions with other ECM molecules, modifications with glycosaminoglycans, and cell-adhesive activities, and were then complemented with immunohistochemical analysis. We identified 16 ECM proteins, of which seven were localized in basement membrane (BM) zones. The identification of these previously unknown BM proteins allowed us to construct a body map of BM proteins, which represents the comprehensive immunohistochemistry-based expression profiles of the tissue-specific customization of BMs.
4. Papilin in development; a pericellular protein with a homology to the ADAMTS metalloproteinases. Papilin is an **extracellular matrix** glycoprotein that we have found to be involved in, (1) thin matrix layers during gastrulation, (2) matrix associated with wandering, phagocytic hemocytes, (3) basement membranes and (4) space-filling matrix during Drosophila development. Determination of its cDNA sequence led to the identification of Caenorhabditis and mammalian papilins. A distinctly conserved 'papilin cassette' of domains at the amino-end of papilins is also the carboxyl-end of the ADAMTS subgroup of secreted, matrix-associated metalloproteinases; this cassette contains one thrombospondin type 1 (TSR) domain, a specific cysteine-rich domain and several partial TSR domains. In vitro, papilin non-competitively inhibits procollagen N-proteinase, an ADAMTS metalloproteinase. Inhibiting papilin synthesis in Drosophila or Caenorhabditis causes defective cell arrangements and embryonic death. Ectopic expression of papilin in Drosophila causes lethal abnormalities in muscle, Malpighian tubule and trachea formation. We suggest that papilin influences cell rearrangements and may modulate metalloproteinases during organogenesis.

#### Component

1. **extracellular matrix**
2. proteinaceous **extracellular matrix**

---

## Kirrel3

- NCBI Gene
- GeneRIF
- Pubmed

| Search Term | Rank | Fields |
| --- | --- | --- |
| [Ff]ilopodia | 1 | PubMed(1); GeneRIFs(1) |
| [Mm]igration | 6 | Process(1); PubMed(2); GeneRIFs(1) |

  
hide/show details for Kirrel3

### [Ff]ilopodia

#### PubMed

1. The intellectual disability gene Kirrel3 regulates target-specific mossy fiber synapse development in the hippocampus. Synaptic target specificity, whereby neurons make distinct types of synapses with different target cells, is critical for brain function, yet the mechanisms driving it are poorly understood. In this study, we demonstrate Kirrel3 regulates target-specific synapse formation at hippocampal mossy fiber (MF) synapses, which connect dentate granule (DG) neurons to both CA3 and GABAergic neurons. Here, we show Kirrel3 is required for formation of MF **filopodia**; the structures that give rise to DG-GABA synapses and that regulate feed-forward inhibition of CA3 neurons. Consequently, loss of Kirrel3 robustly increases CA3 neuron activity in developing mice. Alterations in the Kirrel3 gene are repeatedly associated with intellectual disabilities, but the role of Kirrel3 at synapses remained largely unknown. Our findings demonstrate that subtle synaptic changes during development impact circuit function and provide the first insight toward understanding the cellular basis of Kirrel3-dependent neurodevelopmental disorders.

#### GeneRIFs

1. Kirrel3 is required for formation of hippocampal mossy fiber **filopodia**; the structures that give rise to dentate granule-GABA synapses and that regulate feed-forward inhibition of CA3 neurons.

### [Mm]igration

#### Process

1. neuron **migration**

#### PubMed

1. Role of Neph2 in pontine nuclei formation in the developing hindbrain. Nuclei are anatomical units of the central nervous system (CNS). Their formation sets the structural basis for the functional organization of the brain, a process known as nucleogenesis. In the present study, we investigated the role of the transmembrane immunoglobulin superfamily molecule Neph2 in the nucleogenesis of the pontine nucleus (PN). Neph2 expression is turned on in migrating PN neurons only after they enter the presumptive nuclear region. Neph2 knockdown disrupted the nuclear organization of PN presumably by changing the migratory behavior of PN neurons inside the nuclear region. Moreover, overexpression of the cytoplasmic region of Neph2, which can sequester intracellular signaling of endogenous Neph2, resulted in similar phenotypes. Overall, these results suggest Neph2 is involved in the nucleogenesis of the PN through the control of neuronal **migration** inside the nucleus.
2. Role of Neph2 in pontine nuclei formation in the developing hindbrain. Nuclei are anatomical units of the central nervous system (CNS). Their formation sets the structural basis for the functional organization of the brain, a process known as nucleogenesis. In the present study, we investigated the role of the transmembrane immunoglobulin superfamily molecule Neph2 in the nucleogenesis of the pontine nucleus (PN). Neph2 expression is turned on in migrating PN neurons only after they enter the presumptive nuclear region. Neph2 knockdown disrupted the nuclear organization of PN presumably by changing the migratory behavior of PN neurons inside the nuclear region. Moreover, overexpression of the cytoplasmic region of Neph2, which can sequester intracellular signaling of endogenous Neph2, resulted in similar phenotypes. Overall, these results suggest Neph2 is involved in the nucleogenesis of the PN through the control of neuronal **migration** inside the nucleus.

#### GeneRIFs

1. Results suggest Neph2 is involved in the nucleogenesis of the pontine nucleus through the control of neuronal **migration** inside the nucleus.

---

## Syde1

- NCBI Gene
- GeneRIF
- Pubmed

| Search Term | Rank | Fields |
| --- | --- | --- |
| [Mm]igration | 6 | Process(1) |

  
hide/show details for Syde1

### [Mm]igration

#### Process

1. positive regulation of trophoblast cell **migration**

---

## Gmip

- NCBI Gene
- GeneRIF
- Pubmed

| Search Term | Rank | Fields |
| --- | --- | --- |
| [Mm]igration | 6 | PubMed(1); GeneRIFs(1) |

  
hide/show details for Gmip

### [Mm]igration

#### PubMed

1. Speed control for neuronal **migration** in the postnatal brain by Gmip-mediated local inactivation of RhoA. Throughout life, new neurons generated in the ventricular-subventricular zone take the long journey to the olfactory bulb. The intracellular mechanisms that precisely control the neurons' **migration** speed, enabling their well-organized movement, remain unclear. Rho signalling is known to affect the morphology and movement of various cell types, including neurons. Here we identify Gem-interacting protein (Gmip), a RhoA-specific GTPase-activating protein, as a key factor in saltatory neuronal **migration**. RhoA is activated at the proximal leading process of migrating neurons, where Gmip is also localized and negatively regulates RhoA. Gmip controls the saltatory movement of neurons that regulate their **migration** speed and 'stop' positions in the olfactory bulb, thereby altering the neural circuitry. This study demonstrates that Gmip serves as a brake for the RhoA-mediated movement of neuronal somata, and highlights the significance of speed control in the well-organized neuronal **migration** and the maintenance of neuronal circuits in the postnatal brain.

#### GeneRIFs

1. Gmip serves as a brake for the RhoA-mediated movement of neuronal somata, highlighting the significance of speed control in the well-organized neuronal **migration** and the maintenance of neuronal circuits in the postnatal brain.

---

## Arhgap29

- NCBI Gene
- GeneRIF
- Pubmed

| Search Term | Rank | Fields |
| --- | --- | --- |
| [Mm]igration | 6 | PubMed(1) |
| ECM | 7 | PubMed(1) |
| [Ee]xtracellular [Mm]atrix | 8 | PubMed(1) |
| [Mm]etastasis | 12 | PubMed(1) |

  
hide/show details for Arhgap29

### [Mm]igration

#### PubMed

1. KIF14 negatively regulates Rap1a-Radil signaling during breast cancer progression. The small GTPase Rap1 regulates inside-out integrin activation and thereby influences cell adhesion, **migration**, and polarity. Several Rap1 effectors have been described to mediate the cellular effects of Rap1 in a context-dependent manner. Radil is emerging as an important Rap effector implicated in cell spreading and **migration**, but the molecular mechanisms underlying its functions are unclear. We report here that the kinesin KIF14 associates with the PDZ domain of Radil and negatively regulates Rap1-mediated inside-out integrin activation by tethering Radil on microtubules. The depletion of KIF14 led to increased cell spreading, altered focal adhesion dynamics, and inhibition of cell **migration** and invasion. We also show that Radil is important for breast cancer cell proliferation and for metastasis in mice. Our findings provide evidence that the concurrent up-regulation of Rap1 activity and increased KIF14 levels in several cancers is needed to reach optimal levels of Rap1-Radil signaling, integrin activation, and cell-matrix adhesiveness required for tumor progression.

### ECM

#### PubMed

1. Blood vessel tubulogenesis requires Rasip1 regulation of GTPase signaling. Cardiovascular function depends on patent blood vessel formation by endothelial cells (ECs). However, the mechanisms underlying vascular "tubulogenesis" are only beginning to be unraveled. We show that endothelial tubulogenesis requires the Ras interacting protein 1, Rasip1, and its binding partner, the RhoGAP Arhgap29. Mice lacking Rasip1 fail to form patent lumens in all blood vessels, including the early endocardial tube. Rasipl null angioblasts fail to properly localize the polarity determinant Par3 and display defective cell polarity, resulting in mislocalized junctional complexes and loss of adhesion to extracellular matrix (**ECM**). Similarly, depletion of either Rasip1 or Arhgap29 in cultured ECs blocks in vitro lumen formation, fundamentally alters the cytoskeleton, and reduces integrin-dependent adhesion to **ECM**. These defects result from increased RhoA/ROCK/myosin II activity and blockade of Cdc42 and Rac1 signaling. This study identifies Rasip1 as a unique, endothelial-specific regulator of Rho GTPase signaling, which is essential for blood vessel morphogenesis.

### [Ee]xtracellular [Mm]atrix

#### PubMed

1. Blood vessel tubulogenesis requires Rasip1 regulation of GTPase signaling. Cardiovascular function depends on patent blood vessel formation by endothelial cells (ECs). However, the mechanisms underlying vascular "tubulogenesis" are only beginning to be unraveled. We show that endothelial tubulogenesis requires the Ras interacting protein 1, Rasip1, and its binding partner, the RhoGAP Arhgap29. Mice lacking Rasip1 fail to form patent lumens in all blood vessels, including the early endocardial tube. Rasipl null angioblasts fail to properly localize the polarity determinant Par3 and display defective cell polarity, resulting in mislocalized junctional complexes and loss of adhesion to **extracellular matrix** (ECM). Similarly, depletion of either Rasip1 or Arhgap29 in cultured ECs blocks in vitro lumen formation, fundamentally alters the cytoskeleton, and reduces integrin-dependent adhesion to ECM. These defects result from increased RhoA/ROCK/myosin II activity and blockade of Cdc42 and Rac1 signaling. This study identifies Rasip1 as a unique, endothelial-specific regulator of Rho GTPase signaling, which is essential for blood vessel morphogenesis.

### [Mm]etastasis

#### PubMed

1. KIF14 negatively regulates Rap1a-Radil signaling during breast cancer progression. The small GTPase Rap1 regulates inside-out integrin activation and thereby influences cell adhesion, migration, and polarity. Several Rap1 effectors have been described to mediate the cellular effects of Rap1 in a context-dependent manner. Radil is emerging as an important Rap effector implicated in cell spreading and migration, but the molecular mechanisms underlying its functions are unclear. We report here that the kinesin KIF14 associates with the PDZ domain of Radil and negatively regulates Rap1-mediated inside-out integrin activation by tethering Radil on microtubules. The depletion of KIF14 led to increased cell spreading, altered focal adhesion dynamics, and inhibition of cell migration and invasion. We also show that Radil is important for breast cancer cell proliferation and for **metastasis** in mice. Our findings provide evidence that the concurrent up-regulation of Rap1 activity and increased KIF14 levels in several cancers is needed to reach optimal levels of Rap1-Radil signaling, integrin activation, and cell-matrix adhesiveness required for tumor progression.

---

## Arhgap12

- NCBI Gene
- GeneRIF
- Pubmed

| Search Term | Rank | Fields |
| --- | --- | --- |
| [Ee]xtracellular [Mm]atrix | 8 | GeneRIFs(1) |
| [Ii]nvasive | 10 | PubMed(1) |

  
hide/show details for Arhgap12

### [Ee]xtracellular [Mm]atrix

#### GeneRIFs

1. ARHGAP12 inactivates RAC1, thereby impairing cell motility, invasion and adhesion to the **extracellular matrix**. The gene is transcriptionally suppressed by Hepatocyte Growth Factor in human and murine cell lines.

### [Ii]nvasive

#### PubMed

1. Met-driven **invasive** growth involves transcriptional regulation of Arhgap12. **Invasive** growth is a complex biological program triggered by hepatocyte growth factor (HGF) through its tyrosine kinase receptor encoded by the Met proto-oncogene. The program involves-besides proliferation-cell dissociation, motility and **invasive**ness, controlled by intracellular signals impinging on PI3K and on the small G-proteins of the Rac/Rho family. The mechanism(s) unbalancing Rac/Rho activation are still not completely clarified. Here, we describe a functional link between HGF and Arhgap12, a gene encoding a previously uncharacterized protein of the RhoGAP family. We identified Arhgap12 as a transcriptional target of HGF, through a novel gene trapping strategy. We found that Arhgap12 mRNA and protein are robustly suppressed by HGF treatment, but not by serum. Arhgap12 displayed GTPase activating protein (GAP) activity towards Rac1 and, upon overexpression, impaired cell scattering, invasion and adhesion to fibronectin in response to HGF. Consistently, Arhgap12 silencing by RNA interference selectively increased the scatter and adhesion responses. These data show that HGF-driven **invasive** growth involves transcriptional regulation of a Rac1-specific GAP.

---

## Arhgap15

- NCBI Gene
- GeneRIF
- Pubmed

| Search Term | Rank | Fields |
| --- | --- | --- |
| [Mm]igration | 6 | PubMed(1) |

  
hide/show details for Arhgap15

### [Mm]igration

#### PubMed

1. The RacGAP ArhGAP15 is a master negative regulator of neutrophil functions. In phagocytes, GTPases of the Rac family control crucial antimicrobial functions. The RacGAP ArhGAP15 negatively modulates Rac activity in leukocytes, but its in vivo role in innate immunity remains largely unknown. Here we show that neutrophils and macrophages derived from mice lacking ArhGAP15 presented higher Rac activity but distinct phenotypes. In macrophages, the loss of ArhGAP15 induced increased cellular elongation and membrane protrusions but did not modify chemotactic responses. Conversely, the lack of ArhGAP15 in neutrophils affected critical Rac-dependent antimicrobial functions, specifically causing enhanced chemotactic responses, straighter directional **migration**, amplified reactive oxygen species production, increased phagocytosis, and improved bacterial killing. In vivo, in a model of severe abdominal sepsis, these effects contributed to increase neutrophil recruitment to the site of infection, thereby limiting bacterial growth, controlling infection spread, reducing systemic inflammation, and ultimately improving survival in ArhGAP15-null mice. Altogether, these results demonstrate the relevance of ArhGAP15 in the selective regulation of multiple neutrophil functions, suggesting that ArhGAP15 targeting might be beneficial in specific pathologic settings like severe sepsis.

---

## Racgap1

- NCBI Gene
- GeneRIF
- Pubmed

| Search Term | Rank | Fields |
| --- | --- | --- |
| [Ff]ilopodia | 1 | PubMed(2) |
| [Mm]igration | 6 | PubMed(3) |
| ECM | 7 | PubMed(1); GeneRIFs(1) |
| [Ee]xtracellular [Mm]atrix | 8 | PubMed(1) |
| [Mm]etastasis | 12 | PubMed(1) |

  
hide/show details for Racgap1

### [Ff]ilopodia

#### PubMed

1. Laminin alpha5 is required for dental epithelium growth and polarity and the development of tooth bud and shape. In tooth development, the oral ectoderm and mesenchyme coordinately and reciprocally interact through the basement membrane for their growth and differentiation to form the proper shape and size of the tooth. Laminin alpha5 subunit-containing laminin-10/11 (LM-511/521) is the major laminin in the tooth germ basement membrane. Here, we have examined the role of laminin alpha5 (Lama5) in tooth development using laminin alpha5-null mouse primary dental epithelium and tooth germ organ cultures. Lama5-null mice develop a small tooth germ with defective cusp formation and have reduced proliferation of dental epithelium. Also, cell polarity and formation of the monolayer of the inner dental epithelium are disturbed. The enamel knot, a signaling center for tooth germ development, is defective, and there is a significant reduction of Shh and Fgf4 expression in the dental epithelium. In the absence of laminin alpha5, the basement membrane in the inner dental epithelium becomes discontinuous. In normal mice, integrin alpha6beta4, a receptor for laminin alpha5, is strongly localized at the basal layer of the epithelium, whereas in mutant mice, integrin alpha6beta4 is expressed around the cell surface. In primary dental epithelium culture, laminin-10/11 promotes cell growth, spreading, and **filopodia**-like microspike formation. This promotion is inhibited by anti-integrin alpha6 and beta4 antibodies and by phosphatidylinositol 3-kinase inhibitors and dominant negative Rho-GTPase family proteins Cdc42 and Rac. In organ culture, anti-integrin alpha6 antibody and wortmannin reduce tooth germ size and shape. Our studies demonstrate that laminin alpha5 is required for the proliferation and polarity of basal epithelial cells and suggest that the interaction between laminin-10/11-integrin alpha6beta4 and the phosphatidylinositol 3-kinase-Cdc42/Rac pathways play an important role in determining the size and shape of tooth germ.
2. Laminin alpha5 is required for dental epithelium growth and polarity and the development of tooth bud and shape. In tooth development, the oral ectoderm and mesenchyme coordinately and reciprocally interact through the basement membrane for their growth and differentiation to form the proper shape and size of the tooth. Laminin alpha5 subunit-containing laminin-10/11 (LM-511/521) is the major laminin in the tooth germ basement membrane. Here, we have examined the role of laminin alpha5 (Lama5) in tooth development using laminin alpha5-null mouse primary dental epithelium and tooth germ organ cultures. Lama5-null mice develop a small tooth germ with defective cusp formation and have reduced proliferation of dental epithelium. Also, cell polarity and formation of the monolayer of the inner dental epithelium are disturbed. The enamel knot, a signaling center for tooth germ development, is defective, and there is a significant reduction of Shh and Fgf4 expression in the dental epithelium. In the absence of laminin alpha5, the basement membrane in the inner dental epithelium becomes discontinuous. In normal mice, integrin alpha6beta4, a receptor for laminin alpha5, is strongly localized at the basal layer of the epithelium, whereas in mutant mice, integrin alpha6beta4 is expressed around the cell surface. In primary dental epithelium culture, laminin-10/11 promotes cell growth, spreading, and **filopodia**-like microspike formation. This promotion is inhibited by anti-integrin alpha6 and beta4 antibodies and by phosphatidylinositol 3-kinase inhibitors and dominant negative Rho-GTPase family proteins Cdc42 and Rac. In organ culture, anti-integrin alpha6 antibody and wortmannin reduce tooth germ size and shape. Our studies demonstrate that laminin alpha5 is required for the proliferation and polarity of basal epithelial cells and suggest that the interaction between laminin-10/11-integrin alpha6beta4 and the phosphatidylinositol 3-kinase-Cdc42/Rac pathways play an important role in determining the size and shape of tooth germ.

### [Mm]igration

#### PubMed

1. TLR4 signaling shapes B cell dynamics via MyD88-dependent pathways and Rac GTPases. B cells use a plethora of TLR to recognize pathogen-derived ligands. These innate signals have an important function in the B cell adaptive immune response and modify their trafficking and tissue location. The direct role of TLR signaling on B cell dynamics nonetheless remains almost entirely unknown. In this study, we used a state-of-the-art two-dimensional model combined with real-time microscopy to study the effect of TLR4 stimulation on mouse B cell motility in response to chemokines. We show that a minimum stimulation period is necessary for TLR4 modification of B cell behavior. TLR4 stimulation increased B cell polarization, **migration**, and directionality; these increases were dependent on the MyD88 signaling pathway and did not require ERK or p38 MAPK activity downstream of TLR4. In addition, TLR4 stimulation enhanced Rac GTPase activity and promoted sustained Rac activation in response to chemokines. These results increase our understanding of the regulation of B cell dynamics by innate signals and the underlying molecular mechanisms.
2. Structure and expression of murine mgcRacGAP: its developmental regulation suggests a role for the Rac/MgcRacGAP signalling pathway in neurogenesis. Rho-family GTPases regulate a wide range of biological functions including cell **migration**, cell adhesion and cell growth. Recently, results from studies in vivo in Drosophila, mouse and humans have demonstrated the involvement of these GTPases in mechanisms controlling neuronal differentiation and the development of the central nervous system (CNS). However, the signalling pathways underlying these functions and the proteins directly regulating RhoGTPases in developing neurons are poorly defined. Here we report the structure and expression pattern of the murine orthologue of mgcRacGAP, a human gene encoding a RacGTPase partner expressed in male germ cells [Touré, Dorseuil, Morin, Timmons, Jegou, Reibel and Gacon (1998) J. Biol. Chem. 273, 6019-6023]. In contrast with that from humans, murine mgcRacGAP encodes two distinct transcripts. Both are developmentally regulated. A 2.2 kb transcript is strongly expressed in mature testis and is up-regulated with spermatogenesis. A 3 kb RNA is predominant in the embryo and is expressed primarily in the CNS during the neurogenic phase, decreasing after birth. In situ hybridization analysis in embryonic-day 14.5 mouse embryos demonstrates a preferential expression of mgcRacGAP in the proliferative ventricular zone of the cortex. In addition to the expression of mgcRacGAP in male germ cells already reported in humans and suggesting an involvement in spermatogenesis, we characterize an embryonic transcript whose expression is closely correlated with neurogenesis. This result addresses the question of the role of Rac/MgcRacGAP pathway in neuronal proliferation.
3. Rac regulates vascular endothelial growth factor stimulated motility. During angiogenesis endothelial cells migrate towards a chemotactic stimulus. Understanding the mechanism of endothelial cell **migration** is critical to the therapeutic manipulation of angiogenesis and ultimately cancer prevention. Vascular endothelial growth factor (VEGF) is a potent chemotactic stimulus of endothelial cells during angiogenesis. The endothelial cell signal transduction pathway of VEGF represents a potential target for cancer therapy, but the mechanisms of post-receptor signal transduction including the roles of rho family GTPases in regulating the cytoskeletal effects of VEGF in endothelial cells are not understood. Here we analyze the mechanisms of cell **migration** in the mouse brain endothelial cell line (bEND3). Stable transfectants containing a tetracycline repressible expression vector were used to induce expression of Rac mutants. Endothelial cell haptotaxis was stimulated by constitutively active V12Rac on collagen and vitronectin coated supports, and chemotaxis was further stimulated by VEGF. Osteopontin coated supports were the most stimulatory to bEND3 haptotaxis, but VEGF was not effective in further increasing **migration** on osteopontin coated supports. Haptotaxis on support coated with collagen, vitronectin, and to a lesser degree osteopontin was inhibited by N17 Rac. N17 Rac expression blocked stimulation of endothelial cell chemotaxis by VEGF. As part of the chemotactic stimulation, VEGF caused a loss of actin organization at areas of cell-cell contact and increased stress fiber expression in endothelial cells which were directed towards pores in the transwell membrane. N17 Rac prevented the stimulation of cell-cell contact disruption and the stress fiber stimulation by VEGF. These data demonstrate two pathways of regulating endothelial cell motility, one in which Rac is activated by matrix/integrin stimulation and is a crucial modulator of endothelial cell haptotaxis. The other pathway, in the presence of osteopontin, is Rac independent. VEGF stimulated chemotaxis, is critically dependent on Rac activation. Osteopontin was a potent matrix activator of motility, and perhaps one explanation for the absence of a VEGF plus osteopontin effect is that osteopontin stimulated motility was inhibitory to the Rac pathway.

### ECM

#### PubMed

1. Directional control of cell motility through focal adhesion positioning and spatial control of Rac activation. Local physical interactions between cells and extracellular matrix (**ECM**) influence directional cell motility that is critical for tissue development, wound repair, and cancer metastasis. Here we test the possibility that the precise spatial positioning of focal adhesions governs the direction in which cells spread and move. NIH 3T3 cells were cultured on circular or linear **ECM** islands, which were created using a microcontact printing technique and were 1 microm wide and of various lengths (1 to 8 microm) and separated by 1 to 4.5 microm wide nonadhesive barrier regions. Cells could be driven proactively to spread and move in particular directions by altering either the interisland spacing or the shape of similar-sized **ECM** islands. Immunofluorescence microscopy confirmed that focal adhesions assembled preferentially above the **ECM** islands, with the greatest staining intensity being observed at adhesion sites along the cell periphery. Rac-FRET analysis of living cells revealed that Rac became activated within 2 min after peripheral membrane extensions adhered to new **ECM** islands, and this activation wave propagated outward in an oriented manner as the cells spread from island to island. A computational model, which incorporates that cells preferentially protrude membrane processes from regions near newly formed focal adhesion contacts, could predict with high accuracy the effects of six different arrangements of micropatterned **ECM** islands on directional cell spreading. Taken together, these results suggest that physical properties of the **ECM** may influence directional cell movement by dictating where cells will form new focal adhesions and activate Rac and, hence, govern where new membrane protrusions will form.

#### GeneRIFs

1. Rac became activated within 2 min after peripheral membrane extensions adhered to new **ECM** islands, and this activation wave propagated outward in an oriented manner as the cells spread from island to island

### [Ee]xtracellular [Mm]atrix

#### PubMed

1. Directional control of cell motility through focal adhesion positioning and spatial control of Rac activation. Local physical interactions between cells and **extracellular matrix** (ECM) influence directional cell motility that is critical for tissue development, wound repair, and cancer metastasis. Here we test the possibility that the precise spatial positioning of focal adhesions governs the direction in which cells spread and move. NIH 3T3 cells were cultured on circular or linear ECM islands, which were created using a microcontact printing technique and were 1 microm wide and of various lengths (1 to 8 microm) and separated by 1 to 4.5 microm wide nonadhesive barrier regions. Cells could be driven proactively to spread and move in particular directions by altering either the interisland spacing or the shape of similar-sized ECM islands. Immunofluorescence microscopy confirmed that focal adhesions assembled preferentially above the ECM islands, with the greatest staining intensity being observed at adhesion sites along the cell periphery. Rac-FRET analysis of living cells revealed that Rac became activated within 2 min after peripheral membrane extensions adhered to new ECM islands, and this activation wave propagated outward in an oriented manner as the cells spread from island to island. A computational model, which incorporates that cells preferentially protrude membrane processes from regions near newly formed focal adhesion contacts, could predict with high accuracy the effects of six different arrangements of micropatterned ECM islands on directional cell spreading. Taken together, these results suggest that physical properties of the ECM may influence directional cell movement by dictating where cells will form new focal adhesions and activate Rac and, hence, govern where new membrane protrusions will form.

### [Mm]etastasis

#### PubMed

1. Directional control of cell motility through focal adhesion positioning and spatial control of Rac activation. Local physical interactions between cells and extracellular matrix (ECM) influence directional cell motility that is critical for tissue development, wound repair, and cancer **metastasis**. Here we test the possibility that the precise spatial positioning of focal adhesions governs the direction in which cells spread and move. NIH 3T3 cells were cultured on circular or linear ECM islands, which were created using a microcontact printing technique and were 1 microm wide and of various lengths (1 to 8 microm) and separated by 1 to 4.5 microm wide nonadhesive barrier regions. Cells could be driven proactively to spread and move in particular directions by altering either the interisland spacing or the shape of similar-sized ECM islands. Immunofluorescence microscopy confirmed that focal adhesions assembled preferentially above the ECM islands, with the greatest staining intensity being observed at adhesion sites along the cell periphery. Rac-FRET analysis of living cells revealed that Rac became activated within 2 min after peripheral membrane extensions adhered to new ECM islands, and this activation wave propagated outward in an oriented manner as the cells spread from island to island. A computational model, which incorporates that cells preferentially protrude membrane processes from regions near newly formed focal adhesion contacts, could predict with high accuracy the effects of six different arrangements of micropatterned ECM islands on directional cell spreading. Taken together, these results suggest that physical properties of the ECM may influence directional cell movement by dictating where cells will form new focal adhesions and activate Rac and, hence, govern where new membrane protrusions will form.

---

## Tec

- NCBI Gene
- GeneRIF
- Pubmed

| Search Term | Rank | Fields |
| --- | --- | --- |
| [Ii]nvasive | 10 | PubMed(2) |

  
hide/show details for Tec

### [Ii]nvasive

#### PubMed

1. The non-receptor tyrosine kinase Tec controls assembly and activity of the noncanonical caspase-8 inflammasome. Tec family kinases are intracellular non-receptor tyrosine kinases implicated in numerous functions, including T cell and B cell regulation. However, a role in microbial pathogenesis has not been described. Here, we identified Tec kinase as a novel key mediator of the inflammatory immune response in macrophages invaded by the human fungal pathogen C. albicans. Tec is required for both activation and assembly of the noncanonical caspase-8, but not of the caspase-1 inflammasome, during infections with fungal but not bacterial pathogens, triggering the antifungal response through IL-1β. Furthermore, we identify dectin-1 as the pathogen recognition receptor being required for Syk-dependent Tec activation. Hence, Tec is a novel innate-specific inflammatory kinase, whose genetic ablation or inhibition by small molecule drugs strongly protects mice from fungal sepsis. These data demonstrate a therapeutic potential for Tec kinase inhibition to combat **invasiv**e microbial infections by attenuating the host inflammatory response.
2. The non-receptor tyrosine kinase Tec controls assembly and activity of the noncanonical caspase-8 inflammasome. Tec family kinases are intracellular non-receptor tyrosine kinases implicated in numerous functions, including T cell and B cell regulation. However, a role in microbial pathogenesis has not been described. Here, we identified Tec kinase as a novel key mediator of the inflammatory immune response in macrophages invaded by the human fungal pathogen C. albicans. Tec is required for both activation and assembly of the noncanonical caspase-8, but not of the caspase-1 inflammasome, during infections with fungal but not bacterial pathogens, triggering the antifungal response through IL-1β. Furthermore, we identify dectin-1 as the pathogen recognition receptor being required for Syk-dependent Tec activation. Hence, Tec is a novel innate-specific inflammatory kinase, whose genetic ablation or inhibition by small molecule drugs strongly protects mice from fungal sepsis. These data demonstrate a therapeutic potential for Tec kinase inhibition to combat **invasiv**e microbial infections by attenuating the host inflammatory response.

---

## Bmx

- NCBI Gene
- GeneRIF
- Pubmed

| Search Term | Rank | Fields |
| --- | --- | --- |
| [Mm]igration | 6 | PubMed(3) |
| [Ee]xtracellular [Mm]atrix | 8 | PubMed(1) |
| [Ii]nvasive | 10 | PubMed(1) |
| [Mm]etastasis | 12 | PubMed(1) |

  
hide/show details for Bmx

### [Mm]igration

#### PubMed

1. Etk/Bmx regulates proteinase-activated-receptor1 (PAR1) in breast cancer invasion: signaling partners, hierarchy and physiological significance. While protease-activated-receptor 1 (PAR(1)) plays a central role in tumor progression, little is known about the cell signaling involved.We show here the impact of PAR(1) cellular activities using both an orthotopic mouse mammary xenograft and a colorectal-liver metastasis model in vivo, with biochemical analyses in vitro. Large and highly vascularized tumors were generated by cells over-expressing wt hPar1, Y397Z hPar1, with persistent signaling, or Y381A hPar1 mutant constructs. In contrast, cells over-expressing the truncated form of hPar1, which lacks the cytoplasmic tail, developed small or no tumors, similar to cells expressing empty vector or control untreated cells. Antibody array membranes revealed essential hPar1 partners including Etk/Bmx and Shc. PAR(1) activation induces Etk/Bmx and Shc binding to the receptor C-tail to form a complex. Y/A mutations in the PAR(1) C-tail did not prevent Shc-PAR(1) association, but enhanced the number of liver metastases compared with the already increased metastases obtained with wt hPar1. We found that Etk/Bmx first binds via the PH domain to a region of seven residues, located between C378-S384 in PAR(1) C-tail, enabling subsequent Shc association. Importantly, expression of the hPar1-7A mutant form (substituted A, residues 378-384), which is incapable of binding Etk/Bmx, resulted in inhibition of invasion through Matrigel-coated membranes. Similarly, knocking down Etk/Bmx inhibited PAR(1)-induced MDA-MB-435 cell **migration**. In addition, intact spheroid morphogenesis of MCF10A cells is markedly disrupted by the ectopic expression of wt hPar1. In contrast, the forced expression of the hPar1-7A mutant results in normal ball-shaped spheroids. Thus, by preventing binding of Etk/Bmx to PAR(1) -C-tail, hPar1 oncogenic properties are abrogated.This is the first demonstration that a cytoplasmic portion of the PAR(1) C-tail functions as a scaffold site. We identify here essential signaling partners, determine the hierarchy of binding and provide a platform for therapeutic vehicles via definition of the critical PAR(1)-associating region in the breast cancer signaling niche.
2. Bmx tyrosine kinase transgene induces skin hyperplasia, inflammatory angiogenesis, and accelerated wound healing. The Bmx gene, a member of the Tec family of nonreceptor protein tyrosine kinases, is expressed in arterial endothelium and in certain hematopoietic and epithelial cells. Previous in vitro studies have implicated Bmx signaling in cell **migration** and survival and suggested that it contributes to the progression of prostate carcinomas. However, the function of Bmx in normal tissues in vivo is unknown. We show here that Bmx expression is induced in skin keratinocytes during wound healing. To analyze the role of Bmx in epidermal keratinocytes in vivo, we generated transgenic mice overexpressing Bmx in the skin. We show that Bmx overexpression accelerates keratinocyte proliferation and wound reepithelialization. Bmx expression also induces chronic inflammation and angiogenesis in the skin, and gene expression profiling suggests that this occurs via cytokine-mediated recruitment of inflammatory cells. Our studies provide the first data on Bmx function in vivo and form the basis of evaluation of its role in epithelial neoplasia.
3. Critical function of Bmx/Etk in ischemia-mediated arteriogenesis and angiogenesis. Bmx/Etk non-receptor tyrosine protein kinase has been implicated in endothelial cell **migration** and tube formation in vitro. However, the role of Bmx in vivo is not known. Bmx is highly induced in the vasculature of ischemic hind limbs. We used both mice with a genetic deletion of Bmx (Bmx-KO mice) and transgenic mice expressing a constitutively active form of Bmx under the endothelial Tie-2 enhancer/promoter (Bmx-SK-Tg mice) to study the role of Bmx in ischemia-mediated arteriogenesis/angiogenesis. In response to ischemia, Bmx-KO mice had markedly reduced, whereas Bmx-SK-Tg mice had enhanced, clinical recovery, limb perfusion, and ischemic reserve capacity when compared with nontransgenic control mice. The functional outcomes in these mice were correlated with ischemia-initiated arteriogenesis, capillary formation, and vessel maturation as well as Bmx-dependent expression/activation of TNF receptor 2- and VEGFR2-mediated (TNFR2/VEGFR2-mediated) angiogenic signaling in both hind limb and bone marrow. More importantly, results of bone marrow transplantation studies showed that Bmx in bone marrow-derived cells plays a critical role in the early phase of ischemic tissue remodeling. Our study provides the first demonstration to our knowledge that Bmx in endothelium and bone marrow plays a critical role in arteriogenesis/angiogenesis in vivo and suggests that Bmx may be a novel target for the treatment of vascular diseases such as coronary artery disease and peripheral arterial disease.

### [Ee]xtracellular [Mm]atrix

#### PubMed

1. Endothelial Bmx tyrosine kinase activity is essential for myocardial hypertrophy and remodeling. Cardiac hypertrophy accompanies many forms of heart disease, including ischemic disease, hypertension, heart failure, and valvular disease, and it is a strong predictor of increased cardiovascular morbidity and mortality. Deletion of bone marrow kinase in chromosome X (Bmx), an arterial nonreceptor tyrosine kinase, has been shown to inhibit cardiac hypertrophy in mice. This finding raised the possibility of therapeutic use of Bmx tyrosine kinase inhibitors, which we have addressed here by analyzing cardiac hypertrophy in gene-targeted mice deficient in Bmx tyrosine kinase activity. We found that angiotensin II (Ang II)-induced cardiac hypertrophy is significantly reduced in mice deficient in Bmx and in mice with inactivated Bmx tyrosine kinase compared with WT mice. Genome-wide transcriptomic profiling showed that Bmx inactivation suppresses myocardial expression of genes related to Ang II-induced inflammatory and **extracellular matrix** responses whereas expression of RNAs encoding mitochondrial proteins after Ang II administration was maintained in Bmx-inactivated hearts. Very little or no Bmx mRNA was expressed in human cardiomyocytes whereas human cardiac endothelial cells expressed abundant amounts. Ang II stimulation of endothelial cells increased Bmx phosphorylation, and Bmx gene silencing inhibited downstream STAT3 signaling, which has been implicated in cardiac hypertrophy. Furthermore, activation of the mechanistic target of rapamycin complex 1 pathway by Ang II treatment was decreased in the Bmx-deficient hearts. Our results demonstrate that inhibition of the cross-talk between endothelial cells and cardiomyocytes by Bmx inactivation suppresses Ang II-induced signals for cardiac hypertrophy. These results suggest that the endothelial Bmx tyrosine kinase could provide a target to attenuate the development of cardiac hypertrophy.

### [Ii]nvasive

#### PubMed

1. Etk/Bmx tyrosine kinase activates Pak1 and regulates tumorigenicity of breast cancer cells. Etk/Bmx, a member of the Tec family of nonreceptor protein-tyrosine kinases, is characterized by an N-terminal pleckstrin homology domain and has been shown to be a downstream effector of phosphatidylinositol 3-kinase. P21-activated kinase 1 (Pak1), another well characterized effector of phosphatidylinositol 3-kinase, has been implicated in the progression of breast cancer cells. In this study, we characterized the role of Etk in mammary development and tumorigenesis and explored the functional interactions between Etk and Pak1. We report that Etk expression is developmentally regulated in the mammary gland. Using transient transfection, coimmunoprecipitation and glutathione S-transferase-pull down assays, we showed that Etk directly associates with Pak1 via its N-terminal pleckstrin homology domain and also phosphorylates Pak1 on tyrosine residues. The expression of wild-type Etk in a non-**invasive** human breast cancer MCF-7 cells significantly increased proliferation and anchorage-independent growth of epithelial cancer cells. Conversely, expression of kinase-inactive mutant Etk-KQ suppressed the proliferation, anchorage-independent growth, and tumorigenicity of human breast cancer MDA-MB435 cells. These results indicate that Pak1 is a target of Etk and that Etk controls the proliferation as well as the anchorage-independent and tumorigenic growth of mammary epithelial cancer cells.

### [Mm]etastasis

#### PubMed

1. Etk/Bmx regulates proteinase-activated-receptor1 (PAR1) in breast cancer invasion: signaling partners, hierarchy and physiological significance. While protease-activated-receptor 1 (PAR(1)) plays a central role in tumor progression, little is known about the cell signaling involved.We show here the impact of PAR(1) cellular activities using both an orthotopic mouse mammary xenograft and a colorectal-liver **metastasis** model in vivo, with biochemical analyses in vitro. Large and highly vascularized tumors were generated by cells over-expressing wt hPar1, Y397Z hPar1, with persistent signaling, or Y381A hPar1 mutant constructs. In contrast, cells over-expressing the truncated form of hPar1, which lacks the cytoplasmic tail, developed small or no tumors, similar to cells expressing empty vector or control untreated cells. Antibody array membranes revealed essential hPar1 partners including Etk/Bmx and Shc. PAR(1) activation induces Etk/Bmx and Shc binding to the receptor C-tail to form a complex. Y/A mutations in the PAR(1) C-tail did not prevent Shc-PAR(1) association, but enhanced the number of liver metastases compared with the already increased metastases obtained with wt hPar1. We found that Etk/Bmx first binds via the PH domain to a region of seven residues, located between C378-S384 in PAR(1) C-tail, enabling subsequent Shc association. Importantly, expression of the hPar1-7A mutant form (substituted A, residues 378-384), which is incapable of binding Etk/Bmx, resulted in inhibition of invasion through Matrigel-coated membranes. Similarly, knocking down Etk/Bmx inhibited PAR(1)-induced MDA-MB-435 cell migration. In addition, intact spheroid morphogenesis of MCF10A cells is markedly disrupted by the ectopic expression of wt hPar1. In contrast, the forced expression of the hPar1-7A mutant results in normal ball-shaped spheroids. Thus, by preventing binding of Etk/Bmx to PAR(1) -C-tail, hPar1 oncogenic properties are abrogated.This is the first demonstration that a cytoplasmic portion of the PAR(1) C-tail functions as a scaffold site. We identify here essential signaling partners, determine the hierarchy of binding and provide a platform for therapeutic vehicles via definition of the critical PAR(1)-associating region in the breast cancer signaling niche.

---

## Txk

- NCBI Gene
- GeneRIF
- Pubmed

| Search Term | Rank | Fields |
| --- | --- | --- |
| [Mm]igration | 6 | PubMed(1) |

  
hide/show details for Txk

### [Mm]igration

#### PubMed

1. A Small Molecule Inhibitor of ITK and RLK Impairs Th1 Differentiation and Prevents Colitis Disease Progression. In T cells, the Tec kinases IL-2-inducible T cell kinase (ITK) and resting lymphocyte kinase (RLK) are activated by TCR stimulation and are required for optimal downstream signaling. Studies of CD4(+) T cells from Itk(-/-) and Itk(-/-)Rlk(-/-) mice have indicated differential roles of ITK and RLK in Th1, Th2, and Th17 differentiation and cytokine production. However, these findings are confounded by the complex T cell developmental defects in these mice. In this study, we examine the consequences of ITK and RLK inhibition using a highly selective and potent small molecule covalent inhibitor PRN694. In vitro Th polarization experiments indicate that PRN694 is a potent inhibitor of Th1 and Th17 differentiation and cytokine production. Using a T cell adoptive transfer model of colitis, we find that in vivo administration of PRN694 markedly reduces disease progression, T cell infiltration into the intestinal lamina propria, and IFN-γ production by colitogenic CD4(+) T cells. Consistent with these findings, Th1 and Th17 cells differentiated in the presence of PRN694 show reduced P-selectin binding and impaired **migratio**n to CXCL11 and CCL20, respectively. Taken together, these data indicate that ITK plus RLK inhibition may have therapeutic potential in Th1-mediated inflammatory diseases.

---

## Itk

- NCBI Gene
- GeneRIF
- Pubmed

| Search Term | Rank | Fields |
| --- | --- | --- |
| [Mm]igration | 6 | PubMed(9); GeneRIFs(3) |

  
hide/show details for Itk

### [Mm]igration

#### PubMed

1. A Small Molecule Inhibitor of ITK and RLK Impairs Th1 Differentiation and Prevents Colitis Disease Progression. In T cells, the Tec kinases IL-2-inducible T cell kinase (ITK) and resting lymphocyte kinase (RLK) are activated by TCR stimulation and are required for optimal downstream signaling. Studies of CD4(+) T cells from Itk(-/-) and Itk(-/-)Rlk(-/-) mice have indicated differential roles of ITK and RLK in Th1, Th2, and Th17 differentiation and cytokine production. However, these findings are confounded by the complex T cell developmental defects in these mice. In this study, we examine the consequences of ITK and RLK inhibition using a highly selective and potent small molecule covalent inhibitor PRN694. In vitro Th polarization experiments indicate that PRN694 is a potent inhibitor of Th1 and Th17 differentiation and cytokine production. Using a T cell adoptive transfer model of colitis, we find that in vivo administration of PRN694 markedly reduces disease progression, T cell infiltration into the intestinal lamina propria, and IFN-γ production by colitogenic CD4(+) T cells. Consistent with these findings, Th1 and Th17 cells differentiated in the presence of PRN694 show reduced P-selectin binding and impaired **migratio**n to CXCL11 and CCL20, respectively. Taken together, these data indicate that ITK plus RLK inhibition may have therapeutic potential in Th1-mediated inflammatory diseases.
2. A Small Molecule Inhibitor of ITK and RLK Impairs Th1 Differentiation and Prevents Colitis Disease Progression. In T cells, the Tec kinases IL-2-inducible T cell kinase (ITK) and resting lymphocyte kinase (RLK) are activated by TCR stimulation and are required for optimal downstream signaling. Studies of CD4(+) T cells from Itk(-/-) and Itk(-/-)Rlk(-/-) mice have indicated differential roles of ITK and RLK in Th1, Th2, and Th17 differentiation and cytokine production. However, these findings are confounded by the complex T cell developmental defects in these mice. In this study, we examine the consequences of ITK and RLK inhibition using a highly selective and potent small molecule covalent inhibitor PRN694. In vitro Th polarization experiments indicate that PRN694 is a potent inhibitor of Th1 and Th17 differentiation and cytokine production. Using a T cell adoptive transfer model of colitis, we find that in vivo administration of PRN694 markedly reduces disease progression, T cell infiltration into the intestinal lamina propria, and IFN-γ production by colitogenic CD4(+) T cells. Consistent with these findings, Th1 and Th17 cells differentiated in the presence of PRN694 show reduced P-selectin binding and impaired **migratio**n to CXCL11 and CCL20, respectively. Taken together, these data indicate that ITK plus RLK inhibition may have therapeutic potential in Th1-mediated inflammatory diseases.
3. The Tec kinase ITK regulates thymic expansion, e**migration**, and maturation of γδ NKT cells. The Tec family tyrosine kinase, Itk, regulates signaling downstream of the TCR. The absence of Itk in CD4(+) T cells results in impaired Th2 responses along with defects in maturation, cytokine production, and survival of iNKT cells. Paradoxically, Itk(-/-) mice have spontaneously elevated serum IgE levels, resulting from an expansion of the Vγ1.1(+)Vδ6.3(+) subset of γδ T cells, known as γδ NKT cells. Comparisons between γδ NKT cells and αβ iNKT cells showed convergence in the pattern of cell surface marker expression, cytokine profiles, and gene expression, suggesting that these two subsets of NKT cells undergo similar differentiation programs. Hepatic γδ NKT cells have an invariant TCR and are derived predominantly from fetal progenitors that expand in the thymus during the first weeks of life. The adult thymus contains these invariant γδ NKT cells plus a heterogeneous population of Vγ1.1(+)Vδ6.3(+) T cells with diverse CDR3 sequences. This latter population, normally excluded from the liver, escapes the thymus and homes to the liver when Itk is absent. In addition, Itk(-/-) γδ NKT cells persistently express high levels of Zbtb16 (PLZF) and Il4, genes that are normally downregulated in the most mature subsets of NKT cells. These data indicate that Itk signaling is required to prevent the expansion of γδ NKT cells in the adult th**ymus, to** block their emigration, and to promote terminal NKT cell maturation.
4. Itk signals promote neuroinflammation by regulating CD4+ T-cell activation and trafficking. Here we demonstrate that interleukin-2-inducible T-cell kinase (Itk) signaling in cluster of differentiation 4-positive (CD4(+)) T cells promotes experimental autoimmune encephalomyelitis (EAE), the animal model of multiple sclerosis (MS). We show that Itk(-/-) mice exhibit reduced disease severity, and transfer of Itk(-/-) CD4(+) T cells into T cell-deficient recipients results in lower disease severity. We observed a significant reduction of CD4(+) T cells in the CNS of Itk(-/-) mice or recipients of Itk(-/-) CD4(+) T cells during EAE, which is consistent with attenuated disease. Itk(-/-) CD4(+) T cells exhibit defective response to myelin antigen stimulation attributable to displacement of filamentous actin from the CD4(+) coreceptor. This results in inadequate trans**migration** of Itk(-/-) CD4(+) T cells into the CNS and across brain endothelial barriers in vitro. Finally, Itk(-/-) CD4(+) T cells show significant reduction in production of T-helper 1 (Th1) and Th17 cytokines and exhibit skewed T effector/T regulatory cell ratios. These results indicate that signaling by Itk promotes autoimmunity and CNS inflammation, suggesting that it may be a viable target for treatment of MS.
5. Asymmetric proteasome segregation as a mechanism for unequal partitioning of the transcription factor T-bet during T lymphocyte division. Polarized segregation of proteins in T cells is thought to play a role in diverse cellular functions including signal transduction, **migration**, and directed secretion of cytokines. Persistence of this polarization can result in asymmetric segregation of fate-determining proteins during cell division, which may enable a T cell to generate diverse progeny. Here, we provide evidence that a lineage-determining transcription factor, T-bet, underwent asymmetric organization in activated T cells preparing to divide and that it was unequally partitioned into the two daughter cells. This unequal acquisition of T-bet appeared to result from its asymmetric destruction during mitosis by virtue of concomitant asymmetric segregation of the proteasome. These results suggest a mechanism by which a cell may unequally localize cellular activities during division, thereby imparting disparity in the abundance of cell fate regulators in the daughter cells.
6. T cell specific adapter protein (TSAd) interacts with Tec kinase ITK to promote CXCL12 induced **migration** of human and murine T cells. The chemokine CXCL12/SDF-1alpha interacts with its G-protein coupled receptor CXCR4 to induce **migration** of lymphoid and endothelial cells. T cell specific adapter protein (TSAd) has been found to promote **migration** of Jurkat T cells through interaction with the G protein beta subunit. However, the molecular mechanisms for how TSAd influences cellular **migration** have not been characterized in detail.We show that TSAd is required for tyrosine phosphorylation of the Lck substrate IL2-inducible T cell kinase (Itk). Presence of Itk Y511 was necessary to boost TSAd's effect on CXCL12 induced **migration** of Jurkat T cells. In addition, TSAd's ability to promote CXCL12-induced actin polymerization and **migration** of Jurkat T lymphocytes was dependent on the Itk-interaction site in the proline-rich region of TSAd. Furthermore, TSAd-deficient murine thymocytes failed to respond to CXCL12 with increased Itk phosphorylation, and displayed reduced actin polymerization and cell **migration** responses.We propose that TSAd, through its interaction with both Itk and Lck, primes Itk for Lck mediated phosphorylation and thereby regulates CXCL12 induced T cell **migration** and actin cytoskeleton rearrangements.
7. Differential sensitivity to Itk kinase signals for T helper 2 cytokine production and chemokine-mediated **migration**. Allergic asthma is dependent on chemokine-mediated Th2 cell **migration** and Th2 cytokine secretion into the lungs. The inducible T cell tyrosine kinase Itk regulates the production of Th2 cytokines as well as **migration** in response to chemokine gradients. Mice lacking Itk are resistant to developing allergic asthma. However, the role of kinase activity of Itk in the development of this disease is unclear. In addition, whether distinct Itk-derived signals lead to T cell **migration** and secretion of Th2 cytokines is also unknown. Using transgenic mice specifically lacking Itk kinase activity, we show that active kinase signaling is required for control of Th2 responses and development of allergic asthma. Moreover, dominant suppression of kinase Itk activity led to normal Th2 responses, but significantly reduced chemokine-mediated **migration**, resulting in prevention of allergic asthma. These observations indicate that signals required for Th2 responses and **migration** are differentially sensitive to Itk activity. Manipulation of Itk's activity can thus provide a new strategy to treat allergic asthma by differentially affecting **migration** of T cells into the lungs, leaving Th2 responses intact.
8. Regulation of CXC chemokine receptor 4-mediated **migration** by the Tec family tyrosine kinase ITK. Chemokines are critical in controlling lymphocyte traffic and **migration**. The CXC chemokine CXCL12/SDF-1alpha interacts with its receptor CXCR4 to induce the **migration** of a number of different cell types. Although an understanding of the physiological functions of this chemokine is emerging, the mechanism by which it regulates T cell **migration** is still unclear. We show here that the Tec family kinase ITK is activated rapidly following CXCL12/SDF-1alpha stimulation, and this requires Src and phosphatidylinositol 3-kinase activities. ITK regulates the ability of CXCL12/SDF-1alpha to induce T cell **migration** as overexpression of wild-type ITK-enhanced **migration**, and T cells lacking ITK exhibit reduced **migration** as well as adhesion in response to CXCL12/SDF-1alpha. Further analysis suggests that ITK may regulate CXCR4-mediated **migration** and adhesion by altering the actin cytoskeleton, as ITK null T cells were significantly defective in CXCL12/SDF-1a-mediated actin polymerization. Our data suggest that ITK may regulate the ability of CXCR4 to induce T cell **migration**.
9. Calmodulin and PI(3,4,5)P₃ cooperatively bind to the Itk pleckstrin homology domain to promote efficient calcium signaling and IL-17A production. Precise regulation of the kinetics and magnitude of Ca(2+) signaling enables this signal to mediate diverse responses, such as cel**l migrati**on, differentiation, vesicular trafficking, and cell death. We showed that the Ca(2+)-binding protein calmodulin (CaM) acted in a positive feedback loop to potentiate Ca(2+) signaling downstream of the Tec kinase family member Itk. Using NMR (nuclear magnetic resonance), we mapped CaM binding to two loops adjacent to the lipid-binding pocket within the Itk pleckstrin homology (PH) domain. The Itk PH domain bound synergistically to Ca(2+)/CaM and the lipid phosphatidylinositol 3,4,5-trisphosphate [PI(3,4,5)P3], such that binding to Ca(2+)/CaM enhanced the binding to PI(3,4,5)P3 and vice versa. Disruption of CaM binding attenuated Itk recruitment to the membrane and diminished release of Ca(2+) from the endoplasmic reticulum. Moreover, disruption of this feedback loop abrogated Itk-dependent production of the proinflammatory cytokine IL-17A (interleukin-17A) by CD4(+) T cells. Additionally, we found that CaM associated with PH domains from other proteins, indicating that CaM may regulate other PH domain-containing proteins.

#### GeneRIFs

1. Itk signaling is required to prevent the expansion of gammadelta natural killer (NK)T cells in the adult thymus, to block their e**migration**, and to promote terminal NKT cell maturation.
2. TSAd, through its interaction with both Itk and Lck, primes Itk for Lck mediated phosphorylation and thereby regulates CXCL12 induced T cell **migration** and actin cytoskeleton rearrangements
3. Data suggest that the Tec family kinase ITK may regulate the ability of CXC chemokine receptor 4 to induce T cell **migration**.

---

## Fgfr4

- NCBI Gene
- GeneRIF
- Pubmed

| Search Term | Rank | Fields |
| --- | --- | --- |
| [Mm]igration | 6 | Process(1); PubMed(6) |
| ECM | 7 | PubMed(2) |
| [Ee]xtracellular [Mm]atrix | 8 | Process(1); PubMed(4) |
| [Ii]nvasive | 10 | PubMed(1) |

  
hide/show details for Fgfr4

### [Mm]igration

#### Process

1. cell **migration**

#### PubMed

1. FGF/FGFR2 signaling regulates the generation and correct positioning of Bergmann glia cells in the developing mouse cerebellum. The normal cellular organization and layering of the vertebrate cerebellum is established during embryonic and early postnatal development by the interplay of a complex array of genetic and signaling pathways. Disruption of these processes and of the proper layering of the cerebellum usually leads to ataxic behaviors. Here, we analyzed the relative contribution of Fibroblast growth factor receptor 2 (FGFR2)-mediated signaling to cerebellar development in conditional Fgfr2 single mutant mice. We show that during embryonic mouse development, Fgfr2 expression is higher in the anterior cerebellar primordium and excluded from the proliferative ventricular neuroepithelium. Consistent with this finding, conditional Fgfr2 single mutant mice display the most prominent defects in the anterior lobules of the adult cerebellum. In this context, FGFR2-mediated signaling is required for the proper generation of Bergmann glia cells and the correct positioning of these cells within the Purkinje cell layer, and for cell survival in the developing cerebellar primordium. Using cerebellar microexplant cultures treated with an FGFR agonist (FGF9) or antagonist (SU5402), we also show that FGF9/FGFR-mediated signaling inhibits the outward **migration** of radial glia and Bergmann glia precursors and cells, and might thus act as a positioning cue for these cells. Altogether, our findings reveal the specific functions of the FGFR2-mediated signaling pathway in the generation and positioning of Bergmann glia cells during cerebellar development in the mouse.
2. The roles of FGF signaling in germ cell **migration** in the mouse. Fibroblast growth factor (FGF) signaling is thought to play a role in germ cell behavior. FGF2 has been reported to be a mitogen for primordial germ cells in vitro, whilst combinations of FGF2, steel factor and LIF cause cultured germ cells to transform into permanent lines of pluripotent cells resembling ES cells. However, the actual function of FGF signaling on the migrating germ cells in vivo is unknown. We show, by RT-PCR analysis of cDNA from purified E10.5 germ cells, that germ cells express two FGF receptors: Fgfr1-IIIc and Fgfr2-IIIb. Second, we show that FGF-mediated activation of the MAP kinase pathway occurs in germ cells during their **migration**, and thus they are potentially direct targets of FGF signaling. Third, we use cultured embryo slices in simple gain-of-function experiments, using FGF ligands, to show that FGF2, a ligand for FGFR1-IIIc, affects motility, whereas FGF7, a ligand for FGFR2-IIIb, affects germ cell numbers. Loss of function, using a specific inhibitor of FGF signaling, causes increased apoptosis and inhibition of cell shape change in the migrating germ cells. Lastly, we confirm in vivo the effects seen in slice cultures in vitro, by examining germ cell positions and numbers in embryos carrying a loss-of-function allele of FGFR2-IIIb. In FGFR2-IIIb(-/-) embryos, germ cell **migration** is unaffected, but the numbers of germ cells are significantly reduced. These data show that a major role of FGF signaling through FGFR2-IIIb is to control germ cell numbers. The data do not discriminate between direct and indirect effects of FGF signaling on germ cells, and both may be involved.
3. Expression of the fibroblast growth factor family and their receptor family genes during mouse brain development. The fibroblast growth factor (FGF) family is composed of nine members and four genes encode protein tyrosine kinase receptors for them. To gain insight into the involvement of FGFs and their receptors in the development of nervous system, their expression in brains of perinatal and adult mice was examined by semi-quantitative reverse transcription-linked polymerase chain reactions and in situ hybridization. Although all the genes, with the exception of FGF-4, were found to be expressed, FGF-3, FGF-6, FGF-7 and FGF-8 genes demonstrated higher expression in the late embryonic stages than in postnatal stages, suggesting that these members are involved in the late stages of brain development. In contrast, expression of FGF-1 and FGF-5 increased after birth. Interestingly, FGF-6 expression in perinatal mice was restricted to the central nervous system and skeltal muscles, with intense signals in the developing cerebrum in embryos but in cerebellum in 5-day-old neonates. Furthermore, FGF-receptor (FGFR)-4, a cognate receptor for FGF-6, demonstrated similar spatiotemporal expression, suggesting that FGF-6 and FGFR-4 plays significant roles in the maturation of nervous system as a ligand-receptor system. The results indicate that individual member of the fibroblast growth factor and their receptor family are expressed either sequentially or simultaneously in brain development, strongly suggesting their involvement in the regulation of a variety of developmental processes of brain, i.e., proliferation and **migration** of neuronal progenitor cells, neuron and glia differentiation, neurite extensions, and synapse formations.
4. Six1 and Six4 homeoproteins are required for Pax3 and Mrf expression during myogenesis in the mouse embryo. In mammals, Six5, Six4 and Six1 genes are co-expressed during mouse myogenesis. Six4 and Six5 single knockout (KO) mice have no developmental defects, while Six1 KO mice die at birth and show multiple organ developmental defects. We have generated Six1Six4 double KO mice and show an aggravation of the phenotype previously reported for the single Six1 KO. Six1Six4 double KO mice are characterized by severe craniofacial and rib defects, and general muscle hypoplasia. At the limb bud level, Six1 and Six4 homeogenes control early steps of myogenic cell delamination and **migration** from the somite through the control of Pax3 gene expression. Impaired in their migratory pathway, cells of the somitic ventrolateral dermomyotome are rerouted, lose their identity and die by apoptosis. At the interlimb level, epaxial Met expression is abolished, while it is preserved in Pax3-deficient embryos. Within the myotome, absence of Six1 and Six4 impairs the expression of the myogenic regulatory factors myogenin and Myod1, and Mrf4 expression becomes undetectable. Myf5 expression is correctly initiated but becomes restricted to the caudal region of each somite. Early syndetomal expression of scleraxis is reduced in the Six1Six4 embryo, while the myotomal expression of Fgfr4 and Fgf8 but not Fgf4 and Fgf6 is maintained. These results highlight the different roles played by Six proteins during skeletal myogenesis.
5. Laminar patterning in the developing neocortex by temporally coordinated fibroblast growth factor signaling. Laminar organization, a fundamental neural architecture in the CNS, is a prominent feature of the neocortex, where the cortical neurons in spatially distinct layers are generated from the common progenitors in a temporally distinct manner during development. Despite many advances in the characterization of the molecular mechanisms of the radial **migration** of cortical neurons, the way in which the early-late temporal sequence of cortical neuron generation is linked with the deep-superficial spatial sequence of cell body positioning remains obscure. Using in vivo electroporation-mediated gene transfer, we show here that the activities mediated by fibroblast growth factor receptors (FGFRs) in cortical progenitors are critical for conferring proper migratory properties on nascent neuronal progeny. Furthermore, we provide supportive evidence that Pea3 subfamily members of Ets (Pea3-Ets) transcription factors mediate the activities of FGFR at the mid to late phase of neocortical development. In addition, using FGF18 knock-out mice, we demonstrate that FGF18 expressed by early-generated cortical neurons in the cortical plate is critical for the expression of Pea3-Ets transcription factors and that FGF18 is sufficient to induce their expressions. Our results thus imply that a feedback mechanism mediated by FGF signaling is involved in setting up the proper laminar positioning of cortical neurons; FGF18 derived from early-generated cortical neurons acts on the cortical progenitors expressing FGFRs and induces the expression of Pea3-Ets transcription factors that, in turn, confer proper migratory behaviors on nascent cortical progeny during the mid to late stages of neocortical development.
6. Neuron-derived FGF9 is essential for scaffold formation of Bergmann radial fibers and **migration** of granule neurons in the cerebellum. Although fibroblast growth factor 9 (FGF9) is widely expressed in the central nervous system (CNS), the function of FGF9 in neural development remains undefined. To address this question, we deleted the Fgf9 gene specifically in the neural tube and demonstrated that FGF9 plays a key role in the postnatal **migration** of cerebellar granule neurons. Fgf9-null mice showed severe ataxia associated with disrupted Bergmann fiber scaffold formation, impaired granule neuron **migration**, and upset Purkinje cell maturation. Ex vivo cultured wildtype or Fgf9-null glia displayed a stellate morphology. Coculture with wildtype neurons, but not Fgf9-deficient neurons, or treating with FGF1 or FGF9 induced the cells to adopt a radial glial morphology. In situ hybridization showed that Fgf9 was expressed in neurons and immunostaining revealed that FGF9 was broadly distributed in both neurons and Bergmann glial radial fibers. Genetic analyses revealed that the FGF9 activities in cerebellar development are primarily transduced by FGF receptors 1 and 2. Furthermore, inhibition of the MAP kinase pathway, but not the PI3K/AKT pathway, abrogated the FGF activity to induce glial morphological changes, suggesting that the activity is mediated by the MAP kinase pathway. This work demonstrates that granule neurons secrete FGF9 to control formation of the Bergmann fiber scaffold, which in turn, guides their own inward **migration** and maturation of Purkinje cells.

### ECM

#### PubMed

1. FGF signalling regulates bone growth through autophagy. Skeletal growth relies on both biosynthetic and catabolic processes. While the role of the former is clearly established, how the latter contributes to growth-promoting pathways is less understood. Macroautophagy, hereafter referred to as autophagy, is a catabolic process that plays a fundamental part in tissue homeostasis. We investigated the role of autophagy during bone growth, which is mediated by chondrocyte rate of proliferation, hypertrophic differentiation and extracellular matrix (**ECM**) deposition in growth plates. Here we show that autophagy is induced in growth-plate chondrocytes during post-natal development and regulates the secretion of type II collagen (Col2), the major component of cartilage **ECM**. Mice lacking the autophagy related gene 7 (Atg7) in chondrocytes experience endoplasmic reticulum storage of type II procollagen (PC2) and defective formation of the Col2 fibrillary network in the **ECM**. Surprisingly, post-natal induction of chondrocyte autophagy is mediated by the growth factor FGF18 through FGFR4 and JNK-dependent activation of the autophagy initiation complex VPS34-beclin-1. Autophagy is completely suppressed in growth plates from Fgf18(-/-) embryos, while Fgf18(+/-) heterozygous and Fgfr4(-/-) mice fail to induce autophagy during post-natal development and show decreased Col2 levels in the growth plate. Strikingly, the Fgf18(+/-) and Fgfr4(-/-) phenotypes can be rescued in vivo by pharmacological activation of autophagy, pointing to autophagy as a novel effector of FGF signalling in bone. These data demonstrate that autophagy is a developmentally regulated process necessary for bone growth, and identify FGF signalling as a crucial regulator of autophagy in chondrocytes.
2. Nell1-deficient mice have reduced expression of extracellular matrix proteins causing cranial and vertebral defects. The mammalian Nell1 gene encodes a protein kinase C-beta1 (PKC-beta1) binding protein that belongs to a new class of cell-signaling molecules controlling cell growth and differentiation. Over-expression of Nell1 in the developing cranial sutures in both human and mouse induces craniosynostosis, the premature fusion of the growing cranial bone fronts. Here, we report the generation, positional cloning and characterization of Nell1(6R), a recessive, neonatal-lethal point mutation in the mouse Nell1 gene, induced by N-ethyl-N-nitrosourea. Nell1(6R) has a T-->A base change that converts a codon for cysteine into a premature stop codon [Cys(502)Ter], resulting in severe truncation of the predicted protein product and marked reduction in steady-state levels of the transcript. In addition to the expected alteration of cranial morphology, Nell1(6R) mutants manifest skeletal defects in the vertebral column and ribcage, revealing a hitherto undefined role for Nell1 in signal transduction in endochondral ossification. Real-time quantitative reverse transcription-PCR assays of 219 genes showed an association between the loss of Nell1 function and reduced expression of genes for extracellular matrix (**ECM**) proteins critical for chondrogenesis and osteogenesis. Several affected genes are involved in the human cartilage disorder Ehlers-Danlos Syndrome and other disorders associated with spinal curvature anomalies. Nell1(6R) mutant mice are a new tool for elucidating basic mechanisms in osteoblast and chrondrocyte differentiation in the developing skull and vertebral column and understanding how perturbations in the production of **ECM** proteins can lead to anomalies in these structures.

### [Ee]xtracellular [Mm]atrix

#### Process

1. regulation of **extracellular matrix** disassembly

#### PubMed

1. FGF signalling regulates bone growth through autophagy. Skeletal growth relies on both biosynthetic and catabolic processes. While the role of the former is clearly established, how the latter contributes to growth-promoting pathways is less understood. Macroautophagy, hereafter referred to as autophagy, is a catabolic process that plays a fundamental part in tissue homeostasis. We investigated the role of autophagy during bone growth, which is mediated by chondrocyte rate of proliferation, hypertrophic differentiation and **extracellular matrix** (ECM) deposition in growth plates. Here we show that autophagy is induced in growth-plate chondrocytes during post-natal development and regulates the secretion of type II collagen (Col2), the major component of cartilage ECM. Mice lacking the autophagy related gene 7 (Atg7) in chondrocytes experience endoplasmic reticulum storage of type II procollagen (PC2) and defective formation of the Col2 fibrillary network in the ECM. Surprisingly, post-natal induction of chondrocyte autophagy is mediated by the growth factor FGF18 through FGFR4 and JNK-dependent activation of the autophagy initiation complex VPS34-beclin-1. Autophagy is completely suppressed in growth plates from Fgf18(-/-) embryos, while Fgf18(+/-) heterozygous and Fgfr4(-/-) mice fail to induce autophagy during post-natal development and show decreased Col2 levels in the growth plate. Strikingly, the Fgf18(+/-) and Fgfr4(-/-) phenotypes can be rescued in vivo by pharmacological activation of autophagy, pointing to autophagy as a novel effector of FGF signalling in bone. These data demonstrate that autophagy is a developmentally regulated process necessary for bone growth, and identify FGF signalling as a crucial regulator of autophagy in chondrocytes.
2. MT1-MMP inactivates ADAM9 to regulate FGFR2 signaling and calvarial osteogenesis. MMP14 encodes a membrane-tethered metalloproteinase MT1-MMP, capable of remodeling the **extracellular matrix** and modulating receptors on the cell surface. Loss of MT1-MMP results in craniofacial abnormalities. Here we show that MT1-MMP forms a complex with FGFR2 and ADAM9 in osteoblasts and proteolytically inactivates ADAM9, hence protecting FGFR2 from ADAM9-mediated ectodomain shedding on the cell surface. In Mmp14-/- osteoblasts, FGF-induced proliferation and downstream signaling are specifically compromised, in conjunction with ADAM9 upregulation and FGFR2 shedding. The retarded parietal growth in Mmp14-/- embryos starts at 15.5 dpc, attributable to the impaired FGFR2 signaling due to increased shedding mediated by ADAM9. Adam9 depletion completely rescues the defective FGFR2 signaling and largely restores calvarial bone growth in Mmp14-/- embryos. These data reveal a regulatory paradigm for FGRF2 signaling and identify MT1-MMP as a critical negative modulator of ADAM9 activity to maintain FGFR2 signaling in calvarial osteogenesis.
3. Nell1-deficient mice have reduced expression of **extracellular matrix** proteins causing cranial and vertebral defects. The mammalian Nell1 gene encodes a protein kinase C-beta1 (PKC-beta1) binding protein that belongs to a new class of cell-signaling molecules controlling cell growth and differentiation. Over-expression of Nell1 in the developing cranial sutures in both human and mouse induces craniosynostosis, the premature fusion of the growing cranial bone fronts. Here, we report the generation, positional cloning and characterization of Nell1(6R), a recessive, neonatal-lethal point mutation in the mouse Nell1 gene, induced by N-ethyl-N-nitrosourea. Nell1(6R) has a T-->A base change that converts a codon for cysteine into a premature stop codon [Cys(502)Ter], resulting in severe truncation of the predicted protein product and marked reduction in steady-state levels of the transcript. In addition to the expected alteration of cranial morphology, Nell1(6R) mutants manifest skeletal defects in the vertebral column and ribcage, revealing a hitherto undefined role for Nell1 in signal transduction in endochondral ossification. Real-time quantitative reverse transcription-PCR assays of 219 genes showed an association between the loss of Nell1 function and reduced expression of genes for **extracellular matrix** (ECM) proteins critical for chondrogenesis and osteogenesis. Several affected genes are involved in the human cartilage disorder Ehlers-Danlos Syndrome and other disorders associated with spinal curvature anomalies. Nell1(6R) mutant mice are a new tool for elucidating basic mechanisms in osteoblast and chrondrocyte differentiation in the developing skull and vertebral column and understanding how perturbations in the production of ECM proteins can lead to anomalies in these structures.
4. Global comparative transcriptome analysis of cartilage formation in vivo. During vertebrate embryogenesis the initial stages of bone formation by endochondral ossification involve the aggregation and proliferation of mesenchymal cells into condensations. Continued growth of the condensations and differentiation of the mesenchymal cells into chondrocytes results in the formation of cartilage templates, or anlagen, which prefigure the shape of the future bones. The chondrocytes in the anlagen further differentiate by undergoing a complex sequence of maturation and hypertrophy, and are eventually replaced by mineralized bone. Regulation of the onset of chondrogenesis is incompletely understood, and would be informed by comprehensive analyses of in vivo gene expression.Tibial and fibular pre-condensed mesenchyme was microdissected from mouse hind limbs at 11.5 dpc, and the corresponding condensations at 12.5 dpc and cartilage anlagen at 13.5 dpc. Total RNA was isolated, and cRNA generated by linear amplification was interrogated using mouse whole genome microarrays. Differential expression was validated by quantitative PCR for Agc1, Bmp8a, Col2a1, Fgfr4, Foxa3, Gdf5, Klf2, Klf4, Lepre1, Ncad, Sox11, and Trpv4. Further, independent validation of the microarray data was achieved by in situ hybridization to analyse the expression of Lepre1, Pcdh8, Sox11, and Trpv4 from 11.5 dpc to 13.5 dpc during mouse hind limb development. We found significant differential expression of 931 genes during these early stages of chondrogenesis. Of these, 380 genes were down-regulated and 551 up-regulated. Our studies characterized the expression pattern of gene families previously associated with chondrogenesis, such as adhesion molecules, secreted signalling molecules, transcription factors, and **extracellular matrix** components. Gene ontology approaches identified 892 differentially expressed genes not previously identified during the initiation of chondrogenesis. These included several Bmp, Gdf, Wnt, Sox and Fox family members.These data represent the first global gene expression profiling analysis of chondrogenic tissues during in vivo development. They identify genes for further study on their functional roles in chondrogenesis, and provide a comprehensive and important resource for future studies on cartilage development and disease.

### [Ii]nvasive

#### PubMed

1. A single nucleotide change in the mouse genome accelerates breast cancer progression. In the growth factor receptor gene FGFR4 the presence of the common single nucleotide polymorphism Arg388 has been associated with progression of various types of cancer including breast cancer. However, a causative relationship is not readily assigned due to genetic heterogeneity in different patient cohorts. To address this issue, we compared the effects of this allele on malignant progression in the WAP-TGFalpha transgenic mouse model of breast cancer. A knock-in strain was generated to introduce an analogous Arg385 allele into the murine FGFR4 gene. Mouse embryonic fibroblasts derived from this strain displayed accelerated cell transformation, with transformed cells exhibiting greater motility and **invasive** behavior. In the in vivo context of TGFalpha-induced mammary carcinogenesis, tumor development and progression was significantly advanced in tumor mass, size, and onset of pulmonary metastases. Our findings definitively identify the FGFR4 Arg388 allele as a functional prognostic marker for breast cancer progression.

---

## Tie1

- NCBI Gene
- GeneRIF
- Pubmed

| Search Term | Rank | Fields |
| --- | --- | --- |
| [Mm]igration | 6 | Process(1); PubMed(10); Conserved Domains(1) |
| [Ee]xtracellular [Mm]atrix | 8 | PubMed(3) |
| [Cc]ell [Ii]nvasion | 13 | PubMed(1) |

  
hide/show details for Tie1

### [Mm]igration

#### Process

1. negative regulation of cell **migration**

#### PubMed

1. Mab21l2 is essential for embryonic heart and liver development. During mouse embryogenesis, proper formation of the heart and liver is especially important and is crucial for embryonic viability. In this study, we showed that Mab21l2 was expressed in the trabecular and compact myocardium, and that deletion of Mab21l2 resulted in a reduction of the trabecular myocardium and thinning of the compact myocardium. Mab21l2-deficient embryonic hearts had decreased expression of genes that regulate cell proliferation and apoptosis of cardiomyocytes. These results show that Mab21l2 functions during heart development by regulating the expression of such genes. Mab21l2 was also expressed in the septum transversum mesenchyme (STM). Epicardial progenitor cells are localized to the anterior surface of the STM (proepicardium), and proepicardial cells migrate onto the surface of the heart and form the epicardium, which plays an important role in heart development. The rest of the STM is essential for the growth and survival of hepatoblasts, which are bipotential progenitors for hepatocytes and cholangiocytes. Proepicardial cells in Mab21l2-deficient embryos had defects in cell proliferation, which led to a small proepicardium, in which α4 integrin expression, which is essential for the **migratio**n of proepicardial cells, was down-regulated, suggesting that defects occurred in its **migratio**n. In Mab21l2-deficient embryos, epicardial formation was defective, suggesting that Mab21l2 plays important roles in epicardial formation through the regulation of the cell proliferation of proepicardial cells and the migratory process of proepicardial cells. Mab21l2-deficient embryos also exhibited hypoplasia of the STM surrounding hepatoblasts and decreased hepatoblast proliferation with a resultant loss of defective morphogenesis of the liver. These findings demonstrate that Mab21l2 plays a crucial role in both heart and liver development through STM formation.
2. Edg-1, the G protein-coupled receptor for sphingosine-1-phosphate, is essential for vascular maturation. Sphingolipid signaling pathways have been implicated in many critical cellular events. Sphingosine-1-phosphate (SPP), a sphingolipid metabolite found in high concentrations in platelets and blood, stimulates members of the endothelial differentiation gene (Edg) family of G protein-coupled receptors and triggers diverse effects, including cell growth, survival, **migration**, and morphogenesis. To determine the in vivo functions of the SPP/Edg signaling pathway, we disrupted the Edg1 gene in mice. Edg1(-/-) mice exhibited embryonic hemorrhage leading to intrauterine death between E12.5 and E14.5. Vasculogenesis and angiogenesis appeared normal in the mutant embryos. However, vascular maturation was incomplete due to a deficiency of vascular smooth muscle cells/pericytes. We also show that Edg-1 mediates an SPP-induced **migration** response that is defective in mutant cells due to an inability to activate the small GTPase, Rac. Our data reveal Edg-1 to be the first G protein-coupled receptor required for blood vessel formation and show that sphingolipid signaling is essential during mammalian development.
3. GPR124, an orphan G protein-coupled receptor, is required for CNS-specific vascularization and establishment of the blood-brain barrier. Every organ in the body requires blood vessels for efficient delivery of oxygen and nutrients, but independent vascular beds are highly specialized to meet the individual needs of specific organs. The vasculature of the brain is tightly sealed, with blood-brain barrier (BBB) properties developing coincident with neural vascularization. G protein-coupled receptor 124 (GPR124) (tumor endothelial marker 5, TEM5), an orphan member of the adhesion family of G protein-coupled receptors, was previously identified on the basis of its overexpression in tumor vasculature. Here, we show that global deletion or endothelial-specific deletion of GPR124 in mice results in embryonic lethality associated with abnormal angiogenesis of the forebrain and spinal cord. Expression of GPR124 was found to be required for invasion and **migration** of blood vessels into neuroepithelium, establishment of BBB properties, and expansion of the cerebral cortex. Thus, GPR124 is an important regulator of neurovasculature development and a potential drug target for cerebrovascular diseases.
4. XIAP-associated factor 1 (XAF1) suppresses angiogenesis in mouse endothelial cells. XIAP-associated factor 1 (XAF1) is a tumor suppressor gene, but its role in angiogenesis is unknown. We investigated whether XAF1 has any antiangiogenesis effect.MS1 (a mouse endothelial cell line) was infected with an adenoviral vector ZD55-XAF1. Controls were uninfected or infected with ZD55-EGFP. Wound healing assay and tube formation assay were used to assess angiogenesis. Cell proliferation was detected by WST-1 assay, and apoptosis was detected by TUNEL and APOPercentagetrade mark assays.ZD55-XAF1 significantly upregulated XAF1, which was associated with decreased cell proliferation, **migration** and tube formation of MS1 cells. Ectopic overexpression of XAF1 induced apoptosis in MS1 and also sensitized cells to 5-fluorouracil-induced apoptosis. A significant decrease in the expression of Tie-1, Ang-1, Ang-2 and c-Myc was observed.Our data suggest that XAF1 possesses a potential antiangiogenesis effect. Suppressed expression of Tie-1, Ang-1, Ang-2 and c-Myc may be mechanistically responsible for the observed antiangiogenesis effect.
5. Lack of pericytes leads to endothelial hyperplasia and abnormal vascular morphogenesis. The association of pericytes (PCs) to newly formed blood vessels has been suggested to regulate endothelial cell (EC) proliferation, survival, **migration**, differentiation, and vascular branching. Here, we addressed these issues using PDGF-B-- and PDGF receptor-beta (PDGFR-beta)--deficient mice as in vivo models of brain angiogenesis in the absence of PCs. Quantitative morphological analysis showed that these mutants have normal microvessel density, length, and number of branch points. However, absence of PCs correlates with endothelial hyperplasia, increased capillary diameter, abnormal EC shape and ultrastructure, changed cellular distribution of certain junctional proteins, and morphological signs of increased transendothelial permeability. Brain endothelial hyperplasia was observed already at embryonic day (E) 11.5 and persisted throughout development. From E 13.5, vascular endothelial growth factor-A (VEGF-A) and other genes responsive to metabolic stress became upregulated, suggesting that the abnormal microvessel architecture has systemic metabolic consequences. VEGF-A upregulation correlated temporally with the occurrence of vascular abnormalities in the placenta and dilation of the heart. Thus, although PC deficiency appears to have direct effects on EC number before E 13.5, the subsequent increased VEGF-A levels may further abrogate microvessel architecture, promote vascular permeability, and contribute to formation of the edematous phenotype observed in late gestation PDGF-B and PDGFR-beta knock out embryos.
6. XIAP-associated factor 1 (XAF1) suppresses angiogenesis in mouse endothelial cells. XIAP-associated factor 1 (XAF1) is a tumor suppressor gene, but its role in angiogenesis is unknown. We investigated whether XAF1 has any antiangiogenesis effect.MS1 (a mouse endothelial cell line) was infected with an adenoviral vector ZD55-XAF1. Controls were uninfected or infected with ZD55-EGFP. Wound healing assay and tube formation assay were used to assess angiogenesis. Cell proliferation was detected by WST-1 assay, and apoptosis was detected by TUNEL and APOPercentagetrade mark assays.ZD55-XAF1 significantly upregulated XAF1, which was associated with decreased cell proliferation, **migration** and tube formation of MS1 cells. Ectopic overexpression of XAF1 induced apoptosis in MS1 and also sensitized cells to 5-fluorouracil-induced apoptosis. A significant decrease in the expression of Tie-1, Ang-1, Ang-2 and c-Myc was observed.Our data suggest that XAF1 possesses a potential antiangiogenesis effect. Suppressed expression of Tie-1, Ang-1, Ang-2 and c-Myc may be mechanistically responsible for the observed antiangiogenesis effect.
7. Fgfr1 regulates patterning of the pharyngeal region. Development of the pharyngeal region depends on the interaction and integration of different cell populations, including surface ectoderm, foregut endoderm, paraxial mesoderm, and neural crest. Mice homozygous for a hypomorphic allele of Fgfr1 have craniofacial defects, some of which appeared to result from a failure in the early development of the second branchial arch. A stream of neural crest cells was found to originate from the rhombomere 4 region and migrate toward the second branchial arch in the mutants. Neural crest cells mostly failed to enter the second arch, however, but accumulated in a region proximal to it. Both rescue of the hypomorphic Fgfr1 allele and inactivation of a conditional Fgfr1 allele specifically in neural crest cells indicated that Fgfr1 regulates the entry of neural crest cells into the second branchial arch non-cell-autonomously. Gene expression in the pharyngeal ectoderm overlying the developing second branchial arch was affected in the hypomorphic Fgfr1 mutants at a stage prior to neural crest entry. Our results indicate that Fgfr1 patterns the pharyngeal region to create a permissive environment for neural crest cell **migration**.
8. Inhibitor of differentiation 1 promotes endothelial survival in a bleomycin model of lung injury in mice. The Id family of genes encodes negative regulators of basic helix-loop-helix transcription factors and has been implicated in diverse cellular processes such as proliferation, apoptosis, differentiation, and **migration**. However, the specific role of Id1 in lung injury has not been investigated. Bleomycin has been widely used to generate animal models of acute lung injury and fibrogenesis. In this study we found that, on bleomycin challenge, Id1 expression was significantly up-regulated in the lungs, predominantly in endothelial cells, as revealed by double immunolabeling and quantitative flow cytometric analysis. Mice with Id1 loss-of-function (Id1(-/-)) displayed increased vascular permeability and endothelial apoptosis in the lungs after bleomycin-induced injury. Cultured Id1(-/-) lung microvascular endothelial cells also showed decreased survival when exposed to bleomycin. We detected a decrease in the level of Bcl-2, a primary anti-apoptotic protein, in Id1(-/-) endothelial cells, suggesting that down-regulated Bcl-2 may promote endothelial apoptosis in the lung. Therefore, we propose that Id1 plays a crucial role in promoting endothelial survival in the adult lung on injury. In addition, bleomycin-exposed Id1(-/-) mice showed increased lung collagen accumulation and fibrogenesis, suggesting that Id1 up-regulation in the lung may play a critical role in lung homeostasis.
9. Tie1 deletion inhibits tumor growth and improves angiopoietin antagonist therapy. The endothelial Tie1 receptor is ligand-less, but interacts with the Tie2 receptor for angiopoietins (Angpt). Angpt2 is expressed in tumor blood vessels, and its blockade inhibits tumor angiogenesis. Here we found that Tie1 deletion from the endothelium of adult mice inhibits tumor angiogenesis and growth by decreasing endothelial cell survival in tumor vessels, without affecting normal vasculature. Treatment with VEGF or VEGFR-2 blocking antibodies similarly reduced tumor angiogenesis and growth; however, no additive inhibition was obtained by targeting both Tie1 and VEGF/VEGFR-2. In contrast, treatment of Tie1-deficient mice with a soluble form of the extracellular domain of Tie2, which blocks Angpt activity, resulted in additive inhibition of tumor growth. Notably, Tie1 deletion decreased sprouting angiogenesis and increased Notch pathway activity in the postnatal retinal vasculature, while pharmacological Notch suppression in the absence of Tie1 promoted retinal hypervasularization. Moreover, substantial additive inhibition of the retinal vascular front **migration** was observed when Angpt2 blocking antibodies were administered to Tie1-deficient pups. Thus, Tie1 regulates tumor angiogenesis, postnatal sprouting angiogenesis, and endothelial cell survival, which are controlled by VEGF, Angpt, and Notch signals. Our results suggest that targeting Tie1 in combination with Angpt/Tie2 has the potential to improve antiangiogenic therapy.
10. Protocadherin 12 deficiency alters morphogenesis and transcriptional profile of the placenta. Protocadherins are transmembrane proteins exhibiting homophilic adhesive activities through their extracellular domain. Protocadherin 12 (Pcdh12) is expressed in angiogenic endothelial cells, mesangial cells of kidney glomeruli, and glycogen cells of the mouse placenta. To get insight into the role of this protein in vivo, we analyzed PCDH12-deficient mice and investigated their placental phenotype. The mice were alive and fertile; however, placental and embryonic sizes were reduced compared with wild-type mice. We observed defects in placental layer segregation and a decreased vascularization of the labyrinth associated with a reduction in cell density in this layer. To understand the molecular events responsible for the phenotypic alterations observed in Pcdh12(-/-) placentas, we analyzed the expression profile of embryonic day 12.5 mutant placentas compared with wild-type placentas, using pangenomic chips: 2,289 genes exhibited statistically significant changes in expressed levels due to loss of PCDH12. Functional grouping of modified genes was obtained by GoMiner software. Gene clusters that contained most of the differentially expressed genes were those involved in tissue morphogenesis and development, angiogenesis, cell-matrix adhesion and **migration**, immune response, and chromatin remodeling. Our data show that loss of PCDH12 leads to morphological alterations of the placenta and to notable changes in its gene expression profile. Specific genes emerging from the microarray screen support the biological modifications observed in PCDH12-deficient placentas.

#### Conserved Domains

1. cd00055: EGF\_Lam; Laminin-type epidermal growth factor-like domain; laminins are the major noncollagenous components of basement membranes that mediate cell adhesion, growth **migration**, and differentiation; the laminin-type epidermal growth factor-like module occurs in ...

### [Ee]xtracellular [Mm]atrix

#### PubMed

1. TIE1 and TIE2 receptor tyrosine kinases inversely regulate embryonic angiogenesis by the mechanism of intussusceptive microvascular growth. As shown previously, TIE1 and TIE2 receptor tyrosine kinases are specifically expressed in endothelial cells during embryonic angiogenesis. A detailed analysis of the vascular malformations of homozygous mice for a targeting mutation of both receptors was performed at the histological and cellular level. The data demonstrate that the TIE1 and TIE2 receptor inversely and concomitantly mediate interactions between endothelial cells with their **extracellular matrix** and with surrounding mesenchymal cells. These interactions are obviously crucial for normal endothelial cell motility and/or attachment and also for recruitment of periendothelial cells. The analysis of the TIE2-deficient embryos demonstrates how these cell/cell- and cell/matrix interactions subsequently influence the formation of normally structured tissue folds that divide the vessel lumen. They are also essential for the formation of vessel loops that compose a new vascular network and for the development of the ventricle in the heart. Fold and loop formation follow the principles of intussusceptive microvascular growth. The localization of the cardiovascular malformations corresponds to the temporal and spatial expression pattern of the TIE2 receptor. Angiopoietin-1, a ligand that activates the TIE2 receptor, is expressed in mesenchymal cells surrounding the endothelium. This local relationship is indicative of a paracrine regulation.
2. Histone deacetylase 7 maintains vascular integrity by repressing matrix metalloproteinase 10. Development and homeostasis of the cardiovascular system require intimate interactions between endothelial and smooth muscle cells, which form a seamless circulatory network. We show that histone deacetylase 7 (HDAC7) is specifically expressed in the vascular endothelium during early embryogenesis, where it maintains vascular integrity by repressing the expression of matrix metalloproteinase (MMP) 10, a secreted endoproteinase that degrades the **extracellular matrix**. Disruption of the HDAC7 gene in mice results in embryonic lethality due to a failure in endothelial cell-cell adhesion and consequent dilatation and rupture of blood vessels. HDAC7 represses MMP10 gene transcription by associating with myocyte enhancer factor-2 (MEF2), a direct activator of MMP10 transcription and essential regulator of blood vessel development. These findings reveal an unexpected and specific role for HDAC7 in the maintenance of vascular integrity and have important implications for understanding the processes of angiogenesis and vascular remodeling during cardiovascular development and disease.
3. The LKLF transcription factor is required for normal tunica media formation and blood vessel stabilization during murine embryogenesis. The transcriptional programs that regulate blood vessel formation are largely unknown. In this paper, we examine the role of the zinc finger transcription factor LKLF in murine blood vessel morphogenesis and homeostasis. By in situ hybridization and immunohistochemistry, we show that LKLF is expressed as early as embryonic day 9.5 (E9.5) in vascular endothelial cells throughout the developing mouse embryo. To better understand the function of LKLF, we used homologous recombination in embryonic stem (ES) cells to generate LKLF-deficient (LKLF-/-) mice. Both angiogenesis and vasculogenesis were normal in the LKLF-/- mice. However, LKLF-/- embryos died between E12.5 and E14.5 from severe intra-embryonic and intra-amniotic hemorrhaging. This bleeding disorder was associated with specific defects in blood vessel morphology. Umbilical veins and arteries in the LKLF-/- embryos displayed an abnormally thin tunica media and aneurysmal dilatation before rupturing into the amniotic cavity. Similarly, vascular smooth muscle cells in the aortae from the LKLF-/- animals displayed a cuboidal morphology and failed to organize into a compact tunica media. Consistent with these findings, electron microscopic analyses demonstrated endothelial cell necrosis, significant reductions in the number of vessel-wall pericytes and differentiating smooth muscle cells, and decreased deposition of **extracellular matrix** in the LKLF-/- vessels. Despite these defects, in situ hybridization demonstrated normal expression of platelet-derived growth factor B, Tie1, Tie2, transforming growth factor beta, and heparin-binding epidermal growth factor in the vasculature of the LKLF-/- embryos. Therefore, LKLF defines a novel transcriptional pathway in which endothelial cells regulate the assembly of the vascular tunica media and concomitant vessel wall stabilization during mammalian embryogenesis.

### [Cc]ell [Ii]nvasion

#### PubMed

1. Thyroid follicle development requires Smad1/5- and endothelial cell-dependent basement membrane assembly. Thyroid follicles, the functional units of the thyroid gland, are delineated by a monolayer of thyrocytes resting on a continuous basement membrane. The developmental mechanisms of folliculogenesis, whereby follicles are formed by the reorganization of a non-structured mass of non-polarized epithelial cells, are largely unknown. Here we show that assembly of the epithelial basement membrane is crucial for folliculogenesis and is controlled by endothelial **cell invasion** and by BMP-Smad signaling in thyrocytes. Thyroid-specific Smad1 and Smad5 double-knockout (Smad1/5(dKO)) mice displayed growth retardation, hypothyroidism and defective follicular architecture. In Smad1/5(dKO) embryonic thyroids, epithelial cells remained associated in large clusters and formed small follicles. Although similar follicular defects are found in Vegfa knockout (Vegfa(KO)) thyroids, Smad1/5(dKO) thyroids had normal endothelial cell density yet impaired endothelial differentiation. Interestingly, both Vegfa(KO) and Smad1/5(dKO) thyroids displayed impaired basement membrane assembly. Furthermore, conditioned medium (CM) from embryonic endothelial progenitor cells (eEPCs) rescued the folliculogenesis defects of both Smad1/5(dKO) and Vegfa(KO) thyroids. Laminin α1, β1 and γ1, abundantly released by eEPCs into CM, were crucial for folliculogenesis. Thus, epithelial Smad signaling and endotheli**al cell invas**ion promote folliculogenesis via assembly of the basement membrane.

---

## Kdr

- NCBI Gene
- GeneRIF
- Pubmed

| Search Term | Rank | Fields |
| --- | --- | --- |
| [Ff]ilopodia | 1 | PubMed(8) |
| [Mm]igration | 6 | Process(5); PubMed(117); GeneRIFs(2) |
| ECM | 7 | PubMed(4) |
| [Ee]xtracellular [Mm]atrix | 8 | PubMed(21); GeneRIFs(1) |
| [Ii]nvasive | 10 | PubMed(11) |
| [Mm]etastasis | 12 | PubMed(19); GeneRIFs(2) |
| [Cc]ell [Ii]nvasion | 13 | PubMed(4) |

  
hide/show details for Kdr

### [Ff]ilopodia

#### PubMed

1. Sema3E-PlexinD1 signaling selectively suppresses disoriented angiogenesis in ischemic retinopathy in mice. During development, the retinal vasculature grows toward hypoxic areas in an organized fashion. By contrast, in ischemic retinopathies, new blood vessels grow out of the retinal surfaces without ameliorating retinal hypoxia. Restoration of proper angiogenic directionality would be of great benefit to reoxygenize the ischemic retina and resolve disease pathogenesis. Here, we show that binding of the semaphorin 3E (Sema3E) ligand to the transmembrane PlexinD1 receptor initiates a signaling pathway that normalizes angiogenic directionality in both developing retinas and ischemic retinopathy. In developing mouse retinas, inhibition of VEGF signaling resulted in downregulation of endothelial PlexinD1 expression, suggesting that astrocyte-derived VEGF normally promotes PlexinD1 expression in growing blood vessels. Neuron-derived Sema3E signaled to PlexinD1 and activated the small GTPase RhoJ in ECs, thereby counteracting VEGF-induced **filopodia** projections and defining the retinal vascular pathfinding. In a mouse model of ischemic retinopathy, enhanced expression of PlexinD1 and RhoJ in extraretinal vessels prevented VEGF-induced disoriented projections of the endothelial **filopodia**. Remarkably, intravitreal administration of Sema3E protein selectively suppressed extraretinal vascular outgrowth without affecting the desired regeneration of the retinal vasculature. Our study suggests a new paradigm for vascular regeneration therapy that guides angiogenesis precisely toward the ischemic retina.
2. NRP1 Regulates CDC42 Activation to Promote **Filopodia** Formation in Endothelial Tip Cells. Sprouting blood vessels are led by **filopodia**-studded endothelial tip cells that respond to angiogenic signals. Mosaic lineage tracing previously revealed that NRP1 is essential for tip cell function, although its mechanistic role in tip cells remains poorly defined. Here, we show that NRP1 is dispensable for genetic tip cell identity. Instead, we find that NRP1 is essential to form the **filopodia**l bursts that distinguish tip cells morphologically from neighboring stalk cells, because it enables the extracellular matrix (ECM)-induced activation of CDC42, a key regulator of **filopodia** formation. Accordingly, NRP1 knockdown and pharmacological CDC42 inhibition similarly impaired **filopodia** formation in vitro and in developing zebrafish in vivo. During mouse retinal angiogenesis, CDC42 inhibition impaired tip cell and vascular network formation, causing defects that resembled those due to loss of ECM-induced, but not VEGF-induced, NRP1 signaling. We conclude that NRP1 enables ECM-induced **filopodia** formation for tip cell function during sprouting angiogenesis.
3. Cdc42 is required for cytoskeletal support of endothelial cell adhesion during blood vessel formation in mice. The Rho family of small GTPases has been shown to be required in endothelial cells (ECs) during blood vessel formation. However, the underlying cellular events controlled by different GTPases remain unclear. Here, we assess the cellular mechanisms by which Cdc42 regulates mammalian vascular morphogenesis and maintenance. In vivo deletion of Cdc42 in embryonic ECs (Cdc42(Tie2KO)) results in blocked lumen formation and endothelial tearing, leading to lethality of mutant embryos by E9-10 due to failed blood circulation. Similarly, inducible deletion of Cdc42 (Cdc42(Cad5KO)) at mid-gestation blocks angiogenic tubulogenesis. By contrast, deletion of Cdc42 in postnatal retinal vessels leads to aberrant vascular remodeling and sprouting, as well as markedly reduced **filopodia** formation. We find that Cdc42 is essential for organization of EC adhesion, as its loss results in disorganized cell-cell junctions and reduced focal adhesions. Endothelial polarity is also rapidly lost upon Cdc42 deletion, as seen by failed localization of apical podocalyxin (PODXL) and basal actin. We link observed failures to a defect in F-actin organization, both in vitro and in vivo, which secondarily impairs EC adhesion and polarity. We also identify Cdc42 effectors Pak2/4 and N-WASP, as well as the actomyosin machinery, to be crucial for EC actin organization. This work supports the notion of Cdc42 as a central regulator of the cellular machinery in ECs that drives blood vessel formation.
4. The Notch ligand Delta-like 4 negatively regulates endothelial tip cell formation and vessel branching. Delta-like 4 (Dll4) is a transmembrane ligand for Notch receptors that is expressed in arterial blood vessels and sprouting endothelial cells. Here we show that Dll4 regulates vessel branching during development by inhibiting endothelial tip cell formation. Heterozygous deletion of dll4 or pharmacological inhibition of Notch signaling using gamma-secretase inhibitor revealed a striking vascular phenotype, with greatly increased numbers of **filopodia**-extending endothelial tip cells and increased expression of tip cell marker genes compared with controls. **Filopodia** extension in dll4(+/-) retinal vessels required the vascular growth factor VEGF and was inhibited when VEGF signaling was blocked. Although VEGF expression was not significantly altered in dll4(+/-) retinas, dll4(+/-) vessels showed increased expression of VEGF receptor 2 and decreased expression of VEGF receptor 1 compared with wild-type, suggesting they could be more responsive to VEGF stimulation. In addition, expression of dll4 in wild-type tip cells was itself decreased when VEGF signaling was blocked, indicating that dll4 may act downstream of VEGF as a "brake" on VEGF-mediated angiogenic sprouting. Taken together, these data reveal Dll4 as a negative regulator of vascular sprouting and vessel branching that is required for normal vascular network formation during development.
5. Serum response factor is required for sprouting angiogenesis and vascular integrity. Serum response factor (SRF) is a transcription factor that controls the expression of cytoskeletal proteins and immediate early genes in different cell types. Here, we found that SRF expression is restricted to endothelial cells (ECs) of small vessels such as capillaries in the mouse embryo. EC-specific Srf deletion led to aneurysms and hemorrhages from 11.5 days of mouse development (E11.5) and lethality at E14.5. Mutant embryos presented a reduced capillary density and defects in EC migration, with fewer numbers of **filopodia** in tip cells and ECs showing defects in actin polymerization and intercellular junctions. We show that SRF is essential for the expression of VE-cadherin and beta-actin in ECs both in vivo and in vitro. Moreover, knockdown of SRF in ECs impaired VEGF- and FGF-induced in vitro angiogenesis. Taken together, our results demonstrate that SRF plays an important role in sprouting angiogenesis and small vessel integrity in the mouse embryo.
6. VEGF receptor 2/-3 heterodimers detected in situ by proximity ligation on angiogenic sprouts. The vascular endothelial growth factors VEGFA and VEGFC are crucial regulators of vascular development. They exert their effects by dimerization and activation of the cognate receptors VEGFR2 and VEGFR3. Here, we have used in situ proximity ligation to detect receptor complexes in intact endothelial cells. We show that both VEGFA and VEGFC potently induce formation of VEGFR2/-3 heterodimers. Receptor heterodimers were found in both developing blood vessels and immature lymphatic structures in embryoid bodies. We present evidence that heterodimers frequently localize to tip cell **filopodia**. Interestingly, in the presence of VEGFC, heterodimers were enriched in the leading tip cells as compared with trailing stalk cells of growing sprouts. Neutralization of VEGFR3 to prevent heterodimer formation in response to VEGFA decreased the extent of angiogenic sprouting. We conclude that VEGFR2/-3 heterodimers on angiogenic sprouts induced by VEGFA or VEGFC may serve to positively regulate angiogenic sprouting.
7. Ephrin-B2 regulates VEGFR2 function in developmental and tumour angiogenesis. The formation and guidance of specialized endothelial tip cells is essential for both developmental and pathological angiogenesis. Notch-1 signalling regulates the generation of tip cells, which respond to gradients of vascular endothelial growth factor (VEGF-A). The molecular cues and signalling pathways that control the guidance of tip cells are poorly understood. Bidirectional signalling by Eph receptors and ephrin ligands represents one of the most important guidance cues involved in axon path finding. Here we show that ephrin-B2 reverse signalling involving PDZ interactions regulates endothelial tip cell guidance to control angiogenic sprouting and branching in physiological and pathological angiogenesis. In vivo, ephrin-B2 PDZ-signalling-deficient mice (ephrin-B2DeltaV) exhibit a reduced number of tip cells with fewer **filopodia**l extensions at the vascular front in the mouse retina. In pathological settings, impaired PDZ signalling decreases tumour vascularization and growth. Mechanistically, we show that ephrin-B2 controls VEGF receptor (VEGFR)-2 internalization and signalling. Importantly, internalization of VEGFR2 is necessary for activation and downstream signalling of the receptor and is required for VEGF-induced tip cell **filopodia**l extension. Together, our results suggest that ephrin-B2 at the tip cell **filopodia** regulates the proper spatial activation of VEGFR2 endocytosis and signalling to direct **filopodia**l extension. Blocking ephrin-B2 reverse signalling may be an attractive alternative or combinatorial anti-angiogenic therapy strategy to disrupt VEGFR2 function in tumour angiogenesis.
8. Spatial regulation of VEGF receptor endocytosis in angiogenesis. Activities as diverse as migration, proliferation and patterning occur simultaneously and in a coordinated fashion during tissue morphogenesis. In the growing vasculature, the formation of motile, invasive and **filopodia**-carrying endothelial sprouts is balanced with the stabilization of blood-transporting vessels. Here, we show that sprouting endothelial cells in the retina have high rates of VEGF uptake, VEGF receptor endocytosis and turnover. These internalization processes are opposed by atypical protein kinase C activity in more stable and mature vessels. aPKC phosphorylates Dab2, a clathrin-associated sorting protein that, together with the transmembrane protein ephrin-B2 and the cell polarity regulator PAR-3, enables VEGF receptor endocytosis and downstream signal transduction. Accordingly, VEGF receptor internalization and the angiogenic growth of vascular beds are defective in loss-of-function mice lacking key components of this regulatory pathway. Our work uncovers how vessel growth is dynamically controlled by local VEGF receptor endocytosis and the activity of cell polarity proteins.

### [Mm]igration

#### Process

1. cell **migration**
2. cell **migration** involved in sprouting angiogenesis
3. positive regulation of cell **migration**
4. positive regulation of endothelial cell **migration**
5. positive regulation of endothelial cell **migration**

#### PubMed

1. Vascular endothelial growth factor A (VEGF-A) is involved in guidance of VEGF receptor-positive cells to the anterior portion of early embryos. The hemangioblast in the mesoderm gives rise to both angioblasts and hematopoietic stem cells. The movement of hemangioblast precursor cells in the fetal trunk is a critical event in early embryogenesis. Vascular endothelial growth factor (VEGF) signaling is likely involved in this **migration** given the partial disturbance of VEGF receptor (VEGFR)-positive cell accumulation and **migration** in VEGFR2 null mice or mice with a truncated VEGFR1. However, it is not clear how the VEGF system regulates this **migration** or its direction. We show here that the expression of VEGF-A is dominant in the anterior portion of the embryo, whereas VEGFR1 and VEGFR2 are expressed in the posterior portion of the embryo. An inhibitor of VEGFR kinase blocked the **migration** of VEGFR-positive cells in a whole-embryo culture system. In addition, VEGFR-positive cells migrated toward a VEGFR1- or VEGFR2-specific ligand in vitro. Furthermore, VEGFR-positive cells derived from wild-type or VEGFR2(+/-) mice moved rapidly anteriorly, whereas cells derived from VEGFR2(+/-) mice carrying a truncated VEGFR1 [VEGFR1(TM-TK)(-/-)] migrated little when injected into wild-type mice. These results suggest that the VEGF-A protein concentrated in the anterior region plays an important role in the guidance of VEGFR-positive cells from the posterior portion to the head region by interacting with VEGFR in the mouse embryo.
2. Mab21l2 is essential for embryonic heart and liver development. During mouse embryogenesis, proper formation of the heart and liver is especially important and is crucial for embryonic viability. In this study, we showed that Mab21l2 was expressed in the trabecular and compact myocardium, and that deletion of Mab21l2 resulted in a reduction of the trabecular myocardium and thinning of the compact myocardium. Mab21l2-deficient embryonic hearts had decreased expression of genes that regulate cell proliferation and apoptosis of cardiomyocytes. These results show that Mab21l2 functions during heart development by regulating the expression of such genes. Mab21l2 was also expressed in the septum transversum mesenchyme (STM). Epicardial progenitor cells are localized to the anterior surface of the STM (proepicardium), and proepicardial cells migrate onto the surface of the heart and form the epicardium, which plays an important role in heart development. The rest of the STM is essential for the growth and survival of hepatoblasts, which are bipotential progenitors for hepatocytes and cholangiocytes. Proepicardial cells in Mab21l2-deficient embryos had defects in cell proliferation, which led to a small proepicardium, in which α4 integrin expression, which is essential for the **migratio**n of proepicardial cells, was down-regulated, suggesting that defects occurred in its **migratio**n. In Mab21l2-deficient embryos, epicardial formation was defective, suggesting that Mab21l2 plays important roles in epicardial formation through the regulation of the cell proliferation of proepicardial cells and the migratory process of proepicardial cells. Mab21l2-deficient embryos also exhibited hypoplasia of the STM surrounding hepatoblasts and decreased hepatoblast proliferation with a resultant loss of defective morphogenesis of the liver. These findings demonstrate that Mab21l2 plays a crucial role in both heart and liver development through STM formation.
3. A novel multistep mechanism for initial lymphangiogenesis in mouse embryos based on ultramicroscopy. During mammalian development, a subpopulation of endothelial cells in the cardinal vein (CV) expresses lymphatic-specific genes and subsequently develops into the first lymphatic structures, collectively termed as lymph sacs. Budding, sprouting and ballooning of lymphatic endothelial cells (LECs) have been proposed to underlie the emergence of LECs from the CV, but the exact mechanisms of lymph vessel formation remain poorly understood. Applying selective plane illumination-based ultramicroscopy to entire wholemount-immunostained mouse embryos, we visualized the complete developing vascular system with cellular resolution. Here, we report emergence of the earliest detectable LECs as strings of loosely connected cells between the CV and superficial venous plexus. Subsequent aggregation of LECs resulted in formation of two distinct, previously unidentified lymphatic structures, the dorsal peripheral longitudinal lymphatic vessel (PLLV) and the ventral primordial thoracic duct (pTD), which at later stages formed a direct contact with the CV. Providing new insights into their function, we found vascular endothelial growth factor C (VEGF-C) and the matrix component CCBE1 indispensable for LEC budding and **migration**. Altogether, we present a significantly more detailed view and novel model of early lymphatic development.
4. VEGF signalling controls GnRH neuron survival via NRP1 independently of KDR and blood vessels. Gonadotropin-releasing hormone (GnRH) neurons are neuroendocrine cells that are born in the nasal placode during embryonic development and migrate through the nose and forebrain to the hypothalamus, where they regulate reproduction. Many molecular pathways that guide their **migration** have been identified, but little is known about the factors that control the survival of the migrating GnRH neurons as they negotiate different environments. We previously reported that the class 3 semaphorin SEMA3A signals through its neuropilin receptors, NRP1 and NRP2, to organise the axons that guide migrating GnRH neurons from their birthplace into the brain. By combining analysis of genetically altered mice with in vitro models, we show here that the alternative neuropilin ligand VEGF164 promotes the survival of migrating GnRH neurons by co-activating the ERK and AKT signalling pathways through NRP1. We also demonstrate that survival signalling relies on neuronal, but not endothelial, NRP1 expression and that it occurs independently of KDR, the main VEGF receptor in blood vessels. Therefore, VEGF164 provides survival signals directly to developing GnRH neurons, independently of its role in blood vessels. Finally, we show that the VEGF164-mediated neuronal survival and SEMA3A-mediated axon guidance cooperate to ensure that migrating GnRH neurons reach the brain. Thus, the loss of both neuropilin ligands leads to an almost complete failure to establish the GnRH neuron system.
5. Loss of Notch signalling induced by Dll4 causes arterial calibre reduction by increasing endothelial cell response to angiogenic stimuli. In the vascular system, Notch receptors and ligands are expressed mainly on arteries, with Delta-like 4 (Dll4) being the only ligand known to be expressed early during the development of arterial endothelial cells and capillaries. Dll4 null embryos die very early in development with severely reduced arterial calibre and lumen and loss of arterial cell identity.The current detailed analysis of these mutants shows that the arterial defect precedes the initiation of blood flow and that the arterial Dll4-/- endothelial cells proliferate and migrate more actively. Dll4-/- mutants reveal a defective basement membrane around the forming aorta and increased endothelial cell **migration** from the dorsal aorta to peripheral regions, which constitute the main causes of arterial lumen reduction in these embryos. The increased proliferation and **migration** of Dll4-/- endothelial cells was found to coincide with increased expression of the receptors VEGFR-2 and Robo4 and with downregulation of the TGF-beta accessory receptor Endoglin.Together, these results strongly suggest that Notch signalling can increase arterial stability and calibre by decreasing the response of arterial endothelial cells to local gradients of pro-angiogenic factors like VEGF.
6. Overexpression of delta-like 4 induces arterialization and attenuates vessel formation in developing mouse embryos. The importance of Notch signaling pathway in the regulation of vascular development and angiogenesis is suggested by the expression of Notch receptors and ligands in vascular endothelial cells (ECs) and the observed vascular phenotypes in mutants of Notch receptors or ligands, especially Dll4. DLL4 is specifically expressed in arterial ECs during development, and haplo-insufficiency is embryonically lethal in mice. To address the role of Dll4 in vascular development, we produced mDll4 conditionally overexpressed transgenic mice that were crossed with constitutive recombinase cre lines. Double transgenic embryos displayed grossly enlarged dorsal aortae (DA) and died before embryonic day 10.5 (E10.5), showing a variable degree of premature arteriovenous fusion. Veins displayed ectopic expression of arterial markers. Other defects included reduced vascular sprouting, EC proliferation, and **migration**. mDll4 overexpression also inhibited VEGF signaling and increased fibronectin accumulation around the vessels. In vitro and in vivo studies of DLL4-FL (Dll4-full-length) in ECs recapitulate many of the mDll4 transgenics findings, including decreased tube formation, reduced vascular branching, fewer vessels, increased pericyte recruitment, and increased fibronectin expression. These results establish the role of Dll4 in arterial identity determination, and regulation of angiogenesis subject to dose and location.
7. Robo1 regulates semaphorin signaling to guide the **migration** of cortical interneurons through the ventral forebrain. Cortical interneurons, generated predominantly in the medial ganglionic eminence, migrate around and avoid the developing striatum in the subpallium en route to the cortex. This is attributable to the chemorepulsive cues of class 3 semaphorins expressed in the striatal mantle and acting through neuropilin (Nrp1 and Nrp2) receptors expressed in these cells. Cortical interneurons also express Robo receptors, and we show here that in mice lacking Robo1, but not Robo2, these cells migrate aberrantly through the striatum. In vitro experiments demonstrated that interneurons lacking Robo1 function are significantly less responsive to the effects of semaphorins. Failure to respond to semaphorin appears to be attributable to a reduction in Nrp1 and PlexinA1 receptors within these cells. Biochemical studies further demonstrated that Robo1 binds directly to Nrp1, but not to semaphorins, and this interaction is mediated by a region contained within its first two Ig domains. Thus, we show for the first time that Robo1 interacts with Nrp1 to modulate semaphorin signaling in the developing forebrain and direct the **migration** of interneurons through the subpallium and into the cortex.
8. Deletion of Cdc42 enhances ADAM17-mediated vascular endothelial growth factor receptor 2 shedding and impairs vascular endothelial cell survival and vasculogenesis. Cdc42 is a Ras-related GTPase that plays an important role in the regulation of a range of cellular functions, including cell **migration**, proliferation, and survival. Consistent with its critical functions in vitro, the inactivation of Cdc42 in mice has been shown to result in embryonic lethality at embryonic day 6.5 (E6.5) before blood vessel formation. To determine the role of Cdc42 in new blood vessel formation, we have generated vascular endothelial cell (EC)-specific Cdc42 knockout mice by crossing Cdc42(flox/flox) mice with Tie2-Cre mice. The deletion of Cdc42 in ECs caused embryonic lethality with vasculogenesis and angiogenesis defects. We observed that Cdc42 is critical for EC **migration** and survival but not for cell cycle progression. Moreover, we found that the inactivation of Cdc42 in ECs decreased the level of vascular endothelial growth factor receptor 2 (VEGFR2) protein on the EC surface and promoted the production of a 75-kDa membrane-associated C-terminal VEGFR2 fragment. Using cultured primary mouse ECs and human umbilical vein ECs, we have demonstrated that the deletion of Cdc42 increased ADAM17-mediated VEGFR2 shedding. Notably, inhibition of ADAM17 or overexpression of VEGFR2 can partially reverse Cdc42 deletion-induced EC apoptosis. These data indicate that Cdc42 is essential for VEGFR2-mediated signal transduction in blood vessel formation.
9. Necl-5/poliovirus receptor interacts with VEGFR2 and regulates VEGF-induced angiogenesis. Vascular endothelial growth factor (VEGF), a major proangiogenic agent, exerts its proangiogenic action by binding to VEGF receptor 2 (VEGFR2), the activity of which is regulated by direct interactions with other cell surface proteins, including integrin α(V)β(3). However, how the interaction between VEGFR2 and integrin α(V)β(3) is regulated is not clear.To investigate whether Necl-5/poliovirus receptor, an immunoglobulin-like molecule that is known to bind integrin α(V)β(3), regulates the interaction between VEGFR2 and integrin α(V)β(3), and to clarify the role of Necl-5 in the VEGF-induced angiogenesis.Necl-5-knockout mice displayed no obvious defect in vascular development; however, recovery of blood flow after hindlimb ischemia and the VEGF-induced neovascularization in implanted Matrigel plugs were impaired in Necl-5-knockout mice. To clarify the mechanism of the regulation of angiogenesis by Necl-5, we investigated the roles of Necl-5 in the VEGF-induced angiogenic responses in vitro. Knockdown of Necl-5 by siRNAs in human umbilical vein endothelial cells (HUVECs) inhibited the VEGF-induced capillary-like network formation on Ma**trigel, m**igration, and proliferation, and conversely, enhanced apoptosis. Coimmunoprecipitation assays showed the interaction of Necl-5 with VEGFR2, and knockdown of Necl-5 prevented the VEGF-induced interaction of integrin α(V)β(3) with VEGFR2. Knockdown of Necl-5 suppressed the VEGFR2-mediated activation of downstream proangiogenic and survival signals, including Rap1, Akt, and endothelial nitric oxide synthase.These results demonstrate the critical role of Necl-5 in angiogenesis and suggest that Necl-5 may regulate the VEGF-induced angiogenesis by controlling the interaction of VEGFR2 with integrin α(v)β(3), and the VEGFR2-mediated Rap1-Akt signaling pathway.
10. Edg-1, the G protein-coupled receptor for sphingosine-1-phosphate, is essential for vascular maturation. Sphingolipid signaling pathways have been implicated in many critical cellular events. Sphingosine-1-phosphate (SPP), a sphingolipid metabolite found in high concentrations in platelets and blood, stimulates members of the endothelial differentiation gene (Edg) family of G protein-coupled receptors and triggers diverse effects, including cell growth, survival, **migration**, and morphogenesis. To determine the in vivo functions of the SPP/Edg signaling pathway, we disrupted the Edg1 gene in mice. Edg1(-/-) mice exhibited embryonic hemorrhage leading to intrauterine death between E12.5 and E14.5. Vasculogenesis and angiogenesis appeared normal in the mutant embryos. However, vascular maturation was incomplete due to a deficiency of vascular smooth muscle cells/pericytes. We also show that Edg-1 mediates an SPP-induced **migration** response that is defective in mutant cells due to an inability to activate the small GTPase, Rac. Our data reveal Edg-1 to be the first G protein-coupled receptor required for blood vessel formation and show that sphingolipid signaling is essential during mammalian development.
11. Formation of the collateral circulation is regulated by vascular endothelial growth factor-A and a disintegrin and metalloprotease family members 10 and 17. The density of native (preexisting) collaterals varies widely and is a significant determinant of variation in severity of stroke, myocardial infarction, and peripheral artery disease. However, little is known about mechanisms responsible for formation of the collateral circulation in healthy tissues.We previously found that variation in vascular endothelial growth factor (VEGF) expression causes differences in collateral density of newborn and adult mice. Herein, we sought to determine mechanisms of collaterogenesis in the embryo and the role of VEGF in this process.Pial collaterals begin forming between embryonic day 13.5 and 14.5 as sprout-like extensions from arterioles of existing cerebral artery trees. Global VEGF-A overexpressing mice (Vegf(hi/+)) formed more, and Vegf(lo/+) formed fewer, collaterals during embryogenesis, in association with differences in vascular patterning. Conditional global reduction of Vegf or Flk1 only during collaterogenesis significantly reduced collateral formation, but now without affecting vascular patterning, and the effects remained in adulthood. Endothelial-specific Vegf reduction had no effect on collaterogenesis. Endothelial-specific reduction of a disintegrin-and-metalloprotease-domain-10 (Adam10) and inhibition of γ-secretase increased collateral formation, consistent with their roles in VEGF-induced Notch1 activation and suppression of prosprouting signals. Endothelial-specific knockdown of Adam17 reduced collateral formation, consistent with its roles in endothelial cell **migratio**n and embryonic vascular stabilization, but not in activation of ligand-bound Notch1. These effects also remained in adulthood.Formation of pial collaterals occurs during a narrow developmental window via a sprouting angiogenesis-like mechanism, requires paracrine VEGF stimulation of fetal liver kinase 1-Notch signaling, and adult collateral number is dependent on embryonic collaterogenesis.
12. Comparative structure-function analysis of VEGFR-1 and VEGFR-2: What have we learned from chimeric systems? Activation of vascular endothelial growth factor receptor-1 and -2 (VEGFR-1 and VEGFR-2) plays a critical role in vasculogenesis and angiogenesis. However, the mechanism by which activation of VEGFRs elicits these cellular events is not fully understood. We recently constructed chimeric receptors containing the extracellular domain of human CSF-1R/c-fms fused with the entire transmembrane and cytoplasmic domains of murine VEGFR-1 and VEGFR-2. Selective activation of chimeric VEGFR-2, but not chimeric VEGFR-1, stimulated endothelial cell growth, **migration**, and differentiation. Stimulation of cells coexpressing chimeric VEGFR-1 and VEGFR-2 suppressed VEGFR-2-mediated endothelial cell growth. Site-directed mutagenesis demonstrated that tyrosines 799 and 1173 are required for VEGFR-2-mediated endothelial cell growth and activation of PI3 kinase. Further site-directed mutagenesis demonstrated that tyrosine 1212, located in the carboxyl tail of VEGFR-2, is required for the ligand-dependent autophosphorylation of the receptor and its ability to activate signaling proteins. Collectively, our results suggest that activation of VEGFR-1 and VEGFR-2 differentially regulates endothelial cell function and angiogenesis. Second, activation of VEGFR-2 is associated with many endothelial cell functions, including cell proliferation, **migration**, and differentiation. Third, activation of PI3 kinase by VEGFR-2 regulates endothelial cell proliferation.
13. The PTEN/PI3K pathway governs normal vascular development and tumor angiogenesis. PTEN is an important tumor suppressor gene. Hereditary mutation of PTEN causes tumor-susceptibility diseases such as Cowden disease. We used the Cre-loxP system to generate an endothelial cell-specific mutation of Pten (Tie2CrePten) in mice. Tie2CrePten(flox/+) mice displayed enhanced tumorigenesis due to an increase in angiogenesis driven by vascular growth factors. This effect was partially dependent on the PI3K subunits p85alpha and p110gamma. In vitro, Tie2CrePten(flox/+) endothelial cells showed enhanced proliferation/**migration**. Tie2CrePten(flox/flox) mice died before embryonic day 11.5 (E11.5) due to bleeding and cardiac failure caused by impaired recruitment of pericytes and vascular smooth muscle cells to blood vessels, and of cardiomyocytes to the endocardium. These phenotypes depend strongly on p110gamma rather than on p85alpha and were associated with decreased expression of Ang-1, VCAM-1, connexin 40, and ephrinB2 but increased expression of Ang-2, VEGF-A, VEGFR1, and VEGFR2. Pten is thus indispensable for normal cardiovascular morphogenesis and post-natal angiogenesis, including tumor angiogenesis.
14. Herbal compound farnesiferol C exerts antiangiogenic and antitumor activity and targets multiple aspects of VEGFR1 (Flt1) or VEGFR2 (Flk1) signaling cascades. Farnesiferol C (FC) is one of the major compounds isolated from Ferula assafoetida, an Asian herbal spice used for cancer treatment as a folk remedy. Here, we examined the hypothesis that novel antiangiogenic activities of FC contribute to anticancer efficacy. In human umbilical vein endothelial cells (HUVEC), exposure to the 10 to 40 mumol/L concentration range of FC inhibited vascular endothelial growth factor (VEGF)-induced cell proliferation, **migration**, invasion, tube formation, and the expression of matrix metalloproteinase-2. In addition, FC inhibited the angiogenic sprouting of VEGF-treated rat aorta in an ex vivo model. Furthermore, FC inhibited the in vivo growth of mouse Lewis lung cancer allograft model by 60% (P < 0.001) at a daily i.p. dosage of 1 mg/kg body weight without any negative effect on the weight of the host mice. Immunohistochemistry staining showed decreased microvessel density (CD34) and proliferative index (Ki-67) without affecting the apoptotic (terminal deoxynucleotidyl transferase-mediated dUTP nick end labeling) index. Mechanistically, FC decreased the binding of VEGF to VEGFR1/Flt-1, but not to VEGFR2/KDR/Flk-1. In terms of early signaling, FC exerted a rapid inhibitory action (examined within 10 minutes) on VEGF-induced autophosphorylation of VEGFR1 without affecting that of VEGFR2. Nevertheless, FC decreased the phosphorylation of most of the kinases downstream of VEGFR2: focal adhesion kinase, Src, extracellular signal-regulated kinase 1/2, p38 mitogen-activated protein kinase, and c-jun-NH(2)-kinase without affecting AKT. Computer simulation suggests that FC may inhibit Src or focal adhesion kinase protein activities directly through its docking to their ATP-binding sites. Taken together, the multitargeting actions of FC, particularly VEGFR1 inhibition, may make it a novel drug candidate to complement current VEGF/VEGFR2-targeting antiangiogenic modalities for cancer.
15. CXCL1 induced by prostaglandin E2 promotes angiogenesis in colorectal cancer. Chronic inflammation is a well-known risk factor for cancer. Proinflammatory mediators such as prostaglandin E2 (PGE2) promote colorectal tumor growth by stimulating angiogenesis, cell invasion, and cell growth, and inhibiting apoptosis. Molecules that regulate tumor-associated angiogenesis provide promising therapeutic targets for treatment of colorectal cancer (CRC) as indicated by the recent development of the novel anti-angiogenic agent bevacizumab (Avastin). However, use of this drug only prolongs survival by several months, highlighting the importance of finding more effective treatment regimens. We report here that PGE2 induces expression of CXCL1 (growth-regulated oncogene alpha), a pro-angiogenic chemokine, in human CRC cells. More importantly, CXCL1 released from carcinoma cells induces microvascular endothelial cell **migration** and tube formation in vitro. Furthermore, PGE2 promotes tumor growth in vivo by induction of CXCL1 expression, which results in increased tumor microvessel formation. These results have potential clinical significance because we found that CXCL1 expression correlates with PGE2 levels in human CRCs. Collectively, our findings show for the first time that CXCL1 is regulated by PGE2 and indicate that CXCL1 inhibitors should be evaluated further as potential anti-angiogenic agents for treatment of CRC.
16. Tetraspanin CD63 promotes vascular endothelial growth factor receptor 2-β1 integrin complex formation, thereby regulating activation and downstream signaling in endothelial cells in vitro and in vivo. CD63 is a member of the transmembrane-4 glycoprotein superfamily (tetraspanins) implicated in the regulation of membrane protein trafficking, leukocyte recruitment, and adhesion processes. We have investigated the involvement of CD63 in endothelial cell (EC) signaling downstream of β1 integrin and VEGF. We report that silencing of CD63 in primary ECs arrested capillary sprouting and tube formation in vitro because of impaired adhesion an**d migrati**on of ECs. Mechanistically, CD63 associated with both β1 integrin and the main VEGF receptor on ECs, VEGFR2. Our data suggest that CD63 serves to bridge between β1 integrin and VEGFR2 because CD63 silencing disrupted VEGFR2-β1 integrin complex formation identified using proximity ligation assays. Signaling downstream of β1 integrin and VEGFR2 was attenuated in CD63-silenced cells, although their cell surface expression levels remained unaffected. CD63 was furthermore required for efficient internalization of VEGFR2 in response to VEGF. Importantly, systemic delivery of VEGF failed to potently induce VEGFR2 phosphorylation and downstream signaling in CD63-deficient mouse lungs. Taken together, our findings demonstrate a previously unrecognized role for CD63 in coordinated integrin and receptor tyrosine kinase signaling in vitro and in vivo.
17. Distinct roles of vascular endothelial growth factor receptor-1- and receptor-2-mediated signaling in T cell priming and Th17 polarization to lipopolysaccharide-containing allergens in the lung. Vascular endothelial growth factor (VEGF) is a key mediator in the development of airway immune dysfunction to inhaled allergens. However, the exact role of its receptors-mediated signaling is controversial. In this study, we evaluated the role of VEGF receptor (VEGFR)-1- and VEGFR-2-mediated signaling in T cell priming and polarization in the context of inhalation of LPS-containing allergens. A murine asthma model of mixed Th1 and Th17 cell responses was generated using intranasal sensitization with LPS-containing allergens. Pharmacologic intervention was performed during sensitization. In vivo production of VEGF and Th1- and Th17-polarizing cytokines (IL-12p70 and IL-6, respectively) were upregulated by airway exposure to LPS. Pharmacological intervention with a VEGFR-2-neutralizing Ab (anti-Flk1 mAb) abolished the production of IL-6 (but not IL-12p70) and the subsequent development of allergen-specific Th17 cell response. On the other hand, blocking VEGFR-1 signaling with a VEGFR-1 antagonist (anti-Flt1 hexapeptide) did not affect the production of IL-12p70 and IL-6. However, blocking VEGFR-1 signaling resulted in T cell tolerance rather than priming, mainly by inhibiting the maturation of lung dendritic cells, and their **migration** into lung-draining lymph nodes. These results suggest that T cell priming to LPS-containing allergens depends on VEGFR-1-mediated signaling, and the subsequent Th17 polarization depends on VEGFR-2 signaling.
18. Endothelial progenitor cells induce transplant arteriosclerosis via VEGFR-1/2 activity. Acute rejection (AR) after organ transplantation results in transplant arteriosclerosis (TA). Endothelial progenitor cells (EPCs) are involved in tissue repair and blood vessel formation but are suspected to be a cause of TA.In this study, we introduced a syngeneic and allogeneic abdominal aortic transplant model with C57BL/6 and BALB/c mice. Syngeneic and allogeneic grafts were histopathologically analyzed after transplantation. Bone marrow-derived EPCs were injected into transplant model animals to observe their distribution and temporal concentration changes. Changes of vascular endothelial growth factor receptor 1 (VEGFR-1), phosphorylated VEGFR-1 (pVEGFR-1), VEGFR-2, pVEGFR-2, protein kinase B (Akt), pAkt, extracellular signal-regulated kinase 1 (Erk1), pErk1 levels in EPCs upon VEGF165 and the VEGFR inhibitor Vandetanib exposure were analyzed in vitro with western blotting.In the allogeneic transplant group, two weeks after transplantation, formations of new intima layers could be observed, and its proliferation gradually increased to four and six weeks post-transplantation (p < 0.05), accompanied by significant arterial stenoses. Exogenous EPCs mainly localized to the damaged sites of the transplant arteries. In vivo, Vandetanib caused a significant dose dependent decrease of transplant hyperplasia (p < 0.05) and inhibited VEGF related prolifera**tion, mig**ration and adhesion of EPCs.Vandetanib treatment can reduce arteriosclerosis induced by abdominal aorta transplantation by blocking VEGFRs in EPCs.
19. Rac1 modulates mammalian lung branching morphogenesis in part through canonical Wnt signaling. Lung branching morphogenesis relies on a number of factors, including proper epithelial cell proliferation and differentiation, cell polarity, and **migration**. Rac1, a small Rho GTPase, orchestrates a number of these cellular processes, including cell proliferation and differentiation, cellular alignment, and polarization. Furthermore, Rac1 modulates both noncanonical and canonical Wnt signaling, important pathways in lung branching morphogenesis. Culture of embryonic mouse lung explants in the presence of the Rac1 inhibitor (NSC23766) resulted in a dose-dependent decrease in branching. Increased cell death and BrdU uptake were notably seen in the mesenchyme, while no direct effect on the epithelium was observed. Moreover, vasculogenesis was impaired following Rac1 inhibition as shown by decreased Vegfa expression and impaired LacZ staining in Flk1-Lacz reporter mice. Rac1 inhibition decreased Fgf10 expression in conjunction with many of its associated factors. Moreover, using the reporter lines TOPGAL and Axin2-LacZ, there was an evident decrease in canonical Wnt signaling in the explants treated with the Rac1 inhibitor. Activation of canonical Wnt pathway using WNT3a or WNT7b only partially rescued the branching inhibition. Moreover, these results were validated on human explants, where Rac1 inhibition resulted in impaired branching and decreased AXIN2 and FGFR2b expression. We therefore conclude that Rac1 regulates lung branching morphogenesis, in part through canonical Wnt signaling. However, the exact mechanisms by which Rac1 interacts with canonical Wnt in human and mouse lung requires further investigation.
20. Heterozygous expression of the oncogenic Pik3ca(H1047R) mutation during murine development results in fatal embryonic and extraembryonic defects. The phosphoinositide 3-kinase (PI3K)/AKT signalling pathway regulates many cellular functions including proliferation, **migration**, survival and protein synthesis. Somatic mutations in PIK3CA, the gene encoding the p110α catalytic subunit of PI3K enzyme, are commonly associated with many human cancers as well as recently being implicated in human overgrowth syndromes. However, it is not clear if such mutations can be inherited through the germline. We have used a novel mouse model with Cre recombinase (Cre)-conditional knock-in of the common H1047R mutation into the endogenous Pik3ca gene. Heterozygous expression of the Pik3ca(H1047R) mutation in the developing mouse embryo resulted in failed 'turning' of the embryo and disrupted vascular remodelling within the embryonic and extraembryonic tissues, leading to lethality prior to E10. As vascular endothelial growth factor A (VEGF-A) signalling was disrupted in these embryos, we used Cre under the control of the Tie2 promoter to target the Pik3ca(H1047R) mutation specifically to endothelial cells. In these embryos turning occurred normally but the vascular remodelling defects and embryonic lethality remained, likely as a result of endothelial hyperproliferation. Our results confirm the lethality associated with heterozygous expression of the Pik3ca(H1047R) mutation during development and likely explain the lack of inherited germline PIK3CA mutations in humans.
21. GPR124, an orphan G protein-coupled receptor, is required for CNS-specific vascularization and establishment of the blood-brain barrier. Every organ in the body requires blood vessels for efficient delivery of oxygen and nutrients, but independent vascular beds are highly specialized to meet the individual needs of specific organs. The vasculature of the brain is tightly sealed, with blood-brain barrier (BBB) properties developing coincident with neural vascularization. G protein-coupled receptor 124 (GPR124) (tumor endothelial marker 5, TEM5), an orphan member of the adhesion family of G protein-coupled receptors, was previously identified on the basis of its overexpression in tumor vasculature. Here, we show that global deletion or endothelial-specific deletion of GPR124 in mice results in embryonic lethality associated with abnormal angiogenesis of the forebrain and spinal cord. Expression of GPR124 was found to be required for invasion and **migration** of blood vessels into neuroepithelium, establishment of BBB properties, and expansion of the cerebral cortex. Thus, GPR124 is an important regulator of neurovasculature development and a potential drug target for cerebrovascular diseases.
22. Identification of Flk-1 target genes in vasculogenesis: Pim-1 is required for endothelial and mural cell differentiation in vitro. The tyrosine kinase receptor fetal liver kinase 1 (Flk-1) plays a crucial role in vasculogenesis and angiogenesis, but its target genes remain elusive. Comparing Flk-1(+/+) with Flk-1(-/-) embryonic stem (ES) cells, we identified transcripts regulated by the vascular endothelial growth factor A (VEGF-A)/Flk-1 pathway at an early stage of their differentiation to endothelial and mural precursors. Further analysis of a number of these genes (Nm23-M1, Nm23-M2, Slug, Set, pp32, Cbp, Ship-1, Btk, and Pim-1) showed that their products were transiently up-regulated in vivo in endothelial cells (ECs) during angiogenesis of the ovary, and their mRNA was rapidly induced in vitro by VEGF-A in human umbilical cord vein endothelial cells (HUVECs). Functional analysis by RNA interference (RNAi) in ES cells induced to differentiate demonstrated that Pim-1 is required for their differentiation into ECs and smooth muscle cells (SMCs). In HUVECs, RNAi showed that Pim-1 is required in ECs for VEGF-A-dependent proliferation and **migration**. The identification of Flk-1 target genes should help in elucidating the molecular pathways that govern the vasculogenesis and angiogenesis processes.
23. SnoN facilitates ALK1-Smad1/5 signaling during embryonic angiogenesis. In endothelial cells, two type I receptors of the transforming growth factor β (TGF-β) family, ALK1 and ALK5, coordinate to regulate embryonic angiogenesis in response to BMP9/10 and TGF-β. Whereas TGF-β binds to and activates ALK5, leading to Smad2/3 phosphorylation and inhibition of endothelial cell proliferation **and migra**tion, BMP9/10 and TGF-β also bind to ALK1, resulting in the activation of Smad1/5. SnoN is a negative regulator of ALK5 signaling through the binding and repression of Smad2/3. Here we uncover a positive role of SnoN in enhancing Smad1/5 activation in endothelial cells to promote angiogenesis. Upon ligand binding, SnoN directly bound to ALK1 on the plasma membrane and facilitated the interaction between ALK1 and Smad1/5, enhancing Smad1/5 phosphorylation. Disruption of this SnoN-Smad interaction impaired Smad1/5 activation and up-regulated Smad2/3 activity. This resulted in defective angiogenesis and arteriovenous malformations, leading to embryonic lethality at E12.5. Thus, SnoN is essential for TGF-β/BMP9-dependent biological processes by its ability to both positively and negatively modulate the activities of Smad-dependent pathways.
24. Redundant role of protein kinase C delta and epsilon during mouse embryonic development. Protein Kinase C delta and epsilon are mediators of important cellular events, such as cell proliferation, **migration** or apoptosis. The formation of blood vessels, i.e., vasculo- and angiogenesis, is a process where these isoforms have also been shown to participate. However, mice deficient in either Protein Kinase C delta or epsilon are viable and therefore their individual contribution to the formation of the vasculature appeared so far dispensable. In this study, we show that double null mutation of Protein Kinase C delta and epsilon causes embryonic lethality at approximately E9.5. At this stage, whole mount staining of the endothelial marker CD31 in double null embryos revealed defective blood vessel formation. Moreover, culture of double deficient mouse allantois showed impaired endothelial cell organization, and analyses of double deficient embryo sections showed dilated vessels, decreased endothelial-specific adherent junctions, and decreased contact of endothelial cells with mural cells. Protein kinase C delta and epsilon also appeared essential for vascular smooth muscle cell differentiation, since α-smooth muscle actin, a classical marker for vascular smooth muscle cells, was almost undetectable in double deficient embryonic aorta at E9.5. Subsequent qPCR analyses showed decreased VE-cadherin, Vegfr2, Cd31, Cdh2, Ets1, and Fli-1, among other angiogenesis related transcripts in double deficient embryos. Taken together, these data suggest for the first time an in vivo redundant role between members of the novel Protein Kinase C subfamily that allows for mutual compensation during mouse embryonic development, with vasculogenesis/angiogenesis as an obvious common function of these two Protein Kinase Cs. Protein Kinase C delta and epsilon might therefore be useful targets for inhibiting vasculo- and/or angiogenesis.
25. The homeobox gene Hex is required in definitive endodermal tissues for normal forebrain, liver and thyroid formation. The homeobox gene Hex is expressed in the anterior visceral endoderm (AVE) and rostral definitive endoderm of early mouse embryos. Later, Hex transcripts are detected in liver, thyroid and endothelial precursor cells. A null mutation was introduced into the Hex locus by homologous recombination in embryonic stem cells. Hex mutant embryos exhibit varying degrees of anterior truncation as well as liver and thyroid dysplasia. The liver diverticulum is formed but **migration** of hepatocytes into the septum transversum fails to occur. Development of the thyroid is arrested at the thyroid bud stage at 9.5 dpc. Brain defects are restricted to the rostral forebrain and have a caudal limit at the zona limitans intrathalamica, the boundary between dorsal and ventral thalamus. Analysis of Hex(-/-) mutants at early stages shows that the prospective forebrain ectoderm is correctly induced and patterned at 7.5 days post coitum (dpc), but subsequently fails to develop. AVE markers are expressed and correctly positioned but development of rostral definitive endoderm is greatly disturbed in Hex(-/-) embryos. Chimeric embryos composed of Hex(-/-) cells developing within a wild-type visceral endoderm show forebrain defects indicating that Hex is required in the definitive endoderm. All together, these results demonstrate that Hex function is essential in definitive endoderm for normal development of the forebrain, liver and thyroid gland.
26. Histone H3 lysine 36 methyltransferase Hypb/Setd2 is required for embryonic vascular remodeling. HYPB is a human histone H3 lysine 36 (H3K36)-specific methyltransferase and acts as the ortholog of yeast Set2. This study explored the physiological function of mammalian HYPB using knockout mice. Homozygous disruption of Hypb impaired H3K36 trimethylation but not mono- or dimethylation, and resulted in embryonic lethality at E10.5-E11.5. Severe vascular defects were observed in the Hypb(-/-) embryo, yolk sac, and placenta. The abnormally dilated capillaries in mutant embryos and yolk sacs could not be remodeled into large blood vessels or intricate networks, and the aberrantly rounded mesodermal cells exhibited weakened interaction with endothelial cells. The embryonic vessels failed to invade the labyrinthine layer of placenta, which impaired the embryonic-maternal vascular connection. These defects could not be rescued by wild-type tetraploid blastocysts, excluding the possibility that they were caused by the extraembryonic tissues. Consistent with these phenotypes, gene expression profiling in wild-type and Hypb(-/-) yolk sacs revealed that the Hypb disruption altered the expression of some genes involved in vascular remodeling. At the cellular level, Hypb(-/-) embryonic stem cell-derived embryonic bodies, as well as in vitro-cultured human endothelial cells with siRNA-mediated suppression of HYPB, showed obvious defects in cell **migration** and invasion during vessel formation, suggesting an intrinsic role of Hypb in vascular development. Taken together, these results indicate that Hypb is required for embryonic vascular remodeling and provide a tool to study the function of H3K36 methylation in vasculogenesis/angiogenesis.
27. Rac1 mediates morphogenetic responses to intercellular signals in the gastrulating mouse embryo. The establishment of the mammalian body plan depends on signal-regulated cell **migration** and adhesion, processes that are controlled by the Rho family of GTPases. Here we use a conditional allele of Rac1, the only Rac gene expressed early in development, to define its roles in the gastrulating mouse embryo. Embryos that lack Rac1 in the epiblast (Rac1Δepi) initiate development normally: the signaling pathways required for gastrulation are active, definitive endoderm and all classes of mesoderm are specified, and the neural plate is formed. After the initiation of gastrulation, Rac1Δepi embryos have an enlarged primitive streak, make only a small amount of paraxial mesoderm, and the lateral anlage of the heart do not fuse at the midline. Because these phenotypes are also seen in Nap1 mutants, we conclude that Rac1 acts upstream of the Nap1/WAVE complex to promot**e migrati**on of the nascent mesoderm. In addition t**o migrati**on phenotypes, Rac1Δepi cells fail to adhere to matrix, which leads to extensive cell death. Cell death is largely rescued in Rac1Δepi mutants that are heterozygous for a null mutation in Pten, providing evidence that Rac1 is required to link signals from the basement membrane to activation of the PI3K-Akt pathway in vivo. Surprisingly, the frequency of apoptosis is greater in the anterior half of the embryo, suggesting that cell survival can be promoted either by matrix adhesion or by signals from the posterior primitive streak. Rac1 also has essential roles in morphogenesis of the posterior notochordal plate (the node) and the midline.
28. Bovine papular stomatitis virus encodes a functionally distinct VEGF that binds both VEGFR-1 and VEGFR-2. Bovine papular stomatitis virus (BPSV), a member of the genus Parapoxvirus, causes proliferative dermatitis in cattle and humans. Other species of the genus cause similar lesions, the nature of which has been attributed, at least in part, to a viral-encoded vascular endothelial growth factor (VEGF) that induces vascularization and dermal oedema through VEGF receptor-2 (VEGFR-2). The results of this study showed that BPSV strain V660 encodes a novel VEGF and that the predicted BPSV protein showed only 33-52% amino acid identity to VEGFs encoded by the other species of the genus. BPSV VEGF showed higher identity to mammalian VEGF-A (51%) than the other parapoxviral VEGFs (31-46%). Assays of the purified BPSV VEGF (BPSVV660VEGF) demonstrated that it was also functionally more similar to VEGF-A, as it showed significant binding to VEGFR-1 and induced monocyte **migration**. Like VEGF-A and the other viral VEGFs, BPSVV660VEGF bound VEGFR-2 with high affinity. Sequence analysis and structural modelling of BPSVV660VEGF revealed specific residues, outside the known receptor-binding face, that are predicted either to influence VEGF structure or to mediate binding directly to the VEGFRs. These results indicate that BPSVV660VEGF is a biologically active member of the VEGF family and that, via its interaction with VEGFR-2, it is likely to contribute to the proliferative and highly vascularized nature of BPSV lesions. This is also the first example of a viral VEGF acting via VEGFR-1 and influencing haematopoietic cell function. These data suggest that BPSVV660VEGF is an evolutionary and functional intermediate between VEGF-A and the other parapoxviral VEGFs.
29. Early endocardial morphogenesis requires Scl/Tal1. The primitive heart tube is composed of an outer myocardial and an inner endocardial layer that will give rise to the cardiac valves and septa. Specification and differentiation of these two cell layers are among the earliest events in heart development, but the embryonic origins and genetic regulation of early endocardial development remain largely undefined. We have analyzed early endocardial development in the zebrafish using time-lapse confocal microscopy and show that the endocardium seems to originate from a region in the lateral plate mesoderm that will give rise to hematopoietic cells of the primitive myeloid lineage. Endocardial precursors appear to rapidly migrate to the site of heart tube formation, where they arrive prior to the bilateral myocardial primordia. Analysis of a newly discovered zebrafish Scl/Tal1 mutant showed an additional and previously undescribed role of this transcription factor during the development of the endocardium. In Scl/Tal1 mutant embryos, endocardial precursors are specified, but **migration** is severely defective and endocardial cells aggregate at the ventricular pole of the heart. We further show that the initial fusion of the bilateral myocardial precursor populations occurs independently of the endocardium and tal1 function. Our results suggest early separation of the two components of the primitive heart tube and imply Scl/Tal1 as an indispensable component of the molecular hierarchy that controls endocardium morphogenesis.
30. Lack of pericytes leads to endothelial hyperplasia and abnormal vascular morphogenesis. The association of pericytes (PCs) to newly formed blood vessels has been suggested to regulate endothelial cell (EC) proliferation, survival, **migration**, differentiation, and vascular branching. Here, we addressed these issues using PDGF-B-- and PDGF receptor-beta (PDGFR-beta)--deficient mice as in vivo models of brain angiogenesis in the absence of PCs. Quantitative morphological analysis showed that these mutants have normal microvessel density, length, and number of branch points. However, absence of PCs correlates with endothelial hyperplasia, increased capillary diameter, abnormal EC shape and ultrastructure, changed cellular distribution of certain junctional proteins, and morphological signs of increased transendothelial permeability. Brain endothelial hyperplasia was observed already at embryonic day (E) 11.5 and persisted throughout development. From E 13.5, vascular endothelial growth factor-A (VEGF-A) and other genes responsive to metabolic stress became upregulated, suggesting that the abnormal microvessel architecture has systemic metabolic consequences. VEGF-A upregulation correlated temporally with the occurrence of vascular abnormalities in the placenta and dilation of the heart. Thus, although PC deficiency appears to have direct effects on EC number before E 13.5, the subsequent increased VEGF-A levels may further abrogate microvessel architecture, promote vascular permeability, and contribute to formation of the edematous phenotype observed in late gestation PDGF-B and PDGFR-beta knock out embryos.
31. Increased hemangioblast commitment, not vascular disorganization, is the primary defect in flt-1 knock-out mice. We previously demonstrated the essential role of the flt-1 gene in regulating the development of the cardiovascular system. While the inactivation of the flt-1 gene leads to a very severe disorganization of the vascular system, the primary defect at the cellular level was unknown. Here we report a surprising finding that it is an increase in the number of endothelial progenitors that leads to the vascular disorganization in flt-1(-/-) mice. At the early primitive streak stage (prior to the formation of blood islands), hemangioblasts are formed much more abundantly in flt-1(-/-) embryos. This increase is primarily due to an alteration in cell fate determination among mesenchymal cells, rather than to increased proliferation, **migration** or reduced apoptosis of flt-1(-/-) hemangioblasts. We further show that the increased population density of hemangioblasts is responsible for the observed vascular disorganization, based on the following observations: (1) both flt-1(-/-) and flt-1(+/+) endothelial cells formed normal vascular channels in chimaeric embryos; (2) wild-type endothelial cells formed abnormal vascular channels when their population density was significantly increased; and (3) in the absence of wild-type endothelial cells, flt-1(-/-) endothelial cells alone could form normal vascular channels when sufficiently diluted in a developing embryo. These results define the primary defect in flt-1(-/-) embryos at the cellular level and demonstrate the importance of population density of progenitor cells in pattern formation.
32. Vascular endothelial growth factor a signaling in the podocyte-endothelial compartment is required for mesangial cell **migration** and survival. The glomerular filtration barrier separates the blood from the urinary space and consists of two major cell types: podocytes and fenestrated endothelial cells. Mesangial cells sit between the capillary loops and provide structural support. Proliferation and loss of mesangial cells both are central findings in a number of renal diseases, including diabetic nephropathy and mesangiolysis, respectively. Using cell-specific gene targeting, it was shown previously that vascular endothelial growth factor A (VEGF-A) production by podocytes is required for glomerular endothelial cell **migration**, differentiation, and survival. For further investigation of the effect of gene dose and VEGF-A knockdown within the glomerulus, mice that carry one hypomorphic VEGF-A allele and one podocyte-specific null VEGF-A allele (VEGFhypo/loxP,Neph-Cre+/-) were generated; in these mice, the "allelic dose" of VEGF-A is intermediate between glomerular-specific heterozygous and null states. VEGFhypo/loxP,Neph-Cre+/- mice die at 3 wk of age from renal failure. Although endothelial cell defects are observed, striking loss of mesangial cells occurs postnatally. In addition, differentiated mesangial cells cannot be found in glomeruli of podocyte-specific null VEGF-A mice (VEGFloxP/loxP,Cre+/-). Together, these results demonstrate a key role for VEGF-A production in the podocyte for mesangial cell survival and differentiation.
33. Serum response factor is required for sprouting angiogenesis and vascular integrity. Serum response factor (SRF) is a transcription factor that controls the expression of cytoskeletal proteins and immediate early genes in different cell types. Here, we found that SRF expression is restricted to endothelial cells (ECs) of small vessels such as capillaries in the mouse embryo. EC-specific Srf deletion led to aneurysms and hemorrhages from 11.5 days of mouse development (E11.5) and lethality at E14.5. Mutant embryos presented a reduced capillary density and defects in EC **migration**, with fewer numbers of filopodia in tip cells and ECs showing defects in actin polymerization and intercellular junctions. We show that SRF is essential for the expression of VE-cadherin and beta-actin in ECs both in vivo and in vitro. Moreover, knockdown of SRF in ECs impaired VEGF- and FGF-induced in vitro angiogenesis. Taken together, our results demonstrate that SRF plays an important role in sprouting angiogenesis and small vessel integrity in the mouse embryo.
34. AIP1 functions as an endogenous inhibitor of VEGFR2-mediated signaling and inflammatory angiogenesis in mice. ASK1-interacting protein-1 (AIP1), a recently identified member of the Ras GTPase-activating protein family, is highly expressed in vascular ECs and regulates EC apoptosis in vitro. However, its function in vivo has not been established. To study this, we generated AIP1-deficient mice (KO mice). Although these mice showed no obvious defects in vascular development, they exhibited dramatically enhanced angiogenesis in 2 models of inflammatory angiogenesis. In one of these models, the enhanced angiogenesis observed in the KO mice was associated with increased VEGF-VEGFR2 signaling. Consistent with this, VEGF-induced ear, cornea, and retina neovascularization were greatly augmented in KO mice and the enhanced retinal angiogenesis was markedly diminished by overexpression of AIP1. In vitro, VEGF-induced EC **migration** was inhibited by AIP1 overexpression, whereas it was augmented by both AIP1 knockout and knockdown, with the enhanced EC **migration** caused by AIP1 knockdown being associated with increased VEGFR2 signaling. We present mechanistic data that suggest AIP1 is recruited to the VEGFR2-PI3K complex, binding to both VEGFR2 and PI3K p85, at a late phase of the VEGF response, and that this leads to inhibition of VEGFR2 signaling. Taken together, our data demonstrate that AIP1 functions as an endogenous inhibitor in VEGFR2-mediated adaptive angiogenesis in mice.
35. Endothelial progenitor cells induce transplant arteriosclerosis via VEGFR-1/2 activity. Acute rejection (AR) after organ transplantation results in transplant arteriosclerosis (TA). Endothelial progenitor cells (EPCs) are involved in tissue repair and blood vessel formation but are suspected to be a cause of TA.In this study, we introduced a syngeneic and allogeneic abdominal aortic transplant model with C57BL/6 and BALB/c mice. Syngeneic and allogeneic grafts were histopathologically analyzed after transplantation. Bone marrow-derived EPCs were injected into transplant model animals to observe their distribution and temporal concentration changes. Changes of vascular endothelial growth factor receptor 1 (VEGFR-1), phosphorylated VEGFR-1 (pVEGFR-1), VEGFR-2, pVEGFR-2, protein kinase B (Akt), pAkt, extracellular signal-regulated kinase 1 (Erk1), pErk1 levels in EPCs upon VEGF165 and the VEGFR inhibitor Vandetanib exposure were analyzed in vitro with western blotting.In the allogeneic transplant group, two weeks after transplantation, formations of new intima layers could be observed, and its proliferation gradually increased to four and six weeks post-transplantation (p < 0.05), accompanied by significant arterial stenoses. Exogenous EPCs mainly localized to the damaged sites of the transplant arteries. In vivo, Vandetanib caused a significant dose dependent decrease of transplant hyperplasia (p < 0.05) and inhibited VEGF related prolifera**tion, mig**ration and adhesion of EPCs.Vandetanib treatment can reduce arteriosclerosis induced by abdominal aorta transplantation by blocking VEGFRs in EPCs.
36. Deletion of Cdc42 enhances ADAM17-mediated vascular endothelial growth factor receptor 2 shedding and impairs vascular endothelial cell survival and vasculogenesis. Cdc42 is a Ras-related GTPase that plays an important role in the regulation of a range of cellular functions, including cell **migration**, proliferation, and survival. Consistent with its critical functions in vitro, the inactivation of Cdc42 in mice has been shown to result in embryonic lethality at embryonic day 6.5 (E6.5) before blood vessel formation. To determine the role of Cdc42 in new blood vessel formation, we have generated vascular endothelial cell (EC)-specific Cdc42 knockout mice by crossing Cdc42(flox/flox) mice with Tie2-Cre mice. The deletion of Cdc42 in ECs caused embryonic lethality with vasculogenesis and angiogenesis defects. We observed that Cdc42 is critical for EC **migration** and survival but not for cell cycle progression. Moreover, we found that the inactivation of Cdc42 in ECs decreased the level of vascular endothelial growth factor receptor 2 (VEGFR2) protein on the EC surface and promoted the production of a 75-kDa membrane-associated C-terminal VEGFR2 fragment. Using cultured primary mouse ECs and human umbilical vein ECs, we have demonstrated that the deletion of Cdc42 increased ADAM17-mediated VEGFR2 shedding. Notably, inhibition of ADAM17 or overexpression of VEGFR2 can partially reverse Cdc42 deletion-induced EC apoptosis. These data indicate that Cdc42 is essential for VEGFR2-mediated signal transduction in blood vessel formation.
37. Tetraspanin CD63 promotes vascular endothelial growth factor receptor 2-β1 integrin complex formation, thereby regulating activation and downstream signaling in endothelial cells in vitro and in vivo. CD63 is a member of the transmembrane-4 glycoprotein superfamily (tetraspanins) implicated in the regulation of membrane protein trafficking, leukocyte recruitment, and adhesion processes. We have investigated the involvement of CD63 in endothelial cell (EC) signaling downstream of β1 integrin and VEGF. We report that silencing of CD63 in primary ECs arrested capillary sprouting and tube formation in vitro because of impaired adhesion an**d migrati**on of ECs. Mechanistically, CD63 associated with both β1 integrin and the main VEGF receptor on ECs, VEGFR2. Our data suggest that CD63 serves to bridge between β1 integrin and VEGFR2 because CD63 silencing disrupted VEGFR2-β1 integrin complex formation identified using proximity ligation assays. Signaling downstream of β1 integrin and VEGFR2 was attenuated in CD63-silenced cells, although their cell surface expression levels remained unaffected. CD63 was furthermore required for efficient internalization of VEGFR2 in response to VEGF. Importantly, systemic delivery of VEGF failed to potently induce VEGFR2 phosphorylation and downstream signaling in CD63-deficient mouse lungs. Taken together, our findings demonstrate a previously unrecognized role for CD63 in coordinated integrin and receptor tyrosine kinase signaling in vitro and in vivo.
38. Necl-5/poliovirus receptor interacts with VEGFR2 and regulates VEGF-induced angiogenesis. Vascular endothelial growth factor (VEGF), a major proangiogenic agent, exerts its proangiogenic action by binding to VEGF receptor 2 (VEGFR2), the activity of which is regulated by direct interactions with other cell surface proteins, including integrin α(V)β(3). However, how the interaction between VEGFR2 and integrin α(V)β(3) is regulated is not clear.To investigate whether Necl-5/poliovirus receptor, an immunoglobulin-like molecule that is known to bind integrin α(V)β(3), regulates the interaction between VEGFR2 and integrin α(V)β(3), and to clarify the role of Necl-5 in the VEGF-induced angiogenesis.Necl-5-knockout mice displayed no obvious defect in vascular development; however, recovery of blood flow after hindlimb ischemia and the VEGF-induced neovascularization in implanted Matrigel plugs were impaired in Necl-5-knockout mice. To clarify the mechanism of the regulation of angiogenesis by Necl-5, we investigated the roles of Necl-5 in the VEGF-induced angiogenic responses in vitro. Knockdown of Necl-5 by siRNAs in human umbilical vein endothelial cells (HUVECs) inhibited the VEGF-induced capillary-like network formation on Ma**trigel, m**igration, and proliferation, and conversely, enhanced apoptosis. Coimmunoprecipitation assays showed the interaction of Necl-5 with VEGFR2, and knockdown of Necl-5 prevented the VEGF-induced interaction of integrin α(V)β(3) with VEGFR2. Knockdown of Necl-5 suppressed the VEGFR2-mediated activation of downstream proangiogenic and survival signals, including Rap1, Akt, and endothelial nitric oxide synthase.These results demonstrate the critical role of Necl-5 in angiogenesis and suggest that Necl-5 may regulate the VEGF-induced angiogenesis by controlling the interaction of VEGFR2 with integrin α(v)β(3), and the VEGFR2-mediated Rap1-Akt signaling pathway.
39. VEGF signalling controls GnRH neuron survival via NRP1 independently of KDR and blood vessels. Gonadotropin-releasing hormone (GnRH) neurons are neuroendocrine cells that are born in the nasal placode during embryonic development and migrate through the nose and forebrain to the hypothalamus, where they regulate reproduction. Many molecular pathways that guide their **migration** have been identified, but little is known about the factors that control the survival of the migrating GnRH neurons as they negotiate different environments. We previously reported that the class 3 semaphorin SEMA3A signals through its neuropilin receptors, NRP1 and NRP2, to organise the axons that guide migrating GnRH neurons from their birthplace into the brain. By combining analysis of genetically altered mice with in vitro models, we show here that the alternative neuropilin ligand VEGF164 promotes the survival of migrating GnRH neurons by co-activating the ERK and AKT signalling pathways through NRP1. We also demonstrate that survival signalling relies on neuronal, but not endothelial, NRP1 expression and that it occurs independently of KDR, the main VEGF receptor in blood vessels. Therefore, VEGF164 provides survival signals directly to developing GnRH neurons, independently of its role in blood vessels. Finally, we show that the VEGF164-mediated neuronal survival and SEMA3A-mediated axon guidance cooperate to ensure that migrating GnRH neurons reach the brain. Thus, the loss of both neuropilin ligands leads to an almost complete failure to establish the GnRH neuron system.
40. Herbal compound farnesiferol C exerts antiangiogenic and antitumor activity and targets multiple aspects of VEGFR1 (Flt1) or VEGFR2 (Flk1) signaling cascades. Farnesiferol C (FC) is one of the major compounds isolated from Ferula assafoetida, an Asian herbal spice used for cancer treatment as a folk remedy. Here, we examined the hypothesis that novel antiangiogenic activities of FC contribute to anticancer efficacy. In human umbilical vein endothelial cells (HUVEC), exposure to the 10 to 40 mumol/L concentration range of FC inhibited vascular endothelial growth factor (VEGF)-induced cell proliferation, **migration**, invasion, tube formation, and the expression of matrix metalloproteinase-2. In addition, FC inhibited the angiogenic sprouting of VEGF-treated rat aorta in an ex vivo model. Furthermore, FC inhibited the in vivo growth of mouse Lewis lung cancer allograft model by 60% (P < 0.001) at a daily i.p. dosage of 1 mg/kg body weight without any negative effect on the weight of the host mice. Immunohistochemistry staining showed decreased microvessel density (CD34) and proliferative index (Ki-67) without affecting the apoptotic (terminal deoxynucleotidyl transferase-mediated dUTP nick end labeling) index. Mechanistically, FC decreased the binding of VEGF to VEGFR1/Flt-1, but not to VEGFR2/KDR/Flk-1. In terms of early signaling, FC exerted a rapid inhibitory action (examined within 10 minutes) on VEGF-induced autophosphorylation of VEGFR1 without affecting that of VEGFR2. Nevertheless, FC decreased the phosphorylation of most of the kinases downstream of VEGFR2: focal adhesion kinase, Src, extracellular signal-regulated kinase 1/2, p38 mitogen-activated protein kinase, and c-jun-NH(2)-kinase without affecting AKT. Computer simulation suggests that FC may inhibit Src or focal adhesion kinase protein activities directly through its docking to their ATP-binding sites. Taken together, the multitargeting actions of FC, particularly VEGFR1 inhibition, may make it a novel drug candidate to complement current VEGF/VEGFR2-targeting antiangiogenic modalities for cancer.
41. Distinct roles of vascular endothelial growth factor receptor-1- and receptor-2-mediated signaling in T cell priming and Th17 polarization to lipopolysaccharide-containing allergens in the lung. Vascular endothelial growth factor (VEGF) is a key mediator in the development of airway immune dysfunction to inhaled allergens. However, the exact role of its receptors-mediated signaling is controversial. In this study, we evaluated the role of VEGF receptor (VEGFR)-1- and VEGFR-2-mediated signaling in T cell priming and polarization in the context of inhalation of LPS-containing allergens. A murine asthma model of mixed Th1 and Th17 cell responses was generated using intranasal sensitization with LPS-containing allergens. Pharmacologic intervention was performed during sensitization. In vivo production of VEGF and Th1- and Th17-polarizing cytokines (IL-12p70 and IL-6, respectively) were upregulated by airway exposure to LPS. Pharmacological intervention with a VEGFR-2-neutralizing Ab (anti-Flk1 mAb) abolished the production of IL-6 (but not IL-12p70) and the subsequent development of allergen-specific Th17 cell response. On the other hand, blocking VEGFR-1 signaling with a VEGFR-1 antagonist (anti-Flt1 hexapeptide) did not affect the production of IL-12p70 and IL-6. However, blocking VEGFR-1 signaling resulted in T cell tolerance rather than priming, mainly by inhibiting the maturation of lung dendritic cells, and their **migration** into lung-draining lymph nodes. These results suggest that T cell priming to LPS-containing allergens depends on VEGFR-1-mediated signaling, and the subsequent Th17 polarization depends on VEGFR-2 signaling.
42. AIP1 functions as an endogenous inhibitor of VEGFR2-mediated signaling and inflammatory angiogenesis in mice. ASK1-interacting protein-1 (AIP1), a recently identified member of the Ras GTPase-activating protein family, is highly expressed in vascular ECs and regulates EC apoptosis in vitro. However, its function in vivo has not been established. To study this, we generated AIP1-deficient mice (KO mice). Although these mice showed no obvious defects in vascular development, they exhibited dramatically enhanced angiogenesis in 2 models of inflammatory angiogenesis. In one of these models, the enhanced angiogenesis observed in the KO mice was associated with increased VEGF-VEGFR2 signaling. Consistent with this, VEGF-induced ear, cornea, and retina neovascularization were greatly augmented in KO mice and the enhanced retinal angiogenesis was markedly diminished by overexpression of AIP1. In vitro, VEGF-induced EC **migration** was inhibited by AIP1 overexpression, whereas it was augmented by both AIP1 knockout and knockdown, with the enhanced EC **migration** caused by AIP1 knockdown being associated with increased VEGFR2 signaling. We present mechanistic data that suggest AIP1 is recruited to the VEGFR2-PI3K complex, binding to both VEGFR2 and PI3K p85, at a late phase of the VEGF response, and that this leads to inhibition of VEGFR2 signaling. Taken together, our data demonstrate that AIP1 functions as an endogenous inhibitor in VEGFR2-mediated adaptive angiogenesis in mice.
43. Bovine papular stomatitis virus encodes a functionally distinct VEGF that binds both VEGFR-1 and VEGFR-2. Bovine papular stomatitis virus (BPSV), a member of the genus Parapoxvirus, causes proliferative dermatitis in cattle and humans. Other species of the genus cause similar lesions, the nature of which has been attributed, at least in part, to a viral-encoded vascular endothelial growth factor (VEGF) that induces vascularization and dermal oedema through VEGF receptor-2 (VEGFR-2). The results of this study showed that BPSV strain V660 encodes a novel VEGF and that the predicted BPSV protein showed only 33-52% amino acid identity to VEGFs encoded by the other species of the genus. BPSV VEGF showed higher identity to mammalian VEGF-A (51%) than the other parapoxviral VEGFs (31-46%). Assays of the purified BPSV VEGF (BPSVV660VEGF) demonstrated that it was also functionally more similar to VEGF-A, as it showed significant binding to VEGFR-1 and induced monocyte **migration**. Like VEGF-A and the other viral VEGFs, BPSVV660VEGF bound VEGFR-2 with high affinity. Sequence analysis and structural modelling of BPSVV660VEGF revealed specific residues, outside the known receptor-binding face, that are predicted either to influence VEGF structure or to mediate binding directly to the VEGFRs. These results indicate that BPSVV660VEGF is a biologically active member of the VEGF family and that, via its interaction with VEGFR-2, it is likely to contribute to the proliferative and highly vascularized nature of BPSV lesions. This is also the first example of a viral VEGF acting via VEGFR-1 and influencing haematopoietic cell function. These data suggest that BPSVV660VEGF is an evolutionary and functional intermediate between VEGF-A and the other parapoxviral VEGFs.
44. Comparative structure-function analysis of VEGFR-1 and VEGFR-2: What have we learned from chimeric systems? Activation of vascular endothelial growth factor receptor-1 and -2 (VEGFR-1 and VEGFR-2) plays a critical role in vasculogenesis and angiogenesis. However, the mechanism by which activation of VEGFRs elicits these cellular events is not fully understood. We recently constructed chimeric receptors containing the extracellular domain of human CSF-1R/c-fms fused with the entire transmembrane and cytoplasmic domains of murine VEGFR-1 and VEGFR-2. Selective activation of chimeric VEGFR-2, but not chimeric VEGFR-1, stimulated endothelial cell growth, **migration**, and differentiation. Stimulation of cells coexpressing chimeric VEGFR-1 and VEGFR-2 suppressed VEGFR-2-mediated endothelial cell growth. Site-directed mutagenesis demonstrated that tyrosines 799 and 1173 are required for VEGFR-2-mediated endothelial cell growth and activation of PI3 kinase. Further site-directed mutagenesis demonstrated that tyrosine 1212, located in the carboxyl tail of VEGFR-2, is required for the ligand-dependent autophosphorylation of the receptor and its ability to activate signaling proteins. Collectively, our results suggest that activation of VEGFR-1 and VEGFR-2 differentially regulates endothelial cell function and angiogenesis. Second, activation of VEGFR-2 is associated with many endothelial cell functions, including cell proliferation, **migration**, and differentiation. Third, activation of PI3 kinase by VEGFR-2 regulates endothelial cell proliferation.
45. Cooperative and redundant roles of VEGFR-2 and VEGFR-3 signaling in adult lymphangiogenesis. Activation of vascular endothelial growth factor (VEGF) receptor-3 (VEGFR-3) by VEGF-C initiates lymphangiogenesis by promoting lymphatic proliferation and **migration**. However, it is unclear whether VEGFR-3 signaling is required beyond these initial stages, namely during the organization of new lymphatic endothelial cells (LECs) into functional capillaries. Furthermore, the role of VEGFR-2, which is also expressed on LECs and binds VEGF-C, is unclear. We addressed these questions by selectively neutralizing VEGFR-3 and/or VEGFR-2 for various time periods in an adult model of lymphangiogenesis in regenerating skin. While blocking either VEGFR-2 or VEGFR-3 with specific antagonist mAbs (DC101 and mF4-31C1, respectively) prior to lymphatic **migration** prevented lymphangiogenesis, blocking VEGFR-3 subsequent to **migration** did not affect organization into functional capillaries, and VEGFR-2 blocking had only a small hindrance on organization. These findings were confirmed in vitro using human LECs and anti-human antagonist mAbs (IMC-1121a and hF4-3C5): both VEGFR-2 and -3 signaling were required for **migration** and proliferation, but tubulogenesis in 3D cultures was unaffected by VEGFR-3 blocking and partially hindered by VEGFR-2 blocking. Furthermore, both in vitro and in vivo, while VEGFR-3 blocking had no effect on LEC organization, coneutralization of VEGFR-2, and VEGFR-3 completely prevented lymphatic organization. Our findings demonstrate that cooperative signaling of VEGFR-2 and -3 is necessary for lymphatic **migration** and proliferation, but VEGFR-3 is redundant with VEGFR-2 for LEC organization into functional capillaries.
46. Ontogeny of semaphorins 3A and 3F and their receptors neuropilins 1 and 2 in the kidney. Semaphorins 3A and 3F are axon guidance proteins during nervous system development. Their expression pattern and function outside the nervous system are unknown. Neuropilin 1 and 2 (NP-1, NP-2) are natural ligands for semaphorins 3A and 3F, respectively. NP-1 is also a co-receptor for vascular endothelial growth factor (VEGF) required for normal vascular development. We showed that VEGF is a direct chemoattractant for glomerular endothelial cells towards developing nephrons. To examine whether semaphorins could modulate VEGF endothelial cell guidance cues in the developing kidney, we studied the expression of semaphorin 3A and semaphorin 3F and their receptors NP-1 and NP-2 in the kidney during ontogeny using Northern blot analysis, in situ hybridization, Western blot analysis and immunohistochemistry. All four genes are developmentally regulated, with abundant expression during organogenesis and downregulation in the adult kidney. Semaphorin 3A and 3F are expressed by podocytes and tubules whereas their receptors NP-1 and NP-2 are localized to endothelial cells. In vitro, renal tubular epithelial cell lines (tsMPT, IRPT and MDCK) and glomerular endothelial cells express both semaphorins and their receptors, suggesting the presence of an autocrine system. The distribution of the receptors NP-1 and NP-2 in endothelial cells and developing vessels is complementary to that of the ligands in adjacent epithelial cells during kidney development. The sum of the guidance cues provided by VEGF and semaphorins 3A and 3F may be important determinants of the pattern of endothelial cell **migration** during kidney morphogenesis.
47. Id1 modulates endothelial progenitor cells function through relieving the E2-2-mediated repression of FGFR1 and VEGFR2 in vitro. The **migration** and proliferation of EPCs are crucial for re-endothelialization in vascular repair and development. Id1 has a regulatory role in the regulation of EPCs **migration** and proliferation. Based on these findings, we hypothesized that Id1 plays a regulatory role in modulating the **migration** and proliferation of EPCs by interaction with other factors. Herein, we report that the Id1 protein and E-box protein E2-2 regulate EPCs function with completely opposite effects. Id1 plays a positive role in the regulation of EPC proliferation and **migration**, while endogenous E2-2 appears to be a negative regulator. Immunoprecipitation and immunofluorescence assay revealed that the Id1 protein interacts and co-localizes with the E2-2 protein in EPCs. Further, endogenous E2-2 protein was found to block EPCs function via the inhibition of FGFR1 and VEGFR2 expression. The overexpression and silencing of Id1 have no direct regulatory role on VEGFR2 and FGFR1 expression. On the other hand, Id1 relieves the E2-2-mediated repression of FGFR1 and VEGFR2 expression to modulate EPCs proliferation, **migration**, and tube formation in vitro. In summary, we demonstrated that Id1 and E2-2 are critical regulators of EPCs function in vitro. Id1 interacts with E2-2 and relieves the E2-2-mediated repression of FGFR1 and VEGFR2 expression to modulate EPCs functions. Id1 and E2-2 may represent novel therapeutic targets for re-endothelialization in vascular damage and repair.
48. Decreased levels of embryonic retinoic acid synthesis accelerate recovery from arterial growth delay in a mouse model of DiGeorge syndrome. Loss of Tbx1 and decrease of retinoic acid (RA) synthesis result in DiGeorge/velocardiofacial syndrome (DGS/VCFS)-like phenotypes in mouse models, including defects in septation of the outflow tract of the heart and anomalies of pharyngeal arch-derived structures including arteries of the head and neck, laryngeal-tracheal cartilage, and thymus/parathyroid. Wild-type levels of T-box transcription factor (Tbx)1 and RA signaling are required for normal pharyngeal arch artery development. Recent studies have shown that reduction of RA or loss of Tbx1 alters the contribution of second heart field (SHF) progenitor cells to the elongating heart tube.Here we tested whether Tbx1 and the RA signaling pathway interact during the deployment of the SHF and formation of the mature aortic arch.Molecular markers of the SHF, neural crest and smooth muscle cells, were analyzed in Raldh2;Tbx1 compound heterozygous mutants. Our results revealed that the SHF and outflow tract develop normally in Raldh2(+/-);Tbx1(+/-) embryos. However, we found that decreased levels of RA accelerate the recovery from arterial growth delay observed in Tbx1(+/-) mutant embryos. This compensation coincides with the differentiation of smooth muscle cells in the 4th pharyngeal arch arteries, and is associated with severity of neural crest cell **migration** defects observed in these mutants.Our data suggest that differences in levels of embryonic RA may contribute to the variability in great artery anomalies observed in DGS/VCFS patients.
49. Vascular endothelial growth factor receptor 2 mediates macrophage infiltration into orthotopic pancreatic tumors in mice. Macrophages are an abundant inflammatory cell type in the tumor microenvironment that can contribute to tumor growth and metastasis. Macrophage recruitment into tumors is mediated by multiple cytokines, including vascular endothelial growth factor (VEGF), which is thought to function primarily through VEGF receptor (VEGFR) 1 expressed on macrophages. Macrophage infiltration is affected by VEGF inhibition. We show that selective inhibition of VEGFR2 reduced macrophage infiltration into orthotopic pancreatic tumors. Our studies show that tumor-associated macrophages express VEGFR2. Furthermore, peritoneal macrophages from tumor-bearing animals express VEGFR2, whereas peritoneal macrophages from non-tumor-bearing animals do not. To our knowledge, this is the first time that tumor-associated macrophages have been shown to express VEGFR2. Additionally, we found that the cytokine pleiotrophin is sufficient to induce VEGFR2 expression on macrophages. Pleiotrophin has previously been shown to induce expression of endothelial cell markers on macrophages and was present in the microenvironment of orthotopic pancreatic tumors. Finally, we show that VEGFR2, when expressed by macrophages, is essential for VEGF-stimulated **migration** of tumor-associated macrophages. In summary, tumor-associated macrophages express VEGFR2, and selective inhibition of VEGFR2 reduces recruitment of macrophages into orthotopic pancreatic tumors. Our results show an underappreciated mechanism of action that may directly contribute to the antitumor activity of angiogenesis inhibitors that block the VEGFR2 pathway.
50. Liver X receptor activation reduces angiogenesis by impairing lipid raft localization and signaling of vascular endothelial growth factor receptor-2. Liver X receptors (LXRα, LXRβ) are master regulators of cholesterol homeostasis. In the endothelium, perturbations of cell cholesterol have an impact on fundamental processes. We, therefore, assessed the effects of LXR activation on endothelial functions related to angiogenesis in vitro and in vivo.LXR agonists (T0901317, GW3965) blunte**d migrati**on, tubulogenesis, and proliferation of human umbilical vein endothelial cells. By affecting endothelial cholesterol homeostasis, LXR activation impaired the compartmentation of vascular endothelial growth factor receptor-2 in lipid rafts/caveolae and led to defective phosphorylation and downstream signaling of vascular endothelial growth factor receptor-2 upon vascular endothelial growth factor-A stimulation. Consistently, the antiangiogenic actions of LXR agonists could be prevented by coadministration of exogenous cholesterol. LXR agonists reduced endothelial sprouting from wild-type but not from LXRα(-/-)/LXRβ(-/-) knockout aortas and blunted the vascularization of implanted angioreactors in vivo. Furthermore, T0901317 reduced the growth of Lewis lung carcinoma grafts in mice by impairing angiogenesis.Pharmacological activation of endothelial LXRs reduces angiogenesis by restraining cholesterol-dependent vascular endothelial growth factor receptor-2 compartmentation and signaling. Thus, administration of LXR agonists could exert therapeutic effects in pathological conditions characterized by uncontrolled angiogenesis.
51. Wnt4 is required for proper male as well as female sexual development. Genes previously implicated in mammalian sexual development have either a male- or female-specific role. The signaling molecule WNT4 has been shown to be important in female sexual development. Lack of Wnt4 gives rise to masculinization of the XX gonad and we showed previously that the role of WNT4 was to inhibit endothelial and steroidogenic cell **migration** into the developing ovary. Here we show that Wnt4 also has a function in the male gonad. We find that Sertoli cell differentiation is compromised in Wnt4 mutant testes and that this defect occurs downstream of the testis-determining gene Sry but upstream of Sox9 and Dhh, two early Sertoli cell markers. Genetic analysis shows that this phenotype is primarily due to the action of WNT4 within the early genital ridge. Analysis of different markers identifies the most striking difference in the genital ridge at early stages of its development between wild-type and Wnt4 mutant embryos to be a significant increase of steroidogenic cells in the Wnt4 -/- gonad. These results identify WNT4 as a new factor involved in the mammalian testis determination pathway and show that genes can have a specific but distinct role in both male and female gonad development.
52. Myocardin-related transcription factors control the motility of epicardium-derived cells and the maturation of coronary vessels. An important pool of cardiovascular progenitor cells arises from the epicardium, a single layer of mesothelium lining the heart. Epicardium-derived progenitor cell (EPDC) formation requires epithelial-to-mesenchymal transition (EMT) and the subsequent **migration** of these cells into the sub-epicardial space. Although some of the physiological signals that promote EMT are understood, the functional mediators of EPDC motility and differentiation are not known. Here, we identify a novel regulatory mechanism of EPDC mobilization. Myocardin-related transcription factor (MRTF)-A and MRTF-B (MKL1 and MKL2, respectively) are enriched in the perinuclear space of epicardial cells during development. Transforming growth factor (TGF)-β signaling and disassembly of cell contacts leads to nuclear accumulation of MRTFs and the activation of the motile gene expression program. Conditional ablation of Mrtfa and Mrtfb specifically in the epicardium disrupts cell **migratio**n and leads to sub-epicardial hemorrhage, partially stemming from the depletion of coronary pericytes. Using lineage-tracing analyses, we demonstrate that sub-epicardial pericytes arise from EPDCs in a process that requires the MRTF-dependent motile gene expression program. These findings provide novel mechanisms linking EPDC motility and differentiation, shed light on the transcriptional control of coronary microvascular maturation and suggest novel therapeutic strategies to manipulate epicardium-derived progenitor cells for cardiac repair.
53. Cell lineage in mammalian craniofacial mesenchyme. We have analysed the contributions of neural crest and mesoderm to mammalian craniofacial mesenchyme and its derivatives by cell lineage tracing experiments in mouse embryos, using the permanent genetic markers Wnt1-cre for neural crest and Mesp1-cre for mesoderm, combined with the Rosa26 reporter. At the end of neural crest cell **migration** (E9.5) the two patterns are reciprocal, with a mutual boundary just posterior to the eye. Mesodermal cells expressing endothelial markers (angioblasts) are found not to respect this boundary; they are associated with the migrating neural crest from the 5-somite stage, and by E9.5 they form a pre-endothelial meshwork throughout the cranial mesenchyme. Mesodermal cells of the myogenic lineage also migrate with neural crest cells, as the branchial arches form. By E17.5 the neural crest-mesoderm boundary in the subectodermal mesenchyme becomes out of register with that of the underlying skeletogenic layer, which is between the frontal and parietal bones. At E13.5 the primordia of these bones lie basolateral to the brain, extending towards the vertex of the skull during the following 4-5 days. We used DiI labelling of the bone primordia in ex-utero E13.5 embryos to distinguish between two possibilities for the origin of the frontal and parietal bones: (1) recruitment from adjacent connective tissue or (2) proliferation of the original primordia. The results clearly demonstrated that the bone primordia extend vertically by intrinsic growth, without detectable recruitment of adjacent mesenchymal cells.
54. Heparan sulfate deficiency disrupts developmental angiogenesis and causes congenital diaphragmatic hernia. Congenital diaphragmatic hernia (CDH) is a common birth malformation with a heterogeneous etiology. In this study, we report that ablation of the heparan sulfate biosynthetic enzyme NDST1 in murine endothelium (Ndst1ECKO mice) disrupted vascular development in the diaphragm, which led to hypoxia as well as subsequent diaphragm hypoplasia and CDH. Intriguingly, the phenotypes displayed in Ndst1ECKO mice resembled the developmental defects observed in slit homolog 3 (Slit3) knockout mice. Furthermore, introduction of a heterozygous mutation in roundabout homolog 4 (Robo4), the gene encoding the cognate receptor of SLIT3, aggravated the defect in vascular development in the diaphragm and CDH. NDST1 deficiency diminished SLIT3, but not ROBO4, binding to endothelial heparan sulfate and attenuated EC **migration** and in vivo neovascularization normally elicited by SLIT3-ROBO4 signaling. Together, these data suggest that heparan sulfate presentation of SLIT3 to ROBO4 facilitates initiation of this signaling cascade. Thus, our results demonstrate that loss of NDST1 causes defective diaphragm vascular development and CDH and that heparan sulfate facilitates angiogenic SLIT3-ROBO4 signaling during vascular development.
55. Dopamine in vivo inhibits VEGF-induced phosphorylation of VEGFR-2, MAPK, and focal adhesion kinase in endothelial cells. Vascular permeability factor (VPF)/VEGF is a potent multifunctional cytokine and growth factor that has critical roles in vasculogenesis and in both physiological and pathological angiogenesis. Because it has been recently shown that the neurotransmitter dopamine at pharmacological dose can inhibit VEGF/VPF-mediated microvascular permeability, proliferation, and **migration** of endothelial cells in vitro, we therefore hypothesized that endogenous dopamine may regulate the actions of VPF/VEGF in vivo. We report that VPF/VEGF-induced phosphorylation of VEGF receptor 2, focal adhesion kinase, and MAPK in the endothelial cells is strikingly increased in both dopamine-depleted and dopamine D(2) receptor knockout mice compared with normal controls, thereby indicating that endogenous dopamine regulate these critical signaling cascades required for the in vivo endothelial functions of VPF/VEGF. Together, these observations provide new mechanistic insight into the dopamine-mediated inhibition of the activities of VPF/VEGF and suggest that endogenous neurotransmitter dopamine might be an important physiological regulator of VPF/VEGF activities in vivo.
56. VEGF-A promotes tissue repair-associated lymphatic vessel formation via VEGFR-2 and the alpha1beta1 and alpha2beta1 integrins. Vascular endothelial growth factor-A (VEGF-A) is strongly up-regulated in wounded cutaneous tissue and promotes repair-associated angiogenesis. However, little is known about its role in lymphatic regeneration of the healing skin. We studied wound healing in transgenic mice that overexpress VEGF-A specifically in the epidermis and in wild-type mice in the absence or presence of inhibitors of VEGF-A signaling. Surprisingly, transgenic overexpression of VEGF-A in the skin promoted lymphangiogenesis at the wound healing site, whereas systemic blockade of VEGFR-2 prevented lymphatic vessel formation. Studies in cultured lymphatic endothelial cells revealed that VEGF-A induced expression of the alpha1 and alpha2 integrins, which promoted their in vitro tube formation and their haptotactic **migration** toward type I collagen. VEGF-A-induced lymphatic endothelial cord formation and haptotactic **migration** were suppressed by anti-alpha1 and anti-alpha2 integrin blocking antibodies, and systemic blockade of the alpha1 and alpha2 integrins inhibited VEGF-A-driven lymphangiogenesis in vivo. We propose that VEGF-A promotes lymphatic vasculature formation via activation of VEGFR-2 and that lineage-specific differences of integrin receptor expression contribute to the distinct dynamics of wound-associated angiogenesis and lymphangiogenesis.
57. Notch regulation of myogenic versus endothelial fates of cells that migrate from the somite to the limb. Multipotent Pax3-positive (Pax3(+)) cells in the somites give rise to skeletal muscle and to cells of the vasculature. We had previously proposed that this cell-fate choice depends on the equilibrium between Pax3 and Foxc2 expression. In this study, we report that the Notch pathway promotes vascular versus skeletal muscle cell fates. Overactivating the Notch pathway specifically in Pax3(+) progenitors, via a conditional Pax3(NICD) allele, results in an increase of the number of smooth muscle and endothelial cells contributing to the aorta. At limb level, Pax3(+) cells in the somite give rise to skeletal muscles and to a subpopulation of endothelial cells in blood vessels of the limb. We now demonstrate that in addition to the inhibitory role of Notch signaling on skeletal muscle cell differentiation, the Notch pathway affects the Pax3:Foxc2 balance and promotes the endothelial versus myogenic cell fate, before **migration** to the limb, in multipotent Pax3(+) cells in the somite of the mouse embryo.
58. VEGF inhibits tumor cell invasion and mesenchymal transition through a MET/VEGFR2 complex. Inhibition of VEGF signaling leads to a proinvasive phenotype in mouse models of glioblastoma multiforme (GBM) and in a subset of GBM patients treated with bevacizumab. Here, we demonstrate that vascular endothelial growth factor (VEGF) directly and negatively regulates tumor cell invasion through enhanced recruitment of the protein tyrosine phosphatase 1B (PTP1B) to a MET/VEGFR2 heterocomplex, thereby suppressing HGF-dependent MET phosphorylation and tumor cell **migration**. Consequently, VEGF blockade restores and increases MET activity in GBM cells in a hypoxia-independent manner, while inducing a program reminiscent of epithelial-to-mesenchymal transition highlighted by a T-cadherin to N-cadherin switch and enhanced mesenchymal features. Inhibition of MET in GBM mouse models blocks mesenchymal transition and invasion provoked by VEGF ablation, resulting in substantial survival benefit.
59. Impaired tumor angiogenesis and VEGF-induced pathway in endothelial CD146 knockout mice. CD146 is a newly identified endothelial biomarker that has been implicated in angiogenesis. Though in vitro angiogenic function of CD146 has been extensively reported, in vivo evidence is still lacking. To address this issue, we generated endothelial-specific CD146 knockout (CD146(EC-KO)) mice using the Tg(Tek-cre) system. Surprisingly, these mice did not exhibit any apparent morphological defects in the development of normal retinal vasculature. To evaluate the role of CD146 in pathological angiogenesis, a xenograft tumor model was used. We found that both tumor volume and vascular density were significantly lower in CD146(EC-KO) mice when compared to WT littermates. Additionally, the ability for sprouting, **migration** and tube formation in response to VEGF treatment was impaired in endothelial cells (ECs) of CD146(EC-KO) mice. Mechanistic studies further confirmed that VEGF-induced VEGFR-2 phosphorylation and AKT/p38 MAPKs/NF-κB activation were inhibited in these CD146-null ECs, which might present the underlying cause for the observed inhibition of tumor angiogenesis in CD146(EC-KO) mice. These results suggest that CD146 plays a redundant role in physiological angiogenic processes, but becomes essential during pathological angiogenesis as observed in tumorigenesis.
60. Semaphorin 3A contributes to distal pulmonary epithelial cell differentiation and lung morphogenesis. Semaphorin 3A (Sema3A) is a neural guidance cue that also mediates cell **migration**, proliferation and apoptosis, and inhibits branching morphogenesis. Because we have shown that genetic deletion of neuropilin-1, which encodes an obligatory Sema3A co-receptor, influences airspace remodeling in the smoke-exposed adult lung, we sought to determine whether genetic deletion of Sema3A altered distal lung structure.To determine whether loss of Sema3A signaling influenced distal lung morphology, we compared pulmonary histology, distal epithelial cell morphology and maturation, and the balance between lung cell proliferation and death, in lungs from mice with a targeted genetic deletion of Sema3A (Sema3A(-/-)) and wild-type (Sema3A(+/+)) littermate controls.Genetic deletion of Sema3A resulted in significant perinatal lethality. At E17.5, lungs from Sema3A(-/-) mice had thickened septae and reduced airspace size. Distal lung epithelial cells had increased intracellular glycogen pools and small multivesicular and lamellar bodies with atypical ultrastructure, as well as reduced expression of type I alveolar epithelial cell markers. Alveolarization was markedly attenuated in lungs from the rare Sema3A(-/-) mice that survived the immediate perinatal period. Furthermore, Sema3A deletion was linked with enhanced postnatal alveolar septal cell death.These data suggest that Sema3A modulates distal pulmonary epithelial cell development and alveolar septation. Defining how Sema3A influences structural plasticity of the developing lung is a critical first step for determining if this pathway can be exploited to develop innovative strategies for repair after acute or chronic lung injury.
61. Notch, IL-1 and leptin crosstalk outcome (NILCO) is critical for leptin-induced proliferation, **migration** and VEGF/VEGFR-2 expression in breast cancer. High levels of pro-angiogenic factors, leptin, IL-1, Notch and VEGF (ligands and receptors), are found in breast cancer, which is commonly correlated with metastasis and lower survival of patients. We have previously reported that leptin induces the growth of breast cancer and the expression of VEGF/VEGFR-2 and IL-1 system. We hypothesized that Notch, IL-1 and leptin crosstalk outcome (NILCO) plays an essential role in the regulation of leptin-mediated induction of proliferation/**migration** and expression of pro-angiogenic molecules in breast cancer. To test this hypothesis, leptin's effects on the expression and activation of Notch signaling pathway and VEGF/VEGFR-2/IL-1 were determined in mouse (4T1, EMT6 and MMT) breast cancer cells. Remarkably, leptin up-regulated Notch1-4/JAG1/Dll-4, Notch target genes: Hey2 and survivin, together with IL-1 and VEGF/VEGFR-2. RNA knockdown and pharmacological inhibitors of leptin signaling significantly abrogated activity of reporter gene-luciferase CSL (RBP-Jk) promoter, showing that it was linked to leptin-activated JAK2/STAT3, MAPK, PI-3K/mTOR, p38 and JNK signaling pathways. Interestingly, leptin upregulatory effects on cell proliferation/**migration** and pro-angiogenic factors Notch, IL-1 and VEGF/VEGFR-2 were abrogated by a γ-secretase inhibitor, DAPT, as well as siRNA against CSL. In addition, blockade of IL-1R tI inhibited leptin-induced Notch, Hey2 and survivin as well as VEGF/VEGFR-2 expression. These data suggest leptin is an inducer of Notch (expression/activation) and IL-1 signaling modulates leptin effects on Notch and VEGF/VEGFR-2. We show for the first time that a novel unveiled crosstalk between Notch, IL-1 and leptin (NILCO) occurs in breast cancer. Leptin induction of proliferation**/migratio**n and upregulation of VEGF/VEGFR-2 in breast cancer cells were related to an intact Notch signaling axis. NILCO could represent the integration of developmental, pro-inflammatory and pro-angiogenic signals critical for leptin-induced cell proliferation**/migratio**n and regulation of VEGF/VEGFR-2 in breast cancer. Targeting NILCO might help to design new pharmacological strategies aimed at controlling breast cancer growth and angiogenesis.
62. Limb bud colonization by somite-derived angioblasts is a crucial step for myoblast e**migration**. We have combined the use of mouse genetic strains and the mouse-into-chicken chimera system to determine precisely the sequence of forelimb colonization by presomitic mesoderm (PSM)-derived myoblasts and angioblasts, and the possible role of this latter cell type in myoblast guidance. By creating a new Flk1/Pax3 double reporter mouse line, we have established the precise timetable for angioblast and myoblast delamination/**migration** from the somite to the limb bud. This timetable was conserved when mouse PSM was grafted into a chicken host, which further validates the experimental model. The use of Pax3(GFP/GFP) knockout mice showed that establishment of vascular endothelial and smooth muscle cells (SMCs) is not compromised by the absence of Pax3. Of note, Pax3(GFP/GFP) knockout mouse PSM-derived cells can contribute to aortic, but not to limb, SMCs that are derived from the somatopleure. Finally, using the Flk1(lacZ)(/)(lacZ) knockout mouse, we show that, in the absence of angioblast and vascular network formation, myoblasts are prevented from migrating into the limb. Taken together, our study establishes for the first time the time schedule for endothelial and skeletal muscle cell colonization in the mouse limb bud and establishes the absolute requirement of endothelial cells for myoblast delamination and **migration** to the limb. It also reveals that cells delaminating from the somites display marked differentiation traits, suggesting that if a common progenitor exists, its lifespan is extremely short and restricted to the somite.
63. BMPs promote proliferation and **migration** of endothelial cells via stimulation of VEGF-A/VEGFR2 and angiopoietin-1/Tie2 signalling. The differentiation, growth, and survival of endothelial cells (ECs) are regulated by multiple signalling pathways, such as vascular endothelial growth factors (VEGFs) and angiopoietins through their receptor tyrosine kinases, VEGF receptor (VEGFR) 2 and Tie2, respectively. Bone morphogenetic proteins (BMPs), members of the transforming growth factor (TGF)-beta family, have been implicated in the development and maintenance of vascular systems. However, their effects on EC proliferation remain to be elucidated. In the present study, we show that BMPs induce the proliferation and **migration** of mouse embryonic stem cell (ESC)-derived endothelial cells (MESECs) and human microvascular endothelial cells (HMECs). Addition of BMP-4 to culture induced significant proliferation and **migration** of both types of ECs. BMP-4 also increased the expression and phosphorylation of VEGFR2 and Tie2. These findings suggest that BMP signalling activates endothelium via activation of VEGF/VEGFR2 and Angiopoietin/Tie2 signalling.
64. Biphasic effect of PTK7 on KDR activity in endothelial cells and angiogenesis. Protein tyrosine kinase 7 (PTK7) is a member of the defective receptor protein tyrosine kinase family which lacks catalytic activity. Expression of PTK7 is increased in various cancers but its role in carcinogenesis is not well understood. We previously showed that disruption of PTK7 function suppresses VEGF-induced angiogenic phenotypes in HUVECs and mice. Here, we investigated molecular mechanisms for modulating VEGF-induced physiological effects by PTK7. Treatment with a high concentration of extracellular domain of PTK7 (soluble PTK7; sPTK7) or knockdown of PTK7 inhibited VEGF-induced phosphorylation of kinase insert domain receptor (KDR) but did not inhibit phosphorylation of fms-related tyrosine kinase 1 (FLT-1) in HUVECs. PTK7, more specifically sPTK7, interacted with KDR but not with FLT-1 in HUVECs and HEK293 cells. In vitro binding assay showed that sPTK7 formed oligomers with the extracellular domain of KDR (sKDR) up to an approximately 1:3 molar ratio, and vice versa. sPTK7 at lower molar ratios than sKDR enhanced the binding of VEGF to sKDR. At the same or higher molar ratios, it reduced the binding of VEGF to sKDR. Increasing concentrations of sPTK7 or increasing levels of PTK7 expression first increased and then decreased VEGF-induced KDR phosphorylation, **migration**, and capillary-like tube formation of HUVECs, as well as in vivo angiogenesis. Taken together, our data demonstrates that PTK7 regulates the activity of KDR biphasically by inducing oligomerization of KDR molecules at lower concentrations and by surrounding KDR molecules at higher concentrations.
65. The adaptor protein shb binds to tyrosine 1175 in vascular endothelial growth factor (VEGF) receptor-2 and regulates VEGF-dependent cellular **migration**. Previous studies have shown that the adaptor protein Shb is involved in receptor tyrosine kinase signaling. In this study, we demonstrate that Shb is phosphorylated in an Src-dependent manner upon vascular endothelial growth factor (VEGF) stimulation using porcine aortic endothelial cells expressing the human VEGF receptor 2 (VEGFR-2) (KDR). In co-immunoprecipitation experiments, we could detect an interaction between Shb and the VEGFR-2 in human telomerase-immortalized microvascular endothelial cells. Furthermore, in a glutathione S-transferase pull-down assay, the Src homology 2 domain of Shb was shown to interact with phosphorylated tyrosine 1175 in the C-terminal tail of VEGFR-2. VEGF-induced Shb phosphorylation was lost in porcine aortic endothelial cells expressing a chimeric murine VEGFR-2 (Flk-1) with a mutation at the corresponding position. Shb expression was specifically decreased by 80%, in a transient manner, by using the short interfering RNA technique. Reduced Shb expression led to a loss of stimulation of phosphatidylinositol 3-kinase, phosphorylation of focal adhesion kinase at tyrosine 576, the generation of focal adhesions, and stress fiber formation in response to VEGF. Furthermore, we show that VEGF-induced **migration** is inhibited in Shb short interfering RNA-treated cells. Our data demonstrate that Shb is important for VEGF signaling in endothelial cells. This is achieved by Shb binding to tyrosine 1175 in the VEGFR-2, which regulates VEGF-induced formation of focal adhesions and cell **migration**, of which the latter occurs in a phosphatidylinositol 3-kinase-dependent manner.
66. Roles of ephrinB ligands and EphB receptors in cardiovascular development: demarcation of arterial/venous domains, vascular morphogenesis, and sprouting angiogenesis. Eph receptor tyrosine kinases and their cell-surface-bound ligands, the ephrins, regulate axon guidance and bundling in the developing brain, control cell **migration** and adhesion, and help patterning the embryo. Here we report that two ephrinB ligands and three EphB receptors are expressed in and regulate the formation of the vascular network. Mice lacking ephrinB2 and a proportion of double mutants deficient in EphB2 and EphB3 receptor signaling die in utero before embryonic day 11.5 (E11.5) because of defects in the remodeling of the embryonic vascular system. Our phenotypic analysis suggests complex interactions and multiple functions of Eph receptors and ephrins in the embryonic vasculature. Interaction between ephrinB2 on arteries and its EphB receptors on veins suggests a role in defining boundaries between arterial and venous domains. Expression of ephrinB1 by arterial and venous endothelial cells and EphB3 by veins and some arteries indicates that endothelial cell-to-cell interactions between ephrins and Eph receptors are not restricted to the border between arteries and veins. Furthermore, expression of ephrinB2 and EphB2 in mesenchyme adjacent to vessels and vascular defects in ephB2/ephB3 double mutants indicate a requirement for ephrin-Eph signaling between endothelial cells and surrounding mesenchymal cells. Finally, ephrinB ligands induce capillary sprouting in vitro with a similar efficiency as angiopoietin-1 (Ang1) and vascular endothelial growth factor (VEGF), demonstrating a stimulatory role of ephrins in the remodeling of the developing vascular system.
67. Ras signaling directs endothelial specification of VEGFR2+ vascular progenitor cells. Vascular endothelial growth factor receptor 2 (VEGFR2) transmits signals of crucial importance to vasculogenesis, including proliferation, **migration**, and differentiation of vascular progenitor cells. Embryonic stem cell-derived VEGFR2(+) mesodermal cells differentiate into mural lineage in the presence of platelet derived growth factor (PDGF)-BB or serum but into endothelial lineage in response to VEGF-A. We found that inhibition of H-Ras function by a farnesyltransferase inhibitor or a knockdown technique results in selective suppression of VEGF-A-induced endothelial specification. Experiments with ex vivo whole-embryo culture as well as analysis of H-ras(-/-) mice also supported this conclusion. Furthermore, expression of a constitutively active H-Ras[G12V] in VEGFR2(+) progenitor cells resulted in endothelial differentiation through the extracellular signal-related kinase (Erk) pathway. Both VEGF-A and PDGF-BB activated Ras in VEGFR2(+) progenitor cells 5 min after treatment. However, VEGF-A, but not PDGF-BB, activated Ras 6-9 h after treatment, preceding the induction of endothelial markers. VEGF-A thus activates temporally distinct Ras-Erk signaling to direct endothelial specification of VEGFR2(+) vascular progenitor cells.
68. VEGF-induced neoangiogenesis is mediated by NAADP and two-pore channel-2-dependent Ca2+ signaling. Vascular endothelial growth factor (VEGF) and its receptors VEGFR1/VEGFR2 play major roles in controlling angiogenesis, including vascularization of solid tumors. Here we describe a specific Ca(2+) signaling pathway linked to the VEGFR2 receptor subtype, controlling the critical angiogenic responses of endothelial cells (ECs) to VEGF. Key steps of this pathway are the involvement of the potent Ca(2+) mobilizing messenger, nicotinic acid adenine-dinucleotide phosphate (NAADP), and the specific engagement of the two-pore channel TPC2 subtype on acidic intracellular Ca(2+) stores, resulting in Ca(2+) release and angiogenic responses. Targeting this intracellular pathway pharmacologically using the NAADP antagonist Ned-19 or genetically using Tpcn2(-/-) mice was found to inhibit angiogenic responses to VEGF in vitro and in vivo. In human umbilical vein endothelial cells (HUVECs) Ned-19 abolished VEGF-induced Ca(2+) release, impairing phosphorylation of ERK1/2, Akt, eNOS, JNK, cell proliferation, cell **migration**, and capillary-like tube formation. Interestingly, Tpcn2 shRNA treatment abolished VEGF-induced Ca(2+) release and capillary-like tube formation. Importantly, in vivo VEGF-induced vessel formation in matrigel plugs in mice was abolished by Ned-19 and, most notably, failed to occur in Tpcn2(-/-) mice, but was unaffected in Tpcn1(-/-) animals. These results demonstrate that a VEGFR2/NAADP/TPC2/Ca(2+) signaling pathway is critical for VEGF-induced angiogenesis in vitro and in vivo. Given that VEGF can elicit both pro- and antiangiogenic responses depending upon the balance of signal transduction pathways activated, targeting specific VEGFR2 downstream signaling pathways could modify this balance, potentially leading to more finely tailored therapeutic strategies.
69. Essential regulation of CNS angiogenesis by the orphan G protein-coupled receptor GPR124. The orphan G protein-coupled receptor (GPCR) GPR124/tumor endothelial marker 5 is highly expressed in central nervous system (CNS) endothelium. Here, we show that complete null or endothelial-specific GPR124 deletion resulted in embryonic lethality from CNS-specific angiogenesis arrest in forebrain and neural tube. Conversely, GPR124 overexpression throughout all adult vascular beds produced CNS-specific hyperproliferative vascular malformations. In vivo, GPR124 functioned cell-autonomously in endothelium to regulate sprouting, **migration**, and developmental expression of the blood-brain barrier marker Glut1, whereas in vitro, GPR124 mediated Cdc42-dependent directional **migration** to forebrain-derived, vascular endothelial growth factor-independent cues. Our results demonstrate CNS-specific angiogenesis regulation by an endothelial receptor and illuminate functions of the poorly understood adhesion GPCR subfamily. Further, the functional tropism of GPR124 marks this receptor as a therapeutic target for CNS-related vascular pathologies.
70. MesP1 is expressed in the heart precursor cells and required for the formation of a single heart tube. The Mesp1 gene encodes the basic HLH protein MesP1 which is expressed in the mesodermal cell lineage during early gastrulation. Disruption of the Mesp1 gene leads to aberrant heart morphogenesis, resulting in cardia bifida. In order to study the defects in Mesp1-expressing cells during gastrulation and in the specification of mesodermal cell lineages, we introduced a (beta)-galactosidase gene (lacZ) under the control of the Mesp1 promoter by homologous recombination. The early expression pattern revealed by (beta)-gal staining in heterozygous embryos was almost identical to that observed by whole mount in situ hybridization. However, the (beta)-gal activity was retained longer than the mRNA signal, which enabled us to follow cell **migration** during gastrulation. In heterozygous embryos, the Mesp1-expressing cells migrated out from the primitive streak and were incorporated into the head mesenchyme and heart field. In contrast, Mesp1-expressing cells in the homozygous deficient embryos stayed in the primitive streak for a longer period of time before departure. The expression of FLK-1, an early marker of endothelial cell precursors including heart precursors, also accumulated abnormally in the posterior region in Mesp1-deficient embryos. In addition, using the Cre-loxP site-specific recombination system, we could determine the lineage of the Mesp1-expressing cells. The first mesodermal cells that ingressed through the primitive streak were incorporated as the mesodermal component of the amnion, and the next mesodermal population mainly contributed to the myocardium of the heart tube but not to the endocardium. These results strongly suggest that MesP1 is expressed in the heart tube precursor cells and is required for mesodermal cells to depart from the primitive streak and to generate a single heart tube.
71. SCL interacts with VEGF to suppress apoptosis at the onset of hematopoiesis. During development, hematopoiesis initiates in the yolk sac through a process that depends on VEGF/Flk1 signaling and on the function of the SCL/Tal1 transcription factor. Here we show that VEGF modifies the developmental potential of primitive erythroid progenitors and prolongs their life span. Furthermore, the survival of yolk sac erythrocytes in vivo depends on the dose of VEGF. Interestingly, in Vegf(lo/lo) embryos carrying a hypomorph allele, Flk1-positive cells reach the yolk sac at E8.5, but are severely compromised in their ability to generate primitive erythroid precursors. These observations indicate that during embryonic development, different thresholds of VEGF are required for the **migration** and clonal expansion of hematopoietic precursors. The near absence of primitive erythroid precursors in Vegf(lo/lo) embryos correlates with low levels of Scl in the yolk sac. Strikingly, gain-of-function of SCL partially complements the hematopoietic defect caused by the hypomorph Vegf(lo) allele, and re-establishes the survival of erythroid cells and the expression of erythroid genes (Gata1 and betaH1). This indicates that SCL functions downstream of VEGF to ensure an expansion of the hematopoietic compartment.
72. Annexin A3 Regulates Early Blood Vessel Formation. Annexins are a large family of calcium binding proteins that associate with cell membrane phospholipids and are involved in various cellular processes including endocytosis, exocytosis and membrane-cytoskeletal organization. Despite studies on numerous Annexin proteins, the function of Annexin A3 (Anxa3) is largely unknown. Our studies identify Anxa3 as a unique marker of the endothelial and myeloid cell lineages of Xenopus laevis during development. Anxa3 transcripts are also detected in endothelial cells (ECs) of zebrafish and mouse embryos, suggesting an important evolutionary function during formation of blood vessels. Indeed, Anxa3 loss-of-function experiments in frog embryos reveal its critical role during the morphogenesis of early blood vessels, as angioblasts in MO injected embryos fail to form vascular cords. Furthermore, in vitro experiments in mammalian cells identify a role for Anxa3 in EC **migration**. Our results are the first to reveal an in vivo function for Anxa3 during vascular development and represent a previously unexplored aspect of annexin biology.
73. Dual roles of Sema6D in cardiac morphogenesis through region-specific association of its receptor, Plexin-A1, with off-track and vascular endothelial growth factor receptor type 2. Semaphorins, originally identified as axon guidance facto s in the nervous system, play integral roles in organogenesis. Here, we demonstrate a critical involvement of Sema6D in cardiac morphogenesis. Ectopic expression of Sema6D o RNA interference against Sema6D induces expansion or narrowing of the ventricular chamber, respectively, during chick embryonic development. Sema6D also exerts region-specific activities on cardiac explants, a **migration**-promoting activity on outgrowing cells from the conotruncal segment, and a **migration**-inhibitory activity on those from the ventricle. Plexin-A1 mediates these activities as the major Sema6D-binding receptor. Plexin-A1 forms a receptor complex with vascular endothelial growth factor receptor type 2 in the conotruncal segment or with Off-track in the ventricle segment; these complexes are responsible for the effects of Sema6D on the respective regions. Thus, the differential association of Plexin-A1 with additional receptor components entitles Sema6D to exert distinct biological activities at adjacent regions. This is crucial for complex cardiac morphogenesis.
74. In vitro hematopoietic and endothelial potential of flk-1(-/-) embryonic stem cells and embryos. Mice deficient in the Flk-1 receptor tyrosine kinase are known to die in utero because of defective vascular and hematopoietic development. Here, we show that flk-1(-/-) embryonic stem cells are nevertheless able to differentiate into hematopoietic and endothelial cells in vitro, although they give rise to a greatly reduced number of blast colonies, a measure of hemangioblast potential. Furthermore, normal numbers of hematopoietic progenitors are found in 7.5-day postcoitum flk-1(-/-) embryos, even though 8. 5-day postcoitum flk-1(-/-) embryos are known to be deficient in such cells. Our results suggest that hematopoietic/endothelial progenitors arise independently of Flk-1, but that their subsequent **migration** and expansion require a Flk-1-mediated signal.
75. Inhibition of multiple vascular endothelial growth factor receptors (VEGFR) blocks lymph node metastases but inhibition of VEGFR-2 is sufficient to sensitize tumor cells to platinum-based chemotherapeutics. Vascular endothelial growth factor receptors (VEGFR) have important roles in cancer, affecting blood and lymphatic vessel functionality as well as tumor cells themselves. We compared the efficacy of a VEGFR tyrosine kinase inhibitor, PTK787/ZK222584 (PTK/ZK), which targets the three VEGFRs, with blocking antibodies directed against VEGFR-2 (DC101) or VEGF-A (Pab85618) in a metastatic melanoma model. Although all inhibitors exerted comparable effects on primary tumor growth, only PTK/ZK significantly reduced lymph node metastasis formation. A comparable decrease in lymphatic vessel density following blockade of VEGFR-2 (DC101) or the three VEGFRs (PTK/ZK) was observed in the metastases. However, the functionality of lymphatics surrounding the primary tumor was more significantly disrupted by PTK/ZK, indicating the importance of multiple VEGFRs in the metastatic process. The antimetastatic properties of PTK/ZK were confirmed in a breast carcinoma model. B16/BL6 tumor cells express VEGF ligands and their receptors. Blockade of a VEGFR-1 autocrine loop with PTK/ZK inhibited tumor cell **migration**. Furthermore, the tumor cells also showed enhanced sensitivity to platinum-based chemotherapy in combination with PTK/ZK, indicating that autocrine VEGFRs are promoting tumor cell **migration** and survival. In summary, our results suggest that, in addition to blocking angiogenesis, combined inhibition of the three VEGFRs may more efficiently target other aspects of tumor pathophysiology, including lymphatic vessel functionality, tumor cell dissemination, survival pathways, and response to chemotherapeutic compounds.
76. Myoferlin regulates vascular endothelial growth factor receptor-2 stability and function. Myoferlin and dysferlin are members of the ferlin family of membrane proteins. Recent studies have shown that mutation or genetic disruption of myoferlin or dysferlin promotes muscular dystrophy-related phenotypes in mice, which are the result of impaired plasma membrane integrity. However, no biological functions have been ascribed to myoferlin in non-muscle tissues. Herein, using a proteomic analysis of endothelial cell (EC) caveolae/lipid raft microdomains we identified myoferlin in these domains and show that myoferlin is highly expressed in ECs and vascular tissues. The loss of myoferlin results in lack of proliferation, **migration**, and nitric oxide (NO) release in response to vascular endothelial growth factor (VEGF). Western blotting and surface biotinylation experiments show that loss of myoferlin reduces the expression level and autophosphorylation of VEGF receptor-2 (VEGFR-2) in native ECs. In a reconstituted cell system, transfection of myoferlin increases VEGFR-2 membrane expression and autophosphorylation in response to VEGF. In vivo, VEGFR-2 levels and VEGF-induced permeability are impaired in myoferlin-deficient mice. Mechanistically, myoferlin forms a complex with dynamin-2 and VEGFR-2, which prevents CBL-dependent VEGFR-2 polyubiquitination and proteasomal degradation. These data are the first to report novel biological activities for myoferlin and reveal the role of membrane integrity to VEGF signaling.
77. Hypoxia-induced mitogenic factor has proangiogenic and proinflammatory effects in the lung via VEGF and VEGF receptor-2. From a mouse model of hypoxia-induced pulmonary hypertension, we previously found a highly upregulated protein in the lung that we named hypoxia-induced mitogenic factor (HIMF), also known as found in inflammatory zone 1 (FIZZ1), and resistin-like molecule alpha (RELMalpha). However, the mechanisms of HIMF in the pulmonary vascular remodeling remain unknown. We now demonstrate that HIMF promoted cell proliferation, **migration**, and the production of vascular endothelial growth factor (VEGF) and monocyte chemotactic protein-1 (MCP-1) in pulmonary endothelial cells as well as the production of reactive oxygen species in murine monocyte/macrophage cells. HIMF-induced CD31-positive cell infiltrate in in vivo Matrigel plugs was significantly suppressed by VEGF receptor-2 (VEGFR2) blockade. In ex vivo studies, HIMF stimulated the production of VEGF, MCP-1, and stromal cell-derived factor-1 (SDF-1) in the lung resident cells, and VEGFR2 neutralization significantly suppressed HIMF-induced MCP-1 and SDF-1 production. Furthermore, intravenous injection of HIMF showed marked increase of CD68-positive inflammatory cells in the lungs, and these events were attenuated by VEGFR2 neutralization. Intravenous injection of HIMF also downregulated the expression of VEGFR2 in the lung. These results suggest that HIMF plays critical roles in pulmonary inflammation as well as angiogenesis.
78. Robo4 is a vascular-specific receptor that inhibits endothelial **migration**. Guidance and patterning of axons are orchestrated by cell-surface receptors and ligands that provide directional cues. Interactions between the Robo receptor and Slit ligand families of proteins initiate signaling cascades that repel axonal outgrowth. Although the vascular and nervous systems grow as parallel networks, the mechanisms by which the vascular endothelial cells are guided to their appropriate positions remain obscure. We have identified a putative Robo homologue, Robo4, based on its differential expression in mutant mice with defects in vascular sprouting. In contrast to known neuronal Robo family members, the arrangement of the extracellular domains of Robo4 diverges significantly from that of all other Robo family members. Moreover, Robo4 is specifically expressed in the vascular endothelium during murine embryonic development. We show that Robo4 binds Slit and inhibits cellular **migration** in a heterologous expression system, analogous to the role of known Robo receptors in the nervous system. Immunoprecipitation studies indicate that Robo4 binds to Mena, a known effector of Robo-Slit signaling. Finally, we show that Robo4 is the only Robo family member expressed in primary endothelial cells and that application of Slit inhibits their **migration**. These data demonstrate that Robo4 is a bona fide member of the Robo family and may provide a repulsive cue to migrating endothelial cells during vascular development.
79. Genetic reduction of vascular endothelial growth factor receptor 2 rescues aberrant angiogenesis caused by epsin deficiency. We previously showed that endothelial epsin deficiency caused elevated vascular endothelial growth factor receptor 2 (VEGFR2) and enhanced VEGF signaling, resulting in aberrant tumor angiogenesis and reduced tumor growth in adult mice. However, direct evidence demonstrating that endothelial epsins regulate angiogenesis specifically through VEGFR2 downregulation is still lacking. In addition, whether the lack of epsins causes abnormal angiogenesis during embryonic development remains unclear.A novel strain of endothelial epsin-deleted mice that are heterozygous for VEGFR2 (Epn1(fl/fl); Epn2(-/-); Flk(fl/+); iCDH5 Cre mice) was created. Analysis of embryos at different developmental stages showed that deletion of epsins caused defective embryonic angiogenesis and retarded embryo development. In vitro angiogenesis assays using isolated primary endothelial cells (ECs) from Epn1(fl/fl); Epn2(-/-); iCDH5 Cre (EC-iDKO) and Epn1(fl/fl); Epn2(-/-); Flk(fl/+); iCDH5 Cre (EC-iDKO-Flk(fl/+)) mice demonstrated that VEGFR2 reduction in epsin-depleted cells was sufficient to restore normal VEGF signaling, EC proliferation, EC **migration**, and EC network formation. These findings were complemented by in vivo wound healing, inflammatory angiogenesis, and tumor angiogenesis assays in which reduction of VEGFR2 was sufficient to rescue abnormal angiogenesis in endothelial epsin-deleted mice.Our results provide the first genetic demonstration that epsins function specifically to downregulate VEGFR2 by mediating activated VEGFR2 internalization and degradation and that genetic reduction of VEGFR2 level protects against excessive angiogenesis caused by epsin loss. Our findings indicate that epsins may be a potential therapeutic target in conditions in which tightly regulated angiogenesis is crucial, such as in diabetic wound healing and tumors.
80. VEGFR-2 inhibition augments cigarette smoke-induced oxidative stress and inflammatory responses leading to endothelial dysfunction. Vascular endothelial growth factor (VEGF) induces phosphorylation of VEGF receptor-2 (VEGFR-2) and activates the downstream signaling pathway resulting in endothelial cell **migration**, proliferation, and survival. Cigarette smoking is associated with abnormal vascular and endothelial function, leading to airspace enlargement. Herein, we investigated the mechanism of cigarette smoke (CS) -induced endothelial dysfunction by studying the VEGF-VEGFR-2 signaling in mouse lung and human endothelial cells. CS exposure caused oxidative stress, as shown by increased levels of 4-hydroxy-2-nonenal-adducts in mouse lung and reactive oxygen species generation in human lung microvascular endothelial cells (HMVEC-Ls). Inhibition of VEGFR-2 by a specific kinase inhibitor (NVP-AAD777) enhanced the CS-induced oxidative stress, causing augmented inflammatory cell influx and proinflammatory mediators release in mouse lung. The levels of endothelial nitric oxide synthase (eNOS) and phosphorylated (p) -eNOS in the lungs of mice exposed to CS and/or treated with VEGFR-2 inhibitor were decreased. CS down-regulated VEGFR-2 expression, eNOS levels, and VEGF-induced VEGFR-2 phosphorylation in HMVEC-Ls, resulting in impaired VEGF-induced endothelial cell **migration** and angiogenesis. Overall, these data show that inhibition of VEGFR-2 augmented CS-induced oxidative stress and inflammatory responses leading to endothelial dysfunction. This explains the mechanism of endothelial dysfunction in smokers and has implications in understanding the pathogenesis of pulmonary and cardiovascular diseases.
81. Anti-vascular endothelial growth factor receptor-2 (Flk-1/KDR) antibody suppresses contact hypersensitivity. The angiogenic mediator vascular endothelial growth factor (VEGF) and its receptors (VEGFRs) have been studied extensively in neoplastic disease and some inflammatory conditions. Contact hypersensitivity (CHS) is a prototypic Langerhans' cell-dependent, T-helper (Th) 1 cell-mediated inflammatory skin disease that is now also thought to involve angiogenic mediators. The purpose of our study was to examine the role of angiogenesis and VEGF in CHS. We demonstrated that VEGF production is up-regulated in murine skin after challenge with dinitrofluorobenzene. Administration of a monoclonal antibody directed against the VEGFR-2 (DC101) resulted in a 28.8% decrease in CHS response (P < 0.001). Examination of the DC101-treated mouse skin 24 h after challenge revealed decreases in dermal inflammatory cellular infiltrates and total vessel area. Furthermore, mRNA and protein of the Th1-type cytokine interferon (IFN)-gamma was significantly down-regulated in skin of DC101-treated animals 24 h after challenge. The results of the study demonstrate that VEGFR-2 blockade significantly reduces vascular enlargement and edema formation and effects IFN-gamma expression in the skin during challenge in CHS. Our findings suggest that DC101 could function by reducing inflammatory cell **migration** and hence IFN-gamma expression during the CHS response.
82. Altered proliferative ability of neuronal progenitors in PlexinA1 mutant mice. Cortical interneurons are generated predominantly in the medial ganglionic eminence (MGE) and migrate through the ventral and dorsal telencephalon before taking their final positions within the developing cortical plate. Previously we demonstrated that interneurons from Robo1 knockout (Robo1(-/-)) mice contain reduced levels of neuropilin 1 (Nrp1) and PlexinA1 receptors, rendering them less responsive to the chemorepulsive actions of semaphorin ligands expressed in the striatum and affecting their course of **migration** (Hernandez-Miranda et al. [2011] J. Neurosci. 31:6174-6187). Earlier studies have highlighted the importance of Nrp1 and Nrp2 in interneuron **migration**, and here we assess the role of PlexinA1 in this process. We observed significantly fewer cells expressing the interneuron markers Gad67 and Lhx6 in the cortex of PlexinA1(-/-) mice compared with wild-type littermates at E14.5 and E18.5. Although the level of apoptosis was similar in the mutant and control forebrain, proliferation was significantly reduced in the former. Furthermore, progenitor cells in the MGE of PlexinA1(-/-) mice appeared to be poorly anchored to the ventricular surface and showed reduced adhesive properties, which may account for the observed reduction in proliferation. Together our data uncover a novel role for PlexinA1 in forebrain development.
83. Multiple developmental roles of VEGF suggested by a LacZ-tagged allele. Vascular endothelial growth factor (VEGF) is an angiogenic factor and a potent stimulator of microvascular permeability. It is a mitogen specific for endothelial cells. The expression of VEGF and its two receptors, Flk-1 and Flt-1, is pivotal for the proper formation of blood vessels in embryogenesis as shown by gene-targeting experiments. Interestingly, the loss of even a single allele of VEGF led to embryonic lethality between day E9.5 and day E10.5 in the mouse. To assess the role of VEGF during embryonic development we decided to tag VEGF expression with LacZ, by inserting an IRES (internal ribosome entry site)-LacZ reporter cassette into the 3' untranslated region of the gene. This alteration enabled us to monitor VEGF expression throughout embryonic development at single-cell resolution. beta-Galactosidase expression from the altered VEGF locus was first observed prior to gastrulation and was detectable at all stages of vascular development in the embryo. Later, the specific cellular distribution and the level of VEGF expression indicated its pleiotropic role in development. High expression levels seemed to be associated with vasculogenesis and permeability, whereas lower levels were associated with angiogenesis and cell **migration**. In addition, we found VEGF expression in a subtype of endothelial cells present in the endocardium. We believe that the LacZ-tagged allele we have generated offers a precise means of detecting VEGF expression under a variety of physiological and pathological conditions.
84. Mechanisms of integrin-vascular endothelial growth factor receptor cross-activation in angiogenesis. The functional responses of endothelial cells are dependent on signaling from peptide growth factors and the cellular adhesion receptors, integrins. These include cell adhesion, **migration**, and proliferation, which, in turn, are essential for more complex processes such as formation of the endothelial tube network during angiogenesis. This study identifies the molecular requirements for the cross-activation between beta3 integrin and tyrosine kinase receptor 2 for vascular endothelial growth factor (VEGF) receptor (VEGFR-2) on endothelium. The relationship between VEGFR-2 and beta3 integrin appears to be synergistic, because VEGFR-2 activation induces beta3 integrin tyrosine phosphorylation, which, in turn, is crucial for VEGF-induced tyrosine phosphorylation of VEGFR-2. We demonstrate here that adhesion- and growth factor-induced beta3 integrin tyrosine phosphorylation are directly mediated by c-Src. VEGF-stimulated recruitment and activation of c-Src and subsequent beta3 integrin tyrosine phosphorylation are critical for interaction between VEGFR-2 and beta3 integrin. Moreover, c-Src mediates growth factor-induced beta3 integrin activation, ligand binding, beta3 integrin-dependent cell adhesion, directional **migration** of endothelial cells, and initiation of angiogenic programming in endothelial cells. Thus, the present study determines the molecular mechanisms and consequences of the synergism between 2 cell surface receptor systems, growth factor receptor and integrins, and opens new avenues for the development of pro- and antiangiogenic strategies.
85. Alzheimer's beta-amyloid peptide blocks vascular endothelial growth factor mediated signaling via direct interaction with VEGFR-2. Beta-amyloid peptides (Abeta) are the major constituents of senile plaques and cerebrovascular deposits in the brains of Alzheimer's disease patients. We have shown previously that soluble forms of Abeta are anti-angiogenic both in vitro and in vivo. However, the mechanism of the anti-angiogenic activity of Abeta peptides is unclear. In this study, we examined the effects of Abeta1-42 on vascular endothelial growth factor receptor 2 (VEGFR-2) signaling, which plays a key role in angiogenesis. Abeta inhibited VEGF-induced **migration** of endothelial cells, as well as VEGF-induced permeability of an in vitro model of the blood brain barrier. Consistently, exogenous VEGF dose-dependently antagonized the anti-angiogenic activity of Abeta in a capillary network assay. Abeta1-42 also blocked VEGF-induced tyrosine phosphorylation of VEGFR-2 in two types of primary endothelial cells, suggesting an antagonistic action of Abeta toward VEGFR-2 signaling in cells. Moreover, Abeta was able to directly interact with the extracellular domain of VEGFR-2 and to compete with the binding of VEGF to its receptor in a cell-free assay. Co-immunoprecipitation experiments confirmed that Abeta can bind VEGFR-2 both in vitro and in vivo. Altogether, our data suggest that Abeta acts as an antagonist of VEGFR-2 and provide a mechanism explaining the anti-angiogenic activity of Abeta peptides.
86. A heat-shock protein axis regulates VEGFR2 proteolysis, blood vessel development and repair. Vascular endothelial growth factor A (VEGF-A) binds to the VEGFR2 receptor tyrosine kinase, regulating endothelial function, vascular physiology and angiogenesis. However, the mechanism underlying VEGFR2 turnover and degradation in this response is unclear. Here, we tested a role for heat-shock proteins in regulating the presentation of VEGFR2 to a degradative pathway. Pharmacological inhibition of HSP90 stimulated VEGFR2 degradation in primary endothelial cells and blocked VEGF-A-stimulated intracellular signaling via VEGFR2. HSP90 inhibition stimulated the formation of a VEGFR2-HSP70 complex. Clathrin-mediated VEGFR2 endocytosis is required for this HSP-linked degradative pathway for targeting VEGFR2 to the endosome-lysosome system. HSP90 perturbation selectively inhibited VEGF-A-stimulated human endothelial cell **migration** in vitro. A mouse femoral artery model showed that HSP90 inhibition also blocked blood vessel repair in vivo consistent with decreased endothelial regeneration. Depletion of either HSP70 or HSP90 caused defects in blood vessel formation in a transgenic zebrafish model. We conclude that perturbation of the HSP70-HSP90 heat-shock protein axis stimulates degradation of endothelial VEGFR2 and modulates VEGF-A-stimulated intracellular signaling, endothelial cell **migration**, blood vessel development and repair.
87. VEGF modulates NMDA receptors activity in cerebellar granule cells through Src-family kinases before synapse formation. NMDA type glutamate receptors (NMDARs) are best known for their role in synaptogenesis and synaptic plasticity. Much less is known about their developmental role before neurons form synapses. We report here that VEGF, which promotes **migration** of granule cells (GCs) during postnatal cerebellar development, enhances NMDAR-mediated currents and Ca(2+) influx in immature GCs before synapse formation. The VEGF receptor Flk1 forms a complex with the NMDAR subunits NR1 and NR2B. In response to VEGF, the number of Flk1/NR2B coclusters on the cell surface increases. Stimulation of Flk1 by VEGF activates Src-family kinases, which increases tyrosine phosphorylation of NR2B. Inhibition of Src-family kinases abolishes the VEGF-dependent NR2B phosphorylation and amplification of NMDAR-mediated currents and Ca(2+) influx in GCs. These findings identify VEGF as a modulator of NMDARs before synapse formation and highlight a link between an activity-independent neurovascular guidance cue (VEGF) and an activity-regulated neurotransmitter receptor (NMDAR).
88. Protein tyrosine phosphatase 1B impairs diabetic wound healing through vascular endothelial growth factor receptor 2 dephosphorylation. Impaired wound healing is a major complication of diabetes mellitus. The mechanisms that govern wound healing, however, are complex and incompletely understood. In the present study, we determined the inhibitory role of protein tyrosine phosphatase 1B (PTP1B) in the process of diabetic wound healing.First, by comparing the wound healing process in PTP1B knockout (PTP1B(-/-)) mice, ob/ob mice and their wild-type littermates in the presence or absence of streptozotocin treatment, we showed that the inhibition of mouse wound healing in streptozotocin-induced diabetic conditions is because of the upregulation and activation of PTP1B. Second, the impaired wound healing in ob/ob mice and streptozotocin-treated wild-type mice was rescued by a PTP1B inhibitor. Third, PTP1B, which is upregulated under hyperglycemic condition, inhibited the tube formation, proliferation, and **migration** of human microvascular endothelial cells induced by vascular endothelial growth factor, whereas this inhibition was largely abolished by the PTP1B inhibitor. Finally, mechanism study further indicated that PTP1B likely suppressed the proliferation, **migration**, and tube formation of vascular endothelial cells through dephosphorylation of vascular endothelial growth factor receptor 2.Our study demonstrated that PTP1B negatively modulated the diabetic wound healing process by dephosphorylating the endothelial cell vascular endothelial growth factor receptor 2 and that the specific inhibitor of PTP1B might serve as a potential novel therapeutic tool for diabetic wound healing.
89. Embryonic death of Mek1-deficient mice reveals a role for this kinase in angiogenesis in the labyrinthine region of the placenta. Mek is a dual-specificity kinase that activates the extracellular-signal-regulated (Erk) mitogen-activated protein (MAP) kinases upon agonist binding to receptors. The Erk MAP kinase cascade is involved in cell-fate determination in many organisms. In mammals, this pathway is proposed to regulate cell growth and differentiation. Genetic studies have shown that although a single mek gene is present in Caenorhabditis elegans, Drosophila and Xenopus, two mek homologs, Mek1 and Mek2, are present in the mammalian cascade. In the present study, we describe a mutant mouse line in which the mek1 gene has been disrupted by insertional mutagenesis. The null mutation was recessive lethal, as the homozygous mutant embryos died at 10.5 days of gestation. Histopathological analyses revealed a reduction in vascularization of the placenta that was due to a marked decrease of vascular endothelial cells in the labyrinthine region. The failure to establish a functional placenta probably explains the death of the mek1-/- embryos. Cell-**migration** assays indicated that mek1-/- fibroblasts could not be induced to migrate by fibronectin, although the levels of Mek2 protein and Erk activation were normal. Re-expression of Mek1 in the mutant mouse embryonic fibroblasts (MEFs) restored their ability to migrate. Our findings provide genetic evidence that establishes the unique role played by Mek1 in signal transduction. They also suggest that mek1 function is required for normal response to angiogenic signals that might promote vascularization of the labyrinthine region of the placenta.
90. Matrix-binding vascular endothelial growth factor (VEGF) isoforms guide granule cell **migration** in the cerebellum via VEGF receptor Flk1. Vascular endothelial growth factor (VEGF) regulates angiogenesis, but also has important, yet poorly characterized roles in neuronal wiring. Using several genetic and in vitro approaches, we discovered a novel role for VEGF in the control of cerebellar granule cell (GC) **migration** from the external granule cell layer (EGL) toward the Purkinje cell layer (PCL). GCs express the VEGF receptor Flk1, and are chemoattracted by VEGF, whose levels are higher in the PCL than EGL. Lowering VEGF levels in mice in vivo or ectopic VEGF expression in the EGL ex vivo perturbs GC **migration**. Using GC-specific Flk1 knock-out mice, we provide for the first time in vivo evidence for a direct chemoattractive effect of VEGF on neurons via Flk1 signaling. Finally, using knock-in mice expressing single VEGF isoforms, we show that pericellular deposition of matrix-bound VEGF isoforms around PC dendrites is necessary for proper GC **migration** in vivo. These findings identify a previously unknown role for VEGF in neuronal **migration**.
91. Perlecan modulates VEGF signaling and is essential for vascularization in endochondral bone formation. Perlecan (Hspg2) is a heparan sulfate proteoglycan expressed in basement membranes and cartilage. Perlecan deficiency (Hspg2(-/-)) in mice and humans causes lethal chondrodysplasia, which indicates that perlecan is essential for cartilage development. However, the function of perlecan in endochondral ossification is not clear. Here, we report the critical role of perlecan in VEGF signaling and angiogenesis in growth plate formation. The Hspg2(-/-) growth plate was significantly wider but shorter due to severely impaired endochondral bone formation. Hypertrophic chondrocytes were differentiated in Hspg2(-/-) growth plates; however, removal of the hypertrophic matrix and calcified cartilage was inhibited. Although the expression of MMP-13, CTGF, and VEGFA was significantly upregulated in Hspg2(-/-) growth plates, vascular invasion into the hypertrophic zone was impaired, which resulted in an almost complete lack of bone marrow and trabecular bone. We demonstrated that cartilage perlecan promoted activation of VEGF/VEGFR by binding to the VEGFR of endothelial cells. Expression of the perlecan transgene specific to the cartilage of Hspg2(-/-) mice rescued their perinatal lethality and growth plate abnormalities, and vascularization into the growth plate was restored, indicating that perlecan in the growth plate, not in endothelial cells, is critical in this process. These results suggest that perlecan in cartilage is required for activating VEGFR signaling of endothelial cells for vascular invasion and for osteoblast **migration** into the growth plate. Thus, perlecan in cartilage plays a critical role in endochondral bone formation by promoting angiogenesis essential for cartilage matrix remodeling and subsequent endochondral bone formation.
92. Vasohibin is up-regulated by VEGF in the retina and suppresses VEGF receptor 2 and retinal neovascularization. Vasohibin is a recently identified protein that is up-regulated in cultured vascular endothelial cells by vascular endothelial growth factor and fibroblast growth factor 2. It inhibits endothelial cell **migration**, proliferation, and tube formation, and suppresses angiogenesis in chick chorioallantoic membrane, after subcutaneous implantation of matrigel, and in a tumor xenograft model. This has led to the hypothesis that vasohibin functions as a negative feedback inhibitor of angiogenesis. In this study, we tested that hypothesis in a well-characterized model of retinal neovascularization. In ischemic retina, increased expression of VEGF was accompanied by elevation of vasohibin mRNA and blocking of the increase in vegf mRNA with vegf siRNA significantly attenuated the rise in vasohibin mRNA. In transgenic mice in which the rhodopsin promoter drives expression of VEGF in the retina, there was also a significant increase in vasohibin mRNA. In mice with ischemic retinopathy, there was increased expression of vasohibin in vascular endothelial cells, and vasohibin knockdown caused an increase in neovascularization. Conversely, intraocular injection of recombinant vasohibin or an adenoviral vector containing a vasohibin expression cassette strongly suppressed retinal neovascularization in mice with ischemic retinopathy. Knockdown of vasohibin mRNA in ischemic retina had no significant effect on vegf or vegf receptor 1 mRNA levels but caused a significant elevation in the level of vegf receptor 2 mRNA. These data support the hypothesis that vasohibin acts as a negative feedback regulator of neovascularization in the retina and suggest that suppression of VEGF receptor 2 may play some role in mediating its activity.
93. VEGF is required for dendritogenesis of newly born olfactory bulb interneurons. The angiogenic factor vascular endothelial growth factor A (VEGF) has been shown to have a role in neurogenesis, but how it affects adult neurogenesis is not fully understood. To delineate a role for VEGF in successive stages of olfactory bulb (OB) neurogenesis, we used a conditional transgenic system to suppress VEGF signaling at the adult mouse sub-ventricular zone (SVZ), rostral migratory stream (RMS) and OB, which constitute the respective sites of birth, the **migration** route, and sites where newly born interneurons mature and integrate within the existing OB circuitry. Following the development of fluorescently tagged adult-born neurons, we show that sequestration of VEGF that is constitutively expressed by distinct types of resident OB neurons greatly impaired dendrite development in incoming SVZ-born neurons. This was evidenced by reduced dendritic spine density of granule cells and significantly shorter and less branched dendrites in periglomerular neurons. Notably, the vasculature and perfusion of the SVZ, RMS and OB were not adversely affected when VEGF suppression was delayed until after birth, thus uncoupling the effect of VEGF on dendritogenesis from its known role in vascular maintenance. Furthermore, a requirement for VEGF was specific to newly born neurons, as already established OB neurons were not damaged by VEGF inhibition. This study thus uncovered a surprising perfusion-independent role of VEGF in the adult brain, namely, an essential role in the maturation of adult-born neurons.
94. Homeodomain factor Nkx2-3 controls regional expression of leukocyte homing coreceptor MAdCAM-1 in specialized endothelial cells of the viscera. Regulated e**migration** of blood-borne leukocytes plays a defining role in lymphoid organ development, immune surveillance, and inflammatory responses. We report here that mice deficient in the homeobox gene Nkx2-3, expressed in developing visceral mesoderm, show a complex intestinal malabsorption phenotype and striking abnormalities of gut-associated lymphoid tissue and spleen suggestive of deranged leukocyte homing. Mutant Peyer's patches were reduced in number and size, intestinal villi contained few IgA(+) plasma cells, and mutant spleens were small and often atrophic, showing fused periarterial lymphoid sheaths, partially merged T and B cell zones, an absent marginal zone, and a dearth of macrophages in red pulp. Semiquantitative RT-PCR analysis and immunohistochemistry revealed down-regulation of mucosal addressin cell adhesion molecule-1 (MAdCAM-1) in endothelial cells in which Nkx2-3 is normally expressed. MAdCAM-1 is a member of the immunoglobulin superfamily, acting as an endothelial cell ligand for leukocyte homing receptors L-selectin and alpha4beta7 integrin. Our data suggest a role for a homeodomain factor in establishing the developmental and positional cues in endothelia that regulate leukocyte homing through local control of cellular adhesion and identify MAdCAM-1 as a candidate target gene of Nkx2-3.
95. Requirement of the transcription factor GATA4 for heart tube formation and ventral morphogenesis. The zinc finger transcription factor GATA4 has been implicated in heart development based on its early expression in precardiogenic splanchnic mesoderm and its ability to activate the expression of a number of cardiac-specific genes. To determine the role of GATA4 in embryogenesis, we generated mice homozygous for a GATA4 null allele. Homozygous GATA4 null mice arrested in development between E7.0 and E9.5 because of severe developmental abnormalities. Mutant embryos most notably lacked a primitive heart tube and foregut and developed partially outside the yolk sac. In the mutants, the two bilaterally symmetric promyocardial primordia failed to migrate ventrally but instead remained lateral and generated two independent heart tubes that contained differentiated cardiomyocytes. We show that these deformities resulted from a general loss in lateral to ventral folding throughout the embryo. GATA4 is most highly expressed within the precardiogenic splanchnic mesoderm at the posterior lip of the anterior intestinal portal, corresponding to the region of the embryo that undergoes ventral fusion. We propose that GATA4 is required for the **migration** or folding morphogenesis of the precardiogenic splanchnic mesodermal cells at the level of the AIP.
96. Slit2 Promotes Angiogenic Activity Via the Robo1-VEGFR2-ERK1/2 Pathway in Both In Vivo and In Vitro Studies. Recent research has provided novel but contrary insight into the function of Slit2-Robo signaling in angiogenesis. Although the role of Robo in choroidal neovascularization (CNV) has been studied, the effect of its ligand, Slit2, on CNV development is unclear. This study investigated the role of endogenous Slit2 in CNV and the possible mechanisms.Laser-induced CNV in Slit2 transgenic and wild-type mice was used to study the effects of endogenous Slit2 on angiogenesis in vivo. Fluorescein angiography was performed to evaluate the leakage area of each lesion. Plasmid-based gene transfer technology was used to increase Slit2 expression and to study its effects on human umbilical vein endothelial cells (HUVECs) in vitro. Cell proliferation, **migration**, and tube formation were assessed. Quantitative real-time PCR and Western blot were used to measure expression in the extracellular signal-related kinase 1/2 (ERK1/2), protein kinase B (AKT), and p38 mitogen-activated protein kinase (p38 MAPK) molecular pathways.Laser treatment led to more CNV and vascular leakage in Slit2 transgenic mice compared with wild-type mice. Upregulation of Slit2, Robo1, VEGF receptor 2 (VEGFR2), and phosphorylated ERK1/2 (p-ERK1/2) were detected in retina and choroidal tissue of laser-treated transgenic mice. After transfection of HUVECs with a Slit2 overexpression plasmid, cell proliferation, **migration**, and tube formation capacities were promoted. Slit2, Robo1, VEGFR2, and p-ERK1/2 were elevated in transfected HUVECs.Slit2 overexpression promoted angiogenic effects in both a laser-induced CNV mouse model and HUVECs and promoted the biological activity of endothelial cells. Slit2 may promote angiogenesis by upregulating Robo1 and activating the VEGFR2-ERK1/2 pathway.
97. Type 1 and 3 inositol trisphosphate receptors are required for extra-embryonic vascular development. The embryonic-maternal interface of the placental labyrinth, allantois, and yolk sac are vital during embryogenesis; however, the precise mechanism underlying the vascularization of these structures remains unknown. Herein we focus on the role of inositol 1,4,5-trisphosphate (IP3) receptors (IP3R), which are intracellular Ca(2+) release channels, in placentation. Double knockout (DKO) of type 1 and 3 IP3Rs (IP3R1 and IP3R3, respectively) in mice resulted in embryonic lethality around embryonic day (E) 11.5. Because IP3R1 and IP3R3 were co-expressed in endothelial cells in the labyrinth, allantois, and yolk sac, we investigated extra-embryonic vascular development in IP3R1- and IP3R3-DKO mice. The formation of chorionic plates and yolk sac vessels seemed dysregulated around the timing of the chorio-allantoic attachment, immediately followed by the disorganization of allantoic vessels, the decreased expression of the spongiotrophoblast cell marker Tpbpa and the growth retardation of the embryos in DKO mice. Fluorescent immunohistochemistry demonstrated downregulation of a vascular endothelial marker, CD31, in labyrinth embryonic vessels and poor elongation of extra-embryonic mesoderm into the labyrinth layer in DKO placenta, whereas the branching of the DKO chorionic trophoblast was initiated. In addition, allantoic and yolk sac vessels in extra-embryonic tissues were less remodeled in DKO mice. In vitro endothelial cord formation and **migration** activities of cultured vascular endothelial cells derived from human umbilical vein were downregulated under the inhibition of IP3R. Our results suggest that IP3R1 and IP3R3 are required for extra-embryonic vascularization in the placenta, allantois, and yolk sac. This is the first demonstration of the essential role of IP3/IP3Rs signaling in the development of the vasculature at the embryonic-maternal interface.
98. Targeting NCK-Mediated Endothelial Cell Front-Rear Polarity Inhibits Neovascularization. Sprouting angiogenesis is a key process driving blood vessel growth in ischemic tissues and an important drug target in a number of diseases, including wet macular degeneration and wound healing. Endothelial cells forming the sprout must develop front-rear polarity to allow sprout extension. The adaptor proteins Nck1 and 2 are known regulators of cytoskeletal dynamics and polarity, but their function in angiogenesis is poorly understood. Here, we show that the Nck adaptors are required for endothelial cell front-rear polarity and **migration** downstream of the angiogenic growth factors VEGF-A and Slit2.Mice carrying inducible, endothelial-specific Nck1/2 deletions fail to develop front-rear polarized vessel sprouts and exhibit severe angiogenesis defects in the postnatal retina and during embryonic development. Inactivation of NCK1 and 2 inhibits polarity by preventing Cdc42 and Pak2 activation by VEGF-A and Slit2. Mechanistically, NCK binding to ROBO1 is required for both Slit2- and VEGF-induced front-rear polarity. Selective inhibition of polarized endothelial cell **migration** by targeting Nck1/2 prevents hypersprouting induced by Notch or Bmp signaling inhibition, and pathological ocular neovascularization and wound healing, as well.These data reveal a novel signal integration mechanism involving NCK1/2, ROBO1/2, and VEGFR2 that controls endothelial cell front-rear polarity during sprouting angiogenesis.
99. VE-statin, an endothelial repressor of smooth muscle cell **migration**. The recruitment and proliferation of smooth muscle cells and pericytes are two key events for the stabilization of newly formed capillaries during angiogenesis and, when out of control in the adult, are the main causes of arteriosclerosis. We have identified a novel gene, named VE-statin for vascular endothelial-statin, which is expressed specifically by endothelial cells of the developing mouse embryo and in the adult, and in early endothelial progenitors. The mouse and human VE-statin genes have been located on chromosome 2 and 9, respectively, they span >10 kbp and are transcribed in two major variants arising from independent initiation sites. The VE-statin transcripts code for a unique protein of 30 kDa that contains a signal peptide and two epidermal growth factor (EGF)-like modules. VE-statin is found in the cellular endoplasmic reticulum and secreted in the cell supernatant. Secreted VE-statin inhibits platelet-derived growth factor (PDGF)-BB-induced smooth muscle cell **migration**, but has no effects on endothelial cell **migration**. VE-statin is the first identified inhibitor of mural cell **migration** specifically produced by endothelial cells.
100. Region-specific Etv2 ablation revealed the critical origin of hemogenic capacity from Hox6-positive caudal-lateral primitive mesoderm. Hematopoietic cells (HPCs) develop from hemogenic endothelial cells (ECs), a specialized type of ECs undergoing hematopoietic transition. However, the mesoderm origin for hemogenic ECs or HPCs has not been clarified. To examine the origin for hemogenic mesoderm, we inactivated Etv2, a master regulator for EC/HPC commitment, in specific regions. Region-specific Etv2 ablation in early mesoderm caused local EC differentiation block, resulting in the loss of specific vascular beds without compensatory **migration** of residual ECs into avascular area. This feature of local EC/HPC differentiation block was correlated to the hemogenic potential of each mesoderm subset. We found that caudal-lateral mesoderm of E7.5-8.5 embryos represent the pre-committed population critical for generating hemogenic ECs. Etv2 ablation in caudal-lateral mesoderm by Hoxb6 Cre or Hoxb6CreER transgene affected vitelline plexus formation and intra-aortic hematopoietic clusters. In differentiated embryonic stem cells, this mesoderm subset marked by Hoxb6-lateral mesoderm promoter showed enriched T lymphopoietic potential among Flk-1(+) cells, which could be regarded as a characteristic for definitive HPCs. These findings indicate that critical mesoderm precursors possibly for definitive type hemogenic ECs are regionally specified in primitive mesoderm, suggesting that Hoxb6(+) caudal-lateral mesoderm represents the critical source of HPCs, which are potentially useful to enrich definitive HPCs from embryonic stem cells.
101. Extracellular SOD-derived H2O2 promotes VEGF signaling in caveolae/lipid rafts and post-ischemic angiogenesis in mice. Reactive oxygen species (ROS), in particular, H(2)O(2), is essential for full activation of VEGF receptor2 (VEGFR2) signaling involved in endothelial cell (EC) proliferation and **migration**. Extracellular superoxide dismutase (ecSOD) is a major secreted extracellular enzyme that catalyzes the dismutation of superoxide to H(2)O(2), and anchors to EC surface through heparin-binding domain (HBD). Mice lacking ecSOD show impaired postnatal angiogenesis. However, it is unknown whether ecSOD-derived H(2)O(2) regulates VEGF signaling. Here we show that gene transfer of ecSOD, but not ecSOD lacking HBD (ecSOD-DeltaHBD), increases H(2)O(2) levels in adductor muscle of mice, and promotes angiogenesis after hindlimb ischemia. Mice lacking ecSOD show reduction of H(2)O(2) in non-ischemic and ischemic limbs. In vitro, overexpression of ecSOD, but not ecSOD-DeltaHBD, in cultured medium in ECs enhances VEGF-induced tyrosine phosphorylation of VEGFR2 (VEGFR2-pY), which is prevented by short-term pretreatment with catalase that scavenges extracellular H(2)O(2). Either exogenous H(2)O(2) (<500 microM), which is diffusible, or nitric oxide donor has no effect on VEGF-induced VEGFR2-pY. These suggest that ecSOD binding to ECs via HBD is required for localized generation of extracellular H(2)O(2) to regulate VEGFR2-pY. Mechanistically, VEGF-induced VEGFR2-pY in caveolae/lipid rafts, but non-lipid rafts, is enhanced by ecSOD, which localizes at lipid rafts via HBD. One of the targets of ROS is protein tyrosine phosphatases (PTPs). ecSOD induces oxidation and inactivation of both PTP1B and DEP1, which negatively regulates VEGFR2-pY, in caveolae/lipid rafts, but not non-lipid rafts. Disruption of caveolae/lipid rafts, or PTPs inhibitor orthovanadate, or siRNAs for PTP1B and DEP1 enhances VEGF-induced VEGFR2-pY, which prevents ecSOD-induced effect. Functionally, ecSOD promotes VEGF-stimulated EC **migration** and proliferation. In summary, extracellular H(2)O(2) generated by ecSOD localized at caveolae/lipid rafts via HBD promotes VEGFR2 signaling via oxidative inactivation of PTPs in these microdomains. Thus, ecSOD is a potential therapeutic target for angiogenesis-dependent cardiovascular diseases.
102. KDR-LacZ-expressing cells are involved in ovarian and testis-specific vascular development, suggesting a role for VEGFA in the regulation of this vasculature. Our objectives were to evaluate kinase insert domain protein receptor (KDR)-β-galactosidase (LacZ) expression as a marker for vascular development during gonadal morphogenesis and to determine whether any novel non-angiogenic KDR-LacZ expression was present in mouse testes or ovaries. Gonads were collected from mice expressing LacZ driven by the Kdr promoter (KDR-LacZ) from embryonic day 11 (E11) through postnatal day 60 (P60). At E11.5, mesonephric cells expressing KDR-LacZ seemed to migrate into the developing testis and surrounded developing seminiferous cords. Cells expressing KDR-LacZ appeared in the ovary with no apparent **migratio**n from the adjacent mesonephros, suggesting a different origin of endothelial cells. Testis organ cultures from E11 mice were treated with 8 μM VEGFR-TKI, a vascular endothelial growth factor A signal transduction inhibitor; subsequently, the amount of KDR-LacZ staining was reduced by 66%-99% (P<0.002), and the ability of KDR-expressing cells to form a densely organized vascular network was inhibited. Novel non-angiogenic KDR-LacZ staining was detected in the testis on specific subsets of germ cells at E16, E17, P4, P20, P30, and P60. In ovaries, staining was present on oocytes within oocyte cysts at E17 and within late secondary follicles of postnatal mice. Thus, KDR is an excellent marker for analyzing vascular development in the gonads. Inhibition of VEGFA signal transduction prevents the development of testis-specific vasculature. Furthermore, non-vascular KDR-LacZ staining suggests that KDR directly affects both spermatogenesis and somatic-oocyte interactions during gametogenesis.
103. Lymphangiogenesis in the developing lung promoted by VEGF-A. Understanding the basic processes of late-stage pulmonary vascular development is essential as this period corresponds to the stage when preterm infants have increased chance of survival. During this period, refinement of the gas exchange unit leads to close apposition of the capillary vasculature and airway epithelium through thinning of the mesenchyme, formation of alveolar septae and functional adaptation of endothelial cells into vessels including pulmonary lymphatics. The pulmonary lymphatic network promotes efficient gas exchange through maintaining interstitial fluid balance. Through conditional transgene regulation, we found that a modest, pathologically relevant increase in vascular endothelial growth factor A (VEGF-A) in distal lung during only the perinatal period adversely affected final refinement of the gas exchange unit. VEGF-A induction disrupted the established vascular network, increased endothelial cell number, altered endothelial ultrastructure and reduced mesenchymal thinning. In addition, VEGF-A induction caused a 3-fold increase in small vessels identified as lymphatics in distal lung. mRNA levels of lymphangiogenic factors VEGF-D/-C were unchanged, while levels of the cognate receptor VEGFR-3 increased. The responses to VEGF-A induction in the perinatal period differ from those during early lung development when endothelial **migration**, but not proliferation altered initial vascular patterning (Akeson, A.L., Greenberg, J.M., Cameron, J.E., Thompson, F.Y., Brooks, S.K., Wiginton, D., Whitsett, J.A., 2003. Temporal and spatial regulation of VEGF-A controls vascular patterning in the embryonic lung. Dev. Biol. 264, 443-455). The late-stage response resembles that of adult lung to VEGF-inducing stimuli including injury and disease. These data suggest that VEGF-A influences the balance between development of blood and lymphatic vasculature during lung organogenesis.
104. MesP1 and MesP2 are essential for the development of cardiac mesoderm. The transcription factors, MesP1 and MesP2, sharing an almost identical bHLH motif, have an overlapping expression pattern during gastrulation and somitogenesis. Inactivation of the Mesp1 gene results in abnormal heart morphogenesis due to defective **migration** of heart precursor cells, but somitogenesis is not disrupted because of normal expression of the Mesp2 gene. To understand the cooperative functions of MesP1 and MesP2, either a deletion or sequential gene targeting strategy was employed to inactivate both genes. The double-knockout (dKO) embryos died around 9.5 days postcoitum (dpc) without developing any posterior structures such as heart, somites or gut. The major defect in this double-knockout embryo was the apparent lack of any mesodermal layer between the endoderm and ectoderm. The abnormal accumulation of cells in the primitive streak indicates a defect in the migratory activity of mesodermal cells. Molecular markers employed to characterize the phenotype revealed a lack of the cranio-cardiac and paraxial mesoderm. However, the axial mesoderm, as indicated by brachyury (T) expression, was initially generated but anterior extension was halted after 8.5 dpc. Interestingly, a headfold-like structure developed with right anterior-posterior polarity; however, the embryos lacked any posterior neural properties. The persistent and widely distributed expression of Cerberus-like-1(Cer1), Lim1 and Otx2 in the anterior endoderm might be responsible for the maintenance of anterior neural marker expression. We also performed a chimera analysis to further study the functions of MesP1 and MesP2 in the development of mesodermal derivatives. In the chimeric embryos, dKO cells were scarcely observed in the anterior-cephalic and heart mesoderm, but they did contribute to the formation of the somites, notochord and gut. These results strongly indicate that the defect in the cranial-cardiac mesoderm is cell-autonomous, whereas the defect in the paraxial mesoderm is a non-cell-autonomous secondary consequence.
105. The spatial and temporal expression patterns of netrin receptors, DCC and neogenin, in the developing mouse retina. Recently it has been demonstrated that the guidance of retinal ganglion cell (rgc) axons through the optic disc is dependent on the DCC/netrin-1 axonal guidance system. To gain further insight into the function of the netrin receptors, DCC and Neogenin, in retinal development we have studied the expression patterns of these receptors in the embryonic mouse retina. Neogenin mRNA was restricted to a single neural cell type, the rgc. However, strong Neogenin mRNA expression was observed in the extending fiber cells of the developing lens suggesting a role for Neogenin in the **migration** events shaping the early lens. Our studies demonstrated that DCC mRNA was expressed at high levels in chains of closely opposed neurons as they migrated towards the emerging mantle layer in the early retina (E12.5-E13.5) suggesting a role for DCC in the **migration** of neurons out of the ventricular zone. DCC protein expression was high on rgc axons as they actively navigated through the optic disc into the optic nerve. At birth, when the majority of rgc axons had projected through the optic disc, DCC protein was no longer detectable on the distal axonal segments within the optic nerve despite significant DCC protein expression on the proximal axonal membranes in the nerve fiber layer. These observations suggest that a localized down-regulation of DCC protein occurs on projecting axonal membranes once the DCC guidance function is no longer required. We also demonstrated that DCC mRNA and protein were expressed by amacrine cells and Müller glial cells while DCC mRNA was detected in horizontal cells. Taken together, these expression patterns suggest a role for DCC in axon outgrowth and/or pathfinding for a variety of retinal neurons and in the **migratio**n of newly born neurons within the developing retina.
106. Afadin: A key molecule essential for structural organization of cell-cell junctions of polarized epithelia during embryogenesis. Afadin is an actin filament-binding protein that binds to nectin, an immunoglobulin-like cell adhesion molecule, and is colocalized with nectin at cadherin-based cell-cell adherens junctions (AJs). To explore the function of afadin in cell-cell adhesion during embryogenesis, we generated afadin(-/-) mice and embryonic stem cells. In wild-type mice at embryonic days 6.5-8.5, afadin was highly expressed in the embryonic ectoderm and the mesoderm, but hardly detected in the extraembryonic regions such as the visceral endoderm. Afadin(-/-) mice showed developmental defects at stages during and after gastrulation, including disorganization of the ectoderm, impaired **migration** of the mesoderm, and loss of somites and other structures derived from both the ectoderm and the mesoderm. Cystic embryoid bodies derived from afadin(-/-) embryonic stem cells showed normal organization of the endoderm but disorganization of the ectoderm. Cell-cell AJs and tight junctions were improperly organized in the ectoderm of afadin(-/-) mice and embryoid bodies. These results indicate that afadin is highly expressed in the ectoderm- derived cells during embryogenesis and plays a key role in proper organization of AJs and tight junctions of the highly expressing cells, which is essential for proper tissue morphogenesis.
107. MULTIMERIN2 impairs tumor angiogenesis and growth by interfering with VEGF-A/VEGFR2 pathway. MULTIMERIN2 (MMRN2), also known as Endoglyx-1, is an extracellular matrix glycoprotein whose function has so far remained elusive. Given its specific localization in tight association with the endothelium we hypothesized that this protein could modulate neo-angiogenesis. By multiple assays we showed that MMRN2 significantly impaired endothelial cell (EC) **migration** and organization of a functional vessel network. The interaction of ECs with MMRN2 induced a striking impairment of VEGFR1 and VEGFR2 activation. We focused our attention on VEGFR2, a chief regulator of angiogenesis, and clarified that MMRN2 interfered with the VEGF/VEGFR2 axis through a direct binding with VEGF-A. This novel interaction was assessed in several assays and the affinity was estimated (Kd ∼50  nM). We next questioned whether the anti-angiogenic properties of MMRN2 could impair tumor growth. Although overexpression of MMRN2 by HT1080 cells did not affect their growth and apoptotic rate in vitro, it remarkably affected their growth in vivo. In fact, MMRN2-positive cells failed to efficiently grow and form well-vascularized tumors; a similar outcome was observed following treatment of established tumors with a MMRN2 adenoviral construct. Tumor-section immunostaining revealed a strong co-localization of VEGF-A with the ectopically expressed MMRN2. These novel findings suggest that VEGF may be sequestered by MMRN2 and be less available for the engagement to the receptors. Taken together these results highlight MMRN2 as a crucial player in the regulation of EC function, neo-angiogenesis and hence tumor growth. We hypothesize that secreted and deposited MMRN2 may function as a homeostatic barrier halting the sprouting of novel vessels, and suggest that these studies may embody the potential for the development of novel tools for cancer treatment.
108. Nonoverlapping expression patterns of ALK1 and ALK5 reveal distinct roles of each receptor in vascular development. Transforming growth factor beta (TGF-beta) transmits signals through a heterotetrameric cell-surface complex of type II (TGFBR2) and type I (activin receptor-like kinase 5, ALK5; TGFBR1) serine/threonine kinase receptors, as well as Smad2/3. We have previously shown that another type I receptor, ALK1 (ACVRL1), can also mediate TGF-beta signals via BMP-activated Smads in vascular endothelial cells (ECs). Our group and others have proposed the hypothesis that two TGF-beta signaling pathways via ALK1 and ALK5 in vascular ECs may play a balancing role for controlling the proliferation and **migration** of ECs during angiogenesis. To address in vivo roles of this balance in vascular development, we have created a knockin mouse line that carries a lacZ reporter in the Alk5 gene locus (Alk5(lacZ)). Throughout development, a well-defined, nonubiquitous expression pattern of Alk5 expression was observed in multiple tissues, and organs. Overall, a high level of Alk5 expression was found in perichondria, periostea, and the mesenchymal layers underlying epithelia in the kidney, lung, and gallbladder. In blood vessels, contrasting to predominant Alk1 expression in arterial endothelium, Alk5 expression was localized in the medial and adventitial layers of blood vessels, but was undetectable in the intimal layer. In addition, although Alk5-null embryos exhibit a defect in the formation of vascular smooth muscle layers, the lumens of blood vessels are generated properly, which stands in contrast to the severe dilation of the vascular lumens in Alk1-null mice. These mutually exclusive expression patterns of Alk1 and Alk5 in blood vessels, as well as the undisturbed formation of the vascular lumens in Alk5-null embryos, suggest that each type I receptor has its own unique functions in vascular development. The Alk5(lacZ) mice will be a valuable resource in identifying the in vivo cellular targets of TGF-beta family signals mediated by Alk5, both during embryonic development as well as in diverse pathological conditions.
109. The role of Notch signaling in diabetic endothelial progenitor cells dysfunction. To investigate the role of Notch signaling pathway in vasculogenic dysfunction of diabetic EPCs (DM-EPCs).The study was performed in mice and diabetes was induced with Streptozotocin. The functional consequences of Notch pathway modulation were studied by assessment of colony forming capacity (EPC colony forming assay), EPC differentiation capacity (% of definitive EPC-CFU (dEPC-CFU)), circulating EPCs (EPC culture assay) and migrated cells (**migration** assay); in the presence of Notch inhibitor (γ-secretase inhibitors (GSI)) compared to control. Notch pathway and VEGF involvement in DM- EPCs were assessed by gene expression (RT-qPCR).DM demonstrated to increase Notch pathway expression in bone marrow (BM) EPCs followed by lower EPC-CFU number, EPCs differentiation capacity, number of circulating EPCs, migrated cells and VEGF expression compared to control (p<0.05). Inhibition of Notch pathway by GSI rescued vasculogenic dysfunction in DM-EPCs as represented by increase in EPC-CFU number, differentiation capacity and number of circulating EPCs (p<0.05).Our findings indicate the involvement of Notch pathway in mediating DM-EPCs dysfunction including less number of EPC-CFU, circulating EPCs and migrated cell number compared to control. Further in vitro inhibition of Notch pathway by GSI rescued DM-EPC dysfunction. Therefore targeting Notch pathway in DM may provide a target to restore DM-EPC dysfunction.
110. Thrombospondin-1 modulates VEGF signaling via CD36 by recruiting SHP-1 to VEGFR2 complex in microvascular endothelial cells. Thrombospondin-1 (TSP-1) inhibits growth factor signaling at the receptor level in microvascular endothelial cells (MVEC), and CD36 has been suggested to be involved in this inhibition, but the mechanisms are not known. We hypothesized that CD36-TSP-1 interaction recruits Src homology 2 domain-containing protein tyrosine phosphatase (SHP)-1 to the vascular endothelial growth factor receptor 2 (VEGFR2) signaling complex and attenuates vascular endothelial growth factor (VEGF) signaling. Western blots of anti-CD36 and anti-VEGFR2 immunoprecipitates from VEGF-treated MVEC showed that exposure of the cells to a recombinant protein containing the CD36 binding domain of thrombospondin-1 (known as the TSR domain) induced association of SHP-1 with the VEGFR2/CD36 signaling complex and thereby suppressed VEGFR2 phosphorylation. SHP-1 phosphatase activity was increased in immunoprecipitated VEGFR2 complexes from TSR-treated cells. Silencing CD36 expression in MVEC by small interfering RNA (siRNA) or genetic deletion of cd36 in mice showed that TSR-induced SHP-1/VEGFR2 complex formation required CD36 in vitro and in vivo. Silencing SHP-1 expression in MVEC by siRNA abrogated TSR-mediated inhibition of VEGFR2 phosphorylation as well as TSR-mediated inhibition of VEGF-induced endothelial cell **migration** and tube formation. These studies reveal a SHP-1-mediated antiangiogenic pathway induced by CD36-TSP-1 interaction that inhibits VEGFR2 signaling and they provide a novel target to modulate angiogenesis therapeutically.
111. Spatial regulation of VEGF receptor endocytosis in angiogenesis. Activities as diverse as **migration**, proliferation and patterning occur simultaneously and in a coordinated fashion during tissue morphogenesis. In the growing vasculature, the formation of motile, invasive and filopodia-carrying endothelial sprouts is balanced with the stabilization of blood-transporting vessels. Here, we show that sprouting endothelial cells in the retina have high rates of VEGF uptake, VEGF receptor endocytosis and turnover. These internalization processes are opposed by atypical protein kinase C activity in more stable and mature vessels. aPKC phosphorylates Dab2, a clathrin-associated sorting protein that, together with the transmembrane protein ephrin-B2 and the cell polarity regulator PAR-3, enables VEGF receptor endocytosis and downstream signal transduction. Accordingly, VEGF receptor internalization and the angiogenic growth of vascular beds are defective in loss-of-function mice lacking key components of this regulatory pathway. Our work uncovers how vessel growth is dynamically controlled by local VEGF receptor endocytosis and the activity of cell polarity proteins.
112. Tissue morphogenesis and vascular stability require the Frem2 protein, product of the mouse myelencephalic blebs gene. Adhesive properties of cells undergoing morphogenetic rearrangements can be regulated either at the cellular level or by altering the environment in which rearrangements occur. Here, we describe the identification of a mutation (my(F11)) in the mouse extracellular matrix component Frem2, and provide evidence that suggests Frem2 expression creates an environment conducive to morphogenetic events. Loss of Frem2 function results in defects in developmental events associated with morphogenetic rearrangements of the vasculature and of tissues arising from all germ layers. The Frem2 transcript is restricted both spatially and temporally and appears in advance of cell rearrangement events. Thus, expression of Frem2 may dynamically alter the extracellular matrix to provide a substrate for cell **migration** and rearrangements during embryogenesis.
113. Loss of late primitive streak mesoderm and interruption of left-right morphogenesis in the Ednrb(s-1Acrg) mutant mouse. This study characterizes defects associated with abnormal mesoderm development in mouse embryos homozygous for the induced Ednrb(s-1Acrg) allele of the piebald deletion complex. The Ednrb(s-1Acrg) deletion results in recessive embryonic lethality and mutant embryos exhibit a truncated posterior body axis. The primitive streak and node become disfigured, consistent with evidence that cell **migration** is impaired in newly formed mesoderm. Additional defects related to mesoderm development include notochord degeneration, somite malformations, and abnormal vascular development. Arrested heart looping morphogenesis and a randomized direction of embryonic turning indicate that left-right development is also perturbed. The expression of nodal and leftb, Tgf-beta-related genes involved in a left-determinant signaling pathway, is variably lost in the left lateral plate mesoderm. Mutational analysis has demonstrated that Fgf8 and Brachyury (T) are required for normal mesoderm and left-right development and the asymmetric expression of nodal and leftb. Fgf8 expression in nascent mesoderm exiting the primitive streak is dramatically reduced in mutant embryos, and diminished T expression accompanies the progressive loss of paraxial, lateral, and primitive streak mesoderm. In contrast, axial mesoderm persists and T and nodal appear to be appropriately expressed in their specific domains in the node and notochord. We propose that this mutation disrupts a morphogenetic pathway, likely involving FGF signaling, important for the development of streak-derived posterior mesoderm and lateral morphogenesis.
114. ARNO regulates VEGF-dependent tissue responses by stabilizing endothelial VEGFR-2 surface expression. The vascular endothelial growth factor (VEGF) stimulates angiogenesis by induction of vessel permeability, proliferation, and **migration** of endothelial cells, an important process in ischaemic diseases. ADP-ribosylation factor (ARF) nucleotide-binding site opener (ARNO) (cytohesin-2) is a guanine exchange factor important for cellular signalling through ARF GTPases. However, a role for ARNO in VEGF-dependent endothelial processes has so far not been documented. Therefore, we investigated whether ARNO has a role in VEGF-dependent activation of endothelial cells and thus vessel permeability.ARNO expression was observed in endothelial cells in vitro by RT-PCR, western blotting, and immunofluorescence as well as ex vivo by immunohistochemical staining of mouse aorta. Treatment with the cytohesin inhibitor SecinH3 or with an ARNO siRNA prevented VEGF-dependent Akt activation, assessed by detection of phosphorylated Akt, and proliferation of endothelial cells in vitro, measured by methylthiazoletetrazolium (MTT) reduction. In addition, ARNO suppression reduced VEGF-induced permeability in vessels of the mouse (C57BL/6) cremaster muscle in vivo, as measured by extravasation of fluorescein isothiocyanate (FITC)-dextran. Moreover, ARNO knock-down accelerated ligand-induced reduction in vascular endothelial growth factor receptor-2 (VEGFR-2) surface expression, internalization, and degradation, as assessed by flow cytometry and western blotting, respectively.Our findings indicate an important and novel role for endothelial ARNO in VEGF-dependent initiation of angiogenesis by regulation of VEGFR-2 internalization in endothelial cells, resulting in the activation of the Akt pathway, vessel permeability, and ultimately endothelial proliferation. Thus, ARNO may be a new essential player in endothelial signalling and angiogenesis.
115. CXXC5 is a transcriptional activator of Flk-1 and mediates bone morphogenic protein-induced endothelial cell differentiation and vessel formation. CXXC5 is a member of a small subset of proteins containing CXXC-type zinc-finger domain. Here, we show that CXXC5 is a transcription factor activating Flk-1, a receptor for vascular endothelial growth factor. CXXC5 and Flk-1 were accumulated in nucli and membrane of mouse embryonic stem cells (mESCs), respectively, during their endothelial differentiation. CXXC5 overexpression induced Flk-1 transcription in both endothelium-differentiated mESCs and human umbilical vein endothelial cells (HUVECs). In vitro DNA binding assay showed direct interaction of CXXC5 on the Flk-1 promoter region, and mutation on its DNA-binding motif abolished transcriptional activity. We showed that bone morphorgenetic protein 4 (BMP4) induced CXXC5 transcription in the cells, and inhibitors of BMP signaling suppressed the CXXC5 induction and the consequent Flk-1 induction by BMP4 treatment. CXXC5 knockdown resulted in suppression of BMP4-induced stress fiber formation (56.8 ± 1.3% decrease, P<0.05) and **migratio**n (54.6 ± 1.9% decrease, P<0.05) in HUVECs. The in vivo roles of CXXC5 in BMP-signaling-specific vascular development and angiogenesis were shown by specific defect of caudal vein plex vessel formation (57.9 ± 11.8% decrease, P<0.05) in cxxc5 morpholino-injected zebrafish embryos and by suppression of BMP4-induced angiogenesis in subcutaneously injected Matrigel plugs in CXXC5(-/-) mice. Overall, CXXC5 is a transcriptional activator for Flk-1, mediating BMP signaling for differentiation a**nd migrat**ion of endothelial cell and vessel formation.
116. PTP1b is a physiologic regulator of vascular endothelial growth factor signaling in endothelial cells. Regulation of vascular endothelial growth factor receptor-2 (VEGFR2) signaling is a control point that determines the extent of vascular tree formation. Recent studies demonstrated an important role played by VEGFR2 endothelial trafficking in control of its activity and suggested the involvement of a phosphotyrosine phosphatase 1b (PTP1b) in this process. This study was designed to define the role of PTP1b in endothelial VEGFR2 signaling and its role in regulation of angiogenesis and arteriogenesis.We generated mice carrying an endothelial-specific deletion of PTP1b and examined the effect of this knockout on VEGF signaling, angiogenesis, and arteriogenesis in vitro and in vivo. PTP1b knockout endothelial cells had increased VEGF-dependent activation of extracellular signal-regulated kinase signaling, sprouting, **migration**, and proliferation compared with controls. Endothelial PTP1b null mice had increased retinal and Matrigel implant angiogenesis and accelerated wound healing, pointing to enhanced angiogenesis. Increased arteriogenesis was demonstrated by observations of faster recovery of arterial blood flow and large numbers of newly formed arterioles in the hindlimb ischemia mouse model. PTP1b endothelial knockout also rescued impaired blood flow recovery after common femoral artery ligation in synectin null mice.PTP1b is a key regulator of endothelial VEGFR2 signaling and plays an important role in regulation of the extent of vascular tree formation.
117. CXCR4 signaling regulates radial glial morphology and cell fate during embryonic spinal cord development. Embryonic meninges secrete the chemokine SDF-1/CXCL12 as a chemotactic guide for migrating neural stem cells, but SDF-1 is not known to directly regulate the functions of radial glia. Recently, the developing meninges have been shown to regulate radial glial function, yet the mechanisms and signals responsible for this phenomenon remain unclear. Moreover, as a nonmigratory cell type, radial glia do not conform to traditional models associated with chemokine signaling in the central nervous system. Using fluorescent transgenes, in vivo genetic manipulations and pharmacological techniques, we demonstrate that SDF-1 derived from the meninges exerts a CXCR4-dependent effect on radial glia. Deletion of CXCR4 expression by radial glia influences their morphology, mitosis, and progression through both oligodendroglial and astroglial lineages. Additionally, disruption of CXCR4 signaling in radial glia has a transient effect on the **migration** of oligodendrocyte progenitors. These data indicate that a specific chemokine signal derived from the meninges has multiple regulatory effects on radial glia.

#### GeneRIFs

1. CXXC5 is a transcriptional activator for Flk-1, mediating BMP signaling for differentiation and **migration** of endothelial cell and vessel formation.
2. cooperative signaling of VEGFR-2 and -3 is necessary for lymphatic **migration** and proliferation, but VEGFR-3 is redundant with VEGFR-2 for LEC organization into functional capillaries

### ECM

#### PubMed

1. Blood vessel tubulogenesis requires Rasip1 regulation of GTPase signaling. Cardiovascular function depends on patent blood vessel formation by endothelial cells (ECs). However, the mechanisms underlying vascular "tubulogenesis" are only beginning to be unraveled. We show that endothelial tubulogenesis requires the Ras interacting protein 1, Rasip1, and its binding partner, the RhoGAP Arhgap29. Mice lacking Rasip1 fail to form patent lumens in all blood vessels, including the early endocardial tube. Rasipl null angioblasts fail to properly localize the polarity determinant Par3 and display defective cell polarity, resulting in mislocalized junctional complexes and loss of adhesion to extracellular matrix (**ECM**). Similarly, depletion of either Rasip1 or Arhgap29 in cultured ECs blocks in vitro lumen formation, fundamentally alters the cytoskeleton, and reduces integrin-dependent adhesion to **ECM**. These defects result from increased RhoA/ROCK/myosin II activity and blockade of Cdc42 and Rac1 signaling. This study identifies Rasip1 as a unique, endothelial-specific regulator of Rho GTPase signaling, which is essential for blood vessel morphogenesis.
2. NRP1 Regulates CDC42 Activation to Promote Filopodia Formation in Endothelial Tip Cells. Sprouting blood vessels are led by filopodia-studded endothelial tip cells that respond to angiogenic signals. Mosaic lineage tracing previously revealed that NRP1 is essential for tip cell function, although its mechanistic role in tip cells remains poorly defined. Here, we show that NRP1 is dispensable for genetic tip cell identity. Instead, we find that NRP1 is essential to form the filopodial bursts that distinguish tip cells morphologically from neighboring stalk cells, because it enables the extracellular matrix (**ECM**)-induced activation of CDC42, a key regulator of filopodia formation. Accordingly, NRP1 knockdown and pharmacological CDC42 inhibition similarly impaired filopodia formation in vitro and in developing zebrafish in vivo. During mouse retinal angiogenesis, CDC42 inhibition impaired tip cell and vascular network formation, causing defects that resembled those due to loss of **ECM**-induced, but not VEGF-induced, NRP1 signaling. We conclude that NRP1 enables **ECM**-induced filopodia formation for tip cell function during sprouting angiogenesis.
3. O-fucosylation of thrombospondin type 1 repeats restricts epithelial to mesenchymal transition (EMT) and maintains epiblast pluripotency during mouse gastrulation. Thrombospondin type 1 repeat (TSR) superfamily members regulate diverse biological activities ranging from cell motility to inhibition of angiogenesis. In this study, we verified that mouse protein O-fucosyltransferase-2 (POFUT2) specifically adds O-fucose to TSRs. Using two Pofut2 gene-trap lines, we demonstrated that O-fucosylation of TSRs was essential for restricting epithelial to mesenchymal transition in the primitive streak, correct patterning of mesoderm, and localization of the definitive endoderm. Although Pofut2 mutant embryos established anterior/posterior polarity, they underwent extensive mesoderm differentiation at the expense of maintaining epiblast pluripotency. Moreover, mesoderm differentiation was biased towards the vascular endothelial cell lineage. Localization of Foxa2 and Cer1 expressing cells within the interior of Pofut2 mutant embryos suggested that POFUT2 activity was also required for the displacement of the primitive endoderm by definitive endoderm. Notably, Nodal, BMP4, Fgf8, and Wnt3 expression were markedly elevated and expanded in Pofut2 mutants, providing evidence that O-fucose modification of TSRs was essential for modulation of growth factor signaling during gastrulation. The ability of Pofut2 mutant embryos to form teratomas comprised of tissues from all three germ layer origins suggested that defects in Pofut2 mutant embryos resulted from abnormalities in the extracellular environment. This prediction is consistent with the observation that POFUT2 targets are constitutive components of the extracellular matrix (**ECM**) or associate with the **ECM**. For this reason, the Pofut2 mutants represent a valuable tool for studying the role of O-fucosylation in **ECM** synthesis and remodeling, and will be a valuable model to study how post-translational modification of **ECM** components regulates the formation of tissue boundaries, cell movements, and signaling.
4. Rasip1 regulates vertebrate vascular endothelial junction stability through Epac1-Rap1 signaling. Establishment and stabilization of endothelial tubes with patent lumens is vital during vertebrate development. Ras-interacting protein 1 (RASIP1) has been described as an essential regulator of de novo lumenogenesis through modulation of endothelial cell (EC) adhesion to the extracellular matrix (**ECM**). Here, we show that in mouse and zebrafish embryos, Rasip1-deficient vessels transition from an angioblast cord to a hollow tube, permit circulation of primitive erythrocytes, but ultimately collapse, leading to hemorrhage and embryonic lethality. Knockdown of RASIP1 does not alter EC-**ECM** adhesion, but causes cell-cell detachment and increases permeability of EC monolayers in vitro. We also found that endogenous RASIP1 in ECs binds Ras-related protein 1 (RAP1), but not Ras homolog gene family member A or cell division control protein 42 homolog. Using an exchange protein directly activated by cyclic adenosine monophosphate 1 (EPAC1)-RAP1-dependent model of nascent junction formation, we demonstrate that a fraction of the RASIP1 protein pool localizes to cell-cell contacts. Loss of RASIP1 phenocopies loss of RAP1 or EPAC1 in ECs by altering junctional actin organization, localization of the actin-bundling protein nonmuscle myosin heavy chain IIB, and junction remodeling. Our data show that RASIP1 regulates the integrity of newly formed blood vessels as an effector of EPAC1-RAP1 signaling.

### [Ee]xtracellular [Mm]atrix

#### PubMed

1. Fibronectin signals through integrin α5β1 to regulate cardiovascular development in a cell type-specific manner. Fibronectin (Fn1) is an evolutionarily conserve**d extracellular matr**ix glycoprotein essential for embryonic development. Global deletion of Fn1 leads to mid-gestation lethality from cardiovascular defects. However, severe morphogenetic defects that occur early in embryogenesis in these embryos precluded assigning a direct role for Fn1 in cardiovascular development. We noticed that Fn1 is expressed in strikingly non-uniform patterns during mouse embryogenesis, and that its expression is particularly enriched in the pharyngeal region corresponding with the pharyngeal arches 3, 4, and 6. This region bears a special importance for the developing cardiovascular system, and we hypothesized that the localized enrichment of Fn1 in the pharyngeal region may be essential for cardiovascular morphogenesis. To test this hypothesis, we ablated Fn1 using the Isl1(Cre) knock-in strain of mice. Deletion of Fn1 using the Isl1(Cre) strain resulted in defective formation of the 4th pharyngeal arch arteries (PAAs), aberrant development of the cardiac outflow tract (OFT), and ventricular septum defects. To determine the cell types responding to Fn1 signaling during cardiovascular development, we deleted a major Fn1 receptor, integrin α5 using the Isl1(Cre) strain, and observed the same spectrum of abnormalities seen in the Fn1 conditional mutants. Additional conditional mutagenesis studies designed to ablate integrin α5 in distinct cell types within the Isl1(+) tissues and their derivatives, suggested that the expression of integrin α5 in the pharyngeal arch mesoderm, endothelium, surface ectoderm and the neural crest were not required for PAA formation. Our studies suggest that an (as yet unknown) integrin α5-dependent signal extrinsic to the pharyngeal endothelium mediates the formation of the 4th PAAs.
2. Thymosin beta 4 is dispensable for murine cardiac development and function. Thymosin beta 4 (Tβ4) is a 43-amino acid factor encoded by an X-linked gene. Recent studies have suggested that Tβ4 is a key factor in cardiac development, growth, disease, epicardial integrity, and blood vessel formation. Cardiac-specific short hairpin (sh)RNA knockdown of tβ4 has been reported to result in embryonic lethality at E14.5-16.5, with severe cardiac and angiogenic defects. However, this shRNA tβ4-knockdown model did not completely abrogate Tβ4 expression. To completely ablate Tβ4 and to rule out the possibility of off-target effects associated with shRNA gene silencing, further studies of global or cardiac-specific knockouts are critical.We examined the role of Tβ4 in developing and adult heart through global and cardiac specific tβ4-knockout mouse models.Global tβ4-knockout mice were born at mendelian ratios and exhibited normal heart and blood vessel formation. Furthermore, in adult global tβ4-knockout mice, cardiac function, capillary density, expression of key cardiac fetal and angiogenic genes, epicardial marker expres**sion, and extracellu**lar matrix deposition were indistinguishable from that of controls. Tissue-specific tβ4-deficient mice, generated by crossing tβ4-floxed mice to Nkx2.5-Cre and αMHC-Cre, were also found to have no phenotype.We conclude that Tβ4 is dispensable for embryonic viability, heart development, coronary vessel development, and adult myocardial function.
3. Vascular endothelial growth factor coordinates islet innervation via vascular scaffolding. Neurovascular alignment is a common anatomical feature of organs, but the mechanisms leading to this arrangement are incompletely understood. Here, we show that vascular endothelial growth factor (VEGF) signaling profoundly affects both vascularization and innervation of the pancreatic islet. In mature islets, nerves are closely associated with capillaries, but the islet vascularization process during embryonic organogenesis significantly precedes islet innervation. Although a simple neuronal meshwork interconnects the developing islet clusters as they begin to form at E14.5, the substantial ingrowth of nerve fibers into islets occurs postnatally, when islet vascularization is already complete. Using genetic mouse models, we demonstrate that VEGF regulates islet innervation indirectly through its effects on intra-islet endothelial cells. Our data indicate that formation of a VEGF-directed, intra-islet vascular plexus is required for development of islet innervation, and that VEGF-induced islet hypervascularization leads to increased nerve fiber ingrowth. Transcriptome analysis of hypervascularized islets revealed an increased expression of **extracellular matrix** components and axon guidance molecules, with these transcripts being enriched in the islet-derived endothelial cell population. We propose a mechanism for coordinated neurovascular development within pancreatic islets, in which endocrine cell-derived VEGF directs the patterning of intra-islet capillaries during embryogenesis, forming a scaffold for the postnatal ingrowth of essential autonomic nerve fibers.
4. The forkhead box m1 transcription factor is essential for embryonic development of pulmonary vasculature. Transgenic and gene knock-out studies demonstrated that the mouse Forkhead Box m1 (Foxm1 or Foxm1b) transcription factor (previously called HFH-11B, Trident, Win, or MPP2) is essential for hepatocyte entry into mitosis during liver development, regeneration, and liver cancer. Targeted deletion of Foxm1 gene in mice produces an embryonic lethal phenotype due to severe abnormalities in the development of liver and heart. In this study, we show for the first time that Foxm1(-/-) lungs exhibit severe hypertrophy of arteriolar smooth muscle cells and defects in the formation of peripheral pulmonary capillaries as evidenced by significant reduction in platelet endothelial cell adhesion molecule 1 staining of the distal lung. Consistent with these findings, significant reduction in proliferation of the embryonic Foxm1(-/-) lung mesenchyme was found, yet proliferation levels were normal in the Foxm1-deficient epithelial cells. Severe abnormalities of the lung vasculature in Foxm1(-/-) embryos were associated with diminished expression of the transforming growth factor beta receptor II, a disintegrin and metalloprotease domain 17 (ADAM-17), vascular endothelial growth factor receptors, Polo-like kinase 1, Aurora B kinase, laminin alpha4 (Lama4), and the Forkhead Box f1 transcription factor. Cotransfection studies demonstrated that Foxm1 stimulates transcription of the Lama4 promoter, and this stimulation requires the Foxm1 binding sites located between -1174 and -1145 bp of the mouse Lama4 promoter. In summary, development of mouse lungs depends on the Foxm1 transcription factor, which regulates expression of genes essential for mesenchyme proliferation, **extracellular matrix** remodeling, and vasculogenesis.
5. Blood vessel tubulogenesis requires Rasip1 regulation of GTPase signaling. Cardiovascular function depends on patent blood vessel formation by endothelial cells (ECs). However, the mechanisms underlying vascular "tubulogenesis" are only beginning to be unraveled. We show that endothelial tubulogenesis requires the Ras interacting protein 1, Rasip1, and its binding partner, the RhoGAP Arhgap29. Mice lacking Rasip1 fail to form patent lumens in all blood vessels, including the early endocardial tube. Rasipl null angioblasts fail to properly localize the polarity determinant Par3 and display defective cell polarity, resulting in mislocalized junctional complexes and loss of adhesion to **extracellular matrix** (ECM). Similarly, depletion of either Rasip1 or Arhgap29 in cultured ECs blocks in vitro lumen formation, fundamentally alters the cytoskeleton, and reduces integrin-dependent adhesion to ECM. These defects result from increased RhoA/ROCK/myosin II activity and blockade of Cdc42 and Rac1 signaling. This study identifies Rasip1 as a unique, endothelial-specific regulator of Rho GTPase signaling, which is essential for blood vessel morphogenesis.
6. NRP1 Regulates CDC42 Activation to Promote Filopodia Formation in Endothelial Tip Cells. Sprouting blood vessels are led by filopodia-studded endothelial tip cells that respond to angiogenic signals. Mosaic lineage tracing previously revealed that NRP1 is essential for tip cell function, although its mechanistic role in tip cells remains poorly defined. Here, we show that NRP1 is dispensable for genetic tip cell identity. Instead, we find that NRP1 is essential to form the filopodial bursts that distinguish tip cells morphologically from neighboring stalk cells, because it enables the **extracellular matrix** (ECM)-induced activation of CDC42, a key regulator of filopodia formation. Accordingly, NRP1 knockdown and pharmacological CDC42 inhibition similarly impaired filopodia formation in vitro and in developing zebrafish in vivo. During mouse retinal angiogenesis, CDC42 inhibition impaired tip cell and vascular network formation, causing defects that resembled those due to loss of ECM-induced, but not VEGF-induced, NRP1 signaling. We conclude that NRP1 enables ECM-induced filopodia formation for tip cell function during sprouting angiogenesis.
7. Endothelial cell laminin isoforms, laminins 8 and 10, play decisive roles in T cell recruitment across the blood-brain barrier in experimental autoimmune encephalomyelitis. An active involvement of blood-brain barrier endothelial cell basement membranes in development of inflammatory lesions in the central nervous system (CNS) has not been considered to date. Here we investigated the molecular composition and possible function of the **extracellular matrix** encountered by extravasating T lymphocytes during experimental autoimmune encephalomyelitis (EAE). Endothelial basement membranes contained laminin 8 (alpha4beta1gamma1) and/or 10 (alpha5beta1gamma1) and their expression was influenced by proinflammatory cytokines or angiostatic agents. T cells emigrating into the CNS during EAE encountered two biochemically distinct basement membranes, the endothelial (containing laminins 8 and 10) and the parenchymal (containing laminins 1 and 2) basement membranes. However, inflammatory cuffs occurred exclusively around endothelial basement membranes containing laminin 8, whereas in the presence of laminin 10 no infiltration was detectable. In vitro assays using encephalitogenic T cell lines revealed adhesion to laminins 8 and 10, whereas binding to laminins 1 and 2 could not be induced. Downregulation of integrin alpha6 on cerebral endothelium at sites of T cell infiltration, plus a high turnover of laminin 8 at these sites, suggested two possible roles for laminin 8 in the endothelial basement membrane: one at the level of the endothelial cells resulting in reduced adhesion and, thereby, increased penetrability of the monolayer; and secondly at the level of the T cells providing direct signals to the transmigrating cells.
8. TIE1 and TIE2 receptor tyrosine kinases inversely regulate embryonic angiogenesis by the mechanism of intussusceptive microvascular growth. As shown previously, TIE1 and TIE2 receptor tyrosine kinases are specifically expressed in endothelial cells during embryonic angiogenesis. A detailed analysis of the vascular malformations of homozygous mice for a targeting mutation of both receptors was performed at the histological and cellular level. The data demonstrate that the TIE1 and TIE2 receptor inversely and concomitantly mediate interactions between endothelial cells with their **extracellular matrix** and with surrounding mesenchymal cells. These interactions are obviously crucial for normal endothelial cell motility and/or attachment and also for recruitment of periendothelial cells. The analysis of the TIE2-deficient embryos demonstrates how these cell/cell- and cell/matrix interactions subsequently influence the formation of normally structured tissue folds that divide the vessel lumen. They are also essential for the formation of vessel loops that compose a new vascular network and for the development of the ventricle in the heart. Fold and loop formation follow the principles of intussusceptive microvascular growth. The localization of the cardiovascular malformations corresponds to the temporal and spatial expression pattern of the TIE2 receptor. Angiopoietin-1, a ligand that activates the TIE2 receptor, is expressed in mesenchymal cells surrounding the endothelium. This local relationship is indicative of a paracrine regulation.
9. Histone deacetylase 7 maintains vascular integrity by repressing matrix metalloproteinase 10. Development and homeostasis of the cardiovascular system require intimate interactions between endothelial and smooth muscle cells, which form a seamless circulatory network. We show that histone deacetylase 7 (HDAC7) is specifically expressed in the vascular endothelium during early embryogenesis, where it maintains vascular integrity by repressing the expression of matrix metalloproteinase (MMP) 10, a secreted endoproteinase that degrades the **extracellular matrix**. Disruption of the HDAC7 gene in mice results in embryonic lethality due to a failure in endothelial cell-cell adhesion and consequent dilatation and rupture of blood vessels. HDAC7 represses MMP10 gene transcription by associating with myocyte enhancer factor-2 (MEF2), a direct activator of MMP10 transcription and essential regulator of blood vessel development. These findings reveal an unexpected and specific role for HDAC7 in the maintenance of vascular integrity and have important implications for understanding the processes of angiogenesis and vascular remodeling during cardiovascular development and disease.
10. The membrane-anchored MMP inhibitor RECK is a key regulator of **extracellular matrix** integrity and angiogenesis. Matrix metalloproteinases (MMPs) are essential for proper **extracellular matrix** remodeling. We previously found that a membrane-anchored glycoprotein, RECK, negatively regulates MMP-9 and inhibits tumor invasion and metastasis. Here we show that RECK regulates two other MMPs, MMP-2 and MT1-MMP, known to be involved in cancer progression, that mice lacking a functional RECK gene die around E10.5 with defects in collagen fibrils, the basal lamina, and vascular development, and that this phenotype is partially suppressed by MMP-2 null mutation. Also, vascular sprouting is dramatically suppressed in tumors derived from RECK-expressing fibrosarcoma cells grown in nude mice. These results support a role for RECK in the regulation of MMP-2 in vivo and implicate RECK downregulation in tumor angiogenesis.
11. Genetic and biochemical evidence that gastrulation defects in Pofut2 mutants result from defects in ADAMTS9 secretion. Protein O-fucosyltransferase 2 (POFUT2) adds O-linked fucose to Thrombospondin Type 1 Repeats (TSR) in 49 potential target proteins. Nearly half the POFUT2 targets belong to the A Disintegrin and Metalloprotease with ThromboSpondin type-1 motifs (ADAMTS) or ADAMTS-like family of proteins. Both the mouse Pofut2 RST434 gene trap allele and the Adamts9 knockout were reported to result in early embryonic lethality, suggesting that defects in Pofut2 mutant embryos could result from loss of O-fucosylation on ADAMTS9. To address this question, we compared the Pofut2 and Adamts9 knockout phenotypes and used Cre-mediated deletion of Pofut2 and Adamts9 to dissect the tissue-specific role of O-fucosylated ADAMTS9 during gastrulation. Disruption of Pofut2 using the knockout (LoxP) or gene trap (RST434) allele, as well as deletion of Adamts9, resulted in disorganized epithelia (epiblast, extraembryonic ectoderm, and visceral endoderm) and blocked mesoderm formation during gastrulation. The similarity between Pofut2 and Adamts9 mutants suggested that disruption of ADAMTS9 function could be responsible for the gastrulation defects observed in Pofut2 mutants. Consistent with this prediction, CRISPR/Cas9 knockout of POFUT2 in HEK293T cells blocked secretion of ADAMTS9. We determined that Adamts9 was dynamically expressed during mouse gastrulation by trophoblast giant cells, parietal endoderm, the most proximal visceral endoderm adjacent to the ectoplacental cone, extraembryonic mesoderm, and anterior primitive streak. Conditional deletion of either Pofut2 or Adamts9 in the epiblast rescues the gastrulation defects, and identified a new role for O-fucosylated ADAMTS9 during morphogenesis of the amnion and axial mesendoderm. Combined, these results suggested that loss of ADAMTS9 function in the extra embryonic tissue is responsible for gastrulation defects in the Pofut2 knockout. We hypothesize that loss of ADAMTS9 function in the most proximal visceral endoderm leads to slippage of the visceral endoderm and altered characteristics of the extraembryonic ectoderm. Consequently, loss of input from the extraembryonic ectoderm and/or compression of the epiblast by Reichert's membrane blocks gastrulation. In the future, the Pofut2 and Adamts9 knockouts will be valuable tools for understanding how local changes in the properties of the **extracellular matrix** influence the organization of tissues during mammalian development.
12. O-fucosylation of thrombospondin type 1 repeats restricts epithelial to mesenchymal transition (EMT) and maintains epiblast pluripotency during mouse gastrulation. Thrombospondin type 1 repeat (TSR) superfamily members regulate diverse biological activities ranging from cell motility to inhibition of angiogenesis. In this study, we verified that mouse protein O-fucosyltransferase-2 (POFUT2) specifically adds O-fucose to TSRs. Using two Pofut2 gene-trap lines, we demonstrated that O-fucosylation of TSRs was essential for restricting epithelial to mesenchymal transition in the primitive streak, correct patterning of mesoderm, and localization of the definitive endoderm. Although Pofut2 mutant embryos established anterior/posterior polarity, they underwent extensive mesoderm differentiation at the expense of maintaining epiblast pluripotency. Moreover, mesoderm differentiation was biased towards the vascular endothelial cell lineage. Localization of Foxa2 and Cer1 expressing cells within the interior of Pofut2 mutant embryos suggested that POFUT2 activity was also required for the displacement of the primitive endoderm by definitive endoderm. Notably, Nodal, BMP4, Fgf8, and Wnt3 expression were markedly elevated and expanded in Pofut2 mutants, providing evidence that O-fucose modification of TSRs was essential for modulation of growth factor signaling during gastrulation. The ability of Pofut2 mutant embryos to form teratomas comprised of tissues from all three germ layer origins suggested that defects in Pofut2 mutant embryos resulted from abnormalities in the extracellular environment. This prediction is consistent with the observation that POFUT2 targets are constitutive components of the **extracellular matrix** (ECM) or associate with the ECM. For this reason, the Pofut2 mutants represent a valuable tool for studying the role of O-fucosylation in ECM synthesis and remodeling, and will be a valuable model to study how post-translational modification of ECM components regulates the formation of tissue boundaries, cell movements, and signaling.
13. A novel Flk1-TVA transgenic mouse model for gene delivery to angiogenic vasculature. The genes that regulate the formation of blood vessels in adult tissues represent promising therapeutic targets because angiogenesis plays a role in many diseases, including cancer. We wished to develop a mouse model allowing characterization of gene function in adult angiogenic vasculature while minimizing effects on embryonic vasculature or adult quiescent vasculature. Here we describe a transgenic mouse model that allows expression of proteins in the endothelial cells of newly forming blood vessels in the adult using a selective retroviral gene delivery system. We generated transgenic mouse lines that express the TVA receptor for the RCAS avian-specific retrovirus from Flk1 gene regulatory elements that drive expression in proliferating endothelial cells. Several of these Flk1-TVA lines expressed TVA mRNA in the embryonic vasculature and TVA protein in new blood vessels growing into subcutaneous **extracellular matrix** implants in adult mice. In a Flk1-TVA line that was crossed with the MMTV-PyMT transgenic mammary tumor model, tumor endothelial cells also expressed the TVA protein. Furthermore, endothelial cells in **extracellular matrix** implants and the tumors of Flk1-TVA mice were susceptible to RCAS infection, as determined by expression of green fluorescent protein encoded by the virus. The Flk1-TVA mouse model in conjunction with the RCAS gene delivery system will be useful to study molecular mechanisms underlying adult forms of angiogenesis.
14. Rasip1 regulates vertebrate vascular endothelial junction stability through Epac1-Rap1 signaling. Establishment and stabilization of endothelial tubes with patent lumens is vital during vertebrate development. Ras-interacting protein 1 (RASIP1) has been described as an essential regulator of de novo lumenogenesis through modulation of endothelial cell (EC) adhesion to the **extracellular matrix** (ECM). Here, we show that in mouse and zebrafish embryos, Rasip1-deficient vessels transition from an angioblast cord to a hollow tube, permit circulation of primitive erythrocytes, but ultimately collapse, leading to hemorrhage and embryonic lethality. Knockdown of RASIP1 does not alter EC-ECM adhesion, but causes cell-cell detachment and increases permeability of EC monolayers in vitro. We also found that endogenous RASIP1 in ECs binds Ras-related protein 1 (RAP1), but not Ras homolog gene family member A or cell division control protein 42 homolog. Using an exchange protein directly activated by cyclic adenosine monophosphate 1 (EPAC1)-RAP1-dependent model of nascent junction formation, we demonstrate that a fraction of the RASIP1 protein pool localizes to cell-cell contacts. Loss of RASIP1 phenocopies loss of RAP1 or EPAC1 in ECs by altering junctional actin organization, localization of the actin-bundling protein nonmuscle myosin heavy chain IIB, and junction remodeling. Our data show that RASIP1 regulates the integrity of newly formed blood vessels as an effector of EPAC1-RAP1 signaling.
15. Sustained VEGF blockade results in microenvironmental sequestration of VEGF by tumors and persistent VEGF receptor-2 activation. Vascular endothelial growth factor (VEGF) blockade has been validated clinically as a treatment for human cancers, yet virtually all patients eventually develop progressive disease during therapy. In order to dissect this phenomenon, we examined the effect of sustained VEGF blockade in a model of advanced pediatric cancer. Treatment of late-stage hepatoblastoma xenografts resulted in the initial collapse of the vasculature and significant tumor regression. However, during sustained treatment, vessels recovered, concurrent with a striking increase in tumor expression of perlecan, a heparan sulfate proteoglycan. Whereas VEGF mRNA was expressed at the periphery of surviving clusters of tumor cells, both secreted VEGF and perlecan accumulated circumferential to central vessels. Vascular expression of heparanase, VEGF receptor-2 ligand binding, and receptor activation were concurrently maintained despite circulating unbound VEGF Trap. Endothelial survival signaling via Akt persisted. These findings provide a novel mechanism for vascular survival during sustained VEGF blockade and indicate a role for **extracellular matrix** molecules that sequester and release biologically active VEGF.
16. VEGF enhancement of osteoclast survival and bone resorption involves VEGF receptor-2 signaling and beta3-integrin. VEGF dependent angiogenesis is required for normal bone development and has been implicated in cancer metastasis to bone. These processes, while dependent on osteoclastic bone resorption, are reportedly mediated by endothelial cells, stromal osteoblasts, chondrocytes, and/or tumor cells. We demonstrate here that VEGF treatment of purified murine bone marrow osteoclast precursors directly enhances their survival, differentiation into mature osteoclasts, and resorptive activity. The actions of VEGF on mature osteoclasts principally involve the receptor VEGFR2 (Flk1, KDR), and the receptor signaling utilizes both the PI3-kinase-->Akt and MEK-->ERK pathways. Increased osteoclast survival and resorptive activity is correlated with VEGF-dependent phosphorylation of multiple downstream targets of activated Akt [glycogen synthase kinase, GSK-3beta; forkhead transcription factor, FKHR; and the Bcl-2 antagonist of cell death, Bad (Ser136)] and activated ERK1/2 [ribosomal S6 kinase, p90RSK; and Bad (Ser112)]. Expression of the VEGFR2 gene increases 20-fold during the 6 day in vitro differentiation of mature osteoclasts from mononuclear precursors, while alternate receptors VEGFR1 and neuropilin-1, decrease 30- and 3-fold respectively. Additionally, VEGF enhancement of osteoclast survival is diminished in cells prepared from beta3 integrin-deficient mice, thus associating VEGF signaling in osteoclasts with their attachment to **extracellular matrix**. Our results indicate that VEGF directly targets osteoclasts, thereby playing a novel role in bone development, angiogenesis, and tumor metastasis.
17. MULTIMERIN2 impairs tumor angiogenesis and growth by interfering with VEGF-A/VEGFR2 pathway. MULTIMERIN2 (MMRN2), also known as Endoglyx-1, is an **extracellular matrix** glycoprotein whose function has so far remained elusive. Given its specific localization in tight association with the endothelium we hypothesized that this protein could modulate neo-angiogenesis. By multiple assays we showed that MMRN2 significantly impaired endothelial cell (EC) migration and organization of a functional vessel network. The interaction of ECs with MMRN2 induced a striking impairment of VEGFR1 and VEGFR2 activation. We focused our attention on VEGFR2, a chief regulator of angiogenesis, and clarified that MMRN2 interfered with the VEGF/VEGFR2 axis through a direct binding with VEGF-A. This novel interaction was assessed in several assays and the affinity was estimated (Kd ∼50  nM). We next questioned whether the anti-angiogenic properties of MMRN2 could impair tumor growth. Although overexpression of MMRN2 by HT1080 cells did not affect their growth and apoptotic rate in vitro, it remarkably affected their growth in vivo. In fact, MMRN2-positive cells failed to efficiently grow and form well-vascularized tumors; a similar outcome was observed following treatment of established tumors with a MMRN2 adenoviral construct. Tumor-section immunostaining revealed a strong co-localization of VEGF-A with the ectopically expressed MMRN2. These novel findings suggest that VEGF may be sequestered by MMRN2 and be less available for the engagement to the receptors. Taken together these results highlight MMRN2 as a crucial player in the regulation of EC function, neo-angiogenesis and hence tumor growth. We hypothesize that secreted and deposited MMRN2 may function as a homeostatic barrier halting the sprouting of novel vessels, and suggest that these studies may embody the potential for the development of novel tools for cancer treatment.
18. Focal adhesion kinase is required for blood vessel morphogenesis. The nonreceptor tyrosine kinase focal adhesion kinase (FAK) is a point of convergence for signals from **extracellular matrix**, soluble factors, and mechanical stimuli. Targeted disruption of the fak gene in mice leads to death at embryonic day 8.5 (E8.5). FAK-/- embryos have severely impaired blood vessel development. Gene expression and in vitro differentiation studies revealed that endothelial cell differentiation was comparable in FAK-/- and wild-type E8.5 embryos. We examined the role of FAK in blood vessel morphogenesis using an in vitro tubulogenesis assay and three different culture systems: FAK+/+ and FAK-/- embryoid bodies, FAK+/+ and FAK-/- endothelial cells, and human umbilical vein endothelial cells expressing antisense FAK, a dominant-negative fragment of FAK, or wild-type FAK. In all of these systems, endothelial cells deficient in FAK expression or function displayed a severely reduced ability to form tubules in Matrigel. These studies demonstrate clearly that the vascular defects in FAK-/- mice result from the inability of FAK-deficient endothelial cells to organize themselves into vascular networks, rather than from defects in tissue-specific differentiation.
19. Tissue morphogenesis and vascular stability require the Frem2 protein, product of the mouse myelencephalic blebs gene. Adhesive properties of cells undergoing morphogenetic rearrangements can be regulated either at the cellular level or by altering the environment in which rearrangements occur. Here, we describe the identification of a mutation (my(F11)) in the mouse **extracellular matrix** component Frem2, and provide evidence that suggests Frem2 expression creates an environment conducive to morphogenetic events. Loss of Frem2 function results in defects in developmental events associated with morphogenetic rearrangements of the vasculature and of tissues arising from all germ layers. The Frem2 transcript is restricted both spatially and temporally and appears in advance of cell rearrangement events. Thus, expression of Frem2 may dynamically alter the **extracellular matrix** to provide a substrate for cell migration and rearrangements during embryogenesis.
20. Blood vessel endothelial VEGFR-2 delays lymphangiogenesis: an endogenous trapping mechanism links lymph- and angiogenesis. Angio- and lymphangiogenesis are inherently related processes. However, how blood and lymphatic vessels regulate each other is unknown. This work introduces a novel mechanism explaining the temporal and spatial relation of blood and lymphatic vessels. Vascular endothelial growth factor-A (VEGF-A) surprisingly reduced VEGF-C in the supernatant of blood vessel endothelial cells, suggesting growth factor (GF) clearance by the growing endothelium. The orientation of lymphatic sprouting toward angiogenic vessels and away from exogenous GFs was VEGF-C dependent. In vivo molecular imaging revealed higher VEGF receptor (R)-2 in angiogenic tips compared with normal vessels. Consistently, lymphatic growth was impeded in the angiogenic front. VEGF-C/R-2 complex in the cytoplasm of VEGF-A-treated endothelium indicated that receptor-mediated internalization causes GF clearance from the **extracellular matrix**. GF clearance by receptor-mediated internalization is a new paradigm explaining various characteristics of lymphatics.
21. Hand2 loss-of-function in Hand1-expressing cells reveals distinct roles in epicardial and coronary vessel development. The basic helix-loop-helix (bHLH) transcription factors Hand1 and Hand2 are essential for embryonic development. Given their requirement for cardiogenesis, it is imperative to determine their impact on cardiovascular function.To deduce the role of Hand2 within the epicardium.We engineered a Hand1 allele expressing Cre recombinase. Cardiac Hand1 expression is largely limited to cells of the primary heart field, overlapping little with Hand2 expression. Hand1 is expressed within the septum transversum, and the Hand1 lineage marks the proepicardial organ and epicardium. To examine Hand factor functional overlap, we conditionally deleted Hand2 from Hand1-expressing cells. Hand2 mutants display defective epicardialization and fail to form coronary arteries, coincident with altered **extracellular matrix** deposition and Pdgfr expression.These data demonstrate a hierarchal relationship whereby transient Hand1 septum transversum expression defines epicardial precursors that are subsequently dependent on Hand2 function.

#### GeneRIFs

1. These findings provide a novel mechanism for vascular survival during sustained VEGF blockade and indicate a role for **extracellular matrix** molecules that sequester and release biologically active VEGF.

### [Ii]nvasive

#### PubMed

1. Blimp1/Prdm1 governs terminal differentiation of endovascular trophoblast giant cells and defines multipotent progenitors in the developing placenta. Developmental arrest of Blimp1/Prdm1 mutant embryos at around embryonic day 10.5 (E10.5) has been attributed to placental disturbances. Here we investigate Blimp1/Prdm1 requirements in the trophoblast cell lineage. Loss of function disrupts specification of the **invasive** spiral artery-associated trophoblast giant cells (SpA-TGCs) surrounding maternal blood vessels and severely compromises the ability of the spongiotrophoblast layer to expand appropriately, secondarily causing collapse of the underlying labyrinth layer. Additionally, we identify a population of proliferating Blimp1(+) diploid cells present within the spongiotrophoblast layer. Lineage tracing experiments exploiting a novel Prdm1.Cre-LacZ allele demonstrate that these Blimp1(+) cells give rise to the mature SpA-TGCs, canal TGCs, and glycogen trophoblasts. In sum, the transcriptional repressor Blimp1/Prdm1 is required for terminal differentiation of SpA-TGCs and defines a lineage-restricted progenitor cell population contributing to placental growth and morphogenesis.
2. Conditional HIF-1 induction produces multistage neovascularization with stage-specific sensitivity to VEGFR inhibitors and myeloid cell independence. Neovascularization is a crucial component of tumor growth and ischemia. Although prior work primarily used disease models, delineation of neovascularization in the absence of disease can reveal intrinsic mechanisms of microvessel regulation amenable to manipulation in illness. We created a conditional model of epithelial HIF-1 induction in adult mice (TetON-HIF-1 mice). Longitudinal photoacoustic microscopy (L-PAM) was coincidentally developed for non**invasive**, label-free serial imaging of red blood cell-perfused vasculature in the same mouse for weeks to months. TetON-HIF-1 mice evidenced 3 stages of neovascularization: development, maintenance, and transgene-dependent regression. Regression occurred despite extensive and tight pericyte coverage. L-PAM mapped microvascular architecture and quantified volumetric changes in neocapillary morphogenesis, arteriovenous remodeling, and microvessel regression. Developmental stage endothelial proliferation down-regulation was associated with a DNA damage checkpoint consisting of p53, p21, and endothelial γ-H2AX induction. The neovasculature was temporally responsive to VEGFR2 immuno-blockade, with the developmental stage sensitive, and the maintenance stage resistant, to DC101 treatment. L-PAM analysis also pinpointed microvessels ablated or resistant to VEGFR2 immuno-blockade. HIF-1-recruited myeloid cells did not mediate VEGFR2 inhibitor resistance. Thus, HIF-1 neovascularization in the absence of disease is self-regulated via cell autonomous endothelial checkpoints, and resistant to angiogenesis inhibitors independent of myeloid cells.
3. VEGF inhibits tumor cell invasion and mesenchymal transition through a MET/VEGFR2 complex. Inhibition of VEGF signaling leads to a pro**invasive** phenotype in mouse models of glioblastoma multiforme (GBM) and in a subset of GBM patients treated with bevacizumab. Here, we demonstrate that vascular endothelial growth factor (VEGF) directly and negatively regulates tumor cell invasion through enhanced recruitment of the protein tyrosine phosphatase 1B (PTP1B) to a MET/VEGFR2 heterocomplex, thereby suppressing HGF-dependent MET phosphorylation and tumor cell migration. Consequently, VEGF blockade restores and increases MET activity in GBM cells in a hypoxia-independent manner, while inducing a program reminiscent of epithelial-to-mesenchymal transition highlighted by a T-cadherin to N-cadherin switch and enhanced mesenchymal features. Inhibition of MET in GBM mouse models blocks mesenchymal transition and invasion provoked by VEGF ablation, resulting in substantial survival benefit.
4. Hyperoxia causes regression of vitreous neovascularization by downregulating VEGF/VEGFR2 pathway. Neovascularization (NV) is a sight-threatening complication of retinal ischemia in diabetes, retinal vein occlusion, and retinopathy of prematurity. Current treatment modalities, including laser photocoagulation and repeated intraocular injection of VEGF antagonists, are **invasive** and not always effective, and may carry side effects. We studied the use of hyperoxia as an alternative therapeutic strategy for regressing established vitreous NV in a mouse model of oxygen-induced ischemic retinopathy.Hyperoxia treatment (HT, 75% oxygen) was initiated on postnatal day (P)17 after the onset of vitreous NV. Immunohistochemistry and quantitative PCR were used to assess retinal vascular changes in relation to apoptosis, and expression of VEGFR2 and inflammatory molecules. Effects of intravitreal injections of VEGF-A, VEGF-E, PlGF-1, and VEGF trap were also studied.HT selectively reduced NV by 70% within 24 hours. It robustly increased the level of cleaved caspase-3 in the vitreous NV between 6 and 18 hours and promoted infiltration of macrophage/microglial cells. The HT-induced apoptosis was preceded by a significant reduction in VEGFR2 expression within the NV and an increase in VEGFR2 within the surrounding neural tissue. Intravitreal VEGF-A and VEGF-E (VEGFR2 agonist) but not PlGF-1 (VEGFR1 agonist) prevented HT-induced apoptosis and regression of NV. In contrast, VEGF trap and VEGFR2 blockers mimicked the effect of HT. However, intravitreal VEGF trap induced increases in inflammatory molecules while HT did not have such unwanted effect.HT may be clinically useful to specifically treat proliferative NV in ischemic retinopathy.
5. R-Ras protein inhibits autophosphorylation of vascular endothelial growth factor receptor 2 in endothelial cells and suppresses receptor activation in tumor vasculature. Abnormal angiogenesis is associated with a broad range of medical conditions, including cancer. The formation of neovasculature with functionally defective blood vessels significantly impacts tumor progression, metastasis, and the efficacy of anticancer therapies. Vascular endothelial growth factor (VEGF) potently induces vascular permeability and vessel growth in the tumor microenvironment, and its inhibition normalizes tumor vasculature. In contrast, the signaling of the small GTPase R-Ras inhibits excessive angiogenic growth and promotes the maturation of regenerating blood vessels. R-Ras signaling counteracts VEGF-induced vessel sprouting, permeability, and **invasive** activities of endothelial cells. In this study, we investigated the effect of R-Ras on VEGF receptor 2 (VEGFR2) activation by VEGF, the key mechanism for angiogenic stimulation. We show that tyrosine phosphorylation of VEGFR2 is significantly elevated in the tumor vasculature and dermal microvessels of VEGF-injected skin in R-Ras knockout mice. In cultured endothelial cells, R-Ras suppressed the internalization of VEGFR2, which is required for full activation of the receptor by VEGF. Consequently, R-Ras strongly suppressed autophosphorylation of the receptor at all five major tyrosine phosphorylation sites. Conversely, silencing of R-Ras resulted in increased VEGFR2 phosphorylation. This effect of R-Ras on VEGFR2 was, at least in part, dependent on vascular endothelial cadherin. These findings identify a novel function of R-Ras to control the response of endothelial cells to VEGF and suggest an underlying mechanism by which R-Ras regulates angiogenesis.
6. Microultrasound molecular imaging of vascular endothelial growth factor receptor 2 in a mouse model of tumor angiogenesis. High-frequency microultrasound imaging of tumor progression in mice enables non**invasive** anatomic and functional imaging at excellent spatial and temporal resolution, although microultrasonography alone does not offer molecular scale data. In the current study, we investigated the use of microbubble ultrasound contrast agents bearing targeting ligands specific for molecular markers of tumor angiogenesis using high-frequency microultrasound imaging. A xenograft tumor model in the mouse was used to image vascular endothelial growth factor receptor 2 (VEGFR-2) expression with microbubbles conjugated to an anti-VEGFR-2 monoclonal antibody or an isotype control. Microultrasound imaging was accomplished at a center frequency of 40 MHz, which provided lateral and axial resolutions of 40 and 90 Im, respectively. The B-mode (two-dimensional mode) acoustic signal from microbubbles bound to the molecular target was determined by an ultrasound-based destruction-subtraction scheme. Quantification of the adherent microbubble fraction in nine tumor-bearing mice revealed significant retention of VEGFR-2-targeted microbubbles relative to control-targeted microbubbles. These data demonstrate that contrast-enhanced microultrasound imaging is a useful method for assessing molecular expression of tumor angiogenesis in mice at high resolution.
7. Notch alters VEGF responsiveness in human and murine endothelial cells by direct regulation of VEGFR-3 expression. The Notch family of cell surface receptors and its ligands are highly conserved proteins that regulate cell fate determination, including those involved in mammalian vascular development. We report that Notch induces VEGFR-3 expression in vitro in human endothelial cells and in vivo in mice. In vitro, Notch in complex with the DNA-binding protein CBF-1/suppressor of hairless/Lag1 (CSL) bound the VEGFR-3 promoter and transactivated VEGFR-3 specifically in endothelial cells. Through induction of VEGFR-3, Notch increased endothelial cell responsiveness to VEGF-C, promoting endothelial cell survival and morphological changes. In vivo, VEGFR-3 was upregulated in endothelial cells with active Notch signaling. Mice heterozygous for null alleles of both Notch1 and VEGFR-3 had significantly reduced viability and displayed midgestational vascular patterning defects analogous to Notch1 nullizygous embryos. We found that Notch1 and Notch4 were expressed in normal and tumor lymphatic endothelial cells and that Notch1 was activated in lymphatic endothelium of **invasive** mammary micropapillary carcinomas. These results demonstrate that Notch1 and VEGFR-3 interact genetically, that Notch directly induces VEGFR-3 in blood endothelial cells to regulate vascular development, and that Notch may function in tumor lymphangiogenesis.
8. Spatial regulation of VEGF receptor endocytosis in angiogenesis. Activities as diverse as migration, proliferation and patterning occur simultaneously and in a coordinated fashion during tissue morphogenesis. In the growing vasculature, the formation of motile, **invasive** and filopodia-carrying endothelial sprouts is balanced with the stabilization of blood-transporting vessels. Here, we show that sprouting endothelial cells in the retina have high rates of VEGF uptake, VEGF receptor endocytosis and turnover. These internalization processes are opposed by atypical protein kinase C activity in more stable and mature vessels. aPKC phosphorylates Dab2, a clathrin-associated sorting protein that, together with the transmembrane protein ephrin-B2 and the cell polarity regulator PAR-3, enables VEGF receptor endocytosis and downstream signal transduction. Accordingly, VEGF receptor internalization and the angiogenic growth of vascular beds are defective in loss-of-function mice lacking key components of this regulatory pathway. Our work uncovers how vessel growth is dynamically controlled by local VEGF receptor endocytosis and the activity of cell polarity proteins.
9. Antitumor activity of BIBF 1120, a triple angiokinase inhibitor, and use of VEGFR2+pTyr+ peripheral blood leukocytes as a pharmacodynamic biomarker in vivo. BIBF 1120 is a potent, orally available triple angiokinase inhibitor that inhibits VEGF receptors (VEGFR) 1, 2, and 3, fibroblast growth factor receptors, and platelet-derived growth factor receptors. This study examined the antitumor effects of BIBF 1120 on hepatocellular carcinoma (HCC) and attempted to identify a pharmacodynamic biomarker for use in early clinical trials.We evaluated the antitumor and antiangiogenic effects of BIBF 1120 against HCC cell line both in vitro and in vivo. For the pharmacodynamic study, the phosphorylation levels of VEGFR2 in VEGF-stimulated peripheral blood leukocytes (PBL) were evaluated in mice inoculated with HCC cells and treated with BIBF 1120.BIBF 1120 (0.01 μmol/L) clearly inhibited the VEGFR2 signaling in vitro. The direct growth inhibitory effects of BIBF 1120 on four HCC cell lines were relatively mild in vitro (IC(50) values: 2-5 μmol/L); however, the oral administration of BIBF 1120 (50 or 100 mg/kg/d) significantly inhibited the tumor growth and angiogenesis in a HepG2 xenograft model. A flow cytometric analysis revealed that BIBF 1120 significantly decreased the phosphotyrosine (pTyr) levels of VEGFR2(+)CD45(dim) PBLs and the percentage of VEGFR2(+)pTyr(+) PBLs in vivo; the latter parameter seemed to be a more feasible pharmacodynamic biomarker.We found that BIBF 1120 exhibited potent antitumor and antiangiogenic activity against HCC and identified VEGFR2(+)pTyr(+) PBLs as a feasible and n**oninvasi**ve pharmacodynamic biomarker in vivo.
10. A new PET tracer specific for vascular endothelial growth factor receptor 2. Non**invasive** positron emission tomography (PET) imaging of vascular endothelial growth factor receptor 2 (VEGFR-2) expression could be a valuable tool for evaluation of patients with a variety of malignancies, and particularly for monitoring those undergoing antiangiogenic therapies that block VEGF/VEGFR-2 function. The aim of this study was to develop a VEGFR-2-specific PET tracer.The D63AE64AE67A mutant of VEGF(121) (VEGF(DEE)) was generated by recombinant DNA technology. VEGF(121) and VEGF(DEE) were purified and conjugated with DOTA for (64)Cu labeling. The DOTA conjugates were tested in vitro for VEGFR-2 specificity and functional activity. In vivo tumor targeting efficacy and pharmacokinetics of (64)Cu-labeled VEGF(121) and VEGF(DEE) were compared using an orthotopic 4T1 murine breast tumor model. Blocking experiments, biodistribution studies, and immunofluorescence staining were carried out to confirm the non**invasive** imaging results.Cell binding assay demonstrated that VEGF(DEE) had about 20-fold lower VEGFR-1 binding affinity and only slightly lower VEGFR-2 binding affinity as compared with VEGF(121). MicroPET imaging studies revealed that both (64)Cu-DOTA-VEGF(121) and (64)Cu-DOTA-VEGF(DEE) had rapid and prominent activity accumulation in VEGFR-2-expressing 4T1 tumors. The renal uptake of (64)Cu-DOTA-VEGF(DEE) was significantly lower than that of (64)Cu-DOTA-VEGF(121) as rodent kidneys expressed high levels of VEGFR-1 based on immunofluorescence staining. Blocking experiments and biodistribution studies confirmed the VEGFR specificity of (64)Cu-DOTA-VEGF(DEE).We have developed a VEGFR-2-specific PET tracer, (64)Cu-DOTA-VEGF(DEE). It has comparable tumor targeting efficacy to (64)Cu-DOTA-VEGF(121) but much reduced renal toxicity. This tracer may be translated into the clinic for imaging tumor angiogenesis and monitoring antiangiogenic treatment efficacy.
11. Monitoring of the biological response to murine hindlimb ischemia with 64Cu-labeled vascular endothelial growth factor-121 positron emission tomography. Vascular endothelial growth factor-121 (VEGF121), an angiogenic protein secreted in response to hypoxic stress, binds to VEGF receptors (VEGFRs) overexpressed on vessels of ischemic tissue. The purpose of this study was to evaluate 64Cu-VEGF121 positron emission tomography for non**invasive** spatial, temporal, and quantitative monitoring of VEGFR2 expression in a murine model of hindlimb ischemia with and without treadmill exercise training.64Cu-labeled VEGF121 and a VEGF mutant were tested for VEGFR2 binding specificity in cell culture. Mice (n=58) underwent unilateral ligation of the femoral artery, and postoperative tissue ischemia was assessed with laser Doppler imaging. Longitudinal VEGFR2 expression in exercised and nonexercised mice was quantified with 64Cu-VEGF121 positron emission tomography at postoperative day 8, 15, 22, and 29 and correlated with postmortem gamma-counting. Hindlimbs were excised for immunohistochemistry, Western blotting, and microvessel density measurements. Compared with the VEGF mutant, VEGF121 showed specific binding to VEGFR2. Perfusion in ischemic hindlimbs fell to 9% of contralateral hindlimb on postoperative day 1 and recovered to 82% on day 29. 64Cu-VEGF121 uptake in ischemic hindlimbs increased significantly (P < 0.001) from a control level of 0.61+/-0.17% ID/g (percentage of injected dose per gram) to 1.62+/-0.35% ID/g at postoperative day 8, gradually decreased over the following 3 weeks (0.59+/-0.14% ID/g at day 29), and correlated with gamma-counting (R2 = 0.99). Compared with nonexercised mice, 64Cu-VEGF121 uptake was increased significantly (P < or = 0.0001) in exercised mice (at day 15, 22, and 29) and correlated with VEGFR2 levels as obtained by Western blotting (R2 = 0.76). Ischemic hindlimb tissue stained positively for VEGFR2. In exercised mice, microvessel density was increased significantly (P<0.001) compared with nonexercised mice.64Cu-VEGF121 positron emission tomography allows longitudinal spatial and quantitative monitoring of VEGFR2 expression in murine hindlimb ischemia and indirectly visualizes enhanced angiogenesis stimulated by treadmill exercise training.

### [Mm]etastasis

#### PubMed

1. Anti-PlGF inhibits growth of VEGF(R)-inhibitor-resistant tumors without affecting healthy vessels. Novel antiangiogenic strategies with complementary mechanisms are needed to maximize efficacy and minimize resistance to current angiogenesis inhibitors. We explored the therapeutic potential and mechanisms of alphaPlGF, an antibody against placental growth factor (PlGF), a VEGF homolog, which regulates the angiogenic switch in disease, but not in health. alphaPlGF inhibited growth and **metastasis** of various tumors, including those resistant to VEGF(R) inhibitors (VEGF(R)Is), and enhanced the efficacy of chemotherapy and VEGF(R)Is. alphaPlGF inhibited angiogenesis, lymphangiogenesis, and tumor cell motility. Distinct from VEGF(R)Is, alphaPlGF prevented infiltration of angiogenic macrophages and severe tumor hypoxia, and thus, did not switch on the angiogenic rescue program responsible for resistance to VEGF(R)Is. Moreover, it did not cause or enhance VEGF(R)I-related side effects. The efficacy and safety of alphaPlGF, its pleiotropic and complementary mechanism to VEGF(R)Is, and the negligible induction of an angiogenic rescue program suggest that alphaPlGF may constitute a novel approach for cancer treatment.
2. The membrane-anchored MMP inhibitor RECK is a key regulator of extracellular matrix integrity and angiogenesis. Matrix metalloproteinases (MMPs) are essential for proper extracellular matrix remodeling. We previously found that a membrane-anchored glycoprotein, RECK, negatively regulates MMP-9 and inhibits tumor invasion and **metastasis**. Here we show that RECK regulates two other MMPs, MMP-2 and MT1-MMP, known to be involved in cancer progression, that mice lacking a functional RECK gene die around E10.5 with defects in collagen fibrils, the basal lamina, and vascular development, and that this phenotype is partially suppressed by MMP-2 null mutation. Also, vascular sprouting is dramatically suppressed in tumors derived from RECK-expressing fibrosarcoma cells grown in nude mice. These results support a role for RECK in the regulation of MMP-2 in vivo and implicate RECK downregulation in tumor angiogenesis.
3. AIP1 Expression in Tumor Niche Suppresses Tumor Progression and **Metastasis**. Studies from tumor cells suggest that tumor-suppressor AIP1 inhibits epithelial-mesenchymal transition (EMT). However, the role of AIP1 in the tumor microenvironment has not been examined. We show that a global or vascular endothelial cell (EC)-specific deletion of the AIP1 gene in mice augments tumor growth and **metastasis** in melanoma and breast cancer models. AIP1-deficient vascular environment not only enhances tumor neovascularization and increases premetastatic niche formation, but also secretes tumor EMT-promoting factors. These effects from AIP1 loss are associated with increased VEGFR2 signaling in the vascular EC and could be abrogated by systemic administration of VEGFR2 kinase inhibitors. Mechanistically, AIP1 blocks VEGFR2-dependent signaling by directly binding to the phosphotyrosine residues within the activation loop of VEGFR2. Our data reveal that AIP1, by inhibiting VEGFR2-dependent signaling in tumor niche, suppresses tumor EMT switch, tumor angiogenesis, and tumor premetastatic niche formation to limit tumor growth and **metastasis**.
4. c-Myc is essential for vasculogenesis and angiogenesis during development and tumor progression. c-Myc promotes cell growth and transformation by ill-defined mechanisms. c-myc(-/-) mice die by embryonic day 10.5 (E10.5) with defects in growth and in cardiac and neural development. Here we report that the lethality of c-myc(-/-) embryos is also associated with profound defects in vasculogenesis and primitive erythropoiesis. Furthermore, c-myc(-/-) embryonic stem (ES) and yolk sac cells are compromised in their differentiative and growth potential. These defects are intrinsic to c-Myc, and are in part associated with a requirement for c-Myc for the expression of vascular endothelial growth factor (VEGF), as VEGF can partially rescue these defects. However, c-Myc is also required for the proper expression of other angiogenic factors in ES and yolk sac cells, including angiopoietin-2, and the angiogenic inhibitors thrombospondin-1 and angiopoietin-1. Finally, c-myc(-/-) ES cells are dramatically impaired in their ability to form tumors in immune-compromised mice, and the small tumors that sometimes develop are poorly vascularized. Therefore, c-Myc function is also necessary for the angiogenic switch that is indispensable for the progression and **metastasis** of tumors. These findings support the model wherein c-Myc promotes cell growth and transformation, as well as vascular and hematopoietic development, by functioning as a master regulator of angiogenic factors.
5. AIP1 Expression in Tumor Niche Suppresses Tumor Progression and **Metastasis**. Studies from tumor cells suggest that tumor-suppressor AIP1 inhibits epithelial-mesenchymal transition (EMT). However, the role of AIP1 in the tumor microenvironment has not been examined. We show that a global or vascular endothelial cell (EC)-specific deletion of the AIP1 gene in mice augments tumor growth and **metastasis** in melanoma and breast cancer models. AIP1-deficient vascular environment not only enhances tumor neovascularization and increases premetastatic niche formation, but also secretes tumor EMT-promoting factors. These effects from AIP1 loss are associated with increased VEGFR2 signaling in the vascular EC and could be abrogated by systemic administration of VEGFR2 kinase inhibitors. Mechanistically, AIP1 blocks VEGFR2-dependent signaling by directly binding to the phosphotyrosine residues within the activation loop of VEGFR2. Our data reveal that AIP1, by inhibiting VEGFR2-dependent signaling in tumor niche, suppresses tumor EMT switch, tumor angiogenesis, and tumor premetastatic niche formation to limit tumor growth and **metastasis**.
6. Vascular endothelial growth factor receptor 2 mediates macrophage infiltration into orthotopic pancreatic tumors in mice. Macrophages are an abundant inflammatory cell type in the tumor microenvironment that can contribute to tumor growth and **metastasis**. Macrophage recruitment into tumors is mediated by multiple cytokines, including vascular endothelial growth factor (VEGF), which is thought to function primarily through VEGF receptor (VEGFR) 1 expressed on macrophages. Macrophage infiltration is affected by VEGF inhibition. We show that selective inhibition of VEGFR2 reduced macrophage infiltration into orthotopic pancreatic tumors. Our studies show that tumor-associated macrophages express VEGFR2. Furthermore, peritoneal macrophages from tumor-bearing animals express VEGFR2, whereas peritoneal macrophages from non-tumor-bearing animals do not. To our knowledge, this is the first time that tumor-associated macrophages have been shown to express VEGFR2. Additionally, we found that the cytokine pleiotrophin is sufficient to induce VEGFR2 expression on macrophages. Pleiotrophin has previously been shown to induce expression of endothelial cell markers on macrophages and was present in the microenvironment of orthotopic pancreatic tumors. Finally, we show that VEGFR2, when expressed by macrophages, is essential for VEGF-stimulated migration of tumor-associated macrophages. In summary, tumor-associated macrophages express VEGFR2, and selective inhibition of VEGFR2 reduces recruitment of macrophages into orthotopic pancreatic tumors. Our results show an underappreciated mechanism of action that may directly contribute to the antitumor activity of angiogenesis inhibitors that block the VEGFR2 pathway.
7. Proprotein convertases promote processing of VEGF-D, a critical step for binding the angiogenic receptor VEGFR-2. Vascular endothelial growth factor (VEGF)-D is a secreted glycoprotein that induces angiogenesis and lymphangiogenesis. It consists of a central domain, containing binding sites for VEGF receptor-2 (VEGFR-2) and VEGFR-3, and N- and C-terminal propeptides. It is secreted from the cell as homodimers of the full-length form that can be proteolytically processed to remove the propeptides. It was recently shown, using adenoviral gene delivery, that fully processed VEGF-D induces angiogenesis in vivo, whereas full-length VEGF-D does not. To better understand these observations, we monitored the effect of VEGF-D processing on receptor binding using a full-length VEGF-D mutant that cannot be processed. This mutant binds VEGFR-2, the receptor signaling for angiogenesis, with approximately 17,000-fold lower affinity than mature VEGF-D, indicating the importance of processing for interaction with this receptor. Further, we show that members of the proprotein convertase (PC) family of proteases promote VEGF-D processing, which facilitates the VEGF-D/VEGFR-2 interaction. The PCs furin and PC5 promote cleavage of both propeptides, whereas PC7 promotes cleavage of the C-terminal propeptide only. The finding that PCs promote activation of VEGF-D and other proteins with roles in cancer such as matrix metalloproteinases, emphasizes the importance of these enzymes as potential regulators of tumor progression and **metastasis**.
8. Suppression of lymphangiogenesis by soluble vascular endothelial growth factor receptor-2 in a mouse lung cancer model. The vascular endothelial growth factor (VEGF) family has a key role in the formation of blood vessels and lymphatics. Among the members of this family, VEGF-C is one of the most important factors involved in lymphangiogenesis via binding with two receptors (vascular endothelial growth factor receptor-2 and -3: VEGFR-2 and VEGFR-3). Soluble VEGFR-2 (sVEGFR-2) has a role in maintaining the alymphatic state of the cornea associated with binding to VEGF-C, and selectively inhibits lymphangiogenesis but not angiogenesis. In this study, we introduced sVEGFR-2 into lung cancer cells and evaluated the influence on tumor progression and on genes regulating lymphatic formation and **metastasis** in vivo. A retroviral vector was used to introduce the sVEGFR-2 gene into Lewis lung carcinoma cells (LLC), which were designated as LLC-sVEGFR-2 cells. Proteins secreted into the culture supernatant by these cells were detected by western blotting using specific antibodies. To examine lymphangiogenesis by primary lung cancer in vivo, LLC-sVEGFR-2 cells were subcutaneously injected into C57BL/6 mice. At 14days after injection, immunohistochemistry was performed using an antibody directed against lymphatic vessel endothelial hyaluronan receptor 1 (LYVE-1), a marker of lymphatics. Expression of mRNA for VEGFR-2, VEGFR-3 and matrix metalloproteinases (MMPs) was also determined by real-time PCR. Furthermore, LLC-sVEGFR-2 cells were directly inoculated into the left lung in C57BL/6 mice and the number of micro-metastases in pulmonary lymph nodes was determined. Introduction of sVEGFR-2 into LLC cells resulted in secretion of sVEGFR-2 protein into the culture supernatant. There were fewer LYVE-1 positive lymphatics after inoculation of LLC-sVEGFR-2 into mice compared with the control group. In addition, VEGFR-2, VEGFR-3, and MMPs gene expression was suppressed in the primary tumors of the LLC-sVEGFR-2 group compared with the control group. Furthermore, there were fewer micro-metastases in the pulmonary lymph nodes of the LLC-sVEGFR-2 group compared with the control group after cells were directly inoculated into the lung. These findings indicate that introduction of sVEGFR-2 suppressed lymphangiogenesis in primary lung cancer and also suppressed lymphogenic **metastasis** by inhibiting VEGF-C, followed by down-regulation of VEGFR-2, VEGFR-3 and MMPs. Accordingly, sVEGFR-2 might be a promising target for treatment of cancer by regulating lymphangiogenesis and lymphogenic **metastasis**.
9. Identification of PDCL3 as a novel chaperone protein involved in the generation of functional VEGF receptor 2. Angiogenesis, a hallmark step in tumor **metastasis** and ocular neovascularization, is driven primarily by the function of VEGF ligand on one of its receptors, VEGF receptor 2 (VEGFR-2). Central to the proliferation and ensuing angiogenesis of endothelial cells, the abundance of VEGFR-2 on the surface of endothelial cells is essential for VEGF to recognize and activate VEGFR-2. We have identified phosducin-like 3 (PDCL3, also known as PhLP2A), through a yeast two-hybrid system, as a novel protein involved in the stabilization of VEGFR-2 by serving as a chaperone. PDCL3 binds to the juxtamembrane domain of VEGFR-2 and controls the abundance of VEGFR-2 by inhibiting its ubiquitination and degradation. PDCL3 increases VEGF-induced tyrosine phosphorylation and is required for VEGFR-2-dependent endothelial capillary tube formation and proliferation. Taken together, our data provide strong evidence for the role of PDCL3 in angiogenesis and establishes the molecular mechanism by which it regulates VEGFR-2 expression and function.
10. Active immunotherapy for mouse breast cancer with irradiated whole-cell vaccine expressing VEGFR2. As tumor-associated antigens are not well characterized for the majority of human tumors, polyvalent vaccines prepared with whole-tumor antigens are an attractive approach for tumor vaccination. Vascular endothelial growth factor receptor-2 (VEGFR2), as a model antigen with which to explore the feasibility of immunotherapy, has shown great promise as a tumor vaccine. However, the efficacy of immunotherapy is often not ideal when used alone. In this study, we explored the therapeutic efficacy of an irradiated AdVEGFR2-infected cell vaccine-based immunotherapy in the weakly immunogenic and highly metastatic 4T1 murine mammary cancer model. An adenovirus encoding the VEGFR2 gene (AdVEGFR2) was constructed. Lethally irradiated, virus-infected 4T1 cells were used as vaccines. Vaccination with lethally irradiated AdVEGFR2-infected 4T1 cells inhibited subsequent tumor growth and pulmonary **metastasis** compared with challenge inoculations. Angiogenesis was inhibited, and the number of CD8+ T lymphocytes was increased within the tumors. Antitumor activity was also caused by the adoptive transfer of isolated spleen lymphocytes. In vitro, the expression of HMGB1 and HSP70 in the AdVEGFR2‑infected 4T1 cells was increased, and was involved in the activation of tumor antigen-speciﬁc T-cell immunity. Our results indicate that the immunotherapy based on irradiated AdVEGFR2-infected whole-cancer cell vaccines may be a potentially effective strategy for 4T1 cancer treatment.
11. Notch, IL-1 and leptin crosstalk outcome (NILCO) is critical for leptin-induced proliferation, migration and VEGF/VEGFR-2 expression in breast cancer. High levels of pro-angiogenic factors, leptin, IL-1, Notch and VEGF (ligands and receptors), are found in breast cancer, which is commonly correlated with **metastasis** and lower survival of patients. We have previously reported that leptin induces the growth of breast cancer and the expression of VEGF/VEGFR-2 and IL-1 system. We hypothesized that Notch, IL-1 and leptin crosstalk outcome (NILCO) plays an essential role in the regulation of leptin-mediated induction of proliferation/migration and expression of pro-angiogenic molecules in breast cancer. To test this hypothesis, leptin's effects on the expression and activation of Notch signaling pathway and VEGF/VEGFR-2/IL-1 were determined in mouse (4T1, EMT6 and MMT) breast cancer cells. Remarkably, leptin up-regulated Notch1-4/JAG1/Dll-4, Notch target genes: Hey2 and survivin, together with IL-1 and VEGF/VEGFR-2. RNA knockdown and pharmacological inhibitors of leptin signaling significantly abrogated activity of reporter gene-luciferase CSL (RBP-Jk) promoter, showing that it was linked to leptin-activated JAK2/STAT3, MAPK, PI-3K/mTOR, p38 and JNK signaling pathways. Interestingly, leptin upregulatory effects on cell proliferation/migration and pro-angiogenic factors Notch, IL-1 and VEGF/VEGFR-2 were abrogated by a γ-secretase inhibitor, DAPT, as well as siRNA against CSL. In addition, blockade of IL-1R tI inhibited leptin-induced Notch, Hey2 and survivin as well as VEGF/VEGFR-2 expression. These data suggest leptin is an inducer of Notch (expression/activation) and IL-1 signaling modulates leptin effects on Notch and VEGF/VEGFR-2. We show for the first time that a novel unveiled crosstalk between Notch, IL-1 and leptin (NILCO) occurs in breast cancer. Leptin induction of proliferation/migration and upregulation of VEGF/VEGFR-2 in breast cancer cells were related to an intact Notch signaling axis. NILCO could represent the integration of developmental, pro-inflammatory and pro-angiogenic signals critical for leptin-induced cell proliferation/migration and regulation of VEGF/VEGFR-2 in breast cancer. Targeting NILCO might help to design new pharmacological strategies aimed at controlling breast cancer growth and angiogenesis.
12. Inhibition of multiple vascular endothelial growth factor receptors (VEGFR) blocks lymph node metastases but inhibition of VEGFR-2 is sufficient to sensitize tumor cells to platinum-based chemotherapeutics. Vascular endothelial growth factor receptors (VEGFR) have important roles in cancer, affecting blood and lymphatic vessel functionality as well as tumor cells themselves. We compared the efficacy of a VEGFR tyrosine kinase inhibitor, PTK787/ZK222584 (PTK/ZK), which targets the three VEGFRs, with blocking antibodies directed against VEGFR-2 (DC101) or VEGF-A (Pab85618) in a metastatic melanoma model. Although all inhibitors exerted comparable effects on primary tumor growth, only PTK/ZK significantly reduced lymph node **metastasis** formation. A comparable decrease in lymphatic vessel density following blockade of VEGFR-2 (DC101) or the three VEGFRs (PTK/ZK) was observed in the metastases. However, the functionality of lymphatics surrounding the primary tumor was more significantly disrupted by PTK/ZK, indicating the importance of multiple VEGFRs in the metastatic process. The antimetastatic properties of PTK/ZK were confirmed in a breast carcinoma model. B16/BL6 tumor cells express VEGF ligands and their receptors. Blockade of a VEGFR-1 autocrine loop with PTK/ZK inhibited tumor cell migration. Furthermore, the tumor cells also showed enhanced sensitivity to platinum-based chemotherapy in combination with PTK/ZK, indicating that autocrine VEGFRs are promoting tumor cell migration and survival. In summary, our results suggest that, in addition to blocking angiogenesis, combined inhibition of the three VEGFRs may more efficiently target other aspects of tumor pathophysiology, including lymphatic vessel functionality, tumor cell dissemination, survival pathways, and response to chemotherapeutic compounds.
13. VEGF enhancement of osteoclast survival and bone resorption involves VEGF receptor-2 signaling and beta3-integrin. VEGF dependent angiogenesis is required for normal bone development and has been implicated in cancer **metastasis** to bone. These processes, while dependent on osteoclastic bone resorption, are reportedly mediated by endothelial cells, stromal osteoblasts, chondrocytes, and/or tumor cells. We demonstrate here that VEGF treatment of purified murine bone marrow osteoclast precursors directly enhances their survival, differentiation into mature osteoclasts, and resorptive activity. The actions of VEGF on mature osteoclasts principally involve the receptor VEGFR2 (Flk1, KDR), and the receptor signaling utilizes both the PI3-kinase-->Akt and MEK-->ERK pathways. Increased osteoclast survival and resorptive activity is correlated with VEGF-dependent phosphorylation of multiple downstream targets of activated Akt [glycogen synthase kinase, GSK-3beta; forkhead transcription factor, FKHR; and the Bcl-2 antagonist of cell death, Bad (Ser136)] and activated ERK1/2 [ribosomal S6 kinase, p90RSK; and Bad (Ser112)]. Expression of the VEGFR2 gene increases 20-fold during the 6 day in vitro differentiation of mature osteoclasts from mononuclear precursors, while alternate receptors VEGFR1 and neuropilin-1, decrease 30- and 3-fold respectively. Additionally, VEGF enhancement of osteoclast survival is diminished in cells prepared from beta3 integrin-deficient mice, thus associating VEGF signaling in osteoclasts with their attachment to extracellular matrix. Our results indicate that VEGF directly targets osteoclasts, thereby playing a novel role in bone development, angiogenesis, and tumor **metastasis**.
14. R-Ras protein inhibits autophosphorylation of vascular endothelial growth factor receptor 2 in endothelial cells and suppresses receptor activation in tumor vasculature. Abnormal angiogenesis is associated with a broad range of medical conditions, including cancer. The formation of neovasculature with functionally defective blood vessels significantly impacts tumor progression, **metastasis**, and the efficacy of anticancer therapies. Vascular endothelial growth factor (VEGF) potently induces vascular permeability and vessel growth in the tumor microenvironment, and its inhibition normalizes tumor vasculature. In contrast, the signaling of the small GTPase R-Ras inhibits excessive angiogenic growth and promotes the maturation of regenerating blood vessels. R-Ras signaling counteracts VEGF-induced vessel sprouting, permeability, and invasive activities of endothelial cells. In this study, we investigated the effect of R-Ras on VEGF receptor 2 (VEGFR2) activation by VEGF, the key mechanism for angiogenic stimulation. We show that tyrosine phosphorylation of VEGFR2 is significantly elevated in the tumor vasculature and dermal microvessels of VEGF-injected skin in R-Ras knockout mice. In cultured endothelial cells, R-Ras suppressed the internalization of VEGFR2, which is required for full activation of the receptor by VEGF. Consequently, R-Ras strongly suppressed autophosphorylation of the receptor at all five major tyrosine phosphorylation sites. Conversely, silencing of R-Ras resulted in increased VEGFR2 phosphorylation. This effect of R-Ras on VEGFR2 was, at least in part, dependent on vascular endothelial cadherin. These findings identify a novel function of R-Ras to control the response of endothelial cells to VEGF and suggest an underlying mechanism by which R-Ras regulates angiogenesis.
15. Mannan-modified adenovirus encoding VEGFR-2 as a vaccine to induce anti-tumor immunity. Dendritic cell (DC) vaccines are a promising immunotherapeutic approach for treatment and prevention of cancer. While this methodology is widely accepted, it also has some limitations. Antigen-presenting cells including DCs express the mannan receptor (MR). The delivery of a mannan-modified tumor antigen to the MR has been demonstrated to be efficient. Vascular endothelial growth factor receptor-2 (VEGFR-2) is mainly responsible for angiogenesis and tumor growth. The goal of our study was to deliver VEGFR-2 to DCs by means of mannan-modified adenovirus.VEGFR-2 recombinant adenovirus modified with oxidized mannan was constructed as a tumor vaccine to immunize mice in vivo. IFN-γ in mouse sera and spleen was detected by ELISA and ELISPOT. The killing activity of cytotoxic T lymphocyte (CTL) against VEGFR-2 was measured with a lactate dehydrogenase assay. Vessel densities in tumor tissues were detected by immunohistochemistry. Flow cytometry was used to test CD4(+) and CD8(+) T-cell counts in tumor tissues.The vaccine exhibited both protective and therapeutic efficacy in the inhibition of tumor growth and markedly prolonged survival in mice. Protection against **metastasi**s was also observed. Furthermore, vaccination led to greater IFN-γ and VEGFR-2-specific CTLs. The specific immunity resulted in the suppression of angiogenesis and an increase in CD8(+) cells in tumor tissues.Oxidized mannan-modified adenovirus expressing VEGFR-2 could extraordinarily stimulate both protective and therapeutic immune response in a mice model. Our data suggest that the combination of cancer immunity and anti-angiogenesis via modified mannan is a promising strategy in tumor prophylaxis and therapy.
16. Social defeat stress promotes tumor growth and angiogenesis by upregulating vascular endothelial growth factor/extracellular signal-regulated kinase/matrix metalloproteinase signaling in a mouse model of lung carcinoma. Numerous epidemiological and experimental animal studies have indicated that chronic psychological stress may promote tumor development. However, the underlying molecular mechanisms by which chronic stress promotes tumorigenesis remain to be fully elucidated and animal models have not yet been well established. In the present study, an established mouse model of repeated social defeat stress (RSDS), was generated and used to investigate the effect of stress on tumor growth and **metastasis**. C57BL/6 mice were exposed to RSDS for 10 days, followed by subcutaneousl inoculation with Lewis lung carcinoma cells for seven days. The tumor weight and volume as well as the number of the lung metastatic nodules were then determined. Vascular endothelial growth factor (VEGF) serum levels were measured using ELISAs. In addition, expression levels of VEGF receptor (VEGFR) and L1 cell adhesion molecule (L1CAM) messenger (m)RNA were confirmed using reverse transcription quantitative polymerase chain reaction. Furthermore, protein expression levels of phosphorlyated extracellular signal-regulated kinase (pERK), matrix metalloproteinase (MMP)-2 and MMP-9 were examined using western blot analysis. The results showed that RSDS significantly increased the weight and the volume of the primary tumor as well as the number of the lung metastatic nodules. Serum VEGF levels were significantly higher in the tumor-stress group compared with those of the unstressed tumor mice. In addition, tumors in stressed animals demonstrated markedly enhanced expression of VEGFR-2 and L1CAM mRNA as well as pERK, MMP-2 and MMP-9 protein expression. In conclusion, these results suggested that RSDS contributed to lung cancer progression, angiogenesis and **metastasis**, which was partially associated with increased VEGF secretion and therefore the activation of the ERK signaling pathway, resulting in the induction of MMP-2 and MMP-9 protein expression.
17. Rap1 promotes VEGFR2 activation and angiogenesis by a mechanism involving integrin αvβ₃. Vascular endothelial growth factor (VEGF) acting through VEGF receptor 2 (VEGFR2) on endothelial cells (ECs) is a key regulator of angiogenesis, a process essential for wound healing and tu**mor metast**asis. Rap1a and Rap1b, 2 highly homologous small G proteins, are both required for angiogenesis in vivo and for normal EC responses to VEGF. Here we sought to determine the mechanism through which Rap1 promotes VEGF-mediated angiogenesis. Using lineage-restricted Rap1-knockout mice we show that Rap1-deficiency in endothelium leads to defective angiogenesis in vivo, in a dose-dependent manner. Using ECs obtained from Rap1-deficient mice we demonstrate that Rap1b promotes VEGF-VEGFR2 kinase activation and regulates integrin activation. Importantly, the Rap1b-dependent VEGF-VEGFR2 activation is in part mediated via integrin α(v)β(3). Furthermore, in an in vivo model of zebrafish angiogenesis, we demonstrate that Rap1b is essential for the sprouting of intersomitic vessels, a process known to be dependent on VEGF signaling. Using 2 distinct pharmacologic VEGFR2 inhibitors we show that Rap1b and VEGFR2 act additively to control angiogenesis in vivo. We conclude that Rap1b promotes VEGF-mediated angiogenesis by promoting VEGFR2 activation in ECs via integrin α(v)β(3). These results provide a novel insight into the role of Rap1 in VEGF signaling in ECs.
18. Motif mimetic of epsin perturbs tumor growth and **metastasis**. Tumor angiogenesis is critical for cancer progression. In multiple murine models, endothelium-specific epsin deficiency abrogates tumor progression by shifting the balance of VEGFR2 signaling toward uncontrolled tumor angiogenesis, resulting in dysfunctional tumor vasculature. Here, we designed a tumor endothelium-targeting chimeric peptide (UPI) for the purpose of inhibiting endogenous tumor endothelial epsins by competitively binding activated VEGFR2. We determined that the UPI peptide specifically targets tumor endothelial VEGFR2 through an unconventional binding mechanism that is driven by unique residues present only in the epsin ubiquitin-interacting motif (UIM) and the VEGFR2 kinase domain. In murine models of neoangiogenesis, UPI peptide increased VEGF-driven angiogenesis and neovascularization but spared quiescent vascular beds. Further, in tumor-bearing mice, UPI peptide markedly impaired functional tumor angiogenesis, tumor growth, and **metastasis**, resulting in a notable increase in survival. Coadministration of UPI peptide with cytotoxic chemotherapeutics further sustained tumor inhibition. Equipped with localized tumor endothelium-specific targeting, our UPI peptide provides potential for an effective and alternative cancer therapy.
19. Isolation of cardiac cells from E8.5 yolk sac by ALCAM (CD166) expression. It is known that the adhesion molecule ALCAM (CD166) mediates **metastasis** of malignant cells and organogenesis in embryos. We show here that embryonic day 8.5 (E8.5) murine yolk sac cells express ALCAM protein and that ALCAM expression can be used to define endothelial and cardiac precursors from hematopoietic precursors in E8.5 yolk sacs. ALCAM high+ cells exclusively give rise to endothelial and cardiac cells in matrigel assays but generate no hematopoietic colonies in methylcellulose assays. ALCAM low+ and ALCAM- populations predominantly give rise to hematopoietic cells in methylcellulose, but do not generate any cell clusters in matrigel. The ALCAM high+ population contains both Flk-1+ and Flk-1- cells. The former population exclusively contains endothelial cells whereas the latter give rise to cardiac cells when cultured on OP9 stromal cells. We also show that cardiac lineage marker genes such as Nkx-2.5, and the endothelial marker VE-cadherin are expressed in the ALCAM high+ fraction, whereas the hematopoietic marker GATA1 and Runx1 are expressed in the ALCAM low+/- fraction. However, we did not detect expression of the cardiac structural protein cTn-T in cells from yolk sac cells until these had had been differentiated on OP9 for 5 days. Altogether, these results indicate that cells retaining a potential to differentiate to the cardiac lineage are present in E8.5 yolk sacs and can be isolated as ALCAM high+, Flk-1- cells. Our report provides novel insights into the origin and differentiation process of cardiac cells in the formation of the circulatory system.

#### GeneRIFs

1. Introduction of sVEGFR-2 suppressed lymphangiogenesis in primary lung cancer and also suppressed lymphogenic **metastasis** by inhibiting VEGF-C, followed by down-regulation of VEGFR-2, VEGFR-3 and MMPs.
2. Our data reveal that AIP1, by inhibiting VEGFR2-dependent signaling in tumor niche, suppresses tumor EMT switch, tumor angiogenesis, and tumor premetastatic niche formation to limit tumor growth and **metastasis**.

### [Cc]ell [Ii]nvasion

#### PubMed

1. CXCL1 induced by prostaglandin E2 promotes angiogenesis in colorectal cancer. Chronic inflammation is a well-known risk factor for cancer. Proinflammatory mediators such as prostaglandin E2 (PGE2) promote colorectal tumor growth by stimulating angiogenesis, **cell invasion**, and cell growth, and inhibiting apoptosis. Molecules that regulate tumor-associated angiogenesis provide promising therapeutic targets for treatment of colorectal cancer (CRC) as indicated by the recent development of the novel anti-angiogenic agent bevacizumab (Avastin). However, use of this drug only prolongs survival by several months, highlighting the importance of finding more effective treatment regimens. We report here that PGE2 induces expression of CXCL1 (growth-regulated oncogene alpha), a pro-angiogenic chemokine, in human CRC cells. More importantly, CXCL1 released from carcinoma cells induces microvascular endothelial cell migration and tube formation in vitro. Furthermore, PGE2 promotes tumor growth in vivo by induction of CXCL1 expression, which results in increased tumor microvessel formation. These results have potential clinical significance because we found that CXCL1 expression correlates with PGE2 levels in human CRCs. Collectively, our findings show for the first time that CXCL1 is regulated by PGE2 and indicate that CXCL1 inhibitors should be evaluated further as potential anti-angiogenic agents for treatment of CRC.
2. Thyroid follicle development requires Smad1/5- and endothelial cell-dependent basement membrane assembly. Thyroid follicles, the functional units of the thyroid gland, are delineated by a monolayer of thyrocytes resting on a continuous basement membrane. The developmental mechanisms of folliculogenesis, whereby follicles are formed by the reorganization of a non-structured mass of non-polarized epithelial cells, are largely unknown. Here we show that assembly of the epithelial basement membrane is crucial for folliculogenesis and is controlled by endothelial **cell invasion** and by BMP-Smad signaling in thyrocytes. Thyroid-specific Smad1 and Smad5 double-knockout (Smad1/5(dKO)) mice displayed growth retardation, hypothyroidism and defective follicular architecture. In Smad1/5(dKO) embryonic thyroids, epithelial cells remained associated in large clusters and formed small follicles. Although similar follicular defects are found in Vegfa knockout (Vegfa(KO)) thyroids, Smad1/5(dKO) thyroids had normal endothelial cell density yet impaired endothelial differentiation. Interestingly, both Vegfa(KO) and Smad1/5(dKO) thyroids displayed impaired basement membrane assembly. Furthermore, conditioned medium (CM) from embryonic endothelial progenitor cells (eEPCs) rescued the folliculogenesis defects of both Smad1/5(dKO) and Vegfa(KO) thyroids. Laminin α1, β1 and γ1, abundantly released by eEPCs into CM, were crucial for folliculogenesis. Thus, epithelial Smad signaling and endotheli**al cell invas**ion promote folliculogenesis via assembly of the basement membrane.
3. VEGF inhibits tumor **cell invasion** and mesenchymal transition through a MET/VEGFR2 complex. Inhibition of VEGF signaling leads to a proinvasive phenotype in mouse models of glioblastoma multiforme (GBM) and in a subset of GBM patients treated with bevacizumab. Here, we demonstrate that vascular endothelial growth factor (VEGF) directly and negatively regulates tumor **cell invasion** through enhanced recruitment of the protein tyrosine phosphatase 1B (PTP1B) to a MET/VEGFR2 heterocomplex, thereby suppressing HGF-dependent MET phosphorylation and tumor cell migration. Consequently, VEGF blockade restores and increases MET activity in GBM cells in a hypoxia-independent manner, while inducing a program reminiscent of epithelial-to-mesenchymal transition highlighted by a T-cadherin to N-cadherin switch and enhanced mesenchymal features. Inhibition of MET in GBM mouse models blocks mesenchymal transition and invasion provoked by VEGF ablation, resulting in substantial survival benefit.
4. Microtumor growth initiates angiogenic sprouting with simultaneous expression of VEGF, VEGF receptor-2, and angiopoietin-2. Tumors have been thought to initiate as avascular aggregates of malignant cells that only later induce vascularization. Recently, this classic concept of tumor angiogenesis has been challenged by the suggestion that tumor cells grow by co-opting preexisting host vessels and thus initiate as well-vascularized tumors without triggering angiogenesis. To discriminate between these two mechanisms, we have used intravital epifluorescence microscopy and multi-photon laser scanning confocal microscopy to visualize C6 microglioma vascularization and tumor cell behavior. To address the mechanisms underlying tumor initiation, we assessed the expression of VEGF, VEGF receptor-2 (VEGFR-2), and angiopoietin-2 (Ang-2), as well as endothelial cell proliferation. We show that multicellular aggregates (< 1 mm(3)) initiate vascular growth by angiogenic sprouting via the simultaneous expression of VEGFR-2 and Ang-2 by host and tumor endothelium. Host blood vessels are not co-opted by tumor cells but rather are used as trails for tumor **cell invasion** of the host tissue. Our data further suggest that the established microvasculature of growing tumors is characterized by a continuous vascular remodeling, putatively mediated by the expression of VEGF and Ang-2. The results of this study suggest a new concept of vascular tumor initiation that may have important implications for the clinical application of antiangiogenic strategies.

---

## Tek

- NCBI Gene
- GeneRIF
- Pubmed

| Search Term | Rank | Fields |
| --- | --- | --- |
| [Ff]ilopodia | 1 | PubMed(2) |
| [Mm]igration | 6 | Process(4); PubMed(33); Conserved Domains(1) |
| ECM | 7 | PubMed(1) |
| [Ee]xtracellular [Mm]atrix | 8 | PubMed(10); GeneRIFs(1) |
| [Ii]nvasive | 10 | PubMed(2); GeneRIFs(1) |
| [Mm]etastasis | 12 | PubMed(4) |
| [Cc]ell [Ii]nvasion | 13 | PubMed(1) |

  
hide/show details for Tek

### [Ff]ilopodia

#### PubMed

1. Serum response factor is required for sprouting angiogenesis and vascular integrity. Serum response factor (SRF) is a transcription factor that controls the expression of cytoskeletal proteins and immediate early genes in different cell types. Here, we found that SRF expression is restricted to endothelial cells (ECs) of small vessels such as capillaries in the mouse embryo. EC-specific Srf deletion led to aneurysms and hemorrhages from 11.5 days of mouse development (E11.5) and lethality at E14.5. Mutant embryos presented a reduced capillary density and defects in EC migration, with fewer numbers of **filopodia** in tip cells and ECs showing defects in actin polymerization and intercellular junctions. We show that SRF is essential for the expression of VE-cadherin and beta-actin in ECs both in vivo and in vitro. Moreover, knockdown of SRF in ECs impaired VEGF- and FGF-induced in vitro angiogenesis. Taken together, our results demonstrate that SRF plays an important role in sprouting angiogenesis and small vessel integrity in the mouse embryo.
2. Angiopoietin-1 promotes lymphatic sprouting and hyperplasia. Angiopoietin 1 (Ang1), a ligand for the receptor tyrosine kinase Tie2, regulates the formation and stabilization of the blood vessel network during embryogenesis. In adults, Ang1 is associated with blood vessel stabilization and recruitment of perivascular cells, whereas Ang2 acts to counter these actions. Recent results from gene-targeted mice have shown that Ang2 is also essential for the proper patterning of lymphatic vessels and that Ang1 can be substituted for this function. In order to characterize the effects of the angiopoietins on lymphatic vessels, we employed viral vectors for overexpression of Ang1 in adult mouse tissues. We found that Ang1 activated lymphatic vessel endothelial proliferation, vessel enlargement, and generation of long endothelial cell **filopodia** that eventually fused, leading to new sprouts and vessel development. Cutaneous lymphatic hyperplasia was also detected in transgenic mice expressing Ang1 in the basal epidermal cells. Tie2 was expressed in the lymphatic endothelial cells and Ang1 stimulation of these cells resulted in up-regulation of vascular endothelial growth factor receptor 3 (VEGFR-3). Furthermore, a soluble form of VEGFR-3 inhibited the observed lymphatic sprouting. Our results reinforce the concept that Ang1 therapy may be useful in settings of tissue edema.

### [Mm]igration

#### Process

1. positive regulation of endothelial cell **migration**
2. positive regulation of endothelial cell **migration**
3. regulation of cell **migration**
4. regulation of cell **migration**

#### PubMed

1. Serum response factor is required for sprouting angiogenesis and vascular integrity. Serum response factor (SRF) is a transcription factor that controls the expression of cytoskeletal proteins and immediate early genes in different cell types. Here, we found that SRF expression is restricted to endothelial cells (ECs) of small vessels such as capillaries in the mouse embryo. EC-specific Srf deletion led to aneurysms and hemorrhages from 11.5 days of mouse development (E11.5) and lethality at E14.5. Mutant embryos presented a reduced capillary density and defects in EC **migration**, with fewer numbers of filopodia in tip cells and ECs showing defects in actin polymerization and intercellular junctions. We show that SRF is essential for the expression of VE-cadherin and beta-actin in ECs both in vivo and in vitro. Moreover, knockdown of SRF in ECs impaired VEGF- and FGF-induced in vitro angiogenesis. Taken together, our results demonstrate that SRF plays an important role in sprouting angiogenesis and small vessel integrity in the mouse embryo.
2. Mab21l2 is essential for embryonic heart and liver development. During mouse embryogenesis, proper formation of the heart and liver is especially important and is crucial for embryonic viability. In this study, we showed that Mab21l2 was expressed in the trabecular and compact myocardium, and that deletion of Mab21l2 resulted in a reduction of the trabecular myocardium and thinning of the compact myocardium. Mab21l2-deficient embryonic hearts had decreased expression of genes that regulate cell proliferation and apoptosis of cardiomyocytes. These results show that Mab21l2 functions during heart development by regulating the expression of such genes. Mab21l2 was also expressed in the septum transversum mesenchyme (STM). Epicardial progenitor cells are localized to the anterior surface of the STM (proepicardium), and proepicardial cells migrate onto the surface of the heart and form the epicardium, which plays an important role in heart development. The rest of the STM is essential for the growth and survival of hepatoblasts, which are bipotential progenitors for hepatocytes and cholangiocytes. Proepicardial cells in Mab21l2-deficient embryos had defects in cell proliferation, which led to a small proepicardium, in which α4 integrin expression, which is essential for the **migratio**n of proepicardial cells, was down-regulated, suggesting that defects occurred in its **migratio**n. In Mab21l2-deficient embryos, epicardial formation was defective, suggesting that Mab21l2 plays important roles in epicardial formation through the regulation of the cell proliferation of proepicardial cells and the migratory process of proepicardial cells. Mab21l2-deficient embryos also exhibited hypoplasia of the STM surrounding hepatoblasts and decreased hepatoblast proliferation with a resultant loss of defective morphogenesis of the liver. These findings demonstrate that Mab21l2 plays a crucial role in both heart and liver development through STM formation.
3. Angiopoietin-1/Tie2 signaling pathway inhibits lipopolysaccharide-induced activation of RAW264.7 macrophage cells. Angiopoietin-1 (Ang1) is a ligand for the endothelial-specific tyrosine kinase receptor Tie2 and has been shown to play an essential role in embryonic vasculature development. There have been many studies about the anti-inflammatory effects of Ang1, most of which focus on endothelium cells. In the present study, we explore the role of Ang1-Tie2 signaling in the activation of macrophages upon lipopolysaccharide (LPS) stimulation. We found that Tie2 receptor is expressed on macrophages and Ang1 could inhibit LPS-induced activation of macrophages, as evidenced by cell **migration** and TNF-alpha production, specifically through Tie2 receptor. We further investigated the mechanism and found that Ang1-Tie2 could block LPS-induced activation of NF-kappaB which has been shown to be necessary for macrophage activation with LPS treatment. Thus, we described, for the first time, the role of Ang1-Tie2 signaling in macrophage activation and the possible mechanisms in response to immune stimulation.
4. Protocadherin 12 deficiency alters morphogenesis and transcriptional profile of the placenta. Protocadherins are transmembrane proteins exhibiting homophilic adhesive activities through their extracellular domain. Protocadherin 12 (Pcdh12) is expressed in angiogenic endothelial cells, mesangial cells of kidney glomeruli, and glycogen cells of the mouse placenta. To get insight into the role of this protein in vivo, we analyzed PCDH12-deficient mice and investigated their placental phenotype. The mice were alive and fertile; however, placental and embryonic sizes were reduced compared with wild-type mice. We observed defects in placental layer segregation and a decreased vascularization of the labyrinth associated with a reduction in cell density in this layer. To understand the molecular events responsible for the phenotypic alterations observed in Pcdh12(-/-) placentas, we analyzed the expression profile of embryonic day 12.5 mutant placentas compared with wild-type placentas, using pangenomic chips: 2,289 genes exhibited statistically significant changes in expressed levels due to loss of PCDH12. Functional grouping of modified genes was obtained by GoMiner software. Gene clusters that contained most of the differentially expressed genes were those involved in tissue morphogenesis and development, angiogenesis, cell-matrix adhesion and **migration**, immune response, and chromatin remodeling. Our data show that loss of PCDH12 leads to morphological alterations of the placenta and to notable changes in its gene expression profile. Specific genes emerging from the microarray screen support the biological modifications observed in PCDH12-deficient placentas.
5. Edg-1, the G protein-coupled receptor for sphingosine-1-phosphate, is essential for vascular maturation. Sphingolipid signaling pathways have been implicated in many critical cellular events. Sphingosine-1-phosphate (SPP), a sphingolipid metabolite found in high concentrations in platelets and blood, stimulates members of the endothelial differentiation gene (Edg) family of G protein-coupled receptors and triggers diverse effects, including cell growth, survival, **migration**, and morphogenesis. To determine the in vivo functions of the SPP/Edg signaling pathway, we disrupted the Edg1 gene in mice. Edg1(-/-) mice exhibited embryonic hemorrhage leading to intrauterine death between E12.5 and E14.5. Vasculogenesis and angiogenesis appeared normal in the mutant embryos. However, vascular maturation was incomplete due to a deficiency of vascular smooth muscle cells/pericytes. We also show that Edg-1 mediates an SPP-induced **migration** response that is defective in mutant cells due to an inability to activate the small GTPase, Rac. Our data reveal Edg-1 to be the first G protein-coupled receptor required for blood vessel formation and show that sphingolipid signaling is essential during mammalian development.
6. Endothelin-2 signaling in the neural retina promotes the endothelial tip cell state and inhibits angiogenesis. Endothelin signaling is required for neural crest **migration** and homeostatic regulation of blood pressure. Here, we report that constitutive overexpression of Endothelin-2 (Edn2) in the mouse retina perturbs vascular development by inhibiting endothelial cell **migration** across the retinal surface and subsequent endothelial cell invasion into the retina. Developing endothelial cells exist in one of two states: tip cells at the growing front and stalk cells in the vascular plexus behind the front. This division of endothelial cell states is one of the central organizing principles of angiogenesis. In the developing retina, Edn2 overexpression leads to overproduction of endothelial tip cells by both morphologic and molecular criteria. Spatially localized overexpression of Edn2 produces a correspondingly localized endothelial response. Edn2 overexpression in the early embryo inhibits vascular development at midgestation, but Edn2 overexpression in developing skin and brain has no discernible effect on vascular structure. Inhibition of retinal angiogenesis by Edn2 requires expression of Endothelin receptor A but not Endothelin receptor B in the neural retina. Taken together, these observations imply that the neural retina responds to Edn2 by synthesizing one or more factors that promote the endothelial tip cell state and inhibit angiogenesis. The response to Edn2 is sufficiently potent that it overrides the activities of other homeostatic regulators of angiogenesis, such as Vegf.
7. Myocardin-related transcription factors control the motility of epicardium-derived cells and the maturation of coronary vessels. An important pool of cardiovascular progenitor cells arises from the epicardium, a single layer of mesothelium lining the heart. Epicardium-derived progenitor cell (EPDC) formation requires epithelial-to-mesenchymal transition (EMT) and the subsequent **migration** of these cells into the sub-epicardial space. Although some of the physiological signals that promote EMT are understood, the functional mediators of EPDC motility and differentiation are not known. Here, we identify a novel regulatory mechanism of EPDC mobilization. Myocardin-related transcription factor (MRTF)-A and MRTF-B (MKL1 and MKL2, respectively) are enriched in the perinuclear space of epicardial cells during development. Transforming growth factor (TGF)-β signaling and disassembly of cell contacts leads to nuclear accumulation of MRTFs and the activation of the motile gene expression program. Conditional ablation of Mrtfa and Mrtfb specifically in the epicardium disrupts cell **migratio**n and leads to sub-epicardial hemorrhage, partially stemming from the depletion of coronary pericytes. Using lineage-tracing analyses, we demonstrate that sub-epicardial pericytes arise from EPDCs in a process that requires the MRTF-dependent motile gene expression program. These findings provide novel mechanisms linking EPDC motility and differentiation, shed light on the transcriptional control of coronary microvascular maturation and suggest novel therapeutic strategies to manipulate epicardium-derived progenitor cells for cardiac repair.
8. GPR124, an orphan G protein-coupled receptor, is required for CNS-specific vascularization and establishment of the blood-brain barrier. Every organ in the body requires blood vessels for efficient delivery of oxygen and nutrients, but independent vascular beds are highly specialized to meet the individual needs of specific organs. The vasculature of the brain is tightly sealed, with blood-brain barrier (BBB) properties developing coincident with neural vascularization. G protein-coupled receptor 124 (GPR124) (tumor endothelial marker 5, TEM5), an orphan member of the adhesion family of G protein-coupled receptors, was previously identified on the basis of its overexpression in tumor vasculature. Here, we show that global deletion or endothelial-specific deletion of GPR124 in mice results in embryonic lethality associated with abnormal angiogenesis of the forebrain and spinal cord. Expression of GPR124 was found to be required for invasion and **migration** of blood vessels into neuroepithelium, establishment of BBB properties, and expansion of the cerebral cortex. Thus, GPR124 is an important regulator of neurovasculature development and a potential drug target for cerebrovascular diseases.
9. SnoN facilitates ALK1-Smad1/5 signaling during embryonic angiogenesis. In endothelial cells, two type I receptors of the transforming growth factor β (TGF-β) family, ALK1 and ALK5, coordinate to regulate embryonic angiogenesis in response to BMP9/10 and TGF-β. Whereas TGF-β binds to and activates ALK5, leading to Smad2/3 phosphorylation and inhibition of endothelial cell proliferation **and migra**tion, BMP9/10 and TGF-β also bind to ALK1, resulting in the activation of Smad1/5. SnoN is a negative regulator of ALK5 signaling through the binding and repression of Smad2/3. Here we uncover a positive role of SnoN in enhancing Smad1/5 activation in endothelial cells to promote angiogenesis. Upon ligand binding, SnoN directly bound to ALK1 on the plasma membrane and facilitated the interaction between ALK1 and Smad1/5, enhancing Smad1/5 phosphorylation. Disruption of this SnoN-Smad interaction impaired Smad1/5 activation and up-regulated Smad2/3 activity. This resulted in defective angiogenesis and arteriovenous malformations, leading to embryonic lethality at E12.5. Thus, SnoN is essential for TGF-β/BMP9-dependent biological processes by its ability to both positively and negatively modulate the activities of Smad-dependent pathways.
10. Homeodomain factor Nkx2-3 controls regional expression of leukocyte homing coreceptor MAdCAM-1 in specialized endothelial cells of the viscera. Regulated e**migration** of blood-borne leukocytes plays a defining role in lymphoid organ development, immune surveillance, and inflammatory responses. We report here that mice deficient in the homeobox gene Nkx2-3, expressed in developing visceral mesoderm, show a complex intestinal malabsorption phenotype and striking abnormalities of gut-associated lymphoid tissue and spleen suggestive of deranged leukocyte homing. Mutant Peyer's patches were reduced in number and size, intestinal villi contained few IgA(+) plasma cells, and mutant spleens were small and often atrophic, showing fused periarterial lymphoid sheaths, partially merged T and B cell zones, an absent marginal zone, and a dearth of macrophages in red pulp. Semiquantitative RT-PCR analysis and immunohistochemistry revealed down-regulation of mucosal addressin cell adhesion molecule-1 (MAdCAM-1) in endothelial cells in which Nkx2-3 is normally expressed. MAdCAM-1 is a member of the immunoglobulin superfamily, acting as an endothelial cell ligand for leukocyte homing receptors L-selectin and alpha4beta7 integrin. Our data suggest a role for a homeodomain factor in establishing the developmental and positional cues in endothelia that regulate leukocyte homing through local control of cellular adhesion and identify MAdCAM-1 as a candidate target gene of Nkx2-3.
11. Angiopoietin-2 regulates cortical neurogenesis in the developing telencephalon. Vascular-specific growth factor angiopoietin-2 (Ang2) is mainly involved during vascular network setup. Recently, Ang2 was suggested to play a role in adult neurogenesis, affecting **migration** and differentiation of adult neuroblasts in vitro. However, to date, no data have reported an effect of Ang2 on neurogenesis during embryonic development. As we detected Ang2 expression in the developing cerebral cortex at embryonic day E14.5 and E16.5, we used in utero electroporation to knock down Ang2 expression in neuronal progenitors located in the cortical ventricular zone (VZ) to examine the role of Ang2 in cortical embryonic neurogenesis. Using this strategy, we showed that radial **migration** from the VZ toward the cortical plate of Ang2-knocked down neurons is altered as well as their morphology. In parallel, we observed a perturbation of intermediate progenitor population and the surrounding vasculature. Taken together, our results show for the first time that, in addition to its role during brain vasculature setup, Ang2 is also involved in embryonic cortical neurogenesis and especially in the radial **migration** of projection neurons.
12. The PTEN/PI3K pathway governs normal vascular development and tumor angiogenesis. PTEN is an important tumor suppressor gene. Hereditary mutation of PTEN causes tumor-susceptibility diseases such as Cowden disease. We used the Cre-loxP system to generate an endothelial cell-specific mutation of Pten (Tie2CrePten) in mice. Tie2CrePten(flox/+) mice displayed enhanced tumorigenesis due to an increase in angiogenesis driven by vascular growth factors. This effect was partially dependent on the PI3K subunits p85alpha and p110gamma. In vitro, Tie2CrePten(flox/+) endothelial cells showed enhanced proliferation/**migration**. Tie2CrePten(flox/flox) mice died before embryonic day 11.5 (E11.5) due to bleeding and cardiac failure caused by impaired recruitment of pericytes and vascular smooth muscle cells to blood vessels, and of cardiomyocytes to the endocardium. These phenotypes depend strongly on p110gamma rather than on p85alpha and were associated with decreased expression of Ang-1, VCAM-1, connexin 40, and ephrinB2 but increased expression of Ang-2, VEGF-A, VEGFR1, and VEGFR2. Pten is thus indispensable for normal cardiovascular morphogenesis and post-natal angiogenesis, including tumor angiogenesis.
13. BMPs promote proliferation and **migration** of endothelial cells via stimulation of VEGF-A/VEGFR2 and angiopoietin-1/Tie2 signalling. The differentiation, growth, and survival of endothelial cells (ECs) are regulated by multiple signalling pathways, such as vascular endothelial growth factors (VEGFs) and angiopoietins through their receptor tyrosine kinases, VEGF receptor (VEGFR) 2 and Tie2, respectively. Bone morphogenetic proteins (BMPs), members of the transforming growth factor (TGF)-beta family, have been implicated in the development and maintenance of vascular systems. However, their effects on EC proliferation remain to be elucidated. In the present study, we show that BMPs induce the proliferation and **migration** of mouse embryonic stem cell (ESC)-derived endothelial cells (MESECs) and human microvascular endothelial cells (HMECs). Addition of BMP-4 to culture induced significant proliferation and **migration** of both types of ECs. BMP-4 also increased the expression and phosphorylation of VEGFR2 and Tie2. These findings suggest that BMP signalling activates endothelium via activation of VEGF/VEGFR2 and Angiopoietin/Tie2 signalling.
14. Embryonic death of Mek1-deficient mice reveals a role for this kinase in angiogenesis in the labyrinthine region of the placenta. Mek is a dual-specificity kinase that activates the extracellular-signal-regulated (Erk) mitogen-activated protein (MAP) kinases upon agonist binding to receptors. The Erk MAP kinase cascade is involved in cell-fate determination in many organisms. In mammals, this pathway is proposed to regulate cell growth and differentiation. Genetic studies have shown that although a single mek gene is present in Caenorhabditis elegans, Drosophila and Xenopus, two mek homologs, Mek1 and Mek2, are present in the mammalian cascade. In the present study, we describe a mutant mouse line in which the mek1 gene has been disrupted by insertional mutagenesis. The null mutation was recessive lethal, as the homozygous mutant embryos died at 10.5 days of gestation. Histopathological analyses revealed a reduction in vascularization of the placenta that was due to a marked decrease of vascular endothelial cells in the labyrinthine region. The failure to establish a functional placenta probably explains the death of the mek1-/- embryos. Cell-**migration** assays indicated that mek1-/- fibroblasts could not be induced to migrate by fibronectin, although the levels of Mek2 protein and Erk activation were normal. Re-expression of Mek1 in the mutant mouse embryonic fibroblasts (MEFs) restored their ability to migrate. Our findings provide genetic evidence that establishes the unique role played by Mek1 in signal transduction. They also suggest that mek1 function is required for normal response to angiogenic signals that might promote vascularization of the labyrinthine region of the placenta.
15. Angiopoietin-1/Tie2 signaling pathway inhibits lipopolysaccharide-induced activation of RAW264.7 macrophage cells. Angiopoietin-1 (Ang1) is a ligand for the endothelial-specific tyrosine kinase receptor Tie2 and has been shown to play an essential role in embryonic vasculature development. There have been many studies about the anti-inflammatory effects of Ang1, most of which focus on endothelium cells. In the present study, we explore the role of Ang1-Tie2 signaling in the activation of macrophages upon lipopolysaccharide (LPS) stimulation. We found that Tie2 receptor is expressed on macrophages and Ang1 could inhibit LPS-induced activation of macrophages, as evidenced by cell **migration** and TNF-alpha production, specifically through Tie2 receptor. We further investigated the mechanism and found that Ang1-Tie2 could block LPS-induced activation of NF-kappaB which has been shown to be necessary for macrophage activation with LPS treatment. Thus, we described, for the first time, the role of Ang1-Tie2 signaling in macrophage activation and the possible mechanisms in response to immune stimulation.
16. BMPs promote proliferation and **migration** of endothelial cells via stimulation of VEGF-A/VEGFR2 and angiopoietin-1/Tie2 signalling. The differentiation, growth, and survival of endothelial cells (ECs) are regulated by multiple signalling pathways, such as vascular endothelial growth factors (VEGFs) and angiopoietins through their receptor tyrosine kinases, VEGF receptor (VEGFR) 2 and Tie2, respectively. Bone morphogenetic proteins (BMPs), members of the transforming growth factor (TGF)-beta family, have been implicated in the development and maintenance of vascular systems. However, their effects on EC proliferation remain to be elucidated. In the present study, we show that BMPs induce the proliferation and **migration** of mouse embryonic stem cell (ESC)-derived endothelial cells (MESECs) and human microvascular endothelial cells (HMECs). Addition of BMP-4 to culture induced significant proliferation and **migration** of both types of ECs. BMP-4 also increased the expression and phosphorylation of VEGFR2 and Tie2. These findings suggest that BMP signalling activates endothelium via activation of VEGF/VEGFR2 and Angiopoietin/Tie2 signalling.
17. CYR61 (CCN1) is essential for placental development and vascular integrity. CYR61 (CCN1) is a member of the CCN family of secreted matricellular proteins that includes connective tissue growth factor (CCN2), NOV (CCN3), WISP-1 (CCN4), WISP-2 (CCN5), and WISP-3 (CCN6). First identified as the product of a growth factor-inducible immediate-early gene, CYR61 is an extracellular matrix-associated angiogenic inducer that functions as a ligand of integrin receptors to promote cell adhesion, **migration**, and proliferation. Aberrant expression of Cyr61 is associated with breast cancer, wound healing, and vascular diseases such as atherosclerosis and restenosis. To understand the functions of CYR61 during development, we have disrupted the Cyr61 gene in mice. We show here that Cyr61-null mice suffer embryonic death: approximately 30% succumbed to a failure in chorioallantoic fusion, and the reminder perished due to placental vascular insufficiency and compromised vessel integrity. These findings establish CYR61 as a novel and essential regulator of vascular development. CYR61 deficiency results in a specific defect in vessel bifurcation (nonsprouting angiogenesis) at the chorioallantoic junction, leading to an undervascularization of the placenta without affecting differentiation of the labyrinthine syncytiotrophoblasts. This unique phenotype is correlated with impaired Vegf-C expression in the allantoic mesoderm, suggesting that CYR61-regulated expression of Vegf-C plays a role in vessel bifurcation. The genetic and molecular basis of vessel bifurcation is presently unknown, and these findings provide new insight into this aspect of angiogenesis.
18. Probing chromatin landscape reveals roles of endocardial TBX20 in septation. Mutations in the T-box transcription factor TBX20 are associated with multiple forms of congenital heart defects, including cardiac septal abnormalities, but our understanding of the contributions of endocardial TBX20 to heart development remains incomplete. Here, we investigated how TBX20 interacts with endocardial gene networks to drive the mesenchymal and myocardial movements that are essential for outflow tract and atrioventricular septation. Selective ablation of Tbx20 in murine endocardial lineages reduced the expression of extracellular matrix and cell **migration** genes that are critical for septation. Using the assay for transposase-accessible chromatin with high-throughput sequencing (ATAC-seq), we identified accessible chromatin within endocardial lineages and intersected these data with TBX20 ChIP-seq and chromatin loop maps to determine that TBX20 binds a conserved long-range enhancer to regulate versican (Vcan) expression. We also observed reduced Vcan expression in Tbx20-deficient mice, supporting a direct role for TBX20 in Vcan regulation. Further, we show that the Vcan enhancer drove reporter gene expression in endocardial lineages in a TBX20-binding site-dependent manner. This work illuminates gene networks that interact with TBX20 to orchestrate cardiac septation and provides insight into the chromatin landscape of endocardial lineages during septation.
19. Angiopoietin/Tie2 pathway mediates type 2 diabetes induced vascular damage after cerebral stroke. We investigated the changes and the molecular mechanisms of cerebral vascular damage after stroke in type-2 diabetic (T2DM) mice. Adult male db/db T2DM and wild-type (WT) mice were subjected to transient middle cerebral artery occlusion (MCAo) and sacrificed 24 hours after MCAo. T2DM-mice exhibited significantly increased blood glucose, brain hemorrhagic rate, mortality and cerebrovascular density, but decreased cerebrovascular diameter, arteriolar density and arterial mural cell numbers in the ischemic brain compared with WT mice. The hemorrhagic rate was significantly correlated with the mortality (r = 0.85). T2DM-mice also exhibited increased blood-brain barrier leakage and concomitantly, increased Angiopoietin2, but decreased Angiopoietin1, Tie2 and tight junction protein expression in the ischemic brain. Angiopoietin1 gene expression also significantly decreased in the common carotid artery (CCA) in T2DM-mice compared with WT mice after stroke. To further test the effects of T2DM on cerebrovascular damage, we performed in vitro studies. The capillary-like tube formation of primary cultured mouse brain endothelial cells (MBECs) significantly increased, but artery cell **migration** in the primary CCA cultures significantly decreased both in Sham and MCAo T2DM-mice compared with the WT mice. Angiopoietin1 treatment significantly increased artery cell **migration** in T2DM-CCA after MCAo. Tie2-FC, a neutralized Tie2 antibody, significantly decreased artery cell **migration** in WT-CCA after MCAo. Therefore, decreased Angiopoietin1/Tie2 and increased Angiopoietin2 expression may contribute to diabetes-induced vascular damage after stroke.
20. Identification of Tek/Tie2 binding partners. Binding to a multifunctional docking site mediates cell survival and **migration**. The Tek/Tie2 receptor tyrosine kinase plays a pivotal role in vascular and hematopoietic development. To study the signal transduction pathways that are mediated by this receptor, we have used the yeast two-hybrid system to identify signaling molecules that associate with the phosphorylated Tek receptor. Using this approach, we demonstrate that five molecules, Grb2, Grb7, Grb14, Shp2, and the p85 subunit of phosphatidylinositol 3-kinase can interact with Tek in a phosphotyrosine-dependent manner through their SH2 domains. Mapping of the binding sites of these molecules on Tek reveals the presence of a multisubstrate docking site in the carboxyl tail of Tek (Tyr(1100)). Mutation of this site abrogates binding of Grb2 and Grb7 to Tek in vivo, and this site is required for tyrosine phosphorylation of Grb7 and p85 in vivo. Furthermore, stimulation of Tek-expressing cells with Angiopoietin-1 results in phosphorylation of both Tek and p85 and in activation of endothelial cell **migration** and survival pathways that are dependent in part on phosphatidylinositol 3-kinase. Taken together, these results demonstrate that Angiopoietin-1-induced signaling from the Tek receptor is mediated by a multifunctional docking site that is responsible for activation of both cell **migration** and cell survival pathways.
21. Restricted expression of the hyaluronan receptor, CD44, during postimplantation mouse embryogenesis suggests key roles in tissue formation and patterning. CD44 is a multifunctional adhesion protein that acts as a major receptor for the hygroscopic extracellular matrix component, hyaluronan. This receptor-ligand binding directly mediates at least some of the cell-cell and cell-matrix interactions ascribed to CD44. Other interactions involving CD44 may be modulated indirectly by its ability to bind growth factors and thereby to promote cell attachment. During vertebrate development, multiple cases of hyaluronan involvement in cell proliferation, cell **migration** and histogenesis have been documented. In addition, there is evidence suggesting a central role for cell surface glycoproteins and proteoglycans in mediating the action of polypeptide growth factors involved in tissue patterning. In view of this, we undertook to investigate expression of the CD44 protein during postimplantation mouse embryogenesis. Between 9.5 and 12.5 days of embryonic development, the predominant form of CD44 protein corresponds to the hyaluronan-binding CD44H form. However, species with a higher M(r) were also detected, implying that CD44 isoforms generated by alternative splicing of CD44 RNA are employed in normal development. Further, we used mouse embryos to perform whole-mount immunohistochemistry and examine the temporal and spatial distribution of this glycoprotein. CD44 is expressed at high levels in the heart, somites and condensing limb-bud mesenchyme at critical stages of morphogenesis. These sites correlate with regions where hyaluronan has been demonstrated to regulate morphogenetic events. Of novel interest, however, is the high expression of CD44 in regions that do not correlate with sites of known hyaluronan-mediated developmental events. These include instructive epithelia participating in epithelial-mesenchymal cell interactions such as the apical ectodermal ridge of the developing limb bud and the odontogenic placodes of the presumptive upper and lower jaws.
22. Angiopoietin/Tie2 signaling transforms capillaries into venules primed for leukocyte trafficking in airway inflammation. Vascular endothelial growth factor (VEGF) is a key angiogenic factor in tumors, but less is known about what drives vascular remodeling in inflammation, where plasma leakage and leukocyte influx are prominent features. In chronic airway inflammation in mice infected by the bacterium Mycoplasma pulmonis (M. pulmonis), the segment of the microvasculature that supports leukocyte adhesion and **migration** expands through remodeling of capillaries into vessels with features of venules. Here, we report that the angiopoietin/Tie2 pathway is an essential driving force for capillary remodeling into venules in M. pulmonis-infected mouse airways. Similar to M. pulmonis infection, systemic overexpression of angiopoietin-1 (Ang1) resulted in remodeling of airway capillaries into venular-like vessels that expressed venous markers like P-selectin, ICAM-1, and EphB4 and were sites of leukocyte adhesion during lipopolysaccharide-induced acute inflammation. Ang1 and Ang2 protein increased in M. pulmonis-infected mouse airways but came from different cellular sources: Ang1 was expressed in infiltrating neutrophils and Ang2 in endothelial cells. Indeed, systemic administration of soluble Tie2 inhibited capillary remodeling, induction of venous markers, and leukocyte influx in M. pulmonis-infected mouse airways. Together, these findings suggest that blockade of the Ang/Tie2 pathway may represent a therapeutic approach in airway inflammation.
23. Msx genes define a population of mural cell precursors required for head blood vessel maturation. Vessels are primarily formed from an inner endothelial layer that is secondarily covered by mural cells, namely vascular smooth muscle cells (VSMCs) in arteries and veins and pericytes in capillaries and veinules. We previously showed that, in the mouse embryo, Msx1(lacZ) and Msx2(lacZ) are expressed in mural cells and in a few endothelial cells. To unravel the role of Msx genes in vascular development, we have inactivated the two Msx genes specifically in mural cells by combining the Msx1(lacZ), Msx2(lox) and Sm22α-Cre alleles. Optical projection tomography demonstrated abnormal branching of the cephalic vessels in E11.5 mutant embryos. The carotid and vertebral arteries showed an increase in caliber that was related to reduced vascular smooth muscle coverage. Taking advantage of a newly constructed Msx1(CreERT2) allele, we demonstrated by lineage tracing that the primary defect lies in a population of VSMC precursors. The abnormal phenotype that ensues is a consequence of impaired BMP signaling in the VSMC precursors that leads to downregulation of the metalloprotease 2 (Mmp2) and Mmp9 genes, which are essential for cell **migratio**n and integration into the mural layer. Improper coverage by VSMCs secondarily leads to incomplete maturation of the endothelial layer. Our results demonstrate that both Msx1 and Msx2 are required for the recruitment of a population of neural crest-derived VSMCs.
24. Identification of ARIA regulating endothelial apoptosis and angiogenesis by modulating proteasomal degradation of cIAP-1 and cIAP-2. Endothelial apoptosis is a pivotal process for angiogenesis during embryogenesis as well as postnatal life. By using a retrovirus-mediated signal sequence trap method, we identified a previously undescribed gene, termed ARIA (apoptosis regulator through modulating IAP expression), which regulates endothelial apoptosis and angiogenesis. ARIA was expressed in blood vessels during mouse embryogenesis, as well as in endothelial cells both in vitro and in vivo. ARIA is a unique protein with no homology to previously reported conserved domain structures. Knockdown of ARIA in HUVECs by using small interfering RNA significantly reduced endothelial apoptosis without affecting either cell **migration** or proliferation. ARIA knockdown significantly increased inhibitor of apoptosis (cIAP)-1 and cIAP-2 protein expression, although their mRNA expression was not changed. Simultaneous knockdown of cIAP-1 and cIAP-2 abolished the antiapoptotic effect of ARIA knockdown. Using yeast 2-hybrid screening, we identified the interaction of ARIA with 20S proteasome subunit alpha-7. Thereafter, we found that cIAP-1 and cIAP-2 were degraded by proteasomes in endothelial cells under normal condition. Overexpression of ARIA significantly reduced cIAP-1 expression, and this reduction was abolished by proteasomal inhibition in BAECs. Also, knockdown of ARIA demonstrated an effect similar to proteasomal inhibition with respect to not only expression but also subcellular localization of cIAP-1 and cIAP-2. In vivo angiogenesis studied by Matrigel-plug assay, mouse ischemic retinopathy model, and tumor xenograft model was significantly enhanced by ARIA knockdown. Together, our data indicate that ARIA is a unique factor regulating endothelial apoptosis, as well as angiogenesis, presumably through modulating proteasomal degradation of cIAP-1 and cIAP-2 in endothelial cells.
25. Tie2-dependent knockout of α6 integrin subunit in mice reduces post-ischaemic angiogenesis. Integrins α6β1 and α6β4 are receptors for laminins, the main components of the basement membrane underlying the endothelial cells. In vitro, α6 integrin subunit (α6) expression at the surface of endothelial cells and their progenitors (EPCs) is up-regulated by pro-angiogenic growth factors and is crucial for adh**esion, mi**gration, and pseudotube formation. We investigated the role for α6 in post-ischaemic vascular repair in vivo.We used the cre-lox system to generate a mouse line with specific α6 gene deletion in Tie2-lineage cells. In a model of hind-limb ischaemia, Tie2-dependent α6 deletion reduced neovessel formation and reperfusion of the ischaemic limb. Concerning the role for α6 in post-ischaemic vasculogenesis, we showed previously that α6 was required for EPC recruitment at the site of ischaemia. Here, we found that α6 deletion also reduced EPC mobilization from the bone marrow after ischaemia. Examination of the ischaemic muscles showed that Tie2-dependent α6 deletion decreased the recruitment of pro-angiogenic Tie2-expressing macrophages. In the Matrigel plug assay, fibroblast growth factor-2-induced vascularization was diminished in mice lacking endothelial α6. To specifically investigate the role for α6 in angiogenesis, aortic rings were embedded in Matrigel or collagen and cultured ex vivo. In Matrigel, neovessel outgrowth from rings lacking α6 was strongly diminished, whereas no genotype-dependent difference occurred for rings in collagen.α6 plays a major role in both post-ischaemic angiogenesis and vasculogenesis.
26. Angiopoietin-2 differentially regulates angiogenesis through TIE2 and integrin signaling. Angiopoietin-2 (ANG-2) is a key regulator of angiogenesis that exerts context-dependent effects on ECs. ANG-2 binds the endothelial-specific receptor tyrosine kinase 2 (TIE2) and acts as a negative regulator of ANG-1/TIE2 signaling during angiogenesis, thereby controlling the responsiveness of ECs to exogenous cytokines. Recent data from tumors indicate that under certain conditions ANG-2 can also promote angiogenesis. However, the molecular mechanisms of dual ANG-2 functions are poorly understood. Here, we identify a model for the opposing roles of ANG-2 in angiogenesis. We found that angiogenesis-activated endothelium harbored a subpopulation of TIE2-negative ECs (TIE2lo). TIE2 expression was downregulated in angiogenic ECs, which abundantly expressed several integrins. ANG-2 bound to these integrins in TIE2lo ECs, subsequently inducing, in a TIE2-independent manner, phosphorylation of the integrin adaptor protein FAK, resulting in RAC1 activation, **migration**, and sprouting angiogenesis. Correspondingly, in vivo ANG-2 blockade interfered with integrin signaling and inhibited FAK phosphorylation and sprouting angiogenesis of TIE2lo ECs. These data establish a contextual model whereby differential TIE2 and integrin expression, binding, and activation control the role of ANG-2 in angiogenesis. The results of this study have immediate translational implications for the therapeutic exploitation of angiopoietin signaling.
27. SDF-1/CXCR4 axis in Tie2-lineage cells including endothelial progenitor cells contributes to bone fracture healing. CXC chemokine receptor 4 (CXCR4) is a specific receptor for stromal-derived-factor 1 (SDF-1). SDF-1/CXCR4 interaction is reported to play an important role in vascular development. On the other hand, the therapeutic potential of endothelial progenitor cells (EPCs) in fracture healing has been demonstrated with mechanistic insight of vasculogenesis/angiogenesis and osteogenesis enhancement at sites of fracture. The purpose of this study was to investigate the influence of the SDF-1/CXCR4 pathway in Tie2-lineage cells (including EPCs) in bone formation. We created CXCR4 gene conditional knockout mice using the Cre/loxP system and set two groups of mice: Tie2-Cre(ER) CXCR4 knockout mice (CXCR4(-/-) ) and wild-type mice (WT). We report here that in vitro, EPCs derived from of CXCR4(-/-) mouse bone marrow demonstrated severe reduction of **migration** activity and EPC colony-forming activity when compared with those derived from WT mouse bone marrow. In vivo, radiological and morphological examinations showed fracture healing delayed in the CXCR4(-/-) group and the relative callus area at weeks 2 and 3 was significantly smaller in CXCR4(-/-) group mice. Quantitative analysis of capillary density at perifracture sites also showed a significant decrease in the CXCR4(-/-) group. Especially, CXCR4(-/-) group mice demonstrated significant early reduction of blood flow recovery at fracture sites compared with the WT group in laser Doppler perfusion imaging analysis. Real-time RT-PCR analysis showed that the gene expressions of angiogenic markers (CD31, VE-cadherin, vascular endothelial growth factor [VEGF]) and osteogenic markers (osteocalcin, collagen 1A1, bone morphogenetic protein 2 [BMP2]) were lower in the CXCR4(-/-) group. In the gain-of-function study, the fracture in the SDF-1 intraperitoneally injected WT group healed significantly faster with enough callus formation compared with the SDF-1 injected CXCR4(-/-) group. We demonstrated that an EPC SDF-1/CXCR4 axis plays an important role in bone fracture healing using Tie2-Cre(ER) CXCR4 conditional knockout mice.
28. LKB1 deficiency in Tie2-Cre-expressing cells impairs ischemia-induced angiogenesis. LKB1 is a tumor suppressor protein whose loss leads to HIF1alpha-mediated activation of a proangiogenic program in intestinal polyps. LKB1 is also protein kinase regulator of AMP-activated protein kinase (AMPK) signaling, which is essential for endothelial cell responses to tissue ischemia. To discern whether LKB1 signaling is either pro- or antiangiogenic, we investigated ischemia-induced revascularization in mice that were deficient for LKB1 in Tie2-Cre-expressing cells. Whereas homozygous deletion of LKB1 led to embryonic lethality, heterozygous LKB1-knock-out (KO) (Lkb1(flox/+);Tie2(Tg/+)) mice were viable. Unchallenged heterozygous LKB1-KO mice displayed normal capillary density, but the revascularization of hind limb following ischemic surgery was significantly impaired as evaluated by laser Doppler flow and capillary density measurements. Reduction of LKB1 in cultured endothelial cells, using either small interfering RNA or an adenovirus expressing nonfunctional kinase-dead LKB1 protein, attenuated endothelial proliferation, **migration**, and differentiation into network structures on Matrigel that was accompanied by diminished AMPK phosphorylation at Thr-172. Conversely, adenovirus-mediated LKB1 overexpression (Ad-LKB1) augmented network structure formation, and this was associated with elevated AMPK phosphorylation. The augmented differentiation of endothelial cells into network structures induced by Ad-LKB1 was abrogated by the co-transduction of a dominant negative mutant of AMPK. These observations suggest that the LKB1-AMPK signaling axis in endothelial cells is a positive regulator of the revascularization response to tissue ischemia.
29. Conditional Tek promoter-driven deletion of arginyltransferase in the germ line causes defects in gametogenesis and early embryonic lethality in mice. Posttranslational protein arginylation mediated by Ate1 is essential for cardiovascular development, actin cytoskeleton functioning, and cell **migration**. Ate1 plays a role in the regulation of cytoskeleton and is essential for cardiovascular development and angiogenesis--capillary remodeling driven by in-tissue **migration** of endothelial cells. To address the role of Ate1 in cytoskeleton-dependent processes and endothelial cell function during development, we produced a conditional mouse knockout with Ate1 deletion driven by Tek endothelial receptor tyrosine kinase promoter expressed in the endothelium and in the germ line. Contrary to expectations, Tek-Ate1 mice were viable and had no visible angiogenesis-related phenotypes; however, these mice showed reproductive defects, with high rates of embryonic lethality in the second generation, at stages much earlier than the complete Ate1 knockout strain. While some of the early lethality originated from the subpopulation of embryos with homozygous Tek-Cre transgene--a problem that has not previously been reported for this commercial mouse strain--a distinct subpopulation of embryos had lethality at early post-implantation stages that could be explained only by a previously unknown defect in gametogenesis originating from Tek-driven Ate1 deletion in premeiotic germs cells. These results demonstrate a novel role of Ate1 in germ cell development.
30. Angiopoietin-2-driven vascular remodeling in airway inflammation. Vascular remodeling is a feature of chronic inflammation during which capillaries transform into venules that expand the region of the vasculature in which leakage and leukocyte e**migration** both occur. Recently, we found that angiopoietin/Tie2 receptor signaling drives the transformation of capillaries into venules at an early stage of the sustained inflammatory response in the airways of mice infected with Mycoplasma pulmonis. However, the precise contributions of both angiopoietin-1 (Ang1) and angiopoietin-2 (Ang2) are not clear. In this study, we sought to determine the contribution of Ang2 to this vascular remodeling. Ang2 mRNA expression levels increased and phosphorylated Tie2 immunoreactivity in mucosal blood vessels decreased, indicative of diminished receptor signaling after infection. Selective inhibition of Ang2 throughout the infection by administration of either of two distinct function-blocking antibodies reduced the suppression of Tie2 phosphorylation and decreased the remodeling of mucosal capillaries into venules, the amount of leukocyte influx, and disease severity. These findings are consistent with Ang2 acting as an antagonist of Tie2 receptors and the reduction of Tie2 phosphorylation in endothelial cells rendering the vasculature more responsive to cytokines that promote both vascular remodeling and the consequences of inflammation after M. pulmonis infection. By blocking such changes, Ang2 inhibitors may prove beneficial in the treatment of sustained inflammation in which vascular remodeling, leakage, and leukocyte influx contribute to its pathophysiology.
31. The synthetic tie2 agonist peptide vasculotide protects against vascular leakage and reduces mortality in murine abdominal sepsis. Angiopoietin-1 (Angpt1), the natural agonist ligand for the endothelial Tie2 receptor, is a non-redundant endothelial survival and vascular stabilization factor that reduces endothelial permeability and inhibits leukocyte-endothelium interactions. Here we evaluate the efficacy of a novel polyethylene glycol (PEG)-clustered Tie2 agonist peptide, Vasculotide (VT), to protect against vascular leakage and mortality in a murine model of polymicrobial abdominal sepsis.Polymicrobial abdominal sepsis in C57BL6 mice was induced by cecal-ligation-and-puncture (CLP). Mice were treated with different dosages of VT or equal volume of phosphate-buffered saline (PBS). Sham-operated animals served as time-matched controls.Systemic administration of VT induced long-lasting Tie2 activation in vivo. VT protected against sepsis-induced endothelial barrier dysfunction, as evidenced by attenuation of vascular leakage and leukocyte trans**migration** into the peritoneal cavity. Histological analysis revealed that VT treatment ameliorated leukocyte infiltration in kidneys of septic mice, probably due to reduced endothelial adhesion molecule expression. VT-driven effects were associated with significantly improved organ function and reduced circulating cytokine levels. The endothelial-specific action of VT was supported by additional in vitro studies showing no effect of VT on either cytokine release from isolated peritoneal macrophages, or migratory capacity of isolated neutrophils. Finally, administration of VT pre-CLP (Hazard Ratio 0.39 [95% Confidence interval 0.19-0.81] P < 0.001) and post-CLP reduced mortality in septic mice (HR 0.22 [95% CI 0.06-0.83] P < 0.05).We provide proof of principle in support of the efficacious use of PEGylated VT, a drug-like Tie2 receptor agonist, to counteract microvascular endothelial barrier dysfunction and reduce mortality in a clinically relevant murine sepsis model. Further studies are needed to pave the road for clinical application of this therapeutic concept.
32. Tie2-dependent deletion of α6 integrin subunit in mice reduces tumor growth and angiogenesis. The α6 integrin subunit (α6) has been implicated in cancer ce**ll migrat**ion and in the progression of several malignancies, but its role in tumor angiogenesis is unclear. In mice, anti-α6 blocking antibodies reduce tumor angiogenesis, whereas Tie1-dependent α6 gene deletion enhances neovessel formation in melanoma and lung carcinoma. To clarify the discrepancy in these results we used the cre-lox system to generate a mouse line, α6fl/fl‑Tie2Cre(+), with α6 gene deletion specifically in Tie2-lineage cells: endothelial cells, pericytes, subsets of hematopoietic stem cells, and Tie2-expressing monocytes/macrophages (TEMs), known for their proangiogenic properties. Loss of α6 expression in α6fl/fl‑Tie2Cre(+) mice reduced tumor growth in a murine B16F10 melanoma model. Immunohistological analysis of the tumors showed that Tie2-dependent α6 gene deletion was associated with reduced tumor vascularization and with reduced infiltration of proangiogenic Tie2-expressing macrophages. These findings demonstrate that α6 integrin subunit plays a major role in tumor angiogenesis and TEM infiltration. Targeting α6 could be used as a strategy to reduce tumor growth.
33. Disruption of Ang-1/Tie-2 signaling contributes to the impaired myocardial vascular maturation and angiogenesis in type II diabetic mice. Microvascular insufficiency represents a major cause of end-organ failure among diabetics. The current studies were undertaken to determine whether dysregulation of the angiopoietins/Tie-2 system would result in an impairment of smooth muscle cell (SMC) recruitment and vascular maturation, which contributes to impaired angiogenesis in diabetes.Tie-2 expression was significantly attenuated, whereas angiopoietin-2 (Ang-2) was increased in db/db mice subjected to myocardial ischemia. Our morphological analysis showed that the number of SMC coverage area per neovessel was significantly reduced in db/db mice. This was accompanied by a significant reduction of myocardial capillary density and arteriole formation. Interestingly, Angiopoietin-1(Ang-1)-induced SMC recruitment and vessel outgrowth were severely impaired in db/db mice. Our in vitro studies further demonstrated that exposure of mouse heart endothelial cells to high glucose resulted in a significant upregulation of Ang-2 and a downregulation of Tie-2 expression. These alterations led to a significant impairment of Ang-1-induced Akt and eNOS phosphorylation, along with a remarkable impairment of Ang-1-induced endothelial cell **migration** and endothelial cell spheroid sprouting. Ang-1 gene transfer restored Tie-2 expression and rescued these abnormalities in diabetes.Our findings underscore the important role of Ang-1-Tie-2 signaling in the diabetes-induced impairment of vascular maturation and angiogenesis.

#### Conserved Domains

1. cd00055: EGF\_Lam; Laminin-type epidermal growth factor-like domain; laminins are the major noncollagenous components of basement membranes that mediate cell adhesion, growth **migration**, and differentiation; the laminin-type epidermal growth factor-like module occurs in ...

### ECM

#### PubMed

1. Extracellular matrix structure and tissue stiffness control postnatal lung development through the lipoprotein receptor-related protein 5/Tie2 signaling system. Physical properties of the tissues and remodeling of extracellular matrix (**ECM**) play an important role in organ development. Recently, we have reported that low-density lipoprotein receptor-related protein (LRP) 5/Tie2 signaling controls postnatal lung development by modulating angiogenesis. Here we show that tissue stiffness modulated by the **ECM** cross-linking enzyme, lysyl oxidase (LOX), regulates postnatal lung development through LRP5-Tie2 signaling. The expression of LRP5 and Tie2 is up-regulated twofold in lung microvascular endothelial cells when cultured on stiff matrix compared to those cultured on soft matrix in vitro. LOX inhibitor, β-aminopropionitrile, disrupts lung **EC**M (collagen I, III, and VI, and elastin) structures, softens neonatal mouse lung tissue by 20%, and down-regulates the expression of LRP5 and Tie2 by 20 and 60%, respectively, which leads to the inhibition of postnatal lung development (30% increase in mean linear intercept, 1.5-fold increase in air space area). Importantly, hyperoxia treatment (Postnatal Days 1-10) disrupts **EC**M structure and stiffens mouse lung tissue by up-regulating LOX activity, thereby increasing LRP5 and Tie2 expression and deregulating alveolar morphogenesis in neonatal mice, which is attenuated by inhibiting LOX activity. These findings suggest that appropriate physical properties of lung tissue are necessary for physiological postnatal lung development, and deregulation of this mechanism contributes to postnatal lung developmental disorders, such as bronchopulmonary dysplasia.

### [Ee]xtracellular [Mm]atrix

#### PubMed

1. Thymosin beta 4 is dispensable for murine cardiac development and function. Thymosin beta 4 (Tβ4) is a 43-amino acid factor encoded by an X-linked gene. Recent studies have suggested that Tβ4 is a key factor in cardiac development, growth, disease, epicardial integrity, and blood vessel formation. Cardiac-specific short hairpin (sh)RNA knockdown of tβ4 has been reported to result in embryonic lethality at E14.5-16.5, with severe cardiac and angiogenic defects. However, this shRNA tβ4-knockdown model did not completely abrogate Tβ4 expression. To completely ablate Tβ4 and to rule out the possibility of off-target effects associated with shRNA gene silencing, further studies of global or cardiac-specific knockouts are critical.We examined the role of Tβ4 in developing and adult heart through global and cardiac specific tβ4-knockout mouse models.Global tβ4-knockout mice were born at mendelian ratios and exhibited normal heart and blood vessel formation. Furthermore, in adult global tβ4-knockout mice, cardiac function, capillary density, expression of key cardiac fetal and angiogenic genes, epicardial marker expres**sion, and extracellu**lar matrix deposition were indistinguishable from that of controls. Tissue-specific tβ4-deficient mice, generated by crossing tβ4-floxed mice to Nkx2.5-Cre and αMHC-Cre, were also found to have no phenotype.We conclude that Tβ4 is dispensable for embryonic viability, heart development, coronary vessel development, and adult myocardial function.
2. Forkhead box transcription factor FoxC1 preserves corneal transparency by regulating vascular growth. Normal vision requires the precise control of vascular growth to maintain corneal transparency. Here we provide evidence for a unique mechanism by which the Forkhead box transcription factor FoxC1 regulates corneal vascular development. Murine Foxc1 is essential for development of the ocular anterior segment, and in humans, mutations have been identified in Axenfeld-Rieger syndrome, a disorder characterized by anterior segment dysgenesis. We show that FOXC1 mutations also lead to corneal angiogenesis, and that mice homozygous for either a global (Foxc1(-/-)) or neural crest (NC)-specific (NC-Foxc1(-/-)) null mutation display excessive growth of corneal blood and lymphatic vessels. This is associated with disorganization of the **extracellular matrix** and increased expression of multiple matrix metalloproteinases. Heterozygous mutants (Foxc1(+/-) and NC-Foxc1(+/-)) exhibit milder phenotypes, such as disrupted limbal vasculature. Moreover, environmental exposure to corneal injury significantly increases growth of both blood and lymphatic vessels in both Foxc1(+/-) and NC-Foxc1(+/-) mice compared with controls. Notably, this amplification of the angiogenic response is abolished by inhibition of VEGF receptor 2. Collectively, these findings identify a role for FoxC1 in inhibiting corneal angiogenesis, thereby maintaining corneal transparency by regulating VEGF signaling.
3. TIE1 and TIE2 receptor tyrosine kinases inversely regulate embryonic angiogenesis by the mechanism of intussusceptive microvascular growth. As shown previously, TIE1 and TIE2 receptor tyrosine kinases are specifically expressed in endothelial cells during embryonic angiogenesis. A detailed analysis of the vascular malformations of homozygous mice for a targeting mutation of both receptors was performed at the histological and cellular level. The data demonstrate that the TIE1 and TIE2 receptor inversely and concomitantly mediate interactions between endothelial cells with their **extracellular matrix** and with surrounding mesenchymal cells. These interactions are obviously crucial for normal endothelial cell motility and/or attachment and also for recruitment of periendothelial cells. The analysis of the TIE2-deficient embryos demonstrates how these cell/cell- and cell/matrix interactions subsequently influence the formation of normally structured tissue folds that divide the vessel lumen. They are also essential for the formation of vessel loops that compose a new vascular network and for the development of the ventricle in the heart. Fold and loop formation follow the principles of intussusceptive microvascular growth. The localization of the cardiovascular malformations corresponds to the temporal and spatial expression pattern of the TIE2 receptor. Angiopoietin-1, a ligand that activates the TIE2 receptor, is expressed in mesenchymal cells surrounding the endothelium. This local relationship is indicative of a paracrine regulation.
4. Histone deacetylase 7 maintains vascular integrity by repressing matrix metalloproteinase 10. Development and homeostasis of the cardiovascular system require intimate interactions between endothelial and smooth muscle cells, which form a seamless circulatory network. We show that histone deacetylase 7 (HDAC7) is specifically expressed in the vascular endothelium during early embryogenesis, where it maintains vascular integrity by repressing the expression of matrix metalloproteinase (MMP) 10, a secreted endoproteinase that degrades the **extracellular matrix**. Disruption of the HDAC7 gene in mice results in embryonic lethality due to a failure in endothelial cell-cell adhesion and consequent dilatation and rupture of blood vessels. HDAC7 represses MMP10 gene transcription by associating with myocyte enhancer factor-2 (MEF2), a direct activator of MMP10 transcription and essential regulator of blood vessel development. These findings reveal an unexpected and specific role for HDAC7 in the maintenance of vascular integrity and have important implications for understanding the processes of angiogenesis and vascular remodeling during cardiovascular development and disease.
5. The LKLF transcription factor is required for normal tunica media formation and blood vessel stabilization during murine embryogenesis. The transcriptional programs that regulate blood vessel formation are largely unknown. In this paper, we examine the role of the zinc finger transcription factor LKLF in murine blood vessel morphogenesis and homeostasis. By in situ hybridization and immunohistochemistry, we show that LKLF is expressed as early as embryonic day 9.5 (E9.5) in vascular endothelial cells throughout the developing mouse embryo. To better understand the function of LKLF, we used homologous recombination in embryonic stem (ES) cells to generate LKLF-deficient (LKLF-/-) mice. Both angiogenesis and vasculogenesis were normal in the LKLF-/- mice. However, LKLF-/- embryos died between E12.5 and E14.5 from severe intra-embryonic and intra-amniotic hemorrhaging. This bleeding disorder was associated with specific defects in blood vessel morphology. Umbilical veins and arteries in the LKLF-/- embryos displayed an abnormally thin tunica media and aneurysmal dilatation before rupturing into the amniotic cavity. Similarly, vascular smooth muscle cells in the aortae from the LKLF-/- animals displayed a cuboidal morphology and failed to organize into a compact tunica media. Consistent with these findings, electron microscopic analyses demonstrated endothelial cell necrosis, significant reductions in the number of vessel-wall pericytes and differentiating smooth muscle cells, and decreased deposition of **extracellular matrix** in the LKLF-/- vessels. Despite these defects, in situ hybridization demonstrated normal expression of platelet-derived growth factor B, Tie1, Tie2, transforming growth factor beta, and heparin-binding epidermal growth factor in the vasculature of the LKLF-/- embryos. Therefore, LKLF defines a novel transcriptional pathway in which endothelial cells regulate the assembly of the vascular tunica media and concomitant vessel wall stabilization during mammalian embryogenesis.
6. CYR61 (CCN1) is essential for placental development and vascular integrity. CYR61 (CCN1) is a member of the CCN family of secreted matricellular proteins that includes connective tissue growth factor (CCN2), NOV (CCN3), WISP-1 (CCN4), WISP-2 (CCN5), and WISP-3 (CCN6). First identified as the product of a growth factor-inducible immediate-early gene, CYR61 is an **extracellular matrix**-associated angiogenic inducer that functions as a ligand of integrin receptors to promote cell adhesion, migration, and proliferation. Aberrant expression of Cyr61 is associated with breast cancer, wound healing, and vascular diseases such as atherosclerosis and restenosis. To understand the functions of CYR61 during development, we have disrupted the Cyr61 gene in mice. We show here that Cyr61-null mice suffer embryonic death: approximately 30% succumbed to a failure in chorioallantoic fusion, and the reminder perished due to placental vascular insufficiency and compromised vessel integrity. These findings establish CYR61 as a novel and essential regulator of vascular development. CYR61 deficiency results in a specific defect in vessel bifurcation (nonsprouting angiogenesis) at the chorioallantoic junction, leading to an undervascularization of the placenta without affecting differentiation of the labyrinthine syncytiotrophoblasts. This unique phenotype is correlated with impaired Vegf-C expression in the allantoic mesoderm, suggesting that CYR61-regulated expression of Vegf-C plays a role in vessel bifurcation. The genetic and molecular basis of vessel bifurcation is presently unknown, and these findings provide new insight into this aspect of angiogenesis.
7. Probing chromatin landscape reveals roles of endocardial TBX20 in septation. Mutations in the T-box transcription factor TBX20 are associated with multiple forms of congenital heart defects, including cardiac septal abnormalities, but our understanding of the contributions of endocardial TBX20 to heart development remains incomplete. Here, we investigated how TBX20 interacts with endocardial gene networks to drive the mesenchymal and myocardial movements that are essential for outflow tract and atrioventricular septation. Selective ablation of Tbx20 in murine endocardial lineages reduced the expression of **extracellular matrix** and cell migration genes that are critical for septation. Using the assay for transposase-accessible chromatin with high-throughput sequencing (ATAC-seq), we identified accessible chromatin within endocardial lineages and intersected these data with TBX20 ChIP-seq and chromatin loop maps to determine that TBX20 binds a conserved long-range enhancer to regulate versican (Vcan) expression. We also observed reduced Vcan expression in Tbx20-deficient mice, supporting a direct role for TBX20 in Vcan regulation. Further, we show that the Vcan enhancer drove reporter gene expression in endocardial lineages in a TBX20-binding site-dependent manner. This work illuminates gene networks that interact with TBX20 to orchestrate cardiac septation and provides insight into the chromatin landscape of endocardial lineages during septation.
8. Defective angiogenesis and fatal embryonic hemorrhage in mice lacking core 1-derived O-glycans. The core 1 beta1-3-galactosyltransferase (T-synthase) transfers Gal from UDP-Gal to GalNAcalpha1-Ser/Thr (Tn antigen) to form the core 1 O-glycan Galbeta1-3GalNAcalpha1-Ser/Thr (T antigen). The T antigen is a precursor for extended and branched O-glycans of largely unknown function. We found that wild-type mice expressed the NeuAcalpha2-3Galbeta1-3GalNAcalpha1-Ser/Thr primarily in endothelial, hematopoietic, and epithelial cells during development. Gene-targeted mice lacking T-synthase instead expressed the nonsialylated Tn antigen in these cells and developed brain hemorrhage that was uniformly fatal by embryonic day 14. T-synthase-deficient brains formed a chaotic microvascular network with distorted capillary lumens and defective association of endothelial cells with pericytes and **extracellular matrix**. These data reveal an unexpected requirement for core 1-derived O-glycans during angiogenesis.
9. Restricted expression of the hyaluronan receptor, CD44, during postimplantation mouse embryogenesis suggests key roles in tissue formation and patterning. CD44 is a multifunctional adhesion protein that acts as a major receptor for the hygroscopic **extracellular matrix** component, hyaluronan. This receptor-ligand binding directly mediates at least some of the cell-cell and cell-matrix interactions ascribed to CD44. Other interactions involving CD44 may be modulated indirectly by its ability to bind growth factors and thereby to promote cell attachment. During vertebrate development, multiple cases of hyaluronan involvement in cell proliferation, cell migration and histogenesis have been documented. In addition, there is evidence suggesting a central role for cell surface glycoproteins and proteoglycans in mediating the action of polypeptide growth factors involved in tissue patterning. In view of this, we undertook to investigate expression of the CD44 protein during postimplantation mouse embryogenesis. Between 9.5 and 12.5 days of embryonic development, the predominant form of CD44 protein corresponds to the hyaluronan-binding CD44H form. However, species with a higher M(r) were also detected, implying that CD44 isoforms generated by alternative splicing of CD44 RNA are employed in normal development. Further, we used mouse embryos to perform whole-mount immunohistochemistry and examine the temporal and spatial distribution of this glycoprotein. CD44 is expressed at high levels in the heart, somites and condensing limb-bud mesenchyme at critical stages of morphogenesis. These sites correlate with regions where hyaluronan has been demonstrated to regulate morphogenetic events. Of novel interest, however, is the high expression of CD44 in regions that do not correlate with sites of known hyaluronan-mediated developmental events. These include instructive epithelia participating in epithelial-mesenchymal cell interactions such as the apical ectodermal ridge of the developing limb bud and the odontogenic placodes of the presumptive upper and lower jaws.
10. **Extracellular matrix** structure and tissue stiffness control postnatal lung development through the lipoprotein receptor-related protein 5/Tie2 signaling system. Physical properties of the tissues and remodeling of **extracellular matrix** (ECM) play an important role in organ development. Recently, we have reported that low-density lipoprotein receptor-related protein (LRP) 5/Tie2 signaling controls postnatal lung development by modulating angiogenesis. Here we show that tissue stiffness modulated by the ECM cross-linking enzyme, lysyl oxidase (LOX), regulates postnatal lung development through LRP5-Tie2 signaling. The expression of LRP5 and Tie2 is up-regulated twofold in lung microvascular endothelial cells when cultured on stiff matrix compared to those cultured on soft matrix in vitro. LOX inhibitor, β-aminopropionitrile, disrupts lung ECM (collagen I, III, and VI, and elastin) structures, softens neonatal mouse lung tissue by 20%, and down-regulates the expression of LRP5 and Tie2 by 20 and 60%, respectively, which leads to the inhibition of postnatal lung development (30% increase in mean linear intercept, 1.5-fold increase in air space area). Importantly, hyperoxia treatment (Postnatal Days 1-10) disrupts ECM structure and stiffens mouse lung tissue by up-regulating LOX activity, thereby increasing LRP5 and Tie2 expression and deregulating alveolar morphogenesis in neonatal mice, which is attenuated by inhibiting LOX activity. These findings suggest that appropriate physical properties of lung tissue are necessary for physiological postnatal lung development, and deregulation of this mechanism contributes to postnatal lung developmental disorders, such as bronchopulmonary dysplasia.

#### GeneRIFs

1. tissue stiffness modulated by the **extracellular matrix** cross-linking enzyme, lysyl oxidase, regulates postnatal lung development through LRP5-Tie2 signaling

### [Ii]nvasive

#### PubMed

1. Tie2-dependent VHL knockdown promotes airway microvascular regeneration and attenuates **invasive** growth of Aspergillus fumigatus. Microvascular ischemia and infections are associated with the development of chronic rejection following lung transplantation. The von Hippel–Lindau protein (VHL) controls protein levels of hypoxia-inducible factors (HIFs), regulates vascular repair, and improves tissue perfusion. Here, we studied the role of VHL in microvascular repair by orthotopically transplanting tracheas into mice with VHL haplodeficiency in Tie2 lineage cells. We showed that VHL haplodeficiency prolonged airway microvascular perfusion and promoted tissue blood flow through the production of the angiogenic factors, SDF-1 and angiopoietin 1. VHL-haplodeficient pulmonary endothelial cells exhibited increased angiogenic activity, resistance to serum deprivation-induced cell death, and enhanced microvascular repair. By contrast, in recipient mice with HIF-1α deficiency in Tie2 lineage cells, microvascular repair was significantly diminished and suggested that recipient-derived HIF-1α normally participates in the repair of alloimmune-mediated microvascular damage. To evaluate the translational impact of our findings, we compared VHL-haplodeficient mice with wild-type controls using a model of Aspergillus airway infection. In 83% of the VHL-haplodeficient recipients, Aspergillus fumigatus was **noninva**sive in contrast to 75% of wild-type mice in which the mold was dee**ply inva**sive. Our study demonstrated that stabilization of HIF-1α in angiogenic cells, through Tie2 cell VHL haplodeficiency, promoted airway microvascular regeneration and vascular normalization and thereby minimized tissue ischemia and hypoxia. By also mitigating the virulence of A. fumigatus, a common pathogen and itself a risk factor for the development of lung transplant rejection, the selective enhancement of HIF-1α expression has the prospect of offering several novel therapeutic effects to transplant recipients.
2. Embryo implantation in mouse: fetomaternal coordination in the pattern of expression of uPA, uPAR, PAI-1 and alpha 2MR/LRP genes. During the process of embryo implantation, trophoblast cells invade deep into uterine stroma and play a key role in establishing fetomaternal exchange of molecules. We have studied the in vivo expression patterns of the molecules of the urokinase system, during the process of mouse embryo implantation and early placentation. The sites of synthesis of urokinase-type plasminogen activator (uPA), uPA-receptor (uPAR), plasminogen activator inhibitor type 1 (PAI-1) and alpha 2-macroglobulin receptor/low density lipoprotein receptor-related protein (alpha 2MR/LRP) transcripts were determined by in situ hybridization. These genes were found to be expressed in a finely regulated pattern. High levels of uPA mRNA were found in **invasive** trophoblast cells, while the same cells did not appear to synthesize PAI-1. Starting from day 6.5, endothelial cells of newly forming vessels also transcribed uPA gene. uPAR and alpha 2MR/LRP were in all stages expressed by decidual tissue, and their expression domains overlapped in large areas. Immunohistochemistry with uPA and PAI-1 antibodies revealed areas of co-localization of these secreted proteins with the expression domains of uPAR and alpha 2MR/LRP, which is of great interest in view of the role of these two receptors in clearing uPA-PAI-1 complexes. In situ zymography demonstrated the presence of active uPA in the ectoplacental cone region at 7.5 and 8.5 days. Our studies outline the expression of a set of functionally related genes that is well coordinated between fetal and maternal tissues. This coordination may model other physiological and pathological **invasive** processes.

#### GeneRIFs

1. Tie2-dependent VHL knockdown promotes airway microvascular regeneration and attenuates **invasive** growth of Aspergillus fumigatus.

### [Mm]etastasis

#### PubMed

1. c-Myc is essential for vasculogenesis and angiogenesis during development and tumor progression. c-Myc promotes cell growth and transformation by ill-defined mechanisms. c-myc(-/-) mice die by embryonic day 10.5 (E10.5) with defects in growth and in cardiac and neural development. Here we report that the lethality of c-myc(-/-) embryos is also associated with profound defects in vasculogenesis and primitive erythropoiesis. Furthermore, c-myc(-/-) embryonic stem (ES) and yolk sac cells are compromised in their differentiative and growth potential. These defects are intrinsic to c-Myc, and are in part associated with a requirement for c-Myc for the expression of vascular endothelial growth factor (VEGF), as VEGF can partially rescue these defects. However, c-Myc is also required for the proper expression of other angiogenic factors in ES and yolk sac cells, including angiopoietin-2, and the angiogenic inhibitors thrombospondin-1 and angiopoietin-1. Finally, c-myc(-/-) ES cells are dramatically impaired in their ability to form tumors in immune-compromised mice, and the small tumors that sometimes develop are poorly vascularized. Therefore, c-Myc function is also necessary for the angiogenic switch that is indispensable for the progression and **metastasis** of tumors. These findings support the model wherein c-Myc promotes cell growth and transformation, as well as vascular and hematopoietic development, by functioning as a master regulator of angiogenic factors.
2. Targeting the ANG2/TIE2 axis inhibits tumor growth and **metastasis** by impairing angiogenesis and disabling rebounds of proangiogenic myeloid cells. Tumor-infiltrating myeloid cells convey proangiogenic programs that counteract the efficacy of antiangiogenic therapy. Here, we show that blocking angiopoietin-2 (ANG2), a TIE2 ligand and angiogenic factor expressed by activated endothelial cells (ECs), regresses the tumor vasculature and inhibits progression of late-stage, metastatic MMTV-PyMT mammary carcinomas and RIP1-Tag2 pancreatic insulinomas. ANG2 blockade did not inhibit recruitment of MRC1(+) TIE2-expressing macrophages (TEMs) but impeded their upregulation of Tie2, association with blood vessels, and ability to restore angiogenesis in tumors. Conditional Tie2 gene knockdown in TEMs was sufficient to decrease tumor angiogenesis. Our findings support a model wherein the ANG2-TIE2 axis mediates cell-to-cell interactions between TEMs and ECs that are important for tumor angiogenesis and can be targeted to induce effective antitumor responses.
3. Tie2 signaling regulates osteoclastogenesis and osteolytic bone invasion of breast cancer. Breast to bone **metastasis** is a common occurrence in the majority of patients with advanced breast cancer. The metastases are often incurable and are associated with bone destruction and high rates of morbidity. Understanding the underlying mechanisms of how metastatic tumor cells induce bone destruction is critically important. We previously reported that Tie2, a receptor tyrosine kinase, is significantly increased in human breast cancer tissues compared with normal and benign breast tumors and regulates tumor angiogenesis. In this study, we identify a new function of Tie2 in osteoclastogenesis and osteolytic bone invasion of breast cancer. Tie2 is present in hematopoietic stem/precursor cells. Genetic deletion of Tie2 or neutralization of Tie2 function using soluble Tie2 receptor impaired osteoclastogenesis in an embryonic stem cell differentiation assay. In contrast, deletion of Tie2 has no effect on osteoblastogenesis. As CD11b myeloid cells have the potential to become osteoclasts and Tie2 is present in a certain population of these cells, we isolated Tie2(+) and Tie2(-) myeloid cells. We observed a significant reduction of osteoclastogenesis in Tie2(-) compared with Tie2(+) CD11b cells. Consistently, neutralization of Tie2 activity in vivo significantly inhibited osteolytic bone invasion and tumor growth in a mammary tumor model, which correlated with a significant reduction of osteoclasts and tumor angiogenesis. Collectively, these data reveal a direct and novel role of Tie2 signaling in osteoclast differentiation. These findings identify Tie2 as a therapeutic target for controlling not only tumor angiogenesis but also osteolytic bone **metastasis** in breast cancer.
4. Functional significance of Tie2 signaling in the adult vasculature. Abundant data now demonstrate that the growth of new blood vessels, termed angiogenesis, plays both pathological and beneficial roles in human disease. Based on these data, a tremendous effort has been undertaken to understand the molecular mechanisms that drive blood vessel growth in adult tissues. Tie2 recently was identified as a receptor tyrosine kinase expressed principally on vascular endothelium. Disrupting Tie2 function in mice resulted in embryonic lethality with defects in embryonic vasculature, suggesting a role in blood vessel maturation and maintenance. Based on these studies, we undertook a series of studies to probe the function of Tie2 in adult vasculature that will form the focus of this chapter. Consistent with a role in blood vessel growth in adult vasculature, Tie2 was upregulated and activated in the endothelium of rat ovary and in healing rat skin wounds, both areas of active angiogenesis. Moreover, Tie2 was upregulated in the endothelium of vascular "hot spots" in human breast cancer specimens. Surprisingly, Tie2 also was expressed and activated in the endothelium of all normal rat tissues examined, suggesting a role in maintenance of adult vasculature. To determine the functional role of Tie2 in tumor vasculature, a soluble Tie2 extracellular domain (ExTek) was designed that blocked the activation of Tie2 by its activating ligand, angiopoietin 1 (Ang1). Administration of recombinant ExTek protein or an ExTek adenovirus inhibited tumor growth and **metastasis** in rodent tumor models, demonstrating a functional role for Tie2 in pathological angiogenesis in adult tissues. To begin to understand the endothelial signaling pathways and cellular responses that mediate Tie2 function, we identified signaling molecules that are recruited to the activated, autophosphorylated Tie2 kinase domain. Two of these molecules, SHP2 and GRB2, are part of the pathway upstream of mitogen-activated protein kinase (MAPK) activation, a pathway that may be responsible for morphogenetic effects of Tie2 on endothelial cells. Another signaling molecule, p85, is responsible for recruitment of phosphatidylinositol 3 kinase (PI3-K) and activation of the Akt/PI3-K pathway. Akt/PI3-K has emerged as a critical pathway downstream of Tie2 that is necessary for cell survival effects as well as for chemotaxis, activation of endothelial nitric oxide synthase, and perhaps for anti-inflammatory effects of Tie2 activation. Taken together, these studies and many others demonstrate that the Tie2 pathway has important functions in adult tissues, in both quiescent vasculature and during angiogenesis, and help to validate the Tie2 pathway as a therapeutic target.

### [Cc]ell [Ii]nvasion

#### PubMed

1. Endothelin-2 signaling in the neural retina promotes the endothelial tip cell state and inhibits angiogenesis. Endothelin signaling is required for neural crest migration and homeostatic regulation of blood pressure. Here, we report that constitutive overexpression of Endothelin-2 (Edn2) in the mouse retina perturbs vascular development by inhibiting endothelial cell migration across the retinal surface and subsequent endothelial **cell invasion** into the retina. Developing endothelial cells exist in one of two states: tip cells at the growing front and stalk cells in the vascular plexus behind the front. This division of endothelial cell states is one of the central organizing principles of angiogenesis. In the developing retina, Edn2 overexpression leads to overproduction of endothelial tip cells by both morphologic and molecular criteria. Spatially localized overexpression of Edn2 produces a correspondingly localized endothelial response. Edn2 overexpression in the early embryo inhibits vascular development at midgestation, but Edn2 overexpression in developing skin and brain has no discernible effect on vascular structure. Inhibition of retinal angiogenesis by Edn2 requires expression of Endothelin receptor A but not Endothelin receptor B in the neural retina. Taken together, these observations imply that the neural retina responds to Edn2 by synthesizing one or more factors that promote the endothelial tip cell state and inhibit angiogenesis. The response to Edn2 is sufficiently potent that it overrides the activities of other homeostatic regulators of angiogenesis, such as Vegf.

---

## Styk1

- NCBI Gene
- GeneRIF
- Pubmed

| Search Term | Rank | Fields |
| --- | --- | --- |
| [Mm]igration | 6 | Process(1); PubMed(1) |
| [Mm]etastasis | 12 | PubMed(2) |

  
hide/show details for Styk1

### [Mm]igration

#### Process

1. cell **migration**

#### PubMed

1. A novel protein tyrosine kinase NOK that shares homology with platelet- derived growth factor/fibroblast growth factor receptors induces tumorigenesis and metastasis in nude mice. Receptor protein tyrosine kinases (RPTKs) play important roles in the regulation of a variety of cellular processes including cell **migration**, proliferation, and protection from apoptosis. Here, we report the identification and characterization of a novel RPTK-like molecule that has a critical role in induction of tumorigenesis and metastasis and is termed Novel Oncogene with Kinase-domain (NOK). NOK contains a putative single transmembrane domain and a conserved intracellular tyrosine kinase domain that shares homology with members of the platelet-derived growth factor/fibroblast growth factor receptor superfamily. NOK was exclusively located in the cytoplasm. NOK mRNAs were detected in limited human organs and expressed with the highest abundance in the prostate. A variety of tumor cells also expressed the NOK mRNAs. We demonstrated that NIH3T3 and BaF3 cells could be strongly transformed by the expression of the NOK gene as examined by colony formation experiment. In addition, BaF3 cells with the stable expression of NOK induced rapid tumorigenesis in nude mice. Interestingly, these NOK-expressing tumor cells could promptly invade and spread into various distinct organs and form metastatic foci, eventually leading to the rapid death of these animals. Moreover, molecular mechanism studies indicated that NOK could concomitantly activate both MAP kinase and phosphatidylinositol 3'-kinases (PI3K) pathways in stable BaF3 cells. Thus, our results both in vitro and in vivo suggest that NOK is a novel oncogene with the capacity of promoting cell transformation, tumorigenesis, and metastasis.

### [Mm]etastasis

#### PubMed

1. A novel protein tyrosine kinase NOK that shares homology with platelet- derived growth factor/fibroblast growth factor receptors induces tumorigenesis and **metastasis** in nude mice. Receptor protein tyrosine kinases (RPTKs) play important roles in the regulation of a variety of cellular processes including cell migration, proliferation, and protection from apoptosis. Here, we report the identification and characterization of a novel RPTK-like molecule that has a critical role in induction of tumorigenesis and **metastasis** and is termed Novel Oncogene with Kinase-domain (NOK). NOK contains a putative single transmembrane domain and a conserved intracellular tyrosine kinase domain that shares homology with members of the platelet-derived growth factor/fibroblast growth factor receptor superfamily. NOK was exclusively located in the cytoplasm. NOK mRNAs were detected in limited human organs and expressed with the highest abundance in the prostate. A variety of tumor cells also expressed the NOK mRNAs. We demonstrated that NIH3T3 and BaF3 cells could be strongly transformed by the expression of the NOK gene as examined by colony formation experiment. In addition, BaF3 cells with the stable expression of NOK induced rapid tumorigenesis in nude mice. Interestingly, these NOK-expressing tumor cells could promptly invade and spread into various distinct organs and form metastatic foci, eventually leading to the rapid death of these animals. Moreover, molecular mechanism studies indicated that NOK could concomitantly activate both MAP kinase and phosphatidylinositol 3'-kinases (PI3K) pathways in stable BaF3 cells. Thus, our results both in vitro and in vivo suggest that NOK is a novel oncogene with the capacity of promoting cell transformation, tumorigenesis, and **metastasis**.
2. Point mutation at single tyrosine residue of novel oncogene NOK abrogates tumorigenesis in nude mice. Receptor protein-tyrosine kinases (RPTKs) are tightly regulated during normal cellular processes including cell growth, differentiation, and metabolism. Recently, a RPTK-like molecule named novel oncogene with kinase-domain (NOK) has been cloned and characterized. Overexpression of NOK caused severe cellular transformation as well as tumorigenesis and **metastasis** in nude mice. In the current study, we generated two tyrosine-->phenylalanine (Y-->F) point mutations (Y327F and Y356F) within the endodomain of NOK that are well conserved in many RPTK subfamilies and are the potential tyrosine phosphorylation sites important for major intracellular signaling. Using BaF3 cells stably expressing the ectodomain of mouse erythropoietin receptor, and the transmembrane and endodomain of NOK (BaF3-E/N), we were able to show that point mutations at either Y327 or Y356 dramatically blocked cellular transformation by NOK as examined by colony formation and cellular DNA synthesis. In addition, tumorigenesis induced by BaF3-E/N was completely abrogated upon the introduction of either single mutation. Importantly, signaling studies revealed that the activation of extracellular signal-regulated kinase was inhibited by Y356F and was significantly reduced by Y327F. Both mutations significantly impaired Akt phosphorylation. Interestingly, both mutations did not affect the kinase activity of NOK. Moreover, apoptotic analysis revealed that both mutations accelerated cell death by activating caspase-3-mediated pathways. Thus, our study shows that these potential tyrosine phosphorylation sites may play critical roles in NOK-mediated tumorigenesis both in vitro and in vivo.

---

## Pdgfrb

- NCBI Gene
- GeneRIF
- Pubmed

| Search Term | Rank | Fields |
| --- | --- | --- |
| [Ff]ilopodia | 1 | PubMed(2) |
| [Mm]igration | 6 | Process(9); PubMed(55); GeneRIFs(8) |
| ECM | 7 | PubMed(3); GeneRIFs(1) |
| [Ee]xtracellular [Mm]atrix | 8 | PubMed(7) |
| [Ii]nvasive | 10 | PubMed(5) |
| [Mm]etastasis | 12 | PubMed(4) |
| [Cc]ell [Ii]nvasion | 13 | PubMed(1) |

  
hide/show details for Pdgfrb

### [Ff]ilopodia

#### PubMed

1. Serum response factor is required for sprouting angiogenesis and vascular integrity. Serum response factor (SRF) is a transcription factor that controls the expression of cytoskeletal proteins and immediate early genes in different cell types. Here, we found that SRF expression is restricted to endothelial cells (ECs) of small vessels such as capillaries in the mouse embryo. EC-specific Srf deletion led to aneurysms and hemorrhages from 11.5 days of mouse development (E11.5) and lethality at E14.5. Mutant embryos presented a reduced capillary density and defects in EC migration, with fewer numbers of **filopodia** in tip cells and ECs showing defects in actin polymerization and intercellular junctions. We show that SRF is essential for the expression of VE-cadherin and beta-actin in ECs both in vivo and in vitro. Moreover, knockdown of SRF in ECs impaired VEGF- and FGF-induced in vitro angiogenesis. Taken together, our results demonstrate that SRF plays an important role in sprouting angiogenesis and small vessel integrity in the mouse embryo.
2. The netrin receptor UNC5B mediates guidance events controlling morphogenesis of the vascular system. Blood vessels and nerves are complex, branched structures that share a high degree of anatomical similarity. Guidance of vessels and nerves has to be exquisitely regulated to ensure proper wiring of both systems. Several regulators of axon guidance have been identified and some of these are also expressed in endothelial cells; however, the extent to which their guidance functions are conserved in the vascular system is still incompletely understood. We show here that the repulsive netrin receptor UNC5B is expressed by endothelial tip cells of the vascular system. Disruption of the Unc5b gene in mice, or of Unc5b or netrin-1a in zebrafish, leads to aberrant extension of endothelial tip cell **filopodia**, excessive vessel branching and abnormal navigation. Netrin-1 causes endothelial **filopodia**l retraction, but only when UNC5B is present. Thus, UNC5B functions as a repulsive netrin receptor in endothelial cells controlling morphogenesis of the vascular system.

### [Mm]igration

#### Process

1. cell **migration**
2. cell **migration** involved in coronary angiogenesis
3. cell **migration** involved in vasculogenesis
4. metanephric mesenchymal cell **migration**
5. positive regulation of cell **migration**
6. positive regulation of metanephric mesenchymal cell **migration** by platelet-derived growth factor receptor-beta signaling pathway
7. positive regulation of smooth muscle cell **migration**
8. positive regulation of smooth muscle cell **migration**
9. positive regulation of smooth muscle cell **migration**

#### PubMed

1. Thymosin β4 is not required for embryonic viability or vascular development. Rossdeutsch et al describe a requirement for thymosin β4 (Tβ4) in vascular development. Impaired mural ce**ll migrat**ion, differentiation, partial embryonic lethality, and hemorrhaging were observed after analysis of 2 lines of mice, one of which was germline null for Tβ4 and another in which Tβ4 was knocked down by endothelial-specific expression of Tβ4 short hairpin RNA. These data are in direct contrast to our published global and cardiac-specific Tβ4-knockout lines. Thus, the role of Tβ4 needs to be clarified to understand its importance in cardiovascular development.To investigate and clarify the role of Tβ4 in vascular smooth muscle cell development and vessel stability.Examination of Tβ4 global knockouts did not demonstrate embryonic hemorrhaging, altered mural cell development, or lethality. Endothelial-specific knockouts also did not exhibit any embryonic lethality and were viable to adulthood.Analysis of our Tβ4 global and cardiac- and endothelial-specific knockout models demonstrated that Tβ4 is dispensable for embryonic viability and vascular development.
2. Serum response factor is required for sprouting angiogenesis and vascular integrity. Serum response factor (SRF) is a transcription factor that controls the expression of cytoskeletal proteins and immediate early genes in different cell types. Here, we found that SRF expression is restricted to endothelial cells (ECs) of small vessels such as capillaries in the mouse embryo. EC-specific Srf deletion led to aneurysms and hemorrhages from 11.5 days of mouse development (E11.5) and lethality at E14.5. Mutant embryos presented a reduced capillary density and defects in EC **migration**, with fewer numbers of filopodia in tip cells and ECs showing defects in actin polymerization and intercellular junctions. We show that SRF is essential for the expression of VE-cadherin and beta-actin in ECs both in vivo and in vitro. Moreover, knockdown of SRF in ECs impaired VEGF- and FGF-induced in vitro angiogenesis. Taken together, our results demonstrate that SRF plays an important role in sprouting angiogenesis and small vessel integrity in the mouse embryo.
3. Msx genes define a population of mural cell precursors required for head blood vessel maturation. Vessels are primarily formed from an inner endothelial layer that is secondarily covered by mural cells, namely vascular smooth muscle cells (VSMCs) in arteries and veins and pericytes in capillaries and veinules. We previously showed that, in the mouse embryo, Msx1(lacZ) and Msx2(lacZ) are expressed in mural cells and in a few endothelial cells. To unravel the role of Msx genes in vascular development, we have inactivated the two Msx genes specifically in mural cells by combining the Msx1(lacZ), Msx2(lox) and Sm22α-Cre alleles. Optical projection tomography demonstrated abnormal branching of the cephalic vessels in E11.5 mutant embryos. The carotid and vertebral arteries showed an increase in caliber that was related to reduced vascular smooth muscle coverage. Taking advantage of a newly constructed Msx1(CreERT2) allele, we demonstrated by lineage tracing that the primary defect lies in a population of VSMC precursors. The abnormal phenotype that ensues is a consequence of impaired BMP signaling in the VSMC precursors that leads to downregulation of the metalloprotease 2 (Mmp2) and Mmp9 genes, which are essential for cell **migratio**n and integration into the mural layer. Improper coverage by VSMCs secondarily leads to incomplete maturation of the endothelial layer. Our results demonstrate that both Msx1 and Msx2 are required for the recruitment of a population of neural crest-derived VSMCs.
4. Loss of Notch signalling induced by Dll4 causes arterial calibre reduction by increasing endothelial cell response to angiogenic stimuli. In the vascular system, Notch receptors and ligands are expressed mainly on arteries, with Delta-like 4 (Dll4) being the only ligand known to be expressed early during the development of arterial endothelial cells and capillaries. Dll4 null embryos die very early in development with severely reduced arterial calibre and lumen and loss of arterial cell identity.The current detailed analysis of these mutants shows that the arterial defect precedes the initiation of blood flow and that the arterial Dll4-/- endothelial cells proliferate and migrate more actively. Dll4-/- mutants reveal a defective basement membrane around the forming aorta and increased endothelial cell **migration** from the dorsal aorta to peripheral regions, which constitute the main causes of arterial lumen reduction in these embryos. The increased proliferation and **migration** of Dll4-/- endothelial cells was found to coincide with increased expression of the receptors VEGFR-2 and Robo4 and with downregulation of the TGF-beta accessory receptor Endoglin.Together, these results strongly suggest that Notch signalling can increase arterial stability and calibre by decreasing the response of arterial endothelial cells to local gradients of pro-angiogenic factors like VEGF.
5. Essential regulation of CNS angiogenesis by the orphan G protein-coupled receptor GPR124. The orphan G protein-coupled receptor (GPCR) GPR124/tumor endothelial marker 5 is highly expressed in central nervous system (CNS) endothelium. Here, we show that complete null or endothelial-specific GPR124 deletion resulted in embryonic lethality from CNS-specific angiogenesis arrest in forebrain and neural tube. Conversely, GPR124 overexpression throughout all adult vascular beds produced CNS-specific hyperproliferative vascular malformations. In vivo, GPR124 functioned cell-autonomously in endothelium to regulate sprouting, **migration**, and developmental expression of the blood-brain barrier marker Glut1, whereas in vitro, GPR124 mediated Cdc42-dependent directional **migration** to forebrain-derived, vascular endothelial growth factor-independent cues. Our results demonstrate CNS-specific angiogenesis regulation by an endothelial receptor and illuminate functions of the poorly understood adhesion GPCR subfamily. Further, the functional tropism of GPR124 marks this receptor as a therapeutic target for CNS-related vascular pathologies.
6. The PTEN/PI3K pathway governs normal vascular development and tumor angiogenesis. PTEN is an important tumor suppressor gene. Hereditary mutation of PTEN causes tumor-susceptibility diseases such as Cowden disease. We used the Cre-loxP system to generate an endothelial cell-specific mutation of Pten (Tie2CrePten) in mice. Tie2CrePten(flox/+) mice displayed enhanced tumorigenesis due to an increase in angiogenesis driven by vascular growth factors. This effect was partially dependent on the PI3K subunits p85alpha and p110gamma. In vitro, Tie2CrePten(flox/+) endothelial cells showed enhanced proliferation/**migration**. Tie2CrePten(flox/flox) mice died before embryonic day 11.5 (E11.5) due to bleeding and cardiac failure caused by impaired recruitment of pericytes and vascular smooth muscle cells to blood vessels, and of cardiomyocytes to the endocardium. These phenotypes depend strongly on p110gamma rather than on p85alpha and were associated with decreased expression of Ang-1, VCAM-1, connexin 40, and ephrinB2 but increased expression of Ang-2, VEGF-A, VEGFR1, and VEGFR2. Pten is thus indispensable for normal cardiovascular morphogenesis and post-natal angiogenesis, including tumor angiogenesis.
7. β1 integrins regulate fibroblast chemotaxis through control of N-WASP stability. Chemotactic **migratio**n of fibroblasts towards growth factors, such as during development and wound healing, requires precise spatial coordination of receptor signalling. However, the mechanisms regulating this remain poorly understood. Here, we demonstrate that β1 integrins are required both for fibroblast chemotaxis towards platelet-derived growth factor (PDGF) and growth factor-induced dorsal ruffling. Mechanistically, we show that β1 integrin stabilises and spatially regulates the actin nucleating endocytic protein neuronal Wiskott–Aldrich syndrome protein (N-WASP) to facilitate PDGF receptor traffic and directed motility. Furthermore, we show that in intact cells, PDGF binding leads to rapid activation of β1 integrin within newly assembled actin-rich membrane ruffles. Active β1 in turn controls assembly of N-WASP complexes with both Cdc42 and WASP-interacting protein (WIP), the latter of which acts to stabilise the N-WASP. Both of these protein complexes are required for PDGF internalisation and fibroblast chemotaxis downstream of β1 integrins. This represents a novel mechanism by which integrins cooperate with growth factor receptors to promote localised signalling and directed cell motility.
8. Myocardin-related transcription factors control the motility of epicardium-derived cells and the maturation of coronary vessels. An important pool of cardiovascular progenitor cells arises from the epicardium, a single layer of mesothelium lining the heart. Epicardium-derived progenitor cell (EPDC) formation requires epithelial-to-mesenchymal transition (EMT) and the subsequent **migration** of these cells into the sub-epicardial space. Although some of the physiological signals that promote EMT are understood, the functional mediators of EPDC motility and differentiation are not known. Here, we identify a novel regulatory mechanism of EPDC mobilization. Myocardin-related transcription factor (MRTF)-A and MRTF-B (MKL1 and MKL2, respectively) are enriched in the perinuclear space of epicardial cells during development. Transforming growth factor (TGF)-β signaling and disassembly of cell contacts leads to nuclear accumulation of MRTFs and the activation of the motile gene expression program. Conditional ablation of Mrtfa and Mrtfb specifically in the epicardium disrupts cell **migratio**n and leads to sub-epicardial hemorrhage, partially stemming from the depletion of coronary pericytes. Using lineage-tracing analyses, we demonstrate that sub-epicardial pericytes arise from EPDCs in a process that requires the MRTF-dependent motile gene expression program. These findings provide novel mechanisms linking EPDC motility and differentiation, shed light on the transcriptional control of coronary microvascular maturation and suggest novel therapeutic strategies to manipulate epicardium-derived progenitor cells for cardiac repair.
9. GPR124, an orphan G protein-coupled receptor, is required for CNS-specific vascularization and establishment of the blood-brain barrier. Every organ in the body requires blood vessels for efficient delivery of oxygen and nutrients, but independent vascular beds are highly specialized to meet the individual needs of specific organs. The vasculature of the brain is tightly sealed, with blood-brain barrier (BBB) properties developing coincident with neural vascularization. G protein-coupled receptor 124 (GPR124) (tumor endothelial marker 5, TEM5), an orphan member of the adhesion family of G protein-coupled receptors, was previously identified on the basis of its overexpression in tumor vasculature. Here, we show that global deletion or endothelial-specific deletion of GPR124 in mice results in embryonic lethality associated with abnormal angiogenesis of the forebrain and spinal cord. Expression of GPR124 was found to be required for invasion and **migration** of blood vessels into neuroepithelium, establishment of BBB properties, and expansion of the cerebral cortex. Thus, GPR124 is an important regulator of neurovasculature development and a potential drug target for cerebrovascular diseases.
10. PTEN tumor suppressor associates with NHERF proteins to attenuate PDGF receptor signaling. PTEN, a tumor suppressor frequently inactivated in many human cancers, directly antagonizes the activity of phosphatidylinositol-3-OH kinase (PI3K) by dephosphorylating phosphoinositides. We show here that PTEN interacts directly with the NHERF1 and NHERF2 (Na+/H+ exchanger regulatory factor) homologous adaptor proteins through the PDZ motif of PTEN and the PDZ1 domain of NHERF1 or both PDZ domains of NHERF2. NHERFs were shown to interact directly with platelet-derived growth factor receptor (PDGFR), and we demonstrate the assembly of a ternary complex between PTEN, NHERFs and PDGFR. The activation of the PI3K pathway after PDGFR stimulation was prolonged in NHERF1(-/-) mouse embryonic fibroblasts as compared to wild-type cells, consistent with defective PTEN recruitment to PDGFR in the absence of NHERF1. Depletion of NHERF2 by small interfering RNA similarly increased PI3K signaling. Phenotypically, the loss of NHERF1 enhanced the PDGF-induced cytoskeletal rearrangements and chemotactic **migration** of the cells. These data indicate that, in normal cells, NHERF proteins recruit PTEN to PDGFR to restrict the activation of the PI3K.
11. Defective N-sulfation of heparan sulfate proteoglycans limits PDGF-BB binding and pericyte recruitment in vascular development. During vascular development, endothelial platelet-derived growth factor B (PDGF-B) is critical for pericyte recruitment. Deletion of the conserved C-terminal heparin-binding motif impairs PDGF-BB retention and pericyte recruitment in vivo, suggesting a potential role for heparan sulfate (HS) in PDGF-BB function during vascular development. We studied the participation of HS chains in pericyte recruitment using two mouse models with altered HS biosynthesis. Reduction of N-sulfation due to deficiency in N-deacetylase/N-sulfotransferase-1 attenuated PDGF-BB binding in vitro, and led to pericyte detachment and delayed pericyte **migration** in vivo. Reduced N-sulfation also impaired PDGF-BB signaling and directed cell **migration**, but not proliferation. In contrast, HS from glucuronyl C5-epimerase mutants, which is extensively N- and 6-O-sulfated, but lacks 2-O-sulfated L-iduronic acid residues, retained PDGF-BB in vitro, and pericyte recruitment in vivo was only transiently delayed. These observations were supported by in vitro characterization of the structural features in HS important for PDGF-BB binding. We conclude that pericyte recruitment requires HS with sufficiently extended and appropriately spaced N-sulfated domains to retain PDGF-BB and activate PDGF receptor beta (PDGFRbeta) signaling, whereas the detailed sequence of monosaccharide and sulfate residues does not appear to be important for this interaction.
12. Lack of pericytes leads to endothelial hyperplasia and abnormal vascular morphogenesis. The association of pericytes (PCs) to newly formed blood vessels has been suggested to regulate endothelial cell (EC) proliferation, survival, **migration**, differentiation, and vascular branching. Here, we addressed these issues using PDGF-B-- and PDGF receptor-beta (PDGFR-beta)--deficient mice as in vivo models of brain angiogenesis in the absence of PCs. Quantitative morphological analysis showed that these mutants have normal microvessel density, length, and number of branch points. However, absence of PCs correlates with endothelial hyperplasia, increased capillary diameter, abnormal EC shape and ultrastructure, changed cellular distribution of certain junctional proteins, and morphological signs of increased transendothelial permeability. Brain endothelial hyperplasia was observed already at embryonic day (E) 11.5 and persisted throughout development. From E 13.5, vascular endothelial growth factor-A (VEGF-A) and other genes responsive to metabolic stress became upregulated, suggesting that the abnormal microvessel architecture has systemic metabolic consequences. VEGF-A upregulation correlated temporally with the occurrence of vascular abnormalities in the placenta and dilation of the heart. Thus, although PC deficiency appears to have direct effects on EC number before E 13.5, the subsequent increased VEGF-A levels may further abrogate microvessel architecture, promote vascular permeability, and contribute to formation of the edematous phenotype observed in late gestation PDGF-B and PDGFR-beta knock out embryos.
13. Involvement of platelet-derived growth factor receptor β in fibrosis through extracellular matrix protein production after ischemic stroke. Fibrosis is concomitant with repair processes following injuries in the central nervous system (CNS). Pericytes are considered as an origin of fibrosis-forming cells in the CNS. Here, we examined whether platelet-derived growth factor receptor β (PDGFRβ), a well-known indispensable molecule f**or migrat**ion, proliferation, and survival of pericytes, was involved in the production of extracellular matrix proteins, fibronectin and collagen type I, which is crucial for fibrosis after ischemic stroke. Immunohistochemistry demonstrated induction of PDGFRβ expression in vascular cells of peri-infarct areas at 3-7days in a mouse stroke model. The PDGFRβ-expressing cells extended from peri-infarct areas toward the ischemic core after day 7 while expressing fibronectin and collagen type I in the infarct areas. In contrast, desmin and α-smooth muscle actin, markers of pericytes, were only expressed in vascular cells. In PDGFRβ heterozygous knockout mice, the expression of fibronectin and collagen type I was attenuated at both mRNA and protein levels with an enlargement of the infarct volume after ischemic stroke compared with that in wild-type littermates. In cultured brain pericytes, the expression of PDGF-B, PDGFRβ, fibronectin, and collagen type I, but not desmin, was significantly increased by serum depletion (SD). The SD-induced upregulation of fibronectin and collagen type I was suppressed by SU11652, an inhibitor of PDGFRβ, while PDGF-B further increased the SD-induced upregulation. In conclusion, the expression level of PDGFRβ may be a crucial determinant of fibrosis after ischemic stroke. Moreover, PDGFRβ signaling participates in the production of fibronectin and collagen type I after ischemic stroke.
14. Deletion of the PDGFR-beta gene affects key fibroblast functions important for wound healing. This study provides new perspectives of the unique aspects of platelet-derived growth factor beta-receptor (PDGFR-beta) signaling and biological responses through the establishment of a mutant mouse strain in which two loxP sequences were inserted into the introns of PDGFR-beta genome sequences. Isolation of skin fibroblasts from the mutant mice and Cre recombinase transfection in vitro induced PDGFR-beta gene deletion (PDGFR-betaDelta/Delta). The resultant depletion of the PDGFR-beta protein significantly attenuated platelet-derived growth factor (PDGF)-BB-induced cell **migration**, proliferation, and protection from H2O2-induced apoptosis of the cultured PDGFR-betaDelta/Delta dermal fibroblasts. PDGF-AA and fetal bovine serum were mitogenic and anti-apoptotic but were unable to induce the **migration** in PDGFR-beta Delta/Delta fibroblasts. Concerning the PDGF signaling, PDGF-BB-induced phosphorylation of Akt, ERK1/2, and JNK, but not p38, decreased in PDGFR-betaDelta/Delta fibroblasts, but PDGF-AA-induced signaling was not altered. Overexpression of the phospholipid phosphatases, SHIP2 and/or PTEN, inhibited PDGF-BB-induced phosphorylation of Akt and ERK1/2 in PDGFR-betaDelta/Delta fibroblasts but did not affect that of JNK and p38. These results indicate that disruption of distinct PDGFR-beta signaling pathways in PDGFR-betaDelta/Delta dermal fibroblasts impaired their proliferation and survival, but completely inhibits migratory response, and that PDGF-BB-induced phosphorylation of Akt and ERK1/2 possibly mediated by PDGFR-alpha is regulated, at least in part, by the lipid phosphatases SHIP2 and/or PTEN. Thus, the PDGFR-beta function on dermal fibroblasts appears to be critical in PDGF-BB action for skin wound healing and is clearly distinctive from that of PDGFR-alpha in the ligand-induced biological responses and the underlying properties of cellular signaling.
15. PDGF receptor-{beta} modulates metanephric mesenchyme chemotaxis induced by PDGF AA. PDGF B chain or PDGF receptor (PDGFR)-beta-deficient (-/-) mice lack mesangial cells. To study responses of alpha- and beta-receptor activation to PDGF ligands, metanephric mesenchymal cells (MMCs) were established from embryonic day E11.5 wild-type (+/+) and -/- mouse embryos. PDGF BB stimulated cell **migration** in +/+ cells, whereas PDGF AA did not. Conversely, PDGF AA was chemotactic for -/- MMCs. The mechanism by which PDGFR-beta inhibited AA-induced **migration** was investigated. PDGF BB, but not PDGF AA, increased intracellular Ca(2+) and the production of reactive oxygen species (ROS) in +/+ cells. Transfection of -/- MMCs with the wild-type beta-receptor restored cell **migration** and ROS generation in response to PDGF BB and inhibited AA-induced **migration**. Inhibition of Ca(2+) signaling facilitated PDGF AA-induced chemotaxis in the wild-type cells. The antioxidant N-acetyl-l-cysteine (NAC) or the NADPH oxidase inhibitor diphenyleneiodonium (DPI) abolished the BB-induced increase in intracellular Ca(2+) concentration, suggesting that ROS act as upstream mediators of Ca(2+) in suppressing PDGF AA-induced **migration**. These data indicate that ROS and Ca(2+) generated by active PDGFR-beta play an essential role in suppressing PDGF AA-induced **migration** in +/+ MMCs. During kidney development, PDGFR beta-mediated ROS generation and Ca(2+) influx suppress PDGF AA-induced chemotaxis in metanephric mesenchyme.
16. Ephrin-B2 controls cell motility and adhesion during blood-vessel-wall assembly. New blood vessels are initially formed through the assembly or sprouting of endothelial cells, but the recruitment of supporting pericytes and vascular smooth muscle cells (mural cells) ensures the formation of a mature and stable vascular network. Defective mural-cell coverage is associated with the poorly organized and leaky vasculature seen in tumors or other human diseases. Here we report that mural cells require ephrin-B2, a ligand for Eph receptor tyrosine kinases, for normal association with small-diameter blood vessels (microvessels). Tissue-specific mutant mice display perinatal lethality; vascular defects in skin, lung, gastrointestinal tract, and kidney glomeruli; and abnormal **migration** of smooth muscle cells to lymphatic capillaries. Cultured ephrin-B2-deficient smooth muscle cells are defective in spreading, focal-adhesion formation, and polarized **migration** and show increased motility. Our results indicate that the role of ephrin-B2 and EphB receptors in these processes involves Crk-p130(CAS) signaling and suggest that ephrin-B2 has some cell-cell-contact-independent functions.
17. Wt1 controls retinoic acid signalling in embryonic epicardium through transcriptional activation of Raldh2. Epicardial-derived signals are key regulators of cardiac embryonic development. An important part of these signals is known to relate to a retinoic acid (RA) receptor-dependent mechanism. RA is a potent morphogen synthesised by Raldh enzymes, Raldh2 being the predominant one in mesodermal tissues. Despite the importance of epicardial retinoid signalling in the heart, the molecular mechanisms controlling cardiac Raldh2 transcription remain unknown. In the current study, we show that Wt1-null epicardial cells display decreased expression of Raldh2 both in vivo and in vitro. Using a RA-responsive reporter, we have confirmed that Wt1-null epicardial cells actually show reduced synthesis of RA. We also demonstrate that Raldh2 is a direct transcriptional target of Wt1 in epicardial cells. A secondary objective of this study was to identify the status of RA-related receptors previously reported to be critical to epicardial biology (PDGFRα,β; RXRα). PDGFRα and PDGFRβ mRNA and protein levels are downregulated in the absence of Wt1, but only Pdgfra expression is rescued by the addition of RA to Wt1-null epicardial cells. RXRα mRNA levels are not affected in Wt1-null epicardial cells. Taken together, our results indicate that Wt1 critically regulates epicardial RA signalling via direct activation of the Raldh2 gene, and identify a role for Wt1 in the regulation of morphogen receptors involved in the prolifera**tion, mig**ration, and differentiation of epicardial and epicardially-derived cells (EPDC).
18. Forced expression of platelet-derived growth factor B in the mouse cerebellar primordium changes cell **migration** during midline fusion and causes cerebellar ectopia. The platelet-derived growth factor (PDGF) and receptors are expressed in the developing central nervous system and in brain tumors. To investigate the role of PDGF during normal cerebellar development, we created transgenic mice where PDGF-B was introduced into the endogenous Engrailed1 locus (En1). These mice expressed PDGF-B in all types of cells that constitute the developing cerebellum, with localized high expression in the ventral midline of the cerebellar anlage. This affected cell **migration** in the midline during fusion of the cerebellar anlage and caused misplacement of midline structures. PDGFR-alpha- and laminin alpha1-positive meningeal cells migrated inwards, attracted by the ectopic transgene expression in the ventral neuroepithelium. Other cells followed the meningeal cells and in the adult mouse, cells from all cortical cell layers were found misplaced in the midline. Moreover, the transgene caused an enhancement of capillary vessels. The findings indicate that normal PDGF signaling is important for proper neural tube fusion. It also illustrates that meningeal structures can influence the process.
19. A gain-of-function mutation in the PDGFR-beta alters the kinetics of injury response in liver and skin. Platelet-derived growth factor (PDGF) isoforms stimulate cell proliferation, **migration** and survival. We recently generated mice carrying a gain-of-function mutation within the activation loop of PDGF beta-receptor (PDGFR-beta D849N). Embryonic fibroblasts derived from these mice show elevated basal phosphorylation and altered kinetics for ligand-induced activation of PDGFR-beta, as well as enhanced proliferation and **migration**. To investigate the effect of this mutation in vivo, we used carbon tetrachloride-induced liver injury as a model system. We observed a higher basal activation of mutant PDGFR-beta in unchallenged livers; however, the difference in activation upon carbon tetrachloride stimulation was lower than expected, an effect that might be explained by a delayed response of the mutated receptor toward reactive oxygen species. Mutant mice showed enhanced proliferation of nonparenchymal liver cells and activation of hepatic stellate cells, leading to a small increase in early fibrosis formation. Another mouse strain lacking the binding site for phosphatidylinositol-3' kinase in PDGFR-beta showed the reverse phenotype. These results suggest an important role for PDGFR-beta signaling in the early injury-response. We confirmed this hypothesis with a second injury model, cutaneous wound healing, where we observed earlier proliferation and formation of granulation tissue in D849N-mutant mice.
20. Role of PDGF-B and PDGFR-beta in recruitment of vascular smooth muscle cells and pericytes during embryonic blood vessel formation in the mouse. Development of a vascular system involves the assembly of two principal cell types - endothelial cells and vascular smooth muscle cells/pericytes (vSMC/PC) - into many different types of blood vessels. Most, if not all, vessels begin as endothelial tubes that subsequently acquire a vSMC/PC coating. We have previously shown that PDGF-B is critically involved in the recruitment of pericytes to brain capillaries and to the kidney glomerular capillary tuft. Here, we used desmin and alpha-smooth muscle actin (ASMA) as markers to analyze vSMC/PC development in PDGF-B-/- and PDGFR-beta-/- embryos. Both mutants showed a site-specific reduction of desmin-positive pericytes and ASMA-positive vSMC. We found that endothelial expression of PDGF-B was restricted to immature capillary endothelial cells and to the endothelium of growing arteries. BrdU labeling showed that PDGFR-beta-positive vSMC/PC progenitors normally proliferate at sites of endothelial PDGF-B expression. In PDGF-B-/- embryos, limb arterial vSMC showed a reduced BrdU-labeling index. This suggests a role of PDGF-B in vSMC/PC cell proliferation during vascular growth. Two modes of vSMC recruitment to newly formed vessels have previously been suggested: (1) de novo formation of vSMC by induction of undifferentiated perivascular mesenchymal cells, and (2) co-**migration** of vSMC from a preexisting pool of vSMC. Our data support both modes of vSMC/PC development and lead to a model in which PDGFR-beta-positive vSMC/PC progenitors initially form around certain vessels by PDGF-B-independent induction. Subsequent angiogenic sprouting and vessel enlargement involves PDGF-B-dependent vSMC/PC progenitor co-**migration** and proliferation, and/or PDGF-B-independent new induction of vSMC/PC, depending on tissue context.
21. Sphingosine-1-Phosphate Induces the **Migration** and Angiogenesis of Epcs Through the Akt Signaling Pathway via Sphingosine-1-Phosphate Receptor 3/Platelet-Derived Growth Factor Receptor-β. Endothelial progenitor cells (EPCs) play a fundamental role in neoangiogenesis and tumor angiogenesis. Through the sphingosine-1-phosphate receptor 3 (S1PR3), sphingosine-1-phosphate (S1P) can stimulate the functional capacity of EPCs. Platelet-derived growth factor receptor-beta (PDGFR-β) contributes to th**e migrati**on and angiogenesis of EPCs. This study aimed to investigate whether S1P induces th**e migrati**on and angiogenesis of EPCs through the S1PR3/PDGFR-β/Akt signaling pathway. We used the Transwell system and the Chemicon In Vitro Angiogenesis Assay Kit with CAY10444 (an S1PR3 antagonist), AG1295 (a PDGFR kinase inhibitor) and sc-221226 (an Akt inhibitor) to examine the role of the S1PR3/PDGFR-β/Akt pathway in the S1Pindu**ced migra**tion and angiogenesis of EPCs.
22. LRP1 regulates architecture of the vascular wall by controlling PDGFRbeta-dependent phosphatidylinositol 3-kinase activation. Low density lipoprotein receptor-related protein 1 (LRP1) protects against atherosclerosis by regulating the activation of platelet-derived growth factor receptor beta (PDGFRbeta) in vascular smooth muscle cells (SMCs). Activated PDGFRbeta undergoes tyrosine phosphorylation and subsequently interacts with various signaling molecules, including phosphatidylinositol 3-kinase (PI3K), which binds to the phosphorylated tyrosine 739/750 residues in mice, and thus regulates actin polymerization and cell movement.In this study, we found disorganized actin in the form of membrane ruffling and enhanced cell **migration** in LRP1-deficient (LRP1-/-) SMCs. Marfan syndrome-like phenotypes such as tortuous aortas, disrupted elastic layers and abnormally activated transforming growth factor beta (TGFbeta) signaling are present in smooth muscle-specific LRP1 knockout (smLRP1-/-) mice. To investigate the role of LRP1-regulated PI3K activation by PDGFRbeta in atherogenesis, we generated a strain of smLRP1-/- mice in which tyrosine 739/750 of the PDGFRbeta had been mutated to phenylalanines (PDGFRbeta F2/F2). Spontaneous atherosclerosis was significantly reduced in the absence of hypercholesterolemia in these mice compared to smLRP1-/- animals that express wild type PDGFR. Normal actin organization was restored and spontaneous SMC **migration** as well as PDGF-BB-induced chemotaxis was dramatically reduced, despite continued overactivation of TGFbeta signaling, as indicated by high levels of nuclear phospho-Smad2.Our data suggest that LRP1 regulates actin organization and cell **migration** by controlling PDGFRbeta-dependent activation of PI3K. TGFbeta activation alone is not sufficient for the expression of the Marfan-like vascular phenotype. Thus, regulation of PI3 Kinase by PDGFRbeta is essential for maintaining vascular integrity, and for the prevention of atherosclerosis as well as Marfan syndrome.
23. Cooperation between the PDGF receptors in cardiac neural crest cell **migration**. Neural crest cells (NCCs) are essential components of the sympathetic nervous system, skin, craniofacial skeleton, and aortic arch. It has been known for many years that perturbation of **migration**, proliferation, and/or differentiation of these cells leads to birth defects such as cleft palate and persistent truncus arteriosus (PTA). Previously, we had shown that disruption of the platelet-derived growth factor receptor (PDGFR) alpha in NCCs resulted in defects in craniofacial and aortic arch development, the latter with variable penetrance. Because we observed ventricular septal defects in embryos that are null for the PDGFRbeta, we hypothesized that both PDGF receptors are involved in NCC formation. Here, we show that both receptors are expressed in cardiac NCCs and that the combined loss of the PDGFRalpha and PDGFRbeta in NCCs resulted in NCC-related heart abnormalities, including PTA and a ventricular septal defect (VSD). Using NCC lineage tracing, we observed that loss of PDGF receptor signaling resulted in reduced NCCs in the conotruncus region, leading to defects in aortic arch septation. These results indicate that while PDGFRalpha plays a predominant role in NCC development, the PDGFRbeta is expressed by and functions in cardiac NCCs. Combined PDGF receptor signaling is required for sufficient recruitment of cardiac NCCs into the conotruncal region and for formation of the aortico-pulmonary and ventricular septum.
24. The dietary flavones apigenin and luteolin impair smooth muscle cell **migration** and VEGF expression through inhibition of PDGFR-beta phosphorylation. Platelet-derived growth factor (PDGF)-dependent recruitment of mural cells such as pericytes and smooth muscle cells plays a central role in the maturation and stabilization of newly formed vasculature during angiogenesis. In this work, we show that the dietary flavones apigenin and luteolin may interfere with this event through their inhibitory effect on PDGF-dependent phosphorylation of PDGF receptor beta (PDGFR-beta) in smooth muscle cells. Inhibition of PDGFR-beta activity by apigenin and luteolin occurred at low concentrations of the molecules and resulted in the inhibition of extracellular signal-regulated kinase and Akt phosphorylation triggered by PDGF, as well as in a marked reduction of the migratory and invasive properties of these cells. Apigenin and luteolin also strongly inhibit the PDGF-dependent increase in vascular endothelial growth factor (VEGF) mRNA levels and the secretion of VEGF by smooth muscle cells as well as vessel formation in the mouse Matrigel plug assay, suggesting that the inhibitory effects of both molecules on smooth muscle cell function result in impaired angiogenesis. Overall, these results identify apigenin and luteolin as dietary-derived inhibitors of PDGFR-beta activity and suggest that this inhibitory effect may contribute to the chemopreventive properties of these molecules.
25. Regulation of chemotaxis by the platelet-derived growth factor receptor-beta. Chemotaxis is an important component of wound healing, development, immunity and metastasis, yet the signalling pathways that mediate chemotaxis are poorly understood. Platelet-derived growth factor (PDGF) acts both as a mitogen and a chemoattractant. Upon stimulation, the tyrosine kinase PDGF receptor-beta (PDGFR-beta) autophosphorylates and forms a complex that includes SII2(Src homology 2)-domain-containing proteins such as the phosphatidylinositol-specific phospholipase C-gamma, Ras-GTPase-activating protein (GAP), and phosphatidylinositol-3-OH kinase. Specific tyrosine-to-phenylalanine substitutions in the PDGFR-beta can prevent binding of one SH2-domain-containing protein without affecting binding of other receptor-associated proteins. Here we use phospholipase C-gamma and PDGFR-beta mutants to map specific tyrosines involved in both positive and negative regulation of chemotaxis towards the PDGF-BB homodimer. Our results indicate that a delicate balance of **migration**-promoting (phospholipase C-gamma and phosphatidylinositol-3-OH kinase) and **migration**-suppressing (GAP) activities are recruited by the PDGFR-beta to drive chemotaxis towards PDGF-BB.
26. Embryonic mouse testis development: role of platelet derived growth factor (PDGF-BB). Platelet-derived growth factors (PDGFs) are paracrine growth factors mediating epithelial-mesenchymal interactions and exerting multiple biological activities which include cell proliferation, motility, and differentiation. As previously demonstrated, PDGFs act during embryonic development and recently, by culturing male genital ridges, we have demonstrated that PDGF-BB is able to support in vitro testicular cord formation. In the present paper, we report that PDGF-BB is present during embryonic testis development and, in organ culture, induces cord formation although with reduced diameters compared with the cords formed in the genital ridges cultured in the presence of HGF. Moreover we have analyzed the roles exerted by this growth factor during the morphogenesis of the testis. We demonstrate by immunohistochemical experiments that PDGF-BB and its receptors are synthesized by the male UGRs isolated from 11.5 and 13.5 dpc embryos and by Western blot that the factor is secreted in a biologically active form by testicular cells isolated from 13.5 dpc embryos. The biological roles of the factor have also been studied and we demonstrate that PDGF-BB acts as a migratory factor for male mesonephric cells whose **migration** is a male specific event necessary for a normal testicular morphogenesis. In addition we demonstrate that during testicular development, PDGF-BB induces testicular cell proliferation being in this way responsible for the increase in size of the testis. Finally we demonstrate that PDGF-BB is able to reorganize dissociated testicular cells inducing the formation of large cellular aggregates. However the structures formed in vitro under PDGF-BB stimulation never had a cord-like morphology similar to the cord-like structures formed in the presence of HGF (Ricci et al., 2002, Mech Dev 118:19-28), suggesting that this factor does not act as a morphogenetic factor during testicular development. All together the data presented in this paper demonstrate that PDGF-BB and its receptors (alpha- and beta-subunits) are present during the crucial ages of embryonic mouse testis morphogenesis and indicate the multiple roles exerted by this factor during the development of the male gonad.
27. Lysyl oxidase oxidizes cell membrane proteins and enhances the chemotactic response of vascular smooth muscle cells. Lysyl oxidase (LOX) is a potent chemokine inducing the **migration** of varied cell types. Here we demonstrate that inhibition of LOX activity by beta-aminopropionitrile (BAPN) in cultured rat aortic smooth muscle cells (SMCs) reduced the chemotactic response and sensitivity of these cells toward LOX and toward PDGF-BB. The chemotactic activity of PDGF-BB was significantly enhanced in the presence of a non-chemotactic concentration of LOX. We considered the possibility that extracellular LOX may oxidize cell surface proteins, including the PDGF receptor-beta (PDGFR-beta), to affect PDGF-BB-induced chemotaxis. Plasma membranes purified from control SMC contained oxidized PDGFR-beta. The oxidation of this receptor and other membrane proteins was largely prevented in cells preincubated with BAPN. Addition of purified LOX to these cells restored the profile of oxidized proteins toward that of control cells. The high affinity and capacity for the binding of PDGF-BB by cells containing oxidized PDGFR-beta was diminished by approximately 2-fold when compared with cells in which oxidation by LOX was prevented by BAPN. Phosphorylated members of the PDGFR-beta-dependent signal transduction pathway, including PDGFR-beta, SHP2, AKT1, and ERK1/ERK2 (p44/42 MAPK), turned over faster in BAPN-treated than in control SMCs. LOX knock-out mouse embryonic fibroblasts mirrored the effect obtained with SMCs treated with BAPN. These novel findings suggest that LOX activity is essential to generate optimal chemotactic sensitivity of cells to chemoattractants by oxidizing specific cell surface proteins, such as PDGFR-beta.
28. Trkb signaling in pericytes is required for cardiac microvessel stabilization. Pericyte and vascular smooth muscle cell (SMC) recruitment to the developing vasculature is an important step in blood vessel maturation. Brain-derived neurotrophic factor (BDNF), expressed by endothelial cells, activates the receptor tyrosine kinase TrkB to stabilize the cardiac microvasculature in the perinatal period. However, the effects of the BDNF/TrkB signaling on pericytes/SMCs and the mechanisms downstream of TrkB that promote vessel maturation are unknown. To confirm the involvement of TrkB in vessel maturation, we evaluated TrkB deficient (trkb (-/-)) embryos and observed severe cardiac vascular abnormalities leading to lethality in late gestation to early prenatal life. Ultrastructural analysis demonstrates that trkb(-/-) embryos exhibit defects in endothelial cell integrity and perivascular edema. As TrkB is selectively expressed by pericytes and SMCs in the developing cardiac vasculature, we generated mice deficient in TrkB in these cells. Mice with TrkB deficiency in perivascular cells exhibit reduced pericyte/SMC coverage of the cardiac microvasculature, abnormal endothelial cell ultrastructure, and increased vascular permeability. To dissect biological actions and the signaling pathways downstream of TrkB in pericytes/SMCs, human umbilical SMCs were treated with BDNF. This induced membranous protrusions and cell **migration**, events dependent on myosin light chain phosphorylation. Moreover, inhibition of Rho GTPase and the Rho-associated protein kinase (ROCK) prevented membrane protrusion and myosin light chain phosphorylation in response to BDNF. These results suggest an important role for BDNF in regulating **migration** of TrkB-expressing pericytes/SMCs to promote cardiac blood vessel ensheathment and functional integrity during development.
29. The lipoprotein receptor LRP1 modulates sphingosine-1-phosphate signaling and is essential for vascular development. Low density lipoprotein receptor-related protein 1 (LRP1) is indispensable for embryonic development. Comparing different genetically engineered mouse models, we found that expression of Lrp1 is essential in the embryo proper. Loss of LRP1 leads to lethal vascular defects with lack of proper investment with mural cells of both large and small vessels. We further demonstrate that LRP1 modulates Gi-dependent sphingosine-1-phosphate (S1P) signaling and integrates S1P and PDGF-BB signaling pathways, which are both crucial for mural cell recruitment, via its intracellular domain. Loss of LRP1 leads to a lack of S1P-dependent inhibition of RAC1 and loss of constraint of PDGF-BB-induced cell **migration**. Our studies thus identify LRP1 as a novel player in angiogenesis and in the recruitment and maintenance of mural cells. Moreover, they reveal an unexpected link between lipoprotein receptor and sphingolipid signaling that, in addition to angiogenesis during embryonic development, is of potential importance for other targets of these pathways, such as tumor angiogenesis and inflammatory processes.
30. The chemotactic response to PDGF-BB: evidence of a role for Ras. The PDGF receptor-beta mediates both mitogenic and chemotactic responses to PDGF-BB. Although the role of Ras in tyrosine kinase-mediated mitogenesis has been characterized extensively, its role in PDGF-stimulated chemotaxis has not been defined. Using cells expressing a dominant-negative ras, we find that Ras inhibition suppresses **migration** toward PDGF-BB. Overexpression of either Ras-GTPase activating protein (Ras-GAP) or a Ras guanine releasing factor (GRF) also inhibited PDGF-stimulated chemotaxis. In addition, cells producing excess constitutively active Ras failed to migrate toward PDGF-BB, consistent with the observation that either excess ligand or excess signaling intermediate can suppress the chemotactic response. These results suggest that Ras can function in normal cells to support chemotaxis toward PDGF-BB and that either too little or too much Ras activity can abrogate the chemotactic response. In contrast to Ras overexpression, cells producing excess constitutively active Raf, a downstream effector of Ras, did migrate toward PDGF-BB. Cells expressing dominant-negative Ras were able to migrate toward soluble fibronectin demonstrating that these cells retained the ability to migrate. These results suggest that Ras is an intermediate in PDGF-stimulated chemotaxis but may not be required for fibronectin-stimulated cell motility.
31. The functional PDGFbeta receptor-S1P1 receptor signaling complex is involved in regulating **migration** of mouse embryonic fibroblasts in response to platelet derived growth factor. We report here that mouse embryonic fibroblasts (MEF) express a functional PDGFbeta receptor-S1P(1) receptor complex. The S1P(1) receptor is constitutively active and functions to enhance PDGF-stimulated **migration** of MEF. This was based on three pieces of evidence. Firstly, the S1P(1) receptor and PDGFbeta receptor are co-immunoprecipitated from cell lysates using anti-PDGFbeta receptor antibody. These findings suggest that the receptors form a complex in MEF. Secondly, inverse agonism of the S1P(1) receptor with SB649146 to eliminate the constitutive activity of the S1P(1) receptor reduced the PDGF-induced activation of p42/p44 MAPK in MEF. Thirdly, SB649146 inhibited the **migration** of MEF in response to the selective S1P(1) receptor agonist, SEW2871 or PDGF. In contrast, S1P inhibited PDGF-stimulated MEF **migration**, possibly mediated by the inhibitory S1P(2) receptor. These findings resolve an important issue regarding the functional role of the S1P(1) receptor in regulating MEF **migration** and suggest an important role within the context of PDGFbeta receptor-S1P(1) receptor complex signaling.
32. The Hedgehog pathway promotes blood-brain barrier integrity and CNS immune quiescence. The blood-brain barrier (BBB) is composed of tightly bound endothelial cells (ECs) and perivascular astrocytes that regulate central nervous system (CNS) homeostasis. We showed that astrocytes secrete Sonic hedgehog and that BBB ECs express Hedgehog (Hh) receptors, which together promote BBB formation and integrity during embryonic development and adulthood. Using pharmacological inhibition and genetic inactivation of the Hh signaling pathway in ECs, we also demonstrated a critical role of the Hh pathway in promoting the immune quiescence of BBB ECs by decreasing the expression of proinflammatory mediators and the adhesion and **migration** of leukocytes, in vivo and in vitro. Overall, the Hh pathway provides a barrier-promoting effect and an endogenous anti-inflammatory balance to CNS-directed immune attacks, as occurs in multiple sclerosis.
33. **Migration** of dorsal aorta mesenchymal stem cells induced by mouse embryonic circulation. Mesenchymal stem cells (MSCs) represent powerful tools for regenerative medicine for their differentiation and **migration** capacity. However, ontogeny and **migration** of MSCs in mammalian mid-gestation conceptus is poorly understood. We identified canonical MSCs in the mouse embryonic day (E) 11.5 dorsal aorta (DA). They possessed homogenous immunophenotype (CD45(-)CD31(-)Flk-1(-)CD44(+)CD29(+)), expressed perivascular markers (α-SMA(+)NG2(+)PDGFRβ(+)PDGFRα(+)), and had tri-lineage differentiation potential (osteoblasts, adipocytes, and chondrocytes). Of interest, MSCs were also detected in E12.5-E13.5 embryonic circulation, 24 hr later than in DA, suggesti**ng migrat**ion like hematopoietic stem cells. Functionally, E12.5 embryonic blood could trigger efficie**nt migrat**ion of DA-MSCs through platelet-derived growth factor (PDGF) receptor-, transforming growth factor-beta receptor-, but not basic fibroblast growth factor receptor-mediated signaling. Moreover, downstream JNK and AKT signaling pathway played important roles in embryonic blood- or PDGF-mediat**ed migrat**ion of DA-derived MSCs. Taken together, these results revealed that clonal MSCs developed in the mouse DA. More importantly, the embryonic circulation, in addition to its conventional transporting roles, could modula**te migrat**ion of MSC during early embryogenesis.
34. Platelet-derived growth factor receptor beta signaling is required for efficient epicardial cell **migration** and development of two distinct coronary vascular smooth muscle cell populations. The epicardium plays an essential role in coronary artery formation and myocardial development, but signals controlling the development and differentiation of this tissue are not well understood. To investigate the role of platelet-derived growth factor receptor (PDGFR)beta in development of epicardial-derived vascular smooth muscle cells (VSMCs), we examined PDGFRbeta(-/-) and PDGFRbeta epicardial mutant hearts. We found that PDGFRbeta(-/-) hearts failed to form dominant coronary vessels on the ventral heart surface, had a thinned myocardium, and completely lacked coronary VSMCs (cVSMCs). This constellation of defects was consistent with a primary defect in the epicardium. To verify that these defects were specific to epicardial derivatives, we generated mice with an epicardial deletion of PDGFRbeta that resulted in reduced cVSMCs distal to the aorta. The regional absence of cVSMCs suggested that cVSMCs could arise from 2 sources, epicardial and nonepicardial, and that both were dependent on PDGFRbeta. In the absence of PDGFRbeta signaling, epicardial cells adopted an irregular actin cytoskeleton, leading to aberrant **migration** of epicardial cells into the myocardium in vivo. In addition, PDGF receptor stimulation promoted epicardial cell **migration**, and PDGFRbeta-driven phosphoinositide 3'-kinase signaling was critical for this process. Our data demonstrate that PDGFRbeta is required for the formation of 2 distinct cVSMC populations and that loss of PDGFRbeta-PI3K signaling disrupts epicardial cell **migration**.
35. Potentiation of platelet-derived growth factor receptor-β signaling mediated by integrin-associated MFG-E8. Pericytes/pericyte precursors produce milk fat globule-associated protein with epidermal growth factor and factor VIII-like domains (MFG-E8) in vivo, and this α(v) integrin ligand enhances angiogenesis in tumors and in oxygen-induced retinopathy in mice. Inhibition of MFG-E8 production or function attenuates platelet-derived growth factor-BB (PDGF-BB)-induce**d migrati**on of pericyte/pericyte precursor-like 10T1/2 cells in vitro. Herein, we describe mechanisms by which MFG-E8 modulates PDGF-BB:PDGF receptor β (PDGFRβ) signaling in 10T1/2 cells.Small interfering RNA depletion of MFG-E8 from 10T1/2 cells or antibody inhibition of MFG-E8 action enhanced PDGF-BB-dependent degradation of PDGFRβ and attenuated signaling. Coimmunoprecipitation revealed transient association of MFG-E8 with PDGFRβ in PDGF-BB-treated 10T1/2 cells and reduced PDGFRβ-focal adhesion kinase association in MFG-E8-depleted cells. Confocal microscopy demonstrated that MFG-E8 binding to 10T1/2 cells was RGD motif and α(v) dependent but PDGF-BB treatment independent, whereas colocalization of MFG-E8 with PDGFRβ was enhanced by PDGF-BB. Ubiquitination of PDGFRβ was also increased in MFG-E8 small interfering RNA-transfected cells.Integrin α(v)-bound MFG-E8 associates with PDGFRβ and focal adhesion kinase after PDGF-BB treatment, results in cell surface retention of PDGFRβ, delays receptor degradation, potentiates downstream signaling, **and enhan**ces migration of 10T1/2 cells. MFG-E8 may promote angiogenesis, in part, via cell autonomous actions on pericytes or pericyte precursors that result in enhanced PDGF-BB:PDGFRβ signaling mediated via integrin-growth factor receptor cross-talk.
36. Site-selective regulation of platelet-derived growth factor beta receptor tyrosine phosphorylation by T-cell protein tyrosine phosphatase. The platelet-derived growth factor (PDGF) beta receptor mediates mitogenic and chemotactic signals. Like other tyrosine kinase receptors, the PDGF beta receptor is negatively regulated by protein tyrosine phosphatases (PTPs). To explore whether T-cell PTP (TC-PTP) negatively regulates the PDGF beta receptor, we compared PDGF beta receptor tyrosine phosphorylation in wild-type and TC-PTP knockout (ko) mouse embryos. PDGF beta receptors were hyperphosphorylated in TC-PTP ko embryos. Fivefold-higher ligand-induced receptor phosphorylation was observed in TC-PTP ko mouse embryo fibroblasts (MEFs) as well. Reexpression of TC-PTP partly abolished this difference. As determined with site-specific phosphotyrosine antibodies, the extent of hyperphosphorylation varied among different autophosphorylation sites. The phospholipase Cgamma1 binding site Y1021, previously implicated in chemotaxis, displayed the largest increase in phosphorylation. The increase in Y1021 phosphorylation was accompanied by increased phospholipase Cgamma1 activity and migratory hyperresponsiveness to PDGF. PDGF beta receptor tyrosine phosphorylation in PTP-1B ko MEFs but not in PTPepsilon ko MEFs was also higher than that in control cells. This increase occurred with a site distribution different from that seen after TC-PTP depletion. PDGF-induced **migration** was not increased in PTP-1B ko cells. In summary, our findings identify TC-PTP as a previously unrecognized negative regulator of PDGF beta receptor signaling and support the general notion that PTPs display site selectivity in their action on tyrosine kinase receptors.
37. Epicardial-derived cell epithelial-to-mesenchymal transition and fate specification require PDGF receptor signaling. In early heart development, platelet-derived growth factor (PDGF) receptor expression in the heart ventricles is restricted to the epicardium. Previously, we showed that PDGFRβ is required for coronary vascular smooth muscle cell (cVSMC) development, but a role for PDGFRα has not been identified. Therefore, we investigated the combined and independent roles of these receptors in epicardial development.To understand the contribution of PDGF receptors in epicardial development and epicardial-derived cell fate determination.By generating mice with epicardial-specific deletion of the PDGF receptors, we found that epicardial epithelial-to-mesenchymal transition (EMT) was defective. Sox9, an SRY-related transcription factor, was reduced in PDGF receptor-deficient epicardial cells, and overexpression of Sox9 restored epicardia**l migrati**on, actin reorganization, and EMT gene expression profiles. The failure of epicardial EMT resulted in hearts that lacked epicardial-derived cardiac fibroblasts and cVSMC. Loss of PDGFRα resulted in a specific disruption of cardiac fibroblast development, whereas cVSMC development was unperturbed.Signaling through both PDGF receptors is necessary for epicardial EMT and formation of epicardial-mesenchymal derivatives. PDGF receptors also have independent functions in the development of specific epicardial-derived cell fates.
38. Mitogenic signaling via platelet-derived growth factor beta in metanephric mesenchymal cells. Mice deficient in either platelet-derived growth factor (PDGF) B chain or PDGF receptor (PDGFR) beta lack mesangial cells. PDGF stimulates proliferation and **migration** of metanephric mesenchymal cells, from which mesangial cells are derived. Binding of PDGF to PDGFR-beta induces autophosphorylation at specific tyrosine residues and activates various effector proteins, including phosphatidylinositol-3-kinase (PI3-K). This study explored the role of PI 3-K and reactive oxygen species (ROS) in PDGF-mediated signaling using cells established from wild-type and PDGFR-beta -/- metanephric blastemas at 11.5 days post-conception. PDGF-induced effects that were dependent on PI3-K activation were determined using PDGFR-beta -/- cells made to express "add-back" mutant PDGFR-beta capable of binding PI3-K. We found that PDGF is mitogenic for mesenchymal cells expressing PDGFR-beta, and PI3-K is an important regulator of PDGF-induced DNA synthesis. Activation of ERK1/2 is partially dependent on PI3-K, and both the PI3-K and MEK-ERK1/2 pathways contribute to PI3-K-dependent mitogenesis. In addition, PDGF-induced DNA synthesis in wild-type cells was found to be dependent on ROS that are generated downstream of PI3-K activation. Using antisense oligonucleotides and small interfering RNA, we determined that the NAD(P)H oxidase Nox4 produces these ROS that activate Akt and the MEK-ERK1/2 mitogenic cascade. In conclusion, the present study demonstrates Nox4 involvement in PDGF-induced DNA synthesis in metanephric mesenchymal cells and provides the first evidence that PDGF-induced PI3-K activity enhances production of ROS by Nox4.
39. PDGF-BB modulates hematopoiesis and tumor angiogenesis by inducing erythropoietin production in stromal cells. The platelet-derived growth factor (PDGF) signaling system contributes to tumor angiogenesis and vascular remodeling. Here we show in mouse tumor models that PDGF-BB induces erythropoietin (EPO) mRNA and protein expression by targeting stromal and perivascular cells that express PDGF receptor-β (PDGFR-β). Tumor-derived PDGF-BB promoted tumor growth, angiogenesis and extramedullary hematopoiesis at least in part through modulation of EPO expression. Moreover, adenoviral delivery of PDGF-BB to tumor-free mice increased both EPO production and erythropoiesis, as well as protecting from irradiation-induced anemia. At the molecular level, we show that the PDGF-BB-PDGFR-bβ signaling system activates the EPO promoter, acting in part through transcriptional regulation by the transcription factor Atf3, possibly through its association with two additional transcription factors, c-Jun and Sp1. Our findings suggest that PDGF-BB-induced EPO promotes tumor growth through two mechanisms: first, paracrine stimulation of tumor angiogenesis by direct induction of endothelial cell proliferatio**n, migrat**ion, sprouting and tube formation, and second, endocrine stimulation of extramedullary hematopoiesis leading to increased oxygen perfusion and protection against tumor-associated anemia.
40. Blockade of platelet-derived growth factor or its receptors transiently delays but does not prevent fibrous cap formation in ApoE null mice. Platelet-derived growth factor (PDGF) is a potent stimulant of smooth muscle cell **migration** and proliferation in culture. To test the role of PDGF in the accumulation of smooth muscle cells in vivo, we evaluated ApoE -/- mice that develop complex lesions of atherosclerosis. Fetal liver cells from PDGF-B-deficient embryos were used to replace the circulating cells of lethally irradiated ApoE -/- mice. One month after transplant, all monocytes in PDGF-B -/- chimeras are of donor origin (lack PDGF), and no PDGF-BB is detected in circulating platelets, primary sources of PDGF in lesions. Although lesion volumes are comparable in the PDGF-B +/+ and -/- chimeras at 35 weeks, lesions in PDGF-B -/- chimeras contain mostly macrophages, appear less mature, and have a reduced frequency of fibrous cap formation as compared with PDGF-B +/+ chimeras. However, after 45 weeks, smooth muscle cell accumulation in fibrous caps is indistinguishable in the two groups. Comparison of elicited peritoneal macrophages by RNase protection assay shows an altered cytokine and cytokine receptor profile in PDGF-B -/- chimeras. ApoE -/- mice were also treated for up to 50 weeks with a PDGF receptor antagonist that blocks all three PDGF receptor dimers. Blockade of the PDGF receptors similarly delays, but does not prevent, accumulation of smooth muscle and fibrous cap formation. Thus, elimination of PDGF-B from circulating cells or blockade of PDGF receptors does not appear sufficient to prevent smooth muscle accumulation in advanced lesions of atherosclerosis.
41. Chimera analysis supports a predominant role of PDGFRbeta in promoting smooth-muscle cell chemotaxis after arterial injury. The carotid artery shows a common response to many forms of injury, including a rapid activation of smooth muscle cell (SMC) proliferation in the media and **migration** of SMCs into the intima to form a neointima. Platelet-derived growth factor (PDGF) is believed to play a role in this response to injury, but it has proven difficult to distinguish whether it is stimulating cell **migration** or cell proliferation, and whether the action is direct or indirect. To determine this, we created chimeric mice composed of both wild-type (WT) and marked PDGF receptor beta (PDGFRbeta)-deficient cells, and determined the consequences of PDGFRbeta expression for SMC participation in response to ligation of the left common carotid artery. The proportion of PDGFRbeta-/- SMCs increased 4.5-fold in the media and decreased 1.8-fold during formation of the neointima, consistent with **migration** of WT SMCs out of the media and into the intima, leaving the PDGFRbeta-/- cells behind. The fibrotic reaction in the adventitia, which does not involve cell **migration**, did not result in any change in relative abundance of WT and PDGFRbeta-deficient fibroblasts. We conclude that the most significant direct role of PDGFRbeta is to mediate responses that involve cell **migration** rather than proliferation.
42. Cell **migration** is regulated by platelet-derived growth factor receptor endocytosis. Cell **migration** requires spatial and temporal processes that detect and transfer extracellular stimuli into intracellular signals. The platelet-derived growth factor (PDGF) receptor is a cell surface receptor on fibroblasts that regulates proliferation and chemotaxis in response to PDGF. How the PDGF signal is transmitted accurately through the receptor into cells is an unresolved question. Here, we report a new intracellular signaling pathway by which DOCK4, a Rac1 guanine exchange factor, and Dynamin regulate cell **migration** by PDGF receptor endocytosis. We showed by a series of biochemical and microscopy techniques that Grb2 serves as an adaptor protein in the formation of a ternary complex between the PDGF receptor, DOCK4, and Dynamin, which is formed at the leading edge of cells. We found that this ternary complex regulates PDGF-dependent cell **migration** by promoting PDGF receptor endocytosis and Rac1 activation at the cell membrane. This study revealed a new mechanism by which cell **migration** is regulated by PDGF receptor endocytosis.
43. Radial construction of an arterial wall. Some of the most serious diseases involve altered size and structure of the arterial wall. Elucidating how arterial walls are built could aid understanding of these diseases, but little is known about how concentric layers of muscle cells and the outer adventitial layer are assembled and patterned around endothelial tubes. Using histochemical, clonal, and genetic analysis in mice, here we show that the pulmonary artery wall is constructed radially, from the inside out, by two separate but coordinated processes. One is sequential induction of successive cell layers from surrounding mesenchyme. The other is controlled invasion of outer layers by inner layer cells through developmentally regulated cell reorientation and radial **migration**. We propose that a radial signal gradient controls these processes and provide evidence that PDGF-B and at least one other signal contribute. Modulation of such radial signaling pathways may underlie vessel-specific differences and pathological changes in arterial wall size and structure.
44. Effects of osmotic stress on the activity of MAPKs and PDGFR-beta-mediated signal transduction in NIH-3T3 fibroblasts. Signaling in cell proliferation, cell **migration**, and apoptosis is highly affected by osmotic stress and changes in cell volume, although the mechanisms underlying the significance of cell volume as a signal in cell growth and death are poorly understood. In this study, we used NIH-3T3 fibroblasts in a serum- and nutrient-free inorganic medium (300 mosM) to analyze the effects of osmotic stress on MAPK activity and PDGF receptor (PDGFR)-beta-mediated signal transduction. We found that hypoosmolarity (cell swelling at 211 mosM) induced the phosphorylation and nuclear translocation of ERK1/2, most likely via a pathway independent of PDGFR-beta and MEK1/2. Conversely, hyperosmolarity (cell shrinkage at 582 mosM) moved nuclear and phosphorylated ERK1/2 to the cytoplasm and induced the phosphorylation and nuclear translocation of p38 and phosphorylation of JNK1/2. In a series of parallel experiments, hypoosmolarity did not affect PDGF-BB-induced activation of PDGFR-beta, whereas hyperosmolarity strongly inhibited ligand-dependent PDGFR-beta activation as well as downstream mitogenic signal components of the receptor, including Akt and the MEK1/2-ERK1/2 pathway. Based on these results, we conclude that ligand-dependent activation of PDGFR-beta and its downstream effectors Akt, MEK1/2, and ERK1/2 is strongly modulated (inhibited) by hyperosmotic cell shrinkage, whereas cell swelling does not seem to affect the activation of the receptor but rather to activate ERK1/2 via a different mechanism. It is thus likely that cell swelling via activation of ERK1/2 and cell shrinkage via activation of the p38 and JNK pathway and inhibition of the PDGFR signaling pathway may act as key players in the regulation of tissue homeostasis.
45. Notch signaling is required for the formation of mesangial cells from a stromal mesenchyme precursor during kidney development. Mesangial cells are specialized pericyte/smooth muscle cells that surround and constrain the vascular network within the glomerulus of the kidney. They are derived from the stromal mesenchyme, a progenitor population distinct from nephron stem cells. Whether mesangial cells have a distinct origin from vascular smooth muscle cells (VSMCs) and the pathways that govern their specification are unknown. Here we show that Notch signaling in stromal progenitors is essential for mesangial cell formation but is dispensable for the smooth muscle and interstitial cell lineages. Deletion of RBPjk, the common DNA-binding partner of all active Notch receptors, with Foxd1(tgCre) results in glomerular aneurysm and perinatal death from kidney failure. This defect occurs early in glomerular development as stromal-derived, desmin-positive cells fail to coalesce near forming nephrons and thus do not invade the vascular cleft of the S-shaped body. This is in contrast to other mutants in which the loss of the mesangium was due to **migration** defects, and suggests that loss of Notch signaling results in a failure to specify this population from the stroma. Interestingly, Pdgfrb-positive VSMCs do not enter the vascular cleft and cannot rescue the mesangial deficiency. Notch1 and Notch2 act redundantly through γ-secretase and RBPjk in this process, as individual mutants have mesangial cells at birth. Together, these data demonstrate a unique origin of mesangial cells and demonstrate a novel, redundant function for Notch receptors in mesangial cell specification, proliferation or survival during kidney development.
46. Binding of Cbl to a phospholipase Cgamma1-docking site on platelet-derived growth factor receptor beta provides a dual mechanism of negative regulation. Ubiquitin conjugation to receptor tyrosine kinases is a critical biochemical step in attenuating their signaling through lysosomal degradation. Our previous studies have established Cbl as an E3 ubiquitin ligase for ubiquitinylation and degradation of platelet-derived growth factor receptor (PDGFR) alpha and PDGFRbeta. However, the role of endogenous Cbl in PDGFR regulation and the molecular mechanisms of this regulation remain unclear. Here, we demonstrate that endogenous Cbl is essential for ligand-induced ubiquitinylation and degradation of PDGFRbeta; this involves the Cbl TKB domain binding to PDGFRbeta phosphotyrosine 1021, a known phospholipase C (PLC) gamma1 SH2 domain-binding site. Lack of Cbl or ablation of the Cbl-binding site on PDGFRbeta impedes receptor sorting to the lysosome. Cbl-deficient cells also show more PDGF-induced PLCgamma1 association with PDGFRbeta and enhanced PLC-mediated cell **migration**. Thus, Cbl-dependent negative regulation of PDGFRbeta involves a dual mechanism that concurrently promotes ubiquitin-dependent lysosomal sorting of the receptor and competitively reduces the recruitment of a positive mediator of receptor signaling.
47. Expression and role of PDGF-BB and PDGFR-beta during testis morphogenesis in the mouse embryo. The role played by PDGF in testis morphogenesis is still incompletely understood. The present study investigates the expression and potential role of platelet-derived growth factor-BB (PDGF-BB) and its receptor, PDGF receptor beta (PDGFR-beta), during mouse testis cord formation, and the possibility that the growth factor may be involved in the **migration** to the gonad of mesenchymal cells of mesonephric origin. Studies from this laboratory have previously shown that mesenchymal cells that migrate from the mesonephros into the gonad, to form peritubular myoid cells and most of the intertubular cells, can be identified by the presence on their surface of the p75 neurotrophin receptor (p75NTR), and can be isolated to near-purity by immunomagnetic selection with anti-p75NTR antibody. We show here that mesonephric p75NTR(+) cells also bear the PDGFR-beta, and are able to migrate and proliferate in vitro in response to PDGF-BB. PDGF-BB is expressed at higher levels in male than female developing gonads, suggesting a role for this factor in testis development. Such a role is further supported by the observation that addition of PDGF-BB to serum-free medium is sufficient to allow organ-cultured male 11.5 days post-coitum urogenital ridges to form testis cords. Finally, we show that mesonephric cell motility and growth induced by exposure to PDGF-BB involve mitogen-activated protein kinases (MAPK) and phosphatidylinositol-3 kinase (PI3-K) pathways, as MAPK inhibitor U0126 and PI3K inhibitor Ly294002 inhibit **migration** and proliferation in vitro assays. The present findings support the hypothesis that the PDGF/PDGFR system plays a key role in testis morphogenesis in the mouse embryo.
48. Inhibitory effects of Robo2 on nephrin: a crosstalk between positive and negative signals regulating podocyte structure. Robo2 is the cell surface receptor for the repulsive guidance cue Slit and is involved in axon guidance and neuronal **migration**. Nephrin is a podocyte slit-diaphragm protein that functions in the kidney glomerular filtration barrier. Here, we report that Robo2 is expressed at the basal surface of mouse podocytes and colocalizes with nephrin. Biochemical studies indicate that Robo2 forms a complex with nephrin in the kidney through adaptor protein Nck. In contrast to the role of nephrin that promotes actin polymerization, Slit2-Robo2 signaling inhibits nephrin-induced actin polymerization. In addition, the amount of F-actin associated with nephrin is increased in Robo2 knockout mice that develop an altered podocyte foot process structure. Genetic interaction study further reveals that loss of Robo2 alleviates the abnormal podocyte structural phenotype in nephrin null mice. These results suggest that Robo2 signaling acts as a negative regulator on nephrin to influence podocyte foot process architecture.
49. Characterization of neuroprogenitor cells expressing the PDGF beta-receptor within the subventricular zone of postnatal mice. We report a considerable number of cells in the ventricular and the subventricular zones (SVZ) of newborn mice to stain positive for the PDGF beta-receptor (PDGFRB). Many of them also stained for nestin and/or GFAP but less frequently for the neuroblast marker doublecortin and for the mitotic marker Ki-67. The SVZ of mice with nestin-Cre conditional deletion of PDGFRB expressed the receptor only on blood vessels and was devoid of any morphological abnormality. PDGFRB(-/-) neurospheres showed a higher rate of apoptosis without any significant decrease in proliferation. They demonstrated reduced capacities of **migration** and neuronal differentiation in response to not only PDGF-BB but also bFGF. Furthermore, the PDGFR kinase inhibitor STI571 blocked the effects of bFGF in control neurosphere cultures. bFGF increased the activity of the PDGFRB promoter as well as the expression and phosphorylation of PDGFRB. These results suggest the presence of the signaling convergence between PDGF and FGF. PDGFRB is needed for survival, and the effects of bFGF in **migration** and neural differentiation of the cells may be potentiated by induction of PDGFRB.
50. Myocardin regulates vascular response to injury through miR-24/-29a and platelet-derived growth factor receptor-β. Myocardin, a potent transcriptional coactivator of serum response factor, is involved in vascular development and promotes a contractile smooth muscle phenotype. Myocardin levels are reduced during vascular injury, in association with phenotypic switching of smooth muscle cells (SMCs). However, the direct role of myocardin in vascular disease is unclear.We show that re-expression of myocardin prevents the vascular injury response in murine carotid arteries, with reduced neointima formation due to decreased SMC **migratio**n and proliferation. Myocardin reduced SMC **migratio**n by downregulating platelet-derived growth factor receptor-β (PDGFRB) expression. Pdgfrb was regulated by myocardin-induced miR-24 and miR-29a expression, and antagonizing these microRNAs restored SM**C migrati**on. Furthermore, using miR-24 and miR-29a mimics, we demonstrated that miR-29a directly regulates Pdgfrb expression at the 3' untranslated region while miR-24 has an indirect effect on Pdgfrb levels. Myocardin heterozygous-null mice showed an augmented neointima formation with increased SM**C migrati**on and proliferation, demonstrating that endogenous levels of myocardin are a critical regulator of vessel injury responses.Our results extend the function of myocardin from a developmental role to a pivotal regulator of SMC phenotype in response to injury, and this transcriptional coactivator may be an attractive target for novel therapeutic strategies in vascular disease.
51. Platelet-derived growth factor receptor beta-subtype regulates proliferation and **migration** of gonocytes. Proliferation and **migration** of gonocytes, the precursors of spermatogonial stem cells, to the germline niche in the basal membrane of the seminiferous tubules, are two crucial events that take place between postnatal d 0.5 (P0.5) and P5.0 in the mouse and involve a selection of the cells that are committed to the germline stem cells lineage. Here we show that from embryonic d 18.0 (E18) and up to P5, the gonocytes express platelet-derived growth factor (PDGF) receptor beta-subtype (PDGFR-beta) and that during the same time period, the Sertoli cells express PDGF-B and PDGF-D, both ligands for PDGFR-beta. Inhibition of the PDGFR-beta tyrosine kinase activity during the first five postnatal days provokes a profound reduction of gonocyte number through inhibition of their proliferation and induction of apoptosis. Moreover, we found that PDGFR-beta ligands are chemotactic for gonocytes. These data suggest that PDGFR-beta activation has the remarkable capability to drive the selection, survival, and **migration** of the gonocytes from the center of the seminiferous tubules to the testicular germline niche on the basal membrane.
52. Coordinate regulation by diethylstilbestrol of the platelet-derived growth factor-A (PDGF-A) and -B chains and the PDGF receptor alpha- and beta-subunits in the mouse uterus and vagina: potential mediators of estrogen action. The effects of estrogen on the reproductive tract involve cell proliferation, **migration**, and differentiation, which need to be well coordinated. Polypeptide growth factors are believed to play a vital role in a number of these cellular processes. Among the growth factors now documented to be associated with estrogen action are epidermal growth factor, transforming growth factor-alpha (TGF alpha), transforming growth factor-beta 1 (TGF beta 1), TGF beta 2, TGF beta 3, and insulin-like growth factor-I (IGF-I). Platelet-derived growth factors (PDGFs) are also potent mitogens, which consist of two peptide chains, denoted A and B, that dimerize to isoforms (PDGF-AA, -AB, and -BB) which differ in their functional properties, secretory behavior, receptor binding, and physiological effects. To study the role of the PDGF-A and -B chains and the PDGF receptor subunits, alpha and beta, during estrogen action in the mouse reproductive tract, time-dependent changes in the expression of these genes were examined by Northern and in situ RNA analyses and by immunohistochemistry after a single treatment of immature CD-1 (17- to 19-day-old) mice with the synthetic estrogen, diethylstilbestrol (DES). Our results demonstrate estrogen modulation of the expression of messenger RNA (mRNA) and protein for the PDGF ligands and receptors in both the uterus and vagina of the mouse. Northern and in situ RNA analyses demonstrate time-dependent estrogen induction of the mRNA levels for these genes in both tissues within 3 h after treatment. However, distinctive mRNA expression profiles for the PDGF ligand and receptor genes are exhibited by the uterus and vagina in response to DES, especially in that the induction of transcripts for PDGF-A and both receptor subunits is more transient in the vagina than in the uterus. Steroid specificity studies demonstrate predominant estrogen-specific regulation of mRNA induction for these genes. Analysis of cell-specific RNA expression by in situ hybridization reveals prominent induction of transcripts for the PDGF chains and receptor subunits in the uterine and vaginal epithelium after estrogen treatment, although enhanced expression of mRNA is also noted in the stroma, particularly for the PDGF receptor subunit genes. Cellular localization of the PDGF ligand and receptor protein molecules by immunohistochemistry detected significant immunostaining for all of these proteins in both the uterus and vagina of control animals. After DES treatment, the uterus exhibits a significant decrease in the level of PDGF ligand and receptor proteins immunostained within 6 h, whereas less dramatic effects ar observed in the vagina.(ABSTRACT TRUNCATED AT 400 WORDS)
53. Platelet-derived growth factor-beta receptor activation is essential for fibroblast and pericyte recruitment during cutaneous wound healing. Connective tissue remodeling provides mammals with a rapid mechanism to repair wounds after injury. Inappropriate activation of this reparative process leads to scarring and fibrosis. Here, we studied the effects of platelet-derived growth factor receptor-beta blockade in vivo using the platelet-derived growth factor receptor (PDGFR)-beta inhibitor imatinib mesylate on tissue repair. After 7 days, healing of wounds was delayed with significantly reduced wound closure and concomitant reduction in myofibroblast frequency, expression of fibronectin ED-A, and collagen type I. Using a collagen type I transgenic reporter mouse, we showed that inhibiting PDGFR-beta activation restricted the distribution of collagen-synthesizing cells to wound margins and dramatically reduced cell proliferation in vivo. By 14 days, treated wounds were fully closed. Blocking PDGFR-beta signaling did not prevent the differentiation of myofibroblasts in vitro but potently inhibited fibroblast proliferation and **migration**. In addition, PDGFR-beta inhibition in vivo was accompanied by abnormal microvascular morphogenesis reminiscent of that observed in PDGFR-beta-/- mice with significantly reduced immunostaining of the pericyte marker NG2. Imatinib treatment also inhibited pericyte proliferation and **migration** in vitro. This study highlights the significance of PDGFR-beta signaling for the recruitment, proliferation, and functional activities of fibro-blasts and pericytes during the early phases of wound healing.
54. Pdgfra and Pdgfrb genetically interact during craniofacial development. One of the most prevalent congenital birth defects is cleft palate. The palatal skeleton is derived from the cranial neural crest and platelet-derived growth factors (Pdgf) are critical in palatogenesis. Of the two Pdgf receptors, pdgfra is required for neural crest **migration** and palatogenesis. However, the role pdgfrb plays in the neural crest, or whether pdgfra and pdgfrb interact during palatogenesis is unclear.We find that pdgfrb is dispensable for craniofacial development in zebrafish. However, the palatal defect in pdgfra;pdgfrb double mutants is significantly more severe than in pdgfra single mutants. Data in mouse suggest this interaction is conserved and that neural crest requires both genes. In zebrafish, pdgfra and pdgfrb are both expressed by neural crest within the pharyngeal arches, and pharmacological analyses demonstrate Pdgf signaling is required at these times. While neither proliferation nor cell death appears affected, time-lapsed confocal analysis of pdgfra;pdgfrb mutants shows a failure of proper neural crest condensation during palatogenesis.We provide data showing that pdgfra and pdgfrb interact during palatogenesis in both zebrafish and mouse. In zebrafish, this interaction affects proper condensation of maxillary neural crest cells, revealing a previously unknown interaction between Pdgfra and Pdgfrb during palate formation. Developmental Dynamics 245:641-652, 2016. © 2016 Wiley Periodicals, Inc.
55. Stimulation of platelet-derived growth factor receptor beta (PDGFRbeta) activates ADAM17 and promotes metalloproteinase-dependent cross-talk between the PDGFRbeta and epidermal growth factor receptor (EGFR) signaling pathways. Binding of the platelet-derived growth factor (PDGF)-B to its receptor PDGFRbeta promotes proliferation, **migration**, and recruitment of pericytes and smooth muscle cells to endothelial cells, serving to stabilize developing blood vessels. The main goals of this study were to determine whether the extracellular domain of the PDGFRbeta can be proteolytically released from cell membranes and, if so, to identify the responsible sheddase and determine whether activation of the PDGFRbeta stimulates its shedding and potentially that of other membrane proteins. We found that the PDGFRbeta is shed from cells by a metalloproteinase and used loss-of-function experiments to identify ADAM10 as the sheddase responsible for constitutive and ionomycin-stimulated processing of the PDGFRbeta. Moreover, we showed that ligand-dependent activation of the PDGFRbeta does not trigger its own shedding by ADAM10, but instead it stimulates ADAM17 and shedding of substrates of ADAM17, including tumor necrosis factor alpha and transforming growth factor alpha. Finally, we demonstrated that treatment of mouse embryonic fibroblasts with PDGF-B triggers a metalloproteinase-dependent cross-talk between the PDGFRbeta and the epidermal growth factor receptor (EGFR)/ERK1/2 signaling axis that is also critical for PDGF-B-stimulated cell **migration**, most likely via ADAM17-dependent release and activation of ligands of the EGFR. This study identifies the principal sheddase for the PDGFRbeta and provides new insights into the mechanism of PDGFRbeta-dependent signal transduction and cross-talk with the EGFR.

#### GeneRIFs

1. The role of the S1PR3/PDGFR-beta/Akt pathway in sphingosine-1-phosphate -induced endothelial progenitor cells **migration** and angiogenesis
2. LRP1 regulates actin organization and cell **migration** by controlling PDGFRbeta-dependent activation of PI3K
3. Results reveal a new mechanism by which cell **migration** is regulated by PDGFbeta receptor endocytosis.
4. Data suggest that PDGFR-beta activation has the remarkable capability to drive the selection, survival, and **migration** of the gonocytes from the center of the seminiferous tubules to the testicular germline niche on the basal membrane.
5. Apigenin and luteolin impair smooth muscle cell **migration** and VEGF expression through inhibition of PDGFR-beta phosphorylation.
6. PDGFRbeta is required for the formation of 2 distinct vascular smooth muscle cell populations; loss of PDGFRbeta-phosphoinositide 3'-kinase signaling disrupts epicardial cell **migration**.
7. S1P1 receptor functions to enhance PDGF-stimulated **migration** of embryonic fibroblasts and is mediated by the PDGFbeta receptor-S1P1 receptor complex
8. The most significant direct role of PDGFRbeta is to mediate responses that involve cell **migration** rather than proliferation.

### ECM

#### PubMed

1. ADAMTS9-Mediated Extracellular Matrix Dynamics Regulates Umbilical Cord Vascular Smooth Muscle Differentiation and Rotation. Despite the significance for fetal nourishment in mammals, mechanisms of umbilical cord vascular growth remain poorly understood. Here, the secreted metalloprotease ADAMTS9 is shown to be necessary for murine umbilical cord vascular development. Restricting it to the cell surface using a gene trap allele, Adamts9(Gt), impaired umbilical vessel elongation and radial growth via reduced versican proteolysis and accumulation of extracellular matrix (**ECM**). Both Adamts9(Gt) and conditional Adamts9 deletion revealed that ADAMTS9 produced by mesenchymal cells acted non-autonomously to regulate smooth muscle cell (SMC) proliferation, differentiation, and orthogonal reorientation during growth of the umbilical vasculature. In Adamts9(Gt/Gt), we observed interference with PDGFRβ signaling via the mitogen-activated protein kinase/extracellular signal-regulated kinase (MAPK/ERK) pathway, which regulates cytoskeletal dynamics during SMC rotation. In addition, we observed disrupted Shh signaling and perturbed orientation of the mesenchymal primary cilium. Thus, **EC**M dynamics is a major influence on umbilical vascular SMC fate, with ADAMTS9 acting as its principal mediator.
2. Wnt signaling regulates smooth muscle precursor development in the mouse lung via a tenascin C/PDGFR pathway. Paracrine signaling from lung epithelium to the surrounding mesenchyme is important for lung SMC development and function and is a contributing factor in an array of pulmonary diseases such as bronchopulmonary dysplasia, pulmonary hypertension, and asthma. Wnt7b, which is exclusively expressed in the lung epithelium, is important for lung vascular smooth muscle integrity, but the underlying mechanism by which Wnt signaling regulates lung SMC development is unclear. In this report, we have demonstrated that Wnt7b regulates a program of mesenchymal differentiation in the mouse lung that is essential for SMC development. Genetic loss-of-function studies showed that Wnt7b and beta-catenin were required for expression of Pdgfralpha and Pdgfrbeta and proliferation in pulmonary SMC precursors. In contrast, gain-of-function studies showed that activation of Wnt signaling increased the expression of both Pdgfralpha and Pdgfrbeta as well as the proliferation of SMC precursors. We further showed that the effect on Pdgfr expression was, in part, mediated by direct transcriptional regulation of the **ECM** protein tenascin C (Tnc), which was necessary and sufficient for Pdgfralpha/beta expression in lung explants. Moreover, this pathway was highly upregulated in a mouse model of asthma and in lung tissue from patients with pulmonary hypertension. Together, these data define a Wnt/Tnc/Pdgfr signaling axis that is critical for smooth muscle development and disease progression in the lung.
3. Disruption of miR-29 Leads to Aberrant Differentiation of Smooth Muscle Cells Selectively Associated with Distal Lung Vasculature. Differentiation of lung vascular smooth muscle cells (vSMCs) is tightly regulated during development or in response to challenges in a vessel specific manner. Aberrant vSMCs specifically associated with distal pulmonary arteries have been implicated in the pathogenesis of respiratory diseases, such as pulmonary arterial hypertension (PAH), a progressive and fatal disease, with no effective treatment. Therefore, it is highly relevant to understand the underlying mechanisms of lung vSMC differentiation. miRNAs are known to play critical roles in vSMC maturation and function of systemic vessels; however, little is known regarding the role of miRNAs in lung vSMCs. Here, we report that miR-29 family members are the most abundant miRNAs in adult mouse lungs. Moreover, high levels of miR-29 expression are selectively associated with vSMCs of distal vessels in both mouse and human lungs. Furthermore, we have shown that disruption of miR-29 in vivo leads to immature/synthetic vSMC phenotype specifically associated with distal lung vasculature, at least partially due to the derepression of KLF4, components of the PDGF pathway and **ECM**-related genes associated with synthetic phenotype. Moreover, we found that expression of FBXO32 in vSMCs is significantly upregulated in the distal vasculature of miR-29 null lungs. This indicates a potential important role of miR-29 in smooth muscle cell function by regulating FBXO32 and SMC protein degradation. These results are strongly supported by findings of a cell autonomous role of endogenous miR-29 in promoting SMC differentiation in vitro. Together, our findings suggested a vessel specific role of miR-29 in vSMC differentiation and function by targeting several key negative regulators.

#### GeneRIFs

1. This study demonstrated that PDGFRbeta-expressing cells appear to arise from vascular wall pericytes in peri-infarct areas and then extend toward the ischemic core while producing **ECM** proteins, fibronectin and collagen type I, after MCAO.

### [Ee]xtracellular [Mm]atrix

#### PubMed

1. ADAMTS9-Mediated **Extracellular Matrix** Dynamics Regulates Umbilical Cord Vascular Smooth Muscle Differentiation and Rotation. Despite the significance for fetal nourishment in mammals, mechanisms of umbilical cord vascular growth remain poorly understood. Here, the secreted metalloprotease ADAMTS9 is shown to be necessary for murine umbilical cord vascular development. Restricting it to the cell surface using a gene trap allele, Adamts9(Gt), impaired umbilical vessel elongation and radial growth via reduced versican proteolysis and accumulation of **extracellular matrix** (ECM). Both Adamts9(Gt) and conditional Adamts9 deletion revealed that ADAMTS9 produced by mesenchymal cells acted non-autonomously to regulate smooth muscle cell (SMC) proliferation, differentiation, and orthogonal reorientation during growth of the umbilical vasculature. In Adamts9(Gt/Gt), we observed interference with PDGFRβ signaling via the mitogen-activated protein kinase/extracellular signal-regulated kinase (MAPK/ERK) pathway, which regulates cytoskeletal dynamics during SMC rotation. In addition, we observed disrupted Shh signaling and perturbed orientation of the mesenchymal primary cilium. Thus, ECM dynamics is a major influence on umbilical vascular SMC fate, with ADAMTS9 acting as its principal mediator.
2. Epithelial cell integrin β1 is required for developmental angiogenesis in the pituitary gland. As a key component of the vertebrate neuroendocrine system, the pituitary gland relies on the progressive and coordinated development of distinct hormone-producing cell types and an invading vascular network. The molecular mechanisms that drive formation of the pituitary vasculature, which is necessary for regulated synthesis and secretion of hormones that maintain homeostasis, metabolism, and endocrine function, remain poorly understood. Here, we report that expression of integrin β1 in embryonic pituitary epithelial cells is required for angiogenesis in the developing mouse pituitary gland. Deletion of pituitary epithelial integrin β1 before the onset of angiogenesis resulted in failure of invading endothelial cells to recruit pericytes efficiently, whereas deletion later in embryogenesis led to decreased vascular density and lumen formation. In both cases, lack of epithelial integrin β1 was associated with a complete absence of vasculature in the pituitary gland at birth. Within pituitary epithelial cells, integrin β1 directs a large transcriptional program that includes components of **the extracellular m**atrix and associated signaling factors that are linked to the observed non-cell-autonomous effects on angiogenesis. We conclude that epithelial integrin β1 functions as a critical and canonical regulator of developmental angiogenesis in the pituitary gland, thus providing insight into the long-standing systems biology conundrum of how vascular invasion is coordinated with tissue development.
3. Involvement of platelet-derived growth factor receptor β in fibrosis through **extracellular matri**x protein production after ischemic stroke. Fibrosis is concomitant with repair processes following injuries in the central nervous system (CNS). Pericytes are considered as an origin of fibrosis-forming cells in the CNS. Here, we examined whether platelet-derived growth factor receptor β (PDGFRβ), a well-known indispensable molecule for migration, proliferation, and survival of pericytes, was involved in the production **of extracellular mat**rix proteins, fibronectin and collagen type I, which is crucial for fibrosis after ischemic stroke. Immunohistochemistry demonstrated induction of PDGFRβ expression in vascular cells of peri-infarct areas at 3-7days in a mouse stroke model. The PDGFRβ-expressing cells extended from peri-infarct areas toward the ischemic core after day 7 while expressing fibronectin and collagen type I in the infarct areas. In contrast, desmin and α-smooth muscle actin, markers of pericytes, were only expressed in vascular cells. In PDGFRβ heterozygous knockout mice, the expression of fibronectin and collagen type I was attenuated at both mRNA and protein levels with an enlargement of the infarct volume after ischemic stroke compared with that in wild-type littermates. In cultured brain pericytes, the expression of PDGF-B, PDGFRβ, fibronectin, and collagen type I, but not desmin, was significantly increased by serum depletion (SD). The SD-induced upregulation of fibronectin and collagen type I was suppressed by SU11652, an inhibitor of PDGFRβ, while PDGF-B further increased the SD-induced upregulation. In conclusion, the expression level of PDGFRβ may be a crucial determinant of fibrosis after ischemic stroke. Moreover, PDGFRβ signaling participates in the production of fibronectin and collagen type I after ischemic stroke.
4. Transcriptional targets of TWIST1 in the cranial mesoderm regulate cell-matrix interactions and mesenchyme maintenance. TWIST1, a basic helix-loop-helix transcription factor is essential for the development of cranial mesoderm and cranial neural crest-derived craniofacial structures. We have previously shown that, in the absence of TWIST1, cells within the cranial mesoderm adopt an abnormal epithelial configuration via a process reminiscent of a mesenchymal to epithelial transition (MET). Here, we show by gene expression analysis that loss of TWIST1 in the cranial mesoderm is accompanied by a reduction in the expression of genes that are associated with cell-**extracellular matrix** interactions and the acquisition of mesenchymal characteristics. By comparing the transcriptional profiles of cranial mesoderm-specific Twist1 loss-of-function mutant and control mouse embryos, we identified a set of genes that are both TWIST1-dependent and predominantly expressed in the mesoderm. ChIP-seq was used to identify TWIST1-binding sites in an in vitro model of a TWIST1-dependent mesenchymal cell state, and the data were combined with the transcriptome data to identify potential target genes. Three direct transcriptional targets of TWIST1 (Ddr2, Pcolce and Tgfbi) were validated by ChIP-PCR using mouse embryonic tissues and by luciferase assays. Our findings reveal that the mesenchymal properties of the cranial mesoderm are likely to be regulated by a network of TWIST1 targets that influences the **extracellular matrix** and cell-matrix interactions, and collectively they are required for the morphogenesis of the craniofacial structures.
5. Of mice and Marfan: genetic linkage analyses of the fibrillin genes, Fbn1 and Fbn2, in the mouse genome. The fibrillin genes, FBN1 and FBN2, encode large **extracellular matrix** glycoproteins involved in the structure and function of microfibrils. Mutations in FBN1 are found in patients with Marfan syndrome, a heritable connective tissue disease that primarily affects the cardiovascular, ocular, and skeletal systems. We extended the studies of these genes by determining their chromosomal position in the mouse genome. Restriction fragment length polymorphisms (RFLPs) between the progenitors of an interspecific backcross involving AEJ/Gn and Mus spretus mice were used to establish the segregation patterns of the murine homologs, Fbn1 and Fbn2, in the backcross progeny. The results position Fbn1 between the B2m and Illa genes on mouse Chromosome (Chr) 2 and establish its candidacy for the Tight skin (Tsk) mutation. The results position Fbn2 between the D18Mit35 and Pdgfrb loci in the central region of mouse Chr 18. Fbn2 maps near three mutations [bouncy (bc), plucked (pk), and shaker with syndactyly (sy)] and may be a candidate for the pk mutation.
6. Platelet-derived growth factor beta receptor regulates interstitial fluid homeostasis through phosphatidylinositol-3' kinase signaling. Platelet-derived growth factor (PDGF) isoforms lead to mitogenic, survival, and chemotactic responses in a variety of mesenchymal cell types during development and in the adult. We have studied the importance of phosphatidylinositol-3' kinase (PI3K) signaling in these responses by mutating the PI3K-binding sites in the PDGF-beta receptor by gene targeting in embryonic stem cells. Homozygous mutant mice developed normally; however, cells derived from the mutants were less chemotactic and had largely lost their ability to contract collagen gels in response to PDGF. Injection of a mast cell degranulating agent in mice led to a decrease in interstitial fluid pressure resulting in edema formation. In contrast to wild-type mice, mutant mice were unable to normalize the pressure after treatment with PDGF. Taken together, these observations suggest a function for PDGF signaling through PI3K in interstitial fluid homeostasis by modulating the tension between cells and **extracellular matrix** structures.
7. FOXD1 promotes nephron progenitor differentiation by repressing decorin in the embryonic kidney. Forkhead transcription factors are essential for diverse processes in early embryonic development and organogenesis. Foxd1 is required during kidney development and its inactivation results in failure of nephron progenitor cell differentiation. Foxd1 is expressed in interstitial cells adjacent to nephron progenitor cells, suggesting an essential role for the progenitor cell niche in nephrogenesis. To better understand how cortical interstitial cells in general, and FOXD1 in particular, influence the progenitor cell niche, we examined the differentiation states of two progenitor cell subtypes in Foxd1(-/-) tissue. We found that although nephron progenitor cells are retained in a primitive CITED1-expressing compartment, cortical interstitial cells prematurely differentiate. To identify pathways regulated by FOXD1, we screened for target genes by comparison of Foxd1 null and wild-type tissues. We found that the gene encoding the small leucine-rich proteoglycan decorin (DCN) is repressed by FOXD1 in cortical interstitial cells, and we show that compound genetic inactivation of Dcn partially rescues the failure of progenitor cell differentiation in the Foxd1 null. We demonstrate that DCN antagonizes BMP/SMAD signaling, which is required for the transition of CITED1-expressing nephron progenitor cells to a state that is primed for WNT-induced epithelial differentiation. On the basis of these studies, we propose a mechanism for progenitor cell retention in the Foxd1 null in which misexpressed DCN produced by prematurely differentiated interstitial cells accumulates in the **extracellular matrix**, inhibiting BMP7-mediated transition of nephron progenitor cells to a compartment in which they can respond to epithelial induction signals.

### [Ii]nvasive

#### PubMed

1. MT1-MMP promotes vascular smooth muscle dedifferentiation through LRP1 processing. At sites of vessel-wall injury, vascular smooth muscle cells (VSMCs) can dedifferentiate to express an **invasive** and proliferative phenotype, which contributes to the development of neointimal lesions and vascular disorders. Herein, we demonstrate that the loss of the VSMC differentiated phenotype, as the repression of contractile-protein expression, is correlated with a dramatic upregulation of the membrane-anchored matrix metalloproteinase MT1-MMP (also known as MMP14 and membrane-type 1 matrix metalloproteinase). Matrix metalloproteinase (MMP) inhibitors or MT1-MMP deficiency led to attenuated VSMC dedifferentiation, whereas the phenotypic switch was re-engaged following the restoration of MT1-MMP activity in MT1-MMP(-/-) cells. MT1-MMP-dependent dedifferentiation was mediated by the PDGF-BB-PDGFRbeta pathway in parallel with the proteolytic processing of the multifunctional LDL receptor-related protein LRP1 and the dynamic internalization of a PDGFRbeta-beta3-integrin-MT1-MMP-LRP1 multi-component complex. Importantly, LRP1 silencing allowed the PDGF-BB-induced dedifferentiation program to proceed in the absence of MT1-MMP activity, supporting the role of unprocessed LRP1 as a gatekeeper of VSMC differentiation. Hence, MT1-MMP and LRP1 serve as a new effector-target-molecule axis that controls the PDGF-BB-PDGFRbeta-dependent VSMC phenotype and function.
2. The dietary flavones apigenin and luteolin impair smooth muscle cell migration and VEGF expression through inhibition of PDGFR-beta phosphorylation. Platelet-derived growth factor (PDGF)-dependent recruitment of mural cells such as pericytes and smooth muscle cells plays a central role in the maturation and stabilization of newly formed vasculature during angiogenesis. In this work, we show that the dietary flavones apigenin and luteolin may interfere with this event through their inhibitory effect on PDGF-dependent phosphorylation of PDGF receptor beta (PDGFR-beta) in smooth muscle cells. Inhibition of PDGFR-beta activity by apigenin and luteolin occurred at low concentrations of the molecules and resulted in the inhibition of extracellular signal-regulated kinase and Akt phosphorylation triggered by PDGF, as well as in a marked reduction of the migratory and **invasive** properties of these cells. Apigenin and luteolin also strongly inhibit the PDGF-dependent increase in vascular endothelial growth factor (VEGF) mRNA levels and the secretion of VEGF by smooth muscle cells as well as vessel formation in the mouse Matrigel plug assay, suggesting that the inhibitory effects of both molecules on smooth muscle cell function result in impaired angiogenesis. Overall, these results identify apigenin and luteolin as dietary-derived inhibitors of PDGFR-beta activity and suggest that this inhibitory effect may contribute to the chemopreventive properties of these molecules.
3. Autocrine PDGFR signaling promotes mammary cancer metastasis. Metastasis is the major cause of cancer morbidity, but strategies for direct interference with invasion processes are lacking. Dedifferentiated, late-stage tumor cells secrete multiple factors that represent attractive targets for therapeutic intervention. Here we show that metastatic potential of oncogenic mammary epithelial cells requires an autocrine PDGF/PDGFR loop, which is established as a consequence of TGF-beta-induced epithelial-mesenchymal transition (EMT), a faithful in vitro correlate of metastasis. The cooperation of autocrine PDGFR signaling with oncogenic Ras hyperactivates PI3K and is required for survival during EMT. Autocrine PDGFR signaling also contributes to maintenance of EMT, possibly through activation of STAT1 and other distinct pathways. Inhibition of PDGFR signaling interfered with EMT and caused apoptosis in murine and human mammary carcinoma cell lines. Consequently, overexpression of a dominant-negative PDGFR or application of the established cancer drug STI571 interfered with experimental metastasis in mice. Similarly, in mouse mammary tumor virus-Neu (MMTV-Neu) transgenic mice, TGF-beta enhanced metastasis of mammary tumors, induced EMT, and elevated PDGFR signaling. Finally, expression of PDGFRalpha and -beta correlated with **invasive** behavior in human mammary carcinomas. Thus, autocrine PDGFR signaling plays an essential role during cancer progression, suggesting a novel application of STI571 to therapeutically interfere with metastasis.
4. Protocadherin 12 (VE-cadherin 2) is expressed in endothelial, trophoblast, and mesangial cells. Protocadherin 12 protein (PCDH12, VE-cadherin 2) is a cell adhesion molecule that has been isolated from endothelial cells. Here, we have used Northern and Western blots, immunohistology, and flow cytometry to examine the distribution of PCDH12 in mouse tissues. It is an N-glycosylated protein of 150-kDa mass. In the endothelium, PCDH12 immunoreactivity was variable and dependent upon the vascular bed. In both the embryo and embryonic stem cell differentiation system, signals were localized in vasculogenic rather than angiogenic endothelium. In addition, the protein was strongly expressed in a subset of **invasive** cells of the placenta, which were identified as glycogen-rich trophoblasts. In adult mice, strong PCDH12 signals were observed in mesangial cells of kidney glomeruli whereas expression was not detected in other types of perivascular cells. As opposed to most protocadherins, PCDH12 is not expressed in early embryonic (day 12.5) and adult brains. As a first approach to obtain insight into PCDH12 function, we produced transgenic mice deficient in PCDH12, which were viable and fertile. They did not display any obvious histomorphological defects. We conclude that PCDH12 has a unique expression pattern and that its deficiency does not lead to conspicuous abnormalities. Moreover, PCDH12 is the first specific marker for both glycogen-rich trophoblasts and mesangial cells.
5. Pericyte-fibroblast transition promotes tumor growth and metastasis. Vascular pericytes, an important cellular component in the tumor microenvironment, are often associated with tumor vasculatures, and their functions in cancer invasion and metastasis are poorly understood. Here we show that PDGF-BB induces pericyte-fibroblast transition (PFT), which significantly contributes to tumor invasion and metastasis. Gain- and loss-of-function experiments demonstrate that PDGF-BB-PDGFRβ signaling promotes PFT both in vitro and in in vivo tumors. Genome-wide expression analysis indicates that PDGF-BB-activated pericytes acquire mesenchymal progenitor features. Pharmacological inhibition and genetic deletion of PDGFRβ ablate the PDGF-BB-induced PFT. Genetic tracing of pericytes with two independent mouse strains, TN-AP-CreERT2:R26R-tdTomato and NG2-CreERT2:R26R-tdTomato, shows that PFT cells gain stromal fibroblast and myofibroblast markers in tumors. Importantly, coimplantation of PFT cells with les**s-invasi**ve tumor cells in mice markedly promotes tumor dissemination and invasion, leading to an increased number of circulating tumor cells and metastasis. Our findings reveal a mechanism of vascular pericytes in PDGF-BB-promoted cancer invasion and metastasis by inducing PFT, and thus targeting PFT may offer a new treatment option of cancer metastasis.

### [Mm]etastasis

#### PubMed

1. Regulation of chemotaxis by the platelet-derived growth factor receptor-beta. Chemotaxis is an important component of wound healing, development, immunity and **metastasis**, yet the signalling pathways that mediate chemotaxis are poorly understood. Platelet-derived growth factor (PDGF) acts both as a mitogen and a chemoattractant. Upon stimulation, the tyrosine kinase PDGF receptor-beta (PDGFR-beta) autophosphorylates and forms a complex that includes SII2(Src homology 2)-domain-containing proteins such as the phosphatidylinositol-specific phospholipase C-gamma, Ras-GTPase-activating protein (GAP), and phosphatidylinositol-3-OH kinase. Specific tyrosine-to-phenylalanine substitutions in the PDGFR-beta can prevent binding of one SH2-domain-containing protein without affecting binding of other receptor-associated proteins. Here we use phospholipase C-gamma and PDGFR-beta mutants to map specific tyrosines involved in both positive and negative regulation of chemotaxis towards the PDGF-BB homodimer. Our results indicate that a delicate balance of migration-promoting (phospholipase C-gamma and phosphatidylinositol-3-OH kinase) and migration-suppressing (GAP) activities are recruited by the PDGFR-beta to drive chemotaxis towards PDGF-BB.
2. PDGFRB promotes liver **metastasis** formation of mesenchymal-like colorectal tumor cells. In epithelial tumors, the platelet-derived growth factor receptor B (PDGFRB) is mainly expressed by stromal cells of mesenchymal origin. Tumor cells may also acquire PDGFRB expression following epithelial-to-mesenchymal transition (EMT), which occurs during **metastasis** formation. Little is known about PDGFRB signaling in colorectal tumor cells. We studied the relationship between PDGFRB expression, EMT, and **metastasis** in human colorectal cancer (CRC) cohorts by analysis of gene expression profiles. PDGFRB expression in primary CRC was correlated with short disease-free and overall survival. PDGFRB was co-expressed with genes involved in platelet activation, transforming growth factor beta (TGFB) signaling, and EMT in three CRC cohorts. PDGFRB was expressed in mesenchymal-like tumor cell lines in vitro and stimulated invasion and liver **metastasis** formation in mice. Platelets, a major source of PDGF, preferentially bound to tumor cells in a non-activated state. Platelet activation caused robust PDGFRB tyrosine phosphorylation on tumor cells in vitro and in liver sinusoids in vivo. Platelets also release TGFB, which is a potent inducer of EMT. Inhibition of TGFB signaling in tumor cells caused partial reversion of the mesenchymal phenotype and strongly reduced PDGFRB expression and PDGF-stimulated tumor cell invasion. These results suggest that PDGFRB may contribute to the aggressive phenotype of colorectal tumors with mesenchymal properties, most likely downstream of platelet activation and TGFB signaling.
3. Autocrine PDGFR signaling promotes mammary cancer **metastasis**. **Metastasis** is the major cause of cancer morbidity, but strategies for direct interference with invasion processes are lacking. Dedifferentiated, late-stage tumor cells secrete multiple factors that represent attractive targets for therapeutic intervention. Here we show that metastatic potential of oncogenic mammary epithelial cells requires an autocrine PDGF/PDGFR loop, which is established as a consequence of TGF-beta-induced epithelial-mesenchymal transition (EMT), a faithful in vitro correlate of **metastasis**. The cooperation of autocrine PDGFR signaling with oncogenic Ras hyperactivates PI3K and is required for survival during EMT. Autocrine PDGFR signaling also contributes to maintenance of EMT, possibly through activation of STAT1 and other distinct pathways. Inhibition of PDGFR signaling interfered with EMT and caused apoptosis in murine and human mammary carcinoma cell lines. Consequently, overexpression of a dominant-negative PDGFR or application of the established cancer drug STI571 interfered with experimental **metastasis** in mice. Similarly, in mouse mammary tumor virus-Neu (MMTV-Neu) transgenic mice, TGF-beta enhanced **metastasis** of mammary tumors, induced EMT, and elevated PDGFR signaling. Finally, expression of PDGFRalpha and -beta correlated with invasive behavior in human mammary carcinomas. Thus, autocrine PDGFR signaling plays an essential role during cancer progression, suggesting a novel application of STI571 to therapeutically interfere with **metastasis**.
4. Pericyte-fibroblast transition promotes tumor growth and **metastasis**. Vascular pericytes, an important cellular component in the tumor microenvironment, are often associated with tumor vasculatures, and their functions in cancer invasion and **metastasis** are poorly understood. Here we show that PDGF-BB induces pericyte-fibroblast transition (PFT), which significantly contributes to tumor invasion and **metastasis**. Gain- and loss-of-function experiments demonstrate that PDGF-BB-PDGFRβ signaling promotes PFT both in vitro and in in vivo tumors. Genome-wide expression analysis indicates that PDGF-BB-activated pericytes acquire mesenchymal progenitor features. Pharmacological inhibition and genetic deletion of PDGFRβ ablate the PDGF-BB-induced PFT. Genetic tracing of pericytes with two independent mouse strains, TN-AP-CreERT2:R26R-tdTomato and NG2-CreERT2:R26R-tdTomato, shows that PFT cells gain stromal fibroblast and myofibroblast markers in tumors. Importantly, coimplantation of PFT cells with less-invasive tumor cells in mice markedly promotes tumor dissemination and invasion, leading to an increased number of circulating tumor cells an**d metastas**is. Our findings reveal a mechanism of vascular pericytes in PDGF-BB-promoted cancer invasion an**d metastas**is by inducing PFT, and thus targeting PFT may offer a new treatment option of cance**r metastas**is.

### [Cc]ell [Ii]nvasion

#### PubMed

1. PDGFRB promotes liver metastasis formation of mesenchymal-like colorectal tumor cells. In epithelial tumors, the platelet-derived growth factor receptor B (PDGFRB) is mainly expressed by stromal cells of mesenchymal origin. Tumor cells may also acquire PDGFRB expression following epithelial-to-mesenchymal transition (EMT), which occurs during metastasis formation. Little is known about PDGFRB signaling in colorectal tumor cells. We studied the relationship between PDGFRB expression, EMT, and metastasis in human colorectal cancer (CRC) cohorts by analysis of gene expression profiles. PDGFRB expression in primary CRC was correlated with short disease-free and overall survival. PDGFRB was co-expressed with genes involved in platelet activation, transforming growth factor beta (TGFB) signaling, and EMT in three CRC cohorts. PDGFRB was expressed in mesenchymal-like tumor cell lines in vitro and stimulated invasion and liver metastasis formation in mice. Platelets, a major source of PDGF, preferentially bound to tumor cells in a non-activated state. Platelet activation caused robust PDGFRB tyrosine phosphorylation on tumor cells in vitro and in liver sinusoids in vivo. Platelets also release TGFB, which is a potent inducer of EMT. Inhibition of TGFB signaling in tumor cells caused partial reversion of the mesenchymal phenotype and strongly reduced PDGFRB expression and PDGF-stimulated tumor **cell invasion**. These results suggest that PDGFRB may contribute to the aggressive phenotype of colorectal tumors with mesenchymal properties, most likely downstream of platelet activation and TGFB signaling.

---

## Fgfr1

- NCBI Gene
- GeneRIF
- Pubmed

| Search Term | Rank | Fields |
| --- | --- | --- |
| [Ff]ilopodia | 1 | PubMed(1) |
| [Mm]igration | 6 | Process(2); PubMed(47); GeneRIFs(5) |
| ECM | 7 | PubMed(2) |
| [Ee]xtracellular [Mm]atrix | 8 | PubMed(12) |
| [Ii]nvasive | 10 | PubMed(5); GeneRIFs(1) |
| [Mm]etastasis | 12 | PubMed(5) |
| [Cc]ell [Ii]nvasion | 13 | PubMed(1) |

  
hide/show details for Fgfr1

### [Ff]ilopodia

#### PubMed

1. The F-BAR protein NOSTRIN participates in FGF signal transduction and vascular development. F-BAR proteins are multivalent adaptors that link plasma membrane and cytoskeleton and coordinate cellular processes such as membrane protrusion and migration. Yet, little is known about the function of F-BAR proteins in vivo. Here we report, that the F-BAR protein NOSTRIN is necessary for proper vascular development in zebrafish and postnatal retinal angiogenesis in mice. The loss of NOSTRIN impacts on the migration of endothelial tip cells and leads to a reduction of tip cell **filopodia** number and length. NOSTRIN forms a complex with the GTPase Rac1 and its exchange factor Sos1 and overexpression of NOSTRIN in cells induces Rac1 activation. Furthermore, NOSTRIN is required for fibroblast growth factor 2 dependent activation of Rac1 in primary endothelial cells and the angiogenic response to fibroblast growth factor 2 in the in vivo matrigel plug assay. We propose a novel regulatory circuit, in which NOSTRIN assembles a signalling complex containing FGFR1, Rac1 and Sos1 thereby facilitating the activation of Rac1 in endothelial cells during developmental angiogenesis.

### [Mm]igration

#### Process

1. motogenic signaling involved in postnatal olfactory bulb interneuron **migration**
2. positive regulation of fibroblast **migration**

#### PubMed

1. Id1 modulates endothelial progenitor cells function through relieving the E2-2-mediated repression of FGFR1 and VEGFR2 in vitro. The **migration** and proliferation of EPCs are crucial for re-endothelialization in vascular repair and development. Id1 has a regulatory role in the regulation of EPCs **migration** and proliferation. Based on these findings, we hypothesized that Id1 plays a regulatory role in modulating the **migration** and proliferation of EPCs by interaction with other factors. Herein, we report that the Id1 protein and E-box protein E2-2 regulate EPCs function with completely opposite effects. Id1 plays a positive role in the regulation of EPC proliferation and **migration**, while endogenous E2-2 appears to be a negative regulator. Immunoprecipitation and immunofluorescence assay revealed that the Id1 protein interacts and co-localizes with the E2-2 protein in EPCs. Further, endogenous E2-2 protein was found to block EPCs function via the inhibition of FGFR1 and VEGFR2 expression. The overexpression and silencing of Id1 have no direct regulatory role on VEGFR2 and FGFR1 expression. On the other hand, Id1 relieves the E2-2-mediated repression of FGFR1 and VEGFR2 expression to modulate EPCs proliferation, **migration**, and tube formation in vitro. In summary, we demonstrated that Id1 and E2-2 are critical regulators of EPCs function in vitro. Id1 interacts with E2-2 and relieves the E2-2-mediated repression of FGFR1 and VEGFR2 expression to modulate EPCs functions. Id1 and E2-2 may represent novel therapeutic targets for re-endothelialization in vascular damage and repair.
2. Fgfr1 regulates patterning of the pharyngeal region. Development of the pharyngeal region depends on the interaction and integration of different cell populations, including surface ectoderm, foregut endoderm, paraxial mesoderm, and neural crest. Mice homozygous for a hypomorphic allele of Fgfr1 have craniofacial defects, some of which appeared to result from a failure in the early development of the second branchial arch. A stream of neural crest cells was found to originate from the rhombomere 4 region and migrate toward the second branchial arch in the mutants. Neural crest cells mostly failed to enter the second arch, however, but accumulated in a region proximal to it. Both rescue of the hypomorphic Fgfr1 allele and inactivation of a conditional Fgfr1 allele specifically in neural crest cells indicated that Fgfr1 regulates the entry of neural crest cells into the second branchial arch non-cell-autonomously. Gene expression in the pharyngeal ectoderm overlying the developing second branchial arch was affected in the hypomorphic Fgfr1 mutants at a stage prior to neural crest entry. Our results indicate that Fgfr1 patterns the pharyngeal region to create a permissive environment for neural crest cell **migration**.
3. Expression of the fibroblast growth factor family and their receptor family genes during mouse brain development. The fibroblast growth factor (FGF) family is composed of nine members and four genes encode protein tyrosine kinase receptors for them. To gain insight into the involvement of FGFs and their receptors in the development of nervous system, their expression in brains of perinatal and adult mice was examined by semi-quantitative reverse transcription-linked polymerase chain reactions and in situ hybridization. Although all the genes, with the exception of FGF-4, were found to be expressed, FGF-3, FGF-6, FGF-7 and FGF-8 genes demonstrated higher expression in the late embryonic stages than in postnatal stages, suggesting that these members are involved in the late stages of brain development. In contrast, expression of FGF-1 and FGF-5 increased after birth. Interestingly, FGF-6 expression in perinatal mice was restricted to the central nervous system and skeltal muscles, with intense signals in the developing cerebrum in embryos but in cerebellum in 5-day-old neonates. Furthermore, FGF-receptor (FGFR)-4, a cognate receptor for FGF-6, demonstrated similar spatiotemporal expression, suggesting that FGF-6 and FGFR-4 plays significant roles in the maturation of nervous system as a ligand-receptor system. The results indicate that individual member of the fibroblast growth factor and their receptor family are expressed either sequentially or simultaneously in brain development, strongly suggesting their involvement in the regulation of a variety of developmental processes of brain, i.e., proliferation and **migration** of neuronal progenitor cells, neuron and glia differentiation, neurite extensions, and synapse formations.
4. Cripto is required for mesoderm and endoderm cell allocation during mouse gastrulation. During mouse gastrulation, cells in the primitive streak undergo epithelial-mesenchymal transformation and the resulting mesenchymal cells migrate out laterally to form mesoderm and definitive endoderm across the entire embryonic cylinder. The mechanisms underlying mesoderm and endoderm specification, **migration**, and allocation are poorly understood. In this study, we focused on the function of mouse Cripto, a member of the EGF-CFC gene family that is highly expressed in the primitive streak and migrating mesoderm cells on embryonic day 6.5. Conditional inactivation of Cripto during gastrulation leads to varied defects in mesoderm and endoderm development. Mutant embryos display accumulation of mesenchymal cells around the shortened primitive streak indicating a functional requirement of Cripto during the formation of mesoderm layer in gastrulation. In addition, some mutant embryos showed poor formation and abnormal allocation of definitive endoderm cells on embryonic day 7.5. Consistently, many mutant embryos that survived to embryonic day 8.5 displayed defects in ventral closure of the gut endoderm causing cardia bifida. Detailed analyses revealed that both the Fgf8-Fgfr1 pathway and p38 MAP kinase activation are partially affected by the loss of Cripto function. These results demonstrate a critical role for Cripto during mouse gastrulation, especially in mesoderm and endoderm formation and allocation.
5. Fibronectin induces endothelial cell **migration** through β1 integrin and Src-dependent phosphorylation of fibroblast growth factor receptor-1 at tyrosines 653/654 and 766. The extracellular matrix microenvironment regulates cell phenotype and function. One mechanism by which this is achieved is the transactivation of receptor tyrosine kinases by specific matrix molecules. Here, we demonstrate that the provisional matrix protein, fibronectin (FN), activates fibroblast growth factor (FGF) receptor-1 (FGFR1) independent of FGF ligand in liver endothelial cells. FN activation of FGFR1 requires β1 integrin, as evidenced by neutralizing antibody and siRNA-based studies. Complementary genetic and pharmacologic approaches identify that the non-receptor tyrosine kinase Src is required for FN transactivation of FGFR1. Whereas FGF ligand-induced phosphorylation of FGFR1 preferentially activates ERK, FN-induced phosphorylation of FGFR1 preferentially activates AKT, indicating differential downstream signaling of FGFR1 in response to alternate stimuli. Mutation analysis of known tyrosine residues of FGFR1 reveals that tyrosine 653/654 and 766 residues are required for FN-FGFR1 activation of AKT and chemotaxis. Thus, our study mechanistically dissects a new signaling pathway by which FN achieves endothelial cell chemotaxis, demonstrates how differential phosphorylation profiles of FGFR1 can achieve alternate downstream signals, and, more broadly, highlights the diversity of mechanisms by which the extracellular matrix microenvironment regulates cell behavior through transactivation of receptor tyrosine kinases.
6. Twist function is required for the morphogenesis of the cephalic neural tube and the differentiation of the cranial neural crest cells in the mouse embryo. Loss of Twist function in the cranial mesenchyme of the mouse embryo causes failure of closure of the cephalic neural tube and malformation of the branchial arches. In the Twist(-/-) embryo, the expression of molecular markers that signify dorsal forebrain tissues is either absent or reduced, but those associated with ventral tissues display expanded domains of expression. Dorsoventral organization of the mid- and hindbrain and the anterior-posterior pattern of the neural tube are not affected. In the Twist(-/-) embryo, neural crest cells stray from the subectodermal migratory path and the late-migrating subpopulation invades the cell-free zone separating streams of cells going to the first and second branchial arches. Cell transplantation studies reveal that Twist activity is required in the cranial mesenchyme for directing the **migration** of the neural crest cells, as well as in the neural crest cells within the first branchial arch to achieve correct localization. Twist is also required for the proper differentiation of the first arch tissues into bone, muscle, and teeth.
7. Changes in Sef levels influence auditory brainstem development and function. During development of the CNS, secreted morphogens of the fibroblast growth factor (FGF) family have multiple effects on cell division, **migration**, and survival depending on where, when, and how much FGF signal is received. The consequences of misregulating the FGF pathway were studied in a mouse with decreased levels of the FGF antagonist Sef. To uncover effects in the nervous system, we focused on the auditory system, which is accessible to physiological analysis. We found that the mitogen-activated protein kinase pathway is active in the rhombic lip, a germinal zone that generates diverse types of neurons, including the cochlear nucleus complex of the auditory system. Sef is expressed immediately adjacent to the rhombic lip, overlapping with FGF15 and FGFR1, which is also present in the lip itself. This pattern suggests that Sef may normally function in non-rhombic lip cells and prevent them from responding to FGF ligand in the vicinity. Consistent with this idea, overexpression of Sef in chicks decreased the size of the auditory nuclei. Cochlear nucleus defects were also apparent in mice with reduced levels of Sef, with 13% exhibiting grossly dysmorphic cochlear nuclei and 26% showing decreased amounts of GFAP in the cochlear nucleus. Additional evidence for cochlear nucleus defects was obtained by electrophysiological analysis of Sef mutant mice, which have normal auditory thresholds but abnormal auditory brainstem responses. These results show both increases and decreases in Sef levels affect the assembly and function of the auditory brainstem.
8. Midline radial glia translocation and corpus callosum formation require FGF signaling. Midline astroglia in the cerebral cortex develop earlier than other astrocytes through mechanisms that are still unknown. We show that radial glia in dorsomedial cortex retract their apical endfeet at midneurogenesis and translocate to the overlaying pia, forming the indusium griseum. These cells require the fibroblast growth factor receptor 1 (Fgfr1) gene for their precocious somal translocation to the dorsal midline, as demonstrated by inactivating the Fgfr1 gene in radial glial cells and by RNAi knockdown of Fgfr1 in vivo. Dysfunctional astroglial **migration** underlies the callosal dysgenesis in conditional Fgfr1 knockout mice, suggesting that precise targeting of astroglia to the cortex has unexpected roles in axon guidance. FGF signaling is sufficient to induce somal translocation of radial glial cells throughout the cortex; furthermore, the targeting of astroglia to dorsolateral cortex requires FGFr2 signaling after neurogenesis. Hence, FGFs have an important role in the transition from radial glia to astrocytes by stimulating somal translocation of radial glial cells.
9. The roles of FGF signaling in germ cell **migration** in the mouse. Fibroblast growth factor (FGF) signaling is thought to play a role in germ cell behavior. FGF2 has been reported to be a mitogen for primordial germ cells in vitro, whilst combinations of FGF2, steel factor and LIF cause cultured germ cells to transform into permanent lines of pluripotent cells resembling ES cells. However, the actual function of FGF signaling on the migrating germ cells in vivo is unknown. We show, by RT-PCR analysis of cDNA from purified E10.5 germ cells, that germ cells express two FGF receptors: Fgfr1-IIIc and Fgfr2-IIIb. Second, we show that FGF-mediated activation of the MAP kinase pathway occurs in germ cells during their **migration**, and thus they are potentially direct targets of FGF signaling. Third, we use cultured embryo slices in simple gain-of-function experiments, using FGF ligands, to show that FGF2, a ligand for FGFR1-IIIc, affects motility, whereas FGF7, a ligand for FGFR2-IIIb, affects germ cell numbers. Loss of function, using a specific inhibitor of FGF signaling, causes increased apoptosis and inhibition of cell shape change in the migrating germ cells. Lastly, we confirm in vivo the effects seen in slice cultures in vitro, by examining germ cell positions and numbers in embryos carrying a loss-of-function allele of FGFR2-IIIb. In FGFR2-IIIb(-/-) embryos, germ cell **migration** is unaffected, but the numbers of germ cells are significantly reduced. These data show that a major role of FGF signaling through FGFR2-IIIb is to control germ cell numbers. The data do not discriminate between direct and indirect effects of FGF signaling on germ cells, and both may be involved.
10. Loss of late primitive streak mesoderm and interruption of left-right morphogenesis in the Ednrb(s-1Acrg) mutant mouse. This study characterizes defects associated with abnormal mesoderm development in mouse embryos homozygous for the induced Ednrb(s-1Acrg) allele of the piebald deletion complex. The Ednrb(s-1Acrg) deletion results in recessive embryonic lethality and mutant embryos exhibit a truncated posterior body axis. The primitive streak and node become disfigured, consistent with evidence that cell **migration** is impaired in newly formed mesoderm. Additional defects related to mesoderm development include notochord degeneration, somite malformations, and abnormal vascular development. Arrested heart looping morphogenesis and a randomized direction of embryonic turning indicate that left-right development is also perturbed. The expression of nodal and leftb, Tgf-beta-related genes involved in a left-determinant signaling pathway, is variably lost in the left lateral plate mesoderm. Mutational analysis has demonstrated that Fgf8 and Brachyury (T) are required for normal mesoderm and left-right development and the asymmetric expression of nodal and leftb. Fgf8 expression in nascent mesoderm exiting the primitive streak is dramatically reduced in mutant embryos, and diminished T expression accompanies the progressive loss of paraxial, lateral, and primitive streak mesoderm. In contrast, axial mesoderm persists and T and nodal appear to be appropriately expressed in their specific domains in the node and notochord. We propose that this mutation disrupts a morphogenetic pathway, likely involving FGF signaling, important for the development of streak-derived posterior mesoderm and lateral morphogenesis.
11. Laminar patterning in the developing neocortex by temporally coordinated fibroblast growth factor signaling. Laminar organization, a fundamental neural architecture in the CNS, is a prominent feature of the neocortex, where the cortical neurons in spatially distinct layers are generated from the common progenitors in a temporally distinct manner during development. Despite many advances in the characterization of the molecular mechanisms of the radial **migration** of cortical neurons, the way in which the early-late temporal sequence of cortical neuron generation is linked with the deep-superficial spatial sequence of cell body positioning remains obscure. Using in vivo electroporation-mediated gene transfer, we show here that the activities mediated by fibroblast growth factor receptors (FGFRs) in cortical progenitors are critical for conferring proper migratory properties on nascent neuronal progeny. Furthermore, we provide supportive evidence that Pea3 subfamily members of Ets (Pea3-Ets) transcription factors mediate the activities of FGFR at the mid to late phase of neocortical development. In addition, using FGF18 knock-out mice, we demonstrate that FGF18 expressed by early-generated cortical neurons in the cortical plate is critical for the expression of Pea3-Ets transcription factors and that FGF18 is sufficient to induce their expressions. Our results thus imply that a feedback mechanism mediated by FGF signaling is involved in setting up the proper laminar positioning of cortical neurons; FGF18 derived from early-generated cortical neurons acts on the cortical progenitors expressing FGFRs and induces the expression of Pea3-Ets transcription factors that, in turn, confer proper migratory behaviors on nascent cortical progeny during the mid to late stages of neocortical development.
12. Neuron-derived FGF9 is essential for scaffold formation of Bergmann radial fibers and **migration** of granule neurons in the cerebellum. Although fibroblast growth factor 9 (FGF9) is widely expressed in the central nervous system (CNS), the function of FGF9 in neural development remains undefined. To address this question, we deleted the Fgf9 gene specifically in the neural tube and demonstrated that FGF9 plays a key role in the postnatal **migration** of cerebellar granule neurons. Fgf9-null mice showed severe ataxia associated with disrupted Bergmann fiber scaffold formation, impaired granule neuron **migration**, and upset Purkinje cell maturation. Ex vivo cultured wildtype or Fgf9-null glia displayed a stellate morphology. Coculture with wildtype neurons, but not Fgf9-deficient neurons, or treating with FGF1 or FGF9 induced the cells to adopt a radial glial morphology. In situ hybridization showed that Fgf9 was expressed in neurons and immunostaining revealed that FGF9 was broadly distributed in both neurons and Bergmann glial radial fibers. Genetic analyses revealed that the FGF9 activities in cerebellar development are primarily transduced by FGF receptors 1 and 2. Furthermore, inhibition of the MAP kinase pathway, but not the PI3K/AKT pathway, abrogated the FGF activity to induce glial morphological changes, suggesting that the activity is mediated by the MAP kinase pathway. This work demonstrates that granule neurons secrete FGF9 to control formation of the Bergmann fiber scaffold, which in turn, guides their own inward **migration** and maturation of Purkinje cells.
13. Cubilin, a high affinity receptor for fibroblast growth factor 8, is required for cell survival in the developing vertebrate head. Cubilin (Cubn) is a multiligand endocytic receptor critical for the intestinal absorption of vitamin B12 and renal protein reabsorption. During mouse development, Cubn is expressed in both embryonic and extra-embryonic tissues, and Cubn gene inactivation results in early embryo lethality most likely due to the impairment of the function of extra-embryonic Cubn. Here, we focus on the developmental role of Cubn expressed in the embryonic head. We report that Cubn is a novel, interspecies-conserved Fgf receptor. Epiblast-specific inactivation of Cubn in the mouse embryo as well as Cubn silencing in the anterior head of frog or the cephalic neural crest of chick embryos show that Cubn is required during early somite stages to convey survival signals in the developing vertebrate head. Surface plasmon resonance analysis reveals that fibroblast growth factor 8 (Fgf8), a key mediator of cell survival, **migration**, proliferation, and patterning in the developing head, is a high affinity ligand for Cubn. Cell uptake studies show that binding to Cubn is necessary for the phosphorylation of the Fgf signaling mediators MAPK and Smad1. Although Cubn may not form stable ternary complexes with Fgf receptors (FgfRs), it acts together with and/or is necessary for optimal FgfR activity. We propose that plasma membrane binding of Fgf8, and most likely of the Fgf8 family members Fgf17 and Fgf18, to Cubn improves Fgf ligand endocytosis and availability to FgfRs, thus modulating Fgf signaling activity.
14. FGF/FGFR2 signaling regulates the generation and correct positioning of Bergmann glia cells in the developing mouse cerebellum. The normal cellular organization and layering of the vertebrate cerebellum is established during embryonic and early postnatal development by the interplay of a complex array of genetic and signaling pathways. Disruption of these processes and of the proper layering of the cerebellum usually leads to ataxic behaviors. Here, we analyzed the relative contribution of Fibroblast growth factor receptor 2 (FGFR2)-mediated signaling to cerebellar development in conditional Fgfr2 single mutant mice. We show that during embryonic mouse development, Fgfr2 expression is higher in the anterior cerebellar primordium and excluded from the proliferative ventricular neuroepithelium. Consistent with this finding, conditional Fgfr2 single mutant mice display the most prominent defects in the anterior lobules of the adult cerebellum. In this context, FGFR2-mediated signaling is required for the proper generation of Bergmann glia cells and the correct positioning of these cells within the Purkinje cell layer, and for cell survival in the developing cerebellar primordium. Using cerebellar microexplant cultures treated with an FGFR agonist (FGF9) or antagonist (SU5402), we also show that FGF9/FGFR-mediated signaling inhibits the outward **migration** of radial glia and Bergmann glia precursors and cells, and might thus act as a positioning cue for these cells. Altogether, our findings reveal the specific functions of the FGFR2-mediated signaling pathway in the generation and positioning of Bergmann glia cells during cerebellar development in the mouse.
15. Id1 modulates endothelial progenitor cells function through relieving the E2-2-mediated repression of FGFR1 and VEGFR2 in vitro. The **migration** and proliferation of EPCs are crucial for re-endothelialization in vascular repair and development. Id1 has a regulatory role in the regulation of EPCs **migration** and proliferation. Based on these findings, we hypothesized that Id1 plays a regulatory role in modulating the **migration** and proliferation of EPCs by interaction with other factors. Herein, we report that the Id1 protein and E-box protein E2-2 regulate EPCs function with completely opposite effects. Id1 plays a positive role in the regulation of EPC proliferation and **migration**, while endogenous E2-2 appears to be a negative regulator. Immunoprecipitation and immunofluorescence assay revealed that the Id1 protein interacts and co-localizes with the E2-2 protein in EPCs. Further, endogenous E2-2 protein was found to block EPCs function via the inhibition of FGFR1 and VEGFR2 expression. The overexpression and silencing of Id1 have no direct regulatory role on VEGFR2 and FGFR1 expression. On the other hand, Id1 relieves the E2-2-mediated repression of FGFR1 and VEGFR2 expression to modulate EPCs proliferation, **migration**, and tube formation in vitro. In summary, we demonstrated that Id1 and E2-2 are critical regulators of EPCs function in vitro. Id1 interacts with E2-2 and relieves the E2-2-mediated repression of FGFR1 and VEGFR2 expression to modulate EPCs functions. Id1 and E2-2 may represent novel therapeutic targets for re-endothelialization in vascular damage and repair.
16. Fibronectin induces endothelial cell **migration** through β1 integrin and Src-dependent phosphorylation of fibroblast growth factor receptor-1 at tyrosines 653/654 and 766. The extracellular matrix microenvironment regulates cell phenotype and function. One mechanism by which this is achieved is the transactivation of receptor tyrosine kinases by specific matrix molecules. Here, we demonstrate that the provisional matrix protein, fibronectin (FN), activates fibroblast growth factor (FGF) receptor-1 (FGFR1) independent of FGF ligand in liver endothelial cells. FN activation of FGFR1 requires β1 integrin, as evidenced by neutralizing antibody and siRNA-based studies. Complementary genetic and pharmacologic approaches identify that the non-receptor tyrosine kinase Src is required for FN transactivation of FGFR1. Whereas FGF ligand-induced phosphorylation of FGFR1 preferentially activates ERK, FN-induced phosphorylation of FGFR1 preferentially activates AKT, indicating differential downstream signaling of FGFR1 in response to alternate stimuli. Mutation analysis of known tyrosine residues of FGFR1 reveals that tyrosine 653/654 and 766 residues are required for FN-FGFR1 activation of AKT and chemotaxis. Thus, our study mechanistically dissects a new signaling pathway by which FN achieves endothelial cell chemotaxis, demonstrates how differential phosphorylation profiles of FGFR1 can achieve alternate downstream signals, and, more broadly, highlights the diversity of mechanisms by which the extracellular matrix microenvironment regulates cell behavior through transactivation of receptor tyrosine kinases.
17. The roles of FGF signaling in germ cell **migration** in the mouse. Fibroblast growth factor (FGF) signaling is thought to play a role in germ cell behavior. FGF2 has been reported to be a mitogen for primordial germ cells in vitro, whilst combinations of FGF2, steel factor and LIF cause cultured germ cells to transform into permanent lines of pluripotent cells resembling ES cells. However, the actual function of FGF signaling on the migrating germ cells in vivo is unknown. We show, by RT-PCR analysis of cDNA from purified E10.5 germ cells, that germ cells express two FGF receptors: Fgfr1-IIIc and Fgfr2-IIIb. Second, we show that FGF-mediated activation of the MAP kinase pathway occurs in germ cells during their **migration**, and thus they are potentially direct targets of FGF signaling. Third, we use cultured embryo slices in simple gain-of-function experiments, using FGF ligands, to show that FGF2, a ligand for FGFR1-IIIc, affects motility, whereas FGF7, a ligand for FGFR2-IIIb, affects germ cell numbers. Loss of function, using a specific inhibitor of FGF signaling, causes increased apoptosis and inhibition of cell shape change in the migrating germ cells. Lastly, we confirm in vivo the effects seen in slice cultures in vitro, by examining germ cell positions and numbers in embryos carrying a loss-of-function allele of FGFR2-IIIb. In FGFR2-IIIb(-/-) embryos, germ cell **migration** is unaffected, but the numbers of germ cells are significantly reduced. These data show that a major role of FGF signaling through FGFR2-IIIb is to control germ cell numbers. The data do not discriminate between direct and indirect effects of FGF signaling on germ cells, and both may be involved.
18. Fgfr1 regulates patterning of the pharyngeal region. Development of the pharyngeal region depends on the interaction and integration of different cell populations, including surface ectoderm, foregut endoderm, paraxial mesoderm, and neural crest. Mice homozygous for a hypomorphic allele of Fgfr1 have craniofacial defects, some of which appeared to result from a failure in the early development of the second branchial arch. A stream of neural crest cells was found to originate from the rhombomere 4 region and migrate toward the second branchial arch in the mutants. Neural crest cells mostly failed to enter the second arch, however, but accumulated in a region proximal to it. Both rescue of the hypomorphic Fgfr1 allele and inactivation of a conditional Fgfr1 allele specifically in neural crest cells indicated that Fgfr1 regulates the entry of neural crest cells into the second branchial arch non-cell-autonomously. Gene expression in the pharyngeal ectoderm overlying the developing second branchial arch was affected in the hypomorphic Fgfr1 mutants at a stage prior to neural crest entry. Our results indicate that Fgfr1 patterns the pharyngeal region to create a permissive environment for neural crest cell **migration**.
19. Midline radial glia translocation and corpus callosum formation require FGF signaling. Midline astroglia in the cerebral cortex develop earlier than other astrocytes through mechanisms that are still unknown. We show that radial glia in dorsomedial cortex retract their apical endfeet at midneurogenesis and translocate to the overlaying pia, forming the indusium griseum. These cells require the fibroblast growth factor receptor 1 (Fgfr1) gene for their precocious somal translocation to the dorsal midline, as demonstrated by inactivating the Fgfr1 gene in radial glial cells and by RNAi knockdown of Fgfr1 in vivo. Dysfunctional astroglial **migration** underlies the callosal dysgenesis in conditional Fgfr1 knockout mice, suggesting that precise targeting of astroglia to the cortex has unexpected roles in axon guidance. FGF signaling is sufficient to induce somal translocation of radial glial cells throughout the cortex; furthermore, the targeting of astroglia to dorsolateral cortex requires FGFr2 signaling after neurogenesis. Hence, FGFs have an important role in the transition from radial glia to astrocytes by stimulating somal translocation of radial glial cells.
20. FGF signaling facilitates postinjury recovery of mouse hematopoietic system. Previous studies have shown that fibroblast growth factor (FGF) signaling promotes hematopoietic stem and progenitor cell (HSPC) expansion in vitro. However, it is unknown whether FGF promotes HSPC expansion in vivo. Here we examined FGF receptor 1 (FGFR1) expression and investigated its in vivo function in HSPCs. Conditional knockout (CKO) of Fgfr1 did not affect phenotypical number of HSPCs and homeostatic hematopoiesis, but led to a reduced engraftment only in the secondary transplantation. When treated with 5-fluorouracil (5FU), the Fgfr1 CKO mice showed defects in both proliferation and subsequent mobilization of HSPCs. We identified megakaryocytes (Mks) as a major resource for FGF production, and further discovered a novel mechanism by which Mks underwent FGF-FGFR signaling dependent expansion to accelerate rapid FGF production under stress. Within HSPCs, we observed an up-regulation of nuclear factor κB and CXCR4, a receptor for the chemoattractant SDF-1, in response to bone marrow damage only in control but not in Fgfr1 CKO model, accounting for the corresponding defects in proliferation and **migratio**n of HSPCs. This study provides the first in vivo evidence that FGF signaling facilitates postinjury recovery of the mouse hematopoietic system by promoting proliferation and facilitating mobilization of HSPCs.
21. FGF10/FGFR2b signaling is essential for cardiac fibroblast development and growth of the myocardium. The epicardium serves as a source of growth factors that regulate myocardial proliferation and as a source of epicardial-derived cells (EPDC), which give rise to interstitial cardiac fibroblasts and perivascular cells. These progenitors populate the compact myocardium to become part of the mature coronary vasculature and fibrous skeleton of the heart. Little is known about the mechanisms that regulate EPDC **migration** into the myocardium or the functions carried out by these cells once they enter the myocardium. However, it has been proposed that cardiac fibroblasts are important for growth of the heart during late gestation and are a source of homeostatic factors in the adult. Here, we identify a myocardial to epicardial fibroblast growth factor (FGF) signal, mediated by FGF10 and FGFR2b, that is essential for movement of cardiac fibroblasts into the compact myocardium. Inactivation of this signaling pathway results in fewer epicardial derived cells within the compact myocardium, decreased myocardial proliferation and a resulting smaller thin-walled heart.
22. Fibroblast growth factor 8 signaling through fibroblast growth factor receptor 1 is required for the emergence of gonadotropin-releasing hormone neurons. GnRH neurons are essential for the onset and maintenance of reproduction. Mutations in both fibroblast growth factor receptor (Fgfr1) and Fgf8 have been shown to cause Kallmann syndrome, a disease characterized by hypogonadotropic hypogonadism and anosmia, indicating that FGF signaling is indispensable for the formation of a functional GnRH system. Presently it is unclear which stage of GnRH neuronal development is most impacted by FGF signaling deficiency. GnRH neurons express both FGFR1 and -3; thus, it is also unclear whether FGFR1 or FGFR3 contributes directly to GnRH system development. In this study, we examined the developing GnRH system in mice deficient in FGF8, FGFR1, or FGFR3 to elucidate the individual contribution of these FGF signaling components. Our results show that the early emergence of GnRH neurons from the embryonic olfactory placode requires FGF8 signaling, which is mediated through FGFR1, not FGFR3. These data provide compelling evidence that the developing GnRH system is exquisitely sensitive to reduced levels of FGF signaling. Furthermore, Kallmann syndrome stemming from FGF signaling deficiency may be due primarily to defects in early GnRH neuronal development prior to their **migration** into the forebrain.
23. Fibroblast growth factor 10 alters the balance between goblet and Paneth cells in the adult mouse small intestine. Intestinal epithelial cell renewal relies on the right balance of epithelial cell **migration**, proliferation, differentiation, and apoptosis. Intestinal epithelial cells consist of absorptive and secretory lineage. The latter is comprised of goblet, Paneth, and enteroendocrine cells. Fibroblast growth factor 10 (FGF10) plays a central role in epithelial cell proliferation, survival, and differentiation in several organs. The expression pattern of FGF10 and its receptors in both human and mouse intestine and their role in small intestine have yet to be investigated. First, we analyzed the expression of FGF10, FGFR1, and FGFR2, in the human ileum and throughout the adult mouse small intestine. We found that FGF10, FGFR1b, and FGFR2b are expressed in the human ileum as well as in the mouse small intestine. We then used transgenic mouse models to overexpress Fgf10 and a soluble form of Fgfr2b, to study the impact of gain or loss of Fgf signaling in the adult small intestine. We demonstrated that overexpression of Fgf10 in vivo and in vitro induces goblet cell differentiation while decreasing Paneth cells. Moreover, FGF10 decreases stem cell markers such as Lgr5, Lrig1, Hopx, Ascl2, and Sox9. FGF10 inhibited Hes1 expression in vitro, suggesting that FGF10 induces goblet cell differentiation likely through the inhibition of Notch signaling. Interestingly, Fgf10 overexpression for 3 days in vivo and in vitro increased the number of Mmp7/Muc2 double-positive cells, suggesting that goblet cells replace Paneth cells. Further studies are needed to determine the mechanism by which Fgf10 alters cell differentiation in the small intestine.
24. Syndecan-3 and syndecan-4 specifically mark skeletal muscle satellite cells and are implicated in satellite cell maintenance and muscle regeneration. Myogenesis in the embryo and the adult mammal consists of a highly organized and regulated sequence of cellular processes to form or repair muscle tissue that include cell proliferation, **migration**, and differentiation. Data from cell culture and in vivo experiments implicate both FGFs and HGF as critical regulators of these processes. Both factors require heparan sulfate glycosaminoglycans for signaling from their respective receptors. Since syndecans, a family of cell-surface transmembrane heparan sulfate proteoglycans (HSPGs) are implicated in FGF signaling and skeletal muscle differentiation, we examined the expression of syndecans 1-4 in embryonic, fetal, postnatal, and adult muscle tissue, as well as on primary adult muscle fiber cultures. We show that syndecan-1, -3, and -4 are expressed in developing skeletal muscle tissue and that syndecan-3 and -4 expression is highly restricted in adult skeletal muscle to cells retaining myogenic capacity. These two HSPGs appear to be expressed exclusively and universally on quiescent adult satellite cells in adult skeletal muscle tissue, suggesting a role for HSPGs in satellite cell maintenance or activation. Once activated, all satellite cells maintain expression of syndecan-3 and syndecan-4 for at least 96 h, also implicating these HSPGs in muscle regeneration. Inhibition of HSPG sulfation by treatment of intact myofibers with chlorate results in delayed proliferation and altered MyoD expression, demonstrating that heparan sulfate is required for proper progression of the early satellite cell myogenic program. These data suggest that, in addition to providing potentially useful new markers for satellite cells, syndecan-3 and syndecan-4 may play important regulatory roles in satellite cell maintenance, activation, proliferation, and differentiation during skeletal muscle regeneration.
25. Sef inhibits fibroblast growth factor signaling by inhibiting FGFR1 tyrosine phosphorylation and subsequent ERK activation. Signaling through fibroblast growth factor receptors (FGFRs) is essential for many cellular processes including proliferation and **migration** as well as differentiation events such as angiogenesis, osteogenesis, and chondrogenesis. Recently, genetic screens in Drosophila and gene expression screens in zebrafish have resulted in the identification of several feedback inhibitors of FGF signaling. One of these, Sef (similar expression to fgf genes), encodes a transmembrane protein that belongs to the FGF synexpression group. Here we show that like zebrafish Sef (zSef), mouse Sef (mSef) interacts with FGFR1 and that the cytoplasmic domain of mSef mediates this interaction. Overexpression of mSef in NIH3T3 cells results in a decrease in FGF-induced cell proliferation associated with a decrease in Tyr phosphorylation of FGFR1 and FRS2. As a consequence, there is a reduction in the phosphorylation of Raf-1 at Ser(338), MEK1/2 at Ser(217) and Ser(221), and ERK1/2 at Thr(202) and Tyr(204). Furthermore, mSef inhibits ERK activation mediated by a constitutively activated FGFR1 but not by a constitutively active Ras and decreases FGF but not PDGF-mediated activation of Akt. These results indicate that Sef exerts its inhibitory effects at the level of FGFR and upstream of Ras providing an additional level of negative regulation of FGF signaling.
26. Anosmin-1 over-expression increases adult neurogenesis in the subventricular zone and neuroblast **migration** to the olfactory bulb. New subventricular zone (SVZ)-derived neuroblasts that migrate via the rostral migratory stream are continuously added to the olfactory bulb (OB) of the adult rodent brain. Anosmin-1 (A1) is an extracellular matrix protein that binds to FGF receptor 1 (FGFR1) to exert its biological effects. When mutated as in Kallmann syndrome patients, A1 is associated with severe OB morphogenesis defects leading to anosmia and hypogonadotropic hypogonadism. Here, we show that A1 over-expression in adult mice strongly increases proliferation in the SVZ, mainly with symmetrical divisions, and produces substantial morphological changes in the normal SVZ architecture, where we also report the presence of FGFR1 in almost all SVZ cells. Interestingly, for the first time we show FGFR1 expression in the basal body of primary cilia in neural progenitor cells. Additionally, we have found that A1 over-expression also enhances neuroblast motility, mainly through FGFR1 activity. Together, these changes lead to a selective increase in several GABAergic interneuron populations in different OB layers. These specific alterations in the OB would be sufficient to disrupt the normal processing of sensory information and consequently alter olfactory memory. In summary, this work shows that FGFR1-mediated A1 activity plays a crucial role in the continuous remodelling of the adult OB.
27. ERK1/2 signaling is essential for the chemoattraction exerted by human FGF2 and human anosmin-1 on newborn rat and mouse OPCs via FGFR1. Signaling through fibroblast growth factor receptors (FGFRs) is essential for many cellular processes including proliferation and **migration**, as well as differentiation events such as myelination. Anosmin-1 is an extracellular matrix (ECM) glycoprotein that interacts with the fibroblast growth factor receptor 1 (FGFR1) to exert its biological actions through this receptor, although the intracellular pathways underlying anosmin-1 signaling remain largely unknown. This protein is defective in the X-linked form of Kallmann syndrome (KS) and has a prominent role in the **migration** of neuronal and oligodendroglial precursors. We have shown that anosmin-1 exerts a chemotactic effect via FGFR1 on neuronal precursors from the subventricular zone (SVZ) and the essential role of the ERK1/2 signaling. We report here the positive chemotactic effect of FGF2 and anosmin-1 on rat and mouse postnatal OPCs via FGFR1. The same effect was observed with the truncated N-terminal region of anosmin-1 (A1Nt). The introduction in anosmin-1 of the missense mutation F517L found in patients suffering from KS annulled the chemotactic activity; however, the mutant form carrying the disease-causing mutation E514K also found in KS patients, behaved as the wild-type protein. The chemoattraction exhibited by FGF2 and anosmin-1 on OPCs was blocked by the mitogen-activated protein kinase (MAPK) inhibitor U0126, suggesting that the activation of the ERK1/2 MAPK signaling pathway following interaction with the FGFR1 is necessary for FGF2 and anosmin-1 to exert their chemotactic effect. In fact, both proteins were able to induce the phosphorylation of the ERK1/2 kinases after the activation of the FGFR1 receptor.
28. Murine fibroblast growth factor receptor 1alpha isoforms mediate node regression and are essential for posterior mesoderm development. Alternative splicing in the fibroblast growth factor receptor 1 (Fgfr1) locus generates a variety of splicing isoforms, including FGFR1alpha isoforms, which contain three immunoglobulin-like loops in the extracellular domain of the receptor. It has been previously shown that embryos carrying targeted disruptions of all major isoforms die during gastrulation, displaying severe growth retardation and defective mesodermal structures. Here we selectively disrupted the FGFR1alpha isoforms and found that they play an essential role in posterior mesoderm formation during gastrulation. We show that the mutant embryos lack caudal somites, develop spina bifida, and die at 9.5-12.5 days of embryonic development because they are unable to establish embryonic circulation. The primary defect is a failure of axial mesoderm cell **migration** toward the posterior portions of the embryos during gastrulation, as revealed by regional marker analysis and DiI labeling. In contrast, the anterior **migration** of the notochord is unaffected and the embryonic structures rostral to the forelimb are relatively normal. These data demonstrate that FGF/FGFR1alpha signals are posteriorizing factors that control node regression and posterior embryonic development.
29. The F-BAR protein NOSTRIN participates in FGF signal transduction and vascular development. F-BAR proteins are multivalent adaptors that link plasma membrane and cytoskeleton and coordinate cellular processes such as membrane protrusion and **migration**. Yet, little is known about the function of F-BAR proteins in vivo. Here we report, that the F-BAR protein NOSTRIN is necessary for proper vascular development in zebrafish and postnatal retinal angiogenesis in mice. The loss of NOSTRIN impacts on the **migration** of endothelial tip cells and leads to a reduction of tip cell filopodia number and length. NOSTRIN forms a complex with the GTPase Rac1 and its exchange factor Sos1 and overexpression of NOSTRIN in cells induces Rac1 activation. Furthermore, NOSTRIN is required for fibroblast growth factor 2 dependent activation of Rac1 in primary endothelial cells and the angiogenic response to fibroblast growth factor 2 in the in vivo matrigel plug assay. We propose a novel regulatory circuit, in which NOSTRIN assembles a signalling complex containing FGFR1, Rac1 and Sos1 thereby facilitating the activation of Rac1 in endothelial cells during developmental angiogenesis.
30. SOX3 activity during pharyngeal segmentation is required for craniofacial morphogenesis. Craniofacial development is a complex multi-step process leading to the morphogenesis of the face and sense organs, and to that of the neck, including the anteriormost part of the respiratory and digestive apparatus and associated endocrine glands. In vertebrates, the process is initiated by the formation of the pharyngeal arches from ectoderm, endoderm and mesoderm. These arches are then populated by neural crest cells, which originate from the central nervous system. We show here that, in mouse, there is a requirement for the HMG box factor SOX3 during the earliest stage of pharyngeal development: the formation of the pharyngeal pouches that segment the pharyngeal region by individualising each arch. In Sox3-null mutants, these pouches are expanded at the detriment of the second pharyngeal arch. As a consequence, neural crest cell **migration** and ectoderm-derived epibranchial placode development are affected, leading to craniofacial defects. We also show that Sox3 genetically interacts both with FgfR1 and with Sox2, another member of the Soxb1 family, to fulfil its function in the pharyngeal region. Although the importance of the neural crest has long been recognised, our studies highlight the equally crucial role of the pharyngeal region in craniofacial morphogenesis. They also give insight into the formation of pharyngeal pouches, of which little is known in vertebrates. Finally, this work introduces two new players in craniofacial development - SOX3 and SOX2.
31. Increased expression of FGF1-mediated signaling molecules in adipose tissue of obese mice. Fibroblast growth factors (FGFs) are pleiotropic growth factors that control cell proliferation, **migration**, and differentiation. Herein, we evaluated whether visceral adiposity of mice is accompanied by the alteration of signaling molecules mediated by fibroblast growth factor receptor 1 (FGFR1) induced by using two different male C57BL/6J mice models of obesity namely high-fat diet (HFD)-induced obesity for 12 weeks or mice with genetic deletion of leptin (ob/ob). Both HFD-fed and ob/ob mice exhibited significantly higher messenger RNA (mRNA) levels of FGF1, cyclin D (cycD), transcription factor E2F1, peroxisome proliferator-activated receptor-gamma 2 (PPAR-γ2), CCAAT-enhancer-binding protein alpha (C/EBPα), and adipocyte protein 2 (aP2) genes in their epididymal adipose tissues compared to those of the normal diet (ND)-fed and lean control mice, respectively. In addition, immunoblot analyses of the epididymal adipose tissues revealed that both mice exposed to HFD and ob/ob mice exhibited elevated phosphorylation of FGFR1, extracellular-signal-regulated kinase (ERK), and retinoblastoma (Rb) proteins. These data support the notion that FGF1-mediated signaling represents an important signaling cascade related to adipogenesis, at least partially, among other known signaling pathways. These new findings regarding the molecular mechanisms controlling adipose tissue plasticity provide a novel insight about the functional network with potential therapeutic application against obesity.
32. Differential fibroblast growth factor 8 (FGF8)-mediated autoregulation of its cognate receptors, Fgfr1 and Fgfr3, in neuronal cell lines. Fibroblast growth factors (FGFs) mediate a vast range of CNS developmental processes including neural induction, proliferation, **migration**, and cell survival. Despite the critical role of FGF signaling for normal CNS development, few reports describe the mechanisms that regulate FGF receptor gene expression in the brain. We tested whether FGF8 could autoregulate two of its cognate receptors, Fgfr1 and Fgfr3, in three murine cell lines with different lineages: fibroblast-derived cells (3T3 cells), neuronal cells derived from hippocampus (HT-22 cells), and neuroendocrine cells derived from hypothalamic gonadotropin-releasing hormone (GnRH) neurons (GT1-7 cells). GnRH is produced by neurons in the hypothalamus and is absolutely required for reproductive competence in vertebrate animals. Several lines of evidence strongly suggest that Fgf8 is critical for normal development of the GnRH system, therefore, the GT1-7 cells provided us with an additional endpoint, Gnrh gene expression and promoter activity, to assess potential downstream consequences of FGF8-induced modulation of FGF receptor levels. Results from this study suggest that the autoregulation of its cognate receptor represents a common downstream effect of FGF8. Further, we show that Fgfr1 and Fgfr3 are differentially regulated within the same cell type, implicating these two receptors in different biological roles. Moreover, Fgfr1 and Fgfr3 are differentially regulated among different cell types, suggesting such autoregulation occurs in a cell type-specific fashion. Lastly, we demonstrate that FGF8b decreases Gnrh promoter activity and gene expression, possibly reflecting a downstream consequence of altered FGF receptor populations. Together, our data bring forth the possibility that, in addition to the FGF synexpression group, autoregulation of FGFR expression by FGF8 represents a mechanism by which FGF8 could fine-tune its regulatory actions.
33. Redistribution of cytosolic FGFR1 after induced **migration** of urothelial cells in culture. The distribution of cytosolic fibroblast growth factor receptor 1 (FGFR1) was studied in correlation to cell **migration** in urothelial cell line g/G. Cell motility was analysed with a new method using consecutive series of photographs of cells relocated on CELLocate coverslips and with image analysis software. The results confirmed that FGF1 stimulated cell motility only when cells were grown on collagen I coating. During the transition from sessile to motile cell phenotype a complete redistribution of cytosolic FGFR1 was revealed. In sessile cells, FGFR1 had a filamentous distribution and its location matched cytokeratin 7. In cells of the migrating phenotype, the distribution of FGFR1 was diffuse, mainly located in cytosol. Our data reveal that the location of cytosolic FGFR1 depends on the motile characteristics of the cell. The results also indicate that attachment of cells to collagen I is crucial for the induction of urothelial cell motility with FGF1.
34. Maintenance of vascular integrity in the embryo requires signaling through the fibroblast growth factor receptor. Basic fibroblast growth factor (FGF)-2 is important for vessel formation and/or maintenance of vascular integrity in the embryo. FGF signaling may be mediated through transmembrane tyrosine kinase receptors or directly through intracellular pathways that do not involve receptor activation. To determine the role of receptor-mediated signaling in endothelial cells, an adenovirus encoding truncated FGF receptor (FGFR)-1, under the control of the cytomegalovirus promoter, was expressed in endothelial cells. FGF signaling was impaired, as indicated by inhibition of MAPK phosphorylation. Functional consequences included inhibition of endothelial cell **migration** and induction of apoptosis. To address the role of endothelial FGFR signaling in vascular development, recombinant adenovirus encoding a dominant-negative FGFR was injected into the sinus venosus of embryonic day 9.0 cultured mouse embryos. Previous studies demonstrated that transgenes delivered via adenovirus, under the control of the cytomegalovirus promoter, are expressed selectively in the developing vasculature. Embryos expressing a control adenovirus developed normally, whereas those expressing the FGFR-1 mutant exhibited abnormal embryonic and extra-embryonic vascular development. These data demonstrate that FGF, by signaling through the FGFR, plays a pivotal role in the development and maintenance of a mature vascular network in the embryo.
35. Analysis of non-canonical fibroblast growth factor receptor 1 (FGFR1) interaction reveals regulatory and activating domains of neurofascin. Fibroblast growth factor receptors (FGFRs) are important for many different mechanisms, including cell **migration**, proliferation, differentiation, and survival. Here, we show a new link between FGFR1 and the cell adhesion molecule neurofascin, which is important for neurite outgrowth. After overexpression in HEK293 cells, embryonal neurofascin isoform NF166 was able to associate with FGFR1, whereas the adult isoform NF186, differing from NF166 in additional extracellular sequences, was deficient. Pharmacological inhibitors and overexpression of dominant negative components of the FGFR signaling pathway pointed to the activation of FGFR1 after association with neurofascin in neurite outgrowth assays in chick tectal neurons and rat PC12-E2 cells. Both extra- and intracellular domains of embryonal neurofascin isoform NF166 were able to form complexes with FGFR1 independently. However, the cytosolic domain was both necessary and sufficient for the activation of FGFR1. Cytosolic serine residues 56 and 100 were shown to be essential for the neurite outgrowth-promoting activity of neurofascin, whereas both amino acid residues were dispensable for FGFR1 association. In conclusion, the data suggest a neurofascin intracellular domain, which activates FGFR1 for neurite outgrowth, whereas the extracellular domain functions as an additional, regulatory FGFR1 interaction domain in the course of development.
36. Growth/differentiation factor 15 promotes EGFR signalling, and regulates proliferation and **migration** in the hippocampus of neonatal and young adult mice. The activation of epidermal growth factor receptor (EGFR) affects multiple aspects of neural precursor behaviour, including proliferation and **migration**. Telencephalic precursors acquire EGF responsiveness and upregulate EGFR expression at late stages of development. The events regulating this process and its significance are still unclear. We here show that in the developing and postnatal hippocampus (HP), growth/differentiation factor (GDF) 15 and EGFR are co-expressed in primitive precursors as well as in more differentiated cells. We also provide evidence that GDF15 promotes responsiveness to EGF and EGFR expression in hippocampal precursors through a mechanism that requires active CXC chemokine receptor (CXCR) 4. Besides EGFR expression, GDF15 ablation also leads to decreased proliferation and **migration**. In particular, lack of GDF15 impairs both processes in the cornu ammonis (CA) 1 and only proliferation in the dentate gyrus (DG). Importantly, **migration** and proliferation in the mutant HP were altered only perinatally, when EGFR expression was also affected. These data suggest that GDF15 regulates **migration** and proliferation by promoting EGFR signalling in the perinatal HP and represent a first description of a functional role for GDF15 in the developing telencephalon.
37. Prostaglandin E2 regulates angiogenesis via activation of fibroblast growth factor receptor-1. Prostaglandin E(2) (PGE(2)) behaves as a mitogen in epithelial tumor cells as well as in many other cell types. We investigated the actions of PGE(2) on microvascular endothelial cells (capillary venular endothelial cells) with the purpose of delineating the signaling pathway leading to the acquisition of the angiogenic phenotype and to new vessel formation. PGE(2) (100 nM) produced activation of the fibroblast growth factor receptor 1 (FGFR-1), as measured by its phosphorylation, but not of vascular endothelial growth factor receptor 2. PGE(2) stimulated the EP3 subtype receptor, as deduced by abrogation of EP3 Galpha(i) subunit activity through pertussis toxin. Consistent with this result, in human umbilical venular endothelial cells missing the EP3 receptor, PGE(2) did not phosphorylate FGFR-1. Upon binding to its receptor, PGE(2) initiated an autocrine/paracrine signaling cascade involving the intracellular activation of c-Src, activation of matrix metalloproteinase (predominantly MMP2), which in turn caused the mobilization of membrane-anchored fibroblast growth factor-2 (FGF-2). In fact, in cells unable to release FGF-2 the transfection with both FGFR-1 and EP3 did not result in FGFR-1 phosphorylation in response to PGE(2). Relevance for the FGF2-FGFR-1 system was highlighted by confocal analysis, showing receptor internalization after cell exposure to the prostanoid. ERK1/2 appeared to be the distal signal involved, its phosphorylation being sensitive to either cSrc inhibitor or FGFR-1 blocker. Finally, PGE(2) stimulated cell **migration** and capillary formation in aortic rings, which were severely reduced by inhibitors of signaling molecules or by receptor antagonist. In conclusion, this study provides evidence for the involvement of FGFR-1 through FGF2 in eliciting PGE(2) angiogenic responses. This signaling pattern is similar to the autocrine-paracrine mechanism which operates in endothelial cells to support neovascular growth.
38. CD64 distinguishes macrophages from dendritic cells in the gut and reveals the Th1-inducing role of mesenteric lymph node macrophages during colitis. Dendritic cells (DCs) and monocyte-derived macrophages (MΦs) are key components of intestinal immunity. However, the lack of surface markers differentiating MΦs from DCs has hampered understanding of their respective functions. Here, we demonstrate that, using CD64 expression, MΦs can be distinguished from DCs in the intestine of both mice and humans. On that basis, we revisit the phenotype of intestinal DCs in the absence of contaminating MΦs and we delineate a developmental pathway in the healthy intestine that leads from newly extravasated Ly-6C(hi) monocytes to intestinal MΦs. We determine how inflammation impacts this pathway and show that T cell-mediated colitis is associated with massive recruitment of monocytes to the intestine and the mesenteric lymph node (MLN). There, these monocytes differentiate into inflammatory MΦs endowed with phagocytic activity and the ability to produce inducible nitric oxide synthase. In the MLNs, inflammatory MΦs are located in the T-cell zone and trigger the induction of proinflammatory T cells. Finally, T cell-mediated colitis develops irrespective of intesti**nal DC mi**gration, an unexpected finding supporting an important role for MLN-resident inflammatory MΦs in the etiology of T cell-mediated colitis.
39. FGF signaling regulates mesoderm cell fate specification and morphogenetic movement at the primitive streak. Although FGF signaling plays an integral role in the **migration** and patterning of mesoderm at gastrulation, the mechanism and downstream targets of FGF activity have remained elusive. Here, we demonstrate that FGFR1 orchestrates the epithelial to mesenchymal transition and morphogenesis of mesoderm at the primitive streak by controlling Snail and E-cadherin expression. Furthermore, we show that FGFR1 functions in mesoderm cell fate specification by positively regulating Brachyury and Tbx6 expression. Finally, we provide evidence that the attenuation of Wnt3a signaling observed in Fgfr1 -/- embryos can be rescued by lowering E-cadherin levels. We propose that modulation of cytoplasmic beta-catenin levels, associated with FGF-induced downregulation of E-cadherin, provides a molecular link between FGF and Wnt signaling pathways at the streak.
40. Impaired motor coordination and disrupted cerebellar architecture in Fgfr1 and Fgfr2 double knockout mice. Fibroblast growth factor receptor (FGFR) signaling determines the size of the cerebral cortex by regulating the amplification of radial glial stem cells, and participates in the formation of midline glial structures. We show that Fgfr1 and Fgfr2 double knockouts (FGFR DKO) generated by Cre-mediated recombination driven by the human GFAP promoter (hGFAP) have reduced cerebellar size due to reduced proliferation of radial glia and other glial precursors in late embryonic and neonatal FGFR DKO mice. The proliferation of granule cell progenitors (GCPs) in the EGL was also reduced, leading to reduced granule cell numbers. Furthermore, both inward **migration** of granule cells into the inner granule cell layer (IGL) and outward **migration** of GABA interneurons into the molecular layer (ML) were arrested, disrupting layer and lobular morphology. Purkinje neurons and their dendrites, which were not targeted by Cre-mediated recombination of Fgf receptors, were also misplaced in FGFR DKO mice, possibly as a consequence of altered Bergmann glia orientation or reduced granule cell number. Our findings indicate a dual role for FGFR signaling in cerebellar morphogenesis. The first role is to amplify the number of granule neuron precursors in the external granular layer and glial precursor cells throughout the cerebellum. The second is to establish the correct Bergmann glia morphology, which is crucial for granule cell **migration**. The disrupted cerebellar size and laminar architecture resulting from loss of FGFR signaling impair motor learning and coordination in FGFR DKO mice.
41. FGF receptors 1 and 2 are key regulators of keratinocyte **migration** in vitro and in wounded skin. Efficient wound repair is essential for the maintenance of the integrity of the skin. The repair process is controlled by a variety of growth factors and cytokines, and their abnormal expression or activity can cause healing disorders. Here, we show that wound repair is severely delayed in mice lacking fibroblast growth factor receptors (FGFR) 1 and 2 in keratinocytes. As the underlying mechanism, we identified impaired wound contraction and a delay in re-epithelialization that resulted from impaired keratinocyte **migration** at the wound edge. Scratch wounding and transwell assays demonstrated that FGFR1/2-deficient keratinocytes had a reduced **migration** velocity and impaired directional persistence owing to inefficient formation and turnover of focal adhesions. Underlying this defect, we identified a significant reduction in the expression of major focal adhesion components in the absence of FGFR signaling, resulting in a general migratory deficiency. These results identify FGFs as key regulators of keratinocyte **migration** in wounded skin.
42. Fibroblast growth factor signalling and regional specification of the pharyngeal ectoderm. Branchial arch development involves dynamic interactions between neural crest cells as well as ectodermal, endodermal and mesodermal cell populations. Despite their importance and evolutionary conservation, the intercellular interactions guiding the early development of the branchial arches are still poorly understood. We have here studied fibroblast growth factor (FGF) signalling in early pharyngeal development. In mice homozygous for a hypomorphic allele of Fgfr1, neural crest cells migrating from the hindbrain mostly fail to enter the second branchial arch. This defect is non-cell-autonomous suggesting that Fgfr1 provides a permissive environment for neural crest cell **migration**. Here we demonstrate localized down-regulation of the expression of the FGF responsive gene, Sprouty1 in the epithelium covering the presumptive second branchial arch of hypomorphic Fgfr1 mutants. This appears to result in a failure to establish an ectodermal signalling center expressing Fgf3 and Fgf15. We also studied differentiation of the ectoderm in the second branchial arch region. Development of the geniculate placode as well as the VIIth cranial ganglion is affected in Fgfr1 hypomorphs. Our results suggest that Fgfr1 is important for localized signalling in the pharyngeal ectoderm and consequently for normal tissue interactions in the developing second branchial arch.
43. Macrophages promote fibroblast growth factor receptor-driven tumor cell **migration** and invasion in a CXCR2-dependent manner. Infiltration of immune cells, specifically macrophages, into the tumor microenvironment has been linked to increased mammary tumor formation and progression. Activation of growth factor receptor signaling pathways within mammary epithelial cells, such as the fibroblast growth factor receptor 1 (FGFR1) pathway, induces recruitment of macrophages to the mammary epithelium. These macrophages promote increased epithelial cell proliferation and angiogenesis. However, the specific mechanisms by which these macrophages are regulated by the preneoplastic epithelial cells and the mechanisms of action of the macrophages within the developing FGFR1-driven tumor microenvironment remain unknown. In this study, we show that activation of inducible FGFR1 in mammary glands leads to decreased activity of the TGFβ/Smad3 pathway in macrophages associated with early stage lesions. Further studies show that macrophages have increased expression of inflammatory chemokines that bind Cxcr2 following exposure to conditioned media from mammary epithelial and tumor cells in which the FGF pathway had been activated. The increase in these ligands is inhibited following activation of the TGFβ pathway, suggesting that decreased TGFβ signaling contributes to the upregulation of these chemokines. Using coculture studies, we further show that macrophages are capable of promoting epithelial and tumor ce**ll migrat**ion and invasion through activation of Cxcr2. These results indicate that macrophage-derived Cxcr2 ligands may be important for promoting mammary tumor formation regulated by FGFR signaling. Furthermore, these results suggest that targeting Cxcr2 may represent a novel therapeutic strategy for breast cancers that are associated with high levels of infiltrating macrophages.
44. Impaired cerebral cortex development and blood pressure regulation in FGF-2-deficient mice. Fibroblast growth factor-2 (FGF-2) has been implicated in various signaling processes which control embryonic growth and differentiation, adult physiology and pathology. To analyze the in vivo functions of this signaling molecule, the FGF-2 gene was inactivated by homologous recombination in mouse embryonic stem cells. FGF-2-deficient mice are viable, but display cerebral cortex defects at birth. Bromodeoxyuridine pulse labeling of embryos showed that proliferation of neuronal progenitors is normal, whereas a fraction of them fail to colonize their target layers in the cerebral cortex. A corresponding reduction in parvalbumin-positive neurons is observed in adult cortical layers. Neuronal defects are not limited to the cerebral cortex, as ectopic parvalbumin-positive neurons are present in the hippocampal commissure and neuronal deficiencies are observed in the cervical spinal cord. Physiological studies showed that FGF-2-deficient adult mice are hypotensive. They respond normally to angiotensin II-induced hypertension, whereas neural regulation of blood pressure by the baroreceptor reflex is impaired. The present genetic study establishes that FGF-2 participates in controlling fates, **migration** and differentiation of neuronal cells, whereas it is not essential for their proliferation. The observed autonomic dysfunction in FGF-2-deficient adult mice uncovers more general roles in neural development and function.
45. Fibroblast growth factor receptors (FGFRs) and their roles in limb development. Fibroblast growth factor (FGF) receptors constitute a family of four membrane-spanning tyrosine kinases (FGFR1-4) which serve as high-affinity receptors for 17 growth factors (FGF1-17). To study functions of FGF/ FGFR signals in development, mice that carry mutations in each receptor have been created by gene targeting. Analysis of these mutant mice revealed essential functions of FGF receptors in multiple biological processes, including mesoderm induction and patterning, cell growth and **migration**, organ formation and bone growth. In this review we discuss recent work with FGF receptors to illustrate mechanisms, through which the FGF/FGFR signals specify vertebrate limb initiation, outgrowth and patterning.
46. Pleiotropic effects of FGFR1 on cell proliferation, survival, and **migration** in a 3D mammary epithelial cell model. Members of the fibroblast growth factor (FGF) family and the FGF receptors (FGFRs) have been implicated in mediating various aspects of mammary gland development and transformation. To elucidate the molecular mechanisms of FGFR1 action in a context that mimics polarized epithelial cells, we have developed an in vitro three-dimensional HC11 mouse mammary epithelial cell culture model expressing a drug-inducible FGFR1 (iFGFR1). Using this conditional model, iFGFR1 activation in these growth-arrested and polarized mammary acini initially led to reinitiation of cell proliferation, increased survival of luminal cells, and loss of cell polarity, resulting in the disruption of acinar structures characterized by the absence of an empty lumen. iFGFR1 activation also resulted in a gain of invasive properties and the induction of matrix metalloproteinase 3 (MMP-3), causing the cleavage of E-cadherin and increased expression of smooth muscle actin and vimentin. The addition of a pan MMP inhibitor abolished these phenotypes but did not prevent the effects of iFGFR1 on cell proliferation or survival.
47. Expression of FGF receptors 1, 2, 3 in the embryonic and postnatal mouse brain compared with Pdgfralpha, Olig2 and Plp/dm20: implications for oligodendrocyte development. Fibroblast growth factors (FGF) receptors FgfR1, FgfR2 and FgfR3 are differentially regulated during oligodendrocyte (OL) maturation in vitro: FgfR3 is expressed by OL progenitors whereas FgfR2 is expressed by differentiated OLs [Mol Cell Neurosci 1996;7:263-275], and we have recently shown that FgfR3 is required for the timely differentiation of OLs in vivo [J Neurosci 2003;23:883-894]. Here we have used in situ hybridization to investigate the expression patterns of FgfR1-3 and compare them to the putative OL progenitor markers Olig2, Pdgfralpha and Plp/dm20 as a function of development in vivo, in particular at sites of OL specification, **migration** or differentiation in the mouse forebrain and cerebellum. We show that at early stages FgfR1-3 expression overlaps with that of Olig2 in the embryonic ventricular zone of the lateral and medial ganglionic eminences. Further, a scattered population of cells expressing FgfR3 (but not FgfR1 or FgfR2) in the ventral telencephalon appear to arise from the ventricular zone, and at later stages are found more dorsally in the cortex, in an overall pattern similar to Olig2 and/or Pdgfralpha. Postnatal expression of FgfR2 increases with age, more prominently in specific regions, including the cortical and cerebellar white matter and optic nerve. Thus, the differential expression pattern of FgfR2 and FgfR3 observed in vivo suggests that their expression is developmentally regulated in a manner consistent with the pattern of their expression in culture. These data provide further insights into role of FgfRs in OL development, and they emphasize that these receptors are positioned both spatially and temporally to impact OL generation in vivo.

#### GeneRIFs

1. FGF receptors 1 and 2 are key regulators of keratinocyte **migration** in vitro and in wounded skin.
2. Fibronectin induces endothelial cell **migration** through beta1 integrin and Src-dependent phosphorylation of fibroblast growth factor receptor-1 at tyrosines 653/654 and 766.
3. FGFR1 has roles in cell proliferation, survival, and **migration** in mouse mammary epithelium
4. FGF-mediated activation of the MAP kinase pathway occurs in germ cells during their **migration**, FGF2, a ligand for FGFR1-IIIc, affects motility, whereas FGF7, a ligand for FGFR2-IIIb, affects germ cell numbers.
5. Fgfr1 patterns the pharyngeal region to create a permissive environment for neural crest cell **migration**.

### ECM

#### PubMed

1. Nell1-deficient mice have reduced expression of extracellular matrix proteins causing cranial and vertebral defects. The mammalian Nell1 gene encodes a protein kinase C-beta1 (PKC-beta1) binding protein that belongs to a new class of cell-signaling molecules controlling cell growth and differentiation. Over-expression of Nell1 in the developing cranial sutures in both human and mouse induces craniosynostosis, the premature fusion of the growing cranial bone fronts. Here, we report the generation, positional cloning and characterization of Nell1(6R), a recessive, neonatal-lethal point mutation in the mouse Nell1 gene, induced by N-ethyl-N-nitrosourea. Nell1(6R) has a T-->A base change that converts a codon for cysteine into a premature stop codon [Cys(502)Ter], resulting in severe truncation of the predicted protein product and marked reduction in steady-state levels of the transcript. In addition to the expected alteration of cranial morphology, Nell1(6R) mutants manifest skeletal defects in the vertebral column and ribcage, revealing a hitherto undefined role for Nell1 in signal transduction in endochondral ossification. Real-time quantitative reverse transcription-PCR assays of 219 genes showed an association between the loss of Nell1 function and reduced expression of genes for extracellular matrix (**ECM**) proteins critical for chondrogenesis and osteogenesis. Several affected genes are involved in the human cartilage disorder Ehlers-Danlos Syndrome and other disorders associated with spinal curvature anomalies. Nell1(6R) mutant mice are a new tool for elucidating basic mechanisms in osteoblast and chrondrocyte differentiation in the developing skull and vertebral column and understanding how perturbations in the production of **ECM** proteins can lead to anomalies in these structures.
2. ERK1/2 signaling is essential for the chemoattraction exerted by human FGF2 and human anosmin-1 on newborn rat and mouse OPCs via FGFR1. Signaling through fibroblast growth factor receptors (FGFRs) is essential for many cellular processes including proliferation and migration, as well as differentiation events such as myelination. Anosmin-1 is an extracellular matrix (**ECM**) glycoprotein that interacts with the fibroblast growth factor receptor 1 (FGFR1) to exert its biological actions through this receptor, although the intracellular pathways underlying anosmin-1 signaling remain largely unknown. This protein is defective in the X-linked form of Kallmann syndrome (KS) and has a prominent role in the migration of neuronal and oligodendroglial precursors. We have shown that anosmin-1 exerts a chemotactic effect via FGFR1 on neuronal precursors from the subventricular zone (SVZ) and the essential role of the ERK1/2 signaling. We report here the positive chemotactic effect of FGF2 and anosmin-1 on rat and mouse postnatal OPCs via FGFR1. The same effect was observed with the truncated N-terminal region of anosmin-1 (A1Nt). The introduction in anosmin-1 of the missense mutation F517L found in patients suffering from KS annulled the chemotactic activity; however, the mutant form carrying the disease-causing mutation E514K also found in KS patients, behaved as the wild-type protein. The chemoattraction exhibited by FGF2 and anosmin-1 on OPCs was blocked by the mitogen-activated protein kinase (MAPK) inhibitor U0126, suggesting that the activation of the ERK1/2 MAPK signaling pathway following interaction with the FGFR1 is necessary for FGF2 and anosmin-1 to exert their chemotactic effect. In fact, both proteins were able to induce the phosphorylation of the ERK1/2 kinases after the activation of the FGFR1 receptor.

### [Ee]xtracellular [Mm]atrix

#### PubMed

1. beta1 integrin is necessary for ureteric bud branching morphogenesis and maintenance of collecting duct structural integrity. The kidney collecting system develops from branching morphogenesis of the ureteric bud (UB). This process requires signaling by growth factors such as glial cell line derived neurotrophic factor (GDNF) and fibroblast growth factors (FGFs) as well as cell **extracellular matrix** interactions mediated by integrins. The importance of integrin signaling in UB development was investigated by deleting integrin beta1 at initiation (E10.5) and late (E18.5) stages of development. Deletion at E10.5 resulted in a severe branching morphogenesis phenotype. Deletion at E18.5 did not alter renal development but predisposed the collecting system to severe injury following ureteric obstruction. beta1 integrin was required for renal tubular epithelial cells to mediate GDNF- and FGF-dependent signaling despite normal receptor localization and activation in vitro. Aberrations in the same signaling molecules were present in the beta1-null UBs in vivo. Thus beta1 integrins can regulate organ branching morphogenesis during development by mediating growth-factor-dependent signaling in addition to their well-defined role as adhesion receptors.
2. MT1-MMP inactivates ADAM9 to regulate FGFR2 signaling and calvarial osteogenesis. MMP14 encodes a membrane-tethered metalloproteinase MT1-MMP, capable of remodeling the **extracellular matrix** and modulating receptors on the cell surface. Loss of MT1-MMP results in craniofacial abnormalities. Here we show that MT1-MMP forms a complex with FGFR2 and ADAM9 in osteoblasts and proteolytically inactivates ADAM9, hence protecting FGFR2 from ADAM9-mediated ectodomain shedding on the cell surface. In Mmp14-/- osteoblasts, FGF-induced proliferation and downstream signaling are specifically compromised, in conjunction with ADAM9 upregulation and FGFR2 shedding. The retarded parietal growth in Mmp14-/- embryos starts at 15.5 dpc, attributable to the impaired FGFR2 signaling due to increased shedding mediated by ADAM9. Adam9 depletion completely rescues the defective FGFR2 signaling and largely restores calvarial bone growth in Mmp14-/- embryos. These data reveal a regulatory paradigm for FGRF2 signaling and identify MT1-MMP as a critical negative modulator of ADAM9 activity to maintain FGFR2 signaling in calvarial osteogenesis.
3. Fibronectin induces endothelial cell migration through β1 integrin and Src-dependent phosphorylation of fibroblast growth factor receptor-1 at tyrosines 653/654 and 766. The **extracellular matri**x microenvironment regulates cell phenotype and function. One mechanism by which this is achieved is the transactivation of receptor tyrosine kinases by specific matrix molecules. Here, we demonstrate that the provisional matrix protein, fibronectin (FN), activates fibroblast growth factor (FGF) receptor-1 (FGFR1) independent of FGF ligand in liver endothelial cells. FN activation of FGFR1 requires β1 integrin, as evidenced by neutralizing antibody and siRNA-based studies. Complementary genetic and pharmacologic approaches identify that the non-receptor tyrosine kinase Src is required for FN transactivation of FGFR1. Whereas FGF ligand-induced phosphorylation of FGFR1 preferentially activates ERK, FN-induced phosphorylation of FGFR1 preferentially activates AKT, indicating differential downstream signaling of FGFR1 in response to alternate stimuli. Mutation analysis of known tyrosine residues of FGFR1 reveals that tyrosine 653/654 and 766 residues are required for FN-FGFR1 activation of AKT and chemotaxis. Thus, our study mechanistically dissects a new signaling pathway by which FN achieves endothelial cell chemotaxis, demonstrates how differential phosphorylation profiles of FGFR1 can achieve alternate downstream signals, and, more broadly, highlights the diversity of mechanisms by which th**e extracellular matr**ix microenvironment regulates cell behavior through transactivation of receptor tyrosine kinases.
4. Nell1-deficient mice have reduced expression of **extracellular matrix** proteins causing cranial and vertebral defects. The mammalian Nell1 gene encodes a protein kinase C-beta1 (PKC-beta1) binding protein that belongs to a new class of cell-signaling molecules controlling cell growth and differentiation. Over-expression of Nell1 in the developing cranial sutures in both human and mouse induces craniosynostosis, the premature fusion of the growing cranial bone fronts. Here, we report the generation, positional cloning and characterization of Nell1(6R), a recessive, neonatal-lethal point mutation in the mouse Nell1 gene, induced by N-ethyl-N-nitrosourea. Nell1(6R) has a T-->A base change that converts a codon for cysteine into a premature stop codon [Cys(502)Ter], resulting in severe truncation of the predicted protein product and marked reduction in steady-state levels of the transcript. In addition to the expected alteration of cranial morphology, Nell1(6R) mutants manifest skeletal defects in the vertebral column and ribcage, revealing a hitherto undefined role for Nell1 in signal transduction in endochondral ossification. Real-time quantitative reverse transcription-PCR assays of 219 genes showed an association between the loss of Nell1 function and reduced expression of genes for **extracellular matrix** (ECM) proteins critical for chondrogenesis and osteogenesis. Several affected genes are involved in the human cartilage disorder Ehlers-Danlos Syndrome and other disorders associated with spinal curvature anomalies. Nell1(6R) mutant mice are a new tool for elucidating basic mechanisms in osteoblast and chrondrocyte differentiation in the developing skull and vertebral column and understanding how perturbations in the production of ECM proteins can lead to anomalies in these structures.
5. An FGF autocrine loop initiated in second heart field mesoderm regulates morphogenesis at the arterial pole of the heart. In order to understand how secreted signals regulate complex morphogenetic events, it is crucial to identify their cellular targets. By conditional inactivation of Fgfr1 and Fgfr2 and overexpression of the FGF antagonist sprouty 2 in different cell types, we have dissected the role of FGF signaling during heart outflow tract development in mouse. Contrary to expectation, cardiac neural crest and endothelial cells are not primary paracrine targets. FGF signaling within second heart field mesoderm is required for remodeling of the outflow tract: when disrupted, outflow myocardium fails to produce **extracellular matrix** and TGFbeta and BMP signals essential for endothelial cell transformation and invasion of cardiac neural crest. We conclude that an autocrine regulatory loop, initiated by the reception of FGF signals by the mesoderm, regulates correct morphogenesis at the arterial pole of the heart. These findings provide new insight into how FGF signaling regulates context-dependent cellular responses during development.
6. Fibronectin induces endothelial cell migration through β1 integrin and Src-dependent phosphorylation of fibroblast growth factor receptor-1 at tyrosines 653/654 and 766. The **extracellular matri**x microenvironment regulates cell phenotype and function. One mechanism by which this is achieved is the transactivation of receptor tyrosine kinases by specific matrix molecules. Here, we demonstrate that the provisional matrix protein, fibronectin (FN), activates fibroblast growth factor (FGF) receptor-1 (FGFR1) independent of FGF ligand in liver endothelial cells. FN activation of FGFR1 requires β1 integrin, as evidenced by neutralizing antibody and siRNA-based studies. Complementary genetic and pharmacologic approaches identify that the non-receptor tyrosine kinase Src is required for FN transactivation of FGFR1. Whereas FGF ligand-induced phosphorylation of FGFR1 preferentially activates ERK, FN-induced phosphorylation of FGFR1 preferentially activates AKT, indicating differential downstream signaling of FGFR1 in response to alternate stimuli. Mutation analysis of known tyrosine residues of FGFR1 reveals that tyrosine 653/654 and 766 residues are required for FN-FGFR1 activation of AKT and chemotaxis. Thus, our study mechanistically dissects a new signaling pathway by which FN achieves endothelial cell chemotaxis, demonstrates how differential phosphorylation profiles of FGFR1 can achieve alternate downstream signals, and, more broadly, highlights the diversity of mechanisms by which th**e extracellular matr**ix microenvironment regulates cell behavior through transactivation of receptor tyrosine kinases.
7. FGF9 monomer-dimer equilibrium regulates **extracellular matrix** affinity and tissue diffusion. The spontaneous dominant mouse mutant, Elbow knee synostosis (Eks), shows elbow and knee joint synosotsis, and premature fusion of cranial sutures. Here we identify a missense mutation in the Fgf9 gene that is responsible for the Eks mutation. Through investigation of the pathogenic mechanisms of joint and suture synostosis in Eks mice, we identify a key molecular mechanism that regulates FGF9 signaling in developing tissues. We show that the Eks mutation prevents homodimerization of the FGF9 protein and that monomeric FGF9 binds to heparin with a lower affinity than dimeric FGF9. These biochemical defects result in increased diffusion of the altered FGF9 protein (FGF9(Eks)) through developing tissues, leading to ectopic FGF9 signaling and repression of joint and suture development. We propose a mechanism in which the range of FGF9 signaling in developing tissues is limited by its ability to homodimerize and its affinity for **extracellular matrix** heparan sulfate proteoglycans.
8. Anosmin-1 over-expression increases adult neurogenesis in the subventricular zone and neuroblast migration to the olfactory bulb. New subventricular zone (SVZ)-derived neuroblasts that migrate via the rostral migratory stream are continuously added to the olfactory bulb (OB) of the adult rodent brain. Anosmin-1 (A1) is an **extracellular matrix** protein that binds to FGF receptor 1 (FGFR1) to exert its biological effects. When mutated as in Kallmann syndrome patients, A1 is associated with severe OB morphogenesis defects leading to anosmia and hypogonadotropic hypogonadism. Here, we show that A1 over-expression in adult mice strongly increases proliferation in the SVZ, mainly with symmetrical divisions, and produces substantial morphological changes in the normal SVZ architecture, where we also report the presence of FGFR1 in almost all SVZ cells. Interestingly, for the first time we show FGFR1 expression in the basal body of primary cilia in neural progenitor cells. Additionally, we have found that A1 over-expression also enhances neuroblast motility, mainly through FGFR1 activity. Together, these changes lead to a selective increase in several GABAergic interneuron populations in different OB layers. These specific alterations in the OB would be sufficient to disrupt the normal processing of sensory information and consequently alter olfactory memory. In summary, this work shows that FGFR1-mediated A1 activity plays a crucial role in the continuous remodelling of the adult OB.
9. ERK1/2 signaling is essential for the chemoattraction exerted by human FGF2 and human anosmin-1 on newborn rat and mouse OPCs via FGFR1. Signaling through fibroblast growth factor receptors (FGFRs) is essential for many cellular processes including proliferation and migration, as well as differentiation events such as myelination. Anosmin-1 is an **extracellular matrix** (ECM) glycoprotein that interacts with the fibroblast growth factor receptor 1 (FGFR1) to exert its biological actions through this receptor, although the intracellular pathways underlying anosmin-1 signaling remain largely unknown. This protein is defective in the X-linked form of Kallmann syndrome (KS) and has a prominent role in the migration of neuronal and oligodendroglial precursors. We have shown that anosmin-1 exerts a chemotactic effect via FGFR1 on neuronal precursors from the subventricular zone (SVZ) and the essential role of the ERK1/2 signaling. We report here the positive chemotactic effect of FGF2 and anosmin-1 on rat and mouse postnatal OPCs via FGFR1. The same effect was observed with the truncated N-terminal region of anosmin-1 (A1Nt). The introduction in anosmin-1 of the missense mutation F517L found in patients suffering from KS annulled the chemotactic activity; however, the mutant form carrying the disease-causing mutation E514K also found in KS patients, behaved as the wild-type protein. The chemoattraction exhibited by FGF2 and anosmin-1 on OPCs was blocked by the mitogen-activated protein kinase (MAPK) inhibitor U0126, suggesting that the activation of the ERK1/2 MAPK signaling pathway following interaction with the FGFR1 is necessary for FGF2 and anosmin-1 to exert their chemotactic effect. In fact, both proteins were able to induce the phosphorylation of the ERK1/2 kinases after the activation of the FGFR1 receptor.
10. 2-O Heparan Sulfate Sulfation by Hs2st Is Required for Erk/Mapk Signalling Activation at the Mid-Gestational Mouse Telencephalic Midline. Heparan sulfate (HS) is a linear carbohydrate composed of polymerized uronate-glucosamine disaccharide units that decorates cell surface and secreted glycoproteins in the **extracellular matrix**. In mammals HS is subjected to differential sulfation by fifteen different heparan sulfotransferase (HST) enzymes of which Hs2st uniquely catalyzes the sulfation of the 2-O position of the uronate in HS. HS sulfation is postulated to be important for regulation of signaling pathways by facilitating the interaction of HS with signaling proteins including those of the Fibroblast Growth Factor (Fgf) family which signal through phosphorylation of extracellular signal-regulated kinases Erk1/2. In the developing mouse telencephalon Fgf2 signaling regulates proliferation and neurogenesis. Loss of Hs2st function phenocopies the thinned cerebral cortex of mutant mice in which Fgf2 or Erk1/2 function are abrogated, suggesting the hypothesis that 2-O-sulfated HS structures play a specific role in Fgf2/Erk signaling pathway in this context in vivo. This study investigated the molecular role of 2-O sulfation in Fgf2/Erk signaling in the developing telencephalic midline midway through mouse embryogenesis at E12.5. We examined the expression of Hs2st, Fgf2, and Erk1/2 activity in wild-type and Hs2st-/- mice. We found that Hs2st is expressed at high levels at the midline correlating with high levels of Erk1/2 activation and Erk1/2 activation was drastically reduced in the Hs2st-/- mutant at the rostral telencephalic midline. We also found that 2-O sulfation is specifically required for the binding of Fgf2 protein to Fgfr1, its major cell-surface receptor at the rostral telencephalic midline. We conclude that 2-O sulfated HS structures generated by Hs2st are needed to form productive signaling complexes between HS, Fgf2 and Fgfr1 that activate Erk1/2 at the midline. Overall, our data suggest the interesting possibility that differential expression of Hs2st targets the rostral telencephalic midline for high levels of Erk signaling by increasing the sensitivity of cells to an Fgf2 signal that is rather more widespread.
11. Fibroblast growth factor receptor 1 is a key inhibitor of TGFβ signaling in the endothelium. Abnormal vascular homeostasis can lead to increased proliferation of smooth muscle cells and deposition of **extracellular matri**x, resulting in neointima formation, which contributes to vascular lumen narrowing, a pathology that underlies diseases including transplant vasculopathy, the recurrence of stenosis, and atherosclerosis. Growth of neointima is in part due to endothelial-to-mesenchymal transition (EndMT), a transforming growth factor-β (TGFβ)-driven process, which leads to increased numbers of smooth muscle cells and fibroblasts and deposition **of extracellular mat**rix. We reported that endothelial cell-specific knockout of fibroblast growth factor receptor 1 (FGFR1) led to activation of TGFβ signaling and development of EndMT in vitro and in vivo. Furthermore, EndMT in human diseased vasculature correlated with decreased abundance of FGFR1. These findings identify FGFR1 as the key regulator of TGFβ signaling and EndMT development.
12. Distinct roles of fibroblast growth factor receptor 1 and 2 in regulating cell survival and epithelial-mesenchymal transition. Two related receptor tyrosine kinases (RTKs), fibroblast growth factor receptor 1 and 2 (FGFR1 and FGFR2), exert distinct effects during carcinogenesis. To examine FGFR1 and FGFR2 signaling in polarized epithelia, we have developed an in vitro three-dimensional HC11 mouse mammary epithelial cell culture model combined with a chemically inducible FGFR (iFGFR) dimerization system. Although activation of both RTKs led to reinitiation of cell proliferation and loss of cell polarity, only iFGFR1 activation induced cell survival and epithelial to mesenchymal transition. In contrast, iFGFR2 activation induced cell apoptosis even in the cells in direct contact with the **extracellular matrix**. Activation of iFGFR2, but not iFGFR1, led to rapid receptor down-regulation and transient activation of downstream signaling, which were partially rescued by Cbl small interfering RNA knockdown or the proteasome inhibitor lactacystin. Importantly, inhibition of proteasome activity in iFGFR2-activated structures led to epithelial to mesenchymal transition and invasive phenotypes resembling those observed after iFGFR1 activation. These studies demonstrate, for the first time, that the duration of downstream signaling determines the distinct phenotypes mediated by very homologous RTKs in three-dimensional cultures.

### [Ii]nvasive

#### PubMed

1. Magnetic thermoablation stimuli alter BCL2 and FGF-R1 but not HSP70 expression profiles in BT474 breast tumors. Magnetically induced heating of magnetic nanoparticles (MNP) in an alternating magnetic field (AMF) is a promising minimal **invasive** tool for localized tumor treatment that eradicates tumor cells by applying thermal stress. While temperatures between 42°C and 45°C induce apoptosis and sensitize the cells for chemo- and radiation therapies when applied for at least 30 minutes, temperatures above 50°C, so-called thermoablative temperatures, rapidly induce irreversible cell damage resulting in necrosis. Since only little is known concerning the protein expression of anti-apoptotic B-cell lymphoma 2 (BCL2), fibroblast growth factor receptor 1 (FGF-R1), and heat shock protein (HSP70) after short-time magnetic thermoablative tumor treatment, these relevant tumor proteins were investigated by immunohistochemistry (IHC) in a human BT474 breast cancer mouse xenograft model. In the investigated sample groups, the application of thermoablative temperatures (<2 minutes) led to a downregulation of BCL2 and FGF-R1 on the protein level while the level of HSP70 remained unchanged. Coincidently, the tumor tissue was damaged by heat, resulting in large apoptotic and necrotic areas in regions with high MNP concentration. Taken together, thermoablative heating induced via magnetic methods can reduce the expression of tumor-related proteins and locally inactivate tumor tissue, leading to a prospectively reduced tumorigenicity of cancerous tissues. The presented data allow a deeper insight into the molecular mechanisms in relation to magnetic thermoablative tumor treatments with the aim of further improvements.
2. Distinct roles of fibroblast growth factor receptor 1 and 2 in regulating cell survival and epithelial-mesenchymal transition. Two related receptor tyrosine kinases (RTKs), fibroblast growth factor receptor 1 and 2 (FGFR1 and FGFR2), exert distinct effects during carcinogenesis. To examine FGFR1 and FGFR2 signaling in polarized epithelia, we have developed an in vitro three-dimensional HC11 mouse mammary epithelial cell culture model combined with a chemically inducible FGFR (iFGFR) dimerization system. Although activation of both RTKs led to reinitiation of cell proliferation and loss of cell polarity, only iFGFR1 activation induced cell survival and epithelial to mesenchymal transition. In contrast, iFGFR2 activation induced cell apoptosis even in the cells in direct contact with the extracellular matrix. Activation of iFGFR2, but not iFGFR1, led to rapid receptor down-regulation and transient activation of downstream signaling, which were partially rescued by Cbl small interfering RNA knockdown or the proteasome inhibitor lactacystin. Importantly, inhibition of proteasome activity in iFGFR2-activated structures led to epithelial to mesenchymal transition and **invasive** phenotypes resembling those observed after iFGFR1 activation. These studies demonstrate, for the first time, that the duration of downstream signaling determines the distinct phenotypes mediated by very homologous RTKs in three-dimensional cultures.
3. Pleiotropic effects of FGFR1 on cell proliferation, survival, and migration in a 3D mammary epithelial cell model. Members of the fibroblast growth factor (FGF) family and the FGF receptors (FGFRs) have been implicated in mediating various aspects of mammary gland development and transformation. To elucidate the molecular mechanisms of FGFR1 action in a context that mimics polarized epithelial cells, we have developed an in vitro three-dimensional HC11 mouse mammary epithelial cell culture model expressing a drug-inducible FGFR1 (iFGFR1). Using this conditional model, iFGFR1 activation in these growth-arrested and polarized mammary acini initially led to reinitiation of cell proliferation, increased survival of luminal cells, and loss of cell polarity, resulting in the disruption of acinar structures characterized by the absence of an empty lumen. iFGFR1 activation also resulted in a gain of **invasive** properties and the induction of matrix metalloproteinase 3 (MMP-3), causing the cleavage of E-cadherin and increased expression of smooth muscle actin and vimentin. The addition of a pan MMP inhibitor abolished these phenotypes but did not prevent the effects of iFGFR1 on cell proliferation or survival.
4. Molecular Checkpoint Decisions Made by Subverted Vascular Niche Transform Indolent Tumor Cells into Chemoresistant Cancer Stem Cells. Tumor-associated endothelial cells (TECs) regulate tumor cell aggressiveness. However, the core mechanism by which TECs confer stem cell-like activity to indolent tumors is unknown. Here, we used in vivo murine and human tumor models to identify the tumor-suppressive checkpoint role of TEC-expressed insulin growth factor (IGF) binding protein-7 (IGFBP7/angiomodulin). During tumorigenesis, IGFBP7 blocks IGF1 and inhibits expansion and aggresiveness of tumor stem-like cells (TSCs) expressing IGF1 receptor (IGF1R). However, chemotherapy triggers TECs to suppress IGFBP7, and this stimulates IGF1R(+) TSCs to express FGF4, inducing a feedforward FGFR1-ETS2 angiocrine cascade that obviates TEC IGFBP7. Thus, loss of IGFBP7 and upregulation of IGF1 activates the FGF4-FGFR1-ETS2 pathway in TECs and converts naive tumor cells to chemoresistant TSCs, thereby facilitating their **invasiv**eness and progression.
5. Inducible dimerization of FGFR1: development of a mouse model to analyze progressive transformation of the mammary gland. To develop an inducible and progressive model of mammary gland tumorigenesis, transgenic mice were generated with a mouse mammary tumor virus-long terminal repeat-driven, conditional, fibroblast growth factor (FGF)-independent FGF receptor (FGFR)1 (iFGFR1) that can be induced to dimerize with the drug AP20187. Treatment of transgenic mice with AP20187 resulted in iFGFR1 tyrosine phosphorylation, increased proliferation, activation of mitogen-activated protein kinase and Akt, and lateral budding. Lateral buds appeared as early as 3 d after AP20187 treatment and initially consisted of bilayered epithelial cells and displayed apical and basolateral polarity appeared after 13 d of AP20187 treatment. **Invasive** lesions characterized by multicell-layered lateral buds, decreased myoepithelium, increased vascular branching, and loss of cell polarity were observed after 2-4 wk of treatment. These data indicate that acute iFGFR1 signaling results in increased lateral budding of the mammary ductal epithelium, and that sustained activation induces alveolar hyperplasia and **invasive** lesions.

#### GeneRIFs

1. FGFR1 signaling results in increased lateral budding of the mammary ductal epithelium, and sustained activation induces alveolar hyperplasia and **invasive** lesions.

### [Mm]etastasis

#### PubMed

1. FGFR1 is essential for prostate cancer progression and **metastasis**. The fibroblast growth factor receptor 1 (FGFR1) is ectopically expressed in prostate carcinoma cells, but its functional contributions are undefined. In this study, we report the evaluation of a tissue-specific conditional deletion mutant generated in an ARR2PBi(Pbsn)-Cre/TRAMP/fgfr1(loxP/loxP) transgenic mouse model of prostate cancer. Mice lacking fgfr1, in prostate cells developed smaller tumors that also included distinct cancer foci still expressing fgfr1 indicating focal escape from gene excision. Tumors with confirmed fgfr1 deletion exhibited increased foci of early, well-differentiated cancer and phyllodes-type tumors, and tumors that escaped fgfr1 deletion primarily exhibited a poorly differentiated phenotype. Consistent with these phenotypes, mice carrying the fgfr1 null allele survived significantly longer than those without fgfr1 deletion. Most interestingly, all metastases were primarily negative for the fgfr1 null allele, exhibited high FGFR1 expression, and a neuroendocrine phenotype regardless of fgfr1 status in the primary tumors. Together, these results suggest a critical and permissive role of ectopic FGFR1 signaling in prostate tumorigenesis and particularly in mechanisms of **metastasis**.
2. Blocking Fibroblast Growth Factor receptor signaling inhibits tumor growth, lymphangiogenesis, and **metastasis**. Fibroblast Growth Factor receptor (FGFR) activity plays crucial roles in tumor growth and patient survival. However, FGF (Fibroblast Growth Factor) signaling as a target for cancer therapy has been under-investigated compared to other receptor tyrosine kinases. Here, we studied the effect of FGFR signaling inhibition on tumor growth, **metastasis** and lymphangiogenesis by expressing a dominant negative FGFR (FGFR-2DN) in an orthotopic mouse mammary 66c14 carcinoma model. We show that FGFR-2DN-expressing 66c14 cells proliferate in vitro slower than controls. 66c14 tumor outgrowth and lung metastatic foci are reduced in mice implanted with FGFR-2DN-expressing cells, which also exhibited better overall survival. We found 66c14 cells in the lumen of tumor lymphatic vessels and in lymph nodes. FGFR-2DN-expressing tumors exhibited a decrease in VEGFR-3 (Vascular Endothelial Growth Factor Receptor-3) or podoplanin-positive lymphatic vessels, an increase in isolated intratumoral lymphatic endothelial cells and a reduction in VEGF-C (Vascular Endothelial Growth Factor-C) mRNA expression. FGFs may act in an autocrine manner as the inhibition of FGFR signaling in tumor cells suppresses VEGF-C expression in a COX-2 (cyclooxygenase-2) or HIF1-α (hypoxia-inducible factor-1 α) independent manner. FGFs may also act in a paracrine manner on tumor lymphatics by inducing expression of pro-lymphangiogenic molecules such as VEGFR-3, integrin α9, prox1 and netrin-1. Finally, in vitro lymphangiogenesis is impeded in the presence of FGFR-2DN 66c14 cells. These data confirm that both FGF and VEGF signaling are necessary for the maintenance of vascular morphogenesis and provide evidence that targeting FGFR signaling may be an interesting approach to inhibit tumor lymphangiogenesis and metastatic spread.
3. Fibroblast growth factor receptor splice variants are stable markers of oncogenic transforming growth factor β1 signaling in metastatic breast cancers. Epithelial-mesenchymal transition (EMT) and mesenchymal-epithelial transition (MET) facilitate breast cancer (BC) **metastasi**s; however, stable molecular changes that result as a consequence of these processes remain poorly defined. Therefore, with the hope of targeting unique aspects of metastatic tumor outgrowth, we sought to identify molecular markers that could identify tumor cells that had completed the EMT:MET cycle.An in vivo reporter system for epithelial cadherin (E-cad) expression was used to quantify its regulation in metastatic BC cells during primary and metastatic tumor growth. Exogenous addition of transforming growth factor β1 (TGF-β1) was used to induce EMT in an in situ model of BC. Microarray analysis was employed to examine gene expression changes in cells chronically treated with and withdrawn from TGF-β1, thus completing one full EMT:MET cycle. Changes in fibroblast growth factor receptor type 1 (FGFR1) isoform expression were validated using PCR analyses of patient-derived tumor tissues versus matched normal tissues. FGFR1 gene expression was manipulated using short hairpin RNA depletion and cDNA rescue. Preclinical pharmacological inhibition of FGFR kinase was employed using the orally available compound BGJ-398.Metastatic BC cells undergo spontaneous downregulation of E-cad during primary tumor growth, and its expression subsequently returns following initiation of metastatic outgrowth. Exogenous exposure to TGF-β1 was sufficient to drive **the metas**tasis of an otherwise in situ model of BC and was similarly associated with a depletion and return of E-cad expression during metastatic progression. BC cells treated and withdrawn from TGF-β stably upregulate a truncated FGFR1-β splice variant that lacks the outermost extracellular immunoglobulin domain. Identification of this FGFR1 splice variant was verified in metastatic human BC cell lines and patient-derived tumor samples. Expression of FGFR1-β was also dominant in a model of metastatic outgrowth where depletion of FGFR1 and pharmacologic inhibition of FGFR kinase activity both inhibited pulmonary tumor outgrowth. Highlighting the dichotomous nature of FGFR splice variants and recombinant expression of full-length FGFR1-α also blocked pulmonary tumor outgrowth.The results of our study strongly suggest that FGFR1-β is required for the pulmonary outgrowth of metastatic BC. Moreover, FGFR1 isoform expression can be used as a predictive biomarker for therapeutic application of its kinase inhibitors.
4. FGFR1-WNT-TGF-β signaling in prostate cancer mouse models recapitulates human reactive stroma. The reactive stroma surrounding tumor lesions performs critical roles ranging from supporting tumor cell proliferation to inducing tumorigenesis and **metastasi**s. Therefore, it is critical to understand the cellular components and signaling control mechanisms that underlie the etiology of reactive stroma. Previous studies have individually implicated fibroblast growth factor receptor 1 (FGFR1) and canonical WNT/β-catenin signaling in prostate cancer progression and the initiation and maintenance of a reactive stroma; however, both pathways are frequently found to be coactivated in cancer tissue. Using autochthonous transgenic mouse models for inducible FGFR1 (JOCK1) and prostate-specific and ubiquitously expressed inducible β-catenin (Pro-Cat and Ubi-Cat, respectively) and bigenic crosses between these lines (Pro-Cat × JOCK1 and Ubi-Cat × JOCK1), we describe WNT-induced synergistic acceleration of FGFR1-driven adenocarcinoma, associated with a pronounced fibroblastic reactive stroma activation surrounding prostatic intraepithelial neoplasia (mPIN) lesions found both in in situ and reconstitution assays. Both mouse and human reactive stroma exhibited increased transforming growth factor-β (TGF-β) signaling adjacent to pathologic lesions likely contributing to invasion. Furthermore, elevated stromal TGF-β signaling was associated with higher Gleason scores in archived human biopsies, mirroring murine patterns. Our findings establish the importance of the FGFR1-WNT-TGF-β signaling axes as driving forces behind reactive stroma in aggressive prostate adenocarcinomas, deepening their relevance as therapeutic targets.
5. Conditional activation of fibroblast growth factor receptor (FGFR) 1, but not FGFR2, in prostate cancer cells leads to increased osteopontin induction, extracellular signal-regulated kinase activation, and in vivo proliferation. Changes in the fibroblast growth factor receptor (FGFR) axis are often associated with prostate cancer (CaP) progression. We have used chemically induced dimerization (CID) to elucidate the individual contributions of FGFR1 and FGFR2 to tumor etiology. Novel CaP cell lines stably expressing CID/AP20187-inducible FGFR1 (iFGFR1) and iFGFR2 were made using the tumorigenic transgenic adenocarcinoma of the murine prostate (TRAMP)-derived clone, TRAMP-C2N (C2N), to generate C2N.iFGFR1 or C2N.iFGFR2 cells. To test the effects of iFGFR activation on tumor growth, mice bearing s.c. C2N.iFGFR1- or C2N.iFGFR2-derived tumors were treated biweekly with CID. Activation of iFGFR1 led to rapid tumor growth as a result of increased proliferation. In contrast, expression of iFGFR2 inhibited tumor growth. Furthermore, we have ascertained that FGFR1 activation appears to be most important during the early stages of tumor development, but once established, tumors become rapidly CID independent. In these C2N-based lines, quantitative signaling differences were seen between the two receptors, with iFGFR1 leading to more robust extracellular signal-regulated kinase activation. Additionally, activation of iFGFR1, but not iFGFR2, led to strong up-regulation of osteopontin, a secreted glycoprotein involved in integrin activation and associated with CaP progression and **metastasis**. These studies support the hypothesis that observed changes in the FGFR axis in mammals during CaP progression are causally important.

### [Cc]ell [Ii]nvasion

#### PubMed

1. The FGF-BMP signaling axis regulates outflow tract valve primordium formation by promoting cushion neural crest cell differentiation. Heart valves develop from precursor structures called cardiac cushions, an endothelial-lined cardiac jelly that resides in the inner side of the heart tube. The cushions are then invaded by cells from different sources, undergo a series of complicated and poorly understood remodeling processes, and give rise to valves. Disruption of the fibroblast growth factor (FGF) signaling axis impairs morphogenesis of the outflow tract (OFT). Yet, whether FGF signaling regulates OFT valve formation is unknown.To study how OFT valve formation is regulated and how aberrant cell signaling causes valve defects.By using mouse genetic manipulation, cell lineage tracing, ex vivo heart culture, and molecular biology approaches, we demonstrated that FGF signaling in the OFT myocardium upregulated Bmp4 expression, which then enhanced smooth muscle differentiation of neural crest cells (NCCs) in the cushion. FGF signaling also promoted OFT myocardial **cell invasion** to the cushion. Disrupting FGF signaling interrupted cushion remodeling with reduced NCCs differentiation into smooth muscle and less cardiomyocyte invasion and resulted in malformed OFT valves.The results demonstrate a novel mechanism by which the FGF-BMP signaling axis regulates formation of OFT valve primordia by controlling smooth muscle differentiation of cushion NCCs.

---

## Fgfr3

- NCBI Gene
- GeneRIF
- Pubmed

| Search Term | Rank | Fields |
| --- | --- | --- |
| [Mm]igration | 6 | PubMed(17) |
| ECM | 7 | PubMed(4) |
| [Ee]xtracellular [Mm]atrix | 8 | PubMed(15) |
| [Ii]nvasive | 10 | PubMed(3) |
| [Mm]etastasis | 12 | PubMed(1) |

  
hide/show details for Fgfr3

### [Mm]igration

#### PubMed

1. Expression of the fibroblast growth factor family and their receptor family genes during mouse brain development. The fibroblast growth factor (FGF) family is composed of nine members and four genes encode protein tyrosine kinase receptors for them. To gain insight into the involvement of FGFs and their receptors in the development of nervous system, their expression in brains of perinatal and adult mice was examined by semi-quantitative reverse transcription-linked polymerase chain reactions and in situ hybridization. Although all the genes, with the exception of FGF-4, were found to be expressed, FGF-3, FGF-6, FGF-7 and FGF-8 genes demonstrated higher expression in the late embryonic stages than in postnatal stages, suggesting that these members are involved in the late stages of brain development. In contrast, expression of FGF-1 and FGF-5 increased after birth. Interestingly, FGF-6 expression in perinatal mice was restricted to the central nervous system and skeltal muscles, with intense signals in the developing cerebrum in embryos but in cerebellum in 5-day-old neonates. Furthermore, FGF-receptor (FGFR)-4, a cognate receptor for FGF-6, demonstrated similar spatiotemporal expression, suggesting that FGF-6 and FGFR-4 plays significant roles in the maturation of nervous system as a ligand-receptor system. The results indicate that individual member of the fibroblast growth factor and their receptor family are expressed either sequentially or simultaneously in brain development, strongly suggesting their involvement in the regulation of a variety of developmental processes of brain, i.e., proliferation and **migration** of neuronal progenitor cells, neuron and glia differentiation, neurite extensions, and synapse formations.
2. Fibroblast growth factor 8 signaling through fibroblast growth factor receptor 1 is required for the emergence of gonadotropin-releasing hormone neurons. GnRH neurons are essential for the onset and maintenance of reproduction. Mutations in both fibroblast growth factor receptor (Fgfr1) and Fgf8 have been shown to cause Kallmann syndrome, a disease characterized by hypogonadotropic hypogonadism and anosmia, indicating that FGF signaling is indispensable for the formation of a functional GnRH system. Presently it is unclear which stage of GnRH neuronal development is most impacted by FGF signaling deficiency. GnRH neurons express both FGFR1 and -3; thus, it is also unclear whether FGFR1 or FGFR3 contributes directly to GnRH system development. In this study, we examined the developing GnRH system in mice deficient in FGF8, FGFR1, or FGFR3 to elucidate the individual contribution of these FGF signaling components. Our results show that the early emergence of GnRH neurons from the embryonic olfactory placode requires FGF8 signaling, which is mediated through FGFR1, not FGFR3. These data provide compelling evidence that the developing GnRH system is exquisitely sensitive to reduced levels of FGF signaling. Furthermore, Kallmann syndrome stemming from FGF signaling deficiency may be due primarily to defects in early GnRH neuronal development prior to their **migration** into the forebrain.
3. Fgfr1 regulates patterning of the pharyngeal region. Development of the pharyngeal region depends on the interaction and integration of different cell populations, including surface ectoderm, foregut endoderm, paraxial mesoderm, and neural crest. Mice homozygous for a hypomorphic allele of Fgfr1 have craniofacial defects, some of which appeared to result from a failure in the early development of the second branchial arch. A stream of neural crest cells was found to originate from the rhombomere 4 region and migrate toward the second branchial arch in the mutants. Neural crest cells mostly failed to enter the second arch, however, but accumulated in a region proximal to it. Both rescue of the hypomorphic Fgfr1 allele and inactivation of a conditional Fgfr1 allele specifically in neural crest cells indicated that Fgfr1 regulates the entry of neural crest cells into the second branchial arch non-cell-autonomously. Gene expression in the pharyngeal ectoderm overlying the developing second branchial arch was affected in the hypomorphic Fgfr1 mutants at a stage prior to neural crest entry. Our results indicate that Fgfr1 patterns the pharyngeal region to create a permissive environment for neural crest cell **migration**.
4. The roles of FGF signaling in germ cell **migration** in the mouse. Fibroblast growth factor (FGF) signaling is thought to play a role in germ cell behavior. FGF2 has been reported to be a mitogen for primordial germ cells in vitro, whilst combinations of FGF2, steel factor and LIF cause cultured germ cells to transform into permanent lines of pluripotent cells resembling ES cells. However, the actual function of FGF signaling on the migrating germ cells in vivo is unknown. We show, by RT-PCR analysis of cDNA from purified E10.5 germ cells, that germ cells express two FGF receptors: Fgfr1-IIIc and Fgfr2-IIIb. Second, we show that FGF-mediated activation of the MAP kinase pathway occurs in germ cells during their **migration**, and thus they are potentially direct targets of FGF signaling. Third, we use cultured embryo slices in simple gain-of-function experiments, using FGF ligands, to show that FGF2, a ligand for FGFR1-IIIc, affects motility, whereas FGF7, a ligand for FGFR2-IIIb, affects germ cell numbers. Loss of function, using a specific inhibitor of FGF signaling, causes increased apoptosis and inhibition of cell shape change in the migrating germ cells. Lastly, we confirm in vivo the effects seen in slice cultures in vitro, by examining germ cell positions and numbers in embryos carrying a loss-of-function allele of FGFR2-IIIb. In FGFR2-IIIb(-/-) embryos, germ cell **migration** is unaffected, but the numbers of germ cells are significantly reduced. These data show that a major role of FGF signaling through FGFR2-IIIb is to control germ cell numbers. The data do not discriminate between direct and indirect effects of FGF signaling on germ cells, and both may be involved.
5. Differential fibroblast growth factor 8 (FGF8)-mediated autoregulation of its cognate receptors, Fgfr1 and Fgfr3, in neuronal cell lines. Fibroblast growth factors (FGFs) mediate a vast range of CNS developmental processes including neural induction, proliferation, **migration**, and cell survival. Despite the critical role of FGF signaling for normal CNS development, few reports describe the mechanisms that regulate FGF receptor gene expression in the brain. We tested whether FGF8 could autoregulate two of its cognate receptors, Fgfr1 and Fgfr3, in three murine cell lines with different lineages: fibroblast-derived cells (3T3 cells), neuronal cells derived from hippocampus (HT-22 cells), and neuroendocrine cells derived from hypothalamic gonadotropin-releasing hormone (GnRH) neurons (GT1-7 cells). GnRH is produced by neurons in the hypothalamus and is absolutely required for reproductive competence in vertebrate animals. Several lines of evidence strongly suggest that Fgf8 is critical for normal development of the GnRH system, therefore, the GT1-7 cells provided us with an additional endpoint, Gnrh gene expression and promoter activity, to assess potential downstream consequences of FGF8-induced modulation of FGF receptor levels. Results from this study suggest that the autoregulation of its cognate receptor represents a common downstream effect of FGF8. Further, we show that Fgfr1 and Fgfr3 are differentially regulated within the same cell type, implicating these two receptors in different biological roles. Moreover, Fgfr1 and Fgfr3 are differentially regulated among different cell types, suggesting such autoregulation occurs in a cell type-specific fashion. Lastly, we demonstrate that FGF8b decreases Gnrh promoter activity and gene expression, possibly reflecting a downstream consequence of altered FGF receptor populations. Together, our data bring forth the possibility that, in addition to the FGF synexpression group, autoregulation of FGFR expression by FGF8 represents a mechanism by which FGF8 could fine-tune its regulatory actions.
6. Fibroblast growth factor signalling and regional specification of the pharyngeal ectoderm. Branchial arch development involves dynamic interactions between neural crest cells as well as ectodermal, endodermal and mesodermal cell populations. Despite their importance and evolutionary conservation, the intercellular interactions guiding the early development of the branchial arches are still poorly understood. We have here studied fibroblast growth factor (FGF) signalling in early pharyngeal development. In mice homozygous for a hypomorphic allele of Fgfr1, neural crest cells migrating from the hindbrain mostly fail to enter the second branchial arch. This defect is non-cell-autonomous suggesting that Fgfr1 provides a permissive environment for neural crest cell **migration**. Here we demonstrate localized down-regulation of the expression of the FGF responsive gene, Sprouty1 in the epithelium covering the presumptive second branchial arch of hypomorphic Fgfr1 mutants. This appears to result in a failure to establish an ectodermal signalling center expressing Fgf3 and Fgf15. We also studied differentiation of the ectoderm in the second branchial arch region. Development of the geniculate placode as well as the VIIth cranial ganglion is affected in Fgfr1 hypomorphs. Our results suggest that Fgfr1 is important for localized signalling in the pharyngeal ectoderm and consequently for normal tissue interactions in the developing second branchial arch.
7. Laminar patterning in the developing neocortex by temporally coordinated fibroblast growth factor signaling. Laminar organization, a fundamental neural architecture in the CNS, is a prominent feature of the neocortex, where the cortical neurons in spatially distinct layers are generated from the common progenitors in a temporally distinct manner during development. Despite many advances in the characterization of the molecular mechanisms of the radial **migration** of cortical neurons, the way in which the early-late temporal sequence of cortical neuron generation is linked with the deep-superficial spatial sequence of cell body positioning remains obscure. Using in vivo electroporation-mediated gene transfer, we show here that the activities mediated by fibroblast growth factor receptors (FGFRs) in cortical progenitors are critical for conferring proper migratory properties on nascent neuronal progeny. Furthermore, we provide supportive evidence that Pea3 subfamily members of Ets (Pea3-Ets) transcription factors mediate the activities of FGFR at the mid to late phase of neocortical development. In addition, using FGF18 knock-out mice, we demonstrate that FGF18 expressed by early-generated cortical neurons in the cortical plate is critical for the expression of Pea3-Ets transcription factors and that FGF18 is sufficient to induce their expressions. Our results thus imply that a feedback mechanism mediated by FGF signaling is involved in setting up the proper laminar positioning of cortical neurons; FGF18 derived from early-generated cortical neurons acts on the cortical progenitors expressing FGFRs and induces the expression of Pea3-Ets transcription factors that, in turn, confer proper migratory behaviors on nascent cortical progeny during the mid to late stages of neocortical development.
8. Neuron-derived FGF9 is essential for scaffold formation of Bergmann radial fibers and **migration** of granule neurons in the cerebellum. Although fibroblast growth factor 9 (FGF9) is widely expressed in the central nervous system (CNS), the function of FGF9 in neural development remains undefined. To address this question, we deleted the Fgf9 gene specifically in the neural tube and demonstrated that FGF9 plays a key role in the postnatal **migration** of cerebellar granule neurons. Fgf9-null mice showed severe ataxia associated with disrupted Bergmann fiber scaffold formation, impaired granule neuron **migration**, and upset Purkinje cell maturation. Ex vivo cultured wildtype or Fgf9-null glia displayed a stellate morphology. Coculture with wildtype neurons, but not Fgf9-deficient neurons, or treating with FGF1 or FGF9 induced the cells to adopt a radial glial morphology. In situ hybridization showed that Fgf9 was expressed in neurons and immunostaining revealed that FGF9 was broadly distributed in both neurons and Bergmann glial radial fibers. Genetic analyses revealed that the FGF9 activities in cerebellar development are primarily transduced by FGF receptors 1 and 2. Furthermore, inhibition of the MAP kinase pathway, but not the PI3K/AKT pathway, abrogated the FGF activity to induce glial morphological changes, suggesting that the activity is mediated by the MAP kinase pathway. This work demonstrates that granule neurons secrete FGF9 to control formation of the Bergmann fiber scaffold, which in turn, guides their own inward **migration** and maturation of Purkinje cells.
9. Cubilin, a high affinity receptor for fibroblast growth factor 8, is required for cell survival in the developing vertebrate head. Cubilin (Cubn) is a multiligand endocytic receptor critical for the intestinal absorption of vitamin B12 and renal protein reabsorption. During mouse development, Cubn is expressed in both embryonic and extra-embryonic tissues, and Cubn gene inactivation results in early embryo lethality most likely due to the impairment of the function of extra-embryonic Cubn. Here, we focus on the developmental role of Cubn expressed in the embryonic head. We report that Cubn is a novel, interspecies-conserved Fgf receptor. Epiblast-specific inactivation of Cubn in the mouse embryo as well as Cubn silencing in the anterior head of frog or the cephalic neural crest of chick embryos show that Cubn is required during early somite stages to convey survival signals in the developing vertebrate head. Surface plasmon resonance analysis reveals that fibroblast growth factor 8 (Fgf8), a key mediator of cell survival, **migration**, proliferation, and patterning in the developing head, is a high affinity ligand for Cubn. Cell uptake studies show that binding to Cubn is necessary for the phosphorylation of the Fgf signaling mediators MAPK and Smad1. Although Cubn may not form stable ternary complexes with Fgf receptors (FgfRs), it acts together with and/or is necessary for optimal FgfR activity. We propose that plasma membrane binding of Fgf8, and most likely of the Fgf8 family members Fgf17 and Fgf18, to Cubn improves Fgf ligand endocytosis and availability to FgfRs, thus modulating Fgf signaling activity.
10. Expression of FGF receptors 1, 2, 3 in the embryonic and postnatal mouse brain compared with Pdgfralpha, Olig2 and Plp/dm20: implications for oligodendrocyte development. Fibroblast growth factors (FGF) receptors FgfR1, FgfR2 and FgfR3 are differentially regulated during oligodendrocyte (OL) maturation in vitro: FgfR3 is expressed by OL progenitors whereas FgfR2 is expressed by differentiated OLs [Mol Cell Neurosci 1996;7:263-275], and we have recently shown that FgfR3 is required for the timely differentiation of OLs in vivo [J Neurosci 2003;23:883-894]. Here we have used in situ hybridization to investigate the expression patterns of FgfR1-3 and compare them to the putative OL progenitor markers Olig2, Pdgfralpha and Plp/dm20 as a function of development in vivo, in particular at sites of OL specification, **migration** or differentiation in the mouse forebrain and cerebellum. We show that at early stages FgfR1-3 expression overlaps with that of Olig2 in the embryonic ventricular zone of the lateral and medial ganglionic eminences. Further, a scattered population of cells expressing FgfR3 (but not FgfR1 or FgfR2) in the ventral telencephalon appear to arise from the ventricular zone, and at later stages are found more dorsally in the cortex, in an overall pattern similar to Olig2 and/or Pdgfralpha. Postnatal expression of FgfR2 increases with age, more prominently in specific regions, including the cortical and cerebellar white matter and optic nerve. Thus, the differential expression pattern of FgfR2 and FgfR3 observed in vivo suggests that their expression is developmentally regulated in a manner consistent with the pattern of their expression in culture. These data provide further insights into role of FgfRs in OL development, and they emphasize that these receptors are positioned both spatially and temporally to impact OL generation in vivo.
11. FGF/FGFR2 signaling regulates the generation and correct positioning of Bergmann glia cells in the developing mouse cerebellum. The normal cellular organization and layering of the vertebrate cerebellum is established during embryonic and early postnatal development by the interplay of a complex array of genetic and signaling pathways. Disruption of these processes and of the proper layering of the cerebellum usually leads to ataxic behaviors. Here, we analyzed the relative contribution of Fibroblast growth factor receptor 2 (FGFR2)-mediated signaling to cerebellar development in conditional Fgfr2 single mutant mice. We show that during embryonic mouse development, Fgfr2 expression is higher in the anterior cerebellar primordium and excluded from the proliferative ventricular neuroepithelium. Consistent with this finding, conditional Fgfr2 single mutant mice display the most prominent defects in the anterior lobules of the adult cerebellum. In this context, FGFR2-mediated signaling is required for the proper generation of Bergmann glia cells and the correct positioning of these cells within the Purkinje cell layer, and for cell survival in the developing cerebellar primordium. Using cerebellar microexplant cultures treated with an FGFR agonist (FGF9) or antagonist (SU5402), we also show that FGF9/FGFR-mediated signaling inhibits the outward **migration** of radial glia and Bergmann glia precursors and cells, and might thus act as a positioning cue for these cells. Altogether, our findings reveal the specific functions of the FGFR2-mediated signaling pathway in the generation and positioning of Bergmann glia cells during cerebellar development in the mouse.
12. Differential fibroblast growth factor 8 (FGF8)-mediated autoregulation of its cognate receptors, Fgfr1 and Fgfr3, in neuronal cell lines. Fibroblast growth factors (FGFs) mediate a vast range of CNS developmental processes including neural induction, proliferation, **migration**, and cell survival. Despite the critical role of FGF signaling for normal CNS development, few reports describe the mechanisms that regulate FGF receptor gene expression in the brain. We tested whether FGF8 could autoregulate two of its cognate receptors, Fgfr1 and Fgfr3, in three murine cell lines with different lineages: fibroblast-derived cells (3T3 cells), neuronal cells derived from hippocampus (HT-22 cells), and neuroendocrine cells derived from hypothalamic gonadotropin-releasing hormone (GnRH) neurons (GT1-7 cells). GnRH is produced by neurons in the hypothalamus and is absolutely required for reproductive competence in vertebrate animals. Several lines of evidence strongly suggest that Fgf8 is critical for normal development of the GnRH system, therefore, the GT1-7 cells provided us with an additional endpoint, Gnrh gene expression and promoter activity, to assess potential downstream consequences of FGF8-induced modulation of FGF receptor levels. Results from this study suggest that the autoregulation of its cognate receptor represents a common downstream effect of FGF8. Further, we show that Fgfr1 and Fgfr3 are differentially regulated within the same cell type, implicating these two receptors in different biological roles. Moreover, Fgfr1 and Fgfr3 are differentially regulated among different cell types, suggesting such autoregulation occurs in a cell type-specific fashion. Lastly, we demonstrate that FGF8b decreases Gnrh promoter activity and gene expression, possibly reflecting a downstream consequence of altered FGF receptor populations. Together, our data bring forth the possibility that, in addition to the FGF synexpression group, autoregulation of FGFR expression by FGF8 represents a mechanism by which FGF8 could fine-tune its regulatory actions.
13. Autocrine and paracrine mechanisms regulating primordial germ cell proliferation. Although several mitogens and survival factors have been previously shown to act on primordial germ cells (PGCs) in culture, it is not clear whether they are responsible for controlling proliferation of PGCs in the embryo. We show here that during their migratory phase, PGCs do not express FGF-4, FGF-8, or FGF-17, but these FGFs are expressed by neighboring cells. Thus, any FGF action on migrating PGCs would appear to be through a paracrine mechanism. We found that after entering into the gonads, PGCs start to express FGF-4 and FGF-8. On this basis, we hypothesize that FGF signaling is involved in both a paracrine manner in initiating PGC proliferation during their **migration** and an autocrine manner in sustaining PGC proliferation after their arrival in the gonads. We then studied the role of soluble stem cell factor (SCF), which acts as a survival factor or a mitogen in culture, to determine whether it interacts with FGFs. We found that SCF has a complex effect on PGC proliferation. On one hand, soluble SCF promoted PGC proliferation synergistically with FGF in the absence of membrane-bound SCF. Conversely, soluble SCF inhibited FGF-stimulated proliferation of PGCs in the presence of membrane-bound SCF. We account for these findings in a model involving regulation of PGC proliferation, in which SCF modulates the response to FGFs.
14. Interaction of fibroblast growth factor receptor 3 and the adapter protein SH2-B. A role in STAT5 activation. Fibroblast growth factor receptor 3 (FGFR3) influences a diverse array of biological processes, including cell growth, differentiation, and **migration**. Activating mutations in FGFR3 are associated with multiple myeloma, cervical carcinoma, and bladder cancer. To identify proteins that interact with FGFR3 and which may mediate FGFR3-dependent signaling, a yeast two-hybrid screen was employed using the cytoplasmic kinase domain of FGFR3 as bait. We identified the adapter protein SH2-B as an FGFR3-interacting protein. Coimmunoprecipitation experiments demonstrate binding of the SH2-B beta isoform to FGFR3 in 293T cells. Tyrosine phosphorylation of SH2-B beta was observed when coexpressed with activated FGFR3 mutants such as the weakly activated mutant N540K or the strongly activated mutant K650E, both associated with human developmental syndromes. The extent of tyrosine phosphorylation of SH2-B beta correlates with receptor activation, suggesting that FGFR3 activation mediates tyrosine phosphorylation of SH2-B beta. Furthermore, two tyrosine phosphorylation sites of FGFR3, Tyr-724 and Tyr-760, are required for optimal binding of the Src homology-2 (SH2) domain of SH2-B beta. We also demonstrate the phosphorylation and nuclear translocation of Stat5 by activated FGFR3, which increases in response to overexpression of SH2-B beta. Taken together, our results identify SH2-B beta as a novel FGFR3 binding partner that mediates signal transduction.
15. Prox1 promotes lineage-specific expression of fibroblast growth factor (FGF) receptor-3 in lymphatic endothelium: a role for FGF signaling in lymphangiogenesis. Fibroblast growth factors play important roles in angiogenesis, but their functions in lymphangiogenesis remain poorly understood. The homeodomain transcription factor Prox1 is essential for development of the lymphatic system by specifying lymphatic endothelial cell (LEC) fate. Here, we identify fibroblast growth factor (FGF) receptor (FGFR)-3 as a novel Prox1 target gene. Ectopic overexpression of Prox1 in blood vascular endothelial cells up-regulates FGFR-3. Prox1 induces the expression of the IIIc isoform, which we also found to be the major isoform of FGFR-3 expressed in LECs. This transcriptional activation is mediated by a direct binding of Prox1 to newly identified Prox1-response elements in the FGFR-3 promoter. Consistently, FGFR-3 is up-regulated in Prox1-positive newly formed lymphatic vessels during embryogenesis and its lymphatic-specific expression is maintained throughout development. We also found that FGF-1 and FGF-2 promote proliferation, **migration**, and survival of cultured LECs without involvement of vascular endothelial cell growth factor receptor-3. We show that FGF-2 binds to low- and high-affinity receptors on LECs and is efficiently internalized and processed. Moreover, functional inhibition of FGFR-3 using small interfering RNA represses LEC proliferation. Together, these results indicate that FGFR-3 is an initial target of Prox1 during the lymphatic cell fate specification and that FGF signaling may play an important role in lymphatic vessel development.
16. The extracellular matrix molecule tenascin C modulates expression levels and territories of key patterning genes during spinal cord astrocyte specification. The generation of astrocytes during the development of the mammalian spinal cord is poorly understood. Here, we demonstrate for the first time that the extracellular matrix glycoprotein tenascin C regulates the expression of key patterning genes during late embryonic spinal cord development, leading to a timely maturation of gliogenic neural precursor cells. We first show that tenascin C is expressed by gliogenic neural precursor cells during late embryonic development. The loss of tenascin C leads to a sustained generation and delayed **migration** of Fgfr3-expressing immature astrocytes in vivo. Consistent with an increased generation of astroglial cells, we documented an increased number of GFAP-positive astrocytes at later stages. Mechanistically, we could demonstrate an upregulation and domain shift of the patterning genes Nkx6.1 and Nkx2.2 in vivo. In addition, sulfatase 1, a known downstream target of Nkx2.2 in the ventral spinal cord, was also upregulated. Sulfatase 1 regulates growth factor signalling by cleaving sulphate residues from heparan sulphate proteoglycans. Consistent with this function, we observed changes in both FGF2 and EGF responsiveness of spinal cord neural precursor cells. Taken together, our data implicate Tnc in the regulation of proliferation and lineage progression of astroglial progenitors in specific domains of the developing spinal cord.
17. Control of astrocyte progenitor specification, **migration** and maturation by Nkx6.1 homeodomain transcription factor. Although astrocytes are the most abundant cell type in the central nervous system (CNS), little is known about their molecular specification and differentiation. It has previously been reported that transcription factor Nkx6.1 is expressed in neuroepithelial cells that give rise to astrocyte precursors in the ventral spinal cord. In the present study, we systematically investigated the function of Nkx6.1 in astrocyte development using both conventional and conditional Nkx6.1 mutant mice. At early postnatal stages, Nkx6.1 was expressed in a subpopulation of astrocytes in the ventral spinal cord. In the conventional Nkx6.1KO spinal cord, the initial specification of astrocyte progenitors was affected by the mutation, and subsequent **migration** and differentiation were disrupted in newborn mice. In addition, the development of VA2 subtype astrocytes was also inhibited in the white matter. Further studies with Nkx6.1 conditional mutants revealed significantly delayed differentiation and disorganized arrangement of fibrous astrocytes in the ventral white matter. Together, our studies indicate that Nkx6.1 plays a vital role in astrocyte specification and differentiation in the ventral spinal cord.

### ECM

#### PubMed

1. FGF signalling regulates bone growth through autophagy. Skeletal growth relies on both biosynthetic and catabolic processes. While the role of the former is clearly established, how the latter contributes to growth-promoting pathways is less understood. Macroautophagy, hereafter referred to as autophagy, is a catabolic process that plays a fundamental part in tissue homeostasis. We investigated the role of autophagy during bone growth, which is mediated by chondrocyte rate of proliferation, hypertrophic differentiation and extracellular matrix (**ECM**) deposition in growth plates. Here we show that autophagy is induced in growth-plate chondrocytes during post-natal development and regulates the secretion of type II collagen (Col2), the major component of cartilage **ECM**. Mice lacking the autophagy related gene 7 (Atg7) in chondrocytes experience endoplasmic reticulum storage of type II procollagen (PC2) and defective formation of the Col2 fibrillary network in the **ECM**. Surprisingly, post-natal induction of chondrocyte autophagy is mediated by the growth factor FGF18 through FGFR4 and JNK-dependent activation of the autophagy initiation complex VPS34-beclin-1. Autophagy is completely suppressed in growth plates from Fgf18(-/-) embryos, while Fgf18(+/-) heterozygous and Fgfr4(-/-) mice fail to induce autophagy during post-natal development and show decreased Col2 levels in the growth plate. Strikingly, the Fgf18(+/-) and Fgfr4(-/-) phenotypes can be rescued in vivo by pharmacological activation of autophagy, pointing to autophagy as a novel effector of FGF signalling in bone. These data demonstrate that autophagy is a developmentally regulated process necessary for bone growth, and identify FGF signalling as a crucial regulator of autophagy in chondrocytes.
2. Nell1-deficient mice have reduced expression of extracellular matrix proteins causing cranial and vertebral defects. The mammalian Nell1 gene encodes a protein kinase C-beta1 (PKC-beta1) binding protein that belongs to a new class of cell-signaling molecules controlling cell growth and differentiation. Over-expression of Nell1 in the developing cranial sutures in both human and mouse induces craniosynostosis, the premature fusion of the growing cranial bone fronts. Here, we report the generation, positional cloning and characterization of Nell1(6R), a recessive, neonatal-lethal point mutation in the mouse Nell1 gene, induced by N-ethyl-N-nitrosourea. Nell1(6R) has a T-->A base change that converts a codon for cysteine into a premature stop codon [Cys(502)Ter], resulting in severe truncation of the predicted protein product and marked reduction in steady-state levels of the transcript. In addition to the expected alteration of cranial morphology, Nell1(6R) mutants manifest skeletal defects in the vertebral column and ribcage, revealing a hitherto undefined role for Nell1 in signal transduction in endochondral ossification. Real-time quantitative reverse transcription-PCR assays of 219 genes showed an association between the loss of Nell1 function and reduced expression of genes for extracellular matrix (**ECM**) proteins critical for chondrogenesis and osteogenesis. Several affected genes are involved in the human cartilage disorder Ehlers-Danlos Syndrome and other disorders associated with spinal curvature anomalies. Nell1(6R) mutant mice are a new tool for elucidating basic mechanisms in osteoblast and chrondrocyte differentiation in the developing skull and vertebral column and understanding how perturbations in the production of **ECM** proteins can lead to anomalies in these structures.
3. Proteoglycan desulfation determines the efficiency of chondrocyte autophagy and the extent of FGF signaling during endochondral ossification. Cartilage extracellular matrix (**ECM**) contains large amounts of proteoglycans made of a protein core decorated by highly sulfated sugar chains, the glycosaminoglycans (GAGs). GAGs desulfation, a necessary step for their degradation, is exerted by sulfatases that are activated by another enzyme, Sulfatase-Modifying Factor 1 (SUMF1), whose inactivation in humans leads to severe skeletal abnormalities. We show here that despite being expressed in both osteoblasts and chondrocytes Sumf1 does not affect osteoblast differentiation. Conversely, in chondrocytes it favors **ECM** production and autophagy and promotes proliferation and differentiation by limiting FGF signaling. Thus, proteoglycan desulfation is a critical regulator of chondrogenesis.
4. Depletion of cartilage collagen fibrils in mice carrying a dominant negative Col2a1 transgene affects chondrocyte differentiation. We have generated transgenic mice harboring the deletion of exon 48 in the mouse alpha1(II) procollagen gene (Col2a1). This was the first dominant negative mutation identified in the human alpha1(II) procollagen gene (COL2A1). Patients carrying a single allele with this mutation suffer from a severe skeletal disorder called spondyloepiphyseal dysplasia congenita (SED). Transgenic mice phenotype was neonatally lethal with severe respiratory failure, short bones, and cleft palate. Transgene mRNA was expressed at high levels. Growth plate cartilage of transgenic mice presented morphological abnormalities and reduced number of collagen type II fibrils. Chondrocytes carrying the mutation showed altered expression of several differentiation markers, like fibroblast growth factor receptor 3 (Fgfr3), Indian hedgehog (Ihh), runx2, cyclin-dependent kinase inhibitor P21CIP/WAF (Cdkn1a), and collagen type X (Col10a1), suggesting that a defective extracellular matrix (**ECM**) depleted of collagen fibrils affects chondrocytes differentiation and that this defect participates in the reduced endochondral bone growth observed in chondrodysplasias caused by mutations in COL2A1.

### [Ee]xtracellular [Mm]atrix

#### PubMed

1. FGF9 monomer-dimer equilibrium regulates **extracellular matrix** affinity and tissue diffusion. The spontaneous dominant mouse mutant, Elbow knee synostosis (Eks), shows elbow and knee joint synosotsis, and premature fusion of cranial sutures. Here we identify a missense mutation in the Fgf9 gene that is responsible for the Eks mutation. Through investigation of the pathogenic mechanisms of joint and suture synostosis in Eks mice, we identify a key molecular mechanism that regulates FGF9 signaling in developing tissues. We show that the Eks mutation prevents homodimerization of the FGF9 protein and that monomeric FGF9 binds to heparin with a lower affinity than dimeric FGF9. These biochemical defects result in increased diffusion of the altered FGF9 protein (FGF9(Eks)) through developing tissues, leading to ectopic FGF9 signaling and repression of joint and suture development. We propose a mechanism in which the range of FGF9 signaling in developing tissues is limited by its ability to homodimerize and its affinity for **extracellular matrix** heparan sulfate proteoglycans.
2. FGF signalling regulates bone growth through autophagy. Skeletal growth relies on both biosynthetic and catabolic processes. While the role of the former is clearly established, how the latter contributes to growth-promoting pathways is less understood. Macroautophagy, hereafter referred to as autophagy, is a catabolic process that plays a fundamental part in tissue homeostasis. We investigated the role of autophagy during bone growth, which is mediated by chondrocyte rate of proliferation, hypertrophic differentiation and **extracellular matrix** (ECM) deposition in growth plates. Here we show that autophagy is induced in growth-plate chondrocytes during post-natal development and regulates the secretion of type II collagen (Col2), the major component of cartilage ECM. Mice lacking the autophagy related gene 7 (Atg7) in chondrocytes experience endoplasmic reticulum storage of type II procollagen (PC2) and defective formation of the Col2 fibrillary network in the ECM. Surprisingly, post-natal induction of chondrocyte autophagy is mediated by the growth factor FGF18 through FGFR4 and JNK-dependent activation of the autophagy initiation complex VPS34-beclin-1. Autophagy is completely suppressed in growth plates from Fgf18(-/-) embryos, while Fgf18(+/-) heterozygous and Fgfr4(-/-) mice fail to induce autophagy during post-natal development and show decreased Col2 levels in the growth plate. Strikingly, the Fgf18(+/-) and Fgfr4(-/-) phenotypes can be rescued in vivo by pharmacological activation of autophagy, pointing to autophagy as a novel effector of FGF signalling in bone. These data demonstrate that autophagy is a developmentally regulated process necessary for bone growth, and identify FGF signalling as a crucial regulator of autophagy in chondrocytes.
3. MT1-MMP inactivates ADAM9 to regulate FGFR2 signaling and calvarial osteogenesis. MMP14 encodes a membrane-tethered metalloproteinase MT1-MMP, capable of remodeling the **extracellular matrix** and modulating receptors on the cell surface. Loss of MT1-MMP results in craniofacial abnormalities. Here we show that MT1-MMP forms a complex with FGFR2 and ADAM9 in osteoblasts and proteolytically inactivates ADAM9, hence protecting FGFR2 from ADAM9-mediated ectodomain shedding on the cell surface. In Mmp14-/- osteoblasts, FGF-induced proliferation and downstream signaling are specifically compromised, in conjunction with ADAM9 upregulation and FGFR2 shedding. The retarded parietal growth in Mmp14-/- embryos starts at 15.5 dpc, attributable to the impaired FGFR2 signaling due to increased shedding mediated by ADAM9. Adam9 depletion completely rescues the defective FGFR2 signaling and largely restores calvarial bone growth in Mmp14-/- embryos. These data reveal a regulatory paradigm for FGRF2 signaling and identify MT1-MMP as a critical negative modulator of ADAM9 activity to maintain FGFR2 signaling in calvarial osteogenesis.
4. Nell1-deficient mice have reduced expression of **extracellular matrix** proteins causing cranial and vertebral defects. The mammalian Nell1 gene encodes a protein kinase C-beta1 (PKC-beta1) binding protein that belongs to a new class of cell-signaling molecules controlling cell growth and differentiation. Over-expression of Nell1 in the developing cranial sutures in both human and mouse induces craniosynostosis, the premature fusion of the growing cranial bone fronts. Here, we report the generation, positional cloning and characterization of Nell1(6R), a recessive, neonatal-lethal point mutation in the mouse Nell1 gene, induced by N-ethyl-N-nitrosourea. Nell1(6R) has a T-->A base change that converts a codon for cysteine into a premature stop codon [Cys(502)Ter], resulting in severe truncation of the predicted protein product and marked reduction in steady-state levels of the transcript. In addition to the expected alteration of cranial morphology, Nell1(6R) mutants manifest skeletal defects in the vertebral column and ribcage, revealing a hitherto undefined role for Nell1 in signal transduction in endochondral ossification. Real-time quantitative reverse transcription-PCR assays of 219 genes showed an association between the loss of Nell1 function and reduced expression of genes for **extracellular matrix** (ECM) proteins critical for chondrogenesis and osteogenesis. Several affected genes are involved in the human cartilage disorder Ehlers-Danlos Syndrome and other disorders associated with spinal curvature anomalies. Nell1(6R) mutant mice are a new tool for elucidating basic mechanisms in osteoblast and chrondrocyte differentiation in the developing skull and vertebral column and understanding how perturbations in the production of ECM proteins can lead to anomalies in these structures.
5. Perlecan is essential for cartilage and cephalic development. Perlecan, a large, multi-domain, heparan sulfate proteoglycan originally identified in basement membrane, interacts with **extracellular matrix** proteins, growth factors and receptors, and influences cellular signalling. Perlecan is present in a variety of basement membranes and in other **extracellular matrix** structures. We have disrupted the gene encoding perlecan (Hspg2) in mice. Approximately 40% of Hspg2-/- mice died at embryonic day (E) 10.5 with defective cephalic development. The remaining Hspg2-/- mice died just after birth with skeletal dysplasia characterized by micromelia with broad and bowed long bones, narrow thorax and craniofacial abnormalities. Only 6% of Hspg2-/- mice developed both exencephaly and chondrodysplasia. Hspg2-/- cartilage showed severe disorganization of the columnar structures of chondrocytes and defective endochondral ossification. Hspg2-/- cartilage matrix contained reduced and disorganized collagen fibrils and glycosaminoglycans, suggesting that perlecan has an important role in matrix structure. In Hspg2-/- cartilage, proliferation of chondrocytes was reduced and the prehypertrophic zone was diminished. The abnormal phenotypes of the Hspg2-/- skeleton are similar to those of thanatophoric dysplasia (TD) type I, which is caused by activating mutations in FGFR3 (refs 7, 8, 9), and to those of Fgfr3 gain-of-function mice. Our findings suggest that these molecules affect similar signalling pathways.
6. The transcription factors L-Sox5 and Sox6 are essential for cartilage formation. L-Sox5 and Sox6 are highly identical Sry-related transcription factors coexpressed in cartilage. Whereas Sox5 and Sox6 single null mice are born with mild skeletal abnormalities, Sox5; Sox6 double null fetuses die with a severe, generalized chondrodysplasia. In these double mutants, chondroblasts poorly differentiate. They express the genes for all essential cartilage **extracellular matrix** components at low or undetectable levels and initiate proliferation after a long delay. All cartilages are thus **extracellular matrix** deficient and remain rudimentary. While chondroblasts in the center of cartilages ultimately activate prehypertrophic chondrocyte markers, epiphyseal chondroblasts ectopically activate hypertrophic chondrocyte markers. Thick intramembranous bone collars develop, but the formation of cartilage growth plates and endochondral bones is disrupted. L-Sox5 and Sox6 are thus redundant, potent enhancers of chondroblast functions, thereby essential for endochondral skeleton formation.
7. Aggrecan is required for growth plate cytoarchitecture and differentiation. The proteoglycan aggrecan is a prominent component of the **extracellular matrix** in growth plate cartilage. A naturally occurring, recessive, perinatally lethal mutation in the aggrecan core protein gene, cmd(bc) (Acan(cmd-Bc)), that deletes the entire protein-coding sequence provided a model in which to characterize the phenotypic and morphologic effects of aggrecan deletion on skeletal development. We also generated a novel transgenic mouse, Tg(COL2A1-ACAN), that has the chick ACAN coding sequence driven by the mouse COL2A1 promoter to enable the production of cmd(bc)/cmd(bc); Tg(COL2A1-ACAN) rescue embryos. These were used to assess the impact of aggrecan on growth plate organization, chondrocyte survival and proliferation, and the expression of mRNAs encoding chondrocyte differentiation markers and growth factors. Homozygous mutant (cmd(bc)/cmd(bc)) embryos exhibited severe defects in all skeletal elements with deformed and shortened (50%) limb elements. Expression of aggrecan in rescue embryos reversed the skeletal defects to varying degrees with a 20% increase in limb element length and near-full reversal (80%) of size and diameter of the ribcage and vertebrae. Aggrecan-null growth plates were devoid of matrix and lacked chondrocyte organization and differentiation, while those of the rescue embryos exhibited matrix production concomitant with partial zonation of chondrocytes having proliferative and hypertrophic morphologies. Deformation of the trachea, likely the cause of the mutation's lethality, was reduced in the rescue embryos. Aggrecan-null embryos also had abnormal patterns of COL10A1, SOX9, IHH, PTCH1, and FGFR3 mRNA expression in the growth plate. Expression of chick aggrecan in the rescue embryos notably increased COLX expression, accompanied by the reappearance of a hypertrophic zone and IHH expression. Significantly, in transgenic rescue embryos, the cell death and decreased proliferation phenotypes exhibited by the mutants were reversed; both were restored to wild-type levels. These findings suggest that aggrecan has a major role in regulating the expression of key growth factors and signaling molecules during development of cartilaginous tissue and is essential for proper chondrocyte organization, morphology, and survival during embryonic limb development.
8. Proteoglycan desulfation determines the efficiency of chondrocyte autophagy and the extent of FGF signaling during endochondral ossification. Cartilage **extracellular matrix** (ECM) contains large amounts of proteoglycans made of a protein core decorated by highly sulfated sugar chains, the glycosaminoglycans (GAGs). GAGs desulfation, a necessary step for their degradation, is exerted by sulfatases that are activated by another enzyme, Sulfatase-Modifying Factor 1 (SUMF1), whose inactivation in humans leads to severe skeletal abnormalities. We show here that despite being expressed in both osteoblasts and chondrocytes Sumf1 does not affect osteoblast differentiation. Conversely, in chondrocytes it favors ECM production and autophagy and promotes proliferation and differentiation by limiting FGF signaling. Thus, proteoglycan desulfation is a critical regulator of chondrogenesis.
9. Collagen XXVII organises the pericellular matrix in the growth plate. In order to characterise the function of the novel fibrillar type XXVII collagen, a series of mice expressing mutant forms of the collagen were investigated. Mice harboring a glycine to cysteine substitution in the collagenous domain were phenotypically normal when heterozygote and displayed a mild disruption of growth plate architecture in the homozygous state. Mice expressing an 87 amino acid deletion in the collagenous domain of collagen XXVII were phenotypically normal as heterozygotes whereas homozygotes exhibited a severe chondrodysplasia and died perinatally from a lung defect. Animals expressing the 87 amino acid deletion targeted specifically to cartilage were viable but severely dwarfed. The pericellular matrix of proliferative chondrocytes was disrupted and the proliferative cells exhibited a decreased tendency to flatten and form vertical columns. Collagen XXVII plays an important structural role in the pericellular **extracellular matrix** of the growth plate and is required for the organisation of the proliferative zone.
10. Tissue-specific calibration of **extracellular matrix** material properties by transforming growth factor-β and Runx2 in bone is required for hearing. Physical cues, such as **extracellular matri**x stiffness, direct cell differentiation and support tissue-specific function. Perturbation of these cues underlies diverse pathologies, including osteoarthritis, cardiovascular disease and cancer. However, the molecular mechanisms that establish tissue-specific material properties and link them to healthy tissue function are unknown. We show that Runx2, a key lineage-specific transcription factor, regulates the material properties of bone matrix through the same transforming growth factor-β (TGFβ)-responsive pathway that controls osteoblast differentiation. Deregulated TGFβ or Runx2 function compromises the distinctly hard cochlear bone matrix and causes hearing loss, as seen in human cleidocranial dysplasia. In Runx2+/⁻ mice, inhibition of TGFβ signalling rescues both the material properties of the defective matrix, and hearing. This study elucidates the unknown cause of hearing loss in cleidocranial dysplasia, and demonstrates that a molecular pathway controlling cell differentiation also defines material propert**ies of extracellular** matrix. Furthermore, our results suggest that the careful regulation of these properties is essential for healthy tissue function.
11. Depletion of cartilage collagen fibrils in mice carrying a dominant negative Col2a1 transgene affects chondrocyte differentiation. We have generated transgenic mice harboring the deletion of exon 48 in the mouse alpha1(II) procollagen gene (Col2a1). This was the first dominant negative mutation identified in the human alpha1(II) procollagen gene (COL2A1). Patients carrying a single allele with this mutation suffer from a severe skeletal disorder called spondyloepiphyseal dysplasia congenita (SED). Transgenic mice phenotype was neonatally lethal with severe respiratory failure, short bones, and cleft palate. Transgene mRNA was expressed at high levels. Growth plate cartilage of transgenic mice presented morphological abnormalities and reduced number of collagen type II fibrils. Chondrocytes carrying the mutation showed altered expression of several differentiation markers, like fibroblast growth factor receptor 3 (Fgfr3), Indian hedgehog (Ihh), runx2, cyclin-dependent kinase inhibitor P21CIP/WAF (Cdkn1a), and collagen type X (Col10a1), suggesting that a defective **extracellular matrix** (ECM) depleted of collagen fibrils affects chondrocytes differentiation and that this defect participates in the reduced endochondral bone growth observed in chondrodysplasias caused by mutations in COL2A1.
12. Sulfatases are determinants of alveolar formation. Alveolar formation or alveolarization is orchestrated by a finely regulated and complex interaction between growth factors and **extracellular matrix** proteins. The lung parenchyma contains various **extracellular matrix** proteins including proteoglycans, which are composed of glycosaminoglycans (GAGs) linked to a protein core. Although GAGs are known to regulate growth factor distribution and activity according to their degree of sulfation the role of sulfated GAG in the respiratory system is not well understood. The degree of sulfation of GAGs is regulated in part, by sulfatases that remove sulfate groups. In vertebrates, the enzyme Sulfatase-Modifying Factor 1 (Sumf1) activates all sulfatases. Here we utilized mice lacking Sumf1(-/-) to study the importance of proteoglycan desulfation in lung development. The Sumf1(-/-) mice have normal lungs up until the onset of alveolarization at post-natal day 5 (P5). We detected increased deposition of sulfated GAG throughout the lung parenchyma and a decrease in alveolar septa formation. Moreover, stereological analysis showed that the alveolar volume is 20% larger in Sumf1(-/-) as compared to wild type (WT) mice at P10 and P30. Additionally, pulmonary function test was consistent with increased alveolar volume. Genetic experiments demonstrate that in Sumf1(-/-) mice arrest of alveolarization is independent of fibroblast growth factor signaling. In turn, the Sumf1(-/-) mice have increased transforming growth factor β (TGFβ) signaling and in vivo injection of TGFβ neutralizing antibody leads to normalization of alveolarization. Thus, absence of sulfatase activity increases sulfated GAG deposition in the lungs causing deregulation of TGFβ signaling and arrest of alveolarization.
13. Identification of novel SHOX target genes in the developing limb using a transgenic mouse model. Deficiency of the human short stature homeobox-containing gene (SHOX) has been identified in several disorders characterized by reduced height and skeletal anomalies such as Turner syndrome, Léri-Weill dyschondrosteosis and Langer mesomelic dysplasia as well as isolated short stature. SHOX acts as a transcription factor during limb development and is expressed in chondrocytes of the growth plates. Although highly conserved in vertebrates, rodents lack a SHOX orthologue. This offers the unique opportunity to analyze the effects of human SHOX expression in transgenic mice. We have generated a mouse expressing the human SHOXa cDNA under the control of a murine Col2a1 promoter and enhancer (Tg(Col2a1-SHOX)). SHOX and marker gene expression as well as skeletal phenotypes were characterized in two transgenic lines. No significant skeletal anomalies were found in transgenic compared to wildtype mice. Quantitative and in situ hybridization analyses revealed that Tg(Col2a1-SHOX), however, affected **extracellular matri**x gene expression during early limb development, suggesting a role for SHOX in growth plate assembly and **extracellular matri**x composition during long bone development. For instance, we could show that the connective tissue growth factor gene Ctgf, a gene involved in chondrogenic and angiogenic differentiation, is transcriptionally regulated by SHOX in transgenic mice. This finding was confirmed in human NHDF and U2OS cells and chicken micromass culture, demonstrating the value of the SHOX-transgenic mouse for the characterization of SHOX-dependent genes and pathways in early limb development.
14. The **extracellular matrix** molecule tenascin C modulates expression levels and territories of key patterning genes during spinal cord astrocyte specification. The generation of astrocytes during the development of the mammalian spinal cord is poorly understood. Here, we demonstrate for the first time that the **extracellular matrix** glycoprotein tenascin C regulates the expression of key patterning genes during late embryonic spinal cord development, leading to a timely maturation of gliogenic neural precursor cells. We first show that tenascin C is expressed by gliogenic neural precursor cells during late embryonic development. The loss of tenascin C leads to a sustained generation and delayed migration of Fgfr3-expressing immature astrocytes in vivo. Consistent with an increased generation of astroglial cells, we documented an increased number of GFAP-positive astrocytes at later stages. Mechanistically, we could demonstrate an upregulation and domain shift of the patterning genes Nkx6.1 and Nkx2.2 in vivo. In addition, sulfatase 1, a known downstream target of Nkx2.2 in the ventral spinal cord, was also upregulated. Sulfatase 1 regulates growth factor signalling by cleaving sulphate residues from heparan sulphate proteoglycans. Consistent with this function, we observed changes in both FGF2 and EGF responsiveness of spinal cord neural precursor cells. Taken together, our data implicate Tnc in the regulation of proliferation and lineage progression of astroglial progenitors in specific domains of the developing spinal cord.
15. Fibroblast growth factor (FGF) 18 signals through FGF receptor 3 to promote chondrogenesis. Signaling by fibroblast growth factor (FGF) 18 and FGF receptor 3 (FGFR3) have been shown to regulate proliferation, differentiation, and matrix production of articular and growth plate chondrocytes in vivo and in vitro. Notably, the congenital absence of either FGF18 or FGFR3 resulted in similar expansion of the growth plates of fetal mice and the addition of FGF18 to human articular chondrocytes in culture enhanced proliferation and matrix production. Based on these and other experiments it has been proposed that FGF18 signals through FGFR3 to promote cartilage production by chondrocytes. Its role in chondrogenesis remains to be defined. In the current work we used the limb buds of FGFR3(+/+) and FGFR3(-/-) embryonic mice as a source of mesenchymal cells to determine how FGF18 signaling affects chondrogenesis. Confocal laser-scanning microscopy demonstrated impaired cartilage nodule formation in the FGFR3(-/-) cultures. Potential contributing factors to the phenotype were identified as impaired mitogenic response to FGF18, decreased production of type II collagen and proteoglycan in response to FGF18 stimulation, impaired interactions with the **extracellular matrix** resulting from altered integrin receptor expression, and altered expression of FGFR1 and FGFR2. The data identified FGF18 as a selective ligand for FGFR3 in limb bud mesenchymal cells, which suppressed proliferation and promoted their differentiation and production of cartilage matrix. This work, thus, identifies FGF18 and FGFR3 as potential molecular targets for intervention in tissue engineering aimed at cartilage repair and regeneration of damaged cartilage.

### [Ii]nvasive

#### PubMed

1. Absence of FGFR3 mutations in urinary bladder tumours of rats and mice treated with N-butyl-N-(-4-hydroxybutyl)nitrosamine. Frequent activating mutations of FGFR3 (fibroblast growth factor receptor 3) are found in human urothelial cell carcinomas, particularly in superficial papillary tumours (in 74%-84% of pTaG1-G2), but not in carcinomas in situ (CIS) and at a low rate in **invasive** tumours (in 16%-21% of pT1-4). In mice and rats, BBN (N-butyl-N-(4-hydroxybutyl)nitrosamine) specifically induces bladder tumours. In rats, superficial papillary tumours are mostly observed. In mice, tumour progression follows the CIS pathway: CIS are first observed, followed by tumours that invade surrounding muscle. Therefore, we looked for FGFR3 mutations in these two animal models of bladder cancer. Only the FGFR3b isoform is expressed in human urothelium and derived tumours. We identified the FGFR3b isoform in rats for the first time and showed that this is the main isoform expressed in the bladder urothelium and derived carcinomas in mice and rats, as in humans. SSCP and sequence analysis of FGFR3b showed sequence changes (polymorphisms or silent mutations) in four BBN-induced rat and mouse bladder tumours. The absence of activating mutations of FGFR3 in the mouse model was in agreement with the fact that mouse BBN-induced bladder tumour progression mimics the CIS pathway. The absence of FGFR3 mutations in the rat bladder tumours suggests that, at least at the genetic level, rat superficial papillary tumours differ from their human counterparts.
2. A Modeling Approach to Explain Mutually Exclusive and Co-Occurring Genetic Alterations in Bladder Tumorigenesis. Relationships between genetic alterations, such as co-occurrence or mutual exclusivity, are often observed in cancer, where their understanding may provide new insights into etiology and clinical management. In this study, we combined statistical analyses and computational modeling to explain patterns of genetic alterations seen in 178 patients with bladder tumors (either muscle-**invasive** or non-muscle-**invasive**). A statistical analysis on frequently altered genes identified pair associations, including co-occurrence or mutual exclusivity. Focusing on genetic alterations of protein-coding genes involved in growth factor receptor signaling, cell cycle, and apoptosis entry, we complemented this analysis with a literature search to focus on nine pairs of genetic alterations of our dataset, with subsequent verification in three other datasets available publicly. To understand the reasons and contexts of these patterns of associations while accounting for the dynamics of associated signaling pathways, we built a logical model. This model was validated first on published mutant mice data, then used to study patterns and to draw conclusions on counter-intuitive observations, allowing one to formulate predictions about conditions where combining genetic alterations benefits tumorigenesis. For example, while CDKN2A homozygous deletions occur in a context of FGFR3-activating mutations, our model suggests that additional PIK3CA mutation or p21CIP deletion would greatly favor **invasive**ness. Furthermore, the model sheds light on the temporal orders of gene alterations, for example, showing how mutual exclusivity of FGFR3 and TP53 mutations is interpretable if FGFR3 is mutated first. Overall, our work shows how to predict combinations of the major gene alterations leading to **invasive**ness through two main progression pathways in bladder cancer.
3. Modelling bladder cancer in mice: opportunities and challenges. The prognosis and treatment of bladder cancer have improved little in the past 20 years. Bladder cancer remains a debilitating and often fatal disease, and is among the most costly cancers to treat. The generation of informative mouse models has the potential to improve our understanding of bladder cancer progression, as well as to affect its diagnosis and treatment. However, relatively few mouse models of bladder cancer have been described, and in particular, few that develop **invasive** cancer phenotypes. This Review focuses on opportunities for improving the landscape of mouse models of bladder cancer.

### [Mm]etastasis

#### PubMed

1. Rapid induction of lung adenocarcinoma by fibroblast growth factor 9 signaling through FGF receptor 3. Fibroblast growth factors (FGF) are expressed in many non-small cell lung carcinoma (NSCLC) primary tumors and derived cell lines, and mutations in FGF receptor 3 (FGFR3) have been identified in human lung adenocarcinoma. FGF9 has been implicated in the pathogenesis of NSCLC by synergizing with EGFR pathways or by providing an escape pathway mediating resistance to EGFR inhibition. To model pathogenic mechanisms mediated by FGF signals, we have established a mouse model in which FGF9 expression can be induced in adult lung epithelium. Here, we show that induced expression of FGF9 in adult lung leads to the rapid proliferation of distal airway epithelial cells that express the stem cell marker, Sca-1, and the proximal and distal epithelial markers, Sftpc and CC10, the rapid formation of Sftpc-positive adenocarcinomas, and eventual **metastasis** in some mice. Furthermore, we have identified FGFR3 as the obligate receptor mediating the FGF9 oncogenic signal. These results identify an FGF9-FGFR3 signal as a primary oncogenic pathway for lung adenocarcinoma and suggest that this pathway could be exploited for customized therapeutic applications for both primary tumors and those that have acquired resistance to inhibition of other signaling pathways.

---

## Csf1r

- NCBI Gene
- GeneRIF
- Pubmed

| Search Term | Rank | Fields |
| --- | --- | --- |
| [Mm]igration | 6 | Process(1); PubMed(12); GeneRIFs(1) |
| [Ii]nvasive | 10 | PubMed(1); GeneRIFs(1) |
| [Mm]etastasis | 12 | PubMed(3) |
| [Cc]ell [Ii]nvasion | 13 | PubMed(3) |

  
hide/show details for Csf1r

### [Mm]igration

#### Process

1. positive regulation of cell **migration**

#### PubMed

1. Neural progenitor cells orchestrate microglia **migration** and positioning into the developing cortex. Microglia are observed in the early developing forebrain and contribute to the regulation of neurogenesis through still unravelled mechanisms. In the developing cerebral cortex, microglia cluster in the ventricular/subventricular zone (VZ/SVZ), a region containing Cxcl12-expressing basal progenitors (BPs). Here we show that the ablation of BP as well as genetic loss of Cxcl12 affect microglia recruitment into the SVZ. Ectopic Cxcl12 expression or pharmacological blockage of CxcR4 further supports that Cxcl12/CxcR4 signalling is involved in microglial recruitment during cortical development. Furthermore, we found that cell death in the developing forebrain triggers microglial proliferation and that this is mediated by the release of macrophage **migration** inhibitory factor (MIF). Finally, we show that the depletion of microglia in mice lacking receptor for colony-stimulating factor-1 (Csf-1R) reduces BPs into the cerebral cortex.
2. M-CSF induces the stable interaction of cFms with alphaVbeta3 integrin in osteoclasts. The macrophage colony stimulating factor receptor (cFms) and alpha(V)beta(3) integrin are both abundantly expressed and play critical roles in the differentiation, survival and **migration** of osteoclasts. We have previously demonstrated that cross-talk between cFms- and alpha(V)beta(3)-mediated signaling pathways regulated the cytoskeletal organization required for osteoclast **migration**. To investigate the nature of interaction between the two receptors, we sequentially used anion-exchange chromatography and immunoprecipitation to purify alpha(V)beta(3)-associated protein complexes. We have demonstrated that cFms stably associated with alpha(V)beta(3) in osteoclasts during adhesion, and that the association was induced by macrophage colony stimulating factor (M-CSF) stimulation. However, the kinetics of association of alpha(V)beta(3) and cFms did not correlate with the kinetics of tyrosine phosphorylation of cFms. Instead, maximally observed alpha(V)beta(3)/cFms association was after the peak of cFms tyrosine phosphorylation and correlated inversely with the total amount of cFms remaining. Furthermore, the complex containing cFms and alpha(V)beta(3) also contained a number of other signaling molecules including Pyk2, p130(Cas) and c-Cbl, known downstream regulators of the integrin-mediated signaling pathways in osteoclasts. In the presence of M-CSF, co-localization of alpha(V)beta(3) integrin and cFms was identified in the podosomal actin ring of the osteoclast during adhesion on glass. Interestingly, co-localization of both receptors was not found in the sealing zone, but in punctate structures associated with adhesion- or transcytosis-like structures in osteoclasts on bone. Taken together, we suggest that the association of alpha(V)beta(3) and cFms could be the result of signaling following tyrosine phosphorylation of cFms. The recruitment of cFms to alpha(V)beta(3) integrin may be an integral part of a larger signaling complex via which both of adhesion- and growth factor receptors coordinately regulate osteoclast adhesion, motility and membrane trafficking.
3. Adaptor protein Lnk inhibits c-Fms-mediated macrophage function. The M-CSFR (c-Fms) participates in proliferation, differentiation, and survival of macrophages and is involved in the regulation of distinct macrophage functions. Interaction with the ligand M-CSF results in phosphorylation of tyrosine residues on c-Fms, thereby creating binding sites for molecules containing SH2 domains. Lnk is a SH2 domain adaptor protein that negatively regulates hematopoietic cytokine receptors. Here, we show that Lnk binds to c-Fms. Biological and functional effects of this interaction were examined in macrophages from Lnk-deficient (KO) and WT mice. Clonogenic assays demonstrated an elevated number of M-CFUs in the bone marrow of Lnk KO mice. Furthermore, the M-CSF-induced phosphorylation of Akt in Lnk KO macrophages was increased and prolonged, whereas phosphorylation of Erk was diminished. Zymosan-stimulated production of ROS was increased dramatically in a M-CSF-dependent manner in Lnk KO macrophages. Lastly, Lnk inhibited M-CSF-induced **migration** of macrophages. In summary, we show that Lnk binds to c-Fms and can blunt M-CSF stimulation. Modulation of levels of Lnk in macrophages may provide a unique therapeutic approach to increase innate host defenses.
4. IL-34 and M-CSF share the receptor Fms but are not identical in biological activity and signal activation. Macrophage colony-stimulating factor (M-CSF) regulates the production, survival and function of macrophages through Fms, the receptor tyrosine kinase. Recently, interleukin-34 (IL-34), which shares no sequence homology with M-CSF, was identified as an alternative Fms ligand. Here, we provide the first evidence that these ligands indeed resemble but are not necessarily identical in biological activity and signal activation. In culture systems tested, IL-34 and M-CSF showed an equivalent ability to support cell growth or survival. However, they were different in the ability to induce the production of chemokines such as MCP-1 and eotaxin-2 in primary macrophages, the morphological change in TF-1-fms cells and the **migration** of J774A.1 cells. Importantly, IL-34 induced a stronger but transient tyrosine phosphorylation of Fms and downstream molecules, and rapidly downregulated Fms. Even in the comparison of active domains, these ligands showed no sequence homology including the position of cysteines. Interestingly, an anti-Fms monoclonal antibody (Mab) blocked both IL-34-Fms and M-CSF-Fms binding, but another MAb blocked only M-CSF-Fms binding. These results suggested that IL-34 and M-CSF differed in their structure and Fms domains that they bound, which caused different bioactivities and signal activation kinetics/strength. Our findings indicate that macrophage phenotype and function are differentially regulated even at the level of the single receptor, Fms.
5. Microglial stimulation of glioblastoma invasion involves epidermal growth factor receptor (EGFR) and colony stimulating factor 1 receptor (CSF-1R) signaling. Glioblastoma multiforme is a deadly cancer for which current treatment options are limited. The ability of glioblastoma tumor cells to infiltrate the surrounding brain parenchyma critically limits the effectiveness of current treatments. We investigated how microglia, the resident macrophages of the brain, stimulate glioblastoma cell invasion. We first examined the ability of normal microglia from C57Bl/6J mice to stimulate GL261 glioblastoma cell invasion in vitro. We found that microglia stimulate the invasion of GL261 glioblastoma cells by approximately eightfold in an in vitro invasion assay. Pharmacological inhibition of epidermal growth factor receptor (EGFR) strongly inhibited microglia-stimulated invasion. Furthermore, blockade of colony stimulating factor 1 receptor (CSF-1R) signaling using ribonucleic acid (RNA) interference or pharmacological inhibitors completely inhibited microglial enhancement of glioblastoma invasion. GL261 cells were found to constitutively secrete CSF-1, the levels of which were unaffected by epidermal growth factor (EGF) stimulation, EGFR inhibition or coculture with microglia. CSF-1 only stimulated microglia invasion, whereas EGF only stimulated glioblastoma cell **migration**, demonstrating a synergistic interaction between these two cell types. Finally, using PLX3397 (a CSF-1R inhibitor that can cross the blood-brain barrier) in live animals, we discovered that blockade of CSF-1R signaling in vivo reduced the number of tumor-associated microglia and glioblastoma invasion. These data indicate that glioblastoma and microglia interactions mediated by EGF and CSF-1 can enhance glioblastoma invasion and demonstrate the possibility of inhibiting glioblastoma invasion by targeting glioblastoma-associated microglia via inhibition of the CSF-1R.
6. Induction of STAP-1 promotes neurotoxic activation of microglia. Activated microglia contribute to neurodegenerative processes in the brain and the retina. Via DNA-microarray analysis, we have previously identified up-regulation of several immune-related genes in the dystrophic retina of retinoschisin-deficient (Rs1h(-/Y)) mice. Here we report a strong overexpression of transcripts for the signal-transducing adaptor protein-1 (STAP-1) in isolated Rs1h(-/Y) microglia. Furthermore, STAP-1 expression was induced in activated bone marrow-derived macrophages as well as LPS-, interferon-gamma-, and CpG-stimulated myeloid cell lines. Ectopic expression of STAP-1 in BV-2 microglia changed the morphology and cytoskeletal organization of the cells and transformed ramified cells to an activated state. STAP-1 overexpression also leads to an interaction with the M-CSF receptor/c-Fms diminishing its ligand-dependent phosphorylation. Finally, STAP-1 expressing cells showed strongly reduced **migration** with increased cytotoxicity against 661W photoreceptor like cells. Taken together, our study implicates a previously unknown role of STAP-1 in pro-inflammatory microglia activation potentially contributing to neuronal apoptosis and degeneration.
7. CSF1R signaling blockade stanches tumor-infiltrating myeloid cells and improves the efficacy of radiotherapy in prostate cancer. Radiotherapy is used to treat many types of cancer, but many treated patients relapse with local tumor recurrence. Tumor-infiltrating myeloid cells (TIM), including CD11b (ITGAM)(+)F4/80 (EMR1)+ tumor-associated macrophages (TAM), and CD11b(+)Gr-1 (LY6G)+ myeloid-derived suppressor cells (MDSC), respond to cancer-related stresses and play critical roles in promoting tumor angiogenesis, tissue remodeling, and immunosuppression. In this report, we used a prostate cancer model to investigate the effects of irradiation on TAMs and MDSCs in tumor-bearing animals. Unexpectedly, when primary tumor sites were irradiated, we observed a systemic increase of MDSCs in spleen, lung, lymph nodes, and peripheral blood. Cytokine analysis showed that the macrophage colony-stimulating factor CSF1 increased by two-fold in irradiated tumors. Enhanced macrophage **migration** induced by conditioned media from irradiated tumor cells was completely blocked by a selective inhibitor of CSF1R. These findings were confirmed in patients with prostate cancer, where serum levels of CSF1 increased after radiotherapy. Mechanistic investigations revealed the recruitment of the DNA damage-induced kinase ABL1 into cell nuclei where it bound the CSF1 gene promoter and enhanced CSF1 gene transcription. When added to radiotherapy, a selective inhibitor of CSF1R suppressed tumor growth more effectively than irradiation alone. Our results highlight the importance of CSF1/CSF1R signaling in the recruitment of TIMs that can limit the efficacy of radiotherapy. Furthermore, they suggest that CSF1 inhibitors should be evaluated in clinical trials in combination with radiotherapy as a strategy to improve outcomes.
8. STAP-2 regulates c-Fms/M-CSF receptor signaling in murine macrophage Raw 264.7 cells. Signal-transducing adaptor protein-2 (STAP-2) is a recently identified adaptor protein as a c-Fms/M-CSF receptor-interacting protein and constitutively expressed in macrophages. Our previous studies also revealed that STAP-2 binds to MyD88 and IKK-alpha/beta, and modulates NF-kappaB signaling in macrophages. In the present study, we examined physiological roles of the interaction between STAP-2 and c-Fms in Raw 264.7 macrophage cells. Our immunoprecipitation has revealed that c-Fms directly interacts with the PH domain of STAP-2 independently on M-CSF-stimulation. Ectopic expression of STAP-2 markedly suppressed M-CSF-induced tyrosine phosphorylation of c-Fms as well as activation of Akt and extracellular signal regulated kinase. In addition, Raw 264.7 cells over-expressing STAP-2 showed impaired **migration** in response to M-CSF and wound-healing process. Taken together, our findings demonstrate that STAP-2 directly binds to c-Fms and interferes with the PI3K signaling, which leads to macrophage motility, in Raw 264.7 cells.
9. Donor colonic CD103+ dendritic cells determine the severity of acute graft-versus-host disease. The primacy of the gastrointestinal (GI) tract in dictating the outcome of graft-versus-host disease (GVHD) is broadly accepted; however, the mechanisms controlling this effect are poorly understood. Here, we demonstrate that GVHD markedly enhances alloantigen presentation within the mesenteric lymph nodes (mLNs), mediated by donor CD103(+)CD11b(-) dendritic cells (DCs) that migrate from the colon under the influence of CCR7. Expansion and differentiation of donor T cells specifically within the mLNs is driven by profound levels of alloantigen, IL-12, and IL-6 promoted by Toll-like receptor (TLR) and receptor for advanced glycation end products (RAGE) signals. Critically, alloantigen presentation in the mLNs imprints gut-homing integrin signatures on donor T cells, leading to their e**migration** into the GI tract where they mediate fulminant disease. These data identify a critical, anatomically distinct, donor DC subset that amplifies GVHD. We thus highlight multiple therapeutic targets and the ability of GVHD, once initiated by recipient antigen-presenting cells, to generate a profound, localized, and lethal feed-forward cascade of donor DC-mediated indirect alloantigen presentation and cytokine secretion within the GI tract.
10. Prostaglandin E2 transactivates the colony-stimulating factor-1 receptor and synergizes with colony-stimulating factor-1 in the induction of macrophage **migration** via the mitogen-activated protein kinase ERK1/2. Prostaglandin E2 (PGE2), a key mediator of immunity, inflammation, and cancer, acts through 4 G-protein-coupled E-prostanoid receptors (EPs 1-4). Crosstalk between EPs and receptor tyrosine kinases also occurs. Colony-stimulating factor-1 receptor (CSF-1R) is an RTK that sustains the survival, proliferation, and motility of monocytes/macrophages, which are an essential component of innate immunity and cancer development. The aim of this study was to investigate on a possible crosstalk between EP and CSF-1R. In BAC1.2F5 and RAW264.7 murine macrophages, CSF-1 (EC₅₀ = 18.1 and 10.2 ng/ml, respectively) and PGE2 (EC₅₀ = 1.5 and 5.5 nM, respectively) p**romoted m**igration. PGE2 induced rapid CSF-1R phosphorylation that was dependent on Src family kinases (SFKs). CSF-1R inhibition reduced PGE2-elicited ERK1/2 phosphorylation and mac**rophage m**igration, indicating that CSF-1R plays a role in PGE2-mediated immunoregulation. EP4 appeared responsible for functional PGE2/CSF-1R crosstalk. Furthermore, PGE2 synergized with CSF-1 in inducing ERK1/2 phosphorylation and mac**rophage m**igration. ERK1/2 inhibition completely **blocked m**igration induced by the combination CSF-1/PGE2. CSF-1/PGE2 functional interaction with res**pect to m**igration also occurred in bone marrow-derived murine macrophages (EC₅₀ CSF-1, 6.7 ng/ml; EC₅₀ PGE2, 16.7 nM). These results indicated that PGE2 transactivates CSF-1R and synergizes with its signaling at ERK1/2 level in promo**ting macr**ophage migration.
11. Role of colony-stimulating factor-1 in bone metabolism. Colony-stimulating factor-1 (CSF-1) is a cytokine required for proliferation, differentiation, activity, and survival of cells of the mononuclear phagocytic system. The growth factor is synthesized as a soluble, matrix, or membrane associated molecule. The specific functions of these forms are not clear. However, some data suggest a dependence of the development of various populations of tissue macrophages on the locally expressed and presented cytokine. Deficiency in CSF-1, as is the case in the murine mutant strain op/op, results in low numbers of macrophages and monocytes and, most striking, leads to osteopetrosis due to a virtual absence of osteoclasts. Using the op/op mutation as a model, CSF-1 was established as one of the growth factors for osteoclasts. The expression of CSF-1 receptors, encoded by the proto-oncogene c-fms, by osteoclast precursors and osteoclasts, suggested an effect of this cytokine not only during osteoclast formation but also on the mature cells. In fact, CSF-1 was shown to inhibit the resorbing activity, to stimulate **migration**, and to support survival of isolated osteoclasts in vitro. By these actions on cells of the osteoclast lineage, CSF-1 induces recruitment of new osteoclasts, leading to a net increase of bone resorption, and might govern the spatial distribution of resorption sites within the bone. During these processes, locally expressed and presented forms of the growth factor may play a crucial role, as will be discussed in this article.
12. c-Fms signaling mediates neurofibromatosis Type-1 osteoclast gain-in-functions. Skeletal abnormalities including osteoporosis and osteopenia occur frequently in both pediatric and adult neurofibromatosis type 1 (NF1) patients. NF1 (Nf1) haploinsufficient osteoclasts and osteoclast progenitors derived from both NF1 patients and Nf1(+/-) mice exhibit increased differentiation, **migration**, and bone resorptive capacity in vitro, mediated by hyperactivation of p21(Ras) in response to limiting concentrations of macrophage-colony stimulating factor (M-CSF). Here, we show that M-CSF binding to its receptor, c-Fms, results in increased c-Fms activation in Nf1(+/) (-) osteoclast progenitors, mediating multiple gain-in-functions through the downstream effectors Erk1/2 and p90RSK. PLX3397, a potent and selective c-Fms inhibitor, attenuated M-CSF mediated Nf1(+/-) osteoclast **migration** by 50%, adhesion by 70%, and pit formation by 60%. In vivo, we administered PLX3397 to Nf1(+/-) osteoporotic mice induced by ovariectomy (OVX) and evaluated changes in bone mass and skeletal architecture. We found that PLX3397 prevented bone loss in Nf1(+/-)-OVX mice by reducing osteoclast differentiation and bone resorptive activity in vivo. Collectively, these results implicate the M-CSF/c-Fms signaling axis as a critical pathway underlying the aberrant functioning of Nf1 haploinsufficient osteoclasts and may provide a potential therapeutic target for treating NF1 associated osteoporosis and osteopenia.

#### GeneRIFs

1. Results indicate that prostaglandin E2 activates colony-stimulating factor-1 receptor (CSF-1R) and synergizes with its signaling at mitogen-activated protein kinase ERK1/2 level in promoting macrophage **migration**.

### [Ii]nvasive

#### PubMed

1. cDNA microarray analysis of **invasive** and tumorigenic phenotypes in a breast cancer model. The fms oncogene encodes the macrophage colony-stimulating factor receptor (CSF1R), a transmembrane tyrosine kinase receptor, which is abnormally expressed in breast cancer. Transfection of wild-type CSF1R into HC11 mammary epithelial cells (HC11-CSF1R) renders the transfectants capable of in vitro local invasion and in vivo tumorigenesis. Transfection with CSF1R mutated to express phe at the tyr-721 autophosphorylation site (HC11-CSF1R-721) creates a phenotype that lacks metastastic competence but maintains local **invasive**ness. Conversely, HC11 cells transfected with CSF1R mutated at tyr-807 (HC11-CSF1R-807) retain their metastatic competence, but are not locally **invasive**. Our aims were to determine which genes were differentially expressed with transfection of HC11 with wild-type CSF1R, and to determine the effect of mutation at the autophosphorylation sites on gene expression, using 4.6 K cDNA microarrays. Complementary DNA from HC11, HC11-CSF1R-721 and HC11-CSF1R-807 were each hybridized together with HC11-CSF1R on individual arrays. A principal component spectral method combined with prenormalization procedures was used for sample clustering. Differentially expressed genes were identified by the analysis of variance. Confirmation by Northern blotting was performed for MAP kinase phosphatase-1, WDNM1 (extracellular proteinase inhibitor), Trop 2 (tumor-associated calcium signal transducer-2), procollagen type IV alpha, secretory leukoprotease inhibitor, prenylated snare protein Ykt6, ceruloplasmin and chaperonin 10. Many of these genes have not previously been associated with tumor invasion and metastasis. We have successfully identified genes that can be linked to the **invasive** phenotypes or to tumorigenesis. These genes provide a basis for further studies of metastatic progression and local **invasive**ness, and can be evaluated as therapeutic targets.

#### GeneRIFs

1. Transfection into HC11 mammary epithelial cells with CSF1R mutated to express phe at the tyr-721 autophosphorylation site (HC11-CSF1R-721) creates a phenotype that lacks metastastic competence but maintains local **invasive**ness.

### [Mm]etastasis

#### PubMed

1. A c-fms tyrosine kinase inhibitor, Ki20227, suppresses osteoclast differentiation and osteolytic bone destruction in a bone **metastasis** model. In bone metastatic lesions, osteoclasts play a key role in the development of osteolysis. Previous studies have shown that macrophage colony-stimulating factor (M-CSF) is important for the differentiation of osteoclasts. In this study, we investigated whether an inhibitor of M-CSF receptor (c-Fms) suppresses osteoclast-dependent osteolysis in bone metastatic lesions. We developed small molecule inhibitors against ligand-dependent phosphorylation of c-Fms and examined the effects of these compounds on osteolytic bone destruction in a bone **metastasis** model. We discovered a novel quinoline-urea derivative, Ki20227 (N-{4-[(6,7-dimethoxy-4-quinolyl)oxy]-2-methoxyphenyl}-N'-[1-(1,3-thiazole-2-yl)ethyl]urea), which is a c-Fms tyrosine kinase inhibitor. The IC(50)s of Ki20227 to inhibit c-Fms, vascular endothelial growth factor receptor-2 (KDR), stem cell factor receptor (c-Kit), and platelet-derived growth factor receptor beta were found to be 2, 12, 451, and 217 nmol/L, respectively. Ki20227 did not inhibit other kinases tested, such as fms-like tyrosine kinase-3, epidermal growth factor receptor, or c-Src (c-src proto-oncogene product). Ki20227 was also found to inhibit the M-CSF-dependent growth of M-NFS-60 cells but not the M-CSF-independent growth of A375 human melanoma cells in vitro. Furthermore, in an osteoclast-like cell formation assay using mouse bone marrow cells, Ki20227 inhibited the development of tartrate-resistant acid phosphatase-positive osteoclast-like cells in a dose-dependent manner. In in vivo studies, oral administration of Ki20227 suppressed osteoclast-like cell accumulation and bone resorption induced by metastatic tumor cells in nude rats following intracardiac injection of A375 cells. Moreover, Ki20227 decreased the number of tartrate-resistant acid phosphatase-positive osteoclast-like cells on bone surfaces in ovariectomized (ovx) rats. These findings suggest that Ki20227 inhibits osteolytic bone destruction through the suppression of M-CSF-induced osteoclast accumulation in vivo. Therefore, Ki20227 may be a useful therapeutic agent for osteolytic disease associated with bone **metastasis** and other bone diseases.
2. CCL2-induced chemokine cascade promotes breast cancer **metastasis** by enhancing retention of **metastasis**-associated macrophages. Pulmonary **metastasis** of breast cancer cells is promoted by a distinct population of macrophages, **metastasis**-associated macrophages (MAMs), which originate from inflammatory monocytes (IMs) recruited by the CC-chemokine ligand 2 (CCL2). We demonstrate here that, through activation of the CCL2 receptor CCR2, the recruited MAMs secrete another chemokine ligand CCL3. Genetic deletion of CCL3 or its receptor CCR1 in macrophages reduces the number of lung **metastasis** foci, as well as the number of MAMs accumulated in tumor-challenged lung in mice. Adoptive transfer of WT IMs increases the reduced number of lung **metastasis** foci in Ccl3 deficient mice. Mechanistically, Ccr1 deficiency prevents MAM retention in the lung by reducing MAM-cancer cell interactions. These findings collectively indicate that the CCL2-triggered chemokine cascade in macrophages promotes metastatic seeding of breast cancer cells thereby amplifying the pathology already extant in the system. These data suggest that inhibition of CCR1, the distal part of this signaling relay, may have a therapeutic impact in metastatic disease with lower toxicity than blocking upstream targets.
3. cDNA microarray analysis of invasive and tumorigenic phenotypes in a breast cancer model. The fms oncogene encodes the macrophage colony-stimulating factor receptor (CSF1R), a transmembrane tyrosine kinase receptor, which is abnormally expressed in breast cancer. Transfection of wild-type CSF1R into HC11 mammary epithelial cells (HC11-CSF1R) renders the transfectants capable of in vitro local invasion and in vivo tumorigenesis. Transfection with CSF1R mutated to express phe at the tyr-721 autophosphorylation site (HC11-CSF1R-721) creates a phenotype that lacks metastastic competence but maintains local invasiveness. Conversely, HC11 cells transfected with CSF1R mutated at tyr-807 (HC11-CSF1R-807) retain their metastatic competence, but are not locally invasive. Our aims were to determine which genes were differentially expressed with transfection of HC11 with wild-type CSF1R, and to determine the effect of mutation at the autophosphorylation sites on gene expression, using 4.6 K cDNA microarrays. Complementary DNA from HC11, HC11-CSF1R-721 and HC11-CSF1R-807 were each hybridized together with HC11-CSF1R on individual arrays. A principal component spectral method combined with prenormalization procedures was used for sample clustering. Differentially expressed genes were identified by the analysis of variance. Confirmation by Northern blotting was performed for MAP kinase phosphatase-1, WDNM1 (extracellular proteinase inhibitor), Trop 2 (tumor-associated calcium signal transducer-2), procollagen type IV alpha, secretory leukoprotease inhibitor, prenylated snare protein Ykt6, ceruloplasmin and chaperonin 10. Many of these genes have not previously been associated with tumor invasion and **metastasis**. We have successfully identified genes that can be linked to the invasive phenotypes or to tumorigenesis. These genes provide a basis for further studies of metastatic progression and local invasiveness, and can be evaluated as therapeutic targets.

### [Cc]ell [Ii]nvasion

#### PubMed

1. The role of CSF-1 in normal and neoplastic breast physiology. Colony stimulating factor (CSF-1) and its receptor (CSF-1R, product of c-fms proto-oncogene) were initially implicated as essential for normal monocyte development as well as for trophoblastic implantation. However, recent findings have suggested that CSF-1 and CSF-1R might have additional roles in mammary gland development during pregnancy and lactation. Studies with osteopetrotic (op-/op-) mice, which bear a specific mutation that inactivates the CSF-1 gene, demonstrated that op-/op- mothers are incapable of normal milk production due to the incomplete development of their mammary glands during pregnancy. Also, significant increases in the levels of CSF-1 and CSF-1R proteins are observed in the epithelial cells of mammary gland during pregnancy and lactation. In vitro studies investigating the effect of the three major lactogenic hormones (prolactin, insulin, and glucocorticoids) on the expression of CSF-1 and CSF-1R have demonstrated that expression of CSF-1 can be regulated by prolactin and insulin whereas CSF-1R expression is regulated by glucocorticoids. This apparent role for CSF-1/CSF-1R in normal mammary gland development is very intriguing because this receptor/ligand pair has also been found to be important in the biology of breast cancer, where they regulate tumor **cell invasion** by a urokinase-dependent mechanism. This review aims to summarize recent findings on the role of CSF-1 and its receptor in normal and neoplastic mammary development which may elucidate potential relationships of growth factor-induced biological changes in the breast during pregnancy and tumor progression.
2. Phosphorylation of CSF-1R Y721 mediates its association with PI3K to regulate macrophage motility and enhancement of tumor **cell invasion**. Colony stimulating factor-1 (CSF-1) regulates macrophage morphology and motility, as well as mononuclear phagocytic cell proliferation and differentiation. The CSF-1 receptor (CSF-1R) transduces these pleiotropic signals through autophosphorylation of eight intracellular tyrosine residues. We have used a novel bone-marrow-derived macrophage cell line system to examine specific signaling pathways activated by tyrosine-phosphorylated CSF-1R in macrophages. Screening of macrophages expressing a single species of CSF-1R with individual tyrosine-to-phenylalanine residue mutations revealed striking morphological alterations upon mutation of Y721. M⁻/⁻.Y721F cells were apolar and ruffled poorly in response to CSF-1. Y721-P-mediated CSF-1R signaling regulated adhesion and actin polymerization to control macrophage spreading and motility. Moreover, the reduced motility of M⁻/⁻.Y721F macrophages was associated with their reduced capacity to enhance ca**rcinoma cell** invasion. Y721 phosphorylation mediated the direct association of the p85 subunit of phosphoinositide 3-kinase (PI3K) with the CSF-1R, but not that of phospholipase C (PLC) γ2, and induced polarized PtdIns(3,4,5)P₃ production at the putative leading edge, implicating PI3K as a major regulator of CSF-1-induced macrophage motility. The Y721-P-motif-based motility signaling was at least partially independent of both Akt and increased Rac and Cdc42 activation but mediated the rapid and transient association of an unidentified ~170 kDa phosphorylated protein with either Rac-GTP or Cdc42-GTP. These studies identify CSF-1R-Y721-P-PI3K signaling as a major pathway in CSF-1-regulated macrophage motility and provide a starting point for the discovery of the immediate downstream signaling events.
3. Microglial stimulation of glioblastoma invasion involves epidermal growth factor receptor (EGFR) and colony stimulating factor 1 receptor (CSF-1R) signaling. Glioblastoma multiforme is a deadly cancer for which current treatment options are limited. The ability of glioblastoma tumor cells to infiltrate the surrounding brain parenchyma critically limits the effectiveness of current treatments. We investigated how microglia, the resident macrophages of the brain, stimulate glioblastoma **cell invasion**. We first examined the ability of normal microglia from C57Bl/6J mice to stimulate GL261 glioblastoma **cell invasion** in vitro. We found that microglia stimulate the invasion of GL261 glioblastoma cells by approximately eightfold in an in vitro invasion assay. Pharmacological inhibition of epidermal growth factor receptor (EGFR) strongly inhibited microglia-stimulated invasion. Furthermore, blockade of colony stimulating factor 1 receptor (CSF-1R) signaling using ribonucleic acid (RNA) interference or pharmacological inhibitors completely inhibited microglial enhancement of glioblastoma invasion. GL261 cells were found to constitutively secrete CSF-1, the levels of which were unaffected by epidermal growth factor (EGF) stimulation, EGFR inhibition or coculture with microglia. CSF-1 only stimulated microglia invasion, whereas EGF only stimulated glioblastoma cell migration, demonstrating a synergistic interaction between these two cell types. Finally, using PLX3397 (a CSF-1R inhibitor that can cross the blood-brain barrier) in live animals, we discovered that blockade of CSF-1R signaling in vivo reduced the number of tumor-associated microglia and glioblastoma invasion. These data indicate that glioblastoma and microglia interactions mediated by EGF and CSF-1 can enhance glioblastoma invasion and demonstrate the possibility of inhibiting glioblastoma invasion by targeting glioblastoma-associated microglia via inhibition of the CSF-1R.

---

## Flt3

- NCBI Gene
- GeneRIF
- Pubmed

| Search Term | Rank | Fields |
| --- | --- | --- |
| [Mm]igration | 6 | PubMed(4); GeneRIFs(1) |
| [Ee]xtracellular [Mm]atrix | 8 | PubMed(1) |
| [Ii]nvasive | 10 | PubMed(1) |

  
hide/show details for Flt3

### [Mm]igration

#### PubMed

1. The granulocyte colony-stimulating factor receptor is required for the mobilization of murine hematopoietic progenitors into peripheral blood by cyclophosphamide or interleukin-8 but not flt-3 ligand. Hematopoietic progenitor cells (HPC) can be mobilized from the bone marrow into the peripheral circulation in response to a number of stimuli including hematopoietic growth factors, cytotoxic agents, and certain chemokines. Despite significant differences in their biological activities, these stimuli result in the mobilization of HPC with a similar phenotype, suggesting that a common mechanism for mobilization may exist. In this study, the role of granulocyte colony-stimulating factor (G-CSF) in progenitor mobilization was examined using G-CSF receptor (G-CSFR)-deficient mice. In contrast to wild-type mice, no increase in circulating colony-forming cells (CFU-C), CD34+ lineage- progenitors, or day 12 colony-forming unit-spleen progenitors (CFU-S) was detected in G-CSFR-deficient mice after cyclophosphamide administration. This defect was not due to a failure to regenerate HPC following cyclophosphamide administration as the number of CFU-C in the bone marrow of G-CSFR-deficient mice was increased relative to wild-type mice. Likewise, no increase in circulating CFU-C was detected in G-CSFR-deficient mice following interleukin-8 (IL-8) administration. In contrast, mobilization of HPC in response to flt-3 ligand was nearly normal. These results show that the G-CSFR is required for mobilization in response to cyclophosphamide or IL-8 but not flt-3 ligand and suggest that the G-CSFR may play an important and previously unexpected role in HPC **migration**.
2. Internal tandem duplication of Flt3 modulates chemotaxis and survival of hematopoietic cells by SDF1alpha but negatively regulates marrow homing in vivo. We have previously shown that Flt3 ligand (FL)/Flt3 signaling regulates hematopoietic cell **migration** by modulating the SDF1alpha/CXCR4 signaling pathway. Herein, we evaluated whether a functional interaction between SDF1alpha/CXCR4 signaling and internal tandem duplication (ITD) of Flt3 regulates aberrant hematopoietic survival. We also investigated molecular mechanisms responsible for enhanced **migration** to SDF1alpha as a result of ITD-Flt3 expression and whether ITD-Flt3 regulates hematopoietic cell trafficking.Hematopoietic progenitor cell survival was determined using marrow cells retrovirally expressing ITD-Flt3 and stimulated with SDF1alpha. **Migration**, to SDF1alpha adhesion to vascular cell adhesion molecule-1, and in vivo homing were determined using Ba/F3 cells expressing ITD-Flt3 and transfected with dominant negative (DN) H-Ras.Addition of SDF1alpha significantly increased growth factor-independent proliferation of colony-forming unit granulocyte-macrophage induced by ITD-Flt3. Although a negative gradient of SDF1alpha inhibited **migration** regardless of the stimulation, a positive gradient of FL or ITD-Flt3 significantly increased cell **migration** even in the presence of a negative SDF1alpha gradient. Enhanced **migration** induced by ITD-Flt3 was inhibited by DN-H-Ras, whereas overexpression of a constitutive active form of H-Ras in wild-type Flt3-Ba/F3 cells significantly elevated **migration** to SDF1alpha. Despite enhanced **migration** to SDF1alpha, preincubation with FL or ITD-Flt3 overexpression significantly reduced homing of primary mouse bone marrow cells or Ba/F3 cells to bone marrow that was associated with significant reduction in adhesion to vascular cell adhesion molecule-1 and VLA4 expression.Our results suggest that functional interactions between Flt3 and SDF1alpha/CXCR4 regulate oncogenic proliferation and **migration** of hematopoietic cells, which is mediated by Ras, and that Flt3 signaling regulates hematopoietic cell trafficking in vivo.
3. Migratory properties of pulmonary dendritic cells are determined by their developmental lineage. The chemokine receptor, CCR7, directs the **migration** of dendritic cells (DCs) from peripheral tissue to draining lymph nodes (LNs). However, it is unknown whether all pulmonary DCs possess migratory potential. Using novel Ccr7(gfp) reporter mice, we found that Ccr7 is expressed in CD103⁺ and a CD14(med/lo) subset of CD11b(hi) classical (c)DCs but not in monocyte-derived (mo)DCs, including Ly-6C(hi)CD11b(hi) inflammatory DCs and CD14(hi)CD11b(hi) DCs. Consequently, cDCs migrated to lung-draining LNs but moDCs did not. Mice lacking the chemokine receptor, CCR2, also lacked inflammatory DCs in the lung after lipopolysaccharide inhalation but retained normal levels of migratory DCs. Conversely, the lungs of fms-like tyrosine kinase 3 ligand (Flt3L)-deficient mice lacked cDCs but retained moDCs, which were functionally mature but did not express Ccr7 and were uniformly non-migratory. Thus, the migratory properties of pulmonary DCs are determined by their developmental lineage.
4. Differentiation of mouse bone marrow derived stem cells toward microglia-like cells. Microglia, the macrophages of the brain, have been implicated in the causes of neurodegenerative diseases and display a loss of function during aging. Throughout life, microglia are replenished by limited proliferation of resident microglial cells. Replenishment by bone marrow-derived progenitor cells is still under debate. In this context, we investigated the differentiation of mouse microglia from bone marrow (BM) stem cells. Furthermore, we looked at the effects of FMS-like tyrosine kinase 3 ligand (Flt3L), astrocyte-conditioned medium (ACM) and GM-CSF on the differentiation to microglia-like cells.We assessed in vitro-derived microglia differentiation by marker expression (CD11b/CD45, F4/80), but also for the first time for functional performance (phagocytosis, oxidative burst) and in situ **migration** into living brain tissue. Integration, survival and **migration** were assessed in organotypic brain slices.The cells differentiated from mouse BM show function, markers and morphology of primary microglia and migrate into living brain tissue. Flt3L displays a negative effect on differentiation while GM-CSF enhances differentiation.We conclude that in vitro-derived microglia are the phenotypic and functional equivalents to primary microglia and could be used in cell therapy.

#### GeneRIFs

1. functional interactions between Flt3 and SDF1alpha/CXCR4 regulate oncogenic proliferation and **migration** of hematopoietic cells, which is mediated by Ras, and that Flt3 signaling regulates hematopoietic cell trafficking in vivo.

### [Ee]xtracellular [Mm]atrix

#### PubMed

1. Importance of lymph vessels in the transcapillary fluid balance in the gingiva studied in a transgenic mouse model. The gingiva is frequently challenged by oral bacterial products leading to inflammatory responses such as increased fluid filtration and edema formation. The role of initial lymphatics for transcapillary fluid balance in the gingiva is unknown and was therefore investigated in genetically engineered K14-VEGF receptor 3-Ig (K14) lymphedema mice. The mutant mice demonstrated a total lack of lymphatics in the gingiva, whereas lymphatics were found in the submucosal parts of the alveolar mucosa, although they were almost completely absent in the mucosa. In wild-type (WT) mice, lymphatic vessels were detected in mucosal and submucosal parts of the alveolar mucosa. Interstitial fluid pressure (P(if)) measured with micropipettes was increased in the gingiva of K14 mice in the normal situation (P < 0.001) and after inflammation (P < 0.01) induced by lipopolysaccharide from the oral bacteria Porphyromonas gingivalis compared with WT littermates. Fluid volume expansion caused a >75% increase in interstitial fluid volume followed by a drop in P(if) after recovery in both strains. Continuous measurements during the expansion showed an increase in P(if) followed by a decline, suggesting that compliance is increased after the disruption of the **extracellular matrix** during edema formation. In the alveolar mucosa, no strain differences were observed in P(if) in the normal situation or after fluid volume expansion, suggesting that lymph vessels in the mucosa are not critical for tissue fluid regulation in any situation. Our study demonstrates an important role of gingival lymphatics in transcapillary fluid balance in the steady-state condition and during acute perturbations.

### [Ii]nvasive

#### PubMed

1. Luminal bacteria recruit CD103+ dendritic cells into the intestinal epithelium to sample bacterial antigens for presentation. CD103+ dendritic cells (DCs) carry bacteria from the small intestine and can present antigens to T cells. Yet they have not been recorded sampling luminal bacteria or presenting bacterial antigens in mesentery lymph nodes. We used 2-photon microscopy in live Cx3cr1(+/gfp) ×Cd11c-YFP mice to study these processes. At steady state, sparse CD103+ DCs occupied the epithelium. They patrolled among enterocytes while extending dendrites toward the lumen, likely using tight-junction proteins to penetrate the epithelium. Challenge with Salmonella triggered chemokine- and toll-like receptor (TLR)-dependent recruitment of additional DCs from the lamina propria (LP). The DCs efficiently phagocytosed the bacteria using intraepithelial dendrites. No**ninvasiv**e bacteria were similarly sampled. In contrast, CD103+ DCs sampled soluble luminal antigen inefficiently. In mice harboring CD103+ DCs, antigen-specific CD8 T cells were subsequently activated in MLNs. Intestinal CD103+ DCs are therefore equipped with unique mechanisms to independently complete the processes of uptake, transportation, and presentation of bacterial antigens.

---

## Ret

- NCBI Gene
- GeneRIF
- Pubmed

| Search Term | Rank | Fields |
| --- | --- | --- |
| [Mm]igration | 6 | Process(2); PubMed(57); GeneRIFs(3) |
| ECM | 7 | PubMed(1) |
| [Ee]xtracellular [Mm]atrix | 8 | PubMed(5) |
| [Ii]nvasive | 10 | PubMed(1) |
| [Mm]etastasize | 11 | PubMed(1) |
| [Mm]etastasis | 12 | PubMed(2); GeneRIFs(1) |

  
hide/show details for Ret

### [Mm]igration

#### Process

1. neural crest cell **migration**
2. positive regulation of cell **migration**

#### PubMed

1. Aebp2 as an epigenetic regulator for neural crest cells. Aebp2 is a potential targeting protein for the mammalian Polycomb Repression Complex 2 (PRC2). We generated a mutant mouse line disrupting the transcription of Aebp2 to investigate its in vivo roles. Aebp2-mutant homozygotes were embryonic lethal while heterozygotes survived to adulthood with fertility. In developing mouse embryos, Aebp2 is expressed mainly within cells of neural crest origin. In addition, many heterozygotes display a set of phenotypes, enlarged colon and hypopigmentation, similar to those observed in human patients with Hirschsprung's disease and Waardenburg syndrome. These phenotypes are usually caused by the absence of the neural crest-derived ganglia in hindguts and melanocytes. ChIP analyses demonstrated that the majority of the genes involved in the **migration** and development process of neural crest cells are downstream target genes of AEBP2 and PRC2. Furthermore, expression analyses confirmed that some of these genes are indeed affected in the Aebp2 heterozygotes. Taken together, these results suggest that Aebp2 may regulate the **migration** and development of the neural crest cells through the PRC2-mediated epigenetic mechanism.
2. BMP signaling regulates murine enteric nervous system precursor **migration**, neurite fasciculation, and patterning via altered Ncam1 polysialic acid addition. The enteric nervous system (ENS) forms from migrating neural crest-derived precursors that differentiate into neurons and glia, aggregate into ganglion cell clusters, and extend neuronal processes to form a complex interacting network that controls many aspects of intestinal function. Bone morphogenetic proteins (BMPs) have diverse roles in development and influence the differentiation, proliferation, and survival of ENS precursors. We hypothesized that BMP signaling might also be important for the ENS precursor **migration**, ganglion cell aggregation, and neurite fasciculation necessary to form the enteric nervous system. We now demonstrate that BMP signaling restricts murine ENS precursors to the outer bowel wall during **migration**. In addition, blocking BMP signaling causes faster colonization of the murine colon, reduces ganglion cell aggregation, and reduces neurite fasciculation. BMP signaling also influences patterns of neurite extension within the developing bowel wall. These effects on ENS precursor **migration** and neurite fasciculation appear to be mediated at least in part by increased polysialic acid addition to neural cell adhesion molecule (Ncam1) in response to BMP. Removing PSA enzymatically reverses the BMP effects on ENS precursor **migration** and neurite fasciculation. These studies demonstrate several novel roles for BMP signaling and highlight new functions for sialyltransferases in the developing ENS.
3. Enteric nervous system progenitors are coordinately controlled by the G protein-coupled receptor EDNRB and the receptor tyrosine kinase RET. The enteric nervous system (ENS) in vertebrates is derived mainly from vagal neural crest cells that enter the foregut and colonize the entire wall of the gastrointestinal tract. Failure to completely colonize the gut results in the absence of enteric ganglia (Hirschsprung's disease). Two signaling systems mediated by RET and EDNRB have been identified as critical players in enteric neurogenesis. We demonstrate that interaction between these signaling pathways controls ENS development throughout the intestine. Activation of EDNRB specifically enhances the effect of RET signaling on the proliferation of uncommitted ENS progenitors. In addition, we reveal novel antagonistic roles of these pathways on the **migration** of ENS progenitors. Protein kinase A is a key component of the molecular mechanisms that integrate signaling by the two receptors. Our data provide strong evidence that the coordinate and balanced interaction between receptor tyrosine kinases and G protein-coupled receptors controls the development of the nervous system in mammals.
4. Balancing neural crest cell intrinsic processes with those of the microenvironment in Tcof1 haploinsufficient mice enables complete enteric nervous system formation. The enteric nervous system (ENS) comprises a complex neuronal network that regulates peristalsis of the gut wall and secretions into the lumen. The ENS is formed from a multipotent progenitor cell population called the neural crest, which is derived from the neuroepithelium. Neural crest cells (NCCs) migrate over incredible distances to colonize the entire length of the gut and during their **migration** they must survive, proliferate and ultimately differentiate. The absence of an ENS from variable lengths of the colon results in Hirschsprung's disease (HSCR) or colonic aganglionosis. Mutations in about 12 different genes have been identified in HSCR patients but the complex pattern of inheritance and variable penetrance suggests that additional genes or modifiers must be involved in the etiology and pathogenesis of this disease. We discovered that Tcof1 haploinsufficiency in mice models many of the early features of HSCR. Neuroepithelial apoptosis diminished the size of the neural stem cell pool resulting in reduced NCC numbers and their delayed **migration** along the gut from E10.5 to E14.5. Surprisingly however, we observe continued and complete colonization of the entire colon throughout E14.5-E18.5, a period in which the gut is considered to be non- or less-permissive to NCC. Thus, we reveal for the first time that reduced NCC progenitor numbers and delayed **migration** do not unequivocally equate with a predisposition for the pathogenesis of HSCR. In fact, these deficiencies can be overcome by balancing NCC intrinsic processes of proliferation and differentiation with extrinsic influences of the gut microenvironment.
5. Mouse models of Hirschsprung disease and other developmental disorders of the enteric nervous system: Old and new players. Hirschsprung disease (HSCR, intestinal aganglionosis) is a multigenic disorder with variable penetrance and severity that has a general population incidence of 1/5000 live births. Studies using animal models have contributed to our understanding of the developmental origins of HSCR and the genetic complexity of this disease. This review summarizes recent progress in understanding control of enteric nervous system (ENS) development through analyses in mouse models. An overview of signaling pathways that have long been known to control the **migration**, proliferation and differentiation of enteric neural progenitors into and along the developing gut is provided as a framework for the latest information on factors that influence enteric ganglia formation and maintenance. Newly identified genes and additional factors beyond discrete genes that contribute to ENS pathology including regulatory sequences, miRNAs and environmental factors are also introduced. Finally, because HSCR has become a paradigm for complex oligogenic diseases with non-Mendelian inheritance, the importance of gene interactions, modifier genes, and initial studies on genetic background effects are outlined.
6. Phactr4 regulates directional **migration** of enteric neural crest through PP1, integrin signaling, and cofilin activity. Hirschsprung disease (HSCR) is caused by a reduction of enteric neural crest cells (ENCCs) in the gut and gastrointestinal blockage. Knowledge of the genetics underlying HSCR is incomplete, particularly genes that control cellular behaviors of ENCC **migration**. Here we report a novel regulator of ENCC **migration** in mice. Disruption of the Phactr4 gene causes an embryonic gastrointestinal defect due to colon hypoganglionosis, which resembles human HSCR. Time-lapse imaging of ENCCs within the embryonic gut demonstrates a collective cell **migration** defect. Mutant ENCCs show undirected cellular protrusions and disrupted directional and chain **migration**. Phactr4 acts cell-autonomously in ENCCs and colocalizes with integrin and cofilin at cell protrusions. Mechanistically, we show that Phactr4 negatively regulates integrin signaling through the RHO/ROCK pathway and coordinates protein phosphatase 1 (PP1) with cofilin activity to regulate cytoskeletal dynamics. Strikingly, lamellipodia formation and in vivo ENCC chain **migration** defects are rescued by inhibition of ROCK or integrin function. Our results demonstrate a previously unknown pathway in ENCC collective **migration** in vivo and provide new candidate genes for human genetic studies of HSCR.
7. Hand2 is necessary for terminal differentiation of enteric neurons from crest-derived precursors but not for their **migration** into the gut or for formation of glia. Hand genes encode basic helix-loop-helix transcription factors that are expressed in the developing gut, where their function is unknown. We now report that enteric Hand2 expression is limited to crest-derived cells, whereas Hand1 expression is restricted to muscle and interstitial cells of Cajal. Hand2 is developmentally regulated and is intranuclear in precursors but cytoplasmic in neurons. Neurons develop in explants from wild-type but not Hand2(-/-) bowel, although, in both, crest-derived cells are present and glia arise. Similarly, small interfering RNA (siRNA) silencing of Hand2 in enteric crest-derived cells prevents neuronal development. Terminally differentiated enteric neurons do not develop after conditional inactivation of Hand2 in migrating crest-derived cells; nevertheless, conditional Hand2 inactivation does not prevent precursors from expressing early neural markers. We suggest that enteric neuronal development occurs in stages and that Hand2 expression is required for terminal differentiation but not for precursors to enter the neuronal lineage.
8. Ongoing roles of Phox2 homeodomain transcription factors during neuronal differentiation. Transcriptional determinants of neuronal identity often stay expressed after their downstream genetic program is launched. Whether this maintenance of expression plays a role is for the most part unknown. Here, we address this question for the paralogous paired-like homeobox genes Phox2a and Phox2b, which specify several classes of visceral neurons at the progenitor stage in the central and peripheral nervous systems. By temporally controlled inactivation of Phox2b, we find that the gene, which is required in ventral neural progenitors of the hindbrain for the production of branchio-visceral motoneuronal precursors, is also required in these post-mitotic precursors to maintain their molecular signature - including downstream transcription factors - and allow their tangential **migration** and the histogenesis of the corresponding nuclei. Similarly, maintenance of noradrenergic differentiation during embryogenesis requires ongoing expression of Phox2b in sympathetic ganglia, and of Phox2a in the main noradrenergic center, the locus coeruleus. These data illustrate cases where the neuronal differentiation program does not unfold as a transcriptional `cascade' whereby downstream events are irreversibly triggered by an upstream regulator, but instead require continuous transcriptional input from it.
9. GFR alpha3, a component of the artemin receptor, is required for **migration** and survival of the superior cervical ganglion. GFR alpha3 is a component of the receptor for the neurotrophic factor artemin. The role of GFR alpha3 in nervous system development was examined by generating mice in which the Gfr alpha3 gene was disrupted. The Gfr alpha3-/- mice exhibited severe defects in the superior cervical ganglion (SCG), whereas other ganglia appeared normal. SCG precursor cells in the mutant embryos failed to migrate to the correct position, and they subsequently failed to innervate the target organs. In wild-type embryos, Gfr alpha3 was expressed in migrating SCG precursors, and artemin was expressed in and near the SCG. After birth, SCG neurons in the mutant mice underwent progressive cell death. These observations suggest that GFR alpha3-mediated signaling is required both for the rostral **migration** of SCG precursors and for the survival of mature SCG neurons.
10. Sox11 regulates survival and axonal growth of embryonic sensory neurons. Sensory neurons transduce various stimuli including temperature, pain, and touch from the periphery to the central nervous system. Sensory neuron development is governed by a combination of extracellular cues and specific gene expression. We demonstrated that the transcription factor Sox11 was highly expressed in the developing sensory neurons. To test the function of Sox11, we used a knockin mouse model where the entire coding region of Sox11 was replaced by a LacZ reporter. The ablation of Sox11 caused severe reduction in sensory neuron survival in the trigeminal and dorsal root ganglia, although it did not affect **migration** of neural crest cells or acquisition of major sensory neuron subtypes. We further demonstrated that ablating Sox11 caused an arrest of axonal outgrowth in vivo and in vitro. This defect could not be fully rescued by blocking cell death. Our data suggest that Sox11 is a key regulator of sensory neuron development.
11. Neural precursor death is central to the pathogenesis of intestinal aganglionosis in Ret hypomorphic mice. The RET tyrosine kinase is required for the **migration**, proliferation, and survival of the enteric neural crest-derived cells (ENCCs) that form the enteric nervous system (ENS). Hypomorphic RET alleles cause intestinal aganglionosis [Hirschsprung disease (HSCR)], in which delayed **migration** and successive nonapoptotic ENCC death are considered to be major contributory factors. The significance of ENCC death in intestinal aganglionosis, however, has remained unclear. We show that elevated expression of Bcl-xL inhibits ENCC death in both Ret-null and hypomorphic states. However, the rescued Ret-null mice showed ENS malfunction with reduced nitric oxide synthase expression in colonic neurons, revealing the requirement of RET for neuronal differentiation. In contrast, the inhibition of cell death allows morphologically and functionally normal ENS formation in Ret hypomorphic mice. These results indicate that ENCC death is a principal cause of intestinal aganglionosis in a Ret hypomorphic state, and suggest that the inhibition of cell death is a route to the prevention of HSCR.
12. Effects of different regions of the developing gut on the **migration** of enteric neural crest-derived cells: a role for Sema3A, but not Sema3F. The enteric nervous system arises from vagal (caudal hindbrain) and sacral level neural crest-derived cells that migrate into and along the developing gut. Data from previous studies have suggested that (i) there may be gradients along the gut that induce the caudally directed **migration** of vagal enteric neural precursors (ENPs), (ii) exposure to the caecum might alter the migratory ability of vagal ENPs and (iii) Sema3A might regulate the entry into the hindgut of ENPs derived from sacral neural crest. Using co-cultures we show that there is no detectable gradient of chemoattractive molecules along the pre-caecal gut that specifically promotes the caudally directed **migration** of vagal ENPs, although vagal ENPs migrate faster caudally than rostrally along explants of hindgut. Exposure to the caecum did not alter the rate at which ENPs colonized explants of hindgut, but it did alter the ability of ENPs to colonize the midgut. The co-cultures also revealed that there is localized expression of a repulsive cue in the distal hindgut, which might delay the entry of sacral ENPs. We show that Sema3A is expressed by the hindgut mesenchyme and its receptor, neuropilin-1, is expressed by migrating ENPs. Furthermore, there is premature entry of sacral ENPs and extrinsic axons into the distal hindgut of fetal mice lacking Sema3A. These data show that Sema3A expressed by the distal hindgut regulates the entry of sacral ENPs and extrinsic axons into the hindgut. ENPs did not express neuropilin-2 and there was no detectable change in the timetable by which ENPs colonize the gut in mice lacking neuropilin-2.
13. Absence of the vagus nerve in the stomach of Tbx1-/- mutant mice. Tbx1 is a member of the Tbox family of binding domain transcription factors. TBX1 maps within the region of chromosome 22q11 deleted in humans with DiGeorge syndrome (DGS), a common genetic disorder characterized by numerous physical manifestations including craniofacial and cardiac anomalies. Mice with homozygous null mutations in Tbx1 phenocopy this disorder and have defects including abnormal cranial ganglia formation and cardiac neural crest cell **migration**. These defects prompted us to investigate whether extrinsic vagus nerve or intrinsic enteric nervous system abnormalities are prevalent in the gastrointestinal tract of Tbx1 mutant mice.We used in situ hybridization for Ret, and immunohistochemical staining for neurofilament, HuC/D and βIII-tubulin to study cranial ganglia, vagus nerve, and enteric nervous system development in Tbx1 mutant and control mice.In Tbx1(-/-) embryos, cranial ganglia of the glossopharyngeal (IXth) and vagus (Xth) nerves were malformed and abnormally fused. In the gastrointestinal tract, the vagus nerves adjacent to the esophagus were severely hypoplastic and they did not extend beyond the gastro-esophageal junction nor project branches within the stomach wall, as was observed in Tbx1(+/+) mice.Although cranial ganglia morphology appeared normal in Tbx1(+/-) mice, these animals had a spectrum of stomach vagus innervation defects ranging from mild to severe. In all Tbx1 genotypes, the intrinsic enteric nervous system developed normally. The deficit in vagal innervation of the stomach in mice mutant for a gene implicated in DGS raises the possibility that similar defects may underlie a number of as yet unidentified/unreported congenital disorders affecting gastrointestinal function.
14. RET signaling is essential for **migration**, axonal growth and axon guidance of developing sympathetic neurons. Sympathetic axons use blood vessels as an intermediate path to reach their final target tissues. The initial contact between differentiating sympathetic neurons and blood vessels occurs following the primary sympathetic chain formation, where precursors of sympathetic neurons migrate and project axons along or toward blood vessels. We demonstrate that, in Ret-deficient mice, neuronal precursors throughout the entire sympathetic nervous system fail to migrate and project axons properly. These primary deficits lead to mis-routing of sympathetic nerve trunks and accelerated cell death of sympathetic neurons later in development. Artemin is expressed in blood vessels during periods of early sympathetic differentiation, and can promote and attract axonal growth of the sympathetic ganglion in vitro. This analysis identifies RET and artemin as central regulators of early sympathetic innervation.
15. Loss of the Prader-Willi syndrome protein necdin causes defective **migration**, axonal outgrowth, and survival of embryonic sympathetic neurons. Prader-Willi syndrome is a neurodevelopmental disorder marked by abnormalities in feeding, drinking, thermoregulation, intestinal motility, and reproduction, suggesting disruption of the autonomic nervous system. Necdin, one of several proteins genetically inactivated in individuals with Prader-Willi syndrome, is important for the differentiation of central and sensory neurons. We now show that formation, **migration**, and survival of sympathetic superior cervical ganglion neurons are impaired in Ndn-null embryos. We observed reduced innervation of superior cervical ganglion target organs, including the submandibular gland, parotid gland, and nasal mucosa. While the formation of other sympathetic chain ganglia is unaffected, axonal extension is impaired throughout the sympathetic nervous system. These results demonstrate a novel role for necdin in cellular **migration**, in addition to its roles in survival and axon outgrowth. Furthermore, reduced sympathetic function provides a plausible explanation for deficiencies of salivary gland function in individuals with congenital necdin deficiency consequent to Prader-Willi syndrome.
16. Retinoic acid regulates murine enteric nervous system precursor proliferation, enhances neuronal precursor differentiation, and reduces neurite growth in vitro. Enteric nervous system (ENS) precursors undergo a complex process of cell **migration**, proliferation, and differentiation to form an integrated network of neurons and glia within the bowel wall. Although retinoids regulate ENS development, molecular and cellular mechanisms of retinoid effects on the ENS are not well understood. We hypothesized that retinoids might directly affect ENS precursor differentiation and proliferation, and tested that hypothesis using immunoselected fetal ENS precursors in primary culture. We now demonstrate that all retinoid receptors and many retinoid biosynthetic enzymes are present in the fetal bowel at about the time that migrating ENS precursors reach the distal bowel. We further demonstrate that retinoic acid (RA) enhances proliferation of subsets of ENS precursors in a time-dependent fashion and increases neuronal differentiation. Surprisingly, however, enteric neurons that develop in retinoid deficient media have dramatically longer neurites than those exposed to RA. This difference in neurite growth correlates with increased RhoA protein at the neurite tip, decreased Smurf1 (a protein that targets RhoA for degradation), and dramatically decreased Smurf1 mRNA in response to RA. Collectively these data demonstrate diverse effects of RA on ENS precursor development and suggest that altered fetal retinoid availability or metabolism could contribute to intestinal motility disorders.
17. Tcof1 acts as a modifier of Pax3 during enteric nervous system development and in the pathogenesis of colonic aganglionosis. Hirschsprung disease (HSCR) is a human congenital disorder, defined by the absence of ganglia from variable lengths of the colon. These ganglia comprise the enteric nervous system (ENS) and are derived from migratory neural crest cells (NCCs). The inheritance of HSCR is complex, often non-Mendelian and characterized by variable penetrance. Although extensive research has identified many key players in the pathogenesis of Hirschsprung disease, a large number of cases remain genetically undefined. Therefore, additional unidentified genes or modifiers must contribute to the etiology and pathogenesis of Hirschsprung disease. We have discovered that Tcof1 may be one such modifier. Haploinsufficiency of Tcof1 in mice results in a reduction of vagal NCCs and their delayed **migration** along the length of the gut during early development. This alone, however, is not sufficient to cause colonic aganglionosis as alterations in the balance of NCC proliferation and differentiation ensures NCC colonize the entire length of the gut of Tcof1(+/-) mice by E18.5. In contrast, Tcof1 haploinsufficiency is able to sensitize Pax3(+/-) mice to colonic aganglionosis. Although, Pax3 heterozygous mice do not show ENS defects, compound Pax3;Tcof1 heterozygous mice exhibit cumulative apoptosis which severely reduces the NCC population that migrates into the foregut. In addition, the proliferative capacity of these NCC is also diminished. Taken together with the opposing effects of Pax3 and Tcof1 on NCC differentiation, the synergistic haploinsufficiency of Tcof1 and Pax3 results in colonic aganglionosis in mice and may contribute to the pathogenesis of Hirschsprung disease.
18. GDNF is required for neural colonization of the pancreas. The mammalian pancreas is densely innervated by both the sympathetic and parasympathetic nervous systems, which control exocrine and endocrine secretion. During embryonic development, neural crest cells migrating in a rostrocaudal direction populate the gut, giving rise to neural progenitor cells. Recent studies in mice have shown that neural crest cells enter the pancreatic epithelium at E11.5. However, the cues that guide the **migration** of neural progenitors into the pancreas are poorly defined. In this study we identify glial cell line-derived neurotrophic factor (GDNF) as a key player in this process. GDNF displays a dynamic expression pattern during embryonic development that parallels the chronology of **migration** and differentiation of neural crest derivatives in the pancreas. Conditional inactivation of Gdnf in the pancreatic epithelium results in a dramatic loss of neuronal and glial cells and in reduced parasympathetic innervation in the pancreas. Importantly, the innervation of other regions of the gut remains unaffected. Analysis of Gdnf mutant mouse embryos and ex vivo experiments indicate that GDNF produced in the pancreas acts as a neurotrophic factor for gut-resident neural progenitor cells. Our data further show that exogenous GDNF promotes the proliferation of pancreatic progenitor cells in organ culture. In summary, our results point to GDNF as crucial for the development of the intrinsic innervation of the pancreas.
19. Apolipoprotein B is a new target of the GDNF/RET and ET-3/EDNRB signalling pathways. GDNF/RET and Endothelin-3 (ET-3)/EDNRB regulate survival, differentiation, **migration**, and proliferation of neural crest-derived cells. Although several RET and EDNRB signalling mediators have been characterized, most of the genes targeted by these two pathways are still largely unknown. We focused our study on apolipoprotein B (APOB) as a novel target gene of the RET and EDNRB pathways, based on previous data obtained using a Caenorhabditis elegans strain mutant for the homologue of mammalian ECE1.Molecular and cellular studies of Apob were performed in the murine Neuro2a cells, an in vitro model for studying neural crest-derived cell development, along with a mouse knock-in for the Hirschsprung-associated mutation Ret(C620R). Silencing for Apob and Ret has been performed via shRNA.GDNF/RET and ET-3/EDNRB cooperated in inducing neuronal differentiation resulting in Apob activation in Neuro2a cell line. Apob expression was downregulated in mouse embryos homozygous for the Ret(C620R) mutation and presenting a severe Hirschsprung phenotype. Ret silencing prevented Apob expression increase. MAPK P38 kinase activation evoked Apob expression via GDNF/RET signalling in Neuro2a cells. A p53-dependent repressor element in Apob promoter resulted in a reduced Apob expression. Silencing of Apob reduced HuD protein expression.Apob is a novel downstream target of the RET/EDNRB pathways with a role in neuronal survival and maintenance, as indicated by its effect on HuD expression. Our data provide a conceptual framework to investigate and establish the role of APOB gene in severe gut dysmotility.
20. Vascularisation is not necessary for gut colonisation by enteric neural crest cells. The vasculature and nervous system share striking similarities in their networked, tree-like architecture and in the way they are super-imposed in mature organs. It has previously been suggested that the intestinal microvasculature network directs the **migration** of enteric neural crest cells (ENCC) along the gut to promote the formation of the enteric nervous system (ENS). To investigate the inter-relationship of migrating ENCC, ENS formation and gut vascular development we combined fate-mapping of ENCC with immunolabelling and intravascular dye injection to visualise nascent blood vessel networks. We found that the enteric and vascular networks initially had very distinct patterns of development. In the foregut, ENCC migrated through areas devoid of established vascular networks. In vessel-rich areas, such as the midgut and hindgut, the distribution of migrating ENCC did not support the idea that these cells followed a pre-established vascular network. Moreover, when gut vascular development was impaired, either genetically in Vegfa(120/120) or Tie2-Cre;Nrp1(fl/-) mice or using an in vitro Wnt1-Cre;Rosa26(Yfp/+) mouse model of ENS development, ENCC still colonised the entire length of the gut, including the terminal hindgut. These results demonstrate that blood vessel networks are not necessary to guide migrating ENCC during ENS development. Conversely, in miRet(51) mice, which lack ENS in the hindgut, the vascular network in this region appeared to be normal suggesting that in early development both networks form independently of each other.
21. Reduced endothelin converting enzyme-1 and endothelin-3 mRNA in the developing bowel of male mice may increase expressivity and penetrance of Hirschsprung disease-like distal intestinal aganglionosis. Hirschsprung disease (distal intestinal aganglionosis, HSCR) is a multigenic disorder with incomplete penetrance, variable expressivity, and a strong male gender bias. Recent studies demonstrated that these genetic patterns arise because gene interactions determine whether enteric nervous system (ENS) precursors successfully proliferate and migrate into the distal bowel. We now demonstrate that male gender bias in the extent of distal intestinal aganglionosis occurs in mice with Ret dominant-negative mutations (RetDN) that mimic human HSCR. We hypothesized that male gender bias could result from reduced expression of a gene already known to be essential for ENS development. Using quantitative real-time polymerase chain reaction (PCR) we demonstrated reduced levels of endothelin converting enzyme-1 and endothelin-3 mRNA in the male mouse bowel at the time that ENS precursors migrate into the colon. Other HSCR-associated genes are expressed at comparable levels in male and female mice. Testosterone and Mullerian inhibiting substance had no deleterious effect on ENS precursor development, but adding EDN3 peptide to E11.5 male RetDN heterozygous mouse gut explants in organ culture significantly increased the rate of ENS precursor **migration** through the bowel.
22. Neural precursor death is central to the pathogenesis of intestinal aganglionosis in Ret hypomorphic mice. The RET tyrosine kinase is required for the **migration**, proliferation, and survival of the enteric neural crest-derived cells (ENCCs) that form the enteric nervous system (ENS). Hypomorphic RET alleles cause intestinal aganglionosis [Hirschsprung disease (HSCR)], in which delayed **migration** and successive nonapoptotic ENCC death are considered to be major contributory factors. The significance of ENCC death in intestinal aganglionosis, however, has remained unclear. We show that elevated expression of Bcl-xL inhibits ENCC death in both Ret-null and hypomorphic states. However, the rescued Ret-null mice showed ENS malfunction with reduced nitric oxide synthase expression in colonic neurons, revealing the requirement of RET for neuronal differentiation. In contrast, the inhibition of cell death allows morphologically and functionally normal ENS formation in Ret hypomorphic mice. These results indicate that ENCC death is a principal cause of intestinal aganglionosis in a Ret hypomorphic state, and suggest that the inhibition of cell death is a route to the prevention of HSCR.
23. Prokineticin-1 (Prok-1) works coordinately with glial cell line-derived neurotrophic factor (GDNF) to mediate proliferation and differentiation of enteric neural crest cells. Enteric neural crest cells (NCC) are multipotent progenitors which give rise to neurons and glia of the enteric nervous system (ENS) during fetal development. Glial cell line-derived neurotrophic factor (GDNF)/RET receptor tyrosine kinase (Ret) signaling is indispensable for their survival, **migration** and differentiation. Using microarray analysis and isolated NCCs, we found that 45 genes were differentially expressed after GDNF treatment (16 h), 29 of them were up-regulated including 8 previously undescribed genes. Prokineticin receptor 1 (PK-R1), a receptor for Prokineticins (Prok), was identified in our screen and shown to be consistently up-regulated by GDNF in enteric NCCs. Further, PK-R1 was persistently expressed at a lower level in the enteric ganglions of the c-Ret deficient mice when compared to that of the wild-type littermates. Subsequent functional analysis showed that GDNF potentiated the proliferative and differentiation effects of Prok-1 by up-regulating PK-R1 expression in enteric NCCs. In addition, expression analysis and gene knock-down experiments indicated that Prok-1 and GDNF signalings shared some common downstream targets. More importantly, Prok-1 could induce both proliferation and expression of differentiation markers of c-Ret deficient NCCs, suggesting that Prok-1 may also provide a complementary pathway to GDNF signaling. Taken together, these findings provide evidence that Prok-1 crosstalks with GDNF/Ret signaling and probably provides an additional layer of signaling refinement to maintain proliferation and differentiation of enteric NCCs.
24. Trans-mesenteric neural crest cells are the principal source of the colonic enteric nervous system. Cell **migration** is fundamental to organogenesis. During development, the enteric neural crest cells (ENCCs) that give rise to the enteric nervous system (ENS) migrate and colonize the entire length of the gut, which undergoes substantial growth and morphological rearrangement. How ENCCs adapt to such changes during **migration**, however, is not fully understood. Using time-lapse imaging analyses of mouse ENCCs, we show that a population of ENCCs crosses from the midgut to the hindgut via the mesentery during a developmental time period in which these gut regions are transiently juxtaposed, and that such 'trans-mesenteric' ENCCs constitute a large part of the hindgut ENS. This migratory process requires GDNF signaling, and evidence suggests that impaired trans-mesenteric **migration** of ENCCs may underlie the pathogenesis of Hirschsprung disease (intestinal aganglionosis). The discovery of this trans-mesenteric ENCC population provides a basis for improving our understanding of ENS development and pathogenesis.
25. GDNF and GFRalpha1 promote differentiation and tangential **migration** of cortical GABAergic neurons. Cortical GABAergic neurons are generated in the ventral telencephalon and migrate dorsally into the cortex following a tangential path. GDNF signaling via GFRalpha1 was found to promote the differentiation of ventral precursors into GABAergic cells, enhancing their neuronal morphology and motility. GDNF stimulated axonal growth in cortical GABAergic neurons and acted as a potent chemoattractant of GABAergic cells. These effects required GFRalpha1 but neither RET nor NCAM, the two transmembrane signaling receptors known for GDNF. Mutant mice lacking GDNF or GFRalpha1, but neither RET nor NCAM, showed reduced numbers of GABAergic cells in the cerebral cortex and hippocampus. We conclude that one of the normal functions of GDNF signaling via GFRalpha1 in the developing brain is to promote the differentiation and **migration** of cortical GABAergic neurons. The lack of involvement of RET or NCAM in these processes suggests the existence of additional transmembrane effectors for GDNF.
26. Vitamin A facilitates enteric nervous system precursor **migration** by reducing Pten accumulation. Hirschsprung disease is a serious disorder of enteric nervous system (ENS) development caused by the failure of ENS precursor **migration** into the distal bowel. We now demonstrate that retinoic acid (RA) is crucial for GDNF-induced ENS precursor **migration**, cell polarization and lamellipodia formation, and that vitamin A depletion causes distal bowel aganglionosis in serum retinol-binding-protein-deficient (Rbp4(-/-)) mice. Ret heterozygosity increases the incidence and severity of distal bowel aganglionosis induced by vitamin A deficiency in Rbp4(-/-) animals. Furthermore, RA reduces phosphatase and tensin homolog (Pten) accumulation in migrating cells, whereas Pten overexpression slows ENS precursor **migration**. Collectively, these data support the hypothesis that vitamin A deficiency is a non-genetic risk factor that increases Hirschsprung disease penetrance and expressivity, suggesting that some cases of Hirschsprung disease might be preventable by optimizing maternal nutrition.
27. Actin depolymerizing factors cofilin1 and destrin are required for ureteric bud branching morphogenesis. The actin depolymerizing factors (ADFs) play important roles in several cellular processes that require cytoskeletal rearrangements, such as cell **migration**, but little is known about the in vivo functions of ADFs in developmental events like branching morphogenesis. While the molecular control of ureteric bud (UB) branching during kidney development has been extensively studied, the detailed cellular events underlying this process remain poorly understood. To gain insight into the role of actin cytoskeletal dynamics during renal branching morphogenesis, we studied the functional requirements for the closely related ADFs cofilin1 (Cfl1) and destrin (Dstn) during mouse development. Either deletion of Cfl1 in UB epithelium or an inactivating mutation in Dstn has no effect on renal morphogenesis, but simultaneous lack of both genes arrests branching morphogenesis at an early stage, revealing considerable functional overlap between cofilin1 and destrin. Lack of Cfl1 and Dstn in the UB causes accumulation of filamentous actin, disruption of normal epithelial organization, and defects in cell **migration**. Animals with less severe combinations of mutant Cfl1 and Dstn alleles, which retain one wild-type Cfl1 or Dstn allele, display abnormalities including ureter duplication, renal hypoplasia, and abnormal kidney shape. The results indicate that ADF activity, provided by either cofilin1 or destrin, is essential in UB epithelial cells for normal growth and branching.
28. Nkx6.1 controls **migration** and axon pathfinding of cranial branchio-motoneurons. As many studies have focused on the mechanisms of motoneuron specification, little is known about the factors that control the subsequent development of postmitotic motoneurons. Previously, we showed that the transcription factor Nkx6.1 is required for the early specification of somatic motoneuron progenitors in the spinal cord. Our present analysis of hindbrain motoneuron development in Nkx6.1-deficient mouse embryos reveals that the early specification of branchio-motoneurons is independent of Nkx6.1 function, but that it is required for their subsequent development. In Nkx6.1 mutant mice, we observed defects in the **migration**, as well as in the axon projections of branchio-motoneurons. A detailed analysis of the migratory defect in facial branchio-motoneurons reveals ectopic expression of the cell surface receptors Ret and Unc5h3 in premigratory neurons, but no changes in the rhombomeric environment. Taken together, our findings demonstrate a requirement for Nkx6.1 in the development of postmitotic motoneurons, and suggest a cell-autonomous function in the control of branchio-motoneuron **migration**.
29. Diminished Ret expression compromises neuronal survival in the colon and causes intestinal aganglionosis in mice. Mutations in the RET gene are the primary cause of Hirschsprung disease (HSCR), or congenital intestinal aganglionosis. However, how RET malfunction leads to HSCR is not known. It has recently been shown that glial cell line-derived neurotrophic factor (GDNF) family receptor alpha1 (GFRalpha1), which binds to GDNF and activates RET, is essential for the survival of enteric neurons. In this study, we investigated Ret regulation of enteric neuron survival and its potential involvement in HSCR. Conditional ablation of Ret in postmigratory enteric neurons caused widespread neuronal death in the colon, which led to colonic aganglionosis. To further examine this finding, we generated a mouse model for HSCR by reducing Ret expression levels. These mice recapitulated the genetic and phenotypic features of HSCR and developed colonic aganglionosis due to impaired **migration** and successive death of enteric neural crest-derived cells. Death of enteric neurons was also induced in the colon, where reduction of Ret expression was induced after the period of enteric neural crest cell **migration**, indicating that diminished Ret expression directly affected the survival of colonic neurons. Thus, enteric neuron survival is sensitive to RET dosage, and cell death is potentially involved in the etiology of HSCR.
30. Reelin signaling is necessary for a specific step in the **migration** of hindbrain efferent neurons. The cytoarchitecture of the hindbrain results from precise and co-ordinated sequences of neuronal **migration**s. Here, we show that reelin, an extracellular matrix protein involved in neuronal **migration** during CNS development, is necessary for an early, specific step in the **migration** of several hindbrain nuclei. We identified two cell populations not previously known to be affected in reeler mutants that show a common migratory defect: the olivocochlear efferent neurons and the facial visceral motor nucleus. In control embryos, these cells migrate first toward a lateral position within the neural tube, and then parallel to the glial cell processes, to a ventral position where they settle close to the pial surface. In reeler mutants, the first **migration** is not affected, but the neurons are unable to reach the pial surface and remain in an ectopic position. Indeed, this is the first evidence that the **migration** of specific hindbrain nuclei can be divided into two parts: a reelin-independent and a reelin-dependent **migration**. We also show that reelin is expressed at high levels at the final destination of the migratory process, while the reelin intracellular effector Dab1 was expressed by cell groups that included the two populations affected. Mice mutant at the Dab1 locus, called scrambler, exhibit the same phenotype, a failure of final **migration**. However, examination of mice lacking both reelin receptors, ApoER2 and VLDLR, did not reveal the same phenotype, suggesting involvement of an additional reelin-binding receptor. In the hindbrain, reelin signaling might alter the adhesive properties of efferent neurons and their ability to respond to directional cues, as has been suggested for the **migration** of olfactory bulb precursors.
31. T-Box transcription factor Tbx20 regulates a genetic program for cranial motor neuron cell body **migration**. Members of the T-box transcription factor family (Tbx) are associated with several human syndromes during embryogenesis. Nevertheless, their functions within the developing CNS remain poorly characterized. Tbx20 is expressed by migrating branchiomotor/visceromotor (BM/VM) neurons within the hindbrain during neuronal circuit formation. We examined Tbx20 function in BM/VM cells using conditional Tbx20-null mutant mice to delete the gene in neurons. Hindbrain rhombomere patterning and the initial generation of post-mitotic BM/VM neurons were normal in Tbx20 mutants. However, Tbx20 was required for the tangential (caudal) **migration** of facial neurons, the lateral **migration** of trigeminal cells and the trans-median movement of vestibuloacoustic neurons. Facial cell soma **migration** defects were associated with the coordinate downregulation of multiple components of the planar cell polarity pathway including Fzd7, Wnt11, Prickle1, Vang1 and Vang2. Our study suggests that Tbx20 programs a variety of hindbrain motor neurons for **migration**, independent of directionality, and in facial neurons is a positive regulator of the non-canonical Wnt signaling pathway.
32. Requirement of signalling by receptor tyrosine kinase RET for the directed **migration** of enteric nervous system progenitor cells during mammalian embryogenesis. The majority of neurones and glia of the enteric nervous system (ENS) are derived from the vagal neural crest. Shortly after e**migration** from the neural tube, ENS progenitors invade the anterior foregut and, migrating in a rostrocaudal direction, colonise in an orderly fashion the rest of the foregut, the midgut and the hindgut. We provide evidence that activation of the receptor tyrosine kinase RET by glial cell line-derived neurotrophic factor (GDNF) is required for the directional **migration** of ENS progenitors towards and within the gut wall. We find that neural crest-derived cells present within foetal small intestine explants migrate towards an exogenous source of GDNF in a RET-dependent fashion. Consistent with an in vivo role of GDNF in the **migration** of ENS progenitors, we demonstrate that Gdnf is expressed at high levels in the gut of mouse embryos in a spatially and temporally regulated manner. Thus, during invasion of the foregut by vagal-derived neural crest cells, expression of Gdnf was restricted to the mesenchyme of the stomach, ahead of the invading NC cells. Twenty-four hours later and as the ENS progenitors were colonising the midgut, Gdnf expression was upregulated in a more posterior region - the caecum anlage. In further support of a role of endogenous GDNF in enteric neural crest cell **migration**, we find that in explant cultures GDNF produced by caecum is sufficient to attract NC cells residing in more anterior gut segments. In addition, two independently generated loss-of-function alleles of murine Ret, Ret.k- and miRet51, result in characteristic defects of neural crest cell **migration** within the developing gut. Finally, we identify phosphatidylinositol-3 kinase and the mitogen-activated protein kinase signalling pathways as playing crucial roles in the migratory response of enteric neural crest cells to GDNF.
33. Matrix metalloproteinase-2 is involved in the **migration** and network formation of enteric neural crest-derived cells. The enteric nervous system is derived from neural crest cells that emigrate from the hindbrain, enter the foregut and colonise the entire length of the gastrointestinal tract. Previous studies have shown that although enteric neural crest-derived cells migrate in chains, they have the ability to detach from their existing chain in order to join or form a new chain. In this study, the possible role of matrix metalloproteinase-3, -8 and -2/-9 on the **migration** of enteric neural crest-derived cells and the formation of the neural network within the developing gut were examined using specific pharmacological inhibitors. Blocking MMP-2/MMP-9 activity significantly decreased the distance that enteric neural crest-derived cells migrated through the developing gut. Morevover, the reticulated network formed by these cells was less complex. MMP-3 and MMP-8 inhibitors had no effect on neural crest **migration**. Expression studies showed that MMP-2, but not MMP-9, was expressed within the developing mouse gut. Collectively, the data suggest that MMP-2 activity is important for enteric neural crest-derived cell **migration** and the formation of the neural crest network.
34. The mouse Wnt/PCP protein Vangl2 is necessary for **migration** of facial branchiomotor neurons, and functions independently of Dishevelled. During development, facial branchiomotor (FBM) neurons, which innervate muscles in the vertebrate head, migrate caudally and radially within the brainstem to form a motor nucleus at the pial surface. Several components of the Wnt/planar cell polarity (PCP) pathway, including the transmembrane protein Vangl2, regulate caudal **migration** of FBM neurons in zebrafish, but their roles in neuronal **migration** in mouse have not been investigated in detail. Therefore, we analyzed FBM neuron **migration** in mouse looptail (Lp) mutants, in which Vangl2 is inactivated. In Vangl2(Lp/+) and Vangl2(Lp/Lp) embryos, FBM neurons failed to migrate caudally from rhombomere (r) 4 into r6. Although caudal **migration** was largely blocked, many FBM neurons underwent normal radial **migration** to the pial surface of the neural tube. In addition, hindbrain patterning and FBM progenitor specification were intact, and FBM neurons did not transfate into other non-migratory neuron types, indicating a specific effect on caudal **migration**. Since loss-of-function in some zebrafish Wnt/PCP genes does not affect caudal **migration** of FBM neurons, we tested whether this was also the case in mouse. Embryos null for Ptk7, a regulator of PCP signaling, had severe defects in caudal **migration** of FBM neurons. However, FBM neurons migrated normally in Dishevelled (Dvl) 1/2 double mutants, and in zebrafish embryos with disrupted Dvl signaling, suggesting that Dvl function is essentially dispensable for FBM neuron caudal **migration**. Consistent with this, loss of Dvl2 function in Vangl2(Lp/+) embryos did not exacerbate the Vangl2(Lp/+) neuronal **migration** phenotype. These data indicate that caudal **migration** of FBM neurons is regulated by multiple components of the Wnt/PCP pathway, but, importantly, may not require Dishevelled function. Interestingly, genetic-interaction experiments suggest that rostral FBM neuron **migration**, which is normally suppressed, depends upon Dvl function.
35. RET modulates cell adhesion via its cleavage by caspase in sympathetic neurons. RET is a tyrosine kinase receptor involved in numerous cellular mechanisms including proliferation, neuronal navigation, **migration**, and differentiation upon binding with glial cell derived neurotrophic factor family ligands. RET is an atypical tyrosine kinase receptor containing four cadherin domains in its extracellular part. Furthermore, it has been shown to act as a dependence receptor. Such a receptor is active in the absence of ligand, triggering apoptosis through a mechanism that requires receptor intracellular caspase cleavage. However, different data suggest that RET is not always associated with the cell death/survival balance but rather provides positional information. We demonstrate here that caspase cleavage of RET is involved in the regulation of adhesion in sympathetic neurons. The cleavage of RET generates an N-terminal truncated fragment that functions as a cadherin accessory protein, modifying cadherin environment and potentiating cadherin-mediated cell aggregation. Thus, the caspase cleavage of RET generates two RET fragments: one intracellular domain that can trigger cell death in apoptotic permissive settings, and one membrane-anchored ectodomain with cadherin accessory activity. We propose that this latter function may notably be important for the adequate development of the superior cervical ganglion.
36. Control of the migratory pathway of facial branchiomotor neurones. Facial branchiomotor (fbm) neurones undergo a complex **migration** in the segmented mouse hindbrain. They are born in the basal plate of rhombomere (r) 4, migrate caudally through r5, and then dorsally and radially in r6. To study how migrating cells adapt to their changing environment and control their pathway, we have analysed this stereotyped **migration** in wild-type and mutant backgrounds. We show that during their **migration**, fbm neurones regulate the expression of genes encoding the cell membrane proteins TAG-1, Ret and cadherin 8. Specific combinations of these markers are associated with each migratory phase in r4, r5 and r6. In Krox20 and kreisler mutant mouse embryos, both of which lack r5, fbm neurones migrate dorsally into the anteriorly positioned r6 and adopt an r6-specific expression pattern. In embryos deficient for Ebf1, a gene normally expressed in fbm neurones, part of the fbm neurones migrate dorsally within r5. Accordingly, fbm neurones prematurely express a combination of markers characteristic of an r6 location. These data suggest that fbm neurones adapt to their changing environment by switching on and off specific genes, and that Ebf1 is involved in the control of these responses. In addition, they establish a close correlation between the expression pattern of fbm neurones and their migratory behaviour, suggesting that modifications in gene expression participate in the selection of the local migratory pathway.
37. gdnf activates midline repulsion by Semaphorin3B via NCAM during commissural axon guidance. The Neurotrophic factor gdnf plays diverse developmental roles, supporting survival and also acting as a chemoattractant for axon and cel**l migrati**on. We report that in the developing spinal cord, a focal source of gdnf is present in the floor plate (FP) where commissural axons cross the midline. Gdnf has no direct guidance properties but switches on the responsiveness of crossing commissural growth cones to the midline repellent Semaphorin3B by suppressing calpain-mediated processing of the Sema3B signaling coreceptor Plexin-A1. Analysis of single and double mutant mouse models indicates that although gdnf is the principal trigger of Sema3B midline repulsion, it acts with another FP cue, NrCAM. Finally, genetic and in vitro experiments provide evidence that this gdnf effect is RET independent and mediated by NCAM/GFRα1 signaling. This study identifies a regulator of midline crossing and reveals interplays between Semaphorin and gdnf signaling during axon guidance.
38. Altered segmental identity and abnormal **migration** of motor neurons in mice lacking Hoxb-1. Segmentation of the vertebrate hindbrain into rhombomeres is important for the anterior-posterior arrangement of cranial motor nuclei and efferent nerves. Underlying this reiterated organization, Hox genes display segmentally restricted domains of expression, such as expression of Hoxb-1 (refs 5, 6) in rhombomere 4 (r4). Here we report that absence of Hoxb-1 leads to changes in r4 identity. In mutant mouse embryos, molecular markers indicate that patterning of r4 is initiated properly but not maintained. Cellular analysis by DiI tracing reveals that the r4-specific facial branchiomotor (FBM) and contralateral vestibuloacoustic efferent (CVA) neurons are incorrectly specified. In wild-type mice CVA neurons migrate from r4 into the contralateral side, and we found in lineage analysis that FBM neurons migrate from r4 into r5. In mutants, motor neurons differentiate but the CVA and FBM neurons fail to migrate into their proper positions. Instead, they form a motor nucleus which migrates atypically, and there is a subsequent loss of the facial motor nerve. These results demonstrate that, as a part of its role in maintaining rhombomere identity, Hoxb-1 is involved in controlling migratory properties of motor neurons in the hindbrain.
39. Sonic hedgehog regulates the proliferation, differentiation, and **migration** of enteric neural crest cells in gut. Enteric neural crest cells (NCCs) migrate and colonize the entire gut and proliferate and differentiate into neurons and glia of the enteric nervous system in vertebrate embryos. We have investigated the mitogenic and morphogenic functions of Sonic hedgehog (Shh) on enteric NCCs in cell and organ culture. Enteric NCCs expressed Shh receptor Patched and transcripts encoding the Shh signal transducer (Gli1). Shh promoted the proliferation and inhibited the differentiation of NCCs. The pro-neurogenic effect of glial cell line--derived neurotrophic factor (GDNF) on NCCs was abolished by Shh. In gut explants, NCCs migrated from the explants onto the adjacent substratum if GDNF was added, whereas addition of Shh abolished this **migration**. Neuronal differentiation and coalescence of neural crest--derived cells into myenteric plexuses in explants was repressed by the addition of Shh. Our data suggest that Shh controls the proliferation and differentiation of NCCs and modulates the responsiveness of NCCs toward GDNF inductions.
40. Perturbation of hoxb5 signaling in vagal neural crests down-regulates ret leading to intestinal hypoganglionosis in mice. The enteric nervous system (ENS) controls intestinal peristalsis, and defective development of this system results in hypo/aganglionosis, as seen in Hirschsprung's disease. In the embryo, vagal neural crest cells (NCC) migrate and colonize the intestine rostrocaudally then differentiate into the ganglia of the ENS. Vagal NCC express the homeobox gene Hoxb5, a transcriptional activator, in human and mouse, so we used transgenic mice to investigate the function of Hoxb5 and the receptor tyrosine kinase gene Ret, which is affected in many patients with Hirschsprung's disease, in ENS development.We perturbed the Hoxb5 pathway by expressing a chimeric protein enb5, in which the transcription activation domain of Hoxb5 was replaced with the repressor domain of the Drosophila engrailed protein (en), in vagal NCC. This enb5 transcriptional repressor competes with wild-type Hoxb5 for binding to target genes, exerting a dominant negative effect.We observed that 30.6% +/- 2.3% of NCC expressed enb5 and that these enb5-expressing NCC failed to migrate to the distal intestine. A 34%-37% reduction of ganglia (hypoganglionosis) and slow peristalsis and, occasionally, absence of ganglia and intestinal obstruction were observed in enb5-expressing mice. Ret expression was markedly reduced or absent in NCC and ganglia, and enb5 blocked Hoxb5 induction of Ret in neuroblastoma cells.Our data indicate that Ret is a downstream target of Hoxb5 whose perturbation causes Ret haploinsufficiency, impaired NCC **migration**, and hypo/aganglionosis, suggesting that Hoxb5 may contribute to the etiology of Hirschsprung's disease.
41. A semaphorin code defines subpopulations of spinal motor neurons during mouse development. Abstract In the spinal cord, motor neurons (MNs) with similar muscle targets and sensory inputs are grouped together into motor pools. To date, relatively little is known about the molecular mechanisms that control the establishment of pool-specific circuitry. Semaphorins, a large family of secreted and cell surface proteins, are important mediators of developmental processes such as axon guidance and cell **migration**. Here, we used mRNA in situ hybridization to study the expression patterns of semaphorins and their receptors, neuropilins and plexins, in the embryonic mouse spinal cord. Our data show that semaphorins and their receptors are differentially expressed in MNs that lie in distinct locations within the spinal cord. Furthermore, we report a combinatorial expression of class 3 (secreted) semaphorins and their receptors that characterizes distinct motor pools within the brachial and lumbar spinal cord. Finally, we found that a secreted semaphorin, Sema3A, elicits differential collapse responses in topologically distinct subpopulations of spinal MNs. These findings lead us to propose that semaphorins and their receptors might play important roles in the sorting of motor pools and the patterning of their afferent and efferent projections.
42. Targeted mutation of serine 697 in the Ret tyrosine kinase causes **migration** defect of enteric neural crest cells. The RET receptor tyrosine kinase plays a critical role in the development of the enteric nervous system (ENS) and the kidney. Upon glial-cell-line-derived neurotrophic factor (GDNF) stimulation, RET can activate a variety of intracellular signals, including the Ras/mitogen-activated protein kinase, phosphatidylinositol 3-kinase (PI3K)/AKT, and RAC1/JUN NH(2)-terminal kinase (JNK) pathways. We recently demonstrated that the RAC1/JNK pathway is regulated by serine phosphorylation at the juxtamembrane region of RET in a cAMP-dependent manner. To determine the importance of cAMP-dependent modification of the RET signal in vivo, we generated mutant mice in which serine residue 697, a putative protein kinase A (PKA) phosphorylation site, was replaced with alanine (designated S697A mice). Homozygous S697A mutant mice lacked the ENS in the distal colon, resulting from a **migration** defect of enteric neural crest cells (ENCCs). In vitro organ culture showed an impaired chemoattractant response of the mutant ENCCs to GDNF. JNK activation by GDNF but not ERK, AKT and SRC activation was markedly reduced in neurons derived from the mutant mice. The JNK inhibitor SP600125 and the PKA inhibitor KT5720 suppressed **migration** of the ENCCs in cultured guts from wild-type mice to comparable degrees. Thus, these findings indicated that cAMP-dependent modification of RET function regulates the JNK signaling responsible for proper **migration** of the ENCCs in the developing gut.
43. Reciprocal gene replacements reveal unique functions for Phox2 genes during neural differentiation. The paralogous paired-like homeobox genes Phox2a and Phox2b are involved in the development of specific neural subtypes in the central and peripheral nervous systems. The different phenotypes of Phox2 knockout mutants, together with their asynchronous onset of expression, prompted us to generate two knock-in mutant mice, in which Phox2a is replaced by the Phox2b coding sequence, and vice versa. Our results indicate that Phox2a and Phox2b are not functionally equivalent, as only Phox2b can fulfill the role of Phox2a in the structures that depend on both genes. Furthermore, we demonstrate unique roles of Phox2 genes in the differentiation of specific motor neurons. Whereas the oculomotor and the trochlear neurons require Phox2a for their proper development, the **migration** of the facial branchiomotor neurons depends on Phox2b. Therefore, our analysis strongly indicates that biochemical differences between the proteins rather than temporal regulation of their expression account for the specific function of each paralogue.
44. Cell **migration** by a FRS2-adaptor dependent membrane relocation of ret receptors. During development neural progenitor cells migrate with extraordinary precision to inhabit tissues and organs far from their initial position. Little is known about the cellular basis for directional guidance by tyrosine kinase receptors (RTKs). RET is a RTK with important functions in guiding the **migration** of neuronal cells, and RET dysregulation leads to clinical disease such as agangliosis of the colon. We show here that RET **migration** in neuroepitheliomal and non-neuronal cells is elicited by the activation of specific signaling pathways initiated by the competitive recruitment of the FRS2 adaptor molecule to tyrosine 1062 (Y1062) in RET. FRS2 selectively recruited RET to focal complexes and led to activation of SRC family kinases and focal adhesion kinase (FAK). Activation of SRC depended on its direct interaction with RET at a different intracellular tyrosine (Y981) and activation of molecular signaling from these two separate sites in concert regulated **migration**. Our data suggest that an important function for FRS2 is to concentrate RET in membrane foci, leading to an engagement of specific signaling complexes localized in these membrane domains.
45. GDNF signaling levels control **migration** and neuronal differentiation of enteric ganglion precursors. Pleiotropic growth factors play a number of critical roles in continuous processes of embryonic development; however, the mechanisms by which a single regulatory factor is able to orchestrate diverse developmental events remain imperfectly understood. In the development of the enteric nervous system (ENS), myenteric ganglia (MGs) form initially, after which the submucosal ganglia (SMGs) develop by radial inward **migration** of immature ENS precursors from the myenteric layer. Here, we demonstrate that glial cell line-derived neurotrophic factor (GDNF) is essential for the formation not only of the MGs, but the SMGs as well, establishing GDNF as a long-term acting neurotrophic factor for ENS development in a mouse model. GDNF promotes radial **migration** of SMG precursors. Interestingly, premigratory SMG precursors in the myenteric layer were distinguished from the surrounding neuronally differentiating cells by their lower activation of the GDNF-mediated MAPK pathway, suggesting that low activation of GDNF downstream pathways is required for the maintenance of the immature state. ENS precursors devoid of GDNF signaling during midgestation halt their **migration**, survive, and remain in an undifferentiated state over the long-term in vivo. Reactivation of GDNF signaling in these dormant precursors restores their **migration** and neuronal differentiation in gut organ culture. These findings suggest that pleiotropic function of GDNF is at least in part governed by modulating levels of intracellular activation of GDNF downstream pathways; high activation triggers neuronal differentiation, whereas low activation is crucial for the maintenance of progenitor state.
46. C620R mutation of the murine ret proto-oncogene: loss of function effect in homozygotes and possible gain of function effect in heterozygotes. Germline RET mutations are responsible for different inherited disorders: Hirschsprung disease (congenital aganglionic megacolon), caused by loss of function mutations, familial medullary thyroid carcinoma and multiple endocrine neoplasia type 2, caused by gain of function mutations. Intriguingly, some RET mutations, including C620R, are associated with both types of diseases. To investigate the dual role of such RET mutations, a mouse model with a targeted mutation ret(C620R) was generated. ret(C620R/C620R) offspring die during the first postnatal day, and show kidney agenesis and intestinal aganglionosis. Decreased outgrowth of the Ret-positive cells was observed in ret(C620R/C620R) neuronal cell cultures, which is suggestive of an impaired **migration**, proliferation or survival of the Ret-expressing cells. Electronmicroscopy revealed the absence of membrane-bound Ret in ret(C620R/C620R) cells as compared to ret(+/+) and ret(+/C620R) cells. On the other hand, aged ret(+/C620R) mice develop precancerous lesions in the adrenal gland or in the thyroid. Our results suggest that the ret(C620R) mutation has a loss of function effect in homozygotes and exhibits a dominant gain of function effect with low penetrance causing hyperplasia in heterozygotes.
47. Expression of the c-ret proto-oncogene during mouse embryogenesis. The c-ret proto-oncogene encodes a receptor tyrosine kinase whose normal function has yet to be determined. To begin to investigate the potential role of this gene in vertebrate development, we have isolated cDNA clones representing the murine c-ret gene, and have analyzed the pattern of expression during mouse embryogenesis, using northern blotting, in situ hybridization to histological sections and whole-mount hybridization histochemistry. c-ret transcripts were detected beginning at day 8.5 of embryogenesis, and were observed in a number of cell lineages in the developing peripheral and central nervous systems, as well as in the excretory system. In the cranial region at day 8.5-9.5, c-ret mRNA was restricted to a population of neural crest cells migrating from rhombomere 4 and forming the anlage of the facioacoustic ganglion, as well as to a closely associated domain of surface ectoderm and pharyngeal endoderm. At later stages (10.5-14.5 days), c-ret mRNA was observed in all cranial ganglia. In the peripheral nervous system of the trunk, c-ret was expressed in the autonomic ganglia and in subsets of cells in the dorsal root ganglia. In the enteric nervous system, c-ret was expressed in the presumptive enteric neuroblasts of the vagal crest (day 9.0-11.5), and in the myenteric ganglia of the gut (day 13.5-14.5). c-ret mRNA was observed in several regions of the central nervous system, including the undifferentiated neuroepithelial cells of the ventral neural tube (8.5 days), the motor neurons in the spinal cord and the hindbrain (10.5-14.5 days), the embryonic neuroretina (day 13.5) and the layers of the postnatal retina containing ganglion, amacrine and horizontal cells. Outside the nervous system, c-ret was expressed in the nephric (Wolffian) duct at day 8.5-10.5, the ureteric bud epithelium (but not the surrounding metanephric mesenchyme) at day 11.0-11.5, and the growing tips of the renal collecting ducts (but not the previously formed, subcortical portions of the collecting ducts, or the mesenchyme-derived renal vesicles) at day 13.5-17.5. Our results suggest that the c-ret gene may encode the receptor for a factor involved in the proliferation, **migration**, differentiation or survival of a variety of neuronal cell lineages, as well as in inductive interactions during organogenesis of the kidney.
48. The ErbB2 and ErbB3 receptors and their ligand, neuregulin-1, are essential for development of the sympathetic nervous system. Neuregulins (NDF, heregulin, GGF ARIA, or SMDF) are EGF-like growth and differentiation factors that signal through tyrosine kinase receptors of the ErbB family. Here, we report a novel phenotype in mice with targeted mutations in the erbB2, erbB3, or neuregulin-1 genes. These three mutations cause a severe hypoplasia of the primary sympathetic ganglion chain. We provide evidence that **migration** of neural crest cells to the mesenchyme lateral of the dorsal aorta, in which they differentiate into sympathetic neurons, depends on neuregulin-1 and its receptors. Neuregulin-1 is expressed at the origin of neural crest cells. Moreover, a tight link between neuregulin-1 expression, the migratory path, and the target site of sympathogenic neural crest cells is observed. Sympathetic ganglia synthesize catecholamines in the embryo and the adult. Accordingly, catecholamine levels in mutant embryos are severely decreased, and we suggest that the lack of catecholamines contributes to the embryonal lethality of the erbB3 mutant mice. Thus, neuregulin-1, erbB2, and erbB3 are required for the formation of the sympathetic nervous system; the block in development observed in mutant mice is caused by a lack of neural crest precursor cells in the anlage of the primary sympathetic ganglion chain. Together with previous observations, these findings establish the neuregulin signaling system as a key regulator in the development of neural crest cells.
49. Development of cranial parasympathetic ganglia requires sequential actions of GDNF and neurturin. The neurotrophic factors that influence the development and function of the parasympathetic branch of the autonomic nervous system are obscure. Recently, neurturin has been found to provide trophic support to neurons of the cranial parasympathetic ganglion. Here we show that GDNF signaling via the RET/GFR(alpha)1 complex is crucial for the development of cranial parasympathetic ganglia including the submandibular, sphenopalatine and otic ganglia. GDNF is required early for proliferation and/or **migration** of the neuronal precursors for the sphenopalatine and otic ganglia. Neurturin exerts its effect later and is required for further development and maintenance of these neurons. This switch in ligand dependency during development is at least partly governed by the altered expression of GFR(&agr;) receptors, as evidenced by the predominant expression of GFR(&agr;)2 in these neurons after ganglion formation.
50. The tyrosine phosphatase Shp2 (PTPN11) directs Neuregulin-1/ErbB signaling throughout Schwann cell development. The nonreceptor tyrosine phosphatase Shp2 (PTPN11) has been implicated in tyrosine kinase, cytokine, and integrin receptor signaling. We show here that conditional mutation of Shp2 in neural crest cells and in myelinating Schwann cells resulted in deficits in glial development that are remarkably similar to those observed in mice mutant for Neuregulin-1 (Nrg1) or the Nrg1 receptors, ErbB2 and ErbB3. In cultured Shp2 mutant Schwann cells, Nrg1-evoked cellular responses like proliferation and **migration** were virtually abolished, and Nrg1-dependent intracellular signaling was altered. Pharmacological inhibition of Src family kinases mimicked all cellular and biochemical effects of the Shp2 mutation, implicating Src as a primary Shp2 target during Nrg1 signaling. Together, our genetic and biochemical analyses demonstrate that Shp2 is an essential component in the transduction of Nrg1/ErbB signals.
51. Functional loss of semaphorin 3C and/or semaphorin 3D and their epistatic interaction with ret are critical to Hirschsprung disease liability. Innervation of the gut is segmentally lost in Hirschsprung disease (HSCR), a consequence of cell-autonomous and non-autonomous defects in enteric neuronal cell differentiation, proliferation, **migration**, or survival. Rare, high-penetrance coding variants and common, low-penetrance non-coding variants in 13 genes are known to underlie HSCR risk, with the most frequent variants in the ret proto-oncogene (RET). We used a genome-wide association (220 trios) and replication (429 trios) study to reveal a second non-coding variant distal to RET and a non-coding allele on chromosome 7 within the class 3 Semaphorin gene cluster. Analysis in Ret wild-type and Ret-null mice demonstrates specific expression of Sema3a, Sema3c, and Sema3d in the enteric nervous system (ENS). In zebrafish embryos, sema3 knockdowns show reduction of migratory ENS precursors with complete ablation under conjoint ret loss of function. Seven candidate receptors of Sema3 proteins are also expressed within the mouse ENS and their expression is also lost in the ENS of Ret-null embryos. Sequencing of SEMA3A, SEMA3C, and SEMA3D in 254 HSCR-affected subjects followed by in silico protein structure modeling and functional analyses identified five disease-associated alleles with loss-of-function defects in semaphorin dimerization and binding to their cognate neuropilin and plexin receptors. Thus, semaphorin 3C/3D signaling is an evolutionarily conserved regulator of ENS development whose dys-regulation is a cause of enteric aganglionosis.
52. Differential requirement of Tyr1062 multidocking site by RET isoforms to promote neural cell scattering and epithelial cell branching. The receptor tyrosine kinase RET is alternatively spliced to yield two main isoforms, RET9 and RET51, which differ in their carboxyl terminal. Activated RET induces different biological responses such as morphological transformation, neurite outgrowth, proliferation, cell **migration** and branching. The two isoforms have been suggested to have separate intracellular signaling pathways and different roles in mouse development. Here we show that both isoforms are able to induce cell scattering of SK-N-MC neuroepithelioma cell line and branching tubule formation in MDCK cell line. However, the Y1062F mutation, which abrogates the transforming activity of both activated RET isoforms in NIH3T3 cells, does not abolish scattering and branching morphogenesis of RET51, whereas impairs these biological effects of RET9. The GDNF-induced biological effects of RET51 are inhibited by the simultaneous abrogation of both Tyr1062 and Tyr1096 docking sites. Thus, Tyr1096 may substitute the functions of Tyr1062. GRB2 is the only known adaptor protein binding to Tyr1096. Dominant-negative GRB2 expressed in MDCK cells together with RET9 or RET51 significantly reduces branching. Therefore, GRB2 is necessary for RET-mediated branching of MDCK cells.
53. Phenotypes of neural-crest-derived cells in vagal and sacral pathways. Enteric neurons arise from vagal and sacral level neural crest cells. To examine the phenotype of neural-crest-derived cells in vagal and sacral pathways, we used antisera to Sox10, p75, Phox2b, and Hu, and transgenic mice in which the expression of green fluorescent protein was under the control of the Ret promoter. Sox10 was expressed prior to the e**migration** of vagal cells, whereas p75 was expressed shortly after their e**migration**. Most crest-derived cells that emigrated adjacent to somites 1-4 migrated along a pathway that was later followed by the vagus nerve. A sub-population of these vagal cells coalesced to form vagal ganglia, whereas others continued their **migration** towards the heart and gut. Cells that coalesced into vagal ganglia showed a different phenotype from cells in the migratory streams proximal and distal to the ganglia. Only a sub-population of the vagal cells that first entered the foregut expressed Phox2b or Ret. Sacral neural crest cells gave rise to pelvic ganglia and some neurons in the hindgut. The pathways of sacral neural crest cells were examined by using DbetaH-nlacZ mice. Sacral cells appeared to enter the distal hindgut around embryonic day 14.5. Very few of the previously demonstrated, but rare, neurons that were present in the large intestine of Ret null mutants and that presumably arose from the sacral neural crest expressed nitric oxide synthase, unlike their counterparts in Ret heterozygous mice.
54. Lack of an adrenal cortex in Sf1 mutant mice is compatible with the generation and differentiation of chromaffin cells. The diversification of neural-crest-derived sympathoadrenal (SA) progenitor cells into sympathetic neurons and neuroendocrine adrenal chromaffin cells was thought to be largely understood. In-vitro studies with isolated SA progenitor cells had suggested that chromaffin cell differentiation depends crucially on glucocorticoids provided by adrenal cortical cells. However, analysis of mice lacking the glucocorticoid receptor gene had revealed that adrenal chromaffin cells develop mostly normally in these mice. Alternative cues from the adrenal cortex that may promote chromaffin cell determination and differentiation have not been identified. We therefore investigated whether the chromaffin cell phenotype can develop in the absence of an adrenal cortex, using mice deficient for the nuclear orphan receptor steroidogenic factor-1 (SF1), which lack adrenal cortical cells and gonads. We show that in Sf1-/- mice typical chromaffin cells assemble correctly in the suprarenal region adjacent to the suprarenal sympathetic ganglion. The cells display most features of chromaffin cells, including the typical large chromaffin granules. Sf1-/- chromaffin cells are numerically reduced by about 50% compared with the wild type at embryonic day (E) 13.5 and E17.5. This phenotype is not accounted for by reduced survival or cell proliferation beyond E12.5. However, already at E12.5 the 'adrenal' region in Sf1-/- mice is occupied by fewer PHOX2B+ and TH+ SA cells as well as SOX10+ neural crest cells. Our results suggest that cortical cues are not essential for determining chromaffin cell fate, but may be required for proper **migration** of SA progenitors to and/or colonization of the adrenal anlage.
55. Hirschsprung-like disease is exacerbated by reduced de novo GMP synthesis. Hirschsprung disease (HSCR) is a partially penetrant oligogenic birth defect that occurs when enteric nervous system (ENS) precursors fail to colonize the distal bowel during early pregnancy. Genetic defects underlie HSCR, but much of the variability in the occurrence and severity of the birth defect remain unexplained. We hypothesized that nongenetic factors might contribute to disease development. Here we found that mycophenolate, an inhibitor of de novo guanine nucleotide biosynthesis, and 8 other drugs identified in a zebrafish screen impaired ENS development. In mice, mycophenolate treatment selectively impaired ENS precursor proliferation, delayed precursor **migration**, and induced bowel aganglionosis. In 2 different mouse models of HSCR, addition of mycophenolate increased the penetrance and severity of Hirschsprung-like pathology. Mycophenolate treatment also reduced ENS precursor **migration** as well as lamellipodia formation, proliferation, and survival in cultured enteric neural crest–derived cells. Using X-inactivation mosaicism for the purine salvage gene Hprt, we found that reduced ENS precursor proliferation most likely causes mycophenolate-induce**d migrati**on defects and aganglionosis. To the best of our knowledge, mycophenolate is the first medicine identified that causes major ENS malformations and Hirschsprung-like pathology in a mammalian model. These studies demonstrate a critical role for de novo guanine nucleotide biosynthesis in ENS development and suggest that some cases of HSCR may be preventable.
56. Neural cells in the esophagus respond to glial cell line-derived neurotrophic factor and neurturin, and are RET-dependent. Glial cell line-derived neurotrophic factor (GDNF) is expressed in the gastrointestinal tract of the developing mouse and appears to play an important role in the **migration** of enteric neuron precursors into and along the small and large intestines. Two other GDNF family members, neurturin and artemin, are also expressed in the developing gut although artemin is only expressed in the esophagus. We examined the effects of GDNF, neurturin, and artemin on neural crest cell **migration** and neurite outgrowth in explants of mouse esophagus, midgut, and hindgut. Both GDNF and neurturin induced neural crest cell **migration** and neurite outgrowth in all regions examined. In the esophagus, the effect of GDNF on **migration** and neurite outgrowth declined with age between E11.5 and E14.5, but neurturin still had a strong neurite outgrowth effect at E14.5. Artemin did not promote neural **migration** or neurite outgrowth in any region investigated. The effects of GDNF family ligands are mediated by the Ret tyrosine kinase. We examined the density of neurons in the esophagus of Ret-/- mice, which lack neurons in the small and large intestines. The density of esophageal neurons in Ret-/- mice was only about 4% of the density of esophageal neurons in Ret+/- and Ret+/+ mice. These results show that GDNF and neurturin promote **migration** and neurite outgrowth of crest-derived cells in the esophagus as well as the intestine. Moreover, like intestinal neurons, the development of esophageal neurons is largely Ret-dependent.
57. Protein kinase A regulates GDNF/RET-dependent but not GDNF/Ret-independent ureteric bud outgrowth from the Wolffian duct. Embryonic kidney development begins with the outgrowth of the ureteric bud (UB) from the Wolffian duct (WD) into the adjacent metanephric mesenchyme (MM). Both a GDNF-dependent and GDNF-independent (Maeshima et al., 2007) pathway have been identified. In vivo and in vitro, the GDNF-dependent pathway is inhibited by BMPs, one of the factors invoked to explain the limitation of UB formation in the unbudded regions of the WD surrounding the UB. However, the exact mechanism remains unknown. Here a previously described in vitro system that models UB budding from the WD was utilized to study this process. Because Protein kinase A (PKA) activation has been shown to prevent **migration**, morphogenesis and tubulogenesis of epithelial cells (Santos et al., 1993), its activity in budded and non-budded portions of the GDNF-induced WD was analyzed. The level of PKA activity was 15-fold higher in the unbudded portions of the WD compared to budded portions, suggesting that PKA activity plays a key role in controlling the site of UB emergence. Using well-characterized PKA agonists and antagonists, we demonstrated that at various levels of the PKA-signaling hierarchy, PKA regulates UB outgrowth from the WD by suppressing budding events. This process appeared to be PKA-2 isoform specific, and mediated by changes in the duct rather than the surrounding mesenchyme. In addition, it was not due to changes in either the sorting of junctional proteins, cell death, or cell proliferation. Furthermore, the suppressive effect of cAMP on budding did not appear to be mediated by spread to adjacent cells via gap junctions. Conversely, antagonism of PKA activity stimulated UB outgrowth from the WD and resulted in both an increase in the number of buds per unit length of WD as well as a larger surface area per bud. Using microarrays, analysis of gene expression in GDNF-treated WDs in which the PKA pathway had been activated revealed a nearly 14-fold decrease in Ret, a receptor for GDNF. A smaller decrease in GFRα1. a co-receptor for GDNF, was also observed. Using Ret-null WDs, we were able to demonstrate that PKA regulated GDNF-dependent budding but not GDNF-independent pathway for WD budding. We also found that BMP2 was higher in unbudded regions of the GDNF-stimulated WD. Treatment of isolated WDs with BMP2 suppressed budding and resulted in a 3-fold increase in PKA activity. The data suggests that the suppression of budding by BMPs and possibly other factors in non-budded zones of the WD may be regulated in part by increased PKA activity, probably partially through downregulation of Ret/GFRα1 coreceptor expression.

#### GeneRIFs

1. Ret is a downstream target of Hoxb5 whose perturbation causes Ret haploinsufficiency, impaired NCC **migration**, and hypo/aganglionosis, suggesting that Hoxb5 may contribute to the etiology of Hirschsprung's disease.
2. We provide evidence that activation of the receptor tyrosine kinase RET by glial cell line-derived neurotrophic factor (GDNF) is required for the directional **migration** of ENS progenitors towards and within the gut wall.
3. cAMP-dependent modification of RET function regulates the JNK signaling responsible for proper **migration** of the enteric neural crest cells in the developing gut.

### ECM

#### PubMed

1. The **ECM** protein nephronectin promotes kidney development via integrin alpha8beta1-mediated stimulation of Gdnf expression. Development of the metanephric kidney crucially depends on proper interactions between cells and the surrounding extracellular matrix. For example, we showed previously that in the absence of alpha8beta1 integrin, invasion by the ureteric bud into the metanephric mesenchyme is inhibited, resulting in renal agenesis. Here we present genetic evidence that the extracellular matrix protein nephronectin is an essential ligand that engages alpha8beta1 integrin during early kidney development. We show that embryos lacking a functional nephronectin gene frequently display kidney agenesis or hypoplasia, which can be traced to a delay in the invasion of the metanephric mesenchyme by the ureteric bud at an early stage of kidney development. Significantly, we detected no defects in extracellular matrix organization in the nascent kidneys of the nephronectin mutants. Instead, we found that Gdnf expression was dramatically reduced in both nephronectin- and alpha8 integrin-null mutants specifically in the metanephric mesenchyme at the time of ureteric bud invasion. We show that this reduction is sufficient to explain the agenesis and hypoplasia observed in both mutants. Interestingly, the reduction in Gdnf expression is transient, and its resumption presumably enables the nephronectin-deficient ureteric buds to invade the metanephric mesenchyme and begin branching. Our results thus place nephronectin and alpha8beta1 integrin in a pathway that regulates Gdnf expression and is essential for kidney development.

### [Ee]xtracellular [Mm]atrix

#### PubMed

1. Integrin-linked kinase is an adaptor with essential functions during mouse development. The development of multicellular organisms requires integrin-mediated interactions between cells and their extracellular environment. Integrin binding to **extracellular matrix** catalyses assembly of multiprotein complexes, which transduce mechanical and chemical signals that regulate many aspects of cell physiology. Integrin-linked kinase (Ilk) is a multifunctional protein that binds beta-integrin cytoplasmic domains and regulates actin dynamics by recruiting actin binding regulatory proteins such as alpha- and beta-parvin. Ilk has also been shown to possess serine/threonine kinase activity and to phosphorylate signalling proteins such as Akt1 and glycogen synthase kinase 3beta (Gsk3beta) in mammalian cells; however, these functions have been shown by genetic studies not to occur in flies and worms. Here we show that mice carrying point mutations in the proposed autophosphorylation site of the putative kinase domain and in the pleckstrin homology domain are normal. In contrast, mice with point mutations in the conserved lysine residue of the potential ATP-binding site of the kinase domain, which mediates Ilk binding to alpha-parvin, die owing to renal agenesis. Similar renal defects occur in alpha-parvin-null mice. Thus, we provide genetic evidence that the kinase activity of Ilk is dispensable for mammalian development; however, an interaction between Ilk and alpha-parvin is critical for kidney development.
2. The ECM protein nephronectin promotes kidney development via integrin alpha8beta1-mediated stimulation of Gdnf expression. Development of the metanephric kidney crucially depends on proper interactions between cells and the surrounding **extracellular matrix**. For example, we showed previously that in the absence of alpha8beta1 integrin, invasion by the ureteric bud into the metanephric mesenchyme is inhibited, resulting in renal agenesis. Here we present genetic evidence that the **extracellular matrix** protein nephronectin is an essential ligand that engages alpha8beta1 integrin during early kidney development. We show that embryos lacking a functional nephronectin gene frequently display kidney agenesis or hypoplasia, which can be traced to a delay in the invasion of the metanephric mesenchyme by the ureteric bud at an early stage of kidney development. Significantly, we detected no defects in **extracellular matrix** organization in the nascent kidneys of the nephronectin mutants. Instead, we found that Gdnf expression was dramatically reduced in both nephronectin- and alpha8 integrin-null mutants specifically in the metanephric mesenchyme at the time of ureteric bud invasion. We show that this reduction is sufficient to explain the agenesis and hypoplasia observed in both mutants. Interestingly, the reduction in Gdnf expression is transient, and its resumption presumably enables the nephronectin-deficient ureteric buds to invade the metanephric mesenchyme and begin branching. Our results thus place nephronectin and alpha8beta1 integrin in a pathway that regulates Gdnf expression and is essential for kidney development.
3. Reelin signaling is necessary for a specific step in the migration of hindbrain efferent neurons. The cytoarchitecture of the hindbrain results from precise and co-ordinated sequences of neuronal migrations. Here, we show that reelin, an **extracellular matrix** protein involved in neuronal migration during CNS development, is necessary for an early, specific step in the migration of several hindbrain nuclei. We identified two cell populations not previously known to be affected in reeler mutants that show a common migratory defect: the olivocochlear efferent neurons and the facial visceral motor nucleus. In control embryos, these cells migrate first toward a lateral position within the neural tube, and then parallel to the glial cell processes, to a ventral position where they settle close to the pial surface. In reeler mutants, the first migration is not affected, but the neurons are unable to reach the pial surface and remain in an ectopic position. Indeed, this is the first evidence that the migration of specific hindbrain nuclei can be divided into two parts: a reelin-independent and a reelin-dependent migration. We also show that reelin is expressed at high levels at the final destination of the migratory process, while the reelin intracellular effector Dab1 was expressed by cell groups that included the two populations affected. Mice mutant at the Dab1 locus, called scrambler, exhibit the same phenotype, a failure of final migration. However, examination of mice lacking both reelin receptors, ApoER2 and VLDLR, did not reveal the same phenotype, suggesting involvement of an additional reelin-binding receptor. In the hindbrain, reelin signaling might alter the adhesive properties of efferent neurons and their ability to respond to directional cues, as has been suggested for the migration of olfactory bulb precursors.
4. Gene expression in RET/PTC3 and E7 transgenic mouse thyroids: RET/PTC3 but not E7 tumors are partial and transient models of human papillary thyroid cancers. We studied gene expression profiles in two mouse models of human thyroid carcinoma: the Tg-RET/PTC3 (RP3) and Tg-E7 mice. RP3 fusion gene is the most frequent mutation found in the first wave post-Chernobyl papillary thyroid cancers (PTCs). E7 is an oncoprotein derived from the human papillomavirus 16 responsible for most cervical carcinoma in women. Both transgenic mice develop thyroid hyperplasia followed by solid differentiated carcinoma in older animals. To understand the different steps leading to carcinoma, we analyzed thyroid gene expression in both strains at different ages by microarray technology. Important biological processes were differentially regulated in the two tumor types. In E7 thyroids, cell cycle was the most up-regulated process, an observation consistent with the huge size of these tumors. In RP3 thyroids, contrary to E7 tumors, several human PTC characteristics were observed: overexpression of many immune-related genes, regulation of human PTC markers, up-regulation of EGF-like growth factors and significant regulation of angiogenesis and **extracellular matrix** remodeling-related genes. However, similarities were incomplete; they did not concern the overall gene expression and were not conserved in old animals. Therefore, RP3 tumors are partial and transient models of human PTC. They constitute a good model, especially in young animals, to study the respective role of the biological processes shared with human PTC and will allow testing drugs targeting these validated variables.
5. Increased expression of laminin-1 and collagen (IV) subunits in the aganglionic bowel of ls/ls, but not c-ret -/- mice. **Extracellular matrix** molecules, including laminin, affect the development of enteric neurons and accumulate in the aganglionic colon of ls/ls mice. Quantitative Northern analysis revealed that mRNAs encoding the beta 1 and gamma 1 subunits of laminin and collagens alpha 1(IV) and alpha 2(IV) are increased in the colons of ls/ls mice. Transcripts of laminin alpha 1 were evaluated quantitatively with reverse transcription and the competitive polymerase chain reaction (RT-cPCR). The abundance of laminin alpha 1 transcripts was developmentally regulated, but greater in the ls/ls than the wild-type colon at each age examined. In situ hybridization revealed that transcripts in the colon encoding laminin alpha 1 and beta 1 and collagen alpha 2(IV) were initially expressed in the endoderm, but by E15, expression shifted to cells of the colonic mesenchyme (ls/ls > wild type) where crest-derived cells migrate. The expression of laminin alpha 1 was examined in the totally aganglionic intestine of E15 and newborn c-ret -/- mice, to determine whether an increase occurs when neurogenesis fails independently of the ls/ls defect. RT-cPCR revealed no difference from control in mRNA encoding laminin alpha 1 in the c-ret -/- colon in either E15 or newborn animals. The accumulation of immunohistochemically demonstrable laminin that is prominent in the newborn ls/ls colon could not be detected in that of c-ret -/- animals. These observations suggest that transcripts encoding laminin-1 and collagen (IV) are increased in the colon and surrounding pelvic mesenchyme of ls/ls mice because of an intrinsic lesion, rather than a secondary consequence of aganglionosis. The data are compatible with the hypothesis that the increased expression of laminin-1 contributes to the failure of crest-derived cells to complete their colonization of the ls/ls colon.

### [Ii]nvasive

#### PubMed

1. Transgenic mouse models for tumors of melanocytes and retinal pigment epithelium. Cutaneous and ocular melanomas are due to malignant transformation of neural crest-derived melanocytes. The rising incidence of this tumor in humans has stimulated experiments to devise suitable mouse models. In the past years, transgenic mouse lines have been generated using different oncogenes - Ha-ras, SV40 T antigen (Tag), ret - which develop benign lesions of melanocytes, melanoma, and/or eye tumors. Pigment cell tumors in humans, although rather rare, can also develop from the retinal pigment epithelium (RPE), a cell layer of neuroectodermal origin. We, therefore, established transgenic models for this ocular tumor. Regulated by the promoter of tyrosinase-related protein-1 (TRP-1), two oncogenes, ret and SV40 Tag, were targeted to the developing RPE in transgenic mice. The TRP-1/ret transgenic mice displayed microphthalmia and benign tumors of the RPE. Expression of SV40 T antigen (TRP-1/Tag) led to malignant tumors, which were **invasive** and metastasized to inguinal lymph node and spleen.

### [Mm]etastasize

#### PubMed

1. Transgenic mouse models for tumors of melanocytes and retinal pigment epithelium. Cutaneous and ocular melanomas are due to malignant transformation of neural crest-derived melanocytes. The rising incidence of this tumor in humans has stimulated experiments to devise suitable mouse models. In the past years, transgenic mouse lines have been generated using different oncogenes - Ha-ras, SV40 T antigen (Tag), ret - which develop benign lesions of melanocytes, melanoma, and/or eye tumors. Pigment cell tumors in humans, although rather rare, can also develop from the retinal pigment epithelium (RPE), a cell layer of neuroectodermal origin. We, therefore, established transgenic models for this ocular tumor. Regulated by the promoter of tyrosinase-related protein-1 (TRP-1), two oncogenes, ret and SV40 Tag, were targeted to the developing RPE in transgenic mice. The TRP-1/ret transgenic mice displayed microphthalmia and benign tumors of the RPE. Expression of SV40 T antigen (TRP-1/Tag) led to malignant tumors, which were invasive and **metastasize**d to inguinal lymph node and spleen.

### [Mm]etastasis

#### PubMed

1. A novel RET kinase-beta-catenin signaling pathway contributes to tumorigenesis in thyroid carcinoma. The RET receptor tyrosine kinase has essential roles in cell survival, differentiation, and proliferation. Oncogenic activation of RET causes the cancer syndrome multiple endocrine neoplasia type 2 (MEN 2) and is a frequent event in sporadic thyroid carcinomas. However, the molecular mechanisms underlying RET's potent transforming and mitogenic signals are still not clear. Here, we show that nuclear localization of beta-catenin is frequent in both thyroid tumors and their metastases from MEN 2 patients, suggesting a novel mechanism of RET-mediated function through the beta-catenin signaling pathway. We show that RET binds to, and tyrosine phosphorylates, beta-catenin and show that the interaction between RET and beta-catenin can be direct and independent of cytoplasmic kinases, such as SRC. As a result of RET-mediated tyrosine phosphorylation, beta-catenin escapes cytosolic down-regulation by the adenomatous polyposis coli/Axin/glycogen synthase kinase-3 complex and accumulates in the nucleus, where it can stimulate beta-catenin-specific transcriptional programs in a RET-dependent fashion. We show that down-regulation of beta-catenin activity decreases RET-mediated cell proliferation, colony formation, and tumor growth in nude mice. Together, our data show that a beta-catenin-RET kinase pathway is a critical contributor to the development and **metastasis** of human thyroid carcinoma.
2. The tyrosinase related protein-1 (Tyrp1) promoter in transgenic experiments: targeted expression to the retinal pigment epithelium. Transgenic experiments targeting gene expression to the retinal pigment epithelium (RPE) require use of a pigment cell-specific promoter. We have chosen 1.4 kb and 4 kb from the promoter of the tyrosinase-related protein 1 gene (Tyrp1) for RPE-specific expression, since Tyrp1 mRNA and protein are detected already at midgestation in this epithelial layer. In eyes of transgenic embryos, expression of the Tyrp1-lacZ fusion construct led to strong and specific expression of beta-galactosidase in the RPE from day E10.5 onwards. The promoter thus proved useful to target expression of two different oncogenes to the RPE, a constitutively active tyrosine kinase receptor (Rfp/Ret) and SV40 T antigen (Tag). Tyrp1-Rfp/Ret transgenic mice developed microphthalmia, primarily induced by changes in the developing RPE. In addition, Tyrp1-Rfp/Ret expression induced proliferation of RPE cells leading to benign RPE tumors in the adult. Tyrp1-Tag transgenic mice developed malignant eye tumors of RPE origin, which invaded the optic nerve and led to **metastasis** into lymph nodes and spleen.

#### GeneRIFs

1. a beta-catenin-RET kinase pathway is a critical contributor to the development and **metastasis** of thyroid carcinoma

---

## Fgfrl1

- NCBI Gene
- GeneRIF
- Pubmed

| Search Term | Rank | Fields |
| --- | --- | --- |
| [Mm]igration | 6 | PubMed(1) |

  
hide/show details for Fgfrl1

### [Mm]igration

#### PubMed

1. Examination of FGFRL1 as a candidate gene for diaphragmatic defects at chromosome 4p16.3 shows that Fgfrl1 null mice have reduced expression of Tpm3, sarcomere genes and Lrtm1 in the diaphragm. Fgfrl1 (also known as Fgfr5; OMIM 605830) homozygous null mice have thin, amuscular diaphragms and die at birth because of diaphragm hypoplasia. FGFRL1 is located at 4p16.3, and this chromosome region can be deleted in patients with congenital diaphragmatic hernia (CDH). We examined FGFRL1 as a candidate gene for the diaphragmatic defects associated with 4p16.3 deletions and re-sequenced this gene in 54 patients with CDH. We confirmed six known coding single nucleotide polymorphisms (SNPs): c.209G > A (p.Pro20Pro), c.977G > A (p.Pro276Pro), c.1040T > C (p.Asp297Asp), c.1234C > A (p.Pro362Gln), c.1420G > T (p.Arg424Leu), and c.1540C > T (p.Pro464Leu), but we did not identify any gene mutations. We genotyped additional CDH patients for four of these six SNPs, including the three non-synonymous SNPs, to make a total of 200 chromosomes, and found that the allele frequency for the four SNPs, did not differ significantly between patients and normal controls (p > or = 0.05). We then used Affymetrix Genechip Mouse Gene 1.0 ST arrays and found eight genes with significantly reduced expression levels in the diaphragms of Fgfrl1 homozygous null mice when compared with wildtype mice-Tpm3, Fgfrl1 (p = 0.004), Myl2, Lrtm1, Myh4, Myl3, Myh7 and Hephl1. Lrtm1 is closely related to Slit3, a protein associated with herniation of the central tendon of the diaphragm in mice. The Slit proteins are known to regulate axon branching and cell **migration**, and inhibition of Slit3 reduces cell motility and decreases the expression of Rac and Cdc42, two genes that are essential for myoblast fusion. Further studies to determine if Lrtm1 has a similar function to Slit3 and if reduced Fgfrl1 expression can cause diaphragm hypoplasia through a mechanism involving decreased myoblast motility and/or myoblast fusion, seem indicated.

---

## Flt4

- NCBI Gene
- GeneRIF
- Pubmed

| Search Term | Rank | Fields |
| --- | --- | --- |
| [Ff]ilopodia | 1 | PubMed(3) |
| [Mm]igration | 6 | Process(1); PubMed(23); GeneRIFs(2) |
| ECM | 7 | PubMed(1) |
| [Ee]xtracellular [Mm]atrix | 8 | PubMed(6); GeneRIFs(1) |
| [Ii]nvasive | 10 | PubMed(5) |
| [Mm]etastasize | 11 | PubMed(1) |
| [Mm]etastasis | 12 | PubMed(15); GeneRIFs(2) |

  
hide/show details for Flt4

### [Ff]ilopodia

#### PubMed

1. VEGF receptor 2/-3 heterodimers detected in situ by proximity ligation on angiogenic sprouts. The vascular endothelial growth factors VEGFA and VEGFC are crucial regulators of vascular development. They exert their effects by dimerization and activation of the cognate receptors VEGFR2 and VEGFR3. Here, we have used in situ proximity ligation to detect receptor complexes in intact endothelial cells. We show that both VEGFA and VEGFC potently induce formation of VEGFR2/-3 heterodimers. Receptor heterodimers were found in both developing blood vessels and immature lymphatic structures in embryoid bodies. We present evidence that heterodimers frequently localize to tip cell **filopodia**. Interestingly, in the presence of VEGFC, heterodimers were enriched in the leading tip cells as compared with trailing stalk cells of growing sprouts. Neutralization of VEGFR3 to prevent heterodimer formation in response to VEGFA decreased the extent of angiogenic sprouting. We conclude that VEGFR2/-3 heterodimers on angiogenic sprouts induced by VEGFA or VEGFC may serve to positively regulate angiogenic sprouting.
2. Angiopoietin-1 promotes lymphatic sprouting and hyperplasia. Angiopoietin 1 (Ang1), a ligand for the receptor tyrosine kinase Tie2, regulates the formation and stabilization of the blood vessel network during embryogenesis. In adults, Ang1 is associated with blood vessel stabilization and recruitment of perivascular cells, whereas Ang2 acts to counter these actions. Recent results from gene-targeted mice have shown that Ang2 is also essential for the proper patterning of lymphatic vessels and that Ang1 can be substituted for this function. In order to characterize the effects of the angiopoietins on lymphatic vessels, we employed viral vectors for overexpression of Ang1 in adult mouse tissues. We found that Ang1 activated lymphatic vessel endothelial proliferation, vessel enlargement, and generation of long endothelial cell **filopodia** that eventually fused, leading to new sprouts and vessel development. Cutaneous lymphatic hyperplasia was also detected in transgenic mice expressing Ang1 in the basal epidermal cells. Tie2 was expressed in the lymphatic endothelial cells and Ang1 stimulation of these cells resulted in up-regulation of vascular endothelial growth factor receptor 3 (VEGFR-3). Furthermore, a soluble form of VEGFR-3 inhibited the observed lymphatic sprouting. Our results reinforce the concept that Ang1 therapy may be useful in settings of tissue edema.
3. Angiopoietin-1 promotes lymphatic sprouting and hyperplasia. Angiopoietin 1 (Ang1), a ligand for the receptor tyrosine kinase Tie2, regulates the formation and stabilization of the blood vessel network during embryogenesis. In adults, Ang1 is associated with blood vessel stabilization and recruitment of perivascular cells, whereas Ang2 acts to counter these actions. Recent results from gene-targeted mice have shown that Ang2 is also essential for the proper patterning of lymphatic vessels and that Ang1 can be substituted for this function. In order to characterize the effects of the angiopoietins on lymphatic vessels, we employed viral vectors for overexpression of Ang1 in adult mouse tissues. We found that Ang1 activated lymphatic vessel endothelial proliferation, vessel enlargement, and generation of long endothelial cell **filopodia** that eventually fused, leading to new sprouts and vessel development. Cutaneous lymphatic hyperplasia was also detected in transgenic mice expressing Ang1 in the basal epidermal cells. Tie2 was expressed in the lymphatic endothelial cells and Ang1 stimulation of these cells resulted in up-regulation of vascular endothelial growth factor receptor 3 (VEGFR-3). Furthermore, a soluble form of VEGFR-3 inhibited the observed lymphatic sprouting. Our results reinforce the concept that Ang1 therapy may be useful in settings of tissue edema.

### [Mm]igration

#### Process

1. positive regulation of endothelial cell **migration**

#### PubMed

1. Robo1 regulates semaphorin signaling to guide the **migration** of cortical interneurons through the ventral forebrain. Cortical interneurons, generated predominantly in the medial ganglionic eminence, migrate around and avoid the developing striatum in the subpallium en route to the cortex. This is attributable to the chemorepulsive cues of class 3 semaphorins expressed in the striatal mantle and acting through neuropilin (Nrp1 and Nrp2) receptors expressed in these cells. Cortical interneurons also express Robo receptors, and we show here that in mice lacking Robo1, but not Robo2, these cells migrate aberrantly through the striatum. In vitro experiments demonstrated that interneurons lacking Robo1 function are significantly less responsive to the effects of semaphorins. Failure to respond to semaphorin appears to be attributable to a reduction in Nrp1 and PlexinA1 receptors within these cells. Biochemical studies further demonstrated that Robo1 binds directly to Nrp1, but not to semaphorins, and this interaction is mediated by a region contained within its first two Ig domains. Thus, we show for the first time that Robo1 interacts with Nrp1 to modulate semaphorin signaling in the developing forebrain and direct the **migration** of interneurons through the subpallium and into the cortex.
2. Cooperative and redundant roles of VEGFR-2 and VEGFR-3 signaling in adult lymphangiogenesis. Activation of vascular endothelial growth factor (VEGF) receptor-3 (VEGFR-3) by VEGF-C initiates lymphangiogenesis by promoting lymphatic proliferation and **migration**. However, it is unclear whether VEGFR-3 signaling is required beyond these initial stages, namely during the organization of new lymphatic endothelial cells (LECs) into functional capillaries. Furthermore, the role of VEGFR-2, which is also expressed on LECs and binds VEGF-C, is unclear. We addressed these questions by selectively neutralizing VEGFR-3 and/or VEGFR-2 for various time periods in an adult model of lymphangiogenesis in regenerating skin. While blocking either VEGFR-2 or VEGFR-3 with specific antagonist mAbs (DC101 and mF4-31C1, respectively) prior to lymphatic **migration** prevented lymphangiogenesis, blocking VEGFR-3 subsequent to **migration** did not affect organization into functional capillaries, and VEGFR-2 blocking had only a small hindrance on organization. These findings were confirmed in vitro using human LECs and anti-human antagonist mAbs (IMC-1121a and hF4-3C5): both VEGFR-2 and -3 signaling were required for **migration** and proliferation, but tubulogenesis in 3D cultures was unaffected by VEGFR-3 blocking and partially hindered by VEGFR-2 blocking. Furthermore, both in vitro and in vivo, while VEGFR-3 blocking had no effect on LEC organization, coneutralization of VEGFR-2, and VEGFR-3 completely prevented lymphatic organization. Our findings demonstrate that cooperative signaling of VEGFR-2 and -3 is necessary for lymphatic **migration** and proliferation, but VEGFR-3 is redundant with VEGFR-2 for LEC organization into functional capillaries.
3. Heterozygous expression of the oncogenic Pik3ca(H1047R) mutation during murine development results in fatal embryonic and extraembryonic defects. The phosphoinositide 3-kinase (PI3K)/AKT signalling pathway regulates many cellular functions including proliferation, **migration**, survival and protein synthesis. Somatic mutations in PIK3CA, the gene encoding the p110α catalytic subunit of PI3K enzyme, are commonly associated with many human cancers as well as recently being implicated in human overgrowth syndromes. However, it is not clear if such mutations can be inherited through the germline. We have used a novel mouse model with Cre recombinase (Cre)-conditional knock-in of the common H1047R mutation into the endogenous Pik3ca gene. Heterozygous expression of the Pik3ca(H1047R) mutation in the developing mouse embryo resulted in failed 'turning' of the embryo and disrupted vascular remodelling within the embryonic and extraembryonic tissues, leading to lethality prior to E10. As vascular endothelial growth factor A (VEGF-A) signalling was disrupted in these embryos, we used Cre under the control of the Tie2 promoter to target the Pik3ca(H1047R) mutation specifically to endothelial cells. In these embryos turning occurred normally but the vascular remodelling defects and embryonic lethality remained, likely as a result of endothelial hyperproliferation. Our results confirm the lethality associated with heterozygous expression of the Pik3ca(H1047R) mutation during development and likely explain the lack of inherited germline PIK3CA mutations in humans.
4. SnoN facilitates ALK1-Smad1/5 signaling during embryonic angiogenesis. In endothelial cells, two type I receptors of the transforming growth factor β (TGF-β) family, ALK1 and ALK5, coordinate to regulate embryonic angiogenesis in response to BMP9/10 and TGF-β. Whereas TGF-β binds to and activates ALK5, leading to Smad2/3 phosphorylation and inhibition of endothelial cell proliferation **and migra**tion, BMP9/10 and TGF-β also bind to ALK1, resulting in the activation of Smad1/5. SnoN is a negative regulator of ALK5 signaling through the binding and repression of Smad2/3. Here we uncover a positive role of SnoN in enhancing Smad1/5 activation in endothelial cells to promote angiogenesis. Upon ligand binding, SnoN directly bound to ALK1 on the plasma membrane and facilitated the interaction between ALK1 and Smad1/5, enhancing Smad1/5 phosphorylation. Disruption of this SnoN-Smad interaction impaired Smad1/5 activation and up-regulated Smad2/3 activity. This resulted in defective angiogenesis and arteriovenous malformations, leading to embryonic lethality at E12.5. Thus, SnoN is essential for TGF-β/BMP9-dependent biological processes by its ability to both positively and negatively modulate the activities of Smad-dependent pathways.
5. Early endocardial morphogenesis requires Scl/Tal1. The primitive heart tube is composed of an outer myocardial and an inner endocardial layer that will give rise to the cardiac valves and septa. Specification and differentiation of these two cell layers are among the earliest events in heart development, but the embryonic origins and genetic regulation of early endocardial development remain largely undefined. We have analyzed early endocardial development in the zebrafish using time-lapse confocal microscopy and show that the endocardium seems to originate from a region in the lateral plate mesoderm that will give rise to hematopoietic cells of the primitive myeloid lineage. Endocardial precursors appear to rapidly migrate to the site of heart tube formation, where they arrive prior to the bilateral myocardial primordia. Analysis of a newly discovered zebrafish Scl/Tal1 mutant showed an additional and previously undescribed role of this transcription factor during the development of the endocardium. In Scl/Tal1 mutant embryos, endocardial precursors are specified, but **migration** is severely defective and endocardial cells aggregate at the ventricular pole of the heart. We further show that the initial fusion of the bilateral myocardial precursor populations occurs independently of the endocardium and tal1 function. Our results suggest early separation of the two components of the primitive heart tube and imply Scl/Tal1 as an indispensable component of the molecular hierarchy that controls endocardium morphogenesis.
6. A novel multistep mechanism for initial lymphangiogenesis in mouse embryos based on ultramicroscopy. During mammalian development, a subpopulation of endothelial cells in the cardinal vein (CV) expresses lymphatic-specific genes and subsequently develops into the first lymphatic structures, collectively termed as lymph sacs. Budding, sprouting and ballooning of lymphatic endothelial cells (LECs) have been proposed to underlie the emergence of LECs from the CV, but the exact mechanisms of lymph vessel formation remain poorly understood. Applying selective plane illumination-based ultramicroscopy to entire wholemount-immunostained mouse embryos, we visualized the complete developing vascular system with cellular resolution. Here, we report emergence of the earliest detectable LECs as strings of loosely connected cells between the CV and superficial venous plexus. Subsequent aggregation of LECs resulted in formation of two distinct, previously unidentified lymphatic structures, the dorsal peripheral longitudinal lymphatic vessel (PLLV) and the ventral primordial thoracic duct (pTD), which at later stages formed a direct contact with the CV. Providing new insights into their function, we found vascular endothelial growth factor C (VEGF-C) and the matrix component CCBE1 indispensable for LEC budding and **migration**. Altogether, we present a significantly more detailed view and novel model of early lymphatic development.
7. Impaired angiogenesis and altered Notch signaling in mice overexpressing endothelial Egfl7. Epidermal growth factor-like domain 7 (Egfl7) is important for regulating tubulogenesis in zebrafish, but its role in mammals remains unresolved. We show here that endothelial overexpression of Egfl7 in transgenic mice leads to partial lethality, hemorrhaging, and altered cardiac morphogenesis. These defects are accompanied by abnormal vascular patterning and remodeling in both the embryonic and postnatal vasculature. Egfl7 overexpression in the neonatal retina results in a hyperangiogenic response, and EGFL7 knockdown in human primary endothelial cells suppresses endothelial cell proliferation, sprouting, and **migration**. These phenotypes are reminiscent of Notch inhibition. In addition, our results show that EGFL7 and endothelial-specific NOTCH physically interact in vivo and strongly suggest that Egfl7 antagonizes Notch in both the postnatal retina and in primary endothelial cells. Specifically, Egfl7 inhibits Notch reporter activity and down-regulates the level of Notch target genes when overexpressed. In conclusion, we have uncovered a critical role for Egfl7 in vascular development and have shown that some of these functions are mediated through modulation of Notch signaling.
8. Semaphorin 3A contributes to distal pulmonary epithelial cell differentiation and lung morphogenesis. Semaphorin 3A (Sema3A) is a neural guidance cue that also mediates cell **migration**, proliferation and apoptosis, and inhibits branching morphogenesis. Because we have shown that genetic deletion of neuropilin-1, which encodes an obligatory Sema3A co-receptor, influences airspace remodeling in the smoke-exposed adult lung, we sought to determine whether genetic deletion of Sema3A altered distal lung structure.To determine whether loss of Sema3A signaling influenced distal lung morphology, we compared pulmonary histology, distal epithelial cell morphology and maturation, and the balance between lung cell proliferation and death, in lungs from mice with a targeted genetic deletion of Sema3A (Sema3A(-/-)) and wild-type (Sema3A(+/+)) littermate controls.Genetic deletion of Sema3A resulted in significant perinatal lethality. At E17.5, lungs from Sema3A(-/-) mice had thickened septae and reduced airspace size. Distal lung epithelial cells had increased intracellular glycogen pools and small multivesicular and lamellar bodies with atypical ultrastructure, as well as reduced expression of type I alveolar epithelial cell markers. Alveolarization was markedly attenuated in lungs from the rare Sema3A(-/-) mice that survived the immediate perinatal period. Furthermore, Sema3A deletion was linked with enhanced postnatal alveolar septal cell death.These data suggest that Sema3A modulates distal pulmonary epithelial cell development and alveolar septation. Defining how Sema3A influences structural plasticity of the developing lung is a critical first step for determining if this pathway can be exploited to develop innovative strategies for repair after acute or chronic lung injury.
9. Inflammation induces lymphangiogenesis through up-regulation of VEGFR-3 mediated by NF-kappaB and Prox1. The concept of inflammation-induced lymphangiogenesis (ie, formation of new lymphatic vessels) has long been recognized, but the molecular mechanisms remained largely unknown. The 2 primary mediators of lymphangiogenesis are vascular endothelial growth factor receptor-3 (VEGFR-3) and Prox1. The key factors that regulate inflammation-induced transcription are members of the nuclear factor-kappaB (NF-kappaB) family; however, the role of NF-kappaB in regulation of lymphatic-specific genes has not been defined. Here, we identified VEGFR-3 and Prox1 as downstream targets of the NF-kappaB pathway. In vivo time-course analysis of inflammation-induced lymphangiogenesis showed activation of NF-kappaB followed by sequential up-regulation of Prox1 and VEGFR-3 that preceded lymphangiogenesis by 4 and 2 days, respectively. Activation of NF-kappaB by inflammatory stimuli also elevated Prox1 and VEGFR-3 expression in cultured lymphatic endothelial cells, resulting in increased proliferation and **migration**. We also show that Prox1 synergizes with the p50 of NF-kappaB to control VEGFR-3 expression. Collectively, our findings suggest that induction of the NF-kappaB pathway by inflammatory stimuli activates Prox1, and both NF-kappaB and Prox1 activate the VEGFR-3 promoter leading to increased receptor expression in lymphatic endothelial cells. This, in turn, enhances the responsiveness of preexisting lymphatic endothelium to VEGFR-3 binding factors, VEGF-C and VEGF-D, ultimately resulting in robust lymphangiogenesis.
10. Inflammation induces lymphangiogenesis through up-regulation of VEGFR-3 mediated by NF-kappaB and Prox1. The concept of inflammation-induced lymphangiogenesis (ie, formation of new lymphatic vessels) has long been recognized, but the molecular mechanisms remained largely unknown. The 2 primary mediators of lymphangiogenesis are vascular endothelial growth factor receptor-3 (VEGFR-3) and Prox1. The key factors that regulate inflammation-induced transcription are members of the nuclear factor-kappaB (NF-kappaB) family; however, the role of NF-kappaB in regulation of lymphatic-specific genes has not been defined. Here, we identified VEGFR-3 and Prox1 as downstream targets of the NF-kappaB pathway. In vivo time-course analysis of inflammation-induced lymphangiogenesis showed activation of NF-kappaB followed by sequential up-regulation of Prox1 and VEGFR-3 that preceded lymphangiogenesis by 4 and 2 days, respectively. Activation of NF-kappaB by inflammatory stimuli also elevated Prox1 and VEGFR-3 expression in cultured lymphatic endothelial cells, resulting in increased proliferation and **migration**. We also show that Prox1 synergizes with the p50 of NF-kappaB to control VEGFR-3 expression. Collectively, our findings suggest that induction of the NF-kappaB pathway by inflammatory stimuli activates Prox1, and both NF-kappaB and Prox1 activate the VEGFR-3 promoter leading to increased receptor expression in lymphatic endothelial cells. This, in turn, enhances the responsiveness of preexisting lymphatic endothelium to VEGFR-3 binding factors, VEGF-C and VEGF-D, ultimately resulting in robust lymphangiogenesis.
11. Cooperative and redundant roles of VEGFR-2 and VEGFR-3 signaling in adult lymphangiogenesis. Activation of vascular endothelial growth factor (VEGF) receptor-3 (VEGFR-3) by VEGF-C initiates lymphangiogenesis by promoting lymphatic proliferation and **migration**. However, it is unclear whether VEGFR-3 signaling is required beyond these initial stages, namely during the organization of new lymphatic endothelial cells (LECs) into functional capillaries. Furthermore, the role of VEGFR-2, which is also expressed on LECs and binds VEGF-C, is unclear. We addressed these questions by selectively neutralizing VEGFR-3 and/or VEGFR-2 for various time periods in an adult model of lymphangiogenesis in regenerating skin. While blocking either VEGFR-2 or VEGFR-3 with specific antagonist mAbs (DC101 and mF4-31C1, respectively) prior to lymphatic **migration** prevented lymphangiogenesis, blocking VEGFR-3 subsequent to **migration** did not affect organization into functional capillaries, and VEGFR-2 blocking had only a small hindrance on organization. These findings were confirmed in vitro using human LECs and anti-human antagonist mAbs (IMC-1121a and hF4-3C5): both VEGFR-2 and -3 signaling were required for **migration** and proliferation, but tubulogenesis in 3D cultures was unaffected by VEGFR-3 blocking and partially hindered by VEGFR-2 blocking. Furthermore, both in vitro and in vivo, while VEGFR-3 blocking had no effect on LEC organization, coneutralization of VEGFR-2, and VEGFR-3 completely prevented lymphatic organization. Our findings demonstrate that cooperative signaling of VEGFR-2 and -3 is necessary for lymphatic **migration** and proliferation, but VEGFR-3 is redundant with VEGFR-2 for LEC organization into functional capillaries.
12. Tumour necrosis factor superfamily member 15 (Tnfsf15) facilitates lymphangiogenesis via up-regulation of Vegfr3 gene expression in lymphatic endothelial cells. Lymphangiogenesis is essential in embryonic development but is rare in adults. It occurs, however, in many disease conditions including cancers. Vascular endothelial growth factor-C/D (VEGF-C/D) and VEGF receptor-3 (Vegfr3) play a critical role in the regulation of lymphangiogenesis. We investigated how the VEGF-C/Vegfr3 signalling system is regulated by tumour necrosis factor superfamily member 15 (Tnfsf15), an endothelium-derived cytokine. We report here that Tnfsf15, which is known to induce apoptosis in vascular endothelial cells, can promote lymphatic endothelial cell (LEC) growth and **migration**, stimulate lymphangiogenesis, and facilitate lymphatic circulation. Treatment of mouse LECs with Tnfsf15 results in up-regulation of Vegfr3 expression; this can be inhibited by gene silencing of death domain-containing receptor-3 (DR3; Tnfrsf25), a cell surface receptor for Tnfsf15, with siRNA, or by blocking Tnfsf15-DR3 interaction with a Tnfsf15 neutralizing antibody, 4-3H. Additionally, Tnfsf15/DR3 signalling pathways in LECs include activation of NF-κB. Tnfsf15-overexpressing transgenic mice exhibit a marked enhancement of lymph drainage; this is confirmed by treatment of wild-type mice with intraperitoneal injection of recombinant Tnfsf15. Moreover, systemic treatment of pregnant Tnfsf15 transgenic mice with 4-3H leads to inhibition of embryonic lymphangiogenesis. Our data indicate that Tnfsf15, a cytokine produced largely by endothelial cells, facilitates lymphangiogenesis by up-regulating Vegfr3 gene expression in LECs, contributing to the maintenance of the homeostasis of the circulatory system. This finding also suggests that Tnfsf15 may be of potential value as a therapeutic tool for the treatment of lymphoedema.
13. ALK5 phosphorylation of the endoglin cytoplasmic domain regulates Smad1/5/8 signaling and endothelial cell **migration**. Endoglin, an endothelial cell-specific transforming growth factor-beta (TGF-beta) superfamily coreceptor, has an essential role in angiogenesis. Endoglin-null mice have an embryonic lethal phenotype due to defects in angiogenesis and mutations in endoglin result in the vascular disease hereditary hemorrhagic telangiectasia type I. Increased endoglin expression in the proliferating endothelium of tumors has been correlated with metastasis, tumor grade and decreased survival. Although endoglin is thought to regulate TGF-beta superfamily signaling in endothelial cells through regulating the balance between two TGF-beta-responsive pathways, the activin receptor-like kinase 5 (ALK5)/Smad2/3 pathway and the activin receptor-like kinase 1 (ALK1)/Smad1/5/8 pathway, the mechanism by which endoglin regulates angiogenesis has not been defined. Here, we investigate the role of the cytoplasmic domain of endoglin and its phosphorylation by ALK5 in regulating endoglin function in endothelial cells. We demonstrate that the cytoplasmic domain of endoglin is basally phosphorylated by ALK5, primarily on serines 646 and 649, in endothelial cells. Functionally, the loss of phosphorylation at serine 646 resulted in a loss of endoglin-mediated inhibition of Smad1/5/8 signaling in response to TGF-beta and endothelial cell **migration**, whereas loss of phosphorylation at both serines 646 and 649 resulted in a loss of endoglin-mediated inhibition of Smad1/5/8 signaling in response to bone morphogenetic protein-9. Taken together, these results support endoglin phosphorylation by ALK5 as an important mechanism for regulating TGF-beta superfamily signaling and **migration** in endothelial cells.
14. Prox1 induces lymphatic endothelial differentiation via integrin alpha9 and other signaling cascades. During embryonic lymphatic development, a homeobox transcription factor Prox1 plays important roles in sprouting and **migration** of a subpopulation of blood vessel endothelial cells (BECs) toward VEGF-C-expressing cells. However, effects of Prox1 on endothelial cellular behavior remain to be elucidated. Here, we show that Prox1, via induction of integrin alpha9 expression, inhibits sheet formation and stimulates motility of endothelial cells. Prox1-expressing BECs preferentially migrated toward VEGF-C via up-regulation of the expression of integrin alpha9 and VEGF receptor 3 (VEGFR3). In mouse embryos, expression of VEGFR3 and integrin alpha9 is increased in Prox1-expressing lymphatic endothelial cells (LECs) compared with BECs. Knockdown of Prox1 expression in human LECs led to decrease in the expression of integrin alpha9 and VEGFR3, resulting in the decreased chemotaxes toward VEGF-C. These findings suggest that Prox1 plays important roles in conferring and maintaining the characteristics of LECs by modulating multiple signaling cascades and that integrin alpha9 may function as a key regulator of lymphangiogenesis acting downstream of Prox1.
15. Normal dendritic cell mobilization to lymph nodes under conditions of severe lymphatic hypoplasia. To address the requirement for lymphatic capillaries in dendritic cell (DC) mobilization from skin to lymph nodes (LNs), we used mice bearing one inactivated allele of vascular endothelial growth factor receptor 3 (VEGFR3) where skin lymphatic capillaries are reported absent. Unexpectedly, DC mobilization from the back skin to draining LNs was similar in magnitude, and kinetics to control mice and humoral immunity appeared intact. By contrast, DC **migration** from body extremities, including ear and forepaws, was ablated. An evaluation in different regions of skin revealed rare patches of lymphatic capillaries only in body trunk areas where **migration** was intact. That is, whereas the ear skin was totally devoid of lymphatic capillaries, residual capillaries in the back skin were present though retained only at ∼10% normal density. This reduction in density markedly reduced the clearance of soluble tracers, indicating that normal cel**l migrati**on was spared under conditions when lymphatic transport function was poor. Residual lymphatic capillaries expressed slightly higher levels of CCL21 an**d migrati**on of skin DCs to LNs remained dependent on CCR7 in Chy mice. D**C migrati**on from the ear could be rescued by the introduction of a limited number of lymphatic capillaries through skin transplantation. Thus, the development of lymphatic capillaries in the skin of body extremities was more severely impacted by a mutant copy of VEGFR3 than trunk skin, but lymphatic transport function was markedly reduced throughout the skin, demonstrating that even under conditions when a marked loss in lymphatic capillary density reduces lymph transport, D**C migrati**on from skin to LNs remains normal.
16. Endothelial cell adhesion to the extracellular matrix induces c-Src-dependent VEGFR-3 phosphorylation without the activation of the receptor intrinsic kinase activity. Integrins cooperate with growth factor receptors to promote downstream signaling for cell proliferation and **migration**. However, the mechanism of receptor activation is still unknown.To analyze the mechanism of phosphorylation of the vascular endothelial growth factor receptor (VEGFR)-3 by cell adhesion.We show that VEGFR-3 phosphorylation, induced by cell attachment to the extracellular matrix, is independent from the intrinsic kinase activity of the receptor, as evidenced from phosphorylation cell adhesion experiments with a mutant kinase dead receptor or in the presence of the specific kinase inhibitor MAZ 51. Cell adhesion experiments in the presence of the c-Src inhibitor PP2 or in fibroblast triple knockout for c-Src, Yes, and Fyn (SYF) demonstrate that VEGFR-3 phosphorylation, induced by extracellular matrix, is mediated by c-Src. Kinase assays in vitro with recombinant c-Src show that VEGFR-3 is a direct c-Src target and mass spectrometry analysis identified the sites phosphorylated by c-Src as tyrosine 830, 833, 853, 1063, 1333, and 1337, demonstrating that integrin-mediated receptor phosphorylation induces a phosphorylation pattern that is distinct from that induced by growth factors. Furthermore, pull-down assays show that integrin-mediated VEGFR-3 phosphorylation activates the recruitment to the receptor of the adaptor proteins CRKI/II and SHC inducing activation of JNK.These data suggest that cell adhesion to extracellular matrix induces a downstream signaling using the tyrosine kinase receptor VEGFR-3 as scaffold.
17. The chemokine receptors ACKR2 and CCR2 reciprocally regulate lymphatic vessel density. Macrophages regulate lymphatic vasculature development; however, the molecular mechanisms regulating their recruitment to developing, and adult, lymphatic vascular sites are not known. Here, we report that resting mice deficient for the inflammatory chemokine-scavenging receptor, ACKR2, display increased lymphatic vessel density in a range of tissues under resting and regenerating conditions. This appears not to alter dendritic cell **migration** to draining lymph nodes but is associated with enhanced fluid drainage from peripheral tissues and thus with a hypotensive phenotype. Examination of embryonic skin revealed that this lymphatic vessel density phenotype is developmentally established. Further studies indicated that macrophages and the inflammatory CC-chemokine CCL2, which is scavenged by ACKR2, are associated with this phenotype. Accordingly, mice deficient for the CCL2 signalling receptor, CCR2, displayed a reciprocal phenotype of reduced lymphatic vessel density. Further examination revealed that proximity of pro-lymphangiogenic macrophages to developing lymphatic vessel surfaces is increased in ACKR2-deficient mice and reduced in CCR2-deficient mice. Therefore, these receptors regulate vessel density by reciprocally modulating pro-lymphangiogenic macrophage recruitment, and proximity, to developing, resting and regenerating lymphatic vessels.
18. CCBE1 is essential for mammalian lymphatic vascular development and enhances the lymphangiogenic effect of vascular endothelial growth factor-C in vivo. Collagen- and calcium-binding EGF domains 1 (CCBE1) has been associated with Hennekam syndrome, in which patients have lymphedema, lymphangiectasias, and other cardiovascular anomalies. Insight into the molecular role of CCBE1 is completely lacking, and mouse models for the disease do not exist.CCBE1 deficient mice were generated to understand the function of CCBE1 in cardiovascular development, and CCBE1 recombinant protein was used in both in vivo and in vitro settings to gain insight into the molecular function of CCBE1.Phenotypic analysis of murine Ccbe1 mutant embryos showed a complete lack of definitive lymphatic structures, even though Prox1(+) lymphatic endothelial cells get specified within the cardinal vein. Mutant mice die prenatally. Proximity ligation assays indicate that vascular endothelial growth factor receptor 3 activation appears unaltered in mutants. Human CCBE1 protein binds to components of the extracellular matrix in vitro, and CCBE1 protein strongly enhances vascular endothelial growth factor-C-mediated lymphangiogenesis in a corneal micropocket assay.Our data identify CCBE1 as a factor critically required for budding and **migration** of Prox-1(+) lymphatic endothelial cells from the cardinal vein. CCBE1 probably exerts these effects through binding to components of the extracellular matrix. CCBE1 has little lymphangiogenic effect on its own but dramatically enhances the lymphangiogenic effect of vascular endothelial growth factor-C in vivo. Thus, our data suggest CCBE1 to be essential but not sufficient for lymphangiogenesis.
19. Mirtron microRNA-1236 inhibits VEGFR-3 signaling during inflammatory lymphangiogenesis. Vascular endothelial growth factor receptor(VEGFR)-3 is a critical regulator of developmental and adult vasculogenesis and lymphangiogenesis through its interactions with select members of the VEGF family. The goal of this study was to investigate how VEGFR-3 expression is regulated during inflammatory lymphangiogenesis.In this study, we present for the first time evidence that VEGFR-3 can be negatively regulated by a mirtron, hsa-miR-1236 (miR-1236), which is expressed in primary human lymphatic endothelial cells. In human lymphatic endothelial cells, miR-1236 is upregulated in response to IL-1β, a negative regulator of VEGFR-3. miR-1236 binds the 3' untranslated region of Vegfr3, resulting in translational inhibition. Overexpression of miR-1236 significantly decreased expression of VEGFR-3, but not VEGFR-2, in human lymphatic endothelial cells. Compared to a control miR, overexpression of miR-1236 also led to decreased VEGFR-3 signaling. However, VEGFR-2-specific signaling was not affected. miR-1236 can attenuate human lymphatic endothelial cell **migratio**n and tube formation, as well as in vivo lymphangiogenesis.Our data suggest that miR-1236 may function as a negative regulator of VEGFR-3 signaling during inflammatory lymphangiogenesis.
20. VEGFR-3/Flt-4 mediates proliferation and chemotaxis in glial precursor cells. Neuronal and vascular cells share common chemical signals. Vascular endothelial growth factor (VEGF)-C and -D and their receptor VEGFR-3/Flt-4 mediate lymphangiogenesis, but they occur also in the brain. Quantitative RT-PCR of mouse brain tissues and cultivated cells showed that the VEGFR-3 gene is highest transcribed in postnatal brain and in glial precursor cells whereas VEGF-C and -D are variably produced by different neuronal and glial cells. In neurospheres (neural stem cells) VEGFR-3 was induced by differentiation with platelet-derived growth factor (PDGF). In functional studies with an A2B5- and nestin-positive, O4-negative murine glial precursor cell line, VEGF-C and -D stimulated phosphorylation of the kinases Erk1/2; this signal transduction was inhibited by UO126. Both peptides induced the proliferation of glial precursor cells which could be inhibited by UO126. Furthermore, VEGF-D considerably enhanced their **migration** into an open space in a wound-healing assay. These results show that VEGF-C/-D together with its receptor VEGFR-3 provides an auto-/paracrine growth and chemotactic system for glial precursors in the developing brain.
21. H-, N- and Kras cooperatively regulate lymphatic vessel growth by modulating VEGFR3 expression in lymphatic endothelial cells in mice. Mammalian Ras, which is encoded by three independent genes, has been thought to be a versatile component of intracellular signalling. However, when, where and how Ras signalling plays essential roles in development and whether the three Ras genes have overlapping functions in particular cells remain unclear. Here, we show that the three Ras proteins dose-dependently regulate lymphatic vessel growth in mice. We find that lymphatic vessel hypoplasia is a common phenotype in Ras compound knockout mice and that overexpressed normal Ras in an endothelial cell lineage selectively causes lymphatic vessel hyperplasia in vivo. Overexpression of normal Ras in lymphatic endothelial cells leads to sustained MAPK activation, cellular viability and enhanced endothelial network formation under serum-depleted culture conditions in vitro, and knockdown of endogenous Ras in lymphatic endothelial cells impairs cell proliferation, MAPK activation, cell **migration** and endothelial network formation. Ras overexpression and knockdown result in up- and downregulation of vascular endothelial growth factor receptor (VEGFR) 3 expression, respectively, in lymphatic endothelial cells in vitro. The close link between Ras and VEGFR3 in vitro is consistent with the result that Ras knockout and transgenic alleles are genetic modifiers in lymphatic vessel hypoplasia caused by Vegfr3 haploinsufficiency. Our findings demonstrate a cooperative function of the three Ras proteins in normal development, and also provide a novel aspect of VEGFR3 signalling modulated by Ras in lymphangiogenesis.
22. Vascular endothelial growth factor c/vascular endothelial growth factor receptor 3 signaling regulates chemokine gradients and lymphocyte **migration** from tissues to lymphatics. Circulation of leukocytes via blood, tissue and lymph is integral to adaptive immunity. Afferent lymphatics form CCL21 gradients to guide dendritic cells and T cells to lymphatics and then to draining lymph nodes (dLN). Vascular endothelial growth factor C and vascular endothelial growth factor receptor 3 (VEGFR-3) are the major lymphatic growth factor and receptor. We hypothesized these molecules also regulate chemokine gradients and lymphatic **migration**.CD4 T cells were injected into the foot pad or ear pinnae, and **migration** to afferent lymphatics and dLN quantified by flow cytometry or whole mount immunohistochemistry. Vascular endothelial growth factor receptor 3 or its signaling or downstream actions were modified with blocking monoclonal antibodies (mAbs) or other reagents.Anti-VEGFR-3 prevented **migration** of CD4 T cells into lymphatic lumen and significantly decreased the number that migrated to dLN. Anti-VEGFR-3 abolished CCL21 gradients around lymphatics, although CCL21 production was not inhibited. Heparan sulfate (HS), critical to establish CCL21 gradients, was down-regulated around lymphatics by anti-VEGFR-3 and this was dependent on heparanase-mediated degradation. Moreover, a Phosphoinositide 3-kinase (PI3K)α inhibitor disrupted HS and CCL21 gradients, whereas a PI3K activator prevented the effects of anti-VEGFR-3. During contact hypersensitivity, VEGFR-3, CCL21, and HS expression were all attenuated, and anti-heparanase or PI3K activator reversed these effects.Vascular endothelial growth factor C/VEGFR-3 signaling through PI3Kα regulates the activity of heparanase, which modifies HS and CCL21 gradients around lymphatics. The functional and physical linkages of these molecules regulate lymphati**c migrati**on from tissues to dLN. These represent new therapeutic targets to influence immunity and inflammation.
23. Direct transcriptional regulation of neuropilin-2 by COUP-TFII modulates multiple steps in murine lymphatic vessel development. The lymphatic system plays a key role in tissue fluid homeostasis. Lymphatic dysfunction contributes to the pathogenesis of many human diseases, including lymphedema and tumor metastasis. However, the mechanisms regulating lymphangiogenesis remain largely unknown. Here, we show that COUP-TFII (also known as Nr2f2), an orphan member of the nuclear receptor superfamily, mediates both developmental and pathological lymphangiogenesis in mice. Conditional ablation of COUP-TFII at an early embryonic stage resulted in failed formation of pre-lymphatic ECs (pre-LECs) and lymphatic vessels. COUP-TFII deficiency at a late developmental stage resulted in loss of LEC identity, gain of blood EC fate, and impaired lymphatic vessel sprouting. siRNA-mediated downregulation of COUP-TFII in cultured primary human LECs demonstrated that the maintenance of lymphatic identity and VEGF-C-induced lymphangiogenic activity, including cell proliferation and **migration**, are COUP-TFII-dependent and cell-autonomous processes. COUP-TFII enhanced the pro-lymphangiogenic actions of VEGF-C, at least in part by directly stimulating expression of neuropilin-2, a coreceptor for VEGF-C. In addition, COUP-TFII inactivation in a mammary gland mouse tumor model resulted in inhibition of tumor lymphangiogenesis, suggesting that COUP-TFII also regulates neo-lymphangiogenesis in the adult. Thus, COUP-TFII is a critical factor that controls lymphangiogenesis in embryonic development and tumorigenesis in adults.

#### GeneRIFs

1. Vascular endothelial growth factor C/VEGFR-3 signaling modifies HS and CCL21 gradients around lymphatics, regulating lymphocyte **migration**.
2. cooperative signaling of VEGFR-2 and -3 is necessary for lymphatic **migration** and proliferation, but VEGFR-3 is redundant with VEGFR-2 for LEC organization into functional capillaries

### ECM

#### PubMed

1. Emilin1 deficiency causes structural and functional defects of lymphatic vasculature. Lymphatic-vasculature function critically depends on extracellular matrix (**ECM**) and on its connections with lymphatic endothelial cells (LECs). However, the composition and the architecture of **ECM** have not been fully taken into consideration in studying the biology and the pathology of the lymphatic system. EMILIN1, an elastic microfibril-associated protein, is highly expressed by LECs in vitro and colocalizes with lymphatic vessels in several mouse tissues. A comparative study between WT and Emilin1-/- mice highlighted the fact that Emilin1 deficiency in both CD1 and C57BL/6 backgrounds results in hyperplasia, enlargement, and frequently an irregular pattern of superficial and visceral lymphatic vessels and in a significant reduction of anchoring filaments. Emilin1-deficient mice also develop larger lymphangiomas than WT mice. Lymphatic vascular morphological alterations are accompanied by functional defects, such as mild lymphedema, a highly significant drop in lymph drainage, and enhanced lymph leakage. Our findings demonstrate that EMILIN1 is involved in the regulation of the growth and in the maintenance of the integrity of lymphatic vessels, a fundamental requirement for efficient function. The phenotype displayed by Emilin1(-/-) mice is the first abnormal lymphatic phenotype associated with the deficiency of an **ECM** protein and identifies EMILIN1 as a novel local regulator of lymphangiogenesis.

### [Ee]xtracellular [Mm]atrix

#### PubMed

1. Integrin-alpha9 is required for fibronectin matrix assembly during lymphatic valve morphogenesis. Dysfunction of lymphatic valves underlies human lymphedema, yet the process of valve morphogenesis is poorly understood. Here, we show that during embryogenesis, lymphatic valve leaflet formation is initiated by upregulation of integrin-alpha9 expression and deposition of its ligand fibronectin-EIIIA (FN-EIIIA) in the **extracellular matrix**. Endothelial cell-specific deletion of Itga9 (encoding integrin-alpha9) in mouse embryos results in the development of rudimentary valve leaflets characterized by disorganized FN matrix, short cusps, and retrograde lymphatic flow. Similar morphological and functional defects are observed in mice lacking the EIIIA domain of FN. Mechanistically, we demonstrate that in primary human lymphatic endothelial cells, the integrin-alpha9-EIIIA interaction directly regulates FN fibril assembly, which is essential for the formation of the **extracellular matrix** core of valve leaflets. Our findings reveal an important role for integrin-alpha9 signaling during lymphatic valve morphogenesis and implicate it as a candidate gene for primary lymphedema caused by valve defects.
2. Endothelial cell adhesion to the **extracellular matrix** induces c-Src-dependent VEGFR-3 phosphorylation without the activation of the receptor intrinsic kinase activity. Integrins cooperate with growth factor receptors to promote downstream signaling for cell proliferation and migration. However, the mechanism of receptor activation is still unknown.To analyze the mechanism of phosphorylation of the vascular endothelial growth factor receptor (VEGFR)-3 by cell adhesion.We show that VEGFR-3 phosphorylation, induced by cell attachment to the **extracellular matrix**, is independent from the intrinsic kinase activity of the receptor, as evidenced from phosphorylation cell adhesion experiments with a mutant kinase dead receptor or in the presence of the specific kinase inhibitor MAZ 51. Cell adhesion experiments in the presence of the c-Src inhibitor PP2 or in fibroblast triple knockout for c-Src, Yes, and Fyn (SYF) demonstrate that VEGFR-3 phosphorylation, induced by **extracellular matrix**, is mediated by c-Src. Kinase assays in vitro with recombinant c-Src show that VEGFR-3 is a direct c-Src target and mass spectrometry analysis identified the sites phosphorylated by c-Src as tyrosine 830, 833, 853, 1063, 1333, and 1337, demonstrating that integrin-mediated receptor phosphorylation induces a phosphorylation pattern that is distinct from that induced by growth factors. Furthermore, pull-down assays show that integrin-mediated VEGFR-3 phosphorylation activates the recruitment to the receptor of the adaptor proteins CRKI/II and SHC inducing activation of JNK.These data suggest that cell adhesion to **extracellular matrix** induces a downstream signaling using the tyrosine kinase receptor VEGFR-3 as scaffold.
3. CCBE1 is essential for mammalian lymphatic vascular development and enhances the lymphangiogenic effect of vascular endothelial growth factor-C in vivo. Collagen- and calcium-binding EGF domains 1 (CCBE1) has been associated with Hennekam syndrome, in which patients have lymphedema, lymphangiectasias, and other cardiovascular anomalies. Insight into the molecular role of CCBE1 is completely lacking, and mouse models for the disease do not exist.CCBE1 deficient mice were generated to understand the function of CCBE1 in cardiovascular development, and CCBE1 recombinant protein was used in both in vivo and in vitro settings to gain insight into the molecular function of CCBE1.Phenotypic analysis of murine Ccbe1 mutant embryos showed a complete lack of definitive lymphatic structures, even though Prox1(+) lymphatic endothelial cells get specified within the cardinal vein. Mutant mice die prenatally. Proximity ligation assays indicate that vascular endothelial growth factor receptor 3 activation appears unaltered in mutants. Human CCBE1 protein binds to components of the **extracellular matrix** in vitro, and CCBE1 protein strongly enhances vascular endothelial growth factor-C-mediated lymphangiogenesis in a corneal micropocket assay.Our data identify CCBE1 as a factor critically required for budding and migration of Prox-1(+) lymphatic endothelial cells from the cardinal vein. CCBE1 probably exerts these effects through binding to components of the **extracellular matrix**. CCBE1 has little lymphangiogenic effect on its own but dramatically enhances the lymphangiogenic effect of vascular endothelial growth factor-C in vivo. Thus, our data suggest CCBE1 to be essential but not sufficient for lymphangiogenesis.
4. Mesenchymal expression of vascular endothelial growth factors D and A defines vascular patterning in developing lung. The lung has specific vascular patterning requirements for effective gas exchange at birth, including alignment of airways and blood vessels and lymphatic vessels. Vascular endothelial growth factors (VEGF) are potent effectors of vascular development. We examined the temporal and spatial expression of VEGF-D and specific VEGF-A isoforms at each stage of lung development. VEGF-D, expressed only by cadherin-11-positive cells of the mesenchyme, is first detected at embryonic day (E) 13.5, a period of active vasculogenesis. VEGFR-3, its cognate receptor, is detected earlier on days E11.5 to E14.5, in both blood vessels and lymphatic vessels and later, on day E17.5, in only lymphatic vessels. VEGF-A is expressed in the mesenchyme throughout lung development and also by the epithelium midway through organogenesis. Before E14, the predominant forms of VEGF-A are the soluble isoforms, VEGF-A120 and 164. Not until E14.5 do epithelial cells at the tips of expanding airways express VEGF-A, including VEGF-A188, an isoform with high affinity for **extracellular matrix**. Our results demonstrate unique temporal and spatial expression of VEGF-D and specific VEGF-A isoforms during lung development and suggest these related factors have distinct functions in vascular and lymphatic patterning of the lung.
5. Emilin1 deficiency causes structural and functional defects of lymphatic vasculature. Lymphatic-vasculature function critically depends on **extracellular matrix** (ECM) and on its connections with lymphatic endothelial cells (LECs). However, the composition and the architecture of ECM have not been fully taken into consideration in studying the biology and the pathology of the lymphatic system. EMILIN1, an elastic microfibril-associated protein, is highly expressed by LECs in vitro and colocalizes with lymphatic vessels in several mouse tissues. A comparative study between WT and Emilin1-/- mice highlighted the fact that Emilin1 deficiency in both CD1 and C57BL/6 backgrounds results in hyperplasia, enlargement, and frequently an irregular pattern of superficial and visceral lymphatic vessels and in a significant reduction of anchoring filaments. Emilin1-deficient mice also develop larger lymphangiomas than WT mice. Lymphatic vascular morphological alterations are accompanied by functional defects, such as mild lymphedema, a highly significant drop in lymph drainage, and enhanced lymph leakage. Our findings demonstrate that EMILIN1 is involved in the regulation of the growth and in the maintenance of the integrity of lymphatic vessels, a fundamental requirement for efficient function. The phenotype displayed by Emilin1(-/-) mice is the first abnormal lymphatic phenotype associated with the deficiency of an ECM protein and identifies EMILIN1 as a novel local regulator of lymphangiogenesis.
6. Smooth muscle-endothelial cell communication activates Reelin signaling and regulates lymphatic vessel formation. Active lymph transport relies on smooth muscle cell (SMC) contractions around collecting lymphatic vessels, yet regulation of lymphatic vessel wall assembly and lymphatic pumping are poorly understood. Here, we identify Reelin, an **extracellular matrix** glycoprotein previously implicated in central nervous system development, as an important regulator of lymphatic vascular development. Reelin-deficient mice showed abnormal collecting lymphatic vessels, characterized by a reduced number of SMCs, abnormal expression of lymphatic capillary marker lymphatic vessel endothelial hyaluronan receptor 1 (LYVE-1), and impaired function. Furthermore, we show that SMC recruitment to lymphatic vessels stimulated release and proteolytic processing of endothelium-derived Reelin. Lymphatic endothelial cells in turn responded to Reelin by up-regulating monocyte chemotactic protein 1 (MCP1) expression, which suggests an autocrine mechanism for Reelin-mediated control of endothelial factor expression upstream of SMC recruitment. These results uncover a mechanism by which Reelin signaling is activated by communication between the two cell types of the collecting lymphatic vessels--smooth muscle and endothelial cells--and highlight a hitherto unrecognized and important function for SMCs in lymphatic vessel morphogenesis and function.

#### GeneRIFs

1. Cell adhesion to **extracellular matrix** induces a downstream signaling using the tyrosine kinase receptor VEGFR-3 as scaffold.

### [Ii]nvasive

#### PubMed

1. Notch alters VEGF responsiveness in human and murine endothelial cells by direct regulation of VEGFR-3 expression. The Notch family of cell surface receptors and its ligands are highly conserved proteins that regulate cell fate determination, including those involved in mammalian vascular development. We report that Notch induces VEGFR-3 expression in vitro in human endothelial cells and in vivo in mice. In vitro, Notch in complex with the DNA-binding protein CBF-1/suppressor of hairless/Lag1 (CSL) bound the VEGFR-3 promoter and transactivated VEGFR-3 specifically in endothelial cells. Through induction of VEGFR-3, Notch increased endothelial cell responsiveness to VEGF-C, promoting endothelial cell survival and morphological changes. In vivo, VEGFR-3 was upregulated in endothelial cells with active Notch signaling. Mice heterozygous for null alleles of both Notch1 and VEGFR-3 had significantly reduced viability and displayed midgestational vascular patterning defects analogous to Notch1 nullizygous embryos. We found that Notch1 and Notch4 were expressed in normal and tumor lymphatic endothelial cells and that Notch1 was activated in lymphatic endothelium of **invasive** mammary micropapillary carcinomas. These results demonstrate that Notch1 and VEGFR-3 interact genetically, that Notch directly induces VEGFR-3 in blood endothelial cells to regulate vascular development, and that Notch may function in tumor lymphangiogenesis.
2. Notch alters VEGF responsiveness in human and murine endothelial cells by direct regulation of VEGFR-3 expression. The Notch family of cell surface receptors and its ligands are highly conserved proteins that regulate cell fate determination, including those involved in mammalian vascular development. We report that Notch induces VEGFR-3 expression in vitro in human endothelial cells and in vivo in mice. In vitro, Notch in complex with the DNA-binding protein CBF-1/suppressor of hairless/Lag1 (CSL) bound the VEGFR-3 promoter and transactivated VEGFR-3 specifically in endothelial cells. Through induction of VEGFR-3, Notch increased endothelial cell responsiveness to VEGF-C, promoting endothelial cell survival and morphological changes. In vivo, VEGFR-3 was upregulated in endothelial cells with active Notch signaling. Mice heterozygous for null alleles of both Notch1 and VEGFR-3 had significantly reduced viability and displayed midgestational vascular patterning defects analogous to Notch1 nullizygous embryos. We found that Notch1 and Notch4 were expressed in normal and tumor lymphatic endothelial cells and that Notch1 was activated in lymphatic endothelium of **invasive** mammary micropapillary carcinomas. These results demonstrate that Notch1 and VEGFR-3 interact genetically, that Notch directly induces VEGFR-3 in blood endothelial cells to regulate vascular development, and that Notch may function in tumor lymphangiogenesis.
3. In vivo imaging of lymphatic vessels in development, wound healing, inflammation, and tumor metastasis. Lymphatic vessel growth or lymphangiogenesis occurs during embryonic development and wound healing and plays an important role in tumor metastasis and inflammatory diseases. However, the possibility of non**invasive** detection and quantification of lymphangiogenesis has been lacking. Here, we present the Vegfr3(EGFPLuc) mouse model, where an EGFP-luciferase fusion protein, expressed under the endogenous transcriptional control of the Vegfr3 gene, allows the monitoring of physiological and pathological lymphangiogenesis in vivo. We show tracking of lymphatic vessel development during embryogenesis as well as lymphangiogenesis induced by specific growth factors, during wound healing and in contact hypersensitivity (CHS)--induced inflammation where we also monitor down-regulation of lymphangiogenesis by the glucocorticoid dexamethasone. Importantly, the Vegfr3-reporter allowed us to tracking tumor-induced lymphangiogenesis at the tumor periphery and in lymph nodes in association with the metastatic process. This is the first reporter mouse model for luminescence imaging of lymphangiogenesis. It should provide an important tool for studying the involvement of lymphangiogenesis in pathological processes.
4. Soluble vascular endothelial growth factor receptor-3 suppresses lymphangiogenesis and lymphatic metastasis in bladder cancer. Most bladder cancer patients experience lymphatic metastasis in the course of disease progression, yet the relationship between lymphangiogenesis and lymphatic metastasis is not well known. The aim of this study is to elucidate underlying mechanisms of how expanded lymphatic vessels and tumor microenvironment interacts each other and to find effective therapeutic options to inhibit lymphatic metastasis.The orthotopic urinary bladder cancer (OUBC) model was generated by intravesical injection of MBT-2 cell lines. We investigated the angiogenesis, lymphangiogenesis, and CD11b+/CD68+ tumor-associated macrophages (TAM) by using immunofluorescence staining. OUBC displayed a profound lymphangiogenesis and massive infiltration of TAM in primary tumor and lymphatic metastasis in lymph nodes. TAM flocked near lymphatic vessels and express higher levels of VEGF-C/D than CD11b- cells. Because VEGFR-3 was highly expressed in lymphatic vascular endothelial cells, TAM could assist lymphangiogenesis by paracrine manner in bladder tumor. VEGFR-3 expressing adenovirus was administered to block VEGF-C/D signaling pathway and clodronate liposome was used to deplete TAM. The blockade of VEGF-C/D with soluble VEGF receptor-3 markedly inhibited lymphangiogenesis and lymphatic metastasis in OUBC. In addition, the depletion of TAM with clodronate liposome exerted similar effects on OUBC.VEGF-C/D are the main factors of lymphangiogenesis and lymphatic metastasis in bladder cancer. Moreover, TAM plays an important role in these processes by producing VEGF-C/D. The inhibition of lymphangiogenesis could provide another therapeutic target to inhibit lymphatic metastasis and recurrence in patients with **invasive** bladder cancer.
5. Minimally **invasive** quantification of lymph flow in mice and rats by imaging depot clearance of near-infrared albumin. There is a lack of available methods to non**invasive**ly quantify lymphatic function in small experimental animals, a necessity for studies on lymphatic system pathophysiology. We present a new method to quantify lymph flow in mice and rats, based on optically monitoring the depot clearance of near-infrared fluorescently labeled albumin and subsequent calculation of removal rate constants (k). BSA was conjugated with Alexa680 NHS ester and remained stable in protein-rich solutions without free dye dissociation. To assess lymph flow, mice or rats were imaged every 30 or 60 min during a 3- to 6-h period following an intradermal injection of 0.5 or 1 μl Alexa680-albumin. Mice were awake between measurements, whereas rats were anesthetized throughout the experiment. The k, a parameter defined as equivalent to lymph flow, was calculated from the slopes of the resultant log-linear washout curves and averaged -0.40 ± 0.03 and -0.30 ± 0.02%/min for control C57BL/6 and C3H mice, respectively. Local administration of the vasoconstrictor endothelin-1 in mice led to a significant reduction in k, whereas overhydration in rats increased k, reflecting the coupling between capillary filtration and lymph flow. Furthermore, k was 50% of wild type in lymphedema Chy mice where dermal lymphatics are absent. We conclude that lymph flow can be determined as its rate constant k by optical imaging of depot clearance of submicroliter amounts of Alexa680-albumin. The method offers a minimal**ly invas**ive, reproducible, and simple alternative to assess lymphatic function in mice and rats.

### [Mm]etastasize

#### PubMed

1. VEGF-D promotes tumor growth and lymphatic spread in a mouse model of hepatocellular carcinoma. Lymphatic spread is an important clinical determinant for the prognosis of hepatocellular carcinoma (HCC), but little is known about the control of lymphangiogenesis in HCC. We addressed expression and biological role of the pro-(lymph), angiogenic protein VEGF-D in this tumor entity. Using immunohistochemistry and in situ hybridization on specimens of HCC, cirrhotic and normal liver we found abundant expression of VEGF-D exclusively in the tumor cells. The cognate receptor VEGFR-3 was detected on blood and lymphatic vessels. By clinicopathological analysis VEGF-D expression was correlated with pT-stage of the primary, lymph node metastasis and lymphangiosis carcinomatosa. Three out of 4 human HCC cell lines expressed and secreted VEGF-D. To approach its biological function, VEGF-D deficient SKHep-1 cells were stably transfected with VEGF-D cDNA and effects on tumor progression were determined in vivo. Compared to mock-transfected controls, subcutaneous tumors derived from VEGF-D expressing cells were larger and more frequently **metastasize**d to regional lymph nodes. VEGF-D expressing tumors exhibited increased microvessel density and increased abundance of peri- and intratumoral lymphatics, as assessed by immunostaining for CD31 and for LYVE-1 and/or podoplanin, respectively. Furthermore, coexpression of the soluble extracellular VEGFR-3 domain blocked VEGF-D-induced tumor growth and lymphatic spread via reduction of angiogenesis and lymphangiogenesis. In the orthotopic approach, VEGF-D expression resulted in an increased rate of intra- and extrahepatic as well as lymph node metastasis. In conclusion, our study suggests that expression of VEGF-D is involved in growth and lymphatic spread of HCC. Therefore, VEGF-D might represent a therapeutic target in HCC.

### [Mm]etastasis

#### PubMed

1. Blocking Fibroblast Growth Factor receptor signaling inhibits tumor growth, lymphangiogenesis, and **metastasis**. Fibroblast Growth Factor receptor (FGFR) activity plays crucial roles in tumor growth and patient survival. However, FGF (Fibroblast Growth Factor) signaling as a target for cancer therapy has been under-investigated compared to other receptor tyrosine kinases. Here, we studied the effect of FGFR signaling inhibition on tumor growth, **metastasis** and lymphangiogenesis by expressing a dominant negative FGFR (FGFR-2DN) in an orthotopic mouse mammary 66c14 carcinoma model. We show that FGFR-2DN-expressing 66c14 cells proliferate in vitro slower than controls. 66c14 tumor outgrowth and lung metastatic foci are reduced in mice implanted with FGFR-2DN-expressing cells, which also exhibited better overall survival. We found 66c14 cells in the lumen of tumor lymphatic vessels and in lymph nodes. FGFR-2DN-expressing tumors exhibited a decrease in VEGFR-3 (Vascular Endothelial Growth Factor Receptor-3) or podoplanin-positive lymphatic vessels, an increase in isolated intratumoral lymphatic endothelial cells and a reduction in VEGF-C (Vascular Endothelial Growth Factor-C) mRNA expression. FGFs may act in an autocrine manner as the inhibition of FGFR signaling in tumor cells suppresses VEGF-C expression in a COX-2 (cyclooxygenase-2) or HIF1-α (hypoxia-inducible factor-1 α) independent manner. FGFs may also act in a paracrine manner on tumor lymphatics by inducing expression of pro-lymphangiogenic molecules such as VEGFR-3, integrin α9, prox1 and netrin-1. Finally, in vitro lymphangiogenesis is impeded in the presence of FGFR-2DN 66c14 cells. These data confirm that both FGF and VEGF signaling are necessary for the maintenance of vascular morphogenesis and provide evidence that targeting FGFR signaling may be an interesting approach to inhibit tumor lymphangiogenesis and metastatic spread.
2. Blocking Fibroblast Growth Factor receptor signaling inhibits tumor growth, lymphangiogenesis, and **metastasis**. Fibroblast Growth Factor receptor (FGFR) activity plays crucial roles in tumor growth and patient survival. However, FGF (Fibroblast Growth Factor) signaling as a target for cancer therapy has been under-investigated compared to other receptor tyrosine kinases. Here, we studied the effect of FGFR signaling inhibition on tumor growth, **metastasis** and lymphangiogenesis by expressing a dominant negative FGFR (FGFR-2DN) in an orthotopic mouse mammary 66c14 carcinoma model. We show that FGFR-2DN-expressing 66c14 cells proliferate in vitro slower than controls. 66c14 tumor outgrowth and lung metastatic foci are reduced in mice implanted with FGFR-2DN-expressing cells, which also exhibited better overall survival. We found 66c14 cells in the lumen of tumor lymphatic vessels and in lymph nodes. FGFR-2DN-expressing tumors exhibited a decrease in VEGFR-3 (Vascular Endothelial Growth Factor Receptor-3) or podoplanin-positive lymphatic vessels, an increase in isolated intratumoral lymphatic endothelial cells and a reduction in VEGF-C (Vascular Endothelial Growth Factor-C) mRNA expression. FGFs may act in an autocrine manner as the inhibition of FGFR signaling in tumor cells suppresses VEGF-C expression in a COX-2 (cyclooxygenase-2) or HIF1-α (hypoxia-inducible factor-1 α) independent manner. FGFs may also act in a paracrine manner on tumor lymphatics by inducing expression of pro-lymphangiogenic molecules such as VEGFR-3, integrin α9, prox1 and netrin-1. Finally, in vitro lymphangiogenesis is impeded in the presence of FGFR-2DN 66c14 cells. These data confirm that both FGF and VEGF signaling are necessary for the maintenance of vascular morphogenesis and provide evidence that targeting FGFR signaling may be an interesting approach to inhibit tumor lymphangiogenesis and metastatic spread.
3. ALK5 phosphorylation of the endoglin cytoplasmic domain regulates Smad1/5/8 signaling and endothelial cell migration. Endoglin, an endothelial cell-specific transforming growth factor-beta (TGF-beta) superfamily coreceptor, has an essential role in angiogenesis. Endoglin-null mice have an embryonic lethal phenotype due to defects in angiogenesis and mutations in endoglin result in the vascular disease hereditary hemorrhagic telangiectasia type I. Increased endoglin expression in the proliferating endothelium of tumors has been correlated with **metastasis**, tumor grade and decreased survival. Although endoglin is thought to regulate TGF-beta superfamily signaling in endothelial cells through regulating the balance between two TGF-beta-responsive pathways, the activin receptor-like kinase 5 (ALK5)/Smad2/3 pathway and the activin receptor-like kinase 1 (ALK1)/Smad1/5/8 pathway, the mechanism by which endoglin regulates angiogenesis has not been defined. Here, we investigate the role of the cytoplasmic domain of endoglin and its phosphorylation by ALK5 in regulating endoglin function in endothelial cells. We demonstrate that the cytoplasmic domain of endoglin is basally phosphorylated by ALK5, primarily on serines 646 and 649, in endothelial cells. Functionally, the loss of phosphorylation at serine 646 resulted in a loss of endoglin-mediated inhibition of Smad1/5/8 signaling in response to TGF-beta and endothelial cell migration, whereas loss of phosphorylation at both serines 646 and 649 resulted in a loss of endoglin-mediated inhibition of Smad1/5/8 signaling in response to bone morphogenetic protein-9. Taken together, these results support endoglin phosphorylation by ALK5 as an important mechanism for regulating TGF-beta superfamily signaling and migration in endothelial cells.
4. VEGFD regulates blood vascular development by modulating SOX18 activity. Vascular endothelial growth factor-D (VEGFD) is a potent pro-lymphangiogenic molecule during tumor growth and is considered a key therapeutic target to modulate **metastasis**. Despite roles in pathological neo-lymphangiogenesis, the characterization of an endogenous role for VEGFD in vascular development has remained elusive. Here, we used zebrafish to assay for genetic interactions between the Vegf/Vegf-receptor pathway and SoxF transcription factors and identified a specific interaction between Vegfd and Sox18. Double knockdown zebrafish embryos for Sox18/Vegfd and Sox7/Vegfd exhibit defects in arteriovenous differentiation. Supporting this observation, we found that Sox18/Vegfd double but not single knockout mice displayed dramatic vascular development defects. We find that VEGFD-mitogen-activated protein kinase kinase-extracellular signal-regulated kinase signaling modulates SOX18-mediated transcription, functioning at least in part by enhancing nuclear concentration and transcriptional activity in vascular endothelial cells. This work suggests that VEGFD-mediated pathologies include or involve an underlying dysregulation of SOXF-mediated transcriptional networks.
5. Adrenomedullin signaling is necessary for murine lymphatic vascular development. The lymphatic vascular system mediates fluid homeostasis, immune defense, and tumor **metastasis**. Only a handful of genes are known to affect the development of the lymphatic vasculature, and even fewer represent therapeutic targets for lymphatic diseases. Adrenomedullin (AM) is a multifunctional peptide vasodilator that transduces its effects through the calcitonin receptor-like receptor (calcrl) when the receptor is associated with a receptor activity-modifying protein (RAMP2). Here we report on the involvement of these genes in lymphangiogenesis. AM-, calcrl-, or RAMP2-null mice died mid-gestation after development of interstitial lymphedema. This conserved phenotype provided in vivo evidence that these components were required for AM signaling during embryogenesis. A conditional knockout line with loss of calcrl in endothelial cells confirmed an essential role for AM signaling in vascular development. Loss of AM signaling resulted in abnormal jugular lymphatic vessels due to reduction in lymphatic endothelial cell proliferation. Furthermore, AM caused enhanced activation of ERK signaling in human lymphatic versus blood endothelial cells, likely due to induction of CALCRL gene expression by the lymphatic transcriptional regulator Prox1. Collectively, our studies identify a class of genes involved in lymphangiogenesis that represent a pharmacologically tractable system for the treatment of lymphedema or inhibition of tumor **metastasis**.
6. Lymphatic vessels regulate immune microenvironments in human and murine melanoma. Lymphatic remodeling in tumor microenvironments correlates with progression and **metastasis**, and local lymphatic vessels play complex and poorly understood roles in tumor immunity. Tumor lymphangiogenesis is associated with increased immune suppression, yet lymphatic vessels are required for fluid drainage and immune cell trafficking to lymph nodes, where adaptive immune responses are mounted. Here, we examined the contribution of lymphatic drainage to tumor inflammation and immunity using a mouse model that lacks dermal lymphatic vessels (K14-VEGFR3-Ig mice). Melanomas implanted in these mice grew robustly, but exhibited drastically reduced cytokine expression and leukocyte infiltration compared with those implanted in control animals. In the absence of local immune suppression, transferred cytotoxic T cells more effectively controlled tumors in K14-VEGFR3-Ig mice than in control mice. Furthermore, gene expression analysis of human melanoma samples revealed that patient immune parameters are markedly stratified by levels of lymphatic markers. This work suggests that the establishment of tumor-associated inflammation and immunity critically depends on lymphatic vessel remodeling and drainage. Moreover, these results have implications for immunotherapies, the efficacies of which are regulated by the tumor immune microenvironment.
7. In vivo imaging of lymphatic vessels in development, wound healing, inflammation, and tumor **metastasis**. Lymphatic vessel growth or lymphangiogenesis occurs during embryonic development and wound healing and plays an important role in tumor **metastasis** and inflammatory diseases. However, the possibility of noninvasive detection and quantification of lymphangiogenesis has been lacking. Here, we present the Vegfr3(EGFPLuc) mouse model, where an EGFP-luciferase fusion protein, expressed under the endogenous transcriptional control of the Vegfr3 gene, allows the monitoring of physiological and pathological lymphangiogenesis in vivo. We show tracking of lymphatic vessel development during embryogenesis as well as lymphangiogenesis induced by specific growth factors, during wound healing and in contact hypersensitivity (CHS)--induced inflammation where we also monitor down-regulation of lymphangiogenesis by the glucocorticoid dexamethasone. Importantly, the Vegfr3-reporter allowed us to tracking tumor-induced lymphangiogenesis at the tumor periphery and in lymph nodes in association with the metastatic process. This is the first reporter mouse model for luminescence imaging of lymphangiogenesis. It should provide an important tool for studying the involvement of lymphangiogenesis in pathological processes.
8. Deletion of vascular endothelial growth factor C (VEGF-C) and VEGF-D is not equivalent to VEGF receptor 3 deletion in mouse embryos. Lymphatic vessels play an important role in the regulation of tissue fluid balance, immune responses, and fat adsorption and are involved in diseases including lymphedema and tumor **metastasis**. Vascular endothelial growth factor (VEGF) receptor 3 (VEGFR-3) is necessary for development of the blood vasculature during early embryogenesis, but later, VEGFR-3 expression becomes restricted to the lymphatic vasculature. We analyzed mice deficient in both of the known VEGFR-3 ligands, VEGF-C and VEGF-D. Unlike the Vegfr3(-/-) embryos, the Vegfc(-/-); Vegfd(-/-) embryos displayed normal blood vasculature after embryonic day 9.5. Deletion of Vegfr3 in the epiblast, using keratin 19 (K19) Cre, resulted in a phenotype identical to that of the Vegfr3(-/-) embryos, suggesting that this phenotype is due to defects in the embryo proper and not in placental development. Interestingly, the Vegfr3(neo) hypomorphic mutant mice carrying the neomycin cassette between exons 1 and 2 showed defective lymphatic development. Overexpression of human or mouse VEGF-D in the skin, under the K14 promoter, rescued the lymphatic hypoplasia of the Vegfc(+/-) mice in the K14-VEGF-D; Vegfc(+/-) compound mice, suggesting that VEGF-D is functionally redundant with VEGF-C in the stimulation of developmental lymphangiogenesis. Our results suggest VEGF-C- and VEGF-D-independent functions for VEGFR-3 in the early embryo.
9. Suppression of VEGFR-3 signaling inhibits lymph node **metastasis** in gastric cancer. In gastric cancer, lymph node **metastasis** is one of the major prognostic factors and forms the basis for surgical removal of local lymph nodes. Recently, several studies have demonstrated that overexpression of lymphangiogenic growth factor VEGF-C or VEGF-D induces tumor lymphangiogenesis and promotes lymphatic **metastasis** in mouse tumor models. We examined whether these processes could be inhibited in naturally metastatic tumors by blocking of their cognate receptor VEGFR-3 signaling pathway. Using a mouse orthotopic gastric cancer model which has a high frequency of lymph node **metastasis**, we estimated lymphatic vessels in gastric cancers by immunostaining for VEGFR-3 and other specific lymphatic markers, LYVE-1 and prox-1. Then we systemically administered anti-VEGFR-3 blocking antibodies. This treatment resulted in the inhibition of regional lymph node **metastasis** and reduction of lymphatic vessel density in the primary tumors. In addition, increased density of LYVE-1-positive lymphatic vessels of primary tumors was closely correlated with lymph node **metastasis** in human samples of gastric cancer. Antilymphangiogenesis by inhibiting VEGFR-3 signaling could provide a potential strategy for the prevention of lymph node **metastasis** in gastric cancer.
10. Soluble vascular endothelial growth factor receptor-3 suppresses lymphangiogenesis and lymphatic **metastasis** in bladder cancer. Most bladder cancer patients experience lymphatic **metastasis** in the course of disease progression, yet the relationship between lymphangiogenesis and lymphatic **metastasis** is not well known. The aim of this study is to elucidate underlying mechanisms of how expanded lymphatic vessels and tumor microenvironment interacts each other and to find effective therapeutic options to inhibit lymphatic **metastasis**.The orthotopic urinary bladder cancer (OUBC) model was generated by intravesical injection of MBT-2 cell lines. We investigated the angiogenesis, lymphangiogenesis, and CD11b+/CD68+ tumor-associated macrophages (TAM) by using immunofluorescence staining. OUBC displayed a profound lymphangiogenesis and massive infiltration of TAM in primary tumor and lymphatic **metastasis** in lymph nodes. TAM flocked near lymphatic vessels and express higher levels of VEGF-C/D than CD11b- cells. Because VEGFR-3 was highly expressed in lymphatic vascular endothelial cells, TAM could assist lymphangiogenesis by paracrine manner in bladder tumor. VEGFR-3 expressing adenovirus was administered to block VEGF-C/D signaling pathway and clodronate liposome was used to deplete TAM. The blockade of VEGF-C/D with soluble VEGF receptor-3 markedly inhibited lymphangiogenesis and lymphatic **metastasis** in OUBC. In addition, the depletion of TAM with clodronate liposome exerted similar effects on OUBC.VEGF-C/D are the main factors of lymphangiogenesis and lymphatic **metastasis** in bladder cancer. Moreover, TAM plays an important role in these processes by producing VEGF-C/D. The inhibition of lymphangiogenesis could provide another therapeutic target to inhibit lymphatic **metastasis** and recurrence in patients with invasive bladder cancer.
11. Blockade of vascular endothelial growth factor receptor-3 signaling inhibits fibroblast growth factor-2-induced lymphangiogenesis in mouse cornea. Vascular endothelial growth factor receptor-3 (VEGFR-3) is a major mediator of lymphangiogenesis. Recently, VEGFR-3 ligands, VEGF-C, and VEGF-D were reported to promote tumor lymphangiogenesis and lymphatic **metastasis**, and these processes were inhibited by blocking of the VEGFR-3-signaling pathway. Here, we have adapted the mouse corneal angiogenesis assay to study potential lymphangiogenic factors and inhibitors. Immunohistochemical analysis with lymphatic endothelial markers showed that VEGF-C induces lymphatic as well as blood vessel growth in the cornea. By contrast, VEGF induced angiogenesis but not lymphangiogenesis. Fibroblast growth factor-2 (FGF-2) stimulated both lymphangiogenesis and angiogenesis. FGF-2 up-regulated VEGF-C expression in vascular endothelial and perivascular cells. Furthermore, administration of blocking anti-VEGFR-3 antibodies inhibited the FGF-2-induced lymphangiogenesis. These findings show that VEGFR-3 can mediate lymphangiogenesis induced by other growth factors. Because increased expression of FGF-2 and VEGF-C has been associated with lymphatic **metastasis**, our results provide a potential strategy for the inhibition of lymphatic **metastasis** in cancer therapy.
12. Complete and specific inhibition of adult lymphatic regeneration by a novel VEGFR-3 neutralizing antibody. New lymphatic growth may contribute to tumor **metastasis**. Activation of vascular endothelial growth factor receptor 3 (VEGFR-3) by its ligands VEGF-C and -D is necessary for embryonic and tumor lymphangiogenesis. However, the exact role of VEGFR-3 signaling in adult lymphangiogenesis and in lymphatic vessel survival and regeneration is unclear.A novel rat monoclonal antibody to murine VEGFR-3, mF4-31C1, which potently antagonizes the binding of VEGF-C to VEGFR-3, was developed. We tested the effects of systemic mF4-31C1 administration in a mouse tail skin model of lymphatic regeneration, either with or without local overexpression of VEGF-C, and we observed lymphatic and blood vessel regeneration over time using microlymphangiography and immunostaining.Normal mice regenerated complete and functional lymphatic vessels within 60 days of surgery. In athymic mice implanted with VEGF-C-overexpressing human breast carcinoma cells, lymphatic regeneration took place over 25 days and resulted in hyperplastic vessels. Under either condition, no lymphatic regeneration occurred in mice receiving mF4-31C1 during the regeneration period. Blood angiogenesis and preexisting lymphatic vessels were unaffected, both in morphology and in function.Blocking VEGFR-3 completely and specifically prevented both physiologically normal and tumor VEGF-C-enhanced lymphangiogenesis in the adult mouse but had no effect on either blood angiogenesis or the survival or function of existing lymphatic vessels. Thus, targeting VEGFR-3 with specific inhibitors may block new lymphatic growth exclusively.
13. VEGF-D promotes tumor growth and lymphatic spread in a mouse model of hepatocellular carcinoma. Lymphatic spread is an important clinical determinant for the prognosis of hepatocellular carcinoma (HCC), but little is known about the control of lymphangiogenesis in HCC. We addressed expression and biological role of the pro-(lymph), angiogenic protein VEGF-D in this tumor entity. Using immunohistochemistry and in situ hybridization on specimens of HCC, cirrhotic and normal liver we found abundant expression of VEGF-D exclusively in the tumor cells. The cognate receptor VEGFR-3 was detected on blood and lymphatic vessels. By clinicopathological analysis VEGF-D expression was correlated with pT-stage of the primary, lymph node **metastasis** and lymphangiosis carcinomatosa. Three out of 4 human HCC cell lines expressed and secreted VEGF-D. To approach its biological function, VEGF-D deficient SKHep-1 cells were stably transfected with VEGF-D cDNA and effects on tumor progression were determined in vivo. Compared to mock-transfected controls, subcutaneous tumors derived from VEGF-D expressing cells were larger and more frequently metastasized to regional lymph nodes. VEGF-D expressing tumors exhibited increased microvessel density and increased abundance of peri- and intratumoral lymphatics, as assessed by immunostaining for CD31 and for LYVE-1 and/or podoplanin, respectively. Furthermore, coexpression of the soluble extracellular VEGFR-3 domain blocked VEGF-D-induced tumor growth and lymphatic spread via reduction of angiogenesis and lymphangiogenesis. In the orthotopic approach, VEGF-D expression resulted in an increased rate of intra- and extrahepatic as well as lymph node **metastasis**. In conclusion, our study suggests that expression of VEGF-D is involved in growth and lymphatic spread of HCC. Therefore, VEGF-D might represent a therapeutic target in HCC.
14. Direct transcriptional regulation of neuropilin-2 by COUP-TFII modulates multiple steps in murine lymphatic vessel development. The lymphatic system plays a key role in tissue fluid homeostasis. Lymphatic dysfunction contributes to the pathogenesis of many human diseases, including lymphedema and tumor **metastasis**. However, the mechanisms regulating lymphangiogenesis remain largely unknown. Here, we show that COUP-TFII (also known as Nr2f2), an orphan member of the nuclear receptor superfamily, mediates both developmental and pathological lymphangiogenesis in mice. Conditional ablation of COUP-TFII at an early embryonic stage resulted in failed formation of pre-lymphatic ECs (pre-LECs) and lymphatic vessels. COUP-TFII deficiency at a late developmental stage resulted in loss of LEC identity, gain of blood EC fate, and impaired lymphatic vessel sprouting. siRNA-mediated downregulation of COUP-TFII in cultured primary human LECs demonstrated that the maintenance of lymphatic identity and VEGF-C-induced lymphangiogenic activity, including cell proliferation and migration, are COUP-TFII-dependent and cell-autonomous processes. COUP-TFII enhanced the pro-lymphangiogenic actions of VEGF-C, at least in part by directly stimulating expression of neuropilin-2, a coreceptor for VEGF-C. In addition, COUP-TFII inactivation in a mammary gland mouse tumor model resulted in inhibition of tumor lymphangiogenesis, suggesting that COUP-TFII also regulates neo-lymphangiogenesis in the adult. Thus, COUP-TFII is a critical factor that controls lymphangiogenesis in embryonic development and tumorigenesis in adults.
15. Spontaneous corneal hem- and lymphangiogenesis in mice with destrin-mutation depend on VEGFR3 signaling. Lymphangiogenesis, the formation of new lymphatic vessels, is important for tumor **metastasis** and induction of immunity to peripheral antigens including organ transplants. We herein describe a novel mouse model of spontaneous, secondary lymphangiogenesis in the normally avascular cornea. corn1 mice, which suffer from a deletion in the gene encoding the cytoskeletal protein destrin, develop hemangiogenesis as well as spontaneous outgrowth of LYVE-1+++/CD31+ lymphatic vessels into the cornea starting at age 4 weeks. Corneal lymphangiogenesis is delayed in onset, is less intense, and regresses earlier compared with hemangiogenesis. Moreover, the lymphangiogenesis is preceded only by a mild recruitment of CD45+ inflammatory cells into the cornea. In contrast to mice with inflammation-induced hem- and lymphangiogenesis, corn1 mice do not develop breakdown of the blood-aqueous barrier. Finally, in this novel mouse model, a blocking anti-VEGFR3 antibody significantly inhibited not only lymph- but also hemangiogenesis. In summary, destrin deletion has differential effects on spontaneous hem- and lymphangiogenesis in the normally avascular cornea and represents a novel mouse model to study the mechanisms of lymphangiogenesis and to test the antihem- and antilymphangiogenic properties of known or new antiangiogenic agents.

#### GeneRIFs

1. Data show that that OUBC induces profound lymphangiogenesis in the tumor and SLN and substantial lymphatic **metastasis** to SLN through VEGF-C/D-VEGFR-3 signaling pathway.
2. VEGFR3 signaling is needed for lymph node **metastasis** in gastric cancer.

---

## Kit

- NCBI Gene
- GeneRIF
- Pubmed

| Search Term | Rank | Fields |
| --- | --- | --- |
| [Mm]igration | 6 | Process(4); PubMed(102); GeneRIFs(9) |
| ECM | 7 | PubMed(4) |
| [Ee]xtracellular [Mm]atrix | 8 | PubMed(15); GeneRIFs(1) |
| [Ii]nvasive | 10 | PubMed(6) |
| [Mm]etastasis | 12 | PubMed(6); GeneRIFs(1) |

  
hide/show details for Kit

### [Mm]igration

#### Process

1. germ cell **migration**
2. hematopoietic stem cell **migration**
3. melanocyte **migration**
4. positive regulation of cell **migration**

#### PubMed

1. Changes in protooncogene expression correlated with general and sex-specific differentiation in murine primordial germ cells. Primordial germ cells (PGCs) of the mouse undergo key developmental transitions during embryonic days 12-15. On day 12 they complete **migration** into the gonads. They cease mitotic proliferation on day 13 and subsequently enter sex-specific pathways of development. The molecular mechanisms controlling these transitions are poorly understood, yet they are crucial to production of normal gametes later in life. We have used the polymerase chain reaction (PCR) to directly compare levels of expression of several protooncogenes proposed to be involved in control of cell proliferation and differentiation in proliferating and differentiating PCGs of both sexes over a 4 day time course. We report here that mRNA levels for nuclear protooncogenes c-myc, c-fos, and c-jun increase dramatically in both sexes from little or no detectable expression on day 12 to high expression on days 13-15. We observe c-kit message on day 12 in combined PGCs of both sexes, in female but not male PGCs on day 13, and in both sexes on day 14, c-kit mRNA is undetectable on day 15 in either sex, c-mos is not expressed at detectable levels on day 12 in either sex, but increases gradually in female PGCs to very high levels on day 15. In male PGCs, c-mos is expressed at high levels on days 13-15. Our results are consistent with a role for protooncogenes c-myc, c-fos and c-jun in mediating the initial differentiation of PGCs of both sexes that occurs upon colonization of the gonad. Because c-kit and c-mos are expressed differentially in male and female day 13-15 germ cells, they may play roles in initiating or mediating progress along the sex-specific pathways of development that PGCs embark upon at this time.
2. Hand2 is necessary for terminal differentiation of enteric neurons from crest-derived precursors but not for their **migration** into the gut or for formation of glia. Hand genes encode basic helix-loop-helix transcription factors that are expressed in the developing gut, where their function is unknown. We now report that enteric Hand2 expression is limited to crest-derived cells, whereas Hand1 expression is restricted to muscle and interstitial cells of Cajal. Hand2 is developmentally regulated and is intranuclear in precursors but cytoplasmic in neurons. Neurons develop in explants from wild-type but not Hand2(-/-) bowel, although, in both, crest-derived cells are present and glia arise. Similarly, small interfering RNA (siRNA) silencing of Hand2 in enteric crest-derived cells prevents neuronal development. Terminally differentiated enteric neurons do not develop after conditional inactivation of Hand2 in migrating crest-derived cells; nevertheless, conditional Hand2 inactivation does not prevent precursors from expressing early neural markers. We suggest that enteric neuronal development occurs in stages and that Hand2 expression is required for terminal differentiation but not for precursors to enter the neuronal lineage.
3. Mouse primordial germ cells lacking beta1 integrins enter the germline but fail to migrate normally to the gonads. Primordial germ cells are the founder cells of the gametes. They are set aside at the initial stages of gastrulation in mammals, become embedded in the hind-gut endoderm, then actively migrate to the sites of gonad formation. The molecular basis of this **migration** is poorly understood. Here we sought to determine if members of the integrin family of cell surface receptors are required for primordial germ cell **migration**, as integrins have been implicated in the **migration** of several other motile cell types. We have established a line of mice which express green fluorescent protein in germline cells that has enabled us to efficiently purify primordial germ cells at different stages by flow cytometry. We have catalogued the spectrum of integrin subunit expression by primordial germ cells during and after **migration**, using flow cytometry, immunocytochemistry and RT-PCR. Through analysis of integrin beta1(-/-)-->wild-type chimeras, we show that embryonic cells lacking beta1 integrins can enter the germline. However, integrin beta1(-/-) primordial germ cells do not colonize the gonad efficiently. Embryos with targeted deletion of integrin subunit alpha3, alpha6, or alphaV show no major defects in primordial germ cell **migration**. These results demonstrate a role for beta1-containing integrins in the development of the germline, although an equivalent role for \* integrin subunit(s) has yet to be established.
4. Disruption of genital ridge development causes aberrant primordial germ cell proliferation but does not affect their directional **migration**. The directional **migration** and the following development of primordial germ cells (PGCs) during gonad formation are key steps for germline development. It has been proposed that the interaction between germ cells and genital ridge (GR) somatic cells plays essential roles in this process. However, the in vivo functional requirements of GR somatic cells in germ cell development are largely unknown.Wt1 mutation (Wt1(R394W/R394W)) results in GR agenesis through mitotic arrest of coelomic epitheliums. In this study, we employed the GR-deficient mouse model, Wt1(R394W/R394W), to investigate the roles of GR somatic cells in PGC **migration** and proliferation. We found that the number of PGCs was dramatically reduced in GR-deficient embryos at embryonic day (E) 11.5 and E12.5 due to decreased proliferation of PGCs, involving low levels of BMP signaling. In contrast, the germ cells in Wt1(R394W/R394W) embryos were still mitotically active at E13.5, while all the germ cells in control embryos underwent mitotic arrest at this stage. Strikingly, the directional **migration** of PGCs was not affected by the absence of GR somatic cells. Most of the PGCs reached the mesenchyme under the coelomic epithelium at E10.5 and no ectopic PGCs were noted in GR-deficient embryos. However, the precise positioning of PGCs was disrupted.Our work provides in vivo evidence that the proliferation of germ cells is precisely regulated by GR somatic cells during different stages of gonad development. GR somatic cells are probably dispensable for the directional **migration** of PGCs, but they are required for precise positioning of PGCs at the final step of **migration**.
5. Region-specific Etv2 ablation revealed the critical origin of hemogenic capacity from Hox6-positive caudal-lateral primitive mesoderm. Hematopoietic cells (HPCs) develop from hemogenic endothelial cells (ECs), a specialized type of ECs undergoing hematopoietic transition. However, the mesoderm origin for hemogenic ECs or HPCs has not been clarified. To examine the origin for hemogenic mesoderm, we inactivated Etv2, a master regulator for EC/HPC commitment, in specific regions. Region-specific Etv2 ablation in early mesoderm caused local EC differentiation block, resulting in the loss of specific vascular beds without compensatory **migration** of residual ECs into avascular area. This feature of local EC/HPC differentiation block was correlated to the hemogenic potential of each mesoderm subset. We found that caudal-lateral mesoderm of E7.5-8.5 embryos represent the pre-committed population critical for generating hemogenic ECs. Etv2 ablation in caudal-lateral mesoderm by Hoxb6 Cre or Hoxb6CreER transgene affected vitelline plexus formation and intra-aortic hematopoietic clusters. In differentiated embryonic stem cells, this mesoderm subset marked by Hoxb6-lateral mesoderm promoter showed enriched T lymphopoietic potential among Flk-1(+) cells, which could be regarded as a characteristic for definitive HPCs. These findings indicate that critical mesoderm precursors possibly for definitive type hemogenic ECs are regionally specified in primitive mesoderm, suggesting that Hoxb6(+) caudal-lateral mesoderm represents the critical source of HPCs, which are potentially useful to enrich definitive HPCs from embryonic stem cells.
6. The secreted metalloprotease ADAMTS20 is required for melanoblast survival. ADAMTS20 (Adisintegrin-like and metalloprotease domain with thrombospondin type-1 motifs) is a member of a family of secreted metalloproteases that can process a variety of extracellular matrix (ECM) components and secreted molecules. Adamts20 mutations in belted (bt) mice cause white spotting of the dorsal and ventral torso, indicative of defective neural crest (NC)-derived melanoblast development. The expression pattern of Adamts20 in dermal mesenchymal cells adjacent to migrating melanoblasts led us to initially propose that Adamts20 regulated melanoblast **migration**. However, using a Dct-LacZ transgene to track melanoblast development, we determined that melanoblasts were distributed normally in whole mount E12.5 bt/bt embryos, but were specifically reduced in the trunk of E13.5 bt/bt embryos due to a seven-fold higher rate of apoptosis. The melanoblast defect was exacerbated in newborn skin and embryos from bt/bt animals that were also haploinsufficient for Adamts9, a close homolog of Adamts20, indicating that these metalloproteases functionally overlap in melanoblast development. We identified two potential mechanisms by which Adamts20 may regulate melanoblast survival. First, skin explant cultures demonstrated that Adamts20 was required for melanoblasts to respond to soluble Kit ligand (sKitl). In support of this requirement, bt/bt;Kit(tm1Alf)/+ and bt/bt;Kitl(Sl)/+ mice exhibited synergistically increased spotting. Second, ADAMTS20 cleaved the aggregating proteoglycan versican in vitro and was necessary for versican processing in vivo, raising the possibility that versican can participate in melanoblast development. These findings reveal previously unrecognized roles for Adamts proteases in cell survival and in mediating Kit signaling during melanoblast colonization of the skin. Our results have implications not only for understanding mechanisms of NC-derived melanoblast development but also provide insights on novel biological functions of secreted metalloproteases.
7. Mast cell-mediated inflammatory responses require the alpha 2 beta 1 integrin. Although the alpha 2 beta 1 integrin is widely expressed and has been extensively studied, it has not been previously implicated in mast cell biology. We observed that alpha 2 integrin subunit-deficient mice exhibited markedly diminished neutrophil and interleukin-6 responses during Listeria monocytogenes- and zymosan-induced peritonitis. Since exudative neutrophils of wild-type mice expressed little alpha 2 beta 1 integrin, it seemed unlikely that this integrin mediated neutrophil **migration** directly. Here, we demonstrate constitutive alpha 2 beta 1 integrin expression on peritoneal mast cells. Although alpha 2-null mice contain normal numbers of peritoneal mast cells, these alpha 2-null cells do not support in vivo mast cell-dependent inflammatory responses. We conclude that alpha 2 beta 1 integrin provides a costimulatory function required for mast cell activation and cytokine production in response to infection.
8. A dynamic code of dorsal neural tube genes regulates the segregation between neurogenic and melanogenic neural crest cells. Understanding when and how multipotent progenitors segregate into diverse fates is a key question during embryonic development. The neural crest (NC) is an exemplary model system with which to investigate the dynamics of progenitor cell specification, as it generates a multitude of derivatives. Based on 'in ovo' lineage analysis, we previously suggested an early fate restriction of premigratory trunk NC to generate neural versus melanogenic fates, yet the timing of fate segregation and the underlying mechanisms remained unknown. Analysis of progenitors expressing a Foxd3 reporter reveals that prospective melanoblasts downregulate Foxd3 and have already segregated from neural lineages before e**migration**. When this downregulation is prevented, late-emigrating avian precursors fail to upregulate the melanogenic markers Mitf and MC/1 and the guidance receptor Ednrb2, generating instead glial cells that express P0 and Fabp. In this context, Foxd3 lies downstream of Snail2 and Sox9, constituting a minimal network upstream of Mitf and Ednrb2 to link melanogenic specification with **migration**. Consistent with the gain-of-function data in avians, loss of Foxd3 function in mouse NC results in ectopic melanogenesis in the dorsal tube and sensory ganglia. Altogether, Foxd3 is part of a dynamically expressed gene network that is necessary and sufficient to regulate fate decisions in premigratory NC. Their timely downregulation in the dorsal neural tube is thus necessary for the switch between neural and melanocytic phases of NC development.
9. Genetic evidence for convergence of c-Kit- and alpha4 integrin-mediated signals on class IA PI-3kinase and the Rac pathway in regulating integrin-directed **migration** in mast cells. Mast cells play a critical role in host defense against a number of pathogens. Increased mast cell infiltration has been described in allergic asthma, in rheumatoid arthritis, and during helminthes infection. Despite the importance of mast cells in allergic disease and defense against infection, little is known about the mechanisms by which mast cells migrate to various tissues under steady state conditions or during infection or inflammation. Here, we show that activation of c-Kit by its ligand, stem cell factor (SCF), cooperates with alpha4 integrin in inducing directed **migration** of mast cells on fibronectin. A reduction in **migration** and activation of a small G protein, Rac, was observed in mast cells derived from class IA phosphoinositide-3 kinase (PI-3kinase)-deficient mice in response to SCF stimulation and in mast cells expressing the dominant-negative Rac (RacN17), as well as in mast cells deficient in the hematopoietic-specific small G protein, Rac2. In addition, a PI-3kinase inhibitor inhibited alpha4- as well as SCF-induced **migration** in a dose-dependent fashion. In contrast, a mitogen-activated protein kinase (MAPK) inhibitor had little effect. Consistent with the pharmacologic results, abrogating the binding of the p85alpha subunit of class IA PI-3kinase to c-Kit also resulted in inhibition of SCF-induced **migration** on fibronectin. These genetic and biochemical data demonstrate that both c-Kit and alpha4 integrin signaling are linked to class IA PI-3kinase and Rac pathways and regulate integrin-directed (haptotactic) **migration** in mast cells.
10. Genetic evidence for critical roles of P38α protein in regulating mast cell differentiation and chemotaxis through distinct mechanisms. Mast cells mediate a range of immune responses. However, the mechanisms that contribute to their development remain poorly understood. Here, using a P38α conditional knockout system, we provide evidence to suggest that P38α plays critical roles in regulating mast cell differentiation a**nd migrat**ion via distinct mechanisms. Induced deletion of P38α in bone marrow cells retards the maturation of mast cells in part by inhibiting the activation of cAMP response element-binding protein and expression of microphthalmia-associated transcription factor, which encourages the generation of basophils over mast cells. In fully differentiated mast cells, absence of P38α inhibits stem cell factor-induced activation of Akt and ERK, which is associated with reduced chemotaxis. In vivo, conditional deletion of P38α results in reduced numbers of mast cells in certain tissues and a failure to reconstitute these cells in W(sh) mice transplanted with P38α(-/-) Lin(-)c-kit(+)Sca-1(+) (LKS(+)) cells. Our findings suggest that P38α plays a dual role in mast cell development by regulating IL-3-induced differentiation of mast cell progenitor cells as well as by regulating stem cell factor-**induced m**igration of fully differentiated mast cells.
11. Pak1 regulates multiple c-Kit mediated Ras-MAPK gain-in-function phenotypes in Nf1+/- mast cells. Neurofibromatosis type 1 (NF1) is a common genetic disorder caused by mutations in the NF1 locus, which encodes neurofibromin, a negative regulator of Ras. Patients with NF1 develop numerous neurofibromas, which contain many inflammatory mast cells that contribute to tumor formation. Subsequent to c-Kit stimulation, signaling from Ras to Rac1/2 to the MAPK pathway appears to be responsible for multiple hyperactive mast cell phenotypes; however, the specific effectors that mediate these functions remain uncertain. p21-activated kinase 1 (Pak1) is a downstream mediator of Rac1/2 that has been implicated as a positive regulator of MAPK pathway members and is a modulator of cell growth and cytoskeletal dynamics. Using an intercross of Pak 1(-/-) mice with Nf1(+/-) mice, we determined that Pak1 regulates hyperactive Ras-dependent proliferation via a Pak1/Erk pathway, whereas a Pak1/p38 pathway is required for the increased **migration** in Nf1(+/-) mast cells. Furthermore, we confirmed that loss of Pak1 corrects the dermal accumulation of Nf1(+/-) mast cells in vivo to levels found in wild-type mice. Thus, Pak1 is a novel mast cell mediator that functions as a key node in the MAPK signaling network and potential therapeutic target in NF1 patients.
12. Distinct roles for the SgIGSF adhesion molecule and c-kit receptor tyrosine kinase in the interaction between mast cells and the mesentery. Intraperitoneal injection of bone marrow-derived mast cells (BMMCs) has therapeutic efficacy against acute bacterial peritonitis. For this role, BMMCs need to settle down the mesentery from the peritoneal cavity. Interaction between BMMCs and the mesentery was examined by using mast cell deficient WBB6F1(F1)-W/Wv [c-kit receptor tyrosine kinase (KIT) mutant], F1-Sl/Sld [KIT ligand stem cell factor mutant], and F1-tg/tg [a practically microphthalmia transcription factor (MITF)-null mutant] mice. Three parameters were measured: the number of BMMCs: (1) developed in the mesentery 5 weeks after intraperitoneal injection into mast cell deficient mice, (2) adhered to mesenteric mesothelial cells, and (3) transmigrated across the mesenteric mesothelial cell monolayer when coculturing both cells for 3 and 18 h, respectively. After intraperitoneal injection, F1-wild type (+/+) BMMCs developed in the mesentery of F1-W/Wv mice but not in that of F1-Sl/Sld mice, while F1-tg/tg BMMCs did not develop, even in the mesentery of WBB6F1-W/Wv mice. In the coculture, WB-W/W BMMCs normally adhered to but poorly transmigrated across F1-+/+ mesothelial cells, and in accordance, F1-+/+ BMMCs normally adhered to but poorly transmigrated across F1-Sl/Sld mesothelial cells. F1-tg/tg BMMCs showed poor adhesion and trans**migration**, but both parameters were partially but significantly improved by ectopic expression of spermatogenic immunoglobulin superfamily (SgIGSF), a mast-cell adhesion molecule critically regulated by MITF. Since F1-tg/tg BMMCs expressed reduced levels of KIT, these results suggested that SgIGSF and KIT independently played a significant role in the trans**migration**. Among three parameters, development of mast cells in the mesentery well correlated with the trans**migration**. This process seemed important for mast cells to settle down from the peritoneal cavity to the mesentery.
13. Pak1 regulates multiple c-Kit mediated Ras-MAPK gain-in-function phenotypes in Nf1+/- mast cells. Neurofibromatosis type 1 (NF1) is a common genetic disorder caused by mutations in the NF1 locus, which encodes neurofibromin, a negative regulator of Ras. Patients with NF1 develop numerous neurofibromas, which contain many inflammatory mast cells that contribute to tumor formation. Subsequent to c-Kit stimulation, signaling from Ras to Rac1/2 to the MAPK pathway appears to be responsible for multiple hyperactive mast cell phenotypes; however, the specific effectors that mediate these functions remain uncertain. p21-activated kinase 1 (Pak1) is a downstream mediator of Rac1/2 that has been implicated as a positive regulator of MAPK pathway members and is a modulator of cell growth and cytoskeletal dynamics. Using an intercross of Pak 1(-/-) mice with Nf1(+/-) mice, we determined that Pak1 regulates hyperactive Ras-dependent proliferation via a Pak1/Erk pathway, whereas a Pak1/p38 pathway is required for the increased **migration** in Nf1(+/-) mast cells. Furthermore, we confirmed that loss of Pak1 corrects the dermal accumulation of Nf1(+/-) mast cells in vivo to levels found in wild-type mice. Thus, Pak1 is a novel mast cell mediator that functions as a key node in the MAPK signaling network and potential therapeutic target in NF1 patients.
14. Genetic evidence for convergence of c-Kit- and alpha4 integrin-mediated signals on class IA PI-3kinase and the Rac pathway in regulating integrin-directed **migration** in mast cells. Mast cells play a critical role in host defense against a number of pathogens. Increased mast cell infiltration has been described in allergic asthma, in rheumatoid arthritis, and during helminthes infection. Despite the importance of mast cells in allergic disease and defense against infection, little is known about the mechanisms by which mast cells migrate to various tissues under steady state conditions or during infection or inflammation. Here, we show that activation of c-Kit by its ligand, stem cell factor (SCF), cooperates with alpha4 integrin in inducing directed **migration** of mast cells on fibronectin. A reduction in **migration** and activation of a small G protein, Rac, was observed in mast cells derived from class IA phosphoinositide-3 kinase (PI-3kinase)-deficient mice in response to SCF stimulation and in mast cells expressing the dominant-negative Rac (RacN17), as well as in mast cells deficient in the hematopoietic-specific small G protein, Rac2. In addition, a PI-3kinase inhibitor inhibited alpha4- as well as SCF-induced **migration** in a dose-dependent fashion. In contrast, a mitogen-activated protein kinase (MAPK) inhibitor had little effect. Consistent with the pharmacologic results, abrogating the binding of the p85alpha subunit of class IA PI-3kinase to c-Kit also resulted in inhibition of SCF-induced **migration** on fibronectin. These genetic and biochemical data demonstrate that both c-Kit and alpha4 integrin signaling are linked to class IA PI-3kinase and Rac pathways and regulate integrin-directed (haptotactic) **migration** in mast cells.
15. Distinct roles for the SgIGSF adhesion molecule and c-kit receptor tyrosine kinase in the interaction between mast cells and the mesentery. Intraperitoneal injection of bone marrow-derived mast cells (BMMCs) has therapeutic efficacy against acute bacterial peritonitis. For this role, BMMCs need to settle down the mesentery from the peritoneal cavity. Interaction between BMMCs and the mesentery was examined by using mast cell deficient WBB6F1(F1)-W/Wv [c-kit receptor tyrosine kinase (KIT) mutant], F1-Sl/Sld [KIT ligand stem cell factor mutant], and F1-tg/tg [a practically microphthalmia transcription factor (MITF)-null mutant] mice. Three parameters were measured: the number of BMMCs: (1) developed in the mesentery 5 weeks after intraperitoneal injection into mast cell deficient mice, (2) adhered to mesenteric mesothelial cells, and (3) transmigrated across the mesenteric mesothelial cell monolayer when coculturing both cells for 3 and 18 h, respectively. After intraperitoneal injection, F1-wild type (+/+) BMMCs developed in the mesentery of F1-W/Wv mice but not in that of F1-Sl/Sld mice, while F1-tg/tg BMMCs did not develop, even in the mesentery of WBB6F1-W/Wv mice. In the coculture, WB-W/W BMMCs normally adhered to but poorly transmigrated across F1-+/+ mesothelial cells, and in accordance, F1-+/+ BMMCs normally adhered to but poorly transmigrated across F1-Sl/Sld mesothelial cells. F1-tg/tg BMMCs showed poor adhesion and trans**migration**, but both parameters were partially but significantly improved by ectopic expression of spermatogenic immunoglobulin superfamily (SgIGSF), a mast-cell adhesion molecule critically regulated by MITF. Since F1-tg/tg BMMCs expressed reduced levels of KIT, these results suggested that SgIGSF and KIT independently played a significant role in the trans**migration**. Among three parameters, development of mast cells in the mesentery well correlated with the trans**migration**. This process seemed important for mast cells to settle down from the peritoneal cavity to the mesentery.
16. Synaptotagmin-2 controls regulated exocytosis but not other secretory responses of mast cells. Mast cell degranulation is a highly regulated, calcium-dependent process, which is important for the acute release of inflammatory mediators during the course of many pathological conditions. We previously found that Synaptotagmin-2, a calcium sensor in neuronal exocytosis, was expressed in a mast cell line. We postulated that this protein may be involved in the control of mast cell-regulated exocytosis, and we generated Synaptotagmin-2 knock-out mice to test our hypothesis. Mast cells from this mutant animal conferred an abnormally decreased passive cutaneous anaphylaxis reaction on mast cell-deficient mice that correlated with a specific defect in mast cell-regulated exocytosis, leaving constitutive exocytosis and nonexocytic mast cell effector responses intact. This defect was not secondary to abnormalities in the development, maturation, **migration**, morphology, synthesis, and storage of inflammatory mediators, or intracellular calcium transients of the mast cells. Unlike neurons, the lack of Synaptotagmin-2 in mast cells was not associated with increased spontaneous exocytosis.
17. A KIT juxtamembrane PY567 -directed pathway provides nonredundant signals for erythroid progenitor cell development and stress erythropoiesis. KITL/KIT can elicit diverse sets of signals within lymphoid, myeloid, mast, and erythroid lineages, and exert distinct effects on growth, survival, **migration**, adhesion, and secretory responses. Presently, we have applied a PY-mutant allele knockin approach to specifically assess possible roles for KIT-PY567 and KIT-PY719 sites, and coupled pathways, during erythropoiesis.Mouse models used to investigate this problem include those harboring knocked-in KIT(Y567F/Y567F), KIT(Y569F/Y569F), KIT(Y719F,Y719F), and KIT(Y567F/Y567F:Y569F/Y569F) alleles. The erythron was stressed by myelosuppression using 5-fluorouracil, and by phenylhydrazine-induced hemolysis. In addition, optimized systems for ex vivo analyses of bone marrow and splenic erythropoiesis were employed to more directly analyze possible stage-specific effects on erythroid cell growth, survival, development and KIT signaling events.In Kit(Y567F/Y567F) mice, steady-state erythropoiesis was unperturbed while recovery from anemia due to 5-fluorouracil or phenylhydrazine was markedly impaired. Deficiencies in erythroid progenitor expansion occurred both in the bone marrow and the spleen. Responses to chronic erythropoietin dosing were also compromised. Ex vivo, Kit(Y567F/Y567F) (pro)erythroblast development was skewed from a Kit(pos)CD71(high) stage toward a subsequent Kit(neg)CD71(high) compartment. Proliferation and, to an extent, survival capacities were also compromised. Similar stage-specific defects existed for erythroid progenitors from Kit(Y567F/Y567F:Y569F/Y569F) but not KIT(Y719F/Y719F) mice. Kit(Y567F/Y567F) erythroblasts were used further to analyze KIT-PY567-dependent signals. MEK-1,2/ERK-1,2 signaling was unaffected while AKT, p70S6K, and especially JNK2/p54 pathways were selectively attenuated.Nonredundant KIT-PY567-directed erythroblast-intrinsic signals are selectively critical for stress erythropoiesis. Investigations also add to an understanding of how KIT directs distinct outcomes among diverse progenitors and lineages.
18. Loss of Wnt5a disrupts primordial germ cell **migration** and male sexual development in mice. Disruptions in the regulatory pathways controlling sex determination and differentiation can cause disorders of sex development, often compromising reproductive function. Although extensive efforts have been channeled into elucidating the regulatory mechanisms controlling the many aspects of sexual differentiation, the majority of disorders of sex development phenotypes are still unexplained at the molecular level. In this study, we have analyzed the potential involvement of Wnt5a in sexual development and show in mice that Wnt5a is male-specifically upregulated within testicular interstitial cells at the onset of gonad differentiation. Homozygous deletion of Wnt5a affected sexual development in male mice, causing testicular hypoplasia and bilateral cryptorchidism despite the Leydig cells producing factors such as Hsd3b1 and Insl3. Additionally, Wnt5a-null embryos of both sexes showed a significant reduction in gonadal germ cell numbers, which was caused by aberrant primordial germ cell **migration** along the hindgut endoderm prior to gonadal colonization. Our results indicate multiple roles for Wnt5a during mammalian reproductive development and help to clarify further the etiology of Robinow syndrome (OMIM 268310), a disease previously linked to the WNT5A pathway.
19. Ectopic c-kit expression affects the fate of melanocyte precursors in Patch mutant embryos. The Patch (Ph) mutation in the mouse, a deletion that includes the gene for PDGFR alpha, is a recessive lethal that exhibits a dominant pigment phenotype in heterozygotes. To assess whether the Ph mutation acts cell-autonomously or non-autonomously on melanocyte development, we have examined the melanogenic potential of neural crest populations from normal and mutant crest cells in vitro and the pattern of dispersal and survival of melanocyte precursors (MPs) in vivo. We report that trunk neural crest cells from homozygous Ph embryos give rise to pigmented melanocytes in vitro in response to Steel factor (SlF). In vivo, homozygous Ph embryos contain a subpopulation of crest-derived cells that express c-kit and tyrosinase-related protein-2 characteristic of MPs. These cells begin to migrate normally on the lateral crest **migration** pathway, but then fail to disperse in the dermal mesenchyme and subsequently disappear. Although dermal mesenchyme is adversely affected in Ph homozygotes, SlF mRNA expression by the cells of the dermatome is normal in Ph embryos when neural crest-derived MPs start to migrate on the lateral pathway. In contrast, mRNA for the SlF receptor, c-kit, was observed to be ectopically expressed in somites and lateral mesenchyme in embryos carrying the Ph mutation. Based on this ectopic expression of c-kit in Ph mutant embryos, and the observed distribution of SlF protein in normal and mutant embryos, we suggest that competition for limited amounts of SlF localized on the lateral neural crest **migration** pathway alters melanocyte dispersal and survival.
20. **Migration** of melanoblasts into the developing murine hair follicle is accompanied by transient c-Kit expression. Disruption of the c-Kit/stem cell factor (SCF) signaling pathway interferes with the survival, **migration**, and differentiation of melanocytes during generation of the hair follicle pigmentary unit. We examined c-Kit, SCF, and S100 (a marker for precursor melanocytic cells) expression, as well as melanoblast/melanocyte ultrastructure, in perinatal C57BL/6 mouse skin. Before the onset of hair bulb melanogenesis (i.e., stages 0-4 of hair follicle morphogenesis), strong c-Kit immunoreactivity (IR) was seen in selected non-melanogenic cells in the developing hair placode and hair plug. Many of these cells were S100-IR and were ultrastructurally identified as melanoblasts with migratory appearance. During the subsequent stages (5 and 6), increasingly dendritic c-Kit-IR cells successively invaded the hair bulb, while S100-IR gradually disappeared from these cells. Towards the completion of hair follicle morphogenesis (stages 7 and 8), several distinct follicular melanocytic cell populations could be defined and consisted broadly of (a) undifferentiated, non-pigmented c-Kit-negative melanoblasts in the outer root sheath and bulge and (b) highly differentiated melanocytes adjacent to the hair follicle dermal papilla above Auber's line. Widespread epithelial SCF-IR was seen throughout hair follicle morphogenesis. These findings suggest that melanoblasts express c-Kit as a prerequisite for **migration** into the SCF-supplying hair follicle epithelium. In addition, differentiated c-Kit-IR melanocytes target the bulb, while non-c-Kit-IR melanoblasts invade the outer root sheath and bulge in fully developed hair follicles.
21. Mast cell-associated TNF promotes dendritic cell **migration**. Mast cells represent a potential source of TNF, a mediator which can enhance dendritic cell (DC) **migration**. Although the importance of mast cell-associated TNF in regulating DC **migration** in vivo is not clear, mast cells and mast cell-derived TNF can contribute to the expression of certain models of contact hypersensitivity (CHS). We found that CHS to FITC was significantly impaired in mast cell-deficient Kit(W-sh/W-sh) or TNF(-/)(-) mice. The reduced expression of CHS in Kit(W-sh/W-sh) mice was fully repaired by local transfer of wild-type bone marrow-derived cultured mast cells (BMCMCs), but was only partially repaired by transfer of TNF(-/)(-) BMCMCs. Thus, mast cells, and mast cell-derived TNF, were required for optimal expression of CHS to FITC. We found that the **migration** of FITC-bearing skin DCs into draining lymph nodes (LNs) 24 h after epicutaneous administration of FITC in naive mice was significantly reduced in mast cell-deficient or TNF(-/)(-) mice, but levels of DC **migration** in these mutant mice increased to greater than wild-type levels by 48 h after FITC sensitization. Mast cell-deficient or TNF(-/)(-) mice also exhibited significantly reduced **migration** of airway DCs to local LNs at 24 h after intranasal challenge with FITC-OVA. **Migration** of FITC-bearing DCs to LNs draining the skin or airways 24 h after sensitization was repaired in Kit(W-sh/W-sh) mice which had been engrafted with wild-type but not TNF(-/)(-) BMCMCs. Our findings indicate that mast cell-associated TNF can contribute significantly to the initial stages of FITC-induced **migration** of cutaneous or airway DCs.
22. From white spots to stem cells: the role of the Kit receptor in mammalian development. The Kit tyrosine kinase membrane receptor is essential for melanogenesis, gametogenesis and hematopoiesis during embryonic development and postnatal life. This review summarizes the genetic evidence implicating Kit and its ligand, Steel factor, in the control of stem cell proliferation, **migration** and survival, with emphasis on mutations in the human and mouse genes.
23. The dominant W42 spotting phenotype results from a missense mutation in the c-kit receptor kinase. The murine white spotting locus (W) is allelic with the proto-oncogene c-kit, which encodes a transmembrane tyrosine protein kinase receptor for an unknown ligand. Mutations at the W locus affect various aspects of hematopoiesis and the proliferation and **migration** of primordial germ cells and melanoblasts during development to varying degrees of severity. The W42 mutation has a particularly severe effect in both the homozygous and the heterozygous states. The molecular basis of the W42 mutation was determined. The c-kit protein products in homozygous mutant mast cells were expressed normally but displayed a defective tyrosine kinase activity in vitro. Nucleotide sequence analysis of mutant complementary DNAs revealed a missense mutation that replaces aspartic acid with asparagine at position 790 in the c-kit protein product. Aspartic acid-790 is a conserved residue in all protein kinases. These results provide an explanation for the dominant nature of the W42 mutation and provide insight into the mechanism of c-kit-mediated signal transduction.
24. Primordial germ cell proliferation is impaired in Fused Toes mutant embryos. Over the first 4 days of their life, primordial germ cells invade the endoderm, migrate into and through the developing hindgut, and traverse to the genital ridge where they cluster and ultimately inhabit the nascent gonad. Specific signal-receptor combinations between primordial germ cells and their immediate environment establish successful **migration** and colonization. Here we demonstrate that disruption of a cluster of six genes on murine chromosome 8, as exemplified by the Fused Toes (Ft) mutant mouse model, results in severely decreased numbers of primordial germ cells within the early gonad. Primordial germ cell **migration** appeared normal within Ft mutant embryos; however, germ cell counts progressively decreased during this time. Although no difference in apoptosis was detected, we report a critical decrease in primordial germ cell proliferation by E12.5. The six genes within the Ft locus include the IrxB cluster (Irx3, -5, -6), Fts, Ftm, and Fto, of which only Ftm, Fto, and Fts are expressed in primordial germ cells of the early gonad. From these studies, we have discovered that the Ft locus on mouse chromosome 8 is associated with cell cycle deficits within the primordial germ cell population that initiates just before translocation into the genital ridge.
25. Fas and c-kit are involved in the control of hair follicle melanocyte apoptosis and **migration** in chemotherapy-induced hair loss. Chemotherapy alters the structure and function of hair follicle melanocytes. Molecular mechanisms controlling melanocyte responses during chemotherapy-induced hair loss, however, remain largely unknown. Using immunohistology and multicolor confocal microscopy, we show here that cyclophosphamide administration to C57BL/6 mice alters the activity and fate of hair follicle melanocytes. After 24-48 h, hair bulb melanocytes expressing Fas undergo apoptosis. The number of apoptotic follicular melanocytes is significantly reduced (p<0.01) in cyclophosphamide-treated Fas knockout mice compared to wild-type controls, suggesting that Fas signaling contributes to chemotherapy-induced melanocyte death. After 3-5 d, surviving hair bulb melanocytes express c-kit receptor, proliferate, and appear to migrate up the outer root sheath. Tyrosinase-positive and melanogenically active cells then appear in the epidermis. By Western blotting and immunohistochemistry, expression levels of the c-kit ligand, stem cell factor, in skin and epidermis are strongly increased after cyclophosphamide treatment. Cyclophosphamide-induced **migration** of the hair follicle melanocytes into epidermis is completely abrogated by administration of c-kit neutralizing antibody. These data suggest that chemotherapy induces a complex response in the hair follicle melanocytes, which includes apoptosis, proliferation, and **migration**. Pharmacologic manipulation of Fas and c-kit signaling pathways might be useful for the correction of skin hyperpigmentation as a side-effect of chemotherapy.
26. Distinctive role of the cKit receptor tyrosine kinase signaling in mammalian melanocytes. The cKit receptor plays a critical role in melanocyte physiology, influencing melanogenesis, proliferation, **migration**, and survival of the pigment-producing cells. However, pathways of cKit-mediated intracellular signaling and molecular mechanisms, which regulate specific cellular responses to the activation of the receptor in melanocytes, remain incompletely understood. Here, by using the genetically altered mouse melanocytes expressing an endogenous, constitutively active mutant (D814Y) cKit receptor, we investigated physiological cellular responses to the ligand-independent activation of the receptor tyrosine kinase. It was anticipated that such activation would either trigger uncontrolled proliferation of the melanocytes or stimulate melanin biosynthesis. In contrast to the expectation, we found that constitutive signaling from the cKit receptor did not stimulate melanogenesis and proliferation, but significantly promoted **migration** of the melanocytes both in vitro and in vivo. We also showed that such signaling is not associated with tumorigenic transformation of the pigment-producing cells. Taken together, our observations suggest that, in mammalian melanocytes, activation of the cKit receptor tyrosine kinase is primarily responsible for transmission of pro-**migration** signals, which may antagonize proliferation and melanogenesis. Our data also provide an additional explanation as to why malignant melanocytes lose cKit expression during melanoma progression.
27. Dystroglycan expression in the mouse cochlea. Viable dominant spotting (W(v)/W(v)) mice have a c-kit gene mutation, which impedes the **migration** of neural crest cells to the developing cochlea where they normally differentiate into intermediate cells (ICs). A prominent pathological feature shared by these mutants and the aging human and gerbil cochlea is thickening of the basement membrane (BM) of strial capillaries. Atrophy of strial capillaries in the aging gerbil has been associated with changes in the expression of dystroglycan (DG), a cell-surface receptor that regulates BM assembly. Here we evaluated the expression of DG in W(v)/W(v) mutant and C57BL/6J wild-type mice to investigate the possible role of ICs in regulating strial capillary BM homeostasis. The DG gene product was identified in lateral wall dissections from both W(v)/W(v) mutant and wild-type mice by reverse transcription-polymerase chain reaction. Subunit-specific antibodies were employed to localize the alpha and beta subunits of the DG heterodimer. Some sites in both wild-type and mutant mice, such as the subepithelial BM lining the scala media and regions of contact between selected epithelial cells, expressed alpha-DG alone. Other sites such as the perineural BM and the perivascular BM subtending strial capillaries and capillaries in the central portion of the auditory nerve coexpressed alpha- and beta-DG. The strong diffuse staining for alpha-DG along the basolateral membrane of strial marginal cells disappeared with advancing strial degeneration in abnormal turns of W(v)/W(v) mutants. Variations in staining intensity for both alpha- and beta-DG also occurred in the subendothelial BM of strial capillaries in turns lacking ICs and appeared to correspond with the degree of capillary atrophy. The results support the possibility that ICs play a role in the homeostasis of the strial capillary BM.
28. IL-33 induces skin inflammation with mast cell and neutrophil activation. Psoriasis is a common chronic autoimmune condition of the skin characterized by hyperplasia of epidermal keratinocytes associated with pro-inflammatory cytokines. IL-33 is a new member of the IL-1 superfamily that signals through the ST2 receptor and was originally defined as an inducer of T helper 2 (Th2) cytokines. Recently, broader immune activatory potential has been defined for IL-33 particularly via mast cell activation and neutrophil **migration**. Here, we show that ST2(-/-) mice exhibit reduced cutaneous inflammatory responses compared with WT mice in a phorbol ester-induced model of skin inflammation. Furthermore, injections of IL-33 into the ears of mice induce an inflammatory skin lesion. This inflammatory response was partially dependent on mast cells as mast cell-deficient mice (Kit(W-sh/W-sh) ) showed delayed responses to IL-33. IL-33 also recruited neutrophils to the ear, an effect mediated in part by increased production of the chemokine KC (CXCL1). Finally, we show that IL-33 expression is up-regulated in the epidermis of clinical psoriatic lesions, compared with healthy skin. These results therefore demonstrate that IL-33 may play a role in psoriasis-like plaque inflammation. IL-33 targeting may provide a new treatment strategy for psoriasis.
29. SWAP-70 regulates c-kit-induced mast cell activation, cell-cell adhesion, and **migration**. SWAP-70, an unusual phosphatidylinositol-3-kinase-dependent protein that interacts with the RhoGTPase Rac, is highly expressed in mast cells. Cultured bone marrow mast cells (BMMC) from SWAP-70(-/-) mice are reduced in FcepsilonRI-triggered degranulation. This report describes the hitherto-unknown role of SWAP-70 in c-kit receptor signaling, a key proliferation and differentiation pathway in mast cells. Consistent with the role of Rac in cell motility and regulation of the actin cytoskeleton, mutant cells show abnormal actin rearrangements and are deficient in **migration** in vitro and in vivo. SWAP-70(-/-) BMMC are impaired in calcium flux, in proper translocation and activity of Akt kinase (required for mast cell activation and survival), and in translocation of Rac1 and Rac2 upon c-kit stimulation. Adhesion to fibronectin is reduced, but homotypic cell association induced through c-kit is strongly increased in SWAP-70(-/-) BMMC. Homotypic association requires extracellular Ca(2+) and depends on the integrin alpha(L)beta(2) (LFA-1). ERK is hyperactivated upon c-kit signaling in adherent and dispersed mutant cells. Together, we suggest that SWAP-70 is an important regulator of specific effector pathways in c-kit signaling, including mast cell activation, **migration**, and cell adhesion.
30. Molecular bases of dominant negative and loss of function mutations at the murine c-kit/white spotting locus: W37, Wv, W41 and W. The proto-oncogene c-kit encodes a transmembrane tyrosine protein kinase receptor for an unknown ligand and is allelic with the murine white-spotting locus (W). Mutations at the W locus affect various aspects of hematopoiesis, the proliferation and **migration** of primordial germ cells and melanoblasts during development. The original W mutation and W37 are severe lethal mutations when homozygous. In the heterozygous state the W mutation has a weak phenotype while W37 has dominant characteristics. Wv and W41 are weak W mutations with dominant characteristics. We have characterized the molecular basis of these four W mutations and determined their effects on mast cell differentiation by using a fibroblast/mast cell co-culture assay. We show that W37, Wv and W41 are the result of missense mutations in the kinase domain of the c-kit coding sequence (W37 E----K at position 582; Wv T----M position 660 and W41 V----M position 831), which affect the c-kit associated tyrosine kinase to varying degrees. The c-kit protein products in homozygous mutant mast cells are expressed normally, although the 160 kd cell membrane form of the c-kitW37 protein displays accelerated turnover characteristics. The W mutation is the result of a 78 amino acid deletion which includes the transmembrane domain of the c-kit protein. A 125 kd c-kit protein was detected in homozygous W/W mast cells which lacks kinase activity and is not expressed on the cell surface.(ABSTRACT TRUNCATED AT 250 WORDS)
31. Mast cell activators: a new class of highly effective vaccine adjuvants. Mast cells (MCs) have recently received recognition as prominent effectors in the regulation of immune cell **migration** to draining lymph nodes and lymphocyte activation. However, their role in the development of humoral immune responses is not clear. Here, we demonstrate that subcutaneous or nasal administration of small-molecule MC activators with vaccine antigens evokes large increases in antigen-specific serum immunoglobulin G (IgG) responses. These responses were MC dependent and correlated with increased dendritic cell and lymphocyte recruitment to draining lymph nodes. Nasal instillation of these formulations also evoked antigen-specific secretory IgA and provided protection against anthrax lethal toxin challenge in vitro and against vaccinia virus infection in vivo. Collectively, these results define the MC as an integral sensory arm of the adaptive immune system. Moreover, they highlight MC activators as a new class of vaccine adjuvants, capable of inducing protective antigen-specific immune responses through needle-free routes of administration.
32. c-kit expression profile and regulatory factors during spermatogonial stem cell differentiation. It has been proven that c-kit is crucial for proliferation, **migration**, survival and maturation of spermatogenic cells. A periodic expression of c-kit is observed from primordial germ cells (PGCs) to spermatogenetic stem cells (SSCs), However, the expression profile of c-kit during the entire spermatogenesis process is still unclear. This study aims to reveal and compare c-kit expression profiles in the SSCs before and after the anticipated differentiation, as well as to examine its relationship with retinoic acid (RA) stimulation.We have found that there are more than 4 transcripts of c-kit expressed in the cell lines and in the testes. The transcripts can be divided into short and long categories. The long transcripts include the full-length canonical c-kit transcript and the 3' end short transcript. Short transcripts include the 3.4 kb short transcript and several truncated transcripts (1.9-3.2 kb). In addition, the 3.4 kb transcript (starting from intron 9 and covering exons 10 ~ 21) is discovered to be specifically expressed in the spermatogonia. The extracellular domain of Kit is obtained in the spermatogonia stage, but the intracellular domain (50 kDa) is constantly expressed in both SSCs and spermatogonia. The c-kit expression profiles in the testis and the spermatogonial stem cell lines vary after RA stimulation. The wave-like changes of the quantitative expression pattern of c-kit (increase initially and decrease afterwards) during the induction process are similar to that of the in vivo male germ cell development process.There are dynamic transcription and translation changes of c-kit before and after SSCs' anticipated differentiation and most importantly, RA is a significant upstream regulatory factor for c-kit expression.
33. Epithelial cell-derived IL-25, but not Th17 cell-derived IL-17 or IL-17F, is crucial for murine asthma. IL-17A, IL-17F, and IL-25 are ligands for IL-17RA. In the current study, we demonstrated that IL-25-deficient mice-but not IL-17A-, IL-17F-, IL-17A/F-, IL-23p19-, or retinoic acid-related orphan receptor (ROR)-γt-deficient mice-showed significant suppression of 1) the number of eosinophils and the levels of proinflammatory mediators in bronchoalveolar lavage fluids, 2) airway hyperresponsiveness to methacholine, and 3) OVA-specific IgG1 and IgE levels in the serum during OVA-induced Th2-type/eosinophilic airway inflammation. The IL-25 deficiency did not affect lung dendritic cell **migratio**n or Ag-specific memory-Th2 cell expansion during Ag sensitization. Adoptive transfer of T cells, mast cells, or bone marrow cells from IL-25-deficient mice revealed that induction of Th2-type/eosinophilic airway inflammation was dependent on activation of lung epithelial cells and eosinophils by IL-25 produced by airway structural cells such as epithelial cells but not by such hematopoietic stem-cell-origin immune cells as T cells and mast cells. Therefore, airway structural cell-derived IL-25-rather than Th17 cell-derived IL-17A and IL-17F-is responsible for induction of local inflammation by promoting activation of lung epithelial cells and eosinophils in the elicitation phase of Th2-type/eosinophilic airway inflammation. It is not required for Ag-specific Th2 cell differentiation in the sensitization phase.
34. The phosphatidyl inositol 3-kinase pathway is central to the pathogenesis of Kit-activated melanoma. Mouse Kit L575P, the ortholog of human KIT L576P, a common KIT mutation found in human melanoma was expressed in an immortalized but non-transformed mouse Ink4a-Arf-deficient melanocyte cell line. The resultant Ink4a-Arf-deficient Kit L575P-expressing melanocytes exhibited increased proliferation, the ability to grow in soft agar, and increased **migration**. When these cells were injected subcutaneously into NOD/SCID/gamma(c) mice, melanomas arose in 5 of 7 (71%) mice. One of seven mice (14%) injected with these cells developed metastatic disease. Evaluation of signal transduction pathways downstream of constitutively activated Kit L575P revealed striking activation of the phosphatidyl inositol 3-kinase (PI3K) pathway. Inhibition of the PI3K pathway pharmacologically or genetically abolished the transformation phenotypes gained by the L575P single mutant. These studies validate this Kit L575P-activated model of melanoma and establish the PI3K pathway as a dominant signaling pathway downstream of Kit in melanoma.
35. Mutations at the W locus affect survival of neural crest-derived melanocytes in the mouse. The development of melanoblasts in normally pigmented and dominant spotting (W) embryos was followed by in situ hybridisation to TRP-2/DT mRNA, which labels migratory melanoblasts from 10 days post coitum. Numerous melanoblasts migrate to the inner ear around 11 days. In contrast, few migratory melanoblasts are associated with the eye or skin at this stage and melanoblasts distribution within the trunk and tail is patchy. The distribution of melanoblasts in 10.5-11-day-old Wv/Wv, Wsh/Wsh and W41/W41 mutants was similar to that in controls but melanoblasts density was lower and by 12 days was severely reduced. These results suggest that mutations of the c-kit receptor tyrosine kinase encoded at the W locus do not alter early **migration** or differentiation of melanoblasts but severely affect melanoblasts survival.
36. Critical roles of c-Kit tyrosine residues 567 and 719 in stem cell factor-induced chemotaxis: contribution of src family kinase and PI3-kinase on calcium mobilization and cell **migration**. Stem cell factor (SCF) has crucial roles in proliferation, survival, and differentiation of hematopoietic stem cells and mast cells through binding to c-Kit receptor (KIT). Chemotaxis is another unique function of SCF. However, little is known about the intracellular signaling pathway of SCF/KIT-mediated cell **migration**. To investigate the signaling cascade, we made a series of 22 KIT mutants, in which tyrosine (Y) residue was substituted for phenylalanine (F) in the cytoplasmic domain, and introduced into BAF3 cells or 293T cells. On stimulation with SCF, BAF3 expressing KIT(WT)(WT) showed cell **migration** and Ca(2+) mobilization. Among 22 YF mutants, Y567F, Y569F, and Y719F showed significantly reduced cell **migration** and Ca(2+) mobilization compared to WT. In Y567F, Lyn activation on SCF stimulation decreased and C-terminal Src kinase (Csk) suppressed KIT-mediated Ca(2+) influx and cell **migration**, suggesting that Y567-mediated Src family kinase (SFK) activation leads to Ca(2+) influx and **migration**. Furthermore, we found that p38 mitogen-activated protein kinase (p38 MAPK) and Erk1/2 were also regulated by Y567/SFK and involved in cell **migration**, and that p38 MAPK induced Ca(2+) influx, thereby leading to Erk1/2 activation. In Y719F, the binding of phosphatidylinositol 3'-kinase (PI3K) to KIT was lost and KIT-mediated cell **migration** and Ca(2+) mobilization were suppressed by PI3K chemical inhibitors or dominant-negative PI3K, suggesting the involvement of Y719-mediated PI3K pathway in cell **migration**. Combination of Csk and the PI3K inhibitor synergistically reduced cell **migration**, suggesting the cooperation of SFK and PI3K. Taken together, these results indicate that 2 major KIT signaling pathways lead to cell **migration**, one is Y567-SFK-p38 MAPK-Ca(2+) influx-Erk and the other is Y719-PI3K-Ca(2+) influx.
37. Impaired mast cell-driven immune responses in mice lacking the transcription factor NFATc2. The three calcium-dependent factors NFATc1, c2, and c3 are expressed in cells of the immune system and play pivotal roles in modulating cellular activation. With regard to NFATc2, it was reported that NFATc2-deficient mice display increased immune responses in several models for infection and allergy in vivo. This led to the assumption that NFATc2 is involved in the maintenance of immune homeostasis. Using the synthetic TLR7 agonist imiquimod as an adjuvant in epicutaneous peptide immunization, we observed that both the inflammatory reaction and the peptide-specific CTL response are severely impaired in NFATc2-deficient mice. Detailed analyses revealed that early production of proinflammatory cytokines, lymph node hypertrophy, and **migration** of Langerhans cells are strongly reduced in NFATc2-deficient animals. With the aid of mast cell-deficient mice and reconstitution experiments using mast cells derived from either NFATc2-deficient mice or wild-type controls, we were able to show that NFATc2 expressed in mast cells is critical for the initiation of inflammation, **migration** of Langerhans cells, and the development of full-blown CTL responses following epicutaneous immunization. Thus, NFATc2 is an important factor controlling mast cell accessory function at the interface of innate and adaptive immunity.
38. Catenary cultures of embryonic gastrointestinal tract support organ morphogenesis, motility, neural crest cell **migration**, and cell differentiation. The embryonic gastrointestinal tract develops from a simple tube into a coiled, flexed, and regionalized structure. The changes in gut morphology coincide with the differentiation of multiple cell types in concentric layers, and include colonization by migratory neuron precursors, and the development of gastrointestinal motility. We describe a reliable method for growing embryonic mouse intestine in vitro by the attachment of segments of intestinal tract by their cut ends, with the intervening region suspended in the culture medium. These are termed "catenary cultures." E11-E11.5 mouse midgut, hindgut, or mid- plus hindgut segments were grown in catenary culture for up to 10 days and their growth, morphology, cell differentiation, ability to support neural precursor **migration**, and contractile activity were assessed. The increase in size of the cultured explants was not large, but morphogenesis proceeded, best exemplified by elongation of the caecum. Cell differentiation also proceeded. In the mucosa, goblet cells differentiated. Muscle layers, characterized by desmin expression, and kit-positive interstitial cells of Cajal differentiated in the correct positions. Where segments initially included neural precursors in a small sub-region, these migrated and proliferated to form uniform neuronal networks throughout the entire explant, and the cells expressed the neuron markers nitric oxide synthase and neuron specific enolase. Gut motility was attained 5-6 days into the culture period, and both contractile- and mixing-type movements were observed. Thus, cell types representative of all three germ layer contributions developed, and in addition, the gut, being mainly free, was able to elongate and bend (unlike on solid support cultures), while retaining its rostrocaudal identity.
39. Melanocyte development in vivo and in neural crest cell cultures: crucial dependence on the Mitf basic-helix-loop-helix-zipper transcription factor. The more than 20 different Mitf mutations in the mouse are all associated with deficiencies in neural crest-derived melanocytes that range from minor functional disturbances with some alleles to complete absence of mature melanocytes with others. In the trunk region of wild-type embryos, Mitf-expressing cells that coexpressed the melanoblast marker Dct and the tyrosine kinase receptor Kit were found in the dorsolateral neural crest **migration** pathway. In contrast, in embryos homozygous for an Mitf allele encoding a non-functional Mitf protein, Mitf-expressing cells were extremely rare, no Dct expression was ever found, and the number of Kit-expressing cells was much reduced. Wild-type neural crest cell cultures rapidly gave rise to cells that expressed Mitf and coexpressed Kit and Dct. With time in culture, Kit expression was increased, and pigmented, dendritic cells developed. Addition of the Kit ligand Mgf or endothelin 3 or a combination of these factors all rapidly increased the number of Dct-positive cells. Cultures from Mitf mutant embryos initially displayed Mitf-positive cells similar in numbers and Kit-expression as did wild-type cultures. However, Kit expression did not increase with time in culture and the mutant cells never responded to Mgf or endothelin 3, did not express Dct, and never showed pigment. In fact, even Mitf expression was rapidly lost. The results suggest that Mitf first plays a role in promoting the transition of precursor cells to melanoblasts and subsequently, by influencing Kit expression, melanoblast survival.
40. Tyrosine kinase-deficient Wv c-kit induces mast cell adhesion and chemotaxis. W/Wv mice are deficient in tissue mast cells, and mast cells cultured from these mice do not proliferate in response to the c-kit ligand, stem cell factor (SCF). In this paper, we report that mouse bone marrow cultured mast cells derived from W/Wv mice do adhere to fibronectin in the presence of SCF and exhibit chemotaxis to SCF, and we explore this model for the understanding of c-kit-mediated signaling pathways. Both in vitro and in vivo (in intact cells) phosphorylation experiments demonstrated a low residual level of W/Wv c-kit protein phosphorylation. SCF-induced responses in W/Wv mast cells were abolished by the tyrosine kinase inhibitor herbimycin A and by the phospatidylinositol 3-kinase (PI 3-kinase) inhibitor wortmannin but were not affected by protein kinase C inhibitors. These observations are consistent with the conclusions that Wv c-kit initiates a signaling process that is PI 3-kinase dependent and that mutated Wv c-kit retains the ability to initiate mast cell adhesion and **migration**.
41. Mast cell FcεRI-induced early growth response 2 regulates CC chemokine ligand 1-dependent CD4+ T cell **migratio**n. Mast cells are well positioned in host tissue for detecting environmental signals, including allergens, leading to activation of the high-affinity IgE receptor FcεRI, and initiating a signaling cascade that perpetuates the production of biologically potent mediators, including chemokines. We have identified a novel target of mast cell FcεRI activity in the transcription factor early growth response 2 (Egr2) and sought to characterize its function therein. Egr2 was transiently activated following FcεRI-mediated signaling, targeted the promoter of the chemokine CCL1, and was critical for allergen-induced mast cell CCL1 production. Egr2-deficient mast cells were incapable of directing CD4(+) T c**ell migra**tion via the CCL1-CCR8 axis. In a model of allergic asthma, reconstitution of mast cell-deficient mice with Egr2-deficient mast cells demonstrated that mast cell Egr2 was essential **for migra**tion of CD4(+) T cells to the inflamed lung. These findings position Egr2 as a critical regulator of mast cell-directed CD4(+) T c**ell migra**tion.
42. SCF/c-kit signaling is required in 12-O-tetradecanoylphorbol-13-acetate-induced **migration** and differentiation of hair follicle melanocytes for epidermal pigmentation. Hair follicle melanocyte stem cells (McSCs) are responsible for hair pigmentation and also function as a major melanocyte reservoir for epidermal pigmentation. However, the molecular mechanism promoting McSCs for epidermal pigmentation remains elusive. 12-O-tetradecanoylphorbol-13-acetate (TPA) mimics key signaling involved in melanocyte growth, **migration** and differentiation. We therefore investigated the molecular basis for the contribution of hair follicle McSCs to epidermal pigmentation using the TPA induction model. We found that repetitive TPA treatment of female C57BL/6 mouse dorsal skin induced epidermal pigmentation by increasing the number of epidermal melanocytes. Particularly, TPA treatment induced McSCs to initiate proliferation, exit the stem cell niche and differentiate. We also demonstrated that TPA promotes melanoblast **migration** and differentiation in vitro. At the molecular level, TPA treatment induced robust expression of stem cell factor (SCF) in keratinocytes and c-kit in melanoblasts and melanocytes. Administration of ACK2, a neutralizing antibody against the Kit receptor, suppressed mouse epidermal pigmentation, decreased the number of epidermal melanocytes, and inhibited melanoblast **migration**. Taken together, our data demonstrate that TPA promotes the expansion, **migration** and differentiation of hair follicle McSCs for mouse epidermal pigmentation. SCF/c-kit signaling was required for TPA-induced **migration** and differentiation of hair follicle melanocytes. Our findings may provide an excellent model to investigate the signaling mechanisms regulating epidermal pigmentation from mouse hair follicle McSCs, and a potential therapeutic option for skin pigmentation disorders.
43. Lnk adaptor protein down-regulates specific Kit-induced signaling pathways in primary mast cells. Stem cell factor (SCF) plays critical roles in proliferation, survival, **migration**, and function of hematopoietic progenitor and mast cells through binding to Kit receptor. Previous studies have implicated the adaptor protein Lnk as an important negative regulator of SCF signaling. However, the molecular mechanism underlying this regulation is unclear. Here, we showed that the Src homology 2 domain (SH2) of Lnk binds directly and preferentially to phosphorylated tyrosine 567 in Kit juxtamembrane domain. Using Lnk(-/-) bone marrow mast cells (BMMCs) transduced with different Lnk proteins, we demonstrated that Lnk down-regulates SCF-induced proliferation with attenuation of mitogen-activated protein kinase (MAPK) and c-jun N-terminal kinase signaling. Furthermore, we showed that Lnk(-/-) BMMCs displayed increased SCF-dependent **migration** compared with wild-type cells, revealing a novel Lnk-mediated inhibitory function. This correlated with enhanced Rac and p38 MAPK activation. Finally, we found that Lnk domains and carboxy-terminal tyrosine contribute differently to inhibition of in vitro expansion of hematopoietic progenitors. Altogether, our results demonstrate that Lnk, through its binding to Kit tyrosine 567, negatively modulates specific SCF-dependent signaling pathways involved in the proliferation and **migration** of primary hematopoietic cells.
44. The c-kit receptor ligand functions as a mast cell chemoattractant. Mast cells accumulate at sites of neovascularization, solid tumors, and many immune reactions. Such accumulation requires directed **migration** of mature mast cells or their precursors. The nature of the chemoattractants that regulate mast cell motility and the identity of the receptors that mediate the chemotactic response are poorly understood. We have tested the ability of stem cell factor (SCF), a mast cell growth factor, to stimulate mast cell **migration**. Our results show that SCF is a potent mast cell attractant that stimulates directional motility of both mucosal and connective tissue-type mast cells. The activity is potentiated by costimulation with interleukin-3 (IL-3), another mast cell chemoattractant. SCF, a known ligand for the c-kit tyrosine kinase receptor, was unable to stimulate motility in W42 mutant mast cells, which have a defective c-kit tyrosine kinase. However, W42 mast cells were still able to migrate in response to IL-3. These results show that SCF is a chemotactic factor as well as a growth factor and that the c-kit receptor can transduce signals leading to both cell proliferation and increased directional cell motility.
45. The pro-apoptotic gene Bax is required for the death of ectopic primordial germ cells during their **migration** in the mouse embryo. In the mouse embryo, significant numbers of primordial germ cells (PGCs) fail to migrate correctly to the genital ridges early in organogenesis. These usually die in ectopic locations. In humans, 50% of pediatric germ line tumors arise outside the gonads, and these are thought to arise from PGCs that fail to die in ectopic locations. We show that the pro-apoptotic gene Bax, previously shown to be required for germ cell death during later stages of their differentiation in the gonads, is also expressed during germ cell **migration**, and is required for the normal death of germ cells left in ectopic locations during and after germ cell **migration**. In addition, we show that Bax is downstream of the known cell survival signaling interaction mediated by the Steel factor/Kit ligand/receptor interaction. Together, these observations identify the major mechanism that removes ectopic germ cells from the embryo at early stages.
46. Mast cells are key promoters of contact allergy that mediate the adjuvant effects of haptens. A prominent feature of sensitizing environmental compounds that cause allergic contact dermatitis is the rapid induction of an innate inflammatory response that seems to provide danger signals for efficient T cell priming. We generated mouse models of mast cell deficiency, mast cell-specific gene inactivation, and mast cell reporter mice for intravital imaging and showed that these adjuvant effects of contact allergens are mediated by mast cells and histamine. Mast cell deficiency resulted in impaired **emigratio**n of skin DCs to the lymph node and contact hypersensitivity was dramatically reduced in the absence of mast cells. In addition, mast cell-specific inactivation of the Il10 gene did not reveal any role for mast cell-derived IL-10 in the regulation of contact allergy. Collectively, we demonstrate that mast cells are essential promoters of contact hypersensitivity, thereby highlighting their potential to promote immune responses to antigens entering via the skin.
47. BALB/3T3 fibroblast-conditioned medium attracts cultured mast cells derived from W/W but not from mi/mi mutant mice, both of which are deficient in mast cells. Proliferation of murine mast cells is induced by both T-cell-derived and fibroblast-derived growth factors. Because the most potent T-cell-derived mast cell growth factor, interleukin-3, promotes the **migration** of mast cells, we investigated whether fibroblast-derived growth factors had the chemoattractive activity as well. Conditioned medium (CM) of BALB/3T3 fibroblasts induced the **migration** of cultured mast cells (CMC) derived from normal (+/+) mice. BALB/3T3-CM contained the mast cell growth factor (MGF)/stem cell factor (SCF)/kit ligand (KL), which is the ligand for the receptor encoded by the W (c-kit) gene. CMC derived from the spleen of W/W mice lack the extracellular domain of the W (c-kit) receptor, and W/W CMC did not proliferate in response to BALB/3T3-CM. However, W/W CMC did migrate normally toward BALB/3T3-CM and, moreover, the antibody to the extracellular domain of the W (c-kit) receptor did not inhibit the chemoattractive activity of +/+ CMC toward BALB/3T3-CM. These results indicated that MGF/SCF/KL itself did not represent the major chemoattractive activity. On the other hand, BALB/3T3-CM induced neither proliferation nor **migration** of CMC derived from mi/mi mice. Both W/W and mi/mi mice are deficient in mast cells, but the present results suggest that the mechanism of the abnormality is different between W/W and mi/mi mice.
48. Androgen-dependent sertoli cell tight junction remodeling is mediated by multiple tight junction components. Sertoli cell tight junctions (SCTJs) of the seminiferous epithelium create a specialized microenvironment in the testis to aid differentiation of spermatocytes and spermatids from spermatogonial stem cells. SCTJs must be chronically broken and rebuilt with high fidelity to allow the trans**migration** of preleptotene spermatocytes from the basal to adluminal epithelial compartment. Impairment of androgen signaling in Sertoli cells perturbs SCTJ remodeling. Claudin (CLDN) 3, a tight junction component under androgen regulation, localizes to newly forming SCTJs and is absent in Sertoli cell androgen receptor knockout (SCARKO) mice. We show here that Cldn3-null mice do not phenocopy SCARKO mice: Cldn3(-/-) mice are fertile, show uninterrupted spermatogenesis, and exhibit fully functional SCTJs based on imaging and small molecule tracer analyses, suggesting that other androgen-regulated genes must contribute to the SCARKO phenotype. To further investigate the SCTJ phenotype observed in SCARKO mutants, we generated a new SCARKO model and extensively analyzed the expression of other tight junction components. In addition to Cldn3, we identified altered expression of several other SCTJ molecules, including down-regulation of Cldn13 and a noncanonical tight junction protein 2 isoform (Tjp2iso3). Chromatin immunoprecipitation was used to demonstrate direct androgen receptor binding to regions of these target genes. Furthermore, we demonstrated that CLDN13 is a constituent of SCTJs and that TJP2iso3 colocalizes with tricellulin, a constituent of tricellular junctions, underscoring the importance of androgen signaling in the regulation of both bicellular and tricellular Sertoli cell tight junctions.
49. The kit ligand encoded at the murine Steel locus: a pleiotropic growth and differentiation factor. The c-kit receptor and its recently identified ligand are allelic with the murine White Spotting and Steel loci, respectively. These observations brought to light the functions of the c-kit receptor system in melanogenesis, gametogenesis and hematopoeisis during embryogenesis and in postnatal life. The recent molecular analysis of several White Spotting and Steel alleles has provided insights into the mechanism of c-kit ligand-mediated processes, including cell proliferation, cell **migration** and cell survival. Furthermore, the availability of the kit ligand has allowed in vitro investigations of the pleiotropic functions of c-kit in development and cell differentiation to be carried out.
50. Fetal liver endothelium regulates the seeding of tissue-resident macrophages. Macrophages are required for normal embryogenesis, tissue homeostasis and immunity against microorganisms and tumours. Adult tissue-resident macrophages largely originate from long-lived, self-renewing embryonic precursors and not from haematopoietic stem-cell activity in the bone marrow. Although fate-mapping studies have uncovered a great amount of detail on the origin and kinetics of fetal macrophage development in the yolk sac and liver, the molecules that govern the tissue-specific **migration** of these cells remain completely unknown. Here we show that an endothelium-specific molecule, plasmalemma vesicle-associated protein (PLVAP), regulates the seeding of fetal monocyte-derived macrophages to tissues in mice. We found that PLVAP-deficient mice have completely normal levels of both yolk-sac- and bone-marrow-derived macrophages, but that fetal liver monocyte-derived macrophage populations were practically missing from tissues. Adult PLVAP-deficient mice show major alterations in macrophage-dependent iron recycling and mammary branching morphogenesis. PLVAP forms diaphragms in the fenestrae of liver sinusoidal endothelium during embryogenesis, interacts with chemoattractants and adhesion molecules and regulates the egress of fetal liver monocytes to the systemic vasculature. Thus, PLVAP selectively controls the exit of macrophage precursors from the fetal liver and, to our knowledge, is the first molecule identified in any organ as regulating the migratory events during embryonic macrophage ontogeny.
51. C1q governs deposition of circulating immune complexes and leukocyte Fcgamma receptors mediate subsequent neutrophil recruitment. Inflammation induced by circulating immunoglobulin G-immune complexes (ICs) characterizes many immune-mediated diseases. In this work, the molecular requirements for the deposition of circulating ICs and subsequent acute leukocyte recruitment in mice were elucidated. We show that after intravenous injection, preformed soluble ICs are rapidly deposited in the postcapillary venules of the cremaster microcirculation, secondary to increased vascular permeability. This deposition is dependent on complement C1q. IC deposition is associated with leukocyte recruitment. Leukocyte rolling, which is mediated by P-selectin in the exteriorized cremaster muscle, is not further increased in response to ICs. In contrast, leukocyte rolling velocity is significantly decreased and leukocyte adhesion is significantly increased in the presence of ICs. The IC-mediated slow leukocyte rolling velocity and subsequent adhesion and e**migration** are dependent on Fcgamma receptors (FcgammaRs), particularly FcgammaRIII, with complement C3 and C5 having no detectable role. These studies suggest a regulatory mechanism of IC deposition and leukocyte trafficking in IC-mediated inflammation requiring C1q and FcgammaRs in sequential, noninteracting roles.
52. Membrane-bound Kit ligand regulates melanocyte adhesion and survival, providing physical interaction with an intraepithelial niche. Morphogenesis, as illustrated by melanocyte **migration** and homing to the skin, requires cadherin adhesion, integrin-dependent **migration** and Kit-ligand growth factor signaling. However, it is not known how Kit ligand regulates integrin or cadherin-dependent intraepidermal melanocyte behavior. To answer this question, we developed specific 2-dimensional (2D) and 3D culture systems analyzing how soluble or immobilized Kit-ligand-regulated melanocyte **migration** on vitronectin and laminin, or within a monolayer of kidney epithelial cells. In a 2D system, soluble Kit ligand stimulated integrin-dependent melanoblast **migration** and chemotaxis and accelerated integrin turnover. In contrast, immobilized, but not soluble, Kit ligand, enhanced integrin-dependent melanocyte spreading on suboptimal laminin concentrations. In 3D, membrane-bound Kit ligand induced intraepithelial melanocyte proliferation, survival, and tight adhesion to epithelial cells, while cleavable Kit ligand was less effective. In contrast, melanocyte motility was independent of membrane-bound Kit ligand, but increased in the presence of the cleavable Kit-ligand isoform. Transmembrane-dimerization or basolateral-targeting mutants of Kit ligand altered intraepithelial melanocyte localization, survival, and adhesion to epithelial cells. These data and the identification of c-kit/Kit-ligand clusters at cell contacts suggest that membrane-bound Kit ligand captures cell surface-expressed c-kit, providing mechanical anchoring and survival signaling within intraepithelial niches, and thereby defining a new mechanism for melanocyte homeostasis and requirement for environmental niches.
53. Mast cell tryptase deficiency attenuates mouse abdominal aortic aneurysm formation. Mast cells (MCs) contribute to the formation of abdominal aortic aneurysms (AAAs) by producing biologically active mediators. Tryptase is the most abundant MC granule protein and participates in MC activation, protease maturation, leukocyte recruitment, and angiogenesis-all processes critical to AAA pathogenesis.To test the hypothesis that tryptase participates directly in AAA formation.Immunohistochemistry demonstrated enhanced tryptase staining in media and adventitia of human and mouse AAA lesions. Serum tryptase levels correlated significantly with the annual expansion rate of AAA before (r = 0.30, P = 0.003) and after (r = 0.29, P = 0.005) adjustment for common AAA risk factors in a patient follow-up study, and associated with risks for later surgical repair or overall mortality before (P = 0.009, P = 0.065) and after (P = 0.004, P = 0.001) the adjustment. Using MC protease-6-deficient mice (Mcpt6(-/-)) and aortic elastase perfusion-induced experimental AAAs, we proved a direct role of this tryptase in AAA pathogenesis. Whereas all wild-type (WT) mice developed AAA at 14 or 56 days postperfusion, Mcpt6(-/-) mice were fully protected. AAA lesions from Mcpt6(-/-) mice had fewer inflammatory and apoptotic cells, and lower chemokine levels, than did those from WT mice. MC from WT mice restored reduced AAA lesions and lesion inflammatory cell content in MC-deficient Kit(W-sh/W-sh) mice, but those prepared from Mcpt6(-/-) mice did not. Mechanistic studies demonstrated that tryptase deficiency affected endothelial cell (EC) chemokine and cytokine expression, monocyte trans**migration**, smooth-muscle cell apoptosis, and MC and AAA lesion cysteinyl cathepsin expression and activities.This study establishes the direct participation of MC tryptase in the pathogenesis of experimental AAAs, and suggests that levels of this protease can serve as a novel biomarker for abdominal aortic expansion.
54. Chemokine (C-C motif) receptor 2 mediates mast cell **migration** to abdominal aortic aneurysm lesions in mice. Mast cells participate importantly in abdominal aortic aneurysms (AAAs) by releasing inflammatory cytokines to promote vascular cell protease expression and arterial wall remodelling. Mast cells accumulate in AAA lesions during disease progression, but the exact chemokines by which mast cells migrate to the site of vascular inflammation remain unknown. This study tested the hypothesis that mast cells use chemokine (C-C motif) receptor 2 (CCR2) for their accumulation in experimental mouse AAA lesions.We generated mast cell and apolipoprotein E double-deficient (Apoe(-/-)Kit(W-sh/W-sh)) mice and found that they were protected from angiotensin II (Ang II) chronic infusion-induced AAAs compared with Apoe(-/-) littermates. Using bone-marrow derived mast cells (BMMC) from Apoe(-/-) mice and CCR2 double-deficient (Apoe(-/-)Ccr2(-/-)) mice, we demonstrated that Apoe(-/-)Kit(W-sh/W-sh) mice receiving BMMC from Apoe(-/-)Ccr2(-/-) mice, but not those from Apoe(-/-) mice, remained protected from AAA formation. Adoptive transfer of BMMC from Apoe(-/-) mice into Apoe(-/-)Kit(W-sh/W-sh) mice also increased lesion content of macrophages, T cells, and MHC class II-positive cells; there was also increased apoptosis, angiogenesis, cell proliferation, elastin fragmentation, and medial smooth muscle cell loss. In contrast, adoptive transfer of BMMC from Apoe(-/-)Ccr2(-/-) mice into Apoe(-/-)Kit(W-sh/W-sh) mice did not affect these variables.The increased AAA formation and associated lesion characteristics in Apoe(-/-)Kit(W-sh/W-sh) mice after receiving BMMC from Apoe(-/-) mice, but not from Apoe(-/-)Ccr2(-/-) mice, suggests that mast cells use CCR2 as the chemokine receptor for their recruitment in Ang II-induced mouse AAA lesions.
55. A dynamic niche provides Kit ligand in a stage-specific manner to the earliest thymocyte progenitors. Thymic T cell development is initiated from bone-marrow-derived multi potent thymus-seeding progenitors. During the early stages of thymocyte differentiation, progenitors become T cell restricted. However, the cellular environments supporting these critical initial stages of T cell development within the thymic cortex are not known. Here we use the dependence of early, c-Kit-expressing thymic progenitors on Kit ligand (KitL) to show that CD4(-)CD8(-)c-Kit(+)CD25(-) DN1-stage progenitors associate with, and depend on, the membrane-bound form of KitL (mKitL) provided by a cortex-specific KitL-expressing vascular endothelial cell (VEC) population. In contrast, the subsequent CD4(-)CD8(-)c-Kit(+)CD25(+) DN2-stage progenitors associate selectively with cortical thymic epithelial cells (cTECs) and depend on cTEC-presented mKitL. These results show that the dynamic process of early thymic progenitor differentiation is paralleled by **migration**-dependent change to the supporting niche, and identify VECs as a thymic niche cell, with mKitL as a critical ligand.
56. Primordial germ cells and gastrointestinal stromal tumors respond distinctly to a cKit overactivating allele. KitL, via its receptor cKit, supports primordial germ cell (PGC) growth, survival, **migration** and reprogramming to pluripotent embryonic germ cells (EGCs). However, the signaling downstream of KitL and its regulation in PGCs remain unclear. A constitutively activating mutation, cKit(V558Δ), causes gain-of-function phenotypes in mast cells and intestines, and gastrointestinal stromal tumors (GISTs) when heterozygous. Unexpectedly, we find that PGC growth is not significantly affected in cKit(V558Δ) heterozygotes, whereas in homozygotes, increased apoptosis and inefficien**t migrati**on lead to the depletion of PGCs. Through genetic studies, we reveal that this oncogenic cKit allele exhibits loss-of-function behavior in PGCs distinct from that in GIST development. Examination of downstream signaling in GISTs from cKit(V558Δ/+) mice confirmed hyperphosphorylation of AKT and ERK, but both remain unperturbed in cKit(V558Δ/+) PGCs and EGCs. In contrast, we find reduced activation of ERK1/2 and JNK1 in cKit(V558Δ) homozygous PGCs and EGCs. Inhibiting JNK, though not ERK1/2, increased apoptosis of wild-type PGCs, but did not further affect the already elevated apoptosis of cKit(V558Δ)(/V558Δ) PGCs. These results demonstrate a cell-context-dependent response to the cKit(V558Δ) mutation. We propose that AKT overload protection and JNK-mediated survival comprise PGC-specific mechanisms for regulating cKit signaling.
57. The c-kit receptor is involved in the adhesion of mouse primordial germ cells to somatic cells in culture. The receptor encoded by the W (c-kit) locus is expressed on the membrane of mouse primordial germ cells, whereas its ligand termed stem cell factor (SCF), encoded by the Sl locus, is expressed on the membrane of somatic cells associated with both the primordial germ cell migratory pathways and homing sites. Using an in vitro short time assay which allows a quantitative measure of adhesion between cells, in the present paper we show that SCF/c-kit interaction can modulate primordial germ cell adhesion to somatic cells. We report that the adhesiveness of 11.5 dpc primordial germ cells to four types of somatic cells in culture (TM4 cells, STO fibroblasts, bone marrow stromal cells and gonadal somatic cells) is significantly reduced by antibodies directed against c-kit receptor or SCF, as well by soluble SCF. This SCF/c-kit mediated adhesion seems independent of SCF-induced tyrosine autophosphorylation of c-kit receptor. Moreover, primordial germ cells showed a poor ability to adhere to a bone marrow stromal cell line carrying the Sl(d) mutation (unable to synthesize membrane-bound SCF). This adhesiveness was not further impaired by anti-c-kit antibody. These results demonstrate that SCF/c-kit interaction contributes to the adhesion of primordial germ cells to somatic cells in culture and suggest that the role played by SCF in promoting survival, proliferation and **migration** of these cells in vitro and in vivo, demonstrated by several studies, might depend on the ability of the membrane-bound form of this cytokine to directly mediate primordial germ cell adhesion to the surrounding somatic cells.
58. A role for hematopoietic stem cells in promoting angiogenesis. Angiogenesis is an important event for embryonic organogenesis as well as for tissue repair in the adult. Here, we show that hematopoietic stem cells (HSCs) play important roles for angiogenesis during embryogenesis. To investigate the role of HSCs in endothelial cell (EC) development, we analyzed AML1-deficient embryos, which lack definitive hematopoiesis. These embryos showed defective angiogenesis in the head and pericardium. Para-aortic splanchnopleural (P-Sp) explant cultures on stromal cells (P-Sp culture) did not generate definitive hematopoietic cells and showed defective angiogenesis in the AML1 null embryo. Disrupted angiogenesis in P-Sp cultures from AML1 null embryos was rescued by addition of HSCs or angiopoietin-1 (Ang1). HSCs, which express Ang1, directly promoted **migration** of ECs in vivo and in vitro. These results indicate that HSCs are critical for angiogenesis.
59. C5a initiates the inflammatory cascade in immune complex peritonitis. Immune complex (IC)-induced inflammation is integral to the pathogenesis of several autoimmune diseases. ICs activate the complement system and interact with IgG FcgammaR. In this study, we demonstrate that activation of the complement system, specifically generation of C5a, initiates the neutrophilic inflammation in IC peritonitis. We show that ablation of C5a receptor signaling abrogates neutrophil recruitment in wild-type mice and prevents the enhancement of neutrophil **migration** seen in FcgammaRIIB(-/-) mice, suggesting that C5aR signaling is the crucial initial event upstream of FcgammaR signaling. We also provide evidence that C5a initiates the inflammatory cascade both directly, through C5aR-mediated effector functions on infiltrating and resident peritoneal cells, and indirectly, through shifting the balance between activating and inhibitory FcgammaRs on resident cells toward an inflammatory phenotype. We conclude that complement activation and C5a generation are prerequisites for IC-induced inflammation through activating FcgammaR, which amplifies complement-induced inflammation in autoimmunity.
60. Zinc-finger transcription factor Slug contributes to the function of the stem cell factor c-kit signaling pathway. The stem cell factor c-kit signaling pathway (SCF/c-kit) has been previously implicated in normal hematopoiesis, melanogenesis, and gametogenesis through the formation and **migration** of c-kit(+) cells. These biologic functions are also determinants in epithelial-mesenchymal transitions during embryonic development governed by the Snail family of transcription factors. Here we show that the activation of c-kit by SCF specifically induces the expression of Slug, a Snail family member. Slug mutant mice have a cell-intrinsic defect with pigment deficiency, gonadal defect, and impairment of hematopoiesis. Kit(+) cells derived from Slug mutant mice exhibit migratory defects similar to those of c-kit(+) cells derived from SCF and c-kit mutant mice. Endogenous Slug is expressed in migratory c-kit(+) cells purified from control mice but is not present in c-kit(+) cells derived from SCF mutant mice or in bone marrow cells from W/W(v) mice, though Slug is present in spleen c-kit(+) cells of W/W(v) (mutants expressing c-kit with reduced surface expression and activity). SCF-induced **migration** was affected in primary c-kit(+) cells purified from Slug-/- mice, providing evidence for a role of Slug in the acquisition of c-kit(+) cells with ability to migrate. Slug may thus be considered a molecular target that contributes to the biologic specificity to the SCF/c-kit signaling pathway, opening up new avenues for stem cell mobilization.
61. Intestinal mast cell progenitors require CD49dbeta7 (alpha4beta7 integrin) for tissue-specific homing. Mast cells (MCs) are centrally important in allergic inflammation of the airways, as well as in the intestinal immune response to helminth infection. A single lineage of bone marrow (BM)-derived progenitors emigrates from the circulation and matures into phenotypically distinct MCs in different tissues. Because the mechanisms of MC progenitor (MCp) homing to peripheral tissues have not been evaluated, we used limiting dilution analysis to measure the concentration of MCp in various tissues of mice deficient for candidate homing molecules. MCp were almost completely absent in the small intestine but were present in the lung, spleen, BM, and large intestine of beta7 integrin-deficient mice (on the C57BL/6 background), indicating that a beta7 integrin is critical for homing of these cells to the small intestine. MCp concentrations were not altered in the tissues of mice deficient in the alphaE integrin (CD103), the beta2 integrin (CD18), or the recombination activating gene (RAG)-2 gene either alone or in combination with the interleukin (IL)-receptor common gamma chain. Therefore, it is the alpha4beta7 integrin and not the alphaEbeta7 integrin that is critical, and lymphocytes and natural killer cells play no role in directing MCp **migration** under basal conditions. When MCp in BALB/c mice were eliminated with sublethal doses of gamma-radiation and then reconstituted with syngeneic BM, the administration of anti-alpha4beta7 integrin, anti-alpha4 integrin, anti-beta7 integrin, or anti-MAdCAM-1 monoclonal antibodies (mAbs) blocked the recovery of MCp in the small intestine. The blocking mAbs could be administered as late as 4 d after BM reconstitution with optimal inhibition, implying that the MCp must arise first in the BM, circulate in the vasculature, and then translocate into the intestine. Inasmuch as MCp are preserved in the lungs of beta7 integrin-deficient and anti-alpha4beta7 integrin-treated mice but not in the small intestine, alpha4beta7 integrin is critical for tissue specific extravasation for localization of MCp in the small intestine, but not the lungs.
62. Tissue-selective mast cell reconstitution and differential lung gene expression in mast cell-deficient Kit(W-sh)/Kit(W-sh) sash mice. Mast cell-deficient Kit(W)/Kit(W-v) mice are an important resource for studying mast cell functions in vivo. However, because they are compound heterozygotes in a mixed genetic background and are infertile, they cannot be crossed easily with other mice.To overcome this limitation, we explored the use of Kit(W-sh)/Kit(W-sh) mice for studying mast cell biology in vivo.These mice are in a C57BL/6 background, are fertile and can be bred directly with other genetically modified mice. Ten-week-old Kit(W-sh)/Kit(W-sh) are profoundly mast cell-deficient. No mast cells are detected in any major organ, including the lung. Gene microarrays detect differential expression of just seven of 16,463 genes in lungs of Kit(W-sh)/Kit(W-sh) mice compared with wild-type mice, indicating that resting mast cells regulate expression of a small set of genes in the normal lung. Injecting 10(7) bone marrow-derived mast cells (BMMC) into tail veins of Kit(W-sh)/Kit(W-sh) mice reconstitutes mast cell populations in lung, stomach, liver, inguinal lymph nodes, and spleen, but not in the tongue, trachea or skin. Injection of BMMC into ear dermis or peritoneum reconstitutes mast cells locally in these tissues. When splenectomized Kit(W-sh)/Kit(W-sh) mice are intravenously injected with BMMC, mast cells circulate longer and are found more often in the liver and inguinal lymph nodes, indicating that the spleen acts as a reservoir for mast cells following injection and limits **migration** to some tissues.In summary, these findings show that mast cell-deficient Kit(W-sh)/Kit(W-sh) mice possess unique attributes that favour their use for studying mast cell functions in vivo.
63. Neural crest cell lineage segregation in the mouse neural tube. Neural crest (NC) cells arise in the dorsal neural tube (NT) and migrate into the embryo to develop into many different cell types. A major unresolved question is when and how the fate of NC cells is decided. There is widespread evidence for multipotential NC cells, whose fates are decided during or after **migration**. There is also some evidence that the NC is already divided into subpopulations of discrete precursors within the NT. We have investigated this question in the mouse embryo. We find that a subpopulation of cells on the most dorsomedial aspect of the NT express the receptor tyrosine kinase Kit (previously known as c-kit), emigrate exclusively into the developing dermis, and then express definitive markers of the melanocyte lineage. These are thus melanocyte progenitor cells. They are generated predominantly at the midbrain-hindbrain junction and cervical trunk, with significant numbers also in lower trunk. Other cells within the dorsal NT are Kit-, migrate ventrally, and, from embryonic day 9.5, express the neurotrophin receptor p75. These cells most likely only give rise to ventral NC derivatives such as neurons and glia. The p75+ cells are located ventrolateral to the Kit+ cells in areas of the NT where these two cell types are found. These data provide direct in vivo evidence for NC lineage segregation within the mouse neural tube.
64. RabGEF1 regulates stem cell factor/c-Kit-mediated signaling events and biological responses in mast cells. We recently reported that RabGEF1 is a negative regulator of high-affinity Fc receptor for IgE (Fc epsilonRI)-dependent mast cell activation and that mice lacking RabGEF1 develop severe skin inflammation and increased numbers of dermal mast cells. To better understand how RabGEF1 can regulate signaling events and biological responses in mast cells, we examined the responses of bone marrow-derived cultured mast cells (BMCMCs) from wild-type (+/+) and Rabgef1 knockout (-/-) mice after stimulation with the c-Kit ligand, stem cell factor (SCF), an important regulator of mast cell development, survival, proliferation, and activation. We found that RabGEF1-deficient mast cells exhibited enhanced and prolonged activation of Ras and extracellular regulated kinase, and significantly elevated IL-6 secretion, after stimulation with SCF. SCF-induced activation of c-Jun N-terminal kinase was increased in Rabgef1-/- BMCMCs, but without corresponding significant increases in SCF-induced **migration** or adhesion. SCF-mediated activation of the survival-enhancing kinase, Akt, also was increased in Rabgef1-/- BMCMCs, and these cells had a survival advantage over their +/+ counterparts in vitro. Despite enhanced Ras activation in the absence of RabGEF1, SCF-induced proliferation was lower in Rabgef1-/- BMCMCs compared with their +/+ counterparts. Finally, we found that c-Kit internalization was delayed in the absence of RabGEF1, probably reflecting a positive role for RabGEF1 in the regulation of endocytic events, and that infection of Rabgef1-/- BMCMCs with a wild-type RabGEF1 lentiviral construct normalized c-Kit internalization to the levels seen in +/+ BMCMCs. Thus, RabGEF1 plays a critical role in the regulation of SCF/c-Kit-mediated signaling events and biological responses in mast cells.
65. In vivo knockdown of cKit impairs neuronal **migration** and axonal extension in the cerebral cortex. The tyrosine kinase receptor cKit and its ligand stem cell factor (SCF) are well known mediators in proliferation, survival, and positive chemotaxis of different cell types in the hematopoietic system. However, and in spite of previous reports showing robust expression of cKit and SCF in the brain during development, their possible function in the cerebral cortex has not been clarified. In this study, embryonic knockdown expression of cKit in the rat cortex by in utero electroporation of specific RNAi resulted in delayed radial **migration** of cortical neurons. In conditional Nestin-cKit KO homozygous mutants, radial **migration** in the cortex was also delayed. The opposite phenotype was observed after overexpressing cKit in the cortex: radial **migration** was accelerated. Callosal fibers electroporated with cKit RNAi were also delayed in their extension within the contralateral cortex and eventually failed to innervate their target area. In vitro experiments showed that, whereas SCF was able to promote **migration** of cortical neurons, it had no effect on cortical neurite outgrowth. In summary, our results demonstrate that (1) cKit is necessary for radial **migration** of cortical neurons, probably through SCF binding and (2) cKit is necessary for the correct formation of the callosal projection, most likely by a mechanism not involving SCF.
66. Characteristics of stria vascularis melanocytes of viable dominant spotting (Wv/Wv) mouse mutants. The Wv mutation lies in the kinase domain of the proto-oncogene c-kit which is expressed in a variety of cells including neural crest derived melanoblasts. The mutation results in the abnormal **migration**, proliferation, survival and/or differentiation of melanoblasts. Viable Dominant Spotting (Wv/Wv) mouse mutants have a white coat due to the absence of melanocytes. The majority of these animals have no melanocytes within the stria vascularis and no endocochlear potential (EP). A proportion of homozygous mutants partially escape the effects of the mutation: 47.2% of pinnae and 21% of vestibular regions were pigmented and 10.8% of ears had an EP. All ears with an EP that were available for histology had some pigmentation of the stria. There was no obvious correlation between external and internal spotting in Wv/Wv mice, and asymmetrical pigmentation of the ears was common. Both light and dark intermediate cells (which are derived from melanocytes) were present in the middle and/or basal turns of these cochlear ducts and they appeared to function normally in enabling the stria to produce an EP (although the EP was usually lower than normal). This suggests that the c-kit gene product is needed only during development of the stria, and not for mature melanocyte function because the melanocytes present in the mutant strias were carrying the mutant version of the c-kit gene. Melanocytes were similar in appearance in controls and mutants, except that fewer melanin granules were observed in the strias of Wv/Wv mice. The observations that strial melanocytes with very few melanin granules in Wv/Wv mutants are able to support EP production, together with previous observations that albino animals with strial melanocytes but no melanin have a normal EP, suggest that melanocytes but not melanin are essential for normal strial function.
67. Actin-dependent lamellipodia formation and microtubule-dependent tail retraction control-directed cell **migration**. Migrating cells are polarized with a protrusive lamella at the cell front followed by the main cell body and a retractable tail at the rear of the cell. The lamella terminates in ruffling lamellipodia that face the direction of **migration**. Although the role of actin in the formation of lamellipodia is well established, it remains unclear to what degree microtubules contribute to this process. Herein, we have studied the contribution of microtubules to cell motility by time-lapse video microscopy on green flourescence protein-actin- and tubulin-green fluorescence protein-transfected melanoma cells. Treatment of cells with either the microtubule-disrupting agent nocodazole or with the stabilizing agent taxol showed decreased ruffling and lamellipodium formation. However, this was not due to an intrinsic inability to form ruffles and lamellipodia because both were restored by stimulation of cells with phorbol 12-myristate 13-acetate in a Rac-dependent manner, and by stem cell factor in melanoblasts expressing the receptor tyrosine kinase c-kit. Although ruffling and lamellipodia were formed without microtubules, the microtubular network was needed for advancement of the cell body and the subsequent retraction of the tail. In conclusion, we demonstrate that the formation of lamellipodia can occur via actin polymerization independently of microtubules, but that microtubules are required for cell **migration**, tail retraction, and modulation of cell adhesion.
68. Mast cells regulate homeostatic intestinal epithelial **migration** and barrier function by a chymase/Mcpt4-dependent mechanism. Altered intestinal barrier function is postulated to be a central predisposing factor to intestinal diseases, including inflammatory bowel diseases and food allergies. However, the mechanisms involved in maintaining homeostatic intestinal barrier integrity remain undefined. In this study, we demonstrate that mice deficient in mast cells (Kit(W-sh/W-sh) [Wsh]) or mast cell chymase (Mcpt4(-/-)) have significantly decreased basal small intestinal permeability compared with wild-type (WT) mice. Altered intestinal barrier function was linked to decreased intestinal epithelial cell **migration** along the villus/crypt axis, altered intestinal morphology, and dysregulated claudin-3 crypt expression. Remarkably, engraftment of Wsh mice with WT but not Mcpt4(-/-) mast cells restored intestinal epithelial cell **migration**, morphology, and intestinal epithelial barrier function. Collectively, these findings identify a mechanism by which mast cells regulate homeostatic intestinal epithelial **migration** and barrier function.
69. Mast cells have a pivotal role in TNF-independent lymph node hypertrophy and the mobilization of Langerhans cells in response to bacterial peptidoglycan. Peptidoglycan (PGN) from Gram-positive bacteria, activates multiple immune effector cells. PGN-induced lymph node (LN) hypertrophy and dendritic cell mobilization in vivo were investigated following PGN injection into the skin. Both LN activation and the **migration** of Langerhans cells (LCs) to draining LNs were dependent on the presence of mast cells as demonstrated using mast cell deficient W/W(v) mice. However, these responses did not require TLR2, TLR4, or MYD88. TNF-deficient mice exhibited normal increases in LN cellularity but significantly reduced LC **migration**. In contrast, responses to IgE-mediated mast cell activation were highly TNF dependent. Complement component C3-deficient mice showed decreased LN hypertrophy and abrogated LC **migration** in response to PGN. These data demonstrate a critical role for mast cells and complement in LN responses to PGN and illustrate a novel TNF-independent mechanism whereby mast cells participate in the initiation of immunity.
70. Protein-tyrosine phosphatase alpha regulates stem cell factor-dependent c-Kit activation and **migration** of mast cells. The role of protein-tyrosine phosphatase alpha (PTPalpha) in mast cell function was investigated in tissues and cells from PTPalpha-deficient mice. Bone marrow-derived mast cells (BMMCs) lacking PTPalpha exhibit defective stem cell factor (SCF)-dependent polarization and **migration**. Investigation of the molecular basis for this reveals that SCF/c-Kit-stimulated activation of the Fyn tyrosine kinase is impaired in PTPalpha(-/-) BMMCs, with a consequent inhibition of site-specific c-Kit phosphorylation at tyrosines 567/569 and 719. Although c-Kit-mediated activation of phosphatidylinositol 3-kinase and Akt is unaffected, profound defects occur in the activation of downstream signaling proteins, including mitogen-activated protein kinases and Rho GTPases. Phosphorylation and interaction of Fyn effectors Gab2 and Shp2, which are linked to Rac/JNK activation in mast cells, are impaired in PTPalpha(-/-) BMMCs. Thus, PTPalpha is required for SCF-induced c-Kit and Fyn activation, and in this way regulates a Fyn-based c-Kit signaling axis (Fyn/Gab2/Shp2/Vav/PAK/Rac/JNK) that mediates mast cell **migration**. These defective signaling events may underlie the altered tissue-resident mast cell populations found in PTPalpha(-/-) mice.
71. Mast cells are crucial for early inflammation, **migration** of Langerhans cells, and CTL responses following topical application of TLR7 ligand in mice. Until recently, IgE-activated mast cells have been regarded merely as effector cells of adaptive immune responses, involved in allergic reactions and mucosal immunity to parasites. Herein, we report that murine dermal mast cells, activated by local administration of a cream containing the synthetic TLR7 ligand imiquimod, are essential to initiate an early inflammatory reaction. The mast-cell-derived cytokines TNF-alpha and IL-1beta play an important role in this process. Furthermore, TLR7-activated mast cells are also able to promote the e**migration** of Langerhans cells, which partly depends on the expression of mast-cell-derived IL-1beta. We have previously shown that TLR7 ligation enhances transcutaneous immunization evoked by topical application of vaccine antigens to the skin, a procedure that directly targets skin-resident antigen-presenting cells. Consequently, we now demonstrate here that the capacity to mount a peptide-specific cytotoxic T-lymphocyte response following transcutaneous immunization using imiquimod as adjuvant is severely impaired in mast-cell-deficient mice. Thus, these findings demonstrate the potent versatility of alternatively activated mast cells at the interface of innate and adaptive immunity.
72. Expression of c-kit encoded at the W locus of mice in developing embryonic germ cells and presumptive melanoblasts. The W locus of mice encodes the c-kit tyrosine kinase receptor. In embryos homozygous for severe W mutations, the number of germ cells does not increase after 8 days of development, melanocytes do not appear, and production of erythrocytes and mast cells is deficient. To gain some insight into the role of the c-kit receptor, we have used in situ hybridization to explore the time period of expression of c-kit transcripts in early germ cells and melanoblasts. At 6 1/2 days of development, expression was not seen in the embryonic cylinder, but did appear in parietal endoderm. Germ cells displayed a low level of c-kit transcripts from their first appearance in the 7 1/2 -day embryo, continuing through early proliferation and **migration** to the gonad. During **migration**, surrounding tissues also expressed c-kit. Expression increased in gonia and then ceased as they became nonproliferative. Expression in presumptive melanoblasts was first seen in the cervical region of 10-day embryos and continued as they spread over the surface of the body, entered the epidermis, and differentiated in hair follicles after birth. The effects of mutations of c-kit on germ cells and melanoblasts can be interpreted as an absence of a proliferative signal shortly after their segregation from other cell types. This signal may be required throughout the proliferative phase of early germ cells [and also in postnatal stages of germ cell development (Manova et al. (1990). Development 110, 1057-1069]. In melanoblasts, c-kit may play a role during both proliferation and differentiation.
73. Mast cell **migration** from the skin to the draining lymph nodes upon ultraviolet irradiation represents a key step in the induction of immune suppression. The UV radiation in sunlight is the primary cause of skin cancer. UV is also immunosuppressive and numerous studies have shown that UV-induced immune suppression is a major risk factor for skin cancer induction. Previous studies demonstrated that dermal mast cells play a critical role in the induction of immune suppression. Mast cell-deficient mice are resistant to the immunosuppressive effects of UV radiation, and UV-induced immune suppression can be restored by injecting bone marrow-derived mast cells into the skin of mast cell- deficient mice. The exact process however, by which mast cells contribute to immune suppression, is not known. In this study, we show that one of the first steps in the induction of immune suppression is mast cell **migration** from the skin to the draining lymph nodes. UV exposure, in a dose-dependent manner, causes a significant increase in lymph node mast cell numbers. When GFP(+) skin was grafted onto mast cell-deficient mice, we found that GFP(+) mast cells preferentially migrated into the lymph nodes draining the skin. The mast cells migrated primarily to the B cell areas of the draining nodes. Mast cells express CXCR4(+) and UV exposure up-regulated the expression of its ligand CXCL12 by lymph node B cells. Treating UV-irradiated mice with a CXCR4 antagonist blocked mast cell **migration** and abrogated UV-induced immune suppression. Our findings indicate that UV-induced mast cell **migration** to draining lymph nodes, mediated by CXCR4 interacting with CXCL12, represents a key early step in UV-induced immune suppression.
74. Lack of neurokinin-1 receptor expression affects tissue mast cell numbers but not their spatial relationship with nerves. A spatial association between mast cells and nerves has been described in both the gastrointestinal and genitourinary tracts. However, the factors that influence the anatomic relationship between mast cells and nerves have not been completely defined. It has been suggested that the high-affinity receptor for substance P [neurokinin-1 (NK1)] might modulate this interaction. We therefore assessed mast cell-nerve relationships in tissues isolated from wild-type and NK1 receptor knockout (NK1-/-) mice. We now report that, in the complete absence of NK1 receptor expression, there is a significant increase in the number of mast cells without a change in the anatomic relationship between mast cell and nerves in stomach and bladder tissues at the light microscopic level. We next determined whether transplanted mast cells would maintain their spatial distribution, number, and contact with nerve elements. For this purpose, mast cell-deficient Kit(W)/Kit(W-v) mice were reconstituted with wild-type or NK1-/- bone marrow. No differences in mast cell-nerve contact were observed. These results suggest that NK1 receptor expression is important in the regulation of the number of mast cells but is not important in the interaction between mast cells and nerves. Furthermore, the interaction between mast cells and nerves is not mediated through NK1 receptor expression on the mast cell. Further studies are needed to determine the molecular pathway involved in mast cell **migration** and interaction with nerve elements, but the model of reconstitution of Kit(W)/Kit(W-v) mice with mast cells derived from different genetically engineered mice is a useful approach to further explore these mechanisms.
75. Spatial and temporal patterns of c-kit-expressing cells in WlacZ/+ and WlacZ/WlacZ mouse embryos. In the mouse, the Kit receptor and its ligand, the stem cell factor (SCF), are encoded at the W/Kit and Steel loci, respectively. The Kit/SCF transduction pathway is involved in promoting cellular **migration**, proliferation and/or survival of melanoblasts, hematopoietic progenitors and primordial germ cells. Furthermore, a functional Kit/SCF pathway is required for the development of interstitial cells of Cajal (ICC) in the small intestine. Whereas all c-kit-expressing cells in embryogenesis were not identified, previous studies clearly demonstrated that the c-kit expression pattern extends well beyond cells known to be affected by W mutations. To investigate further Kit function, we specifically marked the c-kit-expressing cells and followed their fate during embryogenesis. A mutation was introduced by gene targeting at the W/Kit locus in mouse embryonic stem cells. The lacZ reporter gene was inserted into the first exon of c-kit, thus creating a null allele, called WlacZ. The lacZ expression reflects normal expression of the c-kit gene in WlacZ/+ embryos. The comparison of the patterns of lacZ-expressing cells between WlacZ/+ and WlacZ/WlacZ embryos allowed us to detect where and when melanoblasts, primordial germ cells and hematopoietic progenitors failed to survive in the absence of Kit. We also observed that ICC express c-kit during embryogenesis. ICC are found identically in WlacZ/+ and WlacZ/WlacZ embryos. Therefore, ICC do not depend on Kit expression during embryogenesis. These results indicate that the function of the c-kit gene is only required for the postnatal development of the ICC. Unexpected sites of c-kit expression were uncovered in embryos, including endothelial, epithelial and endocrine cells. None of these cells are dependent on Kit expression for their **migration**, proliferation and/or survival during embryogenesis. Nevertheless, we assume that the Kit/SCF pathway could be involved in the growth of transformed endothelial, epithelial and endocrine cells.
76. Loss of SLP-76 expression within myeloid cells confers resistance to neutrophil-mediated tissue damage while maintaining effective bacterial killing. The Src homology 2 domain-containing leukocyte phosphoprotein of 76 kDa (SLP-76) is an adaptor molecule critical for immunoreceptor and integrin signaling in multiple hemopoietic lineages. We showed previously that SLP-76 is required for neutrophil function in vitro, including integrin-induced adhesion and production of reactive oxygen intermediates, and to a lesser extent, FcgammaR-induced calcium flux and reactive oxygen intermediate production. It has been difficult to determine whether SLP-76 regulates neutrophil responses in vivo, because Slp-76(-/-) mice exhibit marked defects in thymocyte and vascular development, as well as platelet and mast cell function. To circumvent these issues, we generated mice with targeted loss of SLP-76 expression within myeloid cells. Neutrophils obtained from these animals failed to respond to integrin activation in vitro, similar to Slp-76(-/-) cells. Despite these abnormalities, SLP-76-deficient neutrophils migrated normally in vivo in response to Staphylococcus aureus infection and efficiently cleared micro-organisms. Interestingly, SLP-76-deficient neutrophils did not induce a robust inflammatory response in the localized Shwartzman reaction. Collectively, these data suggest that disruption of integrin signaling via loss of SLP-76 expression differentially impairs neutrophil functions in vivo, with preservation of **migration** and killing of S. aureus but reduction in LPS-induced tissue damage and vascular injury.
77. Temporal control of neural crest lineage generation by Wnt/β-catenin signaling. Wnt/β-catenin signaling controls multiple steps of neural crest development, ranging from neural crest induction, lineage decisions, to differentiation. In mice, conditional β-catenin inactivation in premigratory neural crest cells abolishes both sensory neuron and melanocyte formation. Intriguingly, the generation of melanocytes is also prevented by activation of β-catenin in the premigratory neural crest, which promotes sensory neurogenesis at the expense of other neural crest derivatives. This raises the question of how Wnt/β-catenin signaling regulates the formation of distinct lineages from the neural crest. Using various Cre lines to conditionally activate β-catenin in neural crest cells at different developmental stages, we show that neural crest cell fate decisions in vivo are subject to temporal control by Wnt/β-catenin. Unlike in premigratory neural crest, β-catenin activation in migratory neural crest cells promotes the formation of ectopic melanoblasts, while the production of most other lineages is suppressed. Ectopic melanoblasts emerge at sites of neural crest target structures and in many tissues usually devoid of neural crest-derived cells. β-catenin activation at later stages in glial progenitors or in melanoblasts does not lead to surplus melanoblasts, indicating a narrow time window of Wnt/β-catenin responsiveness during neural c**rest cell** migration. Thus, neural crest cells appear to be multipotent in vivo both before a**nd after** emigration from the neural tube but adapt their response to extracellular signals in a temporally controlled manner.
78. Stem cell factor and c-kit in the mammalian testis: lessons originating from Mother Nature's gene knockouts. Analysis of mice with naturally occurring mutation in the genes encoding the tyrosine kinase receptor protein, c-kit, and its ligand, stem cell factor (SCF), has implicated these proteins in testicular development and function because of the phenotype of these mice of reduced or absent fertility. Descriptive and functional studies have highlighted the involvement of these proteins in primordial germ cell **migration** and survival, and in spermatogonial proliferation, survival and adhesion. This review summarises the current knowledge regarding functions of c-kit and SCF in the testis and discusses the implications of information gleaned from other biological systems.
79. Mast cell-independent mechanisms of immediate hypersensitivity: a role for platelets. Mast cells have been implicated as the central effectors in allergic responses, yet a fatal anaphylactic response can be induced in mast cell-deficient mice. In this study, we examined the immediate hypersensitivity response in wild-type (WT) and mast cell-deficient mice (W/W(v)) in two different tissues (skin and skeletal muscle). Vascular permeability and leukocyte recruitment were studied after immediate challenge or 4 h postchallenge in OVA-sensitized mice. In skin, immediate challenge induced a significant increase in vascular permeability (75%) within 30 min and was accompanied by increased leukocyte adhesion 4 h postchallenge. In the absence of mast cells, no changes in vascular permeability or leukocyte recruitment were observed in skin. In WT skeletal muscle, immediate challenge induced a rapid increase (80%) in vascular permeability within 5 min and significant leukocyte recruitment after 4 h. Surprisingly, in W/W(v), a gradual increase in vascular permeability was observed, reaching a maximum (50%) within 30 min. Despite the absence of mast cells, subsequent leukocyte e**migration** was similar to that observed in WT mice. Pretreatment with anti-platelet serum in W/W(v) returned Ag-induced vascular permeability and leukocyte recruitment to baseline. Platelets were shown to interact with endothelium in skeletal muscle, but not dermal microvasculature. These data illustrate that mast cells play a prominent role in vascular permeability and leukocyte recruitment in skin in response to Ag, however, in skeletal muscle; these changes can occur in the absence of mast cells, and are mediated, in part, by the presence of platelets.
80. N-cadherin+ HSCs in fetal liver exhibit higher long-term bone marrow reconstitution activity than N-cadherin- HSCs. Adult hematopoietic stem cells (HSCs) are maintained in a microenvironment known as the stem cell niche. The regulation of HSCs in fetal liver (FL) and their niche, however, remains to be elucidated. In this study, we investigated the role of N-cadherin (N-cad) in the maintenance of HSCs during FL hematopoiesis. By using anti-N-cad antibodies (Abs) produced by our laboratory, we detected high N-cad expression in embryonic day 12.5 (E12.5) mouse FL HSCs, but not in E15.5 and E18.5 FL. Immunofluorescence staining revealed that N-cad(+)c-Kit(+) and N-cad(+) endothelial protein C receptor (EPCR)(+) HSCs co-localized with Lyve-1(+) sinusoidal endothelial cells (ECs) in E12.5 FL and that some of these cells also expressed N-cad. However, N-cad(+) HSCs were also observed to detach from the perisinusoidal niche at E15.5 and E18.5, concomitant with a down-regulation of N-cad and an up-regulation of E-cadherin (E-cad) in hepatic cells. Moreover, EPCR(+) long-term (LT)-HSCs were enriched in the N-cad(+)Lin(-)Sca-1(+)c-Kit(+) (LSK) fraction in E12.5 FL, but not in E15.5 or E18.5 FL. In a long-term reconstitution (LTR) activity assay, higher engraftment associated with N-cad(+) LSK cells versus N-cad(-) LSK cells in E12.5 FL when transplanted into lethally irradiated recipient mice. However, the higher engraftment of N-cad(+) LSK cells decreased subsequently in E15.5 and E18.5 FL. It is possible that N-cad expression conferred higher LTR activity to HSCs by facilitating interactions with the perisinusoidal niche, especially at E12.5. The down-regulation of N-cad during FL hematopoiesis may help us better understand the regulation and mobility of HSCs before **migration** into BM.
81. Contiguous patterns of c-kit and steel expression: analysis of mutations at the W and Sl loci. Mutations in either the dominant white-spotting (W) or Steel (Sl) loci of the mouse lead to coat color, primordial germ cell and hematopoietic defects. Consistent with the cell autonomous and microenvironmental nature of W and Sl mutations, respectively, it has recently been shown that W encodes the c-kit receptor tyrosine kinase while Sl encodes a ligand for this receptor. Previous in situ hybridization analysis has shown that both c-kit and steel are expressed in the embryo in anatomical sites known to be affected by W and Sl mutations and in various tissues in which no corresponding phenotype has been described. To investigate the possible involvement of the Kit transduction pathway in developmental processes, we compared the patterns of expression of c-kit and steel in wild-type embryos and in embryos homozygous for severe (lethal) and mild (viable) alleles at the W and Sl loci. In addition, we analyzed the patterns of expression of both genes in adult wild-type and mutant gonads and brain. Both c-kit and steel are contiguously expressed in a wide variety of anatomical locations in both the developing embryo and in the adult. In adult gonads, steel is expressed in the follicular cells of the ovary and in Sertoli cells of the testis, the layers that immediately surround the c-kit expressing germ cells. In adult brain, the complementary patterns are particularly striking in the olfactory bulb, cerebral cortex, hippocampus region and cerebellum. steel expression in brain is probably restricted to neurons in certain areas, while c-kit is expressed in neurons and in some glial cells. Severe mutations in the W or Sl loci result in dramatic reduction or absence of c-kit positive cells in lineages known to be affected by these mutations. In contrast, these mutations do not affect the number or histological organization of c-kit positive cells in the embryonic peripheral or central nervous systems, nor is the number or organization of c-kit positive cells detectably altered in Wv/Wv or Sld/Sld adult brain. Taken together, these results suggest that the Kit signaling pathway is not obligatory for the viability and/or **migration** of most c-kit expressing cells either because of functional redundancy with another signaling pathway or because the Kit pathway is involved in post-developmental processes of mature cells.
82. The kit-ligand (steel factor) and its receptor c-kit/W: pleiotropic roles in gametogenesis and melanogenesis. The c-kit receptor tyrosine kinase belongs to the PDGF/CSF-1/c-kit receptor subfamily. The kit-ligand, KL, also called steel factor, is synthesized from two alternatively spliced mRNAs as transmembrane proteins that can either be proteolytically cleaved to produce soluble forms of KL or can function as cell-associated molecules. The c-kit receptor kinase and KL are encoded at the white spotting (W) and steel (Sl) loci of the mouse, respectively. Mutations at both the W and the Sl locus cause deficiencies in gametogenesis, melanogenesis and hematopoiesis. The c-kit receptor is expressed in the cellular targets of W and Sl mutations, while KL is expressed in their microenvironment. In melanogenesis, c-kit is expressed in melanoblasts from the time they leave the neural crest and expression continues during embryonic development and in the melanocytes of postnatal animals. In gametogenesis c-kit is expressed in primordial germ cells, in spermatogonia, and in primordial and growing oocytes, implying a role at three distinct stages of gametogenesis. Many mutant alleles are known at W and Sl loci and their phenotypes vary in the degree of severity in the different cellular targets of the mutations. While many W and Sl alleles severely affect primordial germ cells (PGC), several mild Sl alleles have weak effects on PGCs and exhibit differential male or female sterility. Steel Panda (Sl(pan)) is a KL expression mutation in which KL RNA transcript levels are reduced in most tissues analyzed. In female Sl(pan)/Sl(pan) mice, ovarian follicle development is arrested at the one layered cuboidal stage as a result of reduced KL expression in follicle cells, indicating a role for c-kit in oocyte growth. Wsh is a c-kit expression mutation, which affects mast cells and melanogenesis. While the mast cell defect results from lack of c-kit expression, the pigmentation deficiency appears to stem from ectopic c-kit receptor expression in the somitic dermatome at the time of **migration** of melanoblasts from the neural crest to the periphery. It is proposed that the ectopic c-kit expression in Wsh mice affects early melanogenesis in a dominant fashion. The "sash" or white belt of Wsh/+ animals and some other mutant mice is explained by the varying density of melanoblasts along the body axis of wild-type embryos.
83. FES kinase participates in KIT-ligand induced chemotaxis. FES is a cytoplasmic tyrosine kinase activated by several membrane receptors, originally identified as a viral oncogene product. We have recently identified FES as a crucial effector of oncogenic KIT mutant receptor. However, FES implication in wild-type KIT receptor function was not addressed. We report here that FES interacts with KIT and is phosphorylated following activation by its ligand SCF. Unlike in the context of oncogenic KIT mutant, FES is not involved in wild-type KIT proliferation signal, or in cell adhesion. Instead, FES is required for SCF-induced chemotaxis. In conclusion, FES kinase is a mediator of wild-type KIT signalling implicated in cell **migration**.
84. Activation of the receptor tyrosine kinase Kit is required for the proliferation of melanoblasts in the mouse embryo. The development of neural crest-derived melanocytes, as well as haematopoietic and germ cells, is affected by mutations of the Kit and Mgf genes, which lead to dominant spotting (W) or steel (Sl) phenotypes. Mgf codes for the ligand of the receptor tyrosine kinase encoded by the Kit locus. KitW-v, a point mutation exerting a dominant negative effect, causes a substantial reduction in tyrosine kinase activity of the Kit receptor and leads to a characteristic pigmentation phenotype, namely dilute coat colour and a white ventral and head spot with reduced pigmentation of the feet and tail in the heterozygous animal, as well as slight anaemia. Homozygous animals lack coat pigmentation and are severely anaemic and infertile. Dct is a marker for cells of the melanoblast lineage. In order to study these cells in detail we have generated transgenic mouse lines carrying the lacZ reporter under the control of the Dct promoter and have used the embryonic expression of the reporter to identify early melanoblasts before they begin to produce pigment. Our transgenic lines have simplified the study of melanoblasts in the mouse embryo, and by crossing our mice with KitW-v mutants we have been able to identify the midgestation stages at which melanoblasts rely critically on Mgf/Kit interactions. We conclude that the survival of immature melanoblasts depends crucially upon Kit signalling up until E11, and later in development Kit plays a vital role in melanoblast proliferation. Our data do not describe a dependence upon Kit for melanoblast **migration** or differentiation.
85. Embryonic RNA expression patterns of the c-kit receptor and its cognate ligand suggest multiple functional roles in mouse development. Mutations at the dominant white spotting (W) and Steel (Sl) loci in mouse exert deleterious effects on three migratory cell lineages (primordial germ cells, melanocytes and hematopoietic stem cells) resulting in loss of pigmentation, reduced fertility and anemia. The W locus encodes the c-kit protein tyrosine kinase (TK) receptor. More recently, the Sl locus has been shown to encode a ligand for c-kit, which is variously known as mast cell growth factor (MGF), stem cell growth factor and c-kit ligand. Here we report an in situ hybridization analysis comparing the expression profiles of MGF and c-kit transcripts during mouse embryogenesis. The data are consistent with the c-kit receptor-ligand complex providing a homing mechanism during stem cell **migration** in early development and in stem cell proliferation, differentiation, or survival in late development. In the nervous system, an unexpected and complex pattern of expression is uncovered that suggests involvement of the W and Sl gene products in the organization of the neural tube and brain.
86. The proto-oncogene c-kit encoding a transmembrane tyrosine kinase receptor maps to the mouse W locus. Mice carrying mutations at the W locus located on chromosome 5 are characterized by severe macrocytic anaemia, lack of hair pigmentation and sterility. Mutations at this locus appear to affect the proliferation and/or **migration** of cells during early embryogenesis and result in an intrinsic defect in the haematopoietic stem cell hierarchy. An understanding of the molecular basis of the complex and pleiotropic phenotype in W mutant mice would thus provide insights into the important developmental processes of gametogenesis, melanogenesis and haematopoiesis. Here we show that the mouse mutant W has a deletion of the c-kit proto-oncogene. Interspecific backcross analysis demonstrates that the W locus is very tightly linked to c-kit and that the two loci cannot be segregated at this level of analysis. c-kit is the cellular homologue of the oncogene v-kit of the HZ4 feline sarcoma virus and encodes a transmembrane protein tyrosine kinase receptor that is structurally similar to the receptors for colony-stimulating factor-1 (CSF-1) and platelet derived growth factor. The co-localization of c-kit with W provides a molecular entry into this important region of the mouse genome. In addition, these observations provide the first example of a germ-line mutation in a mammalian proto-oncogene and implicate the c-kit gene as a candidate for the W locus.
87. Steel factor and c-kit regulate cell-matrix adhesion. Steel (SI) and white spotting (W) loci encode steel factor (c-kit ligand) and the c-kit tyrosine kinase receptor, respectively. Mutations at these loci affect **migration** and differentiation of primordial germ cells, neural crest-derived melanoblasts, and hematopoietic cells. In these processes, cell adhesion molecules are hypothesized to be crucial. We have examined the role of steel factor and c-kit in cell-extracellular matrix adhesion using bone marrow-derived mast cells as a model system. Steel factor stimulates mast cells to bind to fibronectin and, to a lesser extent, to vitronectin, whereas interleukin-3 and interleukin-4, which are also mast cell growth factors, do not. Activation of adhesiveness is transient, occurs at concentrations of steel factor 100-fold lower than required for growth stimulation, and requires the integrin VLA-5. Mast cells from c-kit mutant mice adhere to fibronectin on stimulation with phorbol 12-myristate 13-acetate (PMA), but not on stimulation with steel factor, indicating that stimulation of integrin adhesiveness requires activation of the c-kit protein tyrosine kinase. By contrast, c-kit mutant and wild-type mast cells adhere equally well to COS cells expressing membrane-anchored steel factor, showing that the kinase activity of c-kit is not required for adhesion directly mediated by c-kit. Our findings suggest that regulation of adhesion is an important biologic function of steel factor.
88. Proliferation and **migration** of primordial germ cells in We/We mouse embryos. We have examined the numbers and distribution of primordial germ cells in We/We, We/+, and +/+ mouse embryos using Southern blotting to determine embryo genotypes. At early somite stages (5-7 somites: approximately 8 1/2 days post coitum [dpc]) there are 50 to 100 germ cells in embryos of all genotypes. The number of germ cells in We/+ and +/+ embryos then begins to increase: at later somite stages (17-19 somites: approximately 9 1/2 dpc) they number about 200, and by 10 1/2 dpc there are approximately 725 We/+ and 850 +/+ germ cells. During this time, however, the number of germ cells in We/We embryos remains less than 100. At 8 1/2 dpc, the distribution of germ cells in the hindgut endoderm is the same in all genotypes. By 9 1/2 dpc, 30% of We/We germ cells are found in ectopic sites (allantois and vitelline artery); germ cell distribution along the length of the hindgut appears normal, but germ cells remain confined to the floor of the gut in We/We embryos, rather than being distributed around its circumference as in the other two genotypes. By 10 1/2 days, the **migration** of We/We germ cells through the dorsal mesentery lags behind that of the other genotypes, and a larger proportion remains in the gut wall.
89. The REV7 subunit of DNA polymerase ζ is essential for primordial germ cell maintenance in the mouse. REV7 (also known as MAD2L2 and MAD2B) is involved in DNA repair, cell cycle regulation, gene expression, and carcinogenesis. In vitro studies show that REV7 interacts with several proteins and regulates their function. It has been reported that human REV7 is highly expressed in the adult testis by Northern blot analysis. However, the significance of REV7 in mammalian development has not been elucidated. Here, we present analyses of REV7-deficient (Rev7(-/-)) mice to clarify the significance of Rev7 in mouse development. In WT mice (Rev7(+/+)), Rev7 expression was ubiquitously observed in the embryo and confined to germ cells in the testes after birth. Rev7(-/-) mice exhibited growth retardation and a partial embryonic lethal phenotype. Mice that survived to adulthood were infertile in both sexes and showed germ cell aplasia in the testes and ovaries. Analyses of Rev7(-/-) embryos revealed that primordial germ cells (PGCs) were present at embryonic day 8.5 (E8.5). However, progressive loss of PGCs was observed during **migratio**n, and PGCs were absent in the genital ridges at E13.5. An increase of apoptotic cells was detected not only among PGCs but also in the forebrain of the Rev7(-/-) embryo, whereas cell proliferation was unaffected. Moreover, DNA damage accumulation and increased levels of histone methylation were detected in Rev7(-/-) embryos, and expression of Oct4 and Nanog was deregulated by REV7 deficiency at E8.5. These findings indicate that Rev7 is essential for PGC maintenance by prevention of apoptotic cell death in the mouse.
90. Analyzing mast cell development and function using mice carrying mutations at W/c-kit or Sl/MGF (SCF) loci. Mast cells have been implicated in a wide variety of biological responses, but identifying the nature and importance of the mast cell's specific contributions to these reactions has been difficult. W/Wv mice have mutations affecting the c-kit tyrosine kinase receptor which is encoded at the W locus and which is necessary for normal mast cell development. In W/Wv mice, the cells which ordinarily give rise to normal mast cell populations do not adequately respond to a major **migration**, survival, proliferation and maturation factor expressed in the microenvironments where mast cells ordinarily develop: the c-kit receptor ligand, SCF. As a result, W/Wv mice virtually lack tissue mast cells. However, adoptive transfer to W/Wv mice of immature mast cells derived in vitro from the bone marrow cells of the congenic normal (+/+) mice selectively repairs the mast cell deficiency of the W/Wv recipients. These "mast cell knock-in" mice can be used to analyze the expression of biological responses in tissues which differ only because they do or do not contain populations of mast cells. This approach permits identification and quantification of the specific contributions of the mast cell to biological responses expressed in the skin, gastrointestinal tract and other anatomical sites, and also greatly facilitates analysis of the mechanisms by which mast cells influence these responses.
91. An essential role for platelet-activating factor in activating mast cell **migration** following ultraviolet irradiation. The UVB (290-320 nm) radiation in sunlight is responsible for inducing skin cancer. Exposure to UV radiation is also immunosuppressive, and the systemic immune suppression induced by UV is a well-recognized risk factor for cancer induction. As UVB radiation is absorbed within the upper layers of the skin, indirect mechanisms must play a role in activating systemic immune suppression. One prominent example is mast cell **migration**, which from the skin to the draining LN is an essential step in the cascade of events leading to immune suppression. What triggers mast cell **migration** is not entirely clear. Here, we tested the hypothesis that PAF, a lipid mediator of inflammation produced by the skin in response to UV exposure, is involved. Mast cell-deficient mice (Kit(W-sh/W-sh)) are resistant to the suppressive effect of UV radiation, and reconstituting mast cell-deficient mice with normal bone marrow-derived mast cells restores susceptibility to immunosuppression. However, when mast cells from PAFR-/- mice were used, the reconstituted mice were not susceptible to the suppressive effects of UV. Furthermore, PAFR-/- mice showed impaired UV-induced mast cell **migration** when compared with WT mice. Finally, injecting PAF into WT mice mimicked the effect of UV irradiation and induced mast cell **migration** but not in PAFR-/- mice. Our findings indicate that PAFR binding induces mast cells to migrate from the skin to the LNs, where they mediate immune suppression.
92. Type 1 diabetes in NOD mice unaffected by mast cell deficiency. Mast cells have been invoked as important players in immune responses associated with autoimmune diseases. Based on in vitro studies, or in vivo through the use of Kit mutant mice, mast cells have been suggested to play immunological roles in direct antigen presentation to both CD4(+) and CD8(+) T cells, in the regulation of T-cell and dendritic cell **migration** to lymph nodes, and in Th1 versus Th2 polarization, all of which could significantly impact the immune response against self-antigens in autoimmune disease, including type 1 diabetes (T1D). Until now, the role of mast cells in the onset and incidence of T1D has only been indirectly tested through the use of low-specificity mast cell inhibitors and activators, and published studies reported contrasting results. Our three laboratories have generated independently two strains of mast cell-deficient nonobese diabetic (NOD) mice, NOD.Cpa3(Cre/+) (Heidelberg) and NOD.Kit(W-sh/W-sh) (Leuven and Boston), to address the effects of mast cell deficiency on the development of T1D in the NOD strain. Our collective data demonstrate that both incidence and progression of T1D in NOD mice are independent of mast cells. Moreover, analysis of pancreatic lymph node cells indicated that lack of mast cells has no discernible effect on the autoimmune response, which involves both innate and adaptive immune components. Our results demonstrate that mast cells are not involved in T1D in the NOD strain, making their role in this process nonessential and excluding them as potential therapeutic targets.
93. Altered cell-surface targeting of stem cell factor causes loss of melanocyte precursors in Steel17H mutant mice. The normal products of the murine Steel (Sl) and Dominant white spotting (W) genes are essential for the development of melanocyte precursors, germ cells, and hematopoietic cells. The Sl locus encodes stem cell factor (SCF), which is the ligand of c-kit, a receptor tyrosine kinase encoded by the W locus. One allele of the Sl mutation, Sl17H, exhibits minor hematopoietic defects, sterility only in males, and a complete absence of coat pigmentation. The Sl17H gene encodes SCF protein which exhibits an altered cytoplasmic domain due to a splicing defect. In this paper we analyzed the mechanism by which the pigmentation phenotype in Sl17H mutant mice occurs. We show that in embryos homozygous for Sl17H the number of melanocyte precursors is severely reduced on the lateral neural crest **migration** pathway by e11.5 and can no longer be detected by e13.5 when they would enter the epidermis in wildtype embryos. The reduced number of dispersing melanocyte precursors correlates with a reduction of SCF immunoreactivity in mutant embryos in all tissues examined. Regardless of the reduced amount, functional SCF is present at the cell surface of fibroblasts transfected with Sl17H mutant SCF cDNA. Since SCF immunoreactivity normally accumulates in basolateral compartments of SCF-expressing embryonic epithelial tissues, we analyzed the localization of wildtype and Sl17H mutant SCF protein in transfected epithelial (MDCK) cells in vitro. As expected, wildtype forms of SCF localize to and are secreted from the basolateral compartment. In contrast, mutant forms of SCF, which either lack a membrane anchor or exhibit the Sl17H altered cytoplasmic tail, localize to and are secreted from the apical compartment of the cultured epithelium. We suggest, therefore, that the loss of melanocyte precursors prior to epidermal invasion, and the loss of germ cells from mature testis, can be explained by the inability of Sl17H mutant SCF to be targeted to the basolateral compartment of polarized epithelial keratinocytes and Sertoli cells, respectively.
94. Identification and characterization of stem cells in prepubertal spermatogenesis in mice. The stem cell properties of gonocytes and prospermatogonia at prepubertal stages are still largely unknown: it is not clear whether gonocytes and prospermatogonia are a special cell type or similar to adult undifferentiated spermatogonia. To characterize these cells, we have established transgenic mice carrying EGFP (enhanced green fluorescence protein) cDNA under control of an Oct4 18-kb genomic fragment containing the minimal promoter and proximal and distal enhancers; Oct4 is reported to be expressed in undifferentiated spermatogonia at prepubertal stages. Generation of transgenic mice enabled us to purify gonocytes and prospermatogonia from the somatic cells of the testis. Transplantation studies of testicular cells so far have been done with a mixture of germ cells and somatic cells. This is the first report that establishes how to purify germ cells from total testicular cells, enabling evaluation of cell-autonomous repopulating activity of a subpopulation of prospermatogonia. We show that prospermatogonia differ markedly from adult spermatogonia in both the size of the KIT-negative population and cell cycle characteristics. The GFP(+) KIT(-) fraction of prospermatogonia has much higher repopulating activity than does the GFP(+)KIT(+) population in the adult environment. Interestingly, the GFP(+)KIT(+) population still exhibits repopulating activity, unlike adult KIT-positive spermatogonia. We also show that ALCAM, activated leukocyte cell adhesion molecule, is expressed transiently in gonocytes. Sertoli cells and myoid cells also express ALCAM at the same stage, suggesting that ALCAM may contribute to gonocyte-Sertoli cell adhesion and **migration** of gonoyctes toward the basement membrane.
95. **Migration** and ultrastructural localization of the c-kit receptor protein in spermatogenic cells and spermatozoa of the mouse. The c-kit receptor is a proto-oncogene important in germ cell **migration** and maturation and has also been demonstrated on the acrosomal region of mature sperm. The purpose of the present study was to examine the ultrastructural location of the c-kit receptor in mouse testis and sperm.Testis and sperm from mature male mice were examined for the c-kit receptor utilizing electron microscopy and Western blot analysis techniques. Thin sections of mouse testis and sperm were stained with immunogold-labeled anti-c-kit antibodies. The protein from these testes and sperm was also utilized for Western blot analysis.The c-kit protein was localized within the mouse testes to the type A spermatogonia, the round spermatids, and the mature testicular spermatozoa. The c-kit receptor was noted to migrate from the lumen of the acrosomal vesicles in the early spermatids to the plasma membrane of the late spermatids. It was also noted in the acrosomal region of the testicular spermatozoa, as well as the sperm from the epididymis. Sperm undergoing the acrosome reaction demonstrated association of the c-kit receptor with the plasma membrane of the acrosome, but not on the acrosomal membrane itself. Western blot analysis demonstrated protein bands of 150 kDa in testis and intact sperm.The present study confirms the presence of the c-kit receptor in mouse testis and sperm. It also demonstrates that this receptor is localized to the region of the developing acrosome.
96. TRP-2/DT, a new early melanoblast marker, shows that steel growth factor (c-kit ligand) is a survival factor. We have used a probe derived from TRP-2/DT to detect migratory melanoblasts shortly after they emerge from the neural crest, as early as 10 days post coitum (dpc). TRP-2/DT expression is otherwise restricted to the presumptive pigmented retinal epithelium, the developing telencephalon and the endolymphatic duct. The pattern of steel and c-kit hybridisation in the developing brain differed from that of TRP-2. TRP-1 and tyrosinase probes also detected melanoblasts but were both expressed later in development than TRP-2. We used the TRP-2/DT probe to investigate the way that the Steel-dickie (Sld) mutation interferes with melanocyte development, and found that the membrane-bound steel growth factor which is missing in Sld/Sld mutants is necessary for the survival of melanoblasts but not for their early **migration** and initial differentiation.
97. A mutation in the Kit gene leads to novel gonadal phenotypes in both heterozygous and homozygous mice. The direct sequencing of the Kit cDNA obtained from mutant mice was used to reveal the molecular nature of the W(-3Bao) ENU-induced mutation. There was a T to A transversion at the 441st nucleotide in the W(-3Bao) open reading frame (ORF), which introduced a pre-mature termination codon at residue 147. The gross embryonic development, hematopoiesis and spermatogenesis were examined in the mutant mice. There was no visible difference among the W(-3Bao/+), W(-3Bao/3Bao) and wild type embryos before embryonic day 12.5. W(-3Bao/3Bao) embryos appeared pale after E14.5 and dwarf after E16.5. An extremely low level of hematochrome and large red blood cells were found in W(-3Bao/3Bao) 18.5 days old embryos, leading to the stillbirth of the homozygotes. In 18.5 days old embryos the spermatogonia of W(-3Bao/3Bao) embryos did not migrate to the contorted seminiferous tubules properly, but instead were found in the interstitial tissue. The spermatogonia of W(-3Bao/+) or W(+/+) mice were present in both the interstitial tissue and contorted seminiferous tubules. In the adult male hetereozygotes, there are contorted seminiferous tubules with no spermatogonia, suggesting that the **migration** defect was dominant. In female W(-3Bao/3Bao) ovaries, primordial follicles were absent while primordial follicles appeared clearly in the ovaries of W(-3Bao/+) or W(+/+) mice. With a nonsense mutation in the Kit gene, W(-3Bao/+) mice show white spotting and an abnormal development of the contorted seminiferous tubules and W(-3Bao/3Bao) mice are stillborn due to severe macrocytic anemia, and have abnormal genital glands in both the male and female.
98. Soluble and cell-bound forms of steel factor activity play distinct roles in melanocyte precursor dispersal and survival on the lateral neural crest **migration** pathway. Trunk neural crest cells segregate from the neuroepithelium and enter a '**migration** staging area' lateral to the embryonic neural tube. After some crest cells in the **migration** staging area have begun to migrate on a medial pathway, a subpopulation of crest-derived cells remaining in the **migration** staging area expresses mRNAs for the receptor tyrosine kinase, c-kit, and tyrosinase-related protein-2, both of which are characteristic of melanocyte precursors. These putative melanocyte precursors are subsequently observed on the lateral crest **migration** pathway between the dermatome and overlying epithelium, and then dispersed in nascent dermal mesenchyme. Melanocyte precursors transiently require the c-kit ligand, Steel factor for survival. Although Steel factor mRNA is transiently expressed in the dorsal dermatome before the onset of trunk neural crest cell dispersal on the lateral pathway, it is no longer produced by dermatomal cells when melanocyte precursors have dispersed in the dermal mesenchyme. To assess the role of Steel factor in **migration** of melanocyte precursors on the lateral pathway, we analyzed melanocyte precursor dispersal and fate on the lateral pathway of two different Sl mutants, Sl, a null allele, and Sld, which lacks cell surface-associated Steel factor but produces a soluble form. No melanocyte precursors were detected in the dermatome of embryos homozygous for the Sl allele or in W mutants that lack functional c-kit. In contrast, in embryos homozygous for the Sld allele, melanocyte precursors appeared on the lateral pathway, but subsequently disappear from the dermis. These results suggest that soluble Steel factor is required for melanocyte precursor dispersal on the lateral pathway, or for their initial survival in the **migration** staging area. In contrast, membrane-bound Steel factor appears to promote melanocyte precursor survival in the dermis.
99. Developmental expression of Smoc1 and Smoc2 suggests potential roles in fetal gonad and reproductive tract differentiation. SMOC1 and SMOC2 are matricellular proteins thought to influence growth factor signaling, **migration**, proliferation, and angiogenesis. We examined the expression and regulation of Smoc1 and Smoc2 in fetal gonad/mesonephros complexes to discover possible roles for these genes in gonad and mesonephros development. Smoc1 was upregulated at approximately E10.75 in a center-to-poles wave in pre-Sertoli and pre-granulosa cells and its expression was greatly reduced in Wt1, Sf1, and Fog2 mutants. After E13.5, Smoc1 was downregulated in an anterior-to-posterior wave in granulosa cells but persisted in Sertoli cells, suggesting a sexually dimorphic requirement in supporting cell lineage differentiation. Smoc2 was expressed in Leydig cells, mesonephroi, and Wnt4 mutant ovaries, but not wildtype ovaries. Using organ culture, we determined that Smoc2 expression was dependent on Hedgehog signaling in testes, mesonephroi, and kidneys. Overall, these results demonstrate that SMOC1 and SMOC2 may mediate intercellular signaling and cell type-specific differentiation during gonad and reproductive tract development.
100. c-kit receptor and ligand expression in postnatal development of the mouse cerebellum suggests a function for c-kit in inhibitory interneurons. The c-kit receptor and its cognate ligand, KL, are encoded at the white spotting locus (W) and the steel locus (Sl) of the mouse, respectively. Sl and W mutations affect the same cellular targets in melanogenesis, gametogenesis and hematopoiesis during embryonic development and in adult life. c-kit is expressed in cellular targets of W and Sl mutations, whereas KL is expressed in the microenvironment of these targets. c-kit and KL, however, are also expressed in tissues and cell types that are not targets of W and Sl mutations, including the brain. The cerebellum contains a small number of neural cell types whose developmental origins, pathways of **migration**, and synaptic contacts are known. We have investigated the patterns of expression of the c-kit and KL RNA and protein products in postnatal cerebellar development of the mouse. In the adult cerebellum, c-kit RNA and protein expression was evident in basket, stellate, and Golgi neurons. Most strikingly, the c-kit protein is expressed in the basket cell axons that form "basket" and "pinceau" structures entwining the Purkinje cell soma and the initial segment of the Purkinje cell axon. KL RNA expression was found in Purkinje cells, and the KL protein was detected in Purkinje cell bodies and dendrites. Soluble KL protein was also present in c-kit-expressing basket, stellate, and Golgi cells, presumably as a result of internalization of ligand-receptor complexes. During postnatal development, c-kit and KL RNA and protein expression in Golgi and Purkinje neurons, respectively, was evident by day 0 and persisted subsequently. c-kit expression in basket and stellate cells was detected from their time of birth, starting at day 4. These results suggest a role for the c-kit receptor system in postnatal development of the cerebellum.
101. Mast cells condition dendritic cells to mediate allograft tolerance. Peripheral tolerance orchestrated by regulatory T cells, dendritic cells (DCs), and mast cells (MCs) has been studied in several models including skin allograft tolerance. We now define a role for MCs in controlling DC behavior ("conditioning") to facilitate tolerance. Under tolerant conditions, we show that MCs mediated a marked increase in tumor necrosis factor (TNFα)-dependent accumulation of graft-derived DCs in the dLN compared to nontolerant conditions. This increase of DCs in the dLN is due to the local production of granulocyte macrophage colony-stimulating factor (GM-CSF) by MCs that induces a survival advantage of graft-derived DCs. DCs that migrated to the dLN from the tolerant allograft were tolerogenic; i.e., they dominantly suppress T cell responses and control regional immunity. This study underscores the importance of MCs in conditioning DCs to mediate peripheral tolerance and shows a functional impact of peripherally produced TNFα and GM-CSF on **the migra**tion and function of tolerogenic DCs.
102. Regulation of c-kit+ progenitor cells by stromal cell derived factor-1α in adult murine heart. c-kit-positive cardiac progenitor cells (CPCs) have been proven suitable for stem cell therapy. CPCs marker c-kit and its ligand, the stem cell factor (SCF), are associated with the functions of proliferation and differentiation. In our previous study, we found that stromal cell-derived factor-1α (SDF-1α) could enhance the expression of c-kit. However, the mechanism is unknown.CPCs were isolated from adult mouse hearts, and c-kit-positive CPCs were purified by magnetic-activated c-kit cell sorting magnetic beads. The cells were cultured with SDF-1α, c-kit expression was measured by western blotting and qPCR, the proliferation **and migra**tion of cells were measured by CCK-8 and transwell assay, DNA methyltransferase (DNMT) mRNA were measured by qPCR, global DNMT activity was measured by DNMT activity assay kit, and DNA methylation was analysed using Sequenom's MassARRAY platform. Results showed that SDF-1α could enhance the expression of c-kit, which results in the promoting of c-kit-positive CPCs proliferation **and migr**ation. SDF-1α stimulation inhibited the expression of DNMT1, DNMT3β, and global DNMT activity, which led to significant demethylation in c-kit-positive CPCs.SDF-1α signalling, via CXCR4 activation, up-regulated c-kit expression by inhibiting DNMT1 and DNMT3β expression and global DNMT activity, and by subsequent demethylation of the c-kit gene.

#### GeneRIFs

1. Dynamic process of early thymic progenitor differentiation is paralleled by **migration**-dependent change to the supporting niche, and identify vascular endothelial cells as a thymic niche cell, with mKitL as a critical ligand.
2. SCF/c-kit signaling was required for TPA-induced **migration** and differentiation of hair follicle melanocytes.
3. cKit is necessary for radial **migration** of cortical neurons, probably through stem cell factor binding and cKit is necessary for the correct formation of the callosal projection
4. Lnk, through its binding to Kit tyrosine 567, negatively modulates specific SCF-dependent signaling pathways involved in the proliferation and **migration** of primary hematopoietic cells.
5. Protein-tyrosine phosphatase alpha regulates stem cell factor-dependent c-Kit activation and **migration** of mast cells
6. Fas and c-kit are involved in the control of hair follicle melanocyte apoptosis and **migration** in chemotherapy-induced hair loss.
7. genetic and biochemical data demonstrate that both c-Kit and alpha4 integrin signaling are linked to class IA phosphatidylinositol 3-kinase and Rac pathways and regulate integrin-directed **migration** in mast cells
8. SgIGSF and KIT independently play significant roles in cell **migration**
9. SWAP-70 is an important regulator of specific effector pathways in c-kit signaling, including mast cell activation, **migration**, and cell adhesion

### ECM

#### PubMed

1. TGF-beta-1 up-regulates extra-cellular matrix production in mouse hepatoblasts. Fetal liver is the major embryonic hematopoietic organ and is extrinsically colonized by circulating hematopoietic stem cells (HSCs). Integrin beta-1 expression on HSCs is crucial for colonization, suggesting that interaction of Integrin beta-1 with extra-cellular matrix (**ECM**) factors promotes HSC adherence to fetal liver. However, little is known about how **ECM** production is regulated in fetal liver. Here we used flow cytometry to sort fetal liver compartments and detected **ECM** gene and protein expression predominantly in sorted hepatoblasts. mRNA and protein analysis suggested that TGF-beta-1 expressed by hepatoblasts, sinusoid endothelial cells and hematopoietic cells, binds to the TGF-beta receptor type-2 expressed on hepatoblasts to stimulate **ECM** production. Intra-cardiac injection of TGF-inhibitors into mouse embryos dramatically decreased fetal liver **ECM** gene expression. Taken together, our observations suggest that hepatoblasts predominantly produce **ECM** factors under control of TGF-beta-1 in fetal liver.
2. The secreted metalloprotease ADAMTS20 is required for melanoblast survival. ADAMTS20 (Adisintegrin-like and metalloprotease domain with thrombospondin type-1 motifs) is a member of a family of secreted metalloproteases that can process a variety of extracellular matrix (**ECM**) components and secreted molecules. Adamts20 mutations in belted (bt) mice cause white spotting of the dorsal and ventral torso, indicative of defective neural crest (NC)-derived melanoblast development. The expression pattern of Adamts20 in dermal mesenchymal cells adjacent to migrating melanoblasts led us to initially propose that Adamts20 regulated melanoblast migration. However, using a Dct-LacZ transgene to track melanoblast development, we determined that melanoblasts were distributed normally in whole mount E12.5 bt/bt embryos, but were specifically reduced in the trunk of E13.5 bt/bt embryos due to a seven-fold higher rate of apoptosis. The melanoblast defect was exacerbated in newborn skin and embryos from bt/bt animals that were also haploinsufficient for Adamts9, a close homolog of Adamts20, indicating that these metalloproteases functionally overlap in melanoblast development. We identified two potential mechanisms by which Adamts20 may regulate melanoblast survival. First, skin explant cultures demonstrated that Adamts20 was required for melanoblasts to respond to soluble Kit ligand (sKitl). In support of this requirement, bt/bt;Kit(tm1Alf)/+ and bt/bt;Kitl(Sl)/+ mice exhibited synergistically increased spotting. Second, ADAMTS20 cleaved the aggregating proteoglycan versican in vitro and was necessary for versican processing in vivo, raising the possibility that versican can participate in melanoblast development. These findings reveal previously unrecognized roles for Adamts proteases in cell survival and in mediating Kit signaling during melanoblast colonization of the skin. Our results have implications not only for understanding mechanisms of NC-derived melanoblast development but also provide insights on novel biological functions of secreted metalloproteases.
3. Endothelial protein C receptor-expressing hematopoietic stem cells reside in the perisinusoidal niche in fetal liver. Hematopoietic stem cells (HSCs) are maintained in specialized niches in adult bone marrow. However, niche and HSC maintenance mechanism in fetal liver (FL) still remains unclear. Here, we investigated the niche and the molecular mechanism of HSC maintenance in mouse FL using HSCs expressing endothelial protein C receptor (EPCR). The antiapoptotic effect of activated protein C (APC) on EPCR(+) HSCs and the expression of protease-activated receptor 1 (Par-1) mRNA in these cells suggested the involvement of the cytoprotective APC/EPCR/Par-1 pathway in HSC maintenance. Immunohistochemistry revealed that EPCR(+) cells were localized adjacent to, or integrated in, the Lyve-1(+) sinusoidal network, where APC and extracellular matrix (**ECM**) are abundant, suggesting that HSCs in FL were maintained in the APC- and **ECM**-rich perisinusoidal niche. EPCR(+) HSCs were in a relatively slow cycling state, consistent with their high expression levels of p57 and p18. Furthermore, the long-term reconstitution activity of EPCR(+) HSCs decreased significantly after short culture but not when cocultured with feeder layer of FL-derived Lyve-1(+) cells, which suggests that the maintenance of the self-renewal activity of FL HSCs largely depended on the interaction with the perisinusoidal niche. In conclusion, EPCR(+) HSCs resided in the perisinusoidal niche in mouse FL.
4. Delayed wound healing due to increased interleukin-10 expression in mice with lymphatic dysfunction. Skin wound healing is an interactive process involving soluble mediators, **ECM**, resident cells, and infiltrating cells. Little is known about wound healing in the presence of lymphedema. In this study, we investigated wound healing using kCYC⁺/⁻ mice, which demonstrate severe lymphatic dysfunction. Wound healing was delayed significantly in kCYC⁺/⁻ mice when compared with WT mice. In wounded skin of kCYC⁺/⁻ mice, mast cell numbers were increased compared with WT mice, whereas macrophage numbers were decreased. Moreover, IL-10 expression by mast cells was increased, and expression of bFGF, mainly produced by macrophages, was decreased in wounded skin of kCYC⁺/⁻ mice compared with WT mice. We next crossed kCYC⁺/⁻ mice with IL-10⁻/⁻ mice, which were reported to show accelerated wound closure. In kCYC⁺/⁻ IL-10⁺/⁻ mice, time course of wound healing, numbers of macrophages, and IL-10 mRNA expression levels in wounded skin were comparable with WT IL-10⁺/⁻ mice. Similar results were obtained using a different lymphedema model, in which circumferential skin excision was performed on the tails of mice to remove the superficial lymphatics. In summary, these findings suggest that IL-10 plays an important role in delayed wound healing in the setting of lymphatic dysfunction.

### [Ee]xtracellular [Mm]atrix

#### PubMed

1. The secreted metalloprotease ADAMTS20 is required for melanoblast survival. ADAMTS20 (Adisintegrin-like and metalloprotease domain with thrombospondin type-1 motifs) is a member of a family of secreted metalloproteases that can process a variety of **extracellular matrix** (ECM) components and secreted molecules. Adamts20 mutations in belted (bt) mice cause white spotting of the dorsal and ventral torso, indicative of defective neural crest (NC)-derived melanoblast development. The expression pattern of Adamts20 in dermal mesenchymal cells adjacent to migrating melanoblasts led us to initially propose that Adamts20 regulated melanoblast migration. However, using a Dct-LacZ transgene to track melanoblast development, we determined that melanoblasts were distributed normally in whole mount E12.5 bt/bt embryos, but were specifically reduced in the trunk of E13.5 bt/bt embryos due to a seven-fold higher rate of apoptosis. The melanoblast defect was exacerbated in newborn skin and embryos from bt/bt animals that were also haploinsufficient for Adamts9, a close homolog of Adamts20, indicating that these metalloproteases functionally overlap in melanoblast development. We identified two potential mechanisms by which Adamts20 may regulate melanoblast survival. First, skin explant cultures demonstrated that Adamts20 was required for melanoblasts to respond to soluble Kit ligand (sKitl). In support of this requirement, bt/bt;Kit(tm1Alf)/+ and bt/bt;Kitl(Sl)/+ mice exhibited synergistically increased spotting. Second, ADAMTS20 cleaved the aggregating proteoglycan versican in vitro and was necessary for versican processing in vivo, raising the possibility that versican can participate in melanoblast development. These findings reveal previously unrecognized roles for Adamts proteases in cell survival and in mediating Kit signaling during melanoblast colonization of the skin. Our results have implications not only for understanding mechanisms of NC-derived melanoblast development but also provide insights on novel biological functions of secreted metalloproteases.
2. MMP9 limits apoptosis and stimulates branching morphogenesis during kidney development. Early events in kidney organogenesis involve reciprocal interactions between the ureteric bud and the metanephric mesenchyme, which lead to remodeling of the **extracellular matrix**. This remodeling involves matrix metalloproteases (MMPs), but the specific roles of individual MMPs in kidney development are not completely understood. Here, we analyzed MMP9-deficient mice at the first step of kidney development and found that MMP9 deficiency delayed embryonic kidney maturation and increased apoptosis ex vivo by 2.5-fold. These early defects resulted in a 30% decrease in nephron number, a 20% decrease in adult kidney weight, and altered kidney function and morphology at 12 mo. The membrane form of stem cell factor (SCF) increased, whereas the activated form of the SCF receptor, c-kit, decreased in MMP9-deficient embryonic kidneys. In organotypic culture, MMP9-deficient kidneys failed to secrete SCF, and addition of recombinant SCF partially rescued both apoptosis and the branching defect. In conclusion, these data show that MMP9 protects mesenchymal cells from apoptosis during kidney development and stimulates ureteric bud branching morphogenesis, most likely by releasing the soluble form of SCF, suggesting that normal renal development requires MMP9.
3. Involvement of stem cell factor and c-kit in corneal wound healing in mice. To study the roles played by stem cell factor (SCF) and SCF receptor c-kit in wound healing of corneal epithelial cells.A 2 mm corneal epithelial wound was made in control (WBB6F1(+/+)), SCF (Sl/Sl(d))-, and c-kit (W/W(v)) mutant mice, and the speed of wound healing, 5-bromo-2'-deoxyuridine (BrdU) incorporation, and scanning electron microscopic (SEM) morphology of the corneas were examined. The incorporation of BrdU and the degree of cell attachment in cultured mouse corneal epithelial cells (MCECs) isolated from WBB6F1(+/+), Sl/Sl(d), and W/W(v) mice were examined. Cultured immortalized human corneal epithelial cells (HCECs) were examined by a cell attachment assay after their exposure to anti-SCF antibodies, tyrosine kinase inhibitor (genistein), and competitive Arg-Gly-Asp (RGD) peptide, as well as on cultures treated with **extracellular matrix**.The speed of corneal wound healing was slower in Sl/Sl(d) and W/W(v) mice than in controls (p<0.01) and the speed of healing in Sl/Sl(d) mice recovered after topical application of SCF (8 ng/ml). No significant difference was found in the BrdU incorporation assay either in vivo or in vitro. Loosened epithelial cells were detected at wound margins in W/W(v) mice by SEM. The cell attachment rate was increased by 157% in cells from WBB6F1(+/+) and 252% in Sl/Sl(d) MCECs by recombinant mouse SCF; however, no significant difference was found in W/W(v) MCECs. Anti-SCF antibodies (Ab), genistein, and RGD peptide reduced the percentage of attached HCECs. Anti-SCF Ab inhibited the attachment of HCECs on fibronectin, laminin, or type IV collagen coated dishes.These findings indicate that the SCF/c-kit system may play a role in corneal wound healing through epithelial cell attachment.
4. Mast cells are required for full expression of allergen/SEB-induced skin inflammation. Atopic dermatitis (AD) is a chronic pruritic inflammatory skin disease. We recently described an animal model in which repeated epicutaneous applications of a house dust mite extract and Staphylococcal enterotoxin B induced eczematous skin lesions. In this study we showed that global gene expression patterns are very similar between human AD skin and allergen/staphylococcal enterotoxin B-induced mouse skin lesions, particularly in the expression of genes related to epidermal growth/differentiation, skin barrier, lipid/energy metabolism, immune response, or **extracellular matrix**. In this model, mast cells and T cells, but not B cells or eosinophils, were shown to be required for the full expression of dermatitis, as revealed by reduced skin inflammation and reduced serum IgE levels in mice lacking mast cells or T cells (TCRβ(-/-) or Rag1(-/-)). The clinical severity of dermatitis correlated with the numbers of mast cells, but not eosinophils. Consistent with the idea that T helper type 2 (Th2) cells play a predominant role in allergic diseases, the receptor for the Th2-promoting cytokine thymic stromal lymphopoietin and the high-affinity IgE receptor, FcɛRI, were required to attain maximal clinical scores. Therefore, this clinically relevant model provides mechanistic insights into the pathogenic mechanism of human AD.
5. IL-33 induces IL-13-dependent cutaneous fibrosis. IL-33 is constitutively expressed in epithelial barrier tissues, such as skin. Although increased expression of IL-33/IL-33R has been correlated with fibrotic disorders, such as scleroderma and progressive systemic sclerosis, the direct consequences of IL-33 release in skin has not been reported. To determine the effects of dysregulated IL-33 signaling in skin, we administered IL-33 s.c. and monitored its effects at the injection site. Administration of IL-33 resulted in IL-33R-dependent accumulation of eosinophils, CD3(+) lymphocytes, F4/80(+) mononuclear cells, increased expression of IL-13 mRNA, and the development of cutaneous fibrosis. Consistent with extensive cutaneous tissue remodeling, IL-33 resulted in significant modulation of a number of **extracellular matrix**-associated genes, including collagen VI, collagen III, and tissue inhibitor of metalloproteases-1. We establish that IL-33-induced fibrosis requires IL-13 using IL-13 knockout mice and eosinophils using Delta dblGATA mice. We show that bone marrow-derived eosinophils secrete IL-13 in response to IL-33 stimulation, suggesting that eosinophil-derived IL-13 may promote IL-33-induced cutaneous fibrosis. Collectively, our results identify IL-33 as a previously unrecognized profibrotic mediator in skin and highlight the cellular and molecular pathways by which this pathology develops.
6. Mast cells promote scar remodeling and functional recovery after spinal cord injury via mouse mast cell protease 6. An important barrier for axon regeneration and recovery after traumatic spinal cord injury (SCI) is attributed to the scar that is formed at the lesion site. Here, we investigated the effect of mouse mast cell protease (mMCP) 6, a mast cell (MC)-specific tryptase, on scarring and functional recovery after a spinal cord hemisection injury. Functional recovery was significantly impaired in both MC-deficient and mMCP6-knockout (mMCP6(-/-)) mice after SCI compared with wild-type control mice. This decrease in locomotor performance was associated with an increased lesion size and excessive scarring at the injury site. Axon growth-inhibitory chondroitin sulfate proteoglycans and the **extracellular matrix** components fibronectin, laminin, and collagen IV were significantly up-regulated in MC-deficient and mMCP6(-/-) mice, with an increase in scar volume between 23 and 32%. A degradation assay revealed that mMCP6 directly cleaves fibronectin and collagen IV in vitro In addition, gene expression levels of the scar components fibronectin, aggrecan, and collagen IV were increased up to 6.8-fold in mMCP6(-/-) mice in the subacute phase after injury. These data indicate that endogenous mMCP6 has scar-suppressing properties after SCI via indirect cleavage of axon growth-inhibitory scar components and alteration of the gene expression profile of these factors.-Vangansewinkel, T., Geurts, N., Quanten, K., Nelissen, S., Lemmens, S., Geboes, L., Dooley, D., Vidal, P. M., Pejler, G., Hendrix, S. Mast cells promote scar remodeling and functional recovery after spinal cord injury via mouse mast cell protease 6.
7. Endothelial protein C receptor-expressing hematopoietic stem cells reside in the perisinusoidal niche in fetal liver. Hematopoietic stem cells (HSCs) are maintained in specialized niches in adult bone marrow. However, niche and HSC maintenance mechanism in fetal liver (FL) still remains unclear. Here, we investigated the niche and the molecular mechanism of HSC maintenance in mouse FL using HSCs expressing endothelial protein C receptor (EPCR). The antiapoptotic effect of activated protein C (APC) on EPCR(+) HSCs and the expression of protease-activated receptor 1 (Par-1) mRNA in these cells suggested the involvement of the cytoprotective APC/EPCR/Par-1 pathway in HSC maintenance. Immunohistochemistry revealed that EPCR(+) cells were localized adjacent to, or integrated in, the Lyve-1(+) sinusoidal network, where APC and **extracellular matrix** (ECM) are abundant, suggesting that HSCs in FL were maintained in the APC- and ECM-rich perisinusoidal niche. EPCR(+) HSCs were in a relatively slow cycling state, consistent with their high expression levels of p57 and p18. Furthermore, the long-term reconstitution activity of EPCR(+) HSCs decreased significantly after short culture but not when cocultured with feeder layer of FL-derived Lyve-1(+) cells, which suggests that the maintenance of the self-renewal activity of FL HSCs largely depended on the interaction with the perisinusoidal niche. In conclusion, EPCR(+) HSCs resided in the perisinusoidal niche in mouse FL.
8. Involvement of CD44 in mast cell proliferation during terminal differentiation. By using the recently established culture system that reproduces the terminal differentiation process of connective tissue-type mast cells, we found significant transcriptional induction of CD44. As CD44 is a primary receptor for hyaluronan (HA), which is one of the major **extracellular matrix** components, we investigated the role of CD44 in cutaneous mast cells. When co-cultured with fibroblasts, mouse bone marrow-derived cultured mast cells (BMMCs) were found to form clusters in an HA-dependent manner. As compared with BMMCs derived from the wild-type mice, those from the CD44(-/-) mice exhibited impaired growth during the co-cultured period. Furthermore, in the peritoneal cavities and ear tissues, mature mast cells were fewer in number in the CD44(-/-) mice than in the wild-type mice. We investigated roles of CD44 in mast cell proliferation by reconstituting BMMCs into the tissues of mast cell-deficient, Kit(W)/Kit(W-v) mice, and found that the number of metachromatic cells upon acidic toluidine blue staining in the tissues transplanted with CD44(-/-) BMMCs was not significantly changed for 10 weeks, whereas that in the tissues transplanted with the CD44(+/+) BMMCs was significantly increased. These results suggest that CD44 plays a crucial role in the regulation of the cutaneous mast cell number.
9. Diabetic impairment of C-kit bone marrow stem cells involves the disorders of inflammatory factors, cell adhesion and **extracellular matrix** molecules. Bone marrow stem cells from diabetes mellitus patients exhibit functional impairment, but the relative molecular mechanisms responsible for this impairment are poorly understood. We investigated the mechanisms responsible for diabetes-related functional impairment of bone marrow stem cells by extensively screening the expression levels of inflammatory factors, cell cycle regulating molecules, **extracellular matrix** molecules and adhesion molecules. Bone marrow cells were collected from type 2 diabetic (db/db) and healthy control (db/m+) mice, and c-kit+ stem cells were purified (purity>85%) for experiments. Compared with the healthy control mice, diabetic mice had significantly fewer c-kit+ stem cells, and these cells had a lower potency of endothelial differentiation; however, the production of the angiogenic growth factor VEGF did not differ between groups. A pathway-focused array showed that the c-kit+ stem cells from diabetic mice had up-regulated expression levels of many inflammatory factors, including Tlr4, Cxcl9, Il9, Tgfb1, Il4, and Tnfsf5, but no obvious change in the expression levels of cell cycle molecules. Interestingly, diabetes-related alterations of the **extracellular matrix** and adhesion molecules were varied; Pecam, Mmp10, Lamc1, Itgb7, Mmp9, and Timp4 were up-regulated, but Col11a1, Fn1, Admts2, and Itgav were down-regulated. Some of these changes were also confirmed at the protein level by flow cytometry analysis. In conclusion, c-kit+ bone marrow stem cells from diabetic mice exhibited an extensive enhancement of inflammatory factors and disorders of the **extracellular matrix** and adhesion molecules. Further intervention studies are required to determine the precise role of each molecule in the diabetes-related functional impairment of c-kit+ bone marrow stem cells.
10. Nf1-/- Schwann cell-conditioned medium modulates mast cell degranulation by c-Kit-mediated hyperactivation of phosphatidylinositol 3-kinase. Neurofibromatosis type 1 (NF1) is a common genetic disorder and is characterized by both malignant and nonmalignant neurofibromas, which are composed of Schwann cells, degranulating mast cells, fibroblasts, and **extracellular matrix**. We and others have previously shown that hyperactivation of the c-Kit pathway in an Nf1 haploinsufficient microenvironment is required for both tumor formation and progression. Mast cells play a key role in both tumorigenesis and neoangiogenesis via the production of matrix metalloproteinases, heparin, and a range of different growth factors. In the present study, we show that tumorigenic Schwann cells derived from Nf1(-/-) embryos promote increased degranulation of Nf1(+/-) mast cells compared with wild-type mast cells via the secretion of the Kit ligand. Furthermore, we used genetic intercrosses as well as pharmacological agents to link the hyperactivation of the p21(Ras)-phosphatidylinositol 3-kinase (PI3K) pathway to the increased degranulation of Nf1(+/-) mast cells both in vitro and in vivo. These studies identify the p21(Ras)-PI3K pathway as a major regulator of the gain in Nf1(+/-) mast cell degranulation in neurofibromas. Collectively, these studies identify both c-Kit and PI3K as molecular targets that modulate mast cell functions in cases of NF1.
11. Imatinib upregulates compensatory integrin signaling in a mouse model of gastrointestinal stromal tumor and is more effective when combined with dasatinib. Activating mutations in the Kit receptor tyrosine kinase are associated with gastrointestinal stromal tumor (GIST). Imatinib inhibits Kit and is front-line therapy for GIST. However, imatinib most often elicits a partial response or stable disease, and most GIST patients who initially respond to imatinib eventually acquire resistance. Thus, improved treatment strategies for GIST are needed. We investigated the role of Src family kinases (SFK) in tumorigenesis in a mouse model of human GIST. The SFKs Src and Lyn were active in GIST, and surprisingly, imatinib treatment stimulated their phosphorylation/activation. We show that integrin signaling activates focal adhesion kinase and, consequently, SFKs in GIST and that imatinib enhances integrin signaling, implying a role for the **extracellular matrix** and integrin signaling in tumor maintenance and imatinib resistance. Dasatinib, an inhibitor of SFKs and Kit, inhibited SFK and focal adhesion kinase activation in GIST but also inhibited Kit and Kit-dependent downstream signaling pathways including phosphoinositide 3-kinase and mitogen-activated protein kinase, but not signal transducer and activator of transcription (STAT) signaling. Whereas dasatinib and imatinib alone both produced a minimal histopathologic response, combination therapy improved their efficacy, leading to increased necrosis in GIST. These results highlight the importance of SFK and STAT signaling in GIST and suggest that the clinical efficacy of imatinib may be limited by the stimulation of integrin signaling.
12. Steel factor and c-kit regulate cell-matrix adhesion. Steel (SI) and white spotting (W) loci encode steel factor (c-kit ligand) and the c-kit tyrosine kinase receptor, respectively. Mutations at these loci affect migration and differentiation of primordial germ cells, neural crest-derived melanoblasts, and hematopoietic cells. In these processes, cell adhesion molecules are hypothesized to be crucial. We have examined the role of steel factor and c-kit in cell-**extracellular matrix** adhesion using bone marrow-derived mast cells as a model system. Steel factor stimulates mast cells to bind to fibronectin and, to a lesser extent, to vitronectin, whereas interleukin-3 and interleukin-4, which are also mast cell growth factors, do not. Activation of adhesiveness is transient, occurs at concentrations of steel factor 100-fold lower than required for growth stimulation, and requires the integrin VLA-5. Mast cells from c-kit mutant mice adhere to fibronectin on stimulation with phorbol 12-myristate 13-acetate (PMA), but not on stimulation with steel factor, indicating that stimulation of integrin adhesiveness requires activation of the c-kit protein tyrosine kinase. By contrast, c-kit mutant and wild-type mast cells adhere equally well to COS cells expressing membrane-anchored steel factor, showing that the kinase activity of c-kit is not required for adhesion directly mediated by c-kit. Our findings suggest that regulation of adhesion is an important biologic function of steel factor.
13. Esophageal muscle physiology and morphogenesis require assembly of a collagen XIX-rich basement membrane zone. Collagen XIX is an extremely rare **extracellular matrix** component that localizes to basement membrane zones and is transiently expressed by differentiating muscle cells. Characterization of mice harboring null and structural mutations of the collagen XIX (Col19a1) gene has revealed the critical contribution of this matrix protein to muscle physiology and differentiation. The phenotype includes smooth muscle motor dysfunction and hypertensive sphincter resulting from impaired swallowing-induced, nitric oxide-dependent relaxation of the sphincteric muscle. Muscle dysfunction was correlated with a disorganized matrix and a normal complement of enteric neurons and interstitial cells of Cajal. Mice without collagen XIX exhibit an additional defect, namely impaired smooth-to-skeletal muscle cell conversion in the abdominal segment of the esophagus. This developmental abnormality was accounted for by failed activation of myogenic regulatory factors that normally drive esophageal muscle transdifferentiation. Therefore, these findings identify collagen XIX as the first structural determinant of sphincteric muscle function, and as the first extrinsic factor of skeletal myogenesis in the murine esophagus.
14. Mechanisms of oncostatin M-induced pulmonary inflammation and fibrosis. Oncostatin M (OSM), an IL-6 family cytokine, has been implicated in a number of biological processes including the induction of inflammation and the modulation of **extracellular matrix**. In this study, we demonstrate that OSM is up-regulated in the bronchoalveolar lavage fluid of patients with idiopathic pulmonary fibrosis and scleroderma, and investigate the pathological consequences of excess OSM in the lungs. Delivery of OSM to the lungs of mice results in a significant recruitment of inflammatory cells, as well as a dose-dependent increase in collagen deposition in the lungs, with pathological correlates to characteristic human interstitial lung disease. To better understand the relationship between OSM-induced inflammation and OSM-induced fibrosis, we used genetically modified mice and show that the fibrotic response is largely independent of B and T lymphocytes, eosinophils, and mast cells. We further explored the mechanisms of OSM-induced inflammation and fibrosis using both protein and genomic array approaches, generating a "fibrotic footprint" for OSM that shows modulation of various matrix metalloproteinases, **extracellular matrix** components, and cytokines previously implicated in fibrosis. In particular, although the IL-4/IL-13 and TGF-beta pathways have been shown to be important and intertwined of fibrosis, we show that OSM is capable of inducing lung fibrosis independently of these pathways. The demonstration that OSM is a potent mediator of lung inflammation and **extracellular matrix** accumulation, combined with the up-regulation observed in patients with pulmonary fibrosis, may provide a rationale for therapeutically targeting OSM in human disease.
15. Identification of factors promoting ex vivo maintenance of mouse hematopoietic stem cells by long-term single-cell quantification. The maintenance of hematopoietic stem cells (HSCs) during ex vivo culture is an important prerequisite for their therapeutic manipulation. However, despite intense research, culture conditions for robust maintenance of HSCs are still missing. Cultured HSCs are quickly lost, preventing their improved analysis and manipulation. Identification of novel factors supporting HSC ex vivo maintenance is therefore necessary. Coculture with the AFT024 stroma cell line is capable of maintaining HSCs ex vivo long-term, but the responsible molecular players remain unknown. Here, we use continuous long-term single-cell observation to identify the HSC behavioral signature under supportive or nonsupportive stroma cocultures. We report early HSC survival as a major characteristic of HSC-maintaining conditions. Behavioral screening after manipulation of candidate molecules revealed that the **extracellular matrix** protein dermatopontin (Dpt) is involved in HSC maintenance. DPT knockdown in supportive stroma impaired HSC survival, whereas ectopic expression of the Dpt gene or protein in nonsupportive conditions restored HSC survival. Supplementing defined stroma- and serum-free culture conditions with recombinant DPT protein improved HSC clonogenicity. These findings illustrate a previously uncharacterized role of Dpt in maintaining HSCs ex vivo.

#### GeneRIFs

1. c-kit+ bone marrow stem cells from diabetic mice exhibited an extensive enhancement of inflammatory factors and disorders of the **extracellular matrix** and adhesion molecules.

### [Ii]nvasive

#### PubMed

1. Spontaneous malignant transformation of melanocytes explanted from Wf/Wf mice with a Kit kinase-domain mutation. The W/Kit mouse locus, affecting proliferation and survival of pigment cells, blood cells, and germ cells, is known to encode a tyrosine kinase growth factor receptor and is considered a protooncogene; yet it has not heretofore been causally implicated in any malignancies of those cells. The Wf/Wf mutant mouse coat comprises viable and inviable melanoblast clones, seen ultimately as pigmented and white transverse stripes--the latter more prominent. Judging from the pattern, all clones initially expand, and the inviable ones then undergo programmed cell death prenatally. To observe skin melanocytes of the viable clones during extended proliferation, the cells were explanted from individual young mice. An unusually large number of primary explants failed to survive--a result consistent with a growth handicap. In 3 of the 10 surviving cell lines, many cells spontaneously underwent a series of striking changes with the classic features of transformation. The two transformed lines that have been tested by grafting to immunosuppressed hosts formed undifferentiated **invasive** tumors compatible with malignant amelanotic melanoma. None of our 52 other melanocyte lines of the coisogenic wild-type strain and 13 other natural genotypes have become transformed under the same culture conditions. Molecular analysis of the Wf gene revealed a single change from wild-type: a point mutation affecting the catalytic region in the kinase domain of the Kit protein. The apparent growth disadvantage due to the mutation may allow selection for melanocytes mobilizing more efficient pathways, thus leading to neoplasia. Production of both viable and inviable melanoblast clones is unlikely to be due only to the kinase mutation; possibly the degree, duration, and consistency of expression of this locus may be controlled by cis elements outside the coding region.
2. Absence of Fc epsilonRI alpha chain results in upregulation of Fc gammaRIII-dependent mast cell degranulation and anaphylaxis. Evidence of competition between Fc epsilonRI and Fc gammaRIII for limiting amounts of FcR beta and gamma chains. In mouse mast cells, both Fc epsilonRI and Fc gammaRIII are alpha beta gamma2 tetrameric complexes in which different alpha chains confer IgE or IgG ligand recognition while the signaling FcR beta and gamma chains are identical. We used primarily non**invasive** techniques (changes in body temperature, dye extravasation) to assess systemic anaphylactic responses in nonanesthetized wild-type, Fc epsilonRI alpha chain -/- and FcR gamma chain -/- mice. We confirm that systemic anaphylaxis in mice can be mediated largely through IgG1 and Fc gammaRIII and we provide direct evidence that these responses reflect activation of Fc gammaRIII rather than Fc gammaRI. Furthermore, we show that Fc gammaRIII-dependent responses are more intense in normal than in congenic mast cell-deficient KitW/KitW-v mice, indicating that Fc gammaRIII responses have mast cell-dependent and -independent components. Finally, we demonstrate that the upregulation of cell surface expression of Fc gammaRIII seen in Fc epsilonRI alpha chain -/- mice corresponds to an increased association of Fc gammaRIII alpha chains with FcR beta and gamma chains and is associated with enhanced Fc gammaRIII-dependent mast cell degranulation and systemic anaphylactic responses. Therefore, the phenotype of the Fc epsilonRI alpha chain -/- mice suggests that expression of Fc epsilonRI and Fc gammaRIII is limited by availability of the FcR beta and gamma chains and that, in normal mice, changes in the expression of one receptor (Fc epsilonRI) may influence the expression of functional responses dependent on the other (Fc gammaRIII).
3. Mast cell cathelicidin antimicrobial peptide prevents **invasive** group A Streptococcus infection of the skin. Mast cells (MC) express cathelicidin antimicrobial peptides that act as broad-spectrum antibiotics and influence the immune defense of multiple epithelial surfaces. We hypothesized that MC help protect against skin infection through the expression of cathelicidin. The susceptibility of MC-deficient mice (Kit Wsh(-/-)) to **invasive** group A streptococcus (GAS) was compared with control mice. Following s.c. injection of GAS, MC-deficient mice had 30% larger skin lesions, 80% more lesional bacteria, and 30% more spleens positive for bacteria. In contrast to results obtained when GAS was injected into skin, no significant differences were noted between MC-deficient mice and control mice after GAS was applied topically, indicating that MC activity is most important after barrier penetration. To determine whether these differences were due to MC expression of cathelicidin, MC-deficient mice were reconstituted with MC derived from either wild-type or cathelicidin-deficient (Camp(-/-)) mice and challenged with GAS. Forty-eight hours after bacterial injection, mice that did not receive MC had an average lesion size of 200 mm(2), mice reconstituted with wild-type MC showed lesions comparable to control mice (25 mm(2)), while mice reconstituted with Camp(-/-) MC showed an average lesion size of 120 mm(2). Surface-enhanced laser desorption/ionization time-of-flight mass spectrometry (SELDI-TOF-MS) analysis of cathelicidin peptide purified from mast cells defined this as a unique 28-aa peptide. Combined, these results show that MC confer defense against Gram-positive bacterial infection in the skin, a function mediated in part by the expression of a unique cathelicidin peptide.
4. Distortion product otoacoustic emissions in hearing-impaired mutant mice. The acoustic intermodulation distortion product (2f1-f2) was recorded in the ear canal of two different types of normally hearing mice and in four different types of hearing-impaired mutant mice. In the normally hearing animals, primary tones at levels of 60- to 100-dB SPL evoked distortion product emissions (DP's) at 20-50 dB below the primary levels. In the hearing-impaired mutants the level was dependent on the particular type of auditory dysfunction associated with the mutation. In both the deafness and the viable dominant spotting mutants, where either the whole organ of Corti or the stria vascularis is affected by the mutation, no DP's could be detected. The quivering mutant has a central auditory dysfunction associated with the nuclei of the superior olivary complex and the lateral lemniscus, with apparently normal cochlear function. DP's at levels and thresholds similar to those in normally hearing animals were recorded in quivering mice. The Bronx Waltzer mutant has a full complement of outer hair cells but only about of 20%-25% inner hair cells. DP's of small amplitude were recorded but the thresholds were raised by about 30 dB. The data suggest that the 2f1-f2 emission can be used as a non**invasive** monitor of cochlear function.
5. Endogenous Nodal signaling regulates germ cell potency during mammalian testis development. Germ cells, the embryonic precursors of sperm or oocytes, respond to molecular cues that regulate their sex-specific development in the fetal gonads. In males in particular, the balance between continued proliferation and cell fate commitment is crucial: defects in proliferation result in insufficient spermatogonial stem cells for fertility, but escape from commitment and prolonged pluripotency can cause testicular germ cell tumors. However, the factors that regulate this balance remain unidentified. Here, we show that signaling by the TGFβ morphogen Nodal and its co-receptor Cripto is active during a crucial window of male germ cell development. The Nodal pathway is triggered when somatic signals, including FGF9, induce testicular germ cells to upregulate Cripto. Germ cells of mutant mice with compromised Nodal signaling showed premature differentiation, reduced pluripotency marker expression and a reduced ability to form embryonic germ (EG) cell colonies in vitro. Conversely, human testicular tumors showed upregulation of NODAL and CRIPTO that was proportional to **invasiv**eness and to the number of malignant cells. Thus, Nodal signaling provides a molecular control mechanism that regulates male germ cell potency in normal development and testicular cancer.
6. C-kit signaling promotes proliferation and invasion of colorectal mucinous adenocarcinoma in a murine model. It was reported that the receptor tyrosine kinase (RTK) family often highly expressed in several mucinous carcinomas. In the present study, we established a murine model of colorectal mucinous adenocardinoma (CRMAC) by treating C57 mice [both wild type (WT) and loss-of-function c-kit mutant type (Wads-/-)] with AOM+DSS for 37 weeks and found that c-kit, a member of RTK family, clearly enhanced the tumor cell proliferation by decreasing p53 and increasing cyclin D1 through AKT pathway. Significantly, c-kit strongly promoted tumor cell **invasive**ness by increasing ETV4, which induced MMP7 expression and epithelial-mesenchymal transition (EMT) via ERK pathway. In vitro up- or down-regulating c-kit activation in human colorectal cancer HCT-116 cells further consolidated these results. In conclusion, our data suggested that the c-kit signaling obviously promoted proliferation and invasion of CRMAC. Therefore, targeting the c-kit signaling and its downstream molecules might provide the potential strategies for treatment of patients suffering from CRMAC in the future.

### [Mm]etastasis

#### PubMed

1. Cutting edge: mast cells critically augment myeloid-derived suppressor cell activity. Myeloid-derived suppressor cells (MDSCs) are primarily recognized for their immunosuppressive properties in malignant disease. However, their interaction with other innate immune cells and their regulation of immune responses, such as in parasitic infection, necessitate further characterization. We used our previously published mouse model of MDSC accumulation to examine the immunoregulatory role of MDSCs in B16 melanoma **metastasis** and Nippostrongylus brasiliensis infection. In this study, we demonstrate that the activity of MDSCs is dependent on the immune stimuli and subset induced. Monocytic MDSCs predictably suppressed antitumor immune responses but granulocytic MDSCs surprisingly enhanced the clearance of N. brasiliensis infection. Intriguingly, both results were dependent on MDSC interaction with mast cells (MCs), as demonstrated by adoptive-transfer studies in MC-deficient (Kit(Wsh)(/)(Wsh)) mice. These findings were further supported by ex vivo cocultures of MCs and MDSCs, indicating a synergistic increase in cytokine production. Thus, MCs can enhance both immunosuppressive and immunosupportive functions of MDSCs.
2. Isolation of novel mouse genes that were differentially expressed in W/W(V) mouse fundus. Using cDNA microarray analysis that displays a total of 8734 mouse genes, we have identified 21 known and novel transcripts, including mouse expressed sequence tags (ESTs) that were consistently up- or downregulated in the fundus of wild-type mice and W/W(V) mice, where intramuscular interstitial cells of Cajal (ICC; IC-IM) are lost.By using these tags as part of the full-length mRNA sequence of the murine genes, we screened a mouse-brain cDNA library and the FASTA database (http://www.ebi.ac.uk/fasta33/).Three of the queries were identified as novel mouse genes, whereas six transcripts had their human counterparts that were known to encode functional proteins. Four transcripts, DRIM (downregulated in **metastasis**), SLP8 (tumor antigen), PTK7/CCK4 (receptor protein tyrosine kinase-like molecule-7/colon carcinoma kinase-4), and a novel gene AWMS2 were increased in W/W(V) mice. Expression of another five genes was decreased in W/W(V) mice: BB1 (overexpressed in bladder and breast carcinoma), HTATIP/CPLA2 (HIV-1 TAT interactive protein/cytosolic phospholipase A2), Tenascin-receptor like (Hexabrachion, Cytotactin, Neuronectin, Myotendinous antigen), and two novel transcripts: DRWMS1 and DRWMS2. Molecular profiling generated by cDNA microarray analysis from the fundus of W/W(V) mice and its complete list of full-length cDNAs will be shown on our web site (http://www.yamanashi.ac.jp/).Differential gene comparisons between control and mutant animals with losses in specific populations of ICCs will contribute significantly to our understanding of motility disorders associated with the loss of these cells and electrical slow waves in the gastrointestinal tract.
3. [Effect of c-kit gene mutation on prognosis of gastrointestinal stromal tumor]. To evaluate the clinical signification of c-kit gene mutation in gastrointestinal stromal tumor (GIST) and examine whether the presence of mutation of c-kit gene is important as a prognostic factor.The c-kit mutation had been detected by PCR-SSCP, DNA sequence, statistical comparison were used for the relationship of c-kit gene mutation and clinical pathology, clinical behavior, recurrence, et al.The presence of c-kit mutation correlated with tumor size, proliferating cell nuclear antigen index, mitotic cell number, presence of necrosis, microscopic invasion to adjacent tissues, recurrence and distant **metastasis**. The age, sex, location of tumor, cell type, the presence of hemorrhage, and c-kit expression were independently related to the presence of c-kit mutation.The c-kit gene mutation is an important prognostic factor for GIST.
4. C-kit and its ligand stem cell factor: potential contribution to prostate cancer bone **metastasis**. The tyrosine kinase receptor c-kit and its ligand stem cell factor (SCF) have not been explored in prostate cancer (PC) bone **metastasis**. Herein, we found that three human PC cell lines and bone marrow stromal cells express a membrane-bound SCF isoform and release a soluble SCF. Bone marrow stromal cells revealed strong expression of c-kit, whereas PC cells showed very low levels of the receptor or did not express it all. Using an experimental model of PC bone **metastasis**, we found that intraosseous bone tumors formed by otherwise c-kit-negative PC3 cells strongly expressed c-kit, as demonstrated using immunohistochemical and Western blot analyses. Subcutaneous PC3 tumors were, however, c-kit-negative. Both bone and subcutaneous PC3 tumors were positive for SCF. Immunohistochemical analysis of human specimens revealed that the expression frequency of c-kit in epithelial cells was of 5% in benign prostatic hyperplasia, 14% in primary PC, and 40% in PC bone metastases, suggesting an overall trend of increased c-kit expression in clinical PC progression. Stem cell factor expression frequency was more than 80% in all the cases. Our data suggest that the bone microenvironment up-regulates c-kit expression on PC cells, favoring their intraosseous expansion.
5. Incidence and distribution of experimental metastases in mutant mice with defective organ microenvironments (genotypes Sl/Sld and W/Wv). Mice carrying mutations at the Sl (steel) and W (dominant white spotting) loci develop abnormalities on 3 migratory embryonic stem cell populations: hematopoietic stem cells, neural crest-derived melanocytes, and primordial germ cells. Transplantation experiments have indicated that the Sl locus affects the microenvironment where stem cells migrate, proliferate, and differentiate, while the W locus affects the migratory cells themselves. The Sl locus encodes for a multipotent growth factor known as stem cell factor. The W locus encodes the c-kit protein tyrosine kinase receptor whose ligand is the stem cell factor. We have investigated the incidence and organ distribution of experimental metastases after systemic intra-arterial injection of B16-G3.26 melanoma cells into mutant Sl/Sld and W/Wv mice. Both mutant mouse strains had a markedly lower incidence of ovarian metastases when compared with their congenic +/+ mice. In contrast to the rare colonization of the ovaries, Sl/Sld and W/Wv mice developed metastases in the myocardium, kidney, and stomach--anatomic sites that were infrequently or never affected in their congenic nonmutant mice. The only organs in which the average number of metastatic colonies differed between Sl/Sld and W/Wv mice were the bone marrow and kidneys. The average number of colonized bones per mouse in the Sl/Sld group was 5.0 +/- 3.1 (SD), compared with 12.7 +/- 5.3 in the W/Wv group. The average number of metastatic nodules in the kidneys of Sl/Sld mice was 24.6 +/- 9, while W/Wv mice had 15.5 +/- 2.5. Mutant mice with multiple metastatic nodules in the kidneys, heart, and stomach were also found to have forestomach papillomas, an enlarged duodenum, kidney abnormalities, and small body size. The results of this study provide useful information on potential mechanisms of interaction of metastatic cells with their target organs, and suggest that there are additional organ defects associated with the mutations in the Sl and W loci. They also document the importance of mutant mice in **metastasis** research.
6. Arthritis augments breast cancer **metastasis**: role of mast cells and SCF/c-Kit signaling. Breast cancer remains the second leading cause of cancer-related deaths for women in the United States. **Metastasis** is regulated not only by intrinsic genetic changes in malignant cells, but also by the microenvironment, especially those associated with chronic inflammation. We recently reported that mice with autoimmune arthritis have significantly increased incidence of bone and lung **metastasis** and decreased survival associated with breast cancer. In this study, we evaluated the mechanism underlying the increased **metastasis**.We used two mouse models; one that develops spontaneous autoimmune arthritis (SKG mice) injected with metastatic breast cancer cells (4T1), and another that develops spontaneous breast cancer (MMTV-PyV MT mice) injected with type II collagen to induce autoimmune arthritis. Mast cell levels and **metastasis** were monitored.First, we confirmed that breast tumor-bearing arthritic mice have a significantly higher incidence of bone and lung **metastasis** than do their nonarthritic counterparts. Next, we showed increased recruitment of mast cells within the primary tumor of arthritic mice, which facilitates **metastasis**. Next, we report that arthritic mice without any tumors have higher numbers of mast cells in the bones and lungs, which may be the underlying cause for the enhanced lung and bone metastases observed in the arthritic mice. Next, we showed that once the tumor cells populate the metastatic niches (bones and lungs), they further increase the mast cell population within the niche and assist in enhancing **metastasis**. This may primarily be due to the interaction of c-Kit receptor present on mast cells and stem cell factor (SCF, the ligand for ckit) expressed on tumor cells. Finally, we showed that targeting the SCF/cKit interaction with an anti-ckit antibody reduces the differentiation of mast cells and consequently reduces **metastasis**.This is the first report to show that mast cells may play a critical role in remodeling not only the tumor microenvironment but also the metastatic niche to facilitate efficient **metastasis** through SCF/cKit interaction in breast cancer with arthritis.

#### GeneRIFs

1. High c-kit expression is associated with breast cancer **metastasis**.

---

## Fgfr2

- NCBI Gene
- GeneRIF
- Pubmed

| Search Term | Rank | Fields |
| --- | --- | --- |
| [Ff]ilopodia | 1 | PubMed(1) |
| [Mm]igration | 6 | PubMed(43); GeneRIFs(4) |
| ECM | 7 | PubMed(1) |
| [Ee]xtracellular [Mm]atrix | 8 | PubMed(13); Component(1) |
| [Ii]nvasive | 10 | PubMed(4) |
| [Cc]ell [Ii]nvasion | 13 | PubMed(1) |

  
hide/show details for Fgfr2

### [Ff]ilopodia

#### PubMed

1. Imaging **filopodia** dynamics in the mouse blastocyst. During mammalian development, the first cell lineage diversification event occurs in the blastocyst, when the trophectoderm (TE) and the inner cell mass (ICM) become established. Part of the TE (polar) remains in contact with the ICM and differs from the mural TE (mTE) which is separated from the ICM by a cavity known as the blastocoele. The presence of **filopodia** connecting ICM cells with the distant mural TE cells through the blastocoelic fluid was investigated in this work. We describe two types of actin-based cell projections found in freshly dissected and in vitro cultured expanding blastocysts: abundant short **filopodia** projecting into the blastocoelic cavity that present a continuous undulating behavior; and long, thin traversing **filopodia** connecting the mural TE with the ICM. Videomicroscopy analyses revealed the presence of vesicle-like structures moving along traversing **filopodia** and dynamic cytoskeletal rearrangements. These observations, together with immunolocalization of the FGFR2 and the ErbB3 receptors to these cell extensions, suggest that they display signal transduction activity. We propose that traversing **filopodia** are employed by mitotic mTE cells to receive the required signals for cell division after they become distant to the ICM.

### [Mm]igration

#### PubMed

1. FGF10/FGFR2b signaling is essential for cardiac fibroblast development and growth of the myocardium. The epicardium serves as a source of growth factors that regulate myocardial proliferation and as a source of epicardial-derived cells (EPDC), which give rise to interstitial cardiac fibroblasts and perivascular cells. These progenitors populate the compact myocardium to become part of the mature coronary vasculature and fibrous skeleton of the heart. Little is known about the mechanisms that regulate EPDC **migration** into the myocardium or the functions carried out by these cells once they enter the myocardium. However, it has been proposed that cardiac fibroblasts are important for growth of the heart during late gestation and are a source of homeostatic factors in the adult. Here, we identify a myocardial to epicardial fibroblast growth factor (FGF) signal, mediated by FGF10 and FGFR2b, that is essential for movement of cardiac fibroblasts into the compact myocardium. Inactivation of this signaling pathway results in fewer epicardial derived cells within the compact myocardium, decreased myocardial proliferation and a resulting smaller thin-walled heart.
2. Cell lineage in mammalian craniofacial mesenchyme. We have analysed the contributions of neural crest and mesoderm to mammalian craniofacial mesenchyme and its derivatives by cell lineage tracing experiments in mouse embryos, using the permanent genetic markers Wnt1-cre for neural crest and Mesp1-cre for mesoderm, combined with the Rosa26 reporter. At the end of neural crest cell **migration** (E9.5) the two patterns are reciprocal, with a mutual boundary just posterior to the eye. Mesodermal cells expressing endothelial markers (angioblasts) are found not to respect this boundary; they are associated with the migrating neural crest from the 5-somite stage, and by E9.5 they form a pre-endothelial meshwork throughout the cranial mesenchyme. Mesodermal cells of the myogenic lineage also migrate with neural crest cells, as the branchial arches form. By E17.5 the neural crest-mesoderm boundary in the subectodermal mesenchyme becomes out of register with that of the underlying skeletogenic layer, which is between the frontal and parietal bones. At E13.5 the primordia of these bones lie basolateral to the brain, extending towards the vertex of the skull during the following 4-5 days. We used DiI labelling of the bone primordia in ex-utero E13.5 embryos to distinguish between two possibilities for the origin of the frontal and parietal bones: (1) recruitment from adjacent connective tissue or (2) proliferation of the original primordia. The results clearly demonstrated that the bone primordia extend vertically by intrinsic growth, without detectable recruitment of adjacent mesenchymal cells.
3. Fibroblast growth factor signalling and regional specification of the pharyngeal ectoderm. Branchial arch development involves dynamic interactions between neural crest cells as well as ectodermal, endodermal and mesodermal cell populations. Despite their importance and evolutionary conservation, the intercellular interactions guiding the early development of the branchial arches are still poorly understood. We have here studied fibroblast growth factor (FGF) signalling in early pharyngeal development. In mice homozygous for a hypomorphic allele of Fgfr1, neural crest cells migrating from the hindbrain mostly fail to enter the second branchial arch. This defect is non-cell-autonomous suggesting that Fgfr1 provides a permissive environment for neural crest cell **migration**. Here we demonstrate localized down-regulation of the expression of the FGF responsive gene, Sprouty1 in the epithelium covering the presumptive second branchial arch of hypomorphic Fgfr1 mutants. This appears to result in a failure to establish an ectodermal signalling center expressing Fgf3 and Fgf15. We also studied differentiation of the ectoderm in the second branchial arch region. Development of the geniculate placode as well as the VIIth cranial ganglion is affected in Fgfr1 hypomorphs. Our results suggest that Fgfr1 is important for localized signalling in the pharyngeal ectoderm and consequently for normal tissue interactions in the developing second branchial arch.
4. Impaired motor coordination and disrupted cerebellar architecture in Fgfr1 and Fgfr2 double knockout mice. Fibroblast growth factor receptor (FGFR) signaling determines the size of the cerebral cortex by regulating the amplification of radial glial stem cells, and participates in the formation of midline glial structures. We show that Fgfr1 and Fgfr2 double knockouts (FGFR DKO) generated by Cre-mediated recombination driven by the human GFAP promoter (hGFAP) have reduced cerebellar size due to reduced proliferation of radial glia and other glial precursors in late embryonic and neonatal FGFR DKO mice. The proliferation of granule cell progenitors (GCPs) in the EGL was also reduced, leading to reduced granule cell numbers. Furthermore, both inward **migration** of granule cells into the inner granule cell layer (IGL) and outward **migration** of GABA interneurons into the molecular layer (ML) were arrested, disrupting layer and lobular morphology. Purkinje neurons and their dendrites, which were not targeted by Cre-mediated recombination of Fgf receptors, were also misplaced in FGFR DKO mice, possibly as a consequence of altered Bergmann glia orientation or reduced granule cell number. Our findings indicate a dual role for FGFR signaling in cerebellar morphogenesis. The first role is to amplify the number of granule neuron precursors in the external granular layer and glial precursor cells throughout the cerebellum. The second is to establish the correct Bergmann glia morphology, which is crucial for granule cell **migration**. The disrupted cerebellar size and laminar architecture resulting from loss of FGFR signaling impair motor learning and coordination in FGFR DKO mice.
5. FGF receptors 1 and 2 are key regulators of keratinocyte **migration** in vitro and in wounded skin. Efficient wound repair is essential for the maintenance of the integrity of the skin. The repair process is controlled by a variety of growth factors and cytokines, and their abnormal expression or activity can cause healing disorders. Here, we show that wound repair is severely delayed in mice lacking fibroblast growth factor receptors (FGFR) 1 and 2 in keratinocytes. As the underlying mechanism, we identified impaired wound contraction and a delay in re-epithelialization that resulted from impaired keratinocyte **migration** at the wound edge. Scratch wounding and transwell assays demonstrated that FGFR1/2-deficient keratinocytes had a reduced **migration** velocity and impaired directional persistence owing to inefficient formation and turnover of focal adhesions. Underlying this defect, we identified a significant reduction in the expression of major focal adhesion components in the absence of FGFR signaling, resulting in a general migratory deficiency. These results identify FGFs as key regulators of keratinocyte **migration** in wounded skin.
6. Autocrine and paracrine mechanisms regulating primordial germ cell proliferation. Although several mitogens and survival factors have been previously shown to act on primordial germ cells (PGCs) in culture, it is not clear whether they are responsible for controlling proliferation of PGCs in the embryo. We show here that during their migratory phase, PGCs do not express FGF-4, FGF-8, or FGF-17, but these FGFs are expressed by neighboring cells. Thus, any FGF action on migrating PGCs would appear to be through a paracrine mechanism. We found that after entering into the gonads, PGCs start to express FGF-4 and FGF-8. On this basis, we hypothesize that FGF signaling is involved in both a paracrine manner in initiating PGC proliferation during their **migration** and an autocrine manner in sustaining PGC proliferation after their arrival in the gonads. We then studied the role of soluble stem cell factor (SCF), which acts as a survival factor or a mitogen in culture, to determine whether it interacts with FGFs. We found that SCF has a complex effect on PGC proliferation. On one hand, soluble SCF promoted PGC proliferation synergistically with FGF in the absence of membrane-bound SCF. Conversely, soluble SCF inhibited FGF-stimulated proliferation of PGCs in the presence of membrane-bound SCF. We account for these findings in a model involving regulation of PGC proliferation, in which SCF modulates the response to FGFs.
7. Expression of FGF receptors 1, 2, 3 in the embryonic and postnatal mouse brain compared with Pdgfralpha, Olig2 and Plp/dm20: implications for oligodendrocyte development. Fibroblast growth factors (FGF) receptors FgfR1, FgfR2 and FgfR3 are differentially regulated during oligodendrocyte (OL) maturation in vitro: FgfR3 is expressed by OL progenitors whereas FgfR2 is expressed by differentiated OLs [Mol Cell Neurosci 1996;7:263-275], and we have recently shown that FgfR3 is required for the timely differentiation of OLs in vivo [J Neurosci 2003;23:883-894]. Here we have used in situ hybridization to investigate the expression patterns of FgfR1-3 and compare them to the putative OL progenitor markers Olig2, Pdgfralpha and Plp/dm20 as a function of development in vivo, in particular at sites of OL specification, **migration** or differentiation in the mouse forebrain and cerebellum. We show that at early stages FgfR1-3 expression overlaps with that of Olig2 in the embryonic ventricular zone of the lateral and medial ganglionic eminences. Further, a scattered population of cells expressing FgfR3 (but not FgfR1 or FgfR2) in the ventral telencephalon appear to arise from the ventricular zone, and at later stages are found more dorsally in the cortex, in an overall pattern similar to Olig2 and/or Pdgfralpha. Postnatal expression of FgfR2 increases with age, more prominently in specific regions, including the cortical and cerebellar white matter and optic nerve. Thus, the differential expression pattern of FgfR2 and FgfR3 observed in vivo suggests that their expression is developmentally regulated in a manner consistent with the pattern of their expression in culture. These data provide further insights into role of FgfRs in OL development, and they emphasize that these receptors are positioned both spatially and temporally to impact OL generation in vivo.
8. Fibroblast growth factor receptors (FGFRs) and their roles in limb development. Fibroblast growth factor (FGF) receptors constitute a family of four membrane-spanning tyrosine kinases (FGFR1-4) which serve as high-affinity receptors for 17 growth factors (FGF1-17). To study functions of FGF/ FGFR signals in development, mice that carry mutations in each receptor have been created by gene targeting. Analysis of these mutant mice revealed essential functions of FGF receptors in multiple biological processes, including mesoderm induction and patterning, cell growth and **migration**, organ formation and bone growth. In this review we discuss recent work with FGF receptors to illustrate mechanisms, through which the FGF/FGFR signals specify vertebrate limb initiation, outgrowth and patterning.
9. Twist function is required for the morphogenesis of the cephalic neural tube and the differentiation of the cranial neural crest cells in the mouse embryo. Loss of Twist function in the cranial mesenchyme of the mouse embryo causes failure of closure of the cephalic neural tube and malformation of the branchial arches. In the Twist(-/-) embryo, the expression of molecular markers that signify dorsal forebrain tissues is either absent or reduced, but those associated with ventral tissues display expanded domains of expression. Dorsoventral organization of the mid- and hindbrain and the anterior-posterior pattern of the neural tube are not affected. In the Twist(-/-) embryo, neural crest cells stray from the subectodermal migratory path and the late-migrating subpopulation invades the cell-free zone separating streams of cells going to the first and second branchial arches. Cell transplantation studies reveal that Twist activity is required in the cranial mesenchyme for directing the **migration** of the neural crest cells, as well as in the neural crest cells within the first branchial arch to achieve correct localization. Twist is also required for the proper differentiation of the first arch tissues into bone, muscle, and teeth.
10. Growth/differentiation factor 15 promotes EGFR signalling, and regulates proliferation and **migration** in the hippocampus of neonatal and young adult mice. The activation of epidermal growth factor receptor (EGFR) affects multiple aspects of neural precursor behaviour, including proliferation and **migration**. Telencephalic precursors acquire EGF responsiveness and upregulate EGFR expression at late stages of development. The events regulating this process and its significance are still unclear. We here show that in the developing and postnatal hippocampus (HP), growth/differentiation factor (GDF) 15 and EGFR are co-expressed in primitive precursors as well as in more differentiated cells. We also provide evidence that GDF15 promotes responsiveness to EGF and EGFR expression in hippocampal precursors through a mechanism that requires active CXC chemokine receptor (CXCR) 4. Besides EGFR expression, GDF15 ablation also leads to decreased proliferation and **migration**. In particular, lack of GDF15 impairs both processes in the cornu ammonis (CA) 1 and only proliferation in the dentate gyrus (DG). Importantly, **migration** and proliferation in the mutant HP were altered only perinatally, when EGFR expression was also affected. These data suggest that GDF15 regulates **migration** and proliferation by promoting EGFR signalling in the perinatal HP and represent a first description of a functional role for GDF15 in the developing telencephalon.
11. Fibroblast growth factor 10 alters the balance between goblet and Paneth cells in the adult mouse small intestine. Intestinal epithelial cell renewal relies on the right balance of epithelial cell **migration**, proliferation, differentiation, and apoptosis. Intestinal epithelial cells consist of absorptive and secretory lineage. The latter is comprised of goblet, Paneth, and enteroendocrine cells. Fibroblast growth factor 10 (FGF10) plays a central role in epithelial cell proliferation, survival, and differentiation in several organs. The expression pattern of FGF10 and its receptors in both human and mouse intestine and their role in small intestine have yet to be investigated. First, we analyzed the expression of FGF10, FGFR1, and FGFR2, in the human ileum and throughout the adult mouse small intestine. We found that FGF10, FGFR1b, and FGFR2b are expressed in the human ileum as well as in the mouse small intestine. We then used transgenic mouse models to overexpress Fgf10 and a soluble form of Fgfr2b, to study the impact of gain or loss of Fgf signaling in the adult small intestine. We demonstrated that overexpression of Fgf10 in vivo and in vitro induces goblet cell differentiation while decreasing Paneth cells. Moreover, FGF10 decreases stem cell markers such as Lgr5, Lrig1, Hopx, Ascl2, and Sox9. FGF10 inhibited Hes1 expression in vitro, suggesting that FGF10 induces goblet cell differentiation likely through the inhibition of Notch signaling. Interestingly, Fgf10 overexpression for 3 days in vivo and in vitro increased the number of Mmp7/Muc2 double-positive cells, suggesting that goblet cells replace Paneth cells. Further studies are needed to determine the mechanism by which Fgf10 alters cell differentiation in the small intestine.
12. A novel allele of Alx4 results in reduced Fgf10 expression and failure of eyelid fusion in mice. Normal fusion of developing eyelids requires coordination of inductive signals from the eyelid mesenchyme with **migration** of the periderm cell layer and constriction of the eyelids across the eye. Failure of this process results in an eyelids open at birth (EOB) phenotype in mice. We have identified a novel spontaneous allele of Alx4 that displays EOB, in addition to polydactyly and cranial malformations. Alx4 is expressed in the eyelid mesenchyme prior to and during eyelid fusion in a domain overlapping the expression of genes that also play a role in normal eyelid development. We show that Alx4 mutant mice have reduced expression of Fgf10, a key factor expressed in the mesenchyme that is required for initiation of eyelid fusion by the periderm. This is accompanied by a reduced number of periderm cells expressing phosphorylated c-Jun, consistent with the incomplete ablation of Fgf10 expression. Together, these data demonstrate that eyelid fusion in mice requires the expression of Alx4, accompanied by the loss of normal expression of essential components of the eyelid fusion pathway.
13. Fgfr1 regulates patterning of the pharyngeal region. Development of the pharyngeal region depends on the interaction and integration of different cell populations, including surface ectoderm, foregut endoderm, paraxial mesoderm, and neural crest. Mice homozygous for a hypomorphic allele of Fgfr1 have craniofacial defects, some of which appeared to result from a failure in the early development of the second branchial arch. A stream of neural crest cells was found to originate from the rhombomere 4 region and migrate toward the second branchial arch in the mutants. Neural crest cells mostly failed to enter the second arch, however, but accumulated in a region proximal to it. Both rescue of the hypomorphic Fgfr1 allele and inactivation of a conditional Fgfr1 allele specifically in neural crest cells indicated that Fgfr1 regulates the entry of neural crest cells into the second branchial arch non-cell-autonomously. Gene expression in the pharyngeal ectoderm overlying the developing second branchial arch was affected in the hypomorphic Fgfr1 mutants at a stage prior to neural crest entry. Our results indicate that Fgfr1 patterns the pharyngeal region to create a permissive environment for neural crest cell **migration**.
14. FRS2 alpha 2F/2F mice lack carotid body and exhibit abnormalities of the superior cervical sympathetic ganglion and carotid sinus nerve. The docking protein FRS2 alpha is an important mediator of fibroblast growth factor (FGF)-induced signal transduction, and functions by linking FGF receptors (FGFRs) to a variety of intracellular signaling pathways. We show that the carotid body is absent in FRS2 alpha(2F/2F) mice, in which the Shp2-binding sites of FRS2 alpha are disrupted. We also show that the carotid body rudiment is not formed in the wall of the third arch artery in mutant embryos. In wild-type mice, the superior cervical ganglion of the sympathetic trunk connects to the carotid body in the carotid bifurcation region, and extends thick nerve bundles into the carotid body. In FRS2 alpha(2F/2F) mice, the superior cervical ganglion was present in the lower cervical region as an elongated feature, but failed to undergo cranio-ventral **migration**. In addition, few neuronal processes extended from the ganglion into the carotid bifurcation region. The number of carotid sinus nerve fibers that reached the carotid bifurcation region was markedly decreased, and baroreceptor fibers belonging to the glossopharyngeal nerve were absent from the basal part of the internal carotid artery in FRS2 alpha(2F/2F) mutant mice. In some of the mutant mice (5 out of 14), baroreceptors and some glomus cells were distributed in the wall of the common carotid artery, onto which the sympathetic ganglion abutted. We propose that the sympathetic ganglion provides glomus cell precursors into the third arch artery derivative in the presence of sensory fibers of the glossopharyngeal nerve.
15. Expression of the fibroblast growth factor family and their receptor family genes during mouse brain development. The fibroblast growth factor (FGF) family is composed of nine members and four genes encode protein tyrosine kinase receptors for them. To gain insight into the involvement of FGFs and their receptors in the development of nervous system, their expression in brains of perinatal and adult mice was examined by semi-quantitative reverse transcription-linked polymerase chain reactions and in situ hybridization. Although all the genes, with the exception of FGF-4, were found to be expressed, FGF-3, FGF-6, FGF-7 and FGF-8 genes demonstrated higher expression in the late embryonic stages than in postnatal stages, suggesting that these members are involved in the late stages of brain development. In contrast, expression of FGF-1 and FGF-5 increased after birth. Interestingly, FGF-6 expression in perinatal mice was restricted to the central nervous system and skeltal muscles, with intense signals in the developing cerebrum in embryos but in cerebellum in 5-day-old neonates. Furthermore, FGF-receptor (FGFR)-4, a cognate receptor for FGF-6, demonstrated similar spatiotemporal expression, suggesting that FGF-6 and FGFR-4 plays significant roles in the maturation of nervous system as a ligand-receptor system. The results indicate that individual member of the fibroblast growth factor and their receptor family are expressed either sequentially or simultaneously in brain development, strongly suggesting their involvement in the regulation of a variety of developmental processes of brain, i.e., proliferation and **migration** of neuronal progenitor cells, neuron and glia differentiation, neurite extensions, and synapse formations.
16. Loss of mitogen-activated protein kinase kinase kinase 4 (MAP3K4) reveals a requirement for MAPK signalling in mouse sex determination. Sex determination in mammals is controlled by the presence or absence of the Y-linked gene SRY. In the developing male (XY) gonad, sex-determining region of the Y (SRY) protein acts to up-regulate expression of the related gene, SOX9, a transcriptional regulator that in turn initiates a downstream pathway of testis development, whilst also suppressing ovary development. Despite the requirement for a number of transcription factors and secreted signalling molecules in sex determination, intracellular signalling components functioning in this process have not been defined. Here we report a role for the phylogenetically ancient mitogen-activated protein kinase (MAPK) signalling pathway in mouse sex determination. Using a forward genetic screen, we identified the recessive boygirl (byg) mutation. On the C57BL/6J background, embryos homozygous for byg exhibit consistent XY gonadal sex reversal. The byg mutation is an A to T transversion causing a premature stop codon in the gene encoding MAP3K4 (also known as MEKK4), a mitogen-activated protein kinase kinase kinase. Analysis of XY byg/byg gonads at 11.5 d post coitum reveals a growth deficit and a failure to support mesonephric cell **migration**, both early cellular processes normally associated with testis development. Expression analysis of mutant XY gonads at the same stage also reveals a dramatic reduction in Sox9 and, crucially, Sry at the transcript and protein levels. Moreover, we describe experiments showing the presence of activated MKK4, a direct target of MAP3K4, and activated p38 in the coelomic region of the XY gonad at 11.5 d post coitum, establishing a link between MAPK signalling in proliferating gonadal somatic cells and regulation of Sry expression. Finally, we provide evidence that haploinsufficiency for Map3k4 accounts for T-associated sex reversal (Tas). These data demonstrate that MAP3K4-dependent signalling events are required for normal expression of Sry during testis development, and create a novel entry point into the molecular and cellular mechanisms underlying sex determination in mice and disorders of sexual development in humans.
17. Impaired cerebral cortex development and blood pressure regulation in FGF-2-deficient mice. Fibroblast growth factor-2 (FGF-2) has been implicated in various signaling processes which control embryonic growth and differentiation, adult physiology and pathology. To analyze the in vivo functions of this signaling molecule, the FGF-2 gene was inactivated by homologous recombination in mouse embryonic stem cells. FGF-2-deficient mice are viable, but display cerebral cortex defects at birth. Bromodeoxyuridine pulse labeling of embryos showed that proliferation of neuronal progenitors is normal, whereas a fraction of them fail to colonize their target layers in the cerebral cortex. A corresponding reduction in parvalbumin-positive neurons is observed in adult cortical layers. Neuronal defects are not limited to the cerebral cortex, as ectopic parvalbumin-positive neurons are present in the hippocampal commissure and neuronal deficiencies are observed in the cervical spinal cord. Physiological studies showed that FGF-2-deficient adult mice are hypotensive. They respond normally to angiotensin II-induced hypertension, whereas neural regulation of blood pressure by the baroreceptor reflex is impaired. The present genetic study establishes that FGF-2 participates in controlling fates, **migration** and differentiation of neuronal cells, whereas it is not essential for their proliferation. The observed autonomic dysfunction in FGF-2-deficient adult mice uncovers more general roles in neural development and function.
18. Midline radial glia translocation and corpus callosum formation require FGF signaling. Midline astroglia in the cerebral cortex develop earlier than other astrocytes through mechanisms that are still unknown. We show that radial glia in dorsomedial cortex retract their apical endfeet at midneurogenesis and translocate to the overlaying pia, forming the indusium griseum. These cells require the fibroblast growth factor receptor 1 (Fgfr1) gene for their precocious somal translocation to the dorsal midline, as demonstrated by inactivating the Fgfr1 gene in radial glial cells and by RNAi knockdown of Fgfr1 in vivo. Dysfunctional astroglial **migration** underlies the callosal dysgenesis in conditional Fgfr1 knockout mice, suggesting that precise targeting of astroglia to the cortex has unexpected roles in axon guidance. FGF signaling is sufficient to induce somal translocation of radial glial cells throughout the cortex; furthermore, the targeting of astroglia to dorsolateral cortex requires FGFr2 signaling after neurogenesis. Hence, FGFs have an important role in the transition from radial glia to astrocytes by stimulating somal translocation of radial glial cells.
19. The roles of FGF signaling in germ cell **migration** in the mouse. Fibroblast growth factor (FGF) signaling is thought to play a role in germ cell behavior. FGF2 has been reported to be a mitogen for primordial germ cells in vitro, whilst combinations of FGF2, steel factor and LIF cause cultured germ cells to transform into permanent lines of pluripotent cells resembling ES cells. However, the actual function of FGF signaling on the migrating germ cells in vivo is unknown. We show, by RT-PCR analysis of cDNA from purified E10.5 germ cells, that germ cells express two FGF receptors: Fgfr1-IIIc and Fgfr2-IIIb. Second, we show that FGF-mediated activation of the MAP kinase pathway occurs in germ cells during their **migration**, and thus they are potentially direct targets of FGF signaling. Third, we use cultured embryo slices in simple gain-of-function experiments, using FGF ligands, to show that FGF2, a ligand for FGFR1-IIIc, affects motility, whereas FGF7, a ligand for FGFR2-IIIb, affects germ cell numbers. Loss of function, using a specific inhibitor of FGF signaling, causes increased apoptosis and inhibition of cell shape change in the migrating germ cells. Lastly, we confirm in vivo the effects seen in slice cultures in vitro, by examining germ cell positions and numbers in embryos carrying a loss-of-function allele of FGFR2-IIIb. In FGFR2-IIIb(-/-) embryos, germ cell **migration** is unaffected, but the numbers of germ cells are significantly reduced. These data show that a major role of FGF signaling through FGFR2-IIIb is to control germ cell numbers. The data do not discriminate between direct and indirect effects of FGF signaling on germ cells, and both may be involved.
20. Laminar patterning in the developing neocortex by temporally coordinated fibroblast growth factor signaling. Laminar organization, a fundamental neural architecture in the CNS, is a prominent feature of the neocortex, where the cortical neurons in spatially distinct layers are generated from the common progenitors in a temporally distinct manner during development. Despite many advances in the characterization of the molecular mechanisms of the radial **migration** of cortical neurons, the way in which the early-late temporal sequence of cortical neuron generation is linked with the deep-superficial spatial sequence of cell body positioning remains obscure. Using in vivo electroporation-mediated gene transfer, we show here that the activities mediated by fibroblast growth factor receptors (FGFRs) in cortical progenitors are critical for conferring proper migratory properties on nascent neuronal progeny. Furthermore, we provide supportive evidence that Pea3 subfamily members of Ets (Pea3-Ets) transcription factors mediate the activities of FGFR at the mid to late phase of neocortical development. In addition, using FGF18 knock-out mice, we demonstrate that FGF18 expressed by early-generated cortical neurons in the cortical plate is critical for the expression of Pea3-Ets transcription factors and that FGF18 is sufficient to induce their expressions. Our results thus imply that a feedback mechanism mediated by FGF signaling is involved in setting up the proper laminar positioning of cortical neurons; FGF18 derived from early-generated cortical neurons acts on the cortical progenitors expressing FGFRs and induces the expression of Pea3-Ets transcription factors that, in turn, confer proper migratory behaviors on nascent cortical progeny during the mid to late stages of neocortical development.
21. Neuron-derived FGF9 is essential for scaffold formation of Bergmann radial fibers and **migration** of granule neurons in the cerebellum. Although fibroblast growth factor 9 (FGF9) is widely expressed in the central nervous system (CNS), the function of FGF9 in neural development remains undefined. To address this question, we deleted the Fgf9 gene specifically in the neural tube and demonstrated that FGF9 plays a key role in the postnatal **migration** of cerebellar granule neurons. Fgf9-null mice showed severe ataxia associated with disrupted Bergmann fiber scaffold formation, impaired granule neuron **migration**, and upset Purkinje cell maturation. Ex vivo cultured wildtype or Fgf9-null glia displayed a stellate morphology. Coculture with wildtype neurons, but not Fgf9-deficient neurons, or treating with FGF1 or FGF9 induced the cells to adopt a radial glial morphology. In situ hybridization showed that Fgf9 was expressed in neurons and immunostaining revealed that FGF9 was broadly distributed in both neurons and Bergmann glial radial fibers. Genetic analyses revealed that the FGF9 activities in cerebellar development are primarily transduced by FGF receptors 1 and 2. Furthermore, inhibition of the MAP kinase pathway, but not the PI3K/AKT pathway, abrogated the FGF activity to induce glial morphological changes, suggesting that the activity is mediated by the MAP kinase pathway. This work demonstrates that granule neurons secrete FGF9 to control formation of the Bergmann fiber scaffold, which in turn, guides their own inward **migration** and maturation of Purkinje cells.
22. Cubilin, a high affinity receptor for fibroblast growth factor 8, is required for cell survival in the developing vertebrate head. Cubilin (Cubn) is a multiligand endocytic receptor critical for the intestinal absorption of vitamin B12 and renal protein reabsorption. During mouse development, Cubn is expressed in both embryonic and extra-embryonic tissues, and Cubn gene inactivation results in early embryo lethality most likely due to the impairment of the function of extra-embryonic Cubn. Here, we focus on the developmental role of Cubn expressed in the embryonic head. We report that Cubn is a novel, interspecies-conserved Fgf receptor. Epiblast-specific inactivation of Cubn in the mouse embryo as well as Cubn silencing in the anterior head of frog or the cephalic neural crest of chick embryos show that Cubn is required during early somite stages to convey survival signals in the developing vertebrate head. Surface plasmon resonance analysis reveals that fibroblast growth factor 8 (Fgf8), a key mediator of cell survival, **migration**, proliferation, and patterning in the developing head, is a high affinity ligand for Cubn. Cell uptake studies show that binding to Cubn is necessary for the phosphorylation of the Fgf signaling mediators MAPK and Smad1. Although Cubn may not form stable ternary complexes with Fgf receptors (FgfRs), it acts together with and/or is necessary for optimal FgfR activity. We propose that plasma membrane binding of Fgf8, and most likely of the Fgf8 family members Fgf17 and Fgf18, to Cubn improves Fgf ligand endocytosis and availability to FgfRs, thus modulating Fgf signaling activity.
23. FGF/FGFR2 signaling regulates the generation and correct positioning of Bergmann glia cells in the developing mouse cerebellum. The normal cellular organization and layering of the vertebrate cerebellum is established during embryonic and early postnatal development by the interplay of a complex array of genetic and signaling pathways. Disruption of these processes and of the proper layering of the cerebellum usually leads to ataxic behaviors. Here, we analyzed the relative contribution of Fibroblast growth factor receptor 2 (FGFR2)-mediated signaling to cerebellar development in conditional Fgfr2 single mutant mice. We show that during embryonic mouse development, Fgfr2 expression is higher in the anterior cerebellar primordium and excluded from the proliferative ventricular neuroepithelium. Consistent with this finding, conditional Fgfr2 single mutant mice display the most prominent defects in the anterior lobules of the adult cerebellum. In this context, FGFR2-mediated signaling is required for the proper generation of Bergmann glia cells and the correct positioning of these cells within the Purkinje cell layer, and for cell survival in the developing cerebellar primordium. Using cerebellar microexplant cultures treated with an FGFR agonist (FGF9) or antagonist (SU5402), we also show that FGF9/FGFR-mediated signaling inhibits the outward **migration** of radial glia and Bergmann glia precursors and cells, and might thus act as a positioning cue for these cells. Altogether, our findings reveal the specific functions of the FGFR2-mediated signaling pathway in the generation and positioning of Bergmann glia cells during cerebellar development in the mouse.
24. FGF/FGFR2 signaling regulates the generation and correct positioning of Bergmann glia cells in the developing mouse cerebellum. The normal cellular organization and layering of the vertebrate cerebellum is established during embryonic and early postnatal development by the interplay of a complex array of genetic and signaling pathways. Disruption of these processes and of the proper layering of the cerebellum usually leads to ataxic behaviors. Here, we analyzed the relative contribution of Fibroblast growth factor receptor 2 (FGFR2)-mediated signaling to cerebellar development in conditional Fgfr2 single mutant mice. We show that during embryonic mouse development, Fgfr2 expression is higher in the anterior cerebellar primordium and excluded from the proliferative ventricular neuroepithelium. Consistent with this finding, conditional Fgfr2 single mutant mice display the most prominent defects in the anterior lobules of the adult cerebellum. In this context, FGFR2-mediated signaling is required for the proper generation of Bergmann glia cells and the correct positioning of these cells within the Purkinje cell layer, and for cell survival in the developing cerebellar primordium. Using cerebellar microexplant cultures treated with an FGFR agonist (FGF9) or antagonist (SU5402), we also show that FGF9/FGFR-mediated signaling inhibits the outward **migration** of radial glia and Bergmann glia precursors and cells, and might thus act as a positioning cue for these cells. Altogether, our findings reveal the specific functions of the FGFR2-mediated signaling pathway in the generation and positioning of Bergmann glia cells during cerebellar development in the mouse.
25. FGF receptors 1 and 2 are key regulators of keratinocyte **migration** in vitro and in wounded skin. Efficient wound repair is essential for the maintenance of the integrity of the skin. The repair process is controlled by a variety of growth factors and cytokines, and their abnormal expression or activity can cause healing disorders. Here, we show that wound repair is severely delayed in mice lacking fibroblast growth factor receptors (FGFR) 1 and 2 in keratinocytes. As the underlying mechanism, we identified impaired wound contraction and a delay in re-epithelialization that resulted from impaired keratinocyte **migration** at the wound edge. Scratch wounding and transwell assays demonstrated that FGFR1/2-deficient keratinocytes had a reduced **migration** velocity and impaired directional persistence owing to inefficient formation and turnover of focal adhesions. Underlying this defect, we identified a significant reduction in the expression of major focal adhesion components in the absence of FGFR signaling, resulting in a general migratory deficiency. These results identify FGFs as key regulators of keratinocyte **migration** in wounded skin.
26. Impaired motor coordination and disrupted cerebellar architecture in Fgfr1 and Fgfr2 double knockout mice. Fibroblast growth factor receptor (FGFR) signaling determines the size of the cerebral cortex by regulating the amplification of radial glial stem cells, and participates in the formation of midline glial structures. We show that Fgfr1 and Fgfr2 double knockouts (FGFR DKO) generated by Cre-mediated recombination driven by the human GFAP promoter (hGFAP) have reduced cerebellar size due to reduced proliferation of radial glia and other glial precursors in late embryonic and neonatal FGFR DKO mice. The proliferation of granule cell progenitors (GCPs) in the EGL was also reduced, leading to reduced granule cell numbers. Furthermore, both inward **migration** of granule cells into the inner granule cell layer (IGL) and outward **migration** of GABA interneurons into the molecular layer (ML) were arrested, disrupting layer and lobular morphology. Purkinje neurons and their dendrites, which were not targeted by Cre-mediated recombination of Fgf receptors, were also misplaced in FGFR DKO mice, possibly as a consequence of altered Bergmann glia orientation or reduced granule cell number. Our findings indicate a dual role for FGFR signaling in cerebellar morphogenesis. The first role is to amplify the number of granule neuron precursors in the external granular layer and glial precursor cells throughout the cerebellum. The second is to establish the correct Bergmann glia morphology, which is crucial for granule cell **migration**. The disrupted cerebellar size and laminar architecture resulting from loss of FGFR signaling impair motor learning and coordination in FGFR DKO mice.
27. FGF10/FGFR2b signaling is essential for cardiac fibroblast development and growth of the myocardium. The epicardium serves as a source of growth factors that regulate myocardial proliferation and as a source of epicardial-derived cells (EPDC), which give rise to interstitial cardiac fibroblasts and perivascular cells. These progenitors populate the compact myocardium to become part of the mature coronary vasculature and fibrous skeleton of the heart. Little is known about the mechanisms that regulate EPDC **migration** into the myocardium or the functions carried out by these cells once they enter the myocardium. However, it has been proposed that cardiac fibroblasts are important for growth of the heart during late gestation and are a source of homeostatic factors in the adult. Here, we identify a myocardial to epicardial fibroblast growth factor (FGF) signal, mediated by FGF10 and FGFR2b, that is essential for movement of cardiac fibroblasts into the compact myocardium. Inactivation of this signaling pathway results in fewer epicardial derived cells within the compact myocardium, decreased myocardial proliferation and a resulting smaller thin-walled heart.
28. The roles of FGF signaling in germ cell **migration** in the mouse. Fibroblast growth factor (FGF) signaling is thought to play a role in germ cell behavior. FGF2 has been reported to be a mitogen for primordial germ cells in vitro, whilst combinations of FGF2, steel factor and LIF cause cultured germ cells to transform into permanent lines of pluripotent cells resembling ES cells. However, the actual function of FGF signaling on the migrating germ cells in vivo is unknown. We show, by RT-PCR analysis of cDNA from purified E10.5 germ cells, that germ cells express two FGF receptors: Fgfr1-IIIc and Fgfr2-IIIb. Second, we show that FGF-mediated activation of the MAP kinase pathway occurs in germ cells during their **migration**, and thus they are potentially direct targets of FGF signaling. Third, we use cultured embryo slices in simple gain-of-function experiments, using FGF ligands, to show that FGF2, a ligand for FGFR1-IIIc, affects motility, whereas FGF7, a ligand for FGFR2-IIIb, affects germ cell numbers. Loss of function, using a specific inhibitor of FGF signaling, causes increased apoptosis and inhibition of cell shape change in the migrating germ cells. Lastly, we confirm in vivo the effects seen in slice cultures in vitro, by examining germ cell positions and numbers in embryos carrying a loss-of-function allele of FGFR2-IIIb. In FGFR2-IIIb(-/-) embryos, germ cell **migration** is unaffected, but the numbers of germ cells are significantly reduced. These data show that a major role of FGF signaling through FGFR2-IIIb is to control germ cell numbers. The data do not discriminate between direct and indirect effects of FGF signaling on germ cells, and both may be involved.
29. Mesodermal expression of Fgfr2S252W is necessary and sufficient to induce craniosynostosis in a mouse model of Apert syndrome. Coordinated growth of the skull and brain are vital to normal human development. Craniosynostosis, the premature fusion of the calvarial bones of the skull, is a relatively common pediatric disease, occurring in 1 in 2500 births, and requires significant surgical management, especially in syndromic cases. Syndromic craniosynostosis is caused by a variety of genetic lesions, most commonly by activating mutations of FGFRs 1-3, and inactivating mutations of TWIST1. In a mouse model of TWIST1 haploinsufficiency, cell mixing between the neural crest-derived frontal bone and mesoderm-derived parietal bone accompanies coronal suture fusion during embryonic development. However, the relevance of lineage mixing in craniosynostosis induced by activating FGFR mutations is unknown. Here, we demonstrate a novel mechanism of suture fusion in the Apert Fgfr2(S252W) mouse model. Using Cre/lox recombination we simultaneously induce expression of Fgfr2(S252W) and β-galactosidase in either the neural crest or mesoderm of the skull. We show that mutation of the mesoderm alone is necessary and sufficient to cause craniosynostosis, while mutation of the neural crest is neither. The lineage border is not disrupted by aberrant cell **migratio**n during fusion. Instead, the suture mesenchyme itself remains intact and is induced to undergo osteogenesis. We eliminate postulated roles for dura mater or skull base changes in craniosynostosis. The viability of conditionally mutant mice also allows post-natal assessment of other aspects of Apert syndrome.
30. A dual role of FGF10 in proliferation and coordinated **migration** of epithelial leading edge cells during mouse eyelid development. The development of the eyelid requires coordinated cellular processes of proliferation, cell shape changes, **migration** and cell death. Mutant mice deficient in the fibroblast growth factor 10 (Fgf10) gene exhibit open-eyelids at birth. To elucidate the roles of FGF10 during eyelid formation, we examined the expression pattern of Fgf10 during eyelid formation and the phenotype of Fgf10-null eyelids in detail. Fgf10 is expressed by mesenchymal cells just beneath the protruding epidermal cells of the nascent eyelid. However, Fgf10-null epithelial cells running though the eyelid groove do not exhibit typical cuboid shape or sufficient proliferation. Furthermore, peridermal clumps are not maintained on the eyelid leading edge, and epithelial extension does not occur. At the cellular level, the accumulation of actin fibers is not observed in the mutant epithelial leading edge. The expression of activin/inhibin betaB (ActbetaB/Inhbb) and transforming growth factor alpha (Tgfa), previously reported to be crucial for eyelid development, is down-regulated in the mutant leading edge, while the onset of sonic hedgehog (Shh) expression is delayed on the mutant eyelid margin. Explant cultures of mouse eyelid primordia shows that the open-eyelid phenotype of the mutant is reduced by exogenous FGF10 protein, and that the expression of ActbetaB and Tgfa is ectopically induced in the thickened eyelid epithelium by the FGF10 protein. These results indicate a dual role of FGF10 in mouse eyelid development, for both proliferation and coordinated **migration** of eyelid epithelial cells by reorganization of the cytoskeleton, through the regulation of activin, TGFalpha and SHH signaling.
31. Signals via FGF receptor 2 regulate **migration** of endothelial cells. Fibroblast growth factors (FGFs) stimulate angiogenesis, of which signals are transduced via FGF receptor (FGFR) tyrosine kinases. Although FGFR1 is a major receptor in endothelial cells, FGFR2 is frequently detectable in endothelial cells. We have previously demonstrated that the intracellular domain of FGFR1 sufficiently transduced signals leading to proliferation, **migration**, urokinase secretion, and tube formation. However, little is known about the roles of signaling via FGFR2 alone in endothelial cells. Murine brain capillary endothelial cells, denoted IBE cells, express small amounts of IIIc FGFR2, which is not activated by keratinocyte growth factor (KGF). We then transfected the IIIb FGFR2 in these cells. Three stable cell lines expressing IIIb FGFR2 demonstrated chemotaxis toward KGF, but never proliferated, secreted urokinase, or formed tube-like structure by KGF treatment. Weak but sustained activation of mitogen-activated protein kinase (MAPK) was observed in these cells. Chemotaxis toward KGF was significantly attenuated by treatment with PD98059. This is the first demonstration that signaling solely via FGFR2 in endothelial cells only contributes to motility through MAPK.
32. Fkbp52 regulates androgen receptor transactivation activity and male urethra morphogenesis. Hypospadias is a common birth defect in humans, yet its etiology and pattern of onset are largely unknown. Recent studies have shown that male mice with targeted ablation of FK506-binding protein-52 (Fkbp52) develop hypospadias, most likely due to actions of Fkbp52 as a molecular co-chaperone of the androgen receptor (AR). Here, we further dissect the developmental and molecular mechanisms that underlie hypospadias in Fkbp52-deficient mice. Scanning electron microscopy revealed a defect in the elevation of prepucial swelling that led to the onset of the ventral penile cleft. Interestingly, expression of Fkbp52 was highest in the ventral aspect of the developing penis that undergoes fusion of the urethral epithelium. Although in situ hybridization and immunohistochemical analyses suggested that Fkbp52 mutants had a normal urethral epithelium signaling center and epithelial differentiation, a reduced apoptotic cell index at ventral epithelial cells at the site of fusion and a defect of genital mesenchymal cell **migration** were observed. Supplementation of gestating females with excess testosterone partially rescued the hypospadic phenotype in Fkbp52 mutant males, showing that loss of Fkbp52 desensitizes AR to hormonal activation. Direct measurement of AR activity was performed in mouse embryonic fibroblast cells treated with dihydrotestosterone or synthetic agonist R1881. Reduced AR activity at genes controlling sexual dimorphism and cell growth was found in Fkbp52-deficient mouse embryonic fibroblast cells. However, chromatin immunoprecipitation analysis revealed normal occupancy of AR at gene promoters, suggesting that Fkbp52 exerts downstream effects on the transactivation function of AR. Taken together, our data show Fkbp52 to be an important molecular regulator in the androgen-mediated pathway of urethra morphogenesis.
33. Expression of signaling components in embryonic eyelid epithelium. Closure of an epithelium opening is a critical morphogenetic event for development. An excellent example for this process is the transient closure of embryonic eyelid. Eyelid closure requires shape change and **migration** of epithelial cells at the tip of the developing eyelids, and is dictated by numerous signaling pathways. Here we evaluated gene expression in epithelial cells isolated from the tip (leading edge, LE) and inner surface epithelium (IE) of the eyelid from E15.5 mouse fetuses by laser capture microdissection (LCM). We showed that the LE and IE cells are different at E15.5, such that IE had higher expression of muscle specific genes, while LE acquired epithelium identities. Despite their distinct destinies, these cells were overall similar in expression of signaling components for the "eyelid closure pathways". However, while the LE cells had more abundant expression of Fgfr2, Erbb2, Shh, Ptch1 and 2, Smo and Gli2, and Jag1 and Notch1, the IE cells had more abundant expression of Bmp5 and Bmpr1a. In addition, the LE cells had more abundant expression of adenomatosis polyposis coli down-regulated 1 (Apcdd1), but the IE cells had high expression of Dkk2. Our results suggest that the functionally distinct LE and IE cells have also differential expression of signaling molecules that may contribute to the cell-specific responses to morphogenetic signals. The expression pattern suggests that the EGF, Shh and NOTCH pathways are preferentially active in LE cells, the BMP pathways are effective in IE cells, and the Wnt pathway may be repressed in LE and IE cells via different mechanisms.
34. Signaling from fibroblast growth factor receptor 2 in immature hematopoietic cells facilitates donor hematopoiesis after intra-bone marrow-bone marrow transplantation. Fibroblast growth factor (FGF) and FGF receptor (FGFR) are expressed in various cells including endothelial progenitor cells and hematopoietic cells. The interaction between FGF and FGFR is associated with the proliferation, **migration**, and survival of these cells. In this report, we examined the effects of FGFR2 signaling on hematopoiesis in immature hematopoietic cells, using mutant mice in which a constitutively active form of FGFR2 mutant was caused to be overexpressed by the Tie2 promoter (FGFR2 Tg mice). Under normal conditions, hematopoiesis of FGFR2 Tg mice and wild type (Wt) mice do not differ significantly, except for the weight and cell numbers of the thymus. However, the c-kit(+)Sca-1(+)lineage⁻ bone marrow cells (BMCs) of FGFR2 Tg mice facilitate the formation of colony-forming units of culture. When these BMCs were transplanted into the recipient bone marrow (intra-bone marrow-bone marrow transplantation), there was better reconstitution of donor hematopoietic cells. In the in vitro experiment, the c-kit(+)Sca-1(+)lineage⁻ BMCs from FGFR2 Tg mice showed fewer apoptotic cells than those from Wt mice. These results suggest that the antiapoptotic effect of FGFR2 signaling facilitates the hematopoiesis of FGFR2 Tg mice.
35. Intrarenal urothelium proliferation: an unexpected early event following ischemic injury. Identification of renal cell progenitors and recognition of the events contributing to cell regeneration following ischemia-reperfusion injury (IRI) are a major challenge. In a mouse model of unilateral renal IRI, we demonstrated that the first cells to proliferate within injured kidneys were urothelial cells expressing the progenitor cell marker cytokeratin 14. A systematic cutting of the injured kidney revealed that these urothelial cells were located in the deep cortex at the corticomedullary junction in the vicinity of lobar vessels. Contrary to multilayered bladder urothelium, these intrarenal urothelial cells located in the upper part of the medulla constitute a monolayered barrier and express among uroplakins only uroplakin III. However, like bladder progenitors, intrarenal urothelial cells proliferated through a FGF receptor-2 (FGFR2)-mediated process. They strongly expressed FGFR2 and proliferated in vivo after recombinant FGF7 administration to control mice. In addition, IRI led to FGFR phosphorylation together with the selective upregulation of FGF7 and FGF2. Conversely, by day 2 following IRI, renal urothelial cell proliferation was significantly inhibited by FGFR2 antisense oligonucleotide administration into an intrarenal urinary space. Of notice, no significant **migration** of these early dividing urothelial cells was detected in the cortex within 7 days following IRI. Thus our data show that following IRI, proliferation of urothelial cells is mediated by the FGFR2 pathway and precedes tubular cell proliferation, indicating a particular sensitivity of this structure to changes caused by the ischemic process.
36. Keratinocyte growth factor. Keratinocyte growth factor (KGF) is a member of the heparin-binding fibroblast growth factor family (FGF-7) with a distinctive pattern of target-cell specificity. Studies performed in cell culture suggested that KGF was mitogenically active only on epithelial cells, albeit from a variety of tissues. In contrast, KGF was produced solely by cells of mesenchymal origin, leading to the hypothesis that it might function as a paracrine mediator of mesenchymal-epithelial communication. Biochemical analysis and molecular cloning established that the KGF receptor (KGFR) was a tyrosine kinase isoform encoded by the fgfr-2 gene. Many detailed investigations of KGF and KGFR expression in whole tissue and cell lines largely substantiated the pattern initially perceived in vitro of mesenchymal and epithelial distribution, respectively. Moreover, functional assays in organ culture and in vivo and studies of KGF regulation by sex steroid hormones reinforced the idea that KGF acts predominantly on epithelial cells to elicit a variety of responses including proliferation, **migration** and morphogenesis.
37. Flrt2 and Flrt3 have overlapping and non-overlapping expression during craniofacial development. Craniofacial morphogenesis is a complex multi-step process that involves numerous biological processes to coordinate the growth, proliferation, **migration**, and subsequent differentiation of the cranial neural crest cells. Members of the Fibronectin Leucine-Rich Transmembrane (Flrt) gene family have been previously reported to be widely expressed in the developing embryo. We mapped the expression of Flrt2 and Flrt3 at critical stages of craniofacial development and found that, during early craniofacial development, Flrt2 was highly expressed initially in the cranial neural crest cells and Flrt3 in the midbrain. Later both genes were expressed in the developing pharyngeal region. Flrt2 expression predominated in the neural crest-derived mesenchyme in the medial aspect of the developing frontonasal region in close relationships with the expression of Fgfr2, Shh, and Msx1, three genes shown previously to play critical roles in craniofacial development. Flrt2 was also present in the vomero-nasal organ, mandibular primodia, and the posterior aspects of the unfused and fused secondary palatal shelves. Flrt3, however, had a more restrictive expression, being present in the mesenchyme underlying the ectoderm of the medial nasal process and in the mandibular primordium and in regions undergoing outgrowth, in a pattern that overlapped with Bmp4 expression. Both Flrt2 and Flrt3 were later found to be present at sites of epithelial-mesenchymal interactions such as the developing tooth buds, hair follicles, and eye. Together the data suggested important roles for Flrt2 and Flrt3 in mediating events such as NCC **migration**, chondrogenesis and epithelial-mesenchymal interactions during craniofacial development.
38. Craniofacial divergence by distinct prenatal growth patterns in Fgfr2 mutant mice. Differences in cranial morphology arise due to changes in fundamental cell processes like **migration**, proliferation, differentiation and cell death driven by genetic programs. Signaling between fibroblast growth factors (FGFs) and their receptors (FGFRs) affect these processes during head development and mutations in FGFRs result in congenital diseases including FGFR-related craniosynostosis syndromes. Current research in model organisms focuses primarily on how these mutations change cell function local to sutures under the hypothesis that prematurely closing cranial sutures contribute to skull dysmorphogenesis. Though these studies have provided fundamentally important information contributing to the understanding of craniosynostosis conditions, knowledge of changes in cell function local to the sutures leave change in overall three-dimensional cranial morphology largely unexplained. Here we investigate growth of the skull in two inbred mouse models each carrying one of two gain-of-function mutations in FGFR2 on neighboring amino acids (S252W and P253R) that in humans cause Apert syndrome, one of the most severe FGFR-related craniosynostosis syndromes. We examine late embryonic skull development and suture patency in Fgfr2 Apert syndrome mice between embryonic day 17.5 and birth and quantify the effects of these mutations on 3D skull morphology, suture patency and growth.We show in mice what studies in humans can only infer: specific cranial growth deviations occur prenatally and worsen with time in organisms carrying these FGFR2 mutations. We demonstrate that: 1) distinct skull morphologies of each mutation group are established by E17.5; 2) cranial suture patency patterns differ between mice carrying these mutations and their unaffected littermates; 3) the prenatal skull grows differently in each mutation group; and 4) unique Fgfr2-related cranial morphologies are exacerbated by late embryonic growth patterns.Our analysis of mutation-driven changes in cranial growth provides a previously missing piece of knowledge necessary for explaining variation in emergent cranial morphologies and may ultimately be helpful in managing human cases carrying these same mutations. This information is critical to the understanding of craniofacial development, disease and evolution and may contribute to the evaluation of incipient therapeutic strategies.
39. Concerted action of Msx1 and Msx2 in regulating cranial neural crest cell differentiation during frontal bone development. The homeobox genes Msx1 and Msx2 function as transcriptional regulators that control cellular proliferation and differentiation during embryonic development. Mutations in the Msx1 and Msx2 genes in mice disrupt tissue-tissue interactions and cause multiple craniofacial malformations. Although Msx1 and Msx2 are both expressed throughout the entire development of the frontal bone, the frontal bone defect in Msx1 or Msx2 null mutants is rather mild, suggesting the possibility of functional compensation between Msx1 and Msx2 during early frontal bone development. To investigate this hypothesis, we generated Msx1(-/-);Msx2(-/-) mice. These double mutant embryos died at E17 to E18 with no formation of the frontal bone. There was no apparent defect in CNC **migration** into the presumptive frontal bone primordium, but differentiation of the frontal mesenchyme and establishment of the frontal primordium was defective, indicating that Msx1 and Msx2 genes are specifically required for osteogenesis in the cranial neural crest lineage within the frontal bone primordium. Mechanistically, our data suggest that Msx genes are critical for the expression of Runx2 in the frontonasal subpopulation of cranial neural crest cells and for differentiation of the osteogenic lineage. This early function of the Msx genes is likely independent of the Bmp signaling pathway.
40. Stabilized beta-catenin in thymic epithelial cells blocks thymus development and function. Thymic T cell development is dependent on a specialized epithelial microenvironment mainly composed of cortical and medullary thymic epithelial cells (TECs). The molecular programs governing the differentiation and maintenance of TECs remain largely unknown. Wnt signaling is central to the development and maintenance of several organ systems but a specific role of this pathway for thymus organogenesis has not yet been ascertained. In this report, we demonstrate that activation of the canonical Wnt signaling pathway by a stabilizing mutation of beta-catenin targeted exclusively to TECs changes the initial commitment of endodermal epithelia to a thymic cell fate. Consequently, the formation of a correctly composed and organized thymic microenvironment is prevented, thymic im**migration** of hematopoietic precursors is restricted, and intrathymic T cell differentiation is arrested at a very early developmental stage causing severe immunodeficiency. These results suggest that a precise regulation of canonical Wnt signaling in thymic epithelia is essential for normal thymus development and function.
41. Functional proteomics defines the molecular switch underlying FGF receptor trafficking and cellular outputs. The stimulation of fibroblast growth factor receptors (FGFRs) with distinct FGF ligands generates specific cellular responses. However, the mechanisms underlying this paradigm have remained elusive. Here, we show that FGF-7 stimulation leads to FGFR2b degradation and, ultimately, cell proliferation, whereas FGF-10 promotes receptor recycling and cell **migration**. By combining mass-spectrometry-based quantitative proteomics with fluorescence microscopy and biochemical methods, we find that FGF-10 specifically induces the rapid phosphorylation of tyrosine (Y) 734 on FGFR2b, which leads to PI3K and SH3BP4 recruitment. This complex is crucial for FGFR2b recycling and responses, given that FGF-10 stimulation of either FGFR2b\_Y734F mutant- or SH3BP4-depleted cells switches the receptor endocytic route to degradation, resulting in decreased breast cancer cell **migration** and the inhibition of epithelial branching in mouse lung explants. Altogether, these results identify an intriguing ligand-dependent mechanism for the control of receptor fate and cellular outputs that may explain the pathogenic role of deregulated FGFR2b, thus offering therapeutic opportunities.
42. N-cadherin and keratinocyte growth factor receptor mediate the functional interplay between Ki-RASG12V and p53V143A in promoting pancreatic cell **migration**, invasion, and tissue architecture disruption. The genetic basis of pancreatic ductal adenocarcinoma, which constitutes the most common type of pancreatic malignancy, involves the sequential activation of oncogenes and inactivation of tumor suppressor genes. Among the pivotal genetic alterations are Ki-RAS oncogene activation and p53 tumor suppressor gene inactivation. We explain that the combination of these genetic events facilitates pancreatic carcinogenesis as revealed in novel three-dimensional cell (spheroid cyst) culture and in vivo subcutaneous and orthotopic xenotransplantation models. N-cadherin, a member of the classic cadherins important in the regulation of cell-cell adhesion, is induced in the presence of Ki-RAS mutation but subsequently downregulated with the acquisition of p53 mutation as revealed by gene microarrays and corroborated by reverse transcription-PCR and Western blotting. N-cadherin modulates the capacity of pancreatic ductal cells to migrate and invade, in part via complex formation with keratinocyte growth factor receptor and neural cell adhesion molecule and in part via interaction with p120-catenin. However, modulation of these complexes by Ki-RAS and p53 leads to enhanced cell **migration** and invasion. This preferentially induces the downstream effector AKT over mitogen-activated protein kinase to execute changes in cellular behavior. Thus, we are able to define molecules that in part are directly affected by Ki-RAS and p53 during pancreatic ductal carcinogenesis, and this provides a platform for potential new molecularly based therapeutic interventions.
43. Endothelium-targeted overexpression of constitutively active FGF receptor induces cardioprotection in mice myocardial infarction. Fibroblast growth factor receptor (FGFR) is expressed in a variety of cells and is involved in their proliferation/**migration**/survival. To elucidate FGFR-mediated specific action of vascular endothelial cells (ECs) on myocardial ischemia, we generated endothelium-targeted transgenic mice overexpressing constitutively active FGFR2 using Tie2 promoter (FGFR2-Tg). Infarct size, vessel formation and blood perfusion were significantly improved 28 days after myocardial infarction (MI) in FGFR2-Tg, compared with wild-type mice. Aortic ECs isolated from FGFR-Tg showed a marked increase in migratory capacity and tube formation. These in vitro angiogenic activities were blocked by PI3-kinase inhibitor. Whereas, parameters obtained from echocardiography were already improved at three days after MI. Cardiomyocyte apoptosis at the ischemic border zone was decreased in FGFR2-Tg (32.1%, p < 0.05) and cardiac mRNA expression of FGF2 (basic FGF) was also up-regulated (142%, p < 0.05) at 3 days after MI. 1% oxygen-mediated apoptosis was significantly inhibited in FGFR2-Tg-ECs and this inhibition was abolished by PI3-kinase inhibitor. FGFR2-Tg-ECs exposed to 1% oxygen exhibited enhanced phosphorylation of 416-Tyr-Src, 473-Ser-Akt, and HIF1alpha accumulation. The production of FGF2 was enhanced 2.1-fold in FGFR-Tg-ECs under 1% oxygen via the Src/Akt/HIF1alpha pathway, which induced the peri-vessel **migration** of vascular smooth muscle cells (VSMCs) and anti-apoptotic effects on VSMCs and cardiomyocytes. FGF receptor signaling in ECs promoted **migration**, survival and autocrine production of FGF2, leading to reduced infarct size, which is associated with anti-apoptotic action in the early stage and with enhanced angiogenesis in the late stage after MI.

#### GeneRIFs

1. cerebellar microexplant cultures treated with an FGFR agonist or antagonist, we also show that FGF9/FGFR-mediated signaling inhibits the outward **migration** of radial glia and Bergmann glia precursors and cells
2. FGF receptors 1 and 2 are key regulators of keratinocyte **migration** in vitro and in wounded skin.
3. FGF receptor signaling in endothelial cells promoted **migration**, survival and autocrine production of FGF2.
4. FGF-mediated activation of the MAP kinase pathway occurs in germ cells during their **migration**, FGF2, a ligand for FGFR1-IIIc, affects motility, whereas FGF7, a ligand for FGFR2-IIIb, affects germ cell numbers.

### ECM

#### PubMed

1. Nell1-deficient mice have reduced expression of extracellular matrix proteins causing cranial and vertebral defects. The mammalian Nell1 gene encodes a protein kinase C-beta1 (PKC-beta1) binding protein that belongs to a new class of cell-signaling molecules controlling cell growth and differentiation. Over-expression of Nell1 in the developing cranial sutures in both human and mouse induces craniosynostosis, the premature fusion of the growing cranial bone fronts. Here, we report the generation, positional cloning and characterization of Nell1(6R), a recessive, neonatal-lethal point mutation in the mouse Nell1 gene, induced by N-ethyl-N-nitrosourea. Nell1(6R) has a T-->A base change that converts a codon for cysteine into a premature stop codon [Cys(502)Ter], resulting in severe truncation of the predicted protein product and marked reduction in steady-state levels of the transcript. In addition to the expected alteration of cranial morphology, Nell1(6R) mutants manifest skeletal defects in the vertebral column and ribcage, revealing a hitherto undefined role for Nell1 in signal transduction in endochondral ossification. Real-time quantitative reverse transcription-PCR assays of 219 genes showed an association between the loss of Nell1 function and reduced expression of genes for extracellular matrix (**ECM**) proteins critical for chondrogenesis and osteogenesis. Several affected genes are involved in the human cartilage disorder Ehlers-Danlos Syndrome and other disorders associated with spinal curvature anomalies. Nell1(6R) mutant mice are a new tool for elucidating basic mechanisms in osteoblast and chrondrocyte differentiation in the developing skull and vertebral column and understanding how perturbations in the production of **ECM** proteins can lead to anomalies in these structures.

### [Ee]xtracellular [Mm]atrix

#### PubMed

1. beta1 integrin is necessary for ureteric bud branching morphogenesis and maintenance of collecting duct structural integrity. The kidney collecting system develops from branching morphogenesis of the ureteric bud (UB). This process requires signaling by growth factors such as glial cell line derived neurotrophic factor (GDNF) and fibroblast growth factors (FGFs) as well as cell **extracellular matrix** interactions mediated by integrins. The importance of integrin signaling in UB development was investigated by deleting integrin beta1 at initiation (E10.5) and late (E18.5) stages of development. Deletion at E10.5 resulted in a severe branching morphogenesis phenotype. Deletion at E18.5 did not alter renal development but predisposed the collecting system to severe injury following ureteric obstruction. beta1 integrin was required for renal tubular epithelial cells to mediate GDNF- and FGF-dependent signaling despite normal receptor localization and activation in vitro. Aberrations in the same signaling molecules were present in the beta1-null UBs in vivo. Thus beta1 integrins can regulate organ branching morphogenesis during development by mediating growth-factor-dependent signaling in addition to their well-defined role as adhesion receptors.
2. FGF9 monomer-dimer equilibrium regulates **extracellular matrix** affinity and tissue diffusion. The spontaneous dominant mouse mutant, Elbow knee synostosis (Eks), shows elbow and knee joint synosotsis, and premature fusion of cranial sutures. Here we identify a missense mutation in the Fgf9 gene that is responsible for the Eks mutation. Through investigation of the pathogenic mechanisms of joint and suture synostosis in Eks mice, we identify a key molecular mechanism that regulates FGF9 signaling in developing tissues. We show that the Eks mutation prevents homodimerization of the FGF9 protein and that monomeric FGF9 binds to heparin with a lower affinity than dimeric FGF9. These biochemical defects result in increased diffusion of the altered FGF9 protein (FGF9(Eks)) through developing tissues, leading to ectopic FGF9 signaling and repression of joint and suture development. We propose a mechanism in which the range of FGF9 signaling in developing tissues is limited by its ability to homodimerize and its affinity for **extracellular matrix** heparan sulfate proteoglycans.
3. 2-O Heparan Sulfate Sulfation by Hs2st Is Required for Erk/Mapk Signalling Activation at the Mid-Gestational Mouse Telencephalic Midline. Heparan sulfate (HS) is a linear carbohydrate composed of polymerized uronate-glucosamine disaccharide units that decorates cell surface and secreted glycoproteins in the **extracellular matrix**. In mammals HS is subjected to differential sulfation by fifteen different heparan sulfotransferase (HST) enzymes of which Hs2st uniquely catalyzes the sulfation of the 2-O position of the uronate in HS. HS sulfation is postulated to be important for regulation of signaling pathways by facilitating the interaction of HS with signaling proteins including those of the Fibroblast Growth Factor (Fgf) family which signal through phosphorylation of extracellular signal-regulated kinases Erk1/2. In the developing mouse telencephalon Fgf2 signaling regulates proliferation and neurogenesis. Loss of Hs2st function phenocopies the thinned cerebral cortex of mutant mice in which Fgf2 or Erk1/2 function are abrogated, suggesting the hypothesis that 2-O-sulfated HS structures play a specific role in Fgf2/Erk signaling pathway in this context in vivo. This study investigated the molecular role of 2-O sulfation in Fgf2/Erk signaling in the developing telencephalic midline midway through mouse embryogenesis at E12.5. We examined the expression of Hs2st, Fgf2, and Erk1/2 activity in wild-type and Hs2st-/- mice. We found that Hs2st is expressed at high levels at the midline correlating with high levels of Erk1/2 activation and Erk1/2 activation was drastically reduced in the Hs2st-/- mutant at the rostral telencephalic midline. We also found that 2-O sulfation is specifically required for the binding of Fgf2 protein to Fgfr1, its major cell-surface receptor at the rostral telencephalic midline. We conclude that 2-O sulfated HS structures generated by Hs2st are needed to form productive signaling complexes between HS, Fgf2 and Fgfr1 that activate Erk1/2 at the midline. Overall, our data suggest the interesting possibility that differential expression of Hs2st targets the rostral telencephalic midline for high levels of Erk signaling by increasing the sensitivity of cells to an Fgf2 signal that is rather more widespread.
4. MT1-MMP inactivates ADAM9 to regulate FGFR2 signaling and calvarial osteogenesis. MMP14 encodes a membrane-tethered metalloproteinase MT1-MMP, capable of remodeling the **extracellular matrix** and modulating receptors on the cell surface. Loss of MT1-MMP results in craniofacial abnormalities. Here we show that MT1-MMP forms a complex with FGFR2 and ADAM9 in osteoblasts and proteolytically inactivates ADAM9, hence protecting FGFR2 from ADAM9-mediated ectodomain shedding on the cell surface. In Mmp14-/- osteoblasts, FGF-induced proliferation and downstream signaling are specifically compromised, in conjunction with ADAM9 upregulation and FGFR2 shedding. The retarded parietal growth in Mmp14-/- embryos starts at 15.5 dpc, attributable to the impaired FGFR2 signaling due to increased shedding mediated by ADAM9. Adam9 depletion completely rescues the defective FGFR2 signaling and largely restores calvarial bone growth in Mmp14-/- embryos. These data reveal a regulatory paradigm for FGRF2 signaling and identify MT1-MMP as a critical negative modulator of ADAM9 activity to maintain FGFR2 signaling in calvarial osteogenesis.
5. Nell1-deficient mice have reduced expression of **extracellular matrix** proteins causing cranial and vertebral defects. The mammalian Nell1 gene encodes a protein kinase C-beta1 (PKC-beta1) binding protein that belongs to a new class of cell-signaling molecules controlling cell growth and differentiation. Over-expression of Nell1 in the developing cranial sutures in both human and mouse induces craniosynostosis, the premature fusion of the growing cranial bone fronts. Here, we report the generation, positional cloning and characterization of Nell1(6R), a recessive, neonatal-lethal point mutation in the mouse Nell1 gene, induced by N-ethyl-N-nitrosourea. Nell1(6R) has a T-->A base change that converts a codon for cysteine into a premature stop codon [Cys(502)Ter], resulting in severe truncation of the predicted protein product and marked reduction in steady-state levels of the transcript. In addition to the expected alteration of cranial morphology, Nell1(6R) mutants manifest skeletal defects in the vertebral column and ribcage, revealing a hitherto undefined role for Nell1 in signal transduction in endochondral ossification. Real-time quantitative reverse transcription-PCR assays of 219 genes showed an association between the loss of Nell1 function and reduced expression of genes for **extracellular matrix** (ECM) proteins critical for chondrogenesis and osteogenesis. Several affected genes are involved in the human cartilage disorder Ehlers-Danlos Syndrome and other disorders associated with spinal curvature anomalies. Nell1(6R) mutant mice are a new tool for elucidating basic mechanisms in osteoblast and chrondrocyte differentiation in the developing skull and vertebral column and understanding how perturbations in the production of ECM proteins can lead to anomalies in these structures.
6. The mouse KRAB zinc-finger protein CHATO is required in embryonic-derived tissues to control yolk sac and placenta morphogenesis. Yolk sac and placenta are required to sustain embryonic development in mammals, yet our understanding of the genes and processes that control morphogenesis of these extraembryonic tissues is still limited. The chato mutation disrupts ZFP568, a Krüppel-Associated-Box (KRAB) domain Zinc finger protein, and causes a unique set of extraembryonic malformations, including ruffling of the yolk sac membrane, defective extraembryonic mesoderm morphogenesis and vasculogenesis, failure to close the ectoplacental cavity, and incomplete placental development. Phenotypic analysis of chato embryos indicated that ZFP568 does not control proliferation or differentiation of extraembryonic lineages but rather regulates the morphogenetic events that shape extraembryonic tissues. Analysis of chimeric embryos showed that Zfp568 function is required in embryonic-derived lineages, including the extraembryonic mesoderm. Depleting Zfp568 affects the ability of extraembryonic mesoderm cells to migrate. However, explanted Zfp568 mutant cells could migrate properly when plated on appropriate **extracellular matri**x conditions. We show that expression of Fibronectin and Indian Hedgehog are reduced in chato mutant yolk sacs. These data suggest that ZFP568 controls the production of secreted factors required to promote morphogenesis of extraembryonic tissues. Our results support previously undescribed roles of the extraembryonic mesoderm in yolk sac morphogenesis and in the closure of the ectoplacental cavity and identify a novel role of ZFP568 in the development of extraembryonic tissues.
7. An FGF autocrine loop initiated in second heart field mesoderm regulates morphogenesis at the arterial pole of the heart. In order to understand how secreted signals regulate complex morphogenetic events, it is crucial to identify their cellular targets. By conditional inactivation of Fgfr1 and Fgfr2 and overexpression of the FGF antagonist sprouty 2 in different cell types, we have dissected the role of FGF signaling during heart outflow tract development in mouse. Contrary to expectation, cardiac neural crest and endothelial cells are not primary paracrine targets. FGF signaling within second heart field mesoderm is required for remodeling of the outflow tract: when disrupted, outflow myocardium fails to produce **extracellular matrix** and TGFbeta and BMP signals essential for endothelial cell transformation and invasion of cardiac neural crest. We conclude that an autocrine regulatory loop, initiated by the reception of FGF signals by the mesoderm, regulates correct morphogenesis at the arterial pole of the heart. These findings provide new insight into how FGF signaling regulates context-dependent cellular responses during development.
8. Distinct roles of fibroblast growth factor receptor 1 and 2 in regulating cell survival and epithelial-mesenchymal transition. Two related receptor tyrosine kinases (RTKs), fibroblast growth factor receptor 1 and 2 (FGFR1 and FGFR2), exert distinct effects during carcinogenesis. To examine FGFR1 and FGFR2 signaling in polarized epithelia, we have developed an in vitro three-dimensional HC11 mouse mammary epithelial cell culture model combined with a chemically inducible FGFR (iFGFR) dimerization system. Although activation of both RTKs led to reinitiation of cell proliferation and loss of cell polarity, only iFGFR1 activation induced cell survival and epithelial to mesenchymal transition. In contrast, iFGFR2 activation induced cell apoptosis even in the cells in direct contact with the **extracellular matrix**. Activation of iFGFR2, but not iFGFR1, led to rapid receptor down-regulation and transient activation of downstream signaling, which were partially rescued by Cbl small interfering RNA knockdown or the proteasome inhibitor lactacystin. Importantly, inhibition of proteasome activity in iFGFR2-activated structures led to epithelial to mesenchymal transition and invasive phenotypes resembling those observed after iFGFR1 activation. These studies demonstrate, for the first time, that the duration of downstream signaling determines the distinct phenotypes mediated by very homologous RTKs in three-dimensional cultures.
9. MT1-MMP inactivates ADAM9 to regulate FGFR2 signaling and calvarial osteogenesis. MMP14 encodes a membrane-tethered metalloproteinase MT1-MMP, capable of remodeling the **extracellular matrix** and modulating receptors on the cell surface. Loss of MT1-MMP results in craniofacial abnormalities. Here we show that MT1-MMP forms a complex with FGFR2 and ADAM9 in osteoblasts and proteolytically inactivates ADAM9, hence protecting FGFR2 from ADAM9-mediated ectodomain shedding on the cell surface. In Mmp14-/- osteoblasts, FGF-induced proliferation and downstream signaling are specifically compromised, in conjunction with ADAM9 upregulation and FGFR2 shedding. The retarded parietal growth in Mmp14-/- embryos starts at 15.5 dpc, attributable to the impaired FGFR2 signaling due to increased shedding mediated by ADAM9. Adam9 depletion completely rescues the defective FGFR2 signaling and largely restores calvarial bone growth in Mmp14-/- embryos. These data reveal a regulatory paradigm for FGRF2 signaling and identify MT1-MMP as a critical negative modulator of ADAM9 activity to maintain FGFR2 signaling in calvarial osteogenesis.
10. Distinct roles of fibroblast growth factor receptor 1 and 2 in regulating cell survival and epithelial-mesenchymal transition. Two related receptor tyrosine kinases (RTKs), fibroblast growth factor receptor 1 and 2 (FGFR1 and FGFR2), exert distinct effects during carcinogenesis. To examine FGFR1 and FGFR2 signaling in polarized epithelia, we have developed an in vitro three-dimensional HC11 mouse mammary epithelial cell culture model combined with a chemically inducible FGFR (iFGFR) dimerization system. Although activation of both RTKs led to reinitiation of cell proliferation and loss of cell polarity, only iFGFR1 activation induced cell survival and epithelial to mesenchymal transition. In contrast, iFGFR2 activation induced cell apoptosis even in the cells in direct contact with the **extracellular matrix**. Activation of iFGFR2, but not iFGFR1, led to rapid receptor down-regulation and transient activation of downstream signaling, which were partially rescued by Cbl small interfering RNA knockdown or the proteasome inhibitor lactacystin. Importantly, inhibition of proteasome activity in iFGFR2-activated structures led to epithelial to mesenchymal transition and invasive phenotypes resembling those observed after iFGFR1 activation. These studies demonstrate, for the first time, that the duration of downstream signaling determines the distinct phenotypes mediated by very homologous RTKs in three-dimensional cultures.
11. Gene array identification of Ipf1/Pdx1-/- regulated genes in pancreatic progenitor cells. The homeodomain transcription factor IPF1/PDX1 exerts a dual role in the pancreas; Ipf1/Pdx1 global null mutants fail to develop a pancreas whereas conditional inactivation of Ipf1/Pdx1 in beta-cells leads to impaired beta-cell function and diabetes. Although several putative target genes have been linked to the beta-cell function of Ipf1/Pdx1, relatively little is known with respect to genes regulated by IPF1/PDX1 in early pancreatic progenitor cells.Microarray analyses identified a total of 111 genes that were differentially expressed in e10.5 pancreatic buds of Ipf1/Pdx1-/- embryos. The expression of one of these, Spondin 1, which encodes an **extracellular matrix** protein, has not previously been described in the pancreas. Quantitative real-time RT-PCR analyses and immunohistochemical analyses also revealed that the expression of FgfR2IIIb, that encodes the receptor for FGF10, was down-regulated in Ipf1/Pdx1-/- pancreatic progenitor cells.This microarray analysis has identified a number of candidate genes that are differentially expressed in Ipf1/Pdx1-/- pancreatic buds. Several of the differentially expressed genes were known to be important for pancreatic progenitor cell proliferation and differentiation whereas others have not previously been associated with pancreatic development.
12. FGF signaling activates a Sox9-Sox10 pathway for the formation and branching morphogenesis of mouse ocular glands. Murine lacrimal, harderian and meibomian glands develop from the prospective conjunctival and eyelid epithelia and produce secretions that lubricate and protect the ocular surface. Sox9 expression localizes to the presumptive conjunctival epithelium as early as E11.5 and is detected in the lacrimal and harderian glands as they form. Conditional deletion showed that Sox9 is required for the development of the lacrimal and harderian glands and contributes to the formation of the meibomian glands. Sox9 regulates the expression of Sox10 to promote the formation of secretory acinar lobes in the lacrimal gland. Sox9 and FGF signaling were required for the expression of cartilage-associated **extracellular matrix** components during early stage lacrimal gland development. Fgfr2 deletion in the ocular surface epithelium reduced Sox9 and eliminated Sox10 expression. Sox9 deletion from the ectoderm did not affect Fgf10 expression in the adjacent mesenchyme or Fgfr2 expression in the epithelium, but appeared to reduce FGF signaling. Sox9 heterozygotes showed a haploinsufficient phenotype, in which the exorbital branch of the lacrimal gland was absent in most cases. However, enhancement of epithelial FGF signaling by expression of a constitutively active FGF receptor only partially rescued the lacrimal gland defects in Sox9 heterozygotes, suggesting a crucial role of Sox9, downstream of FGF signaling, in regulating lacrimal gland branching and differentiation.
13. Cell surface heparan sulfate chains regulate local reception of FGF signaling in the mouse embryo. Heparan sulfate (HS) proteoglycans modulate the activity of multiple growth factors on the cell surface and **extracellular matrix**. However, it remains unclear how the HS chains control the movement and reception of growth factors into targeted receiving cells during mammalian morphogenetic processes. Here, we found that HS-deficient Ext2 null mutant mouse embryos fail to respond to fibroblast growth factor (FGF) signaling. Marker expression analyses revealed that cell surface-tethered HS chains are crucial for local retention of FGF4 and FGF8 ligands in the extraembryonic ectoderm. Fine chimeric studies with single-cell resolution and expression studies with specific inhibitors for HS movement demonstrated that proteolytic cleavage of HS chains can spread FGF signaling to adjacent cells within a short distance. Together, the results show that spatiotemporal expression of cell surface-tethered HS chains regulate the local reception of FGF-signaling activity during mammalian embryogenesis.

#### Component

1. **extracellular matrix**

### [Ii]nvasive

#### PubMed

1. Distinct roles of fibroblast growth factor receptor 1 and 2 in regulating cell survival and epithelial-mesenchymal transition. Two related receptor tyrosine kinases (RTKs), fibroblast growth factor receptor 1 and 2 (FGFR1 and FGFR2), exert distinct effects during carcinogenesis. To examine FGFR1 and FGFR2 signaling in polarized epithelia, we have developed an in vitro three-dimensional HC11 mouse mammary epithelial cell culture model combined with a chemically inducible FGFR (iFGFR) dimerization system. Although activation of both RTKs led to reinitiation of cell proliferation and loss of cell polarity, only iFGFR1 activation induced cell survival and epithelial to mesenchymal transition. In contrast, iFGFR2 activation induced cell apoptosis even in the cells in direct contact with the extracellular matrix. Activation of iFGFR2, but not iFGFR1, led to rapid receptor down-regulation and transient activation of downstream signaling, which were partially rescued by Cbl small interfering RNA knockdown or the proteasome inhibitor lactacystin. Importantly, inhibition of proteasome activity in iFGFR2-activated structures led to epithelial to mesenchymal transition and **invasive** phenotypes resembling those observed after iFGFR1 activation. These studies demonstrate, for the first time, that the duration of downstream signaling determines the distinct phenotypes mediated by very homologous RTKs in three-dimensional cultures.
2. Distinct roles of fibroblast growth factor receptor 1 and 2 in regulating cell survival and epithelial-mesenchymal transition. Two related receptor tyrosine kinases (RTKs), fibroblast growth factor receptor 1 and 2 (FGFR1 and FGFR2), exert distinct effects during carcinogenesis. To examine FGFR1 and FGFR2 signaling in polarized epithelia, we have developed an in vitro three-dimensional HC11 mouse mammary epithelial cell culture model combined with a chemically inducible FGFR (iFGFR) dimerization system. Although activation of both RTKs led to reinitiation of cell proliferation and loss of cell polarity, only iFGFR1 activation induced cell survival and epithelial to mesenchymal transition. In contrast, iFGFR2 activation induced cell apoptosis even in the cells in direct contact with the extracellular matrix. Activation of iFGFR2, but not iFGFR1, led to rapid receptor down-regulation and transient activation of downstream signaling, which were partially rescued by Cbl small interfering RNA knockdown or the proteasome inhibitor lactacystin. Importantly, inhibition of proteasome activity in iFGFR2-activated structures led to epithelial to mesenchymal transition and **invasive** phenotypes resembling those observed after iFGFR1 activation. These studies demonstrate, for the first time, that the duration of downstream signaling determines the distinct phenotypes mediated by very homologous RTKs in three-dimensional cultures.
3. Interaction between FGFR-2, STAT5, and progesterone receptors in breast cancer. Fibroblast growth factor (FGF) receptor 2 (FGFR-2) polymorphisms have been associated with an increase in estrogen receptor and progesterone receptor (PR)-positive breast cancer risk; however, a clear mechanistic association between FGFR-2 and steroid hormone receptors remains elusive. In previous works, we have shown a cross talk between FGF2 and progestins in mouse mammary carcinomas. To investigate the mechanisms underlying these interactions and to validate our findings in a human setting, we have used T47D human breast cancer cells and human cancer tissue samples. We showed that medroxyprogesterone acetate (MPA) and FGF2 induced cell proliferation and activation of ERK, AKT, and STAT5 in T47D and in murine C4-HI cells. Nuclear interaction between PR, FGFR-2, and STAT5 after MPA and FGF2 treatment was also showed by confocal microscopy and immunoprecipitation. This effect was associated with increased transcription of PRE and/or GAS reporter genes, and of PR/STAT5-regulated genes and proteins. Two antiprogestins and the FGFR inhibitor PD173074, specifically blocked the effects induced by FGF2 or MPA respectively. The presence of PR/FGFR-2/STAT5 complexes bound to the PRE probe was corroborated by using NoShift transcription and chromatin immunoprecipitation of the MYC promoter. Additionally, we showed that T47D cells stably transfected with constitutively active FGFR-2 gave rise to **invasive** carcinomas when transplanted into NOD/SCID mice. Nuclear colocalization between PR and FGFR-2/STAT5 was also observed in human breast cancer tissues. This study represents the first demonstration of a nuclear interaction between FGFR-2 and STAT5, as PR coactivators at the DNA progesterone responsive elements, suggesting that FGFRs are valid therapeutic targets for human breast cancer treatment.
4. Therapeutic effect of nanogel-based delivery of soluble FGFR2 with S252W mutation on craniosynostosis. Apert syndrome is an autosomal dominantly inherited disorder caused by missense mutations in fibroblast growth factor receptor 2 (FGFR2). Surgical procedures are frequently required to reduce morphological and functional defects in patients with Apert syndrome; therefore, the development of non**invasive** procedures to treat Apert syndrome is critical. Here we aimed to clarify the etiological mechanisms of craniosynostosis in mouse models of Apert syndrome and verify the effects of purified soluble FGFR2 harboring the S252W mutation (sFGFR2IIIcS252W) on calvarial sutures in Apert syndrome mice in vitro. We observed increased expression of Fgf10, Esrp1, and Fgfr2IIIb, which are indispensable for epidermal development, in coronal sutures in Apert syndrome mice. Purified sFGFR2IIIcS252W exhibited binding affinity for fibroblast growth factor (Fgf) 2 but also formed heterodimers with FGFR2IIIc, FGFR2IIIcS252W, and FGFR2IIIbS252W. Administration of sFGFR2IIIcS252W also inhibited Fgf2-dependent proliferation, phosphorylation of intracellular signaling molecules, and mineralization of FGFR2S252W-overexpressing MC3T3-E1 osteoblasts. sFGFR2IIIcS252W complexed with nanogels maintained the patency of coronal sutures, whereas synostosis was observed where the nanogel without sFGFR2S252W was applied. Thus, based on our current data, we suggest that increased Fgf10 and Fgfr2IIIb expression may induce the onset of craniosynostosis in patients with Apert syndrome and that the appropriate delivery of purified sFGFR2IIIcS252W could be effective for treating this disorder.

### [Cc]ell [Ii]nvasion

#### PubMed

1. The FGF-BMP signaling axis regulates outflow tract valve primordium formation by promoting cushion neural crest cell differentiation. Heart valves develop from precursor structures called cardiac cushions, an endothelial-lined cardiac jelly that resides in the inner side of the heart tube. The cushions are then invaded by cells from different sources, undergo a series of complicated and poorly understood remodeling processes, and give rise to valves. Disruption of the fibroblast growth factor (FGF) signaling axis impairs morphogenesis of the outflow tract (OFT). Yet, whether FGF signaling regulates OFT valve formation is unknown.To study how OFT valve formation is regulated and how aberrant cell signaling causes valve defects.By using mouse genetic manipulation, cell lineage tracing, ex vivo heart culture, and molecular biology approaches, we demonstrated that FGF signaling in the OFT myocardium upregulated Bmp4 expression, which then enhanced smooth muscle differentiation of neural crest cells (NCCs) in the cushion. FGF signaling also promoted OFT myocardial **cell invasion** to the cushion. Disrupting FGF signaling interrupted cushion remodeling with reduced NCCs differentiation into smooth muscle and less cardiomyocyte invasion and resulted in malformed OFT valves.The results demonstrate a novel mechanism by which the FGF-BMP signaling axis regulates formation of OFT valve primordia by controlling smooth muscle differentiation of cushion NCCs.

---

## Pdgfra

- NCBI Gene
- GeneRIF
- Pubmed

| Search Term | Rank | Fields |
| --- | --- | --- |
| [Ff]ilopodia | 1 | PubMed(1) |
| [Mm]igration | 6 | Process(2); PubMed(69); GeneRIFs(4) |
| ECM | 7 | PubMed(6) |
| [Ee]xtracellular [Mm]atrix | 8 | Process(1); PubMed(17) |
| [Ii]nvasive | 10 | PubMed(8); GeneRIFs(2) |
| [Mm]etastasis | 12 | PubMed(4) |
| [Cc]ell [Ii]nvasion | 13 | PubMed(2) |

  
hide/show details for Pdgfra

### [Ff]ilopodia

#### PubMed

1. Platelet-derived growth factor-mediated signaling through the Shb adaptor protein: effects on cytoskeletal organization. The Src homology (SH) 2 domain adaptor protein Shb has previously been shown to interact with the platelet-derived growth factor (PDGF)-beta receptor. In this study we show an association between Shb and the PDGF-alpha receptor which is mediated by the SH2 domain of Shb and involves tyrosine residue 720 in the kinase insert domain of the receptor. To assess the role of Shb in PDGF-mediated signaling, we have overexpressed wild-type Shb or Shb carrying a mutation (R522K) which renders the SH2 domain inactive, in Patch mouse (PhB) fibroblasts expressing both PDGF receptors (PhB/Ralpha). Overexpression of wild-type Shb, but not the R522K Shb mutant, affected PDGF-mediated reorganization of the cytoskeleton by decreasing membrane ruffle formation and stimulating the generation of **filopodia** relative the parental control cells. In addition, the PDGF-induced receptor-associated phosphatidylinositol 3'-kinase activity and phosphorylation of Akt was similar in both PhB/Ralpha/Shb and PhB/Ralpha/ShbR522K cells compared with the parental control, whereas the activation of Rac in response to PDGF-BB was diminished only in the PhB/Ralpha/Shb cells. We conclude that Shb plays a role in PDGF-dependent regulation of certain cytoskeletal changes by modulating the ability of PDGF to activate Rac.

### [Mm]igration

#### Process

1. cell **migration**
2. positive regulation of cell **migration**

#### PubMed

1. Forced expression of platelet-derived growth factor B in the mouse cerebellar primordium changes cell **migration** during midline fusion and causes cerebellar ectopia. The platelet-derived growth factor (PDGF) and receptors are expressed in the developing central nervous system and in brain tumors. To investigate the role of PDGF during normal cerebellar development, we created transgenic mice where PDGF-B was introduced into the endogenous Engrailed1 locus (En1). These mice expressed PDGF-B in all types of cells that constitute the developing cerebellum, with localized high expression in the ventral midline of the cerebellar anlage. This affected cell **migration** in the midline during fusion of the cerebellar anlage and caused misplacement of midline structures. PDGFR-alpha- and laminin alpha1-positive meningeal cells migrated inwards, attracted by the ectopic transgene expression in the ventral neuroepithelium. Other cells followed the meningeal cells and in the adult mouse, cells from all cortical cell layers were found misplaced in the midline. Moreover, the transgene caused an enhancement of capillary vessels. The findings indicate that normal PDGF signaling is important for proper neural tube fusion. It also illustrates that meningeal structures can influence the process.
2. Wnt4 is required for proper male as well as female sexual development. Genes previously implicated in mammalian sexual development have either a male- or female-specific role. The signaling molecule WNT4 has been shown to be important in female sexual development. Lack of Wnt4 gives rise to masculinization of the XX gonad and we showed previously that the role of WNT4 was to inhibit endothelial and steroidogenic cell **migration** into the developing ovary. Here we show that Wnt4 also has a function in the male gonad. We find that Sertoli cell differentiation is compromised in Wnt4 mutant testes and that this defect occurs downstream of the testis-determining gene Sry but upstream of Sox9 and Dhh, two early Sertoli cell markers. Genetic analysis shows that this phenotype is primarily due to the action of WNT4 within the early genital ridge. Analysis of different markers identifies the most striking difference in the genital ridge at early stages of its development between wild-type and Wnt4 mutant embryos to be a significant increase of steroidogenic cells in the Wnt4 -/- gonad. These results identify WNT4 as a new factor involved in the mammalian testis determination pathway and show that genes can have a specific but distinct role in both male and female gonad development.
3. Stabilized beta-catenin in thymic epithelial cells blocks thymus development and function. Thymic T cell development is dependent on a specialized epithelial microenvironment mainly composed of cortical and medullary thymic epithelial cells (TECs). The molecular programs governing the differentiation and maintenance of TECs remain largely unknown. Wnt signaling is central to the development and maintenance of several organ systems but a specific role of this pathway for thymus organogenesis has not yet been ascertained. In this report, we demonstrate that activation of the canonical Wnt signaling pathway by a stabilizing mutation of beta-catenin targeted exclusively to TECs changes the initial commitment of endodermal epithelia to a thymic cell fate. Consequently, the formation of a correctly composed and organized thymic microenvironment is prevented, thymic im**migration** of hematopoietic precursors is restricted, and intrathymic T cell differentiation is arrested at a very early developmental stage causing severe immunodeficiency. These results suggest that a precise regulation of canonical Wnt signaling in thymic epithelia is essential for normal thymus development and function.
4. Expression of FGF receptors 1, 2, 3 in the embryonic and postnatal mouse brain compared with Pdgfralpha, Olig2 and Plp/dm20: implications for oligodendrocyte development. Fibroblast growth factors (FGF) receptors FgfR1, FgfR2 and FgfR3 are differentially regulated during oligodendrocyte (OL) maturation in vitro: FgfR3 is expressed by OL progenitors whereas FgfR2 is expressed by differentiated OLs [Mol Cell Neurosci 1996;7:263-275], and we have recently shown that FgfR3 is required for the timely differentiation of OLs in vivo [J Neurosci 2003;23:883-894]. Here we have used in situ hybridization to investigate the expression patterns of FgfR1-3 and compare them to the putative OL progenitor markers Olig2, Pdgfralpha and Plp/dm20 as a function of development in vivo, in particular at sites of OL specification, **migration** or differentiation in the mouse forebrain and cerebellum. We show that at early stages FgfR1-3 expression overlaps with that of Olig2 in the embryonic ventricular zone of the lateral and medial ganglionic eminences. Further, a scattered population of cells expressing FgfR3 (but not FgfR1 or FgfR2) in the ventral telencephalon appear to arise from the ventricular zone, and at later stages are found more dorsally in the cortex, in an overall pattern similar to Olig2 and/or Pdgfralpha. Postnatal expression of FgfR2 increases with age, more prominently in specific regions, including the cortical and cerebellar white matter and optic nerve. Thus, the differential expression pattern of FgfR2 and FgfR3 observed in vivo suggests that their expression is developmentally regulated in a manner consistent with the pattern of their expression in culture. These data provide further insights into role of FgfRs in OL development, and they emphasize that these receptors are positioned both spatially and temporally to impact OL generation in vivo.
5. The extracellular matrix molecule tenascin C modulates expression levels and territories of key patterning genes during spinal cord astrocyte specification. The generation of astrocytes during the development of the mammalian spinal cord is poorly understood. Here, we demonstrate for the first time that the extracellular matrix glycoprotein tenascin C regulates the expression of key patterning genes during late embryonic spinal cord development, leading to a timely maturation of gliogenic neural precursor cells. We first show that tenascin C is expressed by gliogenic neural precursor cells during late embryonic development. The loss of tenascin C leads to a sustained generation and delayed **migration** of Fgfr3-expressing immature astrocytes in vivo. Consistent with an increased generation of astroglial cells, we documented an increased number of GFAP-positive astrocytes at later stages. Mechanistically, we could demonstrate an upregulation and domain shift of the patterning genes Nkx6.1 and Nkx2.2 in vivo. In addition, sulfatase 1, a known downstream target of Nkx2.2 in the ventral spinal cord, was also upregulated. Sulfatase 1 regulates growth factor signalling by cleaving sulphate residues from heparan sulphate proteoglycans. Consistent with this function, we observed changes in both FGF2 and EGF responsiveness of spinal cord neural precursor cells. Taken together, our data implicate Tnc in the regulation of proliferation and lineage progression of astroglial progenitors in specific domains of the developing spinal cord.
6. Netrin1 is required for neural and glial precursor **migration**s into the olfactory bulb. Netrin1 (NTN1) deficiency in mouse brain causes defects in axon guidance and cell **migration** during embryonic development. Here we show that NTN1 is required for olfactory bulb (OB) development at late embryogenesis and at early postnatal stages to facilitate the accumulation of proper numbers of granular and glomerular neuron subtypes and oligodendrocytes into the OB. In addition to the analysis of Ntn1-/- mice we made tissue and neurosphere cultures to clarify the role of NTN1 in the anterior forebrain. We propose that a subset of neural progenitors/precursors requires NTN1 to efficiently enter the rostral migratory stream to migrate into the OB. The analysis of postnatal Ntn1-/- OBs revealed a reduction of specific types of interneurons which have been shown to originate from particular subregions of the lateral ventricle walls. Based on Ntn1 expression in ventral parts of the ventricle walls, we observed a decrease in the mainly ventrally derived type II interneurons that express calcium-binding proteins calretinin and calbindin. Instead, no change in the numbers of dorsally derived tyrosine hydroxylase expressing interneurons was detected. In addition to the specific reduction of type II interneurons, our results indicate that NTN1 is required for oligodendroglial **migration** into the OB. Furthermore, we characterised the Ntn1 expressing subpopulation of neurosphere-forming cells from embryonic and adult brain as multipotent and self-renewing. However, NTN1 is dispensable for the proliferation of neurosphere forming progenitor cells and for their differentiation.
7. Transcription factors Sox5 and Sox6 exert direct and indirect influences on oligodendroglial **migration** in spinal cord and forebrain. Transcription factors of the SoxD protein family have previously been shown to prevent precocious specification and terminal differentiation of oligodendrocyte progenitor cells in the developing spinal cord. Using mice with specific deletion of the SoxD proteins Sox5 and Sox6 in the central nervous system, we now show that SoxD proteins additionally influence **migration** of oligodendrocyte progenitors in the spinal cord as well as in the forebrain. In mutant mice, e**migration** of oligodendrocyte progenitors from the ventricular zone and colonization of the mantle zone are significantly delayed probably because of reduced expression of Pdgf receptor alpha and decreased responsiveness toward Pdgf-A as a main migratory cue. In addition to this direct cell-autonomous effect on Pdgf receptor alpha expression, SoxD proteins furthermore promote oligodendroglial **migration** by keeping the cells in an undifferentiated state and preventing a premature loss of their migratory capacity. This indirect effect becomes particularly important during late embryonic and early postnatal phases of oligodendroglial development. Finally, we show that Sox5 and Sox6 cooperate with Sox9 and Sox10 to activate Pdgf receptor alpha expression and thereby maintain oligodendrocyte progenitors in the immature state. This contrasts with their behavior on myelin genes where they antagonize the function of SoxE proteins. It argues that SoxD proteins can function either as repressors or as co-activators of SoxE proteins thereby modulating their function in a stage-specific manner.
8. Pdgfra and Pdgfrb genetically interact during craniofacial development. One of the most prevalent congenital birth defects is cleft palate. The palatal skeleton is derived from the cranial neural crest and platelet-derived growth factors (Pdgf) are critical in palatogenesis. Of the two Pdgf receptors, pdgfra is required for neural crest **migration** and palatogenesis. However, the role pdgfrb plays in the neural crest, or whether pdgfra and pdgfrb interact during palatogenesis is unclear.We find that pdgfrb is dispensable for craniofacial development in zebrafish. However, the palatal defect in pdgfra;pdgfrb double mutants is significantly more severe than in pdgfra single mutants. Data in mouse suggest this interaction is conserved and that neural crest requires both genes. In zebrafish, pdgfra and pdgfrb are both expressed by neural crest within the pharyngeal arches, and pharmacological analyses demonstrate Pdgf signaling is required at these times. While neither proliferation nor cell death appears affected, time-lapsed confocal analysis of pdgfra;pdgfrb mutants shows a failure of proper neural crest condensation during palatogenesis.We provide data showing that pdgfra and pdgfrb interact during palatogenesis in both zebrafish and mouse. In zebrafish, this interaction affects proper condensation of maxillary neural crest cells, revealing a previously unknown interaction between Pdgfra and Pdgfrb during palate formation. Developmental Dynamics 245:641-652, 2016. © 2016 Wiley Periodicals, Inc.
9. Ectopic c-kit expression affects the fate of melanocyte precursors in Patch mutant embryos. The Patch (Ph) mutation in the mouse, a deletion that includes the gene for PDGFR alpha, is a recessive lethal that exhibits a dominant pigment phenotype in heterozygotes. To assess whether the Ph mutation acts cell-autonomously or non-autonomously on melanocyte development, we have examined the melanogenic potential of neural crest populations from normal and mutant crest cells in vitro and the pattern of dispersal and survival of melanocyte precursors (MPs) in vivo. We report that trunk neural crest cells from homozygous Ph embryos give rise to pigmented melanocytes in vitro in response to Steel factor (SlF). In vivo, homozygous Ph embryos contain a subpopulation of crest-derived cells that express c-kit and tyrosinase-related protein-2 characteristic of MPs. These cells begin to migrate normally on the lateral crest **migration** pathway, but then fail to disperse in the dermal mesenchyme and subsequently disappear. Although dermal mesenchyme is adversely affected in Ph homozygotes, SlF mRNA expression by the cells of the dermatome is normal in Ph embryos when neural crest-derived MPs start to migrate on the lateral pathway. In contrast, mRNA for the SlF receptor, c-kit, was observed to be ectopically expressed in somites and lateral mesenchyme in embryos carrying the Ph mutation. Based on this ectopic expression of c-kit in Ph mutant embryos, and the observed distribution of SlF protein in normal and mutant embryos, we suggest that competition for limited amounts of SlF localized on the lateral neural crest **migration** pathway alters melanocyte dispersal and survival.
10. Wt1 controls retinoic acid signalling in embryonic epicardium through transcriptional activation of Raldh2. Epicardial-derived signals are key regulators of cardiac embryonic development. An important part of these signals is known to relate to a retinoic acid (RA) receptor-dependent mechanism. RA is a potent morphogen synthesised by Raldh enzymes, Raldh2 being the predominant one in mesodermal tissues. Despite the importance of epicardial retinoid signalling in the heart, the molecular mechanisms controlling cardiac Raldh2 transcription remain unknown. In the current study, we show that Wt1-null epicardial cells display decreased expression of Raldh2 both in vivo and in vitro. Using a RA-responsive reporter, we have confirmed that Wt1-null epicardial cells actually show reduced synthesis of RA. We also demonstrate that Raldh2 is a direct transcriptional target of Wt1 in epicardial cells. A secondary objective of this study was to identify the status of RA-related receptors previously reported to be critical to epicardial biology (PDGFRα,β; RXRα). PDGFRα and PDGFRβ mRNA and protein levels are downregulated in the absence of Wt1, but only Pdgfra expression is rescued by the addition of RA to Wt1-null epicardial cells. RXRα mRNA levels are not affected in Wt1-null epicardial cells. Taken together, our results indicate that Wt1 critically regulates epicardial RA signalling via direct activation of the Raldh2 gene, and identify a role for Wt1 in the regulation of morphogen receptors involved in the prolifera**tion, mig**ration, and differentiation of epicardial and epicardially-derived cells (EPDC).
11. Cooperation between the PDGF receptors in cardiac neural crest cell **migration**. Neural crest cells (NCCs) are essential components of the sympathetic nervous system, skin, craniofacial skeleton, and aortic arch. It has been known for many years that perturbation of **migration**, proliferation, and/or differentiation of these cells leads to birth defects such as cleft palate and persistent truncus arteriosus (PTA). Previously, we had shown that disruption of the platelet-derived growth factor receptor (PDGFR) alpha in NCCs resulted in defects in craniofacial and aortic arch development, the latter with variable penetrance. Because we observed ventricular septal defects in embryos that are null for the PDGFRbeta, we hypothesized that both PDGF receptors are involved in NCC formation. Here, we show that both receptors are expressed in cardiac NCCs and that the combined loss of the PDGFRalpha and PDGFRbeta in NCCs resulted in NCC-related heart abnormalities, including PTA and a ventricular septal defect (VSD). Using NCC lineage tracing, we observed that loss of PDGF receptor signaling resulted in reduced NCCs in the conotruncus region, leading to defects in aortic arch septation. These results indicate that while PDGFRalpha plays a predominant role in NCC development, the PDGFRbeta is expressed by and functions in cardiac NCCs. Combined PDGF receptor signaling is required for sufficient recruitment of cardiac NCCs into the conotruncal region and for formation of the aortico-pulmonary and ventricular septum.
12. Probing chromatin landscape reveals roles of endocardial TBX20 in septation. Mutations in the T-box transcription factor TBX20 are associated with multiple forms of congenital heart defects, including cardiac septal abnormalities, but our understanding of the contributions of endocardial TBX20 to heart development remains incomplete. Here, we investigated how TBX20 interacts with endocardial gene networks to drive the mesenchymal and myocardial movements that are essential for outflow tract and atrioventricular septation. Selective ablation of Tbx20 in murine endocardial lineages reduced the expression of extracellular matrix and cell **migration** genes that are critical for septation. Using the assay for transposase-accessible chromatin with high-throughput sequencing (ATAC-seq), we identified accessible chromatin within endocardial lineages and intersected these data with TBX20 ChIP-seq and chromatin loop maps to determine that TBX20 binds a conserved long-range enhancer to regulate versican (Vcan) expression. We also observed reduced Vcan expression in Tbx20-deficient mice, supporting a direct role for TBX20 in Vcan regulation. Further, we show that the Vcan enhancer drove reporter gene expression in endocardial lineages in a TBX20-binding site-dependent manner. This work illuminates gene networks that interact with TBX20 to orchestrate cardiac septation and provides insight into the chromatin landscape of endocardial lineages during septation.
13. Oligodendrocyte precursors migrate along vasculature in the developing nervous system. Oligodendrocytes myelinate axons in the central nervous system and develop from oligodendrocyte precursor cells (OPCs) that must first migrate extensively during brain and spinal cord development. We show that OPCs require the vasculature as a physical substrate for **migration**. We observed that OPCs of the embryonic mouse brain and spinal cord, as well as the human cortex, emerge from progenitor domains and associate with the abluminal endothelial surface of nearby blood vessels. Migrating OPCs crawl along and jump between vessels. OPC **migration** in vivo was disrupted in mice with defective vascular architecture but was normal in mice lacking pericytes. Thus, physical interactions with the vascular endothelium are required for OPC **migration**. We identify Wnt-Cxcr4 (chemokine receptor 4) signaling in regulation of OPC-endothelial interactions and propose that this signaling coordinates OPC **migration** with differentiation.
14. Embryonic mouse testis development: role of platelet derived growth factor (PDGF-BB). Platelet-derived growth factors (PDGFs) are paracrine growth factors mediating epithelial-mesenchymal interactions and exerting multiple biological activities which include cell proliferation, motility, and differentiation. As previously demonstrated, PDGFs act during embryonic development and recently, by culturing male genital ridges, we have demonstrated that PDGF-BB is able to support in vitro testicular cord formation. In the present paper, we report that PDGF-BB is present during embryonic testis development and, in organ culture, induces cord formation although with reduced diameters compared with the cords formed in the genital ridges cultured in the presence of HGF. Moreover we have analyzed the roles exerted by this growth factor during the morphogenesis of the testis. We demonstrate by immunohistochemical experiments that PDGF-BB and its receptors are synthesized by the male UGRs isolated from 11.5 and 13.5 dpc embryos and by Western blot that the factor is secreted in a biologically active form by testicular cells isolated from 13.5 dpc embryos. The biological roles of the factor have also been studied and we demonstrate that PDGF-BB acts as a migratory factor for male mesonephric cells whose **migration** is a male specific event necessary for a normal testicular morphogenesis. In addition we demonstrate that during testicular development, PDGF-BB induces testicular cell proliferation being in this way responsible for the increase in size of the testis. Finally we demonstrate that PDGF-BB is able to reorganize dissociated testicular cells inducing the formation of large cellular aggregates. However the structures formed in vitro under PDGF-BB stimulation never had a cord-like morphology similar to the cord-like structures formed in the presence of HGF (Ricci et al., 2002, Mech Dev 118:19-28), suggesting that this factor does not act as a morphogenetic factor during testicular development. All together the data presented in this paper demonstrate that PDGF-BB and its receptors (alpha- and beta-subunits) are present during the crucial ages of embryonic mouse testis morphogenesis and indicate the multiple roles exerted by this factor during the development of the male gonad.
15. Shp2-dependent ERK signaling is essential for induction of Bergmann glia and foliation of the cerebellum. Folding of the cortex and the persistence of radial glia (RG)-like cells called Bergmann glia (BG) are hallmarks of the mammalian cerebellum. Similar to basal RG in the embryonic neocortex, BG maintain only basal processes and continuously express neural stem cell markers. Past studies had focused on the function of BG in granule cell **migration** and how granule cell progenitors (GCP) regulate cerebellar foliation. The molecular control of BG generation and its role in cerebellar foliation are less understood. Here, we have analyzed the function of the protein tyrosine phosphatase Shp2 in mice by deleting its gene Ptpn11 in the entire cerebellum or selectively in the GCP lineage. Deleting Ptpn11 in the entire cerebellum by En1-cre blocks transformation of RG into BG but preserves other major cerebellar cell types. In the absence of BG, inward invagination of GCP persists but is uncoupled from the folding of the Purkinje cell layer and the basement membrane, leading to disorganized lamination and an absence of cerebellar folia. In contrast, removing Ptpn11 in the GCP lineage by Atoh1-cre has no effect on cerebellar development, indicating that Shp2 is not cell autonomously required in GCP. Furthermore, we demonstrate that Ptpn11 interacts with Fgf8 and is essential for ERK activation in RG and nascent BG. Finally, expressing constitutively active MEK1 rescues BG formation and cerebellar foliation in Shp2-deficient cerebella. Our results demonstrate an essential role of Shp2 in BG specification via fibroblast growth factor/extracellular signal-regulated protein kinase signaling, and reveal a crucial function of BG in organizing cerebellar foliation.
16. Afadin: A key molecule essential for structural organization of cell-cell junctions of polarized epithelia during embryogenesis. Afadin is an actin filament-binding protein that binds to nectin, an immunoglobulin-like cell adhesion molecule, and is colocalized with nectin at cadherin-based cell-cell adherens junctions (AJs). To explore the function of afadin in cell-cell adhesion during embryogenesis, we generated afadin(-/-) mice and embryonic stem cells. In wild-type mice at embryonic days 6.5-8.5, afadin was highly expressed in the embryonic ectoderm and the mesoderm, but hardly detected in the extraembryonic regions such as the visceral endoderm. Afadin(-/-) mice showed developmental defects at stages during and after gastrulation, including disorganization of the ectoderm, impaired **migration** of the mesoderm, and loss of somites and other structures derived from both the ectoderm and the mesoderm. Cystic embryoid bodies derived from afadin(-/-) embryonic stem cells showed normal organization of the endoderm but disorganization of the ectoderm. Cell-cell AJs and tight junctions were improperly organized in the ectoderm of afadin(-/-) mice and embryoid bodies. These results indicate that afadin is highly expressed in the ectoderm- derived cells during embryogenesis and plays a key role in proper organization of AJs and tight junctions of the highly expressing cells, which is essential for proper tissue morphogenesis.
17. Different timings of Dicer deletion affect neurogenesis and gliogenesis in the developing mouse central nervous system. MicroRNAs, processed by the RNAase III enzyme Dicer, are approximately 22 nucleotide endogenous noncoding small RNAs. The function of Dicer in the mouse central nervous system (CNS) development is not well understood. Here, we show that specifically deleting Dicer expression in the CNS and in the cerebral cortex using two Cre lines results in reduced progenitor numbers, abnormal neuronal differentiation, and thinner cortical wall. Incomplete Dicer deletion during early embryonic stages contributes to normal development of early-born neurons in the cortex and motor neurons in the spinal cord. However, at late embryonic stages when Dicer is completely ablated in the CNS, the **migration** of late-born neurons in the cortex and oligodendrocyte precursor expansion and differentiation in the spinal cord are greatly affected. Our studies of different timings of Dicer deletion demonstrate the importance of the Dicer-mediated microRNA pathway in regulating distinct phases of neurogenesis and gliogenesis during the CNS development.
18. Endothelin-2 signaling in the neural retina promotes the endothelial tip cell state and inhibits angiogenesis. Endothelin signaling is required for neural crest **migration** and homeostatic regulation of blood pressure. Here, we report that constitutive overexpression of Endothelin-2 (Edn2) in the mouse retina perturbs vascular development by inhibiting endothelial cell **migration** across the retinal surface and subsequent endothelial cell invasion into the retina. Developing endothelial cells exist in one of two states: tip cells at the growing front and stalk cells in the vascular plexus behind the front. This division of endothelial cell states is one of the central organizing principles of angiogenesis. In the developing retina, Edn2 overexpression leads to overproduction of endothelial tip cells by both morphologic and molecular criteria. Spatially localized overexpression of Edn2 produces a correspondingly localized endothelial response. Edn2 overexpression in the early embryo inhibits vascular development at midgestation, but Edn2 overexpression in developing skin and brain has no discernible effect on vascular structure. Inhibition of retinal angiogenesis by Edn2 requires expression of Endothelin receptor A but not Endothelin receptor B in the neural retina. Taken together, these observations imply that the neural retina responds to Edn2 by synthesizing one or more factors that promote the endothelial tip cell state and inhibit angiogenesis. The response to Edn2 is sufficiently potent that it overrides the activities of other homeostatic regulators of angiogenesis, such as Vegf.
19. Platelet-derived growth factor receptor beta signaling is required for efficient epicardial cell **migration** and development of two distinct coronary vascular smooth muscle cell populations. The epicardium plays an essential role in coronary artery formation and myocardial development, but signals controlling the development and differentiation of this tissue are not well understood. To investigate the role of platelet-derived growth factor receptor (PDGFR)beta in development of epicardial-derived vascular smooth muscle cells (VSMCs), we examined PDGFRbeta(-/-) and PDGFRbeta epicardial mutant hearts. We found that PDGFRbeta(-/-) hearts failed to form dominant coronary vessels on the ventral heart surface, had a thinned myocardium, and completely lacked coronary VSMCs (cVSMCs). This constellation of defects was consistent with a primary defect in the epicardium. To verify that these defects were specific to epicardial derivatives, we generated mice with an epicardial deletion of PDGFRbeta that resulted in reduced cVSMCs distal to the aorta. The regional absence of cVSMCs suggested that cVSMCs could arise from 2 sources, epicardial and nonepicardial, and that both were dependent on PDGFRbeta. In the absence of PDGFRbeta signaling, epicardial cells adopted an irregular actin cytoskeleton, leading to aberrant **migration** of epicardial cells into the myocardium in vivo. In addition, PDGF receptor stimulation promoted epicardial cell **migration**, and PDGFRbeta-driven phosphoinositide 3'-kinase signaling was critical for this process. Our data demonstrate that PDGFRbeta is required for the formation of 2 distinct cVSMC populations and that loss of PDGFRbeta-PI3K signaling disrupts epicardial cell **migration**.
20. Epicardial-derived cell epithelial-to-mesenchymal transition and fate specification require PDGF receptor signaling. In early heart development, platelet-derived growth factor (PDGF) receptor expression in the heart ventricles is restricted to the epicardium. Previously, we showed that PDGFRβ is required for coronary vascular smooth muscle cell (cVSMC) development, but a role for PDGFRα has not been identified. Therefore, we investigated the combined and independent roles of these receptors in epicardial development.To understand the contribution of PDGF receptors in epicardial development and epicardial-derived cell fate determination.By generating mice with epicardial-specific deletion of the PDGF receptors, we found that epicardial epithelial-to-mesenchymal transition (EMT) was defective. Sox9, an SRY-related transcription factor, was reduced in PDGF receptor-deficient epicardial cells, and overexpression of Sox9 restored epicardia**l migrati**on, actin reorganization, and EMT gene expression profiles. The failure of epicardial EMT resulted in hearts that lacked epicardial-derived cardiac fibroblasts and cVSMC. Loss of PDGFRα resulted in a specific disruption of cardiac fibroblast development, whereas cVSMC development was unperturbed.Signaling through both PDGF receptors is necessary for epicardial EMT and formation of epicardial-mesenchymal derivatives. PDGF receptors also have independent functions in the development of specific epicardial-derived cell fates.
21. Radial construction of an arterial wall. Some of the most serious diseases involve altered size and structure of the arterial wall. Elucidating how arterial walls are built could aid understanding of these diseases, but little is known about how concentric layers of muscle cells and the outer adventitial layer are assembled and patterned around endothelial tubes. Using histochemical, clonal, and genetic analysis in mice, here we show that the pulmonary artery wall is constructed radially, from the inside out, by two separate but coordinated processes. One is sequential induction of successive cell layers from surrounding mesenchyme. The other is controlled invasion of outer layers by inner layer cells through developmentally regulated cell reorientation and radial **migration**. We propose that a radial signal gradient controls these processes and provide evidence that PDGF-B and at least one other signal contribute. Modulation of such radial signaling pathways may underlie vessel-specific differences and pathological changes in arterial wall size and structure.
22. CXCR4 signaling regulates radial glial morphology and cell fate during embryonic spinal cord development. Embryonic meninges secrete the chemokine SDF-1/CXCL12 as a chemotactic guide for migrating neural stem cells, but SDF-1 is not known to directly regulate the functions of radial glia. Recently, the developing meninges have been shown to regulate radial glial function, yet the mechanisms and signals responsible for this phenomenon remain unclear. Moreover, as a nonmigratory cell type, radial glia do not conform to traditional models associated with chemokine signaling in the central nervous system. Using fluorescent transgenes, in vivo genetic manipulations and pharmacological techniques, we demonstrate that SDF-1 derived from the meninges exerts a CXCR4-dependent effect on radial glia. Deletion of CXCR4 expression by radial glia influences their morphology, mitosis, and progression through both oligodendroglial and astroglial lineages. Additionally, disruption of CXCR4 signaling in radial glia has a transient effect on the **migration** of oligodendrocyte progenitors. These data indicate that a specific chemokine signal derived from the meninges has multiple regulatory effects on radial glia.
23. Coordinate regulation by diethylstilbestrol of the platelet-derived growth factor-A (PDGF-A) and -B chains and the PDGF receptor alpha- and beta-subunits in the mouse uterus and vagina: potential mediators of estrogen action. The effects of estrogen on the reproductive tract involve cell proliferation, **migration**, and differentiation, which need to be well coordinated. Polypeptide growth factors are believed to play a vital role in a number of these cellular processes. Among the growth factors now documented to be associated with estrogen action are epidermal growth factor, transforming growth factor-alpha (TGF alpha), transforming growth factor-beta 1 (TGF beta 1), TGF beta 2, TGF beta 3, and insulin-like growth factor-I (IGF-I). Platelet-derived growth factors (PDGFs) are also potent mitogens, which consist of two peptide chains, denoted A and B, that dimerize to isoforms (PDGF-AA, -AB, and -BB) which differ in their functional properties, secretory behavior, receptor binding, and physiological effects. To study the role of the PDGF-A and -B chains and the PDGF receptor subunits, alpha and beta, during estrogen action in the mouse reproductive tract, time-dependent changes in the expression of these genes were examined by Northern and in situ RNA analyses and by immunohistochemistry after a single treatment of immature CD-1 (17- to 19-day-old) mice with the synthetic estrogen, diethylstilbestrol (DES). Our results demonstrate estrogen modulation of the expression of messenger RNA (mRNA) and protein for the PDGF ligands and receptors in both the uterus and vagina of the mouse. Northern and in situ RNA analyses demonstrate time-dependent estrogen induction of the mRNA levels for these genes in both tissues within 3 h after treatment. However, distinctive mRNA expression profiles for the PDGF ligand and receptor genes are exhibited by the uterus and vagina in response to DES, especially in that the induction of transcripts for PDGF-A and both receptor subunits is more transient in the vagina than in the uterus. Steroid specificity studies demonstrate predominant estrogen-specific regulation of mRNA induction for these genes. Analysis of cell-specific RNA expression by in situ hybridization reveals prominent induction of transcripts for the PDGF chains and receptor subunits in the uterine and vaginal epithelium after estrogen treatment, although enhanced expression of mRNA is also noted in the stroma, particularly for the PDGF receptor subunit genes. Cellular localization of the PDGF ligand and receptor protein molecules by immunohistochemistry detected significant immunostaining for all of these proteins in both the uterus and vagina of control animals. After DES treatment, the uterus exhibits a significant decrease in the level of PDGF ligand and receptor proteins immunostained within 6 h, whereas less dramatic effects ar observed in the vagina.(ABSTRACT TRUNCATED AT 400 WORDS)
24. Cooperation between the PDGF receptors in cardiac neural crest cell **migration**. Neural crest cells (NCCs) are essential components of the sympathetic nervous system, skin, craniofacial skeleton, and aortic arch. It has been known for many years that perturbation of **migration**, proliferation, and/or differentiation of these cells leads to birth defects such as cleft palate and persistent truncus arteriosus (PTA). Previously, we had shown that disruption of the platelet-derived growth factor receptor (PDGFR) alpha in NCCs resulted in defects in craniofacial and aortic arch development, the latter with variable penetrance. Because we observed ventricular septal defects in embryos that are null for the PDGFRbeta, we hypothesized that both PDGF receptors are involved in NCC formation. Here, we show that both receptors are expressed in cardiac NCCs and that the combined loss of the PDGFRalpha and PDGFRbeta in NCCs resulted in NCC-related heart abnormalities, including PTA and a ventricular septal defect (VSD). Using NCC lineage tracing, we observed that loss of PDGF receptor signaling resulted in reduced NCCs in the conotruncus region, leading to defects in aortic arch septation. These results indicate that while PDGFRalpha plays a predominant role in NCC development, the PDGFRbeta is expressed by and functions in cardiac NCCs. Combined PDGF receptor signaling is required for sufficient recruitment of cardiac NCCs into the conotruncal region and for formation of the aortico-pulmonary and ventricular septum.
25. Endothelial and steroidogenic cell **migration** are regulated by WNT4 in the developing mammalian gonad. The signalling molecule WNT4 has been associated with sex reversal phenotypes in mammals. Here we show that the role of WNT4 in gonad development is to pattern the sex-specific vasculature and to regulate steroidogenic cell recruitment. Vascular formation and steroid production in the mammalian gonad occur in a sex-specific manner. During testis development, endothelial cells migrate from the mesonephros into the gonad to form a coelomic blood vessel. Leydig cells differentiate and produce steroid hormones a day later. Neither of these events occurs in the XX gonad. We show that WNT4 represses mesonephric endothelial and steroidogenic cell **migration** in the XX gonad, preventing the formation of a male-specific coelomic blood vessel and the production of steroids. In the XY gonad, Wnt4 expression is downregulated after sex determination. Transgenic misexpression of Wnt4 in the embryonic testis did not inhibit coelomic vessel formation but vascular pattern was affected. Leydig cell differentiation was not affected in these transgenic animals and our data implies that Wnt4 does not regulate steroidogenic cell differentiation but represses the **migration** of steroidogenic adrenal precursors into the gonad. These studies provide a model for understanding how the same signalling molecule can act on two different cell types to coordinate sex development.
26. The mesenchymal alpha11beta1 integrin attenuates PDGF-BB-stimulated chemotaxis of embryonic fibroblasts on collagens. alpha11beta1 constitutes the most recent addition to the integrin family and has been shown to display a binding preference for interstitial collagens found in mesenchymal tissues. We have previously observed that when alpha11beta1 integrin is expressed in cells lacking endogenous collagen receptors, it can mediate PDGF-BB-dependent chemotaxis on collagen I in vitro. To determine in which cells PDGF and alpha11beta1 might cooperate in regulating cell **migration** in vivo, we studied in detail the expression and distribution of alpha11 integrin chain in mouse embryos and tested the ability of PDGF isoforms to stimulate the alpha11beta1-mediated cell **migration** of embryonic fibroblasts. Full-length mouse alpha11 cDNA was sequenced and antibodies were raised to deduced alpha11 integrin amino acid sequence. In the embryonic mouse head, alpha11 protein and RNA were localized to ectomesenchymally derived cells. In the periodontal ligament, alpha11beta1 was expressed as the only detectable collagen-binding integrin, and alpha11beta1 is thus a major receptor for cell **migration** and matrix organization in this cell population. In the remainder of the embryo, the alpha11 chain was expressed in a subset of mesenchymal cells including tendon/ligament fibroblasts, perichondrial cells, and intestinal villi fibroblasts. Most of the alpha11-expressing cells also expressed the alpha2 integrin chain, but no detectable overlap was found with the alpha1 integrin chain. In cells expressing multiple collagen receptors, these might function to promote a more stable cell adhesion and render the cells more resistant to chemotactic stimuli. Wild-type embryonic fibroblasts activated mainly the PDGF beta receptor in response to PDGF-BB and migrated on collagens I, II, III, IV, V, and XI in response to PDGF-BB in vitro, whereas mutant fibroblasts that lacked alpha11beta1 in their collagen receptor repertoire showed a stronger chemotactic response on collagens when stimulated with PDGF-BB. In the cellular context of embryonic fibroblasts, alpha11beta1 is thus anti-migratory. We speculate that the PDGF BB-dependent cell **migration** of mesenchymal cells is tightly regulated by the collagen receptor repertoire, and disturbances of this repertoire might lead to unregulated cell **migration** that could affect normal embryonic development and tissue structure.
27. ERK1 and ERK2 are required for radial glial maintenance and cortical lamination. ERK1/2 is involved in a variety of cellular processes during development, but the functions of these isoforms in brain development remain to be determined. Here, we generated double knockout (DKO) mice to study the individual and combined roles of ERK1 and ERK2 during cortical development. Mice deficient in Erk2, and more dramatically in the DKOs, displayed proliferation defects in late radial glial progenitors within the ventricular zone, and a severe disruption of lamination in the cerebral cortex. Immunohistochemical analyses revealed that late-generated cortical neurons were misplaced and failed to migrate the upper cortical layers in DKO mice. Moreover, these mice displayed fewer radial glial fibers, which provide architectural guides for radially migrating neurons. These results suggest that extracellular signal-regulated kinase signaling is essential for the expansion of the radial glial population and for the maintenance of radial glial scaffolding. Tangential **migration** of interneurons and oligodendrocytes from the ganglionic eminences (GE) to the dorsal cortex was more severely impaired in DKO mice than in mice deficient for Erk2 alone, because of reduced progenitor proliferation in the GE of the ventral telencephalon. These data demonstrate functional overlaps between ERK1 and ERK2 and indicate that extracellular signal-regulated kinase signaling plays a crucial role in cortical development.
28. Netrin-1 is required for the normal development of spinal cord oligodendrocytes. Successful CNS myelination is dependent on the correct localization of oligodendrocytes and their interactions with adjacent axons. In the spinal cord, oligodendrocyte precursors originate at the ventral midline and subsequently migrate to the white matter where they mature. In vitro studies suggest this dispersal is mediated by the guidance molecule netrin-1. Here, we show that in the spinal cord of netrin-1 mutant mice, oligodendrocyte precursors failed to disperse from the ventral midline as a consequence of a lack of polarization and directional **migration**. The lack of netrin-1 also resulted in an overall reduction of oligodendrocyte lineage cells that was independent of the failure of initial dispersal. Oligodendrocyte precursors injected into presumptive white matter underwent extensive radial **migration** and expansion in wild-type but not netrin-1 mutant hosts. These data indicate that netrin-1 is crucial for both the initial dispersal of spinal cord oligodendrocyte precursors and their subsequent development in the presumptive white matter.
29. Altered development of spinal cord in the mouse mutant (Patch) lacking the PDGF receptor alpha-subunit gene. The platelet-derived growth factor receptor alpha subunit (PDGFR alpha) is expressed by glial precursors, glial cells, and some peripheral neurons during normal rodent development. Its ligands are expressed ubiquitously in neurons, including sensory and motor neurons. Thus, neuronally secreted PDGF-A may play a paracrine role in the development of both glial cells and peripheral neurons. The Patch (Ph) mutation, which is a deletion of the PDGFR alpha, is a homozygous embryonic lethal mutation in the mouse. Previously, several developmental abnormalities, including deficiencies in connective tissues in many organs, aberrant neural crest cell **migration**, and defects in non-neuronal derivatives of crest cells, have been shown to be associated with the Patch mutation. However, whether and the extent to which motor and sensory neurons are affected by the mutation are not known. Here, we have examined the survival and/or morphological differentiation of spinal motor and sensory (dorsal root ganglion) neurons during the period of naturally occurring cell death, i.e., between E14 and E18, in control and Ph/Ph mice. The results show a 65-70% decrease in motor and sensory neuron numbers in Ph/Ph mice, compared to controls, at all stages examined. Furthermore, motoneurons in Ph/Ph mice were significantly smaller than those in controls. Because of the bidirectional nature of neuron-glial cell interactions, these results suggest that PDGFR alpha plays an important role in glial cell development and, thus, indirectly in neuronal cell development or, alternatively, that PDGF and the PDGFR alpha are directly involved in peripheral neuron survival and development by an autocrine/paracrine mechanism.
30. Haploinsufficiency of the mouse Forkhead Box f1 gene causes defects in gall bladder development. The forkhead box f1 (Foxf1) transcription factor is expressed in the visceral (splanchnic) mesoderm, which is involved in mesenchymal-epithelial signaling required for development of organs derived from foregut endoderm such as lung, liver, gall bladder, and pancreas. Our previous studies demonstrated that haploinsufficiency of the Foxf1 gene caused pulmonary abnormalities with perinatal lethality from lung hemorrhage in a subset of Foxf1+/- newborn mice. During mouse embryonic development, the liver and biliary primordium emerges from the foregut endoderm, invades the septum transversum mesenchyme, and receives inductive signaling originating from both the septum transversum and cardiac mesenchyme. In this study, we show that Foxf1 is expressed in embryonic septum transversum and gall bladder mesenchyme. Foxf1+/- gall bladders were significantly smaller and had severe structural abnormalities characterized by a deficient external smooth muscle cell layer, reduction in mesenchymal cell number, and in some cases, lack of a discernible biliary epithelial cell layer. This Foxf1+/- phenotype correlates with decreased expression of vascular cell adhesion molecule-1 (VCAM-1), alpha(5) integrin, platelet-derived growth factor receptor alpha (PDGFRalpha) and hepatocyte growth factor (HGF) genes, all of which are critical for cell adhesion, **migration**, and mesenchymal cell differentiation.
31. SoxD proteins influence multiple stages of oligodendrocyte development and modulate SoxE protein function. The myelin-forming oligodendrocytes are an excellent model to study transcriptional regulation of specification events, lineage progression, and terminal differentiation in the central nervous system. Here, we show that the group D Sox transcription factors Sox5 and Sox6 jointly and cell-autonomously regulate several stages of oligodendrocyte development in the mouse spinal cord. They repress specification and terminal differentiation and influence **migration** patterns. As a consequence, oligodendrocyte precursors and terminally differentiating oligodendrocytes appear precociously in spinal cords deficient for both Sox proteins. Sox5 and Sox6 have opposite functions than the group E Sox proteins Sox9 and Sox10, which promote oligodendrocyte specification and terminal differentiation. Both genetic as well as molecular evidence suggests that Sox5 and Sox6 directly interfere with the function of group E Sox proteins. Our studies reveal a complex regulatory network between different groups of Sox proteins that is essential for proper progression of oligodendrocyte development.
32. Sox9 and Sox10 influence survival and **migration** of oligodendrocyte precursors in the spinal cord by regulating PDGF receptor alpha expression. Specification of the myelin-forming oligodendrocytes of the central nervous system requires the Sox9 transcription factor, whereas terminal differentiation depends on the closely related Sox10. Between specification and terminal differentiation, Sox9 and Sox10 are co-expressed in oligodendrocyte precursors and are believed to exert additional functions. To identify such functions, we have deleted Sox9 specifically in already specified oligodendrocyte precursors of the spinal cord. In the absence of Sox9, oligodendrocyte precursors developed normally and started terminal differentiation on schedule. However, when Sox10 was additionally deleted, oligodendrocyte precursors exhibited an altered **migration** pattern and were present in reduced numbers because of increased apoptosis rates. Remaining precursors continued to express many characteristic oligodendroglial markers. Aberrant expression of astrocytic and neuronal markers was not observed. Strikingly, we failed to detect PDGF receptor alpha expression in the mutant oligodendrocyte precursors, arguing that PDGF receptor alpha is under transcriptional control of Sox9 and Sox10. Altered PDGF receptor alpha expression is furthermore sufficient to explain the observed phenotype, as PDGF is both an important survival factor and migratory cue for oligodendrocyte precursors. We thus conclude that Sox9 and Sox10 are required in a functionally redundant manner in oligodendrocyte precursors for PDGF-dependent survival and **migration**.
33. Gli1 is not required for Pdgfralpha expression during mouse embryonic development. Pdgfra is expressed in the mesenchyme of multiple organs during embryonic development and Pdgfralpha is involved in cell proliferation, differentiation, **migration**, and apoptosis in many tissues. A fine-tuned regulation of gene transcription is required to achieve these effects. To investigate if the Shh signaling pathway is involved in the tightly regulated Pdgfra expression during embryogenesis, we systematically compared Gli1 and Pdgfralpha mRNA expression patterns in vivo from mouse embryonic day 9.5 to 14.5. We found that an initial partly overlapping expression of Gli1 and Pdgfralpha in the mesenchyme of foregut and somites was changed to different expression patterns when the mesenchyme differentiated into specialized structures such as intestinal villi and chondrocytes. Gli1 and Pdgfra were also expressed differently in the developing lung, heart, central nervous system, skin, tooth, and eye. Importantly, neither Pdgfralpha mRNA patterns nor levels were altered in Ihh mutant embryos although Gli1 and Ptc mRNA levels were dramatically reduced. Our results demonstrate that Gli1 is not required to induce Pdgfra expression during embryonic bone development, and are consistent with previous findings that Pdgfralpha and Hh pathways serve different functions in, e.g., bone, gut, and lung development. However, we cannot exclude the possibility that Glis can have more complex regulatory effects on Pdgfra gene activity, nor can we exclude such effects in pathological conditions.
34. The primitive endoderm lineage of the mouse blastocyst: sequential transcription factor activation and regulation of differentiation by Sox17. Cells of the primitive endoderm (PrE) and the pluripotent epiblast (EPI), the two lineages specified within the inner cell mass (ICM) of the mouse blastocyst stage embryo, are segregated into adjacent tissue layers by the end of the preimplantation period. The PrE layer which emerges as a polarized epithelium adjacent to the blastocoel, with a basement membrane separating it from the EPI, has two derivatives, the visceral and parietal endoderm. In this study we have investigated the localization of two transcriptional regulators of the SOX family, SOX17 and SOX7, within the PrE and its derivatives. We noted that SOX17 was first detected in a salt-and-pepper distribution within the ICM, subsequently becoming restricted to the nascent PrE epithelium. This dynamic distribution of SOX17 resembled the localization of GATA6 and GATA4, two other PrE lineage-specific transcription factors. By contrast, SOX7 was only detected in PrE cells positioned in contact with the blastocoel, raising the possibility that these cells are molecularly distinct. Our observations support a model of sequential GATA6 > SOX17 > GATA4 > SOX7 transcription factor activation within the PrE lineage, perhaps correlating with the consecutive periods of cell lineage 'naïvete', commitment and sorting. Furthermore our data suggest that co-expression of SOX17 and SOX7 within sorted PrE cells could account for the absence of a detectable phenotype of Sox17 mutant blastocysts. However, analysis of implantation-delayed blastocysts, revealed a role for SOX17 in the maintenance of PrE epithelial integrity, with the absence of SOX17 leading to premature delamination and **migratio**n of parietal endoderm.
35. Cell autonomous requirement for PDGFRalpha in populations of cranial and cardiac neural crest cells. Cardiac and cephalic neural crest cells (NCCs) are essential components of the craniofacial and aortic arch mesenchyme. Genetic disruption of the platelet-derived growth factor receptor alpha (PDGFRalpha) results in defects in multiple tissues in the mouse, including neural crest derivatives contributing to the frontonasal process and the aortic arch. Using chimeric analysis, we show that loss of the receptor in NCCs renders them inefficient at contributing to the cranial mesenchyme. Conditional gene ablation in NCCs results in neonatal lethality because of aortic arch defects and a severely cleft palate. The conotruncal defects are first observed at E11.5 and are consistent with aberrant NCC development in the third, fourth and sixth branchial arches, while the bone malformations present in the frontonasal process and skull coincide with defects of NCCs from the first to third branchial arches. Changes in cell proliferation, **migration**, or survival were not observed in PDGFRalpha NCC conditional embryos, suggesting that the PDGFRalpha may play a role in a later stage of NCC development. Our results demonstrate that the PDGFRalpha plays an essential, cell-autonomous role in the development of cardiac and cephalic NCCs and provides a model for the study of aberrant NCC development.
36. Slit2 regulates the dispersal of oligodendrocyte precursor cells via Fyn/RhoA signaling. Oligodendrocyte precursor cells (OPCs) are a unique type of glia that are responsible for the myelination of the central nervous system. OPC **migration** is important for myelin formation during central nervous system development and repair. However, the precise extracellular and intracellular mechanisms that regulate OPC **migration** remain elusive. Slits were reported to regulate neurodevelopmental processes such as **migration**, adhesion, axon guidance, and elongation through binding to roundabout receptors (Robos). However, the potential roles of Slits/Robos in oligodendrocytes remain unknown. In this study, Slit2 was found to be involved in regulating the dispersal of OPCs through the association between Robo1 and Fyn. Initially, we examined the expression of Robos in OPCs both in vitro and in vivo. Subsequently, the Boyden chamber assay showed that Slit2 could inhibit OPC **migration**. RoboN, a specific inhibitor of Robos, could significantly attenuate this effect. The effects were confirmed through the explant **migration** assay. Furthermore, treating OPCs with Slit2 protein deactivated Fyn and increased the level of activated RhoA-GTP. Finally, Fyn was found to form complexes with Robo1, but this association was decreased after Slit2 stimulation. Thus, we demonstrate for the first time that Slit2 regulates the dispersal of oligodendrocyte precursor cells through Fyn and RhoA signaling.
37. The PDGF alpha receptor is required for neural crest cell development and for normal patterning of the somites. Platelet-derived growth factors (PDGFs) have been implicated in the control of cell proliferation, survival and **migration**. Patch mutant mice harbor a deletion including the PDGF alpha receptor gene and exhibit defects of neural crest origin which affect pigmentation in heterozygotes and cranial bones in homozygotes. To verify the role of the PDGF alphaR gene during development, mice carrying a targeted null mutation were generated. No pigmentation phenotype was observed in heterozygotes. Homozygotes die during embryonic development and exhibit incomplete cephalic closure similar to that observed in a subset of Patch mutants. In addition, increased apoptosis was observed on pathways followed by migrating neural crest cells. However, alterations in mutant vertebrae, ribs and sternum were also observed, which appear to stem from a deficiency in myotome formation. These results indicate that PDGFs may exert their functions during early embryogenesis by affecting cell survival and patterning.
38. Rib truncations and fusions in the Sp2H mouse reveal a role for Pax3 in specification of the ventro-lateral and posterior parts of the somite. The splotch (Pax3) mouse mutant serves as a model for developmental defects of several types, including defective **migration** of dermomyotomal cells to form the limb musculature. Here, we describe abnormalities of the ribs, neural arches, and acromion in Sp2H homozygous embryos, indicating a widespread dependence of lateral somite development on Pax3 function. Moreover, the intercostal and body wall muscles, derivatives of the ventrolateral myotome, are also abnormal in Sp2H homozygotes. Pax3 is expressed in the dermomyotome, but not in either the sclerotome or the myotome, raising the possibility that Pax3-dependent inductive influences from the dermomyotome are necessary for early specification of lateral sclerotome and myotome. Support for this idea comes from analysis of gene expression markers of lateral sclerotome (tenascin-C and scleraxis) and myotome (myogenin, MyoD, and Myf5). All exhibit ventrally truncated domains of expression in Sp2H homozygotes, potentially accounting for the rib and intercostal muscle truncations. In contrast, the medial sclerotomal marker Pax1 is expressed normally in mutant embryos, arguing that Pax3 is not required for development of the medial sclerotome. Most of the somitic markers show ectopic expression in anteroposterior and mediolateral dimensions, suggesting a loss of definition of somite boundaries in splotch and explaining the rib and muscle fusions. An exception is Myf5, which is not ectopically expressed in Sp2H homozygotes, consistent with the previous suggestion that Pax3 and Myf5 function in different pathways of skeletal myogenesis. PDGFalpha and its receptor are candidates for mediating signalling between myotome and sclerotome. We find that both genes are misexpressed in Sp2H embryos, suggesting that PDGFalpha/PDGFRalpha may function downstream of Pax3, accounting for the close similarities between the splotch and Patch mutant phenotypes. Our findings point to additional regulatory functions for the Pax3 transcription factor, apart from those already demonstrated for development of the neural tube, neural crest, and dermomyotome.
39. Complementary roles for Nkx6 and Nkx2 class proteins in the establishment of motoneuron identity in the hindbrain. The genetic program that underlies the generation of visceral motoneurons in the developing hindbrain remains poorly defined. We have examined the role of Nkx6 and Nkx2 class homeodomain proteins in this process, and provide evidence that these proteins mediate complementary roles in the specification of visceral motoneuron fate. The expression of Nkx2.2 in hindbrain progenitor cells is sufficient to mediate the activation of Phox2b, a homeodomain protein required for the generation of hindbrain visceral motoneurons. The redundant activities of Nkx6.1 and Nkx6.2, in turn, are dispensable for visceral motoneuron generation but are necessary to prevent these cells from adopting a parallel program of interneuron differentiation. The expression of Nkx6.1 and Nkx6.2 is further maintained in differentiating visceral motoneurons, and consistent with this the **migration** and axonal projection properties of visceral motoneurons are impaired in mice lacking Nkx6.1 and/or Nkx6.2 function. Our analysis provides insight also into the role of Nkx6 proteins in the generation of somatic motoneurons. Studies in the spinal cord have shown that Nkx6.1 and Nkx6.2 are required for the generation of somatic motoneurons, and that the loss of motoneurons at this level correlates with the extinguished expression of the motoneuron determinant Olig2. Unexpectedly, we find that the initial expression of Olig2 is left intact in the caudal hindbrain of Nkx6.1/Nkx6.2 compound mutants, and despite this, all somatic motoneurons are missing. These data argue against models in which Nkx6 proteins and Olig2 operate in a linear pathway, and instead indicate a parallel requirement for these proteins in the progression of somatic motoneuron differentiation. Thus, both visceral and somatic motoneuron differentiation appear to rely on the combined activity of cell intrinsic determinants, rather than on a single key determinant of neuronal cell fate.
40. Cyclin dependent kinase 5 is required for the normal development of oligodendrocytes and myelin formation. The development of oligodendrocytes, the myelinating cells of the vertebrate CNS, is regulated by a cohort of growth factors and transcription factors. Less is known about the signaling pathways that integrate extracellular signals with intracellular transcriptional regulators to control oligodendrocyte development. Cyclin dependent kinase 5 (Cdk5) and its co-activators play critical roles in the regulation of neuronal differentiation, cortical lamination, neuronal cell **migration** and axon outgrowth. Here we demonstrate a previously unrecognized function of Cdk5 in regulating oligodendrocyte maturation and myelination. During late embryonic development Cdk5 null animals displayed a reduction in the number of MBP+ cells in the spinal cord, but no difference in the number of OPCs. To determine whether the reduction of oligodendrocytes reflected a cell-intrinsic loss of Cdk5, it was selectively deleted from Olig1+ oligodendrocyte lineage cells. In Olig1(Cre/+); Cdk5(fl/fl) conditional mutants, reduced levels of expression of MBP and PLP mRNA were observed throughout the CNS and ultrastructural analyses demonstrated a significant reduction in the proportion of myelinated axons in the optic nerve and spinal cord. Pharmacological inhibition or RNAi knockdown of Cdk5 in vitro resulted in the reduction in oligodendrocyte maturation, but had no effect on OPC cell proliferation. Conversely, over-expression of Cdk5 promoted oligodendrocyte maturation and enhanced process outgrowth. Consistent with this data, Cdk5(-/-) oligodendrocytes developed significantly fewer primary processes and branches than control cells. Together, these findings suggest that Cdk5 function as a signaling integrator to regulate oligodendrocyte maturation and myelination.
41. Pdgfr-alpha mediates testis cord organization and fetal Leydig cell development in the XY gonad. During testis development, the rapid morphological changes initiated by Sry require the coordinate integration of many signaling pathways. Based on the established role of the platelet-derived growth factor (PDGF) family of ligands and receptors in **migration**, proliferation, and differentiation of cells in various organ systems, we have investigated the role of PDGF in testis organogenesis. Analysis of expression patterns and characterization of the gonad phenotype in Pdgfr-alpha(-/-) embryos identified PDGFR-alpha as a critical mediator of signaling in the early testis at multiple steps of testis development. Pdgfr-alpha(-/-) XY gonads displayed disruptions in the organization of the vasculature and in the partitioning of interstitial and testis cord compartments. Closer examination revealed severe reductions in characteristic XY proliferation, mesonephric cell **migration**, and fetal Leydig cell differentiation. This work identifies PDGF signaling through the alpha receptor as an important event downstream of Sry in testis organogenesis and Leydig cell differentiation.
42. Hedgehog-responsive mesenchymal clusters direct patterning and emergence of intestinal villi. In the adult intestine, an organized array of finger-like projections, called villi, provide an enormous epithelial surface area for absorptive function. Villi first emerge at embryonic day (E) 14.5 from a previously flat luminal surface. Here, we analyze the cell biology of villus formation and examine the role of paracrine epithelial Hedgehog (Hh) signals in this process. We find that, before villus emergence, tight clusters of Hh-responsive mesenchymal cells form just beneath the epithelium. Cluster formation is dynamic; clusters first form dorsally and anteriorly and spread circumferentially and posteriorly. Statistical analysis of cluster distribution reveals a patterned array; with time, new clusters form in spaces between existing clusters, promoting approximately four rounds of villus emergence by E18.5. Cells within mesenchymal clusters express Patched1 and Gli1, as well as Pdgfrα, a receptor previously shown to participate in villus development. BrdU-labeling experiments show that clusters form by **migratio**n and aggregation of Hh-responsive cells. Inhibition of Hh signaling prevents cluster formation and villus development, but does not prevent emergence of villi in areas where clusters have already formed. Conversely, increasing Hh signaling increases the size of villus clusters and results in exceptionally wide villi. We conclude that Hh signals dictate the initial aspects of the formation of each villus by controlling mesenchymal cluster aggregation and regulating cluster size.
43. Cell-surface glycoprotein of oligodendrocyte progenitors involved in **migration**. Myelination by oligodendrocytes in the CNS involves the **migration** to and recognition and ensheathment of axons. These distinct developmental phases of myelination are assumed to involve the interplay of a precisely regulated set of cell adhesion molecules expressed by both neurons and glial cells. These molecules remain largely unelucidated. In this paper we have identified a large (330 kDa) glycoprotein expressed by murine oligodendrocyte progenitor cells in vitro and in vivo that is downregulated as oligodendrocytes mature. Antigen-positive oligodendrocyte progenitor cells purified by panning develop into myelin-associated glycoprotein-positive oligodendrocytes and also adhere to cultured neurons. Polyclonal antibodies directed against the protein reduce the **migration** of oligodendrocyte progenitor cells. The observations suggest that the AN2 antigen may play a role in early stages of myelination.
44. A role for PDGF-C/PDGFRα signaling in the formation of the meningeal basement membranes surrounding the cerebral cortex. Platelet-derived growth factor-C (PDGF-C) is one of three known ligands for the tyrosine kinase receptor PDGFRα. Analysis ofPdgfcnull mice has demonstrated roles for PDGF-C in palate closure and the formation of cerebral ventricles, but redundancy with other PDGFRα ligands might obscure additional functions. In search of further developmental roles for PDGF-C, we generated mice that were double mutants forPdgfc(-/-)andPdgfra(GFP/+) These mice display a range of severe phenotypes including spina bifida, lung emphysema, abnormal meninges and neuronal ov**er-migrat**ion in the cerebral cortex. We focused our analysis on the central nervous system (CNS), where PDGF-C was identified as a critical factor for the formation of meninges and assembly of the glia limitans basement membrane. We also present expression data onPdgfa,PdgfcandPdgfrain the cerebral cortex and microarray data on cerebral meninges.
45. Abnormal skeletogenesis occurs coincident with increased apoptosis in the Splotch (Sp2H) mutant: putative roles for Pax3 and PDGFRalpha in rib patterning. Although the importance of the transcription factor Pax3 has been identified in many embryological processes including neural crest cell **migration**, neural tube closure, and limb muscle formation, its role in proper formation of the ribs has not been well characterized. We have used the Splotch mouse which has a mutation in the Pax3 gene to determine what role Pax3 may play in rib morphogenesis. Homozygous Splotch embryos collected from days 13.5 to 16.5 of gestation displayed severe rib abnormalities including fusions at both the proximal and distal regions. Given its expression in the dermomyotome, we sought to determine when Pax3 expression in the Splotch mutant initially becomes abnormal and which rib segment progenitors may be affected. Prior to somite differentiation at 9.5 dpc (days post coitum), Pax3 is normally expressed in the somites; after differentiation, however, Pax3 expression is diminished in Splotch embryos. We also determined that significantly increased levels of apoptosis occur in the thoracic region by 11.0 dpc relative to wild-type littermates. Because Patch mouse mutants which fail to express PDGF alpha-receptor also have rib abnormalities, we sought to determine whether Pax3 may influence the expression of this receptor. By in situ hybridization, we determined that initially the expression of PDGFRalpha is normal in Splotch mutants at 10.0 dpc, but decreases by 12.5 dpc in the thoracic somite region, suggesting that Pax3 may act upstream of PDGFRalpha. Taken together, our results show that Pax3 expression is important for normal rib development and that increased apoptosis occurs in the thoracic muscles. We suggest that Pax3 may influence the expression of PDGFRalpha, and that the reduced and/or deficient thoracic muscles may contribute to the resulting rib abnormalities.
46. PDGFR-β Plays a Key Role in the Ectopic **Migratio**n of Neuroblasts in Cerebral Stroke. The neuroprotective agents and induction of endogenous neurogenesis remain to be the urgent issues to be established for the care of cerebral stroke. Platelet-derived growth factor receptor beta (PDGFR-β) is mainly expressed in neural stem/progenitor cells (NSPCs), neurons and vascular pericytes of the brain; however, the role in pathological neurogenesis remains elusive. To this end, we examined the role of PDGFR-β in t**he migrat**ion and proliferation of NSPCs after stroke. A transient middle cerebral-arterial occlusion (MCAO) was introduced into the mice with conditional Pdgfrb-gene inactivation, including N-PRβ-KO mice where the Pdgfrb-gene was mostly inactivated in the brain except that in vascular pericytes, and E-PRβ-KO mice with tamoxifen-induced systemic Pdgfrb-gene inactivation. **The migr**ation of the DCX(+) neuroblasts from the subventricular zone toward the ischemic core was highly increased in N-PRβ-KO, but not in E-PRβ-KO as compared to Pdgfrb-gene preserving control mice. We showed that CXCL12, a potent chemoattractant for CXCR4-expressing NSPCs, was upregulated in the ischemic lesion of N-PRβ-KO mice. Furthermore, integrin α3 intrinsically expressed in NSPCs that critically mediates extracellular matrix-d**ependent** migration, was upregulated in N-PRβ-KO after MCAO. NSPCs isolated from N-PRβ-KO rapidly migrated on the surface coated with collagen type IV or fibronectin that are abundant in vascular niche and ischemic core. PDGFR-β was suggested to be critically involved in pathological neurogenesis through the regulation of lesion-derived chemoattractant as well as intrinsic signal of NSPCs, and we believe that a coordinated regulation of these molecular events may be able to improve neurogenesis in injured brain for further functional recovery.
47. **Migration** of oligodendrocyte progenitor cells is controlled by transforming growth factor β family proteins during corticogenesis. During embryonic development oligodendrocyte precursor cells (OPCs) are generated first in the ventral forebrain and migrate dorsally to occupy the cortex. The molecular cues that guide this migratory route are currently completely unknown. Here, we show that bone morphogenetic protein-4 (Bmp4), Bmp7, and Tgfβ1 produced by the meninges and pericytes repelled ventral OPCs into the cortex at mouse embryonic stages. Ectopic activation of Bmp or Tgfβ1 signaling before the entrance of OPCs into the cortex hindered O**PC migrat**ion into the cortical areas. OPCs without Smad4 signaling molecules also failed to migrate into the cortex efficiently and formed heterotopia in ventral areas. O**PC migrat**ion into the cortex was also dramatically reduced by conditional inhibition of Tgfβ1 or Bmp expression from mesenchymal cells. The data suggest that mesenchymal Tgfβ family proteins pro**mote migr**ation of ventral OPCs into the cortex during corticogenesis.
48. PDGF-A and PDGFR-alpha regulate tooth formation via autocrine mechanism during mandibular morphogenesis in vitro. Platelet-derived growth factor A (PDGF-A) binding to the PDGF receptor alpha (PDGFR-alpha) mediates signal transduction processes related to DNA synthesis, cell **migration**s, cytodifferentiation, and wound healing. Recent studies indicate that PDGFR-alpha functions during cranial neural crest cell **migration**s and first branchial arch morphogenesis (Stephenson et al. [1991] Proc. Natl. Acad. Sci. USA 88:6-10; Morrison-Graham et al. [1992] Development 115:133-142; Hu et al. [1995] Int. J. Dev. Biol. 39:939-945; Soriano [1997] Development 124:2691-2700). The present studies were designed to test the hypothesis that PDGF-A, interacts with its cognate receptor PDGFR-alpha via an autocrine mechanism that regulates the timing, rates, and size of embryonic mouse tooth morphogenesis. Both PDGF-A and PDGFR-alpha transcripts were coordinately expressed in mandibular prominences prior to and during tooth formation using reverse transcriptase-polymerase chain reaction (RT-PCR). During the dental lamina stage, ligand and receptor were present in both enamel organ epithelium and adjacent mesenchymal cells. During the bud stage, ligand and receptor were localized mainly to the enamel organ epithelium. Exogenous PDGF-A at 20 ng/ml enhanced tooth development to reach the cap stage with increased tooth size (P < 0.05) using embryonic day (E)10 mandibular explants cultured in serumless, chemically defined medium. A significant increase in DNA synthesis was observed within enamel organ epithelium at E10+4 when the mandibular explants were treated with PDGF-A at 20 ng/ml. These data suggest that PDGF-A and its cognate receptor (PDGFR-alpha) regulate the size and stage of tooth development via an autocrine mechanism during odontogenesis in vitro.
49. PDGFR alpha expression during mouse embryogenesis: immunolocalization analyzed by whole-mount immunohistostaining using the monoclonal anti-mouse PDGFR alpha antibody APA5. We investigated the cells that express platelet-derived growth factor receptor alpha (PDGFR alpha) during mouse embryogenesis. PDGFR alpha expression has been identified by in situ hybridization or immunohistochemistry using polyclonal antibodies on tissue sectins. Because no immunostaining study using whole-mount specimens has been published to date, we established a new monoclonal antibody (MAb), APA5, for this purpose. Our results differed in that APA5 stained only the paraxial mesoderm, whereas other investigators concluded that most if not all mesodermal cells expressed PDGFR alpha. Moreover, we did not find PDGFR alpha expression in embryonic erythrocytes, which have been previously suggested to express PDGFR alpha. On the basis of our present results, we wish to revise the proposed PDGFR alpha expression as follows. At the pregastrulation stage, PDGFR alpha is expressed only in primitive endoderm, particularly that in the ectoplacental cone. On gastrulation, it is expressed at high levels in the paraxial mesoderm. This expression continues after its differentiation into the somite. Along with the differentiation and **migration** of the sclerotome, PDGFR alpha + cells begin to become distributed throughout the embryonal mesenchyme. During organogenesis, particularly intense staining is detected in regions of epithelial and mesenchymal interaction, such as the tooth bud and bronchi. In addition to mesodermal derivatives, the developing lens, apical ectodermal ridge, glial precursor, cardiac valves, and choroid plexus express PDGFR alpha. Our results with whole-mount immunostaining show that PDGFR alpha is abundantly expressed and may play important roles during embryogenesis.
50. ILEI requires oncogenic Ras for the epithelial to mesenchymal transition of hepatocytes and liver carcinoma progression. In human hepatocellular carcinoma (HCC), epithelial to mesenchymal transition (EMT) correlates with aggressiveness of tumors and poor survival. We employed a model of EMT based on immortalized p19(ARF) null hepatocytes (MIM), which display tumor growth upon expression of oncogenic Ras and undergo EMT through the synergism of Ras and transforming growth factor (TGF)-beta. Here, we show that the interleukin-related protein interleukin-like EMT inducer (ILEI), a novel EMT-, tumor- and metastasis-inducing protein, cooperates with oncogenic Ras to cause TGF-beta-independent EMT. Ras-transformed MIM hepatocytes overexpressing ILEI showed cytoplasmic E-cadherin, loss of ZO-1 and induction of alpha-smooth muscle actin as well as platelet-derived growth factor (PDGF)/PDGF-R isoforms. As shown by dominant-negative PDGF-R expression in these cells, ILEI-induced PDGF signaling was required for enhanced cell **migration**, nuclear accumulation of beta-catenin, nuclear pY-Stat3 and accelerated growth of lung metastases. In MIM hepatocytes expressing the Ras mutant V12-C40, ILEI collaborated with PI3K signaling resulting in tumor formation without EMT. Clinically, human HCC samples showed granular or cytoplasmic localization of ILEI correlating with well and poorly differentiated tumors, respectively. In conclusion, these data indicate that ILEI requires cooperation with oncogenic Ras to govern hepatocellular EMT through mechanisms involving PDGF-R/beta-catenin and PDGF-R/Stat3 signaling.
51. Directional cell **migration** and chemotaxis in wound healing response to PDGF-AA are coordinated by the primary cilium in fibroblasts. Cell motility and **migration** play pivotal roles in numerous physiological and pathophysiological processes including development and tissue repair. Cell **migration** is regulated through external stimuli such as platelet-derived growth factor-AA (PDGF-AA), a key regulator in directional cell **migration** during embryonic development and a chemoattractant during postnatal migratory responses including wound healing. We previously showed that PDGFRalpha signaling is coordinated by the primary cilium in quiescent cells. However, little is known about the function of the primary cilium in cell **migration**. Here we used micropipette analysis to show that a normal chemosensory response to PDGF-AA in fibroblasts requires the primary cilium. In vitro and in vivo wound healing assays revealed that in ORPK mouse (IFT88(Tg737Rpw)) fibroblasts, where ciliary assembly is defective, chemotaxis towards PDGF-AA is absent, leading to unregulated high speed and uncontrolled directional cell displacement during wound closure, with subsequent defects in wound healing. These data suggest that in coordination with cytoskeletal reorganization, the fibroblast primary cilium functions via ciliary PDGFRalpha signaling to monitor directional movement during wound healing.
52. PDGF-AA promotes osteogenic differentiation and **migration** of mesenchymal stem cell by down-regulating PDGFRα and derepressing BMP-Smad1/5/8 signaling. Platelet-derived growth factors (PDGFs) play important roles in skeletal development and bone fracture healing, yet how PDGFs execute their functions remains incompletely understood. Here we show that PDGF-AA, but not -AB or -BB, could activate the BMP-Smad1/5/8 pathway in mesenchymal stem cells (MSCs), which requires BMPRIA as well as PDGFRα. PDGF-AA promotes MSC osteogenic differentiation through the BMP-Smad1/5/8-Runx2/Osx axis and MS**C migrati**on via the BMP-Smad1/5/8-Twist1/Atf4 axis. Mechanistic studies show that PDGF-AA activates BMP-Smad1/5/8 signaling by feedback down-regulating PDGFRα, which frees BMPRI and allows for BMPRI-BMPRII complex formation to activate smad1/5/8, using BMP molecules in the microenvironment. This study unravels a physical and functional interaction between PDGFRα and BMPRI, which plays an important role in MSC differentiation **and migra**tion, and establishes a link between PDGF-AA and BMPs pathways, two essential regulators of embryonic development and tissue homeostasis.
53. AN2/NG2 protein-expressing glial progenitor cells in the murine CNS: isolation, differentiation, and association with radial glia. During early neural development, the lineage specification of initially pluripotent progenitor cells is associated with proliferation, differentiation, and **migration**. Oligodendroglial progenitor cells migrate from their sites of origin to reach the axons that they will myelinate. We have described a cell-surface protein, AN2, expressed by oligodendroglial progenitor cells in vitro and showed that antibodies against AN2 inhibited the **migration** of cultured primary oligodendroglial progenitor cells, suggesting that the AN2 antigen plays a role in their **migration**. Recently, results from MALDI mass spectroscopy showed that AN2 is the mouse homologue of the rat NG2 protein. In this study, we have analyzed cells staining with AN2 antibodies during development and in the adult murine central nervous system (CNS), carried out double stainings with antibodies against NG2, and investigated the differentiation potential of cells in vitro after isolation from early postnatal brain using AN2 antibodies. AN2 and NG2 antibodies stained totally overlapping populations of cells in the CNS. AN2/NG2 expressing cells in embryonic and postnatal brain expressed the PDGF-alpha-receptor and in postnatal brain exhibited electrophysiological properties typical of glial progenitor cells. Cells isolated from early postnatal brain using AN2 monoclonal antibody developed into oligodendrocytes in low serum medium or into astrocytes in the presence of fetal calf serum. In the embryonic spinal cord, cells staining with AN2 antibodies were found closely apposed to radial glial cells, suggesting that glial precursors, like neurons, may use radial glia as scaffolds for **migration**.
54. A critical role for PDGFRα signaling in medial nasal process development. The primitive face is composed of neural crest cell (NCC) derived prominences. The medial nasal processes (MNP) give rise to the upper lip and vomeronasal organ, and are essential for normal craniofacial development, but the mechanism of MNP development remains largely unknown. PDGFRα signaling is known to be critical for NCC development and craniofacial morphogenesis. In this study, we show that PDGFRα is required for MNP development by maintaining t**he migrat**ion of progenitor neural crest cells (NCCs) and the proliferation of MNP cells. Further investigations reveal that PI3K/Akt and Rac1 signaling mediate PDGFRα function during MNP development. We thus establish PDGFRα as a novel regulator of MNP development and elucidate the roles of its downstream signaling pathways at cellular and molecular levels.
55. Time-specific blockade of PDGFR with Imatinib (Glivec®) causes cataract and disruption of lens fiber cells in neonatal mice. This study aimed at investigating the response of lens epithelial cells in postnatal mice to Imatinib (Glivec®, a potent inhibitor of platelet-derived growth factor receptor (PDGFR)) treatment. Mouse eyes were sampled 10 days after administration of Imatinib (0.5 mg·g(-1)·day(-1)) for 3 days, at either 7, 14, or 21 days postpartum. Structural changes of lens were revealed by routine H.E. staining. Levels of proliferation and apoptosis were revealed by BrdU incorporation and terminal deoxynucleotidyl transferase dUTP nick end labeling (TUNEL) assay, respectively, and immunofluorescent staining with anti-PDGFRα antibody was carried out on the sections of eyeball. PDGFRα and p-PDGFRαprotein levels were evaluated by Western blot. Our results indicated that administration of Imatinib led to blockade of PDGFR signaling. Formation of cataracts was found only in those mice where treatment started from 7 days postpartum (P7), but was not observed in those samples from P14 nor P21. Fiber cells were disorganized in cataract lens core as observed histologicall**y, and mi**gration of epithelial cells was also inhibited. No apoptosis was detected with the TUNEL method. Our results indicated blockade of PDGFR at the neonatal stage (P7) would lead to cataracts and lens fiber cells disorganization, suggesting that PDGFR signaling plays a time-specific and crucial role in the postnatal development of lens in the mouse, and also may provide a new approach to produce a congenital cataract animal model.
56. Genetic Ablation of PDGF-Dependent Signaling Pathways Abolishes Vascular Remodeling and Experimental Pulmonary Hypertension. Despite modern therapies, pulmonary arterial hypertension (PAH) harbors a high mortality. Vascular remodeling is a hallmark of the disease. Recent clinical studies revealed that antiremodeling approaches with tyrosine-kinase inhibitors such as imatinib are effective, but its applicability is limited by significant side effects. Although imatinib has multiple targets, expression analyses support a role for platelet-derived growth factor (PDGF) in the pathobiology of the disease. However, its precise role and downstream signaling events have not been established.Patients with PAH exhibit enhanced expression and phosphorylation of β PDGF receptor (βPDGFR) in remodeled pulmonary arterioles, particularly at the binding sites for phophatidyl-inositol-3-kinase and PLCγ at tyrosine residues 751 and 1021, respectively. These signaling molecules were identified as critical downstream mediators of βPDGFR-mediated proliferation **and migra**tion of pulmonary arterial smooth muscle cells. We, therefore, investigated mice expressing a mutated βPDGFR that is unable to recruit phophatidyl-inositol-3-kinase and PLCγ (βPDGFR(F3/F3)). PDGF-dependent Erk1/2 and Akt phosphorylation, cyclin D1 induction, and prolifer**ation, mi**gration, and protection against apoptosis were abolished in βPDGFR(F3/F3) pulmonary arterial smooth muscle cells. On exposure to chronic hypoxia, vascular remodeling of pulmonary arteries was blunted in βPDGFR(F3/F3) mice compared with wild-type littermates. These alterations led to protection from hypoxia-induced PAH and right ventricular hypertrophy.By means of a genetic approach, our data provide definite evidence that the activated βPDGFR is a key contributor to pulmonary vascular remodeling and PAH. Selective disruption of PDGF-dependent phophatidyl-inositol-3-kinase and PLCγ activity is sufficient to abolish these pathogenic responses in vivo, identifying these signaling events as valuable targets for antiremodeling strategies in PAH.
57. The T-box transcription factor Eomesodermin is essential for AVE induction in the mouse embryo. Reciprocal inductive interactions between the embryonic and extraembryonic tissues establish the anterior-posterior (AP) axis of the early mouse embryo. The anterior visceral endoderm (AVE) signaling center emerges at the distal tip of the embryo at embryonic day 5.5 and translocates to the prospective anterior side of the embryo. The process of AVE induction and **migration** are poorly understood. Here we demonstrate that the T-box gene Eomesodermin (Eomes) plays an essential role in AVE recruitment, in part by directly activating the homeobox transcription factor Lhx1. Thus, Eomes function in the visceral endoderm (VE) initiates an instructive transcriptional program controlling AP identity.
58. Ets1 is required for proper **migration** and differentiation of the cardiac neural crest. Defects in cardiac neural crest lead to congenital heart disease through failure of cardiac outflow tract and ventricular septation. In this report, we demonstrate a previously unappreciated role for the transcription factor Ets1 in the regulation of cardiac neural crest development. When bred onto a C57BL/6 genetic background, Ets1(-/-) mice have a nearly complete perinatal lethality. Histologic examination of Ets1(-/-) embryos revealed a membranous ventricular septal defect and an abnormal nodule of cartilage within the heart. Lineage-tracing experiments in Ets1(-/-) mice demonstrated that cells of the neural crest lineage form this cartilage nodule and do not complete their **migration** to the proximal aspects of the outflow tract endocardial cushions, resulting in the failure of membranous interventricular septum formation. Given previous studies demonstrating that the MEK/ERK pathway directly regulates Ets1 activity, we cultured embryonic hearts in the presence of the MEK inhibitor U0126 and found that U0126 induced intra-cardiac cartilage formation, suggesting the involvement of a MEK/ERK/Ets1 pathway in blocking chondrocyte differentiation of cardiac neural crest. Taken together, these results demonstrate that Ets1 is required to direct the proper **migration** and differentiation of cardiac neural crest in the formation of the interventricular septum, and therefore could play a role in the etiology of human congenital heart disease.
59. PDGFRα signaling in the primary cilium regulates NHE1-dependent fibroblast **migratio**n via coordinated differential activity of MEK1/2-ERK1/2-p90RSK and AKT signaling pathways. In fibroblasts, platelet-derived growth factor receptor alpha (PDGFRα) is upregulated during growth arrest and compartmentalized to the primary cilium. PDGF-AA mediated activation of the dimerized ciliary receptor produces a phosphorylation cascade through the PI3K-AKT and MEK1/2-ERK1/2 pathways leading to the activation of the Na(+)/H(+) exchanger, NHE1, cytoplasmic alkalinization and actin nucleation at the lamellipodium that supports directional cel**l migrati**on. We here show that AKT and MEK1/2-ERK1/2-p90(RSK) inhibition reduced PDGF-AA-induced cel**l migrati**on by distinct mechanisms: AKT inhibition reduced NHE1 activity by blocking the translocation of NHE1 to the cell membrane. MEK1/2 inhibition did not affect NHE1 activity but influenced NHE1 localization, causing NHE1 to localize discontinuously in patches along the plasma membrane, rather than preferentially at the lamellipodium. We also provide direct evidence of NHE1 translocation through the cytoplasm to the leading edge. In conclusion, signals initiated at the primary cilium through the PDGFRαα cascade reorganize the cytoskeleton to regulate c**ell migra**tion differentially through the AKT and the MEK1/2-ERK1/2-p90(RSK) pathways. The AKT pathway is necessary for initiation of NHE1 translocation, presumably in vesicles, to the leading edge and for its activation. In contrast, the MEK1/2-ERK1/2-p90(RSK) pathway controls the spatial organization of NHE1 translocation and incorporation, and therefore specifies the direction of the leading edge formation.
60. Expression of the human PAC1 receptor leads to dose-dependent hydrocephalus-related abnormalities in mice. Hydrocephalus is a common and potentially devastating birth defect affecting the CNS, and its relationship with G protein-coupled receptors (GPCRs) is unknown. We have expressed 2, 4, or 6 copies of a GPCR--the human PAC1 receptor with a 130-kb transgene in the mouse nervous system in a pattern closely resembling that of the endogenous gene. Consistent with PAC1 actions, PKA and PKC activity were elevated in the brains of Tg mice. Remarkably, Tg mice developed dose-dependent hydrocephalus-like characteristics, including enlarged third and lateral ventricles and reduced cerebral cortex, corpus callosum, and subcommissural organ (SCO). Neuronal proliferation and apoptosis were implicated in hydrocephalus, and we observed significantly reduced neuronal proliferation and massively increased neuronal apoptosis in the developing cortex and SCO of Tg embryos, while neurite outgrowth and neuronal **migration** in vitro remain uncompromised. Ventricular ependymal cilia are crucial for directing cerebrospinal fluid flow, and ependyma of Tg mice exhibited disrupted cilia with increased phospho-CREB immunoreactivity. These data demonstrate that altered neuronal proliferation/apoptosis and disrupted ependymal cilia are the main factors contributing to hydrocephalus in PAC1-overexpressing mice. This is the first report to our knowledge demonstrating that misregulation of GPCRs can be involved in hydrocephalus-related neurodevelopmental disorders.
61. Essential role of Sox9 in the pathway that controls formation of cardiac valves and septa. Epithelial-mesenchymal transformation is a critical developmental process reiterated in multiple organs throughout embryogenesis. Formation of endocardial cushions, primordia of valves and septa, is a classic example of epithelial-mesenchymal transformation. Several gene mutations are known to affect cardiac valve formation. Sox9 is activated when endocardial endothelial cells undergo mesenchymal transformation and migrate into an extracellular matrix, called cardiac jelly, to form endocardial cushions. In Sox9-null mutants, endocardial cushions are markedly hypoplastic. In these mutants, Nfatc1 is ectopically expressed and no longer restricted to endothelial cells. Further, Sox9-deficient endocardial mesenchymal cells fail to express ErbB3, which is required for endocardial cushion cell differentiation and proliferation. Our results reveal a succession of molecular steps in the pathway of endocardial cushion development. We propose that loss of Sox9 inhibits epithelial-mesenchymal transformation after delamination and initial **migration**, but before definitive mesenchymal transformation.
62. An activating mutation in the PDGF receptor alpha results in embryonic lethality caused by malformation of the vascular system. Platelet-derived growth factors (PDGF) and their receptors control cell proliferation, survival, and **migration**. To test the influence of an oncogenic mutation to embryonic development, a transgenic mouse line expressing PDGFRalpha (D842V) was established and analyzed. Most of the embryos die on embryonic day 12.5 due to massive hemorrhages in the trunk. In mesenchymal cells of mutant animals, proliferation is decreased while apoptosis is increased. Further analyses reveal that the aortic blood vessels are enlarged showing a reduced numbers of vascular smooth muscle cells (vSMC) around the aorta. We hypothesize that the process of aortic wall formation is impaired, leading to subsequent rupture and leakage of the blood vessel resulting in death of the embryos. We speculate that misexpression of PDGFRalpha in SMCs causes failure of vSMC recruitment to the aorta.
63. Cardiac malformations in Pdgfralpha mutant embryos are associated with increased expression of WT1 and Nkx2.5 in the second heart field. Platelet-derived growth factor receptor alpha (Pdgfralpha) identifies cardiac progenitor cells in the posterior part of the second heart field. We aim to elucidate the role of Pdgfralpha in this region. Hearts of Pdgfralpha-deficient mouse embryos (E9.5-E14.5) showed cardiac malformations consisting of atrial and sinus venosus myocardium hypoplasia, including venous valves and sinoatrial node. In vivo staining for Nkx2.5 showed increased myocardial expression in Pdgfralpha mutants, confirmed by Western blot analysis. Due to hypoplasia of the primary atrial septum, mesenchymal cap, and dorsal mesenchymal protrusion, the atrioventricular septal complex failed to fuse. Impaired epicardial development and severe blebbing coincided with diminished **migration** of epicardium-derived cells and myocardial thinning, which could be linked to increased WT1 and altered alpha4-integrin expression. Our data provide novel insight for a possible role for Pdgfralpha in transduction pathways that lead to repression of Nkx2.5 and WT1 during development of posterior heart field-derived cardiac structures.
64. Brg1-dependent chromatin remodelling is not essentially required during oligodendroglial differentiation. Myelinating Schwann cells in the vertebrate peripheral nervous system rely on Brg1 (Smarca4) for terminal differentiation. Brg1 serves as central ATP-hydrolyzing subunit of the chromatin remodelling BAF complexes and is recruited during myelination as part of these complexes by the transcription factor Sox10 in Schwann cells. Here, we analyzed the role of Brg1 during development of myelinating oligodendrocytes in the CNS of the mouse. Following Brg1 deletion in oligodendrocyte precursors, these cells showed normal survival, proliferation, and **migration**. A mild but significant reduction in the number of oligodendrocytes with myelin gene expression in the absence of Brg1 points to a contribution to oligodendroglial differentiation but also shows that the role of Brg1 is much less prominent than during Schwann cell differentiation. Additionally, we failed to obtain evidence for a genetic interaction between Brg1 and Sox10 comparable with the one in Schwann cells. This argues that similarities exist between the regulatory networks and mechanisms in both types of myelinating glia but that the exact mode of action and the relevance of functional interactions differ, pointing to a surprising degree of variability in the control of myelination.
65. Neuregulin-1/ErbB4 signaling controls the **migration** of oligodendrocyte precursor cells during development. During embryonic development, the oligodendrocyte precursors (OPCs) are generated in specific oligodendrogliogenic sites within the neural tube and migrate to colonize the entire CNS. Different factors have been shown to influence the OPC **migration** and differentiation, including morphogens, growth factors, chemotropic molecules, and extracellular matrix proteins. Neuregulins have been shown to influence the **migration** of neuronal precursors as well as the movement and differentiation of Schwann cells for peripheral myelination, but their role in the motility of OPCs has not been explored. In the present study, we have used the optic nerve as an experimental model to examine the function of Nrg1 and its ErbB4 receptor in the **migration** of OPCs in the developing embryo. In vitro experiments revealed that Nrg1 is a potent chemoattractant for the first wave of OPCs, and that this effect is mediated via ErbB4 receptor. In contrast, OPCs colonizing the optic nerve at postnatal stages (PDGFRα(+)-OPCs) does not respond to Nrg1-chemoattraction. We also found that mouse embryos lacking ErbB4 display deficits in early OPC **migratio**n away from different oligodendrogliogenic regions in vivo. The present findings reveal a new role for Nrg1/ErbB4 signaling in regulating OPC **migratio**n selectively during early stages of CNS development.
66. Disruption of PDGFRalpha-initiated PI3K activation and **migration** of somite derivatives leads to spina bifida. Spina bifida, or failure of the vertebrae to close at the midline, is a common congenital malformation in humans that is often synonymous with neural tube defects (NTDs). However, it is likely that other etiologies exist. Genetic disruption of platelet-derived growth factor receptor (PDGFR) alpha results in spina bifida, but the underlying mechanism has not been identified. To elucidate the cause of this birth defect in PDGFRalpha mutant embryos, we examined the developmental processes involved in vertebrae formation. Exposure of chick embryos to the PDGFR inhibitor imatinib mesylate resulted in spina bifida in the absence of NTDs. We next examined embryos with a tissue-specific deletion of the receptor. We found that loss of the receptor from chondrocytes did not recapitulate the spina bifida phenotype. By contrast, loss of the receptor from all sclerotome and dermatome derivatives or disruption of PDGFRalpha-driven phosphatidyl-inositol 3' kinase (PI3K) activity resulted in spina bifida. Furthermore, we identified a **migration** defect in the sclerotome as the cause of the abnormal vertebral development. We found that primary cells from these mice exhibited defects in PAK1 activation and paxillin localization. Taken together, these results indicate that PDGFRalpha downstream effectors, especially PI3K, are essential for cell **migration** of a somite-derived dorsal mesenchyme and disruption of receptor signaling in these cells leads to spina bifida.
67. LIFR beta plays a major role in neuronal identity determination and glial differentiation in the mouse facial nucleus. In the hindbrain, generation of the facial nucleus involves complex developmental processes that will lead to the formation of a structure composed of motor neurons, astrocytes and oligodendrocytes. The implication of LIF-related cytokines in the development of this nucleus came to light with the analysis of mice mutant for the receptor of these cytokines, LIFR beta, which exhibit a massive loss of facial branchiomotor (fbm) neurons at birth and a severe decrease in GFAP expression, a marker of astrocytes. To uncover the cellular mechanisms regulated by LIFR beta during facial nucleus development, we first analyzed its expression pattern in the hindbrain. lifr beta is first expressed at E11.5 in the hindbrain neuroepithelium. The receptor is absent during the **migration** of fbm post-mitotic neurons but is strongly expressed when fbm neurons have reached rhombomere 6 at E12.5, and its expression is maintained until E18.5. From the analysis of lifr beta mutant embryos, we established that LIFR beta is necessary for fbm neurons' identity determination. We also show that LIFR beta is implicated in astrocyte and oligodendrocyte differentiation, specifically within the facial nucleus.
68. Analysis of Nsdhl-deficient embryos reveals a role for Hedgehog signaling in early placental development. The X-linked Nsdhl gene encodes a sterol dehydrogenase involved in cholesterol biosynthesis. Mutations in this gene cause the male lethal phenotypes in human CHILD syndrome and bare patches (Bpa) mice. Affected male embryos for several mutant Nsdhl alleles die in mid-gestation with a thin and poorly vascularized placental labyrinth. The timing and specific abnormalities noted suggest a defect in one or more developmental signaling pathways as a possible mechanism. Here, we examined the possible involvement of the hedgehog signaling pathway in the placental pathology of Nsdhl mutants using a transgenic mouse line (Ptch1(tm1Mps)) that contains a lacZ reporter under the control of the promoter for Ptch1, the gene that encodes the major hedgehog receptor. We demonstrate expression of Ptch1 in allantoic mesoderm of the placenta from wild-type mid-gestation embryos. The evidence suggests that the signaling is induced by Indian hedgehog that is produced by distal (ectoplacental) visceral endoderm cells that migrate into the allantoic mesoderm before embryonic day 10.0. Using a ubiquitously expressed, X-linked lacZ transgene that undergoes normal X-inactivation, we demonstrate that the placental defects in Nsdhl/+ female embryos are non-cell autonomous. Further, affected placentas from mutant Nsdhl(Bpa-8H) male embryos demonstrate markedly decreased or no Ptch1-lacZ staining and no **migration** of Ihh expressing cells into the developing placenta. These data strongly implicate the hedgehog signaling pathway in the pathogenesis of the placental defects in NSDHL deficiency and provide evidence for a role for the hedgehog pathway in the development of a functional mammalian placenta.
69. A role for CXCR4 signaling in survival and **migration** of neural and oligodendrocyte precursors. Oligodendrocyte development is controlled by a number of survival and migratory factors. The present study shows that signaling of CXCR4 receptor by the chemokine CXCL12 regulates survival and **migration** of neural precursors (NP) as well as oligodendrocyte progenitors (OP). CXCR4 is expressed by E14 striatal NP and OP generated by neurospheres. In CXCR4-defective mice, the number of NP in neurosphere outgrowth was twofold less than in wild-type (WT) mice; NP radial cell **migration** was also decreased. In contrast, the addition of CXCL12 to WT NP increased radial **migration** from the sphere in a dose-dependent manner with a maximal response at 200 nM. When oligodendrocytes differentiated in neurosphere outgrowth, CXCR4 was downregulated. OP isolated from newborn brain coexpressed CXCR4 with platelet-derived growth factor receptor-alpha (PDGFR alpha) or chondroitin sulfate proteoglycan; receptor expression also decreased during differentiation in vitro. Neonatal OP showed a peak migratory response to 20 nM of CXCL12 in chemotactic chambers, a **migration** inhibited by a CXCR4 antagonist and anti-CXCL12 antibody. In the embryonic spinal cord, the number of OP-expressing PDGFR alpha was reduced more than twofold in CXCR4-defective mice compared with WT and the ratio of ventral to dorsal OP was significantly increased. This indicates a defect in OP survival and their dorsal **migration** from the ventral cord region, probably because CXCR4(-/-) OP are unable to respond to CXCL12 made by vascular endothelia and the pia mater. We propose that CXCR4 signaling regulate survival and outward chemotactic **migration** of OP during embryonic and postnatal CNS development.

#### GeneRIFs

1. Data indicate that PDGF-AA promotes mesenchymal stem cel **migration** via bone morphogenetic protein 2 (BMP2)-smad proteins smad1/5/8 activation requires lysosome-mediated degradation of PDGF alpha Receptor (PDGFRalpha).
2. PDGFRalpha signaling in the primary cilium regulates NHE1-dependent fibroblast **migration** via coordinated differential activity of MEK1/2-ERK1/2-p90RSK and AKT signaling pathways.
3. Sox9 and Sox10 are required in a functionally redundant manner in oligodendrocyte precursors for PDGF-dependent survival and **migration**.
4. These results indicate that PDGFRalpha downstream effectors, especially PI3K, are essential for cell **migration** of a somite-derived dorsal mesenchyme and disruption of receptor signaling in these cells leads to spina bifida.

### ECM

#### PubMed

1. Wnt signaling regulates smooth muscle precursor development in the mouse lung via a tenascin C/PDGFR pathway. Paracrine signaling from lung epithelium to the surrounding mesenchyme is important for lung SMC development and function and is a contributing factor in an array of pulmonary diseases such as bronchopulmonary dysplasia, pulmonary hypertension, and asthma. Wnt7b, which is exclusively expressed in the lung epithelium, is important for lung vascular smooth muscle integrity, but the underlying mechanism by which Wnt signaling regulates lung SMC development is unclear. In this report, we have demonstrated that Wnt7b regulates a program of mesenchymal differentiation in the mouse lung that is essential for SMC development. Genetic loss-of-function studies showed that Wnt7b and beta-catenin were required for expression of Pdgfralpha and Pdgfrbeta and proliferation in pulmonary SMC precursors. In contrast, gain-of-function studies showed that activation of Wnt signaling increased the expression of both Pdgfralpha and Pdgfrbeta as well as the proliferation of SMC precursors. We further showed that the effect on Pdgfr expression was, in part, mediated by direct transcriptional regulation of the **ECM** protein tenascin C (Tnc), which was necessary and sufficient for Pdgfralpha/beta expression in lung explants. Moreover, this pathway was highly upregulated in a mouse model of asthma and in lung tissue from patients with pulmonary hypertension. Together, these data define a Wnt/Tnc/Pdgfr signaling axis that is critical for smooth muscle development and disease progression in the lung.
2. Distinct fibroblast lineages determine dermal architecture in skin development and repair. Fibroblasts are the major mesenchymal cell type in connective tissue and deposit the collagen and elastic fibres of the extracellular matrix (**ECM**). Even within a single tissue, fibroblasts exhibit considerable functional diversity, but it is not known whether this reflects the existence of a differentiation hierarchy or is a response to different environmental factors. Here we show, using transplantation assays and lineage tracing in mice, that the fibroblasts of skin connective tissue arise from two distinct lineages. One forms the upper dermis, including the dermal papilla that regulates hair growth and the arrector pili muscle, which controls piloerection. The other forms the lower dermis, including the reticular fibroblasts that synthesize the bulk of the fibrillar **ECM**, and the preadipocytes and adipocytes of the hypodermis. The upper lineage is required for hair follicle formation. In wounded adult skin, the initial wave of dermal repair is mediated by the lower lineage and upper dermal fibroblasts are recruited only during re-epithelialization. Epidermal β-catenin activation stimulates the expansion of the upper dermal lineage, rendering wounds permissive for hair follicle formation. Our findings explain why wounding is linked to formation of **EC**M-rich scar tissue that lacks hair follicles. They also form a platform for discovering fibroblast lineages in other tissues and for examining fibroblast changes in ageing and disease.
3. Epidermal β-catenin activation remodels the dermis via paracrine signalling to distinct fibroblast lineages. Sustained epidermal Wnt/β-catenin signalling expands the stem cell compartment and induces ectopic hair follicles (EFs). This is accompanied by extensive fibroblast proliferation and extracellular matrix **(E**CM) remodelling in the underlying dermis. Here we show that epidermal Hedgehog (Hh) and Transforming growth factor-beta (TGF-β) signalling mediate the dermal changes. Pharmacological inhibition or genetic deletion of these pathways prevents β-catenin-induced dermal reprogramming and EF formation. Epidermal Shh stimulates proliferation of the papillary fibroblast lineage, whereas TGF-β2 controls proliferation, differentiation **an**d ECM production by reticular fibroblasts. Hh inhibitors do not affect TGF-β target gene expression in reticular fibroblasts, and TGF-β inhibition does not prevent Hh target gene induction in papillary fibroblasts. However, when Hh signalling is inhibited the reticular dermis does not respond to epidermal β-catenin activation. We conclude that the dermal response to epidermal Wnt/β-catenin signalling depends on distinct fibroblast lineages responding to different paracrine signals.
4. PDGFRα plays a crucial role in connective tissue remodeling. Platelet derived growth factor (PDGF) plays a pivotal role in the remodeling of connective tissues. Emerging data indicate the distinctive role of PDGF receptor-α (PDGFRα) in this process. In the present study, the Pdgfra gene was systemically inactivated in adult mouse (α-KO mouse), and the role of PDGFRα was examined in the subcutaneously implanted sponge matrices. PDGFRα expressed in the fibroblasts of Pdgfra-preserving control mice (Flox mice), was significantly reduced in the sponges in α-KO mice. Neovascularized areas were largely suppressed in the α-KO mice than in the Flox mice, whereas the other parameters related to the blood vessels and endothelial cells were similar. The deposition of collagen and fibronectin and the expression of collagen 1a1 and 3a1 genes were significantly reduced in α-KO mice. There was a significantly decrease in the number and dividing fibroblasts in the α-KO mice, and those of macrophages were similar between the two genotypes. Hepatocyte growth factor (Hgf) gene expression was suppressed in Pdgfra-inactivated fibroblasts and connective tissue. The findings implicate the role of PDGFRα**-de**pendent ECM and HGF production in fibroblasts that promotes the remodeling of connective tissue and suggest that PDGFRα may be a relevant target to regulate connective tissue remodeling.
5. PDGFRα signaling drives adipose tissue fibrosis by targeting progenitor cell plasticity. Fibrosis is a common disease process in which profibrotic cells disturb organ function by secreting disorganized extracellular matrix **(EC**M). Adipose tissue fibrosis occurs during obesity and is associated with metabolic dysfunction, but how profibrotic cells originate is still being elucidated. Here, we use a developmental model to investigate perivascular cells in white adipose tissue (WAT) and their potential to cause organ fibrosis. We show that a Nestin-Cre transgene targets perivascular cells (adventitial cells and pericyte-like cells) in WAT, and Nestin-GFP specifically labels pericyte-like cells. Activation of PDGFRα signaling in perivascular cells causes them to transition int**o E**CM-synthesizing profibrotic cells. Before this transition occurs, PDGFRα signaling up-regulates mTOR signaling and ribosome biogenesis pathways and perturbs the expression of a network of epigenetically imprinted genes that have been implicated in cell growth and tissue homeostasis. Isolated Nestin-GFP(+) cells differentiate into adipocytes ex vivo and form WAT when transplanted into recipient mice. However, PDGFRα signaling opposes adipogenesis and generates profibrotic cells instead, which leads to fibrotic WAT in transplant experiments. These results identify perivascular cells as fibro/adipogenic progenitors in WAT and show that PDGFRα targets progenitor cell plasticity as a profibrotic mechanism.
6. Protein kinase C-delta transactivates platelet-derived growth factor receptor-alpha in mechanical strain-induced collagenase 3 (matrix metalloproteinase-13) expression by osteoblast-like cells. Matrix metalloproteinase-13 (MMP-13, or collagenase 3) has been shown to degrade intact collagen and to participate in situations where rapid and effective remodeling of collagenous **ECM** is required. Mechanical strain induction of MMP-13 is an example of how osteoblasts respond to high mechanical forces and participate in the bone-remodeling mechanism. Using MC3T3-E1 osteoblast-like cells, we dissected the signaling molecules involved in MMP-13 induction by mechanical strain. Reverse transcription-PCR and zymogram analysis showed that platelet-derived growth factor receptor (PDGFR) inhibitor, AG1296, inhibited the mechanical strain-induced MMP-13 gene and activity. However, the induction was not affected by anti-PDGF-AA serum. Immunoblot analysis revealed time-dependent phosphorylation of PDGFR-alpha up to 2.7-fold increases within 3 min under strain. Transfection with shPDGFR-alpha (at 4 and 8 microg/ml) abolished PDGFR-alpha and reduced MMP-13 expression. Moreover, time-dependent recruitments of phosphoinositide 3-kinase (PI3K) by PDGFR-alpha were detected by immunoprecipitation with anti-PDGFR-alpha serum followed by immunoblot with anti-PI3K serum. AG1296 inhibited PDGFR-alpha/PI3K aggregation and Akt phosphorylation. Interestingly, protein kinase C-delta (PKC-delta) inhibitor, rottlerin, inhibited not only PDGFR-alpha/PI3K aggregation but PDGFR-alpha phosphorylation. The sequential activations were further confirmed by mutants DeltaPKC-delta, DeltaAkt, and DeltaERK1. Consistently, the primary mouse osteoblast cells used the same identified signaling molecules to express MMP-13 under mechanical strain. These results demonstrate that, in osteoblast-like cells, the MMP-13 induction by mechanical strain requires the transactivation of PDGFR-alpha by PKC-delta and the cross-talk between PDGFR-alpha/PI3K/Akt and MEK/ERK pathways.

### [Ee]xtracellular [Mm]atrix

#### Process

1. **extracellular matrix** organization

#### PubMed

1. The **extracellular matrix** molecule tenascin C modulates expression levels and territories of key patterning genes during spinal cord astrocyte specification. The generation of astrocytes during the development of the mammalian spinal cord is poorly understood. Here, we demonstrate for the first time that the **extracellular matrix** glycoprotein tenascin C regulates the expression of key patterning genes during late embryonic spinal cord development, leading to a timely maturation of gliogenic neural precursor cells. We first show that tenascin C is expressed by gliogenic neural precursor cells during late embryonic development. The loss of tenascin C leads to a sustained generation and delayed migration of Fgfr3-expressing immature astrocytes in vivo. Consistent with an increased generation of astroglial cells, we documented an increased number of GFAP-positive astrocytes at later stages. Mechanistically, we could demonstrate an upregulation and domain shift of the patterning genes Nkx6.1 and Nkx2.2 in vivo. In addition, sulfatase 1, a known downstream target of Nkx2.2 in the ventral spinal cord, was also upregulated. Sulfatase 1 regulates growth factor signalling by cleaving sulphate residues from heparan sulphate proteoglycans. Consistent with this function, we observed changes in both FGF2 and EGF responsiveness of spinal cord neural precursor cells. Taken together, our data implicate Tnc in the regulation of proliferation and lineage progression of astroglial progenitors in specific domains of the developing spinal cord.
2. Fibronectin signals through integrin α5β1 to regulate cardiovascular development in a cell type-specific manner. Fibronectin (Fn1) is an evolutionarily conserve**d extracellular matr**ix glycoprotein essential for embryonic development. Global deletion of Fn1 leads to mid-gestation lethality from cardiovascular defects. However, severe morphogenetic defects that occur early in embryogenesis in these embryos precluded assigning a direct role for Fn1 in cardiovascular development. We noticed that Fn1 is expressed in strikingly non-uniform patterns during mouse embryogenesis, and that its expression is particularly enriched in the pharyngeal region corresponding with the pharyngeal arches 3, 4, and 6. This region bears a special importance for the developing cardiovascular system, and we hypothesized that the localized enrichment of Fn1 in the pharyngeal region may be essential for cardiovascular morphogenesis. To test this hypothesis, we ablated Fn1 using the Isl1(Cre) knock-in strain of mice. Deletion of Fn1 using the Isl1(Cre) strain resulted in defective formation of the 4th pharyngeal arch arteries (PAAs), aberrant development of the cardiac outflow tract (OFT), and ventricular septum defects. To determine the cell types responding to Fn1 signaling during cardiovascular development, we deleted a major Fn1 receptor, integrin α5 using the Isl1(Cre) strain, and observed the same spectrum of abnormalities seen in the Fn1 conditional mutants. Additional conditional mutagenesis studies designed to ablate integrin α5 in distinct cell types within the Isl1(+) tissues and their derivatives, suggested that the expression of integrin α5 in the pharyngeal arch mesoderm, endothelium, surface ectoderm and the neural crest were not required for PAA formation. Our studies suggest that an (as yet unknown) integrin α5-dependent signal extrinsic to the pharyngeal endothelium mediates the formation of the 4th PAAs.
3. Probing chromatin landscape reveals roles of endocardial TBX20 in septation. Mutations in the T-box transcription factor TBX20 are associated with multiple forms of congenital heart defects, including cardiac septal abnormalities, but our understanding of the contributions of endocardial TBX20 to heart development remains incomplete. Here, we investigated how TBX20 interacts with endocardial gene networks to drive the mesenchymal and myocardial movements that are essential for outflow tract and atrioventricular septation. Selective ablation of Tbx20 in murine endocardial lineages reduced the expression of **extracellular matrix** and cell migration genes that are critical for septation. Using the assay for transposase-accessible chromatin with high-throughput sequencing (ATAC-seq), we identified accessible chromatin within endocardial lineages and intersected these data with TBX20 ChIP-seq and chromatin loop maps to determine that TBX20 binds a conserved long-range enhancer to regulate versican (Vcan) expression. We also observed reduced Vcan expression in Tbx20-deficient mice, supporting a direct role for TBX20 in Vcan regulation. Further, we show that the Vcan enhancer drove reporter gene expression in endocardial lineages in a TBX20-binding site-dependent manner. This work illuminates gene networks that interact with TBX20 to orchestrate cardiac septation and provides insight into the chromatin landscape of endocardial lineages during septation.
4. Distinct fibroblast lineages determine dermal architecture in skin development and repair. Fibroblasts are the major mesenchymal cell type in connective tissue and deposit the collagen and elastic fibres of the **extracellular matrix** (ECM). Even within a single tissue, fibroblasts exhibit considerable functional diversity, but it is not known whether this reflects the existence of a differentiation hierarchy or is a response to different environmental factors. Here we show, using transplantation assays and lineage tracing in mice, that the fibroblasts of skin connective tissue arise from two distinct lineages. One forms the upper dermis, including the dermal papilla that regulates hair growth and the arrector pili muscle, which controls piloerection. The other forms the lower dermis, including the reticular fibroblasts that synthesize the bulk of the fibrillar ECM, and the preadipocytes and adipocytes of the hypodermis. The upper lineage is required for hair follicle formation. In wounded adult skin, the initial wave of dermal repair is mediated by the lower lineage and upper dermal fibroblasts are recruited only during re-epithelialization. Epidermal β-catenin activation stimulates the expansion of the upper dermal lineage, rendering wounds permissive for hair follicle formation. Our findings explain why wounding is linked to formation of ECM-rich scar tissue that lacks hair follicles. They also form a platform for discovering fibroblast lineages in other tissues and for examining fibroblast changes in ageing and disease.
5. Hand2 loss-of-function in Hand1-expressing cells reveals distinct roles in epicardial and coronary vessel development. The basic helix-loop-helix (bHLH) transcription factors Hand1 and Hand2 are essential for embryonic development. Given their requirement for cardiogenesis, it is imperative to determine their impact on cardiovascular function.To deduce the role of Hand2 within the epicardium.We engineered a Hand1 allele expressing Cre recombinase. Cardiac Hand1 expression is largely limited to cells of the primary heart field, overlapping little with Hand2 expression. Hand1 is expressed within the septum transversum, and the Hand1 lineage marks the proepicardial organ and epicardium. To examine Hand factor functional overlap, we conditionally deleted Hand2 from Hand1-expressing cells. Hand2 mutants display defective epicardialization and fail to form coronary arteries, coincident with altered **extracellular matrix** deposition and Pdgfr expression.These data demonstrate a hierarchal relationship whereby transient Hand1 septum transversum expression defines epicardial precursors that are subsequently dependent on Hand2 function.
6. Epidermal β-catenin activation remodels the dermis via paracrine signalling to distinct fibroblast lineages. Sustained epidermal Wnt/β-catenin signalling expands the stem cell compartment and induces ectopic hair follicles (EFs). This is accompanied by extensive fibroblast proliferation an**d extracellular matr**ix (ECM) remodelling in the underlying dermis. Here we show that epidermal Hedgehog (Hh) and Transforming growth factor-beta (TGF-β) signalling mediate the dermal changes. Pharmacological inhibition or genetic deletion of these pathways prevents β-catenin-induced dermal reprogramming and EF formation. Epidermal Shh stimulates proliferation of the papillary fibroblast lineage, whereas TGF-β2 controls proliferation, differentiation and ECM production by reticular fibroblasts. Hh inhibitors do not affect TGF-β target gene expression in reticular fibroblasts, and TGF-β inhibition does not prevent Hh target gene induction in papillary fibroblasts. However, when Hh signalling is inhibited the reticular dermis does not respond to epidermal β-catenin activation. We conclude that the dermal response to epidermal Wnt/β-catenin signalling depends on distinct fibroblast lineages responding to different paracrine signals.
7. Reprogramming adult dermis to a neonatal state through epidermal activation of β-catenin. Hair follicle formation depends on reciprocal epidermal-dermal interactions and occurs during skin development, but not in adult life. This suggests that the properties of dermal fibroblasts change during postnatal development. To examine this, we used a PdgfraEGFP mouse line to isolate GFP-positive fibroblasts from neonatal skin, adult telogen and anagen skin and adult skin in which ectopic hair follicles had been induced by transgenic epidermal activation of β-catenin (EF skin). We also isolated epidermal cells from each mouse. The gene expression profile of EF epidermis was most similar to that of anagen epidermis, consistent with activation of β-catenin signalling. By contrast, adult dermis with ectopic hair follicles more closely resembled neonatal dermis than adult telogen or anagen dermis. In particular, genes associated with mitosis were upregulated a**nd extracellular mat**rix-associated genes were downregulated in neonatal and EF fibroblasts. We confirmed that sustained epidermal β-catenin activation stimulated fibroblasts to proliferate to reach the high cell density of neonatal skin. In addition, **the extracellular ma**trix was comprehensively remodelled, with mature collagen being replaced by collagen subtypes normally present only in developing skin. The changes in proliferation **and extracellular ma**trix composition originated from a specific subpopulation of fibroblasts located beneath the sebaceous gland. Our results show that adult dermis is an unexpectedly plastic tissue that can be reprogrammed to acquire the molecular, cellular and structural characteristics of neonatal dermis in response to cues from the overlying epidermis.
8. Regulation of PDGFC signalling and **extracellular matrix** composition by FREM1 in mice. Fras1-related **extracellular matrix** protein 1 (FREM1) is required for epidermal adhesion during embryogenesis, and mice lacking the gene develop fetal skin blisters and a range of other developmental defects. Mutations in members of the FRAS/FREM gene family cause diseases of the Fraser syndrome spectrum. Embryonic epidermal blistering is also observed in mice lacking PdgfC and its receptor, PDGFRα. In this article, we show that FREM1 binds to PDGFC and that this interaction regulates signalling downstream of PDGFRα. Fibroblasts from Frem1-mutant mice respond to PDGFC stimulation, but with a shorter duration and amplitude than do wild-type cells. Significantly, PDGFC-stimulated expression of the metalloproteinase inhibitor Timp1 is reduced in cells with Frem1 mutations, leading to reduced basement membrane collagen I deposition. These results show that the physical interaction of FREM1 with PDGFC can regulate remodelling of th**e extracellular matr**ix downstream of PDGFRα. We propose that loss of FREM1 function promotes epidermal blistering in Fraser syndrome as a consequence of reduced PDGFC activity, in addition to its stabilising role in the basement membrane.
9. PDGFR-β Plays a Key Role in the Ectopic Migration of Neuroblasts in Cerebral Stroke. The neuroprotective agents and induction of endogenous neurogenesis remain to be the urgent issues to be established for the care of cerebral stroke. Platelet-derived growth factor receptor beta (PDGFR-β) is mainly expressed in neural stem/progenitor cells (NSPCs), neurons and vascular pericytes of the brain; however, the role in pathological neurogenesis remains elusive. To this end, we examined the role of PDGFR-β in the migration and proliferation of NSPCs after stroke. A transient middle cerebral-arterial occlusion (MCAO) was introduced into the mice with conditional Pdgfrb-gene inactivation, including N-PRβ-KO mice where the Pdgfrb-gene was mostly inactivated in the brain except that in vascular pericytes, and E-PRβ-KO mice with tamoxifen-induced systemic Pdgfrb-gene inactivation. The migration of the DCX(+) neuroblasts from the subventricular zone toward the ischemic core was highly increased in N-PRβ-KO, but not in E-PRβ-KO as compared to Pdgfrb-gene preserving control mice. We showed that CXCL12, a potent chemoattractant for CXCR4-expressing NSPCs, was upregulated in the ischemic lesion of N-PRβ-KO mice. Furthermore, integrin α3 intrinsically expressed in NSPCs that critically **mediates extracellul**ar matrix-dependent migration, was upregulated in N-PRβ-KO after MCAO. NSPCs isolated from N-PRβ-KO rapidly migrated on the surface coated with collagen type IV or fibronectin that are abundant in vascular niche and ischemic core. PDGFR-β was suggested to be critically involved in pathological neurogenesis through the regulation of lesion-derived chemoattractant as well as intrinsic signal of NSPCs, and we believe that a coordinated regulation of these molecular events may be able to improve neurogenesis in injured brain for further functional recovery.
10. PDGFR-alpha signaling is critical for tooth cusp and palate morphogenesis. Platelet-derived growth factor receptor alpha (PDGFR-alpha) and PDGF ligands are key regulators for embryonic development. Although Pdgfralpha is spatially expressed in the cranial neural crest (CNC)-derived odontogenic mesenchyme, mice deficient for Pdgfralpha are embryonic lethal, making it impossible to investigate the functional significance of PDGF signaling in regulating the fate of CNC cells during tooth morphogenesis. Taking advantage of the kidney capsule assay, we investigated the biological function of PDGF signaling in regulating tooth morphogenesis. Pdgfralpha and Pdgfa are specifically and consistently expressed in the CNC-derived odontogenic mesenchyme and the dental epithelium, respectively, throughout all stages of tooth development, suggesting a paracrine function of PDGF signaling in regulating tooth morphogenesis. Highly concentrated expression patterns of Pdgfralpha and Pdgfa are associated with the developing dental cusp, suggesting possible functional importance of PDGF signaling in regulating cusp formation. Loss of the Pdgfralpha gene does not affect proper odontoblasts proliferation and differentiation in the CNC-derived odontogenic mesenchyme but perturbs the formation of **extracellular matrix** and the organization of odontoblast cells at the forming cusp area, resulting in dental cusp growth defect. Pdgfralpha-/- mice have complete cleft palate. We show that the cleft palate in Pdgfralpha mutant mice results from an **extracellular matrix** defect within the CNC-derived palatal mesenchyme. The midline epithelium of the mutant palatal shelf remains functionally competent to mediate palatal fusion once the palatal shelves are placed in close contact in vitro. Collectively, our data suggests that PDGFRalpha and PDGFA are critical regulators for the continued epithelial-mesenchymal interaction during tooth and palate morphogenesis. Disruption of PDGFRalpha signaling disturbs the growth of dental cusp and interferes with the critical extension of palatal shelf during craniofacial development.
11. Neurofibromin modulation of ras activity is required for normal endocardial-mesenchymal transformation in the developing heart. Endocardial cushions are the precursors of the cardiac valves and form by a process of epithelial-mesenchymal transformation. Secreted growth factors from myocardium induce endocardial cells to transform into mesenchyme and invade the overlying **extracellular matrix**. Here, we show that the product of the Nf1 neurofibromatosis gene is required to regulate this event. In the absence of neurofibromin, mouse embryo hearts develop overabundant endocardial cushions due to hyperproliferation and lack of normal apoptosis. Neurofibromin deficiency in explant cultures is reproduced by activation of ras signaling pathways, and the Nf1(-/-) mutant phenotype is prevented by inhibiting ras in vitro. These results indicate that neurofibromin normally acts to modulate epithelial-mesenchymal transformation and proliferation in the developing heart by down regulating ras activity.
12. PDGFRα signaling drives adipose tissue fibrosis by targeting progenitor cell plasticity. Fibrosis is a common disease process in which profibrotic cells disturb organ function by secreting disorganized **extracellular matri**x (ECM). Adipose tissue fibrosis occurs during obesity and is associated with metabolic dysfunction, but how profibrotic cells originate is still being elucidated. Here, we use a developmental model to investigate perivascular cells in white adipose tissue (WAT) and their potential to cause organ fibrosis. We show that a Nestin-Cre transgene targets perivascular cells (adventitial cells and pericyte-like cells) in WAT, and Nestin-GFP specifically labels pericyte-like cells. Activation of PDGFRα signaling in perivascular cells causes them to transition into ECM-synthesizing profibrotic cells. Before this transition occurs, PDGFRα signaling up-regulates mTOR signaling and ribosome biogenesis pathways and perturbs the expression of a network of epigenetically imprinted genes that have been implicated in cell growth and tissue homeostasis. Isolated Nestin-GFP(+) cells differentiate into adipocytes ex vivo and form WAT when transplanted into recipient mice. However, PDGFRα signaling opposes adipogenesis and generates profibrotic cells instead, which leads to fibrotic WAT in transplant experiments. These results identify perivascular cells as fibro/adipogenic progenitors in WAT and show that PDGFRα targets progenitor cell plasticity as a profibrotic mechanism.
13. Essential role of Sox9 in the pathway that controls formation of cardiac valves and septa. Epithelial-mesenchymal transformation is a critical developmental process reiterated in multiple organs throughout embryogenesis. Formation of endocardial cushions, primordia of valves and septa, is a classic example of epithelial-mesenchymal transformation. Several gene mutations are known to affect cardiac valve formation. Sox9 is activated when endocardial endothelial cells undergo mesenchymal transformation and migrate into an **extracellular matrix**, called cardiac jelly, to form endocardial cushions. In Sox9-null mutants, endocardial cushions are markedly hypoplastic. In these mutants, Nfatc1 is ectopically expressed and no longer restricted to endothelial cells. Further, Sox9-deficient endocardial mesenchymal cells fail to express ErbB3, which is required for endocardial cushion cell differentiation and proliferation. Our results reveal a succession of molecular steps in the pathway of endocardial cushion development. We propose that loss of Sox9 inhibits epithelial-mesenchymal transformation after delamination and initial migration, but before definitive mesenchymal transformation.
14. Neuregulin-1/ErbB4 signaling controls the migration of oligodendrocyte precursor cells during development. During embryonic development, the oligodendrocyte precursors (OPCs) are generated in specific oligodendrogliogenic sites within the neural tube and migrate to colonize the entire CNS. Different factors have been shown to influence the OPC migration and differentiation, including morphogens, growth factors, chemotropic molecules, and **extracellular matrix** proteins. Neuregulins have been shown to influence the migration of neuronal precursors as well as the movement and differentiation of Schwann cells for peripheral myelination, but their role in the motility of OPCs has not been explored. In the present study, we have used the optic nerve as an experimental model to examine the function of Nrg1 and its ErbB4 receptor in the migration of OPCs in the developing embryo. In vitro experiments revealed that Nrg1 is a potent chemoattractant for the first wave of OPCs, and that this effect is mediated via ErbB4 receptor. In contrast, OPCs colonizing the optic nerve at postnatal stages (PDGFRα(+)-OPCs) does not respond to Nrg1-chemoattraction. We also found that mouse embryos lacking ErbB4 display deficits in early OPC migration away from different oligodendrogliogenic regions in vivo. The present findings reveal a new role for Nrg1/ErbB4 signaling in regulating OPC migration selectively during early stages of CNS development.
15. Paracrine activation of hepatic stellate cells in platelet-derived growth factor C transgenic mice: evidence for stromal induction of hepatocellular carcinoma. Cirrhosis is the primary risk factor for the development of hepatocellular carcinoma (HCC), yet the mechanisms by which cirrhosis predisposes to carcinogenesis are poorly understood. Using a mouse model that recapitulates many aspects of the pathophysiology of human liver disease, we explored the mechanisms by which changes in the liver microenvironment induce dysplasia and HCC. Hepatic expression of platelet-derived growth factor C (PDGF-C) induces progressive fibrosis, chronic inflammation, neoangiogenesis and sinusoidal congestion, as well as global changes in gene expression. Using reporter mice, immunofluorescence, immunohistochemistry and liver cell isolation, we demonstrate that receptors for PDGF-CC are localized on hepatic stellate cells (HSCs), which proliferate, and transform into myofibroblast-like cells that deposit **extracellular matrix** and lead to production of growth factors and cytokines. We demonstrate induction of cytokine genes at 2 months, and stromal cell-derived hepatocyte growth factors that coincide with the onset of dysplasia at 4 months. Our results support a paracrine signaling model wherein hepatocyte-derived PDGF-C stimulates widespread HSC activation throughout the liver leading to chronic inflammation, liver injury and architectural changes. These complex changes to the liver microenvironment precede the development of HCC. Further, increased PDGF-CC levels were observed in livers of patients with nonalcoholic fatty steatohepatitis and correlate with the stage of disease, suggesting a role for this growth factor in chronic liver disease in humans. PDGF-C transgenic mice provide a unique model for the in vivo study of tumor-stromal interactions in the liver.
16. Fibroblast growth factor signaling in myofibroblasts differs from lipofibroblasts during alveolar septation in mice. Pulmonary alveolar fibroblasts produce **extracellular matrix** in a temporally and spatially regulated pattern to yield a durable yet pliable gas-exchange surface. Proliferation ensures a sufficient complement of cells, but they must differentiate into functionally distinct subtypes: contractile myofibroblasts (MF), which generate elastin and regulate air-flow at the alveolar ducts, and, in mice and rats, lipofibroblasts (LF), which store neutral lipids. PDGF-A is required but acts in conjunction with other differentiation factors arising from adjacent epithelia or within fibroblasts. We hypothesized that FGF receptor (FGFR) expression and function vary for MF and LF and contributes to their divergent differentiation. Whereas approximately half of the FGFR3 was extracellular in MF, FGFR2 and FGFR4 were primarily intracellular. Intracellular FGFR3 localized to the multivesicular body, and its abundance may be modified by Sprouty and interaction with heat shock protein-90. FGF18 mRNA is more abundant in MF, whereas FGF10 mRNA predominated in LF, which also express FGFR1 IIIb, a receptor for FGF10. FGF18 diminished fibroblast proliferation and was chemotactic for cultured fibroblasts. Although PDGF receptor-α (PDGFR-α) primarily signals through phosphoinositide 3-kinase and Akt, p42/p44 MAP kinase (Erk1/2), a major signaling pathway for FGFRs, influenced the abundance of cell-surface PDGFR-α. Observing different FGFR and ligand profiles in MF and LF is consistent with their divergent differentiation although both subpopulations express PDGFR-α. These studies also emphasize the importance of particular cellular locations of FGFR3 and PDGFR-α, which may modify their effects during alveolar development or repair.
17. Role for ephrinB2 in postnatal lung alveolar development and elastic matrix integrity. Alveoli are formed in the lung by the insertion of secondary tissue folds, termed septa, which are subsequently remodeled to form the mature alveolar wall. Secondary septation requires interplay between three cell types: endothelial cells forming capillaries, contractile interstitial myofibroblasts, and epithelial cells. Here, we report that postnatal lung alveolization critically requires ephrinB2, a ligand for Eph receptor tyrosine kinases expressed by the microvasculature. Mice homozygous for the hypomorphic knockin allele ephrinB2DeltaV/DeltaV, encoding mutant ephrinB2 with a disrupted C-terminal PDZ interaction motif, show severe postnatal lung defects including an almost complete absence of lung alveoli and abnormal and disorganized elastic matrix. Lung alveolar formation is not sensitive to loss of ephrinB2 cytoplasmic tyrosine phosphorylation sites. Postnatal day 1 mutant lungs show **extracellular matrix** alterations without differences in proportions of major distal cell populations. We conclude that lung alveolar formation relies on endothelial ephrinB2 function.

### [Ii]nvasive

#### PubMed

1. Links between alpha-catenin, NF-kappaB, and squamous cell carcinoma in skin. Cancers display a diverse set of cellular defects, which are thought to be elicited by multiple genetic mutations. In this study, we show that when a single adherens junction protein, alpha-catenin, is removed by conditional targeting, the entire skin epidermis systematically transforms to a hyperproliferative, **invasive** tissue replete with inflammation. Transcriptional profiling and biochemical analyses reveal that alpha-catenin ablation is accompanied by activation of NF-kappaB and its proinflammatory target genes, along with genes involved in proliferation, wound healing, angiogenesis, and metastasis. Many of these alterations occur in vitro and in the embryo, and thus seem at least partly to be intrinsic to the loss of alpha-catenin. We show that reductions in alpha-catenin, activation of NF-kappaB, and inflammation are common features of human squamous cell carcinomas of the skin.
2. Autocrine PDGFR signaling promotes mammary cancer metastasis. Metastasis is the major cause of cancer morbidity, but strategies for direct interference with invasion processes are lacking. Dedifferentiated, late-stage tumor cells secrete multiple factors that represent attractive targets for therapeutic intervention. Here we show that metastatic potential of oncogenic mammary epithelial cells requires an autocrine PDGF/PDGFR loop, which is established as a consequence of TGF-beta-induced epithelial-mesenchymal transition (EMT), a faithful in vitro correlate of metastasis. The cooperation of autocrine PDGFR signaling with oncogenic Ras hyperactivates PI3K and is required for survival during EMT. Autocrine PDGFR signaling also contributes to maintenance of EMT, possibly through activation of STAT1 and other distinct pathways. Inhibition of PDGFR signaling interfered with EMT and caused apoptosis in murine and human mammary carcinoma cell lines. Consequently, overexpression of a dominant-negative PDGFR or application of the established cancer drug STI571 interfered with experimental metastasis in mice. Similarly, in mouse mammary tumor virus-Neu (MMTV-Neu) transgenic mice, TGF-beta enhanced metastasis of mammary tumors, induced EMT, and elevated PDGFR signaling. Finally, expression of PDGFRalpha and -beta correlated with **invasive** behavior in human mammary carcinomas. Thus, autocrine PDGFR signaling plays an essential role during cancer progression, suggesting a novel application of STI571 to therapeutically interfere with metastasis.
3. Autocrine PDGFR signaling promotes mammary cancer metastasis. Metastasis is the major cause of cancer morbidity, but strategies for direct interference with invasion processes are lacking. Dedifferentiated, late-stage tumor cells secrete multiple factors that represent attractive targets for therapeutic intervention. Here we show that metastatic potential of oncogenic mammary epithelial cells requires an autocrine PDGF/PDGFR loop, which is established as a consequence of TGF-beta-induced epithelial-mesenchymal transition (EMT), a faithful in vitro correlate of metastasis. The cooperation of autocrine PDGFR signaling with oncogenic Ras hyperactivates PI3K and is required for survival during EMT. Autocrine PDGFR signaling also contributes to maintenance of EMT, possibly through activation of STAT1 and other distinct pathways. Inhibition of PDGFR signaling interfered with EMT and caused apoptosis in murine and human mammary carcinoma cell lines. Consequently, overexpression of a dominant-negative PDGFR or application of the established cancer drug STI571 interfered with experimental metastasis in mice. Similarly, in mouse mammary tumor virus-Neu (MMTV-Neu) transgenic mice, TGF-beta enhanced metastasis of mammary tumors, induced EMT, and elevated PDGFR signaling. Finally, expression of PDGFRalpha and -beta correlated with **invasive** behavior in human mammary carcinomas. Thus, autocrine PDGFR signaling plays an essential role during cancer progression, suggesting a novel application of STI571 to therapeutically interfere with metastasis.
4. Ganglioside GD3 Enhances **Invasive**ness of Gliomas by Forming a Complex with Platelet-derived Growth Factor Receptor α and Yes Kinase. There have been a few studies on the ganglioside expression in human glioma tissues. However, the role of these gangliosides such as GD3 and GD2 has not been well understood. In this study we employed a genetically engineered mouse model of glioma to clarify the functions of GD3 in gliomas. Forced expression of platelet-derived growth factor B in cultured astrocytes derived from p53-deficient mice resulted in the expression of GD3 and GD2. GD3-positive astrocytes exhibited increased cell growth and invasion activities along with elevated phosphorylation of Akt and Yes kinase. By enzyme-mediated activation of radical sources reaction and mass spectrometry, we identified PDGF receptor α (PDGFRα) as a GD3-associated molecule. GD3-positive astrocytes showed a significant amount of PDGFRα in glycolipid-enriched microdomains/rafts compared with GD3-negative cells. Src kinase family Yes was co-precipitated with PDGFRα, and its pivotal role in the increased cell invasion of GD3-positive astrocytes was demonstrated by silencing with anti-Yes siRNA. Direct association between PDGFRα and GD3 was also shown, suggesting that GD3 forms ternary complex with PDGFRα and Yes. The fact that GD3, PDGFRα, and activated Yes were colocalized in lamellipodia and the edge of tumors in cultured cells and glioma tissues, respectively, suggests that GD3 induced by platelet-derived growth factor B enhances PDGF signals in glycolipid-enriched microdomain/rafts, leading to the promotion of malignant phenotypes such as cell proliferation and invasion in gliomas.
5. Glioma initiating cells contribute to malignant transformation of host glial cells during tumor tissue remodeling via PDGF signaling. Glioma initiating cells (GICs) play important roles in tumor initiation and progression. However, interactions between tumor cells and host cells of local tumor microenvironment are kept largely unknown. Besides GICs and their progeny cells, whether adjacent normal glial cells contribute to tumorigenesis during glioma tissue remodeling deserves further investigation.Red fluorescence protein (RFP) gene was stably transfected into human GIC cells lines SU3 and U87, then were transplanted intracerebrally into athymic nude mice with whole-body green fluorescence protein (GFP) expression. The interactions between GICs and host cells in vivo were observed during tissue remodeling processes initiated by hGICs. The biological characteristics of host glial cells with high proliferation capability cloned from the xenograft were further assayed.In a SU3 initiated dual-fluorescence xenograft glioma model, part of host cells cloned from the intracerebral tumors were found acquiring the capability of unlimited proliferation. PCR and FISH results indicated that malignant transformed cells were derived from host cells; cell surface marker analysis showed these cells expressed murine oligodendrocyte specific marker CNP, and oligodendrocyte progenitor cells (OPCs) specific markers PDGFR-α and NG2. Chromosomal analysis showed these cells were super tetraploid. In vivo studies showed they behaved with high **invasiv**eness activity and nearly 100% tumorigenic ratio. Compared with SU3 cells with higher PDGF-B expression, GICs derived from U87 cells with low level of PDGF-B expression failed to induce host cell transformation.Primary high **invasiv**e GICs SU3 contribute to transformation of adjacent normal host glial cells in local tumor microenvironment possibly via PDGF/PDGFR signaling activation, which deserved further investigation.
6. Nf1 limits epicardial derivative expansion by regulating epithelial to mesenchymal transition and proliferation. The epicardium is the primary source of coronary vascular smooth muscle cells (cVSMCs) and fibroblasts that reside in the compact myocardium. To form these epicardial-derived cells (EPDCs), the epicardium undergoes the process of epithelial to mesenchymal transition (EMT). Although several signaling pathways have been identified that disrupt EMT, no pathway has been reported that restricts this developmental process. Here, we identify neurofibromin 1 (Nf1) as a key mediator of epicardial EMT. To determine the function of Nf1 during epicardial EMT and the formation of epicardial derivatives, cardiac fibroblasts and cVSMCs, we generated mice with a tissue-specific deletion of Nf1 in the epicardium. We found that mutant epicardial cells transitioned more readily to mesenchymal cells in vitro and in vivo. The mesothelial epicardium lost epithelial gene expression and became more **invasive**. Using lineage tracing of EPDCs, we found that the process of EMT occurred earlier in Nf1 mutant hearts, with an increase in epicardial cells entering the compact myocardium. Moreover, loss of Nf1 caused increased EPDC proliferation and resulted in more cardiac fibroblasts and cVSMCs. Finally, we were able to partially reverse the excessive EMT caused by loss of Nf1 by disrupting Pdgfrα expression in the epicardium. Conversely, Nf1 activation was able to inhibit PDGF-induced epicardial EMT. Our results demonstrate a regulatory role for Nf1 during epicardial EMT and provide insights into the susceptibility of patients with disrupted NF1 signaling to cardiovascular disease.
7. Abnormal development of NG2+PDGFR-α+ neural progenitor cells leads to neonatal hydrocephalus in a ciliopathy mouse model. Hydrocephalus is a common neurological disorder that leads to expansion of the cerebral ventricles and is associated with a high rate of morbidity and mortality. Most neonatal cases are of unknown etiology and are likely to have complex inheritance involving multiple genes and environmental factors. Identifying molecular mechanisms for neonatal hydrocephalus and developing no**ninvasiv**e treatment modalities are high priorities. Here we use a hydrocephalic mouse model of the human ciliopathy Bardet-Biedl Syndrome (BBS) and identify a role for neural progenitors in the pathogenesis of neonatal hydrocephalus. We found that hydrocephalus in this mouse model is caused by aberrant platelet-derived growth factor receptor α (PDGFR-α) signaling, resulting in increased apoptosis and impaired proliferation of chondroitin sulfate proteoglycan 4 (also known as neuron-glial antigen 2 or NG2)(+)PDGFR-α(+) neural progenitors. Targeting this pathway with lithium treatment rescued NG2(+)PDGFR-α(+) progenitor cell proliferation in BBS mutant mice, reducing their ventricular volume. Our findings demonstrate that neural progenitors are crucial in the pathogenesis of neonatal hydrocephalus, and we identify new therapeutic targets for this common neurological disorder.
8. Adult interfollicular tumour-initiating cells are reprogrammed into an embryonic hair follicle progenitor-like fate during basal cell carcinoma initiation. Basal cell carcinoma, the most frequent human skin cancer, arises from activating hedgehog (HH) pathway mutations; however, little is known about the temporal changes that occur in tumour-initiating cells from the first oncogenic hit to the development of **invasive** cancer. Using an inducible mouse model enabling the expression of a constitutively active Smoothened mutant (SmoM2) in the adult epidermis, we carried out transcriptional profiling of SmoM2-expressing cells at different times during cancer initiation. We found that tumour-initiating cells are massively reprogrammed into a fate resembling that of embryonic hair follicle progenitors (EHFPs). Wnt/ β-catenin signalling was very rapidly activated following SmoM2 expression in adult epidermis and coincided with the expression of EHFP markers. Deletion of β-catenin in adult SmoM2-expressing cells prevents EHFP reprogramming and tumour initiation. Finally, human basal cell carcinomas also express genes of the Wnt signalling and EHFP signatures.

#### GeneRIFs

1. Ganglioside GD3 enhances **invasive**ness of gliomas by forming a complex with PDGFRalpha and Yes Kinase.
2. Primary high **invasive** Glioma initiating cells SU3 contribute to transformation of adjacent normal host glial cells in local tumor microenvironment possibly via PDGF/PDGFR signaling activation.

### [Mm]etastasis

#### PubMed

1. Links between alpha-catenin, NF-kappaB, and squamous cell carcinoma in skin. Cancers display a diverse set of cellular defects, which are thought to be elicited by multiple genetic mutations. In this study, we show that when a single adherens junction protein, alpha-catenin, is removed by conditional targeting, the entire skin epidermis systematically transforms to a hyperproliferative, invasive tissue replete with inflammation. Transcriptional profiling and biochemical analyses reveal that alpha-catenin ablation is accompanied by activation of NF-kappaB and its proinflammatory target genes, along with genes involved in proliferation, wound healing, angiogenesis, and **metastasis**. Many of these alterations occur in vitro and in the embryo, and thus seem at least partly to be intrinsic to the loss of alpha-catenin. We show that reductions in alpha-catenin, activation of NF-kappaB, and inflammation are common features of human squamous cell carcinomas of the skin.
2. Autocrine PDGFR signaling promotes mammary cancer **metastasis**. **Metastasis** is the major cause of cancer morbidity, but strategies for direct interference with invasion processes are lacking. Dedifferentiated, late-stage tumor cells secrete multiple factors that represent attractive targets for therapeutic intervention. Here we show that metastatic potential of oncogenic mammary epithelial cells requires an autocrine PDGF/PDGFR loop, which is established as a consequence of TGF-beta-induced epithelial-mesenchymal transition (EMT), a faithful in vitro correlate of **metastasis**. The cooperation of autocrine PDGFR signaling with oncogenic Ras hyperactivates PI3K and is required for survival during EMT. Autocrine PDGFR signaling also contributes to maintenance of EMT, possibly through activation of STAT1 and other distinct pathways. Inhibition of PDGFR signaling interfered with EMT and caused apoptosis in murine and human mammary carcinoma cell lines. Consequently, overexpression of a dominant-negative PDGFR or application of the established cancer drug STI571 interfered with experimental **metastasis** in mice. Similarly, in mouse mammary tumor virus-Neu (MMTV-Neu) transgenic mice, TGF-beta enhanced **metastasis** of mammary tumors, induced EMT, and elevated PDGFR signaling. Finally, expression of PDGFRalpha and -beta correlated with invasive behavior in human mammary carcinomas. Thus, autocrine PDGFR signaling plays an essential role during cancer progression, suggesting a novel application of STI571 to therapeutically interfere with **metastasis**.
3. Autocrine PDGFR signaling promotes mammary cancer **metastasis**. **Metastasis** is the major cause of cancer morbidity, but strategies for direct interference with invasion processes are lacking. Dedifferentiated, late-stage tumor cells secrete multiple factors that represent attractive targets for therapeutic intervention. Here we show that metastatic potential of oncogenic mammary epithelial cells requires an autocrine PDGF/PDGFR loop, which is established as a consequence of TGF-beta-induced epithelial-mesenchymal transition (EMT), a faithful in vitro correlate of **metastasis**. The cooperation of autocrine PDGFR signaling with oncogenic Ras hyperactivates PI3K and is required for survival during EMT. Autocrine PDGFR signaling also contributes to maintenance of EMT, possibly through activation of STAT1 and other distinct pathways. Inhibition of PDGFR signaling interfered with EMT and caused apoptosis in murine and human mammary carcinoma cell lines. Consequently, overexpression of a dominant-negative PDGFR or application of the established cancer drug STI571 interfered with experimental **metastasis** in mice. Similarly, in mouse mammary tumor virus-Neu (MMTV-Neu) transgenic mice, TGF-beta enhanced **metastasis** of mammary tumors, induced EMT, and elevated PDGFR signaling. Finally, expression of PDGFRalpha and -beta correlated with invasive behavior in human mammary carcinomas. Thus, autocrine PDGFR signaling plays an essential role during cancer progression, suggesting a novel application of STI571 to therapeutically interfere with **metastasis**.
4. ILEI requires oncogenic Ras for the epithelial to mesenchymal transition of hepatocytes and liver carcinoma progression. In human hepatocellular carcinoma (HCC), epithelial to mesenchymal transition (EMT) correlates with aggressiveness of tumors and poor survival. We employed a model of EMT based on immortalized p19(ARF) null hepatocytes (MIM), which display tumor growth upon expression of oncogenic Ras and undergo EMT through the synergism of Ras and transforming growth factor (TGF)-beta. Here, we show that the interleukin-related protein interleukin-like EMT inducer (ILEI), a novel EMT-, tumor- and **metastasis**-inducing protein, cooperates with oncogenic Ras to cause TGF-beta-independent EMT. Ras-transformed MIM hepatocytes overexpressing ILEI showed cytoplasmic E-cadherin, loss of ZO-1 and induction of alpha-smooth muscle actin as well as platelet-derived growth factor (PDGF)/PDGF-R isoforms. As shown by dominant-negative PDGF-R expression in these cells, ILEI-induced PDGF signaling was required for enhanced cell migration, nuclear accumulation of beta-catenin, nuclear pY-Stat3 and accelerated growth of lung metastases. In MIM hepatocytes expressing the Ras mutant V12-C40, ILEI collaborated with PI3K signaling resulting in tumor formation without EMT. Clinically, human HCC samples showed granular or cytoplasmic localization of ILEI correlating with well and poorly differentiated tumors, respectively. In conclusion, these data indicate that ILEI requires cooperation with oncogenic Ras to govern hepatocellular EMT through mechanisms involving PDGF-R/beta-catenin and PDGF-R/Stat3 signaling.

### [Cc]ell [Ii]nvasion

#### PubMed

1. Endothelin-2 signaling in the neural retina promotes the endothelial tip cell state and inhibits angiogenesis. Endothelin signaling is required for neural crest migration and homeostatic regulation of blood pressure. Here, we report that constitutive overexpression of Endothelin-2 (Edn2) in the mouse retina perturbs vascular development by inhibiting endothelial cell migration across the retinal surface and subsequent endothelial **cell invasion** into the retina. Developing endothelial cells exist in one of two states: tip cells at the growing front and stalk cells in the vascular plexus behind the front. This division of endothelial cell states is one of the central organizing principles of angiogenesis. In the developing retina, Edn2 overexpression leads to overproduction of endothelial tip cells by both morphologic and molecular criteria. Spatially localized overexpression of Edn2 produces a correspondingly localized endothelial response. Edn2 overexpression in the early embryo inhibits vascular development at midgestation, but Edn2 overexpression in developing skin and brain has no discernible effect on vascular structure. Inhibition of retinal angiogenesis by Edn2 requires expression of Endothelin receptor A but not Endothelin receptor B in the neural retina. Taken together, these observations imply that the neural retina responds to Edn2 by synthesizing one or more factors that promote the endothelial tip cell state and inhibit angiogenesis. The response to Edn2 is sufficiently potent that it overrides the activities of other homeostatic regulators of angiogenesis, such as Vegf.
2. Ganglioside GD3 Enhances Invasiveness of Gliomas by Forming a Complex with Platelet-derived Growth Factor Receptor α and Yes Kinase. There have been a few studies on the ganglioside expression in human glioma tissues. However, the role of these gangliosides such as GD3 and GD2 has not been well understood. In this study we employed a genetically engineered mouse model of glioma to clarify the functions of GD3 in gliomas. Forced expression of platelet-derived growth factor B in cultured astrocytes derived from p53-deficient mice resulted in the expression of GD3 and GD2. GD3-positive astrocytes exhibited increased cell growth and invasion activities along with elevated phosphorylation of Akt and Yes kinase. By enzyme-mediated activation of radical sources reaction and mass spectrometry, we identified PDGF receptor α (PDGFRα) as a GD3-associated molecule. GD3-positive astrocytes showed a significant amount of PDGFRα in glycolipid-enriched microdomains/rafts compared with GD3-negative cells. Src kinase family Yes was co-precipitated with PDGFRα, and its pivotal role in the incre**ased cell inv**asion of GD3-positive astrocytes was demonstrated by silencing with anti-Yes siRNA. Direct association between PDGFRα and GD3 was also shown, suggesting that GD3 forms ternary complex with PDGFRα and Yes. The fact that GD3, PDGFRα, and activated Yes were colocalized in lamellipodia and the edge of tumors in cultured cells and glioma tissues, respectively, suggests that GD3 induced by platelet-derived growth factor B enhances PDGF signals in glycolipid-enriched microdomain/rafts, leading to the promotion of malignant phenotypes such as cell proliferation and invasion in gliomas.

---

## Tpbg

- NCBI Gene
- GeneRIF
- Pubmed

| Search Term | Rank | Fields |
| --- | --- | --- |
| [Mm]igration | 6 | PubMed(1); GeneRIFs(1) |
| [Mm]etastasis | 12 | PubMed(3) |

  
hide/show details for Tpbg

### [Mm]igration

#### PubMed

1. Embryonic expression of murine 5T4 oncofoetal antigen is associated with morphogenetic events at implantation and in developing epithelia. Overexpression of 5T4 oncofoetal antigen, an early marker of ES cell differentiation, in vitro increases cellular motility and decreases adhesion, properties relevant to development and cancer. Embryonic expression of m5T4 antigen is first detected on trophectoderm at implantation and is restricted to extra-embryonic tissues to embryonic day (E) 11.5. In the embryo, significant m5T4 expression is detected at E12.5 in hindbrain roofplate and in various epithelia derived from all germ layers. In keratin 14-expressing epithelia, there is a congruent 5T4 expression pattern with many of these cells being Ki-67 positive. In brain, expression is observed in roofplate, ependymal layers, choroid plexus, and subventricular zones of lateral ventricles at E14.5. By E17.5, expression is decreased in the subventricular zone with further restriction to choroid plexus in adult brain. Our data demonstrate a limited 5T4 expression profile during embryogenesis associated with actively cycling, undifferentiated epithelial progenitor cells that may contribute to their **migration**.

#### GeneRIFs

1. A limited expression profile of 5T4 may contribute to the **migration** of actively cycling, undifferentiated epithelial progenitor cells during embryogenesis

### [Mm]etastasis

#### PubMed

1. The 5T4 oncofoetal antigen is an early differentiation marker of mouse ES cells and its absence is a useful means to assess pluripotency. 5T4 oncotrophoblast antigen is a transmembrane glycoprotein expressed by trophoblast and many carcinomas but not most normal adult tissues. Results from overexpression of human and mouse 5T4 cDNA in cell lines are consistent with it having an influence on adhesion, shape and motility. We show that murine embryonic stem cell lines are 5T4 negative but that there is rapid up regulation of protein and transcripts upon differentiation, including derivatives of each primary germ layer, as evidenced by cell surface FACS, western and RT-PCR analyses. The kinetics of differentiation and 5T4 expression are closely correlated, with early events linking 5T4 expression to changes in motility and morphology. Comparison of 5T4 expression with other ES cell transcript (Oct 3/4; Rex-1) and antigen markers (Forsmann, SSEA-1) establishes 5T4 as a useful marker for the non-destructive detection of early differentiation of ES cells. For example, 'undifferentiated' ES phenotype defined as SSEA-1 positive and 5T4 negative is seven times more efficient at chimera formation than SSEA-1-positive/5T4-positive cells. Thus, 5T4 glycoprotein expression is associated with early differentiative events of ES cells involving altered motility, and it has useful practical consequences for assessing ES potency and studying similar processes in development and **metastasis**.
2. CXCR4 mediated chemotaxis is regulated by 5T4 oncofetal glycoprotein in mouse embryonic cells. 5T4 oncofetal molecules are highly expressed during development and upregulated in cancer while showing only low levels in some adult tissues. Upregulation of 5T4 expression is a marker of loss of pluripotency in the early differentiation of embryonic stem (ES) cells and forms an integrated component of an epithelial-mesenchymal transition, a process important during embryonic development and metastatic spread of epithelial tumors. Investigation of the transcriptional changes in early ES differentiation showed upregulation of CXCL12 and down-regulation of a cell surface protease, CD26, which cleaves this chemokine. CXCL12 binds to the widely expressed CXCR4 and regulates key aspects of development, stem cell motility and tumour **metastasis** to tissues with high levels of CXCL12. We show that the 5T4 glycoprotein is required for optimal functional cell surface expression of the chemokine receptor CXCR4 and CXCL12 mediated chemotaxis in differentiating murine embryonic stem cells and embryo fibroblasts (MEF). Cell surface expression of 5T4 and CXCR4 molecules is co-localized in differentiating ES cells and MEF. By contrast, differentiating ES and MEF derived from 5T4 knockout (KO) mice show only intracellular CXCR4 expression but infection with adenovirus encoding mouse 5T4 restores CXCL12 chemotaxis and surface co-localization with 5T4 molecules. A series of chimeric constructs with interchanged domains of 5T4 and the glycoprotein CD44 were used to map the 5T4 sequences relevant for CXCR4 membrane expression and function in 5T4KO MEF. These data identified the 5T4 transmembrane domain as sufficient and necessary to enable CXCR4 cell surface expression and chemotaxis. Furthermore, some monoclonal antibodies against m5T4 can inhibit CXCL12 chemotaxis of differentiating ES cells and MEF which is not mediated by simple antigenic modulation. Collectively, these data support a molecular interaction of 5T4 and CXCR4 occurring at the cell surface which directly facilitates the biological response to CXCL12. The regulation of CXCR4 surface expression by 5T4 molecules is a novel means to control responses to the chemokine CXCL12 for example during embryogenesis but can also be selected to advantage the spread of a 5T4 positive tumor from its primary site.
3. Organisation of the mouse and human 5T4 oncofoetal leucine-rich glycoprotein genes and expression in foetal and adult murine tissues. The human 5T4 oncotrophoblast leucine-rich glycoprotein may contribute to the process of placentation or **metastasis** by modulating cell adhesion, shape and motility. To understand better the role of 5T4 in development and cancer, the gene structure has been elucidated from both human and mouse genomic clones and mRNA expression has been studied in foetal and adult mouse tissues. The protein coding region is located within the second of two exons, the first exon comprising solely of 5'-untranslated region. Upstream there are no TATA or CAAT boxes, but there are a number of potential Sp1 binding sites. The murine and human proteins show a homologous domain organisation of the leucine rich repeats (LRR) and associated N- and C-terminal flanking regions, although the hydrophilic sequence which intervenes between the two LRR domains contains six additional amino acids in the mouse. The signal peptide, transmembrane region and cytoplasmic tail sequences are identical as are 6 out of the 7 potential N-linked glycosylation sites. Mouse 5T4 transcripts are abundant in placenta and also highly expressed in embryos while in adult tissues transcripts are restricted to brain and ovary. These patterns of expression and the genomic organisation are discussed in relation to possible function and other recently described LRR containing proteins.

---

## Arc

- NCBI Gene
- GeneRIF
- Pubmed

| Search Term | Rank | Fields |
| --- | --- | --- |
| [Mm]igration | 6 | Process(1); PubMed(1) |

  
hide/show details for Arc

### [Mm]igration

#### Process

1. cell **migration**

#### PubMed

1. TSHZ1-dependent gene regulation is essential for olfactory bulb development and olfaction. The olfactory bulb (OB) receives odor information from the olfactory epithelium and relays this to the olfactory cortex. Using a mouse model, we found that development and maturation of OB interneurons depends on the zinc finger homeodomain factor teashirt zinc finger family member 1 (TSHZ1). In mice lacking TSHZ1, neuroblasts exhibited a normal tangential **migration** to the OB; however, upon arrival to the OB, the neuroblasts were distributed aberrantly within the radial dimension, and many immature neuroblasts failed to exit the rostral migratory stream. Conditional deletion of Tshz1 in mice resulted in OB hypoplasia and severe olfactory deficits. We therefore investigated olfaction in human subjects from families with congenital aural atresia that were heterozygous for TSHZ1 loss-of-function mutations. These individuals displayed hyposmia, which is characterized by impaired odor discrimination and reduced olfactory sensitivity. Microarray analysis, in situ hybridization, and ChIP revealed that TSHZ1 bound to and regulated expression of the gene encoding prokineticin receptor 2 (PROKR2), a G protein–coupled receptor essential for OB development. Mutations in PROKR2 lead to Kallmann syndrome, characterized by anosmia and hypogonadotrophic hypogonadism. Our data indicate that TSHZ1 is a key regulator of mammalian OB development and function and controls the expression of molecules involved in human Kallmann syndrome.

---

## Hsd3b3

- NCBI Gene
- GeneRIF
- Pubmed

| Search Term | Rank | Fields |
| --- | --- | --- |
| [Ii]nvasive | 10 | PubMed(1) |

  
hide/show details for Hsd3b3

### [Ii]nvasive

#### PubMed

1. In vivo and in vitro investigations of heterozygous nebulin knock-out mice disclose a mild skeletal muscle phenotype. Nemaline myopathy is the most common congenital skeletal muscle disease, and mutations in the nebulin gene account for 50% of all cases. Recent studies suggest that the disease severity might be related to the nebulin expression levels. Considering that mutations in the nebulin gene are typically recessive, one would expect that a single functional nebulin allele would maintain nebulin protein expression which would result in preserved skeletal muscle function. We investigated skeletal muscle function of heterozygous nebulin knock-out (i.e., nebulin(+/-)) mice using a multidisciplinary approach including protein and gene expression analysis and combined in vivo and in vitro force measurements. Skeletal muscle anatomy and energy metabolism were studied strictly non-**invasive**ly using magnetic resonance imaging and 31P-magnetic resonance spectroscopy. Maximal force production was reduced by around 16% in isolated muscle of nebulin(+/-) mice while in vivo force generating capacity was preserved. Muscle weakness was associated with a shift toward a slower proteomic phenotype, but was not related to nebulin protein deficiency or to an impaired energy metabolism. Further studies would be warranted in order to determine the mechanisms leading to a mild skeletal muscle phenotype resulting from the expression of a single nebulin allele.

---

## Nsdhl

- NCBI Gene
- GeneRIF
- Pubmed

| Search Term | Rank | Fields |
| --- | --- | --- |
| [Mm]igration | 6 | PubMed(4) |

  
hide/show details for Nsdhl

### [Mm]igration

#### PubMed

1. Analysis of hedgehog signaling in cerebellar granule cell precursors in a conditional Nsdhl allele demonstrates an essential role for cholesterol in postnatal CNS development. NSDHL is a 3β-hydroxysterol dehydrogenase that is involved in the removal of two C-4 methyl groups in one of the later steps of cholesterol biosynthesis. Mutations in the gene encoding the enzyme are responsible for the X-linked, male lethal mouse mutations bare patches and striated, as well as most cases of human CHILD syndrome. Rare, hypomorphic NSDHL mutations are also associated with X-linked intellectual disability in males with CK syndrome. Since hemizygous male mice with Nsdhl mutations die by midgestation, we generated a conditional targeted Nsdhl mutation (Nsdhl(tm1.1Hrm)) to investigate the essential role of cholesterol in the early postnatal CNS. Ablation of Nsdhl in radial glia using GFAP-cre resulted in live-born, normal appearing affected male pups. However, the pups develop overt ataxia by postnatal day 8-10 and die shortly thereafter. Histological abnormalities include progressive loss of cortical and hippocampal neurons, as well as deficits in the proliferation and **migratio**n of cerebellar granule precursors and subsequent massive apoptosis of the cerebellar cortex. We replicated the granule cell precursor proliferation defect in vitro and demonstrate that it results from defective signaling by SHH. Furthermore, this defect is almost completely rescued by supplementation of the culture media with exogenous cholesterol, while methylsterol accumulation above the enzymatic block appears to be associated with increased cell death. These data support the absolute requirement for cholesterol synthesis in situ once the blood-brain-barrier forms and cholesterol transport to the fetus is abolished. They further emphasize the complex ramifications of cholesterogenic enzyme deficiency on cellular metabolism.
2. Analysis of Nsdhl-deficient embryos reveals a role for Hedgehog signaling in early placental development. The X-linked Nsdhl gene encodes a sterol dehydrogenase involved in cholesterol biosynthesis. Mutations in this gene cause the male lethal phenotypes in human CHILD syndrome and bare patches (Bpa) mice. Affected male embryos for several mutant Nsdhl alleles die in mid-gestation with a thin and poorly vascularized placental labyrinth. The timing and specific abnormalities noted suggest a defect in one or more developmental signaling pathways as a possible mechanism. Here, we examined the possible involvement of the hedgehog signaling pathway in the placental pathology of Nsdhl mutants using a transgenic mouse line (Ptch1(tm1Mps)) that contains a lacZ reporter under the control of the promoter for Ptch1, the gene that encodes the major hedgehog receptor. We demonstrate expression of Ptch1 in allantoic mesoderm of the placenta from wild-type mid-gestation embryos. The evidence suggests that the signaling is induced by Indian hedgehog that is produced by distal (ectoplacental) visceral endoderm cells that migrate into the allantoic mesoderm before embryonic day 10.0. Using a ubiquitously expressed, X-linked lacZ transgene that undergoes normal X-inactivation, we demonstrate that the placental defects in Nsdhl/+ female embryos are non-cell autonomous. Further, affected placentas from mutant Nsdhl(Bpa-8H) male embryos demonstrate markedly decreased or no Ptch1-lacZ staining and no **migration** of Ihh expressing cells into the developing placenta. These data strongly implicate the hedgehog signaling pathway in the pathogenesis of the placental defects in NSDHL deficiency and provide evidence for a role for the hedgehog pathway in the development of a functional mammalian placenta.
3. Analysis of hedgehog signaling in cerebellar granule cell precursors in a conditional Nsdhl allele demonstrates an essential role for cholesterol in postnatal CNS development. NSDHL is a 3β-hydroxysterol dehydrogenase that is involved in the removal of two C-4 methyl groups in one of the later steps of cholesterol biosynthesis. Mutations in the gene encoding the enzyme are responsible for the X-linked, male lethal mouse mutations bare patches and striated, as well as most cases of human CHILD syndrome. Rare, hypomorphic NSDHL mutations are also associated with X-linked intellectual disability in males with CK syndrome. Since hemizygous male mice with Nsdhl mutations die by midgestation, we generated a conditional targeted Nsdhl mutation (Nsdhl(tm1.1Hrm)) to investigate the essential role of cholesterol in the early postnatal CNS. Ablation of Nsdhl in radial glia using GFAP-cre resulted in live-born, normal appearing affected male pups. However, the pups develop overt ataxia by postnatal day 8-10 and die shortly thereafter. Histological abnormalities include progressive loss of cortical and hippocampal neurons, as well as deficits in the proliferation and **migratio**n of cerebellar granule precursors and subsequent massive apoptosis of the cerebellar cortex. We replicated the granule cell precursor proliferation defect in vitro and demonstrate that it results from defective signaling by SHH. Furthermore, this defect is almost completely rescued by supplementation of the culture media with exogenous cholesterol, while methylsterol accumulation above the enzymatic block appears to be associated with increased cell death. These data support the absolute requirement for cholesterol synthesis in situ once the blood-brain-barrier forms and cholesterol transport to the fetus is abolished. They further emphasize the complex ramifications of cholesterogenic enzyme deficiency on cellular metabolism.
4. Analysis of Nsdhl-deficient embryos reveals a role for Hedgehog signaling in early placental development. The X-linked Nsdhl gene encodes a sterol dehydrogenase involved in cholesterol biosynthesis. Mutations in this gene cause the male lethal phenotypes in human CHILD syndrome and bare patches (Bpa) mice. Affected male embryos for several mutant Nsdhl alleles die in mid-gestation with a thin and poorly vascularized placental labyrinth. The timing and specific abnormalities noted suggest a defect in one or more developmental signaling pathways as a possible mechanism. Here, we examined the possible involvement of the hedgehog signaling pathway in the placental pathology of Nsdhl mutants using a transgenic mouse line (Ptch1(tm1Mps)) that contains a lacZ reporter under the control of the promoter for Ptch1, the gene that encodes the major hedgehog receptor. We demonstrate expression of Ptch1 in allantoic mesoderm of the placenta from wild-type mid-gestation embryos. The evidence suggests that the signaling is induced by Indian hedgehog that is produced by distal (ectoplacental) visceral endoderm cells that migrate into the allantoic mesoderm before embryonic day 10.0. Using a ubiquitously expressed, X-linked lacZ transgene that undergoes normal X-inactivation, we demonstrate that the placental defects in Nsdhl/+ female embryos are non-cell autonomous. Further, affected placentas from mutant Nsdhl(Bpa-8H) male embryos demonstrate markedly decreased or no Ptch1-lacZ staining and no **migration** of Ihh expressing cells into the developing placenta. These data strongly implicate the hedgehog signaling pathway in the pathogenesis of the placental defects in NSDHL deficiency and provide evidence for a role for the hedgehog pathway in the development of a functional mammalian placenta.

---

## Hsd3b1

- NCBI Gene
- GeneRIF
- Pubmed

| Search Term | Rank | Fields |
| --- | --- | --- |
| [Mm]igration | 6 | PubMed(4) |

  
hide/show details for Hsd3b1

### [Mm]igration

#### PubMed

1. Loss of Wnt5a disrupts primordial germ cell **migration** and male sexual development in mice. Disruptions in the regulatory pathways controlling sex determination and differentiation can cause disorders of sex development, often compromising reproductive function. Although extensive efforts have been channeled into elucidating the regulatory mechanisms controlling the many aspects of sexual differentiation, the majority of disorders of sex development phenotypes are still unexplained at the molecular level. In this study, we have analyzed the potential involvement of Wnt5a in sexual development and show in mice that Wnt5a is male-specifically upregulated within testicular interstitial cells at the onset of gonad differentiation. Homozygous deletion of Wnt5a affected sexual development in male mice, causing testicular hypoplasia and bilateral cryptorchidism despite the Leydig cells producing factors such as Hsd3b1 and Insl3. Additionally, Wnt5a-null embryos of both sexes showed a significant reduction in gonadal germ cell numbers, which was caused by aberrant primordial germ cell **migration** along the hindgut endoderm prior to gonadal colonization. Our results indicate multiple roles for Wnt5a during mammalian reproductive development and help to clarify further the etiology of Robinow syndrome (OMIM 268310), a disease previously linked to the WNT5A pathway.
2. Wnt-4 deficiency alters mouse adrenal cortex function, reducing aldosterone production. Wnt-4 is a signaling factor with multiple roles in organogenesis, a deficiency that leads to abnormal development of the kidney, pituitary gland, female reproductive system, and mammary gland. Wnt-4 is expressed in the cortical region of the developing adrenal gland from embryonic d 11.5 onward, especially in the outermost part. Expression of Cyp11B2 and preadipocyte factor 1 is lowered in the glands of Wnt-4 mutant animals, resulting in significantly reduced aldosterone production in the newborn mutants, suggesting that Wnt-4 may be needed for proper formation of the zona glomerulosa. On the other hand, both proopiomelanocortin-derived peptide beta-endorphin and corticosterone concentration levels are elevated in Wnt-deficient mice, and the expression of Cyp17 is altered in Wnt-4 mutant females, so that it mimics the pattern specific for males. Finally, some cells that are positive for Cyp21, which is normally expressed only in the adrenal gland, are found in the gonads of Wnt-4-deficient embryos, indicating that Wnt-4 may play a role in cell **migration** or in the sorting of adrenal and gonadal cells during early development. In summary, these results point to a role for Wnt-4 in adrenal gland development and function.
3. Sprouty2 is involved in male sex organogenesis by controlling fibroblast growth factor 9-induced mesonephric cell **migration** to the developing testis. Fibroblast growth factor 9 (FGF9) signal has a role in organogenesis of the mammalian testis by controlling **migration** of mesonephric cells to the XY gonad, but neither it nor the FGF receptors is expressed sex-specifically. Of the Sprouty genes encoding antagonists of receptor tyrosine kinases including FGFr, mSprouty2 expression was confined to the developing testis and mesonephros. Gain of SPROUTY2 function in the male genital ridge and mesonephros malformed the vas deferens and epididymis, and diminished the number of seminiferous tubules and interstitium associating with reduced mesonephric cell **migration** and Fgf9 expression in embryonic testis, whereas exogenous FGF9 signaling recovered mesonephric cell **migration** inhibited by SPROUTY2. These phenotypes associated also with the decreased expression of Sox9, Desert hedgehog, Hsd3beta, Platelet/endothelial cell adhesion molecule, and alpha-smooth muscle actin, which are markers of the Sertoli, Leydig, endothelial, and peritubular myoid cells of the developing testis. Based on these data, we propose that the Sprouty proteins are involved normally in mediating the sexually dimorphic signaling of FGF9 and controlling cell **migration** from the mesonephros during testis development.
4. Mutation of ARX causes abnormal development of forebrain and testes in mice and X-linked lissencephaly with abnormal genitalia in humans. Male embryonic mice with mutations in the X-linked aristaless-related homeobox gene (Arx) developed with small brains due to suppressed proliferation and regional deficiencies in the forebrain. These mice also showed aberrant **migration** and differentiation of interneurons containing gamma-aminobutyric acid (GABAergic interneurons) in the ganglionic eminence and neocortex as well as abnormal testicular differentiation. These characteristics recapitulate some of the clinical features of X-linked lissencephaly with abnormal genitalia (XLAG) in humans. We found multiple loss-of-function mutations in ARX in individuals affected with XLAG and in some female relatives, and conclude that mutation of ARX causes XLAG. The present report is, to our knowledge, the first to use phenotypic analysis of a knockout mouse to identify a gene associated with an X-linked human brain malformation.

---

## Slc6a9

- NCBI Gene
- GeneRIF
- Pubmed

| Search Term | Rank | Fields |
| --- | --- | --- |
| [Mm]igration | 6 | PubMed(1) |
| [Ee]xtracellular [Mm]atrix | 8 | PubMed(1) |

  
hide/show details for Slc6a9

### [Mm]igration

#### PubMed

1. BARHL2 differentially regulates the development of retinal amacrine and ganglion neurons. Through transcriptional regulations, the BarH family of homeodomain proteins play essential roles in cell fate specification, cell differentiation, **migration**, and survival. Barhl2, a member of the Barh gene family, is expressed in retinal ganglion cells (RGCs), amacrine cells (ACs), and horizontal cells. Here, to investigate the role of Barhl2 in retinal development, Barhl2-deficient mice were generated. Analysis of AC subtypes in Barhl2-deficient retinas suggests that Barhl2 plays a critical role in AC subtype determination. A significant reduction of glycinergic and GABAergic ACs with a substantial increase in the number of cholinergic ACs was observed in Barhl2-null retinas. Barhl2 is also critical for the development of a normal complement of RGCs. Barhl2 deficiency resulted in a 35% increase in RGCs undergoing apoptosis during development. Genetic analysis revealed that Barhl2 functions downstream of the Atoh7-Pou4f3 regulatory pathway and regulates the maturation and/or survival of RGCs. Thus, BARHL2 appears to have numerous roles in retinal development, including regulating neuronal subtype specification, differentiation, and survival.

### [Ee]xtracellular [Mm]atrix

#### PubMed

1. Cell volume regulation is initiated in mouse oocytes after ovulation. Fertilized mouse eggs regulate their size principally by accumulating glycine as an intracellular osmolyte using the GLYT1 (SLC6A9) transporter, a mechanism of cell volume homeostasis apparently unique to early embryos before the morula stage. However, nothing was known of cell volume regulation in oocytes before fertilization. We show here that GLYT1 is quiescent in mouse germinal-vesicle-stage oocytes but becomes fully activated within hours after ovulation is triggered. This initiates accumulation of substantial amounts of intracellular glycine in oocytes during meiotic progression, reaching a maximal level in mature eggs. Measurements of endogenous free glycine showed that there were nearly undetectable levels in ovarian germinal-vesicle-stage oocytes, but high levels were present in mature ovulated eggs and in preimplantation embryos through the two-cell stage, but not in morulae. Furthermore, intracellular glycine was regulated in response to changes in external tonicity in eggs and embryos through the two-cell stage, but not in oocytes or embryos after the two-cell stage. Before activation of GLYT1, oocytes were unable to independently regulate their volume. As GLYT1 became active, however, oocyte volume decreased substantially and oocytes gained the ability to regulate their size, which required GLYT1 activity. Before ovulation, oocyte size was instead determined by a strong adhesion to the rigid **extracellular matrix** of the oocyte, the zona pellucida, which was released coincident with GLYT1 activation. The ability to acutely regulate cell size is thus acquired by the oocyte only after ovulation, when it first develops glycine-dependent cell volume regulation.

---

## Bach1

- NCBI Gene
- GeneRIF
- Pubmed

| Search Term | Rank | Fields |
| --- | --- | --- |
| [Mm]igration | 6 | PubMed(1) |
| [Ee]xtracellular [Mm]atrix | 8 | PubMed(2) |
| [Mm]etastasis | 12 | PubMed(1); GeneRIFs(1) |

  
hide/show details for Bach1

### [Mm]igration

#### PubMed

1. Bach1 Represses Wnt/β-Catenin Signaling and Angiogenesis. Wnt/β-catenin signaling has an important role in the angiogenic activity of endothelial cells (ECs). Bach1 is a transcription factor and is expressed in ECs, but whether Bach1 regulates angiogenesis is unknown.This study evaluated the role of Bach1 in angiogenesis and Wnt/β-catenin signaling.Hind-limb ischemia was surgically induced in Bach1(-/-) mice and their wild-type littermates and in C57BL/6J mice treated with adenoviruses coding for Bach1 or GFP. Lack of Bach1 expression was associated with significant increases in perfusion and vascular density and in the expression of proangiogenic cytokines in the ischemic hindlimb of mice, with enhancement of the angiogenic activity of ECs (eg, tube formatio**n, migrat**ion, and proliferation). Bach1 overexpression impaired angiogenesis in mice with hind-limb ischemia and inhibited Wnt3a-stimulated angiogenic response and the expression of Wnt/β-catenin target genes, such as interleukin-8 and vascular endothelial growth factor, in human umbilical vein ECs. Interleukin-8 and vascular endothelial growth factor were responsible for the antiangiogenic response of Bach1. Immunoprecipitation and GST pull-down assessments indicated that Bach1 binds directly to TCF4 and reduces the interaction of β-catenin with TCF4. Bach1 overexpression reduces the interaction between p300/CBP and β-catenin, as well as β-catenin acetylation, and chromatin immunoprecipitation experiments confirmed that Bach1 occupies the TCF4-binding site of the interleukin-8 promoter and recruits histone deacetylase 1 to the interleukin-8 promoter in human umbilical vein ECs.Bach1 suppresses angiogenesis after ischemic injury and impairs Wnt/β-catenin signaling by disrupting the interaction between β-catenin and TCF4 and by recruiting histone deacetylase 1 to the promoter of TCF4-targeted genes.

### [Ee]xtracellular [Mm]atrix

#### PubMed

1. Developmental gene expression profiling of mammalian, fetal orofacial tissue. The embryonic orofacial region is an excellent developmental paradigm that has revealed the centrality of numerous genes encoding proteins with diverse and important biological functions in embryonic growth and morphogenesis. DNA microarray technology presents an efficient means of acquiring novel and valuable information regarding the expression, regulation, and function of a panoply of genes involved in mammalian orofacial development.To identify differentially expressed genes during mammalian orofacial ontogenesis, the transcript profiles of GD-12, GD-13, and GD-14 murine orofacial tissue were compared utilizing GeneChip arrays from Affymetrix. Changes in gene expression were verified by TaqMan quantitative real-time PCR. Cluster analysis of the microarray data was done with the GeneCluster 2.0 Data Mining Tool and the GeneSpring software.Expression of >50% of the approximately 12,000 genes and expressed sequence tags examined in this study was detected in GD-12, GD-13, and GD-14 murine orofacial tissues and the expression of several hundred genes was up- and downregulated in the developing orofacial tissue from GD-12 to GD-13, as well as from GD-13 to GD-14. Such differential gene expression represents changes in the expression of genes encoding growth factors and signaling molecules; transcription factors; and proteins involved in epithelial-mesenchymal interactions, **extracellular matrix** synthesis, cell adhesion, proliferation, differentiation, and apoptosis. Following cluster analysis of the microarray data, eight distinct patterns of gene expression during murine orofacial ontogenesis were selected for graphic presentation of gene expression patterns.This gene expression profiling study identifies a number of potentially unique developmental participants and serves as a valuable aid in deciphering the complex molecular mechanisms crucial for mammalian orofacial development.
2. Bach1 deficiency reduces severity of osteoarthritis through upregulation of heme oxygenase-1. BTB and CNC homology 1 (Bach1) is a transcriptional repressor of Heme oxygenase-1 (HO-1), which is cytoprotective through its antioxidant effects. The objective of this study was to define the role of Bach1 in cartilage homeostasis and osteoarthritis (OA) development using in vitro models and Bach1 (-/-) mice.HO-1 expression in Bach1 (-/-) mice was analyzed by real-time PCR, immunohistochemistry and immunoblotting. Knee joints from Bach1 (-/-) and wild-type mice with age-related OA and surgically-induced OA were evaluated by OA scoring systems. Levels of autophagy proteins and superoxide dismutase 2 (SOD2) were determined by immunohistochemistry. The relationship between HO-1 and the protective effects for OA was determined in chondrocytes treated with small interfering RNA (siRNA) targeting HO-1 gene.HO-1 expression decreased with aging in articular cartilages and menisci of mouse knees. Bach1 (-/-) mice showed reduced severity of age-related OA and surgically-induced OA compared with wild-type mice. Microtubule-associated protein 1 light chain 3 (LC3), autophagy marker, and SOD2 were increased in articular cartilage of Bach1 (-/-) mice compared with wild-type mice. Interleukin-1β (IL-1β) induced a significant increase in Adamts-5 in wild-type chondrocytes but not in Bach1(-/-) chondrocytes. The expression of SOD2 and the suppression of apoptosis in Bach1 (-/-) chondrocytes were mediated by HO-1.Bach1 deficiency reduces the severity of OA-like changes. This may be due to maintenance of cartilage homeostasis and joint health by antioxidant effects through HO-1 and downregulation o**f extracellular matr**ix degrading enzymes. These results suggest that inactivation of Bach1 is a novel target and signaling pathway in OA prevention.

### [Mm]etastasis

#### PubMed

1. Transcriptional network analysis identifies BACH1 as a master regulator of breast cancer bone **metastasis**. The application of functional genomic analysis of breast cancer **metastasis** has led to the identification of a growing number of organ-specific **metastasis** genes, which often function in concert to facilitate different steps of the metastatic cascade. However, the gene regulatory network that controls the expression of these **metastasis** genes remains largely unknown. Here, we demonstrate a computational approach for the deconvolution of transcriptional networks to discover master regulators of breast cancer bone **metastasis**. Several known regulators of breast cancer bone **metastasis** such as Smad4 and HIF1 were identified in our analysis. Experimental validation of the networks revealed BACH1, a basic leucine zipper transcription factor, as the common regulator of several functional **metastasis** genes, including MMP1 and CXCR4. Ectopic expression of BACH1 enhanced the malignance of breast cancer cells, and conversely, BACH1 knockdown significantly reduced bone **metastasis**. The expression of BACH1 and its target genes was linked to the higher risk of breast cancer recurrence in patients. This study established BACH1 as the master regulator of breast cancer bone **metastasis** and provided a paradigm to identify molecular determinants in complex pathological processes.

#### GeneRIFs

1. Transcriptional network analysis identifies BACH1 as a master regulator of breast cancer bone **metastasis**

---

## Adgre5

- NCBI Gene
- GeneRIF
- Pubmed

| Search Term | Rank | Fields |
| --- | --- | --- |
| [Mm]igration | 6 | PubMed(6); GeneRIFs(1) |
| [Ee]xtracellular [Mm]atrix | 8 | PubMed(1) |

  
hide/show details for Adgre5

### [Mm]igration

#### PubMed

1. Insm1 promotes endocrine cell differentiation by modulating the expression of a network of genes that includes Neurog3 and Ripply3. Insulinoma associated 1 (Insm1) plays an important role in regulating the development of cells in the central and peripheral nervous systems, olfactory epithelium and endocrine pancreas. To better define the role of Insm1 in pancreatic endocrine cell development we generated mice with an Insm1(GFPCre) reporter allele and used them to study Insm1-expressing and null populations. Endocrine progenitor cells lacking Insm1 were less differentiated and exhibited broad defects in hormone production, cell proliferation and cell **migration**. Embryos lacking Insm1 contained greater amounts of a non-coding Neurog3 mRNA splice variant and had fewer Neurog3/Insm1 co-expressing progenitor cells, suggesting that Insm1 positively regulates Neurog3. Moreover, endocrine progenitor cells that express either high or low levels of Pdx1, and thus may be biased towards the formation of specific cell lineages, exhibited cell type-specific differences in the genes regulated by Insm1. Analysis of the function of Ripply3, an Insm1-regulated gene enriched in the Pdx1-high cell population, revealed that it negatively regulates the proliferation of early endocrine cells. Taken together, these findings indicate that in developing pancreatic endocrine cells Insm1 promotes the transition from a ductal progenitor to a committed endocrine cell by repressing a progenitor cell program and activating genes essential for RNA splicing, cell **migration**, controlled cellular proliferation, vasculogenesis, extracellular matrix and hormone secretion.
2. The epidermal growth factor-seven transmembrane (EGF-TM7) receptor CD97 is required for neutrophil **migration** and host defense. The epidermal growth factor-seven transmembrane (EGF-TM7) family is a group of seven-span transmembrane receptors predominantly expressed by cells of the immune system. Family members CD97, EGF module-containing mucin-like receptor (EMR) 1, EMR2, EMR3, EMR4, and EGF-TM7-latrophilin-related protein are characterized by an extended extracellular region with a variable number of N-terminal EGF-like domains. EGF-TM7 receptors bind cellular ligands as demonstrated by the interaction of CD97 with decay accelerating factor (CD55) and dermatan sulfate. Investigating the effect of newly generated mAb on the **migration** of neutrophilic granulocytes, we here report for the first time in vivo data on the function of CD97. In dextran sulfate sodium-induced experimental colitis, we show that homing of adoptively transferred neutrophils to the colon was significantly delayed when cells were preincubated with CD97 mAb. The consequences of this defect in neutrophil **migration** for host defense are demonstrated in a murine model of Streptococcus pneumoniae-induced pneumonia. Mice treated with CD97 mAb to EGF domain 1 (1B2) and EGF domain 3 (1C5) displayed a reduced granulocytic inflammatory infiltrate at 20 h after inoculation. This was associated with a significantly enhanced outgrowth of bacteria in the lungs at 44 h and a strongly diminished survival. Together, these findings indicate an essential role for CD97 in the **migration** of neutrophils.
3. Differential role of CD97 in interleukin-8-induced and granulocyte-colony stimulating factor-induced hematopoietic stem and progenitor cell mobilization. CD97 is broadly expressed on hematopoietic cells and is involved in neutrophil **migration**. Since neutrophils are key regulators in HSC/HPC mobilization, we studied a possible role for CD97 in interleukin-8 and granulocyte-colony stimulating factor-induced HSC/HPC mobilization. Mobilization was absent in mice receiving CD97 mAb followed by interleukin-8, while granulocyte-colony stimulating factor-induced mobilization remained unaltered following anti-CD97 administration. Furthermore, combined administration of CD97 mAb and IL-8 induced a significant reduction in the neutrophilic compartment. We hypothesize that the absence of interleukin-8-induced HSC/HPC mobilization after CD97 mAb administration is due to its effect on neutrophil function.
4. The EGF-TM7 family: a postgenomic view. With the human and mouse genome projects now completed, the receptor repertoire of mammalian cells has finally been elucidated. The EGF-TM7 receptors are a family of class B seven-span transmembrane (TM7) receptors predominantly expressed by cells of the immune system. Within the large TM7 superfamily, the molecular structure and ligand-binding properties of EGF-TM7 receptors are unique. Derived from the processing of a single polypeptide, they are expressed at the cell surface as heterodimers consisting of a large extracellular region associated with a TM7 moiety. Through a variable number of N-terminal epidermal growth factor (EGF)-like domains, EGF-TM7 receptors interact with cellular ligands such as CD55 and chondroitin sulfate. Recent in vivo studies demonstrate a role of the EGF-TM7 receptor CD97 in leukocyte **migration**. The different number of EGF-TM7 genes in man compared with mice, the chimeric nature of EMR2 and the inactivation of human EMR4 point toward a still-evolving receptor family. Here we discuss the currently available information on this intriguing receptor family.
5. Improved antibacterial host defense and altered peripheral granulocyte homeostasis in mice lacking the adhesion class G protein receptor CD97. CD97 is a member of the adhesion family of G protein-coupled receptors. Alternatively spliced forms of CD97 bind integrins alpha5beta1 and alphavbeta3, decay accelerating factor, or dermatan sulfate. CD97 is expressed on myeloid cells at high levels and a variety of other cell types at lower levels. Little is known about the physiological function of CD97. To begin dissecting the function of CD97, we evaluated the immune response of CD97 null mice to systemic infection by Listeria monocytogenes. CD97 null mice were significantly more resistant to listeriosis than matched wild-type mice. A major determinant of the difference in survival appeared to be the comparatively more robust accumulation of granulocytes in the blood and in infected livers of CD97 null mice within 18 h of inoculation, correlating with a decrease in the number of bacteria. CD97 null mice also displayed a mild granulocytosis in the nonchallenged state. Because there is a strong suggestion that CD97 functions in an adhesive capacity, we examined the migratory properties of granulocytes in CD97 null mice. In chimeric animals, CD97 null and wild-type granulocytes migrated similarly, as determined by inflammation-induced e**migration** from the bone marrow and accumulation in the peritoneum. Granulocyte development in the bone marrow of CD97 null mice was comparable to that of wild-type mice, and CD97 deficiency did not appear to stimulate granulocytosis secondary to peripheral inflammation and resultant granulocyte colony-stimulating factor induction, unlike various other models of adhesion deficiencies. Our results suggest that CD97 plays a role in peripheral granulocyte homeostasis.
6. Analysis of CD97 expression and manipulation: antibody treatment but not gene targeting curtails granulocyte **migration**. The heptahelical receptor CD97 is a defining member of the EGF-TM7 family of adhesion class receptors. In both humans and mice, CD97 isoforms are expressed with variable numbers of tandemly arranged N-terminal epidermal growth factor-like domains that facilitate interactions with distinct cellular ligands. Results from treatment of mice with mAbs in various disease models have suggested a role for CD97 in leukocyte trafficking. Here, we aimed to thoroughly characterize the expression profile of CD97, and delineate its biological function. To this end, we applied a novel polyclonal Ab, which is the first antiserum suitable for immunohistochemistry, and combined this analysis with the study of Cd97-lacZ knock-in mice. We show that similar to the situation in humans, hematopoietic, epithelial, endothelial, muscle, and fat cells expressed CD97. Despite this broad expression pattern, the Cd97(-/-) mouse that we created had no overt phenotype, except for a mild granulocytosis. Furthermore, granulocyte accumulation at sites of inflammation was normal in the absence of CD97. Interestingly, application of CD97 mAbs blocked granulocyte trafficking after thioglycollate-induced peritonitis in wild-type but not in knock-out mice. Hence, we conclude that CD97 mAbs actively induce an inhibitory effect that disturbs normal granulocyte trafficking, which is not perturbed by the absence of the molecule.

#### GeneRIFs

1. In vivo findings in experimental colitis and pneumococcal pneumonia indicate an essential role for CD97 in the **migration** of neutrophils.

### [Ee]xtracellular [Mm]atrix

#### PubMed

1. Insm1 promotes endocrine cell differentiation by modulating the expression of a network of genes that includes Neurog3 and Ripply3. Insulinoma associated 1 (Insm1) plays an important role in regulating the development of cells in the central and peripheral nervous systems, olfactory epithelium and endocrine pancreas. To better define the role of Insm1 in pancreatic endocrine cell development we generated mice with an Insm1(GFPCre) reporter allele and used them to study Insm1-expressing and null populations. Endocrine progenitor cells lacking Insm1 were less differentiated and exhibited broad defects in hormone production, cell proliferation and cell migration. Embryos lacking Insm1 contained greater amounts of a non-coding Neurog3 mRNA splice variant and had fewer Neurog3/Insm1 co-expressing progenitor cells, suggesting that Insm1 positively regulates Neurog3. Moreover, endocrine progenitor cells that express either high or low levels of Pdx1, and thus may be biased towards the formation of specific cell lineages, exhibited cell type-specific differences in the genes regulated by Insm1. Analysis of the function of Ripply3, an Insm1-regulated gene enriched in the Pdx1-high cell population, revealed that it negatively regulates the proliferation of early endocrine cells. Taken together, these findings indicate that in developing pancreatic endocrine cells Insm1 promotes the transition from a ductal progenitor to a committed endocrine cell by repressing a progenitor cell program and activating genes essential for RNA splicing, cell migration, controlled cellular proliferation, vasculogenesis, **extracellular matrix** and hormone secretion.

---

## Adgre1

- NCBI Gene
- GeneRIF
- Pubmed

| Search Term | Rank | Fields |
| --- | --- | --- |
| [Mm]igration | 6 | PubMed(11) |
| ECM | 7 | PubMed(1) |
| [Mm]etastasis | 12 | PubMed(1) |

  
hide/show details for Adgre1

### [Mm]igration

#### PubMed

1. The EGF-TM7 family: a postgenomic view. With the human and mouse genome projects now completed, the receptor repertoire of mammalian cells has finally been elucidated. The EGF-TM7 receptors are a family of class B seven-span transmembrane (TM7) receptors predominantly expressed by cells of the immune system. Within the large TM7 superfamily, the molecular structure and ligand-binding properties of EGF-TM7 receptors are unique. Derived from the processing of a single polypeptide, they are expressed at the cell surface as heterodimers consisting of a large extracellular region associated with a TM7 moiety. Through a variable number of N-terminal epidermal growth factor (EGF)-like domains, EGF-TM7 receptors interact with cellular ligands such as CD55 and chondroitin sulfate. Recent in vivo studies demonstrate a role of the EGF-TM7 receptor CD97 in leukocyte **migration**. The different number of EGF-TM7 genes in man compared with mice, the chimeric nature of EMR2 and the inactivation of human EMR4 point toward a still-evolving receptor family. Here we discuss the currently available information on this intriguing receptor family.
2. Homeodomain factor Nkx2-3 controls regional expression of leukocyte homing coreceptor MAdCAM-1 in specialized endothelial cells of the viscera. Regulated e**migration** of blood-borne leukocytes plays a defining role in lymphoid organ development, immune surveillance, and inflammatory responses. We report here that mice deficient in the homeobox gene Nkx2-3, expressed in developing visceral mesoderm, show a complex intestinal malabsorption phenotype and striking abnormalities of gut-associated lymphoid tissue and spleen suggestive of deranged leukocyte homing. Mutant Peyer's patches were reduced in number and size, intestinal villi contained few IgA(+) plasma cells, and mutant spleens were small and often atrophic, showing fused periarterial lymphoid sheaths, partially merged T and B cell zones, an absent marginal zone, and a dearth of macrophages in red pulp. Semiquantitative RT-PCR analysis and immunohistochemistry revealed down-regulation of mucosal addressin cell adhesion molecule-1 (MAdCAM-1) in endothelial cells in which Nkx2-3 is normally expressed. MAdCAM-1 is a member of the immunoglobulin superfamily, acting as an endothelial cell ligand for leukocyte homing receptors L-selectin and alpha4beta7 integrin. Our data suggest a role for a homeodomain factor in establishing the developmental and positional cues in endothelia that regulate leukocyte homing through local control of cellular adhesion and identify MAdCAM-1 as a candidate target gene of Nkx2-3.
3. Fetal liver endothelium regulates the seeding of tissue-resident macrophages. Macrophages are required for normal embryogenesis, tissue homeostasis and immunity against microorganisms and tumours. Adult tissue-resident macrophages largely originate from long-lived, self-renewing embryonic precursors and not from haematopoietic stem-cell activity in the bone marrow. Although fate-mapping studies have uncovered a great amount of detail on the origin and kinetics of fetal macrophage development in the yolk sac and liver, the molecules that govern the tissue-specific **migration** of these cells remain completely unknown. Here we show that an endothelium-specific molecule, plasmalemma vesicle-associated protein (PLVAP), regulates the seeding of fetal monocyte-derived macrophages to tissues in mice. We found that PLVAP-deficient mice have completely normal levels of both yolk-sac- and bone-marrow-derived macrophages, but that fetal liver monocyte-derived macrophage populations were practically missing from tissues. Adult PLVAP-deficient mice show major alterations in macrophage-dependent iron recycling and mammary branching morphogenesis. PLVAP forms diaphragms in the fenestrae of liver sinusoidal endothelium during embryogenesis, interacts with chemoattractants and adhesion molecules and regulates the egress of fetal liver monocytes to the systemic vasculature. Thus, PLVAP selectively controls the exit of macrophage precursors from the fetal liver and, to our knowledge, is the first molecule identified in any organ as regulating the migratory events during embryonic macrophage ontogeny.
4. Neural progenitor cells orchestrate microglia **migration** and positioning into the developing cortex. Microglia are observed in the early developing forebrain and contribute to the regulation of neurogenesis through still unravelled mechanisms. In the developing cerebral cortex, microglia cluster in the ventricular/subventricular zone (VZ/SVZ), a region containing Cxcl12-expressing basal progenitors (BPs). Here we show that the ablation of BP as well as genetic loss of Cxcl12 affect microglia recruitment into the SVZ. Ectopic Cxcl12 expression or pharmacological blockage of CxcR4 further supports that Cxcl12/CxcR4 signalling is involved in microglial recruitment during cortical development. Furthermore, we found that cell death in the developing forebrain triggers microglial proliferation and that this is mediated by the release of macrophage **migration** inhibitory factor (MIF). Finally, we show that the depletion of microglia in mice lacking receptor for colony-stimulating factor-1 (Csf-1R) reduces BPs into the cerebral cortex.
5. The chemokine receptors ACKR2 and CCR2 reciprocally regulate lymphatic vessel density. Macrophages regulate lymphatic vasculature development; however, the molecular mechanisms regulating their recruitment to developing, and adult, lymphatic vascular sites are not known. Here, we report that resting mice deficient for the inflammatory chemokine-scavenging receptor, ACKR2, display increased lymphatic vessel density in a range of tissues under resting and regenerating conditions. This appears not to alter dendritic cell **migration** to draining lymph nodes but is associated with enhanced fluid drainage from peripheral tissues and thus with a hypotensive phenotype. Examination of embryonic skin revealed that this lymphatic vessel density phenotype is developmentally established. Further studies indicated that macrophages and the inflammatory CC-chemokine CCL2, which is scavenged by ACKR2, are associated with this phenotype. Accordingly, mice deficient for the CCL2 signalling receptor, CCR2, displayed a reciprocal phenotype of reduced lymphatic vessel density. Further examination revealed that proximity of pro-lymphangiogenic macrophages to developing lymphatic vessel surfaces is increased in ACKR2-deficient mice and reduced in CCR2-deficient mice. Therefore, these receptors regulate vessel density by reciprocally modulating pro-lymphangiogenic macrophage recruitment, and proximity, to developing, resting and regenerating lymphatic vessels.
6. Runx1 modulates adult hair follicle stem cell emergence and maintenance from distinct embryonic skin compartments. Runx1 controls hematopoietic stem cell emergence and hair follicle stem cell (HFSC) activation and proliferation in adult skin. Here we use lineage tracing and mouse genetic manipulation to address the role of Runx1 in the embryonic development of HFSCs. We find Runx1 is expressed in distinct classes of embryonic skin precursors for short-term HF progenitors, adult HFSCs, and mesenchymal progenitors. Runx1 acts in the embryonic epithelium for timely emergence of adult HFSCs and short-term progenitors, but is dispensable for both of them. In contrast, Runx1 is strictly needed in the embryonic mesenchyme for proper adult HFSC differentiation and long-term skin integrity. Our data implicate Runx1 in epithelial cell adhesion and **migration** and in regulation of paracrine epithelial-mesenchymal cross talk. The latter involves Lef1 and Wnt signaling modulation in opposing directions from two distinct skin compartments. Thus, a master regulator of hematopoiesis also controls HFSC emergence and maintenance via modulation of bidirectional cross talking between nascent stem cells and their niche.
7. Neural crest cell lineage segregation in the mouse neural tube. Neural crest (NC) cells arise in the dorsal neural tube (NT) and migrate into the embryo to develop into many different cell types. A major unresolved question is when and how the fate of NC cells is decided. There is widespread evidence for multipotential NC cells, whose fates are decided during or after **migration**. There is also some evidence that the NC is already divided into subpopulations of discrete precursors within the NT. We have investigated this question in the mouse embryo. We find that a subpopulation of cells on the most dorsomedial aspect of the NT express the receptor tyrosine kinase Kit (previously known as c-kit), emigrate exclusively into the developing dermis, and then express definitive markers of the melanocyte lineage. These are thus melanocyte progenitor cells. They are generated predominantly at the midbrain-hindbrain junction and cervical trunk, with significant numbers also in lower trunk. Other cells within the dorsal NT are Kit-, migrate ventrally, and, from embryonic day 9.5, express the neurotrophin receptor p75. These cells most likely only give rise to ventral NC derivatives such as neurons and glia. The p75+ cells are located ventrolateral to the Kit+ cells in areas of the NT where these two cell types are found. These data provide direct in vivo evidence for NC lineage segregation within the mouse neural tube.
8. KLEIP deficiency in mice causes progressive corneal neovascular dystrophy. The BTB-kelch protein KLEIP/KLHL20 is an actin binding protein that regulates cell-cell contact formation and cell **migration**. The aim of our study was to characterize KLEIP's function in ocular health and disease in mice.KLEIP(-/-) mice were generated, and corneas were examined histologically and stained for keratin-1, loricrin, keratin-12, keratin-14, CD31, LYVE-1, F4/80, E-cadherin, and Ki67. Corneal abrasions were performed after eyelid opening.Corneas of KLEIP(+/+) and KLEIP(-/-) mice were indistinguishable at birth. After eyelid opening corneal epithelial hyperplasia started to manifest in KLEIP(-/-) mice, showing a progressive epithelial metaplasia leading to total corneal opacity. In KLEIP(-/-) mice the initial stratified squamous corneal epithelium was altered to an epidermal histo-architecture showing several superficial keratinized cells, cell infiltrations into the stroma, and several apoptotic cells. Skin markers keratin 1 and loricrin were positive, and surface disease was accompanied by deep stromal vascularization. Expression analysis for E-cadherin in KLEIP(-/-) corneas showed acellular areas in the squamous epithelium, indicating a progressive fragile corneal integrity. Removal of the virgin epithelium accelerated strongly development of the epithelial and stromal alterations, identifying mechanical injuries as the major trigger for corneal dystrophy formation and scarification in KLEIP(-/-) mice.The data identify KLEIP as an important molecule regulating corneal epithelial integrity.
9. Netrin-4 induces lymphangiogenesis in vivo. Netrin-4, a laminin-related secreted protein is an axon guidance cue recently shown essential outside of the nervous system, regulating mammary and lung morphogenesis as well as blood vascular development. Here, we show that Netrin-4, at physiologic doses, induces proliferation, **migration**, adhesion, tube formation and survival of human lymphatic endothelial cells in vitro comparable to well-characterized lymphangiogenic factors fibroblast growth factor-2 (FGF-2), hepatocyte growth factor (HGF), vascular endothelial growth factor-A (VEGF-A), and vascular endothelial growth factor-C (VEGF-C). Netrin-4 stimulates phosphorylation of intracellular signaling components Akt, Erk and S6, and their specific inhibition antagonizes Netrin-4-induced proliferation. Although Netrin receptors Unc5B and neogenin, are expressed by human lymphatic endothelial cells, suppression of either or both does not suppress Netrin-4-promoted in vitro effects. In vivo, Netrin-4 induces growth of lymphatic and blood vessels in the skin of transgenic mice and in breast tumors. Its overexpression in human and mouse mammary carcinoma cancer cells leads to enhanced metastasis. Finally, Netrin-4 stimulates in vitro and in vivo lymphatic permeability by activating small GTPases and Src family kinases/FAK, and down-regulating tight junction proteins. Together, these data provide evidence that Netrin-4 is a lymphangiogenic factor contributing to tumor dissemination and represents a potential target to inhibit metastasis formation.
10. Apoptotic cells enhance sphingosine-1-phosphate receptor 1 dependent macrophage **migration**. The lipid sphingosine-1-phosphate (S1P) is a chemokine for a variety of immune cells including lymphocytes and monocytes. **Migration** toward S1P is determined by the S1P receptor expression profile, with S1PR1/3 (where S1PR is S1P receptor) stimulating and S1PR2 attenuating **migration**. However, the impact and physiological significance of S1P-induced **migration** of macrophages is largely unclear. We observed that alternative activation of human macrophages, by IL-4 or apoptotic cells (ACs), enhanced S1PR1 expression. Moreover, ACs provoked macrophage **migration** toward S1P in an S1PR1-dependent manner as confirmed by pharmacological receptor inhibition and S1PR1-deficient murine macrophages. In a mouse model of resolving peritoneal inflammation, F4/80-driven deletion of S1PR1 reduced postinflammatory macrophage e**migration** from inflammatory sites. S1PR1 expression on macrophages might, therefore, be relevant for restoring tissue homeostasis during the resolution of inflammation.
11. TGFbeta2 in corneal morphogenesis during mouse embryonic development. To examine the roles of TGFbeta isoforms on corneal morphogenesis, the eyes of mice that lack TGFbetas were analyzed at different developmental stages for cell proliferation, **migration** and apoptosis, and for expression patterns of keratin 12, lumican, keratocan and collagen I. Among the three Tgfb(-/-) mice, only Tgfb2(-/-) mice have abnormal ocular morphogenesis characterized by thin corneal stroma, absence of corneal endothelium, fusion of cornea to lens (a Peters'-like anomaly phenotype), and accumulation of hyaline cells in vitreous. In Tgfb2(-/-) mice, fewer keratocytes were found in stroma that has a decreased accumulation of ECM; for example, lumican, keratocan and collagen I were greatly diminished. The absence of TGFbeta2 did not compromise cell proliferation, nor enhance apoptosis. The thinner stroma resulting from decreased ECM synthesis may account for the decreased cell number in the stroma of Tgfb2 null mice. Keratin 12 expression was not altered in Tgfb2(-/-) mice, implicating normal corneal type epithelial differentiation. Delayed appearance of macrophages in ocular tissues was observed in Tgfb2(-/-) mice. Malfunctioning macrophages may account for accumulation of cell mass in vitreous of Tgfb2 null mice.

### ECM

#### PubMed

1. TGFbeta2 in corneal morphogenesis during mouse embryonic development. To examine the roles of TGFbeta isoforms on corneal morphogenesis, the eyes of mice that lack TGFbetas were analyzed at different developmental stages for cell proliferation, migration and apoptosis, and for expression patterns of keratin 12, lumican, keratocan and collagen I. Among the three Tgfb(-/-) mice, only Tgfb2(-/-) mice have abnormal ocular morphogenesis characterized by thin corneal stroma, absence of corneal endothelium, fusion of cornea to lens (a Peters'-like anomaly phenotype), and accumulation of hyaline cells in vitreous. In Tgfb2(-/-) mice, fewer keratocytes were found in stroma that has a decreased accumulation of **ECM**; for example, lumican, keratocan and collagen I were greatly diminished. The absence of TGFbeta2 did not compromise cell proliferation, nor enhance apoptosis. The thinner stroma resulting from decreased **ECM** synthesis may account for the decreased cell number in the stroma of Tgfb2 null mice. Keratin 12 expression was not altered in Tgfb2(-/-) mice, implicating normal corneal type epithelial differentiation. Delayed appearance of macrophages in ocular tissues was observed in Tgfb2(-/-) mice. Malfunctioning macrophages may account for accumulation of cell mass in vitreous of Tgfb2 null mice.

### [Mm]etastasis

#### PubMed

1. Netrin-4 induces lymphangiogenesis in vivo. Netrin-4, a laminin-related secreted protein is an axon guidance cue recently shown essential outside of the nervous system, regulating mammary and lung morphogenesis as well as blood vascular development. Here, we show that Netrin-4, at physiologic doses, induces proliferation, migration, adhesion, tube formation and survival of human lymphatic endothelial cells in vitro comparable to well-characterized lymphangiogenic factors fibroblast growth factor-2 (FGF-2), hepatocyte growth factor (HGF), vascular endothelial growth factor-A (VEGF-A), and vascular endothelial growth factor-C (VEGF-C). Netrin-4 stimulates phosphorylation of intracellular signaling components Akt, Erk and S6, and their specific inhibition antagonizes Netrin-4-induced proliferation. Although Netrin receptors Unc5B and neogenin, are expressed by human lymphatic endothelial cells, suppression of either or both does not suppress Netrin-4-promoted in vitro effects. In vivo, Netrin-4 induces growth of lymphatic and blood vessels in the skin of transgenic mice and in breast tumors. Its overexpression in human and mouse mammary carcinoma cancer cells leads to enhanced **metastasis**. Finally, Netrin-4 stimulates in vitro and in vivo lymphatic permeability by activating small GTPases and Src family kinases/FAK, and down-regulating tight junction proteins. Together, these data provide evidence that Netrin-4 is a lymphangiogenic factor contributing to tumor dissemination and represents a potential target to inhibit **metastasis** formation.

---

## Smyd1

- NCBI Gene
- GeneRIF
- Pubmed

| Search Term | Rank | Fields |
| --- | --- | --- |
| [Mm]igration | 6 | PubMed(2) |
| [Ee]xtracellular [Mm]atrix | 8 | PubMed(1) |

  
hide/show details for Smyd1

### [Mm]igration

#### PubMed

1. Arid3b is essential for second heart field cell deployment and heart patterning. Arid3b, a member of the conserved ARID family of transcription factors, is essential for mouse embryonic development but its precise roles are poorly understood. Here, we show that Arid3b is expressed in the myocardium of the tubular heart and in second heart field progenitors. Arid3b-deficient embryos show cardiac abnormalities, including a notable shortening of the poles, absence of myocardial differentiation and altered patterning of the atrioventricular canal, which also lacks epithelial-to-mesenchymal transition. Proliferation and death of progenitors as well as early patterning of the heart appear normal. However, DiI labelling of second heart field progenitors revealed a defect in the addition of cells to the heart. RNA microarray analysis uncovered a set of differentially expressed genes in Arid3b-deficient tissues, including Bhlhb2, a regulator of cardiomyocyte differentiation, and Lims2, a gene involved in cell **migration**. Arid3b is thus required for heart development by regulating the motility and differentiation of heart progenitors. These findings identify Arid3b as a candidate gene involved in the aetiology of human congenital malformations.
2. skNAC and Smyd1 in transcriptional control. Skeletal and heart muscle-specific variant of the alpha subunit of nascent polypeptide associated complex (skNAC) is exclusively found in striated muscle cells. Its function, however, is largely unknown. Previous reports could demonstrate that skNAC binds to Smyd1 (SET and MYND domain containing protein 1). The facts that (a) SET domains have histone methyltransferase activity, and (b) MYND domains are known recruiters of histone deacetylases (HDACs), implicate the skNAC-Smyd1 complex in transcriptional control. To study potential target genes, we carried out cDNA microarray analysis on differentiating C2C12 myoblasts in which expression of the skNAC gene had been knocked down. We found and confirmed a series of targets, specifically genes encoding regulators of inflammation, cellular metabolism, and cell **migration**. Mechanistically, as shown by Western blot, ELISA, and ChIP analysis at selected promoter regions, transcriptional control by skNAC-Smyd1 appears to be exerted at least in part by affecting a series of histone modifications, specifically H3K4 di- and trimethylation and potentially also histone acetylation. Taken together, our data suggest that the skNAC-Smyd1 complex is involved in transcriptional regulation both via the control of histone methylation and histone (de)acetylation.

### [Ee]xtracellular [Mm]atrix

#### PubMed

1. Deletion of G protein-coupled receptor 48 leads to ocular anterior segment dysgenesis (ASD) through down-regulation of Pitx2. The development of the anterior segment of the mammalian eye is critical for normal ocular function, whereas abnormal development can cause glaucoma, a leading cause of blindness in the world. We report that orphan G protein-coupled receptor 48 (Gpr48/LGR4) plays an important role in the development of the anterior segment structure. Disruption of Gpr48 causes a wide spectrum of anterior segment dysgenesis (ASD), including microphthalmia, iris hypoplasia, irdiocorneal angle malformation, cornea dysgenesis, and cataract. Detailed analyses reveal that defective iris myogenesis and ocular **extracellular matrix** homeostasis are detected at early postnatal stages of eye development, whereas ganglion cell loss, inner nuclear layer thinness, and early onset of glaucoma were detected in 6-month-old Gpr48(-/-) mice. To determine the molecular mechanism of ASD caused by the deletion of Gpr48, we performed gene expression analyses and revealed dramatic down-regulation of Pitx2 in homozygous knockout mice. In vitro studies with the constitutively active Gpr48 mutant receptor demonstrate that Pitx2 is a direct target of the Gpr48-mediated cAMP-CREB signaling pathway in regulating anterior segment development, suggesting a role of Gpr48 as a potential therapeutic target of ASD.

---

## Klk6

- NCBI Gene
- GeneRIF
- Pubmed

| Search Term | Rank | Fields |
| --- | --- | --- |
| [Mm]igration | 6 | PubMed(1); GeneRIFs(1) |
| [Mm]etastasis | 12 | PubMed(1) |

  
hide/show details for Klk6

### [Mm]igration

#### PubMed

1. Kallikrein-related peptidase 8-dependent skin wound healing is associated with upregulation of kallikrein-related peptidase 6 and PAR2. Kallikrein-related peptidase 8 (KLK8) is believed to be involved in the maintenance of skin homeostasis and pathogenesis of inflammatory skin diseases. Although previous studies have shown that KLK8 is expressed around incisional wounds, the exact role of KLK8 in wound healing remains obscure. In the present study, we compared wound healing in wild-type (WT) and Klk8 gene-disrupted (kallikrein-related peptidase 8 knockout, Klk8(-/-)) mouse skin. Wound healing in Klk8(-/-) mice was hampered with defective keratinocyte proliferation, differentiation, and **migration** in the early stages of wound healing. Compared with the prominent induction of Klk6 and protease-activated receptor 2 (PAR2) messenger RNA, and protein in WT mice after wounding, a much lower increase was observed in Klk8(-/-) skin. After skin wounding in WT mice, increased Klk6 was detected from the upper stratum spinosum to the stratum corneum. Moreover, in WT mice, Klk6 protein was processed. PAR2 was diffusely expressed in the cytoplasm of the stratum spinosum at day 7 post wounding in WT mice. These results suggest that Klk8 is involved in the proliferation and **migration** of keratinocytes through the upregulation and activation of Klk6 in the early stages of wound healing, and possibly in keratinocyte differentiation associated with the upregulation and activation of PAR2 in the late stages of wound healing.

#### GeneRIFs

1. Klk8 is involved in the proliferation and **migration** of keratinocytes through Klk6 in the early stages of wound healing, and possibly in keratinocyte differentiation associated with the upregulation of PAR2 in the late stages of wound healing.

### [Mm]etastasis

#### PubMed

1. Human tissue kallikreins: physiologic roles and applications in cancer. Tissue kallikreins are members of the S1 family (clan SA) of trypsin-like serine proteases and are present in at least six mammalian orders. In humans, tissue kallikreins (hK) are encoded by 15 structurally similar, steroid hormone-regulated genes (KLK) that colocalize to chromosome 19q13.4, representing the largest cluster of contiguous protease genes in the entire genome. hKs are widely expressed in diverse tissues and implicated in a range of normal physiologic functions from the regulation of blood pressure and electrolyte balance to tissue remodeling, prohormone processing, neural plasticity, and skin desquamation. Several lines of evidence suggest that hKs may be involved in cascade reactions and that cross-talk may exist with proteases of other catalytic classes. The proteolytic activity of hKs is regulated in several ways including zymogen activation, endogenous inhibitors, such as serpins, and via internal (auto)cleavage leading to inactivation. Dysregulated hK expression is associated with multiple diseases, primarily cancer. As a consequence, many kallikreins, in addition to hK3/PSA, have been identified as promising diagnostic and/or prognostic biomarkers for several cancer types, including ovarian, breast, and prostate. Recent data also suggest that hKs may be causally involved in carcinogenesis, particularly in tumor **metastasis** and invasion, and, thus, may represent attractive drug targets to consider for therapeutic intervention.

---

## Ctsg

- NCBI Gene
- GeneRIF
- Pubmed

| Search Term | Rank | Fields |
| --- | --- | --- |
| [Ee]xtracellular [Mm]atrix | 8 | PubMed(2); Component(1) |
| [Mm]etastasis | 12 | PubMed(2) |

  
hide/show details for Ctsg

### [Ee]xtracellular [Mm]atrix

#### PubMed

1. Cathepsin G activity lowers plasma LDL and reduces atherosclerosis. Cathepsin G (CatG), a serine protease present in mast cells and neutrophils, can produce angiotensin-II (Ang-II) and degrade elastin. Here we demonstrate increased CatG expression in smooth muscle cells (SMCs), endothelial cells (ECs), macrophages, and T cells from human atherosclerotic lesions. In low-density lipoprotein (LDL) receptor-deficient (Ldlr(-/-)) mice, the absence of CatG reduces arterial wall elastin degradation and attenuates early atherosclerosis when mice consume a Western diet for 3months. When mice consume this diet for 6months, however, CatG deficiency exacerbates atherosclerosis in aortic arch without affecting lesion inflammatory cell content or **extracellular matrix** accumulation, but raises plasma total cholesterol and LDL levels without affecting high-density lipoprotein (HDL) or triglyceride levels. Patients with atherosclerosis also have significantly reduced plasma CatG levels that correlate inversely with total cholesterol (r=-0.535, P<0.0001) and LDL cholesterol (r=-0.559, P<0.0001), but not with HDL cholesterol (P=0.901) or triglycerides (P=0.186). Such inverse correlations with total cholesterol (r=-0.504, P<0.0001) and LDL cholesterol (r=-0.502, P<0.0001) remain significant after adjusting for lipid lowering treatments among this patient population. Human CatG degrades purified human LDL, but not HDL. This study suggests that CatG promotes early atherogenesis through its elastinolytic activity, but suppresses late progression of atherosclerosis by degrading LDL without affecting HDL or triglycerides.
2. Cathepsin G deficiency decreases complexity of atherosclerotic lesions in apolipoprotein E-deficient mice. Cathepsin G is a serine protease with a broad range of catalytic activities, including production of angiotensin II, degradation of **extracellular matrix** and cell-cell junctions, modulation of chemotactic responses, and induction of apoptosis. Cathepsin G mRNA expression is increased in human coronary atheroma vs. the normal vessel. To assess whether cathepsin G modulates atherosclerosis, cathepsin G knockout (Cstg(-/-)) mice were bred with apolipoprotein E knockout (Apoe(-/-)) mice to obtain Ctsg(+/-)Apoe(-/-) and Ctsg(+/+)Apoe(-/-) mice. Heterozygous cathepsin G deficiency led to a 70% decrease in cathepsin G activity in bone marrow cells, but this reduced activity did not impair generation of angiotensin II in bone marrow-derived macrophages (BMDM). Atherosclerotic lesions were compared in male Cstg(+/-)Apoe(-/-) and Cstg(+/+)Apoe(-/-) mice after 8 wk on a high-fat diet. Plasma cholesterol levels and cholesterol distribution within serum lipoprotein fractions did not differ between genotypes nor did the atherosclerotic lesion areas in either the aortic root or aortic arch. Cstg(+/-)Apoe(-/-) mice, however, showed a lower percentage of complex lesions within the aortic root and a smaller number of apoptotic cells compared with Cstg(+/+)Apoe(-/-) littermates. Furthermore, apoptotic Cstg(-/-) BMDM were more efficiently engulfed by phagocytic BMDM than were apoptotic Ctsg(+/+) BMDM. Thus cathepsin G activity may impair efferocytosis, which could lead to an accumulation of lesion-associated apoptotic cells and the accelerated progression of early atherosclerotic lesions to more complex lesions in Apoe(-/-) mice.

#### Component

1. **extracellular matrix**

### [Mm]etastasis

#### PubMed

1. Cathepsin G-mediated enhanced TGF-beta signaling promotes angiogenesis via upregulation of VEGF and MCP-1. Transforming growth factor (TGF)-beta signaling makes a significant contribution to the pathogenesis of breast cancer bone **metastasis**. In other tumor types, TGF-beta has been shown to promote tumor vascularity. Here, we report that inhibition of TGF-beta significantly reduces microvessel density in mammary tumor-induced bone lesions, mediated by decreased expression of both vascular endothelial growth factor (VEGF) and monocyte chemotactic protein (MCP)-1, both known angiogenic factors. Cathepsin G upregulation at the tumor-bone interface has been linked to increased TGF-beta signaling, and we also report that inhibition of Cathepsin G reduced tumor vascularity, as well as VEGF and MCP-1 expression.
2. Cathepsin G enhances mammary tumor-induced osteolysis by generating soluble receptor activator of nuclear factor-kappaB ligand. Breast cancer commonly causes osteolytic metastases in bone, a process that is dependent on tumor-stromal interaction. Proteases play an important role in modulating tumor-stromal interactions in a manner that favors tumor establishment and progression. Whereas several studies have examined the role of proteases in modulating the bone microenvironment, little is currently known about their role in tumor-bone interaction during osteolytic **metastasis**. In cancer-induced osteolytic lesions, cleavage of receptor activator of nuclear factor-kappaB ligand (RANKL) to a soluble version (sRANKL) is critical for widespread osteoclast activation. Using a mouse model that mimics osteolytic changes associated with breast cancer-induced bone metastases, we identified cathepsin G, cathepsin K, matrix metalloproteinase (MMP)-9, and MMP13 to be proteases that are up-regulated at the tumor-bone interface using comparative cDNA microarray analysis and quantitative reverse transcription-PCR. Moreover, we showed that cathepsin G is capable of shedding the extracellular domain of RANKL, generating active sRANKL that is capable of inducing differentiation and activation of osteoclast precursors. The major source of cathepsin G at the tumor-bone interface seems to be osteoclasts that up-regulate production of cathepsin G via interaction with tumor cells. Furthermore, we showed that in vitro osteoclastogenesis is reduced by inhibition of cathepsin G in a coculture model and that in vivo inhibition of cathepsin G reduces mammary tumor-induced osteolysis. Together, our data indicate that cathepsin G activity at the tumor-bone interface plays an important role in mammary tumor-induced osteolysis and suggest that cathepsin G is a potentially novel therapeutic target in the treatment of breast cancer bone **metastasis**.

---

## Mcpt4

- NCBI Gene
- GeneRIF
- Pubmed

| Search Term | Rank | Fields |
| --- | --- | --- |
| [Mm]igration | 6 | PubMed(2) |
| ECM | 7 | PubMed(1) |
| [Ee]xtracellular [Mm]atrix | 8 | PubMed(2); GeneRIFs(1) |

  
hide/show details for Mcpt4

### [Mm]igration

#### PubMed

1. Mast cells regulate homeostatic intestinal epithelial **migration** and barrier function by a chymase/Mcpt4-dependent mechanism. Altered intestinal barrier function is postulated to be a central predisposing factor to intestinal diseases, including inflammatory bowel diseases and food allergies. However, the mechanisms involved in maintaining homeostatic intestinal barrier integrity remain undefined. In this study, we demonstrate that mice deficient in mast cells (Kit(W-sh/W-sh) [Wsh]) or mast cell chymase (Mcpt4(-/-)) have significantly decreased basal small intestinal permeability compared with wild-type (WT) mice. Altered intestinal barrier function was linked to decreased intestinal epithelial cell **migration** along the villus/crypt axis, altered intestinal morphology, and dysregulated claudin-3 crypt expression. Remarkably, engraftment of Wsh mice with WT but not Mcpt4(-/-) mast cells restored intestinal epithelial cell **migration**, morphology, and intestinal epithelial barrier function. Collectively, these findings identify a mechanism by which mast cells regulate homeostatic intestinal epithelial **migration** and barrier function.
2. Mast cells regulate homeostatic intestinal epithelial **migration** and barrier function by a chymase/Mcpt4-dependent mechanism. Altered intestinal barrier function is postulated to be a central predisposing factor to intestinal diseases, including inflammatory bowel diseases and food allergies. However, the mechanisms involved in maintaining homeostatic intestinal barrier integrity remain undefined. In this study, we demonstrate that mice deficient in mast cells (Kit(W-sh/W-sh) [Wsh]) or mast cell chymase (Mcpt4(-/-)) have significantly decreased basal small intestinal permeability compared with wild-type (WT) mice. Altered intestinal barrier function was linked to decreased intestinal epithelial cell **migration** along the villus/crypt axis, altered intestinal morphology, and dysregulated claudin-3 crypt expression. Remarkably, engraftment of Wsh mice with WT but not Mcpt4(-/-) mast cells restored intestinal epithelial cell **migration**, morphology, and intestinal epithelial barrier function. Collectively, these findings identify a mechanism by which mast cells regulate homeostatic intestinal epithelial **migration** and barrier function.

### ECM

#### PubMed

1. Altered spatiotemporal expression of collagen types I, III, IV, and VI in Lpar3-deficient peri-implantation mouse uterus. Lpar3 is upregulated in the preimplantation uterus, and deletion of Lpar3 leads to delayed uterine receptivity in mice. Microarray analysis revealed that there was higher expression of Col3a1 and Col6a3 in the Preimplantation Day 3.5 Lpar3(-/-) uterus compared to Day 3.5 wild-type (WT) uterus. Since extracellular matrix (**ECM**) remodeling is indispensable during embryo implantation, and dynamic spatiotemporal alteration of specific collagen types is part of this process, this study aimed to characterize the expression of four main uterine collagen types: fibril-forming collagen (COL) I and COL III, basement membrane COL IV, and microfibrillar COL VI in the peri-implantation WT and Lpar3(-/-) uterus. An observed delay of COL III and COL VI clearance in the Lpar3(-/-) uterus may be associated with higher preimplantation expression of Col3a1 and Col6a3. There was also delayed clearance of COL I and delayed deposition of COL IV in the decidual zone in the Lpar3(-/-) uterus. These changes were different from the effects of 17beta-estradiol and progesterone on uterine collagen expression in ovariectomized WT uterus, indicating that the altered collagen expression in Lpar3(-/-) uterus is unlikely to be a result of alterations in ovarian hormones. Decreased expression of several genes encoding matrix-degrading metallo- and serine proteinases was observed in the Lpar3(-/-) uterus. These results demonstrate that pathways downstream of LPA3 are involved in the dynamic remodeling of **ECM** in the peri-implantation uterus.

### [Ee]xtracellular [Mm]atrix

#### PubMed

1. Dual targets for mouse mast cell protease-4 in mediating tissue damage in experimental bullous pemphigoid. Mouse mast cell protease-4 (mMCP-4) has been linked to autoimmune and inflammatory diseases, although the exact mechanisms underlying its role in these pathological conditions remain unclear. Here, we have found that mMCP-4 is critical in a mouse model of the autoimmune skin blistering disease bullous pemphigoid (BP). Mice lacking mMCP-4 were resistant to experimental BP. Complement activation, mast cell (MC) degranulation, and the early phase of neutrophil (PMN) recruitment occurred comparably in mMCP-4(-/-) and WT mice. However, without mMCP-4, activation of matrix metalloproteinase (MMP)-9 was impaired in cultured mMCP-4(-/-) MCs and in the skin of pathogenic IgG-injected mMCP-4(-/-) mice. MMP-9 activation was not fully restored by local reconstitution with WT or mMCP-4(-/-) PMNs. Local reconstitution with mMCP-4(+/+) MCs, but not with mMCP-4(-/-) MCs, restored blistering, MMP-9 activation, and PMN recruitment in mMCP-4(-/-) mice. mMCP-4 also degraded the hemidesmosomal transmembrane protein BP180 both in the skin and in vitro. These results demonstrate that mMCP-4 plays two different roles in the pathogenesis of experimental BP, by both activating MMP-9 and by cleaving BP180, leading to injury of the hemidesmosomes and **extracellular matrix** of the basement membrane zone.
2. Altered spatiotemporal expression of collagen types I, III, IV, and VI in Lpar3-deficient peri-implantation mouse uterus. Lpar3 is upregulated in the preimplantation uterus, and deletion of Lpar3 leads to delayed uterine receptivity in mice. Microarray analysis revealed that there was higher expression of Col3a1 and Col6a3 in the Preimplantation Day 3.5 Lpar3(-/-) uterus compared to Day 3.5 wild-type (WT) uterus. Since **extracellular matrix** (ECM) remodeling is indispensable during embryo implantation, and dynamic spatiotemporal alteration of specific collagen types is part of this process, this study aimed to characterize the expression of four main uterine collagen types: fibril-forming collagen (COL) I and COL III, basement membrane COL IV, and microfibrillar COL VI in the peri-implantation WT and Lpar3(-/-) uterus. An observed delay of COL III and COL VI clearance in the Lpar3(-/-) uterus may be associated with higher preimplantation expression of Col3a1 and Col6a3. There was also delayed clearance of COL I and delayed deposition of COL IV in the decidual zone in the Lpar3(-/-) uterus. These changes were different from the effects of 17beta-estradiol and progesterone on uterine collagen expression in ovariectomized WT uterus, indicating that the altered collagen expression in Lpar3(-/-) uterus is unlikely to be a result of alterations in ovarian hormones. Decreased expression of several genes encoding matrix-degrading metallo- and serine proteinases was observed in the Lpar3(-/-) uterus. These results demonstrate that pathways downstream of LPA3 are involved in the dynamic remodeling of ECM in the peri-implantation uterus.

#### GeneRIFs

1. mMCP-4 plays two different roles in the pathogenesis of experimental BP, by both activating MMP-9 and by cleaving BP180, leading to injury of the hemidesmosomes and **extracellular matrix** of the basement membrane zone.

---

## Gzmb

- NCBI Gene
- GeneRIF
- Pubmed

| Search Term | Rank | Fields |
| --- | --- | --- |
| [Mm]igration | 6 | PubMed(2) |
| ECM | 7 | PubMed(5); GeneRIFs(1) |
| [Ee]xtracellular [Mm]atrix | 8 | PubMed(8); GeneRIFs(1); Summary(1) |

  
hide/show details for Gzmb

### [Mm]igration

#### PubMed

1. Granzyme B promotes cytotoxic lymphocyte trans**migration** via basement membrane remodeling. Granzyme B (GzmB) is a protease with a well-characterized intracellular role in targeted destruction of compromised cells by cytotoxic lymphocytes. However, GzmB also cleaves extracellular matrix components, suggesting that it influences the interplay between cytotoxic lymphocytes and their environment. Here, we show that GzmB-null effector T cells and natural killer (NK) cells exhibited a cell-autonomous homing deficit in mouse models of inflammation and Ectromelia virus infection. Intravital imaging of effector T cells in inflamed cremaster muscle venules revealed that GzmB-null cells adhered normally to the vessel wall and could extend lamellipodia through it but did not cross it efficiently. In vit**ro migrat**ion assays showed that active GzmB was released from migrating cytotoxic lymphocytes and enabled chemokine-driven movement through basement membranes. Finally, proteomic analysis demonstrated that GzmB cleaved basement membrane constituents. Our results highlight an important role for GzmB in expediting cytotoxic lymphocyte diapedesis via basement membrane remodeling.
2. Granzyme B-Mediated Activation-Induced Death of CD4+ T Cells Inhibits Murine Acute Graft-versus-Host Disease. Granzyme B (GzmB) has previously been shown to be critical for CD8(+) T cell-mediated graft-versus-host disease (GVHD) but dispensable for GVHD mediated by CD4(+) T cells. However, previous studies used high doses of CD4(+) T cells in MHC-mismatched models that caused rapid and lethal GVHD. Because of the hyperacute lethality, it is possible that the role of GzmB was concealed by the system. Therefore, in this study, we have titrated down the T cell dose to precisely determine the contribution of GzmB in GVHD mediated by CD4(+)CD25(-) T cells. Surprisingly, we have found that GzmB(-/-)CD4(+)CD25(-) T cells cause more severe GVHD compared with wild-type CD4(+)CD25(-) T cells in both MHC-matched and mismatched models. Mechanistic analyses reveal that although GzmB does not affect donor T cell engraftment, proliferation or tissue-specific **migration**, GzmB(-/-) CD4(+)CD25(-) T cells exhibit significantly enhanced expansion because of GzmB-mediated activation-induced cell death of wild-type CD4(+)CD25(-) T cells. As a result of enhanced expansion, GzmB(-/-) T cells produced higher amounts of proinflammatory cytokines (e.g., TNF-α and IFN-γ) that may contribute to the exacerbated GVHD. These results reveal that GzmB diminishes the ability of CD4(+) T cells to cause acute GVHD, which contradicts its established role in CD8(+) T cells. The differential roles suggest that targeting GzmB in selected T cell subsets may provide a strategy to control GVHD.

### ECM

#### PubMed

1. Granzyme B degrades extracellular matrix and contributes to delayed wound closure in apolipoprotein E knockout mice. Chronic inflammation and excessive protease activity have a major role in the persistence of non-healing wounds. Granzyme B (GzmB) is a serine protease expressed during chronic inflammation that, in conjunction with perforin, has a well-established role in initiating apoptotic cell death. GzmB is also capable of acting extracellularly, independent of perforin and can degrade several extracellular matrix (**ECM**) proteins that are critical during wound healing. We used apolipoprotein E (ApoE) knockout (AKO) mice as a novel model of chronic inflammation and impaired wound healing to investigate the role of GzmB in chronic wounds. Wild-type and AKO mice were grown to 7 weeks (young) or 37 weeks (old) of age on a regular chow or high-fat diet (HFD), given a 1-cm diameter full thickness wound on their mid dorsum and allowed to heal for 16 days. Old AKO mice fed a HFD exhibited reduced wound closure, delayed contraction, chronic inflammation and altered **ECM** remodeling. Conversely, GzmB/ApoE double knockout mice displayed improved wound closure and contraction rates. In addition, murine GzmB was found to degrade both fibronectin and vitronectin derived from healthy mouse granulation tissue. In addition, GzmB-mediated degradation of fibronectin generated a fragment similar in size to that observed in non-healing mouse wounds. These results provide the first direct evidence that GzmB contributes to chronic wound healing in part through degradation of **ECM**.
2. Granzyme B expression by CD8+ T cells is required for the development of experimental cerebral malaria. Parasite burden predicts disease severity in malaria and risk of death in cerebral malaria patients. In murine experimental cerebral malaria (**ECM**), parasite burden and CD8(+) T cells promote disease by mechanisms that are not fully understood. We found that the majority of brain-recruited CD8(+) T cells expressed granzyme B (GzmB). Furthermore, gzmB(-/-) mice harbored reduced parasite numbers in the brain as a consequence of enhanced antiparasitic CD4(+) T cell responses and were protected from **ECM**. We showed in these **ECM**-resistant mice that adoptively transferred, Ag-specific CD8(+) T cells migrated to the brain, but did not induce **ECM** until a critical Ag threshold was reached. **ECM** induction was exquisitely dependent on Ag-specific CD8(+) T cell-derived perforin and GzmB, but not IFN-γ. In wild-type mice, full activation of brain-recruited CD8(+) T cells also depended on a critical number of parasites in this tissue, which in turn, was sustained by these tissue-recruited cells. Thus, an interdependent relationship between parasite burden and CD8(+) T cells dictates the onset of perforin/GzmB-mediated **EC**M.
3. Granzyme B mediates both direct and indirect cleavage of extracellular matrix in skin after chronic low-dose ultraviolet light irradiation. Extracellular matrix (**ECM**) degradation is a hallmark of many chronic inflammatory diseases that can lead to a loss of function, aging, and disease progression. Ultraviolet light (UV) irradiation from the sun is widely considered as the major cause of visible human skin aging, causing increased inflammation and enhanced **ECM** degradation. Granzyme B (GzmB), a serine protease that is expressed by a variety of cells, accumulates in the extracellular milieu during chronic inflammation and cleaves a number of **ECM** proteins. We hypothesized that GzmB contributes to **ECM** degradation in the skin after UV irradiation through both direct cleavage of **ECM** proteins and indirectly through the induction of other proteinases. Wild-type and GzmB-knockout mice were repeatedly exposed to minimal erythemal doses of solar-simulated UV irradiation for 20 weeks. GzmB expression was significantly increased in wild-type treated skin compared to nonirradiated controls, colocalizing to keratinocytes and to an increased mast cell population. GzmB deficiency significantly protected against the formation of wrinkles and the loss of dermal collagen density, which was related to the cleavage of decorin, an abundant proteoglycan involved in collagen fibrillogenesis and integrity. GzmB also cleaved fibronectin, and GzmB-mediated fibronectin fragments increased the expression of collagen-degrading matrix metalloproteinase-1 (MMP-1) in fibroblasts. Collectively, these findings indicate a significant role for GzmB in **EC**M degradation that may have implications in many age-related chronic inflammatory diseases.
4. Granzyme B releases vascular endothelial growth factor from extracellular matrix and induces vascular permeability. The formation of unstable, leaky neovessels underlies the pathogenesis of many chronic inflammatory diseases. Granzyme B (GZMB) is an immune-derived serine protease that accumulates in the extracellular matrix (**ECM**) during chronic inflammation and is capable of cleaving fibronectin (FN). Vascular endothelial growth factor (VEGF) is a potent vascular permeabilizing agent that is sequestered in the **ECM** through its interaction with FN. As GZMB levels are elevated in chronic inflammatory diseases that are associated with increased vascular permeability, the role of GZMB in the regulation of VEGF bioavailability and vascular permeability were assessed. GZMB was added to either VEGF bound to FN or VEGF bound to endothelial cell (EC)-derived **ECM**. Supernatants containing released VEGF were assessed to determine VEGF activity by treating EC and evaluating VEGF receptor-2 (VEGFR2) phosphorylation. GZMB released VEGF from both FN and from EC-derived matrix, whereas GZMB inhibition prevented FN cleavage and VEGF release. GZMB-mediated VEGF release resulted in significant phosphorylation of VEGFR2. The role of GZMB-mediated VEGF release in altering vascular permeability was also assessed in vivo using Miles/Evans blue permeability assay. GZMB induced a significant VEGF-dependent increase in vascular permeability in vivo that was reduced in the presence of an anti-VEGF-neutralizing antibody. Inflammatory-mediated vascular leakage was also assessed in GZMB-KO mice using a delayed-type hypersensitivity model. GZMB-KO mice exhibited reduced microvascular leakage compared with C57\B6 controls. GZMB increases vascular permeability in part through the proteolytic release of **ECM**-sequestered VEGF, leading to VEGFR2 activation and increased vascular permeability in vivo. These findings present a novel role for GZMB as a modulator of vascular response during chronic inflammation.
5. Granzyme B contributes to extracellular matrix remodeling and skin aging in apolipoprotein E knockout mice. Apolipoprotein E knockout (apoE-KO) mice have been utilized for decades as a model of atherosclerosis. However, in addition to atherosclerosis, apoE-KO mice develop extensive cutaneous xanthomatosis, accelerated skin aging and frailty when fed a high fat diet. Granzyme B (GrB) is a pro-apoptotic serine protease that has recently been shown to exhibit extracellular proteolytic activity in certain pathologies. In the present study, the role of GrB in skin aging and pathology was assessed using the apoE-KO model of skin aging. Male C57BL/6 wild type and apoE-KO mice were grown for 0, 5, 15 or 30 weeks on either a high fat (21.2% fat, 0.2% cholesterol) or regular chow diet (7% fat). ApoE/GrB double knockout (DKO) mice were also generated and assessed after being fed either diet for 30 weeks. Skin was removed from the mid to lower back and examined for age-related changes such as hair loss, skin thinning and collagen remodeling and disorganization. ApoE-KO mice exhibited signs of frailty, hair graying, hair loss, skin thinning, loss of collagen density and increased skin pathologies featuring collagen remodeling and reduced decorin compared to wild type controls. These phenotypes occurred earlier and were more severe when fed a high fat diet. In addition, we also observed increased GrB expression in proximity to areas of decorin degradation and reduced collagen density in the skin of apoE-KO mice. DKO mice exhibited protection against skin thinning, **ECM** degradation and loss of dermal collagen density. In summary, our results provide novel insights into the effects of a high fat diet and apoE deficiency on skin aging and pathology and suggest a role for GrB in age-related skin thinning and frailty.

#### GeneRIFs

1. Data suggest that an interdependent relationship between parasite burden and CD8(+) T cells dictates the onset of perforin/GzmB-mediated **ECM**.

### [Ee]xtracellular [Mm]atrix

#### PubMed

1. Granzyme B degrades **extracellular matrix** and contributes to delayed wound closure in apolipoprotein E knockout mice. Chronic inflammation and excessive protease activity have a major role in the persistence of non-healing wounds. Granzyme B (GzmB) is a serine protease expressed during chronic inflammation that, in conjunction with perforin, has a well-established role in initiating apoptotic cell death. GzmB is also capable of acting extracellularly, independent of perforin and can degrade several **extracellular matrix** (ECM) proteins that are critical during wound healing. We used apolipoprotein E (ApoE) knockout (AKO) mice as a novel model of chronic inflammation and impaired wound healing to investigate the role of GzmB in chronic wounds. Wild-type and AKO mice were grown to 7 weeks (young) or 37 weeks (old) of age on a regular chow or high-fat diet (HFD), given a 1-cm diameter full thickness wound on their mid dorsum and allowed to heal for 16 days. Old AKO mice fed a HFD exhibited reduced wound closure, delayed contraction, chronic inflammation and altered ECM remodeling. Conversely, GzmB/ApoE double knockout mice displayed improved wound closure and contraction rates. In addition, murine GzmB was found to degrade both fibronectin and vitronectin derived from healthy mouse granulation tissue. In addition, GzmB-mediated degradation of fibronectin generated a fragment similar in size to that observed in non-healing mouse wounds. These results provide the first direct evidence that GzmB contributes to chronic wound healing in part through degradation of ECM.
2. Perforin and granzyme B have separate and distinct roles during atherosclerotic plaque development in apolipoprotein E knockout mice. The granzyme B/perforincytotoxic pathway is a well established mechanism of initiating target cell apoptosis. Previous studies have suggested a role for the granzyme B/perforin cytotoxic pathway in vulnerable atherosclerotic plaque formation. In the present study, granzyme B deficiency resulted in reduced atherosclerotic plaque development in the descending aortas of apolipoprotein E knockout mice fed a high fat diet for 30 weeks while perforindeficiency resulted in greater reduction in plaque development with significantly less plaque area than granzyme Bdeficient mice. In contrast to the descending aorta, no significant change in plaque size was observed in aortic roots from either granzyme Bdeficient or perforindeficient apolipoprotein E knockout mice. However, atherosclerotic plaques in the aortic roots did exhibit significantly more collagen in granzyme B, but not perforin deficient mice. Together these results suggest significant, yet separate roles for granzyme B and perforin in the pathogenesis of atherosclerosis that go beyond the traditional apoptotic pathway with additional implications in plaque development, stability and remodelling of **extracellular matrix**.
3. Perforin-independent extracellular granzyme B activity contributes to abdominal aortic aneurysm. Granzyme B (GZMB) is a serine protease that is abundantly expressed in advanced human atherosclerotic lesions and may contribute to plaque instability. Perforin is a pore-forming protein that facilitates GZMB internalization and the induction of apoptosis. Recently a perforin-independent, extracellular role for GZMB has been proposed. In the current study, the role of GZMB in abdominal aortic aneurysm (AAA) was assessed. Apolipoprotein E (APOE)(-/-) x GZMB(-/-) and APOE(-/-) x perforin(-/-) double knockout (GDKO, PDKO) mice were generated to test whether GZMB exerted a causative role in aneurysm formation. To induce aneurysm, mice were given angiotensin II (1000 ng/kg/min) for 28 days. GZMB was found to be abundant in both murine and human AAA specimens. GZMB deficiency was associated with a decrease in AAA and increased survival compared with APOE-KO and PDKO mice. Although AAA rupture was observed frequently in APOE-KO (46.7%; n = 15) and PDKO (43.3%; n = 16) mice, rupture was rarely observed in GDKO (7.1%; n = 14) mice. APOE-KO mice exhibited reduced fibrillin-1 staining compared with GDKO mice, whereas in vitro protease assays demonstrated that fibrillin-1 is a substrate of GZMB. As perforin deficiency did not affect the outcome, our results suggest that GZMB contributes to AAA pathogenesis via a perforin-independent mechanism involving **extracellular matrix** degradation and subsequent loss of vessel wall integrity.
4. Granzyme B promotes cytotoxic lymphocyte transmigration via basement membrane remodeling. Granzyme B (GzmB) is a protease with a well-characterized intracellular role in targeted destruction of compromised cells by cytotoxic lymphocytes. However, GzmB also cleaves **extracellular matrix** components, suggesting that it influences the interplay between cytotoxic lymphocytes and their environment. Here, we show that GzmB-null effector T cells and natural killer (NK) cells exhibited a cell-autonomous homing deficit in mouse models of inflammation and Ectromelia virus infection. Intravital imaging of effector T cells in inflamed cremaster muscle venules revealed that GzmB-null cells adhered normally to the vessel wall and could extend lamellipodia through it but did not cross it efficiently. In vitro migration assays showed that active GzmB was released from migrating cytotoxic lymphocytes and enabled chemokine-driven movement through basement membranes. Finally, proteomic analysis demonstrated that GzmB cleaved basement membrane constituents. Our results highlight an important role for GzmB in expediting cytotoxic lymphocyte diapedesis via basement membrane remodeling.
5. Granzyme B mediates both direct and indirect cleavage of **extracellular matrix** in skin after chronic low-dose ultraviolet light irradiation. **Extracellular matrix** (ECM) degradation is a hallmark of many chronic inflammatory diseases that can lead to a loss of function, aging, and disease progression. Ultraviolet light (UV) irradiation from the sun is widely considered as the major cause of visible human skin aging, causing increased inflammation and enhanced ECM degradation. Granzyme B (GzmB), a serine protease that is expressed by a variety of cells, accumulates in the extracellular milieu during chronic inflammation and cleaves a number of ECM proteins. We hypothesized that GzmB contributes to ECM degradation in the skin after UV irradiation through both direct cleavage of ECM proteins and indirectly through the induction of other proteinases. Wild-type and GzmB-knockout mice were repeatedly exposed to minimal erythemal doses of solar-simulated UV irradiation for 20 weeks. GzmB expression was significantly increased in wild-type treated skin compared to nonirradiated controls, colocalizing to keratinocytes and to an increased mast cell population. GzmB deficiency significantly protected against the formation of wrinkles and the loss of dermal collagen density, which was related to the cleavage of decorin, an abundant proteoglycan involved in collagen fibrillogenesis and integrity. GzmB also cleaved fibronectin, and GzmB-mediated fibronectin fragments increased the expression of collagen-degrading matrix metalloproteinase-1 (MMP-1) in fibroblasts. Collectively, these findings indicate a significant role for GzmB in ECM degradation that may have implications in many age-related chronic inflammatory diseases.
6. Granzyme B releases vascular endothelial growth factor from **extracellular matrix** and induces vascular permeability. The formation of unstable, leaky neovessels underlies the pathogenesis of many chronic inflammatory diseases. Granzyme B (GZMB) is an immune-derived serine protease that accumulates in the **extracellular matrix** (ECM) during chronic inflammation and is capable of cleaving fibronectin (FN). Vascular endothelial growth factor (VEGF) is a potent vascular permeabilizing agent that is sequestered in the ECM through its interaction with FN. As GZMB levels are elevated in chronic inflammatory diseases that are associated with increased vascular permeability, the role of GZMB in the regulation of VEGF bioavailability and vascular permeability were assessed. GZMB was added to either VEGF bound to FN or VEGF bound to endothelial cell (EC)-derived ECM. Supernatants containing released VEGF were assessed to determine VEGF activity by treating EC and evaluating VEGF receptor-2 (VEGFR2) phosphorylation. GZMB released VEGF from both FN and from EC-derived matrix, whereas GZMB inhibition prevented FN cleavage and VEGF release. GZMB-mediated VEGF release resulted in significant phosphorylation of VEGFR2. The role of GZMB-mediated VEGF release in altering vascular permeability was also assessed in vivo using Miles/Evans blue permeability assay. GZMB induced a significant VEGF-dependent increase in vascular permeability in vivo that was reduced in the presence of an anti-VEGF-neutralizing antibody. Inflammatory-mediated vascular leakage was also assessed in GZMB-KO mice using a delayed-type hypersensitivity model. GZMB-KO mice exhibited reduced microvascular leakage compared with C57\B6 controls. GZMB increases vascular permeability in part through the proteolytic release of ECM-sequestered VEGF, leading to VEGFR2 activation and increased vascular permeability in vivo. These findings present a novel role for GZMB as a modulator of vascular response during chronic inflammation.
7. Granzyme B in injury, inflammation, and repair. Fragile skin and susceptibility to skin tearing are major problems among the elderly and can be complicated further by impaired wound healing. Non-healing wounds fail to progress through the normal stages of healing and enter a state of chronic inflammation featuring increased proteolytic activity. Increased expression of the serine protease granzyme B is observed during prolonged inflammation and is implicated in the pathogenesis of several chronic inflammatory diseases. Although its role in cytotoxic lymphocyte-mediated apoptosis is well established, granzyme B can also degrade **extracellular matrix** proteins and alter inflammation if present in the extracellular milieu. The present review focuses on the emerging evidence for the involvement of granzyme B in chronic inflammation, impaired wound healing, and age-related skin fragility.
8. Granzyme B contributes to **extracellular matrix** remodeling and skin aging in apolipoprotein E knockout mice. Apolipoprotein E knockout (apoE-KO) mice have been utilized for decades as a model of atherosclerosis. However, in addition to atherosclerosis, apoE-KO mice develop extensive cutaneous xanthomatosis, accelerated skin aging and frailty when fed a high fat diet. Granzyme B (GrB) is a pro-apoptotic serine protease that has recently been shown to exhibit extracellular proteolytic activity in certain pathologies. In the present study, the role of GrB in skin aging and pathology was assessed using the apoE-KO model of skin aging. Male C57BL/6 wild type and apoE-KO mice were grown for 0, 5, 15 or 30 weeks on either a high fat (21.2% fat, 0.2% cholesterol) or regular chow diet (7% fat). ApoE/GrB double knockout (DKO) mice were also generated and assessed after being fed either diet for 30 weeks. Skin was removed from the mid to lower back and examined for age-related changes such as hair loss, skin thinning and collagen remodeling and disorganization. ApoE-KO mice exhibited signs of frailty, hair graying, hair loss, skin thinning, loss of collagen density and increased skin pathologies featuring collagen remodeling and reduced decorin compared to wild type controls. These phenotypes occurred earlier and were more severe when fed a high fat diet. In addition, we also observed increased GrB expression in proximity to areas of decorin degradation and reduced collagen density in the skin of apoE-KO mice. DKO mice exhibited protection against skin thinning, ECM degradation and loss of dermal collagen density. In summary, our results provide novel insights into the effects of a high fat diet and apoE deficiency on skin aging and pathology and suggest a role for GrB in age-related skin thinning and frailty.

#### GeneRIFs

1. These findings indicate a significant role for GzmB in **extracellular matrix** degradation that may have implications in many age-related chronic inflammatory diseases.

#### Summary

1. This gene encodes a member of the granzyme subfamily of proteins, part of the peptidase S1 family of serine proteases. The encoded preproprotein is secreted by natural killer (NK) cells and cytotoxic T lymphocytes (CTLs) and proteolytically processed to generate the active protease, which induces target cell apoptosis. This protein also processes cytokines and degrades **extracellular matrix** proteins, and these roles are implicated in chronic inflammation and wound healing. Mice lacking a functional copy of this gene exhibit impaired immune cell-mediated cytolysis. [provided by RefSeq, Sep 2016]

---

## Mcpt1

- NCBI Gene
- GeneRIF
- Pubmed

| Search Term | Rank | Fields |
| --- | --- | --- |
| [Mm]igration | 6 | PubMed(1) |

  
hide/show details for Mcpt1

### [Mm]igration

#### PubMed

1. CD8 T cells are involved in skeletal muscle regeneration through facilitating MCP-1 secretion and Gr1(high) macrophage infiltration. Inflammatory microenvironments play a key role in skeletal muscle regeneration. The infiltration of CD8 T cells into injured muscle has been reported. However, the role of CD8 T cells during skeletal muscle regeneration remains unclear. In this study, we used cardiotoxin-induced mouse skeletal muscle injury/regeneration model to investigate the role of CD8 T cells. Muscle regeneration was impaired and matrix deposit was increased in CD8α-deficient mice compared with wild-type (WT) mice whose CD8 T cells were infiltrated into damaged muscle after cardiotoxin injection. Adoptive transfer of CD8 T cells to CD8α-deficient mice improved muscle regeneration and inhibited matrix remodeling. Compared with WT mice, CD8α deficiency limited the recruitment of Gr1(high) macrophages (MPs) into muscle, resulting in the reduction of satellite cell number. The expression of MCP-1 (MCP-1/CCL2), which regulates t**he migrat**ion of Gr1(high) MPs, was reduced in CD8α-deficient mice compared with WT mice. Coculture CD8 T cells with MPs promoted MCP-1 secretion. The i.m. injection of MCP-1 markedly promoted the recruitment of Gr1(high) MPs and improved muscle regeneration in CD8α-deficient mice. We conclude that CD8 T cells are involved in skeletal muscle regeneration by regulating the secretion of MCP-1 to recruit Gr1(high) MPs, which facilitate myoblast proliferation.

---

## Mcpt8

- NCBI Gene
- GeneRIF
- Pubmed

| Search Term | Rank | Fields |
| --- | --- | --- |
| [Mm]igration | 6 | PubMed(1) |
| [Ee]xtracellular [Mm]atrix | 8 | PubMed(1) |

  
hide/show details for Mcpt8

### [Mm]igration

#### PubMed

1. IgE-activated basophils regulate eosinophil tissue entry by modulating endothelial function. Vertebrate immunity has evolved a modular architecture in response to perturbations. Allergic inflammation represents such a module, with signature features of antigen-specific IgE and tissue eosinophilia, although the cellular and molecular circuitry coupling these responses remains unclear. Here, we use genetic and imaging approaches in models of IgE-dependent eosinophilic dermatitis to demonstrate a requisite role for basophils. After antigenic inflammation, basophils initiate trans**migration** like other granulocytes but, upon activation via their high-affinity IgE receptor, alter their migratory kinetics to persist at the endothelium. Prolonged basophil-endothelial interactions, in part dependent on activation of focal adhesion kinases, promote delivery of basophil-derived IL-4 to the endothelium and subsequent induction of endothelial vascular cell adhesion molecule-1 (VCAM-1), which is required for eosinophil accumulation. Thus, basophils are gatekeepers that link adaptive immunity with innate effector programs by altering access to tissue sites by activation-induced interactions with the endothelium.

### [Ee]xtracellular [Mm]atrix

#### PubMed

1. **Extracellular matrix** secretion by cardiac fibroblasts: role of microRNA-29b and microRNA-30c. MicroRNAs (miRNAs), in particular miR-29b and miR-30c, have been implicated as important regulators of cardiac fibrosis.To perform a proteomics comparison of miRNA effects on **extracellular matrix** secretion by cardiac fibroblasts.Mouse cardiac fibroblasts were transfected with pre-/anti-miR of miR-29b and miR-30c, and their conditioned medium was analyzed by mass spectrometry. miR-29b targeted a cadre of proteins involved in fibrosis, including multiple collagens, matrix metalloproteinases, and leukemia inhibitory factor, insulin-like growth factor 1, and pentraxin 3, 3 predicted targets of miR-29b. miR-29b also attenuated the cardiac fibroblast response to transforming growth factor-β. In contrast, miR-30c had little effect on **extracellular matri**x production but opposite effects regarding leukemia inhibitory factor and insulin-like growth factor 1. Both miRNAs indirectly affected cardiac myocytes. On transfection with pre-miR-29b, the conditioned medium of cardiac fibroblasts lost its ability to support adhesion of rat ventricular myocytes and led to a significant reduction of cardiac myocyte proteins (α-actinin, cardiac myosin-binding protein C, and cardiac troponin I). Similarly, cardiomyocytes derived from mouse embryonic stem cells atrophied under pre-miR-29 conditioned medium, whereas pre-miR-30c conditioned medium had a prohypertrophic effect. Levels of miR-29a, miR-29c, and miR-30c, but not miR-29b, were significantly reduced in a mouse model of pathological but not physiological hypertrophy. Treatment with antagomiRs to miR-29b induced excess fibrosis after aortic constriction without overt deterioration in cardiac function.Our proteomic analysis revealed novel molecular targets of miRNAs that are linked to a fibrogenic cardiac phenotype. Such comprehensive screening methods are essential to define the concerted actions of miRNAs in cardiovascular disease.

---

## Cma1

- NCBI Gene
- GeneRIF
- Pubmed

| Search Term | Rank | Fields |
| --- | --- | --- |
| [Mm]igration | 6 | PubMed(4); GeneRIFs(1) |
| ECM | 7 | PubMed(1) |
| [Ee]xtracellular [Mm]atrix | 8 | PubMed(1); Component(1); Summary(1) |

  
hide/show details for Cma1

### [Mm]igration

#### PubMed

1. Emx1-lineage progenitors differentially contribute to neural diversity in the striatum and amygdala. In the developing mammalian basal telencephalon, neural progenitors from the subpallium generate the majority of inhibitory medium spiny neurons (MSNs) in the striatum, while both pallial- and subpallial-derived progenitors contribute to excitatory and inhibitory neuronal diversity in the amygdala. Using a combination of approaches, including genetic fate mapping, cell birth dating, cell **migration** assays, and electrophysiology, we find that cells derived from the Emx1 lineage contribute to two distinct neuronal populations in the mature basal forebrain: inhibitory MSNs in the striatum and functionally distinct subclasses of excitatory neurons in the amygdala. Our cell birth-dating studies reveal that these two populations are born at different times during early neurogenesis, with the amygdala population born before the MSNs. In the striatum, Emx1-lineage neurons represent a unique subpopulation of MSNs: they are disproportionately localized to the dorsal striatum, are found in dopamine receiving, reelin-positive patches, and are born throughout striatal neurogenesis. In addition, our data suggest that a subpopulation of these Emx1-lineage cells originate in the pallium and subsequently migrate to the developing striatum and amygdala. Our intersectional fate-mapping analysis further reveals that Emx1-lineage cells that coexpress Dlx exclusively generate MSNs but do not contribute to the excitatory neurons in the amygdala. Thus, both the timing of neurogenesis and differential combinatorial gene expression appear to be key determinants of striatal versus amygdala fate decisions of Emx1-lineage cells.
2. Eosinophil **migration** induced by mast cell chymase is mediated by extracellular signal-regulated kinase pathway. Mast cell chymase is known to induce eosinophil **migration** in vivo and in vitro. In the present study, we investigated possible involvement of mitogen-activated protein (MAP) kinases; extracellular signal-regulated kinase (ERK), c-Jun amino-terminal kinase (JNK), and p38, in the chymase-induced eosinophil **migration**. Human chymase induced a rapid phosphorylation of ERK1/2 and p38 in human eosinophilic leukemia EoL-1 cells, while no phosphorylation was detected in JNK. The chymase-induced phosphorylation of ERK and p38 was inhibited by pertussis toxin. Similar results were obtained in the experiments using mouse chymase and eosinophils. U0126 (the inhibitor for MAP/ERK kinase) suppressed chymase-induced **migration** of EoL-1 cells and mouse eosinophils. However, SB203580 (p38 inhibitor) and SP600125 (JNK inhibitor) showed little effect on the **migration**. It is suggested therefore that chymase activates ERK and p38 probably through G-protein-coupled receptor, and that ERK but not p38 cascade may have a crucial role in chymase-induced **migration** of eosinophils.
3. Eosinophil **migration** induced by mast cell chymase is mediated by extracellular signal-regulated kinase pathway. Mast cell chymase is known to induce eosinophil **migration** in vivo and in vitro. In the present study, we investigated possible involvement of mitogen-activated protein (MAP) kinases; extracellular signal-regulated kinase (ERK), c-Jun amino-terminal kinase (JNK), and p38, in the chymase-induced eosinophil **migration**. Human chymase induced a rapid phosphorylation of ERK1/2 and p38 in human eosinophilic leukemia EoL-1 cells, while no phosphorylation was detected in JNK. The chymase-induced phosphorylation of ERK and p38 was inhibited by pertussis toxin. Similar results were obtained in the experiments using mouse chymase and eosinophils. U0126 (the inhibitor for MAP/ERK kinase) suppressed chymase-induced **migration** of EoL-1 cells and mouse eosinophils. However, SB203580 (p38 inhibitor) and SP600125 (JNK inhibitor) showed little effect on the **migration**. It is suggested therefore that chymase activates ERK and p38 probably through G-protein-coupled receptor, and that ERK but not p38 cascade may have a crucial role in chymase-induced **migration** of eosinophils.
4. Pulmonary mastocytosis and enhanced lung inflammation in mice heterozygous null for the Foxf1 gene. The Forkhead Box f1 (Foxf1) transcriptional factor (previously known as HFH-8 or Freac-1) is expressed in endothelial and smooth muscle cells in the embryonic and adult lung. To assess effects of Foxf1 during lung injury, we used CCl(4) and butylated hydroxytoluene (BHT) injury models. Foxf1(+/-) mice developed severe airway obstruction and bronchial edema, associated with increased numbers of pulmonary mast cells and increased mast cell degranulation after injury. Pulmonary inflammation in Foxf1(+/-) mice was associated with diminished expression of Foxf1, increased mast cell tryptase, and increased expression of CXCL12, the latter being essential for mast cell **migration** and chemotaxis. After ovalbumin (OVA) sensitization and OVA challenge, increased lung inflammation, airway hyperresponsiveness to methacholine, and elevated expression of CXCL12 were observed in Foxf1(+/-) mice. During lung development, Foxf1(+/-) embryos displayed a marked increase in pulmonary mast cells before birth, and this was associated with increased CXCL12 levels in the lung. Expression of a doxycycline-inducible Foxf1 dominant-negative transgene in primary cultures of lung endothelial cells increased CXCL12 expression in vitro. Foxf1 haploinsufficiency caused pulmonary mastocytosis and enhanced pulmonary inflammation after chemically induced or allergen-mediated lung injury, indicating an important role for Foxf1 in the pathogenesis of pulmonary inflammatory responses.

#### GeneRIFs

1. mast cell chymase activates ERK and p38 probably through G-protein-coupled receptor, and the ERK but not p38 cascade may have a crucial role in chymase-induced **migration** of eosinophils

### ECM

#### PubMed

1. Altered spatiotemporal expression of collagen types I, III, IV, and VI in Lpar3-deficient peri-implantation mouse uterus. Lpar3 is upregulated in the preimplantation uterus, and deletion of Lpar3 leads to delayed uterine receptivity in mice. Microarray analysis revealed that there was higher expression of Col3a1 and Col6a3 in the Preimplantation Day 3.5 Lpar3(-/-) uterus compared to Day 3.5 wild-type (WT) uterus. Since extracellular matrix (**ECM**) remodeling is indispensable during embryo implantation, and dynamic spatiotemporal alteration of specific collagen types is part of this process, this study aimed to characterize the expression of four main uterine collagen types: fibril-forming collagen (COL) I and COL III, basement membrane COL IV, and microfibrillar COL VI in the peri-implantation WT and Lpar3(-/-) uterus. An observed delay of COL III and COL VI clearance in the Lpar3(-/-) uterus may be associated with higher preimplantation expression of Col3a1 and Col6a3. There was also delayed clearance of COL I and delayed deposition of COL IV in the decidual zone in the Lpar3(-/-) uterus. These changes were different from the effects of 17beta-estradiol and progesterone on uterine collagen expression in ovariectomized WT uterus, indicating that the altered collagen expression in Lpar3(-/-) uterus is unlikely to be a result of alterations in ovarian hormones. Decreased expression of several genes encoding matrix-degrading metallo- and serine proteinases was observed in the Lpar3(-/-) uterus. These results demonstrate that pathways downstream of LPA3 are involved in the dynamic remodeling of **ECM** in the peri-implantation uterus.

### [Ee]xtracellular [Mm]atrix

#### PubMed

1. Altered spatiotemporal expression of collagen types I, III, IV, and VI in Lpar3-deficient peri-implantation mouse uterus. Lpar3 is upregulated in the preimplantation uterus, and deletion of Lpar3 leads to delayed uterine receptivity in mice. Microarray analysis revealed that there was higher expression of Col3a1 and Col6a3 in the Preimplantation Day 3.5 Lpar3(-/-) uterus compared to Day 3.5 wild-type (WT) uterus. Since **extracellular matrix** (ECM) remodeling is indispensable during embryo implantation, and dynamic spatiotemporal alteration of specific collagen types is part of this process, this study aimed to characterize the expression of four main uterine collagen types: fibril-forming collagen (COL) I and COL III, basement membrane COL IV, and microfibrillar COL VI in the peri-implantation WT and Lpar3(-/-) uterus. An observed delay of COL III and COL VI clearance in the Lpar3(-/-) uterus may be associated with higher preimplantation expression of Col3a1 and Col6a3. There was also delayed clearance of COL I and delayed deposition of COL IV in the decidual zone in the Lpar3(-/-) uterus. These changes were different from the effects of 17beta-estradiol and progesterone on uterine collagen expression in ovariectomized WT uterus, indicating that the altered collagen expression in Lpar3(-/-) uterus is unlikely to be a result of alterations in ovarian hormones. Decreased expression of several genes encoding matrix-degrading metallo- and serine proteinases was observed in the Lpar3(-/-) uterus. These results demonstrate that pathways downstream of LPA3 are involved in the dynamic remodeling of ECM in the peri-implantation uterus.

#### Component

1. **extracellular matrix**

#### Summary

1. This gene encodes a serine protease that belongs to the peptidase family S1. It is expressed in mast cells and is thought to function in the degradation of the **extracellular matrix**, the regulation of submucosal gland secretion, and the generation of vasoactive peptides. The encoded preproprotein undergoes proteolytic processing to generate a functional enzyme with elastase-like activity. Mice lacking the encoded protein exhibit significant attenuation of ischemia-reperfusion injury of the skeletal muscle. This gene is located in a cluster of related mast cell protease genes on chromosome 14. [provided by RefSeq, Nov 2015]

---

## Egfbp2

- NCBI Gene
- GeneRIF
- Pubmed

| Search Term | Rank | Fields |
| --- | --- | --- |
| [Mm]etastasis | 12 | PubMed(1) |

  
hide/show details for Egfbp2

### [Mm]etastasis

#### PubMed

1. Human tissue kallikreins: physiologic roles and applications in cancer. Tissue kallikreins are members of the S1 family (clan SA) of trypsin-like serine proteases and are present in at least six mammalian orders. In humans, tissue kallikreins (hK) are encoded by 15 structurally similar, steroid hormone-regulated genes (KLK) that colocalize to chromosome 19q13.4, representing the largest cluster of contiguous protease genes in the entire genome. hKs are widely expressed in diverse tissues and implicated in a range of normal physiologic functions from the regulation of blood pressure and electrolyte balance to tissue remodeling, prohormone processing, neural plasticity, and skin desquamation. Several lines of evidence suggest that hKs may be involved in cascade reactions and that cross-talk may exist with proteases of other catalytic classes. The proteolytic activity of hKs is regulated in several ways including zymogen activation, endogenous inhibitors, such as serpins, and via internal (auto)cleavage leading to inactivation. Dysregulated hK expression is associated with multiple diseases, primarily cancer. As a consequence, many kallikreins, in addition to hK3/PSA, have been identified as promising diagnostic and/or prognostic biomarkers for several cancer types, including ovarian, breast, and prostate. Recent data also suggest that hKs may be causally involved in carcinogenesis, particularly in tumor **metastasis** and invasion, and, thus, may represent attractive drug targets to consider for therapeutic intervention.

---

## Klk1b9

- NCBI Gene
- GeneRIF
- Pubmed

| Search Term | Rank | Fields |
| --- | --- | --- |
| ECM | 7 | PubMed(1) |
| [Ee]xtracellular [Mm]atrix | 8 | PubMed(1) |

  
hide/show details for Klk1b9

### ECM

#### PubMed

1. Temporal and tissue-specific expression of kallikrein (Klk) genes and identification of a novel Klk messenger ribonucleic acid transcript during early development in the mouse. The kallikreins are a multigene family of serine proteases that act on a diverse number of substrates, including several growth factors and extracellular matrix (**ECM**) glycoproteins and proteinases. Recently, this family has been implicated in the process of early development and embryo implantation. In this study, we used reverse transcription-polymerase chain reaction with gene-specific primers and Southern hybridization to elucidate the temporal and tissue-specific expression patterns of the mouse kallikreins Klk1, Klk3, Klk5, Klk9, and Klk21 during early development in the embryo, uterus, and decidua. We observed the expression of Klk1 (tissue kallikrein), Klk3 (gamma-nerve growth factor), Klk9 (epidermal growth factor-binding protein), and Klk21 in the early conceptus (until the 2-cell stage). Only Klk21 continued to be expressed in the blastocyst until Day 7.5 of pregnancy. Expression of Klk9 reappeared at Day 7.5 and was consistently detected until Day 11, the last day studied; Klk1 was again expressed in the embryo from Day 9.5, with decreased levels by Day 11. In contrast, in the uterus or decidua, there was no expression of Klk1 until Day 7.5, when mRNA transcripts were abundant; transcripts then decreased in the Day 9.5 and Day 11 uterus. Expression of Klk21 in the uterus and decidua displayed a similar pattern but was detected at much lower levels. Interestingly, a novel Klk21-like mRNA was also detected in uterine tissue samples but not in embryonic samples; Klk3, Klk5, and Klk9 were not consistently expressed in the uterus or decidua over this time. This is the first report of the expression of specific kallikreins during early development. The distinct gene- and tissue-specific expression patterns presented in this study, in conjunction with the well-characterized roles of kallikreins in regulation of protein activation, **ECM** degradation, and proliferative events, suggests the involvement of the kallikrein gene family during early development.

### [Ee]xtracellular [Mm]atrix

#### PubMed

1. Temporal and tissue-specific expression of kallikrein (Klk) genes and identification of a novel Klk messenger ribonucleic acid transcript during early development in the mouse. The kallikreins are a multigene family of serine proteases that act on a diverse number of substrates, including several growth factors and **extracellular matrix** (ECM) glycoproteins and proteinases. Recently, this family has been implicated in the process of early development and embryo implantation. In this study, we used reverse transcription-polymerase chain reaction with gene-specific primers and Southern hybridization to elucidate the temporal and tissue-specific expression patterns of the mouse kallikreins Klk1, Klk3, Klk5, Klk9, and Klk21 during early development in the embryo, uterus, and decidua. We observed the expression of Klk1 (tissue kallikrein), Klk3 (gamma-nerve growth factor), Klk9 (epidermal growth factor-binding protein), and Klk21 in the early conceptus (until the 2-cell stage). Only Klk21 continued to be expressed in the blastocyst until Day 7.5 of pregnancy. Expression of Klk9 reappeared at Day 7.5 and was consistently detected until Day 11, the last day studied; Klk1 was again expressed in the embryo from Day 9.5, with decreased levels by Day 11. In contrast, in the uterus or decidua, there was no expression of Klk1 until Day 7.5, when mRNA transcripts were abundant; transcripts then decreased in the Day 9.5 and Day 11 uterus. Expression of Klk21 in the uterus and decidua displayed a similar pattern but was detected at much lower levels. Interestingly, a novel Klk21-like mRNA was also detected in uterine tissue samples but not in embryonic samples; Klk3, Klk5, and Klk9 were not consistently expressed in the uterus or decidua over this time. This is the first report of the expression of specific kallikreins during early development. The distinct gene- and tissue-specific expression patterns presented in this study, in conjunction with the well-characterized roles of kallikreins in regulation of protein activation, ECM degradation, and proliferative events, suggests the involvement of the kallikrein gene family during early development.

---

## Klk1b5

- NCBI Gene
- GeneRIF
- Pubmed

| Search Term | Rank | Fields |
| --- | --- | --- |
| ECM | 7 | PubMed(1) |
| [Ee]xtracellular [Mm]atrix | 8 | PubMed(2) |

  
hide/show details for Klk1b5

### ECM

#### PubMed

1. Temporal and tissue-specific expression of kallikrein (Klk) genes and identification of a novel Klk messenger ribonucleic acid transcript during early development in the mouse. The kallikreins are a multigene family of serine proteases that act on a diverse number of substrates, including several growth factors and extracellular matrix (**ECM**) glycoproteins and proteinases. Recently, this family has been implicated in the process of early development and embryo implantation. In this study, we used reverse transcription-polymerase chain reaction with gene-specific primers and Southern hybridization to elucidate the temporal and tissue-specific expression patterns of the mouse kallikreins Klk1, Klk3, Klk5, Klk9, and Klk21 during early development in the embryo, uterus, and decidua. We observed the expression of Klk1 (tissue kallikrein), Klk3 (gamma-nerve growth factor), Klk9 (epidermal growth factor-binding protein), and Klk21 in the early conceptus (until the 2-cell stage). Only Klk21 continued to be expressed in the blastocyst until Day 7.5 of pregnancy. Expression of Klk9 reappeared at Day 7.5 and was consistently detected until Day 11, the last day studied; Klk1 was again expressed in the embryo from Day 9.5, with decreased levels by Day 11. In contrast, in the uterus or decidua, there was no expression of Klk1 until Day 7.5, when mRNA transcripts were abundant; transcripts then decreased in the Day 9.5 and Day 11 uterus. Expression of Klk21 in the uterus and decidua displayed a similar pattern but was detected at much lower levels. Interestingly, a novel Klk21-like mRNA was also detected in uterine tissue samples but not in embryonic samples; Klk3, Klk5, and Klk9 were not consistently expressed in the uterus or decidua over this time. This is the first report of the expression of specific kallikreins during early development. The distinct gene- and tissue-specific expression patterns presented in this study, in conjunction with the well-characterized roles of kallikreins in regulation of protein activation, **ECM** degradation, and proliferative events, suggests the involvement of the kallikrein gene family during early development.

### [Ee]xtracellular [Mm]atrix

#### PubMed

1. Temporal and tissue-specific expression of kallikrein (Klk) genes and identification of a novel Klk messenger ribonucleic acid transcript during early development in the mouse. The kallikreins are a multigene family of serine proteases that act on a diverse number of substrates, including several growth factors and **extracellular matrix** (ECM) glycoproteins and proteinases. Recently, this family has been implicated in the process of early development and embryo implantation. In this study, we used reverse transcription-polymerase chain reaction with gene-specific primers and Southern hybridization to elucidate the temporal and tissue-specific expression patterns of the mouse kallikreins Klk1, Klk3, Klk5, Klk9, and Klk21 during early development in the embryo, uterus, and decidua. We observed the expression of Klk1 (tissue kallikrein), Klk3 (gamma-nerve growth factor), Klk9 (epidermal growth factor-binding protein), and Klk21 in the early conceptus (until the 2-cell stage). Only Klk21 continued to be expressed in the blastocyst until Day 7.5 of pregnancy. Expression of Klk9 reappeared at Day 7.5 and was consistently detected until Day 11, the last day studied; Klk1 was again expressed in the embryo from Day 9.5, with decreased levels by Day 11. In contrast, in the uterus or decidua, there was no expression of Klk1 until Day 7.5, when mRNA transcripts were abundant; transcripts then decreased in the Day 9.5 and Day 11 uterus. Expression of Klk21 in the uterus and decidua displayed a similar pattern but was detected at much lower levels. Interestingly, a novel Klk21-like mRNA was also detected in uterine tissue samples but not in embryonic samples; Klk3, Klk5, and Klk9 were not consistently expressed in the uterus or decidua over this time. This is the first report of the expression of specific kallikreins during early development. The distinct gene- and tissue-specific expression patterns presented in this study, in conjunction with the well-characterized roles of kallikreins in regulation of protein activation, ECM degradation, and proliferative events, suggests the involvement of the kallikrein gene family during early development.
2. MiR-382 targeting of kallikrein 5 contributes to renal inner medullary interstitial fibrosis. Previously we have shown that microRNA miR-382 can facilitate loss of renal epithelial characteristics in cultured cells. This study examined the in vivo role of miR-382 in the development of renal interstitial fibrosis in a mouse model. Unilateral ureteral obstruction was used to induce renal interstitial fibrosis in mice. With 3 days of unilateral ureteral obstruction, expression of miR-382 in the obstructed kidney was increased severalfold compared with sham-operated controls. Intravenous delivery of locked nucleic acid-modified anti-miR-382 blocked the increase in miR-382 expression and significantly reduced inner medullary fibrosis. Expression of predicted miR-382 target kallikrein 5, a proteolytic enzyme capable of degrading several **extracellular matrix** proteins, was reduced with unilateral ureteral obstruction. Anti-miR-382 treatment prevented the reduction of kallikrein 5 in the inner medulla. Furthermore, the protective effect of the anti-miR-382 treatment against fibrosis was abolished by renal knockdown of kallikrein 5. Targeting of kallikrein 5 by miR-382 was confirmed by 3'-untranslated region luciferase assay. These data support a completely novel mechanism in which miR-382 targets kallikrein 5 and contributes to the development of renal inner medullary interstitial fibrosis. The study provided the first demonstration of an in vivo functional role of miR-382 in any species and any organ system.

---

## St14

- NCBI Gene
- GeneRIF
- Pubmed

| Search Term | Rank | Fields |
| --- | --- | --- |
| [Mm]igration | 6 | Process(1); PubMed(6); GeneRIFs(1) |
| [Ee]xtracellular [Mm]atrix | 8 | PubMed(1) |
| [Ii]nvasive | 10 | PubMed(2) |
| [Mm]etastasis | 12 | PubMed(3) |

  
hide/show details for St14

### [Mm]igration

#### Process

1. cell **migration**

#### PubMed

1. Matriptase is required for the active form of hepatocyte growth factor induced Met, focal adhesion kinase and protein kinase B activation on neural stem/progenitor cell motility. Hepatocyte growth factor (HGF) is a chemoattractant and inducer for neural stem/progenitor (NS/P) cell **migration**. Although the type II transmembrane serine protease, matriptase (MTP) is an activator of the latent HGF, MTP is indispensable on NS/P cell motility induced by the active form of HGF. This suggests that MTP's action on NS/P cell motility involves mechanisms other than proteolytic activation of HGF. In the present study, we investigate the role of MTP in HGF-stimulated signaling events. Using specific inhibitors of phosphatidylinositol-3-kinase (PI3K), protein kinase B (Akt) or focal adhesion kinase (FAK), we demonstrated that in NS/P cells HGF-activated c-Met induces PI3k-Akt signaling which then leads to FAK activation. This signaling pathway ultimately induces MMP2 expression and NS/P cell motility. Knocking down of MTP in NS/P cells with specific siRNA impaired HGF-stimulation of c-Met, Akt and FAK activation, blocked HGF-induced production of MMP2 and inhibited HGF-stimulated NS/P cell motility. MTP-knockdown NS/P cells cultured in the presence of recombinant protein of MTP protease domain or transfected with the full-length wild-type but not the protease-defected MTP restored HGF-responsive events in NS/P cells. In addition to functioning as HGF activator, our data revealed novel function of MTP on HGF-stimulated c-Met signaling activation.
2. Genetic reduction of matriptase-1 expression is associated with a reduction in the aggressive phenotype of prostate cancer cells in vitro and in vivo. Matriptase-1 has been implicated as playing an important role in various types of cancer progression through many different cancer related pathways. In the current study we assessed the efficacy of targeting matriptase-1 using ribozyme technology in vitro and in vivo.Matriptase-1 expression was reduced in the PC-3 and DU-145 cell line using hammerhead ribozyme transgenes. In vitro assays were set up to assess changes in growth, invasion, adhesion and **migration** in these cells. In vivo tumour development model was also used to examine the efficacy of targeting matriptase-1 in a living environment.The in vitro results suggest an overall reduction in the aggressive nature of the two cell lines (PC-3 and DU-145) when matriptase-1 levels are reduced, with properties such as growth, invasiveness and **migration** all being reduced (in most cases a greater than 50% reduction in **migration** and invasion compared to the control was observed), though strangely an increase in adhesion is seen in the PC-3 knockout. The in vitro data is strongly backed up by the results of the in vivo work which demonstrates matriptase-1 deficient cells have a substantially reduced ability to grow and develop in vivo compared to control cells when explanted into nude mice, with significant differences in growth and development (P < or = 0.05) being seen after 7 days, and highly significant differences (p < or = 0.001) after 15 days.Together this data strongly implicates matriptase-1 as playing a vital role in the aggressive nature and progression of prostate cancer.
3. Soluble epithin/PRSS14 secreted from cancer cells contains active angiogenic potential. Epithin (PRSS14/matriptase/ST14), a type II membrane protein, is involved in progression of epithelial cancers and metastasis as well as in the normal epidermal barrier function. When activated, it translocates into the cell-cell contacts and sheds into media. In order to understand the specific mechanism during tumor progression, we tested the angiogenic potential of secreted form of epithin. Epithin produced from the cancer cells shed more in hypoxia and induced motility of endothelial cells. Epithin enhanced the **migration** and invasion of mouse and bovine endothelial cells without cell proliferation. Furthermore, soluble epithin induced endothelial differentiation in the assay of the human endothelial microvessel-like tube formation and in that of the chicken chorioallantoic membrane. The knock-down of epithin in the 427 thymoma cell line abolished the protease activity of secreted epithin fraction, reduced the invasion of endothelial cells through matrigel, and tube formation activity. Only specific antibodies abolished the **migration** of endothelial cell and the vessel morphogenesis, suggesting that epithin specifically functions in these systems. Therefore, we propose that the secreted epithin in the hypoxic cancer microenvironment plays a role as a proangiogenic factor, and can be modulated with specific antibodies.
4. Proteomic-based detection of a protein cluster dysregulated during cardiovascular development identifies biomarkers of congenital heart defects. Cardiovascular development is vital for embryonic survival and growth. Early gestation embryo loss or malformation has been linked to yolk sac vasculopathy and congenital heart defects (CHDs). However, the molecular pathways that underlie these structural defects in humans remain largely unknown hindering the development of molecular-based diagnostic tools and novel therapies.Murine embryos were exposed to high glucose, a condition known to induce cardiovascular defects in both animal models and humans. We further employed a mass spectrometry-based proteomics approach to identify proteins differentially expressed in embryos with defects from those with normal cardiovascular development. The proteins detected by mass spectrometry (WNT16, ST14, Pcsk1, Jumonji, Morca2a, TRPC5, and others) were validated by Western blotting and immunoflorescent staining of the yolk sac and heart. The proteins within the proteomic dataset clustered to adhesion/**migration**, differentiation, transport, and insulin signaling pathways. A functional role for several proteins (WNT16, ADAM15 and NOGO-A/B) was demonstrated in an ex vivo model of heart development. Additionally, a successful application of a cluster of protein biomarkers (WNT16, ST14 and Pcsk1) as a prenatal screen for CHDs was confirmed in a study of human amniotic fluid (AF) samples from women carrying normal fetuses and those with CHDs.The novel finding that WNT16, ST14 and Pcsk1 protein levels increase in fetuses with CHDs suggests that these proteins may play a role in the etiology of human CHDs. The information gained through this bed-side to bench translational approach contributes to a more complete understanding of the protein pathways dysregulated during cardiovascular development and provides novel avenues for diagnostic and therapeutic interventions, beneficial to fetuses at risk for CHDs.
5. Endogenous expression of matriptase in neural progenitor cells promotes cell **migration** and neuron differentiation. Recent studies show that type II transmembrane serine proteases play important roles in diverse cellular activities and pathological processes. Their expression and functions in the central nervous system, however, are largely unexplored. In this study, we show that the expression of one such member, matriptase (MTP), was cell type-restricted and primarily expressed in neural progenitor (NP) cells and neurons. Blocking MTP expression or MTP activity prevented NP cell traverse of reconstituted basement membrane, whereas overexpression of MTP promoted it. The NP cell mobilization induced by either vascular endothelial growth factor or hepatocyte growth factor was also impaired by knocking down MTP expression. MTP acts upstream of matrix metalloproteinase 2 in promoting NP cell mobility. In embryonic stem cell differentiation to neural cells, MTP knockdown had no effect on entry of embryonic stem cells into the neural lineage. High MTP expression or activity, however, shifts the population dynamics from NP cells toward neurons to favor neuronal differentiation. This is the first report to demonstrate the direct involvement of type II transmembrane serine protease in NP cell function.
6. Epithin, a target of transforming growth factor-beta signaling, mediates epithelial-mesenchymal transition. The epithelial-derived type II transmembrane serine protease epithin has been shown to be upregulated in a variety of cancer cell lines and tumor tissues, and its upregulation correlates well with tumor progression in many cases. However, little is known regarding the regulation of its expression and the mechanism of its roles in tumor progression. Here, we show that transforming growth factor-beta (TGF-beta), a potent inducer of epithelial-mesenchymal transition (EMT) in tumor progression, upregulates epithin, and that epithin plays a critical role in TGF-beta-induced EMT. Forced overexpression of epithin induced EMT to exhibit characteristic morphological changes, alternations in EMT-related proteins and enhanced cell motility. Conversely, shRNA-mediated knockdown of endogenous epithin inhibited TGF-beta-induced expression of mesenchymal markers and morphological changes. Furthermore, TGF-beta-induced cell **migration** and invasion were significantly impaired by epithin knockdown. In addition, we demonstrate that TGF-beta upregulates epithin transcriptionally via the Smad2/Smad4-mediated pathway. These results suggest that epithin is a key mediator of TGF-beta-induced EMT in tumor progression.

#### GeneRIFs

1. Endogenous expression of matriptase in neural progenitor cells promotes cell **migration** and neuron differentiation.

### [Ee]xtracellular [Mm]atrix

#### PubMed

1. Inhibition of tumor invasion by genomic down-regulation of matriptase through suppression of activation of receptor-bound pro-urokinase. Urokinase-type plasminogen activator (uPA) degrades the **extracellular matrix** and plays critical roles in tumor invasion and metastasis. Matriptase, a membrane-bound serine protease, was shown to activate uPA in a uPA receptor-free, solution-based study. We now investigate whether matriptase affects activation of receptor-bound uPA and contributes to the invasiveness of HRA human ovarian cancer cells in vitro and tumor behavior in nude mice. Here we show the following. 1) uPA expression was effectively stimulated by TGF-beta1 in HRA cells. 2) Antisense (AS)-matriptase transfection achieved a marked inhibition of receptor-bound pro-uPA activation without altering expression of uPA and uPA receptor mRNA and proteins, irrespective of whether cells were stimulated with TGF-beta1. 3) Tumor cell receptor-bound pro-uPA could be efficiently cleaved by matriptase to generate enzymatically active two-chain uPA. Thus, matriptase can substitute for plasmin in the proteolytic activation of pro-uPA to enzymatically active uPA. 4) The AS-matriptase-treated cells had a decreased ability to invade an **extracellular matrix** layer, as compared with control cells. 5) When the AS-matriptase-treated cells were injected intraperitoneally into nude mice, the mice developed smaller tumors. Our data identify a novel role for matriptase for activation of receptor-bound uPA.

### [Ii]nvasive

#### PubMed

1. Genetic reduction of matriptase-1 expression is associated with a reduction in the aggressive phenotype of prostate cancer cells in vitro and in vivo. Matriptase-1 has been implicated as playing an important role in various types of cancer progression through many different cancer related pathways. In the current study we assessed the efficacy of targeting matriptase-1 using ribozyme technology in vitro and in vivo.Matriptase-1 expression was reduced in the PC-3 and DU-145 cell line using hammerhead ribozyme transgenes. In vitro assays were set up to assess changes in growth, invasion, adhesion and migration in these cells. In vivo tumour development model was also used to examine the efficacy of targeting matriptase-1 in a living environment.The in vitro results suggest an overall reduction in the aggressive nature of the two cell lines (PC-3 and DU-145) when matriptase-1 levels are reduced, with properties such as growth, **invasive**ness and migration all being reduced (in most cases a greater than 50% reduction in migration and invasion compared to the control was observed), though strangely an increase in adhesion is seen in the PC-3 knockout. The in vitro data is strongly backed up by the results of the in vivo work which demonstrates matriptase-1 deficient cells have a substantially reduced ability to grow and develop in vivo compared to control cells when explanted into nude mice, with significant differences in growth and development (P < or = 0.05) being seen after 7 days, and highly significant differences (p < or = 0.001) after 15 days.Together this data strongly implicates matriptase-1 as playing a vital role in the aggressive nature and progression of prostate cancer.
2. Inhibition of tumor invasion by genomic down-regulation of matriptase through suppression of activation of receptor-bound pro-urokinase. Urokinase-type plasminogen activator (uPA) degrades the extracellular matrix and plays critical roles in tumor invasion and metastasis. Matriptase, a membrane-bound serine protease, was shown to activate uPA in a uPA receptor-free, solution-based study. We now investigate whether matriptase affects activation of receptor-bound uPA and contributes to the **invasive**ness of HRA human ovarian cancer cells in vitro and tumor behavior in nude mice. Here we show the following. 1) uPA expression was effectively stimulated by TGF-beta1 in HRA cells. 2) Antisense (AS)-matriptase transfection achieved a marked inhibition of receptor-bound pro-uPA activation without altering expression of uPA and uPA receptor mRNA and proteins, irrespective of whether cells were stimulated with TGF-beta1. 3) Tumor cell receptor-bound pro-uPA could be efficiently cleaved by matriptase to generate enzymatically active two-chain uPA. Thus, matriptase can substitute for plasmin in the proteolytic activation of pro-uPA to enzymatically active uPA. 4) The AS-matriptase-treated cells had a decreased ability to invade an extracellular matrix layer, as compared with control cells. 5) When the AS-matriptase-treated cells were injected intraperitoneally into nude mice, the mice developed smaller tumors. Our data identify a novel role for matriptase for activation of receptor-bound uPA.

### [Mm]etastasis

#### PubMed

1. Soluble epithin/PRSS14 secreted from cancer cells contains active angiogenic potential. Epithin (PRSS14/matriptase/ST14), a type II membrane protein, is involved in progression of epithelial cancers and **metastasis** as well as in the normal epidermal barrier function. When activated, it translocates into the cell-cell contacts and sheds into media. In order to understand the specific mechanism during tumor progression, we tested the angiogenic potential of secreted form of epithin. Epithin produced from the cancer cells shed more in hypoxia and induced motility of endothelial cells. Epithin enhanced the migration and invasion of mouse and bovine endothelial cells without cell proliferation. Furthermore, soluble epithin induced endothelial differentiation in the assay of the human endothelial microvessel-like tube formation and in that of the chicken chorioallantoic membrane. The knock-down of epithin in the 427 thymoma cell line abolished the protease activity of secreted epithin fraction, reduced the invasion of endothelial cells through matrigel, and tube formation activity. Only specific antibodies abolished the migration of endothelial cell and the vessel morphogenesis, suggesting that epithin specifically functions in these systems. Therefore, we propose that the secreted epithin in the hypoxic cancer microenvironment plays a role as a proangiogenic factor, and can be modulated with specific antibodies.
2. Shedding of epithin/PRSS14 is induced by TGF-β and mediated by tumor necrosis factor-α converting enzyme. Epithin/PRSS14, a type II transmembrane serine protease, plays critical roles in cance**r metastas**is. Previously, we have reported that epithin/PRSS14 undergoes ectodomain shedding in response to phorbol myristate acetate (PMA) stimulation. In this study, we show that transforming growth factor-β (TGF-β) induces rapid epithin/PRSS14 shedding through receptor mediated pathway in 427.1.86 thymoma cells. Tumor necrosis factor-α converting enzyme (TACE) is responsible for this shedding. Amino acid sequence encompassing the putative shedding cleavage site of epithin/PRSS14 exhibit strong homology to the cleavage site of l-selectin, a known TACE substrate. TACE inhibitor, TAPI-0 and TACE siRNA greatly reduced TGF-β-induced epithin/PRSS14 shedding. TGF-β treatment induces translocation of intracellular pool of TACE to the membrane where epithin/PRSS14 resides. These findings suggest that TGF-β induces epithin/PRSS14 shedding by mediating translocation of epithin/PRSS14 sheddase, TACE, to the membrane.
3. Inhibition of tumor invasion by genomic down-regulation of matriptase through suppression of activation of receptor-bound pro-urokinase. Urokinase-type plasminogen activator (uPA) degrades the extracellular matrix and plays critical roles in tumor invasion and **metastasis**. Matriptase, a membrane-bound serine protease, was shown to activate uPA in a uPA receptor-free, solution-based study. We now investigate whether matriptase affects activation of receptor-bound uPA and contributes to the invasiveness of HRA human ovarian cancer cells in vitro and tumor behavior in nude mice. Here we show the following. 1) uPA expression was effectively stimulated by TGF-beta1 in HRA cells. 2) Antisense (AS)-matriptase transfection achieved a marked inhibition of receptor-bound pro-uPA activation without altering expression of uPA and uPA receptor mRNA and proteins, irrespective of whether cells were stimulated with TGF-beta1. 3) Tumor cell receptor-bound pro-uPA could be efficiently cleaved by matriptase to generate enzymatically active two-chain uPA. Thus, matriptase can substitute for plasmin in the proteolytic activation of pro-uPA to enzymatically active uPA. 4) The AS-matriptase-treated cells had a decreased ability to invade an extracellular matrix layer, as compared with control cells. 5) When the AS-matriptase-treated cells were injected intraperitoneally into nude mice, the mice developed smaller tumors. Our data identify a novel role for matriptase for activation of receptor-bound uPA.

---

## Gzma

- NCBI Gene
- GeneRIF
- Pubmed

| Search Term | Rank | Fields |
| --- | --- | --- |
| [Mm]igration | 6 | PubMed(1) |

  
hide/show details for Gzma

### [Mm]igration

#### PubMed

1. Granzyme A Is Required for Regulatory T-Cell Mediated Prevention of Gastrointestinal Graft-versus-Host Disease. In our previous work we could identify defects in human regulatory T cells (Tregs) likely favoring the development of graft-versus-host disease (GvHD) following allogeneic stem cell transplantation (SCT). Treg transcriptome analyses comparing GvHD and immune tolerant patients uncovered regulated gene transcripts highly relevant for Treg cell function. Moreover, granzyme A (GZMA) also showed a significant lower expression at the protein level in Tregs of GvHD patients. GZMA induces cytolysis in a perforin-dependent, FAS-FASL independent manner and represents a cell-contact dependent mechanism for Tregs to control immune responses. We therefore analyzed the functional role of GZMA in a murine standard model for GvHD. For this purpose, adoptively transferred CD4+CD25+ Tregs from gzmA-/- mice were analyzed in comparison to their wild type counterparts for their capability to prevent murine GvHD. GzmA-/- Tregs home efficiently to secondary lymphoid organs and do not show phenotypic alterations with respect to activation and **migration** properties to inflammatory sites. Whereas gzmA-/- Tregs are highly suppressive in vitro, Tregs require GZMA to rescue hosts from murine GvHD, especially regarding gastrointestinal target organ damage. We herewith identify GZMA as critical effector molecule of human Treg function for gastrointestinal immune response in an experimental GvHD model.

---

## Zap70

- NCBI Gene
- GeneRIF
- Pubmed

| Search Term | Rank | Fields |
| --- | --- | --- |
| [Ff]ilopodia | 1 | PubMed(1) |
| [Mm]igration | 6 | Process(1); PubMed(5); GeneRIFs(2) |

  
hide/show details for Zap70

### [Ff]ilopodia

#### PubMed

1. The immune molecule CD3zeta and its downstream effectors ZAP-70/Syk mediate ephrin signaling in neurons to regulate early neuritogenesis. Recent studies have highlighted the key role of the immune protein CD3ζ in the maturation of neuronal circuits in the CNS. Yet, the upstream signals that might recruit and activate CD3ζ in neurons are still unknown. In this study, we show that CD3ζ functions early in neuronal development and we identify ephrinA1-dependent EphA4 receptor activation as an upstream regulator of CD3ζ. When newly born neurons are still spherical, before neurite extension, we found a transient CD3ζ aggregation at the cell periphery matching the initiation site of the future neurite. This accumulation of CD3ζ correlated with a stimulatory effe**ct on fil**opodia extension via a Rho-GEF Vav2 pathway and a repression of neurite outgrowth. Conversely, cultured neurons lacking CD3ζ isolated from CD3ζ(-/-) mice showed a decreased nu**mber of f**ilopodia and an enhanced neurite number. Stimulation with ephrinA1 induces the translocation of both CD3ζ and its activated effector molecules, ZAP-70/Syk tyrosine kinases, to EphA4 receptor clusters. EphrinA1-induced growth cone collapse was abrogated in CD3ζ(-/-) neurons and was markedly reduced by ZAP-70/Syk inhibition. Moreover, ephrinA1-induced ZAP-70/Syk activation was inhibited in CD3ζ(-/-) neurons. Altogether, our data suggest that CD3ζ mediates the ZAP-70/Syk kinase activation triggered by ephrinA-activated pathway to regulate early neuronal morphogenesis.

### [Mm]igration

#### Process

1. cell **migration**

#### PubMed

1. CBAP functions as a novel component in chemokine-induced ZAP70-mediated T-cell adhesion and **migration**. Activated chemokine receptor initiates inside-out signaling to transiently trigger activation of integrins, a process involving multiple components that have not been fully characterized. Here we report that GM-CSF/IL-3/IL-5 receptor common beta-chain-associated protein (CBAP) is required to optimize this inside-out signaling and activation of integrins. First, knockdown of CBAP expression in human Jurkat T cells caused attenuated CXC chemokine ligand-12 (CXCL12)-induced cell **migration** and integrin α4β1- and αLβ2-mediated cell adhesion in vitro, which could be rescued sufficiently upon expression of murine CBAP proteins. Freshly isolated CBAP-deficient primary T cells also exhibited diminution of chemotaxis toward CC chemokine ligand-21 (CCL21) and CXCL12, and these chemokines-induced T-cell adhesions in vitro. Adoptive transfer of isolated naive T cells demonstrated that CBAP deficiency significantly reduced lymph node homing ability in vivo. Final**ly, migra**tion of T cell-receptor-activated T cells induced by inflammatory chemokines was also attenuated in CBAP-deficient cells. Further analyses revealed that CBAP constitutively associated with both integrin β1 and ZAP70 and that CBAP is required for chemokine-induced initial binding of the talin-Vav1 complex to integrin β1 and to facilitate subsequent ZAP70-mediated dissociation of the talin-Vav1 complex and Vav1 phosphorylation. Within such an integrin signaling complex, CBAP likely functions as an adaptor and ultimately leads to activation of both integrin α4β1 and Rac1. Taken together, our data suggest that CBAP indeed can function as a novel signaling component within the ZAP70/Vav1/talin complex and plays an important role in regulating chemokine-promoted T-cell trafficking.
2. Intrathymic progenitor cell transplantation across histocompatibility barriers results in the persistence of early thymic progenitors and T-cell differentiation. Donor hematopoietic stem cells (HSCs) can correct T-cell deficiencies in patients with severe combined immunodeficiency by replacing resident thymus cells. However, as those progenitors that naturally migrate to the thymus are not capable of supporting long-term thymopoiesis, a successful transplant is thought to require the ongoing **migration** of donor progenitors. We previously showed that the forced intrathymic administration of histocompatible HSCs can sustain long-term thymopoiesis in ZAP-70-immunodeficient mice. However, it is not known whether T-cell reconstitution across histocompatibility barriers is modulated by intrathymic vs intravenous administration of HSCs. In the absence of conditioning, long-term thymopoiesis by semiallogeneic progenitors was detected in mice transplanted via the intrathymic, but not the intravenous, route. In intrathymic-transplanted mice, ongoing thymopoiesis was associated with a 10-fold higher level of early thymic progenitors (ETPs). The enhanced reconstitution capacity of these intrathymic-derived ETPs was corroborated by their significantly augmented myeloid lineage potential compared with endogenous ETPs. Notably, though, myeloablative conditioning resulted in a reduced expansion of intrathymic-administered donor ETPs. Thus, in the absence of conditioning, the forced thymic entry of HSCs results in a sustained T-cell development across histocompatibility barriers, highlighting the capacity of the thymus to support cells with long-term renewal potential.
3. ZAP-70 promotes the infiltration of malignant B-lymphocytes into the bone marrow by enhancing signaling and **migration** after CXCR4 stimulation. ZAP-70 in chronic lymphocytic leukemia (CLL) is associated with enhanced response to microenvironmental stimuli. We analyzed the functional consequences of ZAP-70 ectopic expression in malignant B-cells in a xenograft mouse model of disseminated B-cell leukemia. Mice injected with B-cells expressing ZAP-70 showed a prominently higher infiltration of the bone marrow. In vitro analysis of the response of malignant B-cells to CXCL12, the main attracting chemokine regulating trafficking of lymphocytes to the bone marrow, or to bone marrow stromal cells, revealed that ZAP-70 induces an increased response in terms of signaling and **migration**. These effects are probably mediated by direct participation of ZAP-70 in CXCL12-CXCR4 signaling since CXCR4 stimulation led to activation of ZAP-70 and downstream signaling pathways, such as MAPK and Akt, whereas ZAP-70 did not alter the expression of the CXCR4 receptor. In addition, subclones of primary CLL cells with high expression of ZAP-70 also showed increased migrative capacity toward CXCL12. Neutralization of CXCR4 with a monoclonal antibody resulted in impaired in vitro responses to CXCL12 and bone marrow stromal cells. We conclude that ZAP-70 enhances the **migration** of malignant B-cells into the supportive microenvironment found in the bone marrow mainly by enhancing signaling and **migration** after CXCR4 stimulation.
4. MIF interacts with CXCR7 to promote receptor internalization, ERK1/2 and ZAP-70 signaling, and lymphocyte chemotaxis. Macrophage **migration**-inhibitory factor (MIF) is a pleiotropic cytokine with chemokine-like functions and is a mediator in numerous inflammatory conditions. Depending on the context, MIF signals through 1 or more of its receptors cluster of differentiation (CD)74, CXC-motif chemokine receptor (CXCR)2, and CXCR4. In addition, heteromeric receptor complexes have been identified. We characterized the atypical chemokine receptor CXCR7 as a novel receptor for MIF. MIF promoted human CXCR7 internalization up to 40%, peaking at 50-400 nM and 30 min, but CXCR7 internalization by MIF was not dependent on CXCR4. Yet, by coimmunoprecipitation, fluorescence microscopy, and a proximity ligation assay, CXCR7 was found to engage in MIF receptor complexes with CXCR4 and CD74, both after ectopic overexpression and in endogenous conditions in a human B-cell line. Receptor competition binding and coimmunoprecipitation studies combined with sulfo-SBED-biotin-transfer provided evidence for a direct interaction between MIF and CXCR7. Finally, we demonstrated MIF/CXCR7-mediated functional responses. Blockade of CXCR7 suppressed MIF-mediated ERK- and zeta-chain-associated protein kinase (ZAP)-70 activation (from 2.1- to 1.2-fold and from 2.5- to 1.6-fold, respectively) and fully abrogated primary murine B-cell chemotaxis triggered by MIF, but not by CXCL12. B cells from Cxcr7(-/-) mice exhibited an ablated trans**migration** response to MIF, indicating that CXCR7 is essential for MIF-promoted B-cell **migration**. Our findings provide biochemical and functional evidence that MIF is an alternative ligand of CXCR7 and suggest a functional role of the MIF-CXCR7 axis in B-lymphocyte **migration**.
5. MIF promotes B cell chemotaxis through the receptors CXCR4 and CD74 and ZAP-70 signaling. Macrophage **migration** inhibitory factor (MIF) is a proinflammatory cytokine with chemokine-like functions that plays a pivotal role in the pathogenesis of inflammatory diseases by promoting leukocyte recruitment. We showed that MIF promotes the atherogenic recruitment of monocytes and T cells through its receptors CXCR2 and CXCR4. Effects of MIF on B cell recruitment have not been addressed. In this study, we tested the involvement of MIF in B cell chemotaxis and studied the underlying mechanism. We show that MIF promotes primary murine B cell chemotaxis in a dose-dependent manner, comparable to the B cell chemokines CXCL13 and CXCL12. Splenic B cells express CXCR4 and the receptor CD74 but not CXCR2. Inhibition of CXCR4 or CD74 or a genetic deficiency of Cd74 in primary B cells fully abrogated MIF-mediated B cell **migration**, implying cooperative involvement of both receptors. MIF stimulation of B cells resulted in a rapid increase in intracellular Ca(2+) mobilization and F-actin polymerization. Intriguingly, the tyrosine kinase ZAP-70 was activated upon MIF and CXCL12 treatment in a CXCR4- and CD74-dependent manner. Pharmacological inhibition of ZAP-70 resulted in abrogation of primary B cell **migration**. Functional involvement of ZAP-70 was confirmed by small interfering RNA-mediated knockdown in Ramos B cell **migration**. Finally, primary B cells from ZAP-70 gene-deficient mice exhibited ablated trans**migration** in response to MIF or CXCL12. We conclude that MIF promotes the **migration** of B cells through a ZAP-70-dependent pathway mediated by cooperative engagement of CXCR4 and CD74. The data also suggest that MIF may contribute to B cell recruitment in vivo (e.g., in B cell-related immune disorders).

#### GeneRIFs

1. ZAP-70 enhances the **migration** of malignant B-cells into the supportive microenvironment found in the bone marrow mainly by enhancing signaling and **migration** after CXCR4 stimulation.
2. MIF promotes the **migration** of B cells through a ZAP-70-dependent pathway mediated by cooperative engagement of CXCR4 and CD74.

---

## Syk

- NCBI Gene
- GeneRIF
- Pubmed

| Search Term | Rank | Fields |
| --- | --- | --- |
| [Ff]ilopodia | 1 | PubMed(3) |
| [Ii]nvadopodia | 3 | PubMed(1) |
| [Mm]igration | 6 | Process(1); PubMed(22); GeneRIFs(5) |
| [Ee]xtracellular [Mm]atrix | 8 | PubMed(4) |
| [Ii]nvasive | 10 | PubMed(2); GeneRIFs(1) |
| [Mm]etastasis | 12 | PubMed(3) |

  
hide/show details for Syk

### [Ff]ilopodia

#### PubMed

1. Complement receptor-3 negatively regulates the phagocytosis of degenerated myelin through tyrosine kinase Syk and cofilin. Intact myelin, which normally surrounds axons, breaks down in Wallerian degeneration following axonal injury and during neurodegenerative diseases such as multiple sclerosis. Clearance of degenerated myelin by phagocytosis is essential since myelin impedes repair and exacerbates damage. CR3 (complement receptor-3) is a principal phagocytic receptor in myelin phagocytosis. We studied how tyrosine kinase Syk (spleen tyrosine kinase) and cofilin control phagocytosis of degenerated myelin by CR3 in microglia and macrophages. Syk is a non-receptor tyrosine kinase that CR3 recruits to convey cellular functions. Cofilin is an actin-depolymerizing protein that controls F-actin (filamentous actin) remodeling (i.e., disassembly and reassembly) by shifting between active unphosphorylated and inactive phosphorylated states.Syk was continuously activated during prolonged phagocytosis. Phagocytosis increased when Syk activity and expression were reduced, suggesting that normally Syk down regulates CR3-mediated myelin phagocytosis. Levels of inactive p-cofilin (phosphorylated cofilin) decreased transiently during prolonged phagocytosis. In contrast, p-cofilin levels decreased continuously when Syk activity and expression were continuously reduced, suggesting that normally Syk advances the inactive state of cofilin. Observations also revealed inverse relationships between levels of phagocytosis and levels of inactive p-cofilin, suggesting that active unphosphorylated cofilin advances phagocytosis. Active cofilin could advance phagocytosis by promoting F-actin remodeling, which supports the production of membrane protrusions (e.g., **filopodia**), which, as we also revealed, are instrumental in myelin phagocytosis.CR3 both activates and downregulates myelin phagocytosis at the same time. Activation was previously documented. We presently demonstrate that downregulation is mediated through Syk, which advances the inactive phosphorylated state of cofilin. Self-negative control of phagocytosis by the phagocytic receptor can be useful in protecting phagocytes from excessive phagocytosis (i.e., "overeating") during extended exposure to particles that are destined for ingestion.
2. Coordinate interactions of Csk, Src, and Syk kinases with [alpha]IIb[beta]3 initiate integrin signaling to the cytoskeleton. Integrins regulate cell adhesion and motility through tyrosine kinases, but initiation of this process is poorly understood. We find here that Src associates constitutively with integrin alphaIIbbeta3 in platelets. Platelet adhesion to fibrinogen caused a rapid increase in alphaIIbbeta3-associated Src activity, and active Src localized to **filopodia** and cell edges. Csk, which negatively regulates Src by phosphorylating Tyr-529, was also constitutively associated with alphaIIbbeta3. However, fibrinogen binding caused Csk to dissociate from alphaIIbbeta3, concomitant with dephosphorylation of Src Tyr-529 and phosphorylation of Src activation loop Tyr-418. In contrast to the behavior of Src and Csk, Syk was associated with alphaIIbbeta3 only after fibrinogen binding. Platelets multiply deficient in Src, Hck, Fgr, and Lyn, or normal platelets treated with Src kinase inhibitors failed to spread on fibrinogen. Inhibition of Src kinases blocked Syk activation and inhibited phosphorylation of Syk substrates (Vav1, Vav3, SLP-76) implicated in cytoskeletal regulation. Syk-deficient platelets exhibited Src activation upon adhesion to fibrinogen, but no spreading or phosphorylation of Vav1, Vav3, and SLP-76. These studies establish that platelet spreading on fibrinogen requires sequential activation of Src and Syk in proximity to alphaIIbbeta3, thus providing a paradigm for initiation of integrin signaling to the actin cytoskeleton.
3. Laminin stimulates spreading of platelets through integrin alpha6beta1-dependent activation of GPVI. The extracellular matrix protein, laminin, supports platelet adhesion through binding to integrin alpha6beta1 In the present study, we demonstrate that human laminin, purified from placenta, also stimulates formation of **filopodia** and lamellipodia in human and mouse platelets through a pathway that is dependent on alpha6beta1 and the collagen receptor GPVI. The integrin alpha6beta1 is essential for adhesion to laminin, as demonstrated using an alpha6-blocking antibody, whereas GPVI is dispensable for this response, as shown using "knockout" mouse platelets. On the other hand, lamellipodia formation on laminin is completely inhibited in the absence of GPVI, although **filopodia** formation remains and is presumably mediated via alpha6beta1 Lamellipodia and **filopodia** formation are inhibited in Syk-deficient platelets, demonstrating a key role for the kinase in signaling downstream of GPVI and integrin alpha6beta1 GPVI was confirmed as a receptor for laminin using surface plasmon resonance spectroscopy and by demonstration of lamellipodia formation on laminin in the presence of collagenase. These results identify GPVI as a novel receptor for laminin and support a model in which integrin alpha6beta1 brings laminin to GPVI, which in turn mediates lamellipodia formation. We speculate that laminin contributes to platelet spreading in vivo through a direct interaction with GPVI.

### [Ii]nvadopodia

#### PubMed

1. Tumor suppressor function of Syk in human MCF10A in vitro and normal mouse mammary epithelium in vivo. The normal function of Syk in epithelium of the developing or adult breast is not known, however, Syk suppresses tumor growth, invasion, and metastasis in breast cancer cells. Here, we demonstrate that in the mouse mammary gland, loss of one Syk allele profoundly increases proliferation and ductal branching and invasion of epithelial cells through the mammary fat pad during puberty. Mammary carcinomas develop by one year. Syk also suppresses proliferation and invasion in vitro. siRNA or shRNA knockdown of Syk in MCF10A breast epithelial cells dramatically increased proliferation, anchorage independent growth, cellular motility, and invasion, with formation of functional, extracellular matrix-degrading **invadopodia**. Morphological and gene microarray analysis following Syk knockdown revealed a loss of luminal and differentiated epithelial features with epithelial to mesenchymal transition and a gain in **invadopodia**l cell surface markers CD44, CD49F, and MMP14. These results support the role of Syk in limiting proliferation and invasion of epithelial cells during normal morphogenesis, and emphasize the critical role of Syk as a tumor suppressor for breast cancer. The question of breast cancer risk following systemic anti-Syk therapy is raised since only partial loss of Syk was sufficient to induce mammary carcinomas.

### [Mm]igration

#### Process

1. cell **migration**

#### PubMed

1. Rac2 specificity in macrophage integrin signaling: potential role for Syk kinase. Herein we report that, despite the similarity of Rac2 to Rac1 (92% amino acid identity), macrophages derived from Rac2-/- mice, which continue to express Rac1, display a marked defect in alphavbeta3/alphavbeta5 and alpha4beta1 integrin-directed **migration** measured on vitronectin and fibronectin fragments (FN-H296), respectively. In contrast, mouse embryo fibroblasts derived from the Rac2 knockout mice utilize Rac1 for **migration** via alphavbeta3/alphavbeta5 and alpha4beta1. The genetic reconstitution of bone marrow-derived macrophages (BMM) with Rac2 restores the integrin-dependent **migration** of Rac2-deficient macrophages on vitronectin (VN) and FN-H296. The levels of GTP-Rac2 generated upon specific integrin engagement in wild type macrophages parallels the phenotypic defect observed in Rac2-deficient macrophages; i.e. FN-H296, alpha4beta1 > VN, alphavbeta3/alphavbeta5 > FN-CH271, alpha5beta1 > intact FN. In a COS7 cell system, the expression of Syk kinase alone is sufficient to convert the alpha4beta1 **migration** response to Rac2 dependence. Therefore, we present the first evidence that the alpha4beta1 receptor in blood cells has evolved a Syk-Rac2 signaling axis to transmit signals required for integrin-directed **migration** suggesting that Syk kinase in part encodes myeloid Rac2 specificity in vivo.
2. Critical roles for Rac1 and Rac2 GTPases in B cell development and signaling. The Rac1 guanosine triphosphatase (GTPase) has been implicated in multiple cellular functions, including actin dynamics, proliferation, apoptosis, adhesion, and **migration** resulting from signaling by multiple receptors, including the B cell antigen receptor (BCR). We used conditional gene targeting to generate mice with specific Rac1 deficiency in the B cell lineage. In the absence of both Rac1 and the highly related Rac2, B cell development was almost completely blocked. Both GTPases were required to transduce BCR signals leading to proliferation, survival and up-regulation of BAFF-R, a receptor for BAFF, a key survival molecule required for B cell development and maintenance.
3. Role of the docking protein Gab2 in beta(1)-integrin signaling pathway-mediated hematopoietic cell adhesion and **migration**. Gab2, a newly identified pleckstrin homology domain-containing docking protein, is a major binding protein of SHP-2 tyrosine phosphatase in interleukin (IL)-3-stimulated hematopoietic cells. Its signaling mechanism remains largely unknown. We report here an important regulatory role for Gab2 in beta(1) integrin signaling pathway that mediates hematopoietic cell adhesion and **migration**. Cross-linking of the beta(1) integrin on Ba/F3 cells induced rapid tyrosine phosphorylation of Gab2 and its association with Syk kinase, SHP-2 phosphatase, and the p85 subunit of phosphatidylinositol (PI)-3 kinase. In addition, Gab2 was also constitutively associated with SHP-1 phosphatase via its C-terminal Src homology 2 domain. Overexpression of the pleckstrin homology domain or a mutant Gab2 molecule lacking SHP-2 binding sites resulted in significant reductions in Ba/F3 cell adhesion and **migration**. Biochemical analyses revealed that enforced expression of Gab2 mutant molecules dramatically reduced beta(1)-integrin ligation-triggered PI3 kinase activation, whereas Erk kinase activation remained unaltered. Furthermore, transduction of primary hematopoietic progenitor cells from viable motheaten mice with these mutant Gab2 molecules also significantly ameliorated their enhanced **migration** capacity associated with the SHP1 gene mutation. Taken together, these results suggest an important signaling role for Gab2 in regulating hematopoietic cell adhesion and **migration**.
4. A novel Rac-dependent checkpoint in B cell development controls entry into the splenic white pulp and cell survival. Rac1 and Rac2 GTPases transduce signals from multiple receptors leading to cell **migration**, adhesion, proliferation, and survival. In the absence of Rac1 and Rac2, B cell development is arrested at an IgD- transitional B cell stage that we term transitional type 0 (T0). We show that T0 cells cannot enter the white pulp of the spleen until they mature into the T1 and T2 stages, and that this entry into the white pulp requires integrin and chemokine receptor signaling and is required for cell survival. In the absence of Rac1 and Rac2, transitional B cells are unable to migrate in response to chemokines and cannot enter the splenic white pulp. We propose that loss of Rac1 and Rac2 causes arrest at the T0 stage at least in part because transitional B cells need to migrate into the white pulp to receive survival signals. Finally, we show that in the absence of Syk, a kinase that transduces B cell antigen receptor signals required for positive selection, development is arrested at the same T0 stage, with transitional B cells excluded from the white pulp. Thus, these studies identify a novel developmental checkpoint that coincides with B cell positive selection.
5. Rac2 specificity in macrophage integrin signaling: potential role for Syk kinase. Herein we report that, despite the similarity of Rac2 to Rac1 (92% amino acid identity), macrophages derived from Rac2-/- mice, which continue to express Rac1, display a marked defect in alphavbeta3/alphavbeta5 and alpha4beta1 integrin-directed **migration** measured on vitronectin and fibronectin fragments (FN-H296), respectively. In contrast, mouse embryo fibroblasts derived from the Rac2 knockout mice utilize Rac1 for **migration** via alphavbeta3/alphavbeta5 and alpha4beta1. The genetic reconstitution of bone marrow-derived macrophages (BMM) with Rac2 restores the integrin-dependent **migration** of Rac2-deficient macrophages on vitronectin (VN) and FN-H296. The levels of GTP-Rac2 generated upon specific integrin engagement in wild type macrophages parallels the phenotypic defect observed in Rac2-deficient macrophages; i.e. FN-H296, alpha4beta1 > VN, alphavbeta3/alphavbeta5 > FN-CH271, alpha5beta1 > intact FN. In a COS7 cell system, the expression of Syk kinase alone is sufficient to convert the alpha4beta1 **migration** response to Rac2 dependence. Therefore, we present the first evidence that the alpha4beta1 receptor in blood cells has evolved a Syk-Rac2 signaling axis to transmit signals required for integrin-directed **migration** suggesting that Syk kinase in part encodes myeloid Rac2 specificity in vivo.
6. Critical role of Src-Syk-PLC{gamma}2 signaling in megakaryocyte **migration** and thrombopoiesis. **Migration** of megakaryocytes (MKs) from the proliferative osteoblastic niche to the capillary-rich vascular niche is essential for proplatelet formation and platelet release. In this study, we explore the role of surface glycoprotein receptors and signaling proteins in regulating MK **migration** and platelet recovery after immune-induced thrombocytopenia. We show that spreading and **migration** of mouse primary bone marrow-derived MKs on a fibronectin matrix are abolished by the Src family kinases inhibitor PP1, the Syk kinase inhibitor R406 and the integrin alphaIIbbeta3 antagonist lotrafiban. We also demonstrate that these responses are inhibited in primary phospholipase C gamma2 (PLCgamma2)-deficient MKs. Conversely, MK spreading and **migration** were unaltered in the absence of the collagen receptor, the glycoprotein VI-FcRgamma-chain complex. We previously reported a correlation between a defect in MK **migration** and platelet recovery in the absence of platelet endothelial cell adhesion molecule-1 and the tyrosine phosphatase CD148. This correlation also holds for mice deficient in PLCgamma2. This study identifies a model in which integrin signaling via Src family kinases and Syk kinase to PLCgamma2 is required for MK spreading, **migration**, and platelet formation.
7. C-type lectin receptors differentially induce th17 cells and vaccine immunity to the endemic mycosis of North America. Vaccine immunity to the endemic mycoses of North America requires Th17 cells, but the pattern recognition receptors and signaling pathways that drive these protective responses have not been defined. We show that C-type lectin receptors exert divergent contributions to the development of antifungal Th17 cells and vaccine resistance against Blastomyces dermatitidis, Histoplasma capsulatum, and Coccidioides posadasii. Acquired immunity to B. dermatitidis requires Dectin-2, whereas vaccination against H. capsulatum and C. posadasii infection depends on innate sensing by Dectin-1 and Dectin-2, but not Mincle. Tracking Ag-specific T cells in vivo established that the Card9 signaling pathway acts indispensably and exclusively on differentiation of Th17 cells, while leaving intact their activation, proliferation, survival, and **migration**. Whereas Card9 signaling is essential, C-type lectin receptors offer distinct and divergent contributions to vaccine immunity against these endemic fungal pathogens. Our work provides new insight into innate immune mechanisms that drive vaccine immunity and Th17 cells.
8. Syk is required for monocyte/macrophage chemotaxis to CX3CL1 (Fractalkine). CX3CL1 (fractalkine), the only member of the delta subclass of chemokines, is a known chemotactic factor for monocytes/macrophages as well as NK cells and T lymphocytes. In several pathologies, excessive production of CX3CL1 at specific sites leads primarily to monocyte/macrophage recruitment, which causes tissue and vascular damage. Despite their clinical relevance, the mechanisms underlying monocyte/macrophage chemotaxis to CX3CL1 remain poorly documented. The present report addresses this issue and identifies cell signaling crucial for this process. Using the murine monocyte/macrophage RAW cell line, we show that CX3CL1 treatment elicits a rapid and transient increase in F-actin and the formation of F-actin-enriched cell protrusions. CX3CL1 also triggers tyrosine phosphorylation of proteins localized in those protrusions. The protein tyrosine kinase Syk is activated upon CX3CL1 treatment, and reduction of Syk expression using RNA-mediated interference results in a specific and massive impairment of RAW cell **migration** to CX3CL1. Similar results are obtained using the Syk inhibitor, piceatannol. Cells with reduced Syk expression also exhibit a major defect in CX3CL1-induced cytoskeletal remodeling. These data suggest that in monocytes/macrophages, Syk is essential for proper reorganization of the actin cytoskeleton in response to CX3CL1 and is therefore required for cell chemotaxis to CX3CL1.
9. The Vav binding site of the non-receptor tyrosine kinase Syk at Tyr 348 is critical for beta2 integrin (CD11/CD18)-mediated neutrophil **migration**. Leukocyte adhesion via beta(2) integrins (CD11/CD18) activates the tyrosine kinase Syk. We found that Syk was enriched at the lamellipodium during N-formyl-Met-Leu-Phe-induced **migration** of neutrophil-like differentiated HL-60 cells. Here, Syk colocalized with Vav, a guanine nucleotide exchange factor for Rac and Cdc42. The enrichment of Syk at the lamellipodium and its colocalization with Vav were absent upon expression of a Syk kinase-dead mutant (Syk K402R) or a Syk mutant lacking the binding site of Vav (Syk Y348F). Live cell imaging revealed that both mutations resulted in excessive lamellipodium formation and severely compromised **migration** compared with control cells. Similar results were obtained upon down-regulation of Syk by RNA interference (RNAi) technique as well as in Syk(-/-) neutrophils from wild-type mice reconstituted with Syk(-/-) bone marrow. A pivotal role of Syk in vivo was demonstrated in the Arthus reaction, where neutrophil extravasation, edema formation, and hemorrhage were profoundly diminished in Syk(-/-) bone marrow chimeras compared with those in control animals. In the inflamed cremaster muscle, Syk(-/-) neutrophils revealed a defect in adhesion and **migration**. These findings indicate that Syk is critical for beta(2) integrin-mediated neutrophil **migration** in vitro and plays a fundamental role in neutrophil recruitment during the inflammatory response in vivo.
10. Syk regulates multiple signaling pathways leading to CX3CL1 chemotaxis in macrophages. Several studies have clearly established the importance of the interaction between macrophages and CX3CL1 in the progression of disease. A previous study demonstrated that Syk was required for CX3CL1-mediated actin polymerization and chemotaxis. Here, we delineated the signaling cascade of Syk-mediated cell **migration** in response to CX3CL1. Inhibition of Syk in bone marrow-derived macrophages or reduction of Syk expression using siRNA in RAW/LR5 cells indicated that Syk was required for the activation of PI3K, Cdc42, and Rac1. Also, reduction in WASP or WAVE2 levels, common downstream effectors of Cdc42 or Rac1, resulted in impaired cell **migration** to CX3CL1. Syk indirectly regulated WASP tyrosine phosphorylation through Cdc42 activation. Altogether, our data identify that Syk mediated chemotaxis toward CX3CL1 by regulating both Rac1/WAVE2 and Cdc42/WASP pathways, whereas Src family kinases were required for proper WASP tyrosine phosphorylation.
11. Regulation of developmental lymphangiogenesis by Syk(+) leukocytes. Lymphatic vessels are essential for tissue homeostasis and immune surveillance and contribute to pathological conditions. Lymphatic endothelium differentiates from veins and forms an independent vascular tree with only few connections to the venous circulation. Failure of blood and lymphatic vessel separation results in hemorrhage and edema. VEGF-C and -D are strong inducers of lymphangiogenesis and have essential (VEGF-C) and modulatory (VEGF-D) roles during developmental lymphangiogenesis. We describe here a myeloid population that is defined by expression of the tyrosine kinase Syk, comprises largely M2-polarized mononuclear cells, and robustly expresses angiogenic factors, including VEGF-C/-D and chemokines. These cells stimulate lymphangiogenesis in vivo. Deletion of Syk causes increased chemotractant production, enhanced trans**migration**, and accumulation in the skin. Ensuing lymphatic hyperplasia and vessel dilation cause the formation of blood-lymphatic shunts. This mechanism does not involve circulating endothelial progenitor cells and demonstrates the potential of hematopoietic cells to control developmental lymphangiogenesis.
12. Macrophages generate reactive oxygen species in response to minimally oxidized low-density lipoprotein: toll-like receptor 4- and spleen tyrosine kinase-dependent activation of NADPH oxidase 2. Oxidative modification of low-density lipoprotein (LDL) plays a causative role in the development of atherosclerosis. In this study, we demonstrate that minimally oxidized LDL (mmLDL) stimulates intracellular reactive oxygen species (ROS) generation in macrophages through NADPH oxidase 2 (gp91phox/Nox2), which, in turn, induces production of RANTES and **migration** of smooth muscle cells. Peritoneal macrophages from gp91phox/Nox2(-/-) mice or J774 macrophages in which Nox2 was knocked down by small interfering RNA failed to generate ROS in response to mmLDL. Because mmLDL-induced cytoskeletal changes were dependent on Toll-like receptor (TLR)4, we analyzed ROS generation in peritoneal macrophages from wild-type, TLR4(-/-), or MyD88(-/-) mice and found that mmLDL-mediated ROS was generated in a TLR4-dependent, but MyD88-independent, manner. Furthermore, we found that ROS generation required the recruitment and activation of spleen tyrosine kinase (Syk) and that mmLDL also induced phospholipase PLCgamma1 phosphorylation and protein kinase C membrane translocation. Importantly, the phospholipase Cgamma1 phosphorylation was reduced in J774 cells expressing Syk-specific short hairpin RNA. Nox2 modulated mmLDL activation of macrophages by regulating the expression of proinflammatory cytokines interleukin-1beta, interleukin-6, and RANTES. We showed that purified RANTES was able to stimulate **migration** of mouse aortic smooth muscle cells and addition of neutralizing antibody against RANTES abolished the **migration** of mouse aortic smooth muscle cells stimulated by mmLDL-stimulated macrophages. These results suggest that mmLDL induces generation of ROS through sequential activation of TLR4, Syk, phospholipase Cgamma1, protein kinase C, and gp91phox/Nox2 and thereby stimulates expression of proinflammatory cytokines. These data help explain mechanisms by which endogenous ligands, such as mmLDL, can induce TLR4-dependent, proatherogenic activation of macrophages.
13. Inhibition of beta 2 integrin receptor and Syk kinase signaling in monocytes by the Src family kinase Fgr. While beta 2 integrin ligand-receptor recognition interactions are well characterized, less is known about how these events trigger signal transduction cascades to regulate the transition from tethering to firm adhesion, spreading, and transendothelial **migration**. We have identified critical positive and negative regulatory components of this cascade in monocytes. Whereas the Syk tyrosine kinase is essential for beta 2 integrin signaling and cell spreading, the Src family kinase Fgr is a negative regulator of this pathway. Fgr selectively inhibits beta 2 but not beta 1 integrin signaling and Syk kinase function via a direct association between the Fgr SH2 domain and Syk tyrosine Y342. The inhibitory effects of Fgr are independent of its kinase activity, are dose dependent, and can be overcome by chemokines and inflammatory mediators.
14. Syk is required for integrin signaling in neutrophils. The Syk tyrosine kinase plays a critical role in the signaling machinery of various receptors of the adaptive immune system. Here we show that Syk is also an essential component of integrin signaling in neutrophils. syk(-/-) neutrophils failed to undergo respiratory burst, degranulation, or spreading in response to proinflammatory stimuli while adherent to immobilized integrin ligands or when stimulated by direct crosslinking of integrins. Signaling from the beta(1), beta(2), or beta(3) integrins was defective in syk(-/-) cells. Syk colocalized with CD18 during cell spreading and initiated downstream signaling events leading to actin polymerization. Surprisingly, these defects in integrin-mediated activation did not impair the integrin-dependent in vitro or in vivo **migration** of syk(-/-) neutrophils or of cells deficient in Src-family kinases. Thus, integrins use different signaling mechanisms to support **migration** and adherent activation.
15. **Migration** inhibition of mammary epithelial cells by Syk is blocked in the presence of DDR1 receptors. The non-receptor tyrosine kinase Syk is a well-characterized hematopoietic signal transducer, which is also expressed in non-hematopoietic cells. In epithelial cells, the function of Syk is not wholly known. It interacts with the receptor tyrosine kinase DDR1 and is frequently lost from metastatic mammary tumors. Here, using genetic tracing, we demonstrate Syk expression in murine mammary epithelium, myoepithelium and skin epithelium, but not in intestinal or lung epithelia. Investigating possible functions of Syk, we found a substantial suppression of cell mobility that depended on Syk kinase activity in trans-well **migration** and wounding assays. Co-expression of DDR1 resulted in constitutive interaction and strong activation of Syk kinase. Most importantly, Syk-mediated **migration** inhibition was blocked in the presence of DDR1, while conversely DDR1 knockdown restored **migration** inhibition. Our study identifies Syk as a potent inhibitor of epithelial **migration** and describes a first functional consequence of the interaction with the collagen receptor DDR1.
16. G-protein-coupled receptor signaling in Syk-deficient neutrophils and mast cells. The Syk tyrosine kinase is essential for immunoreceptor and multiple integrin functions as well as being implicated in signaling from G-protein-coupled receptors (GPCR) in cell lines, transfection systems, and pharmacologic studies. In contrast, using Syk-deficient primary cells, we show here that Syk does not play a major functional role in chemoattractant/chemokine signaling in neutrophils and mast cells. syk(-/-) neutrophils showed normal respiratory burst and degranulation in response to the bacterial peptide formyl-Met-Leu-Phe (fMLP). The **migration** of neutrophils toward fMLP was similarly not affected by the syk(-/-) mutation. fMLP initiated normal Ca(2+)-signal, activation of the extracellular signal-related kinase (ERK) and p38 mitogen-activated protein (MAP) kinase cascades, and polymerization of cellular actin in the absence of Syk. syk(-/-) and wild-type neutrophils also responded similarly to LTB(4), C5a, and the chemokines macrophage inflammatory protein-1 (MIP-1)alpha or MIP-2, both in functional assays and in intracellular signaling mechanisms. Furthermore, bone marrow-derived syk(-/-) mast cells showed normal activation of the Akt, ERK, and p38 MAP kinase pathways when stimulated by the GPCR ligand adenosine. We conclude that, in contrast to previous reports, Syk does not play a major role in GPCR signaling.
17. Syk-mediated translocation of PI3Kdelta to the leading edge controls lamellipodium formation and **migration** of leukocytes. The non-receptor tyrosine kinase Syk is mainly expressed in the hematopoietic system and plays an essential role in beta(2) integrin-mediated leukocyte activation. To elucidate the signaling pathway downstream of Syk during beta2 integrin (CD11/CD18)-mediated **migration** and extravasation of polymorphonuclear neutrophils (PMN), we generated neutrophil-like differentiated HL-60 (dHL-60) cells expressing a fluorescently tagged Syk mutant lacking the tyrosine residue at the position 323 (Syk-Tyr323) that is known to be required for the binding of the regulatory subunit p85 of the phosphatidylinositol 3-kinase (PI3K) class I(A). Syk-Tyr323 was found to be critical for the enrichment of the catalytic subunit p110delta of PI3K class I(A) as well as for the generation of PI3K products at the leading edge of the majority of polarized cells. In accordance, the translocation of PI3K p110delta to the leading edge was diminished in Syk deficient murine PMN. Moreover, the expression of EGFP-Syk Y323F interfered with proper cell polarization and it impaired efficient **migration** of dHL-60 cells. In agreement with a major role of beta2 integrins in the recruitment of phagocytic cells to sites of lesion, mice with a Syk-deficient hematopoietic system demonstrated impaired PMN infiltration into the wounded tissue that was associated with prolonged cutaneous wound healing. These data imply a novel role of Syk via PI3K p110delta signaling for beta2 integrin-mediated **migration** which is a prerequisite for efficient PMN recruitment in vivo.
18. Induced Syk deletion leads to suppressed allergic responses but has no effect on neutrophil or monocyte **migration** in vivo. The spleen tyrosine kinase (Syk) is a key mediator of immunoreceptor signaling in immune cells. Thus, interfering with the function of Syk by genetic deletion or pharmacological inhibition might influence a variety of allergic and autoimmune processes. Since conventional Syk knockout mice are not viable, studies addressing the effect of Syk deletion in adult animals have been limited. To further explore functions of Syk in animal models of allergy and to shed light on the role of Syk in the in vivo **migration** of neutrophils and monocytes, we generated inducible Syk knockout mice. These mice harbor a floxed Syk gene and a tamoxifen-inducible Cre recombinase under the control of the ubiquitously active Rosa26-promoter. Thus, treatment of mice with tamoxifen leads to the deletion of Syk in all organs. Syk-deleted mice were analyzed in mast cell-dependent models and in models focusing on neutrophil and monocyte **migration**. We show that Syk deletion in adult mice reduces inflammatory responses in mast cell-driven animal models of allergy and asthma but has no effect on the **migration** of neutrophils and monocytes. Therefore, the inducible Syk knockout mice presented here provide a valuable tool to further explore the role of Syk in disease-related animal models.
19. Tyrosine kinase Btk regulates E-selectin-mediated integrin activation and neutrophil recruitment by controlling phospholipase C (PLC) gamma2 and PI3Kgamma pathways. Selectins mediate leukocyte rolling, trigger beta(2)-integrin activation, and promote leukocyte recruitment into inflamed tissue. E-selectin binding to P-selectin glycoprotein ligand 1 (PSGL-1) leads to activation of an immunoreceptor tyrosine-based activation motif (ITAM)-dependent pathway, which in turn activates the spleen tyrosine kinase (Syk). However, the signaling pathway linking Syk to integrin activation after E-selectin engagement is unknown. To identify the pathway, we used different gene-deficient mice in autoperfused flow chamber, intravital microscopy, peritonitis, and biochemical studies. We report here that the signaling pathway downstream of Syk divides into a phospholipase C (PLC) gamma2- and phosphoinositide 3-kinase (PI3K) gamma-dependent pathway. The Tec family kinase Bruton tyrosine kinase (Btk) is required for activating both pathways, generating inositol-3,4,5-trisphosphate (IP(3)), and inducing E-selectin-mediated slow rolling. Inhibition of this signal-transduction pathway diminished Galpha(i)-independent leukocyte adhesion to and trans**migration** through endothelial cells in inflamed postcapillary venules of the cremaster. Galpha(i)-independent neutrophil recruitment into the inflamed peritoneal cavity was reduced in Btk(-/-) and Plcg2(-/-) mice. Our data demonstrate the functional importance of this newly identified signaling pathway mediated by E-selectin engagement.
20. Endothelial cell-derived CD95 ligand serves as a chemokine in induction of neutrophil slow rolling and adhesion. Integrin activation is crucial for the regulation of leukocyte rolling, adhesion and trans-vessel **migration** during inflammation and occurs by engagement of myeloid cells through factors presented by inflamed vessels. However, endothelial-dependent mechanisms of myeloid cell recruitment are not fully understood. Here we show using an autoperfused flow chamber assay of whole blood neutrophils and intravital microscopy of the inflamed cremaster muscle that CD95 mediates leukocyte slow rolling, adhesion and trans**migration** upon binding of CD95-ligand (CD95L) that is presented by endothelial cells. In myeloid cells, CD95 triggers activation of Syk-Btk/PLCγ2/Rap1 signaling that ultimately leads to integrin activation. Excitingly, CD95-deficient myeloid cells exhibit impaired bacterial clearance in an animal model of sepsis induced by cecal ligation and puncture (CLP). Our data identify the cellular and molecular mechanisms underlying the chemoattractant effect of endothelial cell-derived CD95L in induction of neutrophil recruitment and support the use of therapeutic inhibition of CD95's activity in inflammatory diseases.
21. Pyk2 is required for neutrophil degranulation and host defense responses to bacterial infection. The appropriate regulation of neutrophil activation is critical for maintaining host defense and limiting inflammation. Polymorphonuclear neutrophils (PMNs) express a number of cytoplasmic tyrosine kinases that regulate signaling pathways leading to activation. One of the most highly expressed, but least studied, kinases in PMNs is proline rich kinase 2 (Pyk2). By analogy to the related focal adhesion kinase, Pyk2 has been implicated in regulating PMN adhesion and **migration**; however, its physiologic function has yet to be described. Using pyk2(-/-) mice, we found that this kinase was required for integrin-mediated degranulation responses, but was not involved in adhesion-induced cell spreading or activation of superoxide production. Pyk2-deficient PMNs also manifested reduced **migration** on fibrinogen-coated surfaces. The absence of Pyk2 resulted in a severe reduction in paxillin and Vav phosphorylation following integrin ligation, which likely accounts for the poor degranulation and cell **migration**. Pyk2(-/-) mice were unable to efficiently clear infection with Staphylococcus aureus in a skin abscess model, owing in part to the poor release of granule contents at the site of infection. However, Pyk2-deficient PMNs responded normally to soluble agonists, demonstrating that this kinase functions mainly in the integrin pathway. These data demonstrate the unrealized physiologic role of this kinase in regulating the adhesion-mediated release of PMN granule contents.
22. CLEC-2 and Syk in the megakaryocytic/platelet lineage are essential for development. The C-type lectin receptor CLEC-2 signals through a pathway that is critically dependent on the tyrosine kinase Syk. We show that homozygous loss of either protein results in defects in brain vascular and lymphatic development, lung inflation, and perinatal lethality. Furthermore, we find that conditional deletion of Syk in the hematopoietic lineage, or conditional deletion of CLEC-2 or Syk in the megakaryocyte/platelet lineage, also causes defects in brain vascular and lymphatic development, although the mice are viable. In contrast, conditional deletion of Syk in other hematopoietic lineages had no effect on viability or brain vasculature and lymphatic development. We show that platelets, but not platelet releasate, modulate the **migration** and intercellular adhesion of lymphatic endothelial cells through a pathway that depends on CLEC-2 and Syk. These studies found that megakaryocyte/platelet expression of CLEC-2 and Syk is required for normal brain vasculature and lymphatic development and that platelet CLEC-2 and Syk directly modulate lymphatic endothelial cell behavior in vitro.

#### GeneRIFs

1. Data identifyies Syk as a potent inhibitor of epithelial **migration** and describes a first functional consequence of the interaction with the collagen receptor DDR1.
2. Syk deletion in adult mice reduces inflammatory responses in mast cell-driven animal models of allergy and asthma but has no effect on the **migration** of neutrophils and monocytes
3. Integrin alphaIIbbeta3 signaling via Src family kinases and Syk kinase to PLCgamma2 is required for megakaryocyte spreading, **migration**, and platelet formation.
4. Syk plays a crucial role in multiple integrin-mediated effector functions of the innate immune system, but it is dispensable for integrin-dependent neutrophil **migration** both in vitro and in vivo.
5. evidence that the alpha4beta1 receptor in blood cells has evolved a Syk-Rac2 signaling axis to transmit signals required for integrin-directed **migration** suggesting that Syk kinase in part encodes myeloid Rac2 specificity in vivo

### [Ee]xtracellular [Mm]atrix

#### PubMed

1. Tumor suppressor function of Syk in human MCF10A in vitro and normal mouse mammary epithelium in vivo. The normal function of Syk in epithelium of the developing or adult breast is not known, however, Syk suppresses tumor growth, invasion, and metastasis in breast cancer cells. Here, we demonstrate that in the mouse mammary gland, loss of one Syk allele profoundly increases proliferation and ductal branching and invasion of epithelial cells through the mammary fat pad during puberty. Mammary carcinomas develop by one year. Syk also suppresses proliferation and invasion in vitro. siRNA or shRNA knockdown of Syk in MCF10A breast epithelial cells dramatically increased proliferation, anchorage independent growth, cellular motility, and invasion, with formation of functional, **extracellular matrix**-degrading invadopodia. Morphological and gene microarray analysis following Syk knockdown revealed a loss of luminal and differentiated epithelial features with epithelial to mesenchymal transition and a gain in invadopodial cell surface markers CD44, CD49F, and MMP14. These results support the role of Syk in limiting proliferation and invasion of epithelial cells during normal morphogenesis, and emphasize the critical role of Syk as a tumor suppressor for breast cancer. The question of breast cancer risk following systemic anti-Syk therapy is raised since only partial loss of Syk was sufficient to induce mammary carcinomas.
2. Spleen tyrosine kinase suppresses osteoblastic differentiation through MAPK and PKCα. Spleen tyrosine kinase (Syk) is a non-receptor protein kinase present in abundance in a wide range of hematopoietic cells. Syk reportedly plays a crucial role in immune signaling in B cells and cells bearing Fcγ-activation receptors. The role of syk in osteoblastic differentiation has not been well elucidated. We report herein the role of syk in osteoblastic differentiation. We investigated the effects of two syk inhibitors on osteoblastic differentiation in mouse preosteoblastic MC3T3-E1 cells and bone marrow stromal ST2 cells. Expression of syk was detected in these two cell lines. Two syk inhibitors stimulated mRNA expression of osteoblastic markers (ALP, Runx2, Osterix). Mineralization o**f extracellular matr**ix was also promoted by treatment with syk inhibitors. Knockdown of Syk caused increased mRNA expression of osteoblastic markers. In addition, syk inhibitor and knockdown of Syk suppressed phosphorylation of mitogen-activated protein kinase (MAPK) and protein kinase Cα (PKCα). Our results indicate that syk might regulate osteoblastic differentiation through MAPK and PKCα.
3. Analysis of mouse LMIR5/CLM-7 as an activating receptor: differential regulation of LMIR5/CLM-7 in mouse versus human cells. We have analyzed leukocyte mono-Ig-like receptor 5 (LMIR5) as an activating receptor among paired LMIRs. Mouse LMIR5 (mLMIR5) is expressed in myeloid cells such as mast cells, granulocytes, macrophages, and dendritic cells. Cross-linking of transduced mLMIR5 in bone marrow-derived mast cells (BMMCs) caused activation events, including cytokine production, cell survival, degranulation, and adhesion to the **extracellular matrix**. mLMIR5 associated with DAP12 and to a lesser extent with DAP10, and mLMIR5-mediated functions of BMMCs were strongly inhibited by DAP12 deficiency. Importantly, cross-linking of endogenous mLMIR5 induced Syk-dependent activation of fetal liver-derived mast cells. Unlike mLMIR5, cross-linking of human LMIR5 (hLMIR5) induced cytokine production of BMMCs even in the absence of both DAP12 and DAP10, suggesting the existence of unidentified adaptors. Interestingly, hLMIR5 possessed a tyrosine residue (Y188) in the cytoplasmic region. Signaling via Y188 phosphorylation played a predominant role in hLMIR5-mediated cytokine production in DAP12-deficient, but not wild-type BMMCs. In addition, experiments using DAP10/DAP12 double-deficient BMMCs suggested the existence of Y188 phoshorylation-dependent and -independent signals from unidentified adaptors. Collectively, although both mouse and human LMIR5 play activatory roles in innate immunity cells, the functions of LMIR5 were differentially regulated in mouse versus human cells.
4. Laminin stimulates spreading of platelets through integrin alpha6beta1-dependent activation of GPVI. The **extracellular matrix** protein, laminin, supports platelet adhesion through binding to integrin alpha6beta1 In the present study, we demonstrate that human laminin, purified from placenta, also stimulates formation of filopodia and lamellipodia in human and mouse platelets through a pathway that is dependent on alpha6beta1 and the collagen receptor GPVI. The integrin alpha6beta1 is essential for adhesion to laminin, as demonstrated using an alpha6-blocking antibody, whereas GPVI is dispensable for this response, as shown using "knockout" mouse platelets. On the other hand, lamellipodia formation on laminin is completely inhibited in the absence of GPVI, although filopodia formation remains and is presumably mediated via alpha6beta1 Lamellipodia and filopodia formation are inhibited in Syk-deficient platelets, demonstrating a key role for the kinase in signaling downstream of GPVI and integrin alpha6beta1 GPVI was confirmed as a receptor for laminin using surface plasmon resonance spectroscopy and by demonstration of lamellipodia formation on laminin in the presence of collagenase. These results identify GPVI as a novel receptor for laminin and support a model in which integrin alpha6beta1 brings laminin to GPVI, which in turn mediates lamellipodia formation. We speculate that laminin contributes to platelet spreading in vivo through a direct interaction with GPVI.

### [Ii]nvasive

#### PubMed

1. Class IA phosphoinositide 3-kinase β and δ regulate neutrophil oxidase activation in response to Aspergillus fumigatus hyphae. An effective immune response to the ubiquitous fungus Aspergillus fumigatus is dependent upon production of reactive oxygen species (ROS) by the NADPH oxidase. This is evidenced by the acute sensitivity of oxidase-deficient humans and mice t**o invasi**ve aspergillosis. Neutrophils are recruited to the lungs shortly postinfection and respond by phagocytosing conidia and mediating extracellular killing of germinated hyphae in a ROS-dependent manner. However, the signaling mechanisms regulating the generation of ROS in response to hyphae are poorly understood. PI3Ks are important regulators of numerous cellular processes, with much recent work describing unique roles for the different class I PI3K isoforms. We showed by live-cell imaging that the lipid products of class I PI3Ks accumulated at the hyphal-bound neutrophil plasma membrane. Further, we used pharmacological and genetic approaches to demonstrate essential, but overlapping, roles for PI3Kβ and PI3Kδ in the ROS and spreading responses of murine neutrophils to Aspergillus hyphae. Hyphal-induced ROS responses were substantially inhibited by deletion of the common β2-integrin subunit CD18, with only a minor, redundant role for Dectin-1. However, addition of soluble algal glucans plus the genetic deletion of CD18 were required to significantly inhibit activation of the PI3K-effector protein kinase B. Hyphal ROS responses were also totally dependent on the presence of Syk, but not its ITAM-containing adaptor proteins FcRγ or DAP12, and the Vav family of Rac-guanine nucleotide exchange factors. These results start to define the signaling network controlling neutrophil ROS responses to A. fumigatus hyphae.
2. Development of a metastatic fluorescent Lewis Lung carcinoma mouse model: identification of mRNAs and microRNAs involved in tumor invasion. Cancer metastasis is the foremost cause of death in cancer patients. A series of observable pathological changes takes place during progression and metastasis of cancer, but the underlying genetic changes remain unclear. Therefore, new approaches are required, including insights from cancer mouse models. To examine the mechanisms involved in tumor metastasis, we first generated a stably transfected Lewis Lung carcinoma cell line expressing a far-red fluorescent protein, called Katushka. After in vivo growth in syngeneic mice, two fluorescent Lewis Lung cancer subpopulations were isolated from primary tumors and lung metastases. The metastasis-derived cells exhibited a significant improvement in in vitro **invasive** activity compared to the primary tumor-derived cells, using a quantitative invasion chamber assay. Moreover, expression levels of 84 tumor metastasis-related mRNAs, 88 cancer-related microRNAs as well as Dicer and Drosha were determined using RT-qPCR. Compared to the primary Lewis Lung carcinoma subculture, the metastasis-derived cells exhibited statistically significantly increased mRNA levels for several matrix metalloproteinases as well as hepatocyte growth factor (HGF) and spleen tyrosine kinase (SYK). A modest decrease in Drosha and Dicer mRNA levels was accompanied by significant downregulation of ten microRNAs, including miR-9 and miR-203, in the lung metastatic Lewis Lung carcinoma cell culture. Thus, a tool for cancer metastasis studies has been established and the model is well suited for the identification of novel microRNAs and mRNAs involved in malignant progression. Our results suggest that increases in metalloproteinase expression and impairment of microRNA processing are involved in the acquirement of metastatic ability.

#### GeneRIFs

1. Upregulation of SYK is associated with neoplasm **invasive**ness in lung cancer.

### [Mm]etastasis

#### PubMed

1. Targeted downregulation of platelet CLEC-2 occurs through Syk-independent internalization. Platelet aggregation at sites of vascular injury is not only essential for hemostasis, but may also cause acute ischemic disease states such as myocardial infarction or stroke. The hemi-immunoreceptor tyrosine-based activation motif-containing C-type lectinlike receptor 2 (CLEC-2) mediates powerful platelet activation through a Src- and spleen tyrosine kinase (Syk)-dependent tyrosine phosphorylation cascade. Thereby, CLEC-2 not only contributes to thrombus formation and stabilization but also plays a central role in blood-lymphatic vessel development, tumor **metastasis**, and prevention of inflammatory bleeding, making it a potential pharmacologic target to modulate these processes. We have previously shown that injection of the anti-CLEC-2 antibody, INU1, results in virtually complete immunodepletion of platelet CLEC-2 in mice, which is, however, preceded by a severe transient thrombocytopenia thereby limiting its potential therapeutic use. The mechanisms underlying this targeted CLEC-2 downregulation have remained elusive. Here, we show that INU1-induced CLEC-2 immunodepletion occurs through Src-family kinase-dependent receptor internalization in vitro and in vivo, presumably followed by intracellular degradation. In mice with platelet-specific Syk deficiency, INU1-induced CLEC-2 internalization/degradation was fully preserved whereas the associated thrombocytopenia was largely prevented. These results show for the first time that CLEC-2 can be downregulated from the platelet surface through internalization in vitro and in vivo and that this can be mechanistically uncoupled from the associated antibody-induced thrombocytopenia.
2. Tumor suppressor function of Syk in human MCF10A in vitro and normal mouse mammary epithelium in vivo. The normal function of Syk in epithelium of the developing or adult breast is not known, however, Syk suppresses tumor growth, invasion, and **metastasis** in breast cancer cells. Here, we demonstrate that in the mouse mammary gland, loss of one Syk allele profoundly increases proliferation and ductal branching and invasion of epithelial cells through the mammary fat pad during puberty. Mammary carcinomas develop by one year. Syk also suppresses proliferation and invasion in vitro. siRNA or shRNA knockdown of Syk in MCF10A breast epithelial cells dramatically increased proliferation, anchorage independent growth, cellular motility, and invasion, with formation of functional, extracellular matrix-degrading invadopodia. Morphological and gene microarray analysis following Syk knockdown revealed a loss of luminal and differentiated epithelial features with epithelial to mesenchymal transition and a gain in invadopodial cell surface markers CD44, CD49F, and MMP14. These results support the role of Syk in limiting proliferation and invasion of epithelial cells during normal morphogenesis, and emphasize the critical role of Syk as a tumor suppressor for breast cancer. The question of breast cancer risk following systemic anti-Syk therapy is raised since only partial loss of Syk was sufficient to induce mammary carcinomas.
3. Development of a metastatic fluorescent Lewis Lung carcinoma mouse model: identification of mRNAs and microRNAs involved in tumor invasion. Cancer **metastasis** is the foremost cause of death in cancer patients. A series of observable pathological changes takes place during progression and **metastasis** of cancer, but the underlying genetic changes remain unclear. Therefore, new approaches are required, including insights from cancer mouse models. To examine the mechanisms involved in tumor **metastasis**, we first generated a stably transfected Lewis Lung carcinoma cell line expressing a far-red fluorescent protein, called Katushka. After in vivo growth in syngeneic mice, two fluorescent Lewis Lung cancer subpopulations were isolated from primary tumors and lung metastases. The **metastasis**-derived cells exhibited a significant improvement in in vitro invasive activity compared to the primary tumor-derived cells, using a quantitative invasion chamber assay. Moreover, expression levels of 84 tumor **metastasis**-related mRNAs, 88 cancer-related microRNAs as well as Dicer and Drosha were determined using RT-qPCR. Compared to the primary Lewis Lung carcinoma subculture, the **metastasis**-derived cells exhibited statistically significantly increased mRNA levels for several matrix metalloproteinases as well as hepatocyte growth factor (HGF) and spleen tyrosine kinase (SYK). A modest decrease in Drosha and Dicer mRNA levels was accompanied by significant downregulation of ten microRNAs, including miR-9 and miR-203, in the lung metastatic Lewis Lung carcinoma cell culture. Thus, a tool for cancer **metastasis** studies has been established and the model is well suited for the identification of novel microRNAs and mRNAs involved in malignant progression. Our results suggest that increases in metalloproteinase expression and impairment of microRNA processing are involved in the acquirement of metastatic ability.

---

## Tnk2

- NCBI Gene
- GeneRIF
- Pubmed

| Search Term | Rank | Fields |
| --- | --- | --- |
| [Mm]igration | 6 | Process(1) |

  
hide/show details for Tnk2

### [Mm]igration

#### Process

1. cell **migration**

---

## Tnk1

- NCBI Gene
- GeneRIF
- Pubmed

| Search Term | Rank | Fields |
| --- | --- | --- |
| [Mm]igration | 6 | Process(1) |

  
hide/show details for Tnk1

### [Mm]igration

#### Process

1. cell **migration**

---

## Mypn

- NCBI Gene
- GeneRIF
- Pubmed

| Search Term | Rank | Fields |
| --- | --- | --- |
| [Mm]igration | 6 | Process(1) |

  
hide/show details for Mypn

### [Mm]igration

#### Process

1. regulation of cell **migration**

---

## Nexn

- NCBI Gene
- GeneRIF
- Pubmed

| Search Term | Rank | Fields |
| --- | --- | --- |
| [Mm]igration | 6 | Process(2) |

  
hide/show details for Nexn

### [Mm]igration

#### Process

1. regulation of cell **migration**
2. regulation of cell **migration**

---

## Zfp64

- NCBI Gene
- GeneRIF
- Pubmed

| Search Term | Rank | Fields |
| --- | --- | --- |
| [Ee]xtracellular [Mm]atrix | 8 | PubMed(1) |

  
hide/show details for Zfp64

### [Ee]xtracellular [Mm]atrix

#### PubMed

1. Identification of tooth-specific downstream targets of Runx2. Odontogenesis or tooth development is a highly regulated process that involves complex epithelial-mesenchymal signaling interactions that lead to cuspal morphogenesis, cell differentiation and the subsequent formation of the specialized matrices of enamel, dentin, cementum and bone. Although studies on tooth epithelial-mesenchymal signaling interactions have greatly increased our understanding of molecules that regulate tooth initiation and early morphogenesis (review: Jernvall and Thesleff, Mech. Dev. 92 (2000) 19), the precise nature of the molecular events controlling late morphogenesis and terminal cytodifferentiation is not known. We have recently reported a unique phenotype involving dentition in mice lacking a functional Runx2 gene (D'Souza et al., Development 126 (1999) 2911). The markedly hypoplastic tooth organs as well as defects in the maturation of ameloblasts and odontoblasts point to an important and non-redundant role for Runx2 in tooth morphogenesis and cytodifferentiation. In order to identify genes that are affected by the absence of Runx2, a cDNA library was generated from Runx2(-/-) and Runx2(+/+) first molar organs. Thus far, our analysis has revealed several tooth-specific downstream target genes of Runx2 that include **extracellular matrix** proteins, kinases, receptors, growth factors, mitochondrial proteins and transcription molecules. Sequence analysis of 61 differentially expressed genes revealed that 96.03% of the clones matched previously described genes in the GenBank/EBML database and 3.96% did not match any entries in the database. Our preliminary expression analysis of one of the differentially expressed clones which encodes for a zinc finger transcription factor termed Zfp reveals that the gene is temporally regulated during tooth development. In conclusion, we have successfully generated a library enriched in genes expressed in Runx2(+/+) molar tooth organs and performed preliminary studies to assess the role of Zfp in tooth development.

---

## Zfp335

- NCBI Gene
- GeneRIF
- Pubmed

| Search Term | Rank | Fields |
| --- | --- | --- |
| [Mm]igration | 6 | PubMed(1) |

  
hide/show details for Zfp335

### [Mm]igration

#### PubMed

1. p35 regulates the CRM1-dependent nucleocytoplasmic shuttling of nuclear hormone receptor coregulator-interacting factor 1 (NIF-1). Cyclin-dependent kinase 5 (Cdk5) is a proline-directed serine/threonine kinase, which plays critical roles in a wide spectrum of neuronal functions including neuronal survival, neurite outgrowth, and synapse development and plasticity. Cdk5 activity is controlled by its specific activators: p35 or p39. While knockout studies reveal that Cdk5/p35 is critical for neuronal **migration** during early brain development, functions of Cdk5/p35 have been unraveled through the identification of the interacting proteins of p35, most of which are Cdk5/p35 substrates. However, it remains unclear whether p35 can regulate neuronal functions independent of Cdk5 activity. Here, we report that a nuclear protein, nuclear hormone receptor coregulator (NRC)-interacting factor 1 (NIF-1), is a new interacting partner of p35. Interestingly, p35 regulates the functions of NIF-1 independent of Cdk5 activity. NIF-1 was initially discovered as a transcriptional regulator that enhances the transcriptional activity of nuclear hormone receptors. Our results show that p35 interacts with NIF-1 and regulates its nucleocytoplasmic trafficking via the nuclear export pathway. Furthermore, we identified a nuclear export signal on p35; mutation of this site or blockade of the CRM1/exportin-dependent nuclear export pathway resulted in the nuclear accumulation of p35. Intriguingly, blocking the nuclear export of p35 attenuated the nuclear accumulation of NIF-1. These findings reveal a new p35-dependent mechanism in transcriptional regulation that involves the nucleocytoplasmic shuttling of transcription regulators.

---

## Ces1d

- NCBI Gene
- GeneRIF
- Pubmed

| Search Term | Rank | Fields |
| --- | --- | --- |
| [Mm]igration | 6 | PubMed(1) |

  
hide/show details for Ces1d

### [Mm]igration

#### PubMed

1. Prox1 ablation in hepatic progenitors causes defective hepatocyte specification and increases biliary cell commitment. The liver has multiple functions that preserve homeostasis. Liver diseases are debilitating, costly and often result in death. Elucidating the developmental mechanisms that establish the liver's architecture or generate the cellular diversity of this organ should help advance the prevention, diagnosis and treatment of hepatic diseases. We previously reported that **migration** of early hepatic precursors away from the gut epithelium requires the activity of the homeobox gene Prox1. Here, we show that Prox1 is a novel regulator of cell differentiation and morphogenesis during hepatogenesis. Prox1 ablation in bipotent hepatoblasts dramatically reduced the expression of multiple hepatocyte genes and led to very defective hepatocyte morphogenesis. As a result, abnormal epithelial structures expressing hepatocyte and cholangiocyte markers or resembling ectopic bile ducts developed in the Prox1-deficient liver parenchyma. By contrast, excessive commitment of hepatoblasts into cholangiocytes, premature intrahepatic bile duct morphogenesis, and biliary hyperplasia occurred in periportal areas of Prox1-deficient livers. Together, these abnormalities indicate that Prox1 activity is necessary to correctly allocate cell fates in liver precursors. These results increase our understanding of differentiation anomalies in pathological conditions and will contribute to improving stem cell protocols in which differentiation is directed towards hepatocytes and cholangiocytes.

---

## Ces2a

- NCBI Gene
- GeneRIF
- Pubmed

| Search Term | Rank | Fields |
| --- | --- | --- |
| [Ii]nvasive | 10 | PubMed(1) |

  
hide/show details for Ces2a

### [Ii]nvasive

#### PubMed

1. Fgf10 is an oncogene activated by MMTV insertional mutagenesis in mouse mammary tumors and overexpressed in a subset of human breast carcinomas. Mouse mammary tumor virus (MMTV) infection causes a high incidence of murine mammary carcinomas by insertion of its proviral DNA in the genome of mammary epithelial cells. Retroviral insertion can activate flanking proto-oncogenes by a process called insertional mutagenesis. By sequencing the DNA adjacent to MMTV proviral insertions in mammary tumors from BALB/c mice infected with C3H-MMTV, we have found a common MMTV insertion site in the Fgf10 locus. RT-PCR studies showed that Fgf10 is expressed only in those tumors harboring a MMTV proviral insertion in this locus, suggesting that Fgf10 is a proto-oncogene. The oncogenicity of Fgf10 was evaluated in vivo by subcutaneous transplantation of retrovirally transduced HC11 mammary epithelial cells into BALB/c mice. Highly vascularized **invasive** subcutaneous tumors developed indicating that Fgf10 can act as an oncogene. A survey of primary human breast carcinomas revealed strongly elevated Fgf10 mRNA levels in approximately 10% of the tumors tested, suggesting that Fgf10 may also be involved in oncogenicity of a subset of human breast cancers.

---

## Hic1

- NCBI Gene
- GeneRIF
- Pubmed

| Search Term | Rank | Fields |
| --- | --- | --- |
| [Mm]igration | 6 | PubMed(1) |

  
hide/show details for Hic1

### [Mm]igration

#### PubMed

1. A microarray analysis of the XX Wnt4 mutant gonad targeted at the identification of genes involved in testis vascular differentiation. One of the earliest morphological changes during testicular differentiation is the establishment of an XY specific vasculature. The testis vascular system is derived from mesonephric endothelial cells that migrate into the gonad. In the XX gonad, mesonephric cell **migration** and testis vascular development are inhibited by WNT4 signaling. In Wnt4 mutant XX gonads, endothelial cells migrate from the mesonephros and form a male-like coelomic vessel. Interestingly, this process occurs in the absence of other obvious features of testis differentiation, suggesting that Wnt4 specifically inhibits XY vascular development. Consequently, the XX Wnt4 mutant mice presented an opportunity to focus a gene expression screen on the processes of mesonephric cell **migration** and testicular vascular development. We compared differences in gene expression between XY Wnt4+/+ and XX Wnt4+/+ gonads and between XX Wnt4+/+ and XX Wnt4+/+ gonads to identify sets of genes similarly upregulated in wildtype XY gonads and XX mutant gonads or upregulated in XX gonads as compared to XY gonads and XX mutant gonads. We show that several genes identified in the first set are expressed in vascular domains, and have predicted functions related to cell **migration** or vascular development. However, the expression patterns and known functions of other genes are not consistent with roles in these processes. This screen has identified candidates for regulation of sex specific vascular development, and has implicated a role for WNT4 signaling in the development of Sertoli and germ cell lineages not immediately obvious from previous phenotypic analyses.

---

## Sp1

- NCBI Gene
- GeneRIF
- Pubmed

| Search Term | Rank | Fields |
| --- | --- | --- |
| [Mm]igration | 6 | PubMed(4); GeneRIFs(1) |
| ECM | 7 | PubMed(2) |
| [Ee]xtracellular [Mm]atrix | 8 | PubMed(8) |
| [Ii]nvasive | 10 | PubMed(2) |
| [Mm]etastasis | 12 | PubMed(6); GeneRIFs(2) |
| [Cc]ell [Ii]nvasion | 13 | PubMed(1); GeneRIFs(1) |

  
hide/show details for Sp1

### [Mm]igration

#### PubMed

1. Macrophage **migration** arrest due to a winning balance of Rac2/Sp1 repression over β-catenin-induced PLD expression. Monocytes and neutrophils infiltrate into tissues during inflammation and stay for extended periods of time until the initial insult is resolved or sometimes remain even longer in the case of chronic inflammation. The mechanism as to why phagocytes become immobilized after the initial cell **migratio**n event is not understood completely. Here, we show that overexpression or hyperactivation of Rac2 decreases sustained chemotactic responses of macrophages to MCP-1/CCL2. The resulting leukocyte arrest is not caused by a diminished availability of the cytokine receptor CCR2 that remains intact during MCP-1 stimulation. We show a novel mechanism that links the Rac2-dependent arrest of chemotaxis to decreased expression of PLD2 through the transcription regulator Sp1. Prolonged Rac2 activity leads to nuclear overactivation of Sp1, which acts as a repressor for PLD2. Also, another signaling component plays a regulatory role: β-catenin. Although early times of stimulation (≈ 20 min) with MCP-1/CCL2 resulted in activation of β-catenin with a positive effect on PLD2, after ≈ 3 h of stimulation, the levels of β-catenin were reduced and not able to prevent the negative effect of Rac2 on PLD2 activity. This is a novel molecular mechanism underlying immobilization of monocyte/mac**rophage m**igration that is important for the physiological maintenance of leukocytes at the site of inflammation. If this immobilization is prolonged enough, it could lead to chronic inflammation.
2. Macrophage **migration** arrest due to a winning balance of Rac2/Sp1 repression over β-catenin-induced PLD expression. Monocytes and neutrophils infiltrate into tissues during inflammation and stay for extended periods of time until the initial insult is resolved or sometimes remain even longer in the case of chronic inflammation. The mechanism as to why phagocytes become immobilized after the initial cell **migratio**n event is not understood completely. Here, we show that overexpression or hyperactivation of Rac2 decreases sustained chemotactic responses of macrophages to MCP-1/CCL2. The resulting leukocyte arrest is not caused by a diminished availability of the cytokine receptor CCR2 that remains intact during MCP-1 stimulation. We show a novel mechanism that links the Rac2-dependent arrest of chemotaxis to decreased expression of PLD2 through the transcription regulator Sp1. Prolonged Rac2 activity leads to nuclear overactivation of Sp1, which acts as a repressor for PLD2. Also, another signaling component plays a regulatory role: β-catenin. Although early times of stimulation (≈ 20 min) with MCP-1/CCL2 resulted in activation of β-catenin with a positive effect on PLD2, after ≈ 3 h of stimulation, the levels of β-catenin were reduced and not able to prevent the negative effect of Rac2 on PLD2 activity. This is a novel molecular mechanism underlying immobilization of monocyte/mac**rophage m**igration that is important for the physiological maintenance of leukocytes at the site of inflammation. If this immobilization is prolonged enough, it could lead to chronic inflammation.
3. Sp1 expression regulates lung tumor progression. The role of specificity protein 1 (Sp1) in controlling gene expression in lung tumor development and metastasis is not well understood. In this study, we showed that the Sp1 level was highly increased and required for lung tumor growth in transgenic mice bearing Kras-induced lung tumors under the control of doxycycline. Furthermore, the Sp1 level was highly upregulated in lung adenocarcinoma cells with low invasiveness and in patients with stage I lung cancer. We also demonstrated that Sp1 was downregulated in lung adenocarcinoma cells with high invasiveness and in patients with stage IV lung adenocarcinoma. Moreover, Sp1 inversely regulated **migration**, invasion and metastasis of lung adenocarcinoma cells in vivo. In addition, a decrease in the Sp1 level in highly invasive lung adenocarcinoma cells resulted from instability of the Sp1 protein. Furthermore, overexpression of Sp1 in highly invasive lung adenocarcinoma cells increased expression of E-cadherin, a suppressor of metastasis, and attenuated the translocation of β-catenin into the cellular nucleus that leads to tumor malignancy. Taken together, Sp1 level accumulated strongly in early stage and then declined in late stage, which is important for lung cancer cell proliferation and metastasis during tumorigenesis.
4. Sp1, acetylated histone-3 and p300 regulate TRAIL transcription: mechanisms of PDGF-BB-mediated VSMC proliferation and **migration**. We recently reported that TNF-related apoptosis-inducing ligand (TRAIL) is important in atherogenesis, since it can induce vascular smooth muscle cell (VSMC) proliferation and arterial thickening following injury. Here we show the first demonstrate that TRAIL siRNA reduces platelet-derived growth factor-BB (PDGF-BB)-stimulated VSMC proliferation and **migration**. PDGF-BB-inducible VSMC proliferation was completely inhibited in VSMCs isolated from aortas of TRAIL(-/-) mice; whereas inducible **migration** was blocked compared to control VSMCs. TRAIL transcriptional control mediating this response is not established. TRAIL mRNA, protein and promoter activity was increased by PDGF-BB and subsequently inhibited by dominant-negative Sp1, suggesting that the transcription factor Sp1 plays a role. Sp1 bound multiple Sp1 sites on the TRAIL promoter, including two established (Sp1-1 and -2) and two novel Sp1-5/6 and -7 sites. PDGF-BB-inducible TRAIL promoter activity by Sp1 was mediated through these sites, since transverse mutations to each abolished inducible activity. PDGF-BB stimulation increased acetylation of histone-3 (ac-H3) and expression of the transcriptional co-activator p300, implicating chromatin remodelling. p300 overexpression increased TRAIL promoter activity, which was blocked by dominant-negative Sp1. Furthermore, PDGF-BB treatment increased the physical interaction of Sp1, p300 and ac-H3, while chromatin immunoprecipitation studies revealed Sp1, p300 and ac-H3 enrichment on the TRAIL promoter. Taken together, our studies demonstrate for the first time that PDGF-BB-induced TRAIL transcriptional activity requires the cooperation of Sp1, ac-H3 and p300, mediating increased expression of TRAIL which is important for VSMC proliferation and **migration**. Our findings have the promising potential for targeting TRAIL as a new therapeutic for vascular proliferative disorders.

#### GeneRIFs

1. Studies demonstrate for the first time that PDGF-BB-induced TRAIL transcriptional activity requires the cooperation of Sp1, ac-H3 and p300, mediating increased expression of TRAIL which is important for VSMC proliferation and **migration**.

### ECM

#### PubMed

1. Antifibrotic effect via the regulation of transcription factor Sp1 in lung fibrosis. The aim of this study is to evaluate the antifibrotic effect of ring-type Sp1 decoy oligonucleotides (ODNs) through blocking the transcription of transforming growth factor (TGF)-beta1 and its downstream target genes. In this experiment, the expression of TGF-beta1, metalloproteinase (MMP)-13, and fibronectin was decreased in the group with the treatment of the ring-type Sp1 decoy ODNs. Also, alpha-smooth muscle actin positive bronchial lining cells and alveolar epithelial cells were observed, especially around the lesions of extracellular matrix (**ECM**) deposition. These findings provide evidences for the finding of pulmonary epithelial-mesenchymal transition (EMT) and the effectiveness of Sp1 transcription factor as a target for the gene therapy on lung fibrosis.
2. miR-204 regulates epithelial-mesenchymal transition by targeting SP1 in the tubular epithelial cells after acute kidney injury induced by ischemia-reperfusion. Acute kidney injury (AKI) is a disease where kidney function is lost almost instantaneously; it can develop very rapidly over few hours to maximum of few days. Despite the advent of technology, the clinical management against this disease is very poor, and most of the time it is life-threatening. AKI has been actively regulated by extracellular matrix proteins (**ECM**), however, its underlying mechanism of regulation during AKI progression is very poorly understood. In this study, we explored the integrated network of mRNA and microRNAs (miRNAs) that maintains the progression of **ECM** after induction of AKI by lethal ischemia. To identify key regulators of **ECM**, we screened large number of transcriptomes using laser capture microdissection (LCM) technique in addition to microarray and RT-qPCR. Our result clearly showed that 9 miRNAs including miR-21, miR-483, miR-5115, miR-204e, miR-128, miR-181c, miR-203, miR-204 and miR-204c were highly regulated, out of which miR-204 expression change (decrease) was most drastic during ischemia/reperfusion. Detail mechanistic study utilizing combined experimental and computational approach revealed that TGF-β signaling pathway was potentially modulated by deregulated miRNA-204 through SP1, where the TGF-β signaling pathway plays a vital role **in** ECM regulation. Apart from targeting SP1 and antagonizing epithelial-mesenchymal transition (EMT) signaling our result also showed that miR-204 protects interstitial tissue of renal tubules from chronic fibrotic change. Altogether our study provides sufficient details of how miRNA mediat**ed** ECM regulation occur during AKI, which can be effectively utilized in future for better AKI management and diagnosis.

### [Ee]xtracellular [Mm]atrix

#### PubMed

1. Down-regulation of tissue inhibitor of metalloproteinase-3 (TIMP-3) expression is necessary for adipocyte differentiation. Matrix metalloproteinase activity is essential for proper **extracellular matrix** remodeling that takes place during adipose tissue formation. Four tissue inhibitors of matrix metalloproteinases (TIMPs) regulate their activity. However, the role of TIMPs in adipocyte differentiation is poorly understood. We found that the expression of all TIMPs was modified during adipocyte differentiation, but that of TIMP-3 was distinguished by its extreme down-regulation. TIMP-3 expression was closely linked to the differentiation process. Indeed, it remained low during the adipocyte differentiation but increased when cell differentiation was prevented. We identified the transcription factor Sp1 as being responsible for the regulation of TIMP-3 expression during adipocyte differentiation. Overexpression of TIMP-3 reduced adipocyte differentiation, underlining its active role in this process. TIMP-3 overexpression decreased the expression of the early and obligate key inductors of adipogenesis Krüppel-like factor 4 (Klf4), early growth response 2 (Egr2/Krox20), and CAAT/enhancer-binding protein beta (C/EBPbeta). Our results indicate that during preadipocyte differentiation, the Sp1-dependent decrease in TIMP-3 expression is required for the successful implementation of the adipocyte differentiation program.
2. Down-regulation of tissue inhibitor of metalloproteinase-3 (TIMP-3) expression is necessary for adipocyte differentiation. Matrix metalloproteinase activity is essential for proper **extracellular matrix** remodeling that takes place during adipose tissue formation. Four tissue inhibitors of matrix metalloproteinases (TIMPs) regulate their activity. However, the role of TIMPs in adipocyte differentiation is poorly understood. We found that the expression of all TIMPs was modified during adipocyte differentiation, but that of TIMP-3 was distinguished by its extreme down-regulation. TIMP-3 expression was closely linked to the differentiation process. Indeed, it remained low during the adipocyte differentiation but increased when cell differentiation was prevented. We identified the transcription factor Sp1 as being responsible for the regulation of TIMP-3 expression during adipocyte differentiation. Overexpression of TIMP-3 reduced adipocyte differentiation, underlining its active role in this process. TIMP-3 overexpression decreased the expression of the early and obligate key inductors of adipogenesis Krüppel-like factor 4 (Klf4), early growth response 2 (Egr2/Krox20), and CAAT/enhancer-binding protein beta (C/EBPbeta). Our results indicate that during preadipocyte differentiation, the Sp1-dependent decrease in TIMP-3 expression is required for the successful implementation of the adipocyte differentiation program.
3. Antifibrotic effect via the regulation of transcription factor Sp1 in lung fibrosis. The aim of this study is to evaluate the antifibrotic effect of ring-type Sp1 decoy oligonucleotides (ODNs) through blocking the transcription of transforming growth factor (TGF)-beta1 and its downstream target genes. In this experiment, the expression of TGF-beta1, metalloproteinase (MMP)-13, and fibronectin was decreased in the group with the treatment of the ring-type Sp1 decoy ODNs. Also, alpha-smooth muscle actin positive bronchial lining cells and alveolar epithelial cells were observed, especially around the lesions of **extracellular matrix** (ECM) deposition. These findings provide evidences for the finding of pulmonary epithelial-mesenchymal transition (EMT) and the effectiveness of Sp1 transcription factor as a target for the gene therapy on lung fibrosis.
4. Sp proteins and Runx2 mediate regulation of matrix gla protein (MGP) expression by parathyroid hormone. As part of its catabolic action in bone, parathyroid hormone (PTH) inhibits **extracellular matrix** mineralization. We previously showed that PTH dose-dependently induces matrix gla protein (MGP) expression in osteoblasts and this induction is at least partially responsible for PTH-mediated inhibition of mineralization. Recently, we identified PKA and ERK/MAPK as the key signaling pathways involved in PTH regulation of MGP expression. The goal of this study was to further characterize the mechanism by which PTH stimulates expression of MGP. Deletion analysis of the murine Mgp gene promoter identified a PTH-responsive region between -173 bp and-49 bp. Using gel-mobility shift assays we found that Sp1/Sp3, and Runx2 bind to distinct sites within this region. Mutation of either the Sp or the Runx2 site reduced MGP induction by PTH, while mutation of both sites completely abolished PTH responsiveness. Overexpression of Runx2 or Sp1 activated the Mgp reporter, while Sp3 was a dose-dependent repressor of Sp1 and PTH-induced MGP expression. Collectively, these data show that PTH regulates MGP gene transcription in osteoblasts through altered activities of Sp and Runx2 transcription factors.
5. miR-204 regulates epithelial-mesenchymal transition by targeting SP1 in the tubular epithelial cells after acute kidney injury induced by ischemia-reperfusion. Acute kidney injury (AKI) is a disease where kidney function is lost almost instantaneously; it can develop very rapidly over few hours to maximum of few days. Despite the advent of technology, the clinical management against this disease is very poor, and most of the time it is life-threatening. AKI has been actively regulated by **extracellular matrix** proteins (ECM), however, its underlying mechanism of regulation during AKI progression is very poorly understood. In this study, we explored the integrated network of mRNA and microRNAs (miRNAs) that maintains the progression of ECM after induction of AKI by lethal ischemia. To identify key regulators of ECM, we screened large number of transcriptomes using laser capture microdissection (LCM) technique in addition to microarray and RT-qPCR. Our result clearly showed that 9 miRNAs including miR-21, miR-483, miR-5115, miR-204e, miR-128, miR-181c, miR-203, miR-204 and miR-204c were highly regulated, out of which miR-204 expression change (decrease) was most drastic during ischemia/reperfusion. Detail mechanistic study utilizing combined experimental and computational approach revealed that TGF-β signaling pathway was potentially modulated by deregulated miRNA-204 through SP1, where the TGF-β signaling pathway plays a vital role in ECM regulation. Apart from targeting SP1 and antagonizing epithelial-mesenchymal transition (EMT) signaling our result also showed that miR-204 protects interstitial tissue of renal tubules from chronic fibrotic change. Altogether our study provides sufficient details of how miRNA mediated ECM regulation occur during AKI, which can be effectively utilized in future for better AKI management and diagnosis.
6. miR-29 Regulates Type VII Collagen in Recessive Dystrophic Epidermolysis Bullosa. Recessive dystrophic epidermolysis bullosa (RDEB) is a complex inherited skin disorder caused by loss-of-function mutations in the COL7A1 gene. For an effective treatment of this disorder to be realized, both a thorough understanding of the regulation of COL7A1 and an understanding of the underlying nature of the complications of RDEB are needed. Currently, both posttranscriptional regulation of COL7A1 and the underlying causes of fibrosis in RDEB patients are poorly understood. Here, we describe a mechanism of regulation, to our knowledge previously unknown, by which micro RNA-29 (miR-29) regulates COL7A1 in a complex network: both directly through targeting its 3' untranslated region at two distinct seed regions and indirectly through targeting an essential transcription factor required for basal COL7A1 expression, SP1. In turn, miR-29 itself is regulated by SP1 activity and transforming growth factor-β signaling. RDEB mice express high levels of transforming growth factor-β and significantly lower miR-29 compared with wild-type cohorts. The sustained decrease in miR-29 in RDEB skin leads to an increase of miR-29 target genes expressed in the skin, including profibroti**c extracellular matr**ix collagens. Collectively, we identify miR-29 as an important factor in both regulating COL7A1 and inhibiting transforming growth factor-β-mediated fibrosis.
7. Sp1 upregulates the proximal promoter activity of the mouse collagen α1(XI) gene (Col11a1) in chondrocytes. Type XI collagen is a cartilage-specific **extracellular matri**x, and is important for collagen fibril formation and skeletal morphogenesis. We have previously reported that NF-Y regulated the proximal promoter activity of the mouse collagen α1(XI) gene (Col11a1) in chondrocytes (Hida et. al. In Vitro Cell. Dev. Biol. Anim. 2014). However, the mechanism of the Col11a1 gene regulation in chondrocytes has not been fully elucidated. In this study, we further characterized the proximal promoter activity of the mouse Col11a1 gene in chondrocytes. Cell transfection experiments with deletion and mutation constructs indicated that the downstream region of the NF-Y binding site (-116 to +1) is also necessary to regulate the proximal promoter activity of the mouse Col11a1 gene. This minimal promoter region has no TATA box and GC-rich sequence; we therefore examined whether the GC-rich sequence (-96 to -67) is necessary for the transcription regulation of the Col11a1 gene. Luciferase assays using a series of mutation constructs exhibited that the GC-rich sequence is a critical element of Col11a1 promoter activity in chondrocytes. Moreover, in silico analysis of this region suggested that one of the most effective candidates was transcription factor Sp1. Consistent with the prediction, overexpression of Sp1 significantly increased the promoter activity. Furthermore, knockdown of Sp1 expression by siRNA transfection suppressed the proximal promoter activity and the expression of endogenous transcript of the mouse Col11a1 gene. Taken together, these results indicate that the transcription factor Sp1 upregulates the proximal promoter activity of the mouse Col11a1 gene in chondrocytes.
8. Myocardin-related transcription factor-A complexes activate type I collagen expression in lung fibroblasts. Pulmonary fibrosis is characterized by the excessive deposition of a collagen-rich **extracellular matrix**. The accumulation of collagen within the lung interstitium leads to impaired respiratory function. Furthermore, smooth muscle actin-positive myofibroblasts within the fibrotic lung contribute to disease progression. Because collagen and smooth muscle cell α-actin are coordinately expressed in the setting of fibrosis, the hypothesis was tested that specific transcriptional regulators of the myocardin family might also regulate collagen gene expression in myofibroblasts. Myocardin-related transcription factors (MRTFs), through their interaction with the serum-response factor (SRF) on CArG box regulatory elements (CC(A/T)6GG), are important regulators of myofibroblast differentiation. MRTF-A transactivated type I collagen gene reporters as much as 100-fold in lung myofibroblasts. Loss of functional MRTF-A using either a dominant negative MRTF-A isoform, shRNA targeting MRTF-A, or genetic deletion of MRTF-A in lung fibroblasts significantly disrupted type I collagen synthesis relative to controls. Analysis of the COL1A2 proximal promoter revealed a noncanonical CArG box (CCAAACTTGG), flanked by several Sp1 sites important for MRTF-A activation. Chromatin immunoprecipitation experiments confirmed the co-localization of MRTF-A, SRF, and Sp1 bound to the same region of the COL1A2 promoter. Mutagenesis of either the noncanonical CArG box or the Sp1 sites significantly disrupted MRTF-A activation of COL1A2. Together, our findings show that MRTF-A is an important regulator of collagen synthesis in lung fibroblasts and exhibits a dependence on both SRF and Sp1 function to enhance collagen expression.

### [Ii]nvasive

#### PubMed

1. Chronic exposure to 12-O-tetradecanoylphorbol-13-acetate represses sod2 induction in vivo: the negative role of p50. It is well documented that the tumor promoter 12-O-tetradecanoylphorbol-13-acetate (TPA) can activate manganese superoxide dismutase (MnSOD) expression. However, it is unclear how repeated exposure to TPA following a single application of tumor initiator 7,12-dimethylbenz-(a)-anthracene causes tumor development. We generated transgenic mice expressing human MnSOD promoter- and enhancer-driven luciferase reporter gene and used a non-**invasive** imaging system to investigate the effects of TPA on MnSOD expression in vivo. Our data indicate that TPA initially activates MnSOD expression, but this positive effect declines after repeated applications. Changes in MnSOD expression in vivo were verified by measuring MnSOD mRNA and protein levels. Using chromatin immunoprecipitation coupled to Western analysis of the transcription factors known to be essential for the constitutive and TPA-induced transcription of MnSOD, we found that TPA treatment leads to both activation and inactivation of MnSOD gene transcription. During the activation phase, the levels of p50, p65, specificity protein 1 (Sp1) and nucleophosmin (NPM) increase after TPA treatments. Sustained treatments with TPA lead to further increase of p50 but not p65, Sp1 or NPM, suggesting that excess p50 may have inhibitory effects leading to the suppression of MnSOD. Alteration of p50 levels by expressing p50 cDNA or p50 small interfering RNA in mouse epithelial (JB6) cells confirms that p50 is inhibitory to MnSOD transcription. These findings identify p50 as having a negative effect on MnSOD induction upon repeated applications of TPA and provide an insight into a cause for the reduction of MnSOD expression during early stages of skin carcinogenesis.
2. Sp1 expression regulates lung tumor progression. The role of specificity protein 1 (Sp1) in controlling gene expression in lung tumor development and metastasis is not well understood. In this study, we showed that the Sp1 level was highly increased and required for lung tumor growth in transgenic mice bearing Kras-induced lung tumors under the control of doxycycline. Furthermore, the Sp1 level was highly upregulated in lung adenocarcinoma cells with low **invasive**ness and in patients with stage I lung cancer. We also demonstrated that Sp1 was downregulated in lung adenocarcinoma cells with high **invasive**ness and in patients with stage IV lung adenocarcinoma. Moreover, Sp1 inversely regulated migration, invasion and metastasis of lung adenocarcinoma cells in vivo. In addition, a decrease in the Sp1 level in highly **invasive** lung adenocarcinoma cells resulted from instability of the Sp1 protein. Furthermore, overexpression of Sp1 in highly **invasive** lung adenocarcinoma cells increased expression of E-cadherin, a suppressor of metastasis, and attenuated the translocation of β-catenin into the cellular nucleus that leads to tumor malignancy. Taken together, Sp1 level accumulated strongly in early stage and then declined in late stage, which is important for lung cancer cell proliferation and metastasis during tumorigenesis.

### [Mm]etastasis

#### PubMed

1. Differential regulation of GPR54 transcription by specificity protein-1 and partial estrogen response element in mouse pituitary cells. Precise spatial and temporal expression of the recently identified G-protein coupled receptor GPR54 is critical for proper reproductive function and **metastasis** suppression. However, regulatory factors that control GPR54 expression remain unknown. Thus, the identification of these cis-acting DNA elements can provide insight into the role of GPR54 in reproduction and cancer. Using luciferase reporter, electrophoretic mobility shift, and chromatin immunoprecipitation assays, we demonstrate that three SP1 sites and a partial estrogen response element modulate mouse GPR54 (mGPR54) promoter activity. Supporting experiments show transcription factor SP1 binds directly to the mGPR54 promoter region and activates gene expression. In conclusion, these novel findings now identify factors that regulate activity of the mGPR54 promoter, and these factors are highly conserved across multiple mammalian species.
2. Sp1 expression regulates lung tumor progression. The role of specificity protein 1 (Sp1) in controlling gene expression in lung tumor development and **metastasis** is not well understood. In this study, we showed that the Sp1 level was highly increased and required for lung tumor growth in transgenic mice bearing Kras-induced lung tumors under the control of doxycycline. Furthermore, the Sp1 level was highly upregulated in lung adenocarcinoma cells with low invasiveness and in patients with stage I lung cancer. We also demonstrated that Sp1 was downregulated in lung adenocarcinoma cells with high invasiveness and in patients with stage IV lung adenocarcinoma. Moreover, Sp1 inversely regulated migration, invasion and **metastasis** of lung adenocarcinoma cells in vivo. In addition, a decrease in the Sp1 level in highly invasive lung adenocarcinoma cells resulted from instability of the Sp1 protein. Furthermore, overexpression of Sp1 in highly invasive lung adenocarcinoma cells increased expression of E-cadherin, a suppressor of **metastasis**, and attenuated the translocation of β-catenin into the cellular nucleus that leads to tumor malignancy. Taken together, Sp1 level accumulated strongly in early stage and then declined in late stage, which is important for lung cancer cell proliferation and **metastasi**s during tumorigenesis.
3. Regulation of embryonic kidney branching morphogenesis and glomerular development by KISS1 receptor (Gpr54) through NFAT2- and Sp1-mediated Bmp7 expression. G-protein-coupled receptor 54 (Gpr54, KISS1 receptor) plays critical roles in puberty regulation, tumor **metastasis** suppression, and vasoconstriction. Bone morphogenetic protein-7 (Bmp7) is required for kidney organogenesis. However, whether Gpr54 is involved in embryonic kidney development and how Bmp7 expression is regulated in the kidney are largely unknown. Here we report that Gpr54 deletion leads to kidney branching morphogenesis and glomerular development retardation in embryonic kidneys in vivo and in explanted kidneys in vitro. Gpr54 inactivation results in a high risk of low glomerular number in adult kidneys. Gpr54 is expressed in condensed mesenchyme at E12.5 and epithelial cells of proximal and distal tubules and collecting ducts at E17.5 and P0 mouse kidney. Deletion of Gpr54 decreases Bmp7 expression and Smad1 phosphorylation in the developing kidney. Using chromatin immunoprecipitation and luciferase assays, we demonstrate that Gpr54 regulates NFAT2- and Sp1-mediated Bmp7 transcription. Furthermore, we show that NFAT2 cooperates with Sp1 to promote Bmp7 transcription activation. Together, these data suggest that Gpr54 regulates Bmp7 expression through NFAT2 and Sp1 and plays an important role in embryonic kidney branching morphogenesis and glomerular development.
4. v-Src-mediated down-regulation of SSeCKS **metastasis** suppressor gene promoter by the recruitment of HDAC1 into a USF1-Sp1-Sp3 complex. SSeCKS (Src-suppressed C kinase substrate), also called gravin/AKAP12, is a large scaffolding protein with **metastasis** suppressor activity. Two major isoforms of SSeCKS are expressed in most cell and tissue types under the control of two independent promoters, designated alpha and beta, separated by 68 kb. SSeCKS transcript and protein levels are severely decreased in Src- and Ras-transformed fibroblasts and in many epithelial tumors. By dissecting its promoters with progressive deletion analysis, we identified the sequence between -106 and -49 in the alpha proximal promoter as the minimal v-Src-responsive element, which contains E- and GC-boxes bound by USF1 and Sp1/Sp3, respectively. Both E- and GC-boxes are crucial for v-Src-responsive and basal promoter activities. v-Src does not alter USF1 binding levels at the E-box, but it increases Sp1/Sp3 binding to the GC-box despite no change in their cellular protein abundance. SSeCKS alpha and beta transcript levels in v-Src/3T3 cells can be restored by treatment with the histone deacetylase inhibitor, trichostatin A, but not with the DNA demethylation agent, 5-azacytidine. Chromatin changes are found only on the alpha promoter even though the beta proximal promoter contains a similar E- and GC-box arrangement. Recruitment of HDAC1 is necessary and sufficient to cause repression of alpha proximal promoter activity, and the addition of Sp1 and/or Sp3 potentiates the repression. Our data suggest that suppression of the beta promoter is facilitated by Src-induced changes in the alpha promoter chromatinization mediated by a USF1-Sp1-Sp3 complex.
5. PIASy mediates hypoxia-induced SIRT1 transcriptional repression and epithelial-to-mesenchymal transition in ovarian cancer cells. Epithelial-mesenchymal transition (EMT) has an essential role in organogenesis and contributes to a host of pathologies, including carcinogenesis. Hypoxia (low oxygen supply) aids tumor **metastasis** in part by promoting EMT in cancer cells. The underlying mechanism whereby hypoxia orchestrates EMT remains poorly defined. Here we report that SIRT1, a multifaceted player in tumorigenesis, opposed ovarian cancer **metastasis** in vitro and in vivo by impeding EMT. Hypoxic stress downregulated the expression of SIRT1, primarily at the transcriptional level, by reducing the occupancy of the transcriptional activator Sp1 on the proximal promoter of the SIRT1 gene in a SUMOylation-dependent manner. Further analysis revealed that the SUMO E3 ligase PIASy (also known as PIAS4) was induced by hypoxia and prevented Sp1 from binding to the SIRT1 promoter. Conversely, knockdown of PIASy by small interfering RNA (siRNA) restored Sp1 binding and SIRT1 expression in cancer cells challenged with hypobaric hypoxia, reversed cancer cell EMT, and attenuated **metastasis** in vivo in nude mice. Importantly, analysis of human ovarian tumor specimens indicated that PIASy expression was positively, whereas SIRT1 expression was inversely, correlated with cancer aggressiveness. In summary, our work has identified a new pathway that links downregulation of SIRT1 to hypoxia-induced EMT in ovarian cancer cells and, as such, sheds light on the development of novel anti-tumor therapeutics.
6. HER-2/neu represses the **metastasis** suppressor RECK via ERK and Sp transcription factors to promote cell invasion. Matrix metalloproteinase (MMP) inhibitory proteins may negatively regulate MMP activity to suppress tumor **metastasis**. In this study, we demonstrate that the HER-2/neu oncogene inhibits the expression of the MMP inhibitor RECK to promote cell invasion. RECK was inhibited via transcriptional repression in B104-1-1 cells, which express constitutively active HER-2/neu. Overexpression of HER-2/neu in NIH/3T3 or HaCaT cells also suppressed RECK expression. Deletion and mutation assays showed that HER-2/neu repressed RECK via the Sp1-binding site localized in the -82/-71 region from the translational start site. DNA affinity precipitation and chromatin immunoprecipitation assays indicated that binding of Sp1 and Sp3 to this consensus site was increased in B104-1-1 cells. We also found that HER-2/neu inhibited RECK via the ERK signaling pathway. Sp1 proteins phosphorylated at Thr453 and Thr739 by ERK bound preferentially to the RECK promoter, and this binding was reversed by HER-2/neu and ERK inhibitors. Furthermore, our data indicate that HER-2/neu obviously increased HDAC1 binding to the Sp1-binding site localized in the -82/-71 region of the RECK promoter. The histone deacetylase inhibitor trichostatin A reversed HER-2/neu-induced inhibition of RECK. HER-2/neu activation was associated with increased MMP-9 secretion and activation. Re-expression of RECK in HER-2/neu-overexpressing cells inhibited MMP-9 secretion and cell invasion. Taken together, our results suggest that HER-2/neu induces the binding of Sp proteins and HDAC1 to the RECK promoter to inhibit RECK expression and to promote cell invasion. Restoration of RECK provides a novel strategy for the inhibition of HER-2/neu-induced **metastasis**.

#### GeneRIFs

1. Sp1 level accumulated strongly in early stage and then declined in late stage, which is important for lung cancer cell proliferation and **metastasis** during tumorigenesis.
2. v-Src-mediated down-regulation of SSeCKS **metastasis** suppressor gene promoter by the recruitment of HDAC1 into a USF1-Sp1-Sp3 complex.

### [Cc]ell [Ii]nvasion

#### PubMed

1. HER-2/neu represses the metastasis suppressor RECK via ERK and Sp transcription factors to promote **cell invasion**. Matrix metalloproteinase (MMP) inhibitory proteins may negatively regulate MMP activity to suppress tumor metastasis. In this study, we demonstrate that the HER-2/neu oncogene inhibits the expression of the MMP inhibitor RECK to promote **cell invasion**. RECK was inhibited via transcriptional repression in B104-1-1 cells, which express constitutively active HER-2/neu. Overexpression of HER-2/neu in NIH/3T3 or HaCaT cells also suppressed RECK expression. Deletion and mutation assays showed that HER-2/neu repressed RECK via the Sp1-binding site localized in the -82/-71 region from the translational start site. DNA affinity precipitation and chromatin immunoprecipitation assays indicated that binding of Sp1 and Sp3 to this consensus site was increased in B104-1-1 cells. We also found that HER-2/neu inhibited RECK via the ERK signaling pathway. Sp1 proteins phosphorylated at Thr453 and Thr739 by ERK bound preferentially to the RECK promoter, and this binding was reversed by HER-2/neu and ERK inhibitors. Furthermore, our data indicate that HER-2/neu obviously increased HDAC1 binding to the Sp1-binding site localized in the -82/-71 region of the RECK promoter. The histone deacetylase inhibitor trichostatin A reversed HER-2/neu-induced inhibition of RECK. HER-2/neu activation was associated with increased MMP-9 secretion and activation. Re-expression of RECK in HER-2/neu-overexpressing cells inhibited MMP-9 secretion and **cell invasion**. Taken together, our results suggest that HER-2/neu induces the binding of Sp proteins and HDAC1 to the RECK promoter to inhibit RECK expression and to promote **cell invasion**. Restoration of RECK provides a novel strategy for the inhibition of HER-2/neu-induced metastasis.

#### GeneRIFs

1. HER-2/neu induces the binding of Sp proteins and HDAC1 to the RECK promoter to inhibit RECK expression and to promote **cell invasion**

---

## Sp3

- NCBI Gene
- GeneRIF
- Pubmed

| Search Term | Rank | Fields |
| --- | --- | --- |
| [Ee]xtracellular [Mm]atrix | 8 | PubMed(2) |
| [Mm]etastasis | 12 | PubMed(2); GeneRIFs(1) |

  
hide/show details for Sp3

### [Ee]xtracellular [Mm]atrix

#### PubMed

1. Sp proteins and Runx2 mediate regulation of matrix gla protein (MGP) expression by parathyroid hormone. As part of its catabolic action in bone, parathyroid hormone (PTH) inhibits **extracellular matrix** mineralization. We previously showed that PTH dose-dependently induces matrix gla protein (MGP) expression in osteoblasts and this induction is at least partially responsible for PTH-mediated inhibition of mineralization. Recently, we identified PKA and ERK/MAPK as the key signaling pathways involved in PTH regulation of MGP expression. The goal of this study was to further characterize the mechanism by which PTH stimulates expression of MGP. Deletion analysis of the murine Mgp gene promoter identified a PTH-responsive region between -173 bp and-49 bp. Using gel-mobility shift assays we found that Sp1/Sp3, and Runx2 bind to distinct sites within this region. Mutation of either the Sp or the Runx2 site reduced MGP induction by PTH, while mutation of both sites completely abolished PTH responsiveness. Overexpression of Runx2 or Sp1 activated the Mgp reporter, while Sp3 was a dose-dependent repressor of Sp1 and PTH-induced MGP expression. Collectively, these data show that PTH regulates MGP gene transcription in osteoblasts through altered activities of Sp and Runx2 transcription factors.
2. Sp proteins and Runx2 mediate regulation of matrix gla protein (MGP) expression by parathyroid hormone. As part of its catabolic action in bone, parathyroid hormone (PTH) inhibits **extracellular matrix** mineralization. We previously showed that PTH dose-dependently induces matrix gla protein (MGP) expression in osteoblasts and this induction is at least partially responsible for PTH-mediated inhibition of mineralization. Recently, we identified PKA and ERK/MAPK as the key signaling pathways involved in PTH regulation of MGP expression. The goal of this study was to further characterize the mechanism by which PTH stimulates expression of MGP. Deletion analysis of the murine Mgp gene promoter identified a PTH-responsive region between -173 bp and-49 bp. Using gel-mobility shift assays we found that Sp1/Sp3, and Runx2 bind to distinct sites within this region. Mutation of either the Sp or the Runx2 site reduced MGP induction by PTH, while mutation of both sites completely abolished PTH responsiveness. Overexpression of Runx2 or Sp1 activated the Mgp reporter, while Sp3 was a dose-dependent repressor of Sp1 and PTH-induced MGP expression. Collectively, these data show that PTH regulates MGP gene transcription in osteoblasts through altered activities of Sp and Runx2 transcription factors.

### [Mm]etastasis

#### PubMed

1. v-Src-mediated down-regulation of SSeCKS **metastasis** suppressor gene promoter by the recruitment of HDAC1 into a USF1-Sp1-Sp3 complex. SSeCKS (Src-suppressed C kinase substrate), also called gravin/AKAP12, is a large scaffolding protein with **metastasis** suppressor activity. Two major isoforms of SSeCKS are expressed in most cell and tissue types under the control of two independent promoters, designated alpha and beta, separated by 68 kb. SSeCKS transcript and protein levels are severely decreased in Src- and Ras-transformed fibroblasts and in many epithelial tumors. By dissecting its promoters with progressive deletion analysis, we identified the sequence between -106 and -49 in the alpha proximal promoter as the minimal v-Src-responsive element, which contains E- and GC-boxes bound by USF1 and Sp1/Sp3, respectively. Both E- and GC-boxes are crucial for v-Src-responsive and basal promoter activities. v-Src does not alter USF1 binding levels at the E-box, but it increases Sp1/Sp3 binding to the GC-box despite no change in their cellular protein abundance. SSeCKS alpha and beta transcript levels in v-Src/3T3 cells can be restored by treatment with the histone deacetylase inhibitor, trichostatin A, but not with the DNA demethylation agent, 5-azacytidine. Chromatin changes are found only on the alpha promoter even though the beta proximal promoter contains a similar E- and GC-box arrangement. Recruitment of HDAC1 is necessary and sufficient to cause repression of alpha proximal promoter activity, and the addition of Sp1 and/or Sp3 potentiates the repression. Our data suggest that suppression of the beta promoter is facilitated by Src-induced changes in the alpha promoter chromatinization mediated by a USF1-Sp1-Sp3 complex.
2. v-Src-mediated down-regulation of SSeCKS **metastasis** suppressor gene promoter by the recruitment of HDAC1 into a USF1-Sp1-Sp3 complex. SSeCKS (Src-suppressed C kinase substrate), also called gravin/AKAP12, is a large scaffolding protein with **metastasis** suppressor activity. Two major isoforms of SSeCKS are expressed in most cell and tissue types under the control of two independent promoters, designated alpha and beta, separated by 68 kb. SSeCKS transcript and protein levels are severely decreased in Src- and Ras-transformed fibroblasts and in many epithelial tumors. By dissecting its promoters with progressive deletion analysis, we identified the sequence between -106 and -49 in the alpha proximal promoter as the minimal v-Src-responsive element, which contains E- and GC-boxes bound by USF1 and Sp1/Sp3, respectively. Both E- and GC-boxes are crucial for v-Src-responsive and basal promoter activities. v-Src does not alter USF1 binding levels at the E-box, but it increases Sp1/Sp3 binding to the GC-box despite no change in their cellular protein abundance. SSeCKS alpha and beta transcript levels in v-Src/3T3 cells can be restored by treatment with the histone deacetylase inhibitor, trichostatin A, but not with the DNA demethylation agent, 5-azacytidine. Chromatin changes are found only on the alpha promoter even though the beta proximal promoter contains a similar E- and GC-box arrangement. Recruitment of HDAC1 is necessary and sufficient to cause repression of alpha proximal promoter activity, and the addition of Sp1 and/or Sp3 potentiates the repression. Our data suggest that suppression of the beta promoter is facilitated by Src-induced changes in the alpha promoter chromatinization mediated by a USF1-Sp1-Sp3 complex.

#### GeneRIFs

1. v-Src-mediated down-regulation of SSeCKS **metastasis** suppressor gene promoter by the recruitment of HDAC1 into a USF1-Sp1-Sp3 complex.

---

## Klf5

- NCBI Gene
- GeneRIF
- Pubmed

| Search Term | Rank | Fields |
| --- | --- | --- |
| [Mm]igration | 6 | PubMed(6); GeneRIFs(2) |
| ECM | 7 | PubMed(1) |
| [Ee]xtracellular [Mm]atrix | 8 | PubMed(2) |

  
hide/show details for Klf5

### [Mm]igration

#### PubMed

1. Krüppel-like factor 5 controls keratinocyte **migratio**n via the integrin-linked kinase. **Migratio**n of epithelial cells is critical for normal homeostasis in gut and skin, but the factors regulating this process are not completely understood. The zinc finger transcription factor Klf5 (IKLF; BTEB2) is highly expressed in proliferating cells of esophagus, skin, and other organs. We hypothesized that Klf5 regulates keratinocyte **migratio**n via the integrin-linked kinase (ILK), which, like Klf5, is localized to basal keratinocytes. We stably transduced mouse primary esophageal keratinocytes to overexpress Klf5 or small interfering RNA against Klf5. Klf5 overexpression in keratinocytes increased **migratio**n and correlated directly with ILK expression and activation. ILK expression restored migratory capacity in keratinocytes with suppression of Klf5, whereas ILK small interfering RNA blocked the increased **migratio**n resulting from Klf5 overexpression. By chromatin immunoprecipitation, electromobility shift assay, and luciferase reporter assays, we confirmed that ILK was a direct target for Klf5. In addition, Klf5 induced the activation of the ILK targets Cdc42 and myosin light chain, which are critical for cell **migratio**n and motility but not Rac1, AKT, or GSK3beta. Overall, these results demonstrate that Klf5 is a key regulator of cell **migratio**n via ILK and provide new insight into the regulation of epithelial cell **migratio**n.
2. Krüppel-like factor 5 controls keratinocyte **migratio**n via the integrin-linked kinase. **Migratio**n of epithelial cells is critical for normal homeostasis in gut and skin, but the factors regulating this process are not completely understood. The zinc finger transcription factor Klf5 (IKLF; BTEB2) is highly expressed in proliferating cells of esophagus, skin, and other organs. We hypothesized that Klf5 regulates keratinocyte **migratio**n via the integrin-linked kinase (ILK), which, like Klf5, is localized to basal keratinocytes. We stably transduced mouse primary esophageal keratinocytes to overexpress Klf5 or small interfering RNA against Klf5. Klf5 overexpression in keratinocytes increased **migratio**n and correlated directly with ILK expression and activation. ILK expression restored migratory capacity in keratinocytes with suppression of Klf5, whereas ILK small interfering RNA blocked the increased **migratio**n resulting from Klf5 overexpression. By chromatin immunoprecipitation, electromobility shift assay, and luciferase reporter assays, we confirmed that ILK was a direct target for Klf5. In addition, Klf5 induced the activation of the ILK targets Cdc42 and myosin light chain, which are critical for cell **migratio**n and motility but not Rac1, AKT, or GSK3beta. Overall, these results demonstrate that Klf5 is a key regulator of cell **migratio**n via ILK and provide new insight into the regulation of epithelial cell **migratio**n.
3. Krüppel-like factor 5 is important for maintenance of crypt architecture and barrier function in mouse intestine. Krüppel-like factor 5 (KLF5) is transcription factor that is expressed by dividing epithelial cells of the intestinal epithelium. KLF5 promotes proliferation in vitro and in vivo and is induced by mitogens and various stress stimuli. To study the role of KLF5 in intestinal epithelial homeostasis, we examined the phenotype of mice with conditional deletion of Klf5 in the gut.Mice were generated with intestinal-specific deletion of Klf5 (Vil-Cre;Klf5fl/fl). Morphologic changes in the small intestine and colon were examined by immunohistochemistry, immunoblotting, and real-time polymerase chain reaction.Klf5 mutant mice were born at a normal Mendelian ratio but had high mortality compared with controls. Complete deletion of Klf5 from the intestinal mucosa resulted in neonatal lethality that corresponded with an absence of epithelial proliferation. Variegated intestinal-specific deletion of Klf5 in adult mice resulted in morphologic changes that included a regenerative phenotype, impaired barrier function, and inflammation. Adult mutant mice exhibited defects in epithelial differentiation an**d migrati**on. These changes were associated with reduced expression of Caudal type homeobox (Cdx) 1, Cdx2, and Eph and ephrin signaling proteins. Concomitantly, Wnt signaling to β-catenin was reduced. Proliferation in regenerative crypts was associated with increased expression of the progenitor cell marker Sox9.Deletion of Klf5 in the gut epithelium of mice demonstrated that KLF5 maintains epithelial proliferation, differentiation, and cell positioning along the crypt radial axis. Morphologic changes that occur with deletion of Klf5 are associated with disruption of canonical Wnt signaling and increased expression of Sox9.
4. Krüppel-like factor 5 protects against dextran sulfate sodium-induced colonic injury in mice by promoting epithelial repair. Krüppel-like factor 5 (KLF5) is a transcription factor that promotes proliferation, is highly expressed in dividing crypt cells of the gastrointestinal epithelium, and is induced by various stress stimuli. We sought to determine the role of KLF5 in colonic inflammation and recovery by studying mice with dextran sulfate sodium (DSS)-induced colitis.Wild-type (WT) and Klf5(+/-) mice were given DSS in the drinking water to induce colitis. For recovery experiments, mice were given normal drinking water for 5 days after DSS administration. The extent of colitis was determined using established clinical and histological scoring systems. Immunohistochemical and immunoblotting analyses were used to examine proliferation**, migrati**on, and expression of the epidermal growth factor receptor.Klf5 expression was increased in colonic tissues of WT mice given DSS; induction of Klf5 was downstream of mitogen-activated protein kinase signaling. In DSS-induced colitis, Klf5(+/-) mice exhibited greater sensitivity to DSS than WT mice, with significantly higher clinical and histological colitis scores. In recovery experiments, Klf5(+/-) mice showed poor recovery, with continued weight loss and higher mortality than WT mice. Klf5(+/-) mice from the recovery period had reduced epithelial proliferation and cel**l migrati**on at sites of ulceration compared to WT mice; these reductions correlated with reduced expression of epidermal growth factor receptor.Epithelial repair is an important aspect of recovery from DSS-induced colitis. The transcription factor KLF5 regulates mucosal healing through its effects on epithelial proliferation an**d migrati**on.
5. KLF5 inhibits angiogenesis in PTEN-deficient prostate cancer by attenuating AKT activation and subsequent HIF1α accumulation. KLF5 is a basic transcriptional factor that regulates multiple physiopathological processes. Our recent study showed that deletion of Klf5 in mouse prostate promotes tumorigenesis initiated by the deletion of Pten. While molecular characterization of Klf5-null tumors suggested that angiogenesis was partially responsible for tumor promotion, the precise function and mechanism of KLF5 deletion in prostate tumor angiogenesis remain unclear.Applying histological staining to Pten-null mouse prostates, we observed that deletion of Klf5 significantly increased the number of microvessels, accompanied by the upregulation of multiple angiogenesis-related genes based on microarray analysis with MetaCore software. In human umbilical vein endothelial cells (HuVECs), tube formation and **migratio**n, both of which are indicators of angiogenic activities, were decreased by conditioned media from PC-3 and DU 145 human prostate cancer cells with KLF5 overexpression, but increased by media from cells with KLF5 knockdown. HIF1α, a key angiogenesis inducer, was upregulated by KLF5 loss at the protein but not the mRNA level in both mouse tissues and human cell lines, as determined by immunohistochemical staining, real-time RT-PCR and Western blotting. Consistently, KLF5 loss also upregulated VEGF and PDGF, two pro-angiogenic mediators of HIF1α function, as analyzed by immunohistochemical staining in mouse tissues and ELISA in conditioned media. Mechanistically, AKT activity, which caused the accumulation of HIF1α, was increased by KLF5 knockout or knockdown but decreased by KLF5 overexpression. PI3K/AKT inhibitors consistently abolished the effects of KLF5 knockdown on angiogenic activity, HIF1α accumulation, and VEGF and PDGF expression.KLF5 loss enhances tumor angiogenesis by attenuating PI3K/AKT signaling and subsequent accumulation of HIF1α in PTEN deficient prostate tumors.
6. Krüppel-like factor 5 protects against murine colitis and activates JAK-STAT signaling in vivo. Inflammatory bowel disease (IBD), which is characterized by chronic or recurring inflammation of the gastrointestinal tract, affects 1.4 million persons in the United States alone. KLF5, a Krüppel-like factor (KLF) family member, is expressed within the epithelia of the gastrointestinal tract and has been implicated in rapid cell proliferation**, migrati**on, and remodeling in a number of tissues. Given these functions, we hypothesized that constitutive Klf5 expression would protect against the development of colitis in vivo. To examine the role of KLF5 in vivo, we used the Villin promoter to target Klf5 to the entire horizontal axis of the small intestine and colon. Villin-Klf5 transgenic mice were born at normal Mendelian ratios and appeared grossly normal to at least 1 year of age. Surprisingly, there were no significant changes in cell proliferation or in the differentiation of any of the intestinal lineages within the duodenum, jejunum, ileum, and colon of Villin-Klf5 mice, compared to littermate controls. However, when Villin-Klf5 mice were treated with dextran sodium sulfate (DSS) to induce colitis, they developed less colonic injury and significantly reduced disease activity scores than littermate controls. The mechanism for this decreased injury may come via JAK-STAT signaling, the activation of which was increased in colonic mucosa of DSS treated Villin-Klf5 mice compared to controls. Thus, KLF5 and its downstream mediators may provide therapeutic targets and disease markers for IBD or other diseases characterized by injury and disruption of intestinal epithelia.

#### GeneRIFs

1. Kruppel-like factor 5 regulates mucosal healing through its effects on epithelial proliferation and **migration**.
2. Klf5 induced the activation of the ILK targets Cdc42 and myosin light chain, which are critical for cell **migration** and motility

### ECM

#### PubMed

1. Critical role of Klf5 in regulating gene expression during post-eyelid opening maturation of mouse corneas. Klf5 plays an important role in maturation and maintenance of the mouse ocular surface. Here, we quantify WT and Klf5-conditional null (Klf5CN) corneal gene expression, identify Klf5-target genes and compare them with the previously identified Klf4-target genes to understand the molecular basis for non-redundant functions of Klf4 and Klf5 in the cornea.Postnatal day-11 (PN11) and PN56 WT and Klf5CN corneal transcriptomes were quantified by microarrays to compare gene expression in maturing WT corneas, identify Klf5-target genes, and compare corneal Klf4- and Klf5-target genes. Whole-mount corneal immunofluorescent staining was employed to examine CD45+ cell influx and neovascularization. Effect of Klf5 on expression of desmosomal components was studied by immunofluorescent staining and transient co-transfection assays. Expression of 714 and 753 genes was increased, and 299 and 210 genes decreased in PN11 and PN56 Klf5CN corneas, respectively, with 366 concordant increases and 72 concordant decreases. PN56 Klf5CN corneas shared 241 increases and 98 decreases with those previously described in Klf4CN corneas. Xenobiotic metabolism related pathways were enriched among genes decreased in Klf5CN corneas. Expression of angiogenesis and immune response-related genes was elevated, consistent with neovascularization and CD45+ cell influx in Klf5CN corneas. Expression of 1574 genes was increased and 1915 genes decreased in WT PN56 compared with PN11 corneas. Expression of **ECM**-associated genes decreased, while that of solute carrier family members increased in WT PN56 compared with PN11 corneas. Dsg1a, Dsg1b and Dsp were down-regulated in Klf5CN corneas and their corresponding promoter activities were stimulated by Klf5 in transient co-transfection assays.Differences between PN11 and PN56 corneal Klf5-target genes reveal dynamic changes in functions of Klf5 during corneal maturation. Klf5 contributes to corneal epithelial homeostasis by regulating the expression of desmosomal components. Klf4- and Klf5-target genes are largely distinct, consistent with their non-redundant roles in the mouse cornea.

### [Ee]xtracellular [Mm]atrix

#### PubMed

1. Ezh2-mediated repression of a transcriptional pathway upstream of Mmp9 maintains integrity of the developing vasculature. Maintenance of vascular integrity is required for embryogenesis and organ homeostasis. However, the gene expression programs that stabilize blood vessels are poorly understood. Here, we show that the histone methyltransferase Ezh2 maintains integrity of the developing vasculature by repressing a transcriptional program that activates expression of Mmp9. Inactivation of Ezh2 in developing mouse endothelium caused embryonic lethality with compromised vascular integrity and increased **extracellular matrix** degradation. Genome-wide approaches showed that Ezh2 targets Mmp9 and its activators Fosl1 and Klf5. In addition, we uncovered Creb3l1 as an Ezh2 target that directly activates Mmp9 gene expression in the endothelium. Furthermore, genetic inactivation of Mmp9 rescued vascular integrity defects in Ezh2-deficient embryos. Thus, epigenetic repression of Creb3l1, Fosl1, Klf5 and Mmp9 by Ezh2 in endothelial cells maintains the integrity of the developing vasculature, potentially linking this transcriptional network to diseases with compromised vascular integrity.
2. KLF5/BTEB2, a Krüppel-like zinc-finger type transcription factor, mediates both smooth muscle cell activation and cardiac hypertrophy. Cardiac and vascular biology need to be approached interactively because they share many common biological features as seen in activation of the local renin-angiotensin system, angiogenesis, and **extracellular matri**x production. We previously reported KLF5/BTEB2, a Krüppel-like zinc-finger type transcription factor, to activate various gene promoters that are activated in phenotypically modulated smooth muscle cells, such as a nonmuscle type myosin heavy chain gene SMemb, plasminogen activator inhibitor-1 (PAI-1), iNOS, PDGF-A, Egr-1 and VEGF receptors at least in vitro. KLF5/BTEB2 mRNA levels are downregulated with vascular development but upregulated in neointima that is produced in response to vascular injury. Mitogenic stimulation activates KLF5/BTEB2 gene expression through MEK1 and Egr-1. Chromatin immunoprecipitation assay showed KLF5/BTEB2 to be induced and to bind the promoter of the PDGF-A gene in response to angiotensin II stimulation. In order to define the role of KLF5/BTEB2 in cardiovascular remodeling, we targeted the KLF5/BTEB2 gene in mice. Homozygous mice resulted in early embryonic lethality whereas heterozygous mice were apparently normal. However, in response to external stress, arteries of heterozygotes exhibited diminished levels of smooth muscle and adventitial cell activation. Furthermore, cardiac fibrosis and hypertrophy induced by continuous angiotensin II infusion. We also found that RARa binds KLF5/BTEB2, and that Am80, a potent synthetic RAR agonist, inhibits angiotensin II-induced cardiac hypertrophy. These results indicate that KLF5/BTEB2 is an essential transcription factor that causes not only smooth muscle phenotypic modulation but also cardiac hypertrophy and fibrosis.

---

## Klf10

- NCBI Gene
- GeneRIF
- Pubmed

| Search Term | Rank | Fields |
| --- | --- | --- |
| [Mm]igration | 6 | PubMed(1) |
| [Ee]xtracellular [Mm]atrix | 8 | PubMed(1) |
| [Mm]etastasis | 12 | PubMed(1); GeneRIFs(1) |
| [Cc]ell [Ii]nvasion | 13 | PubMed(1) |

  
hide/show details for Klf10

### [Mm]igration

#### PubMed

1. Bone marrow-derived Kruppel-like factor 10 controls reendothelialization in response to arterial injury. The objective of this study was to investigate the role of Kruppel-like factor (KLF) 10, a zinc-finger transcription factor, in bone marrow (BM)-derived cell responses to arterial endothelial injury. Accumulating evidence indicates that BM-derived progenitors are recruited to sites of vascular injury and contribute to endothelial repair.In response to carotid artery endothelial denudation, KLF10 mRNA expression was markedly increased in both BM and circulating lin(-) progenitor cells. To examine the specific role of KLF10 in arterial reendothelialization, we used 2 models of endothelial denudation (wire- and thermal-induced injury) of the carotid artery in wild-type (WT) and KLF10(-/-) mice. WT mice displayed higher areas of reendothelialization compared with KLF10(-/-) mice after endothelial injury using either method. BM transplant studies revealed that reconstitution of KLF10(-/-) mice with WT BM fully rescued the defect in reendothelialization and increased lin(-)CD34(+)kinase insert domain receptor(+) progenitors in the blood and injured carotid arteries. Conversely, reconstitution of WT mice with KLF10(-/-) BM recapitulated the defects in reendothelialization and peripheral cell progenitors. The media from cultured KLF10(-)/(-) BM progenitors was markedly inefficient in promoting endothelial cell growth and **migration** compared with the media from WT progenitors, indicative of defective paracrine trophic effects from KLF10(-)/(-) BM progenitors. Finally, BM-derived KLF10(-/-) lin(-) progenitors from reconstituted mice had reduced CXC-chemokine receptor 4 expression and impaired migratory responses.Collectively, these observations demonstrate a protective role for BM-derived KLF10 in paracrine and homing responses important for arterial endothelial injury and highlight KLF10 as a possible therapeutic target to promote endothelial repair in vascular disease states.

### [Ee]xtracellular [Mm]atrix

#### PubMed

1. TIEG1-null tenocytes display age-dependent differences in their gene expression, adhesion, spreading and proliferation properties. The remodeling of **extracellular matrix** is a crucial mechanism in tendon development and the proliferation of fibroblasts is a key factor in this process. The purpose of this study was to further elucidate the role of TIEG1 in mediating important tenocyte properties throughout the aging process. Wildtype and TIEG1 knockout tenocytes adhesion, spreading and proliferation were characterized on different substrates (fibronectin, collagen type I, gelatin and laminin) and the expression levels of various genes known to be involved with tendon development were analyzed by RT-PCR. The experiments revealed age-dependent and substrate-dependent properties for both wildtype and TIEG1 knockout tenocytes. Taken together, our results indicate an important role for TIEG1 in regulating tenocytes adhesion, spreading, and proliferation throughout the aging process. Understanding the basic mechanisms of TIEG1 in tenocytes may provide valuable information for treating multiple tendon disorders.

### [Mm]etastasis

#### PubMed

1. TIEG1 inhibits breast cancer invasion and **metastasis** by inhibition of epidermal growth factor receptor (EGFR) transcription and the EGFR signaling pathway. TIEG1 can induce apoptosis of cancer cells, but its role in inhibiting invasion and **metastasis** has not been reported and is unclear. In this study, we find that decreased TIEG1 expression is associated with increased human epidermal growth factor receptor (EGFR) expression in breast cancer tissues and cell lines. TIEG1 plays an important role in suppressing transcription of EGFR by directly binding to the EGFR promoter. While overexpression of TIEG1 attenuates EGFR expression, knockdown of TIEG1 stimulates EGFR expression. Furthermore, TIEG1 and HDAC1 form a complex, which binds to Sp1 sites on the EGFR promoter and inhibits its transcription by suppressing histone acetylation. TIEG1 significantly inhibits breast cancer cell invasion, suppresses mammary tumorigenesis in xenografts in mice, and decreases lung **metastasis** by inhibition of EGFR gene transcription and the EGFR signaling pathway. Therefore, TIEG1 is an anti**metastasis** gene product; regulation of EGFR expression by TIEG1 may be part of an integral signaling pathway that determines and explains breast cancer invasion and **metastasis**.

#### GeneRIFs

1. Tieg1 suppresses mammary tumorigenesis in xenografts in mice, and decreases lung **metastasis** by inhibition of Egfr gene transcription and the Egfr signaling pathway.

### [Cc]ell [Ii]nvasion

#### PubMed

1. TIEG1 inhibits breast cancer invasion and metastasis by inhibition of epidermal growth factor receptor (EGFR) transcription and the EGFR signaling pathway. TIEG1 can induce apoptosis of cancer cells, but its role in inhibiting invasion and metastasis has not been reported and is unclear. In this study, we find that decreased TIEG1 expression is associated with increased human epidermal growth factor receptor (EGFR) expression in breast cancer tissues and cell lines. TIEG1 plays an important role in suppressing transcription of EGFR by directly binding to the EGFR promoter. While overexpression of TIEG1 attenuates EGFR expression, knockdown of TIEG1 stimulates EGFR expression. Furthermore, TIEG1 and HDAC1 form a complex, which binds to Sp1 sites on the EGFR promoter and inhibits its transcription by suppressing histone acetylation. TIEG1 significantly inhibits breast cancer **cell invasion**, suppresses mammary tumorigenesis in xenografts in mice, and decreases lung metastasis by inhibition of EGFR gene transcription and the EGFR signaling pathway. Therefore, TIEG1 is an antimetastasis gene product; regulation of EGFR expression by TIEG1 may be part of an integral signaling pathway that determines and explains breast cancer invasion and metastasis.

---

## Scaf11

- NCBI Gene
- GeneRIF
- Pubmed

| Search Term | Rank | Fields |
| --- | --- | --- |
| [Ii]nvasive | 10 | PubMed(1) |

  
hide/show details for Scaf11

### [Ii]nvasive

#### PubMed

1. Cytoplasmic LPS activates caspase-11: implications in TLR4-independent endotoxic shock. Inflammatory caspases, such as caspase-1 and -11, mediate innate immune detection of pathogens. Caspase-11 induces pyroptosis, a form of programmed cell death, and specifically defends against bacterial pathogens that invade the cytosol. During endotoxemia, however, excessive caspase-11 activation causes shock. We report that contamination of the cytoplasm by lipopolysaccharide (LPS) is the signal that triggers caspase-11 activation in mice. Specifically, caspase-11 responds to penta- and hexa-acylated lipid A, whereas tetra-acylated lipid A is not detected, providing a mechanism of evasion for cytosol-**invasive** Francisella. Priming the caspase-11 pathway in vivo resulted in extreme sensitivity to subsequent LPS challenge in both wild-type and Tlr4-deficient mice, whereas Casp11-deficient mice were relatively resistant. Together, our data reveal a new pathway for detecting cytoplasmic LPS.

---

## Akap12

- NCBI Gene
- GeneRIF
- Pubmed

| Search Term | Rank | Fields |
| --- | --- | --- |
| [Ff]ilopodia | 1 | PubMed(1); GeneRIFs(1) |
| [Mm]igration | 6 | PubMed(1) |
| [Ii]nvasive | 10 | PubMed(1) |
| [Mm]etastasis | 12 | PubMed(9); GeneRIFs(2) |

  
hide/show details for Akap12

### [Ff]ilopodia

#### PubMed

1. Suppression of chemotaxis by SSeCKS via scaffolding of phosphoinositol phosphates and the recruitment of the Cdc42 GEF, Frabin, to the leading edge. Chemotaxis is controlled by interactions between receptors, Rho-family GTPases, phosphatidylinositol 3-kinases, and cytoskeleton remodeling proteins. We investigated how the metastasis suppressor, SSeCKS, attenuates chemotaxis. Chemotaxis activity inversely correlated with SSeCKS levels in mouse embryo fibroblasts (MEF), DU145 and MDA-MB-231 cancer cells. SSeCKS loss induced chemotactic velocity and linear directionality, correlating with replacement of leading edge lamellipodia with fascin-enriched **filopodia**-like extensions, the formation of thickened longitudinal F-actin stress fibers reaching to **filopodia**l tips, relative enrichments at the leading edge of phosphatidylinositol (3,4,5)P3 (PIP3), Akt, PKC-ζ, Cdc42-GTP and active Src (SrcpoY416), and a loss of Rac1. Leading edge lamellipodia and chemotaxis inhibition in SSeCKS-null MEF could be restored by full-length SSeCKS or SSeCKS deleted of its Src-binding domain (ΔSrc), but not by SSeCKS deleted of its three MARCKS (myristylated alanine-rich C kinase substrate) polybasic domains (ΔPBD), which bind PIP2 and PIP3. The enrichment of activated Cdc42 in SSeCKS-null leading ed**ge filopo**dia correlated with recruitment of the Cdc42-specific guanine nucleotide exchange factor, Frabin, likely recruited via multiple PIP2/3-binding domains. Frabin knockdown in SSeCKS-null MEF restores leading edge lamellipodia and chemotaxis inhibition. However, SSeCKS failed to co-immunoprecipitate with Rac1, Cdc42 or Frabin. Consistent with the notion that chemotaxis is controlled by SSeCKS-PIP (vs. -Src) scaffolding activity, constitutively-active phosphatidylinositol 3-kinase could override the ability of the Src inhibitor, SKI-606, to suppress chemotaxis a**nd filopo**dial enrichment of Frabin in SSeCKS-null MEF. Our data suggest a role for SSeCKS in controlling Rac1 vs. Cdc42-induced cellular dynamics at the leading chemotactic edge through the scaffolding of phospholipids and signal mediators, and through the reorganization of the actin cytoskeleton controlling directional movement.

#### GeneRIFs

1. The enrichment of activated Cdc42 in SSeCKS-null leading edge **filopodia** correlated with recruitment of the Cdc42-specific guanine nucleotide exchange factor, Frabin.

### [Mm]igration

#### PubMed

1. A role for SSeCKS, a major protein kinase C substrate with tumour suppressor activity, in cytoskeletal architecture, formation of migratory processes, and cell **migration** during embryogenesis. SSeCKS is a major protein kinase C substrate which has tumour suppressor activity in models of src- and ras-induced oncogenic transformation. The mitogenic regulatory activity of SSeCKS is likely manifested by its ability to bind key signalling proteins such as protein kinases C and A and calmodulin, and to control actin-based cytoskeletal architecture. Rat SSeCKS shares extensive homology with human Gravin, an autoantigen in myasthenia gravis that encodes kinase scaffolding functions and whose expression pattern in fibroblasts and nerves suggests a role in cell motility. Here, we analyse the expression of SSeCKS and Gravin in rodent and human fibroblast and epithelial cell lines using antibodies specific or crossreactive for SSeCKS or Gravin. SSeCKS expression was then analysed in developing mouse embryos and in adult tissues. In the foetal mouse, early SSeCKS protein expression (E10-11) is focused in the loose mesenchyme, luminal surface of the neural tube, notochord, early heart and pericardium, urogenital ridge, and dorsal and ventral sections of limb buds. In later stages (E12-14), SSeCKS is widely expressed in mesenchymal cells but is absent in the spinal ganglia. By E15, SSeCKS expression is ubiquitous, although the staining pattern varies from being striated within smooth muscle sarcomeres to filamentous in mesenchymal and select epithelial cells. In the adult mouse, SSeCKS staining is relatively ubiquitous, with highest expression in the gonads, smooth and cardiac muscle, lung, brain and heart. High expression is also detected in fibroblasts and nerve fibres as well as in more specialized cells such as glomerular mesangial cells and testicular Sertoli cells. SSeCKS expression in the rat testes correlates with the induction of puberty, and in mature mouse spermatozoa, SSeCKS is found in peripheral acrosome membranes and in a helix-like winding pattern within the midsection. Periodic enrichments of SSeCKS are found in sperm midsections and in developing axons, suggesting a role in architectural infrastructure. As with Gravin, high SSeCKS expression is absent in most epithelial cells; however, in contrast to Gravin, SSeCKS is expressed in Purkinje cells, cardiac muscle, macrophages and hepatic stellate cells, indicating overlapping yet distinct patterns of tissue expression in the SSeCKS/Gravin family. The data suggest roles for SSeCKS in the control of cytoskeletal and tissue architecture, formation of migratory processes and cell **migration** during embryogenesis.

### [Ii]nvasive

#### PubMed

1. SERPINB5 and AKAP12 - expression and promoter methylation of metastasis suppressor genes in pancreatic ductal adenocarcinoma. Early metastasis and infiltration are survival limiting characteristics of pancreatic ductal adenocarcinoma (PDAC). Thus, PDAC is likely to harbor alterations in metastasis suppressor genes that may provide novel diagnostic and therapeutic opportunities. This study investigates a panel of metastasis suppressor genes in correlation to PDAC phenotype and examines promoter methylation for regulatory influence on metastasis suppressor gene expression and for its potential as a diagnostic tool.Metastatic and **invasive** potential of 16 PDAC cell lines were quantified in an orthotopic mouse model and mRNA expression of 11 metastasis suppressor genes determined by quantitative RT-PCR. Analysis for promoter methylation was performed using methylation specific PCR and bisulfite sequencing PCR. Protein expression was determined by Western blot.In general, higher metastasis suppressor gene mRNA expression was not consistent with less aggressive phenotypes of PDAC. Instead, mRNA overexpression of several metastasis suppressor genes was found in PDAC cell lines vs. normal pancreatic RNA. Of the investigated metastasis suppressor genes, only higher AKAP12 mRNA expression was correlated with decreased metastasis (P < 0.05) and invasion scores (P < 0.01) while higher SERPINB5 mRNA expression was correlated with increased metastasis scores (P < 0.05). Both genes' promoters showed methylation, but only increased SERPINB5 methylation was associated with loss of mRNA and protein expression (P < 0.05). SERPINB5 methylation was also directly correlated to decreased metastasis scores (P < 0.05).AKAP12 mRNA expression was correlated to attenuated **invasive** and metastatic potential and may be associated with less aggressive phenotypes of PDAC while no such evidence was obtained for the remaining metastasis suppressor genes. Increased SERPINB5 mRNA expression was correlated to increased metastasis and mRNA expression was regulated by methylation. Thus, SERPINB5 methylation was directly correlated to metastasis scores and may provide a diagnostic tool for PDAC.

### [Mm]etastasis

#### PubMed

1. v-Src-mediated down-regulation of SSeCKS **metastasis** suppressor gene promoter by the recruitment of HDAC1 into a USF1-Sp1-Sp3 complex. SSeCKS (Src-suppressed C kinase substrate), also called gravin/AKAP12, is a large scaffolding protein with **metastasis** suppressor activity. Two major isoforms of SSeCKS are expressed in most cell and tissue types under the control of two independent promoters, designated alpha and beta, separated by 68 kb. SSeCKS transcript and protein levels are severely decreased in Src- and Ras-transformed fibroblasts and in many epithelial tumors. By dissecting its promoters with progressive deletion analysis, we identified the sequence between -106 and -49 in the alpha proximal promoter as the minimal v-Src-responsive element, which contains E- and GC-boxes bound by USF1 and Sp1/Sp3, respectively. Both E- and GC-boxes are crucial for v-Src-responsive and basal promoter activities. v-Src does not alter USF1 binding levels at the E-box, but it increases Sp1/Sp3 binding to the GC-box despite no change in their cellular protein abundance. SSeCKS alpha and beta transcript levels in v-Src/3T3 cells can be restored by treatment with the histone deacetylase inhibitor, trichostatin A, but not with the DNA demethylation agent, 5-azacytidine. Chromatin changes are found only on the alpha promoter even though the beta proximal promoter contains a similar E- and GC-box arrangement. Recruitment of HDAC1 is necessary and sufficient to cause repression of alpha proximal promoter activity, and the addition of Sp1 and/or Sp3 potentiates the repression. Our data suggest that suppression of the beta promoter is facilitated by Src-induced changes in the alpha promoter chromatinization mediated by a USF1-Sp1-Sp3 complex.
2. v-Src-mediated down-regulation of SSeCKS **metastasis** suppressor gene promoter by the recruitment of HDAC1 into a USF1-Sp1-Sp3 complex. SSeCKS (Src-suppressed C kinase substrate), also called gravin/AKAP12, is a large scaffolding protein with **metastasis** suppressor activity. Two major isoforms of SSeCKS are expressed in most cell and tissue types under the control of two independent promoters, designated alpha and beta, separated by 68 kb. SSeCKS transcript and protein levels are severely decreased in Src- and Ras-transformed fibroblasts and in many epithelial tumors. By dissecting its promoters with progressive deletion analysis, we identified the sequence between -106 and -49 in the alpha proximal promoter as the minimal v-Src-responsive element, which contains E- and GC-boxes bound by USF1 and Sp1/Sp3, respectively. Both E- and GC-boxes are crucial for v-Src-responsive and basal promoter activities. v-Src does not alter USF1 binding levels at the E-box, but it increases Sp1/Sp3 binding to the GC-box despite no change in their cellular protein abundance. SSeCKS alpha and beta transcript levels in v-Src/3T3 cells can be restored by treatment with the histone deacetylase inhibitor, trichostatin A, but not with the DNA demethylation agent, 5-azacytidine. Chromatin changes are found only on the alpha promoter even though the beta proximal promoter contains a similar E- and GC-box arrangement. Recruitment of HDAC1 is necessary and sufficient to cause repression of alpha proximal promoter activity, and the addition of Sp1 and/or Sp3 potentiates the repression. Our data suggest that suppression of the beta promoter is facilitated by Src-induced changes in the alpha promoter chromatinization mediated by a USF1-Sp1-Sp3 complex.
3. A transgenic mouse model for early prostate **metastasis** to lymph nodes. The emergence of recurrent, metastatic prostate cancer following the failure of androgen-deprivation therapy represents the lethal phenotype of this disease. However, little is known regarding the genes and pathways that regulate this metastatic process, and moreover, it is unclear whether **metastasis** is an early or late event. The individual genetic loss of the **metastasis** suppressor, SSeCKS/Gravin/AKAP12 or Rb, genes that are downregulated or deleted in human prostate cancer, results in prostatic hyperplasia. Here, we show that the combined loss of Akap12 and Rb results in prostatic intraepithelial neoplasia (PIN) that fails to progress to malignancy after 18 months. Strikingly, 83% of mice with PIN lesions exhibited metastases to draining lymph nodes, marked by relatively differentiated tumor cells expressing markers of basal (p63, cytokeratin 14) and luminal (cytokeratin 8 and androgen receptor) epithelial cells, although none expressed the basal marker, cytokeratin 5. The finding that PIN lesions contain increased numbers of p63/AR-positive, cytokeratin 5-negative basal cells compared with WT or Akap12-/- prostate lobes suggests that these transitional cells may be the source of the lymph node metastases. Taken together, these data suggest that in the context of Rb loss, Akap12 suppresses the oncogenic proliferation and early metastatic spread of basal-luminal prostate tumor cells.
4. SERPINB5 and AKAP12 - expression and promoter methylation of **metastasis** suppressor genes in pancreatic ductal adenocarcinoma. Early **metastasis** and infiltration are survival limiting characteristics of pancreatic ductal adenocarcinoma (PDAC). Thus, PDAC is likely to harbor alterations in **metastasis** suppressor genes that may provide novel diagnostic and therapeutic opportunities. This study investigates a panel of **metastasis** suppressor genes in correlation to PDAC phenotype and examines promoter methylation for regulatory influence on **metastasis** suppressor gene expression and for its potential as a diagnostic tool.Metastatic and invasive potential of 16 PDAC cell lines were quantified in an orthotopic mouse model and mRNA expression of 11 **metastasis** suppressor genes determined by quantitative RT-PCR. Analysis for promoter methylation was performed using methylation specific PCR and bisulfite sequencing PCR. Protein expression was determined by Western blot.In general, higher **metastasis** suppressor gene mRNA expression was not consistent with less aggressive phenotypes of PDAC. Instead, mRNA overexpression of several **metastasis** suppressor genes was found in PDAC cell lines vs. normal pancreatic RNA. Of the investigated **metastasis** suppressor genes, only higher AKAP12 mRNA expression was correlated with decreased **metastasis** (P < 0.05) and invasion scores (P < 0.01) while higher SERPINB5 mRNA expression was correlated with increased **metastasis** scores (P < 0.05). Both genes' promoters showed methylation, but only increased SERPINB5 methylation was associated with loss of mRNA and protein expression (P < 0.05). SERPINB5 methylation was also directly correlated to decreased **metastasis** scores (P < 0.05).AKAP12 mRNA expression was correlated to attenuated invasive and metastatic potential and may be associated with less aggressive phenotypes of PDAC while no such evidence was obtained for the remaining **metastasis** suppressor genes. Increased SERPINB5 mRNA expression was correlated to increased **metastasis** and mRNA expression was regulated by methylation. Thus, SERPINB5 methylation was directly correlated to **metastasis** scores and may provide a diagnostic tool for PDAC.
5. Suppression of chemotaxis by SSeCKS via scaffolding of phosphoinositol phosphates and the recruitment of the Cdc42 GEF, Frabin, to the leading edge. Chemotaxis is controlled by interactions between receptors, Rho-family GTPases, phosphatidylinositol 3-kinases, and cytoskeleton remodeling proteins. We investigated how the **metastasis** suppressor, SSeCKS, attenuates chemotaxis. Chemotaxis activity inversely correlated with SSeCKS levels in mouse embryo fibroblasts (MEF), DU145 and MDA-MB-231 cancer cells. SSeCKS loss induced chemotactic velocity and linear directionality, correlating with replacement of leading edge lamellipodia with fascin-enriched filopodia-like extensions, the formation of thickened longitudinal F-actin stress fibers reaching to filopodial tips, relative enrichments at the leading edge of phosphatidylinositol (3,4,5)P3 (PIP3), Akt, PKC-ζ, Cdc42-GTP and active Src (SrcpoY416), and a loss of Rac1. Leading edge lamellipodia and chemotaxis inhibition in SSeCKS-null MEF could be restored by full-length SSeCKS or SSeCKS deleted of its Src-binding domain (ΔSrc), but not by SSeCKS deleted of its three MARCKS (myristylated alanine-rich C kinase substrate) polybasic domains (ΔPBD), which bind PIP2 and PIP3. The enrichment of activated Cdc42 in SSeCKS-null leading edge filopodia correlated with recruitment of the Cdc42-specific guanine nucleotide exchange factor, Frabin, likely recruited via multiple PIP2/3-binding domains. Frabin knockdown in SSeCKS-null MEF restores leading edge lamellipodia and chemotaxis inhibition. However, SSeCKS failed to co-immunoprecipitate with Rac1, Cdc42 or Frabin. Consistent with the notion that chemotaxis is controlled by SSeCKS-PIP (vs. -Src) scaffolding activity, constitutively-active phosphatidylinositol 3-kinase could override the ability of the Src inhibitor, SKI-606, to suppress chemotaxis and filopodial enrichment of Frabin in SSeCKS-null MEF. Our data suggest a role for SSeCKS in controlling Rac1 vs. Cdc42-induced cellular dynamics at the leading chemotactic edge through the scaffolding of phospholipids and signal mediators, and through the reorganization of the actin cytoskeleton controlling directional movement.
6. Carcinogen-induced squamous papillomas and oncogenic progression in the absence of the SSeCKS/AKAP12 **metastasis** suppressor correlate with FAK upregulation. The ability of SSeCKS/Gravin/AKAP12 (SSeCKS) to negatively regulate cell cycle progression is thought to relate to its spatiotemporal scaffolding activity for key signaling molecules such as protein kinase A and C, calmodulin and cyclins. SSeCKS is downregulated upon progression to malignancy in many cancer types, including melanoma and nonmelanoma skin cancer. The forced re-expression of SSeCKS is especially potent in suppressing **metastasis** through the inhibition of VEGF-mediated neovascularization. We have previously shown that SSeCKS-null (KO) mice exhibit hyperplasia and focal dysplasia in the prostate marked by activated Akt. To address whether KO mice exhibit increased skin carcinogenesis, WT and KO C57BL/6 mice were treated topically with 12-O-tetradecanoylphorbol-13-acetate and 7,12-dimethylbenzanthracene. Compared to WT mice, KO mice developed squamous papillomas more rapidly and in greater numbers and also exhibited significantly increased progression to squamous cell carcinoma. Untreated KO epidermal layers were thicker than those in age-matched WT mice and exhibited significantly increased levels of FAK and phospho-ERK1/2, known mediators of carcinogen-induced squamous papilloma progression to carcinoma. Compared to protein levels in WT mouse embryo fibroblasts (MEF), SSeCKS levels were increased in FAK-null cells, whereas FAK levels were increased in SSeCKS-null cells. RNAi studies in WT MEF cells suggest that SSeCKS and FAK attenuate each other's expression. Our study implicates a role for SSeCKS in preventing of skin cancer progression possibly through negatively regulating FAK expression.
7. Rb-dependent cellular senescence, multinucleation and susceptibility to oncogenic transformation through PKC scaffolding by SSeCKS/AKAP12. A subset of AKAPs (A Kinase Anchoring Proteins) regulate signaling and cytoskeletal pathways through the spaciotemporal scaffolding of multiple protein kinases (PK) such as PKC and PKA, and associations with the plasma membrane and the actin-based cytoskeleton. SSeCKS/Gravin/Akap12 expression is severely downregulated in many advanced cancers and exhibits tumor- and **metastasis**-suppressing activity. akap12-null (KO) mice develop prostatic hyperplasia with focal dysplasia, but the precise mechanism how Akap12 prevents oncogenic progression remains unclear. Here, we show that KO mouse embryonic fibroblasts (MEF) exhibit premature senescence marked by polyploidy and multinucleation, and by increased susceptibility to oncogenic transformation. Although p53 and Rb pathways are activated in the absence of Akap12, senescence is dependent on Rb. Senescence is driven by the activation of PKCα, which induces p16(Ink4a)/Rb through a MEK-dependent downregulation of Id1, and PKCδ, which downregulates Lats1/Warts, a mitotic exit network kinase required for cytokinesis. Our data strongly suggest that Akap12 controls Rb-mediated cell aging and oncogenic progression by directly scaffolding and attenuating PKCα/δ.
8. Control of protein kinase C activity, phorbol ester-induced cytoskeletal remodeling, and cell survival signals by the scaffolding protein SSeCKS/GRAVIN/AKAP12. The product of the SSeCKS/GRAVIN/AKAP12 gene ("SSeCKS") is a major protein kinase (PK) C substrate that exhibits tumor- and **metastasis**-suppressing activity likely through its ability to scaffold multiple signaling mediators such as PKC, PKA, cyclins, calmodulin, and Src. Although SSeCKS and PKCα bind phosphatidylserine, we demonstrate that phosphatidylserine-independent binding of PKC by SSeCKS is facilitated by two homologous SSeCKS motifs, EG(I/V)(T/S)XWXSFK(K/R)(M/L)VTP(K/R)K(K/R)X(K/R)XXXEXXXE(E/D) (amino acids 592-620 and 741-769). SSeCKS binding to PKCα decreased kinase activity and was dependent on the two PKC-binding motifs. SSeCKS scaffolding of PKC was increased in confluent cell cultures, correlating with significantly increased SSeCKS protein levels and decreased PKCα activity, suggesting a role for SSeCKS in suppressing PKC activation during contact inhibition. SSeCKS-null mouse embryo fibroblasts displayed increased relative basal and phorbol ester (phorbol 12-myristate 13-acetate)-induced PKC activity but were defective in phorbol 12-myristate 13-acetate-induced actin cytoskeletal reorganization and cell shape change; these responses could be rescued by the forced expression of full-length SSeCKS but not by an SSeCKS variant deleted of its PKC-binding domains. Finally, the PKC binding sites in SSeCKS were required to restore cell rounding and/or decreased apoptosis in phorbol ester-treated LNCaP, LNCaP-C4-2, and MAT-LyLu prostate cancer cells. Thus, PKC-mediated remodeling of the actin cytoskeleton is likely regulated by the ability of SSeCKS to control PKC signaling and activity through a direct scaffolding function.
9. Loss of the SSeCKS/Gravin/AKAP12 gene results in prostatic hyperplasia. SSeCKS/Gravin/AKAP12 (SSeCKS) is a kinase scaffolding protein that encodes **metastasis**-suppressor activity through the suppression of Src-mediated oncogenic signaling and vascular endothelial growth factor expression. SSeCKS expression is down-regulated in Src- and Ras-transformed fibroblasts, in human cancer cell lines and in several types of human cancer, including prostate. Normal human and mouse prostates express abundant SSeCKS in secretory epithelial cells and, to a lesser extent, in the surrounding mesenchyme. Here, we show that the loss of SSeCKS results in prostatic hyperplasia in the anterior and ventral lobes as well as increased levels of apoptosis throughout the prostate. Dysplastic foci were observed less frequently but were associated with the loss of E-cadherin staining and the loss of high molecular weight cytokeratin-positive basal epithelial cells. SSeCKS-null prostate tissues expressed significantly higher relative levels of AKT(poS473) compared with wild-type controls, suggesting that SSeCKS attenuates phosphatidylinositol-3-OH kinase signaling. The data suggest that SSeCKS-null mice have increased susceptibility for oncogenic transformation in the prostate.

#### GeneRIFs

1. SERPINB5 and AKAP12 may have a role in increased **metastasis** in pancreatic ductal adenocarcinoma
2. v-Src-mediated down-regulation of SSeCKS **metastasis** suppressor gene promoter by the recruitment of HDAC1 into a USF1-Sp1-Sp3 complex.

---

## Rtn1

- NCBI Gene
- GeneRIF
- Pubmed

| Search Term | Rank | Fields |
| --- | --- | --- |
| [Ee]xtracellular [Mm]atrix | 8 | PubMed(1) |

  
hide/show details for Rtn1

### [Ee]xtracellular [Mm]atrix

#### PubMed

1. Gene array identification of Ipf1/Pdx1-/- regulated genes in pancreatic progenitor cells. The homeodomain transcription factor IPF1/PDX1 exerts a dual role in the pancreas; Ipf1/Pdx1 global null mutants fail to develop a pancreas whereas conditional inactivation of Ipf1/Pdx1 in beta-cells leads to impaired beta-cell function and diabetes. Although several putative target genes have been linked to the beta-cell function of Ipf1/Pdx1, relatively little is known with respect to genes regulated by IPF1/PDX1 in early pancreatic progenitor cells.Microarray analyses identified a total of 111 genes that were differentially expressed in e10.5 pancreatic buds of Ipf1/Pdx1-/- embryos. The expression of one of these, Spondin 1, which encodes an **extracellular matrix** protein, has not previously been described in the pancreas. Quantitative real-time RT-PCR analyses and immunohistochemical analyses also revealed that the expression of FgfR2IIIb, that encodes the receptor for FGF10, was down-regulated in Ipf1/Pdx1-/- pancreatic progenitor cells.This microarray analysis has identified a number of candidate genes that are differentially expressed in Ipf1/Pdx1-/- pancreatic buds. Several of the differentially expressed genes were known to be important for pancreatic progenitor cell proliferation and differentiation whereas others have not previously been associated with pancreatic development.

---

## Map2

- NCBI Gene
- GeneRIF
- Pubmed

| Search Term | Rank | Fields |
| --- | --- | --- |
| [Ff]ilopodia | 1 | PubMed(2) |
| [Mm]igration | 6 | PubMed(83) |
| ECM | 7 | PubMed(1) |
| [Ee]xtracellular [Mm]atrix | 8 | PubMed(8) |
| [Ii]nvasive | 10 | GeneRIFs(1) |
| [Mm]etastasis | 12 | PubMed(1) |

  
hide/show details for Map2

### [Ff]ilopodia

#### PubMed

1. Sarm1, a negative regulator of innate immunity, interacts with syndecan-2 and regulates neuronal morphology. Dendritic arborization is a critical neuronal differentiation process. Here, we demonstrate that syndecan-2 (Sdc2), a synaptic heparan sulfate proteoglycan that triggers dendritic **filopodia** and spine formation, regulates dendritic arborization in cultured hippocampal neurons. This process is controlled by sterile α and TIR motif-containing 1 protein (Sarm1), a negative regulator of Toll-like receptor 3 (TLR3) in innate immunity signaling. We show that Sarm1 interacts with and receives signal from Sdc2 and controls dendritic arborization through the MKK4-JNK pathway. In Sarm1 knockdown mice, dendritic arbors of neurons were less complex than those of wild-type littermates. In addition to acting downstream of Sdc2, Sarm1 is expressed earlier than Sdc2, which suggests that it has multiple roles in neuronal morphogenesis. Specifically, it is required for proper initiation and elongation of dendrites, axonal outgrowth, and neuronal polarization. These functions likely involve Sarm1-mediated regulation of microtubule stability, as Sarm1 influenced tubulin acetylation. This study thus reveals the molecular mechanism underlying the action of Sarm1 in neuronal morphogenesis.
2. The F-BAR domain of srGAP2 induces membrane protrusions required for neuronal migration and morphogenesis. During brain development, proper neuronal migration and morphogenesis is critical for the establishment of functional neural circuits. Here we report that srGAP2 negatively regulates neuronal migration and induces neurite outgrowth and branching through the ability of its F-BAR domain to induce **filopodia**-like membrane protrusions resembling those induced by I-BAR domains in vivo and in vitro. Previous work has suggested that in nonneuronal cells **filopodia** dynamics decrease the rate of cell migration and the persistence of leading edge protrusions. srGAP2 knockdown reduces leading process branching and increases the rate of neuronal migration in vivo. Overexpression of srGAP2 or its F-BAR domain has the opposite effects, increasing leading process branching and decreasing migration. These results suggest that F-BAR domains are functionally diverse and highlight the functional importance of proteins directly regulating membrane deformation for proper neuronal migration and morphogenesis.

### [Mm]igration

#### PubMed

1. The zinc finger transcription factor RP58 negatively regulates Rnd2 for the control of neuronal **migration** during cerebral cortical development. The zinc finger transcription factor RP58 (also known as ZNF238) regulates neurogenesis of the mouse neocortex and cerebellum (Okado et al. 2009; Xiang et al. 2011; Baubet et al. 2012; Ohtaka-Maruyama et al. 2013), but its mechanism of action remains unclear. In this study, we report a cell-autonomous function for RP58 during the differentiation of embryonic cortical projection neurons via its activities as a transcriptional repressor. Disruption of RP58 expression alters the differentiation of immature neurons and impairs their **migration** and positioning within the mouse cerebral cortex. Loss of RP58 within the embryonic cortex also leads to elevated mRNA for Rnd2, a member of the Rnd family of atypical RhoA-like GTPase proteins important for cortical neuron **migration** (Heng et al. 2008). Mechanistically, RP58 represses transcription of Rnd2 via binding to a 3'-regulatory enhancer in a sequence-specific fashion. Using reporter assays, we found that RP58 repression of Rnd2 is competed by proneural basic helix-loop-helix transcriptional activators. Finally, our rescue experiments revealed that negative regulation of Rnd2 by RP58 was important for cortical cell **migration** in vivo. Taken together, these studies demonstrate that RP58 is a key player in the transcriptional control of cell **migration** in the developing cerebral cortex.
2. Receptor protein tyrosine phosphatases are expressed by cycling retinal progenitor cells and involved in neuronal development of mouse retina. Receptor protein tyrosine phosphatases (RPTPs) appear to coordinate many aspects of neural development, including cell proliferation, **migration** and differentiation. Here we investigated potential roles of RPTPs in the developing mouse retina. Using a degenerate oligonucleotide-based reverse transcription polymerase chain reaction approach, we identified 11 different RPTPs in the retina at embryonic stage 13 (E13). Subsequently, the expression patterns of RPTPkappa, RPTPJ, RPTPRR, RPTPsigma, RPTPepsilon and RPTPgamma in the retina from embryonic stages to adult were analyzed in detail using quantitative real-time-PCR, in situ hybridization, immunohistochemistry and Western blotting. At E13, all six RPTPs are expressed in actively cycling retinal progenitor cells and postmitotic newborn retinal neurons. With ongoing maturation, RPTPkappa, RPTPJ, RPTPRR, RPTPsigma, RPTPepsilon and RPTPgamma display a different spatiotemporal regulation of mRNAs and proteins in the pre- and postnatal retina. Finally, in adulthood these six RPTPs localize to distinct cellular compartments of multiple retinal neurons. Additional studies in RPTPgamma(-/-) and RPTPbeta/zeta(-/-) (also known as PTPRZ1, RPTPbeta or RPTPzeta) mice at postnatal stage P1 reveal no apparent differences in retinal laminar organization or in the expression pattern of specific retinal cell-type markers when compared with wild type. However, in RPTPbeta/zeta(-/-) retinas, immunoreactivity of vimentin, a marker of Müller glial cells, is selectively reduced and the morphology of vimentin-immunoreactive radial processes of Müller cells is considerably disturbed. Our results suggest distinct roles of RPTPs in cell proliferation and establishing phenotypes of different retinal cells during retinogenesis as well as later in the maintenance of mature retina.
3. Targeted deletion of the mitogen-activated protein kinase kinase 4 gene in the nervous system causes severe brain developmental defects and premature death. The c-Jun NH2-terminal protein kinase (JNK) is a mitogen-activated protein kinase (MAPK) involved in the regulation of various physiological processes. Its activity is increased upon phosphorylation by the MAPK kinases MKK4 and MKK7. The early embryonic death of mice lacking an mkk4 or mkk7 gene has provided genetic evidence that MKK4 and MKK7 have nonredundant functions in vivo. To elucidate the physiological role of MKK4, we generated a novel mouse model in which the mkk4 gene could be specifically deleted in the brain. At birth, the mutant mice were indistinguishable from their control littermates, but they stopped growing a few days later and died prematurely, displaying severe neurological defects. Decreased JNK activity in the absence of MKK4 correlated with impaired phosphorylation of a subset of physiologically relevant JNK substrates and with altered gene expression. These defects resulted in the misalignment of the Purkinje cells in the cerebellum and delayed radial **migration** in the cerebral cortex. Together, our data demonstrate for the first time that MKK4 is an essential activator of JNK required for the normal development of the brain.
4. Identification of a novel brain-specific and Reelin-regulated gene that encodes a protein colocalized with synapsin. We carried out a screening of genes that are differentially expressed in normal mice and reeler mutants and are characterized by abnormal neuronal **migration** and neurite deployment due to defective Reelin signalling. A novel gene, provisionally named C61, was overexpressed in Reelin-deficient embryonic mouse brain RNA. C61 encodes a 3.7 kb mRNA that is brain specific and developmentally regulated, with predominant expression in differentiating neurons. The predicted protein is 664 amino acids long, and contains LAG1 and Ezrin/Radixin/Moesin-Myosin-Filament motifs, suggesting that it may function as an intracellular adaptor. From E14.5 to birth, C61 was highly expressed in all neuronal differentiation fields, with the highest signal in the telencephalic cortical plate and mitral cells in the olfactory bulb. When expressed as a GFP fusion protein in transfected non-neuronal cells and primary neurons, this protein localizes, respectively, to the nuclear membrane or axonal outgrowths, indicating a function in axonal traffic or signalling.
5. Dlx transcription factors promote **migration** through repression of axon and dendrite growth. In the mouse telencephalon, Dlx homeobox transcription factors are essential for the tangential **migration** of subpallial-derived GABAergic interneurons to neocortex. However, the mechanisms underlying this process are poorly understood. Here, we demonstrate that Dlx1/2 has a central role in restraining neurite growth of subpallial-derived immature interneurons at a stage when they migrate tangentially to cortex. In Dlx1-/-;Dlx2-/- mutants, neurite length is increased and cells fail to migrate. In Dlx1-/-;Dlx2+/- mutants, while the tangential **migration** of immature interneurons appears normal, they develop dendritic and axonal processes with increased length and decreased branching, and have deficits in their neocortical laminar positions. Thus, Dlx1/2 is required for coordinating programs of neurite maturation and **migration**. In this regard, we provide genetic evidence that in immature interneurons Dlx1/2 repression of the p21-activated serine/threonine kinase PAK3, a downstream effector of the Rho family of GTPases, is critical in restraining neurite growth and promoting tangential **migration**.
6. Inactivation of aPKClambda results in the loss of adherens junctions in neuroepithelial cells without affecting neurogenesis in mouse neocortex. In developing mammalian telencephalon, the loss of adherens junctions and cell cycle exit represent crucial steps in the differentiation of neuroepithelial cells into neurons, but the relationship between these cellular events remains obscure. Atypical protein kinase C (aPKC) is known to contribute to junction formation in epithelial cells and to cell fate determination for Drosophila neuroblasts. To elucidate the functions of aPKClambda, one out of two aPKC members, in mouse neocortical neurogenesis, a Nestin-Cre mediated conditional gene targeting system was employed. In conditional aPKClambda knockout mice, neuroepithelial cells of the neocortical region lost aPKClambda protein at embryonic day 15 and demonstrated a loss of adherens junctions, retraction of apical processes and impaired interkinetic nuclear **migration** that resulted in disordered neuroepithelial tissue architecture. These results are evidence that aPKClambda is indispensable for the maintenance of adherens junctions and may function in the regulation of adherens junction integrity upon differentiation of neuroepithelial cells into neurons. In spite of the loss of adherens junctions in the neuroepithelium of conditional aPKClambda knockout mice, neurons were produced at a normal rate. Therefore, we concluded that, at least in the later stages of neurogenesis, regulation of cell cycle exit is independent of adherens junctions.
7. Cyclin-dependent kinase 5 is essential for neuronal cell cycle arrest and differentiation. Cyclin-dependent kinase 5 (Cdk5) is a serine/threonine kinase with significant homology to cell cycle-related Cdks but is not believed to be active in a typical cell cycle. In Cdk5-deficient embryos and Cdk5 chimeras, **migration** and survival of postmitotic neurons is compromised in a cell-autonomous manner. In the present study, we show that loss of Cdk5 leads to both failure of neuronal differentiation and loss of cell cycle control. Using specific cytoskeletal proteins as indices of neuronal differentiation, we find that Cdk5-deficient neurons are significantly arrested or delayed in their developmental program both in vivo and in vitro. For example, immunocytochemistry of embryonic day 16 (E16) cortex reveals that the expression of microtubule-associated protein 2c (Map-2c), a marker of mature neurons, is nearly absent in Cdk5(-/-) cells that have migrated to the cortical plate while these same cells continue to express nestin. Similarly, in vitro, Map-2-positive cells are rare in cultures from E16 Cdk5(-/-) embryos. Cell cycle control is also deficient in Cdk5(-/-) cells. In vivo, neurons engaged in cell cycle activities are found in the cortical plate, and, in vitro, class III beta-tubulin-positive cells continue to label with bromodeoxyuridine even after 5 d of incubation. Transfection of a wild-type Cdk5 construct reveals that cell cycle control can be regained in Cdk5(-/-) cells by overexpression of Cdk5. These data indicate that Cdk5 is necessary for both neuronal differentiation and cell cycle inhibition.
8. Close homolog of L1 modulates area-specific neuronal positioning and dendrite orientation in the cerebral cortex. We show that the neural cell recognition molecule Close Homolog of L1 (CHL1) is required for neuronal positioning and dendritic growth of pyramidal neurons in the posterior region of the developing mouse neocortex. CHL1 was expressed in pyramidal neurons in a high-caudal to low-rostral gradient within the developing cortex. Deep layer pyramidal neurons of CHL1-minus mice were shifted to lower laminar positions in the visual and somatosensory cortex and developed misoriented, often inverted apical dendrites. Impaired **migration** of CHL1-minus cortical neurons was suggested by strikingly slower rates of radial **migration** in cortical slices, failure to potentiate integrin-dependent haptotactic cell **migration** in vitro, and accumulation of migratory cells in the intermediate and ventricular/subventricular zones in vivo. The restriction of CHL1 expression and effects of its deletion in posterior neocortical areas suggests that CHL1 may regulate area-specific neuronal connectivity and, by extension, function in the visual and somatosensory cortex.
9. Involvement of the myelin-associated inhibitor Nogo-A in early cortical development and neuronal maturation. Nogo-A is a myelin-associated protein expressed by neurons and myelinating mature oligodendrocytes in the central nervous system. Although most research has focused on the participation of Nogo-A in the prevention of axonal regeneration and plasticity in the adult, little attention has been paid to the putative functions of Nogo-A during embryonic development. Here we examined the general pattern and cell-specific distribution of Nogo-A in the prenatal mouse telencephalon. In addition, we studied the development of the major axon tracts and radial and tangential **migration** in Nogo-A/B/C knockout mice. The pattern of Nogo-A showed distinct distribution in radial glia and postmitotic neurons, in which it is particularly enriched in developing axons. Similarly, Nogo-A was enriched at the leading process of tangentially migrating interneurons but not detectable in radial migrating neurons. Although a low level of Nogo-A appears to be on the surface of many cortical neurons, most proteins have intracellular localization. In Nogo-deficient background, neurons displayed early polarization and increased branching in vitro, probably reflecting a cell-intrinsic role of Nogo proteins in branching reduction, and early tangential **migration** was delayed. On the basis of these observations, we propose that Nogo proteins, particularly Nogo-A, are involved in multiple processes during cortical development.
10. The protocadherin gene Celsr3 is required for interneuron **migration** in the mouse forebrain. Interneurons are extremely diverse in the mammalian brain and provide an essential balance for functional neural circuitry. The vast majority of murine cortical interneurons are generated in the subpallium and migrate tangentially over a long distance to acquire their final positions. By using a mouse line with a deletion of the Celsr3 (Flamingo, or Fmi1) gene and a knock-in of the green fluorescent protein reporter, we find that Celsr3, a member of the nonclustered protocadherin (Pcdh) family, is predominantly expressed in the cortical interneurons in adults and in the interneuron germinal zones in embryos. We show that Celsr3 is crucial for interneuron **migration** in the developing mouse forebrain. Specifically, in Celsr3 knockout mice, calretinin-positive interneurons are reduced in the developing neocortex, accumulated in the corticostriatal boundary, and increased in the striatum. Moreover, the laminar distribution of cortical calbindin-positive cells is altered. Finally, we found that expression patterns of NRG1 (neuregulin-1) and its receptor ErbB4, which are essential for interneuron **migration**, are changed in Celsr3 mutants. These results demonstrate that the protocadherin Celsr3 gene is essential for both tangential and radial interneuron **migration**s in a class-specific manner.
11. Tbr1 regulates differentiation of the preplate and layer 6. During corticogenesis, early-born neurons of the preplate and layer 6 are important for guiding subsequent neuronal **migration**s and axonal projections. Tbr1 is a putative transcription factor that is highly expressed in glutamatergic early-born cortical neurons. In Tbr1-deficient mice, these early-born neurons had molecular and functional defects. Cajal-Retzius cells expressed decreased levels of Reelin, resulting in a reeler-like cortical **migration** disorder. Impaired subplate differentiation was associated with ectopic projection of thalamocortical fibers into the basal telencephalon. Layer 6 defects contributed to errors in the thalamocortical, corticothalamic, and callosal projections. These results show that Tbr1 is a common genetic determinant for the differentiation of early-born glutamatergic neocortical neurons and provide insights into the functions of these neurons as regulators of cortical development.
12. Expression of Disabled 1 suppresses astroglial differentiation in neural stem cells. Disabled 1 (Dab1), a cytoplasmic adaptor protein expressed predominantly in the CNS, transduces a Reelin-initiated signaling that controls neuronal **migration** and positioning during brain development. To determine the role of Dab1 in neural stem cell (NSC) differentiation, we established a culture of neurospheres derived from the embryonic forebrain of the Dab1(-/-) mice, yotari. Differentiating Dab1(-/-) neurospheres exhibited a higher expression of GFAP, an astrocytic marker, at the expense of neuronal markers. Under Dab1-deficient condition, the expression of NeuroD, a transcription factor for neuronal differentiation, was decreased and the JAK-STAT pathway was evidently increased during differentiation of NSC, suggesting the possible involvement of Dab1 in astrocyte differentiation via JAK-STAT pathway. Notably, expression of neural and glial markers and the level of JAK-STAT signaling molecules were not changed in differentiating NSC by Reelin treatment, indicating that differentiation of NSC is Reelin-independent. Immunohistochemical analyses showed a decrease in the number of neurons and an increase in the number of GFAP-positive cells in developing yotari brains. Our results suggest that Dab1 participates in the differentiation of NSCs into a specific cell lineage, thereby maintaining a balance between neurogenesis and gliogenesis.
13. The transcriptional repressor RP58 is crucial for cell-division patterning and neuronal survival in the developing cortex. The neocortex and the hippocampus comprise several specific layers containing distinct neurons that originate from progenitors at specific development times, under the control of an adequate cell-division patterning mechanism. Although many molecules are known to regulate this cell-division patterning process, its details are not well understood. Here, we show that, in the developing cerebral cortex, the RP58 transcription repressor protein was expressed both in postmitotic glutamatergic projection neurons and in their progenitor cells, but not in GABAergic interneurons. Targeted deletion of the RP58 gene led to dysplasia of the neocortex and of the hippocampus, reduction of the number of mature cortical neurons, and defects of laminar organization, which reflect abnormal neuronal **migration** within the cortical plate. We demonstrate an impairment of the cell-division patterning during the late embryonic stage and an enhancement of apoptosis of the postmitotic neurons in the RP58-deficient cortex. These results suggest that RP58 controls cell division of progenitor cells and regulates the survival of postmitotic cortical neurons.
14. The Rb/E2F pathway modulates neurogenesis through direct regulation of the Dlx1/Dlx2 bigene cluster. During brain morphogenesis, the mechanisms through which the cell cycle machinery integrates with differentiation signals remain elusive. Here we show that the Rb/E2F pathway regulates key aspects of differentiation and **migration** through direct control of the Dlx1 and Dlx2 homeodomain proteins, required for interneuron specification. Rb deficiency results in a dramatic reduction of Dlx1 and Dlx2 gene expression manifested by loss of interneuron subtypes and severe **migration** defects in the mouse brain. The Rb/E2F pathway modulates Dlx1/Dlx2 regulation through direct interaction with a Dlx forebrain-specific enhancer, I12b, and the Dlx1/Dlx2 proximal promoter regions, through repressor E2F sites both in vitro and in vivo. In the absence of Rb, we demonstrate that repressor E2Fs inhibit Dlx transcription at the Dlx1/Dlx2 promoters and Dlx1/2-I12b enhancer to suppress differentiation. Our findings support a model whereby the cell cycle machinery not only controls cell division but also modulates neuronal differentiation and **migration** through direct regulation of the Dlx1/Dlx2 bigene cluster during embryonic development.
15. Dlx5 and Dlx6 regulate the development of parvalbumin-expressing cortical interneurons. Dlx5 and Dlx6 homeobox genes are expressed in developing and mature cortical interneurons. Simultaneous deletion of Dlx5 and 6 results in exencephaly of the anterior brain; despite this defect, prenatal basal ganglia differentiation appeared largely intact, while tangential **migration** of Lhx6(+) and Mafb(+) interneurons to the cortex was reduced and disordered. The **migration** deficits were associated with reduced CXCR4 expression. Transplantation of mutant immature interneurons into a wild-type brain demonstrated that loss of either Dlx5 or Dlx5&6 preferentially reduced the number of mature parvalbumin(+) interneurons; those parvalbumin(+) interneurons that were present had increased dendritic branching. Dlx5/6(+/-) mice, which appear normal histologically, show spontaneous electrographic seizures and reduced power of gamma oscillations. Thus, Dlx5&6 appeared to be required for development and function of somal innervating (parvalbumin(+)) neocortical interneurons. This contrasts with Dlx1, whose function is required for dendrite innervating (calretinin(+), somatostatin(+), and neuropeptide Y(+)) interneurons (Cobos et al., 2005).
16. Gene deletion mutants reveal a role for semaphorin receptors of the plexin-B family in mechanisms underlying corticogenesis. Semaphorins and their receptors, plexins, are emerging as key regulators of various aspects of neural and nonneural development. Semaphorin 4D (Sema4D) and B-type plexins demonstrate distinct expression patterns over critical time windows during the development of the murine neocortex. Here, analysis of mice genetically lacking plexin-B1 or plexin-B2 revealed the significance of Sema4D-plexin-B signaling in cortical development. Deficiency of plexin-B2 resulted in abnormal cortical layering and defective **migration** and differentiation of several subtypes of cortical neurons, including Cajal-Retzius cells, GABAergic interneurons, and principal cells in vivo. In contrast, a lack of plexin-B1 did not impact on cortical development in vivo. In various ex vivo assays on embryonic forebrain, Sema4D enhanced the radial and tangential **migration** of developing neurons in a plexin-B2-dependent manner. These results suggest that Sema4D-plexin-B2 interactions regulate mechanisms underlying cell specification, differentiation, and **migration** during corticogenesis.
17. Dicer is required for proliferation, viability, **migration** and differentiation in corticoneurogenesis. In mice, microRNAs (miRNAs) are required for embryonic viability, and previous reports implicate miRNA participation in brain cortical neurogenesis. Here, we provide a more comprehensive analysis of miRNA involvement in cortical brain development. To accomplish this we used mice in which Dicer, the RNase III enzyme necessary for canonical miRNA biogenesis, is depleted from Nestin-expressing progenitors and progeny cells. We systematically assessed how Dicer depletion impacts proliferation, cell death, **migration** and differentiation in the developing brain. Using markers for proliferation and in vivo labeling with thymidine analogs, we found reduced numbers of proliferating cells, and altered cell cycle kinetics from embryonic day 15.5 (E15.5). Progenitor cells were distributed aberrantly throughout the cortex rather than restricted to the ventricular and subventricular zones. Activated Caspase3 was elevated, reflecting increased cortical cell death as early as E15.5. Cajal-Retzius-positive cells were more numerous at E15.5 and were dysmorphic relative to control cortices. Consistent with this, Reelin levels were enhanced. Doublecortin and Rnd2 were also increased and showed altered distribution, supporting a strong regulatory role for miRNAs in both early and late neuronal **migration**. In addition, GFAP staining at E15.5 was more intense and disorganized throughout the cortex with Dicer depletion. These results significantly extend earlier works, and emphasize the impact of miRNAs on neural progenitor cell proliferation, apoptosis, **migration**, and differentiation in the developing mammalian brain.
18. MACF1 regulates the **migration** of pyramidal neurons via microtubule dynamics and GSK-3 signaling. Neuronal **migration** and subsequent differentiation play critical roles for establishing functional neural circuitry in the developing brain. However, the molecular mechanisms that regulate these processes are poorly understood. Here, we show that microtubule actin crosslinking factor 1 (MACF1) determines neuronal positioning by regulating microtubule dynamics and mediating GSK-3 signaling during brain development. First, using MACF1 floxed allele mice and in utero gene manipulation, we find that MACF1 deletion suppresses **migration** of cortical pyramidal neurons and results in aberrant neuronal positioning in the developing brain. The cell autonomous deficit in **migration** is associated with abnormal dynamics of leading processes and centrosomes. Furthermore, microtubule stability is severely damaged in neurons lacking MACF1, resulting in abnormal microtubule dynamics. Finally, MACF1 interacts with and mediates GSK-3 signaling in developing neurons. Our findings establish a cellular mechanism underlying neuronal **migration** and provide insights into the regulation of cytoskeleton dynamics in developing neurons.
19. Transient inhibition of the ERK pathway prevents cerebellar developmental defects and improves long-term motor functions in murine models of neurofibromatosis type 1. Individuals with neurofibromatosis type 1 (NF1) frequently exhibit cognitive and motor impairments and characteristics of autism. The cerebellum plays a critical role in motor control, cognition, and social interaction, suggesting that cerebellar defects likely contribute to NF1-associated neurodevelopmental disorders. Here we show that Nf1 inactivation during early, but not late stages of cerebellar development, disrupts neuronal lamination, which is partially caused by overproduction of glia and subsequent disruption of the Bergmann glia (BG) scaffold. Specific Nf1 inactivation in glutamatergic neuronal precursors causes premature differentiation of granule cell (GC) precursors and ectopic production of unipolar brush cells (UBCs), indirectly disrupting neuronal **migration**. Transient MEK inhibition during a neonatal window prevents cerebellar developmental defects and improves long-term motor performance of Nf1-deficient mice. This study reveals essential roles of Nf1 in GC/UBC **migration** by generating correct numbers of glia and controlling GC/UBC fate-specification/differentiation, identifying a therapeutic prevention strategy for multiple NF1-associcated developmental abnormalities.
20. Phosphorylation of Neurogenin2 specifies the **migration** properties and the dendritic morphology of pyramidal neurons in the neocortex. The molecular mechanisms specifying the dendritic morphology of different neuronal subtypes are poorly understood. Here we demonstrate that the bHLH transcription factor Neurogenin2 (Ngn2) is both necessary and sufficient for specifying the dendritic morphology of pyramidal neurons in vivo by specifying the polarity of its leading process during the initiation of radial **migration**. The ability of Ngn2 to promote a polarized leading process outgrowth requires the phosphorylation of a single tyrosine residue at position 241, an event that is neither involved in Ngn2 direct transactivation properties nor its proneural function. Interestingly, the **migration** defect observed in the Ngn2 knockout mouse and in progenitors expressing the Ngn2(Y241F) mutation can be rescued by inhibiting the activity of the small-GTPase RhoA in cortical progenitors. Our results demonstrate that Ngn2 coordinates the acquisition of the radial **migration** properties and the unipolar dendritic morphology characterizing pyramidal neurons through molecular mechanisms distinct from those mediating its proneural activity.
21. Hand2 is necessary for terminal differentiation of enteric neurons from crest-derived precursors but not for their **migration** into the gut or for formation of glia. Hand genes encode basic helix-loop-helix transcription factors that are expressed in the developing gut, where their function is unknown. We now report that enteric Hand2 expression is limited to crest-derived cells, whereas Hand1 expression is restricted to muscle and interstitial cells of Cajal. Hand2 is developmentally regulated and is intranuclear in precursors but cytoplasmic in neurons. Neurons develop in explants from wild-type but not Hand2(-/-) bowel, although, in both, crest-derived cells are present and glia arise. Similarly, small interfering RNA (siRNA) silencing of Hand2 in enteric crest-derived cells prevents neuronal development. Terminally differentiated enteric neurons do not develop after conditional inactivation of Hand2 in migrating crest-derived cells; nevertheless, conditional Hand2 inactivation does not prevent precursors from expressing early neural markers. We suggest that enteric neuronal development occurs in stages and that Hand2 expression is required for terminal differentiation but not for precursors to enter the neuronal lineage.
22. MAPK-upstream protein kinase (MUK) regulates the radial **migration** of immature neurons in telencephalon of mouse embryo. The radial **migration** of differentiating neurons provides an essential step in the generation of laminated neocortex, although its molecular mechanism is not fully understood. We show that the protein levels of a JNK activator kinase, MUK/DLK/ZPK, and JNK activity increase potently and temporally in newly generated neurons in developing mouse telencephalon during radial **migration**. The ectopic expression of MUK/DLK/ZPK in neural precursor cells in utero impairs radial **migration**, whereas it allows these cells to leave the ventricular zone and differentiate into neural cells. The MUK/DLK/ZPK protein is associated with dotted structures that are frequently located along microtubules and with Golgi apparatus in cultured embryonic cortical cells. In COS-1 cells, MUK/DLK/ZPK overexpression impairs the radial organization of microtubules without massive depolymerization. These results suggest that MUK/DLK/ZPK and JNK regulate radial cell **migration** via microtubule-based events.
23. Ephrin signalling controls brain size by regulating apoptosis of neural progenitors. Mechanisms controlling brain size include the regulation of neural progenitor cell proliferation, differentiation, survival and **migration**. Here we show that ephrin-A/EphA receptor signalling plays a key role in controlling the size of the mouse cerebral cortex by regulating cortical progenitor cell apoptosis. In vivo gain of EphA receptor function, achieved through ectopic expression of ephrin-A5 in early cortical progenitors expressing EphA7, caused a transient wave of neural progenitor cell apoptosis, resulting in premature depletion of progenitors and a subsequent dramatic decrease in cortical size. In vitro treatment with soluble ephrin-A ligands similarly induced the rapid death of cultured dissociated cortical progenitors in a caspase-3-dependent manner, thereby confirming a direct effect of ephrin/Eph signalling on apoptotic cascades. Conversely, in vivo loss of EphA function, achieved through EphA7 gene disruption, caused a reduction in apoptosis occurring normally in forebrain neural progenitors, resulting in an increase in cortical size and, in extreme cases, exencephalic forebrain overgrowth. Together, these results identify ephrin/Eph signalling as a physiological trigger for apoptosis that can alter brain size and shape by regulating the number of neural progenitors.
24. Ccm3, a gene associated with cerebral cavernous malformations, is required for neuronal **migration**. Loss of function of cerebral cavernous malformation 3 (CCM3) results in an autosomal dominant cerebrovascular disorder. Here, we uncover a developmental role for CCM3 in regulating neuronal **migration** in the neocortex. Using cell type-specific gene inactivation in mice, we show that CCM3 has both cell autonomous and cell non-autonomous functions in neural progenitors and is specifically required in radial glia and newly born pyramidal neurons migrating through the subventricular zone, but not in those migrating through the cortical plate. Loss of CCM3 function leads to RhoA activation, alterations in the actin and microtubule cytoskeleton affecting neuronal morphology, and abnormalities in laminar positioning of primarily late-born neurons, indicating CCM3 involvement in radial glia-dependent locomotion and possible interaction with the Cdk5/RhoA pathway. Thus, we identify a novel cytoplasmic regulator of neuronal **migration** and demonstrate that its inactivation in radial glia progenitors and nascent neurons produces severe malformations of cortical development.
25. Regulation of neural progenitor cell motility by ceramide and potential implications for mouse brain development. We provide evidence that the sphingolipid ceramide, in addition to its pro-apoptotic function, regulates neural progenitor (NP) motility in vitro and brain development in vivo. Ceramide (N-palmitoyl d-erythro sphingosine and N-oleoyl d-erythro sphingosine) and the ceramide analog N-oleoyl serinol (S18) stimulate **migration** of NPs in scratch (wounding) **migration** assays. Sphingolipid depletion by inhibition of de novo ceramide biosynthesis, or ceramide inactivation using an anti-ceramide antibody, obliterates NP motility, which is restored by ceramide or S18. These results suggest that ceramide is crucial for NP motility. Wounding of the NP monolayer activates neutral sphingomyelinase indicating that ceramide is generated from sphingomyelin. In membrane processes, ceramide is co-distributed with its binding partner atypical protein kinase C zeta/lambda (aPKC), and Cdc42, alpha/beta-tubulin, and beta-catenin, three proteins involved in aPKC-dependent regulation of cell polarity and motility. Sphingolipid depletion by myriocin prevents membrane translocation of aPKC and Cdc42, which is restored by ceramide or S18. These results suggest that ceramide-mediated membrane association of aPKC/Cdc42 is important for NP motility. In vivo, sphingolipid depletion leads to ectopic localization of mitotic or post-mitotic neural cells in the embryonic brain, while S18 restores the normal brain organization. In summary, our study provides novel evidence that ceramide is critical for NP motility and polarity in vitro and in vivo.
26. α2-chimaerin controls neuronal **migratio**n and functioning of the cerebral cortex through CRMP-2. Disrupted cortical neuronal **migratio**n is associated with epileptic seizures and developmental delay. However, the molecular mechanism by which disruptions of early cortical development result in neurological symptoms is poorly understood. Here we report α2-chimaerin as a key regulator of cortical neurona**l migrati**on and function. In utero suppression of α2-chimaerin arrested neuron**al migrat**ion at the multipolar stage, leading to accumulation of ectopic neurons in the subcortical region. Mice with su**ch migrat**ion defects showed an imbalance between excitation and inhibition in local cortical circuitry and greater susceptibility to convulsant-induced seizures. We further show that α2-chimaerin regulates bipolar transition and neuro**nal migra**tion through modulating the activity of CRMP-2, a microtubule-associated protein. These findings establish a new α2-chimaerin-dependent mechanism underlying neur**onal migr**ation and proper functioning of the cerebral cortex and provide insights into the pathogenesis of seizure-related neurodevelopmental disorders.
27. Regulation of apoptosis and neurite extension by FKBP38 is required for neural tube formation in the mouse. FKBP38 (also known as FKBP8) is a transmembrane chaperone protein that inhibits apoptosis by recruiting the anti-apoptotic proteins Bcl-2 and Bcl-x(L) to mitochondria. We have now generated mice harboring a loss-of-function mutation in Fkbp38. The Fkbp38(-/-) mice die soon after birth manifesting defects in neural tube closure in the thoraco-lumbar-sacral region (spina bifida) as well as skeletal defects including scoliosis, rib deformities, club foot and curled tail. The neuroepithelium is disorganized and that formation of dorsal root ganglia is defective in Fkbp38(-/-) embryos, likely as a result of an increased frequency of apoptosis and aberrant **migration** of neuronal cells. Furthermore, the extension of nerve fibers in the spinal cord is abnormal in the mutant embryos. To explore the mechanisms underlying these characteristics, we screened for proteins that interact with FKBP38 in the yeast two-hybrid system and thereby identified protrudin, a protein that promotes process formation by regulating membrane trafficking. Protrudin was found to be hyperphosphorylated in the brain of Fkbp38(-/-) mice, suggesting that FKBP38 regulates protrudin-dependent membrane recycling and neurite outgrowth. Together, our findings suggest that FKBP38 is required for neuroectodermal organization during neural tube formation as a result of its anti-apoptotic activity and regulation of neurite extension.
28. Expression of MUK/DLK/ZPK, an activator of the JNK pathway, in the nervous systems of the developing mouse embryo. C-Jun N-terminal kinase (JNK) is implicated in regulating the various cellular events during neural development that include differentiation, apoptosis and **migration**. MUK/DLK/ZPK is a MAP kinase kinase kinase (MAPKKK) enzyme that activates JNK via MAP kinase kinases (MAPKK) such as MKK7. We show here that the expression of MUK/DLK/ZPK protein in the developing mouse embryo is almost totally specific for the neural tissues, including central, peripheral, and autonomic nervous systems. The only obvious exception is the liver, in which the protein is temporally expressed at around E11. The expression becomes obvious in the neurons of the brain and neural crest tissues at embryonic day 10 (E10) after neuron production is initiated. By E14, MUK/DLK/ZPK proteins are found in various neural tissues including the brain, spinal cord, sensory ganglia (such as trigeminal and dorsal root ganglia), and the sympathetic and visceral nerves. The localization of MUK/DLK/ZPK protein in neural cells almost consistently overlapped that of betaIII-tubulin, a neuron specific tubulin isoform, and both proteins were more concentrated in axons than in cell bodies and dendrites. The intensely overlapping localization of betaIII-tubulin and MUK/DLK/ZPK indicated that this protein kinase is tightly associated with the microtubules of neurons.
29. The Rho-GTPase cdc42 regulates neural progenitor fate at the apical surface. Stem cell persistence into adulthood requires self-renewal from early developmental stages. In the developing mouse brain, only apical progenitors located at the ventricle are self-renewing, whereas basal progenitors gradually deplete. However, nothing is known about the mechanisms regulating the fundamental difference between these progenitors. Here we show that the conditional deletion of the small Rho-GTPase cdc42 at different stages of neurogenesis in mouse telencephalon results in an immediate increase in basal mitoses. Whereas cdc42-deficient progenitors have normal cell cycle length, orientation of cell division and basement membrane contact, the apical location of the Par complex and adherens junctions are gradually lost, leading to an increasing failure of apically directed interkinetic nuclear **migration**. These cells then undergo mitoses at basal positions and acquire the fate of basal progenitors. Thus, cdc42 has a crucial role at the apical pole of progenitors, thereby regulating the position of mitoses and cell fate.
30. Pax6 regulates regional development and neuronal **migration** in the cerebral cortex. Mutations in the Pax6 gene disrupt telencephalic development, resulting in a thin cortical plate, expansion of proliferative layers, and the absence of the olfactory bulb. The primary defect in the neuronal cell population of the developing cerebral cortex was analysed by using mouse chimeras containing a mixture of wild-type and Pax6-deficient cells. The chimeric analysis shows that Pax6 influences cellular activity throughout corticogenesis. At early stages, Pax6-deficient and wildtype cells segregate into exclusive patches, indicating an inability of different cell genotypes to interact. At later stages, cells are sorted further based on telencephalic domains. Pax6-deficient cells are specifically reduced in the mediocaudal domain of the dorsal telencephalon, indicating a role in regionalization. In addition, Pax6 regulates the process of radial **migration** of neuronal precursors. Loss of Pax6 particularly affects movement of neuronal precursors at the subventricular zone/intermediate zone boundary at a transitional migratory phase essential for entry into the intermediate zone. We suggest that the primary role of Pax6 is the continual regulation of cell surface properties responsible for both cellular identity and radial **migration**, defects of which cause regional cell sorting and abnormalities of **migration** in chimeras.
31. COUP-TFI is required for the formation of commissural projections in the forebrain by regulating axonal growth. The transcription factor COUP-TFI (NR2F1), an orphan member of the nuclear receptor superfamily, is an important regulator of neurogenesis, cellular differentiation and cell **migration**. In the forebrain, COUP-TFI controls the connectivity between thalamus and cortex and neuronal tangential **migration** in the basal telencephalon. Here, we show that COUP-TFI is required for proper axonal growth and guidance of all major forebrain commissures. Fibres of the corpus callosum, the hippocampal commissure and the anterior commissure project aberrantly and fail to cross the midline in COUP-TFI null mutants. Moreover, hippocampal neurons lacking COUP-TFI have a defect in neurite outgrowth and show an abnormal axonal morphology. To search for downstream effectors, we used microarray analysis and showed that, in the absence of COUP-TFI, expression of various cytoskeleton molecules involved in neuronal morphogenesis is affected. Diminished protein levels of the microtubule-associated protein MAP1B and increased levels of the GTP-binding protein RND2 were confirmed in the developing cortex in vivo and in primary hippocampal neurons in vitro. Therefore, based on morphological studies, gene expression profiling and primary cultured neurons, the present data uncover a previously unappreciated intrinsic role for COUP-TFI in axonal growth in vivo and supply one of the premises for COUP-TFI coordination of neuronal morphogenesis in the developing forebrain.
32. Different timings of Dicer deletion affect neurogenesis and gliogenesis in the developing mouse central nervous system. MicroRNAs, processed by the RNAase III enzyme Dicer, are approximately 22 nucleotide endogenous noncoding small RNAs. The function of Dicer in the mouse central nervous system (CNS) development is not well understood. Here, we show that specifically deleting Dicer expression in the CNS and in the cerebral cortex using two Cre lines results in reduced progenitor numbers, abnormal neuronal differentiation, and thinner cortical wall. Incomplete Dicer deletion during early embryonic stages contributes to normal development of early-born neurons in the cortex and motor neurons in the spinal cord. However, at late embryonic stages when Dicer is completely ablated in the CNS, the **migration** of late-born neurons in the cortex and oligodendrocyte precursor expansion and differentiation in the spinal cord are greatly affected. Our studies of different timings of Dicer deletion demonstrate the importance of the Dicer-mediated microRNA pathway in regulating distinct phases of neurogenesis and gliogenesis during the CNS development.
33. Frizzled-3 is required for the development of major fiber tracts in the rostral CNS. Many ligand/receptor families are known to contribute to axonal growth and targeting. Thus far, there have been no reports implicating Wnts and Frizzleds in this process, despite their large numbers and widespread expression within the CNS. In this study, we show that targeted deletion of the mouse fz3 gene leads to severe defects in several major axon tracts within the forebrain. In particular, fz3(-/-) mice show a complete loss of the thalamocortical, corticothalamic, and nigrostriatal tracts and of the anterior commissure, and they show a variable loss of the corpus callosum. Peripheral nerve fibers and major axon tracts in the more caudal regions of the CNS are mostly or completely unaffected. Cell proliferation in the ventricular zone and cell **migration** to the developing cortex proceed normally until at least embryonic day 14. Extensive cell death in the fz3(-/-) striatum occurs late in gestation, perhaps secondary to the nearly complete absence of long-range connections. In contrast, there is little cell death, as assayed by terminal deoxynucleotidyl transferase-mediated biotinylated UTP nick end labeling, in the cortex. These data provide the first link between Frizzled signaling and axonal development.
34. Cell **migration** from the ganglionic eminences is required for the development of hippocampal GABAergic interneurons. GABAergic interneurons have major roles in hippocampal function and dysfunction. Here we provide evidence that, in mice, virtually all of these cells originate from progenitors in the basal telencephalon. Immature interneurons tangentially migrate from the basal telencephalon through the neocortex to take up their final positions in the hippocampus. Disrupting differentiation in the embryonic basal telencephalon (lateral and medial ganglionic eminences) through loss of Dlx1/2 homeobox function blocks the **migration** of virtually all GABAergic interneurons to the hippocampus. On the other hand, disrupting specification of the medial ganglionic eminence through loss of Nkx2.1 homeobox function depletes the hippocampus of a distinct subset of hippocampal interneurons. Loss of hippocampal interneurons does not appear to have major effects on the early development of hippocampal projection neurons nor on the pathfinding of afferrent tracts.
35. LPIAT1 regulates arachidonic acid content in phosphatidylinositol and is required for cortical lamination in mice. Dietary arachidonic acid (AA) has roles in growth, neuronal development, and cognitive function in infants. AA is remarkably enriched in phosphatidylinositol (PI), an important constituent of biological membranes in mammals; however, the physiological significance of AA-containing PI remains unknown. In an RNA interference-based genetic screen using Caenorhabditis elegans, we recently cloned mboa-7 as an acyltransferase that selectively incorporates AA into PI. Here we show that lysophosphatidylinositol acyltransferase 1 (LPIAT1, also known as MBOAT7), the closest mammalian homologue, plays a crucial role in brain development in mice. Lpiat1(-/-) mice show almost no LPIAT activity with arachidonoyl-CoA as an acyl donor and show reduced AA contents in PI and PI phosphates. Lpiat1(-/-) mice die within a month and show atrophy of the cerebral cortex and hippocampus. Immunohistochemical analysis reveals disordered cortical lamination and delayed neuronal **migration** in the cortex of E18.5 Lpiat1(-/-) mice. LPIAT1 deficiency also causes disordered neuronal processes in the cortex and reduced neurite outgrowth in vitro. Taken together, these results demonstrate that AA-containing PI/PI phosphates play an important role in normal cortical lamination during brain development in mice.
36. Myocardin-related transcription factors regulate the Cdk5/Pctaire1 kinase cascade to control neurite outgrowth, neuronal **migration** and brain development. Numerous motile cell functions depend on signaling from the cytoskeleton to the nucleus. Myocardin-related transcription factors (MRTFs) translocate to the nucleus in response to actin polymerization and cooperate with serum response factor (Srf) to regulate the expression of genes encoding actin and other components of the cytoskeleton. Here, we show that MRTF-A (Mkl1) and MRTF-B (Mkl2) redundantly control neuronal **migration** and neurite outgrowth during mouse brain development. Conditional deletion of the genes encoding these Srf coactivators disrupts the formation of multiple brain structures, reflecting a failure in neuronal actin polymerization and cytoskeletal assembly. These abnormalities were accompanied by dysregulation of the actin-severing protein gelsolin and Pctaire1 (Cdk16) kinase, which cooperates with Cdk5 to initiate a kinase cascade that governs cytoskeletal rearrangements essential for neuron **migration** and neurite outgrowth. Thus, the MRTF/Srf partnership interlinks two key signaling pathways that control actin treadmilling and neuronal maturation, thereby fulfilling a regulatory loop that couples cytoskeletal dynamics to nuclear gene transcription during brain development.
37. Upregulation of neurovascular communication through filamin abrogation promotes ectopic periventricular neurogenesis. Neuronal fate-restricted intermediate progenitors (IPs) are derived from the multipotent radial glia (RGs) and serve as the direct precursors for cerebral cortical neurons, but factors that control their neurogenic plasticity remain elusive. Here we report that IPs' neuron production is enhanced by abrogating filamin function, leading to the generation of periventricular neurons independent of normal neocortical neurogenesis and neuronal **migration**. Loss of Flna in neural progenitor cells (NPCs) led RGs to undergo changes resembling epithelial-mesenchymal transition (EMT) along with exuberant angiogenesis that together changed the microenvironment and increased neurogenesis of IPs. We show that by collaborating with β-arrestin, Flna maintains the homeostatic signaling between the vasculature and NPCs, and loss of this function results in escalated Vegfa and Igf2 signaling, which exacerbates both EMT and angiogenesis to further potentiate IPs' neurogenesis. These results suggest that the neurogenic potential of IPs may be boosted in vivo by manipulating Flna-mediated neurovascular communication.
38. Characterization of neogenin-expressing neural progenitor populations and migrating neuroblasts in the embryonic mouse forebrain. Many studies have demonstrated a role for netrin-1-deleted in colorectal cancer (DCC) interactions in both axon guidance and neuronal **migration**. Neogenin, a member of the DCC receptor family, has recently been shown to be a chemorepulsive axon guidance receptor for the repulsive guidance molecule (RGM) family of guidance cues [Rajagopalan S, Deitinghoff L, Davis D, Conrad S, Skutella T, Chedotal A, Mueller B, Strittmatter S (2004) Neogenin mediates the action of repulsive guidance molecule. Nat Cell Biol 6:755-762]. Here we show that neogenin is present on neural progenitors, including neurogenic radial glia, in the embryonic mouse forebrain suggesting that neogenin expression is a hallmark of neural progenitor populations. Neogenin-positive progenitors were isolated from embryonic day 14.5 forebrain using flow cytometry and cultured as neurospheres. Neogenin-positive progenitors gave rise to neurospheres displaying a high proliferative and neurogenic potential. In contrast, neogenin-negative forebrain cells did not produce long-term neurosphere cultures and did not possess a significant neurogenic potential. These observations argue strongly for a role for neogenin in neural progenitor biology. In addition, we also observed neogenin on parvalbumin- and calbindin-positive interneuron neuroblasts that were migrating through the medial and lateral ganglionic eminences, suggesting a role for neogenin in tangential **migration**. Therefore, neogenin may be a multi-functional receptor regulating both progenitor activity and neuroblast **migration** in the embryonic forebrain.
39. Transcriptional regulation of cortical neuron **migration** by POU domain factors. The identification of pathways mediated by the kinase Cdk5 and the ligand reelin has provided a conceptual framework for exploring the molecular mechanisms underlying proper lamination of the developing mammalian cerebral cortex. In this report, we identify a component of the regulation of Cdk5-mediated cortical lamination by genetic analysis of the roles of the class III POU domain transcription factors, Brn-1 and Brn-2, expressed during the development of the forebrain and coexpressed in most layer II-V cortical neurons. Brn-1 and Brn-2 appear to critically control the initiation of radial **migration**, redundantly regulating the cell-autonomous expression of the p35 and p39 regulatory subunits of Cdk5 in migrating cortical neurons, with Brn-1(-/-)/Brn-2(-/-) mice exhibiting cortical inversion.
40. The c-Jun N-terminal kinase activator dual leucine zipper kinase regulates axon growth and neuronal **migration** in the developing cerebral cortex. Mammalian corticogenesis substantially depends on **migration** and axon projection of newborn neurons that are coordinated by a yet unidentified molecular mechanism. Dual leucine zipper kinase (DLK) induces activation of c-Jun N-terminal kinase (JNK), a molecule that regulates morphogenesis in various organisms. We show here, using gene targeting in mice, that DLK is indispensable for establishing axon tracts, especially those originating from neocortical pyramidal neurons of the cerebrum. Direct and quantitative analysis of radial **migration** of pyramidal neurons using slice culture and a time-lapse imaging system revealed that acceleration around the subplate was affected by DLK gene disruption and by administration of a JNK inhibitor. Phosphorylation of JNK substrates, including c-Jun and doublecortin, and of JNK itself at the activation loop were partially affected in brains of DLK-deficient mouse embryos. These data suggest that DLK plays a significant role in the coordinated regulation of radial **migration** and axon projection by modulating JNK activity.
41. Vinculin knockout results in heart and brain defects during embryonic development. The vinculin gene codes for a cytoskeletal protein, found in focal adhesion plaques and in cell-cell adherens junctions. Vinculin was inactivated by homologous recombination using a targeting vector in embryonic stem (ES) cells. The heterozygous ES cells were introduced into mice by established procedures to produce heterozygous animals that were normal and fertile. No homozygous vinculin-/- embryos were born and analyses during the gestational period showed that the vinculin null embryos were small and abnormal from day E8 but some survived until E10. The most prominent defect was lack of midline fusion of the rostral neural tube, producing a cranial bilobular appearance and attenuation of cranial and spinal nerve development. Heart development was curtailed at E9.5, with severely reduced and akinetic myocardial and endocardial structures. Mutant embryos were 30-40% smaller, somites and limbs were retarded and ectodermal tissues were sparse and fragile. Fibroblasts (MEF) isolated from mutant embryos were shown to have reduced adhesion to fibronectin, vitronectin, laminin and collagen compared to wild-type levels. In addition, **migration** rates over these substrata were two-fold higher and the level of focal adhesion kinase (FAK) activity was three-fold higher. We conclude that vinculin is necessary for normal embryonic development, probably because of its role in the regulation of cell adhesion and locomotion, cell behaviors essential for normal embryonic morphogenesis, although specific roles in neural and cardiac development cannot be ruled out.
42. Peripheral contributions to olfactory bulb cell populations (**migration**s towards the olfactory bulb). The olfactory system represents one of the most suitable models to study interactions between the peripheral and central nervous systems. The developing olfactory epithelium (olfactory placode and pit) gives rise to several cell populations that migrate towards the telencephalic vesicle. One of these cell populations, called the Migratory Mass (MM), accompanies the first emerging olfactory axons from the olfactory placode, but the fate of these cells and their contribution to the Olfactory Bulb (OB) populations has not been properly addressed. To asses this issue we performed ultrasound-guided in utero retroviral injections at embryonic day (E) 11 revealing the MM as an early source of Olfactory Ensheathing Cells in later postnatal stages. Employing a wide number of antibodies to identify the nature of the infected cells we described that those cells generated within the MM at E11 belong to different cell populations both in the mesenchyma, where they envelop olfactory axons and express the most common glial markers, and in the olfactory bulb, where they are restricted to the Olfactory Nerve and Glomerular layers. Thus, the data reveal the existence of a novel progenitor class within the MM, potentially derived from the olfactory placode which gives rise to different neural cell population including some CNS neurons, glia and olfactory ensheathing cells.
43. Expression of liver X receptor beta is essential for formation of superficial cortical layers and **migration** of later-born neurons. Liver X receptor (LXR) beta regulates cholesterol levels in the brain and is essential for maintenance of motor neurons in the spinal cord and dopaminergic neurons in the substantia nigra. Here, we have examined the expression pattern of LXRbeta protein in the cerebral cortex and looked for defects in cortical development in LXRbeta knockout (LXRbeta(-/-)) mice. LXRbeta protein was widely expressed in the mouse brain at later embryonic stages, and the expression pattern in the cerebral cortex was developmentally regulated. In normal postnatal mice, LXRbeta was localized mainly in the upper layers of the cerebral cortex. In LXRbeta(-/-) mice layers II and III were thinner with fewer neurons. Layer I was slightly thicker, whereas layers IV-VI were essentially normal. Consistent with this finding, Brn2 and NeuN expression were decreased in the upper layers in the LXRbeta(-/-) neonatal cortex. The number of S-phase progenitor cells in the cortex between embryonic day (E) 12.5 to E16.5, was similar in WT and LXRbeta(-/-) littermates but BrdU birth dating revealed that late-generated neurons labeled by BrdU injections administered at E14.5 or E16.5, and destined to cortical layers II/III, were disorganized and failed to migrate. The defect in **migration** appears to be caused by a reduction in the number of vertical processes emanating from the radial glia. These processes are the architectural guides for later-born migrating neurons. Taken together, these findings suggest that LXRbeta expression in the cerebral cortex is involved in cortex lamination and is essential for the **migration** of late-generated neocortical neurons.
44. Tangential **migration** and proliferation of intermediate progenitors of GABAergic neurons in the mouse telencephalon. In the embryonic neocortex, neuronal precursors are generated in the ventricular zone (VZ) and accumulate in the cortical plate. Recently, the subventricular zone (SVZ) of the embryonic neocortex was recognized as an additional neurogenic site for both principal excitatory neurons and GABAergic inhibitory neurons. To gain insight into the neurogenesis of GABAergic neurons in the SVZ, we investigated the characteristics of intermediate progenitors of GABAergic neurons (IPGNs) in mouse neocortex by immunohistochemistry, immunocytochemistry, single-cell RT-PCR and single-cell array analysis. IPGNs were identified by their expression of some neuronal and cell cycle markers. Moreover, we investigated the origins of the neocortical IPGNs by Cre-loxP fate mapping in transgenic mice and the transduction of part of the telencephalic VZ by Cre-reporter plasmids, and found them in the medial and lateral ganglionic eminence. Therefore, they must migrate tangentially within the telencephalon to reach the neocortex. Cell-lineage analysis by simple-retrovirus transduction revealed that the neocortical IPGNs self-renew and give rise to a small number of neocortical GABAergic neurons and to a large number of granule and periglomerular cells in the olfactory bulb. IPGNs are maintained in the neocortex and may act as progenitors for adult neurogenesis.
45. The lack of Emx2 causes impairment of Reelin signaling and defects of neuronal **migration** in the developing cerebral cortex. Neocorticogenesis in mice homozygous for an Emx2 null allele is the topic of this article. The development of both main components of neocortex, primordial plexiform layer derivatives and cortical plate, was analyzed, paying special attention to radial **migration** of neurons forming the cortical plate. The products of the Reelin gene, normally playing a key role in orchestrating radial **migration** of these neurons, display normal distribution at the beginning of the cortical neuronogenesis but are absent in the neocortical marginal zone of the mutant mice at the time when the cortical plate is laid down. As a consequence, the development of radial glia is impaired, and neurons making up the cortical plate display abnormal **migration** patterns. In addition, restricted defects along the rostrocaudal and the mediolateral axes are present in the subplate, suggesting an Emx2-specific role in priming the proper development of this layer.
46. Presenilin-1 regulates neuronal differentiation during neurogenesis. Mutations in Presenilin-1 (PS1) are a major cause of familial Alzheimer's disease. Our previous studies showed that PS1 is required for murine neural development. Here we report that lack of PS1 leads to premature differentiation of neural progenitor cells, indicating a role for PS1 in a cell fate decision between postmitotic neurons and neural progenitor cells. Neural proliferation and apoptotic cell death during neurogenesis are unaltered in PS1(-/-) mice, suggesting that the reduction in the neural progenitor cells observed in the PS1(-/-) brain is due to premature differentiation of progenitor cells, rather than to increased apoptotic cell death or decreased cell proliferation. In addition, the premature neuronal differentiation in the PS1(-/-) brain is associated with aberrant neuronal **migration** and disorganization of the laminar architecture of the developing cerebral hemisphere. In the ventricular zone of PS1(-/-) mice, expression of the Notch1 downstream effector gene Hes5 is reduced and expression of the Notch1 ligand Dll1 is elevated, whereas expression of Notch1 is unchanged. The level of Dll1 transcripts is also increased in the presomitic mesoderm of PS1(-/-) embryos, while the level of Notch1 transcripts is unchanged, in contrast to a previous report (Wong et al., 1997, Nature 387, 288-292). These results provide direct evidence that PS1 controls neuronal differentiation in association with the downregulation of Notch signalling during neurogenesis.
47. Cortical **migration** defects in mice expressing A-RAF from the B-RAF locus. We have previously shown that mice lacking the protein kinase B-RAF have defects in both neural and endothelial cell lineages and die around embryonic day 12 (E12). To delineate the function of B-RAF in the brain, B-RAF KIN/KIN mice lacking B-RAF and expressing A-RAF under the control of the B-RAF locus were created. B-RAF KIN/KIN embryos displayed no vascular defects, no endothelial and neuronal apoptosis, or gross developmental abnormalities, and a significant proportion of these animals survived for up to 8 weeks. Cell proliferation in the neocortex was reduced from E14.5 onwards. Newborn cortical neurons were impaired in their **migration** toward the cortical plate, causing a depletion of Brn-2-expressing pyramidal neurons in layers II, III, and V of the postnatal cortex. Our data reveal that B-RAF is an important mediator of neuronal survival, **migration**, and dendrite formation and that A-RAF cannot fully compensate for these functions.
48. DCAMKL1 encodes a protein kinase with homology to doublecortin that regulates microtubule polymerization. Doublecortin (DCX) is a microtubule-associated protein required for neuronal **migration** to the cerebral cortex. DCAMKL1 consists of an N terminus that is 65% similar to DCX throughout the entire length of DCX, but also contains an additional 360 amino acid C-terminal domain encoding a putative Ca(2+)/calmodulin-dependent protein kinase. The homology to DCX suggested that DCAMKL1 may regulate microtubules, as well as mediate a phosphorylation-dependent signal transduction pathway. Here we show that DCAMKL1 is expressed throughout the CNS and PNS in migrating neuronal populations and overlaps in its expression with DCX and microtubules. Purified DCAMKL1 associates with microtubules and stimulates polymerization of purified tubulin and the formation of aster-like microtubule structures. Overexpressed DCAMKL1 leads to striking microtubule bundling in cell lines and cultured primary neural cells. Time-lapse imaging of cells transfected with a DCAMKL1-green fluorescent protein fusion protein shows that the microtubules associated with the protein remain dynamic. DCAMKL1 also encodes a functional kinase capable of phosphorylating myelin basic protein and itself. However, elimination of the kinase activity of DCAMKL1 has no detectable effect on its microtubule polymerization activity. Because DCAMKL1 is coexpressed with DCX, the two proteins form a potentially mutually regulatory network linking calcium signaling and microtubule dynamics.
49. The F-BAR domain of srGAP2 induces membrane protrusions required for neuronal **migration** and morphogenesis. During brain development, proper neuronal **migration** and morphogenesis is critical for the establishment of functional neural circuits. Here we report that srGAP2 negatively regulates neuronal **migration** and induces neurite outgrowth and branching through the ability of its F-BAR domain to induce filopodia-like membrane protrusions resembling those induced by I-BAR domains in vivo and in vitro. Previous work has suggested that in nonneuronal cells filopodia dynamics decrease the rate of cell **migration** and the persistence of leading edge protrusions. srGAP2 knockdown reduces leading process branching and increases the rate of neuronal **migration** in vivo. Overexpression of srGAP2 or its F-BAR domain has the opposite effects, increasing leading process branching and decreasing **migration**. These results suggest that F-BAR domains are functionally diverse and highlight the functional importance of proteins directly regulating membrane deformation for proper neuronal **migration** and morphogenesis.
50. Expression of connexins in embryonic mouse neocortical development. During embryonic development, young neurons migrate from the ventricular zone to the cortical plate of the cerebral cortex. Disturbances in this neuronal **migration** have been associated with numerous diseases such as mental retardation, double cortex, Down syndrome, and epilepsy. One possible cause of these neuropathologies is an aberration in normal gap junctional communication. At least 20 connexin (Cx) genes encode gap junction proteins in mice and humans. A proper understanding of the role of specific connexins in the developing brain requires the characterization of their spatial and temporal pattern of expression. In the current study we performed all the experiments on mouse developing cortex at embryonic days (E) 14, 16, and 18, timepoints that are highly active with regard to cortical development. Using reverse transcription-polymerase chain reaction, Western blot analysis, and immunohistochemistry, we found that among the family of gap junction proteins, Cx26, Cx36, Cx37, Cx43, and Cx45 were expressed in the developing cortex of mice, Cx30 and Cx32 were absent, while Cx40 was expressed at a very low level. Our results demonstrate that Cx26 and Cx37 were evenly distributed in the cortical layers of developing brain, while Cx36 and Cx43 were more abundant in the ventricular zone and cortical plate. Cx45 distribution appeared to be more abundant at E18 compared to the other timepoints (E14 and E16). Thus, the present study provides identification and the distribution pattern for Cxs associated with cortical development during normal neuronal **migration**.
51. AMP-activated protein kinase (AMPK) activity is not required for neuronal development but regulates axogenesis during metabolic stress. Mammalian brain connectivity requires the coordinated production and **migration** of billions of neurons and the formation of axons and dendrites. The LKB1/Par4 kinase is required for axon formation during cortical development in vivo partially through its ability to activate SAD-A/B kinases. LKB1 is a master kinase phosphorylating and activating at least 11 other serine/threonine kinases including the metabolic sensor AMP-activated protein kinase (AMPK), which defines this branch of the kinome. A recent study using a gene-trap allele of the β1 regulatory subunit of AMPK suggested that AMPK catalytic activity is required for proper brain development including neurogenesis and neuronal survival. We used a genetic loss-of-function approach producing AMPKα1/α2-null cortical neurons to demonstrate that AMPK catalytic activity is not required for cortical neurogenesis, neuron**al migrat**ion, polarization, or survival. However, we found that application of metformin or AICAR, potent AMPK activators, inhibit axogenesis and axon growth in an AMPK-dependent manner. We show that inhibition of axon growth mediated by AMPK overactivation requires TSC1/2-mediated inhibition of the mammalian target of rapamycin (mTOR) signaling pathway. Our results demonstrate that AMPK catalytic activity is not required for early neural development in vivo but its overactivation during metabolic stress impairs neuronal polarization in a mTOR-dependent manner.
52. EGF transactivation of Trk receptors regulates the **migration** of newborn cortical neurons. The development of neuronal networks in the neocortex depends on control mechanisms for mitosis and **migration** that allow newborn neurons to find their accurate position. Multiple mitogens, neurotrophic factors, guidance molecules and their corresponding receptors are involved in this process, but the mechanisms by which these signals are integrated are only poorly understood. We found that TrkB and TrkC, the receptors for brain-derived neurotrophic factor (BDNF) and neurotrophin-3 (NT-3), are activated by epidermal growth factor receptor (EGFR) signaling rather than by BDNF or NT-3 in embryonic mouse cortical precursor cells. This transactivation event regulated **migration** of early neuronal cells to their final position in the developing cortex. Transactivation by EGF led to membrane translocation of TrkB, promoting its signaling responsiveness. Our results provide genetic evidence that TrkB and TrkC activation in early cortical neurons do not depend on BDNF and NT-3, but instead on transactivation by EGFR signaling.
53. The expression pattern of a novel gene encoding brain-fatty acid binding protein correlates with neuronal and glial cell development. Fatty acid binding proteins (FABPs) are a multigene family of small intracellular proteins that bind hydrophobic ligands. In this report we describe the cloning and expression pattern of a novel member of this gene family that is specifically expressed in the developing and adult nervous system and thus was designated brain (B)-FABP. B-FABP is closely related to heart (H)-FABP with 67% amino acid identity. B-FABP expression was first detected at mouse embryonic day 10 in neuroepithelial cells and its pattern correlates with early neuronal differentiation. Upon further development, B-FABP was confined to radial glial cells and immature astrocytes. B-FABP mRNA and protein were found in glial cells of the peripheral nervous system such as satellite cells of spinal and cranial ganglia and ensheathing cells of the olfactory nerve layer from as early as embryonic day 11 until adulthood. In the adult mouse brain, B-FABP was found in the glia limitans, in radial glial cells of the hippocampal dentate gyrus and Bergman glial cells. These findings suggest a function of B-FABP during neurogenesis or neuronal **migration** in the developing nervous system. The partially overlapping expression pattern with that of cellular retinoid binding proteins suggests that B-FABP is involved in the metabolism of a so far unknown hydrophobic ligand with potential morphogenic activity during CNS development.
54. Type I phosphatidylinositol 4-phosphate 5-kinase γ is required for neuronal **migratio**n in the mouse developing cerebral cortex. Type I phosphatidylinositol 4-phosphate 5-kinase (PIP5KI)γ is one of the phosphoinositide kinases that produce phosphatidylinositol 4,5-bisphosphate, which is a critical regulator of cell adhesion formation, actin dynamics and membrane trafficking. Here, we examined the functional roles of PIP5KIγ in radial neuron**al migrat**ion during cortical formation. Reverse transcription-polymerase chain reaction analysis revealed that PIP5KIγ\_v2/v6 and PIP5KIγ\_v3 were expressed throughout cortical development with distinct expression patterns. In situ hybridisation analysis showed that PIP5KIγ mRNA was expressed throughout the cortical layers. Immunohistochemical analysis revealed that PIP5KIγ was localised in a punctate manner in the radial glia and migrating neuroblasts. Knockdown of PIP5KIγ using in utero electroporation disturbed the radial n**euronal m**igration and recruitment of talin and focal adhesion kinase to puncta beneath the plasma membrane. The same inhibitory effect on n**euronal m**igration was observed by overexpression of a catalytically inactive mutant of PIP5KIγ\_v2 but not PIP5KIγ\_v1 or PIP5KIγ\_v3. These findings suggest an essential role of PIP5KIγ, particularly PIP5KIγ\_i2, **in neuro**nal migration, possibly through recruitment of adhesion components to the plasma membrane.
55. Role of presenilin-1 in cortical lamination and survival of Cajal-Retzius neurons. Presenilin-1 (PS1), the major causative gene of familial Alzheimer disease, regulates neuronal differentiation and Notch signaling during early neural development. To investigate the role of PS1 in neuronal **migration** and cortical lamination of the postnatal brain, we circumvented the perinatal lethality of PS1-null mice by generating a conditional knockout (cKO) mouse in which PS1 inactivation is restricted to neural progenitor cells (NPCs) and NPC-derived neurons and glia. BrdU birthdating analysis revealed that many late-born neurons fail to migrate beyond the early-born neurons to arrive at their appropriate positions in the superficial layer, while the **migration** of the early-born neurons is largely normal. The **migration** defect of late-born neurons coincides with the progressive reduction of radial glia in PS1 cKO mice. In contrast to the premature loss of Cajal-Retzius (CR) neurons in PS1-null mice, generation and survival of CR neurons are unaffected in PS1 cKO mice. Furthermore, the number of proliferating meningeal cells, which have been shown to be important for the survival of CR neurons, is increased in PS1-null mice but not in PS1 cKO mice. These findings show a cell-autonomous role for PS1 in cortical lamination and radial glial development, and a non-cell-autonomous role for PS1 in CR neuron survival.
56. Fibroblast growth factor 13 is a microtubule-stabilizing protein regulating neuronal polarization and **migration**. Secretory fibroblast growth factors (FGFs) and their receptors are known for their regulatory function in the early stages of neural development. FGF13, a nonsecretory protein of the FGF family, is expressed in cerebral cortical neurons during development and is a candidate gene for syndromal and nonspecific forms of X-chromosome-linked mental retardation (XLMR). However, its function during development remains unclear. We show that FGF13 acts intracellularly as a microtubule-stabilizing protein required for axon and leading process development and neuronal **migration** in the cerebral cortex. FGF13 is enriched in axonal growth cones and interacts directly with microtubules. Furthermore, FGF13 polymerizes tubulins and stabilizes microtubules. The loss of FGF13 impairs neuronal polarization and increases the branching of axons and leading processes. Genetic deletion of FGF13 in mice results in neuronal **migration** defects in both the neocortex and the hippocampus. FGF13-deficient mice also exhibit weakened learning and memory, which is correlated to XLMR patients' intellectual disability.
57. Rescue of ataxia and preplate splitting by ectopic expression of Reelin in reeler mice. The gene mutated in reeler (reelin) encodes a protein secreted by neurons in the developing brain that controls laminar positioning of migrating cells in the CNS by an unknown mechanism. To investigate Reelin function, we used the nestin promoter to express Reelin ectopically in the ventricular zone and other brain regions in transgenic mice. In the presence of the endogenous protein, ectopic Reelin did not alter cell **migration** in the neocortex or the cerebellum. However, in the reeler background, ectopic Reelin induced tyrosine phosphorylation of Dab-1 in the ventricular zone and rescued some, but not all, of the neuroanatomic and behavioral abnormalities characteristic of reeler. These results indicate that Reelin does not function simply as a positional signal. Rather, it appears to participate in multiple events critical for neuronal **migration** and cell positioning.
58. Stage-specific modulation of cortical neuronal development by Mmu-miR-134. To realize the potential of microRNAs (miRs) as fine-tuning regulators of embryonic neuronal differentiation, it is critical to define their developmental function. Mmu-miR-134 (miR-134) is a powerful inducer of pluripotent stem cell differentiation. However, its functional role during embryonic, neuronal development is unknown. We demonstrate that mature, miR-134 transcript levels elevate during embryonic, neuronal differentiation in vitro and in vivo. To define the developmental targets and function of miR-134, we identified multiple brain-expressed targets including the neural progenitor cell-enriched, bone morphogenetic protein (BMP) antagonist Chordin-like 1 (Chrdl-1) and the postmitotic, neuron-specific, microtubule-associated protein, Doublecortin (Dcx). We show that, through interaction with Dcx and/or Chrdl-1, miR-134 has stage-specific effects on cortical progenitors, migratory neurons, and differentiated neurons. In neural progenitors, miR-134 promotes cell proliferation and counteracts Chrdl-1-induced apoptosis and Dcx-induced differentiation in vitro. In neurons, miR-134 reduces cell **migration** in vitro and in vivo in a Dcx-dependent manner. In differentiating neurons, miR-134 modulates process outgrowth in response to exogenous BMP-4 in a noggin-reversible manner. Taken together, we present Dcx and Chrdl-1 as new regulatory targets of miR-134 during embryonic, mouse, cortical, and neuronal differentiation and show a novel and previously undiscovered role for miR-134 in the stage-specific modulation of cortical development.
59. Connective tissue growth factor: a novel marker of layer VII neurons in the rat cerebral cortex. Connective tissue growth factor (CTGF) belongs to a family of secreted, extracellular matrix-associated proteins that are involved in the regulation of cellular functions such as adhesion, **migration**, mitogenesis, differentiation and survival. Recent studies have also suggested the up-regulation of CTGF in response to trauma, scar formation and excitotoxicity in the CNS. To further elucidate the localization and regulation of this molecule in the rat brain we performed in situ hybridization experiments and found a very strong and selective expression of CTGF messenger ribonucleic acid (mRNA) on the band of layer VII neurons throughout the adult cerebral cortex. Similarly strong neuronal expression was also present in the dorsal endopiriform nucleus, extending rostrally from the ventrocaudal cortical layer VII, and in the deep layers of the olfactory glomeruli and the accessory olfactory nucleus. Double in situ hybridization confirmed selective CTGF mRNA expression on a subpopulation (approximately 35%) of microtubule-associated protein 2 mRNA-positive neurons in the cortical layer VII and the dorsal endopiriform nucleus. The nucleus of lateral olfactory tract showed moderate signal intensity; other parts of the forebrain, mesencephalon and brain stem only revealed a very weak level of CTGF mRNA expression. Non-neuronal expression was rare, considerably weaker than on cortical layer VII neurons, and normally associated with blood vessels. Developmental analysis of CTGF mRNA expression in embryonic and postnatal mouse also showed a moderately late onset at embryonic day 16-18, and confirmed the presence of CTGF mRNA in cortical layer VII in a second rodent species. Interestingly, injury experiments using direct cerebral trauma or injection of excitotoxic kainic acid into rat brain failed to up-regulate CTGF mRNA after injury and during the ensuing period of neuronal cell death, gliosis and neural scar tissue formation. Altogether, the current data suggest a constitutive role of CTGF, particularly in the adult cerebral cortex. In view of the strong ascending projections of subplate neurons into cortical layer 1, this molecule may be involved in the modulation of synaptic input to apical dendrites of pyramidal neurons.
60. Control of hippocampal morphogenesis and neuronal differentiation by the LIM homeobox gene Lhx5. The mammalian hippocampus contains the neural circuitry that is crucial for cognitive functions such as learning and memory. The development of such circuitry is dependent on the generation and correct placement of the appropriate number and types of neurons. Mice lacking function of the LIM homeobox gene Lhx5 showed a defect in hippocampus development. Hippocampal neural precursor cells were specified and proliferated, but many of them failed to either exit the cell cycle or to differentiate and migrate properly. Lhx5 is therefore essential for the regulation of precursor cell proliferation and the control of neuronal differentiation and **migration** during hippocampal development.
61. Complete loss of Ndel1 results in neuronal **migration** defects and early embryonic lethality. Regulation of cytoplasmic dynein and microtubule dynamics is crucial for both mitotic cell division and neuronal **migration**. NDEL1 was identified as a protein interacting with LIS1, the protein product of a gene mutated in the lissencephaly. To elucidate NDEL1 function in vivo, we generated null and hypomorphic alleles of Ndel1 in mice by targeted gene disruption. Ndel1(-/-) mice were embryonic lethal at the peri-implantation stage like null mutants of Lis1 and cytoplasmic dynein heavy chain. In addition, Ndel1(-/-) blastocysts failed to grow in culture and exhibited a cell proliferation defect in inner cell mass. Although Ndel1(+/-) mice displayed no obvious phenotypes, further reduction of NDEL1 by making null/hypomorph compound heterozygotes (Ndel1(cko/-)) resulted in histological defects consistent with mild neuronal **migration** defects. Double Lis1(cko/+)-Ndel1(+/-) mice or Lis1(+/-)-Ndel1(+/-) mice displayed more severe neuronal **migration** defects than Lis1(cko/+)-Ndel1(+/)(+) mice or Lis1(+/-)-Ndel1(+/+) mice, respectively. We demonstrated distinct abnormalities in microtubule organization and similar defects in the distribution of beta-COP-positive vesicles (to assess dynein function) between Ndel1 or Lis1-null MEFs, as well as similar neuronal **migration** defects in Ndel1- or Lis1-null granule cells. Rescue of these defects in mouse embryonic fibroblasts and granule cells by overexpressing LIS1, NDEL1, or NDE1 suggest that NDEL1, LIS1, and NDE1 act in a common pathway to regulate dynein but each has distinct roles in the regulation of microtubule organization and neuronal **migration**.
62. Increased expression of MAP2 inhibits melanoma cell proliferation, invasion and tumor growth in vitro and in vivo. Malignant melanoma (MM) is characterized by aggressive metastasis and high mortality rate. Microtubule-associated proteins 2 (MAP2) is expressed abundantly in majority of melanocytic nevi and primary melanomas, but absent in metastatic melanomas. To determine whether MAP2 correlates with tumor progression of MM, we investigated the effects of MAP2 inhibition on the biological behaviour of metastatic melanoma in vitro and in vivo. Our results demonstrated that adenovirus-mediated MAP2 induced apoptotic cell death and cell cycle arrest in metastatic human and mouse melanoma cell lines in vitro, and substantially inhibited the growth of melanomas in nude mice in vivo. In addition, intracellular expression of MAP2 was found to induce the morphologic alteration, suppress the **migration** and invasion and affect the assembly, stabilization and bundling of microtubules in melanoma cells. This is the first study that MAP2 expression significantly inhibits the growth of MM in vivo. Our results suggest that MAP2 may serve as a promising molecular target for therapy and chemoprevention of MM in humans.
63. Multimodal tangential **migration** of neocortical GABAergic neurons independent of GPI-anchored proteins. Neuronal **migration** is crucial for the construction of neuronal architecture such as layers and nuclei. Most inhibitory interneurons in the neocortex derive from the basal forebrain and migrate tangentially; however, little is known about the mode of **migration** of these neurons in the cortex. We used glutamate decarboxylase (Gad)67-green fluorescent protein (GFP) knock-in embryonic mice with expression of GFP in gamma-aminobutyric acid (GABA)-ergic neurons and performed time-lapse analysis. In coronal slices, many GFP-positive neurons in the lower intermediate zone (IZ) and subventricular zone (SVZ) showed robust tangential **migration** from lateral to medial cortex, while others showed radial and non-radial **migration** mostly towards the pial surface. In flat-mount preparations, GFP-positive neurons of the marginal zone (MZ) showed multidirectional tangential **migration**. Some of these neurons descended toward the cortical plate (CP). Intracortical **migration** of these neurons was largely unaffected by a treatment that cleaves glycosylphosphatidylinositol (GPI) anchors. These findings suggest that tangential **migration** of cortical interneurons from lateral to medial cortex predominantly occurs in the IZ/SVZ and raise the possibility that a part of the pial surface-directed neurons in the IZ/SVZ reach the MZ, whereby they spread into the whole area of the cortex. At least a part of these neurons may descend toward the CP. Our results also suggest that intracortical **migration** of GABAergic neurons occurs independent of GPI-anchored proteins.
64. Endothelial VEGF sculpts cortical cytoarchitecture. Current models of brain development support the view that VEGF, a signaling protein secreted by neuronal cells, regulates angiogenesis and neuronal development. Here we demonstrate an autonomous and pivotal role for endothelial cell-derived VEGF that has far-reaching consequences for mouse brain development. Selective deletion of Vegf from endothelial cells resulted in impaired angiogenesis and marked perturbation of cortical cytoarchitecture. Abnormal cell clusters or heterotopias were detected in the marginal zone, and disorganization of cortical cells induced several malformations, including aberrant cortical lamination. Critical events during brain development-neuronal proliferation, differentiation, and **migration** were significantly affected. In addition, axonal tracts in the telencephalon were severely defective in the absence of endothelial VEGF. The unique roles of endothelial VEGF cannot be compensated by neuronal VEGF and underscores the high functional significance of endothelial VEGF for cerebral cortex development and from disease perspectives.
65. Gamma protocadherins are required for survival of spinal interneurons. The murine genome contains approximately 70 protocadherin (Pcdh) genes. Many are expressed in the nervous system, suggesting that Pcdhs may specify neuronal connectivity. Here, we analyze the 22 contiguous genes of the Pcdh-gamma cluster. Individual neurons express subsets of Pcdh-gamma genes. Pcdh-gamma proteins are present in most neurons and associated with, but not confined to, synapses. Early steps in neuronal **migration**, axon outgrowth, and synapse formation proceed in mutant mice lacking all 22 Pcdh-gamma genes. At late embryonic stages, however, dramatic neurodegeneration leads to neonatal death. In mutant spinal cord, many interneurons are lost, but sensory and motor neurons are relatively spared. In cultures from mutant spinal cord, neurons differentiate and form synapses but then die. Thus, Pcdh-gamma genes are dispensable for at least some aspects of connectivity but required for survival of specific neuronal types.
66. Synergistic effects of MAP2 and MAP1B knockout in neuronal **migration**, dendritic outgrowth, and microtubule organization. MAP1B and MAP2 are major members of neuronal microtubule-associated proteins (MAPs). To gain insights into the function of MAP2 in vivo, we generated MAP2-deficient (map2(-/-)) mice. They developed without any apparent abnormalities, which indicates that MAP2 is dispensable in mouse survival. Because previous reports suggest a functional redundancy among MAPs, we next generated mice lacking both MAP2 and MAP1B to test their possible synergistic functions in vivo. Map2(-/-)map1b(-/-) mice died in their perinatal period. They showed not only fiber tract malformations but also disrupted cortical patterning caused by retarded neuronal **migration**. In spite of this, their cortical layer maintained an "inside-out" pattern. Detailed observation of primary cultures of hippocampal neurons from map2(-/-)map1b(-/-) mice revealed inhibited microtubule bundling and neurite elongation. In these neurons, synergistic effects caused by the loss of MAP2 and MAP1B were more apparent in dendrites than in axons. The spacing of microtubules was reduced significantly in map2(-/-)map1b(-/-) mice in vitro and in vivo. These results suggest that MAP2 and MAP1B have overlapping functions in neuronal **migration** and neurite outgrowth by organizing microtubules in developing neurons both for axonal and dendritic morphogenesis but more dominantly for dendritic morphogenesis.
67. Collapsin response mediator protein 1 mediates reelin signaling in cortical neuronal **migration**. Collapsin response mediator protein 1 (CRMP1) is one of the CRMP family members that mediates signal transduction of axon guidance molecules. Here, we show evidence that CRMP1 is involved in Reelin (Reln) signaling to regulate neuronal **migration** in the cerebral cortex. In crmp1-/- mice, radial **migration** of cortical neurons was retarded. This phenotype was not observed in the sema3A-/- and crmp1+/-;sema3A+/- cortices. However, CRMP1 was colocalized with disabled-1 (Dab1), an adaptor protein in Reln signaling. In the Reln(rl/rl) cortex, CRMP1 and Dab1 were expressed at a higher level, yet tyrosine phosphorylated at a lower level. Loss of crmp1 in a dab1 heterozygous background led to the disruption of hippocampal lamination, a Reeler-like phenotype. In addition to axon guidance, CRMP1 regulates neuronal **migration** by mediating Reln signaling.
68. From the Cover: Indispensability of the glutamate transporters GLAST and GLT1 to brain development. Previous in vitro studies have shown that the neurotransmitter glutamate is important in brain development. Paradoxically, loss-of-function mouse models of glutamatergic signaling that are generated by genetic deletion of glutamate receptors or glutamate release show normal brain assembly. We examined the direct consequences on brain development of extracellular glutamate buildup due to the depletion of the glutamate transporters GLAST and GLT1. GLAST/GLT1 double knockout mice show multiple brain defects, including cortical, hippocampal, and olfactory bulb disorganization with perinatal mortality. Here, we report abnormal formation of the neocortex in GLAST/GLT1 mutants. Several essential aspects of neuronal development, such as stem cell proliferation, radial **migration**, neuronal differentiation, and survival of SP neurons, were impaired. These results provide direct in vivo evidence that GLAST and GLT1 are necessary for brain development through regulation of extracellular glutamate concentration and show that an important mechanism is likely to be maintenance of glutamate-mediated synaptic transmission.
69. Reelin regulates cadherin function via Dab1/Rap1 to control neuronal **migration** and lamination in the neocortex. Neuronal **migration** is critical for establishing neocortical cell layers and **migration** defects can cause neurological and psychiatric diseases. Recent studies show that radially migrating neocortical neurons use glia-dependent and glia-independent modes of **migration**, but the signaling pathways that control different **migration** modes and the transitions between them are poorly defined. Here, we show that Dab1, an essential component of the reelin pathway, is required in radially migrating neurons for glia-independent somal translocation, but not for glia-guided locomotion. During **migration**, Dab1 acts in translocating neurons to stabilize their leading processes in a Rap1-dependent manner. Rap1, in turn, controls cadherin function to regulate somal translocation. Furthermore, cell-autonomous neuronal deficits in somal translocation are sufficient to cause severe neocortical lamination defects. Thus, we define the cellular mechanism of reelin function during radial **migration**, elucidate the molecular pathway downstream of Dab1 during somal translocation, and establish the importance of glia-independent motility in neocortical development.
70. Reeler/Disabled-like disruption of neuronal **migration** in knockout mice lacking the VLDL receptor and ApoE receptor 2. Layering of neurons in the cerebral cortex and cerebellum requires Reelin, an extracellular matrix protein, and mammalian Disabled (mDab1), a cytosolic protein that activates tyrosine kinases. Here, we report the requirement for two other proteins, cell surface receptors termed very low density lipoprotein receptor (VLDLR) and apolipoprotein E receptor 2 (ApoER2). Both receptors can bind mDab1 on their cytoplasmic tails and are expressed in cortical and cerebellar layers adjacent to layers that express Reelin. mDab1 expression is upregulated in knockout mice that lack both VLDLR and ApoER2. Inversion of cortical layers and absence of cerebellar foliation in these animals precisely mimic the phenotype of mice lacking Reelin or mDab1. These findings suggest that VLDLR and ApoER2 participate in transmitting the extracellular Reelin signal to intracellular signaling processes initiated by mDab1.
71. Receptor protein tyrosine phosphatase alpha is essential for hippocampal neuronal **migration** and long-term potentiation. Despite clear indications of their importance in lower organisms, the contributions of protein tyrosine phosphatases (PTPs) to development or function of the mammalian nervous system have been poorly explored. In vitro studies have indicated that receptor protein tyrosine phosphatase alpha (RPTPalpha) regulates SRC family kinases, potassium channels and NMDA receptors. Here, we report that absence of RPTPalpha compromises correct positioning of pyramidal neurons during development of mouse hippocampus. Thus, RPTPalpha is a novel member of the functional class of genes that control radial neuronal **migration**. The migratory abnormality likely results from a radial glial dysfunction rather than from a neuron-autonomous defect. In spite of this aberrant development, basic synaptic transmission from the Schaffer collateral pathway to CA1 pyramidal neurons remains intact in Ptpra(-/-) mice. However, these synapses are unable to undergo long-term potentiation. Mice lacking RPTPalpha also underperform in the radial-arm water-maze test. These studies identify RPTPalpha as a key mediator of neuronal **migration** and synaptic plasticity.
72. TrkB regulates neocortex formation through the Shc/PLCgamma-mediated control of neuronal **migration**. The generation of complex neuronal structures, such as the neocortex, requires accurate positioning of neurons and glia within the structure, followed by differentiation, formation of neuronal connections, and myelination. To understand the importance of TrkB signaling during these events, we have used conditional and knockin mutagenesis of the TrkB neurotrophin receptor, and we now show that this tyrosine kinase receptor, through docking sites for the Shc/FRS2 adaptors and phospholipase Cgamma (PLCgamma), coordinates these events in the cerebral cortex by (1) controlling cortical stratification through the timing of neuronal **migration** during cortex formation, and (2) regulating both neuronal and oligodendrocyte differentiation. These results provide genetic evidence that TrkB regulates important functions throughout the formation of the cerebral cortex via recruitment of the Shc/FRS2 adaptors and PLCgamma.
73. Ectopic HOXA5 expression results in abnormal differentiation, **migration** and p53-independent cell death of superficial dorsal horn neurons. Previously, we reported a line of mice (Hoxa5SV2) that ectopically expresses HOXA5 in the developing cervical and brachial dorsal spinal cord. Animals from this line exhibited a clear loss of cells in the outer lamina of the mature dorsal horn that coincided with an adult phenotype of sensory and motor defects of the forelimb. In this report, we examined the etiology of lost dorsal horn cells. Cells normally fated to populate the outer laminae I-III of the dorsal horn migrated inappropriately, as the percentage of laterally positioned cells in the dorsal horn was significantly reduced in Hoxa5SV2 transgenics. Apoptosis was a major cause of cell loss while proliferation of neurons was not affected in Hoxa5SV2 animals. Although Hoxa5 has been shown in vitro to regulate p53 expression and cause p53-dependent apoptosis, p53 was not required in vivo for the inappropriate apoptosis seen in Hoxa5SV2 mice, or for the normal death of motor neurons. Normal apoptosis is not dependent on Hoxa5, as the level of ventral horn motor neuron apoptosis was not changed in Hoxa5 null animals. As a possible cause of aberrant **migration** and/or apoptosis of dorsal neurons, misexpression of cell type markers was demonstrated. Further, the expression pattern of laminar markers was altered and sensory fibers aberrantly penetrated the outer lamina of mutants. Our evidence suggests that the loss of dorsal horn neurons in Hoxa5SV2 mutants was due to misexpression of dorsal horn neuronal markers, aberrant **migration**, and inappropriate apoptosis.
74. Trip6 promotes dendritic morphogenesis through dephosphorylated GRIP1-dependent myosin VI and F-actin organization. Thyroid receptor-interacting protein 6 (Trip6), a multifunctional protein belonging to the zyxin family of LIM proteins, is involved in various physiological and pathological processes, including cell **migration** and tumorigenesis. However, the role of Trip6 in neurons remains unknown. Here, we show that Trip6 is expressed in mouse hippocampal neurons and promotes dendritic morphogenesis. Through interaction with the glutamate receptor-interacting protein 1 (GRIP1) and myosin VI, Trip6 is crucial for the total dendritic length and the number of primary dendrites in cultured hippocampal neurons. Trip6 depletion reduces F-actin content and impairs dendritic morphology, and this phenocopies GRIP1 or myosin VI knockdown. Furthermore, phosphorylation of GRIP1(956T) by AKT1 inhibits the interaction between GRIP1 and myosin VI, but facilitates GRIP1 binding to 14-3-3 protein, which is required for regulating F-actin organization and dendritic morphogenesis. Thus, the Trip6-GRIP1-myosin VI interaction and its regulation on F-actin network play a significant role in dendritic morphogenesis.
75. EphA7 signaling guides cortical dendritic development and spine maturation. The process by which excitatory neurons are generated and mature during the development of the cerebral cortex occurs in a stereotyped manner; coordinated neuronal birth, **migration**, and differentiation during embryonic and early postnatal life are prerequisites for selective synaptic connections that mediate meaningful neurotransmission in maturity. Normal cortical function depends upon the proper elaboration of neurons, including the initial extension of cellular processes that lead to the formation of axons and dendrites and the subsequent maturation of synapses. Here, we examine the role of cell-based signaling via the receptor tyrosine kinase EphA7 in guiding the extension and maturation of cortical dendrites. EphA7, localized to dendritic shafts and spines of pyramidal cells, is uniquely expressed during cortical neuronal development. On patterned substrates, EphA7 signaling restricts dendritic extent, with Src and Tsc1 serving as downstream mediators. Perturbation of EphA7 signaling in vitro and in vivo alters dendritic elaboration: Dendrites are longer and more complex when EphA7 is absent and are shorter and simpler when EphA7 is ectopically expressed. Later in neuronal maturation, EphA7 influences protrusions from dendritic shafts and the assembling of synaptic components. Indeed, synaptic function relies on EphA7; the electrophysiological maturation of pyramidal neurons is delayed in cultures lacking EphA7, indicating that EphA7 enhances synaptic function. These results provide evidence of roles for Eph signaling, first in limiting the elaboration of cortical neuronal dendrites and then in coordinating the maturation and function of synapses.
76. Inactivation of Arx, the murine ortholog of the X-linked lissencephaly with ambiguous genitalia gene, leads to severe disorganization of the ventral telencephalon with impaired neuronal **migration** and differentiation. ARX loss-of-function mutations cause X-linked lissencephaly with ambiguous genitalia (XLAG), a severe neurological condition that results in profound brain malformations, including microcephaly, absence of corpus callosum, and impairment of the basal ganglia. Despite such dramatic defects, their nature and origin remain largely unknown. Here, we used Arx mutant mice as a model to characterize the cellular and molecular mechanisms underlying the basal ganglia alterations. In these animals, the early differentiation of this tissue appeared normal, whereas subsequent differentiation was impaired, leading to the periventricular accumulation of immature neurons in both the lateral ganglionic eminence and medial ganglionic eminence (MGE). Both tangential **migration** toward the cortex and striatum and radial **migration** to the globus pallidus and striatum were greatly reduced in the mutants, causing a periventricular accumulation of NPY+ or calretinin+ neurons in the MGE. Arx mutant neurons retained their differentiation potential in vitro but exhibited deficits in morphology and **migration** ability. These findings imply that cell-autonomous defects in **migration** underlie the neuronal localization defects. Furthermore, Arx mutants lacked a large fraction of cholinergic neurons and displayed a strong impairment of thalamocortical projections, in which major axon fiber tracts failed to traverse the basal ganglia. Altogether, these results highlight the critical functions of Arx in promoting neural **migration** and regulating basal ganglia differentiation in mice, consistent with the phenotype of XLAG patients.
77. Cdk5 is required for multipolar-to-bipolar transition during radial neuronal **migration** and proper dendrite development of pyramidal neurons in the cerebral cortex. The mammalian cerebral cortex consists of six layers that are generated via coordinated neuronal **migration** during the embryonic period. Recent studies identified specific phases of radial **migration** of cortical neurons. After the final division, neurons transform from a multipolar to a bipolar shape within the subventricular zone-intermediate zone (SVZ-IZ) and then migrate along radial glial fibres. Mice lacking Cdk5 exhibit abnormal corticogenesis owing to neuronal **migration** defects. When we introduced GFP into migrating neurons at E14.5 by in utero electroporation, we observed migrating neurons in wild-type but not in Cdk5(-/-) embryos after 3-4 days. Introduction of the dominant-negative form of Cdk5 into the wild-type migrating neurons confirmed specific impairment of the multipolar-to-bipolar transition within the SVZ-IZ in a cell-autonomous manner. Cortex-specific Cdk5 conditional knockout mice showed inverted layering of the cerebral cortex and the layer V and callosal neurons, but not layer VI neurons, had severely impaired dendritic morphology. The amount of the dendritic protein Map2 was decreased in the cerebral cortex of Cdk5-deficient mice, and the axonal trajectory of cortical neurons within the cortex was also abnormal. These results indicate that Cdk5 is required for proper multipolar-to-bipolar transition, and a deficiency of Cdk5 results in abnormal morphology of pyramidal neurons. In addition, proper radial neuronal **migration** generates an inside-out pattern of cerebral cortex formation and normal axonal trajectories of cortical pyramidal neurons.
78. SOX5 postmitotically regulates **migration**, postmigratory differentiation, and projections of subplate and deep-layer neocortical neurons. Neocortical projection neurons exhibit layer-specific molecular profiles and axonal connections. Here we show that the molecular identities of early-born subplate and deep-layer neurons are not acquired solely during generation or shortly thereafter but undergo progressive postmitotic refinement mediated by SOX5. Fezf2 and Bcl11b, transiently expressed in all subtypes of newly postmigratory early-born neurons, are subsequently downregulated in layer 6 and subplate neurons, thereby establishing their layer 5-enriched postnatal patterns. In Sox5-null mice, this downregulation is disrupted, and layer 6 and subplate neurons maintain an immature differentiation state, abnormally expressing these genes postnatally. Consistent with this disruption, SOX5 binds and represses a conserved enhancer near Fezf2. The Sox5-null neocortex exhibits failed preplate partition and laminar inversion of early-born neurons, loss of layer 5 subcerebral axons, and misrouting of subplate and layer 6 corticothalamic axons to the hypothalamus. Thus, SOX5 postmitotically regulates the **migration**, postmigratory differentiation, and subcortical projections of subplate and deep-layer neurons.
79. Lmx1b controls the differentiation and **migration** of the superficial dorsal horn neurons of the spinal cord. The differentiation and **migration** of superficial dorsal horn neurons and subsequent ingrowth of cutaneous afferents are crucial events in the formation of somatosensory circuitry in the dorsal spinal cord. We report that the differentiation and **migration** of the superficial dorsal horn neurons are regulated by the LIM homeobox gene Lmx1b, and its downstream targets Rnx and Drg11, two transcription factors implicated in the development of dorsal horn circuitry. An analysis of Lmx1b mutants shows that Lmx1b normally acts to maintain the expression of the Ebf genes and to repress the Zic genes. Lmx1b mutants also exhibit the disruption of the cutaneous afferent ingrowth, suggesting that the dorsal horn cells might provide important cues guiding sensory axons into the dorsal spinal cord. Our results thus indicate that Lmx1b has a pivotal role in genetic cascades that control the assembly of circuitry in the superficial dorsal horn.
80. C3G regulates cortical neuron **migration**, preplate splitting and radial glial cell attachment. Neuronal **migration** is integral to the development of the cerebral cortex and higher brain function. Cortical neuron **migration** defects lead to mental disorders such as lissencephaly and epilepsy. Interaction of neurons with their extracellular environment regulates cortical neuron **migration** through cell surface receptors. However, it is unclear how the signals from extracellular matrix proteins are transduced intracellularly. We report here that mouse embryos lacking the Ras family guanine nucleotide exchange factor, C3G (Rapgef1, Grf2), exhibit a cortical neuron **migration** defect resulting in a failure to split the preplate into marginal zone and subplate and a failure to form a cortical plate. C3G-deficient cortical neurons fail to migrate. Instead, they arrest in a multipolar state and accumulate below the preplate. The basement membrane is disrupted and radial glial processes are disorganised and lack attachment in C3G-deficient brains. C3G is activated in response to reelin in cortical neurons, which, in turn, leads to activation of the small GTPase Rap1. In C3G-deficient cells, Rap1 GTP loading in response to reelin stimulation is reduced. In conclusion, the Ras family regulator C3G is essential for two aspects of cortex development, namely radial glial attachment and neuronal **migration**.
81. Absence of Cajal-Retzius cells and subplate neurons associated with defects of tangential cell **migration** from ganglionic eminence in Emx1/2 double mutant cerebral cortex. Emx1 and Emx2, mouse orthologs of the Drosophila head gap gene, ems, are expressed during corticogenesis. Emx2 null mutants exhibit mild defects in cortical lamination. Segregation of differentiating neurons from proliferative cells is normal for the most part, however, reelin-positive Cajal-Retzius cells are lost by the late embryonic period. Additionally, late-born cortical plate neurons display abnormal position. These types of lamination defects are subtle in the Emx1 mutant cortex. In the present study we show that Emx1 and Emx2 double mutant neocortex is much more severely affected. Thickness of the cerebral wall was diminished with the decrease in cell number. Bromodeoxyuridine uptake in the germinal zone was nearly normal; moreover, no apparent increase in cell death or tetraploid cell number was observed. However, tangential **migration** of cells from the ganglionic eminence into the neocortex was greatly inhibited. The wild-type ganglionic eminence cells transplanted into Emx1/2-double mutant telencephalon did not move to the cortex. MAP2-positive neuronal bodies and RC2-positive radial glial cells emerged normally, but the laminar structure subsequently formed was completely abnormal. Furthermore, both corticofugal and corticopetal fibers were predominantly absent in the cortex. Most importantly, neither Cajal-Retzius cells nor subplate neurons were found throughout E11.5-E18.5. Thus, this investigation suggests that laminar organization in the cortex or the production of Cajal-Retzius cells and subplate neurons is interrelated to the tangential movement of cells from the ganglionic eminence under the control of Emx1 and Emx2.
82. Perinatal lethality of microtubule-associated protein 1B-deficient mice expressing alternative isoforms of the protein at low levels. Microtubule-associated protein 1B (MAP1B) has been implicated in axogenesis in cultured cells. To gain insight into the functions that MAP1B plays in vivo, we analyzed a strain of Map1B mutant mice generated by a gene trapping approach. Homozygous mice die on the first day after birth, probably due to a severe abnormal development of the nervous system. They present alterations in the structure of several brain regions. The normal Map1B gene yields different protein isoforms from alternatively spliced transcripts. The smaller isoforms were present in wild type, hetero-, and homozygous mice, but their expression was higher in the mutants than in the wild-type. Moreover, trace amounts of MAP1B protein were also observed in Map1B homozygous mutants, indicating an alternative splicing around the gene trap insertion. Thus, the Map1B gene trapped mutation reported in this work did not generated a null mutant, but a mouse with a drastic deficiency in MAP1B expression. Analyses of these mice indicate the presence of several neural defects and suggest the participation of MAP1B in neuronal **migration**.
83. Reelin stabilizes the actin cytoskeleton of neuronal processes by inducing n-cofilin phosphorylation at serine3. The extracellular matrix protein Reelin, secreted by Cajal-Retzius cells in the marginal zone of the cortex, controls the radial **migration** of cortical neurons. Reelin signaling involves the lipoprotein receptors apolipoprotein E receptor 2 (ApoER2) and very low density lipoprotein receptor (VLDLR), the adapter protein Disabled1 (Dab1), and phosphatidylinositol-3-kinase (PI3K). Eventually, Reelin signaling acts on the cytoskeleton; however, these effects on cytoskeletal organization have remained elusive. In Reelin-deficient mutant mice, most cortical neurons are unable to migrate to their destinations, suggesting a role for Reelin signaling in the dynamic cytoskeletal reorganization that is required for neurons to migrate. Here, we show that Reelin signaling leads to serine3 phosphorylation of n-cofilin, an actin-depolymerizing protein that promotes the disassembly of F-actin. Phosphorylation at serine3 renders n-cofilin unable to depolymerize F-actin, thereby stabilizing the cytoskeleton. We provide evidence for ApoER2, Dab1, Src family kinases (SFKs), and PI3K to be involved in n-cofilin serine3 phosphorylation. Phosphorylation of n-cofilin takes place in the leading processes of migrating neurons as they approach the Reelin-containing marginal zone. Immunostaining for phospho-cofilin in dissociated reeler neurons is significantly increased after incubation in Reelin-containing medium compared with control medium. In a stripe choice assay, neuronal processes are stable on Reelin-coated stripes but grow on control stripes by forming lamellipodia. These novel findings suggest that Reelin-induced stabilization of neuronal processes anchors them to the marginal zone which appears to be required for the directional **migration** process.

### ECM

#### PubMed

1. Overcoming neurite-inhibitory chondroitin sulfate proteoglycans in the astrocyte matrix. Acute trauma to the central nervous system (CNS) can result in permanent damage and loss of function related to the poor regeneration of injured axons. Injured axons encounter several barriers to regeneration, such as the glial scar at the injury site. The glial scar contains extracellular matrix (**ECM**) macromolecules deposited by reactive astrocytes in response to injury. The scar **ECM** is rich in chondroitin sulfate proteoglycans (CSPGs), macromolecules that inhibit axonal growth. CSPGs consist of a core protein with attachment sites for glycosaminoglycan (GAG) chains. An extensive literature demonstrates that enzymatic removal of the GAG chains by chondroitinase ABC permits some axonal regrowth; however, the remaining intact core proteins also possess inhibitory domains. Because metalloproteinases can degrade core proteins of CSPGs, we have evaluated five matrix metalloproteinases (MMPs) and a related protease-a disintegrin and metalloproteinase with thrombospondin motifs-4 (ADAMTS-4)-for their capacity to overcome CSPG inhibition of neuritic growth in culture. The metalloproteinases were selected for their known expression after CNS injuries. Of the MMPs, MMP-3, -7 and -8 reduced or abolished inhibition of neurite outgrowth on a purified CSPG substrate and on an astrocyte-derived **ECM**. ADAMTS-4 also attenuated CSPG inhibition of neurites and had the additional benefits of neither degrading laminin nor causing neurotoxicity. The efficacy of ADAMTS-4 matched that of blocking the EGFR signaling previously reported to mediate CSPG inhibition. These findings highlight ADAMTS-4 as a superior protease for overcoming CSPG inhibition of axonal regeneration in the CNS.

### [Ee]xtracellular [Mm]atrix

#### PubMed

1. p190 RhoGAP is the principal Src substrate in brain and regulates axon outgrowth, guidance and fasciculation. The Src tyrosine kinases have been implicated in several aspects of neural development and nervous system function; however, their relevant substrates in brain and their mechanism of action in neurons remain to be established clearly. Here we identify the potent Rho regulatory protein, p190 RhoGAP (GTPase-activating protein), as the principal Src substrate detected in the developing and mature nervous system. We also find that mice lacking functional p190 RhoGAP exhibit defects in axon guidance and fasciculation. p190 RhoGAP is co-enriched with F-actin in the distal tips of axons, and overexpressing p190 RhoGAP in neuroblastoma cells promotes extensive neurite outgrowth, indicating that p190 RhoGAP may be an important regulator of Rho-mediated actin reorganization in neuronal growth cones. p190 RhoGAP transduces signals downstream of cell-surface adhesion molecules, and we find that p190-RhoGAP-mediated neurite outgrowth is promoted by the **extracellular matrix** protein laminin. Together with the fact that mice lacking neural adhesion molecules or Src kinases also exhibit defects in axon outgrowth, guidance and fasciculation, our results suggest that p190 RhoGAP mediates a Src-dependent adhesion signal for neuritogenesis to the actin cytoskeleton through the Rho GTPase.
2. Connective tissue growth factor: a novel marker of layer VII neurons in the rat cerebral cortex. Connective tissue growth factor (CTGF) belongs to a family of secreted, **extracellular matrix**-associated proteins that are involved in the regulation of cellular functions such as adhesion, migration, mitogenesis, differentiation and survival. Recent studies have also suggested the up-regulation of CTGF in response to trauma, scar formation and excitotoxicity in the CNS. To further elucidate the localization and regulation of this molecule in the rat brain we performed in situ hybridization experiments and found a very strong and selective expression of CTGF messenger ribonucleic acid (mRNA) on the band of layer VII neurons throughout the adult cerebral cortex. Similarly strong neuronal expression was also present in the dorsal endopiriform nucleus, extending rostrally from the ventrocaudal cortical layer VII, and in the deep layers of the olfactory glomeruli and the accessory olfactory nucleus. Double in situ hybridization confirmed selective CTGF mRNA expression on a subpopulation (approximately 35%) of microtubule-associated protein 2 mRNA-positive neurons in the cortical layer VII and the dorsal endopiriform nucleus. The nucleus of lateral olfactory tract showed moderate signal intensity; other parts of the forebrain, mesencephalon and brain stem only revealed a very weak level of CTGF mRNA expression. Non-neuronal expression was rare, considerably weaker than on cortical layer VII neurons, and normally associated with blood vessels. Developmental analysis of CTGF mRNA expression in embryonic and postnatal mouse also showed a moderately late onset at embryonic day 16-18, and confirmed the presence of CTGF mRNA in cortical layer VII in a second rodent species. Interestingly, injury experiments using direct cerebral trauma or injection of excitotoxic kainic acid into rat brain failed to up-regulate CTGF mRNA after injury and during the ensuing period of neuronal cell death, gliosis and neural scar tissue formation. Altogether, the current data suggest a constitutive role of CTGF, particularly in the adult cerebral cortex. In view of the strong ascending projections of subplate neurons into cortical layer 1, this molecule may be involved in the modulation of synaptic input to apical dendrites of pyramidal neurons.
3. Reeler/Disabled-like disruption of neuronal migration in knockout mice lacking the VLDL receptor and ApoE receptor 2. Layering of neurons in the cerebral cortex and cerebellum requires Reelin, an **extracellular matrix** protein, and mammalian Disabled (mDab1), a cytosolic protein that activates tyrosine kinases. Here, we report the requirement for two other proteins, cell surface receptors termed very low density lipoprotein receptor (VLDLR) and apolipoprotein E receptor 2 (ApoER2). Both receptors can bind mDab1 on their cytoplasmic tails and are expressed in cortical and cerebellar layers adjacent to layers that express Reelin. mDab1 expression is upregulated in knockout mice that lack both VLDLR and ApoER2. Inversion of cortical layers and absence of cerebellar foliation in these animals precisely mimic the phenotype of mice lacking Reelin or mDab1. These findings suggest that VLDLR and ApoER2 participate in transmitting the extracellular Reelin signal to intracellular signaling processes initiated by mDab1.
4. Deconstructing the perineuronal net: cellular contributions and molecular composition of the neuronal **extracellular matrix**. Perineuronal nets (PNNs) are lattice-like substructures of the neural **extracellular matrix** that enwrap particular populations of neurons throughout the central nervous system. Previous work suggests that this structure plays a major role in modulating developmental neural plasticity and brain maturation. Understanding the precise role of these structures has been hampered by incomplete comprehension of their molecular composition and cellular contributions to their formation, which is studied herein using primary cortical cell cultures. By defining culture conditions to reduce (cytosine-β-d-arabinofuranoside/AraC addition) or virtually eliminate (elevated potassium chloride (KCl) and AraC application) glia, PNN components impacted by this cell type were identified. Effects of depolarizing KCl concentrations alone were also assessed. Our work identified aggrecan as the primary neuronal component of the PNN and its expression was dramatically up-regulated by both depolarization and glial cell inhibition and additionally, the development of aggrecan-positive PNNs was accelerated. Surprisingly, most of the other PNN components tested were made in a glial-dependent manner in our culture system. Interestingly, in the absence of these glial-derived components, an aggrecan- and hyaluronan-reactive PNN developed, demonstrating that these two components are sufficient for base PNN assembly. Other components were expressed in a glial-dependent manner. Overall, this work provides deeper insight into the complex interplay between neurons and glia in the formation of the PNN and improves our understanding of the molecular composition of these structures.
5. C3G regulates cortical neuron migration, preplate splitting and radial glial cell attachment. Neuronal migration is integral to the development of the cerebral cortex and higher brain function. Cortical neuron migration defects lead to mental disorders such as lissencephaly and epilepsy. Interaction of neurons with their extracellular environment regulates cortical neuron migration through cell surface receptors. However, it is unclear how the signals from **extracellular matrix** proteins are transduced intracellularly. We report here that mouse embryos lacking the Ras family guanine nucleotide exchange factor, C3G (Rapgef1, Grf2), exhibit a cortical neuron migration defect resulting in a failure to split the preplate into marginal zone and subplate and a failure to form a cortical plate. C3G-deficient cortical neurons fail to migrate. Instead, they arrest in a multipolar state and accumulate below the preplate. The basement membrane is disrupted and radial glial processes are disorganised and lack attachment in C3G-deficient brains. C3G is activated in response to reelin in cortical neurons, which, in turn, leads to activation of the small GTPase Rap1. In C3G-deficient cells, Rap1 GTP loading in response to reelin stimulation is reduced. In conclusion, the Ras family regulator C3G is essential for two aspects of cortex development, namely radial glial attachment and neuronal migration.
6. Reelin stabilizes the actin cytoskeleton of neuronal processes by inducing n-cofilin phosphorylation at serine3. The **extracellular matrix** protein Reelin, secreted by Cajal-Retzius cells in the marginal zone of the cortex, controls the radial migration of cortical neurons. Reelin signaling involves the lipoprotein receptors apolipoprotein E receptor 2 (ApoER2) and very low density lipoprotein receptor (VLDLR), the adapter protein Disabled1 (Dab1), and phosphatidylinositol-3-kinase (PI3K). Eventually, Reelin signaling acts on the cytoskeleton; however, these effects on cytoskeletal organization have remained elusive. In Reelin-deficient mutant mice, most cortical neurons are unable to migrate to their destinations, suggesting a role for Reelin signaling in the dynamic cytoskeletal reorganization that is required for neurons to migrate. Here, we show that Reelin signaling leads to serine3 phosphorylation of n-cofilin, an actin-depolymerizing protein that promotes the disassembly of F-actin. Phosphorylation at serine3 renders n-cofilin unable to depolymerize F-actin, thereby stabilizing the cytoskeleton. We provide evidence for ApoER2, Dab1, Src family kinases (SFKs), and PI3K to be involved in n-cofilin serine3 phosphorylation. Phosphorylation of n-cofilin takes place in the leading processes of migrating neurons as they approach the Reelin-containing marginal zone. Immunostaining for phospho-cofilin in dissociated reeler neurons is significantly increased after incubation in Reelin-containing medium compared with control medium. In a stripe choice assay, neuronal processes are stable on Reelin-coated stripes but grow on control stripes by forming lamellipodia. These novel findings suggest that Reelin-induced stabilization of neuronal processes anchors them to the marginal zone which appears to be required for the directional migration process.
7. Overcoming neurite-inhibitory chondroitin sulfate proteoglycans in the astrocyte matrix. Acute trauma to the central nervous system (CNS) can result in permanent damage and loss of function related to the poor regeneration of injured axons. Injured axons encounter several barriers to regeneration, such as the glial scar at the injury site. The glial scar contains **extracellular matrix** (ECM) macromolecules deposited by reactive astrocytes in response to injury. The scar ECM is rich in chondroitin sulfate proteoglycans (CSPGs), macromolecules that inhibit axonal growth. CSPGs consist of a core protein with attachment sites for glycosaminoglycan (GAG) chains. An extensive literature demonstrates that enzymatic removal of the GAG chains by chondroitinase ABC permits some axonal regrowth; however, the remaining intact core proteins also possess inhibitory domains. Because metalloproteinases can degrade core proteins of CSPGs, we have evaluated five matrix metalloproteinases (MMPs) and a related protease-a disintegrin and metalloproteinase with thrombospondin motifs-4 (ADAMTS-4)-for their capacity to overcome CSPG inhibition of neuritic growth in culture. The metalloproteinases were selected for their known expression after CNS injuries. Of the MMPs, MMP-3, -7 and -8 reduced or abolished inhibition of neurite outgrowth on a purified CSPG substrate and on an astrocyte-derived ECM. ADAMTS-4 also attenuated CSPG inhibition of neurites and had the additional benefits of neither degrading laminin nor causing neurotoxicity. The efficacy of ADAMTS-4 matched that of blocking the EGFR signaling previously reported to mediate CSPG inhibition. These findings highlight ADAMTS-4 as a superior protease for overcoming CSPG inhibition of axonal regeneration in the CNS.
8. BDNF regulates reelin expression and Cajal-Retzius cell development in the cerebral cortex. Cajal-Retzius (CR) cells of the cerebral cortex express receptors for the neurotrophin brain-derived neurotrophic factor (BDNF) and downregulate expression of the **extracellular matrix** protein Reelin during early postnatal development, coincident with the onset of cortical BDNF expression. During this period, mice lacking BDNF have elevated levels of Reelin in CR cells. Acute BDNF stimulation of cortical neuron cultures and overexpression of BDNF in the developing brain of transgenic mice prior to the onset of endogenous production causes a profound, dose-dependent reduction of Reelin expression in CR cells. In addition, overexpression of BDNF produces gaps and heterotopias in the marginal zone and disorganization and aggregation of cortical CR cells and induces several other malformations, including aberrant cortical lamination, similar to the phenotype of reeler mutant mice, which lack Reelin. These results demonstrate a role for BDNF on cortical CR cells and identify Reelin as a direct effector of this neurotrophin during brain development.

### [Ii]nvasive

#### GeneRIFs

1. Increased MAP2 expression in malignant melanoma cells significantly inhibits tumor cell growth and neoplasm **invasive**ness, and induces apoptosis and cell cycle arrest.

### [Mm]etastasis

#### PubMed

1. Increased expression of MAP2 inhibits melanoma cell proliferation, invasion and tumor growth in vitro and in vivo. Malignant melanoma (MM) is characterized by aggressive **metastasis** and high mortality rate. Microtubule-associated proteins 2 (MAP2) is expressed abundantly in majority of melanocytic nevi and primary melanomas, but absent in metastatic melanomas. To determine whether MAP2 correlates with tumor progression of MM, we investigated the effects of MAP2 inhibition on the biological behaviour of metastatic melanoma in vitro and in vivo. Our results demonstrated that adenovirus-mediated MAP2 induced apoptotic cell death and cell cycle arrest in metastatic human and mouse melanoma cell lines in vitro, and substantially inhibited the growth of melanomas in nude mice in vivo. In addition, intracellular expression of MAP2 was found to induce the morphologic alteration, suppress the migration and invasion and affect the assembly, stabilization and bundling of microtubules in melanoma cells. This is the first study that MAP2 expression significantly inhibits the growth of MM in vivo. Our results suggest that MAP2 may serve as a promising molecular target for therapy and chemoprevention of MM in humans.

---

## Cmya5

- NCBI Gene
- GeneRIF
- Pubmed

| Search Term | Rank | Fields |
| --- | --- | --- |
| [Ii]nvasive | 10 | PubMed(1) |

  
hide/show details for Cmya5

### [Ii]nvasive

#### PubMed

1. Real-time evaluation of p53 oscillatory behavior in vivo using bioluminescent imaging. p53 is a key mediator of cellular response to stress, and, although its function has been carefully evaluated in vitro, non**invasive** evaluation of the transcriptional activity of p53 in live animals has not been reported. To this end, we developed a transgenic mouse model wherein the firefly luciferase gene expression was dependent on the p53-responsive P2 promoter from the murine double minute 2 (MDM2) gene. Bioluminescence activity following ionizing radiation was shown to be dose, time, and p53 dependent. In addition, expression of both p53 and its activated form as well as the expression of p53 target genes (MDM2 and p21) correlated with bioluminescence activity. Temporal evaluation of p53 activity following ionizing radiation showed a distinct oscillatory pattern, which confirmed the oscillations observed previously in cultured cells. In addition, the kinetics of oscillations were altered by pretreatment with radiation-modifying agents. These results show the use of this mouse model in enhancing our understanding of the transcriptional role of p53 in vivo.

---

## Rtn4

- NCBI Gene
- GeneRIF
- Pubmed

| Search Term | Rank | Fields |
| --- | --- | --- |
| [Ff]ilopodia | 1 | PubMed(3) |
| [Mm]igration | 6 | Process(3); PubMed(14); GeneRIFs(4) |
| [Ee]xtracellular [Mm]atrix | 8 | PubMed(1) |

  
hide/show details for Rtn4

### [Ff]ilopodia

#### PubMed

1. GPR50 interacts with neuronal NOGO-A and affects neurite outgrowth. G protein-coupled receptors (GPCRs) form a link between the cell and their environment when signalling pathways are activated upon ligand binding. However, the ligands and functions for many GPCRs remain to be determined. We sought to understand the function of one such orphan, G protein-coupled receptor 50 (GPR50), through identification of protein interactors. GPR50 was previously discovered as a candidate gene for psychiatric illness and lipid metabolism. Here, we identified neurite outgrowth inhibitor NOGO-A as an interacting partner of GPR50 by yeast two-hybrid studies. We confirmed the interaction in mammalian cells and found an enrichment of both Gpr50 and neuronal Nogo-A at the synapse. In contrast to neuronal NOGO-A overexpression, overexpression of GPR50 increased neurite length and **filopodia**- and lamellipodia-like structures in differentiated Neuroscreen-1 cells. The results are markedly similar to a recent study in Nogo-A KO mice and support the involvement of GPR50 in mental disorders with links to several disease mechanisms.
2. Nogo-A is a negative regulator of CNS angiogenesis. Nogo-A is an important axonal growth inhibitor in the adult and developing CNS. In vitro, Nogo-A has been shown to inhibit migration and cell spreading of neuronal and nonneuronal cell types. Here, we studied in vivo and in vitro effects of Nogo-A on vascular endothelial cells during angiogenesis of the early postnatal brain and retina in which Nogo-A is expressed by many types of neurons. Genetic ablation or virus-mediated knock down of Nogo-A or neutralization of Nogo-A with an antibody caused a marked increase in the blood vessel density in vivo. In culture, Nogo-A inhibited spreading, migration, and sprouting of primary brain microvascular endothelial cells (MVECs) in a dose-dependent manner and induced the retraction of MVEC lamellipodia and **filopodia**. Mechanistically, we show that only the Nogo-A-specific Delta 20 domain exerts inhibitory effects on MVECs, but the Nogo-66 fragment, an inhibitory domain common to Nogo-A, -B, and -C, does not. Furthermore, the action of Nogo-A Delta 20 on MVECs required the intracellular activation of the Ras homolog gene family, member A (Rho-A)-associated, coiled-coil containing protein kinase (ROCK)-Myosin II pathway. The inhibitory effects of early postnatal brain membranes or cultured neurons on MVECs were relieved significantly by anti-Nogo-A antibodies. These findings identify Nogo-A as an important negative regulator of developmental angiogenesis in the CNS. They may have important implications in CNS pathologies involving angiogenesis such as stroke, brain tumors, and retinopathies.
3. Dendritic spine alterations in neocortical pyramidal neurons following postnatal neuronal Nogo-A knockdown. The myelin-associated protein Nogo-A is a well-known inhibitor of axonal regeneration and compensatory plasticity, yet functions of neuronal Nogo-A are not as clear. The present study examined the effects of decreased levels of neuronal Nogo-A on dendritic spines of developing neocortical neurons. Decreased Nogo-A levels in these neurons resulted in lowered spine density and an increase in **filopodia**l type protrusions. These results suggest a role for neuronal Nogo-A in maintaining a spine phenotype in neocortical pyramidal cells.

### [Mm]igration

#### Process

1. cerebral cortex radial glia guided **migration**
2. positive regulation of epithelial cell **migration**
3. regulation of cell **migration**

#### PubMed

1. Proteomic-based detection of a protein cluster dysregulated during cardiovascular development identifies biomarkers of congenital heart defects. Cardiovascular development is vital for embryonic survival and growth. Early gestation embryo loss or malformation has been linked to yolk sac vasculopathy and congenital heart defects (CHDs). However, the molecular pathways that underlie these structural defects in humans remain largely unknown hindering the development of molecular-based diagnostic tools and novel therapies.Murine embryos were exposed to high glucose, a condition known to induce cardiovascular defects in both animal models and humans. We further employed a mass spectrometry-based proteomics approach to identify proteins differentially expressed in embryos with defects from those with normal cardiovascular development. The proteins detected by mass spectrometry (WNT16, ST14, Pcsk1, Jumonji, Morca2a, TRPC5, and others) were validated by Western blotting and immunoflorescent staining of the yolk sac and heart. The proteins within the proteomic dataset clustered to adhesion/**migration**, differentiation, transport, and insulin signaling pathways. A functional role for several proteins (WNT16, ADAM15 and NOGO-A/B) was demonstrated in an ex vivo model of heart development. Additionally, a successful application of a cluster of protein biomarkers (WNT16, ST14 and Pcsk1) as a prenatal screen for CHDs was confirmed in a study of human amniotic fluid (AF) samples from women carrying normal fetuses and those with CHDs.The novel finding that WNT16, ST14 and Pcsk1 protein levels increase in fetuses with CHDs suggests that these proteins may play a role in the etiology of human CHDs. The information gained through this bed-side to bench translational approach contributes to a more complete understanding of the protein pathways dysregulated during cardiovascular development and provides novel avenues for diagnostic and therapeutic interventions, beneficial to fetuses at risk for CHDs.
2. Nogo-a regulates neural precursor **migration** in the embryonic mouse cortex. Although Nogo-A has been intensively studied for its inhibitory effect on axonal regeneration in the adult central nervous system, little is known about its function during brain development. In the embryonic mouse cortex, Nogo-A is expressed by radial precursor/glial cells and by tangentially migrating as well as postmigratory neurons. We studied radially migrating neuroblasts in wild-type and Nogo-A knockout (KO) mouse embryos. In vitro analysis showed that Nogo-A and its receptor components NgR, Lingo-1, TROY, and p75 are expressed in cells emigrating from embryonic forebrain-derived neurospheres. Live imaging revealed an increased cell motility when Nogo-A was knocked out or blocked with antibodies. Antibodies blocking NgR or Lingo-1 showed the same motility-enhancing effect supporting a direct role of surface Nogo-A on **migration**. Bromodeoxyuridine (BrdU) labeling of embryonic day (E)15.5 embryos demonstrated that Nogo-A influences the radial **migration** of neuronal precursors. At E17.5, the normal transient accumulation of radially migrating precursors within the subventricular zone was not detectable in the Nogo-A KO mouse cortex. At E19, **migration** to the upper cortical layers was disturbed. These findings suggest that Nogo-A and its receptor complex play a role in the interplay of adhesive and repulsive cell interactions in radial **migration** during cortical development.
3. Involvement of the myelin-associated inhibitor Nogo-A in early cortical development and neuronal maturation. Nogo-A is a myelin-associated protein expressed by neurons and myelinating mature oligodendrocytes in the central nervous system. Although most research has focused on the participation of Nogo-A in the prevention of axonal regeneration and plasticity in the adult, little attention has been paid to the putative functions of Nogo-A during embryonic development. Here we examined the general pattern and cell-specific distribution of Nogo-A in the prenatal mouse telencephalon. In addition, we studied the development of the major axon tracts and radial and tangential **migration** in Nogo-A/B/C knockout mice. The pattern of Nogo-A showed distinct distribution in radial glia and postmitotic neurons, in which it is particularly enriched in developing axons. Similarly, Nogo-A was enriched at the leading process of tangentially migrating interneurons but not detectable in radial migrating neurons. Although a low level of Nogo-A appears to be on the surface of many cortical neurons, most proteins have intracellular localization. In Nogo-deficient background, neurons displayed early polarization and increased branching in vitro, probably reflecting a cell-intrinsic role of Nogo proteins in branching reduction, and early tangential **migration** was delayed. On the basis of these observations, we propose that Nogo proteins, particularly Nogo-A, are involved in multiple processes during cortical development.
4. Nogo-a regulates neural precursor **migration** in the embryonic mouse cortex. Although Nogo-A has been intensively studied for its inhibitory effect on axonal regeneration in the adult central nervous system, little is known about its function during brain development. In the embryonic mouse cortex, Nogo-A is expressed by radial precursor/glial cells and by tangentially migrating as well as postmigratory neurons. We studied radially migrating neuroblasts in wild-type and Nogo-A knockout (KO) mouse embryos. In vitro analysis showed that Nogo-A and its receptor components NgR, Lingo-1, TROY, and p75 are expressed in cells emigrating from embryonic forebrain-derived neurospheres. Live imaging revealed an increased cell motility when Nogo-A was knocked out or blocked with antibodies. Antibodies blocking NgR or Lingo-1 showed the same motility-enhancing effect supporting a direct role of surface Nogo-A on **migration**. Bromodeoxyuridine (BrdU) labeling of embryonic day (E)15.5 embryos demonstrated that Nogo-A influences the radial **migration** of neuronal precursors. At E17.5, the normal transient accumulation of radially migrating precursors within the subventricular zone was not detectable in the Nogo-A KO mouse cortex. At E19, **migration** to the upper cortical layers was disturbed. These findings suggest that Nogo-A and its receptor complex play a role in the interplay of adhesive and repulsive cell interactions in radial **migration** during cortical development.
5. Involvement of the myelin-associated inhibitor Nogo-A in early cortical development and neuronal maturation. Nogo-A is a myelin-associated protein expressed by neurons and myelinating mature oligodendrocytes in the central nervous system. Although most research has focused on the participation of Nogo-A in the prevention of axonal regeneration and plasticity in the adult, little attention has been paid to the putative functions of Nogo-A during embryonic development. Here we examined the general pattern and cell-specific distribution of Nogo-A in the prenatal mouse telencephalon. In addition, we studied the development of the major axon tracts and radial and tangential **migration** in Nogo-A/B/C knockout mice. The pattern of Nogo-A showed distinct distribution in radial glia and postmitotic neurons, in which it is particularly enriched in developing axons. Similarly, Nogo-A was enriched at the leading process of tangentially migrating interneurons but not detectable in radial migrating neurons. Although a low level of Nogo-A appears to be on the surface of many cortical neurons, most proteins have intracellular localization. In Nogo-deficient background, neurons displayed early polarization and increased branching in vitro, probably reflecting a cell-intrinsic role of Nogo proteins in branching reduction, and early tangential **migration** was delayed. On the basis of these observations, we propose that Nogo proteins, particularly Nogo-A, are involved in multiple processes during cortical development.
6. The Nogo/Nogo Receptor (NgR) Signal Is Involved in Neuroinflammation through the Regulation of Microglial Inflammatory Activation. Microglia have been proposed to play a pivotal role in the inflammation response of the CNS by expressing a range of proinflammatory enzymes and cytokines under pathological stimulus. Our previous study has confirmed that Nogo receptor (NgR), an axon outgrowth inhibition receptor, is also expressed on microglia and regulates cell adhesion and **migration** behavior in vitro. In the present study, we further investigated the proinflammatory effects and possible mechanisms of Nogo on microglia in vitro. In this study, Nogo peptide, Nogo-P4, a 25-amino acid core inhibitory peptide sequence of Nogo-66, was used. We found that Nogo-P4 was able to induce the expression of inducible nitric-oxide synthase and cyclooxygenase-2 and the release of proinflammatory cytokines, including IL-1β, TNF-α, NO, and prostaglandin E2 in microglia, which could be reversed by NEP1-40 (Nogo-66(1-40) antagonist peptide), phosphatidylinositol-specificphospholipase C, or NgR siRNA treatment. After Nogo-P4 stimulated microglia, the phosphorylation levels of NF-κB and STAT3 were increased obviously, which further mediated microglia expressing proinflammatory factors induced by Nogo-P4. Taken together, we concluded that Nogo peptide could directly take part in CNS inflammatory process by influencing the expression of proinflammatory factors in microglia, which were related to the NF-κB and STAT3 signal pathways. Besides neurite outgrowth restriction, the Nogo/NgR signal might be involved in multiple processes in various inflammation-associated CNS diseases.
7. Nogo-A is a negative regulator of CNS angiogenesis. Nogo-A is an important axonal growth inhibitor in the adult and developing CNS. In vitro, Nogo-A has been shown to inhibit **migration** and cell spreading of neuronal and nonneuronal cell types. Here, we studied in vivo and in vitro effects of Nogo-A on vascular endothelial cells during angiogenesis of the early postnatal brain and retina in which Nogo-A is expressed by many types of neurons. Genetic ablation or virus-mediated knock down of Nogo-A or neutralization of Nogo-A with an antibody caused a marked increase in the blood vessel density in vivo. In culture, Nogo-A inhibited spreading, **migration**, and sprouting of primary brain microvascular endothelial cells (MVECs) in a dose-dependent manner and induced the retraction of MVEC lamellipodia and filopodia. Mechanistically, we show that only the Nogo-A-specific Delta 20 domain exerts inhibitory effects on MVECs, but the Nogo-66 fragment, an inhibitory domain common to Nogo-A, -B, and -C, does not. Furthermore, the action of Nogo-A Delta 20 on MVECs required the intracellular activation of the Ras homolog gene family, member A (Rho-A)-associated, coiled-coil containing protein kinase (ROCK)-Myosin II pathway. The inhibitory effects of early postnatal brain membranes or cultured neurons on MVECs were relieved significantly by anti-Nogo-A antibodies. These findings identify Nogo-A as an important negative regulator of developmental angiogenesis in the CNS. They may have important implications in CNS pathologies involving angiogenesis such as stroke, brain tumors, and retinopathies.
8. The ER structural protein Rtn4A stabilizes and enhances signaling through the receptor tyrosine kinase ErbB3. ErbB3 and ErbB4 are receptor tyrosine kinases that are activated by the neuregulin (NRG) family of growth factors. These receptors govern various developmental processes, and their dysregulation contributes to several human disease states. The abundance of ErbB3 and ErbB4, and thus signaling through these receptors, is limited by the E3 ubiquitin ligase Nrdp1, which targets ErbB3 and ErbB4 for degradation. Reticulons are proteins that influence the morphology of the endoplasmic reticulum (ER) by promoting the formation of tubules, a response of cells to some stressors. We found that the ER structural protein reticulon 4A (Rtn4A, also known as Nogo-A) increased ErbB3 abundance and proliferative signaling by suppressing Nrdp1 function. Rtn4A interacted with Nrdp1 and stabilized ErbB3 in an Nrdp1-dependent manner. Rtn4A overexpression induced the redistribution of Nrdp1 from a cytosolic or perinuclear localization to ER tubules. Rtn4A knockdown in human breast tumor cells decreased ErbB3 abundance, NRG-stimulated signaling, and cellular proliferation and **migration**. Because proteins destined for the plasma membrane are primarily synthesized in the sheet portions of the ER, our observations suggest that Rtn4A counteracts the Nrdp1-mediated degradation of ErbB3 by sequestering the ubiquitin ligase into ER tubules. The involvement of a reticulon suggests a molecular link between ER structure and the sensitivity of cells to receptor tyrosine kinase-mediated survival signals at the cell surface.
9. A new role for Nogo as a regulator of vascular remodeling. Although Nogo-A has been identified in the central nervous system as an inhibitor of axonal regeneration, the peripheral roles of Nogo isoforms remain virtually unknown. Here, using a proteomic analysis to identify proteins enriched in caveolae and/or lipid rafts (CEM/LR), we show that Nogo-B is highly expressed in cultured endothelial and smooth muscle cells, as well as in intact blood vessels. The N terminus of Nogo-B promotes the **migration** of endothelial cells but inhibits the **migration** of vascular smooth muscle (VSM) cells, processes necessary for vascular remodeling. Vascular injury in Nogo-A/B-deficient mice promotes exaggerated neointimal proliferation, and adenoviral-mediated gene transfer of Nogo-B rescues the abnormal vascular expansion in those knockout mice. Our discovery that Nogo-B is a regulator of vascular homeostasis and remodeling broadens the functional scope of this family of proteins.
10. Nogo-A downregulation improves insulin secretion in mice. Type 2 diabetes (T2D) is characterized by β-cell dysfunction and the subsequent depletion of insulin production, usually in a context of increased peripheral insulin resistance. T2D patients are routinely treated with oral antidiabetic agents such as sulfonylureas or dipeptidyl peptidase-4 antagonists, which promote glucose- and incretin-dependent insulin secretion, respectively. Interestingly, insulin secretion may also be induced by neural stimulation. Here we report the expression of Nogo-A in β-cells. Nogo-A is a membrane protein that inhibits neurite outgrowth and cel**l migrati**on in the central nervous system. We observed that Nogo-A-deficient mice display improved insulin secretion and glucose clearance. This was associated with a stronger parasympathetic input and higher sensitivity of β-cells to the cholinergic analog carbachol. Insulin secretion was also improved in diabetic db/db mice treated with neutralizing antibody against Nogo-A. Together, these findings suggest that promoting the vagal stimulation of insulin secretion through the selective inhibition of Nogo-A could be a novel therapeutic approach in T2D.
11. Distinct roles of Nogo-a and Nogo receptor 1 in the homeostatic regulation of adult neural stem cell function and neuroblast **migration**. In the adult mammalian subventricular zone (SVZ), GFAP-positive neural stem cells (NSCs) generate neuroblasts that migrate tangentially along the rostral migratory stream (RMS) toward the olfactory bulb (OB). In the mouse brain, we found that the plasticity inhibitors Nogo-A and Nogo receptor 1 (NgR1) are differentially expressed in the SVZ-OB system, in which Nogo-A identifies immature neuroblasts and NgR1 germinal astrocytes. We therefore examined the role of Nogo-A and NgR1 in the regulation of neurogenesis. Pharmacological experiments show that Nogo-66/NgR1 interaction reduces the proliferation of NSCs. This is consistent with a negative-feedback loop, in which newly generated neurons modulate cell division of SVZ stem cells. Moreover, the Nogo-A-Δ20 domain promotes neuroblast **migratio**n toward the OB through activation of the Rho/ROCK (Rho-associated, coiled-coil containing protein kinase) pathway, without the participation of NgR1. Our findings reveal a new unprecedented function for Nogo-A and NgR1 in the homeostatic regulation of the pace of neurogenesis in the adult mouse SVZ and in the **migratio**n of neuroblasts along the RMS.
12. Nogo-B regulates **migration** and contraction of airway smooth muscle cells by decreasing ARPC 2/3 and increasing MYL-9 expression. Abnormal proliferation, apoptosis, **migration** and contraction of airway smooth muscle (ASM) cells in airway remodeling in asthma are basically excessive repair responses to a network of inflammatory mediators such as PDGF, but the mechanisms of such responses remain unclear. Nogo-B, a member of the reticulum family 4(RTN4), is known to play a key role in arteriogenesis and tissue repair. Further studies are needed to elucidate the role of Nogo-B in airway smooth muscle abnormalities.A mouse model of chronic asthma was established by repeated OVA inhalation and subjected to Nogo-B expression analysis using immunohistochemistry and Western Blotting. Then, primary human bronchial smooth muscle cells (HBSMCs) were cultured in vitro and a siRNA interference was performed to knockdown the expression of Nogo-B in the cells. The effects of Nogo-B inhibition on PDGF-induced HBSMCs proliferation, **migration** and contraction were evaluated. Finally, a proteomic analysis was conducted to unveil the underlying mechanisms responsible for the function of Nogo-B.Total Nogo-B expression was approximately 3.08-fold lower in chronic asthmatic mice compared to naïve mice, which was obvious in the smooth muscle layer of the airways. Interference of Nogo-B expression by siRNA resulted nearly 96% reduction in mRNA in cultured HBSMCs. In addition, knockdown of Nogo-B using specific siRNA significantly decreased PDGF-induced **migratio**n of HBSMCs by 2.3-fold, and increased the cellular contraction by 16% compared to negative controls, but had limited effects on PDGF-induced proliferation. Furthermore, using proteomic analysis, we demonstrate that the expression of actin related protein 2/3 complex subunit 5 (ARPC 2/3) decreased and, myosin regulatory light chain 9 isoform a (MYL-9) increased after Nogo-B knockdown.These data define a novel role for Nogo-B in airway remodeling in chronic asthma. Endogenous Nogo-B, which may exert its effects through ARPC 2/3 and MYL-9, is necessary for the **migratio**n and contraction of airway smooth muscle cells.
13. Expression of SKAP-HOM in DCs is required for an optimal immune response in vivo. The cytosolic adaptor molecule SKAP-HOM, similar to the T cell-specific homologue SKAP55, interacts directly with ADAP, and both molecules are involved in inside-out signaling. Previous studies have shown that in the absence of SKAP-HOM, antigen receptor-triggered integrin-mediated adhesion is impaired severely in B cells but not in T cells. In addition, loss of SKAP-HOM results in a less severe clinical course of EAE. DCs are the most potent APCs and express SKAP-HOM. However, the role of SKAP-HOM in DCs remains unknown. Here, we assessed whether the reduced severity of EAE observed in SKAP-HOM-deficient mice is at least partially a result of an impaired cooperation between APCs and T cells. We demonstrate that **migration** of LC in vivo and the spontaneous motility of BMDCs in vitro are increased in the absence of SKAP-HOM. In contrast, triggering of the integrin results in a drastic decrease of DC motility and in enhanced actin polymerization in SKAP-HOM-deficient DCs. Furthermore, the antigen-dependent conjugate formed between wild-type T cells and SKAP-HOM(-/-) DCs is delayed in comparison with wild-type DCs. Strikingly, fewer antigen-specific T cells are induced by immunization with SKAP-HOM(-/-) BMDCs as compared with wild-type BMDCs in vivo. Thus, these findings suggest that SKAP-HOM expression in DCs is required for the induction of an optimal immune response.
14. Endothelial reticulon-4B (Nogo-B) regulates ICAM-1-mediated leukocyte trans**migration** and acute inflammation. The reticulon (Rtn) family of proteins are localized primarily to the endoplasmic reticulum (ER) of most cells. The Rtn-4 family, (aka Nogo) consists of 3 splice variants of a common gene called Rtn-4A, Rtn-4B, and Rtn-4C. Recently, we identified the Rtn-4B (Nogo-B) protein in endothelial and smooth muscle cells of the vessel wall, and showed that Nogo-B is a regulator of cell **migration** in vitro and vascular remodeling and angiogenesis in vivo. However, the role of Nogo-B in inflammation is still largely unknown. In the present study, we use 2 models of inflammation to show that endothelial Nogo-B regulates leukocyte trans**migration** and intercellular adhesion molecule-1 (ICAM-1)-dependent signaling. Mice lacking Nogo-A/B have a marked reduction in neutrophil and monocyte recruitment to sites of inflammation, while Nogo-A/B(-/-) mice engrafted with wild-type (WT) bone marrow still exhibit impaired inflammation compared with WT mice engrafted with Nogo-A/B(-/-) bone marrow, arguing for a critical role of host Nogo in this response. Using human leukocytes and endothelial cells, we show mechanistically that the silencing of Nogo-B with small interfering RNA (siRNA) impairs the trans**migration** of neutrophils and reduces ICAM-1-stimulated phosphorylation of vascular endothelial-cell cadherin (VE-cadherin). Our results reveal a novel role of endothelial Nogo-B in basic immune functions and provide a key link in the molecular network governing endothelial-cell regulation of diapedesis.

#### GeneRIFs

1. findings reveal a new function for Nogo-A and NgR1 in the homeostatic regulation of the pace of neurogenesis and neuroblast **migration**.
2. Endogenous Nogo-B, which may exert its effects through ARPC 2/3 and MYL-9, is necessary for the **migration** and contraction of airway smooth muscle cells.
3. use 2 models of inflammation to show that endothelial Nogo-B regulates leukocyte trans**migration** and intercellular adhesion molecule-1 (ICAM-1)-dependent signaling
4. This study suggested that Nogo-A and its receptor complex play a role in the interplay of adhesive and repulsive cell interactions in radial **migration** during cortical development.

### [Ee]xtracellular [Mm]atrix

#### PubMed

1. The N-terminal domain of Nogo-A inhibits cell adhesion and axonal outgrowth by an integrin-specific mechanism. Myelin-derived Nogo-A protein limits axonal growth after CNS injury. One domain binds to the Nogo-66 receptor to inhibit axonal outgrowth, whereas a second domain, Amino-Nogo, inhibits axonal outgrowth and cell adhesion through unknown mechanisms. Here, we show that Amino-Nogo inhibition depends strictly on the composition of the **extracellular matrix**, suggesting that Amino-Nogo inhibits the function of certain integrins. Amino-Nogo inhibition can be partially overcome by antibodies that activate integrin beta1 or by the addition of Mn2+, an integrin activator. Furthermore, Amino-Nogo reduces focal adhesion kinase activation by fibronectin. Analysis of various cell lines reveals that alpha(v)beta3, alpha5, and alpha4 integrins are sensitive to Amino-Nogo, but alpha6 integrin is not. Both alpha(v) and alpha5 integrins have widespread expression in adult brain and are found in axonal growth cones. Thus, inhibition of integrin signaling by Amino-Nogo contributes to the failure of CNS axon regeneration.

---

## Gprin1

- NCBI Gene
- GeneRIF
- Pubmed

| Search Term | Rank | Fields |
| --- | --- | --- |
| [Mm]igration | 6 | PubMed(1) |

  
hide/show details for Gprin1

### [Mm]igration

#### PubMed

1. Dynamic expression patterns of G protein-regulated inducer of neurite outgrowth 1 (GRIN1) and its colocalization with Galphao implicate significant roles of Galphao-GRIN1 signaling in nervous system. GRIN1 (Gprin 1) is a signaling molecule coexpression of which with constitutively active form of Galphao can stimulate neurite extensions in Neuro2a cells, yet its in vivo roles remain elusive. Here, we examine expression profiles of GRIN1 during mouse development by in situ hybridization (ISH) and immunohistochemistry. ISH analysis revealed that GRIN1 expression was limited to the nervous system at all developmental stages tested: in the central nervous system, GRIN1 expression occurred within the entire embryonic mantle zones, while it became restricted to sets of nuclei at postnatal to adult stages. Immunohistochemistry using a GRIN1-specific antibody demonstrated that GRIN1 colocalized with Galphao at neuronal dendrites and axons, but it was not detected in glial cells. These results suggest that Galphao-GRIN1 pathway could mediate significant roles in neuronal **migration** and differentiation at embryonic stages and exert functions in wiring and/or maintenance of specific neural circuitries at postnatal to adult stages.

---
